# Supplementary material for: A catalog of the diversity and ubiquity of bacterial microcompartments
Source: Nat Commun. 2021 Jun 21;12:3809. doi: 10.1038/s41467-021-24126-4 (PMC8217296; doi:10.1038/s41467-021-24126-4)
Supplement: Supplementary file 1 — Supplementary Information [file 41467_2021_24126_MOESM1_ESM.pdf]

A Catalog of the Diversity and Ubiquity of Bacterial Microcompartments

Markus Sutter, Matthew R. Melnicki, Frederik Schulz, Tanja Woyke, Cheryl A. Kerfeld

Supplementary Information

	total #	ARO	BUF1	BUF1B	BUF2	BUF3	EUT1	EUT2A	EUT2B	EUT2C	EUT2D	EUT2E	EUT2F	EUT2G	EUT2H	EUT2I	EUT2x	EUT3	GRM1A	GRM2	GRM3A	GRM3B	GRM4	GRM5	GRM6	GRMguf	MIC1	MUF1	PDU1AB	PDU1C	PDU1D	PDU1E	PDU1F	PVM	PVMlike	SPU1	SPU4
ARO	17				2																						3										
BUF1	46					2		2	14							7			4												4						
BUF1B	55									11		1													1										4		
BUF2	8	2																																			
BUF3	10		2						2										1												1						2
EUT1	1408						2													68		28	4						817			1					
EUT2A	177		2																19											10		13			9		
EUT2B	147		14			2			6					14					17					4	1						27				8		
EUT2C	88			11								2					8														1						4
EUT2D	28										18								14							3					15						
EUT2E	75			1						2		10				1	3																				1
EUT2F	59																		1														37				
EUT2G	29								14					2										1							10						
EUT2H	38																8																				
EUT2I	162		7									1				24			36		2			1							3				4		
EUT2x	24									8		3			8																1						2
EUT3	21																		4							4					2						
GRM1A	297		4			1		19	17		14		1			36		4	4		9			1	3	11		5		1	14	8			19		2
GRM2	261						68													2		17	1						71								
GRM3A	84															2			9		2											2			3		
GRM3B	94						28													17									2								
GRM4	8						4													1									4								
GRM5	216								4					1		1			1					2							1						
GRM6	19			1					1										3												2	1			1		
GRMguf	18										3							4	11												1						
MIC1	46	3																									4										
MUF1	7																		5																		
PDU1AB	1315						817													71		2	4						18			1					
PDU1C	89							10											1													1					
PDU1D	183		4			1			27	1	15			10		3	1	2	14					1	2	1					8						
PDU1E	46						1	13											8		2				1				1	1							
PDU1F	44												37																								
PVM	305																																	10			
PVMlike	57			4				9	8							4			19		3				1												
SPU1	65																																			6	
SPU4	34					2				4		1					2		2																		2

Supplementary Table 1. Co-occurrence of BMC types. Green and dark green colors indicate 50% or higher co-occurrence of the BMC type on the left with the corresponding second type in the rows. BMC types with less than 3 total co-occurrences are not shown for clarity.

# genomes					
1	BUF1	BUF3	EUT2	GRM1	PDU1
3	BUF1	EUT2	GRM1		
1	BUF1	EUT2	PDU1		
1	EUT1	GRM2	GRM4		
43	EUT1	GRM2	PDU1		
3	EUT1	GRM4	PDU1		
1	EUT2	GRM1	GRM5		
1	EUT2	GRM1	GRM6		
6	EUT2	GRM1	GRMguf		
7	EUT2	GRM1	PDU1		
16	EUT2	GRM1	PVMlike		
1	GRM1	GRM3	PVMlike		
1	GRM1	GRM6	PDU1		
1	GRM1	GRM6	PVMlike		
1	GRM1	GRMguf	PDU1		

Supplementary Table 2. Co-occurrence loci for distinct BMC functional types in genomes that contain three or more BMC types. Number of genomes vs the types found in them. Duplicate BMC types are not counted, colors are highlighting the different general BMC types.

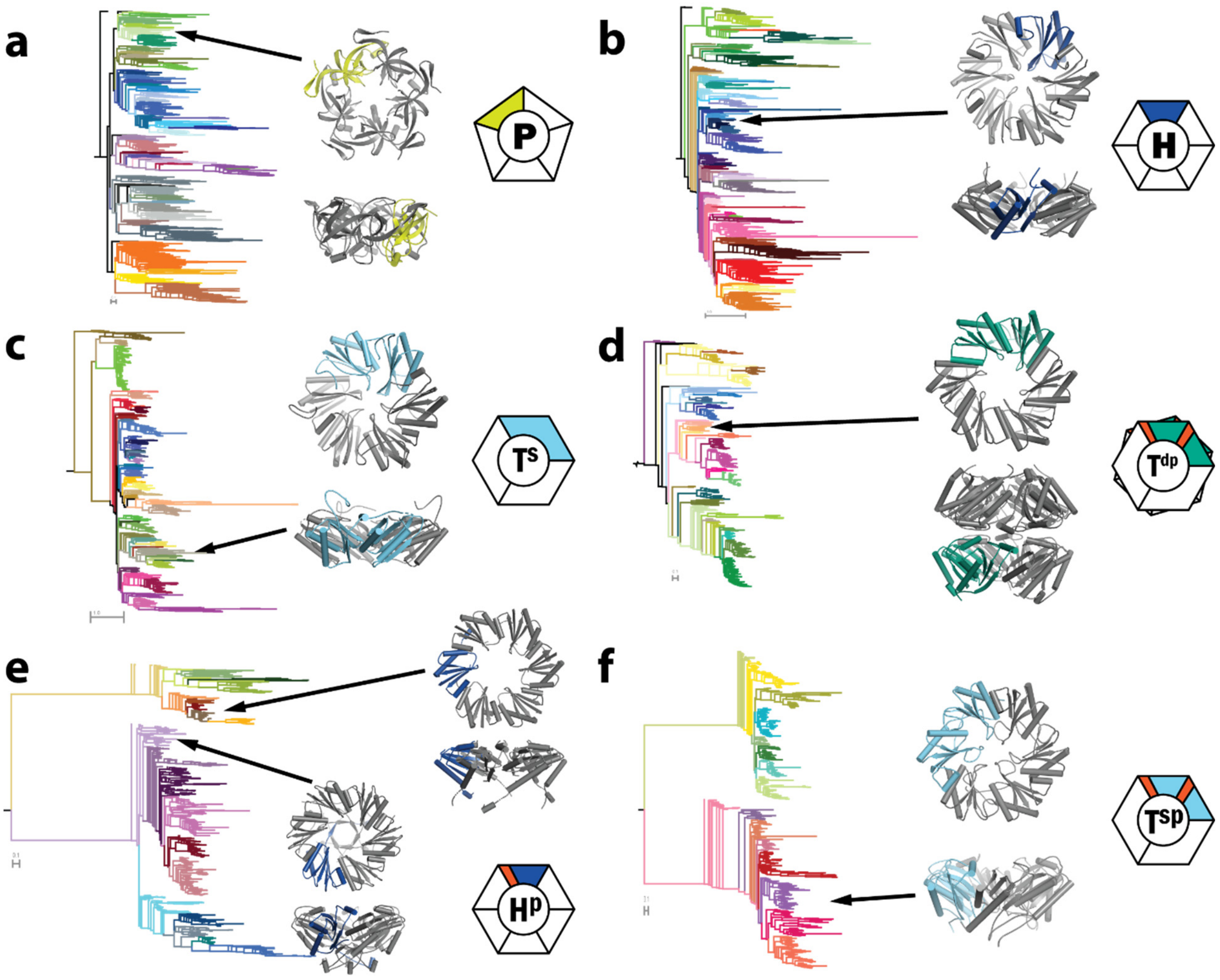

Supplementary Fig. 1. BMC shell protein phylogenetic trees with representative protein structures. **a**, BMC-P: Hoch_5814 PDB ID 5V74 (HO complete shell). **b**, BMC-H: Hoch_5815, PDB ID 5V74 (HO complete shell). **c**, BMC-T^{s}: Hoch_5812, PDB ID 5DIH. **d**, BMC-T^{dp} : Hoch_5816, PDB ID 5V75. **e**, BMC-H^{p} : (top): GrpU, PDB ID 4OLO; (bottom): EutS, PDB ID 4AXI. **f**, BMC-Tsp: EutL, PDB ID 3GFH.

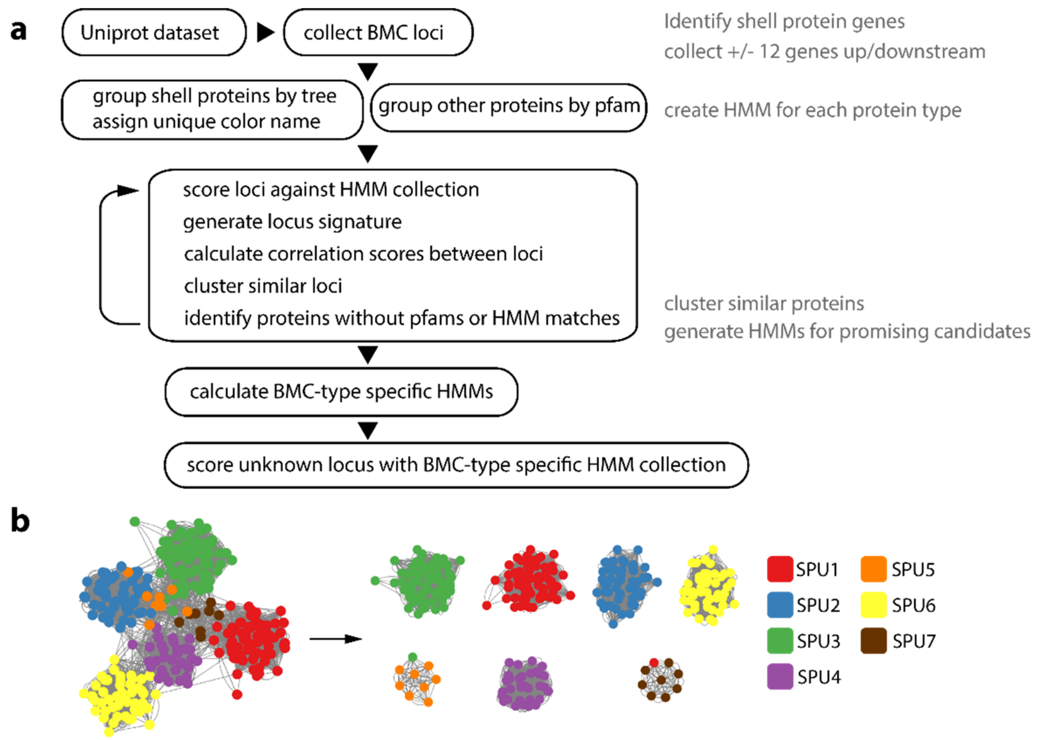

Supplementary Fig. 2. BMC classification methodology and clustering example. **a**, Flowchart of the methodology of BMC locus classification. **b**, Example of clustering by locus scoring, shown for all SPU (sugar phosphate utilization) BMC types: Force directed layout (left) and MCL clustering result (right) shows consistency of the BMC type clusters.

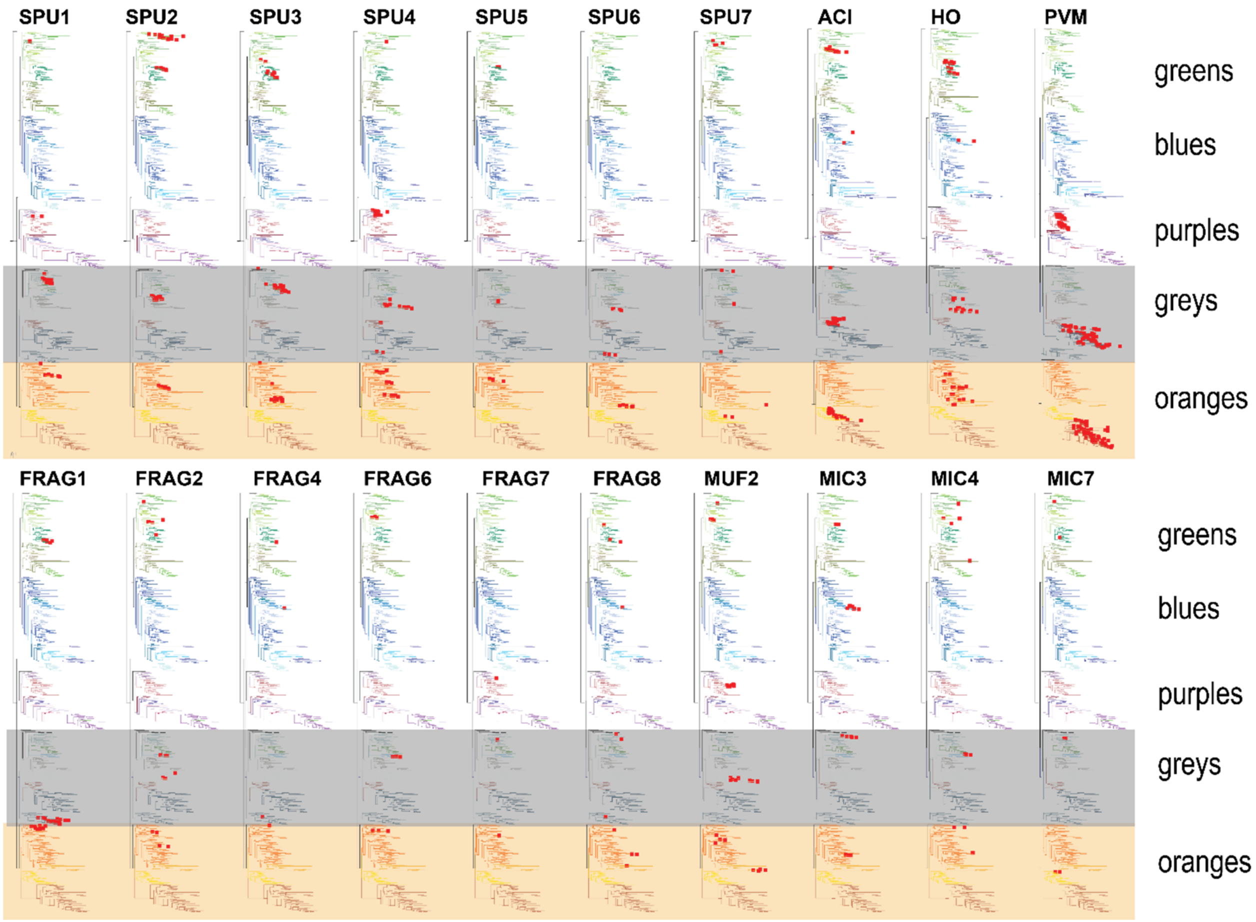

Supplementary Fig. 3. Distribution of BMC-P proteins in BMC types that have characteristic BMC-P triplets. In BMC types with at least three distinct BMC-P, one member each is from the grey (shaded) and orange (shaded) major clades and a third from the green/blue/purple clades. Positions of the BMC-P on the tree for each type are marked with red squares.

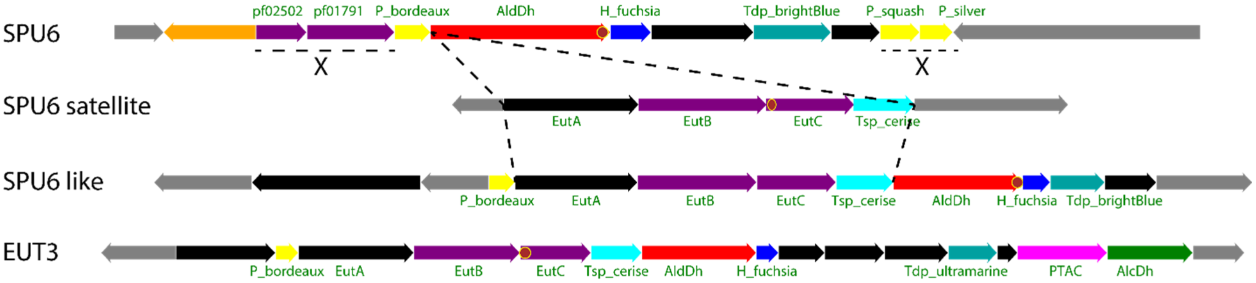

Supplementary Fig. 4. Locus diagrams comparing the SPU6 and EUT3 loci. Locus diagrams of an SPU6 locus, its satellite, a SPU6 like locus and a EUT3 locus. The satellite of SPU6 inserting into the main locus would result in a SPU6 like locus. The genes for SPU signature enzymes pf02502 and pf01791 as well as two BMC-P would be deleted. Unlabeled genes in black are conserved among loci, in grey non-conserved proteins.

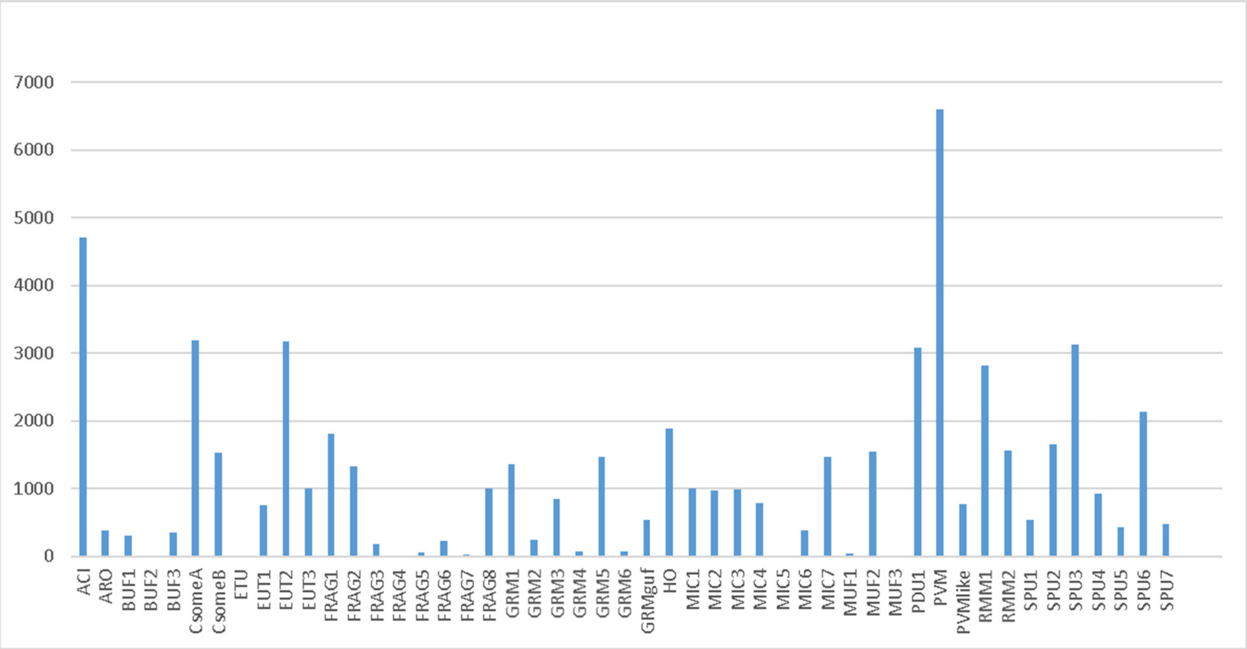

Supplementary Fig. 5. Presence of BMC types in metagenomes. Y-axis represents the number of metagenomes where both the BMC-H and BMC-P of a certain type are found as top hits of an HMM search.

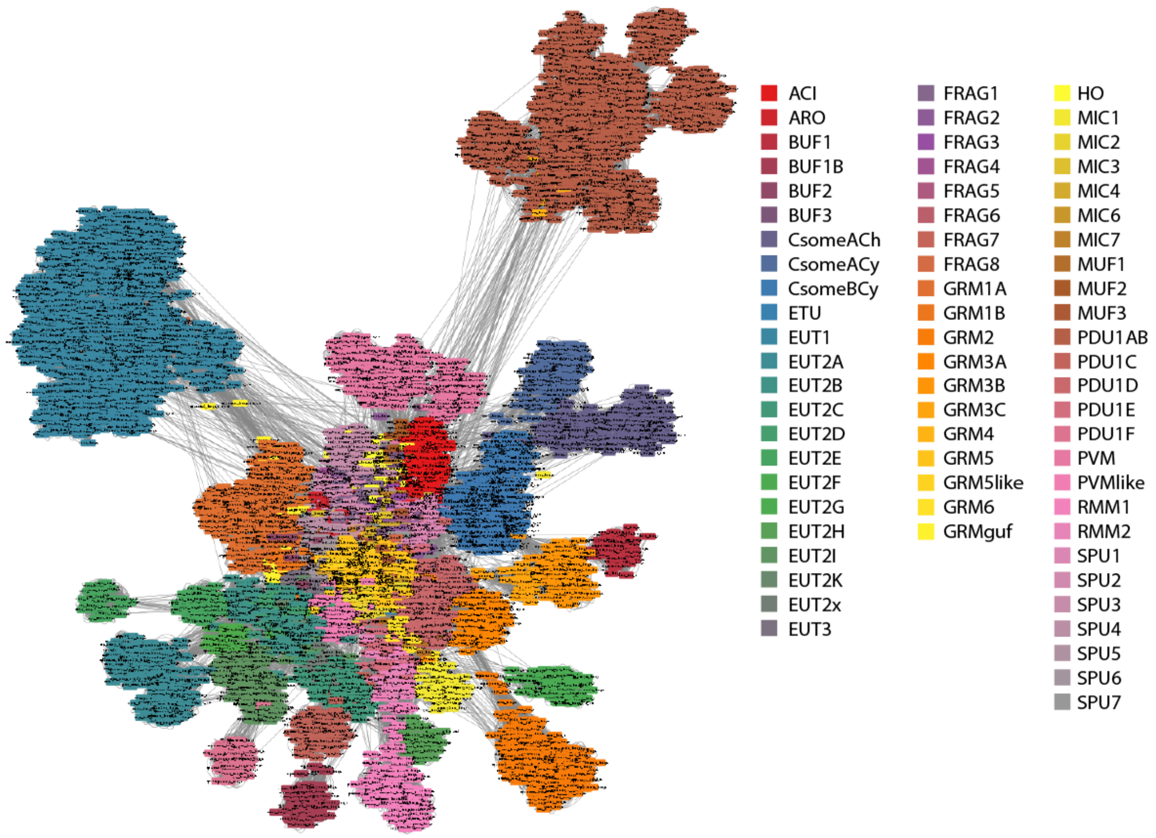

Supplementary Fig. 6. Visualization of all vs all BMC type scoring in a force-directed layout. Colors represent different BMC types/subtypes according to legend.
