# Supplementary material for: A catalog of the diversity and ubiquity of bacterial microcompartments
Source: Nat Commun. 2021 Jun 21;12:3809. doi: 10.1038/s41467-021-24126-4 (PMC8217296; doi:10.1038/s41467-021-24126-4)
Supplement: Supplementary file 2 — Description of Additional Supplementary Files [file 41467_2021_24126_MOESM2_ESM.pdf]

Description of Additional Supplementary Files

File Name: Supplementary Data 1
Description: High resolution shell protein trees. PDF versions of the BMC shell protein trees in Figure 3

File Name: Supplementary Data 2
Description: BMC types overview. Detailed description of BMC loci with example locus diagrams

File Name: Supplementary Data 3
Description: Locus diagrams of all BMC loci identified in this study. Searchable PDF of locus diagrams

File Name: Supplementary Data 4
Description: Shell protein tree xml files. XML format version of the shell protein trees in Figure 3

File Name: Supplementary Data 5
Description: HMM library of BMC locus proteins

File Name: Supplementary Data 6
Description: Table of the description of the HMM names used in the HMM library in Supplementary Data 5
