# Supplementary material for: A catalog of the diversity and ubiquity of bacterial microcompartments
Source: Nat Commun. 2021 Jun 21;12:3809. doi: 10.1038/s41467-021-24126-4 (PMC8217296; doi:10.1038/s41467-021-24126-4)
Supplement: Supplementary file 9 — Reporting Summary [file 41467_2021_24126_MOESM9_ESM.pdf]

nature research

Corresponding author(s): Cheryl A. Kerfeld

Last updated by author(s): May 25, 2021

Reporting Summary

Nature Research wishes to improve the reproducibility of the work that we publish. This form provides structure for consistency and transparency in reporting. For further information on Nature Research policies, see our Editorial Policies and the Editorial Policy Checklist.

Statistics

For all statistical analyses, confirm that the following items are present in the figure legend, table legend, main text, or Methods section.

n/a	Confirmed	
x	☐	The exact sample size (n) for each experimental group/condition, given as a discrete number and unit of measurement
x	☐	A statement on whether measurements were taken from distinct samples or whether the same sample was measured repeatedly
x	☐	The statistical test(s) used AND whether they are one- or two-sided<br>*Only common tests should be described solely by name; describe more complex techniques in the Methods section.*
x	☐	A description of all covariates tested
x	☐	A description of any assumptions or corrections, such as tests of normality and adjustment for multiple comparisons
x	☐	A full description of the statistical parameters including central tendency (e.g. means) or other basic estimates (e.g. regression coefficient) AND variation (e.g. standard deviation) or associated estimates of uncertainty (e.g. confidence intervals)
x	☐	For null hypothesis testing, the test statistic (e.g. F, t, r) with confidence intervals, effect sizes, degrees of freedom and P value noted<br>*Give P values as exact values whenever suitable.*
x	☐	For Bayesian analysis, information on the choice of priors and Markov chain Monte Carlo settings
x	☐	For hierarchical and complex designs, identification of the appropriate level for tests and full reporting of outcomes
x	☐	Estimates of effect sizes (e.g. Cohen's d, Pearson's r), indicating how they were calculated

Our web collection on statistics for biologists contains articles on many of the points above.

Software and code

Policy information about availability of computer code

Data collection	no software used for data collection
Data analysis	The following software was used for data analysis / visualization: HMMER 3.1b2 , Cytoscape 3.7.2, clustalw 2.1, trimAl 1.2rev59, usearch v11.0.667, Archaeopteryx 0.9920, RAxML-NG 0.6.0, BMGE v1.12, FastTree2, ete3, MAFFT-linsi v7.294b, pymol 2.3.0a0, Excel 2016, scripts found on https://github.com/markussutter/bmc_loci

For manuscripts utilizing custom algorithms or software that are central to the research but not yet described in published literature, software must be made available to editors and reviewers. We strongly encourage code deposition in a community repository (e.g. GitHub). See the Nature Research guidelines for submitting code & software for further information.

Data

Policy information about availability of data

All manuscripts must include a data availability statement. This statement should provide the following information, where applicable:

- Accession codes, unique identifiers, or web links for publicly available datasets
- A list of figures that have associated raw data
- A description of any restrictions on data availability

All sequence and metadata was obtained from public databases (Uniprot, ENA, IMG/M). All discussed BMC types are found as locus diagrams in Supplementary Data: Locus diagrams and the individual unique GeneIDs can be derived from the diagrams. An annotated version of the phylogenomic trees in Fig. 3 can be found in Supplementary Data: High resolution shell protein trees. The HMM collection is found as a compressed file in Supplementary Data: HMM library, a description of the HMM names used in those is found as Supplementary Table: HMM names table. Any additional data is available from the corresponding author upon reasonable request.

Field-specific reporting

Please select the one below that is the best fit for your research. If you are not sure, read the appropriate sections before making your selection.

☐ Life sciences ☐ Behavioural & social sciences ☒ Ecological, evolutionary & environmental sciences

For a reference copy of the document with all sections, see nature.com/documents/nr-reporting-summary-flat.pdf

Ecological, evolutionary & environmental sciences study design

All studies must disclose on these points even when the disclosure is negative.

Study description	Protein sequence data and metadata obtained from the UniprotKB database was analyzed with various sequence alignment and classification tools
Research sample	protein sequence data based on the UniprotKB database (https://www.uniprot.org/)
Sampling strategy	all shell protein hits in UniprotKB according to methods section (pfam and interpro identifiers) and genomic surrounding +/-12 proteins
Data collection	database download using search terms described in methods section
Timing and spatial scale	March 2020
Data exclusions	incomplete BMC loci from incomplete sequencing were excluded manually
Reproducibility	an iterative process was used to establish consistent and reproducible BMC type assignment
Randomization	Not applicable, data was clustered into different BMC types as described in methods section with manual supervision
Blinding	Not applicable, removing BMC type HMMs for one type will cause those types to be assigned to the next closest BMC type

Did the study involve field work? ☐ Yes ☒ No

Reporting for specific materials, systems and methods

We require information from authors about some types of materials, experimental systems and methods used in many studies. Here, indicate whether each material, system or method listed is relevant to your study. If you are not sure if a list item applies to your research, read the appropriate section before selecting a response.

Materials & experimental systems

n/a	Involved in the study	
☒	☐	Antibodies
☒	☐	Eukaryotic cell lines
☒	☐	Palaeontology and archaeology
☒	☐	Animals and other organisms
☒	☐	Human research participants
☒	☐	Clinical data
☒	☐	Dual use research of concern

Methods

n/a	Involved in the study	
☒	☐	ChIP-seq
☒	☐	Flow cytometry
☒	☐	MRI-based neuroimaging
