# Supplementary material for: A catalog of the diversity and ubiquity of bacterial microcompartments
Source: Nat Commun. 2021 Jun 21;12:3809. doi: 10.1038/s41467-021-24126-4 (PMC8217296; doi:10.1038/s41467-021-24126-4)
Supplement: Supplementary file 5 — Supplementary Data 3 [file Supplementary_Data_3_Locus_diagrams.pdf › Supplementary_Data_3_Locus_diagrams.pdf]

# A Catalog of the Diversity and Ubiquity of Bacterial Microcompartments

Markus Sutter, Matthew R. Melnicki, Frederik Schulz, Tanja Woyke, Cheryl A. Kerfeld

## Supplementary Data: Locus Diagrams

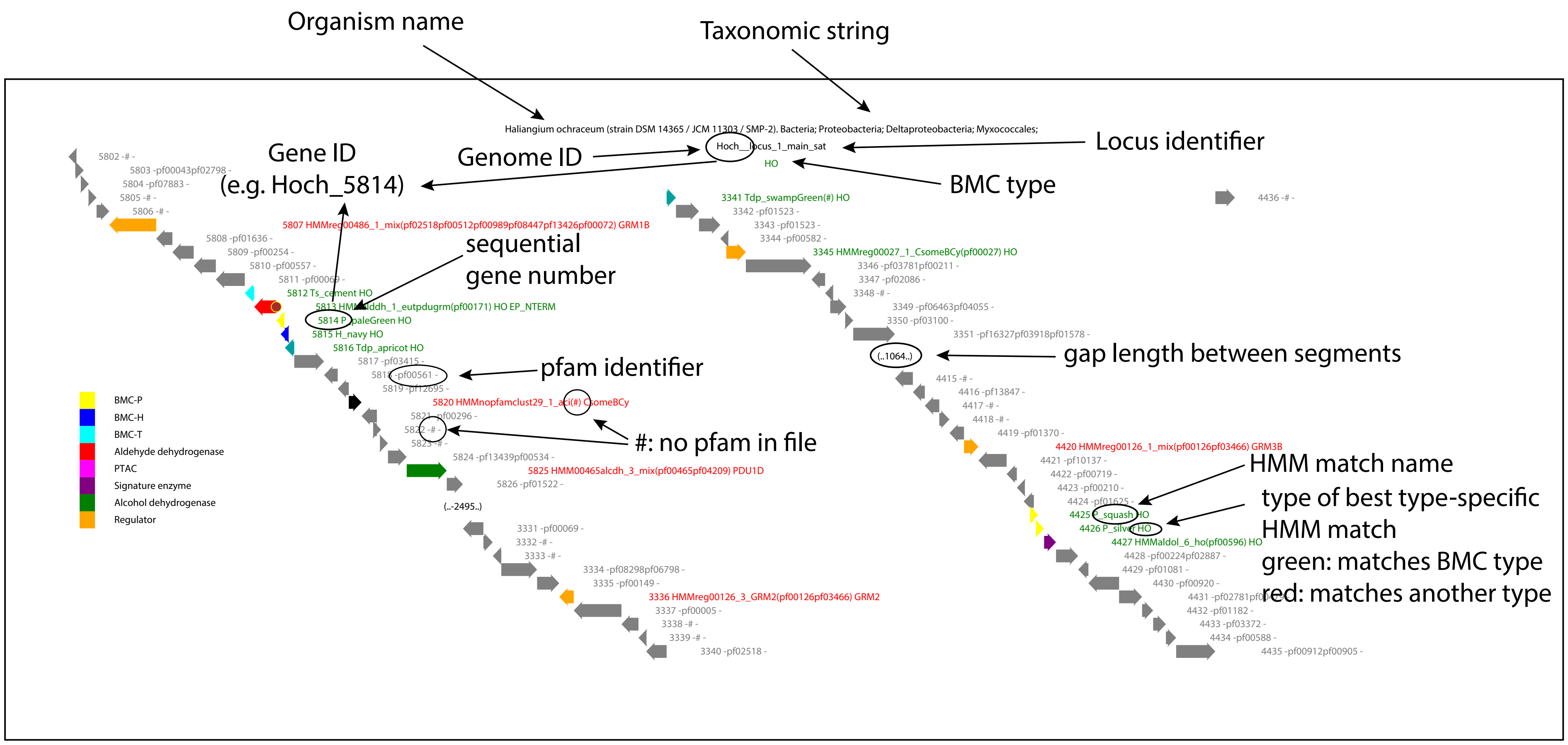

Acidobacteria bacterium RIFCSPHIGHO2_02_FULL_67_57. Bacteria; Acidobacteria.

A3B65__locus_1_main_sat

ACI

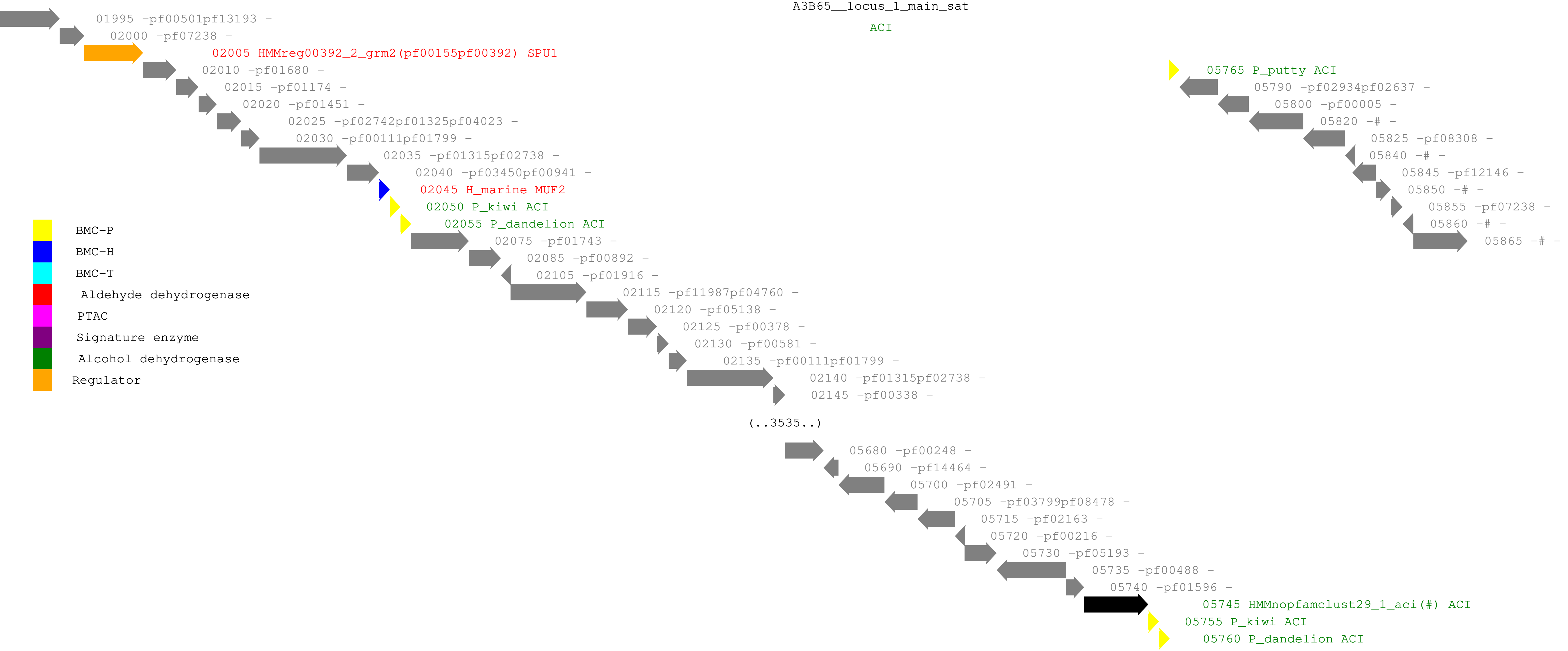

Acidobacteria bacterium RIFCSPHIGHO2_12_FULL_67_30. Bacteria; Acidobacteria.

A3D93__locus_1

ACI

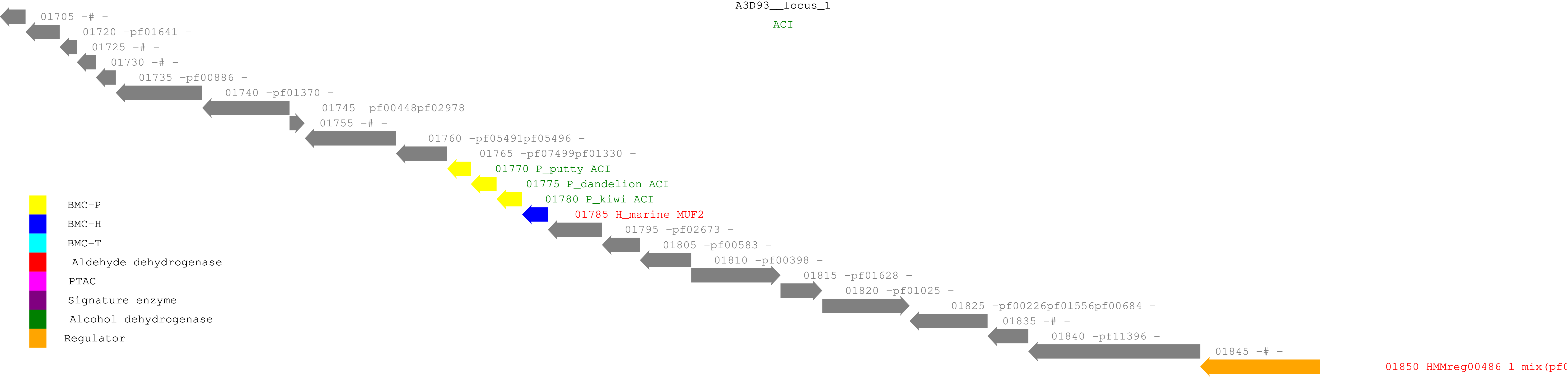

Acidobacteria bacterium RIFCSPLOWO2_12_FULL_66_21. Bacteria; Acidobacteria.

A3G21__locus_1_main_sat

ACI

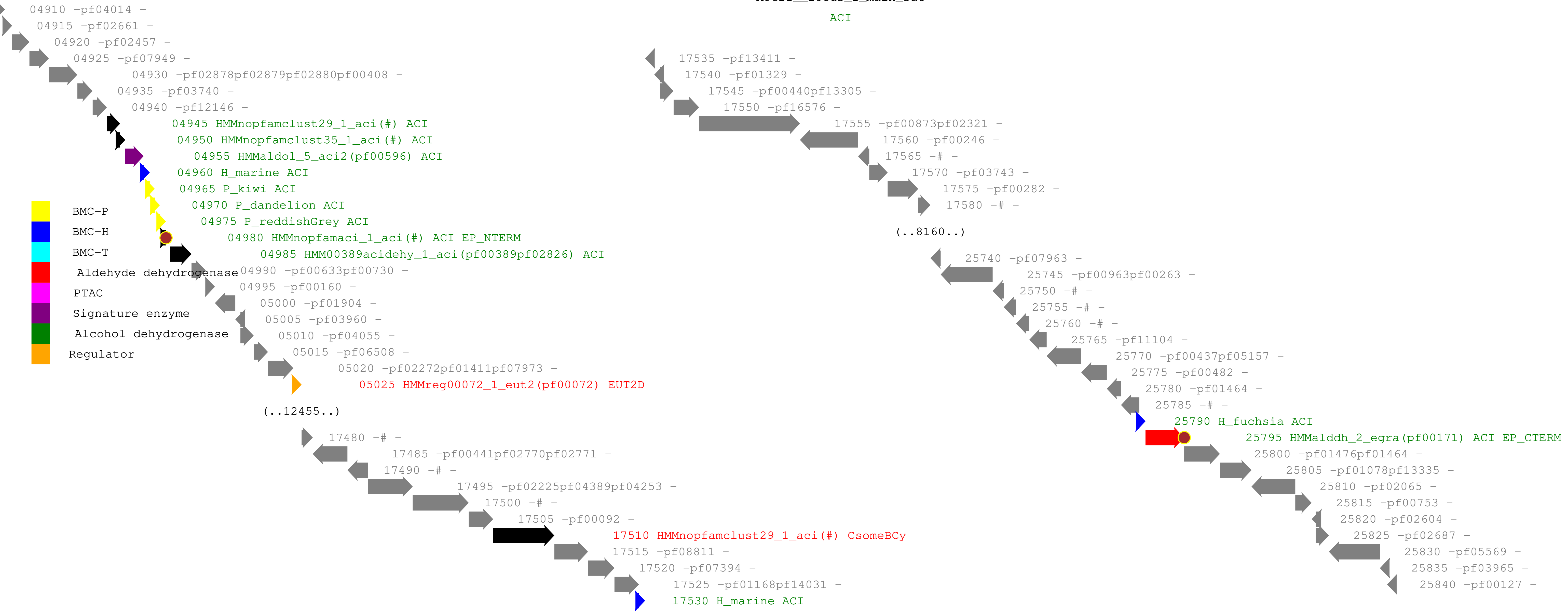

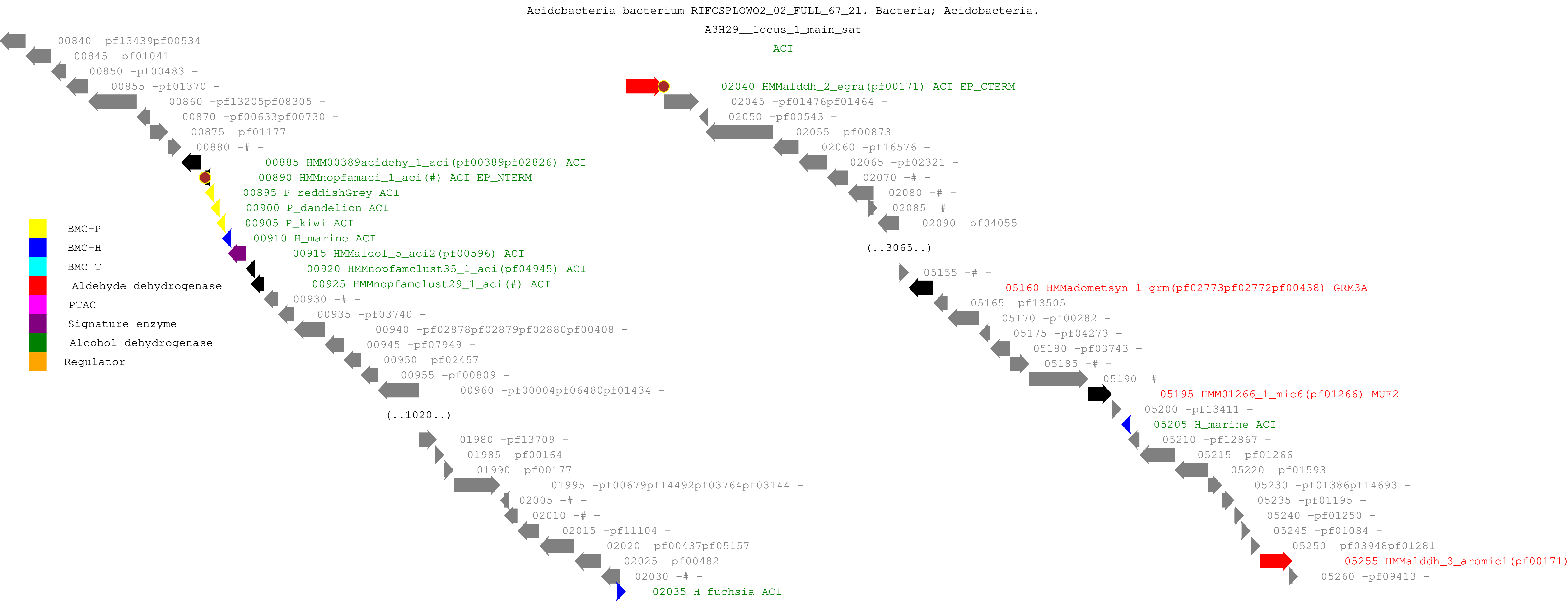
Acidobacteria bacterium RIFCSPLOWO2_02_FULL_67_21. Bacteria; Acidobacteria.
A3H29__locus_1_main_sat
ACI
00840 -pf13439pf00534 -
00845 -pf01041 -
00850 -pf00483 -
00855 -pf01370 -
00860 -pf13205pf08305 -
00870 -pf00633pf00730 -
00875 -pf01177 -
00880 -# -
00885 HMM00389acidehy_1_aci(pf00389pf02826) ACI
00890 HMMnopfamaci_1_aci(#) ACI EP_NTERM
00895 P_reddishGrey ACI
00900 P_dandelion ACI
00905 P_kiwi ACI
00910 H_marine ACI
00915 HMMaldol_5_aci2(pf00596) ACI
00920 HMMnopfamclust35_1_aci(pf04945) ACI
00925 HMMnopfamclust29_1_aci(#) ACI
00930 -# -
00935 -pf03740 -
00940 -pf02878pf02879pf02880pf00408 -
00945 -pf07949 -
00950 -pf02457 -
00955 -pf00809 -
00960 -pf00004pf06480pf01434 -
(..1020..)
01980 -pf13709 -
01985 -pf00164 -
01990 -pf00177 -
01995 -pf00679pf14492pf03764pf03144 -
02005 -# -
02010 -# -
02015 -pf11104 -
02020 -pf00437pf05157 -
02025 -pf00482 -
02030 -# -
02035 H_fuchsia ACI
BMC-P
BMC-H
BMC-T
Aldehyde dehydrogenase
PTAC
Signature enzyme
Alcohol dehydrogenase
Regulator
02040 HMMaldh_2_egra(pf00171) ACI EP_CTERM
02045 -pf01476pf01464 -
02050 -pf00543 -
02055 -pf00873 -
02060 -pf16576 -
02065 -pf02321 -
02070 -# -
02080 -# -
02085 -# -
02090 -pf04055 -
(..3065..)
05155 -# -
05160 HMMadometsyn_1_grm(pf02773pf02772pf00438) GRM3A
05165 -pf13505 -
05170 -pf00282 -
05175 -pf04273 -
05180 -pf03743 -
05185 -# -
05190 -# -
05195 HMM01266_1_mic6(pf01266) MUF2
05200 -pf13411 -
05205 H_marine ACI
05210 -pf12867 -
05215 -pf01266 -
05220 -pf01593 -
05230 -pf01386pf14693 -
05235 -pf01195 -
05240 -pf01250 -
05245 -pf01084 -
05250 -pf03948pf01281 -
05255 HMMaldh_3_aromic1(pf00171)
05260 -pf09413 -

Acidobacteria bacterium RIFCSPLOWO2_02_FULL_64_15. Bacteria; Acidobacteria.

A3H95__locus_1_main_sat

ACI

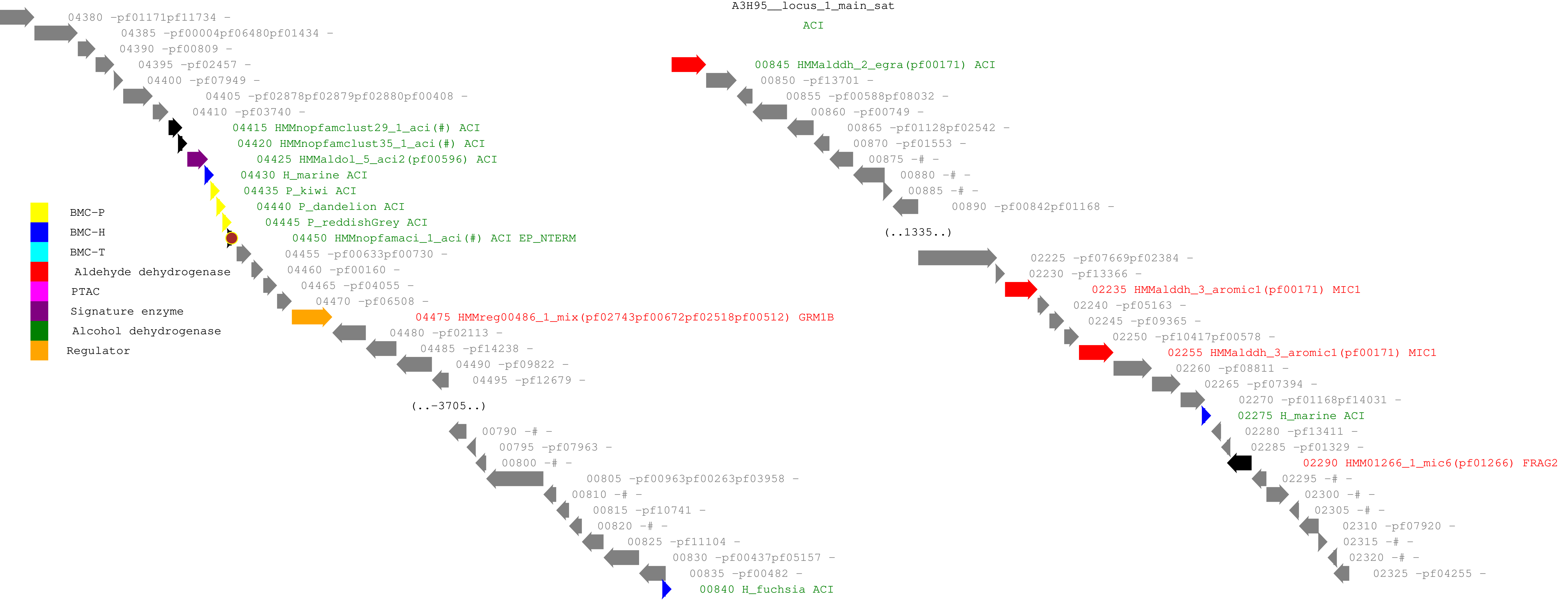

Acidobacteria bacterium RIFCSPLOWO2_02_FULL_65_29. Bacteria; Acidobacteria.

A3H97__locus_1_main_sat

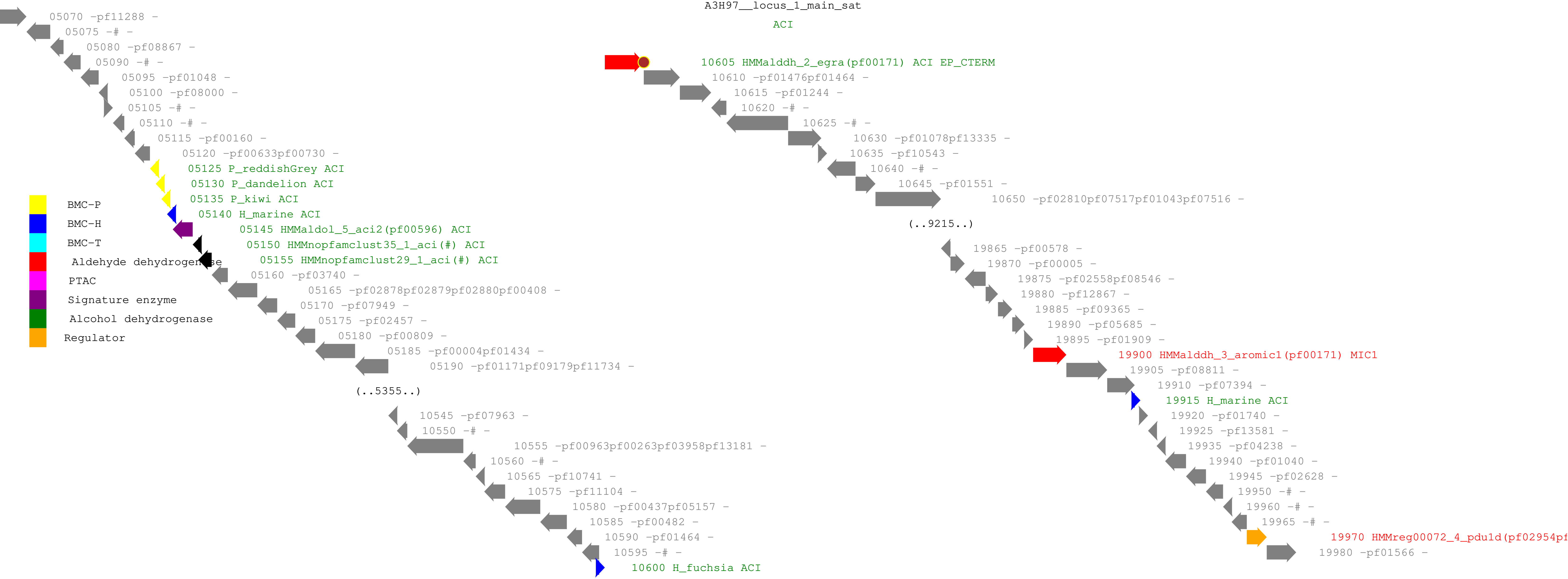

Acidobacteria bacterium RIFCSPLOWO2_02_FULL_68_18. Bacteria; Acidobacteria.

A3I61__locus_1_main_sat

ACI

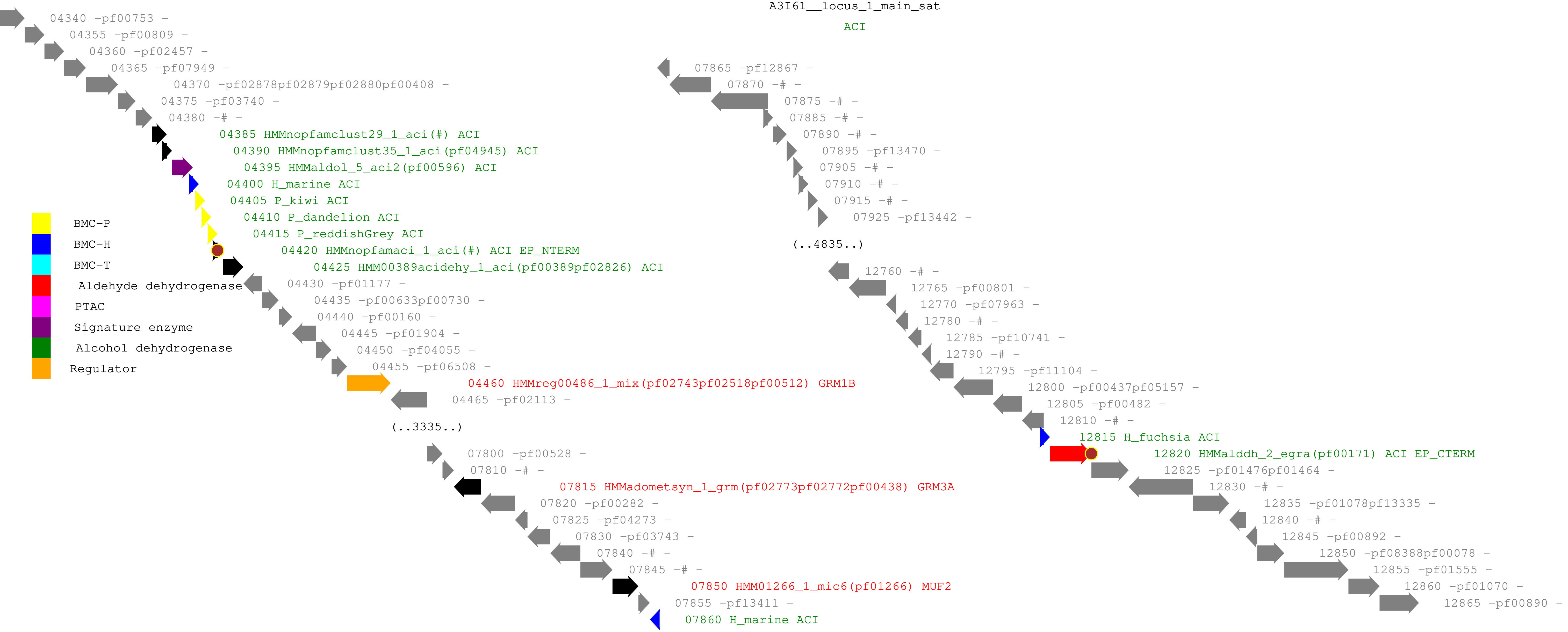

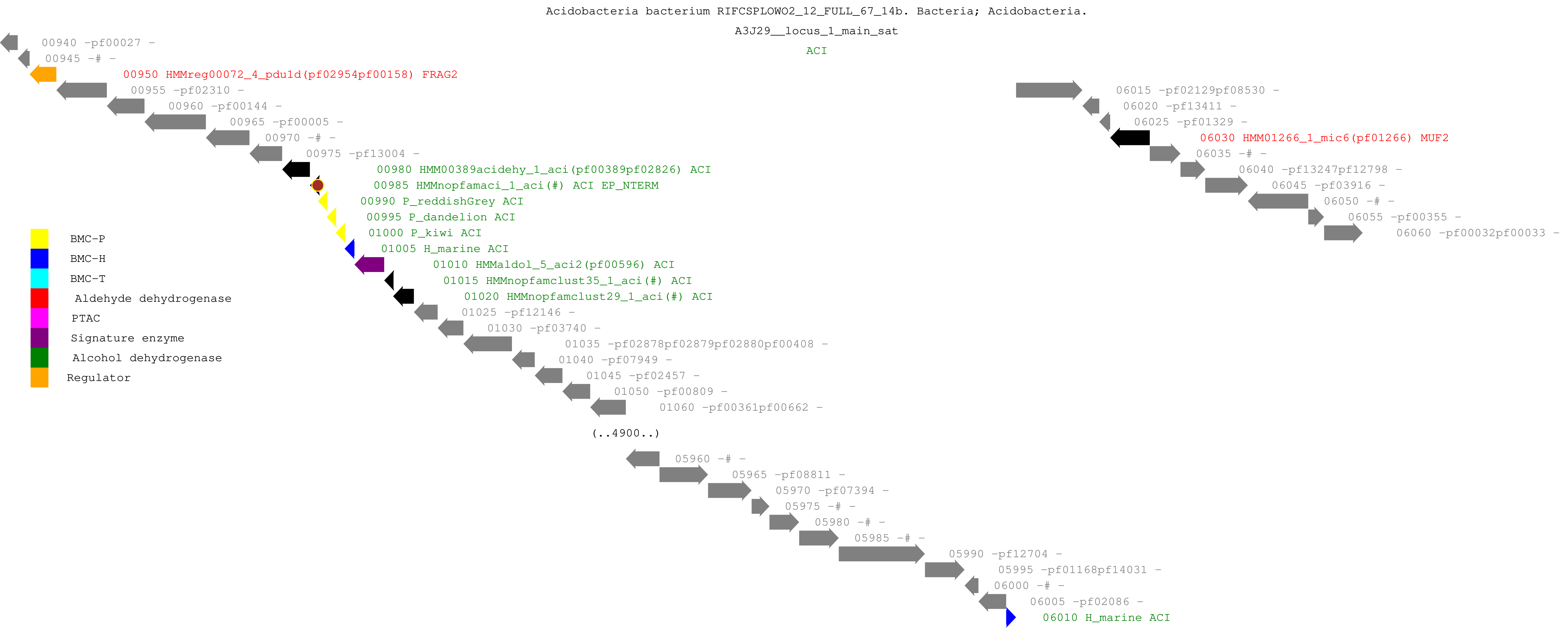

Acidobacteria bacterium RIFCSPLOWO2_12_FULL_67_14b. Bacteria; Acidobacteria.
A3J29__locus_1_main_sat
ACI
00940 -pf00027 -
00945 -# -
00950 HMMreg00072_4_pdu1d(pf02954pf00158) FRAG2
00955 -pf02310 -
00960 -pf00144 -
00965 -pf00005 -
00970 -# -
00975 -pf13004 -
00980 HMM00389acidehy_1_aci(pf00389pf02826) ACI
00985 HMMnopfamaci_1_aci(#) ACI EP_NTERM
00990 P_reddishGrey ACI
00995 P_dandelion ACI
01000 P_kiwi ACI
01005 H_marine ACI
01010 HMMaldol_5_aci2(pf00596) ACI
01015 HMMnopfamclust35_1_aci(#) ACI
01020 HMMnopfamclust29_1_aci(#) ACI
01025 -pf12146 -
01030 -pf03740 -
01035 -pf02878pf02879pf02880pf00408 -
01040 -pf07949 -
01045 -pf02457 -
01050 -pf00809 -
01060 -pf00361pf00662 -
(..4900..)
05960 -# -
05965 -pf08811 -
05970 -pf07394 -
05975 -# -
05980 -# -
05985 -# -
05990 -pf12704 -
05995 -pf01168pf14031 -
06000 -# -
06005 -pf02086 -
06010 H_marine ACI
06015 -pf02129pf08530 -
06020 -pf13411 -
06025 -pf01329 -
06030 HMM01266_1_mic6(pf01266) MUF2
06035 -# -
06040 -pf13247pf12798 -
06045 -pf03916 -
06050 -# -
06055 -pf00355 -
06060 -pf00032pf00033 -
BMC-P
BMC-H
BMC-T
Aldehyde dehydrogenase
PTAC
Signature enzyme
Alcohol dehydrogenase
Regulator

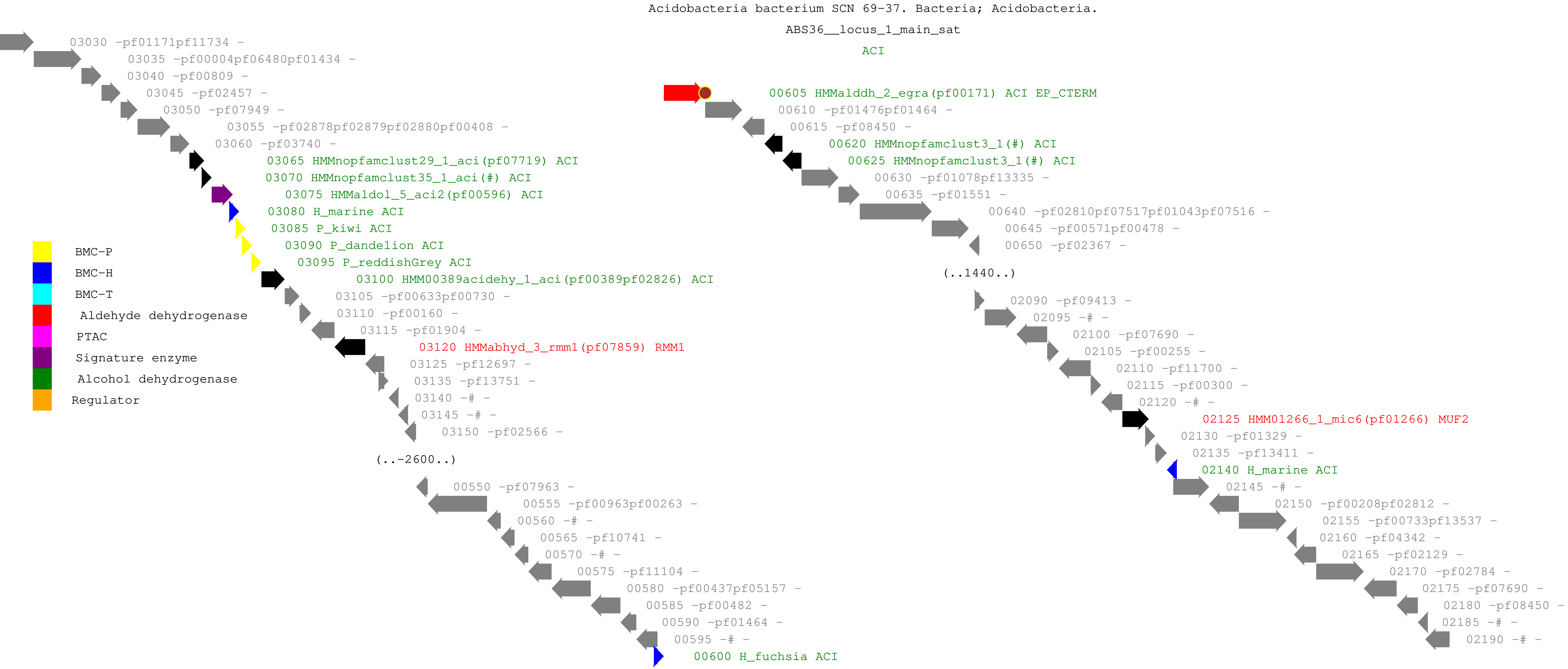

Acidobacteria bacterium SCN 69-37. Bacteria; Acidobacteria.
ABS36__locus_1_main_sat
ACI
03030 -pf01171pf11734 -
03035 -pf00004pf06480pf01434 -
03040 -pf00809 -
03045 -pf02457 -
03050 -pf07949 -
03055 -pf02878pf02879pf02880pf00408 -
03060 -pf03740 -
03065 HMMnopfamclust29_1_aci(pf07719) ACI
03070 HMMnopfamclust35_1_aci(#) ACI
03075 HMMaldol_5_aci2(pf00596) ACI
03080 H_marine ACI
03085 P_kiwi ACI
03090 P_dandelion ACI
03095 P_reddishGrey ACI
03100 HMM00389acidehy_1_aci(pf00389pf02826) ACI
03105 -pf00633pf00730 -
03110 -pf00160 -
03115 -pf01904 -
03120 HMMabhyd_3_rmm1(pf07859) RMM1
03125 -pf12697 -
03135 -pf13751 -
03140 -# -
03145 -# -
03150 -pf02566 -
(..-2600..)
00550 -pf07963 -
00555 -pf00963pf00263 -
00560 -# -
00565 -pf10741 -
00570 -# -
00575 -pf11104 -
00580 -pf00437pf05157 -
00585 -pf00482 -
00590 -pf01464 -
00595 -# -
00600 H_fuchsia ACI
00605 HMMalddh_2_egra(pf00171) ACI EP_CTERM
00610 -pf01476pf01464 -
00615 -pf08450 -
00620 HMMnopfamclust3_1(#) ACI
00625 HMMnopfamclust3_1(#) ACI
00630 -pf01078pf13335 -
00635 -pf01551 -
00640 -pf02810pf07517pf01043pf07516 -
00645 -pf00571pf00478 -
00650 -pf02367 -
(..1440..)
02090 -pf09413 -
02095 -# -
02100 -pf07690 -
02105 -pf00255 -
02110 -pf11700 -
02115 -pf00300 -
02120 -# -
02125 HMM01266_1_mic6(pf01266) MUF2
02130 -pf01329 -
02135 -pf13411 -
02140 H_marine ACI
02145 -# -
02150 -pf00208pf02812 -
02155 -pf00733pf13537 -
02160 -pf04342 -
02165 -pf02129 -
02170 -pf02784 -
02175 -pf07690 -
02180 -pf08450 -
02185 -# -
02190 -# -
BMC-P
BMC-H
BMC-T
Aldehyde dehydrogenase
PTAC
Signature enzyme
Alcohol dehydrogenase
Regulator

Solibacter usitatus (strain Ellin6076). Bacteria; Acidobacteria; Solibacteres; Solibacterales;

Acid__locus_1_main_sat

ACI

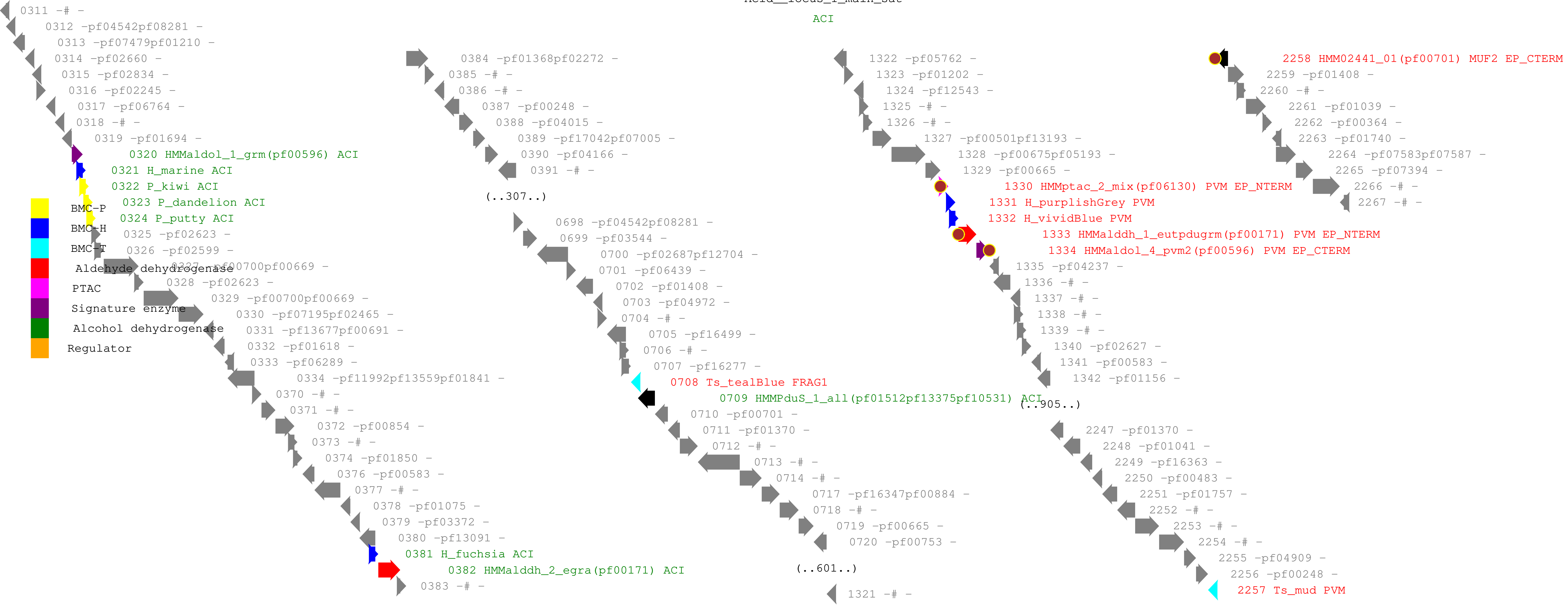

Acidobacteria bacterium 13_1_20CM_2_65_9. Bacteria; Acidobacteria.

AUF76__locus_1_main_sat

ACI

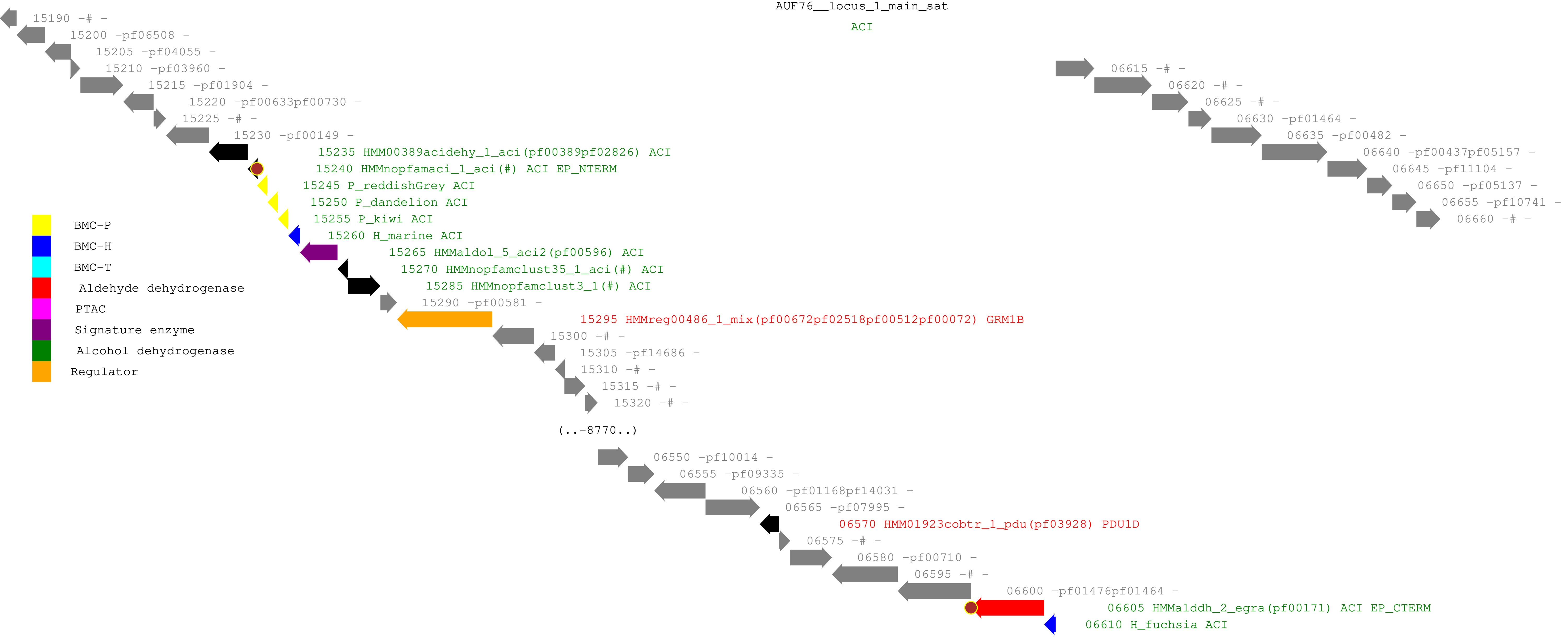

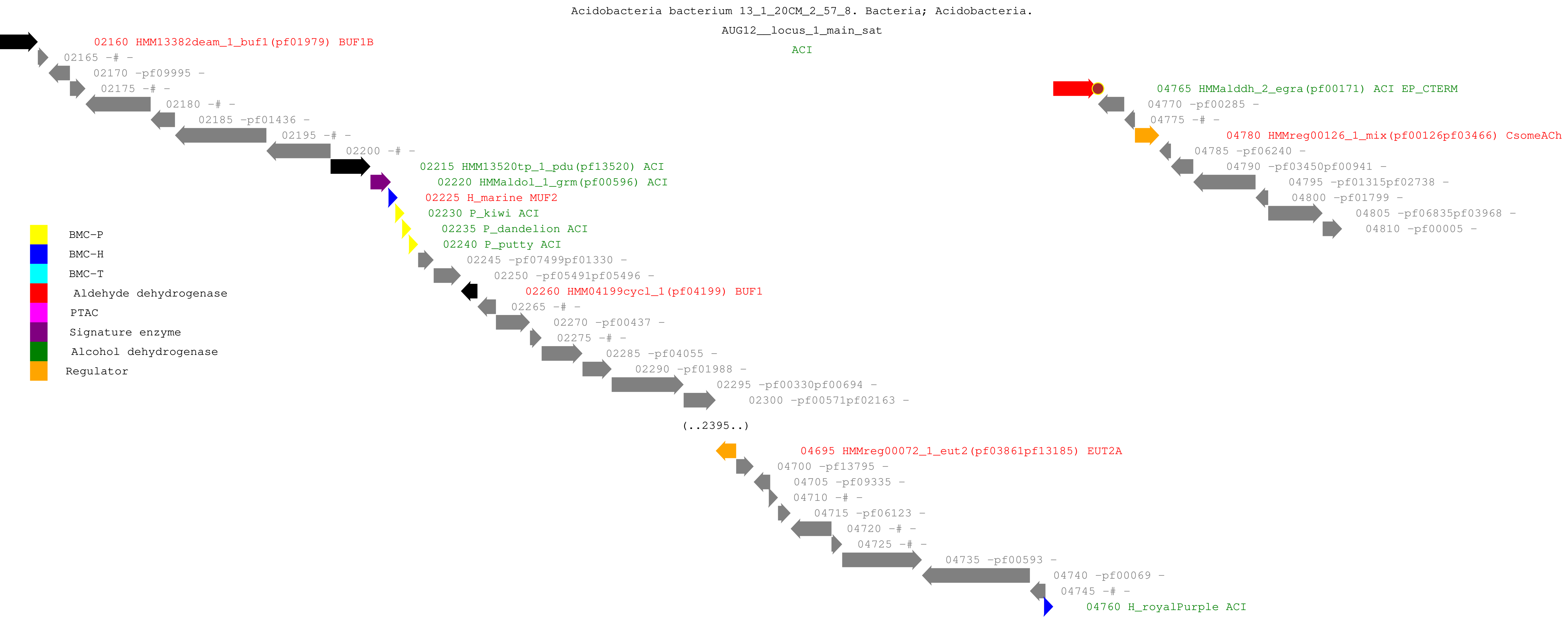
Acidobacteria bacterium 13_1_20CM_2_57_8. Bacteria; Acidobacteria.
AUG12__locus_1_main_sat
ACI
02160 HMM13382deam_1_buf1(pf01979) BUF1B
02165 -# -
02170 -pf09995 -
02175 -# -
02180 -# -
02185 -pf01436 -
02195 -# -
02200 -# -
02215 HMM13520tp_1_pdu(pf13520) ACI
02220 HMMaldol_1_grm(pf00596) ACI
02225 H_marine MUF2
02230 P_kiwi ACI
02235 P_dandelion ACI
02240 P_putty ACI
02245 -pf07499pf01330 -
02250 -pf05491pf05496 -
02260 HMM04199cycl_1(pf04199) BUF1
02265 -# -
02270 -pf00437 -
02275 -# -
02285 -pf04055 -
02290 -pf01988 -
02295 -pf00330pf00694 -
02300 -pf00571pf02163 -
(..2395..)
04695 HMMreg00072_1_eut2(pf03861pf13185) EUT2A
04700 -pf13795 -
04705 -pf09335 -
04710 -# -
04715 -pf06123 -
04720 -# -
04725 -# -
04735 -pf00593 -
04740 -pf00069 -
04745 -# -
04760 H_royalPurple ACI
04765 HMMalddh_2_egra(pf00171) ACI EP_CTERM
04770 -pf00285 -
04775 -# -
04780 HMMreg00126_1_mix(pf00126pf03466) CsomeACh
04785 -pf06240 -
04790 -pf03450pf00941 -
04795 -pf01315pf02738 -
04800 -pf01799 -
04805 -pf06835pf03968 -
04810 -pf00005 -
BMC-P
BMC-H
BMC-T
Aldehyde dehydrogenase
PTAC
Signature enzyme
Alcohol dehydrogenase
Regulator

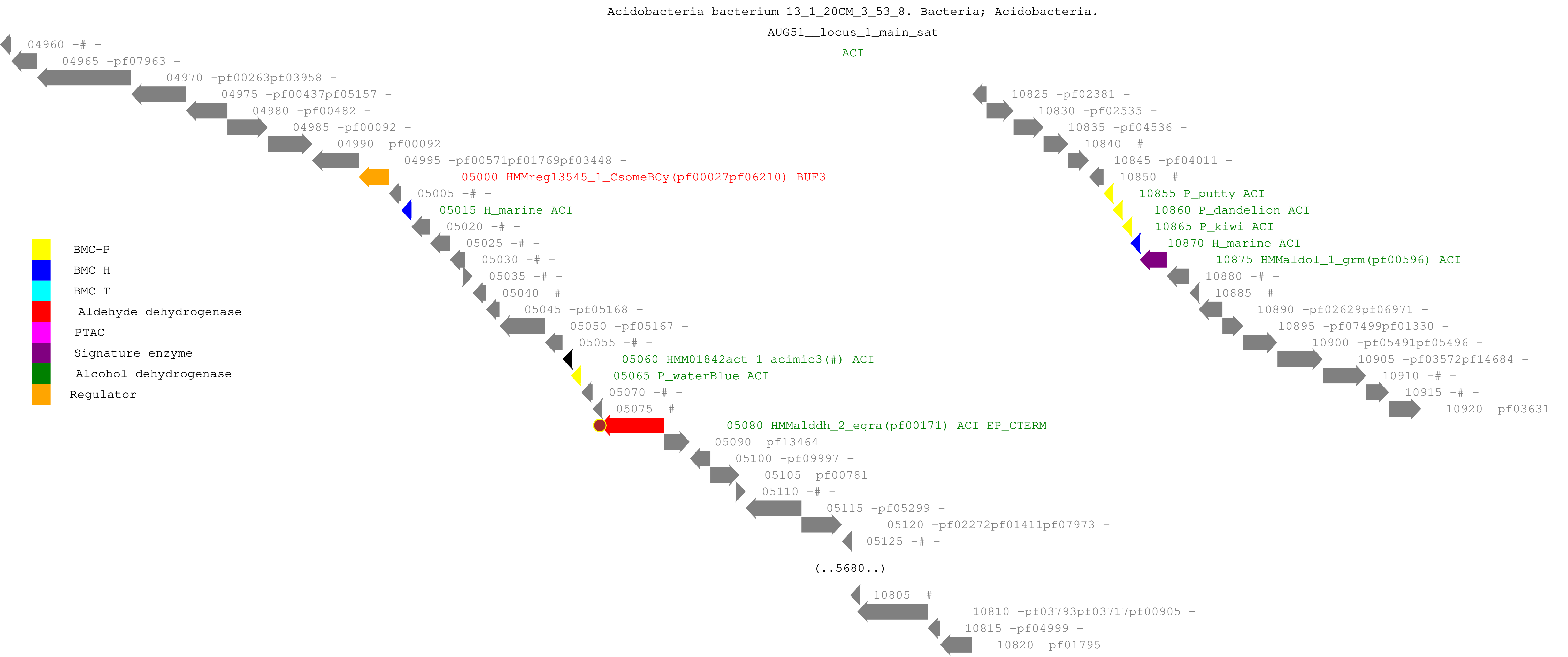
Acidobacteria bacterium 13_1_20CM_3_53_8. Bacteria; Acidobacteria.
AUG51__locus_1_main_sat
ACI
04960 -# -
04965 -pf07963 -
04970 -pf00263pf03958 -
04975 -pf00437pf05157 -
04980 -pf00482 -
04985 -pf00092 -
04990 -pf00092 -
04995 -pf00571pf01769pf03448 -
05000 HMMreg13545_1_CsomeBCy(pf00027pf06210) BUF3
05005 -# -
05015 H_marine ACI
05020 -# -
05025 -# -
05030 -# -
05035 -# -
05040 -# -
05045 -pf05168 -
05050 -pf05167 -
05055 -# -
05060 HMM01842act_1_acimic3(#) ACI
05065 P_waterBlue ACI
05070 -# -
05075 -# -
05080 HMMaldd h_2_egra(pf00171) ACI EP_CTERM
05090 -pf13464 -
05100 -pf09997 -
05105 -pf00781 -
05110 -# -
05115 -pf05299 -
05120 -pf02272pf01411pf07973 -
05125 -# -
(..5680..)
10805 -# -
10810 -pf03793pf03717pf00905 -
10815 -pf04999 -
10820 -pf01795 -
10825 -pf02381 -
10830 -pf02535 -
10835 -pf04536 -
10840 -# -
10845 -pf04011 -
10850 -# -
10855 P_putty ACI
10860 P_dandelion ACI
10865 P_kiwi ACI
10870 H_marine ACI
10875 HMMaldol_1_grm(pf00596) ACI
10880 -# -
10885 -# -
10890 -pf02629pf06971 -
10895 -pf07499pf01330 -
10900 -pf05491pf05496 -
10905 -pf03572pf14684 -
10910 -# -
10915 -# -
10920 -pf03631 -
BMC-P
BMC-H
BMC-T
Aldehyde dehydrogenase
PTAC
Signature enzyme
Alcohol dehydrogenase
Regulator

Acidobacteria bacterium 13_1_40CM_56_16. Bacteria; Acidobacteria.

AUH28__locus_1_main_sat

ACI

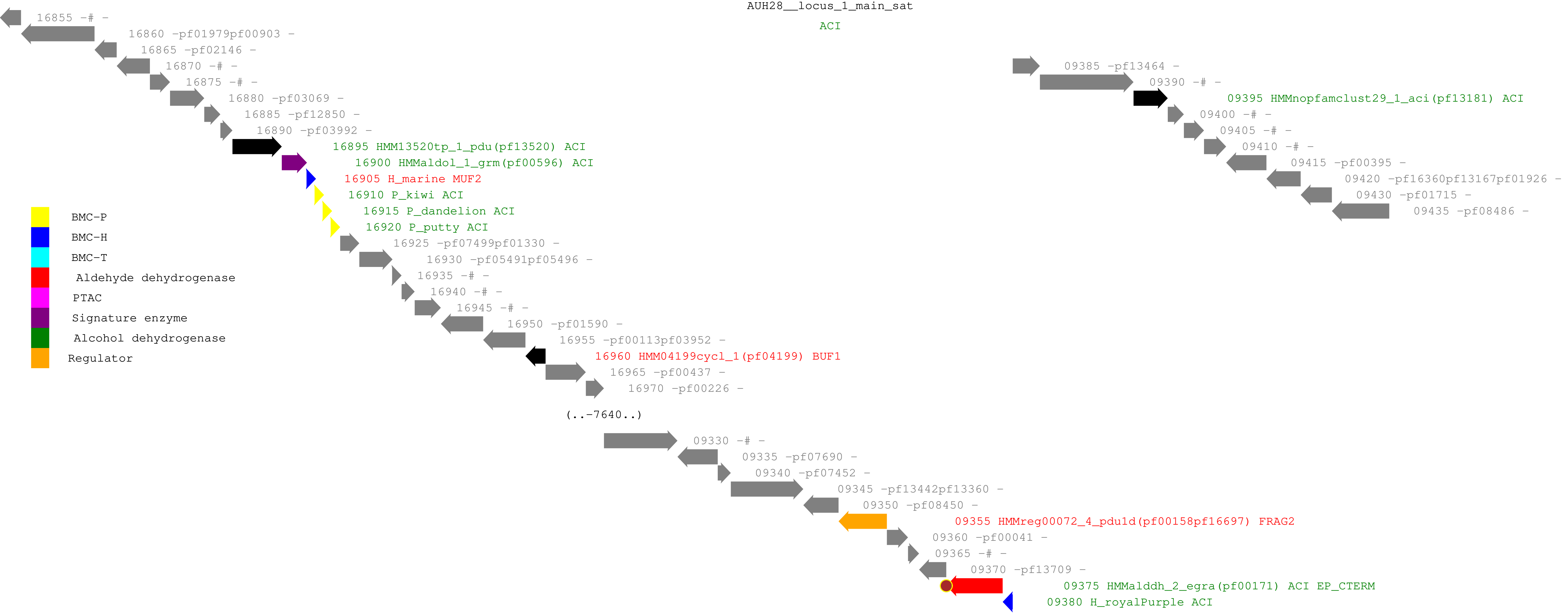

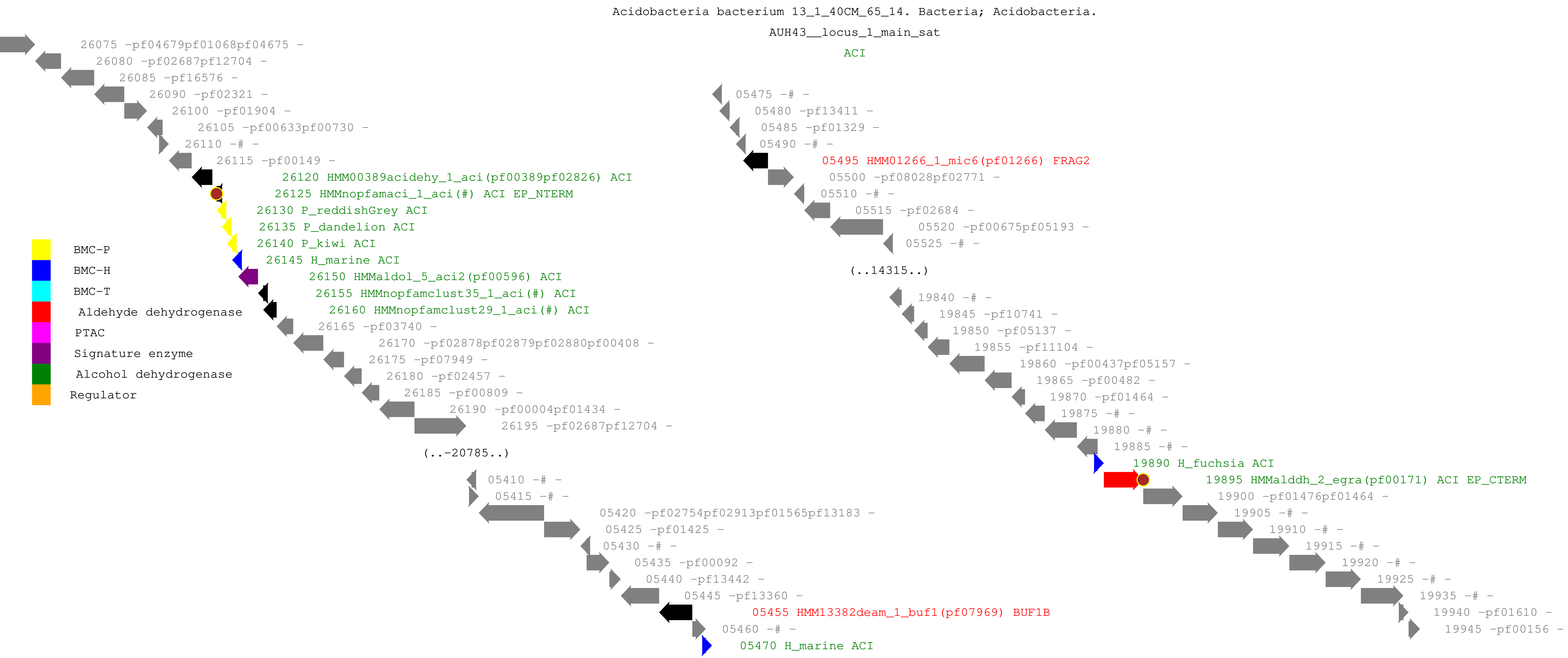
Acidobacteria bacterium 13_1_40CM_65_14. Bacteria; Acidobacteria.
AUH43__locus_1_main_sat
ACI
26075 -pf04679pf01068pf04675 -
26080 -pf02687pf12704 -
26085 -pf16576 -
26090 -pf02321 -
26100 -pf01904 -
26105 -pf00633pf00730 -
26110 -# -
26115 -pf00149 -
26120 HMM00389acidehy_1_aci(pf00389pf02826) ACI
26125 HMMnopfamaci_1_aci(#) ACI EP_NTERM
26130 P_reddishGrey ACI
26135 P_dandelion ACI
26140 P_kiwi ACI
26145 H_marine ACI
26150 HMMaldol_5_aci2(pf00596) ACI
26155 HMMnopfamclust35_1_aci(#) ACI
26160 HMMnopfamclust29_1_aci(#) ACI
26165 -pf03740 -
26170 -pf02878pf02879pf02880pf00408 -
26175 -pf07949 -
26180 -pf02457 -
26185 -pf00809 -
26190 -pf00004pf01434 -
26195 -pf02687pf12704 -
(..-20785..)
05410 -# -
05415 -# -
05420 -pf02754pf02913pf01565pf13183 -
05425 -pf01425 -
05430 -# -
05435 -pf00092 -
05440 -pf13442 -
05445 -pf13360 -
05455 HMM13382deam_1_buf1(pf07969) BUF1B
05460 -# -
05470 H_marine ACI
BMC-P
BMC-H
BMC-T
Aldehyde dehydrogenase
PTAC
Signature enzyme
Alcohol dehydrogenase
Regulator
05475 -# -
05480 -pf13411 -
05485 -pf01329 -
05490 -# -
05495 HMM01266_1_mic6(pf01266) FRAG2
05500 -pf08028pf02771 -
05510 -# -
05515 -pf02684 -
05520 -pf00675pf05193 -
05525 -# -
(..14315..)
19840 -# -
19845 -pf10741 -
19850 -pf05137 -
19855 -pf11104 -
19860 -pf00437pf05157 -
19865 -pf00482 -
19870 -pf01464 -
19875 -# -
19880 -# -
19885 -# -
19890 H_fuchsia ACI
19895 HMMalddh_2_egra(pf00171) ACI EP_CTERM
19900 -pf01476pf01464 -
19905 -# -
19910 -# -
19915 -# -
19920 -# -
19925 -# -
19935 -# -
19940 -pf01610 -
19945 -pf00156 -

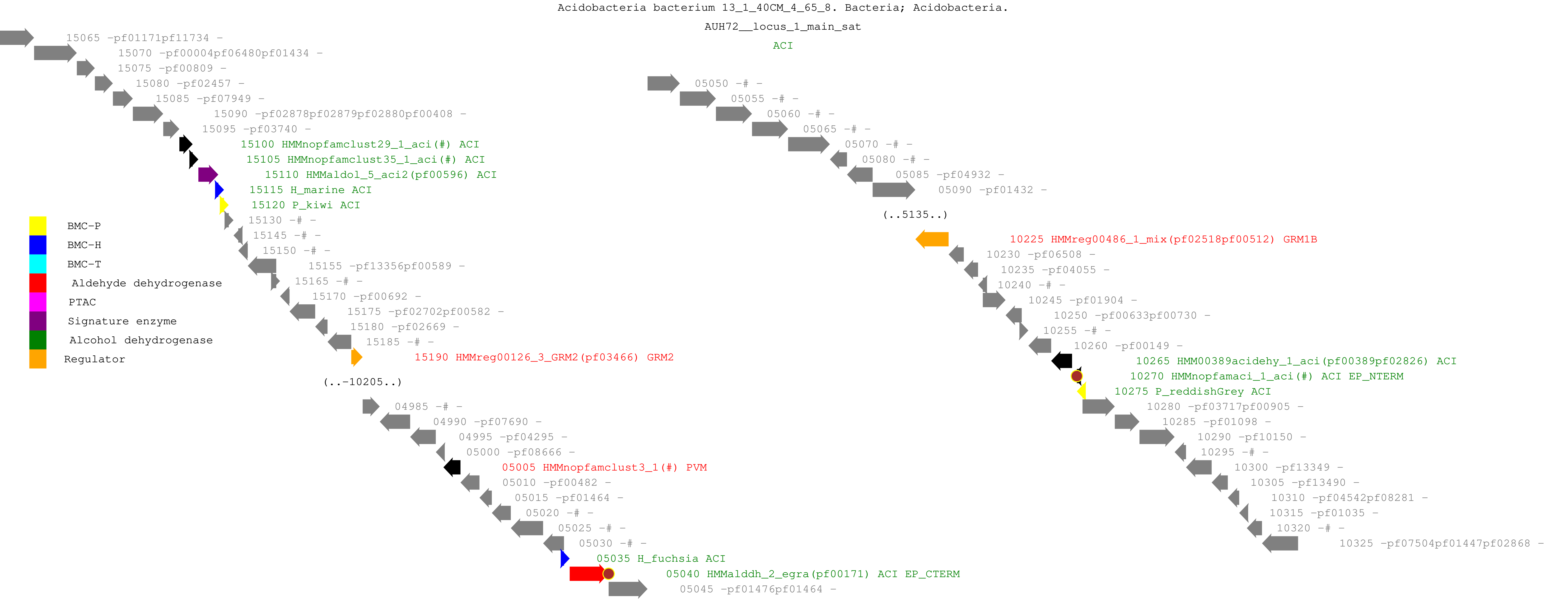

Acidobacteria bacterium 13_1_40CM_4_65_8. Bacteria; Acidobacteria.
AUH72__locus_1_main_sat
ACI
15065 -pf01171pf11734 -
15070 -pf00004pf06480pf01434 -
15075 -pf00809 -
15080 -pf02457 -
15085 -pf07949 -
15090 -pf02878pf02879pf02880pf00408 -
15095 -pf03740 -
15100 HMMnopfamclust29_1_aci(#) ACI
15105 HMMnopfamclust35_1_aci(#) ACI
15110 HMMaldol_5_aci2(pf00596) ACI
15115 H_marine ACI
15120 P_kiwi ACI
15130 -# -
15145 -# -
15150 -# -
15155 -pf13356pf00589 -
15165 -# -
15170 -pf00692 -
15175 -pf02702pf00582 -
15180 -pf02669 -
15185 -# -
15190 HMMreg00126_3_GRM2(pf03466) GRM2
(..-10205..)
04985 -# -
04990 -pf07690 -
04995 -pf04295 -
05000 -pf08666 -
05005 HMMnopfamclust3_1(#) PVM
05010 -pf00482 -
05015 -pf01464 -
05020 -# -
05025 -# -
05030 -# -
05035 H_fuchsia ACI
05040 HMMaldh_2_egra(pf00171) ACI EP_CTERM
05045 -pf01476pf01464 -
05050 -# -
05055 -# -
05060 -# -
05065 -# -
05070 -# -
05080 -# -
05085 -pf04932 -
05090 -pf01432 -
(..5135..)
10225 HMMreg00486_1_mix(pf02518pf00512) GRM1B
10230 -pf06508 -
10235 -pf04055 -
10240 -# -
10245 -pf01904 -
10250 -pf00633pf00730 -
10255 -# -
10260 -pf00149 -
10265 HMM00389acidehy_1_aci(pf00389pf02826) ACI
10270 HMMnopfamaci_1_aci(#) ACI EP_NTERM
10275 P_reddishGrey ACI
10280 -pf03717pf00905 -
10285 -pf01098 -
10290 -pf10150 -
10295 -# -
10300 -pf13349 -
10305 -pf13490 -
10310 -pf04542pf08281 -
10315 -pf01035 -
10320 -# -
10325 -pf07504pf01447pf02868 -
BMC-P
BMC-H
BMC-T
Aldehyde dehydrogenase
PTAC
Signature enzyme
Alcohol dehydrogenase
Regulator

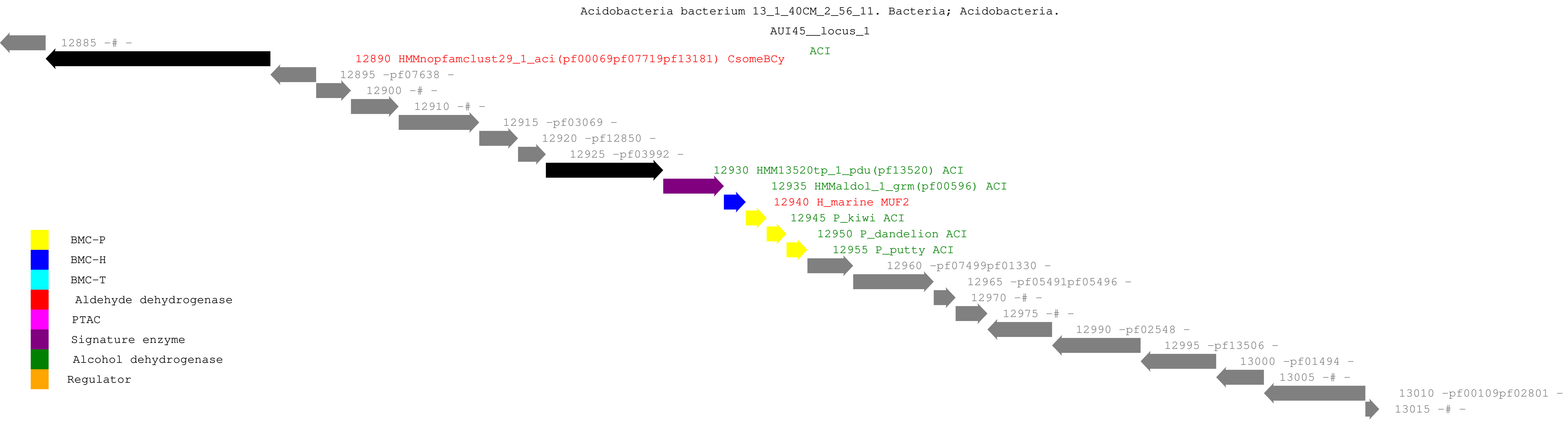
Acidobacteria bacterium 13_1_40CM_2_56_11. Bacteria; Acidobacteria.
AUI45__locus_1
ACI
12885 -# -
12890 HMMnopfamclust29_1_aci(pf00069pf07719pf13181) CsomeBCy
12895 -pf07638 -
12900 -# -
12910 -# -
12915 -pf03069 -
12920 -pf12850 -
12925 -pf03992 -
12930 HMM13520tp_1_pdu(pf13520) ACI
12935 HMMaldol_1_grm(pf00596) ACI
12940 H_marine MUF2
12945 P_kiwi ACI
12950 P_dandelion ACI
12955 P_putty ACI
12960 -pf07499pf01330 -
12965 -pf05491pf05496 -
12970 -# -
12975 -# -
12990 -pf02548 -
12995 -pf13506 -
13000 -pf01494 -
13005 -# -
13010 -pf00109pf02801 -
13015 -# -
BMC-P
BMC-H
BMC-T
Aldehyde dehydrogenase
PTAC
Signature enzyme
Alcohol dehydrogenase
Regulator

Acidobacteria bacterium 13_1_40CM_2_64_6. Bacteria; Acidobacteria.

AUI64__locus_1_main_sat

ACI

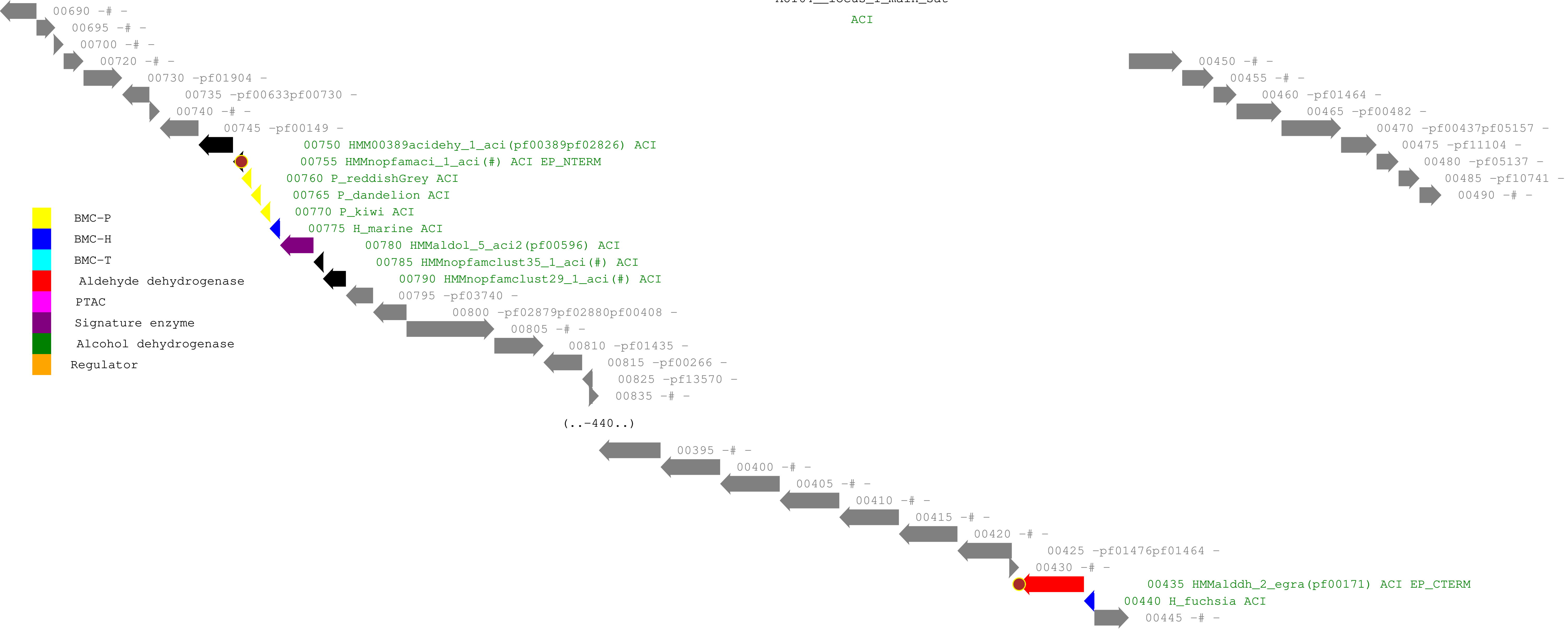

Acidobacteria bacterium 13_1_40CM_3_65_5. Bacteria; Acidobacteria.

AUJ01__locus_1_main_sat

ACI

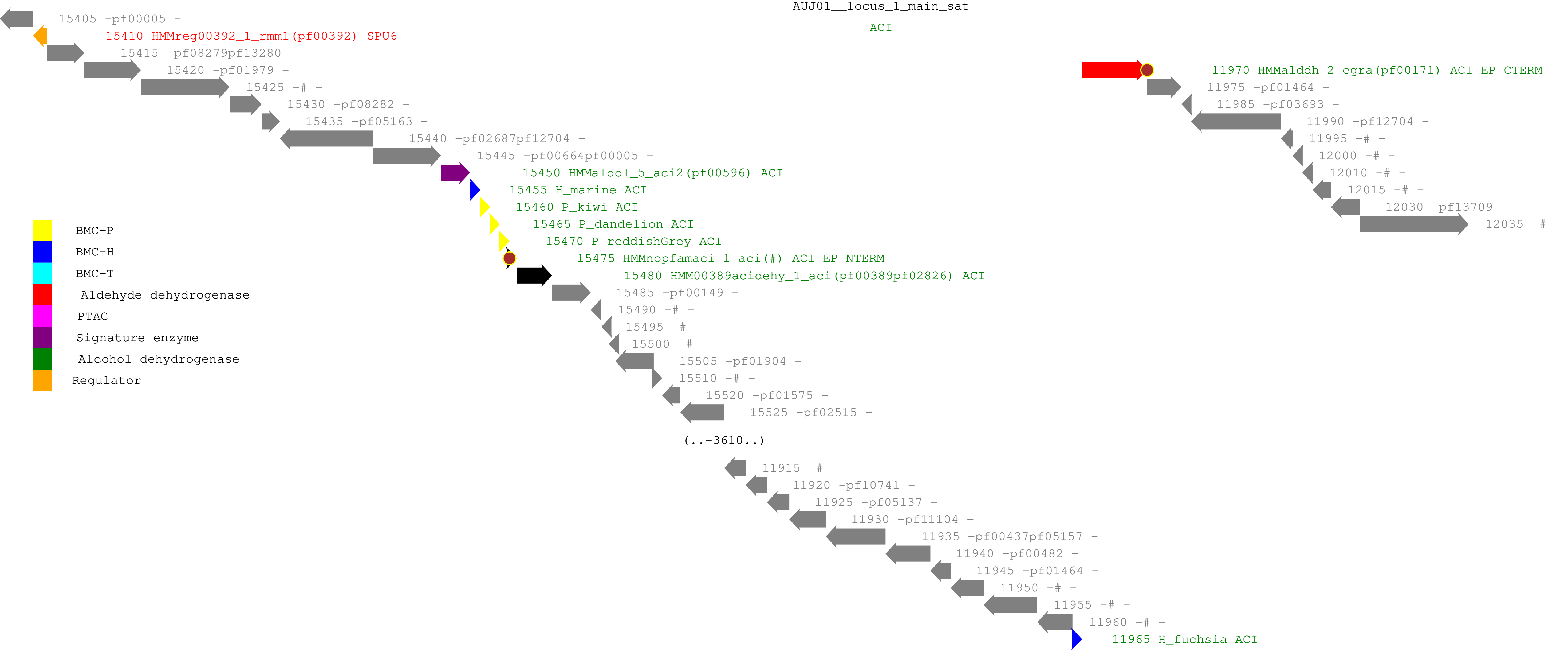

Acidobacteria bacterium 37-65-4. Bacteria; Acidobacteria.

B7X11__locus_1_main_sat

ACI

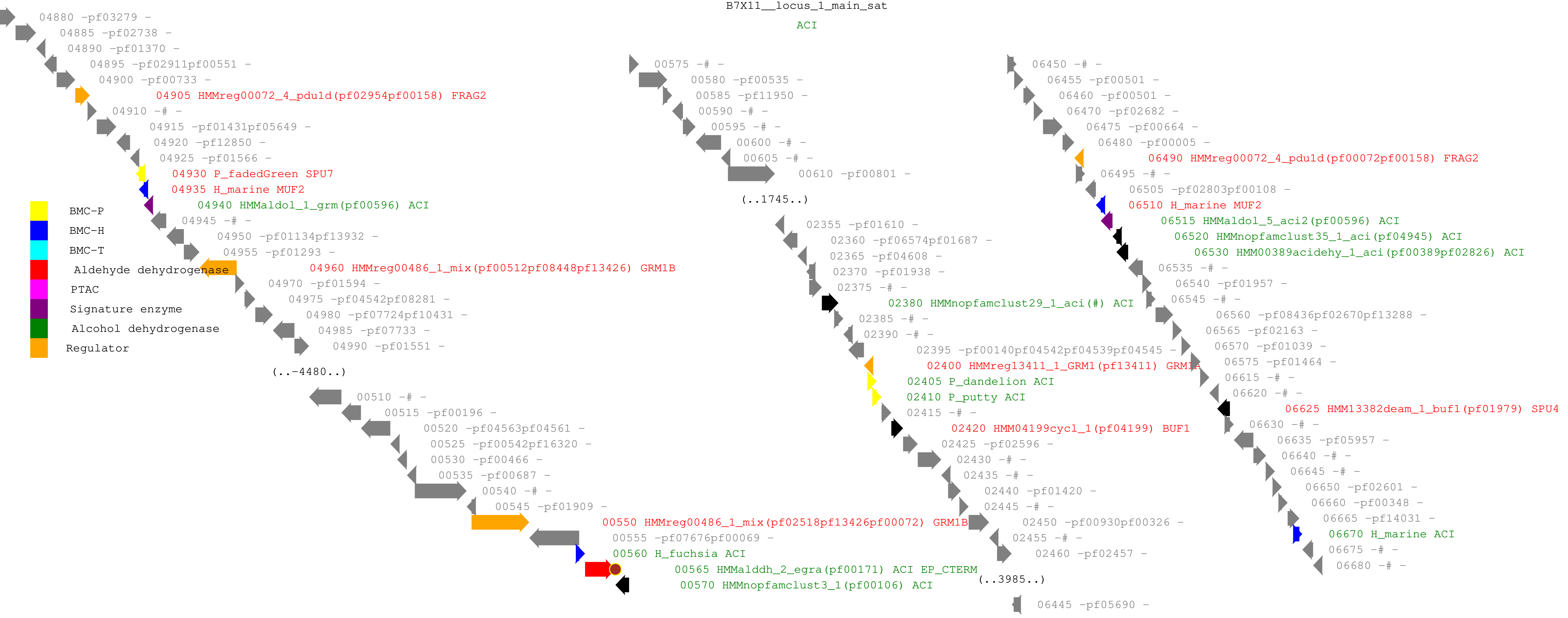

Acidobacteria bacterium. Bacteria; Acidobacteria.

C5B44__locus_1_main_sat

ACI

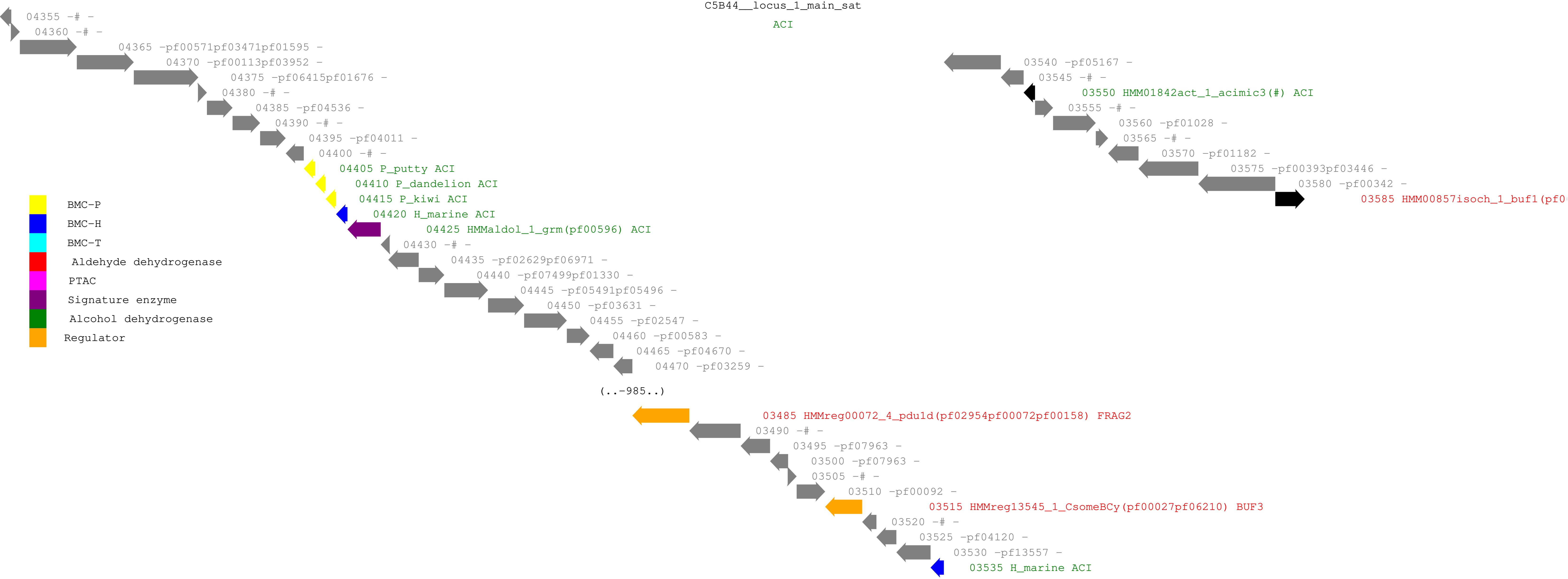

Solibacteres bacterium. Bacteria; Acidobacteria; Solibacteres.

C5B51__locus_1_main_sat

ACI

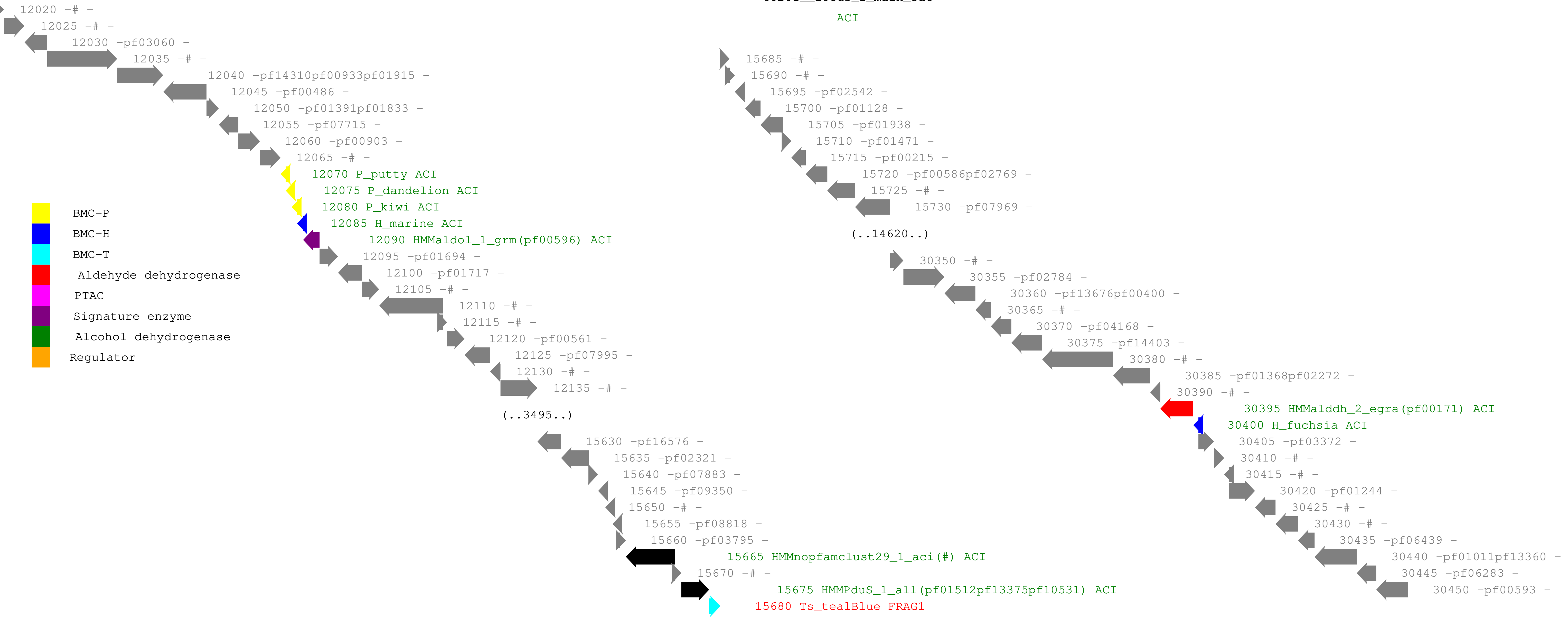

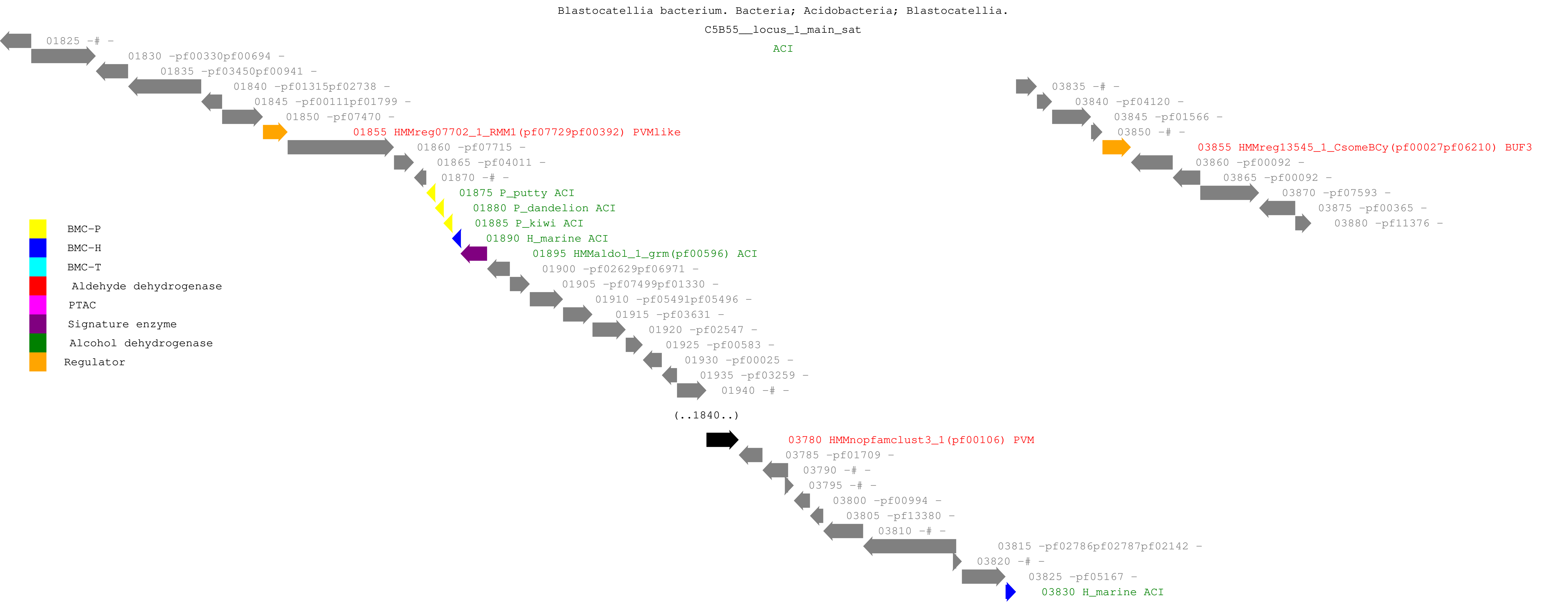

Blastocatellia bacterium. Bacteria; Acidobacteria; Blastocatellia.
C5B55__locus_1_main_sat
ACI
01825 -# -
01830 -pf00330pf00694 -
01835 -pf03450pf00941 -
01840 -pf01315pf02738 -
01845 -pf00111pf01799 -
01850 -pf07470 -
01855 HMMreg07702_1_RMM1(pf07729pf00392) PVMlike
01860 -pf07715 -
01865 -pf04011 -
01870 -# -
01875 P_putty ACI
01880 P_dandelion ACI
01885 P_kiwi ACI
01890 H_marine ACI
01895 HMMaldol_1_grm(pf00596) ACI
01900 -pf02629pf06971 -
01905 -pf07499pf01330 -
01910 -pf05491pf05496 -
01915 -pf03631 -
01920 -pf02547 -
01925 -pf00583 -
01930 -pf00025 -
01935 -pf03259 -
01940 -# -
(..1840..)
03780 HMMnopfamclust3_1(pf00106) PVM
03785 -pf01709 -
03790 -# -
03795 -# -
03800 -pf00994 -
03805 -pf13380 -
03810 -# -
03815 -pf02786pf02787pf02142 -
03820 -# -
03825 -pf05167 -
03830 H_marine ACI
03835 -# -
03840 -pf04120 -
03845 -pf01566 -
03850 -# -
03855 HMMreg13545_1_CsomeBCy(pf00027pf06210) BUF3
03860 -pf00092 -
03865 -pf00092 -
03870 -pf07593 -
03875 -pf00365 -
03880 -pf11376 -
BMC-P
BMC-H
BMC-T
Aldehyde dehydrogenase
PTAC
Signature enzyme
Alcohol dehydrogenase
Regulator

Acidobacterium sp. Bacteria; Acidobacteria; Acidobacteriales; Acidobacteriaceae;

CK533__locus_1_main_sat

ACI

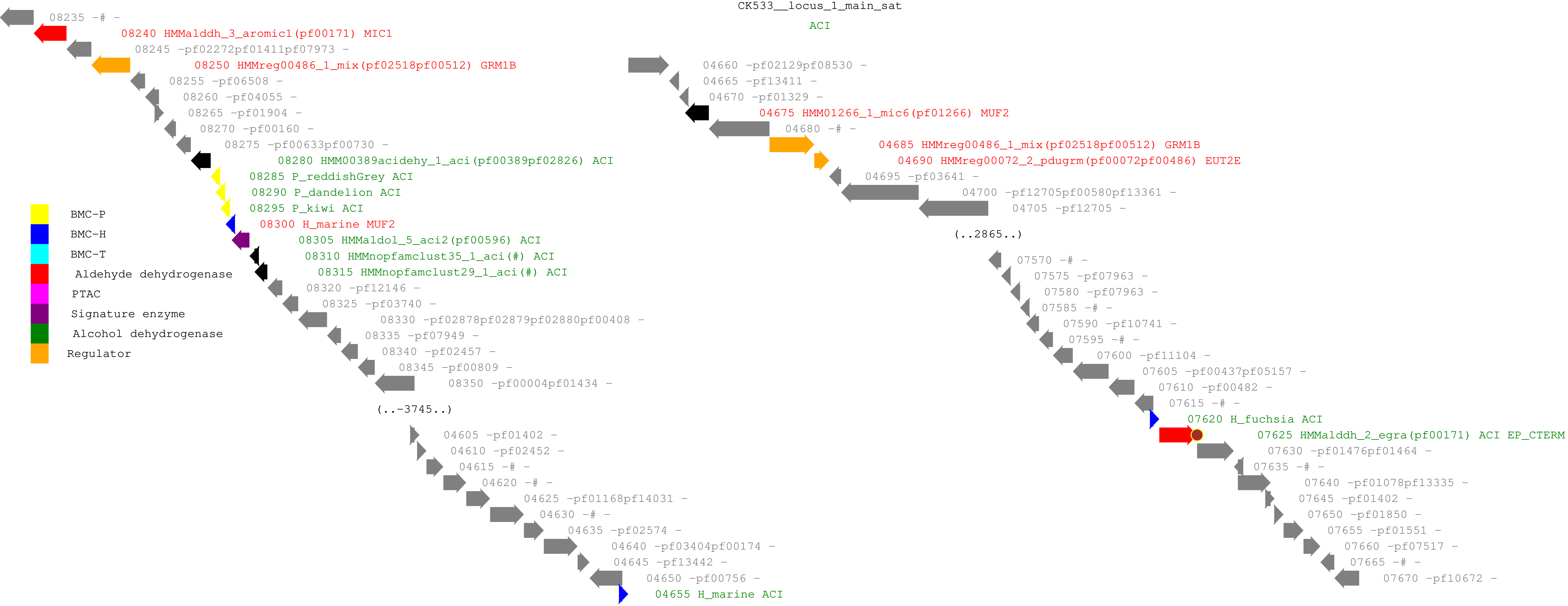

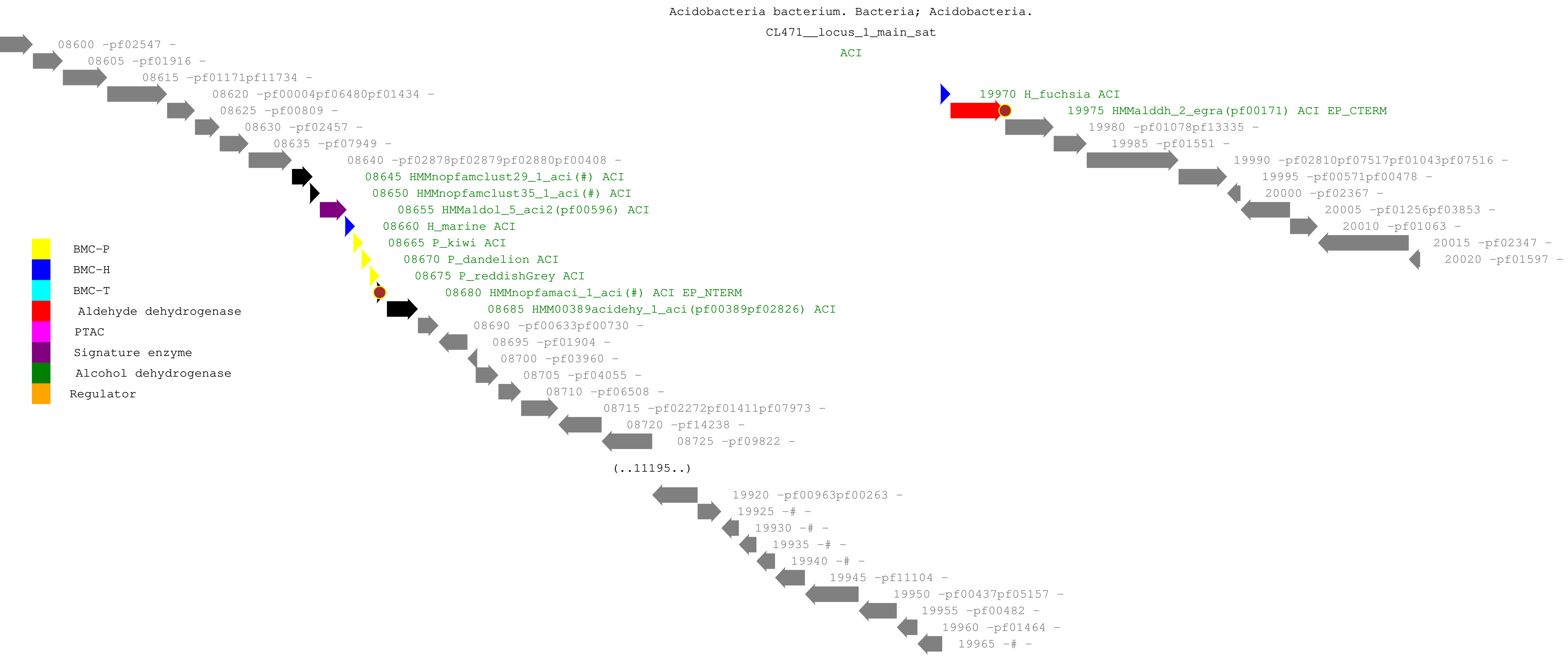

Acidobacteria bacterium. Bacteria; Acidobacteria.
CL471__locus_1_main_sat
ACI
08600 -pf02547 -
08605 -pf01916 -
08615 -pf01171pf11734 -
08620 -pf00004pf06480pf01434 -
08625 -pf00809 -
08630 -pf02457 -
08635 -pf07949 -
08640 -pf02878pf02879pf02880pf00408 -
08645 HMMnopfamclust29_1_aci(#) ACI
08650 HMMnopfamclust35_1_aci(#) ACI
08655 HMMaldol_5_aci2(pf00596) ACI
08660 H_marine ACI
08665 P_kiwi ACI
08670 P_dandelion ACI
08675 P_reddishGrey ACI
08680 HMMnopfamaci_1_aci(#) ACI EP_NTERM
08685 HMM00389acidehy_1_aci(pf00389pf02826) ACI
08690 -pf00633pf00730 -
08695 -pf01904 -
08700 -pf03960 -
08705 -pf04055 -
08710 -pf06508 -
08715 -pf02272pf01411pf07973 -
08720 -pf14238 -
08725 -pf09822 -
(..11195..)
19920 -pf00963pf00263 -
19925 -# -
19930 -# -
19935 -# -
19940 -# -
19945 -pf11104 -
19950 -pf00437pf05157 -
19955 -pf00482 -
19960 -pf01464 -
19965 -# -
19970 H_fuchsia ACI
19975 HMMalddh_2_egra(pf00171) ACI EP_CTERM
19980 -pf01078pf13335 -
19985 -pf01551 -
19990 -pf02810pf07517pf01043pf07516 -
19995 -pf00571pf00478 -
20000 -pf02367 -
20005 -pf01256pf03853 -
20010 -pf01063 -
20015 -pf02347 -
20020 -pf01597 -
BMC-P
BMC-H
BMC-T
Aldehyde dehydrogenase
PTAC
Signature enzyme
Alcohol dehydrogenase
Regulator

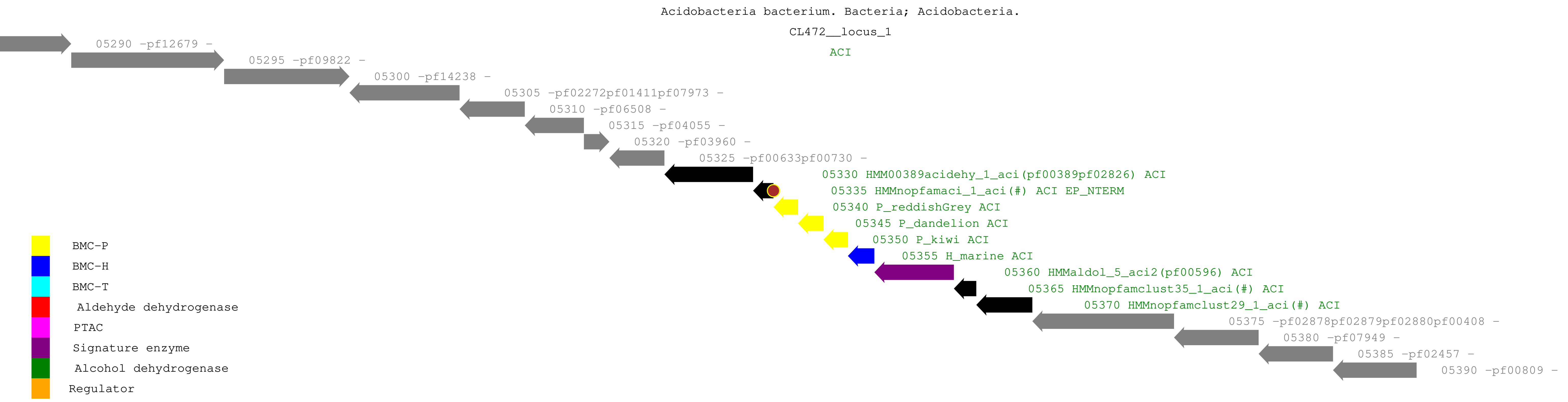

Acidobacteria bacterium. Bacteria; Acidobacteria.
CL472__locus_1
ACI
05290 -pf12679 -
05295 -pf09822 -
05300 -pf14238 -
05305 -pf02272pf01411pf07973 -
05310 -pf06508 -
05315 -pf04055 -
05320 -pf03960 -
05325 -pf00633pf00730 -
05330 HMM00389acidehy_1_aci(pf00389pf02826) ACI
05335 HMMnopfamaci_1_aci(#) ACI EP_NTERM
05340 P_reddishGrey ACI
05345 P_dandelion ACI
05350 P_kiwi ACI
05355 H_marine ACI
05360 HMMaldol_5_aci2(pf00596) ACI
05365 HMMnopfamclust35_1_aci(#) ACI
05370 HMMnopfamclust29_1_aci(#) ACI
05375 -pf02878pf02879pf02880pf00408 -
05380 -pf07949 -
05385 -pf02457 -
05390 -pf00809 -
BMC-P
BMC-H
BMC-T
Aldehyde dehydrogenase
PTAC
Signature enzyme
Alcohol dehydrogenase
Regulator

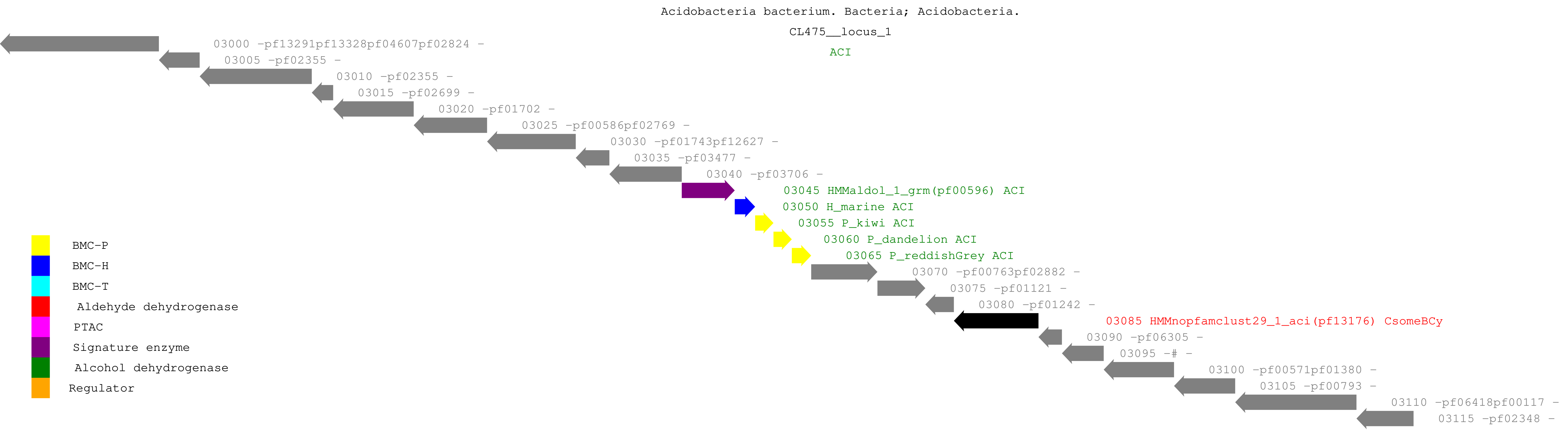
Acidobacteria bacterium. Bacteria; Acidobacteria.
CL475__locus_1
ACI
03000 -pf13291pf13328pf04607pf02824 -
03005 -pf02355 -
03010 -pf02355 -
03015 -pf02699 -
03020 -pf01702 -
03025 -pf00586pf02769 -
03030 -pf01743pf12627 -
03035 -pf03477 -
03040 -pf03706 -
03045 HMMaldol_1_grm(pf00596) ACI
03050 H_marine ACI
03055 P_kiwi ACI
03060 P_dandelion ACI
03065 P_reddishGrey ACI
03070 -pf00763pf02882 -
03075 -pf01121 -
03080 -pf01242 -
03085 HMMnopfamclust29_1_aci(pf13176) CsomeBCy
03090 -pf06305 -
03095 -# -
03100 -pf00571pf01380 -
03105 -pf00793 -
03110 -pf06418pf00117 -
03115 -pf02348 -
BMC-P
BMC-H
BMC-T
Aldehyde dehydrogenase
PTAC
Signature enzyme
Alcohol dehydrogenase
Regulator

Acidobacteria bacterium. Bacteria; Acidobacteria.

CL476__locus_1_main_sat

ACI

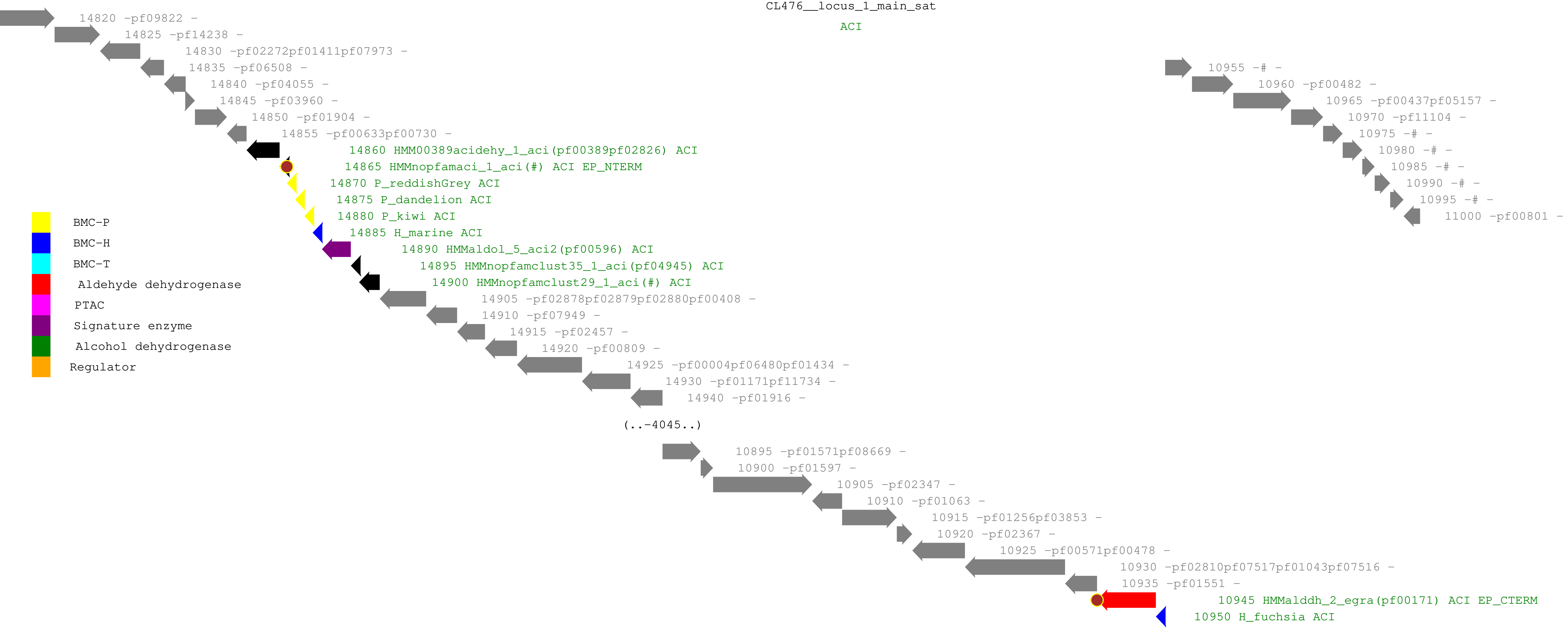

Acidobacteria bacterium. Bacteria; Acidobacteria.

CL477__locus_1_main_sat

ACI

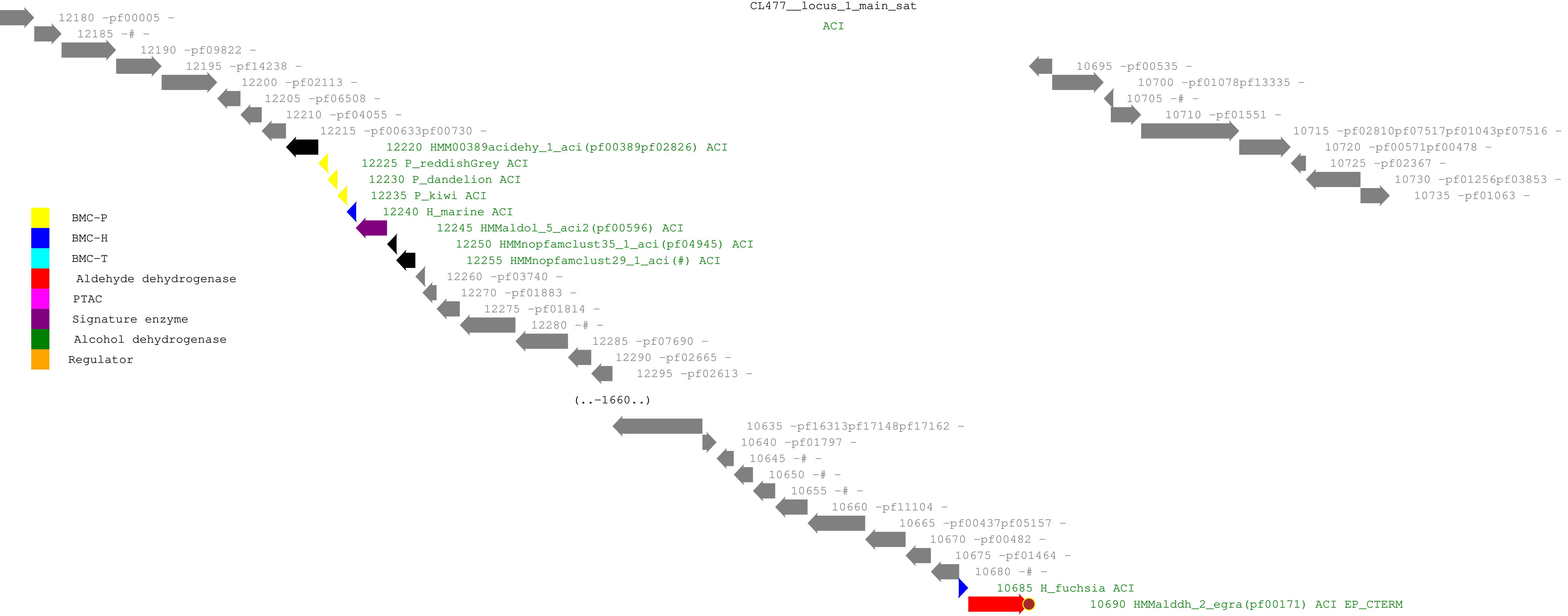

Acidobacteria bacterium. Bacteria; Acidobacteria.

CL478__locus_1_main_sat

ACI

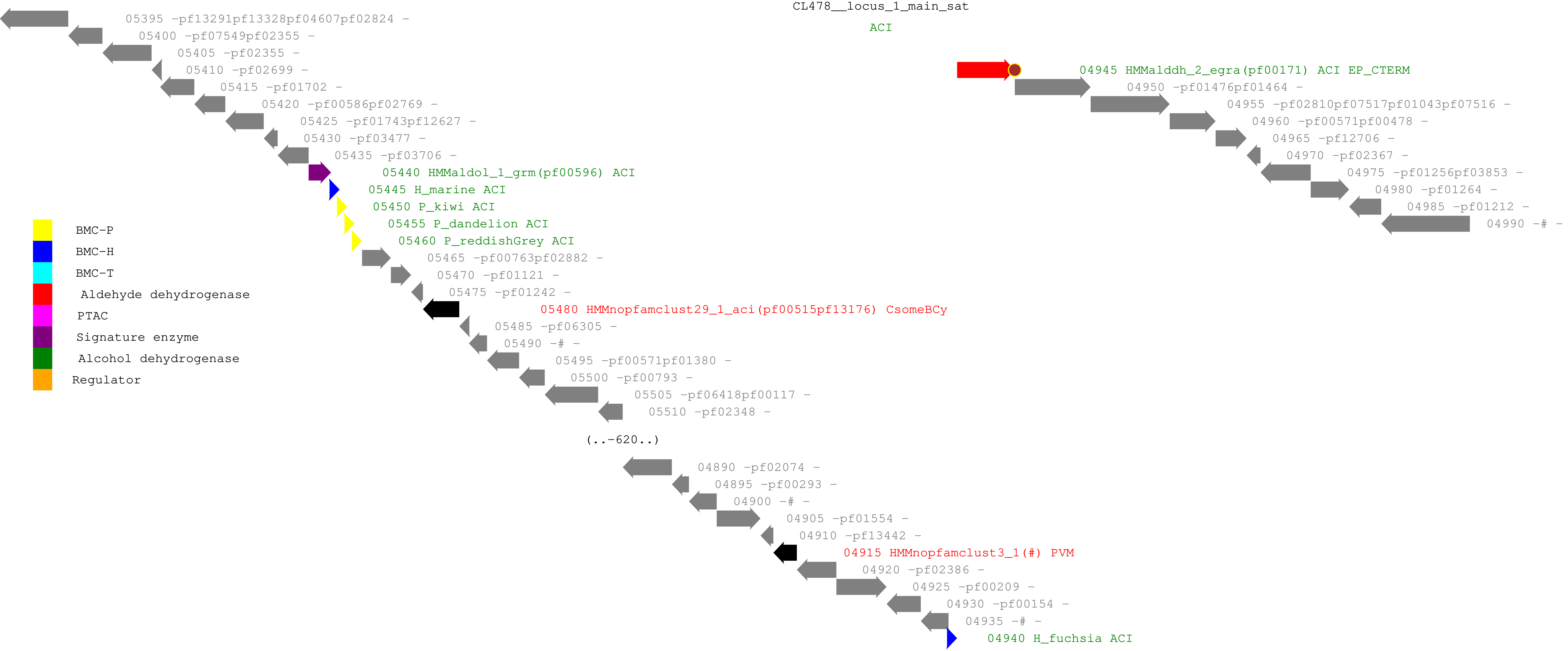

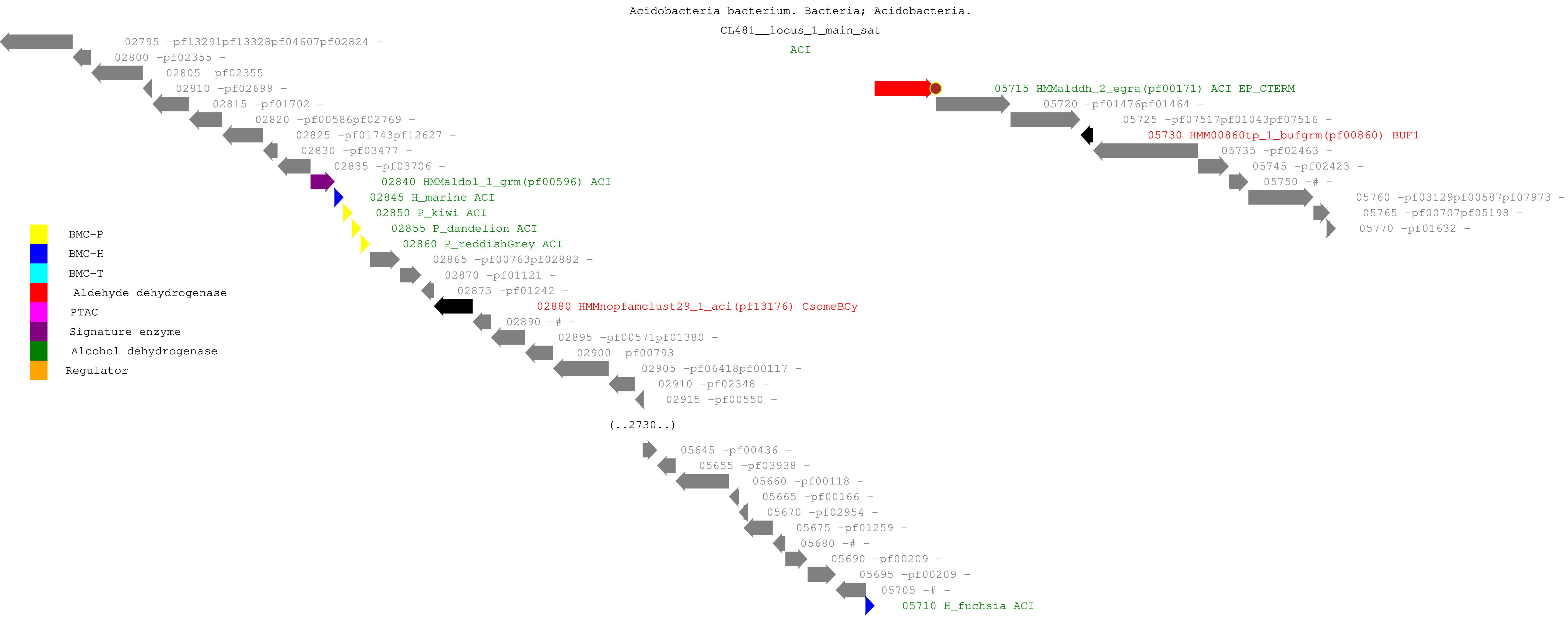
Acidobacteria bacterium. Bacteria; Acidobacteria.
CL481__locus_1_main_sat
ACI
02795 -pf13291pf13328pf04607pf02824 -
02800 -pf02355 -
02805 -pf02355 -
02810 -pf02699 -
02815 -pf01702 -
02820 -pf00586pf02769 -
02825 -pf01743pf12627 -
02830 -pf03477 -
02835 -pf03706 -
02840 HMMaldol_1_grm(pf00596) ACI
02845 H_marine ACI
02850 P_kiwi ACI
02855 P_dandelion ACI
02860 P_reddishGrey ACI
02865 -pf00763pf02882 -
02870 -pf01121 -
02875 -pf01242 -
02880 HMMnopfamclust29_1_aci(pf13176) CsomeBCy
02890 -# -
02895 -pf00571pf01380 -
02900 -pf00793 -
02905 -pf06418pf00117 -
02910 -pf02348 -
02915 -pf00550 -
(..2730..)
05645 -pf00436 -
05655 -pf03938 -
05660 -pf00118 -
05665 -pf00166 -
05670 -pf02954 -
05675 -pf01259 -
05680 -# -
05690 -pf00209 -
05695 -pf00209 -
05705 -# -
05710 H_fuchsia ACI
05715 HMMalddh_2_egra(pf00171) ACI EP_CTERM
05720 -pf01476pf01464 -
05725 -pf07517pf01043pf07516 -
05730 HMM00860tp_1_bufgrm(pf00860) BUF1
05735 -pf02463 -
05745 -pf02423 -
05750 -# -
05760 -pf03129pf00587pf07973 -
05765 -pf00707pf05198 -
05770 -pf01632 -
BMC-P
BMC-H
BMC-T
Aldehyde dehydrogenase
PTAC
Signature enzyme
Alcohol dehydrogenase
Regulator

Acidobacteria bacterium. Bacteria; Acidobacteria.

CL482__locus_1

ACI

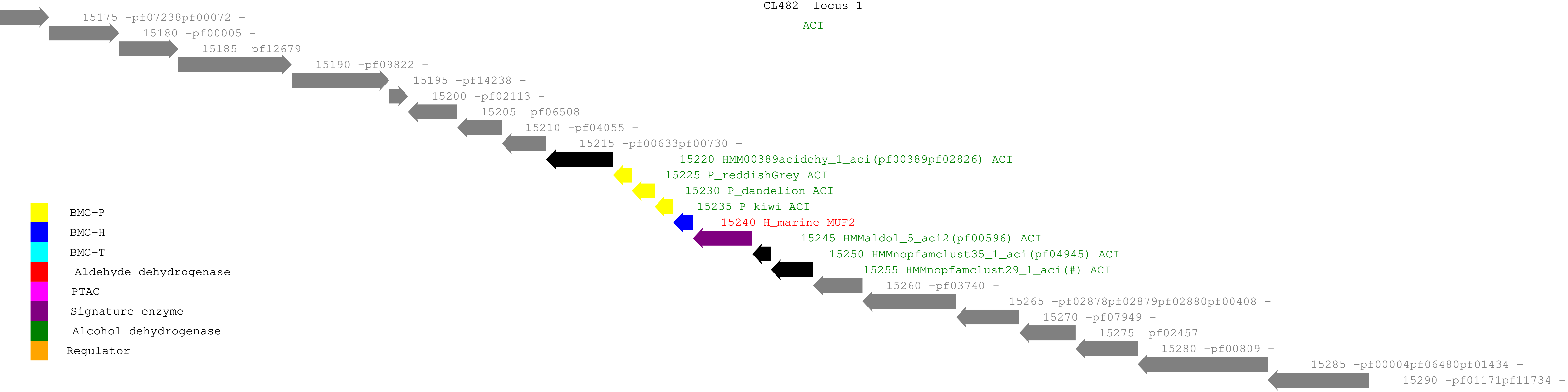

Acidobacteria bacterium. Bacteria; Acidobacteria.

CL483__locus_1_main_sat

ACI

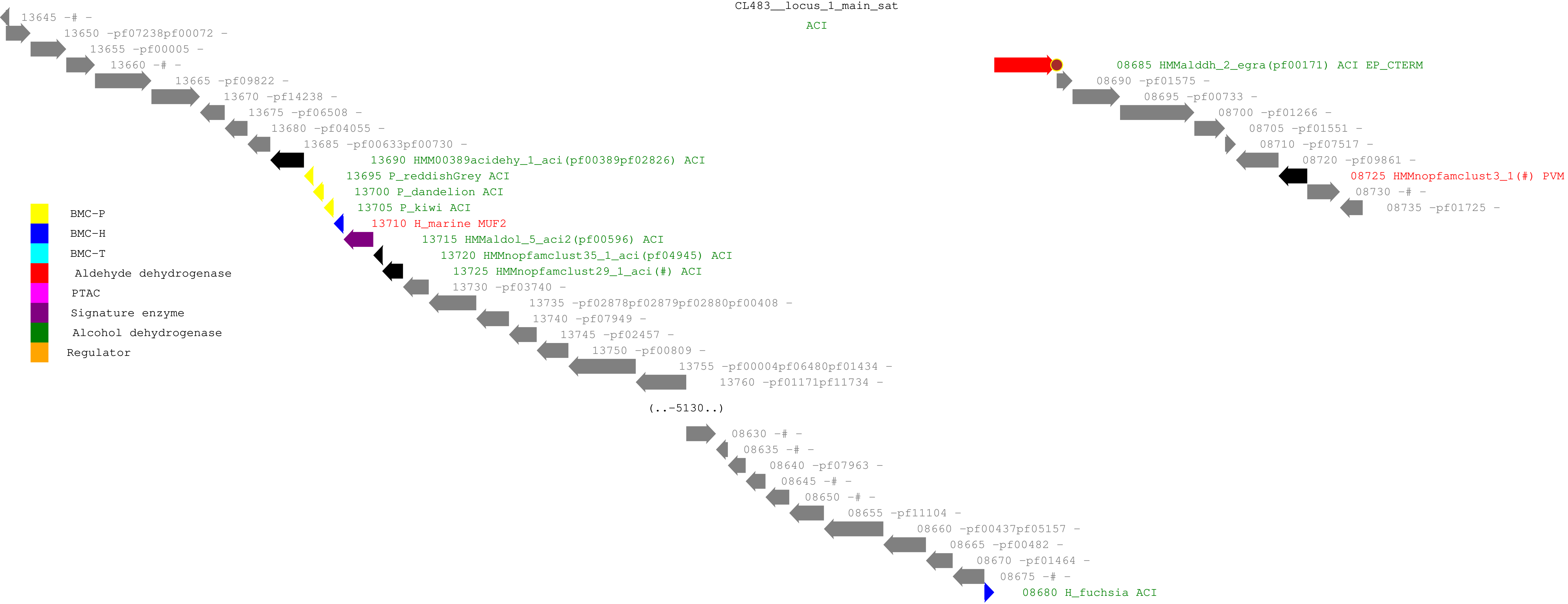

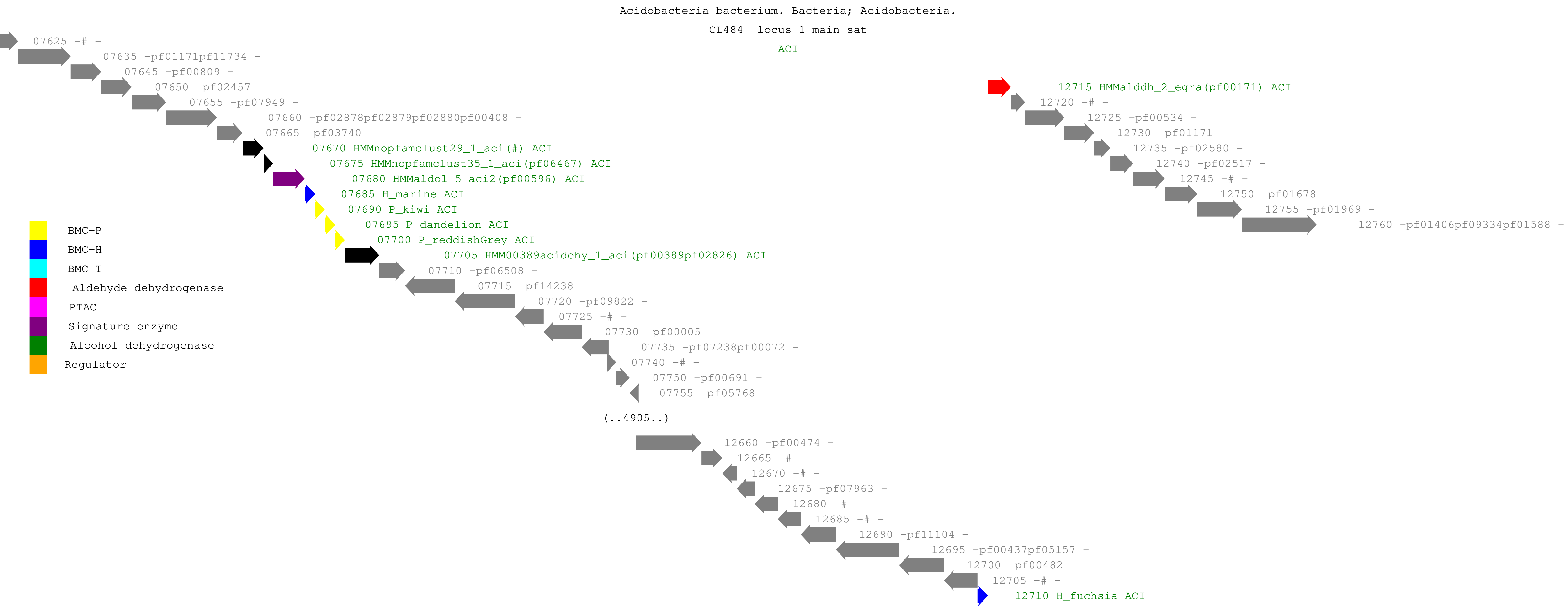
Acidobacteria bacterium. Bacteria; Acidobacteria.
CL484__locus_1_main_sat
ACI
07625 -# -
07635 -pf01171pf11734 -
07645 -pf00809 -
07650 -pf02457 -
07655 -pf07949 -
07660 -pf02878pf02879pf02880pf00408 -
07665 -pf03740 -
07670 HMMnopfamclust29_1_aci(#) ACI
07675 HMMnopfamclust35_1_aci(pf06467) ACI
07680 HMMaldol_5_aci2(pf00596) ACI
07685 H_marine ACI
07690 P_kiwi ACI
07695 P_dandelion ACI
07700 P_reddishGrey ACI
07705 HMM00389acidehy_1_aci(pf00389pf02826) ACI
07710 -pf06508 -
07715 -pf14238 -
07720 -pf09822 -
07725 -# -
07730 -pf00005 -
07735 -pf07238pf00072 -
07740 -# -
07750 -pf00691 -
07755 -pf05768 -
(..4905..)
12660 -pf00474 -
12665 -# -
12670 -# -
12675 -pf07963 -
12680 -# -
12685 -# -
12690 -pf11104 -
12695 -pf00437pf05157 -
12700 -pf00482 -
12705 -# -
12710 H_fuchsia ACI
12715 HMMalddh_2_egra(pf00171) ACI
12720 -# -
12725 -pf00534 -
12730 -pf01171 -
12735 -pf02580 -
12740 -pf02517 -
12745 -# -
12750 -pf01678 -
12755 -pf01969 -
12760 -pf01406pf09334pf01588 -
BMC-P
BMC-H
BMC-T
Aldehyde dehydrogenase
PTAC
Signature enzyme
Alcohol dehydrogenase
Regulator

Acidobacteria bacterium. Bacteria; Acidobacteria.

CL485__locus_fragmented

ACI

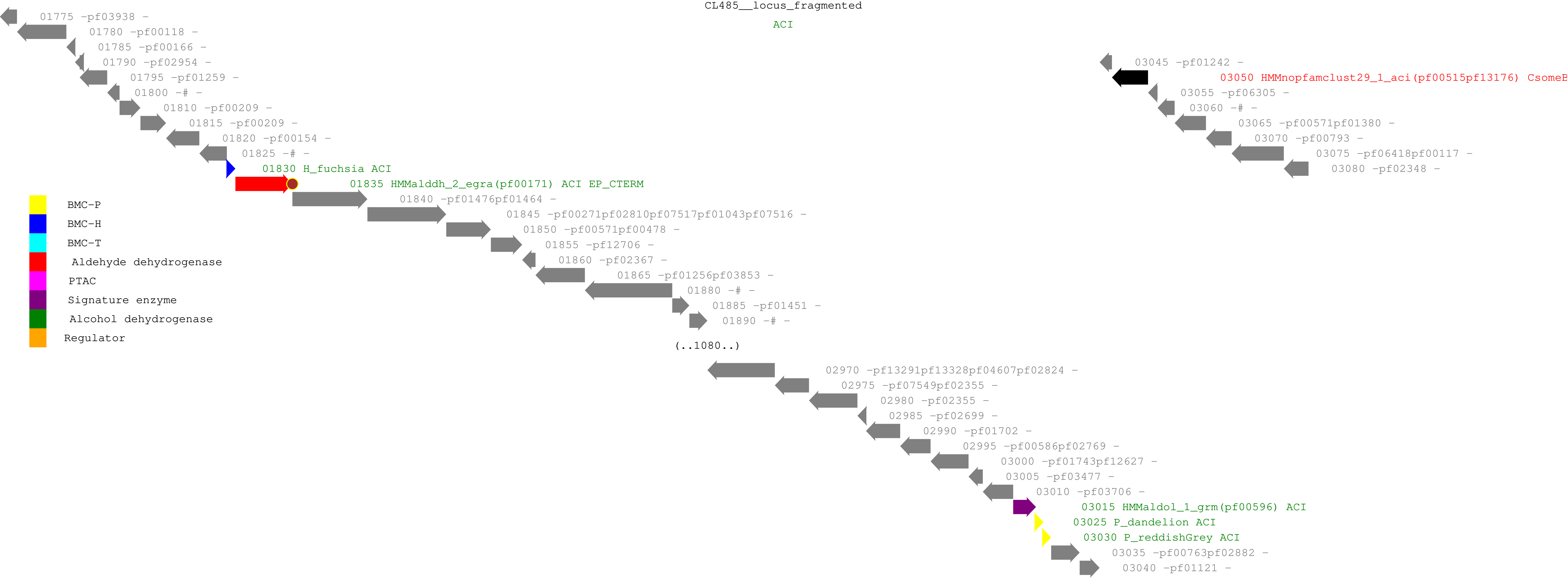

Acidobacteria bacterium. Bacteria; Acidobacteria.

CL486__locus_1_main_sat

ACI

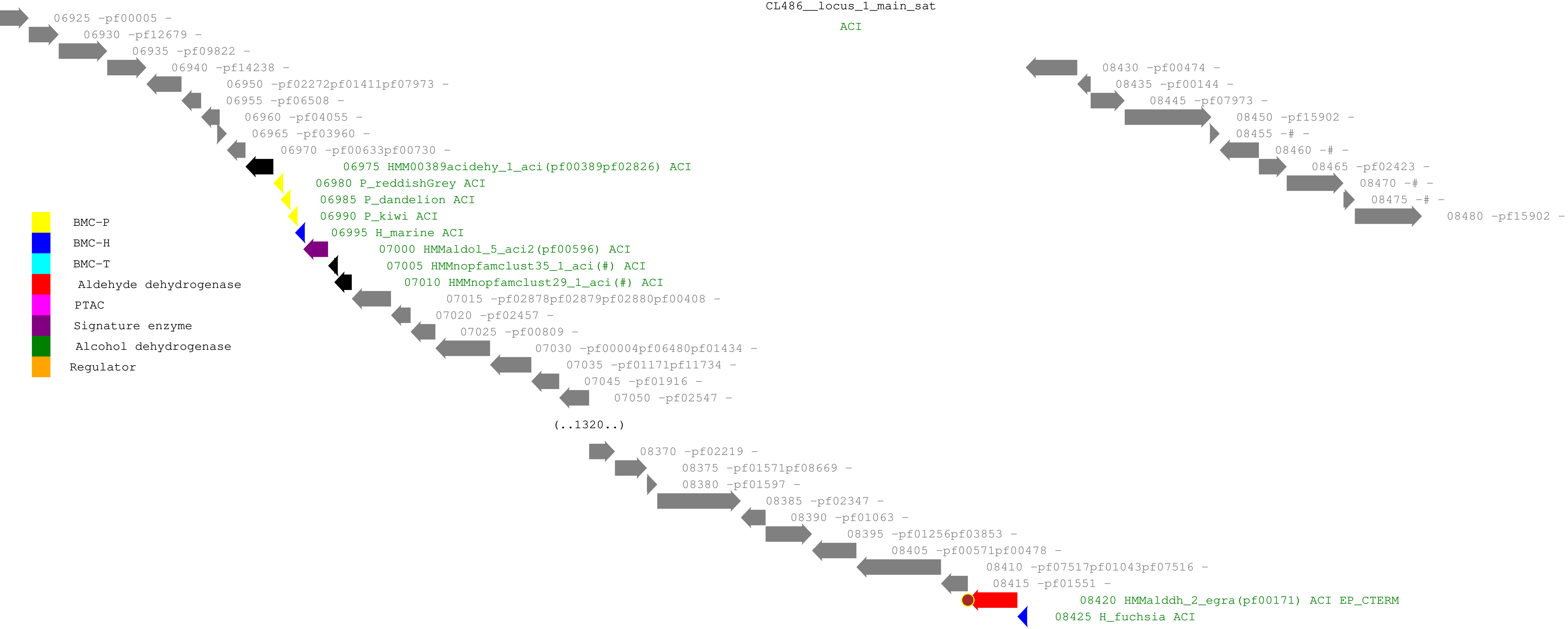

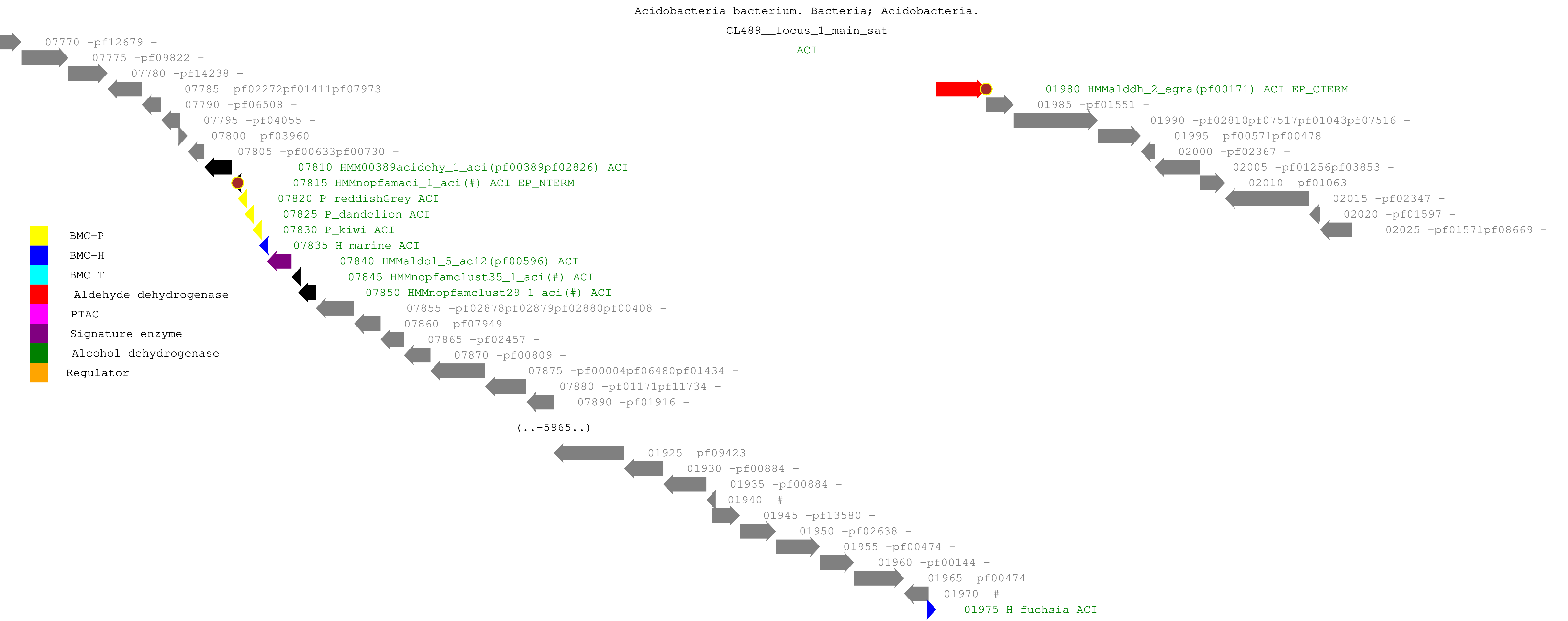

Acidobacteria bacterium. Bacteria; Acidobacteria.
CL489__locus_1_main_sat
ACI
07770 -pf12679 -
07775 -pf09822 -
07780 -pf14238 -
07785 -pf02272pf01411pf07973 -
07790 -pf06508 -
07795 -pf04055 -
07800 -pf03960 -
07805 -pf00633pf00730 -
07810 HMM00389acidehy_1_aci(pf00389pf02826) ACI
07815 HMMnopfamaci_1_aci(#) ACI EP_NTERM
07820 P_reddishGrey ACI
07825 P_dandelion ACI
07830 P_kiwi ACI
07835 H_marine ACI
07840 HMMaldol_5_aci2(pf00596) ACI
07845 HMMnopfamclust35_1_aci(#) ACI
07850 HMMnopfamclust29_1_aci(#) ACI
07855 -pf02878pf02879pf02880pf00408 -
07860 -pf07949 -
07865 -pf02457 -
07870 -pf00809 -
07875 -pf00004pf06480pf01434 -
07880 -pf01171pf11734 -
07890 -pf01916 -
(..-5965..)
01925 -pf09423 -
01930 -pf00884 -
01935 -pf00884 -
01940 -# -
01945 -pf13580 -
01950 -pf02638 -
01955 -pf00474 -
01960 -pf00144 -
01965 -pf00474 -
01970 -# -
01975 H_fuchsia ACI
01980 HMMalddh_2_egra(pf00171) ACI EP_CTERM
01985 -pf01551 -
01990 -pf02810pf07517pf01043pf07516 -
01995 -pf00571pf00478 -
02000 -pf02367 -
02005 -pf01256pf03853 -
02010 -pf01063 -
02015 -pf02347 -
02020 -pf01597 -
02025 -pf01571pf08669 -
BMC-P
BMC-H
BMC-T
Aldehyde dehydrogenase
PTAC
Signature enzyme
Alcohol dehydrogenase
Regulator

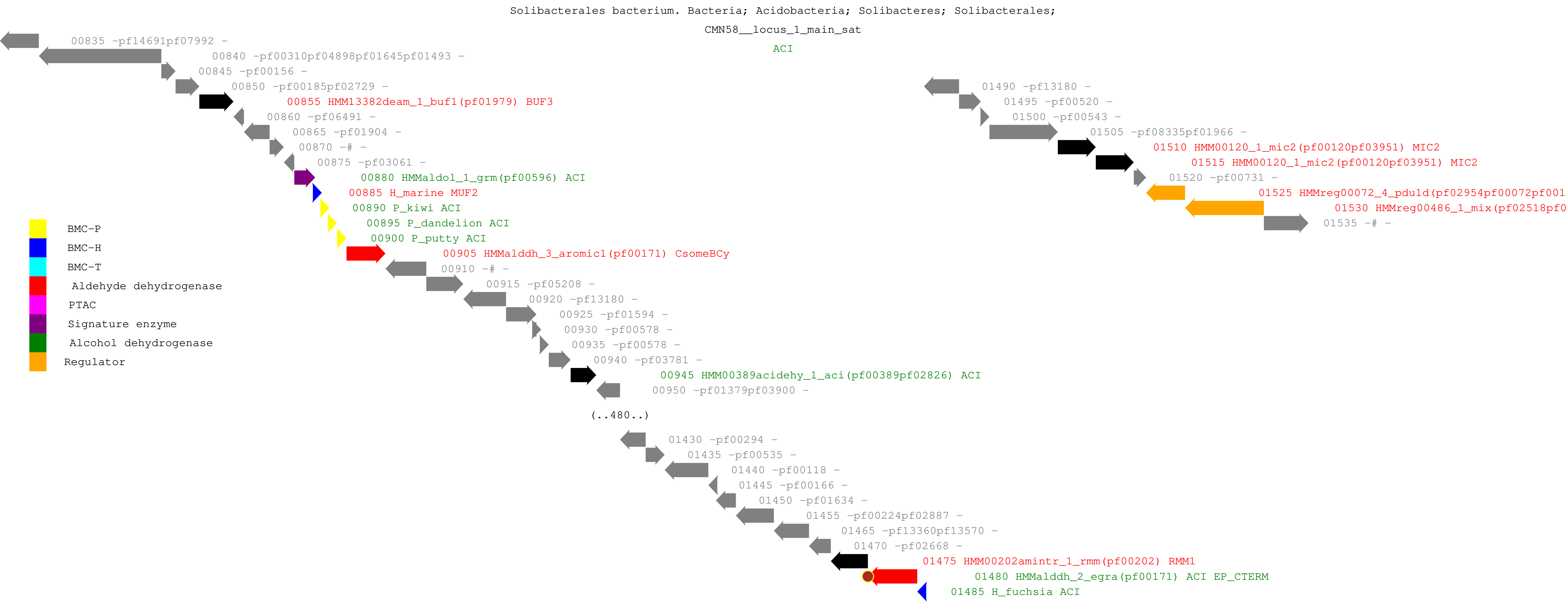

Solibacterales bacterium. Bacteria; Acidobacteria; Solibacteres; Solibacterales;
CMN58__locus_1_main_sat
ACI
00835 -pf14691pf07992 -
00840 -pf00310pf04898pf01645pf01493 -
00845 -pf00156 -
00850 -pf00185pf02729 -
00855 HMM13382deam_1_buf1(pf01979) BUF3
00860 -pf06491 -
00865 -pf01904 -
00870 -# -
00875 -pf03061 -
00880 HMMaldol_1_grm(pf00596) ACI
00885 H_marine MUF2
00890 P_kiwi ACI
00895 P_dandelion ACI
00900 P_putty ACI
00905 HMMalddh_3_aromic1(pf00171) CsomeBCy
00910 -# -
00915 -pf05208 -
00920 -pf13180 -
00925 -pf01594 -
00930 -pf00578 -
00935 -pf00578 -
00940 -pf03781 -
00945 HMM00389acidehy_1_aci(pf00389pf02826) ACI
00950 -pf01379pf03900 -
(..480..)
01430 -pf00294 -
01435 -pf00535 -
01440 -pf00118 -
01445 -pf00166 -
01450 -pf01634 -
01455 -pf00224pf02887 -
01465 -pf13360pf13570 -
01470 -pf02668 -
01475 HMM00202amintr_1_rmm(pf00202) RMM1
01480 HMMalddh_2_egra(pf00171) ACI EP_CTERM
01485 H_fuchsia ACI
01490 -pf13180 -
01495 -pf00520 -
01500 -pf00543 -
01505 -pf08335pf01966 -
01510 HMM00120_1_mic2(pf00120pf03951) MIC2
01515 HMM00120_1_mic2(pf00120pf03951) MIC2
01520 -pf00731 -
01525 HMMreg00072_4_pdu1d(pf02954pf00072pf001
01530 HMMreg00486_1_mix(pf02518pf0
01535 -# -
BMC-P
BMC-H
BMC-T
Aldehyde dehydrogenase
PTAC
Signature enzyme
Alcohol dehydrogenase
Regulator

Woeseiaceae bacterium. Bacteria; Proteobacteria; Gammaproteobacteria; Chromatiales;

CMP01__locus_1_main_sat

ACI

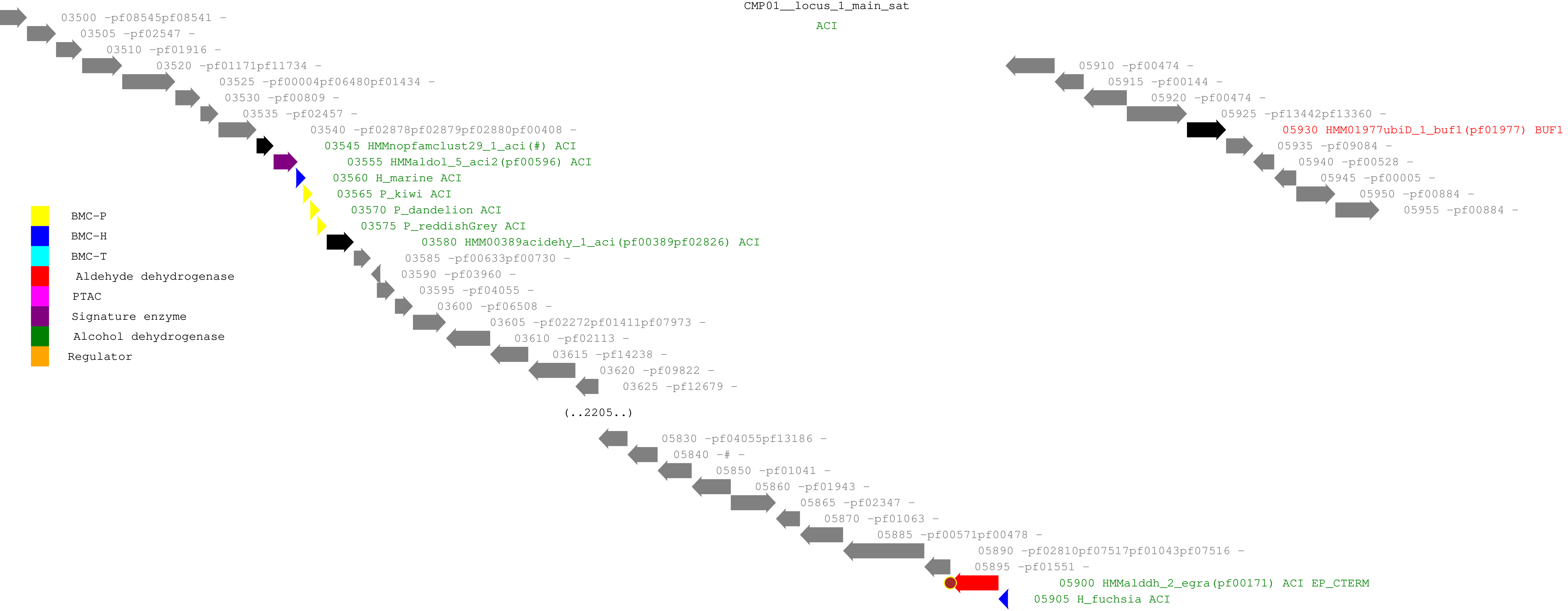

Acidobacteria bacterium CG_4_9_14_3_um_filter_49_7. Bacteria; Acidobacteria.

CO090__locus_1_main_sat

ACI

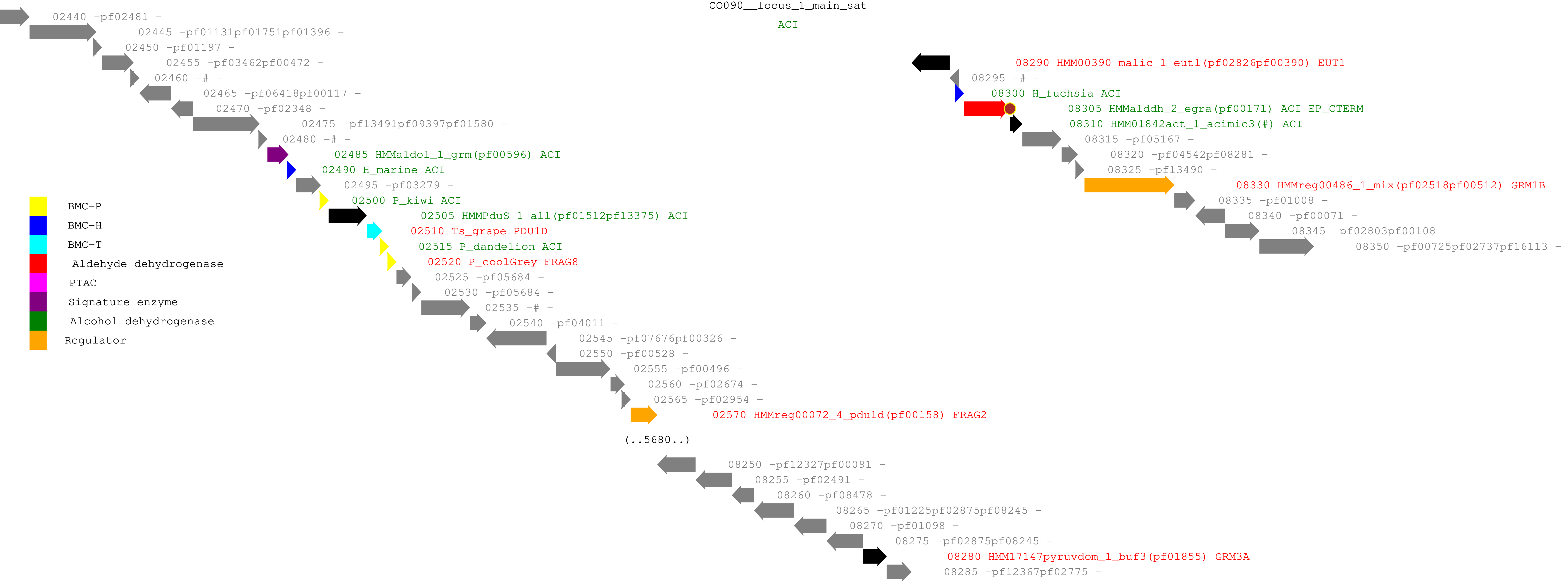

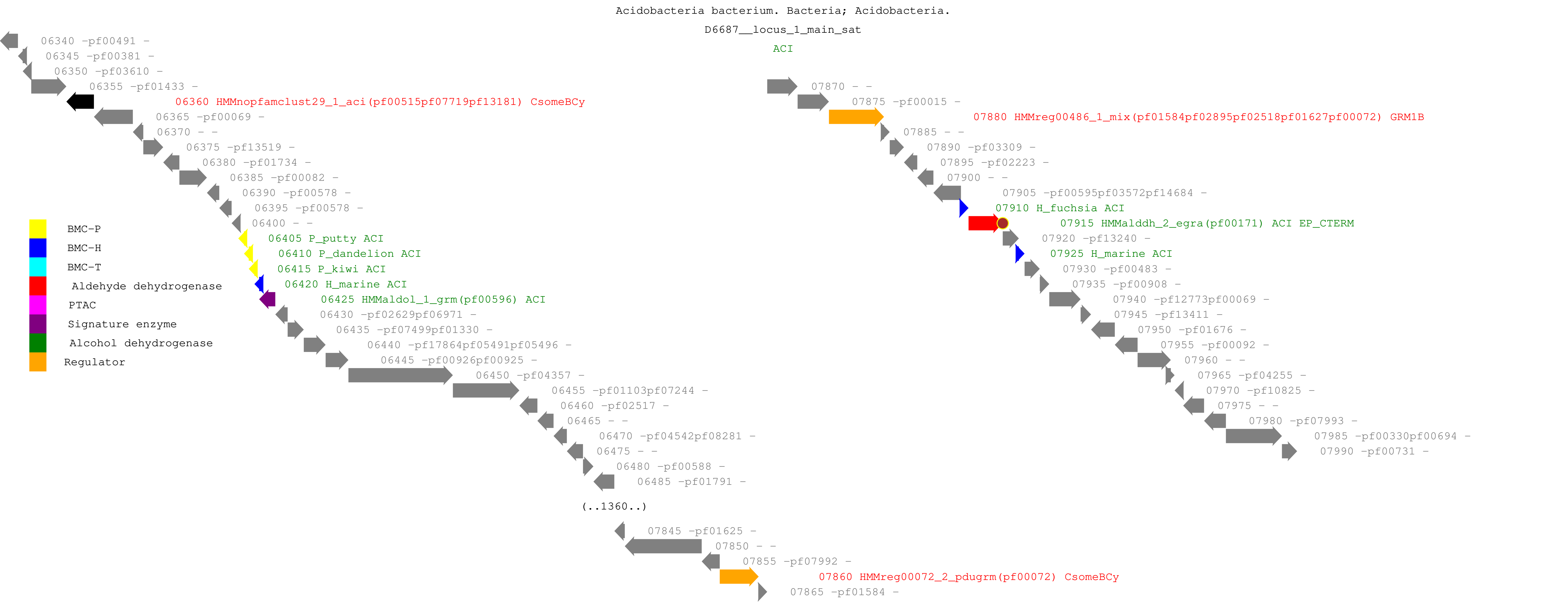
Acidobacteria bacterium. Bacteria; Acidobacteria.
D6687__locus_1_main_sat
ACI
06340 -pf00491 -
06345 -pf00381 -
06350 -pf03610 -
06355 -pf01433 -
06360 HMMnopfamclust29_1_aci(pf00515pf07719pf13181) CsomeBCy
06365 -pf00069 -
06370 - -
06375 -pf13519 -
06380 -pf01734 -
06385 -pf00082 -
06390 -pf00578 -
06395 -pf00578 -
06400 - -
06405 P_putty ACI
06410 P_dandelion ACI
06415 P_kiwi ACI
06420 H_marine ACI
06425 HMMaldol_1_grm(pf00596) ACI
06430 -pf02629pf06971 -
06435 -pf07499pf01330 -
06440 -pf17864pf05491pf05496 -
06445 -pf00926pf00925 -
06450 -pf04357 -
06455 -pf01103pf07244 -
06460 -pf02517 -
06465 - -
06470 -pf04542pf08281 -
06475 - -
06480 -pf00588 -
06485 -pf01791 -
(..1360..)
07845 -pf01625 -
07850 - -
07855 -pf07992 -
07860 HMMreg00072_2_pdugrm(pf00072) CsomeBCy
07865 -pf01584 -
07870 - -
07875 -pf00015 -
07880 HMMreg00486_1_mix(pf01584pf02895pf02518pf01627pf00072) GRM1B
07885 - -
07890 -pf03309 -
07895 -pf02223 -
07900 - -
07905 -pf00595pf03572pf14684 -
07910 H_fuchsia ACI
07915 HMMalddh_2_egra(pf00171) ACI EP_CTERM
07920 -pf13240 -
07925 H_marine ACI
07930 -pf00483 -
07935 -pf00908 -
07940 -pf12773pf00069 -
07945 -pf13411 -
07950 -pf01676 -
07955 -pf00092 -
07960 - -
07965 -pf04255 -
07970 -pf10825 -
07975 - -
07980 -pf07993 -
07985 -pf00330pf00694 -
07990 -pf00731 -
BMC-P
BMC-H
BMC-T
Aldehyde dehydrogenase
PTAC
Signature enzyme
Alcohol dehydrogenase
Regulator

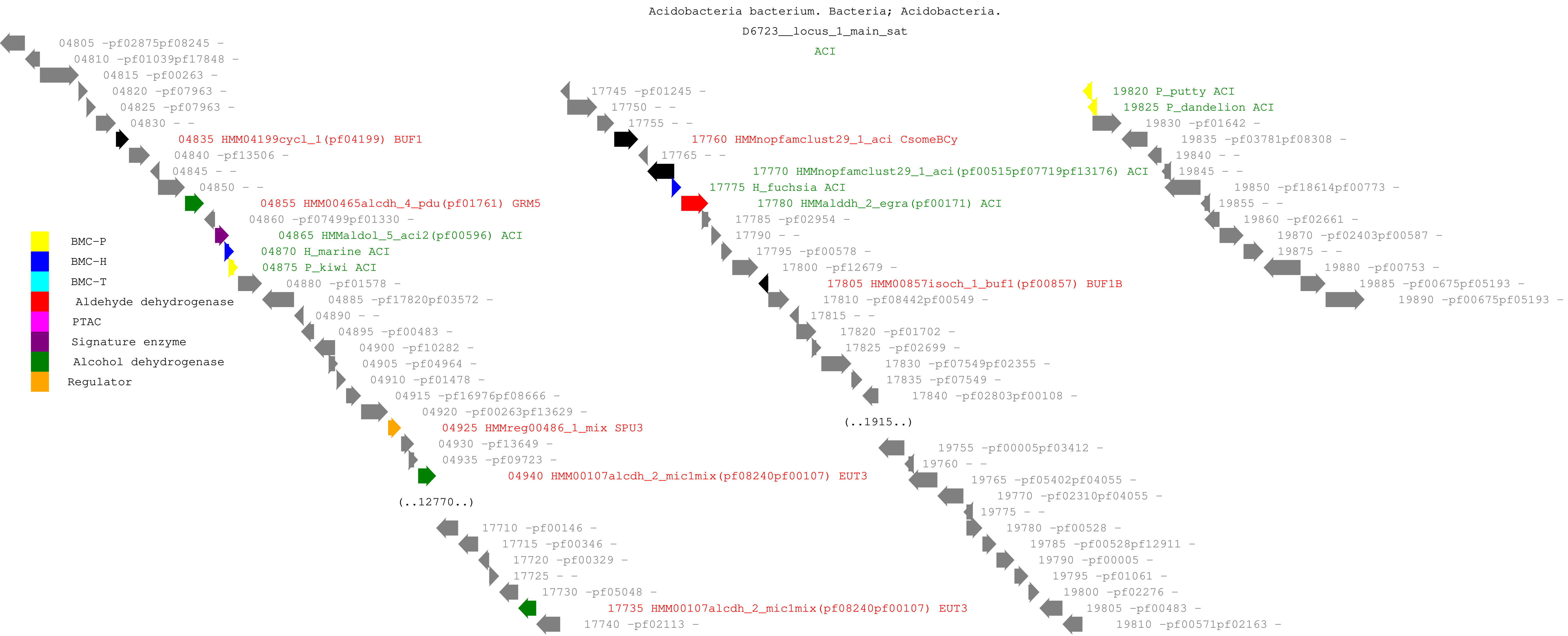

Acidobacteria bacterium. Bacteria; Acidobacteria.
D6723__locus_1_main_sat
ACI
04805 -pf02875pf08245 -
04810 -pf01039pf17848 -
04815 -pf00263 -
04820 -pf07963 -
04825 -pf07963 -
04830 - -
04835 HMM04199cycl_1(pf04199) BUF1
04840 -pf13506 -
04845 - -
04850 - -
04855 HMM00465alcdh_4_pdu(pf01761) GRM5
04860 -pf07499pf01330 -
04865 HMMaldol_5_aci2(pf00596) ACI
04870 H_marine ACI
04875 P_kiwi ACI
04880 -pf01578 -
04885 -pf17820pf03572 -
04890 - -
04895 -pf00483 -
04900 -pf10282 -
04905 -pf04964 -
04910 -pf01478 -
04915 -pf16976pf08666 -
04920 -pf00263pf13629 -
04925 HMMreg00486_1_mix SPU3
04930 -pf13649 -
04935 -pf09723 -
04940 HMM00107alcdh_2_mic1mix(pf08240pf00107) EUT3
(..12770..)
17710 -pf00146 -
17715 -pf00346 -
17720 -pf00329 -
17725 - -
17730 -pf05048 -
17735 HMM00107alcdh_2_mic1mix(pf08240pf00107) EUT3
17740 -pf02113 -
17745 -pf01245 -
17750 - -
17755 - -
17760 HMMnopfamclust29_1_aci CsomeBCy
17765 - -
17770 HMMnopfamclust29_1_aci(pf00515pf07719pf13176) ACI
17775 H_fuchsia ACI
17780 HMMaldh_2_egra(pf00171) ACI
17785 -pf02954 -
17790 - -
17795 -pf00578 -
17800 -pf12679 -
17805 HMM00857isoch_1_buf1(pf00857) BUF1B
17810 -pf08442pf00549 -
17815 - -
17820 -pf01702 -
17825 -pf02699 -
17830 -pf07549pf02355 -
17835 -pf07549 -
17840 -pf02803pf00108 -
(..1915..)
19755 -pf00005pf03412 -
19760 - -
19765 -pf05402pf04055 -
19770 -pf02310pf04055 -
19775 - -
19780 -pf00528 -
19785 -pf00528pf12911 -
19790 -pf00005 -
19795 -pf01061 -
19800 -pf02276 -
19805 -pf00483 -
19810 -pf00571pf02163 -
19820 P_putty ACI
19825 P_dandelion ACI
19830 -pf01642 -
19835 -pf03781pf08308 -
19840 - -
19845 - -
19850 -pf18614pf00773 -
19855 - -
19860 -pf02661 -
19870 -pf02403pf00587 -
19875 - -
19880 -pf00753 -
19885 -pf00675pf05193 -
19890 -pf00675pf05193 -
BMC-P
BMC-H
BMC-T
Aldehyde dehydrogenase
PTAC
Signature enzyme
Alcohol dehydrogenase
Regulator

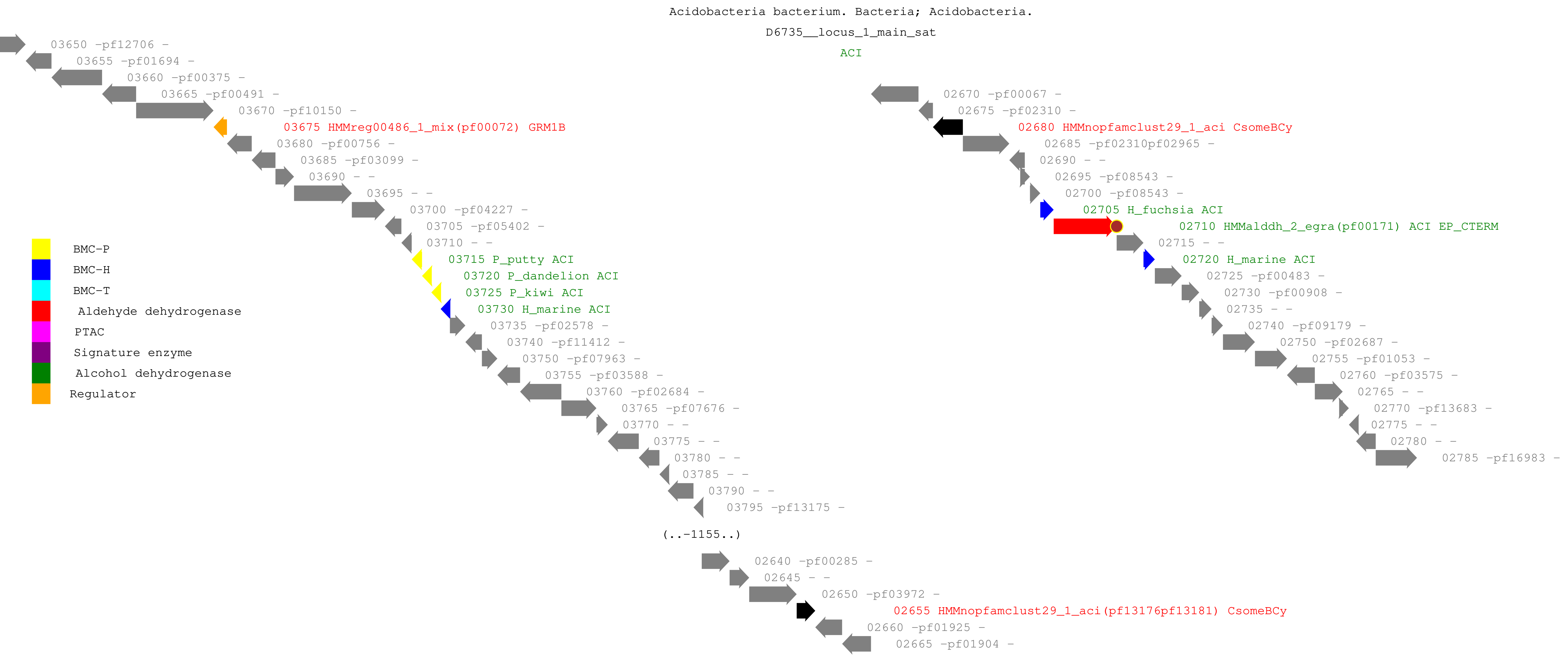

Acidobacteria bacterium. Bacteria; Acidobacteria.
D6735__locus_1_main_sat
ACI
03650 -pf12706 -
03655 -pf01694 -
03660 -pf00375 -
03665 -pf00491 -
03670 -pf10150 -
03675 HMMreg00486_1_mix(pf00072) GRM1B
03680 -pf00756 -
03685 -pf03099 -
03690 - -
03695 - -
03700 -pf04227 -
03705 -pf05402 -
03710 - -
03715 P_putty ACI
03720 P_dandelion ACI
03725 P_kiwi ACI
03730 H_marine ACI
03735 -pf02578 -
03740 -pf11412 -
03750 -pf07963 -
03755 -pf03588 -
03760 -pf02684 -
03765 -pf07676 -
03770 - -
03775 - -
03780 - -
03785 - -
03790 - -
03795 -pf13175 -
(..-1155..)
02640 -pf00285 -
02645 - -
02650 -pf03972 -
02655 HMMnopfamclust29_1_aci(pf13176pf13181) CsomeBCy
02660 -pf01925 -
02665 -pf01904 -
02670 -pf00067 -
02675 -pf02310 -
02680 HMMnopfamclust29_1_aci CsomeBCy
02685 -pf02310pf02965 -
02690 - -
02695 -pf08543 -
02700 -pf08543 -
02705 H_fuchsia ACI
02710 HMMalddh_2_egra(pf00171) ACI EP_CTERM
02715 - -
02720 H_marine ACI
02725 -pf00483 -
02730 -pf00908 -
02735 - -
02740 -pf09179 -
02750 -pf02687 -
02755 -pf01053 -
02760 -pf03575 -
02765 - -
02770 -pf13683 -
02775 - -
02780 - -
02785 -pf16983 -
BMC-P
BMC-H
BMC-T
Aldehyde dehydrogenase
PTAC
Signature enzyme
Alcohol dehydrogenase
Regulator

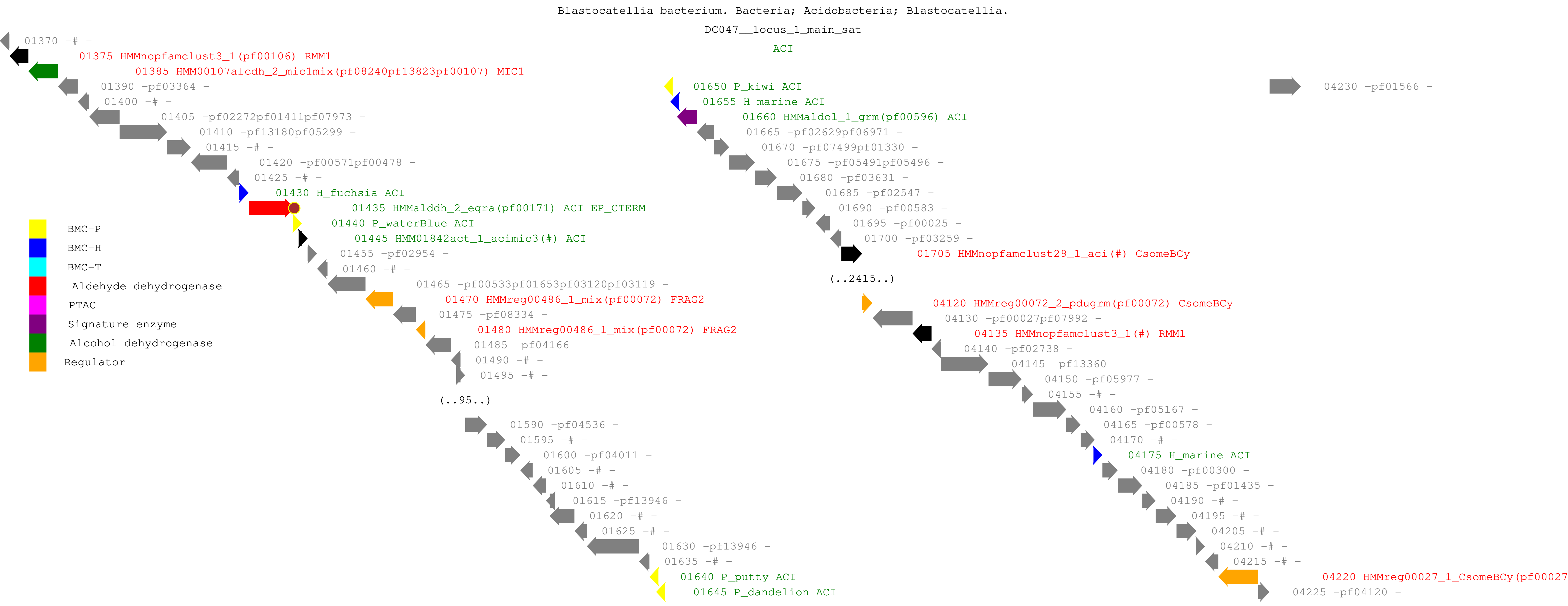

Blastocatellia bacterium. Bacteria; Acidobacteria; Blastocatellia.
DC047__locus_1_main_sat
ACI
01370 -# -
01375 HMMnopfamclust3_1(pf00106) RMM1
01385 HMM00107alcdh_2_mic1mix(pf08240pf13823pf00107) MIC1
01390 -pf03364 -
01400 -# -
01405 -pf02272pf01411pf07973 -
01410 -pf13180pf05299 -
01415 -# -
01420 -pf00571pf00478 -
01425 -# -
01430 H_fuchsia ACI
01435 HMMalddh_2_egra(pf00171) ACI EP_CTERM
01440 P_waterBlue ACI
01445 HMM01842act_1_acimic3(#) ACI
01455 -pf02954 -
01460 -# -
01465 -pf00533pf01653pf03120pf03119 -
01470 HMMreg00486_1_mix(pf00072) FRAG2
01475 -pf08334 -
01480 HMMreg00486_1_mix(pf00072) FRAG2
01485 -pf04166 -
01490 -# -
01495 -# -
(..95..)
01590 -pf04536 -
01595 -# -
01600 -pf04011 -
01605 -# -
01610 -# -
01615 -pf13946 -
01620 -# -
01625 -# -
01630 -pf13946 -
01635 -# -
01640 P_putty ACI
01645 P_dandelion ACI
01650 P_kiwi ACI
01655 H_marine ACI
01660 HMMaldol_1_grm(pf00596) ACI
01665 -pf02629pf06971 -
01670 -pf07499pf01330 -
01675 -pf05491pf05496 -
01680 -pf03631 -
01685 -pf02547 -
01690 -pf00583 -
01695 -pf00025 -
01700 -pf03259 -
01705 HMMnopfamclust29_1_aci(#) CsomeBCy
(..2415..)
04120 HMMreg00072_2_pdugrm(pf00072) CsomeBCy
04130 -pf00027pf07992 -
04135 HMMnopfamclust3_1(#) RMM1
04140 -pf02738 -
04145 -pf13360 -
04150 -pf05977 -
04155 -# -
04160 -pf05167 -
04165 -pf00578 -
04170 -# -
04175 H_marine ACI
04180 -pf00300 -
04185 -pf01435 -
04190 -# -
04195 -# -
04205 -# -
04210 -# -
04215 -# -
04220 HMMreg00027_1_CsomeBCy(pf00027
04225 -pf04120 -
04230 -pf01566 -
BMC-P
BMC-H
BMC-T
Aldehyde dehydrogenase
PTAC
Signature enzyme
Alcohol dehydrogenase
Regulator

Blastocatellia bacterium. Bacteria; Acidobacteria; Blastocatellia.

DC054__locus_1_main_sat

ACI

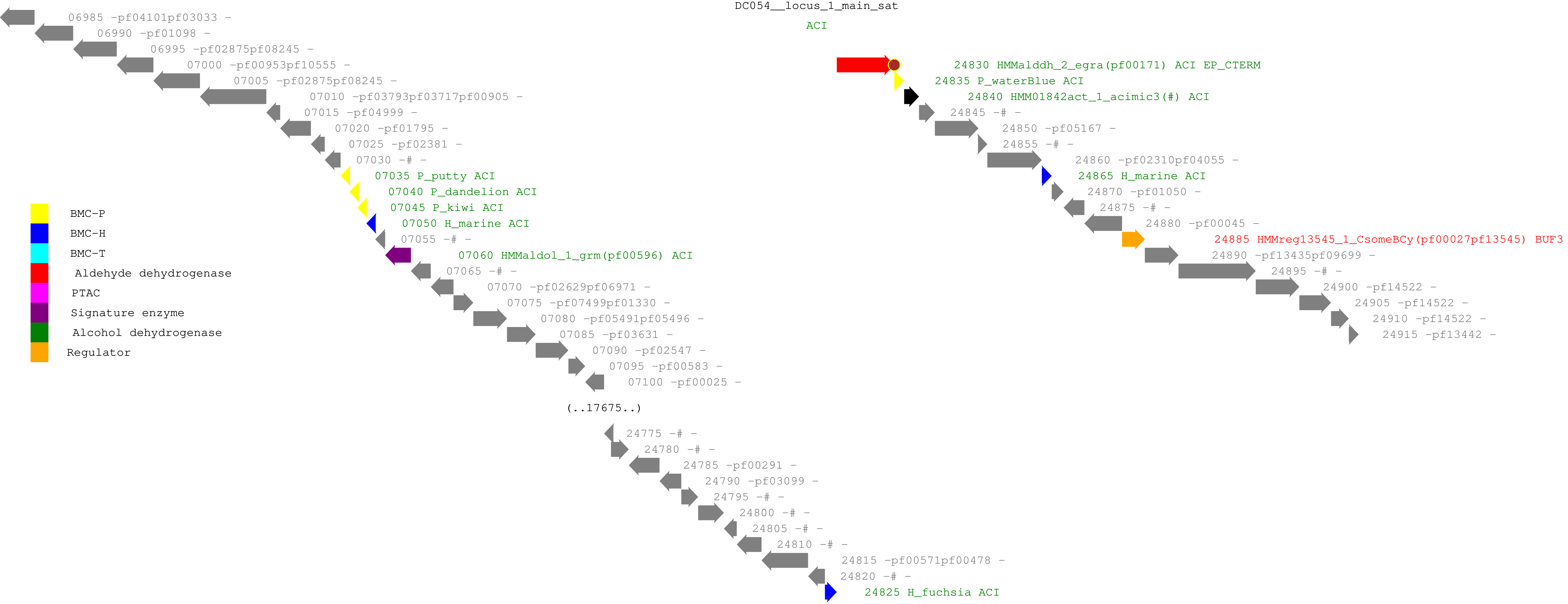

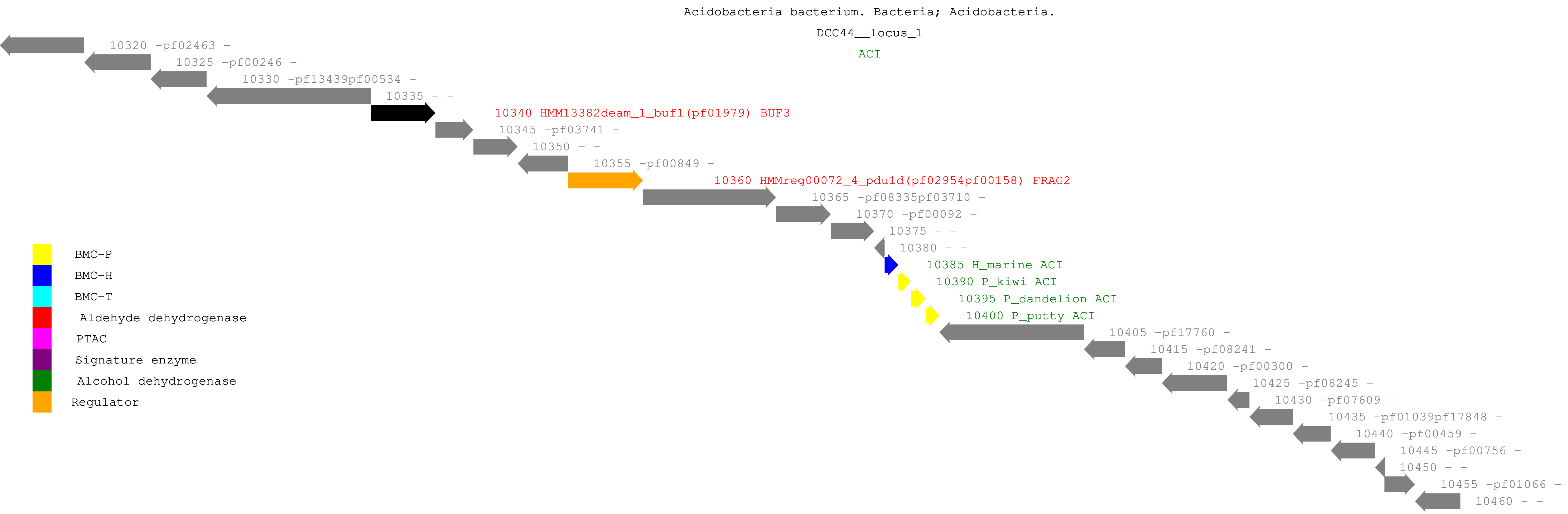

Acidobacteria bacterium. Bacteria; Acidobacteria.
DCC44__locus_1
ACI
10320 -pf02463 -
10325 -pf00246 -
10330 -pf13439pf00534 -
10335 - -
10340 HMM13382deam_1_buf1(pf01979) BUF3
10345 -pf03741 -
10350 - -
10355 -pf00849 -
10360 HMMreg00072_4_pdu1d(pf02954pf00158) FRAG2
10365 -pf08335pf03710 -
10370 -pf00092 -
10375 - -
10380 - -
10385 H_marine ACI
10390 P_kiwi ACI
10395 P_dandelion ACI
10400 P_putty ACI
10405 -pf17760 -
10415 -pf08241 -
10420 -pf00300 -
10425 -pf08245 -
10430 -pf07609 -
10435 -pf01039pf17848 -
10440 -pf00459 -
10445 -pf00756 -
10450 - -
10455 -pf01066 -
10460 - -
BMC-P
BMC-H
BMC-T
Aldehyde dehydrogenase
PTAC
Signature enzyme
Alcohol dehydrogenase
Regulator

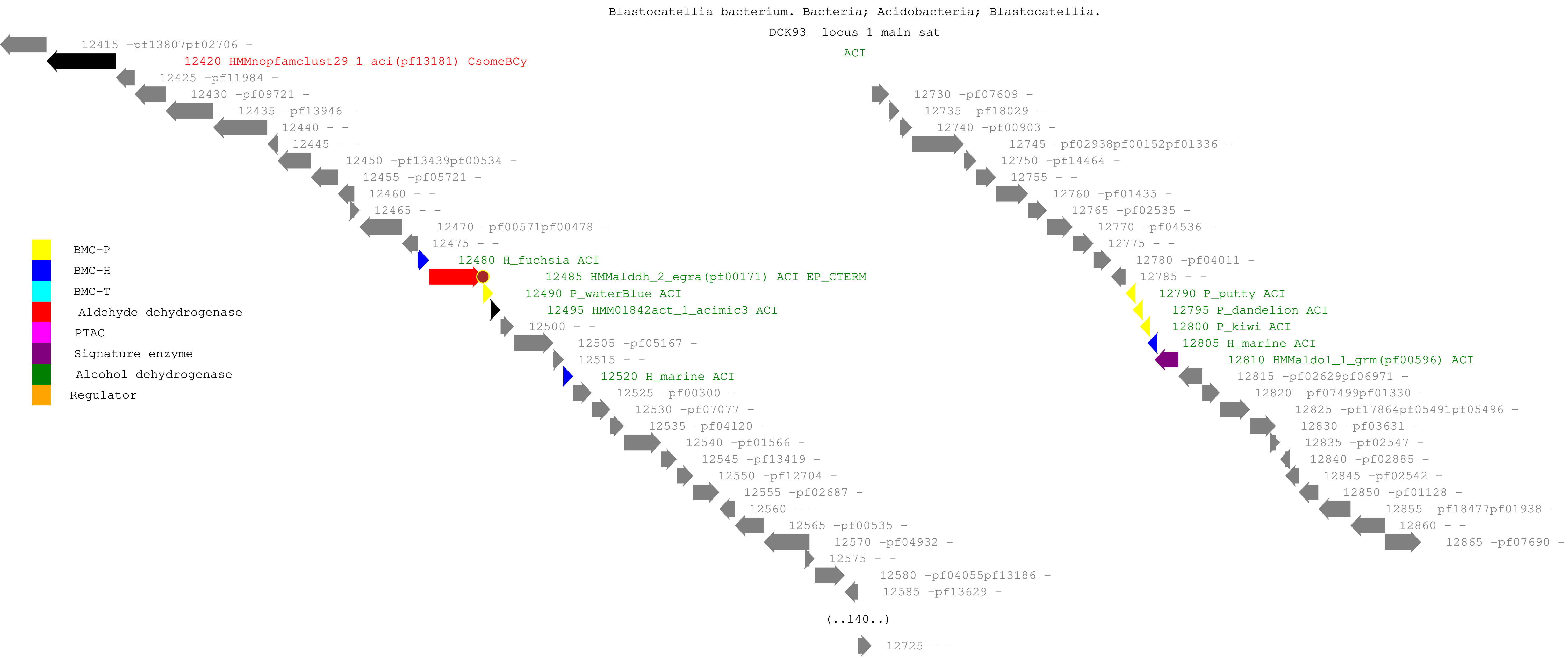
Blastocatellia bacterium. Bacteria; Acidobacteria; Blastocatellia.
DCK93__locus_1_main_sat
ACI
12415 -pf13807pf02706 -
12420 HMMnopfamclust29_1_aci(pf13181) CsomeBCy
12425 -pf11984 -
12430 -pf09721 -
12435 -pf13946 -
12440 - -
12445 - -
12450 -pf13439pf00534 -
12455 -pf05721 -
12460 - -
12465 - -
12470 -pf00571pf00478 -
12475 - -
12480 H_fuchsia ACI
12485 HMMalddh_2_egra(pf00171) ACI EP_CTERM
12490 P_waterBlue ACI
12495 HMM01842act_1_acimic3 ACI
12500 - -
12505 -pf05167 -
12515 - -
12520 H_marine ACI
12525 -pf00300 -
12530 -pf07077 -
12535 -pf04120 -
12540 -pf01566 -
12545 -pf13419 -
12550 -pf12704 -
12555 -pf02687 -
12560 - -
12565 -pf00535 -
12570 -pf04932 -
12575 - -
12580 -pf04055pf13186 -
12585 -pf13629 -
(..140..)
12725 - -
12730 -pf07609 -
12735 -pf18029 -
12740 -pf00903 -
12745 -pf02938pf00152pf01336 -
12750 -pf14464 -
12755 - -
12760 -pf01435 -
12765 -pf02535 -
12770 -pf04536 -
12775 - -
12780 -pf04011 -
12785 - -
12790 P_putty ACI
12795 P_dandelion ACI
12800 P_kiwi ACI
12805 H_marine ACI
12810 HMMaldol_1_grm(pf00596) ACI
12815 -pf02629pf06971 -
12820 -pf07499pf01330 -
12825 -pf17864pf05491pf05496 -
12830 -pf03631 -
12835 -pf02547 -
12840 -pf02885 -
12845 -pf02542 -
12850 -pf01128 -
12855 -pf18477pf01938 -
12860 - -
12865 -pf07690 -
BMC-P
BMC-H
BMC-T
Aldehyde dehydrogenase
PTAC
Signature enzyme
Alcohol dehydrogenase
Regulator

Blastocatellia bacterium. Bacteria; Acidobacteria; Blastocatellia.

DCK99__locus_1_main_sat

ACI

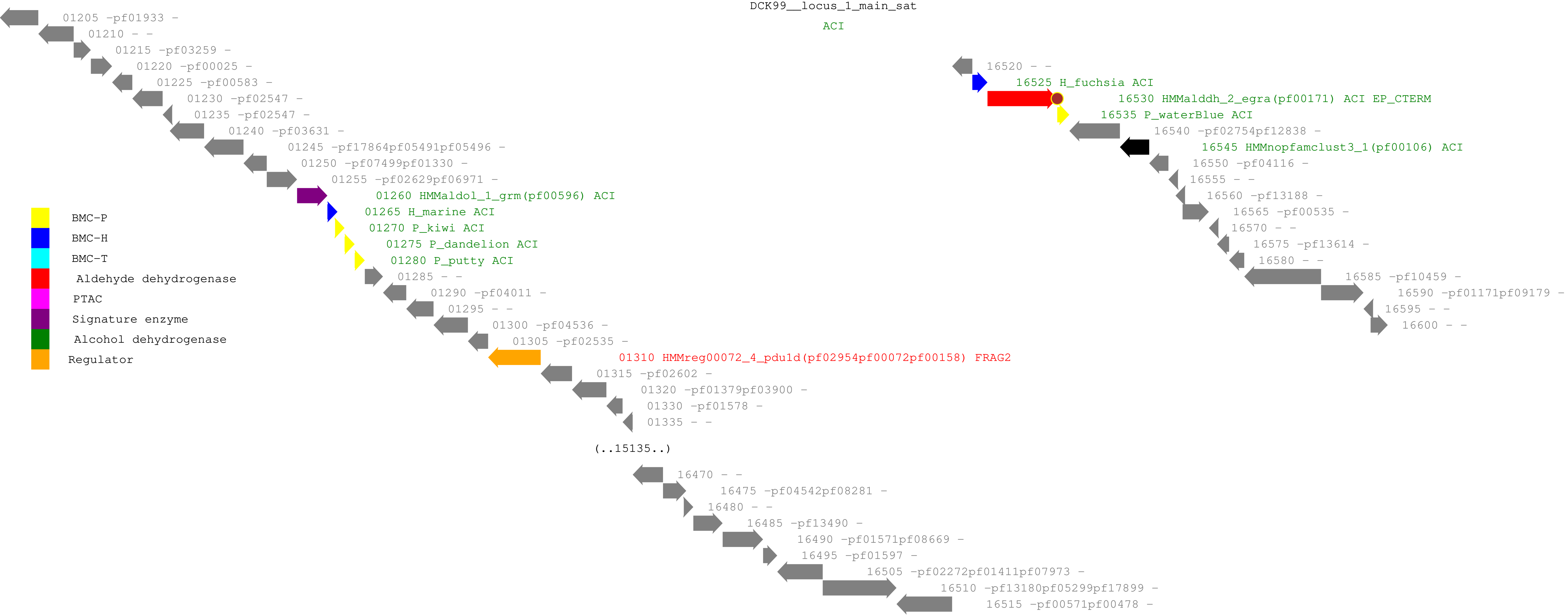

Solibacterales bacterium. Bacteria; Acidobacteria; Solibacteres; Solibacterales;

DCY80__locus_fragmented

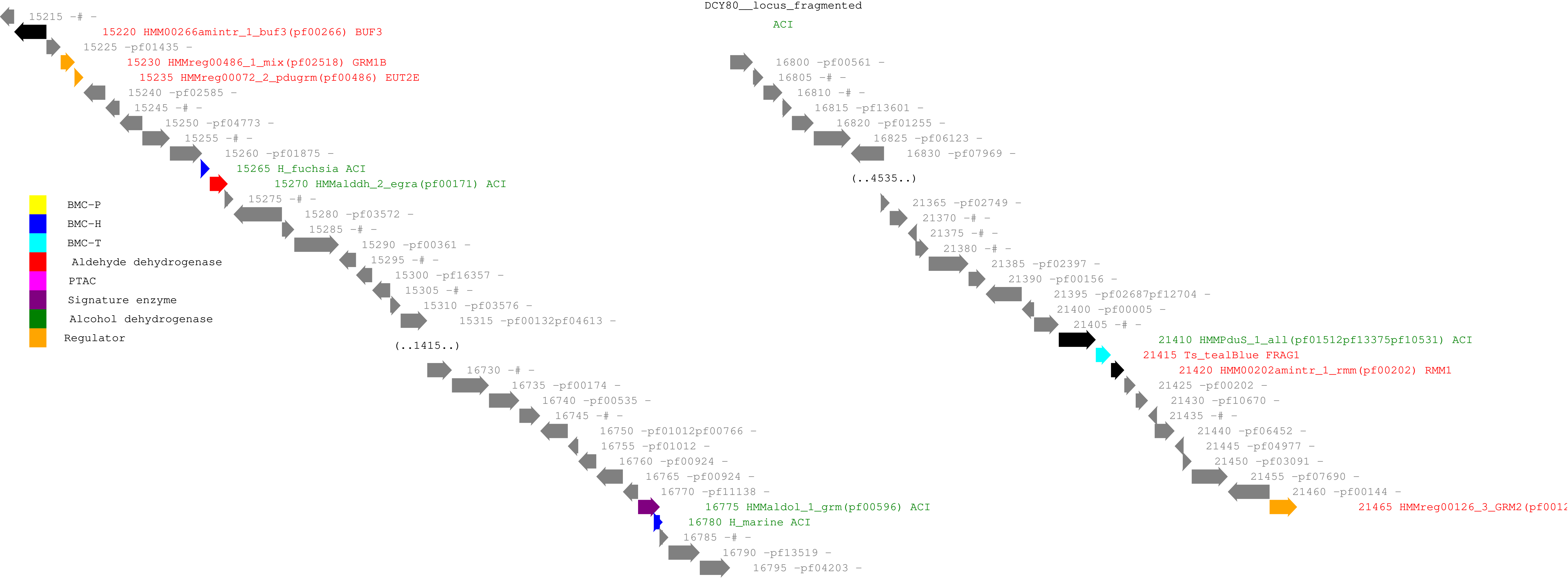

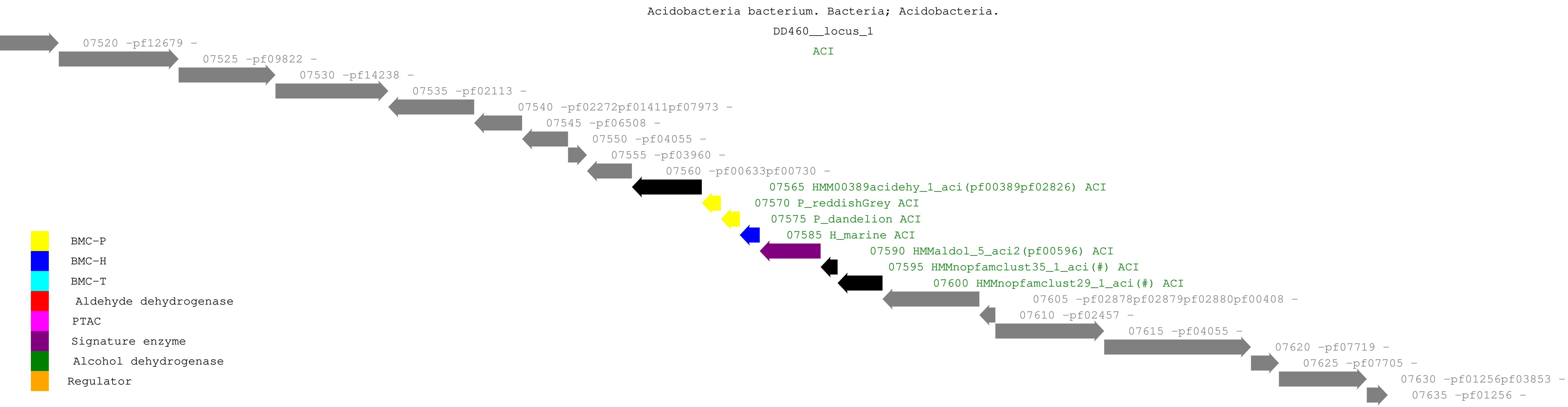
Acidobacteria bacterium. Bacteria; Acidobacteria.
DD460__locus_1
ACI
07520 -pf12679 -
07525 -pf09822 -
07530 -pf14238 -
07535 -pf02113 -
07540 -pf02272pf01411pf07973 -
07545 -pf06508 -
07550 -pf04055 -
07555 -pf03960 -
07560 -pf00633pf00730 -
07565 HMM00389acidehy_1_aci(pf00389pf02826) ACI
07570 P_reddishGrey ACI
07575 P_dandelion ACI
07585 H_marine ACI
07590 HMMaldol_5_aci2(pf00596) ACI
07595 HMMnopfamclust35_1_aci(#) ACI
07600 HMMnopfamclust29_1_aci(#) ACI
07605 -pf02878pf02879pf02880pf00408 -
07610 -pf02457 -
07615 -pf04055 -
07620 -pf07719 -
07625 -pf07705 -
07630 -pf01256pf03853 -
07635 -pf01256 -
BMC-P
BMC-H
BMC-T
Aldehyde dehydrogenase
PTAC
Signature enzyme
Alcohol dehydrogenase
Regulator

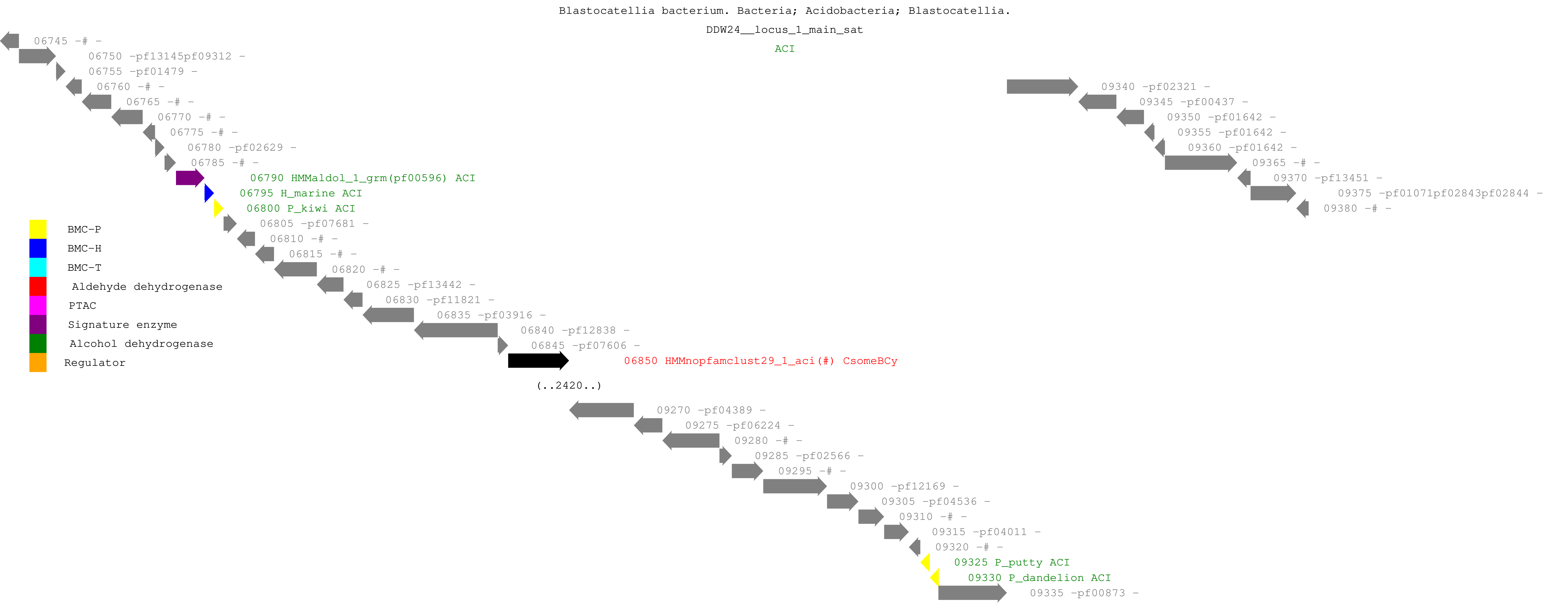
Blastocatellia bacterium. Bacteria; Acidobacteria; Blastocatellia.
DDW24__locus_1_main_sat
ACI
06745 -# -
06750 -pf13145pf09312 -
06755 -pf01479 -
06760 -# -
06765 -# -
06770 -# -
06775 -# -
06780 -pf02629 -
06785 -# -
06790 HMMaldol_1_grm(pf00596) ACI
06795 H_marine ACI
06800 P_kiwi ACI
06805 -pf07681 -
06810 -# -
06815 -# -
06820 -# -
06825 -pf13442 -
06830 -pf11821 -
06835 -pf03916 -
06840 -pf12838 -
06845 -pf07606 -
06850 HMMnopfamclust29_1_aci(#) CsomeBCy
(..2420..)
09270 -pf04389 -
09275 -pf06224 -
09280 -# -
09285 -pf02566 -
09295 -# -
09300 -pf12169 -
09305 -pf04536 -
09310 -# -
09315 -pf04011 -
09320 -# -
09325 P_putty ACI
09330 P_dandelion ACI
09335 -pf00873 -
09340 -pf02321 -
09345 -pf00437 -
09350 -pf01642 -
09355 -pf01642 -
09360 -pf01642 -
09365 -# -
09370 -pf13451 -
09375 -pf01071pf02843pf02844 -
09380 -# -
BMC-P
BMC-H
BMC-T
Aldehyde dehydrogenase
PTAC
Signature enzyme
Alcohol dehydrogenase
Regulator

Blastocatellia bacterium. Bacteria; Acidobacteria; Blastocatellia.

DEA22__locus_1_main_sat

ACI

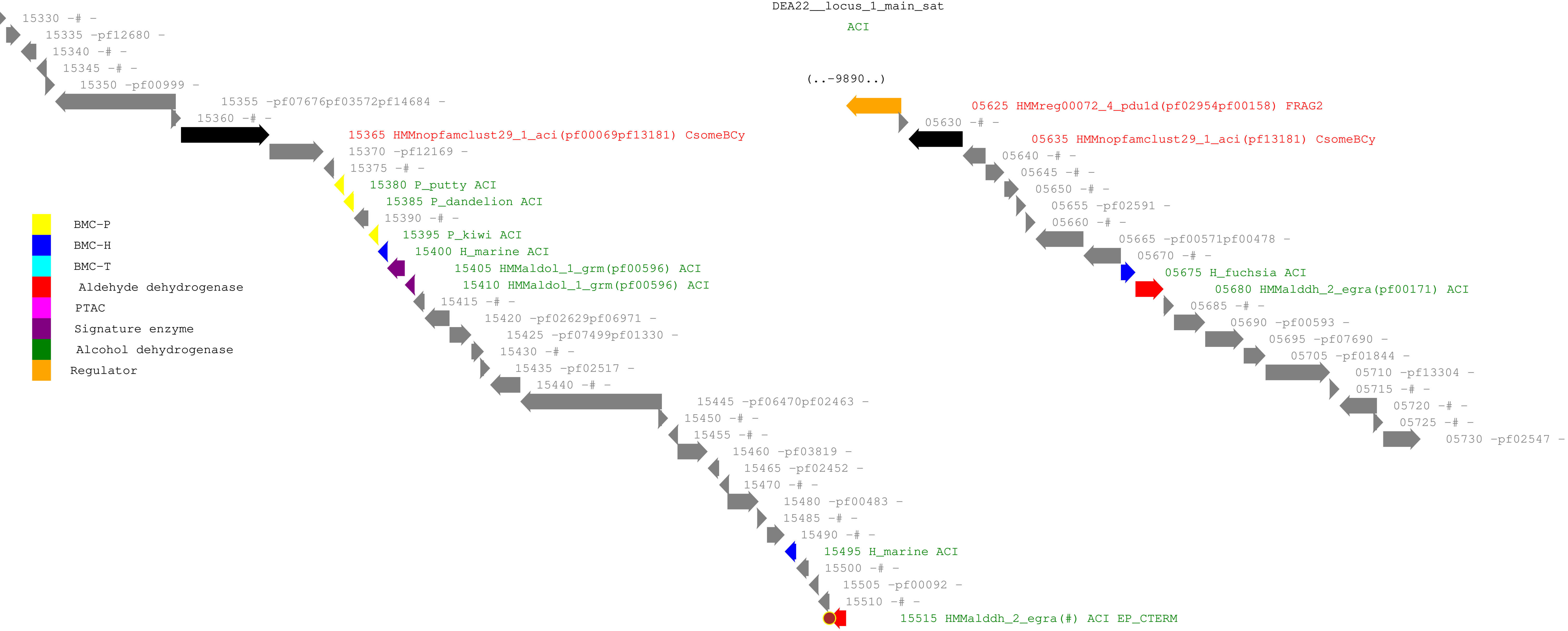

Solibacterales bacterium. Bacteria; Acidobacteria; Solibacteres; Solibacterales;

DEH78__locus_1_main_sat

ACI

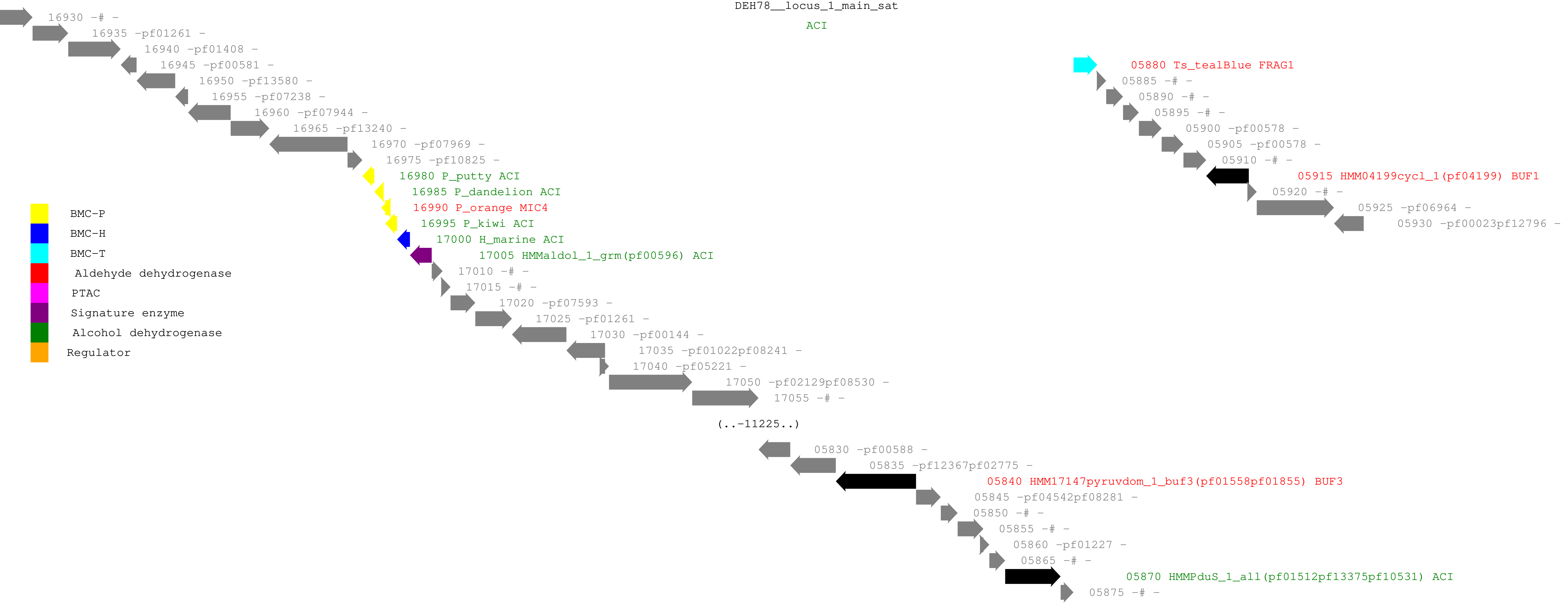

Blastocatellia bacterium. Bacteria; Acidobacteria; Blastocatellia.

DEP46__locus_1_main_sat

ACI

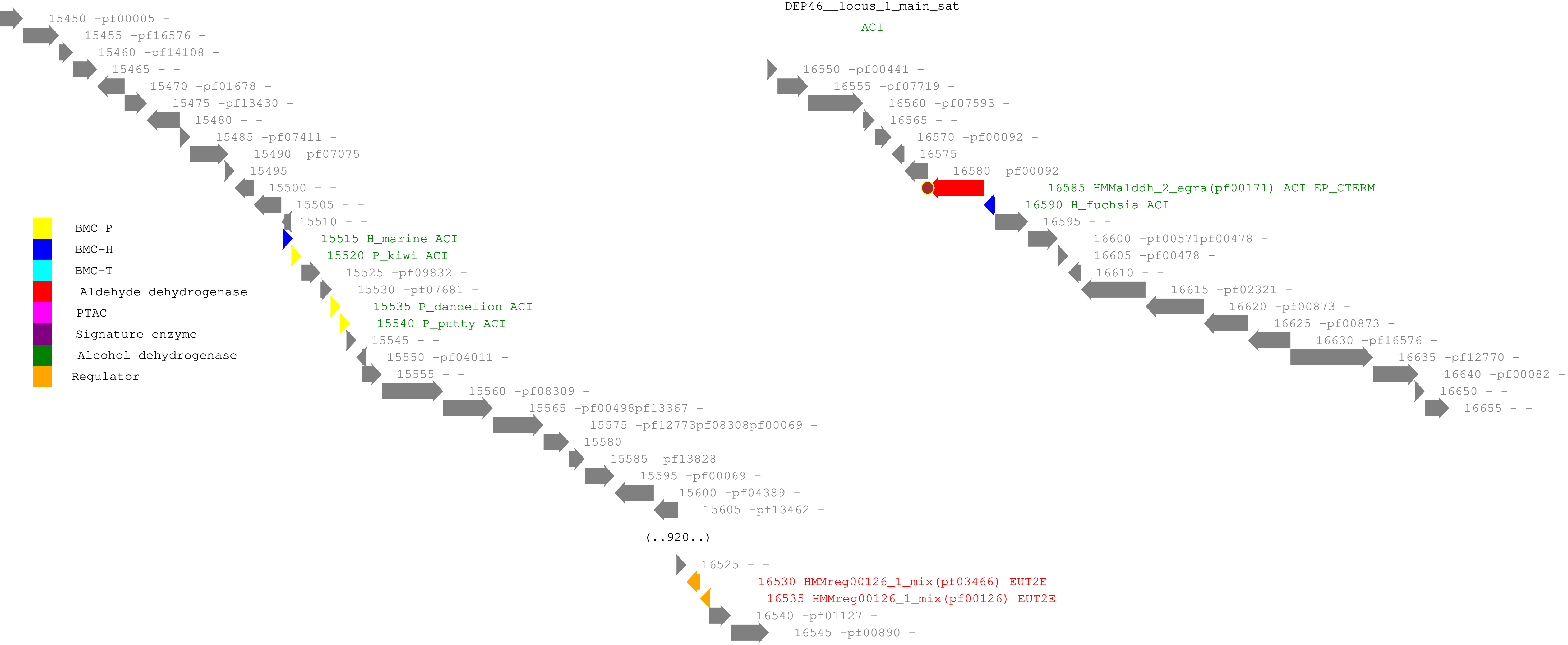

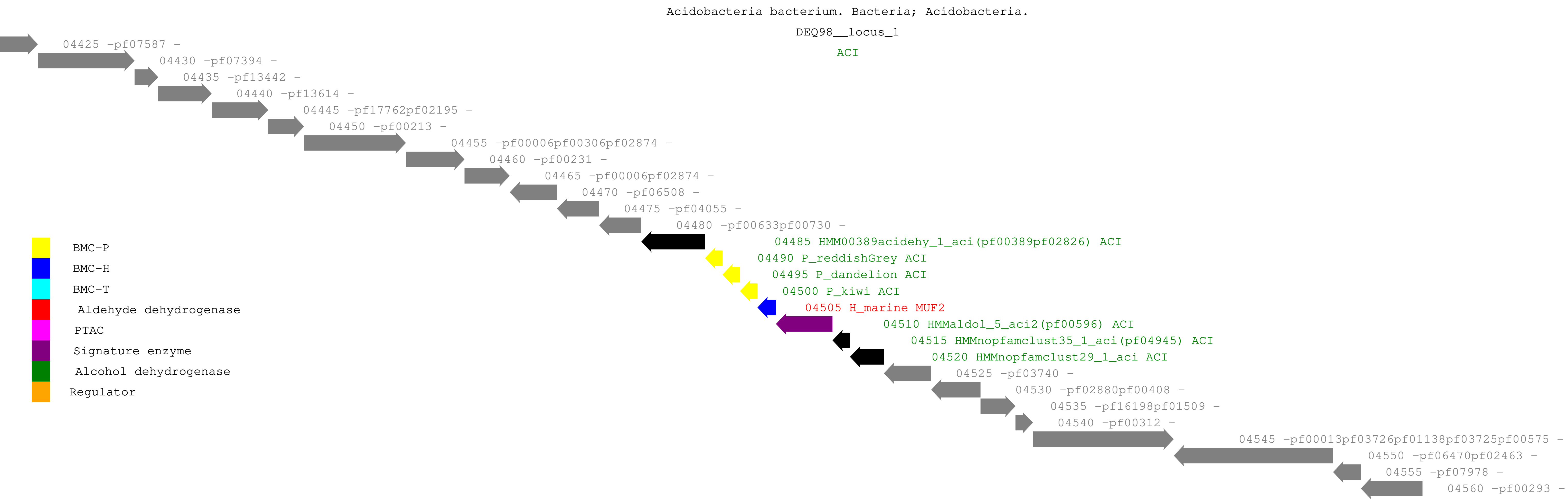
Acidobacteria bacterium. Bacteria; Acidobacteria.
DEQ98__locus_1
ACI
04425 -pf07587 -
04430 -pf07394 -
04435 -pf13442 -
04440 -pf13614 -
04445 -pf17762pf02195 -
04450 -pf00213 -
04455 -pf00006pf00306pf02874 -
04460 -pf00231 -
04465 -pf00006pf02874 -
04470 -pf06508 -
04475 -pf04055 -
04480 -pf00633pf00730 -
04485 HMM00389acidehy_1_aci(pf00389pf02826) ACI
04490 P_reddishGrey ACI
04495 P_dandelion ACI
04500 P_kiwi ACI
04505 H_marine MUF2
04510 HMMaldol_5_aci2(pf00596) ACI
04515 HMMnopfamclust35_1_aci(pf04945) ACI
04520 HMMnopfamclust29_1_aci ACI
04525 -pf03740 -
04530 -pf02880pf00408 -
04535 -pf16198pf01509 -
04540 -pf00312 -
04545 -pf00013pf03726pf01138pf03725pf00575 -
04550 -pf06470pf02463 -
04555 -pf07978 -
04560 -pf00293 -
BMC-P
BMC-H
BMC-T
Aldehyde dehydrogenase
PTAC
Signature enzyme
Alcohol dehydrogenase
Regulator

Blastocatellia bacterium. Bacteria; Acidobacteria; Blastocatellia.

DHU55__locus_1_main_sat

ACI

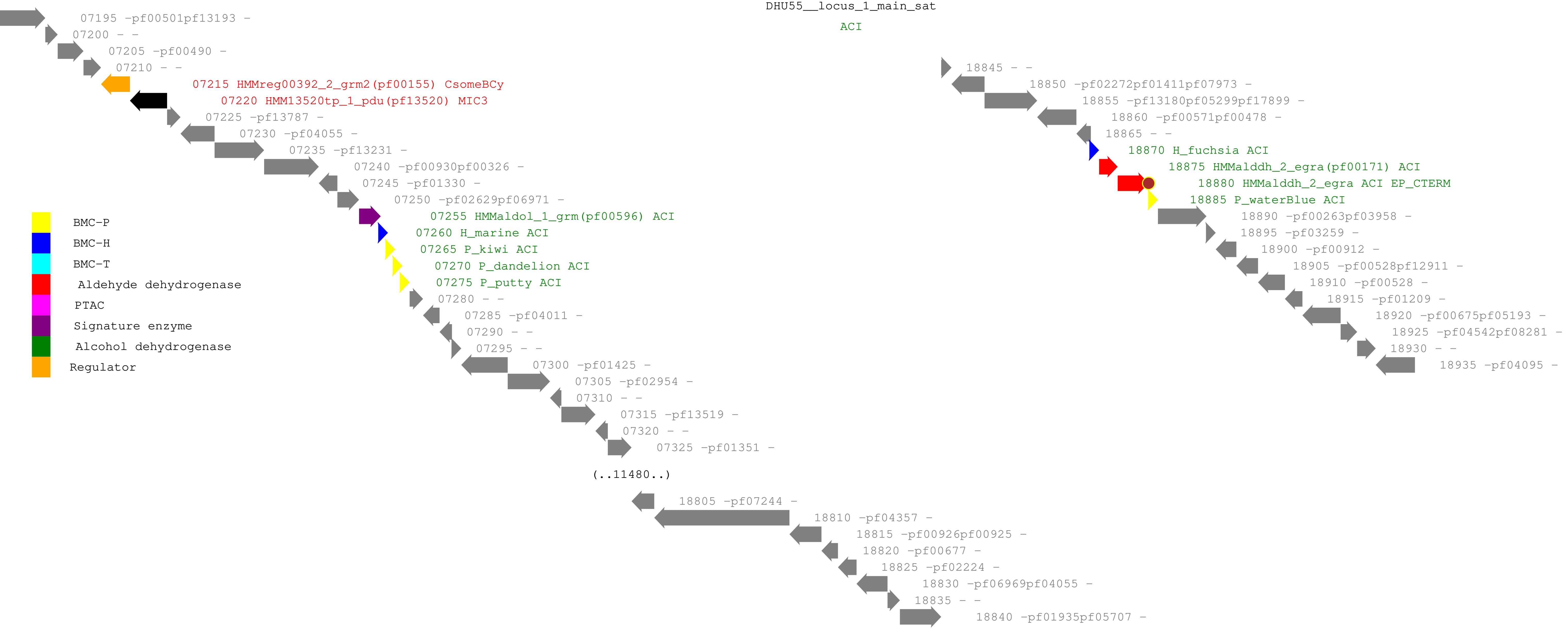

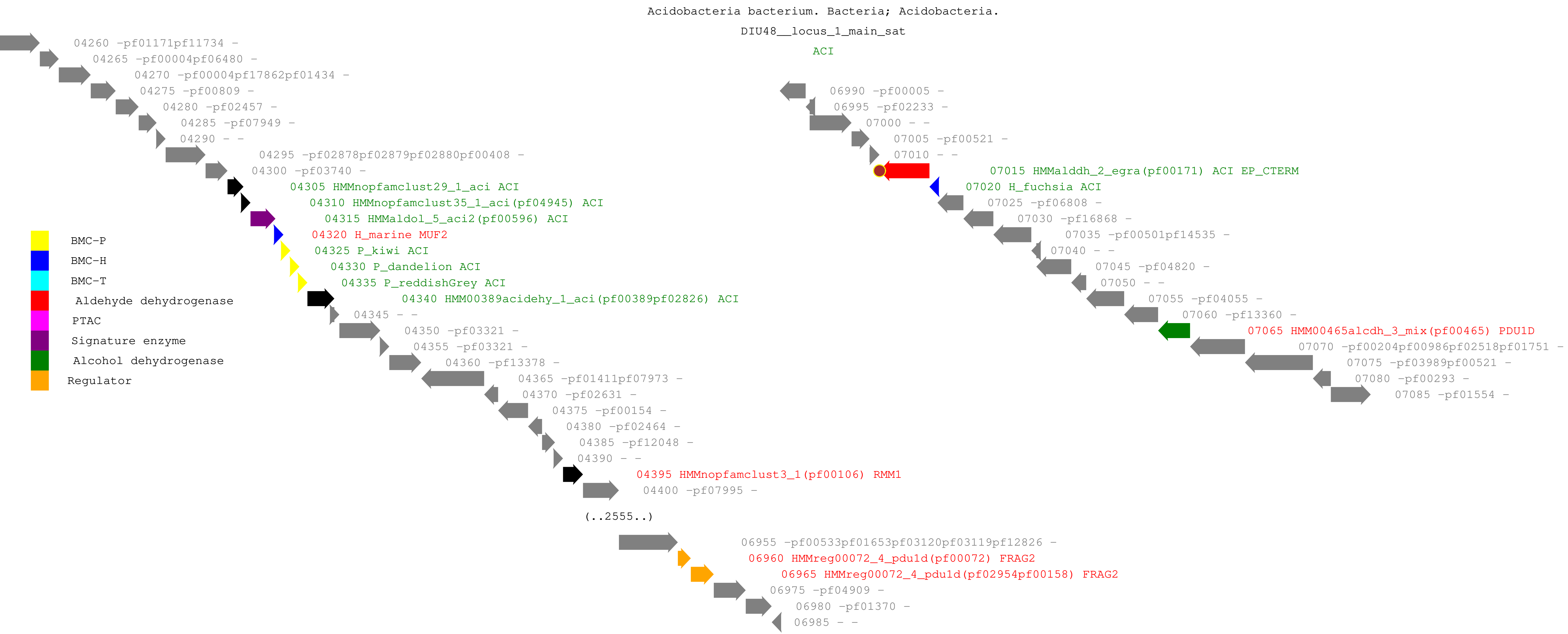
Acidobacteria bacterium. Bacteria; Acidobacteria.
DIU48__locus_1_main_sat
ACI
04260 -pf01171pf11734 -
04265 -pf00004pf06480 -
04270 -pf00004pf17862pf01434 -
04275 -pf00809 -
04280 -pf02457 -
04285 -pf07949 -
04290 - -
04295 -pf02878pf02879pf02880pf00408 -
04300 -pf03740 -
04305 HMMnopfamclust29_1_aci ACI
04310 HMMnopfamclust35_1_aci(pf04945) ACI
04315 HMMaldol_5_aci2(pf00596) ACI
04320 H_marine MUF2
04325 P_kiwi ACI
04330 P_dandelion ACI
04335 P_reddishGrey ACI
04340 HMM00389acidehy_1_aci(pf00389pf02826) ACI
04345 - -
04350 -pf03321 -
04355 -pf03321 -
04360 -pf13378 -
04365 -pf01411pf07973 -
04370 -pf02631 -
04375 -pf00154 -
04380 -pf02464 -
04385 -pf12048 -
04390 - -
04395 HMMnopfamclust3_1(pf00106) RMM1
04400 -pf07995 -
(..2555..)
06955 -pf00533pf01653pf03120pf03119pf12826 -
06960 HMMreg00072_4_pdu1d(pf00072) FRAG2
06965 HMMreg00072_4_pdu1d(pf02954pf00158) FRAG2
06975 -pf04909 -
06980 -pf01370 -
06985 - -
06990 -pf00005 -
06995 -pf02233 -
07000 - -
07005 -pf00521 -
07010 - -
07015 HMMalddh_2_egra(pf00171) ACI EP_CTERM
07020 H_fuchsia ACI
07025 -pf06808 -
07030 -pf16868 -
07035 -pf00501pf14535 -
07040 - -
07045 -pf04820 -
07050 - -
07055 -pf04055 -
07060 -pf13360 -
07065 HMM00465alcdh_3_mix(pf00465) PDU1D
07070 -pf00204pf00986pf02518pf01751 -
07075 -pf03989pf00521 -
07080 -pf00293 -
07085 -pf01554 -
BMC-P
BMC-H
BMC-T
Aldehyde dehydrogenase
PTAC
Signature enzyme
Alcohol dehydrogenase
Regulator

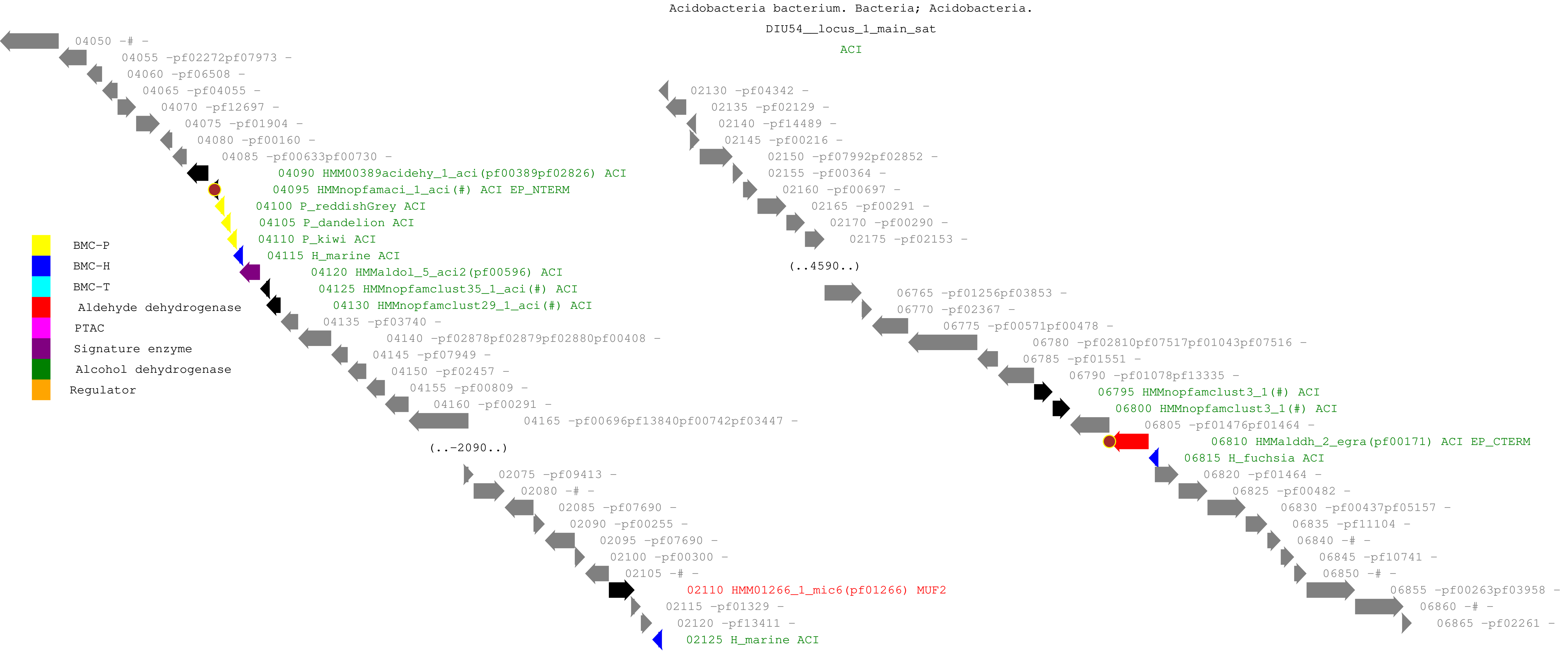
Acidobacteria bacterium. Bacteria; Acidobacteria.
DIU54__locus_1_main_sat
ACI
04050 -# -
04055 -pf02272pf07973 -
04060 -pf06508 -
04065 -pf04055 -
04070 -pf12697 -
04075 -pf01904 -
04080 -pf00160 -
04085 -pf00633pf00730 -
04090 HMM00389acidehy_1_aci(pf00389pf02826) ACI
04095 HMMnopfamaci_1_aci(#) ACI EP_NTERM
04100 P_reddishGrey ACI
04105 P_dandelion ACI
04110 P_kiwi ACI
04115 H_marine ACI
04120 HMMaldol_5_aci2(pf00596) ACI
04125 HMMnopfamclust35_1_aci(#) ACI
04130 HMMnopfamclust29_1_aci(#) ACI
04135 -pf03740 -
04140 -pf02878pf02879pf02880pf00408 -
04145 -pf07949 -
04150 -pf02457 -
04155 -pf00809 -
04160 -pf00291 -
04165 -pf00696pf13840pf00742pf03447 -
(..-2090..)
02075 -pf09413 -
02080 -# -
02085 -pf07690 -
02090 -pf00255 -
02095 -pf07690 -
02100 -pf00300 -
02105 -# -
02110 HMM01266_1_mic6(pf01266) MUF2
02115 -pf01329 -
02120 -pf13411 -
02125 H_marine ACI
BMC-P
BMC-H
BMC-T
Aldehyde dehydrogenase
PTAC
Signature enzyme
Alcohol dehydrogenase
Regulator
02130 -pf04342 -
02135 -pf02129 -
02140 -pf14489 -
02145 -pf00216 -
02150 -pf07992pf02852 -
02155 -pf00364 -
02160 -pf00697 -
02165 -pf00291 -
02170 -pf00290 -
02175 -pf02153 -
(..4590..)
06765 -pf01256pf03853 -
06770 -pf02367 -
06775 -pf00571pf00478 -
06780 -pf02810pf07517pf01043pf07516 -
06785 -pf01551 -
06790 -pf01078pf13335 -
06795 HMMnopfamclust3_1(#) ACI
06800 HMMnopfamclust3_1(#) ACI
06805 -pf01476pf01464 -
06810 HMMalddh_2_egra(pf00171) ACI EP_CTERM
06815 H_fuchsia ACI
06820 -pf01464 -
06825 -pf00482 -
06830 -pf00437pf05157 -
06835 -pf11104 -
06840 -# -
06845 -pf10741 -
06850 -# -
06855 -pf00263pf03958 -
06860 -# -
06865 -pf02261 -

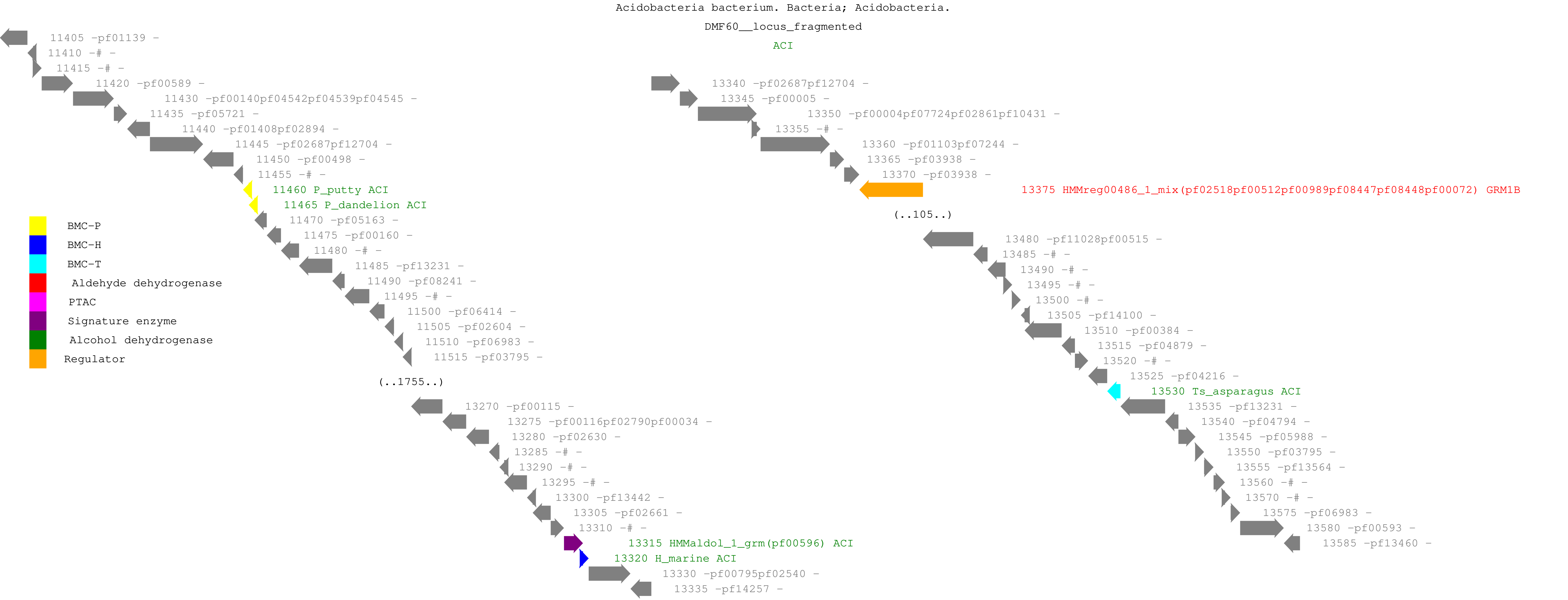
Acidobacteria bacterium. Bacteria; Acidobacteria.
DMF60__locus_fragmented
ACI
11405 -pf01139 -
11410 -# -
11415 -# -
11420 -pf00589 -
11430 -pf00140pf04542pf04539pf04545 -
11435 -pf05721 -
11440 -pf01408pf02894 -
11445 -pf02687pf12704 -
11450 -pf00498 -
11455 -# -
11460 P_putty ACI
11465 P_dandelion ACI
11470 -pf05163 -
11475 -pf00160 -
11480 -# -
11485 -pf13231 -
11490 -pf08241 -
11495 -# -
11500 -pf06414 -
11505 -pf02604 -
11510 -pf06983 -
11515 -pf03795 -
(..1755..)
13270 -pf00115 -
13275 -pf00116pf02790pf00034 -
13280 -pf02630 -
13285 -# -
13290 -# -
13295 -# -
13300 -pf13442 -
13305 -pf02661 -
13310 -# -
13315 HMMaldol_1_grm(pf00596) ACI
13320 H_marine ACI
13330 -pf00795pf02540 -
13335 -pf14257 -
13340 -pf02687pf12704 -
13345 -pf00005 -
13350 -pf00004pf07724pf02861pf10431 -
13355 -# -
13360 -pf01103pf07244 -
13365 -pf03938 -
13370 -pf03938 -
13375 HMMreg00486_1_mix(pf02518pf00512pf00989pf08447pf08448pf00072) GRM1B
(..105..)
13480 -pf11028pf00515 -
13485 -# -
13490 -# -
13495 -# -
13500 -# -
13505 -pf14100 -
13510 -pf00384 -
13515 -pf04879 -
13520 -# -
13525 -pf04216 -
13530 Ts_asparagus ACI
13535 -pf13231 -
13540 -pf04794 -
13545 -pf05988 -
13550 -pf03795 -
13555 -pf13564 -
13560 -# -
13570 -# -
13575 -pf06983 -
13580 -pf00593 -
13585 -pf13460 -
BMC-P
BMC-H
BMC-T
Aldehyde dehydrogenase
PTAC
Signature enzyme
Alcohol dehydrogenase
Regulator

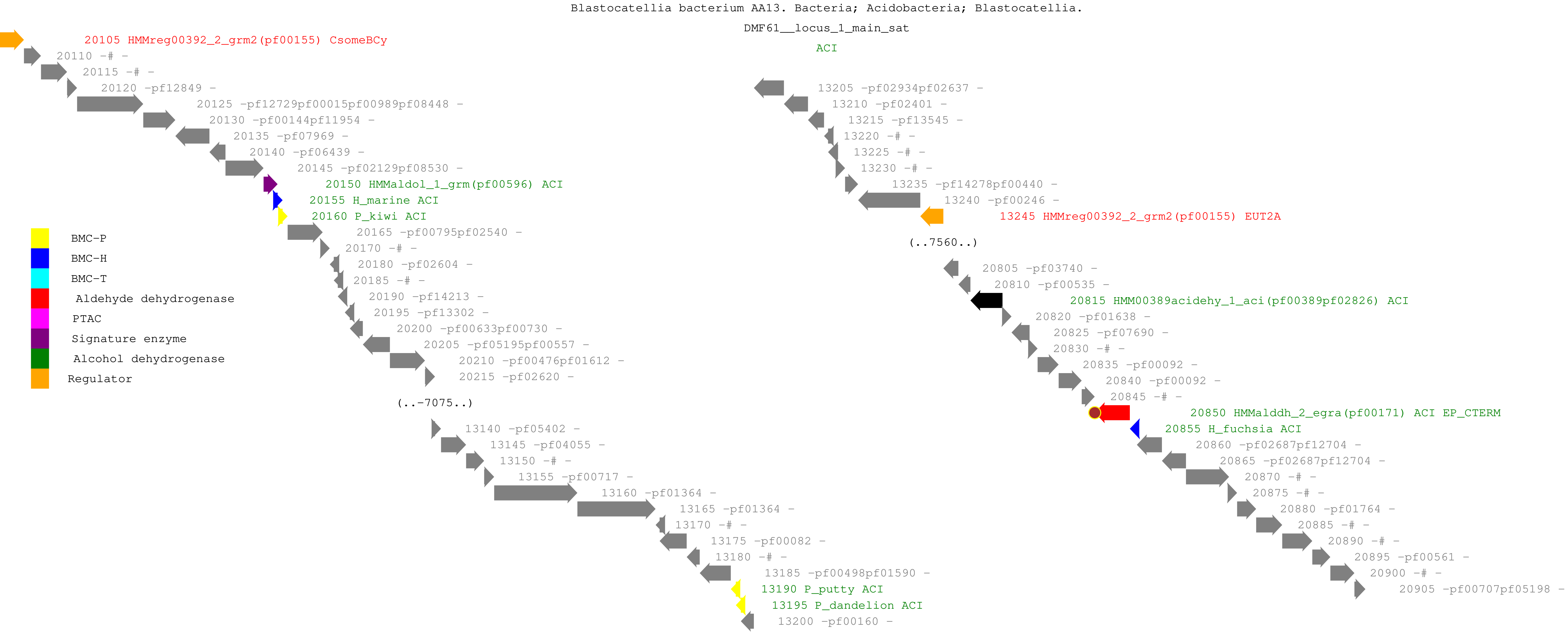

Blastocatellia bacterium AA13. Bacteria; Acidobacteria; Blastocatellia.
DMF61__locus_1_main_sat
ACI
20105 HMMreg00392_2_grm2(pf00155) CsomeBCy
20110 -# -
20115 -# -
20120 -pf12849 -
20125 -pf12729pf00015pf00989pf08448 -
20130 -pf00144pf11954 -
20135 -pf07969 -
20140 -pf06439 -
20145 -pf02129pf08530 -
20150 HMMaldol_1_grm(pf00596) ACI
20155 H_marine ACI
20160 P_kiwi ACI
20165 -pf00795pf02540 -
20170 -# -
20180 -pf02604 -
20185 -# -
20190 -pf14213 -
20195 -pf13302 -
20200 -pf00633pf00730 -
20205 -pf05195pf00557 -
20210 -pf00476pf01612 -
20215 -pf02620 -
(..-7075..)
13140 -pf05402 -
13145 -pf04055 -
13150 -# -
13155 -pf00717 -
13160 -pf01364 -
13165 -pf01364 -
13170 -# -
13175 -pf00082 -
13180 -# -
13185 -pf00498pf01590 -
13190 P_putty ACI
13195 P_dandelion ACI
13200 -pf00160 -
13205 -pf02934pf02637 -
13210 -pf02401 -
13215 -pf13545 -
13220 -# -
13225 -# -
13230 -# -
13235 -pf14278pf00440 -
13240 -pf00246 -
13245 HMMreg00392_2_grm2(pf00155) EUT2A
(..7560..)
20805 -pf03740 -
20810 -pf00535 -
20815 HMM00389acidehy_1_aci(pf00389pf02826) ACI
20820 -pf01638 -
20825 -pf07690 -
20830 -# -
20835 -pf00092 -
20840 -pf00092 -
20845 -# -
20850 HMMalddh_2_egra(pf00171) ACI EP_CTERM
20855 H_fuchsia ACI
20860 -pf02687pf12704 -
20865 -pf02687pf12704 -
20870 -# -
20875 -# -
20880 -pf01764 -
20885 -# -
20890 -# -
20895 -pf00561 -
20900 -# -
20905 -pf00707pf05198 -
BMC-P
BMC-H
BMC-T
Aldehyde dehydrogenase
PTAC
Signature enzyme
Alcohol dehydrogenase
Regulator

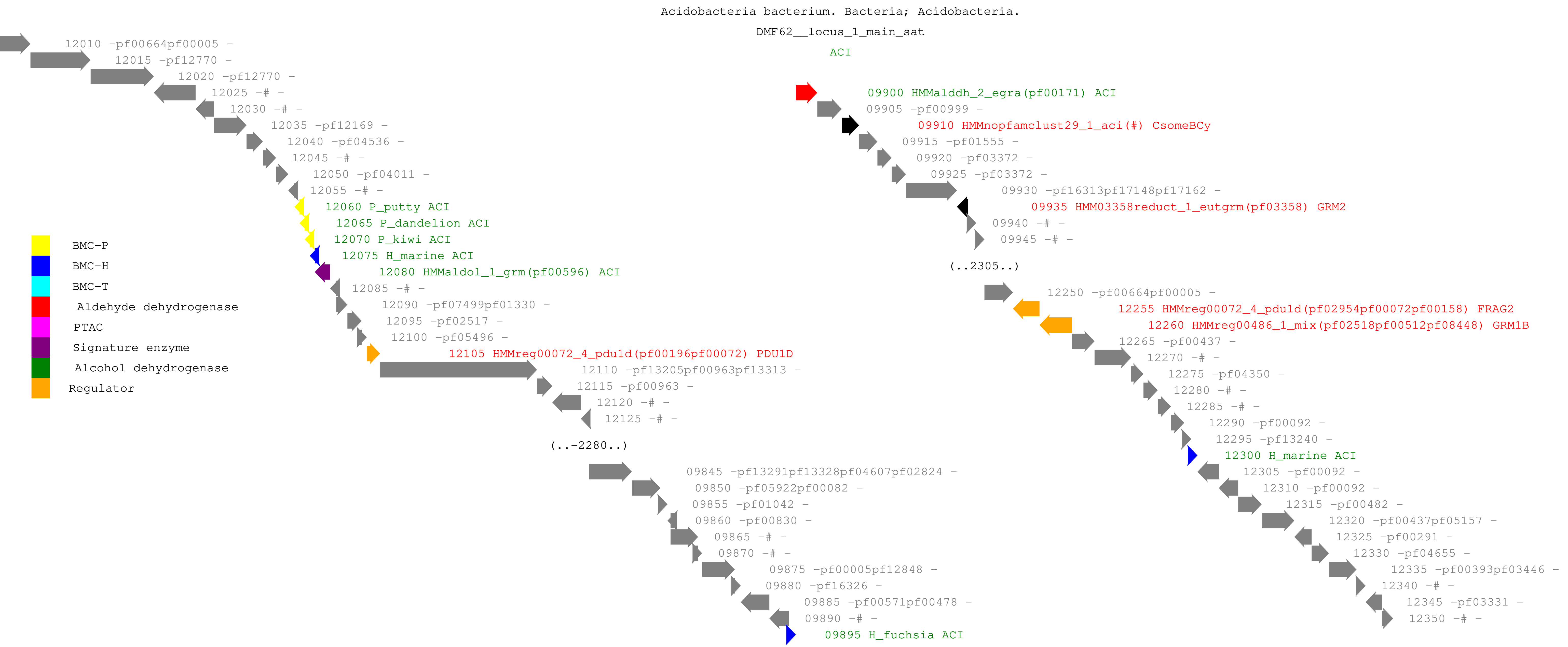
Acidobacteria bacterium. Bacteria; Acidobacteria.
DMF62__locus_1_main_sat
ACI
12010 -pf00664pf00005 -
12015 -pf12770 -
12020 -pf12770 -
12025 -# -
12030 -# -
12035 -pf12169 -
12040 -pf04536 -
12045 -# -
12050 -pf04011 -
12055 -# -
12060 P_putty ACI
12065 P_dandelion ACI
12070 P_kiwi ACI
12075 H_marine ACI
12080 HMMaldol_1_grm(pf00596) ACI
12085 -# -
12090 -pf07499pf01330 -
12095 -pf02517 -
12100 -pf05496 -
12105 HMMreg00072_4_pdu1d(pf00196pf00072) PDU1D
12110 -pf13205pf00963pf13313 -
12115 -pf00963 -
12120 -# -
12125 -# -
(..-2280..)
09845 -pf13291pf13328pf04607pf02824 -
09850 -pf05922pf00082 -
09855 -pf01042 -
09860 -pf00830 -
09865 -# -
09870 -# -
09875 -pf00005pf12848 -
09880 -pf16326 -
09885 -pf00571pf00478 -
09890 -# -
09895 H_fuchsia ACI
09900 HMMaldh_2_egra(pf00171) ACI
09905 -pf00999 -
09910 HMMnopfamclust29_1_aci(#) CsomeBCy
09915 -pf01555 -
09920 -pf03372 -
09925 -pf03372 -
09930 -pf16313pf17148pf17162 -
09935 HMM03358reduct_1_eutgrm(pf03358) GRM2
09940 -# -
09945 -# -
(..2305..)
12250 -pf00664pf00005 -
12255 HMMreg00072_4_pdu1d(pf02954pf00072pf00158) FRAG2
12260 HMMreg00486_1_mix(pf02518pf00512pf08448) GRM1B
12265 -pf00437 -
12270 -# -
12275 -pf04350 -
12280 -# -
12285 -# -
12290 -pf00092 -
12295 -pf13240 -
12300 H_marine ACI
12305 -pf00092 -
12310 -pf00092 -
12315 -pf00482 -
12320 -pf00437pf05157 -
12325 -pf00291 -
12330 -pf04655 -
12335 -pf00393pf03446 -
12340 -# -
12345 -pf03331 -
12350 -# -
BMC-P
BMC-H
BMC-T
Aldehyde dehydrogenase
PTAC
Signature enzyme
Alcohol dehydrogenase
Regulator

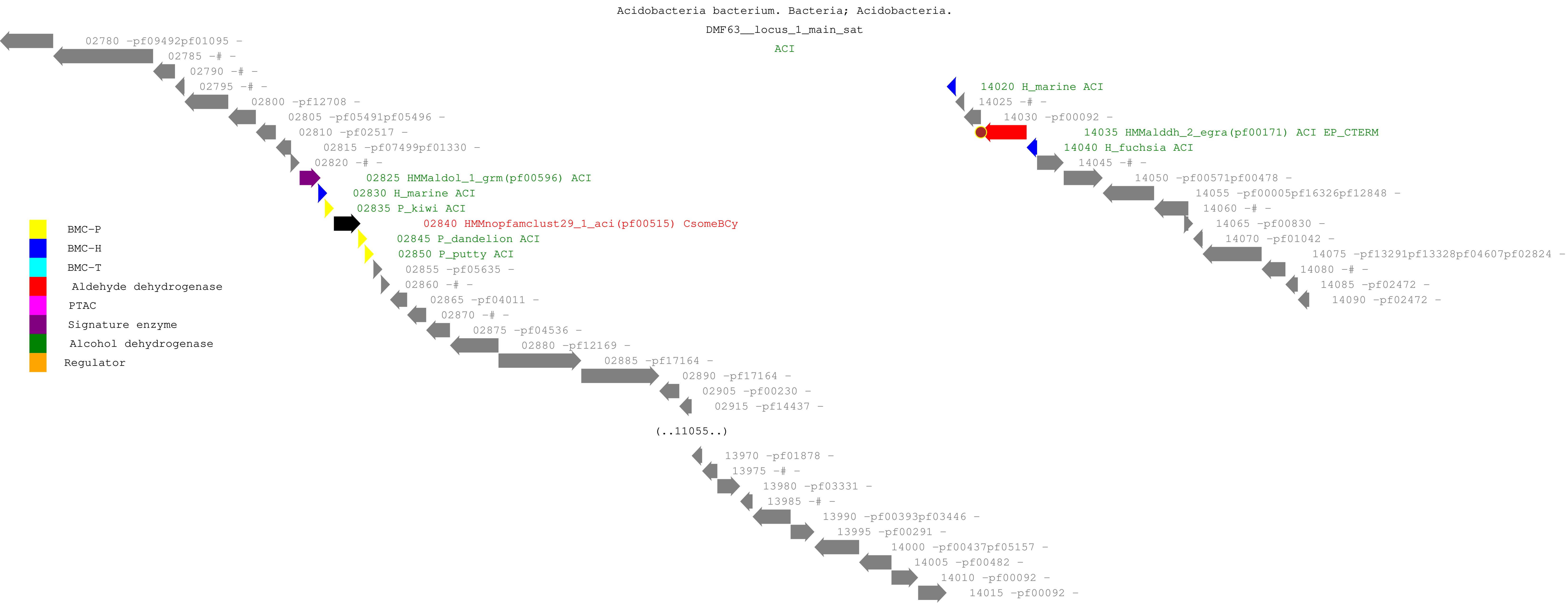
Acidobacteria bacterium. Bacteria; Acidobacteria.
DMF63__locus_1_main_sat
ACI
02780 -pf09492pf01095 -
02785 -# -
02790 -# -
02795 -# -
02800 -pf12708 -
02805 -pf05491pf05496 -
02810 -pf02517 -
02815 -pf07499pf01330 -
02820 -# -
02825 HMMaldol_1_grm(pf00596) ACI
02830 H_marine ACI
02835 P_kiwi ACI
02840 HMMnopfamclust29_1_aci(pf00515) CsomeBCy
02845 P_dandelion ACI
02850 P_putty ACI
02855 -pf05635 -
02860 -# -
02865 -pf04011 -
02870 -# -
02875 -pf04536 -
02880 -pf12169 -
02885 -pf17164 -
02890 -pf17164 -
02905 -pf00230 -
02915 -pf14437 -
(..11055..)
13970 -pf01878 -
13975 -# -
13980 -pf03331 -
13985 -# -
13990 -pf00393pf03446 -
13995 -pf00291 -
14000 -pf00437pf05157 -
14005 -pf00482 -
14010 -pf00092 -
14015 -pf00092 -
14020 H_marine ACI
14025 -# -
14030 -pf00092 -
14035 HMMalddh_2_egra(pf00171) ACI EP_CTERM
14040 H_fuchsia ACI
14045 -# -
14050 -pf00571pf00478 -
14055 -pf00005pf16326pf12848 -
14060 -# -
14065 -pf00830 -
14070 -pf01042 -
14075 -pf13291pf13328pf04607pf02824 -
14080 -# -
14085 -pf02472 -
14090 -pf02472 -
BMC-P
BMC-H
BMC-T
Aldehyde dehydrogenase
PTAC
Signature enzyme
Alcohol dehydrogenase
Regulator

Acidobacteria bacterium. Bacteria; Acidobacteria.

DMF64__locus_1_main_sat

ACI

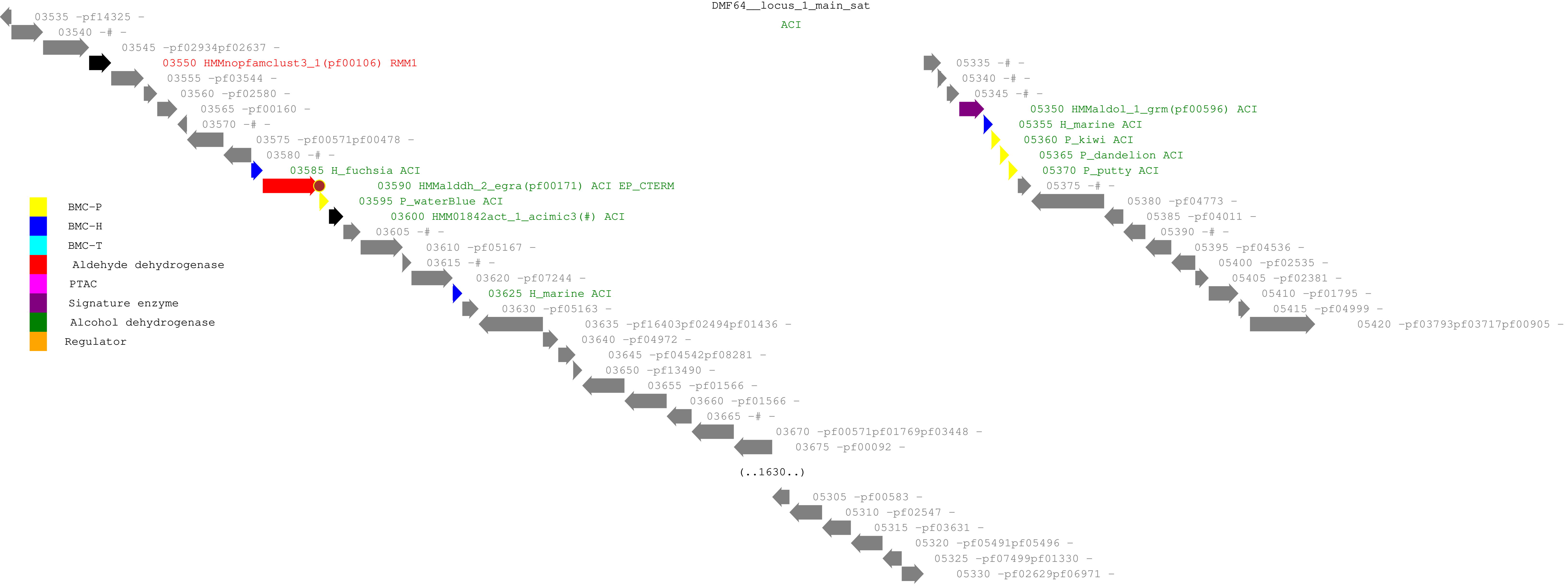

Acidobacteria bacterium. Bacteria; Acidobacteria.

DMF67__locus_1_main_sat

ACI

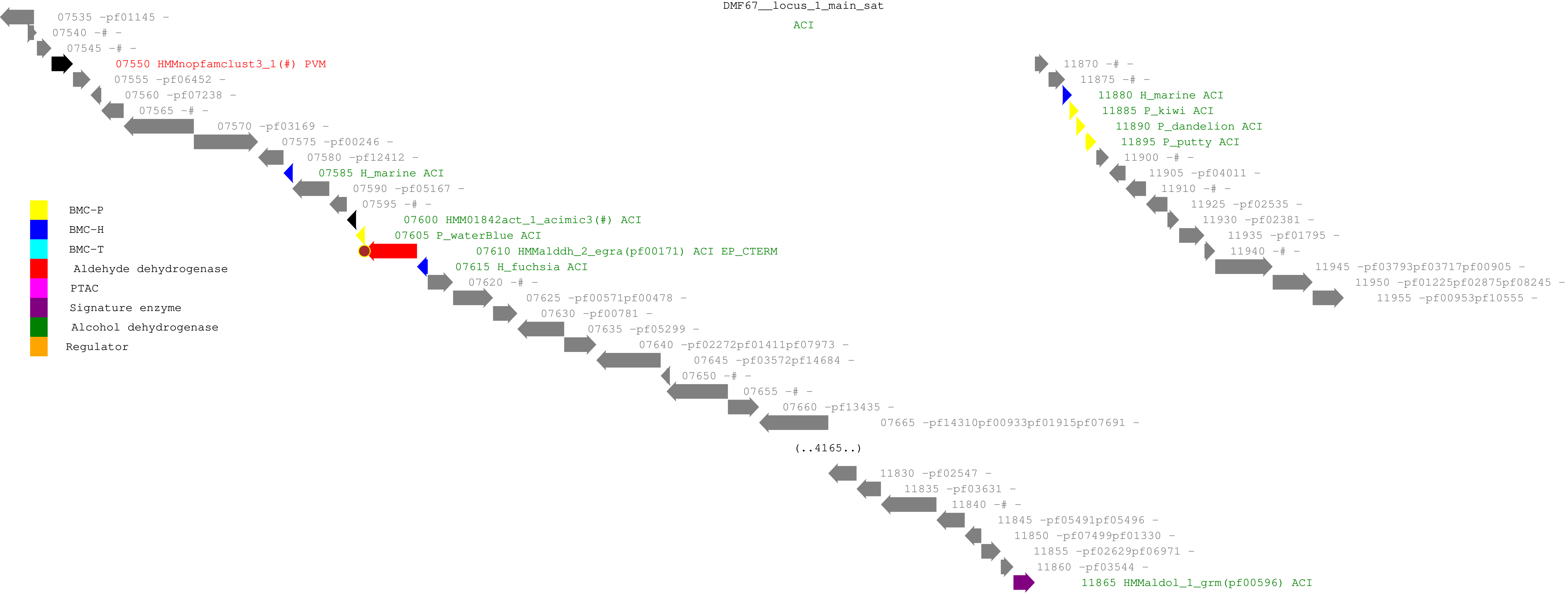

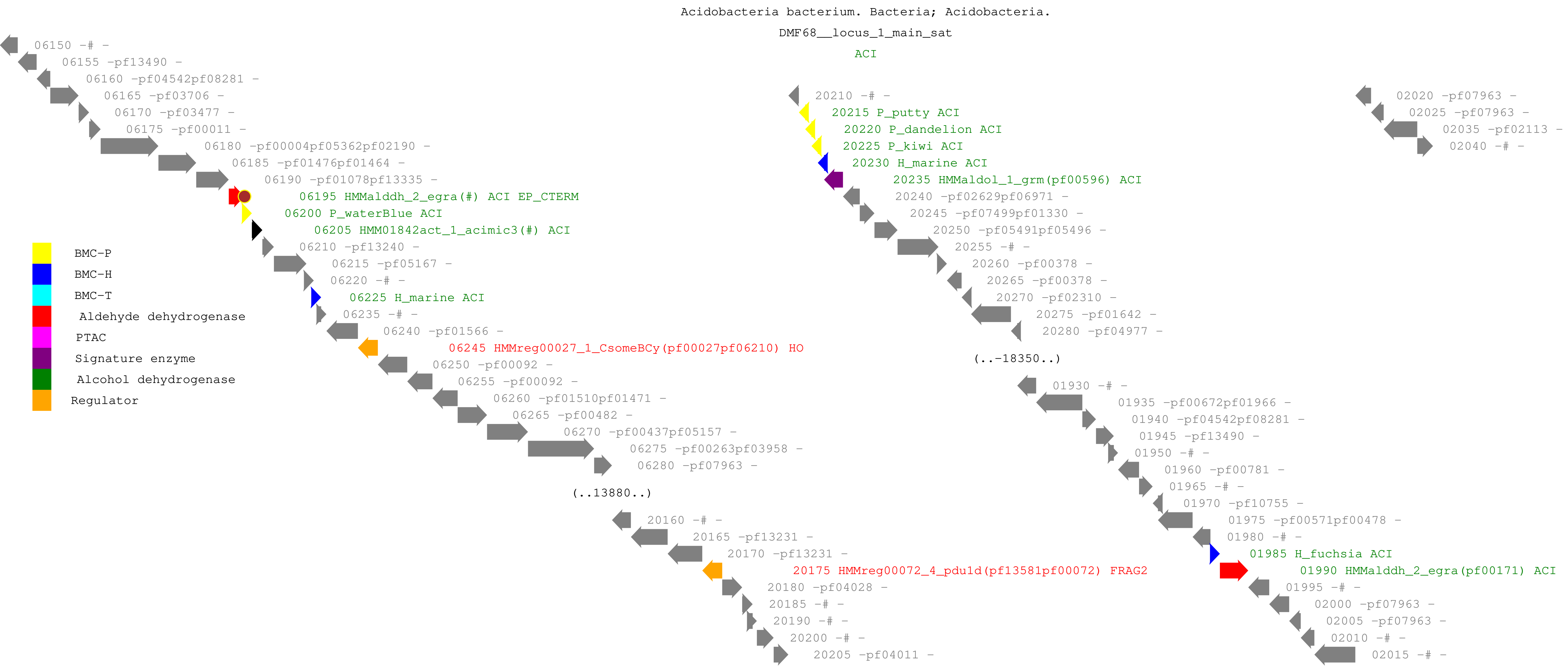

Acidobacteria bacterium. Bacteria; Acidobacteria.
DMF68__locus_1_main_sat
ACI
06150 -# -
06155 -pf13490 -
06160 -pf04542pf08281 -
06165 -pf03706 -
06170 -pf03477 -
06175 -pf00011 -
06180 -pf00004pf05362pf02190 -
06185 -pf01476pf01464 -
06190 -pf01078pf13335 -
06195 HMMaldh_2_egra(#) ACI EP_CTERM
06200 P_waterBlue ACI
06205 HMM01842act_1_acimic3(#) ACI
06210 -pf13240 -
06215 -pf05167 -
06220 -# -
06225 H_marine ACI
06235 -# -
06240 -pf01566 -
06245 HMMreg00027_1_CsomeBCy(pf00027pf06210) HO
06250 -pf00092 -
06255 -pf00092 -
06260 -pf01510pf01471 -
06265 -pf00482 -
06270 -pf00437pf05157 -
06275 -pf00263pf03958 -
06280 -pf07963 -
(..13880..)
20160 -# -
20165 -pf13231 -
20170 -pf13231 -
20175 HMMreg00072_4_pdu1d(pf13581pf00072) FRAG2
20180 -pf04028 -
20185 -# -
20190 -# -
20200 -# -
20205 -pf04011 -
20210 -# -
20215 P_putty ACI
20220 P_dandelion ACI
20225 P_kiwi ACI
20230 H_marine ACI
20235 HMMaldol_1_grm(pf00596) ACI
20240 -pf02629pf06971 -
20245 -pf07499pf01330 -
20250 -pf05491pf05496 -
20255 -# -
20260 -pf00378 -
20265 -pf00378 -
20270 -pf02310 -
20275 -pf01642 -
20280 -pf04977 -
(..-18350..)
01930 -# -
01935 -pf00672pf01966 -
01940 -pf04542pf08281 -
01945 -pf13490 -
01950 -# -
01960 -pf00781 -
01965 -# -
01970 -pf10755 -
01975 -pf00571pf00478 -
01980 -# -
01985 H_fuchsia ACI
01990 HMMaldh_2_egra(pf00171) ACI
01995 -# -
02000 -pf07963 -
02005 -pf07963 -
02010 -# -
02015 -# -
02020 -pf07963 -
02025 -pf07963 -
02035 -pf02113 -
02040 -# -
BMC-P
BMC-H
BMC-T
Aldehyde dehydrogenase
PTAC
Signature enzyme
Alcohol dehydrogenase
Regulator

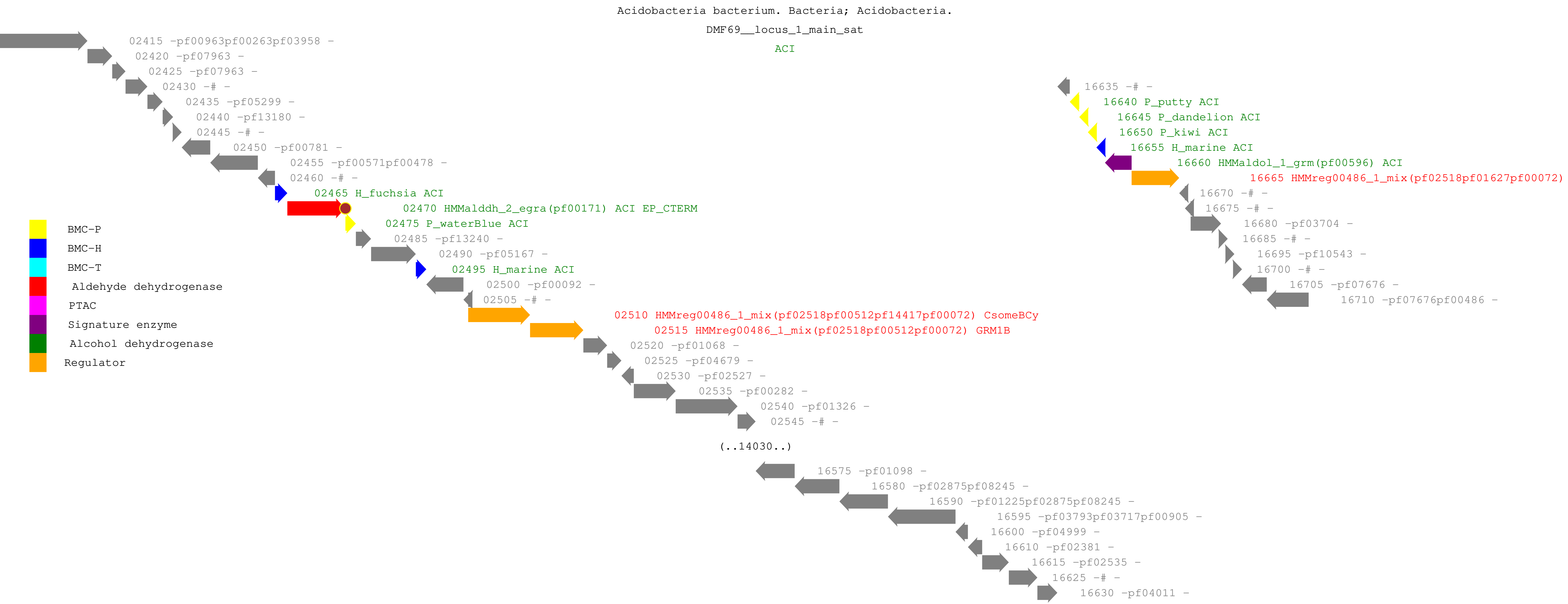

Acidobacteria bacterium. Bacteria; Acidobacteria.
DMF69__locus_1_main_sat
ACI
02415 -pf00963pf00263pf03958 -
02420 -pf07963 -
02425 -pf07963 -
02430 -# -
02435 -pf05299 -
02440 -pf13180 -
02445 -# -
02450 -pf00781 -
02455 -pf00571pf00478 -
02460 -# -
02465 H_fuchsia ACI
02470 HMMaldh_2_egra(pf00171) ACI EP_CTERM
02475 P_waterBlue ACI
02485 -pf13240 -
02490 -pf05167 -
02495 H_marine ACI
02500 -pf00092 -
02505 -# -
02510 HMMreg00486_1_mix(pf02518pf00512pf14417pf00072) CsomeBCy
02515 HMMreg00486_1_mix(pf02518pf00512pf00072) GRM1B
02520 -pf01068 -
02525 -pf04679 -
02530 -pf02527 -
02535 -pf00282 -
02540 -pf01326 -
02545 -# -
(..14030..)
16575 -pf01098 -
16580 -pf02875pf08245 -
16590 -pf01225pf02875pf08245 -
16595 -pf03793pf03717pf00905 -
16600 -pf04999 -
16610 -pf02381 -
16615 -pf02535 -
16625 -# -
16630 -pf04011 -
16635 -# -
16640 P_putty ACI
16645 P_dandelion ACI
16650 P_kiwi ACI
16655 H_marine ACI
16660 HMMaldol_1_grm(pf00596) ACI
16665 HMMreg00486_1_mix(pf02518pf01627pf00072)
16670 -# -
16675 -# -
16680 -pf03704 -
16685 -# -
16695 -pf10543 -
16700 -# -
16705 -pf07676 -
16710 -pf07676pf00486 -
BMC-P
BMC-H
BMC-T
Aldehyde dehydrogenase
PTAC
Signature enzyme
Alcohol dehydrogenase
Regulator

Acidobacteria bacterium. Bacteria; Acidobacteria.

DMF70__locus_1_main_sat

ACI

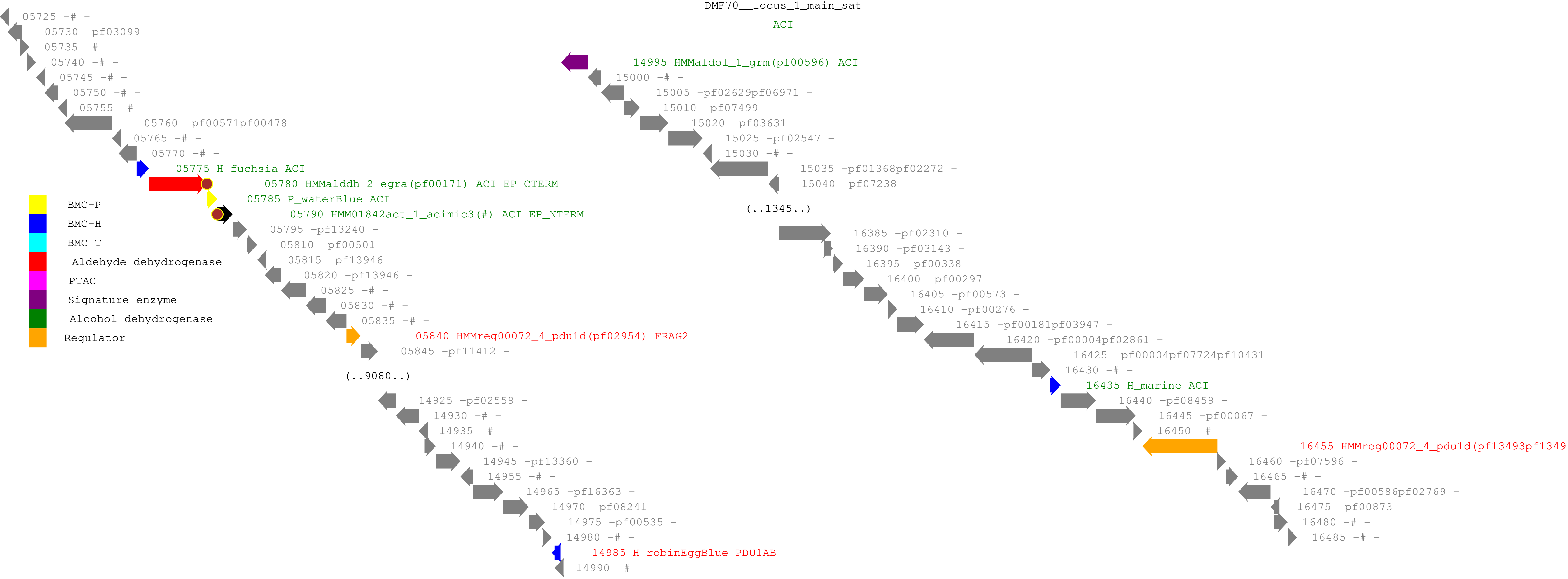

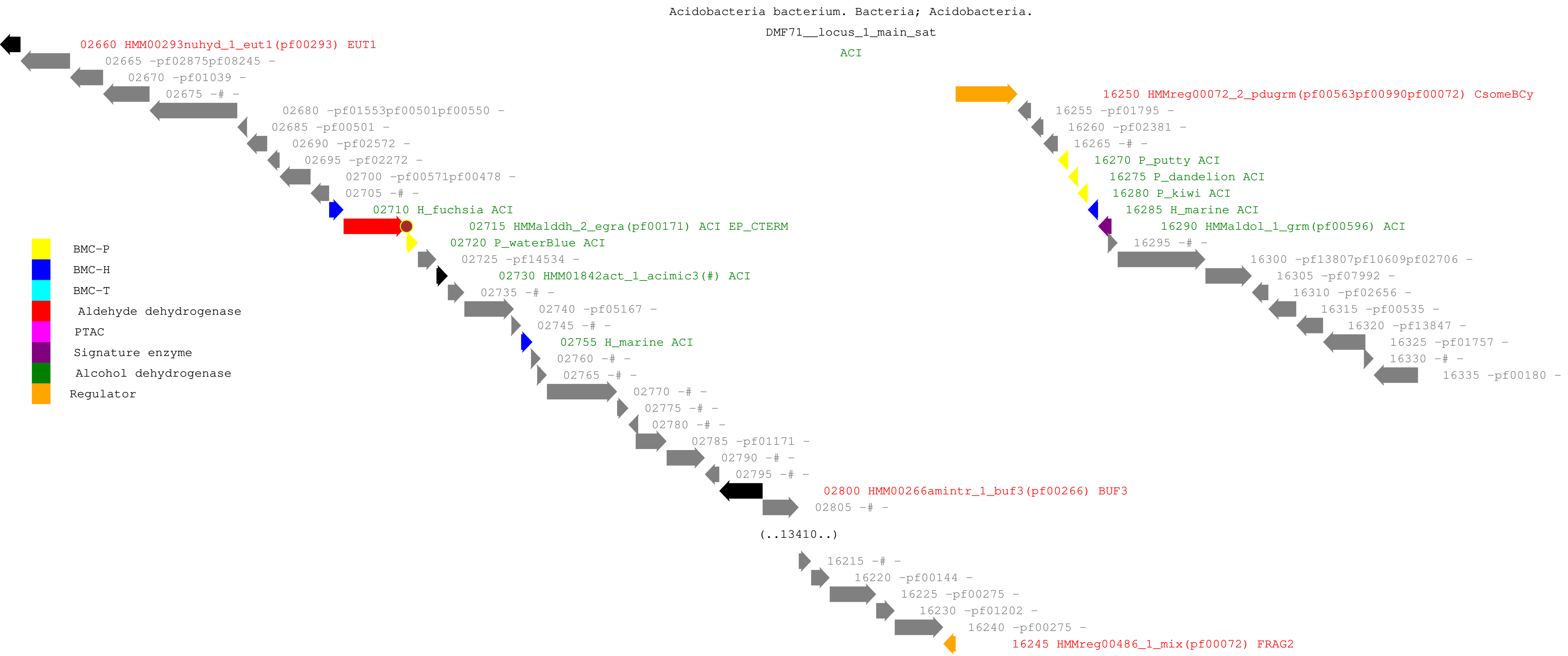
Acidobacteria bacterium. Bacteria; Acidobacteria.
DMF71__locus_1_main_sat
ACI
02660 HMM00293nuhyd_1_eut1(pf00293) EUT1
02665 -pf02875pf08245 -
02670 -pf01039 -
02675 -# -
02680 -pf01553pf00501pf00550 -
02685 -pf00501 -
02690 -pf02572 -
02695 -pf02272 -
02700 -pf00571pf00478 -
02705 -# -
02710 H_fuchsia ACI
02715 HMMalddh_2_egra(pf00171) ACI EP_CTERM
02720 P_waterBlue ACI
02725 -pf14534 -
02730 HMM01842act_1_acimic3(#) ACI
02735 -# -
02740 -pf05167 -
02745 -# -
02755 H_marine ACI
02760 -# -
02765 -# -
02770 -# -
02775 -# -
02780 -# -
02785 -pf01171 -
02790 -# -
02795 -# -
02800 HMM00266amintr_1_buf3(pf00266) BUF3
02805 -# -
(..13410..)
16215 -# -
16220 -pf00144 -
16225 -pf00275 -
16230 -pf01202 -
16240 -pf00275 -
16245 HMMreg00486_1_mix(pf00072) FRAG2
16250 HMMreg00072_2_pdugrm(pf00563pf00990pf00072) CsomeBCy
16255 -pf01795 -
16260 -pf02381 -
16265 -# -
16270 P_putty ACI
16275 P_dandelion ACI
16280 P_kiwi ACI
16285 H_marine ACI
16290 HMMaldol_1_grm(pf00596) ACI
16295 -# -
16300 -pf13807pf10609pf02706 -
16305 -pf07992 -
16310 -pf02656 -
16315 -pf00535 -
16320 -pf13847 -
16325 -pf01757 -
16330 -# -
16335 -pf00180 -
BMC-P
BMC-H
BMC-T
Aldehyde dehydrogenase
PTAC
Signature enzyme
Alcohol dehydrogenase
Regulator

Acidobacteria bacterium. Bacteria; Acidobacteria.

DMF72__locus_1_main_sat

ACI

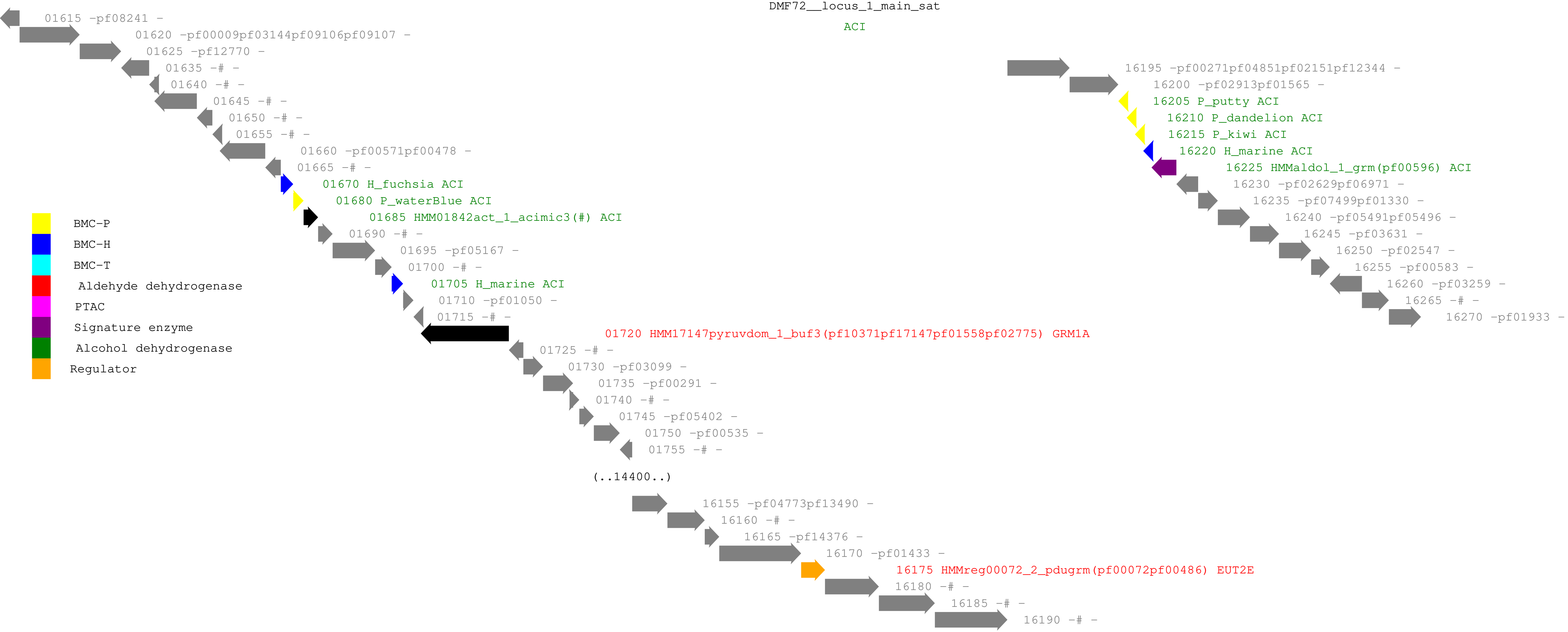

Acidobacteria bacterium. Bacteria; Acidobacteria.

DMF73__locus_1

ACI

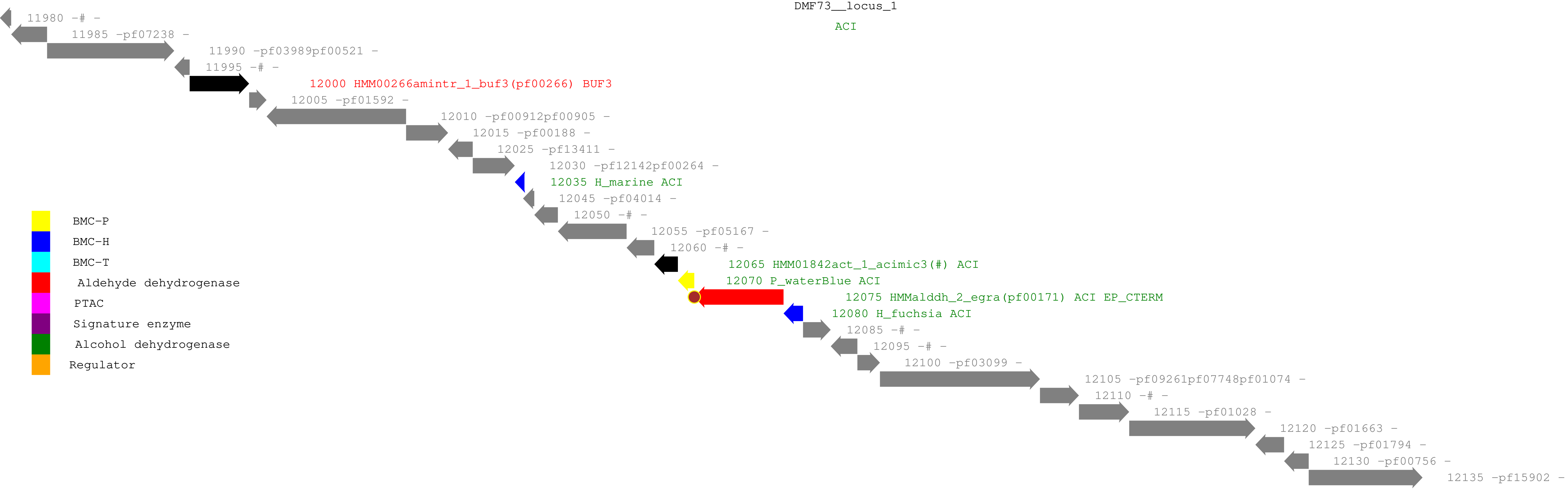

Acidobacteria bacterium. Bacteria; Acidobacteria.

DMF74__locus_1_main_sat

ACI

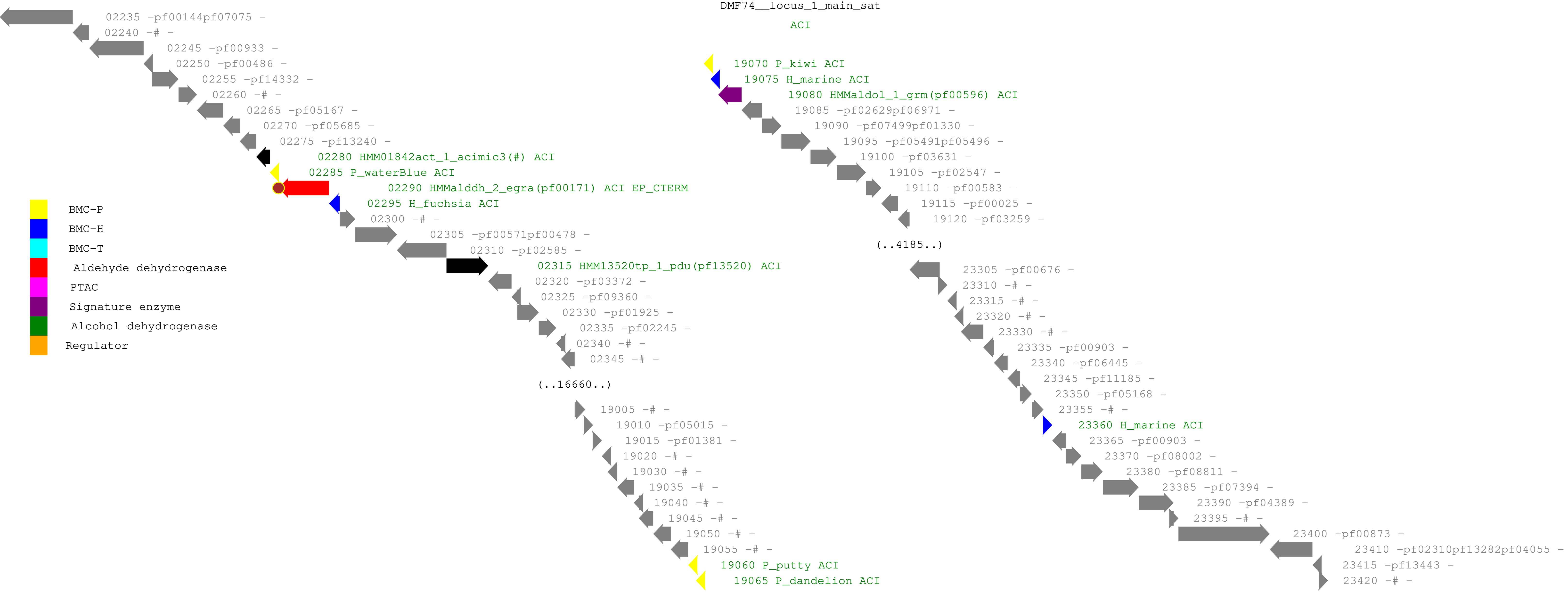

Acidobacteria bacterium. Bacteria; Acidobacteria.

DMF75__locus_1

ACI

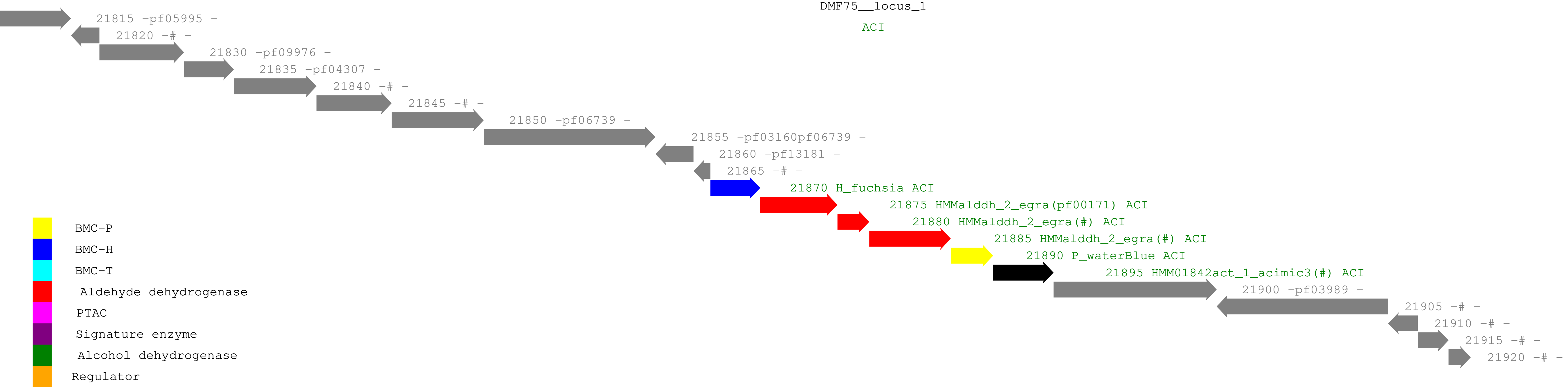

Acidobacteria bacterium. Bacteria; Acidobacteria.

DMF76__locus_1_main_sat

ACI

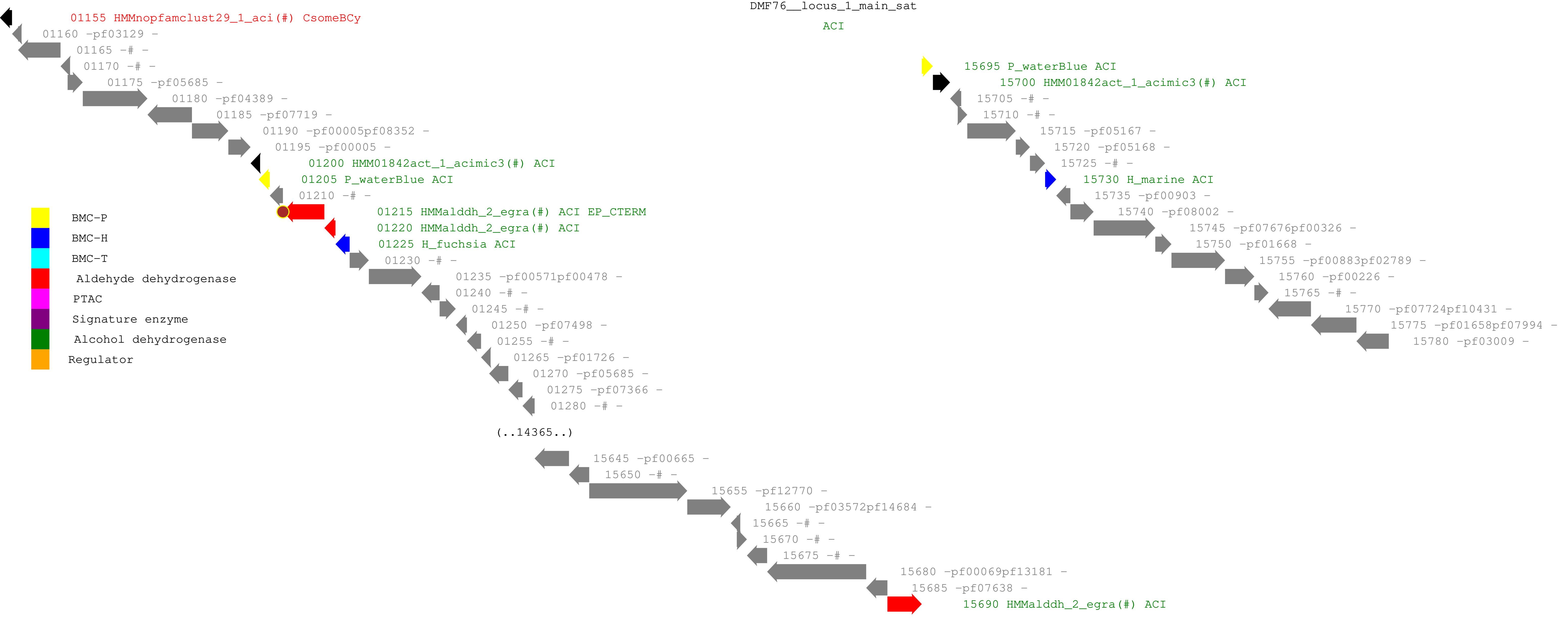

Acidobacteria bacterium. Bacteria; Acidobacteria.

DMF76__locus_3

ACI

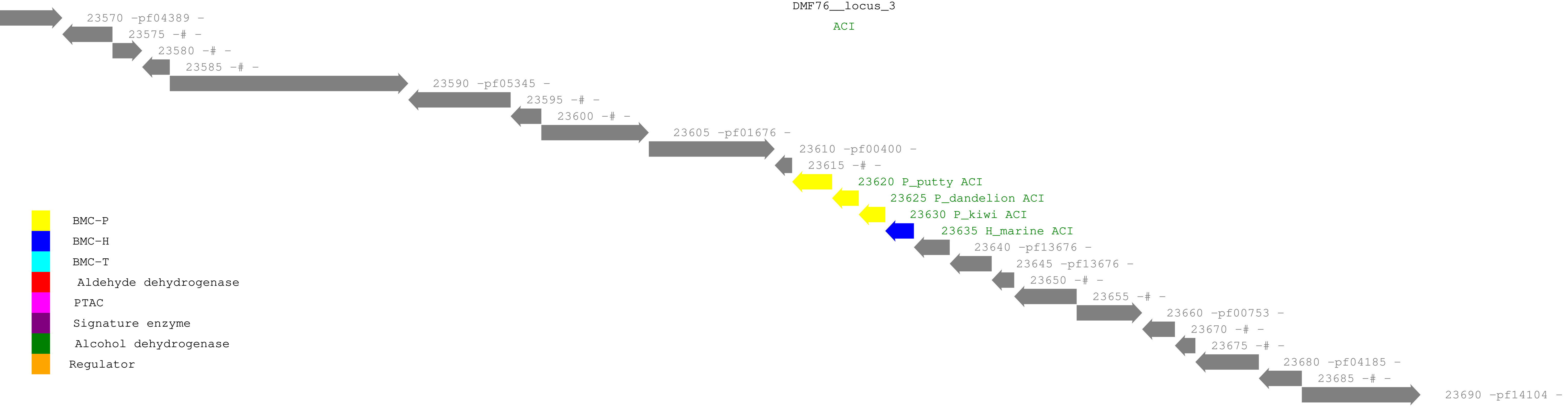

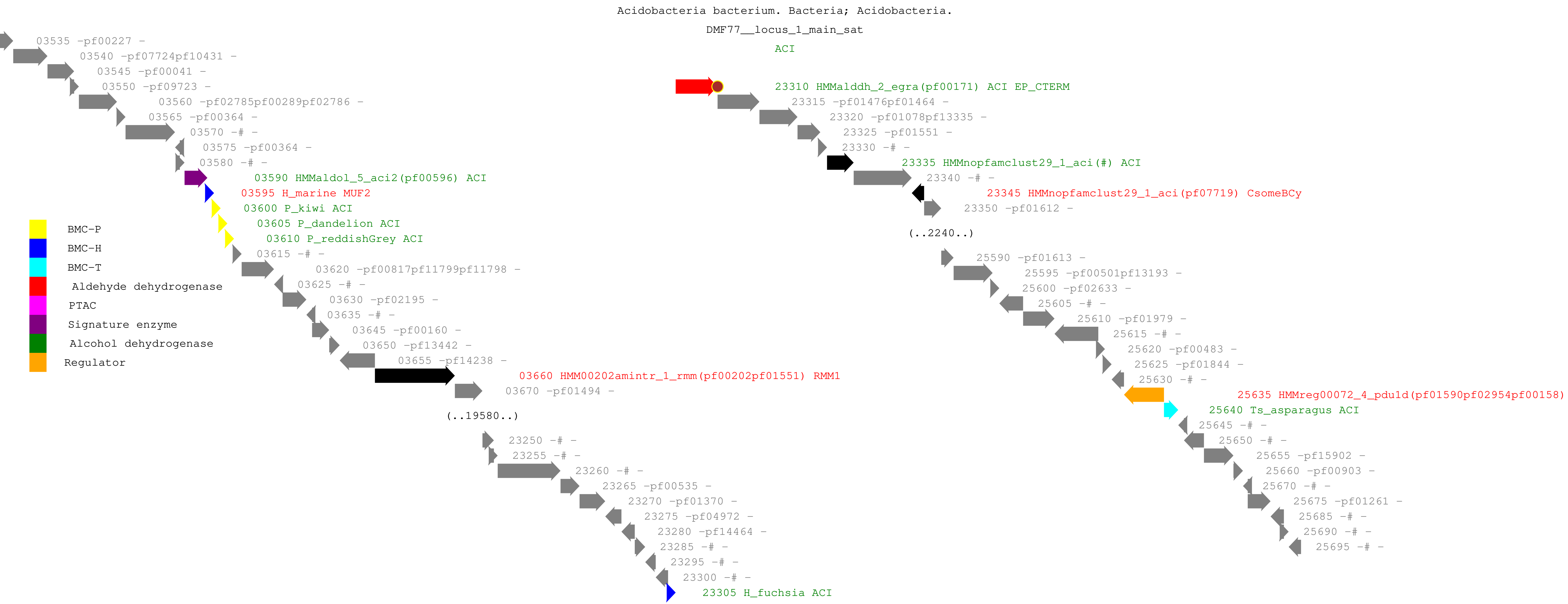
Acidobacteria bacterium. Bacteria; Acidobacteria.
DMF77__locus_1_main_sat
ACI
03535 -pf00227 -
03540 -pf07724pf10431 -
03545 -pf00041 -
03550 -pf09723 -
03560 -pf02785pf00289pf02786 -
03565 -pf00364 -
03570 -# -
03575 -pf00364 -
03580 -# -
03590 HMMaldol_5_aci2(pf00596) ACI
03595 H_marine MUF2
03600 P_kiwi ACI
03605 P_dandelion ACI
03610 P_reddishGrey ACI
03615 -# -
03620 -pf00817pf11799pf11798 -
03625 -# -
03630 -pf02195 -
03635 -# -
03645 -pf00160 -
03650 -pf13442 -
03655 -pf14238 -
03660 HMM00202amintr_1_rmm(pf00202pf01551) RMM1
03670 -pf01494 -
(..19580..)
23250 -# -
23255 -# -
23260 -# -
23265 -pf00535 -
23270 -pf01370 -
23275 -pf04972 -
23280 -pf14464 -
23285 -# -
23295 -# -
23300 -# -
23305 H_fuchsia ACI
23310 HMMalddh_2_egra(pf00171) ACI EP_CTERM
23315 -pf01476pf01464 -
23320 -pf01078pf13335 -
23325 -pf01551 -
23330 -# -
23335 HMMnopfamclust29_1_aci(#) ACI
23340 -# -
23345 HMMnopfamclust29_1_aci(pf07719) CsomeBCy
23350 -pf01612 -
(..2240..)
25590 -pf01613 -
25595 -pf00501pf13193 -
25600 -pf02633 -
25605 -# -
25610 -pf01979 -
25615 -# -
25620 -pf00483 -
25625 -pf01844 -
25630 -# -
25635 HMMreg00072_4_pdu1d(pf01590pf02954pf00158)
25640 Ts_asparagus ACI
25645 -# -
25650 -# -
25655 -pf15902 -
25660 -pf00903 -
25670 -# -
25675 -pf01261 -
25685 -# -
25690 -# -
25695 -# -
BMC-P
BMC-H
BMC-T
Aldehyde dehydrogenase
PTAC
Signature enzyme
Alcohol dehydrogenase
Regulator

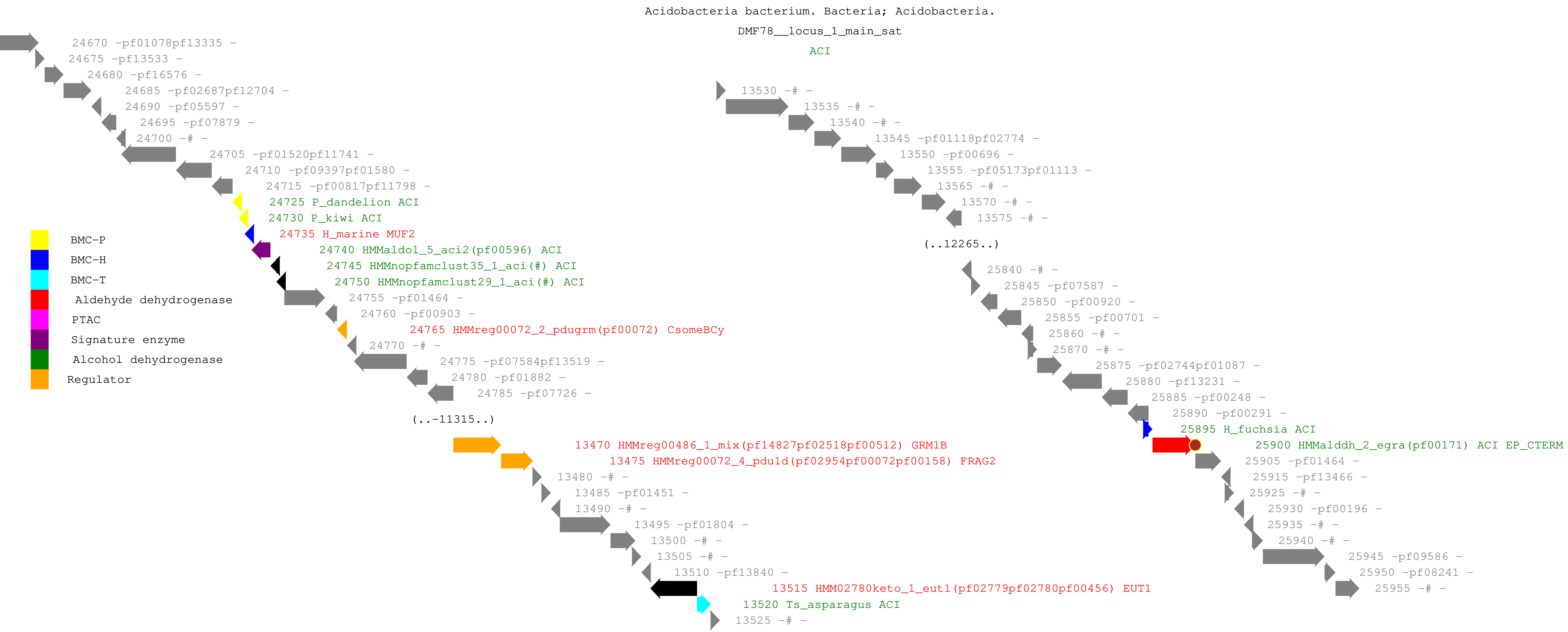

Acidobacteria bacterium. Bacteria; Acidobacteria.
DMF78__locus_1_main_sat
ACI
24670 -pf01078pf13335 -
24675 -pf13533 -
24680 -pf16576 -
24685 -pf02687pf12704 -
24690 -pf05597 -
24695 -pf07879 -
24700 -# -
24705 -pf01520pf11741 -
24710 -pf09397pf01580 -
24715 -pf00817pf11798 -
24725 P_dandelion ACI
24730 P_kiwi ACI
24735 H_marine MUF2
24740 HMMaldol_5_aci2(pf00596) ACI
24745 HMMnopfamclust35_1_aci(#) ACI
24750 HMMnopfamclust29_1_aci(#) ACI
24755 -pf01464 -
24760 -pf00903 -
24765 HMMreg00072_2_pdugrm(pf00072) CsomeBCy
24770 -# -
24775 -pf07584pf13519 -
24780 -pf01882 -
24785 -pf07726 -
(..-11315..)
13470 HMMreg00486_1_mix(pf14827pf02518pf00512) GRM1B
13475 HMMreg00072_4_pdu1d(pf02954pf00072pf00158) FRAG2
13480 -# -
13485 -pf01451 -
13490 -# -
13495 -pf01804 -
13500 -# -
13505 -# -
13510 -pf13840 -
13515 HMM02780keto_1_eut1(pf02779pf02780pf00456) EUT1
13520 Ts_asparagus ACI
13525 -# -
13530 -# -
13535 -# -
13540 -# -
13545 -pf01118pf02774 -
13550 -pf00696 -
13555 -pf05173pf01113 -
13565 -# -
13570 -# -
13575 -# -
(..12265..)
25840 -# -
25845 -pf07587 -
25850 -pf00920 -
25855 -pf00701 -
25860 -# -
25870 -# -
25875 -pf02744pf01087 -
25880 -pf13231 -
25885 -pf00248 -
25890 -pf00291 -
25895 H_fuchsia ACI
25900 HMMalddh_2_egra(pf00171) ACI EP_CTERM
25905 -pf01464 -
25915 -pf13466 -
25925 -# -
25930 -pf00196 -
25935 -# -
25940 -# -
25945 -pf09586 -
25950 -pf08241 -
25955 -# -
BMC-P
BMC-H
BMC-T
Aldehyde dehydrogenase
PTAC
Signature enzyme
Alcohol dehydrogenase
Regulator

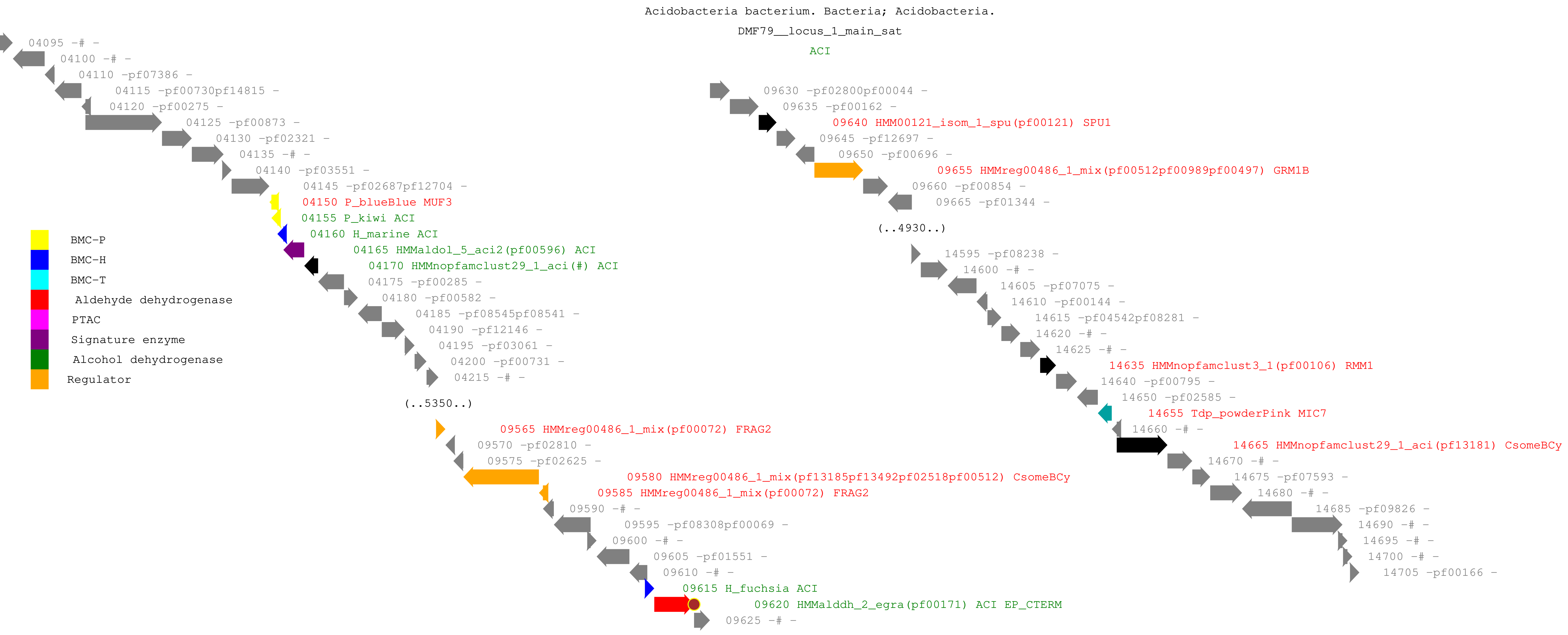
Acidobacteria bacterium. Bacteria; Acidobacteria.
DMF79__locus_1_main_sat
ACI
04095 -# -
04100 -# -
04110 -pf07386 -
04115 -pf00730pf14815 -
04120 -pf00275 -
04125 -pf00873 -
04130 -pf02321 -
04135 -# -
04140 -pf03551 -
04145 -pf02687pf12704 -
04150 P_blueBlue MUF3
04155 P_kiwi ACI
04160 H_marine ACI
04165 HMMaldol_5_aci2(pf00596) ACI
04170 HMMnopfamclust29_1_aci(#) ACI
04175 -pf00285 -
04180 -pf00582 -
04185 -pf08545pf08541 -
04190 -pf12146 -
04195 -pf03061 -
04200 -pf00731 -
04215 -# -
(..5350..)
09565 HMMreg00486_1_mix(pf00072) FRAG2
09570 -pf02810 -
09575 -pf02625 -
09580 HMMreg00486_1_mix(pf13185pf13492pf02518pf00512) CsomeBCy
09585 HMMreg00486_1_mix(pf00072) FRAG2
09590 -# -
09595 -pf08308pf00069 -
09600 -# -
09605 -pf01551 -
09610 -# -
09615 H_fuchsia ACI
09620 HMMaldh_2_egra(pf00171) ACI EP_CTERM
09625 -# -
09630 -pf02800pf00044 -
09635 -pf00162 -
09640 HMM00121_isom_1_spu(pf00121) SPU1
09645 -pf12697 -
09650 -pf00696 -
09655 HMMreg00486_1_mix(pf00512pf00989pf00497) GRM1B
09660 -pf00854 -
09665 -pf01344 -
(..4930..)
14595 -pf08238 -
14600 -# -
14605 -pf07075 -
14610 -pf00144 -
14615 -pf04542pf08281 -
14620 -# -
14625 -# -
14635 HMMnopfamclust3_1(pf00106) RMM1
14640 -pf00795 -
14650 -pf02585 -
14655 Tdp_powderPink MIC7
14660 -# -
14665 HMMnopfamclust29_1_aci(pf13181) CsomeBCy
14670 -# -
14675 -pf07593 -
14680 -# -
14685 -pf09826 -
14690 -# -
14695 -# -
14700 -# -
14705 -pf00166 -
BMC-P
BMC-H
BMC-T
Aldehyde dehydrogenase
PTAC
Signature enzyme
Alcohol dehydrogenase
Regulator

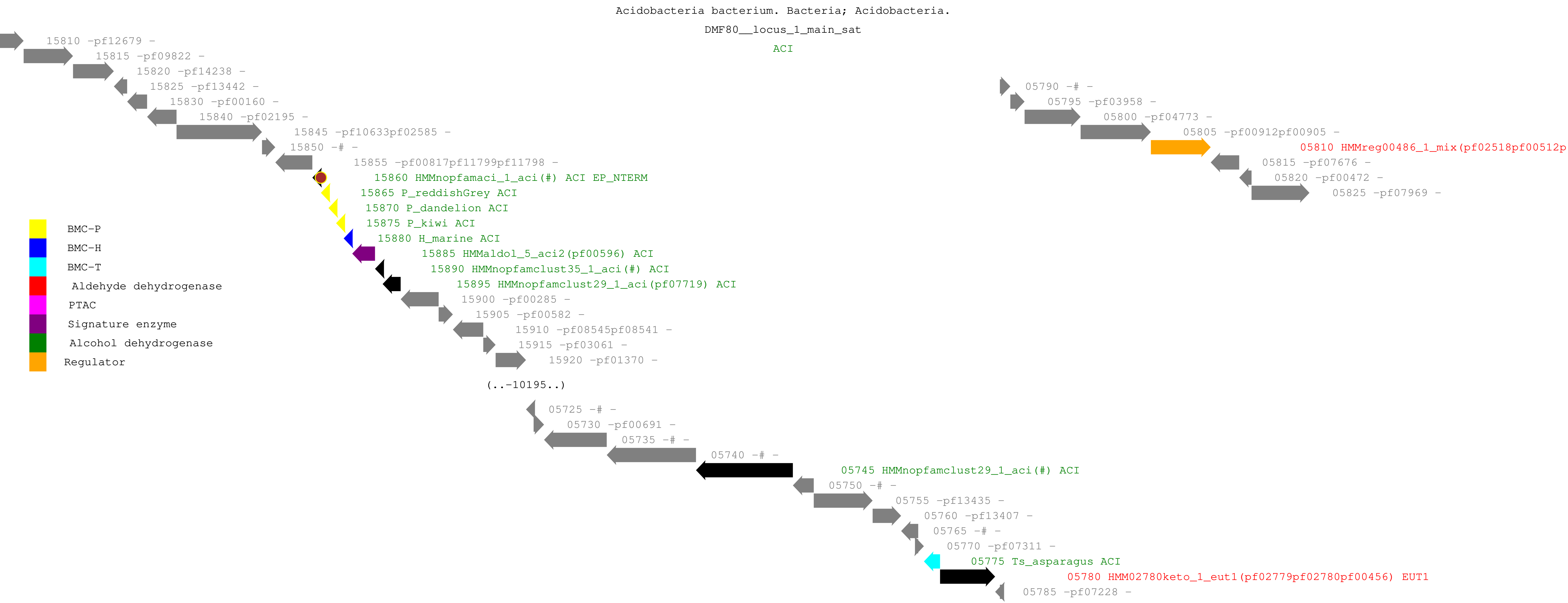
Acidobacteria bacterium. Bacteria; Acidobacteria.
DMF80__locus_1_main_sat
ACI
15810 -pf12679 -
15815 -pf09822 -
15820 -pf14238 -
15825 -pf13442 -
15830 -pf00160 -
15840 -pf02195 -
15845 -pf10633pf02585 -
15850 -# -
15855 -pf00817pf11799pf11798 -
15860 HMMnopfamaci_1_aci(#) ACI EP_NTERM
15865 P_reddishGrey ACI
15870 P_dandelion ACI
15875 P_kiwi ACI
15880 H_marine ACI
15885 HMMaldol_5_aci2(pf00596) ACI
15890 HMMnopfamclust35_1_aci(#) ACI
15895 HMMnopfamclust29_1_aci(pf07719) ACI
15900 -pf00285 -
15905 -pf00582 -
15910 -pf08545pf08541 -
15915 -pf03061 -
15920 -pf01370 -
(..-10195..)
05725 -# -
05730 -pf00691 -
05735 -# -
05740 -# -
05745 HMMnopfamclust29_1_aci(#) ACI
05750 -# -
05755 -pf13435 -
05760 -pf13407 -
05765 -# -
05770 -pf07311 -
05775 Ts_asparagus ACI
05780 HMM02780keto_1_eut1(pf02779pf02780pf00456) EUT1
05785 -pf07228 -
05790 -# -
05795 -pf03958 -
05800 -pf04773 -
05805 -pf00912pf00905 -
05810 HMMreg00486_1_mix(pf02518pf00512p
05815 -pf07676 -
05820 -pf00472 -
05825 -pf07969 -
BMC-P
BMC-H
BMC-T
Aldehyde dehydrogenase
PTAC
Signature enzyme
Alcohol dehydrogenase
Regulator

Acidobacteria bacterium. Bacteria; Acidobacteria.

DMF81__locus_fragmented

ACI

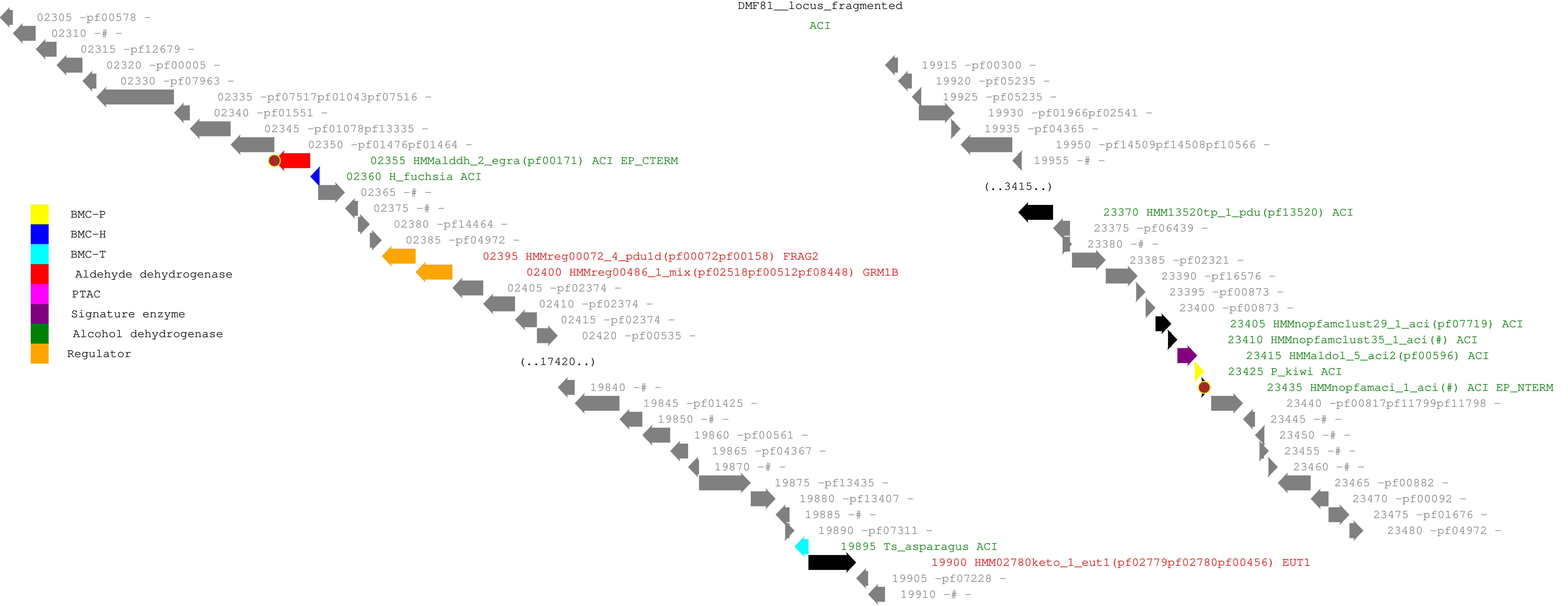

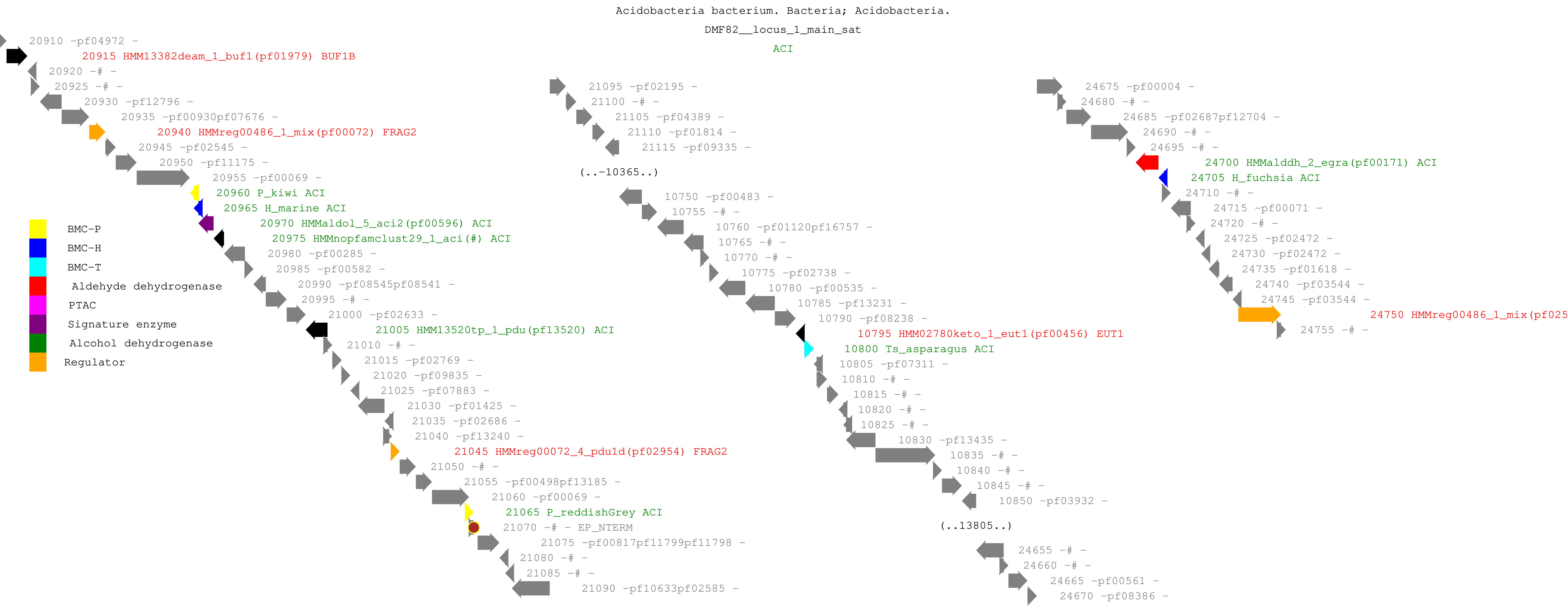
Acidobacteria bacterium. Bacteria; Acidobacteria.
DMF82__locus_1_main_sat
ACI
20910 -pf04972 -
20915 HMM13382deam_1_buf1(pf01979) BUF1B
20920 -# -
20925 -# -
20930 -pf12796 -
20935 -pf00930pf07676 -
20940 HMMreg00486_1_mix(pf00072) FRAG2
20945 -pf02545 -
20950 -pf11175 -
20955 -pf00069 -
20960 P_kiwi ACI
20965 H_marine ACI
20970 HMMaldol_5_aci2(pf00596) ACI
20975 HMMnopfamclust29_1_aci(#) ACI
20980 -pf00285 -
20985 -pf00582 -
20990 -pf08545pf08541 -
20995 -# -
21000 -pf02633 -
21005 HMM13520tp_1_pdu(pf13520) ACI
21010 -# -
21015 -pf02769 -
21020 -pf09835 -
21025 -pf07883 -
21030 -pf01425 -
21035 -pf02686 -
21040 -pf13240 -
21045 HMMreg00072_4_pdu1d(pf02954) FRAG2
21050 -# -
21055 -pf00498pf13185 -
21060 -pf00069 -
21065 P_reddishGrey ACI
21070 -# - EP_NTERM
21075 -pf00817pf11799pf11798 -
21080 -# -
21085 -# -
21090 -pf10633pf02585 -
BMC-P
BMC-H
BMC-T
Aldehyde dehydrogenase
PTAC
Signature enzyme
Alcohol dehydrogenase
Regulator
21095 -pf02195 -
21100 -# -
21105 -pf04389 -
21110 -pf01814 -
21115 -pf09335 -
(..-10365..)
10750 -pf00483 -
10755 -# -
10760 -pf01120pf16757 -
10765 -# -
10770 -# -
10775 -pf02738 -
10780 -pf00535 -
10785 -pf13231 -
10790 -pf08238 -
10795 HMM02780keto_1_eut1(pf00456) EUT1
10800 Ts_asparagus ACI
10805 -pf07311 -
10810 -# -
10815 -# -
10820 -# -
10825 -# -
10830 -pf13435 -
10835 -# -
10840 -# -
10845 -# -
10850 -pf03932 -
(..13805..)
24655 -# -
24660 -# -
24665 -pf00561 -
24670 -pf08386 -
24675 -pf00004 -
24680 -# -
24685 -pf02687pf12704 -
24690 -# -
24695 -# -
24700 HMMalddh_2_egra(pf00171) ACI
24705 H_fuchsia ACI
24710 -# -
24715 -pf00071 -
24720 -# -
24725 -pf02472 -
24730 -pf02472 -
24735 -pf01618 -
24740 -pf03544 -
24745 -pf03544 -
24750 HMMreg00486_1_mix(pf025
24755 -# -

Acidobacteria bacterium. Bacteria; Acidobacteria.

DMF83__locus_1_main_sat

ACI

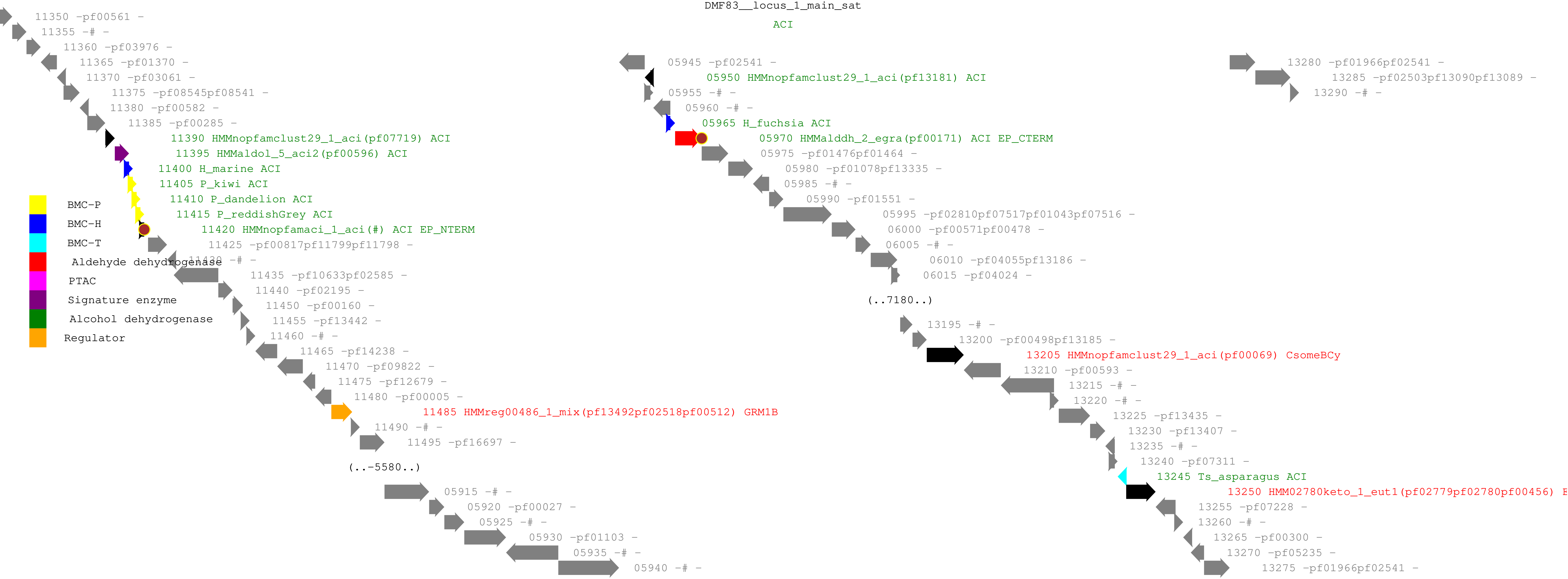

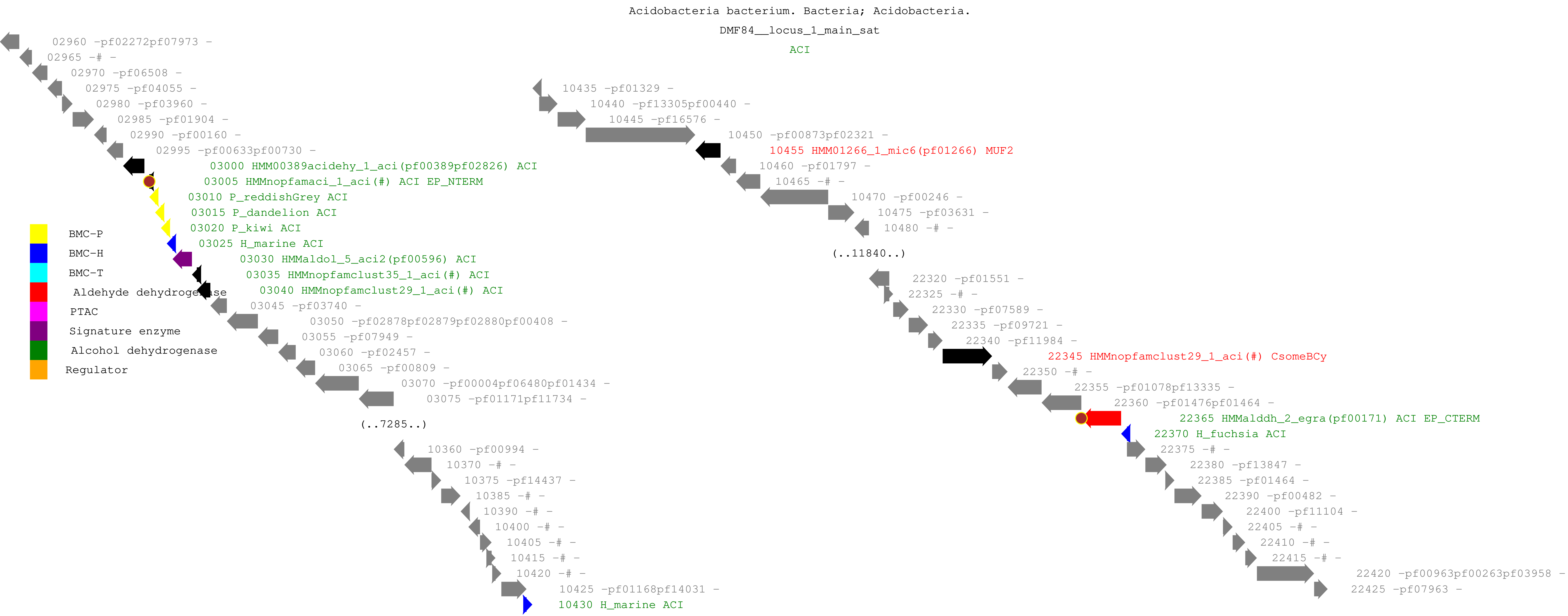
Acidobacteria bacterium. Bacteria; Acidobacteria.
DMF84__locus_1_main_sat
ACI
02960 -pf02272pf07973 -
02965 -# -
02970 -pf06508 -
02975 -pf04055 -
02980 -pf03960 -
02985 -pf01904 -
02990 -pf00160 -
02995 -pf00633pf00730 -
03000 HMM00389acidehy_1_aci(pf00389pf02826) ACI
03005 HMMnopfamaci_1_aci(#) ACI EP_NTERM
03010 P_reddishGrey ACI
03015 P_dandelion ACI
03020 P_kiwi ACI
03025 H_marine ACI
03030 HMMaldol_5_aci2(pf00596) ACI
03035 HMMnopfamclust35_1_aci(#) ACI
03040 HMMnopfamclust29_1_aci(#) ACI
03045 -pf03740 -
03050 -pf02878pf02879pf02880pf00408 -
03055 -pf07949 -
03060 -pf02457 -
03065 -pf00809 -
03070 -pf00004pf06480pf01434 -
03075 -pf01171pf11734 -
(..7285..)
10360 -pf00994 -
10370 -# -
10375 -pf14437 -
10385 -# -
10390 -# -
10400 -# -
10405 -# -
10415 -# -
10420 -# -
10425 -pf01168pf14031 -
10430 H_marine ACI
10435 -pf01329 -
10440 -pf13305pf00440 -
10445 -pf16576 -
10450 -pf00873pf02321 -
10455 HMM01266_1_mic6(pf01266) MUF2
10460 -pf01797 -
10465 -# -
10470 -pf00246 -
10475 -pf03631 -
10480 -# -
(..11840..)
22320 -pf01551 -
22325 -# -
22330 -pf07589 -
22335 -pf09721 -
22340 -pf11984 -
22345 HMMnopfamclust29_1_aci(#) CsomeBCy
22350 -# -
22355 -pf01078pf13335 -
22360 -pf01476pf01464 -
22365 HMMalddh_2_egra(pf00171) ACI EP_CTERM
22370 H_fuchsia ACI
22375 -# -
22380 -pf13847 -
22385 -pf01464 -
22390 -pf00482 -
22400 -pf11104 -
22405 -# -
22410 -# -
22415 -# -
22420 -pf00963pf00263pf03958 -
22425 -pf07963 -
BMC-P
BMC-H
BMC-T
Aldehyde dehydrogenase
PTAC
Signature enzyme
Alcohol dehydrogenase
Regulator

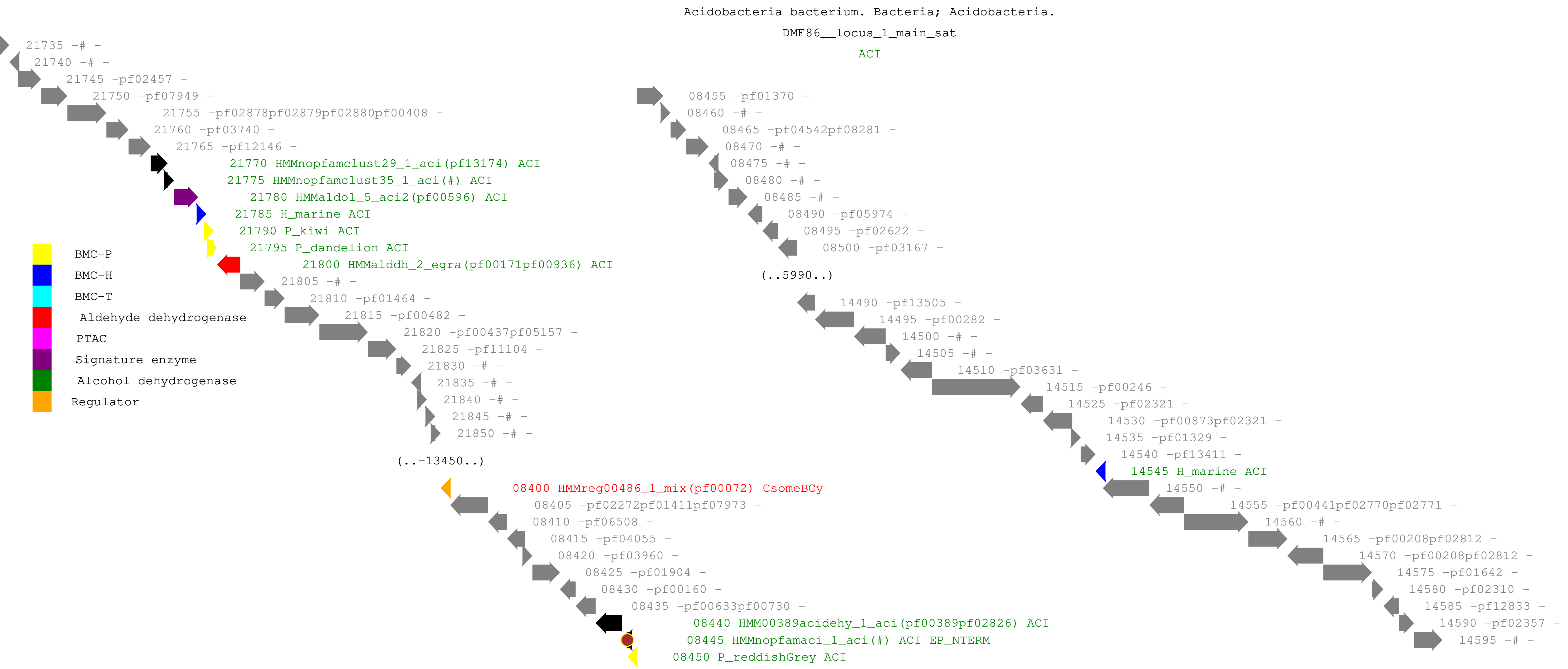
Acidobacteria bacterium. Bacteria; Acidobacteria.
DMF86__locus_1_main_sat
ACI
21735 -# -
21740 -# -
21745 -pf02457 -
21750 -pf07949 -
21755 -pf02878pf02879pf02880pf00408 -
21760 -pf03740 -
21765 -pf12146 -
21770 HMMnopfamclust29_1_aci(pf13174) ACI
21775 HMMnopfamclust35_1_aci(#) ACI
21780 HMMaldol_5_aci2(pf00596) ACI
21785 H_marine ACI
21790 P_kiwi ACI
21795 P_dandelion ACI
21800 HMMalddh_2_egra(pf00171pf00936) ACI
21805 -# -
21810 -pf01464 -
21815 -pf00482 -
21820 -pf00437pf05157 -
21825 -pf11104 -
21830 -# -
21835 -# -
21840 -# -
21845 -# -
21850 -# -
(..-13450..)
08400 HMMreg00486_1_mix(pf00072) CsomeBCy
08405 -pf02272pf01411pf07973 -
08410 -pf06508 -
08415 -pf04055 -
08420 -pf03960 -
08425 -pf01904 -
08430 -pf00160 -
08435 -pf00633pf00730 -
08440 HMM00389acidehy_1_aci(pf00389pf02826) ACI
08445 HMMnopfamaci_1_aci(#) ACI EP_NTERM
08450 P_reddishGrey ACI
08455 -pf01370 -
08460 -# -
08465 -pf04542pf08281 -
08470 -# -
08475 -# -
08480 -# -
08485 -# -
08490 -pf05974 -
08495 -pf02622 -
08500 -pf03167 -
(..5990..)
14490 -pf13505 -
14495 -pf00282 -
14500 -# -
14505 -# -
14510 -pf03631 -
14515 -pf00246 -
14525 -pf02321 -
14530 -pf00873pf02321 -
14535 -pf01329 -
14540 -pf13411 -
14545 H_marine ACI
14550 -# -
14555 -pf00441pf02770pf02771 -
14560 -# -
14565 -pf00208pf02812 -
14570 -pf00208pf02812 -
14575 -pf01642 -
14580 -pf02310 -
14585 -pf12833 -
14590 -pf02357 -
14595 -# -
BMC-P
BMC-H
BMC-T
Aldehyde dehydrogenase
PTAC
Signature enzyme
Alcohol dehydrogenase
Regulator

Acidobacteria bacterium. Bacteria; Acidobacteria.

DMF87__locus_1_main_sat

ACI

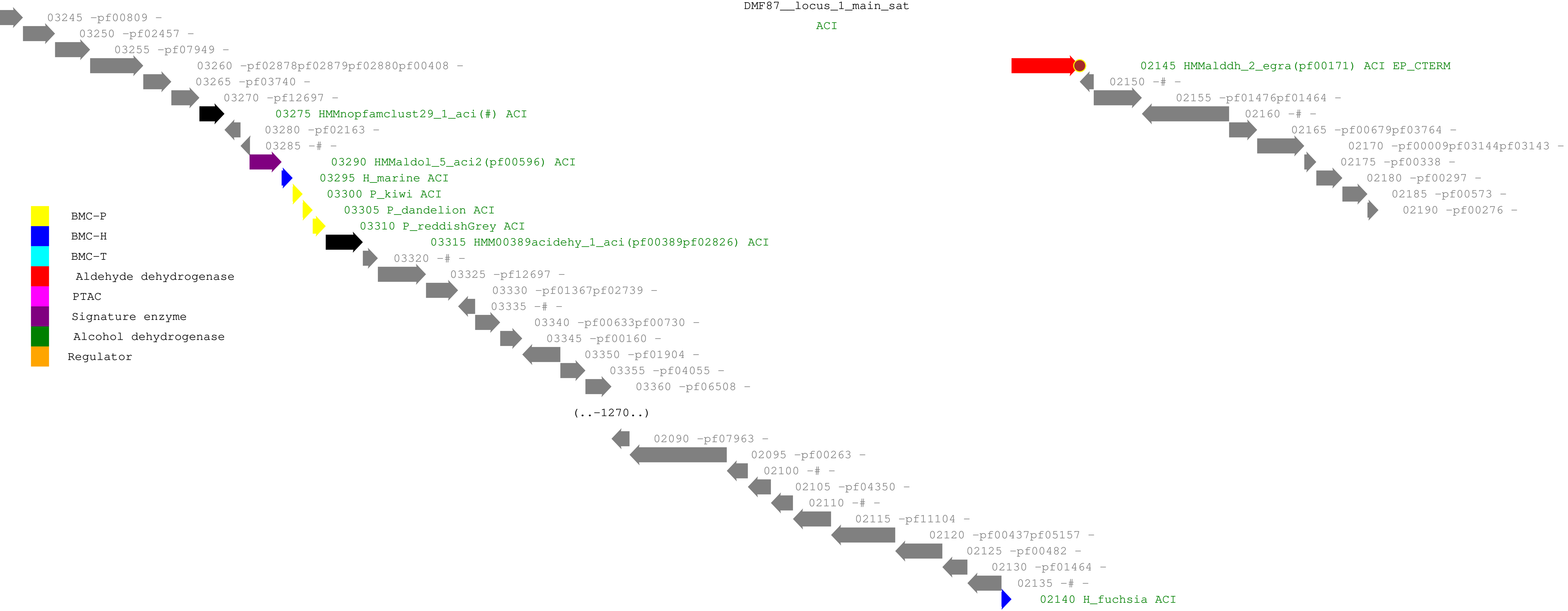

Acidobacteria bacterium. Bacteria; Acidobacteria.

DMF88__locus_fragmented

ACI

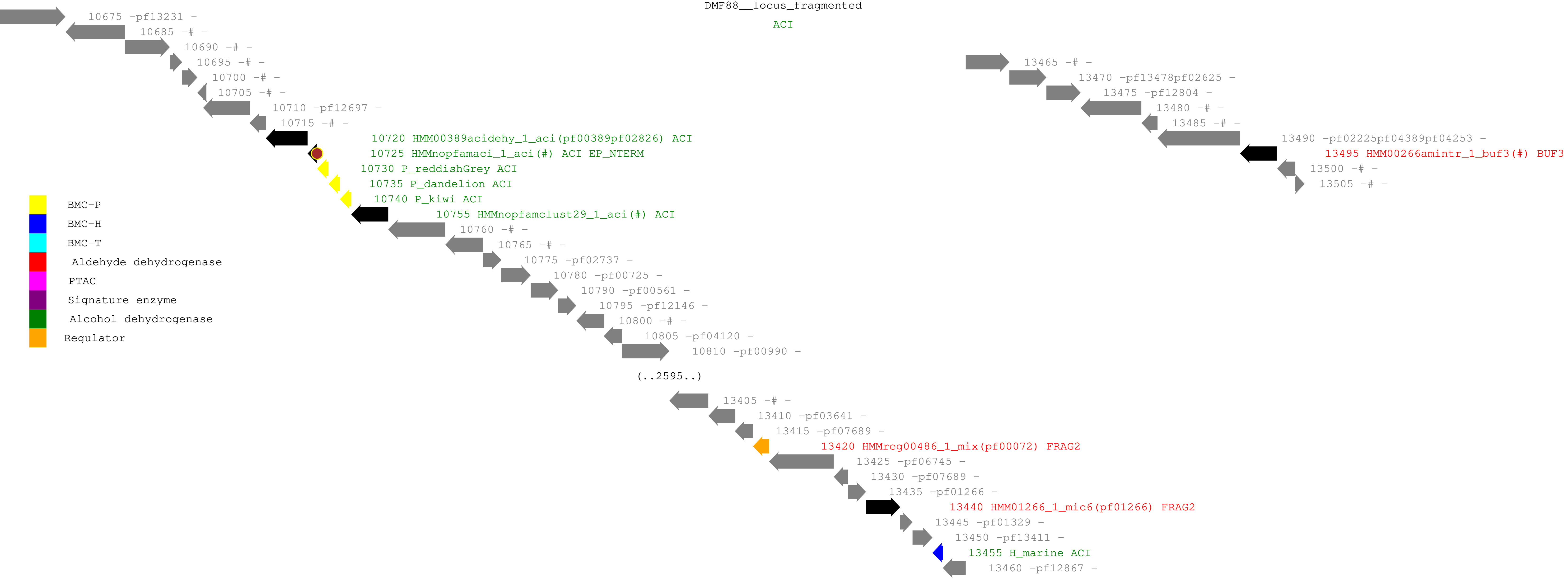

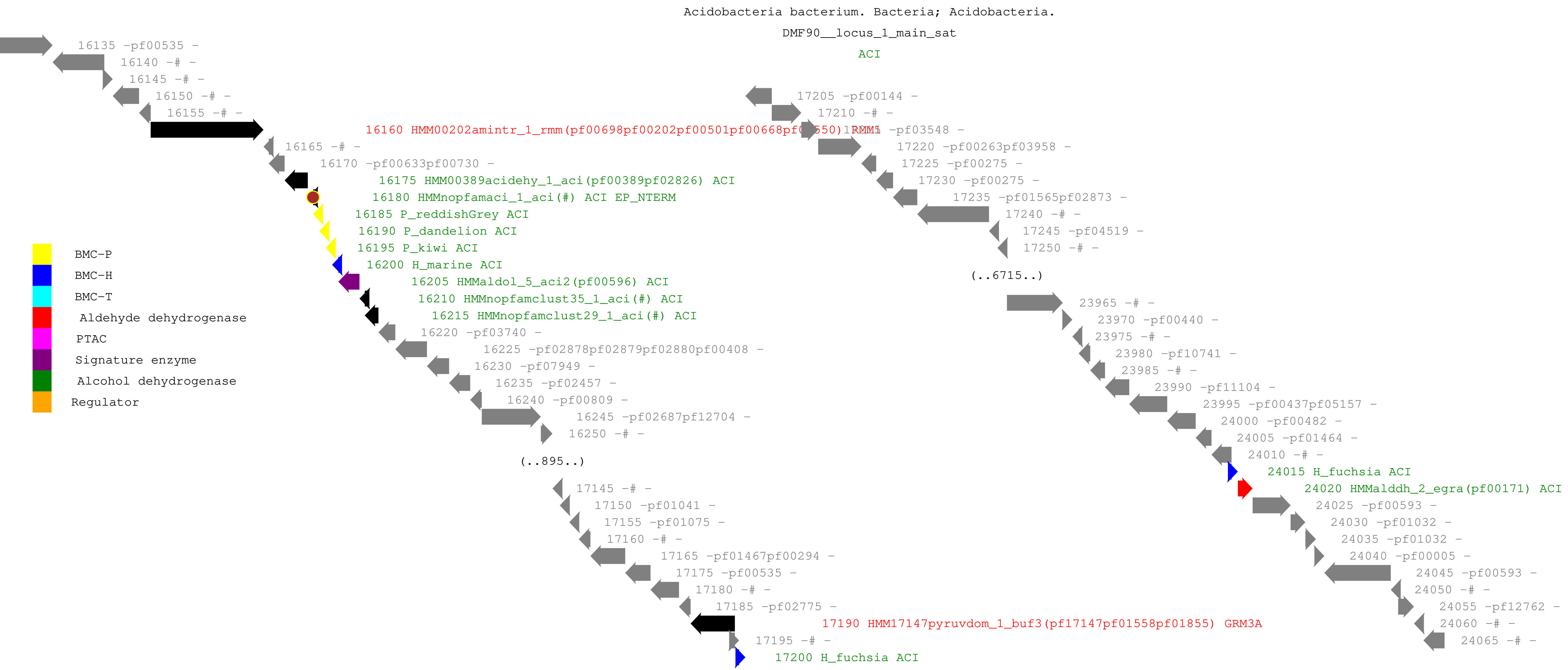

Acidobacteria bacterium. Bacteria; Acidobacteria.
DMF90__locus_1_main_sat
ACI
16135 -pf00535 -
16140 -# -
16145 -# -
16150 -# -
16155 -# -
16160 HMM00202amintr_1_rmm(pf00698pf00202pf00501pf00668pf0 550) RMM1
16165 -# -
16170 -pf00633pf00730 -
16175 HMM00389acidehy_1_aci(pf00389pf02826) ACI
16180 HMMnopfamaci_1_aci(#) ACI EP_NTERM
16185 P_reddishGrey ACI
16190 P_dandelion ACI
16195 P_kiwi ACI
16200 H_marine ACI
16205 HMMaldol_5_aci2(pf00596) ACI
16210 HMMnopfamclust35_1_aci(#) ACI
16215 HMMnopfamclust29_1_aci(#) ACI
16220 -pf03740 -
16225 -pf02878pf02879pf02880pf00408 -
16230 -pf07949 -
16235 -pf02457 -
16240 -pf00809 -
16245 -pf02687pf12704 -
16250 -# -
(..895..)
17145 -# -
17150 -pf01041 -
17155 -pf01075 -
17160 -# -
17165 -pf01467pf00294 -
17175 -pf00535 -
17180 -# -
17185 -pf02775 -
17190 HMM17147pyruvdom_1_buf3(pf17147pf01558pf01855) GRM3A
17195 -# -
17200 H_fuchsia ACI
17205 -pf00144 -
17210 -# -
-pf03548 -
17220 -pf00263pf03958 -
17225 -pf00275 -
17230 -pf00275 -
17235 -pf01565pf02873 -
17240 -# -
17245 -pf04519 -
17250 -# -
(..6715..)
23965 -# -
23970 -pf00440 -
23975 -# -
23980 -pf10741 -
23985 -# -
23990 -pf11104 -
23995 -pf00437pf05157 -
24000 -pf00482 -
24005 -pf01464 -
24010 -# -
24015 H_fuchsia ACI
24020 HMMalddh_2_egra(pf00171) ACI
24025 -pf00593 -
24030 -pf01032 -
24035 -pf01032 -
24040 -pf00005 -
24045 -pf00593 -
24050 -# -
24055 -pf12762 -
24060 -# -
24065 -# -
BMC-P
BMC-H
BMC-T
Aldehyde dehydrogenase
PTAC
Signature enzyme
Alcohol dehydrogenase
Regulator

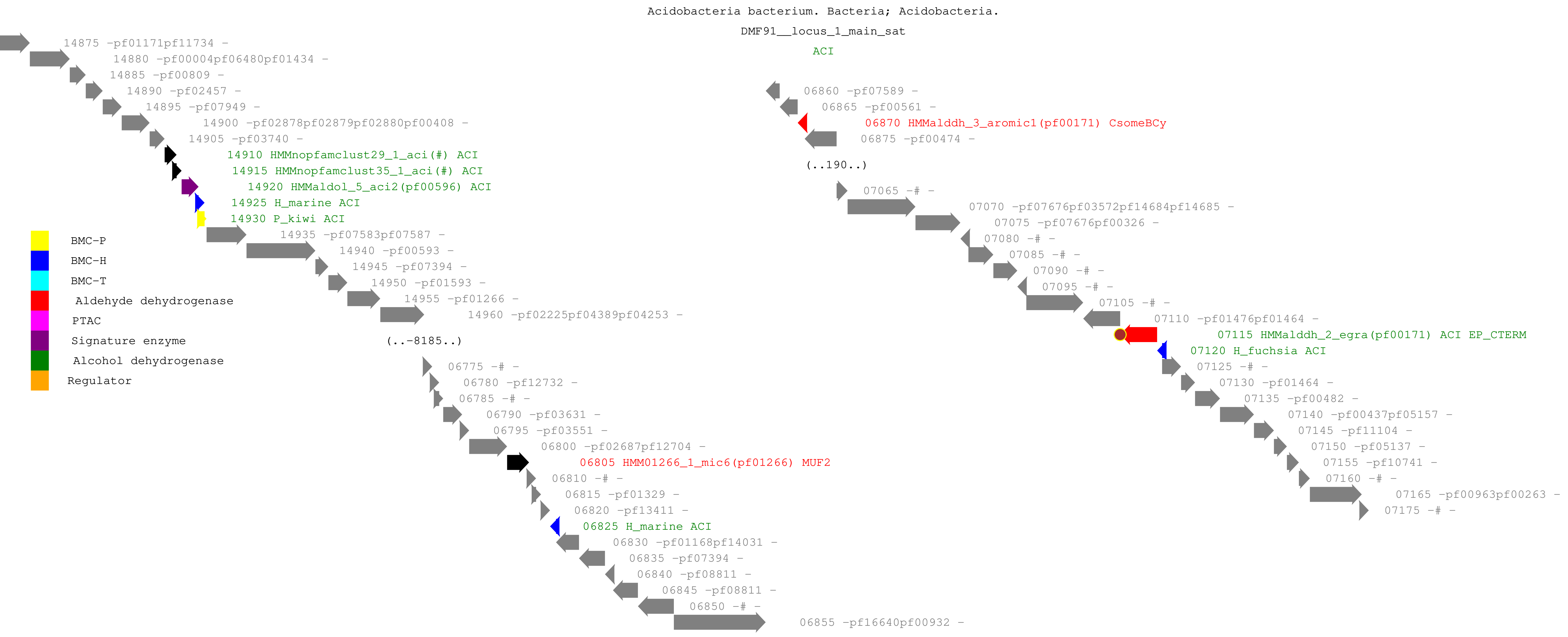
Acidobacteria bacterium. Bacteria; Acidobacteria.
DMF91__locus_1_main_sat
ACI
14875 -pf01171pf11734 -
14880 -pf00004pf06480pf01434 -
14885 -pf00809 -
14890 -pf02457 -
14895 -pf07949 -
14900 -pf02878pf02879pf02880pf00408 -
14905 -pf03740 -
14910 HMMnopfamclust29_1_aci(#) ACI
14915 HMMnopfamclust35_1_aci(#) ACI
14920 HMMaldol_5_aci2(pf00596) ACI
14925 H_marine ACI
14930 P_kiwi ACI
14935 -pf07583pf07587 -
14940 -pf00593 -
14945 -pf07394 -
14950 -pf01593 -
14955 -pf01266 -
14960 -pf02225pf04389pf04253 -
(..-8185..)
06775 -# -
06780 -pf12732 -
06785 -# -
06790 -pf03631 -
06795 -pf03551 -
06800 -pf02687pf12704 -
06805 HMM01266_1_mic6(pf01266) MUF2
06810 -# -
06815 -pf01329 -
06820 -pf13411 -
06825 H_marine ACI
06830 -pf01168pf14031 -
06835 -pf07394 -
06840 -pf08811 -
06845 -pf08811 -
06850 -# -
06855 -pf16640pf00932 -
06860 -pf07589 -
06865 -pf00561 -
06870 HMMalddh_3_aromic1(pf00171) CsomeBCy
06875 -pf00474 -
(..190..)
07065 -# -
07070 -pf07676pf03572pf14684pf14685 -
07075 -pf07676pf00326 -
07080 -# -
07085 -# -
07090 -# -
07095 -# -
07105 -# -
07110 -pf01476pf01464 -
07115 HMMalddh_2_egra(pf00171) ACI EP_CTERM
07120 H_fuchsia ACI
07125 -# -
07130 -pf01464 -
07135 -pf00482 -
07140 -pf00437pf05157 -
07145 -pf11104 -
07150 -pf05137 -
07155 -pf10741 -
07160 -# -
07165 -pf00963pf00263 -
07175 -# -
BMC-P
BMC-H
BMC-T
Aldehyde dehydrogenase
PTAC
Signature enzyme
Alcohol dehydrogenase
Regulator

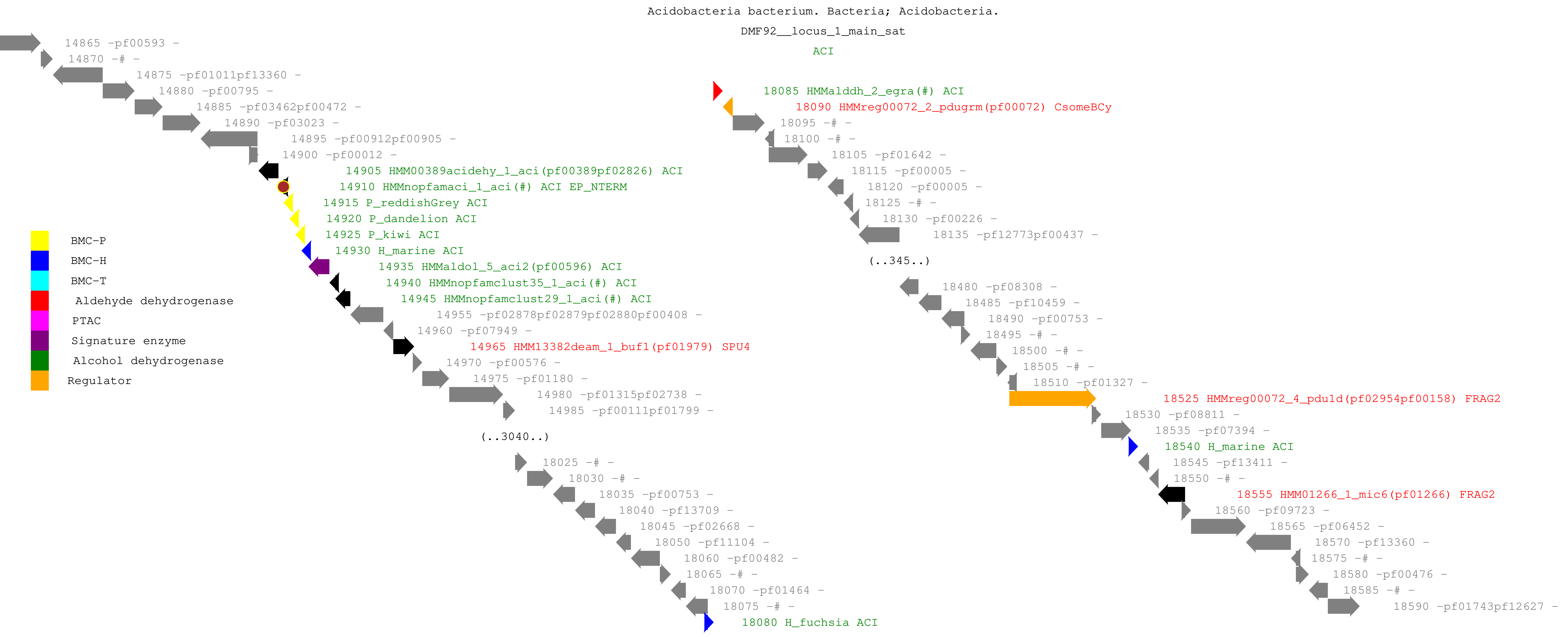

Acidobacteria bacterium. Bacteria; Acidobacteria.
DMF92__locus_1_main_sat
ACI
14865 -pf00593 -
14870 -# -
14875 -pf01011pf13360 -
14880 -pf00795 -
14885 -pf03462pf00472 -
14890 -pf03023 -
14895 -pf00912pf00905 -
14900 -pf00012 -
14905 HMM00389acidehy_1_aci(pf00389pf02826) ACI
14910 HMMnopfamaci_1_aci(#) ACI EP_NTERM
14915 P_reddishGrey ACI
14920 P_dandelion ACI
14925 P_kiwi ACI
14930 H_marine ACI
14935 HMMaldol_5_aci2(pf00596) ACI
14940 HMMnopfamclust35_1_aci(#) ACI
14945 HMMnopfamclust29_1_aci(#) ACI
14955 -pf02878pf02879pf02880pf00408 -
14960 -pf07949 -
14965 HMM13382deam_1_buf1(pf01979) SPU4
14970 -pf00576 -
14975 -pf01180 -
14980 -pf01315pf02738 -
14985 -pf00111pf01799 -
(..3040..)
18025 -# -
18030 -# -
18035 -pf00753 -
18040 -pf13709 -
18045 -pf02668 -
18050 -pf11104 -
18060 -pf00482 -
18065 -# -
18070 -pf01464 -
18075 -# -
18080 H_fuchsia ACI
18085 HMMaldh_2_egra(#) ACI
18090 HMMreg00072_2_pdugrm(pf00072) CsomeBCy
18095 -# -
18100 -# -
18105 -pf01642 -
18115 -pf00005 -
18120 -pf00005 -
18125 -# -
18130 -pf00226 -
18135 -pf12773pf00437 -
(..345..)
18480 -pf08308 -
18485 -pf10459 -
18490 -pf00753 -
18495 -# -
18500 -# -
18505 -# -
18510 -pf01327 -
18525 HMMreg00072_4_pdu1d(pf02954pf00158) FRAG2
18530 -pf08811 -
18535 -pf07394 -
18540 H_marine ACI
18545 -pf13411 -
18550 -# -
18555 HMM01266_1_mic6(pf01266) FRAG2
18560 -pf09723 -
18565 -pf06452 -
18570 -pf13360 -
18575 -# -
18580 -pf00476 -
18585 -# -
18590 -pf01743pf12627 -
BMC-P
BMC-H
BMC-T
Aldehyde dehydrogenase
PTAC
Signature enzyme
Alcohol dehydrogenase
Regulator

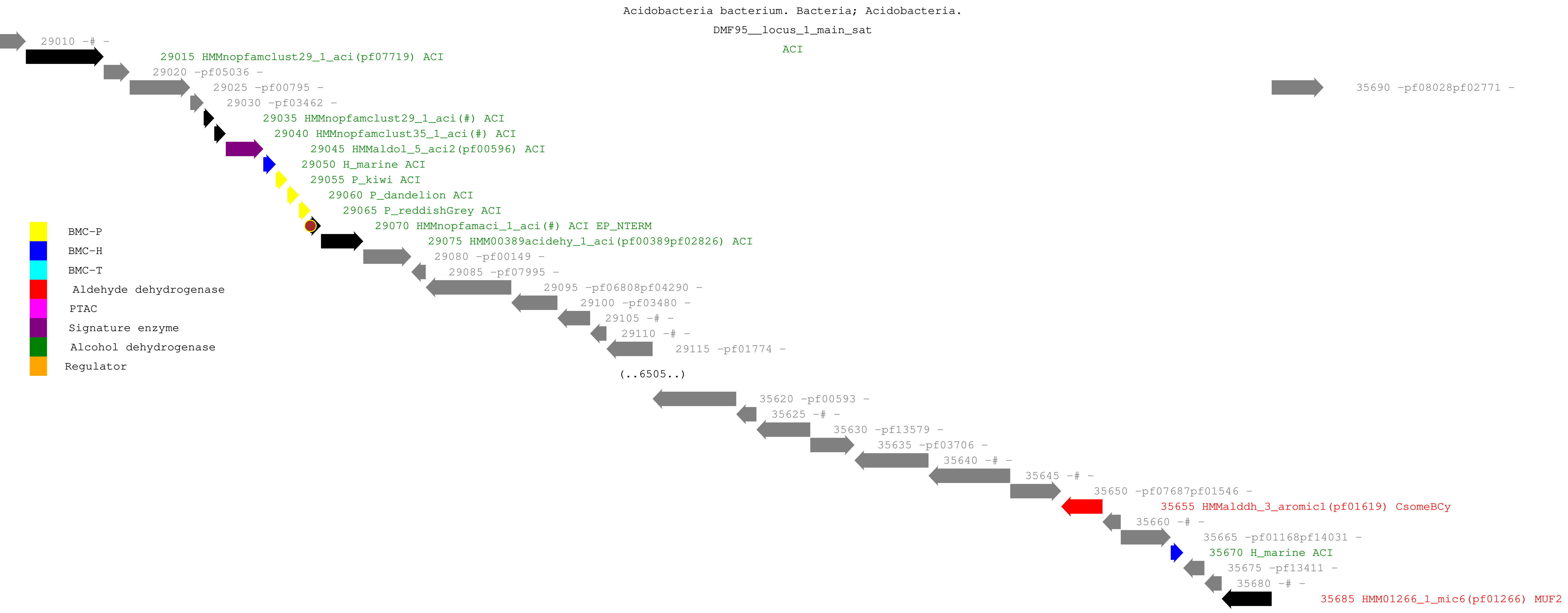

Acidobacteria bacterium. Bacteria; Acidobacteria.
DMF95__locus_1_main_sat
ACI
29010 -# -
29015 HMMnopfamclust29_1_aci(pf07719) ACI
29020 -pf05036 -
29025 -pf00795 -
29030 -pf03462 -
29035 HMMnopfamclust29_1_aci(#) ACI
29040 HMMnopfamclust35_1_aci(#) ACI
29045 HMMaldol_5_aci2(pf00596) ACI
29050 H_marine ACI
29055 P_kiwi ACI
29060 P_dandelion ACI
29065 P_reddishGrey ACI
29070 HMMnopfamaci_1_aci(#) ACI EP_NTERM
29075 HMM00389acidehy_1_aci(pf00389pf02826) ACI
29080 -pf00149 -
29085 -pf07995 -
29095 -pf06808pf04290 -
29100 -pf03480 -
29105 -# -
29110 -# -
29115 -pf01774 -
(..6505..)
35620 -pf00593 -
35625 -# -
35630 -pf13579 -
35635 -pf03706 -
35640 -# -
35645 -# -
35650 -pf07687pf01546 -
35655 HMMaldh_3_aromic1(pf01619) CsomeBCy
35660 -# -
35665 -pf01168pf14031 -
35670 H_marine ACI
35675 -pf13411 -
35680 -# -
35685 HMM01266_1_mic6(pf01266) MUF2
35690 -pf08028pf02771 -
BMC-P
BMC-H
BMC-T
Aldehyde dehydrogenase
PTAC
Signature enzyme
Alcohol dehydrogenase
Regulator

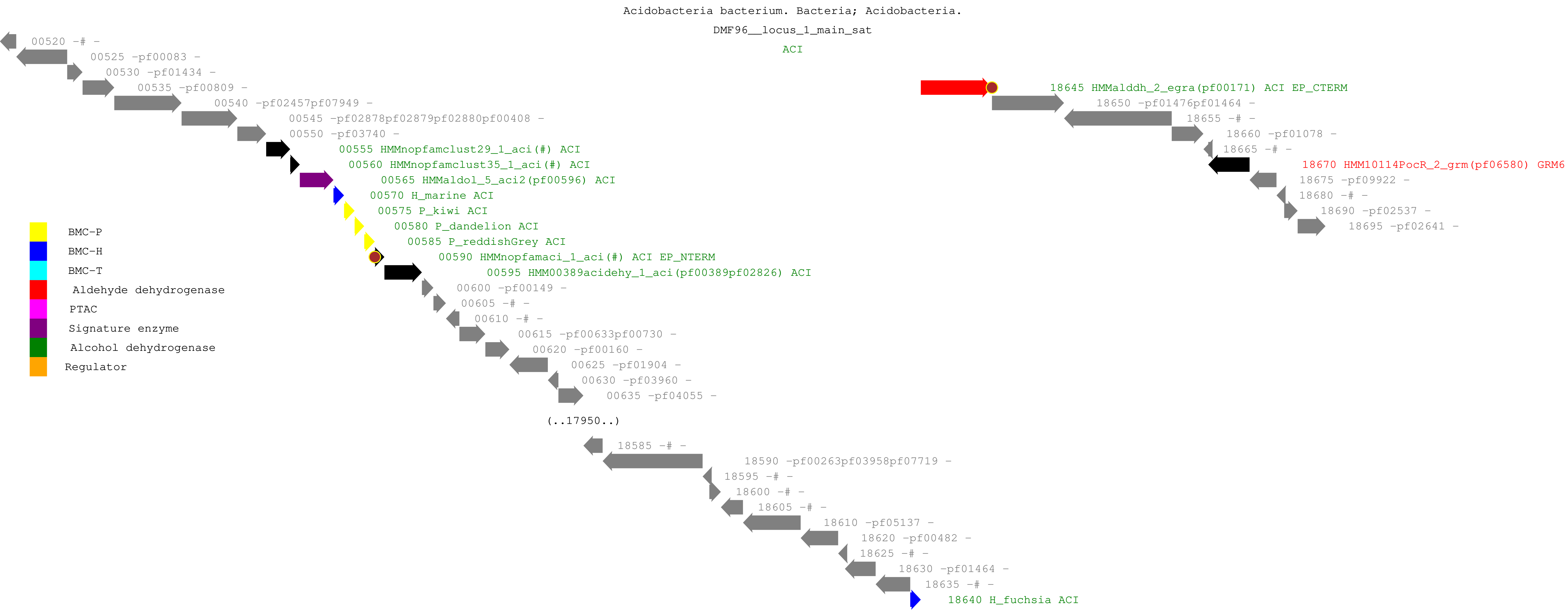
Acidobacteria bacterium. Bacteria; Acidobacteria.
DMF96__locus_1_main_sat
ACI
00520 -# -
00525 -pf00083 -
00530 -pf01434 -
00535 -pf00809 -
00540 -pf02457pf07949 -
00545 -pf02878pf02879pf02880pf00408 -
00550 -pf03740 -
00555 HMMnopfamclust29_1_aci(#) ACI
00560 HMMnopfamclust35_1_aci(#) ACI
00565 HMMaldol_5_aci2(pf00596) ACI
00570 H_marine ACI
00575 P_kiwi ACI
00580 P_dandelion ACI
00585 P_reddishGrey ACI
00590 HMMnopfamaci_1_aci(#) ACI EP_NTERM
00595 HMM00389acidehy_1_aci(pf00389pf02826) ACI
00600 -pf00149 -
00605 -# -
00610 -# -
00615 -pf00633pf00730 -
00620 -pf00160 -
00625 -pf01904 -
00630 -pf03960 -
00635 -pf04055 -
(..17950..)
18585 -# -
18590 -pf00263pf03958pf07719 -
18595 -# -
18600 -# -
18605 -# -
18610 -pf05137 -
18620 -pf00482 -
18625 -# -
18630 -pf01464 -
18635 -# -
18640 H_fuchsia ACI
18645 HMMalddh_2_egra(pf00171) ACI EP_CTERM
18650 -pf01476pf01464 -
18655 -# -
18660 -pf01078 -
18665 -# -
18670 HMM10114PocR_2_grm(pf06580) GRM6
18675 -pf09922 -
18680 -# -
18690 -pf02537 -
18695 -pf02641 -
BMC-P
BMC-H
BMC-T
Aldehyde dehydrogenase
PTAC
Signature enzyme
Alcohol dehydrogenase
Regulator

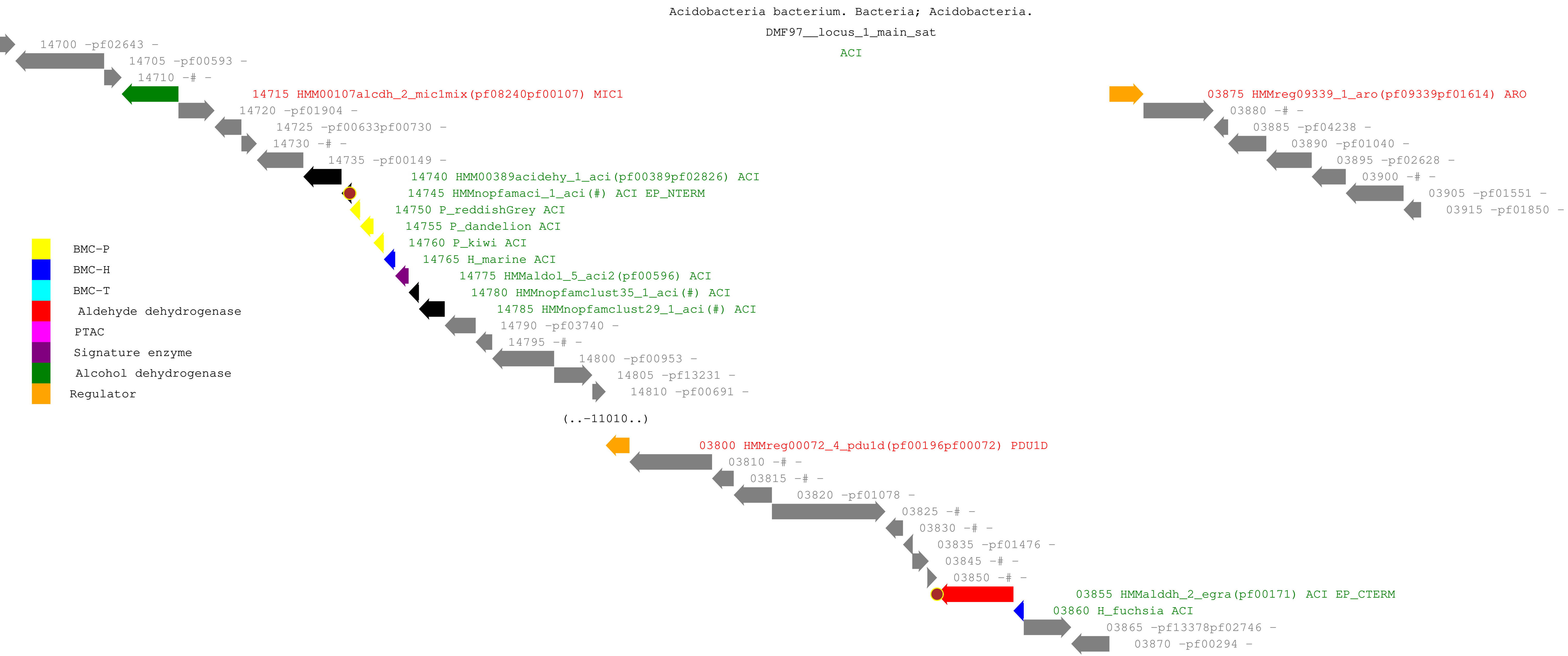
Acidobacteria bacterium. Bacteria; Acidobacteria.
DMF97__locus_1_main_sat
ACI
14700 -pf02643 -
14705 -pf00593 -
14710 -# -
14715 HMM00107alcdh_2_mic1mix(pf08240pf00107) MIC1
14720 -pf01904 -
14725 -pf00633pf00730 -
14730 -# -
14735 -pf00149 -
14740 HMM00389acidehy_1_aci(pf00389pf02826) ACI
14745 HMMnopfamaci_1_aci(#) ACI EP_NTERM
14750 P_reddishGrey ACI
14755 P_dandelion ACI
14760 P_kiwi ACI
14765 H_marine ACI
14775 HMMaldol_5_aci2(pf00596) ACI
14780 HMMnopfamclust35_1_aci(#) ACI
14785 HMMnopfamclust29_1_aci(#) ACI
14790 -pf03740 -
14795 -# -
14800 -pf00953 -
14805 -pf13231 -
14810 -pf00691 -
(..-11010..)
03800 HMMreg00072_4_pdu1d(pf00196pf00072) PDU1D
03810 -# -
03815 -# -
03820 -pf01078 -
03825 -# -
03830 -# -
03835 -pf01476 -
03845 -# -
03850 -# -
03855 HMMalddh_2_egra(pf00171) ACI EP_CTERM
03860 H_fuchsia ACI
03865 -pf13378pf02746 -
03870 -pf00294 -
03875 HMMreg09339_1_aro(pf09339pf01614) ARO
03880 -# -
03885 -pf04238 -
03890 -pf01040 -
03895 -pf02628 -
03900 -# -
03905 -pf01551 -
03915 -pf01850 -
BMC-P
BMC-H
BMC-T
Aldehyde dehydrogenase
PTAC
Signature enzyme
Alcohol dehydrogenase
Regulator

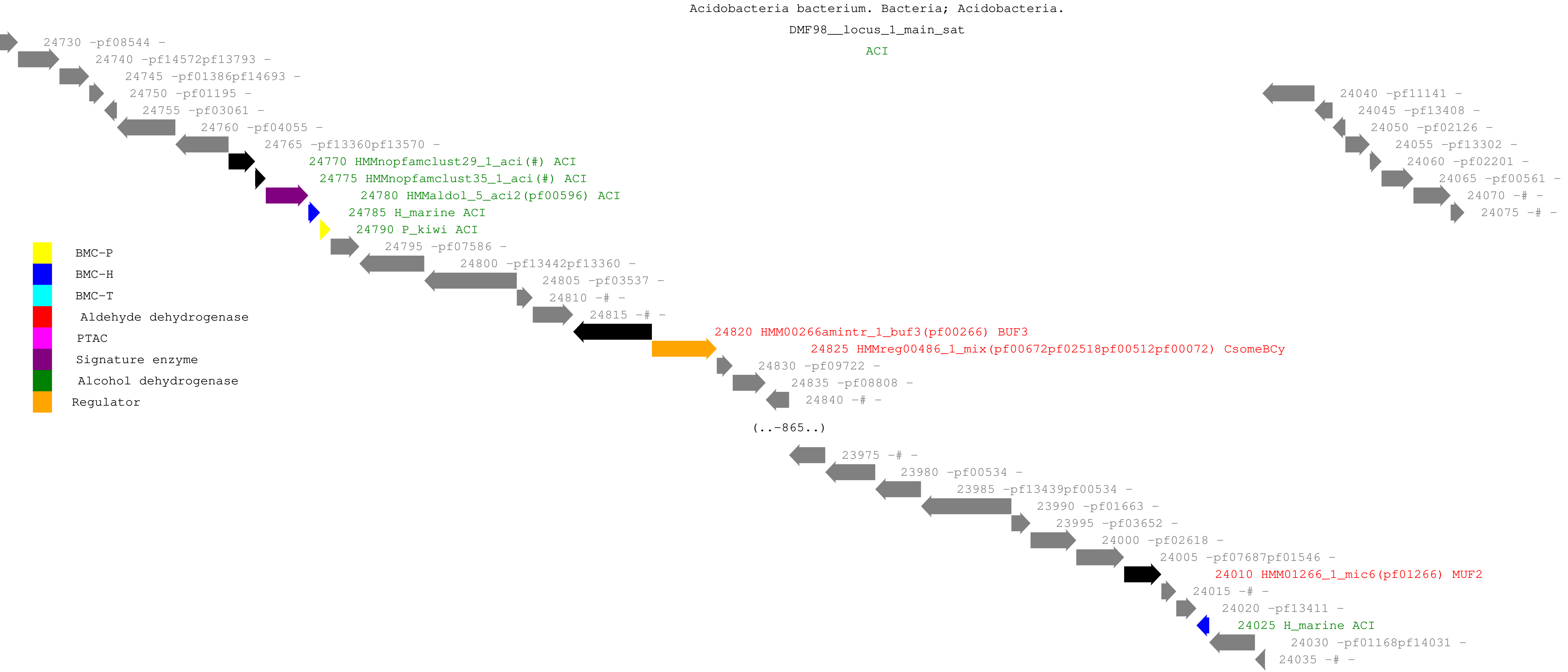
Acidobacteria bacterium. Bacteria; Acidobacteria.
DMF98__locus_1_main_sat
ACI
24730 -pf08544 -
24740 -pf14572pf13793 -
24745 -pf01386pf14693 -
24750 -pf01195 -
24755 -pf03061 -
24760 -pf04055 -
24765 -pf13360pf13570 -
24770 HMMnopfamclust29_1_aci(#) ACI
24775 HMMnopfamclust35_1_aci(#) ACI
24780 HMMaldol_5_aci2(pf00596) ACI
24785 H_marine ACI
24790 P_kiwi ACI
24795 -pf07586 -
24800 -pf13442pf13360 -
24805 -pf03537 -
24810 -# -
24815 -# -
24820 HMM00266amintr_1_buf3(pf00266) BUF3
24825 HMMreg00486_1_mix(pf00672pf02518pf00512pf00072) CsomeBCy
24830 -pf09722 -
24835 -pf08808 -
24840 -# -
(..-865..)
23975 -# -
23980 -pf00534 -
23985 -pf13439pf00534 -
23990 -pf01663 -
23995 -pf03652 -
24000 -pf02618 -
24005 -pf07687pf01546 -
24010 HMM01266_1_mic6(pf01266) MUF2
24015 -# -
24020 -pf13411 -
24025 H_marine ACI
24030 -pf01168pf14031 -
24035 -# -
24040 -pf11141 -
24045 -pf13408 -
24050 -pf02126 -
24055 -pf13302 -
24060 -pf02201 -
24065 -pf00561 -
24070 -# -
24075 -# -
BMC-P
BMC-H
BMC-T
Aldehyde dehydrogenase
PTAC
Signature enzyme
Alcohol dehydrogenase
Regulator

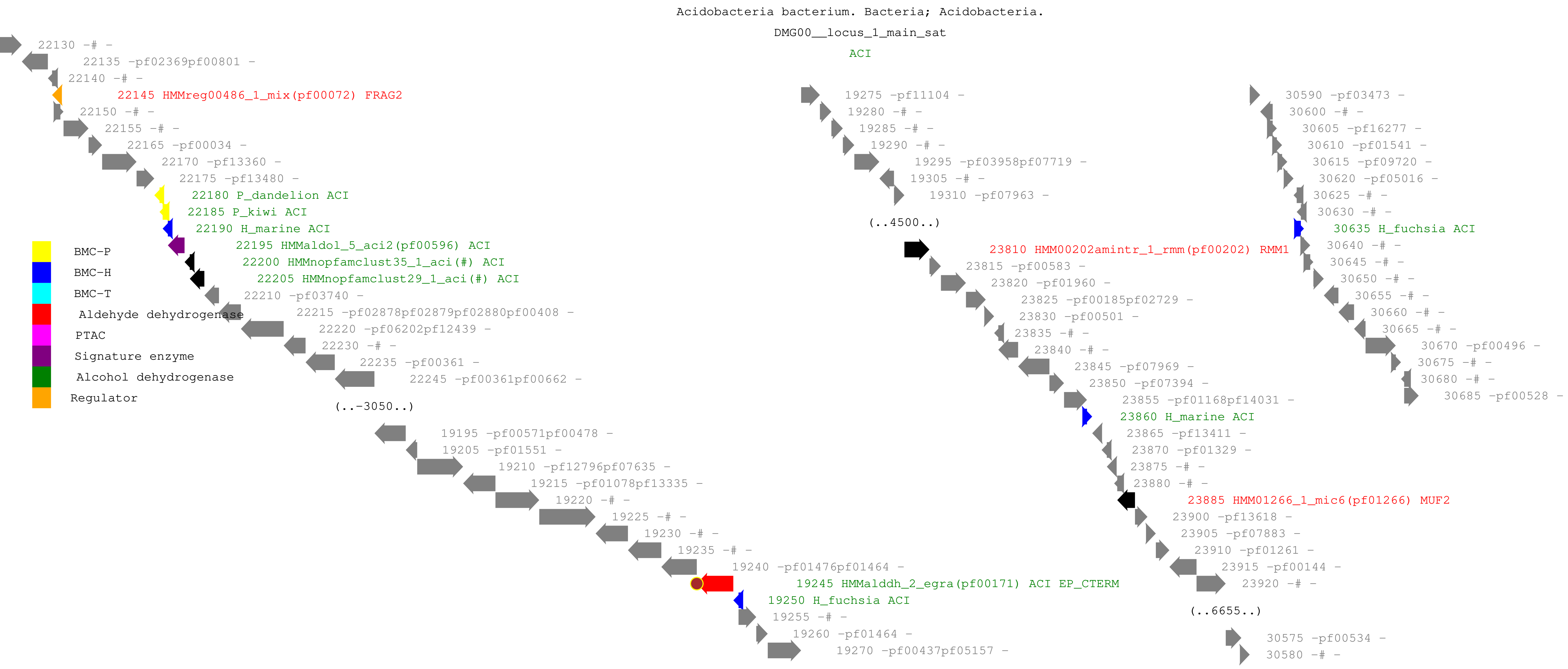

Acidobacteria bacterium. Bacteria; Acidobacteria.
DMG00__locus_1_main_sat
ACI
22130 -# -
22135 -pf02369pf00801 -
22140 -# -
22145 HMMreg00486_1_mix(pf00072) FRAG2
22150 -# -
22155 -# -
22165 -pf00034 -
22170 -pf13360 -
22175 -pf13480 -
22180 P_dandelion ACI
22185 P_kiwi ACI
22190 H_marine ACI
22195 HMMaldol_5_aci2(pf00596) ACI
22200 HMMnopfamclust35_1_aci(#) ACI
22205 HMMnopfamclust29_1_aci(#) ACI
22210 -pf03740 -
22215 -pf02878pf02879pf02880pf00408 -
22220 -pf06202pf12439 -
22230 -# -
22235 -pf00361 -
22245 -pf00361pf00662 -
(..-3050..)
19195 -pf00571pf00478 -
19205 -pf01551 -
19210 -pf12796pf07635 -
19215 -pf01078pf13335 -
19220 -# -
19225 -# -
19230 -# -
19235 -# -
19240 -pf01476pf01464 -
19245 HMMaldh_2_egra(pf00171) ACI EP_CTERM
19250 H_fuchsia ACI
19255 -# -
19260 -pf01464 -
19270 -pf00437pf05157 -
BMC-P
BMC-H
BMC-T
Aldehyde dehydrogenase
PTAC
Signature enzyme
Alcohol dehydrogenase
Regulator
19275 -pf11104 -
19280 -# -
19285 -# -
19290 -# -
19295 -pf03958pf07719 -
19305 -# -
19310 -pf07963 -
(..4500..)
23810 HMM00202amintr_1_rmm(pf00202) RMM1
23815 -pf00583 -
23820 -pf01960 -
23825 -pf00185pf02729 -
23830 -pf00501 -
23835 -# -
23840 -# -
23845 -pf07969 -
23850 -pf07394 -
23855 -pf01168pf14031 -
23860 H_marine ACI
23865 -pf13411 -
23870 -pf01329 -
23875 -# -
23880 -# -
23885 HMM01266_1_mic6(pf01266) MUF2
23900 -pf13618 -
23905 -pf07883 -
23910 -pf01261 -
23915 -pf00144 -
23920 -# -
(..6655..)
30575 -pf00534 -
30580 -# -
30590 -pf03473 -
30600 -# -
30605 -pf16277 -
30610 -pf01541 -
30615 -pf09720 -
30620 -pf05016 -
30625 -# -
30630 -# -
30635 H_fuchsia ACI
30640 -# -
30645 -# -
30650 -# -
30655 -# -
30660 -# -
30665 -# -
30670 -pf00496 -
30675 -# -
30680 -# -
30685 -pf00528 -

Acidobacteria bacterium. Bacteria; Acidobacteria.

DMG01__locus_1_main_sat

ACI

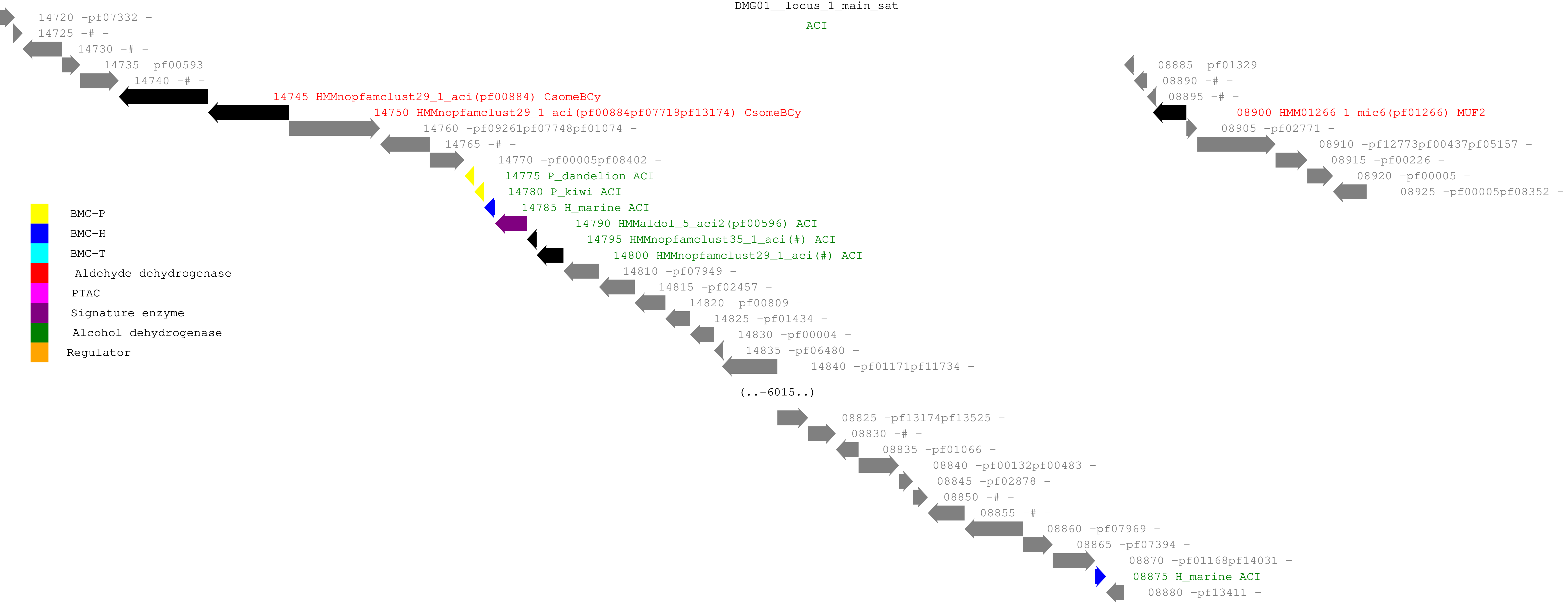

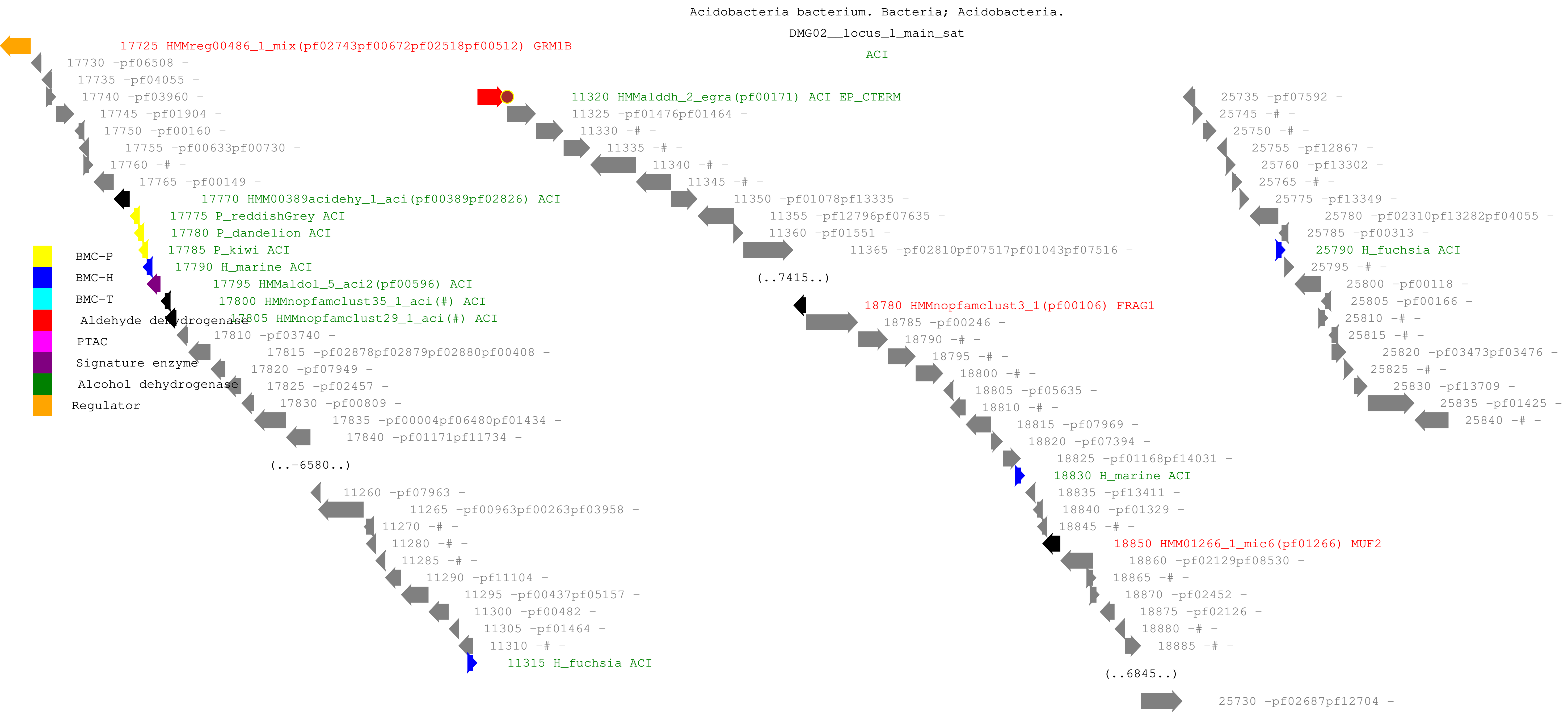
Acidobacteria bacterium. Bacteria; Acidobacteria.
DMG02__locus_1_main_sat
ACI
17725 HMMreg00486_1_mix(pf02743pf00672pf02518pf00512) GRM1B
17730 -pf06508 -
17735 -pf04055 -
17740 -pf03960 -
17745 -pf01904 -
17750 -pf00160 -
17755 -pf00633pf00730 -
17760 -# -
17765 -pf00149 -
17770 HMM00389acidehy_1_aci(pf00389pf02826) ACI
17775 P_reddishGrey ACI
17780 P_dandelion ACI
17785 P_kiwi ACI
17790 H_marine ACI
17795 HMMaldol_5_aci2(pf00596) ACI
17800 HMMnopfamclust35_1_aci(#) ACI
17805 HMMnopfamclust29_1_aci(#) ACI
17810 -pf03740 -
17815 -pf02878pf02879pf02880pf00408 -
17820 -pf07949 -
17825 -pf02457 -
17830 -pf00809 -
17835 -pf00004pf06480pf01434 -
17840 -pf01171pf11734 -
(..-6580..)
11260 -pf07963 -
11265 -pf00963pf00263pf03958 -
11270 -# -
11280 -# -
11285 -# -
11290 -pf11104 -
11295 -pf00437pf05157 -
11300 -pf00482 -
11305 -pf01464 -
11310 -# -
11315 H_fuchsia ACI
BMC-P
BMC-H
BMC-T
Aldehyde dehydrogenase
PTAC
Signature enzyme
Alcohol dehydrogenase
Regulator
11320 HMMalddh_2_egra(pf00171) ACI EP_CTERM
11325 -pf01476pf01464 -
11330 -# -
11335 -# -
11340 -# -
11345 -# -
11350 -pf01078pf13335 -
11355 -pf12796pf07635 -
11360 -pf01551 -
11365 -pf02810pf07517pf01043pf07516 -
(..7415..)
18780 HMMnopfamclust3_1(pf00106) FRAG1
18785 -pf00246 -
18790 -# -
18795 -# -
18800 -# -
18805 -pf05635 -
18810 -# -
18815 -pf07969 -
18820 -pf07394 -
18825 -pf01168pf14031 -
18830 H_marine ACI
18835 -pf13411 -
18840 -pf01329 -
18845 -# -
18850 HMM01266_1_mic6(pf01266) MUF2
18860 -pf02129pf08530 -
18865 -# -
18870 -pf02452 -
18875 -pf02126 -
18880 -# -
18885 -# -
(..6845..)
25730 -pf02687pf12704 -
25735 -pf07592 -
25745 -# -
25750 -# -
25755 -pf12867 -
25760 -pf13302 -
25765 -# -
25775 -pf13349 -
25780 -pf02310pf13282pf04055 -
25785 -pf00313 -
25790 H_fuchsia ACI
25795 -# -
25800 -pf00118 -
25805 -pf00166 -
25810 -# -
25815 -# -
25820 -pf03473pf03476 -
25825 -# -
25830 -pf13709 -
25835 -pf01425 -
25840 -# -

Acidobacteria bacterium. Bacteria; Acidobacteria.

DMG05__locus_1_main_sat

ACI

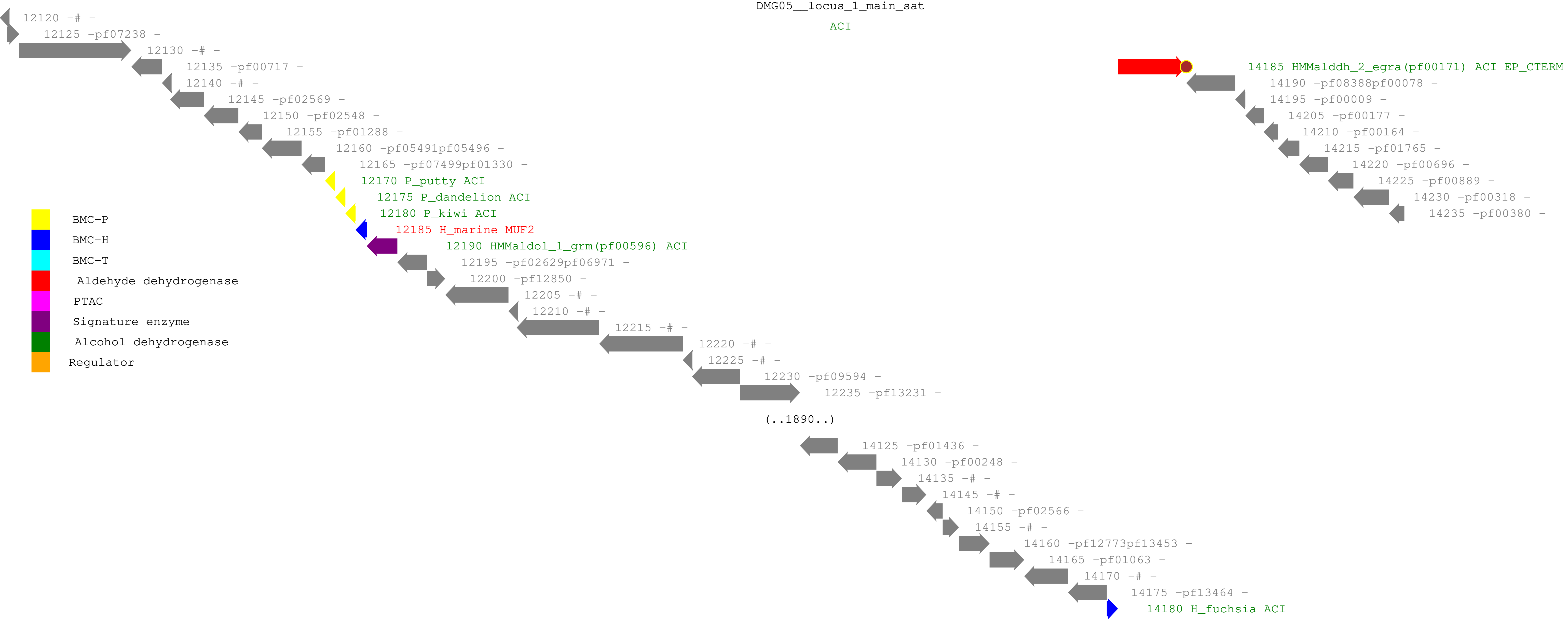

Acidobacteria bacterium. Bacteria; Acidobacteria.

DMG06__locus_1_main_sat

ACI

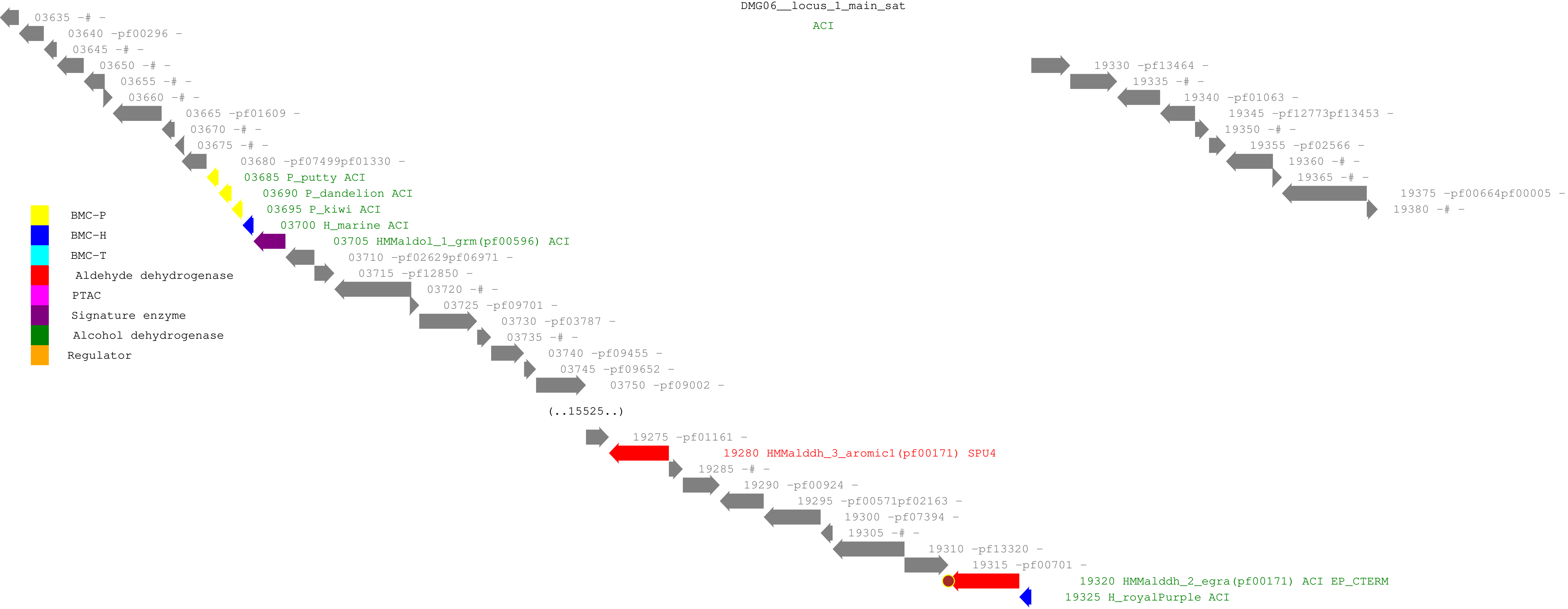

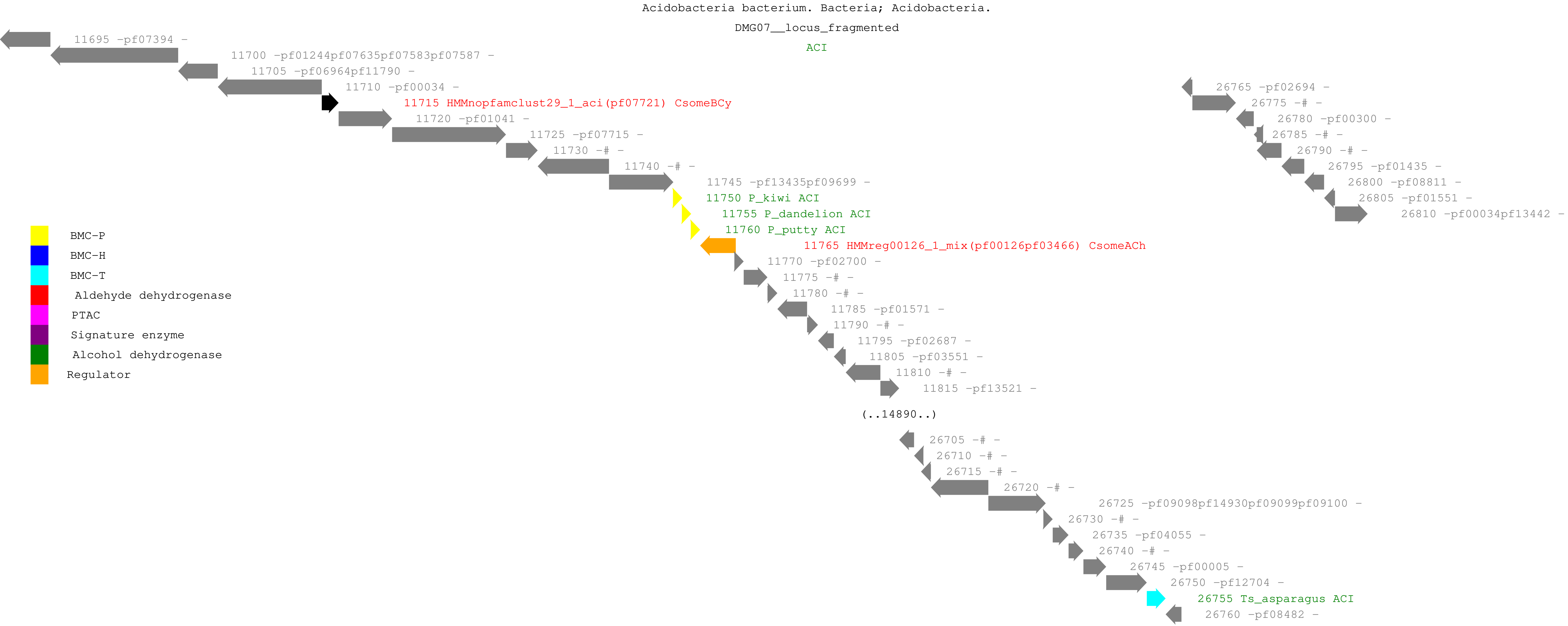
Acidobacteria bacterium. Bacteria; Acidobacteria.
DMG07__locus_fragmented
ACI
11695 -pf07394 -
11700 -pf01244pf07635pf07583pf07587 -
11705 -pf06964pf11790 -
11710 -pf00034 -
11715 HMMnopfamclust29_1_aci(pf07721) CsomeBCy
11720 -pf01041 -
11725 -pf07715 -
11730 -# -
11740 -# -
11745 -pf13435pf09699 -
11750 P_kiwi ACI
11755 P_dandelion ACI
11760 P_putty ACI
11765 HMMreg00126_1_mix(pf00126pf03466) CsomeACh
11770 -pf02700 -
11775 -# -
11780 -# -
11785 -pf01571 -
11790 -# -
11795 -pf02687 -
11805 -pf03551 -
11810 -# -
11815 -pf13521 -
(..14890..)
26705 -# -
26710 -# -
26715 -# -
26720 -# -
26725 -pf09098pf14930pf09099pf09100 -
26730 -# -
26735 -pf04055 -
26740 -# -
26745 -pf00005 -
26750 -pf12704 -
26755 Ts_asparagus ACI
26760 -pf08482 -
26765 -pf02694 -
26775 -# -
26780 -pf00300 -
26785 -# -
26790 -# -
26795 -pf01435 -
26800 -pf08811 -
26805 -pf01551 -
26810 -pf00034pf13442 -
BMC-P
BMC-H
BMC-T
Aldehyde dehydrogenase
PTAC
Signature enzyme
Alcohol dehydrogenase
Regulator

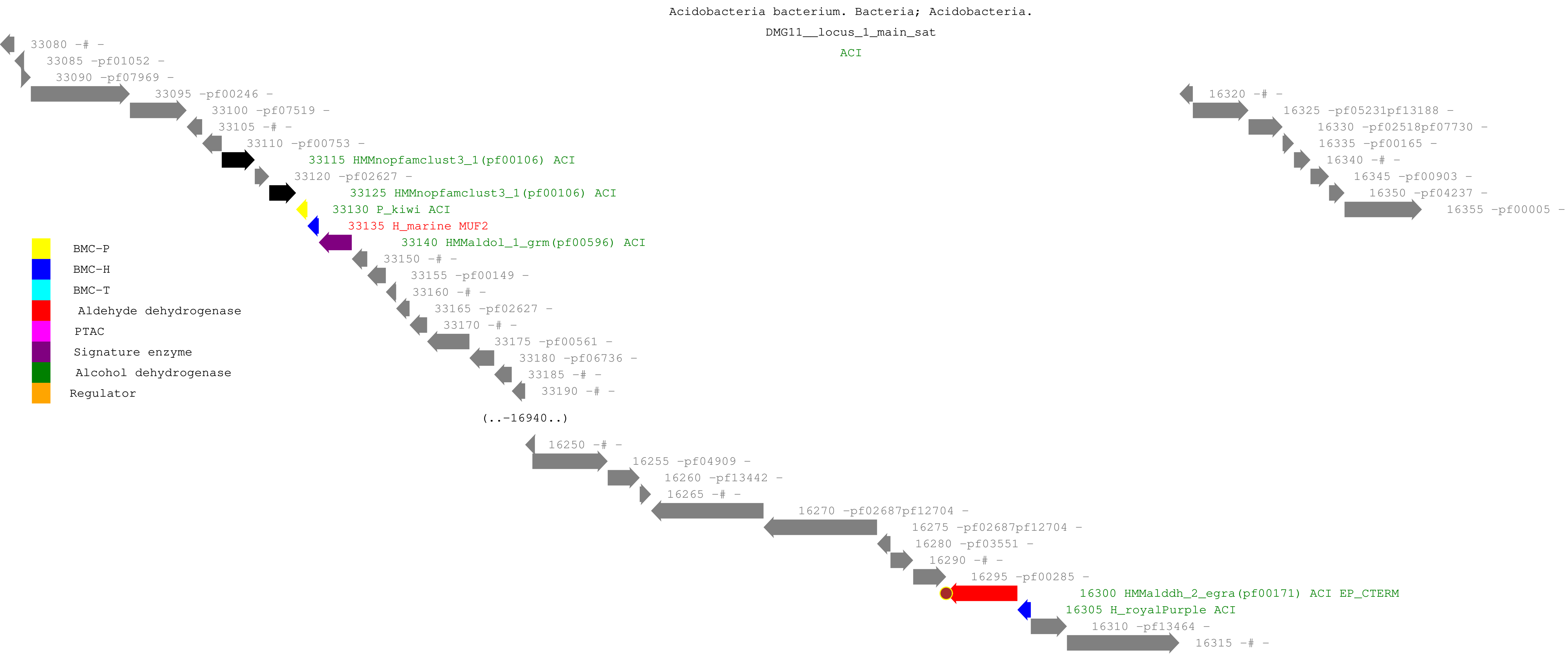
Acidobacteria bacterium. Bacteria; Acidobacteria.
DMG11__locus_1_main_sat
ACI
33080 -# -
33085 -pf01052 -
33090 -pf07969 -
33095 -pf00246 -
33100 -pf07519 -
33105 -# -
33110 -pf00753 -
33115 HMMnopfamclust3_1(pf00106) ACI
33120 -pf02627 -
33125 HMMnopfamclust3_1(pf00106) ACI
33130 P_kiwi ACI
33135 H_marine MUF2
33140 HMMaldol_1_grm(pf00596) ACI
33150 -# -
33155 -pf00149 -
33160 -# -
33165 -pf02627 -
33170 -# -
33175 -pf00561 -
33180 -pf06736 -
33185 -# -
33190 -# -
(..-16940..)
16250 -# -
16255 -pf04909 -
16260 -pf13442 -
16265 -# -
16270 -pf02687pf12704 -
16275 -pf02687pf12704 -
16280 -pf03551 -
16290 -# -
16295 -pf00285 -
16300 HMMaldh_2_egra(pf00171) ACI EP_CTERM
16305 H_royalPurple ACI
16310 -pf13464 -
16315 -# -
16320 -# -
16325 -pf05231pf13188 -
16330 -pf02518pf07730 -
16335 -pf00165 -
16340 -# -
16345 -pf00903 -
16350 -pf04237 -
16355 -pf00005 -
BMC-P
BMC-H
BMC-T
Aldehyde dehydrogenase
PTAC
Signature enzyme
Alcohol dehydrogenase
Regulator

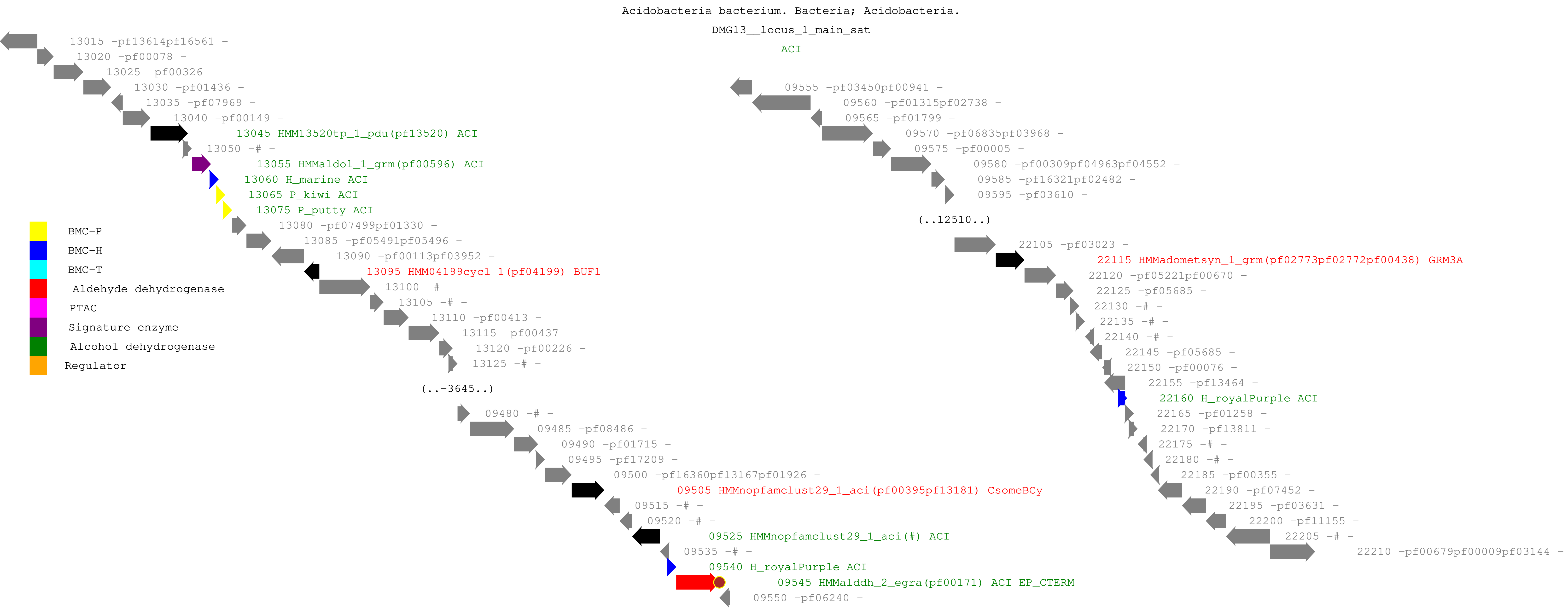
Acidobacteria bacterium. Bacteria; Acidobacteria.
DMG13__locus_1_main_sat
ACI
13015 -pf13614pf16561 -
13020 -pf00078 -
13025 -pf00326 -
13030 -pf01436 -
13035 -pf07969 -
13040 -pf00149 -
13045 HMM13520tp_1_pdu(pf13520) ACI
13050 -# -
13055 HMMaldol_1_grm(pf00596) ACI
13060 H_marine ACI
13065 P_kiwi ACI
13075 P_putty ACI
13080 -pf07499pf01330 -
13085 -pf05491pf05496 -
13090 -pf00113pf03952 -
13095 HMM04199cycl_1(pf04199) BUF1
13100 -# -
13105 -# -
13110 -pf00413 -
13115 -pf00437 -
13120 -pf00226 -
13125 -# -
(..-3645..)
09480 -# -
09485 -pf08486 -
09490 -pf01715 -
09495 -pf17209 -
09500 -pf16360pf13167pf01926 -
09505 HMMnopfamclust29_1_aci(pf00395pf13181) CsomeBCy
09515 -# -
09520 -# -
09525 HMMnopfamclust29_1_aci(#) ACI
09535 -# -
09540 H_royalPurple ACI
09545 HMMalddh_2_egra(pf00171) ACI EP_CTERM
09550 -pf06240 -
09555 -pf03450pf00941 -
09560 -pf01315pf02738 -
09565 -pf01799 -
09570 -pf06835pf03968 -
09575 -pf00005 -
09580 -pf00309pf04963pf04552 -
09585 -pf16321pf02482 -
09595 -pf03610 -
(..12510..)
22105 -pf03023 -
22115 HMMadometsyn_1_grm(pf02773pf02772pf00438) GRM3A
22120 -pf05221pf00670 -
22125 -pf05685 -
22130 -# -
22135 -# -
22140 -# -
22145 -pf05685 -
22150 -pf00076 -
22155 -pf13464 -
22160 H_royalPurple ACI
22165 -pf01258 -
22170 -pf13811 -
22175 -# -
22180 -# -
22185 -pf00355 -
22190 -pf07452 -
22195 -pf03631 -
22200 -pf11155 -
22205 -# -
22210 -pf00679pf00009pf03144 -
BMC-P
BMC-H
BMC-T
Aldehyde dehydrogenase
PTAC
Signature enzyme
Alcohol dehydrogenase
Regulator

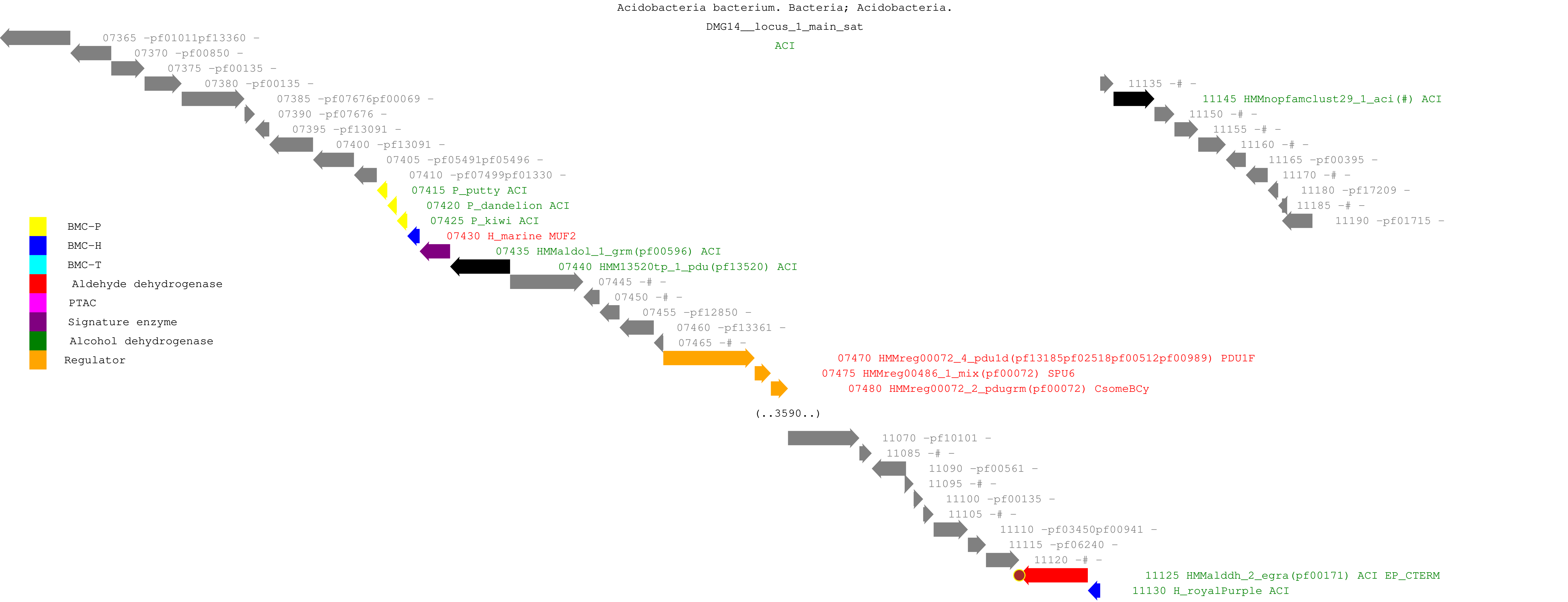

Acidobacteria bacterium. Bacteria; Acidobacteria.
DMG14__locus_1_main_sat
ACI
07365 -pf01011pf13360 -
07370 -pf00850 -
07375 -pf00135 -
07380 -pf00135 -
07385 -pf07676pf00069 -
07390 -pf07676 -
07395 -pf13091 -
07400 -pf13091 -
07405 -pf05491pf05496 -
07410 -pf07499pf01330 -
07415 P_putty ACI
07420 P_dandelion ACI
07425 P_kiwi ACI
07430 H_marine MUF2
07435 HMMaldol_1_grm(pf00596) ACI
07440 HMM13520tp_1_pdu(pf13520) ACI
07445 -# -
07450 -# -
07455 -pf12850 -
07460 -pf13361 -
07465 -# -
07470 HMMreg00072_4_pdu1d(pf13185pf02518pf00512pf00989) PDU1F
07475 HMMreg00486_1_mix(pf00072) SPU6
07480 HMMreg00072_2_pdugrm(pf00072) CsomeBCy
(..3590..)
11070 -pf10101 -
11085 -# -
11090 -pf00561 -
11095 -# -
11100 -pf00135 -
11105 -# -
11110 -pf03450pf00941 -
11115 -pf06240 -
11120 -# -
11125 HMMaldh_2_egra(pf00171) ACI EP_CTERM
11130 H_royalPurple ACI
11135 -# -
11145 HMMnopfamclust29_1_aci(#) ACI
11150 -# -
11155 -# -
11160 -# -
11165 -pf00395 -
11170 -# -
11180 -pf17209 -
11185 -# -
11190 -pf01715 -
BMC-P
BMC-H
BMC-T
Aldehyde dehydrogenase
PTAC
Signature enzyme
Alcohol dehydrogenase
Regulator

Acidobacteria bacterium. Bacteria; Acidobacteria.

DMG15__locus_1_main_sat

ACI

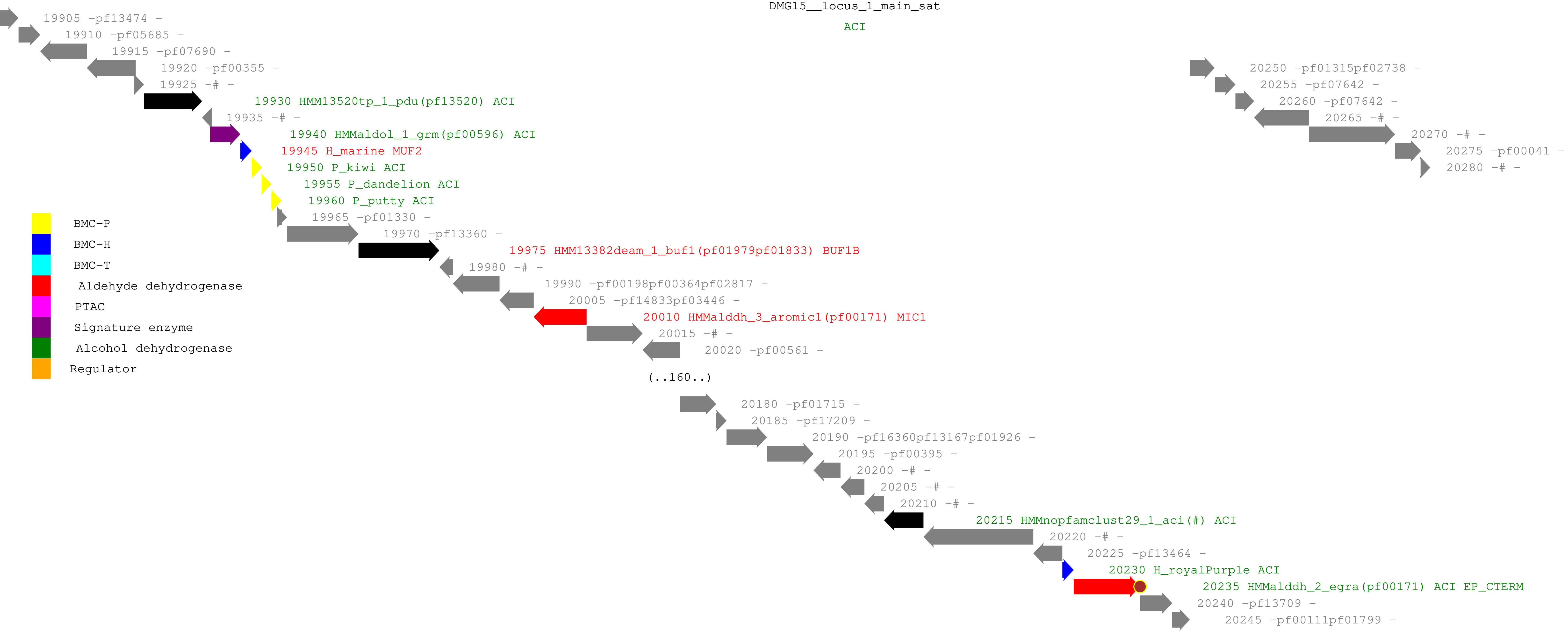

Acidobacteria bacterium. Bacteria; Acidobacteria.

DMG16__locus_1_main_sat

ACI

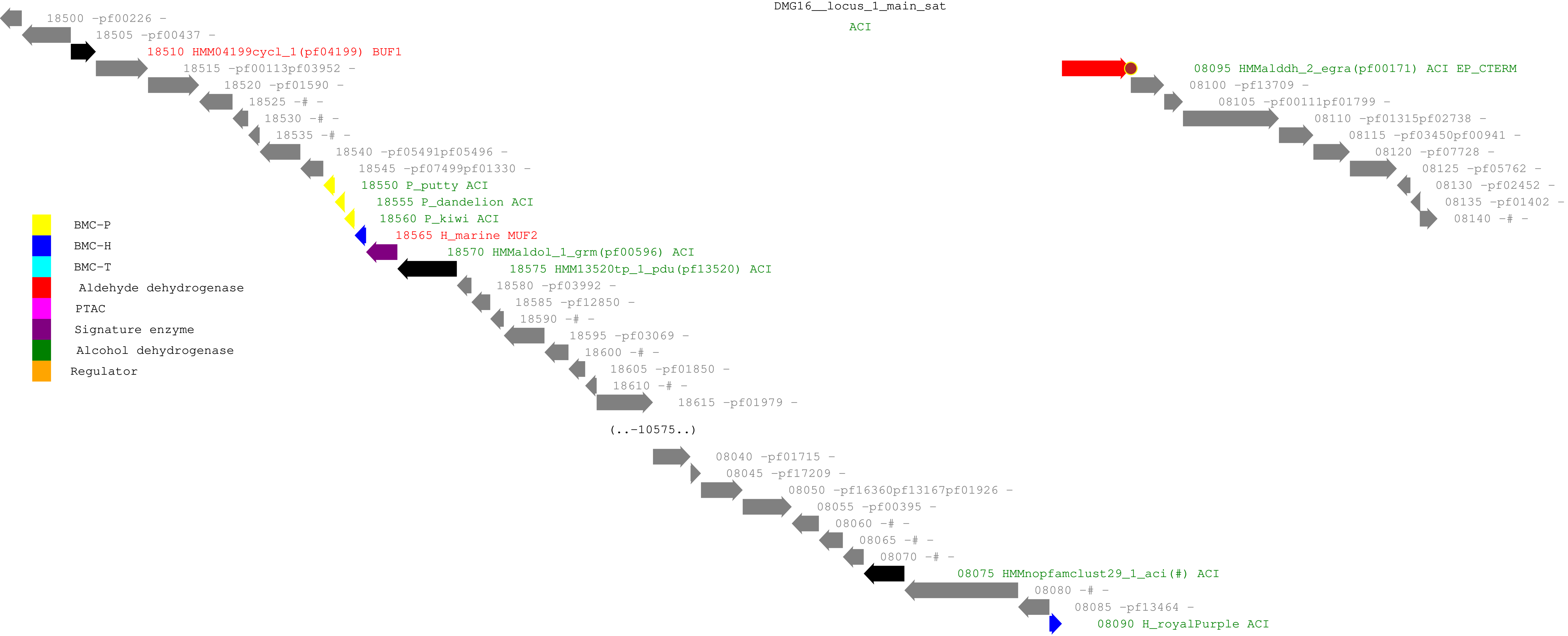

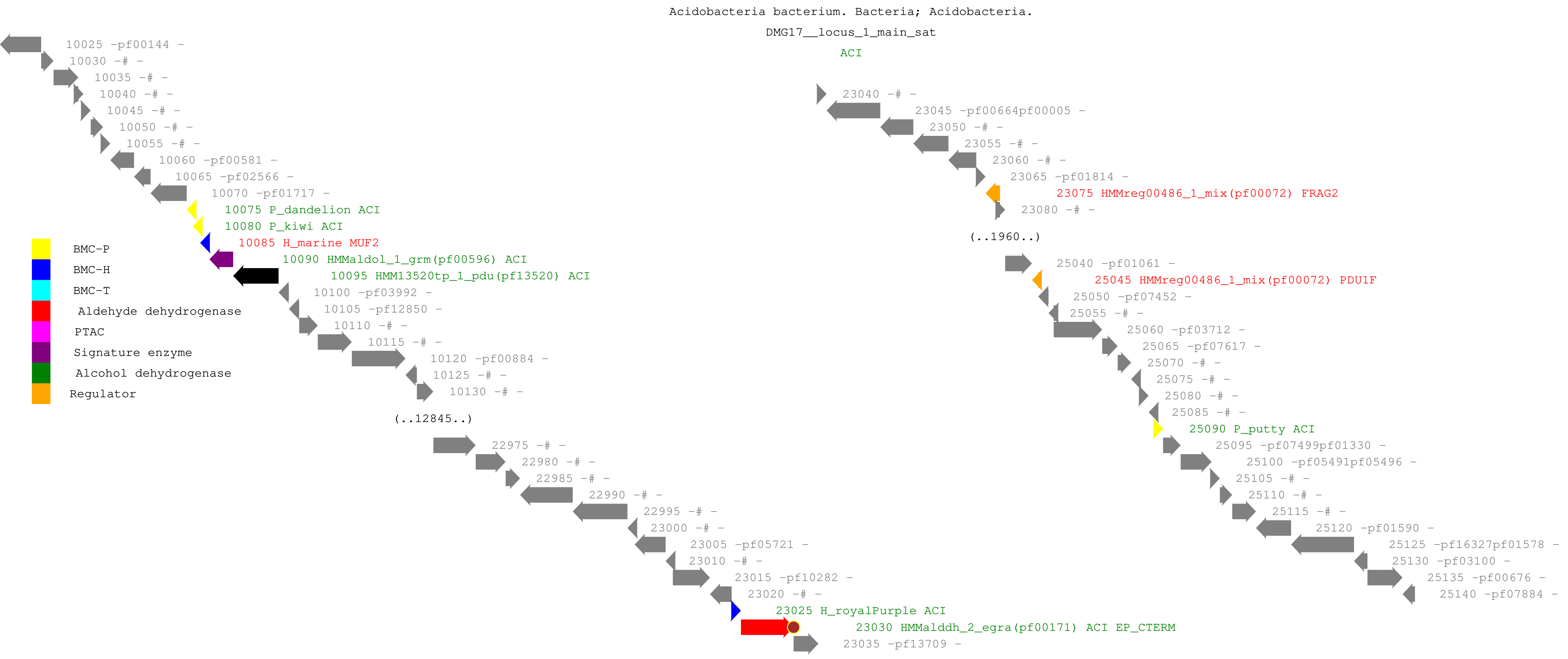

Acidobacteria bacterium. Bacteria; Acidobacteria.
DMG17__locus_1_main_sat
ACI
10025 -pf00144 -
10030 -# -
10035 -# -
10040 -# -
10045 -# -
10050 -# -
10055 -# -
10060 -pf00581 -
10065 -pf02566 -
10070 -pf01717 -
10075 P_dandelion ACI
10080 P_kiwi ACI
10085 H_marine MUF2
10090 HMMaldol_1_grm(pf00596) ACI
10095 HMM13520tp_1_pdu(pf13520) ACI
10100 -pf03992 -
10105 -pf12850 -
10110 -# -
10115 -# -
10120 -pf00884 -
10125 -# -
10130 -# -
(..12845..)
22975 -# -
22980 -# -
22985 -# -
22990 -# -
22995 -# -
23000 -# -
23005 -pf05721 -
23010 -# -
23015 -pf10282 -
23020 -# -
23025 H_royalPurple ACI
23030 HMMalddh_2_egra(pf00171) ACI EP_CTERM
23035 -pf13709 -
23040 -# -
23045 -pf00664pf00005 -
23050 -# -
23055 -# -
23060 -# -
23065 -pf01814 -
23075 HMMreg00486_1_mix(pf00072) FRAG2
23080 -# -
(..1960..)
25040 -pf01061 -
25045 HMMreg00486_1_mix(pf00072) PDU1F
25050 -pf07452 -
25055 -# -
25060 -pf03712 -
25065 -pf07617 -
25070 -# -
25075 -# -
25080 -# -
25085 -# -
25090 P_putty ACI
25095 -pf07499pf01330 -
25100 -pf05491pf05496 -
25105 -# -
25110 -# -
25115 -# -
25120 -pf01590 -
25125 -pf16327pf01578 -
25130 -pf03100 -
25135 -pf00676 -
25140 -pf07884 -
BMC-P
BMC-H
BMC-T
Aldehyde dehydrogenase
PTAC
Signature enzyme
Alcohol dehydrogenase
Regulator

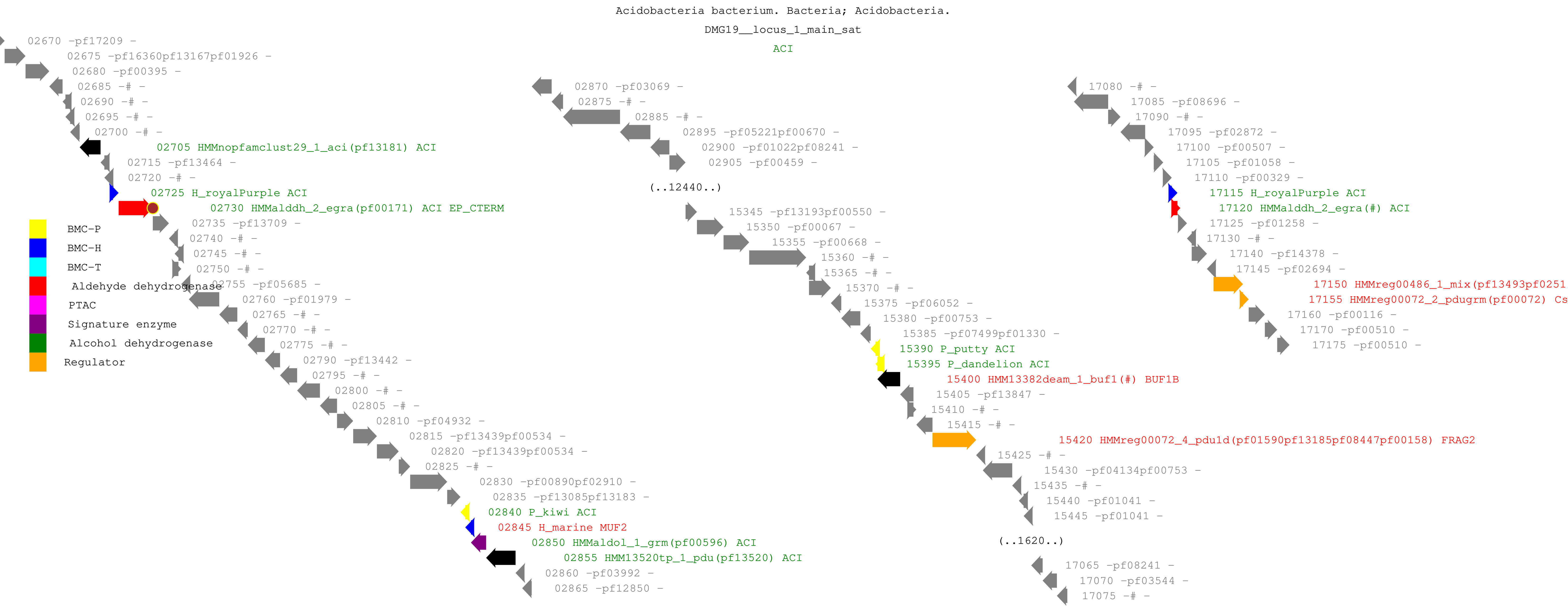

Acidobacteria bacterium. Bacteria; Acidobacteria.
DMG19__locus_1_main_sat
ACI
02670 -pf17209 -
02675 -pf16360pf13167pf01926 -
02680 -pf00395 -
02685 -# -
02690 -# -
02695 -# -
02700 -# -
02705 HMMnopfamclust29_1_aci(pf13181) ACI
02715 -pf13464 -
02720 -# -
02725 H_royalPurple ACI
02730 HMMalddh_2_egra(pf00171) ACI EP_CTERM
02735 -pf13709 -
02740 -# -
02745 -# -
02750 -# -
02755 -pf05685 -
02760 -pf01979 -
02765 -# -
02770 -# -
02775 -# -
02790 -pf13442 -
02795 -# -
02800 -# -
02805 -# -
02810 -pf04932 -
02815 -pf13439pf00534 -
02820 -pf13439pf00534 -
02825 -# -
02830 -pf00890pf02910 -
02835 -pf13085pf13183 -
02840 P_kiwi ACI
02845 H_marine MUF2
02850 HMMaldol_1_grm(pf00596) ACI
02855 HMM13520tp_1_pdu(pf13520) ACI
02860 -pf03992 -
02865 -pf12850 -
BMC-P
BMC-H
BMC-T
Aldehyde dehydrogenase
PTAC
Signature enzyme
Alcohol dehydrogenase
Regulator
02870 -pf03069 -
02875 -# -
02885 -# -
02895 -pf05221pf00670 -
02900 -pf01022pf08241 -
02905 -pf00459 -
(..12440..)
15345 -pf13193pf00550 -
15350 -pf00067 -
15355 -pf00668 -
15360 -# -
15365 -# -
15370 -# -
15375 -pf06052 -
15380 -pf00753 -
15385 -pf07499pf01330 -
15390 P_putty ACI
15395 P_dandelion ACI
15400 HMM13382deam_1_buf1(#) BUF1B
15405 -pf13847 -
15410 -# -
15415 -# -
15420 HMMreg00072_4_pdu1d(pf01590pf13185pf08447pf00158) FRAG2
15425 -# -
15430 -pf04134pf00753 -
15435 -# -
15440 -pf01041 -
15445 -pf01041 -
(..1620..)
17065 -pf08241 -
17070 -pf03544 -
17075 -# -
17080 -# -
17085 -pf08696 -
17090 -# -
17095 -pf02872 -
17100 -pf00507 -
17105 -pf01058 -
17110 -pf00329 -
17115 H_royalPurple ACI
17120 HMMalddh_2_egra(#) ACI
17125 -pf01258 -
17130 -# -
17140 -pf14378 -
17145 -pf02694 -
17150 HMMreg00486_1_mix(pf13493pf0251
17155 HMMreg00072_2_pdugrm(pf00072) Cs
17160 -pf00116 -
17170 -pf00510 -
17175 -pf00510 -

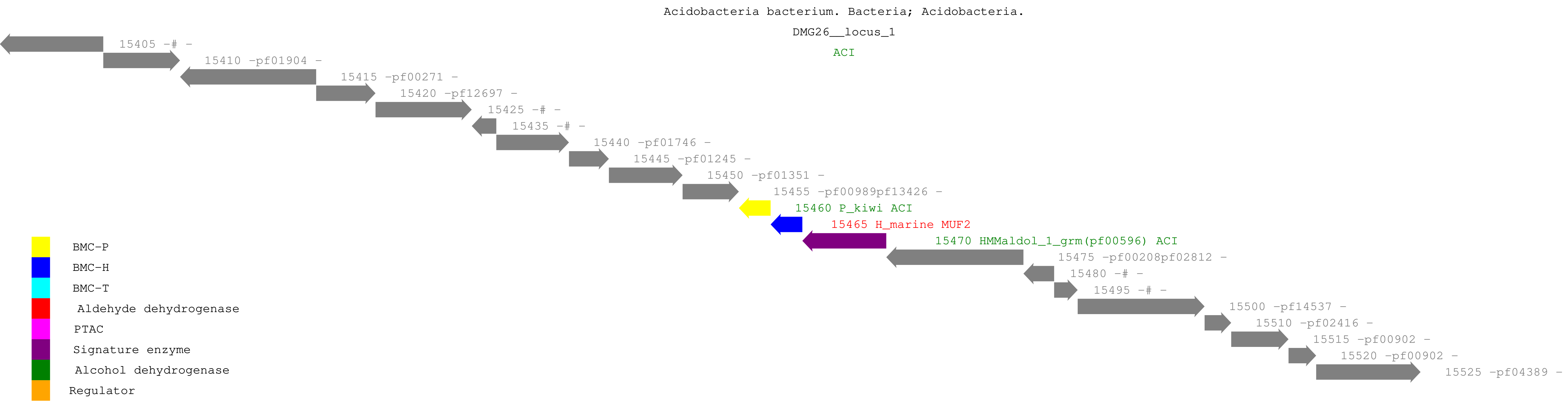
Acidobacteria bacterium. Bacteria; Acidobacteria.
DMG26__locus_1
ACI
15405 -# -
15410 -pf01904 -
15415 -pf00271 -
15420 -pf12697 -
15425 -# -
15435 -# -
15440 -pf01746 -
15445 -pf01245 -
15450 -pf01351 -
15455 -pf00989pf13426 -
15460 P_kiwi ACI
15465 H_marine MUF2
15470 HMMaldol_1_grm(pf00596) ACI
15475 -pf00208pf02812 -
15480 -# -
15495 -# -
15500 -pf14537 -
15510 -pf02416 -
15515 -pf00902 -
15520 -pf00902 -
15525 -pf04389 -
BMC-P
BMC-H
BMC-T
Aldehyde dehydrogenase
PTAC
Signature enzyme
Alcohol dehydrogenase
Regulator

Acidobacteria bacterium. Bacteria; Acidobacteria.

DMG27__locus_1

ACI

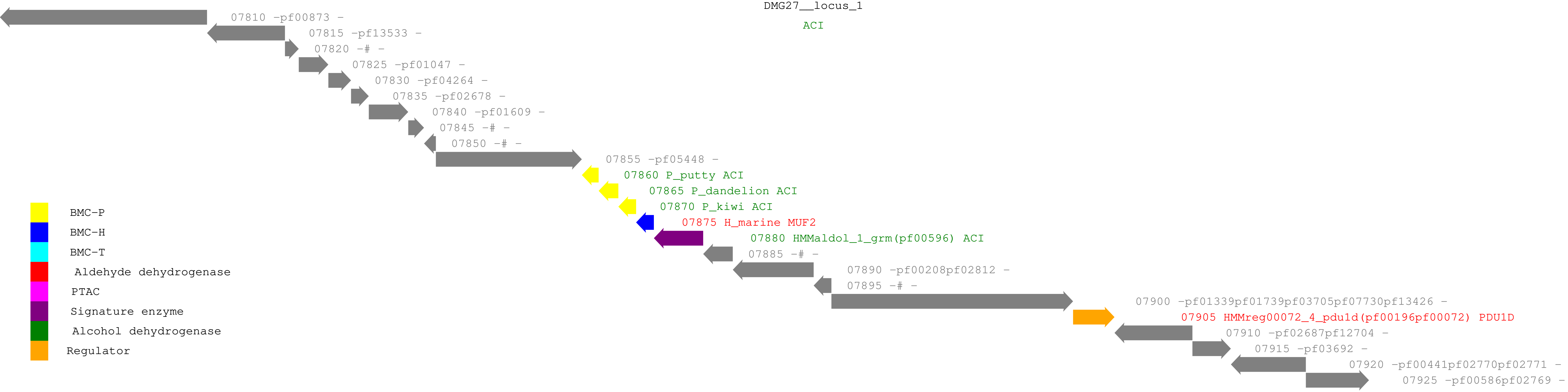

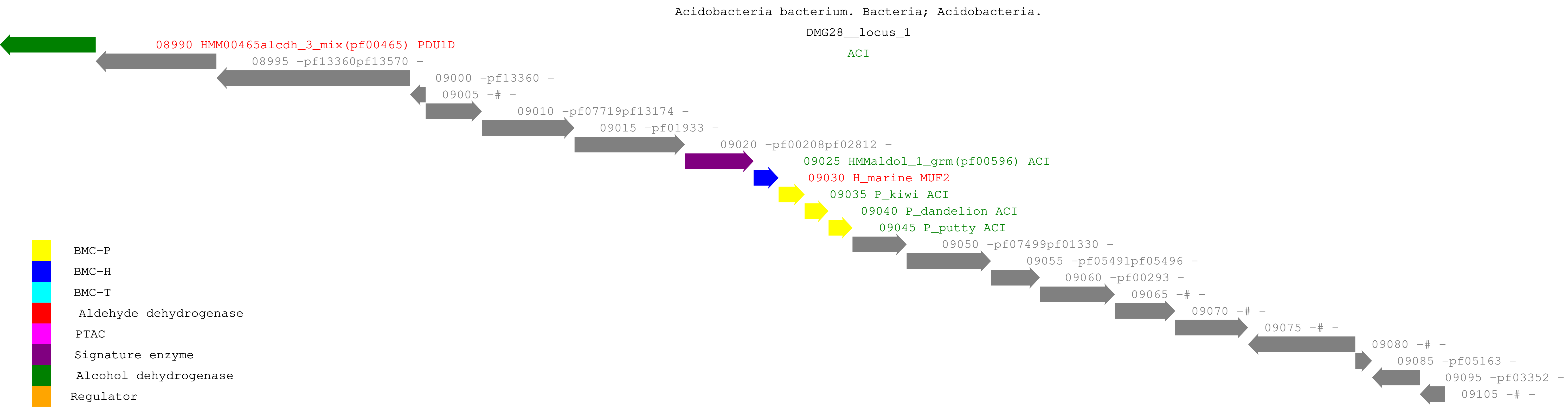
Acidobacteria bacterium. Bacteria; Acidobacteria.
DMG28__locus_1
ACI
08990 HMM00465alcdh_3_mix(pf00465) PDU1D
08995 -pf13360pf13570 -
09000 -pf13360 -
09005 -# -
09010 -pf07719pf13174 -
09015 -pf01933 -
09020 -pf00208pf02812 -
09025 HMMaldol_1_grm(pf00596) ACI
09030 H_marine MUF2
09035 P_kiwi ACI
09040 P_dandelion ACI
09045 P_putty ACI
09050 -pf07499pf01330 -
09055 -pf05491pf05496 -
09060 -pf00293 -
09065 -# -
09070 -# -
09075 -# -
09080 -# -
09085 -pf05163 -
09095 -pf03352 -
09105 -# -
BMC-P
BMC-H
BMC-T
Aldehyde dehydrogenase
PTAC
Signature enzyme
Alcohol dehydrogenase
Regulator

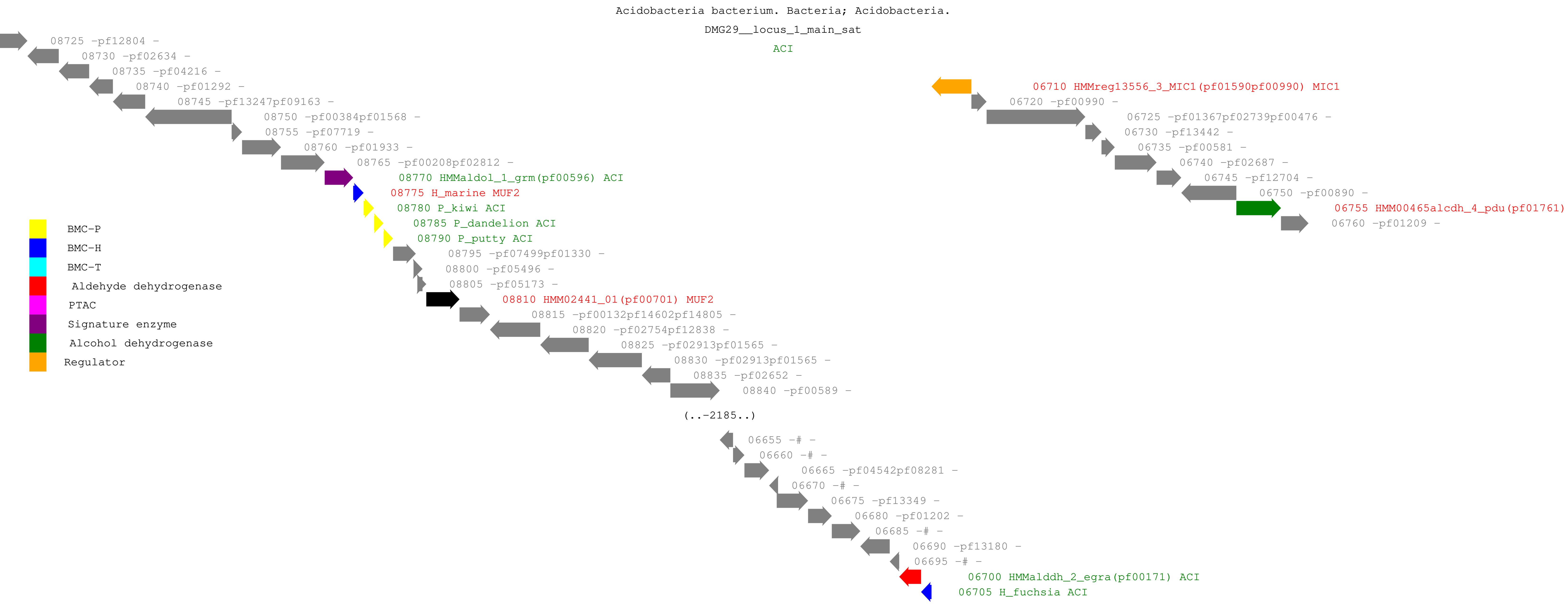
Acidobacteria bacterium. Bacteria; Acidobacteria.
DMG29__locus_1_main_sat
ACI
08725 -pf12804 -
08730 -pf02634 -
08735 -pf04216 -
08740 -pf01292 -
08745 -pf13247pf09163 -
08750 -pf00384pf01568 -
08755 -pf07719 -
08760 -pf01933 -
08765 -pf00208pf02812 -
08770 HMMaldol_1_grm(pf00596) ACI
08775 H_marine MUF2
08780 P_kiwi ACI
08785 P_dandelion ACI
08790 P_putty ACI
08795 -pf07499pf01330 -
08800 -pf05496 -
08805 -pf05173 -
08810 HMM02441_01(pf00701) MUF2
08815 -pf00132pf14602pf14805 -
08820 -pf02754pf12838 -
08825 -pf02913pf01565 -
08830 -pf02913pf01565 -
08835 -pf02652 -
08840 -pf00589 -
(..-2185..)
06655 -# -
06660 -# -
06665 -pf04542pf08281 -
06670 -# -
06675 -pf13349 -
06680 -pf01202 -
06685 -# -
06690 -pf13180 -
06695 -# -
06700 HMMaldh_2_egra(pf00171) ACI
06705 H_fuchsia ACI
06710 HMMreg13556_3_MIC1(pf01590pf00990) MIC1
06720 -pf00990 -
06725 -pf01367pf02739pf00476 -
06730 -pf13442 -
06735 -pf00581 -
06740 -pf02687 -
06745 -pf12704 -
06750 -pf00890 -
06755 HMM00465alcdh_4_pdu(pf01761)
06760 -pf01209 -
BMC-P
BMC-H
BMC-T
Aldehyde dehydrogenase
PTAC
Signature enzyme
Alcohol dehydrogenase
Regulator

Acidobacteria bacterium. Bacteria; Acidobacteria.

DMG57__locus_1_main_sat

ACI

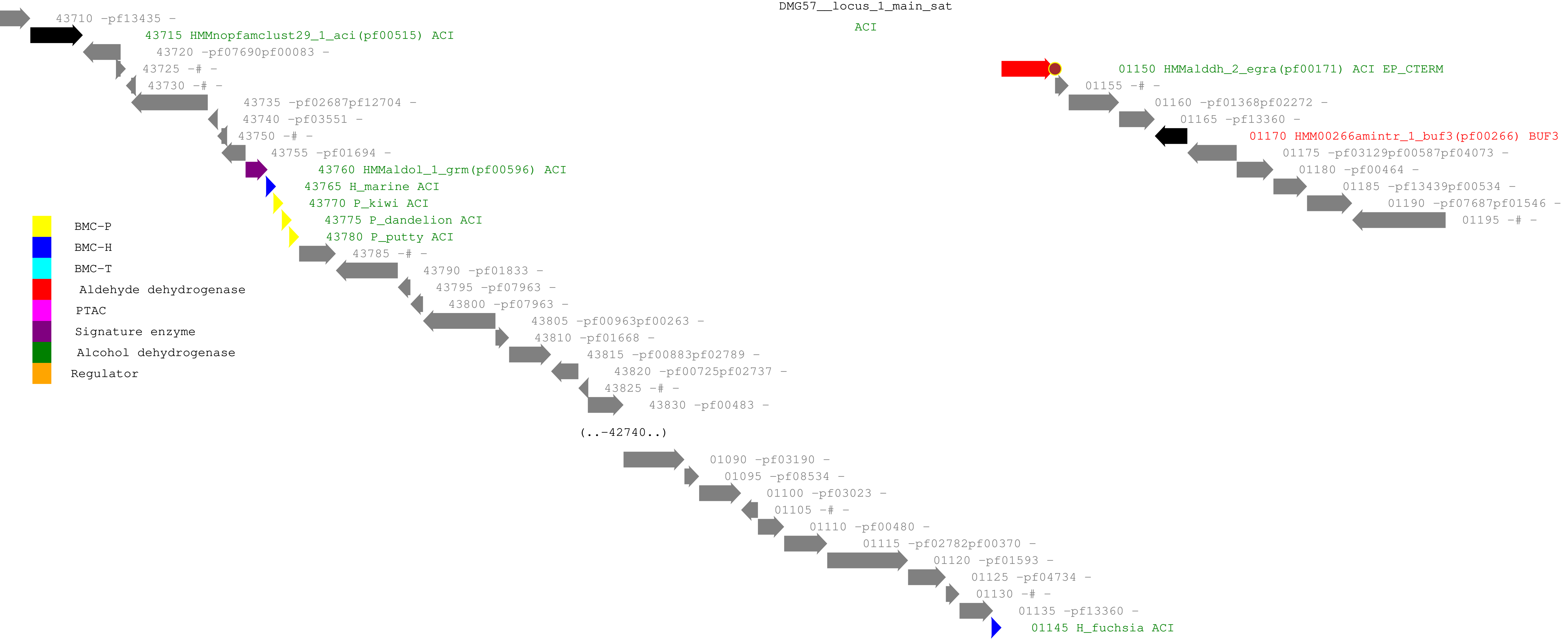

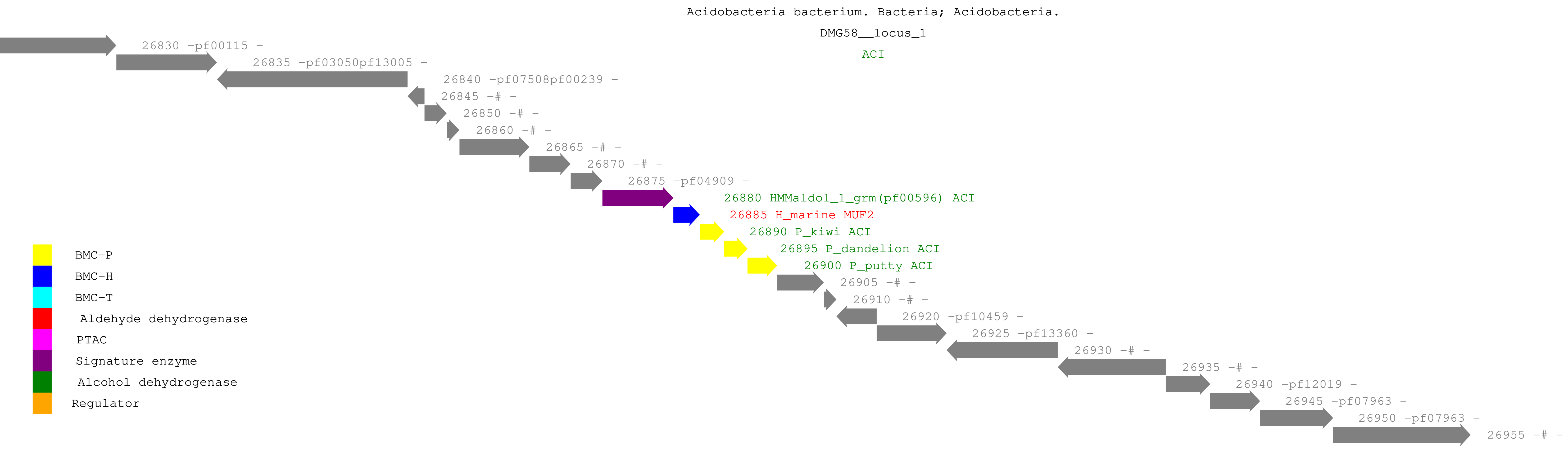
Acidobacteria bacterium. Bacteria; Acidobacteria.
DMG58__locus_1
ACI
26830 -pf00115 -
26835 -pf03050pf13005 -
26840 -pf07508pf00239 -
26845 -# -
26850 -# -
26860 -# -
26865 -# -
26870 -# -
26875 -pf04909 -
26880 HMMaldol_1_grm(pf00596) ACI
26885 H_marine MUF2
26890 P_kiwi ACI
26895 P_dandelion ACI
26900 P_putty ACI
26905 -# -
26910 -# -
26920 -pf10459 -
26925 -pf13360 -
26930 -# -
26935 -# -
26940 -pf12019 -
26945 -pf07963 -
26950 -pf07963 -
26955 -# -
BMC-P
BMC-H
BMC-T
Aldehyde dehydrogenase
PTAC
Signature enzyme
Alcohol dehydrogenase
Regulator

Acidobacteria bacterium. Bacteria; Acidobacteria.

DRJ14__locus_1_main_sat

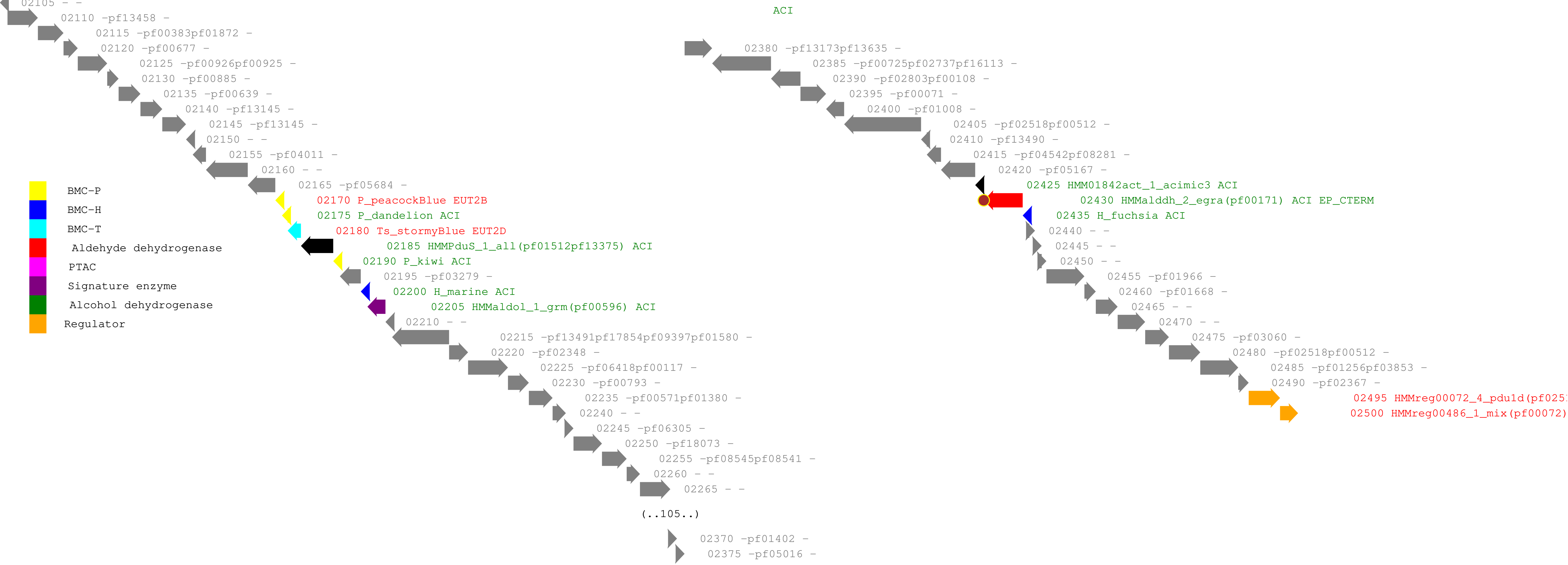

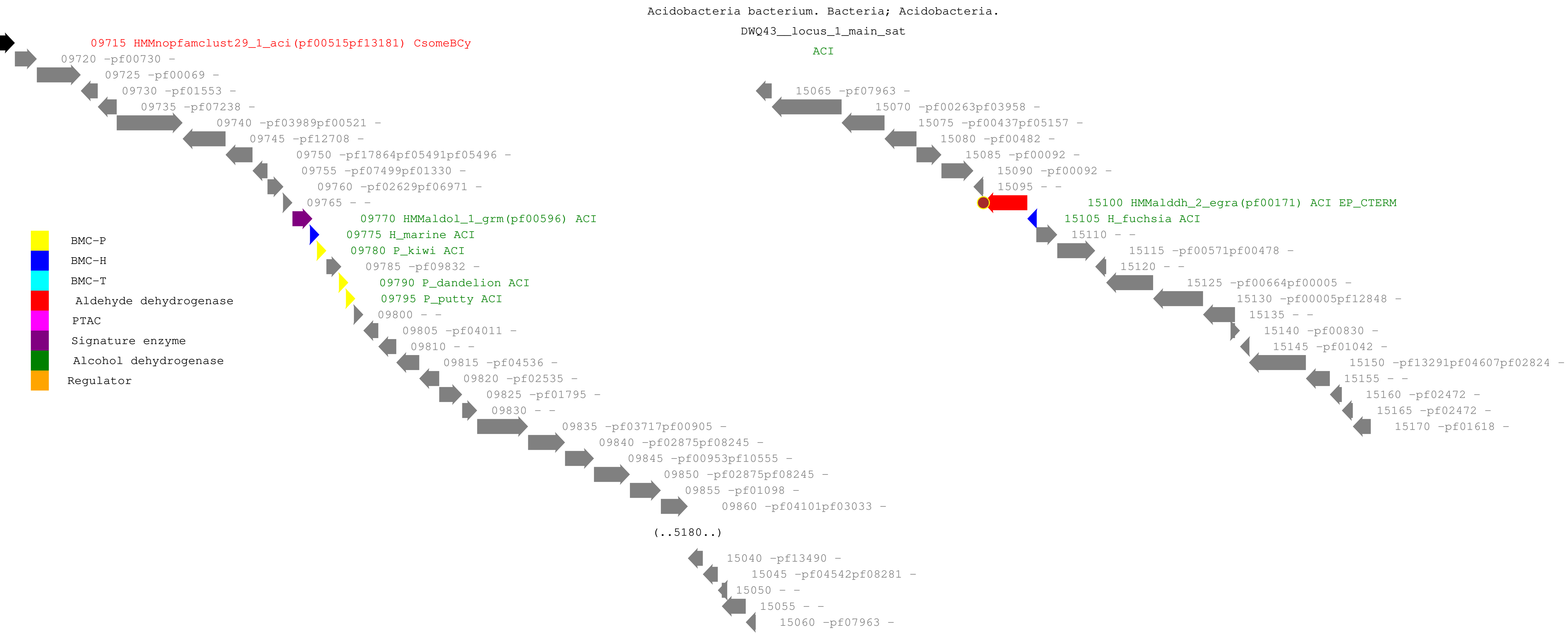
Acidobacteria bacterium. Bacteria; Acidobacteria.
DWQ43__locus_1_main_sat
ACI
09715 HMMnopfamclust29_1_aci(pf00515pf13181) CsomeBCy
09720 -pf00730 -
09725 -pf00069 -
09730 -pf01553 -
09735 -pf07238 -
09740 -pf03989pf00521 -
09745 -pf12708 -
09750 -pf17864pf05491pf05496 -
09755 -pf07499pf01330 -
09760 -pf02629pf06971 -
09765 - -
09770 HMMaldol_1_grm(pf00596) ACI
09775 H_marine ACI
09780 P_kiwi ACI
09785 -pf09832 -
09790 P_dandelion ACI
09795 P_putty ACI
09800 - -
09805 -pf04011 -
09810 - -
09815 -pf04536 -
09820 -pf02535 -
09825 -pf01795 -
09830 - -
09835 -pf03717pf00905 -
09840 -pf02875pf08245 -
09845 -pf00953pf10555 -
09850 -pf02875pf08245 -
09855 -pf01098 -
09860 -pf04101pf03033 -
(..5180..)
15040 -pf13490 -
15045 -pf04542pf08281 -
15050 - -
15055 - -
15060 -pf07963 -
15065 -pf07963 -
15070 -pf00263pf03958 -
15075 -pf00437pf05157 -
15080 -pf00482 -
15085 -pf00092 -
15090 -pf00092 -
15095 - -
15100 HMMalddh_2_egra(pf00171) ACI EP_CTERM
15105 H_fuchsia ACI
15110 - -
15115 -pf00571pf00478 -
15120 - -
15125 -pf00664pf00005 -
15130 -pf00005pf12848 -
15135 - -
15140 -pf00830 -
15145 -pf01042 -
15150 -pf13291pf04607pf02824 -
15155 - -
15160 -pf02472 -
15165 -pf02472 -
15170 -pf01618 -
BMC-P
BMC-H
BMC-T
Aldehyde dehydrogenase
PTAC
Signature enzyme
Alcohol dehydrogenase
Regulator

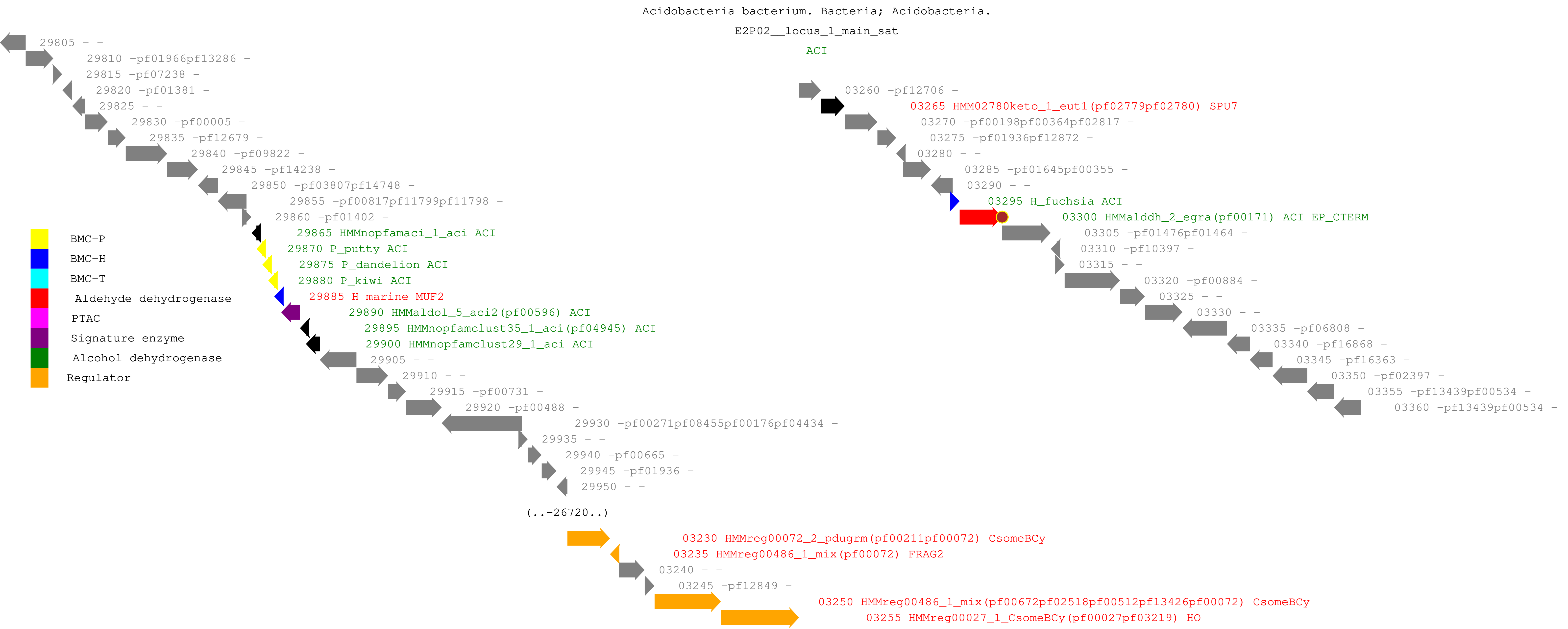
Acidobacteria bacterium. Bacteria; Acidobacteria.
E2P02__locus_1_main_sat
ACI
29805 - -
29810 -pf01966pf13286 -
29815 -pf07238 -
29820 -pf01381 -
29825 - -
29830 -pf00005 -
29835 -pf12679 -
29840 -pf09822 -
29845 -pf14238 -
29850 -pf03807pf14748 -
29855 -pf00817pf11799pf11798 -
29860 -pf01402 -
29865 HMMnopfamaci_1_aci ACI
29870 P_putty ACI
29875 P_dandelion ACI
29880 P_kiwi ACI
29885 H_marine MUF2
29890 HMMaldol_5_aci2(pf00596) ACI
29895 HMMnopfamclust35_1_aci(pf04945) ACI
29900 HMMnopfamclust29_1_aci ACI
29905 - -
29910 - -
29915 -pf00731 -
29920 -pf00488 -
29930 -pf00271pf08455pf00176pf04434 -
29935 - -
29940 -pf00665 -
29945 -pf01936 -
29950 - -
(..-26720..)
03230 HMMreg00072_2_pdugrm(pf00211pf00072) CsomeBCy
03235 HMMreg00486_1_mix(pf00072) FRAG2
03240 - -
03245 -pf12849 -
03250 HMMreg00486_1_mix(pf00672pf02518pf00512pf13426pf00072) CsomeBCy
03255 HMMreg00027_1_CsomeBCy(pf00027pf03219) HO
03260 -pf12706 -
03265 HMM02780keto_1_eut1(pf02779pf02780) SPU7
03270 -pf00198pf00364pf02817 -
03275 -pf01936pf12872 -
03280 - -
03285 -pf01645pf00355 -
03290 - -
03295 H_fuchsia ACI
03300 HMMalddh_2_egra(pf00171) ACI EP_CTERM
03305 -pf01476pf01464 -
03310 -pf10397 -
03315 - -
03320 -pf00884 -
03325 - -
03330 - -
03335 -pf06808 -
03340 -pf16868 -
03345 -pf16363 -
03350 -pf02397 -
03355 -pf13439pf00534 -
03360 -pf13439pf00534 -
BMC-P
BMC-H
BMC-T
Aldehyde dehydrogenase
PTAC
Signature enzyme
Alcohol dehydrogenase
Regulator

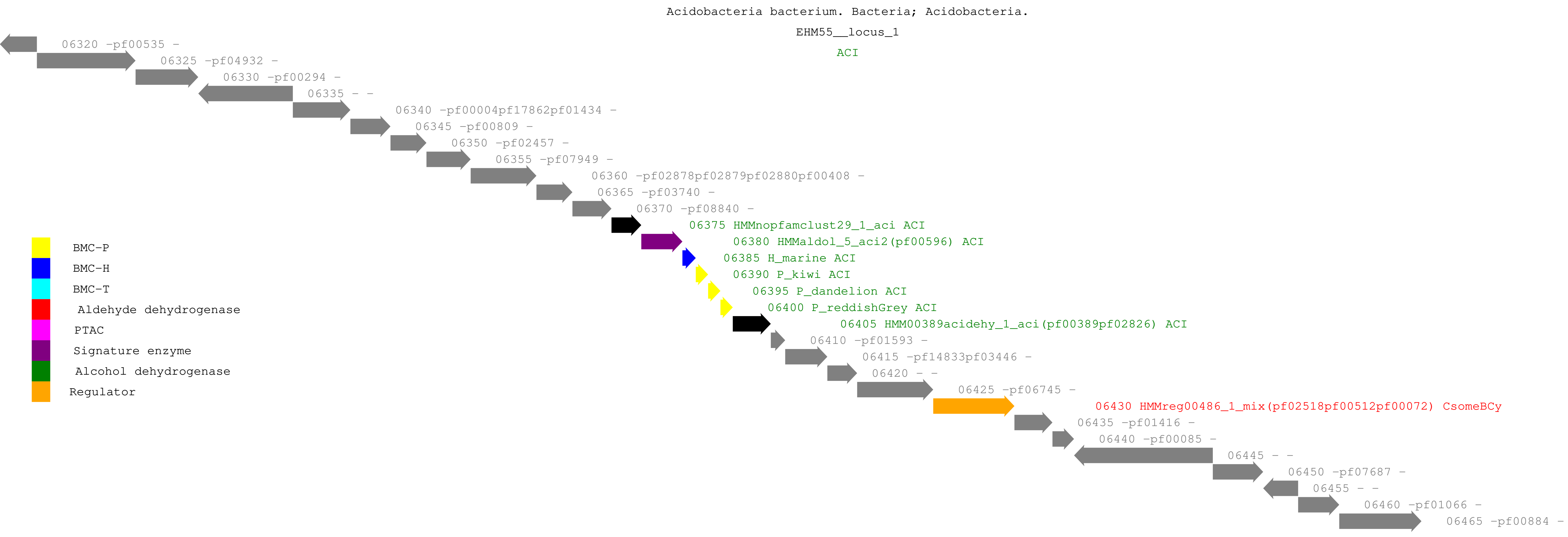

Acidobacteria bacterium. Bacteria; Acidobacteria.
EHM55__locus_1
ACI
06320 -pf00535 -
06325 -pf04932 -
06330 -pf00294 -
06335 - -
06340 -pf00004pf17862pf01434 -
06345 -pf00809 -
06350 -pf02457 -
06355 -pf07949 -
06360 -pf02878pf02879pf02880pf00408 -
06365 -pf03740 -
06370 -pf08840 -
06375 HMMnopfamclust29_1_aci ACI
06380 HMMaldol_5_aci2(pf00596) ACI
06385 H_marine ACI
06390 P_kiwi ACI
06395 P_dandelion ACI
06400 P_reddishGrey ACI
06405 HMM00389acidehy_1_aci(pf00389pf02826) ACI
06410 -pf01593 -
06415 -pf14833pf03446 -
06420 - -
06425 -pf06745 -
06430 HMMreg00486_1_mix(pf02518pf00512pf00072) CsomeBCy
06435 -pf01416 -
06440 -pf00085 -
06445 - -
06450 -pf07687 -
06455 - -
06460 -pf01066 -
06465 -pf00884 -
BMC-P
BMC-H
BMC-T
Aldehyde dehydrogenase
PTAC
Signature enzyme
Alcohol dehydrogenase
Regulator

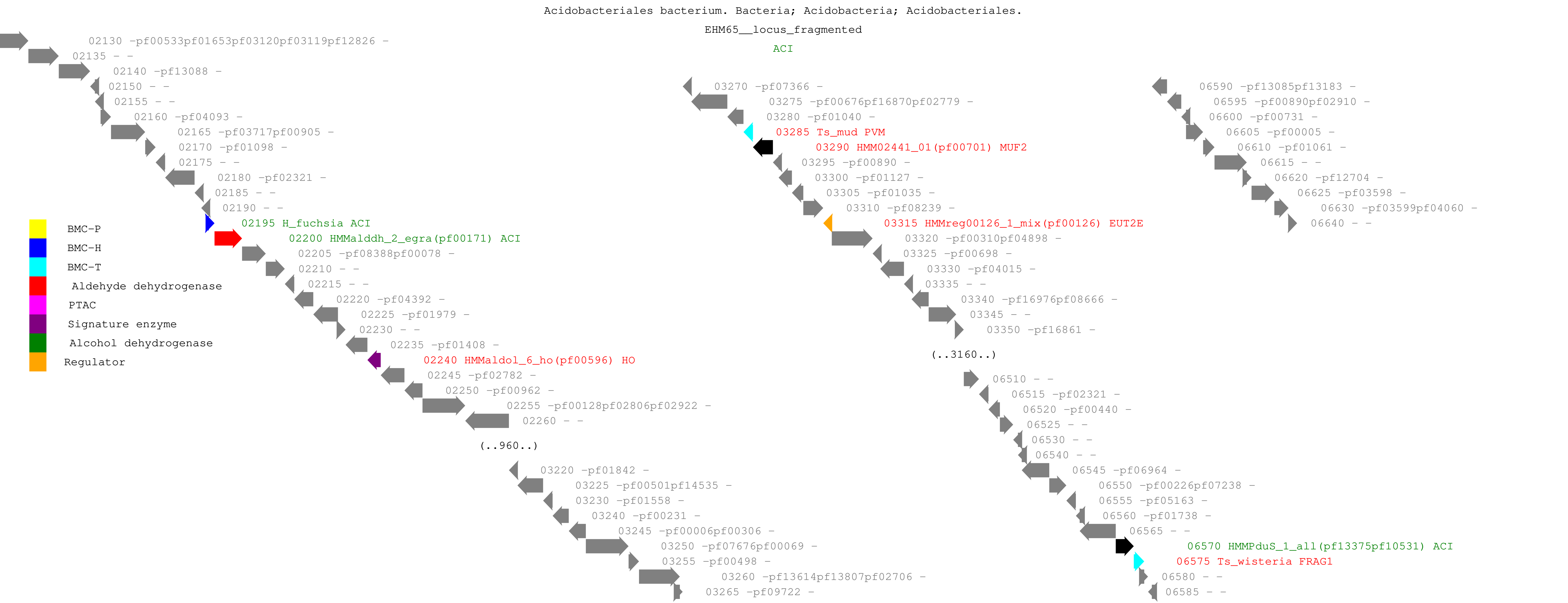

Acidobacteriales bacterium. Bacteria; Acidobacteria; Acidobacteriales.
EHM65__locus_fragmented
ACI
02130 -pf00533pf01653pf03120pf03119pf12826 -
02135 - -
02140 -pf13088 -
02150 - -
02155 - -
02160 -pf04093 -
02165 -pf03717pf00905 -
02170 -pf01098 -
02175 - -
02180 -pf02321 -
02185 - -
02190 - -
02195 H_fuchsia ACI
02200 HMMalddh_2_egra(pf00171) ACI
02205 -pf08388pf00078 -
02210 - -
02215 - -
02220 -pf04392 -
02225 -pf01979 -
02230 - -
02235 -pf01408 -
02240 HMMaldol_6_ho(pf00596) HO
02245 -pf02782 -
02250 -pf00962 -
02255 -pf00128pf02806pf02922 -
02260 - -
(..960..)
03220 -pf01842 -
03225 -pf00501pf14535 -
03230 -pf01558 -
03240 -pf00231 -
03245 -pf00006pf00306 -
03250 -pf07676pf00069 -
03255 -pf00498 -
03260 -pf13614pf13807pf02706 -
03265 -pf09722 -
03270 -pf07366 -
03275 -pf00676pf16870pf02779 -
03280 -pf01040 -
03285 Ts_mud PVM
03290 HMM02441_01(pf00701) MUF2
03295 -pf00890 -
03300 -pf01127 -
03305 -pf01035 -
03310 -pf08239 -
03315 HMMreg00126_1_mix(pf00126) EUT2E
03320 -pf00310pf04898 -
03325 -pf00698 -
03330 -pf04015 -
03335 - -
03340 -pf16976pf08666 -
03345 - -
03350 -pf16861 -
(..3160..)
06510 - -
06515 -pf02321 -
06520 -pf00440 -
06525 - -
06530 - -
06540 - -
06545 -pf06964 -
06550 -pf00226pf07238 -
06555 -pf05163 -
06560 -pf01738 -
06565 - -
06570 HMMPduS_1_all(pf13375pf10531) ACI
06575 Ts_wisteria FRAG1
06580 - -
06585 - -
06590 -pf13085pf13183 -
06595 -pf00890pf02910 -
06600 -pf00731 -
06605 -pf00005 -
06610 -pf01061 -
06615 - -
06620 -pf12704 -
06625 -pf03598 -
06630 -pf03599pf04060 -
06640 - -
BMC-P
BMC-H
BMC-T
Aldehyde dehydrogenase
PTAC
Signature enzyme
Alcohol dehydrogenase
Regulator

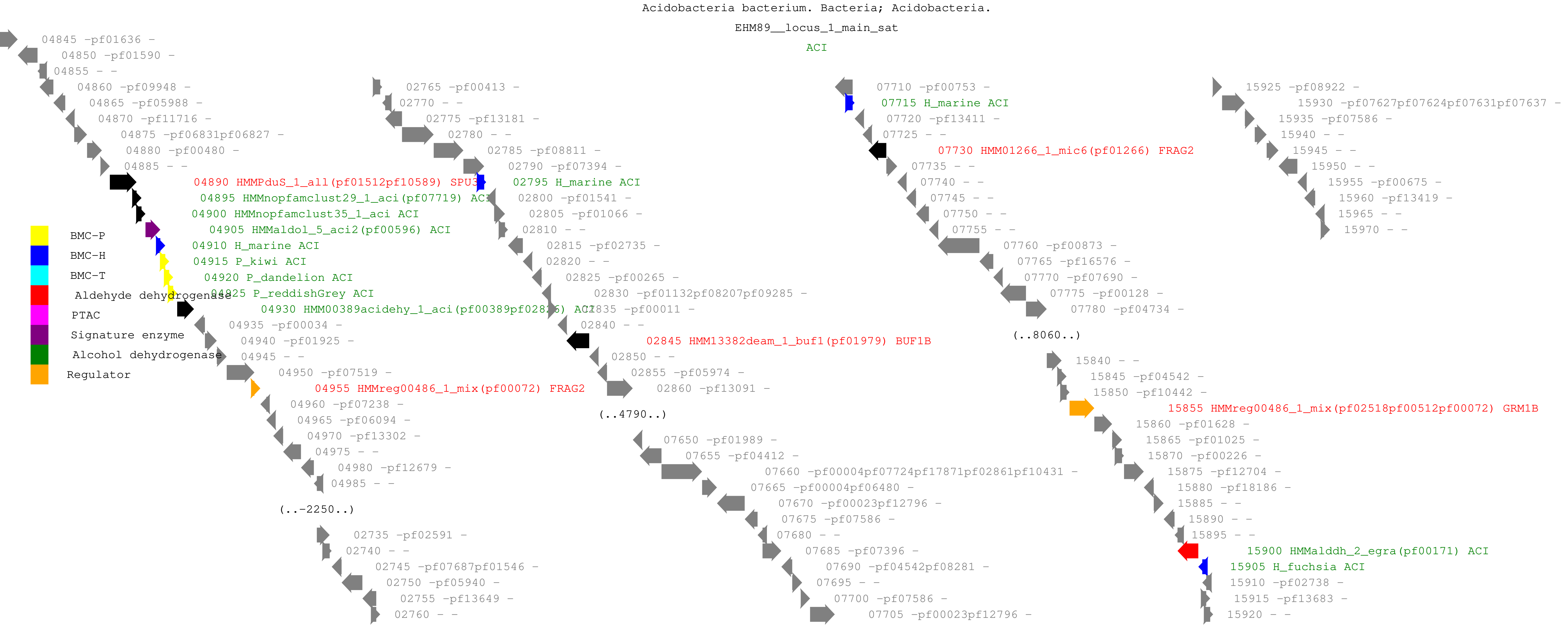
Acidobacteria bacterium. Bacteria; Acidobacteria.
EHM89__locus_1_main_sat
ACI
BMC-P
BMC-H
BMC-T
Aldehyde dehydrogenase
PTAC
Signature enzyme
Alcohol dehydrogenase
Regulator
04845 -pf01636 -
04850 -pf01590 -
04855 - -
04860 -pf09948 -
04865 -pf05988 -
04870 -pf11716 -
04875 -pf06831pf06827 -
04880 -pf00480 -
04885 - -
04890 HMMPduS_1_all(pf01512pf10589) SPU3
04895 HMMnopfamclust29_1_aci(pf07719) ACI
04900 HMMnopfamclust35_1_aci ACI
04905 HMMaldol_5_aci2(pf00596) ACI
04910 H_marine ACI
04915 P_kiwi ACI
04920 P_dandelion ACI
04925 P_reddishGrey ACI
04930 HMM00389acidehy_1_aci(pf00389pf02826) ACI
04935 -pf00034 -
04940 -pf01925 -
04945 - -
04950 -pf07519 -
04955 HMMreg00486_1_mix(pf00072) FRAG2
04960 -pf07238 -
04965 -pf06094 -
04970 -pf13302 -
04975 - -
04980 -pf12679 -
04985 - -
(..-2250..)
02735 -pf02591 -
02740 - -
02745 -pf07687pf01546 -
02750 -pf05940 -
02755 -pf13649 -
02760 - -
02765 -pf00413 -
02770 - -
02775 -pf13181 -
02780 - -
02785 -pf08811 -
02790 -pf07394 -
02795 H_marine ACI
02800 -pf01541 -
02805 -pf01066 -
02810 - -
02815 -pf02735 -
02820 - -
02825 -pf00265 -
02830 -pf01132pf08207pf09285 -
02835 -pf00011 -
02840 - -
02845 HMM13382deam_1_buf1(pf01979) BUF1B
02850 - -
02855 -pf05974 -
02860 -pf13091 -
(..4790..)
07650 -pf01989 -
07655 -pf04412 -
07660 -pf00004pf07724pf17871pf02861pf10431 -
07665 -pf00004pf06480 -
07670 -pf00023pf12796 -
07675 -pf07586 -
07680 - -
07685 -pf07396 -
07690 -pf04542pf08281 -
07695 - -
07700 -pf07586 -
07705 -pf00023pf12796 -
07710 -pf00753 -
07715 H_marine ACI
07720 -pf13411 -
07725 - -
07730 HMM01266_1_mic6(pf01266) FRAG2
07735 - -
07740 - -
07745 - -
07750 - -
07755 - -
07760 -pf00873 -
07765 -pf16576 -
07770 -pf07690 -
07775 -pf00128 -
07780 -pf04734 -
(..8060..)
15840 - -
15845 -pf04542 -
15850 -pf10442 -
15855 HMMreg00486_1_mix(pf02518pf00512pf00072) GRM1B
15860 -pf01628 -
15865 -pf01025 -
15870 -pf00226 -
15875 -pf12704 -
15880 -pf18186 -
15885 - -
15890 - -
15895 - -
15900 HMMalddh_2_egra(pf00171) ACI
15905 H_fuchsia ACI
15910 -pf02738 -
15915 -pf13683 -
15920 - -
15925 -pf08922 -
15930 -pf07627pf07624pf07631pf07637 -
15935 -pf07586 -
15940 - -
15945 - -
15950 - -
15955 -pf00675 -
15960 -pf13419 -
15965 - -
15970 - -

Acidobacteria bacterium. Bacteria; Acidobacteria.

EPN33__locus_1_main_sat

ACI

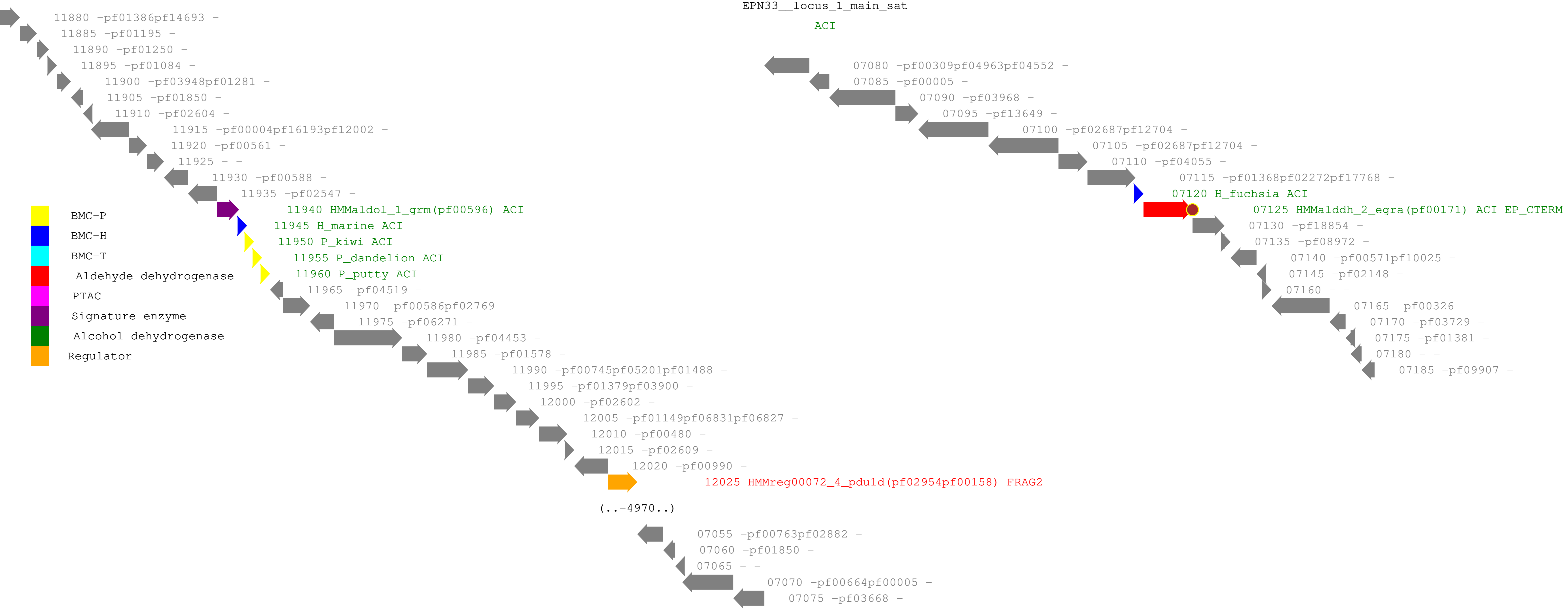

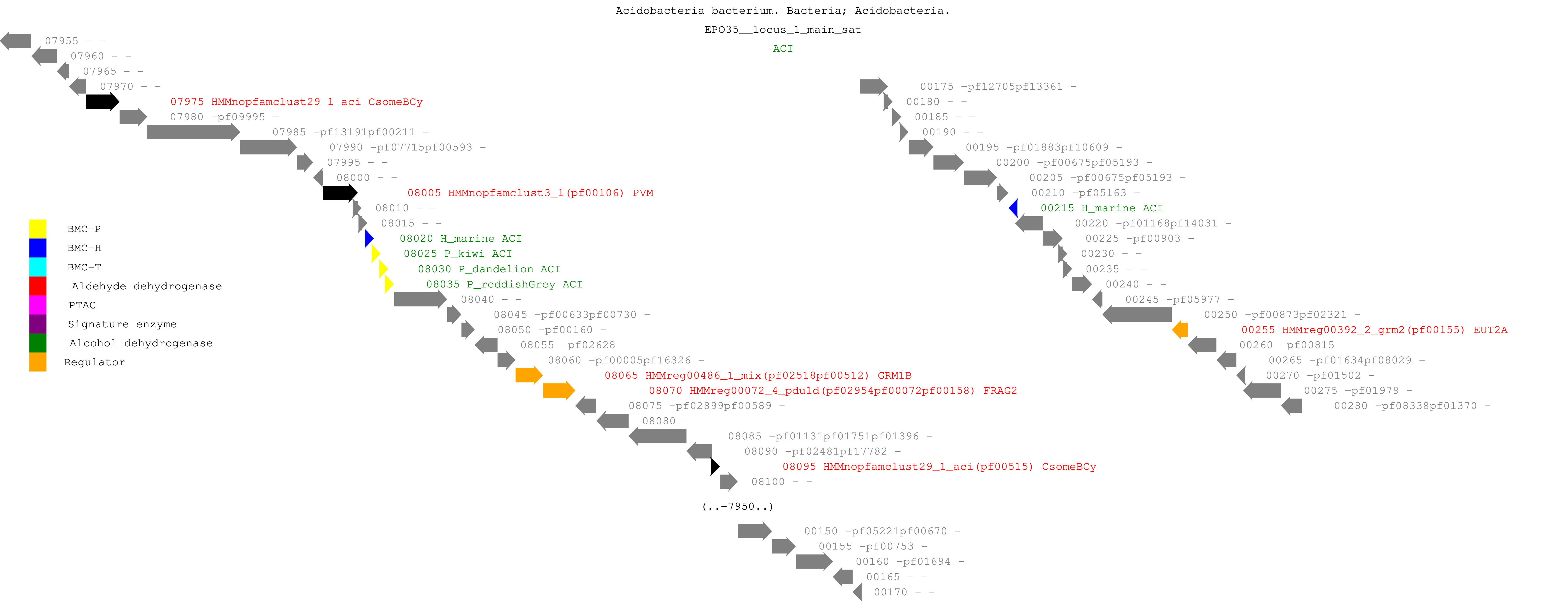

Acidobacteria bacterium. Bacteria; Acidobacteria.
EPO35__locus_1_main_sat
ACI
07955 - -
07960 - -
07965 - -
07970 - -
07975 HMMnopfamclust29_1_aci CsomeBCy
07980 -pf09995 -
07985 -pf13191pf00211 -
07990 -pf07715pf00593 -
07995 - -
08000 - -
08005 HMMnopfamclust3_1(pf00106) PVM
08010 - -
08015 - -
08020 H_marine ACI
08025 P_kiwi ACI
08030 P_dandelion ACI
08035 P_reddishGrey ACI
08040 - -
08045 -pf00633pf00730 -
08050 -pf00160 -
08055 -pf02628 -
08060 -pf00005pf16326 -
08065 HMMreg00486_1_mix(pf02518pf00512) GRM1B
08070 HMMreg00072_4_pdu1d(pf02954pf00072pf00158) FRAG2
08075 -pf02899pf00589 -
08080 - -
08085 -pf01131pf01751pf01396 -
08090 -pf02481pf17782 -
08095 HMMnopfamclust29_1_aci(pf00515) CsomeBCy
08100 - -
(..-7950..)
00150 -pf05221pf00670 -
00155 -pf00753 -
00160 -pf01694 -
00165 - -
00170 - -
00175 -pf12705pf13361 -
00180 - -
00185 - -
00190 - -
00195 -pf01883pf10609 -
00200 -pf00675pf05193 -
00205 -pf00675pf05193 -
00210 -pf05163 -
00215 H_marine ACI
00220 -pf01168pf14031 -
00225 -pf00903 -
00230 - -
00235 - -
00240 - -
00245 -pf05977 -
00250 -pf00873pf02321 -
00255 HMMreg00392_2_grm2(pf00155) EUT2A
00260 -pf00815 -
00265 -pf01634pf08029 -
00270 -pf01502 -
00275 -pf01979 -
00280 -pf08338pf01370 -
BMC-P
BMC-H
BMC-T
Aldehyde dehydrogenase
PTAC
Signature enzyme
Alcohol dehydrogenase
Regulator

Acidobacteria bacterium DSM 100886. Bacteria; Acidobacteria; Acidobacteria subdivision 6.

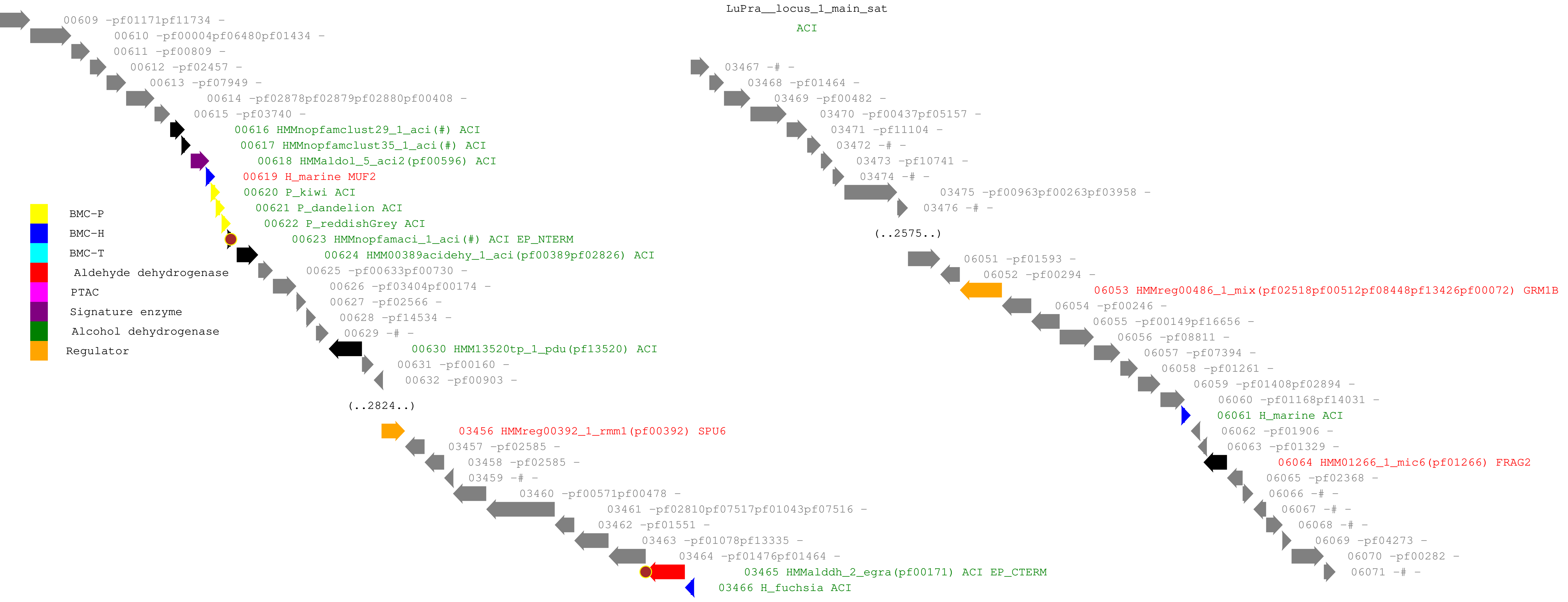

Pyrinomonas methylaliphatogenes. Bacteria; Acidobacteria; Blastocatellia; Pyrinomonas.

PYK22__locus_1_main_sat

ACI

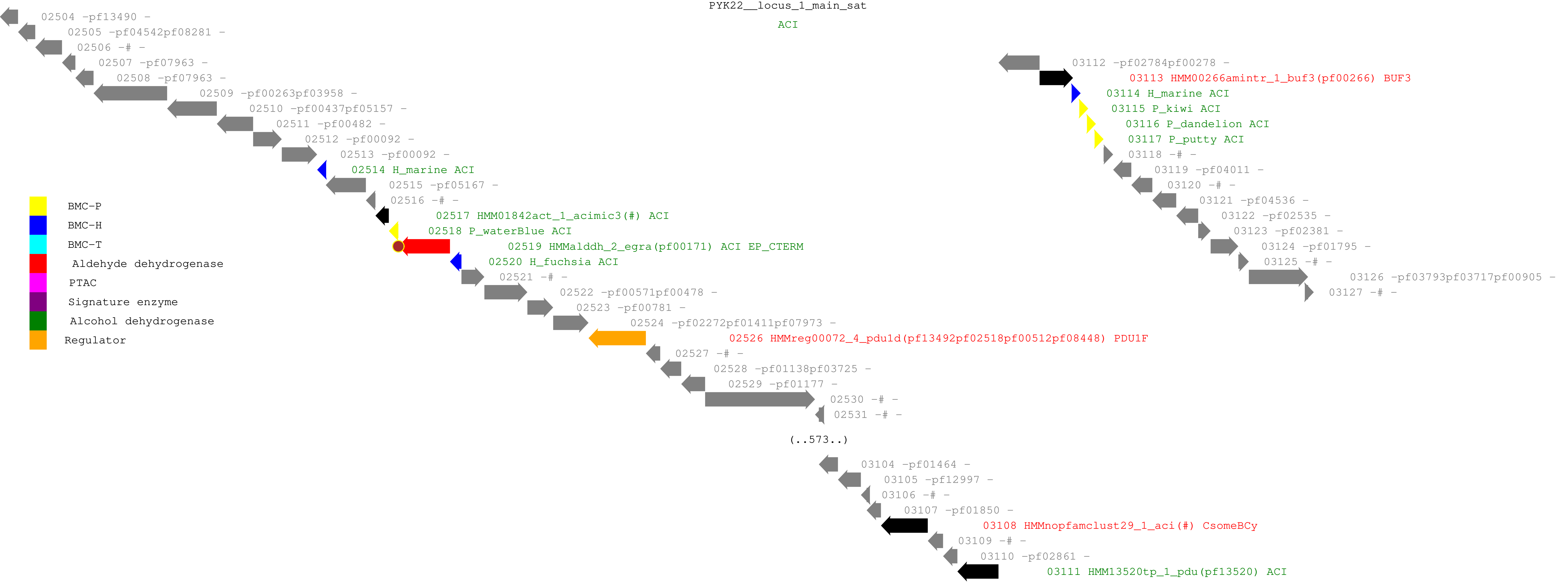

Candidatus Sulfopaludibacter sp. SbA3. Bacteria; Acidobacteria; Solibacteres; Solibacterales;

SBA3__locus_1_main_sat

ACI

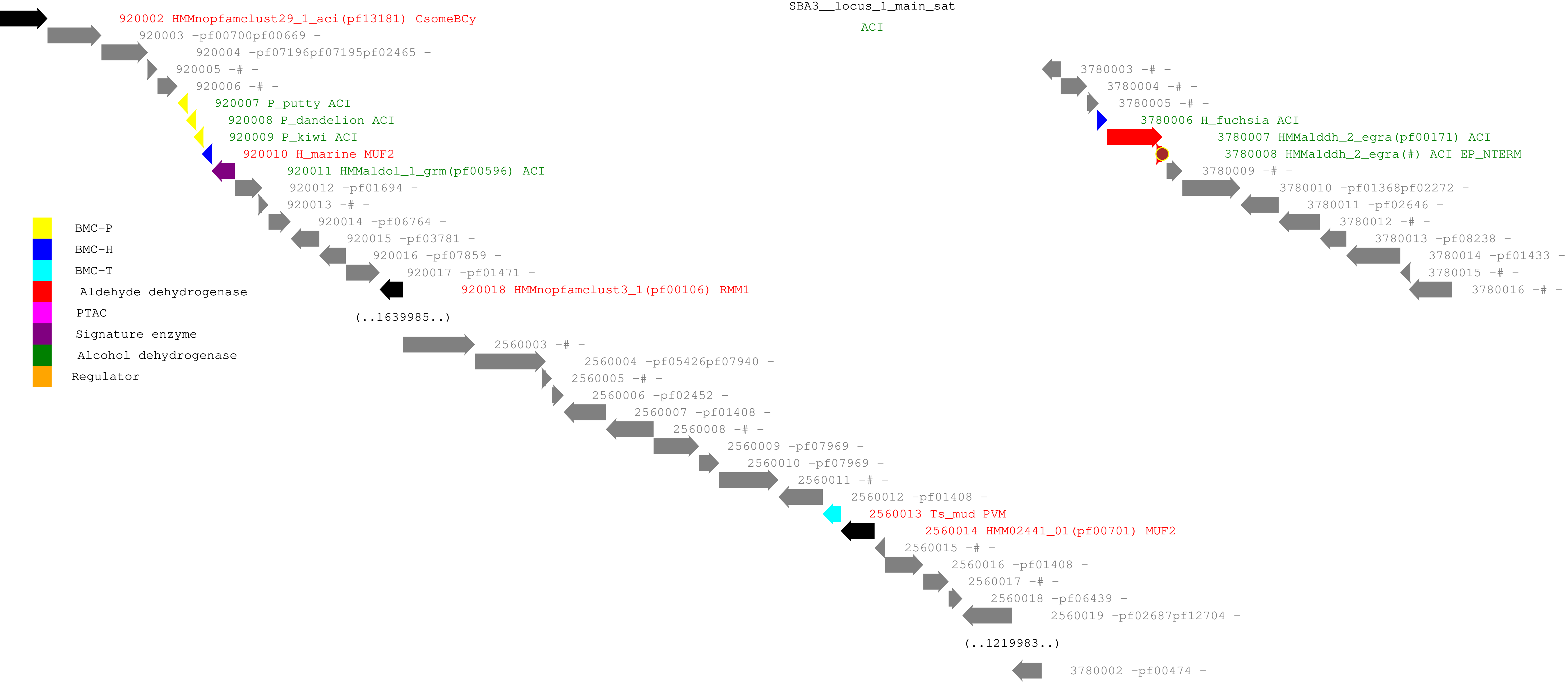

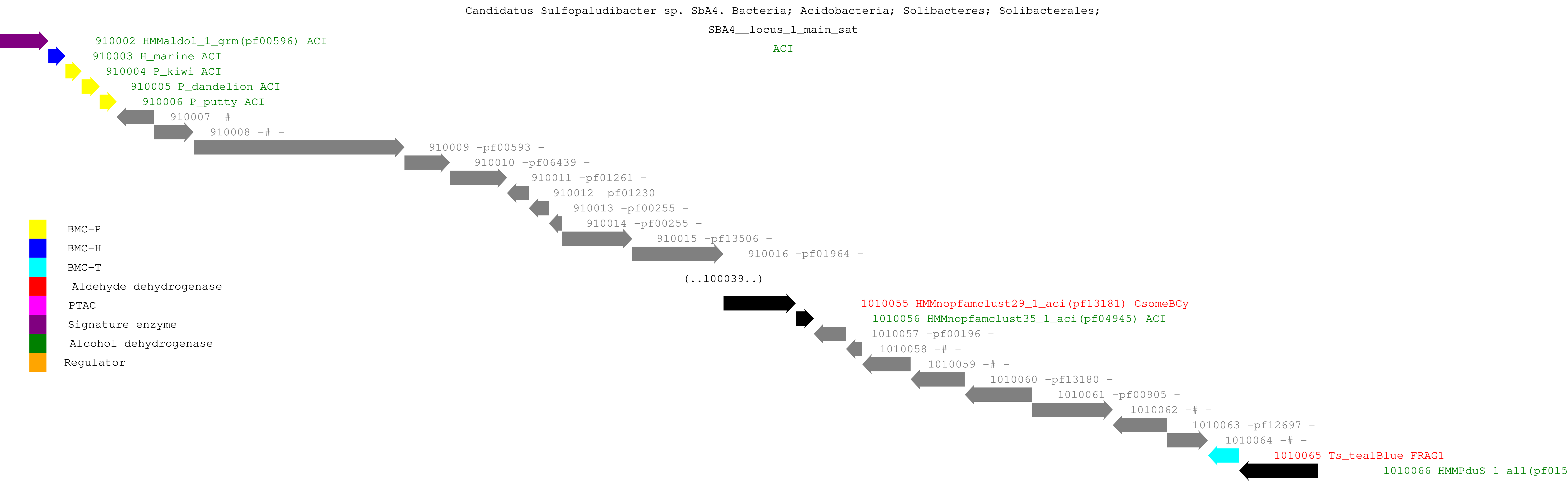
Candidatus Sulfopaludibacter sp. SbA4. Bacteria; Acidobacteria; Solibacteres; Solibacterales;
SBA4__locus_1_main_sat
ACI
910002 HMMaldol_1_grm(pf00596) ACI
910003 H_marine ACI
910004 P_kiwi ACI
910005 P_dandelion ACI
910006 P_putty ACI
910007 -# -
910008 -# -
910009 -pf00593 -
910010 -pf06439 -
910011 -pf01261 -
910012 -pf01230 -
910013 -pf00255 -
910014 -pf00255 -
910015 -pf13506 -
910016 -pf01964 -
(..100039..)
1010055 HMMnopfamclust29_1_aci(pf13181) CsomeBCy
1010056 HMMnopfamclust35_1_aci(pf04945) ACI
1010057 -pf00196 -
1010058 -# -
1010059 -# -
1010060 -pf13180 -
1010061 -pf00905 -
1010062 -# -
1010063 -pf12697 -
1010064 -# -
1010065 Ts_tealBlue FRAG1
1010066 HMMPduS_1_all(pf015
BMC-P
BMC-H
BMC-T
Aldehyde dehydrogenase
PTAC
Signature enzyme
Alcohol dehydrogenase
Regulator

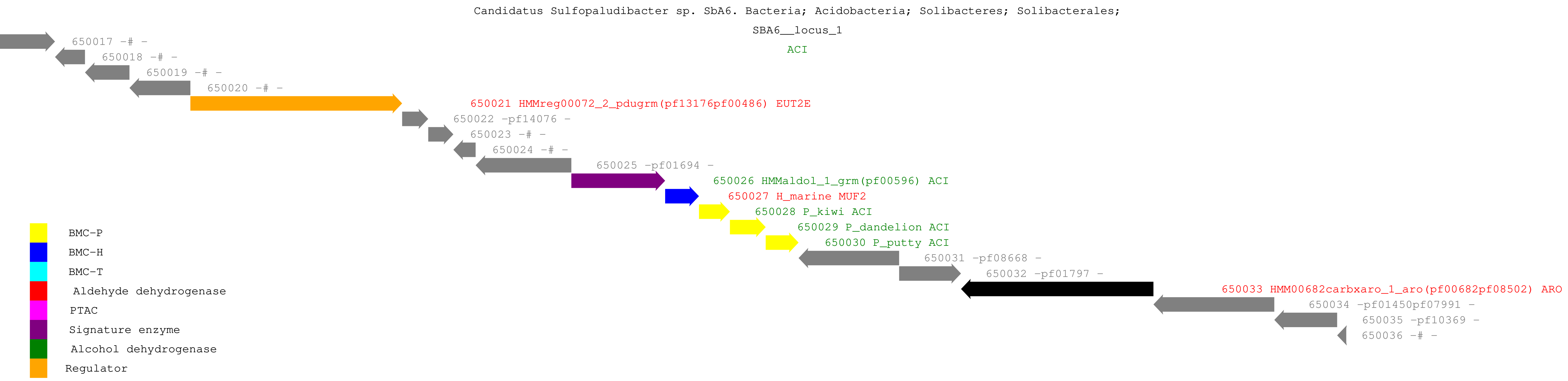

Candidatus Sulfopaludibacter sp. SbA6. Bacteria; Acidobacteria; Solibacteres; Solibacterales;
SBA6__locus_1
ACI
650017 -# -
650018 -# -
650019 -# -
650020 -# -
650021 HMMreg00072_2_pdugrm(pf13176pf00486) EUT2E
650022 -pf14076 -
650023 -# -
650024 -# -
650025 -pf01694 -
650026 HMMaldol_1_grm(pf00596) ACI
650027 H_marine MUF2
650028 P_kiwi ACI
650029 P_dandelion ACI
650030 P_putty ACI
650031 -pf08668 -
650032 -pf01797 -
650033 HMM00682carbxaro_1_aro(pf00682pf08502) ARO
650034 -pf01450pf07991 -
650035 -pf10369 -
650036 -# -
BMC-P
BMC-H
BMC-T
Aldehyde dehydrogenase
PTAC
Signature enzyme
Alcohol dehydrogenase
Regulator

Acidobacteria bacterium OLB17. Bacteria; Acidobacteria.

UZ17_ACD_locus_1_main_sat

ACI

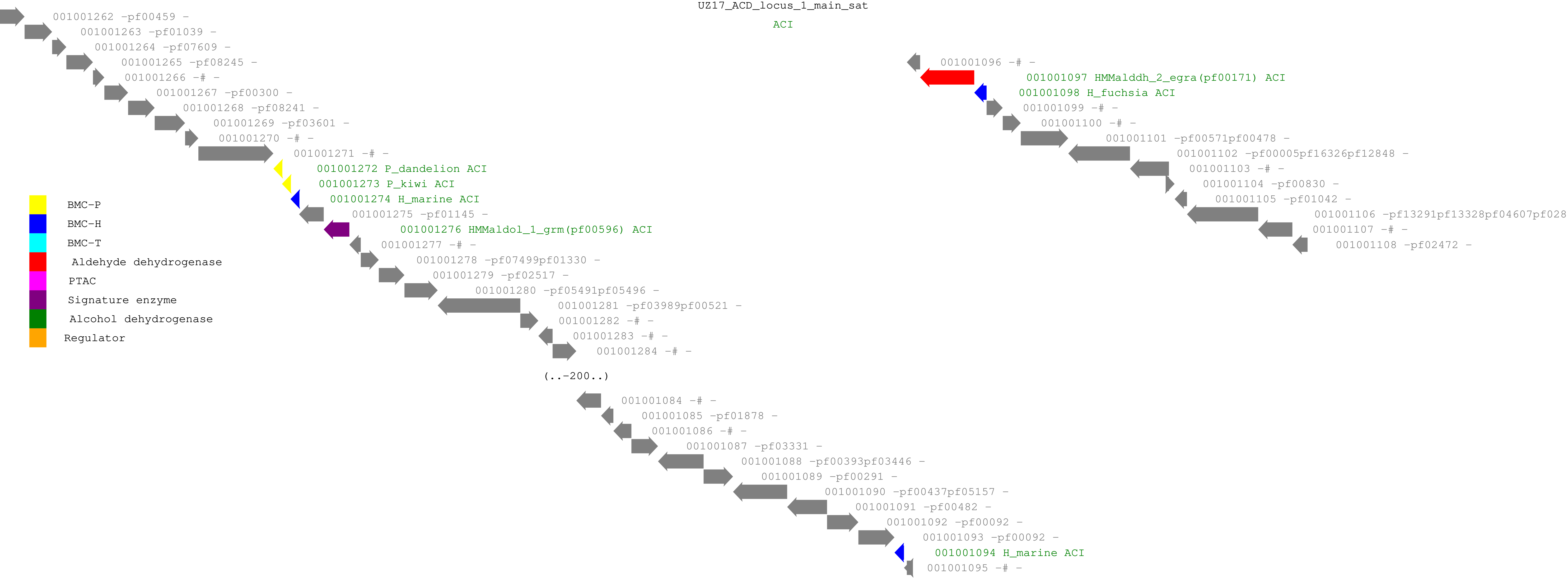

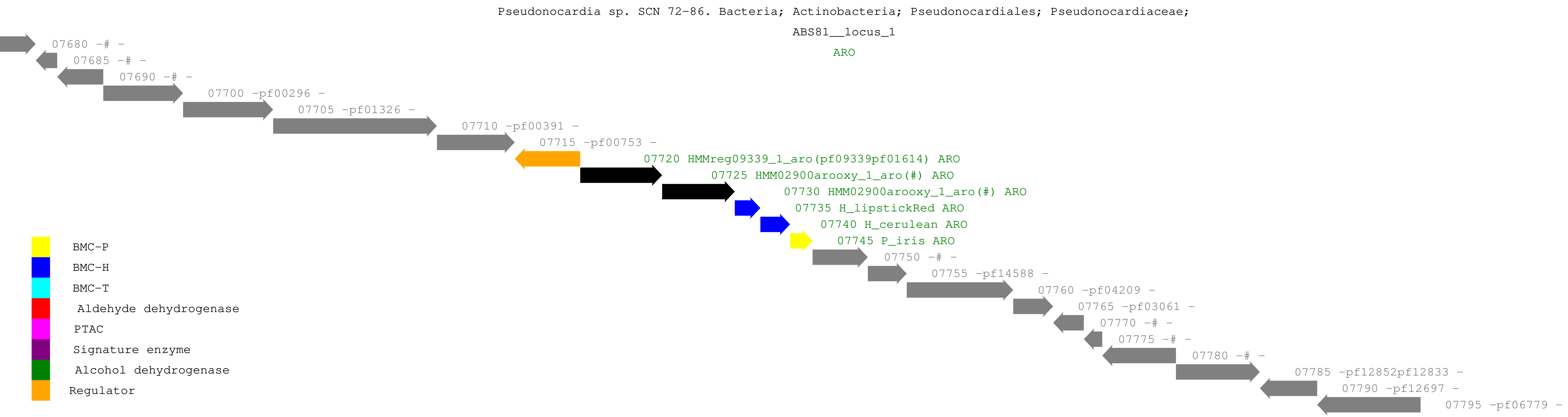
Pseudonocardia sp. SCN 72-86. Bacteria; Actinobacteria; Pseudonocardiales; Pseudonocardiaceae;
ABS81__locus_1
ARO
07680 -# -
07685 -# -
07690 -# -
07700 -pf00296 -
07705 -pf01326 -
07710 -pf00391 -
07715 -pf00753 -
07720 HMMreg09339_1_aro(pf09339pf01614) ARO
07725 HMM02900arooxy_1_aro(#) ARO
07730 HMM02900arooxy_1_aro(#) ARO
07735 H_lipstickRed ARO
07740 H_cerulean ARO
07745 P_iris ARO
07750 -# -
07755 -pf14588 -
07760 -pf04209 -
07765 -pf03061 -
07770 -# -
07775 -# -
07780 -# -
07785 -pf12852pf12833 -
07790 -pf12697 -
07795 -pf06779 -
BMC-P
BMC-H
BMC-T
Aldehyde dehydrogenase
PTAC
Signature enzyme
Alcohol dehydrogenase
Regulator

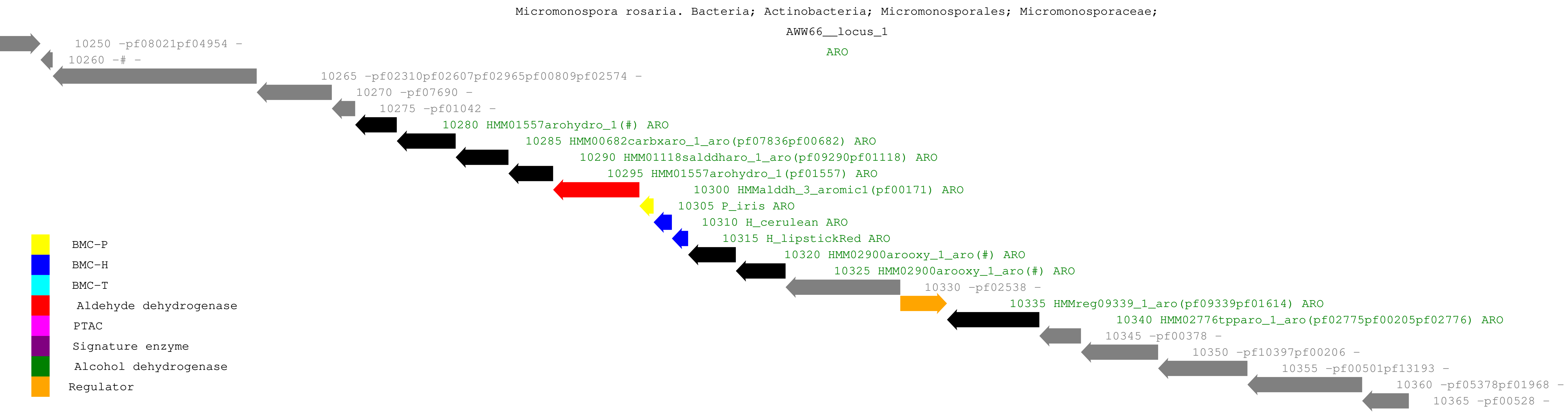
Micromonospora rosaria. Bacteria; Actinobacteria; Micromonosporales; Micromonosporaceae;
AWW66__locus_1
ARO
10250 -pf08021pf04954 -
10260 -# -
10265 -pf02310pf02607pf02965pf00809pf02574 -
10270 -pf07690 -
10275 -pf01042 -
10280 HMM01557arohydro_1(#) ARO
10285 HMM00682carbxaro_1_aro(pf07836pf00682) ARO
10290 HMM01118salddharo_1_aro(pf09290pf01118) ARO
10295 HMM01557arohydro_1(pf01557) ARO
10300 HMMalddh_3_aromic1(pf00171) ARO
10305 P_iris ARO
10310 H_cerulean ARO
10315 H_lipstickRed ARO
10320 HMM02900arooxy_1_aro(#) ARO
10325 HMM02900arooxy_1_aro(#) ARO
10330 -pf02538 -
10335 HMMreg09339_1_aro(pf09339pf01614) ARO
10340 HMM02776tppparo_1_aro(pf02775pf00205pf02776) ARO
10345 -pf00378 -
10350 -pf10397pf00206 -
10355 -pf00501pf13193 -
10360 -pf05378pf01968 -
10365 -pf00528 -
BMC-P
BMC-H
BMC-T
Aldehyde dehydrogenase
PTAC
Signature enzyme
Alcohol dehydrogenase
Regulator

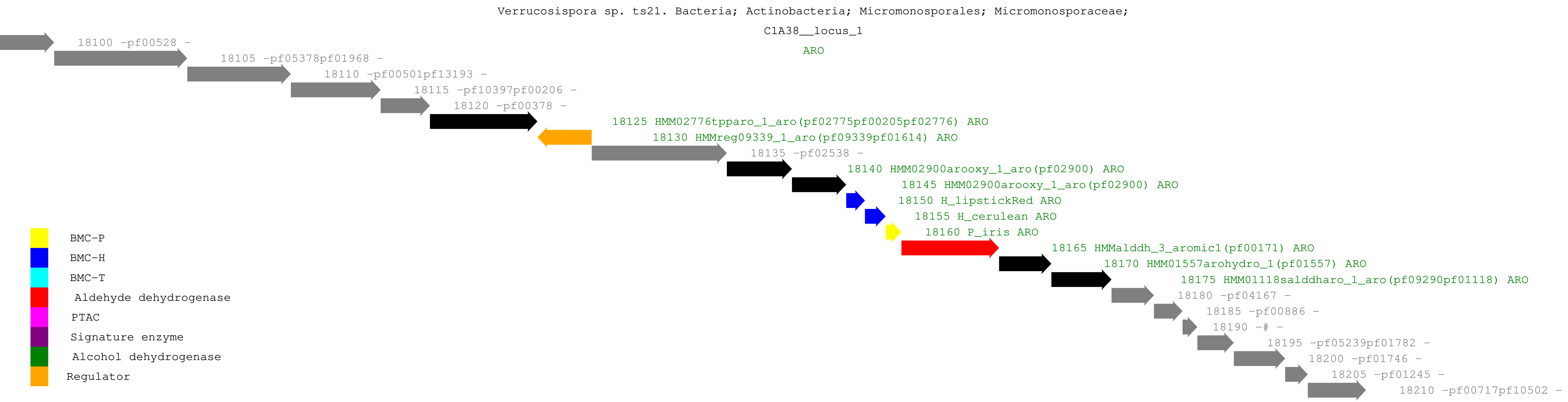

Verrucosispora sp. ts21. Bacteria; Actinobacteria; Micromonosporales; Micromonosporaceae;
C1A38__locus_1
ARO
18100 -pf00528 -
18105 -pf05378pf01968 -
18110 -pf00501pf13193 -
18115 -pf10397pf00206 -
18120 -pf00378 -
18125 HMM02776tpparo_1_aro(pf02775pf00205pf02776) ARO
18130 HMMreg09339_1_aro(pf09339pf01614) ARO
18135 -pf02538 -
18140 HMM02900arooxy_1_aro(pf02900) ARO
18145 HMM02900arooxy_1_aro(pf02900) ARO
18150 H_lipstickRed ARO
18155 H_cerulean ARO
18160 P_iris ARO
18165 HMMalddh_3_aromic1(pf00171) ARO
18170 HMM01557arohydro_1(pf01557) ARO
18175 HMM01118salddharo_1_aro(pf09290pf01118) ARO
18180 -pf04167 -
18185 -pf00886 -
18190 -# -
18195 -pf05239pf01782 -
18200 -pf01746 -
18205 -pf01245 -
18210 -pf00717pf10502 -
BMC-P
BMC-H
BMC-T
Aldehyde dehydrogenase
PTAC
Signature enzyme
Alcohol dehydrogenase
Regulator

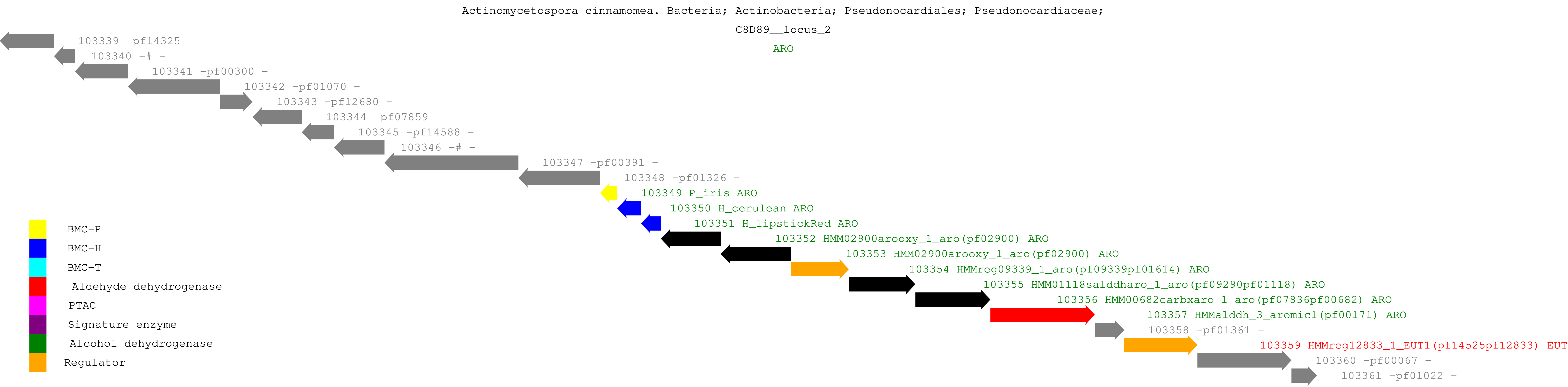
Actinomycetospora cinnamomea. Bacteria; Actinobacteria; Pseudonocardiales; Pseudonocardiaceae;
C8D89__locus_2
ARO
103339 -pf14325 -
103340 -# -
103341 -pf00300 -
103342 -pf01070 -
103343 -pf12680 -
103344 -pf07859 -
103345 -pf14588 -
103346 -# -
103347 -pf00391 -
103348 -pf01326 -
103349 P_iris ARO
103350 H_cerulean ARO
103351 H_lipstickRed ARO
103352 HMM02900arooxy_1_aro(pf02900) ARO
103353 HMM02900arooxy_1_aro(pf02900) ARO
103354 HMMreg09339_1_aro(pf09339pf01614) ARO
103355 HMM01118salddharo_1_aro(pf09290pf01118) ARO
103356 HMM00682carbxaro_1_aro(pf07836pf00682) ARO
103357 HMMaldh_3_aromic1(pf00171) ARO
103358 -pf01361 -
103359 HMMreg12833_1_EUT1(pf14525pf12833) EUT
103360 -pf00067 -
103361 -pf01022 -
BMC-P
BMC-H
BMC-T
Aldehyde dehydrogenase
PTAC
Signature enzyme
Alcohol dehydrogenase
Regulator

Micromonospora sp. CNZ309. Bacteria; Actinobacteria; Micromonosporales; Micromonosporaceae;

CLT72__locus_1

ARO

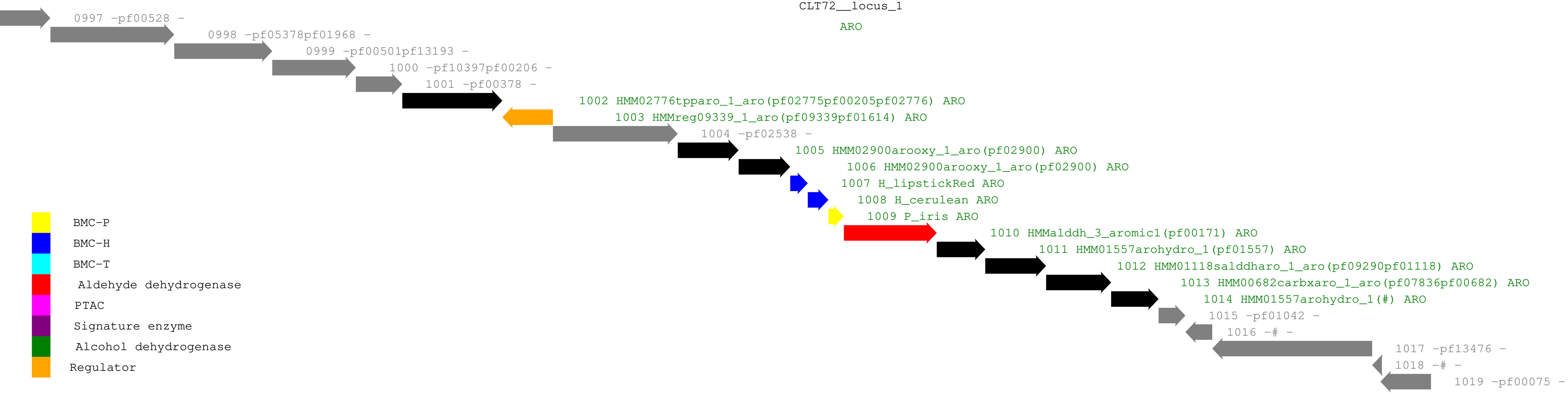

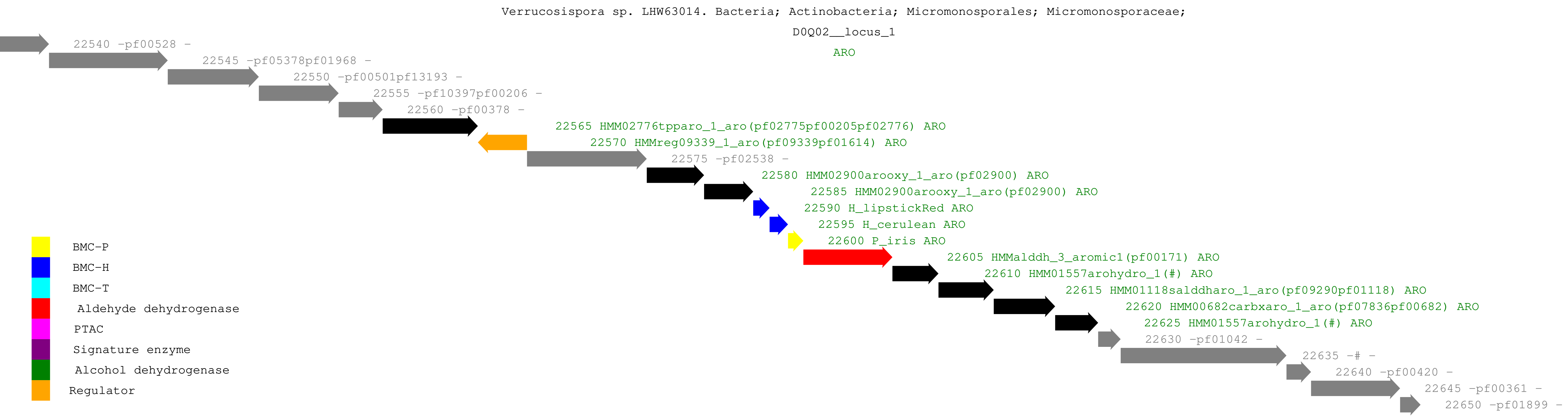

Verrucosispora sp. LHW63014. Bacteria; Actinobacteria; Micromonosporales; Micromonosporaceae;
D0Q02__locus_1
ARO
22540 -pf00528 -
22545 -pf05378pf01968 -
22550 -pf00501pf13193 -
22555 -pf10397pf00206 -
22560 -pf00378 -
22565 HMM02776tpparo_1_aro(pf02775pf00205pf02776) ARO
22570 HMMreg09339_1_aro(pf09339pf01614) ARO
22575 -pf02538 -
22580 HMM02900arooxy_1_aro(pf02900) ARO
22585 HMM02900arooxy_1_aro(pf02900) ARO
22590 H_lipstickRed ARO
22595 H_cerulean ARO
22600 P_iris ARO
22605 HMMalddh_3_aromic1(pf00171) ARO
22610 HMM01557arohydro_1(#) ARO
22615 HMM01118salddharo_1_aro(pf09290pf01118) ARO
22620 HMM00682carbxaro_1_aro(pf07836pf00682) ARO
22625 HMM01557arohydro_1(#) ARO
22630 -pf01042 -
22635 -# -
22640 -pf00420 -
22645 -pf00361 -
22650 -pf01899 -
BMC-P
BMC-H
BMC-T
Aldehyde dehydrogenase
PTAC
Signature enzyme
Alcohol dehydrogenase
Regulator

Micromonospora sp. M71_S20. Bacteria; Actinobacteria; Micromonosporales; Micromonosporaceae;

DER29__locus_1

ARO

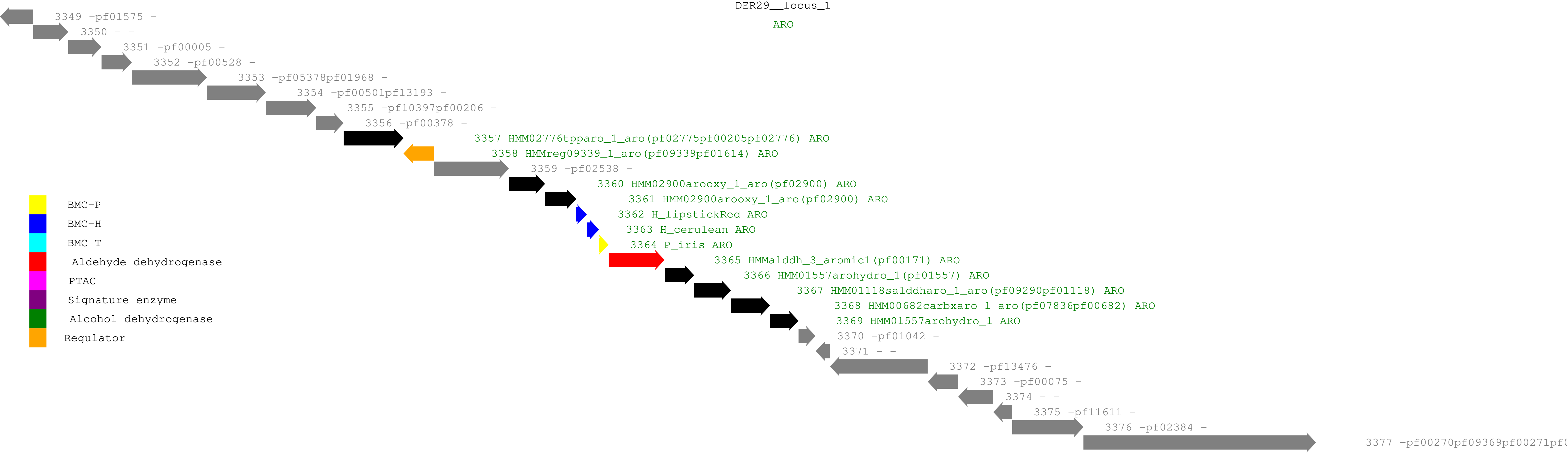

Verrucosispora sp. FIM060022. Bacteria; Actinobacteria; Micromonosporales; Micromonosporaceae;

EG812__locus_1

ARO

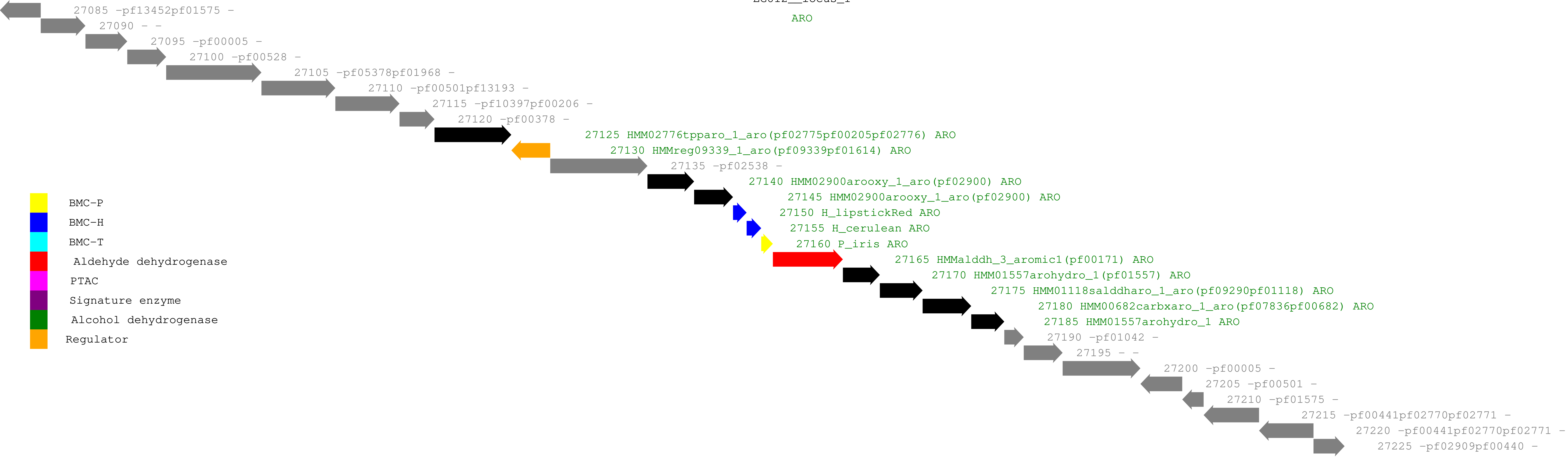

Actinomycetospora succinea. Bacteria; Actinobacteria; Pseudonocardiales; Pseudonocardiaceae;

EV188__locus_1

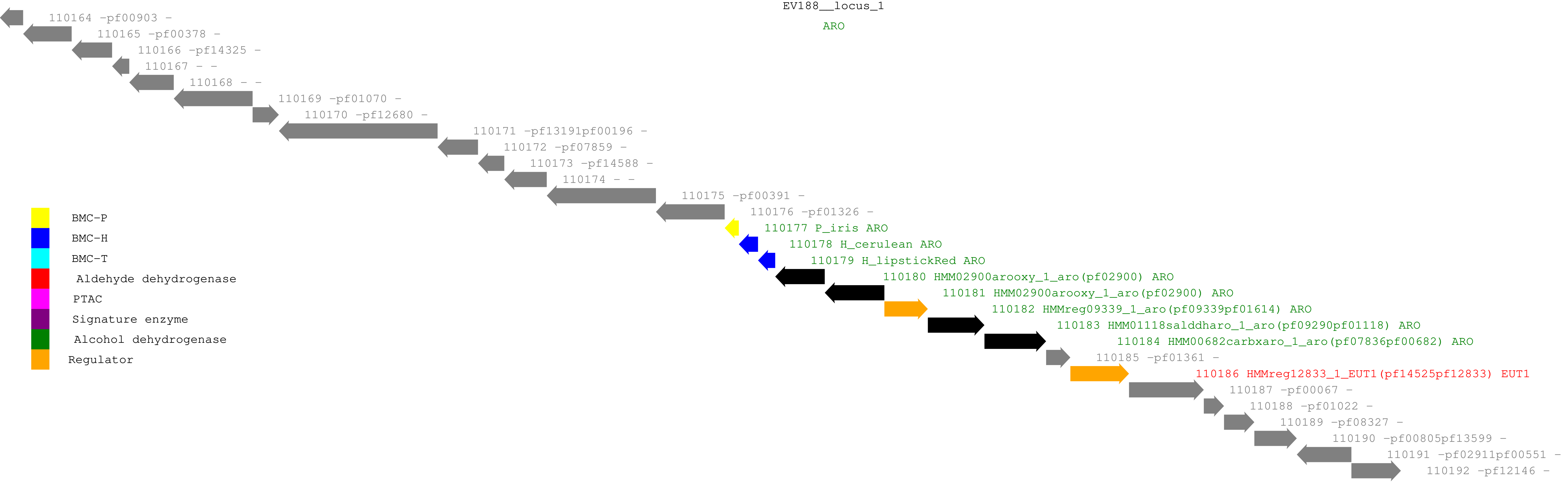

Micromonospora sp. CNZ303. Bacteria; Actinobacteria; Micromonosporales; Micromonosporaceae;

EV374__locus_1

ARO

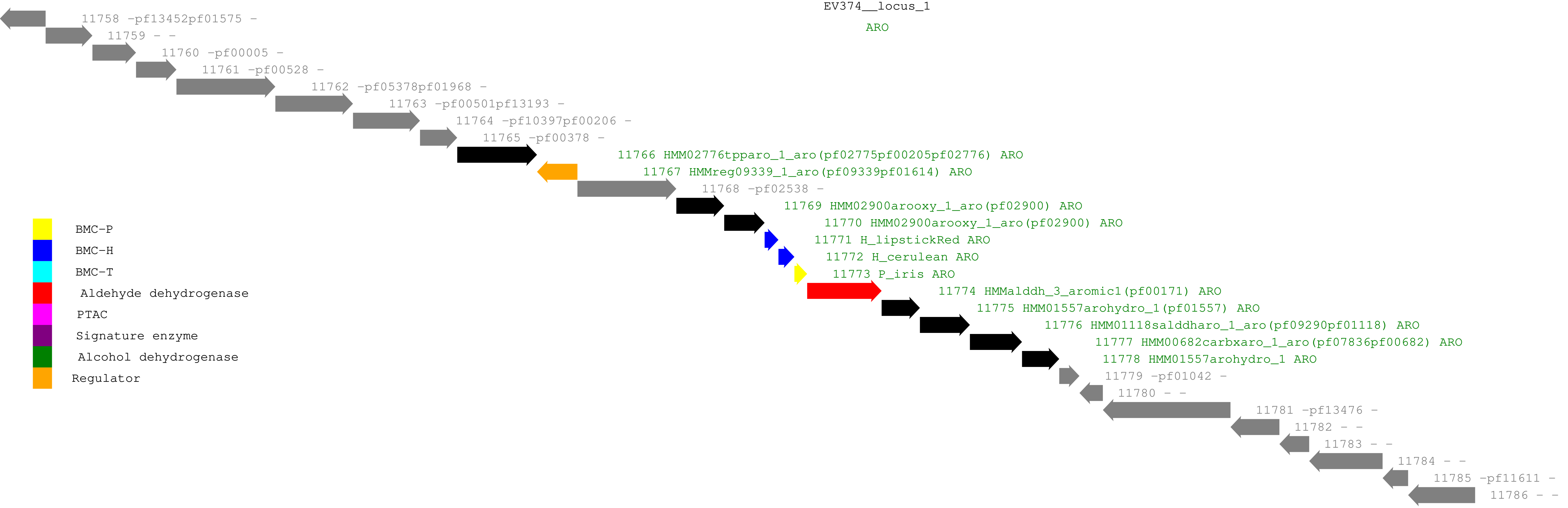

Verrucosispora sp. SN26_14.1. Bacteria; Actinobacteria; Micromonosporales; Micromonosporaceae;

EYA84__locus_2_main_sat

ARO

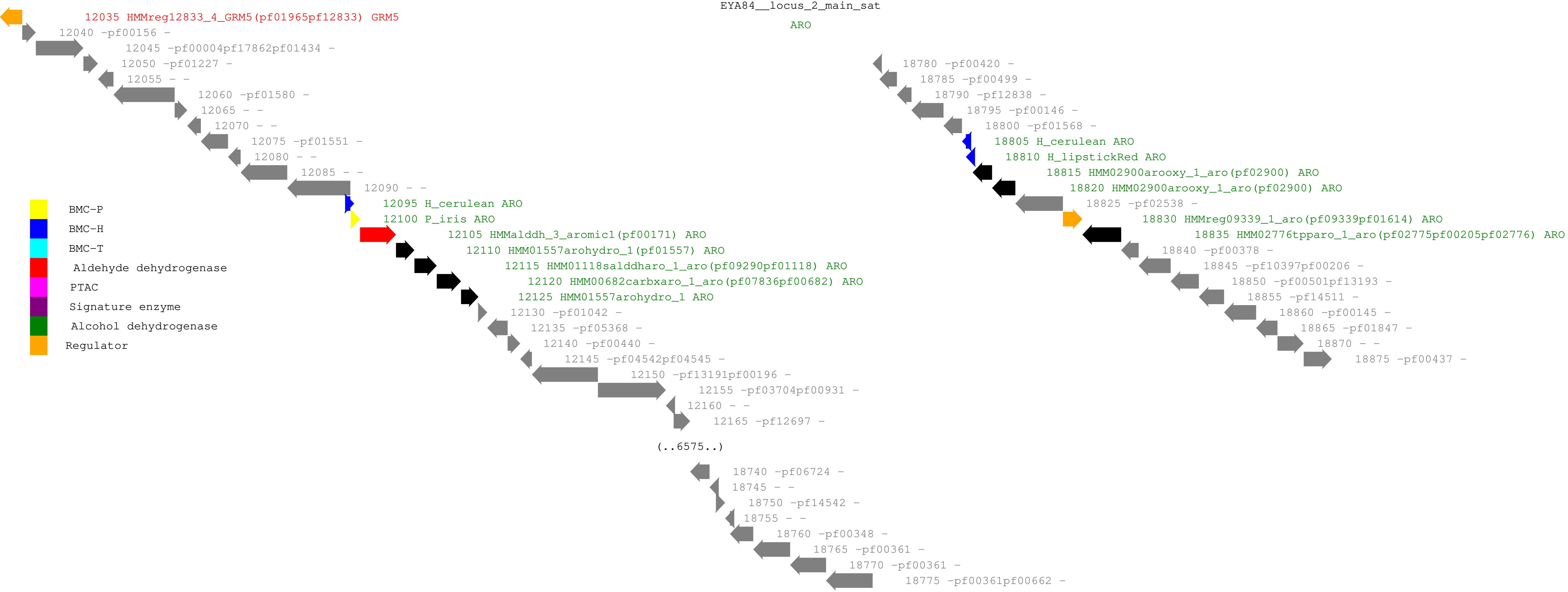

Micromonospora marina. Bacteria; Actinobacteria; Micromonosporales; Micromonosporaceae;

GA0070215__locus_1

ARO

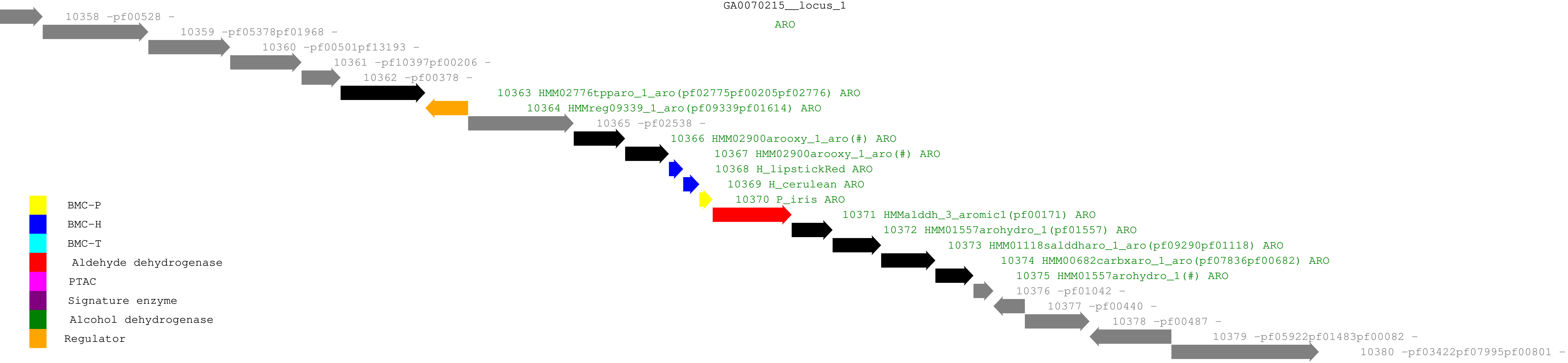

Micromonospora echinofusca. Bacteria; Actinobacteria; Micromonosporales; Micromonosporaceae;

GA0070610__locus_1

ARO

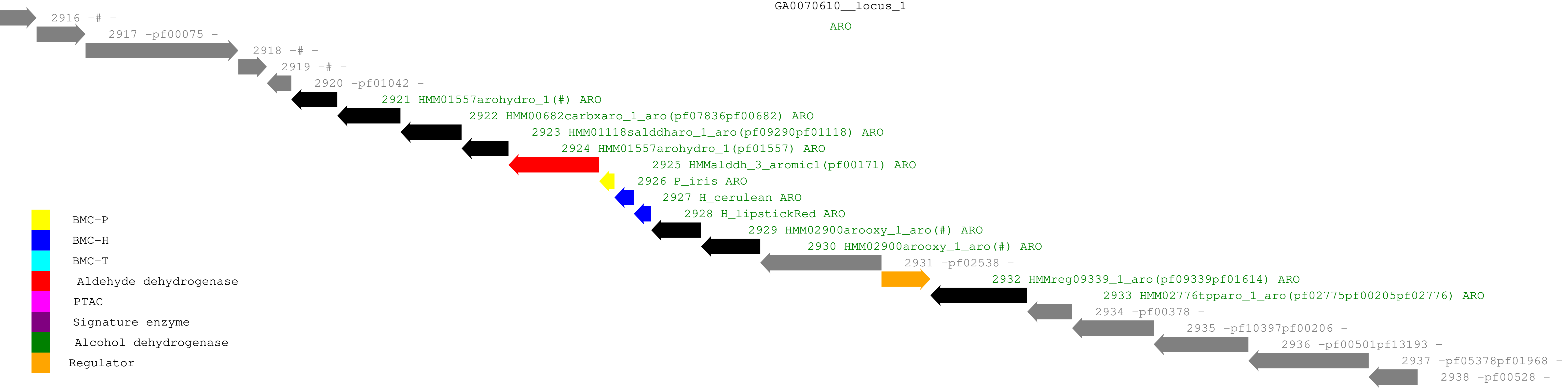

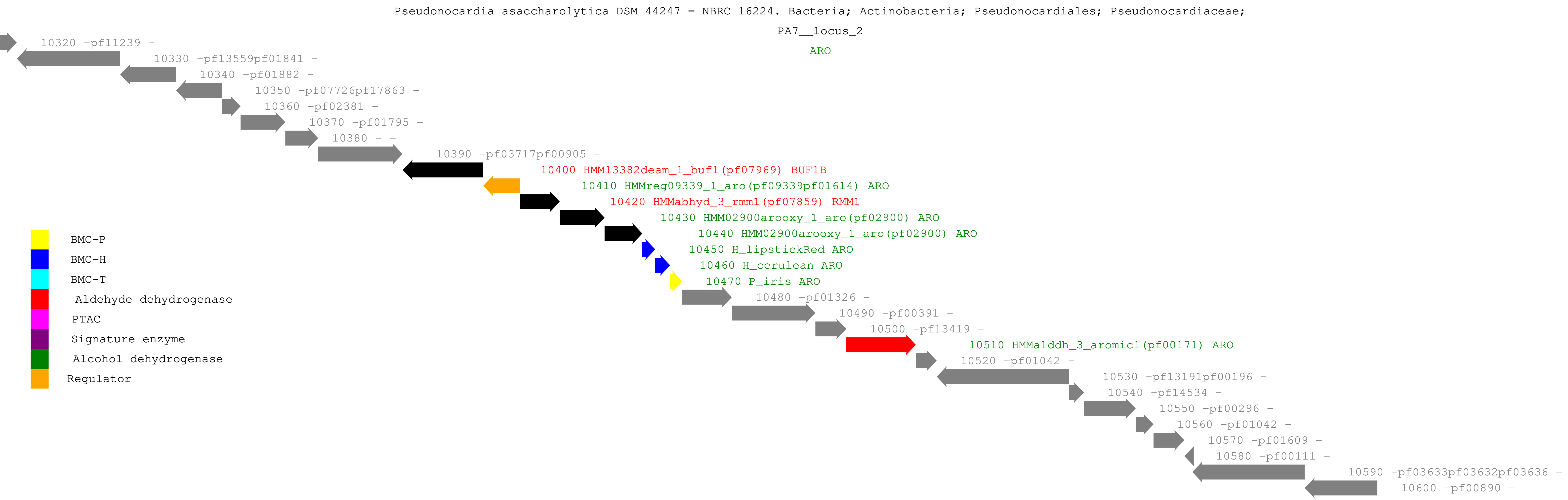
Pseudonocardia asaccharolytica DSM 44247 = NBRC 16224. Bacteria; Actinobacteria; Pseudonocardiales; Pseudonocardiaceae;
PA7__locus_2
ARO
10320 -pf11239 -
10330 -pf13559pf01841 -
10340 -pf01882 -
10350 -pf07726pf17863 -
10360 -pf02381 -
10370 -pf01795 -
10380 - -
10390 -pf03717pf00905 -
10400 HMM13382deam_1_buf1(pf07969) BUF1B
10410 HMMreg09339_1_aro(pf09339pf01614) ARO
10420 HMMabhyd_3_rmm1(pf07859) RMM1
10430 HMM02900arooxy_1_aro(pf02900) ARO
10440 HMM02900arooxy_1_aro(pf02900) ARO
10450 H_lipstickRed ARO
10460 H_cerulean ARO
10470 P_iris ARO
10480 -pf01326 -
10490 -pf00391 -
10500 -pf13419 -
10510 HMMaldh_3_aromic1(pf00171) ARO
10520 -pf01042 -
10530 -pf13191pf00196 -
10540 -pf14534 -
10550 -pf00296 -
10560 -pf01042 -
10570 -pf01609 -
10580 -pf00111 -
10590 -pf03633pf03632pf03636 -
10600 -pf00890 -
BMC-P
BMC-H
BMC-T
Aldehyde dehydrogenase
PTAC
Signature enzyme
Alcohol dehydrogenase
Regulator

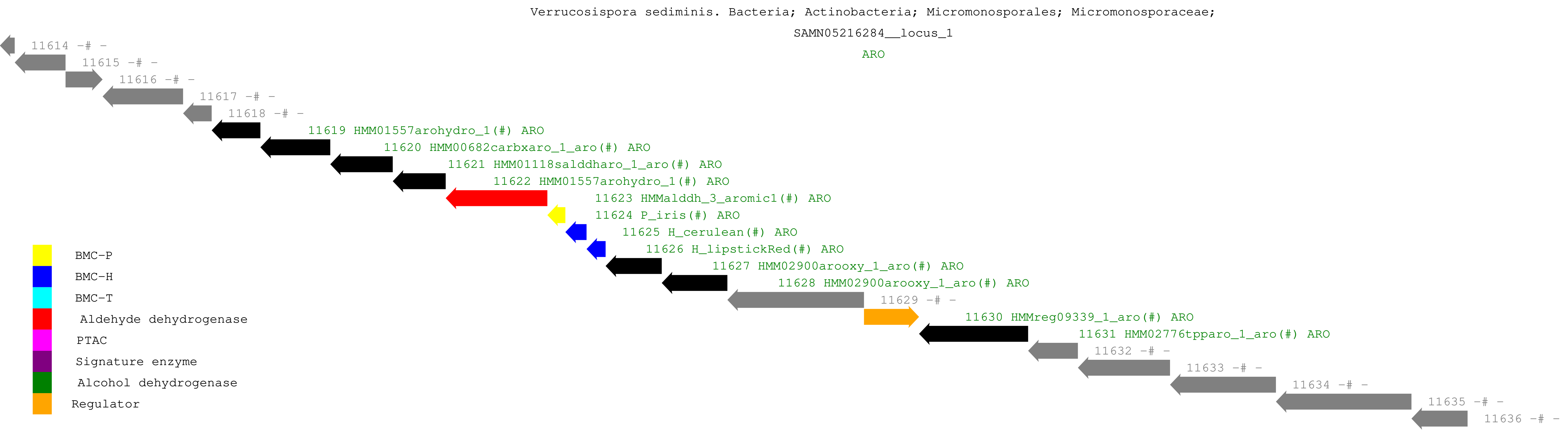
Verrucosispora sediminis. Bacteria; Actinobacteria; Micromonosporales; Micromonosporaceae;
SAMN05216284__locus_1
ARO
11614 -# -
11615 -# -
11616 -# -
11617 -# -
11618 -# -
11619 HMM01557arohydro_1(#) ARO
11620 HMM00682carbxaro_1_aro(#) ARO
11621 HMM01118salddharo_1_aro(#) ARO
11622 HMM01557arohydro_1(#) ARO
11623 HMMalddh_3_aromic1(#) ARO
11624 P_iris(#) ARO
11625 H_cerulean(#) ARO
11626 H_lipstickRed(#) ARO
11627 HMM02900arooxy_1_aro(#) ARO
11628 HMM02900arooxy_1_aro(#) ARO
11629 -# -
11630 HMMreg09339_1_aro(#) ARO
11631 HMM02776tpparo_1_aro(#) ARO
11632 -# -
11633 -# -
11634 -# -
11635 -# -
11636 -# -
BMC-P
BMC-H
BMC-T
Aldehyde dehydrogenase
PTAC
Signature enzyme
Alcohol dehydrogenase
Regulator

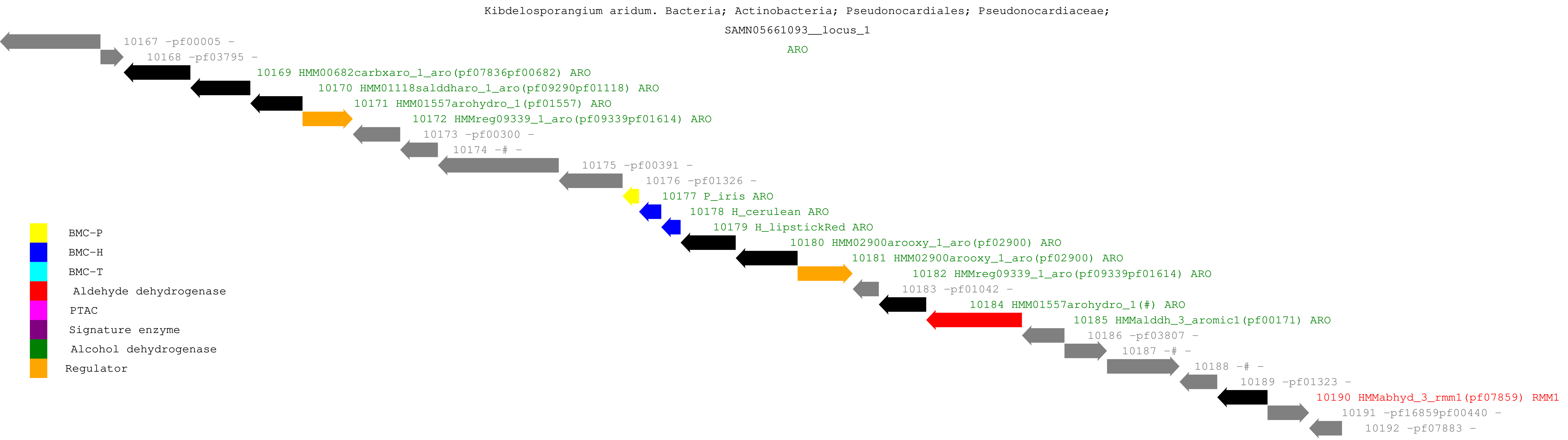

Kibdelosporangium aridum. Bacteria; Actinobacteria; Pseudonocardiales; Pseudonocardiaceae;
SAMN05661093__locus_1
ARO
10167 -pf00005 -
10168 -pf03795 -
10169 HMM00682carbxaro_1_aro(pf07836pf00682) ARO
10170 HMM01118salddharo_1_aro(pf09290pf01118) ARO
10171 HMM01557arohydro_1(pf01557) ARO
10172 HMMreg09339_1_aro(pf09339pf01614) ARO
10173 -pf00300 -
10174 -# -
10175 -pf00391 -
10176 -pf01326 -
10177 P_iris ARO
10178 H_cerulean ARO
10179 H_lipstickRed ARO
10180 HMM02900arooxy_1_aro(pf02900) ARO
10181 HMM02900arooxy_1_aro(pf02900) ARO
10182 HMMreg09339_1_aro(pf09339pf01614) ARO
10183 -pf01042 -
10184 HMM01557arohydro_1(#) ARO
10185 HMMaldh_3_aromic1(pf00171) ARO
10186 -pf03807 -
10187 -# -
10188 -# -
10189 -pf01323 -
10190 HMMabhyd_3_rmm1(pf07859) RMM1
10191 -pf16859pf00440 -
10192 -pf07883 -
BMC-P
BMC-H
BMC-T
Aldehyde dehydrogenase
PTAC
Signature enzyme
Alcohol dehydrogenase
Regulator

Verrucosispora maris (strain AB-18-032). Bacteria; Actinobacteria; Micromonosporales; Micromonosporaceae;

VAB18032__locus_1

ARO

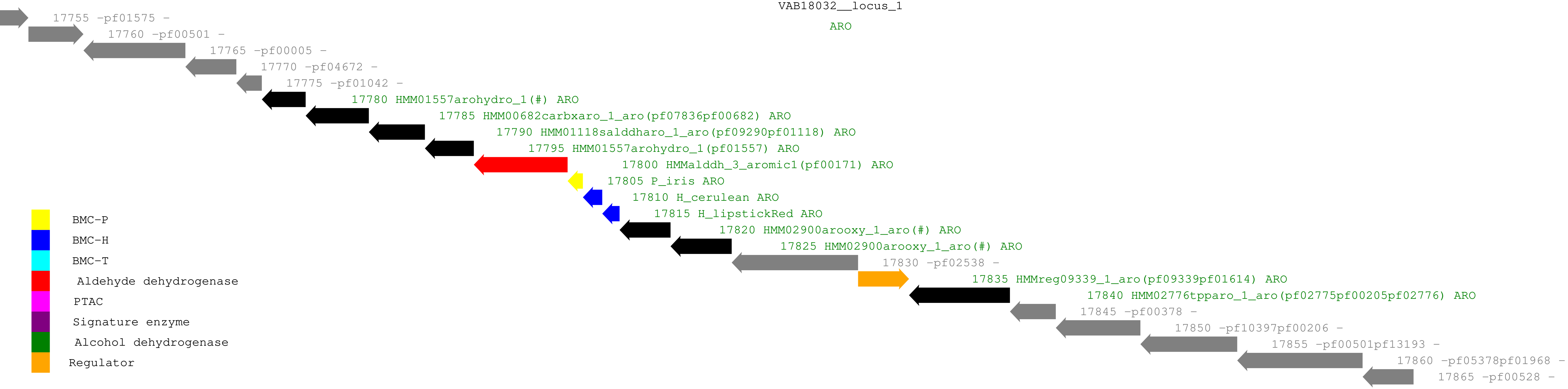

Caloramator mitchellensis. Bacteria; Firmicutes; Clostridia; Clostridiales; Clostridiaceae;

ABG79__locus_1

BUF1

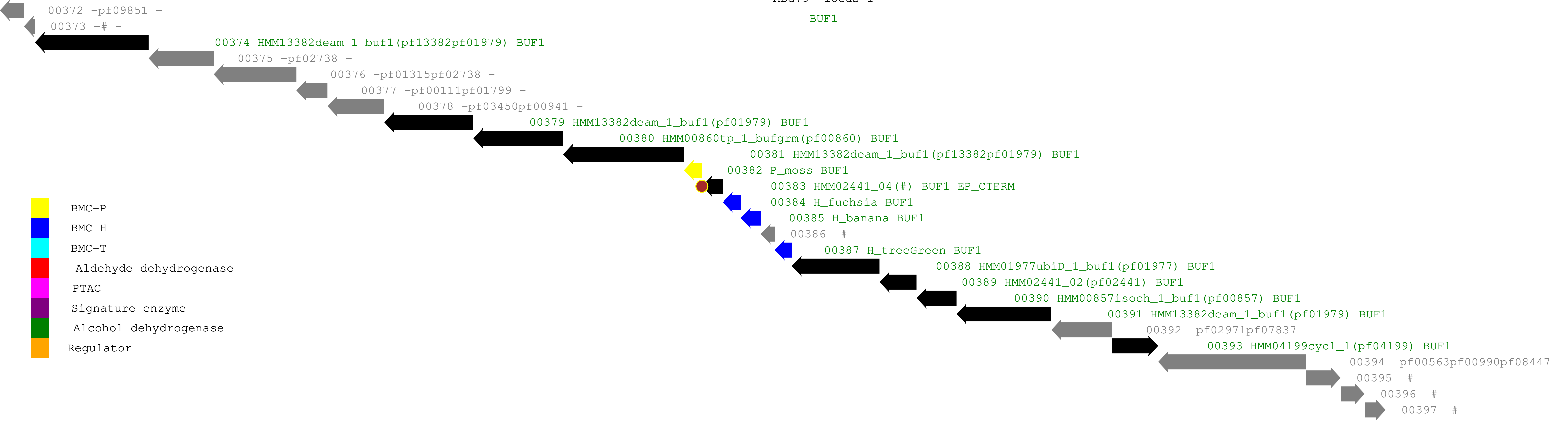

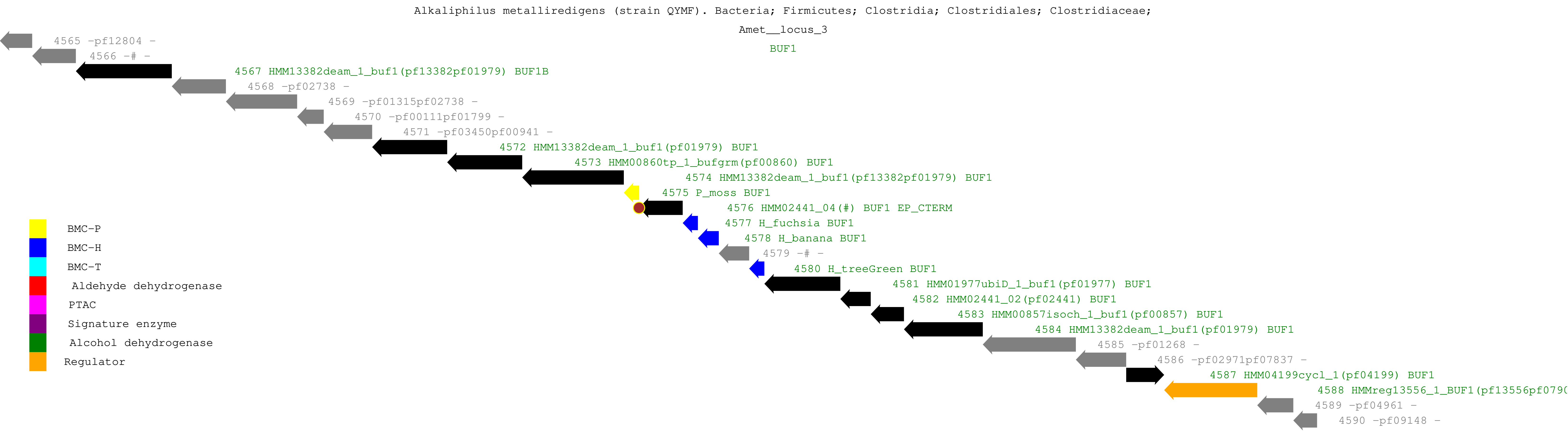

Alkaliphilus metalliredigens (strain QYMF). Bacteria; Firmicutes; Clostridia; Clostridiales; Clostridiaceae;
Amet__locus_3
BUF1
4565 -pf12804 -
4566 -# -
4567 HMM13382deam_1_buf1(pf13382pf01979) BUF1B
4568 -pf02738 -
4569 -pf01315pf02738 -
4570 -pf00111pf01799 -
4571 -pf03450pf00941 -
4572 HMM13382deam_1_buf1(pf01979) BUF1
4573 HMM00860tp_1_bufgrm(pf00860) BUF1
4574 HMM13382deam_1_buf1(pf13382pf01979) BUF1
4575 P_moss BUF1
4576 HMM02441_04(#) BUF1 EP_CTERM
4577 H_fuchsia BUF1
4578 H_banana BUF1
4579 -# -
4580 H_treeGreen BUF1
4581 HMM01977ubiD_1_buf1(pf01977) BUF1
4582 HMM02441_02(pf02441) BUF1
4583 HMM00857isoch_1_buf1(pf00857) BUF1
4584 HMM13382deam_1_buf1(pf01979) BUF1
4585 -pf01268 -
4586 -pf02971pf07837 -
4587 HMM04199cycl_1(pf04199) BUF1
4588 HMMreg13556_1_BUF1(pf13556pf0790
4589 -pf04961 -
4590 -pf09148 -
BMC-P
BMC-H
BMC-T
Aldehyde dehydrogenase
PTAC
Signature enzyme
Alcohol dehydrogenase
Regulator

Thermotalea metallivorans. Bacteria; Firmicutes; Clostridia; Clostridiales; Clostridiaceae;

AN619__locus_1

BUF1

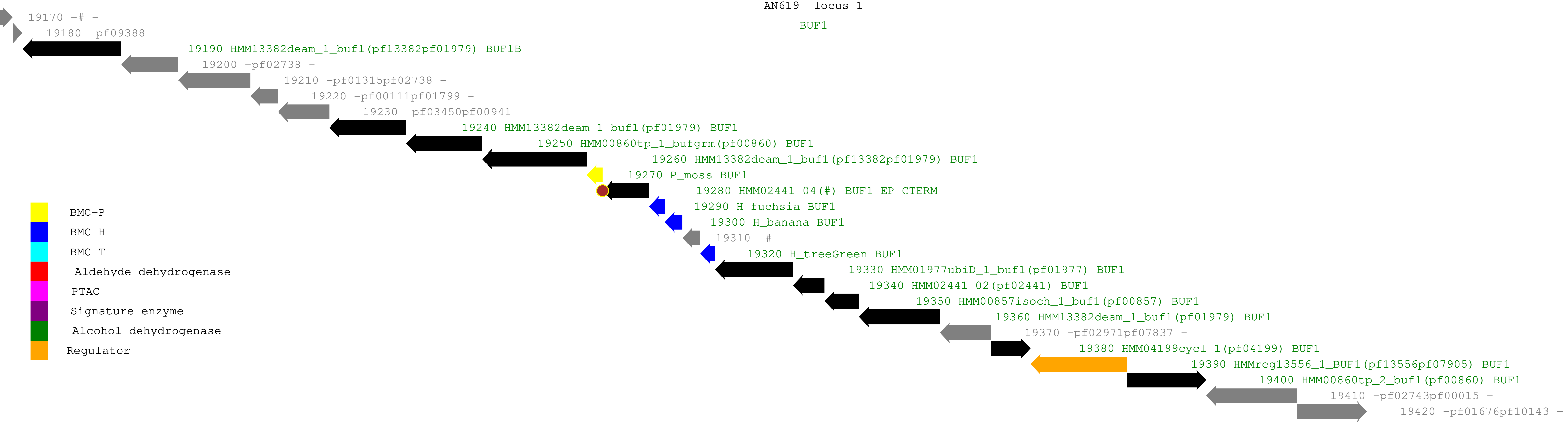

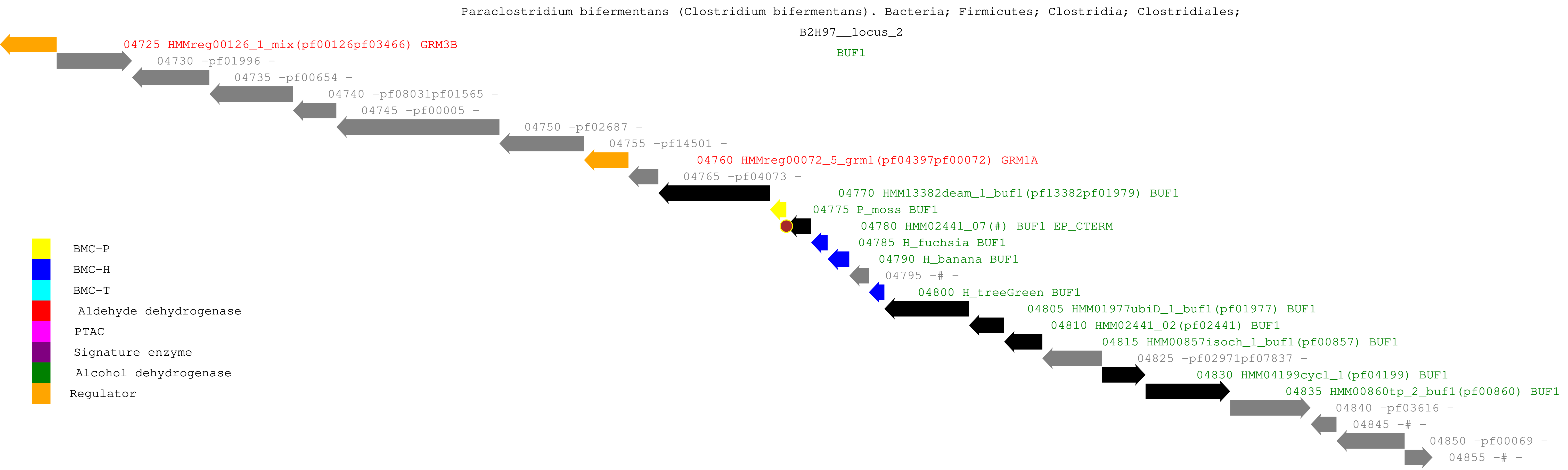
Paraclostridium bifermentans (Clostridium bifermentans). Bacteria; Firmicutes; Clostridia; Clostridiales;
B2H97__locus_2
BUF1
04725 HMMreg00126_1_mix(pf00126pf03466) GRM3B
04730 -pf01996 -
04735 -pf00654 -
04740 -pf08031pf01565 -
04745 -pf00005 -
04750 -pf02687 -
04755 -pf14501 -
04760 HMMreg00072_5_grm1(pf04397pf00072) GRM1A
04765 -pf04073 -
04770 HMM13382deam_1_buf1(pf13382pf01979) BUF1
04775 P_moss BUF1
04780 HMM02441_07(#) BUF1 EP_CTERM
04785 H_fuchsia BUF1
04790 H_banana BUF1
04795 -# -
04800 H_treeGreen BUF1
04805 HMM01977ubiD_1_buf1(pf01977) BUF1
04810 HMM02441_02(pf02441) BUF1
04815 HMM00857isoch_1_buf1(pf00857) BUF1
04825 -pf02971pf07837 -
04830 HMM04199cycl_1(pf04199) BUF1
04835 HMM00860tp_2_buf1(pf00860) BUF1
04840 -pf03616 -
04845 -# -
04850 -pf00069 -
04855 -# -
BMC-P
BMC-H
BMC-T
Aldehyde dehydrogenase
PTAC
Signature enzyme
Alcohol dehydrogenase
Regulator

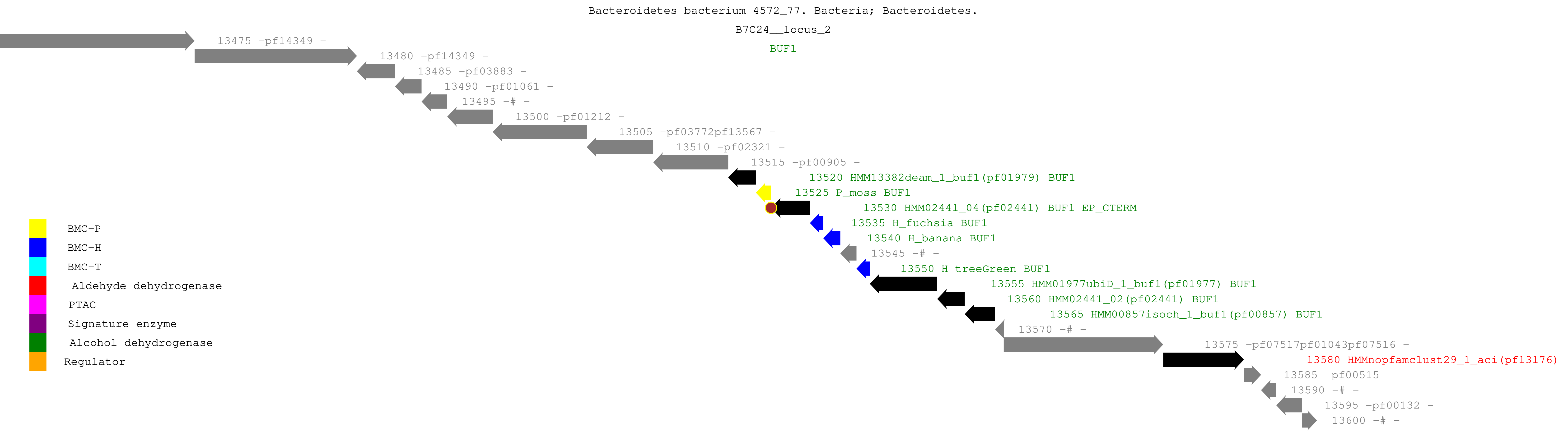

Bacteroidetes bacterium 4572_77. Bacteria; Bacteroidetes.
B7C24__locus_2
BUF1
13475 -pf14349 -
13480 -pf14349 -
13485 -pf03883 -
13490 -pf01061 -
13495 -# -
13500 -pf01212 -
13505 -pf03772pf13567 -
13510 -pf02321 -
13515 -pf00905 -
13520 HMM13382deam_1_buf1(pf01979) BUF1
13525 P_moss BUF1
13530 HMM02441_04(pf02441) BUF1 EP_CTERM
13535 H_fuchsia BUF1
13540 H_banana BUF1
13545 -# -
13550 H_treeGreen BUF1
13555 HMM01977ubiD_1_buf1(pf01977) BUF1
13560 HMM02441_02(pf02441) BUF1
13565 HMM00857isoch_1_buf1(pf00857) BUF1
13570 -# -
13575 -pf07517pf01043pf07516 -
13580 HMMnopfamclust29_1_aci(pf13176)
13585 -pf00515 -
13590 -# -
13595 -pf00132 -
13600 -# -
BMC-P
BMC-H
BMC-T
Aldehyde dehydrogenase
PTAC
Signature enzyme
Alcohol dehydrogenase
Regulator

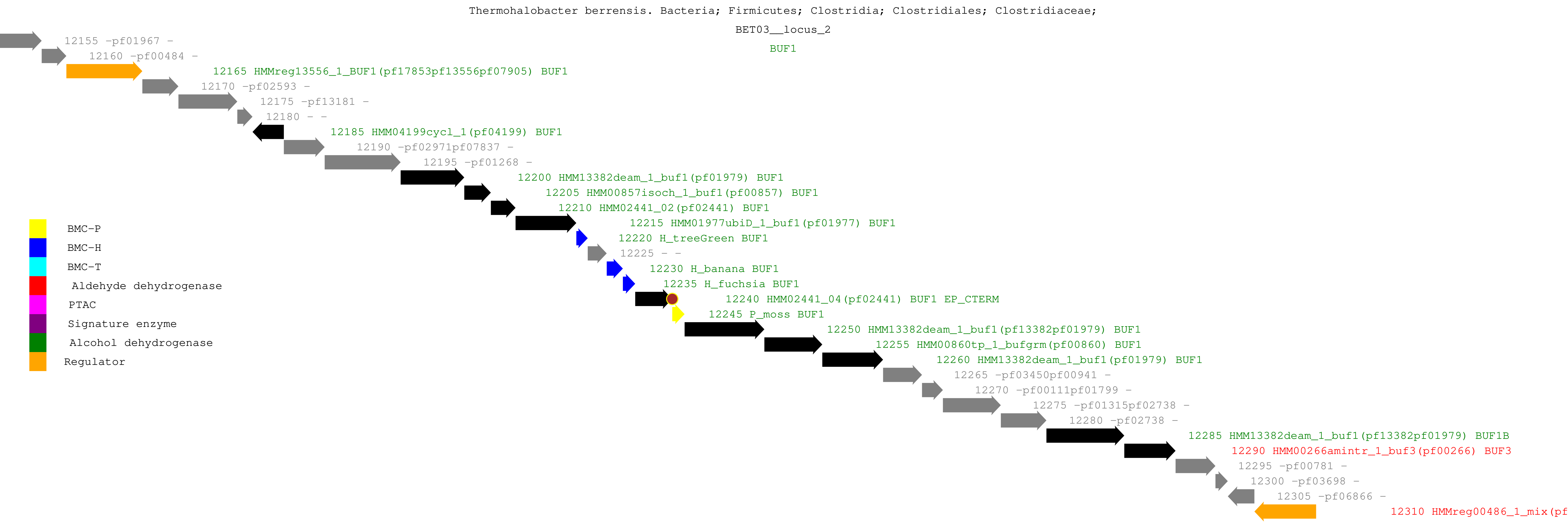
Thermohalobacter berrensis. Bacteria; Firmicutes; Clostridia; Clostridiales; Clostridiaceae;
BET03__locus_2
BUF1
12155 -pf01967 -
12160 -pf00484 -
12165 HMMreg13556_1_BUF1(pf17853pf13556pf07905) BUF1
12170 -pf02593 -
12175 -pf13181 -
12180 - -
12185 HMM04199cycl_1(pf04199) BUF1
12190 -pf02971pf07837 -
12195 -pf01268 -
12200 HMM13382deam_1_buf1(pf01979) BUF1
12205 HMM00857isoch_1_buf1(pf00857) BUF1
12210 HMM02441_02(pf02441) BUF1
12215 HMM01977ubiD_1_buf1(pf01977) BUF1
12220 H_treeGreen BUF1
12225 - -
12230 H_banana BUF1
12235 H_fuchsia BUF1
12240 HMM02441_04(pf02441) BUF1 EP_CTERM
12245 P_moss BUF1
12250 HMM13382deam_1_buf1(pf13382pf01979) BUF1
12255 HMM00860tp_1_bufgrm(pf00860) BUF1
12260 HMM13382deam_1_buf1(pf01979) BUF1
12265 -pf03450pf00941 -
12270 -pf00111pf01799 -
12275 -pf01315pf02738 -
12280 -pf02738 -
12285 HMM13382deam_1_buf1(pf13382pf01979) BUF1B
12290 HMM00266amintr_1_buf3(pf00266) BUF3
12295 -pf00781 -
12300 -pf03698 -
12305 -pf06866 -
12310 HMMreg00486_1_mix(pf
BMC-P
BMC-H
BMC-T
Aldehyde dehydrogenase
PTAC
Signature enzyme
Alcohol dehydrogenase
Regulator

Paraclostridium bifermentans ATCC 19299. Bacteria; Firmicutes; Clostridia; Clostridiales;

C671__locus_2

BUF1

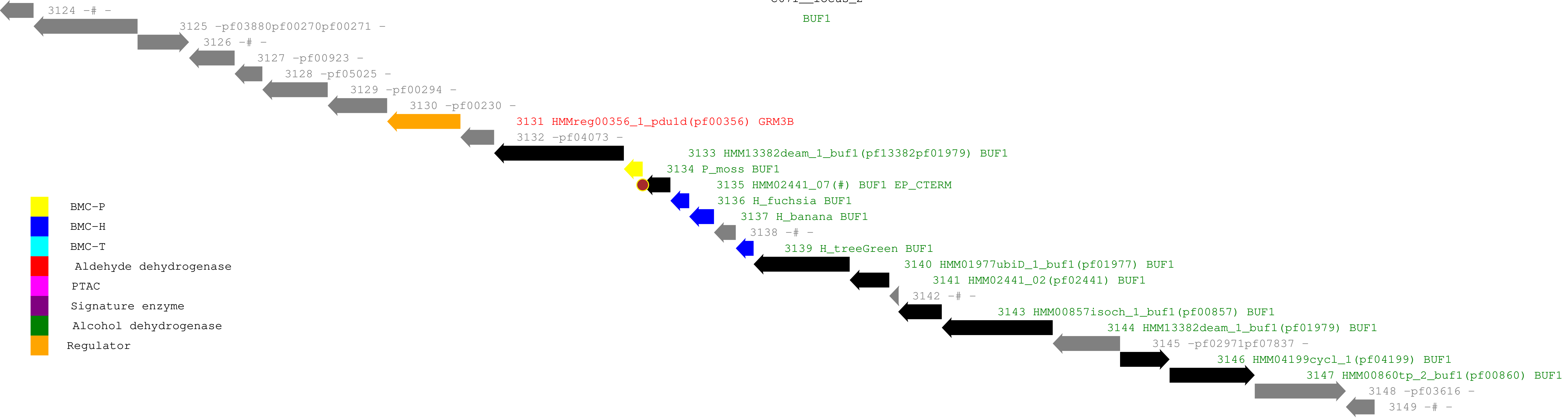

Paraclostridium bifermentans ATCC 638. Bacteria; Firmicutes; Clostridia; Clostridiales;

C672__locus_2

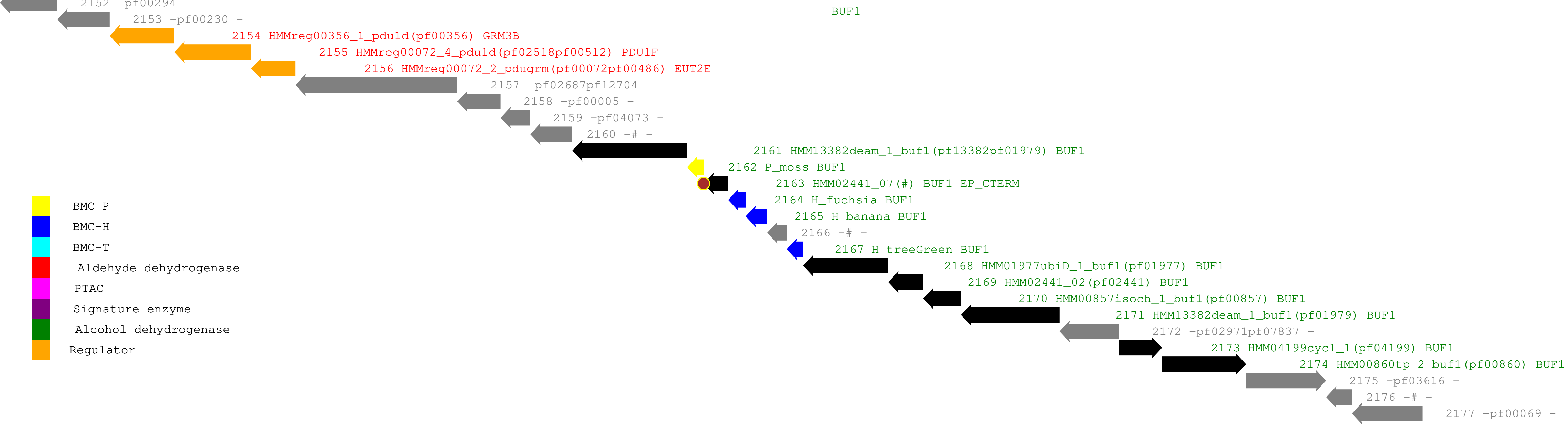

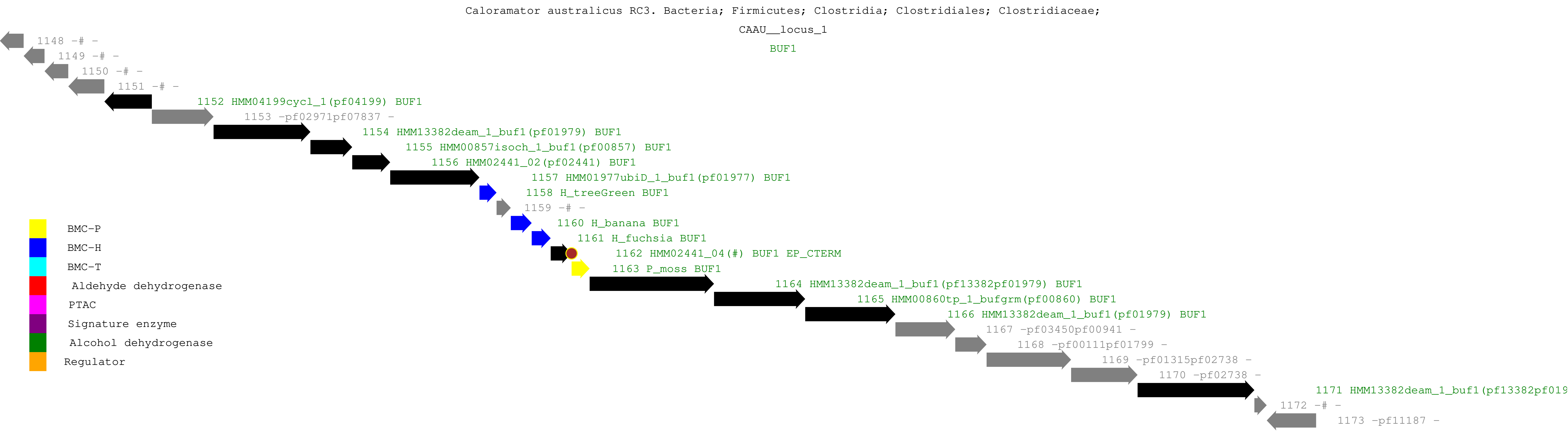
Caloramator australicus RC3. Bacteria; Firmicutes; Clostridia; Clostridiales; Clostridiaceae;
CAAU__locus_1
BUF1
1148 -# -
1149 -# -
1150 -# -
1151 -# -
1152 HMM04199cycl_1(pf04199) BUF1
1153 -pf02971pf07837 -
1154 HMM13382deam_1_buf1(pf01979) BUF1
1155 HMM00857isoch_1_buf1(pf00857) BUF1
1156 HMM02441_02(pf02441) BUF1
1157 HMM01977ubiD_1_buf1(pf01977) BUF1
1158 H_treeGreen BUF1
1159 -# -
1160 H_banana BUF1
1161 H_fuchsia BUF1
1162 HMM02441_04(#) BUF1 EP_CTERM
1163 P_moss BUF1
1164 HMM13382deam_1_buf1(pf13382pf01979) BUF1
1165 HMM00860tp_1_bufgrm(pf00860) BUF1
1166 HMM13382deam_1_buf1(pf01979) BUF1
1167 -pf03450pf00941 -
1168 -pf00111pf01799 -
1169 -pf01315pf02738 -
1170 -pf02738 -
1171 HMM13382deam_1_buf1(pf13382pf019
1172 -# -
1173 -pf11187 -
BMC-P
BMC-H
BMC-T
Aldehyde dehydrogenase
PTAC
Signature enzyme
Alcohol dehydrogenase
Regulator

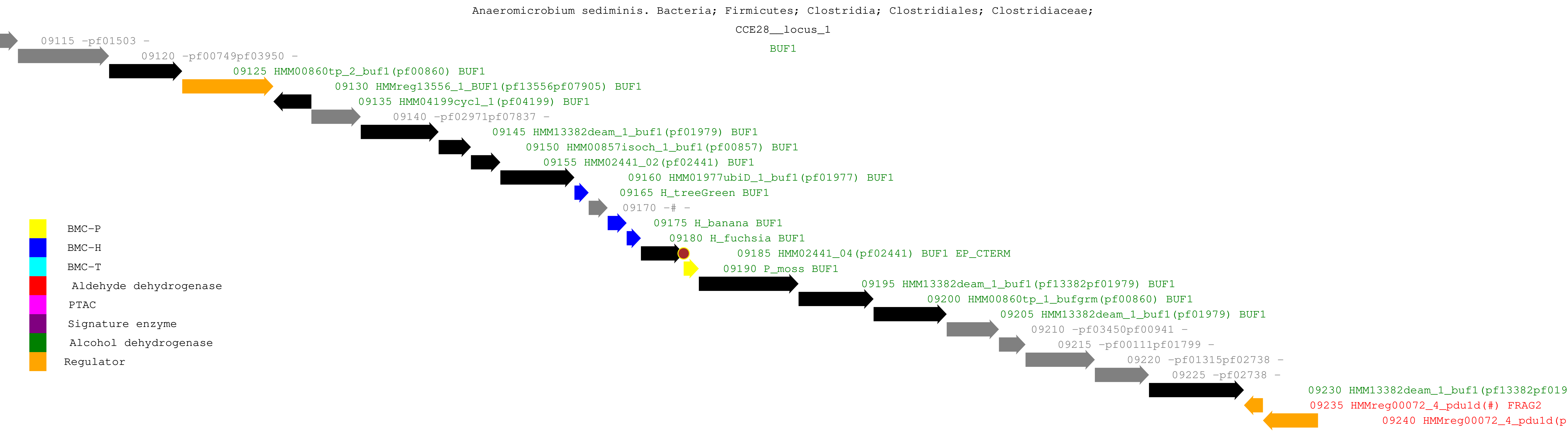

Anaeromicrobium sediminis. Bacteria; Firmicutes; Clostridia; Clostridiales; Clostridiaceae;
CCE28__locus_1
BUF1
09115 -pf01503 -
09120 -pf00749pf03950 -
09125 HMM00860tp_2_buf1(pf00860) BUF1
09130 HMMreg13556_1_BUF1(pf13556pf07905) BUF1
09135 HMM04199cycl_1(pf04199) BUF1
09140 -pf02971pf07837 -
09145 HMM13382deam_1_buf1(pf01979) BUF1
09150 HMM00857isoch_1_buf1(pf00857) BUF1
09155 HMM02441_02(pf02441) BUF1
09160 HMM01977ubiD_1_buf1(pf01977) BUF1
09165 H_treeGreen BUF1
09170 -# -
09175 H_banana BUF1
09180 H_fuchsia BUF1
09185 HMM02441_04(pf02441) BUF1 EP_CTERM
09190 P_moss BUF1
09195 HMM13382deam_1_buf1(pf13382pf01979) BUF1
09200 HMM00860tp_1_bufgrm(pf00860) BUF1
09205 HMM13382deam_1_buf1(pf01979) BUF1
09210 -pf03450pf00941 -
09215 -pf00111pf01799 -
09220 -pf01315pf02738 -
09225 -pf02738 -
09230 HMM13382deam_1_buf1(pf13382pf019
09235 HMMreg00072_4_pdu1d(#) FRAG2
09240 HMMreg00072_4_pdu1d(p
BMC-P
BMC-H
BMC-T
Aldehyde dehydrogenase
PTAC
Signature enzyme
Alcohol dehydrogenase
Regulator

Carboxydothermus hydrogenoformans (strain ATCC BAA-161 / DSM 6008 / Bacteria; Firmicutes; Clostridia; Thermoanaerobacterales;

CHY__locus_1

BUF1

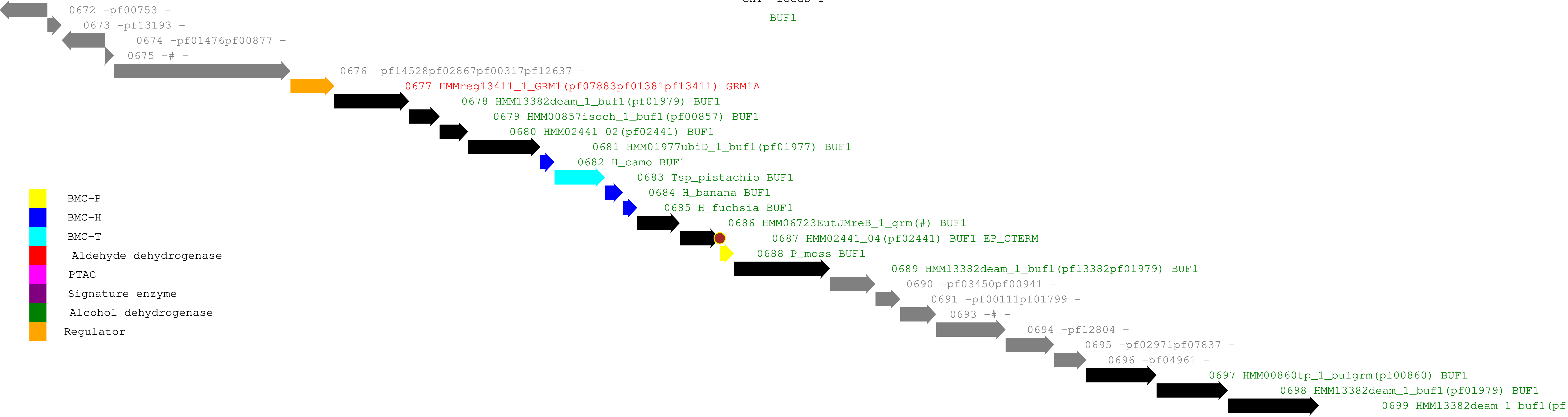

Carboxydothermus islandicus. Bacteria; Firmicutes; Clostridia; Thermoanaerobacterales;

ciss__locus_1

BUF1

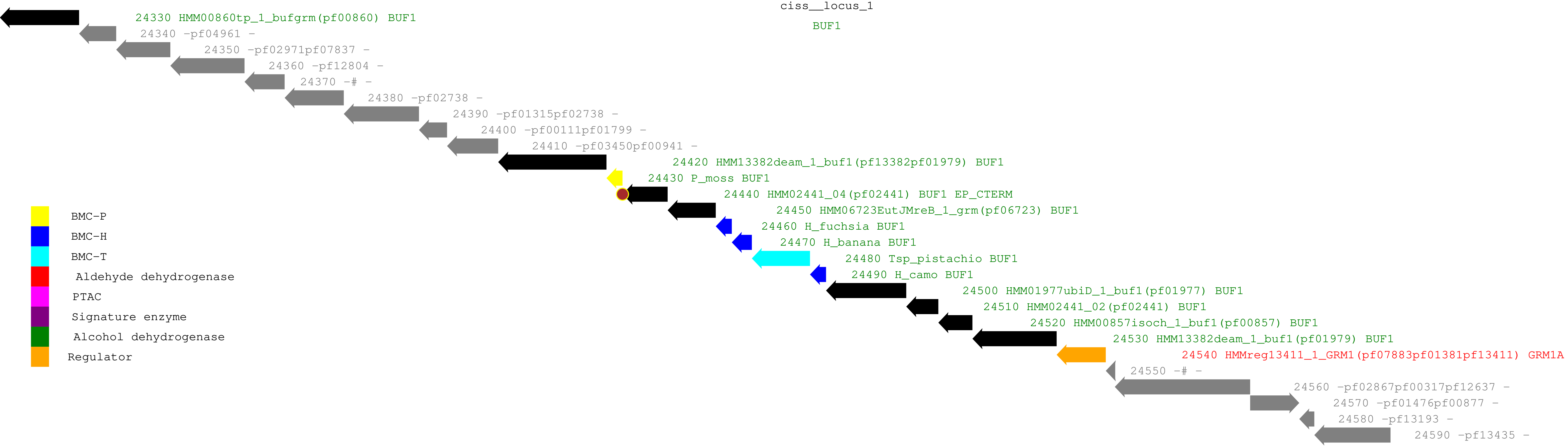

Tissierella sp. P1. Bacteria; Firmicutes; Tissierellia; Tissierellales; Tissierellaceae;

CIW83__locus_2

BUF1

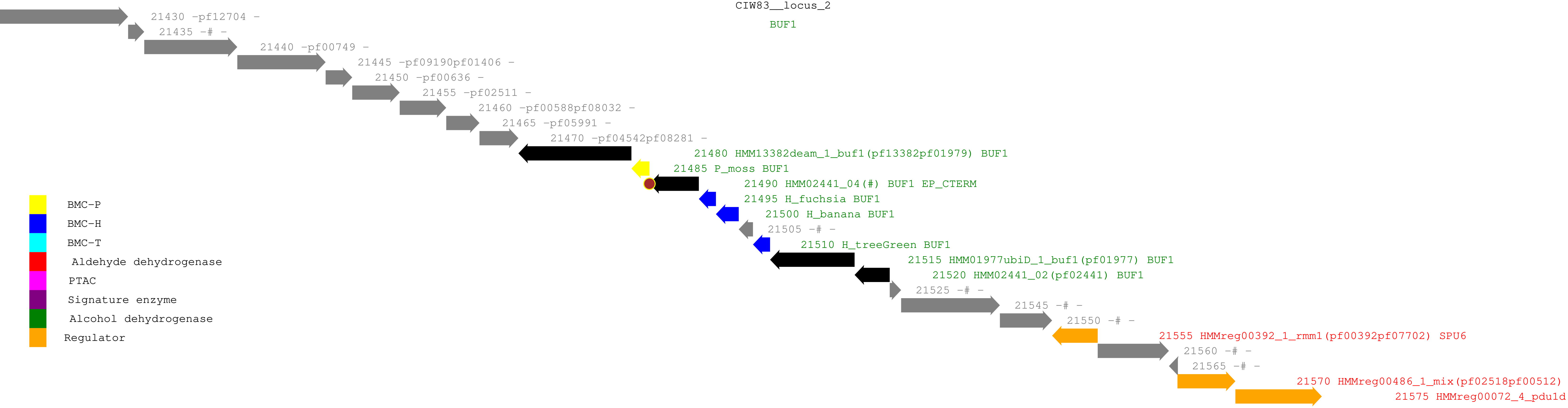

Clostridium cylindrosporum DSM 605. Bacteria; Firmicutes; Clostridia; Clostridiales; Clostridiaceae;

CLCY_4c_locus_1

BUF1

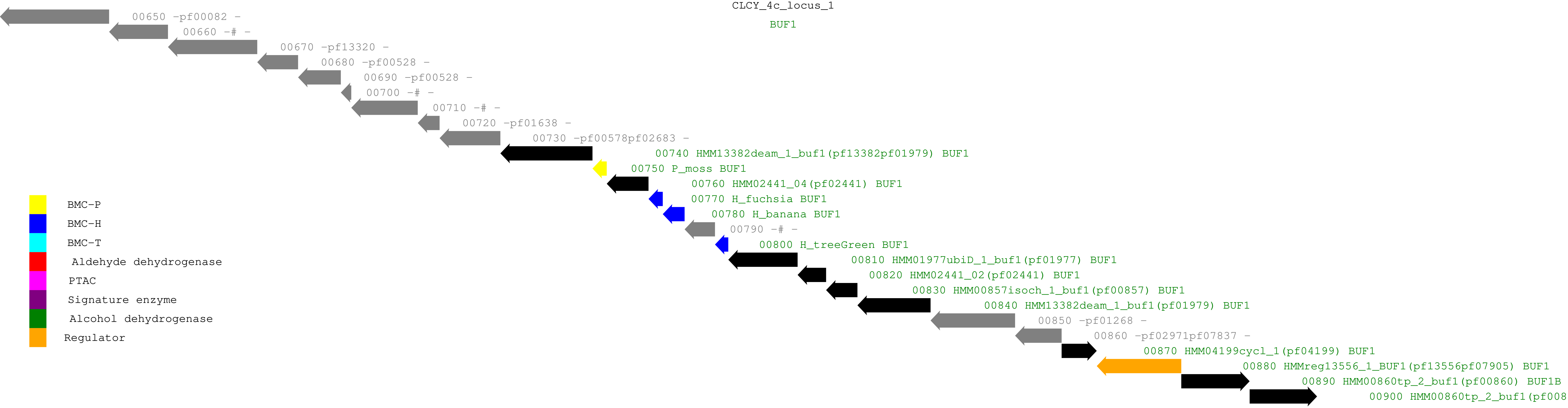

Alkaliphilus oremlandii (strain OhILAs) (Clostridium oremlandii Bacteria; Firmicutes; Clostridia; Clostridiales; Clostridiaceae;

Clos__locus_1

BUF1

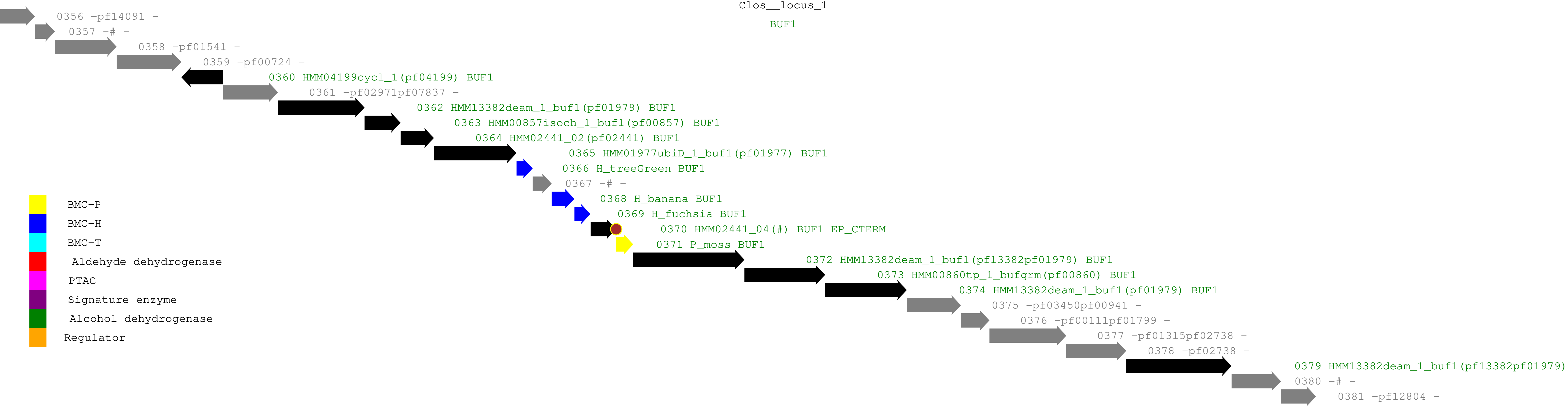

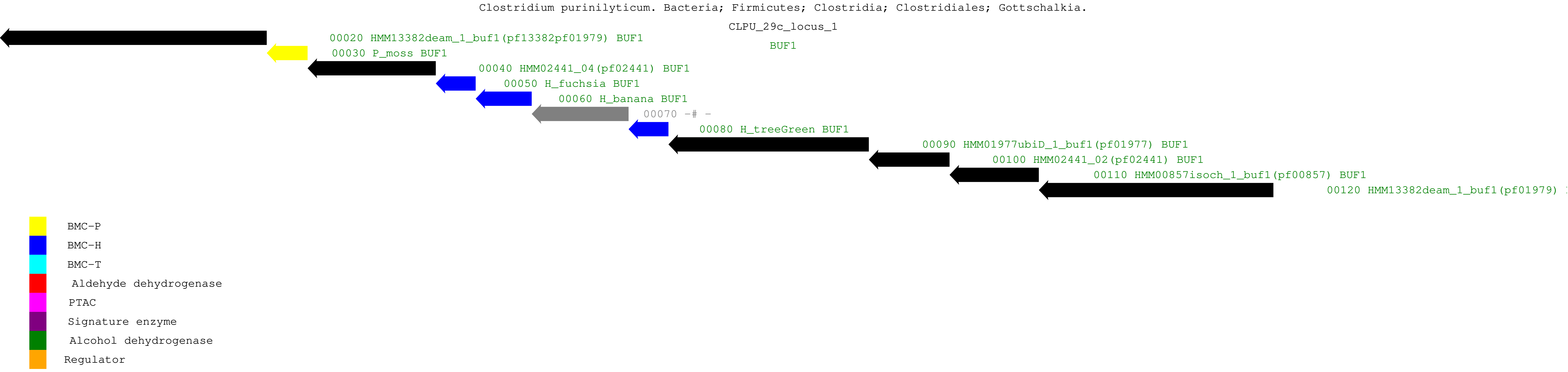
Clostridium purinilyticum. Bacteria; Firmicutes; Clostridia; Clostridiales; Gottschalkia.
CLPU_29c_locus_1
BUF1
00020 HMM13382deam_1_buf1(pf13382pf01979) BUF1
00030 P_moss BUF1
00040 HMM02441_04(pf02441) BUF1
00050 H_fuchsia BUF1
00060 H_banana BUF1
00070 -# -
00080 H_treeGreen BUF1
00090 HMM01977ubiD_1_buf1(pf01977) BUF1
00100 HMM02441_02(pf02441) BUF1
00110 HMM00857isoch_1_buf1(pf00857) BUF1
00120 HMM13382deam_1_buf1(pf01979)
BMC-P
BMC-H
BMC-T
Aldehyde dehydrogenase
PTAC
Signature enzyme
Alcohol dehydrogenase
Regulator

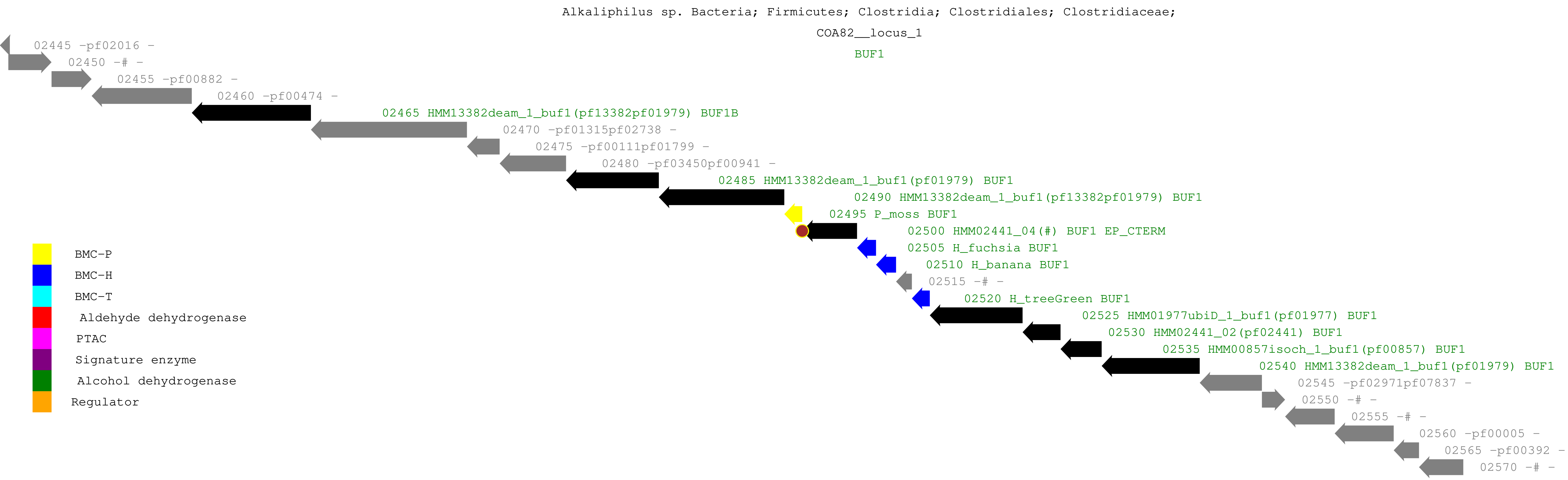
Alkaliphilus sp. Bacteria; Firmicutes; Clostridia; Clostridiales; Clostridiaceae;
COA82__locus_1
BUF1
02445 -pf02016 -
02450 -# -
02455 -pf00882 -
02460 -pf00474 -
02465 HMM13382deam_1_buf1(pf13382pf01979) BUF1B
02470 -pf01315pf02738 -
02475 -pf00111pf01799 -
02480 -pf03450pf00941 -
02485 HMM13382deam_1_buf1(pf01979) BUF1
02490 HMM13382deam_1_buf1(pf13382pf01979) BUF1
02495 P_moss BUF1
02500 HMM02441_04(#) BUF1 EP_CTERM
02505 H_fuchsia BUF1
02510 H_banana BUF1
02515 -# -
02520 H_treeGreen BUF1
02525 HMM01977ubiD_1_buf1(pf01977) BUF1
02530 HMM02441_02(pf02441) BUF1
02535 HMM00857isoch_1_buf1(pf00857) BUF1
02540 HMM13382deam_1_buf1(pf01979) BUF1
02545 -pf02971pf07837 -
02550 -# -
02555 -# -
02560 -pf00005 -
02565 -pf00392 -
02570 -# -
BMC-P
BMC-H
BMC-T
Aldehyde dehydrogenase
PTAC
Signature enzyme
Alcohol dehydrogenase
Regulator

Carboxydothermus pertinax. Bacteria; Firmicutes; Clostridia; Thermoanaerobacterales;

cpu__locus_1

BUF1

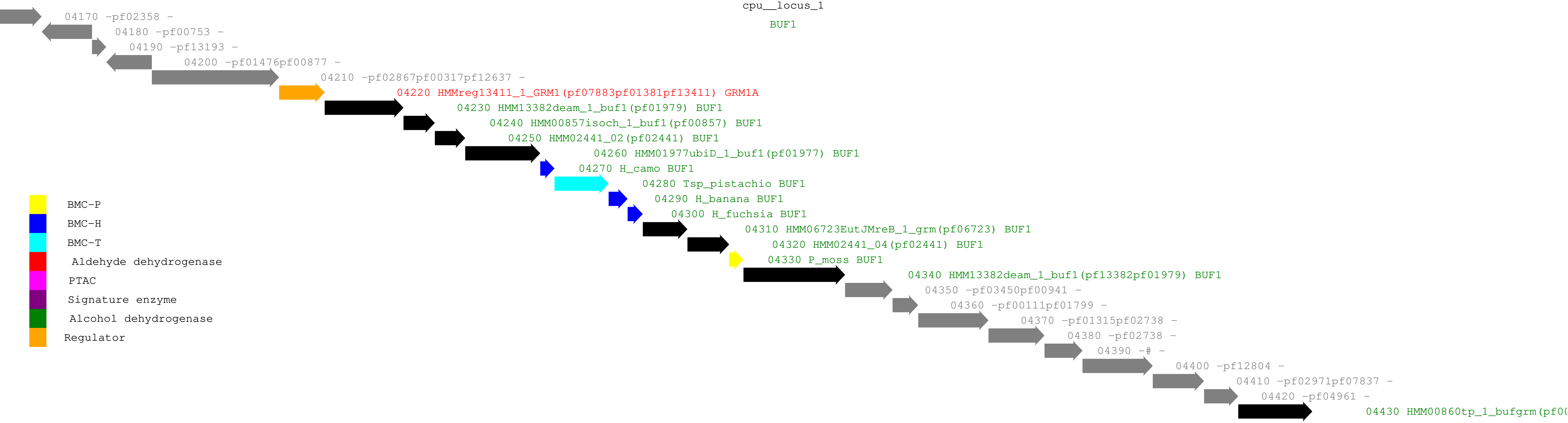

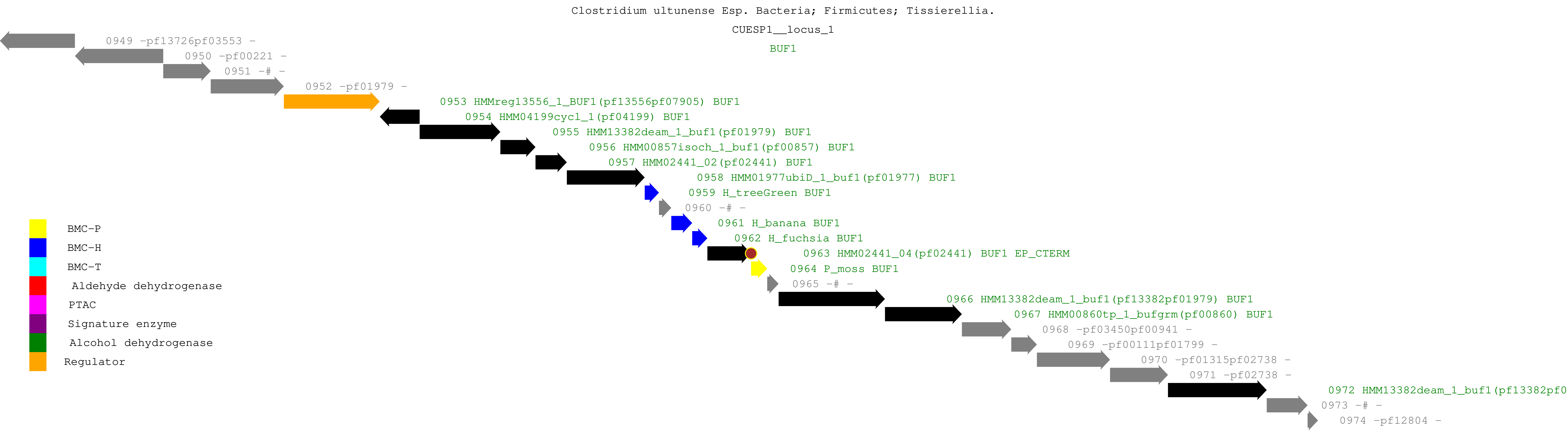

Clostridium ultunense Esp. Bacteria; Firmicutes; Tissierellia.
CUESP1__locus_1
BUF1
0949 -pf13726pf03553 -
0950 -pf00221 -
0951 -# -
0952 -pf01979 -
0953 HMMreg13556_1_BUF1(pf13556pf07905) BUF1
0954 HMM04199cycl_1(pf04199) BUF1
0955 HMM13382deam_1_buf1(pf01979) BUF1
0956 HMM00857isoch_1_buf1(pf00857) BUF1
0957 HMM02441_02(pf02441) BUF1
0958 HMM01977ubiD_1_buf1(pf01977) BUF1
0959 H_treeGreen BUF1
0960 -# -
0961 H_banana BUF1
0962 H_fuchsia BUF1
0963 HMM02441_04(pf02441) BUF1 EP_CTERM
0964 P_moss BUF1
0965 -# -
0966 HMM13382deam_1_buf1(pf13382pf01979) BUF1
0967 HMM00860tp_1_bufgrm(pf00860) BUF1
0968 -pf03450pf00941 -
0969 -pf00111pf01799 -
0970 -pf01315pf02738 -
0971 -pf02738 -
0972 HMM13382deam_1_buf1(pf13382pf0
0973 -# -
0974 -pf12804 -
BMC-P
BMC-H
BMC-T
Aldehyde dehydrogenase
PTAC
Signature enzyme
Alcohol dehydrogenase
Regulator

Clostridium acidurici (strain ATCC 7906 / CIP 104303 / DSM 604 / NCIMB Bacteria; Firmicutes; Clostridia; Clostridiales; Gottschalkia.

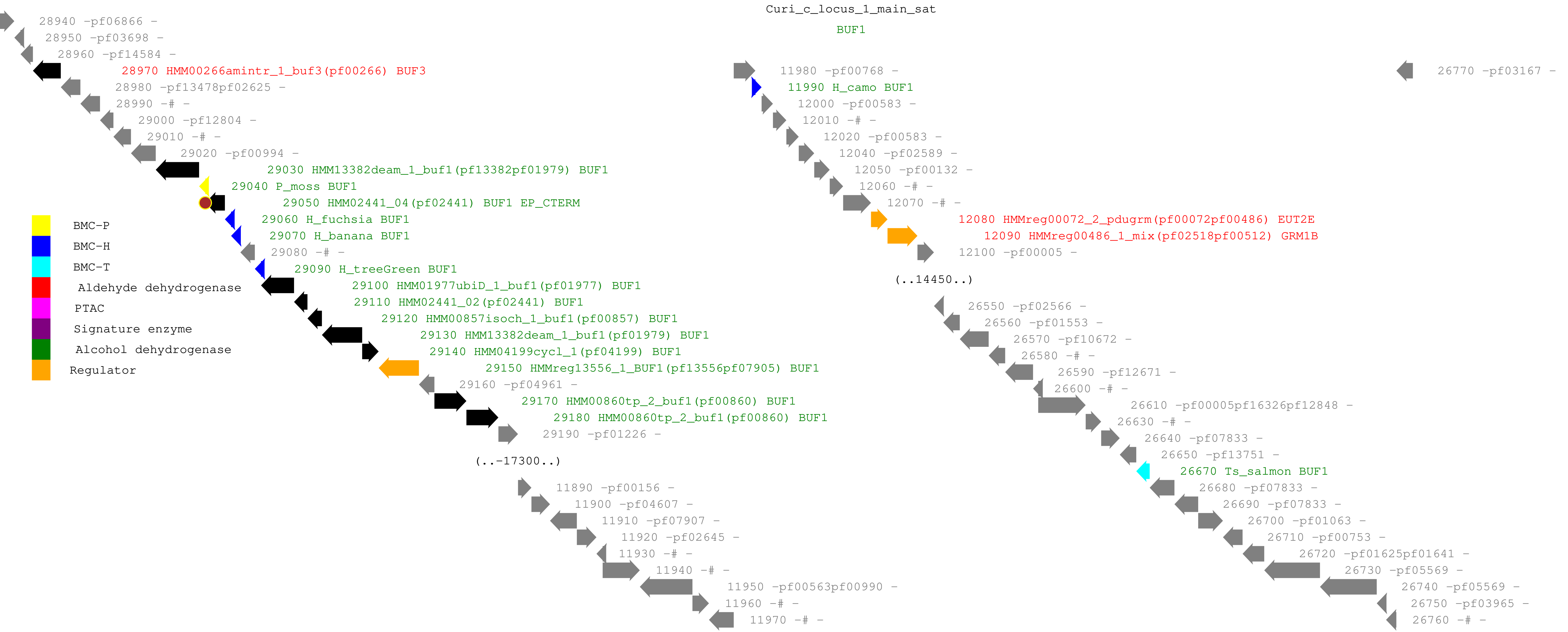

Clostridiaceae bacterium. Bacteria; Firmicutes; Clostridia; Clostridiales; Clostridiaceae.

DHV55__locus_2_main_sat

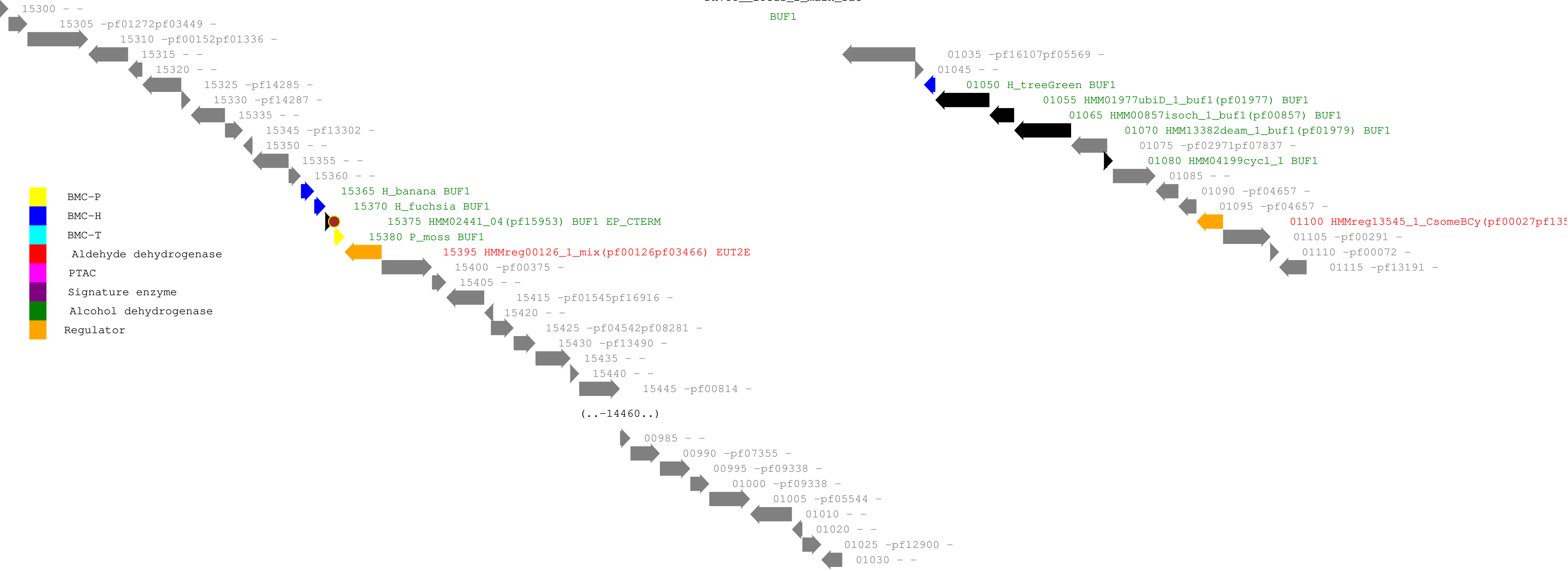

Acinetobacter sp. RIT592. Bacteria; Proteobacteria; Gammaproteobacteria; Pseudomonadales;

DVA85__locus_2

BUF1

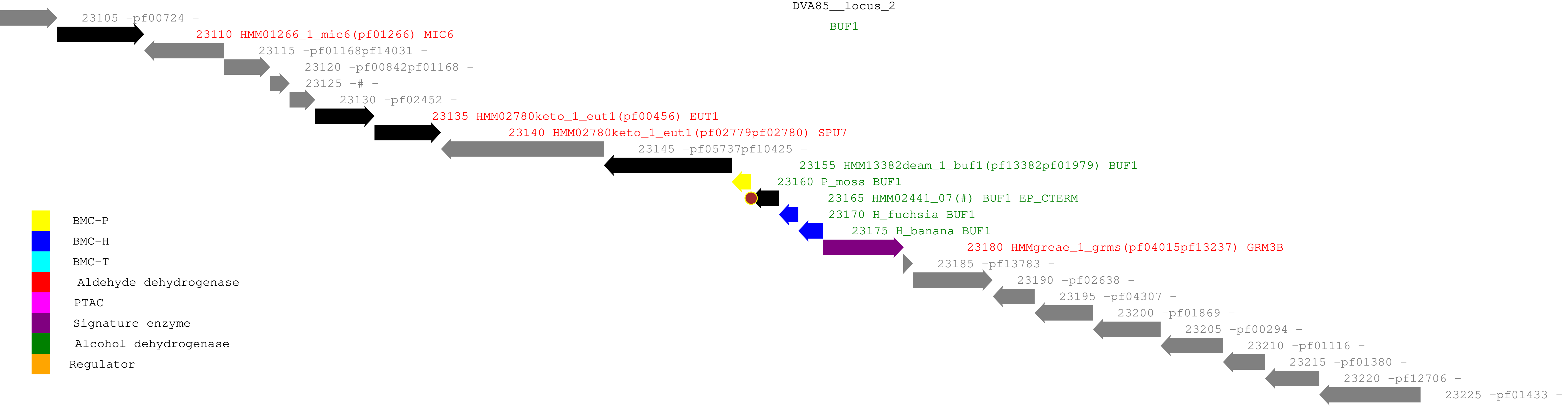

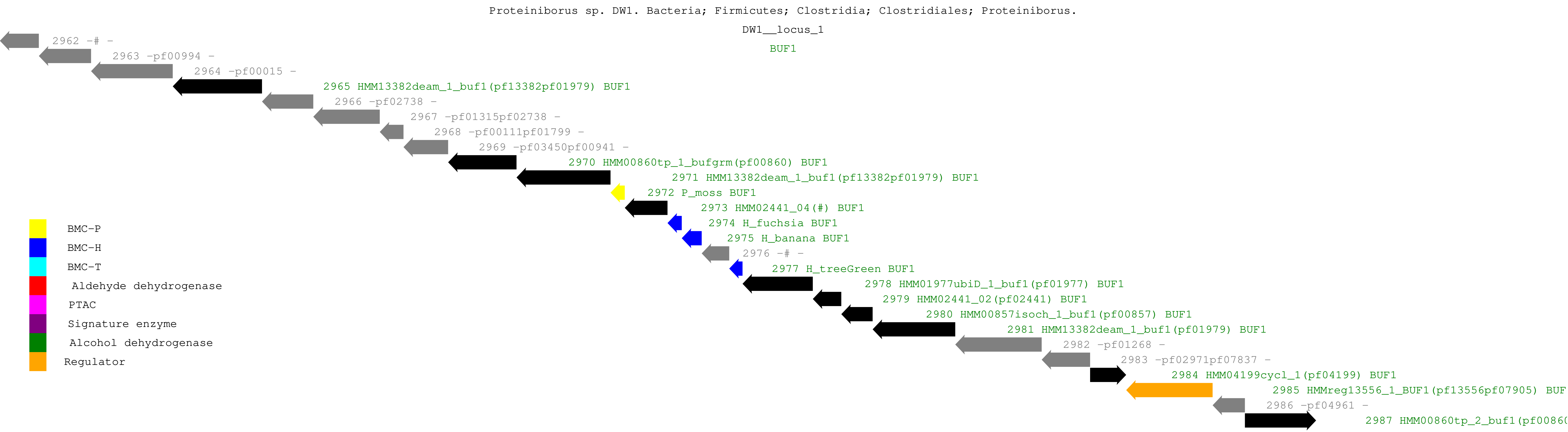
Proteiniborus sp. DW1. Bacteria; Firmicutes; Clostridia; Clostridiales; Proteiniborus.
DW1__locus_1
BUF1
2962 -# -
2963 -pf00994 -
2964 -pf00015 -
2965 HMM13382deam_1_buf1(pf13382pf01979) BUF1
2966 -pf02738 -
2967 -pf01315pf02738 -
2968 -pf00111pf01799 -
2969 -pf03450pf00941 -
2970 HMM00860tp_1_bufgrm(pf00860) BUF1
2971 HMM13382deam_1_buf1(pf13382pf01979) BUF1
2972 P_moss BUF1
2973 HMM02441_04(#) BUF1
2974 H_fuchsia BUF1
2975 H_banana BUF1
2976 -# -
2977 H_treeGreen BUF1
2978 HMM01977ubiD_1_buf1(pf01977) BUF1
2979 HMM02441_02(pf02441) BUF1
2980 HMM00857isoch_1_buf1(pf00857) BUF1
2981 HMM13382deam_1_buf1(pf01979) BUF1
2982 -pf01268 -
2983 -pf02971pf07837 -
2984 HMM04199cycl_1(pf04199) BUF1
2985 HMMreg13556_1_BUF1(pf13556pf07905) BUF
2986 -pf04961 -
2987 HMM00860tp_2_buf1(pf0086
BMC-P
BMC-H
BMC-T
Aldehyde dehydrogenase
PTAC
Signature enzyme
Alcohol dehydrogenase
Regulator

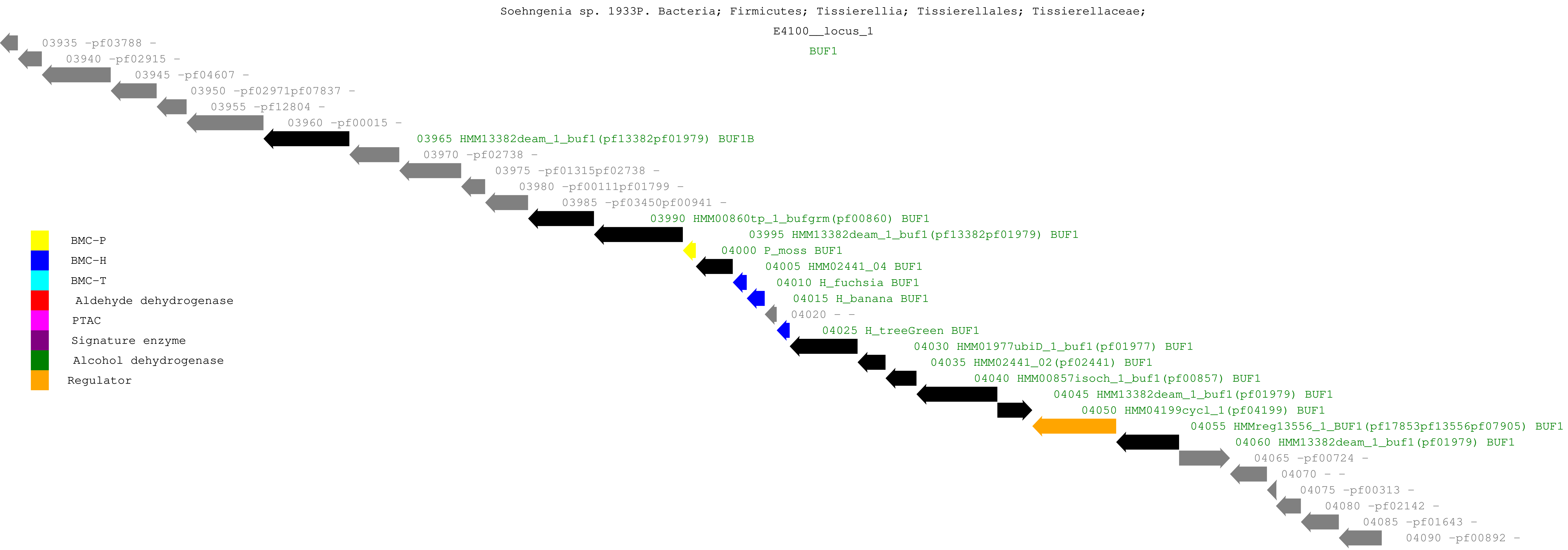
Soehngenia sp. 1933P. Bacteria; Firmicutes; Tissierellia; Tissierellales; Tissierellaceae;
E4100__locus_1
BUF1
03935 -pf03788 -
03940 -pf02915 -
03945 -pf04607 -
03950 -pf02971pf07837 -
03955 -pf12804 -
03960 -pf00015 -
03965 HMM13382deam_1_buf1(pf13382pf01979) BUF1B
03970 -pf02738 -
03975 -pf01315pf02738 -
03980 -pf00111pf01799 -
03985 -pf03450pf00941 -
03990 HMM00860tp_1_bufgrm(pf00860) BUF1
03995 HMM13382deam_1_buf1(pf13382pf01979) BUF1
04000 P_moss BUF1
04005 HMM02441_04 BUF1
04010 H_fuchsia BUF1
04015 H_banana BUF1
04020 - -
04025 H_treeGreen BUF1
04030 HMM01977ubiD_1_buf1(pf01977) BUF1
04035 HMM02441_02(pf02441) BUF1
04040 HMM00857isoch_1_buf1(pf00857) BUF1
04045 HMM13382deam_1_buf1(pf01979) BUF1
04050 HMM04199cycl_1(pf04199) BUF1
04055 HMMreg13556_1_BUF1(pf17853pf13556pf07905) BUF1
04060 HMM13382deam_1_buf1(pf01979) BUF1
04065 -pf00724 -
04070 - -
04075 -pf00313 -
04080 -pf02142 -
04085 -pf01643 -
04090 -pf00892 -
BMC-P
BMC-H
BMC-T
Aldehyde dehydrogenase
PTAC
Signature enzyme
Alcohol dehydrogenase
Regulator

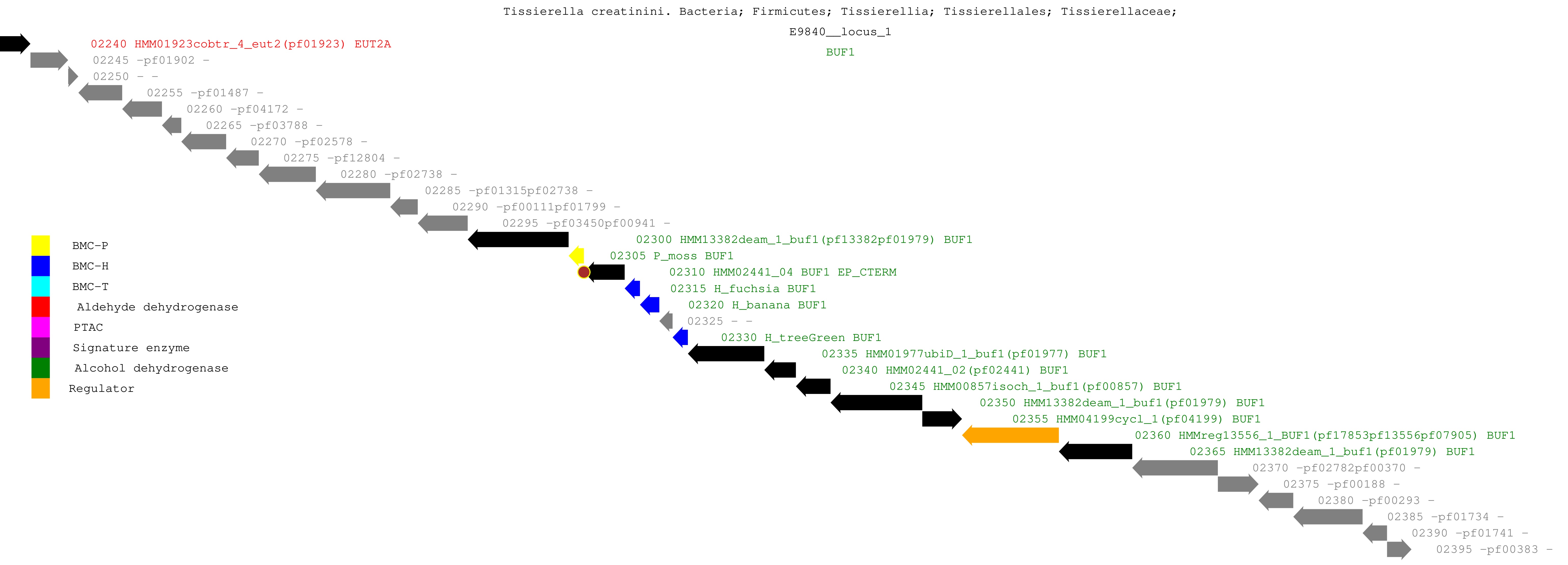
Tissierella creatinini. Bacteria; Firmicutes; Tissierellia; Tissierellales; Tissierellaceae;
E9840__locus_1
BUF1
02240 HMM01923cobtr_4_eut2(pf01923) EUT2A
02245 -pf01902 -
02250 - -
02255 -pf01487 -
02260 -pf04172 -
02265 -pf03788 -
02270 -pf02578 -
02275 -pf12804 -
02280 -pf02738 -
02285 -pf01315pf02738 -
02290 -pf00111pf01799 -
02295 -pf03450pf00941 -
02300 HMM13382deam_1_buf1(pf13382pf01979) BUF1
02305 P_moss BUF1
02310 HMM02441_04 BUF1 EP_CTERM
02315 H_fuchsia BUF1
02320 H_banana BUF1
02325 - -
02330 H_treeGreen BUF1
02335 HMM01977ubiD_1_buf1(pf01977) BUF1
02340 HMM02441_02(pf02441) BUF1
02345 HMM00857isoch_1_buf1(pf00857) BUF1
02350 HMM13382deam_1_buf1(pf01979) BUF1
02355 HMM04199cycl_1(pf04199) BUF1
02360 HMMreg13556_1_BUF1(pf17853pf13556pf07905) BUF1
02365 HMM13382deam_1_buf1(pf01979) BUF1
02370 -pf02782pf00370 -
02375 -pf00188 -
02380 -pf00293 -
02385 -pf01734 -
02390 -pf01741 -
02395 -pf00383 -
BMC-P
BMC-H
BMC-T
Aldehyde dehydrogenase
PTAC
Signature enzyme
Alcohol dehydrogenase
Regulator

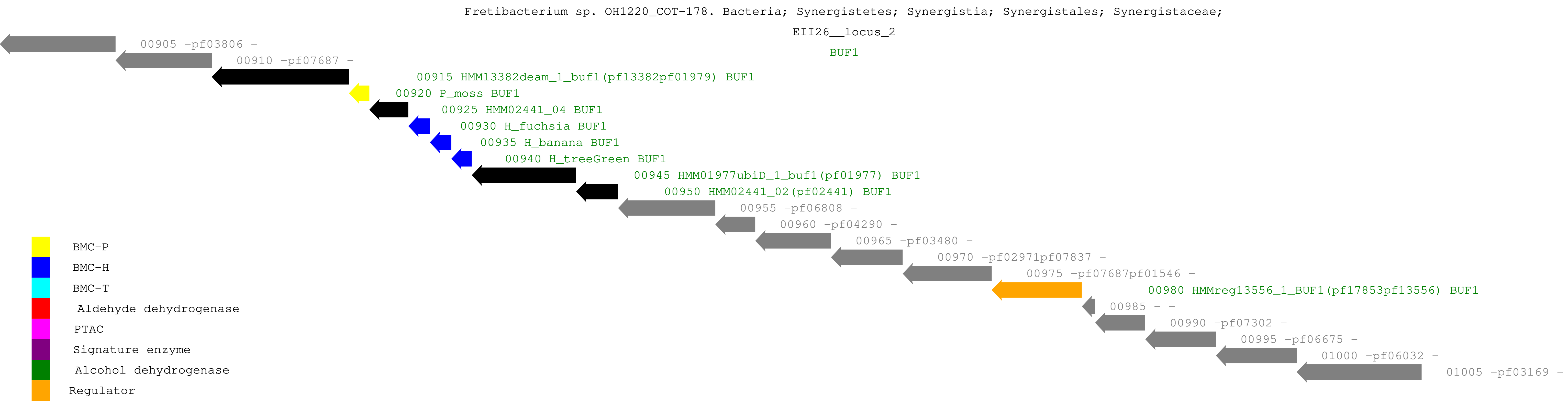
Fretibacterium sp. OH1220_COT-178. Bacteria; Synergistetes; Synergistia; Synergistales; Synergistaceae;
EII26__locus_2
BUF1
00905 -pf03806 -
00910 -pf07687 -
00915 HMM13382deam_1_buf1(pf13382pf01979) BUF1
00920 P_moss BUF1
00925 HMM02441_04 BUF1
00930 H_fuchsia BUF1
00935 H_banana BUF1
00940 H_treeGreen BUF1
00945 HMM01977ubiD_1_buf1(pf01977) BUF1
00950 HMM02441_02(pf02441) BUF1
00955 -pf06808 -
00960 -pf04290 -
00965 -pf03480 -
00970 -pf02971pf07837 -
00975 -pf07687pf01546 -
00980 HMMreg13556_1_BUF1(pf17853pf13556) BUF1
00985 - -
00990 -pf07302 -
00995 -pf06675 -
01000 -pf06032 -
01005 -pf03169 -
BMC-P
BMC-H
BMC-T
Aldehyde dehydrogenase
PTAC
Signature enzyme
Alcohol dehydrogenase
Regulator

Eubacterium angustum. Bacteria; Firmicutes; Clostridia; Clostridiales; Gottschalkia.

EUAN__locus_1_main_sat

BUF1

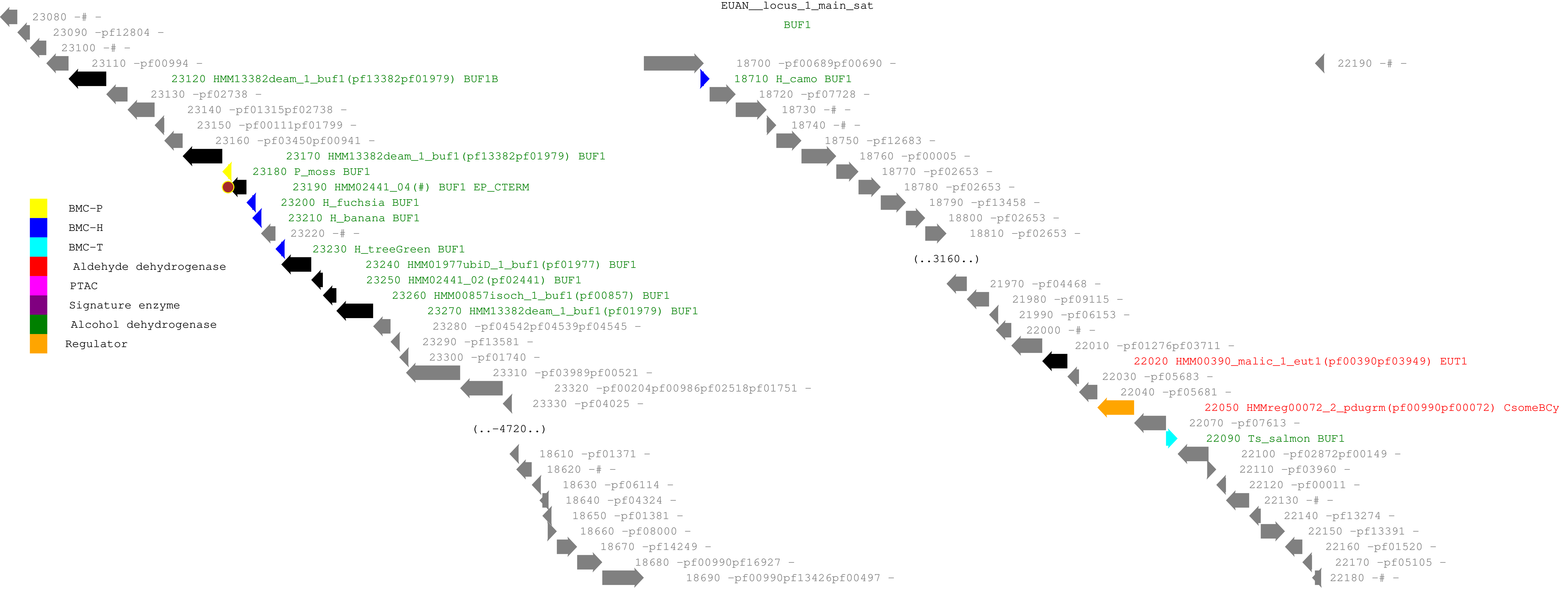

Marinisporobacter balticus. Bacteria; Firmicutes; Clostridia; Clostridiales; Clostridiaceae;

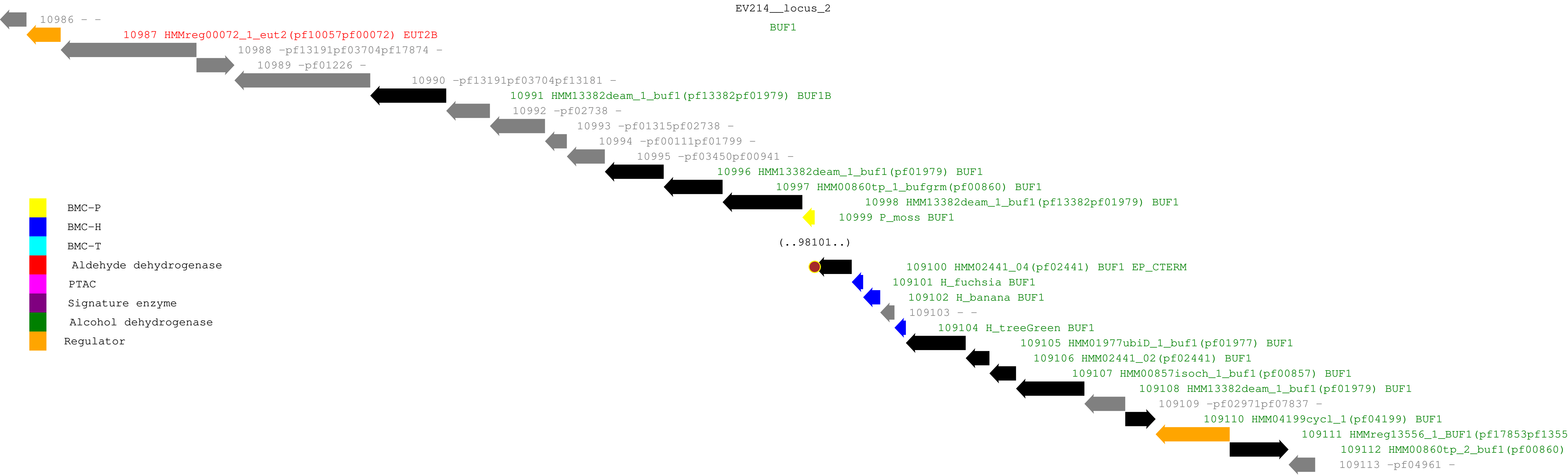

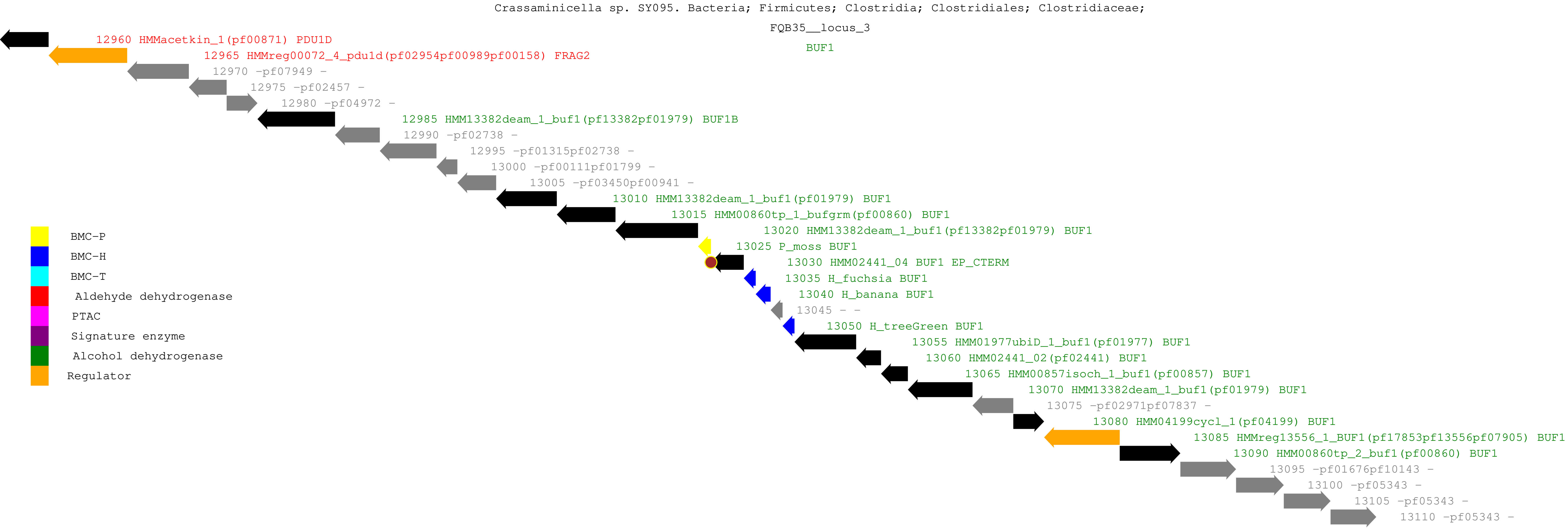
Crassaminicella sp. SY095. Bacteria; Firmicutes; Clostridia; Clostridiales; Clostridiaceae;
FQB35__locus_3
BUF1
12960 HMMacetkin_1(pf00871) PDU1D
12965 HMMreg00072_4_pdu1d(pf02954pf00989pf00158) FRAG2
12970 -pf07949 -
12975 -pf02457 -
12980 -pf04972 -
12985 HMM13382deam_1_buf1(pf13382pf01979) BUF1B
12990 -pf02738 -
12995 -pf01315pf02738 -
13000 -pf00111pf01799 -
13005 -pf03450pf00941 -
13010 HMM13382deam_1_buf1(pf01979) BUF1
13015 HMM00860tp_1_bufgrm(pf00860) BUF1
13020 HMM13382deam_1_buf1(pf13382pf01979) BUF1
13025 P_moss BUF1
13030 HMM02441_04 BUF1 EP_CTERM
13035 H_fuchsia BUF1
13040 H_banana BUF1
13045 - -
13050 H_treeGreen BUF1
13055 HMM01977ubiD_1_buf1(pf01977) BUF1
13060 HMM02441_02(pf02441) BUF1
13065 HMM00857isoch_1_buf1(pf00857) BUF1
13070 HMM13382deam_1_buf1(pf01979) BUF1
13075 -pf02971pf07837 -
13080 HMM04199cycl_1(pf04199) BUF1
13085 HMMreg13556_1_BUF1(pf17853pf13556pf07905) BUF1
13090 HMM00860tp_2_buf1(pf00860) BUF1
13095 -pf01676pf10143 -
13100 -pf05343 -
13105 -pf05343 -
13110 -pf05343 -
BMC-P
BMC-H
BMC-T
Aldehyde dehydrogenase
PTAC
Signature enzyme
Alcohol dehydrogenase
Regulator

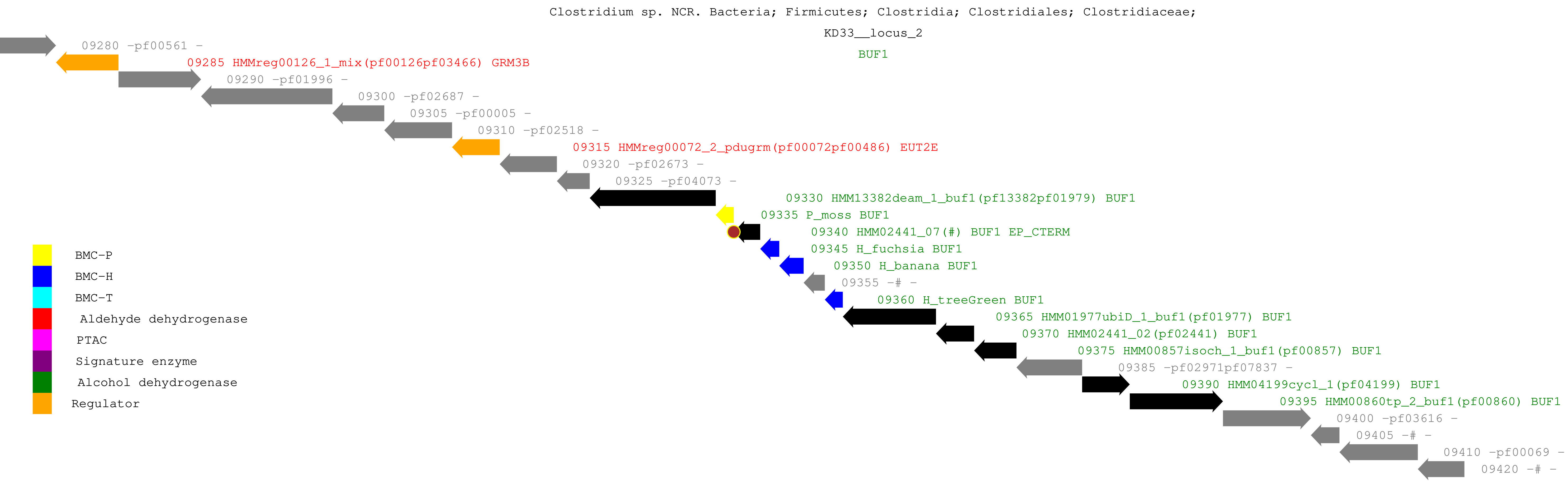
Clostridium sp. NCR. Bacteria; Firmicutes; Clostridia; Clostridiales; Clostridiaceae;
KD33__locus_2
BUF1
09280 -pf00561 -
09285 HMMreg00126_1_mix(pf00126pf03466) GRM3B
09290 -pf01996 -
09300 -pf02687 -
09305 -pf00005 -
09310 -pf02518 -
09315 HMMreg00072_2_pdugrm(pf00072pf00486) EUT2E
09320 -pf02673 -
09325 -pf04073 -
09330 HMM13382deam_1_buf1(pf13382pf01979) BUF1
09335 P_moss BUF1
09340 HMM02441_07(#) BUF1 EP_CTERM
09345 H_fuchsia BUF1
09350 H_banana BUF1
09355 -# -
09360 H_treeGreen BUF1
09365 HMM01977ubiD_1_buf1(pf01977) BUF1
09370 HMM02441_02(pf02441) BUF1
09375 HMM00857isoch_1_buf1(pf00857) BUF1
09385 -pf02971pf07837 -
09390 HMM04199cycl_1(pf04199) BUF1
09395 HMM00860tp_2_buf1(pf00860) BUF1
09400 -pf03616 -
09405 -# -
09410 -pf00069 -
09420 -# -
BMC-P
BMC-H
BMC-T
Aldehyde dehydrogenase
PTAC
Signature enzyme
Alcohol dehydrogenase
Regulator

Tissierella praeacuta. Bacteria; Firmicutes; Tissierellia; Tissierellales; Tissierellaceae;

NCTC11158__locus_1

BUF1

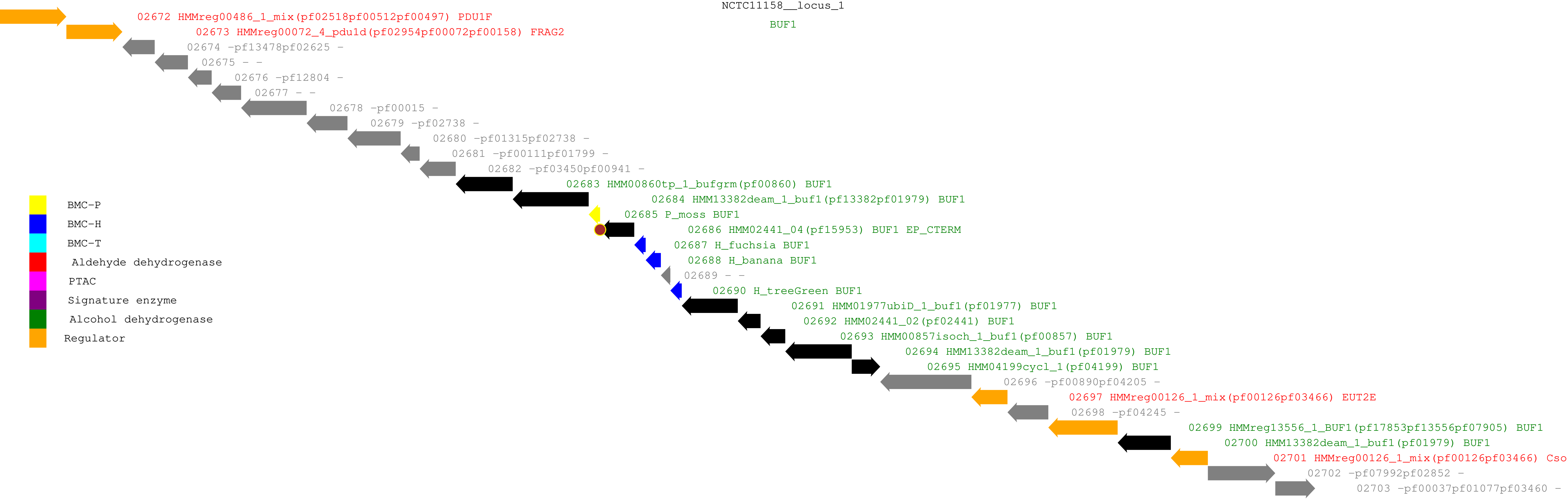

Maledivibacter halophilus. Bacteria; Firmicutes; Clostridia; Clostridiales; Clostridiaceae;

SAMN02194393__locus_3

BUF1

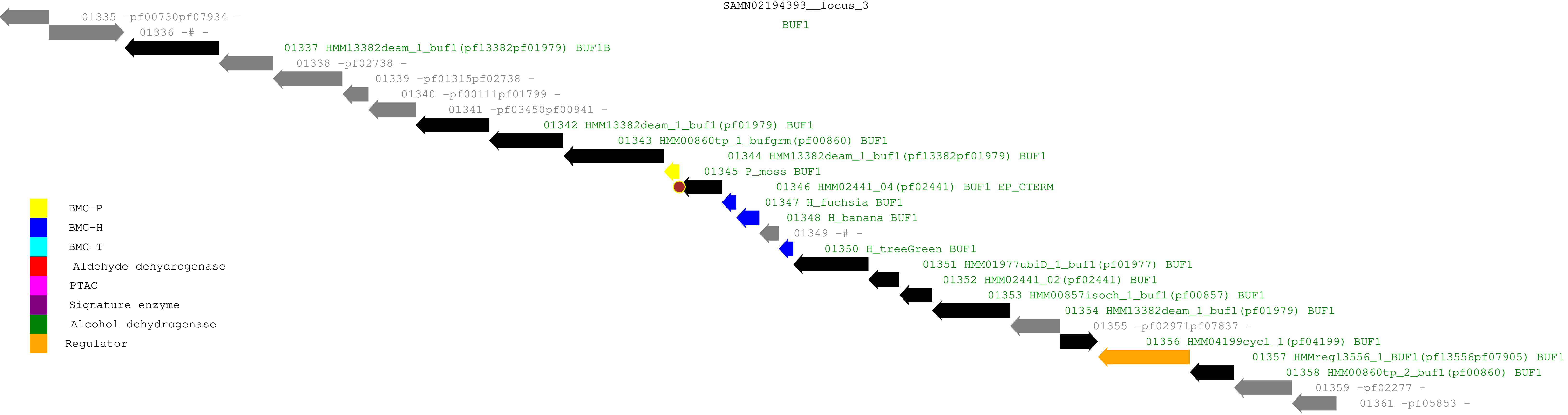

Sporanaerobacter acetigenes DSM 13106. Bacteria; Firmicutes; Tissierellia; Tissierellales; Sporanaerobacter.

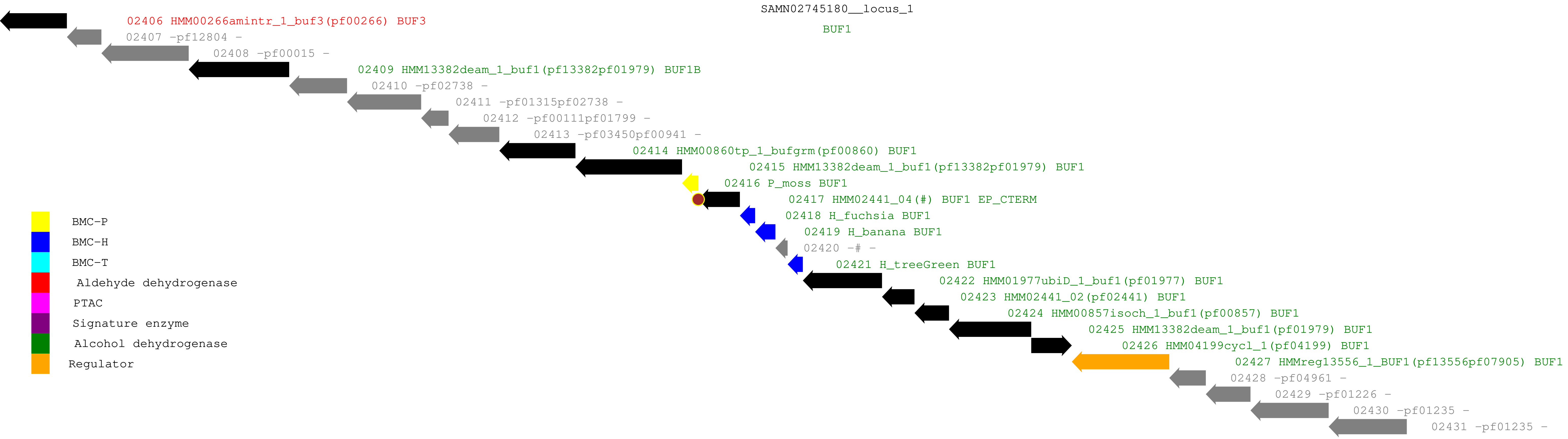

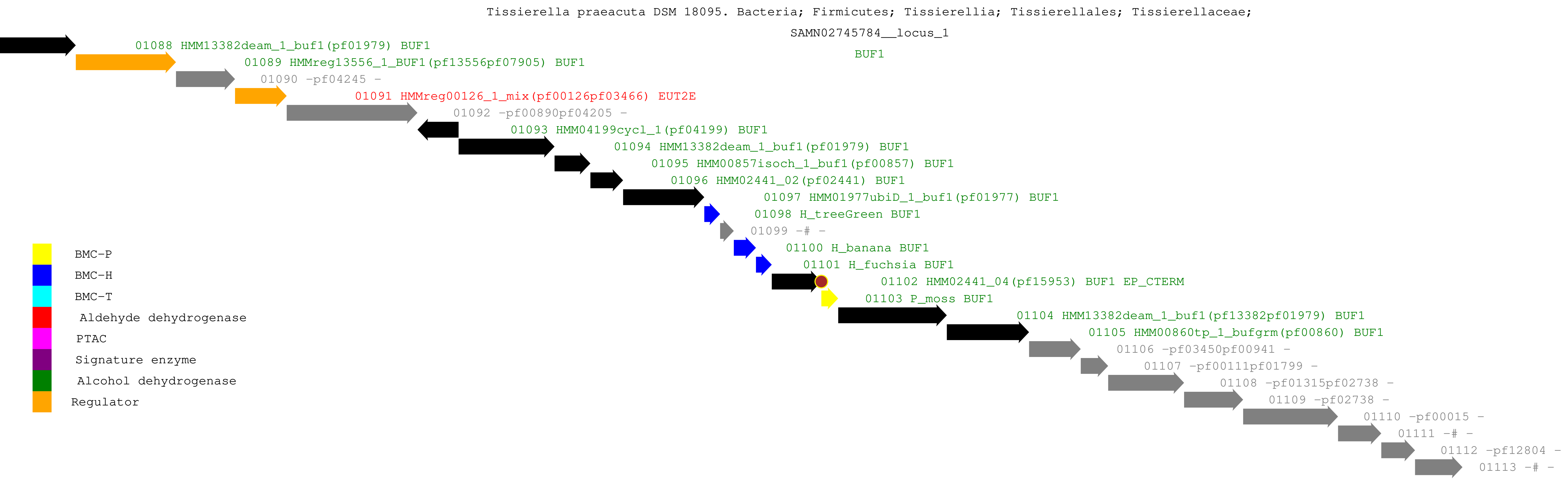
Tissierella praeacuta DSM 18095. Bacteria; Firmicutes; Tissierellia; Tissierellales; Tissierellaceae;
SAMN02745784__locus_1
BUF1
01088 HMM13382deam_1_buf1(pf01979) BUF1
01089 HMMreg13556_1_BUF1(pf13556pf07905) BUF1
01090 -pf04245 -
01091 HMMreg00126_1_mix(pf00126pf03466) EUT2E
01092 -pf00890pf04205 -
01093 HMM04199cycl_1(pf04199) BUF1
01094 HMM13382deam_1_buf1(pf01979) BUF1
01095 HMM00857isoch_1_buf1(pf00857) BUF1
01096 HMM02441_02(pf02441) BUF1
01097 HMM01977ubiD_1_buf1(pf01977) BUF1
01098 H_treeGreen BUF1
01099 -# -
01100 H_banana BUF1
01101 H_fuchsia BUF1
01102 HMM02441_04(pf15953) BUF1 EP_CTERM
01103 P_moss BUF1
01104 HMM13382deam_1_buf1(pf13382pf01979) BUF1
01105 HMM00860tp_1_bufgrm(pf00860) BUF1
01106 -pf03450pf00941 -
01107 -pf00111pf01799 -
01108 -pf01315pf02738 -
01109 -pf02738 -
01110 -pf00015 -
01111 -# -
01112 -pf12804 -
01113 -# -
BMC-P
BMC-H
BMC-T
Aldehyde dehydrogenase
PTAC
Signature enzyme
Alcohol dehydrogenase
Regulator

Acidaminobacter hydrogenoformans DSM 2784. Bacteria; Firmicutes; Clostridia; Clostridiales;

SAMN03080599__locus_1_main_sat

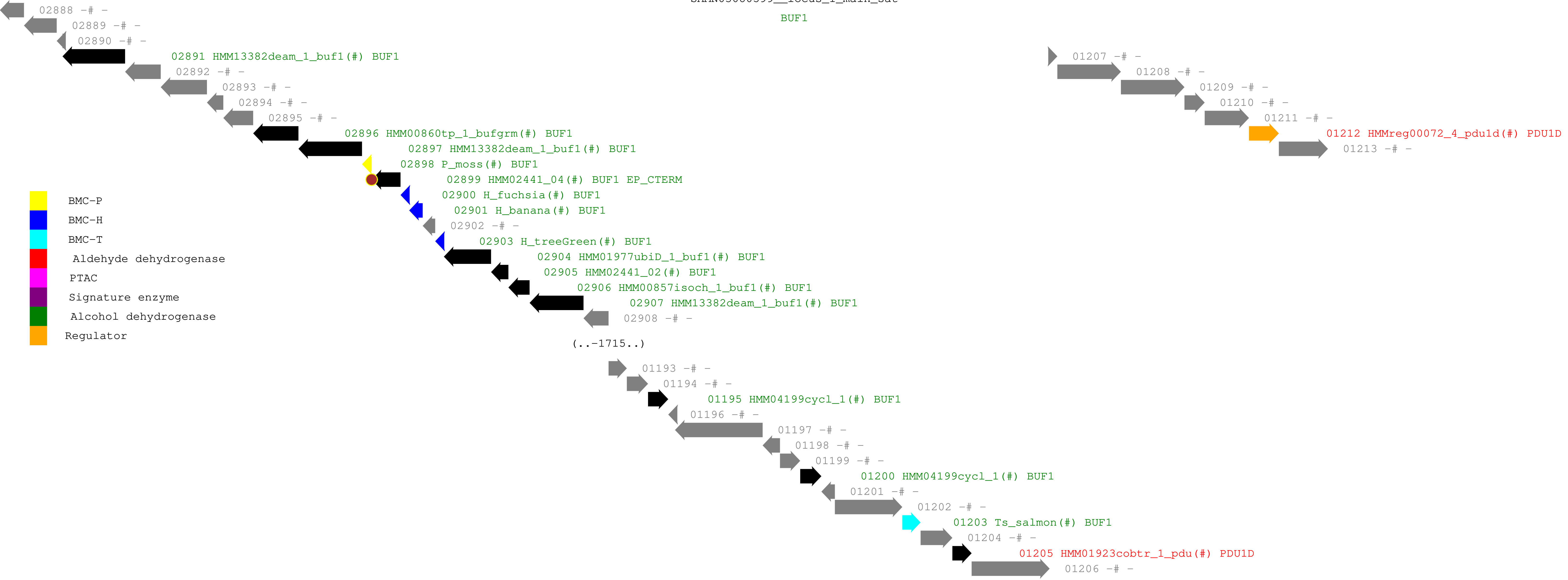

Alkaliphilus peptidifermentans DSM 18978. Bacteria; Firmicutes; Clostridia; Clostridiales; Clostridiaceae;

SAMN03080606__locus_3

BUF1

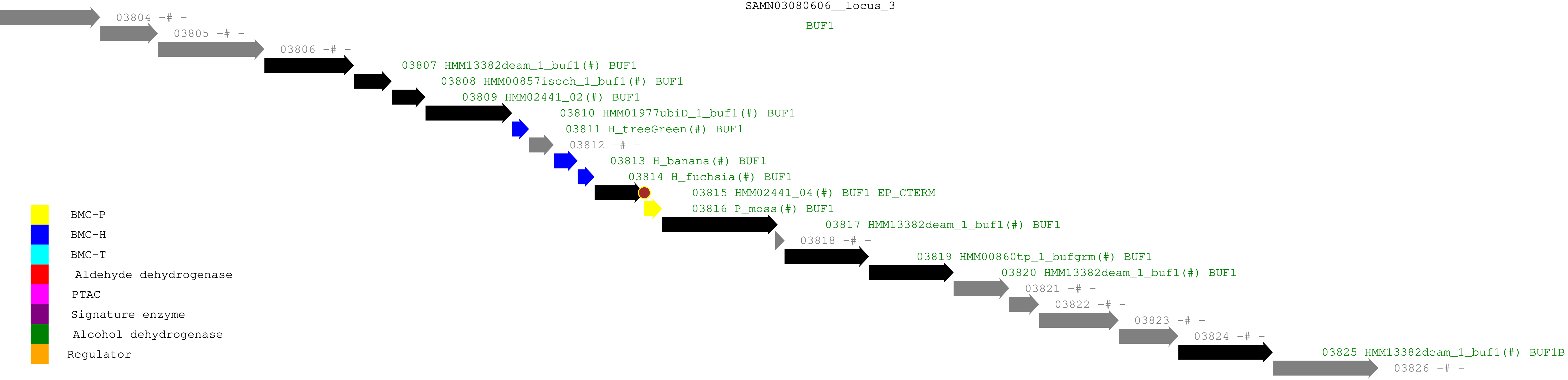

Romboutsia lituseburensis DSM 797. Bacteria; Firmicutes; Clostridia; Clostridiales;

SAMN04515677__locus_2

BUF1

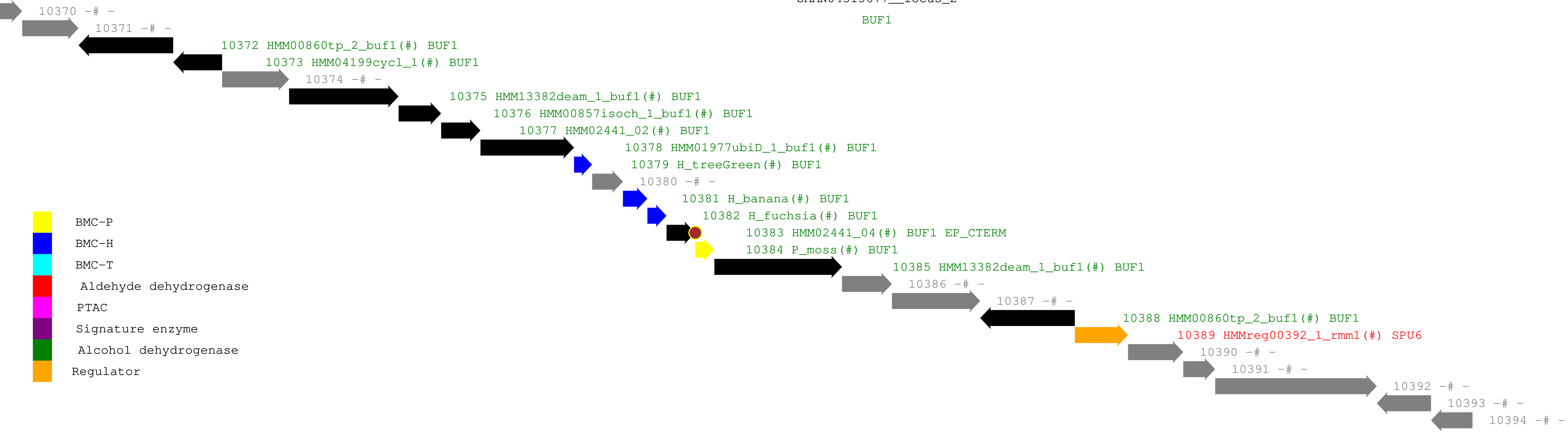

Tindallia californiensis. Bacteria; Firmicutes; Clostridia; Clostridiales; Clostridiaceae;

SAMN05192546__locus_2

BUF1

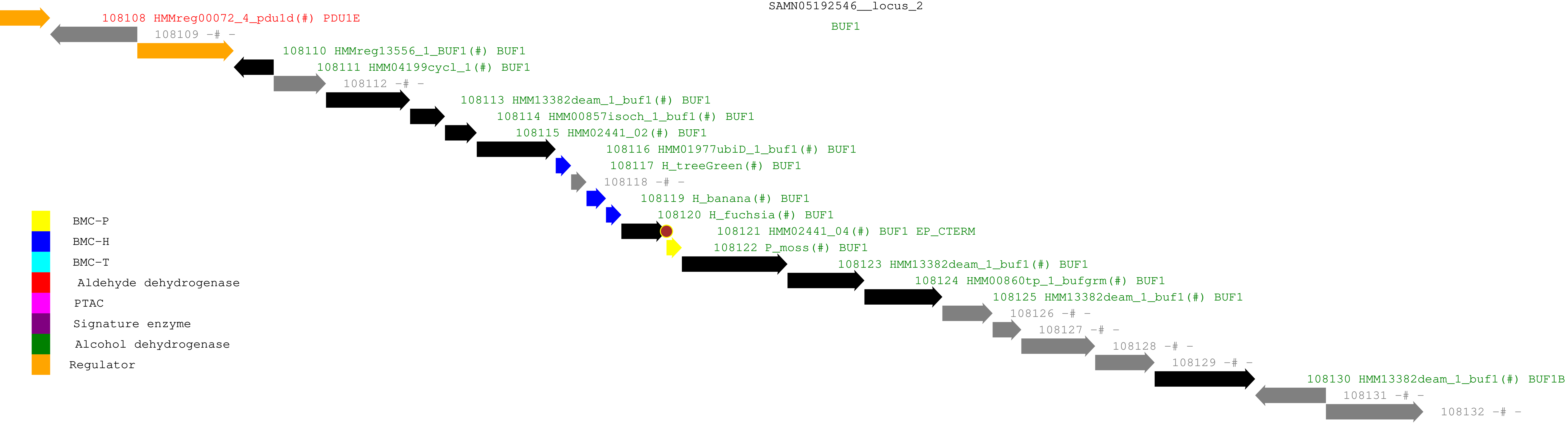

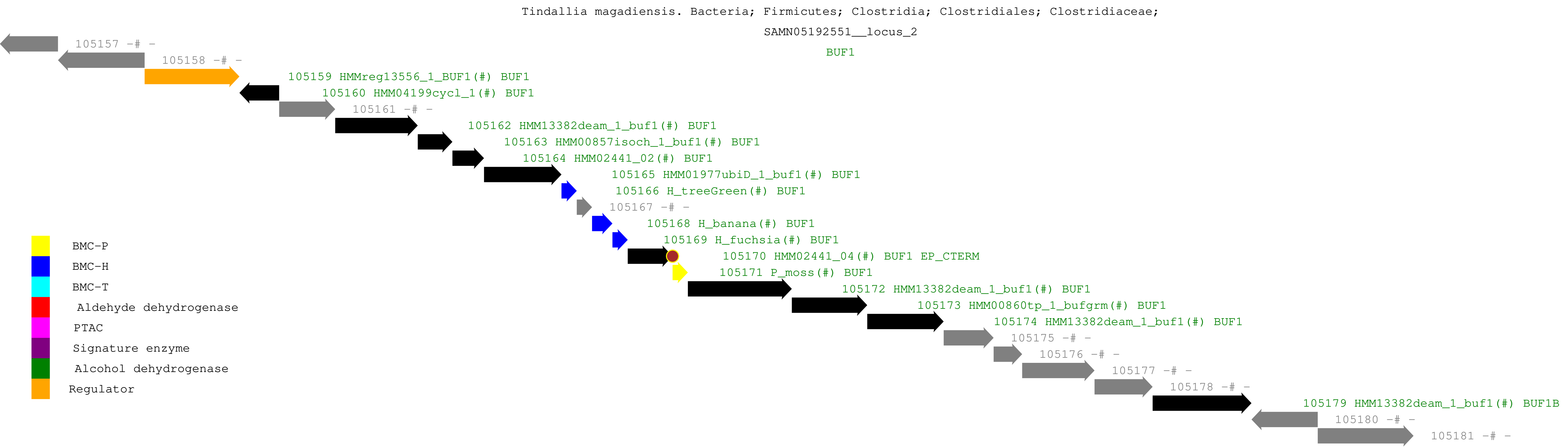
Tindallia magadiensis. Bacteria; Firmicutes; Clostridia; Clostridiales; Clostridiaceae;
SAMN05192551__locus_2
BUF1
105157 -# -
105158 -# -
105159 HMMreg13556_1_BUF1(#) BUF1
105160 HMM04199cycl_1(#) BUF1
105161 -# -
105162 HMM13382deam_1_buf1(#) BUF1
105163 HMM00857isoch_1_buf1(#) BUF1
105164 HMM02441_02(#) BUF1
105165 HMM01977ubiD_1_buf1(#) BUF1
105166 H_treeGreen(#) BUF1
105167 -# -
105168 H_banana(#) BUF1
105169 H_fuchsia(#) BUF1
105170 HMM02441_04(#) BUF1 EP_CTERM
105171 P_moss(#) BUF1
105172 HMM13382deam_1_buf1(#) BUF1
105173 HMM00860tp_1_bufgrm(#) BUF1
105174 HMM13382deam_1_buf1(#) BUF1
105175 -# -
105176 -# -
105177 -# -
105178 -# -
105179 HMM13382deam_1_buf1(#) BUF1B
105180 -# -
105181 -# -
BMC-P
BMC-H
BMC-T
Aldehyde dehydrogenase
PTAC
Signature enzyme
Alcohol dehydrogenase
Regulator

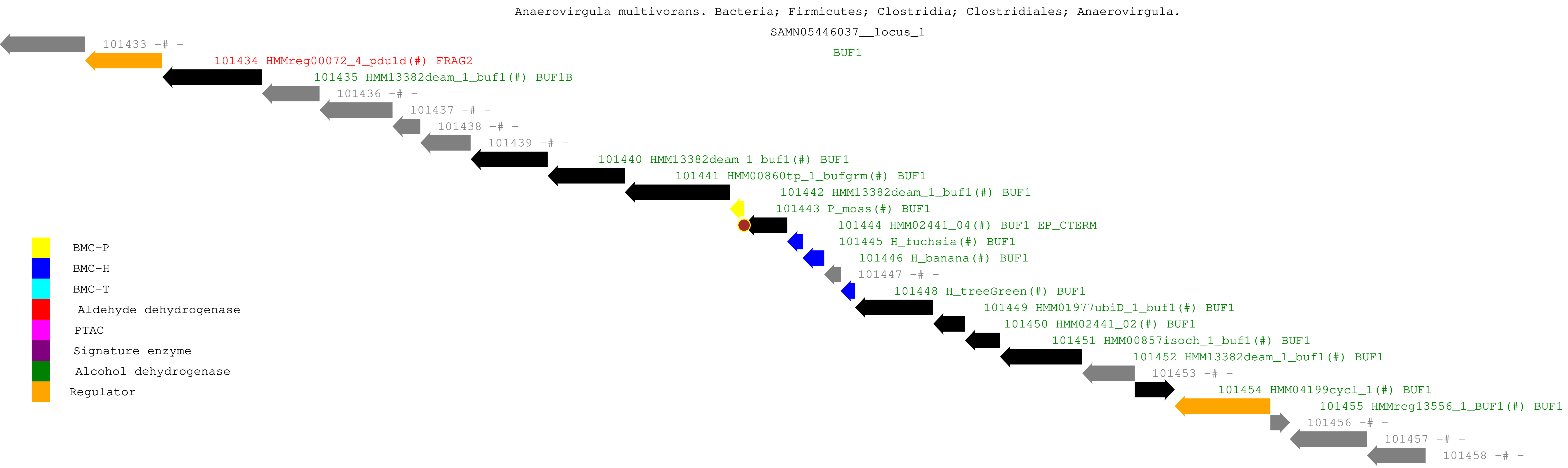
Anaerovirgula multivorans. Bacteria; Firmicutes; Clostridia; Clostridiales; Anaerovirgula.
SAMN05446037__locus_1
BUF1
101433 -# -
101434 HMMreg00072_4_pdu1d(#) FRAG2
101435 HMM13382deam_1_buf1(#) BUF1B
101436 -# -
101437 -# -
101438 -# -
101439 -# -
101440 HMM13382deam_1_buf1(#) BUF1
101441 HMM00860tp_1_bufgrm(#) BUF1
101442 HMM13382deam_1_buf1(#) BUF1
101443 P_moss(#) BUF1
101444 HMM02441_04(#) BUF1 EP_CTERM
101445 H_fuchsia(#) BUF1
101446 H_banana(#) BUF1
101447 -# -
101448 H_treeGreen(#) BUF1
101449 HMM01977ubiD_1_buf1(#) BUF1
101450 HMM02441_02(#) BUF1
101451 HMM00857isoch_1_buf1(#) BUF1
101452 HMM13382deam_1_buf1(#) BUF1
101453 -# -
101454 HMM04199cycl_1(#) BUF1
101455 HMMreg13556_1_BUF1(#) BUF1
101456 -# -
101457 -# -
101458 -# -
BMC-P
BMC-H
BMC-T
Aldehyde dehydrogenase
PTAC
Signature enzyme
Alcohol dehydrogenase
Regulator

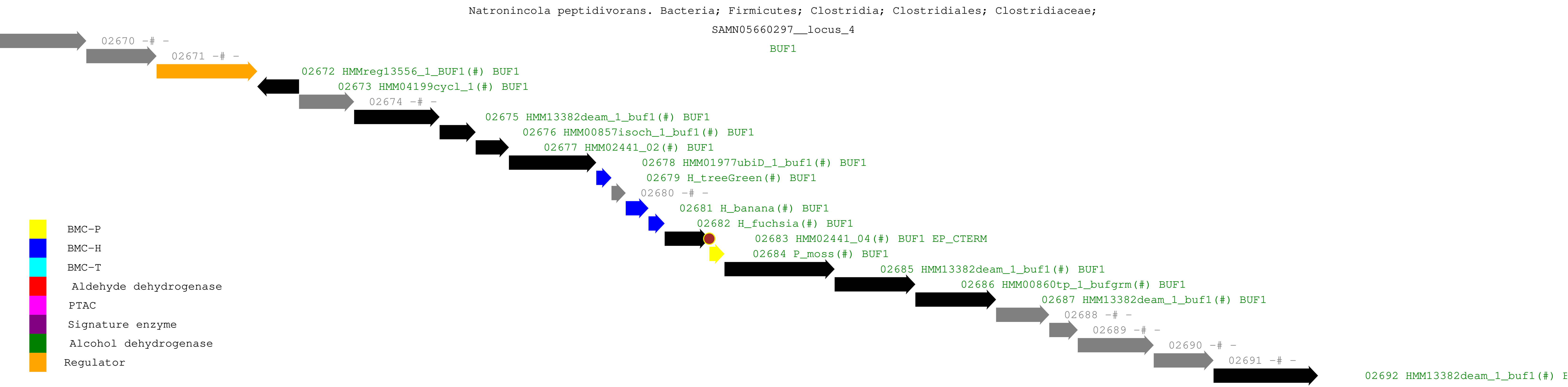

Natronincola peptidivorans. Bacteria; Firmicutes; Clostridia; Clostridiales; Clostridiaceae;
SAMN05660297__locus_4
BUF1
02670 -# -
02671 -# -
02672 HMMreg13556_1_BUF1(#) BUF1
02673 HMM04199cycl_1(#) BUF1
02674 -# -
02675 HMM13382deam_1_buf1(#) BUF1
02676 HMM00857isoch_1_buf1(#) BUF1
02677 HMM02441_02(#) BUF1
02678 HMM01977ubiD_1_buf1(#) BUF1
02679 H_treeGreen(#) BUF1
02680 -# -
02681 H_banana(#) BUF1
02682 H_fuchsia(#) BUF1
02683 HMM02441_04(#) BUF1 EP_CTERM
02684 P_moss(#) BUF1
02685 HMM13382deam_1_buf1(#) BUF1
02686 HMM00860tp_1_bufgrm(#) BUF1
02687 HMM13382deam_1_buf1(#) BUF1
02688 -# -
02689 -# -
02690 -# -
02691 -# -
02692 HMM13382deam_1_buf1(#)
BMC-P
BMC-H
BMC-T
Aldehyde dehydrogenase
PTAC
Signature enzyme
Alcohol dehydrogenase
Regulator

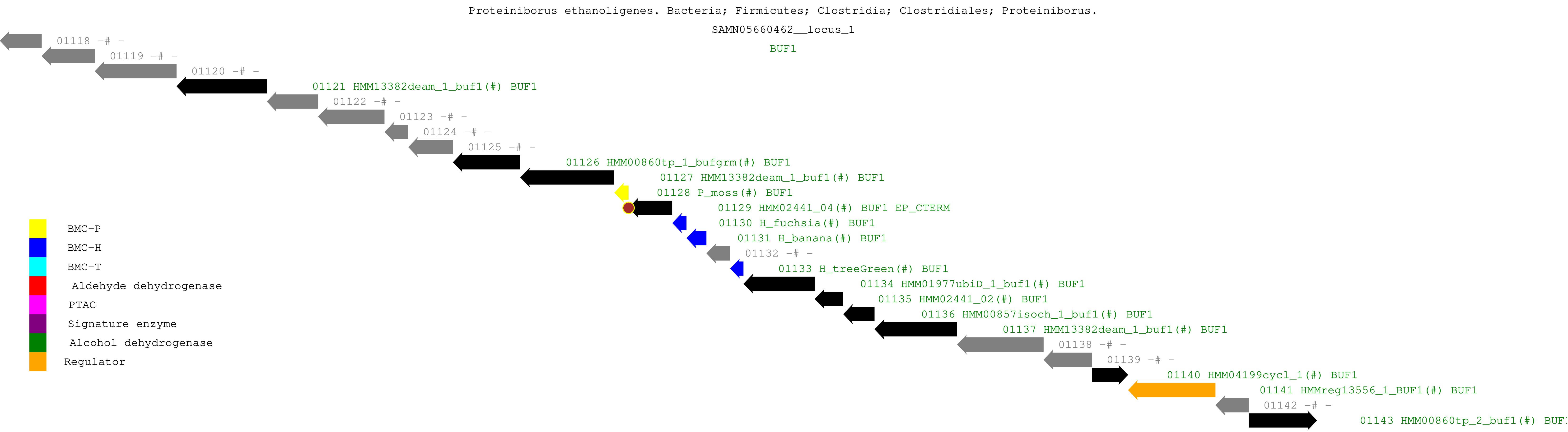
Proteiniborus ethanoligenes. Bacteria; Firmicutes; Clostridia; Clostridiales; Proteiniborus.
SAMN05660462__locus_1
BUF1
01118 -# -
01119 -# -
01120 -# -
01121 HMM13382deam_1_buf1(#) BUF1
01122 -# -
01123 -# -
01124 -# -
01125 -# -
01126 HMM00860tp_1_bufgrm(#) BUF1
01127 HMM13382deam_1_buf1(#) BUF1
01128 P_moss(#) BUF1
01129 HMM02441_04(#) BUF1 EP_CTERM
01130 H_fuchsia(#) BUF1
01131 H_banana(#) BUF1
01132 -# -
01133 H_treeGreen(#) BUF1
01134 HMM01977ubiD_1_buf1(#) BUF1
01135 HMM02441_02(#) BUF1
01136 HMM00857isoch_1_buf1(#) BUF1
01137 HMM13382deam_1_buf1(#) BUF1
01138 -# -
01139 -# -
01140 HMM04199cycl_1(#) BUF1
01141 HMMreg13556_1_BUF1(#) BUF1
01142 -# -
01143 HMM00860tp_2_buf1(#) BUF1
BMC-P
BMC-H
BMC-T
Aldehyde dehydrogenase
PTAC
Signature enzyme
Alcohol dehydrogenase
Regulator

Caloramator fervidus. Bacteria; Firmicutes; Clostridia; Clostridiales; Clostridiaceae;

SAMN05660865__locus_1

BUF1

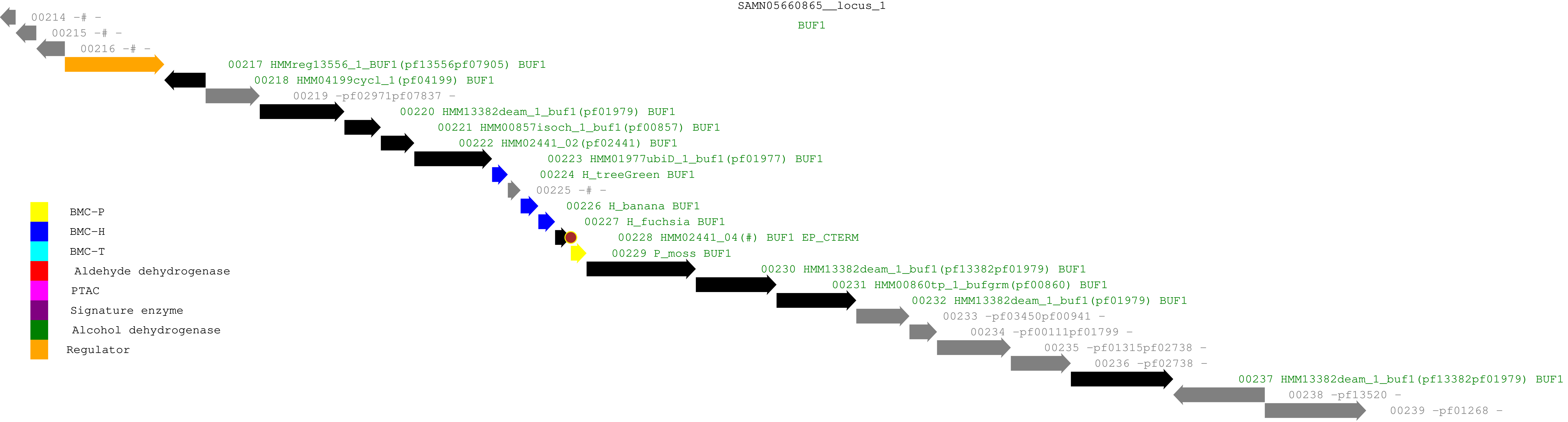

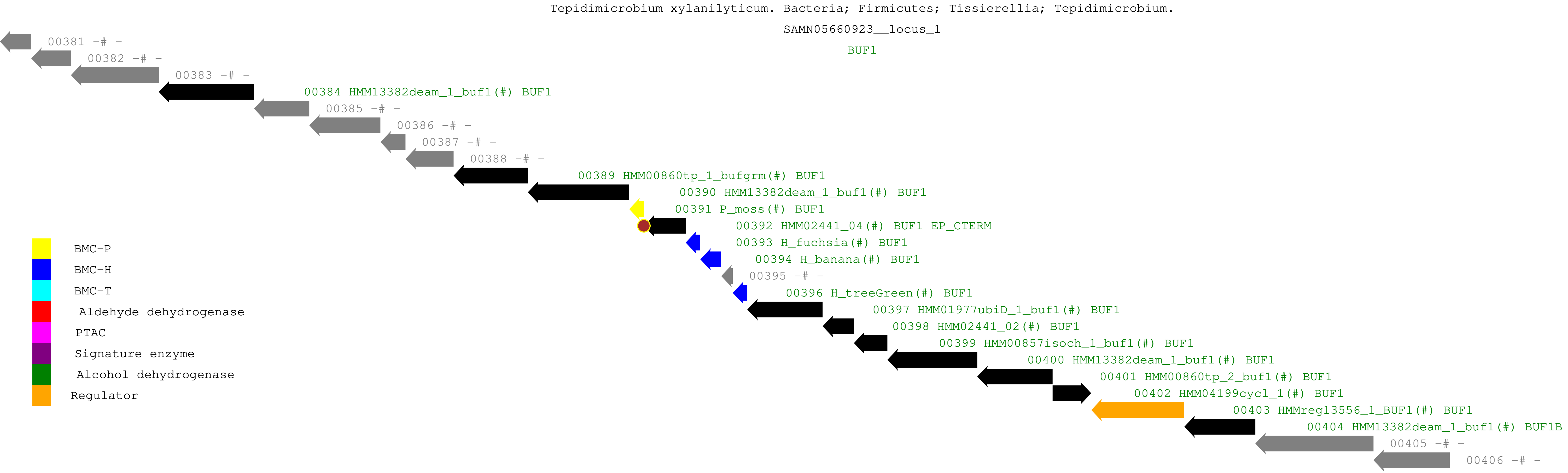
Tepidimicrobium xylanilyticum. Bacteria; Firmicutes; Tissierellia; Tepidimicrobium.
SAMN05660923__locus_1
BUF1
00381 -# -
00382 -# -
00383 -# -
00384 HMM13382deam_1_buf1(#) BUF1
00385 -# -
00386 -# -
00387 -# -
00388 -# -
00389 HMM00860tp_1_bufgrm(#) BUF1
00390 HMM13382deam_1_buf1(#) BUF1
00391 P_moss(#) BUF1
00392 HMM02441_04(#) BUF1 EP_CTERM
00393 H_fuchsia(#) BUF1
00394 H_banana(#) BUF1
00395 -# -
00396 H_treeGreen(#) BUF1
00397 HMM01977ubiD_1_buf1(#) BUF1
00398 HMM02441_02(#) BUF1
00399 HMM00857isoch_1_buf1(#) BUF1
00400 HMM13382deam_1_buf1(#) BUF1
00401 HMM00860tp_2_buf1(#) BUF1
00402 HMM04199cycl_1(#) BUF1
00403 HMMreg13556_1_BUF1(#) BUF1
00404 HMM13382deam_1_buf1(#) BUF1B
00405 -# -
00406 -# -
BMC-P
BMC-H
BMC-T
Aldehyde dehydrogenase
PTAC
Signature enzyme
Alcohol dehydrogenase
Regulator

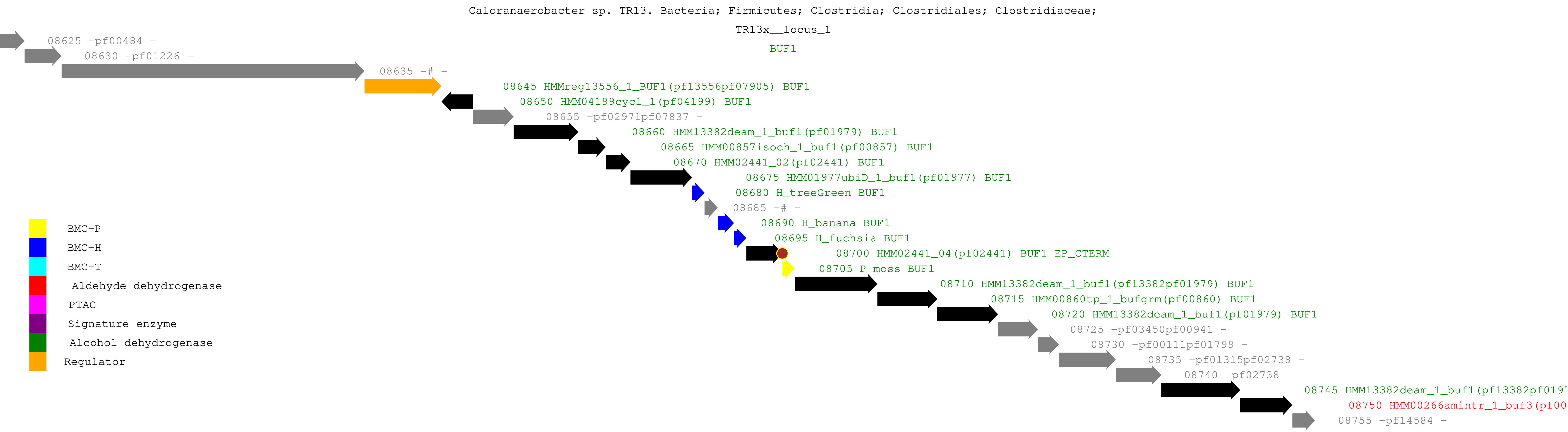

Caloranaerobacter sp. TR13. Bacteria; Firmicutes; Clostridia; Clostridiales; Clostridiaceae;
TR13x__locus_1
BUF1
08625 -pf00484 -
08630 -pf01226 -
08635 -# -
08645 HMMreg13556_1_BUF1(pf13556pf07905) BUF1
08650 HMM04199cycl_1(pf04199) BUF1
08655 -pf02971pf07837 -
08660 HMM13382deam_1_buf1(pf01979) BUF1
08665 HMM00857isoch_1_buf1(pf00857) BUF1
08670 HMM02441_02(pf02441) BUF1
08675 HMM01977ubiD_1_buf1(pf01977) BUF1
08680 H_treeGreen BUF1
08685 -# -
08690 H_banana BUF1
08695 H_fuchsia BUF1
08700 HMM02441_04(pf02441) BUF1 EP_CTERM
08705 P_moss BUF1
08710 HMM13382deam_1_buf1(pf13382pf01979) BUF1
08715 HMM00860tp_1_bufgrm(pf00860) BUF1
08720 HMM13382deam_1_buf1(pf01979) BUF1
08725 -pf03450pf00941 -
08730 -pf00111pf01799 -
08735 -pf01315pf02738 -
08740 -pf02738 -
08745 HMM13382deam_1_buf1(pf13382pf019
08750 HMM00266amintr_1_buf3(pf00
08755 -pf14584 -
BMC-P
BMC-H
BMC-T
Aldehyde dehydrogenase
PTAC
Signature enzyme
Alcohol dehydrogenase
Regulator

Paraclostridium benzoelyticum. Bacteria; Firmicutes; Clostridia; Clostridiales;

VN21__locus_1

BUF1

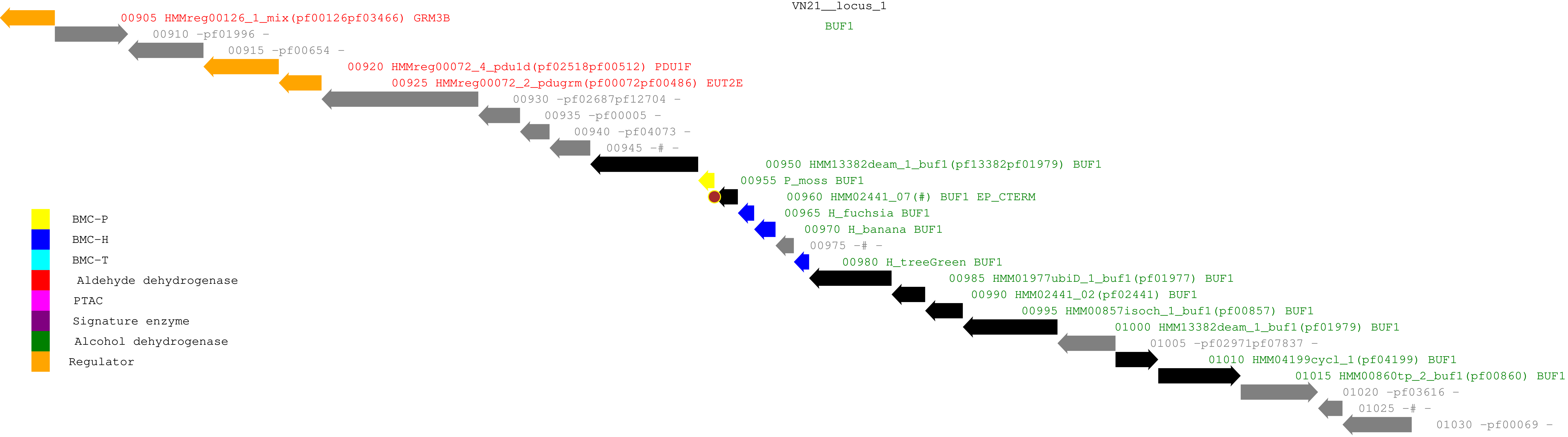

Bacillus oceanisediminis 2691. Bacteria; Firmicutes; Bacilli; Bacillales; Bacillaceae; Bacillus.

A361__locus_1

BUF1B

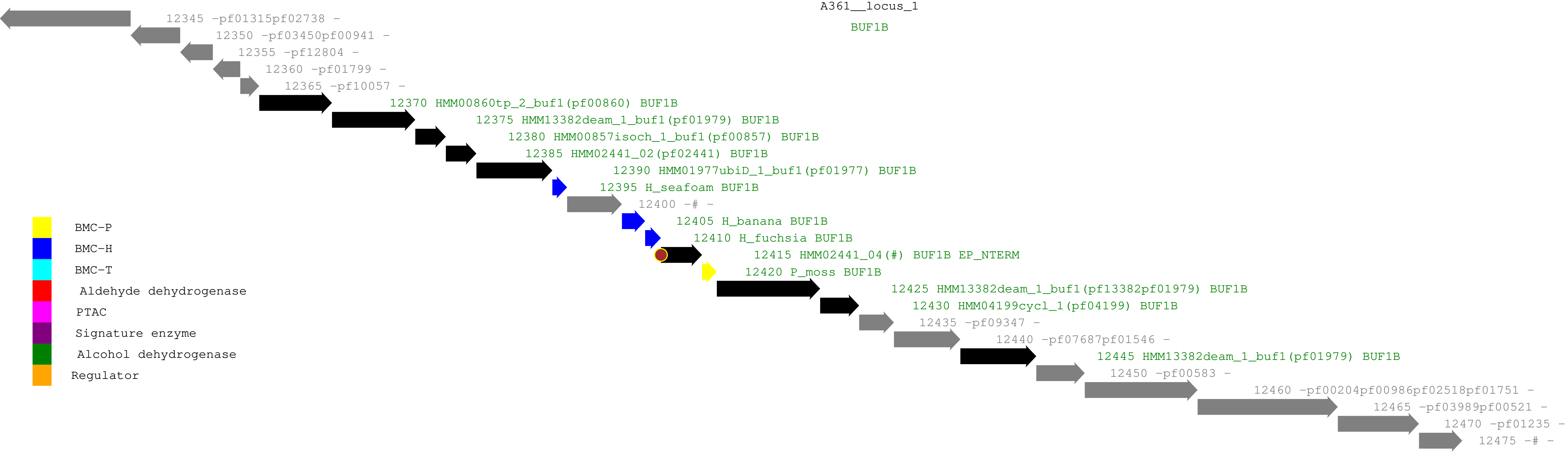

Sporosarcina globispora (Bacillus globisporus). Bacteria; Firmicutes; Bacilli; Bacillales; Planococcaceae;

AF332__locus_2

BUF1B

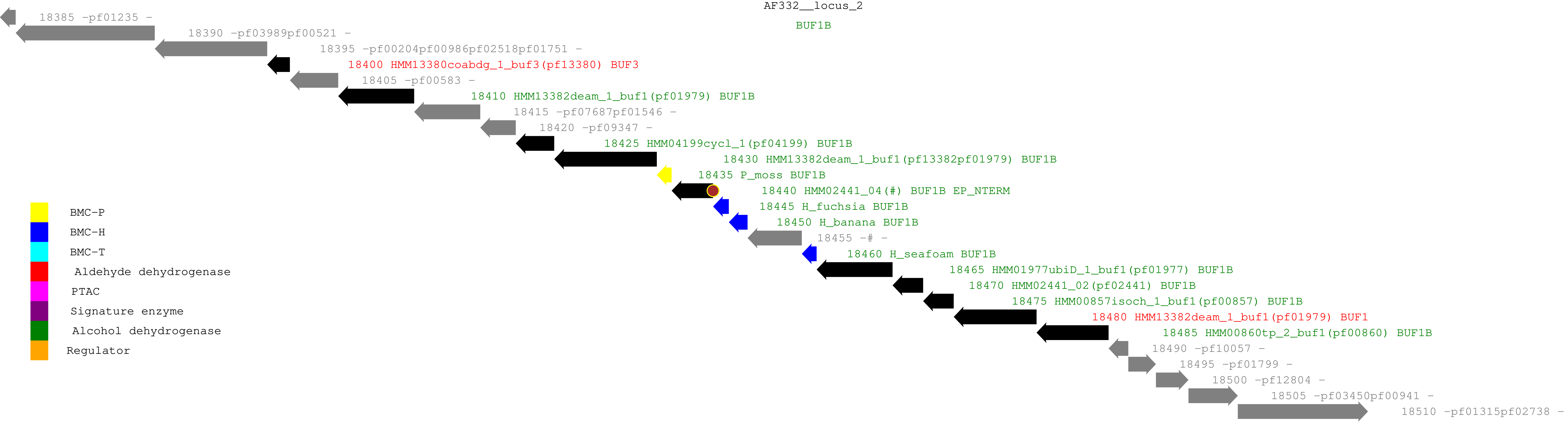

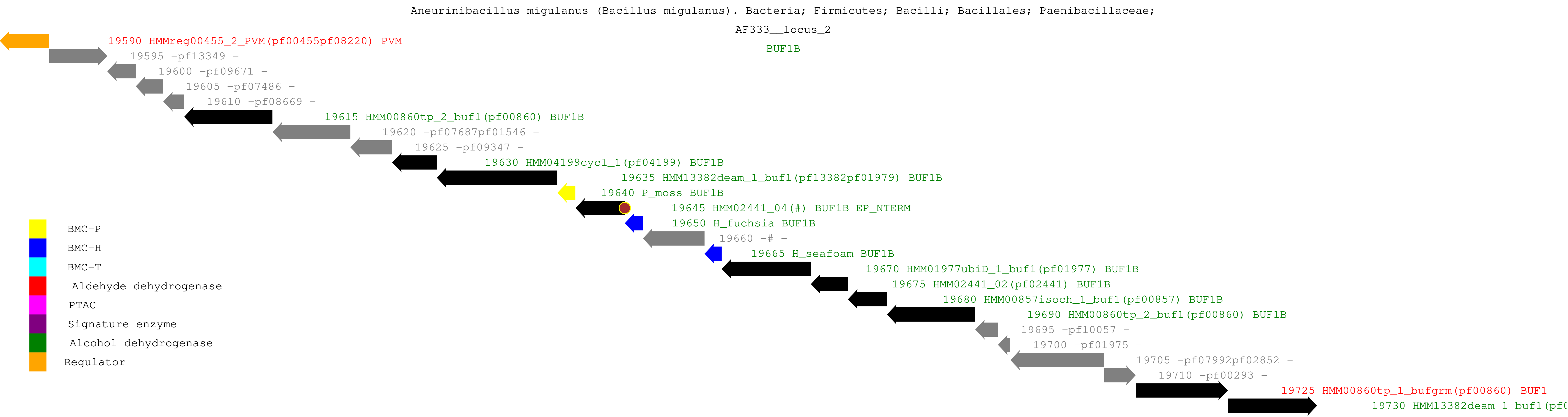
Aneurinibacillus migulanus (Bacillus migulanus). Bacteria; Firmicutes; Bacilli; Bacillales; Paenibacillaceae;
AF333__locus_2
BUF1B
19590 HMMreg00455_2_PVM(pf00455pf08220) PVM
19595 -pf13349 -
19600 -pf09671 -
19605 -pf07486 -
19610 -pf08669 -
19615 HMM00860tp_2_buf1(pf00860) BUF1B
19620 -pf07687pf01546 -
19625 -pf09347 -
19630 HMM04199cycl_1(pf04199) BUF1B
19635 HMM13382deam_1_buf1(pf13382pf01979) BUF1B
19640 P_moss BUF1B
19645 HMM02441_04(#) BUF1B EP_NTERM
19650 H_fuchsia BUF1B
19660 -# -
19665 H_seafoam BUF1B
19670 HMM01977ubiD_1_buf1(pf01977) BUF1B
19675 HMM02441_02(pf02441) BUF1B
19680 HMM00857isoch_1_buf1(pf00857) BUF1B
19690 HMM00860tp_2_buf1(pf00860) BUF1B
19695 -pf10057 -
19700 -pf01975 -
19705 -pf07992pf02852 -
19710 -pf00293 -
19725 HMM00860tp_1_bufgrm(pf00860) BUF1
19730 HMM13382deam_1_buf1(pf0
BMC-P
BMC-H
BMC-T
Aldehyde dehydrogenase
PTAC
Signature enzyme
Alcohol dehydrogenase
Regulator

Bacillus sp. FJAT-21945. Bacteria; Firmicutes; Bacilli; Bacillales; Bacillaceae; Bacillus.

AMS60__locus_1

BUF1B

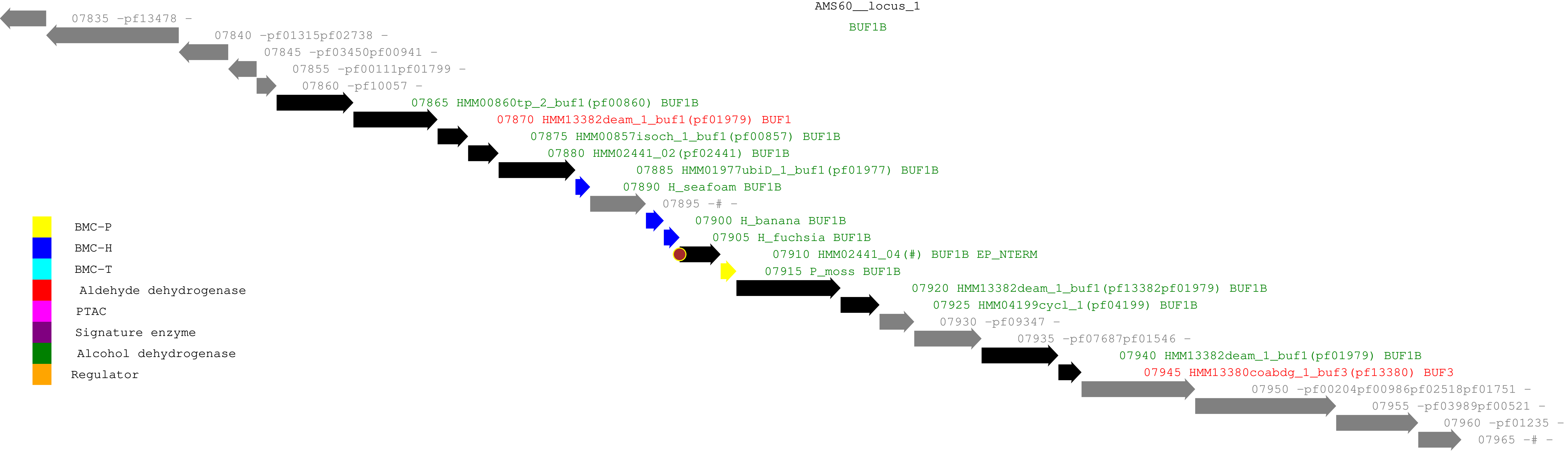

Bacillus solani. Bacteria; Firmicutes; Bacilli; Bacillales; Bacillaceae; Bacillus.

AN957__locus_1

BUF1B

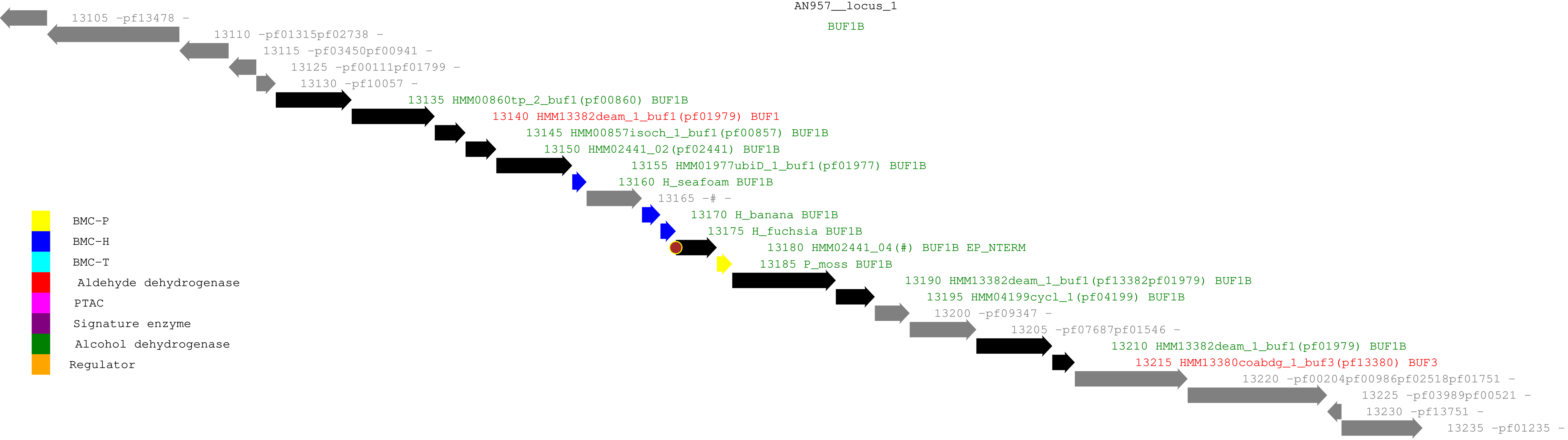

Fictibacillus enclensis. Bacteria; Firmicutes; Bacilli; Bacillales; Bacillaceae; Fictibacillus.

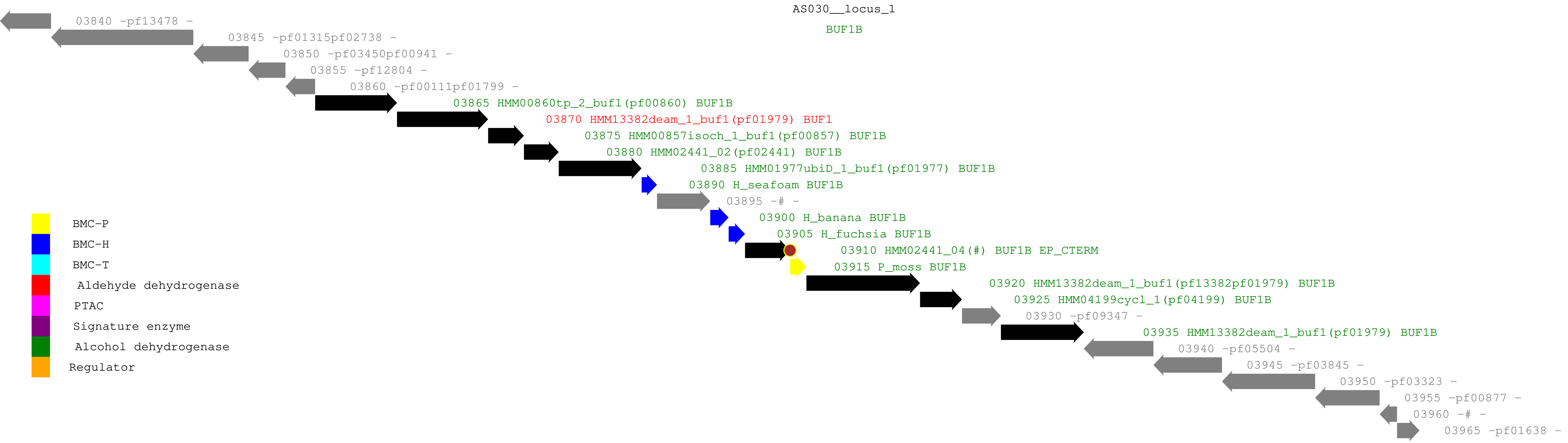

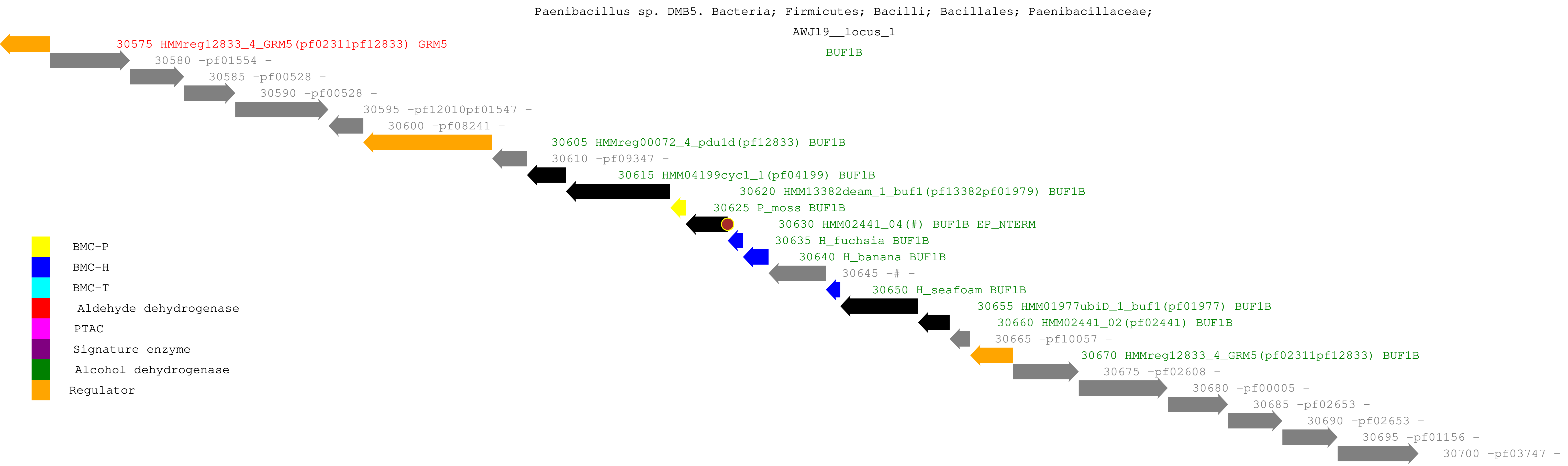

Paenibacillus sp. DMB5. Bacteria; Firmicutes; Bacilli; Bacillales; Paenibacillaceae;
AWJ19__locus_1
BUF1B
30575 HMMreg12833_4_GRM5(pf02311pf12833) GRM5
30580 -pf01554 -
30585 -pf00528 -
30590 -pf00528 -
30595 -pf12010pf01547 -
30600 -pf08241 -
30605 HMMreg00072_4_pdu1d(pf12833) BUF1B
30610 -pf09347 -
30615 HMM04199cycl_1(pf04199) BUF1B
30620 HMM13382deam_1_buf1(pf13382pf01979) BUF1B
30625 P_moss BUF1B
30630 HMM02441_04(#) BUF1B EP_NTERM
30635 H_fuchsia BUF1B
30640 H_banana BUF1B
30645 -# -
30650 H_seafoam BUF1B
30655 HMM01977ubiD_1_buf1(pf01977) BUF1B
30660 HMM02441_02(pf02441) BUF1B
30665 -pf10057 -
30670 HMMreg12833_4_GRM5(pf02311pf12833) BUF1B
30675 -pf02608 -
30680 -pf00005 -
30685 -pf02653 -
30690 -pf02653 -
30695 -pf01156 -
30700 -pf03747 -
BMC-P
BMC-H
BMC-T
Aldehyde dehydrogenase
PTAC
Signature enzyme
Alcohol dehydrogenase
Regulator

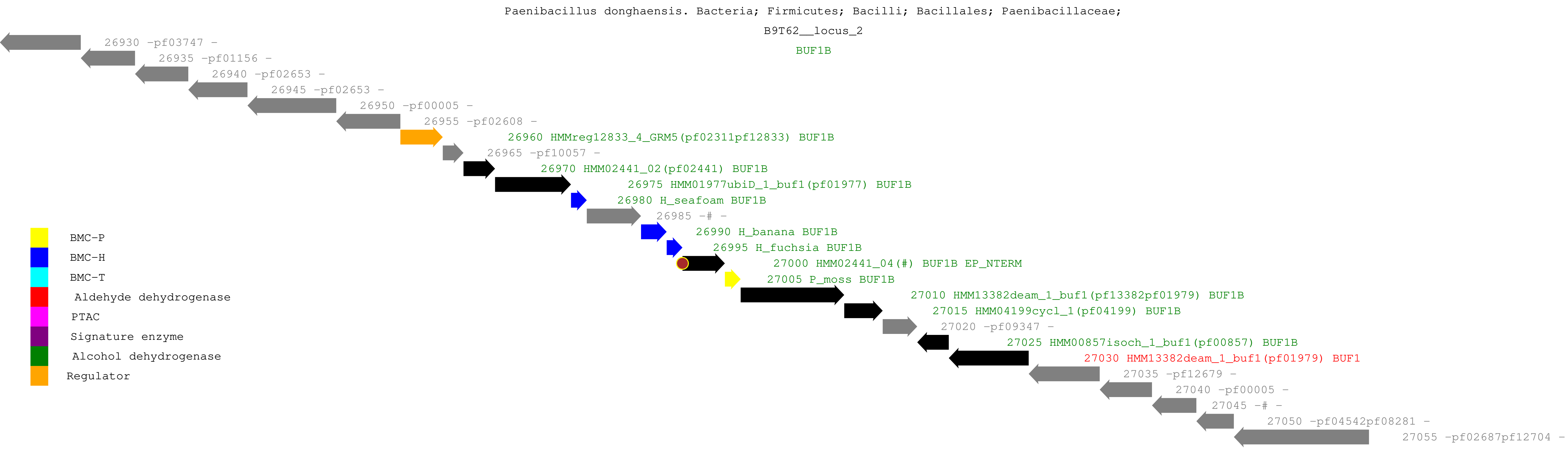
Paenibacillus donghaensis. Bacteria; Firmicutes; Bacilli; Bacillales; Paenibacillaceae;
B9T62__locus_2
BUF1B
26930 -pf03747 -
26935 -pf01156 -
26940 -pf02653 -
26945 -pf02653 -
26950 -pf00005 -
26955 -pf02608 -
26960 HMMreg12833_4_GRM5(pf02311pf12833) BUF1B
26965 -pf10057 -
26970 HMM02441_02(pf02441) BUF1B
26975 HMM01977ubiD_1_buf1(pf01977) BUF1B
26980 H_seafoam BUF1B
26985 -# -
26990 H_banana BUF1B
26995 H_fuchsia BUF1B
27000 HMM02441_04(#) BUF1B EP_NTERM
27005 P_moss BUF1B
27010 HMM13382deam_1_buf1(pf13382pf01979) BUF1B
27015 HMM04199cycl_1(pf04199) BUF1B
27020 -pf09347 -
27025 HMM00857isoch_1_buf1(pf00857) BUF1B
27030 HMM13382deam_1_buf1(pf01979) BUF1
27035 -pf12679 -
27040 -pf00005 -
27045 -# -
27050 -pf04542pf08281 -
27055 -pf02687pf12704 -
BMC-P
BMC-H
BMC-T
Aldehyde dehydrogenase
PTAC
Signature enzyme
Alcohol dehydrogenase
Regulator

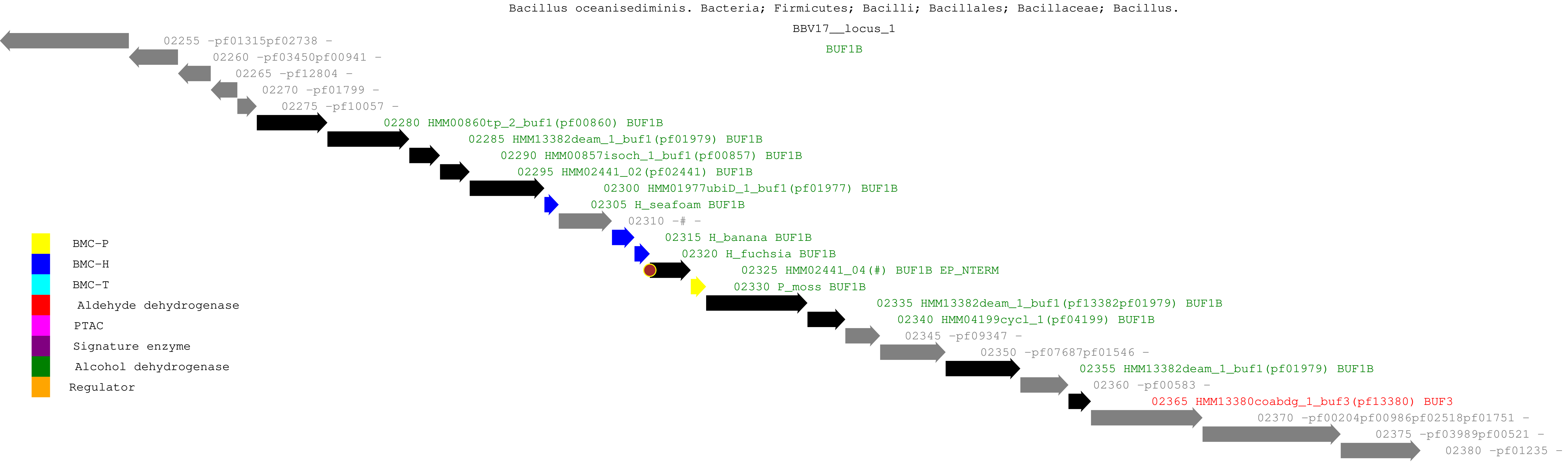
Bacillus oceanisediminis. Bacteria; Firmicutes; Bacilli; Bacillales; Bacillaceae; Bacillus.
BBV17__locus_1
BUF1B
02255 -pf01315pf02738 -
02260 -pf03450pf00941 -
02265 -pf12804 -
02270 -pf01799 -
02275 -pf10057 -
02280 HMM00860tp_2_buf1(pf00860) BUF1B
02285 HMM13382deam_1_buf1(pf01979) BUF1B
02290 HMM00857isoch_1_buf1(pf00857) BUF1B
02295 HMM02441_02(pf02441) BUF1B
02300 HMM01977ubiD_1_buf1(pf01977) BUF1B
02305 H_seafoam BUF1B
02310 -# -
02315 H_banana BUF1B
02320 H_fuchsia BUF1B
02325 HMM02441_04(#) BUF1B EP_NTERM
02330 P_moss BUF1B
02335 HMM13382deam_1_buf1(pf13382pf01979) BUF1B
02340 HMM04199cycl_1(pf04199) BUF1B
02345 -pf09347 -
02350 -pf07687pf01546 -
02355 HMM13382deam_1_buf1(pf01979) BUF1B
02360 -pf00583 -
02365 HMM13380coabdg_1_buf3(pf13380) BUF3
02370 -pf00204pf00986pf02518pf01751 -
02375 -pf03989pf00521 -
02380 -pf01235 -
BMC-P
BMC-H
BMC-T
Aldehyde dehydrogenase
PTAC
Signature enzyme
Alcohol dehydrogenase
Regulator

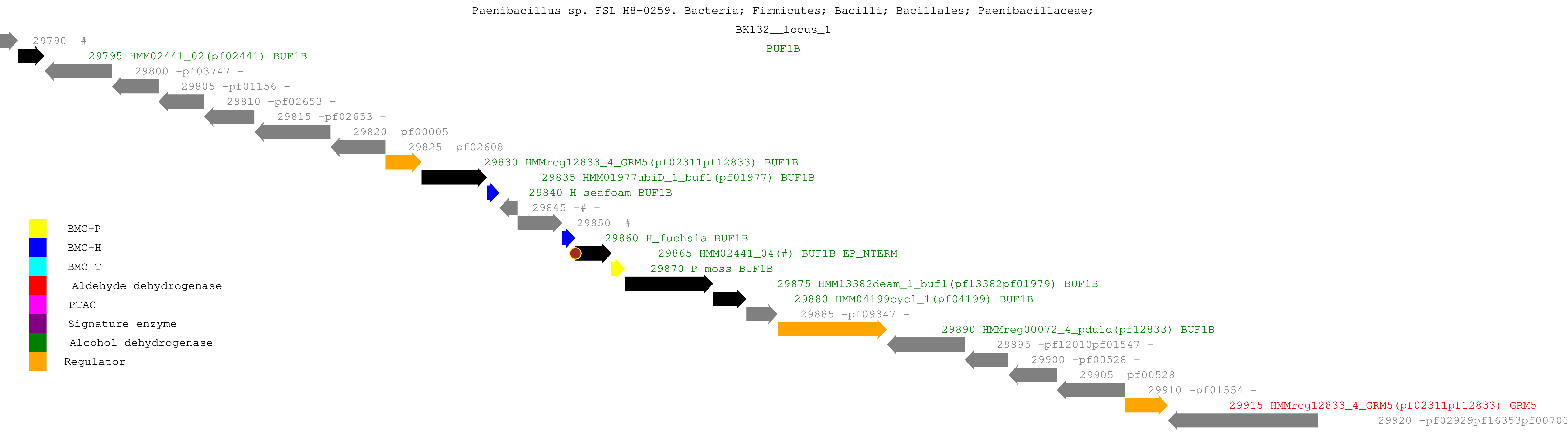
Paenibacillus sp. FSL H8-0259. Bacteria; Firmicutes; Bacilli; Bacillales; Paenibacillaceae;
BK132__locus_1
BUF1B
29790 -# -
29795 HMM02441_02(pf02441) BUF1B
29800 -pf03747 -
29805 -pf01156 -
29810 -pf02653 -
29815 -pf02653 -
29820 -pf00005 -
29825 -pf02608 -
29830 HMMreg12833_4_GRM5(pf02311pf12833) BUF1B
29835 HMM01977ubiD_1_buf1(pf01977) BUF1B
29840 H_seafoam BUF1B
29845 -# -
29850 -# -
29860 H_fuchsia BUF1B
29865 HMM02441_04(#) BUF1B EP_NTERM
29870 P_moss BUF1B
29875 HMM13382deam_1_buf1(pf13382pf01979) BUF1B
29880 HMM04199cycl_1(pf04199) BUF1B
29885 -pf09347 -
29890 HMMreg00072_4_pdu1d(pf12833) BUF1B
29895 -pf12010pf01547 -
29900 -pf00528 -
29905 -pf00528 -
29910 -pf01554 -
29915 HMMreg12833_4_GRM5(pf02311pf12833) GRM5
29920 -pf02929pf16353pf0070
BMC-P
BMC-H
BMC-T
Aldehyde dehydrogenase
PTAC
Signature enzyme
Alcohol dehydrogenase
Regulator

Paenibacillus sp. FSL R5-0490. Bacteria; Firmicutes; Bacilli; Bacillales; Paenibacillaceae;

BK139__locus_1

BUF1B

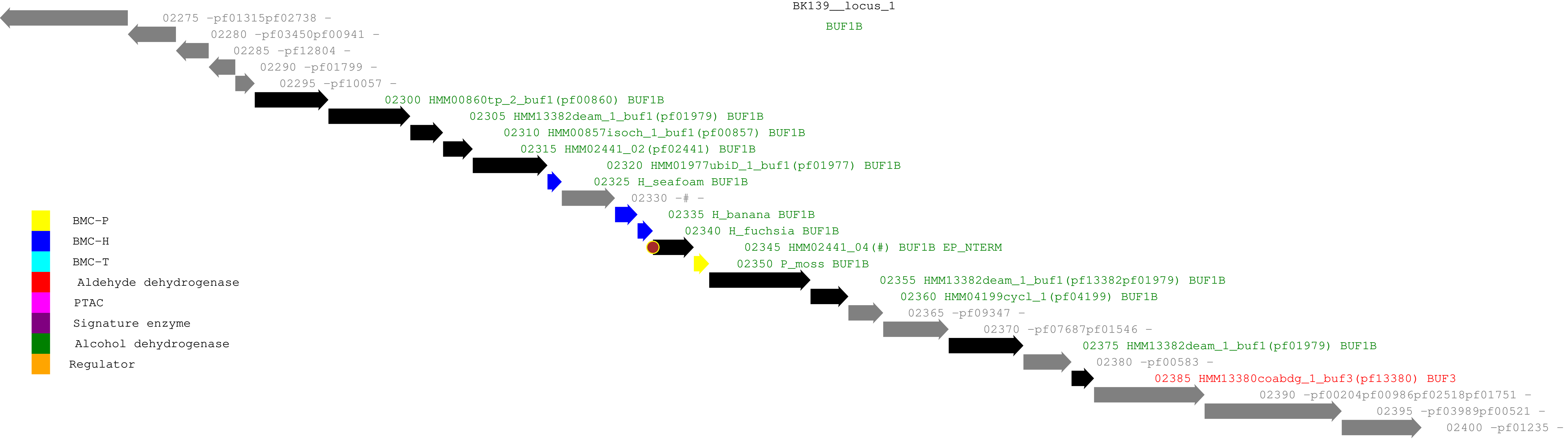

Sulfobacillus benefaciens. Bacteria; Firmicutes; Clostridia; Clostridiales;

C7B46__locus_1

BUF1B

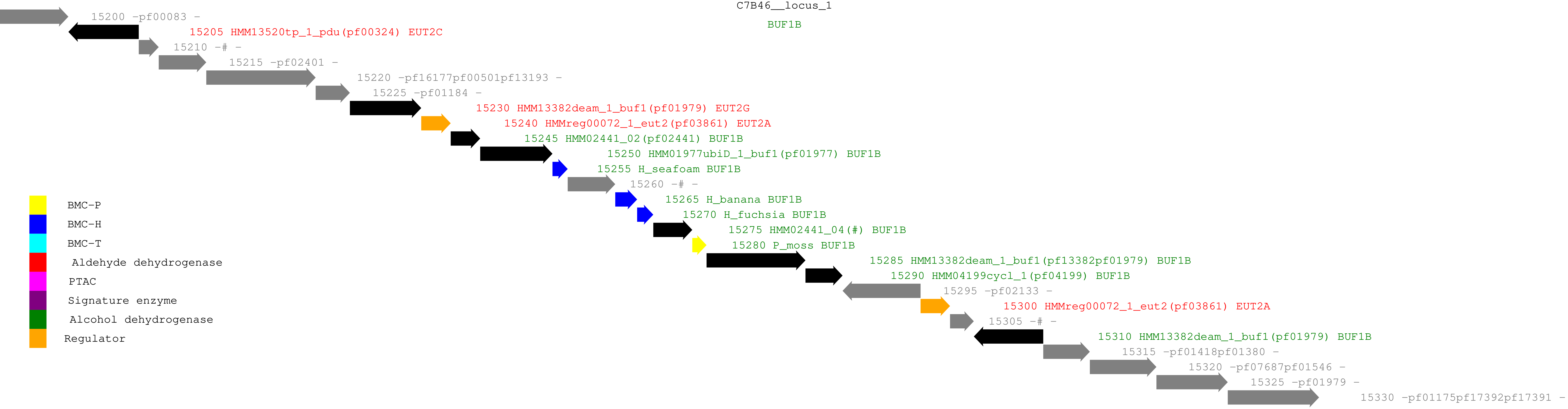

Bacillus sp. 7894-2. Bacteria; Firmicutes; Bacilli; Bacillales; Bacillaceae; Bacillus.

CHI10__locus_1

BUF1B

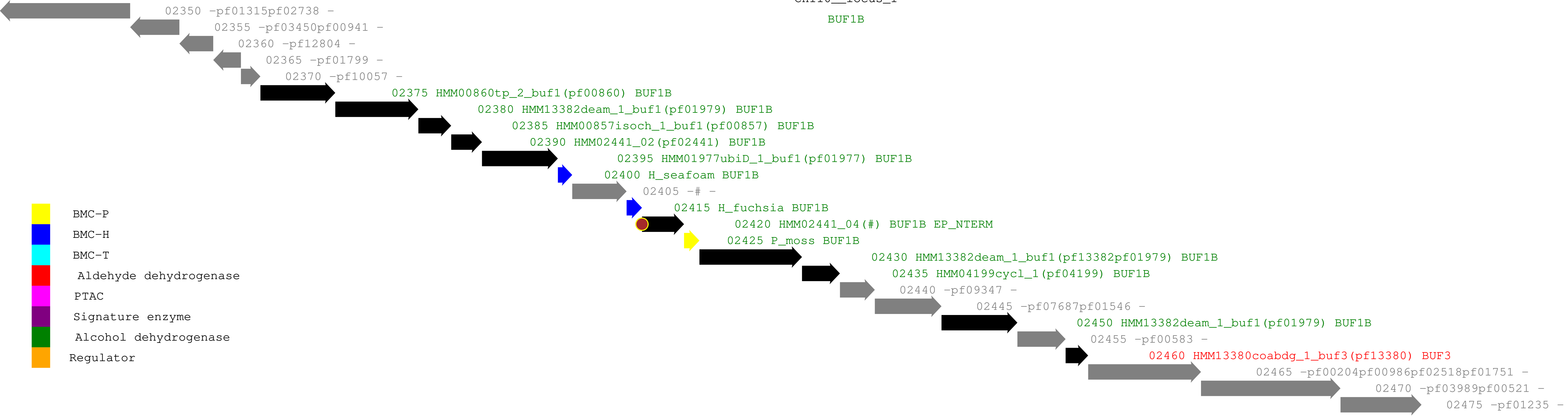

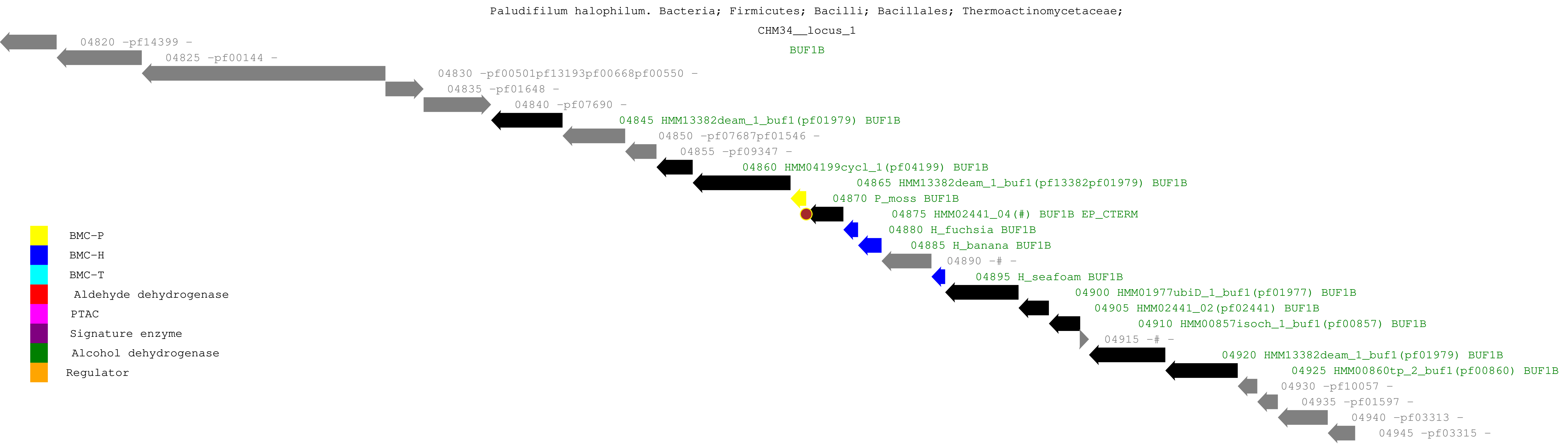

Paludifilum halophilum. Bacteria; Firmicutes; Bacilli; Bacillales; Thermoactinomycetaceae;
CHM34__locus_1
BUF1B
04820 -pf14399 -
04825 -pf00144 -
04830 -pf00501pf13193pf00668pf00550 -
04835 -pf01648 -
04840 -pf07690 -
04845 HMM13382deam_1_buf1(pf01979) BUF1B
04850 -pf07687pf01546 -
04855 -pf09347 -
04860 HMM04199cycl_1(pf04199) BUF1B
04865 HMM13382deam_1_buf1(pf13382pf01979) BUF1B
04870 P_moss BUF1B
04875 HMM02441_04(#) BUF1B EP_CTERM
04880 H_fuchsia BUF1B
04885 H_banana BUF1B
04890 -# -
04895 H_seafoam BUF1B
04900 HMM01977ubiD_1_buf1(pf01977) BUF1B
04905 HMM02441_02(pf02441) BUF1B
04910 HMM00857isoch_1_buf1(pf00857) BUF1B
04915 -# -
04920 HMM13382deam_1_buf1(pf01979) BUF1B
04925 HMM00860tp_2_buf1(pf00860) BUF1B
04930 -pf10057 -
04935 -pf01597 -
04940 -pf03313 -
04945 -pf03315 -
BMC-P
BMC-H
BMC-T
Aldehyde dehydrogenase
PTAC
Signature enzyme
Alcohol dehydrogenase
Regulator

Bacillus mesonae. Bacteria; Firmicutes; Bacilli; Bacillales; Bacillaceae; Bacillus.

CHR53__locus_1

BUF1B

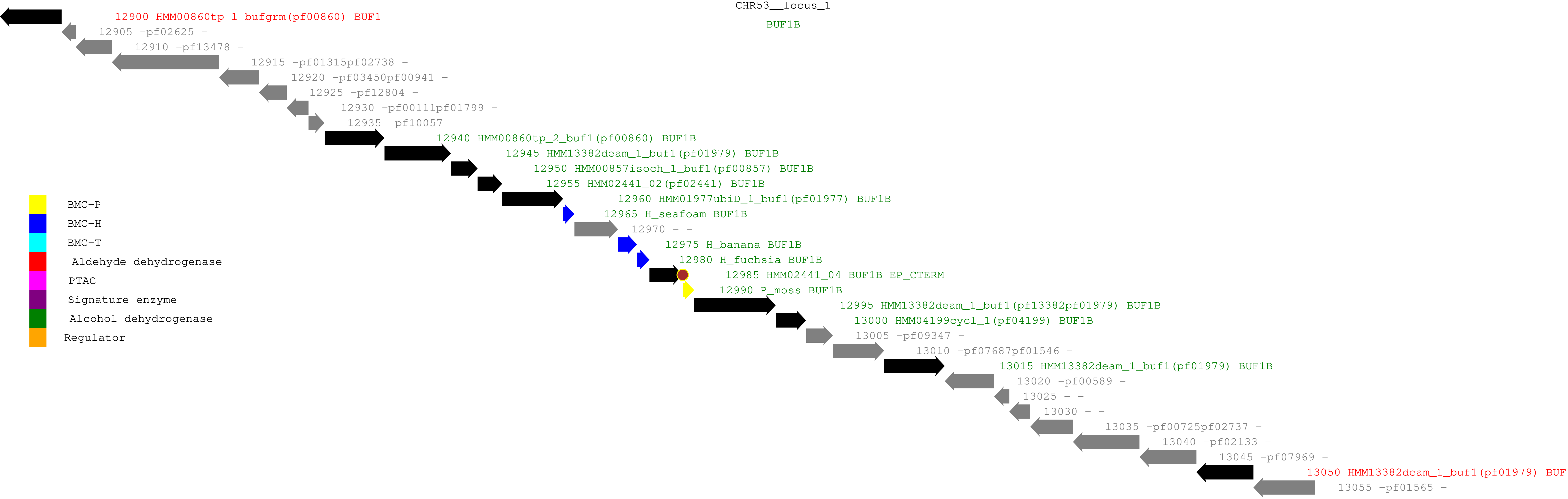

Virgibacillus sp. IO3-P2-C2. Bacteria; Firmicutes; Bacilli; Bacillales; Bacillaceae; Virgibacillus.

CIL03__locus_1

BUF1B

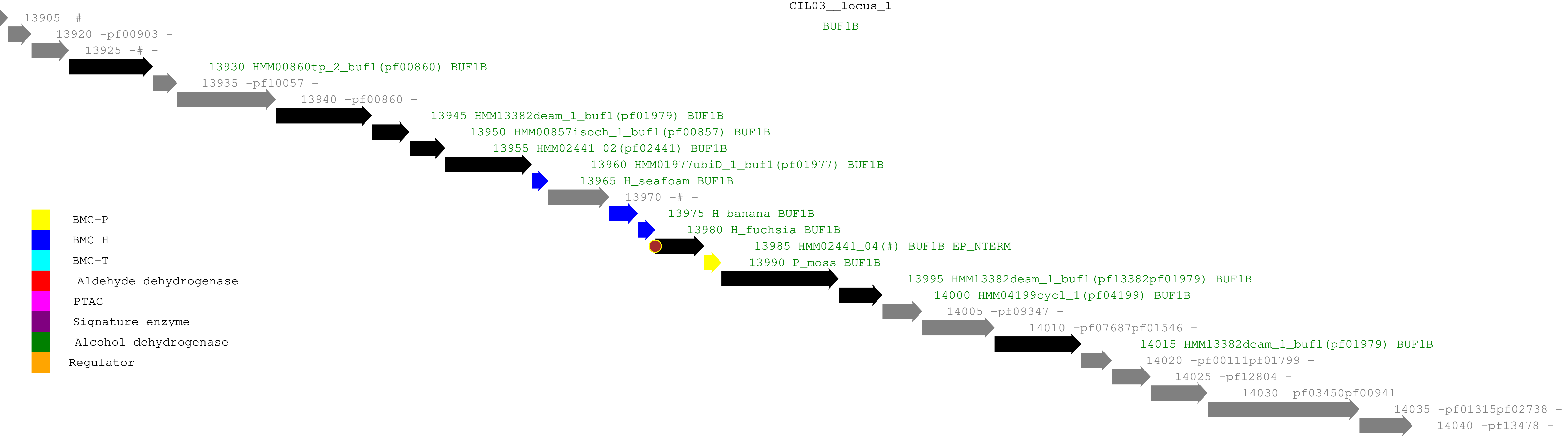

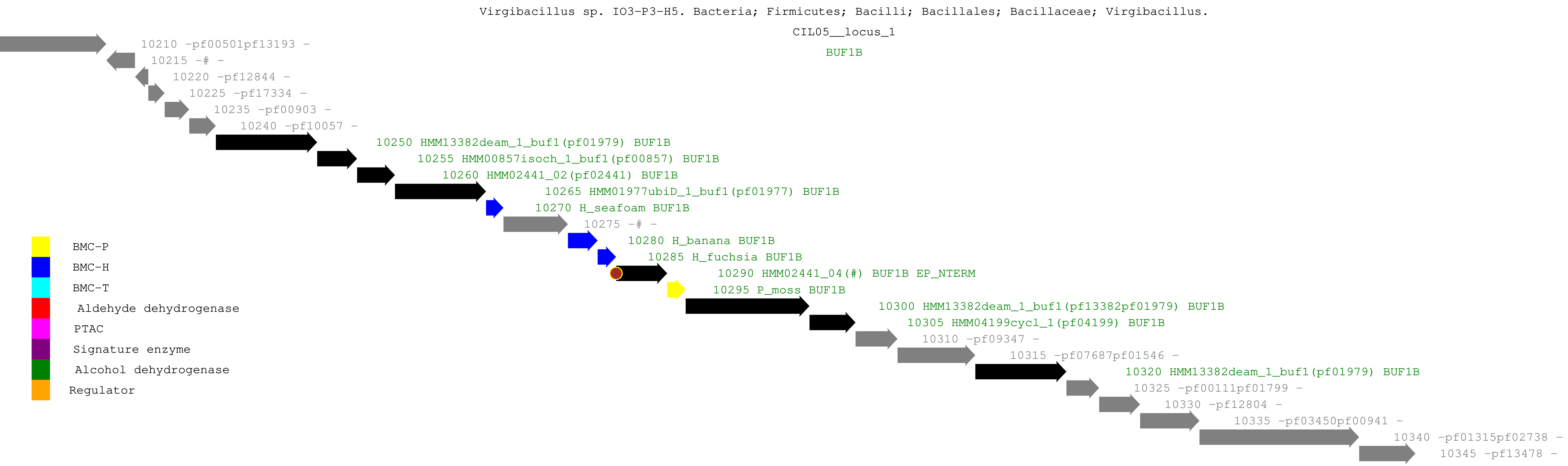
Virgibacillus sp. IO3-P3-H5. Bacteria; Firmicutes; Bacilli; Bacillales; Bacillaceae; Virgibacillus.
CIL05__locus_1
BUF1B
10210 -pf00501pf13193 -
10215 -# -
10220 -pf12844 -
10225 -pf17334 -
10235 -pf00903 -
10240 -pf10057 -
10250 HMM13382deam_1_buf1(pf01979) BUF1B
10255 HMM00857isoch_1_buf1(pf00857) BUF1B
10260 HMM02441_02(pf02441) BUF1B
10265 HMM01977ubiD_1_buf1(pf01977) BUF1B
10270 H_seafoam BUF1B
10275 -# -
10280 H_banana BUF1B
10285 H_fuchsia BUF1B
10290 HMM02441_04(#) BUF1B EP_NTERM
10295 P_moss BUF1B
10300 HMM13382deam_1_buf1(pf13382pf01979) BUF1B
10305 HMM04199cycl_1(pf04199) BUF1B
10310 -pf09347 -
10315 -pf07687pf01546 -
10320 HMM13382deam_1_buf1(pf01979) BUF1B
10325 -pf00111pf01799 -
10330 -pf12804 -
10335 -pf03450pf00941 -
10340 -pf01315pf02738 -
10345 -pf13478 -
BMC-P
BMC-H
BMC-T
Aldehyde dehydrogenase
PTAC
Signature enzyme
Alcohol dehydrogenase
Regulator

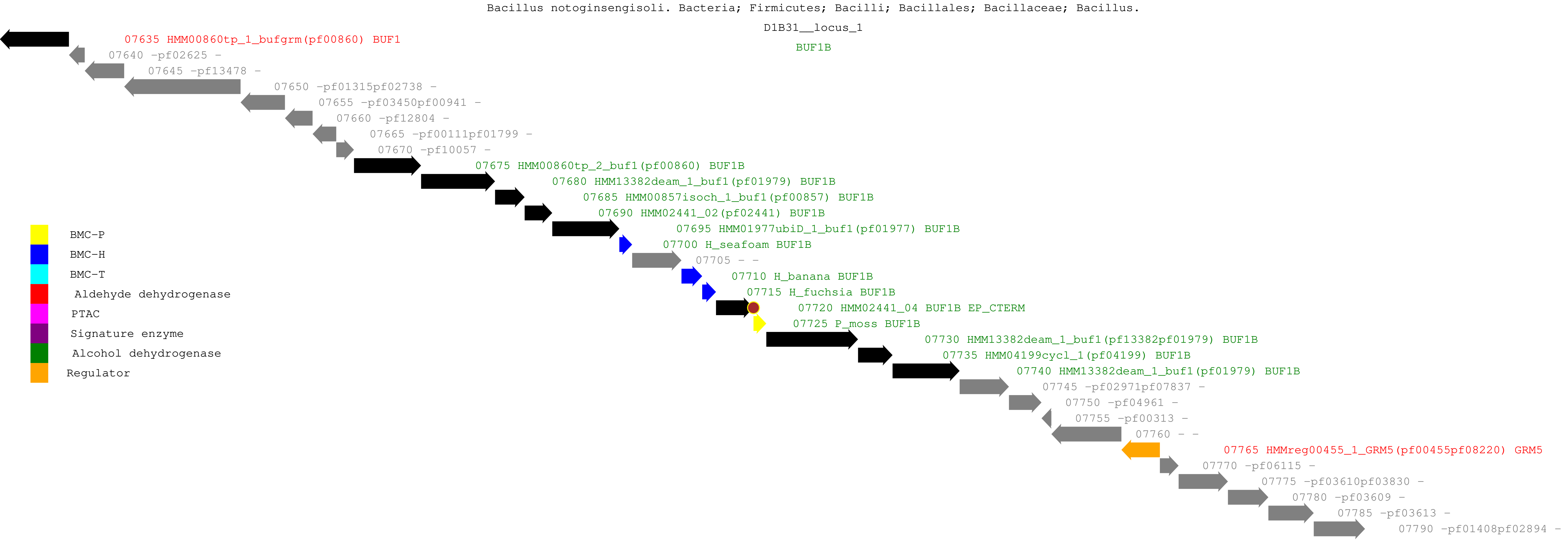
Bacillus notoginsengisoli. Bacteria; Firmicutes; Bacilli; Bacillales; Bacillaceae; Bacillus.
D1B31__locus_1
BUF1B
07635 HMM00860tp_1_bufgrm(pf00860) BUF1
07640 -pf02625 -
07645 -pf13478 -
07650 -pf01315pf02738 -
07655 -pf03450pf00941 -
07660 -pf12804 -
07665 -pf00111pf01799 -
07670 -pf10057 -
07675 HMM00860tp_2_buf1(pf00860) BUF1B
07680 HMM13382deam_1_buf1(pf01979) BUF1B
07685 HMM00857isoch_1_buf1(pf00857) BUF1B
07690 HMM02441_02(pf02441) BUF1B
07695 HMM01977ubiD_1_buf1(pf01977) BUF1B
07700 H_seafoam BUF1B
07705 - -
07710 H_banana BUF1B
07715 H_fuchsia BUF1B
07720 HMM02441_04 BUF1B EP_CTERM
07725 P_moss BUF1B
07730 HMM13382deam_1_buf1(pf13382pf01979) BUF1B
07735 HMM04199cycl_1(pf04199) BUF1B
07740 HMM13382deam_1_buf1(pf01979) BUF1B
07745 -pf02971pf07837 -
07750 -pf04961 -
07755 -pf00313 -
07760 - -
07765 HMMreg00455_1_GRM5(pf00455pf08220) GRM5
07770 -pf06115 -
07775 -pf03610pf03830 -
07780 -pf03609 -
07785 -pf03613 -
07790 -pf01408pf02894 -
BMC-P
BMC-H
BMC-T
Aldehyde dehydrogenase
PTAC
Signature enzyme
Alcohol dehydrogenase
Regulator

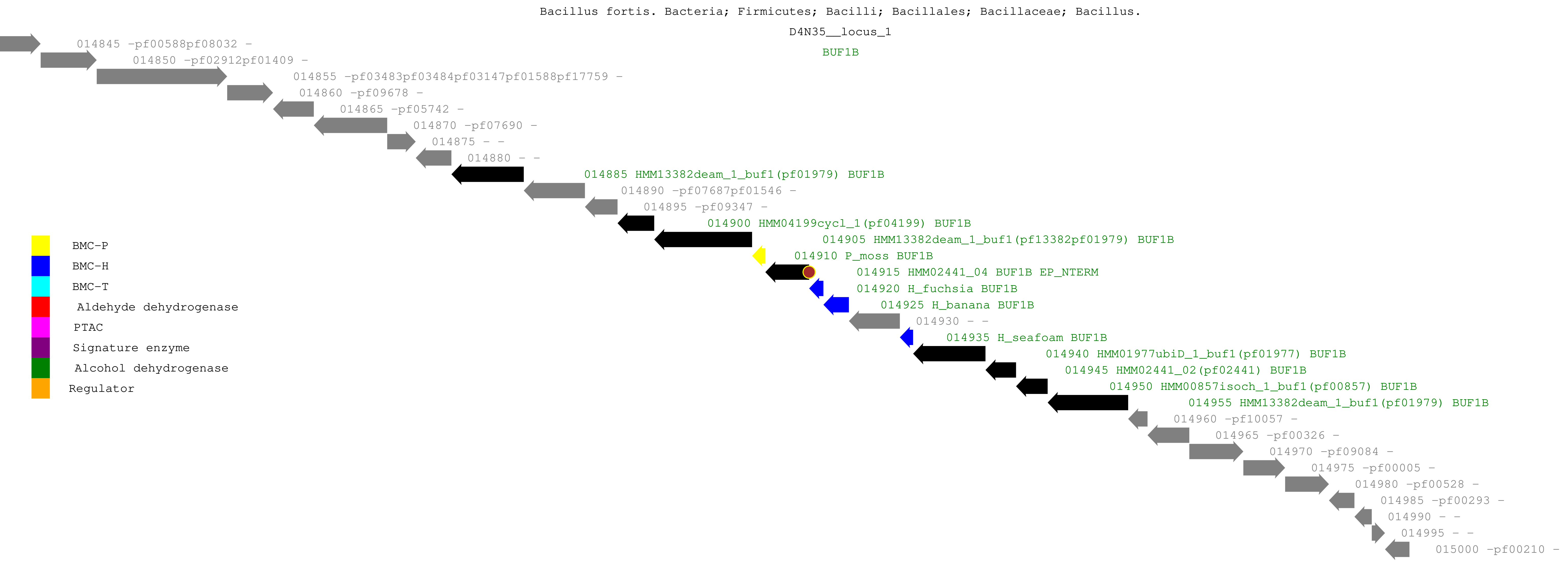
Bacillus fortis. Bacteria; Firmicutes; Bacilli; Bacillales; Bacillaceae; Bacillus.
D4N35__locus_1
BUF1B
014845 -pf00588pf08032 -
014850 -pf02912pf01409 -
014855 -pf03483pf03484pf03147pf01588pf17759 -
014860 -pf09678 -
014865 -pf05742 -
014870 -pf07690 -
014875 - -
014880 - -
014885 HMM13382deam_1_buf1(pf01979) BUF1B
014890 -pf07687pf01546 -
014895 -pf09347 -
014900 HMM04199cycl_1(pf04199) BUF1B
014905 HMM13382deam_1_buf1(pf13382pf01979) BUF1B
014910 P_moss BUF1B
014915 HMM02441_04 BUF1B EP_NTERM
014920 H_fuchsia BUF1B
014925 H_banana BUF1B
014930 - -
014935 H_seafoam BUF1B
014940 HMM01977ubiD_1_buf1(pf01977) BUF1B
014945 HMM02441_02(pf02441) BUF1B
014950 HMM00857isoch_1_buf1(pf00857) BUF1B
014955 HMM13382deam_1_buf1(pf01979) BUF1B
014960 -pf10057 -
014965 -pf00326 -
014970 -pf09084 -
014975 -pf00005 -
014980 -pf00528 -
014985 -pf00293 -
014990 - -
014995 - -
015000 -pf00210 -
BMC-P
BMC-H
BMC-T
Aldehyde dehydrogenase
PTAC
Signature enzyme
Alcohol dehydrogenase
Regulator

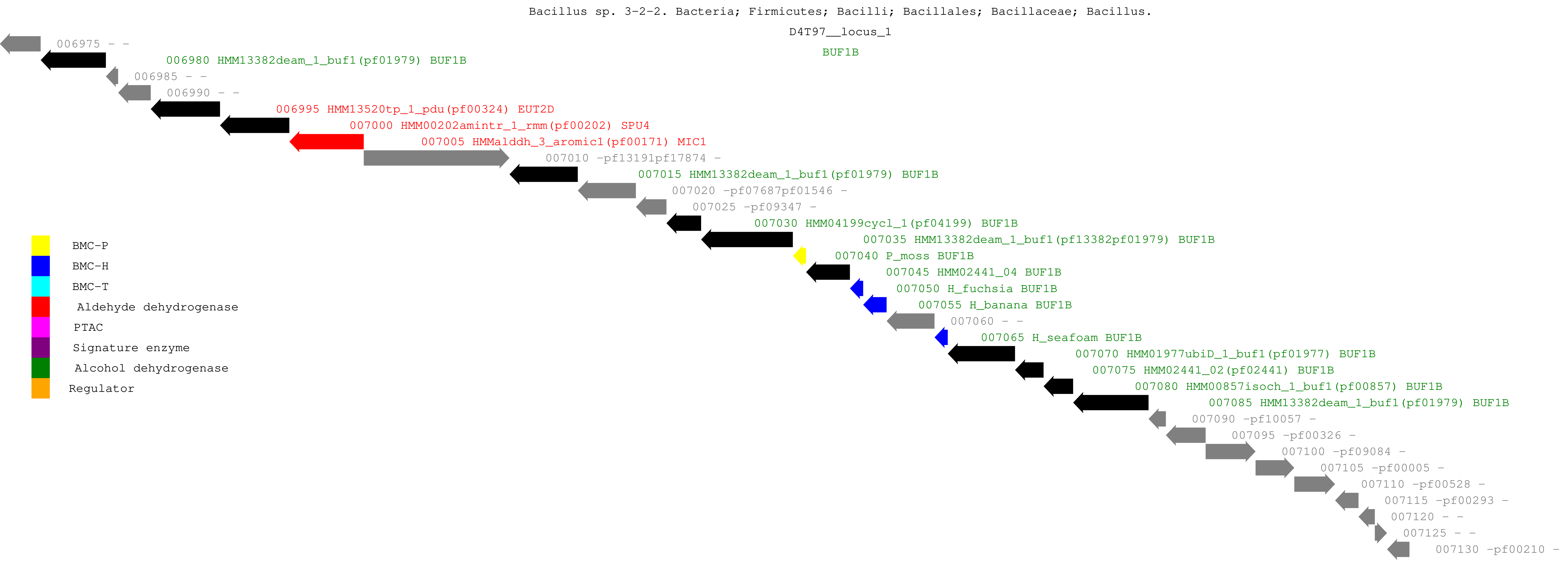

Bacillus sp. 3-2-2. Bacteria; Firmicutes; Bacilli; Bacillales; Bacillaceae; Bacillus.
D4T97__locus_1
BUF1B
006975 - -
006980 HMM13382deam_1_buf1(pf01979) BUF1B
006985 - -
006990 - -
006995 HMM13520tp_1_pdu(pf00324) EUT2D
007000 HMM00202amintr_1_rmm(pf00202) SPU4
007005 HMMaldh_3_aromic1(pf00171) MIC1
007010 -pf13191pf17874 -
007015 HMM13382deam_1_buf1(pf01979) BUF1B
007020 -pf07687pf01546 -
007025 -pf09347 -
007030 HMM04199cycl_1(pf04199) BUF1B
007035 HMM13382deam_1_buf1(pf13382pf01979) BUF1B
007040 P_moss BUF1B
007045 HMM02441_04 BUF1B
007050 H_fuchsia BUF1B
007055 H_banana BUF1B
007060 - -
007065 H_seafoam BUF1B
007070 HMM01977ubiD_1_buf1(pf01977) BUF1B
007075 HMM02441_02(pf02441) BUF1B
007080 HMM00857isoch_1_buf1(pf00857) BUF1B
007085 HMM13382deam_1_buf1(pf01979) BUF1B
007090 -pf10057 -
007095 -pf00326 -
007100 -pf09084 -
007105 -pf00005 -
007110 -pf00528 -
007115 -pf00293 -
007120 - -
007125 - -
007130 -pf00210 -
BMC-P
BMC-H
BMC-T
Aldehyde dehydrogenase
PTAC
Signature enzyme
Alcohol dehydrogenase
Regulator

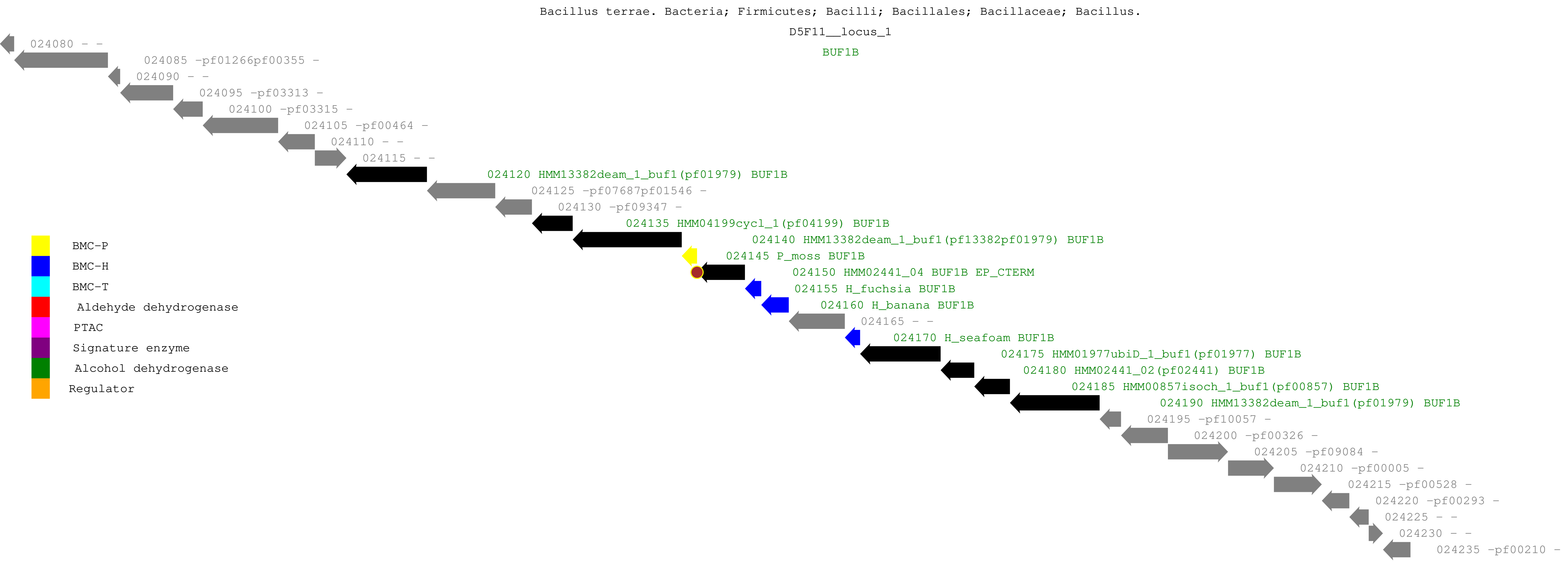
Bacillus terrae. Bacteria; Firmicutes; Bacilli; Bacillales; Bacillaceae; Bacillus.
D5F11__locus_1
BUF1B
024080 - -
024085 -pf01266pf00355 -
024090 - -
024095 -pf03313 -
024100 -pf03315 -
024105 -pf00464 -
024110 - -
024115 - -
024120 HMM13382deam_1_buf1(pf01979) BUF1B
024125 -pf07687pf01546 -
024130 -pf09347 -
024135 HMM04199cycl_1(pf04199) BUF1B
024140 HMM13382deam_1_buf1(pf13382pf01979) BUF1B
024145 P_moss BUF1B
024150 HMM02441_04 BUF1B EP_CTERM
024155 H_fuchsia BUF1B
024160 H_banana BUF1B
024165 - -
024170 H_seafoam BUF1B
024175 HMM01977ubiD_1_buf1(pf01977) BUF1B
024180 HMM02441_02(pf02441) BUF1B
024185 HMM00857isoch_1_buf1(pf00857) BUF1B
024190 HMM13382deam_1_buf1(pf01979) BUF1B
024195 -pf10057 -
024200 -pf00326 -
024205 -pf09084 -
024210 -pf00005 -
024215 -pf00528 -
024220 -pf00293 -
024225 - -
024230 - -
024235 -pf00210 -
BMC-P
BMC-H
BMC-T
Aldehyde dehydrogenase
PTAC
Signature enzyme
Alcohol dehydrogenase
Regulator

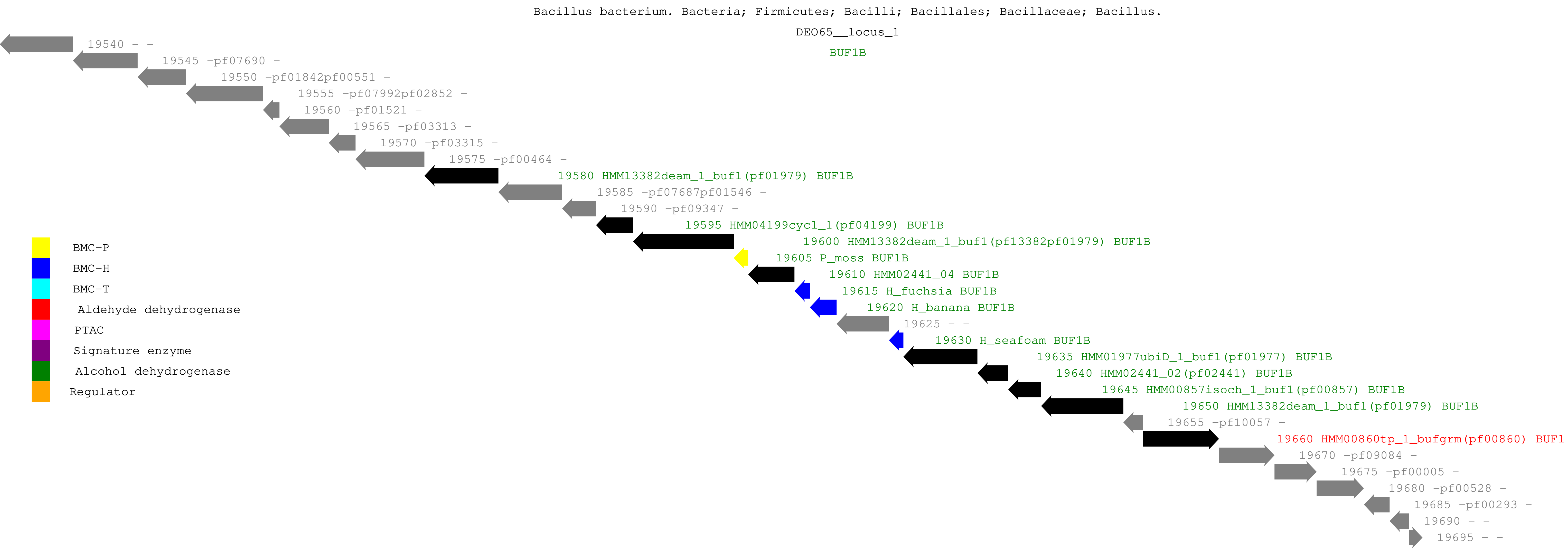
Bacillus bacterium. Bacteria; Firmicutes; Bacilli; Bacillales; Bacillaceae; Bacillus.
DEO65__locus_1
BUF1B
19540 - -
19545 -pf07690 -
19550 -pf01842pf00551 -
19555 -pf07992pf02852 -
19560 -pf01521 -
19565 -pf03313 -
19570 -pf03315 -
19575 -pf00464 -
19580 HMM13382deam_1_buf1(pf01979) BUF1B
19585 -pf07687pf01546 -
19590 -pf09347 -
19595 HMM04199cycl_1(pf04199) BUF1B
19600 HMM13382deam_1_buf1(pf13382pf01979) BUF1B
19605 P_moss BUF1B
19610 HMM02441_04 BUF1B
19615 H_fuchsia BUF1B
19620 H_banana BUF1B
19625 - -
19630 H_seafoam BUF1B
19635 HMM01977ubiD_1_buf1(pf01977) BUF1B
19640 HMM02441_02(pf02441) BUF1B
19645 HMM00857isoch_1_buf1(pf00857) BUF1B
19650 HMM13382deam_1_buf1(pf01979) BUF1B
19655 -pf10057 -
19660 HMM00860tp_1_bufgrm(pf00860) BUF1
19670 -pf09084 -
19675 -pf00005 -
19680 -pf00528 -
19685 -pf00293 -
19690 - -
19695 - -
BMC-P
BMC-H
BMC-T
Aldehyde dehydrogenase
PTAC
Signature enzyme
Alcohol dehydrogenase
Regulator

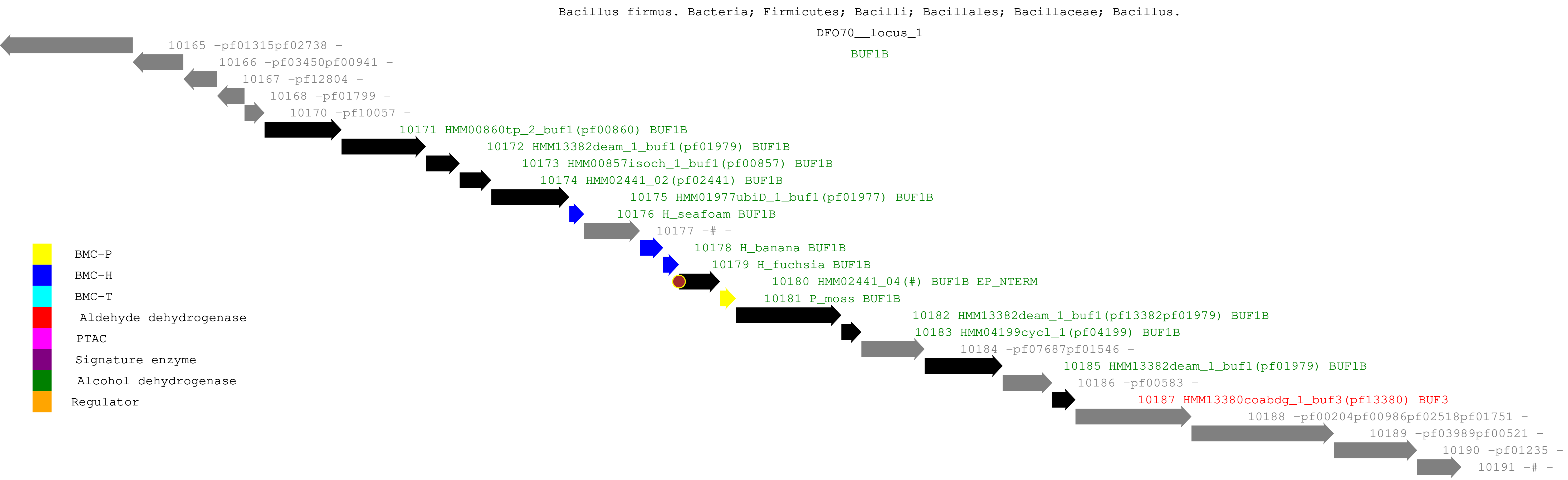

Bacillus firmus. Bacteria; Firmicutes; Bacilli; Bacillales; Bacillaceae; Bacillus.
DFO70__locus_1
BUF1B
10165 -pf01315pf02738 -
10166 -pf03450pf00941 -
10167 -pf12804 -
10168 -pf01799 -
10170 -pf10057 -
10171 HMM00860tp_2_buf1(pf00860) BUF1B
10172 HMM13382deam_1_buf1(pf01979) BUF1B
10173 HMM00857isoch_1_buf1(pf00857) BUF1B
10174 HMM02441_02(pf02441) BUF1B
10175 HMM01977ubiD_1_buf1(pf01977) BUF1B
10176 H_seafoam BUF1B
10177 -# -
10178 H_banana BUF1B
10179 H_fuchsia BUF1B
10180 HMM02441_04(#) BUF1B EP_NTERM
10181 P_moss BUF1B
10182 HMM13382deam_1_buf1(pf13382pf01979) BUF1B
10183 HMM04199cycl_1(pf04199) BUF1B
10184 -pf07687pf01546 -
10185 HMM13382deam_1_buf1(pf01979) BUF1B
10186 -pf00583 -
10187 HMM13380coabdg_1_buf3(pf13380) BUF3
10188 -pf00204pf00986pf02518pf01751 -
10189 -pf03989pf00521 -
10190 -pf01235 -
10191 -# -
BMC-P
BMC-H
BMC-T
Aldehyde dehydrogenase
PTAC
Signature enzyme
Alcohol dehydrogenase
Regulator

Bacillus oceanisediminis. Bacteria; Firmicutes; Bacilli; Bacillales; Bacillaceae; Bacillus.

DFO72__locus_2

BUF1B

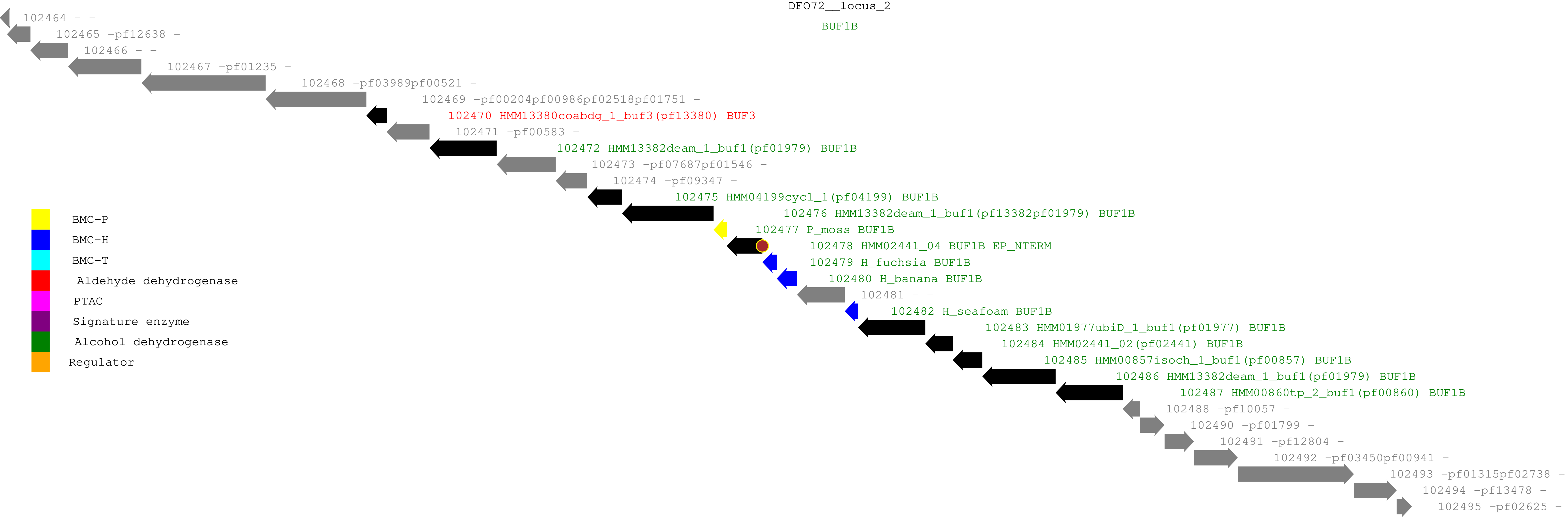

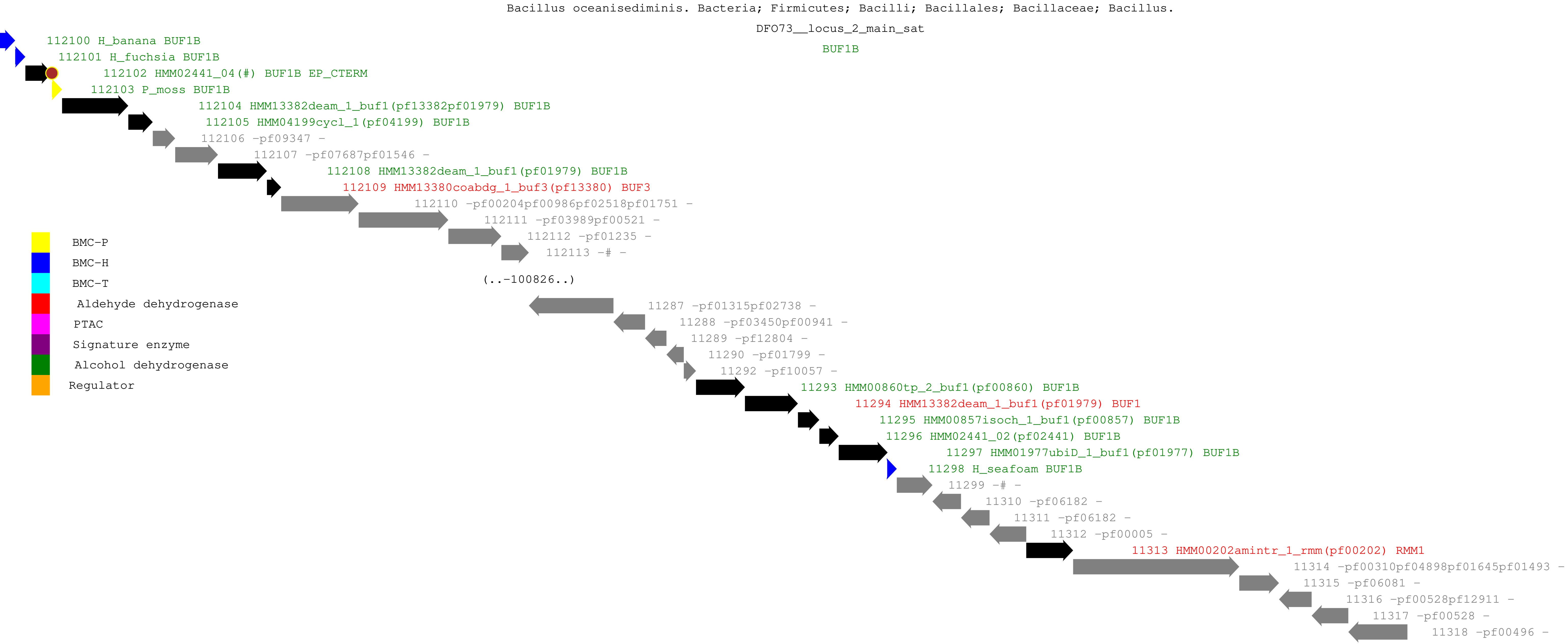
Bacillus oceanisediminis. Bacteria; Firmicutes; Bacilli; Bacillales; Bacillaceae; Bacillus.
DFO73__locus_2_main_sat
BUF1B
112100 H_banana BUF1B
112101 H_fuchsia BUF1B
112102 HMM02441_04(#) BUF1B EP_CTERM
112103 P_moss BUF1B
112104 HMM13382deam_1_buf1(pf13382pf01979) BUF1B
112105 HMM04199cycl_1(pf04199) BUF1B
112106 -pf09347 -
112107 -pf07687pf01546 -
112108 HMM13382deam_1_buf1(pf01979) BUF1B
112109 HMM13380coabdg_1_buf3(pf13380) BUF3
112110 -pf00204pf00986pf02518pf01751 -
112111 -pf03989pf00521 -
112112 -pf01235 -
112113 -# -
(..-100826..)
11287 -pf01315pf02738 -
11288 -pf03450pf00941 -
11289 -pf12804 -
11290 -pf01799 -
11292 -pf10057 -
11293 HMM00860tp_2_buf1(pf00860) BUF1B
11294 HMM13382deam_1_buf1(pf01979) BUF1
11295 HMM00857isoch_1_buf1(pf00857) BUF1B
11296 HMM02441_02(pf02441) BUF1B
11297 HMM01977ubiD_1_buf1(pf01977) BUF1B
11298 H_seafoam BUF1B
11299 -# -
11310 -pf06182 -
11311 -pf06182 -
11312 -pf00005 -
11313 HMM00202amintr_1_rmm(pf00202) RMM1
11314 -pf00310pf04898pf01645pf01493 -
11315 -pf06081 -
11316 -pf00528pf12911 -
11317 -pf00528 -
11318 -pf00496 -
BMC-P
BMC-H
BMC-T
Aldehyde dehydrogenase
PTAC
Signature enzyme
Alcohol dehydrogenase
Regulator

Thermoflavimicrobium sp. FBKL4.011. Bacteria; Firmicutes; Bacilli; Bacillales; Thermoactinomycetaceae;

DL897__locus_1

BUF1B

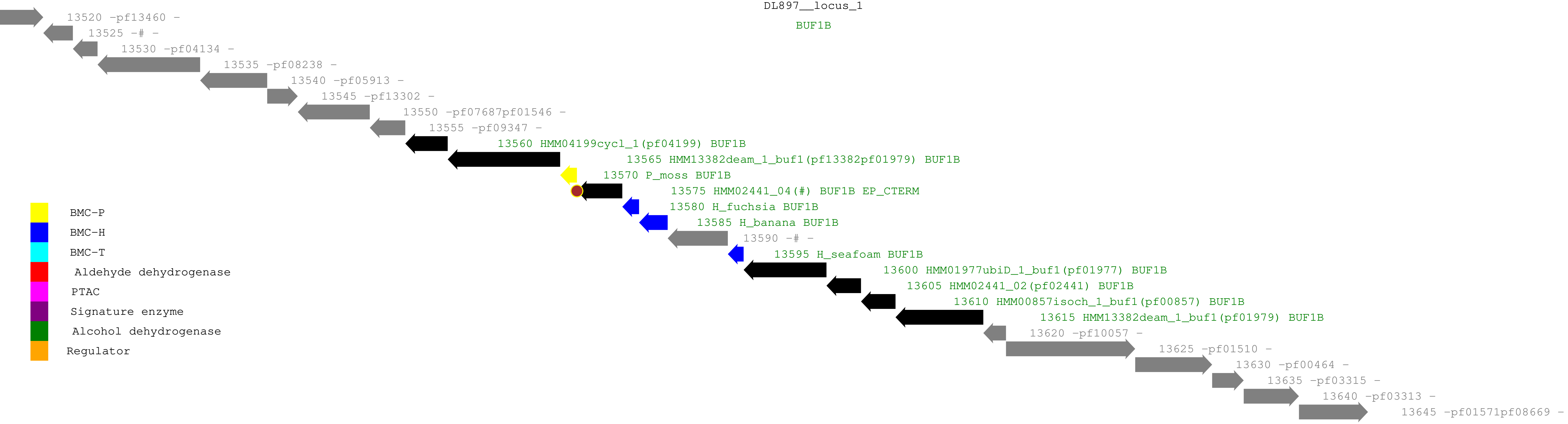

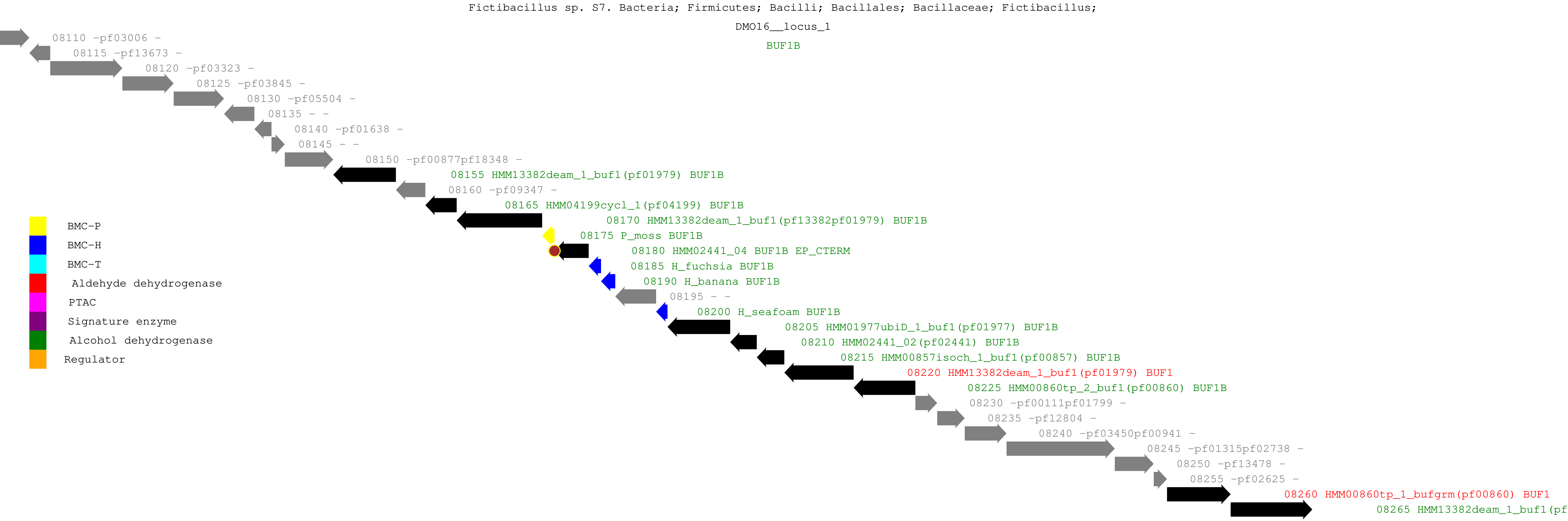
Fictibacillus sp. S7. Bacteria; Firmicutes; Bacilli; Bacillales; Bacillaceae; Fictibacillus;
DMO16__locus_1
BUF1B
08110 -pf03006 -
08115 -pf13673 -
08120 -pf03323 -
08125 -pf03845 -
08130 -pf05504 -
08135 - -
08140 -pf01638 -
08145 - -
08150 -pf00877pf18348 -
08155 HMM13382deam_1_buf1(pf01979) BUF1B
08160 -pf09347 -
08165 HMM04199cycl_1(pf04199) BUF1B
08170 HMM13382deam_1_buf1(pf13382pf01979) BUF1B
08175 P_moss BUF1B
08180 HMM02441_04 BUF1B EP_CTERM
08185 H_fuchsia BUF1B
08190 H_banana BUF1B
08195 - -
08200 H_seafoam BUF1B
08205 HMM01977ubiD_1_buf1(pf01977) BUF1B
08210 HMM02441_02(pf02441) BUF1B
08215 HMM00857isoch_1_buf1(pf00857) BUF1B
08220 HMM13382deam_1_buf1(pf01979) BUF1
08225 HMM00860tp_2_buf1(pf00860) BUF1B
08230 -pf00111pf01799 -
08235 -pf12804 -
08240 -pf03450pf00941 -
08245 -pf01315pf02738 -
08250 -pf13478 -
08255 -pf02625 -
08260 HMM00860tp_1_bufgrm(pf00860) BUF1
08265 HMM13382deam_1_buf1(pf
BMC-P
BMC-H
BMC-T
Aldehyde dehydrogenase
PTAC
Signature enzyme
Alcohol dehydrogenase
Regulator

Bacillus praedii. Bacteria; Firmicutes; Bacilli; Bacillales; Bacillaceae; Bacillus.

E0Y62__locus_2

BUF1B

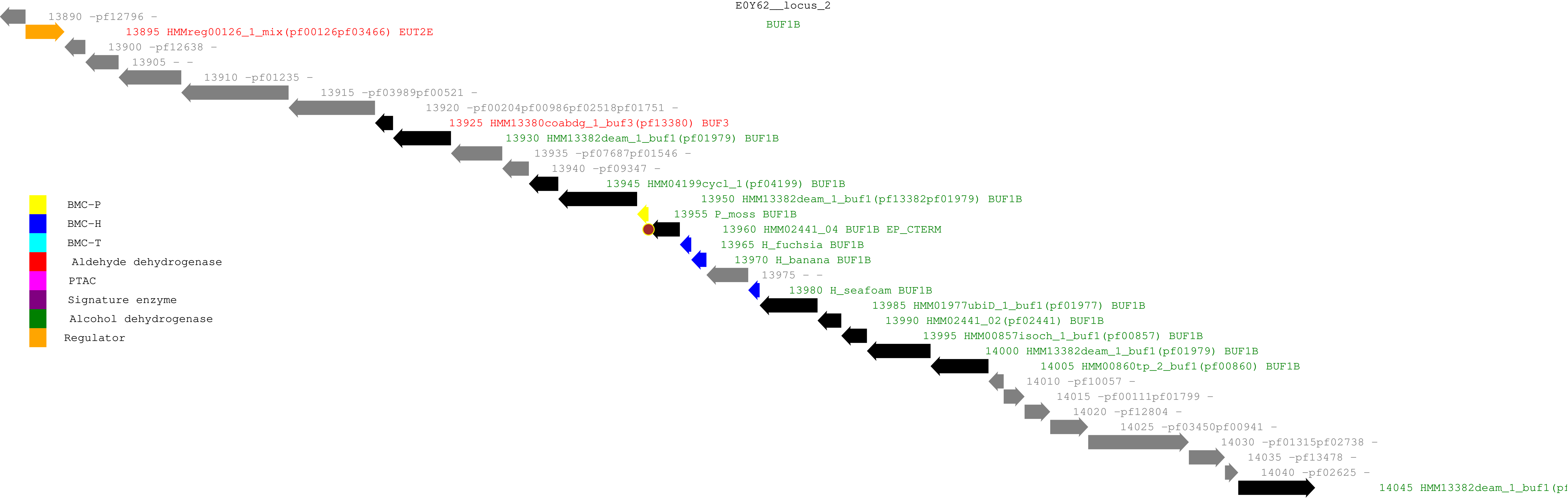

Ammoniphilus sp. CFH 90114. Bacteria; Firmicutes; Bacilli; Bacillales; Paenibacillaceae;

EIZ39__locus_1

BUF1B

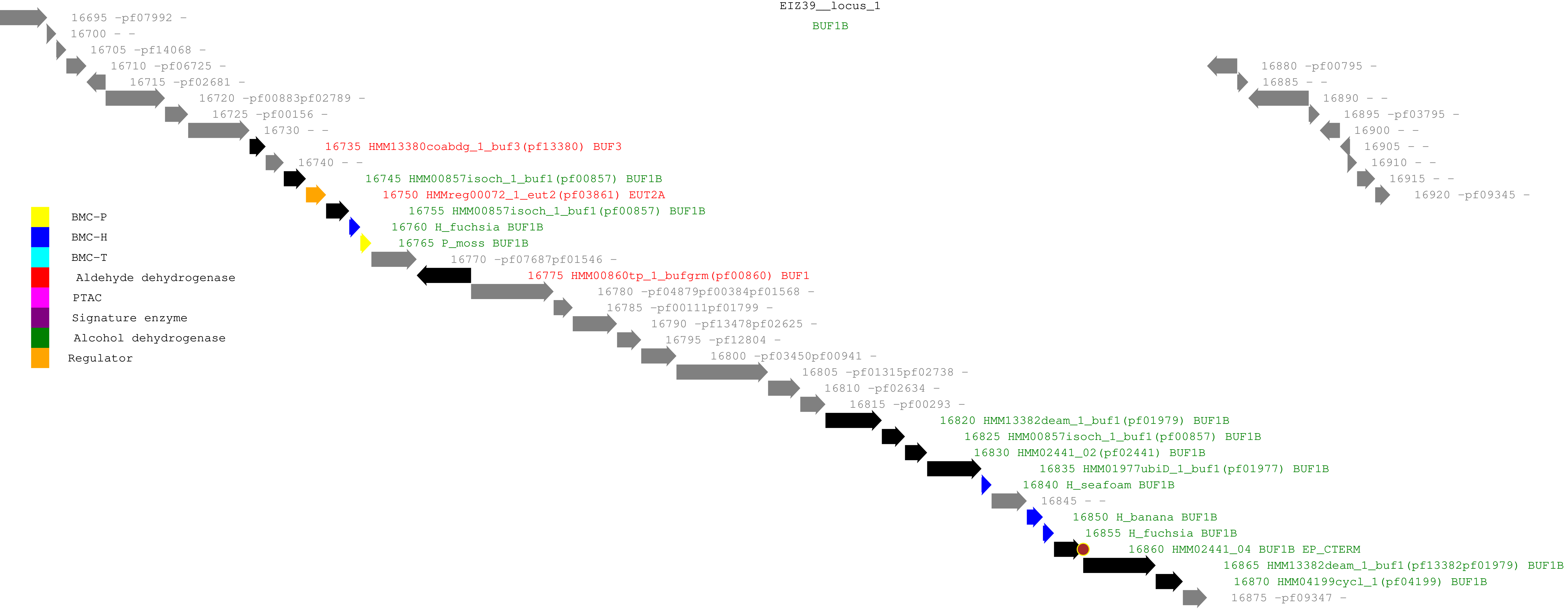

Cohnella sp. AR92. Bacteria; Firmicutes; Bacilli; Bacillales; Paenibacillaceae; Cohnella;

ELR57__locus_1

BUF1B

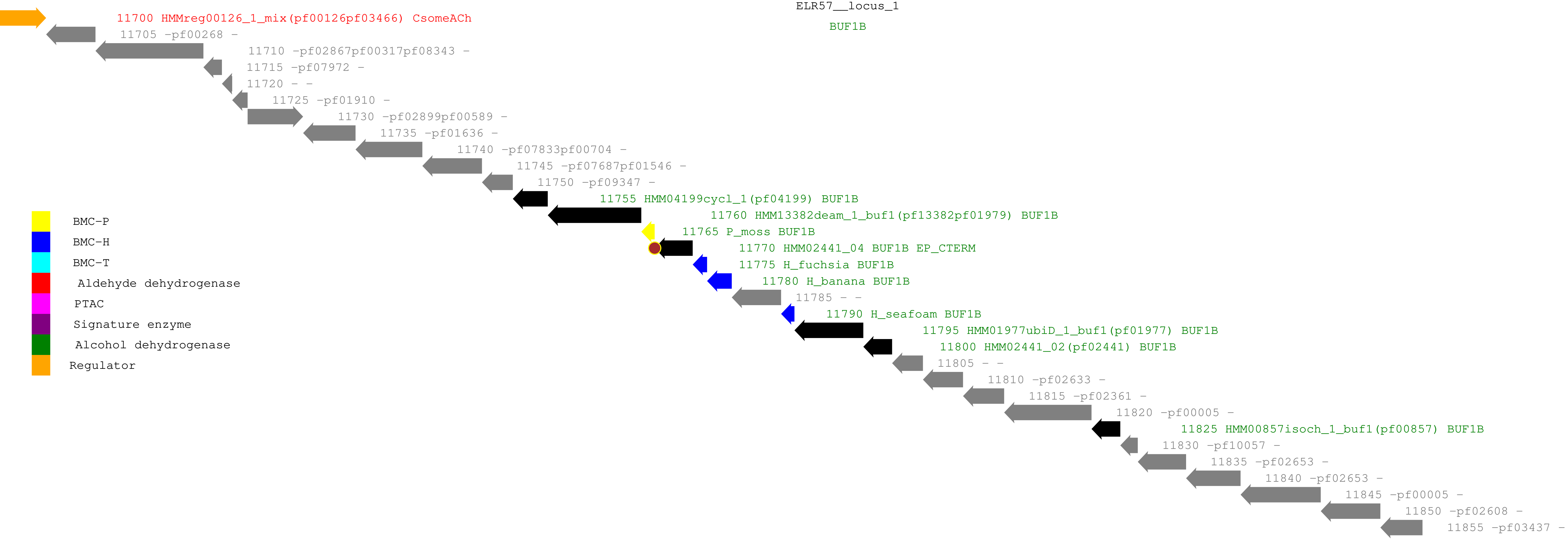

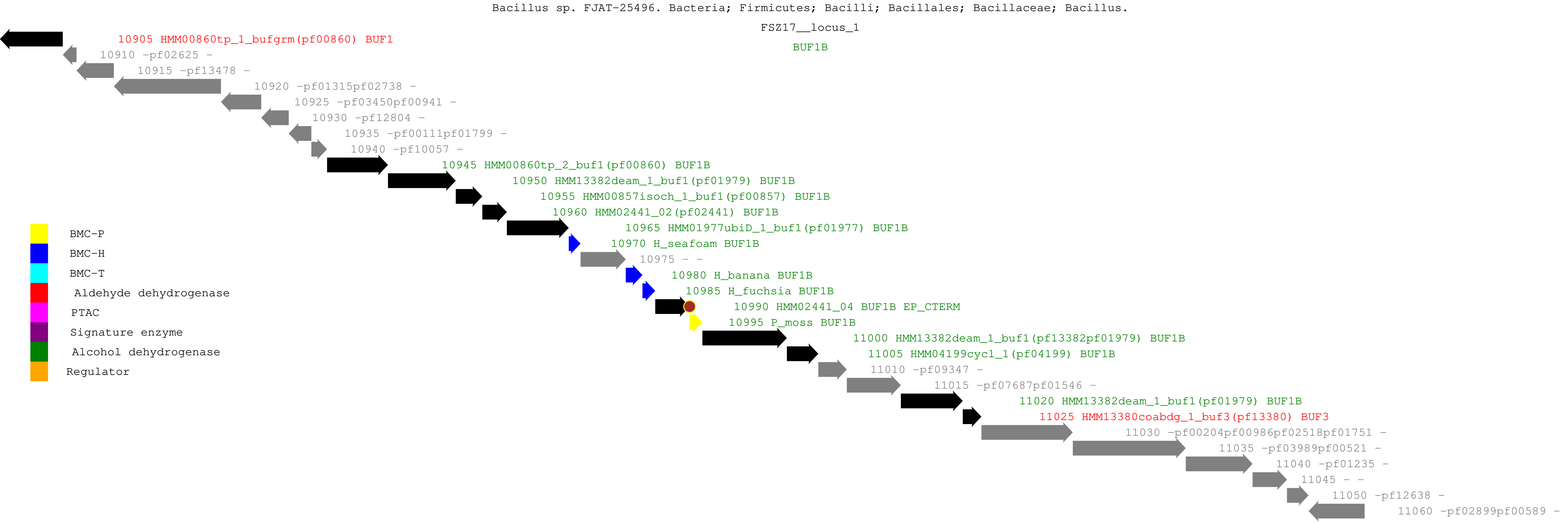

Bacillus sp. FJAT-25496. Bacteria; Firmicutes; Bacilli; Bacillales; Bacillaceae; Bacillus.
FSZ17__locus_1
BUF1B
10905 HMM00860tp_1_bufgrm(pf00860) BUF1
10910 -pf02625 -
10915 -pf13478 -
10920 -pf01315pf02738 -
10925 -pf03450pf00941 -
10930 -pf12804 -
10935 -pf00111pf01799 -
10940 -pf10057 -
10945 HMM00860tp_2_buf1(pf00860) BUF1B
10950 HMM13382deam_1_buf1(pf01979) BUF1B
10955 HMM00857isoch_1_buf1(pf00857) BUF1B
10960 HMM02441_02(pf02441) BUF1B
10965 HMM01977ubiD_1_buf1(pf01977) BUF1B
10970 H_seafoam BUF1B
10975 - -
10980 H_banana BUF1B
10985 H_fuchsia BUF1B
10990 HMM02441_04 BUF1B EP_CTERM
10995 P_moss BUF1B
11000 HMM13382deam_1_buf1(pf13382pf01979) BUF1B
11005 HMM04199cycl_1(pf04199) BUF1B
11010 -pf09347 -
11015 -pf07687pf01546 -
11020 HMM13382deam_1_buf1(pf01979) BUF1B
11025 HMM13380coabdg_1_buf3(pf13380) BUF3
11030 -pf00204pf00986pf02518pf01751 -
11035 -pf03989pf00521 -
11040 -pf01235 -
11045 - -
11050 -pf12638 -
11060 -pf02899pf00589 -
BMC-P
BMC-H
BMC-T
Aldehyde dehydrogenase
PTAC
Signature enzyme
Alcohol dehydrogenase
Regulator

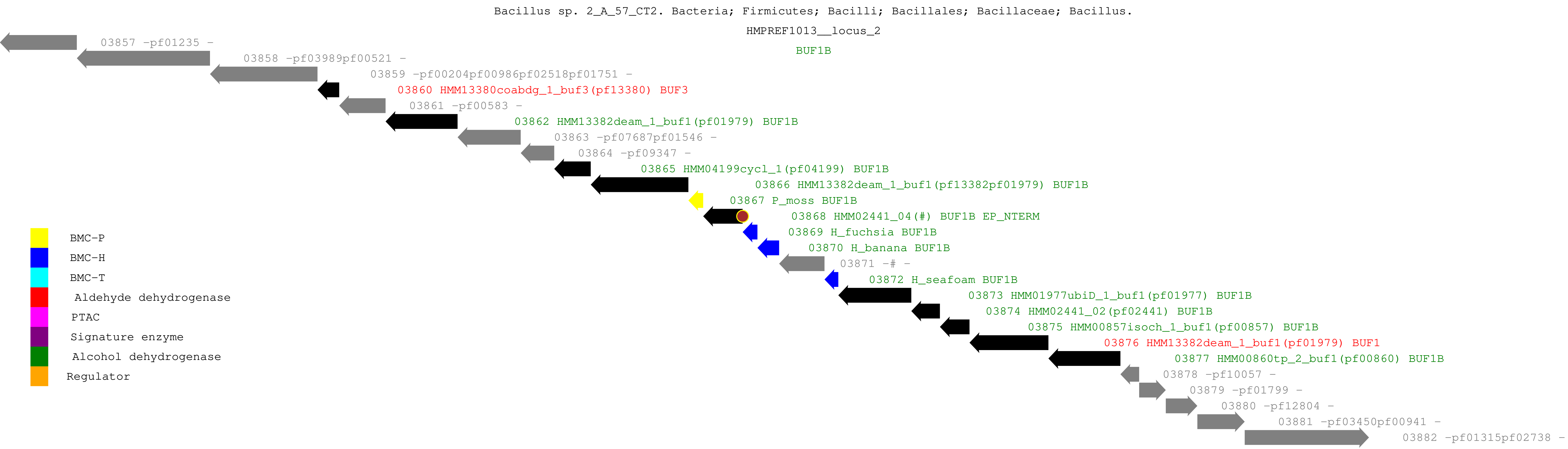
Bacillus sp. 2_A_57_CT2. Bacteria; Firmicutes; Bacilli; Bacillales; Bacillaceae; Bacillus.
HMPREF1013__locus_2
BUF1B
03857 -pf01235 -
03858 -pf03989pf00521 -
03859 -pf00204pf00986pf02518pf01751 -
03860 HMM13380coabdg_1_buf3(pf13380) BUF3
03861 -pf00583 -
03862 HMM13382deam_1_buf1(pf01979) BUF1B
03863 -pf07687pf01546 -
03864 -pf09347 -
03865 HMM04199cycl_1(pf04199) BUF1B
03866 HMM13382deam_1_buf1(pf13382pf01979) BUF1B
03867 P_moss BUF1B
03868 HMM02441_04(#) BUF1B EP_NTERM
03869 H_fuchsia BUF1B
03870 H_banana BUF1B
03871 -# -
03872 H_seafoam BUF1B
03873 HMM01977ubiD_1_buf1(pf01977) BUF1B
03874 HMM02441_02(pf02441) BUF1B
03875 HMM00857isoch_1_buf1(pf00857) BUF1B
03876 HMM13382deam_1_buf1(pf01979) BUF1
03877 HMM00860tp_2_buf1(pf00860) BUF1B
03878 -pf10057 -
03879 -pf01799 -
03880 -pf12804 -
03881 -pf03450pf00941 -
03882 -pf01315pf02738 -
BMC-P
BMC-H
BMC-T
Aldehyde dehydrogenase
PTAC
Signature enzyme
Alcohol dehydrogenase
Regulator

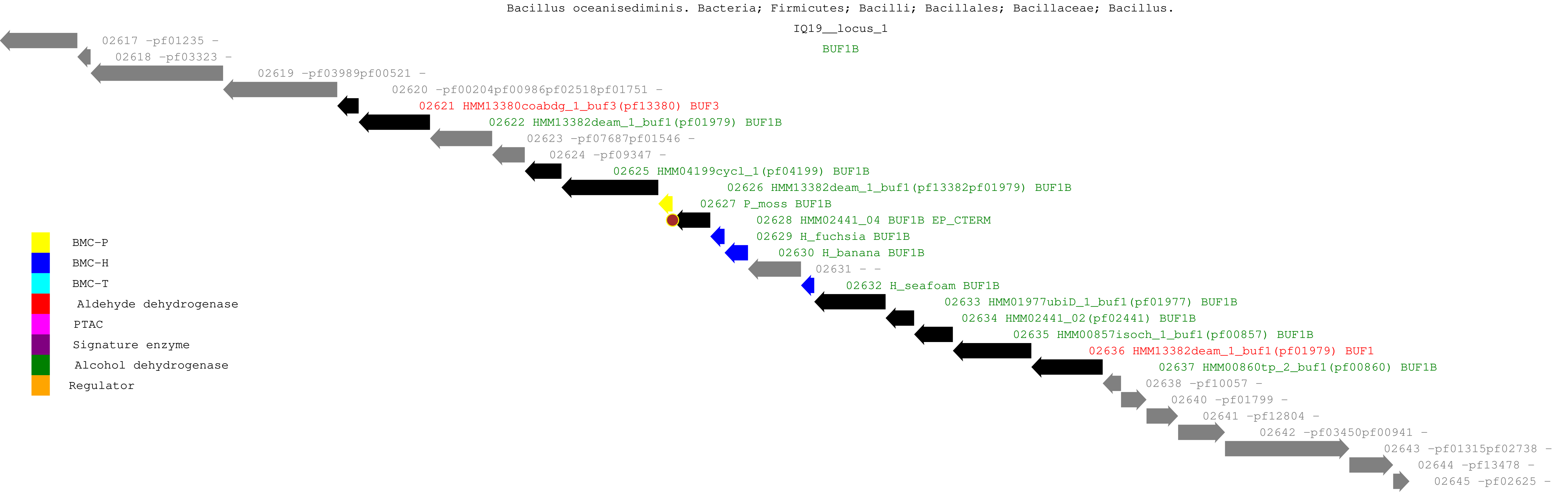

Bacillus oceanisediminis. Bacteria; Firmicutes; Bacilli; Bacillales; Bacillaceae; Bacillus.
IQ19__locus_1
BUF1B
02617 -pf01235 -
02618 -pf03323 -
02619 -pf03989pf00521 -
02620 -pf00204pf00986pf02518pf01751 -
02621 HMM13380coabdg_1_buf3(pf13380) BUF3
02622 HMM13382deam_1_buf1(pf01979) BUF1B
02623 -pf07687pf01546 -
02624 -pf09347 -
02625 HMM04199cycl_1(pf04199) BUF1B
02626 HMM13382deam_1_buf1(pf13382pf01979) BUF1B
02627 P_moss BUF1B
02628 HMM02441_04 BUF1B EP_CTERM
02629 H_fuchsia BUF1B
02630 H_banana BUF1B
02631 - -
02632 H_seafoam BUF1B
02633 HMM01977ubiD_1_buf1(pf01977) BUF1B
02634 HMM02441_02(pf02441) BUF1B
02635 HMM00857isoch_1_buf1(pf00857) BUF1B
02636 HMM13382deam_1_buf1(pf01979) BUF1
02637 HMM00860tp_2_buf1(pf00860) BUF1B
02638 -pf10057 -
02640 -pf01799 -
02641 -pf12804 -
02642 -pf03450pf00941 -
02643 -pf01315pf02738 -
02644 -pf13478 -
02645 -pf02625 -
BMC-P
BMC-H
BMC-T
Aldehyde dehydrogenase
PTAC
Signature enzyme
Alcohol dehydrogenase
Regulator

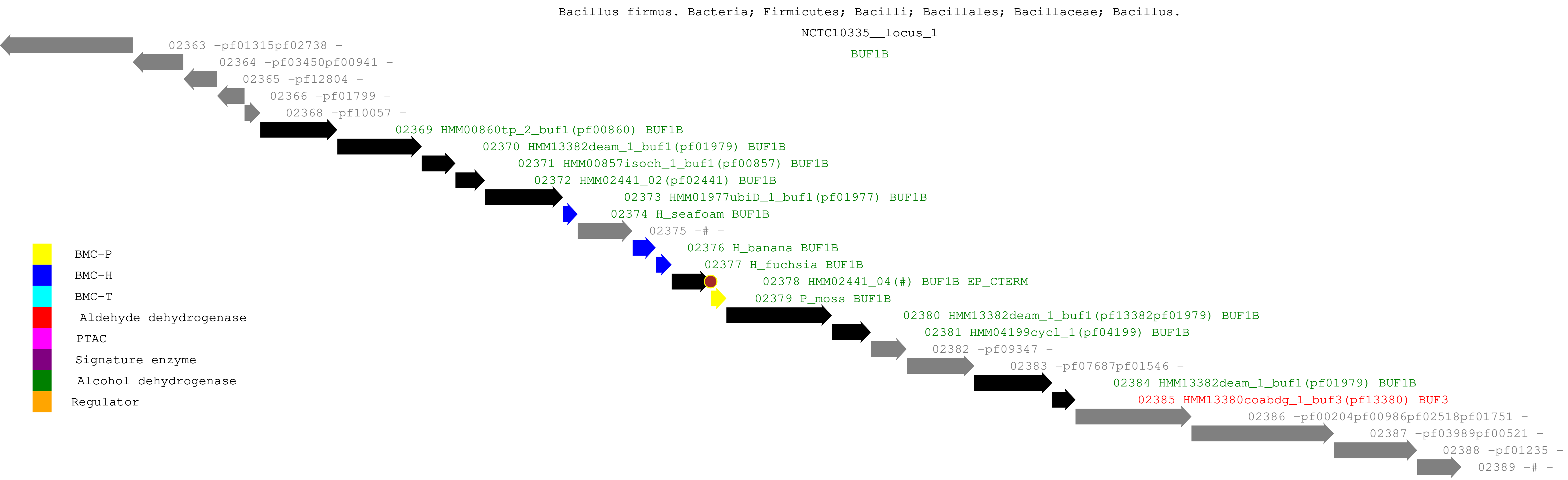
Bacillus firmus. Bacteria; Firmicutes; Bacilli; Bacillales; Bacillaceae; Bacillus.
NCTC10335__locus_1
BUF1B
02363 -pf01315pf02738 -
02364 -pf03450pf00941 -
02365 -pf12804 -
02366 -pf01799 -
02368 -pf10057 -
02369 HMM00860tp_2_buf1(pf00860) BUF1B
02370 HMM13382deam_1_buf1(pf01979) BUF1B
02371 HMM00857isoch_1_buf1(pf00857) BUF1B
02372 HMM02441_02(pf02441) BUF1B
02373 HMM01977ubiD_1_buf1(pf01977) BUF1B
02374 H_seafoam BUF1B
02375 -# -
02376 H_banana BUF1B
02377 H_fuchsia BUF1B
02378 HMM02441_04(#) BUF1B EP_CTERM
02379 P_moss BUF1B
02380 HMM13382deam_1_buf1(pf13382pf01979) BUF1B
02381 HMM04199cycl_1(pf04199) BUF1B
02382 -pf09347 -
02383 -pf07687pf01546 -
02384 HMM13382deam_1_buf1(pf01979) BUF1B
02385 HMM13380coabdg_1_buf3(pf13380) BUF3
02386 -pf00204pf00986pf02518pf01751 -
02387 -pf03989pf00521 -
02388 -pf01235 -
02389 -# -
BMC-P
BMC-H
BMC-T
Aldehyde dehydrogenase
PTAC
Signature enzyme
Alcohol dehydrogenase
Regulator

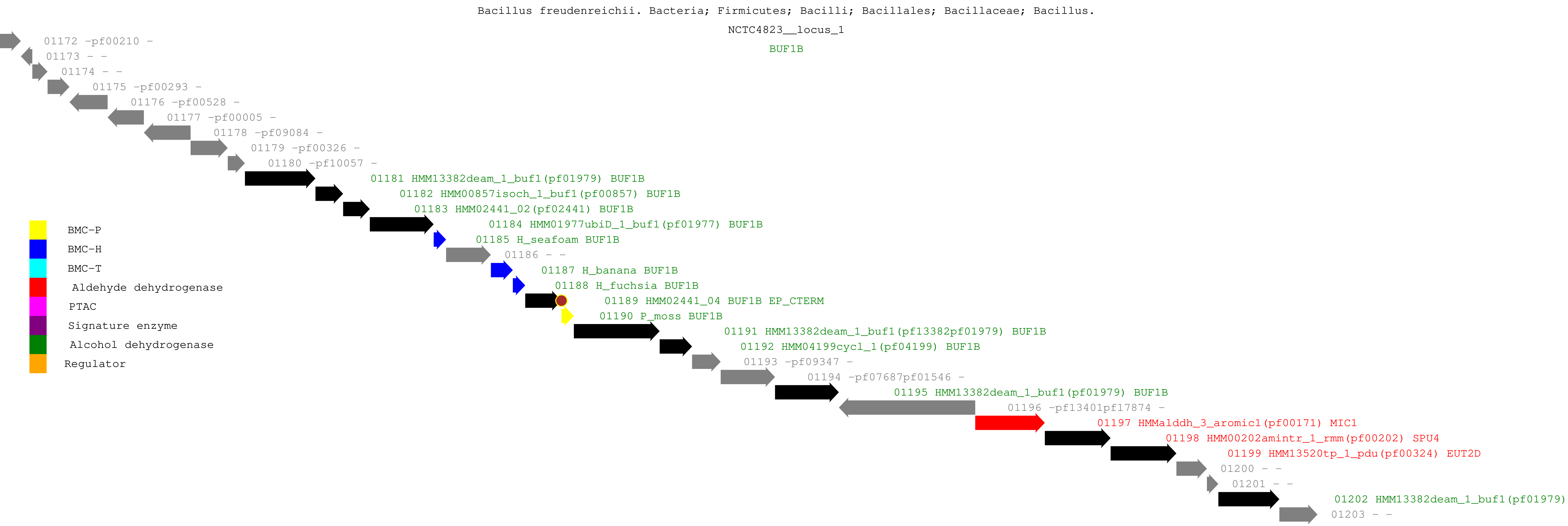
Bacillus freudenreichii. Bacteria; Firmicutes; Bacilli; Bacillales; Bacillaceae; Bacillus.
NCTC4823__locus_1
BUF1B
01172 -pf00210 -
01173 - -
01174 - -
01175 -pf00293 -
01176 -pf00528 -
01177 -pf00005 -
01178 -pf09084 -
01179 -pf00326 -
01180 -pf10057 -
01181 HMM13382deam_1_buf1(pf01979) BUF1B
01182 HMM00857isoch_1_buf1(pf00857) BUF1B
01183 HMM02441_02(pf02441) BUF1B
01184 HMM01977ubiD_1_buf1(pf01977) BUF1B
01185 H_seafoam BUF1B
01186 - -
01187 H_banana BUF1B
01188 H_fuchsia BUF1B
01189 HMM02441_04 BUF1B EP_CTERM
01190 P_moss BUF1B
01191 HMM13382deam_1_buf1(pf13382pf01979) BUF1B
01192 HMM04199cycl_1(pf04199) BUF1B
01193 -pf09347 -
01194 -pf07687pf01546 -
01195 HMM13382deam_1_buf1(pf01979) BUF1B
01196 -pf13401pf17874 -
01197 HMMalddh_3_aromic1(pf00171) MIC1
01198 HMM00202amintr_1_rmm(pf00202) SPU4
01199 HMM13520tp_1_pdu(pf00324) EUT2D
01200 - -
01201 - -
01202 HMM13382deam_1_buf1(pf01979)
01203 - -
BMC-P
BMC-H
BMC-T
Aldehyde dehydrogenase
PTAC
Signature enzyme
Alcohol dehydrogenase
Regulator

Bacillus firmus DS1. Bacteria; Firmicutes; Bacilli; Bacillales; Bacillaceae; Bacillus.

PBF__locus_1

BUF1B

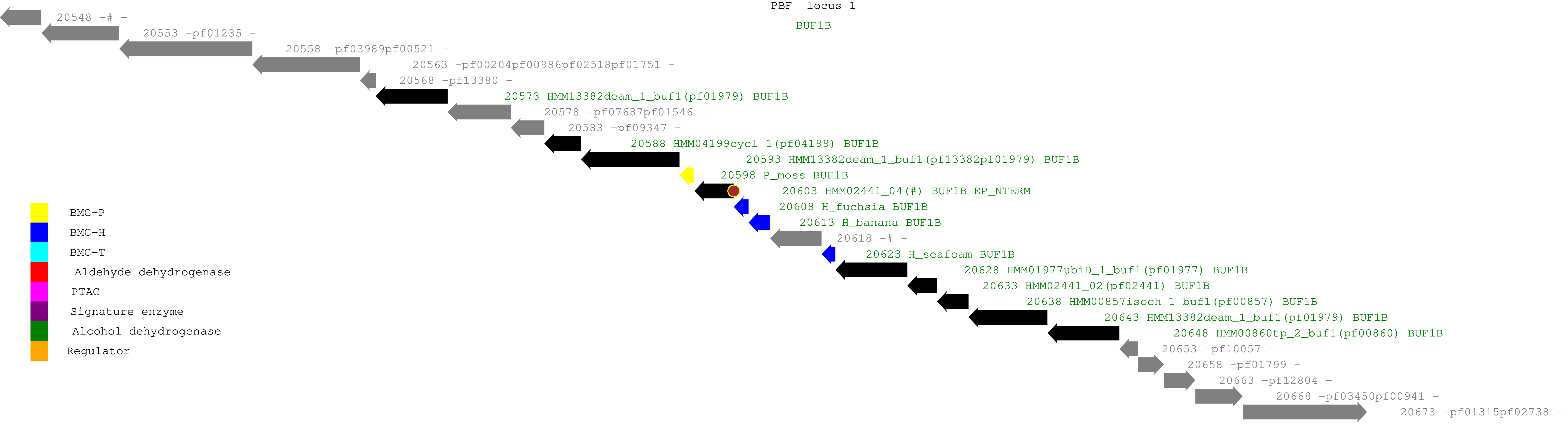

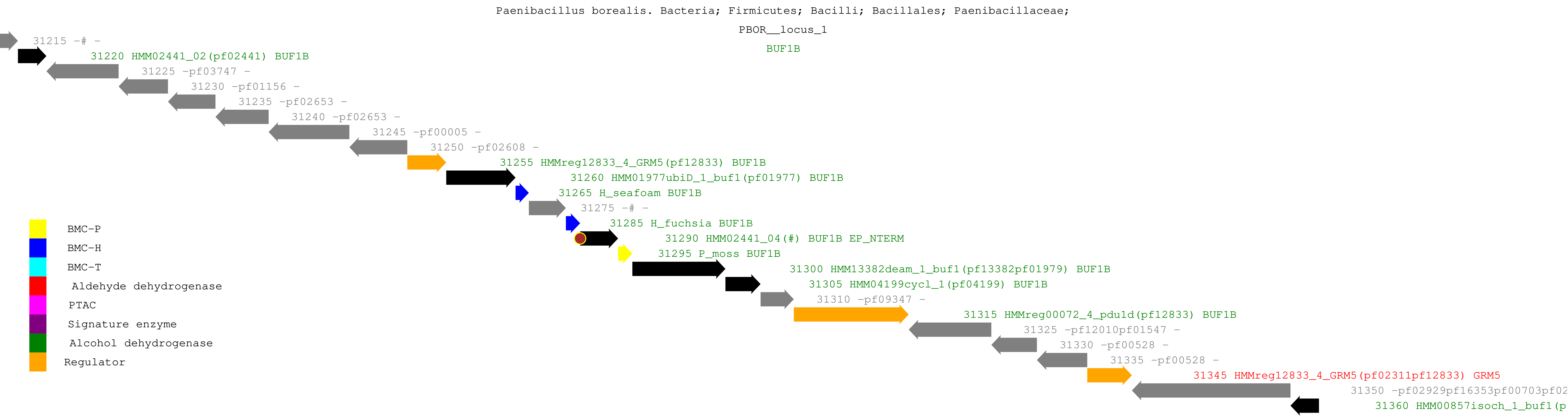
Paenibacillus borealis. Bacteria; Firmicutes; Bacilli; Bacillales; Paenibacillaceae;
PBOR__locus_1
BUF1B
31215 -# -
31220 HMM02441_02(pf02441) BUF1B
31225 -pf03747 -
31230 -pf01156 -
31235 -pf02653 -
31240 -pf02653 -
31245 -pf00005 -
31250 -pf02608 -
31255 HMMreg12833_4_GRM5(pf12833) BUF1B
31260 HMM01977ubiD_1_buf1(pf01977) BUF1B
31265 H_seafoam BUF1B
31275 -# -
31285 H_fuchsia BUF1B
31290 HMM02441_04(#) BUF1B EP_NTERM
31295 P_moss BUF1B
31300 HMM13382deam_1_buf1(pf13382pf01979) BUF1B
31305 HMM04199cycl_1(pf04199) BUF1B
31310 -pf09347 -
31315 HMMreg00072_4_pdu1d(pf12833) BUF1B
31325 -pf12010pf01547 -
31330 -pf00528 -
31335 -pf00528 -
31345 HMMreg12833_4_GRM5(pf02311pf12833) GRM5
31350 -pf02929pf16353pf00703pf02
31360 HMM00857isoch_1_buf1(p
BMC-P
BMC-H
BMC-T
Aldehyde dehydrogenase
PTAC
Signature enzyme
Alcohol dehydrogenase
Regulator

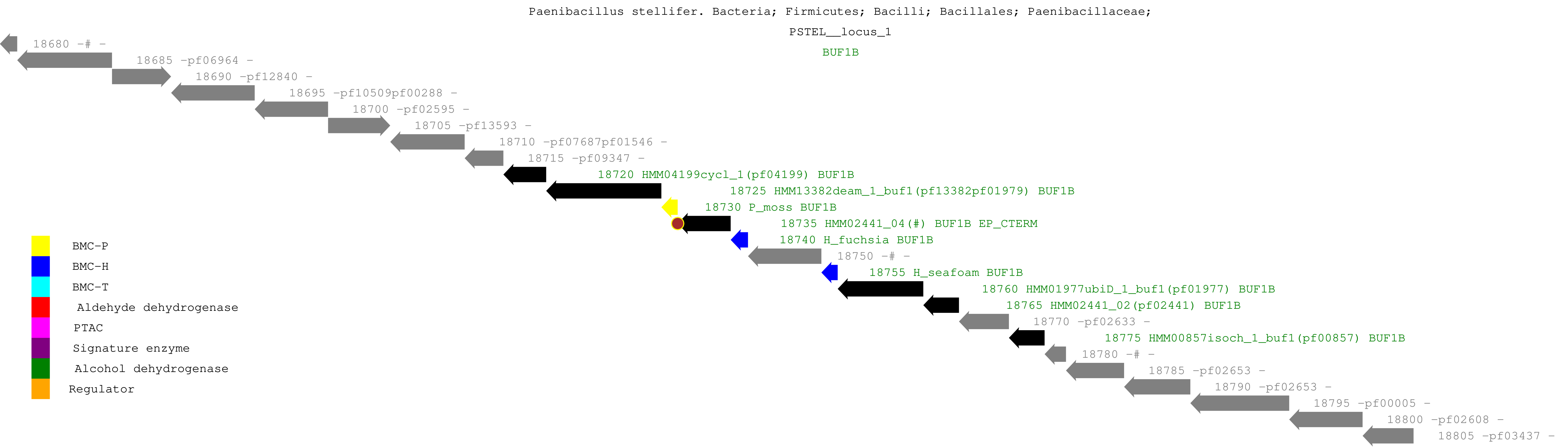
Paenibacillus stellifer. Bacteria; Firmicutes; Bacilli; Bacillales; Paenibacillaceae;
PSTEL__locus_1
BUF1B
18680 -# -
18685 -pf06964 -
18690 -pf12840 -
18695 -pf10509pf00288 -
18700 -pf02595 -
18705 -pf13593 -
18710 -pf07687pf01546 -
18715 -pf09347 -
18720 HMM04199cycl_1(pf04199) BUF1B
18725 HMM13382deam_1_buf1(pf13382pf01979) BUF1B
18730 P_moss BUF1B
18735 HMM02441_04(#) BUF1B EP_CTERM
18740 H_fuchsia BUF1B
18750 -# -
18755 H_seafoam BUF1B
18760 HMM01977ubiD_1_buf1(pf01977) BUF1B
18765 HMM02441_02(pf02441) BUF1B
18770 -pf02633 -
18775 HMM00857isoch_1_buf1(pf00857) BUF1B
18780 -# -
18785 -pf02653 -
18790 -pf02653 -
18795 -pf00005 -
18800 -pf02608 -
18805 -pf03437 -
BMC-P
BMC-H
BMC-T
Aldehyde dehydrogenase
PTAC
Signature enzyme
Alcohol dehydrogenase
Regulator

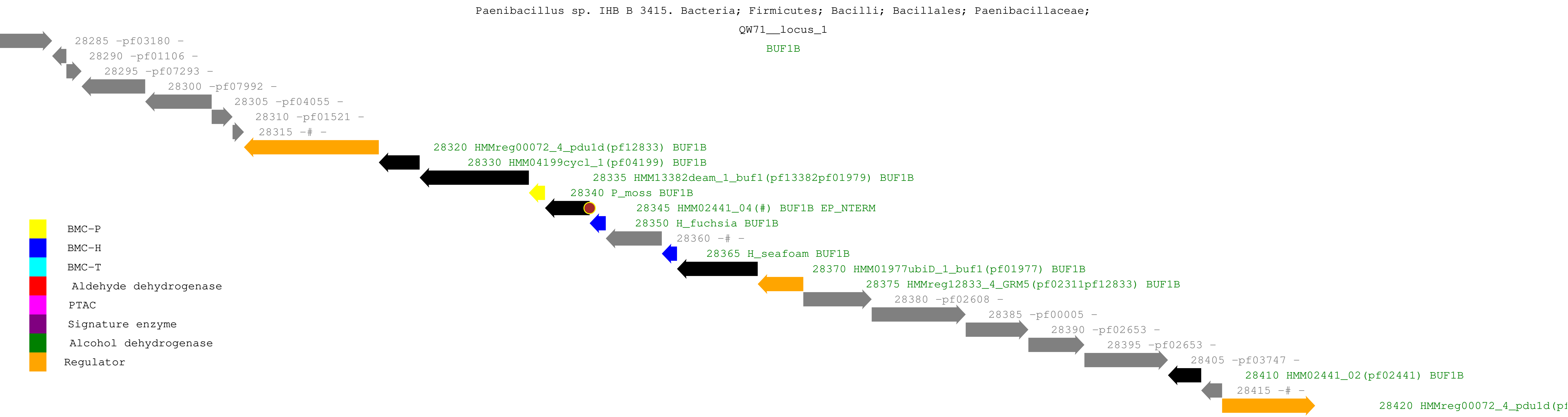
Paenibacillus sp. IHB B 3415. Bacteria; Firmicutes; Bacilli; Bacillales; Paenibacillaceae;
QW71__locus_1
BUF1B
28285 -pf03180 -
28290 -pf01106 -
28295 -pf07293 -
28300 -pf07992 -
28305 -pf04055 -
28310 -pf01521 -
28315 -# -
28320 HMMreg00072_4_pdu1d(pf12833) BUF1B
28330 HMM04199cycl_1(pf04199) BUF1B
28335 HMM13382deam_1_buf1(pf13382pf01979) BUF1B
28340 P_moss BUF1B
28345 HMM02441_04(#) BUF1B EP_NTERM
28350 H_fuchsia BUF1B
28360 -# -
28365 H_seafoam BUF1B
28370 HMM01977ubiD_1_buf1(pf01977) BUF1B
28375 HMMreg12833_4_GRM5(pf02311pf12833) BUF1B
28380 -pf02608 -
28385 -pf00005 -
28390 -pf02653 -
28395 -pf02653 -
28405 -pf03747 -
28410 HMM02441_02(pf02441) BUF1B
28415 -# -
28420 HMMreg00072_4_pdu1d(pf
BMC-P
BMC-H
BMC-T
Aldehyde dehydrogenase
PTAC
Signature enzyme
Alcohol dehydrogenase
Regulator

Paenibacillus sp. FSL R5-0912. Bacteria; Firmicutes; Bacilli; Bacillales; Paenibacillaceae;

R50912__locus_1

BUF1B

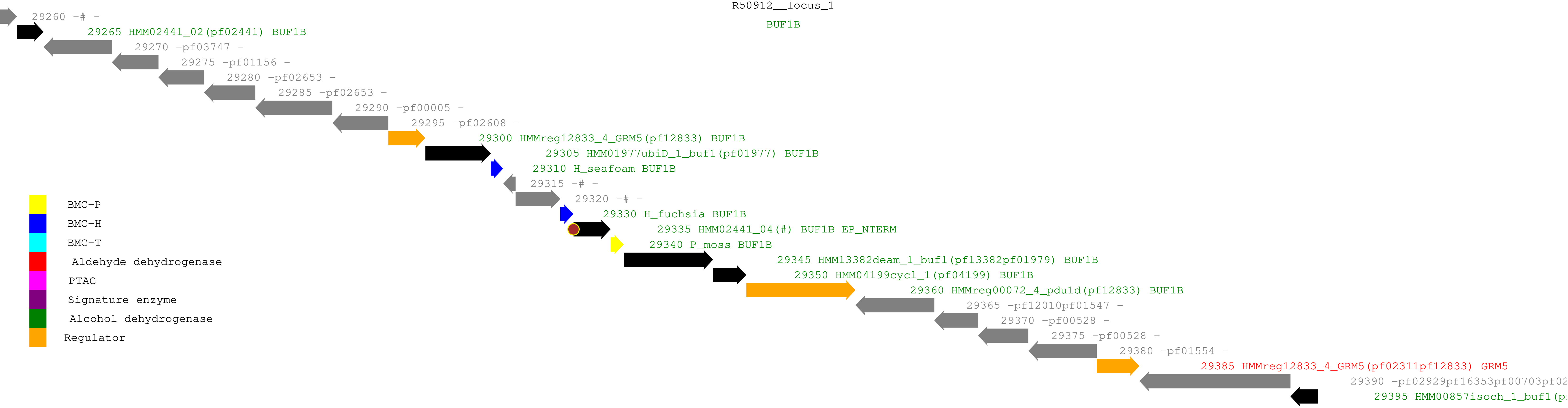

Paenibacillus sp. FSL R7-0331. Bacteria; Firmicutes; Bacilli; Bacillales; Paenibacillaceae;

R70331__locus_1

BUF1B

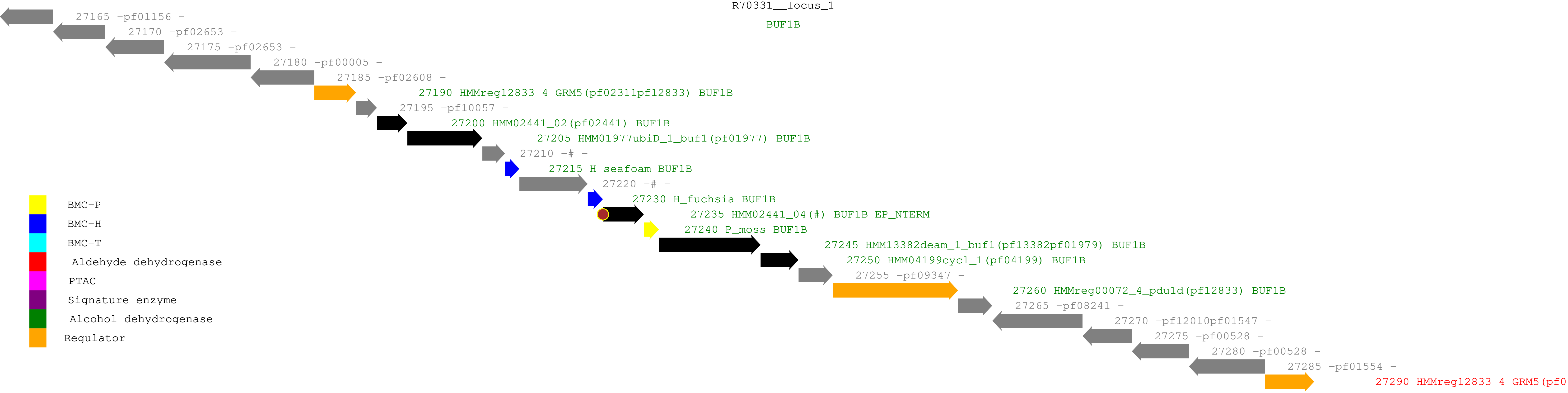

Paenibacillus sp. FSL R7-0273. Bacteria; Firmicutes; Bacilli; Bacillales; Paenibacillaceae;

R70723__locus_1

BUF1B

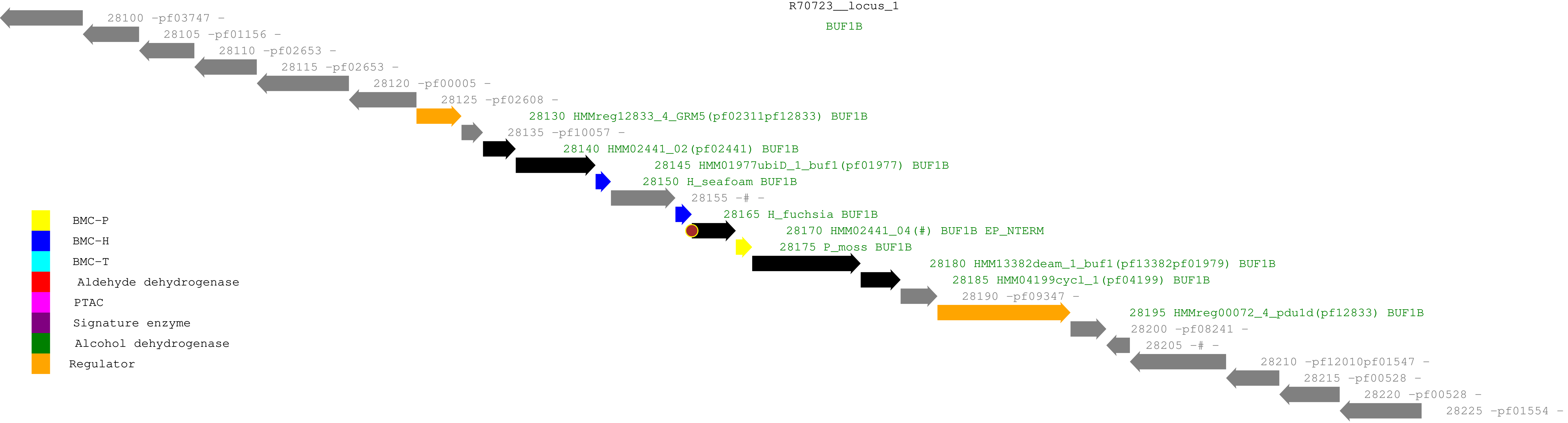

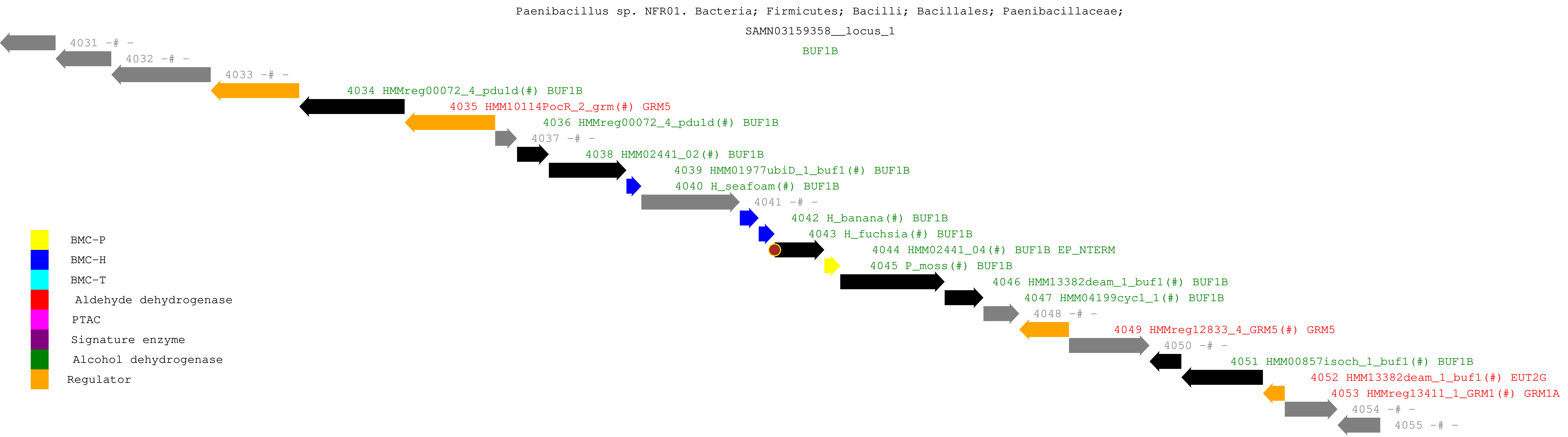

Paenibacillus sp. NFR01. Bacteria; Firmicutes; Bacilli; Bacillales; Paenibacillaceae;
SAMN03159358__locus_1
BUF1B
4031 -# -
4032 -# -
4033 -# -
4034 HMMreg00072_4_pdu1d(#) BUF1B
4035 HMM10114PocR_2_grm(#) GRM5
4036 HMMreg00072_4_pdu1d(#) BUF1B
4037 -# -
4038 HMM02441_02(#) BUF1B
4039 HMM01977ubiD_1_buf1(#) BUF1B
4040 H_seafoam(#) BUF1B
4041 -# -
4042 H_banana(#) BUF1B
4043 H_fuchsia(#) BUF1B
4044 HMM02441_04(#) BUF1B EP_NTERM
4045 P_moss(#) BUF1B
4046 HMM13382deam_1_buf1(#) BUF1B
4047 HMM04199cycl_1(#) BUF1B
4048 -# -
4049 HMMreg12833_4_GRM5(#) GRM5
4050 -# -
4051 HMM00857isoch_1_buf1(#) BUF1B
4052 HMM13382deam_1_buf1(#) EUT2G
4053 HMMreg13411_1_GRM1(#) GRM1A
4054 -# -
4055 -# -
BMC-P
BMC-H
BMC-T
Aldehyde dehydrogenase
PTAC
Signature enzyme
Alcohol dehydrogenase
Regulator

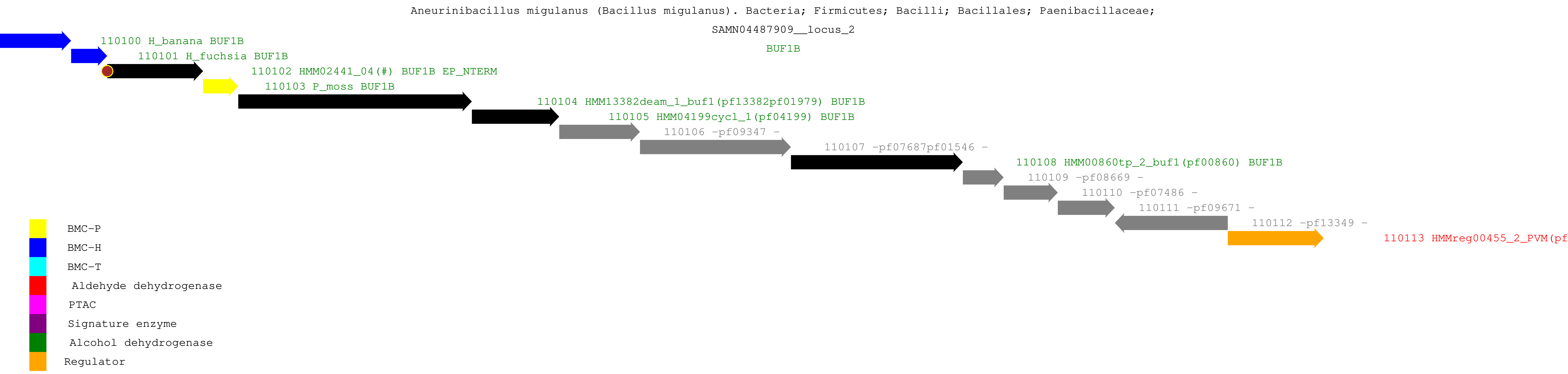

Aneurinibacillus migulanus (Bacillus migulanus). Bacteria; Firmicutes; Bacilli; Bacillales; Paenibacillaceae;
SAMN04487909__locus_2
BUF1B
110100 H_banana BUF1B
110101 H_fuchsia BUF1B
110102 HMM02441_04(#) BUF1B EP_NTERM
110103 P_moss BUF1B
110104 HMM13382deam_1_buf1(pf13382pf01979) BUF1B
110105 HMM04199cycl_1(pf04199) BUF1B
110106 -pf09347 -
110107 -pf07687pf01546 -
110108 HMM00860tp_2_buf1(pf00860) BUF1B
110109 -pf08669 -
110110 -pf07486 -
110111 -pf09671 -
110112 -pf13349 -
110113 HMMreg00455_2_PVM(pf
BMC-P
BMC-H
BMC-T
Aldehyde dehydrogenase
PTAC
Signature enzyme
Alcohol dehydrogenase
Regulator

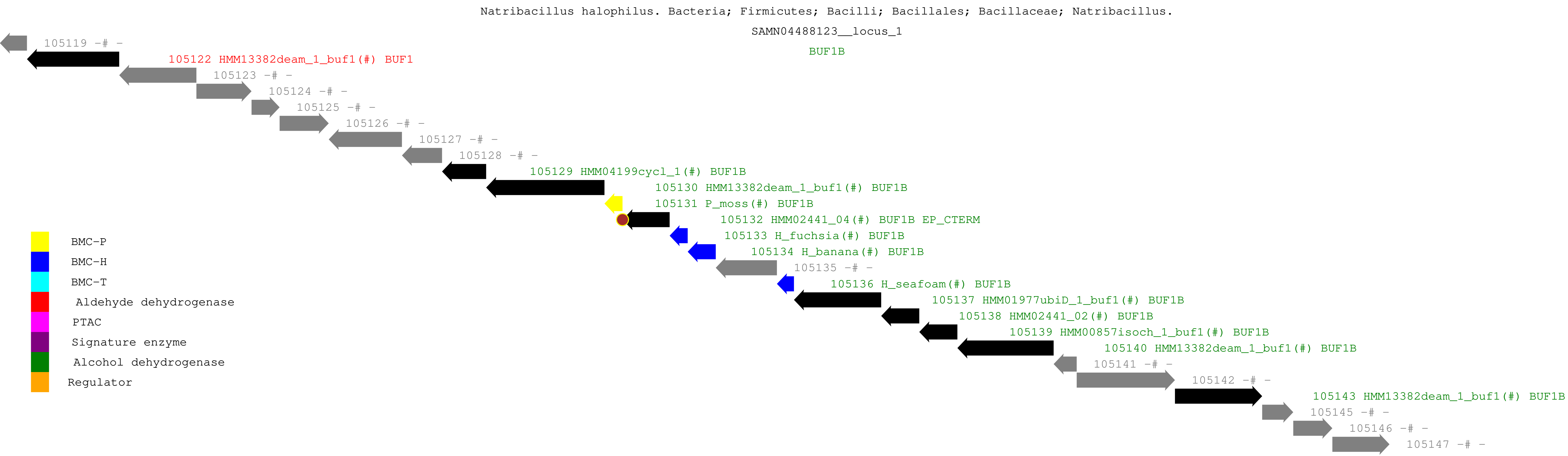
Natribacillus halophilus. Bacteria; Firmicutes; Bacilli; Bacillales; Bacillaceae; Natribacillus.
SAMN04488123__locus_1
BUF1B
105119 -# -
105122 HMM13382deam_1_buf1(#) BUF1
105123 -# -
105124 -# -
105125 -# -
105126 -# -
105127 -# -
105128 -# -
105129 HMM04199cycl_1(#) BUF1B
105130 HMM13382deam_1_buf1(#) BUF1B
105131 P_moss(#) BUF1B
105132 HMM02441_04(#) BUF1B EP_CTERM
105133 H_fuchsia(#) BUF1B
105134 H_banana(#) BUF1B
105135 -# -
105136 H_seafoam(#) BUF1B
105137 HMM01977ubiD_1_buf1(#) BUF1B
105138 HMM02441_02(#) BUF1B
105139 HMM00857isoch_1_buf1(#) BUF1B
105140 HMM13382deam_1_buf1(#) BUF1B
105141 -# -
105142 -# -
105143 HMM13382deam_1_buf1(#) BUF1B
105145 -# -
105146 -# -
105147 -# -
BMC-P
BMC-H
BMC-T
Aldehyde dehydrogenase
PTAC
Signature enzyme
Alcohol dehydrogenase
Regulator

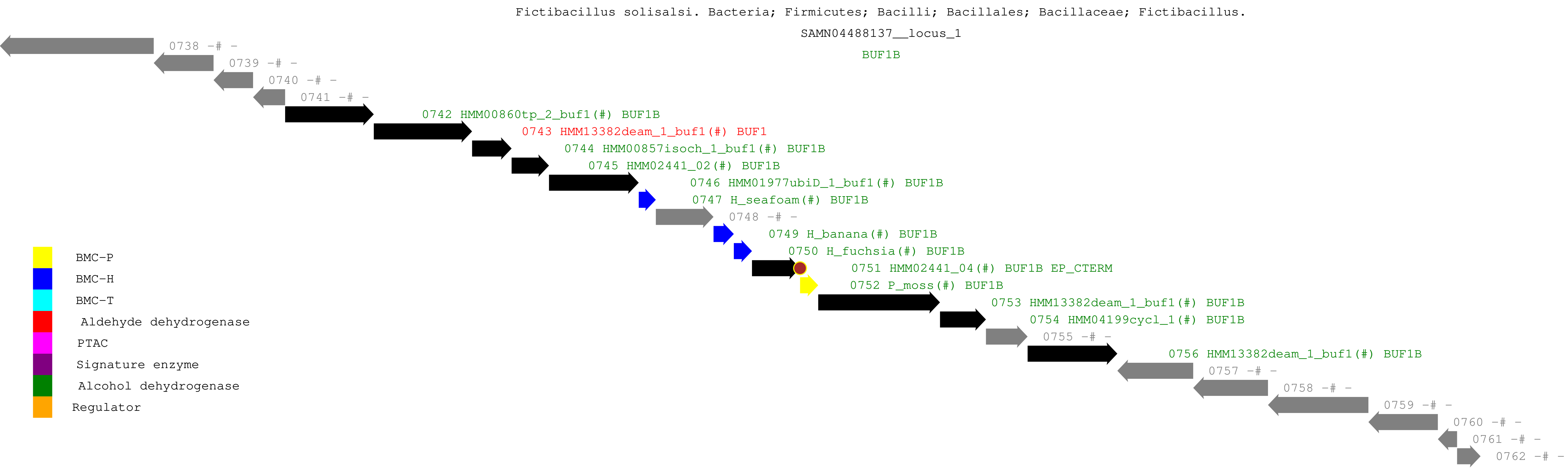

Fictibacillus solisalsi. Bacteria; Firmicutes; Bacilli; Bacillales; Bacillaceae; Fictibacillus.
SAMN04488137__locus_1
BUF1B
0738 -# -
0739 -# -
0740 -# -
0741 -# -
0742 HMM00860tp_2_buf1(#) BUF1B
0743 HMM13382deam_1_buf1(#) BUF1
0744 HMM00857isoch_1_buf1(#) BUF1B
0745 HMM02441_02(#) BUF1B
0746 HMM01977ubiD_1_buf1(#) BUF1B
0747 H_seafoam(#) BUF1B
0748 -# -
0749 H_banana(#) BUF1B
0750 H_fuchsia(#) BUF1B
0751 HMM02441_04(#) BUF1B EP_CTERM
0752 P_moss(#) BUF1B
0753 HMM13382deam_1_buf1(#) BUF1B
0754 HMM04199cycl_1(#) BUF1B
0755 -# -
0756 HMM13382deam_1_buf1(#) BUF1B
0757 -# -
0758 -# -
0759 -# -
0760 -# -
0761 -# -
0762 -# -
BMC-P
BMC-H
BMC-T
Aldehyde dehydrogenase
PTAC
Signature enzyme
Alcohol dehydrogenase
Regulator

Fontibacillus panacisegetis. Bacteria; Firmicutes; Bacilli; Bacillales; Paenibacillaceae;

SAMN04488542__locus_1

BUF1B

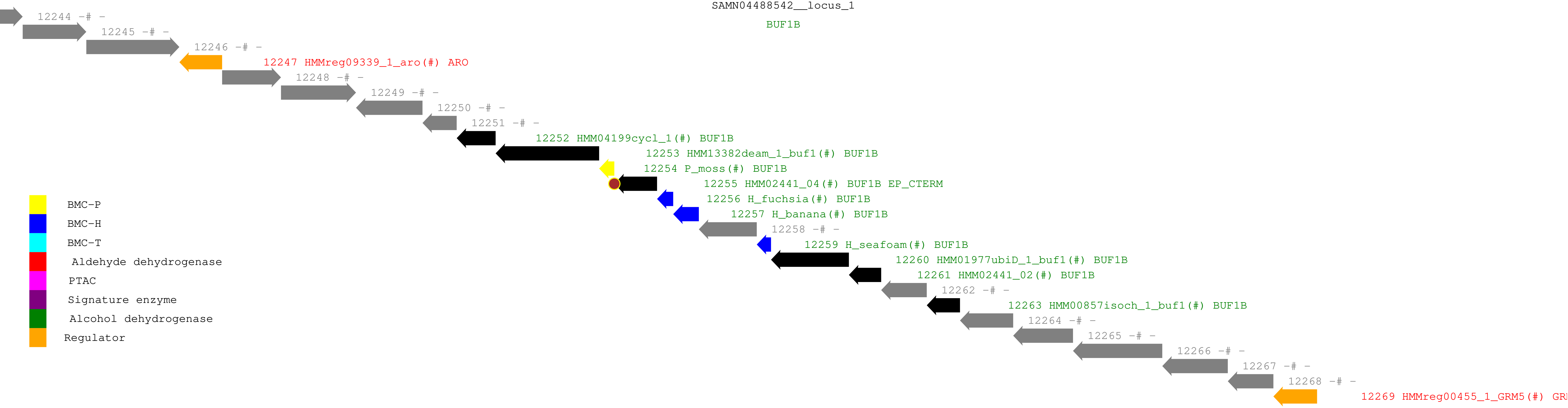

Bacillus sp. cl95. Bacteria; Firmicutes; Bacilli; Bacillales; Bacillaceae; Bacillus.

SAMN04488577__locus_1

BUF1B

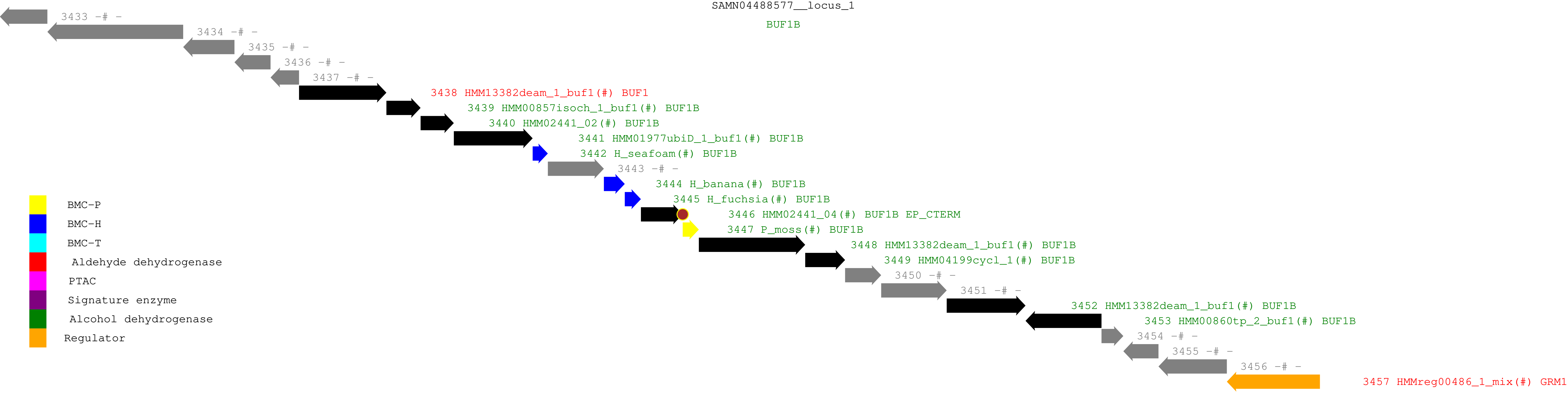

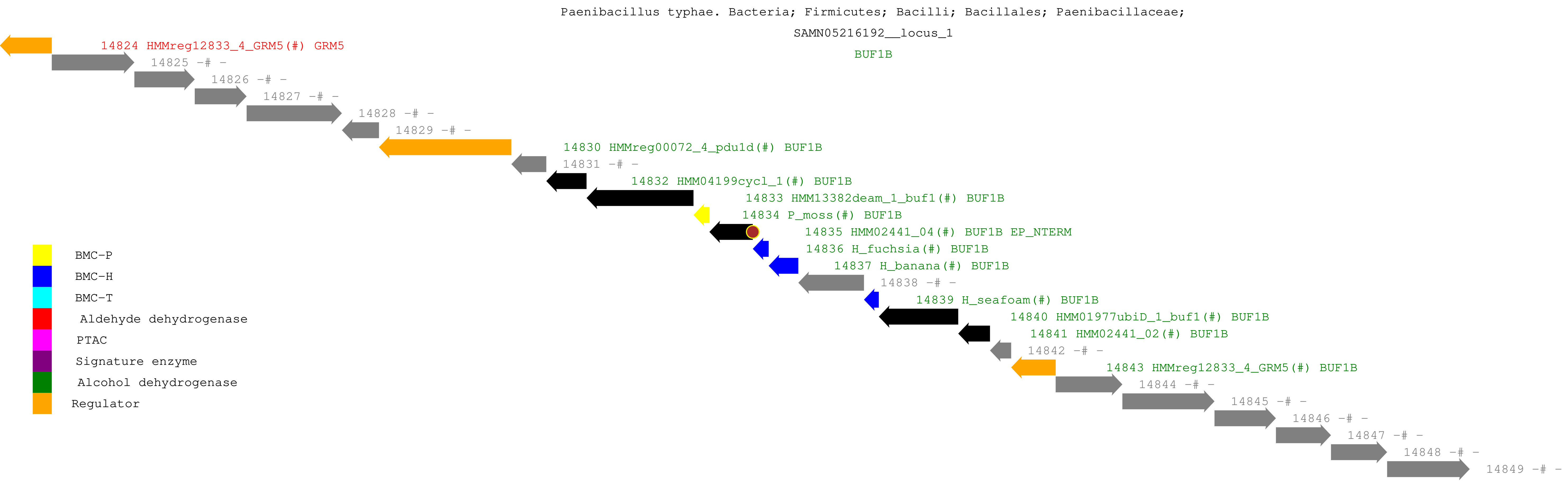
Paenibacillus typhae. Bacteria; Firmicutes; Bacilli; Bacillales; Paenibacillaceae;
SAMN05216192__locus_1
BUF1B
14824 HMMreg12833_4_GRM5(#) GRM5
14825 -# -
14826 -# -
14827 -# -
14828 -# -
14829 -# -
14830 HMMreg00072_4_pdu1d(#) BUF1B
14831 -# -
14832 HMM04199cycl_1(#) BUF1B
14833 HMM13382deam_1_buf1(#) BUF1B
14834 P_moss(#) BUF1B
14835 HMM02441_04(#) BUF1B EP_NTERM
14836 H_fuchsia(#) BUF1B
14837 H_banana(#) BUF1B
14838 -# -
14839 H_seafoam(#) BUF1B
14840 HMM01977ubiD_1_buf1(#) BUF1B
14841 HMM02441_02(#) BUF1B
14842 -# -
14843 HMMreg12833_4_GRM5(#) BUF1B
14844 -# -
14845 -# -
14846 -# -
14847 -# -
14848 -# -
14849 -# -
BMC-P
BMC-H
BMC-T
Aldehyde dehydrogenase
PTAC
Signature enzyme
Alcohol dehydrogenase
Regulator

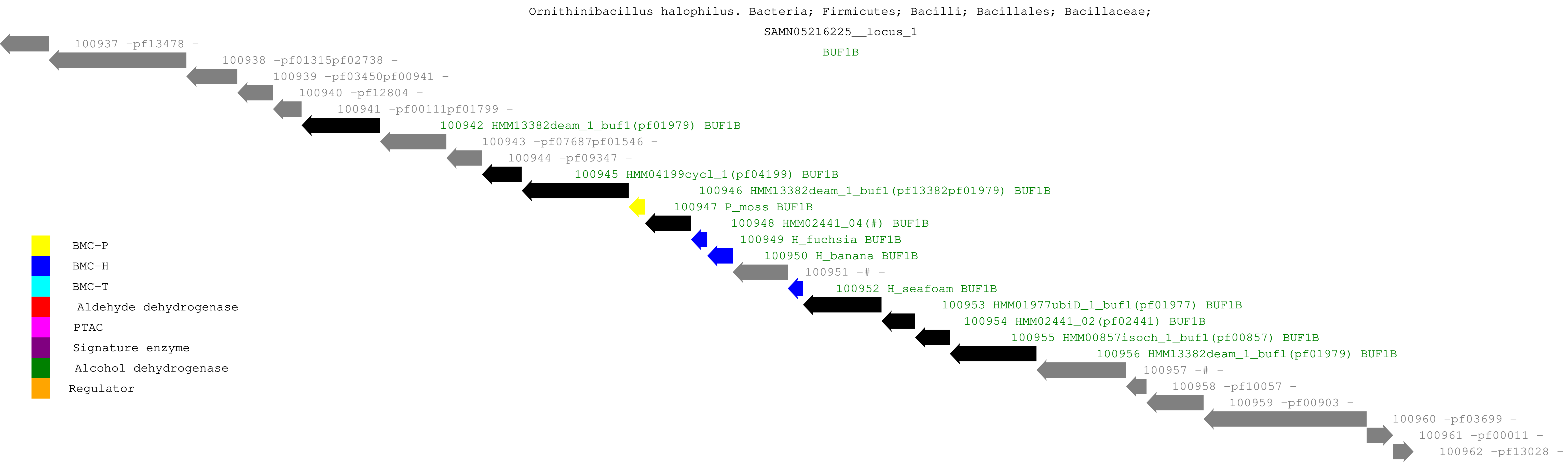
Ornithinibacillus halophilus. Bacteria; Firmicutes; Bacilli; Bacillales; Bacillaceae;
SAMN05216225__locus_1
BUF1B
100937 -pf13478 -
100938 -pf01315pf02738 -
100939 -pf03450pf00941 -
100940 -pf12804 -
100941 -pf00111pf01799 -
100942 HMM13382deam_1_buf1(pf01979) BUF1B
100943 -pf07687pf01546 -
100944 -pf09347 -
100945 HMM04199cycl_1(pf04199) BUF1B
100946 HMM13382deam_1_buf1(pf13382pf01979) BUF1B
100947 P_moss BUF1B
100948 HMM02441_04(#) BUF1B
100949 H_fuchsia BUF1B
100950 H_banana BUF1B
100951 -# -
100952 H_seafoam BUF1B
100953 HMM01977ubiD_1_buf1(pf01977) BUF1B
100954 HMM02441_02(pf02441) BUF1B
100955 HMM00857isoch_1_buf1(pf00857) BUF1B
100956 HMM13382deam_1_buf1(pf01979) BUF1B
100957 -# -
100958 -pf10057 -
100959 -pf00903 -
100960 -pf03699 -
100961 -pf00011 -
100962 -pf13028 -
BMC-P
BMC-H
BMC-T
Aldehyde dehydrogenase
PTAC
Signature enzyme
Alcohol dehydrogenase
Regulator

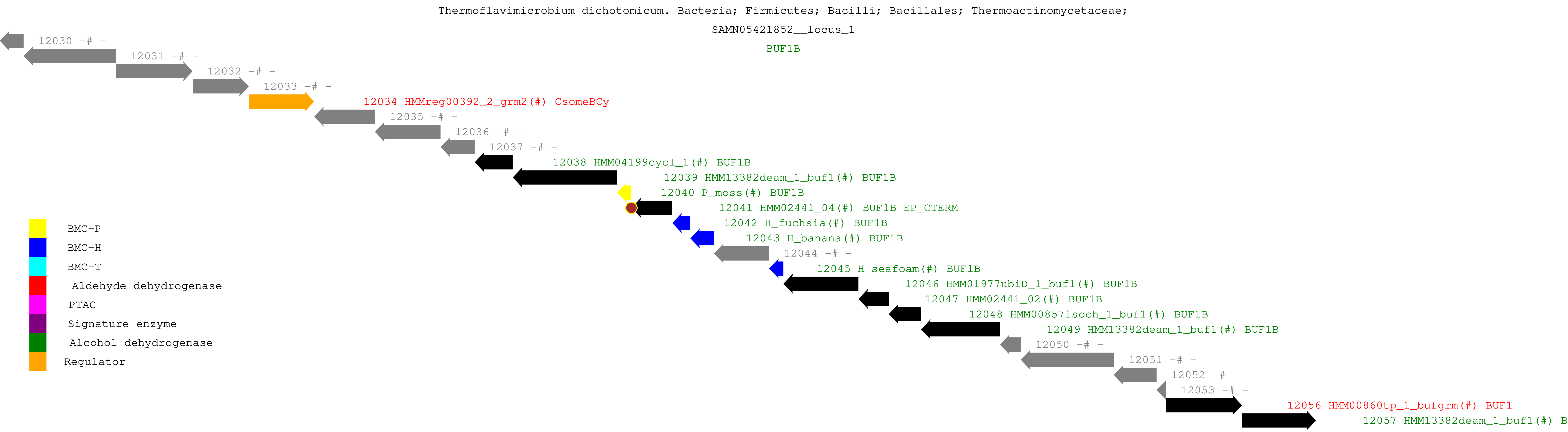

Thermoflavimicrobium dichotomicum. Bacteria; Firmicutes; Bacilli; Bacillales; Thermoactinomycetaceae;
SAMN05421852__locus_1
BUF1B
12030 -# -
12031 -# -
12032 -# -
12033 -# -
12034 HMMreg00392_2_grm2(#) CsomeBCy
12035 -# -
12036 -# -
12037 -# -
12038 HMM04199cycl_1(#) BUF1B
12039 HMM13382deam_1_buf1(#) BUF1B
12040 P_moss(#) BUF1B
12041 HMM02441_04(#) BUF1B EP_CTERM
12042 H_fuchsia(#) BUF1B
12043 H_banana(#) BUF1B
12044 -# -
12045 H_seafoam(#) BUF1B
12046 HMM01977ubiD_1_buf1(#) BUF1B
12047 HMM02441_02(#) BUF1B
12048 HMM00857isoch_1_buf1(#) BUF1B
12049 HMM13382deam_1_buf1(#) BUF1B
12050 -# -
12051 -# -
12052 -# -
12053 -# -
12056 HMM00860tp_1_bufgrm(#) BUF1
12057 HMM13382deam_1_buf1(#) B
BMC-P
BMC-H
BMC-T
Aldehyde dehydrogenase
PTAC
Signature enzyme
Alcohol dehydrogenase
Regulator

Bacillus sp. OV194. Bacteria; Firmicutes; Bacilli; Bacillales; Bacillaceae; Bacillus.

SAMN05428981__locus_1

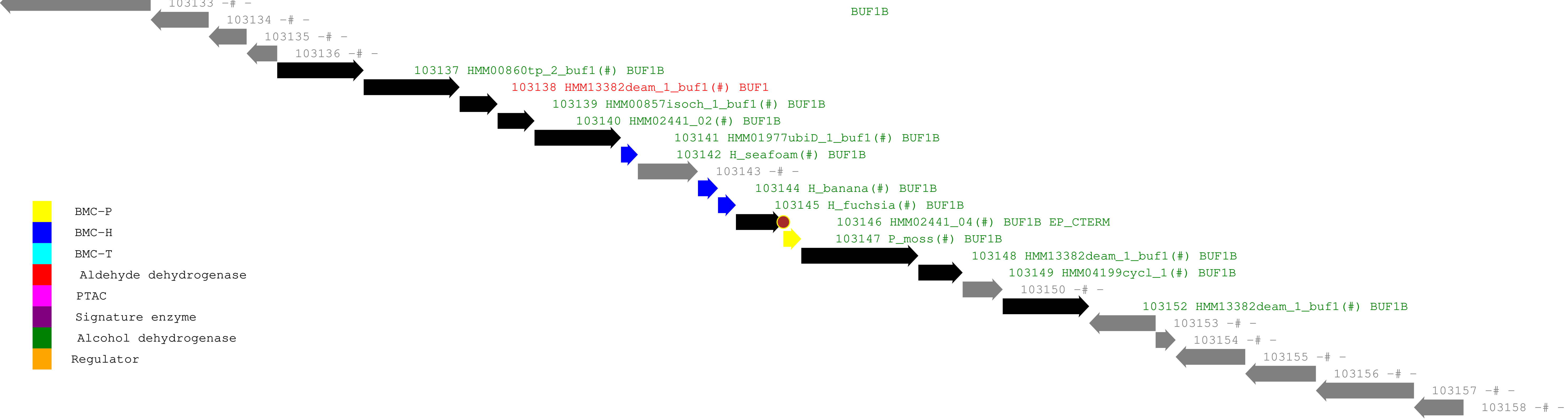

Margininema mesophilum. Bacteria; Firmicutes; Bacilli; Bacillales; Thermoactinomycetaceae;

SAMN05444487__locus_1

BUF1B

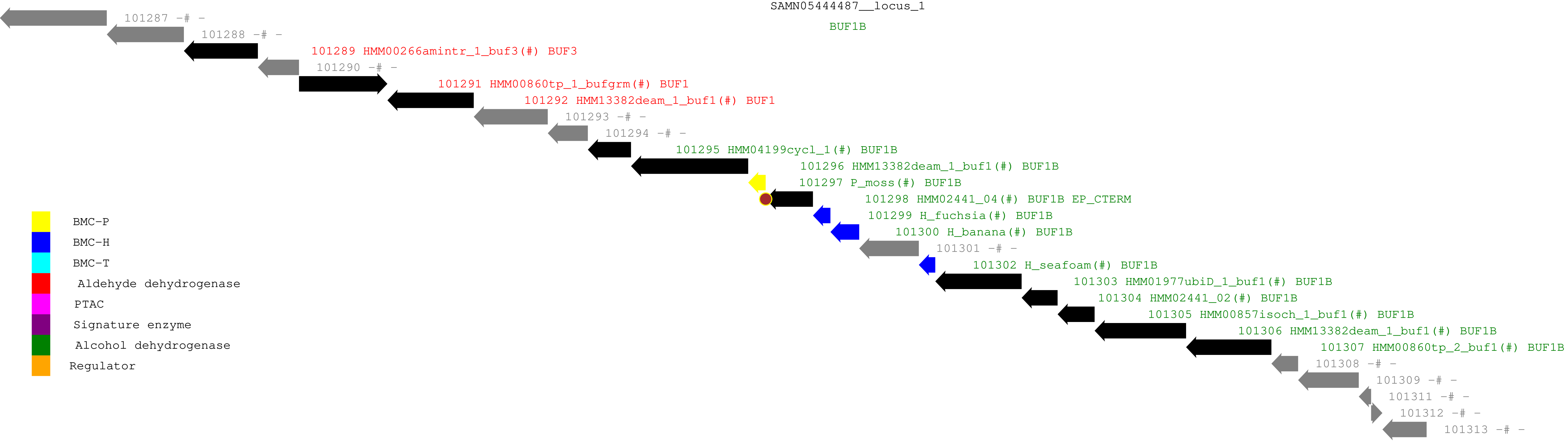

Marininema halotolerans. Bacteria; Firmicutes; Bacilli; Bacillales; Thermoactinomycetaceae;

SAMN05444972__locus_1

BUF1B

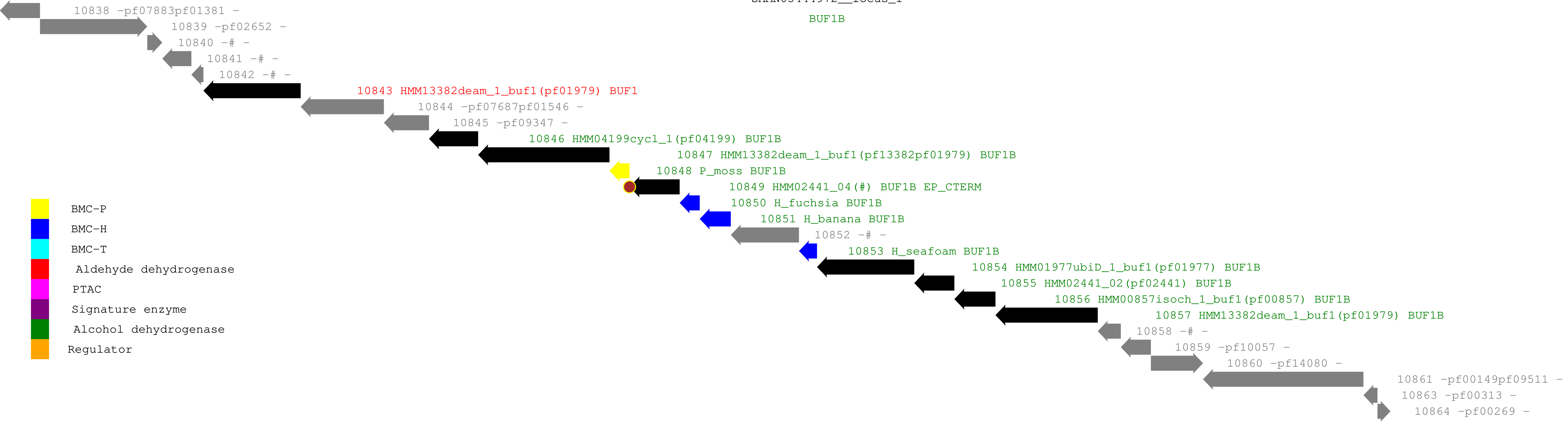

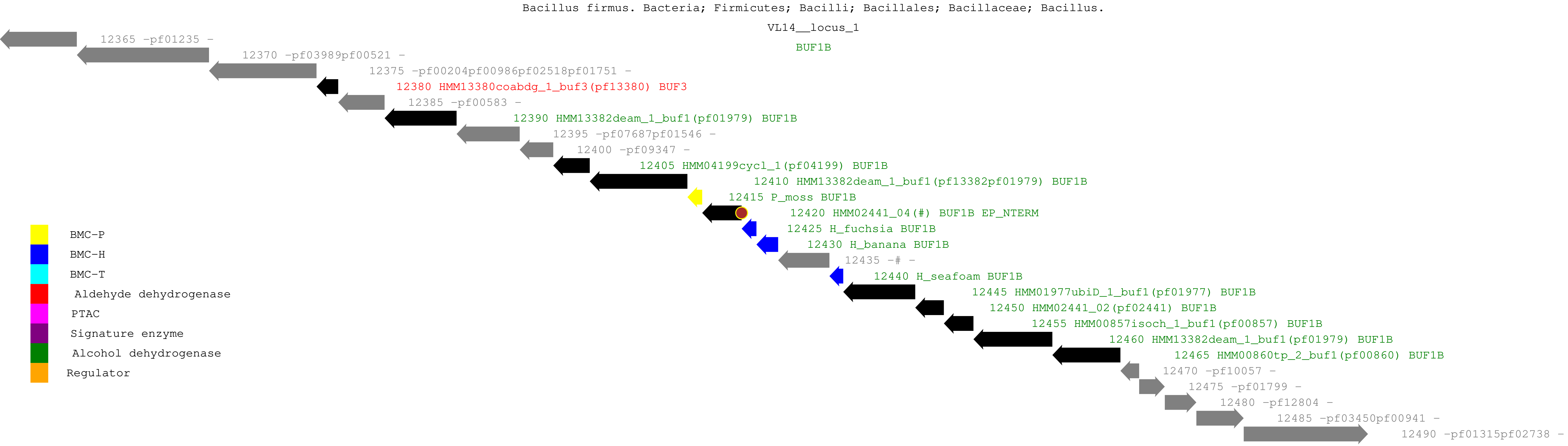

Bacillus firmus. Bacteria; Firmicutes; Bacilli; Bacillales; Bacillaceae; Bacillus.
VL14__locus_1
BUF1B
12365 -pf01235 -
12370 -pf03989pf00521 -
12375 -pf00204pf00986pf02518pf01751 -
12380 HMM13380coabdg_1_buf3(pf13380) BUF3
12385 -pf00583 -
12390 HMM13382deam_1_buf1(pf01979) BUF1B
12395 -pf07687pf01546 -
12400 -pf09347 -
12405 HMM04199cycl_1(pf04199) BUF1B
12410 HMM13382deam_1_buf1(pf13382pf01979) BUF1B
12415 P_moss BUF1B
12420 HMM02441_04(#) BUF1B EP_NTERM
12425 H_fuchsia BUF1B
12430 H_banana BUF1B
12435 -# -
12440 H_seafoam BUF1B
12445 HMM01977ubiD_1_buf1(pf01977) BUF1B
12450 HMM02441_02(pf02441) BUF1B
12455 HMM00857isoch_1_buf1(pf00857) BUF1B
12460 HMM13382deam_1_buf1(pf01979) BUF1B
12465 HMM00860tp_2_buf1(pf00860) BUF1B
12470 -pf10057 -
12475 -pf01799 -
12480 -pf12804 -
12485 -pf03450pf00941 -
12490 -pf01315pf02738 -
BMC-P
BMC-H
BMC-T
Aldehyde dehydrogenase
PTAC
Signature enzyme
Alcohol dehydrogenase
Regulator

Micromonospora sp. TSRI0369. Bacteria; Actinobacteria; Micromonosporales; Micromonosporaceae;

AMK25__locus_1

BUF2

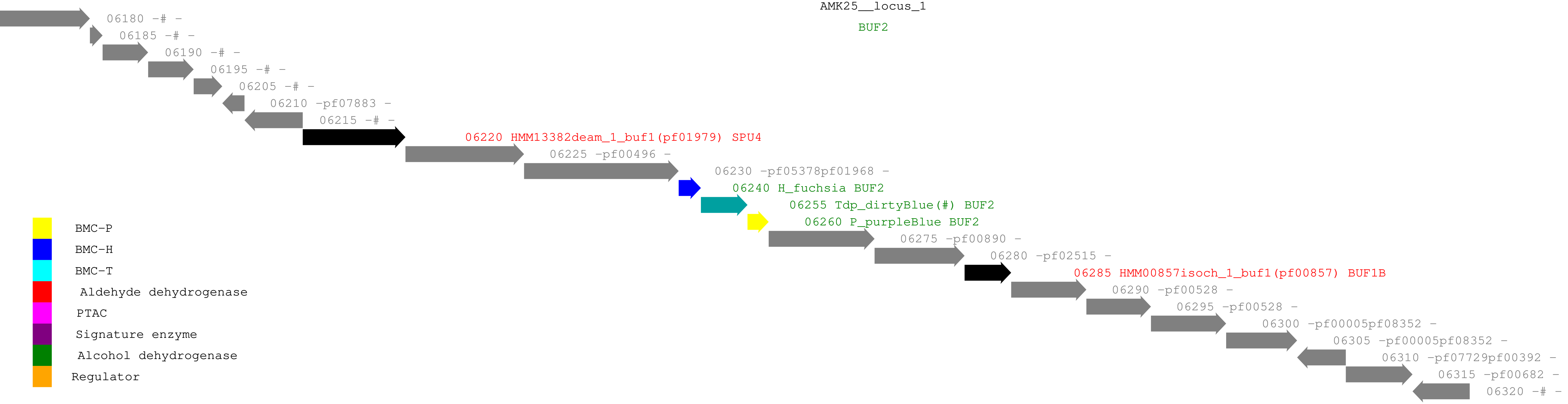

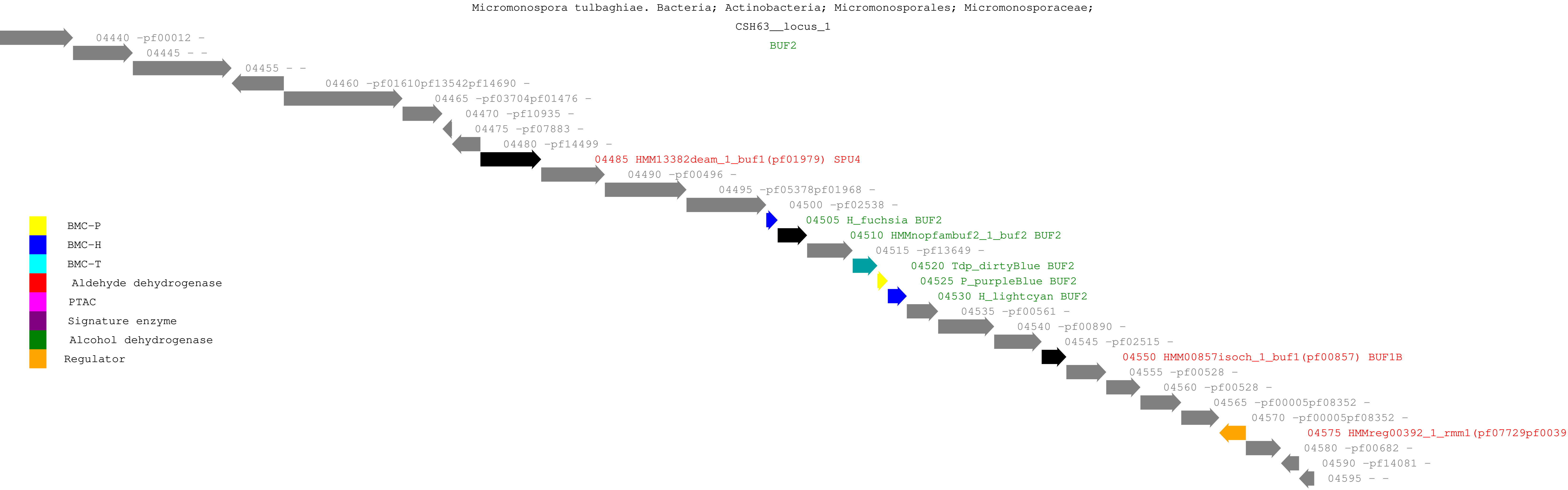
Micromonospora tulbaghiae. Bacteria; Actinobacteria; Micromonosporales; Micromonosporaceae;
CSH63__locus_1
BUF2
04440 -pf00012 -
04445 - -
04455 - -
04460 -pf01610pf13542pf14690 -
04465 -pf03704pf01476 -
04470 -pf10935 -
04475 -pf07883 -
04480 -pf14499 -
04485 HMM13382deam_1_buf1(pf01979) SPU4
04490 -pf00496 -
04495 -pf05378pf01968 -
04500 -pf02538 -
04505 H_fuchsia BUF2
04510 HMMnopfambuf2_1_buf2 BUF2
04515 -pf13649 -
04520 Tdp_dirtyBlue BUF2
04525 P_purpleBlue BUF2
04530 H_lightcyan BUF2
04535 -pf00561 -
04540 -pf00890 -
04545 -pf02515 -
04550 HMM00857isoch_1_buf1(pf00857) BUF1B
04555 -pf00528 -
04560 -pf00528 -
04565 -pf00005pf08352 -
04570 -pf00005pf08352 -
04575 HMMreg00392_1_rmm1(pf07729pf0039
04580 -pf00682 -
04590 -pf14081 -
04595 - -
BMC-P
BMC-H
BMC-T
Aldehyde dehydrogenase
PTAC
Signature enzyme
Alcohol dehydrogenase
Regulator

Verrucosispora sp. LHW63014. Bacteria; Actinobacteria; Micromonosporales; Micromonosporaceae;

D0Q02__locus_2

BUF2

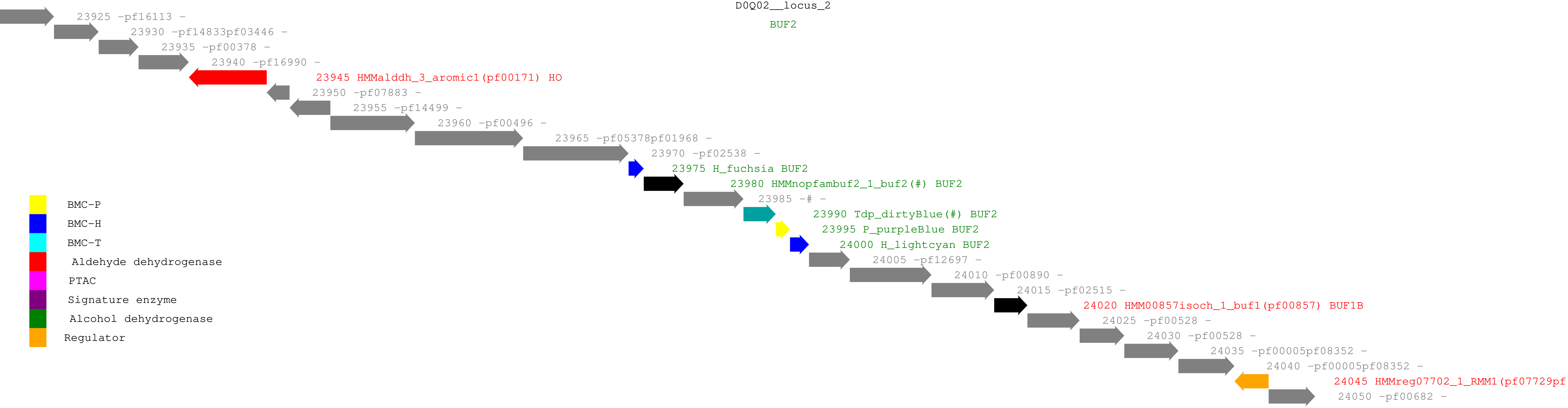

Verrucosispora sp. SN26_14.1. Bacteria; Actinobacteria; Micromonosporales; Micromonosporaceae;

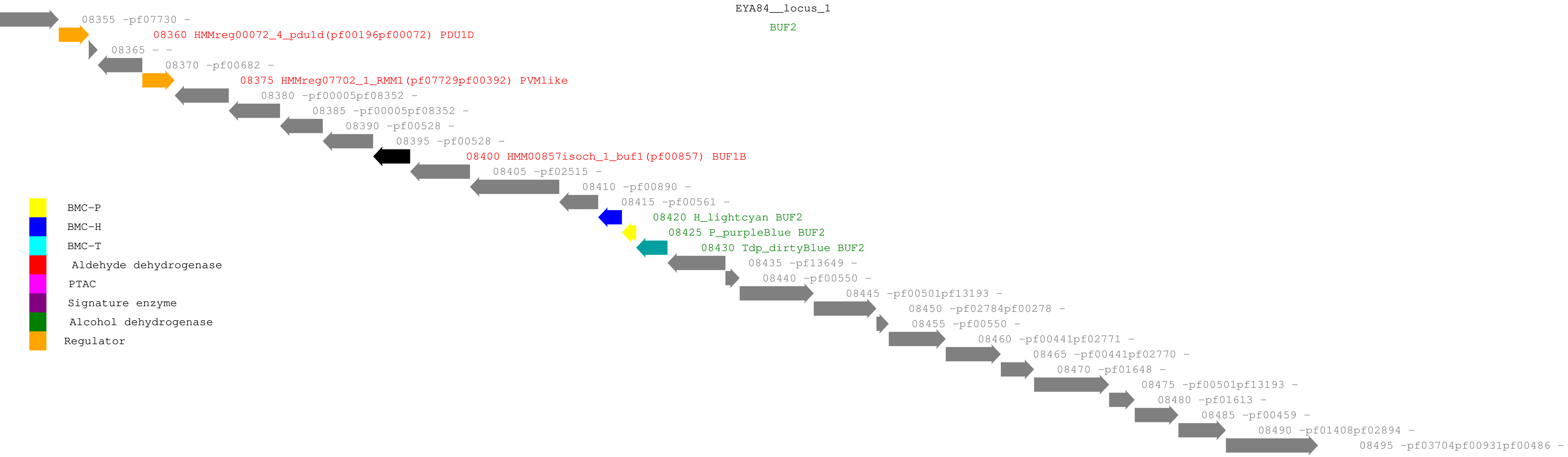

Micromonospora sp. XM-20-01. Bacteria; Actinobacteria; Micromonosporales; Micromonosporaceae;

FJ969__locus_1

BUF2

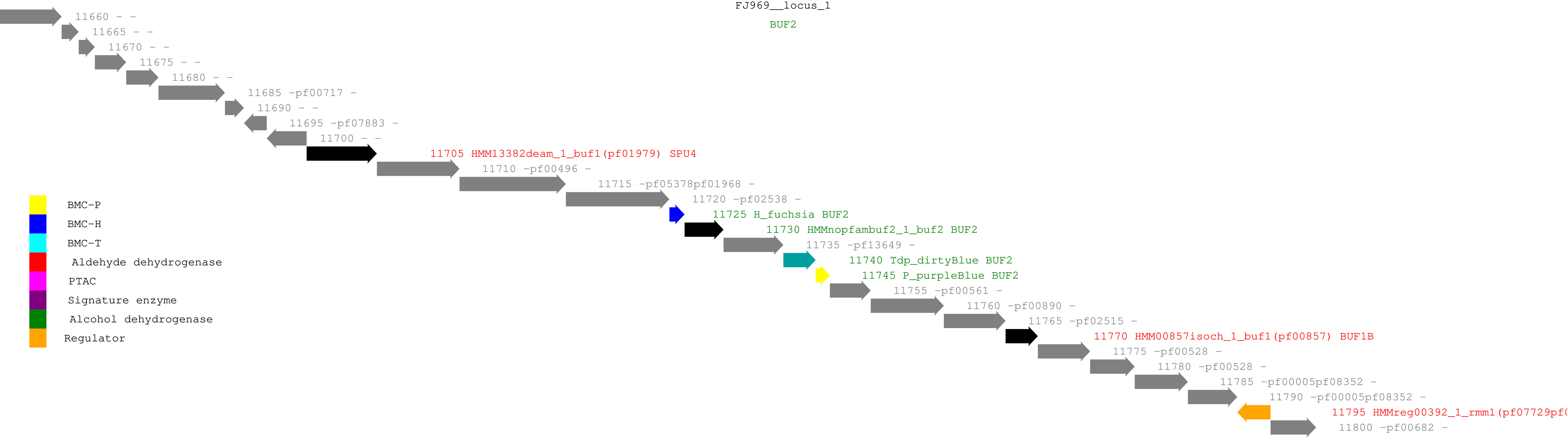

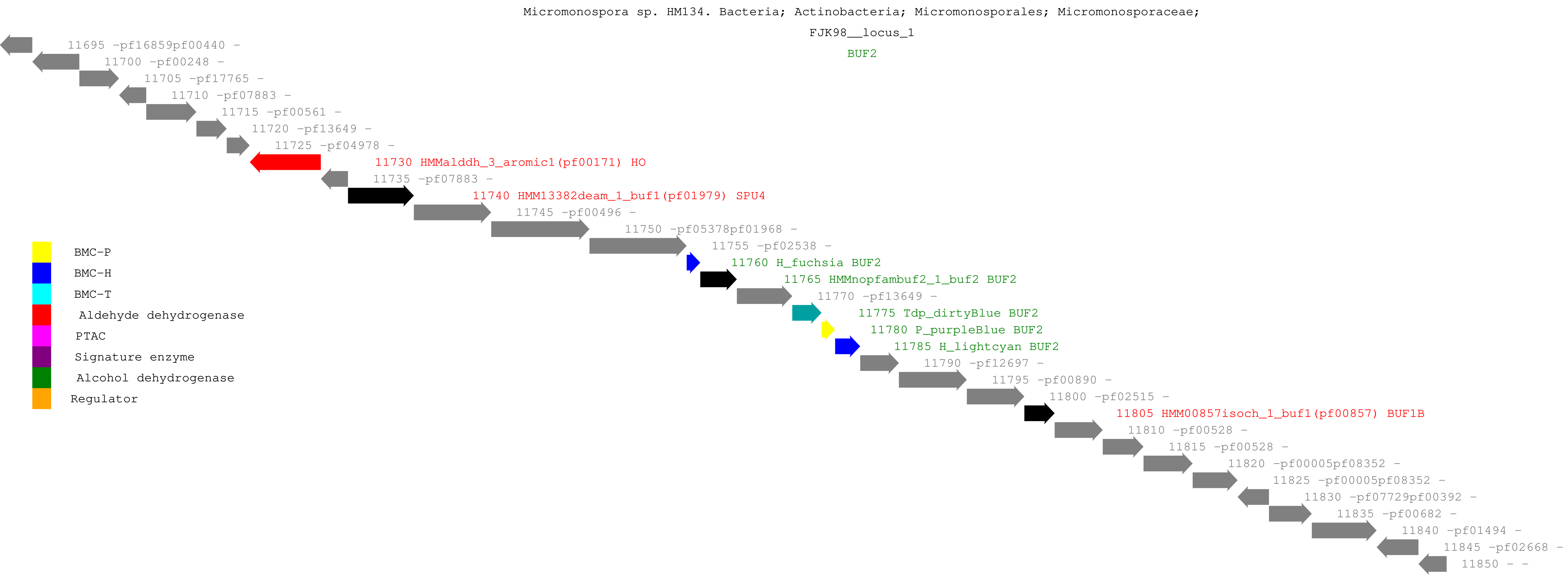
Micromonospora sp. HM134. Bacteria; Actinobacteria; Micromonosporales; Micromonosporaceae;
FJK98__locus_1
BUF2
11695 -pf16859pf00440 -
11700 -pf00248 -
11705 -pf17765 -
11710 -pf07883 -
11715 -pf00561 -
11720 -pf13649 -
11725 -pf04978 -
11730 HMMalddh_3_aromic1(pf00171) HO
11735 -pf07883 -
11740 HMM13382deam_1_buf1(pf01979) SPU4
11745 -pf00496 -
11750 -pf05378pf01968 -
11755 -pf02538 -
11760 H_fuchsia BUF2
11765 HMMnopfambuf2_1_buf2 BUF2
11770 -pf13649 -
11775 Tdp_dirtyBlue BUF2
11780 P_purpleBlue BUF2
11785 H_lightcyan BUF2
11790 -pf12697 -
11795 -pf00890 -
11800 -pf02515 -
11805 HMM00857isoch_1_buf1(pf00857) BUF1B
11810 -pf00528 -
11815 -pf00528 -
11820 -pf00005pf08352 -
11825 -pf00005pf08352 -
11830 -pf07729pf00392 -
11835 -pf00682 -
11840 -pf01494 -
11845 -pf02668 -
11850 - -
BMC-P
BMC-H
BMC-T
Aldehyde dehydrogenase
PTAC
Signature enzyme
Alcohol dehydrogenase
Regulator

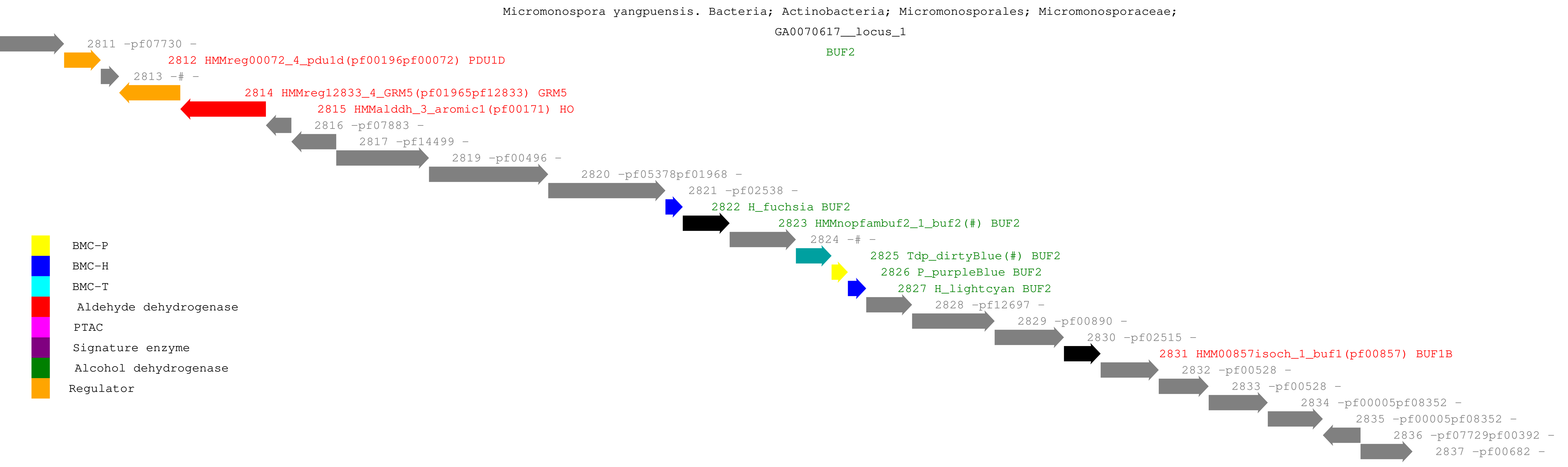
Micromonospora yangpuensis. Bacteria; Actinobacteria; Micromonosporales; Micromonosporaceae;
GA0070617__locus_1
BUF2
2811 -pf07730 -
2812 HMMreg00072_4_pdu1d(pf00196pf00072) PDU1D
2813 -# -
2814 HMMreg12833_4_GRM5(pf01965pf12833) GRM5
2815 HMMalddh_3_aromic1(pf00171) HO
2816 -pf07883 -
2817 -pf14499 -
2819 -pf00496 -
2820 -pf05378pf01968 -
2821 -pf02538 -
2822 H_fuchsia BUF2
2823 HMMnopfambuf2_1_buf2(#) BUF2
2824 -# -
2825 Tdp_dirtyBlue(#) BUF2
2826 P_purpleBlue BUF2
2827 H_lightcyan BUF2
2828 -pf12697 -
2829 -pf00890 -
2830 -pf02515 -
2831 HMM00857isoch_1_buf1(pf00857) BUF1B
2832 -pf00528 -
2833 -pf00528 -
2834 -pf00005pf08352 -
2835 -pf00005pf08352 -
2836 -pf07729pf00392 -
2837 -pf00682 -
BMC-P
BMC-H
BMC-T
Aldehyde dehydrogenase
PTAC
Signature enzyme
Alcohol dehydrogenase
Regulator

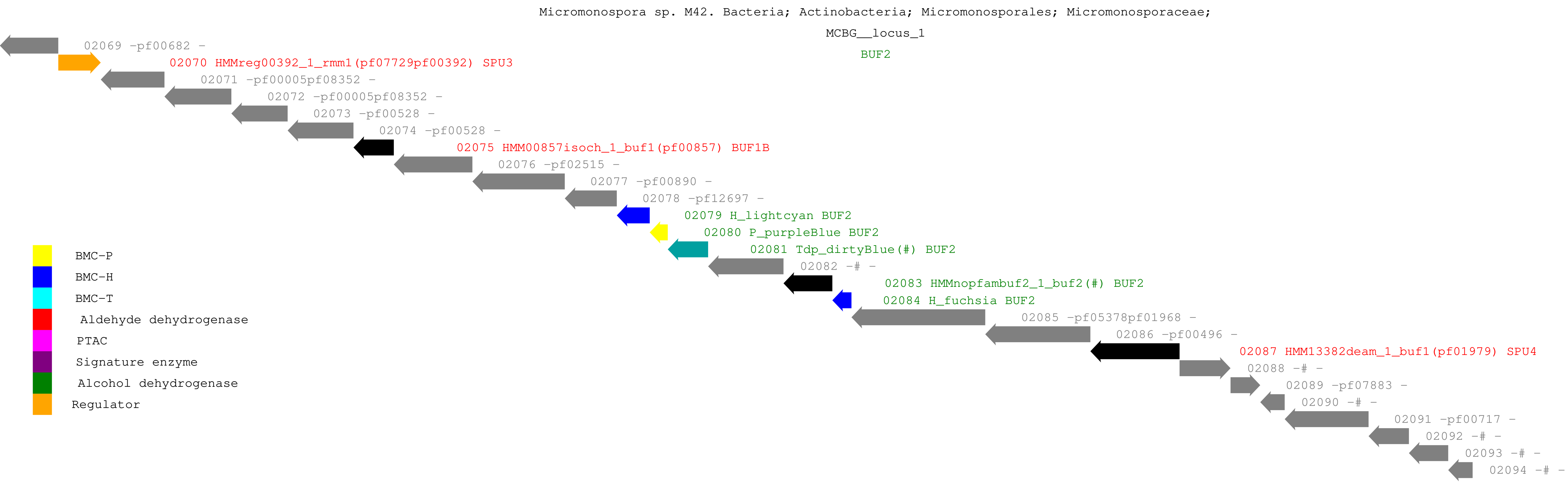
Micromonospora sp. M42. Bacteria; Actinobacteria; Micromonosporales; Micromonosporaceae;
MCBG__locus_1
BUF2
02069 -pf00682 -
02070 HMMreg00392_1_rmm1(pf07729pf00392) SPU3
02071 -pf00005pf08352 -
02072 -pf00005pf08352 -
02073 -pf00528 -
02074 -pf00528 -
02075 HMM00857isoch_1_buf1(pf00857) BUF1B
02076 -pf02515 -
02077 -pf00890 -
02078 -pf12697 -
02079 H_lightcyan BUF2
02080 P_purpleBlue BUF2
02081 Tdp_dirtyBlue(#) BUF2
02082 -# -
02083 HMMnopfambuf2_1_buf2(#) BUF2
02084 H_fuchsia BUF2
02085 -pf05378pf01968 -
02086 -pf00496 -
02087 HMM13382deam_1_buf1(pf01979) SPU4
02088 -# -
02089 -pf07883 -
02090 -# -
02091 -pf00717 -
02092 -# -
02093 -# -
02094 -# -
BMC-P
BMC-H
BMC-T
Aldehyde dehydrogenase
PTAC
Signature enzyme
Alcohol dehydrogenase
Regulator

Clostridia bacterium BRH_c25. Bacteria; Firmicutes; Clostridia.

APF77__locus_1

BUF3

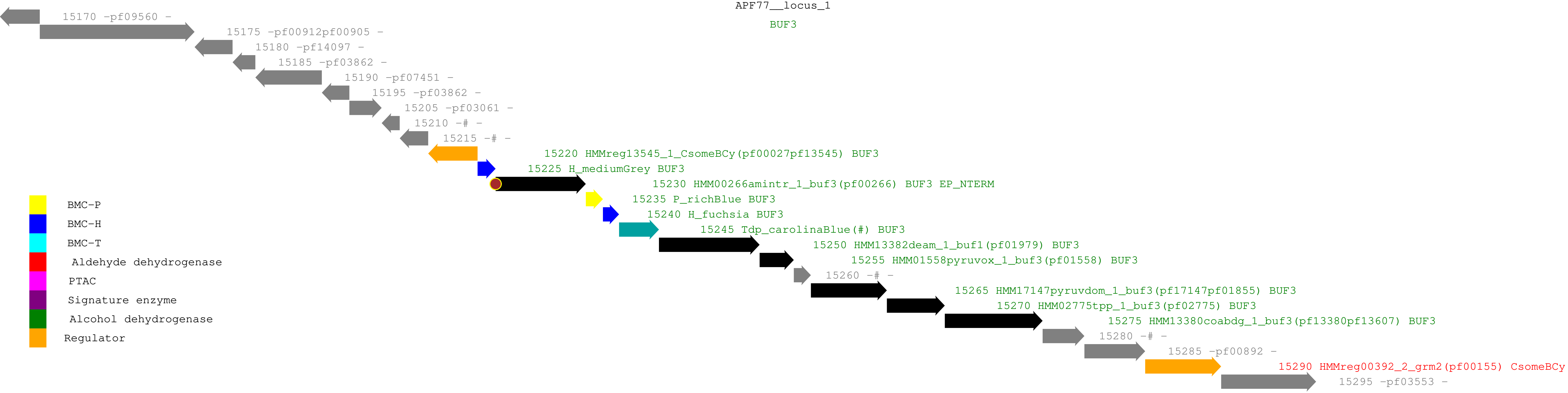

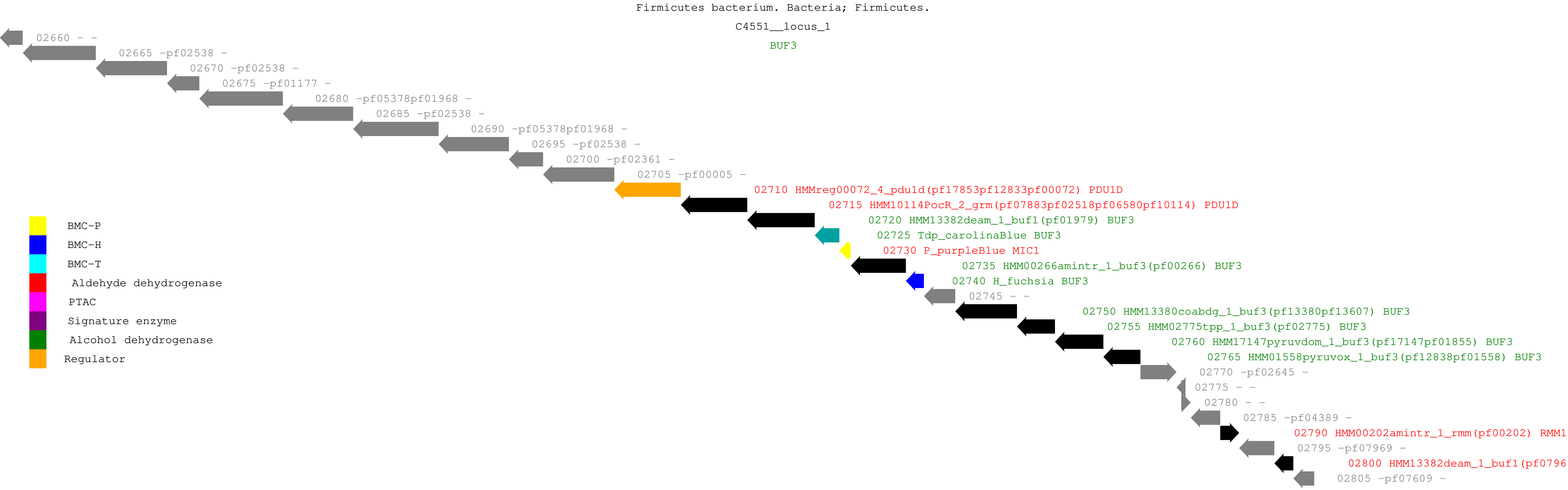

Firmicutes bacterium. Bacteria; Firmicutes.
C4551__locus_1
BUF3
02660 - -
02665 -pf02538 -
02670 -pf02538 -
02675 -pf01177 -
02680 -pf05378pf01968 -
02685 -pf02538 -
02690 -pf05378pf01968 -
02695 -pf02538 -
02700 -pf02361 -
02705 -pf00005 -
02710 HMMreg00072_4_pdu1d(pf17853pf12833pf00072) PDU1D
02715 HMM10114PocR_2_grm(pf07883pf02518pf06580pf10114) PDU1D
02720 HMM13382deam_1_buf1(pf01979) BUF3
02725 Tdp_carolinaBlue BUF3
02730 P_purpleBlue MIC1
02735 HMM00266amintr_1_buf3(pf00266) BUF3
02740 H_fuchsia BUF3
02745 - -
02750 HMM13380coabdg_1_buf3(pf13380pf13607) BUF3
02755 HMM02775tpp_1_buf3(pf02775) BUF3
02760 HMM17147pyruvdom_1_buf3(pf17147pf01855) BUF3
02765 HMM01558pyruvox_1_buf3(pf12838pf01558) BUF3
02770 -pf02645 -
02775 - -
02780 - -
02785 -pf04389 -
02790 HMM00202amintr_1_rmm(pf00202) RMM1
02795 -pf07969 -
02800 HMM13382deam_1_buf1(pf0796
02805 -pf07609 -
BMC-P
BMC-H
BMC-T
Aldehyde dehydrogenase
PTAC
Signature enzyme
Alcohol dehydrogenase
Regulator

Clostridium hylemonae DSM 15053. Bacteria; Firmicutes; Clostridia; Clostridiales; Lachnospiraceae.

CLOHYLEM__locus_1

BUF3

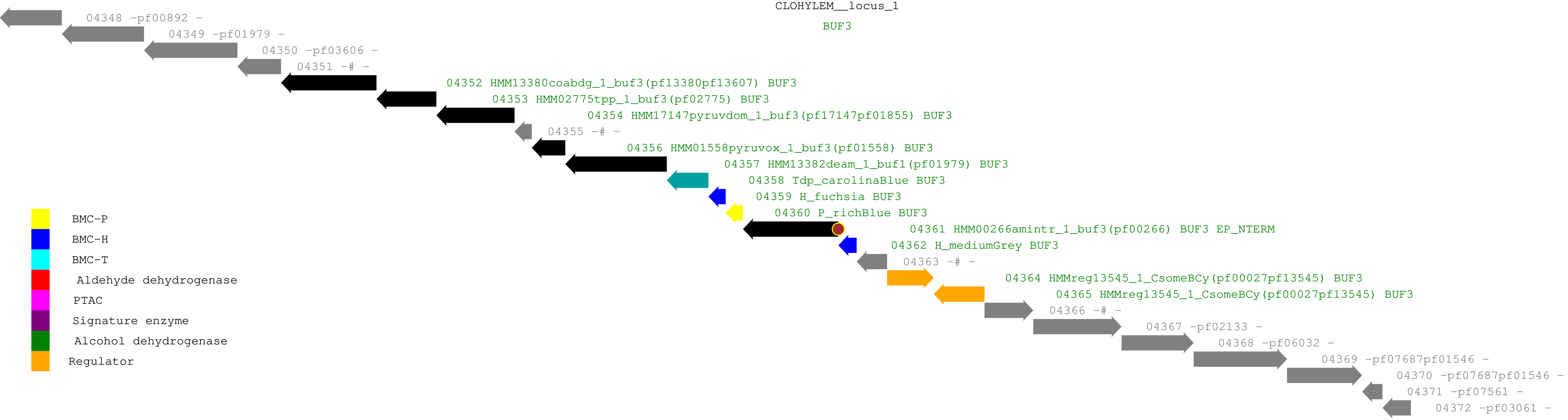

Spirochaetae bacterium HGW-Spirochaetae-7. Bacteria; Spirochaetes.

CVV51__locus_1

BUF3

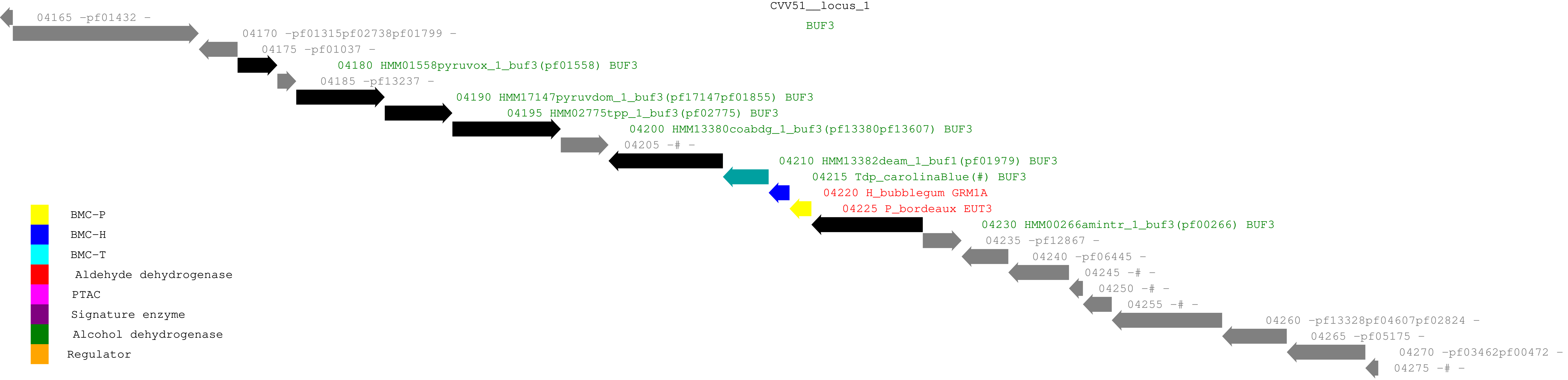

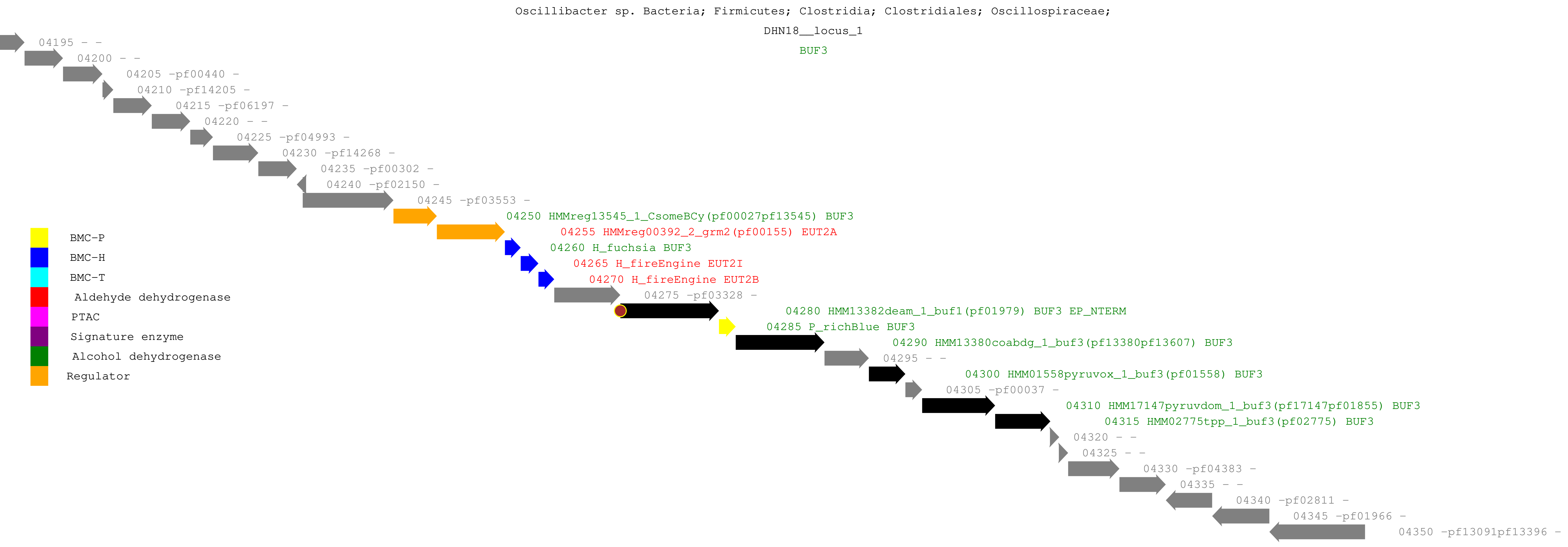
Oscillibacter sp. Bacteria; Firmicutes; Clostridia; Clostridiales; Oscillospiraceae;
DHN18__locus_1
BUF3
04195 - -
04200 - -
04205 -pf00440 -
04210 -pf14205 -
04215 -pf06197 -
04220 - -
04225 -pf04993 -
04230 -pf14268 -
04235 -pf00302 -
04240 -pf02150 -
04245 -pf03553 -
04250 HMMreg13545_1_CsomeBCy(pf00027pf13545) BUF3
04255 HMMreg00392_2_grm2(pf00155) EUT2A
04260 H_fuchsia BUF3
04265 H_fireEngine EUT2I
04270 H_fireEngine EUT2B
04275 -pf03328 -
04280 HMM13382deam_1_buf1(pf01979) BUF3 EP_NTERM
04285 P_richBlue BUF3
04290 HMM13380coabdg_1_buf3(pf13380pf13607) BUF3
04295 - -
04300 HMM01558pyruvox_1_buf3(pf01558) BUF3
04305 -pf00037 -
04310 HMM17147pyruvdom_1_buf3(pf17147pf01855) BUF3
04315 HMM02775tpp_1_buf3(pf02775) BUF3
04320 - -
04325 - -
04330 -pf04383 -
04335 - -
04340 -pf02811 -
04345 -pf01966 -
04350 -pf13091pf13396 -
BMC-P
BMC-H
BMC-T
Aldehyde dehydrogenase
PTAC
Signature enzyme
Alcohol dehydrogenase
Regulator

Clostridiaceae bacterium. Bacteria; Firmicutes; Clostridia; Clostridiales; Clostridiaceae.

DHV55__locus_1

BUF3

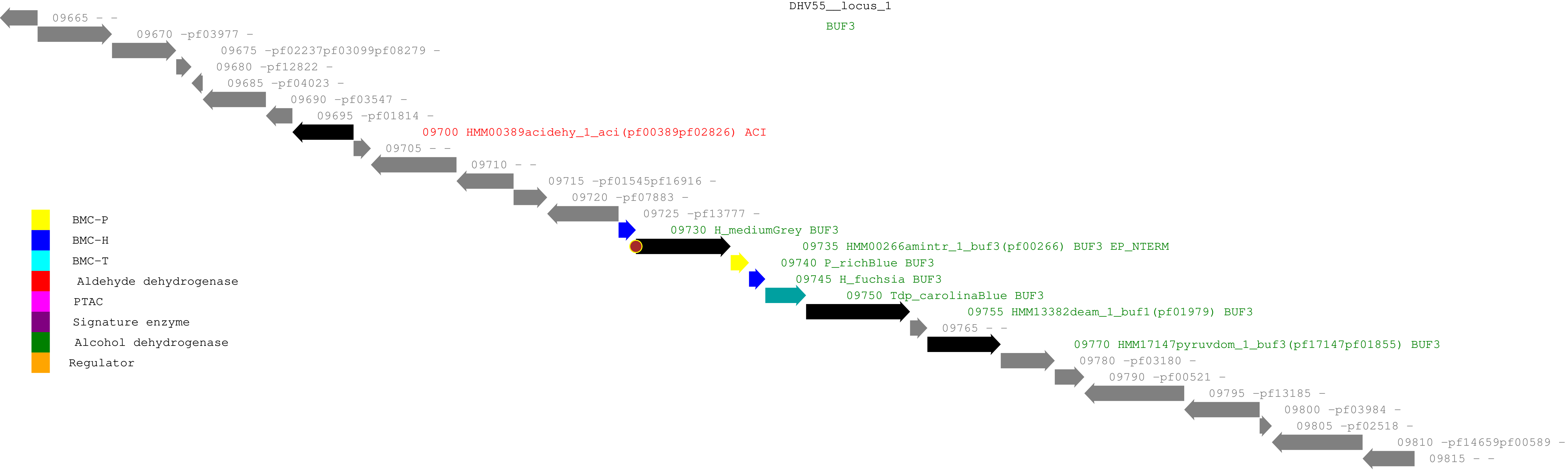

Clostridium sp. AF15-17LB. Bacteria; Firmicutes; Clostridia; Clostridiales; Clostridiaceae;

DWW31__locus_2

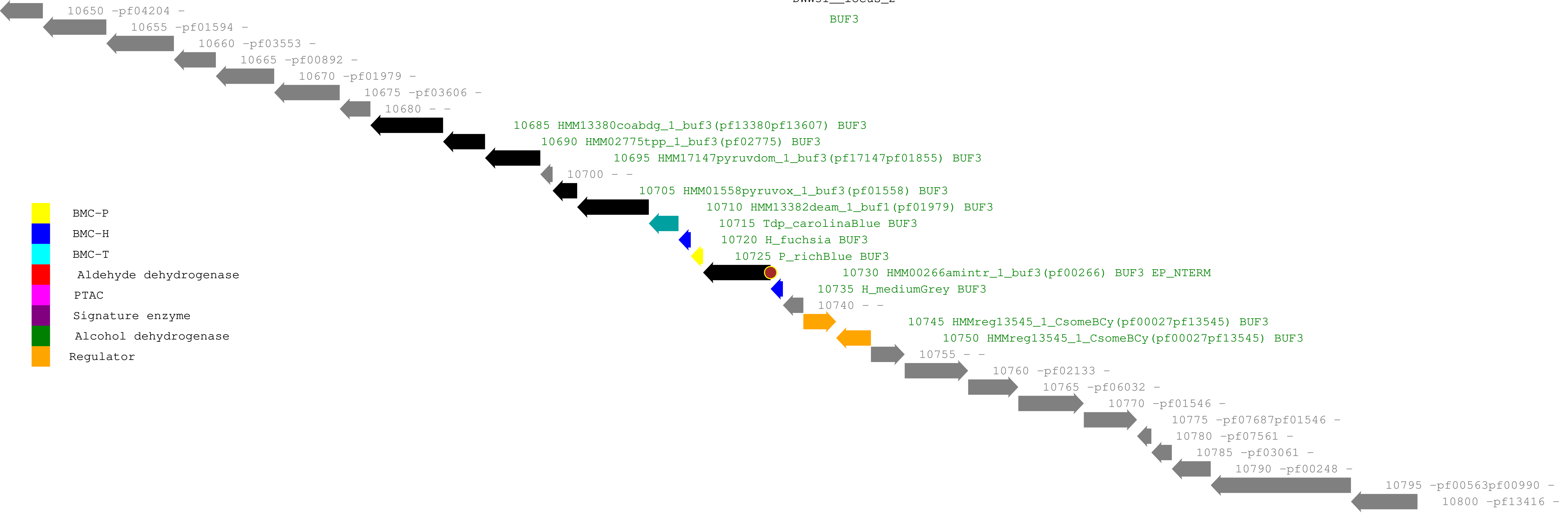

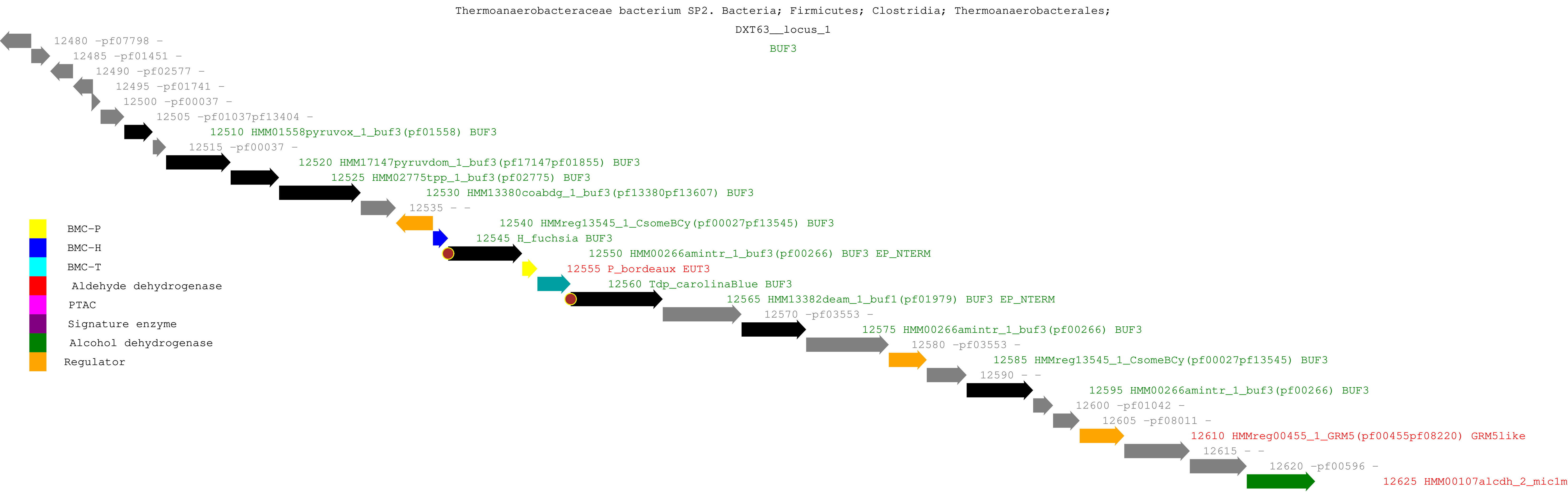
Thermoanaerobacteraceae bacterium SP2. Bacteria; Firmicutes; Clostridia; Thermoanaerobacterales;
DXT63__locus_1
BUF3
12480 -pf07798 -
12485 -pf01451 -
12490 -pf02577 -
12495 -pf01741 -
12500 -pf00037 -
12505 -pf01037pf13404 -
12510 HMM01558pyruvox_1_buf3(pf01558) BUF3
12515 -pf00037 -
12520 HMM17147pyruvdom_1_buf3(pf17147pf01855) BUF3
12525 HMM02775tpp_1_buf3(pf02775) BUF3
12530 HMM13380coabdg_1_buf3(pf13380pf13607) BUF3
12535 - -
12540 HMMreg13545_1_CsomeBCy(pf00027pf13545) BUF3
12545 H_fuchsia BUF3
12550 HMM00266amintr_1_buf3(pf00266) BUF3 EP_NTERM
12555 P_bordeaux EUT3
12560 Tdp_carolinaBlue BUF3
12565 HMM13382deam_1_buf1(pf01979) BUF3 EP_NTERM
12570 -pf03553 -
12575 HMM00266amintr_1_buf3(pf00266) BUF3
12580 -pf03553 -
12585 HMMreg13545_1_CsomeBCy(pf00027pf13545) BUF3
12590 - -
12595 HMM00266amintr_1_buf3(pf00266) BUF3
12600 -pf01042 -
12605 -pf08011 -
12610 HMMreg00455_1_GRM5(pf00455pf08220) GRM5like
12615 - -
12620 -pf00596 -
12625 HMM00107alcdh_2_mic1m
BMC-P
BMC-H
BMC-T
Aldehyde dehydrogenase
PTAC
Signature enzyme
Alcohol dehydrogenase
Regulator

Dorea sp. D27. Bacteria; Firmicutes; Clostridia; Clostridiales; Lachnospiraceae;

HMPREF0980__locus_1

BUF3

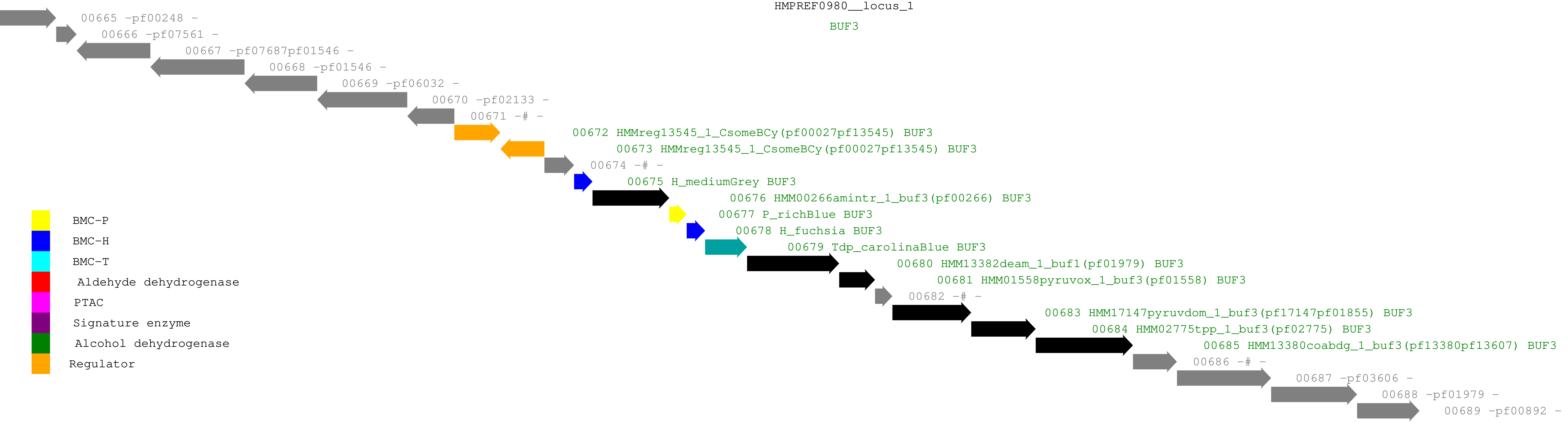

Maledivibacter halophilus. Bacteria; Firmicutes; Clostridia; Clostridiales; Clostridiaceae;

SAMN02194393__locus_1

BUF3

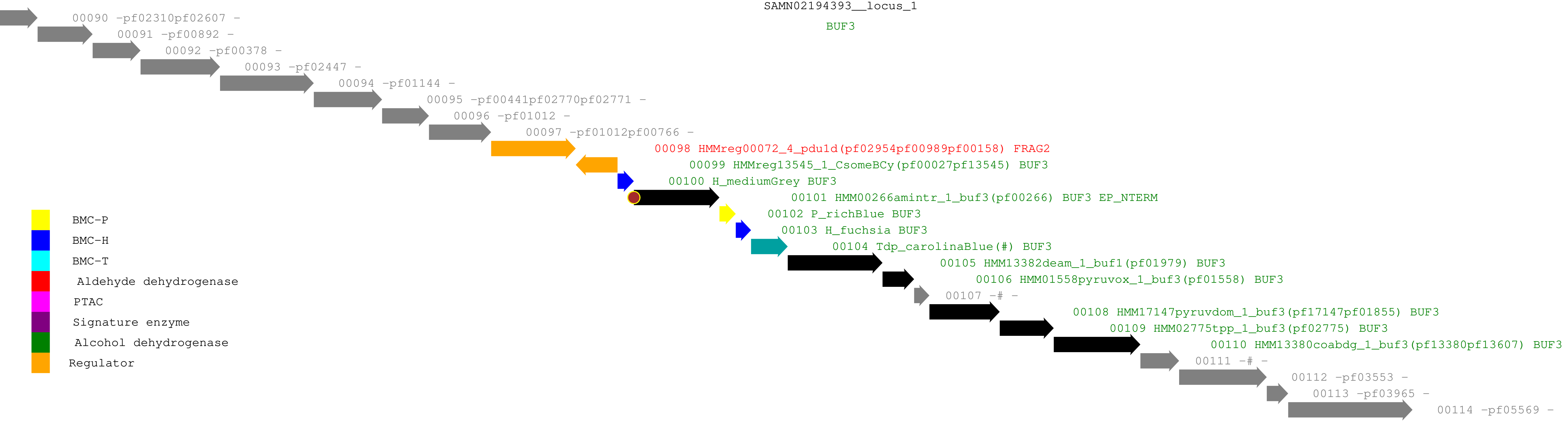

Hydrogenophilales bacterium RIFOXYD1_FULL_62_11. Bacteria; Proteobacteria; Betaproteobacteria; Hydrogenophilales.

A2580__locus_1_main_sat

CsomeACh

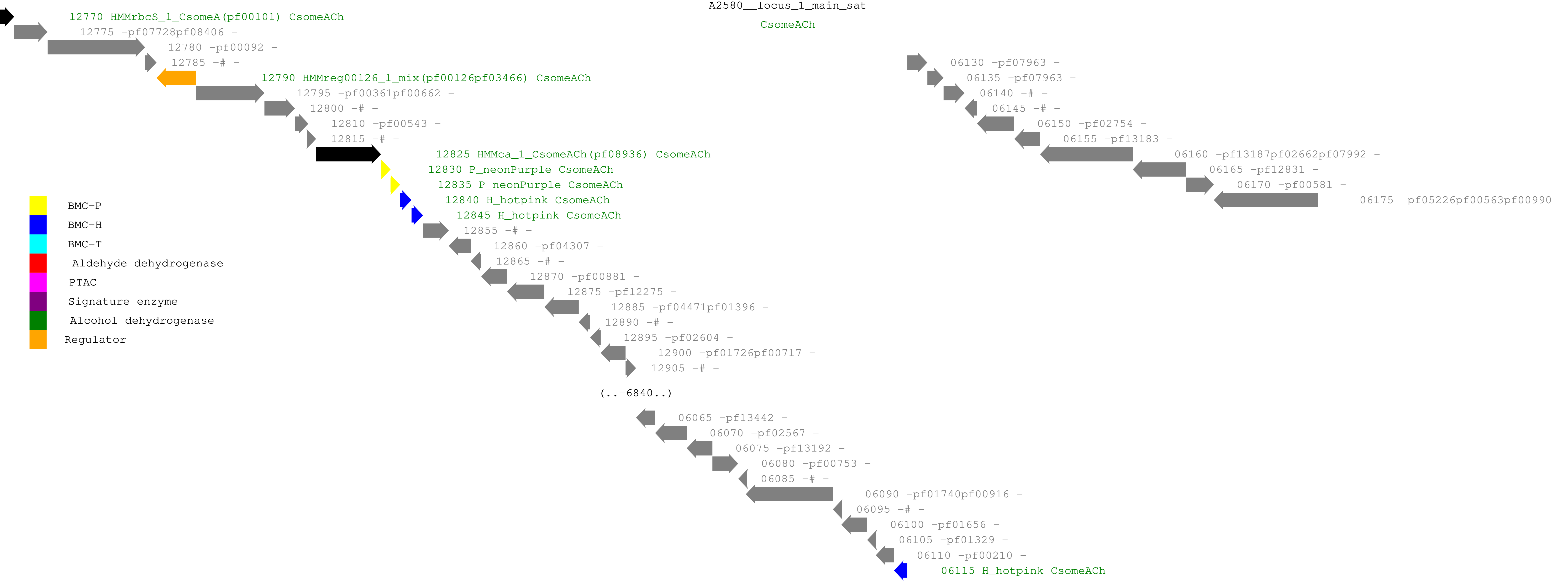

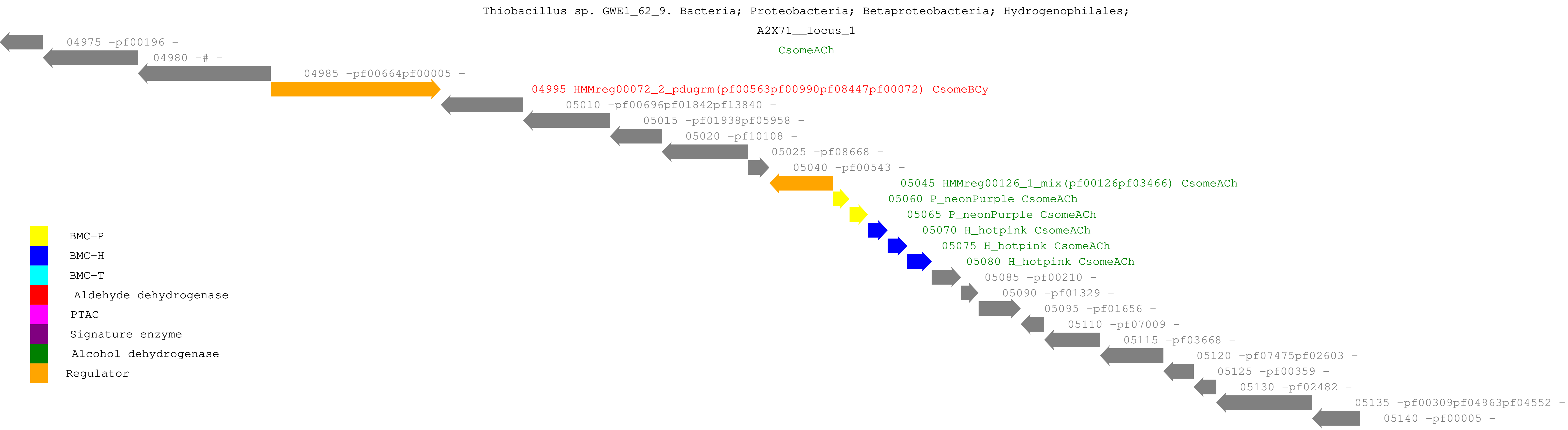

Thiobacillus sp. GWE1_62_9. Bacteria; Proteobacteria; Betaproteobacteria; Hydrogenophilales;
A2X71__locus_1
CsomeACh
04975 -pf00196 -
04980 -# -
04985 -pf00664pf00005 -
04995 HMMreg00072_2_pdugrm(pf00563pf00990pf08447pf00072) CsomeBCy
05010 -pf00696pf01842pf13840 -
05015 -pf01938pf05958 -
05020 -pf10108 -
05025 -pf08668 -
05040 -pf00543 -
05045 HMMreg00126_1_mix(pf00126pf03466) CsomeACh
05060 P_neonPurple CsomeACh
05065 P_neonPurple CsomeACh
05070 H_hotpink CsomeACh
05075 H_hotpink CsomeACh
05080 H_hotpink CsomeACh
05085 -pf00210 -
05090 -pf01329 -
05095 -pf01656 -
05110 -pf07009 -
05115 -pf03668 -
05120 -pf07475pf02603 -
05125 -pf00359 -
05130 -pf02482 -
05135 -pf00309pf04963pf04552 -
05140 -pf00005 -
BMC-P
BMC-H
BMC-T
Aldehyde dehydrogenase
PTAC
Signature enzyme
Alcohol dehydrogenase
Regulator

Acidithiobacillus ferrooxidans (Thiobacillus ferrooxidans). Bacteria; Proteobacteria; Acidithiobacillia; Acidithiobacillales;

A4H96__locus_fragmented

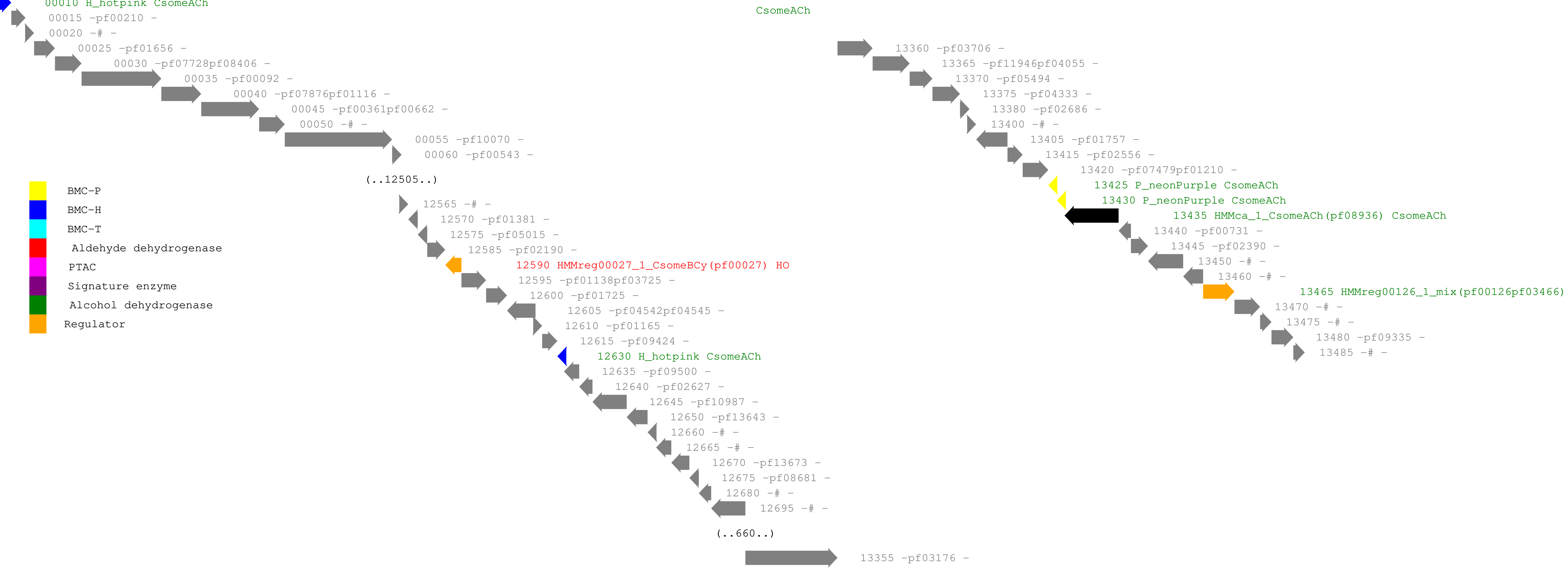

Acidithiobacillus ferrivorans. Bacteria; Proteobacteria; Acidithiobacillia; Acidithiobacillales;

A4U49__locus_1

CsomeACh

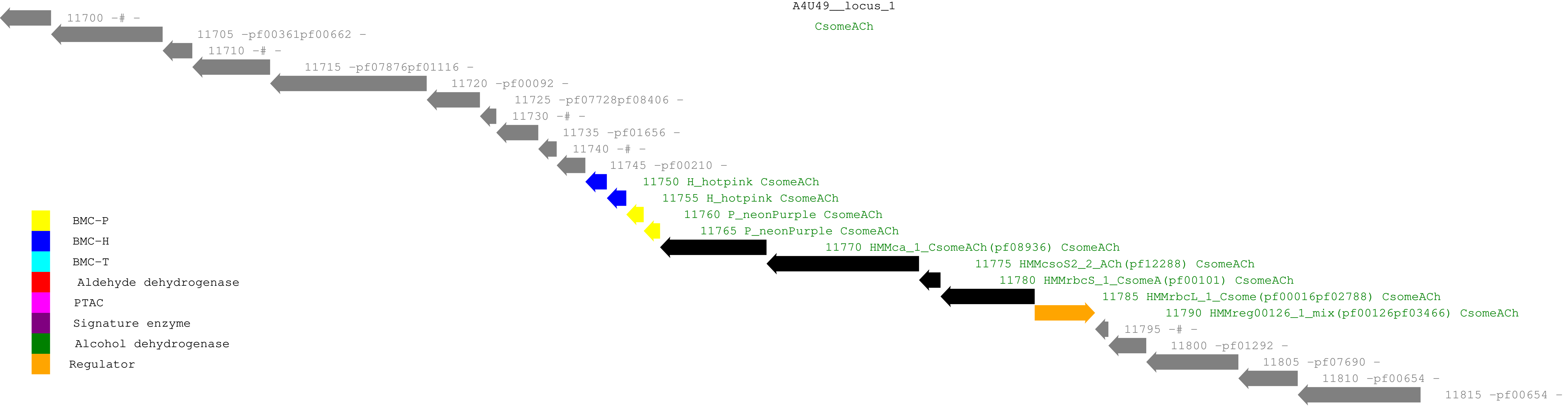

Acidithiobacillus thiooxidans (Thiobacillus thiooxidans). Bacteria; Proteobacteria; Acidithiobacillia; Acidithiobacillales;

A6M23__locus_1_main_sat

CsomeACh

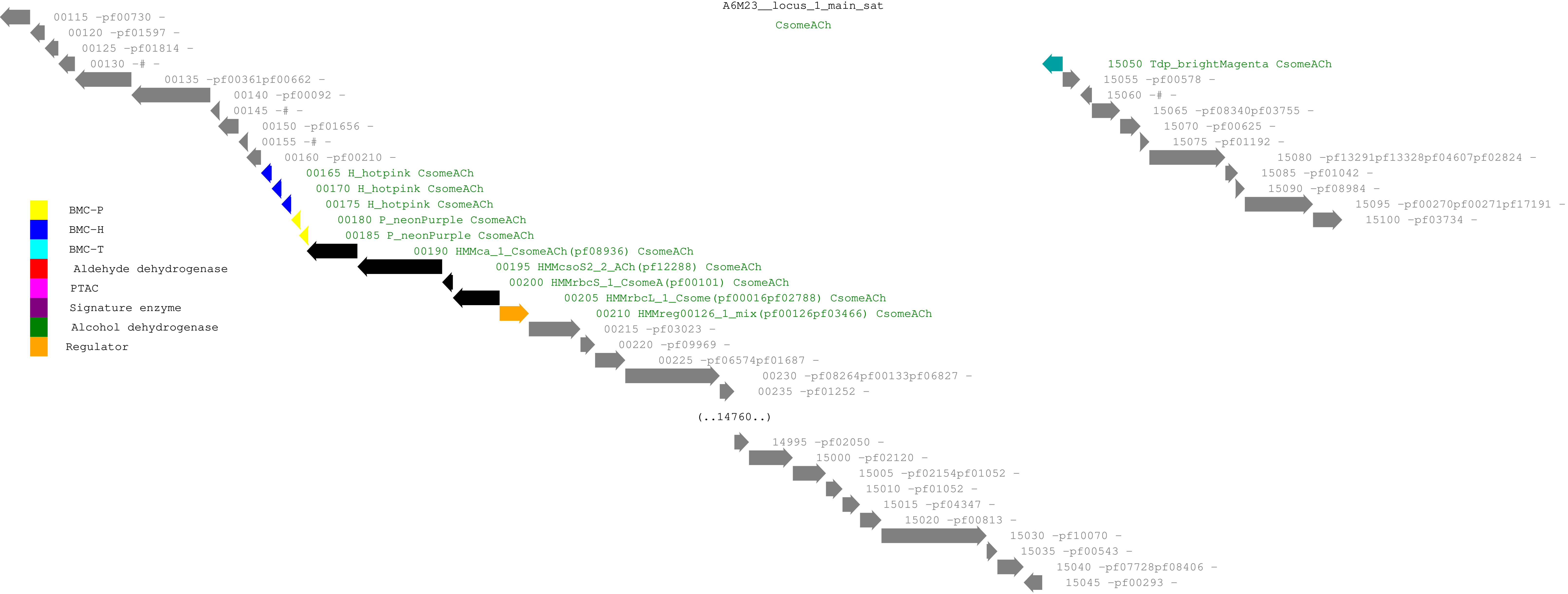

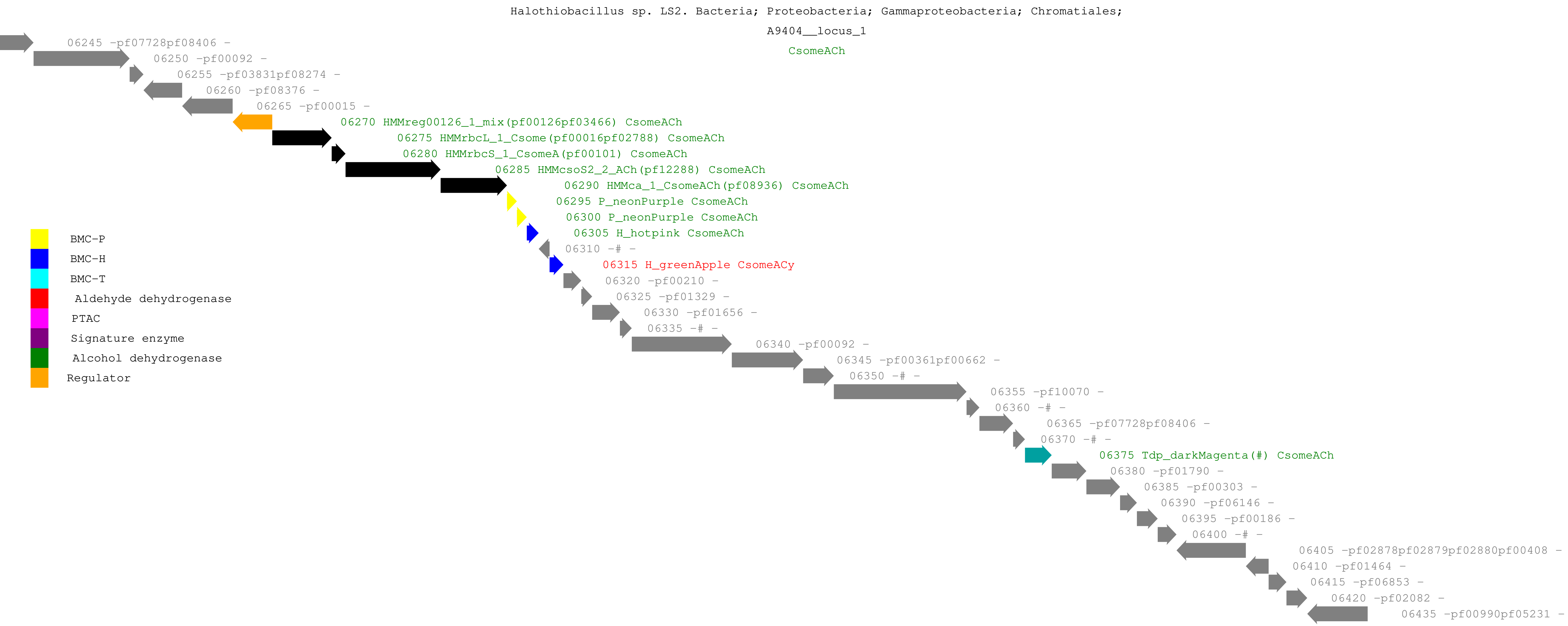
Halothiobacillus sp. LS2. Bacteria; Proteobacteria; Gammaproteobacteria; Chromatiales;
A9404__locus_1
CsomeACh
06245 -pf07728pf08406 -
06250 -pf00092 -
06255 -pf03831pf08274 -
06260 -pf08376 -
06265 -pf00015 -
06270 HMMreg00126_1_mix(pf00126pf03466) CsomeACh
06275 HMMrbcL_1_Csome(pf00016pf02788) CsomeACh
06280 HMMrbcS_1_CsomeA(pf00101) CsomeACh
06285 HMMcsoS2_2_ACh(pf12288) CsomeACh
06290 HMMca_1_CsomeACh(pf08936) CsomeACh
06295 P_neonPurple CsomeACh
06300 P_neonPurple CsomeACh
06305 H_hotpink CsomeACh
06310 -# -
06315 H_greenApple CsomeACy
06320 -pf00210 -
06325 -pf01329 -
06330 -pf01656 -
06335 -# -
06340 -pf00092 -
06345 -pf00361pf00662 -
06350 -# -
06355 -pf10070 -
06360 -# -
06365 -pf07728pf08406 -
06370 -# -
06375 Tdp_darkMagenta(#) CsomeACh
06380 -pf01790 -
06385 -pf00303 -
06390 -pf06146 -
06395 -pf00186 -
06400 -# -
06405 -pf02878pf02879pf02880pf00408 -
06410 -pf01464 -
06415 -pf06853 -
06420 -pf02082 -
06435 -pf00990pf05231 -
BMC-P
BMC-H
BMC-T
Aldehyde dehydrogenase
PTAC
Signature enzyme
Alcohol dehydrogenase
Regulator

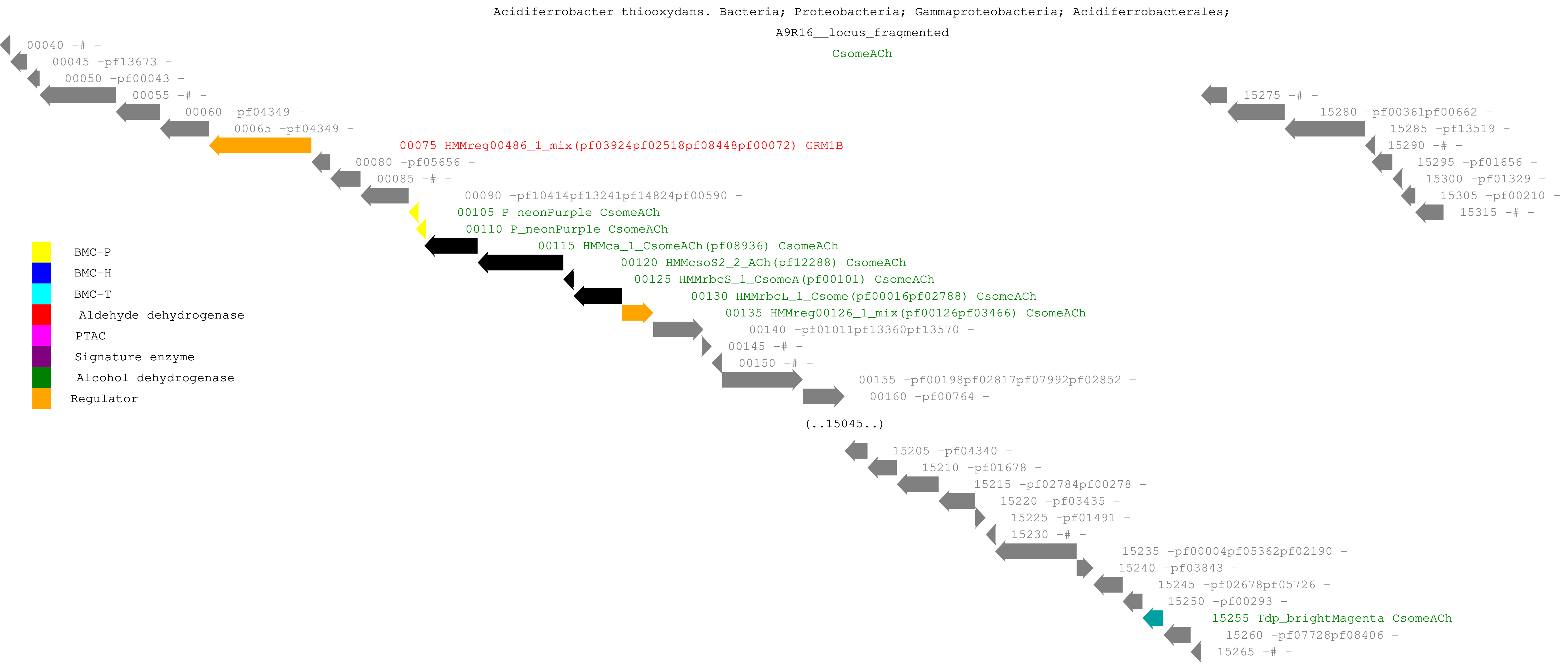
Acidiferrobacter thiooxydans. Bacteria; Proteobacteria; Gammaproteobacteria; Acidiferrobacterales;
A9R16__locus_fragmented
CsomeACh
00040 -# -
00045 -pf13673 -
00050 -pf00043 -
00055 -# -
00060 -pf04349 -
00065 -pf04349 -
00075 HMMreg00486_1_mix(pf03924pf02518pf08448pf00072) GRM1B
00080 -pf05656 -
00085 -# -
00090 -pf10414pf13241pf14824pf00590 -
00105 P_neonPurple CsomeACh
00110 P_neonPurple CsomeACh
00115 HMMca_1_CsomeACh(pf08936) CsomeACh
00120 HMMcsoS2_2_ACh(pf12288) CsomeACh
00125 HMMrbcS_1_CsomeA(pf00101) CsomeACh
00130 HMMrbcL_1_Csome(pf00016pf02788) CsomeACh
00135 HMMreg00126_1_mix(pf00126pf03466) CsomeACh
00140 -pf01011pf13360pf13570 -
00145 -# -
00150 -# -
00155 -pf00198pf02817pf07992pf02852 -
00160 -pf00764 -
(..15045..)
15205 -pf04340 -
15210 -pf01678 -
15215 -pf02784pf00278 -
15220 -pf03435 -
15225 -pf01491 -
15230 -# -
15235 -pf00004pf05362pf02190 -
15240 -pf03843 -
15245 -pf02678pf05726 -
15250 -pf00293 -
15255 Tdp_brightMagenta CsomeACh
15260 -pf07728pf08406 -
15265 -# -
15275 -# -
15280 -pf00361pf00662 -
15285 -pf13519 -
15290 -# -
15295 -pf01656 -
15300 -pf01329 -
15305 -pf00210 -
15315 -# -
BMC-P
BMC-H
BMC-T
Aldehyde dehydrogenase
PTAC
Signature enzyme
Alcohol dehydrogenase
Regulator

Nitrosomonadales bacterium SCN 54-20. Bacteria; Proteobacteria; Betaproteobacteria; Nitrosomonadales.

ABS69__locus_1

CsomeACh

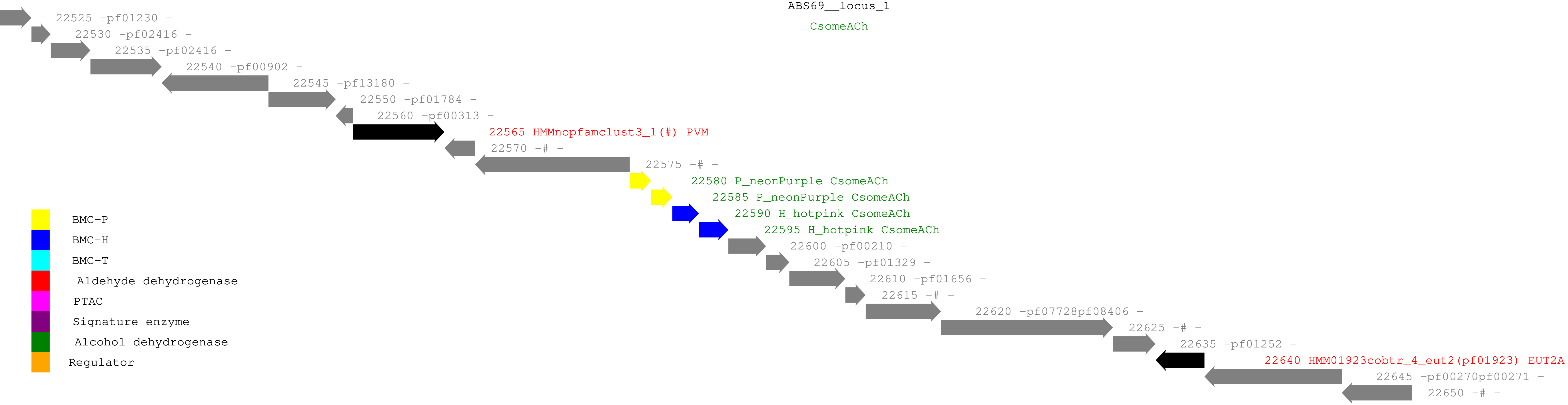

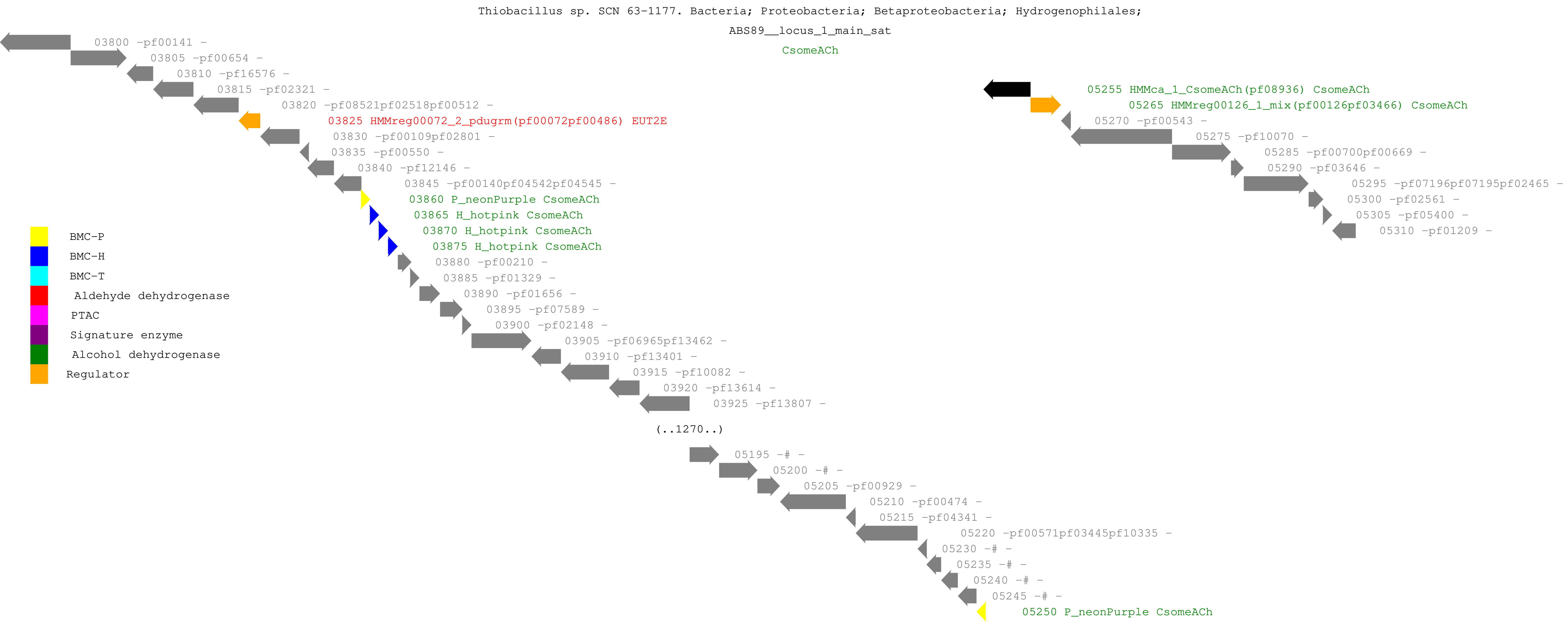
Thiobacillus sp. SCN 63-1177. Bacteria; Proteobacteria; Betaproteobacteria; Hydrogenophilales;
ABS89__locus_1_main_sat
CsomeACh
03800 -pf00141 -
03805 -pf00654 -
03810 -pf16576 -
03815 -pf02321 -
03820 -pf08521pf02518pf00512 -
03825 HMMreg00072_2_pdugrm(pf00072pf00486) EUT2E
03830 -pf00109pf02801 -
03835 -pf00550 -
03840 -pf12146 -
03845 -pf00140pf04542pf04545 -
03860 P_neonPurple CsomeACh
03865 H_hotpink CsomeACh
03870 H_hotpink CsomeACh
03875 H_hotpink CsomeACh
03880 -pf00210 -
03885 -pf01329 -
03890 -pf01656 -
03895 -pf07589 -
03900 -pf02148 -
03905 -pf06965pf13462 -
03910 -pf13401 -
03915 -pf10082 -
03920 -pf13614 -
03925 -pf13807 -
(..1270..)
05195 -# -
05200 -# -
05205 -pf00929 -
05210 -pf00474 -
05215 -pf04341 -
05220 -pf00571pf03445pf10335 -
05230 -# -
05235 -# -
05240 -# -
05245 -# -
05250 P_neonPurple CsomeACh
05255 HMMca_1_CsomeACh(pf08936) CsomeACh
05265 HMMreg00126_1_mix(pf00126pf03466) CsomeACh
05270 -pf00543 -
05275 -pf10070 -
05285 -pf00700pf00669 -
05290 -pf03646 -
05295 -pf07196pf07195pf02465 -
05300 -pf02561 -
05305 -pf05400 -
05310 -pf01209 -
BMC-P
BMC-H
BMC-T
Aldehyde dehydrogenase
PTAC
Signature enzyme
Alcohol dehydrogenase
Regulator

Thiobacillus sp. SCN 63-374. Bacteria; Proteobacteria; Betaproteobacteria; Hydrogenophilales;

ABS92__locus_1_main_sat

CsomeACh

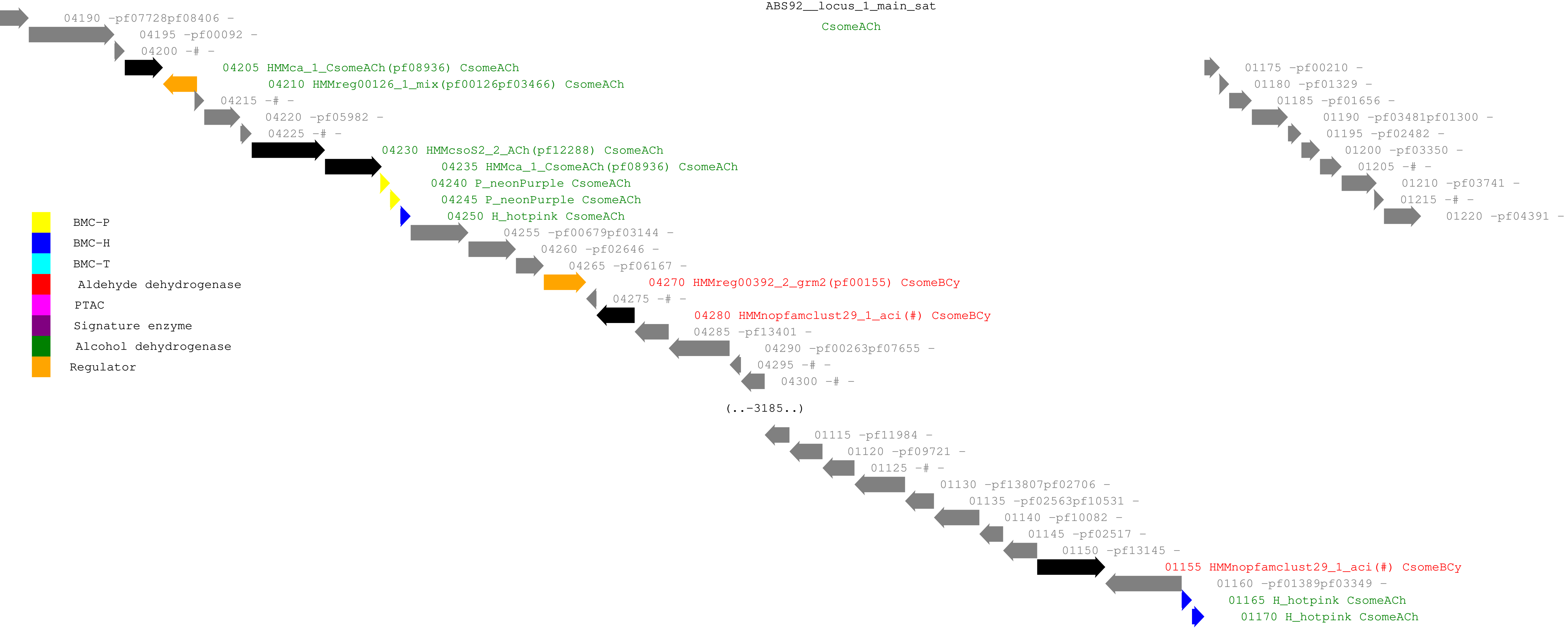

Pseudonocardia sp. SCN 73-27. Bacteria; Actinobacteria; Pseudonocardiales; Pseudonocardiaceae;

ABT15__locus_1

CsomeACh

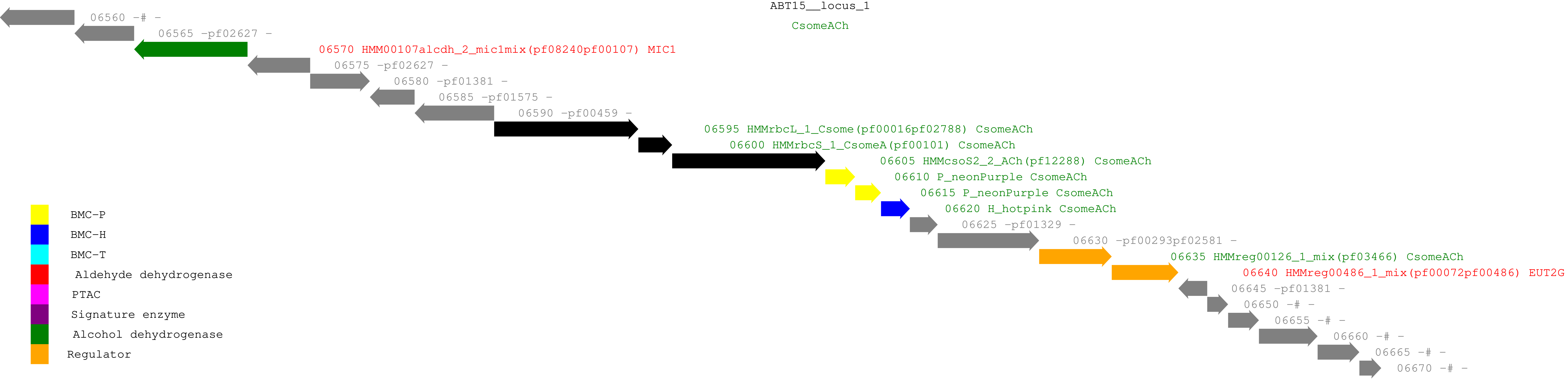

Thiobacillus sp. SCN 65-179. Bacteria; Proteobacteria; Betaproteobacteria; Hydrogenophilales;

ABT21__locus_1

CsomeACh

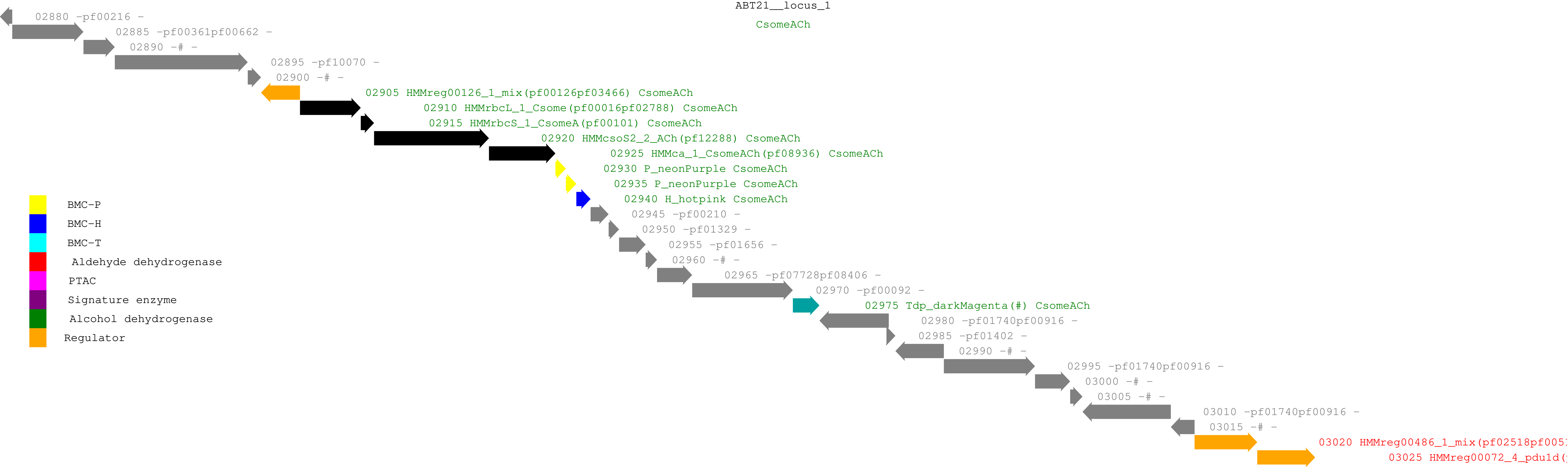

Thiobacillus sp. SCN 63-57. Bacteria; Proteobacteria; Betaproteobacteria; Hydrogenophilales;

ABT23__locus_1

CsomeACh

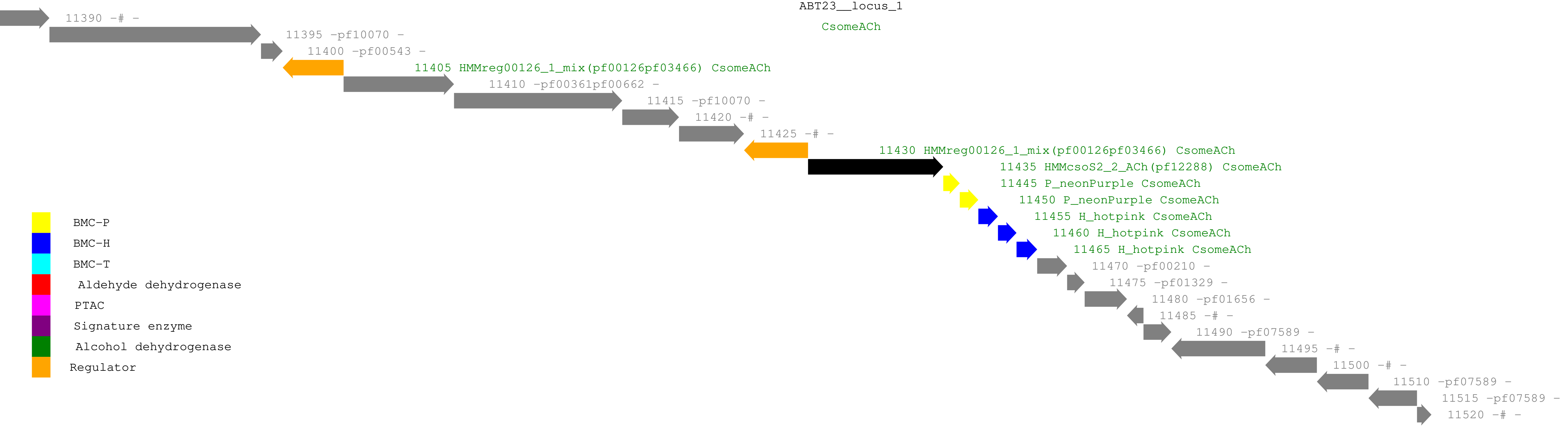

Thiomonas sp. SCN 64-16. Bacteria; Proteobacteria; Betaproteobacteria; Burkholderiales;

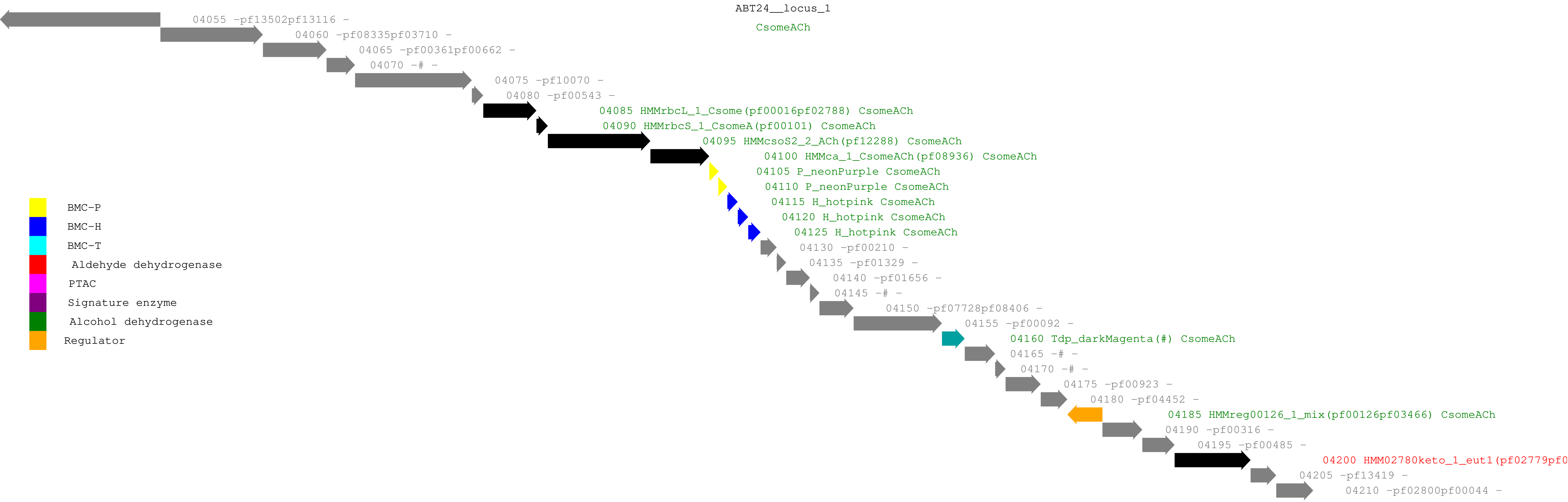

Acidithiobacillus caldus ATCC 51756. Bacteria; Proteobacteria; Acidithiobacillia; Acidithiobacillales;

Acaty_c_locus_1

CsomeACh

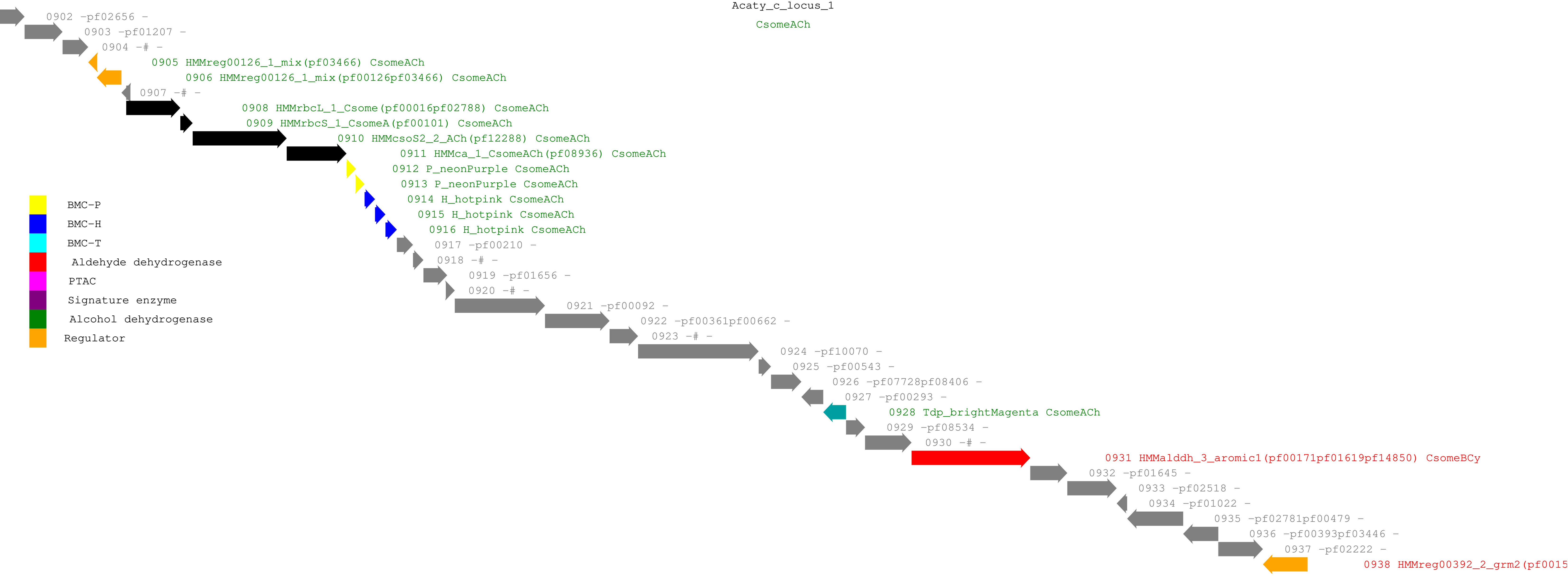

Acidithiobacillus ferrivorans SS3. Bacteria; Proteobacteria; Acidithiobacillia; Acidithiobacillales;

Acife__locus_1

CsomeACh

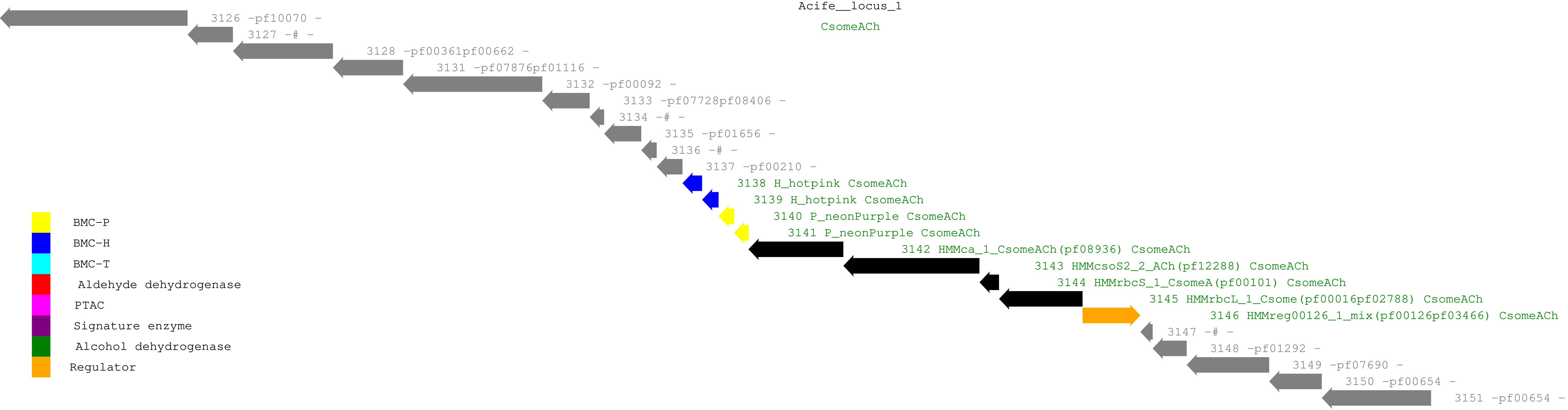

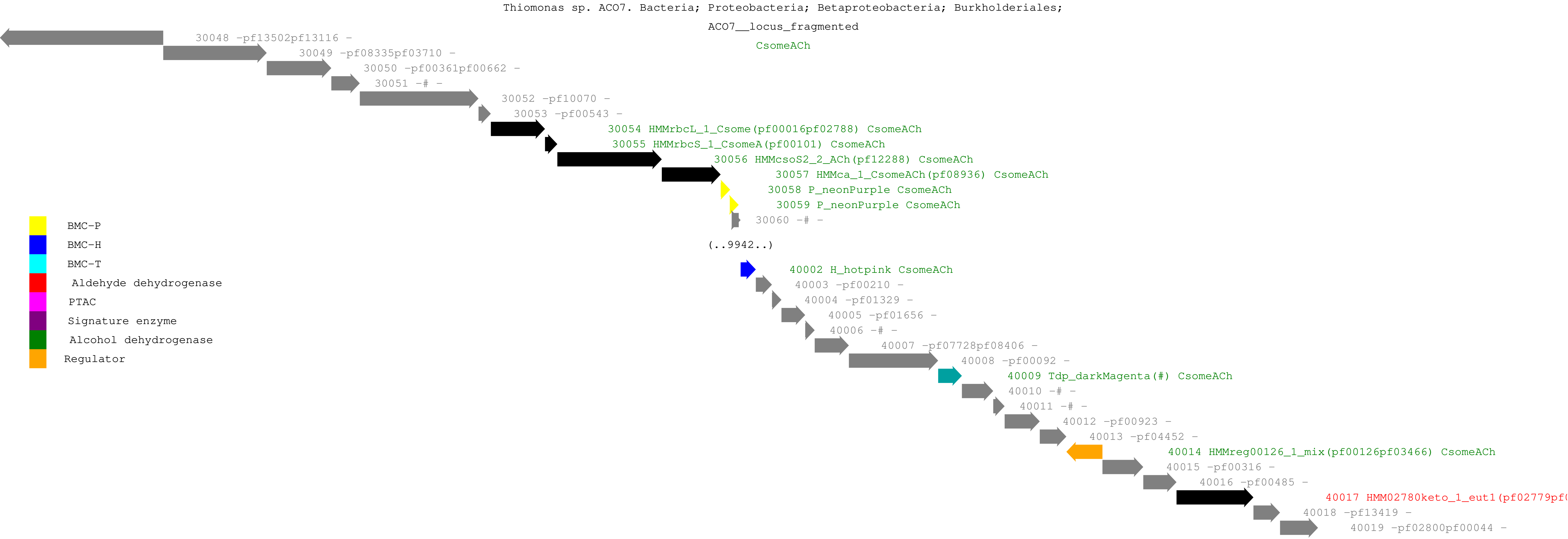
Thiomonas sp. ACO7. Bacteria; Proteobacteria; Betaproteobacteria; Burkholderiales;
ACO7__locus_fragmented
CsomeACh
30048 -pf13502pf13116 -
30049 -pf08335pf03710 -
30050 -pf00361pf00662 -
30051 -# -
30052 -pf10070 -
30053 -pf00543 -
30054 HMMrbcL_1_Csome(pf00016pf02788) CsomeACh
30055 HMMrbcS_1_CsomeA(pf00101) CsomeACh
30056 HMMcsoS2_2_ACh(pf12288) CsomeACh
30057 HMMca_1_CsomeACh(pf08936) CsomeACh
30058 P_neonPurple CsomeACh
30059 P_neonPurple CsomeACh
30060 -# -
(..9942..)
40002 H_hotpink CsomeACh
40003 -pf00210 -
40004 -pf01329 -
40005 -pf01656 -
40006 -# -
40007 -pf07728pf08406 -
40008 -pf00092 -
40009 Tdp_darkMagenta(#) CsomeACh
40010 -# -
40011 -# -
40012 -pf00923 -
40013 -pf04452 -
40014 HMMreg00126_1_mix(pf00126pf03466) CsomeACh
40015 -pf00316 -
40016 -pf00485 -
40017 HMM02780keto_1_eut1(pf02779pf
40018 -pf13419 -
40019 -pf02800pf00044 -
BMC-P
BMC-H
BMC-T
Aldehyde dehydrogenase
PTAC
Signature enzyme
Alcohol dehydrogenase
Regulator

Acidithiobacillus ferrooxidans (strain ATCC 23270 / DSM 14882 / CIP Bacteria; Proteobacteria; Acidithiobacillia; Acidithiobacillales;

AFE__locus_1

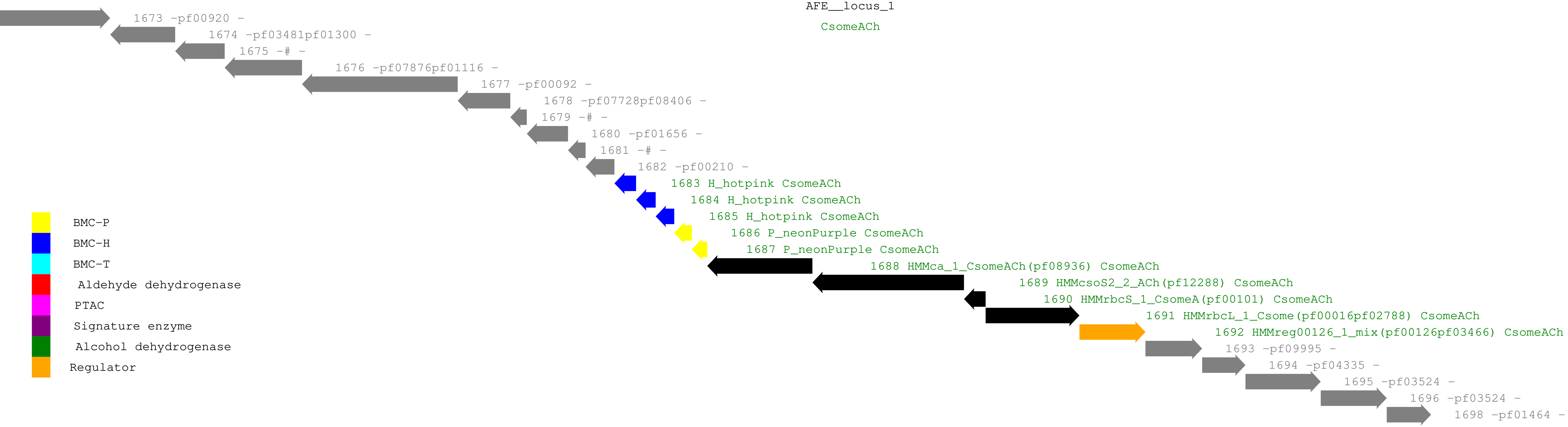

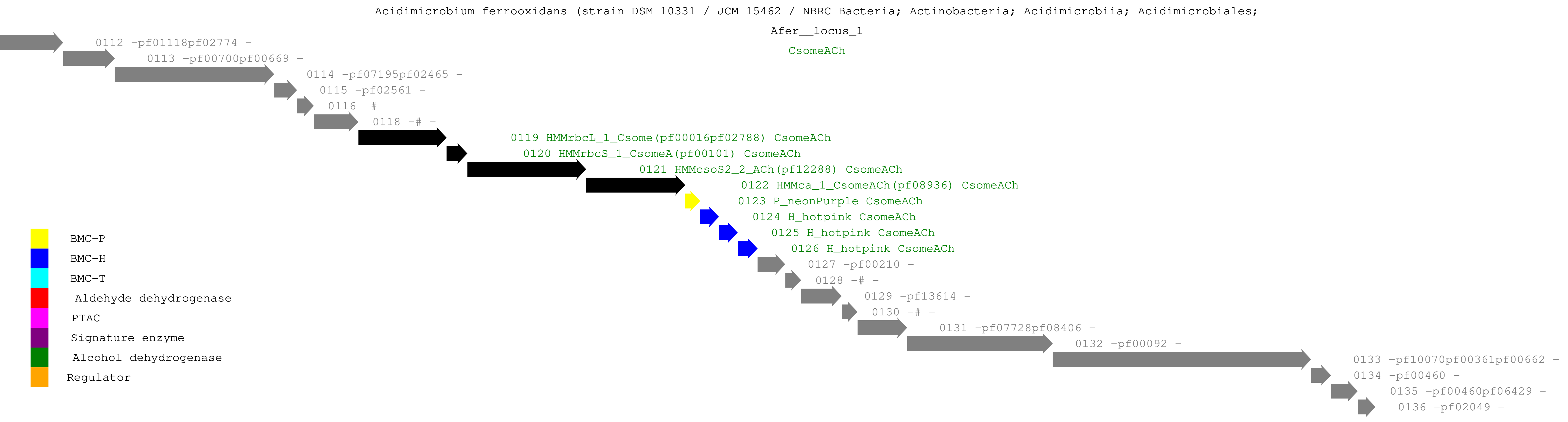
Acidimicrobium ferrooxidans (strain DSM 10331 / JCM 15462 / NBRC Bacteria; Actinobacteria; Acidimicrobiia; Acidimicrobiales;
Afer__locus_1
CsomeACh
0112 -pf01118pf02774 -
0113 -pf00700pf00669 -
0114 -pf07195pf02465 -
0115 -pf02561 -
0116 -# -
0118 -# -
0119 HMMrbcL_1_Csome(pf00016pf02788) CsomeACh
0120 HMMrbcS_1_CsomeA(pf00101) CsomeACh
0121 HMMcsoS2_2_ACh(pf12288) CsomeACh
0122 HMMca_1_CsomeACh(pf08936) CsomeACh
0123 P_neonPurple CsomeACh
0124 H_hotpink CsomeACh
0125 H_hotpink CsomeACh
0126 H_hotpink CsomeACh
0127 -pf00210 -
0128 -# -
0129 -pf13614 -
0130 -# -
0131 -pf07728pf08406 -
0132 -pf00092 -
0133 -pf10070pf00361pf00662 -
0134 -pf00460 -
0135 -pf00460pf06429 -
0136 -pf02049 -
BMC-P
BMC-H
BMC-T
Aldehyde dehydrogenase
PTAC
Signature enzyme
Alcohol dehydrogenase
Regulator

Acidithiobacillus ferridurans. Bacteria; Proteobacteria; Acidithiobacillia; Acidithiobacillales;

AFERRID__locus_1

CsomeACh

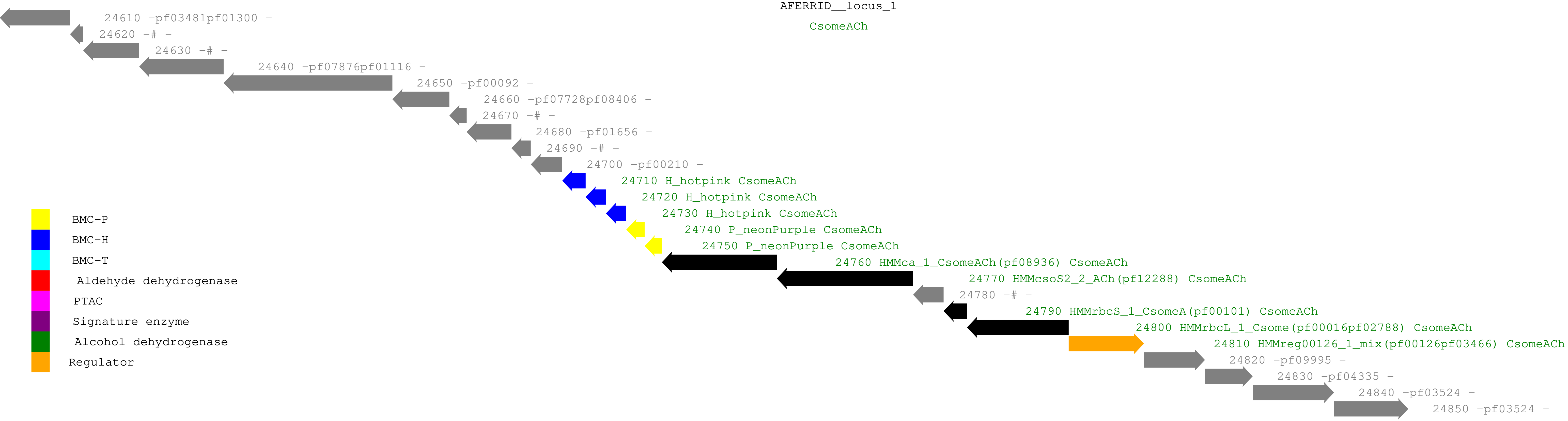

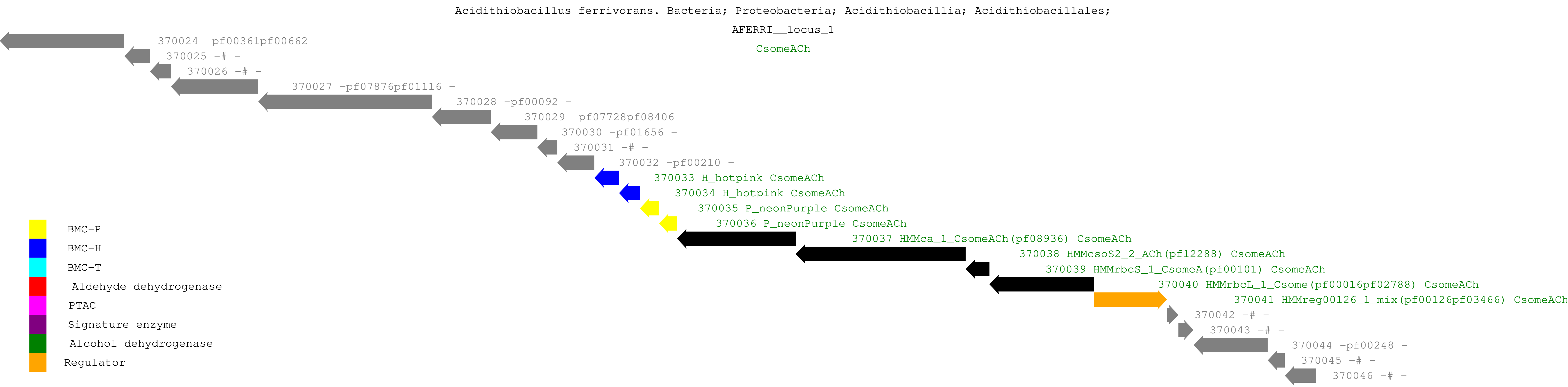
Acidithiobacillus ferrivorans. Bacteria; Proteobacteria; Acidithiobacillia; Acidithiobacillales;
AFERRI__locus_1
CsomeACh
370024 -pf00361pf00662 -
370025 -# -
370026 -# -
370027 -pf07876pf01116 -
370028 -pf00092 -
370029 -pf07728pf08406 -
370030 -pf01656 -
370031 -# -
370032 -pf00210 -
370033 H_hotpink CsomeACh
370034 H_hotpink CsomeACh
370035 P_neonPurple CsomeACh
370036 P_neonPurple CsomeACh
370037 HMMca_1_CsomeACh(pf08936) CsomeACh
370038 HMMcsoS2_2_ACh(pf12288) CsomeACh
370039 HMMrbcS_1_CsomeA(pf00101) CsomeACh
370040 HMMrbcL_1_Csome(pf00016pf02788) CsomeACh
370041 HMMreg00126_1_mix(pf00126pf03466) CsomeACh
370042 -# -
370043 -# -
370044 -pf00248 -
370045 -# -
370046 -# -
BMC-P
BMC-H
BMC-T
Aldehyde dehydrogenase
PTAC
Signature enzyme
Alcohol dehydrogenase
Regulator

Allochromatium vinosum (strain ATCC 17899 / DSM 180 / NBRC 103801 / Bacteria; Proteobacteria; Gammaproteobacteria; Chromatiales;

Alvin__locus_1_main_sat

CsomeACh

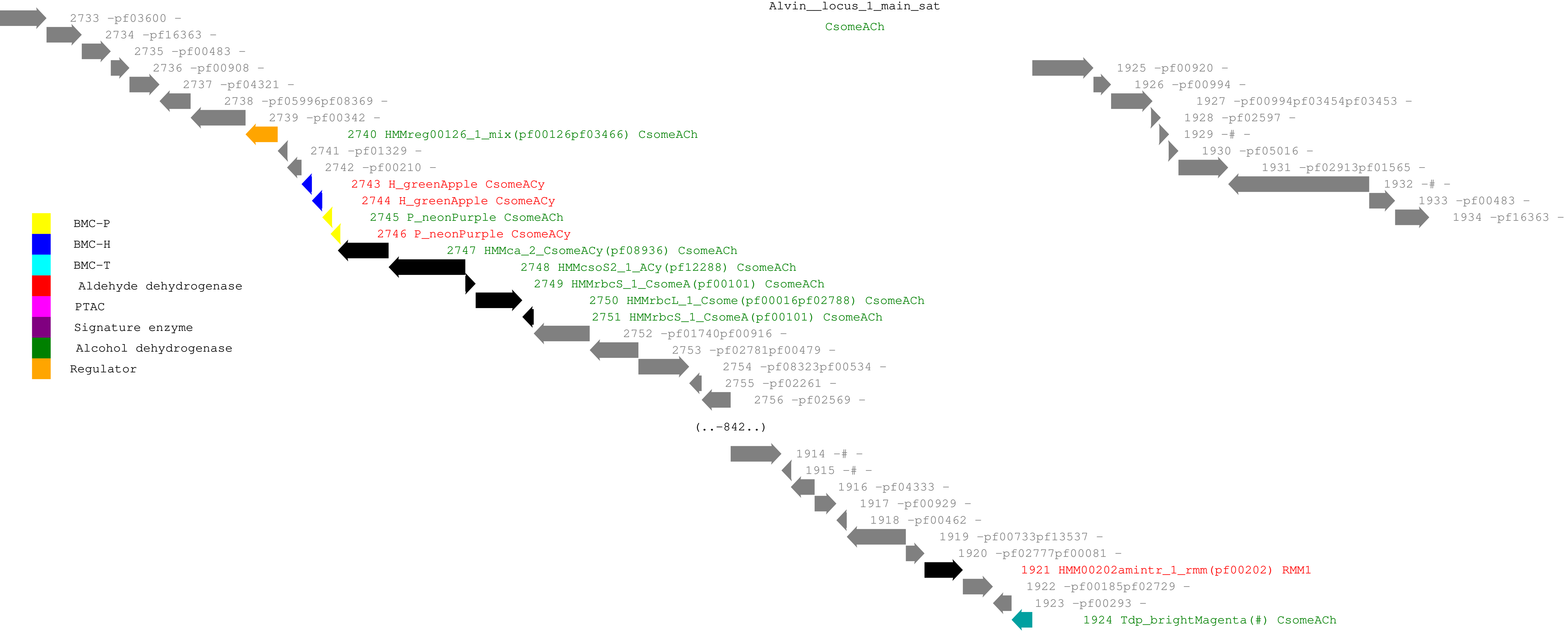

Gammaproteobacteria bacterium SG8_15. Bacteria; Proteobacteria; Gammaproteobacteria.

AMJ55__locus_1_main_sat

CsomeACh

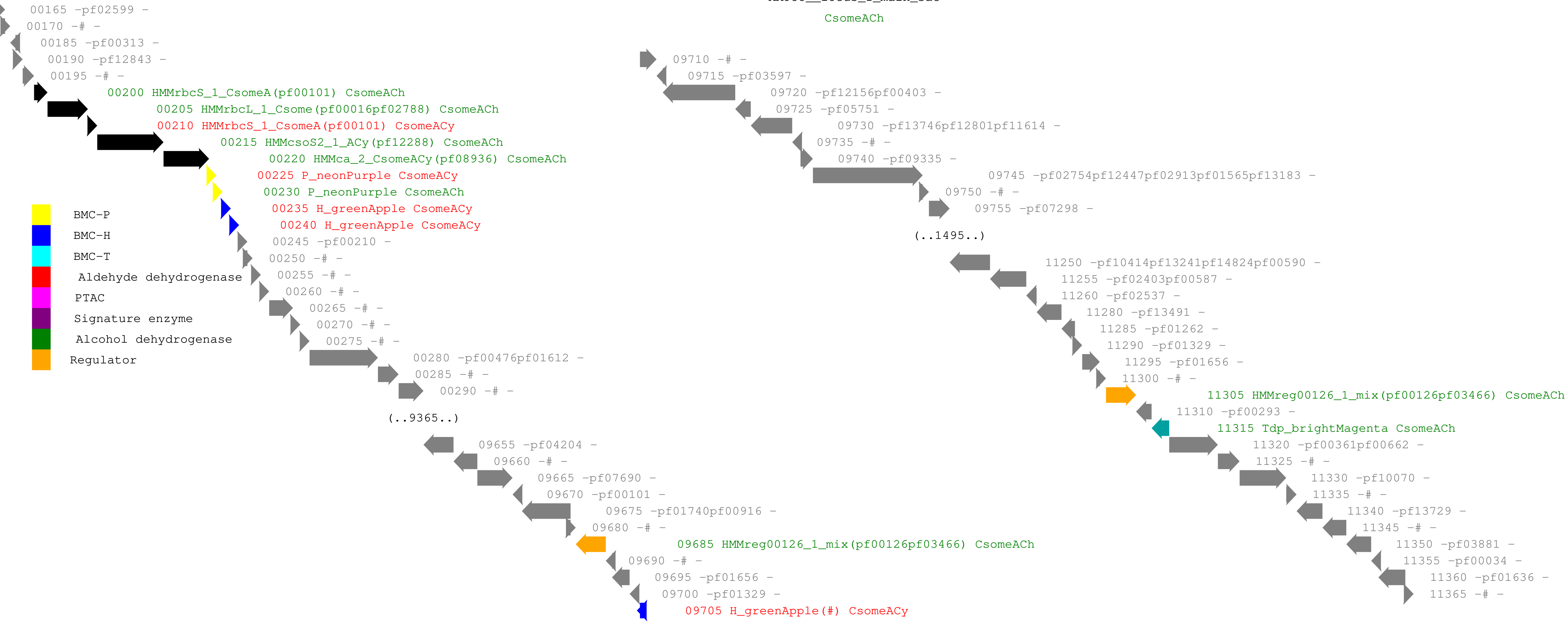

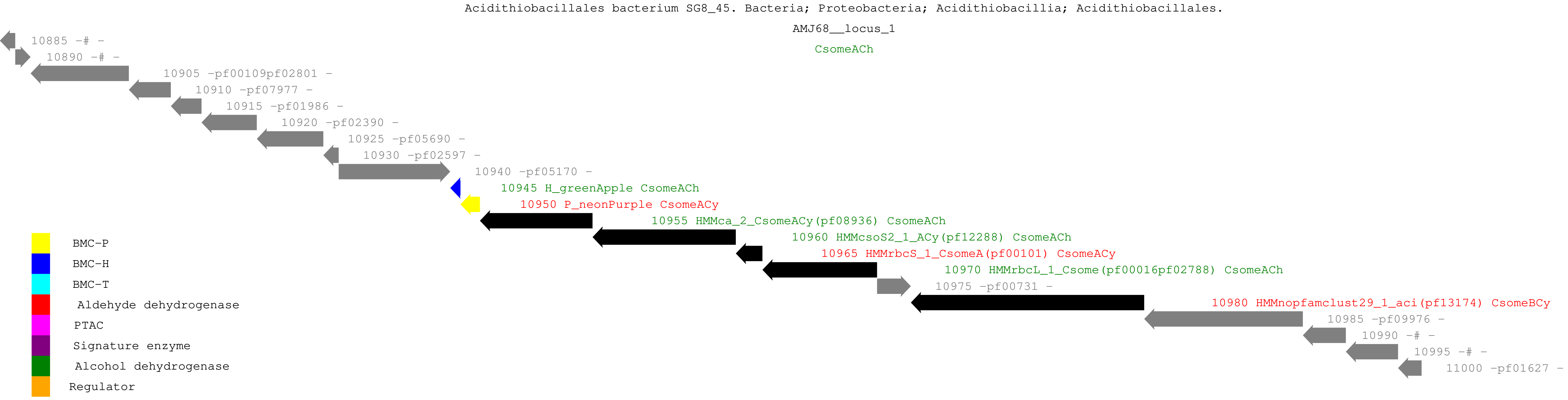
Acidithiobacillales bacterium SG8_45. Bacteria; Proteobacteria; Acidithiobacillia; Acidithiobacillales.
AMJ68__locus_1
CsomeACh
10885 -# -
10890 -# -
10905 -pf00109pf02801 -
10910 -pf07977 -
10915 -pf01986 -
10920 -pf02390 -
10925 -pf05690 -
10930 -pf02597 -
10940 -pf05170 -
10945 H_greenApple CsomeACh
10950 P_neonPurple CsomeACy
10955 HMMca_2_CsomeACy(pf08936) CsomeACh
10960 HMMcsoS2_1_ACy(pf12288) CsomeACh
10965 HMMrbcS_1_CsomeA(pf00101) CsomeACy
10970 HMMrbcL_1_Csome(pf00016pf02788) CsomeACh
10975 -pf00731 -
10980 HMMnopfamclust29_1_aci(pf13174) CsomeBCy
10985 -pf09976 -
10990 -# -
10995 -# -
11000 -pf01627 -
BMC-P
BMC-H
BMC-T
Aldehyde dehydrogenase
PTAC
Signature enzyme
Alcohol dehydrogenase
Regulator

Gammaproteobacteria bacterium SG8_47. Bacteria; Proteobacteria; Gammaproteobacteria.

AMJ69__locus_1

CsomeACh

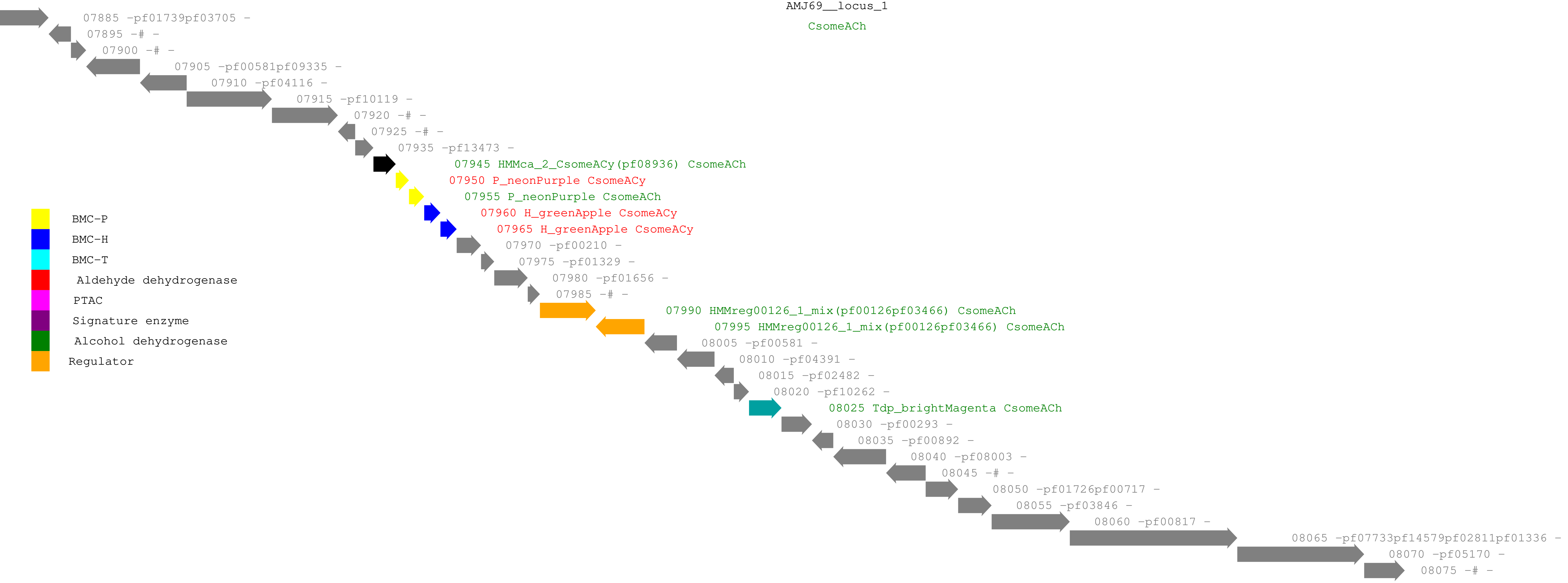

Thiotrichales bacterium SG8_50. Bacteria; Proteobacteria; Gammaproteobacteria; Thiotrichales;

AMS22__locus_1_main_sat

CsomeACh

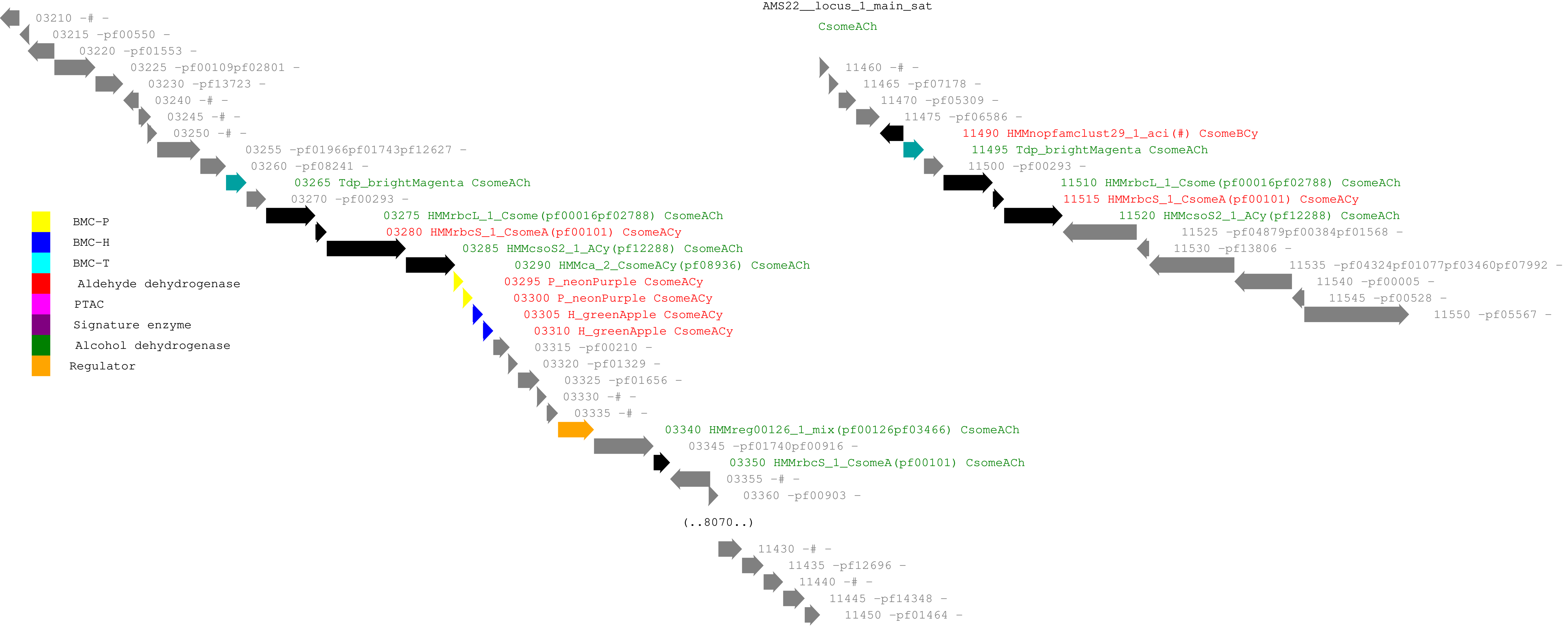

Thiohalorhabdus denitrificans. Bacteria; Proteobacteria; Gammaproteobacteria; Thiohalorhabdus.

AN478__locus_1

CsomeACh

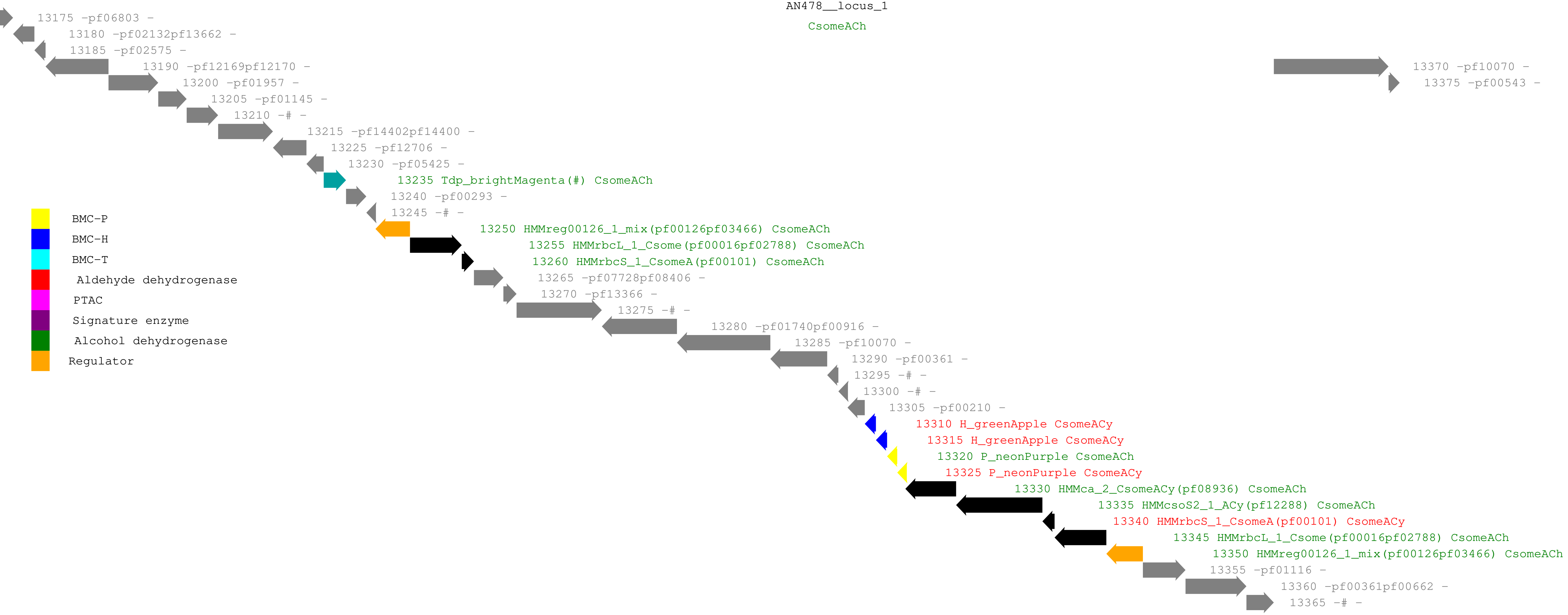

Acidithiobacillus caldus (strain SM-1). Bacteria; Proteobacteria; Acidithiobacillia; Acidithiobacillales;

Atc__locus_1

CsomeACh

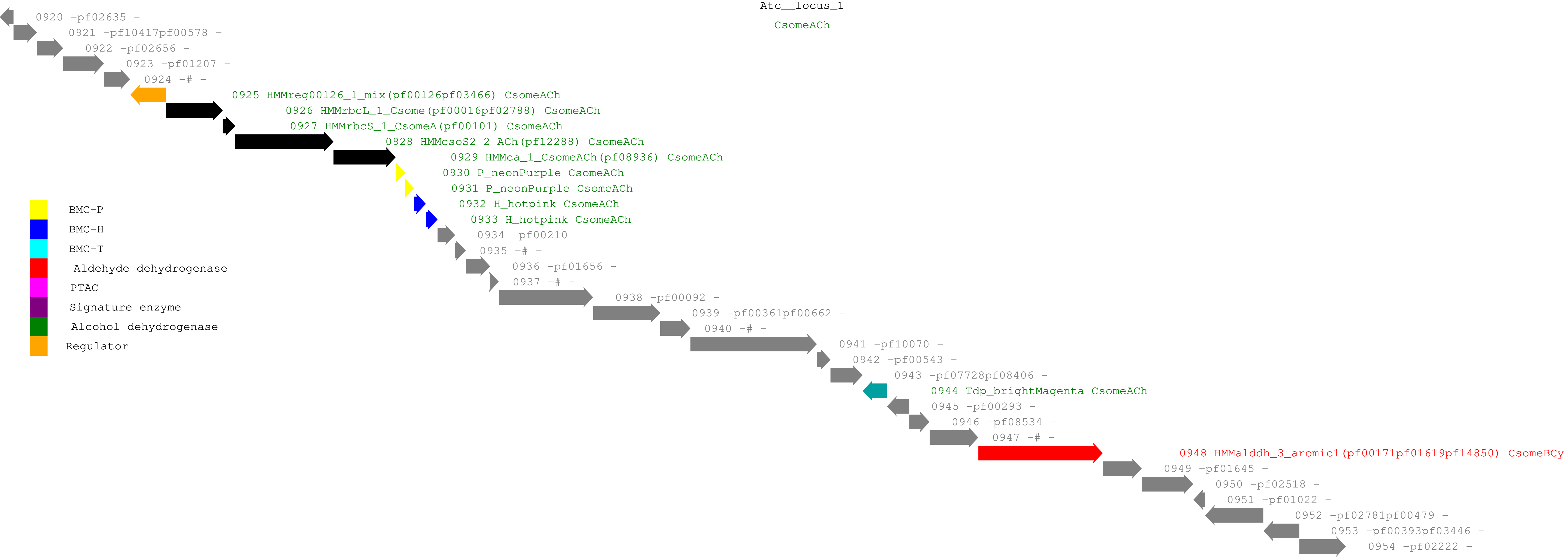

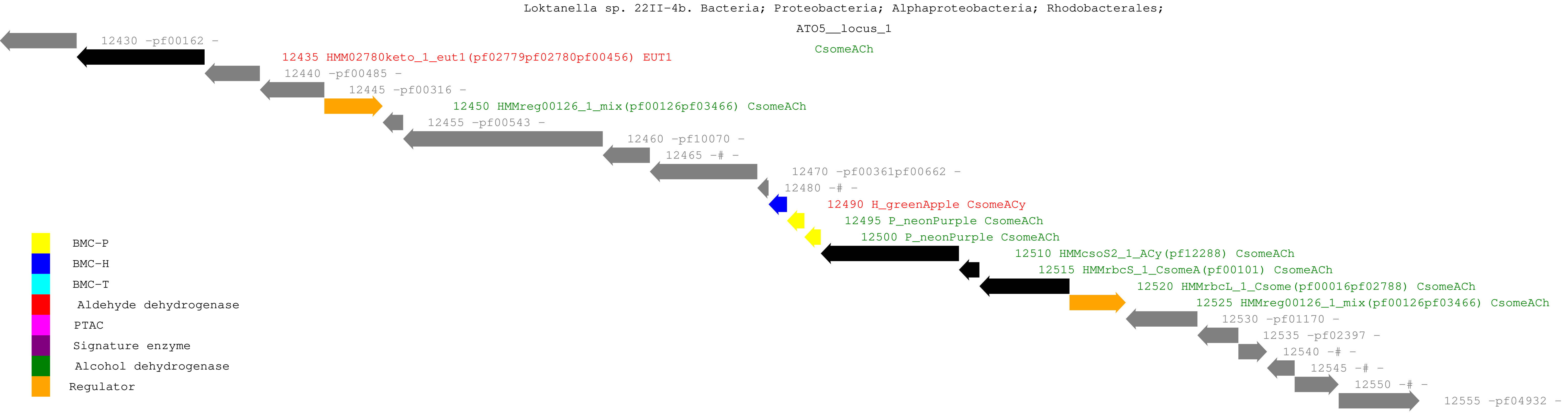
Loktanella sp. 22II-4b. Bacteria; Proteobacteria; Alphaproteobacteria; Rhodobacterales;
ATO5__locus_1
CsomeACh
12430 -pf00162 -
12435 HMM02780keto_1_eut1(pf02779pf02780pf00456) EUT1
12440 -pf00485 -
12445 -pf00316 -
12450 HMMreg00126_1_mix(pf00126pf03466) CsomeACh
12455 -pf00543 -
12460 -pf10070 -
12465 -# -
12470 -pf00361pf00662 -
12480 -# -
12490 H_greenApple CsomeACy
12495 P_neonPurple CsomeACh
12500 P_neonPurple CsomeACh
12510 HMMcsoS2_1_ACy(pf12288) CsomeACh
12515 HMMrbcS_1_CsomeA(pf00101) CsomeACh
12520 HMMrbcL_1_Csome(pf00016pf02788) CsomeACh
12525 HMMreg00126_1_mix(pf00126pf03466) CsomeACh
12530 -pf01170 -
12535 -pf02397 -
12540 -# -
12545 -# -
12550 -# -
12555 -pf04932 -
BMC-P
BMC-H
BMC-T
Aldehyde dehydrogenase
PTAC
Signature enzyme
Alcohol dehydrogenase
Regulator

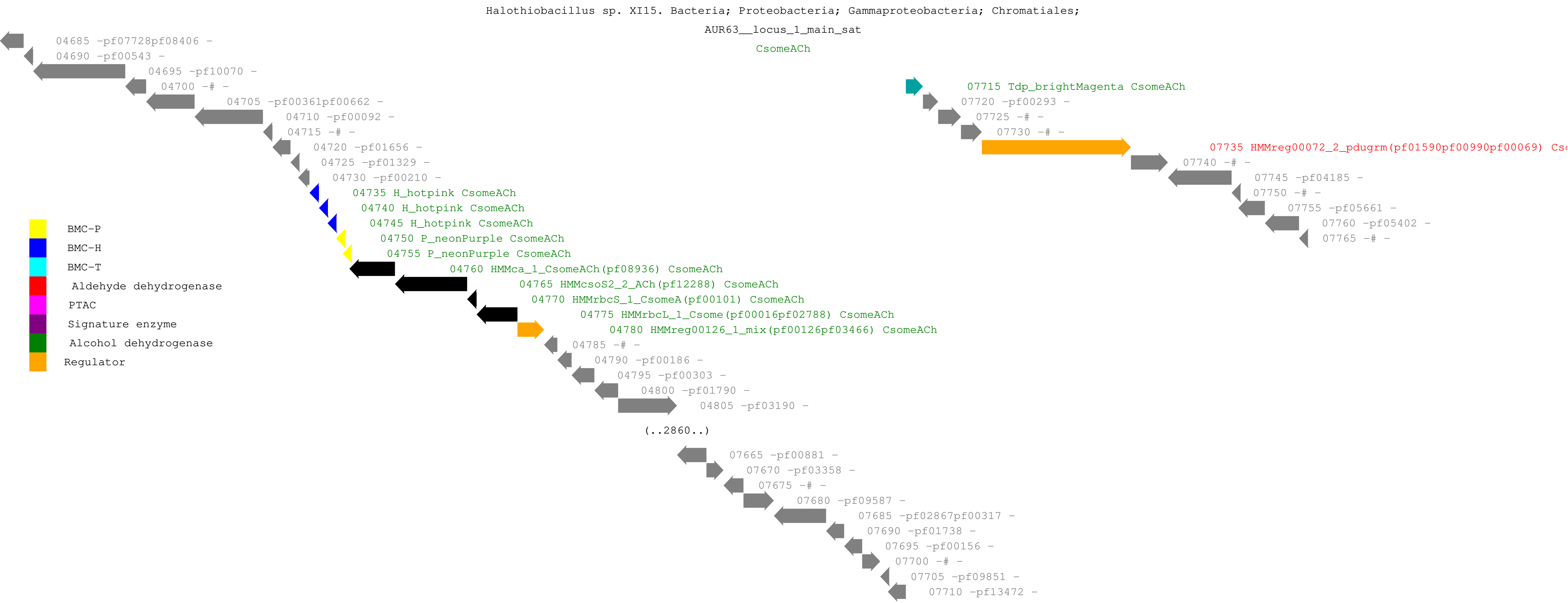

Halothiobacillus sp. XI15. Bacteria; Proteobacteria; Gammaproteobacteria; Chromatiales;
AUR63__locus_1_main_sat
CsomeACh
04685 -pf07728pf08406 -
04690 -pf00543 -
04695 -pf10070 -
04700 -# -
04705 -pf00361pf00662 -
04710 -pf00092 -
04715 -# -
04720 -pf01656 -
04725 -pf01329 -
04730 -pf00210 -
04735 H_hotpink CsomeACh
04740 H_hotpink CsomeACh
04745 H_hotpink CsomeACh
04750 P_neonPurple CsomeACh
04755 P_neonPurple CsomeACh
04760 HMMca_1_CsomeACh(pf08936) CsomeACh
04765 HMMcsoS2_2_ACh(pf12288) CsomeACh
04770 HMMrbcS_1_CsomeA(pf00101) CsomeACh
04775 HMMrbcL_1_Csome(pf00016pf02788) CsomeACh
04780 HMMreg00126_1_mix(pf00126pf03466) CsomeACh
04785 -# -
04790 -pf00186 -
04795 -pf00303 -
04800 -pf01790 -
04805 -pf03190 -
(..2860..)
07665 -pf00881 -
07670 -pf03358 -
07675 -# -
07680 -pf09587 -
07685 -pf02867pf00317 -
07690 -pf01738 -
07695 -pf00156 -
07700 -# -
07705 -pf09851 -
07710 -pf13472 -
07715 Tdp_brightMagenta CsomeACh
07720 -pf00293 -
07725 -# -
07730 -# -
07735 HMMreg00072_2_pdugrm(pf01590pf00990pf00069)
07740 -# -
07745 -pf04185 -
07750 -# -
07755 -pf05661 -
07760 -pf05402 -
07765 -# -
BMC-P
BMC-H
BMC-T
Aldehyde dehydrogenase
PTAC
Signature enzyme
Alcohol dehydrogenase
Regulator

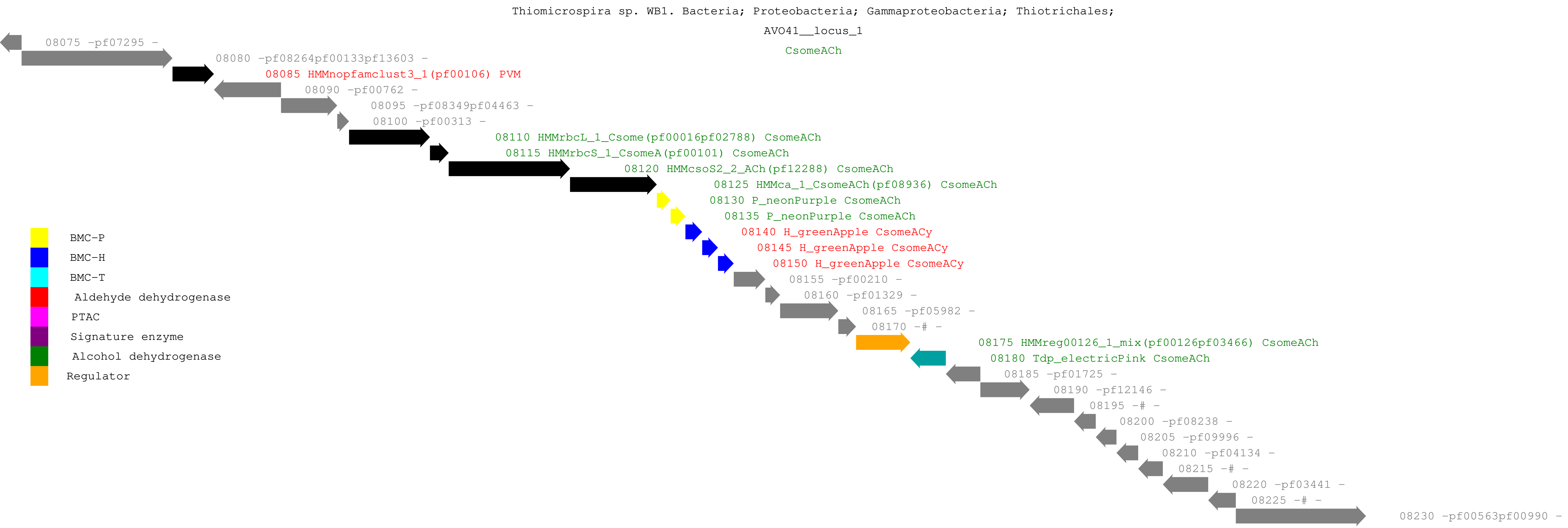
Thiomicrospira sp. WB1. Bacteria; Proteobacteria; Gammaproteobacteria; Thiotrichales;
AVO41__locus_1
CsomeACh
08075 -pf07295 -
08080 -pf08264pf00133pf13603 -
08085 HMMnopfamclust3_1(pf00106) PVM
08090 -pf00762 -
08095 -pf08349pf04463 -
08100 -pf00313 -
08110 HMMrbcL_1_Csome(pf00016pf02788) CsomeACh
08115 HMMrbcS_1_CsomeA(pf00101) CsomeACh
08120 HMMcsoS2_2_ACh(pf12288) CsomeACh
08125 HMMca_1_CsomeACh(pf08936) CsomeACh
08130 P_neonPurple CsomeACh
08135 P_neonPurple CsomeACh
08140 H_greenApple CsomeACy
08145 H_greenApple CsomeACy
08150 H_greenApple CsomeACy
08155 -pf00210 -
08160 -pf01329 -
08165 -pf05982 -
08170 -# -
08175 HMMreg00126_1_mix(pf00126pf03466) CsomeACh
08180 Tdp_electricPink CsomeACh
08185 -pf01725 -
08190 -pf12146 -
08195 -# -
08200 -pf08238 -
08205 -pf09996 -
08210 -pf04134 -
08215 -# -
08220 -pf03441 -
08225 -# -
08230 -pf00563pf00990 -
BMC-P
BMC-H
BMC-T
Aldehyde dehydrogenase
PTAC
Signature enzyme
Alcohol dehydrogenase
Regulator

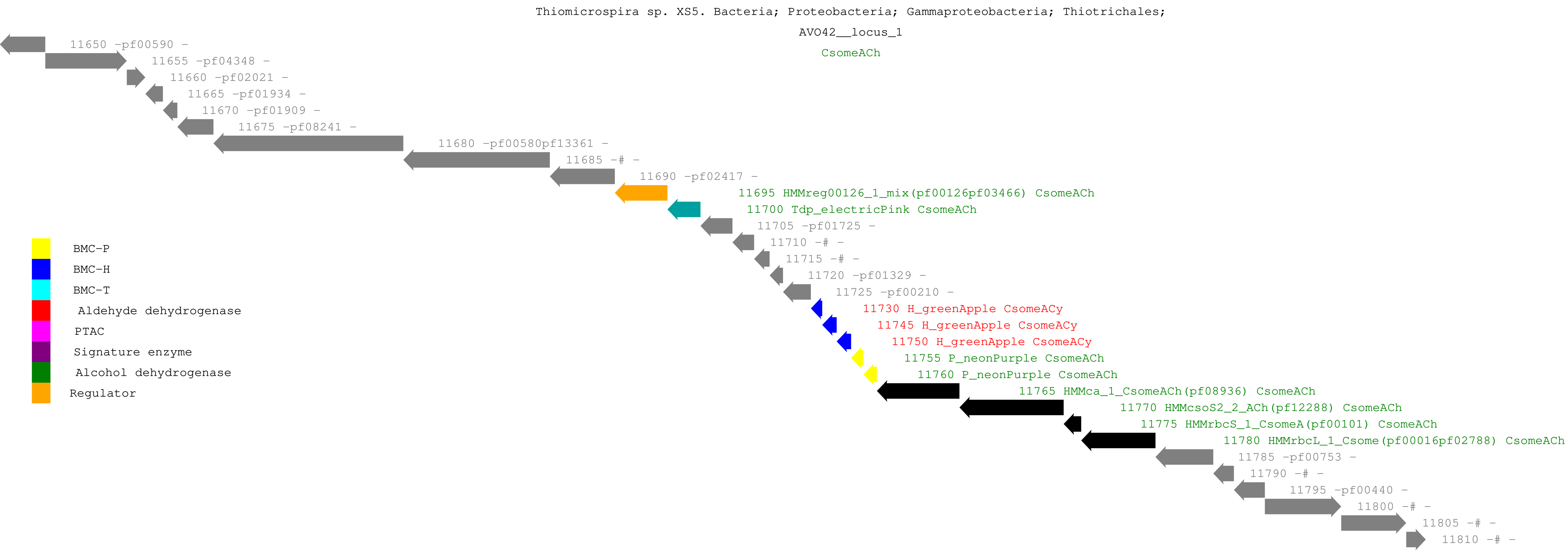
Thiomicrospira sp. XS5. Bacteria; Proteobacteria; Gammaproteobacteria; Thiotrichales;
AVO42__locus_1
CsomeACh
11650 -pf00590 -
11655 -pf04348 -
11660 -pf02021 -
11665 -pf01934 -
11670 -pf01909 -
11675 -pf08241 -
11680 -pf00580pf13361 -
11685 -# -
11690 -pf02417 -
11695 HMMreg00126_1_mix(pf00126pf03466) CsomeACh
11700 Tdp_electricPink CsomeACh
11705 -pf01725 -
11710 -# -
11715 -# -
11720 -pf01329 -
11725 -pf00210 -
11730 H_greenApple CsomeACy
11745 H_greenApple CsomeACy
11750 H_greenApple CsomeACy
11755 P_neonPurple CsomeACh
11760 P_neonPurple CsomeACh
11765 HMMca_1_CsomeACh(pf08936) CsomeACh
11770 HMMcsoS2_2_ACh(pf12288) CsomeACh
11775 HMMrbcS_1_CsomeA(pf00101) CsomeACh
11780 HMMrbcL_1_Csome(pf00016pf02788) CsomeACh
11785 -pf00753 -
11790 -# -
11795 -pf00440 -
11800 -# -
11805 -# -
11810 -# -
BMC-P
BMC-H
BMC-T
Aldehyde dehydrogenase
PTAC
Signature enzyme
Alcohol dehydrogenase
Regulator

Candidatus Gallionella acididurans. Bacteria; Proteobacteria; Betaproteobacteria; Gallionellales;

AWT59__locus_1_main_sat

CsomeACh

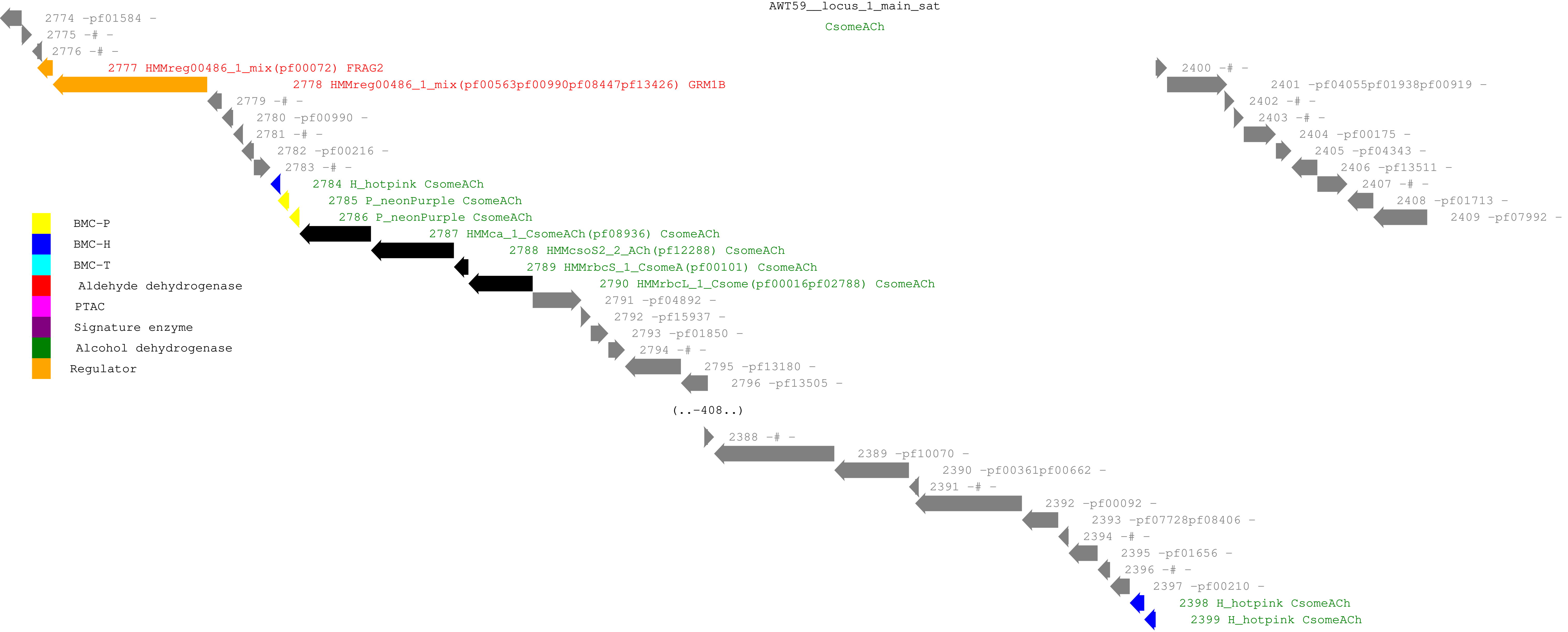

Marichromatium gracile (Chromatium gracile). Bacteria; Proteobacteria; Gammaproteobacteria; Chromatiales;

AY586__locus_1

CsomeACh

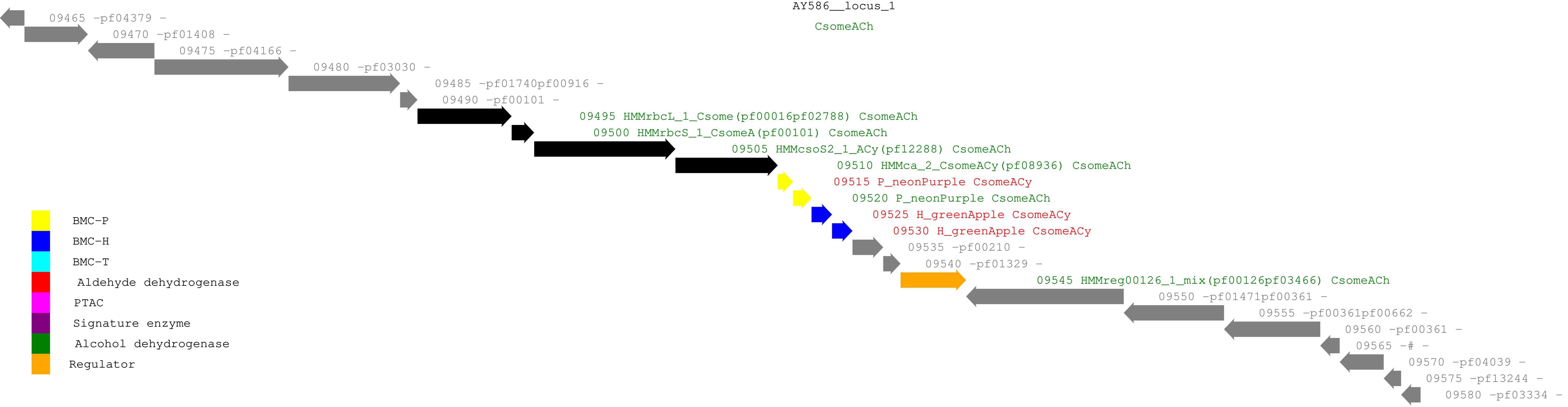

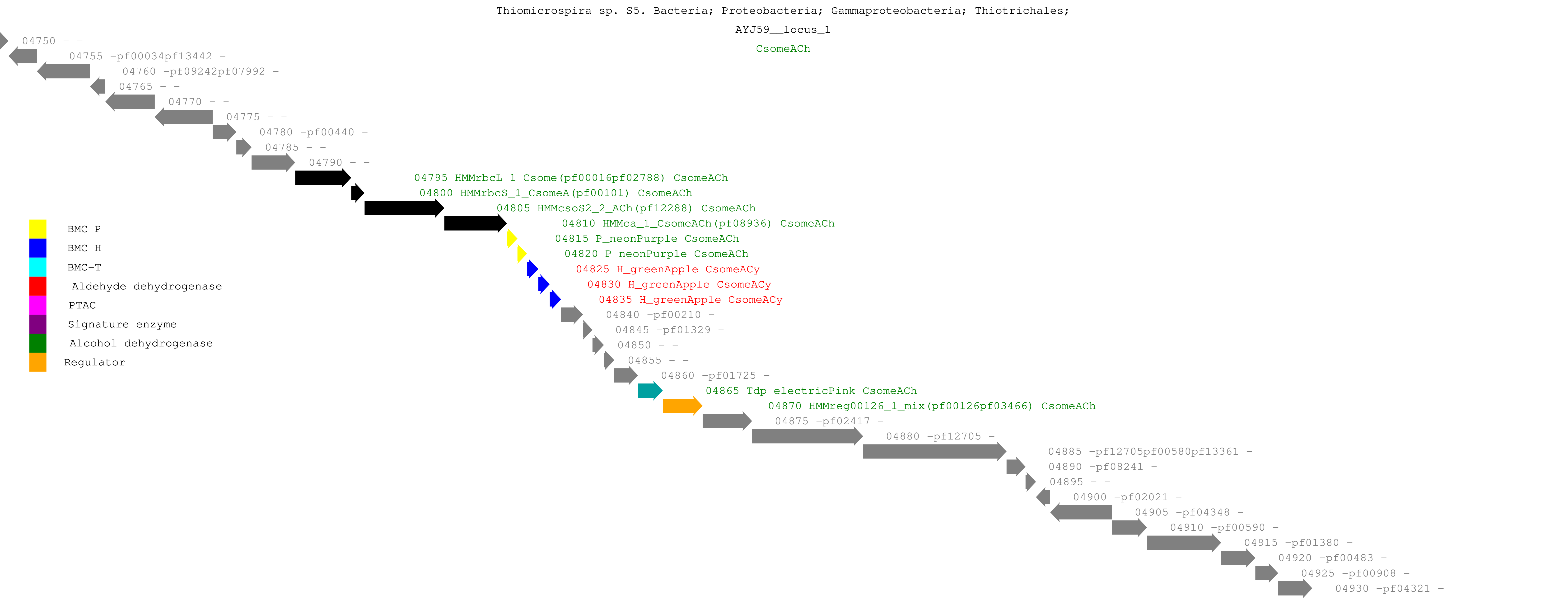
Thiomicrospira sp. S5. Bacteria; Proteobacteria; Gammaproteobacteria; Thiotrichales;
AYJ59__locus_1
CsomeACh
04750 - -
04755 -pf00034pf13442 -
04760 -pf09242pf07992 -
04765 - -
04770 - -
04775 - -
04780 -pf00440 -
04785 - -
04790 - -
04795 HMMrbcL_1_Csome(pf00016pf02788) CsomeACh
04800 HMMrbcS_1_CsomeA(pf00101) CsomeACh
04805 HMMcsoS2_2_ACh(pf12288) CsomeACh
04810 HMMca_1_CsomeACh(pf08936) CsomeACh
04815 P_neonPurple CsomeACh
04820 P_neonPurple CsomeACh
04825 H_greenApple CsomeACy
04830 H_greenApple CsomeACy
04835 H_greenApple CsomeACy
04840 -pf00210 -
04845 -pf01329 -
04850 - -
04855 - -
04860 -pf01725 -
04865 Tdp_electricPink CsomeACh
04870 HMMreg00126_1_mix(pf00126pf03466) CsomeACh
04875 -pf02417 -
04880 -pf12705 -
04885 -pf12705pf00580pf13361 -
04890 -pf08241 -
04895 - -
04900 -pf02021 -
04905 -pf04348 -
04910 -pf00590 -
04915 -pf01380 -
04920 -pf00483 -
04925 -pf00908 -
04930 -pf04321 -
BMC-P
BMC-H
BMC-T
Aldehyde dehydrogenase
PTAC
Signature enzyme
Alcohol dehydrogenase
Regulator

Thioalkalivibrio versutus. Bacteria; Proteobacteria; Gammaproteobacteria; Chromatiales;

B0684__locus_1_main_sat

CsomeACh

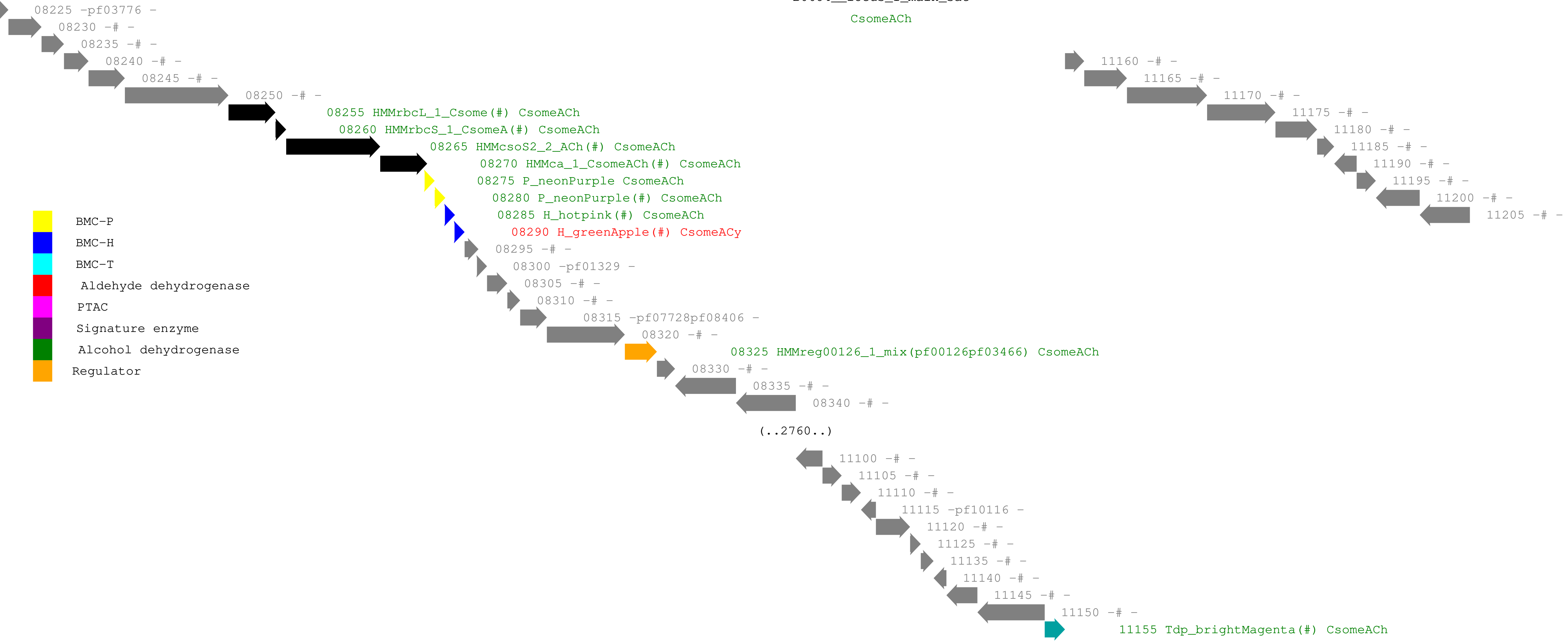

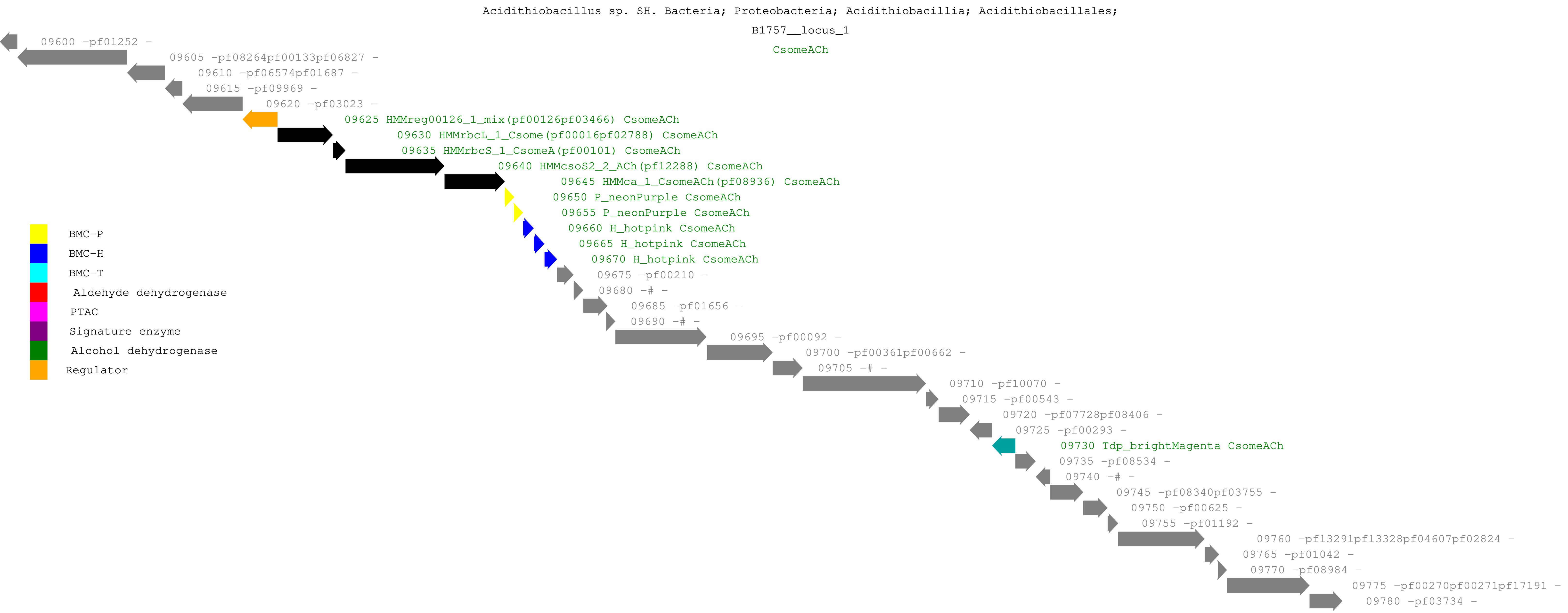

Acidithiobacillus sp. SH. Bacteria; Proteobacteria; Acidithiobacillia; Acidithiobacillales;
B1757__locus_1
CsomeACh
09600 -pf01252 -
09605 -pf08264pf00133pf06827 -
09610 -pf06574pf01687 -
09615 -pf09969 -
09620 -pf03023 -
09625 HMMreg00126_1_mix(pf00126pf03466) CsomeACh
09630 HMMrbcL_1_Csome(pf00016pf02788) CsomeACh
09635 HMMrbcS_1_CsomeA(pf00101) CsomeACh
09640 HMMcsoS2_2_ACh(pf12288) CsomeACh
09645 HMMca_1_CsomeACh(pf08936) CsomeACh
09650 P_neonPurple CsomeACh
09655 P_neonPurple CsomeACh
09660 H_hotpink CsomeACh
09665 H_hotpink CsomeACh
09670 H_hotpink CsomeACh
09675 -pf00210 -
09680 -# -
09685 -pf01656 -
09690 -# -
09695 -pf00092 -
09700 -pf00361pf00662 -
09705 -# -
09710 -pf10070 -
09715 -pf00543 -
09720 -pf07728pf08406 -
09725 -pf00293 -
09730 Tdp_brightMagenta CsomeACh
09735 -pf08534 -
09740 -# -
09745 -pf08340pf03755 -
09750 -pf00625 -
09755 -pf01192 -
09760 -pf13291pf13328pf04607pf02824 -
09765 -pf01042 -
09770 -pf08984 -
09775 -pf00270pf00271pf17191 -
09780 -pf03734 -
BMC-P
BMC-H
BMC-T
Aldehyde dehydrogenase
PTAC
Signature enzyme
Alcohol dehydrogenase
Regulator

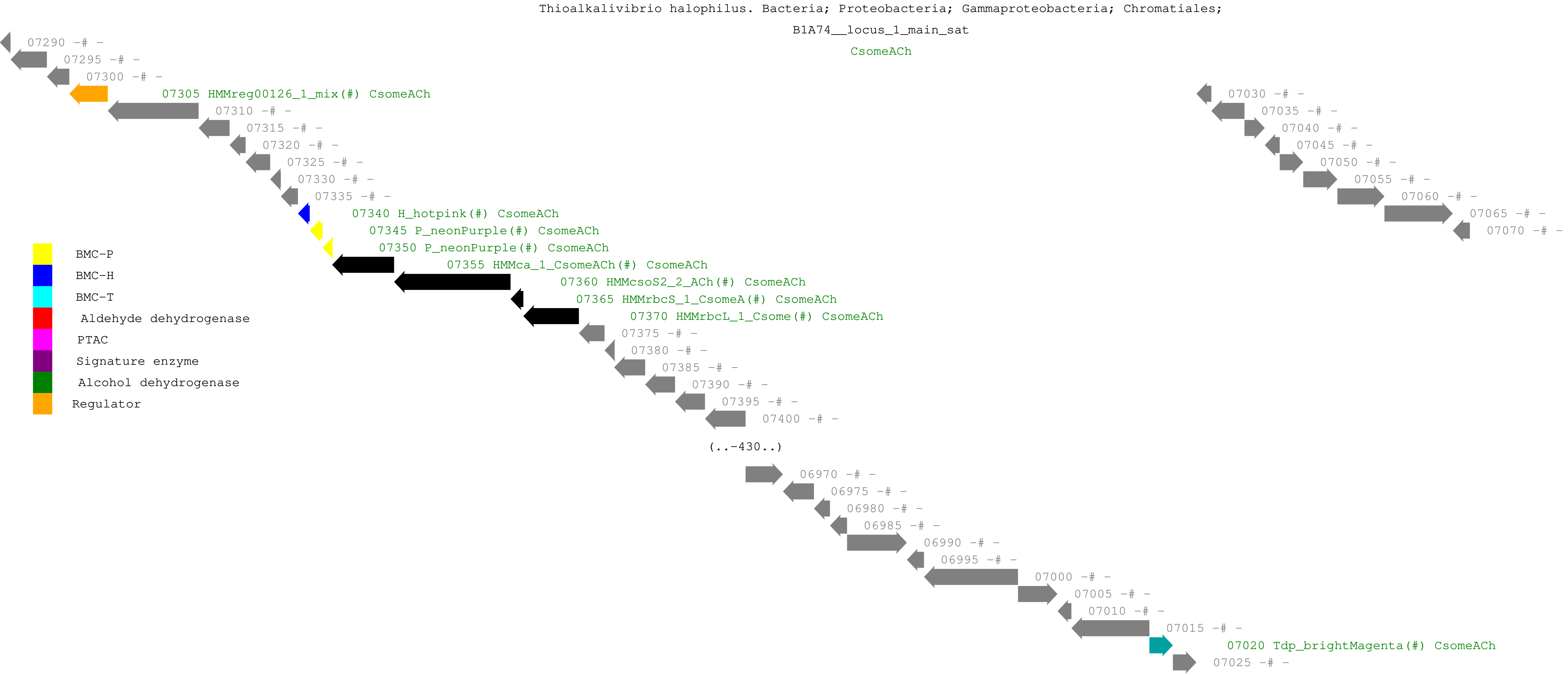
Thioalkalivibrio halophilus. Bacteria; Proteobacteria; Gammaproteobacteria; Chromatiales;
B1A74__locus_1_main_sat
CsomeACh
07290 -# -
07295 -# -
07300 -# -
07305 HMMreg00126_1_mix(#) CsomeACh
07310 -# -
07315 -# -
07320 -# -
07325 -# -
07330 -# -
07335 -# -
07340 H_hotpink(#) CsomeACh
07345 P_neonPurple(#) CsomeACh
07350 P_neonPurple(#) CsomeACh
07355 HMMca_1_CsomeACh(#) CsomeACh
07360 HMMcsoS2_2_ACh(#) CsomeACh
07365 HMMrbcS_1_CsomeA(#) CsomeACh
07370 HMMrbcL_1_Csome(#) CsomeACh
07375 -# -
07380 -# -
07385 -# -
07390 -# -
07395 -# -
07400 -# -
(..-430..)
06970 -# -
06975 -# -
06980 -# -
06985 -# -
06990 -# -
06995 -# -
07000 -# -
07005 -# -
07010 -# -
07015 -# -
07020 Tdp_brightMagenta(#) CsomeACh
07025 -# -
07030 -# -
07035 -# -
07040 -# -
07045 -# -
07050 -# -
07055 -# -
07060 -# -
07065 -# -
07070 -# -
BMC-P
BMC-H
BMC-T
Aldehyde dehydrogenase
PTAC
Signature enzyme
Alcohol dehydrogenase
Regulator

mine drainage metagenome. unclassified sequences; metagenomes; ecological metagenomes.

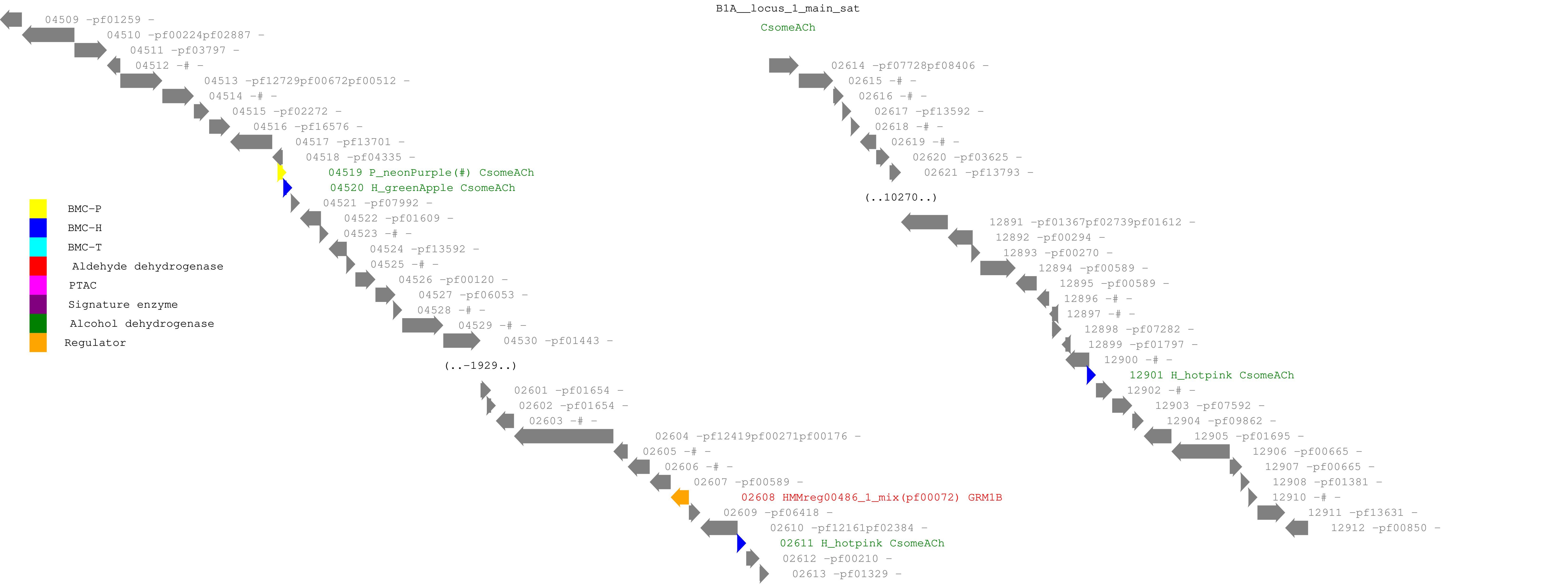

Thioalkalivibrio denitrificans. Bacteria; Proteobacteria; Gammaproteobacteria; Chromatiales;

B1C78__locus_1

CsomeACh

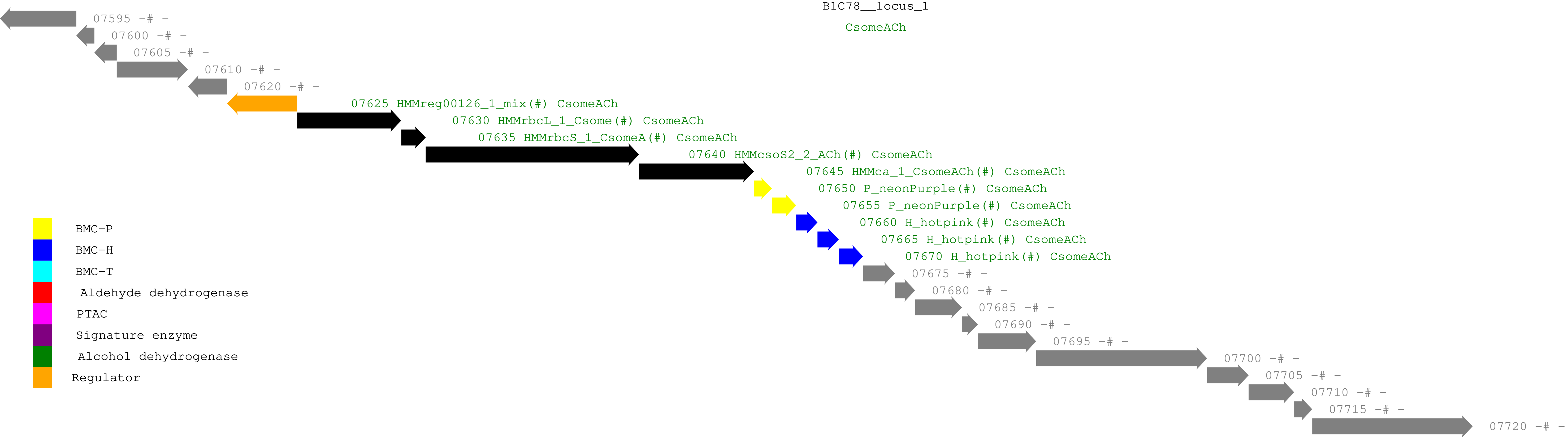

Nitrobacter vulgaris. Bacteria; Proteobacteria; Alphaproteobacteria; Rhizobiales;

B2M20__locus_1

CsomeACh

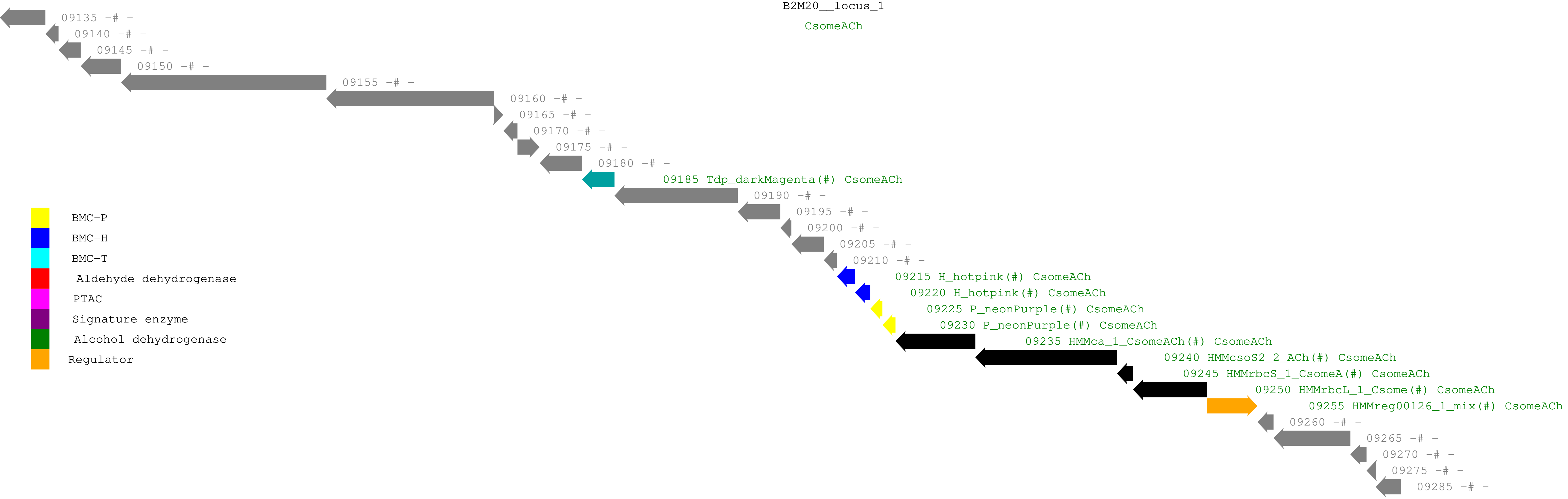

Burkholderiales bacterium 21-58-4. Bacteria; Proteobacteria; Betaproteobacteria; Burkholderiales.

B7X10__locus_1

CsomeACh

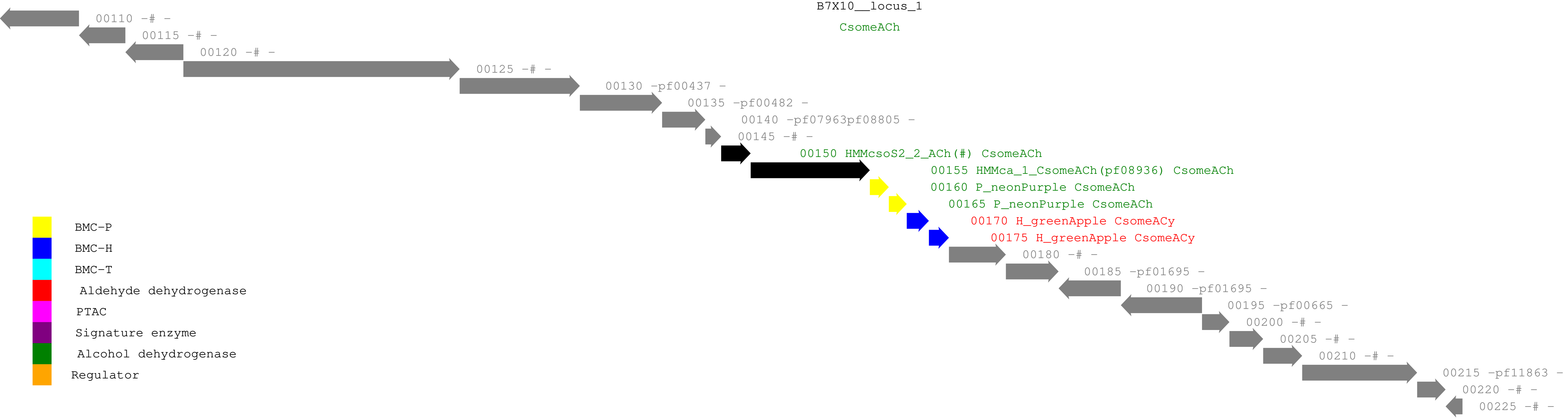

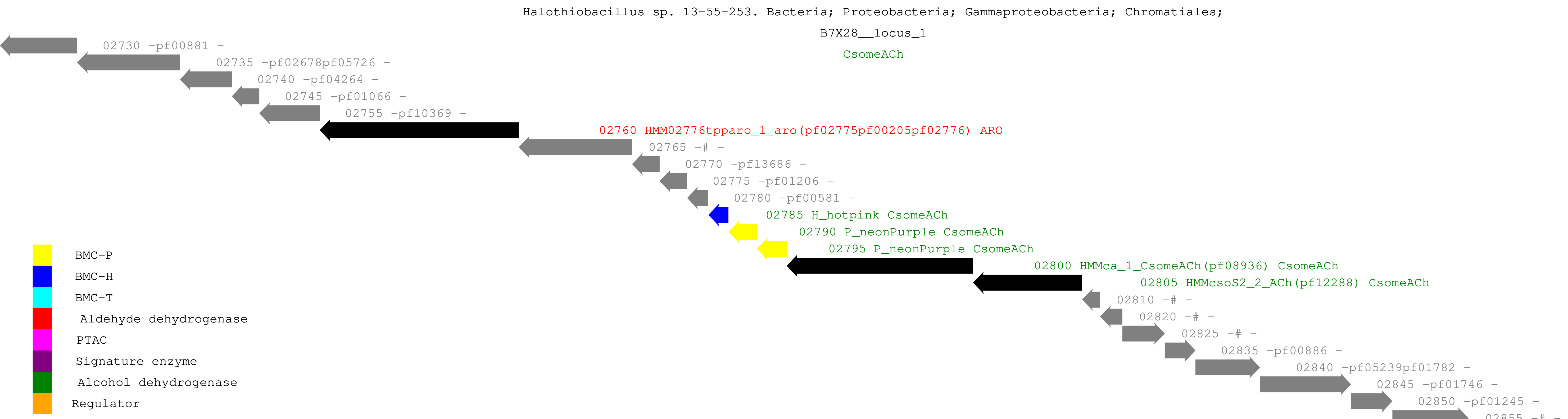
Halothiobacillus sp. 13-55-253. Bacteria; Proteobacteria; Gammaproteobacteria; Chromatiales;
B7X28__locus_1
CsomeACh
02730 -pf00881 -
02735 -pf02678pf05726 -
02740 -pf04264 -
02745 -pf01066 -
02755 -pf10369 -
02760 HMM02776tpparo_1_aro(pf02775pf00205pf02776) ARO
02765 -# -
02770 -pf13686 -
02775 -pf01206 -
02780 -pf00581 -
02785 H_hotpink CsomeACh
02790 P_neonPurple CsomeACh
02795 P_neonPurple CsomeACh
02800 HMMca_1_CsomeACh(pf08936) CsomeACh
02805 HMMcsoS2_2_ACh(pf12288) CsomeACh
02810 -# -
02820 -# -
02825 -# -
02835 -pf00886 -
02840 -pf05239pf01782 -
02845 -pf01746 -
02850 -pf01245 -
02855 -# -
BMC-P
BMC-H
BMC-T
Aldehyde dehydrogenase
PTAC
Signature enzyme
Alcohol dehydrogenase
Regulator

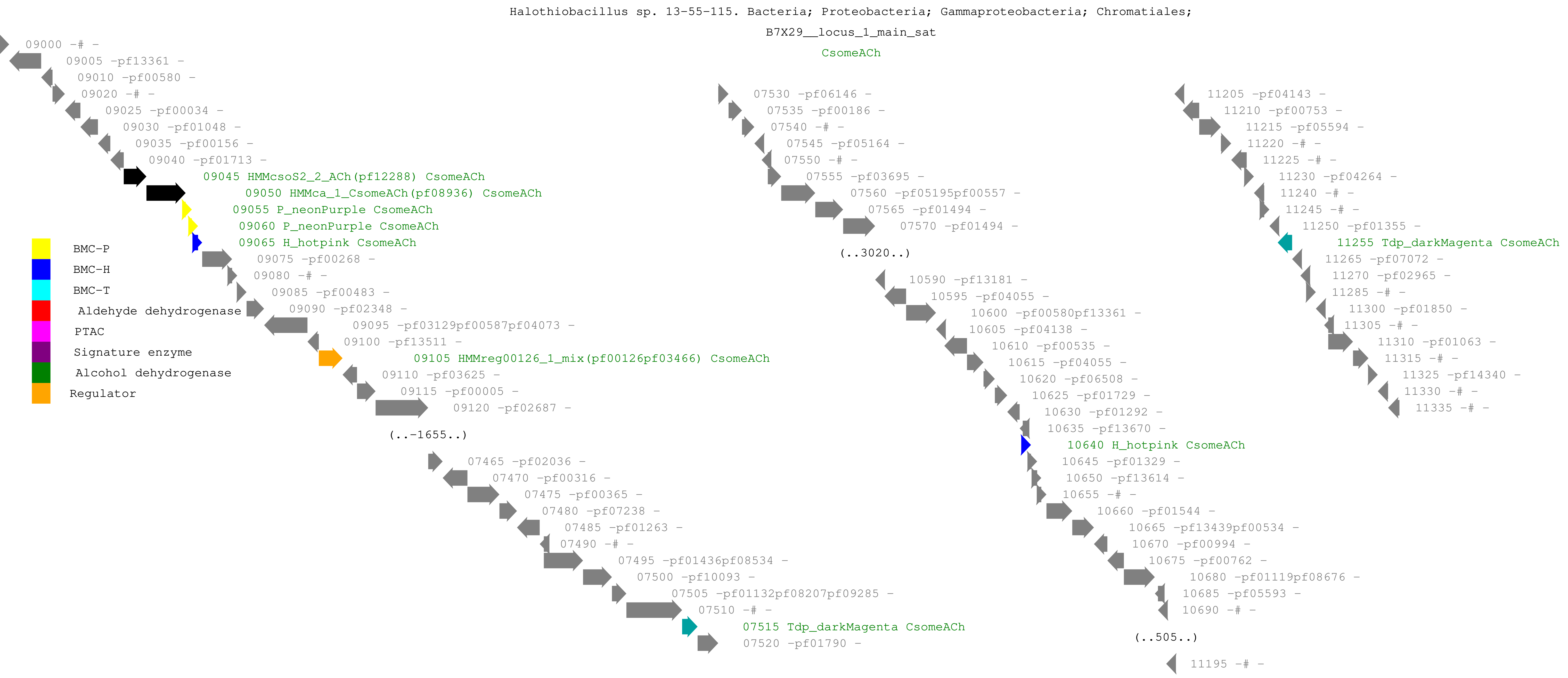
Halothiobacillus sp. 13-55-115. Bacteria; Proteobacteria; Gammaproteobacteria; Chromatiales;
B7X29__locus_1_main_sat
CsomeACh
09000 -# -
09005 -pf13361 -
09010 -pf00580 -
09020 -# -
09025 -pf00034 -
09030 -pf01048 -
09035 -pf00156 -
09040 -pf01713 -
09045 HMMcsoS2_2_ACh(pf12288) CsomeACh
09050 HMMca_1_CsomeACh(pf08936) CsomeACh
09055 P_neonPurple CsomeACh
09060 P_neonPurple CsomeACh
09065 H_hotpink CsomeACh
09075 -pf00268 -
09080 -# -
09085 -pf00483 -
09090 -pf02348 -
09095 -pf03129pf00587pf04073 -
09100 -pf13511 -
09105 HMMreg00126_1_mix(pf00126pf03466) CsomeACh
09110 -pf03625 -
09115 -pf00005 -
09120 -pf02687 -
(..-1655..)
07465 -pf02036 -
07470 -pf00316 -
07475 -pf00365 -
07480 -pf07238 -
07485 -pf01263 -
07490 -# -
07495 -pf01436pf08534 -
07500 -pf10093 -
07505 -pf01132pf08207pf09285 -
07510 -# -
07515 Tdp_darkMagenta CsomeACh
07520 -pf01790 -
BMC-P
BMC-H
BMC-T
Aldehyde dehydrogenase
PTAC
Signature enzyme
Alcohol dehydrogenase
Regulator
07530 -pf06146 -
07535 -pf00186 -
07540 -# -
07545 -pf05164 -
07550 -# -
07555 -pf03695 -
07560 -pf05195pf00557 -
07565 -pf01494 -
07570 -pf01494 -
(..3020..)
10590 -pf13181 -
10595 -pf04055 -
10600 -pf00580pf13361 -
10605 -pf04138 -
10610 -pf00535 -
10615 -pf04055 -
10620 -pf06508 -
10625 -pf01729 -
10630 -pf01292 -
10635 -pf13670 -
10640 H_hotpink CsomeACh
10645 -pf01329 -
10650 -pf13614 -
10655 -# -
10660 -pf01544 -
10665 -pf13439pf00534 -
10670 -pf00994 -
10675 -pf00762 -
10680 -pf01119pf08676 -
10685 -pf05593 -
10690 -# -
(..505..)
11195 -# -
11205 -pf04143 -
11210 -pf00753 -
11215 -pf05594 -
11220 -# -
11225 -# -
11230 -pf04264 -
11240 -# -
11245 -# -
11250 -pf01355 -
11255 Tdp_darkMagenta CsomeACh
11265 -pf07072 -
11270 -pf02965 -
11285 -# -
11300 -pf01850 -
11305 -# -
11310 -pf01063 -
11315 -# -
11325 -pf14340 -
11330 -# -
11335 -# -

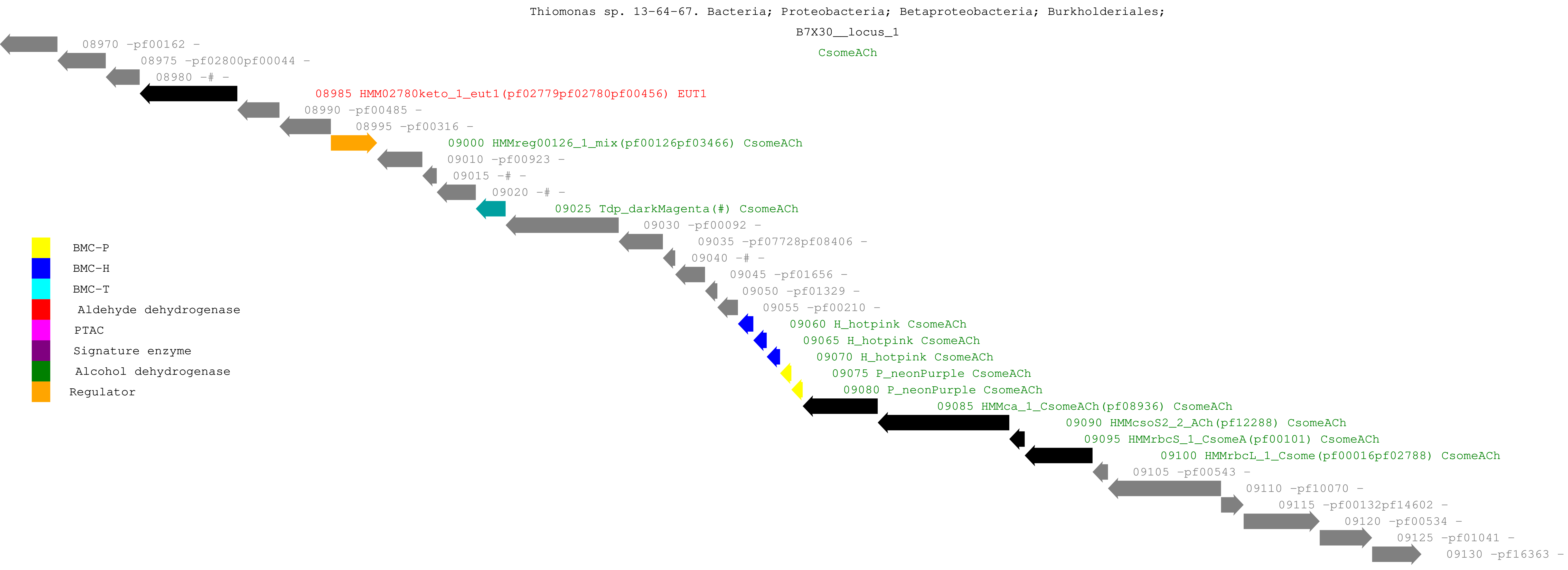
Thiomonas sp. 13-64-67. Bacteria; Proteobacteria; Betaproteobacteria; Burkholderiales;
B7X30__locus_1
CsomeACh
08970 -pf00162 -
08975 -pf02800pf00044 -
08980 -# -
08985 HMM02780keto_1_eut1(pf02779pf02780pf00456) EUT1
08990 -pf00485 -
08995 -pf00316 -
09000 HMMreg00126_1_mix(pf00126pf03466) CsomeACh
09010 -pf00923 -
09015 -# -
09020 -# -
09025 Tdp_darkMagenta(#) CsomeACh
09030 -pf00092 -
09035 -pf07728pf08406 -
09040 -# -
09045 -pf01656 -
09050 -pf01329 -
09055 -pf00210 -
09060 H_hotpink CsomeACh
09065 H_hotpink CsomeACh
09070 H_hotpink CsomeACh
09075 P_neonPurple CsomeACh
09080 P_neonPurple CsomeACh
09085 HMMca_1_CsomeACh(pf08936) CsomeACh
09090 HMMcsoS2_2_ACh(pf12288) CsomeACh
09095 HMMrbcS_1_CsomeA(pf00101) CsomeACh
09100 HMMrbcL_1_Csome(pf00016pf02788) CsomeACh
09105 -pf00543 -
09110 -pf10070 -
09115 -pf00132pf14602 -
09120 -pf00534 -
09125 -pf01041 -
09130 -pf16363 -
BMC-P
BMC-H
BMC-T
Aldehyde dehydrogenase
PTAC
Signature enzyme
Alcohol dehydrogenase
Regulator

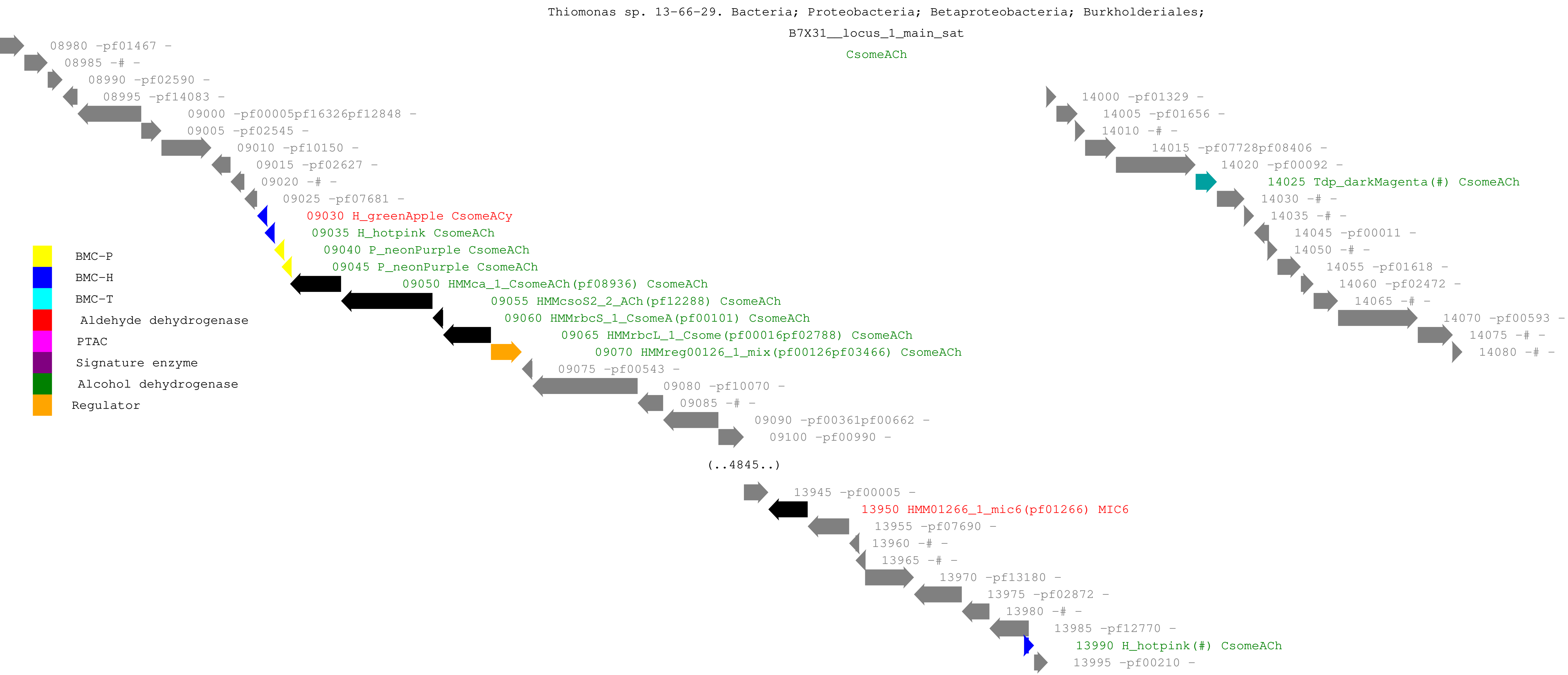
Thiomonas sp. 13-66-29. Bacteria; Proteobacteria; Betaproteobacteria; Burkholderiales;
B7X31__locus_1_main_sat
CsomeACh
08980 -pf01467 -
08985 -# -
08990 -pf02590 -
08995 -pf14083 -
09000 -pf00005pf16326pf12848 -
09005 -pf02545 -
09010 -pf10150 -
09015 -pf02627 -
09020 -# -
09025 -pf07681 -
09030 H_greenApple CsomeACy
09035 H_hotpink CsomeACh
09040 P_neonPurple CsomeACh
09045 P_neonPurple CsomeACh
09050 HMMca_1_CsomeACh(pf08936) CsomeACh
09055 HMMcsoS2_2_ACh(pf12288) CsomeACh
09060 HMMrbcS_1_CsomeA(pf00101) CsomeACh
09065 HMMrbcL_1_Csome(pf00016pf02788) CsomeACh
09070 HMMreg00126_1_mix(pf00126pf03466) CsomeACh
09075 -pf00543 -
09080 -pf10070 -
09085 -# -
09090 -pf00361pf00662 -
09100 -pf00990 -
(..4845..)
13945 -pf00005 -
13950 HMM01266_1_mic6(pf01266) MIC6
13955 -pf07690 -
13960 -# -
13965 -# -
13970 -pf13180 -
13975 -pf02872 -
13980 -# -
13985 -pf12770 -
13990 H_hotpink(#) CsomeACh
13995 -pf00210 -
14000 -pf01329 -
14005 -pf01656 -
14010 -# -
14015 -pf07728pf08406 -
14020 -pf00092 -
14025 Tdp_darkMagenta(#) CsomeACh
14030 -# -
14035 -# -
14045 -pf00011 -
14050 -# -
14055 -pf01618 -
14060 -pf02472 -
14065 -# -
14070 -pf00593 -
14075 -# -
14080 -# -
BMC-P
BMC-H
BMC-T
Aldehyde dehydrogenase
PTAC
Signature enzyme
Alcohol dehydrogenase
Regulator

Thiomonas sp. 14-64-326. Bacteria; Proteobacteria; Betaproteobacteria; Burkholderiales;

B7X36__locus_1

CsomeACh

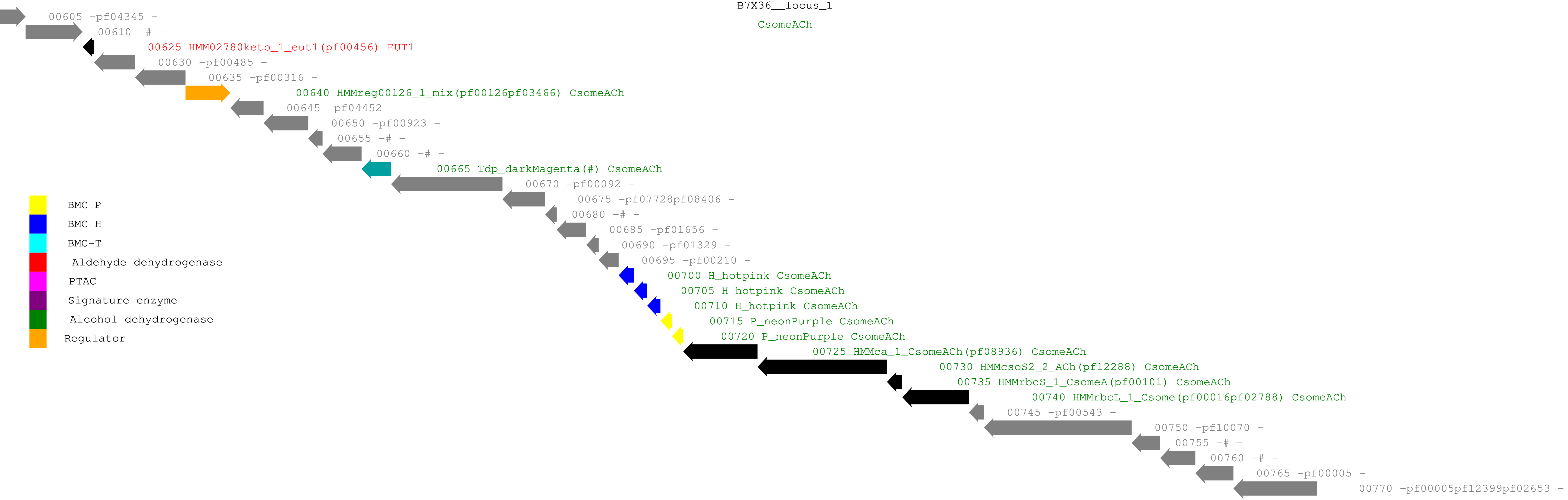

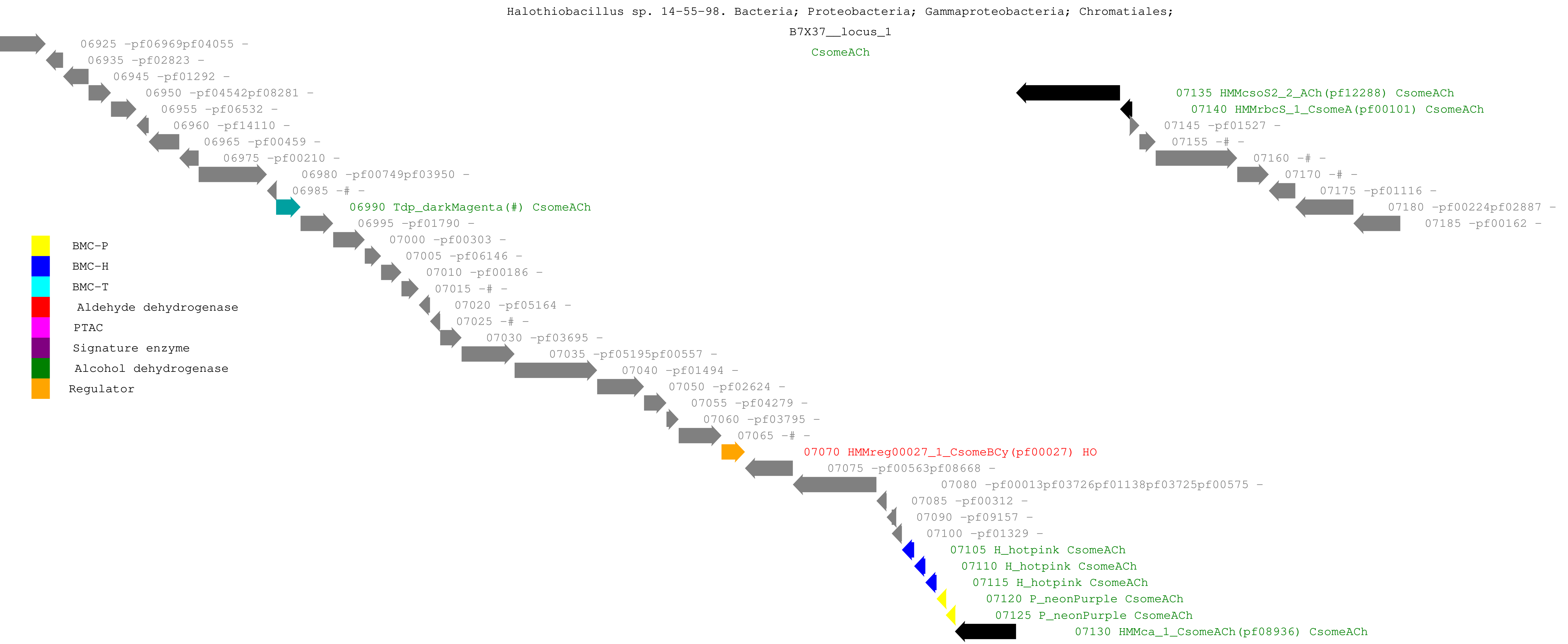
Halothiobacillus sp. 14-55-98. Bacteria; Proteobacteria; Gammaproteobacteria; Chromatiales;
B7X37__locus_1
CsomeACh
06925 -pf06969pf04055 -
06935 -pf02823 -
06945 -pf01292 -
06950 -pf04542pf08281 -
06955 -pf06532 -
06960 -pf14110 -
06965 -pf00459 -
06975 -pf00210 -
06980 -pf00749pf03950 -
06985 -# -
06990 Tdp_darkMagenta(#) CsomeACh
06995 -pf01790 -
07000 -pf00303 -
07005 -pf06146 -
07010 -pf00186 -
07015 -# -
07020 -pf05164 -
07025 -# -
07030 -pf03695 -
07035 -pf05195pf00557 -
07040 -pf01494 -
07050 -pf02624 -
07055 -pf04279 -
07060 -pf03795 -
07065 -# -
07070 HMMreg00027_1_CsomeBCy(pf00027) HO
07075 -pf00563pf08668 -
07080 -pf00013pf03726pf01138pf03725pf00575 -
07085 -pf00312 -
07090 -pf09157 -
07100 -pf01329 -
07105 H_hotpink CsomeACh
07110 H_hotpink CsomeACh
07115 H_hotpink CsomeACh
07120 P_neonPurple CsomeACh
07125 P_neonPurple CsomeACh
07130 HMMca_1_CsomeACh(pf08936) CsomeACh
07135 HMMcsoS2_2_ACh(pf12288) CsomeACh
07140 HMMrbcS_1_CsomeA(pf00101) CsomeACh
07145 -pf01527 -
07155 -# -
07160 -# -
07170 -# -
07175 -pf01116 -
07180 -pf00224pf02887 -
07185 -pf00162 -
BMC-P
BMC-H
BMC-T
Aldehyde dehydrogenase
PTAC
Signature enzyme
Alcohol dehydrogenase
Regulator

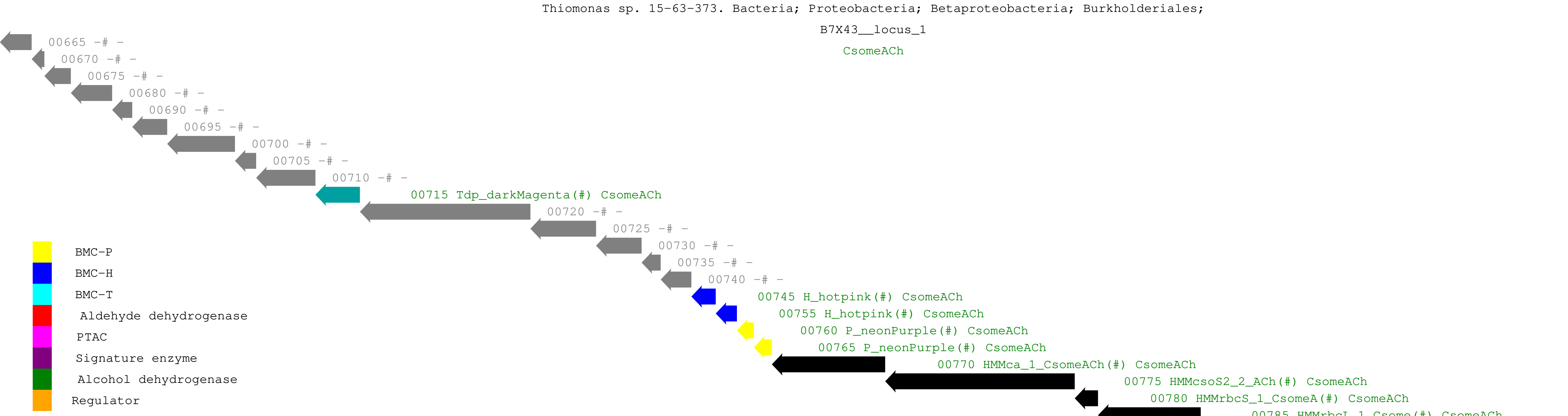
Thiomonas sp. 15-63-373. Bacteria; Proteobacteria; Betaproteobacteria; Burkholderiales;
B7X43__locus_1
CsomeACh
00665 -# -
00670 -# -
00675 -# -
00680 -# -
00690 -# -
00695 -# -
00700 -# -
00705 -# -
00710 -# -
00715 Tdp_darkMagenta(#) CsomeACh
00720 -# -
00725 -# -
00730 -# -
00735 -# -
00740 -# -
00745 H_hotpink(#) CsomeACh
00755 H_hotpink(#) CsomeACh
00760 P_neonPurple(#) CsomeACh
00765 P_neonPurple(#) CsomeACh
00770 HMMca_1_CsomeACh(#) CsomeACh
00775 HMMcsoS2_2_ACh(#) CsomeACh
00780 HMMrbcS_1_CsomeA(#) CsomeACh
00785 HMMrbcL_1_Csome(#) CsomeACh
00790 -# -
00795 -# -
00800 -# -
00805 -# -
00810 -# -
00815 -# -
BMC-P
BMC-H
BMC-T
Aldehyde dehydrogenase
PTAC
Signature enzyme
Alcohol dehydrogenase
Regulator

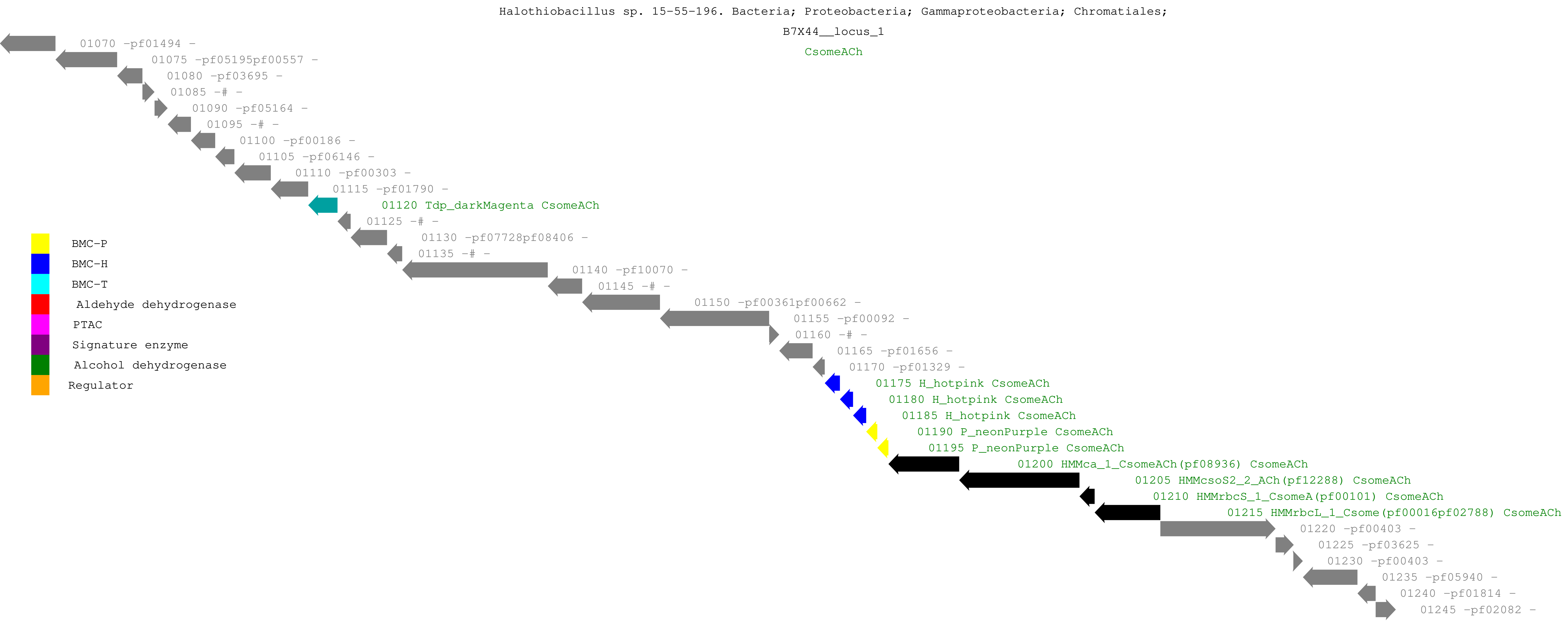
Halothiobacillus sp. 15-55-196. Bacteria; Proteobacteria; Gammaproteobacteria; Chromatiales;
B7X44__locus_1
CsomeACh
01070 -pf01494 -
01075 -pf05195pf00557 -
01080 -pf03695 -
01085 -# -
01090 -pf05164 -
01095 -# -
01100 -pf00186 -
01105 -pf06146 -
01110 -pf00303 -
01115 -pf01790 -
01120 Tdp_darkMagenta CsomeACh
01125 -# -
01130 -pf07728pf08406 -
01135 -# -
01140 -pf10070 -
01145 -# -
01150 -pf00361pf00662 -
01155 -pf00092 -
01160 -# -
01165 -pf01656 -
01170 -pf01329 -
01175 H_hotpink CsomeACh
01180 H_hotpink CsomeACh
01185 H_hotpink CsomeACh
01190 P_neonPurple CsomeACh
01195 P_neonPurple CsomeACh
01200 HMMca_1_CsomeACh(pf08936) CsomeACh
01205 HMMcsoS2_2_ACh(pf12288) CsomeACh
01210 HMMrbcS_1_CsomeA(pf00101) CsomeACh
01215 HMMrbcL_1_Csome(pf00016pf02788) CsomeACh
01220 -pf00403 -
01225 -pf03625 -
01230 -pf00403 -
01235 -pf05940 -
01240 -pf01814 -
01245 -pf02082 -
BMC-P
BMC-H
BMC-T
Aldehyde dehydrogenase
PTAC
Signature enzyme
Alcohol dehydrogenase
Regulator

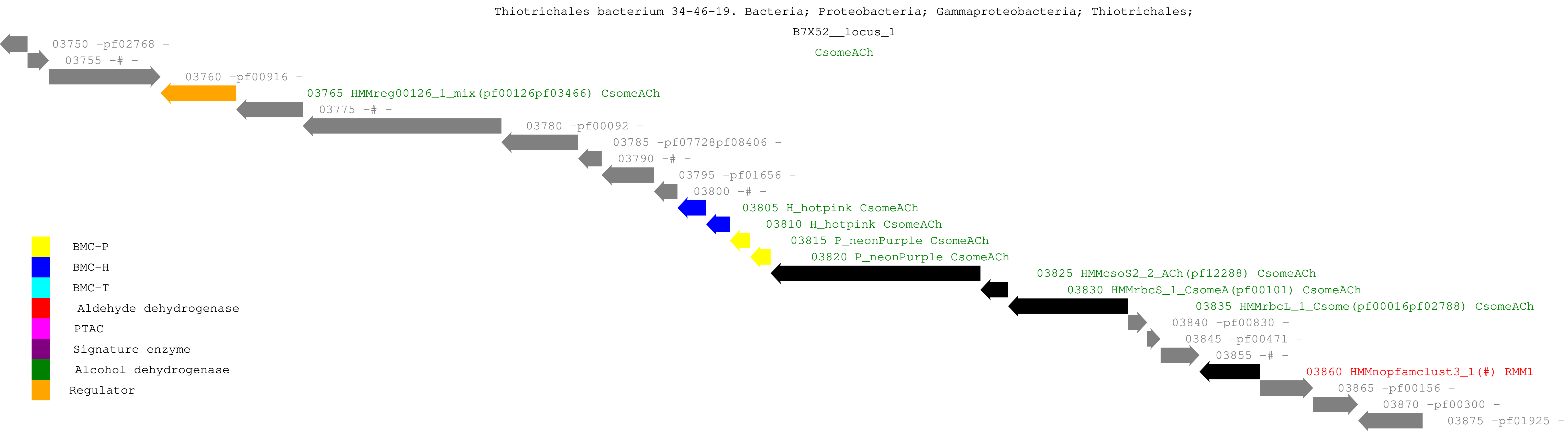
Thiotrichales bacterium 34-46-19. Bacteria; Proteobacteria; Gammaproteobacteria; Thiotrichales;
B7X52__locus_1
CsomeACh
03750 -pf02768 -
03755 -# -
03760 -pf00916 -
03765 HMMreg00126_1_mix(pf00126pf03466) CsomeACh
03775 -# -
03780 -pf00092 -
03785 -pf07728pf08406 -
03790 -# -
03795 -pf01656 -
03800 -# -
03805 H_hotpink CsomeACh
03810 H_hotpink CsomeACh
03815 P_neonPurple CsomeACh
03820 P_neonPurple CsomeACh
03825 HMMcsoS2_2_ACh(pf12288) CsomeACh
03830 HMMrbcS_1_CsomeA(pf00101) CsomeACh
03835 HMMrbcL_1_Csome(pf00016pf02788) CsomeACh
03840 -pf00830 -
03845 -pf00471 -
03855 -# -
03860 HMMnopfamclust3_1(#) RMM1
03865 -pf00156 -
03870 -pf00300 -
03875 -pf01925 -
BMC-P
BMC-H
BMC-T
Aldehyde dehydrogenase
PTAC
Signature enzyme
Alcohol dehydrogenase
Regulator

Hydrogenophilales bacterium 17-61-76. Bacteria; Proteobacteria; Hydrogenophilalia; Hydrogenophilales.

B7X81__locus_1

CsomeACh

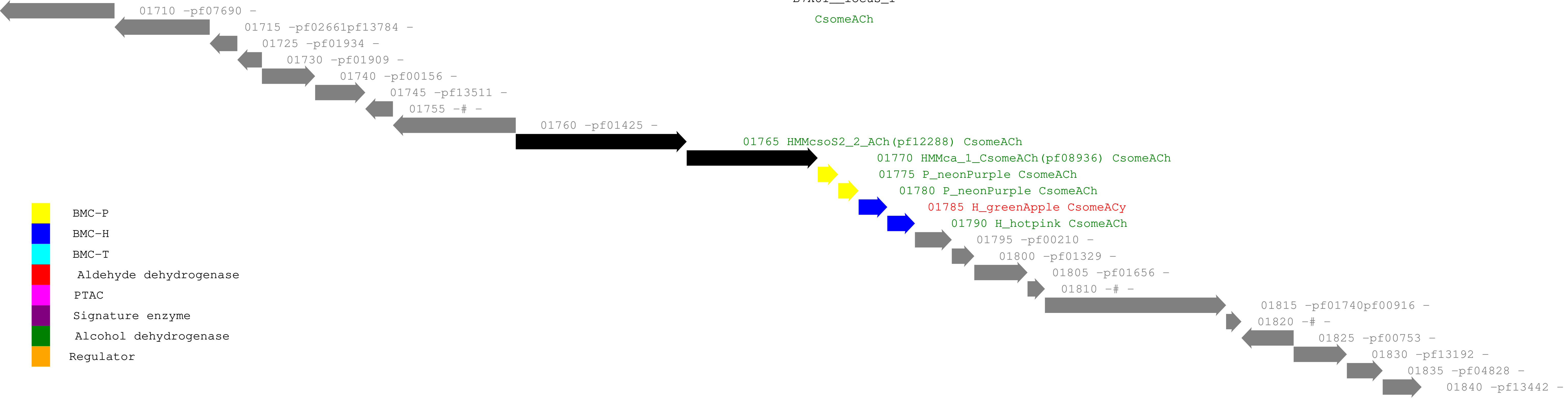

Hydrogenophilales bacterium 17-61-76. Bacteria; Proteobacteria; Hydrogenophilalia; Hydrogenophilales.

B7X81__locus_2

CsomeACh

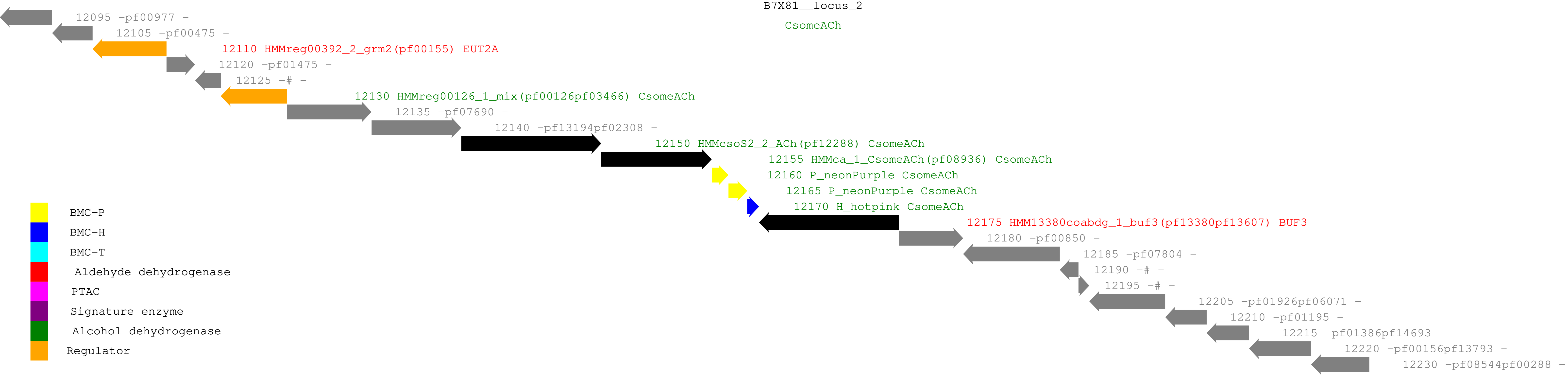

Hydrogenophilales bacterium 17-64-34. Bacteria; Proteobacteria; Hydrogenophilalia; Hydrogenophilales.

B7X87__locus_1

CsomeACh

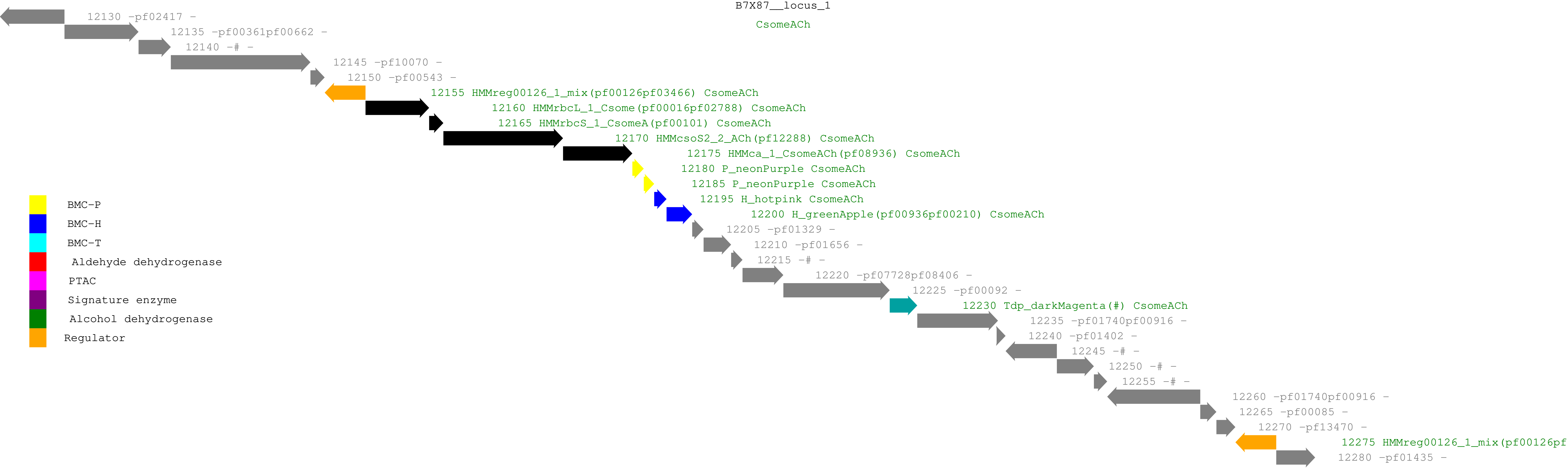

Hydrogenophilales bacterium 17-64-11. Bacteria; Proteobacteria; Hydrogenophilalia; Hydrogenophilales.

B7X91__locus_1

CsomeACh

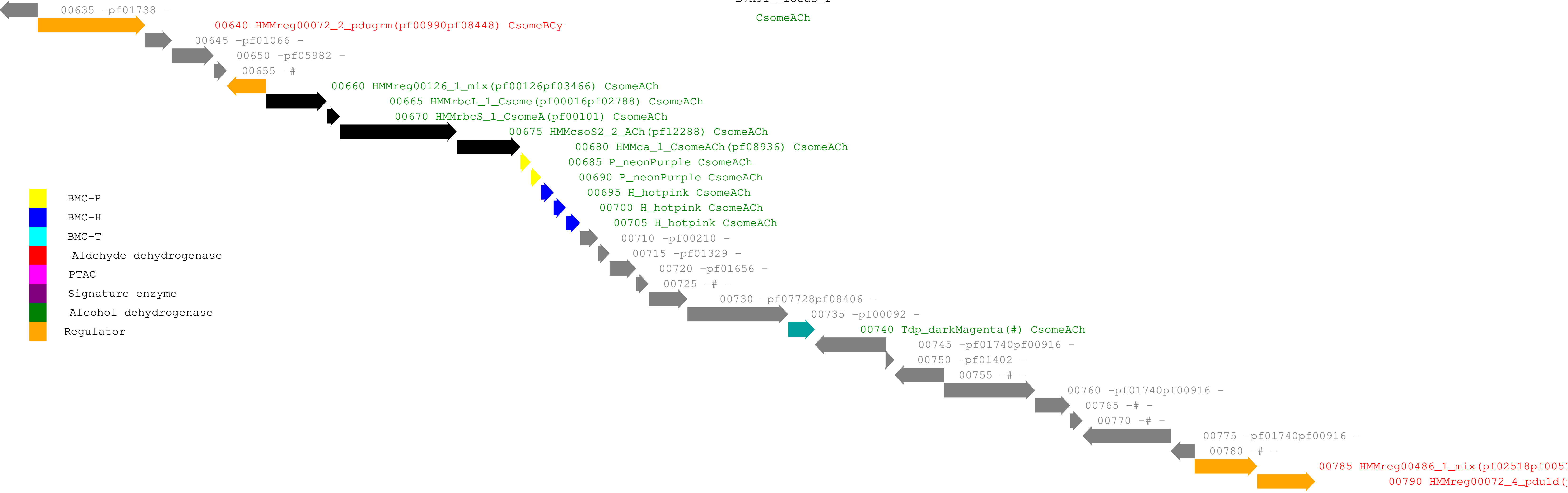

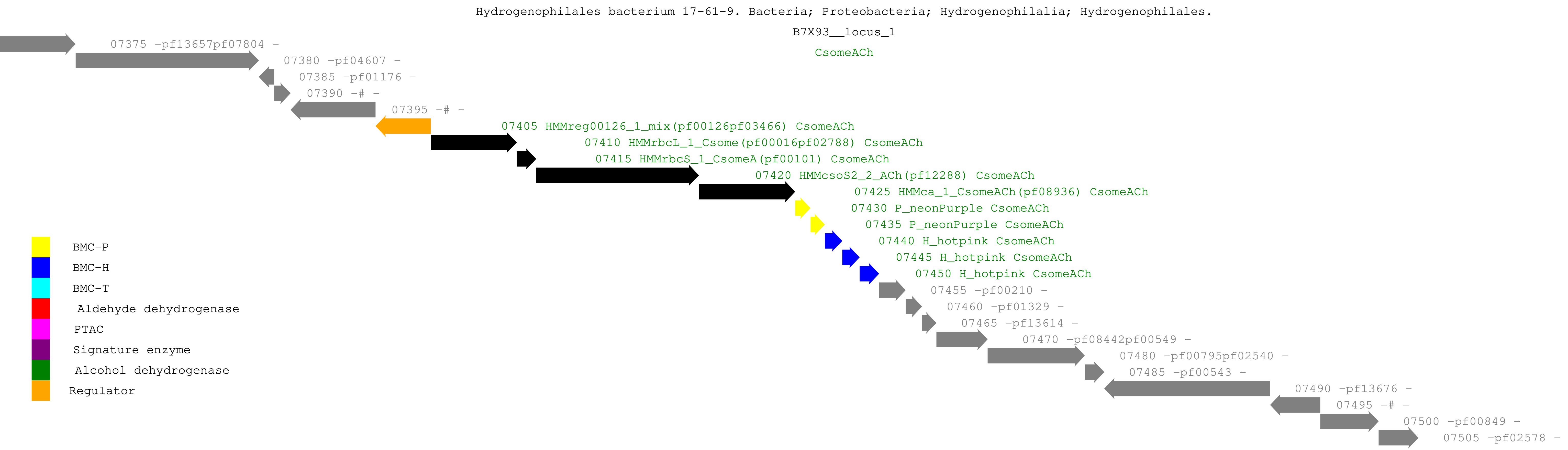
Hydrogenophilales bacterium 17-61-9. Bacteria; Proteobacteria; Hydrogenophilalia; Hydrogenophilales.
B7X93__locus_1
CsomeACh
07375 -pf13657pf07804 -
07380 -pf04607 -
07385 -pf01176 -
07390 -# -
07395 -# -
07405 HMMreg00126_1_mix(pf00126pf03466) CsomeACh
07410 HMMrbcL_1_Csome(pf00016pf02788) CsomeACh
07415 HMMrbcS_1_CsomeA(pf00101) CsomeACh
07420 HMMcsoS2_2_ACh(pf12288) CsomeACh
07425 HMMca_1_CsomeACh(pf08936) CsomeACh
07430 P_neonPurple CsomeACh
07435 P_neonPurple CsomeACh
07440 H_hotpink CsomeACh
07445 H_hotpink CsomeACh
07450 H_hotpink CsomeACh
07455 -pf00210 -
07460 -pf01329 -
07465 -pf13614 -
07470 -pf08442pf00549 -
07480 -pf00795pf02540 -
07485 -pf00543 -
07490 -pf13676 -
07495 -# -
07500 -pf00849 -
07505 -pf02578 -
BMC-P
BMC-H
BMC-T
Aldehyde dehydrogenase
PTAC
Signature enzyme
Alcohol dehydrogenase
Regulator

Halothiobacillus sp. 24-54-40. Bacteria; Proteobacteria; Gammaproteobacteria; Chromatiales;

B7Y07__locus_1

CsomeACh

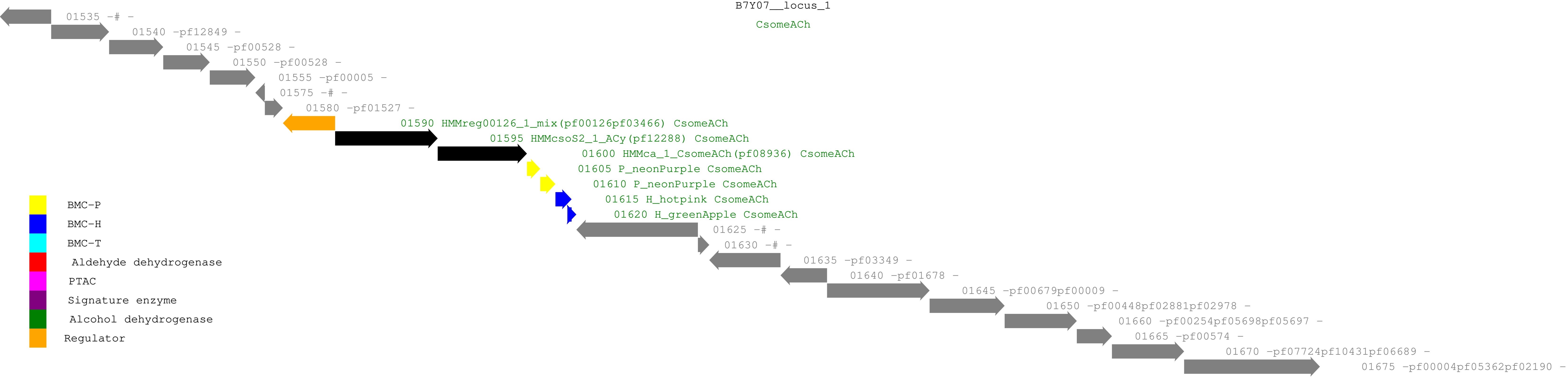

Hydrogenophilales bacterium 16-61-112. Bacteria; Proteobacteria; Hydrogenophilalia; Hydrogenophilales.

B7Y21__locus_1

CsomeACh

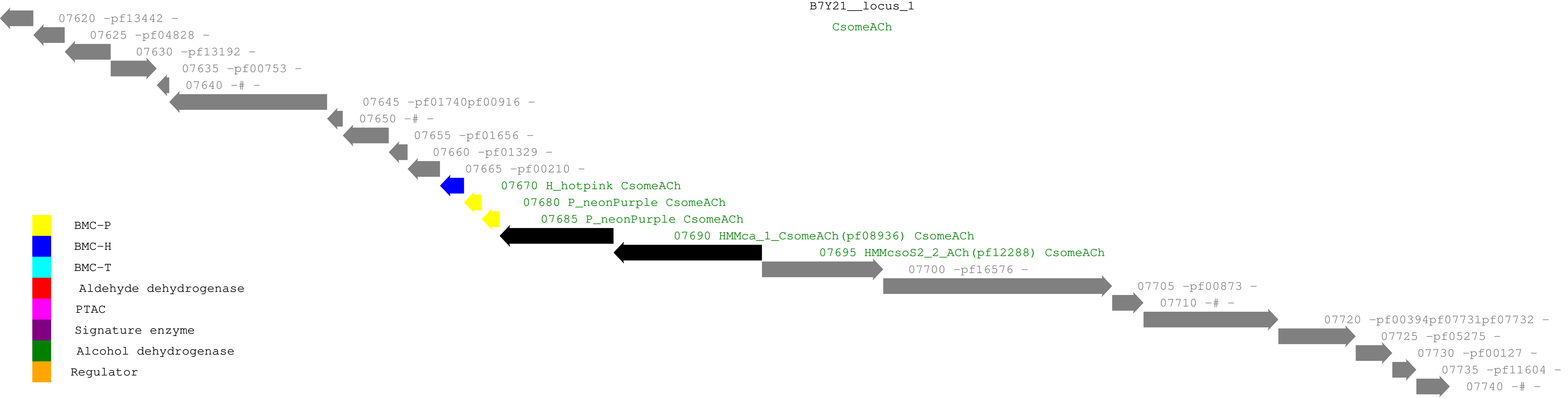

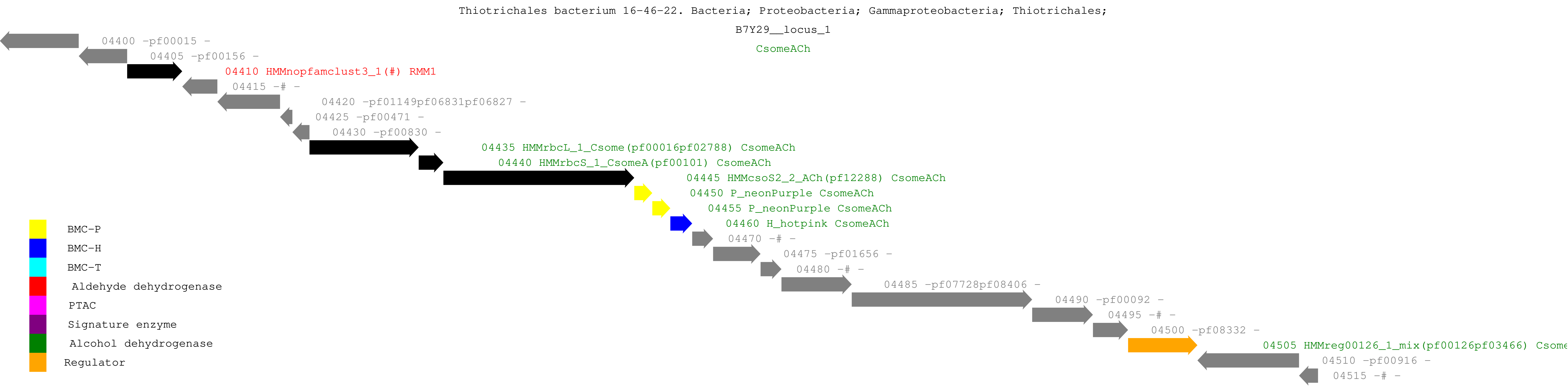
Thiotrichales bacterium 16-46-22. Bacteria; Proteobacteria; Gammaproteobacteria; Thiotrichales;
B7Y29__locus_1
CsomeACh
04400 -pf00015 -
04405 -pf00156 -
04410 HMMnopfamclust3_1(#) RMM1
04415 -# -
04420 -pf01149pf06831pf06827 -
04425 -pf00471 -
04430 -pf00830 -
04435 HMMrbcL_1_Csome(pf00016pf02788) CsomeACh
04440 HMMrbcS_1_CsomeA(pf00101) CsomeACh
04445 HMMcsoS2_2_ACh(pf12288) CsomeACh
04450 P_neonPurple CsomeACh
04455 P_neonPurple CsomeACh
04460 H_hotpink CsomeACh
04470 -# -
04475 -pf01656 -
04480 -# -
04485 -pf07728pf08406 -
04490 -pf00092 -
04495 -# -
04500 -pf08332 -
04505 HMMreg00126_1_mix(pf00126pf03466) Csome
04510 -pf00916 -
04515 -# -
BMC-P
BMC-H
BMC-T
Aldehyde dehydrogenase
PTAC
Signature enzyme
Alcohol dehydrogenase
Regulator

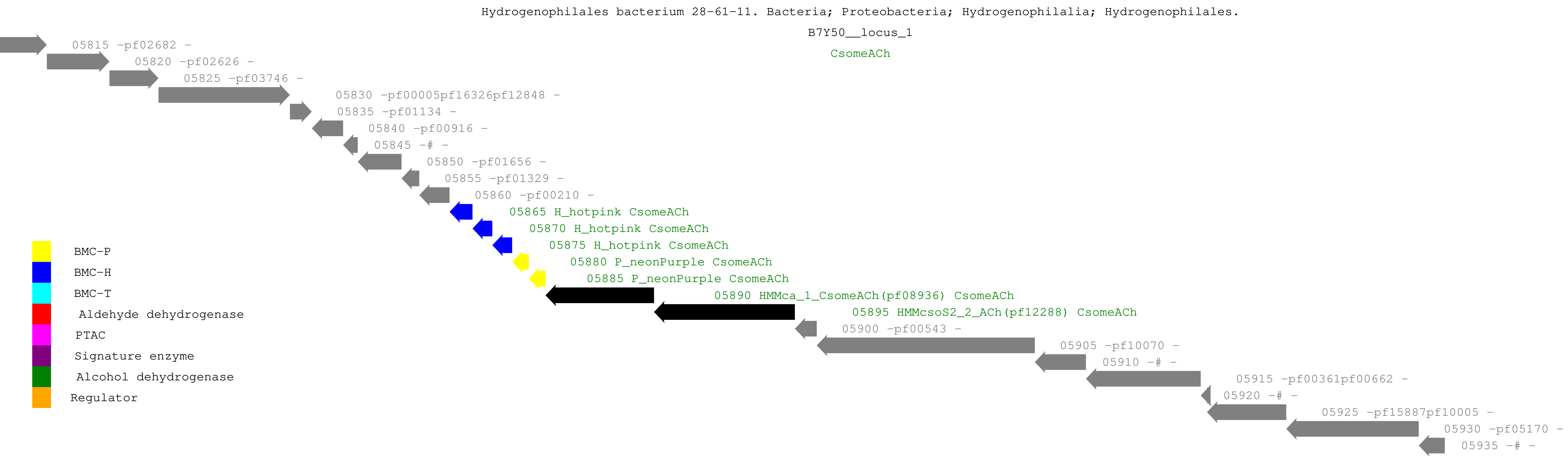

Hydrogenophilales bacterium 28-61-11. Bacteria; Proteobacteria; Hydrogenophilalia; Hydrogenophilales.
B7Y50__locus_1
CsomeACh
05815 -pf02682 -
05820 -pf02626 -
05825 -pf03746 -
05830 -pf00005pf16326pf12848 -
05835 -pf01134 -
05840 -pf00916 -
05845 -# -
05850 -pf01656 -
05855 -pf01329 -
05860 -pf00210 -
05865 H_hotpink CsomeACh
05870 H_hotpink CsomeACh
05875 H_hotpink CsomeACh
05880 P_neonPurple CsomeACh
05885 P_neonPurple CsomeACh
05890 HMMca_1_CsomeACh(pf08936) CsomeACh
05895 HMMcsoS2_2_ACh(pf12288) CsomeACh
05900 -pf00543 -
05905 -pf10070 -
05910 -# -
05915 -pf00361pf00662 -
05920 -# -
05925 -pf15887pf10005 -
05930 -pf05170 -
05935 -# -
BMC-P
BMC-H
BMC-T
Aldehyde dehydrogenase
PTAC
Signature enzyme
Alcohol dehydrogenase
Regulator

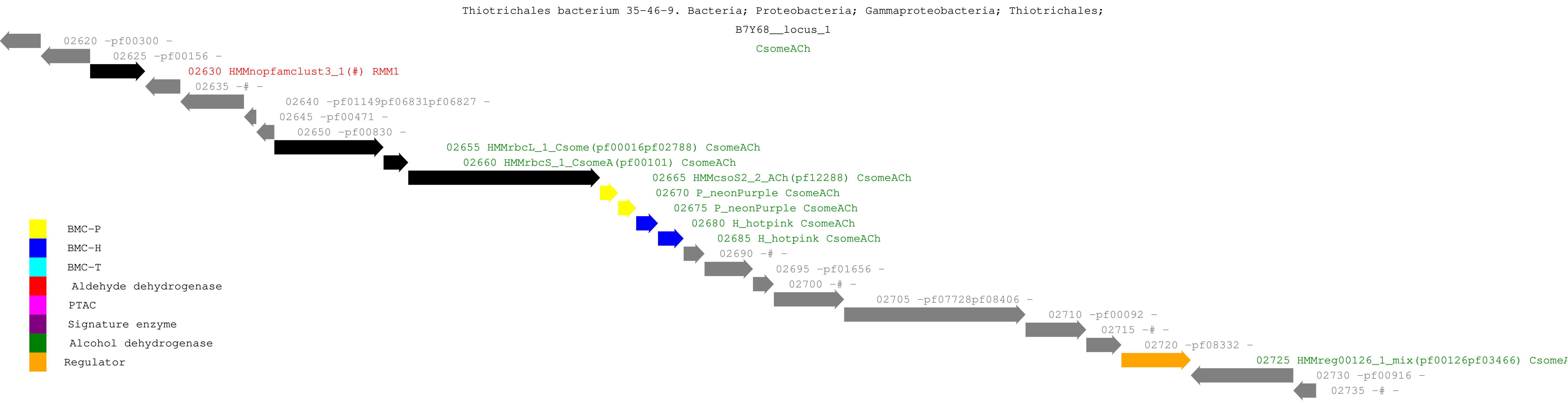
Thiotrichales bacterium 35-46-9. Bacteria; Proteobacteria; Gammaproteobacteria; Thiotrichales;
B7Y68__locus_1
CsomeACh
02620 -pf00300 -
02625 -pf00156 -
02630 HMMnopfamclust3_1(#) RMM1
02635 -# -
02640 -pf01149pf06831pf06827 -
02645 -pf00471 -
02650 -pf00830 -
02655 HMMrbcL_1_Csome(pf00016pf02788) CsomeACh
02660 HMMrbcS_1_CsomeA(pf00101) CsomeACh
02665 HMMcsoS2_2_ACh(pf12288) CsomeACh
02670 P_neonPurple CsomeACh
02675 P_neonPurple CsomeACh
02680 H_hotpink CsomeACh
02685 H_hotpink CsomeACh
02690 -# -
02695 -pf01656 -
02700 -# -
02705 -pf07728pf08406 -
02710 -pf00092 -
02715 -# -
02720 -pf08332 -
02725 HMMreg00126_1_mix(pf00126pf03466) CsomeA
02730 -pf00916 -
02735 -# -
BMC-P
BMC-H
BMC-T
Aldehyde dehydrogenase
PTAC
Signature enzyme
Alcohol dehydrogenase
Regulator

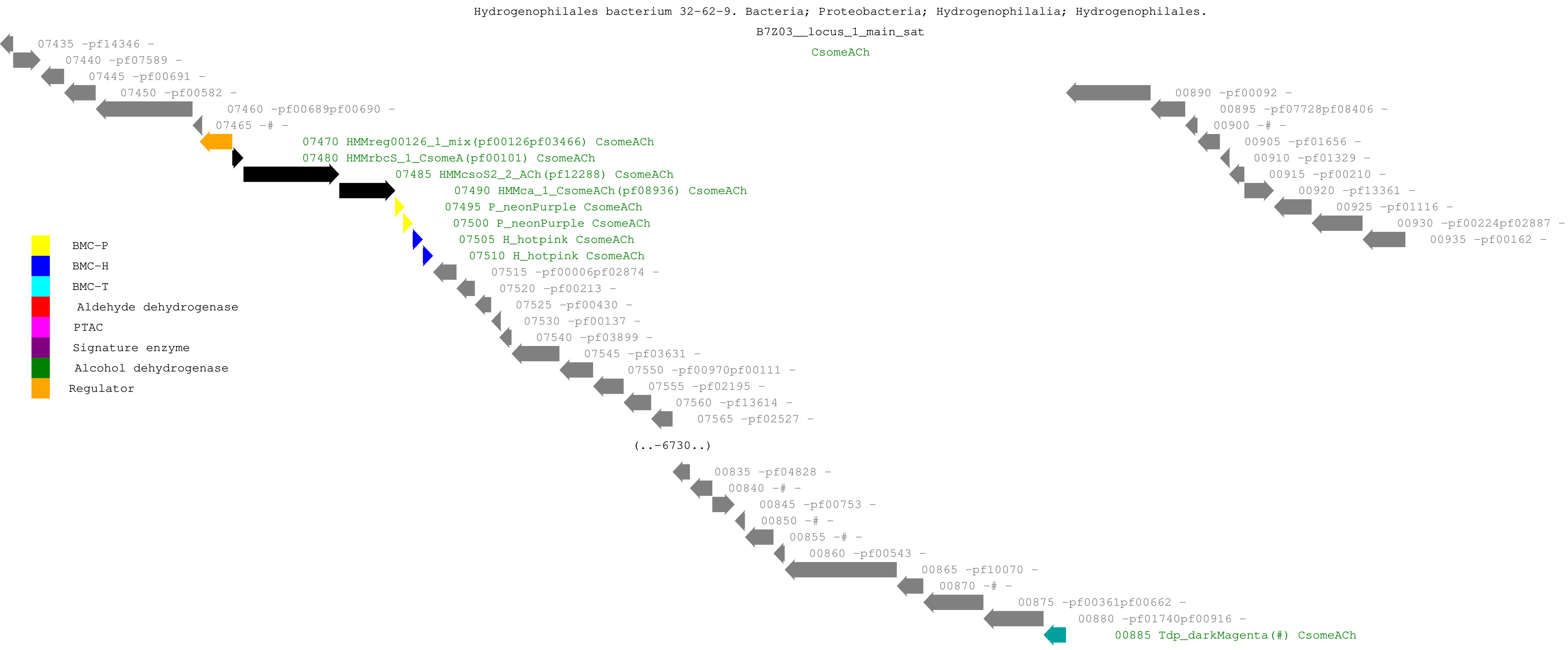
Hydrogenophilales bacterium 32-62-9. Bacteria; Proteobacteria; Hydrogenophilalia; Hydrogenophilales.
B7Z03__locus_1_main_sat
CsomeACh
07435 -pf14346 -
07440 -pf07589 -
07445 -pf00691 -
07450 -pf00582 -
07460 -pf00689pf00690 -
07465 -# -
07470 HMMreg00126_1_mix(pf00126pf03466) CsomeACh
07480 HMMrbcS_1_CsomeA(pf00101) CsomeACh
07485 HMMcsoS2_2_ACh(pf12288) CsomeACh
07490 HMMca_1_CsomeACh(pf08936) CsomeACh
07495 P_neonPurple CsomeACh
07500 P_neonPurple CsomeACh
07505 H_hotpink CsomeACh
07510 H_hotpink CsomeACh
07515 -pf00006pf02874 -
07520 -pf00213 -
07525 -pf00430 -
07530 -pf00137 -
07540 -pf03899 -
07545 -pf03631 -
07550 -pf00970pf00111 -
07555 -pf02195 -
07560 -pf13614 -
07565 -pf02527 -
(..-6730..)
00835 -pf04828 -
00840 -# -
00845 -pf00753 -
00850 -# -
00855 -# -
00860 -pf00543 -
00865 -pf10070 -
00870 -# -
00875 -pf00361pf00662 -
00880 -pf01740pf00916 -
00885 Tdp_darkMagenta(#) CsomeACh
00890 -pf00092 -
00895 -pf07728pf08406 -
00900 -# -
00905 -pf01656 -
00910 -pf01329 -
00915 -pf00210 -
00920 -pf13361 -
00925 -pf01116 -
00930 -pf00224pf02887 -
00935 -pf00162 -
BMC-P
BMC-H
BMC-T
Aldehyde dehydrogenase
PTAC
Signature enzyme
Alcohol dehydrogenase
Regulator

Thiotrichales bacterium 32-46-8. Bacteria; Proteobacteria; Gammaproteobacteria; Thiotrichales;

B7Z05__locus_1

CsomeACh

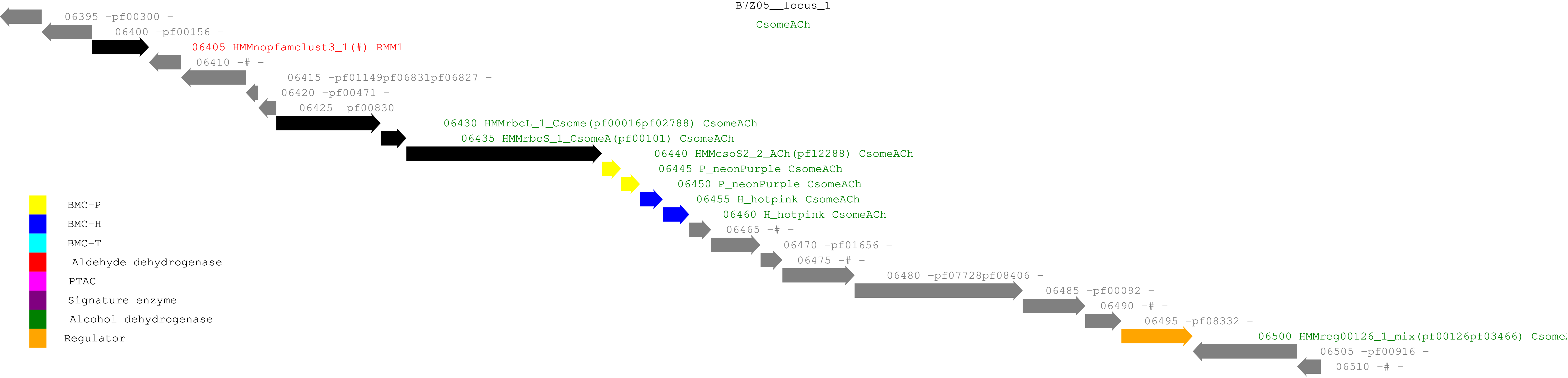

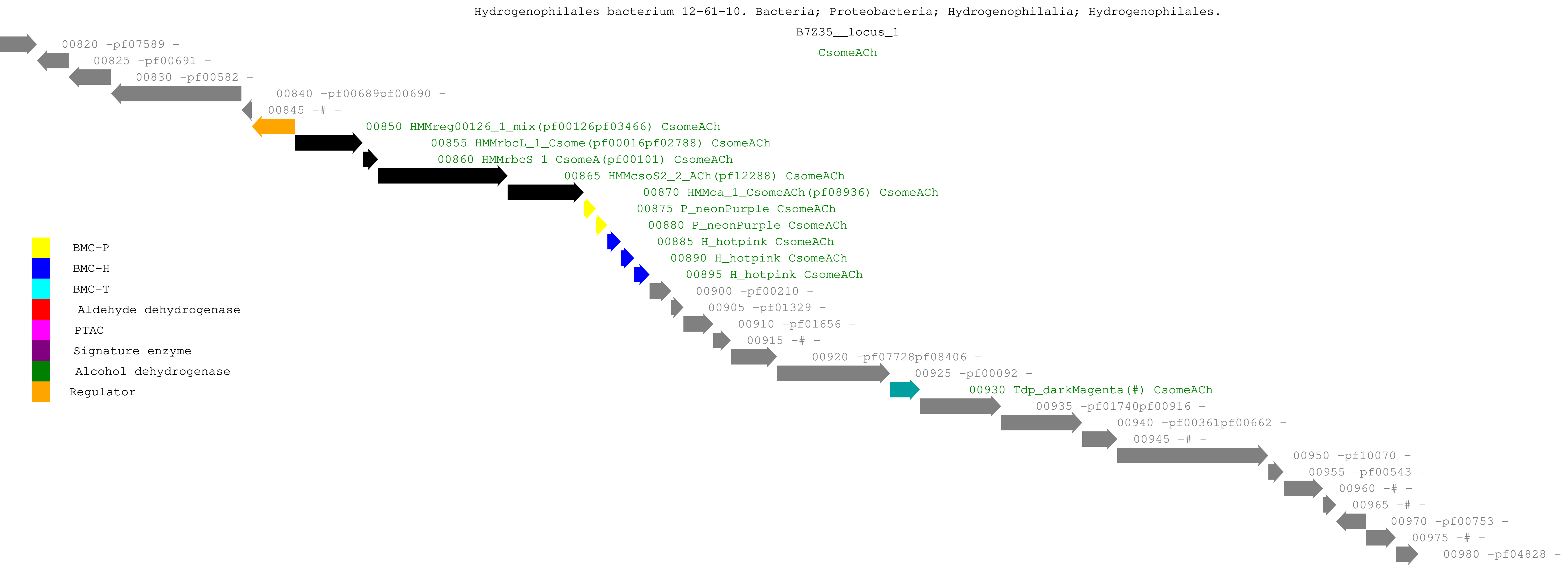

Hydrogenophilales bacterium 12-61-10. Bacteria; Proteobacteria; Hydrogenophilalia; Hydrogenophilales.
B7Z35__locus_1
CsomeACh
00820 -pf07589 -
00825 -pf00691 -
00830 -pf00582 -
00840 -pf00689pf00690 -
00845 -# -
00850 HMMreg00126_1_mix(pf00126pf03466) CsomeACh
00855 HMMrbcL_1_Csome(pf00016pf02788) CsomeACh
00860 HMMrbcS_1_CsomeA(pf00101) CsomeACh
00865 HMMcsoS2_2_ACh(pf12288) CsomeACh
00870 HMMca_1_CsomeACh(pf08936) CsomeACh
00875 P_neonPurple CsomeACh
00880 P_neonPurple CsomeACh
00885 H_hotpink CsomeACh
00890 H_hotpink CsomeACh
00895 H_hotpink CsomeACh
00900 -pf00210 -
00905 -pf01329 -
00910 -pf01656 -
00915 -# -
00920 -pf07728pf08406 -
00925 -pf00092 -
00930 Tdp_darkMagenta(#) CsomeACh
00935 -pf01740pf00916 -
00940 -pf00361pf00662 -
00945 -# -
00950 -pf10070 -
00955 -pf00543 -
00960 -# -
00965 -# -
00970 -pf00753 -
00975 -# -
00980 -pf04828 -
BMC-P
BMC-H
BMC-T
Aldehyde dehydrogenase
PTAC
Signature enzyme
Alcohol dehydrogenase
Regulator

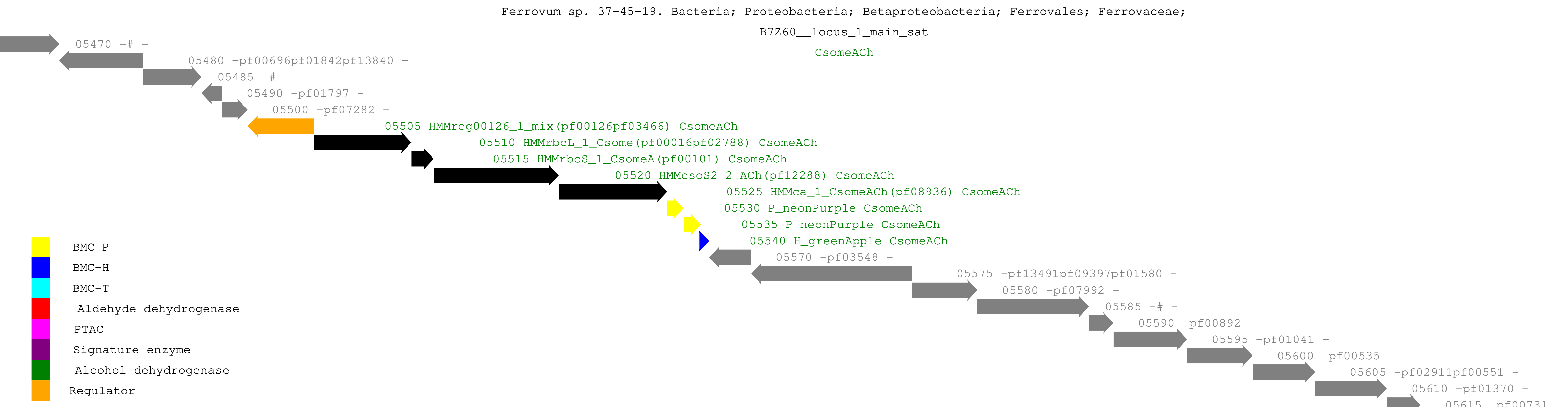
Ferrovum sp. 37-45-19. Bacteria; Proteobacteria; Betaproteobacteria; Ferrovales; Ferrovaceae;
B7Z60__locus_1_main_sat
CsomeACh
05470 -# -
05480 -pf00696pf01842pf13840 -
05485 -# -
05490 -pf01797 -
05500 -pf07282 -
05505 HMMreg00126_1_mix(pf00126pf03466) CsomeACh
05510 HMMrbcL_1_Csome(pf00016pf02788) CsomeACh
05515 HMMrbcS_1_CsomeA(pf00101) CsomeACh
05520 HMMcsoS2_2_ACh(pf12288) CsomeACh
05525 HMMca_1_CsomeACh(pf08936) CsomeACh
05530 P_neonPurple CsomeACh
05535 P_neonPurple CsomeACh
05540 H_greenApple CsomeACh
05570 -pf03548 -
05575 -pf13491pf09397pf01580 -
05580 -pf07992 -
05585 -# -
05590 -pf00892 -
05595 -pf01041 -
05600 -pf00535 -
05605 -pf02911pf00551 -
05610 -pf01370 -
05615 -pf00731 -
BMC-P
BMC-H
BMC-T
Aldehyde dehydrogenase
PTAC
Signature enzyme
Alcohol dehydrogenase
Regulator

Chromatiales bacterium 21-64-14. Bacteria; Proteobacteria; Gammaproteobacteria; Chromatiales;

B7Z66__locus_1

CsomeACh

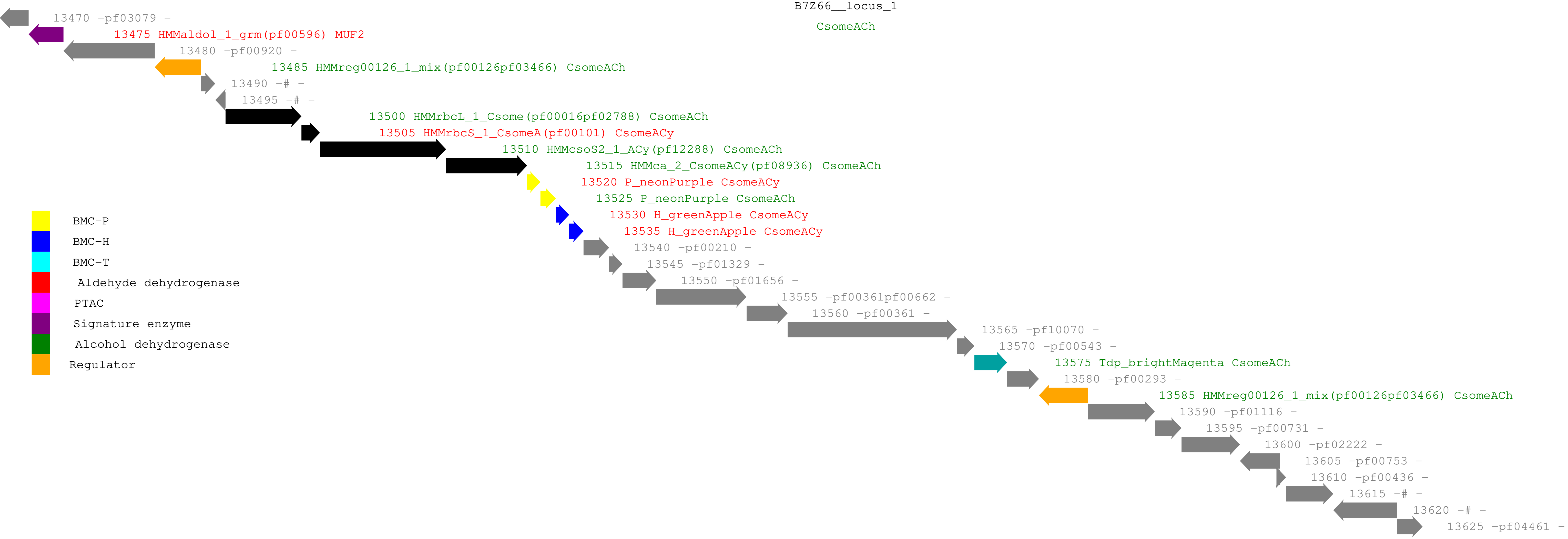

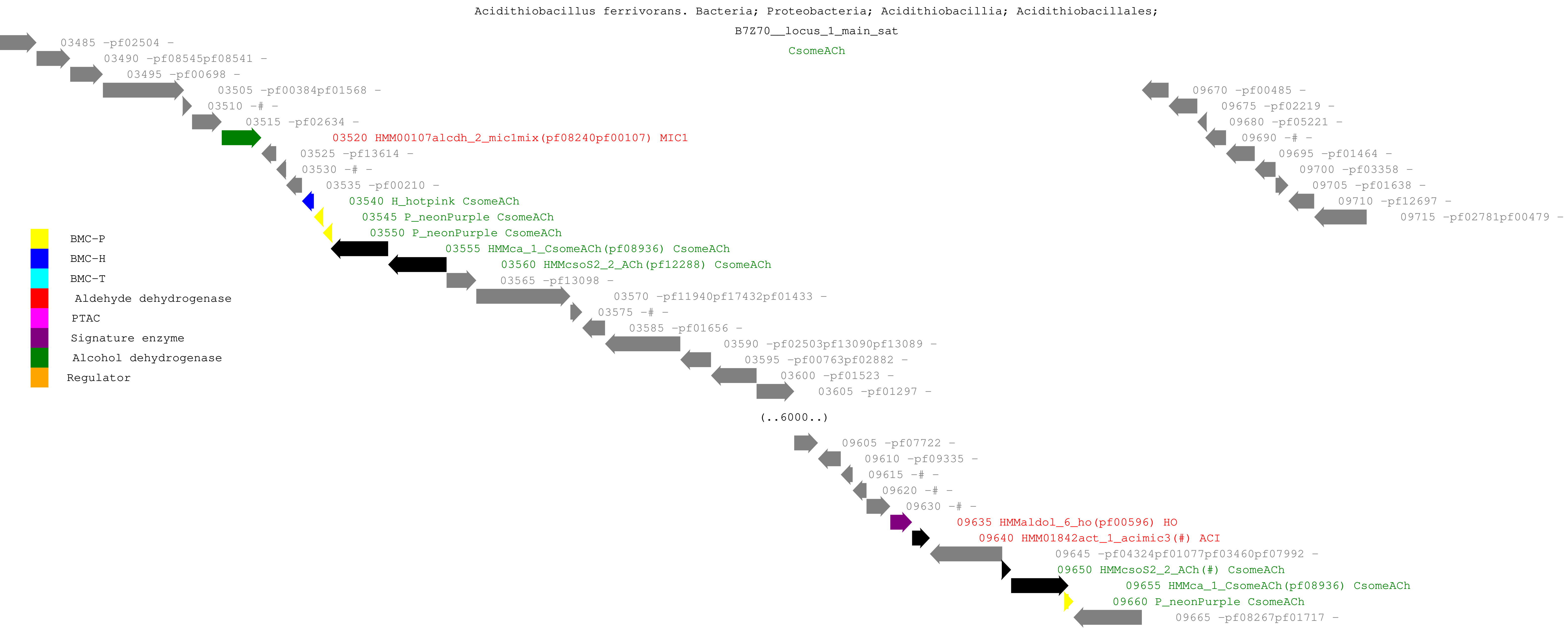

Acidithiobacillus ferrivorans. Bacteria; Proteobacteria; Acidithiobacillia; Acidithiobacillales;
B7Z70__locus_1_main_sat
CsomeACh
03485 -pf02504 -
03490 -pf08545pf08541 -
03495 -pf00698 -
03505 -pf00384pf01568 -
03510 -# -
03515 -pf02634 -
03520 HMM00107alcdh_2_mic1mix(pf08240pf00107) MIC1
03525 -pf13614 -
03530 -# -
03535 -pf00210 -
03540 H_hotpink CsomeACh
03545 P_neonPurple CsomeACh
03550 P_neonPurple CsomeACh
03555 HMMca_1_CsomeACh(pf08936) CsomeACh
03560 HMMcsoS2_2_ACh(pf12288) CsomeACh
03565 -pf13098 -
03570 -pf11940pf17432pf01433 -
03575 -# -
03585 -pf01656 -
03590 -pf02503pf13090pf13089 -
03595 -pf00763pf02882 -
03600 -pf01523 -
03605 -pf01297 -
(..6000..)
09605 -pf07722 -
09610 -pf09335 -
09615 -# -
09620 -# -
09630 -# -
09635 HMMaldol_6_ho(pf00596) HO
09640 HMM01842act_1_acimic3(#) ACI
09645 -pf04324pf01077pf03460pf07992 -
09650 HMMcsoS2_2_ACh(#) CsomeACh
09655 HMMca_1_CsomeACh(pf08936) CsomeACh
09660 P_neonPurple CsomeACh
09665 -pf08267pf01717 -
09670 -pf00485 -
09675 -pf02219 -
09680 -pf05221 -
09690 -# -
09695 -pf01464 -
09700 -pf03358 -
09705 -pf01638 -
09710 -pf12697 -
09715 -pf02781pf00479 -
BMC-P
BMC-H
BMC-T
Aldehyde dehydrogenase
PTAC
Signature enzyme
Alcohol dehydrogenase
Regulator

Rhodospirillales bacterium 20-60-12. Bacteria; Proteobacteria; Alphaproteobacteria; Rhodospirillales;

B7Z78__locus_1

CsomeACh

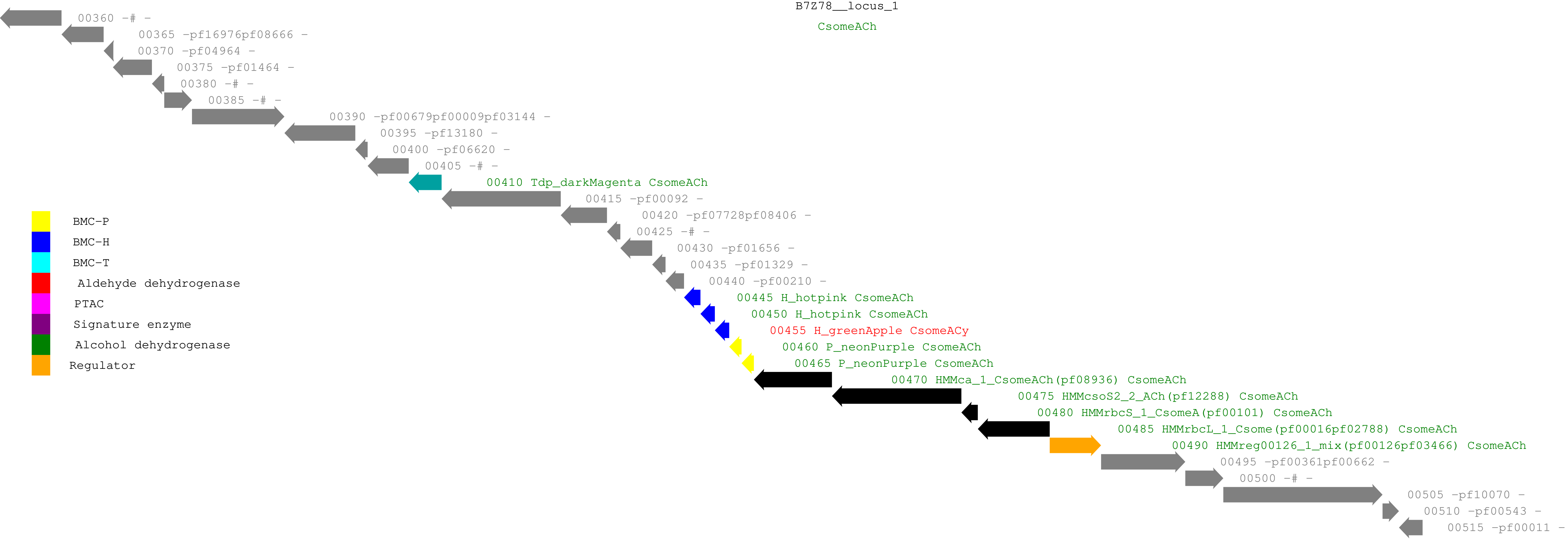

Thiomonas sp. 20-64-5. Bacteria; Proteobacteria; Betaproteobacteria; Burkholderiales;

B7Z83__locus_fragmented

CsomeACh

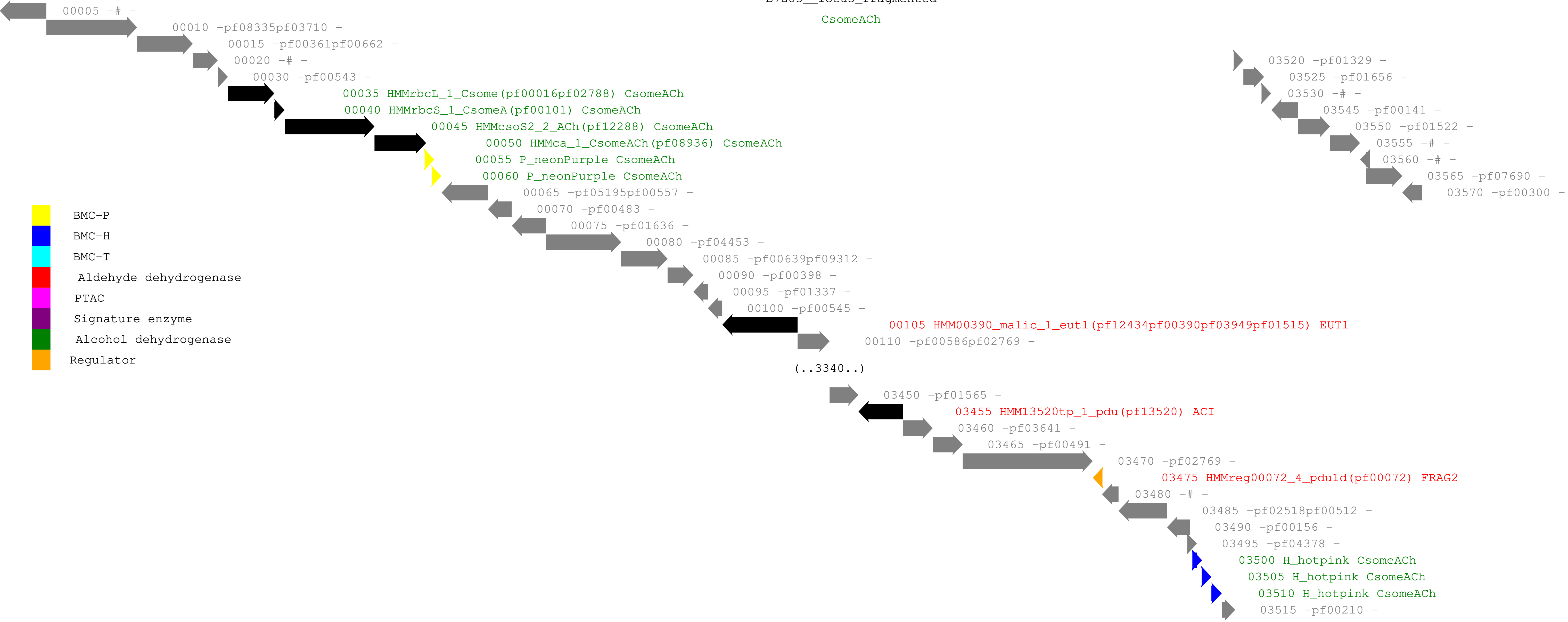

Acidithiobacillus caldus. Bacteria; Proteobacteria; Acidithiobacillia; Acidithiobacillales;

BAE27__locus_fragmented

CsomeACh

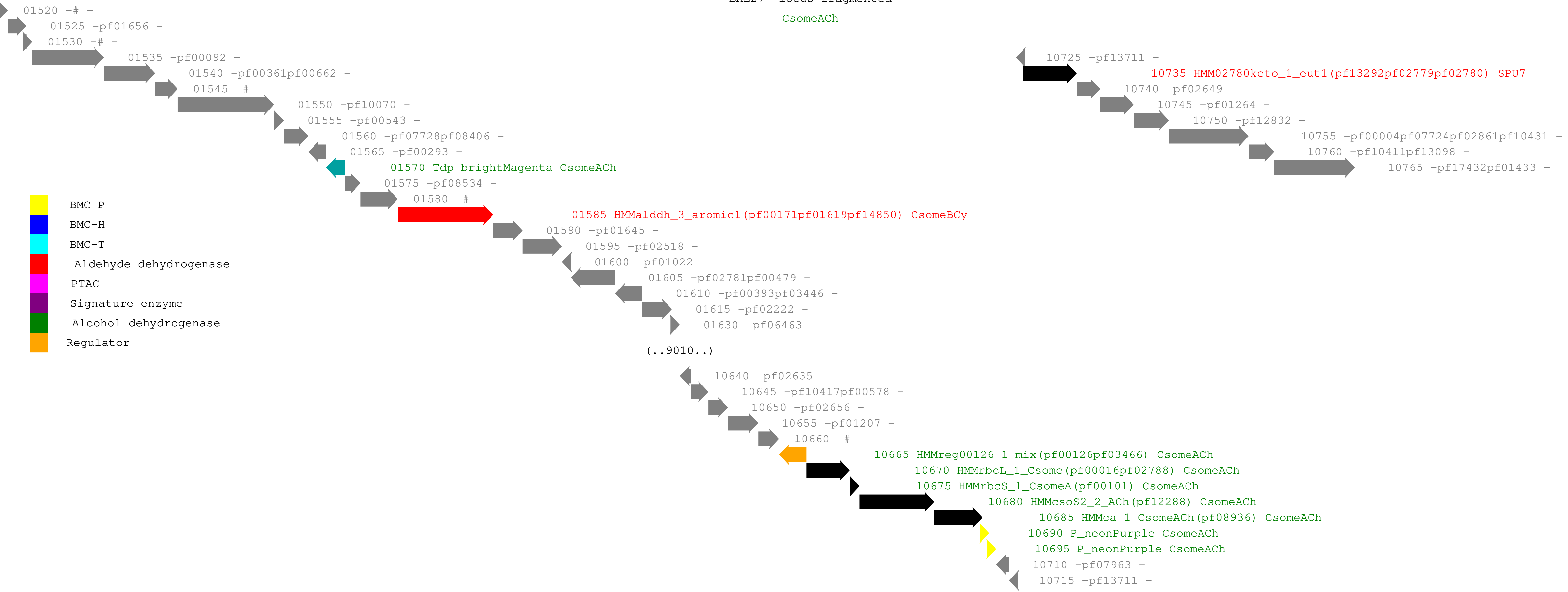

Bradyrhizobium sp. (strain BTAi1 / ATCC BAA-1182). Bacteria; Proteobacteria; Alphaproteobacteria; Rhizobiales;

BBta__locus_1

CsomeACh

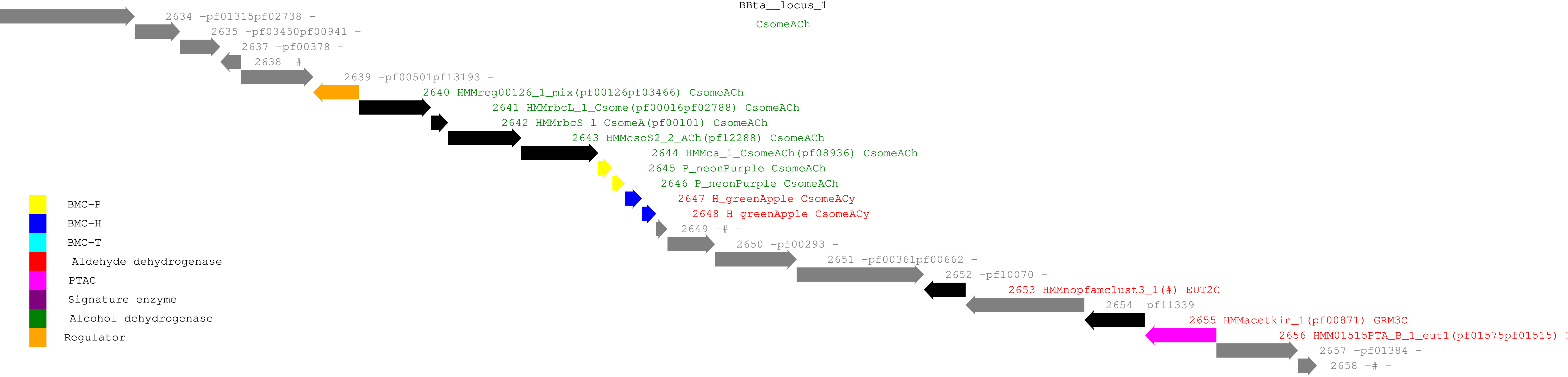

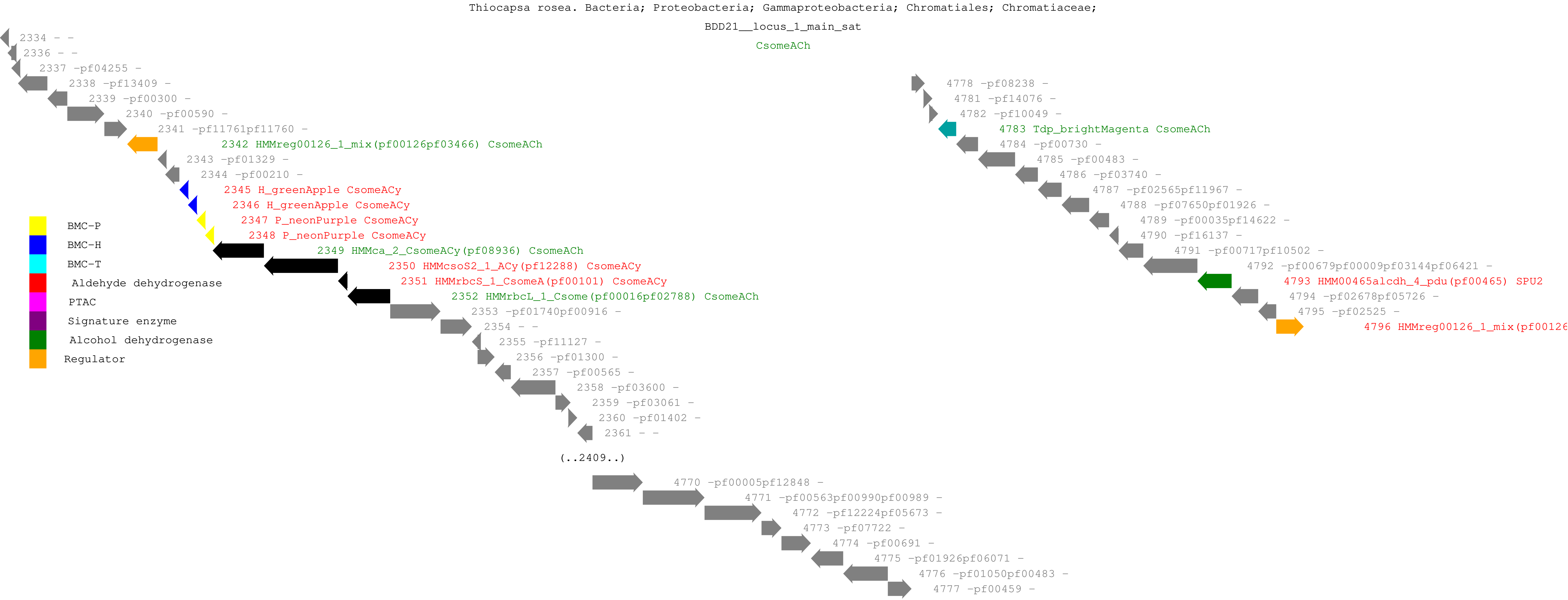

Thiocapsa rosea. Bacteria; Proteobacteria; Gammaproteobacteria; Chromatiales; Chromatiaceae;
BDD21__locus_1_main_sat
CsomeACh
2334 - -
2336 - -
2337 -pf04255 -
2338 -pf13409 -
2339 -pf00300 -
2340 -pf00590 -
2341 -pf11761pf11760 -
2342 HMMreg00126_1_mix(pf00126pf03466) CsomeACh
2343 -pf01329 -
2344 -pf00210 -
2345 H_greenApple CsomeACy
2346 H_greenApple CsomeACy
2347 P_neonPurple CsomeACy
2348 P_neonPurple CsomeACy
2349 HMMca_2_CsomeACy(pf08936) CsomeACh
2350 HMMcsoS2_1_ACy(pf12288) CsomeACy
2351 HMMrbcS_1_CsomeA(pf00101) CsomeACy
2352 HMMrbcL_1_Csome(pf00016pf02788) CsomeACh
2353 -pf01740pf00916 -
2354 - -
2355 -pf11127 -
2356 -pf01300 -
2357 -pf00565 -
2358 -pf03600 -
2359 -pf03061 -
2360 -pf01402 -
2361 - -
(..2409..)
4770 -pf00005pf12848 -
4771 -pf00563pf00990pf00989 -
4772 -pf12224pf05673 -
4773 -pf07722 -
4774 -pf00691 -
4775 -pf01926pf06071 -
4776 -pf01050pf00483 -
4777 -pf00459 -
4778 -pf08238 -
4781 -pf14076 -
4782 -pf10049 -
4783 Tdp_brightMagenta CsomeACh
4784 -pf00730 -
4785 -pf00483 -
4786 -pf03740 -
4787 -pf02565pf11967 -
4788 -pf07650pf01926 -
4789 -pf00035pf14622 -
4790 -pf16137 -
4791 -pf00717pf10502 -
4792 -pf00679pf00009pf03144pf06421 -
4793 HMM00465alcdh_4_pdu(pf00465) SPU2
4794 -pf02678pf05726 -
4795 -pf02525 -
4796 HMMreg00126_1_mix(pf00126
BMC-P
BMC-H
BMC-T
Aldehyde dehydrogenase
PTAC
Signature enzyme
Alcohol dehydrogenase
Regulator

Nitrobacter sp. 62-13. Bacteria; Proteobacteria; Alphaproteobacteria; Rhizobiales;

BGN91__locus_fragmented

CsomeACh

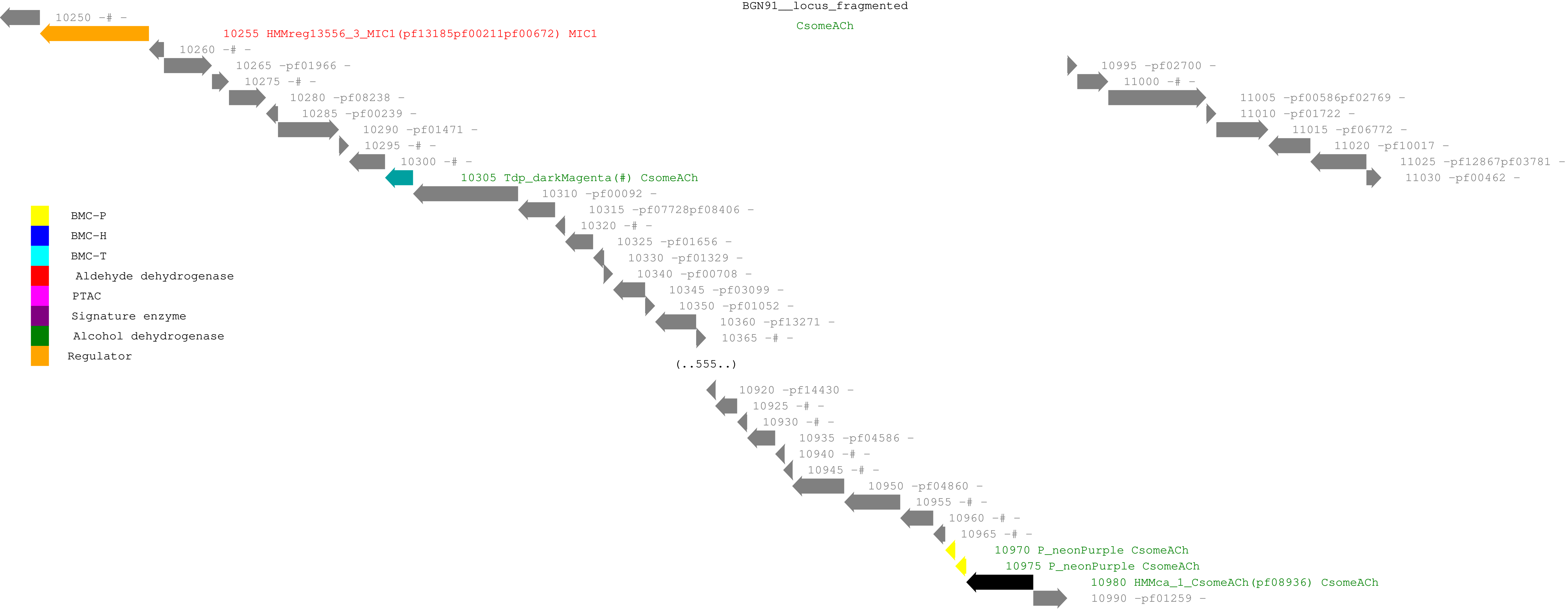

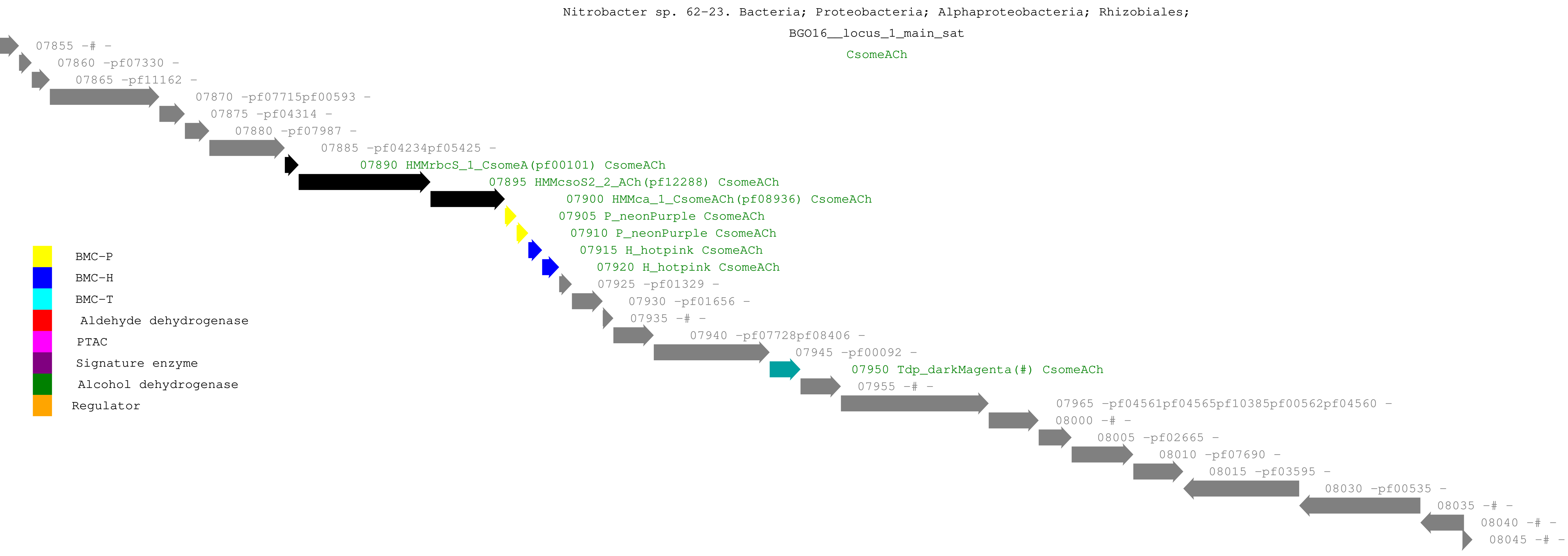

Nitrobacter sp. 62-23. Bacteria; Proteobacteria; Alphaproteobacteria; Rhizobiales;
BGO16__locus_1_main_sat
CsomeACh
07855 -# -
07860 -pf07330 -
07865 -pf11162 -
07870 -pf07715pf00593 -
07875 -pf04314 -
07880 -pf07987 -
07885 -pf04234pf05425 -
07890 HMMrbcS_1_CsomeA(pf00101) CsomeACh
07895 HMMcsoS2_2_ACh(pf12288) CsomeACh
07900 HMMca_1_CsomeACh(pf08936) CsomeACh
07905 P_neonPurple CsomeACh
07910 P_neonPurple CsomeACh
07915 H_hotpink CsomeACh
07920 H_hotpink CsomeACh
07925 -pf01329 -
07930 -pf01656 -
07935 -# -
07940 -pf07728pf08406 -
07945 -pf00092 -
07950 Tdp_darkMagenta(#) CsomeACh
07955 -# -
07965 -pf04561pf04565pf10385pf00562pf04560 -
08000 -# -
08005 -pf02665 -
08010 -pf07690 -
08015 -pf03595 -
08030 -pf00535 -
08035 -# -
08040 -# -
08045 -# -
BMC-P
BMC-H
BMC-T
Aldehyde dehydrogenase
PTAC
Signature enzyme
Alcohol dehydrogenase
Regulator

Thiobacillus sp. 65-1059. Bacteria; Proteobacteria; Betaproteobacteria; Hydrogenophilales;

BGO60__locus_1

CsomeACh

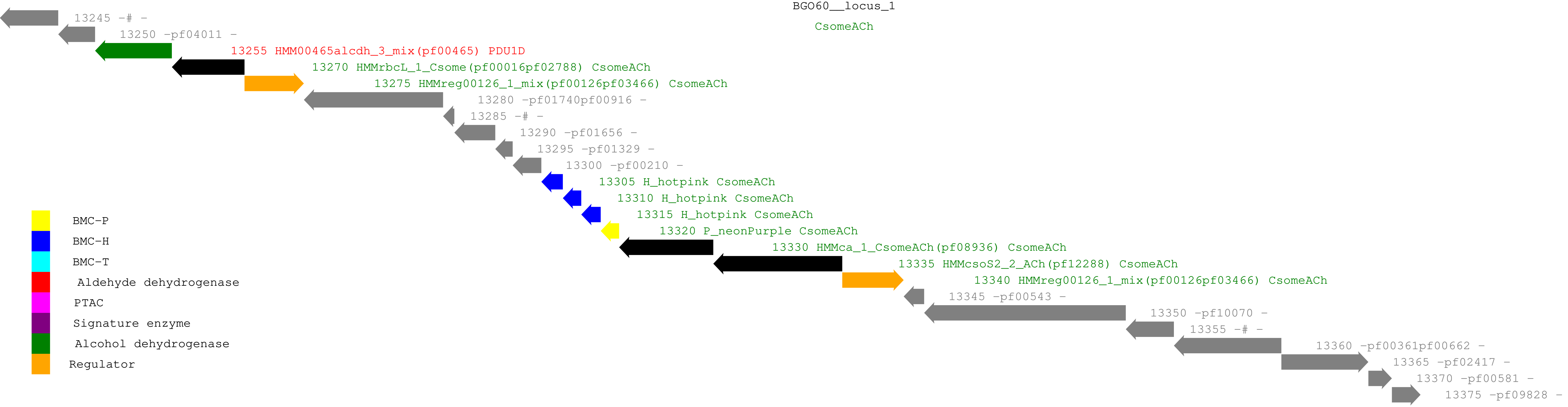

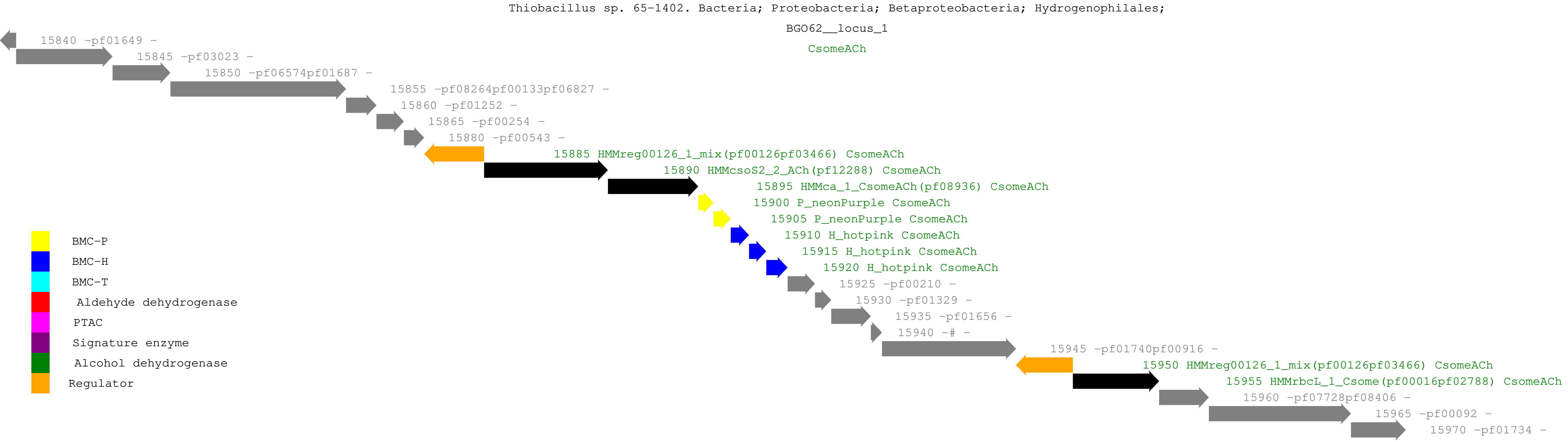

Thiobacillus sp. 65-1402. Bacteria; Proteobacteria; Betaproteobacteria; Hydrogenophilales;
BGO62__locus_1
CsomeACh
15840 -pf01649 -
15845 -pf03023 -
15850 -pf06574pf01687 -
15855 -pf08264pf00133pf06827 -
15860 -pf01252 -
15865 -pf00254 -
15880 -pf00543 -
15885 HMMreg00126_1_mix(pf00126pf03466) CsomeACh
15890 HMMcsoS2_2_ACh(pf12288) CsomeACh
15895 HMMca_1_CsomeACh(pf08936) CsomeACh
15900 P_neonPurple CsomeACh
15905 P_neonPurple CsomeACh
15910 H_hotpink CsomeACh
15915 H_hotpink CsomeACh
15920 H_hotpink CsomeACh
15925 -pf00210 -
15930 -pf01329 -
15935 -pf01656 -
15940 -# -
15945 -pf01740pf00916 -
15950 HMMreg00126_1_mix(pf00126pf03466) CsomeACh
15955 HMMrbcL_1_Csome(pf00016pf02788) CsomeACh
15960 -pf07728pf08406 -
15965 -pf00092 -
15970 -pf01734 -
BMC-P
BMC-H
BMC-T
Aldehyde dehydrogenase
PTAC
Signature enzyme
Alcohol dehydrogenase
Regulator

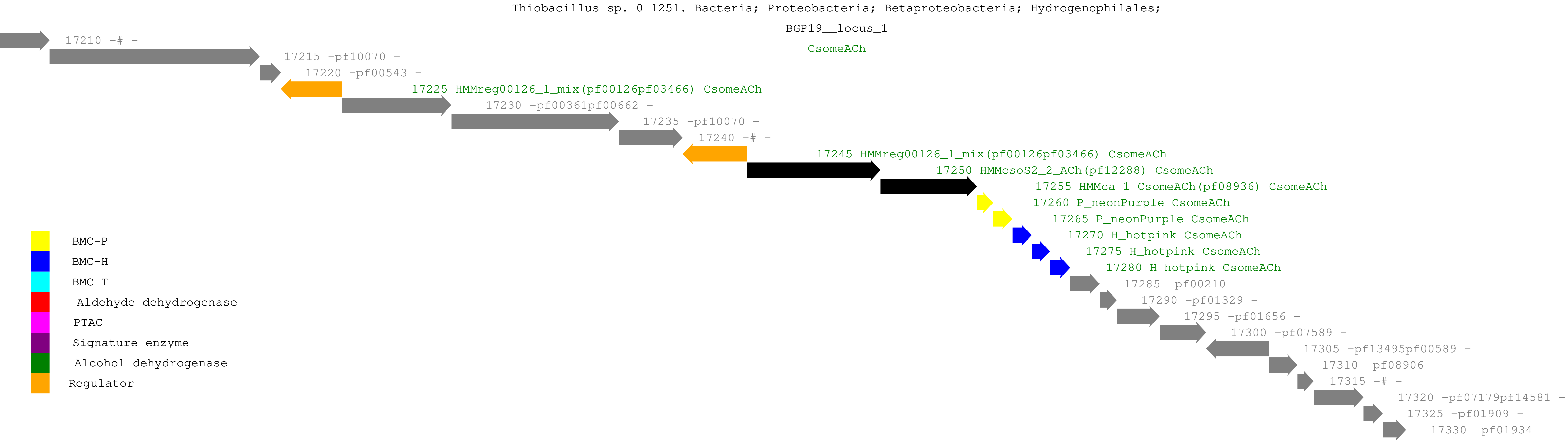

Thiobacillus sp. 0-1251. Bacteria; Proteobacteria; Betaproteobacteria; Hydrogenophilales;
BGP19__locus_1
CsomeACh
17210 -# -
17215 -pf10070 -
17220 -pf00543 -
17225 HMMreg00126_1_mix(pf00126pf03466) CsomeACh
17230 -pf00361pf00662 -
17235 -pf10070 -
17240 -# -
17245 HMMreg00126_1_mix(pf00126pf03466) CsomeACh
17250 HMMcsoS2_2_ACh(pf12288) CsomeACh
17255 HMMca_1_CsomeACh(pf08936) CsomeACh
17260 P_neonPurple CsomeACh
17265 P_neonPurple CsomeACh
17270 H_hotpink CsomeACh
17275 H_hotpink CsomeACh
17280 H_hotpink CsomeACh
17285 -pf00210 -
17290 -pf01329 -
17295 -pf01656 -
17300 -pf07589 -
17305 -pf13495pf00589 -
17310 -pf08906 -
17315 -# -
17320 -pf07179pf14581 -
17325 -pf01909 -
17330 -pf01934 -
BMC-P
BMC-H
BMC-T
Aldehyde dehydrogenase
PTAC
Signature enzyme
Alcohol dehydrogenase
Regulator

Acidihalobacter prosperus. Bacteria; Proteobacteria; Gammaproteobacteria; Chromatiales;

BI364__locus_1

CsomeACh

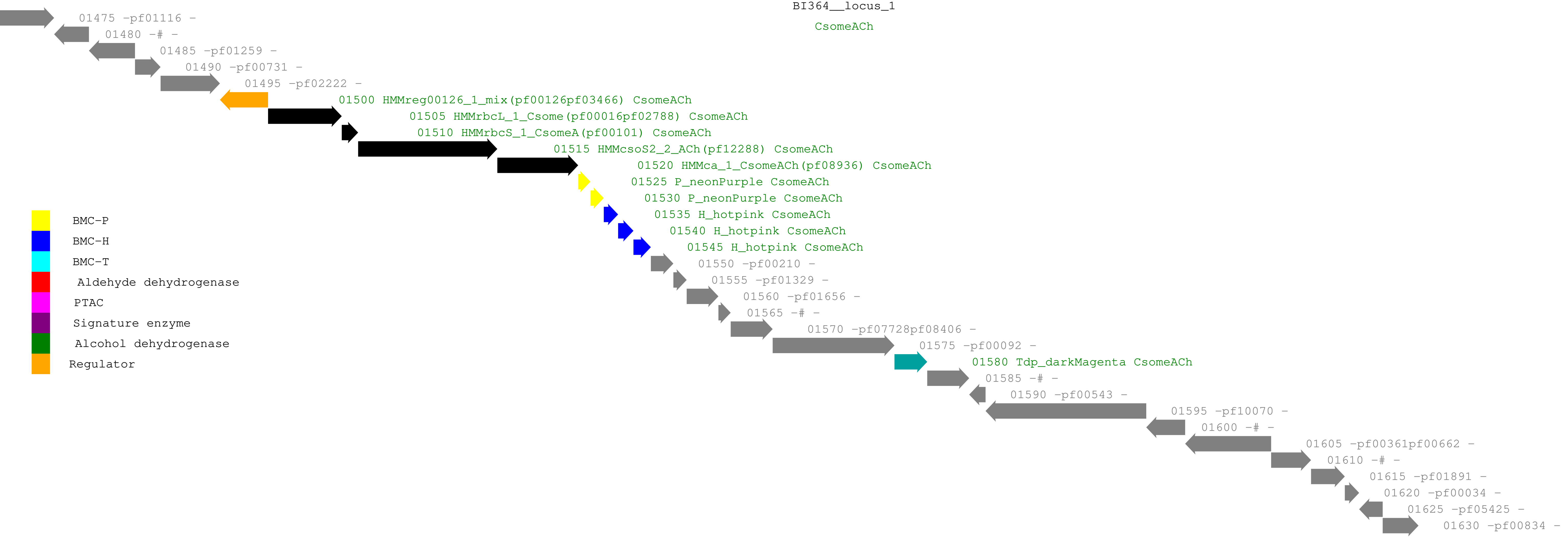

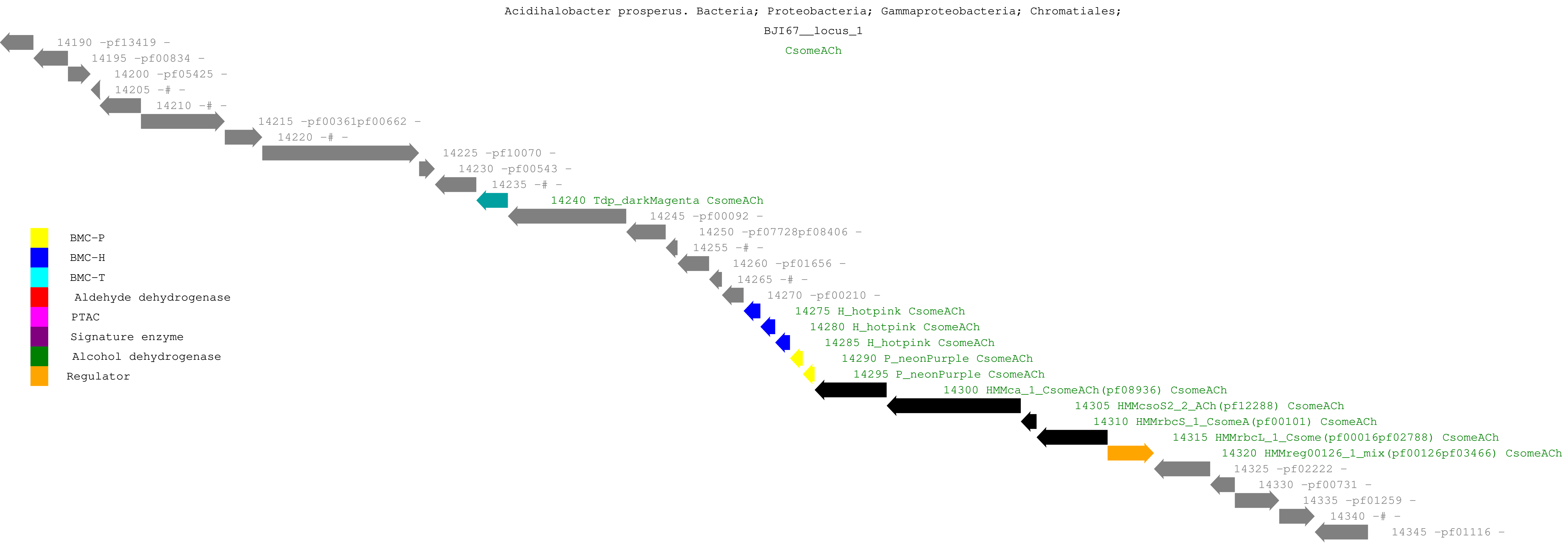
Acidihalobacter prosperus. Bacteria; Proteobacteria; Gammaproteobacteria; Chromatiales;
BJI67__locus_1
CsomeACh
14190 -pf13419 -
14195 -pf00834 -
14200 -pf05425 -
14205 -# -
14210 -# -
14215 -pf00361pf00662 -
14220 -# -
14225 -pf10070 -
14230 -pf00543 -
14235 -# -
14240 Tdp_darkMagenta CsomeACh
14245 -pf00092 -
14250 -pf07728pf08406 -
14255 -# -
14260 -pf01656 -
14265 -# -
14270 -pf00210 -
14275 H_hotpink CsomeACh
14280 H_hotpink CsomeACh
14285 H_hotpink CsomeACh
14290 P_neonPurple CsomeACh
14295 P_neonPurple CsomeACh
14300 HMMca_1_CsomeACh(pf08936) CsomeACh
14305 HMMcsoS2_2_ACh(pf12288) CsomeACh
14310 HMMrbcS_1_CsomeA(pf00101) CsomeACh
14315 HMMrbcL_1_Csome(pf00016pf02788) CsomeACh
14320 HMMreg00126_1_mix(pf00126pf03466) CsomeACh
14325 -pf02222 -
14330 -pf00731 -
14335 -pf01259 -
14340 -# -
14345 -pf01116 -
BMC-P
BMC-H
BMC-T
Aldehyde dehydrogenase
PTAC
Signature enzyme
Alcohol dehydrogenase
Regulator

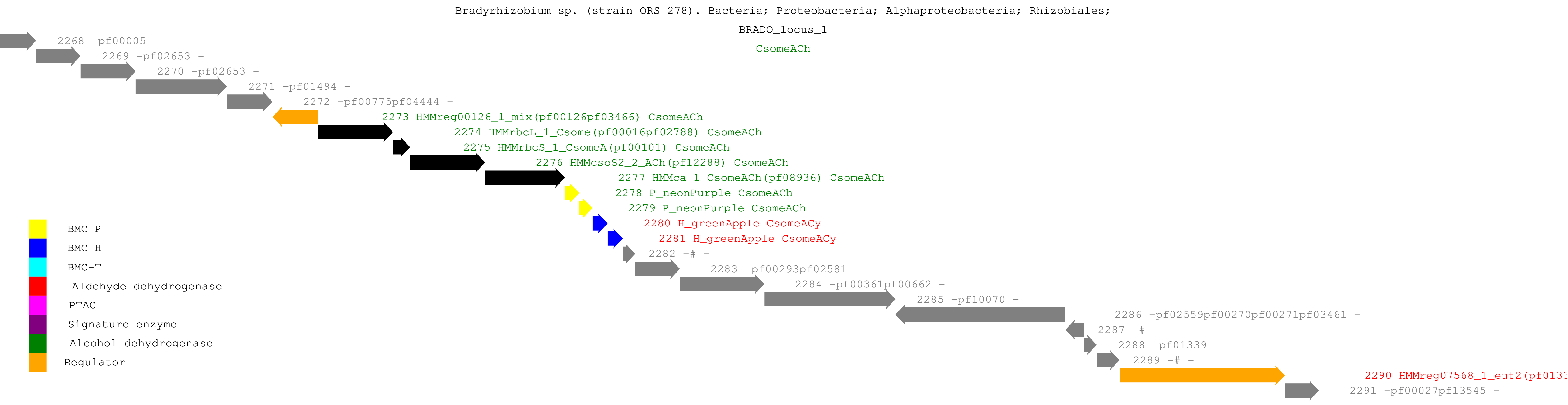
Bradyrhizobium sp. (strain ORS 278). Bacteria; Proteobacteria; Alphaproteobacteria; Rhizobiales;
BRADO_locus_1
CsomeACh
2268 -pf00005 -
2269 -pf02653 -
2270 -pf02653 -
2271 -pf01494 -
2272 -pf00775pf04444 -
2273 HMMreg00126_1_mix(pf00126pf03466) CsomeACh
2274 HMMrbcL_1_Csome(pf00016pf02788) CsomeACh
2275 HMMrbcS_1_CsomeA(pf00101) CsomeACh
2276 HMMcsoS2_2_ACh(pf12288) CsomeACh
2277 HMMca_1_CsomeACh(pf08936) CsomeACh
2278 P_neonPurple CsomeACh
2279 P_neonPurple CsomeACh
2280 H_greenApple CsomeACy
2281 H_greenApple CsomeACy
2282 -# -
2283 -pf00293pf02581 -
2284 -pf00361pf00662 -
2285 -pf10070 -
2286 -pf02559pf00270pf00271pf03461 -
2287 -# -
2288 -pf01339 -
2289 -# -
2290 HMMreg07568_1_eut2(pf0133
2291 -pf00027pf13545 -
BMC-P
BMC-H
BMC-T
Aldehyde dehydrogenase
PTAC
Signature enzyme
Alcohol dehydrogenase
Regulator

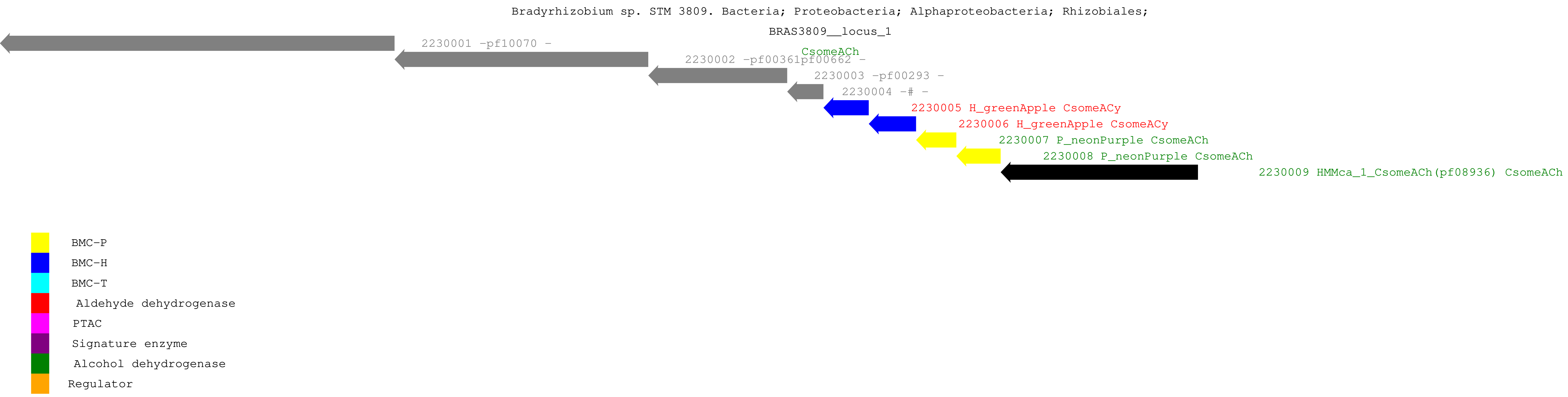
Bradyrhizobium sp. STM 3809. Bacteria; Proteobacteria; Alphaproteobacteria; Rhizobiales;
BRAS3809__locus_1
CsomeACh
2230001 -pf10070 -
2230002 -pf00361pf00662 -
2230003 -pf00293 -
2230004 -# -
2230005 H_greenApple CsomeACy
2230006 H_greenApple CsomeACy
2230007 P_neonPurple CsomeACh
2230008 P_neonPurple CsomeACh
2230009 HMMca_1_CsomeACh(pf08936) CsomeACh
BMC-P
BMC-H
BMC-T
Aldehyde dehydrogenase
PTAC
Signature enzyme
Alcohol dehydrogenase
Regulator

Xuhuaishuia manganoxidans. Bacteria; Proteobacteria; Alphaproteobacteria; Rhodobacterales;

BV394__locus_1

CsomeACh

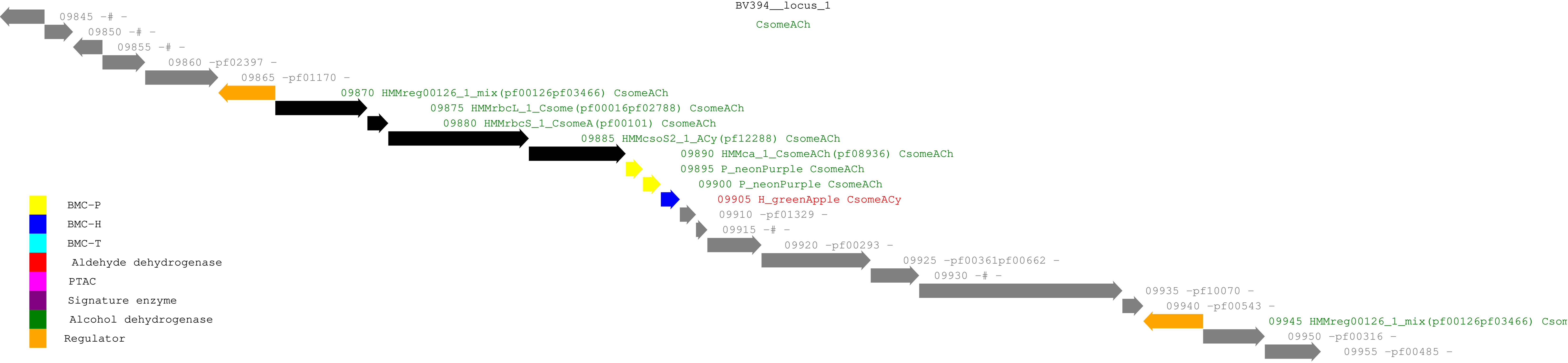

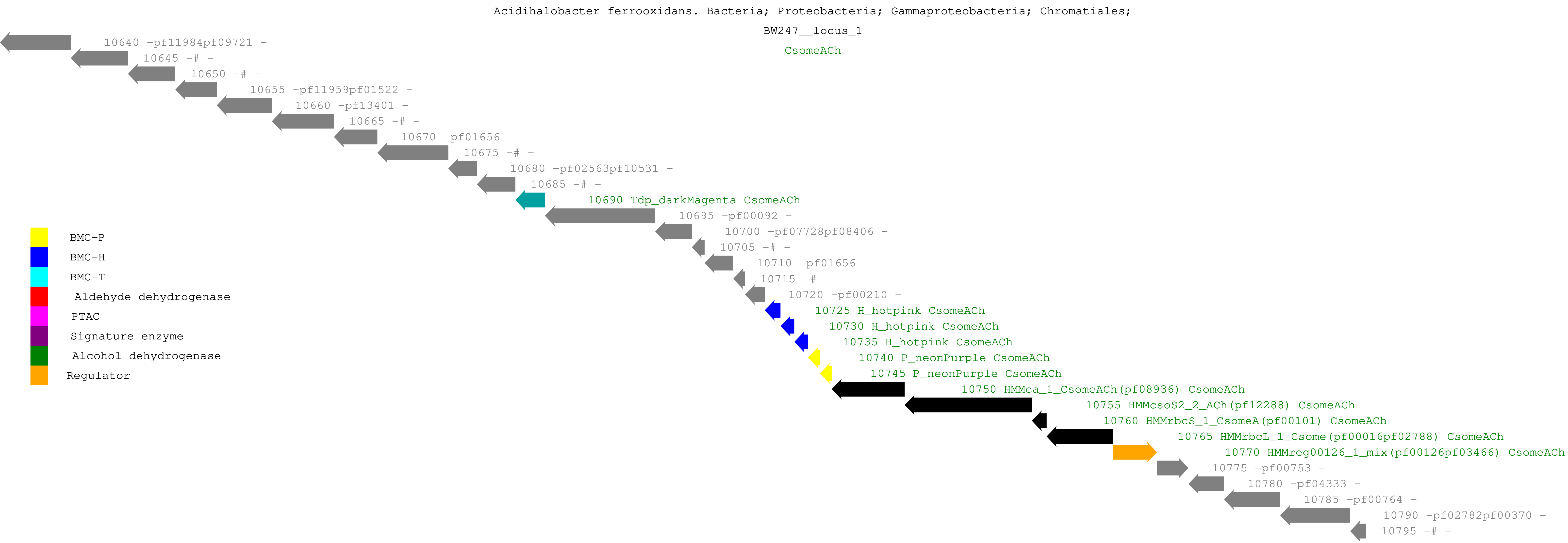

Acidihalobacter ferrooxidans. Bacteria; Proteobacteria; Gammaproteobacteria; Chromatiales;
BW247__locus_1
CsomeACh
10640 -pf11984pf09721 -
10645 -# -
10650 -# -
10655 -pf11959pf01522 -
10660 -pf13401 -
10665 -# -
10670 -pf01656 -
10675 -# -
10680 -pf02563pf10531 -
10685 -# -
10690 Tdp_darkMagenta CsomeACh
10695 -pf00092 -
10700 -pf07728pf08406 -
10705 -# -
10710 -pf01656 -
10715 -# -
10720 -pf00210 -
10725 H_hotpink CsomeACh
10730 H_hotpink CsomeACh
10735 H_hotpink CsomeACh
10740 P_neonPurple CsomeACh
10745 P_neonPurple CsomeACh
10750 HMMca_1_CsomeACh(pf08936) CsomeACh
10755 HMMcsoS2_2_ACh(pf12288) CsomeACh
10760 HMMrbcS_1_CsomeA(pf00101) CsomeACh
10765 HMMrbcL_1_Csome(pf00016pf02788) CsomeACh
10770 HMMreg00126_1_mix(pf00126pf03466) CsomeACh
10775 -pf00753 -
10780 -pf04333 -
10785 -pf00764 -
10790 -pf02782pf00370 -
10795 -# -
BMC-P
BMC-H
BMC-T
Aldehyde dehydrogenase
PTAC
Signature enzyme
Alcohol dehydrogenase
Regulator

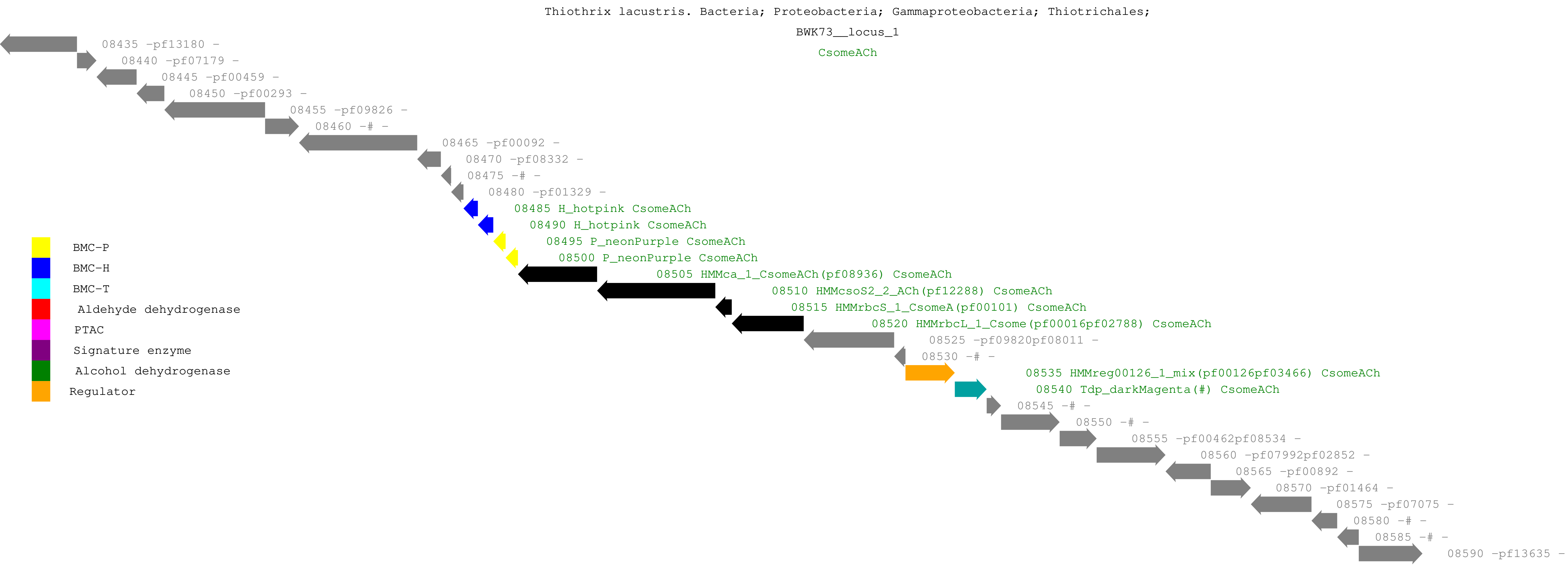
Thiothrix lacustris. Bacteria; Proteobacteria; Gammaproteobacteria; Thiotrichales;
BWK73__locus_1
CsomeACh
08435 -pf13180 -
08440 -pf07179 -
08445 -pf00459 -
08450 -pf00293 -
08455 -pf09826 -
08460 -# -
08465 -pf00092 -
08470 -pf08332 -
08475 -# -
08480 -pf01329 -
08485 H_hotpink CsomeACh
08490 H_hotpink CsomeACh
08495 P_neonPurple CsomeACh
08500 P_neonPurple CsomeACh
08505 HMMca_1_CsomeACh(pf08936) CsomeACh
08510 HMMcsoS2_2_ACh(pf12288) CsomeACh
08515 HMMrbcS_1_CsomeA(pf00101) CsomeACh
08520 HMMrbcL_1_Csome(pf00016pf02788) CsomeACh
08525 -pf09820pf08011 -
08530 -# -
08535 HMMreg00126_1_mix(pf00126pf03466) CsomeACh
08540 Tdp_darkMagenta(#) CsomeACh
08545 -# -
08550 -# -
08555 -pf00462pf08534 -
08560 -pf07992pf02852 -
08565 -pf00892 -
08570 -pf01464 -
08575 -pf07075 -
08580 -# -
08585 -# -
08590 -pf13635 -
BMC-P
BMC-H
BMC-T
Aldehyde dehydrogenase
PTAC
Signature enzyme
Alcohol dehydrogenase
Regulator

Acidithiobacillus ferrooxidans (Thiobacillus ferrooxidans). Bacteria; Proteobacteria; Acidithiobacillia; Acidithiobacillales;

C3R74__locus_1

CsomeACh

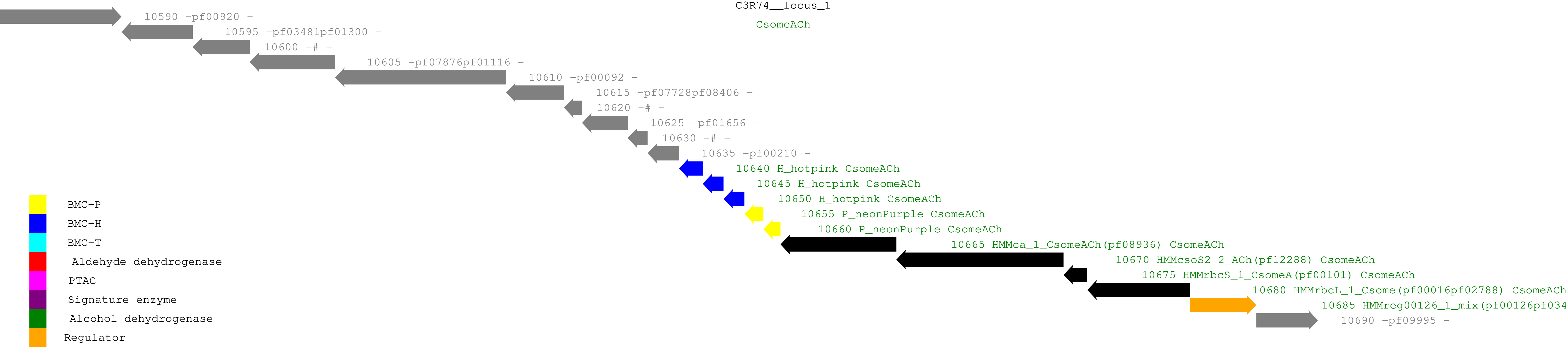

Acidiferrobacter thiooxydans. Bacteria; Proteobacteria; Gammaproteobacteria; Acidiferrobacterales;

C4900__locus_1

CsomeACh

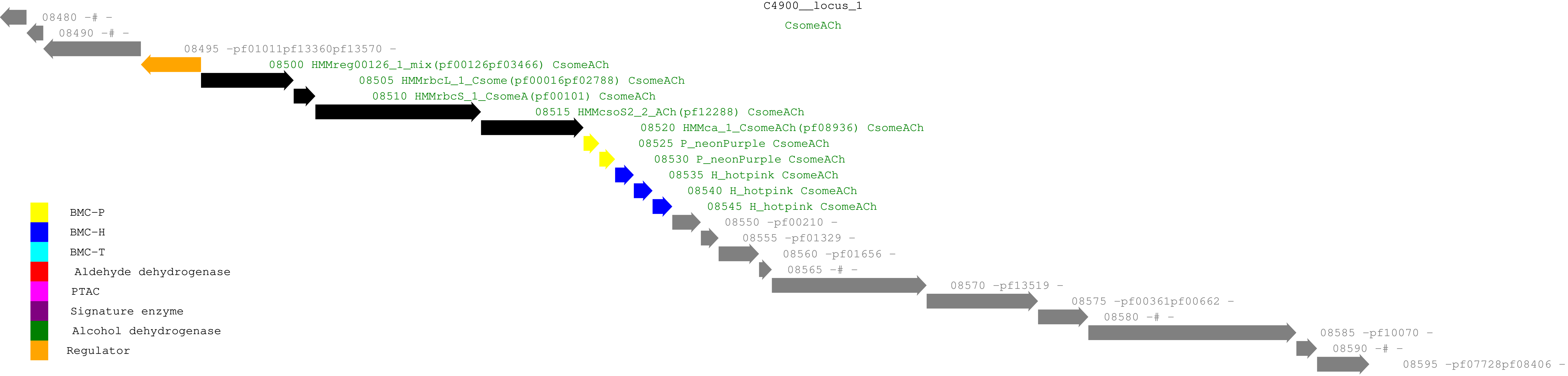

Acidiferrobacter sp. SPIII_3. Bacteria; Proteobacteria; Gammaproteobacteria; Acidiferrobacterales;

C4901__locus_1

CsomeACh

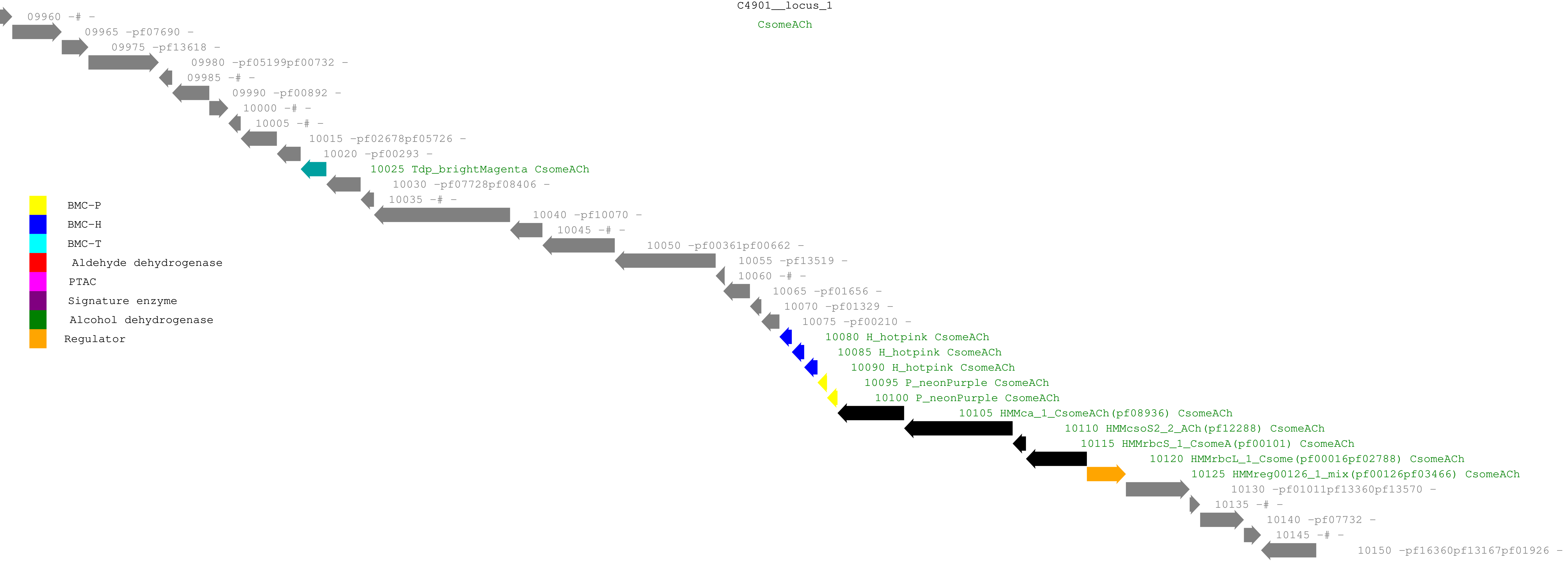

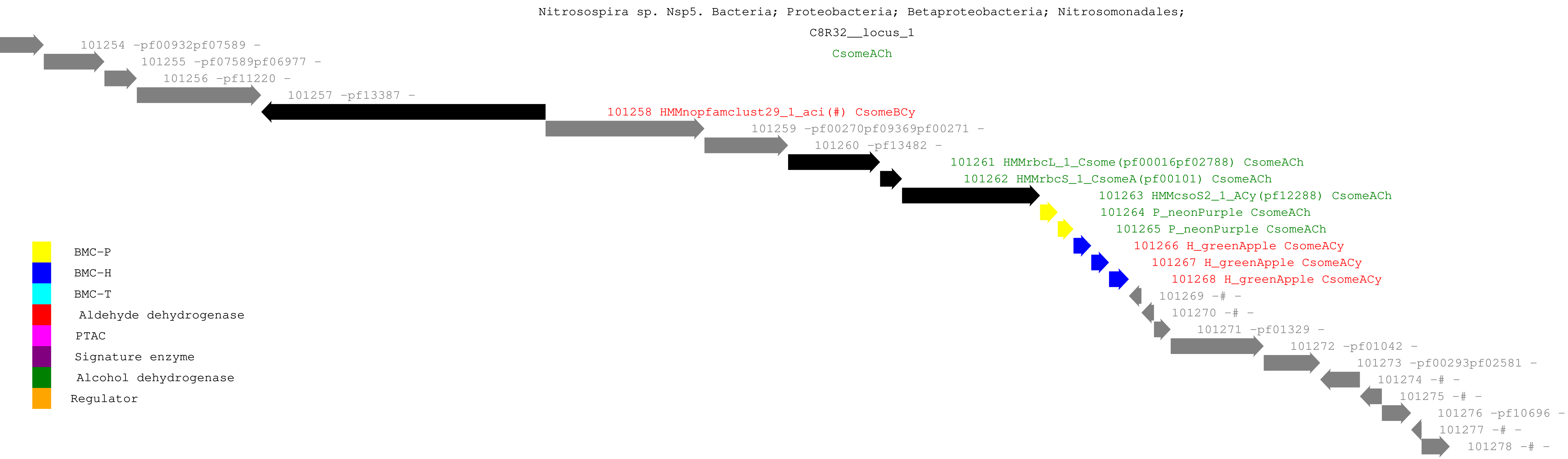
Nitrosospira sp. Nsp5. Bacteria; Proteobacteria; Betaproteobacteria; Nitrosomonadales;
C8R32__locus_1
CsomeACh
101254 -pf00932pf07589 -
101255 -pf07589pf06977 -
101256 -pf11220 -
101257 -pf13387 -
101258 HMMnopfamclust29_1_aci(#) CsomeBCy
101259 -pf00270pf09369pf00271 -
101260 -pf13482 -
101261 HMMrbcL_1_Csome(pf00016pf02788) CsomeACh
101262 HMMrbcS_1_CsomeA(pf00101) CsomeACh
101263 HMMcsoS2_1_ACy(pf12288) CsomeACh
101264 P_neonPurple CsomeACh
101265 P_neonPurple CsomeACh
101266 H_greenApple CsomeACy
101267 H_greenApple CsomeACy
101268 H_greenApple CsomeACy
101269 -# -
101270 -# -
101271 -pf01329 -
101272 -pf01042 -
101273 -pf00293pf02581 -
101274 -# -
101275 -# -
101276 -pf10696 -
101277 -# -
101278 -# -
BMC-P
BMC-H
BMC-T
Aldehyde dehydrogenase
PTAC
Signature enzyme
Alcohol dehydrogenase
Regulator

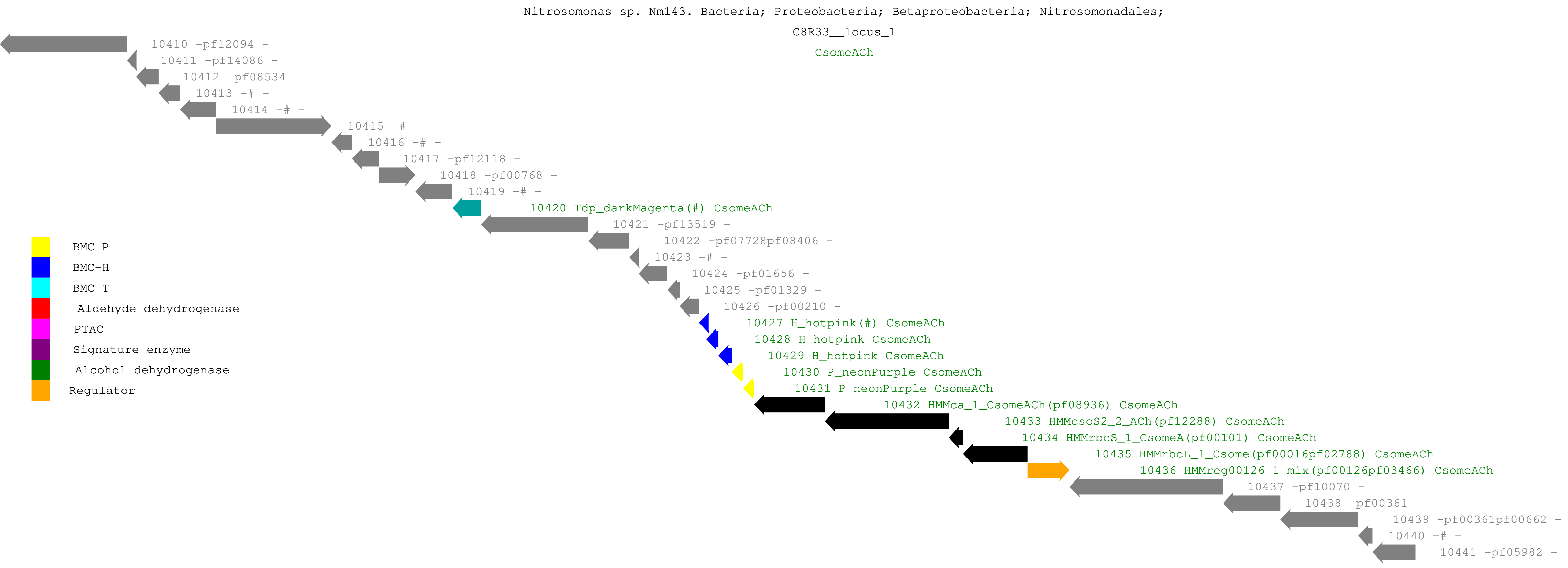
Nitrosomonas sp. Nm143. Bacteria; Proteobacteria; Betaproteobacteria; Nitrosomonadales;
C8R33__locus_1
CsomeACh
10410 -pf12094 -
10411 -pf14086 -
10412 -pf08534 -
10413 -# -
10414 -# -
10415 -# -
10416 -# -
10417 -pf12118 -
10418 -pf00768 -
10419 -# -
10420 Tdp_darkMagenta(#) CsomeACh
10421 -pf13519 -
10422 -pf07728pf08406 -
10423 -# -
10424 -pf01656 -
10425 -pf01329 -
10426 -pf00210 -
10427 H_hotpink(#) CsomeACh
10428 H_hotpink CsomeACh
10429 H_hotpink CsomeACh
10430 P_neonPurple CsomeACh
10431 P_neonPurple CsomeACh
10432 HMMca_1_CsomeACh(pf08936) CsomeACh
10433 HMMcsoS2_2_ACh(pf12288) CsomeACh
10434 HMMrbcS_1_CsomeA(pf00101) CsomeACh
10435 HMMrbcL_1_Csome(pf00016pf02788) CsomeACh
10436 HMMreg00126_1_mix(pf00126pf03466) CsomeACh
10437 -pf10070 -
10438 -pf00361 -
10439 -pf00361pf00662 -
10440 -# -
10441 -pf05982 -
BMC-P
BMC-H
BMC-T
Aldehyde dehydrogenase
PTAC
Signature enzyme
Alcohol dehydrogenase
Regulator

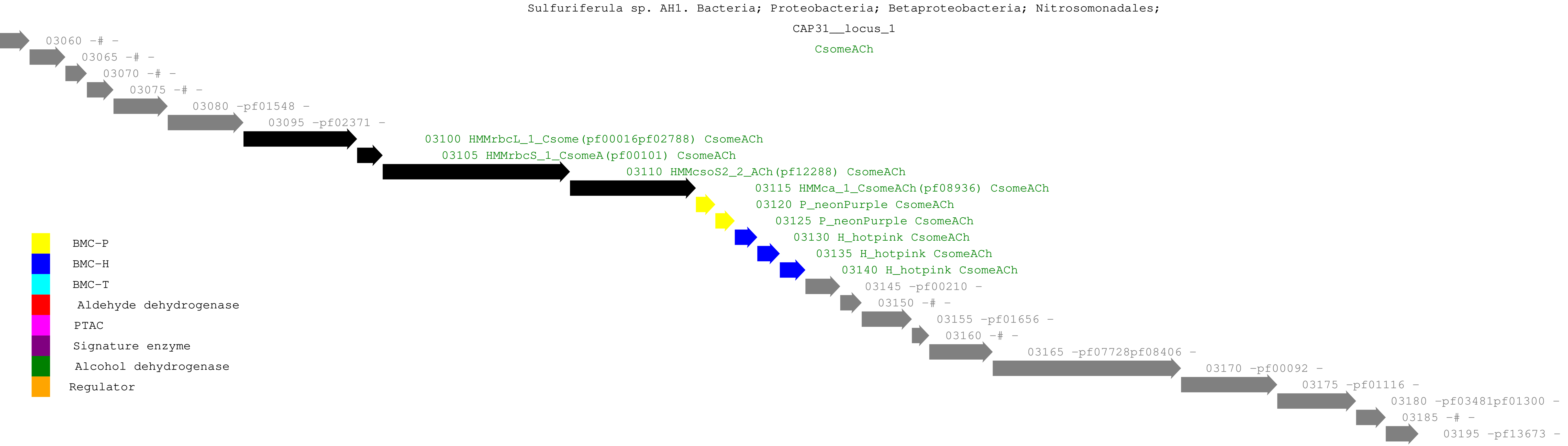
Sulfuriferula sp. AH1. Bacteria; Proteobacteria; Betaproteobacteria; Nitrosomonadales;
CAP31__locus_1
CsomeACh
03060 -# -
03065 -# -
03070 -# -
03075 -# -
03080 -pf01548 -
03095 -pf02371 -
03100 HMMrbcL_1_Csome(pf00016pf02788) CsomeACh
03105 HMMrbcS_1_CsomeA(pf00101) CsomeACh
03110 HMMcsoS2_2_ACh(pf12288) CsomeACh
03115 HMMca_1_CsomeACh(pf08936) CsomeACh
03120 P_neonPurple CsomeACh
03125 P_neonPurple CsomeACh
03130 H_hotpink CsomeACh
03135 H_hotpink CsomeACh
03140 H_hotpink CsomeACh
03145 -pf00210 -
03150 -# -
03155 -pf01656 -
03160 -# -
03165 -pf07728pf08406 -
03170 -pf00092 -
03175 -pf01116 -
03180 -pf03481pf01300 -
03185 -# -
03195 -pf13673 -
BMC-P
BMC-H
BMC-T
Aldehyde dehydrogenase
PTAC
Signature enzyme
Alcohol dehydrogenase
Regulator

mine drainage metagenome. unclassified sequences; metagenomes; ecological metagenomes.

CARN2__locus_1

CsomeACh

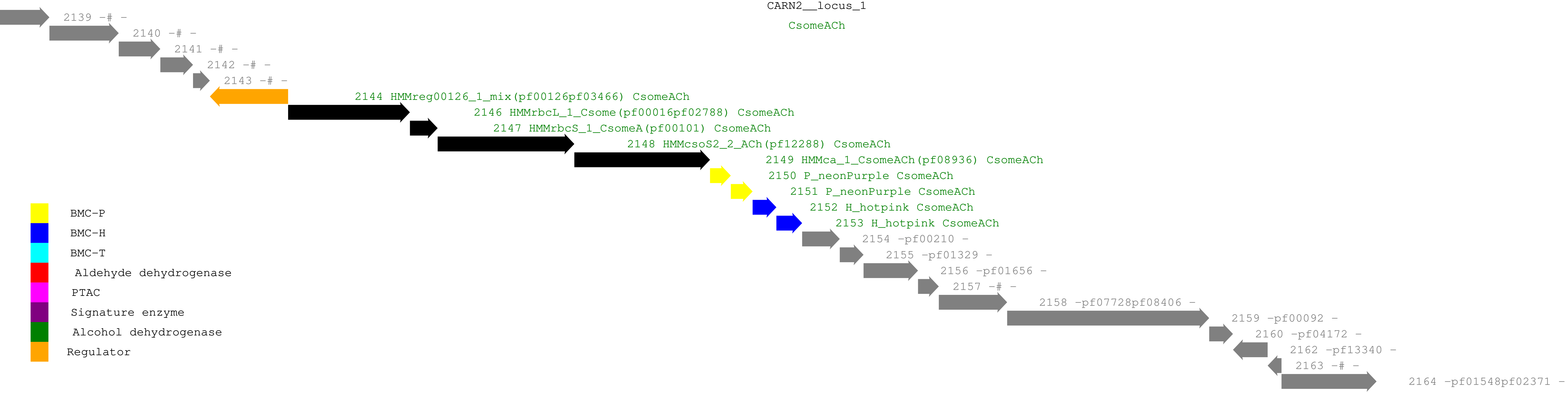

mine drainage metagenome. unclassified sequences; metagenomes; ecological metagenomes.

CARN5__locus_1

CsomeACh

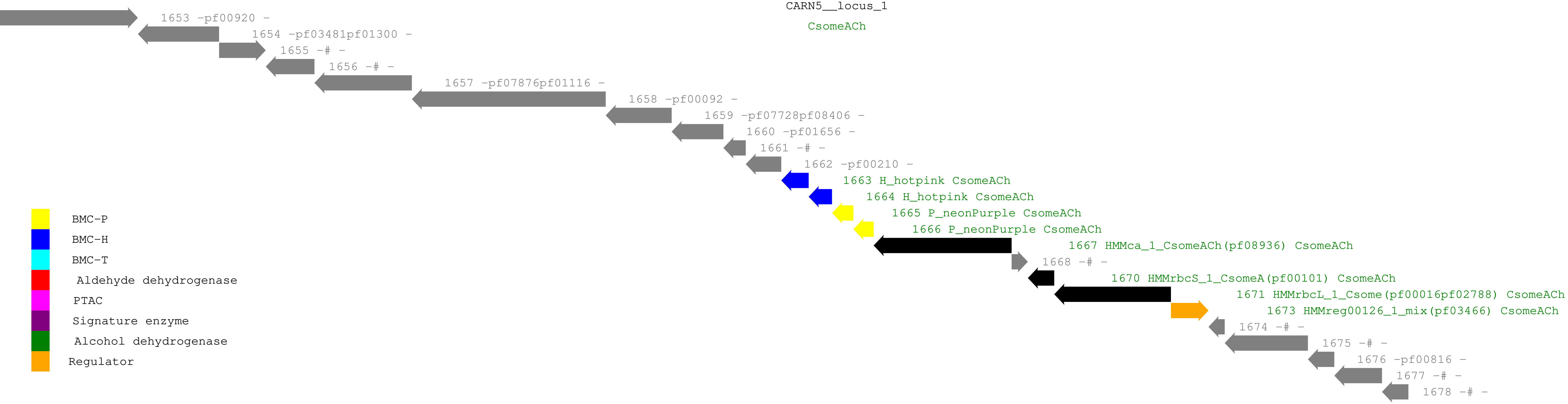

mine drainage metagenome. unclassified sequences; metagenomes; ecological metagenomes.

CARN8__locus_1

CsomeACh

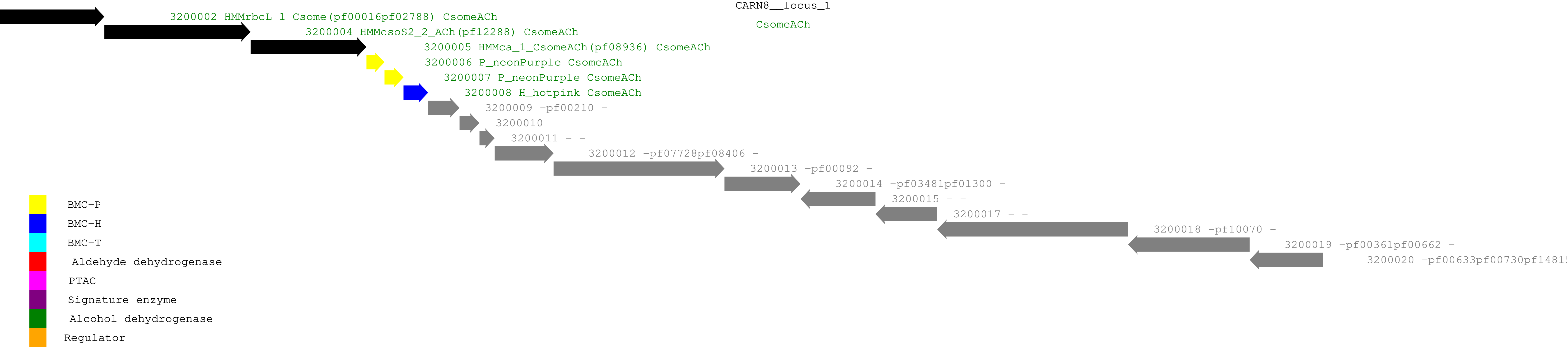

Oceanospirillales bacterium TMED59. Bacteria; Proteobacteria; Gammaproteobacteria; Oceanospirillales;

CBC19__locus_1

CsomeACh

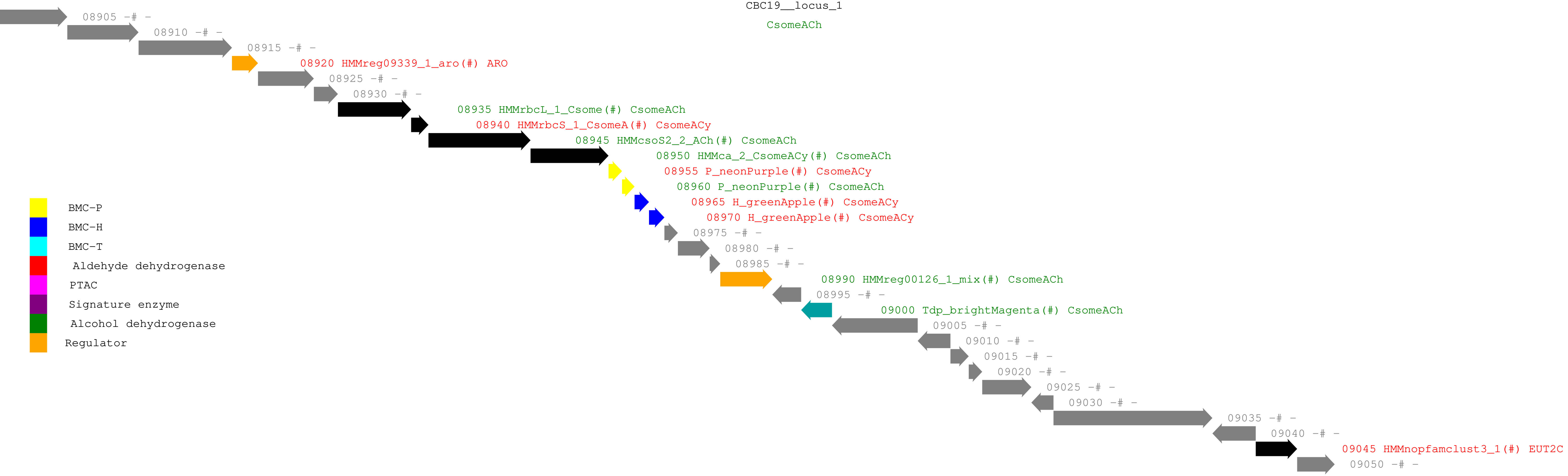

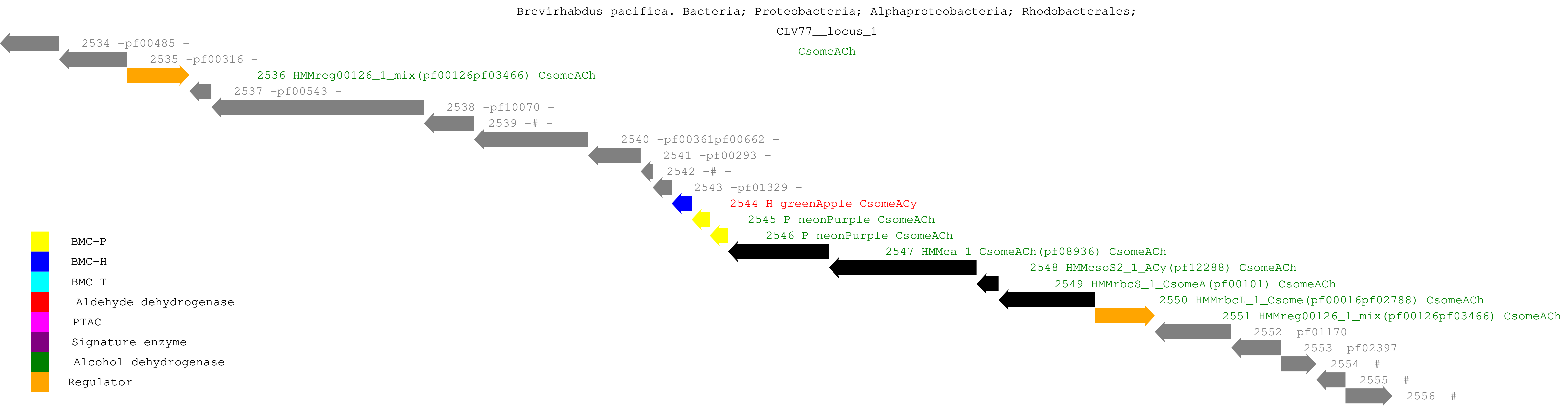
Brevirhabdus pacifica. Bacteria; Proteobacteria; Alphaproteobacteria; Rhodobacterales;
CLV77__locus_1
CsomeACh
2534 -pf00485 -
2535 -pf00316 -
2536 HMMreg00126_1_mix(pf00126pf03466) CsomeACh
2537 -pf00543 -
2538 -pf10070 -
2539 -# -
2540 -pf00361pf00662 -
2541 -pf00293 -
2542 -# -
2543 -pf01329 -
2544 H_greenApple CsomeACy
2545 P_neonPurple CsomeACh
2546 P_neonPurple CsomeACh
2547 HMMca_1_CsomeACh(pf08936) CsomeACh
2548 HMMcsoS2_1_ACy(pf12288) CsomeACh
2549 HMMrbcS_1_CsomeA(pf00101) CsomeACh
2550 HMMrbcL_1_Csome(pf00016pf02788) CsomeACh
2551 HMMreg00126_1_mix(pf00126pf03466) CsomeACh
2552 -pf01170 -
2553 -pf02397 -
2554 -# -
2555 -# -
2556 -# -
BMC-P
BMC-H
BMC-T
Aldehyde dehydrogenase
PTAC
Signature enzyme
Alcohol dehydrogenase
Regulator

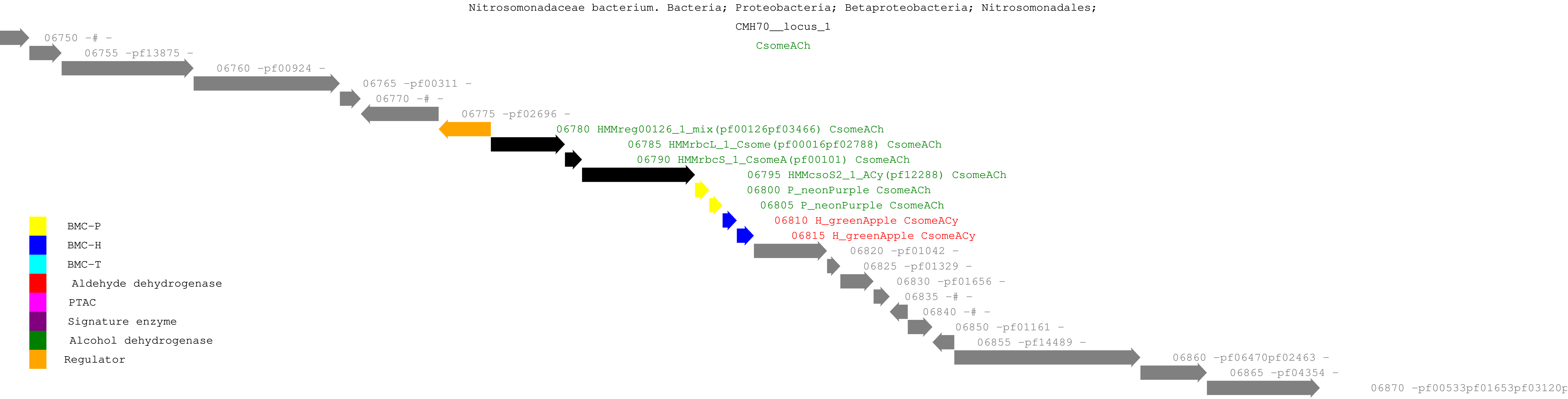
Nitrosomonadaceae bacterium. Bacteria; Proteobacteria; Betaproteobacteria; Nitrosomonadales;
CMH70__locus_1
CsomeACh
06750 -# -
06755 -pf13875 -
06760 -pf00924 -
06765 -pf00311 -
06770 -# -
06775 -pf02696 -
06780 HMMreg00126_1_mix(pf00126pf03466) CsomeACh
06785 HMMrbcL_1_Csome(pf00016pf02788) CsomeACh
06790 HMMrbcS_1_CsomeA(pf00101) CsomeACh
06795 HMMcsoS2_1_ACy(pf12288) CsomeACh
06800 P_neonPurple CsomeACh
06805 P_neonPurple CsomeACh
06810 H_greenApple CsomeACy
06815 H_greenApple CsomeACy
06820 -pf01042 -
06825 -pf01329 -
06830 -pf01656 -
06835 -# -
06840 -# -
06850 -pf01161 -
06855 -pf14489 -
06860 -pf06470pf02463 -
06865 -pf04354 -
06870 -pf00533pf01653pf03120p
BMC-P
BMC-H
BMC-T
Aldehyde dehydrogenase
PTAC
Signature enzyme
Alcohol dehydrogenase
Regulator

Gammaproteobacteria bacterium. Bacteria; Proteobacteria; Gammaproteobacteria.

CMQ72__locus_1

CsomeACh

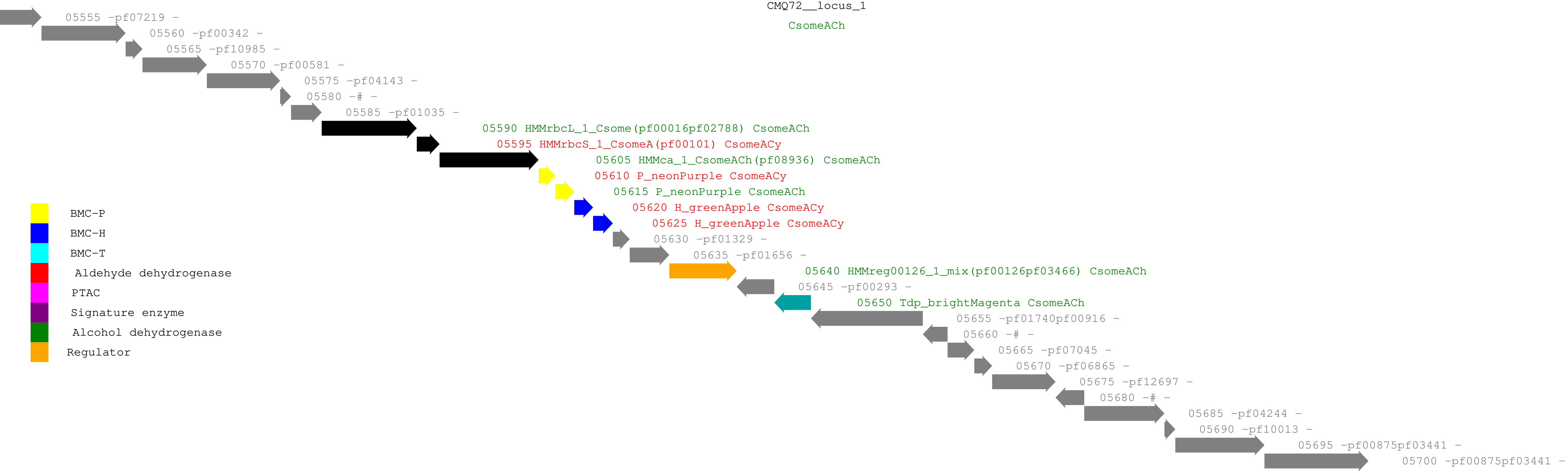

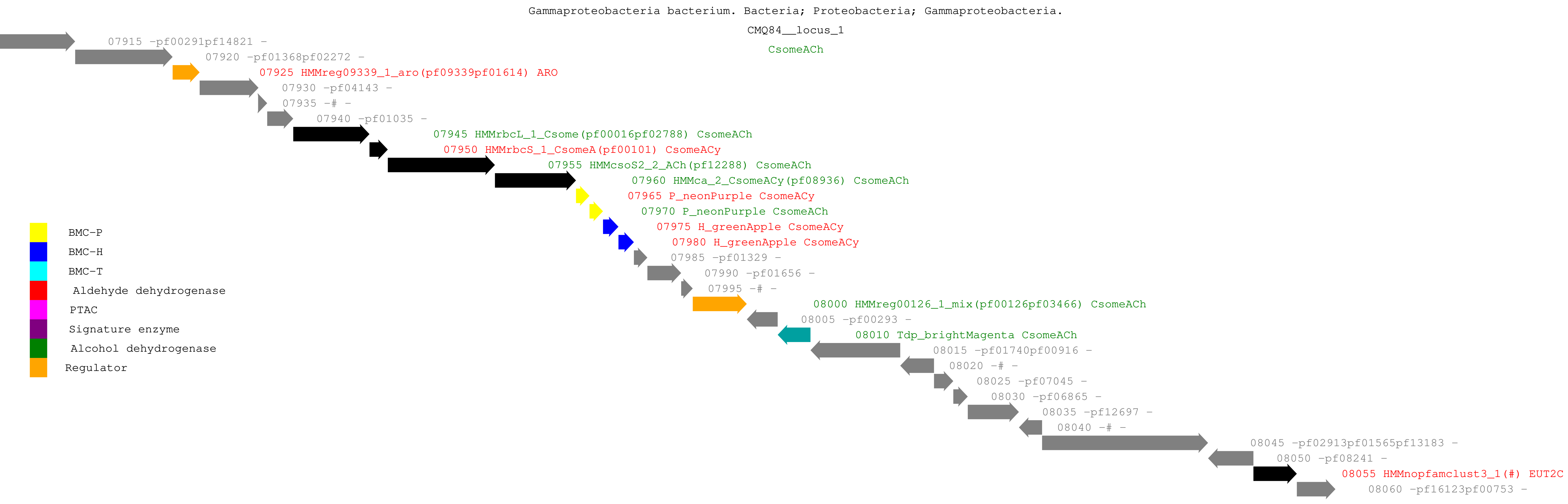
Gammaproteobacteria bacterium. Bacteria; Proteobacteria; Gammaproteobacteria.
CMQ84__locus_1
CsomeACh
07915 -pf00291pf14821 -
07920 -pf01368pf02272 -
07925 HMMreg09339_1_aro(pf09339pf01614) ARO
07930 -pf04143 -
07935 -# -
07940 -pf01035 -
07945 HMMrbcL_1_Csome(pf00016pf02788) CsomeACh
07950 HMMrbcS_1_CsomeA(pf00101) CsomeACy
07955 HMMcsoS2_2_ACh(pf12288) CsomeACh
07960 HMMca_2_CsomeACy(pf08936) CsomeACh
07965 P_neonPurple CsomeACy
07970 P_neonPurple CsomeACh
07975 H_greenApple CsomeACy
07980 H_greenApple CsomeACy
07985 -pf01329 -
07990 -pf01656 -
07995 -# -
08000 HMMreg00126_1_mix(pf00126pf03466) CsomeACh
08005 -pf00293 -
08010 Tdp_brightMagenta CsomeACh
08015 -pf01740pf00916 -
08020 -# -
08025 -pf07045 -
08030 -pf06865 -
08035 -pf12697 -
08040 -# -
08045 -pf02913pf01565pf13183 -
08050 -pf08241 -
08055 HMMnopfamclust3_1(#) EUT2C
08060 -pf16123pf00753 -
BMC-P
BMC-H
BMC-T
Aldehyde dehydrogenase
PTAC
Signature enzyme
Alcohol dehydrogenase
Regulator

Gammaproteobacteria bacterium. Bacteria; Proteobacteria; Gammaproteobacteria.

CMQ88__locus_1

CsomeACh

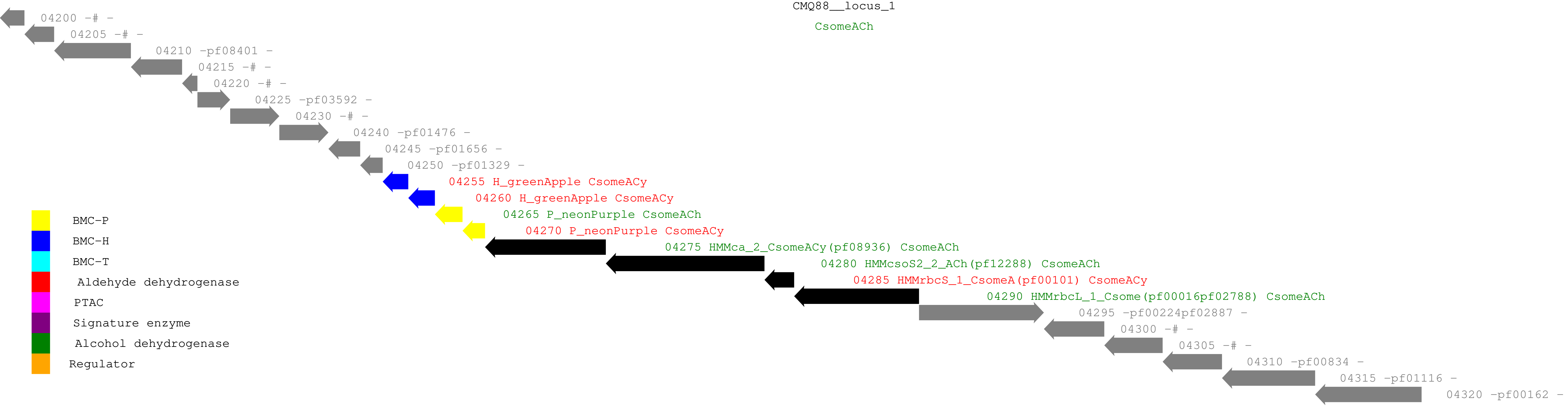

Thiotrichaceae bacterium. Bacteria; Proteobacteria; Gammaproteobacteria; Thiotrichales;

COA54__locus_1

CsomeACh

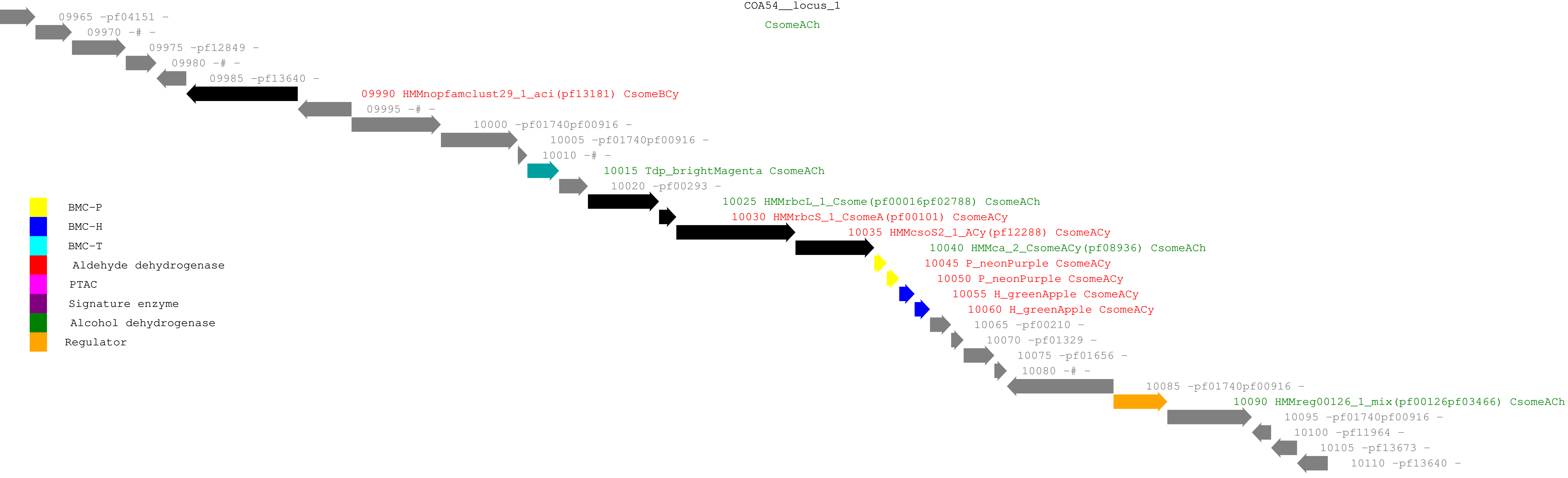

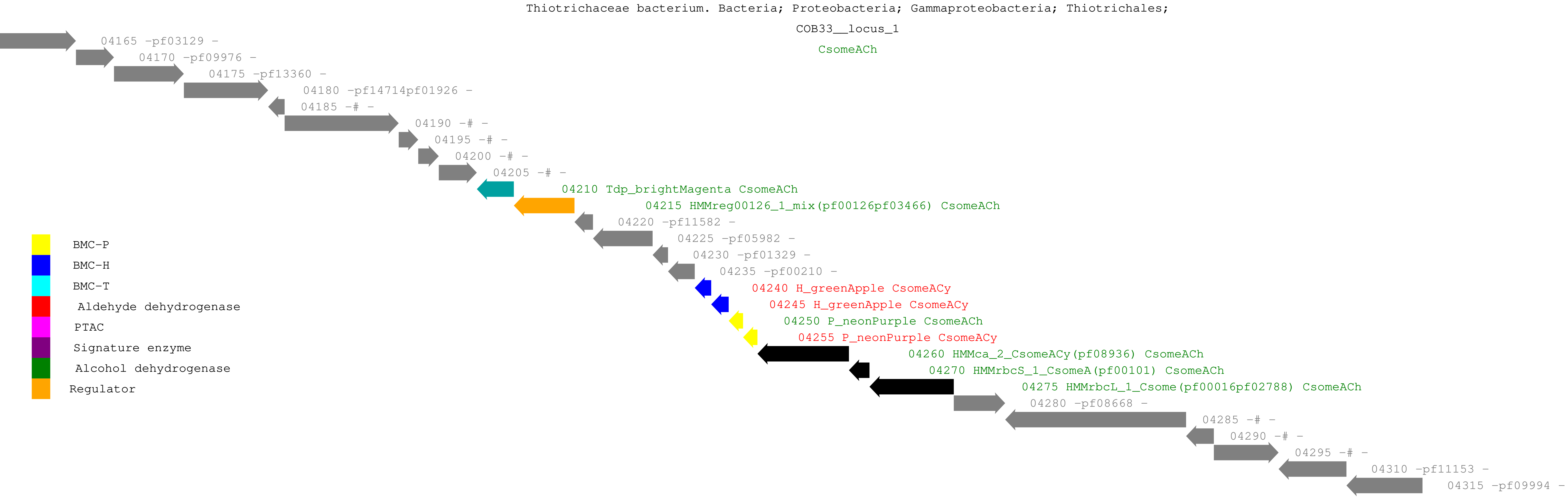
Thiotrichaceae bacterium. Bacteria; Proteobacteria; Gammaproteobacteria; Thiotrichales;
COB33__locus_1
CsomeACh
04165 -pf03129 -
04170 -pf09976 -
04175 -pf13360 -
04180 -pf14714pf01926 -
04185 -# -
04190 -# -
04195 -# -
04200 -# -
04205 -# -
04210 Tdp_brightMagenta CsomeACh
04215 HMMreg00126_1_mix(pf00126pf03466) CsomeACh
04220 -pf11582 -
04225 -pf05982 -
04230 -pf01329 -
04235 -pf00210 -
04240 H_greenApple CsomeACy
04245 H_greenApple CsomeACy
04250 P_neonPurple CsomeACh
04255 P_neonPurple CsomeACy
04260 HMMca_2_CsomeACy(pf08936) CsomeACh
04270 HMMrbcS_1_CsomeA(pf00101) CsomeACh
04275 HMMrbcL_1_Csome(pf00016pf02788) CsomeACh
04280 -pf08668 -
04285 -# -
04290 -# -
04295 -# -
04310 -pf11153 -
04315 -pf09994 -
BMC-P
BMC-H
BMC-T
Aldehyde dehydrogenase
PTAC
Signature enzyme
Alcohol dehydrogenase
Regulator

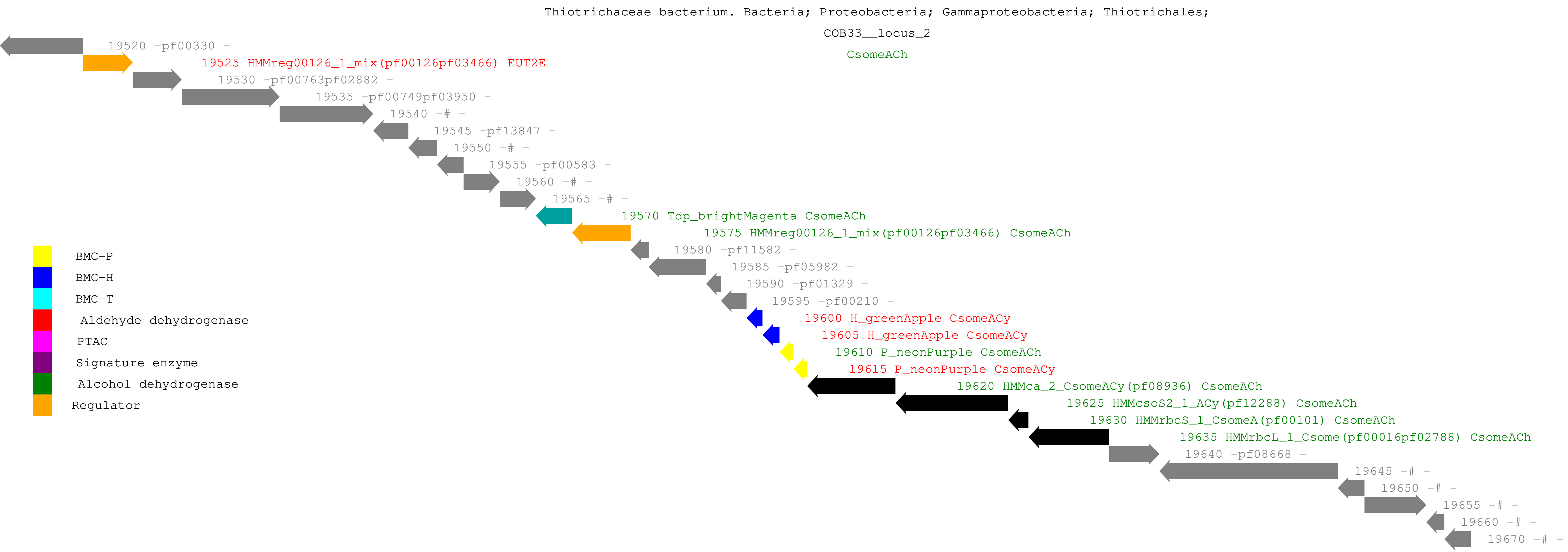

Thiotrichaceae bacterium. Bacteria; Proteobacteria; Gammaproteobacteria; Thiotrichales;
COB33__locus_2
CsomeACh
19520 -pf00330 -
19525 HMMreg00126_1_mix(pf00126pf03466) EUT2E
19530 -pf00763pf02882 -
19535 -pf00749pf03950 -
19540 -# -
19545 -pf13847 -
19550 -# -
19555 -pf00583 -
19560 -# -
19565 -# -
19570 Tdp_brightMagenta CsomeACh
19575 HMMreg00126_1_mix(pf00126pf03466) CsomeACh
19580 -pf11582 -
19585 -pf05982 -
19590 -pf01329 -
19595 -pf00210 -
19600 H_greenApple CsomeACy
19605 H_greenApple CsomeACy
19610 P_neonPurple CsomeACh
19615 P_neonPurple CsomeACy
19620 HMMca_2_CsomeACy(pf08936) CsomeACh
19625 HMMcsoS2_1_ACy(pf12288) CsomeACh
19630 HMMrbcS_1_CsomeA(pf00101) CsomeACh
19635 HMMrbcL_1_Csome(pf00016pf02788) CsomeACh
19640 -pf08668 -
19645 -# -
19650 -# -
19655 -# -
19660 -# -
19670 -# -
BMC-P
BMC-H
BMC-T
Aldehyde dehydrogenase
PTAC
Signature enzyme
Alcohol dehydrogenase
Regulator

Gammaproteobacteria bacterium. Bacteria; Proteobacteria; Gammaproteobacteria.

COC09__locus_1

CsomeACh

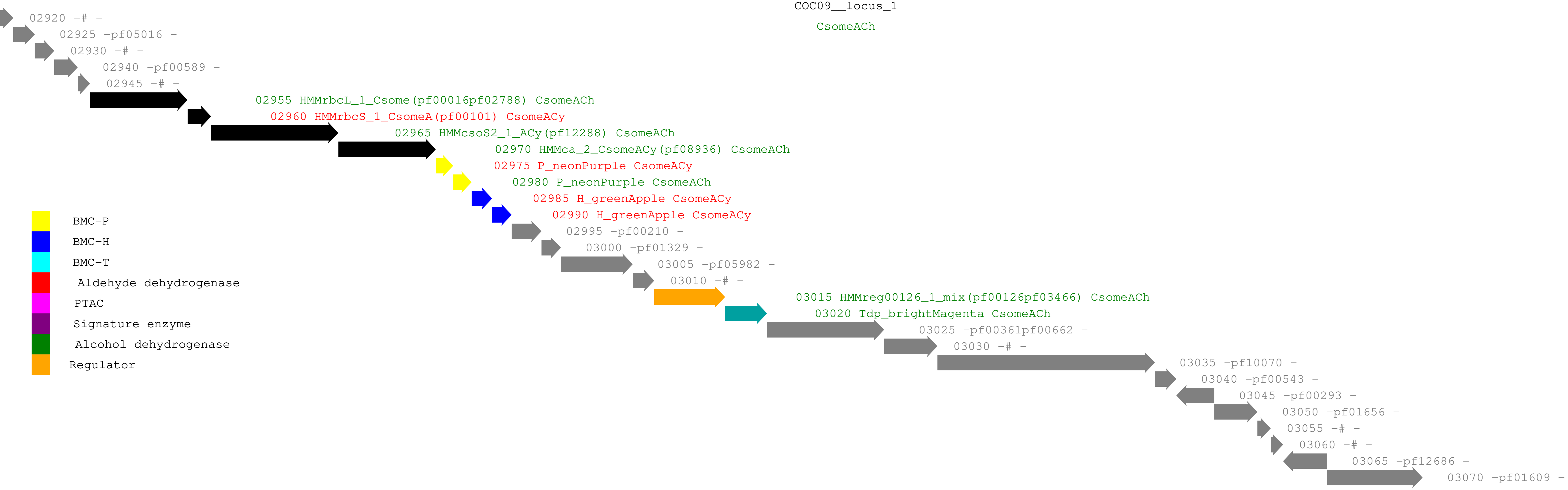

Betaproteobacteria bacterium HGW-Betaproteobacteria-17. Bacteria; Proteobacteria; Betaproteobacteria.

CVU23__locus_1_main_sat

CsomeACh

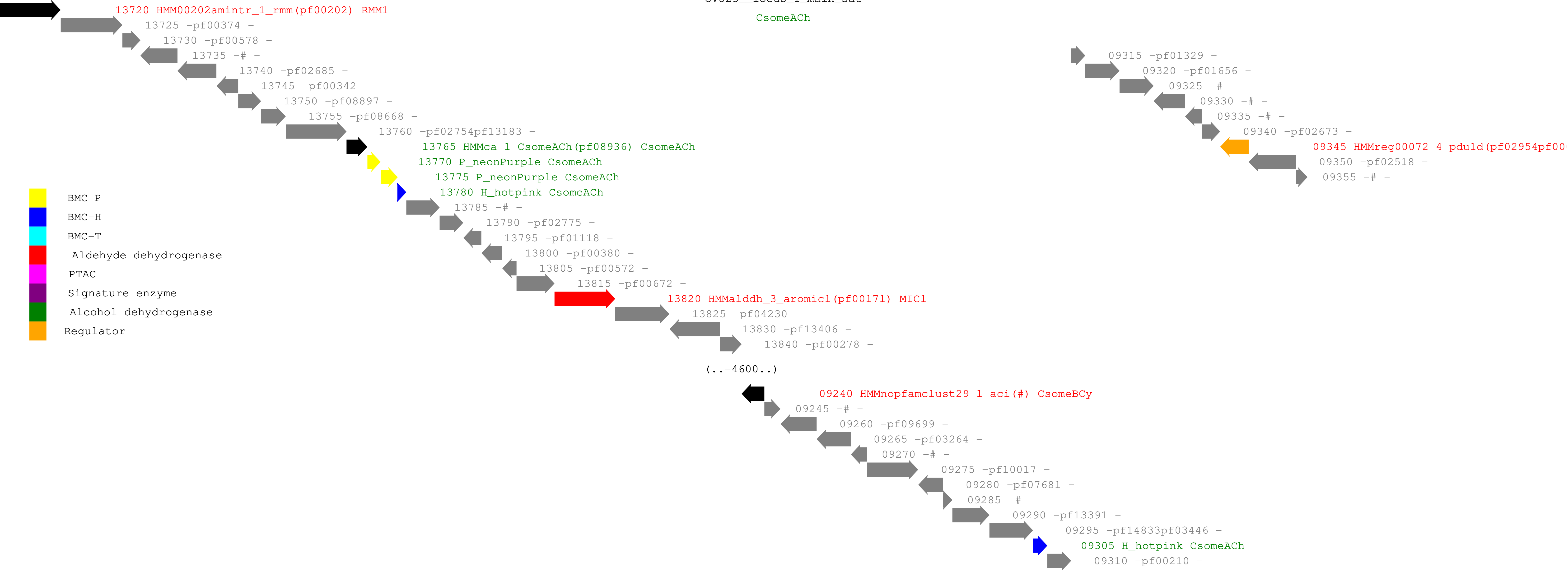

gamma proteobacterium BW-2. Bacteria; Proteobacteria; Gammaproteobacteria.

D5085__locus_1

CsomeACh

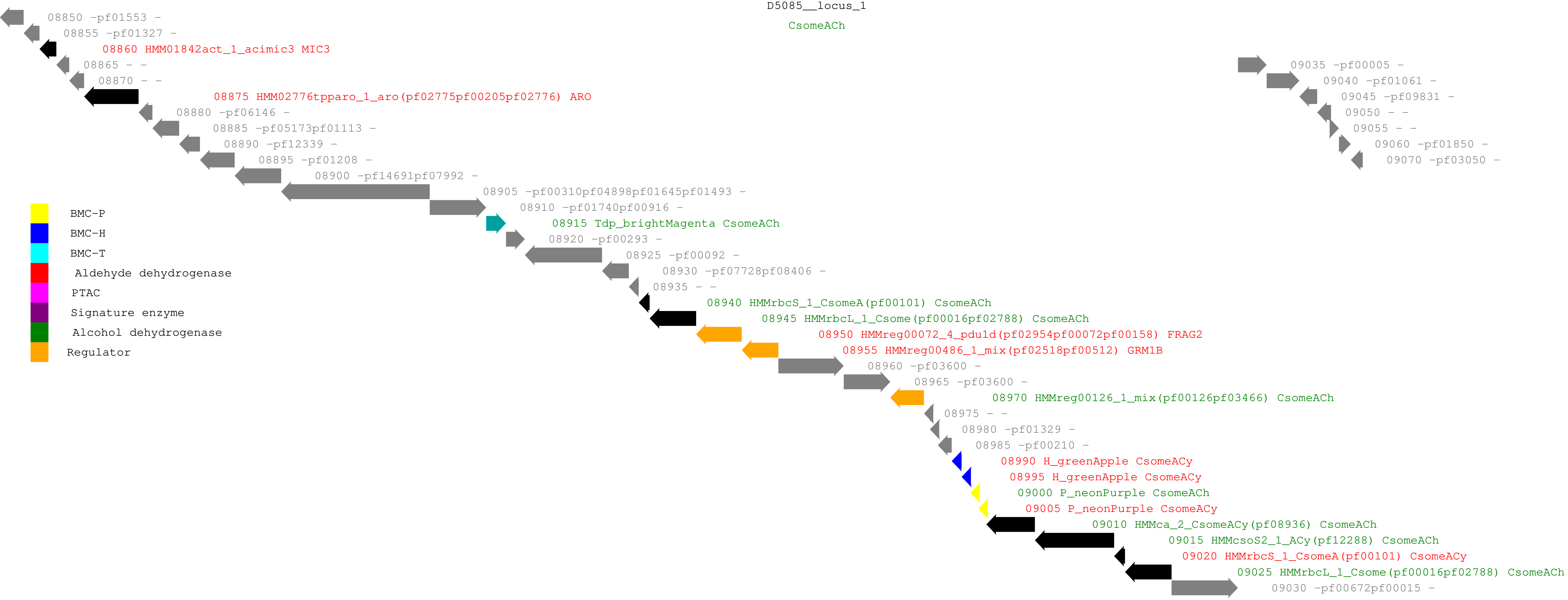

Guyparkeria sp. SCN-R1. Bacteria; Proteobacteria; Gammaproteobacteria; Chromatiales;

D5687__locus_1_main_sat

CsomeACh

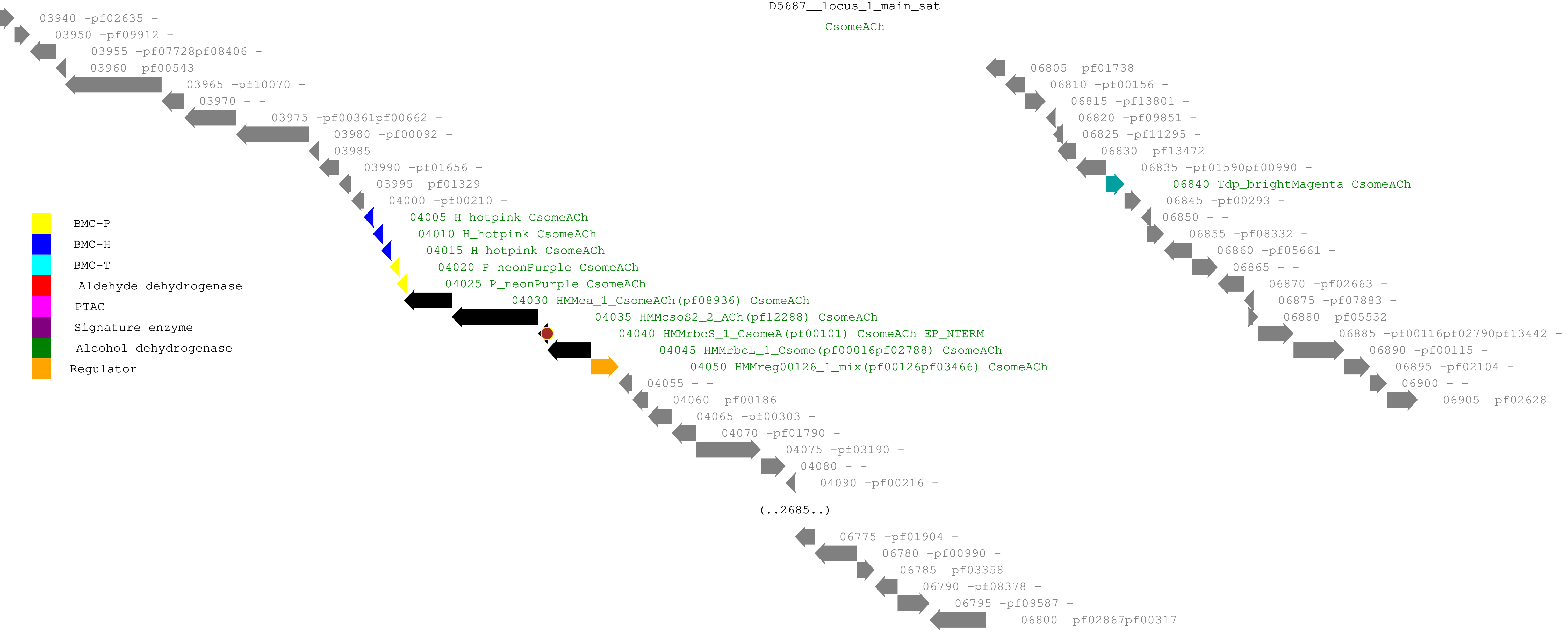

Thiohalobacter thiocyanaticus. Bacteria; Proteobacteria; Gammaproteobacteria; Chromatiales;

D6C00__locus_1

CsomeACh

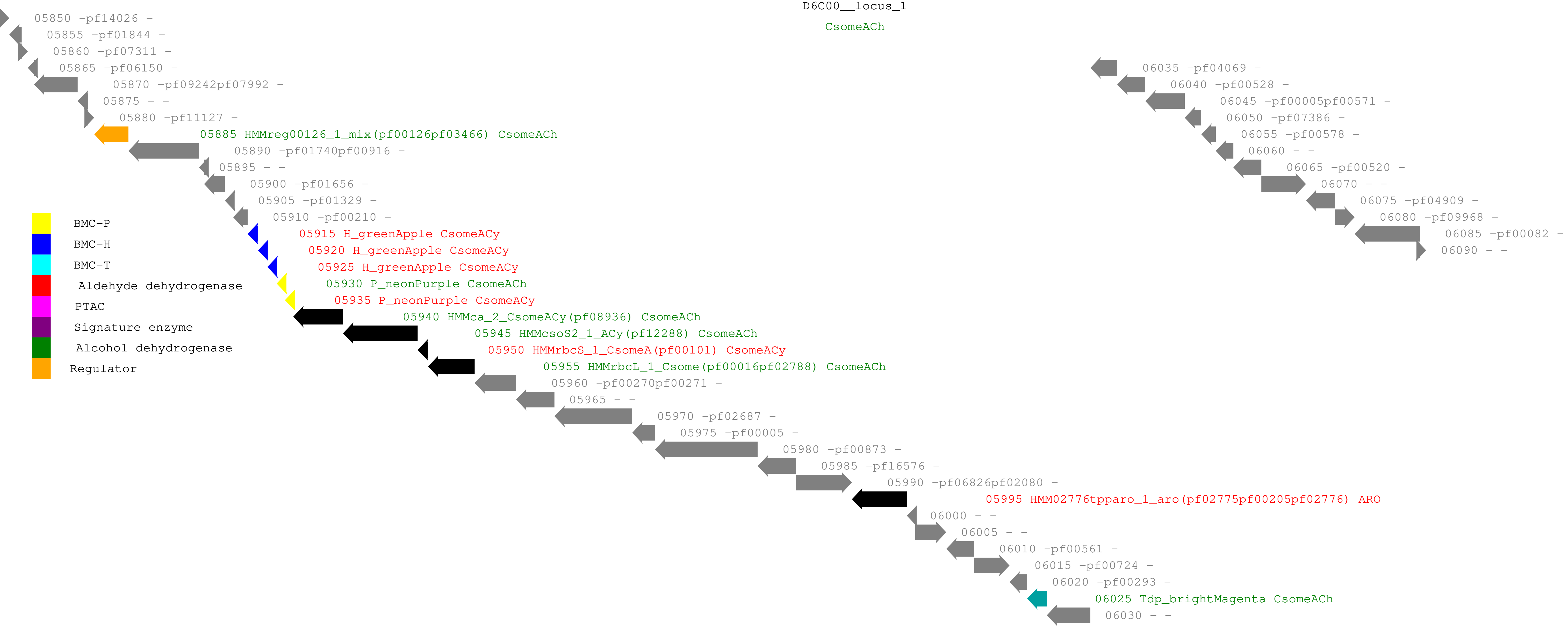

Thiorhodococcus sp. AK35. Bacteria; Proteobacteria; Gammaproteobacteria; Chromatiales;

D779__locus_1

CsomeACh

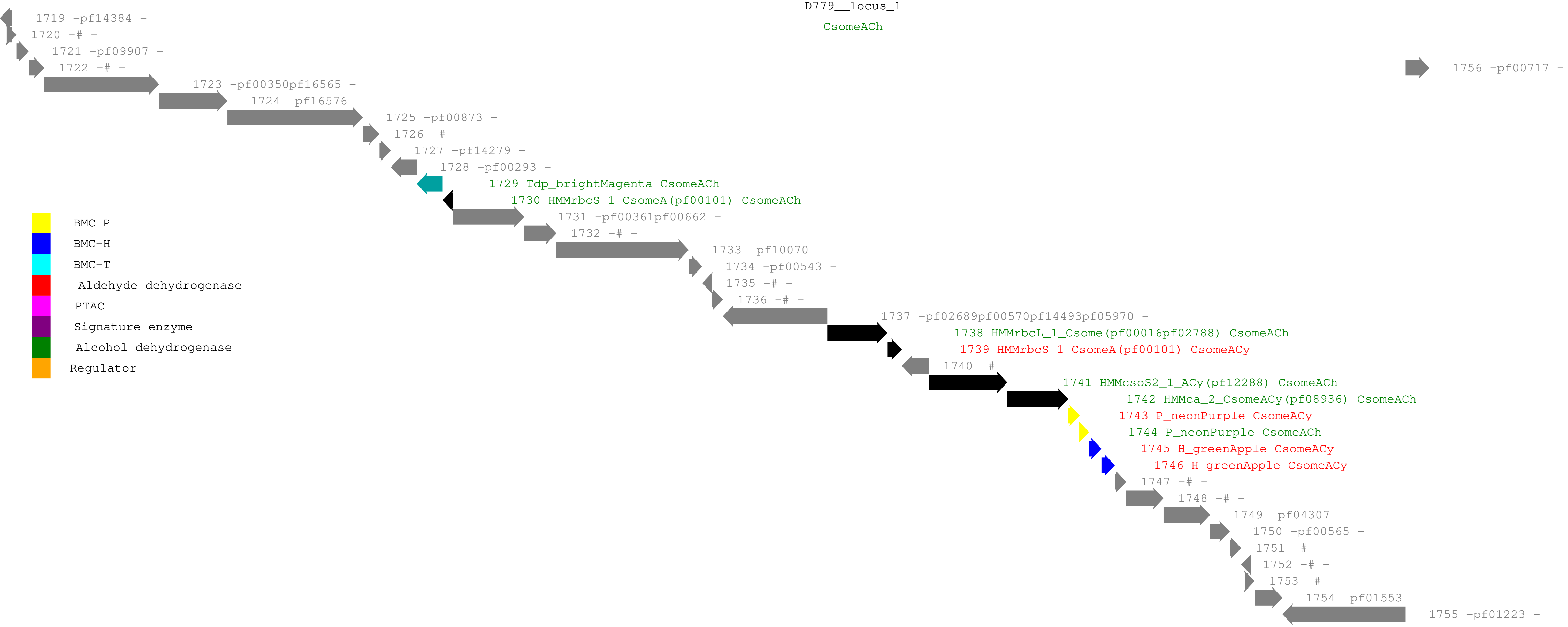

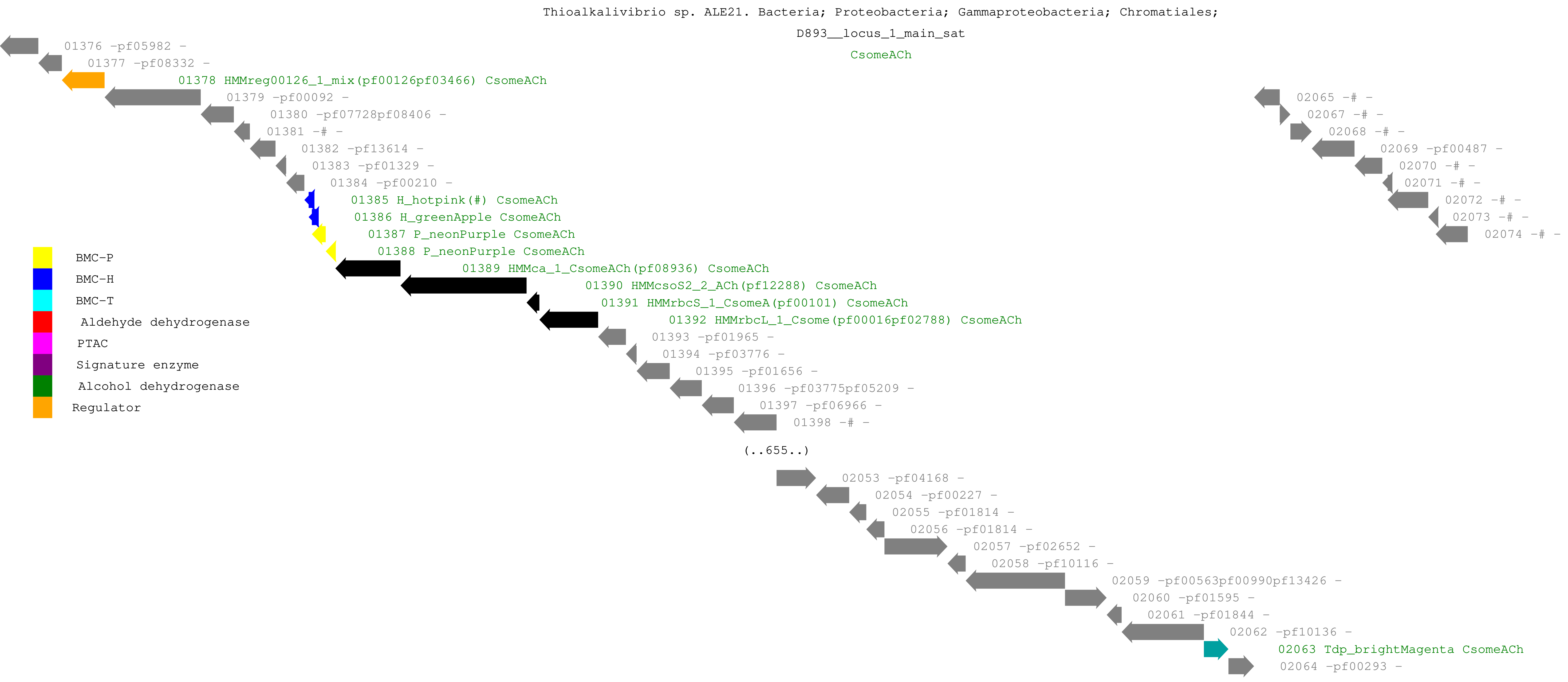
Thioalkalivibrio sp. ALE21. Bacteria; Proteobacteria; Gammaproteobacteria; Chromatiales;
D893__locus_1_main_sat
CsomeACh
01376 -pf05982 -
01377 -pf08332 -
01378 HMMreg00126_1_mix(pf00126pf03466) CsomeACh
01379 -pf00092 -
01380 -pf07728pf08406 -
01381 -# -
01382 -pf13614 -
01383 -pf01329 -
01384 -pf00210 -
01385 H_hotpink(#) CsomeACh
01386 H_greenApple CsomeACh
01387 P_neonPurple CsomeACh
01388 P_neonPurple CsomeACh
01389 HMMca_1_CsomeACh(pf08936) CsomeACh
01390 HMMcsoS2_2_ACh(pf12288) CsomeACh
01391 HMMrbcS_1_CsomeA(pf00101) CsomeACh
01392 HMMrbcL_1_Csome(pf00016pf02788) CsomeACh
01393 -pf01965 -
01394 -pf03776 -
01395 -pf01656 -
01396 -pf03775pf05209 -
01397 -pf06966 -
01398 -# -
(..655..)
02053 -pf04168 -
02054 -pf00227 -
02055 -pf01814 -
02056 -pf01814 -
02057 -pf02652 -
02058 -pf10116 -
02059 -pf00563pf00990pf13426 -
02060 -pf01595 -
02061 -pf01844 -
02062 -pf10136 -
02063 Tdp_brightMagenta CsomeACh
02064 -pf00293 -
02065 -# -
02067 -# -
02068 -# -
02069 -pf00487 -
02070 -# -
02071 -# -
02072 -# -
02073 -# -
02074 -# -
BMC-P
BMC-H
BMC-T
Aldehyde dehydrogenase
PTAC
Signature enzyme
Alcohol dehydrogenase
Regulator

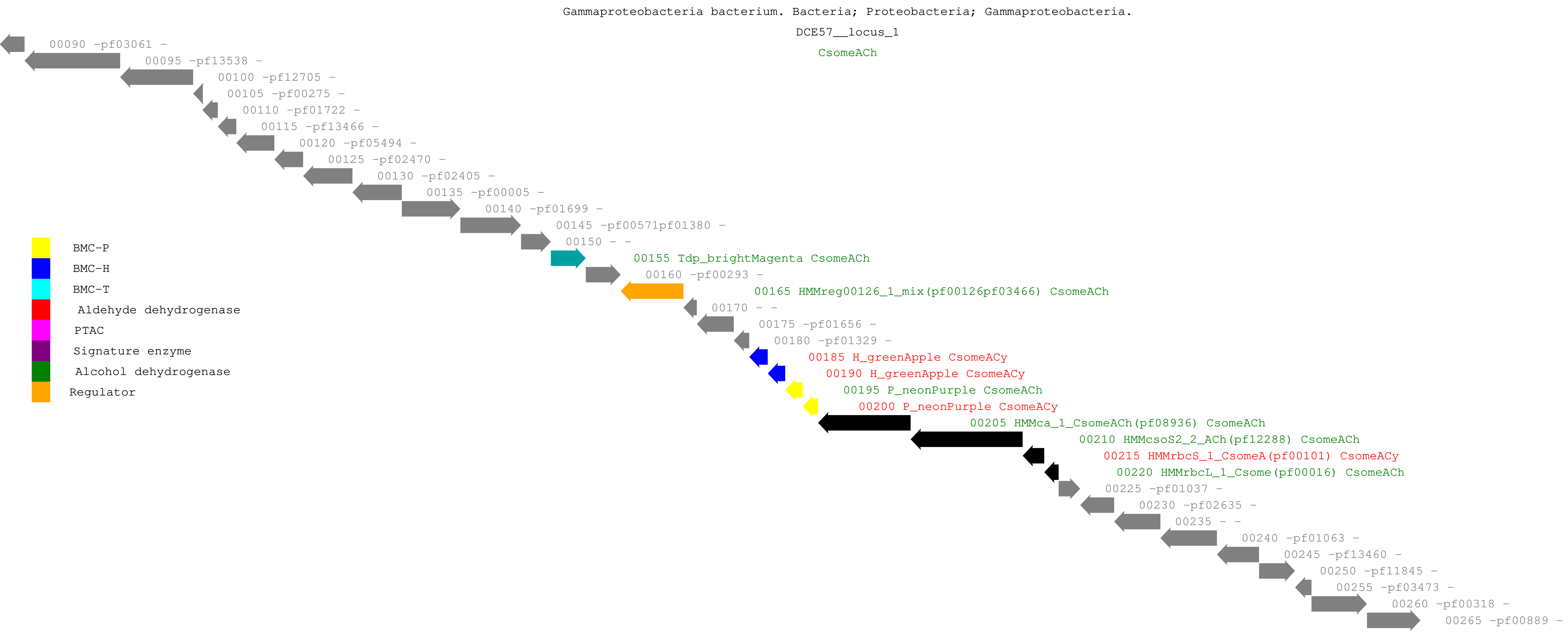
Gammaproteobacteria bacterium. Bacteria; Proteobacteria; Gammaproteobacteria.
DCE57__locus_1
CsomeACh
00090 -pf03061 -
00095 -pf13538 -
00100 -pf12705 -
00105 -pf00275 -
00110 -pf01722 -
00115 -pf13466 -
00120 -pf05494 -
00125 -pf02470 -
00130 -pf02405 -
00135 -pf00005 -
00140 -pf01699 -
00145 -pf00571pf01380 -
00150 - -
00155 Tdp_brightMagenta CsomeACh
00160 -pf00293 -
00165 HMMreg00126_1_mix(pf00126pf03466) CsomeACh
00170 - -
00175 -pf01656 -
00180 -pf01329 -
00185 H_greenApple CsomeACy
00190 H_greenApple CsomeACy
00195 P_neonPurple CsomeACh
00200 P_neonPurple CsomeACy
00205 HMMca_1_CsomeACh(pf08936) CsomeACh
00210 HMMcsoS2_2_ACh(pf12288) CsomeACh
00215 HMMrbcS_1_CsomeA(pf00101) CsomeACy
00220 HMMrbcL_1_Csome(pf00016) CsomeACh
00225 -pf01037 -
00230 -pf02635 -
00235 - -
00240 -pf01063 -
00245 -pf13460 -
00250 -pf11845 -
00255 -pf03473 -
00260 -pf00318 -
00265 -pf00889 -
BMC-P
BMC-H
BMC-T
Aldehyde dehydrogenase
PTAC
Signature enzyme
Alcohol dehydrogenase
Regulator

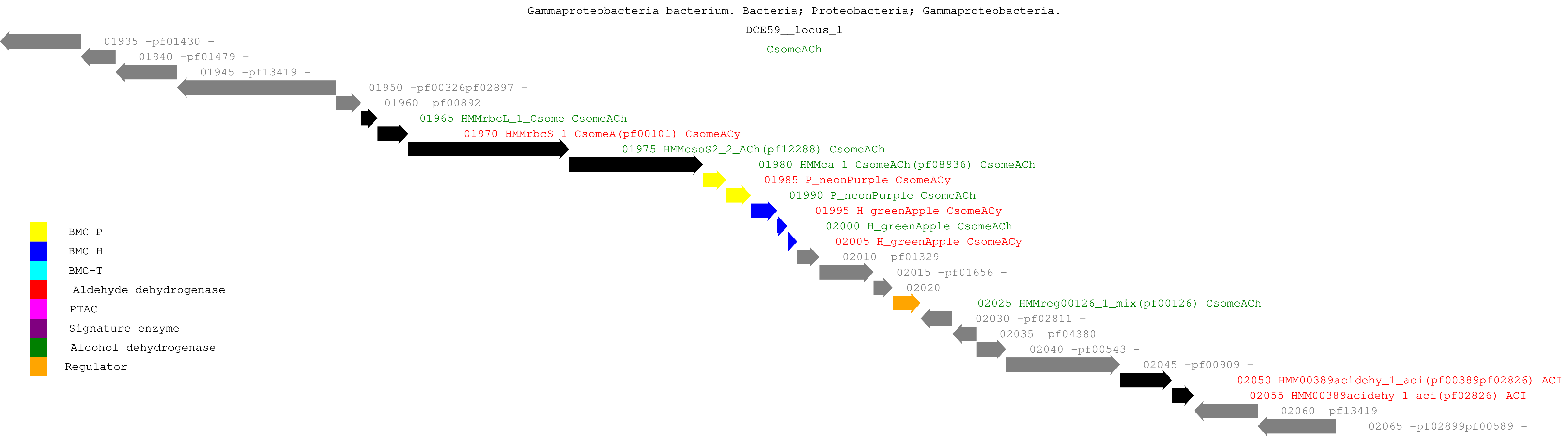
Gammaproteobacteria bacterium. Bacteria; Proteobacteria; Gammaproteobacteria.
DCE59__locus_1
CsomeACh
01935 -pf01430 -
01940 -pf01479 -
01945 -pf13419 -
01950 -pf00326pf02897 -
01960 -pf00892 -
01965 HMMrbcL_1_Csome CsomeACh
01970 HMMrbcS_1_CsomeA(pf00101) CsomeACy
01975 HMMcsoS2_2_ACh(pf12288) CsomeACh
01980 HMMca_1_CsomeACh(pf08936) CsomeACh
01985 P_neonPurple CsomeACy
01990 P_neonPurple CsomeACh
01995 H_greenApple CsomeACy
02000 H_greenApple CsomeACh
02005 H_greenApple CsomeACy
02010 -pf01329 -
02015 -pf01656 -
02020 - -
02025 HMMreg00126_1_mix(pf00126) CsomeACh
02030 -pf02811 -
02035 -pf04380 -
02040 -pf00543 -
02045 -pf00909 -
02050 HMM00389acidehy_1_aci(pf00389pf02826) ACI
02055 HMM00389acidehy_1_aci(pf02826) ACI
02060 -pf13419 -
02065 -pf02899pf00589 -
BMC-P
BMC-H
BMC-T
Aldehyde dehydrogenase
PTAC
Signature enzyme
Alcohol dehydrogenase
Regulator

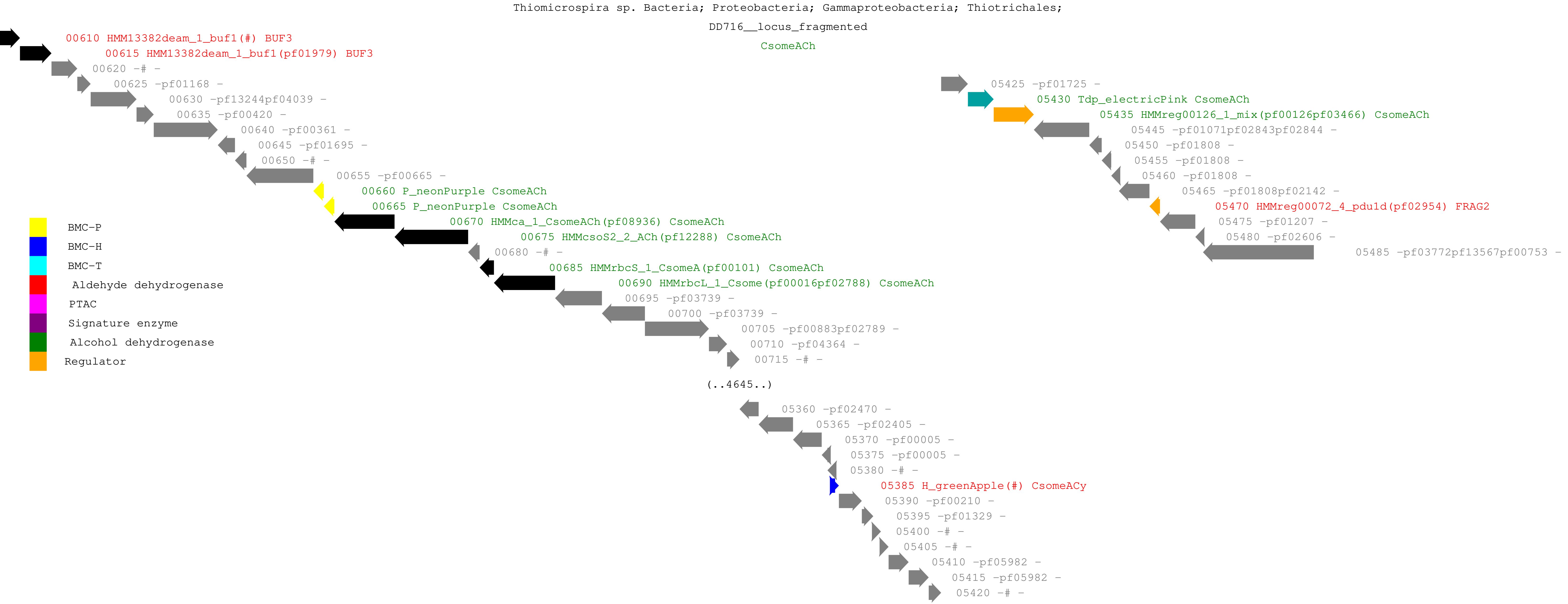
Thiomicrospira sp. Bacteria; Proteobacteria; Gammaproteobacteria; Thiotrichales;
DD716__locus_fragmented
CsomeACh
00610 HMM13382deam_1_buf1(#) BUF3
00615 HMM13382deam_1_buf1(pf01979) BUF3
00620 -# -
00625 -pf01168 -
00630 -pf13244pf04039 -
00635 -pf00420 -
00640 -pf00361 -
00645 -pf01695 -
00650 -# -
00655 -pf00665 -
00660 P_neonPurple CsomeACh
00665 P_neonPurple CsomeACh
00670 HMMca_1_CsomeACh(pf08936) CsomeACh
00675 HMMcsoS2_2_ACh(pf12288) CsomeACh
00680 -# -
00685 HMMrbcS_1_CsomeA(pf00101) CsomeACh
00690 HMMrbcL_1_Csome(pf00016pf02788) CsomeACh
00695 -pf03739 -
00700 -pf03739 -
00705 -pf00883pf02789 -
00710 -pf04364 -
00715 -# -
(..4645..)
05360 -pf02470 -
05365 -pf02405 -
05370 -pf00005 -
05375 -pf00005 -
05380 -# -
05385 H_greenApple(#) CsomeACy
05390 -pf00210 -
05395 -pf01329 -
05400 -# -
05405 -# -
05410 -pf05982 -
05415 -pf05982 -
05420 -# -
05425 -pf01725 -
05430 Tdp_electricPink CsomeACh
05435 HMMreg00126_1_mix(pf00126pf03466) CsomeACh
05445 -pf01071pf02843pf02844 -
05450 -pf01808 -
05455 -pf01808 -
05460 -pf01808 -
05465 -pf01808pf02142 -
05470 HMMreg00072_4_pduld(pf02954) FRAG2
05475 -pf01207 -
05480 -pf02606 -
05485 -pf03772pf13567pf00753 -
BMC-P
BMC-H
BMC-T
Aldehyde dehydrogenase
PTAC
Signature enzyme
Alcohol dehydrogenase
Regulator

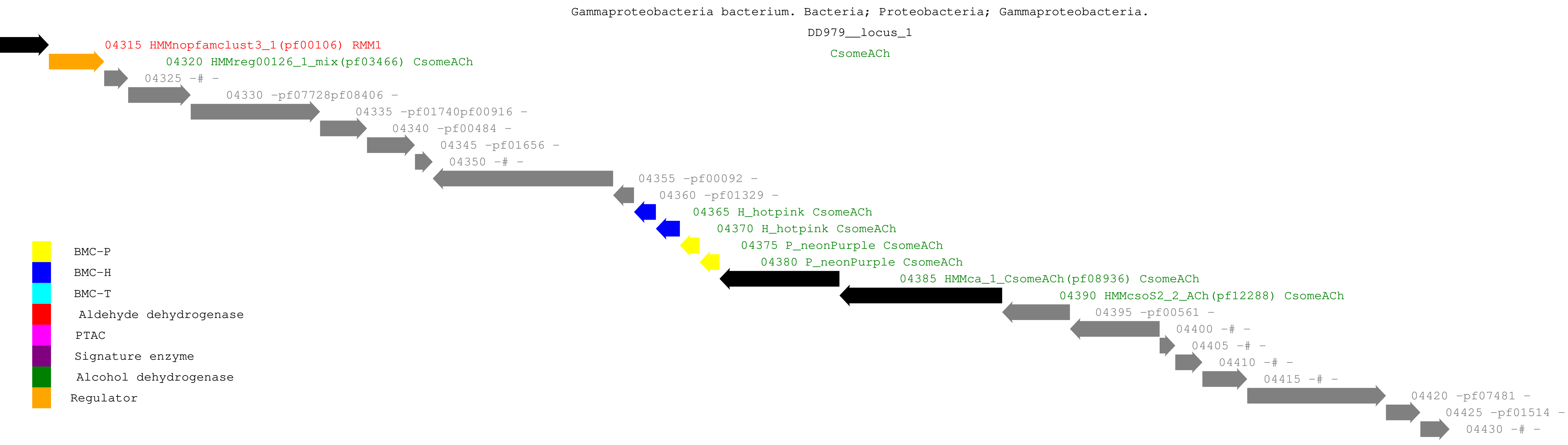

Gammaproteobacteria bacterium. Bacteria; Proteobacteria; Gammaproteobacteria.
DD979__locus_1
CsomeACh
04315 HMMnopfamclust3_1(pf00106) RMM1
04320 HMMreg00126_1_mix(pf03466) CsomeACh
04325 -# -
04330 -pf07728pf08406 -
04335 -pf01740pf00916 -
04340 -pf00484 -
04345 -pf01656 -
04350 -# -
04355 -pf00092 -
04360 -pf01329 -
04365 H_hotpink CsomeACh
04370 H_hotpink CsomeACh
04375 P_neonPurple CsomeACh
04380 P_neonPurple CsomeACh
04385 HMMca_1_CsomeACh(pf08936) CsomeACh
04390 HMMcsoS2_2_ACh(pf12288) CsomeACh
04395 -pf00561 -
04400 -# -
04405 -# -
04410 -# -
04415 -# -
04420 -pf07481 -
04425 -pf01514 -
04430 -# -
BMC-P
BMC-H
BMC-T
Aldehyde dehydrogenase
PTAC
Signature enzyme
Alcohol dehydrogenase
Regulator

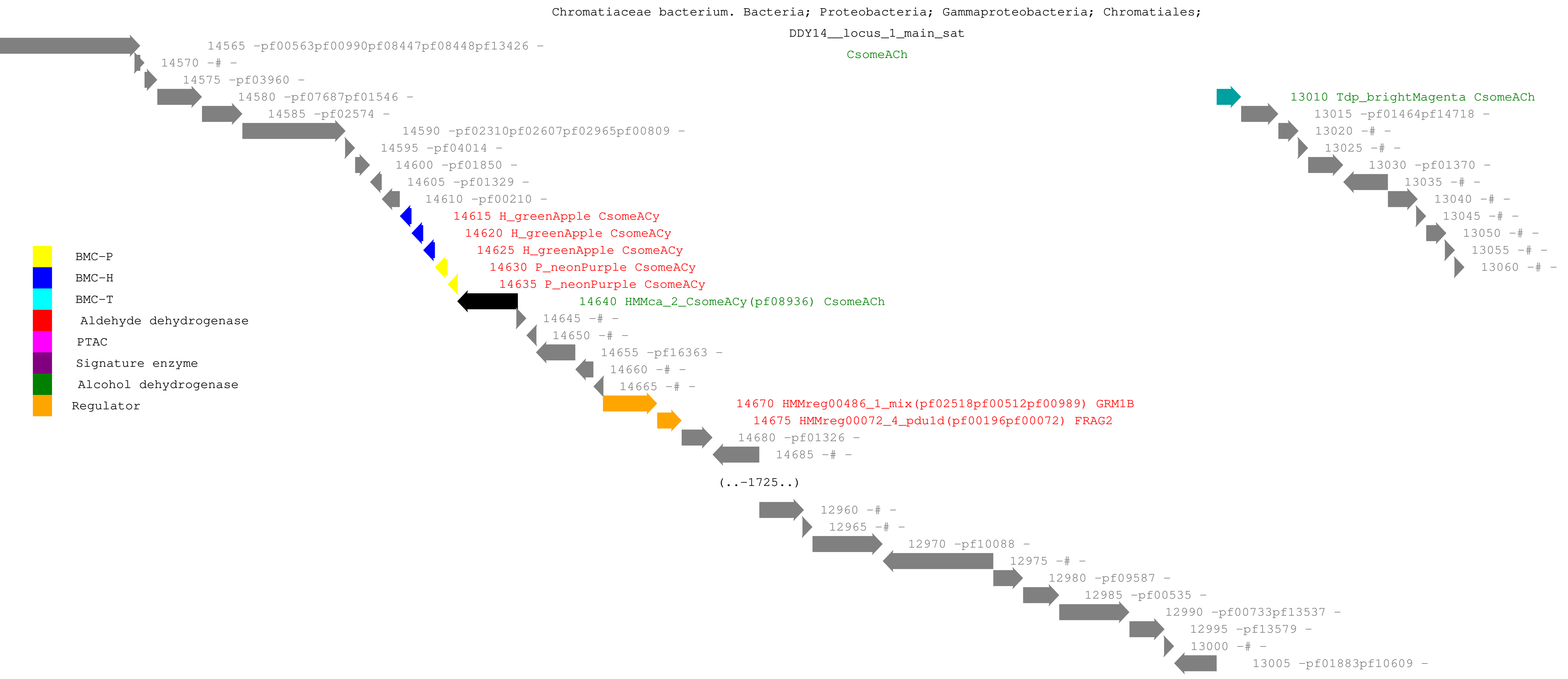
Chromatiaceae bacterium. Bacteria; Proteobacteria; Gammaproteobacteria; Chromatiales;
DDY14__locus_1_main_sat
CsomeACh
14565 -pf00563pf00990pf08447pf08448pf13426 -
14570 -# -
14575 -pf03960 -
14580 -pf07687pf01546 -
14585 -pf02574 -
14590 -pf02310pf02607pf02965pf00809 -
14595 -pf04014 -
14600 -pf01850 -
14605 -pf01329 -
14610 -pf00210 -
14615 H_greenApple CsomeACy
14620 H_greenApple CsomeACy
14625 H_greenApple CsomeACy
14630 P_neonPurple CsomeACy
14635 P_neonPurple CsomeACy
14640 HMMca_2_CsomeACy(pf08936) CsomeACh
14645 -# -
14650 -# -
14655 -pf16363 -
14660 -# -
14665 -# -
14670 HMMreg00486_1_mix(pf02518pf00512pf00989) GRM1B
14675 HMMreg00072_4_pdu1d(pf00196pf00072) FRAG2
14680 -pf01326 -
14685 -# -
(..-1725..)
12960 -# -
12965 -# -
12970 -pf10088 -
12975 -# -
12980 -pf09587 -
12985 -pf00535 -
12990 -pf00733pf13537 -
12995 -pf13579 -
13000 -# -
13005 -pf01883pf10609 -
13010 Tdp_brightMagenta CsomeACh
13015 -pf01464pf14718 -
13020 -# -
13025 -# -
13030 -pf01370 -
13035 -# -
13040 -# -
13045 -# -
13050 -# -
13055 -# -
13060 -# -
BMC-P
BMC-H
BMC-T
Aldehyde dehydrogenase
PTAC
Signature enzyme
Alcohol dehydrogenase
Regulator

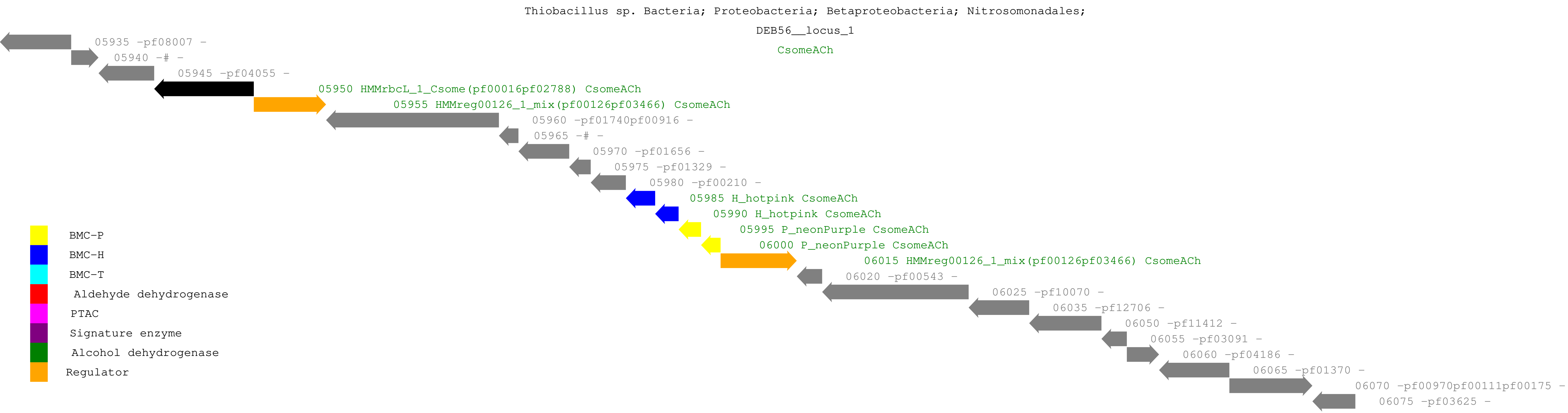

Thiobacillus sp. Bacteria; Proteobacteria; Betaproteobacteria; Nitrosomonadales;
DEB56__locus_1
CsomeACh
05935 -pf08007 -
05940 -# -
05945 -pf04055 -
05950 HMMrbcL_1_Csome(pf00016pf02788) CsomeACh
05955 HMMreg00126_1_mix(pf00126pf03466) CsomeACh
05960 -pf01740pf00916 -
05965 -# -
05970 -pf01656 -
05975 -pf01329 -
05980 -pf00210 -
05985 H_hotpink CsomeACh
05990 H_hotpink CsomeACh
05995 P_neonPurple CsomeACh
06000 P_neonPurple CsomeACh
06015 HMMreg00126_1_mix(pf00126pf03466) CsomeACh
06020 -pf00543 -
06025 -pf10070 -
06035 -pf12706 -
06050 -pf11412 -
06055 -pf03091 -
06060 -pf04186 -
06065 -pf01370 -
06070 -pf00970pf00111pf00175 -
06075 -pf03625 -
BMC-P
BMC-H
BMC-T
Aldehyde dehydrogenase
PTAC
Signature enzyme
Alcohol dehydrogenase
Regulator

Azospira oryzae. Bacteria; Proteobacteria; Betaproteobacteria; Rhodocyclales;

DI596__locus_fragmented

CsomeACh

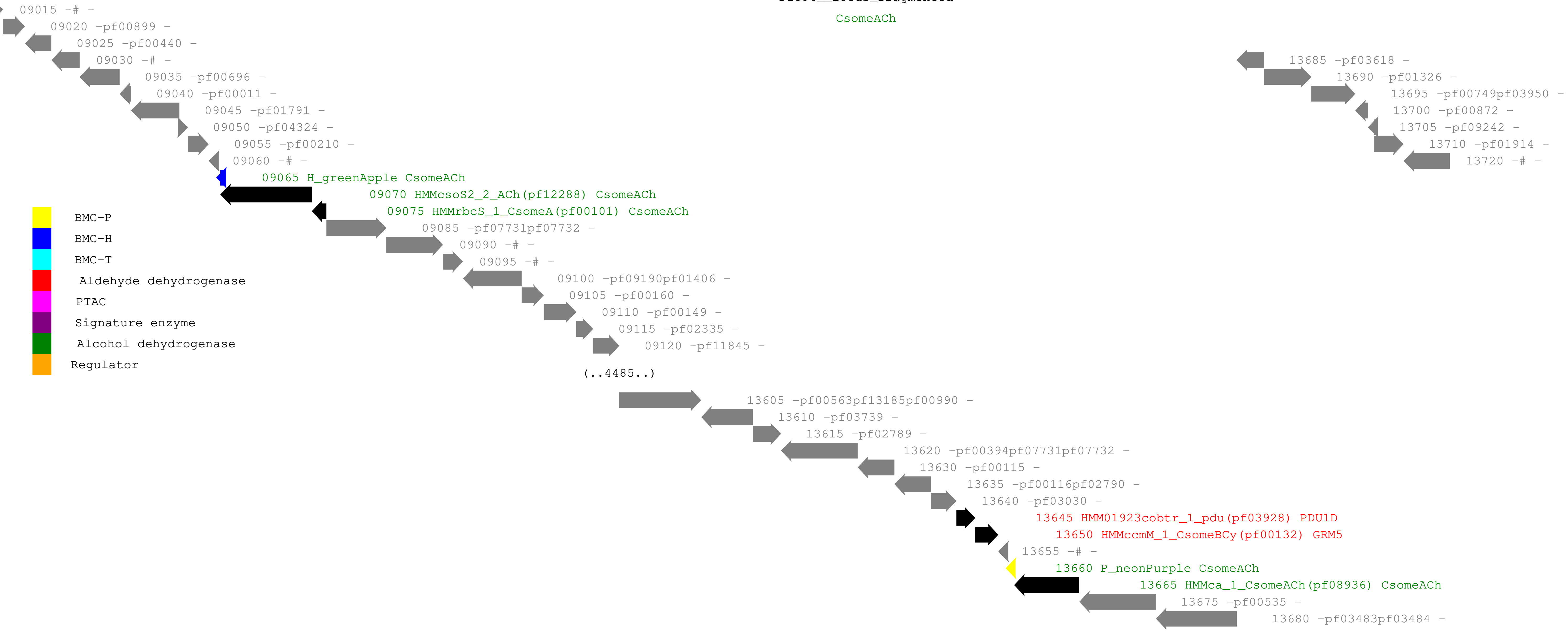

Burkholderiales bacterium. Bacteria; Proteobacteria; Betaproteobacteria; Burkholderiales.

DIT38__locus_1

CsomeACh

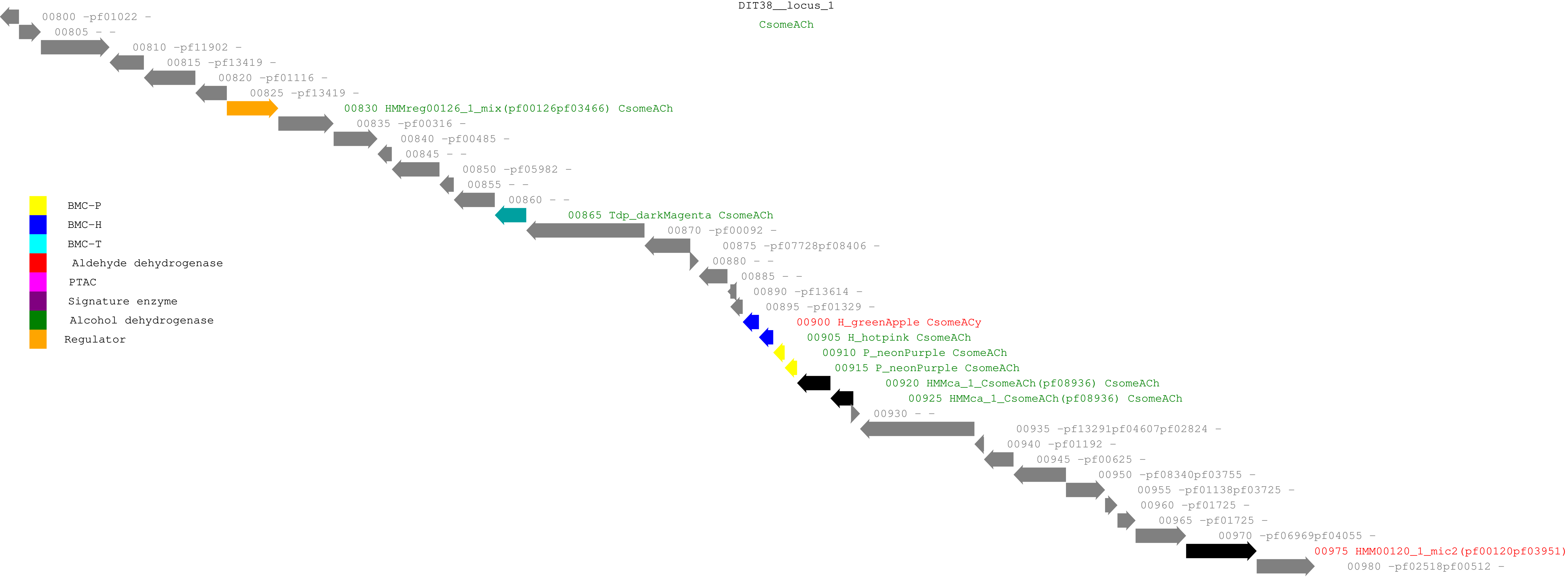

Chromatiaceae bacterium. Bacteria; Proteobacteria; Gammaproteobacteria; Chromatiales; Chromatiaceae.

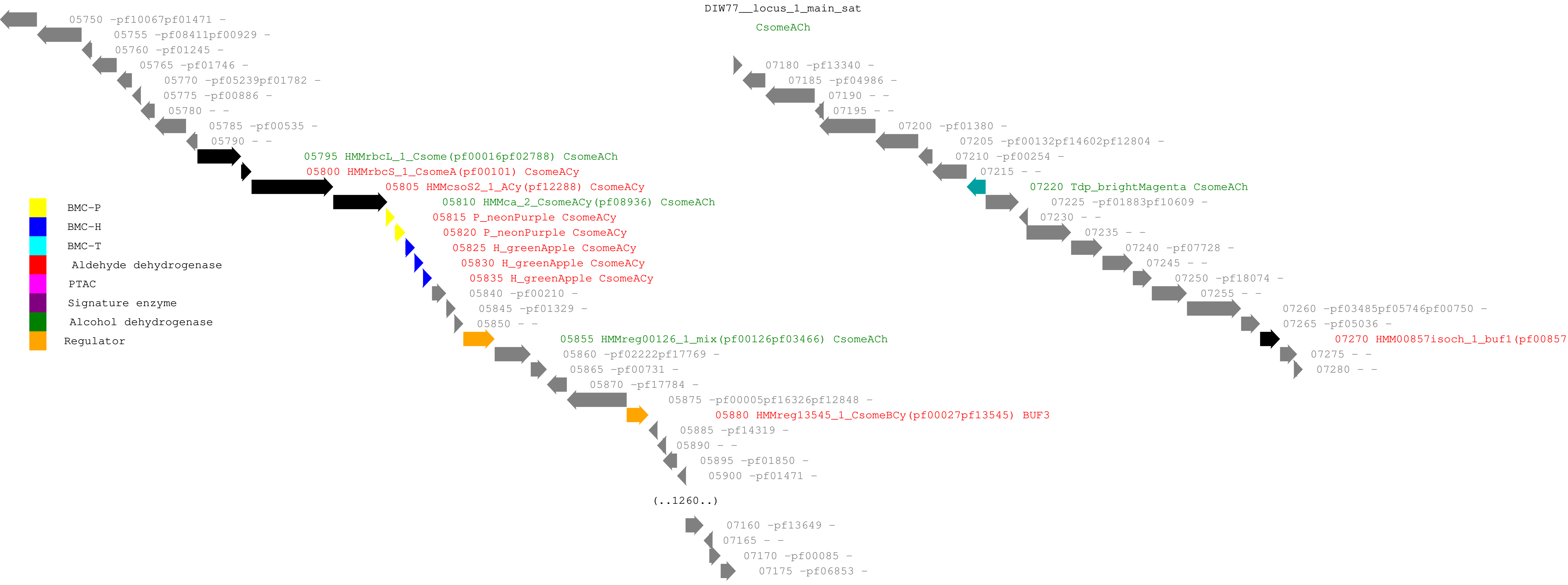

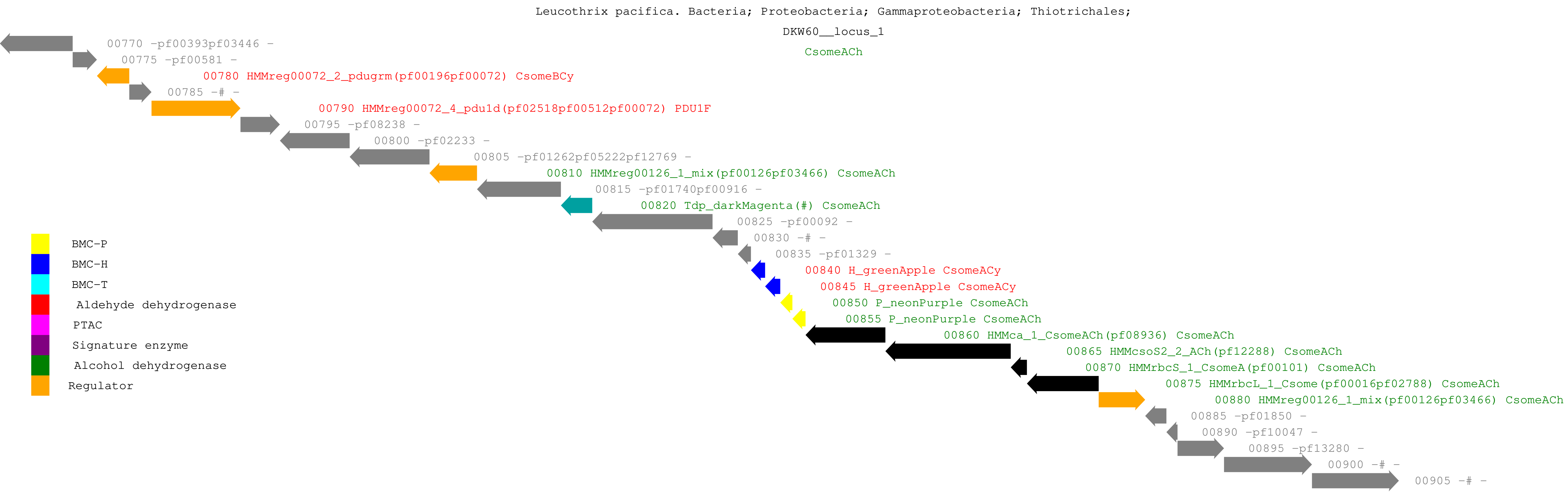

Leucothrix pacifica. Bacteria; Proteobacteria; Gammaproteobacteria; Thiotrichales;
DKW60__locus_1
CsomeACh
00770 -pf00393pf03446 -
00775 -pf00581 -
00780 HMMreg00072_2_pdugrm(pf00196pf00072) CsomeBCy
00785 -# -
00790 HMMreg00072_4_pdu1d(pf02518pf00512pf00072) PDU1F
00795 -pf08238 -
00800 -pf02233 -
00805 -pf01262pf05222pf12769 -
00810 HMMreg00126_1_mix(pf00126pf03466) CsomeACh
00815 -pf01740pf00916 -
00820 Tdp_darkMagenta(#) CsomeACh
00825 -pf00092 -
00830 -# -
00835 -pf01329 -
00840 H_greenApple CsomeACy
00845 H_greenApple CsomeACy
00850 P_neonPurple CsomeACh
00855 P_neonPurple CsomeACh
00860 HMMca_1_CsomeACh(pf08936) CsomeACh
00865 HMMcsoS2_2_ACh(pf12288) CsomeACh
00870 HMMrbcS_1_CsomeA(pf00101) CsomeACh
00875 HMMrbcL_1_Csome(pf00016pf02788) CsomeACh
00880 HMMreg00126_1_mix(pf00126pf03466) CsomeACh
00885 -pf01850 -
00890 -pf10047 -
00895 -pf13280 -
00900 -# -
00905 -# -
BMC-P
BMC-H
BMC-T
Aldehyde dehydrogenase
PTAC
Signature enzyme
Alcohol dehydrogenase
Regulator

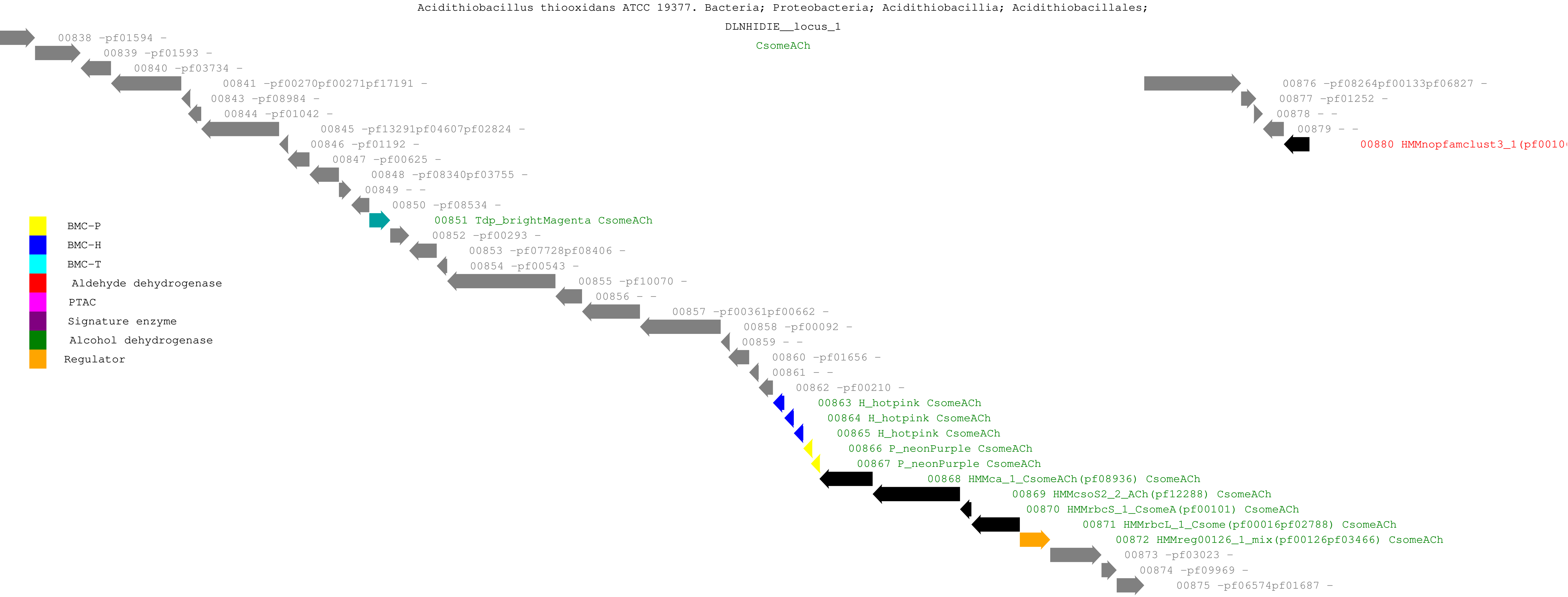

Acidithiobacillus thiooxidans ATCC 19377. Bacteria; Proteobacteria; Acidithiobacillia; Acidithiobacillales;
DLNHIDIE__locus_1
CsomeACh
00838 -pf01594 -
00839 -pf01593 -
00840 -pf03734 -
00841 -pf00270pf00271pf17191 -
00843 -pf08984 -
00844 -pf01042 -
00845 -pf13291pf04607pf02824 -
00846 -pf01192 -
00847 -pf00625 -
00848 -pf08340pf03755 -
00849 - -
00850 -pf08534 -
00851 Tdp_brightMagenta CsomeACh
00852 -pf00293 -
00853 -pf07728pf08406 -
00854 -pf00543 -
00855 -pf10070 -
00856 - -
00857 -pf00361pf00662 -
00858 -pf00092 -
00859 - -
00860 -pf01656 -
00861 - -
00862 -pf00210 -
00863 H_hotpink CsomeACh
00864 H_hotpink CsomeACh
00865 H_hotpink CsomeACh
00866 P_neonPurple CsomeACh
00867 P_neonPurple CsomeACh
00868 HMMca_1_CsomeACh(pf08936) CsomeACh
00869 HMMcsoS2_2_ACh(pf12288) CsomeACh
00870 HMMrbcS_1_CsomeA(pf00101) CsomeACh
00871 HMMrbcL_1_Csome(pf00016pf02788) CsomeACh
00872 HMMreg00126_1_mix(pf00126pf03466) CsomeACh
00873 -pf03023 -
00874 -pf09969 -
00875 -pf06574pf01687 -
00876 -pf08264pf00133pf06827 -
00877 -pf01252 -
00878 - -
00879 - -
00880 HMMnopfamclust3_1(pf0010
BMC-P
BMC-H
BMC-T
Aldehyde dehydrogenase
PTAC
Signature enzyme
Alcohol dehydrogenase
Regulator

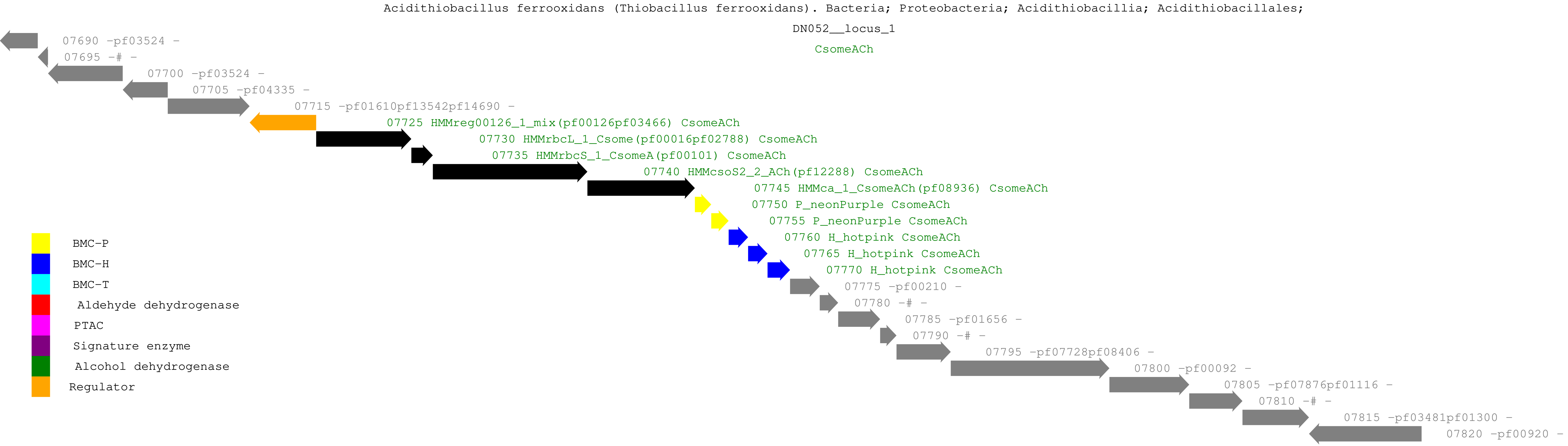
Acidithiobacillus ferrooxidans (Thiobacillus ferrooxidans). Bacteria; Proteobacteria; Acidithiobacillia; Acidithiobacillales;
DN052__locus_1
CsomeACh
07690 -pf03524 -
07695 -# -
07700 -pf03524 -
07705 -pf04335 -
07715 -pf01610pf13542pf14690 -
07725 HMMreg00126_1_mix(pf00126pf03466) CsomeACh
07730 HMMrbcL_1_Csome(pf00016pf02788) CsomeACh
07735 HMMrbcS_1_CsomeA(pf00101) CsomeACh
07740 HMMcsoS2_2_ACh(pf12288) CsomeACh
07745 HMMca_1_CsomeACh(pf08936) CsomeACh
07750 P_neonPurple CsomeACh
07755 P_neonPurple CsomeACh
07760 H_hotpink CsomeACh
07765 H_hotpink CsomeACh
07770 H_hotpink CsomeACh
07775 -pf00210 -
07780 -# -
07785 -pf01656 -
07790 -# -
07795 -pf07728pf08406 -
07800 -pf00092 -
07805 -pf07876pf01116 -
07810 -# -
07815 -pf03481pf01300 -
07820 -pf00920 -
BMC-P
BMC-H
BMC-T
Aldehyde dehydrogenase
PTAC
Signature enzyme
Alcohol dehydrogenase
Regulator

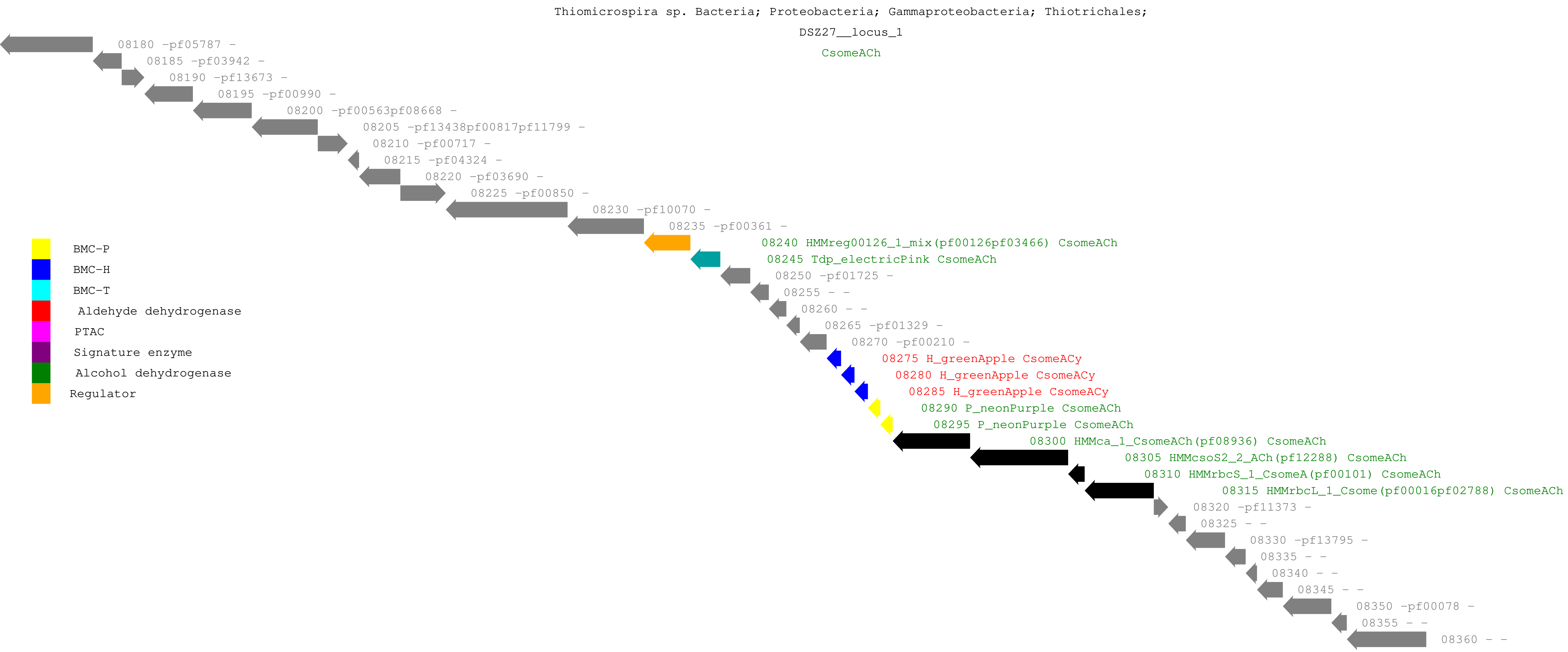
Thiomicrospira sp. Bacteria; Proteobacteria; Gammaproteobacteria; Thiotrichales;
DSZ27__locus_1
CsomeACh
08180 -pf05787 -
08185 -pf03942 -
08190 -pf13673 -
08195 -pf00990 -
08200 -pf00563pf08668 -
08205 -pf13438pf00817pf11799 -
08210 -pf00717 -
08215 -pf04324 -
08220 -pf03690 -
08225 -pf00850 -
08230 -pf10070 -
08235 -pf00361 -
08240 HMMreg00126_1_mix(pf00126pf03466) CsomeACh
08245 Tdp_electricPink CsomeACh
08250 -pf01725 -
08255 - -
08260 - -
08265 -pf01329 -
08270 -pf00210 -
08275 H_greenApple CsomeACy
08280 H_greenApple CsomeACy
08285 H_greenApple CsomeACy
08290 P_neonPurple CsomeACh
08295 P_neonPurple CsomeACh
08300 HMMca_1_CsomeACh(pf08936) CsomeACh
08305 HMMcsoS2_2_ACh(pf12288) CsomeACh
08310 HMMrbcS_1_CsomeA(pf00101) CsomeACh
08315 HMMrbcL_1_Csome(pf00016pf02788) CsomeACh
08320 -pf11373 -
08325 - -
08330 -pf13795 -
08335 - -
08340 - -
08345 - -
08350 -pf00078 -
08355 - -
08360 - -
BMC-P
BMC-H
BMC-T
Aldehyde dehydrogenase
PTAC
Signature enzyme
Alcohol dehydrogenase
Regulator

Burkholderiales bacterium GJ-E10. Bacteria; Proteobacteria; Betaproteobacteria; Burkholderiales.

E10__locus_1

CsomeACh

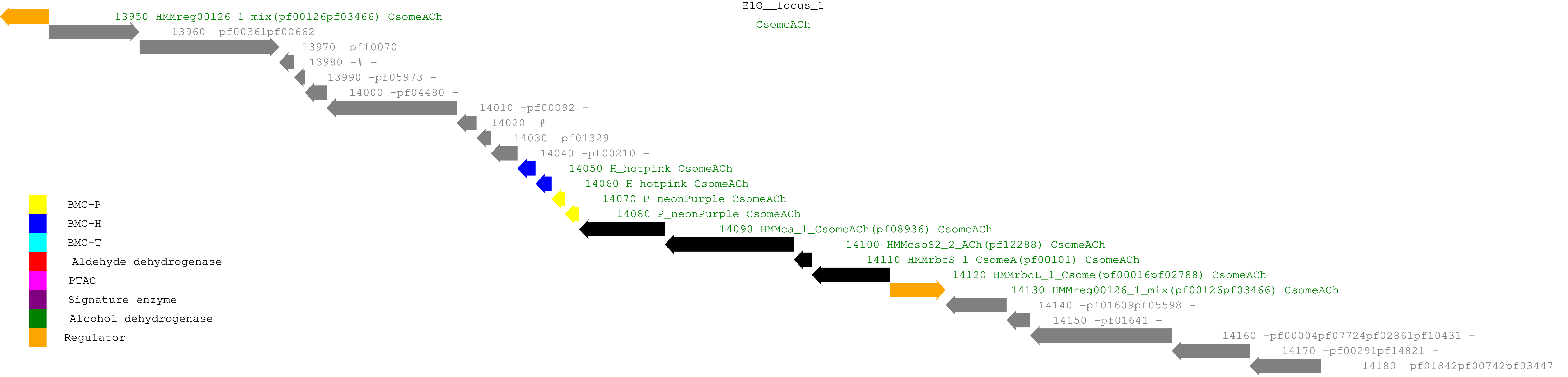

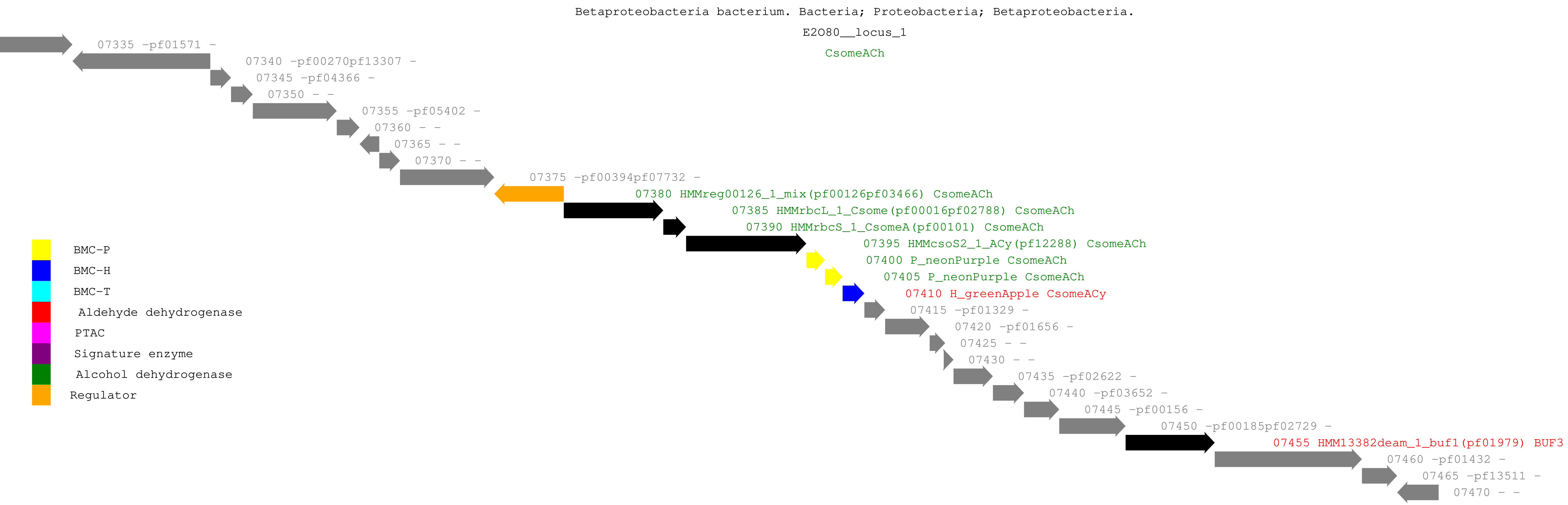

Betaproteobacteria bacterium. Bacteria; Proteobacteria; Betaproteobacteria.
E2O80__locus_1
CsomeACh
07335 -pf01571 -
07340 -pf00270pf13307 -
07345 -pf04366 -
07350 - -
07355 -pf05402 -
07360 - -
07365 - -
07370 - -
07375 -pf00394pf07732 -
07380 HMMreg00126_1_mix(pf00126pf03466) CsomeACh
07385 HMMrbcL_1_Csome(pf00016pf02788) CsomeACh
07390 HMMrbcS_1_CsomeA(pf00101) CsomeACh
07395 HMMcsoS2_1_ACy(pf12288) CsomeACh
07400 P_neonPurple CsomeACh
07405 P_neonPurple CsomeACh
07410 H_greenApple CsomeACy
07415 -pf01329 -
07420 -pf01656 -
07425 - -
07430 - -
07435 -pf02622 -
07440 -pf03652 -
07445 -pf00156 -
07450 -pf00185pf02729 -
07455 HMM13382deam_1_buf1(pf01979) BUF3
07460 -pf01432 -
07465 -pf13511 -
07470 - -
BMC-P
BMC-H
BMC-T
Aldehyde dehydrogenase
PTAC
Signature enzyme
Alcohol dehydrogenase
Regulator

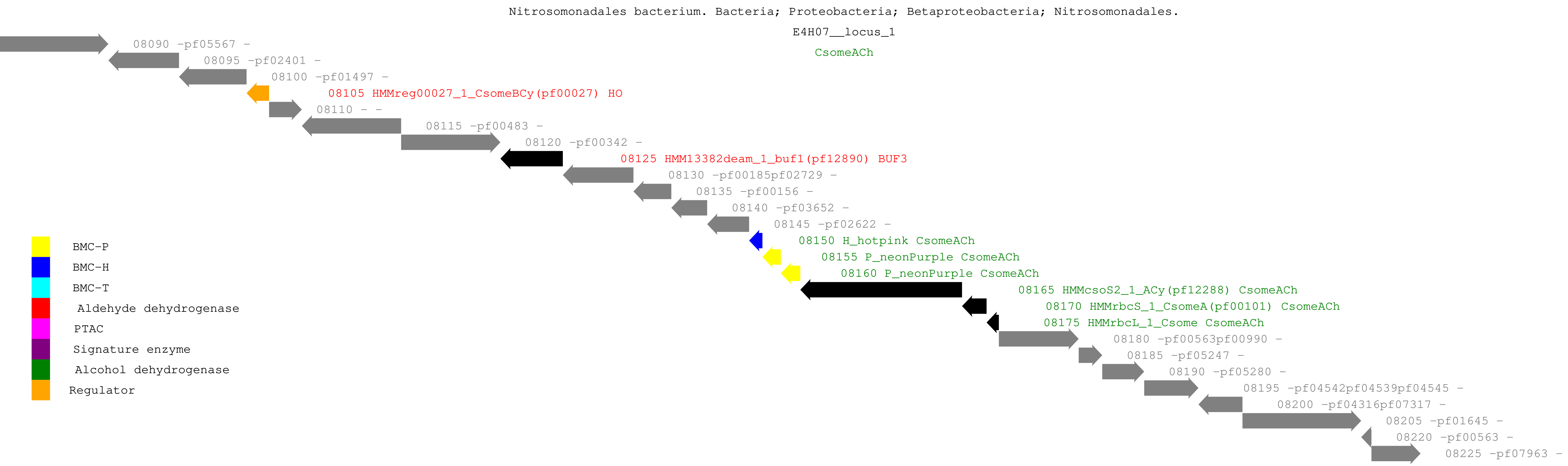
Nitrosomonadales bacterium. Bacteria; Proteobacteria; Betaproteobacteria; Nitrosomonadales.
E4H07__locus_1
CsomeACh
08090 -pf05567 -
08095 -pf02401 -
08100 -pf01497 -
08105 HMMreg00027_1_CsomeBCy(pf00027) HO
08110 - -
08115 -pf00483 -
08120 -pf00342 -
08125 HMM13382deam_1_buf1(pf12890) BUF3
08130 -pf00185pf02729 -
08135 -pf00156 -
08140 -pf03652 -
08145 -pf02622 -
08150 H_hotpink CsomeACh
08155 P_neonPurple CsomeACh
08160 P_neonPurple CsomeACh
08165 HMMcsoS2_1_ACy(pf12288) CsomeACh
08170 HMMrbcS_1_CsomeA(pf00101) CsomeACh
08175 HMMrbcL_1_Csome CsomeACh
08180 -pf00563pf00990 -
08185 -pf05247 -
08190 -pf05280 -
08195 -pf04542pf04539pf04545 -
08200 -pf04316pf07317 -
08205 -pf01645 -
08220 -pf00563 -
08225 -pf07963 -
BMC-P
BMC-H
BMC-T
Aldehyde dehydrogenase
PTAC
Signature enzyme
Alcohol dehydrogenase
Regulator

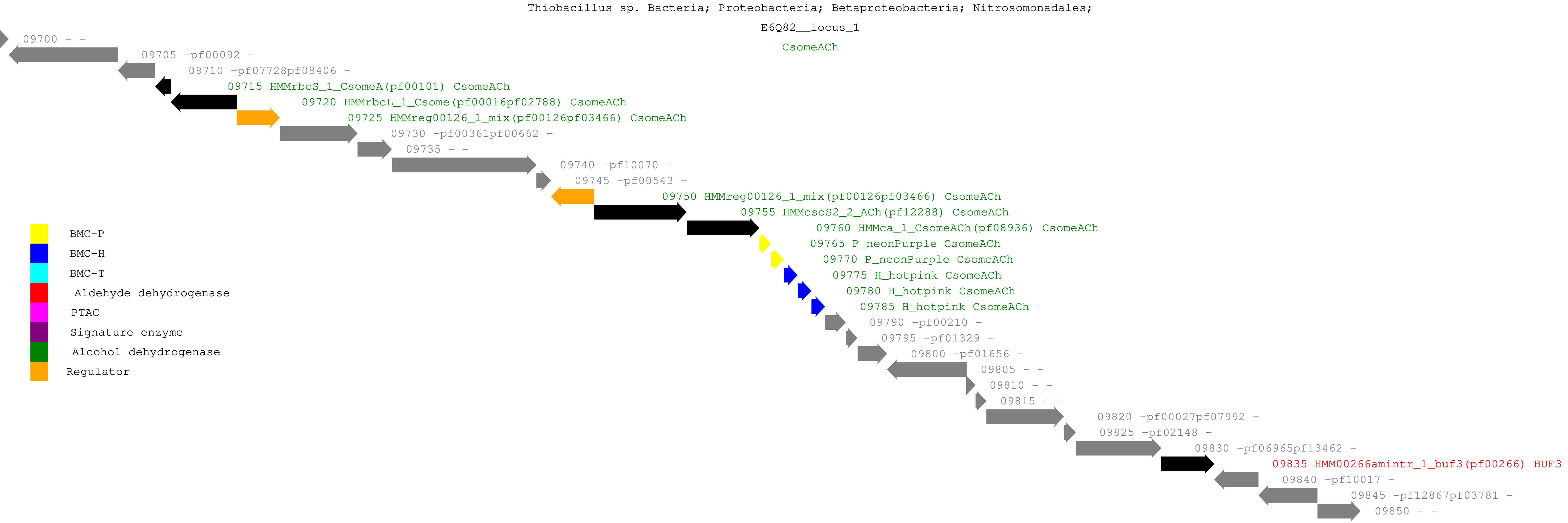
Thiobacillus sp. Bacteria; Proteobacteria; Betaproteobacteria; Nitrosomonadales;
E6Q82__locus_1
CsomeACh
09700 – –
09705 –pf00092 –
09710 –pf07728pf08406 –
09715 HMMrbcS_1_CsomeA(pf00101) CsomeACh
09720 HMMrbcL_1_Csome(pf00016pf02788) CsomeACh
09725 HMMreg00126_1_mix(pf00126pf03466) CsomeACh
09730 –pf00361pf00662 –
09735 – –
09740 –pf10070 –
09745 –pf00543 –
09750 HMMreg00126_1_mix(pf00126pf03466) CsomeACh
09755 HMMcsoS2_2_ACh(pf12288) CsomeACh
09760 HMMca_1_CsomeACh(pf08936) CsomeACh
09765 P_neonPurple CsomeACh
09770 P_neonPurple CsomeACh
09775 H_hotpink CsomeACh
09780 H_hotpink CsomeACh
09785 H_hotpink CsomeACh
09790 –pf00210 –
09795 –pf01329 –
09800 –pf01656 –
09805 – –
09810 – –
09815 – –
09820 –pf00027pf07992 –
09825 –pf02148 –
09830 –pf06965pf13462 –
09835 HMM00266amintr_1_buf3(pf00266) BUF3
09840 –pf10017 –
09845 –pf12867pf03781 –
09850 – –
BMC-P
BMC-H
BMC-T
Aldehyde dehydrogenase
PTAC
Signature enzyme
Alcohol dehydrogenase
Regulator

Acidithiobacillus sulfuriphilus. Bacteria; Proteobacteria; Acidithiobacillia; Acidithiobacillales;

EC580__locus_1

CsomeACh

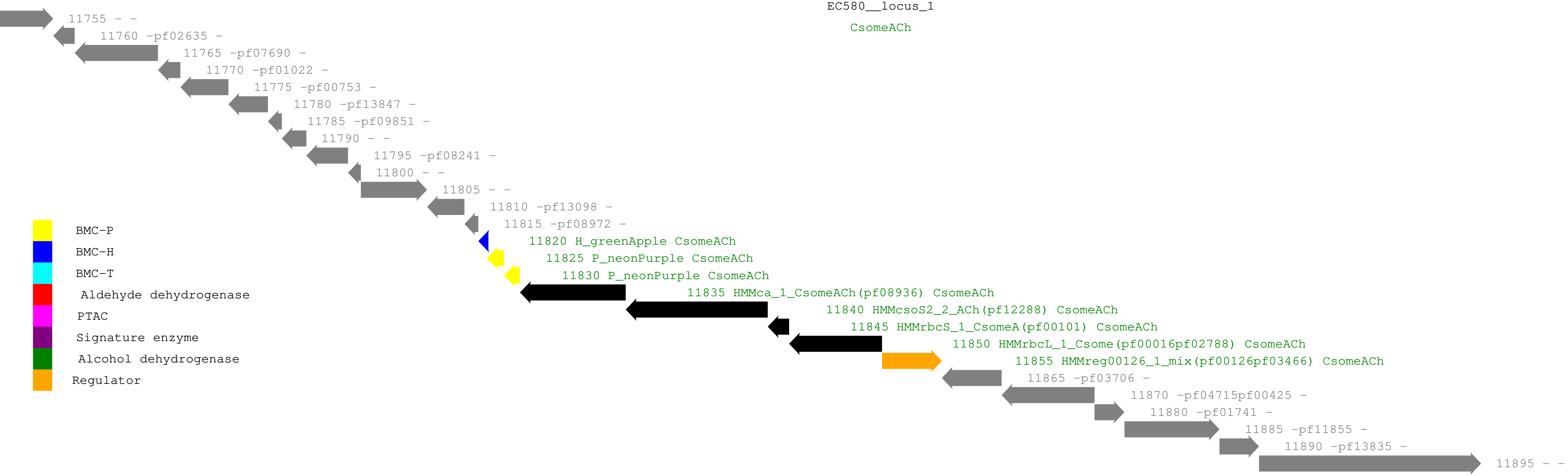

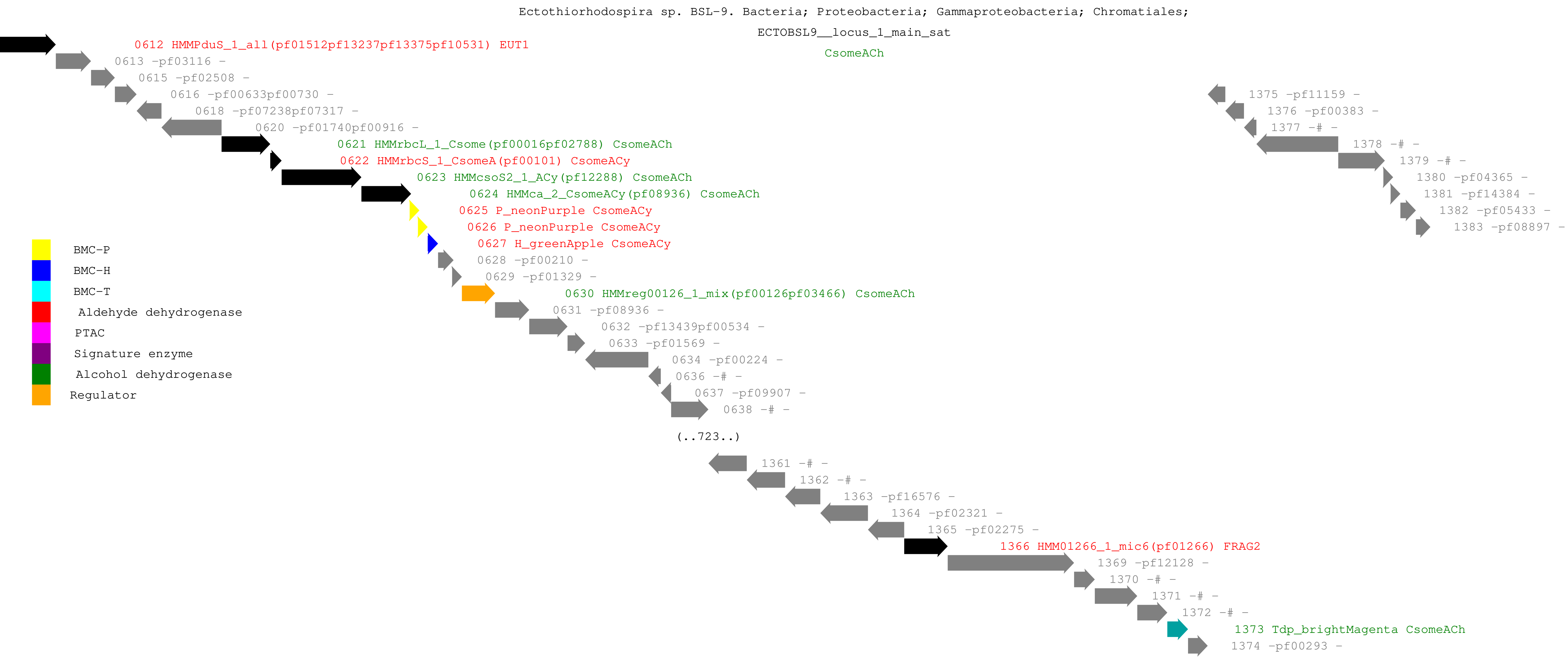
Ectothiorhodospira sp. BSL-9. Bacteria; Proteobacteria; Gammaproteobacteria; Chromatiales;
ECTOBSL9__locus_1_main_sat
CsomeACh
0612 HMMPduS_1_all(pf01512pf13237pf13375pf10531) EUT1
0613 -pf03116 -
0615 -pf02508 -
0616 -pf00633pf00730 -
0618 -pf07238pf07317 -
0620 -pf01740pf00916 -
0621 HMMrbcL_1_Csome(pf00016pf02788) CsomeACh
0622 HMMrbcS_1_CsomeA(pf00101) CsomeACy
0623 HMMcsoS2_1_ACy(pf12288) CsomeACh
0624 HMMca_2_CsomeACy(pf08936) CsomeACh
0625 P_neonPurple CsomeACy
0626 P_neonPurple CsomeACy
0627 H_greenApple CsomeACy
0628 -pf00210 -
0629 -pf01329 -
0630 HMMreg00126_1_mix(pf00126pf03466) CsomeACh
0631 -pf08936 -
0632 -pf13439pf00534 -
0633 -pf01569 -
0634 -pf00224 -
0636 -# -
0637 -pf09907 -
0638 -# -
(..723..)
1361 -# -
1362 -# -
1363 -pf16576 -
1364 -pf02321 -
1365 -pf02275 -
1366 HMM01266_1_mic6(pf01266) FRAG2
1369 -pf12128 -
1370 -# -
1371 -# -
1372 -# -
1373 Tdp_brightMagenta CsomeACh
1374 -pf00293 -
1375 -pf11159 -
1376 -pf00383 -
1377 -# -
1378 -# -
1379 -# -
1380 -pf04365 -
1381 -pf14384 -
1382 -pf05433 -
1383 -pf08897 -
BMC-P
BMC-H
BMC-T
Aldehyde dehydrogenase
PTAC
Signature enzyme
Alcohol dehydrogenase
Regulator

Thiohalophilus thiocyanatoxydans. Bacteria; Proteobacteria; Gammaproteobacteria; Chromatiales;

EDC23__locus_1

CsomeACh

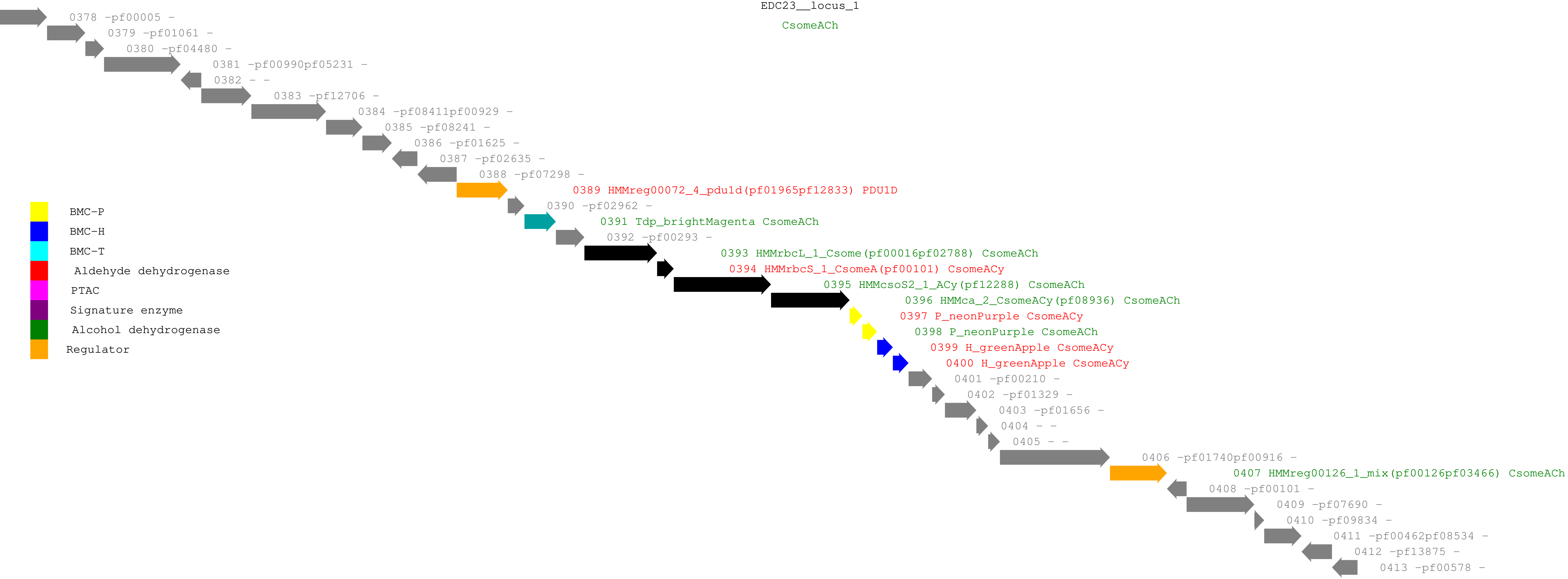

Marichromatium gracile (Chromatium gracile). Bacteria; Proteobacteria; Gammaproteobacteria; Chromatiales; Chromatiaceae;

EDC29__locus_1

CsomeACh

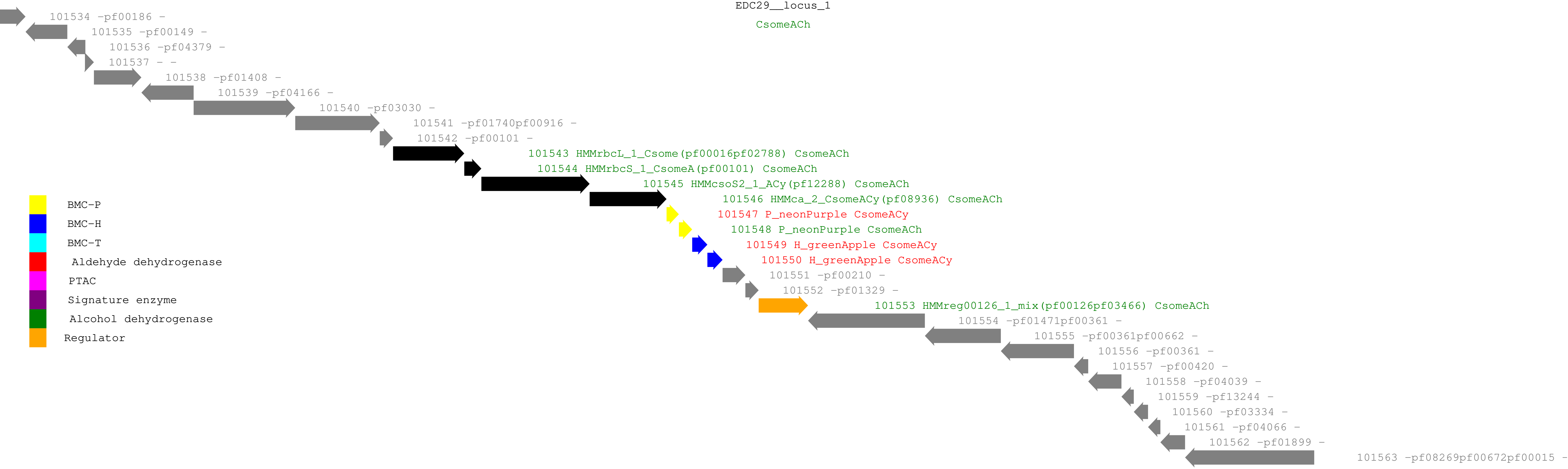

Tepidimonas ignava. Bacteria; Proteobacteria; Betaproteobacteria; Burkholderiales; Tepidimonas.

EDC36__locus_1

CsomeACh

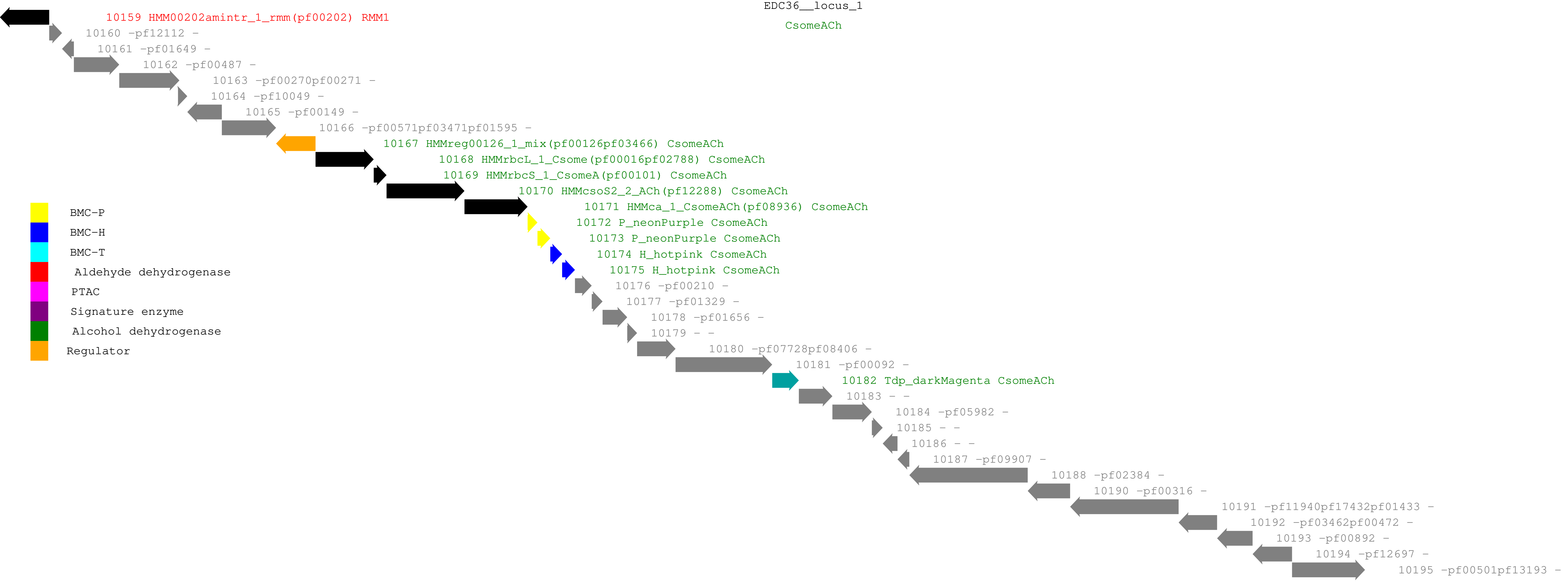

Sulfuritortus calidifontis. Bacteria; Proteobacteria; Betaproteobacteria; Nitrosomonadales;

EDC61__locus_1

CsomeACh

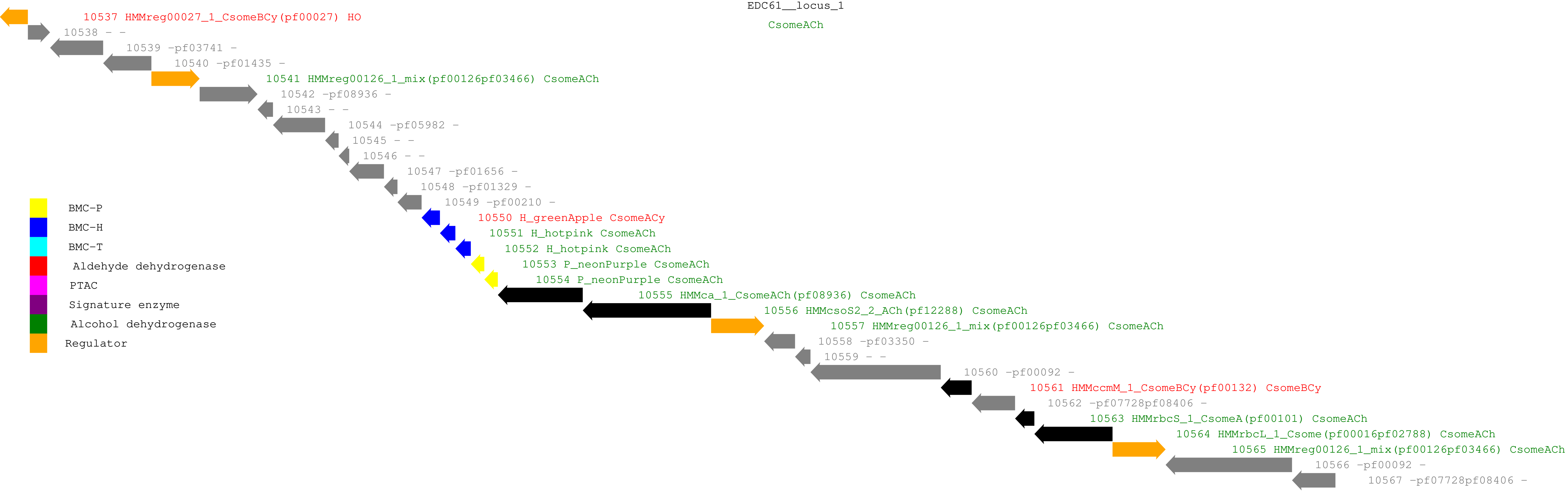

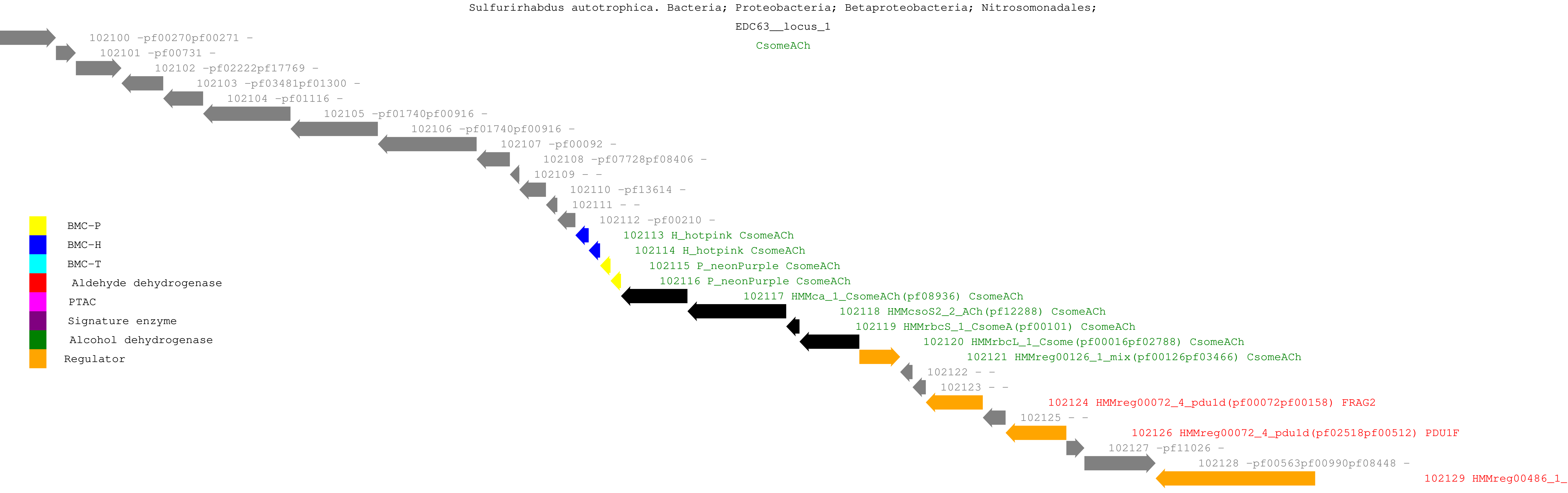

Sulfurirhabdus autotrophica. Bacteria; Proteobacteria; Betaproteobacteria; Nitrosomonadales;
EDC63__locus_1
CsomeACh
102100 -pf00270pf00271 -
102101 -pf00731 -
102102 -pf02222pf17769 -
102103 -pf03481pf01300 -
102104 -pf01116 -
102105 -pf01740pf00916 -
102106 -pf01740pf00916 -
102107 -pf00092 -
102108 -pf07728pf08406 -
102109 - -
102110 -pf13614 -
102111 - -
102112 -pf00210 -
102113 H_hotpink CsomeACh
102114 H_hotpink CsomeACh
102115 P_neonPurple CsomeACh
102116 P_neonPurple CsomeACh
102117 HMMca_1_CsomeACh(pf08936) CsomeACh
102118 HMMcsoS2_2_ACh(pf12288) CsomeACh
102119 HMMrbcS_1_CsomeA(pf00101) CsomeACh
102120 HMMrbcL_1_Csome(pf00016pf02788) CsomeACh
102121 HMMreg00126_1_mix(pf00126pf03466) CsomeACh
102122 - -
102123 - -
102124 HMMreg00072_4_pdu1d(pf00072pf00158) FRAG2
102125 - -
102126 HMMreg00072_4_pdu1d(pf02518pf00512) PDU1F
102127 -pf11026 -
102128 -pf00563pf00990pf08448 -
102129 HMMreg00486_1_
BMC-P
BMC-H
BMC-T
Aldehyde dehydrogenase
PTAC
Signature enzyme
Alcohol dehydrogenase
Regulator

Hydrogenovibrio marinus. Bacteria; Proteobacteria; Gammaproteobacteria; Thiotrichales;

EI16__locus_1

CsomeACh

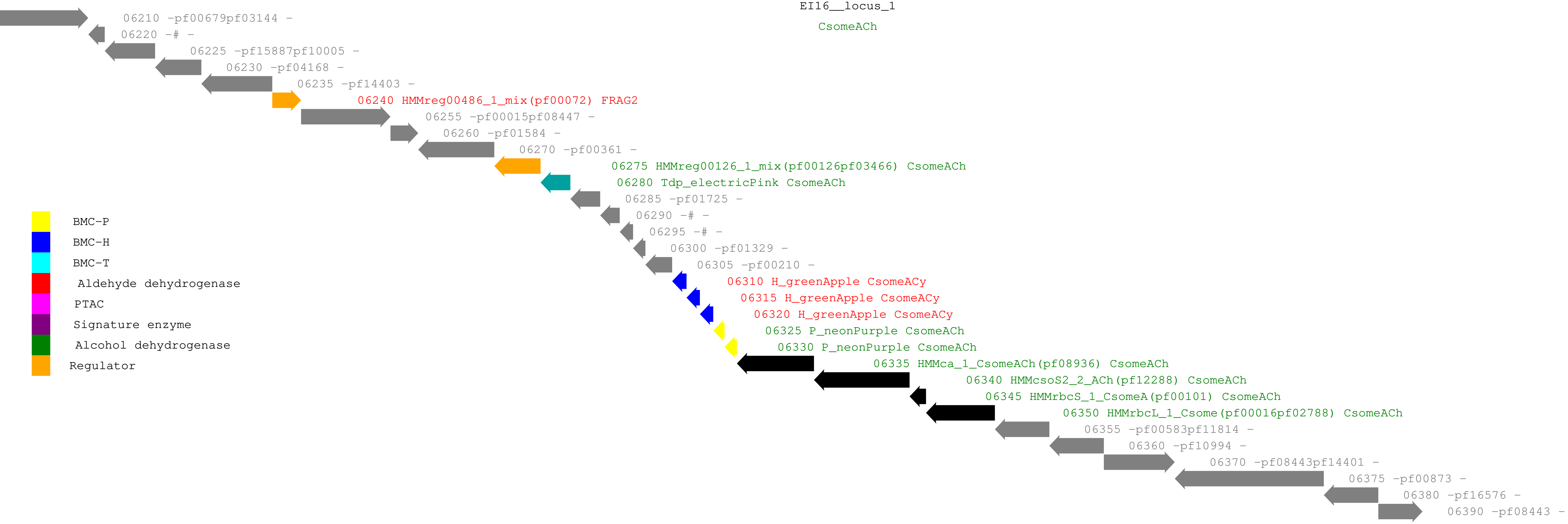

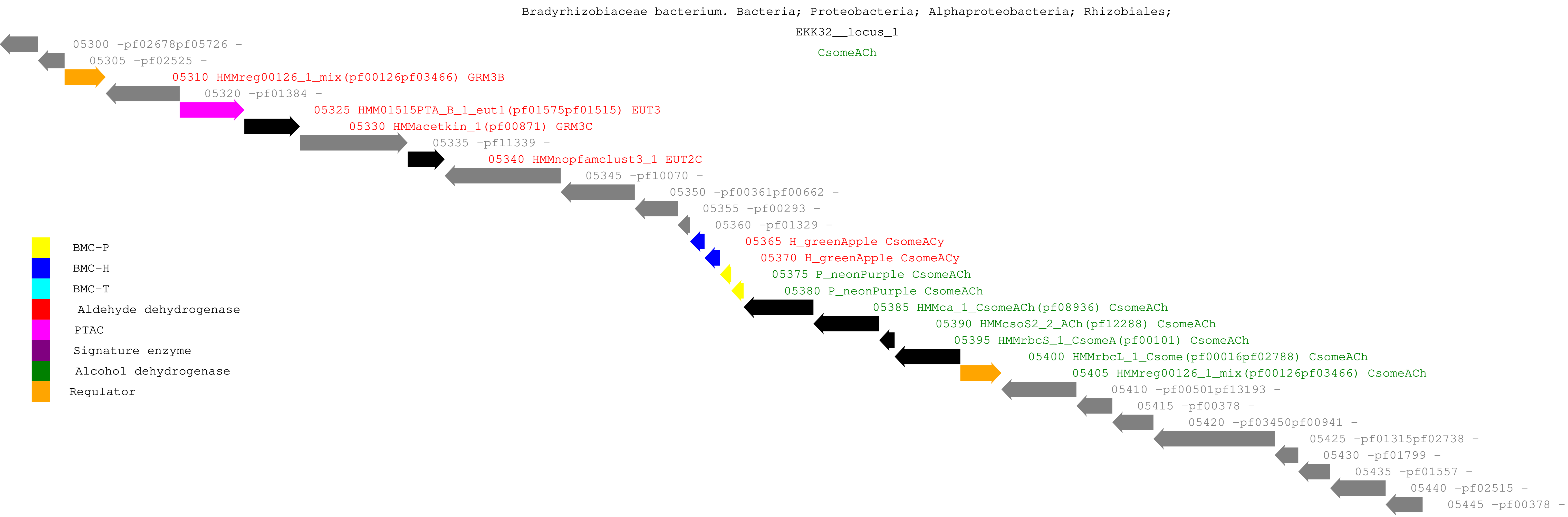
Bradyrhizobiaceae bacterium. Bacteria; Proteobacteria; Alphaproteobacteria; Rhizobiales;
EKK32__locus_1
CsomeACh
05300 -pf02678pf05726 -
05305 -pf02525 -
05310 HMMreg00126_1_mix(pf00126pf03466) GRM3B
05320 -pf01384 -
05325 HMM01515PTA_B_1_eut1(pf01575pf01515) EUT3
05330 HMMacetkin_1(pf00871) GRM3C
05335 -pf11339 -
05340 HMMnopfamclust3_1 EUT2C
05345 -pf10070 -
05350 -pf00361pf00662 -
05355 -pf00293 -
05360 -pf01329 -
05365 H_greenApple CsomeACy
05370 H_greenApple CsomeACy
05375 P_neonPurple CsomeACh
05380 P_neonPurple CsomeACh
05385 HMMca_1_CsomeACh(pf08936) CsomeACh
05390 HMMcsoS2_2_ACh(pf12288) CsomeACh
05395 HMMrbcS_1_CsomeA(pf00101) CsomeACh
05400 HMMrbcL_1_Csome(pf00016pf02788) CsomeACh
05405 HMMreg00126_1_mix(pf00126pf03466) CsomeACh
05410 -pf00501pf13193 -
05415 -pf00378 -
05420 -pf03450pf00941 -
05425 -pf01315pf02738 -
05430 -pf01799 -
05435 -pf01557 -
05440 -pf02515 -
05445 -pf00378 -
BMC-P
BMC-H
BMC-T
Aldehyde dehydrogenase
PTAC
Signature enzyme
Alcohol dehydrogenase
Regulator

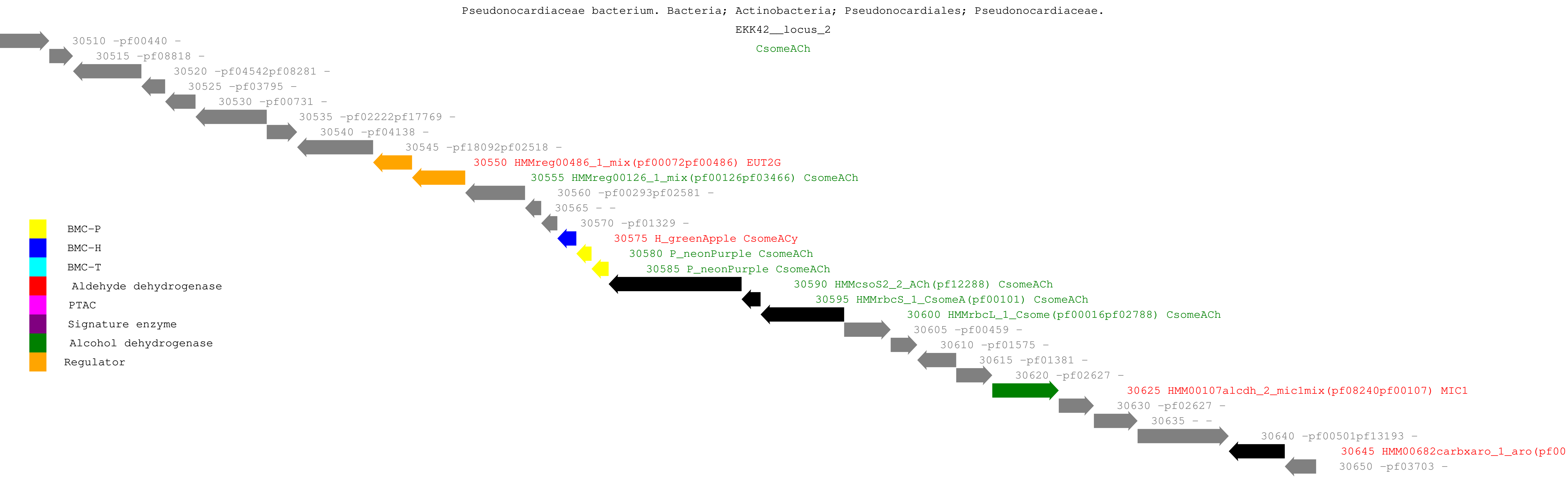
Pseudonocardiaceae bacterium. Bacteria; Actinobacteria; Pseudonocardiales; Pseudonocardiaceae.
EKK42__locus_2
CsomeACh
30510 -pf00440 -
30515 -pf08818 -
30520 -pf04542pf08281 -
30525 -pf03795 -
30530 -pf00731 -
30535 -pf02222pf17769 -
30540 -pf04138 -
30545 -pf18092pf02518 -
30550 HMMreg00486_1_mix(pf00072pf00486) EUT2G
30555 HMMreg00126_1_mix(pf00126pf03466) CsomeACh
30560 -pf00293pf02581 -
30565 - -
30570 -pf01329 -
30575 H_greenApple CsomeACy
30580 P_neonPurple CsomeACh
30585 P_neonPurple CsomeACh
30590 HMMcsoS2_2_ACh(pf12288) CsomeACh
30595 HMMrbcS_1_CsomeA(pf00101) CsomeACh
30600 HMMrbcL_1_Csome(pf00016pf02788) CsomeACh
30605 -pf00459 -
30610 -pf01575 -
30615 -pf01381 -
30620 -pf02627 -
30625 HMM00107alcdh_2_mic1mix(pf08240pf00107) MIC1
30630 -pf02627 -
30635 - -
30640 -pf00501pf13193 -
30645 HMM00682carbxaro_1_aro(pf00
30650 -pf03703 -
BMC-P
BMC-H
BMC-T
Aldehyde dehydrogenase
PTAC
Signature enzyme
Alcohol dehydrogenase
Regulator

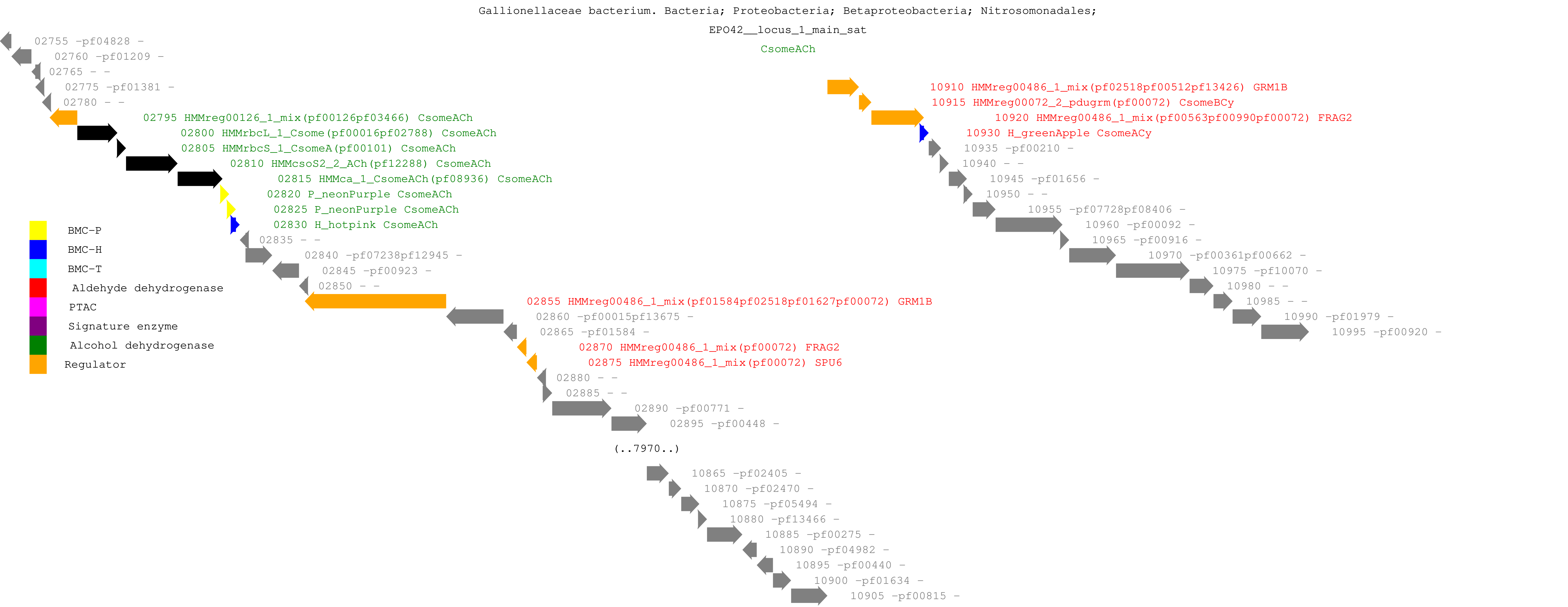
Gallionellaceae bacterium. Bacteria; Proteobacteria; Betaproteobacteria; Nitrosomonadales;
EPO42__locus_1_main_sat
CsomeACh
02755 -pf04828 -
02760 -pf01209 -
02765 - -
02775 -pf01381 -
02780 - -
02795 HMMreg00126_1_mix(pf00126pf03466) CsomeACh
02800 HMMrbcL_1_Csome(pf00016pf02788) CsomeACh
02805 HMMrbcS_1_CsomeA(pf00101) CsomeACh
02810 HMMcsoS2_2_ACh(pf12288) CsomeACh
02815 HMMca_1_CsomeACh(pf08936) CsomeACh
02820 P_neonPurple CsomeACh
02825 P_neonPurple CsomeACh
02830 H_hotpink CsomeACh
02835 - -
02840 -pf07238pf12945 -
02845 -pf00923 -
02850 - -
02855 HMMreg00486_1_mix(pf01584pf02518pf01627pf00072) GRM1B
02860 -pf00015pf13675 -
02865 -pf01584 -
02870 HMMreg00486_1_mix(pf00072) FRAG2
02875 HMMreg00486_1_mix(pf00072) SPU6
02880 - -
02885 - -
02890 -pf00771 -
02895 -pf00448 -
(..7970..)
10865 -pf02405 -
10870 -pf02470 -
10875 -pf05494 -
10880 -pf13466 -
10885 -pf00275 -
10890 -pf04982 -
10895 -pf00440 -
10900 -pf01634 -
10905 -pf00815 -
10910 HMMreg00486_1_mix(pf02518pf00512pf13426) GRM1B
10915 HMMreg00072_2_pdugrm(pf00072) CsomeBCy
10920 HMMreg00486_1_mix(pf00563pf00990pf00072) FRAG2
10930 H_greenApple CsomeACy
10935 -pf00210 -
10940 - -
10945 -pf01656 -
10950 - -
10955 -pf07728pf08406 -
10960 -pf00092 -
10965 -pf00916 -
10970 -pf00361pf00662 -
10975 -pf10070 -
10980 - -
10985 - -
10990 -pf01979 -
10995 -pf00920 -
BMC-P
BMC-H
BMC-T
Aldehyde dehydrogenase
PTAC
Signature enzyme
Alcohol dehydrogenase
Regulator

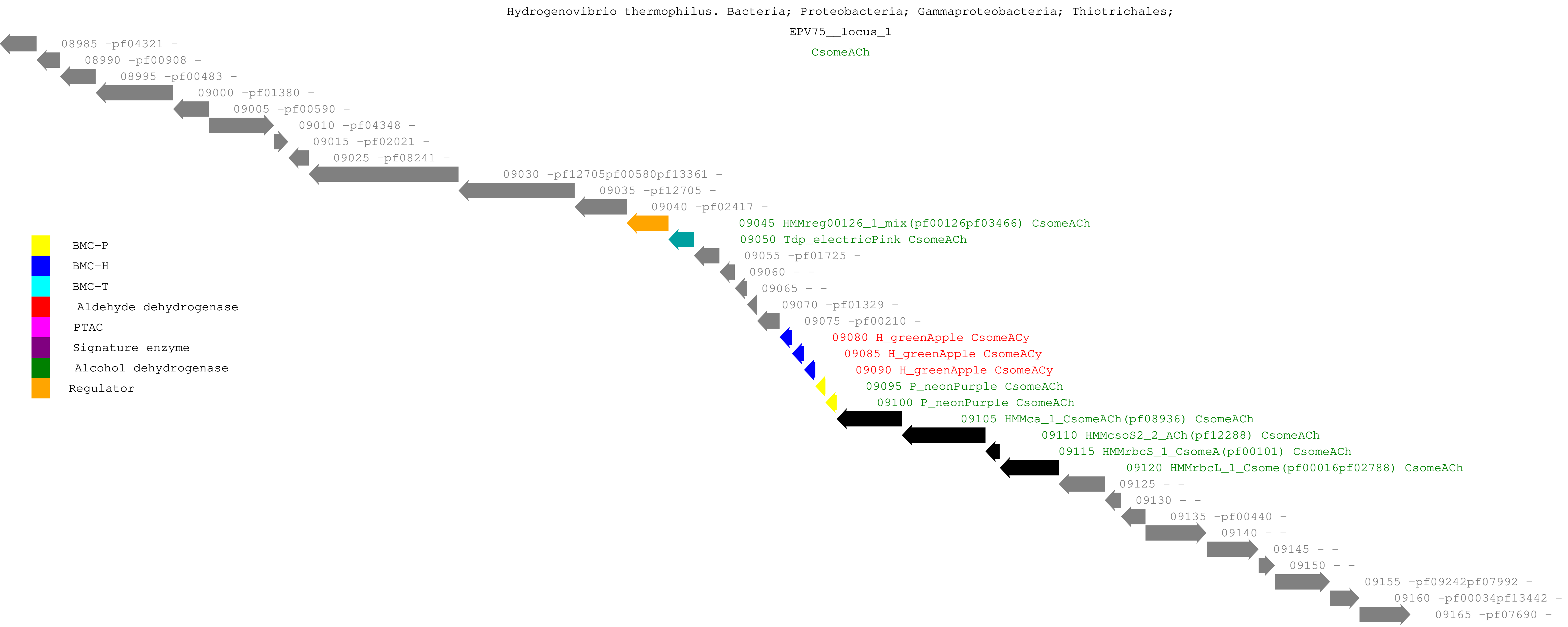
Hydrogenovibrio thermophilus. Bacteria; Proteobacteria; Gammaproteobacteria; Thiotrichales;
EPV75__locus_1
CsomeACh
08985 -pf04321 -
08990 -pf00908 -
08995 -pf00483 -
09000 -pf01380 -
09005 -pf00590 -
09010 -pf04348 -
09015 -pf02021 -
09025 -pf08241 -
09030 -pf12705pf00580pf13361 -
09035 -pf12705 -
09040 -pf02417 -
09045 HMMreg00126_1_mix(pf00126pf03466) CsomeACh
09050 Tdp_electricPink CsomeACh
09055 -pf01725 -
09060 - -
09065 - -
09070 -pf01329 -
09075 -pf00210 -
09080 H_greenApple CsomeACy
09085 H_greenApple CsomeACy
09090 H_greenApple CsomeACy
09095 P_neonPurple CsomeACh
09100 P_neonPurple CsomeACh
09105 HMMca_1_CsomeACh(pf08936) CsomeACh
09110 HMMcsoS2_2_ACh(pf12288) CsomeACh
09115 HMMrbcS_1_CsomeA(pf00101) CsomeACh
09120 HMMrbcL_1_Csome(pf00016pf02788) CsomeACh
09125 - -
09130 - -
09135 -pf00440 -
09140 - -
09145 - -
09150 - -
09155 -pf09242pf07992 -
09160 -pf00034pf13442 -
09165 -pf07690 -
BMC-P
BMC-H
BMC-T
Aldehyde dehydrogenase
PTAC
Signature enzyme
Alcohol dehydrogenase
Regulator

Litoricolaceae bacterium. Bacteria; Proteobacteria; Gammaproteobacteria; Oceanospirillales;

EVA60__locus_1

CsomeACh

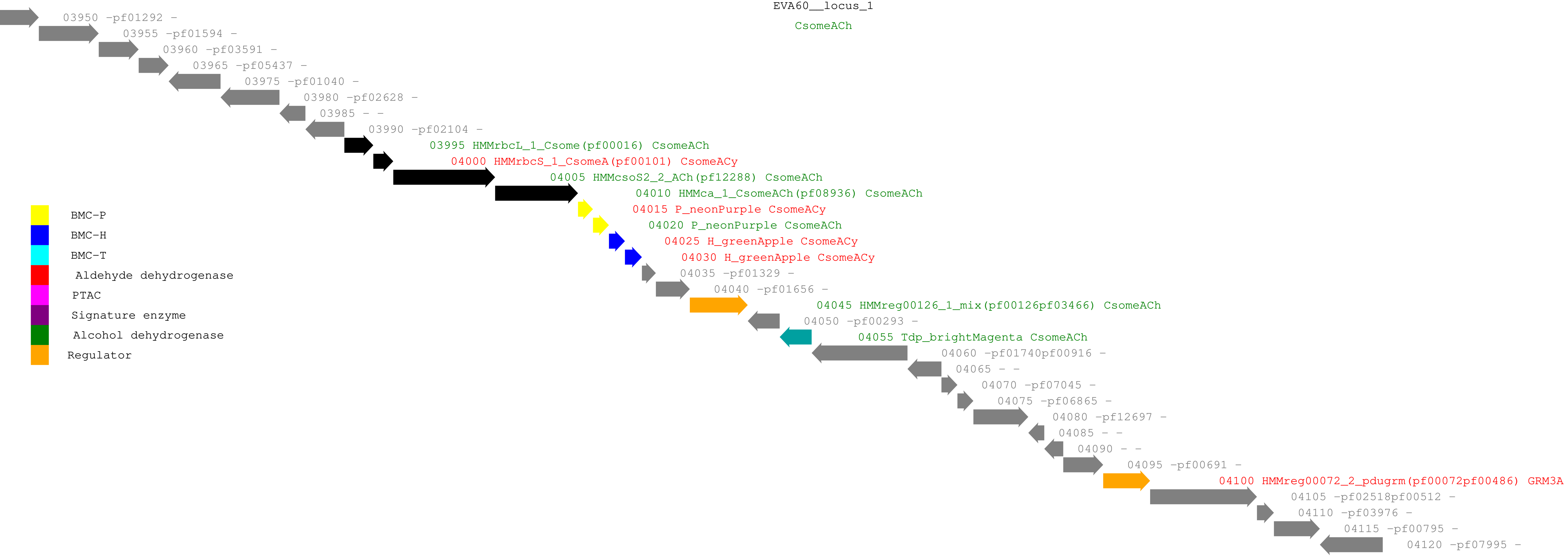

Thiobacillaceae bacterium LSR1. Bacteria; Proteobacteria; Betaproteobacteria; Nitrosomonadales;

EZJ19__locus_1

CsomeACh

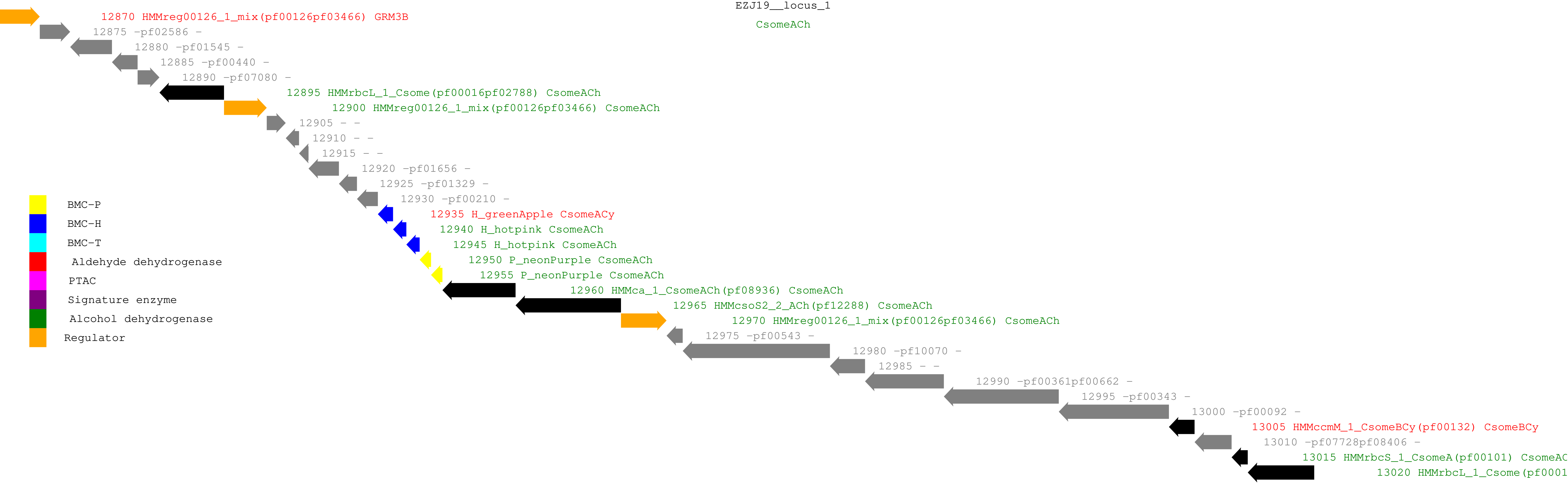

Acidithiobacillus sp. 'AMD consortium'. Bacteria; Proteobacteria; Acidithiobacillia; Acidithiobacillales;

F6A13__locus_1

CsomeACh

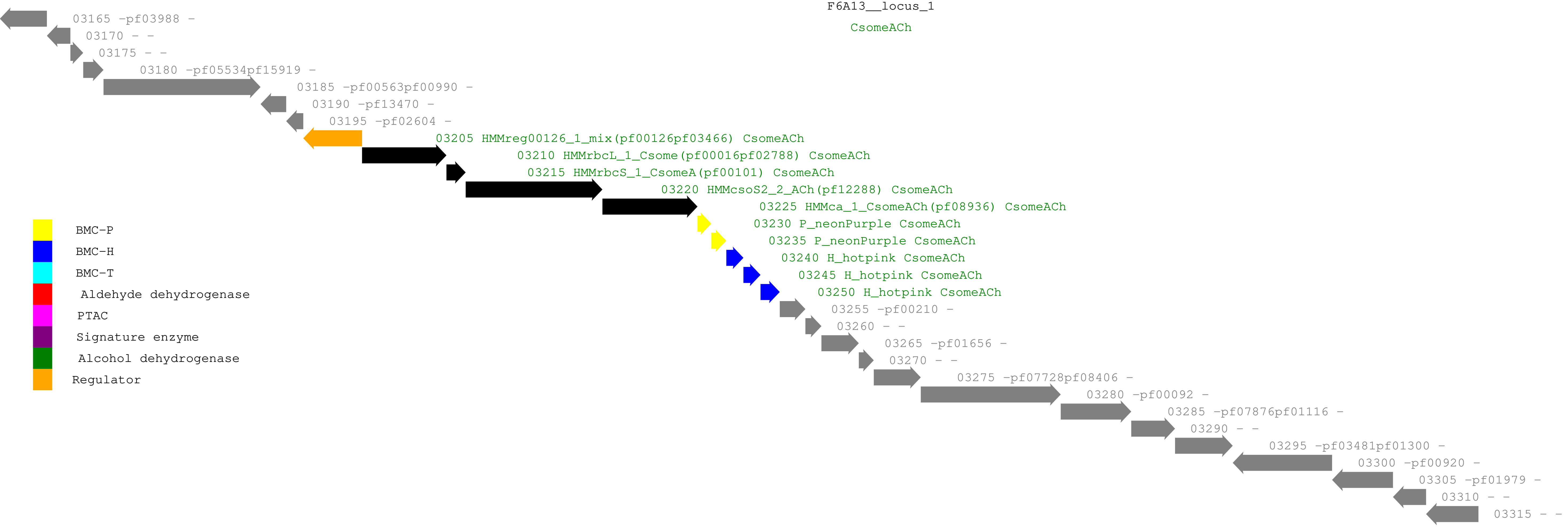

Acidithiobacillus sp. 'AMD consortium'. Bacteria; Proteobacteria; Acidithiobacillia; Acidithiobacillales;

F6A13__locus_2

CsomeACh

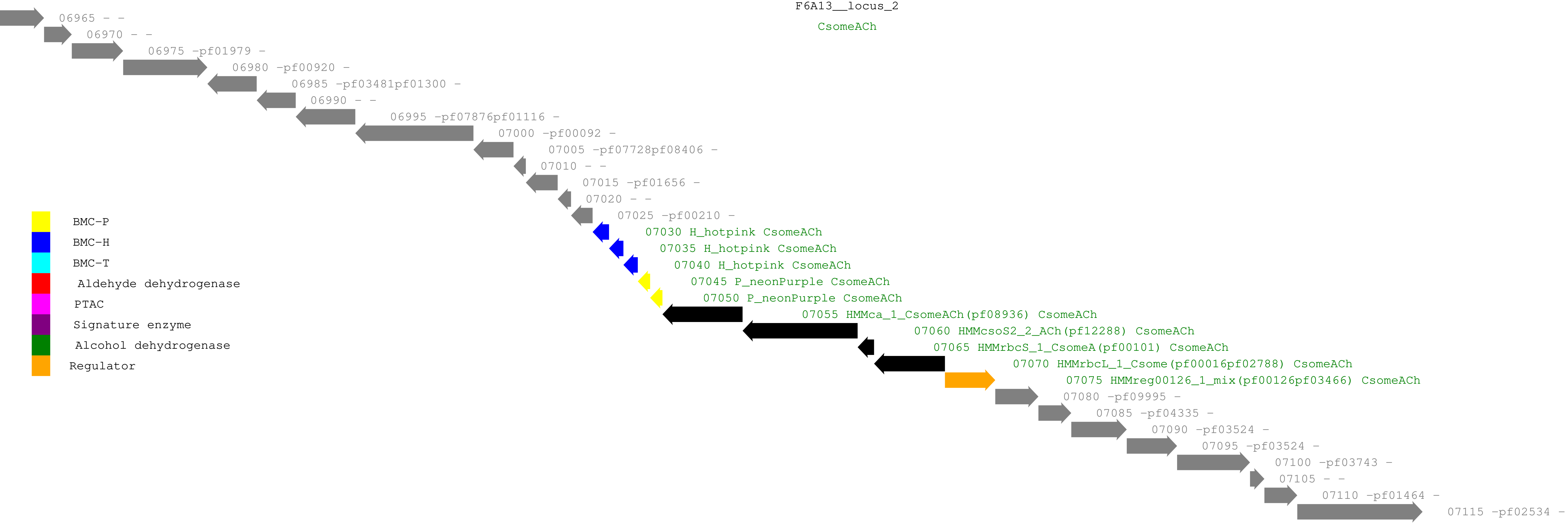

Halothiobacillus sp. SB14A. Bacteria; Proteobacteria; Gammaproteobacteria; Chromatiales;

FAZ79__locus_1_main_sat

CsomeACh

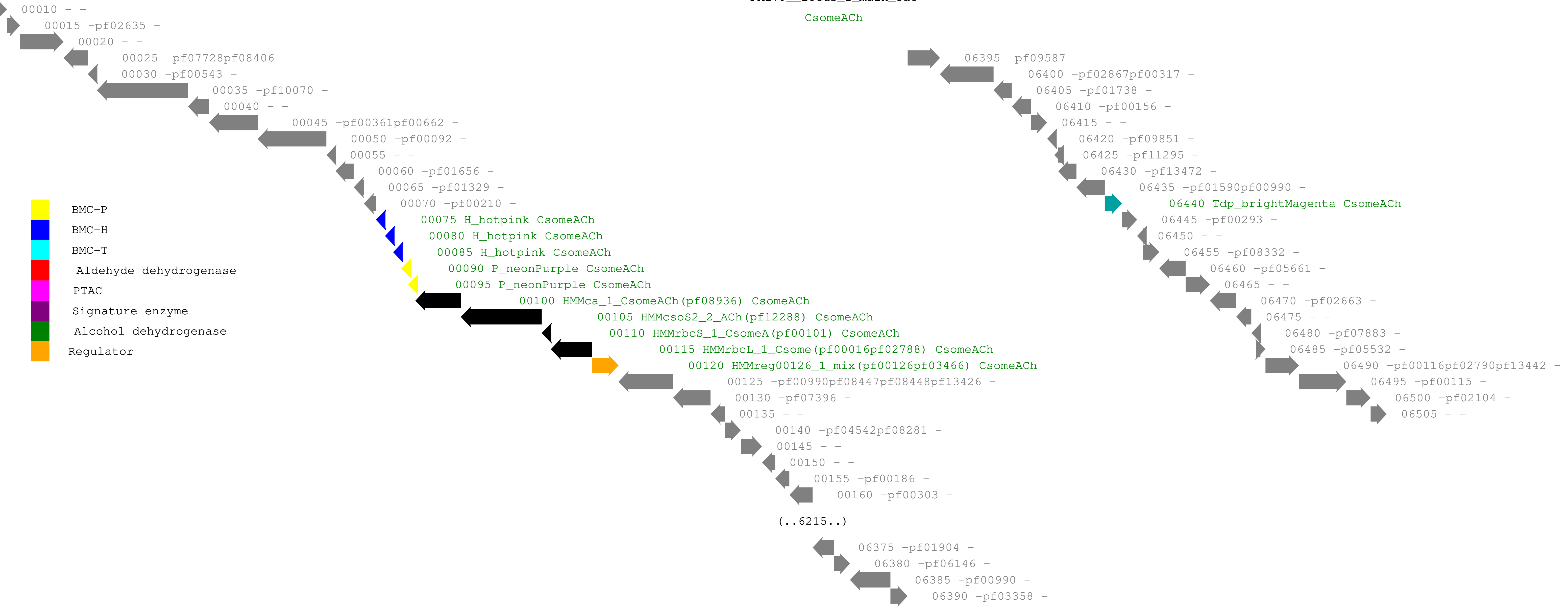

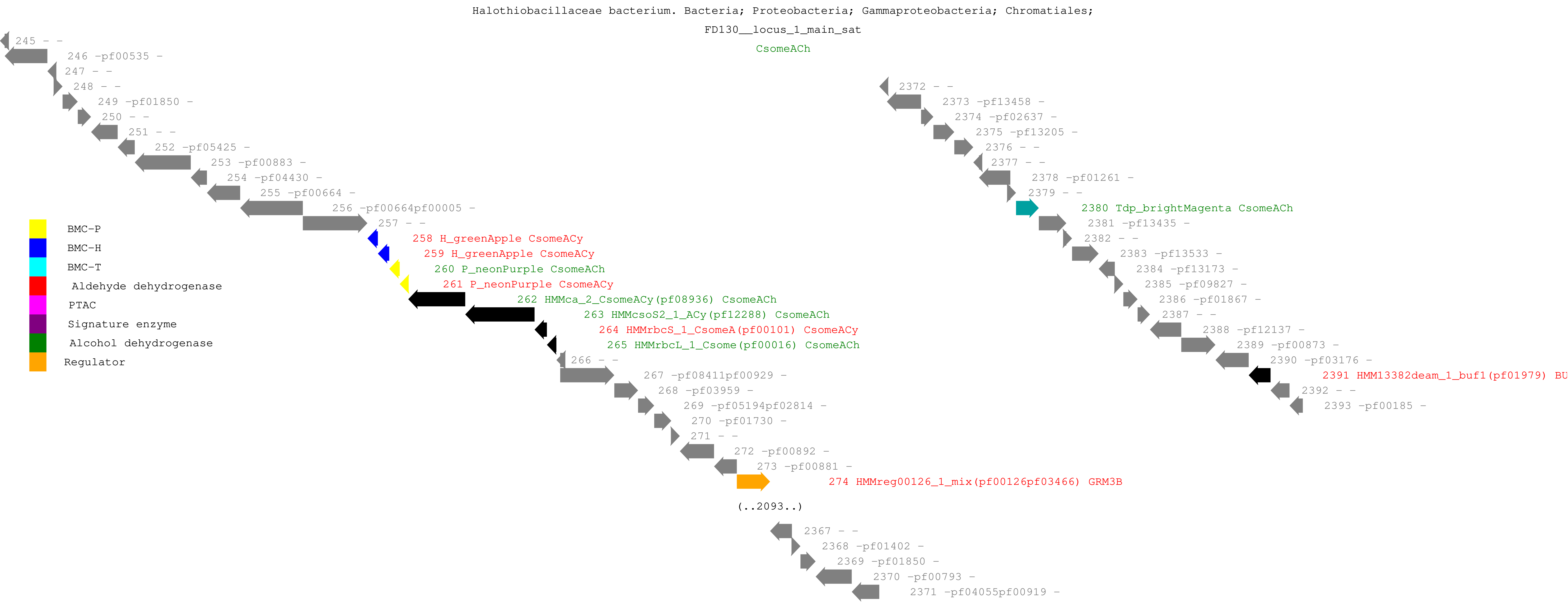

Halothiobacillaceae bacterium. Bacteria; Proteobacteria; Gammaproteobacteria; Chromatiales;
FD130__locus_1_main_sat
CsomeACh
245 - -
246 -pf00535 -
247 - -
248 - -
249 -pf01850 -
250 - -
251 - -
252 -pf05425 -
253 -pf00883 -
254 -pf04430 -
255 -pf00664 -
256 -pf00664pf00005 -
257 - -
258 H_greenApple CsomeACy
259 H_greenApple CsomeACy
260 P_neonPurple CsomeACh
261 P_neonPurple CsomeACy
262 HMMca_2_CsomeACy(pf08936) CsomeACh
263 HMMcsoS2_1_ACy(pf12288) CsomeACh
264 HMMrbcS_1_CsomeA(pf00101) CsomeACy
265 HMMrbcL_1_Csome(pf00016) CsomeACh
266 - -
267 -pf08411pf00929 -
268 -pf03959 -
269 -pf05194pf02814 -
270 -pf01730 -
271 - -
272 -pf00892 -
273 -pf00881 -
274 HMMreg00126_1_mix(pf00126pf03466) GRM3B
(..2093..)
2367 - -
2368 -pf01402 -
2369 -pf01850 -
2370 -pf00793 -
2371 -pf04055pf00919 -
2372 - -
2373 -pf13458 -
2374 -pf02637 -
2375 -pf13205 -
2376 - -
2377 - -
2378 -pf01261 -
2379 - -
2380 Tdp_brightMagenta CsomeACh
2381 -pf13435 -
2382 - -
2383 -pf13533 -
2384 -pf13173 -
2385 -pf09827 -
2386 -pf01867 -
2387 - -
2388 -pf12137 -
2389 -pf00873 -
2390 -pf03176 -
2391 HMM13382deam_1_buf1(pf01979) BU
2392 - -
2393 -pf00185 -
BMC-P
BMC-H
BMC-T
Aldehyde dehydrogenase
PTAC
Signature enzyme
Alcohol dehydrogenase
Regulator

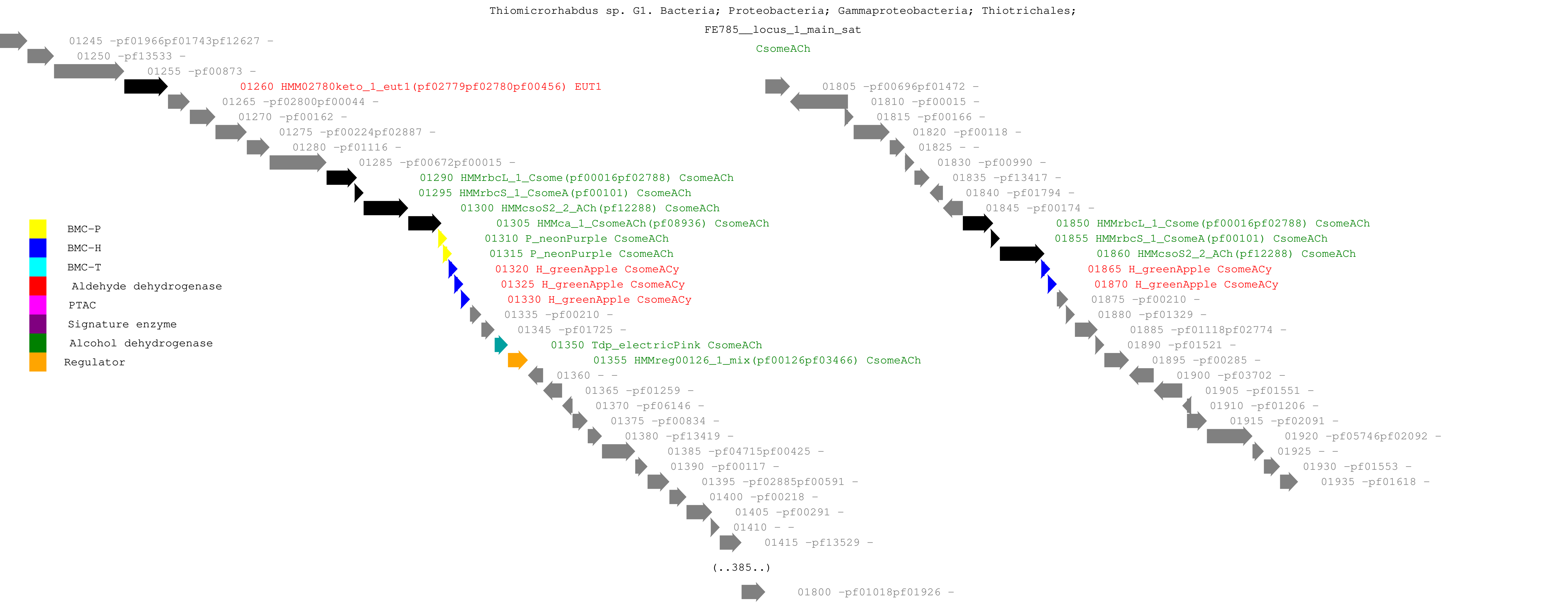
Thiomicrorhabdus sp. G1. Bacteria; Proteobacteria; Gammaproteobacteria; Thiotrichales;
FE785__locus_1_main_sat
CsomeACh
01245 -pf01966pf01743pf12627 -
01250 -pf13533 -
01255 -pf00873 -
01260 HMM02780keto_1_eut1(pf02779pf02780pf00456) EUT1
01265 -pf02800pf00044 -
01270 -pf00162 -
01275 -pf00224pf02887 -
01280 -pf01116 -
01285 -pf00672pf00015 -
01290 HMMrbcL_1_Csome(pf00016pf02788) CsomeACh
01295 HMMrbcS_1_CsomeA(pf00101) CsomeACh
01300 HMMcsoS2_2_ACh(pf12288) CsomeACh
01305 HMMca_1_CsomeACh(pf08936) CsomeACh
01310 P_neonPurple CsomeACh
01315 P_neonPurple CsomeACh
01320 H_greenApple CsomeACy
01325 H_greenApple CsomeACy
01330 H_greenApple CsomeACy
01335 -pf00210 -
01345 -pf01725 -
01350 Tdp_electricPink CsomeACh
01355 HMMreg00126_1_mix(pf00126pf03466) CsomeACh
01360 - -
01365 -pf01259 -
01370 -pf06146 -
01375 -pf00834 -
01380 -pf13419 -
01385 -pf04715pf00425 -
01390 -pf00117 -
01395 -pf02885pf00591 -
01400 -pf00218 -
01405 -pf00291 -
01410 - -
01415 -pf13529 -
(..385..)
01800 -pf01018pf01926 -
01805 -pf00696pf01472 -
01810 -pf00015 -
01815 -pf00166 -
01820 -pf00118 -
01825 - -
01830 -pf00990 -
01835 -pf13417 -
01840 -pf01794 -
01845 -pf00174 -
01850 HMMrbcL_1_Csome(pf00016pf02788) CsomeACh
01855 HMMrbcS_1_CsomeA(pf00101) CsomeACh
01860 HMMcsoS2_2_ACh(pf12288) CsomeACh
01865 H_greenApple CsomeACy
01870 H_greenApple CsomeACy
01875 -pf00210 -
01880 -pf01329 -
01885 -pf01118pf02774 -
01890 -pf01521 -
01895 -pf00285 -
01900 -pf03702 -
01905 -pf01551 -
01910 -pf01206 -
01915 -pf02091 -
01920 -pf05746pf02092 -
01925 - -
01930 -pf01553 -
01935 -pf01618 -
BMC-P
BMC-H
BMC-T
Aldehyde dehydrogenase
PTAC
Signature enzyme
Alcohol dehydrogenase
Regulator

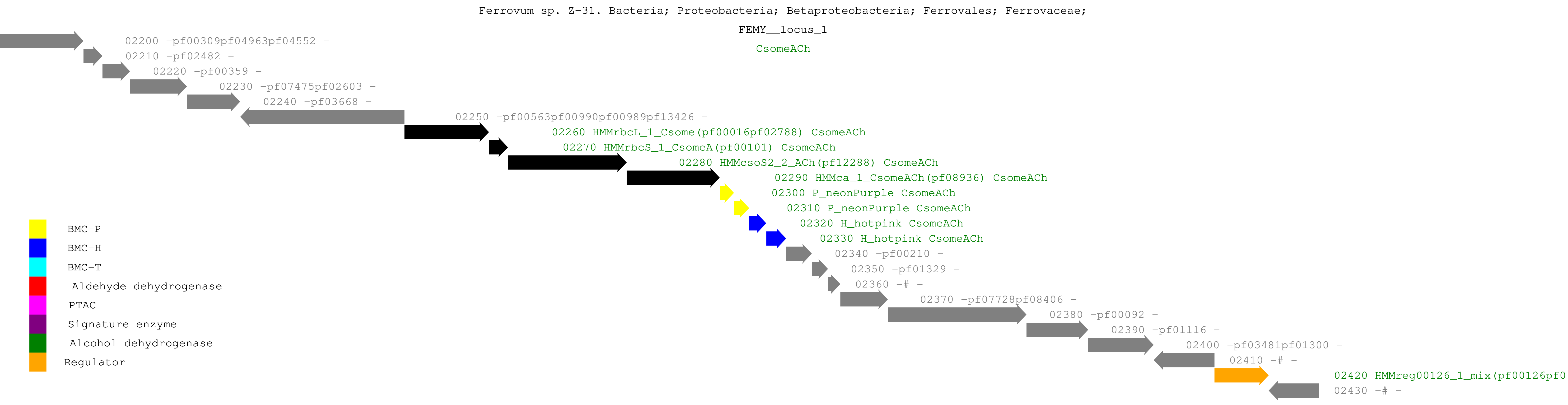

Ferrovum sp. Z-31. Bacteria; Proteobacteria; Betaproteobacteria; Ferrovales; Ferrovaceae;
FEMY__locus_1
CsomeACh
02200 -pf00309pf04963pf04552 -
02210 -pf02482 -
02220 -pf00359 -
02230 -pf07475pf02603 -
02240 -pf03668 -
02250 -pf00563pf00990pf00989pf13426 -
02260 HMMrbcL_1_Csome(pf00016pf02788) CsomeACh
02270 HMMrbcS_1_CsomeA(pf00101) CsomeACh
02280 HMMcsoS2_2_ACh(pf12288) CsomeACh
02290 HMMca_1_CsomeACh(pf08936) CsomeACh
02300 P_neonPurple CsomeACh
02310 P_neonPurple CsomeACh
02320 H_hotpink CsomeACh
02330 H_hotpink CsomeACh
02340 -pf00210 -
02350 -pf01329 -
02360 -# -
02370 -pf07728pf08406 -
02380 -pf00092 -
02390 -pf01116 -
02400 -pf03481pf01300 -
02410 -# -
02420 HMMreg00126_1_mix(pf00126pf0
02430 -# -
BMC-P
BMC-H
BMC-T
Aldehyde dehydrogenase
PTAC
Signature enzyme
Alcohol dehydrogenase
Regulator

Ferrovum sp. JA12. Bacteria; Proteobacteria; Betaproteobacteria; Ferrovales; Ferrovaceae;

FERRO__locus_1

CsomeACh

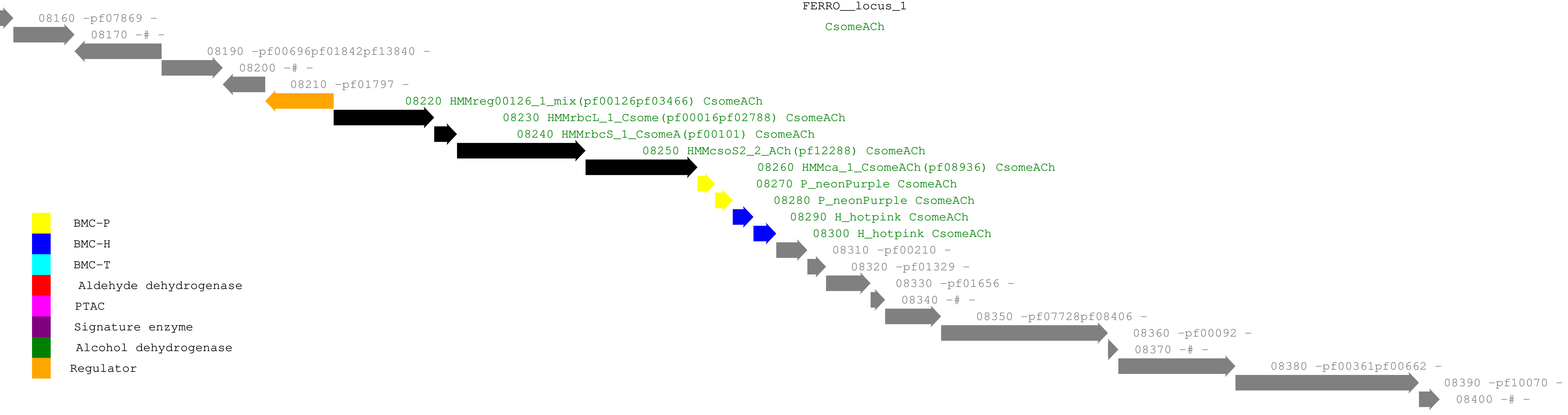

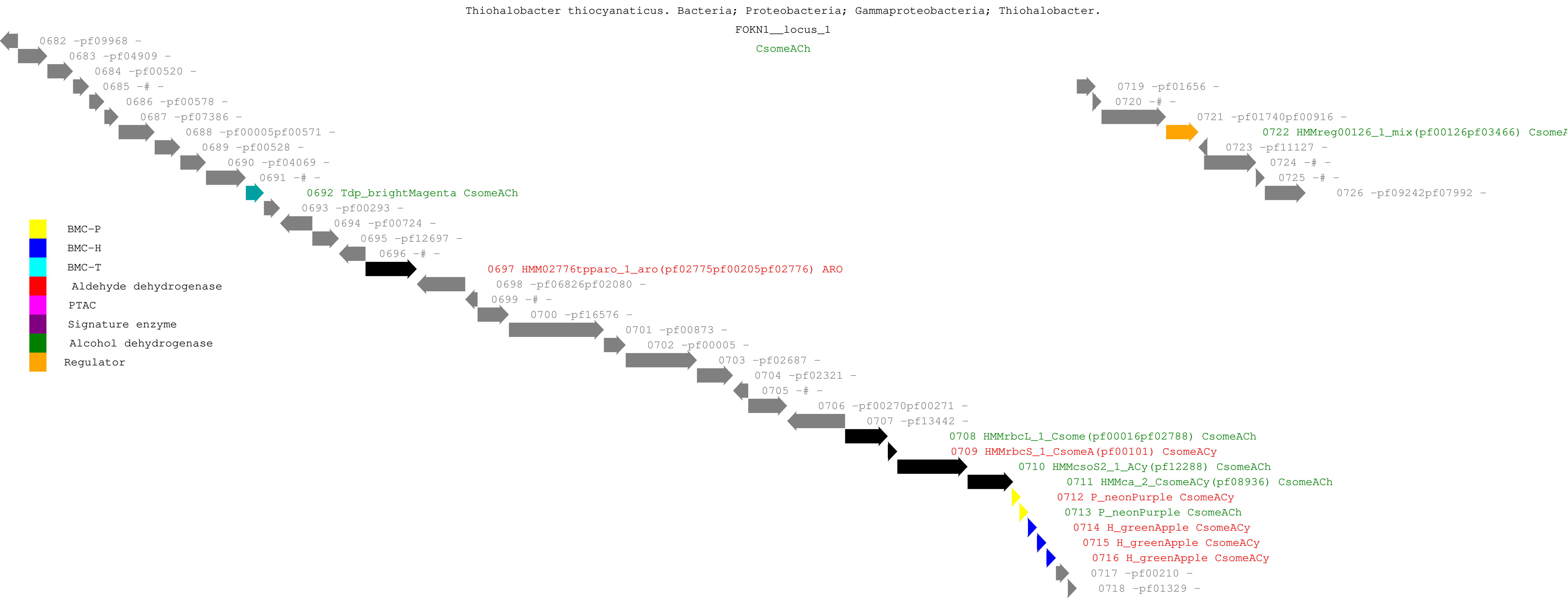
Thiohalobacter thiocyanaticus. Bacteria; Proteobacteria; Gammaproteobacteria; Thiohalobacter.
FOKN1__locus_1
CsomeACh
0682 -pf09968 -
0683 -pf04909 -
0684 -pf00520 -
0685 -# -
0686 -pf00578 -
0687 -pf07386 -
0688 -pf00005pf00571 -
0689 -pf00528 -
0690 -pf04069 -
0691 -# -
0692 Tdp_brightMagenta CsomeACh
0693 -pf00293 -
0694 -pf00724 -
0695 -pf12697 -
0696 -# -
0697 HMM02776tpparo_1_aro(pf02775pf00205pf02776) ARO
0698 -pf06826pf02080 -
0699 -# -
0700 -pf16576 -
0701 -pf00873 -
0702 -pf00005 -
0703 -pf02687 -
0704 -pf02321 -
0705 -# -
0706 -pf00270pf00271 -
0707 -pf13442 -
0708 HMMrbcL_1_Csome(pf00016pf02788) CsomeACh
0709 HMMrbcS_1_CsomeA(pf00101) CsomeACy
0710 HMMcsoS2_1_ACy(pf12288) CsomeACh
0711 HMMca_2_CsomeACy(pf08936) CsomeACh
0712 P_neonPurple CsomeACy
0713 P_neonPurple CsomeACh
0714 H_greenApple CsomeACy
0715 H_greenApple CsomeACy
0716 H_greenApple CsomeACy
0717 -pf00210 -
0718 -pf01329 -
0719 -pf01656 -
0720 -# -
0721 -pf01740pf00916 -
0722 HMMreg00126_1_mix(pf00126pf03466) CsomeA
0723 -pf11127 -
0724 -# -
0725 -# -
0726 -pf09242pf07992 -
BMC-P
BMC-H
BMC-T
Aldehyde dehydrogenase
PTAC
Signature enzyme
Alcohol dehydrogenase
Regulator

Hydrogenophilales bacterium SM250. Bacteria; Proteobacteria; Hydrogenophilalia; Hydrogenophilales.

FR698__locus_1

CsomeACh

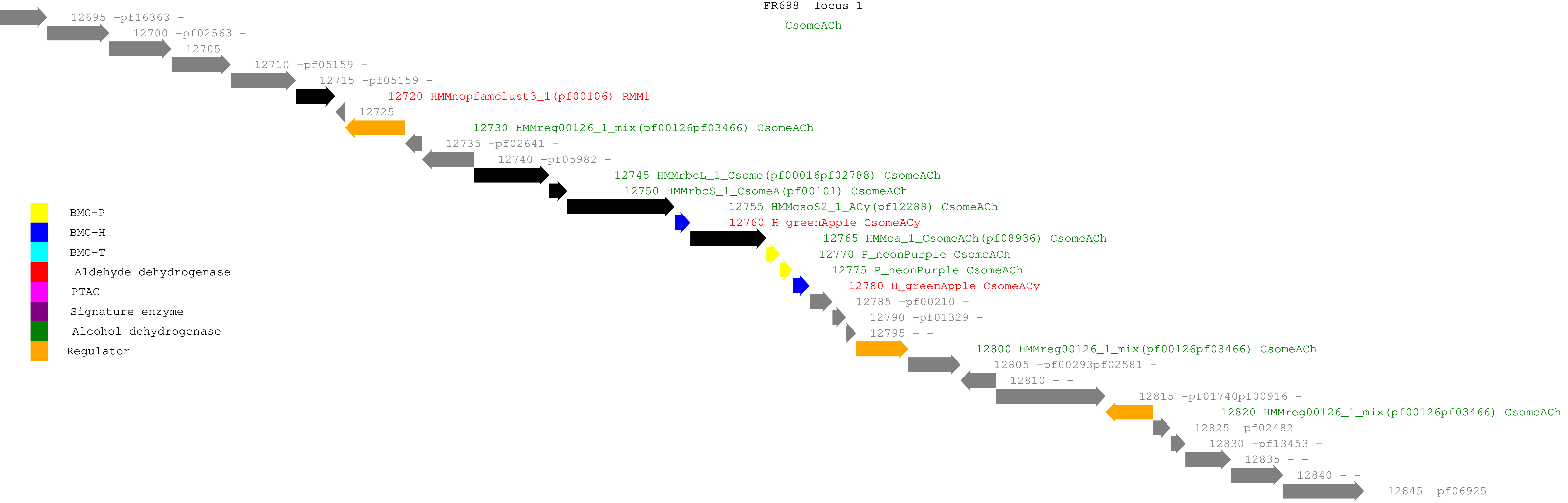

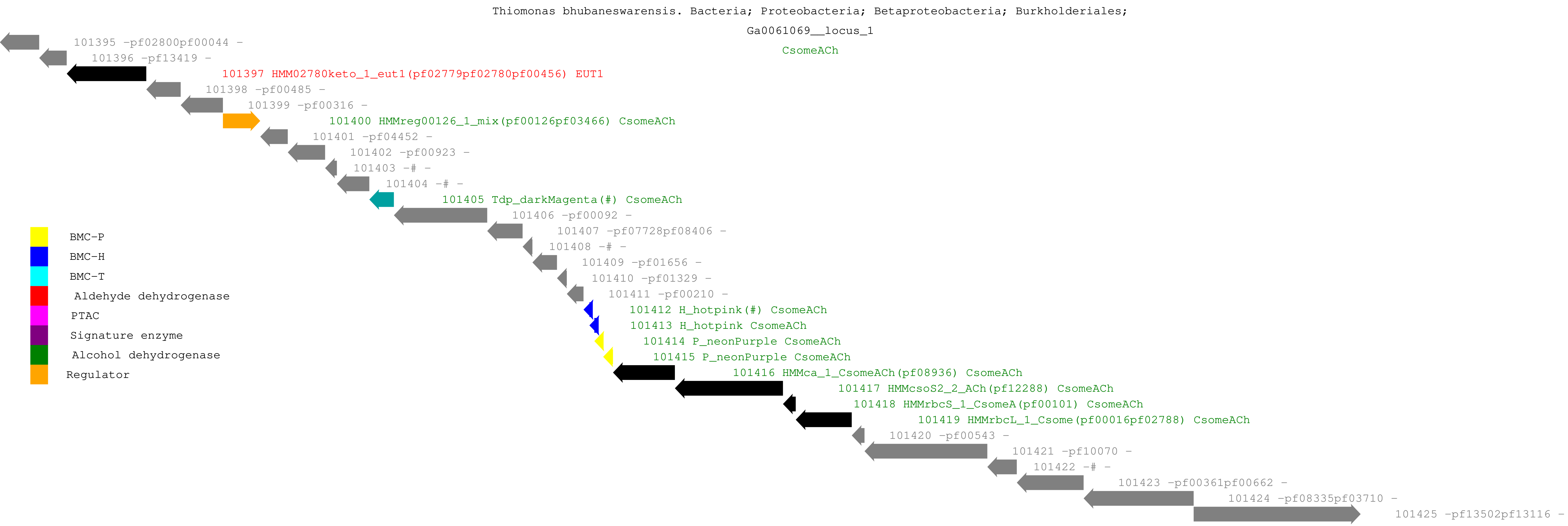
Thiomonas bhubaneswarensis. Bacteria; Proteobacteria; Betaproteobacteria; Burkholderiales;
Ga0061069__locus_1
CsomeACh
101395 -pf02800pf00044 -
101396 -pf13419 -
101397 HMM02780keto_1_eut1(pf02779pf02780pf00456) EUT1
101398 -pf00485 -
101399 -pf00316 -
101400 HMMreg00126_1_mix(pf00126pf03466) CsomeACh
101401 -pf04452 -
101402 -pf00923 -
101403 -# -
101404 -# -
101405 Tdp_darkMagenta(#) CsomeACh
101406 -pf00092 -
101407 -pf07728pf08406 -
101408 -# -
101409 -pf01656 -
101410 -pf01329 -
101411 -pf00210 -
101412 H_hotpink(#) CsomeACh
101413 H_hotpink CsomeACh
101414 P_neonPurple CsomeACh
101415 P_neonPurple CsomeACh
101416 HMMca_1_CsomeACh(pf08936) CsomeACh
101417 HMMcsoS2_2_ACh(pf12288) CsomeACh
101418 HMMrbcS_1_CsomeA(pf00101) CsomeACh
101419 HMMrbcL_1_Csome(pf00016pf02788) CsomeACh
101420 -pf00543 -
101421 -pf10070 -
101422 -# -
101423 -pf00361pf00662 -
101424 -pf08335pf03710 -
101425 -pf13502pf13116 -
BMC-P
BMC-H
BMC-T
Aldehyde dehydrogenase
PTAC
Signature enzyme
Alcohol dehydrogenase
Regulator

mine drainage metagenome. unclassified sequences; metagenomes; ecological metagenomes.

GALL__locus_2

CsomeACh

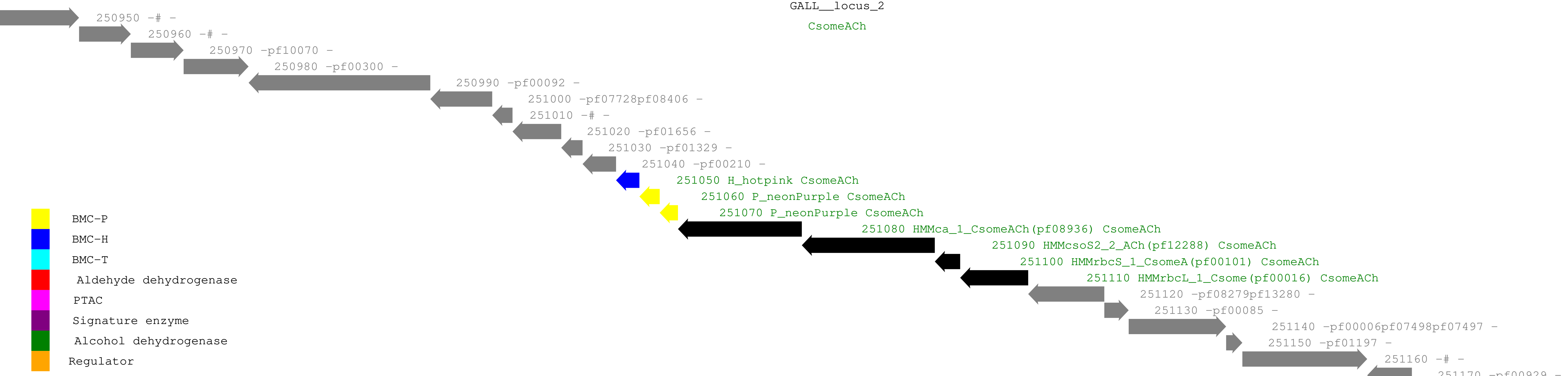

Acidithiobacillus sp. GGI-221. Bacteria; Proteobacteria; Acidithiobacillia; Acidithiobacillales;

GGI1__locus_fragmented

CsomeACh

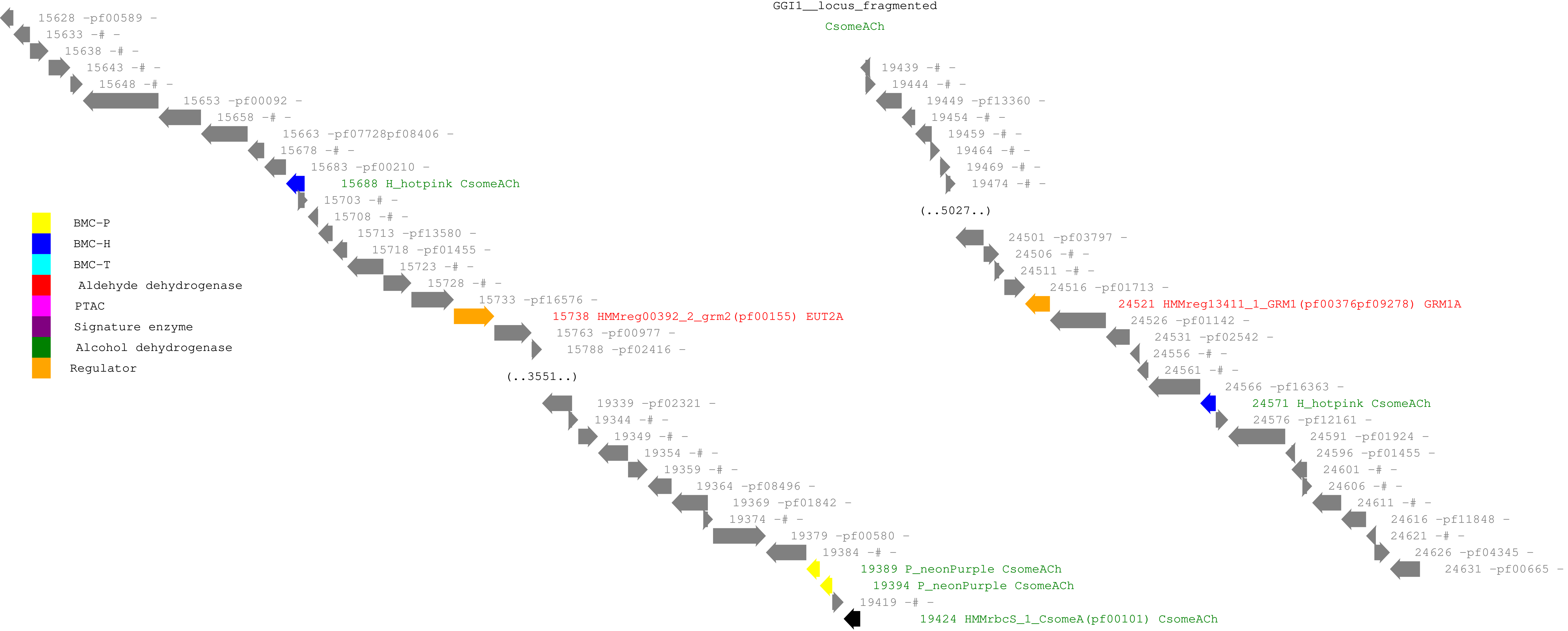

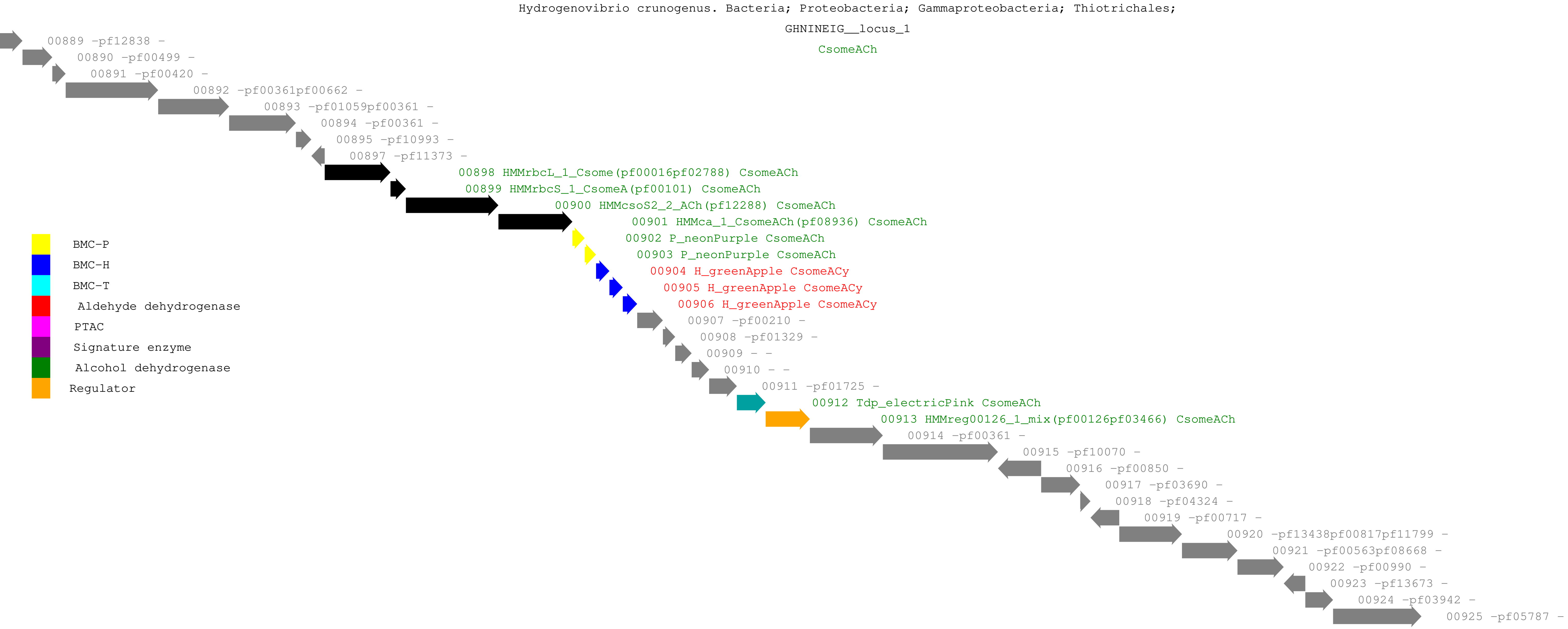
Hydrogenovibrio crunogenus. Bacteria; Proteobacteria; Gammaproteobacteria; Thiotrichales;
GHNINEIG__locus_1
CsomeACh
00889 -pf12838 -
00890 -pf00499 -
00891 -pf00420 -
00892 -pf00361pf00662 -
00893 -pf01059pf00361 -
00894 -pf00361 -
00895 -pf10993 -
00897 -pf11373 -
00898 HMMrbcL_1_Csome(pf00016pf02788) CsomeACh
00899 HMMrbcS_1_CsomeA(pf00101) CsomeACh
00900 HMMcsoS2_2_ACh(pf12288) CsomeACh
00901 HMMca_1_CsomeACh(pf08936) CsomeACh
00902 P_neonPurple CsomeACh
00903 P_neonPurple CsomeACh
00904 H_greenApple CsomeACy
00905 H_greenApple CsomeACy
00906 H_greenApple CsomeACy
00907 -pf00210 -
00908 -pf01329 -
00909 - -
00910 - -
00911 -pf01725 -
00912 Tdp_electricPink CsomeACh
00913 HMMreg00126_1_mix(pf00126pf03466) CsomeACh
00914 -pf00361 -
00915 -pf10070 -
00916 -pf00850 -
00917 -pf03690 -
00918 -pf04324 -
00919 -pf00717 -
00920 -pf13438pf00817pf11799 -
00921 -pf00563pf08668 -
00922 -pf00990 -
00923 -pf13673 -
00924 -pf03942 -
00925 -pf05787 -
BMC-P
BMC-H
BMC-T
Aldehyde dehydrogenase
PTAC
Signature enzyme
Alcohol dehydrogenase
Regulator

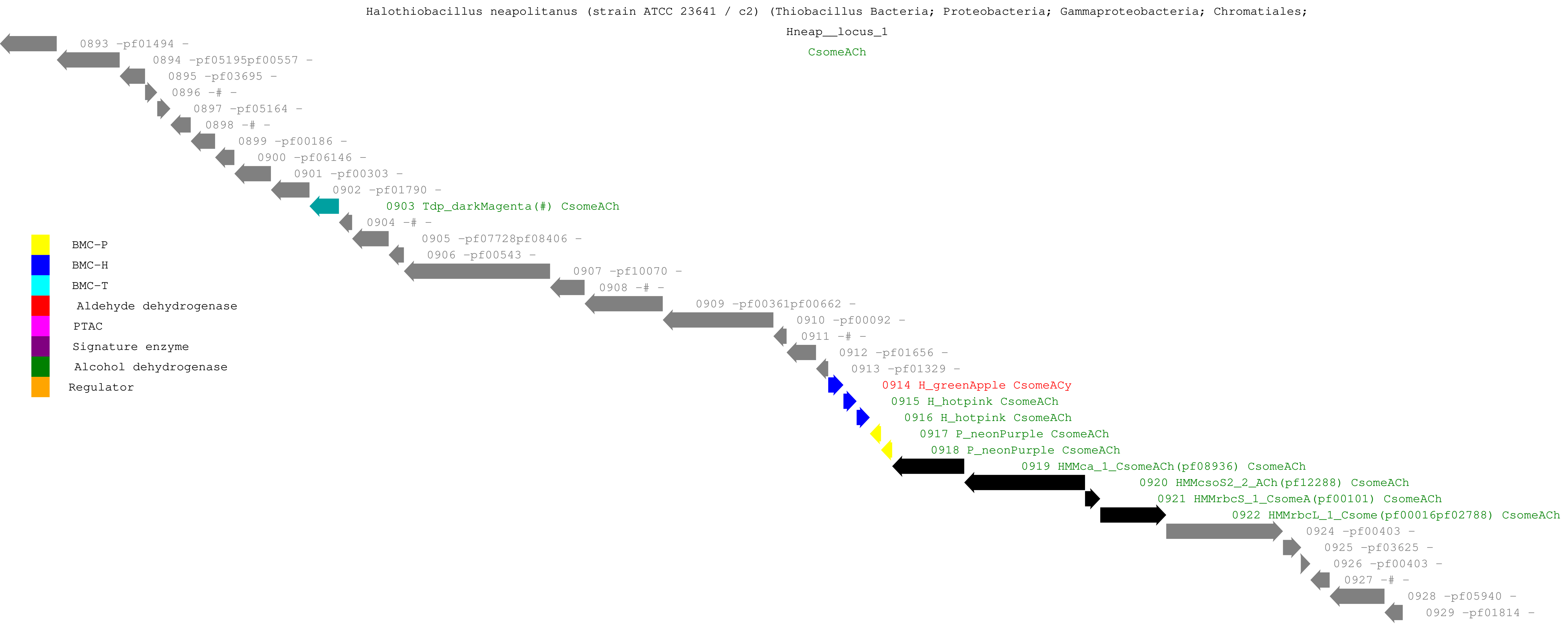
Halothiobacillus neapolitanus (strain ATCC 23641 / c2) (Thiobacillus Bacteria; Proteobacteria; Gammaproteobacteria; Chromatiales;
Hneap__locus_1
CsomeACh
0893 -pf01494 -
0894 -pf05195pf00557 -
0895 -pf03695 -
0896 -# -
0897 -pf05164 -
0898 -# -
0899 -pf00186 -
0900 -pf06146 -
0901 -pf00303 -
0902 -pf01790 -
0903 Tdp_darkMagenta(#) CsomeACh
0904 -# -
0905 -pf07728pf08406 -
0906 -pf00543 -
0907 -pf10070 -
0908 -# -
0909 -pf00361pf00662 -
0910 -pf00092 -
0911 -# -
0912 -pf01656 -
0913 -pf01329 -
0914 H_greenApple CsomeACy
0915 H_hotpink CsomeACh
0916 H_hotpink CsomeACh
0917 P_neonPurple CsomeACh
0918 P_neonPurple CsomeACh
0919 HMMca_1_CsomeACh(pf08936) CsomeACh
0920 HMMcsoS2_2_ACh(pf12288) CsomeACh
0921 HMMrbcS_1_CsomeA(pf00101) CsomeACh
0922 HMMrbcL_1_Csome(pf00016pf02788) CsomeACh
0924 -pf00403 -
0925 -pf03625 -
0926 -pf00403 -
0927 -# -
0928 -pf05940 -
0929 -pf01814 -
BMC-P
BMC-H
BMC-T
Aldehyde dehydrogenase
PTAC
Signature enzyme
Alcohol dehydrogenase
Regulator

Nitrosomonadaceae bacterium. Bacteria; Proteobacteria; Betaproteobacteria; Nitrosomonadales;

LBMAG32__locus_1

CsomeACh

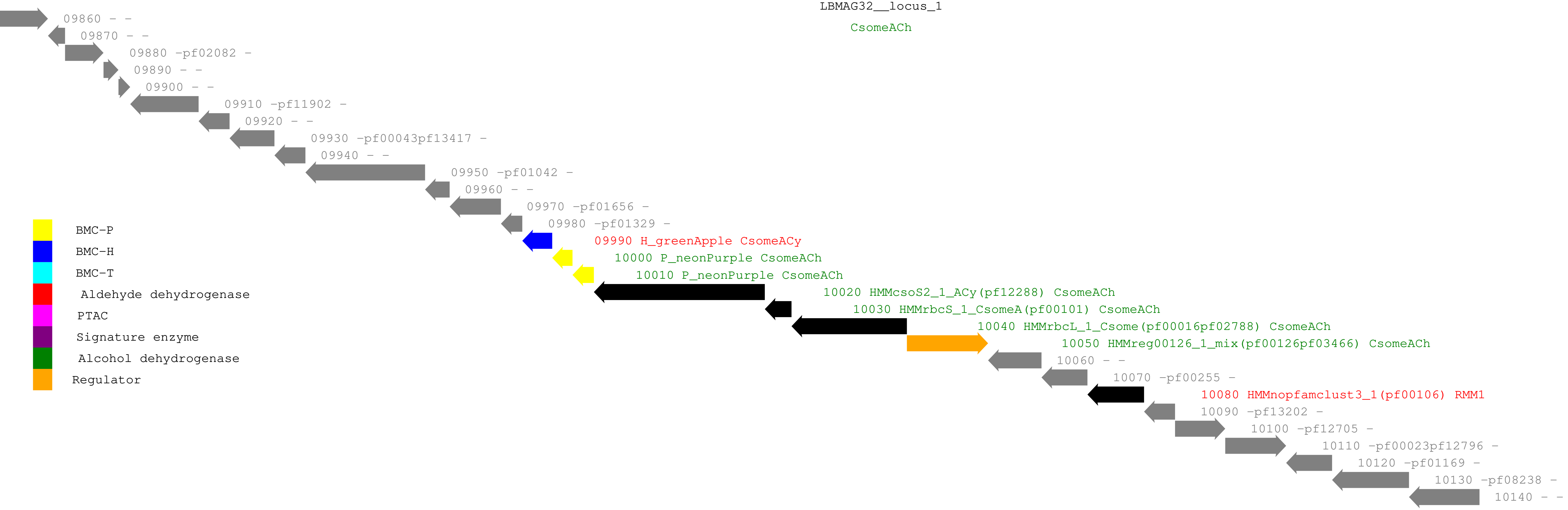

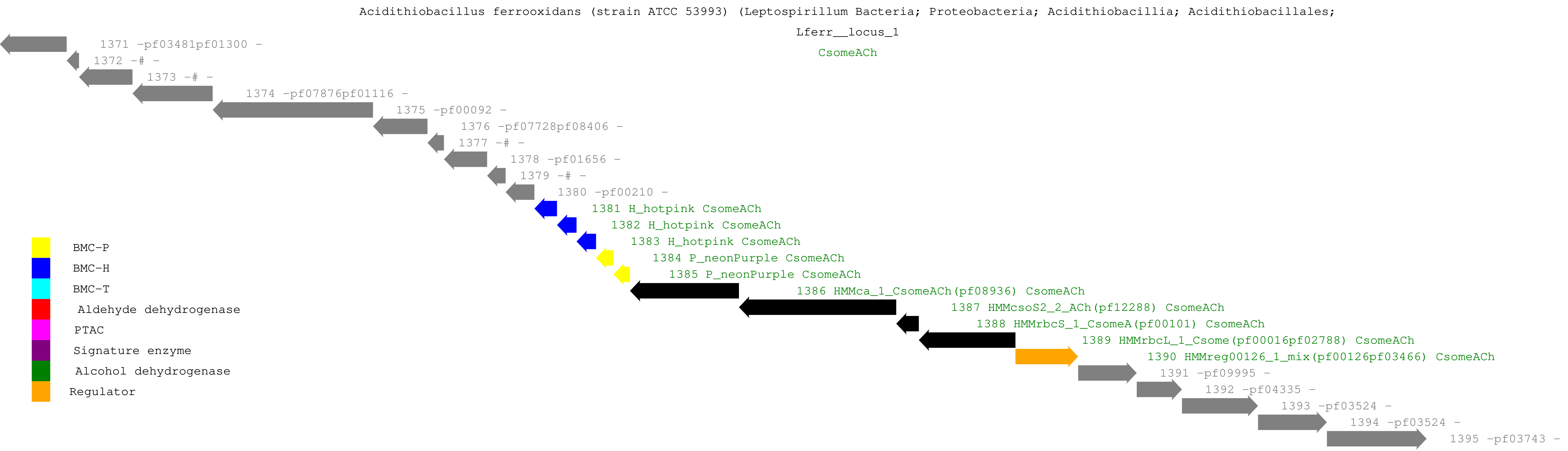
Acidithiobacillus ferrooxidans (strain ATCC 53993) (Leptospirillum Bacteria; Proteobacteria; Acidithiobacillia; Acidithiobacillales;
Lferr__locus_1
CsomeACh
1371 -pf03481pf01300 -
1372 -# -
1373 -# -
1374 -pf07876pf01116 -
1375 -pf00092 -
1376 -pf07728pf08406 -
1377 -# -
1378 -pf01656 -
1379 -# -
1380 -pf00210 -
1381 H_hotpink CsomeACh
1382 H_hotpink CsomeACh
1383 H_hotpink CsomeACh
1384 P_neonPurple CsomeACh
1385 P_neonPurple CsomeACh
1386 HMMca_1_CsomeACh(pf08936) CsomeACh
1387 HMMcsoS2_2_ACh(pf12288) CsomeACh
1388 HMMrbcS_1_CsomeA(pf00101) CsomeACh
1389 HMMrbcL_1_Csome(pf00016pf02788) CsomeACh
1390 HMMreg00126_1_mix(pf00126pf03466) CsomeACh
1391 -pf09995 -
1392 -pf04335 -
1393 -pf03524 -
1394 -pf03524 -
1395 -pf03743 -
BMC-P
BMC-H
BMC-T
Aldehyde dehydrogenase
PTAC
Signature enzyme
Alcohol dehydrogenase
Regulator

Halorhodospira halochloris str. A. Bacteria; Proteobacteria; Gammaproteobacteria; Chromatiales;

M911__locus_1_main_sat

CsomeACh

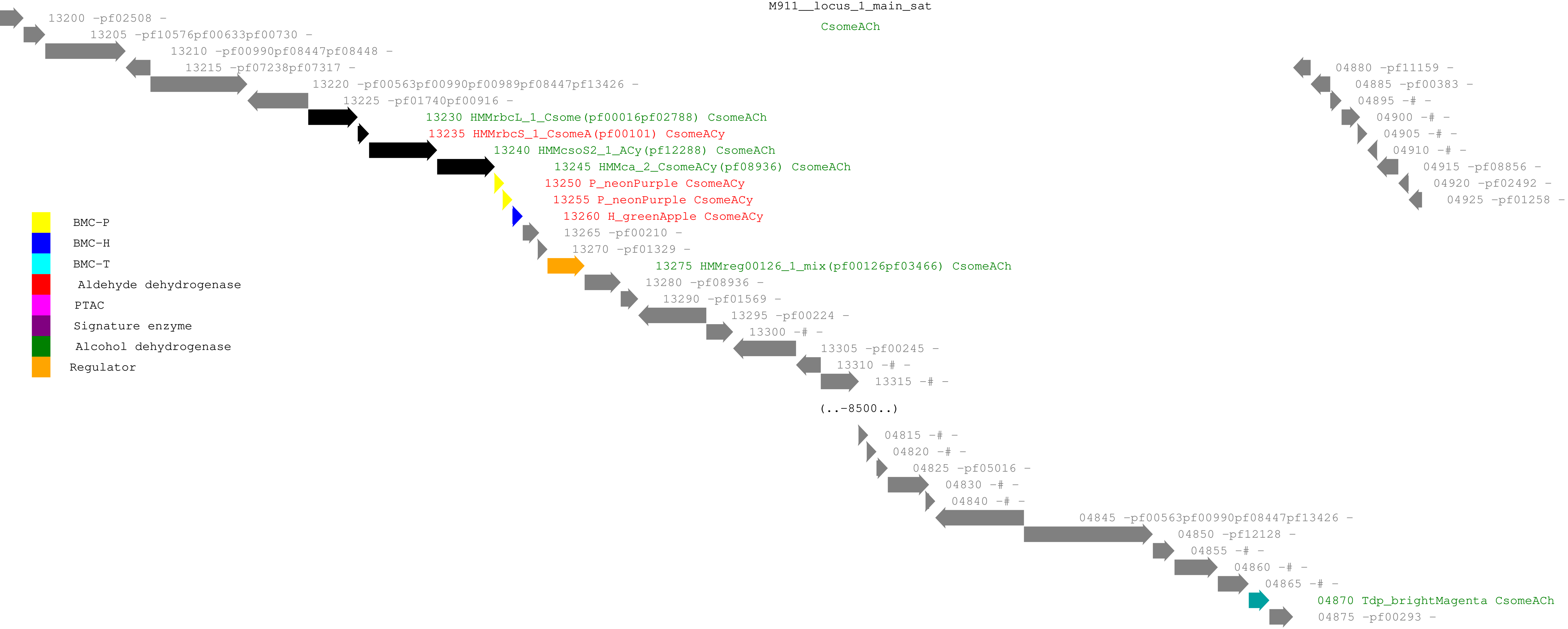

Marichromatium purpuratum 984. Bacteria; Proteobacteria; Gammaproteobacteria; Chromatiales;

MARPU__locus_1

CsomeACh

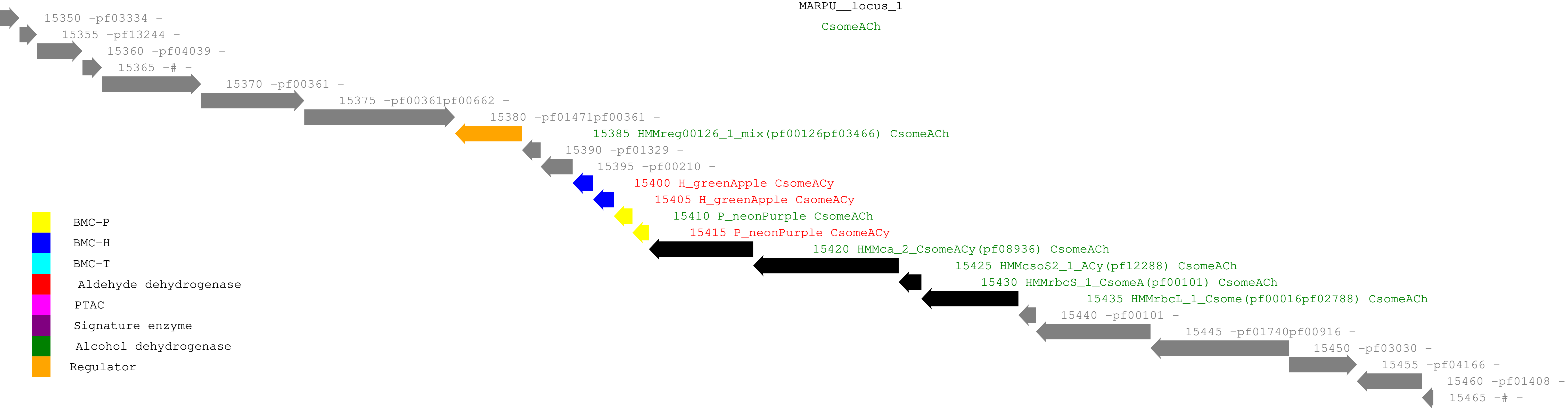

hydrothermal vent metagenome. unclassified sequences; metagenomes; ecological metagenomes.

MNBD_GAMMA04-_locus_1_main_sat

CsomeACh

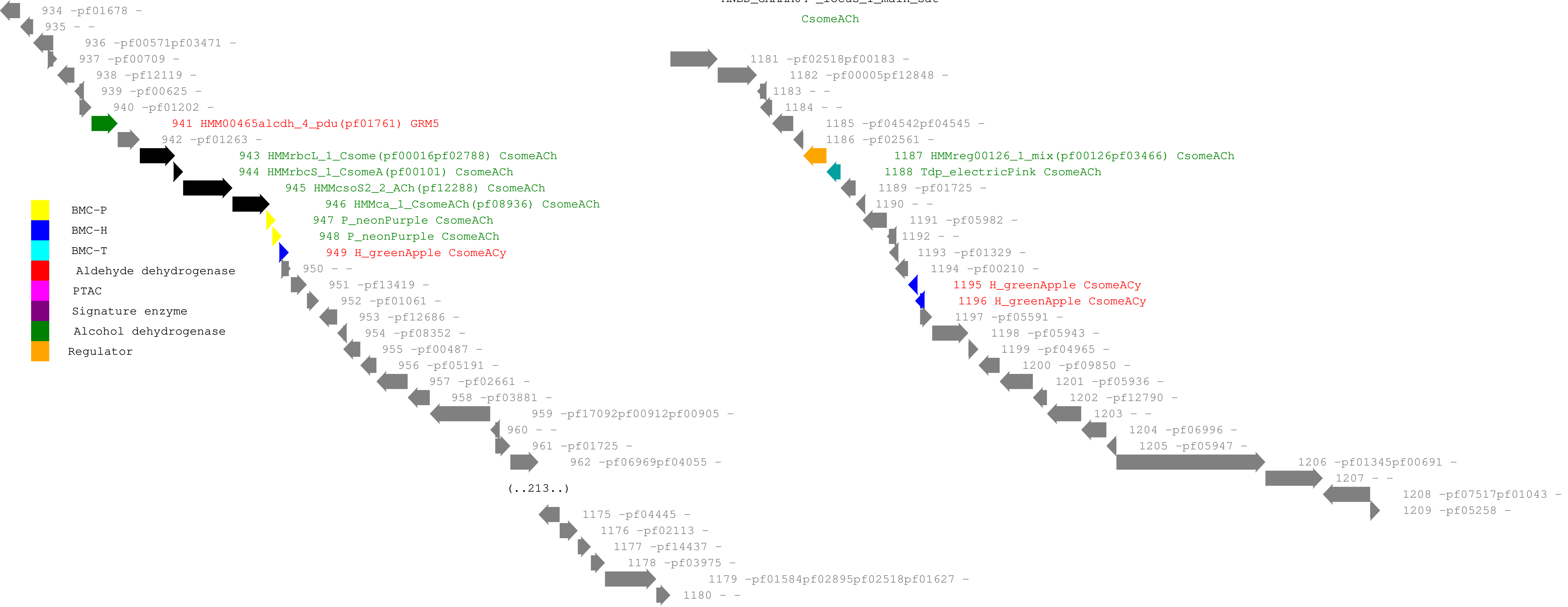

hydrothermal vent metagenome. unclassified sequences; metagenomes; ecological metagenomes.

MNBD_GAMMA16-_locus_1

CsomeACh

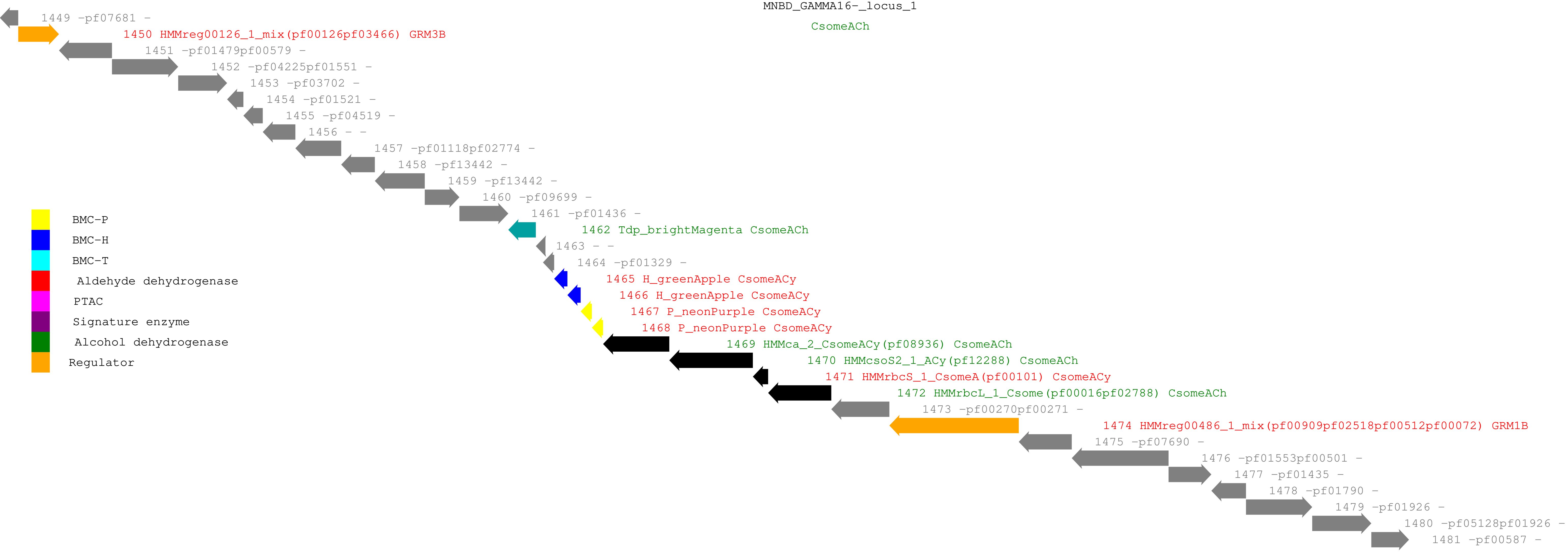

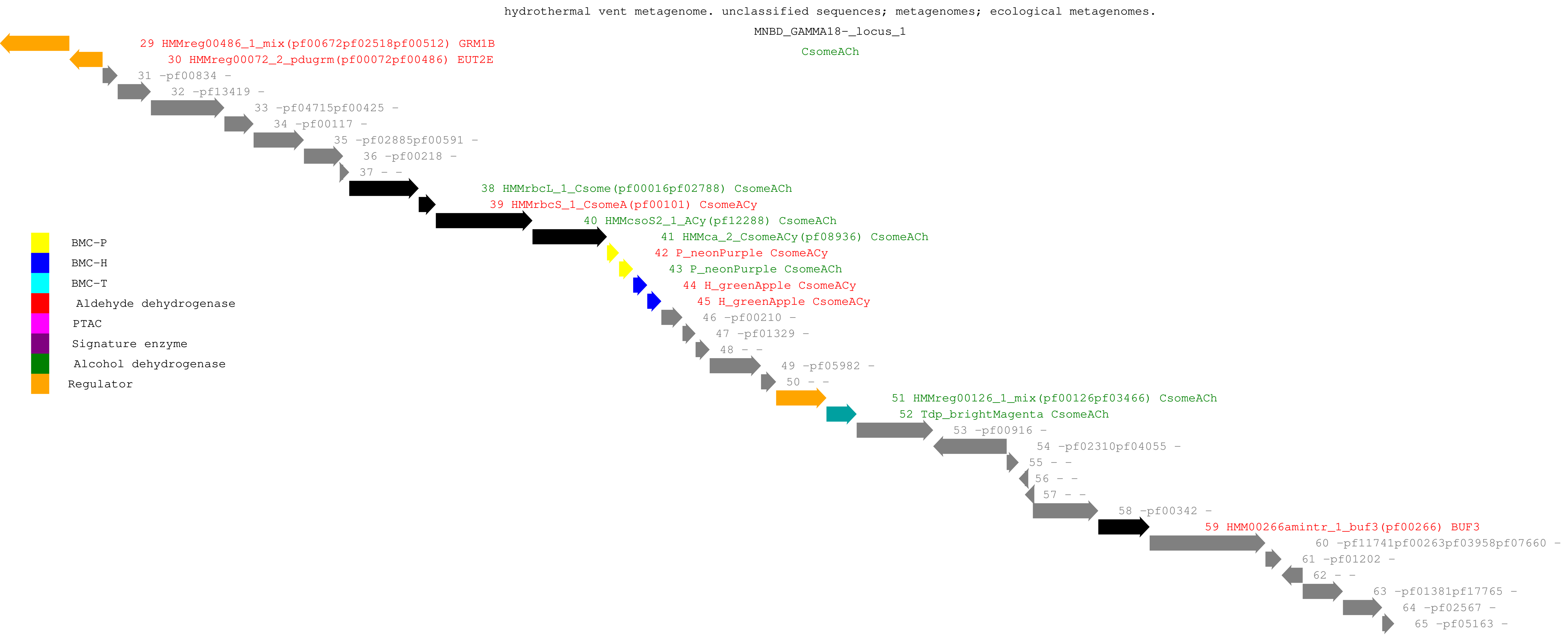
hydrothermal vent metagenome. unclassified sequences; metagenomes; ecological metagenomes.
MNBD_GAMMA18-_locus_1
CsomeACh
29 HMMreg00486_1_mix(pf00672pf02518pf00512) GRM1B
30 HMMreg00072_2_pdugrm(pf00072pf00486) EUT2E
31 -pf00834 -
32 -pf13419 -
33 -pf04715pf00425 -
34 -pf00117 -
35 -pf02885pf00591 -
36 -pf00218 -
37 - -
38 HMMrbcL_1_Csome(pf00016pf02788) CsomeACh
39 HMMrbcS_1_CsomeA(pf00101) CsomeACy
40 HMMcsoS2_1_ACy(pf12288) CsomeACh
41 HMMca_2_CsomeACy(pf08936) CsomeACh
42 P_neonPurple CsomeACy
43 P_neonPurple CsomeACh
44 H_greenApple CsomeACy
45 H_greenApple CsomeACy
46 -pf00210 -
47 -pf01329 -
48 - -
49 -pf05982 -
50 - -
51 HMMreg00126_1_mix(pf00126pf03466) CsomeACh
52 Tdp_brightMagenta CsomeACh
53 -pf00916 -
54 -pf02310pf04055 -
55 - -
56 - -
57 - -
58 -pf00342 -
59 HMM00266amintr_1_buf3(pf00266) BUF3
60 -pf11741pf00263pf03958pf07660 -
61 -pf01202 -
62 - -
63 -pf01381pf17765 -
64 -pf02567 -
65 -pf05163 -
BMC-P
BMC-H
BMC-T
Aldehyde dehydrogenase
PTAC
Signature enzyme
Alcohol dehydrogenase
Regulator

hydrothermal vent metagenome. unclassified sequences; metagenomes; ecological metagenomes.

MNBD_GAMMA20-_locus_1

CsomeACh

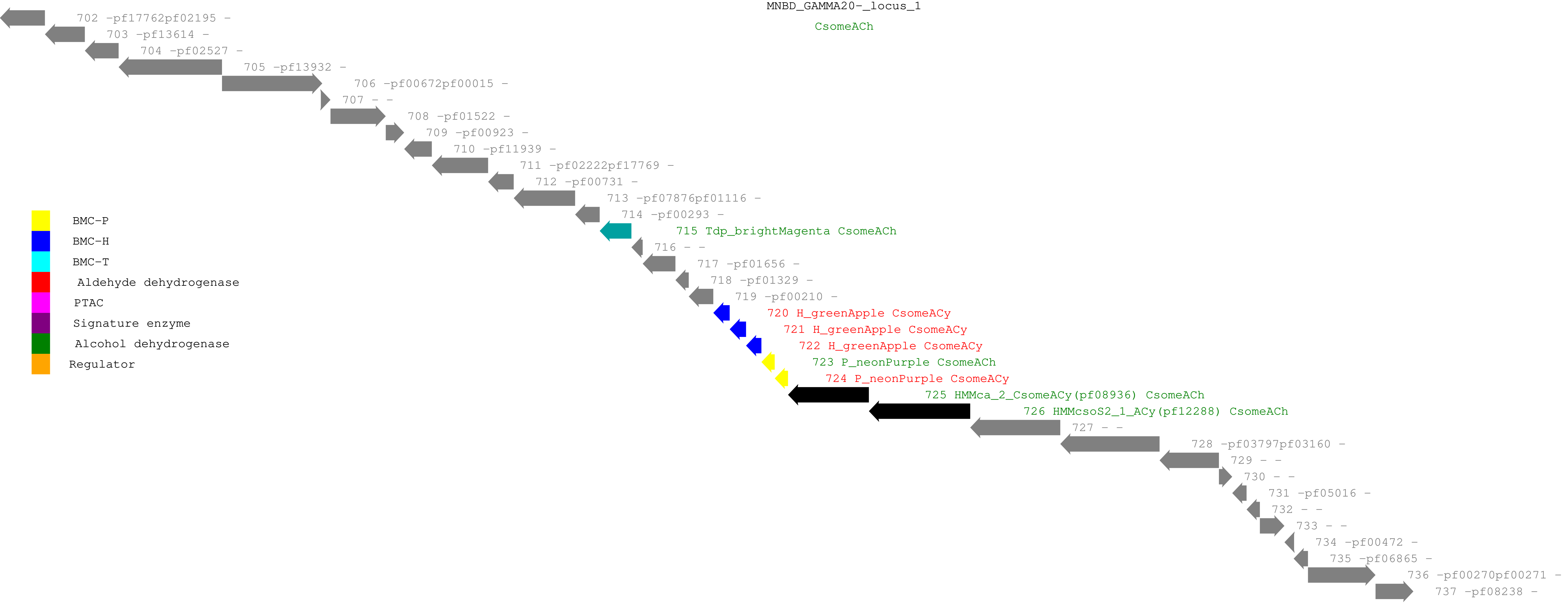

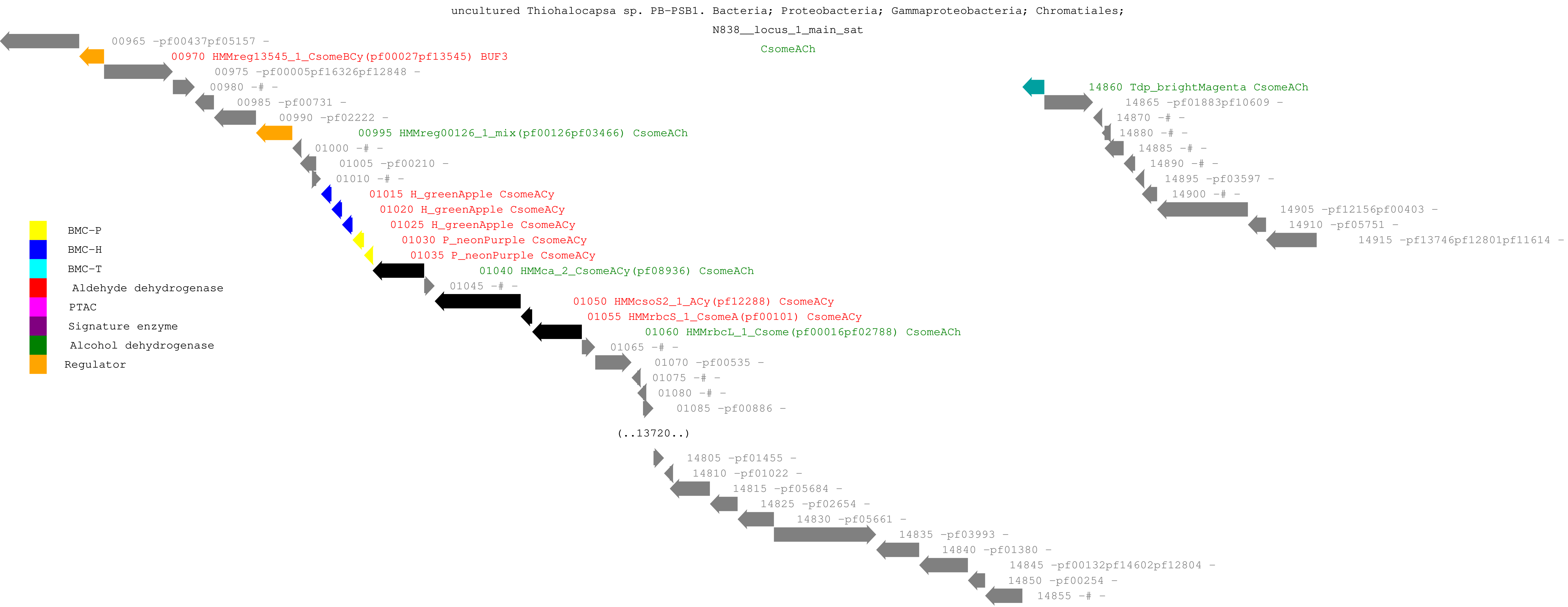
uncultured Thiohalocapsa sp. PB-PSB1. Bacteria; Proteobacteria; Gammaproteobacteria; Chromatiales;
N838__locus_1_main_sat
CsomeACh
00965 -pf00437pf05157 -
00970 HMMreg13545_1_CsomeBCy(pf00027pf13545) BUF3
00975 -pf00005pf16326pf12848 -
00980 -# -
00985 -pf00731 -
00990 -pf02222 -
00995 HMMreg00126_1_mix(pf00126pf03466) CsomeACh
01000 -# -
01005 -pf00210 -
01010 -# -
01015 H_greenApple CsomeACy
01020 H_greenApple CsomeACy
01025 H_greenApple CsomeACy
01030 P_neonPurple CsomeACy
01035 P_neonPurple CsomeACy
01040 HMMca_2_CsomeACy(pf08936) CsomeACh
01045 -# -
01050 HMMcsoS2_1_ACy(pf12288) CsomeACy
01055 HMMrbcS_1_CsomeA(pf00101) CsomeACy
01060 HMMrbcL_1_Csome(pf00016pf02788) CsomeACh
01065 -# -
01070 -pf00535 -
01075 -# -
01080 -# -
01085 -pf00886 -
(..13720..)
14805 -pf01455 -
14810 -pf01022 -
14815 -pf05684 -
14825 -pf02654 -
14830 -pf05661 -
14835 -pf03993 -
14840 -pf01380 -
14845 -pf00132pf14602pf12804 -
14850 -pf00254 -
14855 -# -
14860 Tdp_brightMagenta CsomeACh
14865 -pf01883pf10609 -
14870 -# -
14880 -# -
14885 -# -
14890 -# -
14895 -pf03597 -
14900 -# -
14905 -pf12156pf00403 -
14910 -pf05751 -
14915 -pf13746pf12801pf11614 -
BMC-P
BMC-H
BMC-T
Aldehyde dehydrogenase
PTAC
Signature enzyme
Alcohol dehydrogenase
Regulator

Nitrococcus mobilis Nb-231. Bacteria; Proteobacteria; Gammaproteobacteria; Chromatiales;

NB231__locus_1

CsomeACh

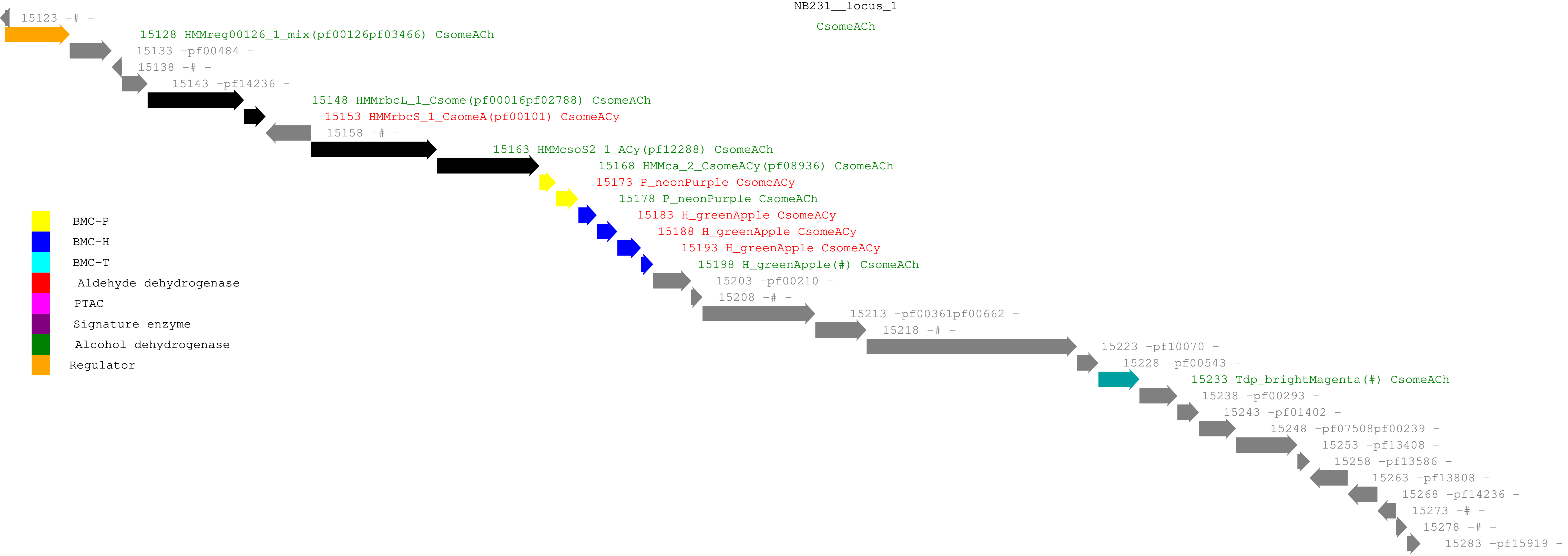

Nitrobacter sp. Nb-311A. Bacteria; Proteobacteria; Alphaproteobacteria; Rhizobiales;

NB311A__locus_1

CsomeACh

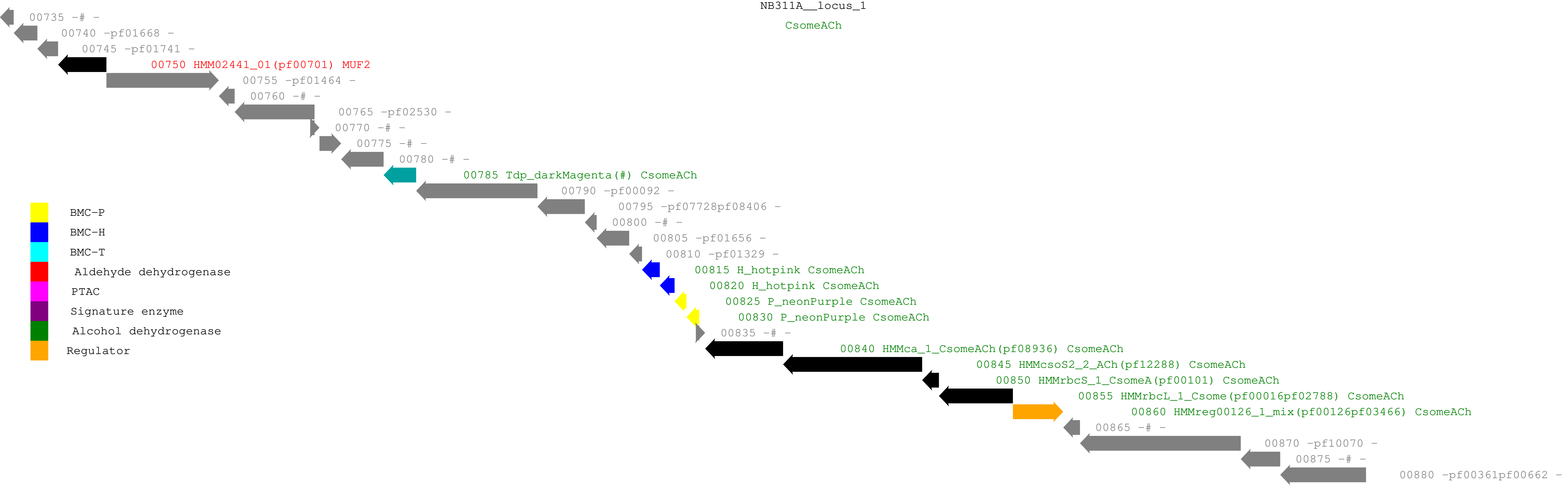

Nitrosomonas eutropha (strain C91). Bacteria; Proteobacteria; Betaproteobacteria; Nitrosomonadales;

Neut__locus_1

CsomeACh

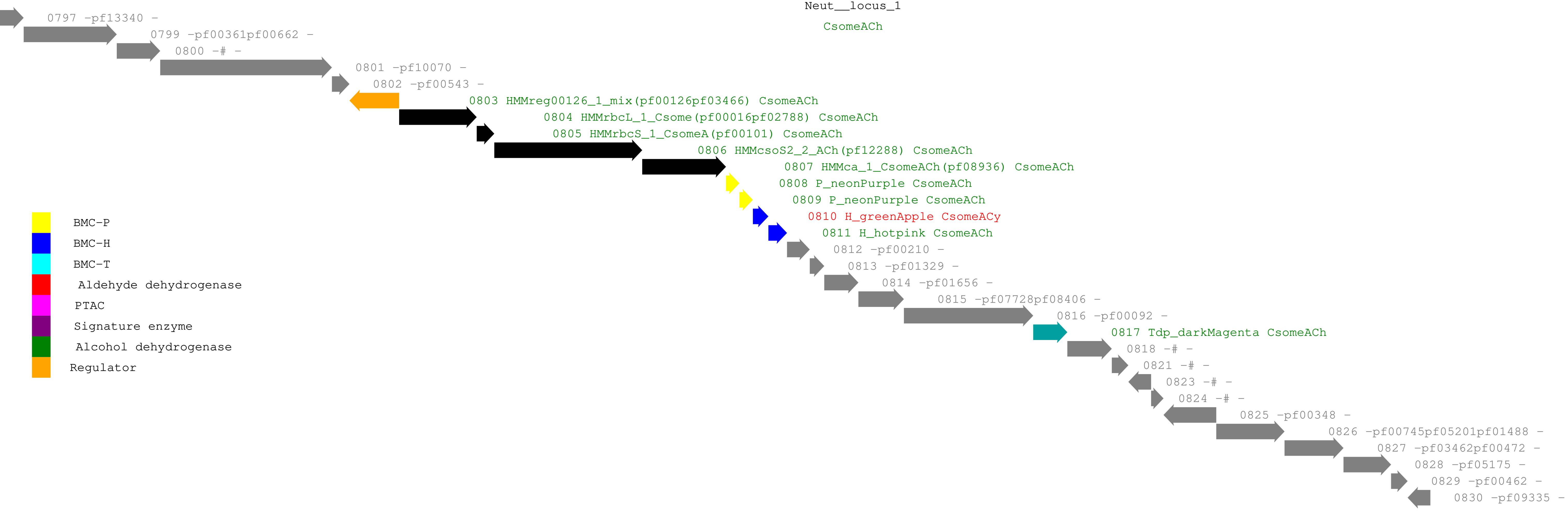

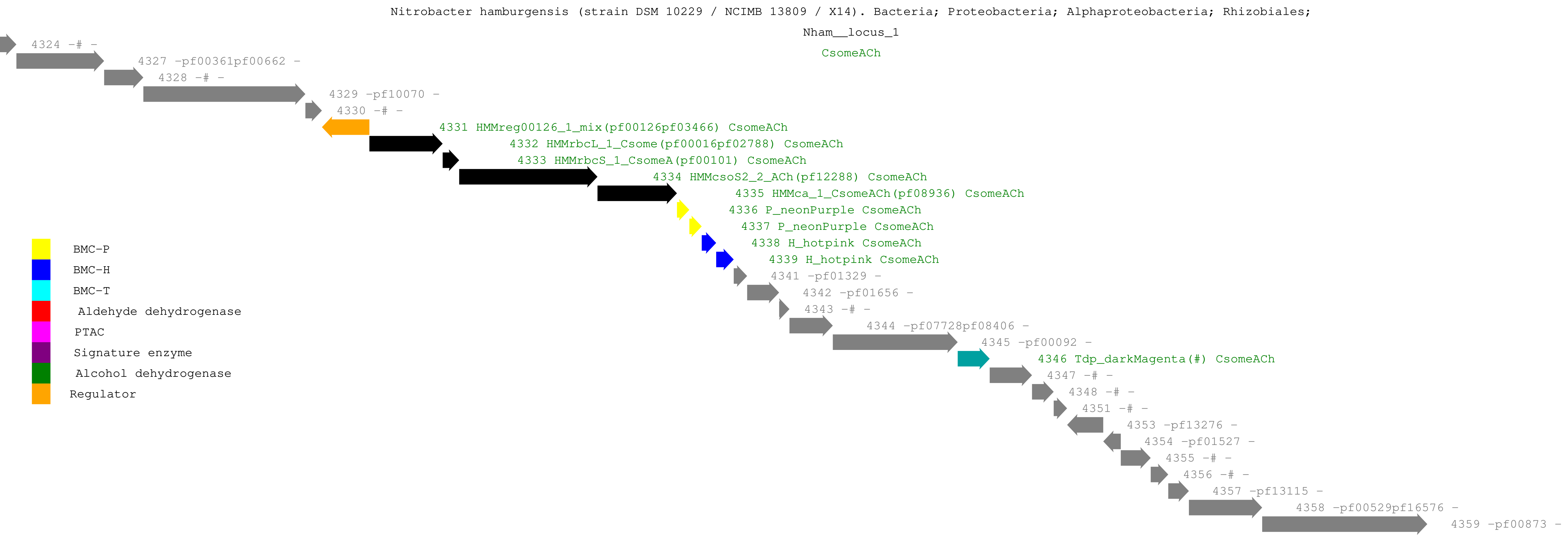

Nitrobacter hamburgensis (strain DSM 10229 / NCIMB 13809 / X14). Bacteria; Proteobacteria; Alphaproteobacteria; Rhizobiales;
Nham__locus_1
CsomeACh
4324 -# -
4327 -pf00361pf00662 -
4328 -# -
4329 -pf10070 -
4330 -# -
4331 HMMreg00126_1_mix(pf00126pf03466) CsomeACh
4332 HMMrbcL_1_Csome(pf00016pf02788) CsomeACh
4333 HMMrbcS_1_CsomeA(pf00101) CsomeACh
4334 HMMcsoS2_2_ACh(pf12288) CsomeACh
4335 HMMca_1_CsomeACh(pf08936) CsomeACh
4336 P_neonPurple CsomeACh
4337 P_neonPurple CsomeACh
4338 H_hotpink CsomeACh
4339 H_hotpink CsomeACh
4341 -pf01329 -
4342 -pf01656 -
4343 -# -
4344 -pf07728pf08406 -
4345 -pf00092 -
4346 Tdp_darkMagenta(#) CsomeACh
4347 -# -
4348 -# -
4351 -# -
4353 -pf13276 -
4354 -pf01527 -
4355 -# -
4356 -# -
4357 -pf13115 -
4358 -pf00529pf16576 -
4359 -pf00873 -
BMC-P
BMC-H
BMC-T
Aldehyde dehydrogenase
PTAC
Signature enzyme
Alcohol dehydrogenase
Regulator

Nitrobacter winogradskyi (Nitrobacter agilis). Bacteria; Proteobacteria; Alphaproteobacteria; Rhizobiales;

NWI01__locus_1

CsomeACh

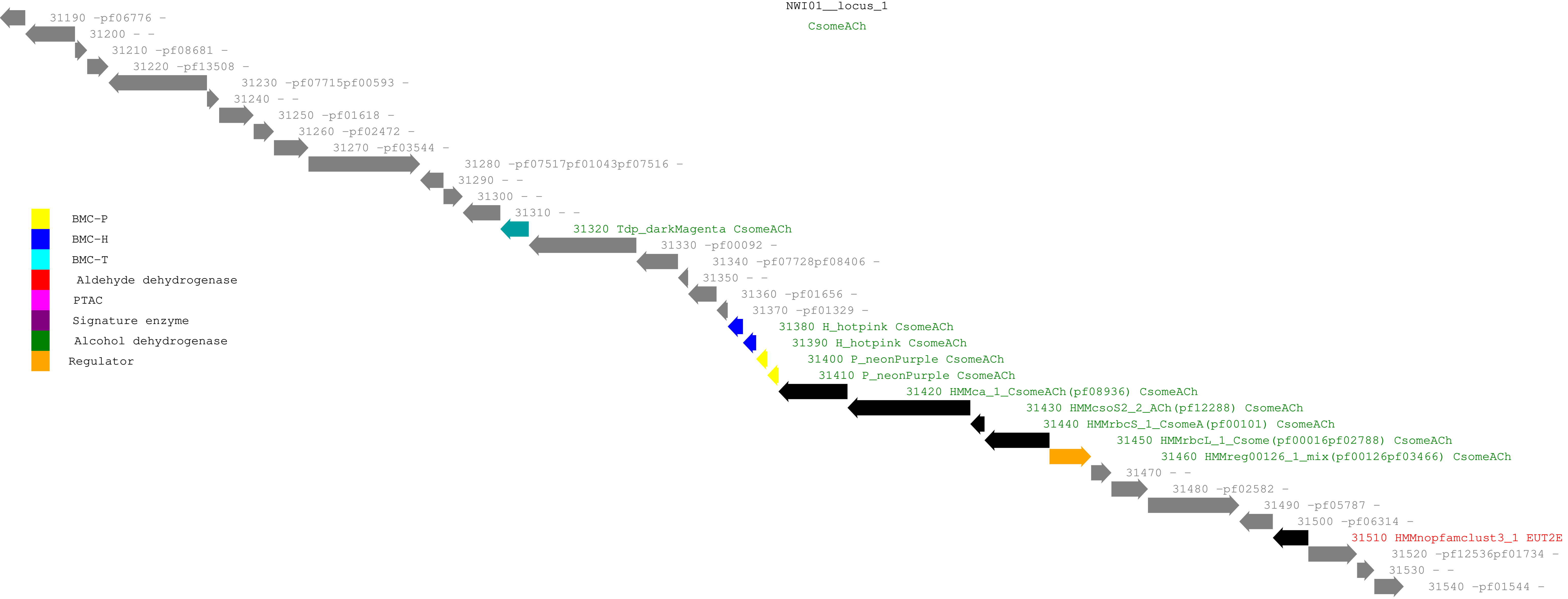

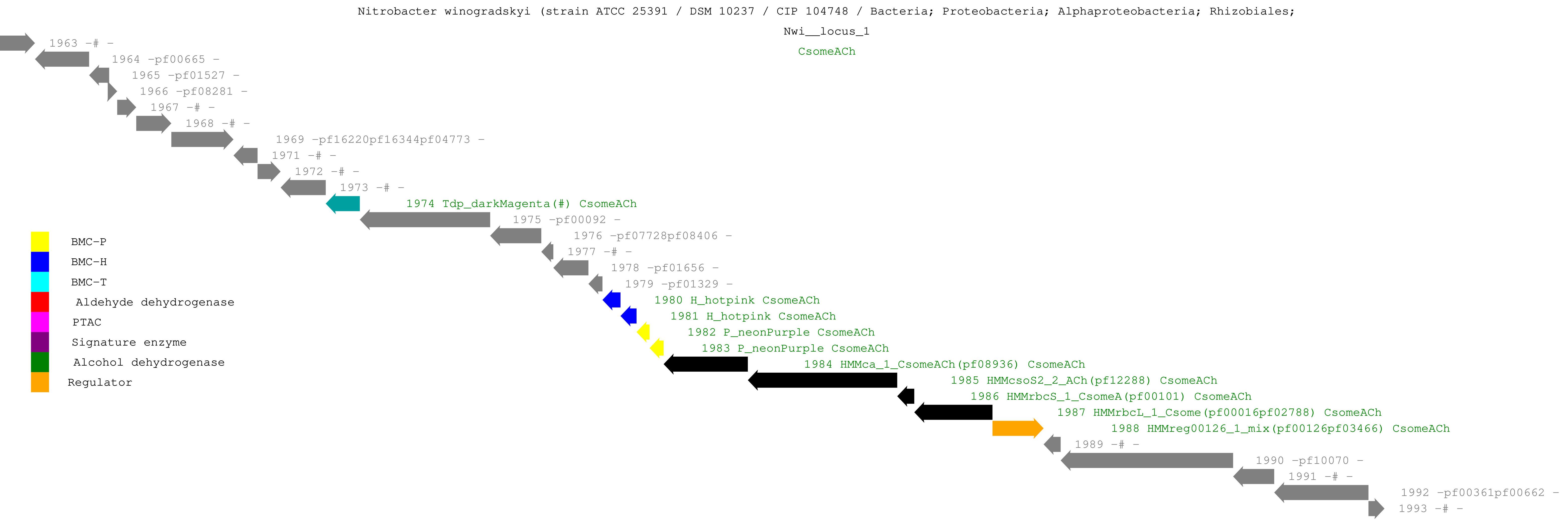

Nitrobacter winogradskyi (strain ATCC 25391 / DSM 10237 / CIP 104748 / Bacteria; Proteobacteria; Alphaproteobacteria; Rhizobiales;
Nwi__locus_1
CsomeACh
1963 -# -
1964 -pf00665 -
1965 -pf01527 -
1966 -pf08281 -
1967 -# -
1968 -# -
1969 -pf16220pf16344pf04773 -
1971 -# -
1972 -# -
1973 -# -
1974 Tdp_darkMagenta(#) CsomeACh
1975 -pf00092 -
1976 -pf07728pf08406 -
1977 -# -
1978 -pf01656 -
1979 -pf01329 -
1980 H_hotpink CsomeACh
1981 H_hotpink CsomeACh
1982 P_neonPurple CsomeACh
1983 P_neonPurple CsomeACh
1984 HMMca_1_CsomeACh(pf08936) CsomeACh
1985 HMMcsoS2_2_ACh(pf12288) CsomeACh
1986 HMMrbcS_1_CsomeA(pf00101) CsomeACh
1987 HMMrbcL_1_Csome(pf00016pf02788) CsomeACh
1988 HMMreg00126_1_mix(pf00126pf03466) CsomeACh
1989 -# -
1990 -pf10070 -
1991 -# -
1992 -pf00361pf00662 -
1993 -# -
BMC-P
BMC-H
BMC-T
Aldehyde dehydrogenase
PTAC
Signature enzyme
Alcohol dehydrogenase
Regulator

Ferrithrix thermotolerans DSM 19514. Bacteria; Actinobacteria; Acidimicrobiia; Acidimicrobiales;

SAMN02745225__locus_1_main_sat

CsomeACh

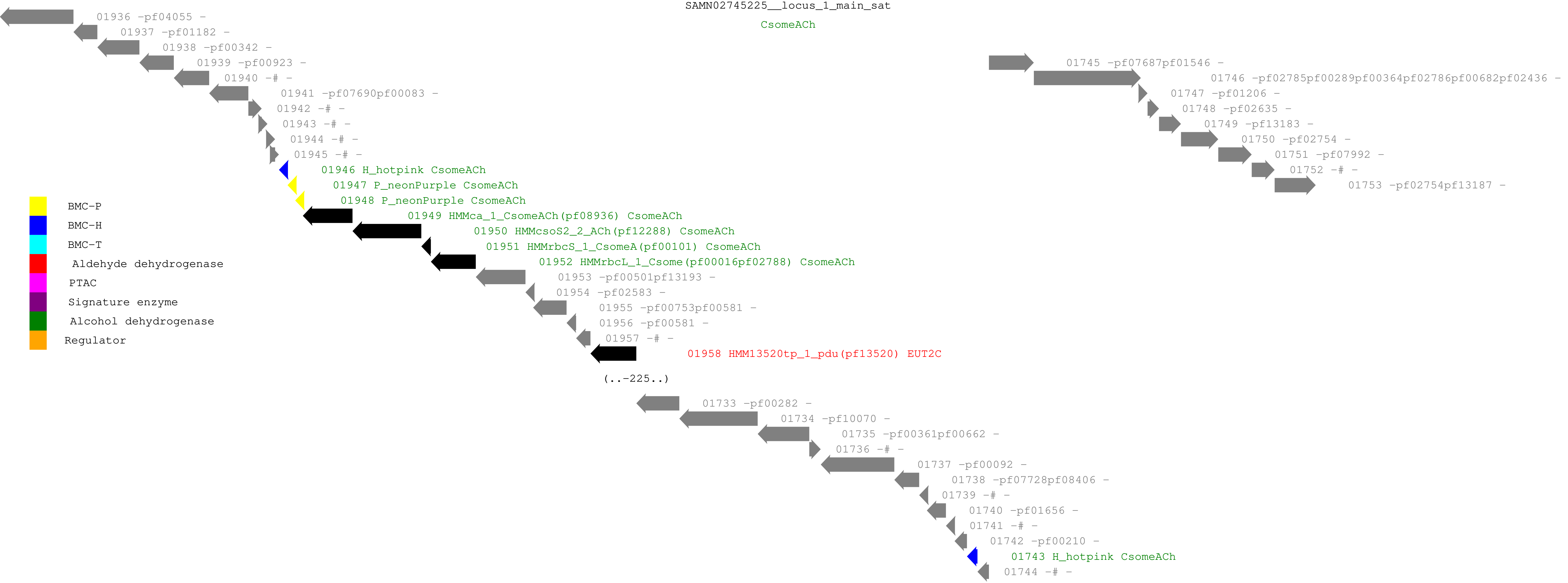

Thiomicrospira sp. ALE5. Bacteria; Proteobacteria; Gammaproteobacteria; Thiotrichales;

SAMN03092900__locus_1

CsomeACh

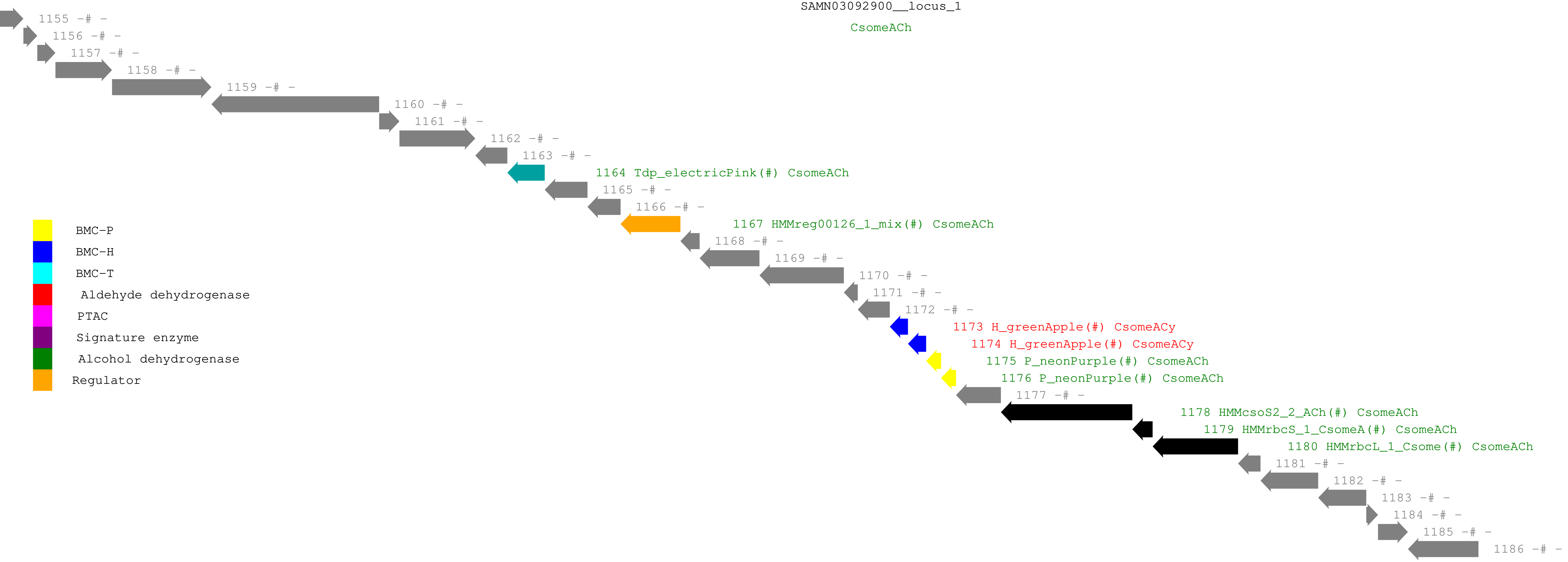

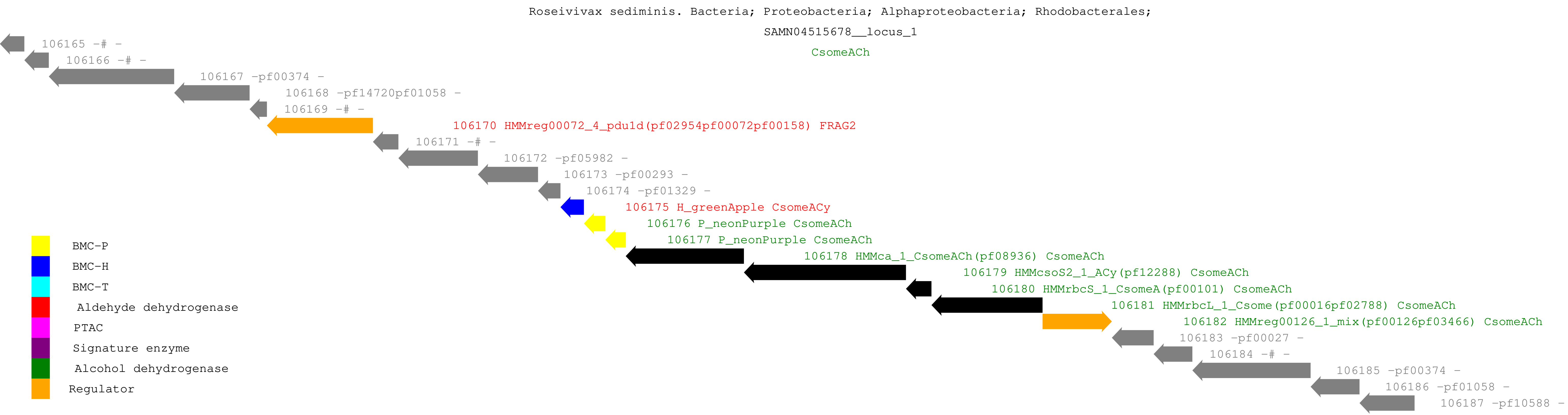

Roseivivax sediminis. Bacteria; Proteobacteria; Alphaproteobacteria; Rhodobacterales;
SAMN04515678__locus_1
CsomeACh
106165 -# -
106166 -# -
106167 -pf00374 -
106168 -pf14720pf01058 -
106169 -# -
106170 HMMreg00072_4_pdu1d(pf02954pf00072pf00158) FRAG2
106171 -# -
106172 -pf05982 -
106173 -pf00293 -
106174 -pf01329 -
106175 H_greenApple CsomeACy
106176 P_neonPurple CsomeACh
106177 P_neonPurple CsomeACh
106178 HMMca_1_CsomeACh(pf08936) CsomeACh
106179 HMMcsoS2_1_ACy(pf12288) CsomeACh
106180 HMMrbcS_1_CsomeA(pf00101) CsomeACh
106181 HMMrbcL_1_Csome(pf00016pf02788) CsomeACh
106182 HMMreg00126_1_mix(pf00126pf03466) CsomeACh
106183 -pf00027 -
106184 -# -
106185 -pf00374 -
106186 -pf01058 -
106187 -pf10588 -
BMC-P
BMC-H
BMC-T
Aldehyde dehydrogenase
PTAC
Signature enzyme
Alcohol dehydrogenase
Regulator

Nitrosomonas eutropha. Bacteria; Proteobacteria; Betaproteobacteria; Nitrosomonadales;

SAMN05216339__locus_fragmented

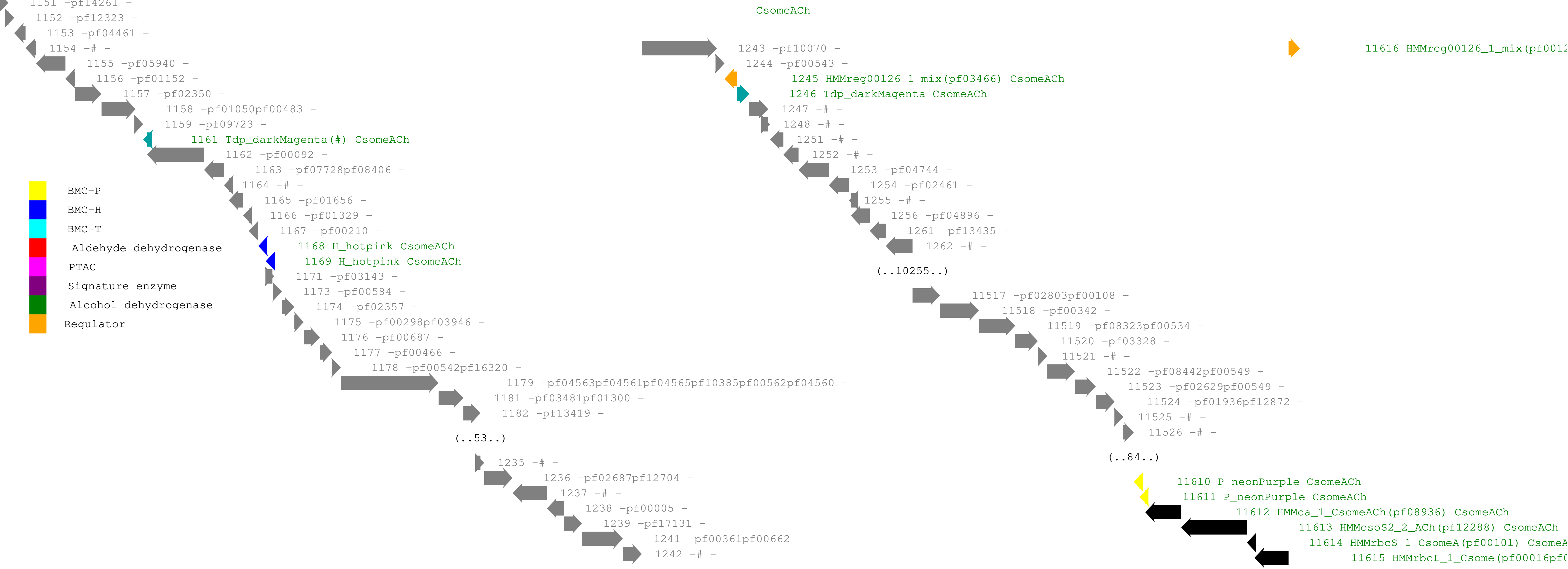

Nitrosomonas eutropha. Bacteria; Proteobacteria; Betaproteobacteria; Nitrosomonadales;

SAMN05216379__locus_1

CsomeACh

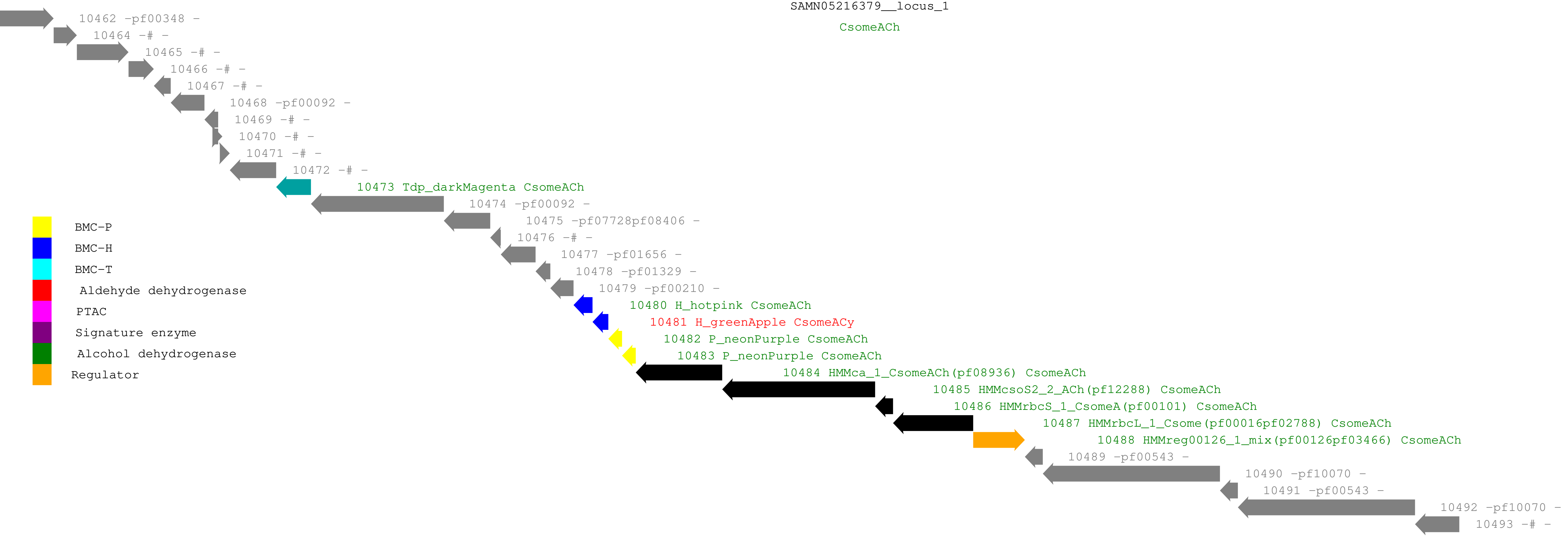

Nitrosospira multiformis. Bacteria; Proteobacteria; Betaproteobacteria; Nitrosomonadales;

SAMN05216402__locus_1

CsomeACh

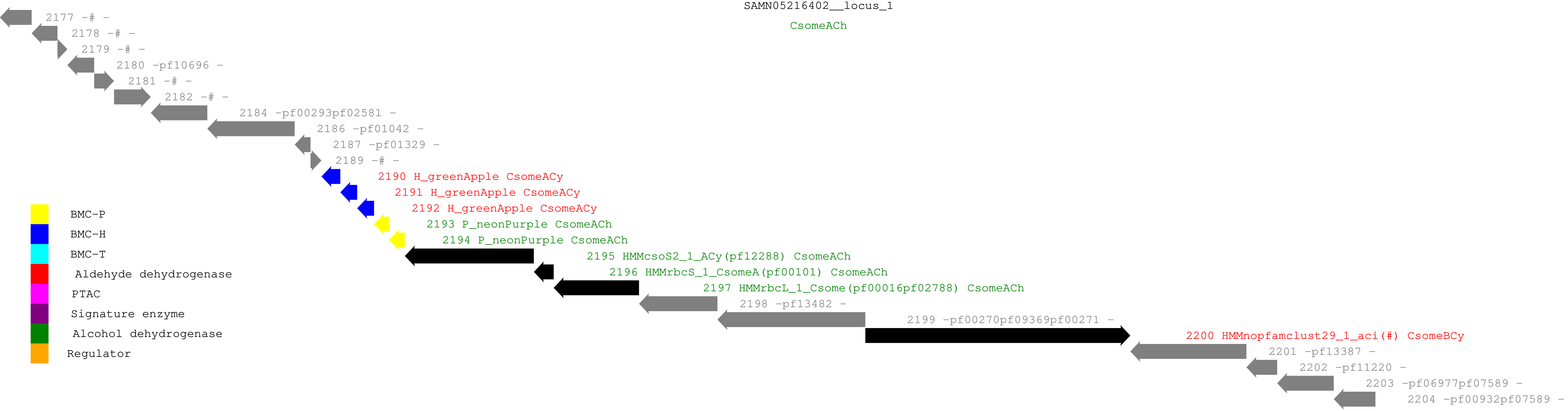

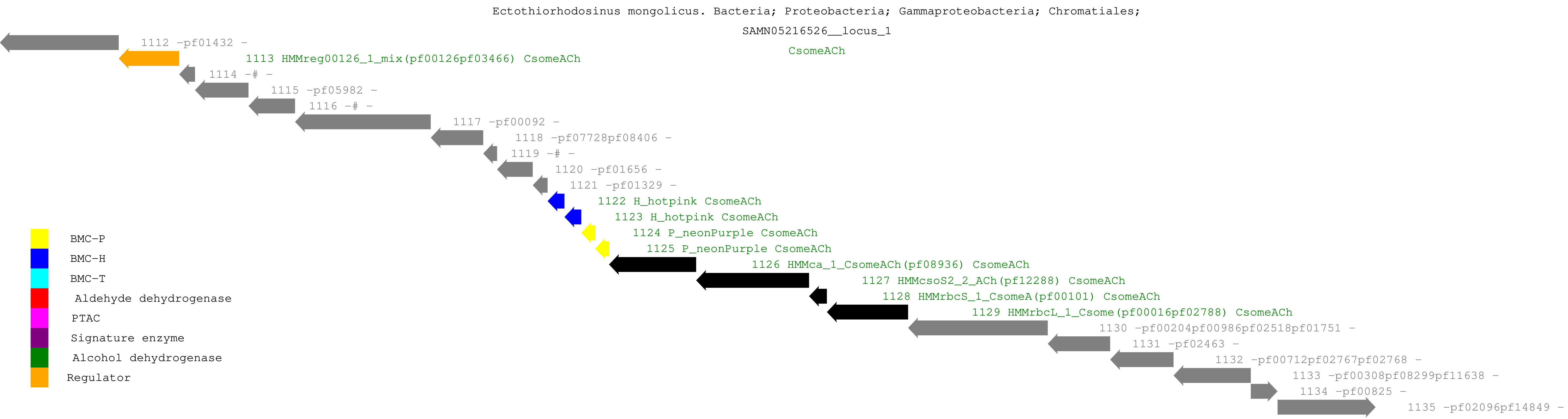
Ectothiorhodosinus mongolicus. Bacteria; Proteobacteria; Gammaproteobacteria; Chromatiales;
SAMN05216526__locus_1
CsomeACh
1112 -pf01432 -
1113 HMMreg00126_1_mix(pf00126pf03466) CsomeACh
1114 -# -
1115 -pf05982 -
1116 -# -
1117 -pf00092 -
1118 -pf07728pf08406 -
1119 -# -
1120 -pf01656 -
1121 -pf01329 -
1122 H_hotpink CsomeACh
1123 H_hotpink CsomeACh
1124 P_neonPurple CsomeACh
1125 P_neonPurple CsomeACh
1126 HMMca_1_CsomeACh(pf08936) CsomeACh
1127 HMMcsoS2_2_ACh(pf12288) CsomeACh
1128 HMMrbcS_1_CsomeA(pf00101) CsomeACh
1129 HMMrbcL_1_Csome(pf00016pf02788) CsomeACh
1130 -pf00204pf00986pf02518pf01751 -
1131 -pf02463 -
1132 -pf00712pf02767pf02768 -
1133 -pf00308pf08299pf11638 -
1134 -pf00825 -
1135 -pf02096pf14849 -
BMC-P
BMC-H
BMC-T
Aldehyde dehydrogenase
PTAC
Signature enzyme
Alcohol dehydrogenase
Regulator

Nitrosomonas sp. Nm51. Bacteria; Proteobacteria; Betaproteobacteria; Nitrosomonadales;

SAMN05421690__locus_1_main_sat

CsomeACh

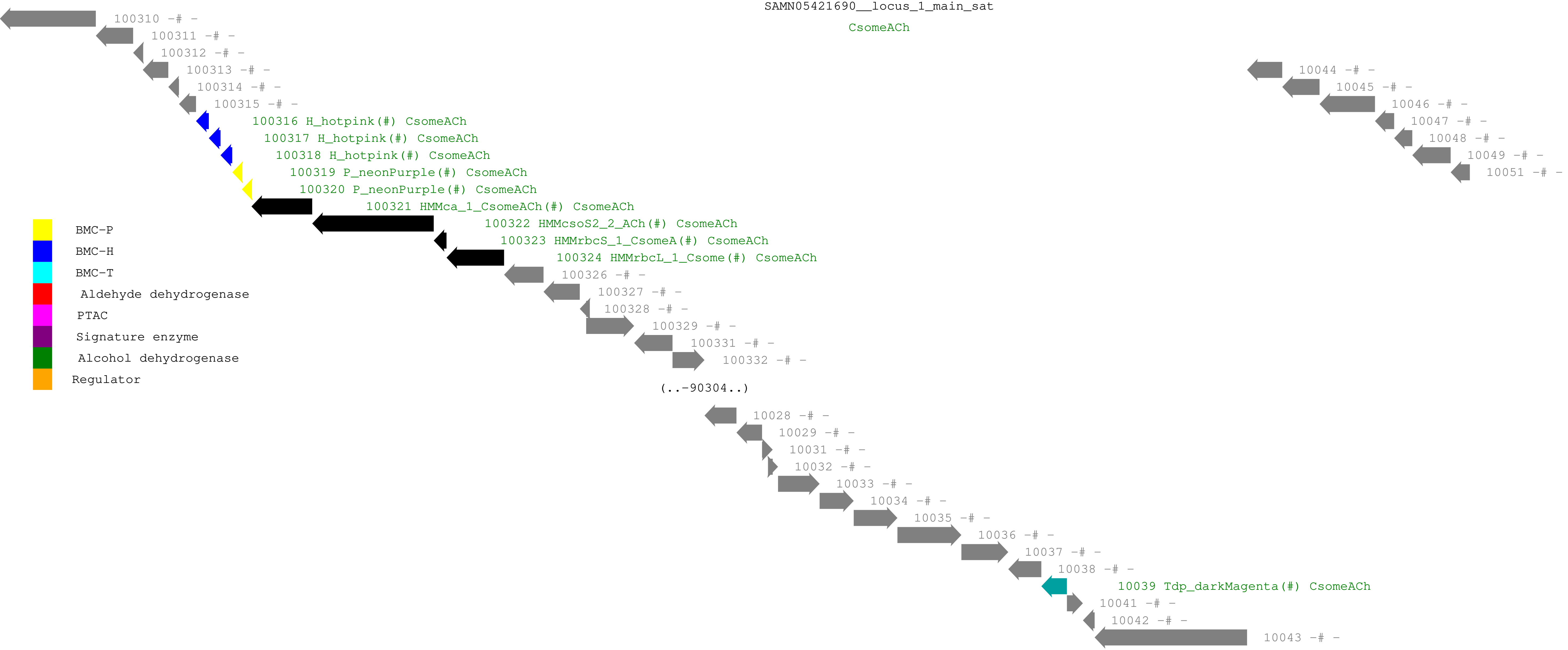

Ectothiorhodospira magna. Bacteria; Proteobacteria; Gammaproteobacteria; Chromatiales;

SAMN05421693__locus_1_main_sat

CsomeACh

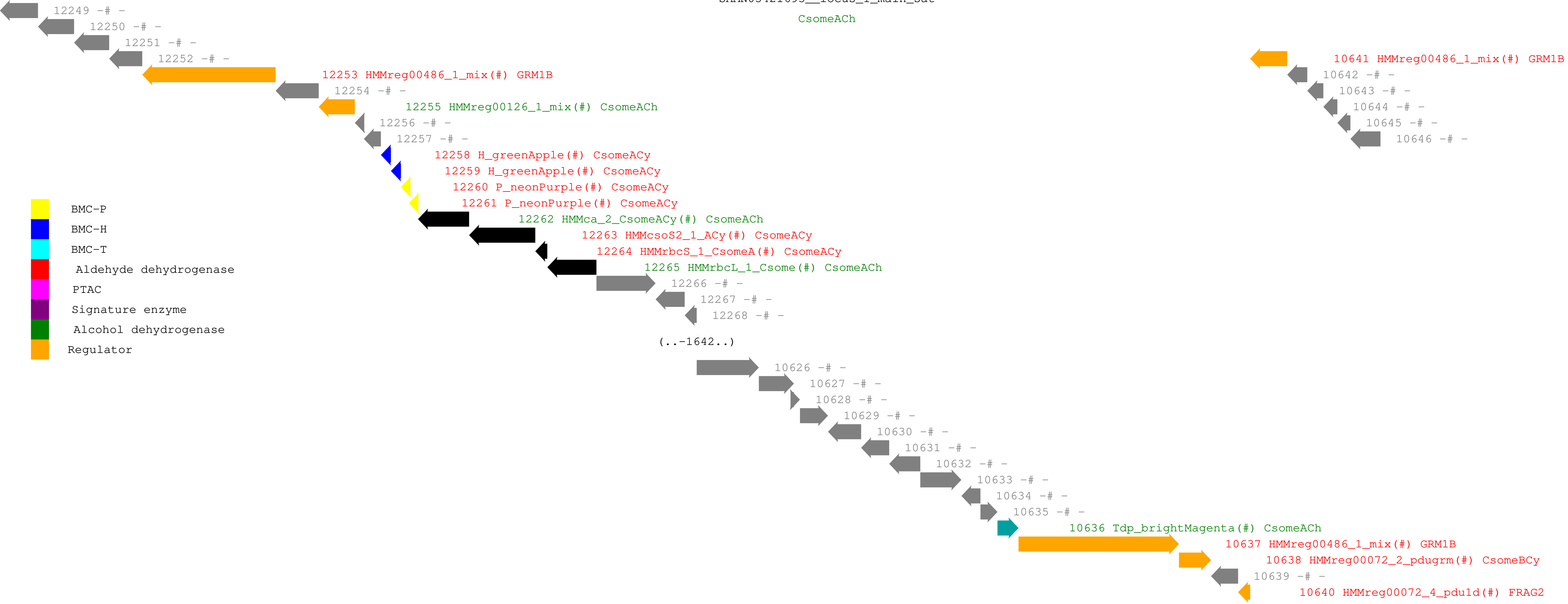

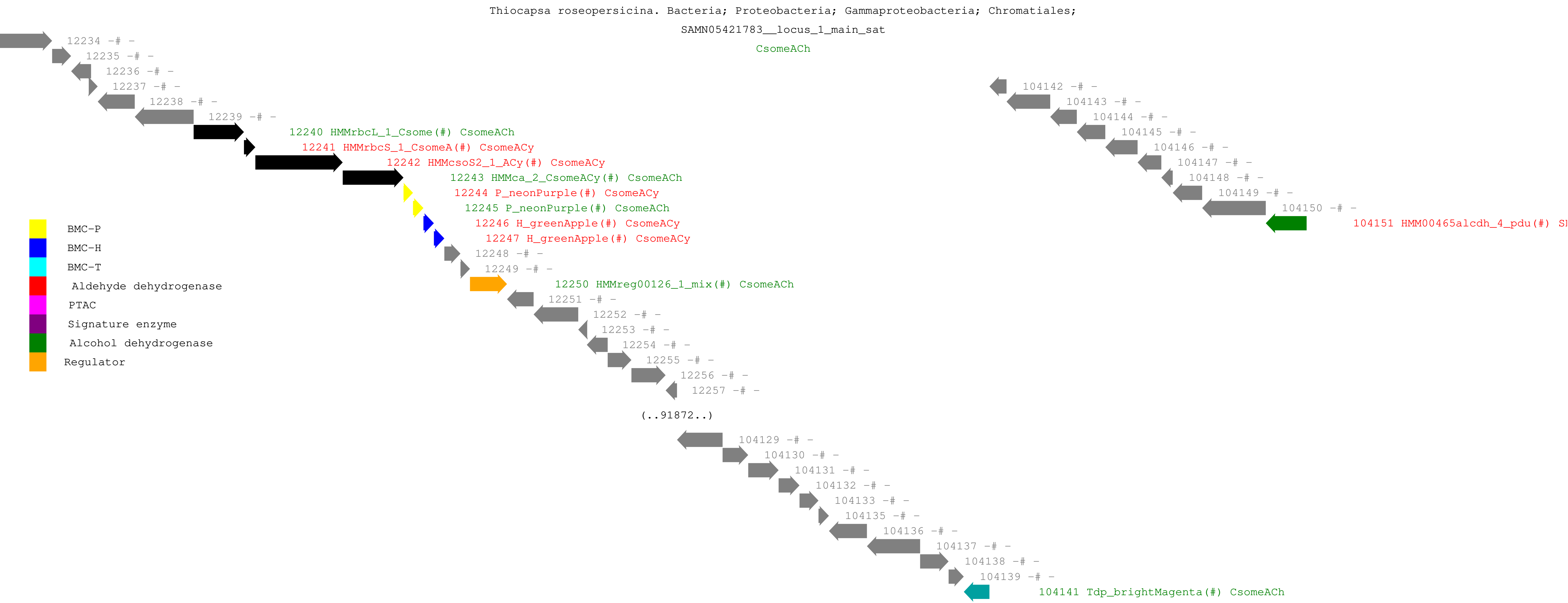
Thiocapsa roseopersicina. Bacteria; Proteobacteria; Gammaproteobacteria; Chromatiales;
SAMN05421783__locus_1_main_sat
CsomeACh
12234 -# -
12235 -# -
12236 -# -
12237 -# -
12238 -# -
12239 -# -
12240 HMMrbcL_1_Csome(#) CsomeACh
12241 HMMrbcS_1_CsomeA(#) CsomeACy
12242 HMMcsoS2_1_ACy(#) CsomeACy
12243 HMMca_2_CsomeACy(#) CsomeACh
12244 P_neonPurple(#) CsomeACy
12245 P_neonPurple(#) CsomeACh
12246 H_greenApple(#) CsomeACy
12247 H_greenApple(#) CsomeACy
12248 -# -
12249 -# -
12250 HMMreg00126_1_mix(#) CsomeACh
12251 -# -
12252 -# -
12253 -# -
12254 -# -
12255 -# -
12256 -# -
12257 -# -
(..91872..)
104129 -# -
104130 -# -
104131 -# -
104132 -# -
104133 -# -
104135 -# -
104136 -# -
104137 -# -
104138 -# -
104139 -# -
104141 Tdp_brightMagenta(#) CsomeACh
104142 -# -
104143 -# -
104144 -# -
104145 -# -
104146 -# -
104147 -# -
104148 -# -
104149 -# -
104150 -# -
104151 HMM00465alcdh_4_pdu(#) SI
BMC-P
BMC-H
BMC-T
Aldehyde dehydrogenase
PTAC
Signature enzyme
Alcohol dehydrogenase
Regulator

Nitrosomonas nitrosa. Bacteria; Proteobacteria; Betaproteobacteria; Nitrosomonadales;

SAMN05421880__locus_1

CsomeACh

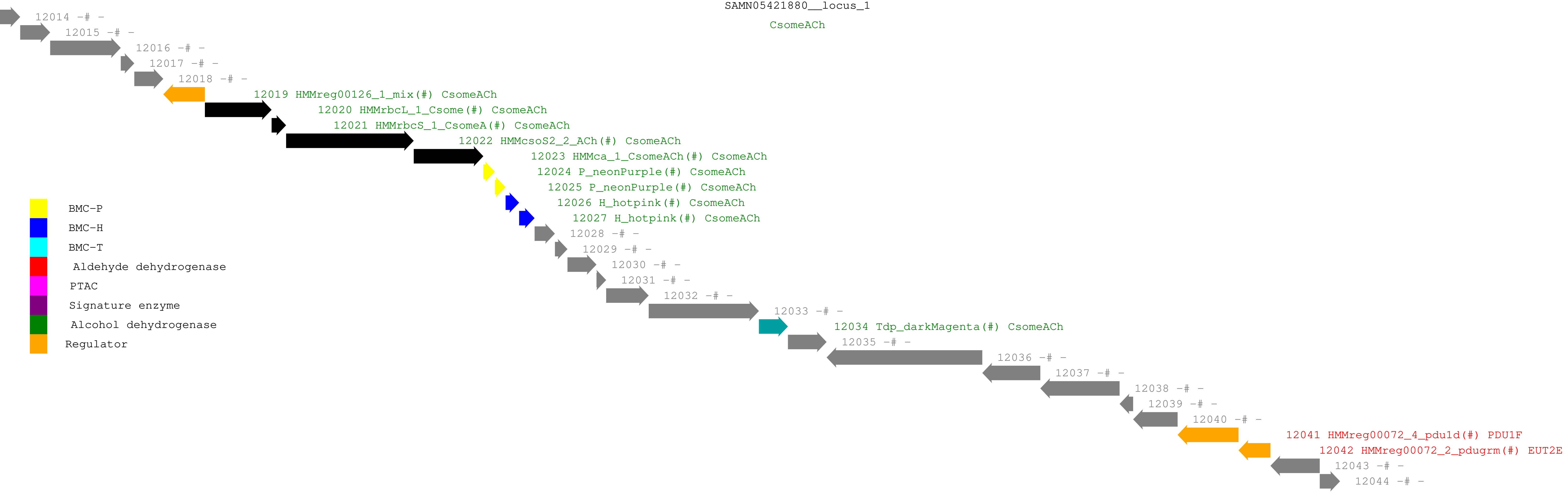

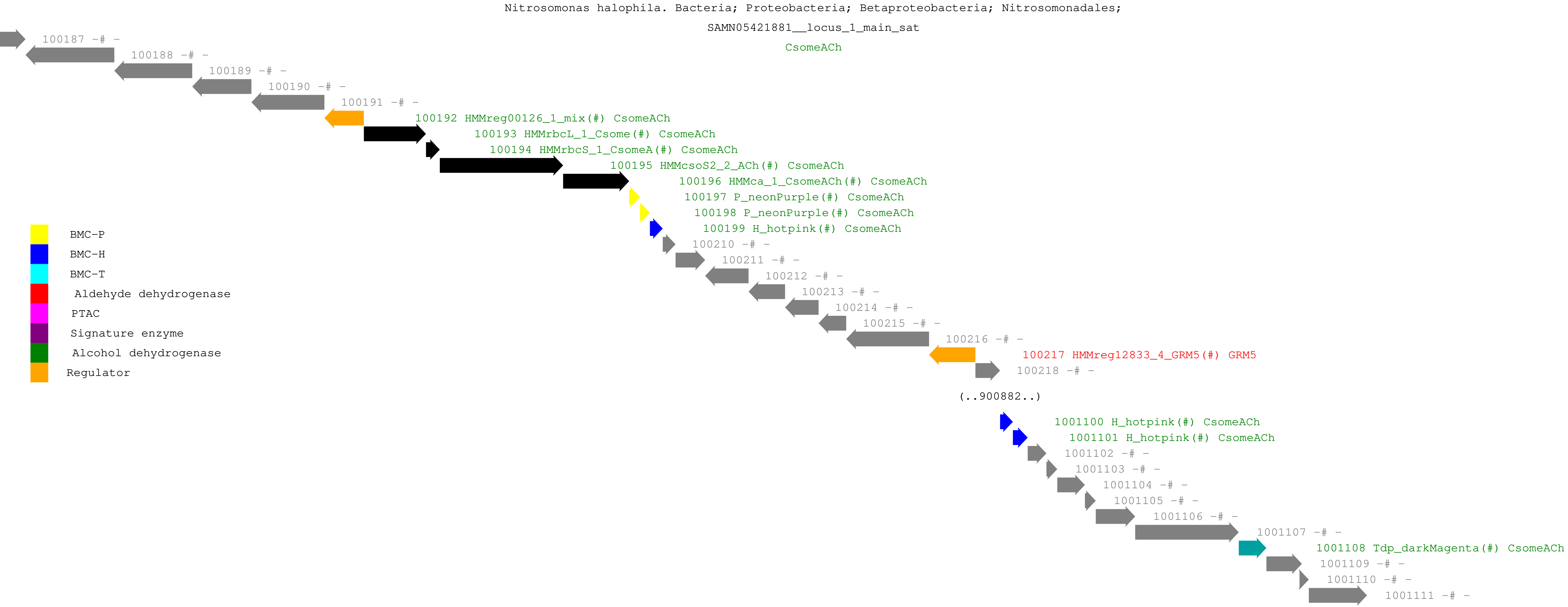

Nitrosomonas halophila. Bacteria; Proteobacteria; Betaproteobacteria; Nitrosomonadales;
SAMN05421881__locus_1_main_sat
CsomeACh
100187 -# -
100188 -# -
100189 -# -
100190 -# -
100191 -# -
100192 HMMreg00126_1_mix(#) CsomeACh
100193 HMMrbcL_1_Csome(#) CsomeACh
100194 HMMrbcS_1_CsomeA(#) CsomeACh
100195 HMMcsoS2_2_ACh(#) CsomeACh
100196 HMMca_1_CsomeACh(#) CsomeACh
100197 P_neonPurple(#) CsomeACh
100198 P_neonPurple(#) CsomeACh
100199 H_hotpink(#) CsomeACh
100210 -# -
100211 -# -
100212 -# -
100213 -# -
100214 -# -
100215 -# -
100216 -# -
100217 HMMreg12833_4_GRM5(#) GRM5
100218 -# -
(..900882..)
1001100 H_hotpink(#) CsomeACh
1001101 H_hotpink(#) CsomeACh
1001102 -# -
1001103 -# -
1001104 -# -
1001105 -# -
1001106 -# -
1001107 -# -
1001108 Tdp_darkMagenta(#) CsomeACh
1001109 -# -
1001110 -# -
1001111 -# -
BMC-P
BMC-H
BMC-T
Aldehyde dehydrogenase
PTAC
Signature enzyme
Alcohol dehydrogenase
Regulator

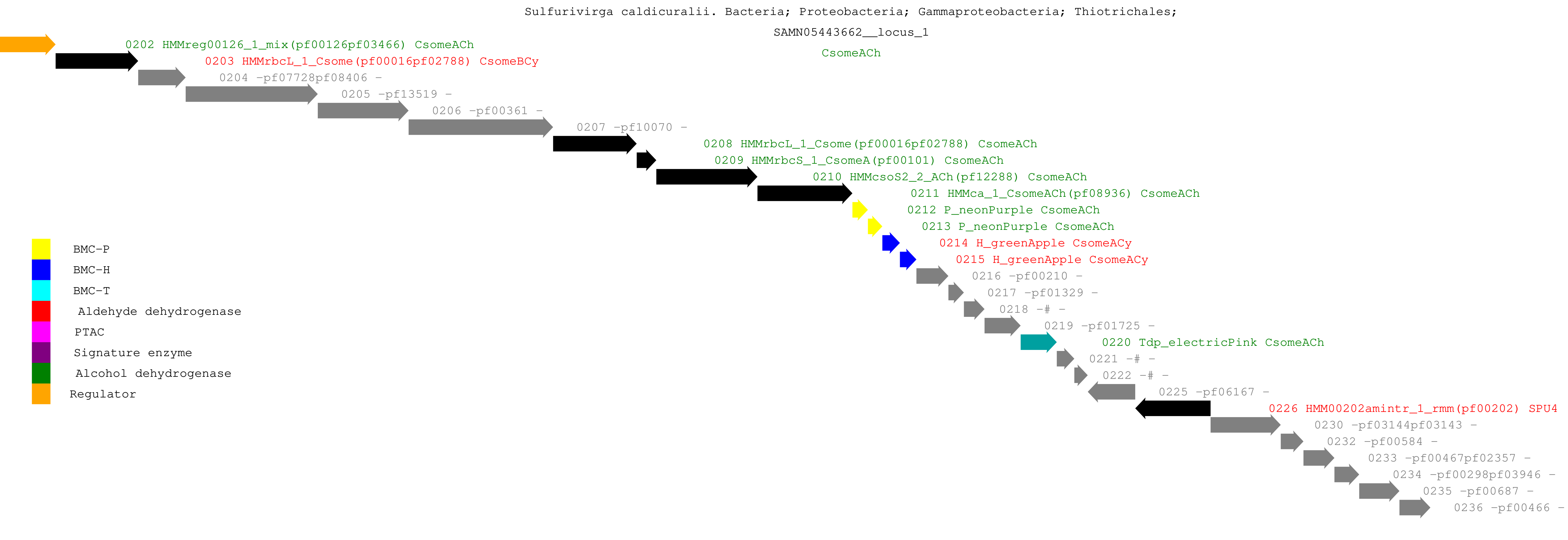
Sulfurivirga caldicuralii. Bacteria; Proteobacteria; Gammaproteobacteria; Thiotrichales;
SAMN05443662__locus_1
CsomeACh
0202 HMMreg00126_1_mix(pf00126pf03466) CsomeACh
0203 HMMrbcL_1_Csome(pf00016pf02788) CsomeBCy
0204 -pf07728pf08406 -
0205 -pf13519 -
0206 -pf00361 -
0207 -pf10070 -
0208 HMMrbcL_1_Csome(pf00016pf02788) CsomeACh
0209 HMMrbcS_1_CsomeA(pf00101) CsomeACh
0210 HMMcsoS2_2_ACh(pf12288) CsomeACh
0211 HMMca_1_CsomeACh(pf08936) CsomeACh
0212 P_neonPurple CsomeACh
0213 P_neonPurple CsomeACh
0214 H_greenApple CsomeACy
0215 H_greenApple CsomeACy
0216 -pf00210 -
0217 -pf01329 -
0218 -# -
0219 -pf01725 -
0220 Tdp_electricPink CsomeACh
0221 -# -
0222 -# -
0225 -pf06167 -
0226 HMM00202amintr_1_rmm(pf00202) SPU4
0230 -pf03144pf03143 -
0232 -pf00584 -
0233 -pf00467pf02357 -
0234 -pf00298pf03946 -
0235 -pf00687 -
0236 -pf00466 -
BMC-P
BMC-H
BMC-T
Aldehyde dehydrogenase
PTAC
Signature enzyme
Alcohol dehydrogenase
Regulator

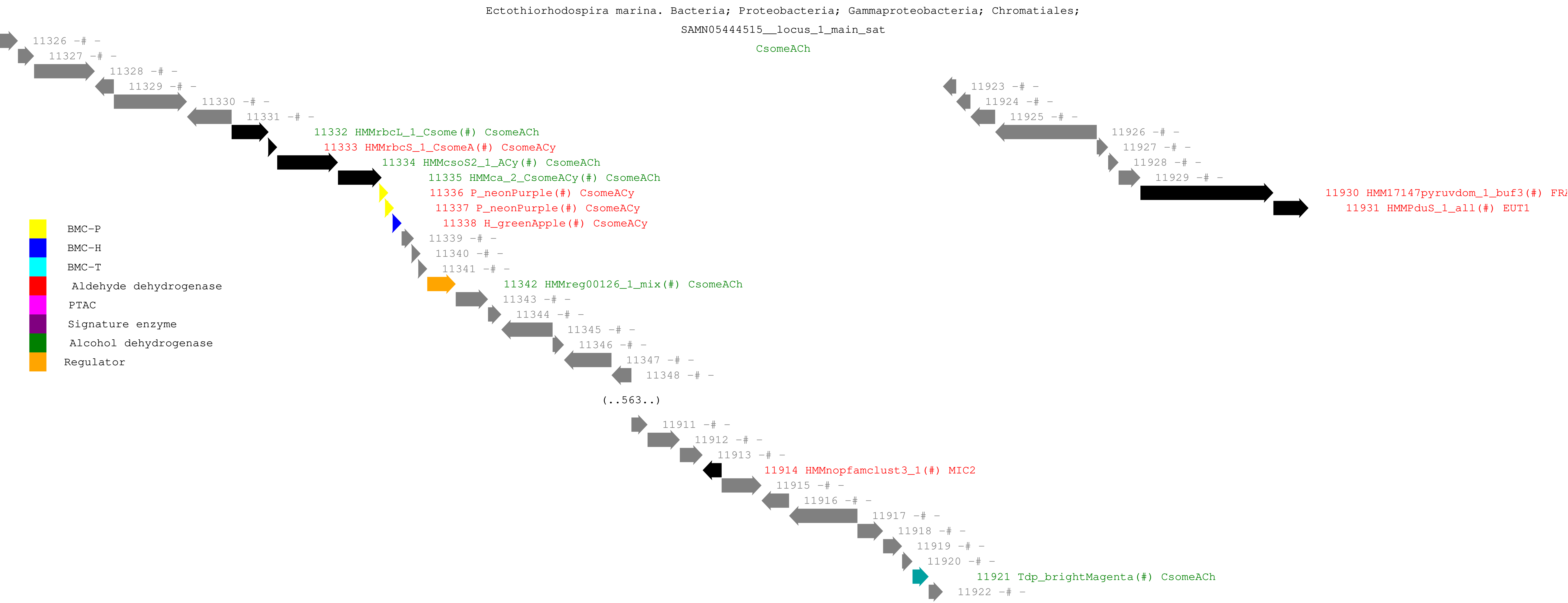
Ectothiorhodospira marina. Bacteria; Proteobacteria; Gammaproteobacteria; Chromatiales;
SAMN05444515__locus_1_main_sat
CsomeACh
11326 -# -
11327 -# -
11328 -# -
11329 -# -
11330 -# -
11331 -# -
11332 HMMrbcL_1_Csome(#) CsomeACh
11333 HMMrbcS_1_CsomeA(#) CsomeACy
11334 HMMcsoS2_1_ACy(#) CsomeACh
11335 HMMca_2_CsomeACy(#) CsomeACh
11336 P_neonPurple(#) CsomeACy
11337 P_neonPurple(#) CsomeACy
11338 H_greenApple(#) CsomeACy
11339 -# -
11340 -# -
11341 -# -
11342 HMMreg00126_1_mix(#) CsomeACh
11343 -# -
11344 -# -
11345 -# -
11346 -# -
11347 -# -
11348 -# -
(..563..)
11911 -# -
11912 -# -
11913 -# -
11914 HMMnopfamclust3_1(#) MIC2
11915 -# -
11916 -# -
11917 -# -
11918 -# -
11919 -# -
11920 -# -
11921 Tdp_brightMagenta(#) CsomeACh
11922 -# -
11923 -# -
11924 -# -
11925 -# -
11926 -# -
11927 -# -
11928 -# -
11929 -# -
11930 HMM17147pyruvdom_1_buf3(#) FR
11931 HMMPduS_1_all(#) EUT1
BMC-P
BMC-H
BMC-T
Aldehyde dehydrogenase
PTAC
Signature enzyme
Alcohol dehydrogenase
Regulator

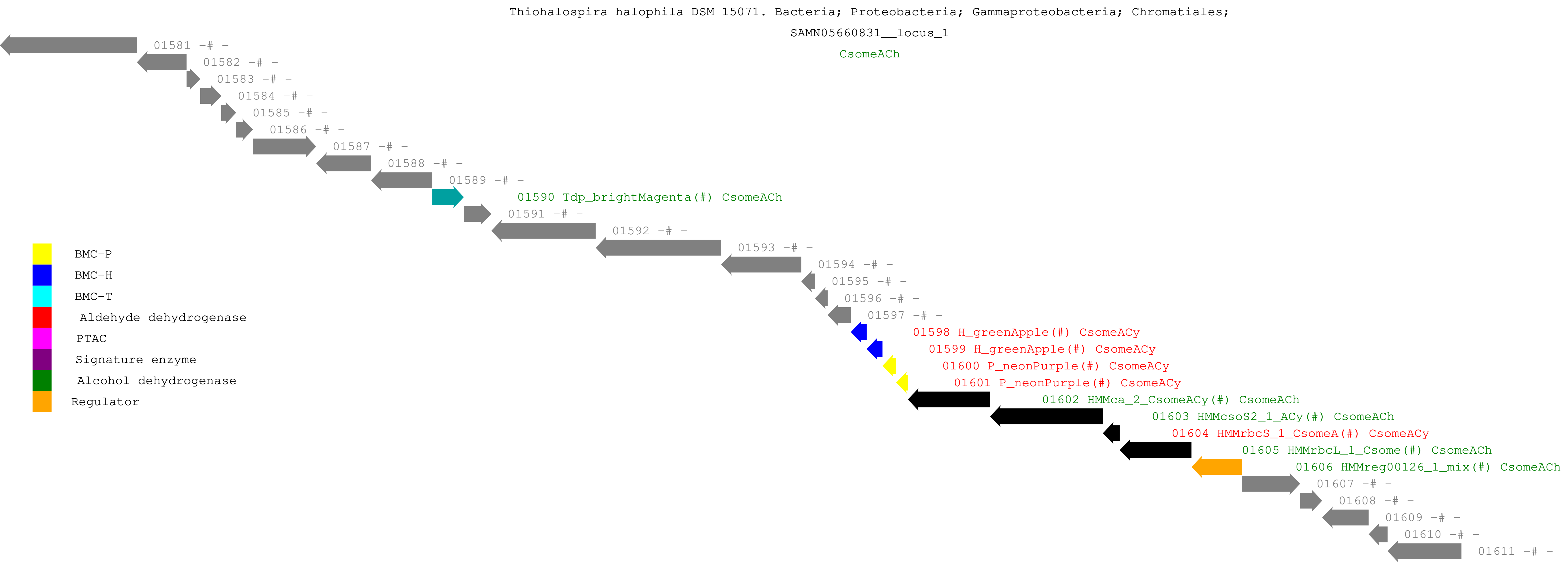
Thiohalospira halophila DSM 15071. Bacteria; Proteobacteria; Gammaproteobacteria; Chromatiales;
SAMN05660831__locus_1
CsomeACh
01581 -# -
01582 -# -
01583 -# -
01584 -# -
01585 -# -
01586 -# -
01587 -# -
01588 -# -
01589 -# -
01590 Tdp_brightMagenta(#) CsomeACh
01591 -# -
01592 -# -
01593 -# -
01594 -# -
01595 -# -
01596 -# -
01597 -# -
01598 H_greenApple(#) CsomeACy
01599 H_greenApple(#) CsomeACy
01600 P_neonPurple(#) CsomeACy
01601 P_neonPurple(#) CsomeACy
01602 HMMca_2_CsomeACy(#) CsomeACh
01603 HMMcsoS2_1_ACy(#) CsomeACh
01604 HMMrbcS_1_CsomeA(#) CsomeACy
01605 HMMrbcL_1_Csome(#) CsomeACh
01606 HMMreg00126_1_mix(#) CsomeACh
01607 -# -
01608 -# -
01609 -# -
01610 -# -
01611 -# -
BMC-P
BMC-H
BMC-T
Aldehyde dehydrogenase
PTAC
Signature enzyme
Alcohol dehydrogenase
Regulator

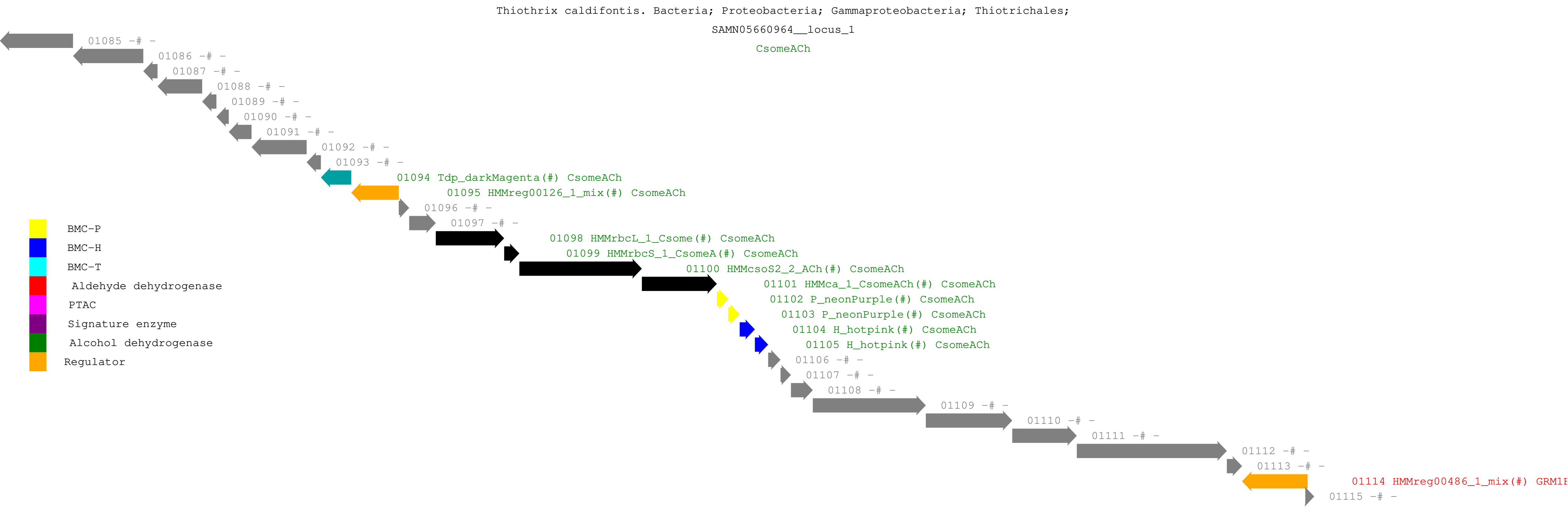
Thiothrix caldifontis. Bacteria; Proteobacteria; Gammaproteobacteria; Thiotrichales;
SAMN05660964__locus_1
CsomeACh
01085 -# -
01086 -# -
01087 -# -
01088 -# -
01089 -# -
01090 -# -
01091 -# -
01092 -# -
01093 -# -
01094 Tdp_darkMagenta(#) CsomeACh
01095 HMMreg00126_1_mix(#) CsomeACh
01096 -# -
01097 -# -
01098 HMMrbcL_1_Csome(#) CsomeACh
01099 HMMrbcS_1_CsomeA(#) CsomeACh
01100 HMMcsoS2_2_ACh(#) CsomeACh
01101 HMMca_1_CsomeACh(#) CsomeACh
01102 P_neonPurple(#) CsomeACh
01103 P_neonPurple(#) CsomeACh
01104 H_hotpink(#) CsomeACh
01105 H_hotpink(#) CsomeACh
01106 -# -
01107 -# -
01108 -# -
01109 -# -
01110 -# -
01111 -# -
01112 -# -
01113 -# -
01114 HMMreg00486_1_mix(#) GRM1
01115 -# -
BMC-P
BMC-H
BMC-T
Aldehyde dehydrogenase
PTAC
Signature enzyme
Alcohol dehydrogenase
Regulator

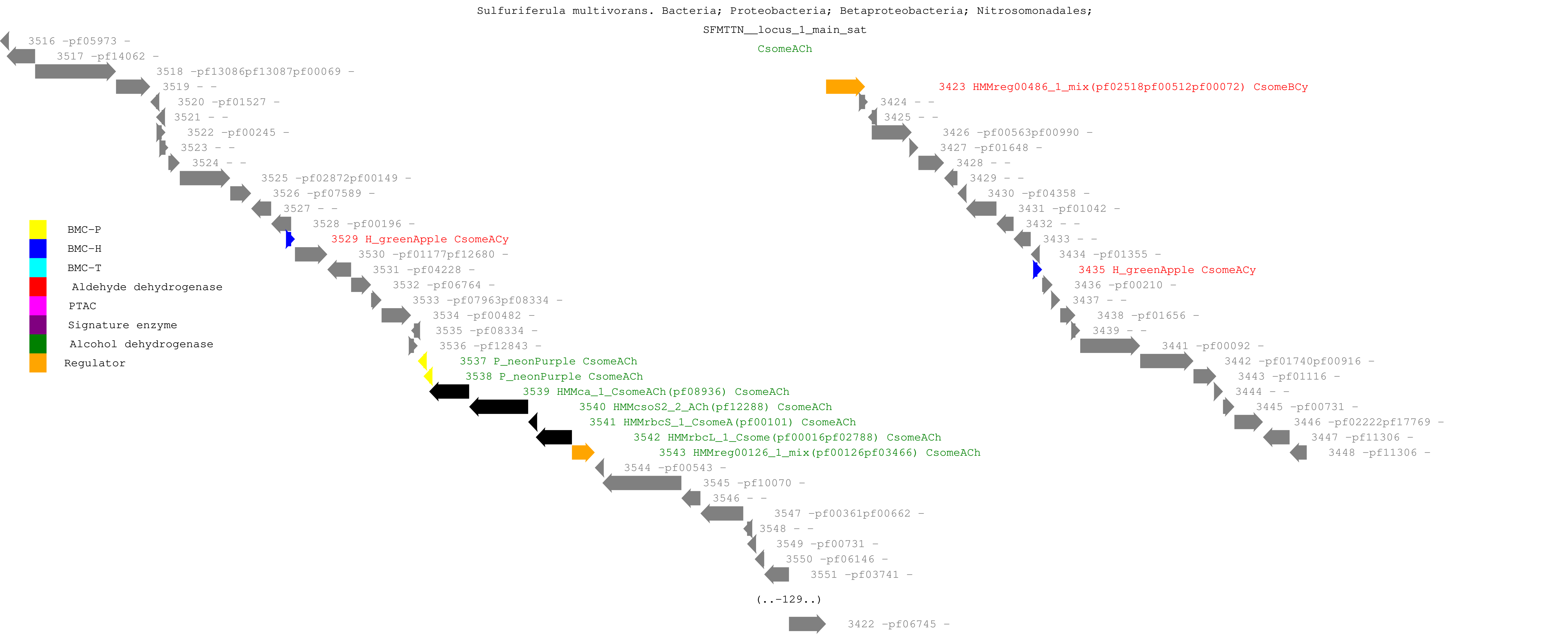

Sulfuriferula multivorans. Bacteria; Proteobacteria; Betaproteobacteria; Nitrosomonadales;
SFMTTN__locus_1_main_sat
CsomeACh
3516 -pf05973 -
3517 -pf14062 -
3518 -pf13086pf13087pf00069 -
3519 - -
3520 -pf01527 -
3521 - -
3522 -pf00245 -
3523 - -
3524 - -
3525 -pf02872pf00149 -
3526 -pf07589 -
3527 - -
3528 -pf00196 -
3529 H_greenApple CsomeACy
3530 -pf01177pf12680 -
3531 -pf04228 -
3532 -pf06764 -
3533 -pf07963pf08334 -
3534 -pf00482 -
3535 -pf08334 -
3536 -pf12843 -
3537 P_neonPurple CsomeACh
3538 P_neonPurple CsomeACh
3539 HMMca_1_CsomeACh(pf08936) CsomeACh
3540 HMMcsoS2_2_ACh(pf12288) CsomeACh
3541 HMMrbcS_1_CsomeA(pf00101) CsomeACh
3542 HMMrbcL_1_Csome(pf00016pf02788) CsomeACh
3543 HMMreg00126_1_mix(pf00126pf03466) CsomeACh
3544 -pf00543 -
3545 -pf10070 -
3546 - -
3547 -pf00361pf00662 -
3548 - -
3549 -pf00731 -
3550 -pf06146 -
3551 -pf03741 -
(..-129..)
3422 -pf06745 -
3423 HMMreg00486_1_mix(pf02518pf00512pf00072) CsomeBCy
3424 - -
3425 - -
3426 -pf00563pf00990 -
3427 -pf01648 -
3428 - -
3429 - -
3430 -pf04358 -
3431 -pf01042 -
3432 - -
3433 - -
3434 -pf01355 -
3435 H_greenApple CsomeACy
3436 -pf00210 -
3437 - -
3438 -pf01656 -
3439 - -
3441 -pf00092 -
3442 -pf01740pf00916 -
3443 -pf01116 -
3444 - -
3445 -pf00731 -
3446 -pf02222pf17769 -
3447 -pf11306 -
3448 -pf11306 -
BMC-P
BMC-H
BMC-T
Aldehyde dehydrogenase
PTAC
Signature enzyme
Alcohol dehydrogenase
Regulator

Comamonadaceae bacterium B1. Bacteria; Proteobacteria; Betaproteobacteria; Burkholderiales;

SMCB__locus_1

CsomeACh

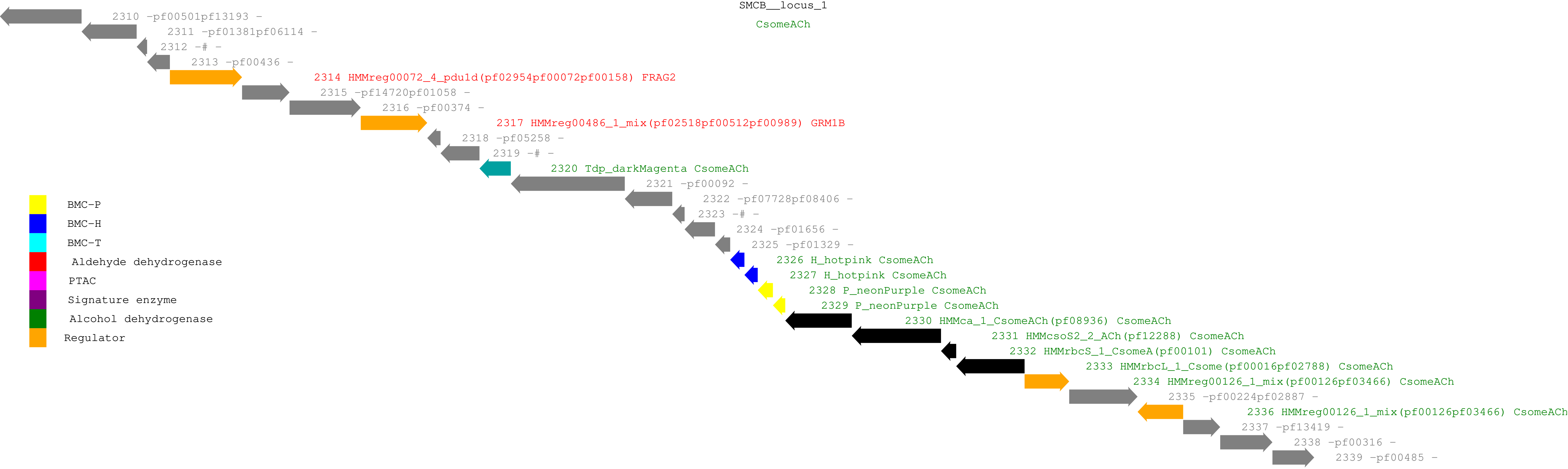

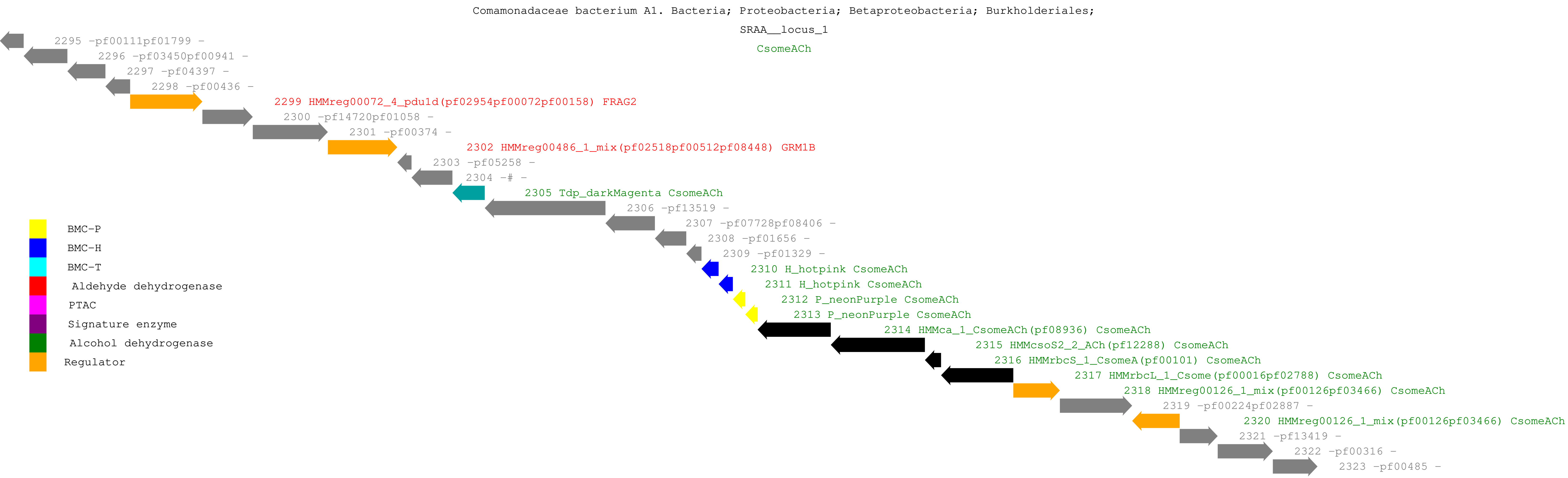
Comamonadaceae bacterium A1. Bacteria; Proteobacteria; Betaproteobacteria; Burkholderiales;
SRAA__locus_1
CsomeACh
2295 -pf00111pf01799 -
2296 -pf03450pf00941 -
2297 -pf04397 -
2298 -pf00436 -
2299 HMMreg00072_4_pdu1d(pf02954pf00072pf00158) FRAG2
2300 -pf14720pf01058 -
2301 -pf00374 -
2302 HMMreg00486_1_mix(pf02518pf00512pf08448) GRM1B
2303 -pf05258 -
2304 -# -
2305 Tdp_darkMagenta CsomeACh
2306 -pf13519 -
2307 -pf07728pf08406 -
2308 -pf01656 -
2309 -pf01329 -
2310 H_hotpink CsomeACh
2311 H_hotpink CsomeACh
2312 P_neonPurple CsomeACh
2313 P_neonPurple CsomeACh
2314 HMMca_1_CsomeACh(pf08936) CsomeACh
2315 HMMcsoS2_2_ACh(pf12288) CsomeACh
2316 HMMrbcS_1_CsomeA(pf00101) CsomeACh
2317 HMMrbcL_1_Csome(pf00016pf02788) CsomeACh
2318 HMMreg00126_1_mix(pf00126pf03466) CsomeACh
2319 -pf00224pf02887 -
2320 HMMreg00126_1_mix(pf00126pf03466) CsomeACh
2321 -pf13419 -
2322 -pf00316 -
2323 -pf00485 -
BMC-P
BMC-H
BMC-T
Aldehyde dehydrogenase
PTAC
Signature enzyme
Alcohol dehydrogenase
Regulator

Tepidimonas alkaliphilus. Bacteria; Proteobacteria; Betaproteobacteria; Burkholderiales; Tepidimonas.

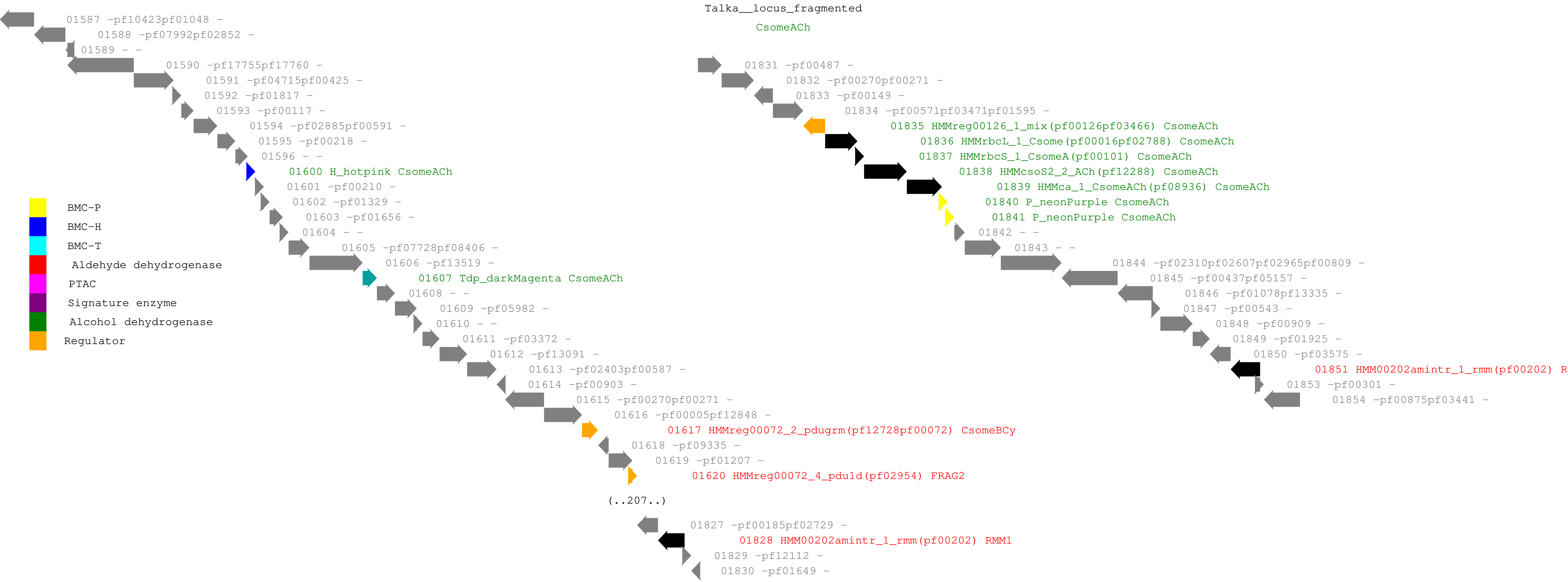

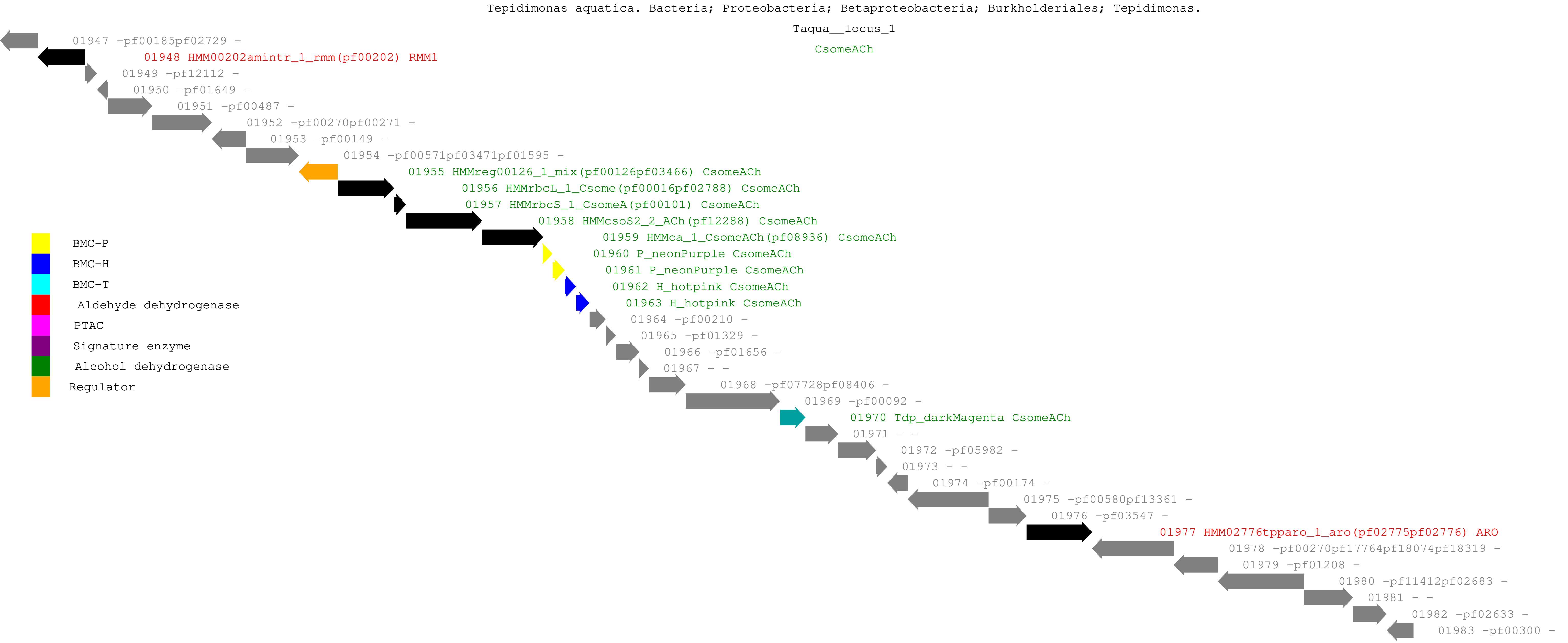
Tepidimonas aquatica. Bacteria; Proteobacteria; Betaproteobacteria; Burkholderiales; Tepidimonas.
Taqua__locus_1
CsomeACh
01947 -pf00185pf02729 -
01948 HMM00202amintr_1_rmm(pf00202) RMM1
01949 -pf12112 -
01950 -pf01649 -
01951 -pf00487 -
01952 -pf00270pf00271 -
01953 -pf00149 -
01954 -pf00571pf03471pf01595 -
01955 HMMreg00126_1_mix(pf00126pf03466) CsomeACh
01956 HMMrbcL_1_Csome(pf00016pf02788) CsomeACh
01957 HMMrbcS_1_CsomeA(pf00101) CsomeACh
01958 HMMcsoS2_2_ACh(pf12288) CsomeACh
01959 HMMca_1_CsomeACh(pf08936) CsomeACh
01960 P_neonPurple CsomeACh
01961 P_neonPurple CsomeACh
01962 H_hotpink CsomeACh
01963 H_hotpink CsomeACh
01964 -pf00210 -
01965 -pf01329 -
01966 -pf01656 -
01967 - -
01968 -pf07728pf08406 -
01969 -pf00092 -
01970 Tdp_darkMagenta CsomeACh
01971 - -
01972 -pf05982 -
01973 - -
01974 -pf00174 -
01975 -pf00580pf13361 -
01976 -pf03547 -
01977 HMM02776tpparo_1_aro(pf02775pf02776) ARO
01978 -pf00270pf17764pf18074pf18319 -
01979 -pf01208 -
01980 -pf11412pf02683 -
01981 - -
01982 -pf02633 -
01983 -pf00300 -
BMC-P
BMC-H
BMC-T
Aldehyde dehydrogenase
PTAC
Signature enzyme
Alcohol dehydrogenase
Regulator

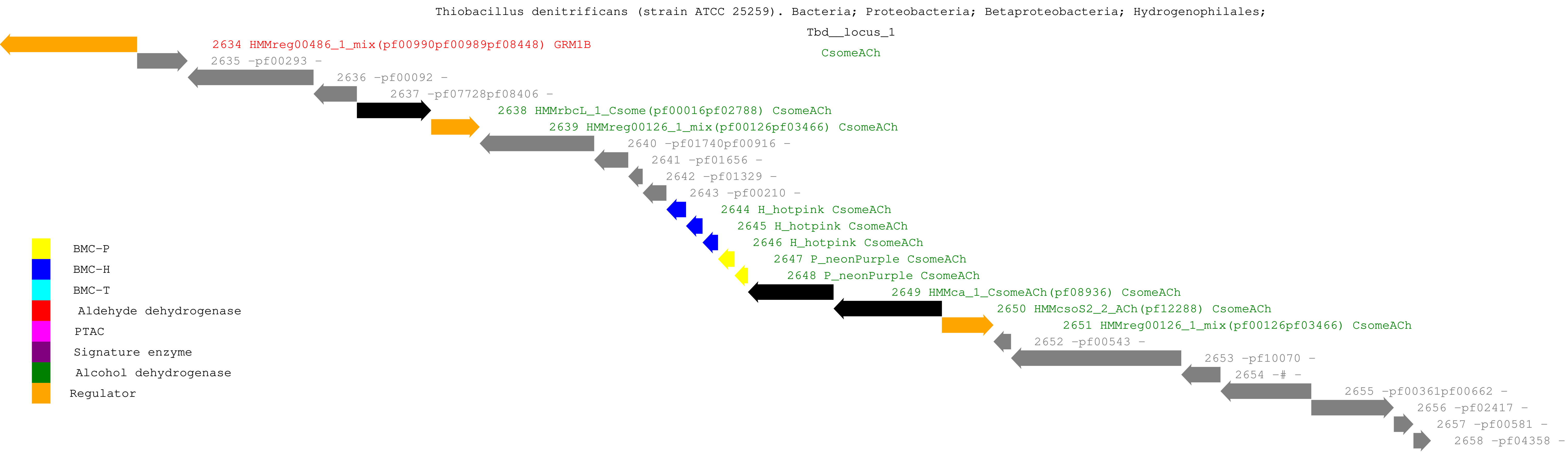
Thiobacillus denitrificans (strain ATCC 25259). Bacteria; Proteobacteria; Betaproteobacteria; Hydrogenophilales;
Tbd__locus_1
CsomeACh
2634 HMMreg00486_1_mix(pf00990pf00989pf08448) GRM1B
2635 -pf00293 -
2636 -pf00092 -
2637 -pf07728pf08406 -
2638 HMMrbcL_1_Csome(pf00016pf02788) CsomeACh
2639 HMMreg00126_1_mix(pf00126pf03466) CsomeACh
2640 -pf01740pf00916 -
2641 -pf01656 -
2642 -pf01329 -
2643 -pf00210 -
2644 H_hotpink CsomeACh
2645 H_hotpink CsomeACh
2646 H_hotpink CsomeACh
2647 P_neonPurple CsomeACh
2648 P_neonPurple CsomeACh
2649 HMMca_1_CsomeACh(pf08936) CsomeACh
2650 HMMcsoS2_2_ACh(pf12288) CsomeACh
2651 HMMreg00126_1_mix(pf00126pf03466) CsomeACh
2652 -pf00543 -
2653 -pf10070 -
2654 -# -
2655 -pf00361pf00662 -
2656 -pf02417 -
2657 -pf00581 -
2658 -pf04358 -
BMC-P
BMC-H
BMC-T
Aldehyde dehydrogenase
PTAC
Signature enzyme
Alcohol dehydrogenase
Regulator

Tepidimonas sp. SPSP-6. Bacteria; Proteobacteria; Betaproteobacteria; Burkholderiales; Tepidimonas;

Tchar__locus_1

CsomeACh

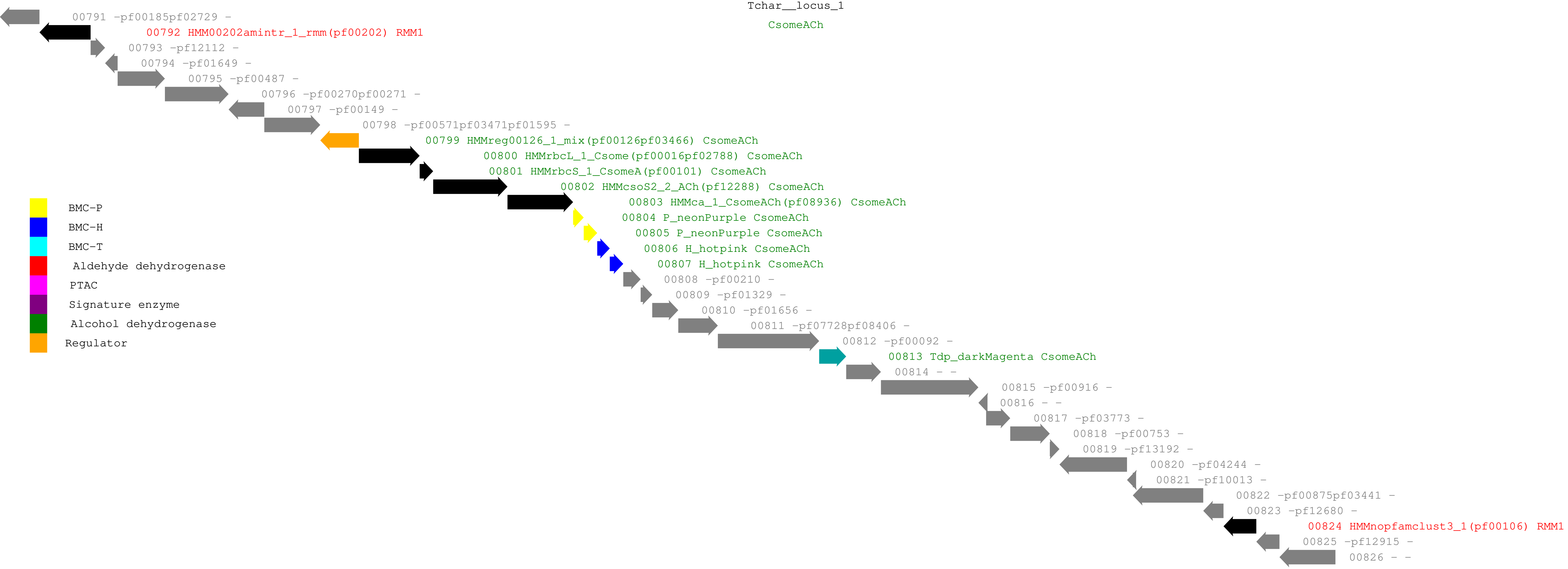

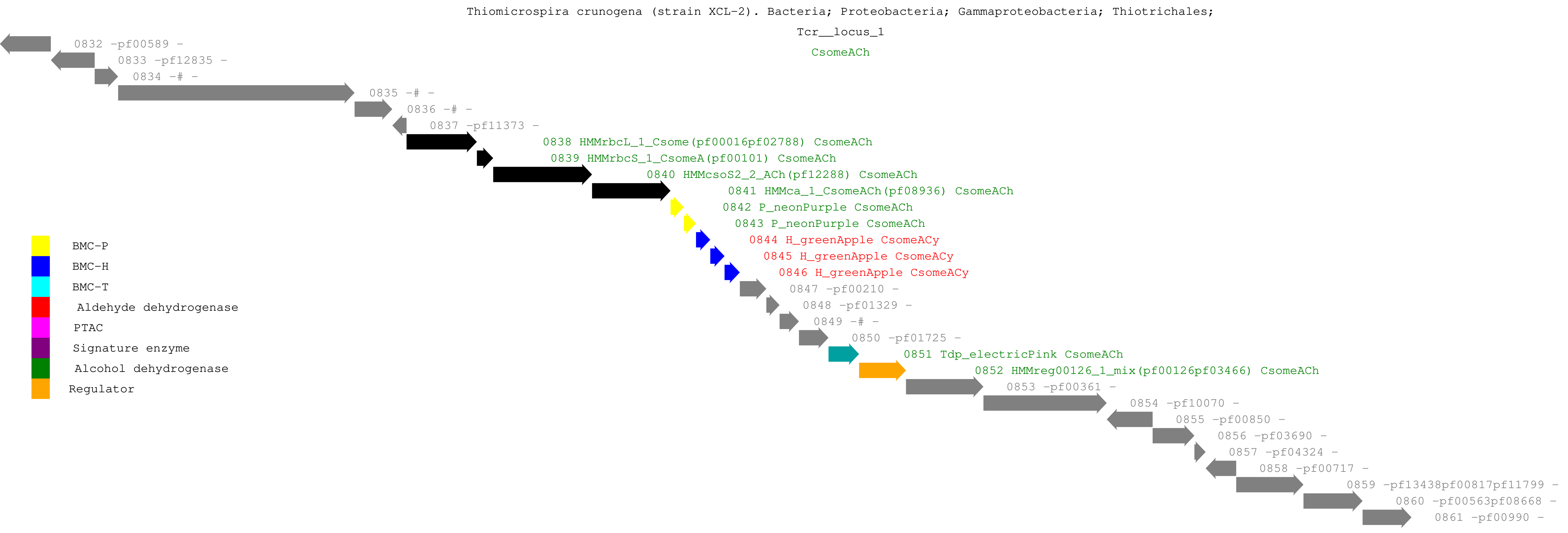
Thiomicrospira crunogena (strain XCL-2). Bacteria; Proteobacteria; Gammaproteobacteria; Thiotrichales;
Tcr__locus_1
CsomeACh
0832 -pf00589 -
0833 -pf12835 -
0834 -# -
0835 -# -
0836 -# -
0837 -pf11373 -
0838 HMMrbcL_1_Csome(pf00016pf02788) CsomeACh
0839 HMMrbcS_1_CsomeA(pf00101) CsomeACh
0840 HMMcsoS2_2_ACh(pf12288) CsomeACh
0841 HMMca_1_CsomeACh(pf08936) CsomeACh
0842 P_neonPurple CsomeACh
0843 P_neonPurple CsomeACh
0844 H_greenApple CsomeACy
0845 H_greenApple CsomeACy
0846 H_greenApple CsomeACy
0847 -pf00210 -
0848 -pf01329 -
0849 -# -
0850 -pf01725 -
0851 Tdp_electricPink CsomeACh
0852 HMMreg00126_1_mix(pf00126pf03466) CsomeACh
0853 -pf00361 -
0854 -pf10070 -
0855 -pf00850 -
0856 -pf03690 -
0857 -pf04324 -
0858 -pf00717 -
0859 -pf13438pf00817pf11799 -
0860 -pf00563pf08668 -
0861 -pf00990 -
BMC-P
BMC-H
BMC-T
Aldehyde dehydrogenase
PTAC
Signature enzyme
Alcohol dehydrogenase
Regulator

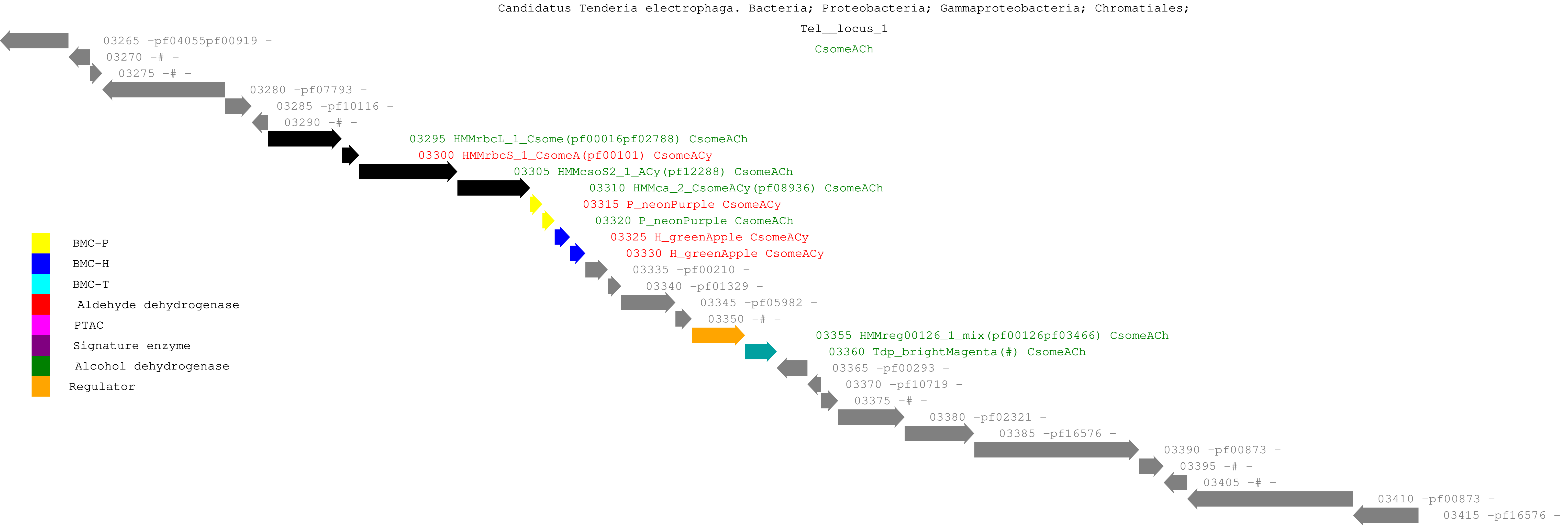

Candidatus Tenderia electrophaga. Bacteria; Proteobacteria; Gammaproteobacteria; Chromatiales;
Tel__locus_1
CsomeACh
03265 -pf04055pf00919 -
03270 -# -
03275 -# -
03280 -pf07793 -
03285 -pf10116 -
03290 -# -
03295 HMMrbcL_1_Csome(pf00016pf02788) CsomeACh
03300 HMMrbcS_1_CsomeA(pf00101) CsomeACy
03305 HMMcsoS2_1_ACy(pf12288) CsomeACh
03310 HMMca_2_CsomeACy(pf08936) CsomeACh
03315 P_neonPurple CsomeACy
03320 P_neonPurple CsomeACh
03325 H_greenApple CsomeACy
03330 H_greenApple CsomeACy
03335 -pf00210 -
03340 -pf01329 -
03345 -pf05982 -
03350 -# -
03355 HMMreg00126_1_mix(pf00126pf03466) CsomeACh
03360 Tdp_brightMagenta(#) CsomeACh
03365 -pf00293 -
03370 -pf10719 -
03375 -# -
03380 -pf02321 -
03385 -pf16576 -
03390 -pf00873 -
03395 -# -
03405 -# -
03410 -pf00873 -
03415 -pf16576 -
BMC-P
BMC-H
BMC-T
Aldehyde dehydrogenase
PTAC
Signature enzyme
Alcohol dehydrogenase
Regulator

Thioalkalivibrio sulfidiphilus (strain HL-EbGR7). Bacteria; Proteobacteria; Gammaproteobacteria; Chromatiales;

Tgr7__locus_1_main_sat

CsomeACh

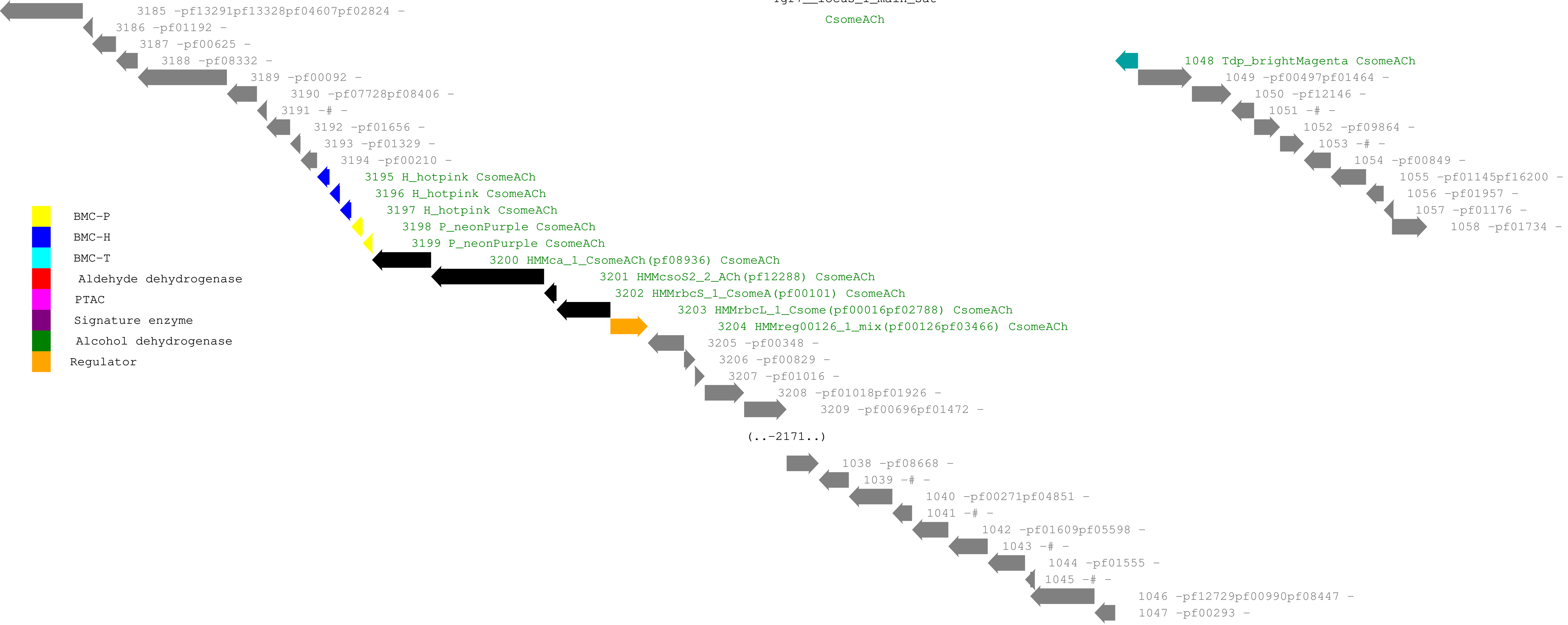

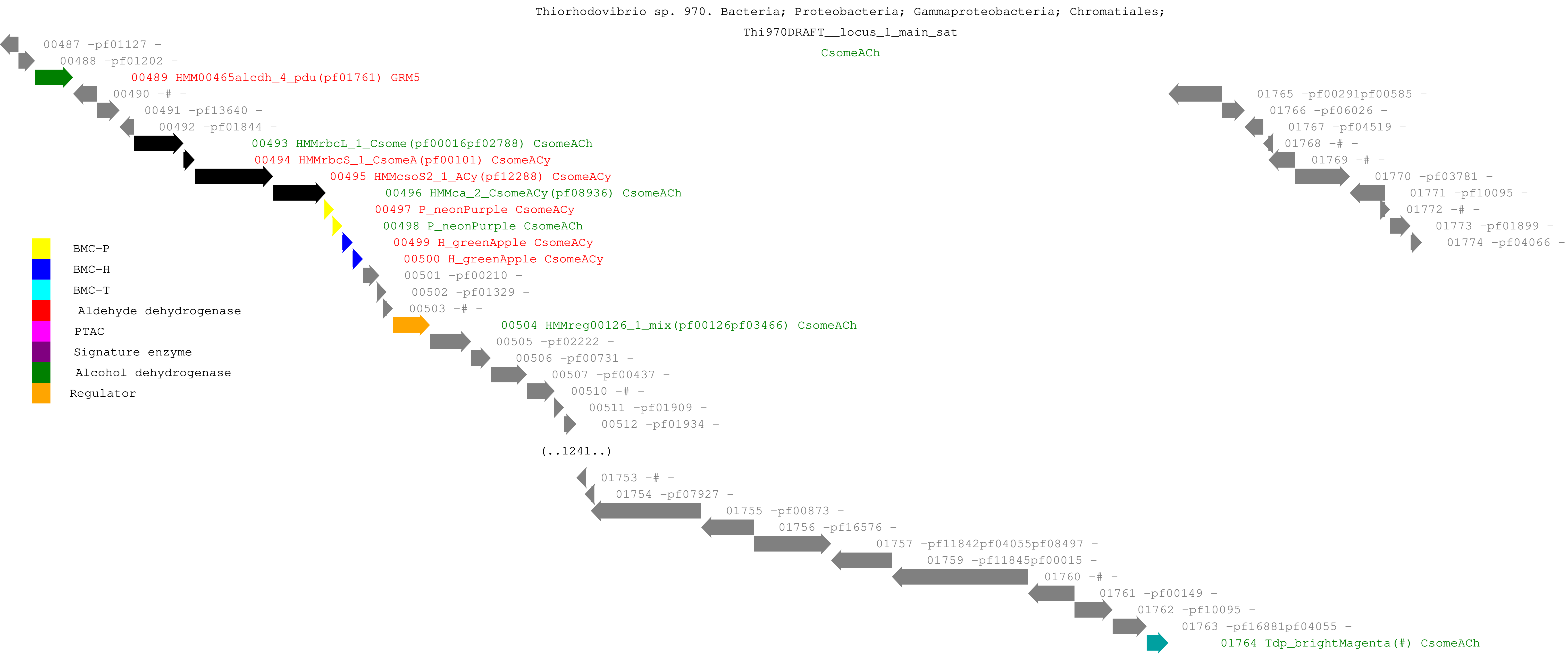
Thiorhodovibrio sp. 970. Bacteria; Proteobacteria; Gammaproteobacteria; Chromatiales;
Thi970DRAFT__locus_1_main_sat
CsomeACh
00487 -pf01127 -
00488 -pf01202 -
00489 HMM00465alcdh_4_pdu(pf01761) GRM5
00490 -# -
00491 -pf13640 -
00492 -pf01844 -
00493 HMMrbcL_1_Csome(pf00016pf02788) CsomeACh
00494 HMMrbcS_1_CsomeA(pf00101) CsomeACy
00495 HMMcsoS2_1_ACy(pf12288) CsomeACy
00496 HMMca_2_CsomeACy(pf08936) CsomeACh
00497 P_neonPurple CsomeACy
00498 P_neonPurple CsomeACh
00499 H_greenApple CsomeACy
00500 H_greenApple CsomeACy
00501 -pf00210 -
00502 -pf01329 -
00503 -# -
00504 HMMreg00126_1_mix(pf00126pf03466) CsomeACh
00505 -pf02222 -
00506 -pf00731 -
00507 -pf00437 -
00510 -# -
00511 -pf01909 -
00512 -pf01934 -
(..1241..)
01753 -# -
01754 -pf07927 -
01755 -pf00873 -
01756 -pf16576 -
01757 -pf11842pf04055pf08497 -
01759 -pf11845pf00015 -
01760 -# -
01761 -pf00149 -
01762 -pf10095 -
01763 -pf16881pf04055 -
01764 Tdp_brightMagenta(#) CsomeACh
01765 -pf00291pf00585 -
01766 -pf06026 -
01767 -pf04519 -
01768 -# -
01769 -# -
01770 -pf03781 -
01771 -pf10095 -
01772 -# -
01773 -pf01899 -
01774 -pf04066 -
BMC-P
BMC-H
BMC-T
Aldehyde dehydrogenase
PTAC
Signature enzyme
Alcohol dehydrogenase
Regulator

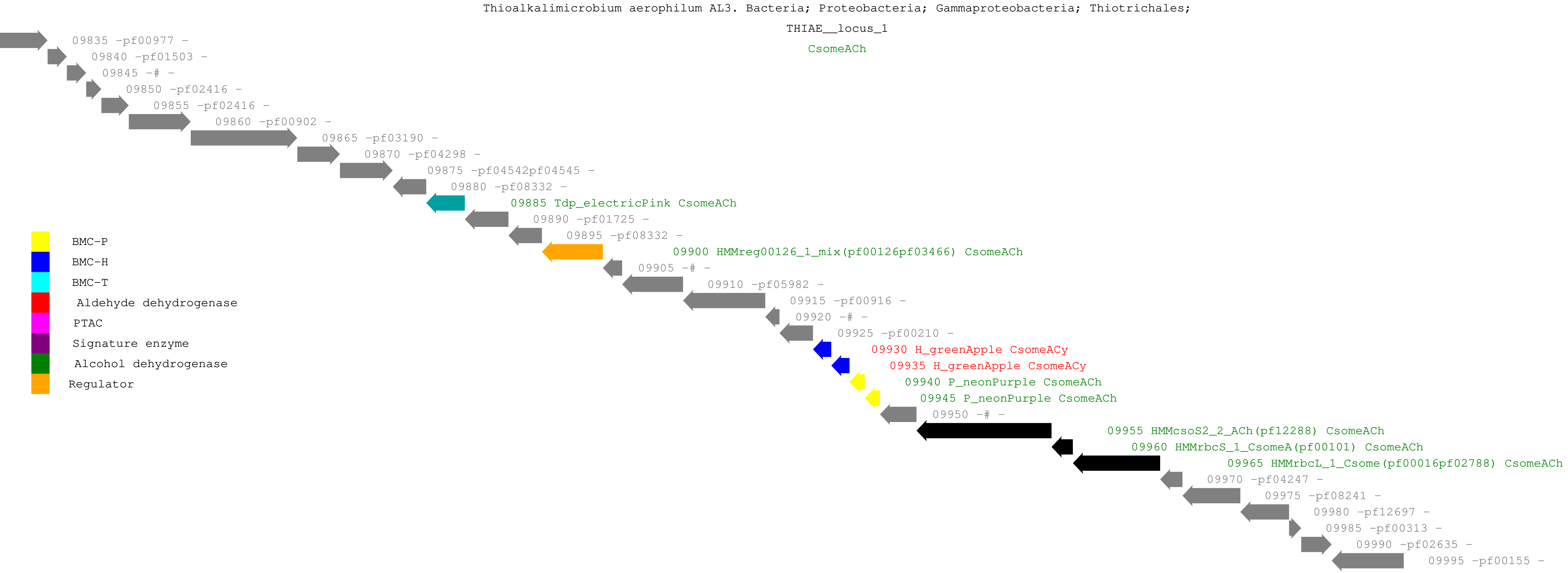
Thioalkalimicrobium aerophilum AL3. Bacteria; Proteobacteria; Gammaproteobacteria; Thiotrichales;
THIAE__locus_1
CsomeACh
09835 -pf00977 -
09840 -pf01503 -
09845 -# -
09850 -pf02416 -
09855 -pf02416 -
09860 -pf00902 -
09865 -pf03190 -
09870 -pf04298 -
09875 -pf04542pf04545 -
09880 -pf08332 -
09885 Tdp_electricPink CsomeACh
09890 -pf01725 -
09895 -pf08332 -
09900 HMMreg00126_1_mix(pf00126pf03466) CsomeACh
09905 -# -
09910 -pf05982 -
09915 -pf00916 -
09920 -# -
09925 -pf00210 -
09930 H_greenApple CsomeACy
09935 H_greenApple CsomeACy
09940 P_neonPurple CsomeACh
09945 P_neonPurple CsomeACh
09950 -# -
09955 HMMcsoS2_2_ACh(pf12288) CsomeACh
09960 HMMrbcS_1_CsomeA(pf00101) CsomeACh
09965 HMMrbcL_1_Csome(pf00016pf02788) CsomeACh
09970 -pf04247 -
09975 -pf08241 -
09980 -pf12697 -
09985 -pf00313 -
09990 -pf02635 -
09995 -pf00155 -
BMC-P
BMC-H
BMC-T
Aldehyde dehydrogenase
PTAC
Signature enzyme
Alcohol dehydrogenase
Regulator

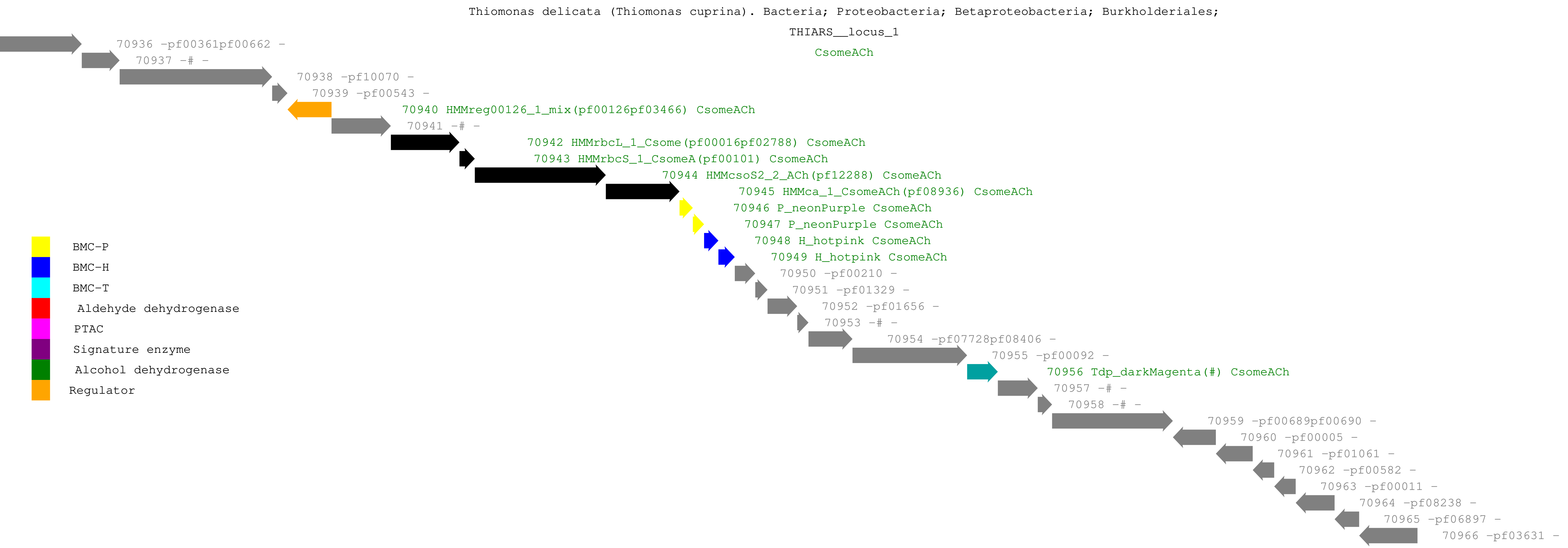
Thiomonas delicata (Thiomonas cuprina). Bacteria; Proteobacteria; Betaproteobacteria; Burkholderiales;
THIARS__locus_1
CsomeACh
70936 -pf00361pf00662 -
70937 -# -
70938 -pf10070 -
70939 -pf00543 -
70940 HMMreg00126_1_mix(pf00126pf03466) CsomeACh
70941 -# -
70942 HMMrbcL_1_Csome(pf00016pf02788) CsomeACh
70943 HMMrbcS_1_CsomeA(pf00101) CsomeACh
70944 HMMcsoS2_2_ACh(pf12288) CsomeACh
70945 HMMca_1_CsomeACh(pf08936) CsomeACh
70946 P_neonPurple CsomeACh
70947 P_neonPurple CsomeACh
70948 H_hotpink CsomeACh
70949 H_hotpink CsomeACh
70950 -pf00210 -
70951 -pf01329 -
70952 -pf01656 -
70953 -# -
70954 -pf07728pf08406 -
70955 -pf00092 -
70956 Tdp_darkMagenta(#) CsomeACh
70957 -# -
70958 -# -
70959 -pf00689pf00690 -
70960 -pf00005 -
70961 -pf01061 -
70962 -pf00582 -
70963 -pf00011 -
70964 -pf08238 -
70965 -pf06897 -
70966 -pf03631 -
BMC-P
BMC-H
BMC-T
Aldehyde dehydrogenase
PTAC
Signature enzyme
Alcohol dehydrogenase
Regulator

Thioalkalimicrobium cyclicum (strain DSM 14477 / JCM 11371 / ALM1). Bacteria; Proteobacteria; Gammaproteobacteria; Thiotrichales;

Thicy__locus_1

CsomeACh

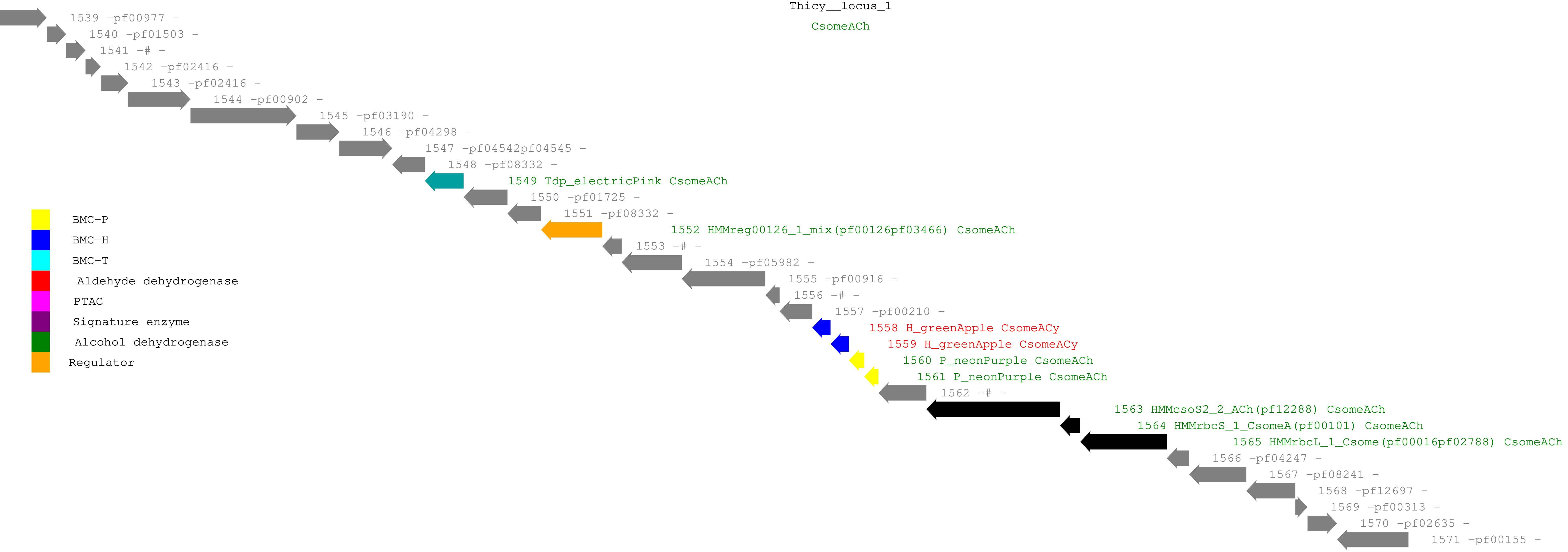

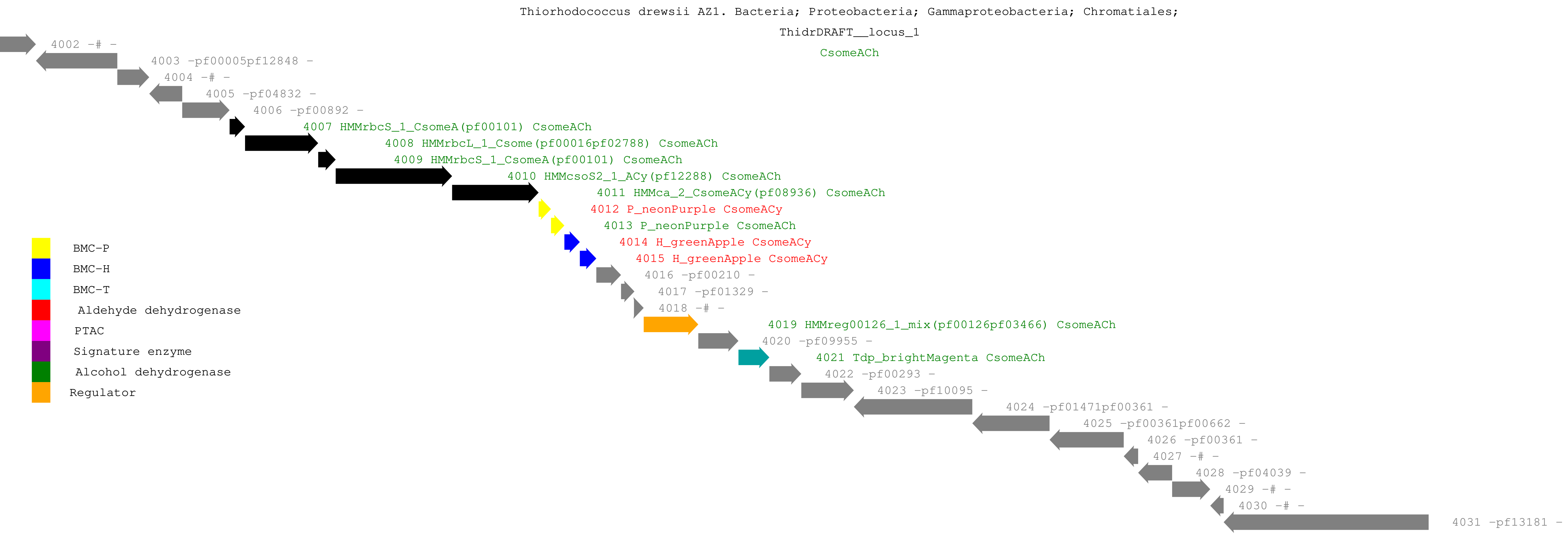

Thiorhodococcus drewsii AZ1. Bacteria; Proteobacteria; Gammaproteobacteria; Chromatiales;
ThidrDRAFT__locus_1
CsomeACh
4002 -# -
4003 -pf00005pf12848 -
4004 -# -
4005 -pf04832 -
4006 -pf00892 -
4007 HMMrbcS_1_CsomeA(pf00101) CsomeACh
4008 HMMrbcL_1_Csome(pf00016pf02788) CsomeACh
4009 HMMrbcS_1_CsomeA(pf00101) CsomeACh
4010 HMMcsoS2_1_ACy(pf12288) CsomeACh
4011 HMMca_2_CsomeACy(pf08936) CsomeACh
4012 P_neonPurple CsomeACy
4013 P_neonPurple CsomeACh
4014 H_greenApple CsomeACy
4015 H_greenApple CsomeACy
4016 -pf00210 -
4017 -pf01329 -
4018 -# -
4019 HMMreg00126_1_mix(pf00126pf03466) CsomeACh
4020 -pf09955 -
4021 Tdp_brightMagenta CsomeACh
4022 -pf00293 -
4023 -pf10095 -
4024 -pf01471pf00361 -
4025 -pf00361pf00662 -
4026 -pf00361 -
4027 -# -
4028 -pf04039 -
4029 -# -
4030 -# -
4031 -pf13181 -
BMC-P
BMC-H
BMC-T
Aldehyde dehydrogenase
PTAC
Signature enzyme
Alcohol dehydrogenase
Regulator

Thiomonas arsenitoxydans (strain DSM 22701 / CIP 110005 / 3As). Bacteria; Proteobacteria; Betaproteobacteria; Burkholderiales;

THI__locus_1

CsomeACh

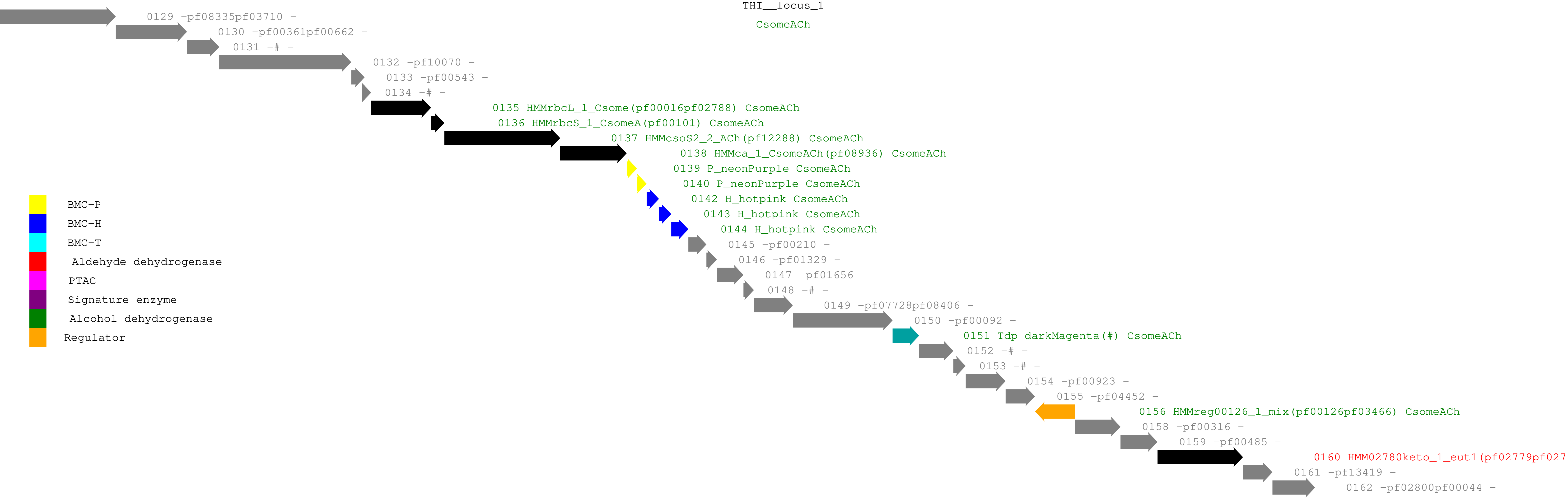

Thiocapsa marina 5811. Bacteria; Proteobacteria; Gammaproteobacteria; Chromatiales;

ThimaDRAFT__locus_1_main_sat

CsomeACh

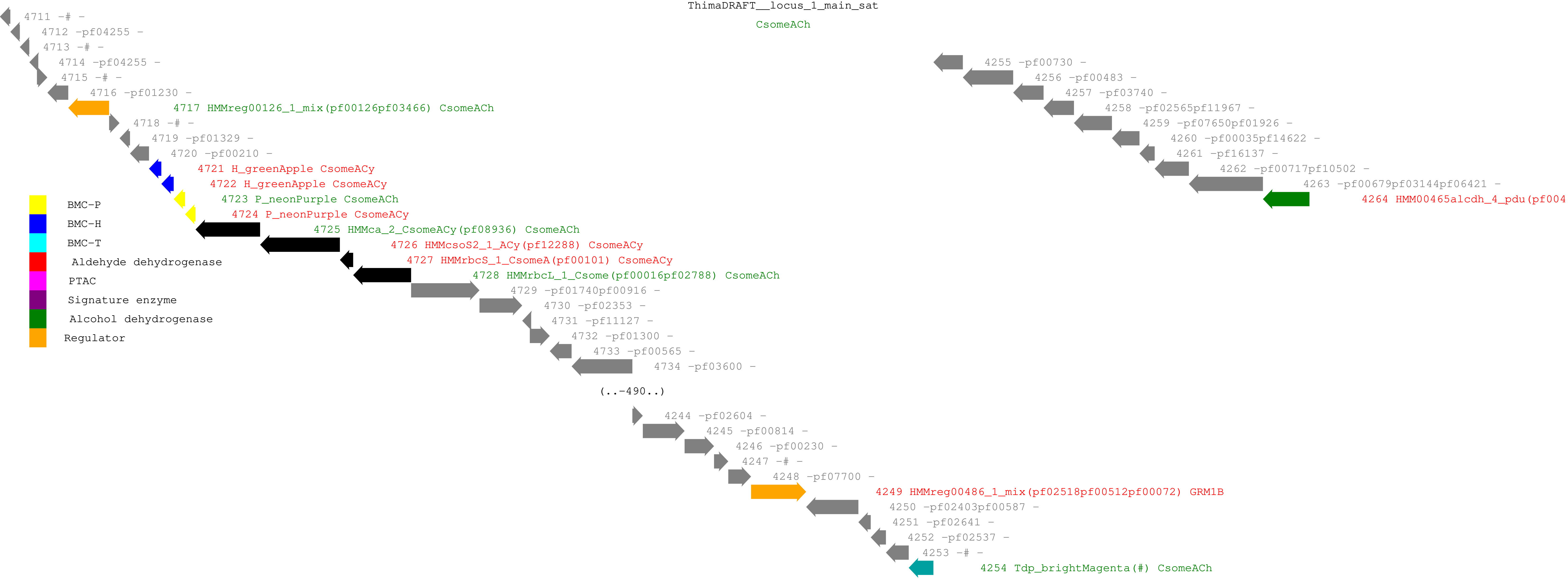

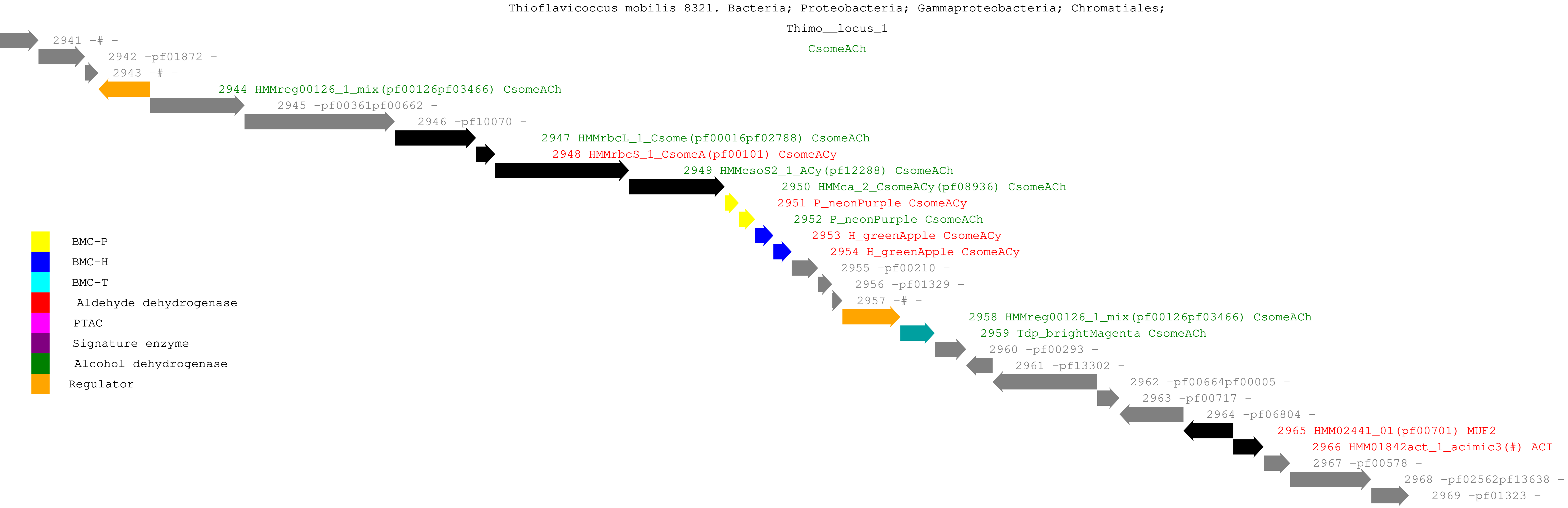

Thioflavicoccus mobilis 8321. Bacteria; Proteobacteria; Gammaproteobacteria; Chromatiales;
Thimo__locus_1
CsomeACh
2941 -# -
2942 -pf01872 -
2943 -# -
2944 HMMreg00126_1_mix(pf00126pf03466) CsomeACh
2945 -pf00361pf00662 -
2946 -pf10070 -
2947 HMMrbcL_1_Csome(pf00016pf02788) CsomeACh
2948 HMMrbcS_1_CsomeA(pf00101) CsomeACy
2949 HMMcsoS2_1_ACy(pf12288) CsomeACh
2950 HMMca_2_CsomeACy(pf08936) CsomeACh
2951 P_neonPurple CsomeACy
2952 P_neonPurple CsomeACh
2953 H_greenApple CsomeACy
2954 H_greenApple CsomeACy
2955 -pf00210 -
2956 -pf01329 -
2957 -# -
2958 HMMreg00126_1_mix(pf00126pf03466) CsomeACh
2959 Tdp_brightMagenta CsomeACh
2960 -pf00293 -
2961 -pf13302 -
2962 -pf00664pf00005 -
2963 -pf00717 -
2964 -pf06804 -
2965 HMM02441_01(pf00701) MUF2
2966 HMM01842act_1_acimic3(#) ACI
2967 -pf00578 -
2968 -pf02562pf13638 -
2969 -pf01323 -
BMC-P
BMC-H
BMC-T
Aldehyde dehydrogenase
PTAC
Signature enzyme
Alcohol dehydrogenase
Regulator

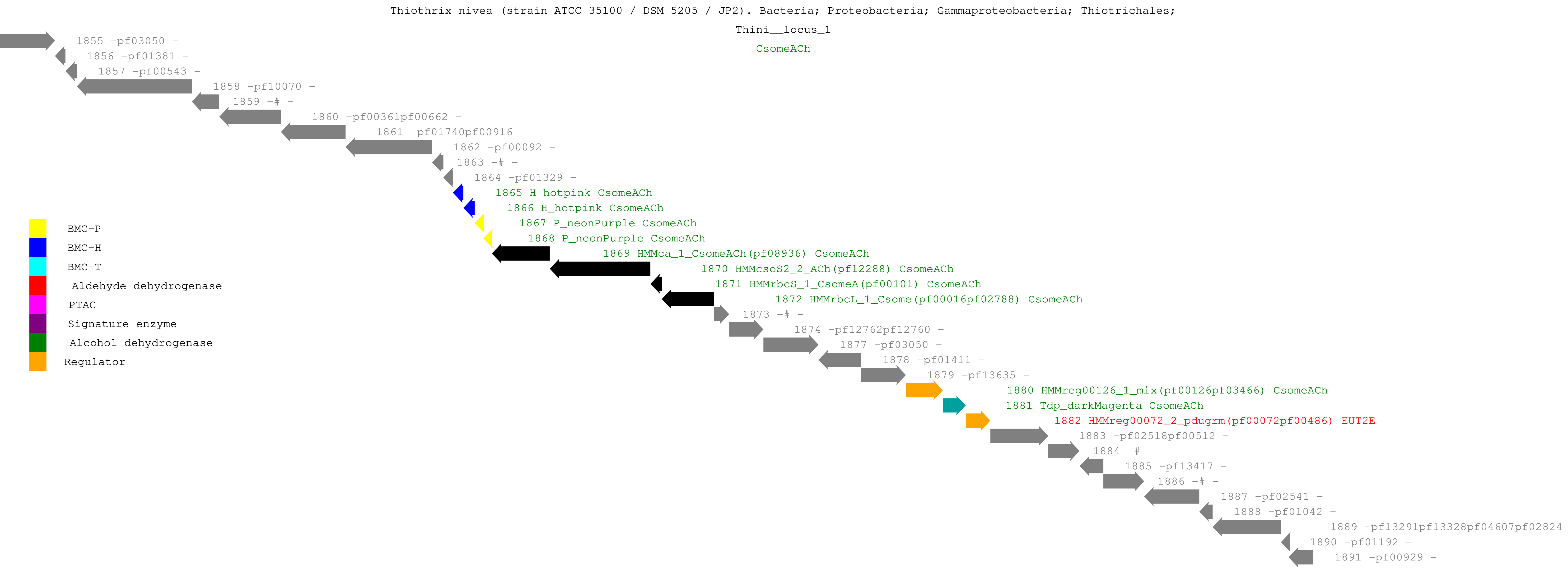
Thiothrix nivea (strain ATCC 35100 / DSM 5205 / JP2). Bacteria; Proteobacteria; Gammaproteobacteria; Thiotrichales;
Thini__locus_1
CsomeACh
1855 -pf03050 -
1856 -pf01381 -
1857 -pf00543 -
1858 -pf10070 -
1859 -# -
1860 -pf00361pf00662 -
1861 -pf01740pf00916 -
1862 -pf00092 -
1863 -# -
1864 -pf01329 -
1865 H_hotpink CsomeACh
1866 H_hotpink CsomeACh
1867 P_neonPurple CsomeACh
1868 P_neonPurple CsomeACh
1869 HMMca_1_CsomeACh(pf08936) CsomeACh
1870 HMMcsoS2_2_ACh(pf12288) CsomeACh
1871 HMMrbcS_1_CsomeA(pf00101) CsomeACh
1872 HMMrbcL_1_Csome(pf00016pf02788) CsomeACh
1873 -# -
1874 -pf12762pf12760 -
1877 -pf03050 -
1878 -pf01411 -
1879 -pf13635 -
1880 HMMreg00126_1_mix(pf00126pf03466) CsomeACh
1881 Tdp_darkMagenta CsomeACh
1882 HMMreg00072_2_pdugrm(pf00072pf00486) EUT2E
1883 -pf02518pf00512 -
1884 -# -
1885 -pf13417 -
1886 -# -
1887 -pf02541 -
1888 -pf01042 -
1889 -pf13291pf13328pf04607pf02824
1890 -pf01192 -
1891 -pf00929 -
BMC-P
BMC-H
BMC-T
Aldehyde dehydrogenase
PTAC
Signature enzyme
Alcohol dehydrogenase
Regulator

Thioalkalivibrio paradoxus ARh 1. Bacteria; Proteobacteria; Gammaproteobacteria; Chromatiales;

THITH__locus_1_main_sat

CsomeACh

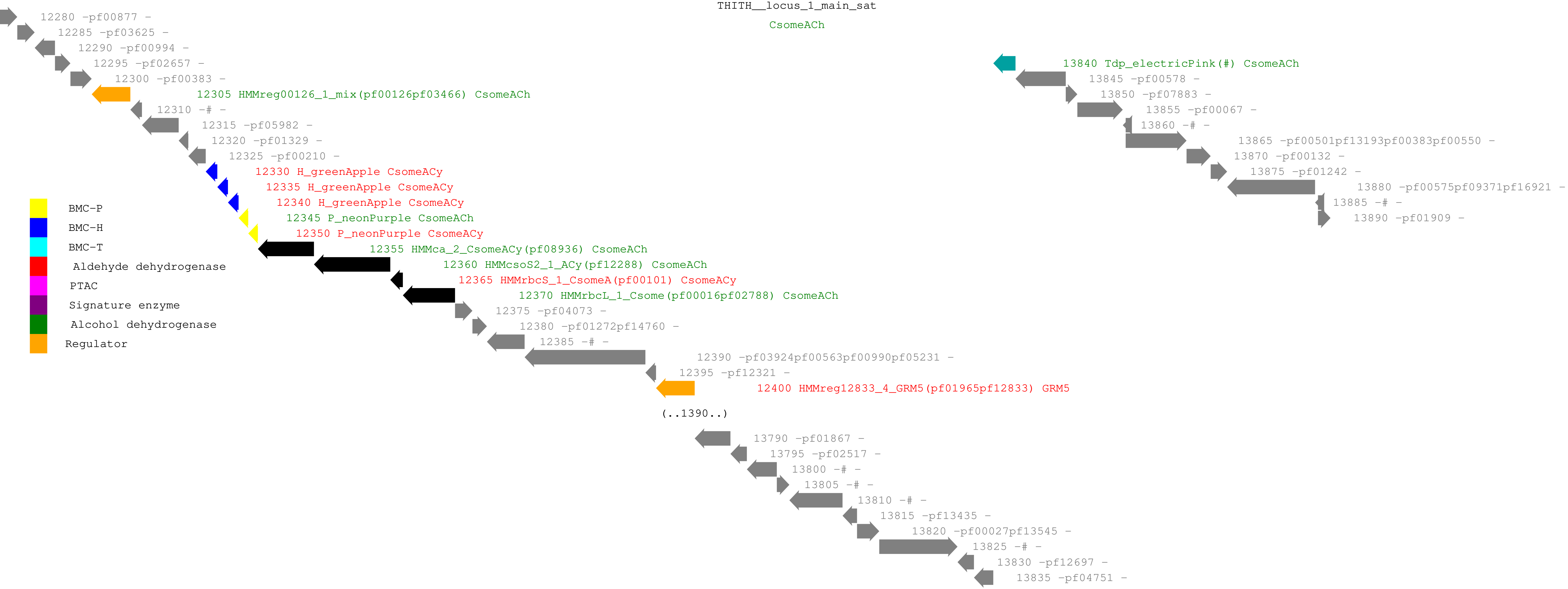

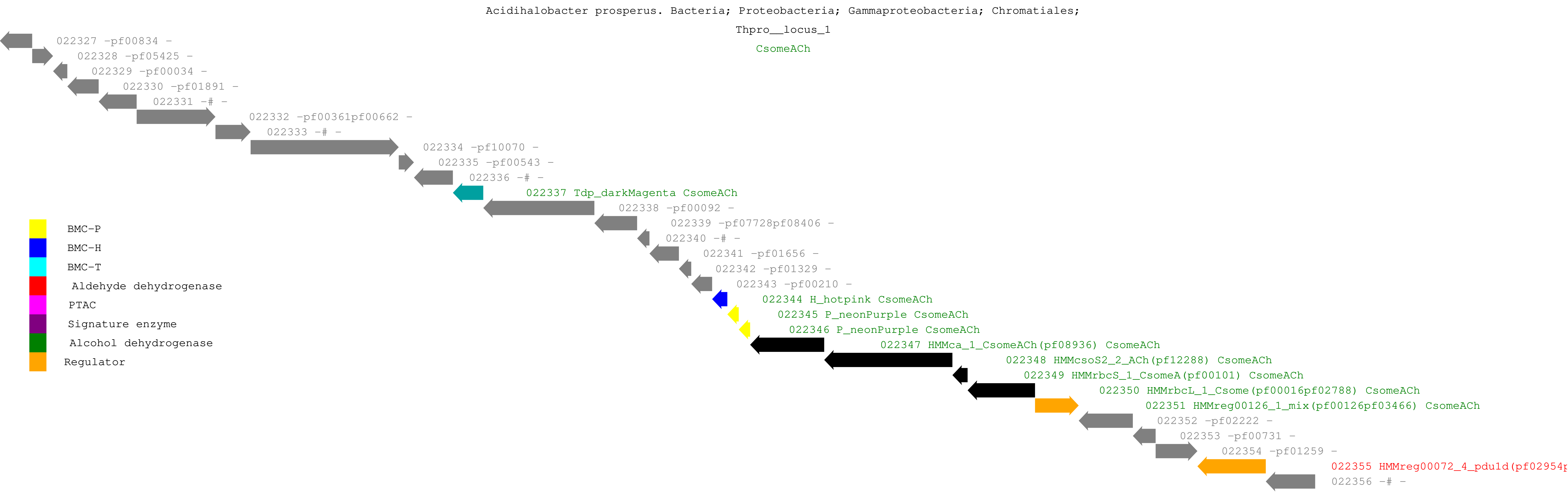
Acidihalobacter prosperus. Bacteria; Proteobacteria; Gammaproteobacteria; Chromatiales;
Thpro__locus_1
CsomeACh
022327 -pf00834 -
022328 -pf05425 -
022329 -pf00034 -
022330 -pf01891 -
022331 -# -
022332 -pf00361pf00662 -
022333 -# -
022334 -pf10070 -
022335 -pf00543 -
022336 -# -
022337 Tdp_darkMagenta CsomeACh
022338 -pf00092 -
022339 -pf07728pf08406 -
022340 -# -
022341 -pf01656 -
022342 -pf01329 -
022343 -pf00210 -
022344 H_hotpink CsomeACh
022345 P_neonPurple CsomeACh
022346 P_neonPurple CsomeACh
022347 HMMca_1_CsomeACh(pf08936) CsomeACh
022348 HMMcsoS2_2_ACh(pf12288) CsomeACh
022349 HMMrbcS_1_CsomeA(pf00101) CsomeACh
022350 HMMrbcL_1_Csome(pf00016pf02788) CsomeACh
022351 HMMreg00126_1_mix(pf00126pf03466) CsomeACh
022352 -pf02222 -
022353 -pf00731 -
022354 -pf01259 -
022355 HMMreg00072_4_pdu1d(pf02954p
022356 -# -
BMC-P
BMC-H
BMC-T
Aldehyde dehydrogenase
PTAC
Signature enzyme
Alcohol dehydrogenase
Regulator

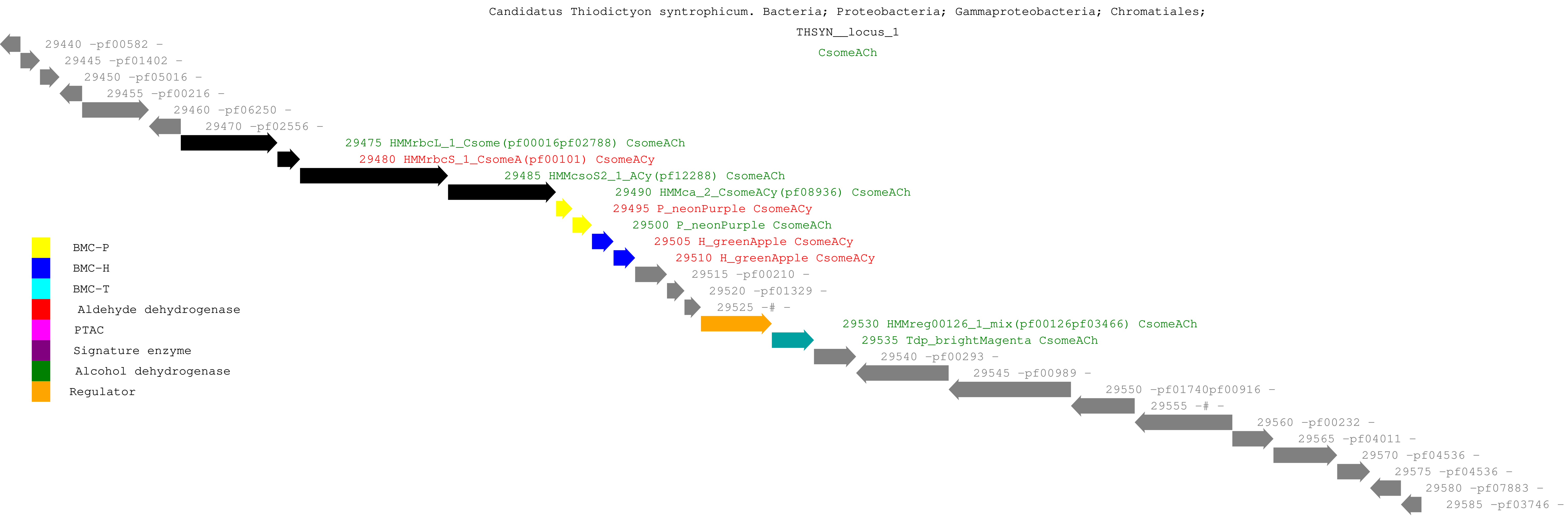
Candidatus Thiodictyon syntrophicum. Bacteria; Proteobacteria; Gammaproteobacteria; Chromatiales;
THSYN__locus_1
CsomeACh
29440 -pf00582 -
29445 -pf01402 -
29450 -pf05016 -
29455 -pf00216 -
29460 -pf06250 -
29470 -pf02556 -
29475 HMMrbcL_1_Csome(pf00016pf02788) CsomeACh
29480 HMMrbcS_1_CsomeA(pf00101) CsomeACy
29485 HMMcsoS2_1_ACy(pf12288) CsomeACh
29490 HMMca_2_CsomeACy(pf08936) CsomeACh
29495 P_neonPurple CsomeACy
29500 P_neonPurple CsomeACh
29505 H_greenApple CsomeACy
29510 H_greenApple CsomeACy
29515 -pf00210 -
29520 -pf01329 -
29525 -# -
29530 HMMreg00126_1_mix(pf00126pf03466) CsomeACh
29535 Tdp_brightMagenta CsomeACh
29540 -pf00293 -
29545 -pf00989 -
29550 -pf01740pf00916 -
29555 -# -
29560 -pf00232 -
29565 -pf04011 -
29570 -pf04536 -
29575 -pf04536 -
29580 -pf07883 -
29585 -pf03746 -
BMC-P
BMC-H
BMC-T
Aldehyde dehydrogenase
PTAC
Signature enzyme
Alcohol dehydrogenase
Regulator

Thiomonas intermedia (strain K12) (Thiobacillus intermedius). Bacteria; Proteobacteria; Betaproteobacteria; Burkholderiales;

Tint__locus_1

CsomeACh

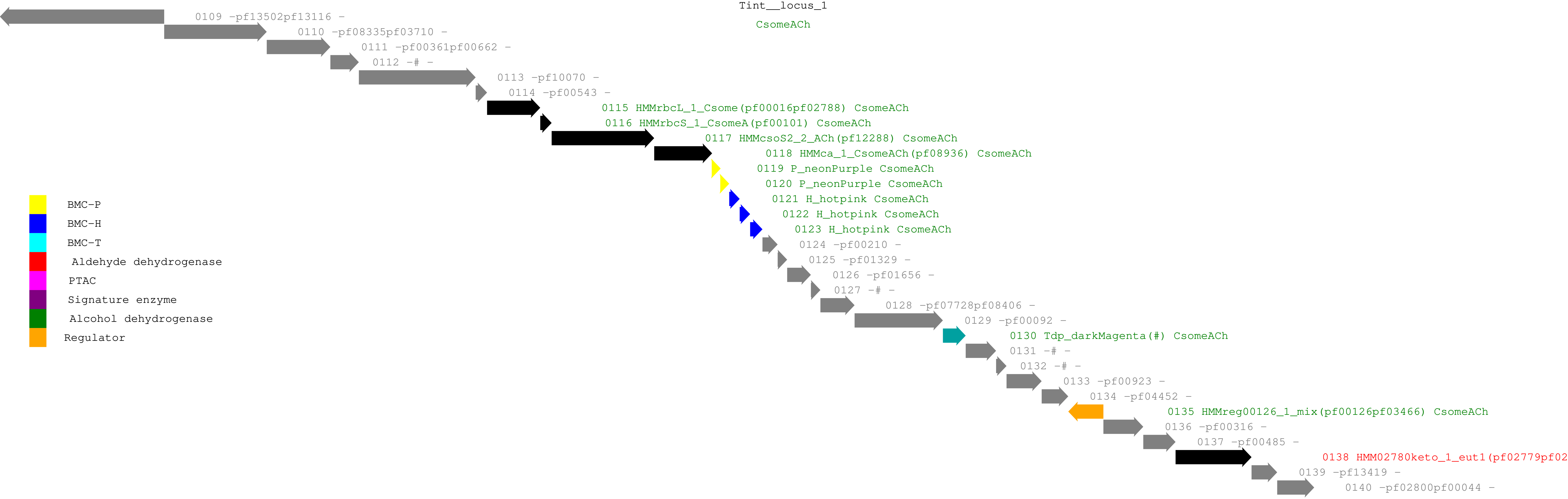

Thioalkalivibrio sp. (strain K90mix). Bacteria; Proteobacteria; Gammaproteobacteria; Chromatiales;

TK90__locus_1_main_sat

CsomeACh

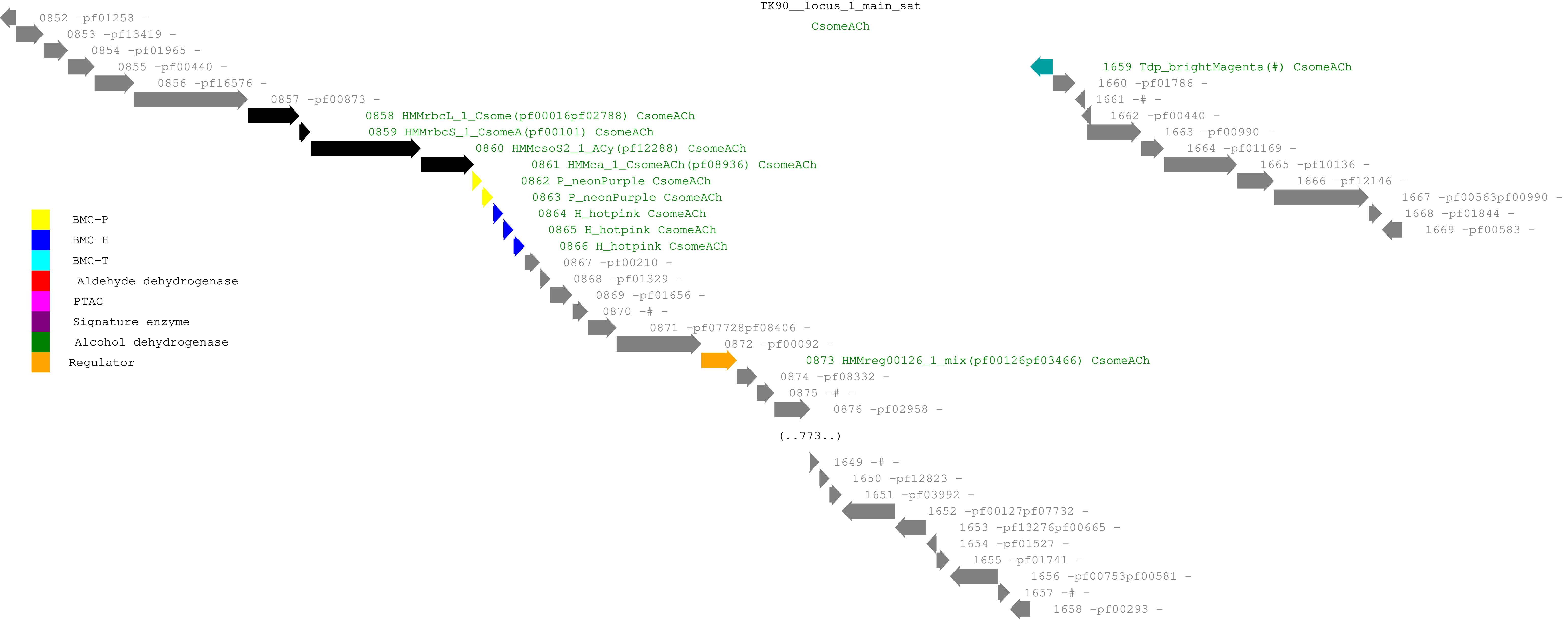

Pseudonocardia sp. N23. Bacteria; Actinobacteria; Pseudonocardiales; Pseudonocardiaceae;

TOK__locus_2

CsomeACh

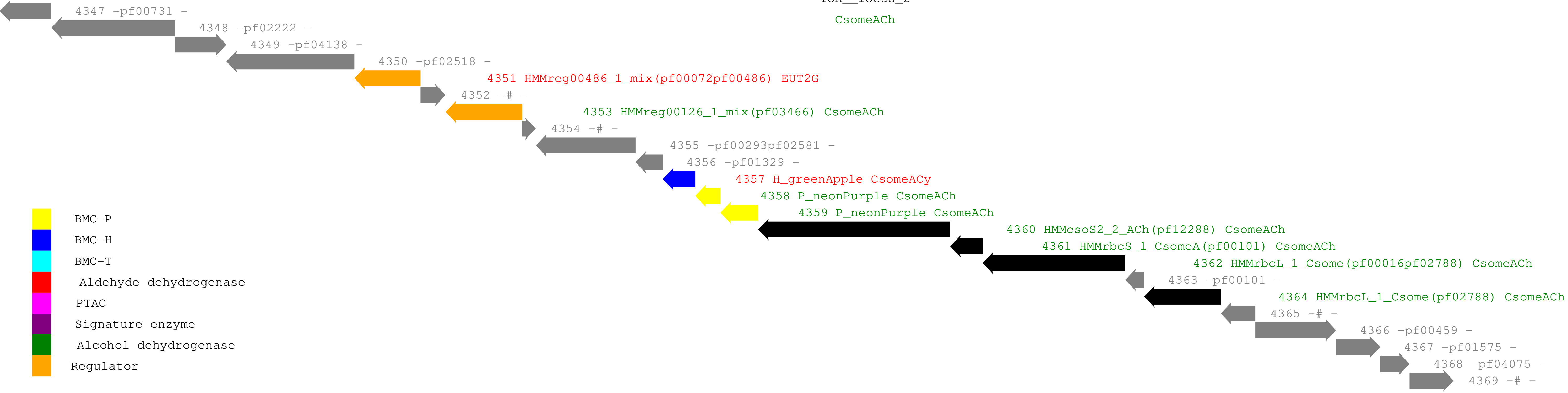

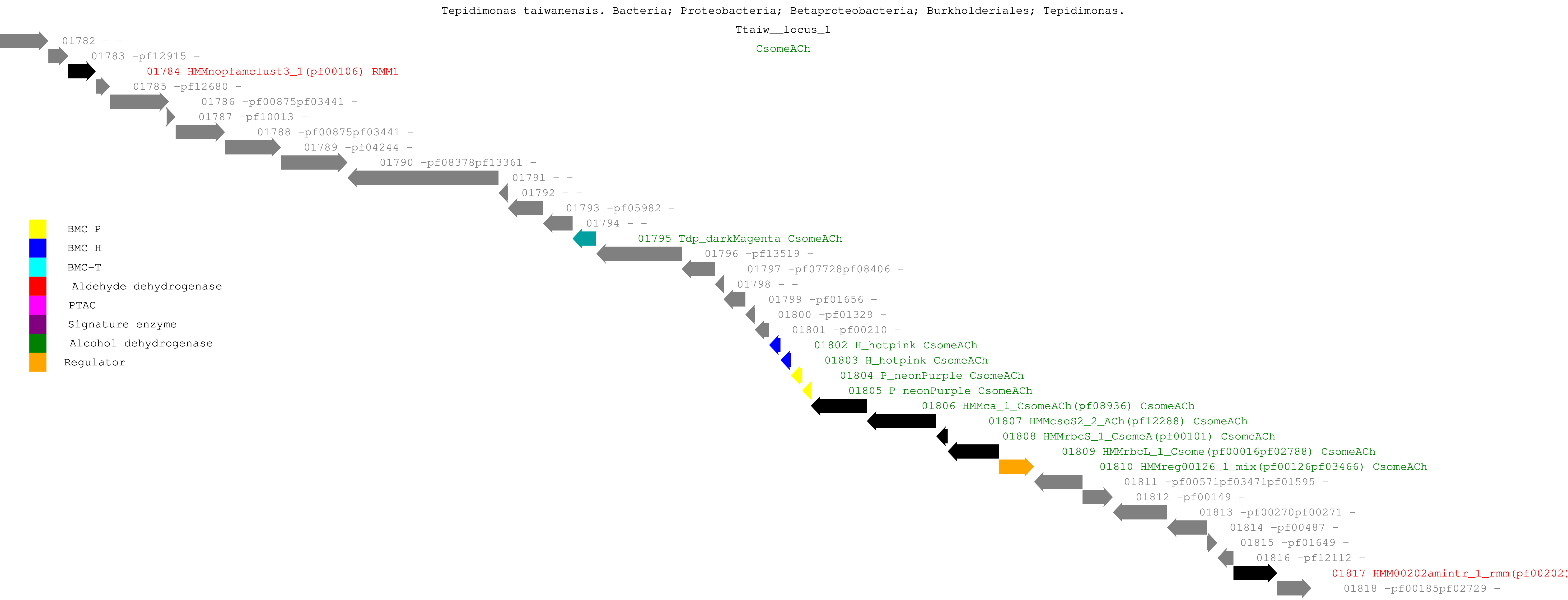

Tepidimonas taiwanensis. Bacteria; Proteobacteria; Betaproteobacteria; Burkholderiales; Tepidimonas.
Ttaiw__locus_1
CsomeACh
01782 - -
01783 -pf12915 -
01784 HMMnopfamclust3_1(pf00106) RMM1
01785 -pf12680 -
01786 -pf00875pf03441 -
01787 -pf10013 -
01788 -pf00875pf03441 -
01789 -pf04244 -
01790 -pf08378pf13361 -
01791 - -
01792 - -
01793 -pf05982 -
01794 - -
01795 Tdp_darkMagenta CsomeACh
01796 -pf13519 -
01797 -pf07728pf08406 -
01798 - -
01799 -pf01656 -
01800 -pf01329 -
01801 -pf00210 -
01802 H_hotpink CsomeACh
01803 H_hotpink CsomeACh
01804 P_neonPurple CsomeACh
01805 P_neonPurple CsomeACh
01806 HMMca_1_CsomeACh(pf08936) CsomeACh
01807 HMMcsoS2_2_ACh(pf12288) CsomeACh
01808 HMMrbcS_1_CsomeA(pf00101) CsomeACh
01809 HMMrbcL_1_Csome(pf00016pf02788) CsomeACh
01810 HMMreg00126_1_mix(pf00126pf03466) CsomeACh
01811 -pf00571pf03471pf01595 -
01812 -pf00149 -
01813 -pf00270pf00271 -
01814 -pf00487 -
01815 -pf01649 -
01816 -pf12112 -
01817 HMM00202amintr_1_rmm(pf00202)
01818 -pf00185pf02729 -
BMC-P
BMC-H
BMC-T
Aldehyde dehydrogenase
PTAC
Signature enzyme
Alcohol dehydrogenase
Regulator

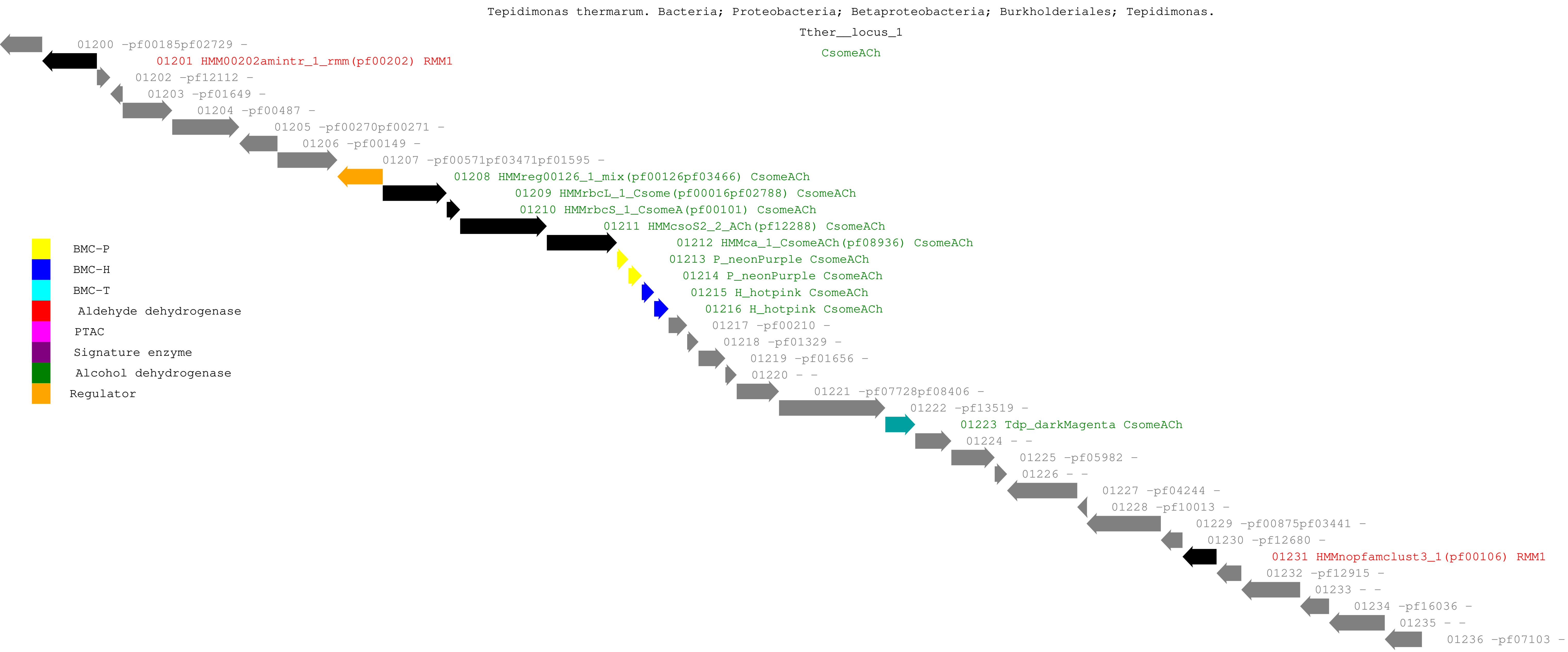
Tepidimonas thermarum. Bacteria; Proteobacteria; Betaproteobacteria; Burkholderiales; Tepidimonas.
Tther__locus_1
CsomeACh
01200 -pf00185pf02729 -
01201 HMM00202amintr_1_rmm(pf00202) RMM1
01202 -pf12112 -
01203 -pf01649 -
01204 -pf00487 -
01205 -pf00270pf00271 -
01206 -pf00149 -
01207 -pf00571pf03471pf01595 -
01208 HMMreg00126_1_mix(pf00126pf03466) CsomeACh
01209 HMMrbcL_1_Csome(pf00016pf02788) CsomeACh
01210 HMMrbcS_1_CsomeA(pf00101) CsomeACh
01211 HMMcsoS2_2_ACh(pf12288) CsomeACh
01212 HMMca_1_CsomeACh(pf08936) CsomeACh
01213 P_neonPurple CsomeACh
01214 P_neonPurple CsomeACh
01215 H_hotpink CsomeACh
01216 H_hotpink CsomeACh
01217 -pf00210 -
01218 -pf01329 -
01219 -pf01656 -
01220 - -
01221 -pf07728pf08406 -
01222 -pf13519 -
01223 Tdp_darkMagenta CsomeACh
01224 - -
01225 -pf05982 -
01226 - -
01227 -pf04244 -
01228 -pf10013 -
01229 -pf00875pf03441 -
01230 -pf12680 -
01231 HMMnopfamclust3_1(pf00106) RMM1
01232 -pf12915 -
01233 - -
01234 -pf16036 -
01235 - -
01236 -pf07103 -
BMC-P
BMC-H
BMC-T
Aldehyde dehydrogenase
PTAC
Signature enzyme
Alcohol dehydrogenase
Regulator

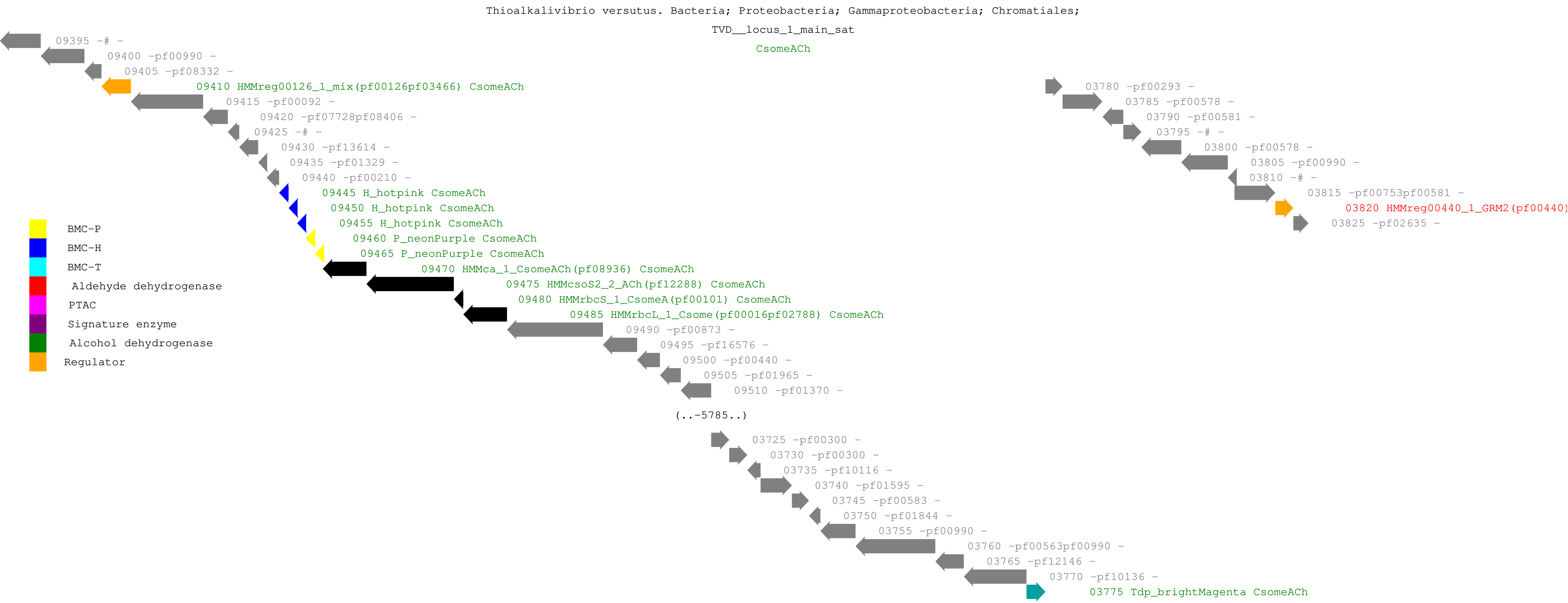

Thioalkalivibrio versutus. Bacteria; Proteobacteria; Gammaproteobacteria; Chromatiales;
TVD__locus_1_main_sat
CsomeACh
09395 -# -
09400 -pf00990 -
09405 -pf08332 -
09410 HMMreg00126_1_mix(pf00126pf03466) CsomeACh
09415 -pf00092 -
09420 -pf07728pf08406 -
09425 -# -
09430 -pf13614 -
09435 -pf01329 -
09440 -pf00210 -
09445 H_hotpink CsomeACh
09450 H_hotpink CsomeACh
09455 H_hotpink CsomeACh
09460 P_neonPurple CsomeACh
09465 P_neonPurple CsomeACh
09470 HMMca_1_CsomeACh(pf08936) CsomeACh
09475 HMMcsoS2_2_ACh(pf12288) CsomeACh
09480 HMMrbcS_1_CsomeA(pf00101) CsomeACh
09485 HMMrbcL_1_Csome(pf00016pf02788) CsomeACh
09490 -pf00873 -
09495 -pf16576 -
09500 -pf00440 -
09505 -pf01965 -
09510 -pf01370 -
(..-5785..)
03725 -pf00300 -
03730 -pf00300 -
03735 -pf10116 -
03740 -pf01595 -
03745 -pf00583 -
03750 -pf01844 -
03755 -pf00990 -
03760 -pf00563pf00990 -
03765 -pf12146 -
03770 -pf10136 -
03775 Tdp_brightMagenta CsomeACh
03780 -pf00293 -
03785 -pf00578 -
03790 -pf00581 -
03795 -# -
03800 -pf00578 -
03805 -pf00990 -
03810 -# -
03815 -pf00753pf00581 -
03820 HMMreg00440_1_GRM2(pf00440)
03825 -pf02635 -
BMC-P
BMC-H
BMC-T
Aldehyde dehydrogenase
PTAC
Signature enzyme
Alcohol dehydrogenase
Regulator

Thioalkalivibrio nitratireducens (strain DSM 14787 / UNIQEM 213 / Bacteria; Proteobacteria; Gammaproteobacteria; Chromatiales;

TVNIR__locus_1_main_sat

CsomeACh

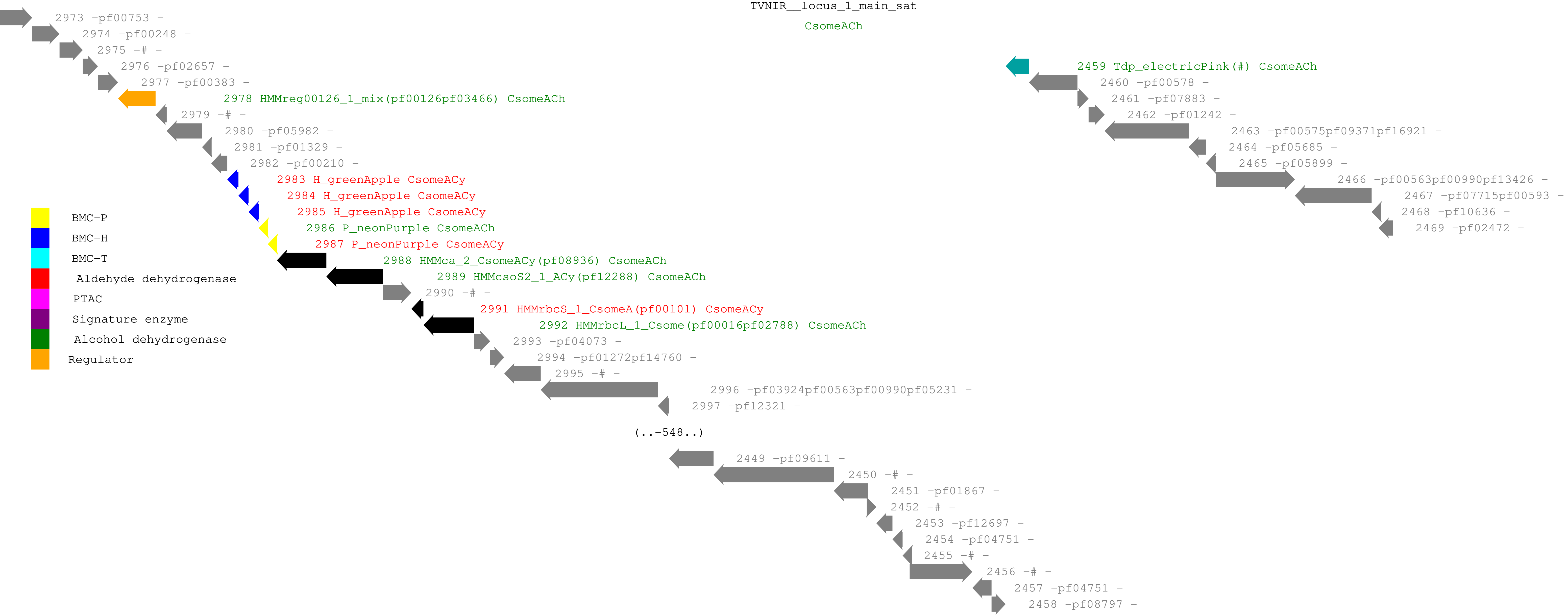

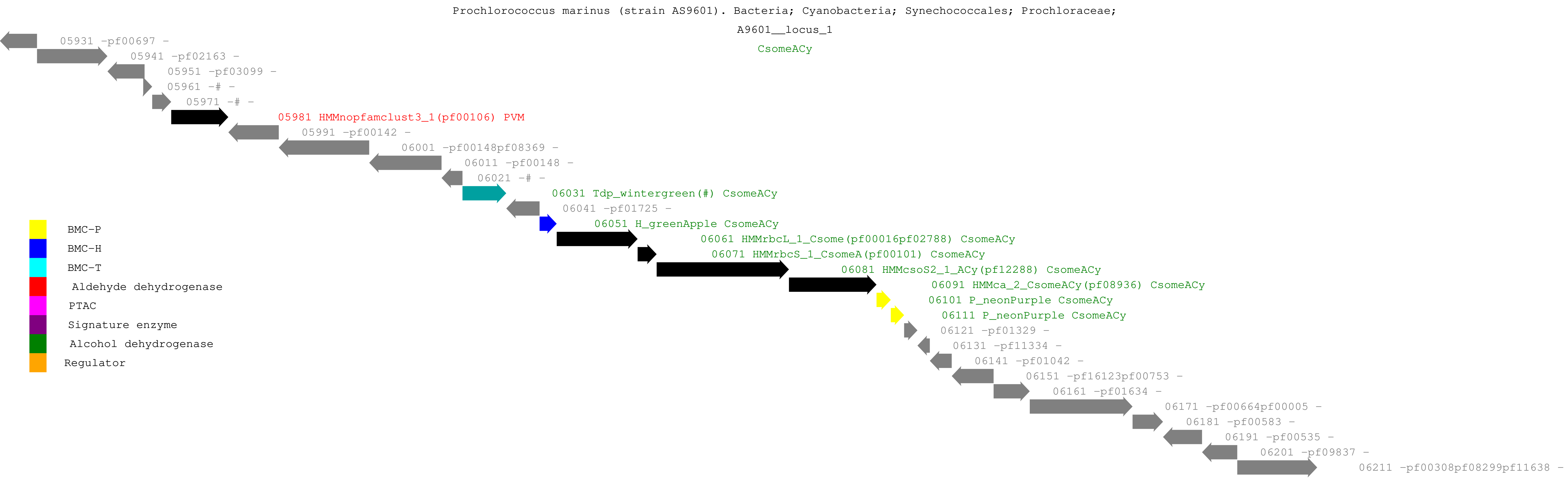

Prochlorococcus marinus (strain AS9601). Bacteria; Cyanobacteria; Synechococcales; Prochloraceae;
A9601__locus_1
CsomeACy
05931 -pf00697 -
05941 -pf02163 -
05951 -pf03099 -
05961 -# -
05971 -# -
05981 HMMnopfamclust3_1(pf00106) PVM
05991 -pf00142 -
06001 -pf00148pf08369 -
06011 -pf00148 -
06021 -# -
06031 Tdp_wintergreen(#) CsomeACy
06041 -pf01725 -
06051 H_greenApple CsomeACy
06061 HMMrbcL_1_Csome(pf00016pf02788) CsomeACy
06071 HMMrbcS_1_CsomeA(pf00101) CsomeACy
06081 HMMcsoS2_1_ACy(pf12288) CsomeACy
06091 HMMca_2_CsomeACy(pf08936) CsomeACy
06101 P_neonPurple CsomeACy
06111 P_neonPurple CsomeACy
06121 -pf01329 -
06131 -pf11334 -
06141 -pf01042 -
06151 -pf16123pf00753 -
06161 -pf01634 -
06171 -pf00664pf00005 -
06181 -pf00583 -
06191 -pf00535 -
06201 -pf09837 -
06211 -pf00308pf08299pf11638 -
BMC-P
BMC-H
BMC-T
Aldehyde dehydrogenase
PTAC
Signature enzyme
Alcohol dehydrogenase
Regulator

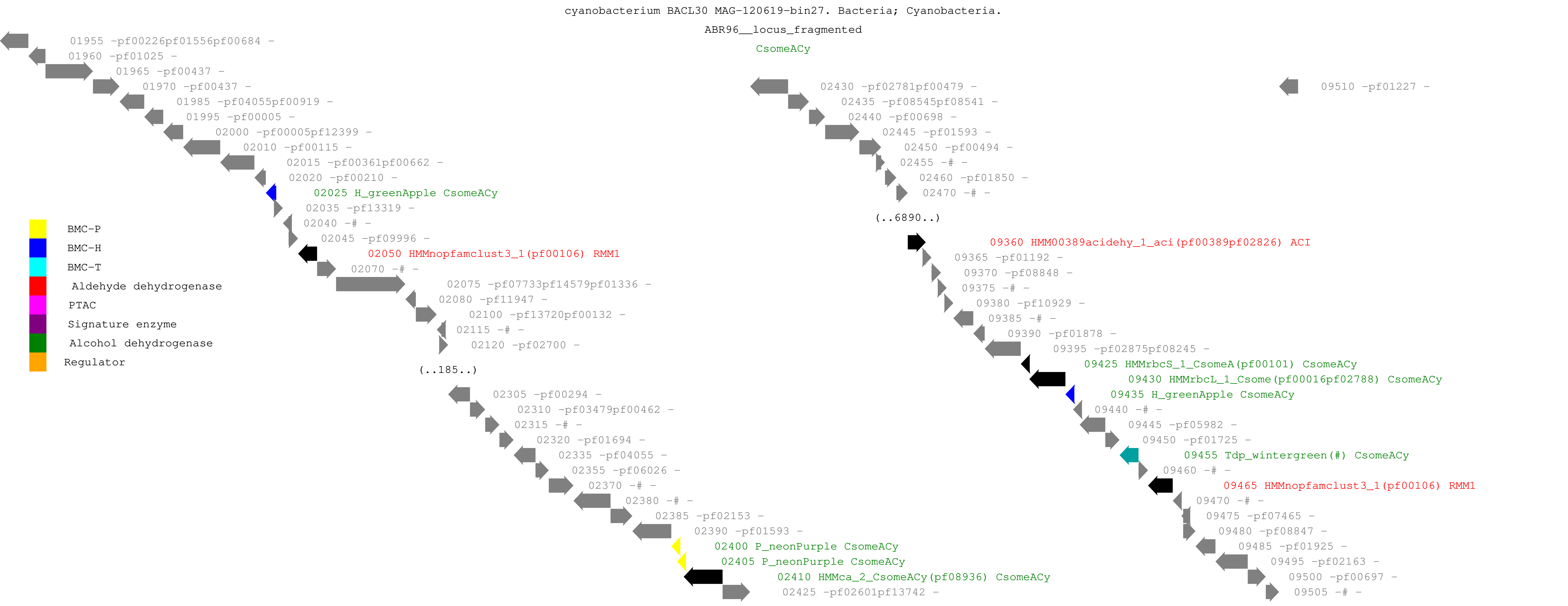
cyanobacterium BACL30 MAG-120619-bin27. Bacteria; Cyanobacteria.
ABR96__locus_fragmented
CsomeACy
01955 -pf00226pf01556pf00684 -
01960 -pf01025 -
01965 -pf00437 -
01970 -pf00437 -
01985 -pf04055pf00919 -
01995 -pf00005 -
02000 -pf00005pf12399 -
02010 -pf00115 -
02015 -pf00361pf00662 -
02020 -pf00210 -
02025 H_greenApple CsomeACy
02035 -pf13319 -
02040 -# -
02045 -pf09996 -
02050 HMMnopfamclust3_1(pf00106) RMM1
02070 -# -
02075 -pf07733pf14579pf01336 -
02080 -pf11947 -
02100 -pf13720pf00132 -
02115 -# -
02120 -pf02700 -
(..185..)
02305 -pf00294 -
02310 -pf03479pf00462 -
02315 -# -
02320 -pf01694 -
02335 -pf04055 -
02355 -pf06026 -
02370 -# -
02380 -# -
02385 -pf02153 -
02390 -pf01593 -
02400 P_neonPurple CsomeACy
02405 P_neonPurple CsomeACy
02410 HMMca_2_CsomeACy(pf08936) CsomeACy
02425 -pf02601pf13742 -
02430 -pf02781pf00479 -
02435 -pf08545pf08541 -
02440 -pf00698 -
02445 -pf01593 -
02450 -pf00494 -
02455 -# -
02460 -pf01850 -
02470 -# -
(..6890..)
09360 HMM00389acidehy_1_aci(pf00389pf02826) ACI
09365 -pf01192 -
09370 -pf08848 -
09375 -# -
09380 -pf10929 -
09385 -# -
09390 -pf01878 -
09395 -pf02875pf08245 -
09425 HMMrbcS_1_CsomeA(pf00101) CsomeACy
09430 HMMrbcL_1_Csome(pf00016pf02788) CsomeACy
09435 H_greenApple CsomeACy
09440 -# -
09445 -pf05982 -
09450 -pf01725 -
09455 Tdp_wintergreen(#) CsomeACy
09460 -# -
09465 HMMnopfamclust3_1(pf00106) RMM1
09470 -# -
09475 -pf07465 -
09480 -pf08847 -
09485 -pf01925 -
09495 -pf02163 -
09500 -pf00697 -
09505 -# -
09510 -pf01227 -
BMC-P
BMC-H
BMC-T
Aldehyde dehydrogenase
PTAC
Signature enzyme
Alcohol dehydrogenase
Regulator

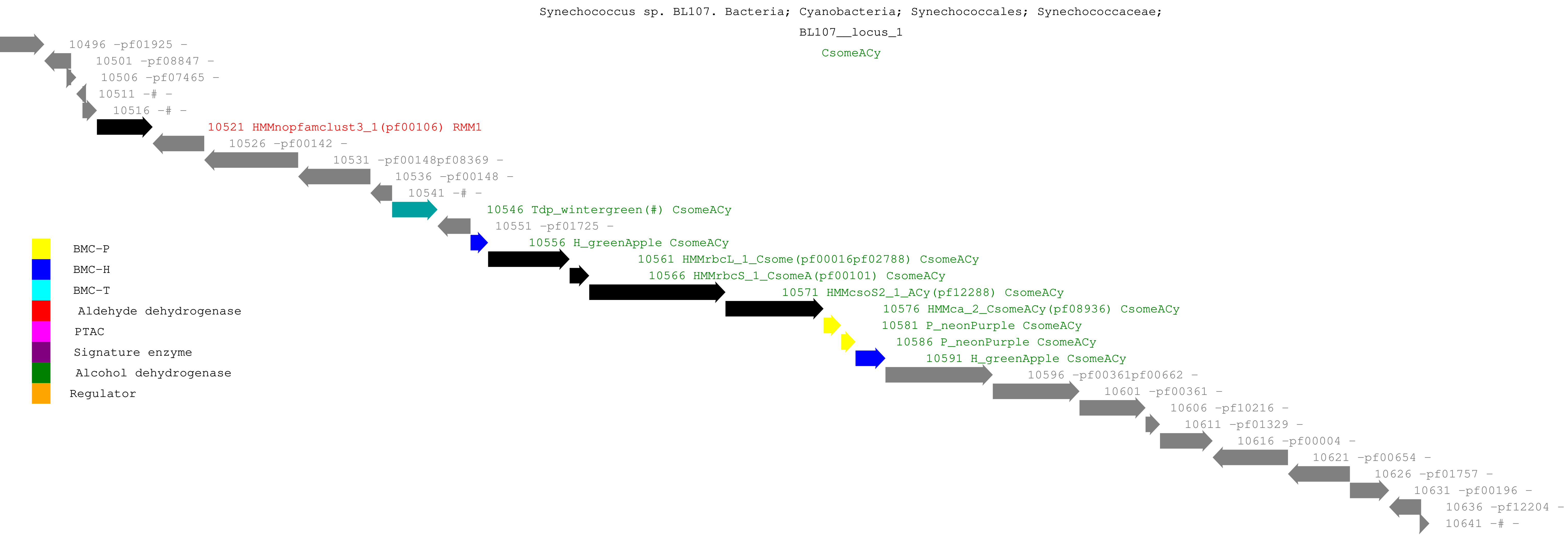
Synechococcus sp. BL107. Bacteria; Cyanobacteria; Synechococcales; Synechococcaceae;
BL107__locus_1
CsomeACy
10496 -pf01925 -
10501 -pf08847 -
10506 -pf07465 -
10511 -# -
10516 -# -
10521 HMMnopfamclust3_1(pf00106) RMM1
10526 -pf00142 -
10531 -pf00148pf08369 -
10536 -pf00148 -
10541 -# -
10546 Tdp_wintergreen(#) CsomeACy
10551 -pf01725 -
10556 H_greenApple CsomeACy
10561 HMMrbcL_1_Csome(pf00016pf02788) CsomeACy
10566 HMMrbcS_1_CsomeA(pf00101) CsomeACy
10571 HMMcsoS2_1_ACy(pf12288) CsomeACy
10576 HMMca_2_CsomeACy(pf08936) CsomeACy
10581 P_neonPurple CsomeACy
10586 P_neonPurple CsomeACy
10591 H_greenApple CsomeACy
10596 -pf00361pf00662 -
10601 -pf00361 -
10606 -pf10216 -
10611 -pf01329 -
10616 -pf00004 -
10621 -pf00654 -
10626 -pf01757 -
10631 -pf00196 -
10636 -pf12204 -
10641 -# -
BMC-P
BMC-H
BMC-T
Aldehyde dehydrogenase
PTAC
Signature enzyme
Alcohol dehydrogenase
Regulator

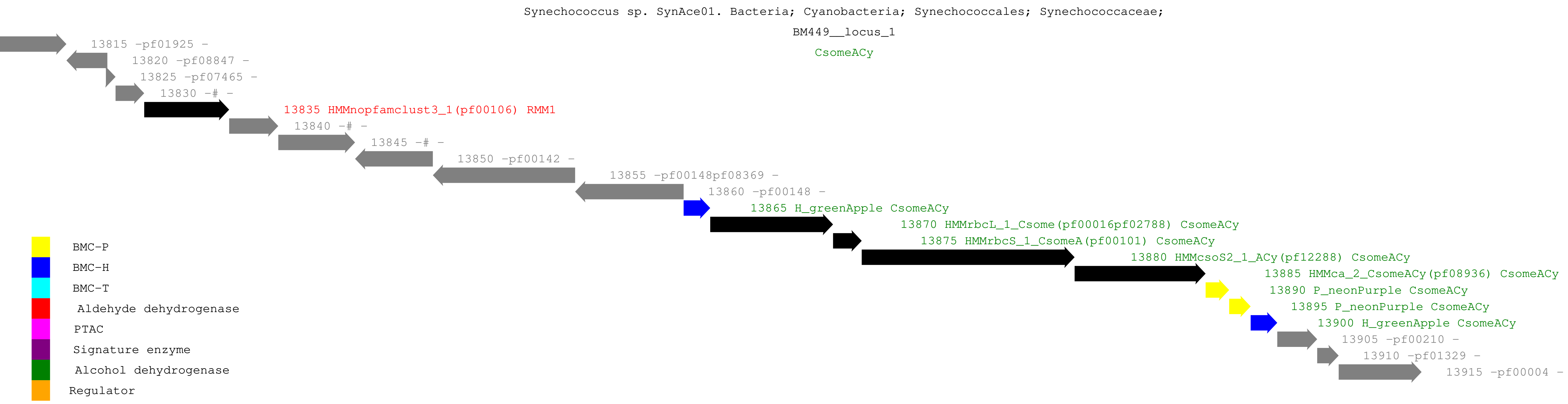
Synechococcus sp. SynAce01. Bacteria; Cyanobacteria; Synechococcales; Synechococcaceae;
BM449__locus_1
CsomeACy
13815 -pf01925 -
13820 -pf08847 -
13825 -pf07465 -
13830 -# -
13835 HMMnopfamclust3_1(pf00106) RMM1
13840 -# -
13845 -# -
13850 -pf00142 -
13855 -pf00148pf08369 -
13860 -pf00148 -
13865 H_greenApple CsomeACy
13870 HMMrbcL_1_Csome(pf00016pf02788) CsomeACy
13875 HMMrbcS_1_CsomeA(pf00101) CsomeACy
13880 HMMcsoS2_1_ACy(pf12288) CsomeACy
13885 HMMca_2_CsomeACy(pf08936) CsomeACy
13890 P_neonPurple CsomeACy
13895 P_neonPurple CsomeACy
13900 H_greenApple CsomeACy
13905 -pf00210 -
13910 -pf01329 -
13915 -pf00004 -
BMC-P
BMC-H
BMC-T
Aldehyde dehydrogenase
PTAC
Signature enzyme
Alcohol dehydrogenase
Regulator

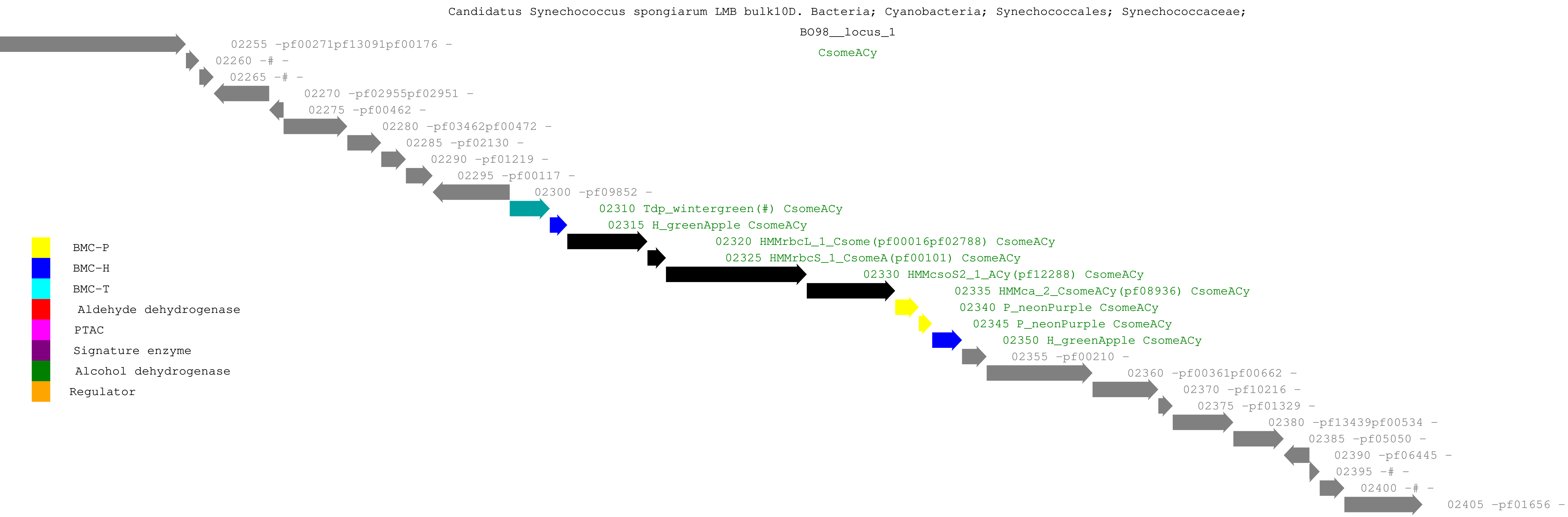
Candidatus Synechococcus spongiarum LMB bulk10D. Bacteria; Cyanobacteria; Synechococcales; Synechococcaceae;
BO98__locus_1
CsomeACy
02255 -pf00271pf13091pf00176 -
02260 -# -
02265 -# -
02270 -pf02955pf02951 -
02275 -pf00462 -
02280 -pf03462pf00472 -
02285 -pf02130 -
02290 -pf01219 -
02295 -pf00117 -
02300 -pf09852 -
02310 Tdp_wintergreen(#) CsomeACy
02315 H_greenApple CsomeACy
02320 HMMrbcL_1_Csome(pf00016pf02788) CsomeACy
02325 HMMrbcS_1_CsomeA(pf00101) CsomeACy
02330 HMMcsoS2_1_ACy(pf12288) CsomeACy
02335 HMMca_2_CsomeACy(pf08936) CsomeACy
02340 P_neonPurple CsomeACy
02345 P_neonPurple CsomeACy
02350 H_greenApple CsomeACy
02355 -pf00210 -
02360 -pf00361pf00662 -
02370 -pf10216 -
02375 -pf01329 -
02380 -pf13439pf00534 -
02385 -pf05050 -
02390 -pf06445 -
02395 -# -
02400 -# -
02405 -pf01656 -
BMC-P
BMC-H
BMC-T
Aldehyde dehydrogenase
PTAC
Signature enzyme
Alcohol dehydrogenase
Regulator

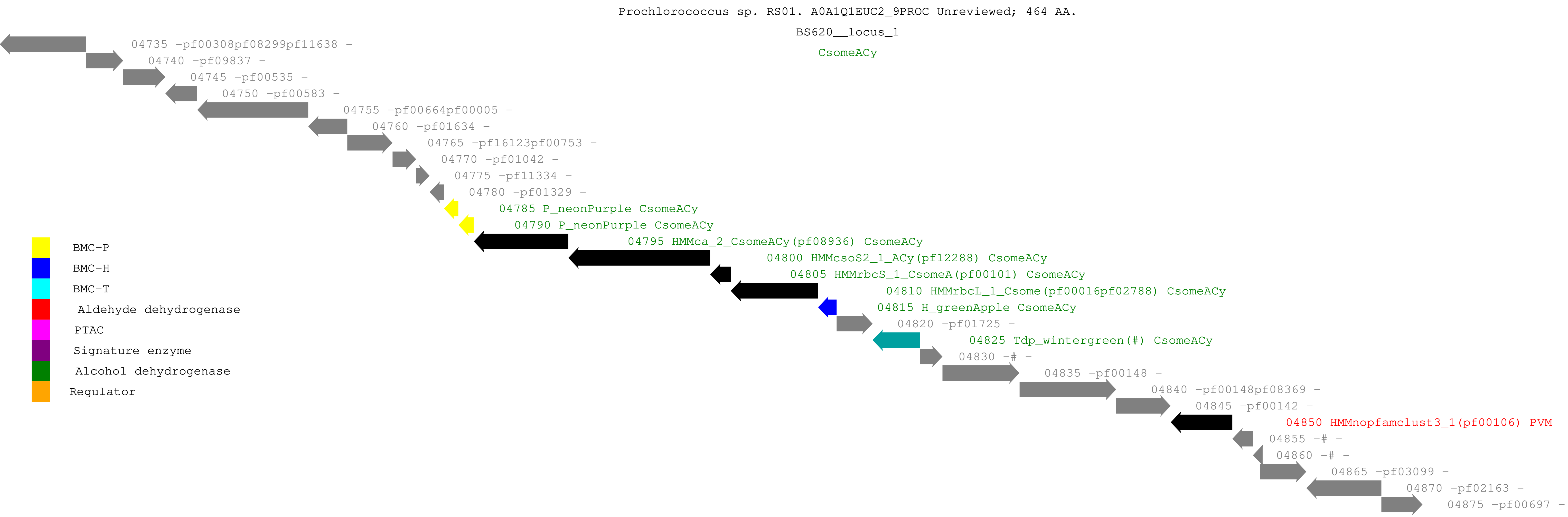

Prochlorococcus sp. RS01. A0A1Q1EUC2_9PROC Unreviewed; 464 AA.
BS620__locus_1
CsomeACy
04735 -pf00308pf08299pf11638 -
04740 -pf09837 -
04745 -pf00535 -
04750 -pf00583 -
04755 -pf00664pf00005 -
04760 -pf01634 -
04765 -pf16123pf00753 -
04770 -pf01042 -
04775 -pf11334 -
04780 -pf01329 -
04785 P_neonPurple CsomeACy
04790 P_neonPurple CsomeACy
04795 HMMca_2_CsomeACy(pf08936) CsomeACy
04800 HMMcsoS2_1_ACy(pf12288) CsomeACy
04805 HMMrbcS_1_CsomeA(pf00101) CsomeACy
04810 HMMrbcL_1_Csome(pf00016pf02788) CsomeACy
04815 H_greenApple CsomeACy
04820 -pf01725 -
04825 Tdp_wintergreen(#) CsomeACy
04830 -# -
04835 -pf00148 -
04840 -pf00148pf08369 -
04845 -pf00142 -
04850 HMMnopfamclust3_1(pf00106) PVM
04855 -# -
04860 -# -
04865 -pf03099 -
04870 -pf02163 -
04875 -pf00697 -
BMC-P
BMC-H
BMC-T
Aldehyde dehydrogenase
PTAC
Signature enzyme
Alcohol dehydrogenase
Regulator

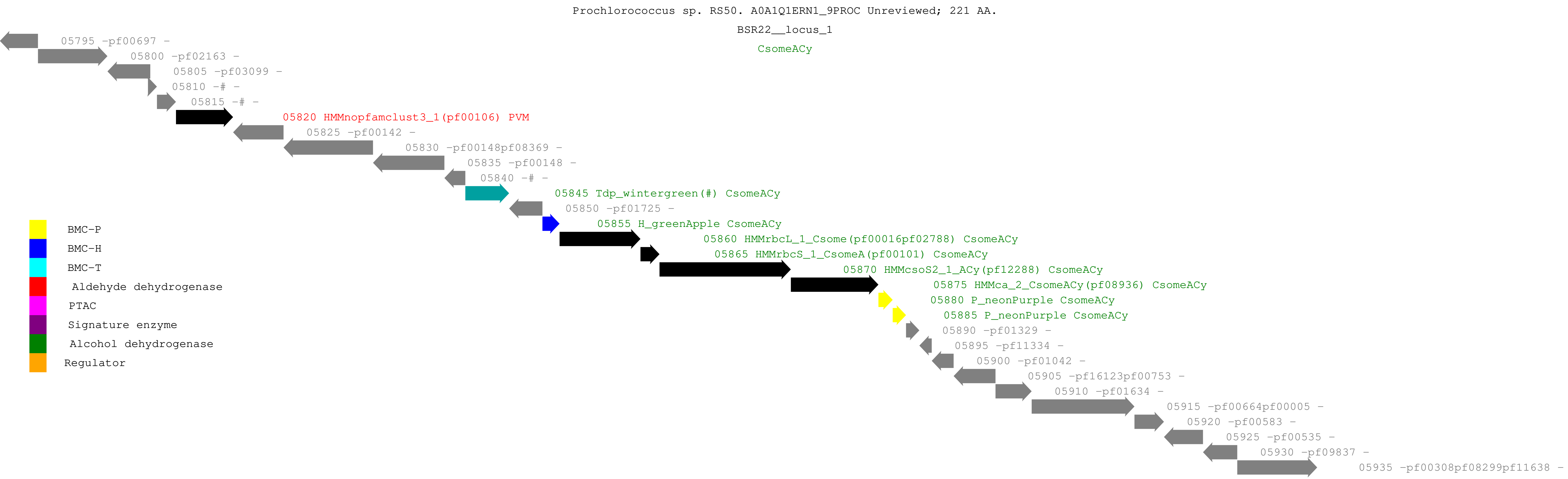
Prochlorococcus sp. RS50. A0A1Q1ERN1_9PROC Unreviewed; 221 AA.
BSR22__locus_1
CsomeACy
05795 -pf00697 -
05800 -pf02163 -
05805 -pf03099 -
05810 -# -
05815 -# -
05820 HMMnopfamclust3_1(pf00106) PVM
05825 -pf00142 -
05830 -pf00148pf08369 -
05835 -pf00148 -
05840 -# -
05845 Tdp_wintergreen(#) CsomeACy
05850 -pf01725 -
05855 H_greenApple CsomeACy
05860 HMMrbcL_1_Csome(pf00016pf02788) CsomeACy
05865 HMMrbcS_1_CsomeA(pf00101) CsomeACy
05870 HMMcsoS2_1_ACy(pf12288) CsomeACy
05875 HMMca_2_CsomeACy(pf08936) CsomeACy
05880 P_neonPurple CsomeACy
05885 P_neonPurple CsomeACy
05890 -pf01329 -
05895 -pf11334 -
05900 -pf01042 -
05905 -pf16123pf00753 -
05910 -pf01634 -
05915 -pf00664pf00005 -
05920 -pf00583 -
05925 -pf00535 -
05930 -pf09837 -
05935 -pf00308pf08299pf11638 -
BMC-P
BMC-H
BMC-T
Aldehyde dehydrogenase
PTAC
Signature enzyme
Alcohol dehydrogenase
Regulator

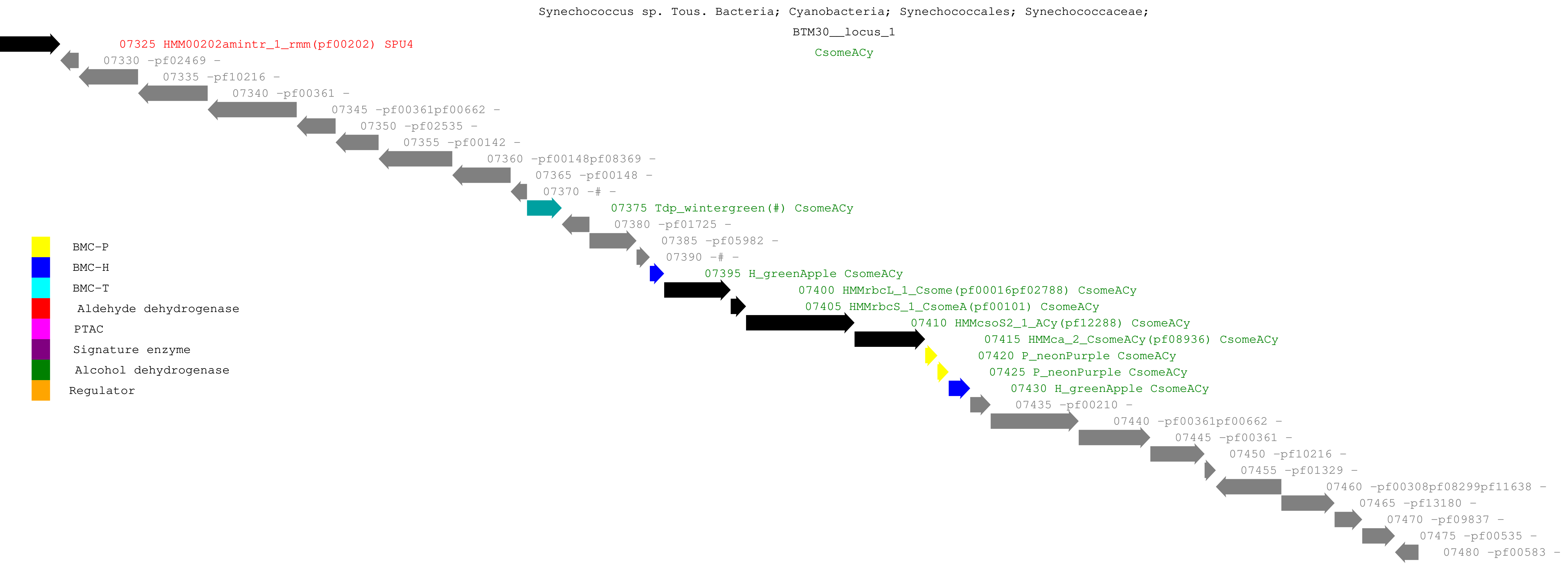
Synechococcus sp. Tous. Bacteria; Cyanobacteria; Synechococcales; Synechococcaceae;
BTM30__locus_1
CsomeACy
07325 HMM00202amintr_1_rmm(pf00202) SPU4
07330 -pf02469 -
07335 -pf10216 -
07340 -pf00361 -
07345 -pf00361pf00662 -
07350 -pf02535 -
07355 -pf00142 -
07360 -pf00148pf08369 -
07365 -pf00148 -
07370 -# -
07375 Tdp_wintergreen(#) CsomeACy
07380 -pf01725 -
07385 -pf05982 -
07390 -# -
07395 H_greenApple CsomeACy
07400 HMMrbcL_1_Csome(pf00016pf02788) CsomeACy
07405 HMMrbcS_1_CsomeA(pf00101) CsomeACy
07410 HMMcsoS2_1_ACy(pf12288) CsomeACy
07415 HMMca_2_CsomeACy(pf08936) CsomeACy
07420 P_neonPurple CsomeACy
07425 P_neonPurple CsomeACy
07430 H_greenApple CsomeACy
07435 -pf00210 -
07440 -pf00361pf00662 -
07445 -pf00361 -
07450 -pf10216 -
07455 -pf01329 -
07460 -pf00308pf08299pf11638 -
07465 -pf13180 -
07470 -pf09837 -
07475 -pf00535 -
07480 -pf00583 -
BMC-P
BMC-H
BMC-T
Aldehyde dehydrogenase
PTAC
Signature enzyme
Alcohol dehydrogenase
Regulator

Candidatus Synechococcus spongiarum LMB bulk15N. Bacteria; Cyanobacteria; Synechococcales; Synechococcaceae;

BV53__locus_1

CsomeACy

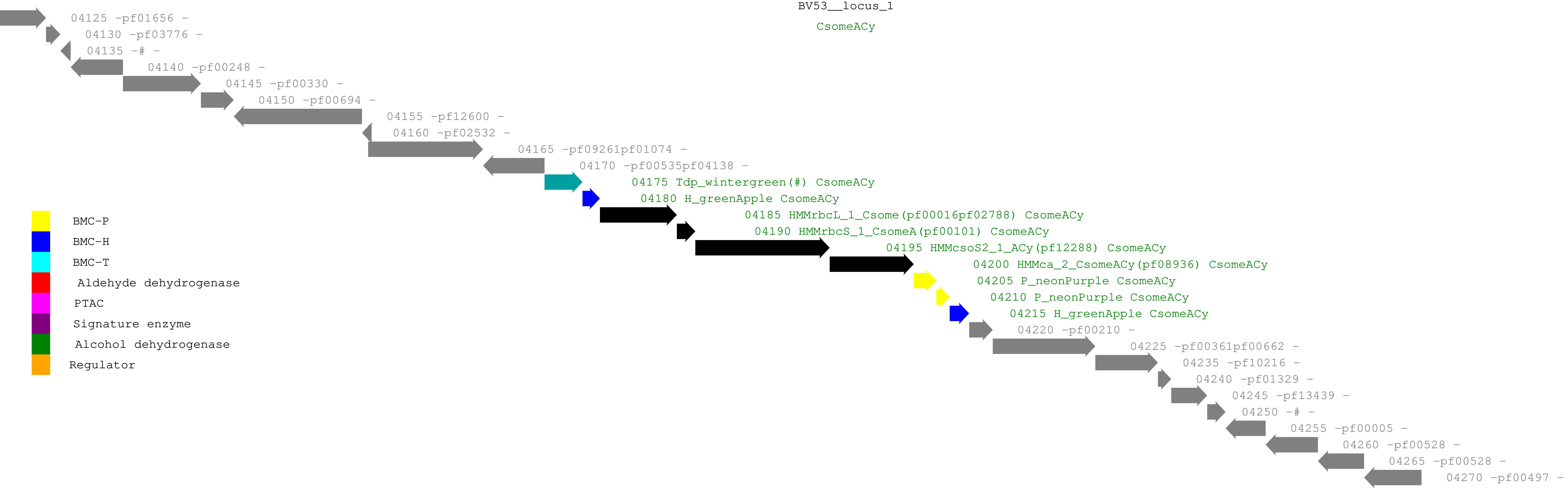

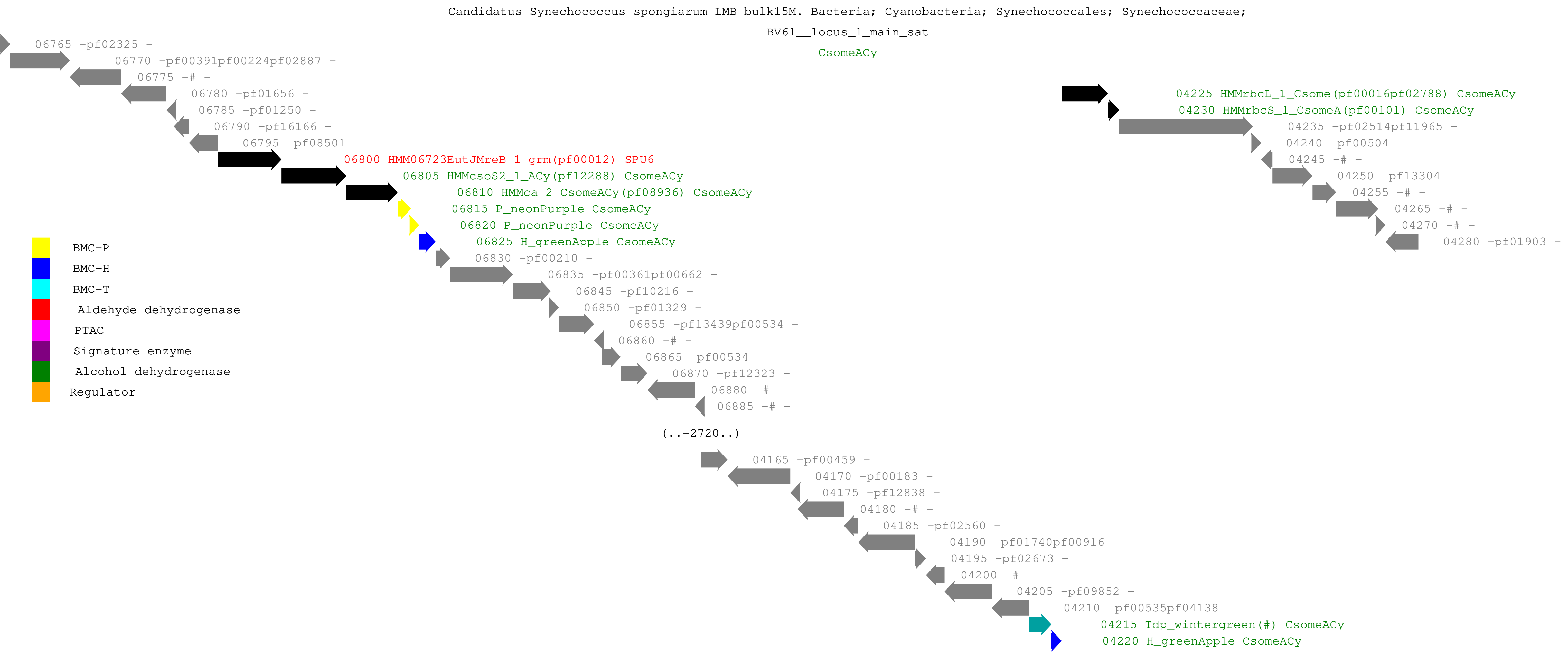
Candidatus Synechococcus spongiarum LMB bulk15M. Bacteria; Cyanobacteria; Synechococcales; Synechococcaceae;
BV61__locus_1_main_sat
CsomeACy
06765 -pf02325 -
06770 -pf00391pf00224pf02887 -
06775 -# -
06780 -pf01656 -
06785 -pf01250 -
06790 -pf16166 -
06795 -pf08501 -
06800 HMM06723EutJMreB_1_grm(pf00012) SPU6
06805 HMMcsoS2_1_ACy(pf12288) CsomeACy
06810 HMMca_2_CsomeACy(pf08936) CsomeACy
06815 P_neonPurple CsomeACy
06820 P_neonPurple CsomeACy
06825 H_greenApple CsomeACy
06830 -pf00210 -
06835 -pf00361pf00662 -
06845 -pf10216 -
06850 -pf01329 -
06855 -pf13439pf00534 -
06860 -# -
06865 -pf00534 -
06870 -pf12323 -
06880 -# -
06885 -# -
(..-2720..)
04165 -pf00459 -
04170 -pf00183 -
04175 -pf12838 -
04180 -# -
04185 -pf02560 -
04190 -pf01740pf00916 -
04195 -pf02673 -
04200 -# -
04205 -pf09852 -
04210 -pf00535pf04138 -
04215 Tdp_wintergreen(#) CsomeACy
04220 H_greenApple CsomeACy
04225 HMMrbcL_1_Csome(pf00016pf02788) CsomeACy
04230 HMMrbcS_1_CsomeA(pf00101) CsomeACy
04235 -pf02514pf11965 -
04240 -pf00504 -
04245 -# -
04250 -pf13304 -
04255 -# -
04265 -# -
04270 -# -
04280 -pf01903 -
BMC-P
BMC-H
BMC-T
Aldehyde dehydrogenase
PTAC
Signature enzyme
Alcohol dehydrogenase
Regulator

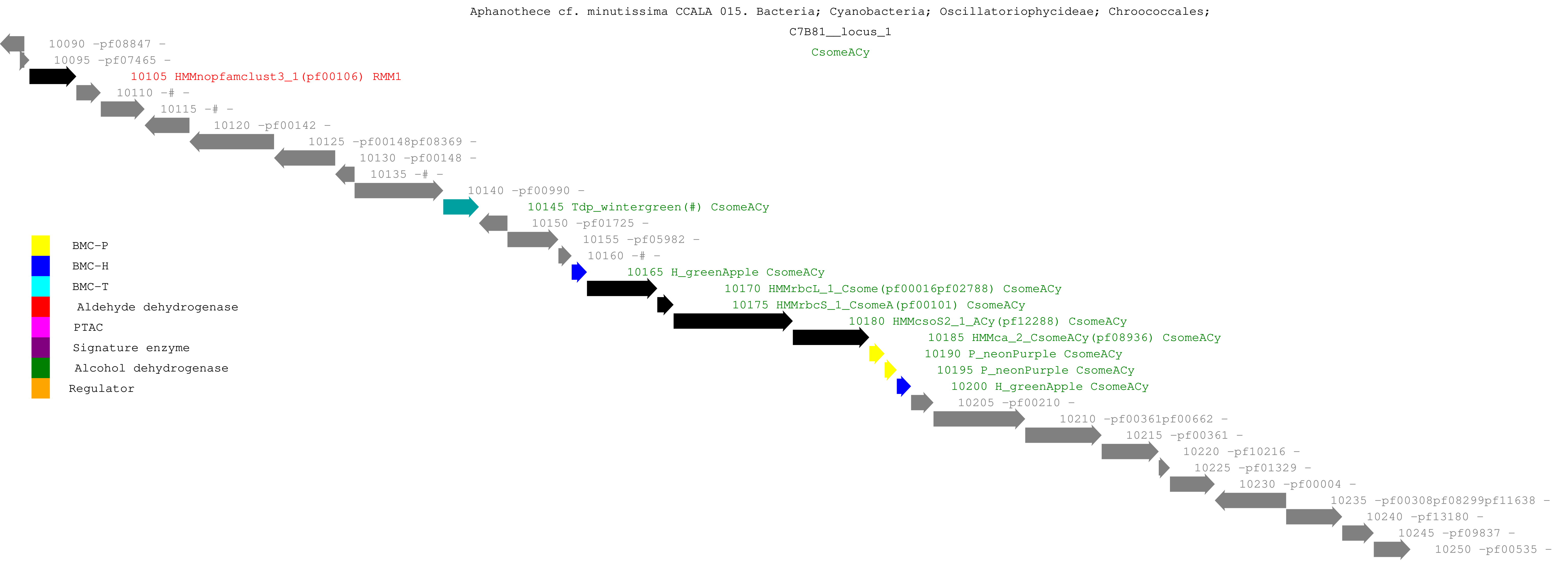
Aphanothece cf. minutissima CCALA 015. Bacteria; Cyanobacteria; Oscillatoriophycideae; Chroococcales;
C7B81__locus_1
CsomeACy
10090 -pf08847 -
10095 -pf07465 -
10105 HMMnopfamclust3_1(pf00106) RMM1
10110 -# -
10115 -# -
10120 -pf00142 -
10125 -pf00148pf08369 -
10130 -pf00148 -
10135 -# -
10140 -pf00990 -
10145 Tdp_wintergreen(#) CsomeACy
10150 -pf01725 -
10155 -pf05982 -
10160 -# -
10165 H_greenApple CsomeACy
10170 HMMrbcL_1_Csome(pf00016pf02788) CsomeACy
10175 HMMrbcS_1_CsomeA(pf00101) CsomeACy
10180 HMMcsoS2_1_ACy(pf12288) CsomeACy
10185 HMMca_2_CsomeACy(pf08936) CsomeACy
10190 P_neonPurple CsomeACy
10195 P_neonPurple CsomeACy
10200 H_greenApple CsomeACy
10205 -pf00210 -
10210 -pf00361pf00662 -
10215 -pf00361 -
10220 -pf10216 -
10225 -pf01329 -
10230 -pf00004 -
10235 -pf00308pf08299pf11638 -
10240 -pf13180 -
10245 -pf09837 -
10250 -pf00535 -
BMC-P
BMC-H
BMC-T
Aldehyde dehydrogenase
PTAC
Signature enzyme
Alcohol dehydrogenase
Regulator

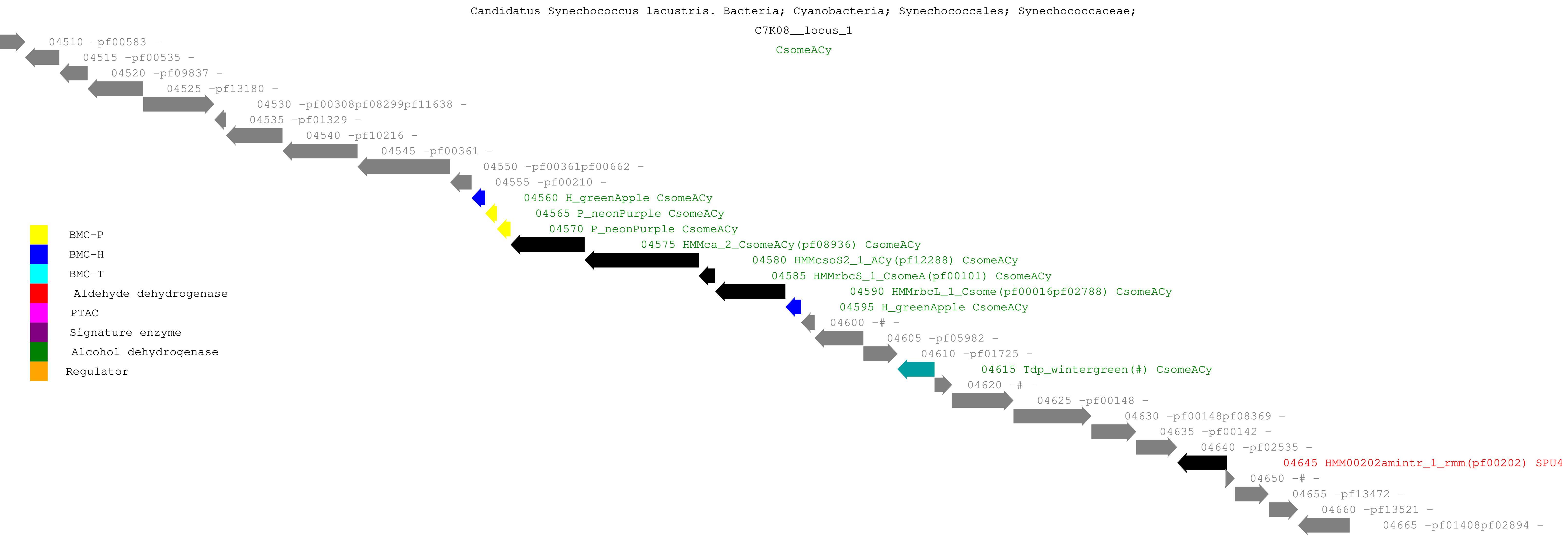
Candidatus Synechococcus lacustris. Bacteria; Cyanobacteria; Synechococcales; Synechococcaceae;
C7K08__locus_1
CsomeACy
04510 -pf00583 -
04515 -pf00535 -
04520 -pf09837 -
04525 -pf13180 -
04530 -pf00308pf08299pf11638 -
04535 -pf01329 -
04540 -pf10216 -
04545 -pf00361 -
04550 -pf00361pf00662 -
04555 -pf00210 -
04560 H_greenApple CsomeACy
04565 P_neonPurple CsomeACy
04570 P_neonPurple CsomeACy
04575 HMMca_2_CsomeACy(pf08936) CsomeACy
04580 HMMcsoS2_1_ACy(pf12288) CsomeACy
04585 HMMrbcS_1_CsomeA(pf00101) CsomeACy
04590 HMMrbcL_1_Csome(pf00016pf02788) CsomeACy
04595 H_greenApple CsomeACy
04600 -# -
04605 -pf05982 -
04610 -pf01725 -
04615 Tdp_wintergreen(#) CsomeACy
04620 -# -
04625 -pf00148 -
04630 -pf00148pf08369 -
04635 -pf00142 -
04640 -pf02535 -
04645 HMM00202amintr_1_rmm(pf00202) SPU4
04650 -# -
04655 -pf13472 -
04660 -pf13521 -
04665 -pf01408pf02894 -
BMC-P
BMC-H
BMC-T
Aldehyde dehydrogenase
PTAC
Signature enzyme
Alcohol dehydrogenase
Regulator

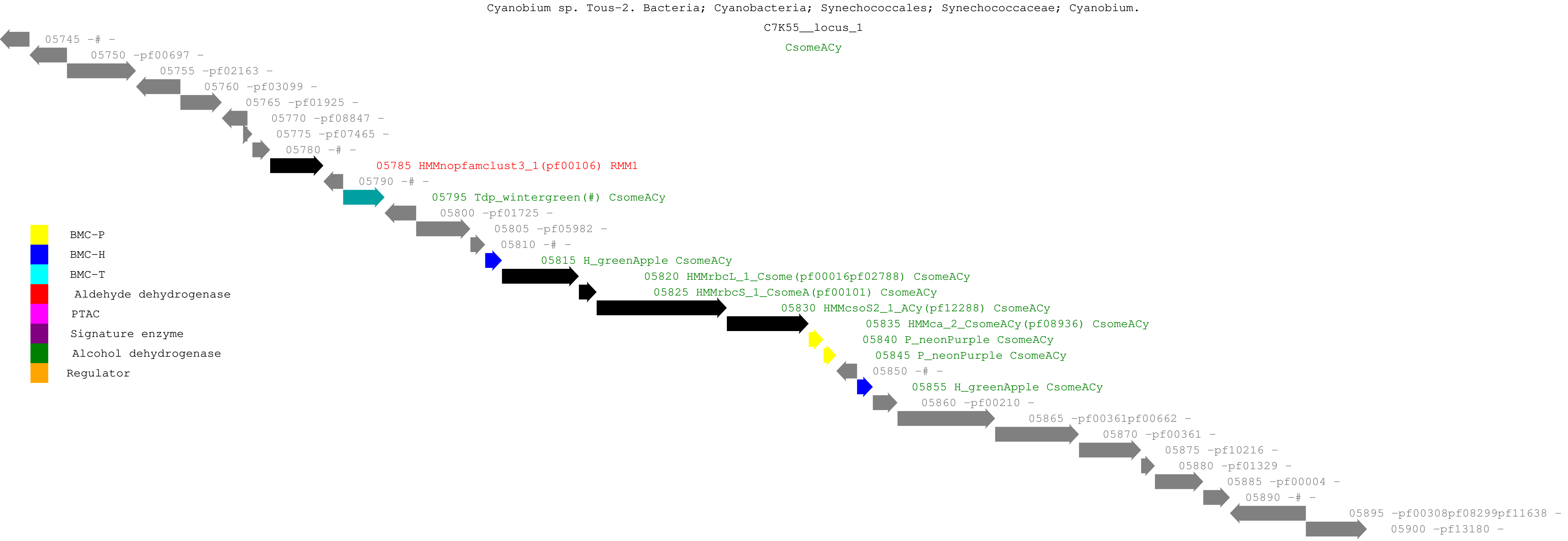

Cyanobium sp. Tous-2. Bacteria; Cyanobacteria; Synechococcales; Synechococcaceae; Cyanobium.
C7K55__locus_1
CsomeACy
05745 -# -
05750 -pf00697 -
05755 -pf02163 -
05760 -pf03099 -
05765 -pf01925 -
05770 -pf08847 -
05775 -pf07465 -
05780 -# -
05785 HMMnopfamclust3_1(pf00106) RMM1
05790 -# -
05795 Tdp_wintergreen(#) CsomeACy
05800 -pf01725 -
05805 -pf05982 -
05810 -# -
05815 H_greenApple CsomeACy
05820 HMMrbcL_1_Csome(pf00016pf02788) CsomeACy
05825 HMMrbcS_1_CsomeA(pf00101) CsomeACy
05830 HMMcsoS2_1_ACy(pf12288) CsomeACy
05835 HMMca_2_CsomeACy(pf08936) CsomeACy
05840 P_neonPurple CsomeACy
05845 P_neonPurple CsomeACy
05850 -# -
05855 H_greenApple CsomeACy
05860 -pf00210 -
05865 -pf00361pf00662 -
05870 -pf00361 -
05875 -pf10216 -
05880 -pf01329 -
05885 -pf00004 -
05890 -# -
05895 -pf00308pf08299pf11638 -
05900 -pf13180 -
BMC-P
BMC-H
BMC-T
Aldehyde dehydrogenase
PTAC
Signature enzyme
Alcohol dehydrogenase
Regulator

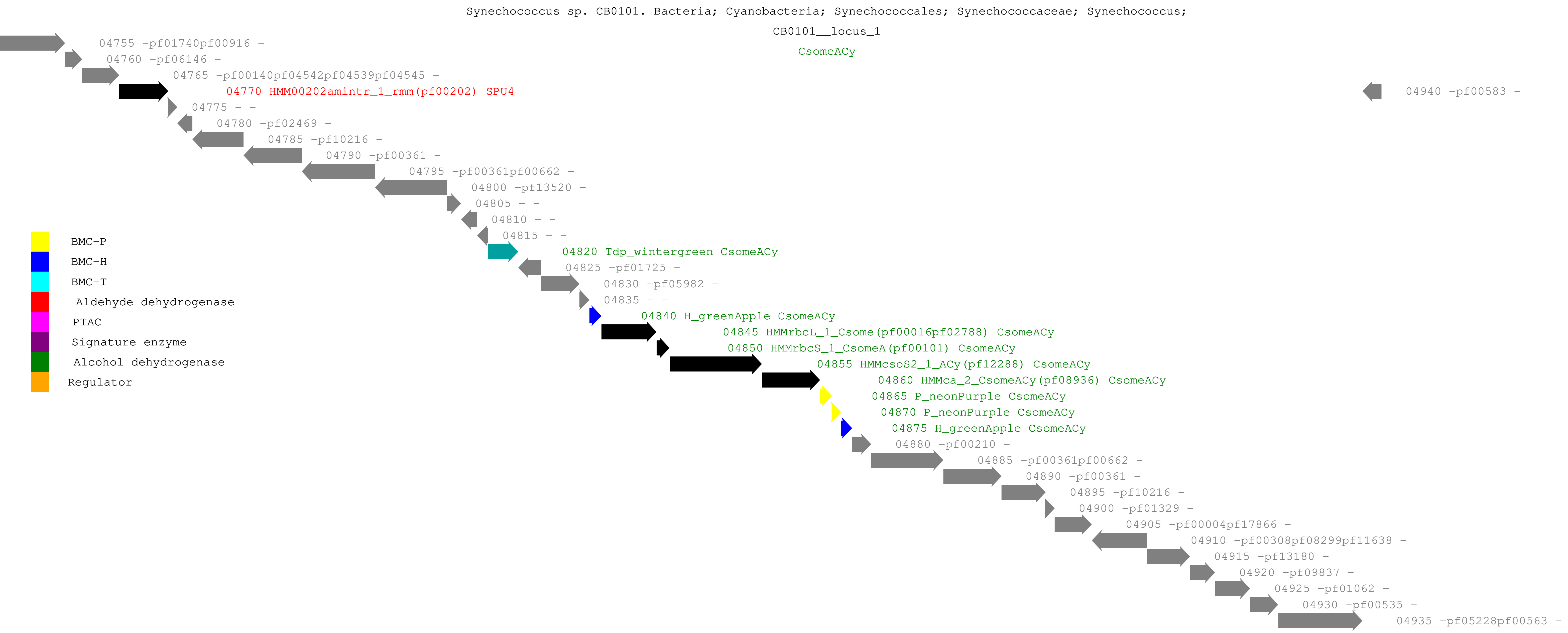
Synechococcus sp. CB0101. Bacteria; Cyanobacteria; Synechococcales; Synechococcaceae; Synechococcus;
CB0101__locus_1
CsomeACy
04755 -pf01740pf00916 -
04760 -pf06146 -
04765 -pf00140pf04542pf04539pf04545 -
04770 HMM00202amintr_1_rmm(pf00202) SPU4
04775 - -
04780 -pf02469 -
04785 -pf10216 -
04790 -pf00361 -
04795 -pf00361pf00662 -
04800 -pf13520 -
04805 - -
04810 - -
04815 - -
04820 Tdp_wintergreen CsomeACy
04825 -pf01725 -
04830 -pf05982 -
04835 - -
04840 H_greenApple CsomeACy
04845 HMMrbcL_1_Csome(pf00016pf02788) CsomeACy
04850 HMMrbcS_1_CsomeA(pf00101) CsomeACy
04855 HMMcsoS2_1_ACy(pf12288) CsomeACy
04860 HMMca_2_CsomeACy(pf08936) CsomeACy
04865 P_neonPurple CsomeACy
04870 P_neonPurple CsomeACy
04875 H_greenApple CsomeACy
04880 -pf00210 -
04885 -pf00361pf00662 -
04890 -pf00361 -
04895 -pf10216 -
04900 -pf01329 -
04905 -pf00004pf17866 -
04910 -pf00308pf08299pf11638 -
04915 -pf13180 -
04920 -pf09837 -
04925 -pf01062 -
04930 -pf00535 -
04935 -pf05228pf00563 -
04940 -pf00583 -
BMC-P
BMC-H
BMC-T
Aldehyde dehydrogenase
PTAC
Signature enzyme
Alcohol dehydrogenase
Regulator

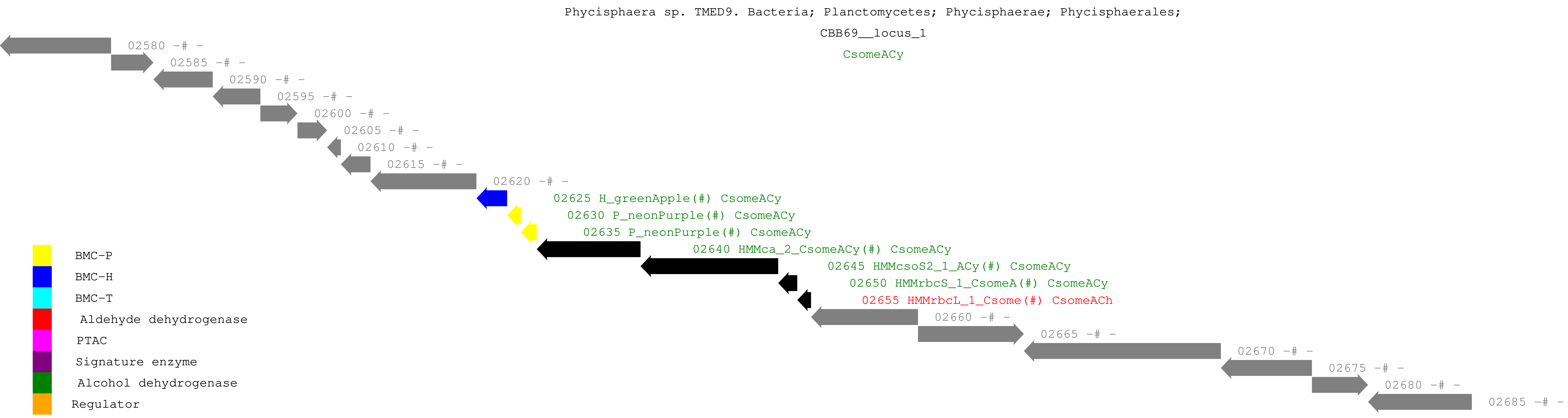
Phycisphaera sp. TMED9. Bacteria; Planctomycetes; Phycisphaerae; Phycisphaerales;
CBB69__locus_1
CsomeACy
02580 -# -
02585 -# -
02590 -# -
02595 -# -
02600 -# -
02605 -# -
02610 -# -
02615 -# -
02620 -# -
02625 H_greenApple(#) CsomeACy
02630 P_neonPurple(#) CsomeACy
02635 P_neonPurple(#) CsomeACy
02640 HMMca_2_CsomeACy(#) CsomeACy
02645 HMMcsoS2_1_ACy(#) CsomeACy
02650 HMMrbcS_1_CsomeA(#) CsomeACy
02655 HMMrbcL_1_Csome(#) CsomeACh
02660 -# -
02665 -# -
02670 -# -
02675 -# -
02680 -# -
02685 -# -
BMC-P
BMC-H
BMC-T
Aldehyde dehydrogenase
PTAC
Signature enzyme
Alcohol dehydrogenase
Regulator

Synechococcus sp. TMED19. Bacteria; Cyanobacteria; Synechococcales; Synechococcaceae;

CBB79__locus_1

CsomeACy

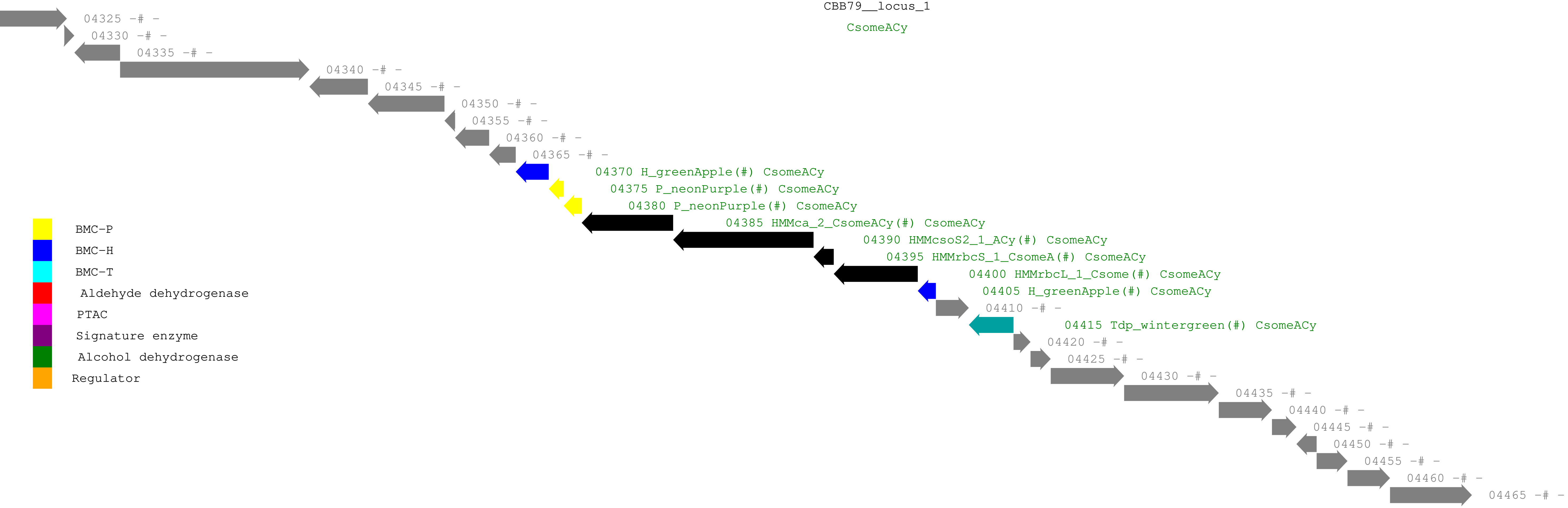

Synechococcus sp. TMED20. Bacteria; Cyanobacteria; Synechococcales; Synechococcaceae; Synechococcus;

CBB80__locus_1

CsomeACy

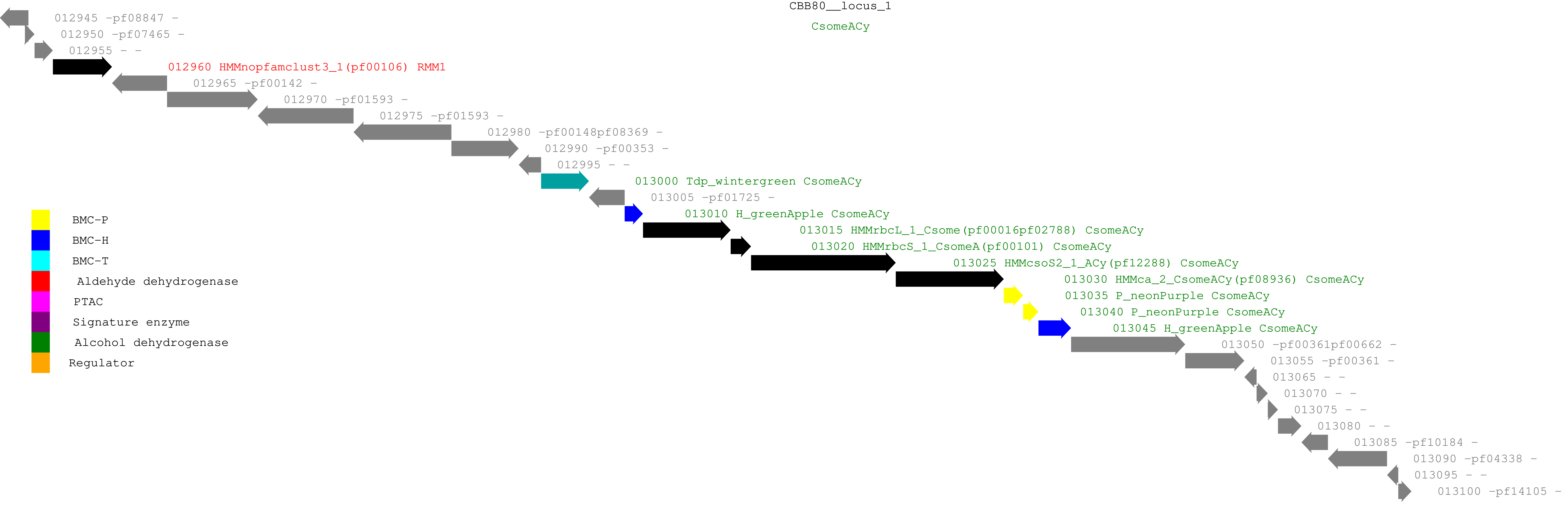

Synechococcus sp. TMED66. Bacteria; Cyanobacteria; Synechococcales; Synechococcaceae;

CBC26__locus_1_main_sat

CsomeACy

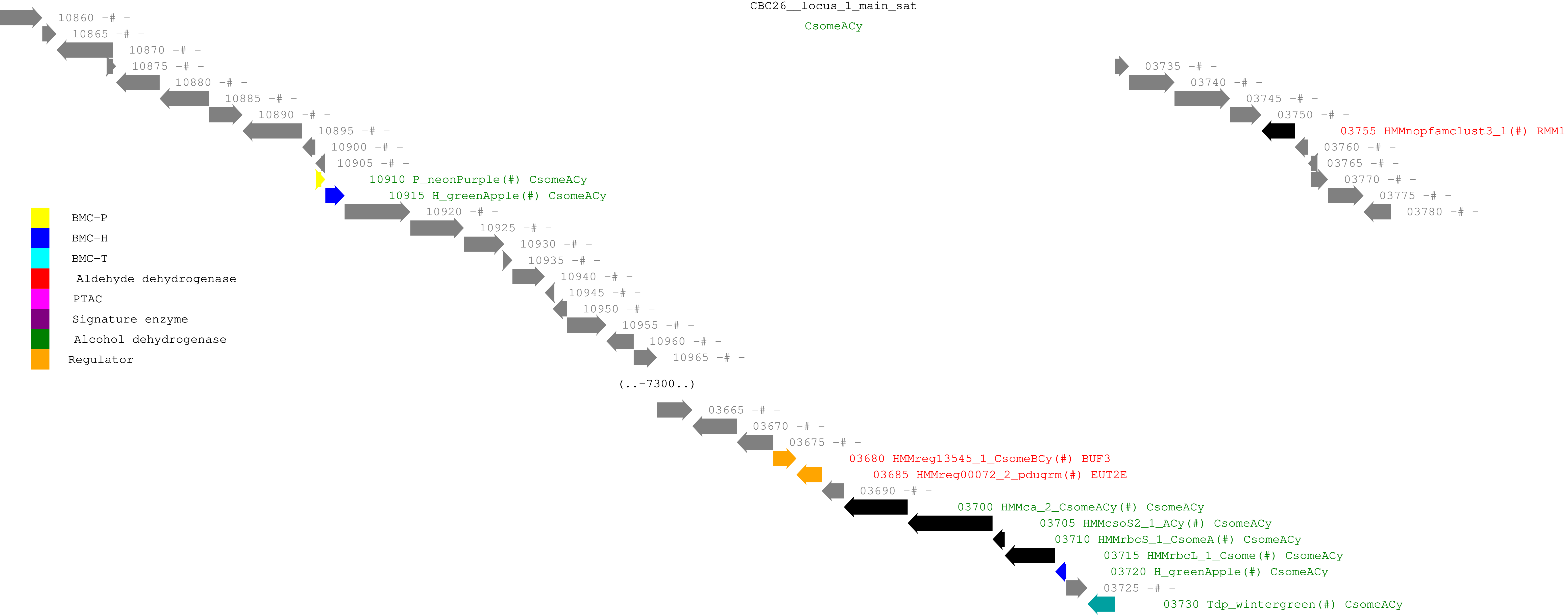

Synechococcus sp. TMED90. Bacteria; Cyanobacteria; Synechococcales; Synechococcaceae;

CBC50__locus_1_main_sat

CsomeACy

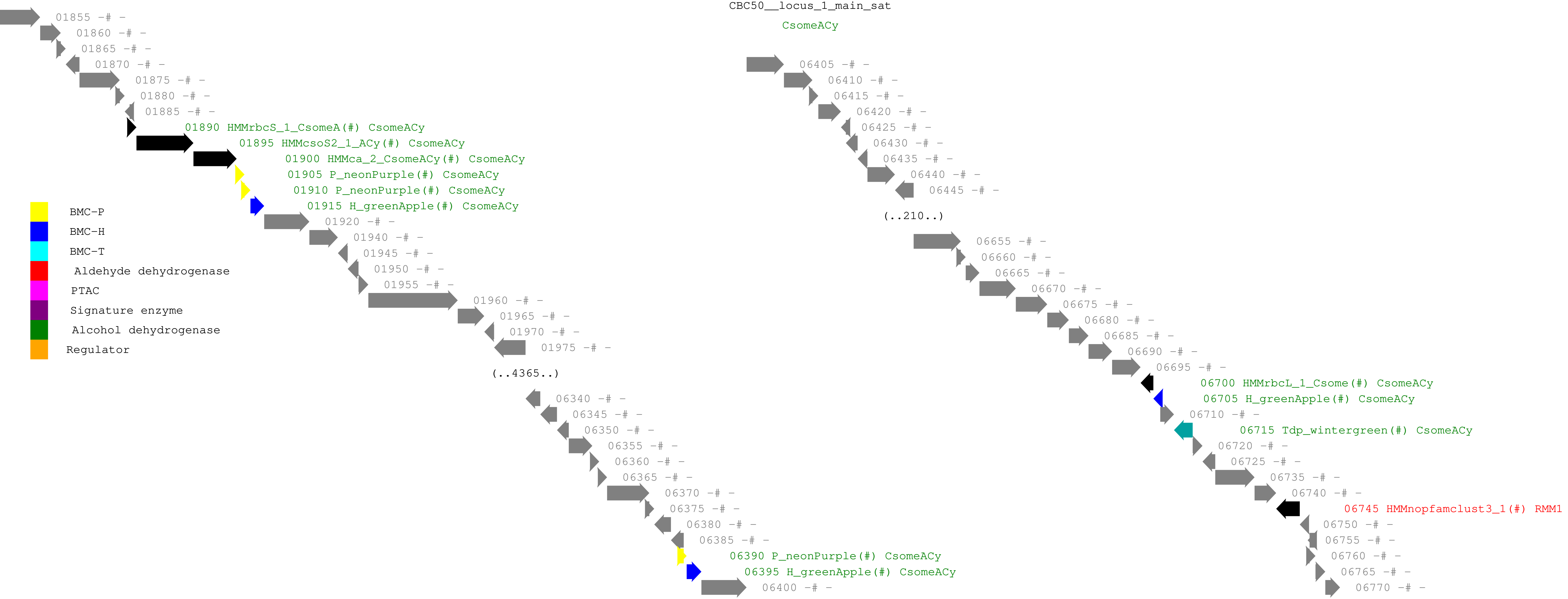

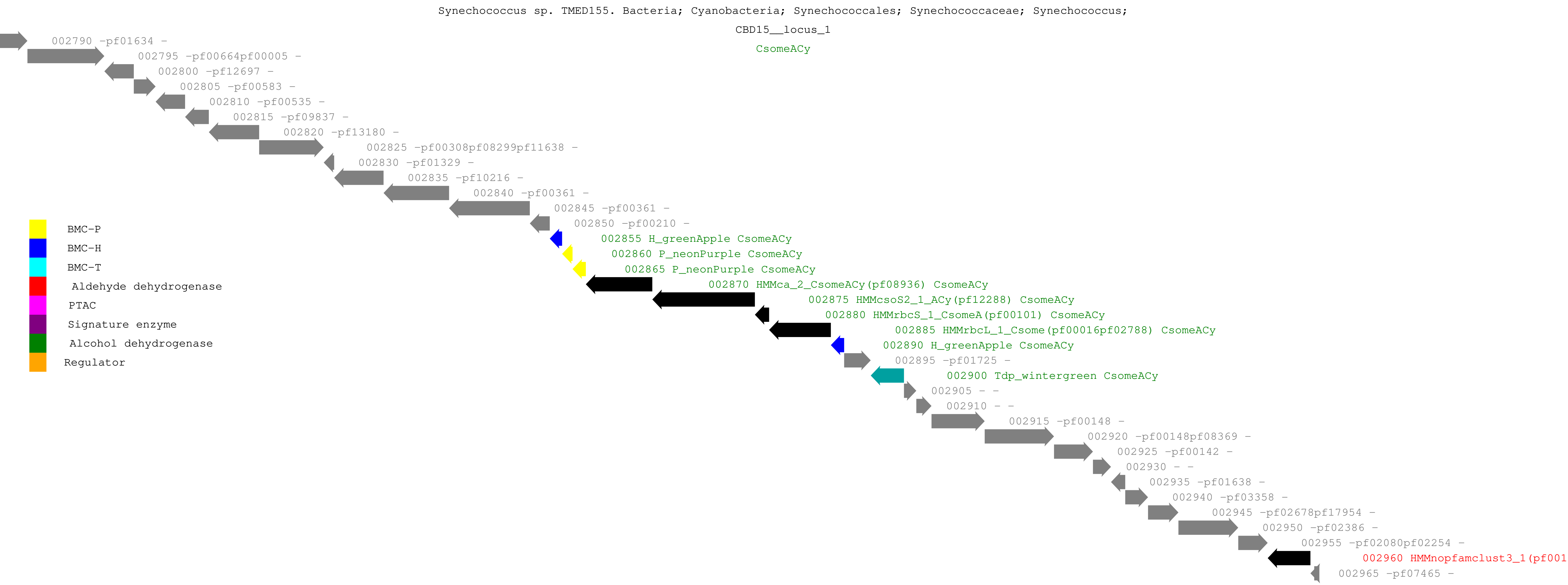
Synechococcus sp. TMED155. Bacteria; Cyanobacteria; Synechococcales; Synechococcaceae; Synechococcus;
CBD15__locus_1
CsomeACy
002790 -pf01634 -
002795 -pf00664pf00005 -
002800 -pf12697 -
002805 -pf00583 -
002810 -pf00535 -
002815 -pf09837 -
002820 -pf13180 -
002825 -pf00308pf08299pf11638 -
002830 -pf01329 -
002835 -pf10216 -
002840 -pf00361 -
002845 -pf00361 -
002850 -pf00210 -
002855 H_greenApple CsomeACy
002860 P_neonPurple CsomeACy
002865 P_neonPurple CsomeACy
002870 HMMca_2_CsomeACy(pf08936) CsomeACy
002875 HMMcsoS2_1_ACy(pf12288) CsomeACy
002880 HMMrbcS_1_CsomeA(pf00101) CsomeACy
002885 HMMrbcL_1_Csome(pf00016pf02788) CsomeACy
002890 H_greenApple CsomeACy
002895 -pf01725 -
002900 Tdp_wintergreen CsomeACy
002905 - -
002910 - -
002915 -pf00148 -
002920 -pf00148pf08369 -
002925 -pf00142 -
002930 - -
002935 -pf01638 -
002940 -pf03358 -
002945 -pf02678pf17954 -
002950 -pf02386 -
002955 -pf02080pf02254 -
002960 HMMnopfamclust3_1(pf001
002965 -pf07465 -
BMC-P
BMC-H
BMC-T
Aldehyde dehydrogenase
PTAC
Signature enzyme
Alcohol dehydrogenase
Regulator

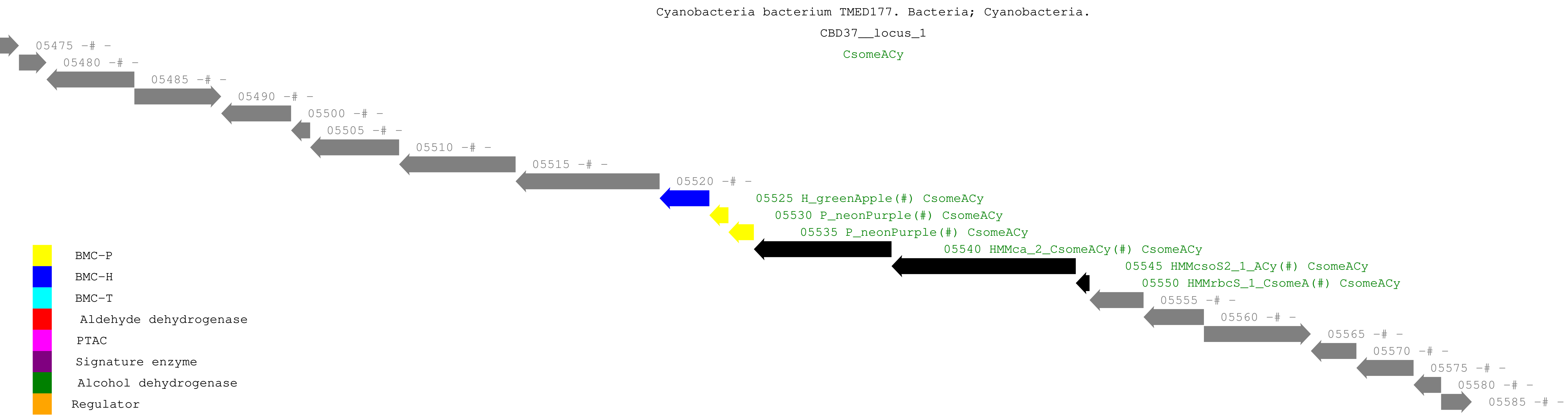
Cyanobacteria bacterium TMED177. Bacteria; Cyanobacteria.
CBD37__locus_1
CsomeACy
05475 -# -
05480 -# -
05485 -# -
05490 -# -
05500 -# -
05505 -# -
05510 -# -
05515 -# -
05520 -# -
05525 H_greenApple(#) CsomeACy
05530 P_neonPurple(#) CsomeACy
05535 P_neonPurple(#) CsomeACy
05540 HMMca_2_CsomeACy(#) CsomeACy
05545 HMMcsoS2_1_ACy(#) CsomeACy
05550 HMMrbcS_1_CsomeA(#) CsomeACy
05555 -# -
05560 -# -
05565 -# -
05570 -# -
05575 -# -
05580 -# -
05585 -# -
BMC-P
BMC-H
BMC-T
Aldehyde dehydrogenase
PTAC
Signature enzyme
Alcohol dehydrogenase
Regulator

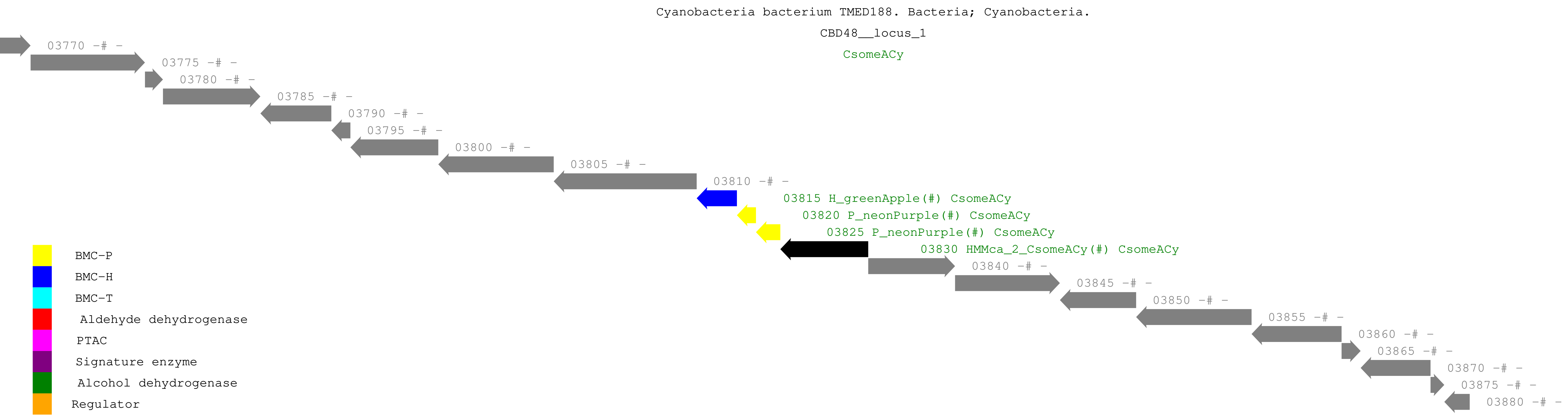
Cyanobacteria bacterium TMED188. Bacteria; Cyanobacteria.
CBD48__locus_1
CsomeACy
03770 -# -
03775 -# -
03780 -# -
03785 -# -
03790 -# -
03795 -# -
03800 -# -
03805 -# -
03810 -# -
03815 H_greenApple(#) CsomeACy
03820 P_neonPurple(#) CsomeACy
03825 P_neonPurple(#) CsomeACy
03830 HMMca_2_CsomeACy(#) CsomeACy
03840 -# -
03845 -# -
03850 -# -
03855 -# -
03860 -# -
03865 -# -
03870 -# -
03875 -# -
03880 -# -
BMC-P
BMC-H
BMC-T
Aldehyde dehydrogenase
PTAC
Signature enzyme
Alcohol dehydrogenase
Regulator

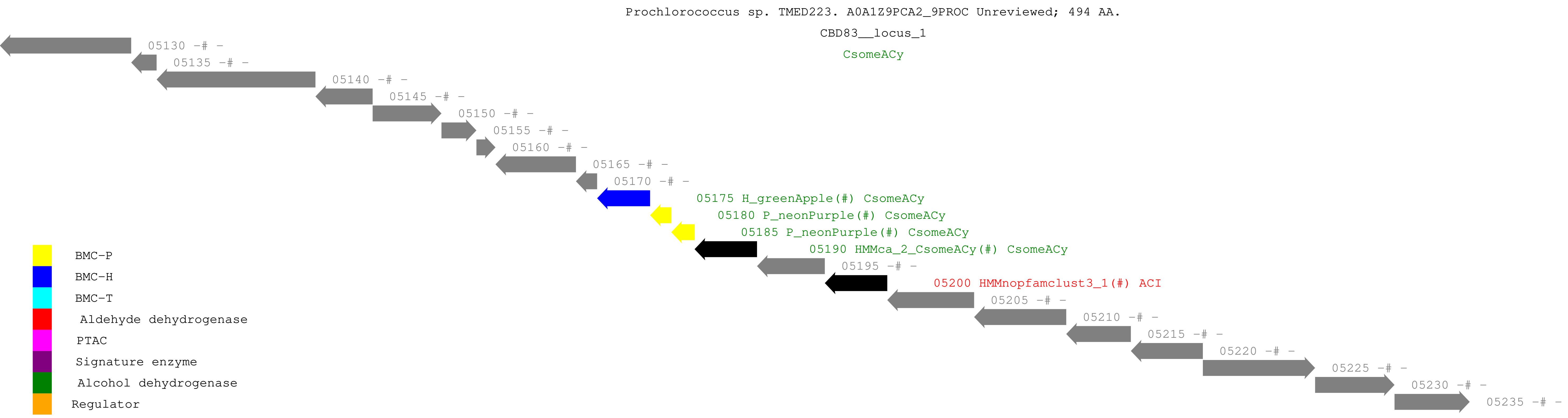
Prochlorococcus sp. TMED223. A0A1Z9PCA2_9PROC Unreviewed; 494 AA.
CBD83__locus_1
CsomeACy
05130 -# -
05135 -# -
05140 -# -
05145 -# -
05150 -# -
05155 -# -
05160 -# -
05165 -# -
05170 -# -
05175 H_greenApple(#) CsomeACy
05180 P_neonPurple(#) CsomeACy
05185 P_neonPurple(#) CsomeACy
05190 HMMca_2_CsomeACy(#) CsomeACy
05195 -# -
05200 HMMnopfamclust3_1(#) ACI
05205 -# -
05210 -# -
05215 -# -
05220 -# -
05225 -# -
05230 -# -
05235 -# -
BMC-P
BMC-H
BMC-T
Aldehyde dehydrogenase
PTAC
Signature enzyme
Alcohol dehydrogenase
Regulator

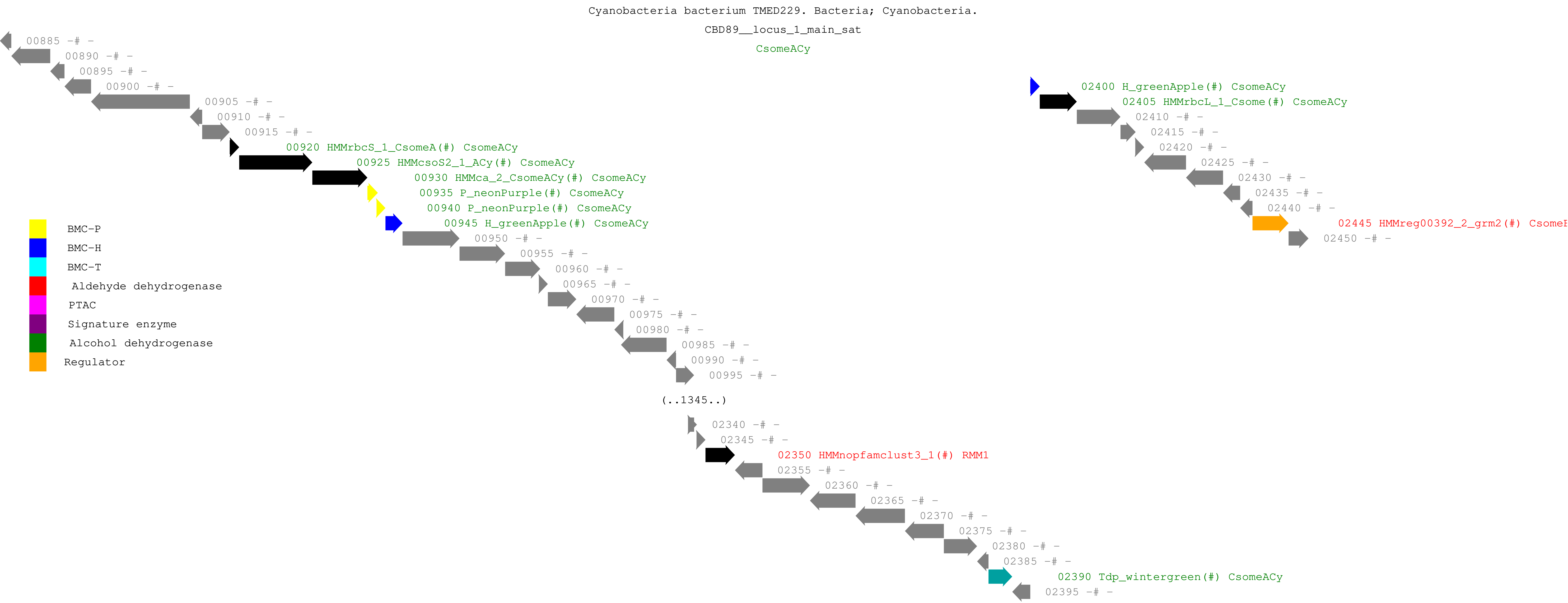
Cyanobacteria bacterium TMED229. Bacteria; Cyanobacteria.
CBD89__locus_1_main_sat
CsomeACy
00885 -# -
00890 -# -
00895 -# -
00900 -# -
00905 -# -
00910 -# -
00915 -# -
00920 HMMrbcS_1_CsomeA(#) CsomeACy
00925 HMMcsoS2_1_ACy(#) CsomeACy
00930 HMMca_2_CsomeACy(#) CsomeACy
00935 P_neonPurple(#) CsomeACy
00940 P_neonPurple(#) CsomeACy
00945 H_greenApple(#) CsomeACy
00950 -# -
00955 -# -
00960 -# -
00965 -# -
00970 -# -
00975 -# -
00980 -# -
00985 -# -
00990 -# -
00995 -# -
(..1345..)
02340 -# -
02345 -# -
02350 HMMnopfamclust3_1(#) RMM1
02355 -# -
02360 -# -
02365 -# -
02370 -# -
02375 -# -
02380 -# -
02385 -# -
02390 Tdp_wintergreen(#) CsomeACy
02395 -# -
02400 H_greenApple(#) CsomeACy
02405 HMMrbcL_1_Csome(#) CsomeACy
02410 -# -
02415 -# -
02420 -# -
02425 -# -
02430 -# -
02435 -# -
02440 -# -
02445 HMMreg00392_2_grm2(#) CsomeE
02450 -# -
BMC-P
BMC-H
BMC-T
Aldehyde dehydrogenase
PTAC
Signature enzyme
Alcohol dehydrogenase
Regulator

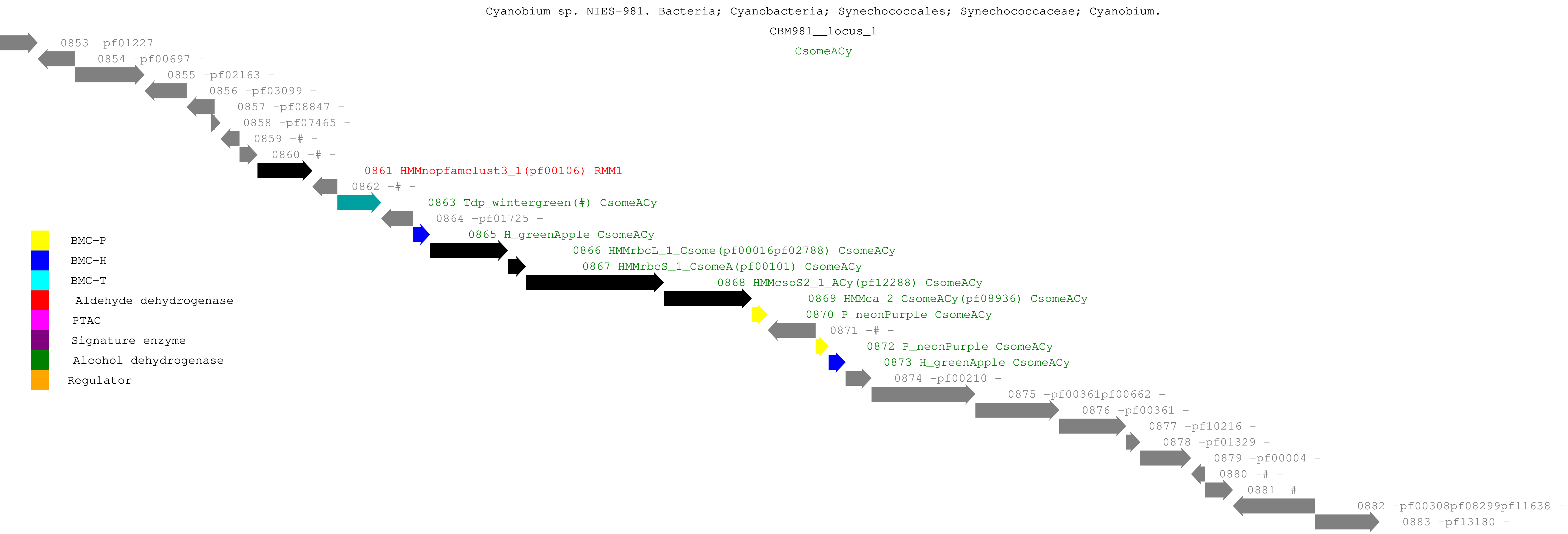

Cyanobium sp. NIES-981. Bacteria; Cyanobacteria; Synechococcales; Synechococcaceae; Cyanobium.
CBM981__locus_1
CsomeACy
0853 -pf01227 -
0854 -pf00697 -
0855 -pf02163 -
0856 -pf03099 -
0857 -pf08847 -
0858 -pf07465 -
0859 -# -
0860 -# -
0861 HMMnopfamclust3_1(pf00106) RMM1
0862 -# -
0863 Tdp_wintergreen(#) CsomeACy
0864 -pf01725 -
0865 H_greenApple CsomeACy
0866 HMMrbcL_1_Csome(pf00016pf02788) CsomeACy
0867 HMMrbcS_1_CsomeA(pf00101) CsomeACy
0868 HMMcsoS2_1_ACy(pf12288) CsomeACy
0869 HMMca_2_CsomeACy(pf08936) CsomeACy
0870 P_neonPurple CsomeACy
0871 -# -
0872 P_neonPurple CsomeACy
0873 H_greenApple CsomeACy
0874 -pf00210 -
0875 -pf00361pf00662 -
0876 -pf00361 -
0877 -pf10216 -
0878 -pf01329 -
0879 -pf00004 -
0880 -# -
0881 -# -
0882 -pf00308pf08299pf11638 -
0883 -pf13180 -
BMC-P
BMC-H
BMC-T
Aldehyde dehydrogenase
PTAC
Signature enzyme
Alcohol dehydrogenase
Regulator

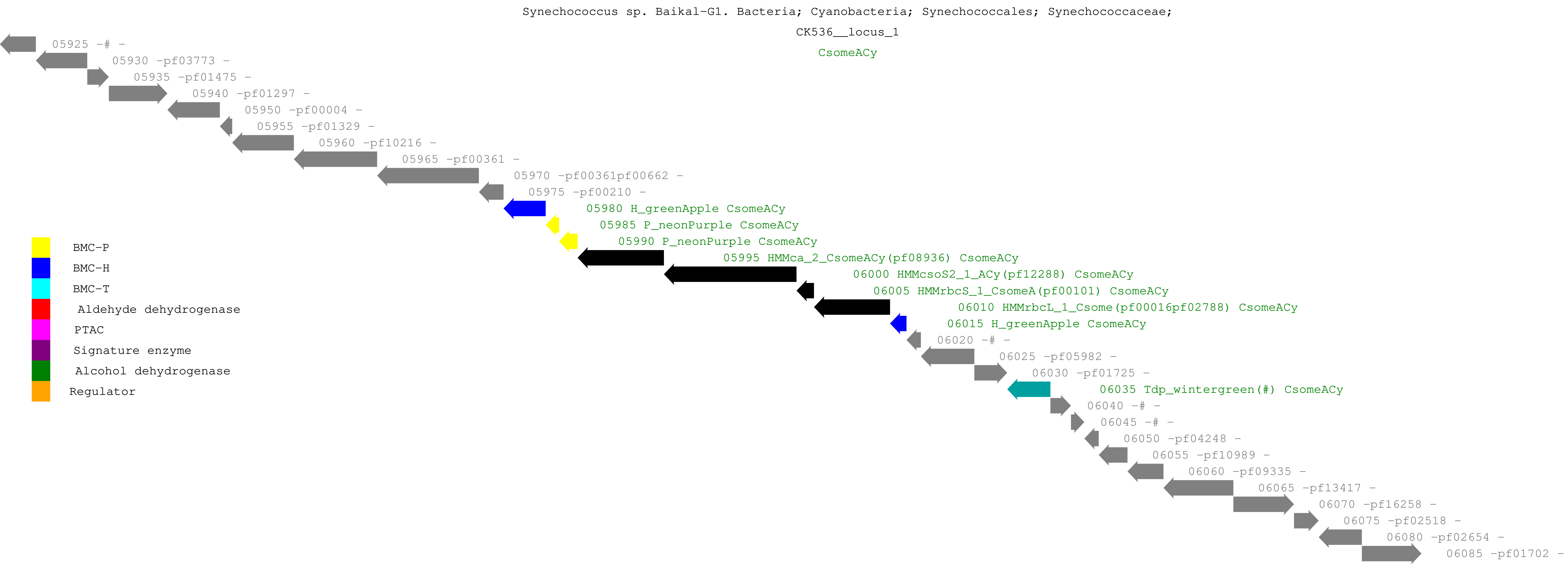
Synechococcus sp. Baikal-G1. Bacteria; Cyanobacteria; Synechococcales; Synechococcaceae;
CK536__locus_1
CsomeACy
05925 -# -
05930 -pf03773 -
05935 -pf01475 -
05940 -pf01297 -
05950 -pf00004 -
05955 -pf01329 -
05960 -pf10216 -
05965 -pf00361 -
05970 -pf00361pf00662 -
05975 -pf00210 -
05980 H_greenApple CsomeACy
05985 P_neonPurple CsomeACy
05990 P_neonPurple CsomeACy
05995 HMMca_2_CsomeACy(pf08936) CsomeACy
06000 HMMcsoS2_1_ACy(pf12288) CsomeACy
06005 HMMrbcS_1_CsomeA(pf00101) CsomeACy
06010 HMMrbcL_1_Csome(pf00016pf02788) CsomeACy
06015 H_greenApple CsomeACy
06020 -# -
06025 -pf05982 -
06030 -pf01725 -
06035 Tdp_wintergreen(#) CsomeACy
06040 -# -
06045 -# -
06050 -pf04248 -
06055 -pf10989 -
06060 -pf09335 -
06065 -pf13417 -
06070 -pf16258 -
06075 -pf02518 -
06080 -pf02654 -
06085 -pf01702 -
BMC-P
BMC-H
BMC-T
Aldehyde dehydrogenase
PTAC
Signature enzyme
Alcohol dehydrogenase
Regulator

Cyanobium sp. Baikal-G2. Bacteria; Cyanobacteria; Synechococcales; Synechococcaceae; Cyanobium.

CK549__locus_1_main_sat

CsomeACy

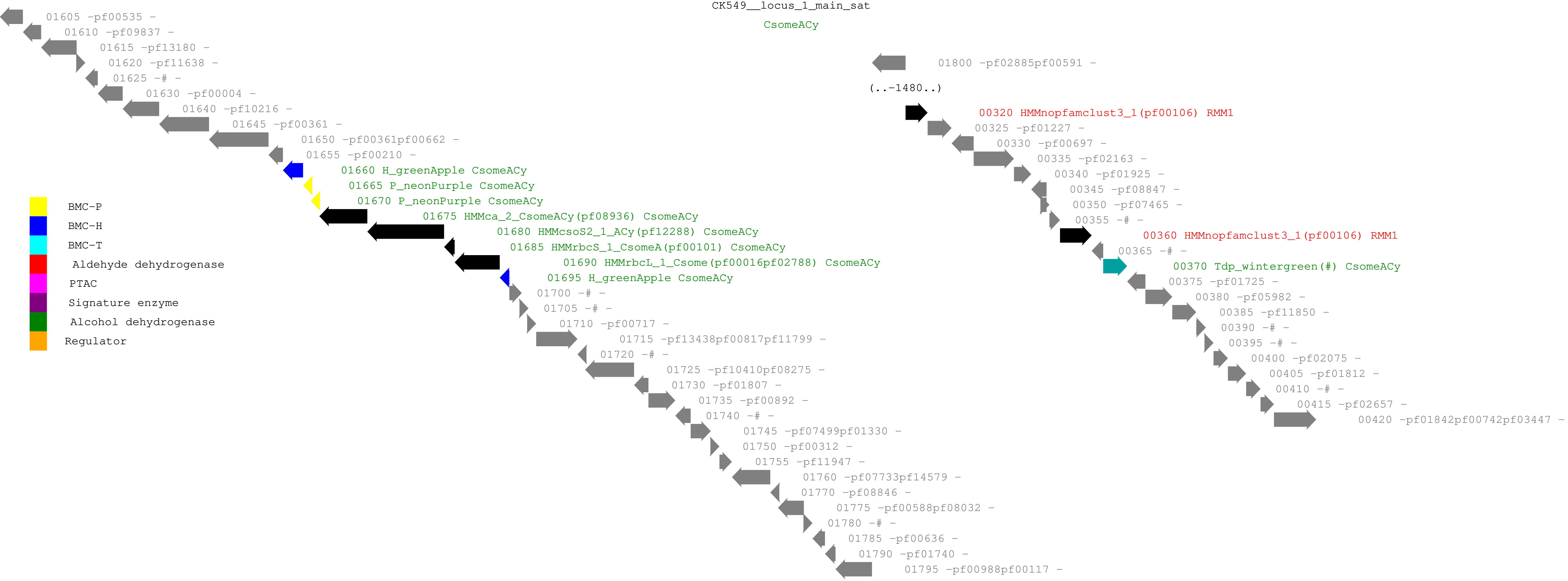

Cyanobium sp. NAT70. Bacteria; Cyanobacteria; Synechococcales; Synechococcaceae; Cyanobium.

CL862__locus_1_main_sat

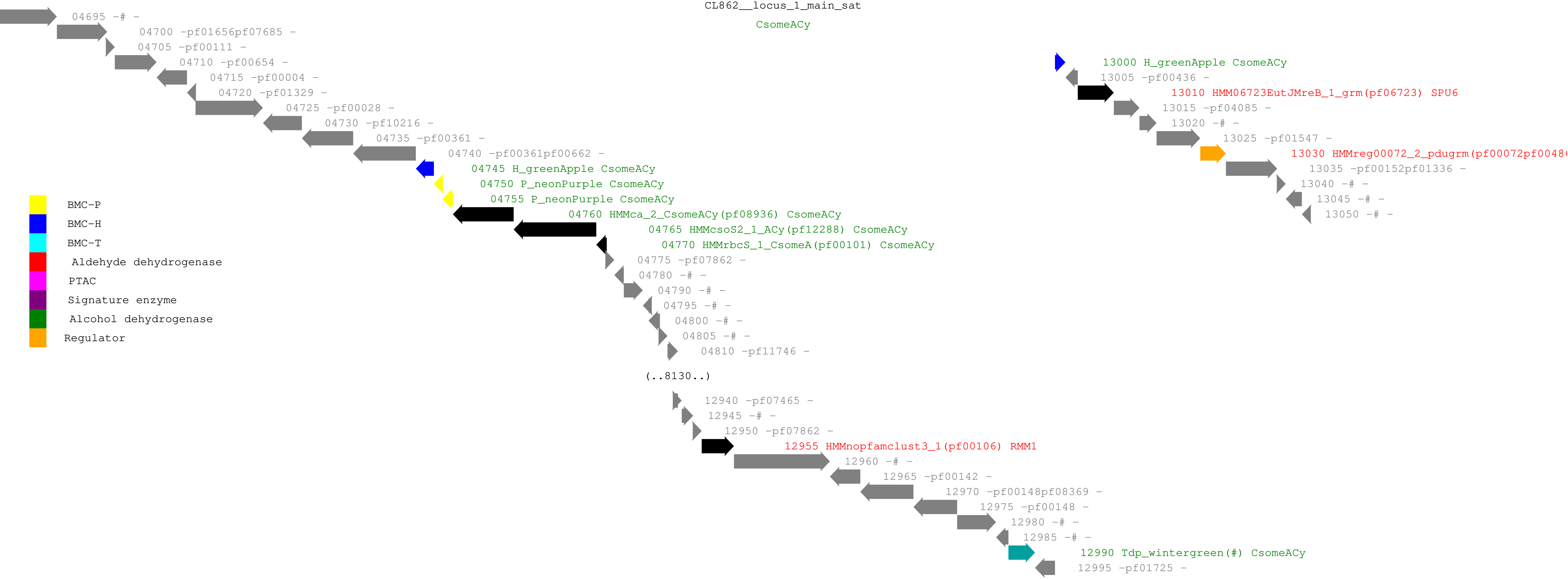

Prochlorococcus sp. MED105. A0A2D7TIP7_9PROC Unreviewed; 112 AA.

CMK47__locus_1

CsomeACy

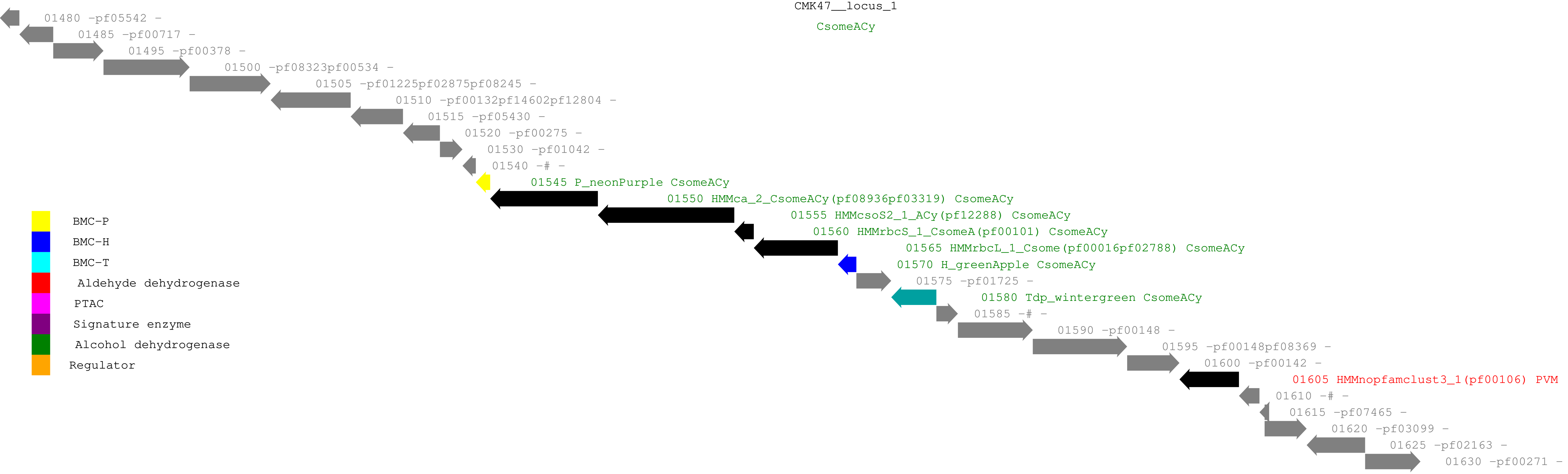

Prochlorococcus sp. MED630. A0A2D7LSD3_9PROC Unreviewed; 90 AA.

CMK48__locus_1

CsomeACy

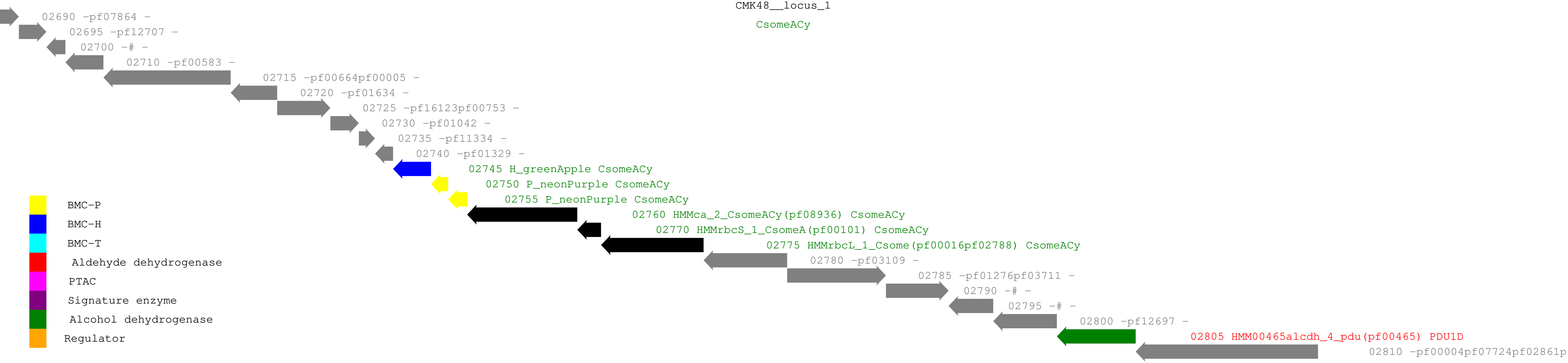

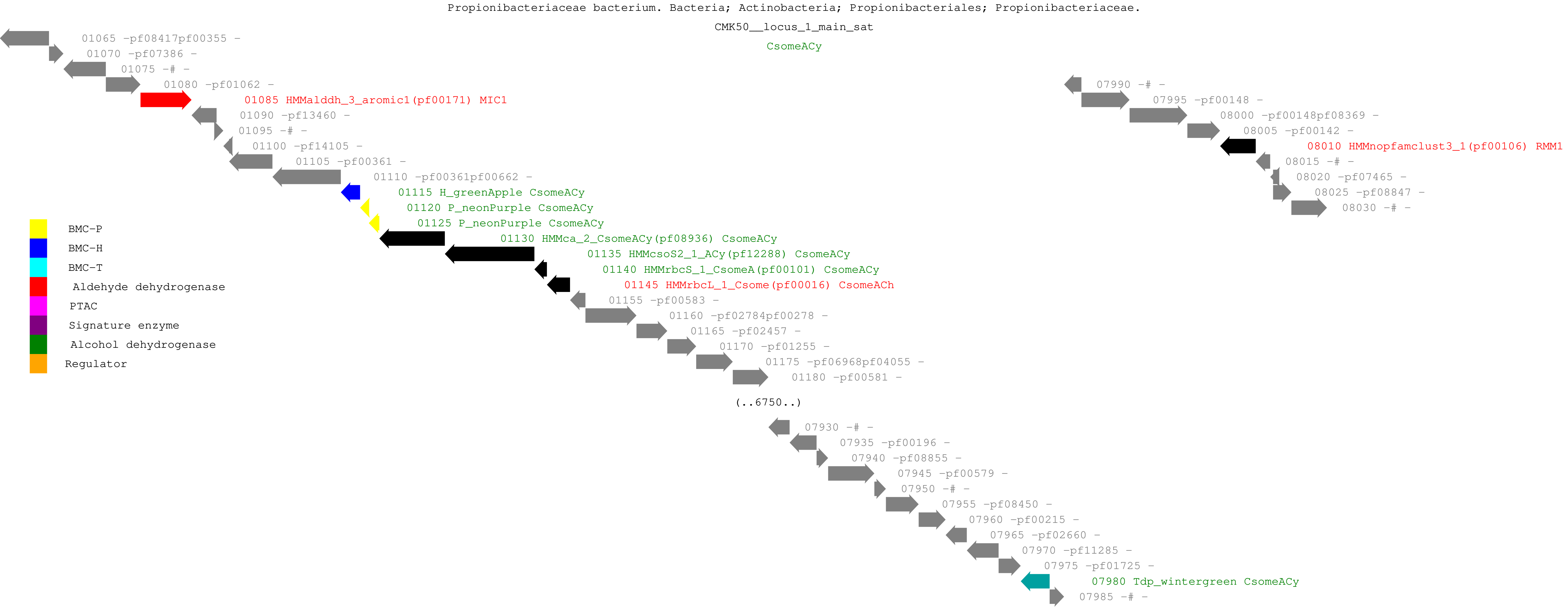
Propionibacteriaceae bacterium. Bacteria; Actinobacteria; Propionibacteriales; Propionibacteriaceae.
CMK50__locus_1_main_sat
CsomeACy
01065 -pf08417pf00355 -
01070 -pf07386 -
01075 -# -
01080 -pf01062 -
01085 HMMalddh_3_aromic1(pf00171) MIC1
01090 -pf13460 -
01095 -# -
01100 -pf14105 -
01105 -pf00361 -
01110 -pf00361pf00662 -
01115 H_greenApple CsomeACy
01120 P_neonPurple CsomeACy
01125 P_neonPurple CsomeACy
01130 HMMca_2_CsomeACy(pf08936) CsomeACy
01135 HMMcsoS2_1_ACy(pf12288) CsomeACy
01140 HMMrbcS_1_CsomeA(pf00101) CsomeACy
01145 HMMrbcL_1_Csome(pf00016) CsomeACh
01155 -pf00583 -
01160 -pf02784pf00278 -
01165 -pf02457 -
01170 -pf01255 -
01175 -pf06968pf04055 -
01180 -pf00581 -
(..6750..)
07930 -# -
07935 -pf00196 -
07940 -pf08855 -
07945 -pf00579 -
07950 -# -
07955 -pf08450 -
07960 -pf00215 -
07965 -pf02660 -
07970 -pf11285 -
07975 -pf01725 -
07980 Tdp_wintergreen CsomeACy
07985 -# -
07990 -# -
07995 -pf00148 -
08000 -pf00148pf08369 -
08005 -pf00142 -
08010 HMMnopfamclust3_1(pf00106) RMM1
08015 -# -
08020 -pf07465 -
08025 -pf08847 -
08030 -# -
BMC-P
BMC-H
BMC-T
Aldehyde dehydrogenase
PTAC
Signature enzyme
Alcohol dehydrogenase
Regulator

Synechococcus sp. ARS1019. Bacteria; Cyanobacteria; Synechococcales; Synechococcaceae;

CMN91__locus_1_main_sat

CsomeACy

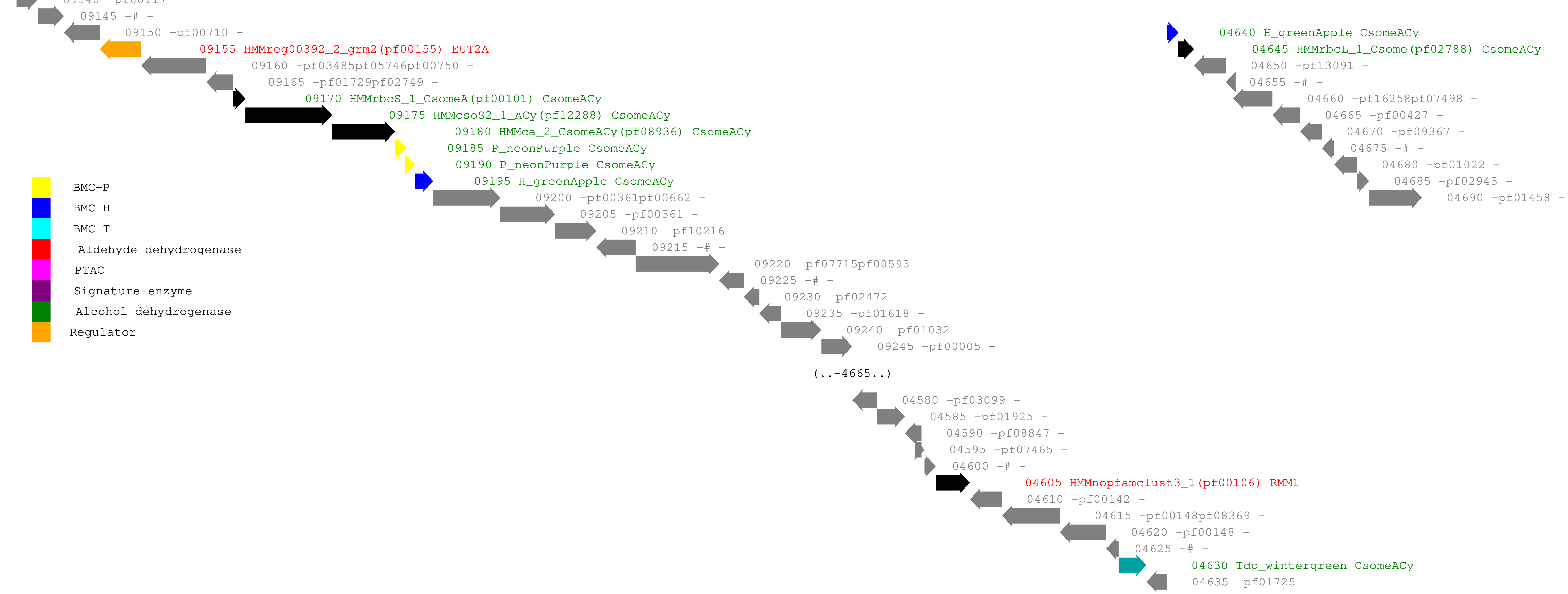

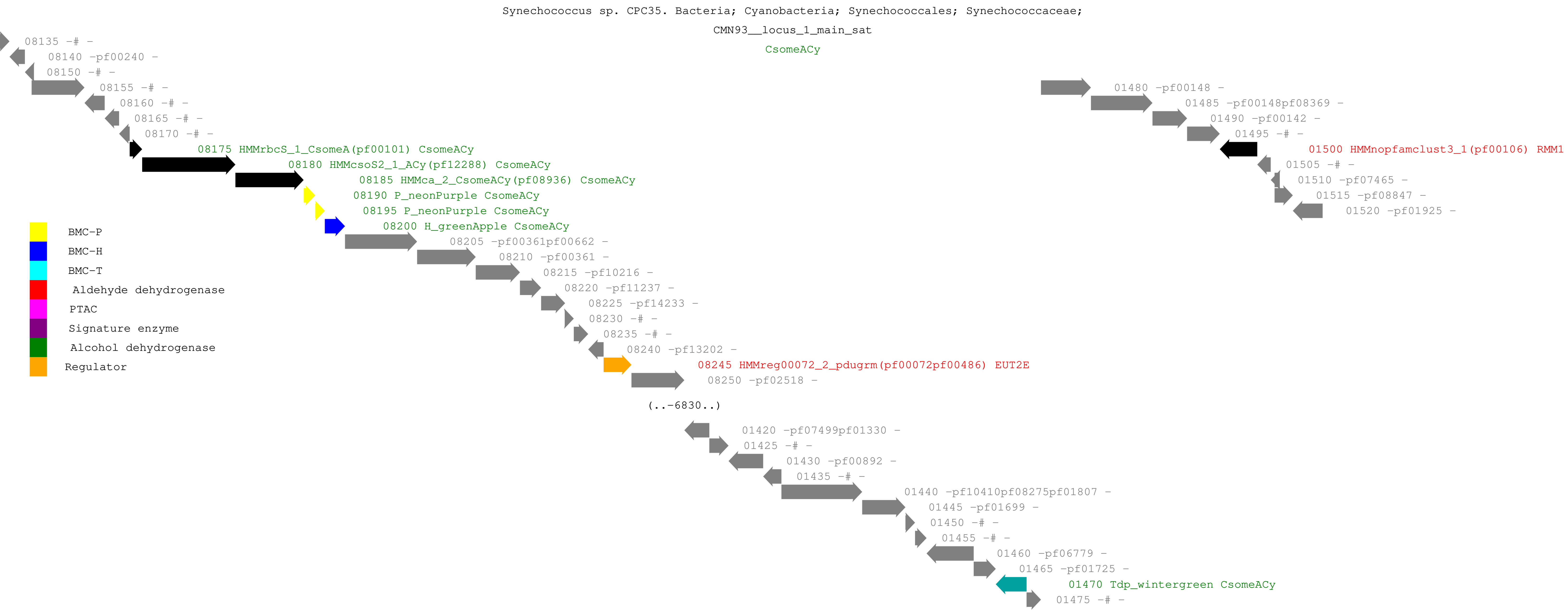

Synechococcus sp. CPC35. Bacteria; Cyanobacteria; Synechococcales; Synechococcaceae;
CMN93__locus_1_main_sat
CsomeACy
08135 -# -
08140 -pf00240 -
08150 -# -
08155 -# -
08160 -# -
08165 -# -
08170 -# -
08175 HMMrbcS_1_CsomeA(pf00101) CsomeACy
08180 HMMcsoS2_1_ACy(pf12288) CsomeACy
08185 HMMca_2_CsomeACy(pf08936) CsomeACy
08190 P_neonPurple CsomeACy
08195 P_neonPurple CsomeACy
08200 H_greenApple CsomeACy
08205 -pf00361pf00662 -
08210 -pf00361 -
08215 -pf10216 -
08220 -pf11237 -
08225 -pf14233 -
08230 -# -
08235 -# -
08240 -pf13202 -
08245 HMMreg00072_2_pdugrm(pf00072pf00486) EUT2E
08250 -pf02518 -
(..-6830..)
01420 -pf07499pf01330 -
01425 -# -
01430 -pf00892 -
01435 -# -
01440 -pf10410pf08275pf01807 -
01445 -pf01699 -
01450 -# -
01455 -# -
01460 -pf06779 -
01465 -pf01725 -
01470 Tdp_wintergreen CsomeACy
01475 -# -
01480 -pf00148 -
01485 -pf00148pf08369 -
01490 -pf00142 -
01495 -# -
01500 HMMnopfamclust3_1(pf00106) RMM1
01505 -# -
01510 -pf07465 -
01515 -pf08847 -
01520 -pf01925 -
BMC-P
BMC-H
BMC-T
Aldehyde dehydrogenase
PTAC
Signature enzyme
Alcohol dehydrogenase
Regulator

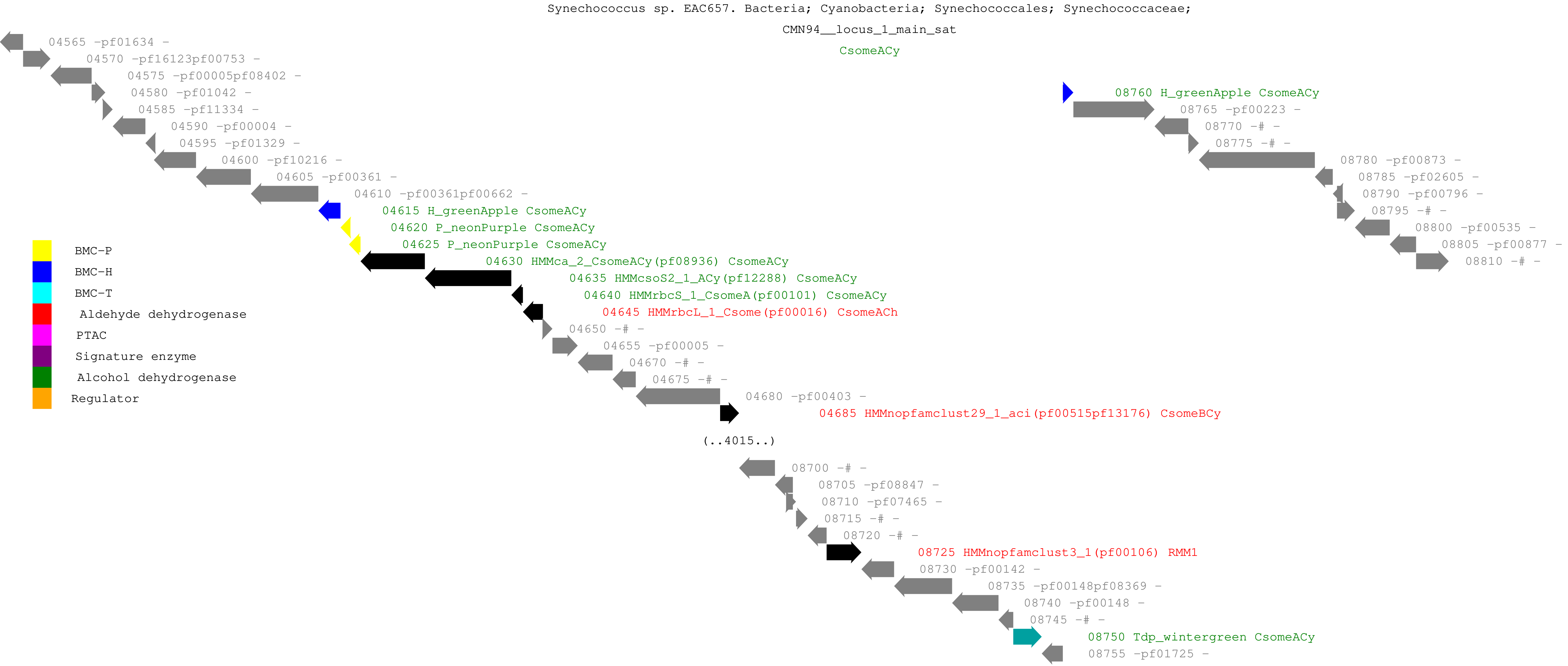
Synechococcus sp. EAC657. Bacteria; Cyanobacteria; Synechococcales; Synechococcaceae;
CMN94__locus_1_main_sat
CsomeACy
04565 -pf01634 -
04570 -pf16123pf00753 -
04575 -pf00005pf08402 -
04580 -pf01042 -
04585 -pf11334 -
04590 -pf00004 -
04595 -pf01329 -
04600 -pf10216 -
04605 -pf00361 -
04610 -pf00361pf00662 -
04615 H_greenApple CsomeACy
04620 P_neonPurple CsomeACy
04625 P_neonPurple CsomeACy
04630 HMMca_2_CsomeACy(pf08936) CsomeACy
04635 HMMcsoS2_1_ACy(pf12288) CsomeACy
04640 HMMrbcS_1_CsomeA(pf00101) CsomeACy
04645 HMMrbcL_1_Csome(pf00016) CsomeACh
04650 -# -
04655 -pf00005 -
04670 -# -
04675 -# -
04680 -pf00403 -
04685 HMMnopfamclust29_1_aci(pf00515pf13176) CsomeBCy
(..4015..)
08700 -# -
08705 -pf08847 -
08710 -pf07465 -
08715 -# -
08720 -# -
08725 HMMnopfamclust3_1(pf00106) RMM1
08730 -pf00142 -
08735 -pf00148pf08369 -
08740 -pf00148 -
08745 -# -
08750 Tdp_wintergreen CsomeACy
08755 -pf01725 -
08760 H_greenApple CsomeACy
08765 -pf00223 -
08770 -# -
08775 -# -
08780 -pf00873 -
08785 -pf02605 -
08790 -pf00796 -
08795 -# -
08800 -pf00535 -
08805 -pf00877 -
08810 -# -
BMC-P
BMC-H
BMC-T
Aldehyde dehydrogenase
PTAC
Signature enzyme
Alcohol dehydrogenase
Regulator

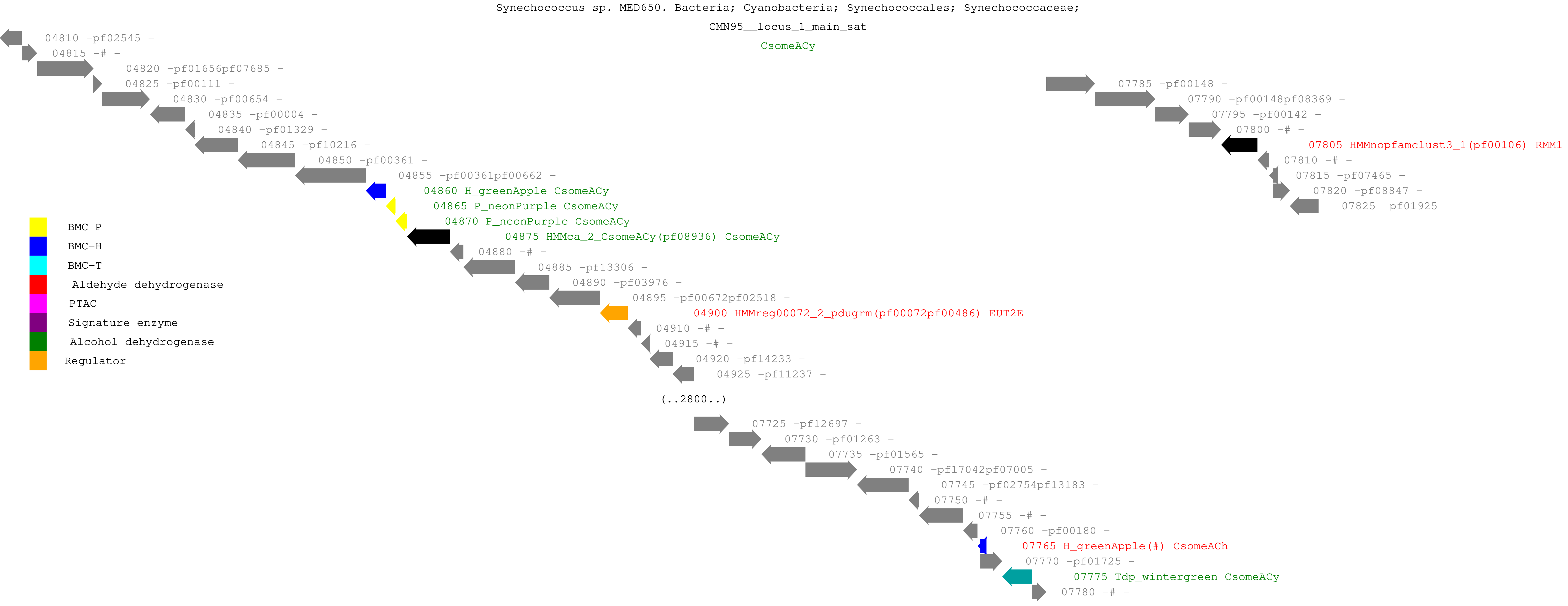

Synechococcus sp. MED650. Bacteria; Cyanobacteria; Synechococcales; Synechococcaceae;
CMN95__locus_1_main_sat
CsomeACy
04810 -pf02545 -
04815 -# -
04820 -pf01656pf07685 -
04825 -pf00111 -
04830 -pf00654 -
04835 -pf00004 -
04840 -pf01329 -
04845 -pf10216 -
04850 -pf00361 -
04855 -pf00361pf00662 -
04860 H_greenApple CsomeACy
04865 P_neonPurple CsomeACy
04870 P_neonPurple CsomeACy
04875 HMMca_2_CsomeACy(pf08936) CsomeACy
04880 -# -
04885 -pf13306 -
04890 -pf03976 -
04895 -pf00672pf02518 -
04900 HMMreg00072_2_pdugrm(pf00072pf00486) EUT2E
04910 -# -
04915 -# -
04920 -pf14233 -
04925 -pf11237 -
(..2800..)
07725 -pf12697 -
07730 -pf01263 -
07735 -pf01565 -
07740 -pf17042pf07005 -
07745 -pf02754pf13183 -
07750 -# -
07755 -# -
07760 -pf00180 -
07765 H_greenApple(#) CsomeACh
07770 -pf01725 -
07775 Tdp_wintergreen CsomeACy
07780 -# -
07785 -pf00148 -
07790 -pf00148pf08369 -
07795 -pf00142 -
07800 -# -
07805 HMMnopfamclust3_1(pf00106) RMM1
07810 -# -
07815 -pf07465 -
07820 -pf08847 -
07825 -pf01925 -
BMC-P
BMC-H
BMC-T
Aldehyde dehydrogenase
PTAC
Signature enzyme
Alcohol dehydrogenase
Regulator

Synechococcus sp. MED850. Bacteria; Cyanobacteria; Synechococcales; Synechococcaceae;

CMN96__locus_1_main_sat

CsomeACy

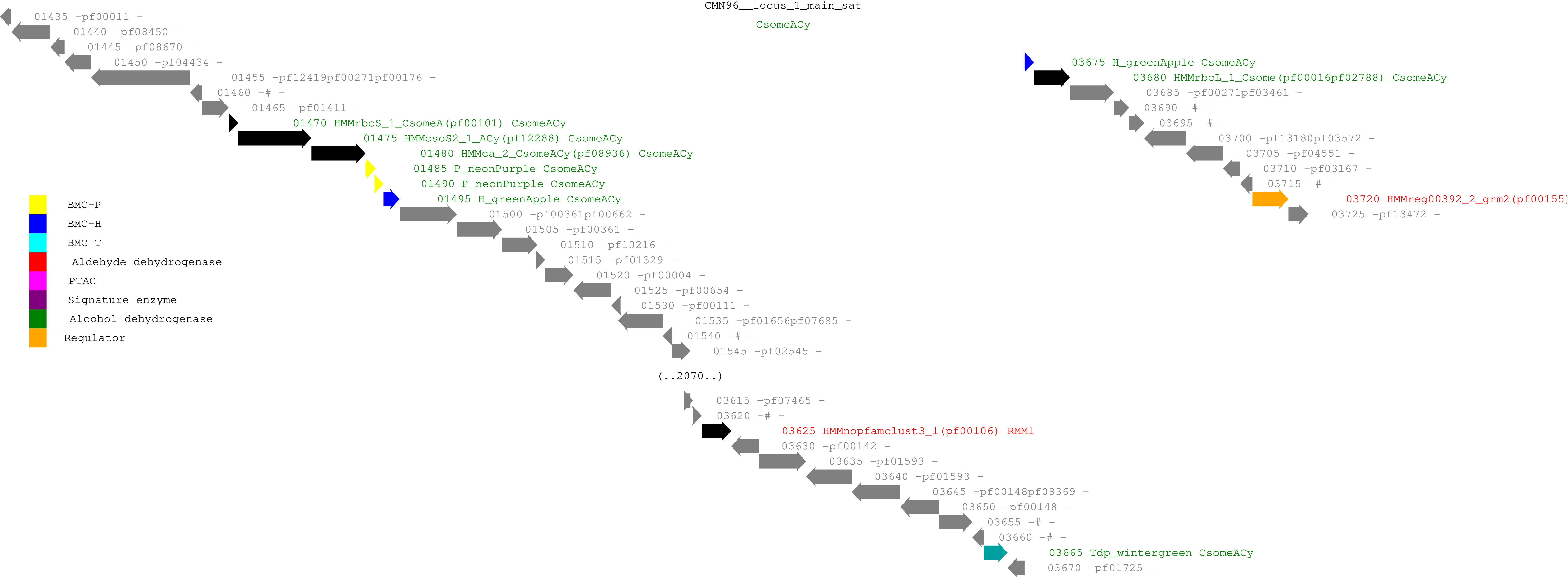

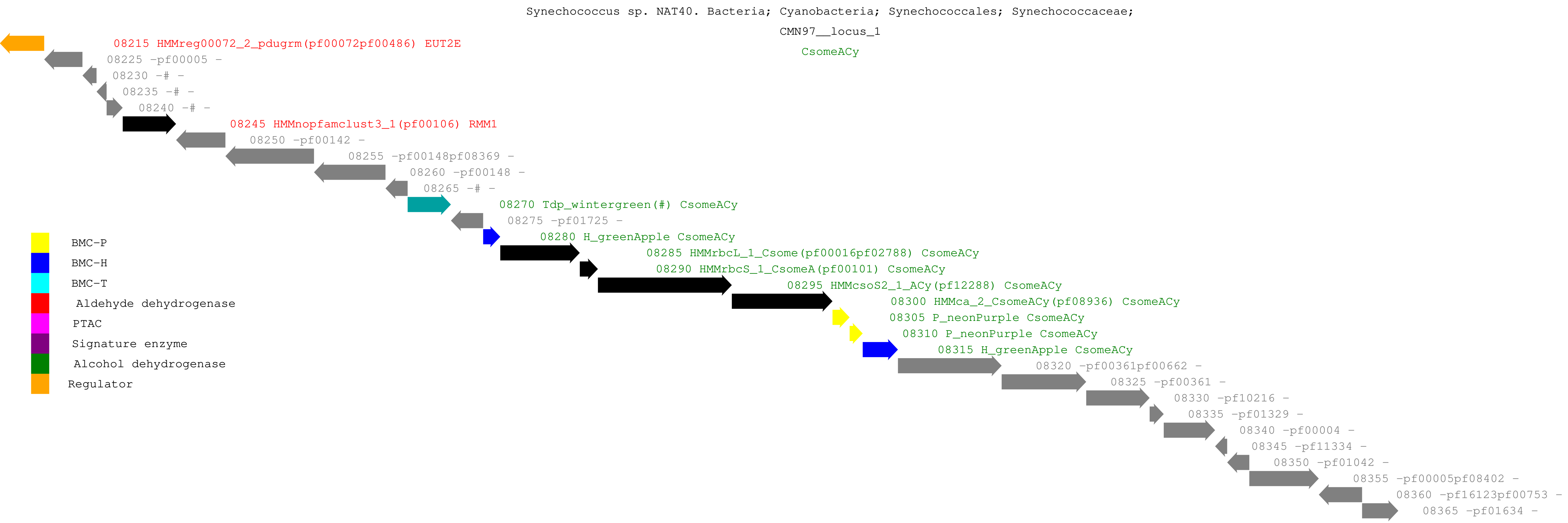

Synechococcus sp. NAT40. Bacteria; Cyanobacteria; Synechococcales; Synechococcaceae;
CMN97__locus_1
CsomeACy
08215 HMMreg00072_2_pdugrm(pf00072pf00486) EUT2E
08225 -pf00005 -
08230 -# -
08235 -# -
08240 -# -
08245 HMMnopfamclust3_1(pf00106) RMM1
08250 -pf00142 -
08255 -pf00148pf08369 -
08260 -pf00148 -
08265 -# -
08270 Tdp_wintergreen(#) CsomeACy
08275 -pf01725 -
08280 H_greenApple CsomeACy
08285 HMMrbcL_1_Csome(pf00016pf02788) CsomeACy
08290 HMMrbcS_1_CsomeA(pf00101) CsomeACy
08295 HMMcsoS2_1_ACy(pf12288) CsomeACy
08300 HMMca_2_CsomeACy(pf08936) CsomeACy
08305 P_neonPurple CsomeACy
08310 P_neonPurple CsomeACy
08315 H_greenApple CsomeACy
08320 -pf00361pf00662 -
08325 -pf00361 -
08330 -pf10216 -
08335 -pf01329 -
08340 -pf00004 -
08345 -pf11334 -
08350 -pf01042 -
08355 -pf00005pf08402 -
08360 -pf16123pf00753 -
08365 -pf01634 -
BMC-P
BMC-H
BMC-T
Aldehyde dehydrogenase
PTAC
Signature enzyme
Alcohol dehydrogenase
Regulator

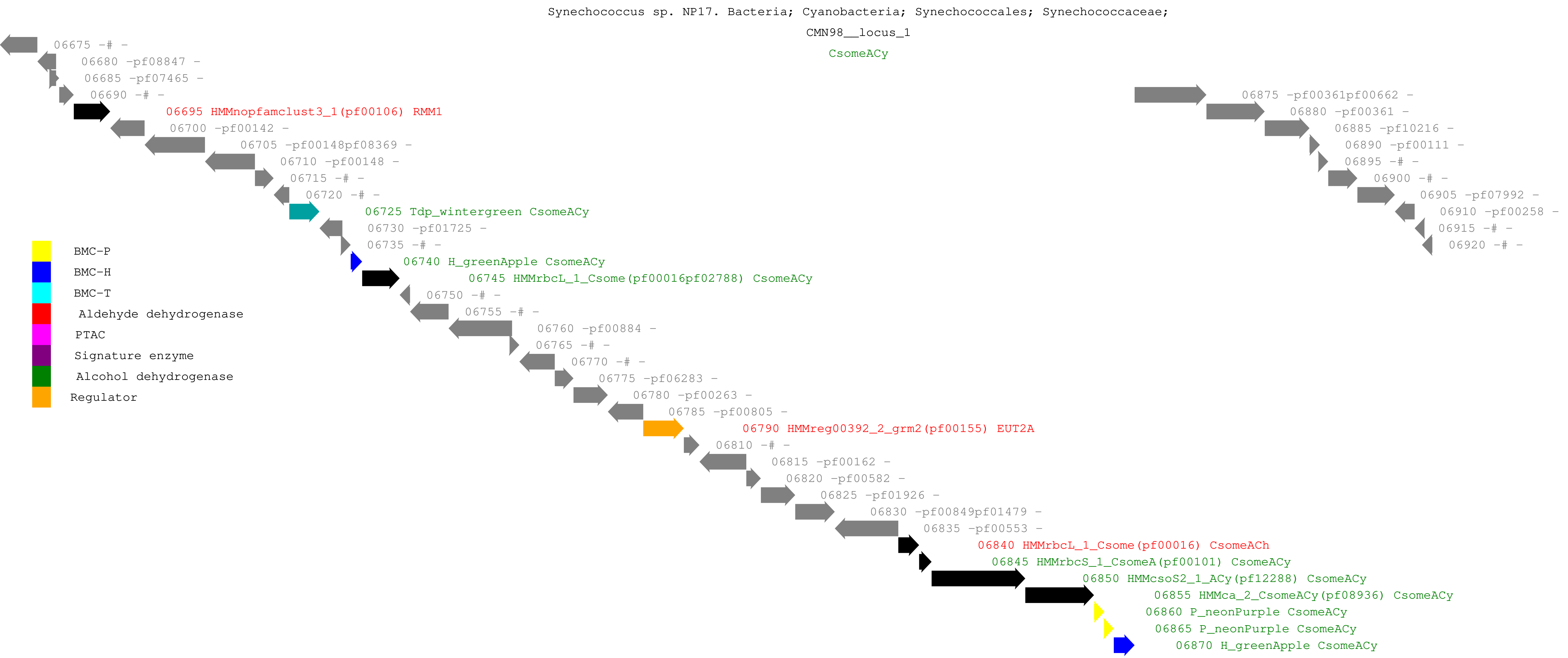
Synechococcus sp. NP17. Bacteria; Cyanobacteria; Synechococcales; Synechococcaceae;
CMN98__locus_1
CsomeACy
06675 -# -
06680 -pf08847 -
06685 -pf07465 -
06690 -# -
06695 HMMnopfamclust3_1(pf00106) RMM1
06700 -pf00142 -
06705 -pf00148pf08369 -
06710 -pf00148 -
06715 -# -
06720 -# -
06725 Tdp_wintergreen CsomeACy
06730 -pf01725 -
06735 -# -
06740 H_greenApple CsomeACy
06745 HMMrbcL_1_Csome(pf00016pf02788) CsomeACy
06750 -# -
06755 -# -
06760 -pf00884 -
06765 -# -
06770 -# -
06775 -pf06283 -
06780 -pf00263 -
06785 -pf00805 -
06790 HMMreg00392_2_grm2(pf00155) EUT2A
06810 -# -
06815 -pf00162 -
06820 -pf00582 -
06825 -pf01926 -
06830 -pf00849pf01479 -
06835 -pf00553 -
06840 HMMrbcL_1_Csome(pf00016) CsomeACh
06845 HMMrbcS_1_CsomeA(pf00101) CsomeACy
06850 HMMcsoS2_1_ACy(pf12288) CsomeACy
06855 HMMca_2_CsomeACy(pf08936) CsomeACy
06860 P_neonPurple CsomeACy
06865 P_neonPurple CsomeACy
06870 H_greenApple CsomeACy
06875 -pf00361pf00662 -
06880 -pf00361 -
06885 -pf10216 -
06890 -pf00111 -
06895 -# -
06900 -# -
06905 -pf07992 -
06910 -pf00258 -
06915 -# -
06920 -# -
BMC-P
BMC-H
BMC-T
Aldehyde dehydrogenase
PTAC
Signature enzyme
Alcohol dehydrogenase
Regulator

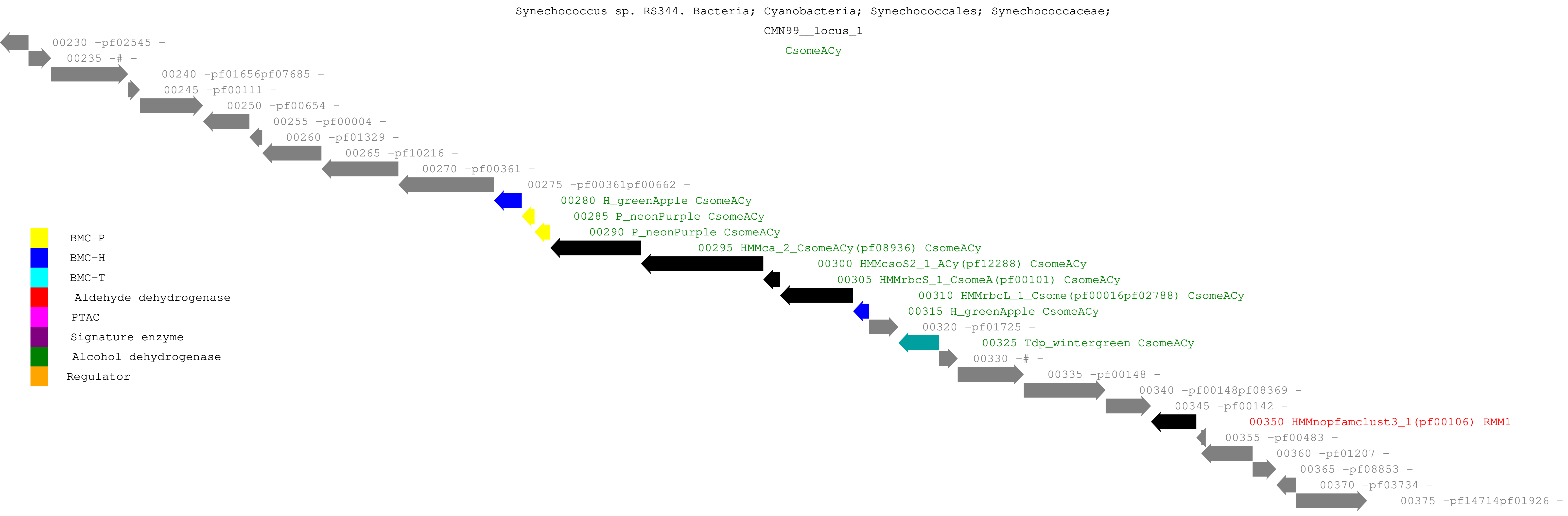
Synechococcus sp. RS344. Bacteria; Cyanobacteria; Synechococcales; Synechococcaceae;
CMN99__locus_1
CsomeACy
00230 -pf02545 -
00235 -# -
00240 -pf01656pf07685 -
00245 -pf00111 -
00250 -pf00654 -
00255 -pf00004 -
00260 -pf01329 -
00265 -pf10216 -
00270 -pf00361 -
00275 -pf00361pf00662 -
00280 H_greenApple CsomeACy
00285 P_neonPurple CsomeACy
00290 P_neonPurple CsomeACy
00295 HMMca_2_CsomeACy(pf08936) CsomeACy
00300 HMMcsoS2_1_ACy(pf12288) CsomeACy
00305 HMMrbcS_1_CsomeA(pf00101) CsomeACy
00310 HMMrbcL_1_Csome(pf00016pf02788) CsomeACy
00315 H_greenApple CsomeACy
00320 -pf01725 -
00325 Tdp_wintergreen CsomeACy
00330 -# -
00335 -pf00148 -
00340 -pf00148pf08369 -
00345 -pf00142 -
00350 HMMnopfamclust3_1(pf00106) RMM1
00355 -pf00483 -
00360 -pf01207 -
00365 -pf08853 -
00370 -pf03734 -
00375 -pf14714pf01926 -
BMC-P
BMC-H
BMC-T
Aldehyde dehydrogenase
PTAC
Signature enzyme
Alcohol dehydrogenase
Regulator

Cyanobium sp. PCC 7001. Bacteria; Cyanobacteria; Synechococcales; Synechococcaceae; Cyanobium.

CPCC7001__locus_fragmented

CsomeACy

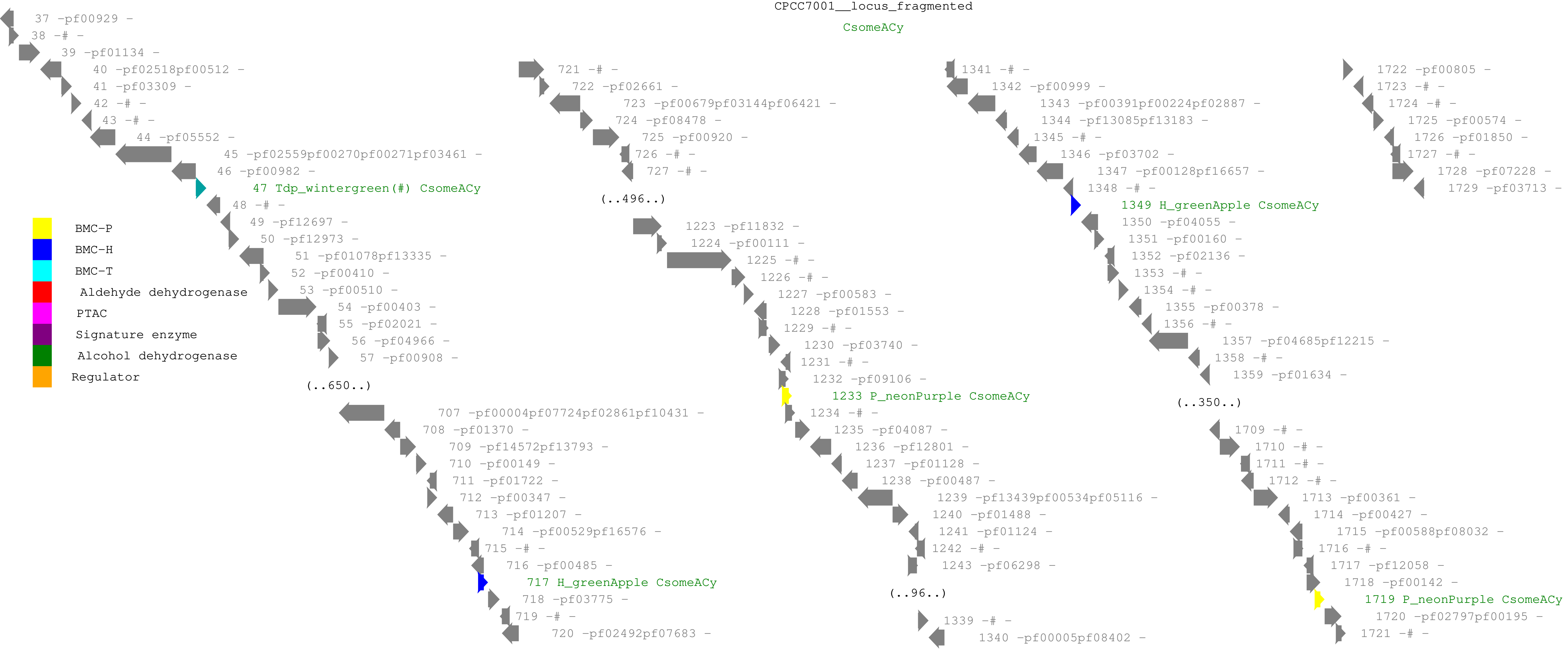

Prochlorococcus marinus str. XMU1401. Bacteria; Cyanobacteria; Synechococcales; Prochloraceae;

CUB78__locus_1

CsomeACy

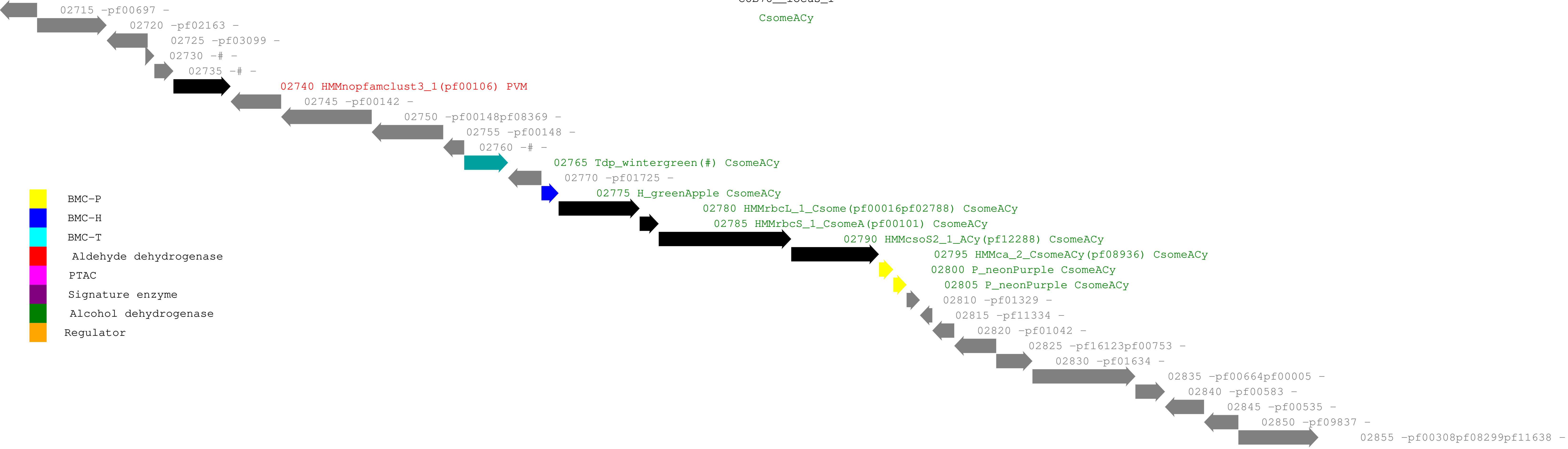

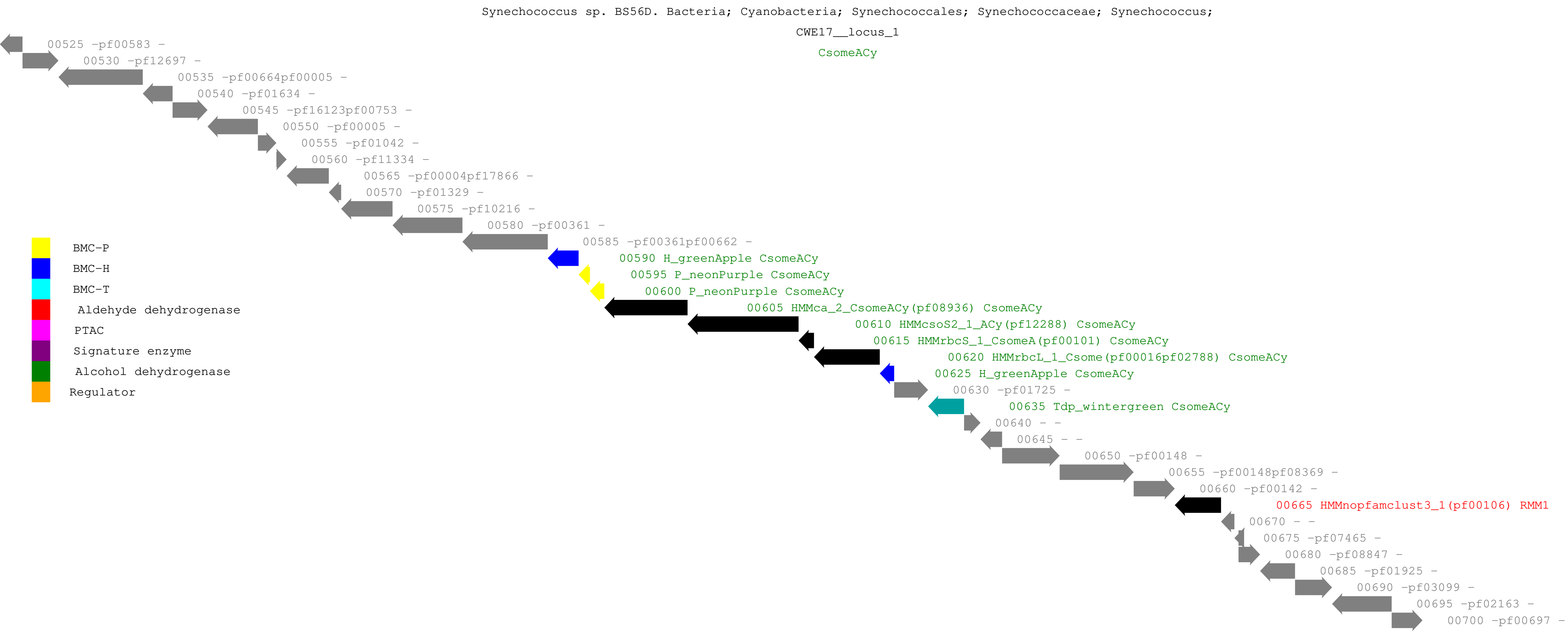
Synechococcus sp. BS56D. Bacteria; Cyanobacteria; Synechococcales; Synechococcaceae; Synechococcus;
CWE17__locus_1
CsomeACy
00525 -pf00583 -
00530 -pf12697 -
00535 -pf00664pf00005 -
00540 -pf01634 -
00545 -pf16123pf00753 -
00550 -pf00005 -
00555 -pf01042 -
00560 -pf11334 -
00565 -pf00004pf17866 -
00570 -pf01329 -
00575 -pf10216 -
00580 -pf00361 -
00585 -pf00361pf00662 -
00590 H_greenApple CsomeACy
00595 P_neonPurple CsomeACy
00600 P_neonPurple CsomeACy
00605 HMMca_2_CsomeACy(pf08936) CsomeACy
00610 HMMcsoS2_1_ACy(pf12288) CsomeACy
00615 HMMrbcS_1_CsomeA(pf00101) CsomeACy
00620 HMMrbcL_1_Csome(pf00016pf02788) CsomeACy
00625 H_greenApple CsomeACy
00630 -pf01725 -
00635 Tdp_wintergreen CsomeACy
00640 - -
00645 - -
00650 -pf00148 -
00655 -pf00148pf08369 -
00660 -pf00142 -
00665 HMMnopfamclust3_1(pf00106) RMM1
00670 - -
00675 -pf07465 -
00680 -pf08847 -
00685 -pf01925 -
00690 -pf03099 -
00695 -pf02163 -
00700 -pf00697 -
BMC-P
BMC-H
BMC-T
Aldehyde dehydrogenase
PTAC
Signature enzyme
Alcohol dehydrogenase
Regulator

Cyanobium gracile (strain ATCC 27147 / PCC 6307). Bacteria; Cyanobacteria; Synechococcales; Synechococcaceae; Cyanobium.

Cyagr__locus_1

CsomeACy

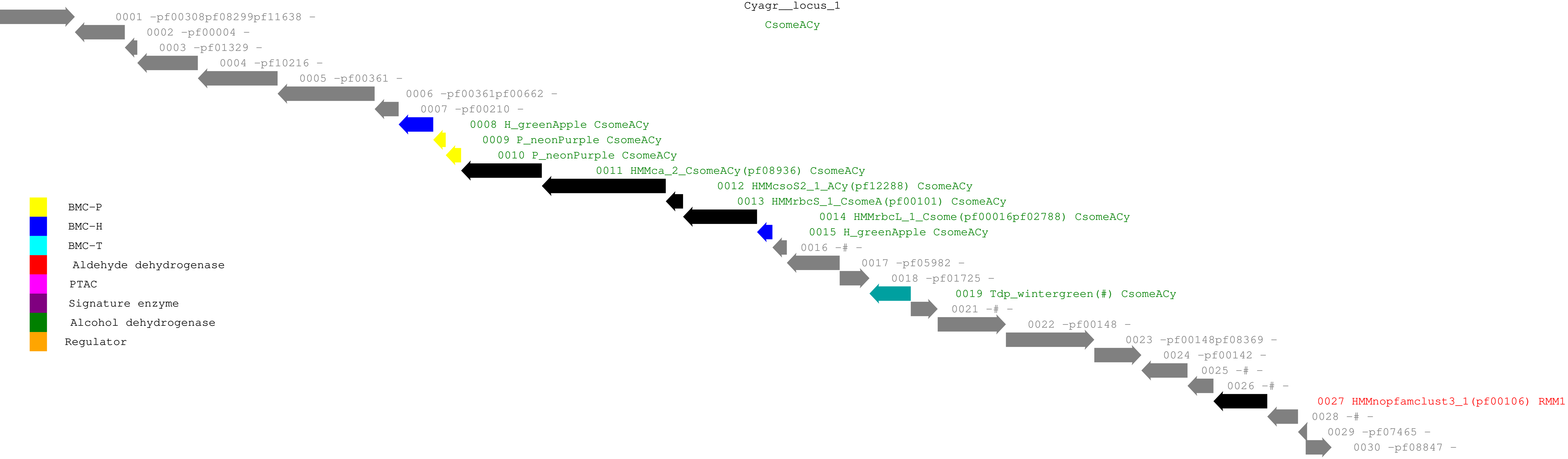

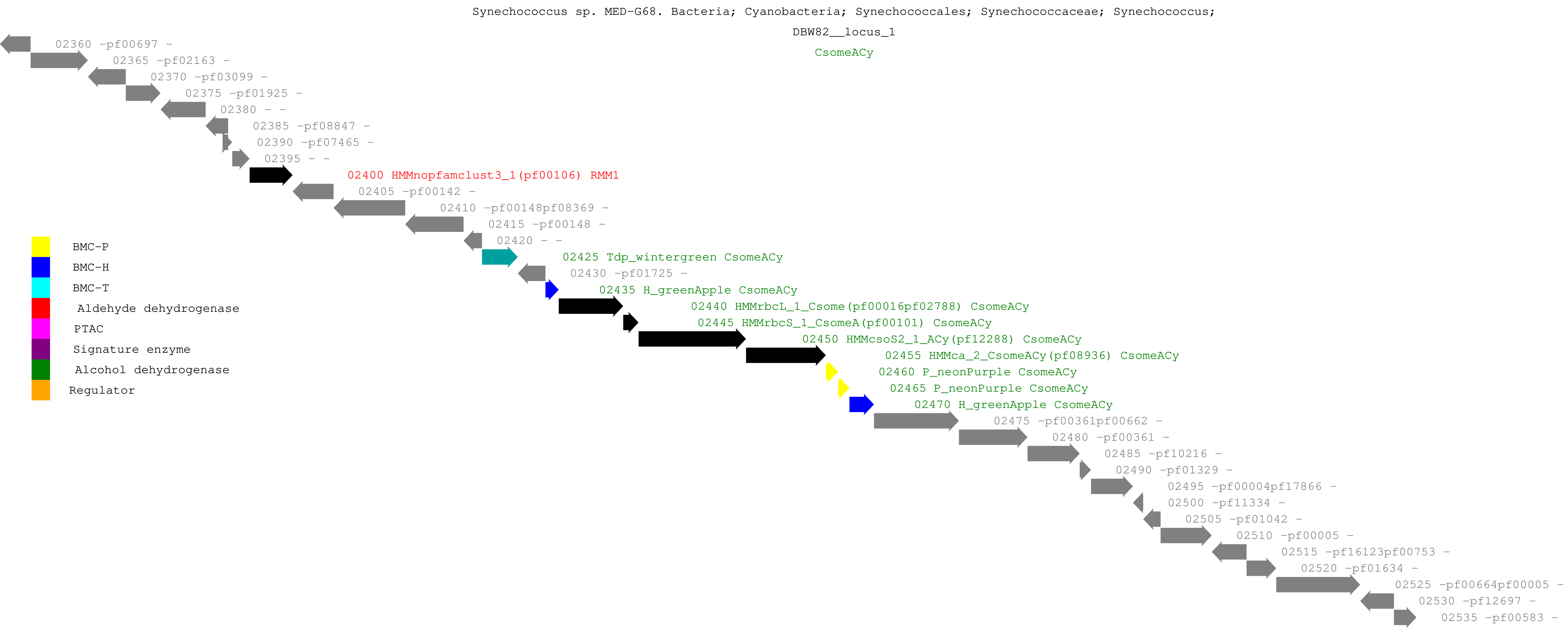
Synechococcus sp. MED-G68. Bacteria; Cyanobacteria; Synechococcales; Synechococcaceae; Synechococcus;
DBW82__locus_1
CsomeACy
02360 -pf00697 -
02365 -pf02163 -
02370 -pf03099 -
02375 -pf01925 -
02380 - -
02385 -pf08847 -
02390 -pf07465 -
02395 - -
02400 HMMnopfamclust3_1(pf00106) RMM1
02405 -pf00142 -
02410 -pf00148pf08369 -
02415 -pf00148 -
02420 - -
02425 Tdp_wintergreen CsomeACy
02430 -pf01725 -
02435 H_greenApple CsomeACy
02440 HMMrbcL_1_Csome(pf00016pf02788) CsomeACy
02445 HMMrbcS_1_CsomeA(pf00101) CsomeACy
02450 HMMcsoS2_1_ACy(pf12288) CsomeACy
02455 HMMca_2_CsomeACy(pf08936) CsomeACy
02460 P_neonPurple CsomeACy
02465 P_neonPurple CsomeACy
02470 H_greenApple CsomeACy
02475 -pf00361pf00662 -
02480 -pf00361 -
02485 -pf10216 -
02490 -pf01329 -
02495 -pf00004pf17866 -
02500 -pf11334 -
02505 -pf01042 -
02510 -pf00005 -
02515 -pf16123pf00753 -
02520 -pf01634 -
02525 -pf00664pf00005 -
02530 -pf12697 -
02535 -pf00583 -
BMC-P
BMC-H
BMC-T
Aldehyde dehydrogenase
PTAC
Signature enzyme
Alcohol dehydrogenase
Regulator

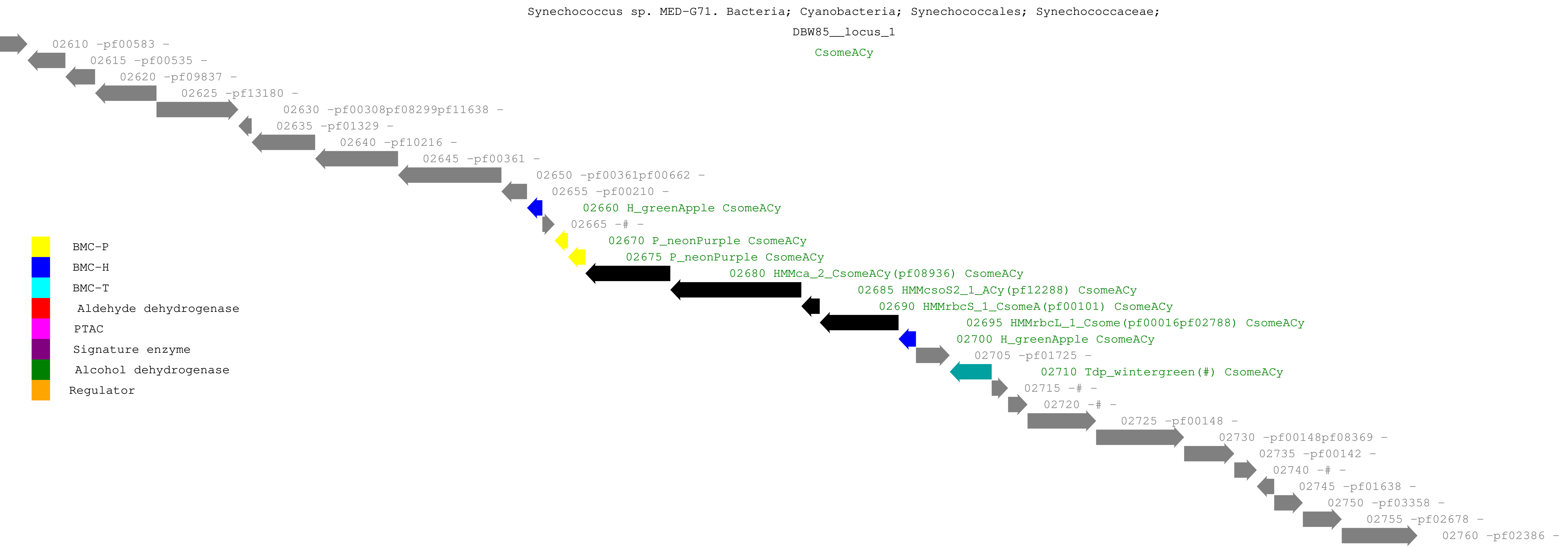
Synechococcus sp. MED-G71. Bacteria; Cyanobacteria; Synechococcales; Synechococcaceae;
DBW85__locus_1
CsomeACy
02610 -pf00583 -
02615 -pf00535 -
02620 -pf09837 -
02625 -pf13180 -
02630 -pf00308pf08299pf11638 -
02635 -pf01329 -
02640 -pf10216 -
02645 -pf00361 -
02650 -pf00361pf00662 -
02655 -pf00210 -
02660 H_greenApple CsomeACy
02665 -# -
02670 P_neonPurple CsomeACy
02675 P_neonPurple CsomeACy
02680 HMMca_2_CsomeACy(pf08936) CsomeACy
02685 HMMcsoS2_1_ACy(pf12288) CsomeACy
02690 HMMrbcS_1_CsomeA(pf00101) CsomeACy
02695 HMMrbcL_1_Csome(pf00016pf02788) CsomeACy
02700 H_greenApple CsomeACy
02705 -pf01725 -
02710 Tdp_wintergreen(#) CsomeACy
02715 -# -
02720 -# -
02725 -pf00148 -
02730 -pf00148pf08369 -
02735 -pf00142 -
02740 -# -
02745 -pf01638 -
02750 -pf03358 -
02755 -pf02678 -
02760 -pf02386 -
BMC-P
BMC-H
BMC-T
Aldehyde dehydrogenase
PTAC
Signature enzyme
Alcohol dehydrogenase
Regulator

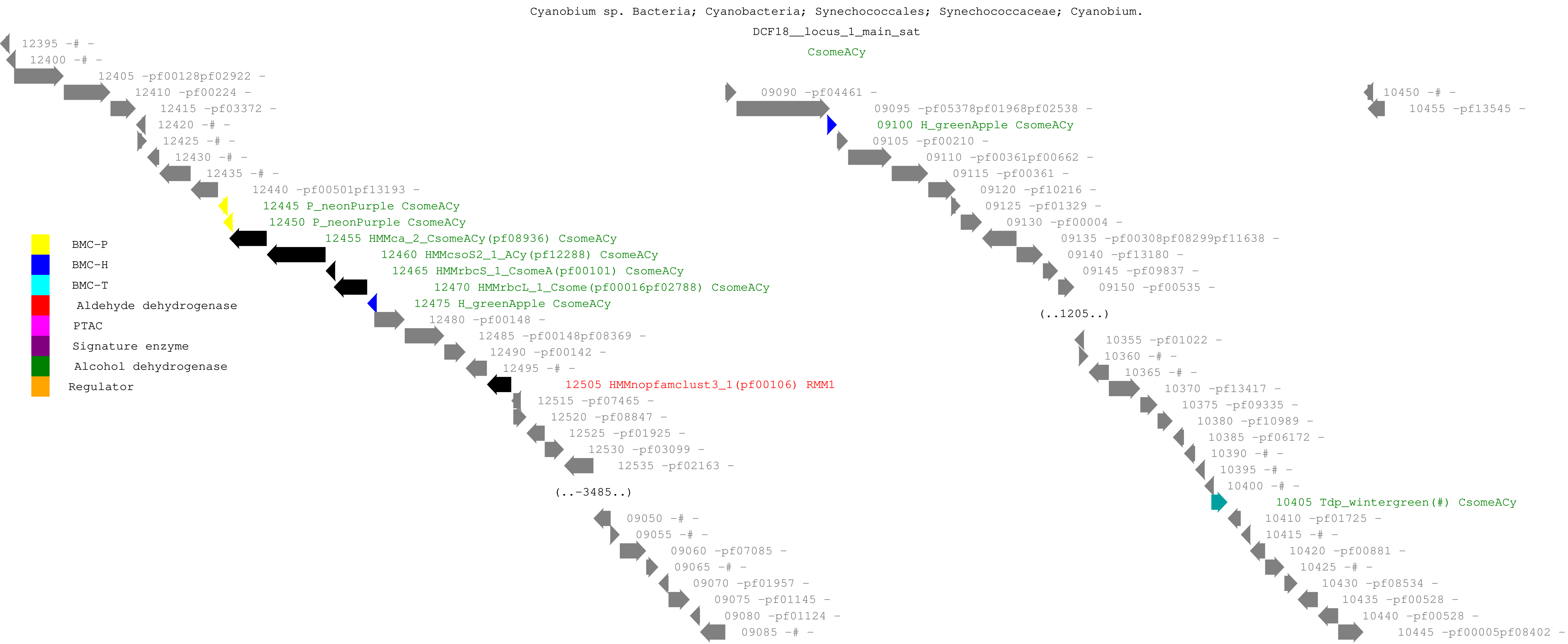
Cyanobium sp. Bacteria; Cyanobacteria; Synechococcales; Synechococcaceae; Cyanobium.
DCF18__locus_1_main_sat
CsomeACy
12395 -# -
12400 -# -
12405 -pf00128pf02922 -
12410 -pf00224 -
12415 -pf03372 -
12420 -# -
12425 -# -
12430 -# -
12435 -# -
12440 -pf00501pf13193 -
12445 P_neonPurple CsomeACy
12450 P_neonPurple CsomeACy
12455 HMMca_2_CsomeACy(pf08936) CsomeACy
12460 HMMcsoS2_1_ACy(pf12288) CsomeACy
12465 HMMrbcS_1_CsomeA(pf00101) CsomeACy
12470 HMMrbcL_1_Csome(pf00016pf02788) CsomeACy
12475 H_greenApple CsomeACy
12480 -pf00148 -
12485 -pf00148pf08369 -
12490 -pf00142 -
12495 -# -
12505 HMMnopfamclust3_1(pf00106) RMM1
12515 -pf07465 -
12520 -pf08847 -
12525 -pf01925 -
12530 -pf03099 -
12535 -pf02163 -
(..-3485..)
09050 -# -
09055 -# -
09060 -pf07085 -
09065 -# -
09070 -pf01957 -
09075 -pf01145 -
09080 -pf01124 -
09085 -# -
09090 -pf04461 -
09095 -pf05378pf01968pf02538 -
09100 H_greenApple CsomeACy
09105 -pf00210 -
09110 -pf00361pf00662 -
09115 -pf00361 -
09120 -pf10216 -
09125 -pf01329 -
09130 -pf00004 -
09135 -pf00308pf08299pf11638 -
09140 -pf13180 -
09145 -pf09837 -
09150 -pf00535 -
(..1205..)
10355 -pf01022 -
10360 -# -
10365 -# -
10370 -pf13417 -
10375 -pf09335 -
10380 -pf10989 -
10385 -pf06172 -
10390 -# -
10395 -# -
10400 -# -
10405 Tdp_wintergreen(#) CsomeACy
10410 -pf01725 -
10415 -# -
10420 -pf00881 -
10425 -# -
10430 -pf08534 -
10435 -pf00528 -
10440 -pf00528 -
10445 -pf00005pf08402 -
10450 -# -
10455 -pf13545 -
BMC-P
BMC-H
BMC-T
Aldehyde dehydrogenase
PTAC
Signature enzyme
Alcohol dehydrogenase
Regulator

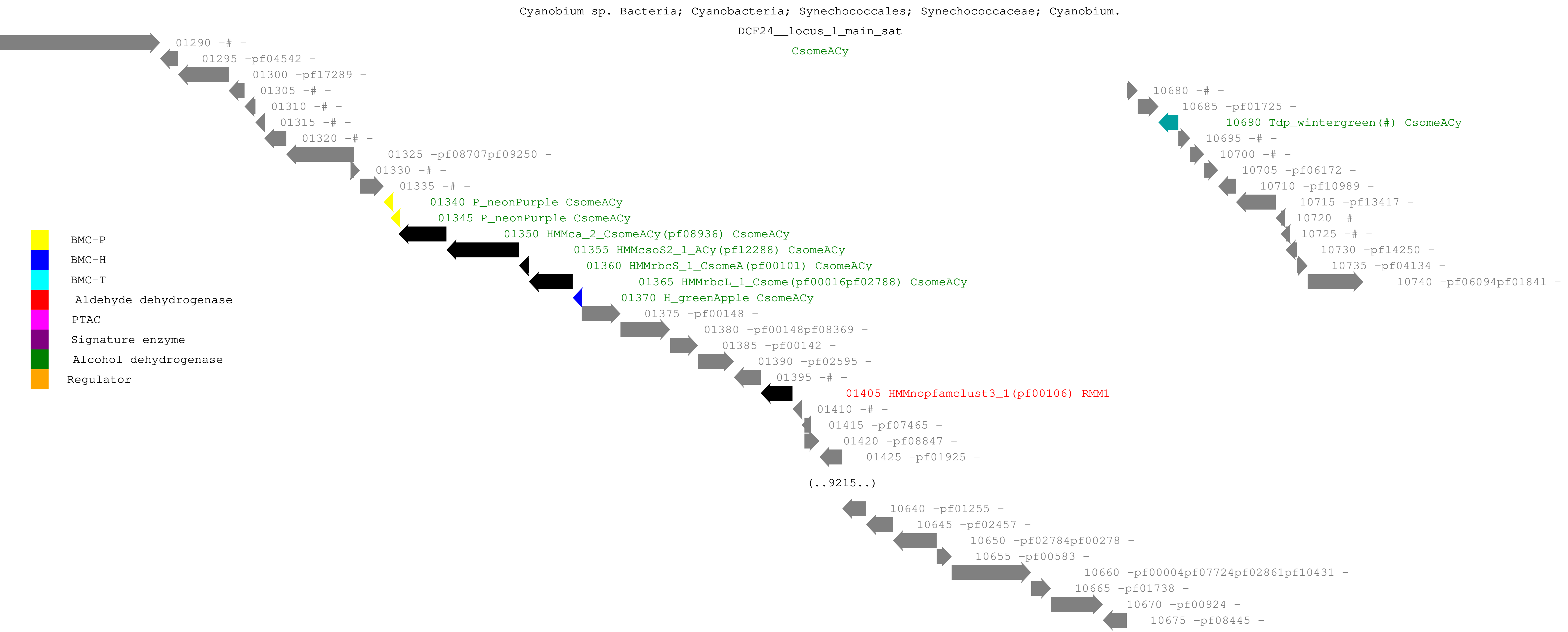
Cyanobium sp. Bacteria; Cyanobacteria; Synechococcales; Synechococcaceae; Cyanobium.
DCF24__locus_1_main_sat
CsomeACy
01290 -# -
01295 -pf04542 -
01300 -pf17289 -
01305 -# -
01310 -# -
01315 -# -
01320 -# -
01325 -pf08707pf09250 -
01330 -# -
01335 -# -
01340 P_neonPurple CsomeACy
01345 P_neonPurple CsomeACy
01350 HMMca_2_CsomeACy(pf08936) CsomeACy
01355 HMMcsoS2_1_ACy(pf12288) CsomeACy
01360 HMMrbcS_1_CsomeA(pf00101) CsomeACy
01365 HMMrbcL_1_Csome(pf00016pf02788) CsomeACy
01370 H_greenApple CsomeACy
01375 -pf00148 -
01380 -pf00148pf08369 -
01385 -pf00142 -
01390 -pf02595 -
01395 -# -
01405 HMMnopfamclust3_1(pf00106) RMM1
01410 -# -
01415 -pf07465 -
01420 -pf08847 -
01425 -pf01925 -
(..9215..)
10640 -pf01255 -
10645 -pf02457 -
10650 -pf02784pf00278 -
10655 -pf00583 -
10660 -pf00004pf07724pf02861pf10431 -
10665 -pf01738 -
10670 -pf00924 -
10675 -pf08445 -
10680 -# -
10685 -pf01725 -
10690 Tdp_wintergreen(#) CsomeACy
10695 -# -
10700 -# -
10705 -pf06172 -
10710 -pf10989 -
10715 -pf13417 -
10720 -# -
10725 -# -
10730 -pf14250 -
10735 -pf04134 -
10740 -pf06094pf01841 -
BMC-P
BMC-H
BMC-T
Aldehyde dehydrogenase
PTAC
Signature enzyme
Alcohol dehydrogenase
Regulator

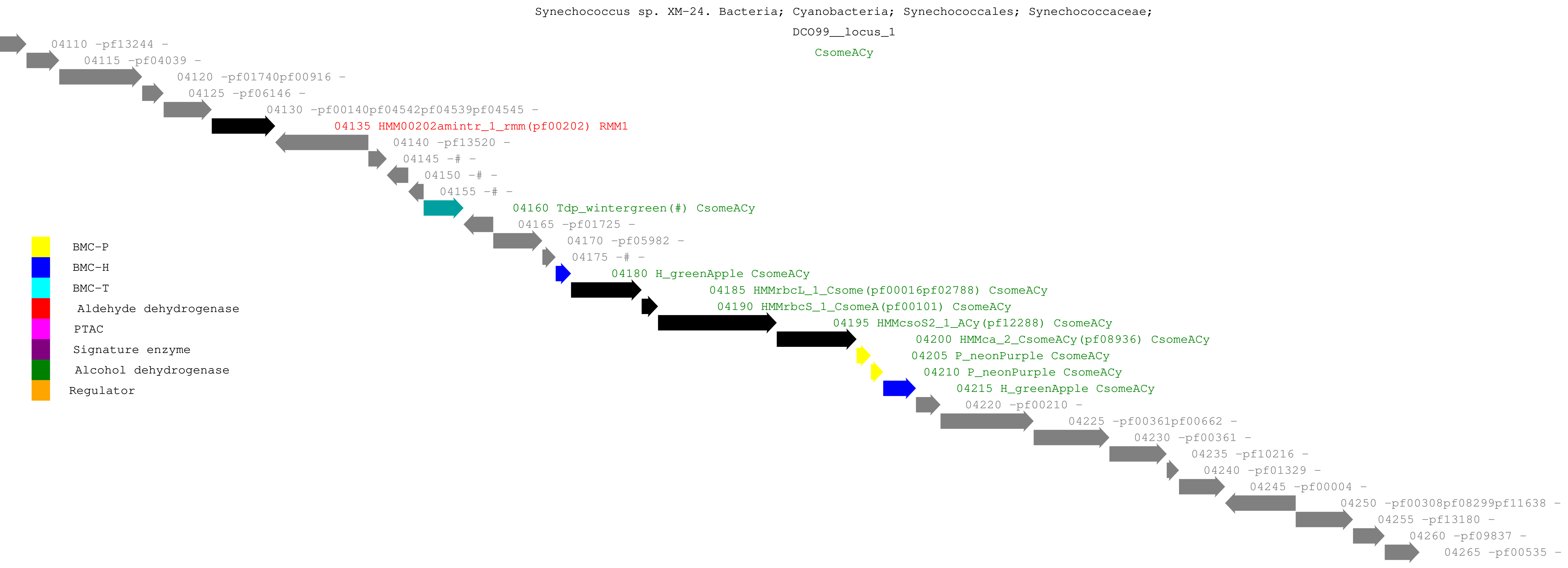
Synechococcus sp. XM-24. Bacteria; Cyanobacteria; Synechococcales; Synechococcaceae;
DCO99__locus_1
CsomeACy
04110 -pf13244 -
04115 -pf04039 -
04120 -pf01740pf00916 -
04125 -pf06146 -
04130 -pf00140pf04542pf04539pf04545 -
04135 HMM00202amintr_1_rmm(pf00202) RMM1
04140 -pf13520 -
04145 -# -
04150 -# -
04155 -# -
04160 Tdp_wintergreen(#) CsomeACy
04165 -pf01725 -
04170 -pf05982 -
04175 -# -
04180 H_greenApple CsomeACy
04185 HMMrbcL_1_Csome(pf00016pf02788) CsomeACy
04190 HMMrbcS_1_CsomeA(pf00101) CsomeACy
04195 HMMcsoS2_1_ACy(pf12288) CsomeACy
04200 HMMca_2_CsomeACy(pf08936) CsomeACy
04205 P_neonPurple CsomeACy
04210 P_neonPurple CsomeACy
04215 H_greenApple CsomeACy
04220 -pf00210 -
04225 -pf00361pf00662 -
04230 -pf00361 -
04235 -pf10216 -
04240 -pf01329 -
04245 -pf00004 -
04250 -pf00308pf08299pf11638 -
04255 -pf13180 -
04260 -pf09837 -
04265 -pf00535 -
BMC-P
BMC-H
BMC-T
Aldehyde dehydrogenase
PTAC
Signature enzyme
Alcohol dehydrogenase
Regulator

Synechococcus sp. UBA8638. Bacteria; Cyanobacteria; Synechococcales; Synechococcaceae;

DD643__locus_fragmented

CsomeACy

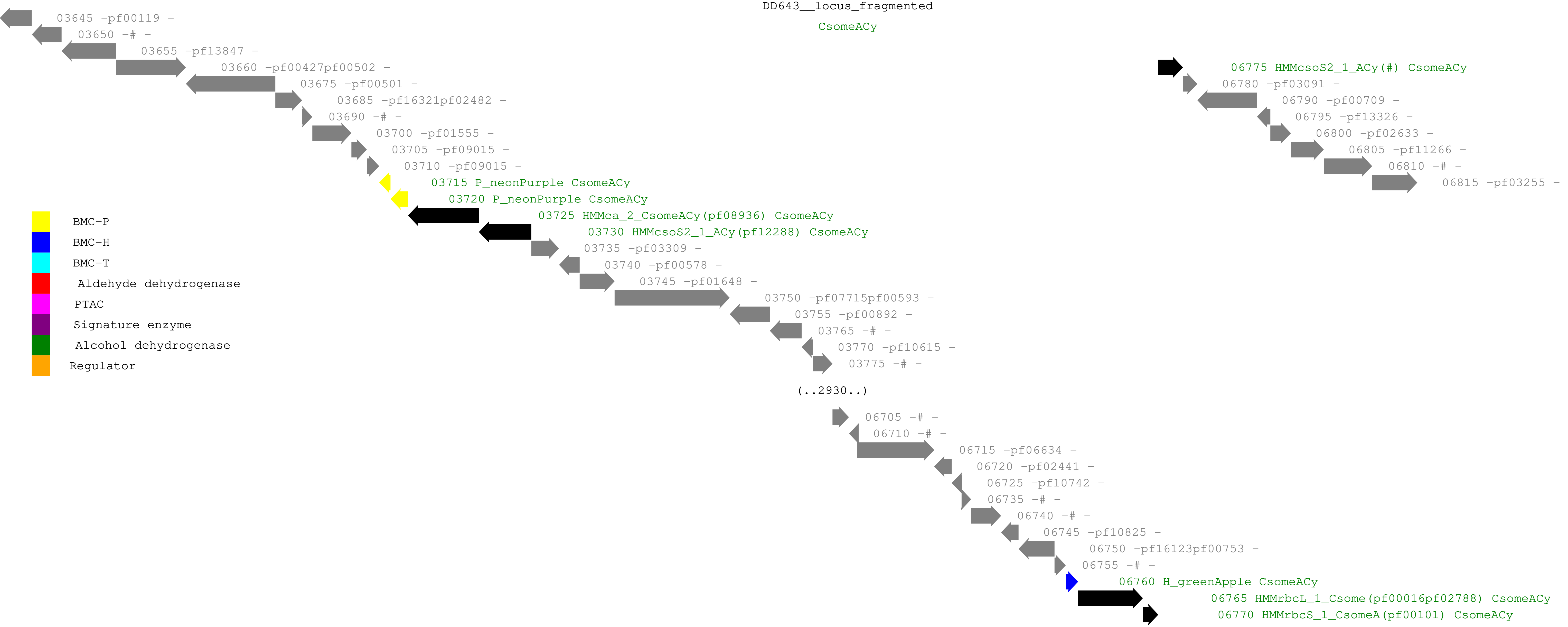

Synechococcales bacterium UBA10510. Bacteria; Cyanobacteria; Synechococcales.

DDY43__locus_1

CsomeACy

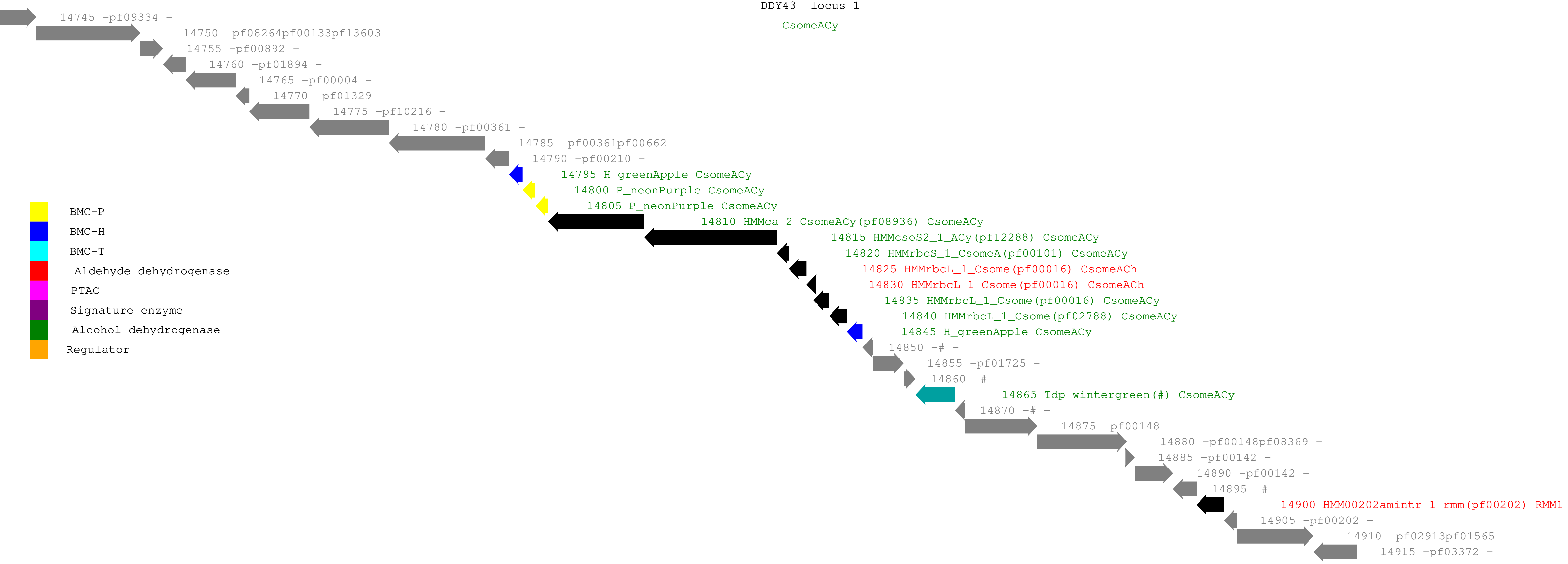

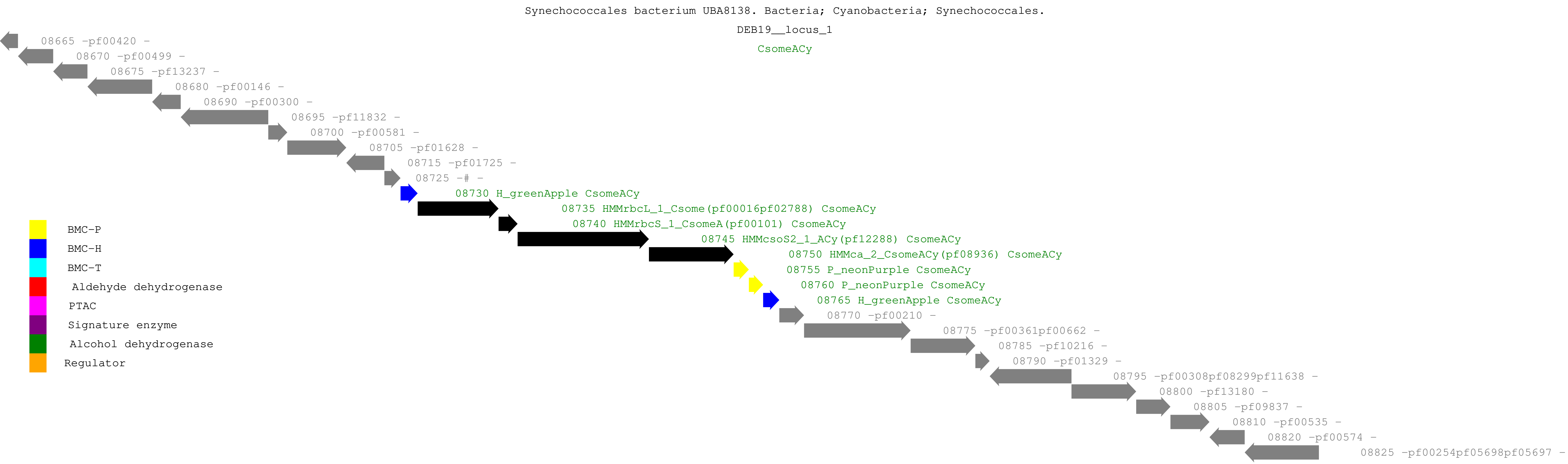
Synechococcales bacterium UBA8138. Bacteria; Cyanobacteria; Synechococcales.
DEB19__locus_1
CsomeACy
08665 -pf00420 -
08670 -pf00499 -
08675 -pf13237 -
08680 -pf00146 -
08690 -pf00300 -
08695 -pf11832 -
08700 -pf00581 -
08705 -pf01628 -
08715 -pf01725 -
08725 -# -
08730 H_greenApple CsomeACy
08735 HMMrbcL_1_Csome(pf00016pf02788) CsomeACy
08740 HMMrbcS_1_CsomeA(pf00101) CsomeACy
08745 HMMcsoS2_1_ACy(pf12288) CsomeACy
08750 HMMca_2_CsomeACy(pf08936) CsomeACy
08755 P_neonPurple CsomeACy
08760 P_neonPurple CsomeACy
08765 H_greenApple CsomeACy
08770 -pf00210 -
08775 -pf00361pf00662 -
08785 -pf10216 -
08790 -pf01329 -
08795 -pf00308pf08299pf11638 -
08800 -pf13180 -
08805 -pf09837 -
08810 -pf00535 -
08820 -pf00574 -
08825 -pf00254pf05698pf05697 -
BMC-P
BMC-H
BMC-T
Aldehyde dehydrogenase
PTAC
Signature enzyme
Alcohol dehydrogenase
Regulator

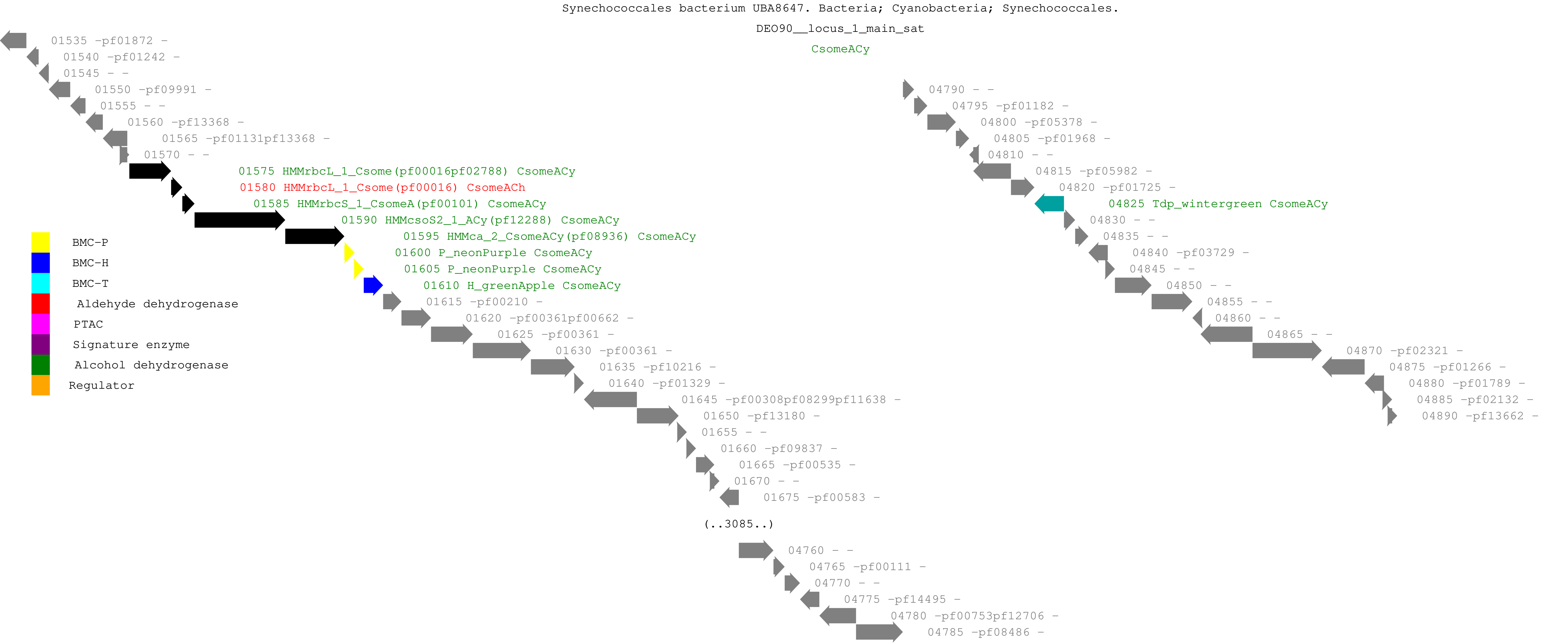
Synechococcales bacterium UBA8647. Bacteria; Cyanobacteria; Synechococcales.
DEO90__locus_1_main_sat
CsomeACy
01535 -pf01872 -
01540 -pf01242 -
01545 - -
01550 -pf09991 -
01555 - -
01560 -pf13368 -
01565 -pf01131pf13368 -
01570 - -
01575 HMMrbcL_1_Csome(pf00016pf02788) CsomeACy
01580 HMMrbcL_1_Csome(pf00016) CsomeACh
01585 HMMrbcS_1_CsomeA(pf00101) CsomeACy
01590 HMMcsoS2_1_ACy(pf12288) CsomeACy
01595 HMMca_2_CsomeACy(pf08936) CsomeACy
01600 P_neonPurple CsomeACy
01605 P_neonPurple CsomeACy
01610 H_greenApple CsomeACy
01615 -pf00210 -
01620 -pf00361pf00662 -
01625 -pf00361 -
01630 -pf00361 -
01635 -pf10216 -
01640 -pf01329 -
01645 -pf00308pf08299pf11638 -
01650 -pf13180 -
01655 - -
01660 -pf09837 -
01665 -pf00535 -
01670 - -
01675 -pf00583 -
(..3085..)
04760 - -
04765 -pf00111 -
04770 - -
04775 -pf14495 -
04780 -pf00753pf12706 -
04785 -pf08486 -
04790 - -
04795 -pf01182 -
04800 -pf05378 -
04805 -pf01968 -
04810 - -
04815 -pf05982 -
04820 -pf01725 -
04825 Tdp_wintergreen CsomeACy
04830 - -
04835 - -
04840 -pf03729 -
04845 - -
04850 - -
04855 - -
04860 - -
04865 - -
04870 -pf02321 -
04875 -pf01266 -
04880 -pf01789 -
04885 -pf02132 -
04890 -pf13662 -
BMC-P
BMC-H
BMC-T
Aldehyde dehydrogenase
PTAC
Signature enzyme
Alcohol dehydrogenase
Regulator

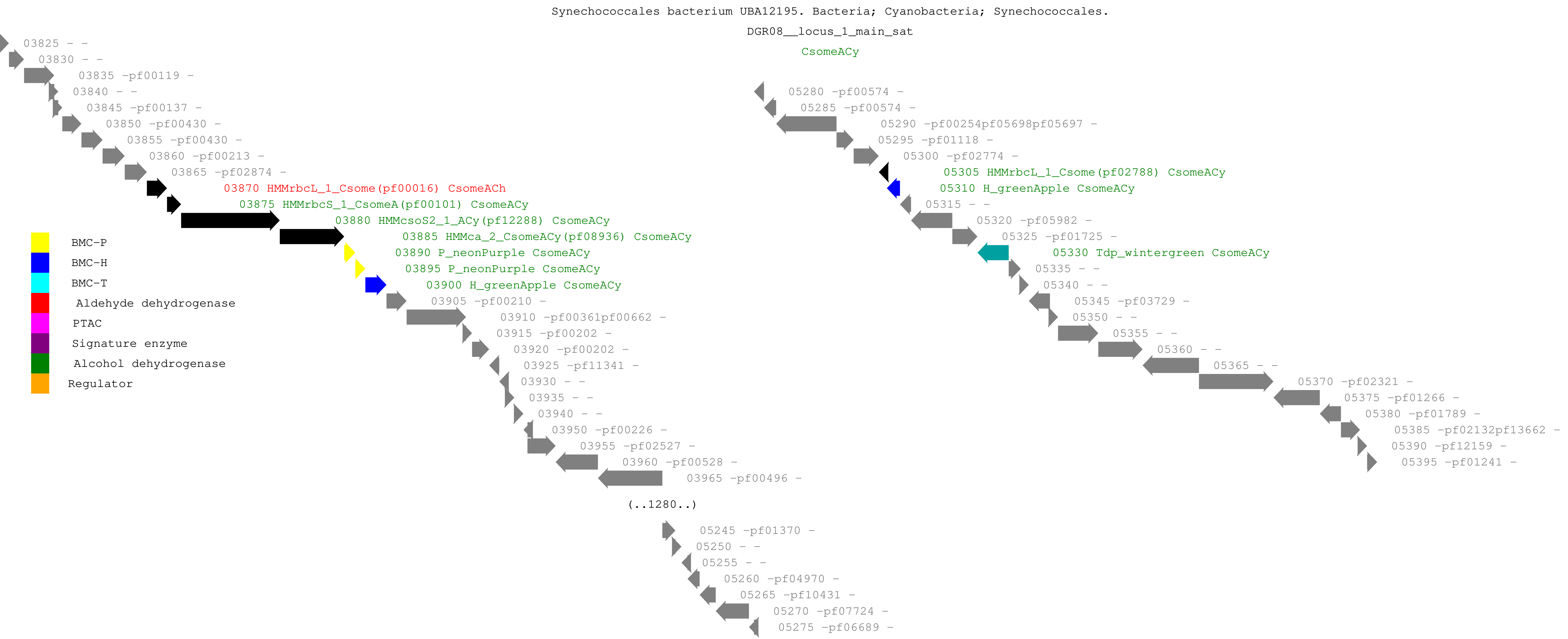

Synechococcales bacterium UBA12195. Bacteria; Cyanobacteria; Synechococcales.
DGR08__locus_1_main_sat
CsomeACy
03825 - -
03830 - -
03835 -pf00119 -
03840 - -
03845 -pf00137 -
03850 -pf00430 -
03855 -pf00430 -
03860 -pf00213 -
03865 -pf02874 -
03870 HMMrbcL_1_Csome(pf00016) CsomeACh
03875 HMMrbcS_1_CsomeA(pf00101) CsomeACy
03880 HMMcsoS2_1_ACy(pf12288) CsomeACy
03885 HMMca_2_CsomeACy(pf08936) CsomeACy
03890 P_neonPurple CsomeACy
03895 P_neonPurple CsomeACy
03900 H_greenApple CsomeACy
03905 -pf00210 -
03910 -pf00361pf00662 -
03915 -pf00202 -
03920 -pf00202 -
03925 -pf11341 -
03930 - -
03935 - -
03940 - -
03950 -pf00226 -
03955 -pf02527 -
03960 -pf00528 -
03965 -pf00496 -
(..1280..)
05245 -pf01370 -
05250 - -
05255 - -
05260 -pf04970 -
05265 -pf10431 -
05270 -pf07724 -
05275 -pf06689 -
05280 -pf00574 -
05285 -pf00574 -
05290 -pf00254pf05698pf05697 -
05295 -pf01118 -
05300 -pf02774 -
05305 HMMrbcL_1_Csome(pf02788) CsomeACy
05310 H_greenApple CsomeACy
05315 - -
05320 -pf05982 -
05325 -pf01725 -
05330 Tdp_wintergreen CsomeACy
05335 - -
05340 - -
05345 -pf03729 -
05350 - -
05355 - -
05360 - -
05365 - -
05370 -pf02321 -
05375 -pf01266 -
05380 -pf01789 -
05385 -pf02132pf13662 -
05390 -pf12159 -
05395 -pf01241 -
BMC-P
BMC-H
BMC-T
Aldehyde dehydrogenase
PTAC
Signature enzyme
Alcohol dehydrogenase
Regulator

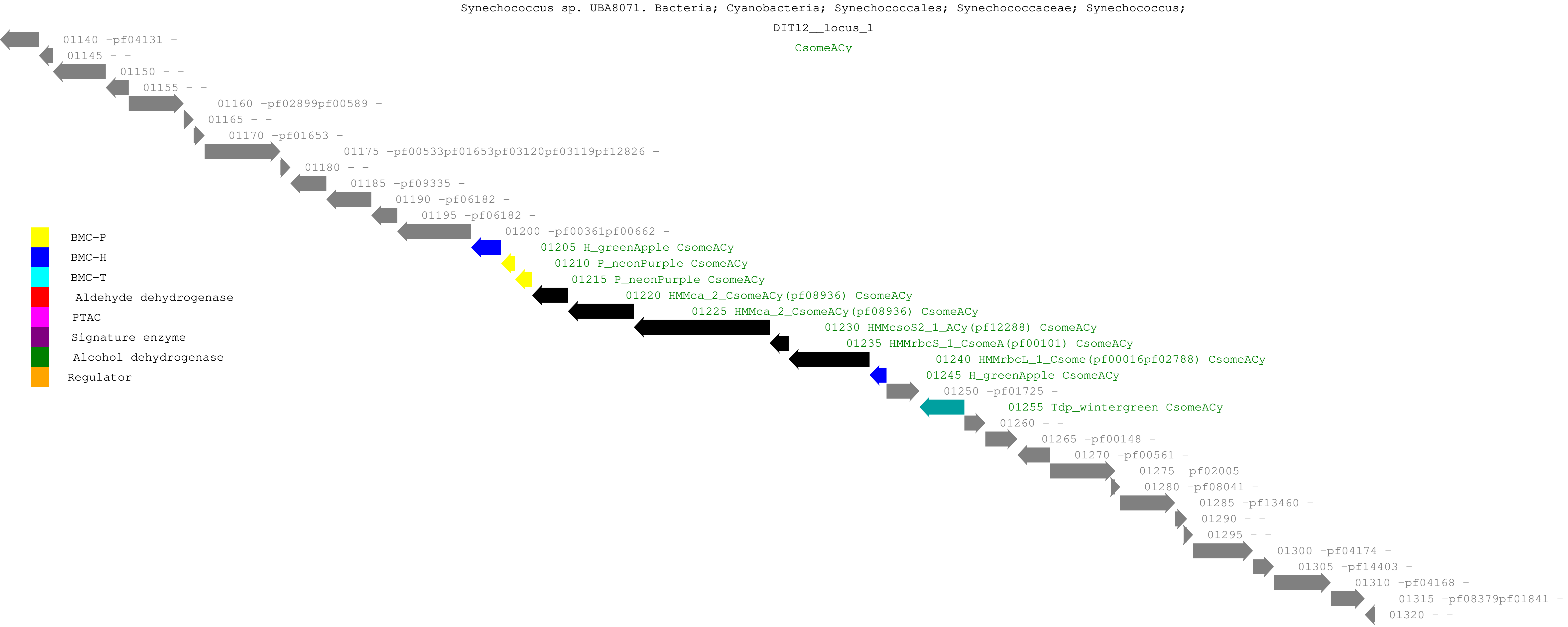
Synechococcus sp. UBA8071. Bacteria; Cyanobacteria; Synechococcales; Synechococcaceae; Synechococcus;
DIT12__locus_1
CsomeACy
01140 -pf04131 -
01145 - -
01150 - -
01155 - -
01160 -pf02899pf00589 -
01165 - -
01170 -pf01653 -
01175 -pf00533pf01653pf03120pf03119pf12826 -
01180 - -
01185 -pf09335 -
01190 -pf06182 -
01195 -pf06182 -
01200 -pf00361pf00662 -
01205 H_greenApple CsomeACy
01210 P_neonPurple CsomeACy
01215 P_neonPurple CsomeACy
01220 HMMca_2_CsomeACy(pf08936) CsomeACy
01225 HMMca_2_CsomeACy(pf08936) CsomeACy
01230 HMMcsoS2_1_ACy(pf12288) CsomeACy
01235 HMMrbcS_1_CsomeA(pf00101) CsomeACy
01240 HMMrbcL_1_Csome(pf00016pf02788) CsomeACy
01245 H_greenApple CsomeACy
01250 -pf01725 -
01255 Tdp_wintergreen CsomeACy
01260 - -
01265 -pf00148 -
01270 -pf00561 -
01275 -pf02005 -
01280 -pf08041 -
01285 -pf13460 -
01290 - -
01295 - -
01300 -pf04174 -
01305 -pf14403 -
01310 -pf04168 -
01315 -pf08379pf01841 -
01320 - -
BMC-P
BMC-H
BMC-T
Aldehyde dehydrogenase
PTAC
Signature enzyme
Alcohol dehydrogenase
Regulator

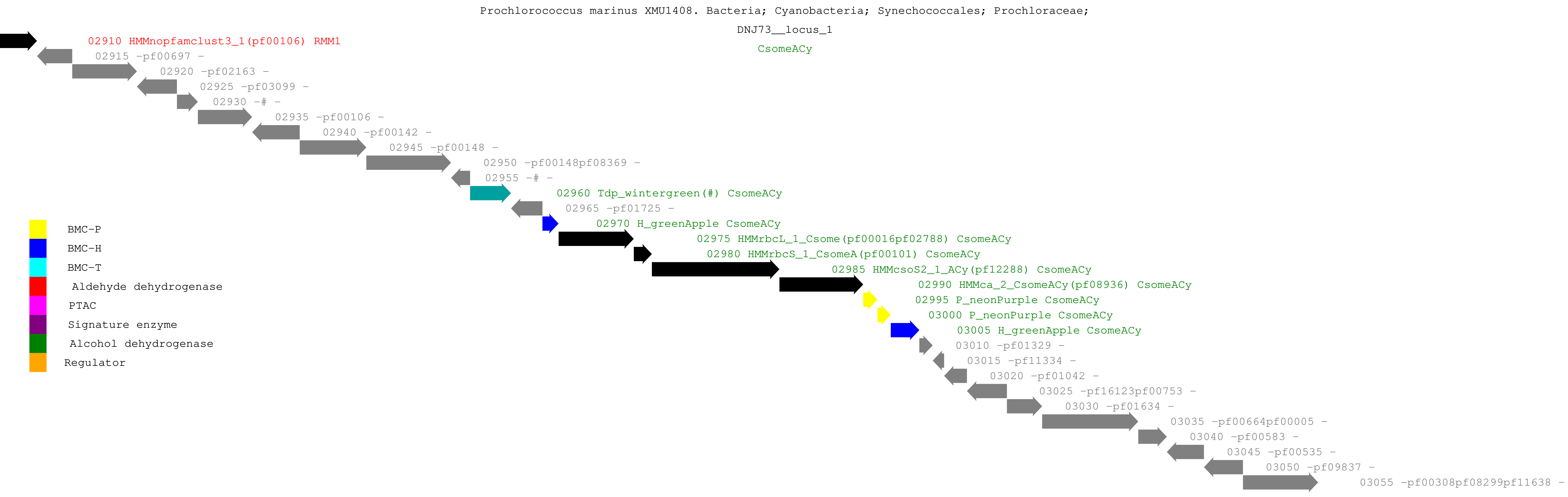
Prochlorococcus marinus XMU1408. Bacteria; Cyanobacteria; Synechococcales; Prochloraceae;
DNJ73__locus_1
CsomeACy
02910 HMMnopfamclust3_1(pf00106) RMM1
02915 -pf00697 -
02920 -pf02163 -
02925 -pf03099 -
02930 -# -
02935 -pf00106 -
02940 -pf00142 -
02945 -pf00148 -
02950 -pf00148pf08369 -
02955 -# -
02960 Tdp_wintergreen(#) CsomeACy
02965 -pf01725 -
02970 H_greenApple CsomeACy
02975 HMMrbcL_1_Csome(pf00016pf02788) CsomeACy
02980 HMMrbcS_1_CsomeA(pf00101) CsomeACy
02985 HMMcsoS2_1_ACy(pf12288) CsomeACy
02990 HMMca_2_CsomeACy(pf08936) CsomeACy
02995 P_neonPurple CsomeACy
03000 P_neonPurple CsomeACy
03005 H_greenApple CsomeACy
03010 -pf01329 -
03015 -pf11334 -
03020 -pf01042 -
03025 -pf16123pf00753 -
03030 -pf01634 -
03035 -pf00664pf00005 -
03040 -pf00583 -
03045 -pf00535 -
03050 -pf09837 -
03055 -pf00308pf08299pf11638 -
BMC-P
BMC-H
BMC-T
Aldehyde dehydrogenase
PTAC
Signature enzyme
Alcohol dehydrogenase
Regulator

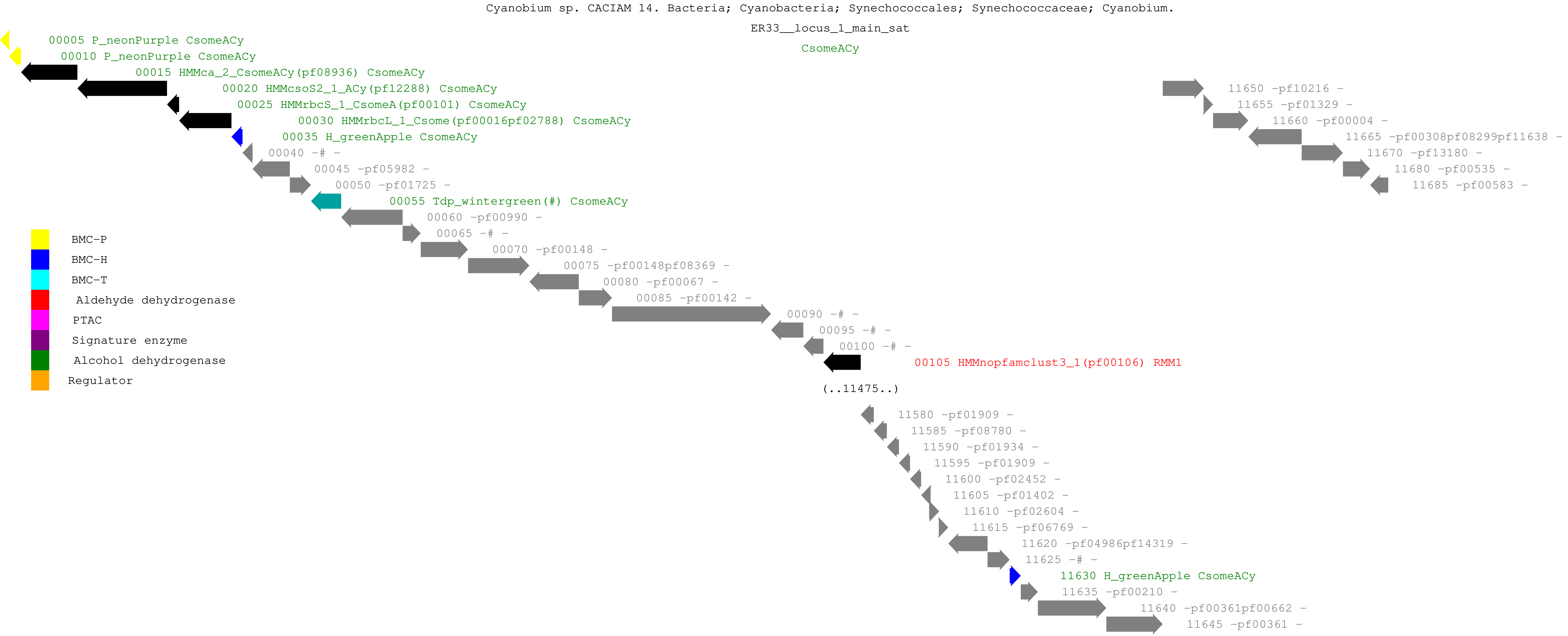

Cyanobium sp. CACIAM 14. Bacteria; Cyanobacteria; Synechococcales; Synechococcaceae; Cyanobium.
ER33__locus_1_main_sat
CsomeACy
00005 P_neonPurple CsomeACy
00010 P_neonPurple CsomeACy
00015 HMMca_2_CsomeACy(pf08936) CsomeACy
00020 HMMcsoS2_1_ACy(pf12288) CsomeACy
00025 HMMrbcS_1_CsomeA(pf00101) CsomeACy
00030 HMMrbcL_1_Csome(pf00016pf02788) CsomeACy
00035 H_greenApple CsomeACy
00040 -# -
00045 -pf05982 -
00050 -pf01725 -
00055 Tdp_wintergreen(#) CsomeACy
00060 -pf00990 -
00065 -# -
00070 -pf00148 -
00075 -pf00148pf08369 -
00080 -pf00067 -
00085 -pf00142 -
00090 -# -
00095 -# -
00100 -# -
00105 HMMnopfamclust3_1(pf00106) RMM1
(..11475..)
11580 -pf01909 -
11585 -pf08780 -
11590 -pf01934 -
11595 -pf01909 -
11600 -pf02452 -
11605 -pf01402 -
11610 -pf02604 -
11615 -pf06769 -
11620 -pf04986pf14319 -
11625 -# -
11630 H_greenApple CsomeACy
11635 -pf00210 -
11640 -pf00361pf00662 -
11645 -pf00361 -
11650 -pf10216 -
11655 -pf01329 -
11660 -pf00004 -
11665 -pf00308pf08299pf11638 -
11670 -pf13180 -
11680 -pf00535 -
11685 -pf00583 -
BMC-P
BMC-H
BMC-T
Aldehyde dehydrogenase
PTAC
Signature enzyme
Alcohol dehydrogenase
Regulator

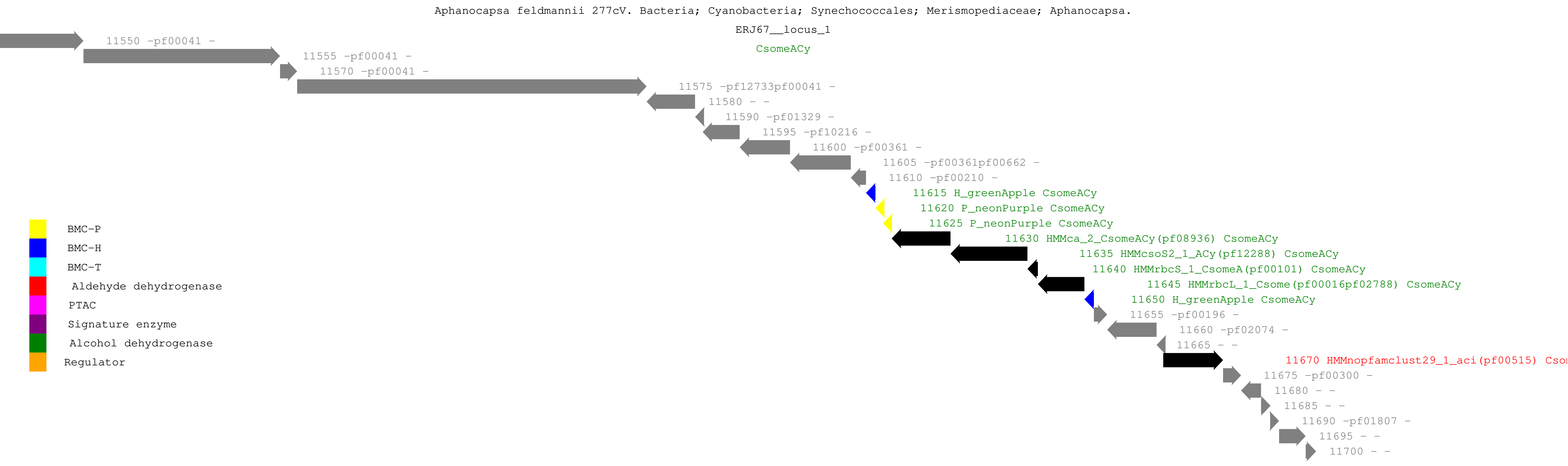

Aphanocapsa feldmannii 277cV. Bacteria; Cyanobacteria; Synechococcales; Merismopediaceae; Aphanocapsa.
ERJ67__locus_1
CsomeACy
11550 -pf00041 -
11555 -pf00041 -
11570 -pf00041 -
11575 -pf12733pf00041 -
11580 - -
11590 -pf01329 -
11595 -pf10216 -
11600 -pf00361 -
11605 -pf00361pf00662 -
11610 -pf00210 -
11615 H_greenApple CsomeACy
11620 P_neonPurple CsomeACy
11625 P_neonPurple CsomeACy
11630 HMMca_2_CsomeACy(pf08936) CsomeACy
11635 HMMcsoS2_1_ACy(pf12288) CsomeACy
11640 HMMrbcS_1_CsomeA(pf00101) CsomeACy
11645 HMMrbcL_1_Csome(pf00016pf02788) CsomeACy
11650 H_greenApple CsomeACy
11655 -pf00196 -
11660 -pf02074 -
11665 - -
11670 HMMnopfamclust29_1_aci(pf00515) Cso
11675 -pf00300 -
11680 - -
11685 - -
11690 -pf01807 -
11695 - -
11700 - -
BMC-P
BMC-H
BMC-T
Aldehyde dehydrogenase
PTAC
Signature enzyme
Alcohol dehydrogenase
Regulator

Aphanocapsa feldmannii 277cI. Bacteria; Cyanobacteria; Synechococcales; Merismopediaceae; Aphanocapsa.

ERJ68__locus_1_main_sat

CsomeACy

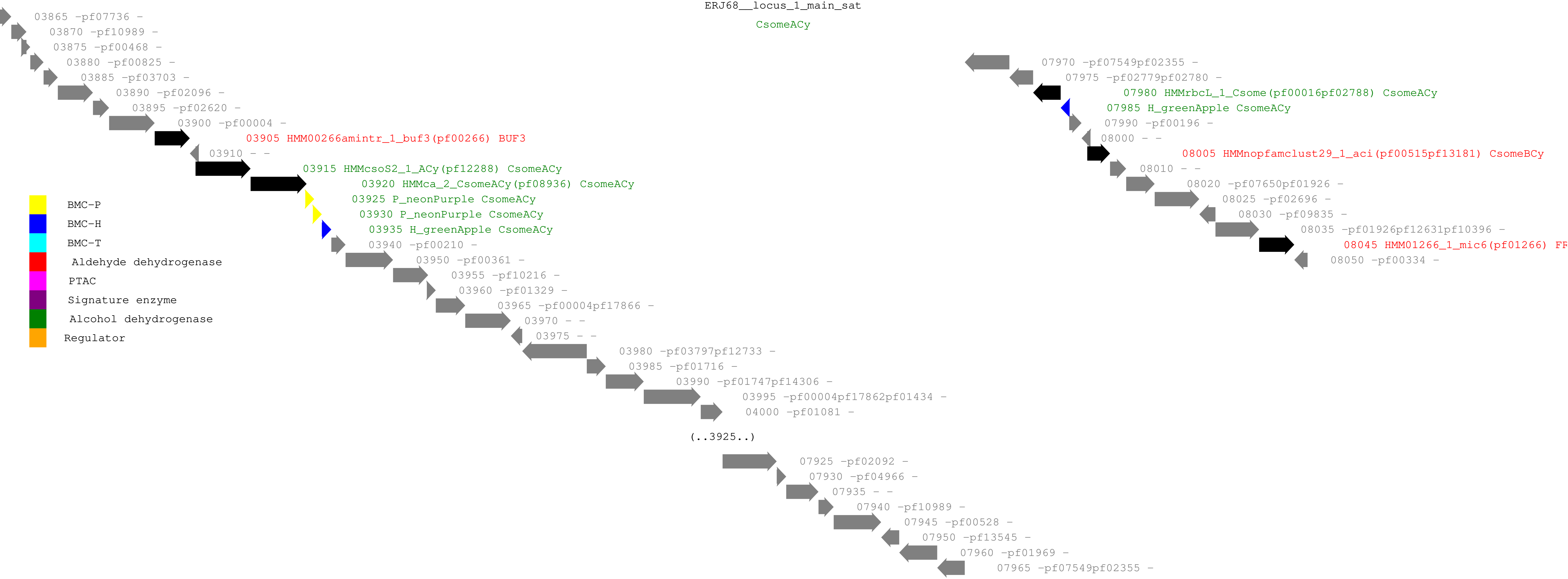

Prochlorococcus marinus str. GP2. Bacteria; Cyanobacteria; Synechococcales; Prochloraceae;

EU91__locus_1

CsomeACy

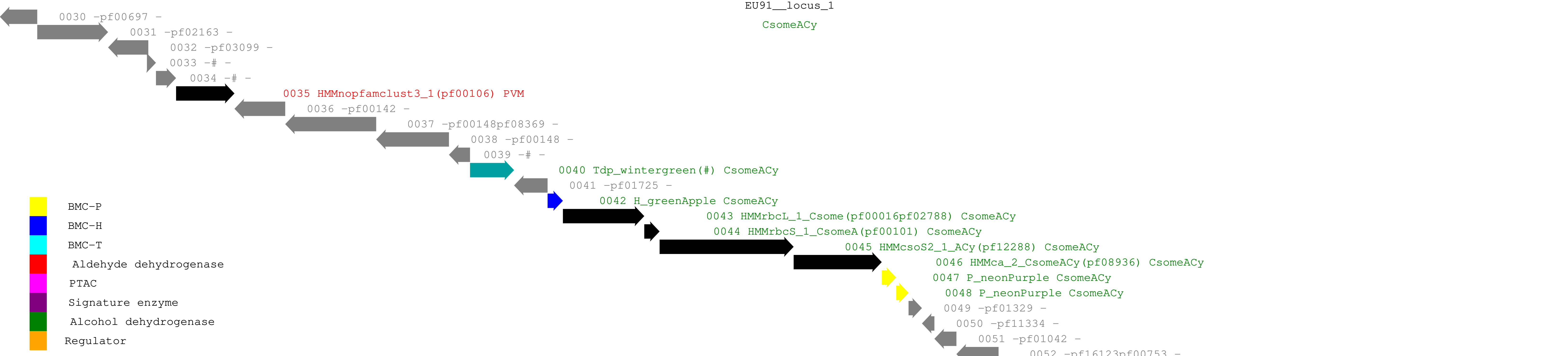

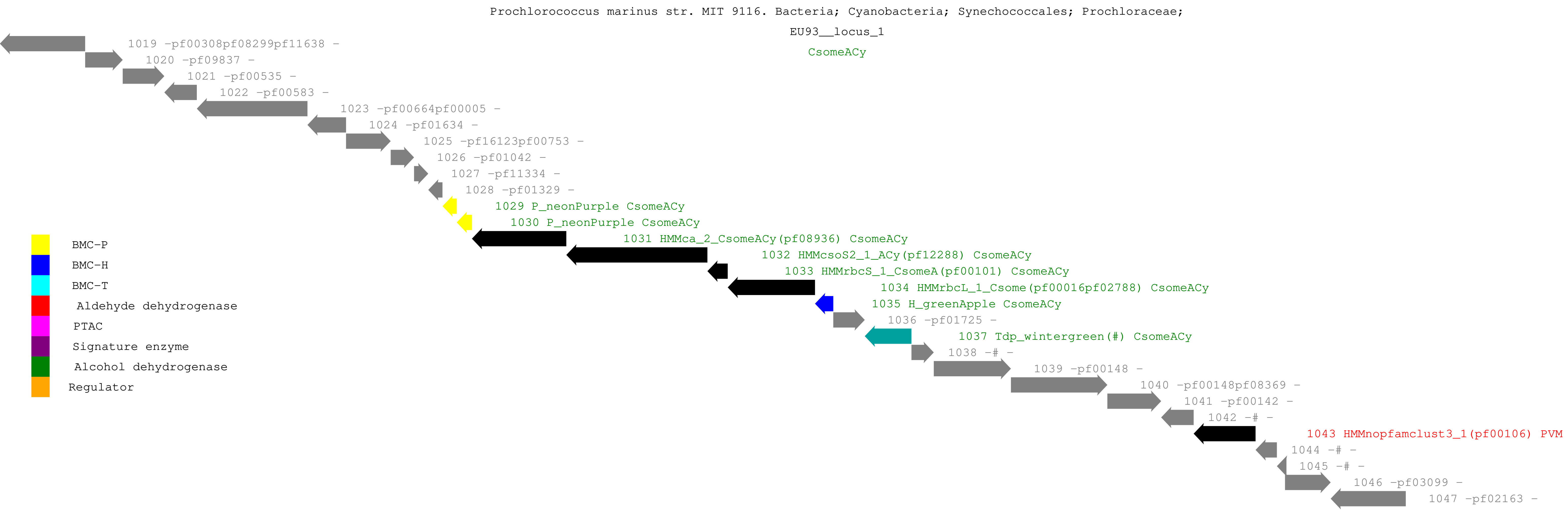

Prochlorococcus marinus str. MIT 9116. Bacteria; Cyanobacteria; Synechococcales; Prochloraceae;
EU93__locus_1
CsomeACy
1019 -pf00308pf08299pf11638 -
1020 -pf09837 -
1021 -pf00535 -
1022 -pf00583 -
1023 -pf00664pf00005 -
1024 -pf01634 -
1025 -pf16123pf00753 -
1026 -pf01042 -
1027 -pf11334 -
1028 -pf01329 -
1029 P_neonPurple CsomeACy
1030 P_neonPurple CsomeACy
1031 HMMca_2_CsomeACy(pf08936) CsomeACy
1032 HMMcsoS2_1_ACy(pf12288) CsomeACy
1033 HMMrbcS_1_CsomeA(pf00101) CsomeACy
1034 HMMrbcL_1_Csome(pf00016pf02788) CsomeACy
1035 H_greenApple CsomeACy
1036 -pf01725 -
1037 Tdp_wintergreen(#) CsomeACy
1038 -# -
1039 -pf00148 -
1040 -pf00148pf08369 -
1041 -pf00142 -
1042 -# -
1043 HMMnopfamclust3_1(pf00106) PVM
1044 -# -
1045 -# -
1046 -pf03099 -
1047 -pf02163 -
BMC-P
BMC-H
BMC-T
Aldehyde dehydrogenase
PTAC
Signature enzyme
Alcohol dehydrogenase
Regulator

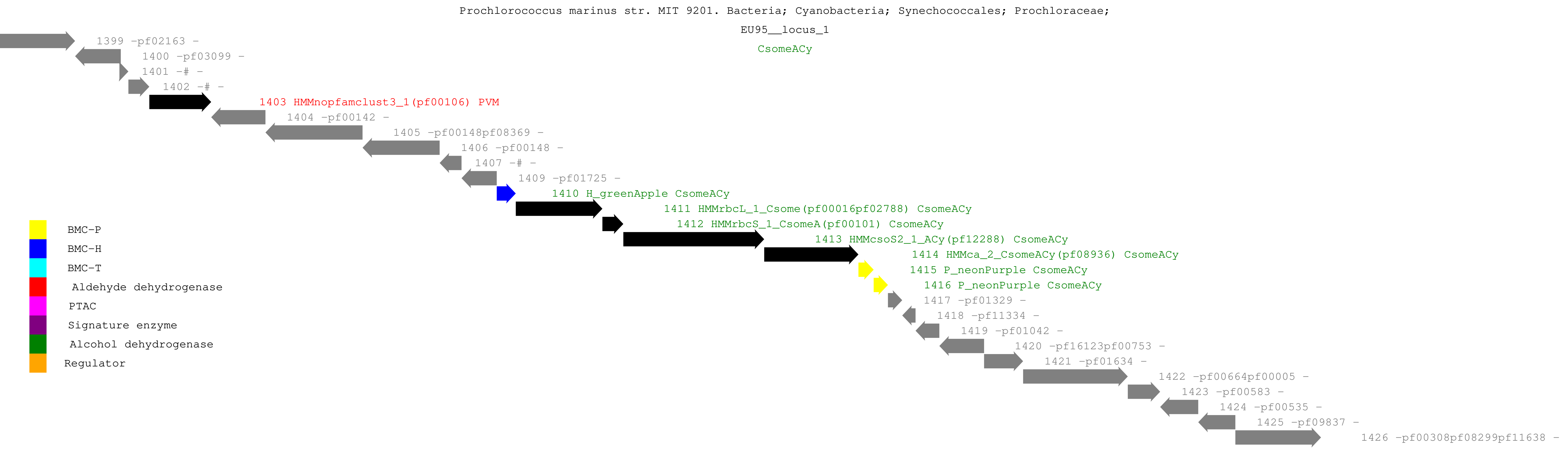

Prochlorococcus marinus str. MIT 9201. Bacteria; Cyanobacteria; Synechococcales; Prochloraceae;
EU95__locus_1
CsomeACy
1399 -pf02163 -
1400 -pf03099 -
1401 -# -
1402 -# -
1403 HMMnopfamclust3_1(pf00106) PVM
1404 -pf00142 -
1405 -pf00148pf08369 -
1406 -pf00148 -
1407 -# -
1409 -pf01725 -
1410 H_greenApple CsomeACy
1411 HMMrbcL_1_Csome(pf00016pf02788) CsomeACy
1412 HMMrbcS_1_CsomeA(pf00101) CsomeACy
1413 HMMcsoS2_1_ACy(pf12288) CsomeACy
1414 HMMca_2_CsomeACy(pf08936) CsomeACy
1415 P_neonPurple CsomeACy
1416 P_neonPurple CsomeACy
1417 -pf01329 -
1418 -pf11334 -
1419 -pf01042 -
1420 -pf16123pf00753 -
1421 -pf01634 -
1422 -pf00664pf00005 -
1423 -pf00583 -
1424 -pf00535 -
1425 -pf09837 -
1426 -pf00308pf08299pf11638 -
BMC-P
BMC-H
BMC-T
Aldehyde dehydrogenase
PTAC
Signature enzyme
Alcohol dehydrogenase
Regulator

Prochlorococcus marinus str. MIT 9302. Bacteria; Cyanobacteria; Synechococcales; Prochloraceae;

EU96__locus_1

CsomeACy

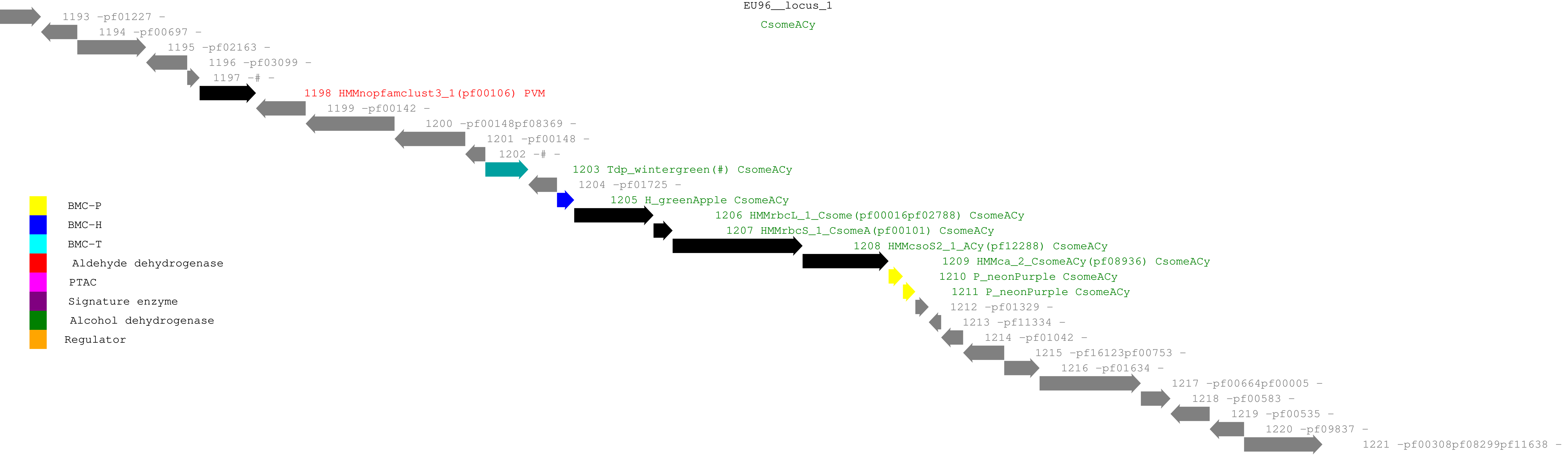

Prochlorococcus marinus str. MIT 9314. Bacteria; Cyanobacteria; Synechococcales; Prochloraceae;

EU98__locus_1

CsomeACy

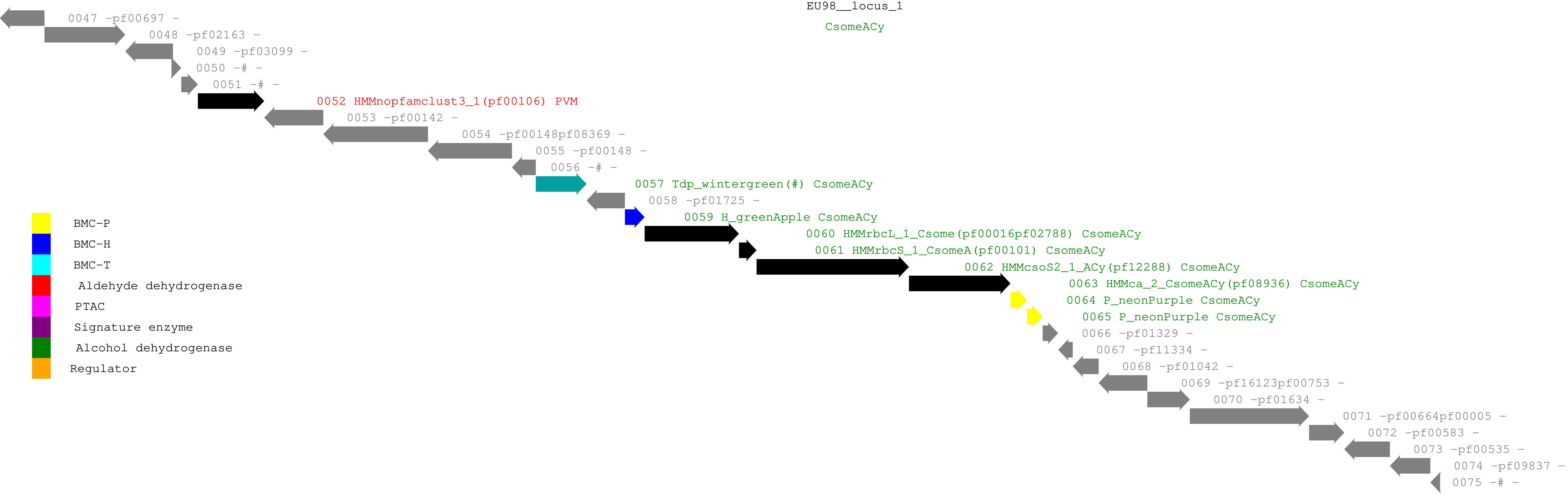

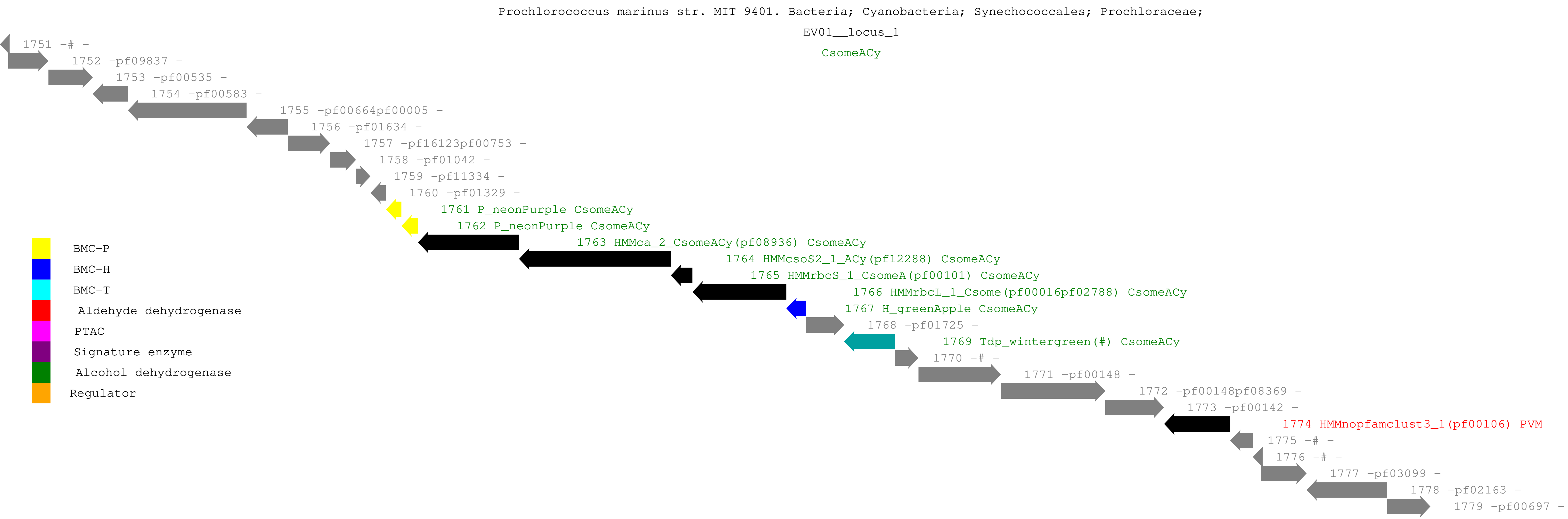
Prochlorococcus marinus str. MIT 9401. Bacteria; Cyanobacteria; Synechococcales; Prochloraceae;
EV01__locus_1
CsomeACy
1751 -# -
1752 -pf09837 -
1753 -pf00535 -
1754 -pf00583 -
1755 -pf00664pf00005 -
1756 -pf01634 -
1757 -pf16123pf00753 -
1758 -pf01042 -
1759 -pf11334 -
1760 -pf01329 -
1761 P_neonPurple CsomeACy
1762 P_neonPurple CsomeACy
1763 HMMca_2_CsomeACy(pf08936) CsomeACy
1764 HMMcsoS2_1_ACy(pf12288) CsomeACy
1765 HMMrbcS_1_CsomeA(pf00101) CsomeACy
1766 HMMrbcL_1_Csome(pf00016pf02788) CsomeACy
1767 H_greenApple CsomeACy
1768 -pf01725 -
1769 Tdp_wintergreen(#) CsomeACy
1770 -# -
1771 -pf00148 -
1772 -pf00148pf08369 -
1773 -pf00142 -
1774 HMMnopfamclust3_1(pf00106) PVM
1775 -# -
1776 -# -
1777 -pf03099 -
1778 -pf02163 -
1779 -pf00697 -
BMC-P
BMC-H
BMC-T
Aldehyde dehydrogenase
PTAC
Signature enzyme
Alcohol dehydrogenase
Regulator

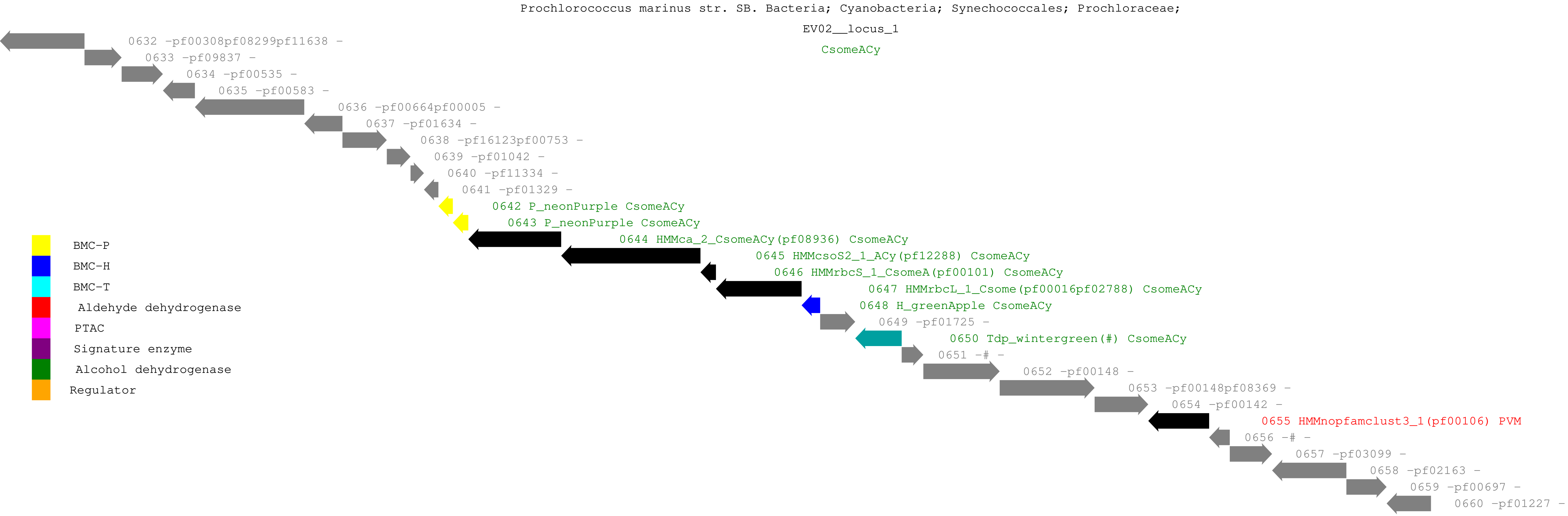

Prochlorococcus marinus str. SB. Bacteria; Cyanobacteria; Synechococcales; Prochloraceae;
EV02__locus_1
CsomeACy
0632 -pf00308pf08299pf11638 -
0633 -pf09837 -
0634 -pf00535 -
0635 -pf00583 -
0636 -pf00664pf00005 -
0637 -pf01634 -
0638 -pf16123pf00753 -
0639 -pf01042 -
0640 -pf11334 -
0641 -pf01329 -
0642 P_neonPurple CsomeACy
0643 P_neonPurple CsomeACy
0644 HMMca_2_CsomeACy(pf08936) CsomeACy
0645 HMMcsoS2_1_ACy(pf12288) CsomeACy
0646 HMMrbcS_1_CsomeA(pf00101) CsomeACy
0647 HMMrbcL_1_Csome(pf00016pf02788) CsomeACy
0648 H_greenApple CsomeACy
0649 -pf01725 -
0650 Tdp_wintergreen(#) CsomeACy
0651 -# -
0652 -pf00148 -
0653 -pf00148pf08369 -
0654 -pf00142 -
0655 HMMnopfamclust3_1(pf00106) PVM
0656 -# -
0657 -pf03099 -
0658 -pf02163 -
0659 -pf00697 -
0660 -pf01227 -
BMC-P
BMC-H
BMC-T
Aldehyde dehydrogenase
PTAC
Signature enzyme
Alcohol dehydrogenase
Regulator

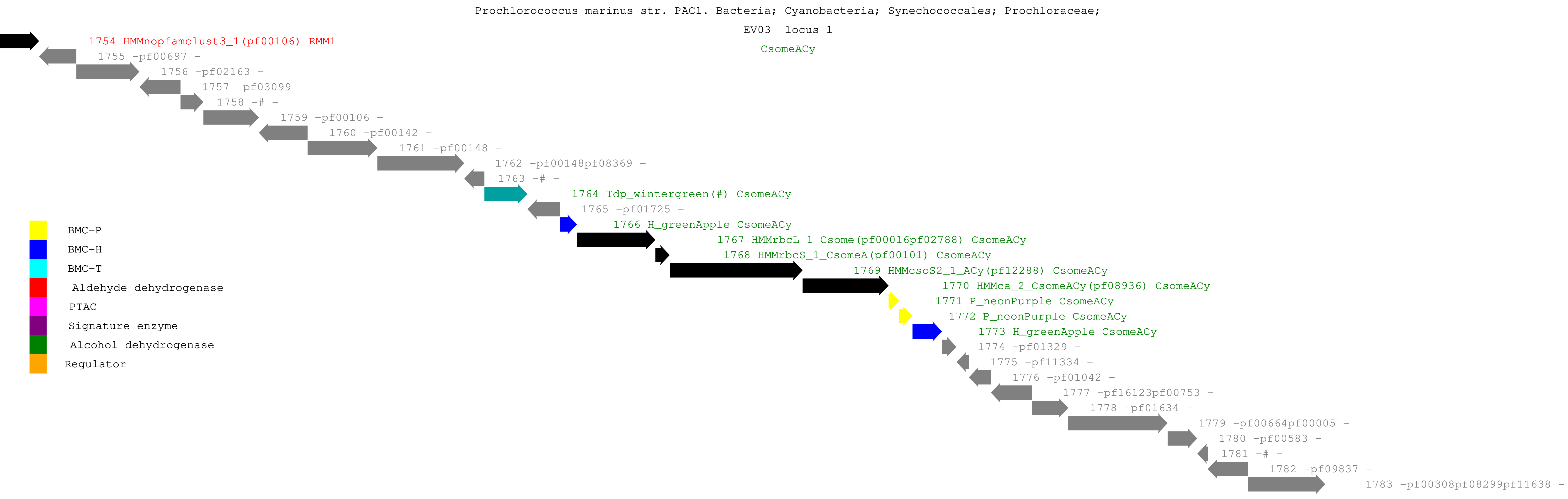
Prochlorococcus marinus str. PAC1. Bacteria; Cyanobacteria; Synechococcales; Prochloraceae;
EV03__locus_1
CsomeACy
1754 HMMnopfamclust3_1(pf00106) RMM1
1755 -pf00697 -
1756 -pf02163 -
1757 -pf03099 -
1758 -# -
1759 -pf00106 -
1760 -pf00142 -
1761 -pf00148 -
1762 -pf00148pf08369 -
1763 -# -
1764 Tdp_wintergreen(#) CsomeACy
1765 -pf01725 -
1766 H_greenApple CsomeACy
1767 HMMrbcL_1_Csome(pf00016pf02788) CsomeACy
1768 HMMrbcS_1_CsomeA(pf00101) CsomeACy
1769 HMMcsoS2_1_ACy(pf12288) CsomeACy
1770 HMMca_2_CsomeACy(pf08936) CsomeACy
1771 P_neonPurple CsomeACy
1772 P_neonPurple CsomeACy
1773 H_greenApple CsomeACy
1774 -pf01329 -
1775 -pf11334 -
1776 -pf01042 -
1777 -pf16123pf00753 -
1778 -pf01634 -
1779 -pf00664pf00005 -
1780 -pf00583 -
1781 -# -
1782 -pf09837 -
1783 -pf00308pf08299pf11638 -
BMC-P
BMC-H
BMC-T
Aldehyde dehydrogenase
PTAC
Signature enzyme
Alcohol dehydrogenase
Regulator

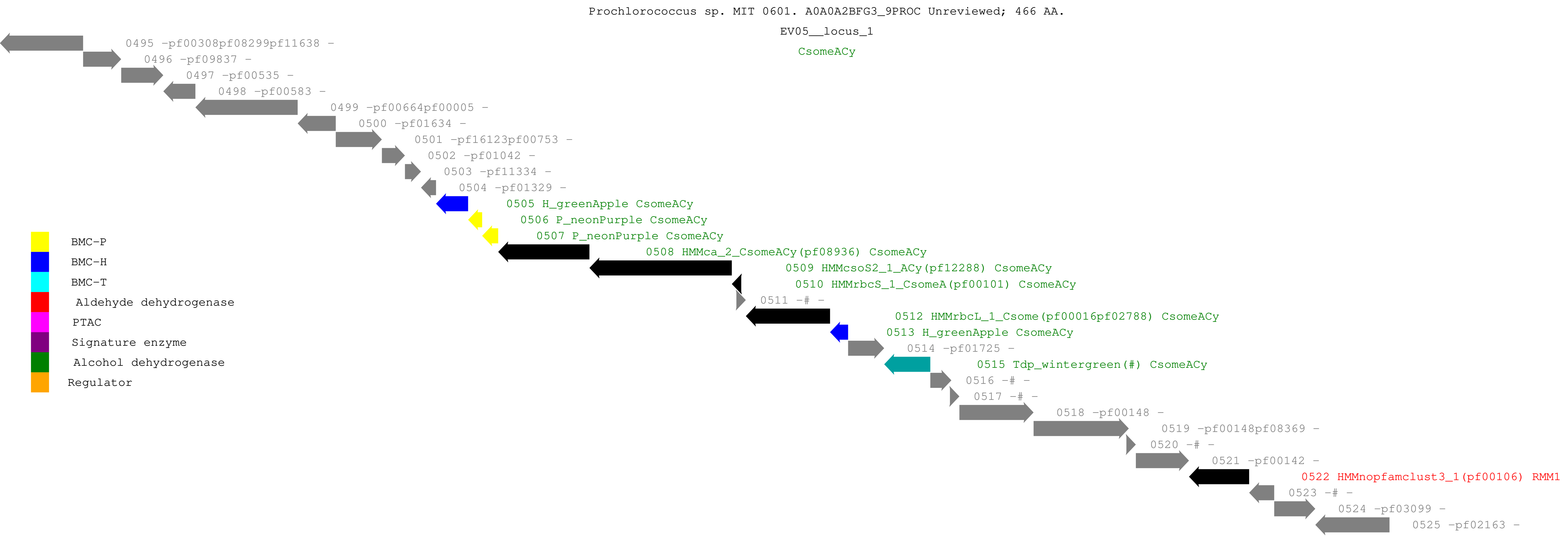
Prochlorococcus sp. MIT 0601. A0A0A2BFG3_9PROC Unreviewed; 466 AA.
EV05__locus_1
CsomeACy
0495 -pf00308pf08299pf11638 -
0496 -pf09837 -
0497 -pf00535 -
0498 -pf00583 -
0499 -pf00664pf00005 -
0500 -pf01634 -
0501 -pf16123pf00753 -
0502 -pf01042 -
0503 -pf11334 -
0504 -pf01329 -
0505 H_greenApple CsomeACy
0506 P_neonPurple CsomeACy
0507 P_neonPurple CsomeACy
0508 HMMca_2_CsomeACy(pf08936) CsomeACy
0509 HMMcsoS2_1_ACy(pf12288) CsomeACy
0510 HMMrbcS_1_CsomeA(pf00101) CsomeACy
0511 -# -
0512 HMMrbcL_1_Csome(pf00016pf02788) CsomeACy
0513 H_greenApple CsomeACy
0514 -pf01725 -
0515 Tdp_wintergreen(#) CsomeACy
0516 -# -
0517 -# -
0518 -pf00148 -
0519 -pf00148pf08369 -
0520 -# -
0521 -pf00142 -
0522 HMMnopfamclust3_1(pf00106) RMM1
0523 -# -
0524 -pf03099 -
0525 -pf02163 -
BMC-P
BMC-H
BMC-T
Aldehyde dehydrogenase
PTAC
Signature enzyme
Alcohol dehydrogenase
Regulator

Prochlorococcus sp. MIT 0602. A0A0A2BQP8_9PROC Unreviewed; 222 AA.

EV06__locus_1

CsomeACy

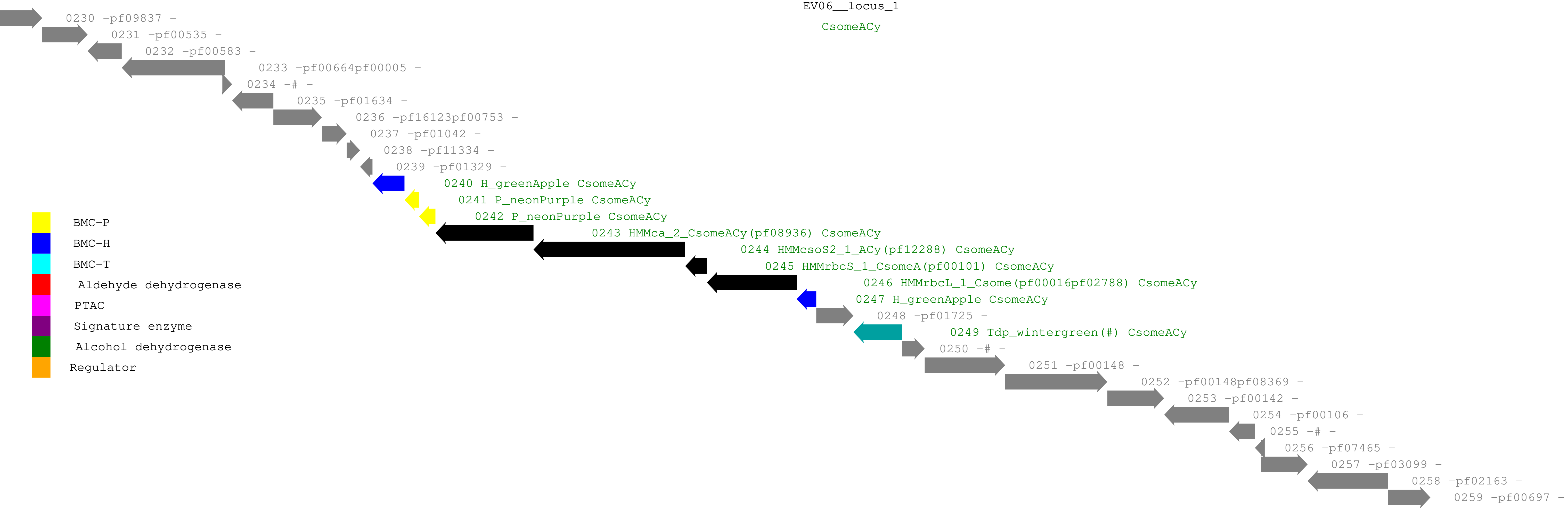

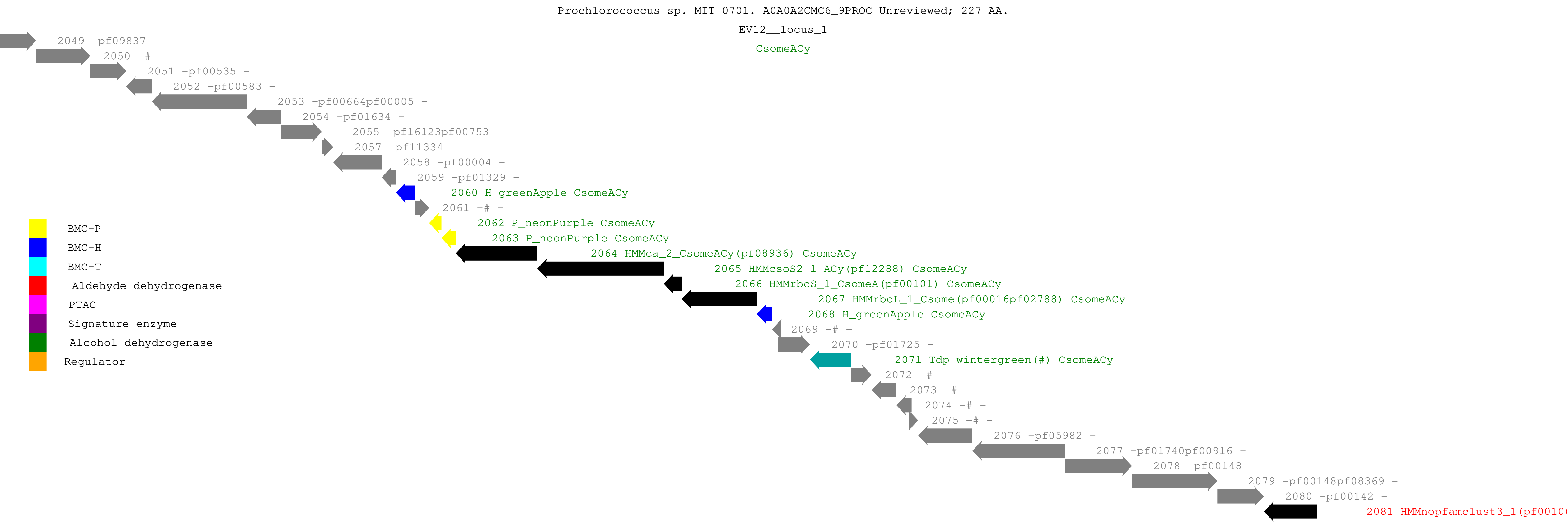
Prochlorococcus sp. MIT 0701. A0A0A2CMC6_9PROC Unreviewed; 227 AA.
EV12__locus_1
CsomeACy
2049 -pf09837 -
2050 -# -
2051 -pf00535 -
2052 -pf00583 -
2053 -pf00664pf00005 -
2054 -pf01634 -
2055 -pf16123pf00753 -
2057 -pf11334 -
2058 -pf00004 -
2059 -pf01329 -
2060 H_greenApple CsomeACy
2061 -# -
2062 P_neonPurple CsomeACy
2063 P_neonPurple CsomeACy
2064 HMMca_2_CsomeACy(pf08936) CsomeACy
2065 HMMcsoS2_1_ACy(pf12288) CsomeACy
2066 HMMrbcS_1_CsomeA(pf00101) CsomeACy
2067 HMMrbcL_1_Csome(pf00016pf02788) CsomeACy
2068 H_greenApple CsomeACy
2069 -# -
2070 -pf01725 -
2071 Tdp_wintergreen(#) CsomeACy
2072 -# -
2073 -# -
2074 -# -
2075 -# -
2076 -pf05982 -
2077 -pf01740pf00916 -
2078 -pf00148 -
2079 -pf00148pf08369 -
2080 -pf00142 -
BMC-P
BMC-H
BMC-T
Aldehyde dehydrogenase
PTAC
Signature enzyme
Alcohol dehydrogenase
Regulator

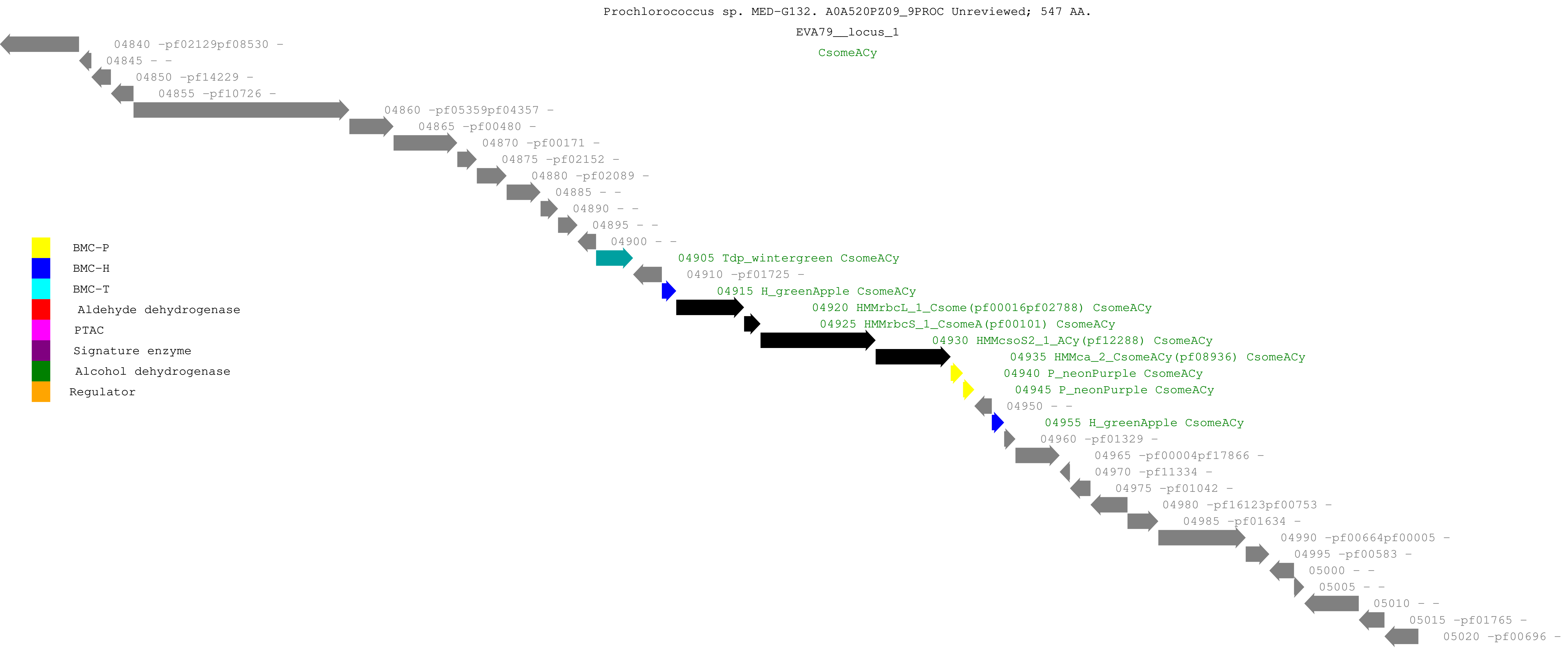
Prochlorococcus sp. MED-G132. A0A520PZ09_9PROC Unreviewed; 547 AA.
EVA79__locus_1
CsomeACy
04840 -pf02129pf08530 -
04845 - -
04850 -pf14229 -
04855 -pf10726 -
04860 -pf05359pf04357 -
04865 -pf00480 -
04870 -pf00171 -
04875 -pf02152 -
04880 -pf02089 -
04885 - -
04890 - -
04895 - -
04900 - -
04905 Tdp_wintergreen CsomeACy
04910 -pf01725 -
04915 H_greenApple CsomeACy
04920 HMMrbcL_1_Csome(pf00016pf02788) CsomeACy
04925 HMMrbcS_1_CsomeA(pf00101) CsomeACy
04930 HMMcsoS2_1_ACy(pf12288) CsomeACy
04935 HMMca_2_CsomeACy(pf08936) CsomeACy
04940 P_neonPurple CsomeACy
04945 P_neonPurple CsomeACy
04950 - -
04955 H_greenApple CsomeACy
04960 -pf01329 -
04965 -pf00004pf17866 -
04970 -pf11334 -
04975 -pf01042 -
04980 -pf16123pf00753 -
04985 -pf01634 -
04990 -pf00664pf00005 -
04995 -pf00583 -
05000 - -
05005 - -
05010 - -
05015 -pf01765 -
05020 -pf00696 -
BMC-P
BMC-H
BMC-T
Aldehyde dehydrogenase
PTAC
Signature enzyme
Alcohol dehydrogenase
Regulator

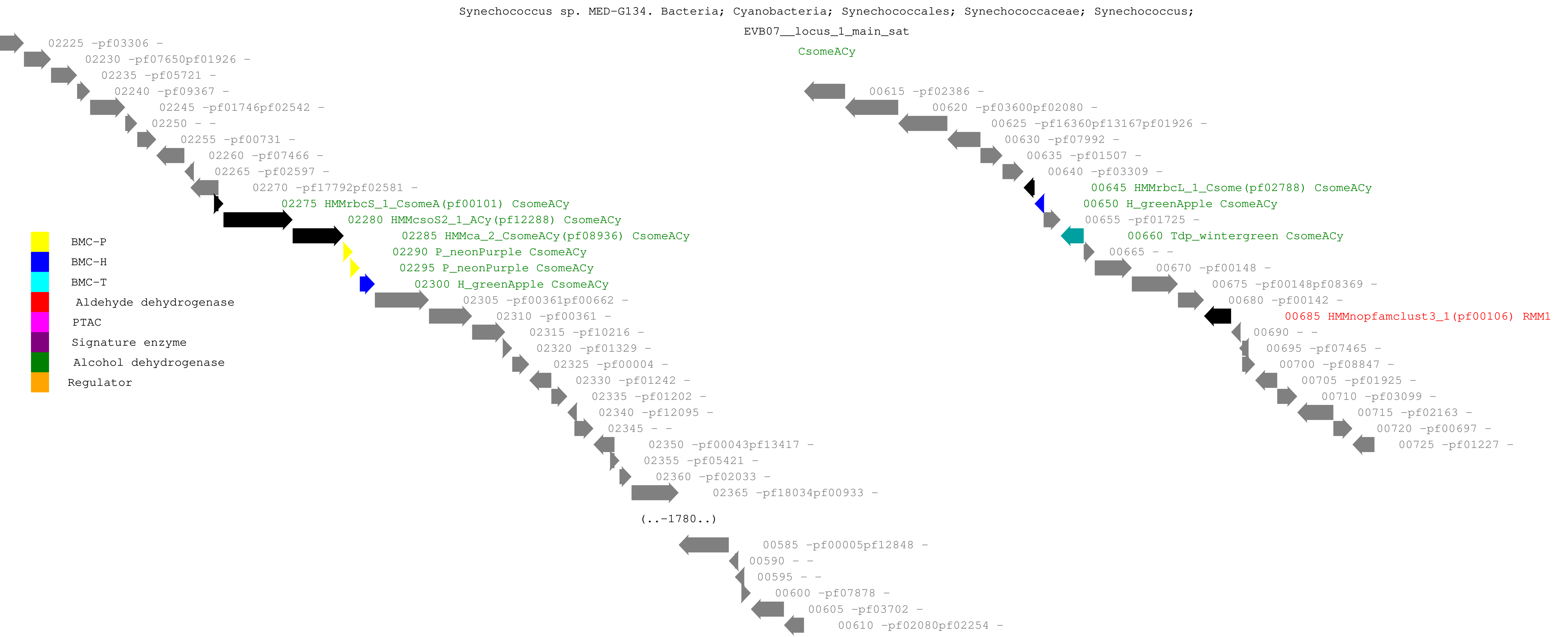

Synechococcus sp. MED-G134. Bacteria; Cyanobacteria; Synechococcales; Synechococcaceae; Synechococcus;
EVB07__locus_1_main_sat
CsomeACy
02225 -pf03306 -
02230 -pf07650pf01926 -
02235 -pf05721 -
02240 -pf09367 -
02245 -pf01746pf02542 -
02250 - -
02255 -pf00731 -
02260 -pf07466 -
02265 -pf02597 -
02270 -pf17792pf02581 -
02275 HMMrbcS_1_CsomeA(pf00101) CsomeACy
02280 HMMcsoS2_1_ACy(pf12288) CsomeACy
02285 HMMca_2_CsomeACy(pf08936) CsomeACy
02290 P_neonPurple CsomeACy
02295 P_neonPurple CsomeACy
02300 H_greenApple CsomeACy
02305 -pf00361pf00662 -
02310 -pf00361 -
02315 -pf10216 -
02320 -pf01329 -
02325 -pf00004 -
02330 -pf01242 -
02335 -pf01202 -
02340 -pf12095 -
02345 - -
02350 -pf00043pf13417 -
02355 -pf05421 -
02360 -pf02033 -
02365 -pf18034pf00933 -
(..-1780..)
00585 -pf00005pf12848 -
00590 - -
00595 - -
00600 -pf07878 -
00605 -pf03702 -
00610 -pf02080pf02254 -
00615 -pf02386 -
00620 -pf03600pf02080 -
00625 -pf16360pf13167pf01926 -
00630 -pf07992 -
00635 -pf01507 -
00640 -pf03309 -
00645 HMMrbcL_1_Csome(pf02788) CsomeACy
00650 H_greenApple CsomeACy
00655 -pf01725 -
00660 Tdp_wintergreen CsomeACy
00665 - -
00670 -pf00148 -
00675 -pf00148pf08369 -
00680 -pf00142 -
00685 HMMnopfamclust3_1(pf00106) RMM1
00690 - -
00695 -pf07465 -
00700 -pf08847 -
00705 -pf01925 -
00710 -pf03099 -
00715 -pf02163 -
00720 -pf00697 -
00725 -pf01227 -
BMC-P
BMC-H
BMC-T
Aldehyde dehydrogenase
PTAC
Signature enzyme
Alcohol dehydrogenase
Regulator

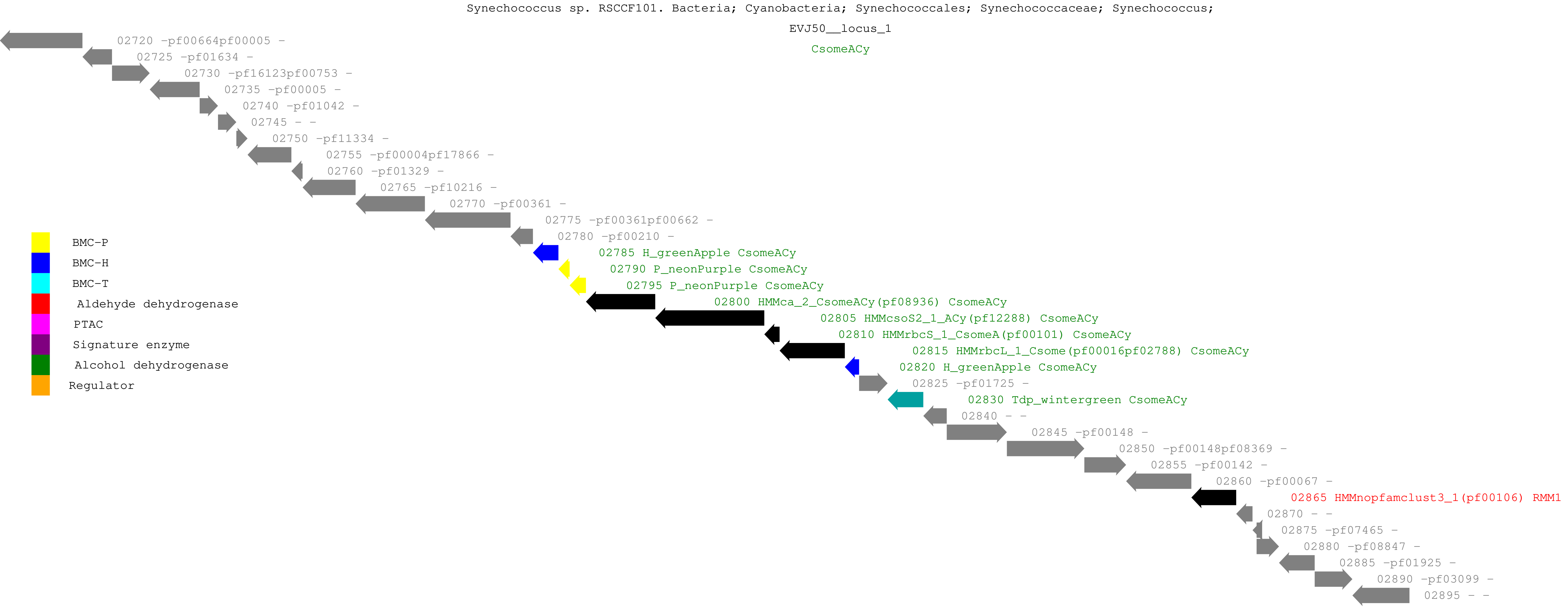

Synechococcus sp. RSCCF101. Bacteria; Cyanobacteria; Synechococcales; Synechococcaceae; Synechococcus;
EVJ50__locus_1
CsomeACy
02720 -pf00664pf00005 -
02725 -pf01634 -
02730 -pf16123pf00753 -
02735 -pf00005 -
02740 -pf01042 -
02745 - -
02750 -pf11334 -
02755 -pf00004pf17866 -
02760 -pf01329 -
02765 -pf10216 -
02770 -pf00361 -
02775 -pf00361pf00662 -
02780 -pf00210 -
02785 H_greenApple CsomeACy
02790 P_neonPurple CsomeACy
02795 P_neonPurple CsomeACy
02800 HMMca_2_CsomeACy(pf08936) CsomeACy
02805 HMMcsoS2_1_ACy(pf12288) CsomeACy
02810 HMMrbcS_1_CsomeA(pf00101) CsomeACy
02815 HMMrbcL_1_Csome(pf00016pf02788) CsomeACy
02820 H_greenApple CsomeACy
02825 -pf01725 -
02830 Tdp_wintergreen CsomeACy
02840 - -
02845 -pf00148 -
02850 -pf00148pf08369 -
02855 -pf00142 -
02860 -pf00067 -
02865 HMMnopfamclust3_1(pf00106) RMM1
02870 - -
02875 -pf07465 -
02880 -pf08847 -
02885 -pf01925 -
02890 -pf03099 -
02895 - -
BMC-P
BMC-H
BMC-T
Aldehyde dehydrogenase
PTAC
Signature enzyme
Alcohol dehydrogenase
Regulator

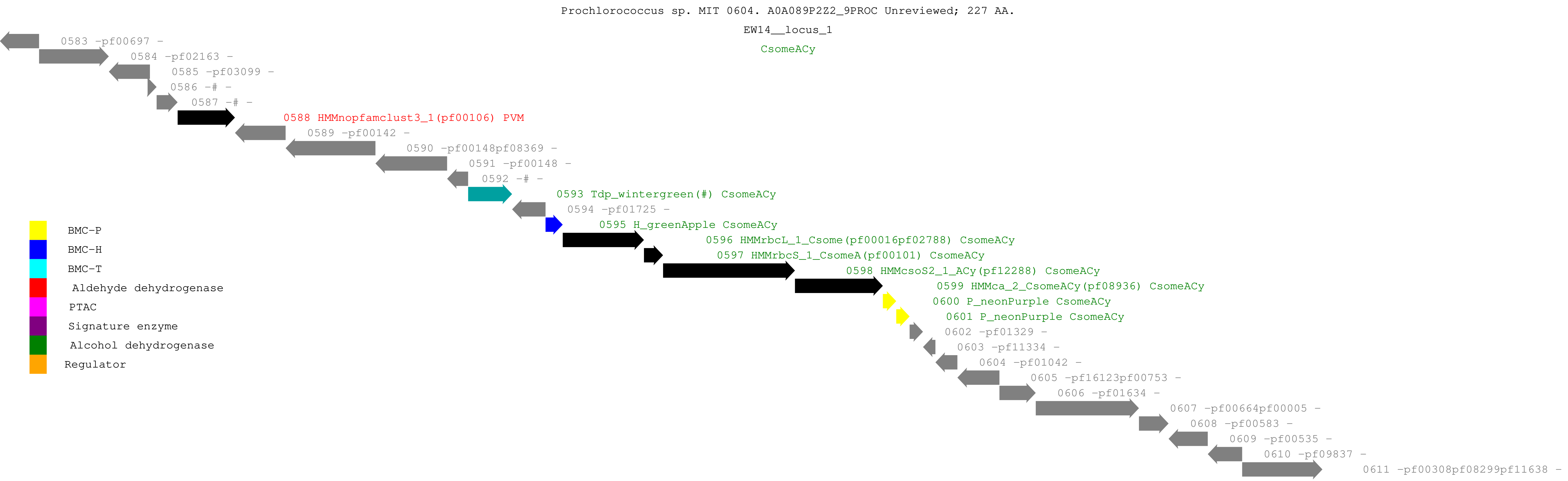
Prochlorococcus sp. MIT 0604. A0A089P2Z2_9PROC Unreviewed; 227 AA.
EW14__locus_1
CsomeACy
0583 -pf00697 -
0584 -pf02163 -
0585 -pf03099 -
0586 -# -
0587 -# -
0588 HMMnopfamclust3_1(pf00106) PVM
0589 -pf00142 -
0590 -pf00148pf08369 -
0591 -pf00148 -
0592 -# -
0593 Tdp_wintergreen(#) CsomeACy
0594 -pf01725 -
0595 H_greenApple CsomeACy
0596 HMMrbcL_1_Csome(pf00016pf02788) CsomeACy
0597 HMMrbcS_1_CsomeA(pf00101) CsomeACy
0598 HMMcsoS2_1_ACy(pf12288) CsomeACy
0599 HMMca_2_CsomeACy(pf08936) CsomeACy
0600 P_neonPurple CsomeACy
0601 P_neonPurple CsomeACy
0602 -pf01329 -
0603 -pf11334 -
0604 -pf01042 -
0605 -pf16123pf00753 -
0606 -pf01634 -
0607 -pf00664pf00005 -
0608 -pf00583 -
0609 -pf00535 -
0610 -pf09837 -
0611 -pf00308pf08299pf11638 -
BMC-P
BMC-H
BMC-T
Aldehyde dehydrogenase
PTAC
Signature enzyme
Alcohol dehydrogenase
Regulator

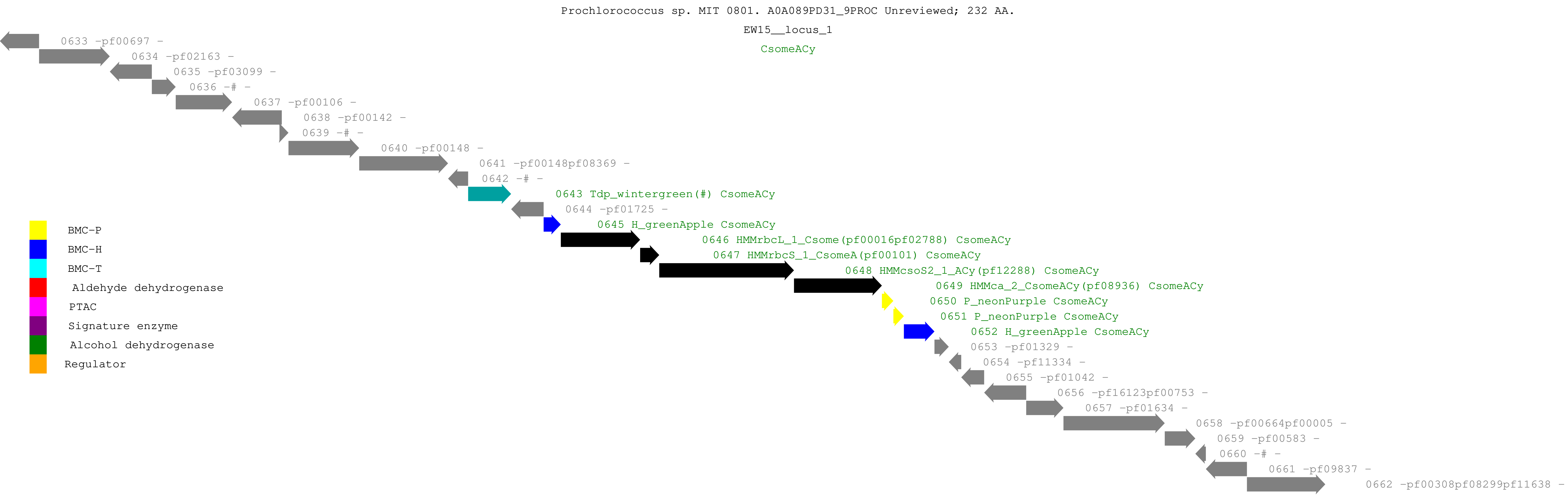

Prochlorococcus sp. MIT 0801. A0A089PD31_9PROC Unreviewed; 232 AA.
EW15__locus_1
CsomeACy
0633 -pf00697 -
0634 -pf02163 -
0635 -pf03099 -
0636 -# -
0637 -pf00106 -
0638 -pf00142 -
0639 -# -
0640 -pf00148 -
0641 -pf00148pf08369 -
0642 -# -
0643 Tdp_wintergreen(#) CsomeACy
0644 -pf01725 -
0645 H_greenApple CsomeACy
0646 HMMrbcL_1_Csome(pf00016pf02788) CsomeACy
0647 HMMrbcS_1_CsomeA(pf00101) CsomeACy
0648 HMMcsoS2_1_ACy(pf12288) CsomeACy
0649 HMMca_2_CsomeACy(pf08936) CsomeACy
0650 P_neonPurple CsomeACy
0651 P_neonPurple CsomeACy
0652 H_greenApple CsomeACy
0653 -pf01329 -
0654 -pf11334 -
0655 -pf01042 -
0656 -pf16123pf00753 -
0657 -pf01634 -
0658 -pf00664pf00005 -
0659 -pf00583 -
0660 -# -
0661 -pf09837 -
0662 -pf00308pf08299pf11638 -
BMC-P
BMC-H
BMC-T
Aldehyde dehydrogenase
PTAC
Signature enzyme
Alcohol dehydrogenase
Regulator

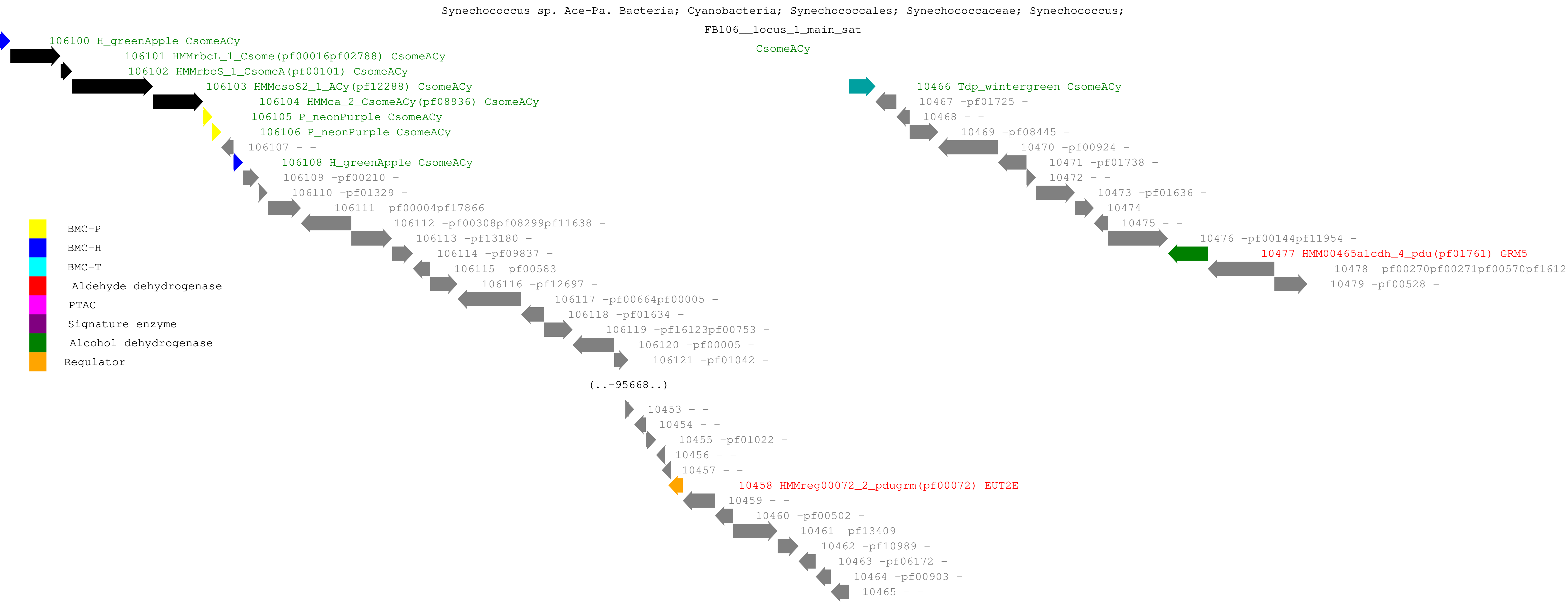
Synechococcus sp. Ace-Pa. Bacteria; Cyanobacteria; Synechococcales; Synechococcaceae; Synechococcus;
FB106__locus_1_main_sat
CsomeACy
106100 H_greenApple CsomeACy
106101 HMMrbcL_1_Csome(pf00016pf02788) CsomeACy
106102 HMMrbcS_1_CsomeA(pf00101) CsomeACy
106103 HMMcsoS2_1_ACy(pf12288) CsomeACy
106104 HMMca_2_CsomeACy(pf08936) CsomeACy
106105 P_neonPurple CsomeACy
106106 P_neonPurple CsomeACy
106107 - -
106108 H_greenApple CsomeACy
106109 -pf00210 -
106110 -pf01329 -
106111 -pf00004pf17866 -
106112 -pf00308pf08299pf11638 -
106113 -pf13180 -
106114 -pf09837 -
106115 -pf00583 -
106116 -pf12697 -
106117 -pf00664pf00005 -
106118 -pf01634 -
106119 -pf16123pf00753 -
106120 -pf00005 -
106121 -pf01042 -
(..-95668..)
10453 - -
10454 - -
10455 -pf01022 -
10456 - -
10457 - -
10458 HMMreg00072_2_pdugrm(pf00072) EUT2E
10459 - -
10460 -pf00502 -
10461 -pf13409 -
10462 -pf10989 -
10463 -pf06172 -
10464 -pf00903 -
10465 - -
10466 Tdp_wintergreen CsomeACy
10467 -pf01725 -
10468 - -
10469 -pf08445 -
10470 -pf00924 -
10471 -pf01738 -
10472 - -
10473 -pf01636 -
10474 - -
10475 - -
10476 -pf00144pf11954 -
10477 HMM00465alcdh_4_pdu(pf01761) GRM5
10478 -pf00270pf00271pf00570pf1612
10479 -pf00528 -
BMC-P
BMC-H
BMC-T
Aldehyde dehydrogenase
PTAC
Signature enzyme
Alcohol dehydrogenase
Regulator

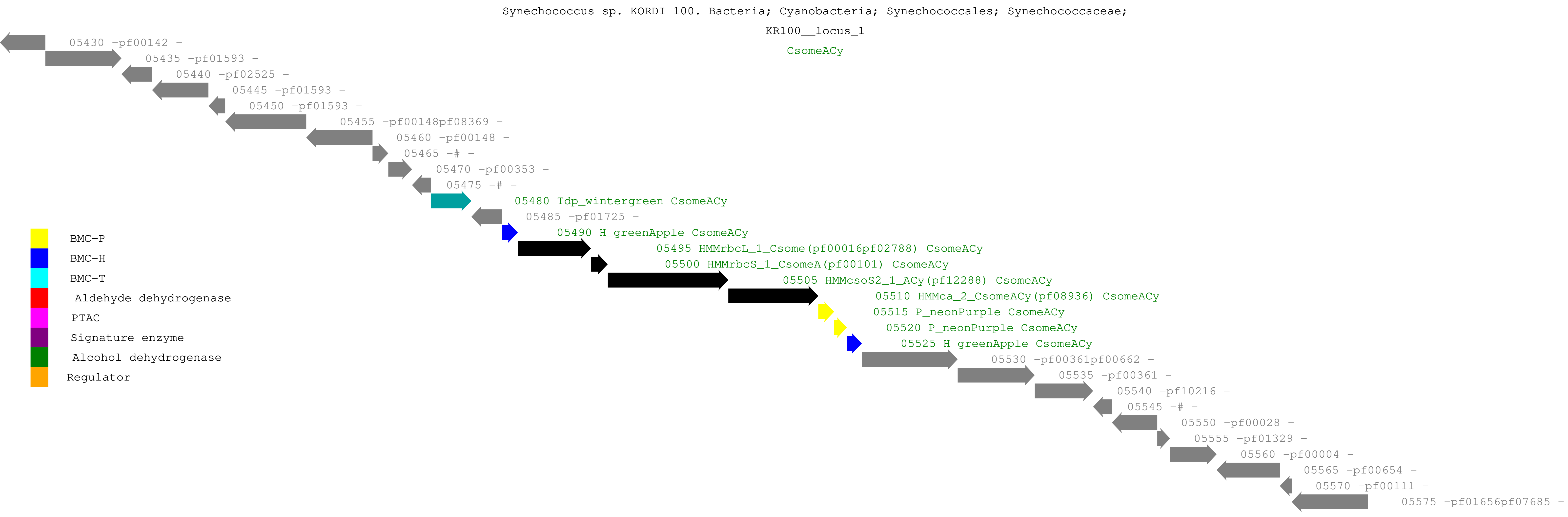
Synechococcus sp. KORDI-100. Bacteria; Cyanobacteria; Synechococcales; Synechococcaceae;
KR100__locus_1
CsomeACy
05430 -pf00142 -
05435 -pf01593 -
05440 -pf02525 -
05445 -pf01593 -
05450 -pf01593 -
05455 -pf00148pf08369 -
05460 -pf00148 -
05465 -# -
05470 -pf00353 -
05475 -# -
05480 Tdp_wintergreen CsomeACy
05485 -pf01725 -
05490 H_greenApple CsomeACy
05495 HMMrbcL_1_Csome(pf00016pf02788) CsomeACy
05500 HMMrbcS_1_CsomeA(pf00101) CsomeACy
05505 HMMcsoS2_1_ACy(pf12288) CsomeACy
05510 HMMca_2_CsomeACy(pf08936) CsomeACy
05515 P_neonPurple CsomeACy
05520 P_neonPurple CsomeACy
05525 H_greenApple CsomeACy
05530 -pf00361pf00662 -
05535 -pf00361 -
05540 -pf10216 -
05545 -# -
05550 -pf00028 -
05555 -pf01329 -
05560 -pf00004 -
05565 -pf00654 -
05570 -pf00111 -
05575 -pf01656pf07685 -
BMC-P
BMC-H
BMC-T
Aldehyde dehydrogenase
PTAC
Signature enzyme
Alcohol dehydrogenase
Regulator

Synechococcus sp. KORDI-49. Bacteria; Cyanobacteria; Synechococcales; Synechococcaceae;

KR49__locus_1

CsomeACy

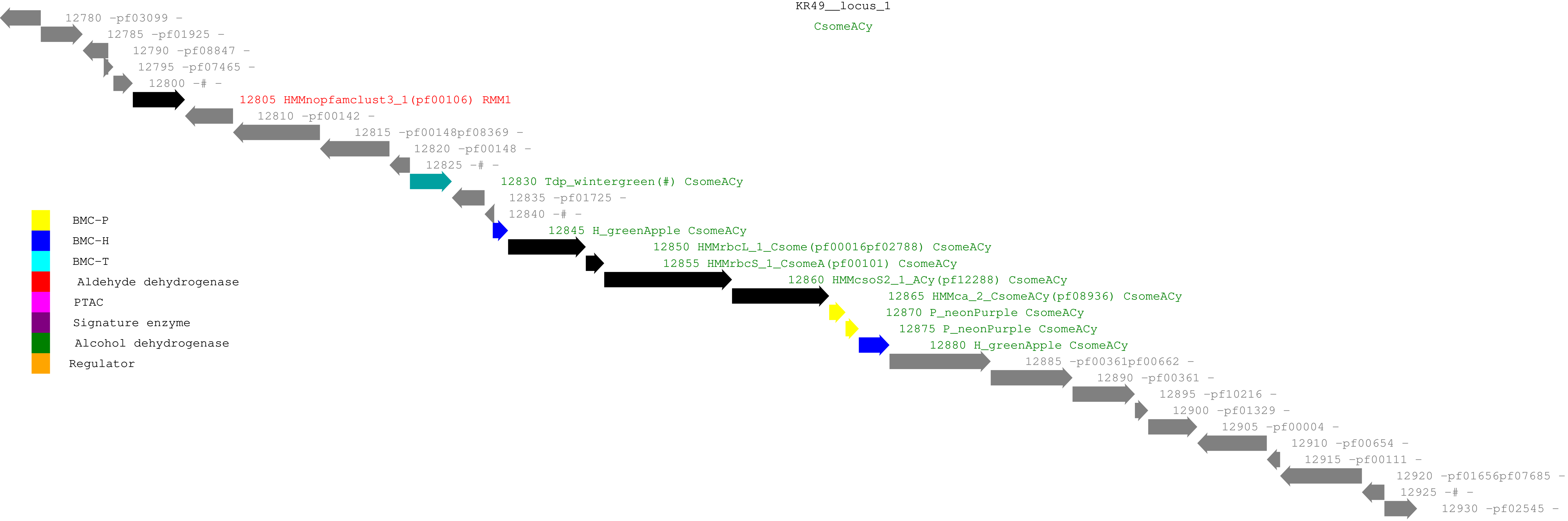

Synechococcus sp. KORDI-52. Bacteria; Cyanobacteria; Synechococcales; Synechococcaceae;

KR52__locus_1

CsomeACy

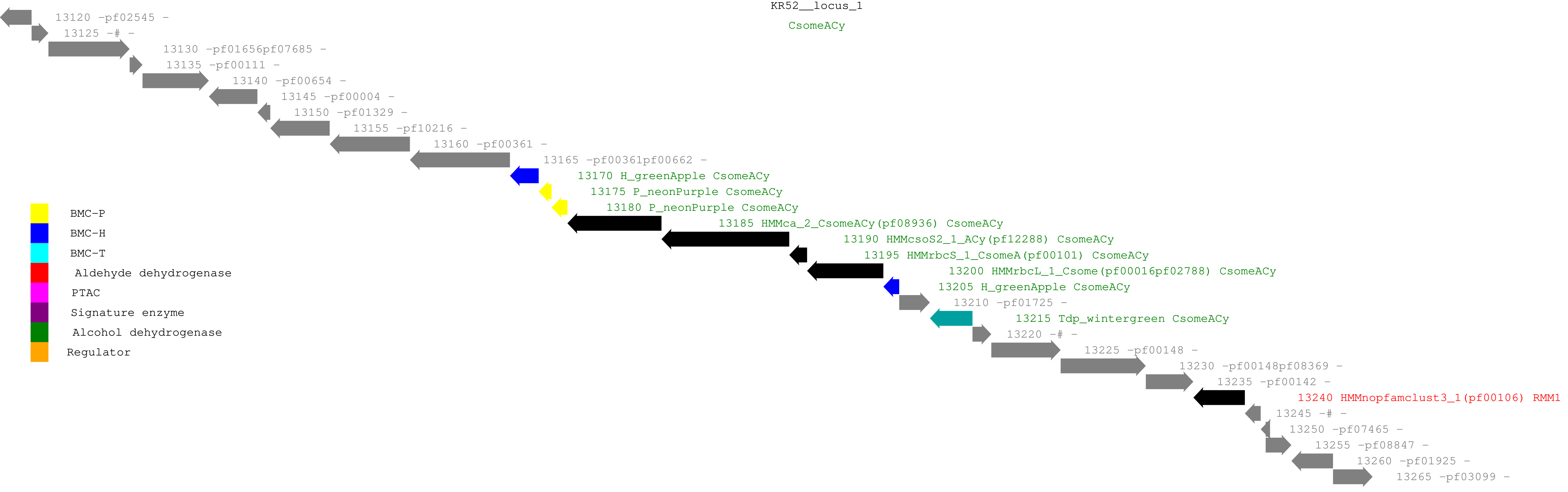

Cyanobium sp. Bacteria; Cyanobacteria; Synechococcales; Synechococcaceae; Cyanobium;

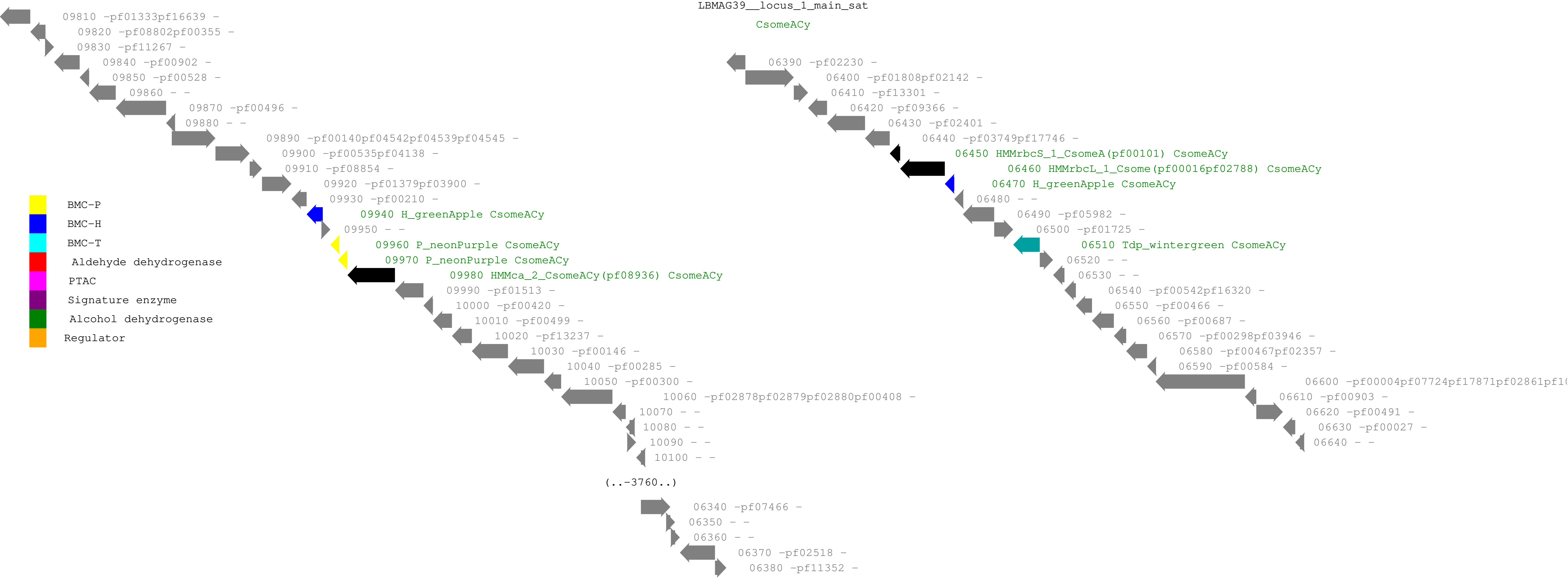

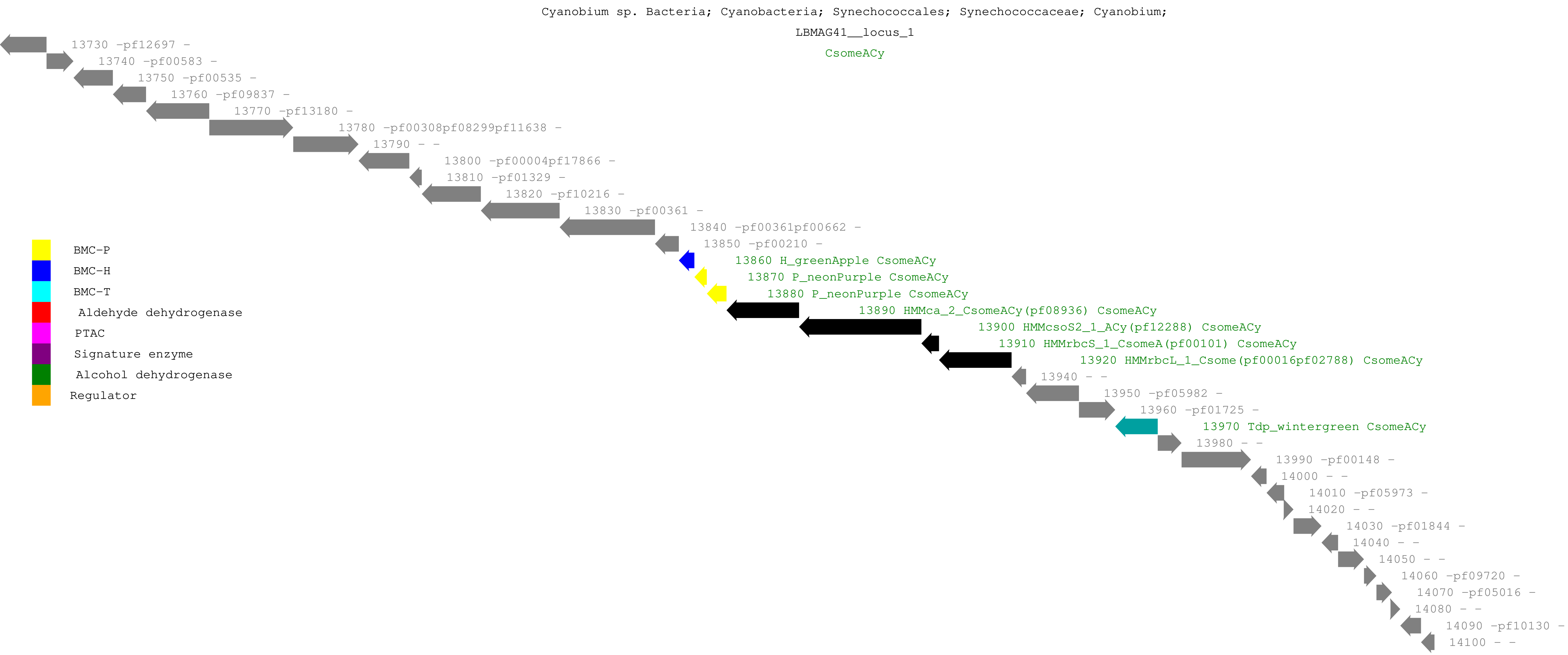
Cyanobium sp. Bacteria; Cyanobacteria; Synechococcales; Synechococcaceae; Cyanobium;
LBMAG41__locus_1
CsomeACy
13730 -pf12697 -
13740 -pf00583 -
13750 -pf00535 -
13760 -pf09837 -
13770 -pf13180 -
13780 -pf00308pf08299pf11638 -
13790 - -
13800 -pf00004pf17866 -
13810 -pf01329 -
13820 -pf10216 -
13830 -pf00361 -
13840 -pf00361pf00662 -
13850 -pf00210 -
13860 H_greenApple CsomeACy
13870 P_neonPurple CsomeACy
13880 P_neonPurple CsomeACy
13890 HMMca_2_CsomeACy(pf08936) CsomeACy
13900 HMMcsoS2_1_ACy(pf12288) CsomeACy
13910 HMMrbcS_1_CsomeA(pf00101) CsomeACy
13920 HMMrbcL_1_Csome(pf00016pf02788) CsomeACy
13940 - -
13950 -pf05982 -
13960 -pf01725 -
13970 Tdp_wintergreen CsomeACy
13980 - -
13990 -pf00148 -
14000 - -
14010 -pf05973 -
14020 - -
14030 -pf01844 -
14040 - -
14050 - -
14060 -pf09720 -
14070 -pf05016 -
14080 - -
14090 -pf10130 -
14100 - -
BMC-P
BMC-H
BMC-T
Aldehyde dehydrogenase
PTAC
Signature enzyme
Alcohol dehydrogenase
Regulator

Synechococcus sp. MIT S9504. Bacteria; Cyanobacteria; Synechococcales; Synechococcaceae;

MITS9504__locus_1

CsomeACy

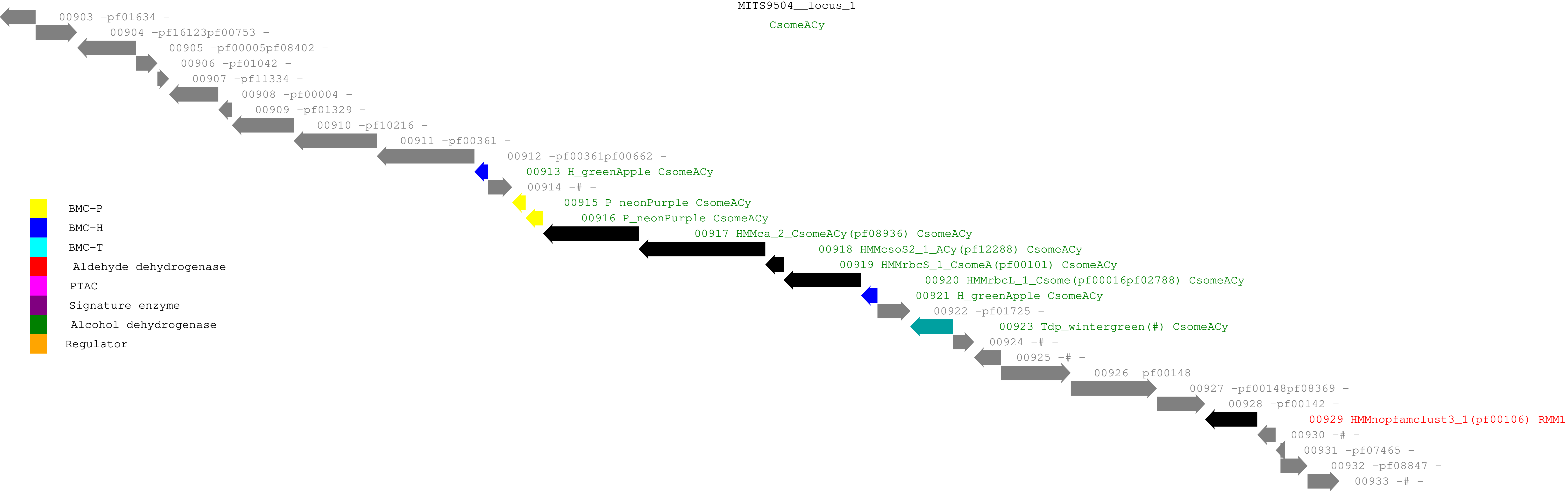

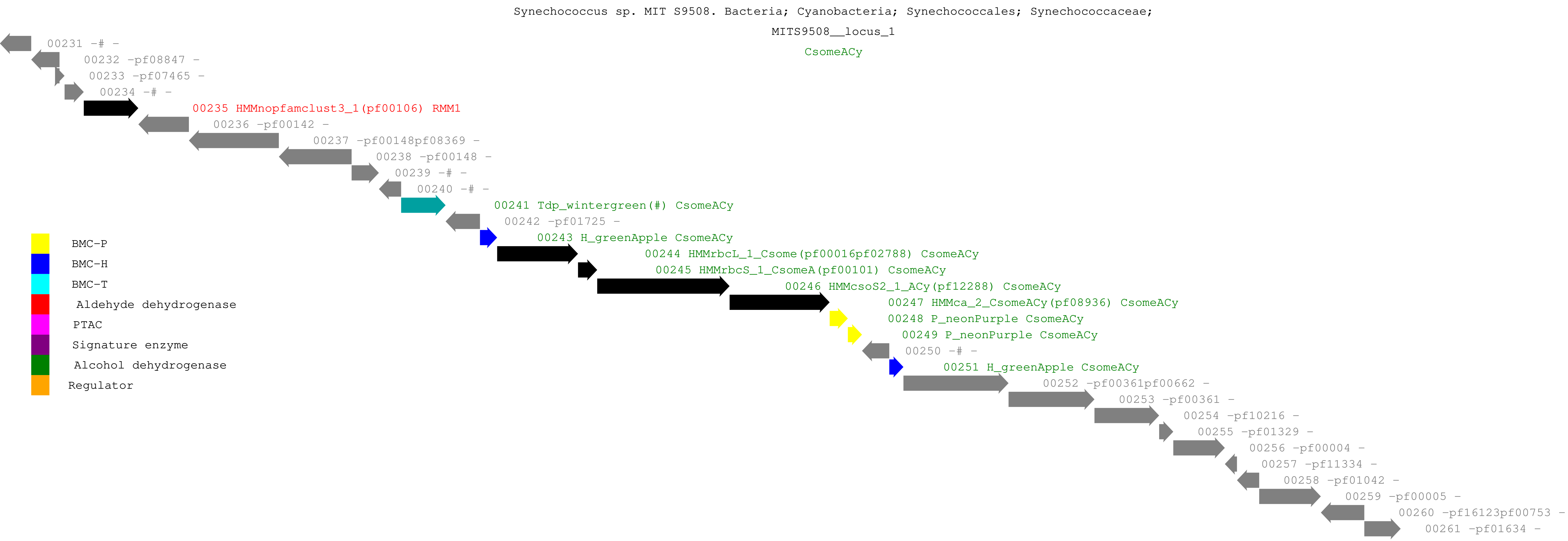
Synechococcus sp. MIT S9508. Bacteria; Cyanobacteria; Synechococcales; Synechococcaceae;
MITS9508__locus_1
CsomeACy
00231 -# -
00232 -pf08847 -
00233 -pf07465 -
00234 -# -
00235 HMMnopfamclust3_1(pf00106) RMM1
00236 -pf00142 -
00237 -pf00148pf08369 -
00238 -pf00148 -
00239 -# -
00240 -# -
00241 Tdp_wintergreen(#) CsomeACy
00242 -pf01725 -
00243 H_greenApple CsomeACy
00244 HMMrbcL_1_Csome(pf00016pf02788) CsomeACy
00245 HMMrbcS_1_CsomeA(pf00101) CsomeACy
00246 HMMcsoS2_1_ACy(pf12288) CsomeACy
00247 HMMca_2_CsomeACy(pf08936) CsomeACy
00248 P_neonPurple CsomeACy
00249 P_neonPurple CsomeACy
00250 -# -
00251 H_greenApple CsomeACy
00252 -pf00361pf00662 -
00253 -pf00361 -
00254 -pf10216 -
00255 -pf01329 -
00256 -pf00004 -
00257 -pf11334 -
00258 -pf01042 -
00259 -pf00005 -
00260 -pf16123pf00753 -
00261 -pf01634 -
BMC-P
BMC-H
BMC-T
Aldehyde dehydrogenase
PTAC
Signature enzyme
Alcohol dehydrogenase
Regulator

Prochlorococcus marinus (strain NATL1A). Bacteria; Cyanobacteria; Synechococcales; Prochloraceae;

NATL1__locus_1

CsomeACy

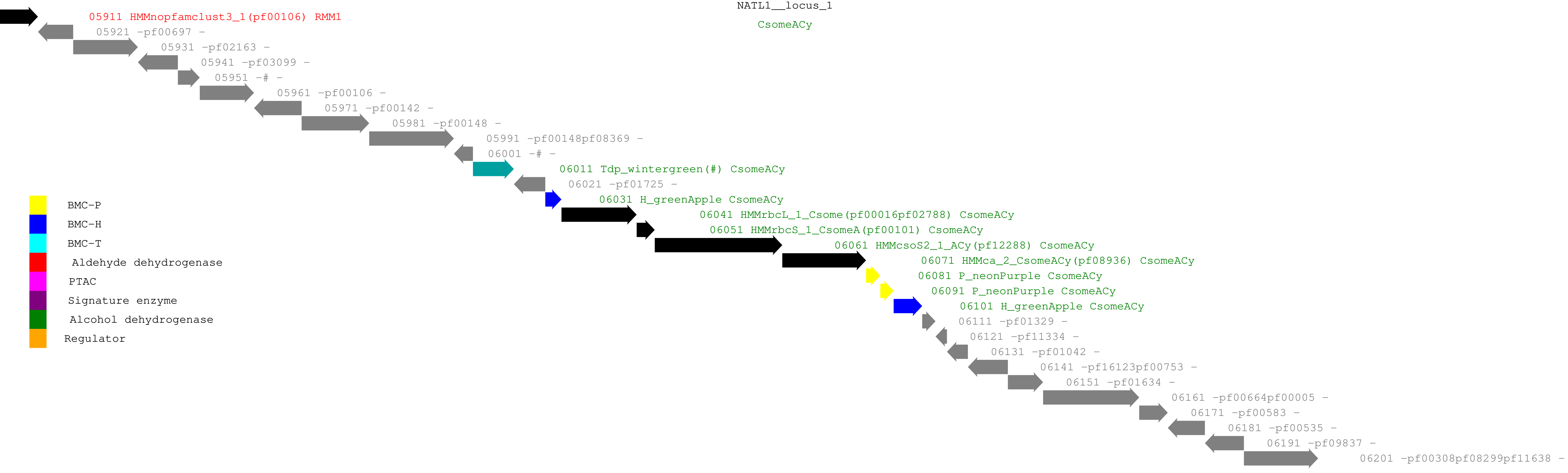

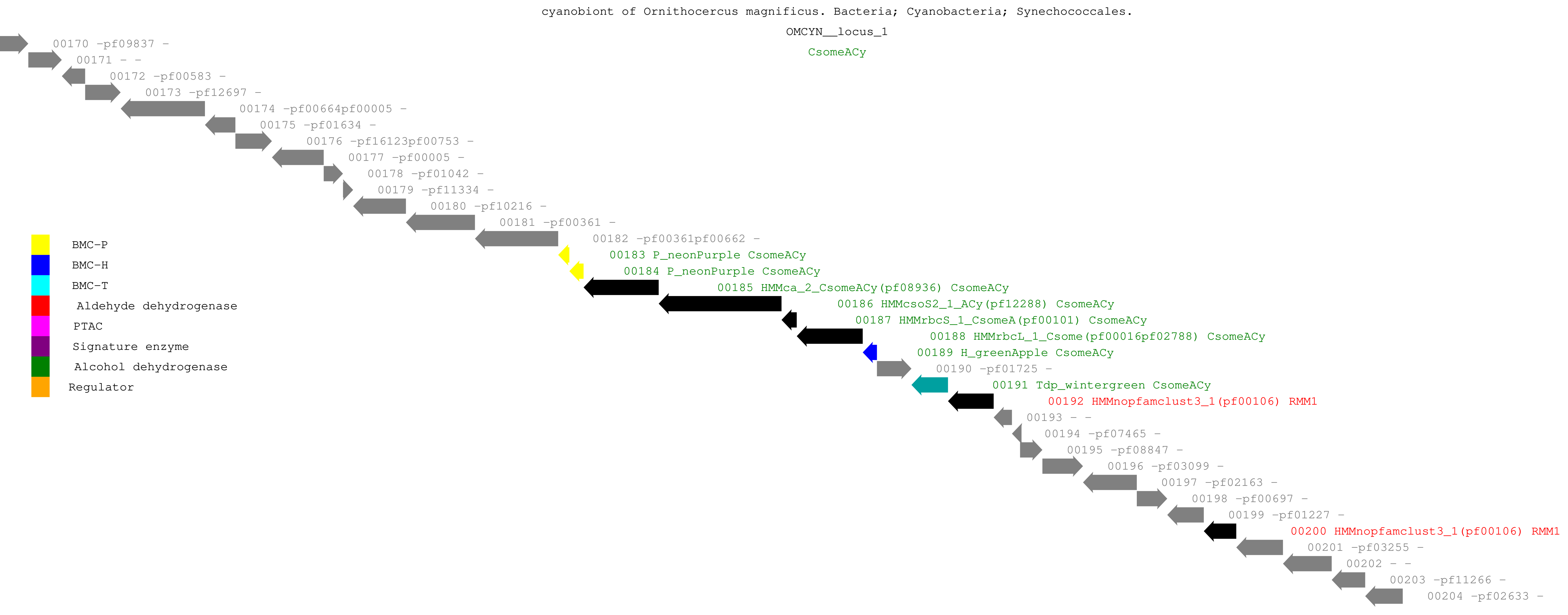
cyanobiont of Ornithocercus magnificus. Bacteria; Cyanobacteria; Synechococcales.
OMCYN__locus_1
CsomeACy
00170 -pf09837 -
00171 - -
00172 -pf00583 -
00173 -pf12697 -
00174 -pf00664pf00005 -
00175 -pf01634 -
00176 -pf16123pf00753 -
00177 -pf00005 -
00178 -pf01042 -
00179 -pf11334 -
00180 -pf10216 -
00181 -pf00361 -
00182 -pf00361pf00662 -
00183 P_neonPurple CsomeACy
00184 P_neonPurple CsomeACy
00185 HMMca_2_CsomeACy(pf08936) CsomeACy
00186 HMMcsoS2_1_ACy(pf12288) CsomeACy
00187 HMMrbcS_1_CsomeA(pf00101) CsomeACy
00188 HMMrbcL_1_Csome(pf00016pf02788) CsomeACy
00189 H_greenApple CsomeACy
00190 -pf01725 -
00191 Tdp_wintergreen CsomeACy
00192 HMMnopfamclust3_1(pf00106) RMM1
00193 - -
00194 -pf07465 -
00195 -pf08847 -
00196 -pf03099 -
00197 -pf02163 -
00198 -pf00697 -
00199 -pf01227 -
00200 HMMnopfamclust3_1(pf00106) RMM1
00201 -pf03255 -
00202 - -
00203 -pf11266 -
00204 -pf02633 -
BMC-P
BMC-H
BMC-T
Aldehyde dehydrogenase
PTAC
Signature enzyme
Alcohol dehydrogenase
Regulator

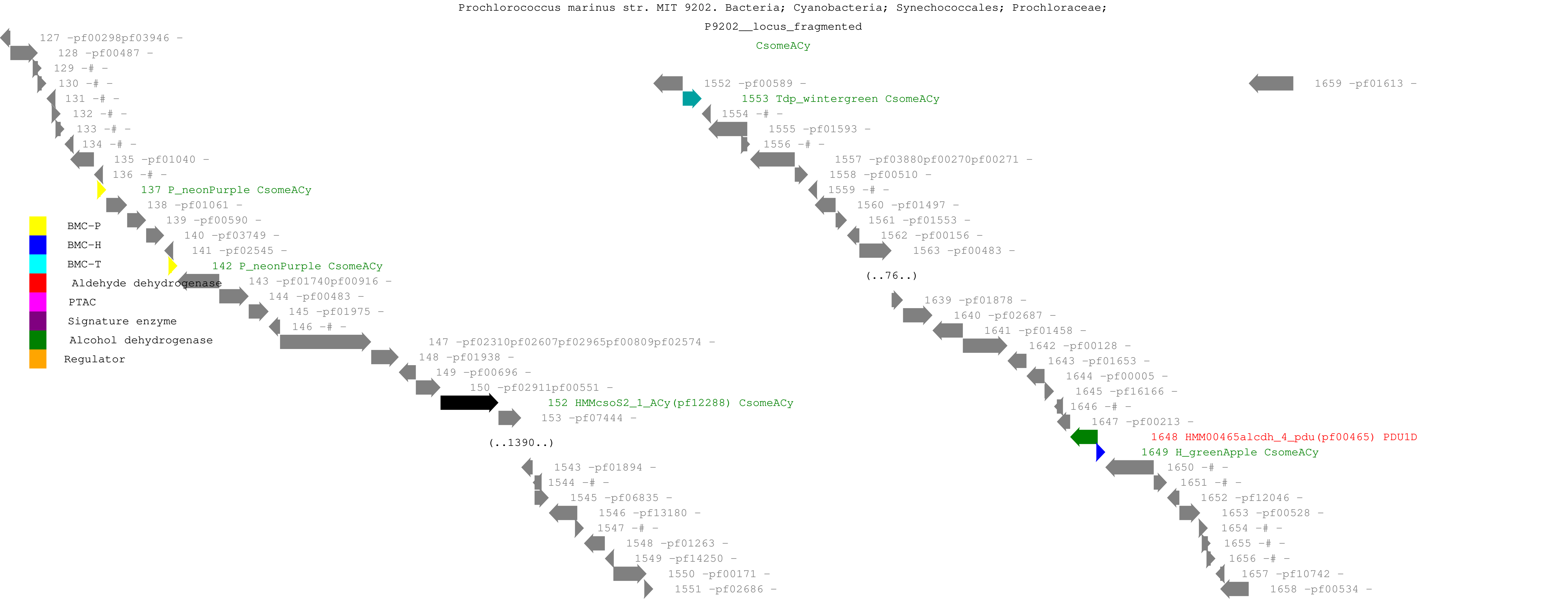

Prochlorococcus marinus str. MIT 9202. Bacteria; Cyanobacteria; Synechococcales; Prochloraceae;
P9202__locus_fragmented
CsomeACy
127 -pf00298pf03946 -
128 -pf00487 -
129 -# -
130 -# -
131 -# -
132 -# -
133 -# -
134 -# -
135 -pf01040 -
136 -# -
137 P_neonPurple CsomeACy
138 -pf01061 -
139 -pf00590 -
140 -pf03749 -
141 -pf02545 -
142 P_neonPurple CsomeACy
143 -pf01740pf00916 -
144 -pf00483 -
145 -pf01975 -
146 -# -
147 -pf02310pf02607pf02965pf00809pf02574 -
148 -pf01938 -
149 -pf00696 -
150 -pf02911pf00551 -
152 HMMcsoS2_1_ACy(pf12288) CsomeACy
153 -pf07444 -
(..1390..)
1543 -pf01894 -
1544 -# -
1545 -pf06835 -
1546 -pf13180 -
1547 -# -
1548 -pf01263 -
1549 -pf14250 -
1550 -pf00171 -
1551 -pf02686 -
1552 -pf00589 -
1553 Tdp_wintergreen CsomeACy
1554 -# -
1555 -pf01593 -
1556 -# -
1557 -pf03880pf00270pf00271 -
1558 -pf00510 -
1559 -# -
1560 -pf01497 -
1561 -pf01553 -
1562 -pf00156 -
1563 -pf00483 -
(..76..)
1639 -pf01878 -
1640 -pf02687 -
1641 -pf01458 -
1642 -pf00128 -
1643 -pf01653 -
1644 -pf00005 -
1645 -pf16166 -
1646 -# -
1647 -pf00213 -
1648 HMM00465alcdh_4_pdu(pf00465) PDU1D
1649 H_greenApple CsomeACy
1650 -# -
1651 -# -
1652 -pf12046 -
1653 -pf00528 -
1654 -# -
1655 -# -
1656 -# -
1657 -pf10742 -
1658 -pf00534 -
1659 -pf01613 -
BMC-P
BMC-H
BMC-T
Aldehyde dehydrogenase
PTAC
Signature enzyme
Alcohol dehydrogenase
Regulator

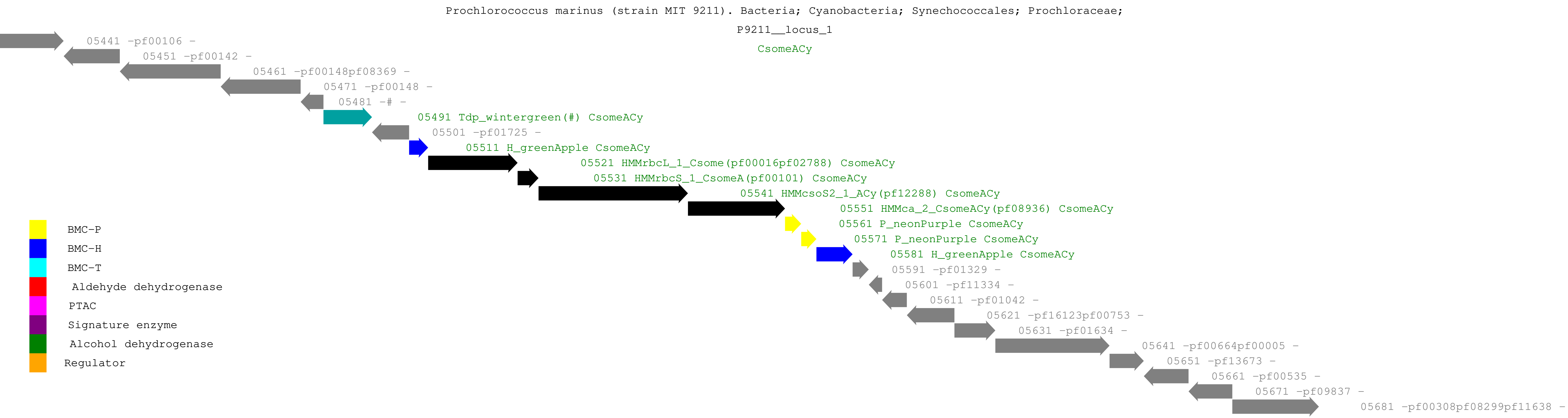

Prochlorococcus marinus (strain MIT 9211). Bacteria; Cyanobacteria; Synechococcales; Prochloraceae;
P9211__locus_1
CsomeACy
05441 -pf00106 -
05451 -pf00142 -
05461 -pf00148pf08369 -
05471 -pf00148 -
05481 -# -
05491 Tdp_wintergreen(#) CsomeACy
05501 -pf01725 -
05511 H_greenApple CsomeACy
05521 HMMrbcL_1_Csome(pf00016pf02788) CsomeACy
05531 HMMrbcS_1_CsomeA(pf00101) CsomeACy
05541 HMMcsoS2_1_ACy(pf12288) CsomeACy
05551 HMMca_2_CsomeACy(pf08936) CsomeACy
05561 P_neonPurple CsomeACy
05571 P_neonPurple CsomeACy
05581 H_greenApple CsomeACy
05591 -pf01329 -
05601 -pf11334 -
05611 -pf01042 -
05621 -pf16123pf00753 -
05631 -pf01634 -
05641 -pf00664pf00005 -
05651 -pf13673 -
05661 -pf00535 -
05671 -pf09837 -
05681 -pf00308pf08299pf11638 -
BMC-P
BMC-H
BMC-T
Aldehyde dehydrogenase
PTAC
Signature enzyme
Alcohol dehydrogenase
Regulator

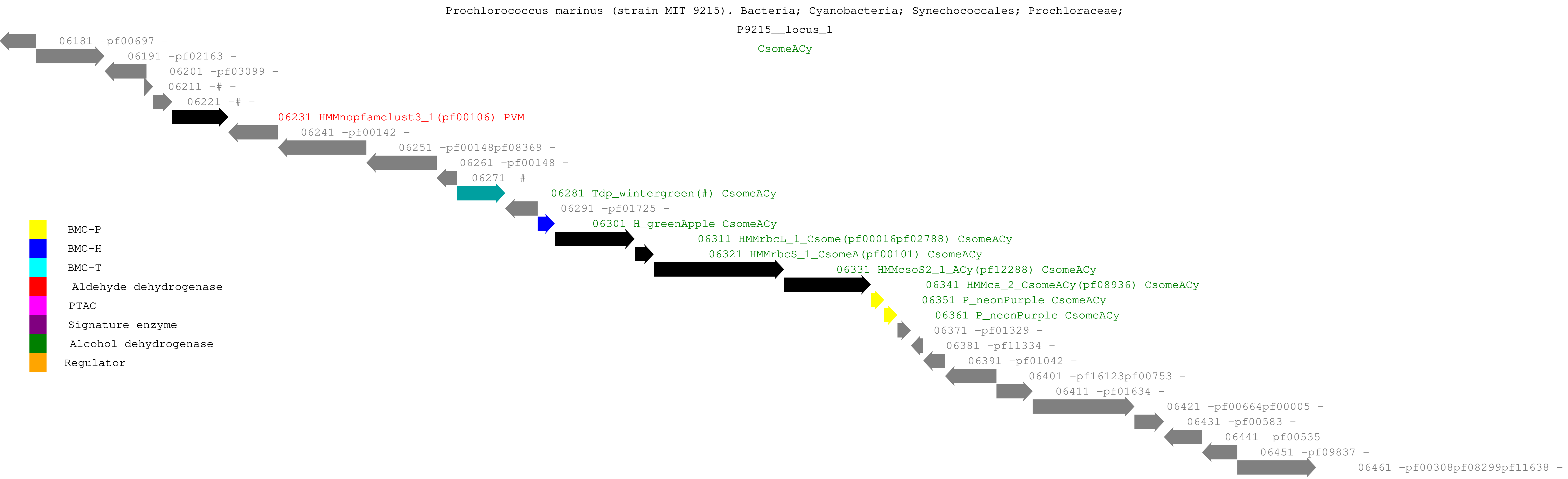
Prochlorococcus marinus (strain MIT 9215). Bacteria; Cyanobacteria; Synechococcales; Prochloraceae;
P9215__locus_1
CsomeACy
06181 -pf00697 -
06191 -pf02163 -
06201 -pf03099 -
06211 -# -
06221 -# -
06231 HMMnopfamclust3_1(pf00106) PVM
06241 -pf00142 -
06251 -pf00148pf08369 -
06261 -pf00148 -
06271 -# -
06281 Tdp_wintergreen(#) CsomeACy
06291 -pf01725 -
06301 H_greenApple CsomeACy
06311 HMMrbcL_1_Csome(pf00016pf02788) CsomeACy
06321 HMMrbcS_1_CsomeA(pf00101) CsomeACy
06331 HMMcsoS2_1_ACy(pf12288) CsomeACy
06341 HMMca_2_CsomeACy(pf08936) CsomeACy
06351 P_neonPurple CsomeACy
06361 P_neonPurple CsomeACy
06371 -pf01329 -
06381 -pf11334 -
06391 -pf01042 -
06401 -pf16123pf00753 -
06411 -pf01634 -
06421 -pf00664pf00005 -
06431 -pf00583 -
06441 -pf00535 -
06451 -pf09837 -
06461 -pf00308pf08299pf11638 -
BMC-P
BMC-H
BMC-T
Aldehyde dehydrogenase
PTAC
Signature enzyme
Alcohol dehydrogenase
Regulator

Prochlorococcus marinus (strain MIT 9301). Bacteria; Cyanobacteria; Synechococcales; Prochloraceae;

P9301__locus_1

CsomeACy

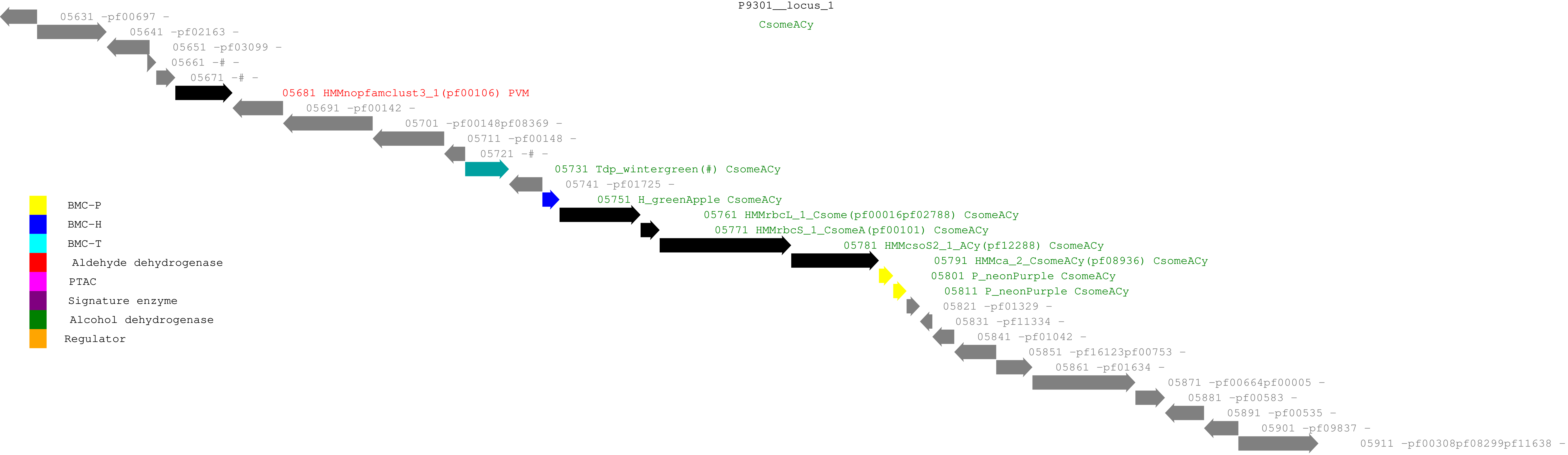

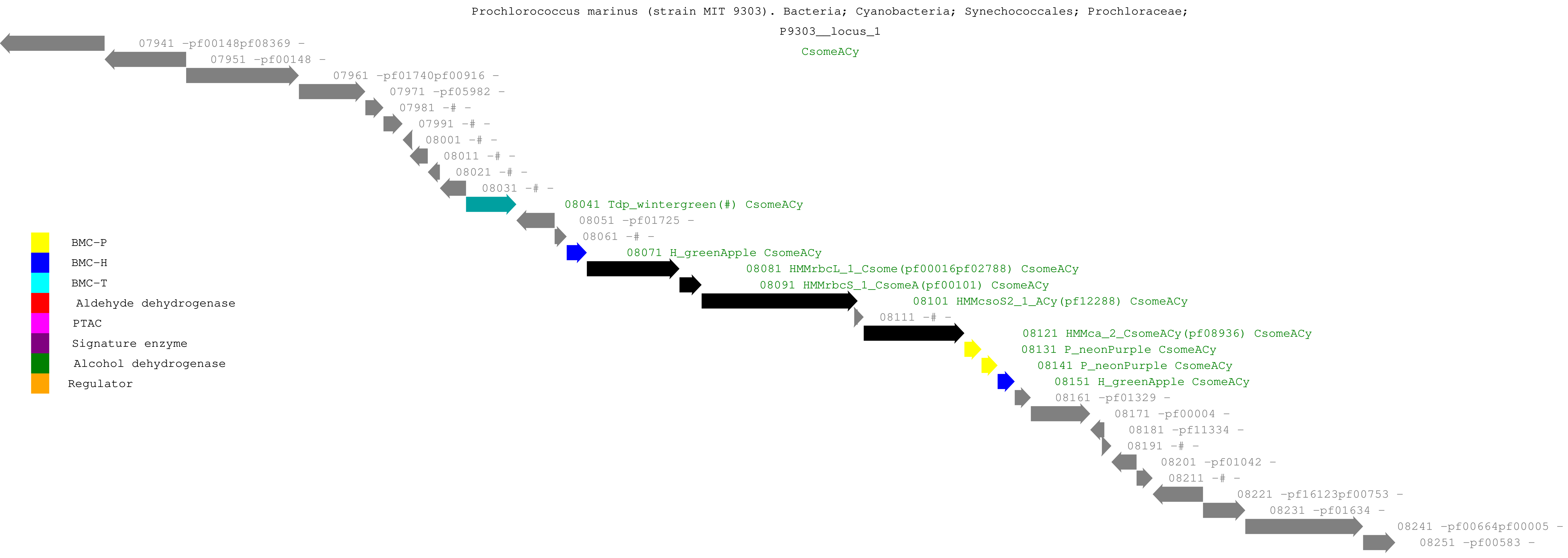
Prochlorococcus marinus (strain MIT 9303). Bacteria; Cyanobacteria; Synechococcales; Prochloraceae;
P9303__locus_1
CsomeACy
07941 -pf00148pf08369 -
07951 -pf00148 -
07961 -pf01740pf00916 -
07971 -pf05982 -
07981 -# -
07991 -# -
08001 -# -
08011 -# -
08021 -# -
08031 -# -
08041 Tdp_wintergreen(#) CsomeACy
08051 -pf01725 -
08061 -# -
08071 H_greenApple CsomeACy
08081 HMMrbcL_1_Csome(pf00016pf02788) CsomeACy
08091 HMMrbcS_1_CsomeA(pf00101) CsomeACy
08101 HMMcsoS2_1_ACy(pf12288) CsomeACy
08111 -# -
08121 HMMca_2_CsomeACy(pf08936) CsomeACy
08131 P_neonPurple CsomeACy
08141 P_neonPurple CsomeACy
08151 H_greenApple CsomeACy
08161 -pf01329 -
08171 -pf00004 -
08181 -pf11334 -
08191 -# -
08201 -pf01042 -
08211 -# -
08221 -pf16123pf00753 -
08231 -pf01634 -
08241 -pf00664pf00005 -
08251 -pf00583 -
BMC-P
BMC-H
BMC-T
Aldehyde dehydrogenase
PTAC
Signature enzyme
Alcohol dehydrogenase
Regulator

Prochlorococcus marinus (strain MIT 9515). Bacteria; Cyanobacteria; Synechococcales; Prochloraceae;

P9515__locus_1

CsomeACy

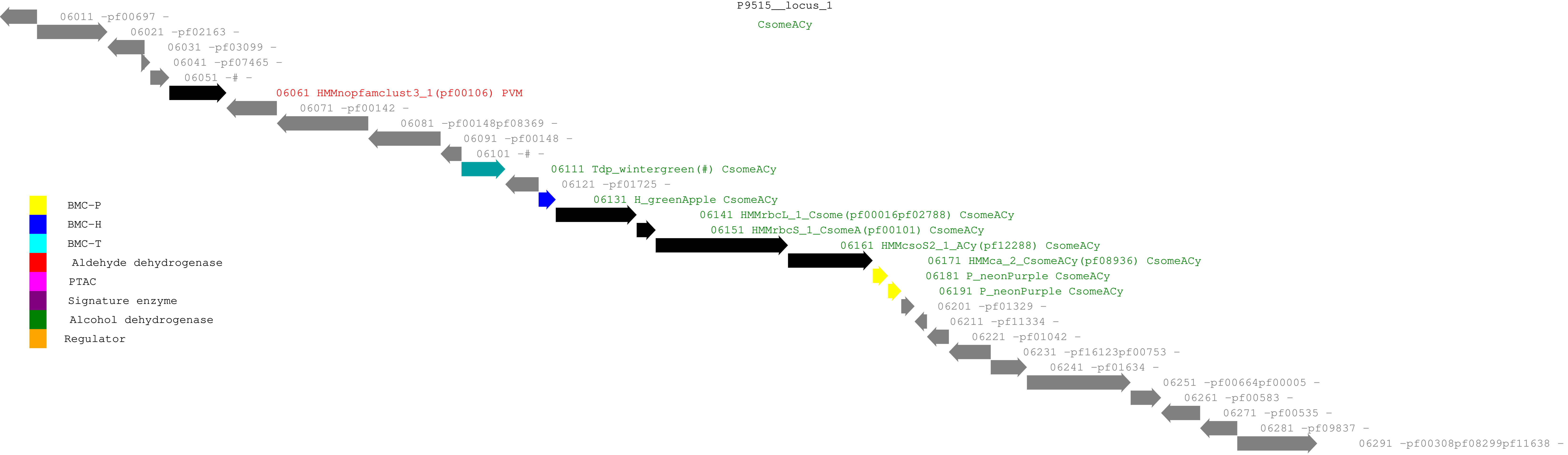

Paulinella chromatophora. Eukaryota; Rhizaria; Cercozoa; Imbricatea; Silicofilosea; Euglyphida;

PCC__locus_1

CsomeACy

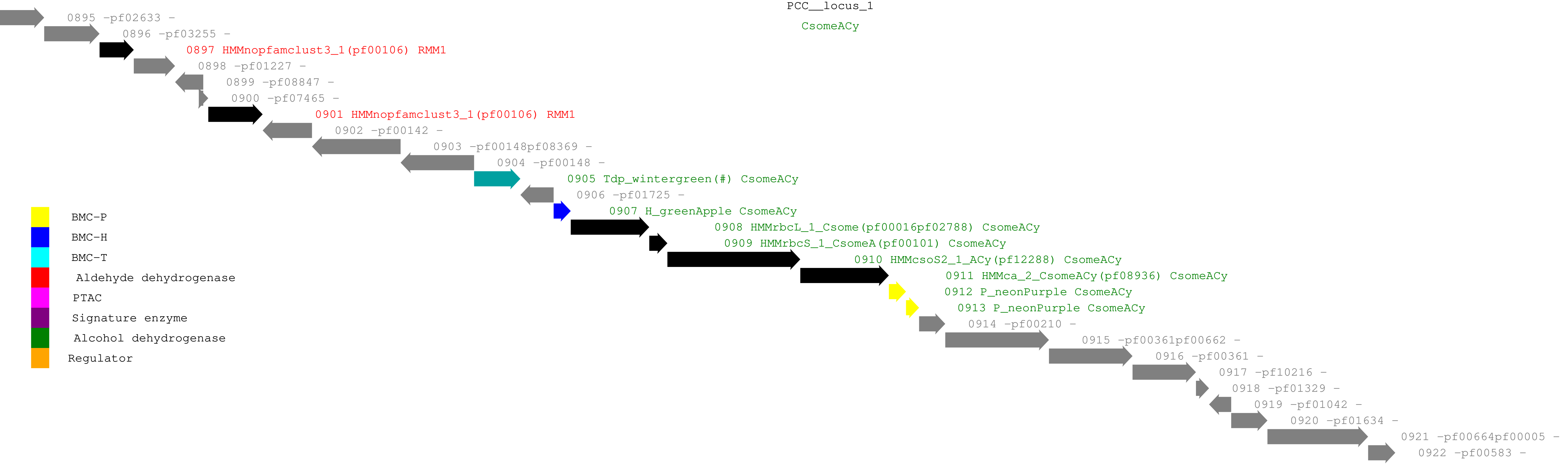

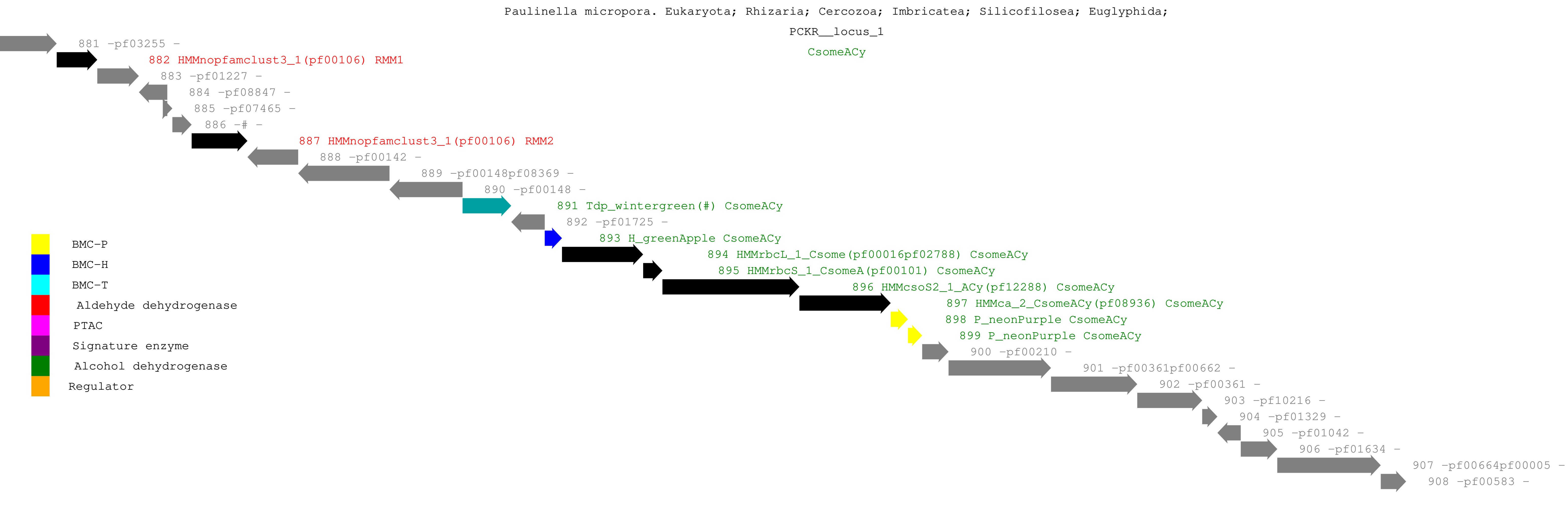
Paulinella micropora. Eukaryota; Rhizaria; Cercozoa; Imbricatea; Silicofilosea; Euglyphida;
PCKR__locus_1
CsomeACy
881 -pf03255 -
882 HMMnopfamclust3_1(pf00106) RMM1
883 -pf01227 -
884 -pf08847 -
885 -pf07465 -
886 -# -
887 HMMnopfamclust3_1(pf00106) RMM2
888 -pf00142 -
889 -pf00148pf08369 -
890 -pf00148 -
891 Tdp_wintergreen(#) CsomeACy
892 -pf01725 -
893 H_greenApple CsomeACy
894 HMMrbcL_1_Csome(pf00016pf02788) CsomeACy
895 HMMrbcS_1_CsomeA(pf00101) CsomeACy
896 HMMcsoS2_1_ACy(pf12288) CsomeACy
897 HMMca_2_CsomeACy(pf08936) CsomeACy
898 P_neonPurple CsomeACy
899 P_neonPurple CsomeACy
900 -pf00210 -
901 -pf00361pf00662 -
902 -pf00361 -
903 -pf10216 -
904 -pf01329 -
905 -pf01042 -
906 -pf01634 -
907 -pf00664pf00005 -
908 -pf00583 -
BMC-P
BMC-H
BMC-T
Aldehyde dehydrogenase
PTAC
Signature enzyme
Alcohol dehydrogenase
Regulator

Prochlorococcus sp. MIT 1303. A0A163G6N1_9PROC Unreviewed; 105 AA.

PMIT1303__locus_1

CsomeACy

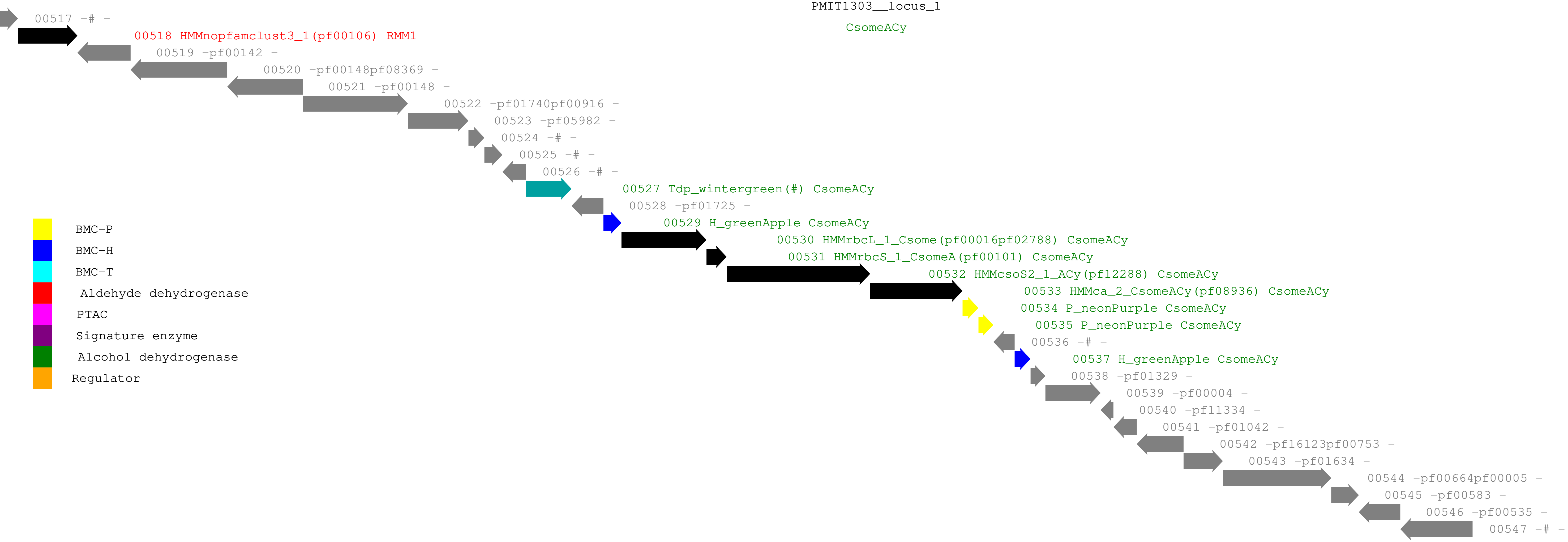

Prochlorococcus sp. MIT 1306. A0A163C295_9PROC Unreviewed; 477 AA.

PMIT1306__locus_1

CsomeACy

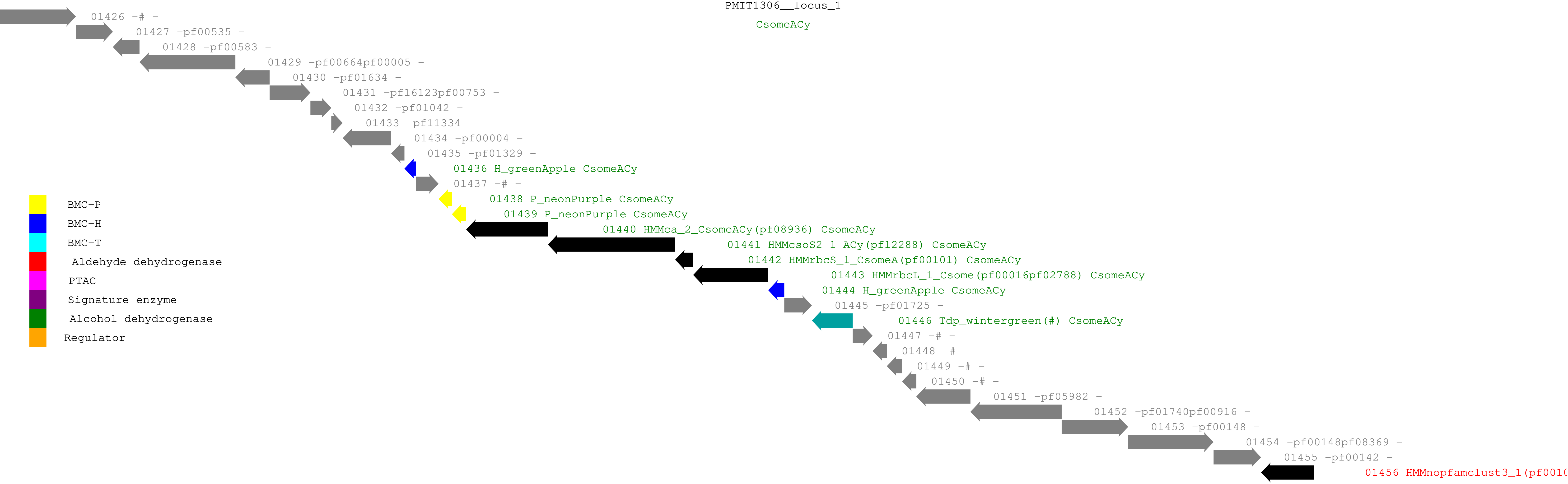

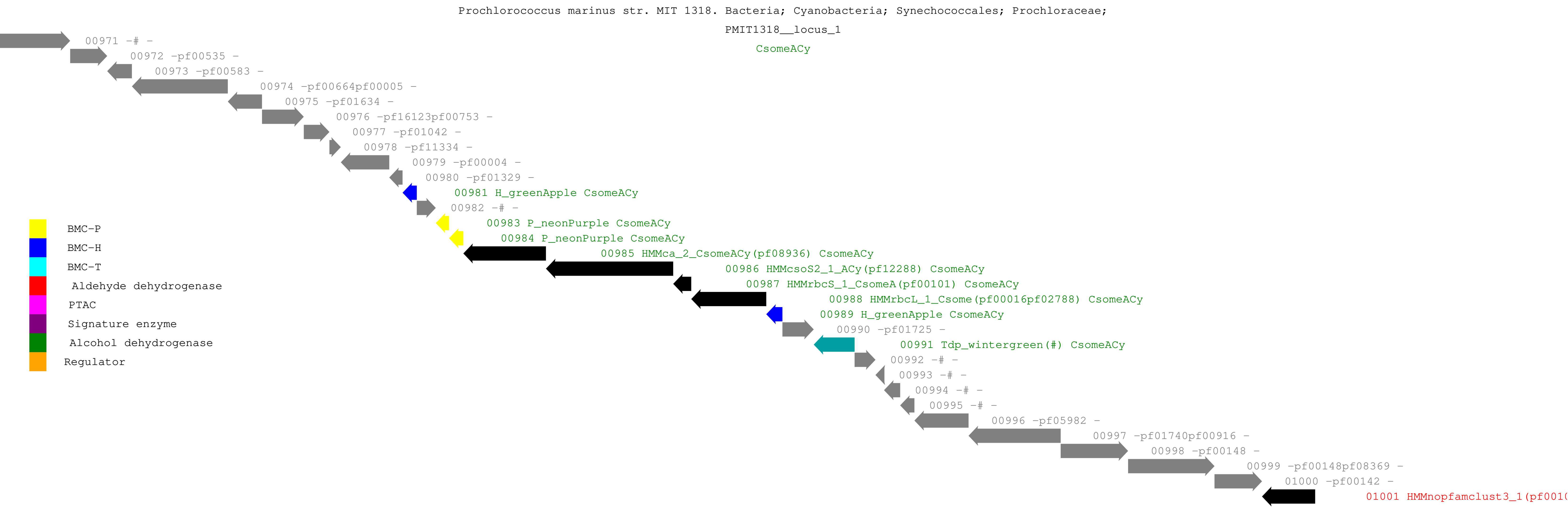
Prochlorococcus marinus str. MIT 1318. Bacteria; Cyanobacteria; Synechococcales; Prochloraceae;
PMIT1318__locus_1
CsomeACy
00971 -# -
00972 -pf00535 -
00973 -pf00583 -
00974 -pf00664pf00005 -
00975 -pf01634 -
00976 -pf16123pf00753 -
00977 -pf01042 -
00978 -pf11334 -
00979 -pf00004 -
00980 -pf01329 -
00981 H_greenApple CsomeACy
00982 -# -
00983 P_neonPurple CsomeACy
00984 P_neonPurple CsomeACy
00985 HMMca_2_CsomeACy(pf08936) CsomeACy
00986 HMMcsoS2_1_ACy(pf12288) CsomeACy
00987 HMMrbcS_1_CsomeA(pf00101) CsomeACy
00988 HMMrbcL_1_Csome(pf00016pf02788) CsomeACy
00989 H_greenApple CsomeACy
00990 -pf01725 -
00991 Tdp_wintergreen(#) CsomeACy
00992 -# -
00993 -# -
00994 -# -
00995 -# -
00996 -pf05982 -
00997 -pf01740pf00916 -
00998 -pf00148 -
00999 -pf00148pf08369 -
01000 -pf00142 -
01001 HMMnopfamclust3_1(pf001
BMC-P
BMC-H
BMC-T
Aldehyde dehydrogenase
PTAC
Signature enzyme
Alcohol dehydrogenase
Regulator

Prochlorococcus marinus str. MIT 1320. Bacteria; Cyanobacteria; Synechococcales; Prochloraceae;

PMIT1320__locus_1

CsomeACy

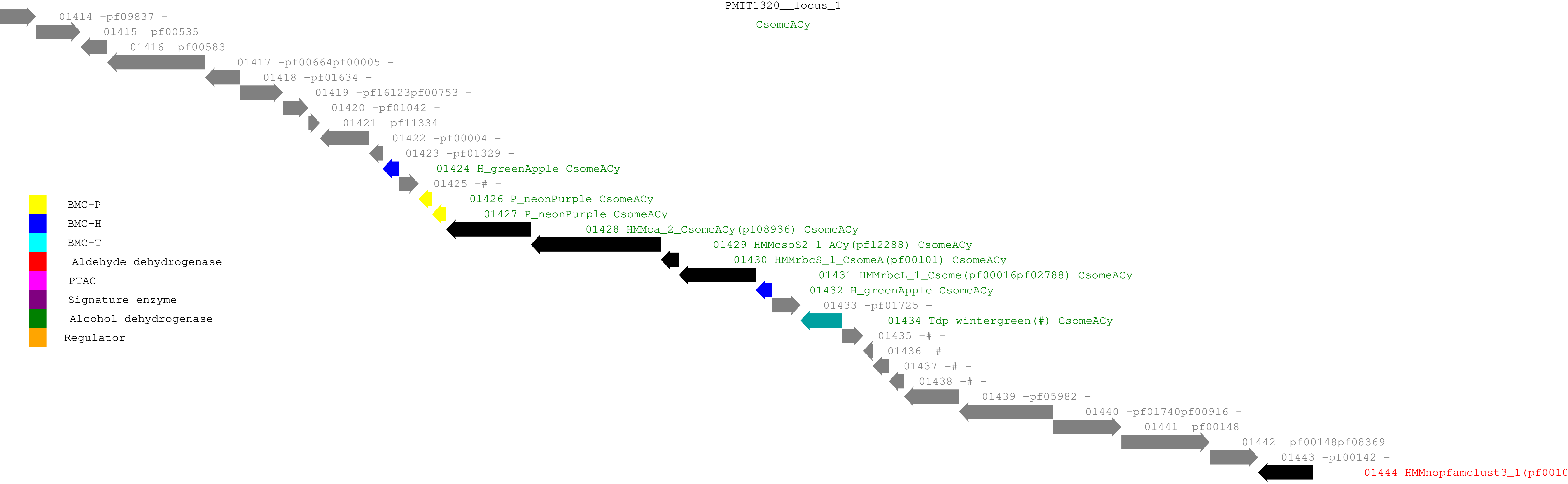

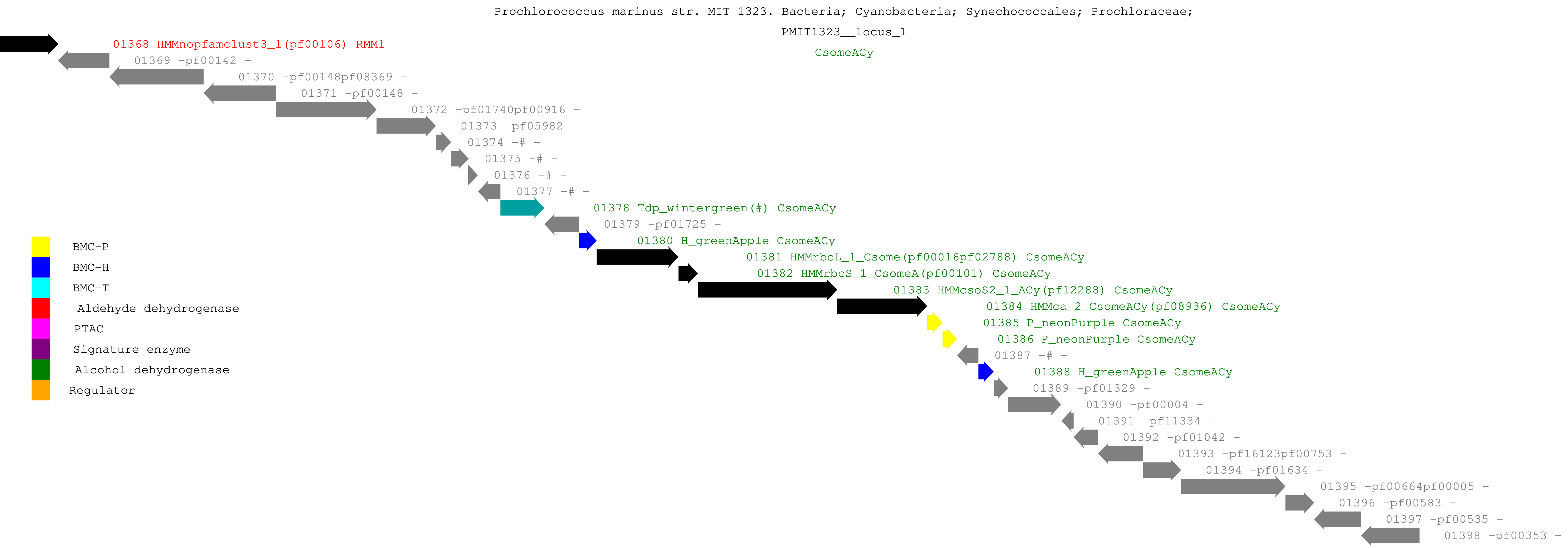
Prochlorococcus marinus str. MIT 1323. Bacteria; Cyanobacteria; Synechococcales; Prochloraceae;
PMIT1323__locus_1
CsomeACy
01368 HMMnopfamclust3_1(pf00106) RMM1
01369 -pf00142 -
01370 -pf00148pf08369 -
01371 -pf00148 -
01372 -pf01740pf00916 -
01373 -pf05982 -
01374 -# -
01375 -# -
01376 -# -
01377 -# -
01378 Tdp_wintergreen(#) CsomeACy
01379 -pf01725 -
01380 H_greenApple CsomeACy
01381 HMMrbcL_1_Csome(pf00016pf02788) CsomeACy
01382 HMMrbcS_1_CsomeA(pf00101) CsomeACy
01383 HMMcsoS2_1_ACy(pf12288) CsomeACy
01384 HMMca_2_CsomeACy(pf08936) CsomeACy
01385 P_neonPurple CsomeACy
01386 P_neonPurple CsomeACy
01387 -# -
01388 H_greenApple CsomeACy
01389 -pf01329 -
01390 -pf00004 -
01391 -pf11334 -
01392 -pf01042 -
01393 -pf16123pf00753 -
01394 -pf01634 -
01395 -pf00664pf00005 -
01396 -pf00583 -
01397 -pf00535 -
01398 -pf00353 -
BMC-P
BMC-H
BMC-T
Aldehyde dehydrogenase
PTAC
Signature enzyme
Alcohol dehydrogenase
Regulator

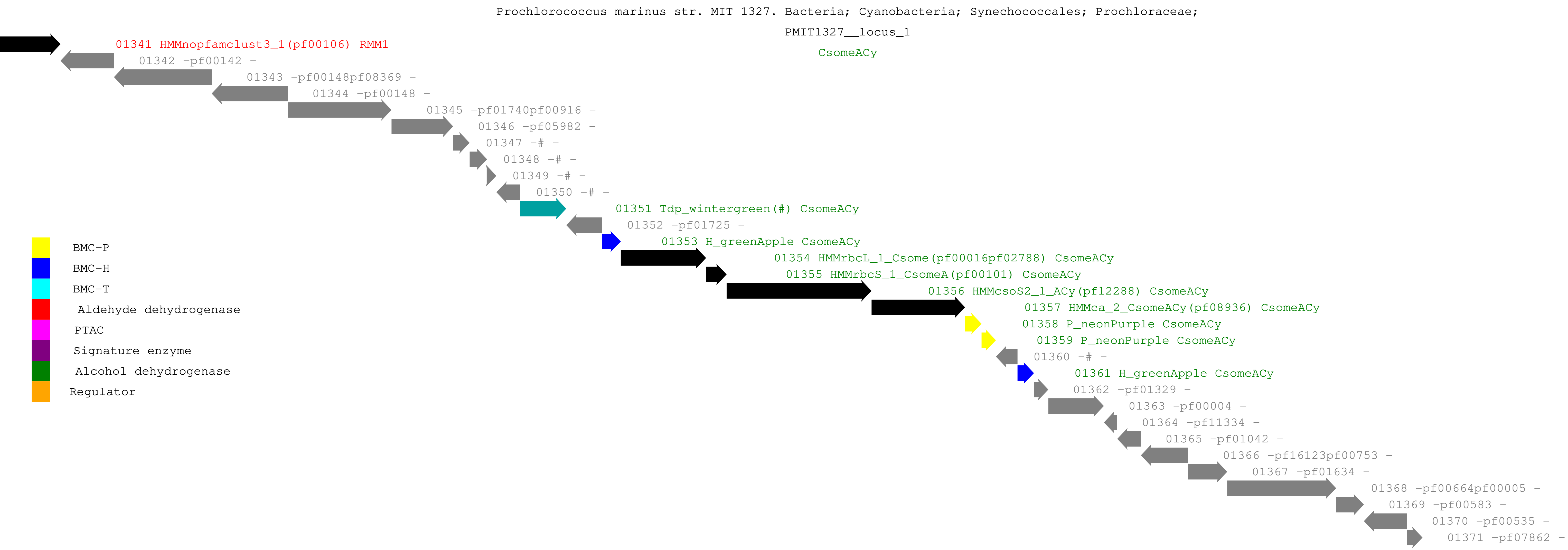
Prochlorococcus marinus str. MIT 1327. Bacteria; Cyanobacteria; Synechococcales; Prochloraceae;
PMIT1327__locus_1
CsomeACy
01341 HMMnopfamclust3_1(pf00106) RMM1
01342 -pf00142 -
01343 -pf00148pf08369 -
01344 -pf00148 -
01345 -pf01740pf00916 -
01346 -pf05982 -
01347 -# -
01348 -# -
01349 -# -
01350 -# -
01351 Tdp_wintergreen(#) CsomeACy
01352 -pf01725 -
01353 H_greenApple CsomeACy
01354 HMMrbcL_1_Csome(pf00016pf02788) CsomeACy
01355 HMMrbcS_1_CsomeA(pf00101) CsomeACy
01356 HMMcsoS2_1_ACy(pf12288) CsomeACy
01357 HMMca_2_CsomeACy(pf08936) CsomeACy
01358 P_neonPurple CsomeACy
01359 P_neonPurple CsomeACy
01360 -# -
01361 H_greenApple CsomeACy
01362 -pf01329 -
01363 -pf00004 -
01364 -pf11334 -
01365 -pf01042 -
01366 -pf16123pf00753 -
01367 -pf01634 -
01368 -pf00664pf00005 -
01369 -pf00583 -
01370 -pf00535 -
01371 -pf07862 -
BMC-P
BMC-H
BMC-T
Aldehyde dehydrogenase
PTAC
Signature enzyme
Alcohol dehydrogenase
Regulator

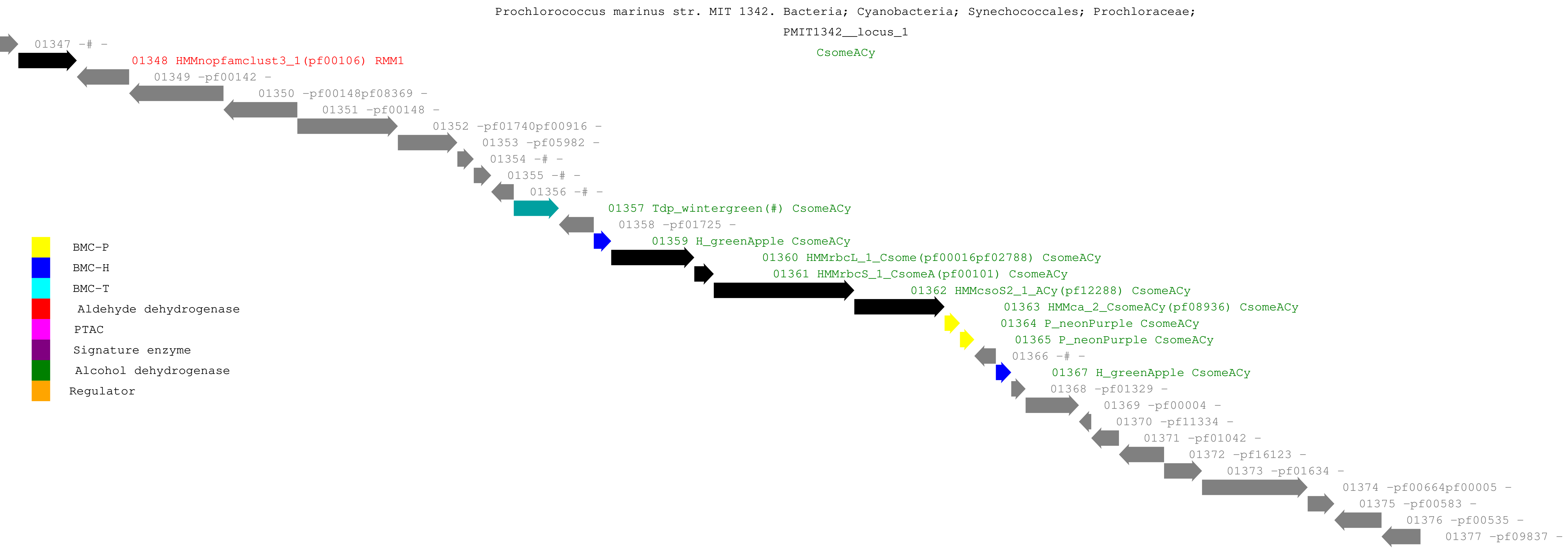
Prochlorococcus marinus str. MIT 1342. Bacteria; Cyanobacteria; Synechococcales; Prochloraceae;
PMIT1342__locus_1
CsomeACy
01347 -# -
01348 HMMnopfamclust3_1(pf00106) RMM1
01349 -pf00142 -
01350 -pf00148pf08369 -
01351 -pf00148 -
01352 -pf01740pf00916 -
01353 -pf05982 -
01354 -# -
01355 -# -
01356 -# -
01357 Tdp_wintergreen(#) CsomeACy
01358 -pf01725 -
01359 H_greenApple CsomeACy
01360 HMMrbcL_1_Csome(pf00016pf02788) CsomeACy
01361 HMMrbcS_1_CsomeA(pf00101) CsomeACy
01362 HMMcsoS2_1_ACy(pf12288) CsomeACy
01363 HMMca_2_CsomeACy(pf08936) CsomeACy
01364 P_neonPurple CsomeACy
01365 P_neonPurple CsomeACy
01366 -# -
01367 H_greenApple CsomeACy
01368 -pf01329 -
01369 -pf00004 -
01370 -pf11334 -
01371 -pf01042 -
01372 -pf16123 -
01373 -pf01634 -
01374 -pf00664pf00005 -
01375 -pf00583 -
01376 -pf00535 -
01377 -pf09837 -
BMC-P
BMC-H
BMC-T
Aldehyde dehydrogenase
PTAC
Signature enzyme
Alcohol dehydrogenase
Regulator

Prochlorococcus marinus subsp. pastoris (strain CCMP1986 / NIES-2087 / Bacteria; Cyanobacteria; Synechococcales; Prochloraceae;

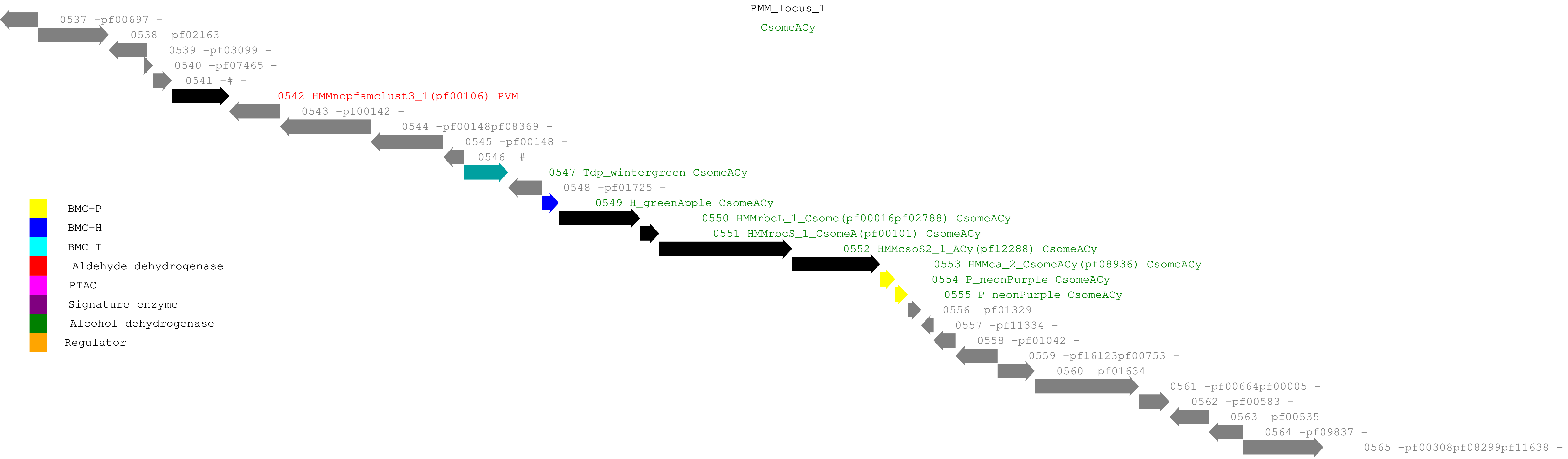

Prochlorococcus marinus (strain NATL2A). Bacteria; Cyanobacteria; Synechococcales; Prochloraceae;

PMN2A__locus_1

CsomeACy

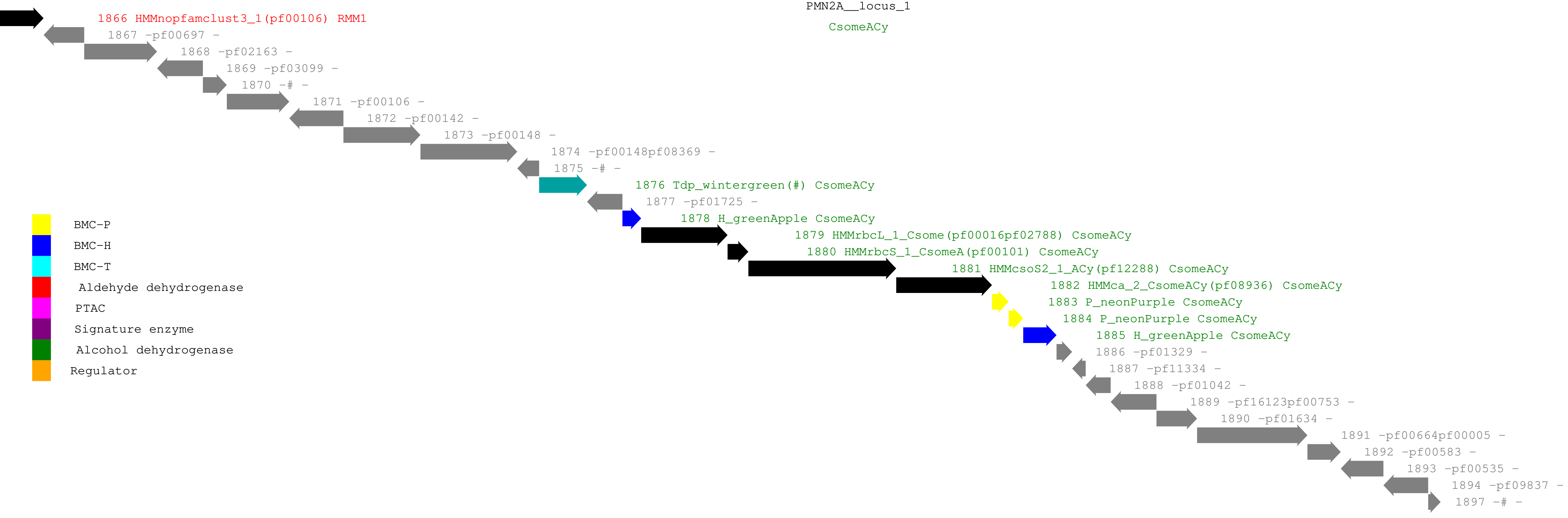

Paulinella micropora. Eukaryota; Rhizaria; Cercozoa; Imbricatea; Silicofilosea; Euglyphida;

PMNZ__locus_1

CsomeACy

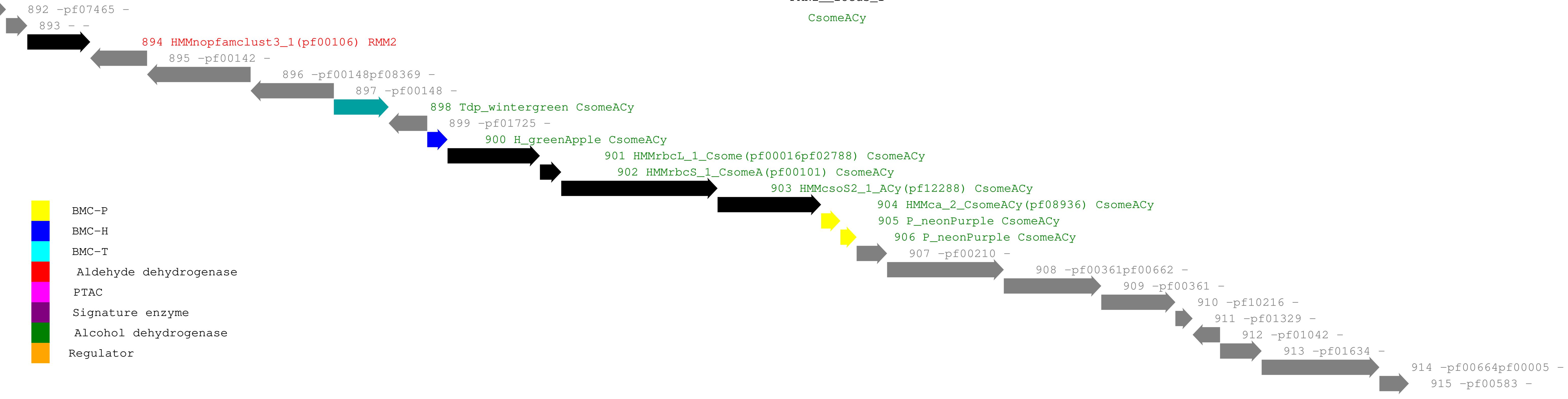

Prochlorococcus marinus (strain MIT 9312). Bacteria; Cyanobacteria; Synechococcales; Prochloraceae;

PMT9312__locus_1

CsomeACy

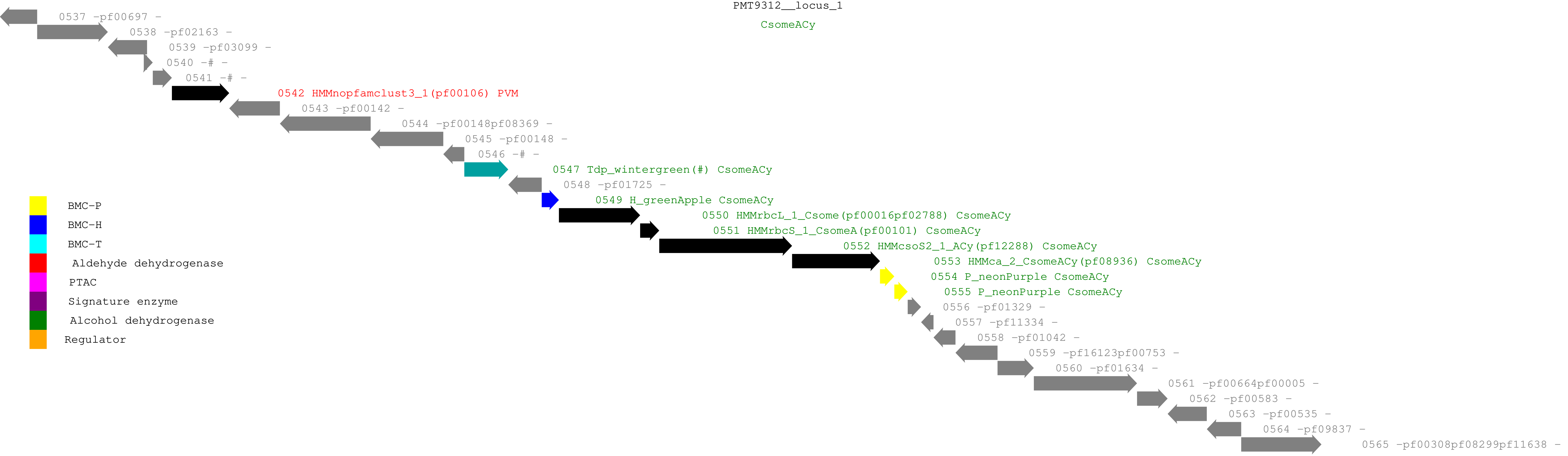

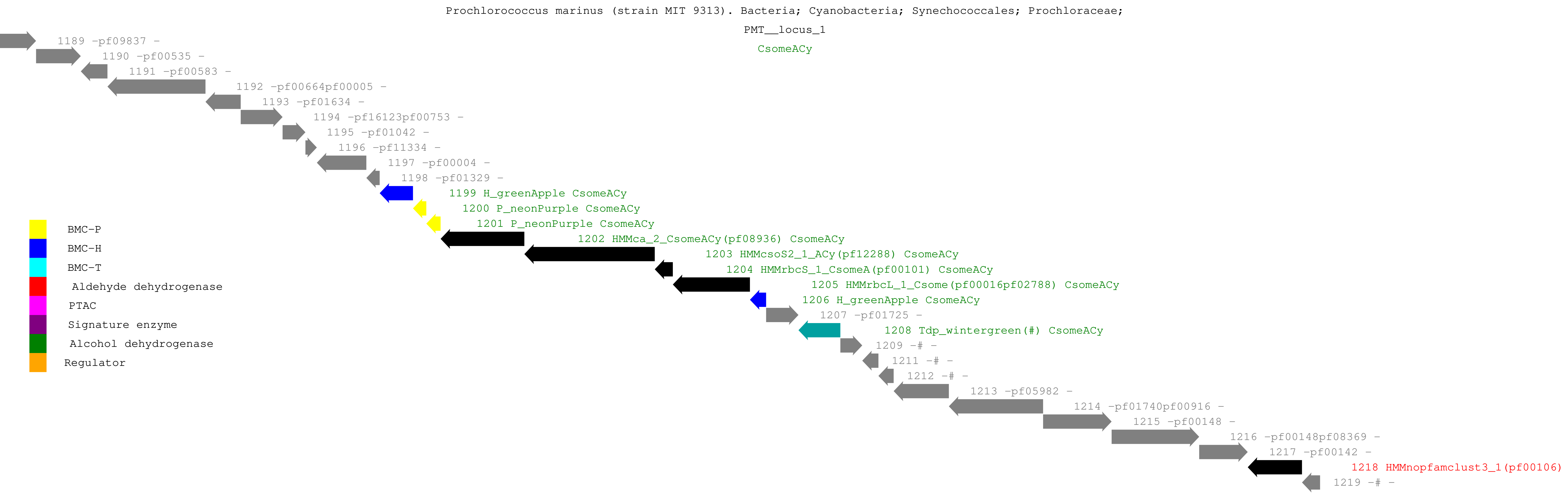
Prochlorococcus marinus (strain MIT 9313). Bacteria; Cyanobacteria; Synechococcales; Prochloraceae;
PMT__locus_1
CsomeACy
1189 -pf09837 -
1190 -pf00535 -
1191 -pf00583 -
1192 -pf00664pf00005 -
1193 -pf01634 -
1194 -pf16123pf00753 -
1195 -pf01042 -
1196 -pf11334 -
1197 -pf00004 -
1198 -pf01329 -
1199 H_greenApple CsomeACy
1200 P_neonPurple CsomeACy
1201 P_neonPurple CsomeACy
1202 HMMca_2_CsomeACy(pf08936) CsomeACy
1203 HMMcsoS2_1_ACy(pf12288) CsomeACy
1204 HMMrbcS_1_CsomeA(pf00101) CsomeACy
1205 HMMrbcL_1_Csome(pf00016pf02788) CsomeACy
1206 H_greenApple CsomeACy
1207 -pf01725 -
1208 Tdp_wintergreen(#) CsomeACy
1209 -# -
1211 -# -
1212 -# -
1213 -pf05982 -
1214 -pf01740pf00916 -
1215 -pf00148 -
1216 -pf00148pf08369 -
1217 -pf00142 -
1218 HMMnopfamclust3_1(pf00106)
1219 -# -
BMC-P
BMC-H
BMC-T
Aldehyde dehydrogenase
PTAC
Signature enzyme
Alcohol dehydrogenase
Regulator

Prochlorococcus marinus (strain SARG / CCMP1375 / SS120). Bacteria; Cyanobacteria; Synechococcales; Prochloraceae;

Pro__locus_1

CsomeACy

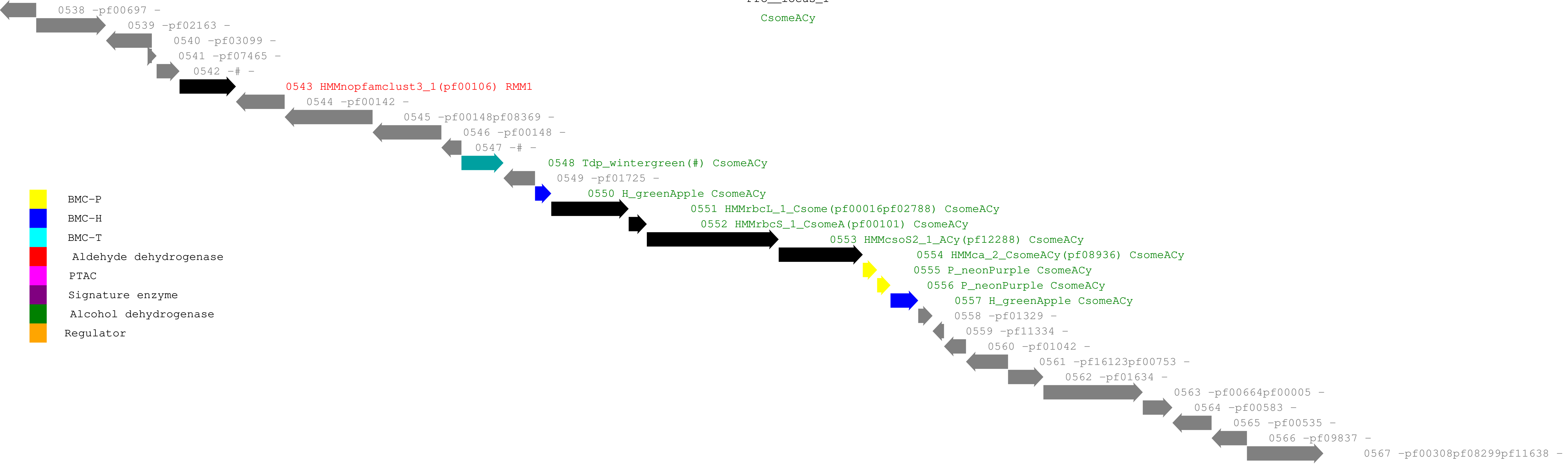

Synechococcus sp. RS9916. Bacteria; Cyanobacteria; Synechococcales; Synechococcaceae;

RS9916__locus_1

CsomeACy

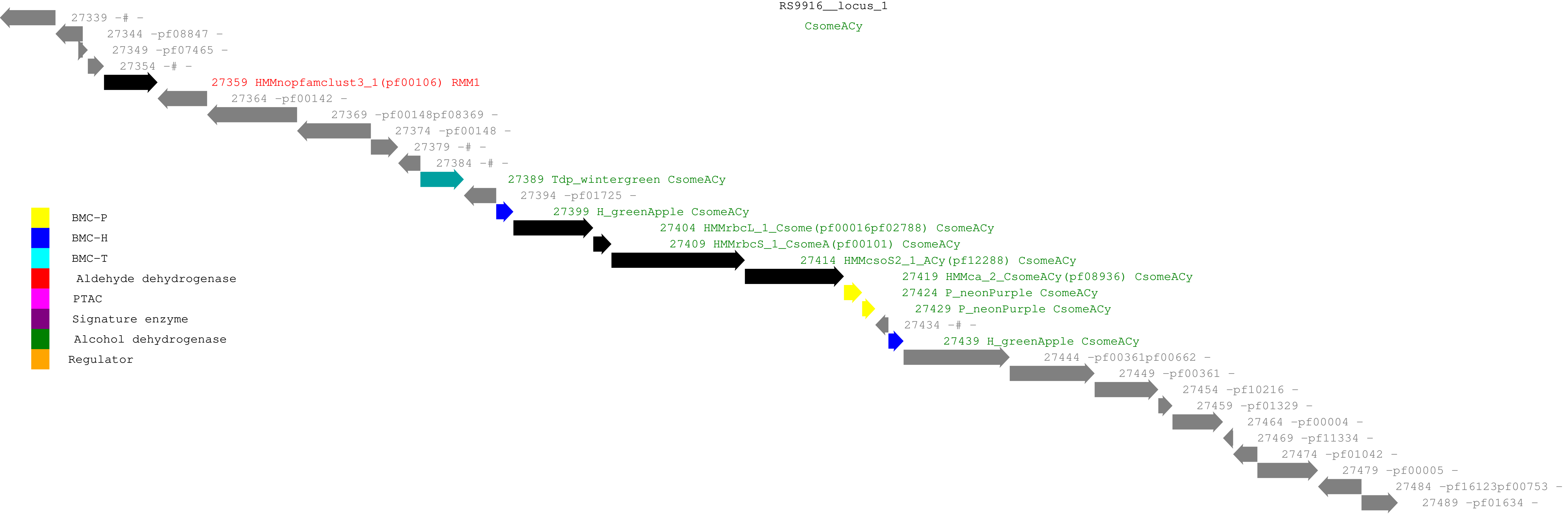

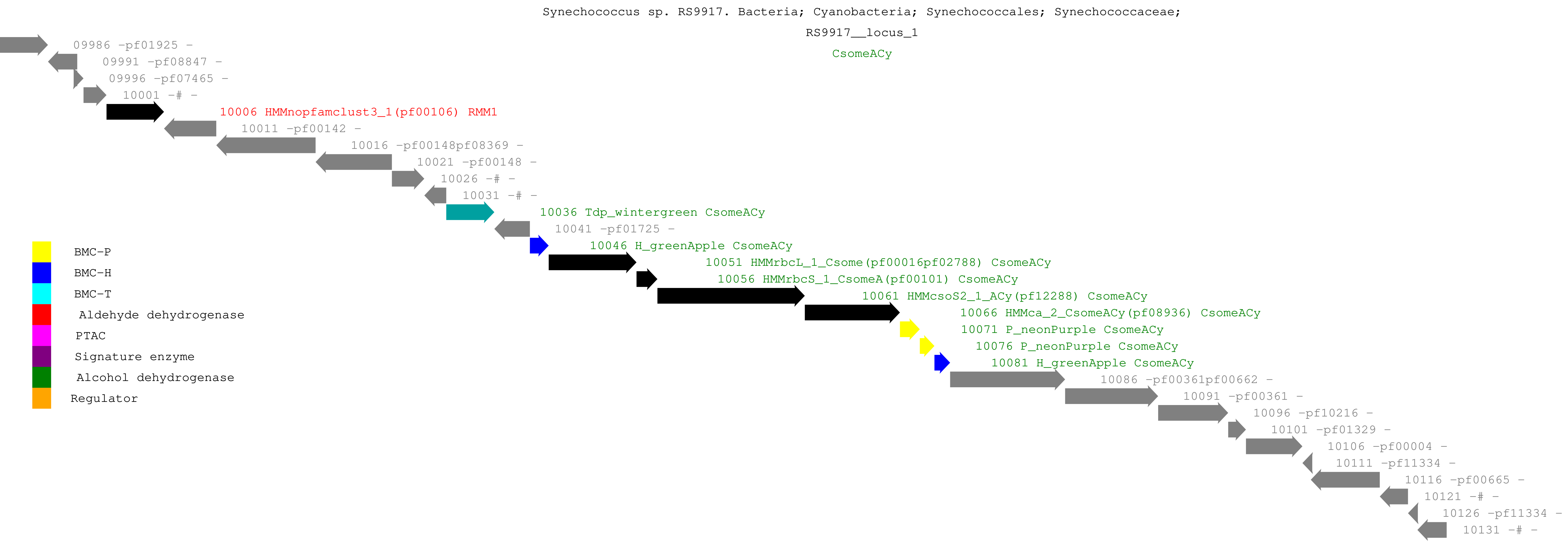

Synechococcus sp. RS9917. Bacteria; Cyanobacteria; Synechococcales; Synechococcaceae;
RS9917__locus_1
CsomeACy
09986 -pf01925 -
09991 -pf08847 -
09996 -pf07465 -
10001 -# -
10006 HMMnopfamclust3_1(pf00106) RMM1
10011 -pf00142 -
10016 -pf00148pf08369 -
10021 -pf00148 -
10026 -# -
10031 -# -
10036 Tdp_wintergreen CsomeACy
10041 -pf01725 -
10046 H_greenApple CsomeACy
10051 HMMrbcL_1_Csome(pf00016pf02788) CsomeACy
10056 HMMrbcS_1_CsomeA(pf00101) CsomeACy
10061 HMMcsoS2_1_ACy(pf12288) CsomeACy
10066 HMMca_2_CsomeACy(pf08936) CsomeACy
10071 P_neonPurple CsomeACy
10076 P_neonPurple CsomeACy
10081 H_greenApple CsomeACy
10086 -pf00361pf00662 -
10091 -pf00361 -
10096 -pf10216 -
10101 -pf01329 -
10106 -pf00004 -
10111 -pf11334 -
10116 -pf00665 -
10121 -# -
10126 -pf11334 -
10131 -# -
BMC-P
BMC-H
BMC-T
Aldehyde dehydrogenase
PTAC
Signature enzyme
Alcohol dehydrogenase
Regulator

Synechococcus sp. WH 8016. Bacteria; Cyanobacteria; Synechococcales; Synechococcaceae;

Syn8016DRAFT__locus_1

CsomeACy

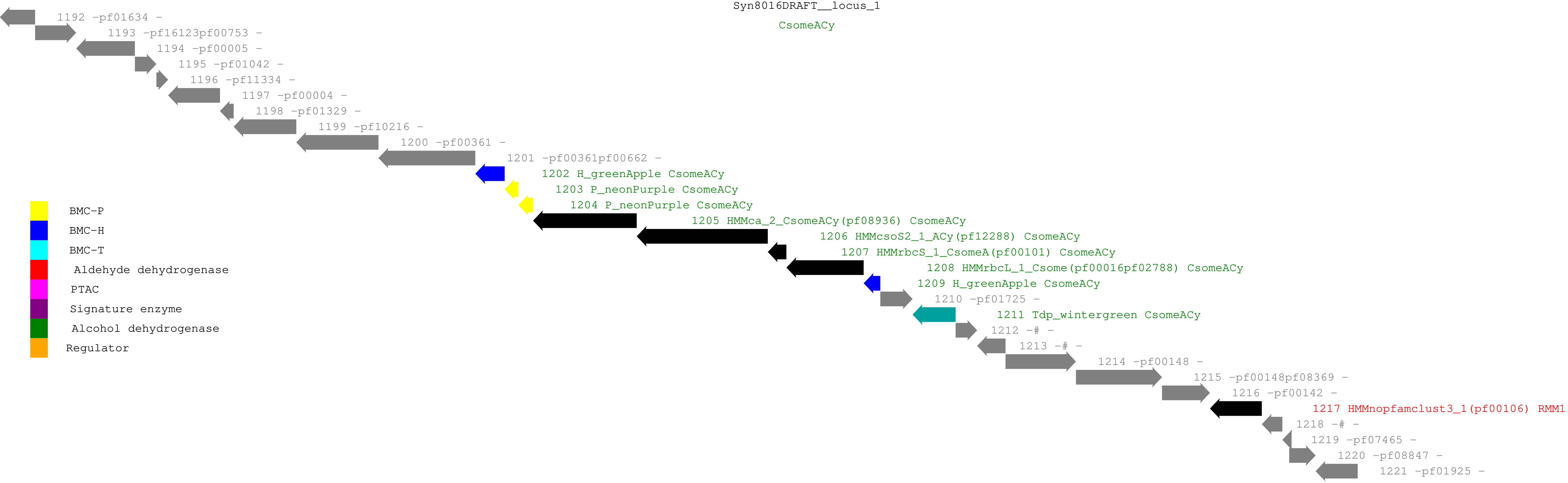

Synechococcus sp. WH 8109. Bacteria; Cyanobacteria; Synechococcales; Synechococcaceae;

Syncc8109__locus_1

CsomeACy

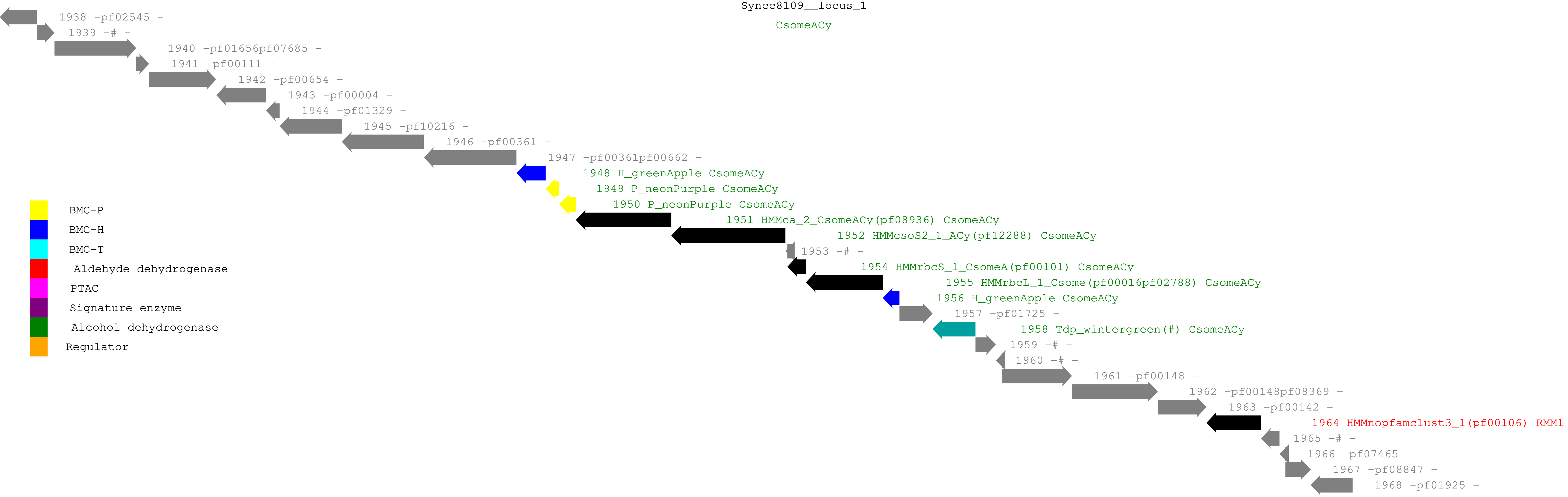

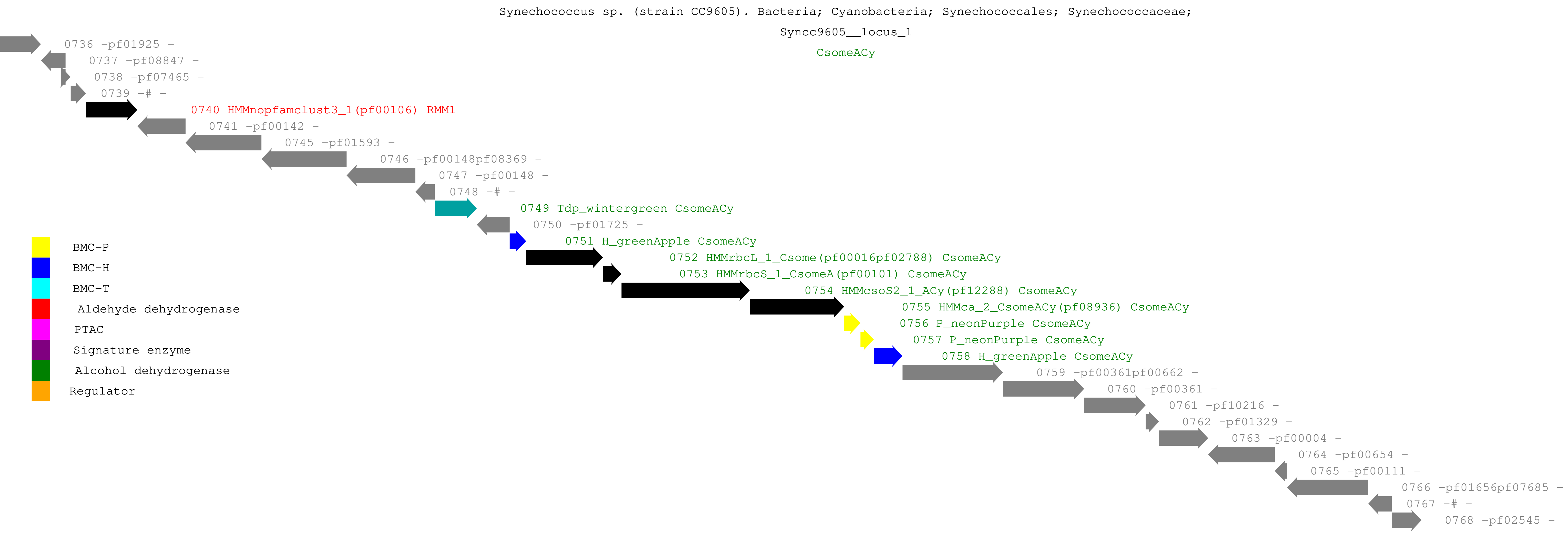
Synechococcus sp. (strain CC9605). Bacteria; Cyanobacteria; Synechococcales; Synechococcaceae;
Syncc9605__locus_1
CsomeACy
0736 -pf01925 -
0737 -pf08847 -
0738 -pf07465 -
0739 -# -
0740 HMMnopfamclust3_1(pf00106) RMM1
0741 -pf00142 -
0745 -pf01593 -
0746 -pf00148pf08369 -
0747 -pf00148 -
0748 -# -
0749 Tdp_wintergreen CsomeACy
0750 -pf01725 -
0751 H_greenApple CsomeACy
0752 HMMrbcL_1_Csome(pf00016pf02788) CsomeACy
0753 HMMrbcS_1_CsomeA(pf00101) CsomeACy
0754 HMMcsoS2_1_ACy(pf12288) CsomeACy
0755 HMMca_2_CsomeACy(pf08936) CsomeACy
0756 P_neonPurple CsomeACy
0757 P_neonPurple CsomeACy
0758 H_greenApple CsomeACy
0759 -pf00361pf00662 -
0760 -pf00361 -
0761 -pf10216 -
0762 -pf01329 -
0763 -pf00004 -
0764 -pf00654 -
0765 -pf00111 -
0766 -pf01656pf07685 -
0767 -# -
0768 -pf02545 -
BMC-P
BMC-H
BMC-T
Aldehyde dehydrogenase
PTAC
Signature enzyme
Alcohol dehydrogenase
Regulator

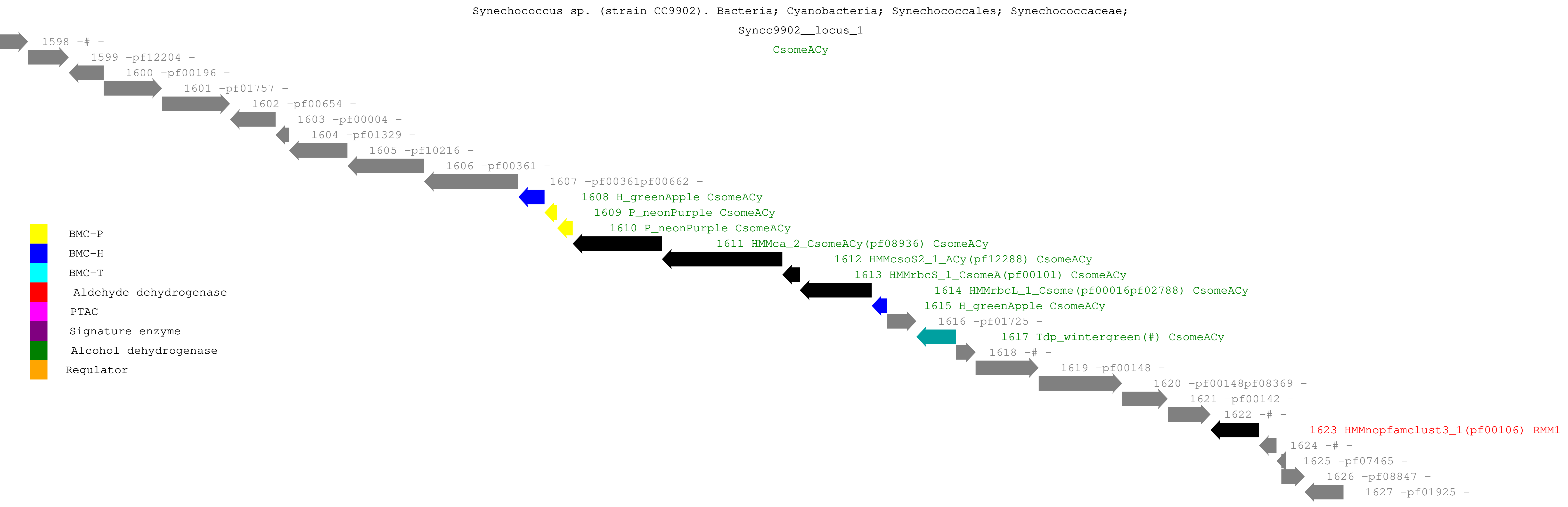
Synechococcus sp. (strain CC9902). Bacteria; Cyanobacteria; Synechococcales; Synechococcaceae;
Syncc9902__locus_1
CsomeACy
1598 -# -
1599 -pf12204 -
1600 -pf00196 -
1601 -pf01757 -
1602 -pf00654 -
1603 -pf00004 -
1604 -pf01329 -
1605 -pf10216 -
1606 -pf00361 -
1607 -pf00361pf00662 -
1608 H_greenApple CsomeACy
1609 P_neonPurple CsomeACy
1610 P_neonPurple CsomeACy
1611 HMMca_2_CsomeACy(pf08936) CsomeACy
1612 HMMcsoS2_1_ACy(pf12288) CsomeACy
1613 HMMrbcS_1_CsomeA(pf00101) CsomeACy
1614 HMMrbcL_1_Csome(pf00016pf02788) CsomeACy
1615 H_greenApple CsomeACy
1616 -pf01725 -
1617 Tdp_wintergreen(#) CsomeACy
1618 -# -
1619 -pf00148 -
1620 -pf00148pf08369 -
1621 -pf00142 -
1622 -# -
1623 HMMnopfamclust3_1(pf00106) RMM1
1624 -# -
1625 -pf07465 -
1626 -pf08847 -
1627 -pf01925 -
BMC-P
BMC-H
BMC-T
Aldehyde dehydrogenase
PTAC
Signature enzyme
Alcohol dehydrogenase
Regulator

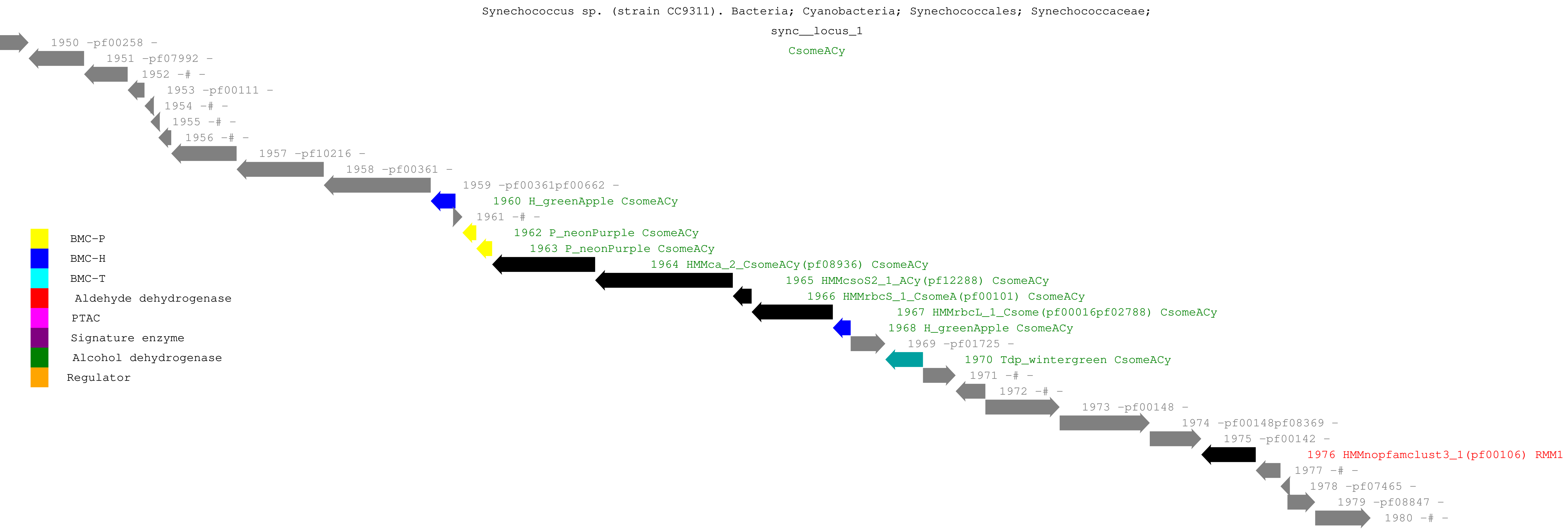
Synechococcus sp. (strain CC9311). Bacteria; Cyanobacteria; Synechococcales; Synechococcaceae;
sync__locus_1
CsomeACy
1950 -pf00258 -
1951 -pf07992 -
1952 -# -
1953 -pf00111 -
1954 -# -
1955 -# -
1956 -# -
1957 -pf10216 -
1958 -pf00361 -
1959 -pf00361pf00662 -
1960 H_greenApple CsomeACy
1961 -# -
1962 P_neonPurple CsomeACy
1963 P_neonPurple CsomeACy
1964 HMMca_2_CsomeACy(pf08936) CsomeACy
1965 HMMcsoS2_1_ACy(pf12288) CsomeACy
1966 HMMrbcS_1_CsomeA(pf00101) CsomeACy
1967 HMMrbcL_1_Csome(pf00016pf02788) CsomeACy
1968 H_greenApple CsomeACy
1969 -pf01725 -
1970 Tdp_wintergreen CsomeACy
1971 -# -
1972 -# -
1973 -pf00148 -
1974 -pf00148pf08369 -
1975 -pf00142 -
1976 HMMnopfamclust3_1(pf00106) RMM1
1977 -# -
1978 -pf07465 -
1979 -pf08847 -
1980 -# -
BMC-P
BMC-H
BMC-T
Aldehyde dehydrogenase
PTAC
Signature enzyme
Alcohol dehydrogenase
Regulator

Synechococcus sp. GFB01. Bacteria; Cyanobacteria; Synechococcales; Synechococcaceae;

SYNGFB01__locus_1_main_sat

CsomeACy

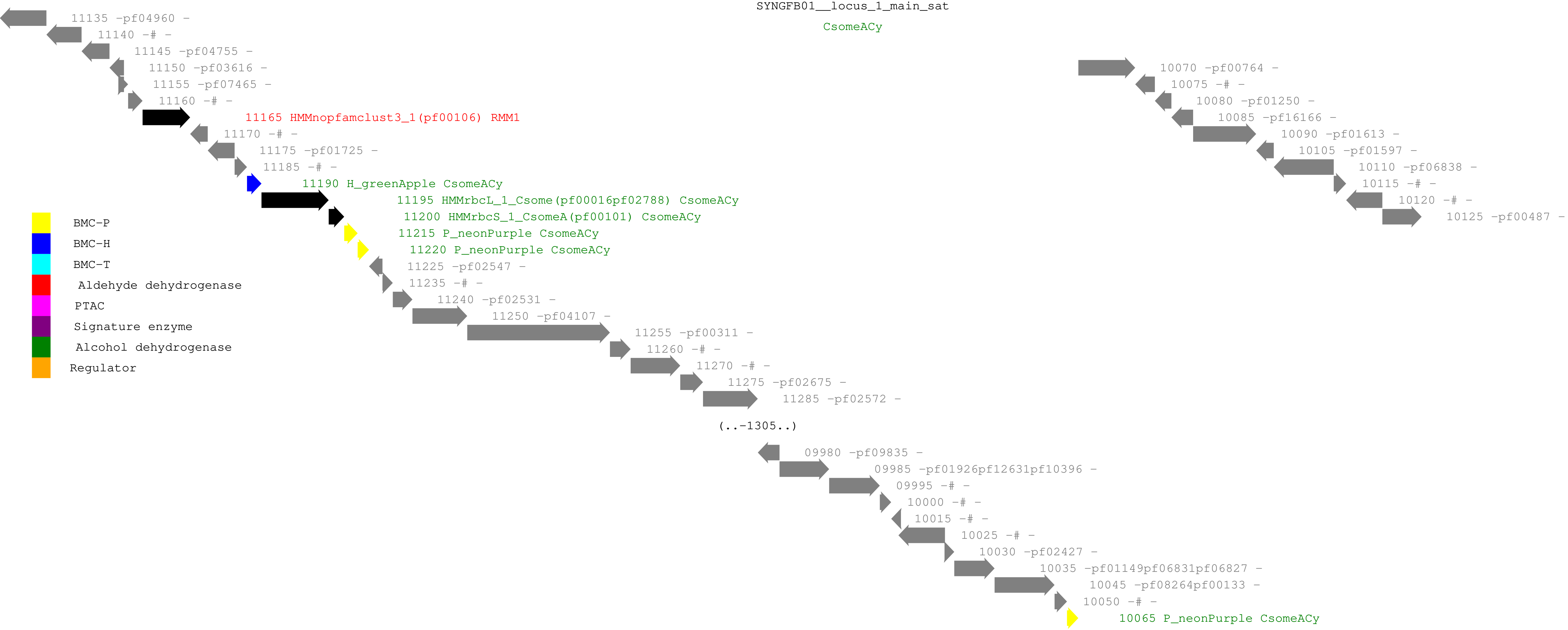

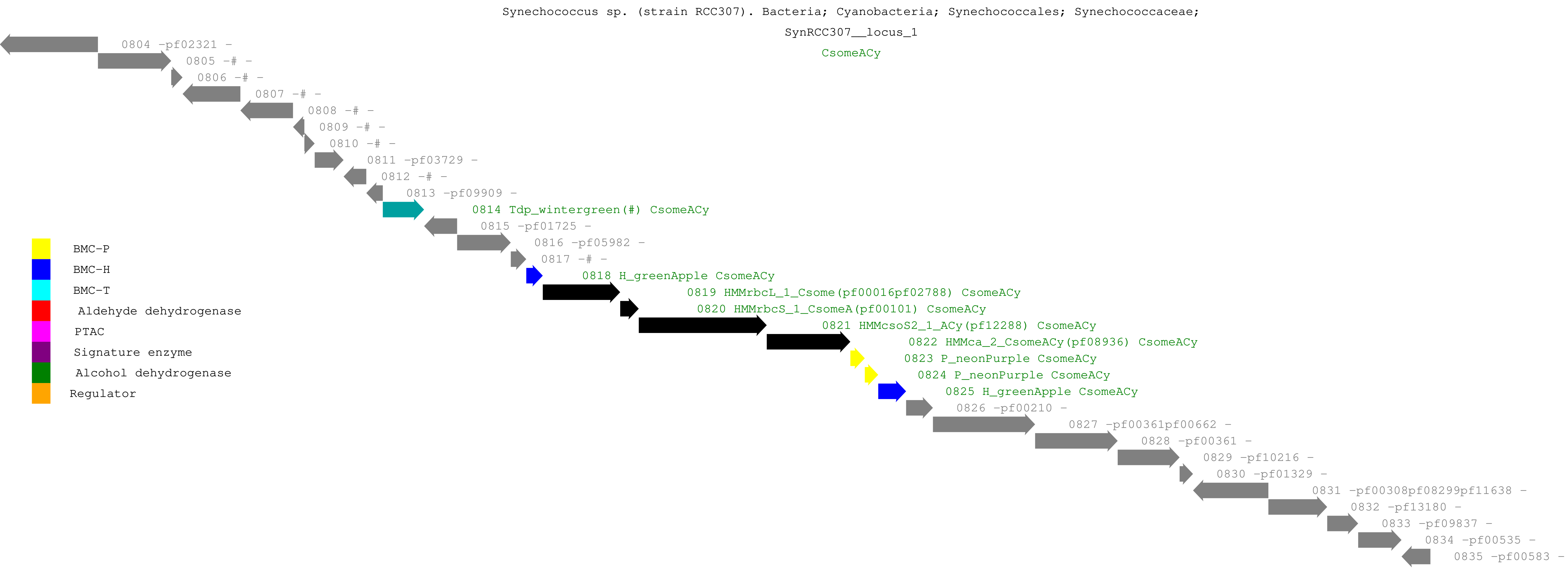
Synechococcus sp. (strain RCC307). Bacteria; Cyanobacteria; Synechococcales; Synechococcaceae;
SynRCC307__locus_1
CsomeACy
0804 -pf02321 -
0805 -# -
0806 -# -
0807 -# -
0808 -# -
0809 -# -
0810 -# -
0811 -pf03729 -
0812 -# -
0813 -pf09909 -
0814 Tdp_wintergreen(#) CsomeACy
0815 -pf01725 -
0816 -pf05982 -
0817 -# -
0818 H_greenApple CsomeACy
0819 HMMrbcL_1_Csome(pf00016pf02788) CsomeACy
0820 HMMrbcS_1_CsomeA(pf00101) CsomeACy
0821 HMMcsoS2_1_ACy(pf12288) CsomeACy
0822 HMMca_2_CsomeACy(pf08936) CsomeACy
0823 P_neonPurple CsomeACy
0824 P_neonPurple CsomeACy
0825 H_greenApple CsomeACy
0826 -pf00210 -
0827 -pf00361pf00662 -
0828 -pf00361 -
0829 -pf10216 -
0830 -pf01329 -
0831 -pf00308pf08299pf11638 -
0832 -pf13180 -
0833 -pf09837 -
0834 -pf00535 -
0835 -pf00583 -
BMC-P
BMC-H
BMC-T
Aldehyde dehydrogenase
PTAC
Signature enzyme
Alcohol dehydrogenase
Regulator

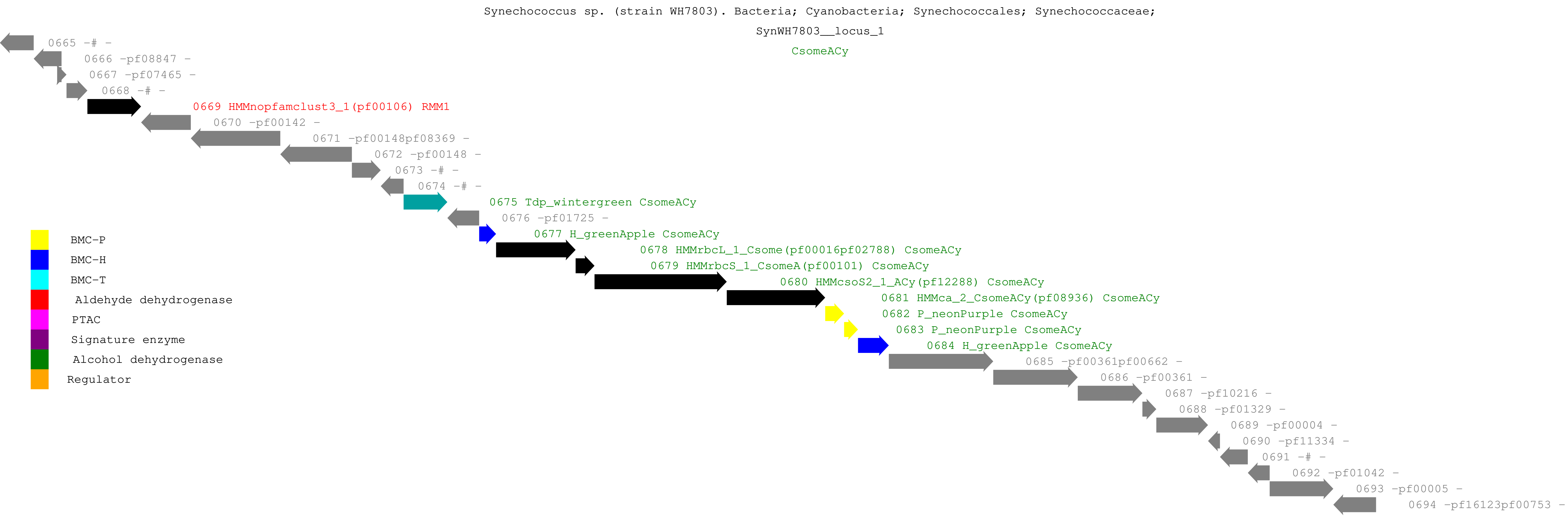
Synechococcus sp. (strain WH7803). Bacteria; Cyanobacteria; Synechococcales; Synechococcaceae;
SynWH7803__locus_1
CsomeACy
0665 -# -
0666 -pf08847 -
0667 -pf07465 -
0668 -# -
0669 HMMnopfamclust3_1(pf00106) RMM1
0670 -pf00142 -
0671 -pf00148pf08369 -
0672 -pf00148 -
0673 -# -
0674 -# -
0675 Tdp_wintergreen CsomeACy
0676 -pf01725 -
0677 H_greenApple CsomeACy
0678 HMMrbcL_1_Csome(pf00016pf02788) CsomeACy
0679 HMMrbcS_1_CsomeA(pf00101) CsomeACy
0680 HMMcsoS2_1_ACy(pf12288) CsomeACy
0681 HMMca_2_CsomeACy(pf08936) CsomeACy
0682 P_neonPurple CsomeACy
0683 P_neonPurple CsomeACy
0684 H_greenApple CsomeACy
0685 -pf00361pf00662 -
0686 -pf00361 -
0687 -pf10216 -
0688 -pf01329 -
0689 -pf00004 -
0690 -pf11334 -
0691 -# -
0692 -pf01042 -
0693 -pf00005 -
0694 -pf16123pf00753 -
BMC-P
BMC-H
BMC-T
Aldehyde dehydrogenase
PTAC
Signature enzyme
Alcohol dehydrogenase
Regulator

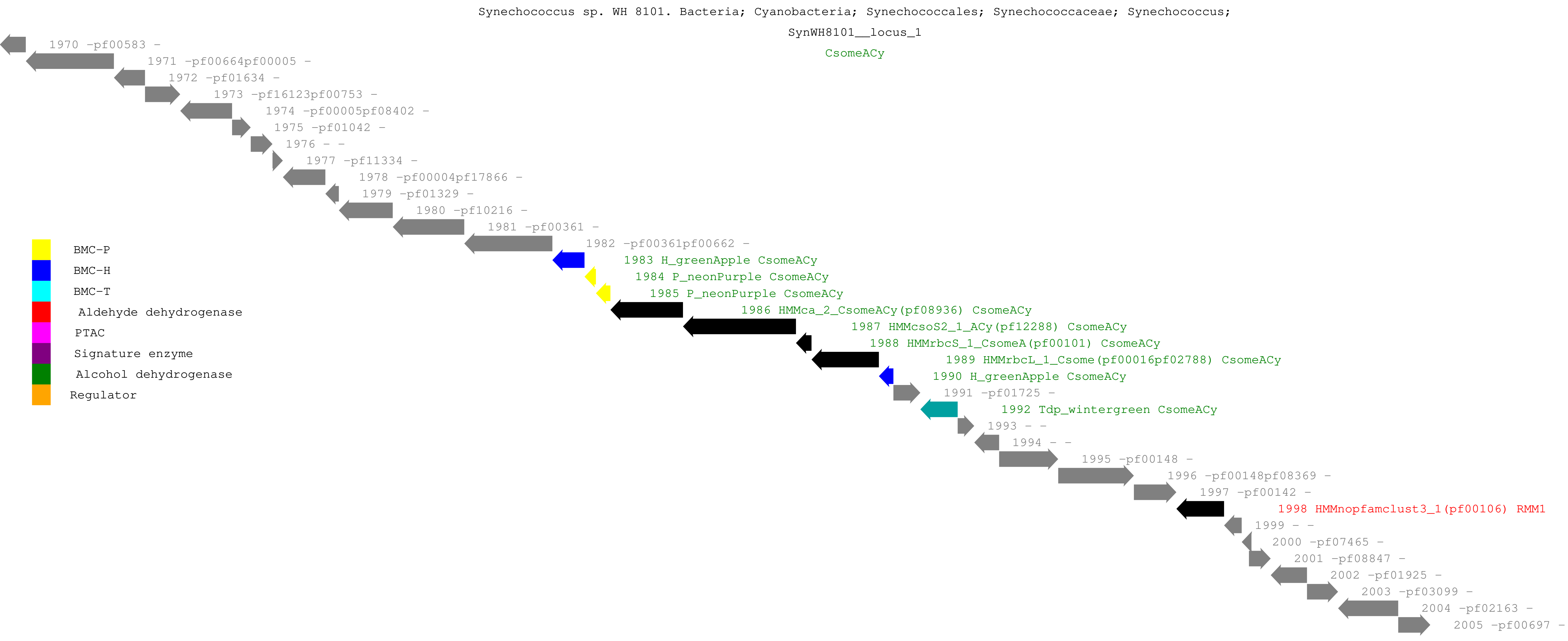
Synechococcus sp. WH 8101. Bacteria; Cyanobacteria; Synechococcales; Synechococcaceae; Synechococcus;
SynWH8101__locus_1
CsomeACy
1970 -pf00583 -
1971 -pf00664pf00005 -
1972 -pf01634 -
1973 -pf16123pf00753 -
1974 -pf00005pf08402 -
1975 -pf01042 -
1976 - -
1977 -pf11334 -
1978 -pf00004pf17866 -
1979 -pf01329 -
1980 -pf10216 -
1981 -pf00361 -
1982 -pf00361pf00662 -
1983 H_greenApple CsomeACy
1984 P_neonPurple CsomeACy
1985 P_neonPurple CsomeACy
1986 HMMca_2_CsomeACy(pf08936) CsomeACy
1987 HMMcsoS2_1_ACy(pf12288) CsomeACy
1988 HMMrbcS_1_CsomeA(pf00101) CsomeACy
1989 HMMrbcL_1_Csome(pf00016pf02788) CsomeACy
1990 H_greenApple CsomeACy
1991 -pf01725 -
1992 Tdp_wintergreen CsomeACy
1993 - -
1994 - -
1995 -pf00148 -
1996 -pf00148pf08369 -
1997 -pf00142 -
1998 HMMnopfamclust3_1(pf00106) RMM1
1999 - -
2000 -pf07465 -
2001 -pf08847 -
2002 -pf01925 -
2003 -pf03099 -
2004 -pf02163 -
2005 -pf00697 -
BMC-P
BMC-H
BMC-T
Aldehyde dehydrogenase
PTAC
Signature enzyme
Alcohol dehydrogenase
Regulator

Synechococcus sp. (strain WH8103). Bacteria; Cyanobacteria; Synechococcales; Synechococcaceae;

SynWH8103__locus_1

CsomeACy

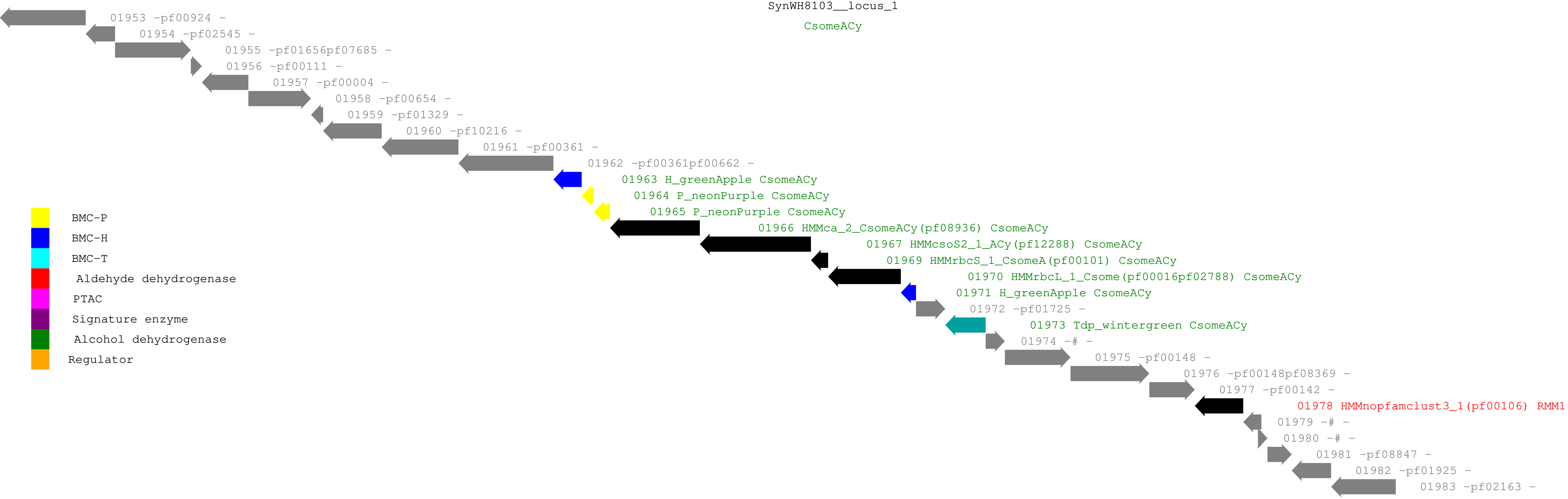

Synechococcus sp. (strain WH8102). Bacteria; Cyanobacteria; Synechococcales; Synechococcaceae;

SYNW_locus_1

CsomeACy

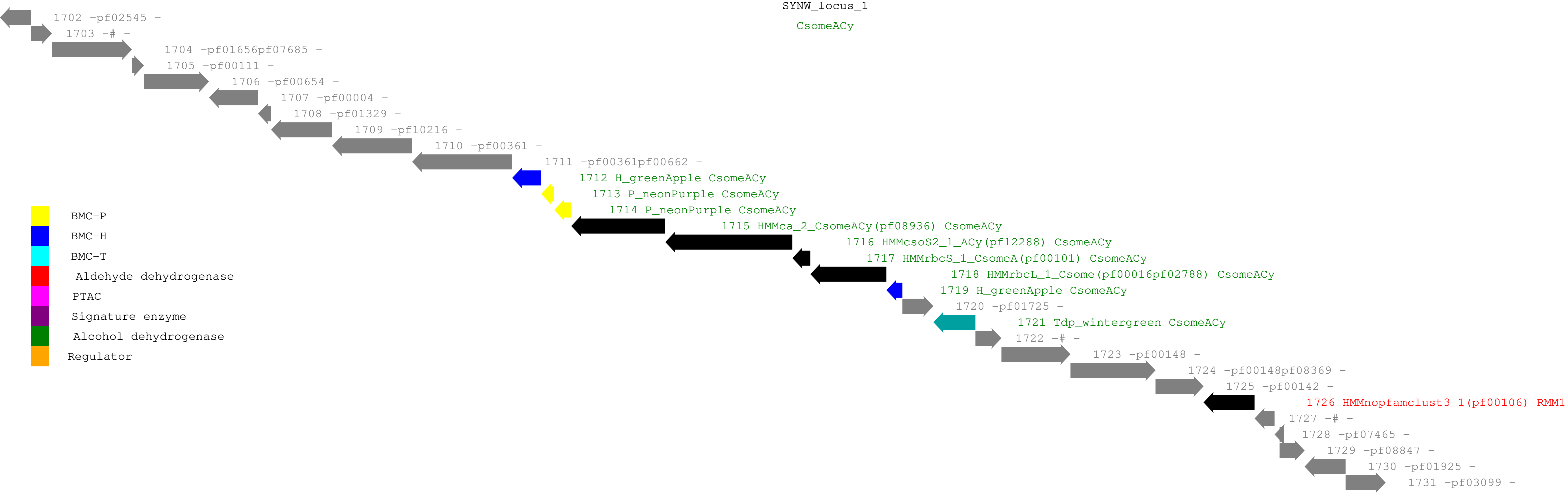

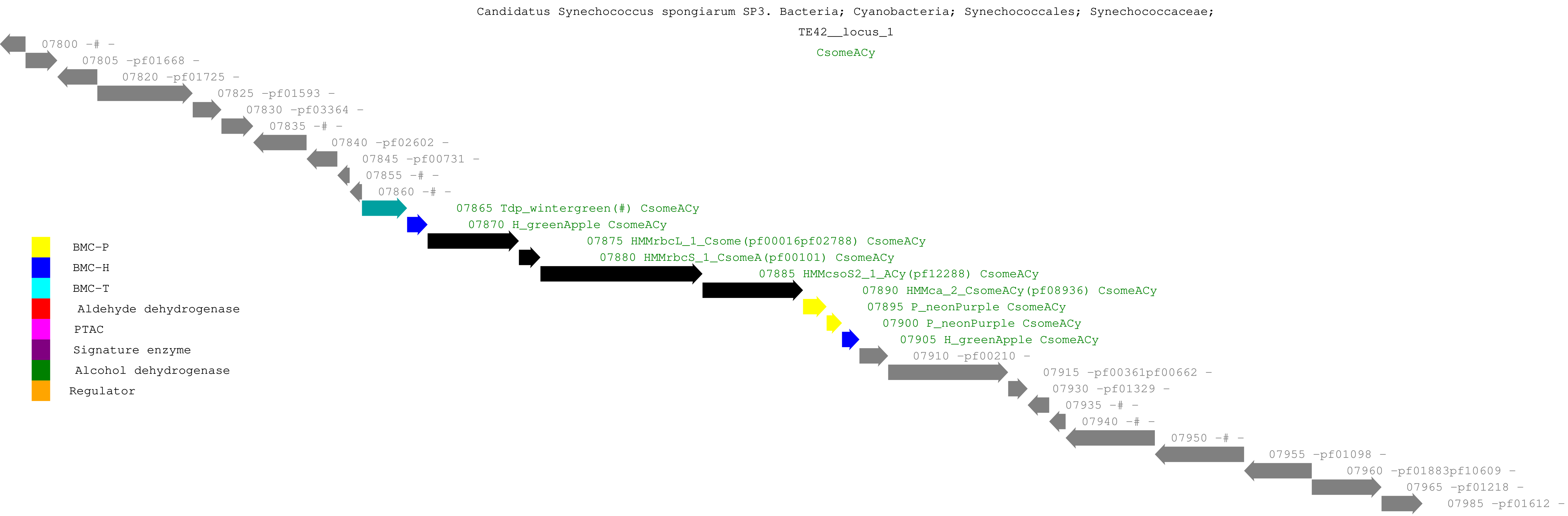

Candidatus Synechococcus spongiarum SP3. Bacteria; Cyanobacteria; Synechococcales; Synechococcaceae;
TE42__locus_1
CsomeACy
07800 -# -
07805 -pf01668 -
07820 -pf01725 -
07825 -pf01593 -
07830 -pf03364 -
07835 -# -
07840 -pf02602 -
07845 -pf00731 -
07855 -# -
07860 -# -
07865 Tdp_wintergreen(#) CsomeACy
07870 H_greenApple CsomeACy
07875 HMMrbcL_1_Csome(pf00016pf02788) CsomeACy
07880 HMMrbcS_1_CsomeA(pf00101) CsomeACy
07885 HMMcsoS2_1_ACy(pf12288) CsomeACy
07890 HMMca_2_CsomeACy(pf08936) CsomeACy
07895 P_neonPurple CsomeACy
07900 P_neonPurple CsomeACy
07905 H_greenApple CsomeACy
07910 -pf00210 -
07915 -pf00361pf00662 -
07930 -pf01329 -
07935 -# -
07940 -# -
07950 -# -
07955 -pf01098 -
07960 -pf01883pf10609 -
07965 -pf01218 -
07985 -pf01612 -
BMC-P
BMC-H
BMC-T
Aldehyde dehydrogenase
PTAC
Signature enzyme
Alcohol dehydrogenase
Regulator

Candidatus Synechococcus spongiarum 142. Bacteria; Cyanobacteria; Synechococcales; Synechococcaceae;

TH68__locus_1

CsomeACy

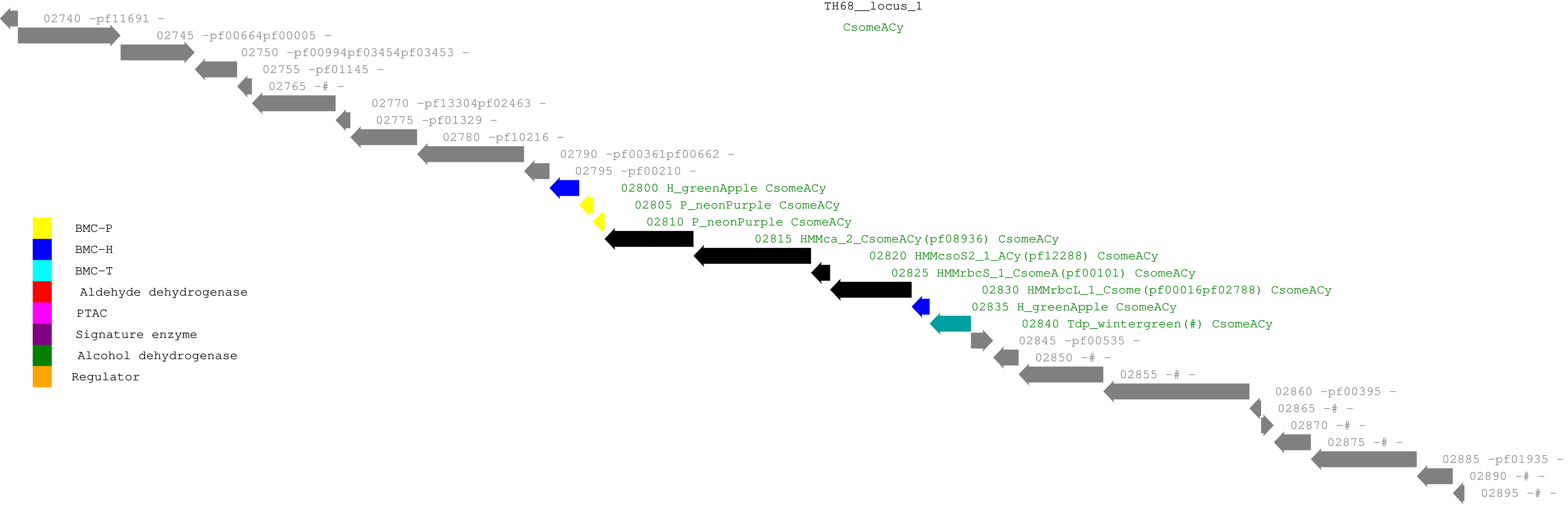

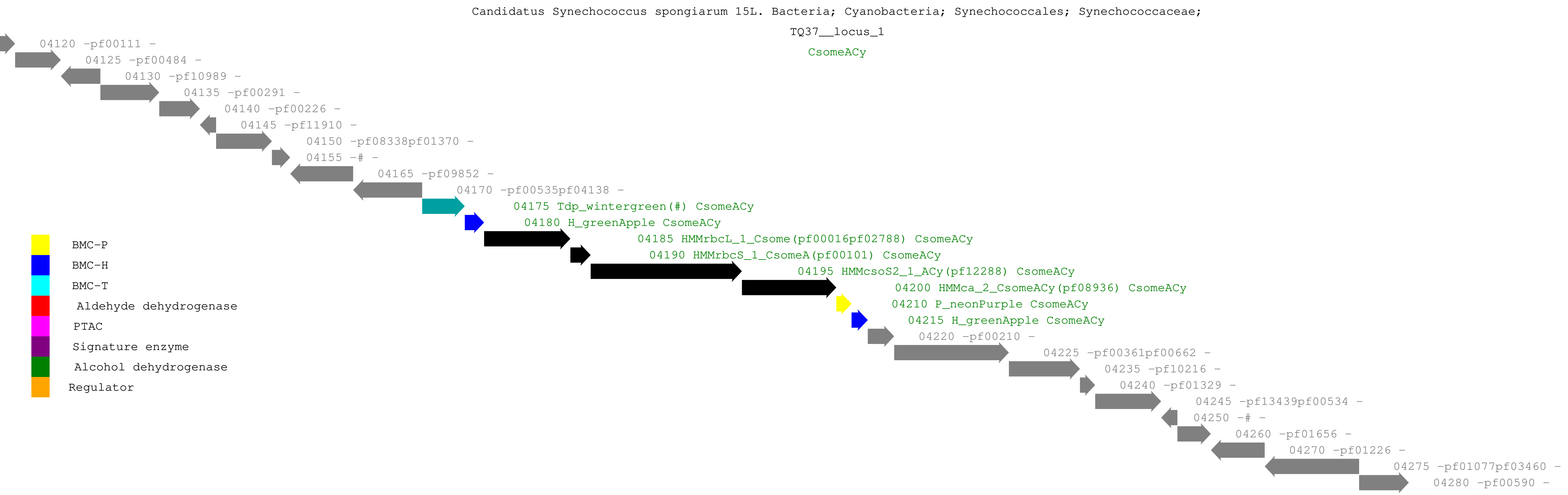
Candidatus Synechococcus spongiarum 15L. Bacteria; Cyanobacteria; Synechococcales; Synechococcaceae;
TQ37__locus_1
CsomeACy
04120 -pf00111 -
04125 -pf00484 -
04130 -pf10989 -
04135 -pf00291 -
04140 -pf00226 -
04145 -pf11910 -
04150 -pf08338pf01370 -
04155 -# -
04165 -pf09852 -
04170 -pf00535pf04138 -
04175 Tdp_wintergreen(#) CsomeACy
04180 H_greenApple CsomeACy
04185 HMMrbcL_1_Csome(pf00016pf02788) CsomeACy
04190 HMMrbcS_1_CsomeA(pf00101) CsomeACy
04195 HMMcsoS2_1_ACy(pf12288) CsomeACy
04200 HMMca_2_CsomeACy(pf08936) CsomeACy
04210 P_neonPurple CsomeACy
04215 H_greenApple CsomeACy
04220 -pf00210 -
04225 -pf00361pf00662 -
04235 -pf10216 -
04240 -pf01329 -
04245 -pf13439pf00534 -
04250 -# -
04260 -pf01656 -
04270 -pf01226 -
04275 -pf01077pf03460 -
04280 -pf00590 -
BMC-P
BMC-H
BMC-T
Aldehyde dehydrogenase
PTAC
Signature enzyme
Alcohol dehydrogenase
Regulator

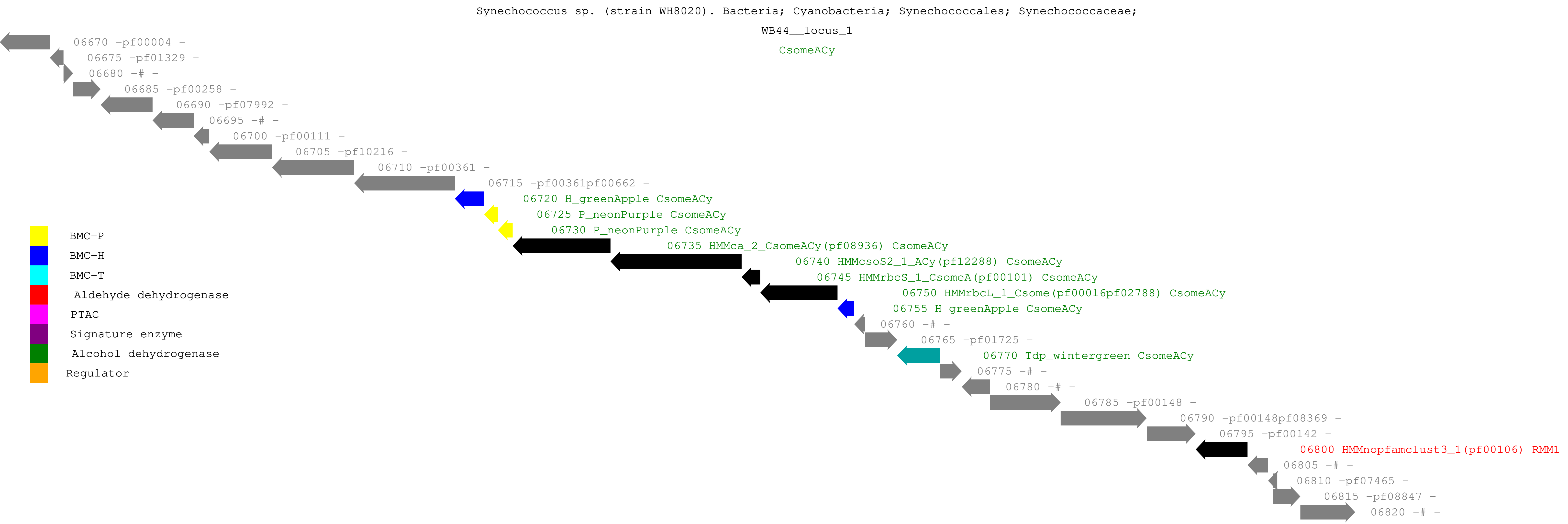
Synechococcus sp. (strain WH8020). Bacteria; Cyanobacteria; Synechococcales; Synechococcaceae;
WB44__locus_1
CsomeACy
06670 -pf00004 -
06675 -pf01329 -
06680 -# -
06685 -pf00258 -
06690 -pf07992 -
06695 -# -
06700 -pf00111 -
06705 -pf10216 -
06710 -pf00361 -
06715 -pf00361pf00662 -
06720 H_greenApple CsomeACy
06725 P_neonPurple CsomeACy
06730 P_neonPurple CsomeACy
06735 HMMca_2_CsomeACy(pf08936) CsomeACy
06740 HMMcsoS2_1_ACy(pf12288) CsomeACy
06745 HMMrbcS_1_CsomeA(pf00101) CsomeACy
06750 HMMrbcL_1_Csome(pf00016pf02788) CsomeACy
06755 H_greenApple CsomeACy
06760 -# -
06765 -pf01725 -
06770 Tdp_wintergreen CsomeACy
06775 -# -
06780 -# -
06785 -pf00148 -
06790 -pf00148pf08369 -
06795 -pf00142 -
06800 HMMnopfamclust3_1(pf00106) RMM1
06805 -# -
06810 -pf07465 -
06815 -pf08847 -
06820 -# -
BMC-P
BMC-H
BMC-T
Aldehyde dehydrogenase
PTAC
Signature enzyme
Alcohol dehydrogenase
Regulator

Synechococcus sp. WH 5701. Bacteria; Cyanobacteria; Synechococcales; Synechococcaceae;

WH5701__locus_1_main_sat

CsomeACy

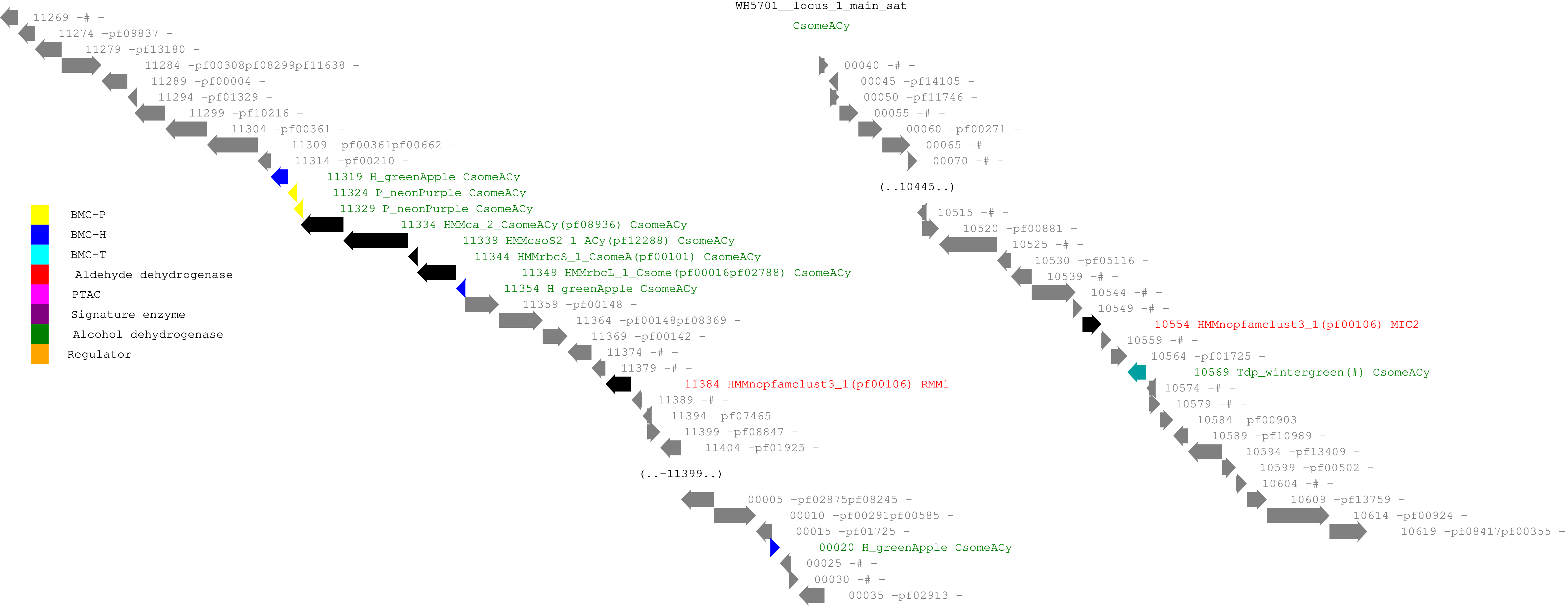

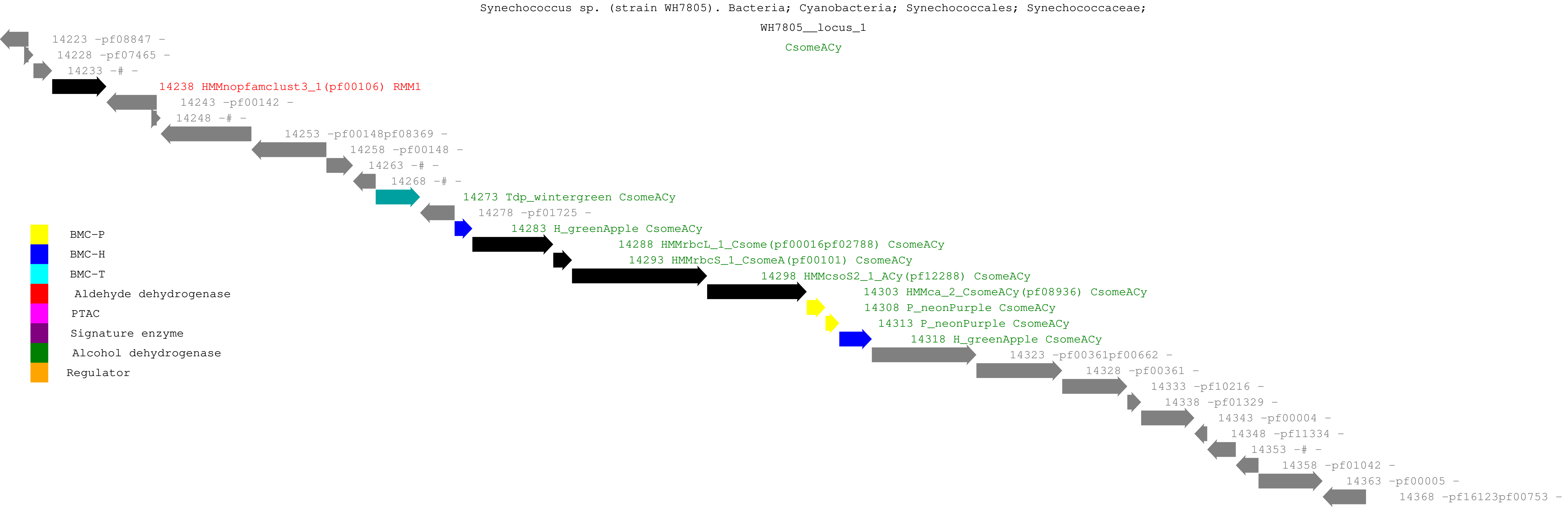

Synechococcus sp. (strain WH7805). Bacteria; Cyanobacteria; Synechococcales; Synechococcaceae;
WH7805__locus_1
CsomeACy
14223 -pf08847 -
14228 -pf07465 -
14233 -# -
14238 HMMnopfamclust3_1(pf00106) RMM1
14243 -pf00142 -
14248 -# -
14253 -pf00148pf08369 -
14258 -pf00148 -
14263 -# -
14268 -# -
14273 Tdp_wintergreen CsomeACy
14278 -pf01725 -
14283 H_greenApple CsomeACy
14288 HMMrbcL_1_Csome(pf00016pf02788) CsomeACy
14293 HMMrbcS_1_CsomeA(pf00101) CsomeACy
14298 HMMcsoS2_1_ACy(pf12288) CsomeACy
14303 HMMca_2_CsomeACy(pf08936) CsomeACy
14308 P_neonPurple CsomeACy
14313 P_neonPurple CsomeACy
14318 H_greenApple CsomeACy
14323 -pf00361pf00662 -
14328 -pf00361 -
14333 -pf10216 -
14338 -pf01329 -
14343 -pf00004 -
14348 -pf11334 -
14353 -# -
14358 -pf01042 -
14363 -pf00005 -
14368 -pf16123pf00753 -
BMC-P
BMC-H
BMC-T
Aldehyde dehydrogenase
PTAC
Signature enzyme
Alcohol dehydrogenase
Regulator

Planktothrix agardhii NIVA-CYA 126/8. Bacteria; Cyanobacteria; Oscillatoriophycideae; Oscillatoriales;

A19Y__locus_1_main_sat

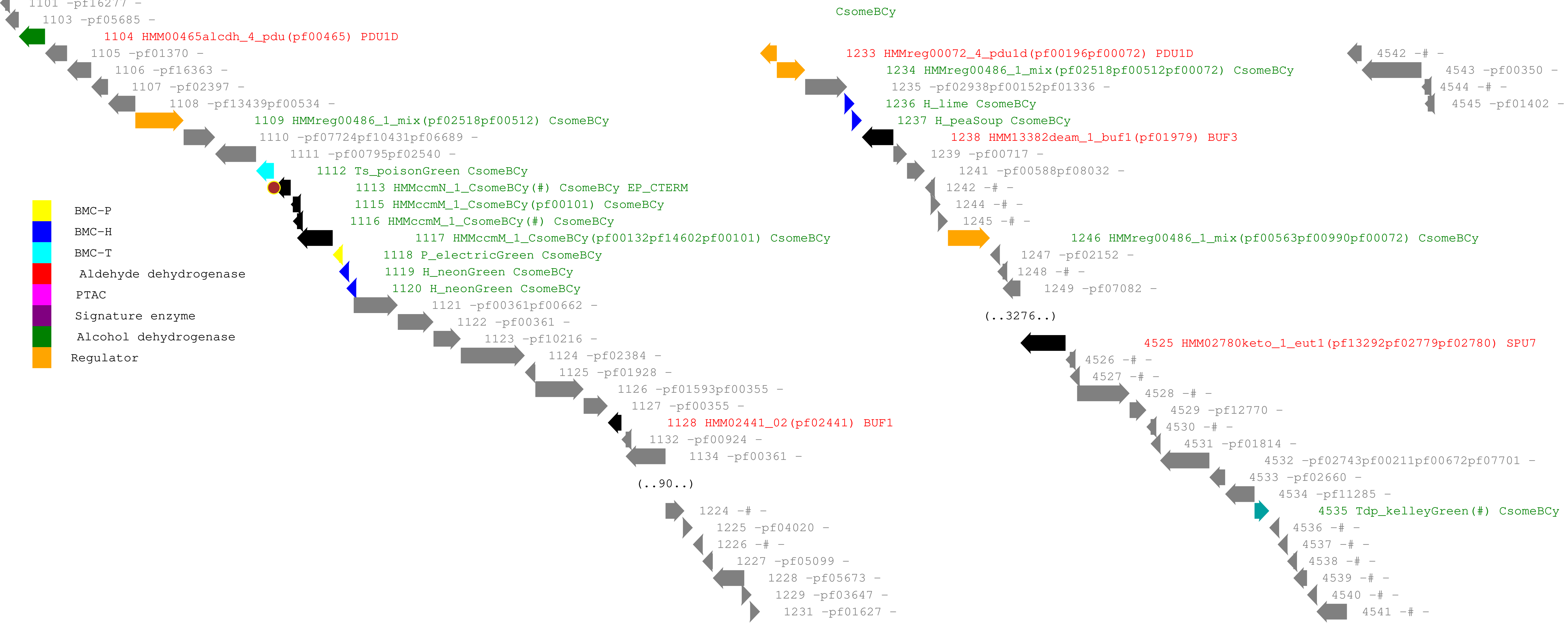

Nodularia spumigena CENA596. Bacteria; Cyanobacteria; Nostocales; Aphanizomenonaceae; Nodularia.

A2T98__locus_1_main_sat

CsomeBCy

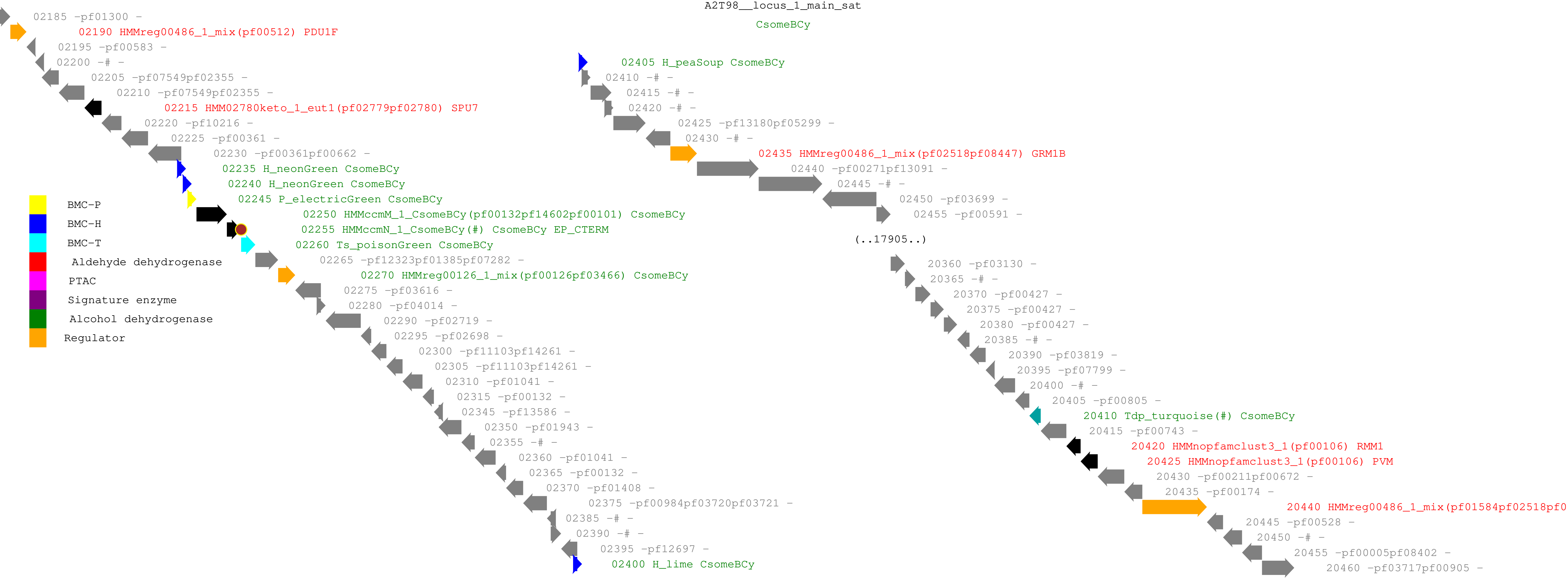

Nostoc sp. KVJ20. Bacteria; Cyanobacteria; Nostocales; Nostocaceae; Nostoc.

A4S05__locus_1_main_sat

CsomeBCy

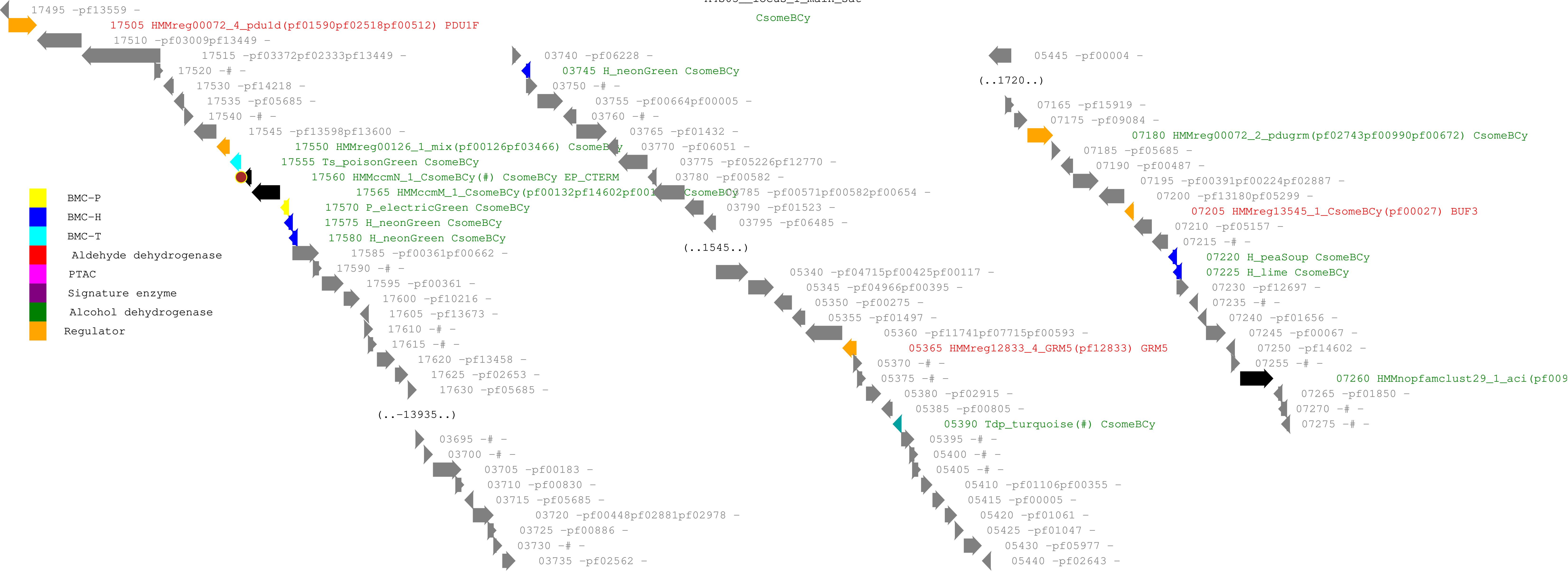

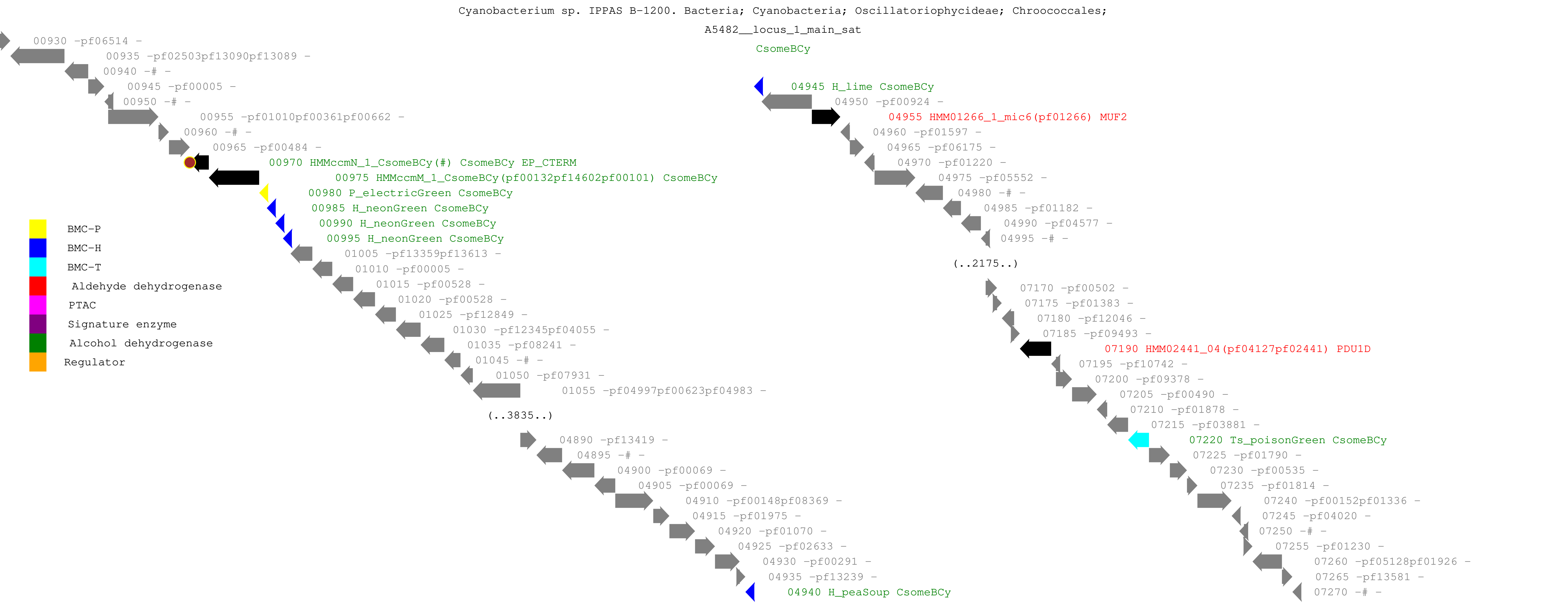
Cyanobacterium sp. IPPAS B-1200. Bacteria; Cyanobacteria; Oscillatoriophycideae; Chroococcales;
A5482__locus_1_main_sat
CsomeBCy
00930 -pf06514 -
00935 -pf02503pf13090pf13089 -
00940 -# -
00945 -pf00005 -
00950 -# -
00955 -pf01010pf00361pf00662 -
00960 -# -
00965 -pf00484 -
00970 HMMccmN_1_CsomeBCy(#) CsomeBCy EP_CTERM
00975 HMMccmM_1_CsomeBCy(pf00132pf14602pf00101) CsomeBCy
00980 P_electricGreen CsomeBCy
00985 H_neonGreen CsomeBCy
00990 H_neonGreen CsomeBCy
00995 H_neonGreen CsomeBCy
01005 -pf13359pf13613 -
01010 -pf00005 -
01015 -pf00528 -
01020 -pf00528 -
01025 -pf12849 -
01030 -pf12345pf04055 -
01035 -pf08241 -
01045 -# -
01050 -pf07931 -
01055 -pf04997pf00623pf04983 -
(..3835..)
04890 -pf13419 -
04895 -# -
04900 -pf00069 -
04905 -pf00069 -
04910 -pf00148pf08369 -
04915 -pf01975 -
04920 -pf01070 -
04925 -pf02633 -
04930 -pf00291 -
04935 -pf13239 -
04940 H_peaSoup CsomeBCy
BMC-P
BMC-H
BMC-T
Aldehyde dehydrogenase
PTAC
Signature enzyme
Alcohol dehydrogenase
Regulator
04945 H_lime CsomeBCy
04950 -pf00924 -
04955 HMM01266_1_mic6(pf01266) MUF2
04960 -pf01597 -
04965 -pf06175 -
04970 -pf01220 -
04975 -pf05552 -
04980 -# -
04985 -pf01182 -
04990 -pf04577 -
04995 -# -
(..2175..)
07170 -pf00502 -
07175 -pf01383 -
07180 -pf12046 -
07185 -pf09493 -
07190 HMM02441_04(pf04127pf02441) PDU1D
07195 -pf10742 -
07200 -pf09378 -
07205 -pf00490 -
07210 -pf01878 -
07215 -pf03881 -
07220 Ts_poisonGreen CsomeBCy
07225 -pf01790 -
07230 -pf00535 -
07235 -pf01814 -
07240 -pf00152pf01336 -
07245 -pf04020 -
07250 -# -
07255 -pf01230 -
07260 -pf05128pf01926 -
07265 -pf13581 -
07270 -# -

Nostoc punctiforme NIES-2108. Bacteria; Cyanobacteria; Nostocales; Nostocaceae; Nostoc.

A6769__locus_1_main_sat

CsomeBCy

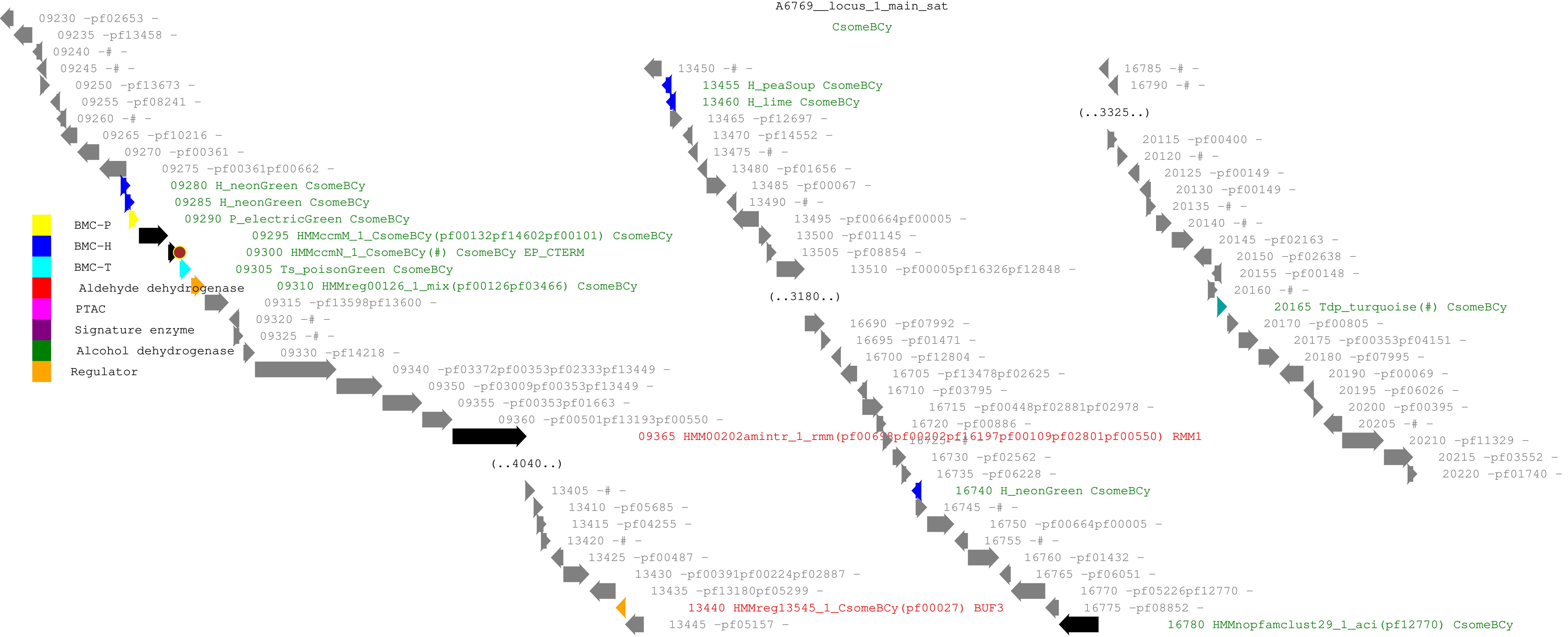

Nostoc minutum NIES-26. Bacteria; Cyanobacteria; Nostocales; Nostocaceae; Nostoc.

A6770__locus_1_main_sat

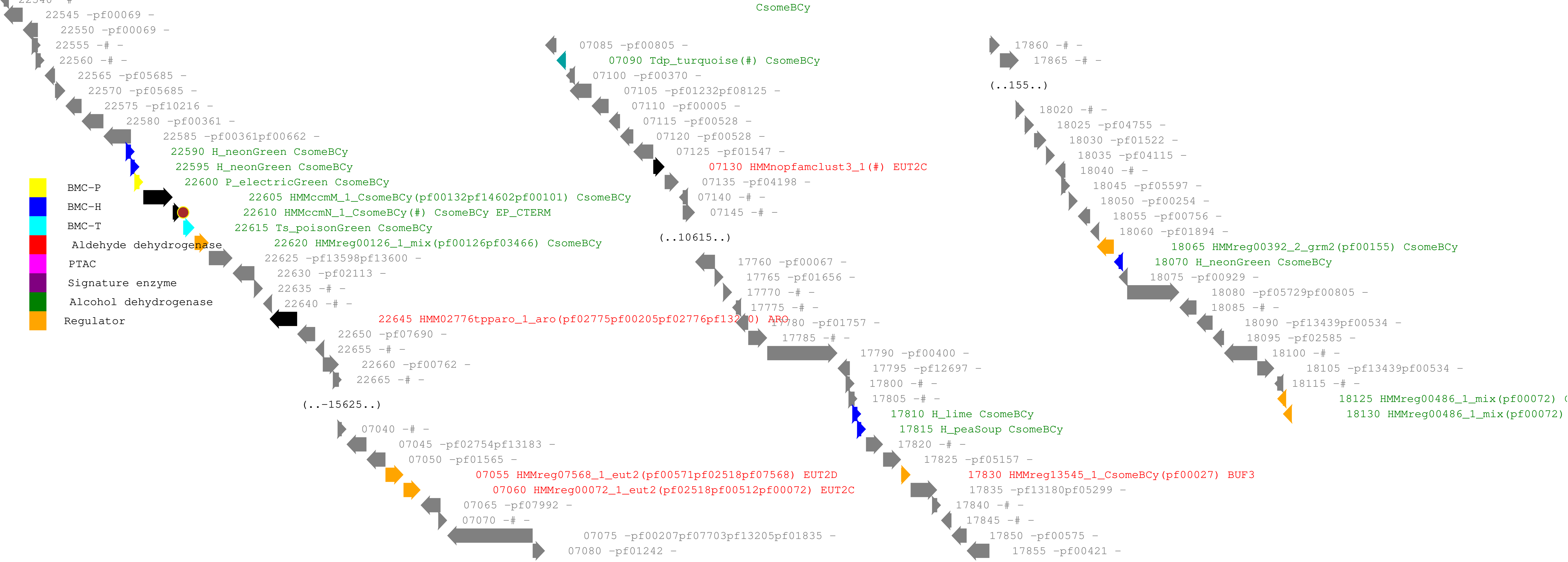

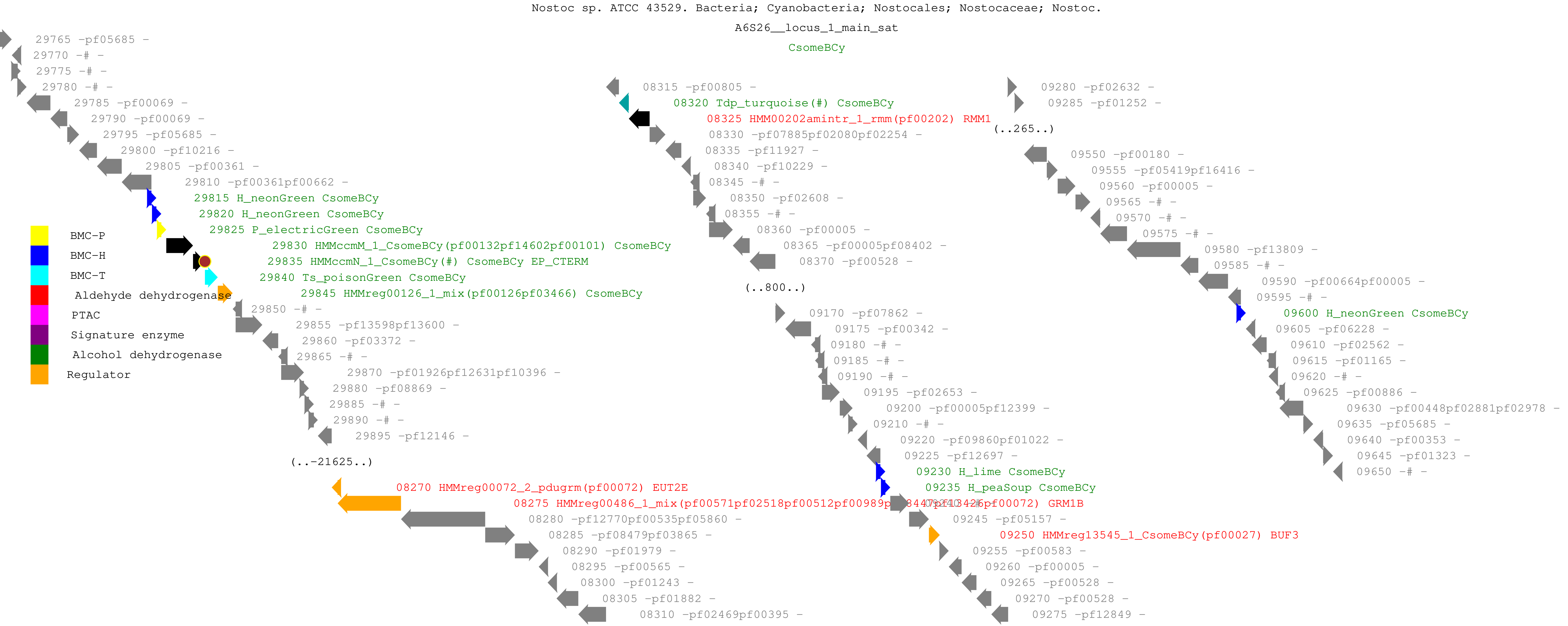
Nostoc sp. ATCC 43529. Bacteria; Cyanobacteria; Nostocales; Nostocaceae; Nostoc.
A6S26__locus_1_main_sat
CsomeBCy
BMC-P
BMC-H
BMC-T
Aldehyde dehydrogenase
PTAC
Signature enzyme
Alcohol dehydrogenase
Regulator
29765 -pf05685 -
29770 -# -
29775 -# -
29780 -# -
29785 -pf00069 -
29790 -pf00069 -
29795 -pf05685 -
29800 -pf10216 -
29805 -pf00361 -
29810 -pf00361pf00662 -
29815 H_neonGreen CsomeBCy
29820 H_neonGreen CsomeBCy
29825 P_electricGreen CsomeBCy
29830 HMMccmM_1_CsomeBCy(pf00132pf14602pf00101) CsomeBCy
29835 HMMccmN_1_CsomeBCy(#) CsomeBCy EP_CTERM
29840 Ts_poisonGreen CsomeBCy
29845 HMMreg00126_1_mix(pf00126pf03466) CsomeBCy
29850 -# -
29855 -pf13598pf13600 -
29860 -pf03372 -
29865 -# -
29870 -pf01926pf12631pf10396 -
29880 -pf08869 -
29885 -# -
29890 -# -
29895 -pf12146 -
(..-21625..)
08270 HMMreg00072_2_pdugrm(pf00072) EUT2E
08275 HMMreg00486_1_mix(pf00571pf02518pf00512pf00989p[illegible]8447pf13426pf00072) GRM1B
08280 -pf12770pf00535pf05860 -
08285 -pf08479pf03865 -
08290 -pf01979 -
08295 -pf00565 -
08300 -pf01243 -
08305 -pf01882 -
08310 -pf02469pf00395 -
08315 -pf00805 -
08320 Tdp_turquoise(#) CsomeBCy
08325 HMM00202amintr_1_rmm(pf00202) RMM1
08330 -pf07885pf02080pf02254 -
08335 -pf11927 -
08340 -pf10229 -
08345 -# -
08350 -pf02608 -
08355 -# -
08360 -pf00005 -
08365 -pf00005pf08402 -
08370 -pf00528 -
(..800..)
09170 -pf07862 -
09175 -pf00342 -
09180 -# -
09185 -# -
09190 -# -
09195 -pf02653 -
09200 -pf00005pf12399 -
09210 -# -
09220 -pf09860pf01022 -
09225 -pf12697 -
09230 H_lime CsomeBCy
09235 H_peaSoup CsomeBCy
09240 -# -
09245 -pf05157 -
09250 HMMreg13545_1_CsomeBCy(pf00027) BUF3
09255 -pf00583 -
09260 -pf00005 -
09265 -pf00528 -
09270 -pf00528 -
09275 -pf12849 -
09280 -pf02632 -
09285 -pf01252 -
(..265..)
09550 -pf00180 -
09555 -pf05419pf16416 -
09560 -pf00005 -
09565 -# -
09570 -# -
09575 -# -
09580 -pf13809 -
09585 -# -
09590 -pf00664pf00005 -
09595 -# -
09600 H_neonGreen CsomeBCy
09605 -pf06228 -
09610 -pf02562 -
09615 -pf01165 -
09620 -# -
09625 -pf00886 -
09630 -pf00448pf02881pf02978 -
09635 -pf05685 -
09640 -pf00353 -
09645 -pf01323 -
09650 -# -

Nostoc sp. ATCC 53789. Bacteria; Cyanobacteria; Nostocales; Nostocaceae; Nostoc.

A6V25__locus_1_main_sat

CsomeBCy

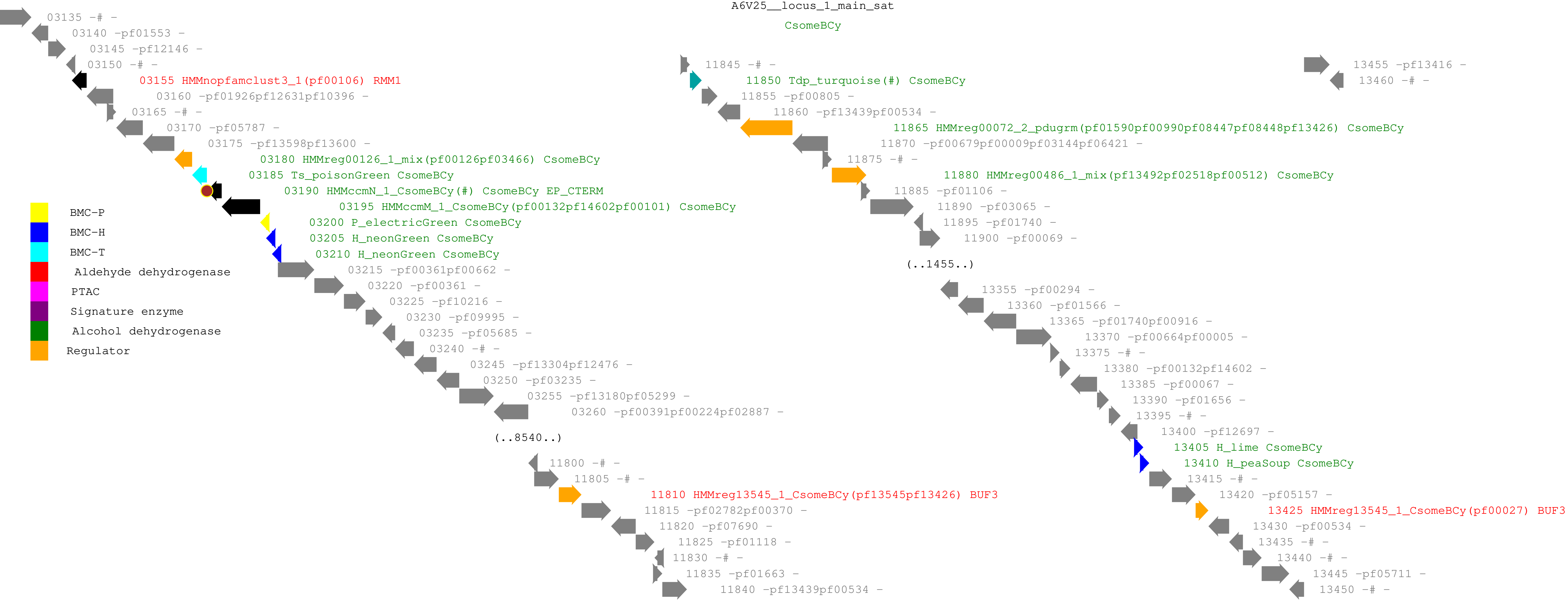

Cyanobacterium sp. HL-69. Bacteria; Cyanobacteria; Oscillatoriophycideae; Chroococcales;

AA637__locus_1_main_sat

CsomeBCy

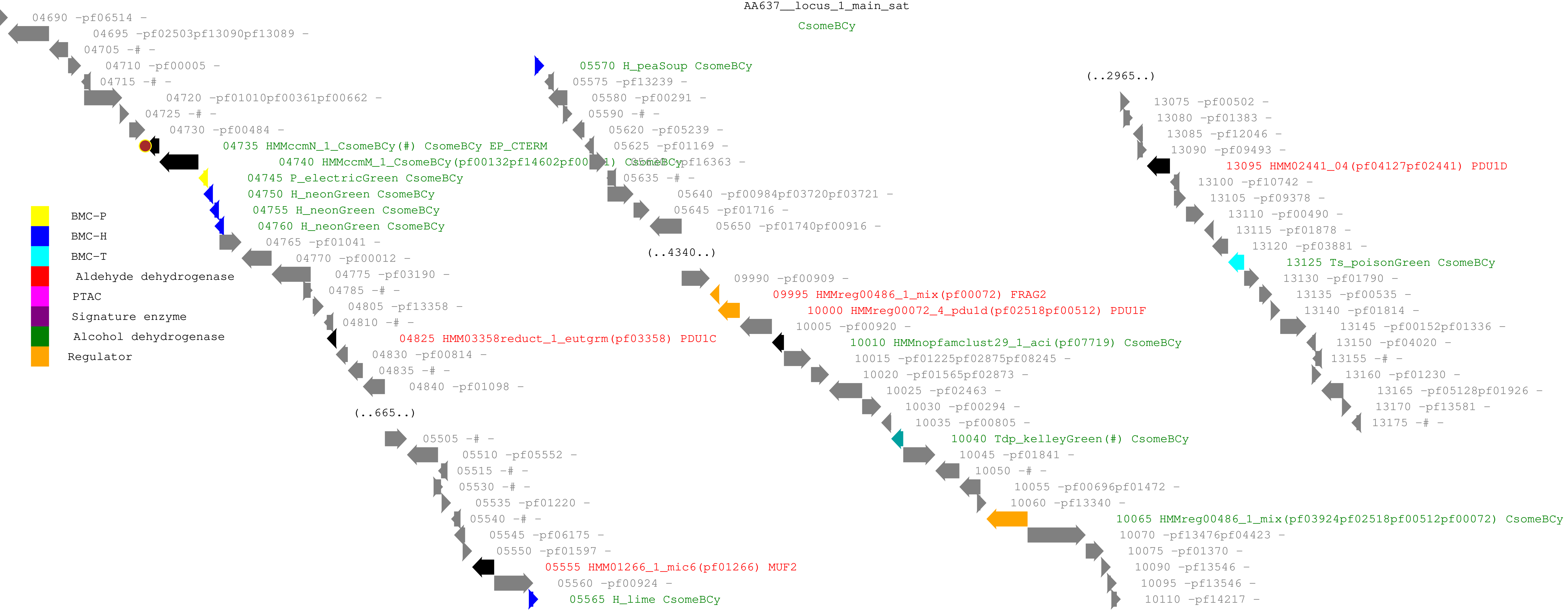

Anabaena sp. WA102. Bacteria; Cyanobacteria; Nostocales; Nostocaceae; Anabaena.

AA650__locus_1_main_sat

CsomeBCy

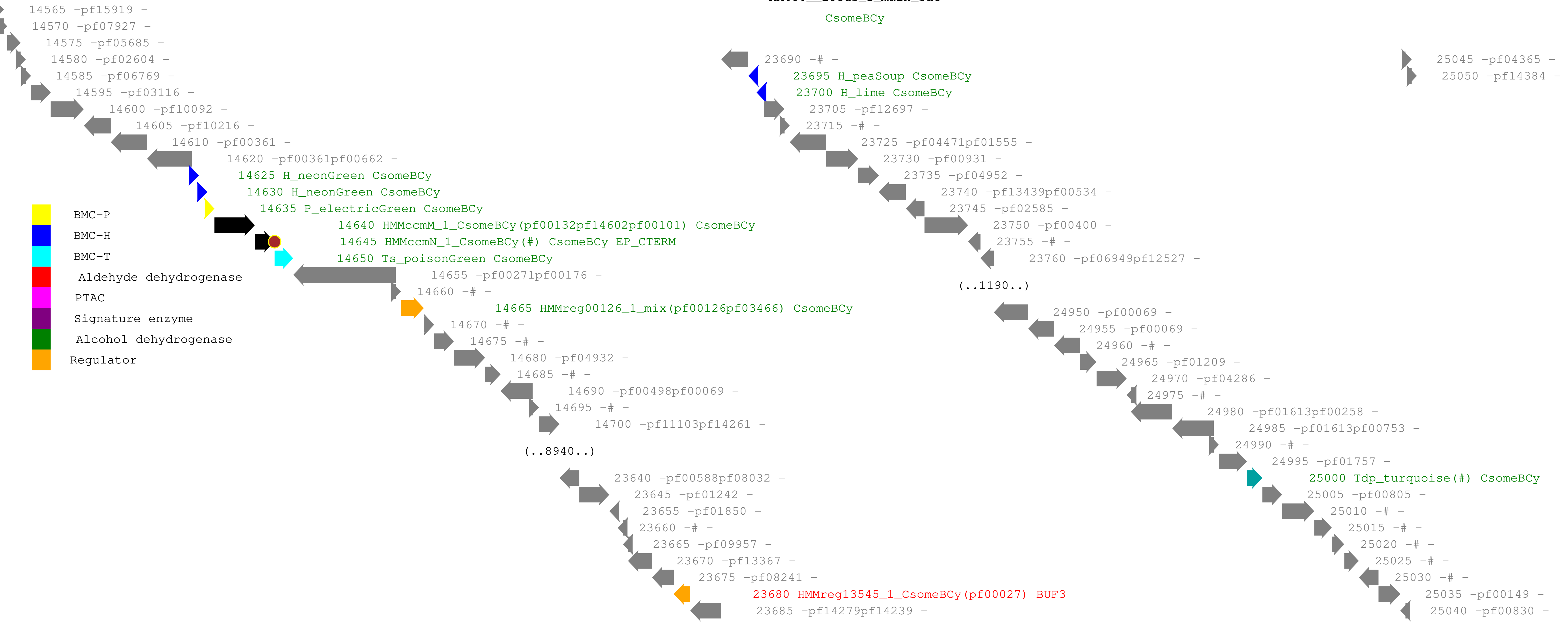

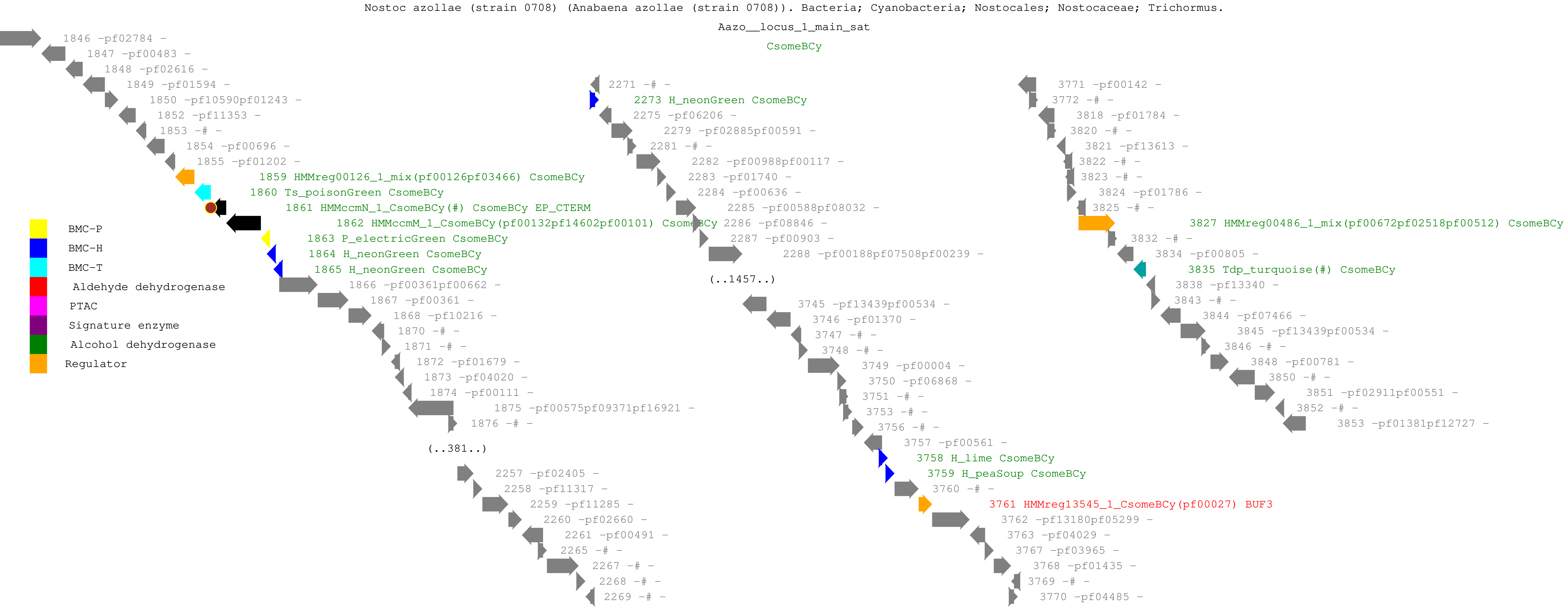

Nostoc azollae (strain 0708) (Anabaena azollae (strain 0708)). Bacteria; Cyanobacteria; Nostocales; Nostocaceae; Trichormus.
Aazo__locus_1_main_sat
CsomeBCy
1846 -pf02784 -
1847 -pf00483 -
1848 -pf02616 -
1849 -pf01594 -
1850 -pf10590pf01243 -
1852 -pf11353 -
1853 -# -
1854 -pf00696 -
1855 -pf01202 -
1859 HMMreg00126_1_mix(pf00126pf03466) CsomeBCy
1860 Ts_poisonGreen CsomeBCy
1861 HMMccmN_1_CsomeBCy(#) CsomeBCy EP_CTERM
1862 HMMccmM_1_CsomeBCy(pf00132pf14602pf00101) CsomeBCy
1863 P_electricGreen CsomeBCy
1864 H_neonGreen CsomeBCy
1865 H_neonGreen CsomeBCy
1866 -pf00361pf00662 -
1867 -pf00361 -
1868 -pf10216 -
1870 -# -
1871 -# -
1872 -pf01679 -
1873 -pf04020 -
1874 -pf00111 -
1875 -pf00575pf09371pf16921 -
1876 -# -
(..381..)
2257 -pf02405 -
2258 -pf11317 -
2259 -pf11285 -
2260 -pf02660 -
2261 -pf00491 -
2265 -# -
2267 -# -
2268 -# -
2269 -# -
2271 -# -
2273 H_neonGreen CsomeBCy
2275 -pf06206 -
2279 -pf02885pf00591 -
2281 -# -
2282 -pf00988pf00117 -
2283 -pf01740 -
2284 -pf00636 -
2285 -pf00588pf08032 -
2286 -pf08846 -
2287 -pf00903 -
2288 -pf00188pf07508pf00239 -
(..1457..)
3745 -pf13439pf00534 -
3746 -pf01370 -
3747 -# -
3748 -# -
3749 -pf00004 -
3750 -pf06868 -
3751 -# -
3753 -# -
3756 -# -
3757 -pf00561 -
3758 H_lime CsomeBCy
3759 H_peaSoup CsomeBCy
3760 -# -
3761 HMMreg13545_1_CsomeBCy(pf00027) BUF3
3762 -pf13180pf05299 -
3763 -pf04029 -
3767 -pf03965 -
3768 -pf01435 -
3769 -# -
3770 -pf04485 -
3771 -pf00142 -
3772 -# -
3818 -pf01784 -
3820 -# -
3821 -pf13613 -
3822 -# -
3823 -# -
3824 -pf01786 -
3825 -# -
3827 HMMreg00486_1_mix(pf00672pf02518pf00512) CsomeBCy
3832 -# -
3834 -pf00805 -
3835 Tdp_turquoise(#) CsomeBCy
3838 -pf13340 -
3843 -# -
3844 -pf07466 -
3845 -pf13439pf00534 -
3846 -# -
3848 -pf00781 -
3850 -# -
3851 -pf02911pf00551 -
3852 -# -
3853 -pf01381pf12727 -
BMC-P
BMC-H
BMC-T
Aldehyde dehydrogenase
PTAC
Signature enzyme
Alcohol dehydrogenase
Regulator

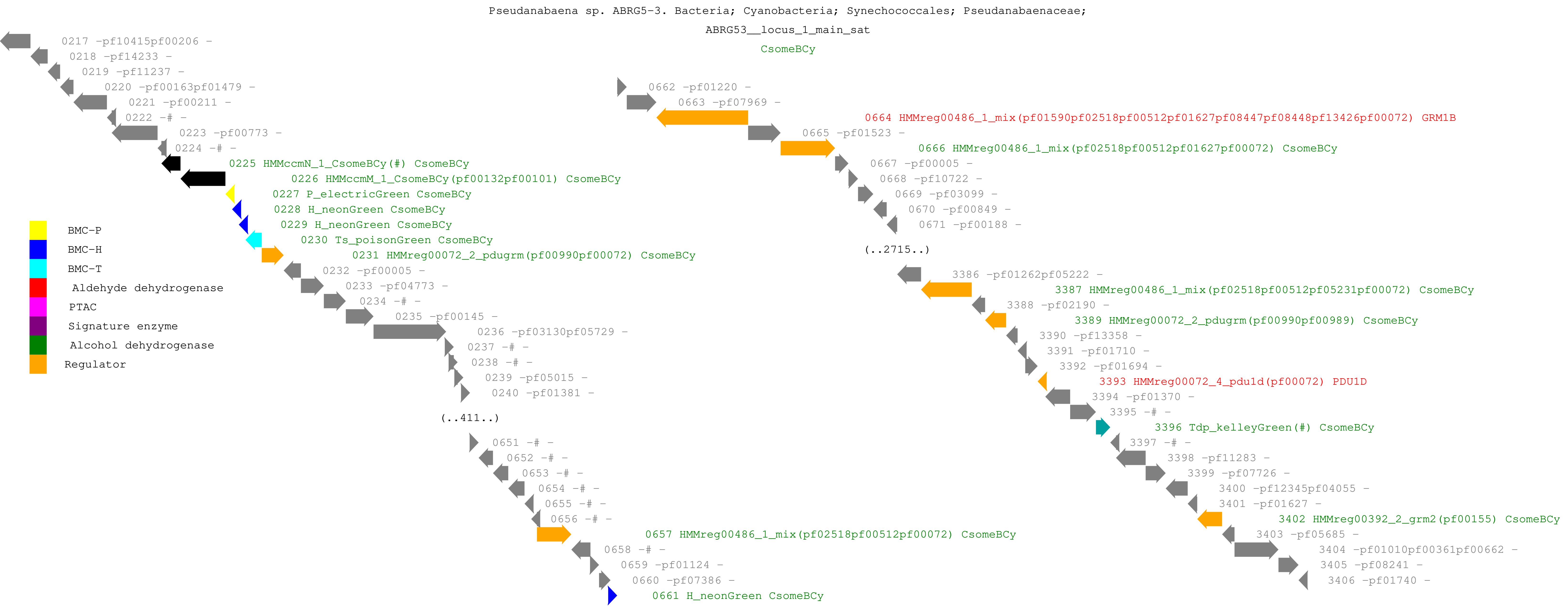

Pseudanabaena sp. ABRG5-3. Bacteria; Cyanobacteria; Synechococcales; Pseudanabaenaceae;
ABRG53__locus_1_main_sat
CsomeBCy
0217 -pf10415pf00206 -
0218 -pf14233 -
0219 -pf11237 -
0220 -pf00163pf01479 -
0221 -pf00211 -
0222 -# -
0223 -pf00773 -
0224 -# -
0225 HMMccmN_1_CsomeBCy(#) CsomeBCy
0226 HMMccmM_1_CsomeBCy(pf00132pf00101) CsomeBCy
0227 P_electricGreen CsomeBCy
0228 H_neonGreen CsomeBCy
0229 H_neonGreen CsomeBCy
0230 Ts_poisonGreen CsomeBCy
0231 HMMreg00072_2_pdugrm(pf00990pf00072) CsomeBCy
0232 -pf00005 -
0233 -pf04773 -
0234 -# -
0235 -pf00145 -
0236 -pf03130pf05729 -
0237 -# -
0238 -# -
0239 -pf05015 -
0240 -pf01381 -
(..411..)
0651 -# -
0652 -# -
0653 -# -
0654 -# -
0655 -# -
0656 -# -
0657 HMMreg00486_1_mix(pf02518pf00512pf00072) CsomeBCy
0658 -# -
0659 -pf01124 -
0660 -pf07386 -
0661 H_neonGreen CsomeBCy
0662 -pf01220 -
0663 -pf07969 -
0664 HMMreg00486_1_mix(pf01590pf02518pf00512pf01627pf08447pf08448pf13426pf00072) GRM1B
0665 -pf01523 -
0666 HMMreg00486_1_mix(pf02518pf00512pf01627pf00072) CsomeBCy
0667 -pf00005 -
0668 -pf10722 -
0669 -pf03099 -
0670 -pf00849 -
0671 -pf00188 -
(..2715..)
3386 -pf01262pf05222 -
3387 HMMreg00486_1_mix(pf02518pf00512pf05231pf00072) CsomeBCy
3388 -pf02190 -
3389 HMMreg00072_2_pdugrm(pf00990pf00989) CsomeBCy
3390 -pf13358 -
3391 -pf01710 -
3392 -pf01694 -
3393 HMMreg00072_4_pdu1d(pf00072) PDU1D
3394 -pf01370 -
3395 -# -
3396 Tdp_kelleyGreen(#) CsomeBCy
3397 -# -
3398 -pf11283 -
3399 -pf07726 -
3400 -pf12345pf04055 -
3401 -pf01627 -
3402 HMMreg00392_2_grm2(pf00155) CsomeBCy
3403 -pf05685 -
3404 -pf01010pf00361pf00662 -
3405 -pf08241 -
3406 -pf01740 -
BMC-P
BMC-H
BMC-T
Aldehyde dehydrogenase
PTAC
Signature enzyme
Alcohol dehydrogenase
Regulator

Nostoc piscinale CENA21. Bacteria; Cyanobacteria; Nostocales; Nostocaceae; Nostoc.

ACX27__locus_1_main_sat

CsomeBCy

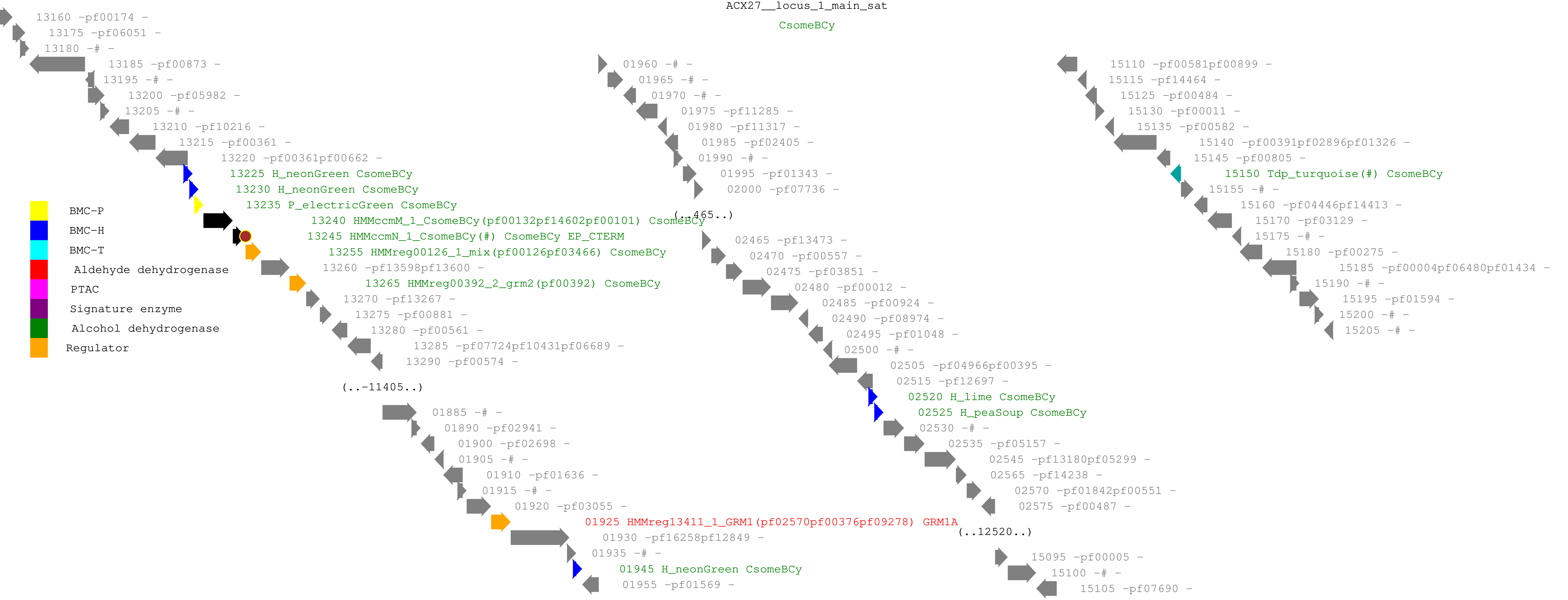

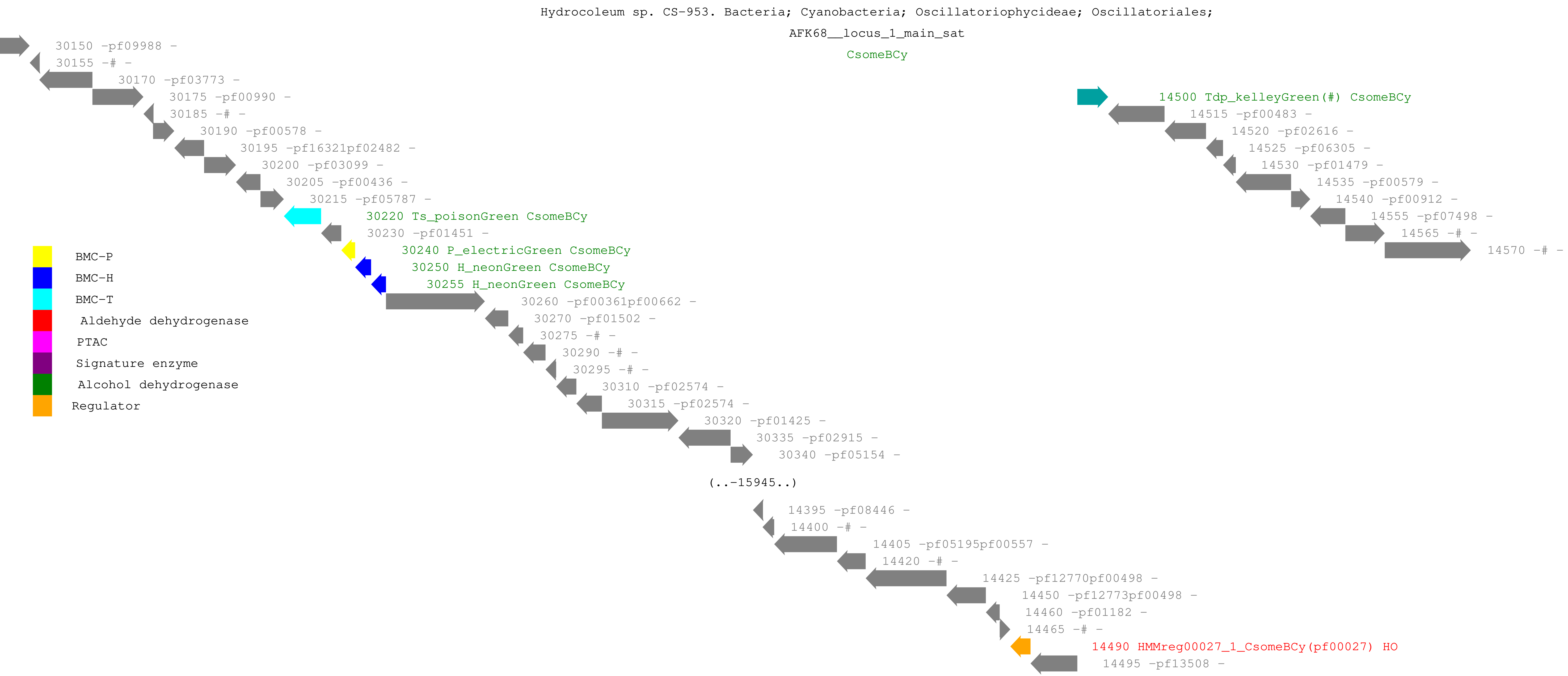
Hydrocoleum sp. CS-953. Bacteria; Cyanobacteria; Oscillatoriophycideae; Oscillatoriales;
AFK68__locus_1_main_sat
CsomeBCy
30150 -pf09988 -
30155 -# -
30170 -pf03773 -
30175 -pf00990 -
30185 -# -
30190 -pf00578 -
30195 -pf16321pf02482 -
30200 -pf03099 -
30205 -pf00436 -
30215 -pf05787 -
30220 Ts_poisonGreen CsomeBCy
30230 -pf01451 -
30240 P_electricGreen CsomeBCy
30250 H_neonGreen CsomeBCy
30255 H_neonGreen CsomeBCy
30260 -pf00361pf00662 -
30270 -pf01502 -
30275 -# -
30290 -# -
30295 -# -
30310 -pf02574 -
30315 -pf02574 -
30320 -pf01425 -
30335 -pf02915 -
30340 -pf05154 -
(..-15945..)
14395 -pf08446 -
14400 -# -
14405 -pf05195pf00557 -
14420 -# -
14425 -pf12770pf00498 -
14450 -pf12773pf00498 -
14460 -pf01182 -
14465 -# -
14490 HMMreg00027_1_CsomeBCy(pf00027) HO
14495 -pf13508 -
14500 Tdp_kelleyGreen(#) CsomeBCy
14515 -pf00483 -
14520 -pf02616 -
14525 -pf06305 -
14530 -pf01479 -
14535 -pf00579 -
14540 -pf00912 -
14555 -pf07498 -
14565 -# -
14570 -# -
BMC-P
BMC-H
BMC-T
Aldehyde dehydrogenase
PTAC
Signature enzyme
Alcohol dehydrogenase
Regulator

Acaryochloris marina (strain MBIC 11017). Bacteria; Cyanobacteria; Synechococcales; Acaryochloridaceae;

AM1__locus_1_main_sat

CsomeBCy

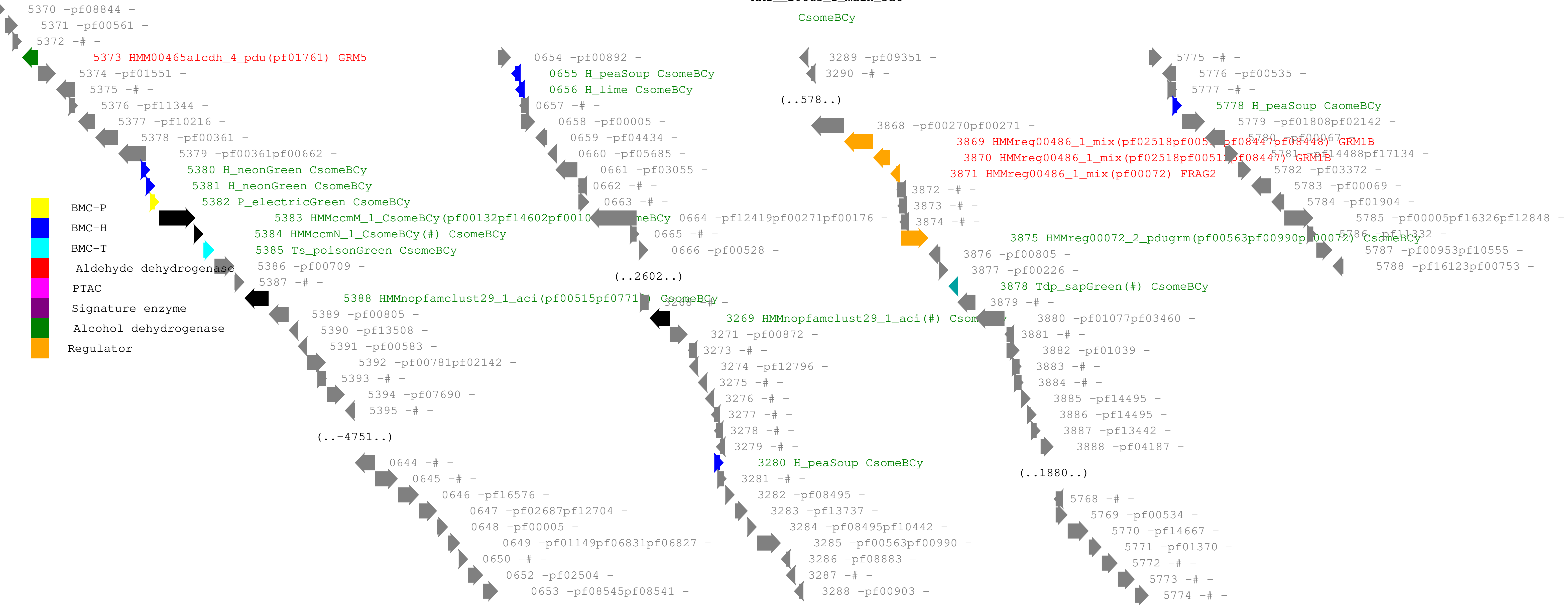

Planktothricoides sp. SR001. Bacteria; Cyanobacteria; Oscillatoriophycideae; Oscillatoriales;

AM228__locus_1_main_sat

CsomeBCy

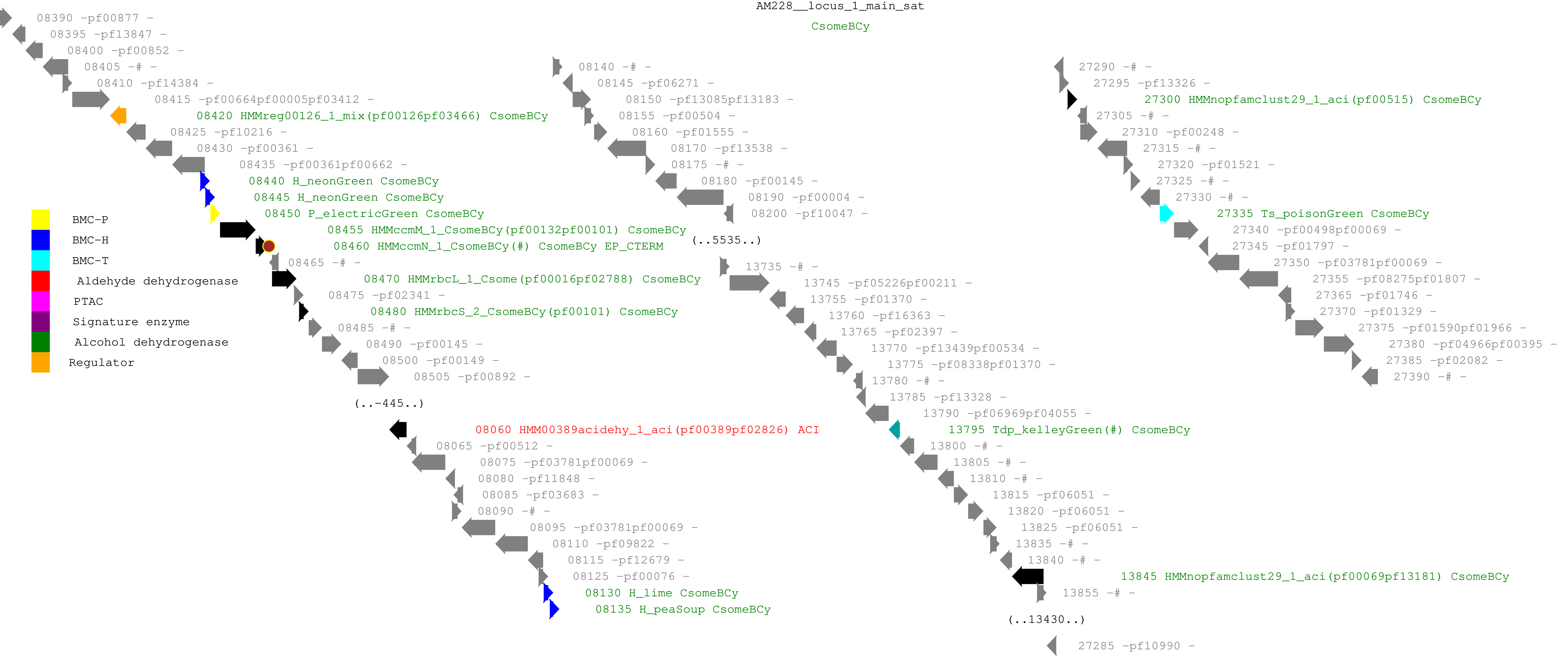

Arthrospira maxima CS-328. Bacteria; Cyanobacteria; Oscillatoriophycideae; Oscillatoriales;

AmaxDRAFT__locus_1_main_sat

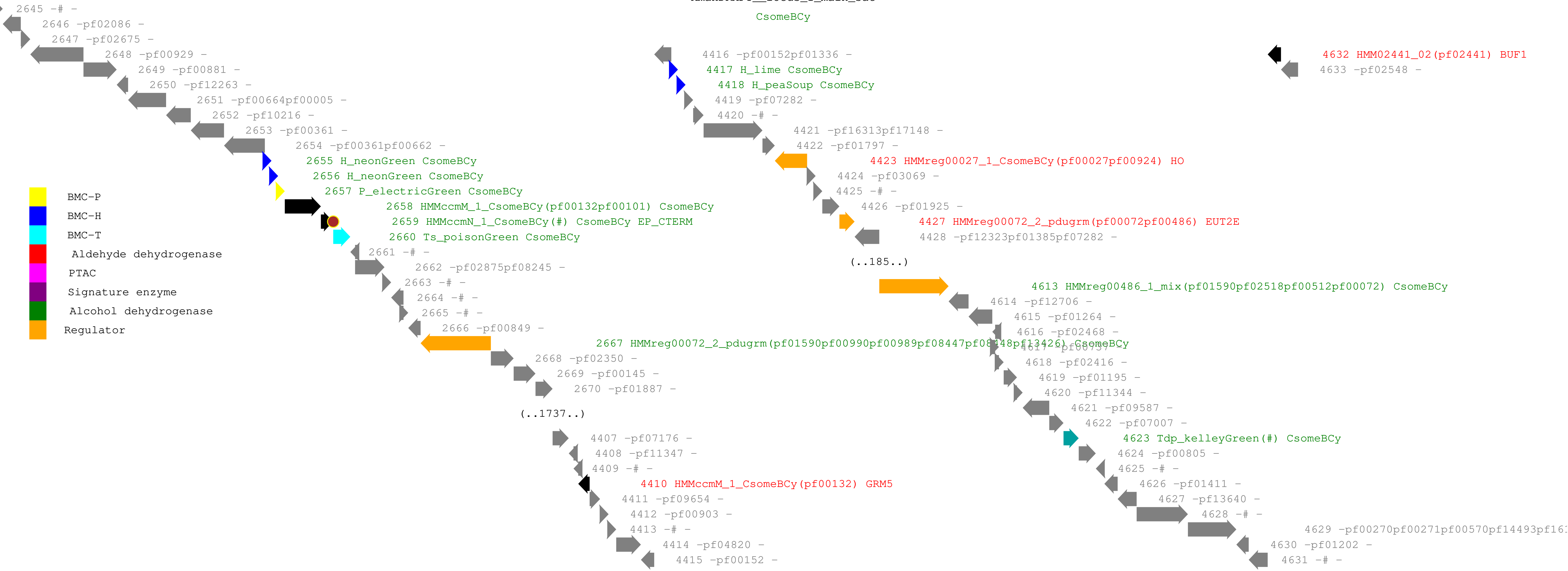

Hapalosiphon sp. MRB220. Bacteria; Cyanobacteria; Nostocales; Hapalosiphonaceae; Hapalosiphon.

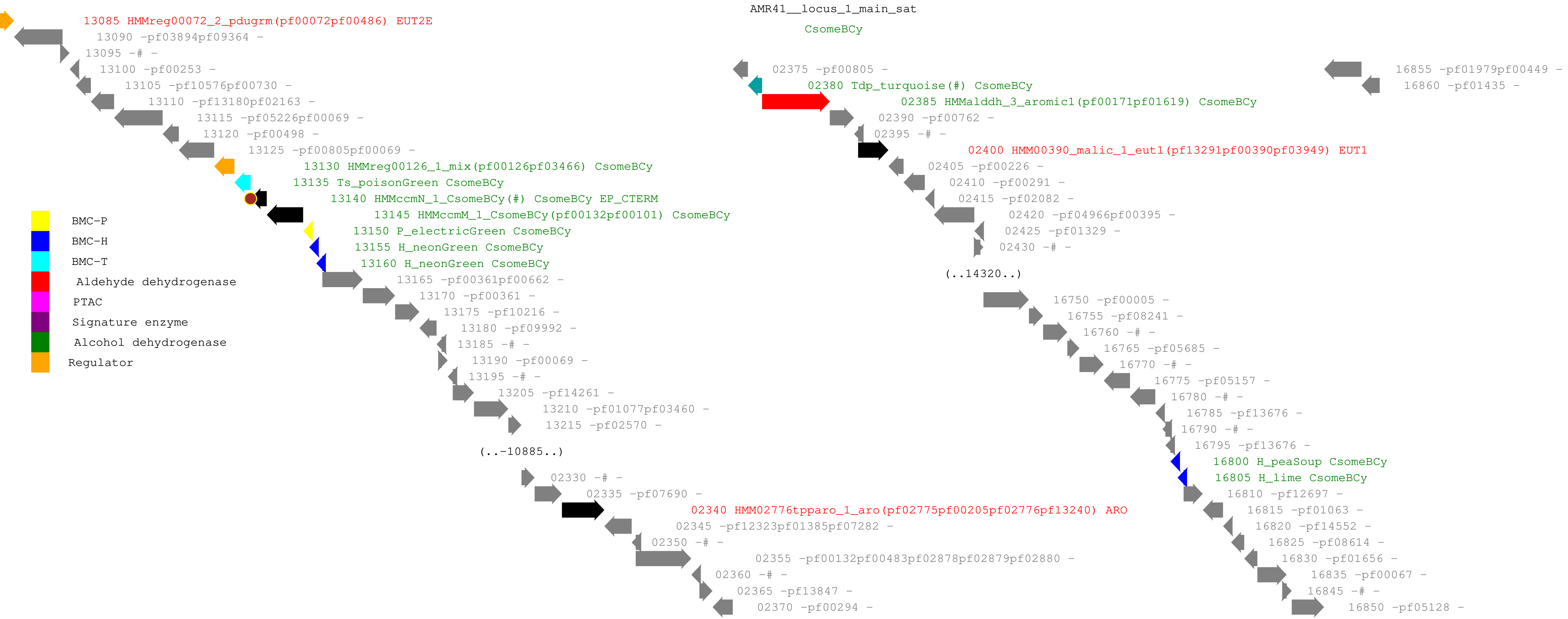

Limnothrix sp. PR1529. Bacteria; Cyanobacteria; Synechococcales; Pseudanabaenaceae;

AMR42__locus_1_main_sat

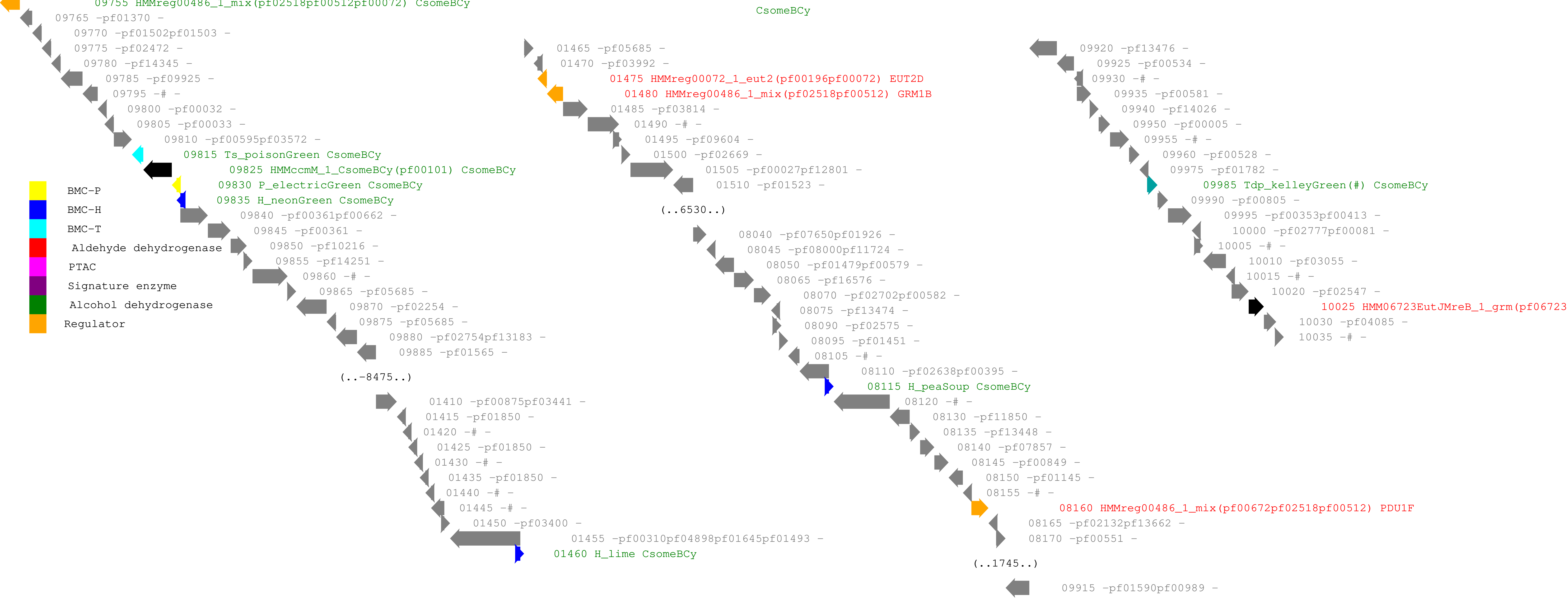

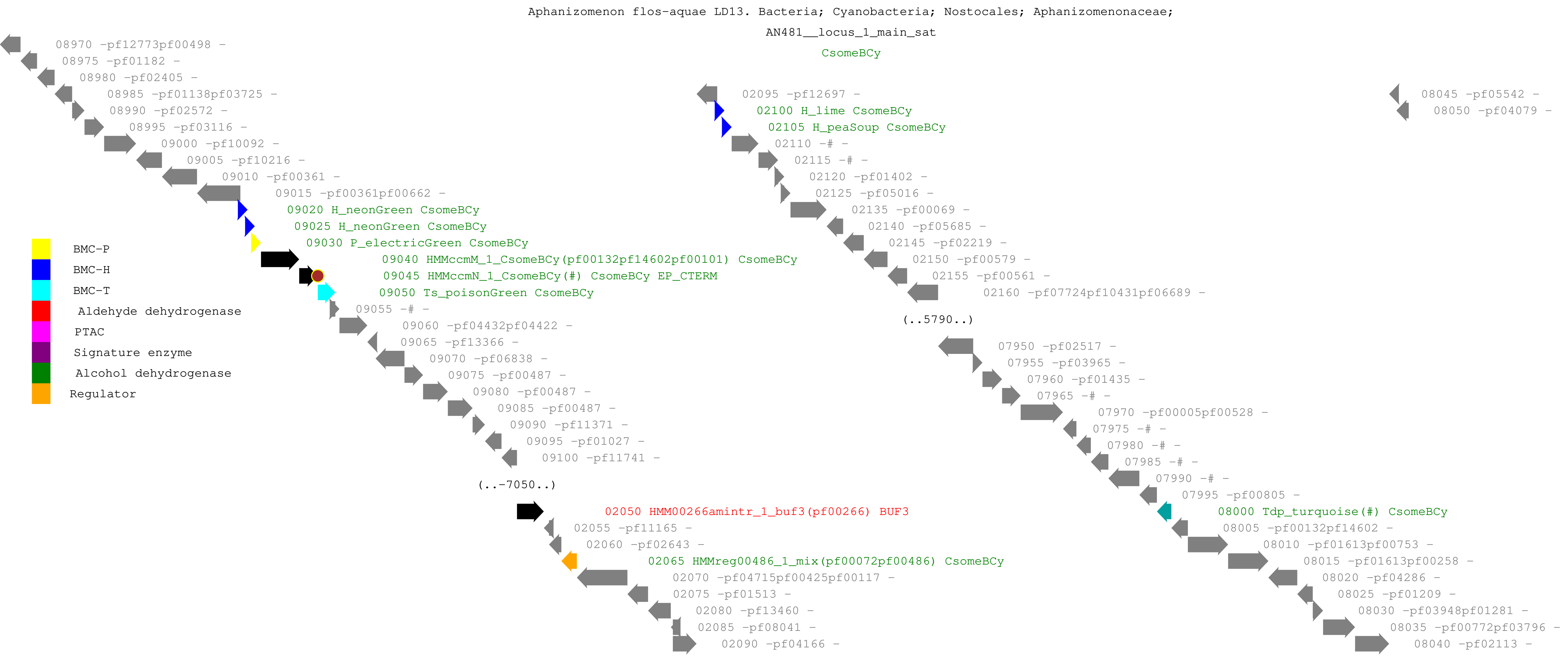
Aphanizomenon flos-aquae LD13. Bacteria; Cyanobacteria; Nostocales; Aphanizomenonaceae;
AN481__locus_1_main_sat
CsomeBCy
08970 -pf12773pf00498 -
08975 -pf01182 -
08980 -pf02405 -
08985 -pf01138pf03725 -
08990 -pf02572 -
08995 -pf03116 -
09000 -pf10092 -
09005 -pf10216 -
09010 -pf00361 -
09015 -pf00361pf00662 -
09020 H_neonGreen CsomeBCy
09025 H_neonGreen CsomeBCy
09030 P_electricGreen CsomeBCy
09040 HMMccmM_1_CsomeBCy(pf00132pf14602pf00101) CsomeBCy
09045 HMMccmN_1_CsomeBCy(#) CsomeBCy EP_CTERM
09050 Ts_poisonGreen CsomeBCy
09055 -# -
09060 -pf04432pf04422 -
09065 -pf13366 -
09070 -pf06838 -
09075 -pf00487 -
09080 -pf00487 -
09085 -pf00487 -
09090 -pf11371 -
09095 -pf01027 -
09100 -pf11741 -
(..-7050..)
02050 HMM00266amintr_1_buf3(pf00266) BUF3
02055 -pf11165 -
02060 -pf02643 -
02065 HMMreg00486_1_mix(pf00072pf00486) CsomeBCy
02070 -pf04715pf00425pf00117 -
02075 -pf01513 -
02080 -pf13460 -
02085 -pf08041 -
02090 -pf04166 -
02095 -pf12697 -
02100 H_lime CsomeBCy
02105 H_peaSoup CsomeBCy
02110 -# -
02115 -# -
02120 -pf01402 -
02125 -pf05016 -
02135 -pf00069 -
02140 -pf05685 -
02145 -pf02219 -
02150 -pf00579 -
02155 -pf00561 -
02160 -pf07724pf10431pf06689 -
(..5790..)
07950 -pf02517 -
07955 -pf03965 -
07960 -pf01435 -
07965 -# -
07970 -pf00005pf00528 -
07975 -# -
07980 -# -
07985 -# -
07990 -# -
07995 -pf00805 -
08000 Tdp_turquoise(#) CsomeBCy
08005 -pf00132pf14602 -
08010 -pf01613pf00753 -
08015 -pf01613pf00258 -
08020 -pf04286 -
08025 -pf01209 -
08030 -pf03948pf01281 -
08035 -pf00772pf03796 -
08040 -pf02113 -
08045 -pf05542 -
08050 -pf04079 -
BMC-P
BMC-H
BMC-T
Aldehyde dehydrogenase
PTAC
Signature enzyme
Alcohol dehydrogenase
Regulator

Anabaena sp. LE011-02. Bacteria; Cyanobacteria; Nostocales; Nostocaceae; Anabaena.

AN482__locus_1_main_sat

CsomeBCy

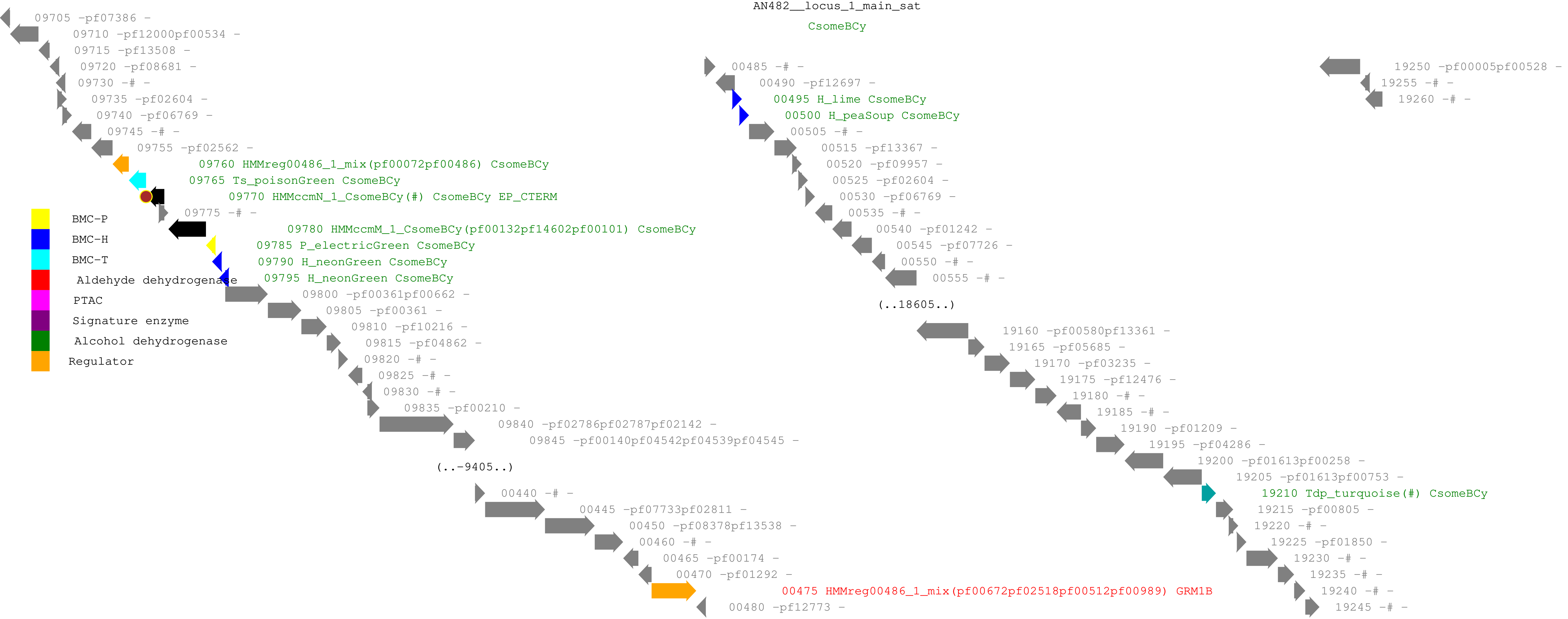

Aphanizomenon flos-aquae WA102. Bacteria; Cyanobacteria; Nostocales; Aphanizomenonaceae;

AN484__locus_1_main_sat

CsomeBCy

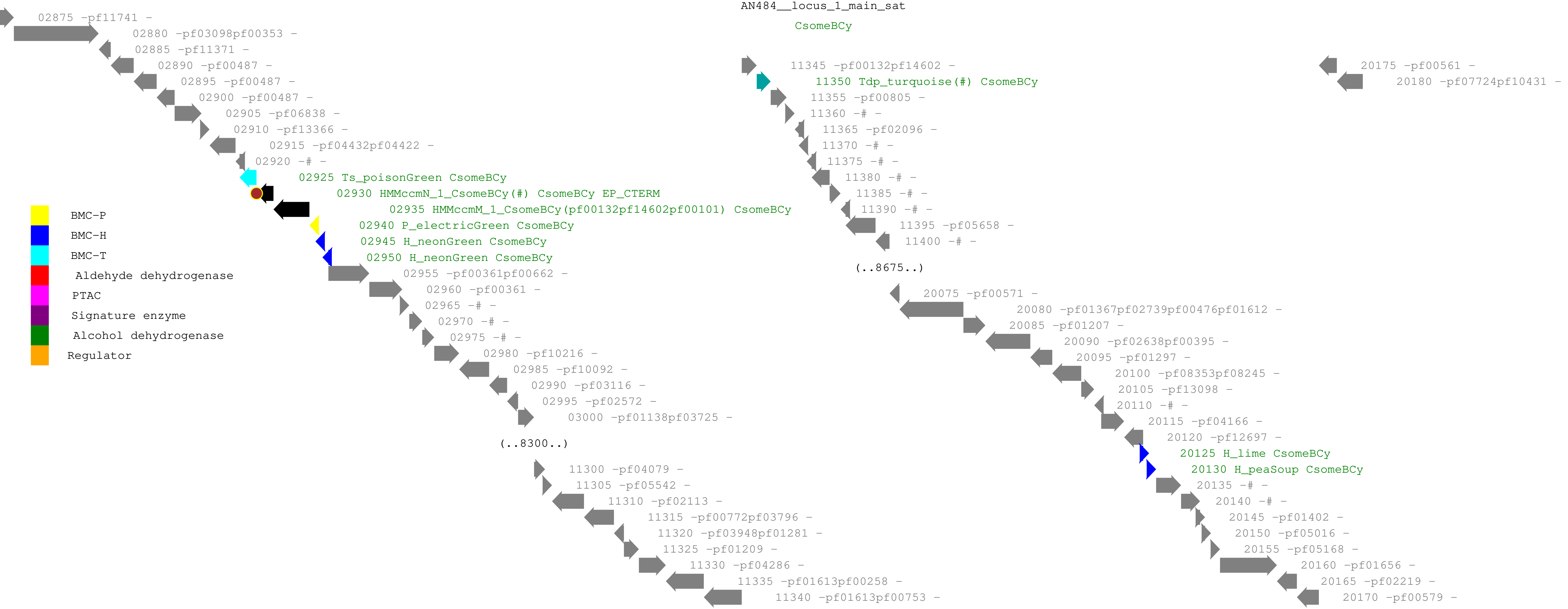

Anabaena sp. MDT14b. Bacteria; Cyanobacteria; Nostocales; Nostocaceae; Anabaena.

AN485__locus_1_main_sat

CsomeBCy

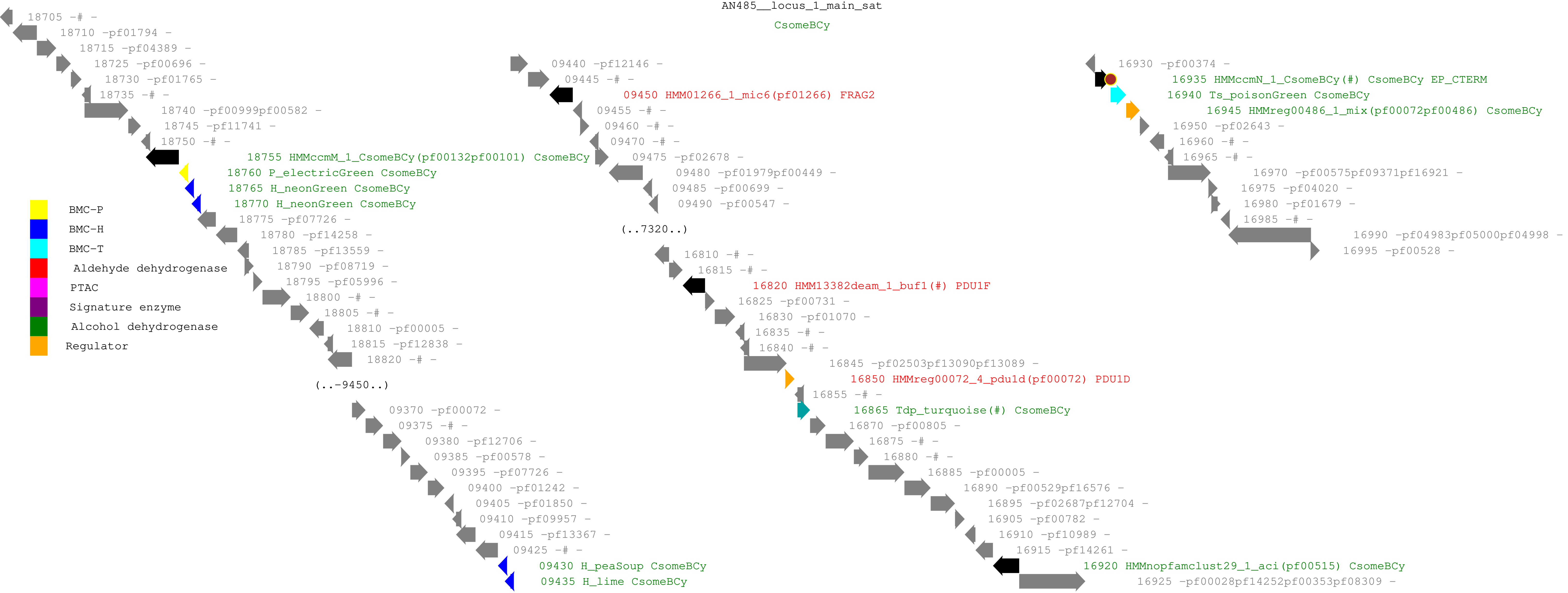

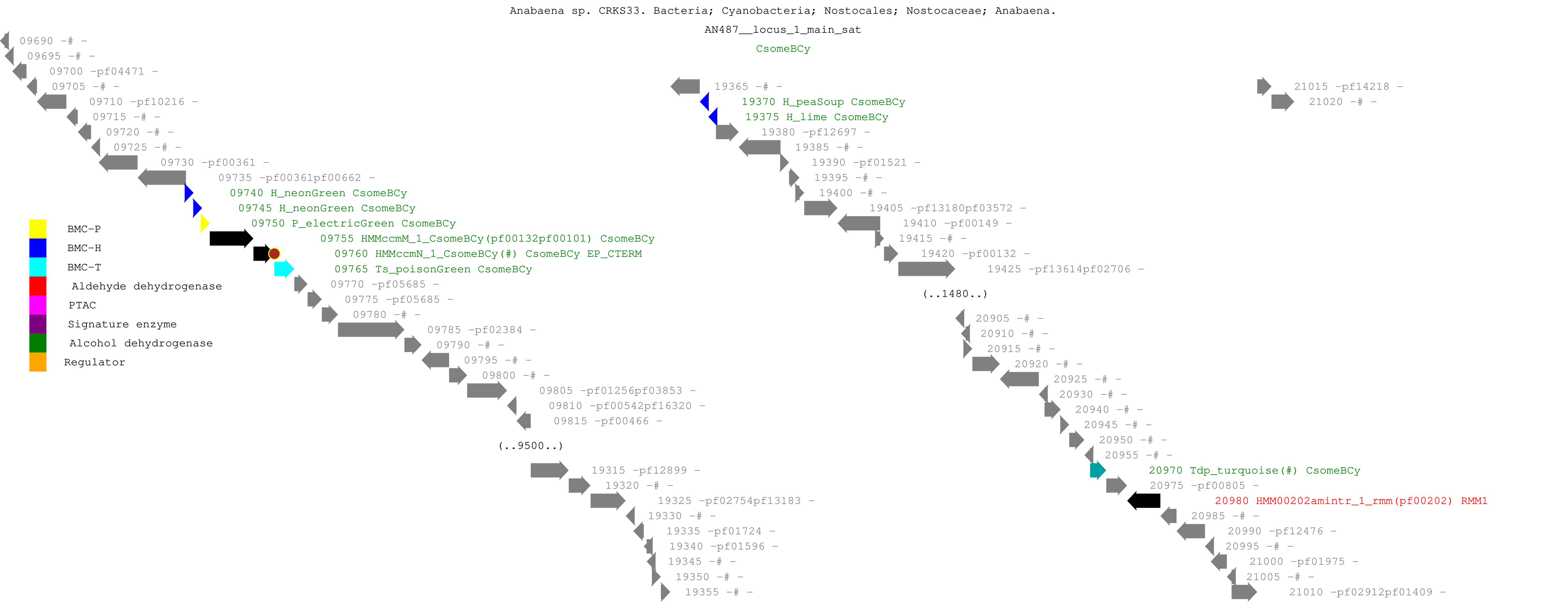

Anabaena sp. CRKS33. Bacteria; Cyanobacteria; Nostocales; Nostocaceae; Anabaena.
AN487__locus_1_main_sat
CsomeBCy
09690 -# -
09695 -# -
09700 -pf04471 -
09705 -# -
09710 -pf10216 -
09715 -# -
09720 -# -
09725 -# -
09730 -pf00361 -
09735 -pf00361pf00662 -
09740 H_neonGreen CsomeBCy
09745 H_neonGreen CsomeBCy
09750 P_electricGreen CsomeBCy
09755 HMMccmM_1_CsomeBCy(pf00132pf00101) CsomeBCy
09760 HMMccmN_1_CsomeBCy(#) CsomeBCy EP_CTERM
09765 Ts_poisonGreen CsomeBCy
09770 -pf05685 -
09775 -pf05685 -
09780 -# -
09785 -pf02384 -
09790 -# -
09795 -# -
09800 -# -
09805 -pf01256pf03853 -
09810 -pf00542pf16320 -
09815 -pf00466 -
(..9500..)
19315 -pf12899 -
19320 -# -
19325 -pf02754pf13183 -
19330 -# -
19335 -pf01724 -
19340 -pf01596 -
19345 -# -
19350 -# -
19355 -# -
19365 -# -
19370 H_peaSoup CsomeBCy
19375 H_lime CsomeBCy
19380 -pf12697 -
19385 -# -
19390 -pf01521 -
19395 -# -
19400 -# -
19405 -pf13180pf03572 -
19410 -pf00149 -
19415 -# -
19420 -pf00132 -
19425 -pf13614pf02706 -
(..1480..)
20905 -# -
20910 -# -
20915 -# -
20920 -# -
20925 -# -
20930 -# -
20940 -# -
20945 -# -
20950 -# -
20955 -# -
20970 Tdp_turquoise(#) CsomeBCy
20975 -pf00805 -
20980 HMM00202amintr_1_rmm(pf00202) RMM1
20985 -# -
20990 -pf12476 -
20995 -# -
21000 -pf01975 -
21005 -# -
21010 -pf02912pf01409 -
21015 -pf14218 -
21020 -# -
BMC-P
BMC-H
BMC-T
Aldehyde dehydrogenase
PTAC
Signature enzyme
Alcohol dehydrogenase
Regulator

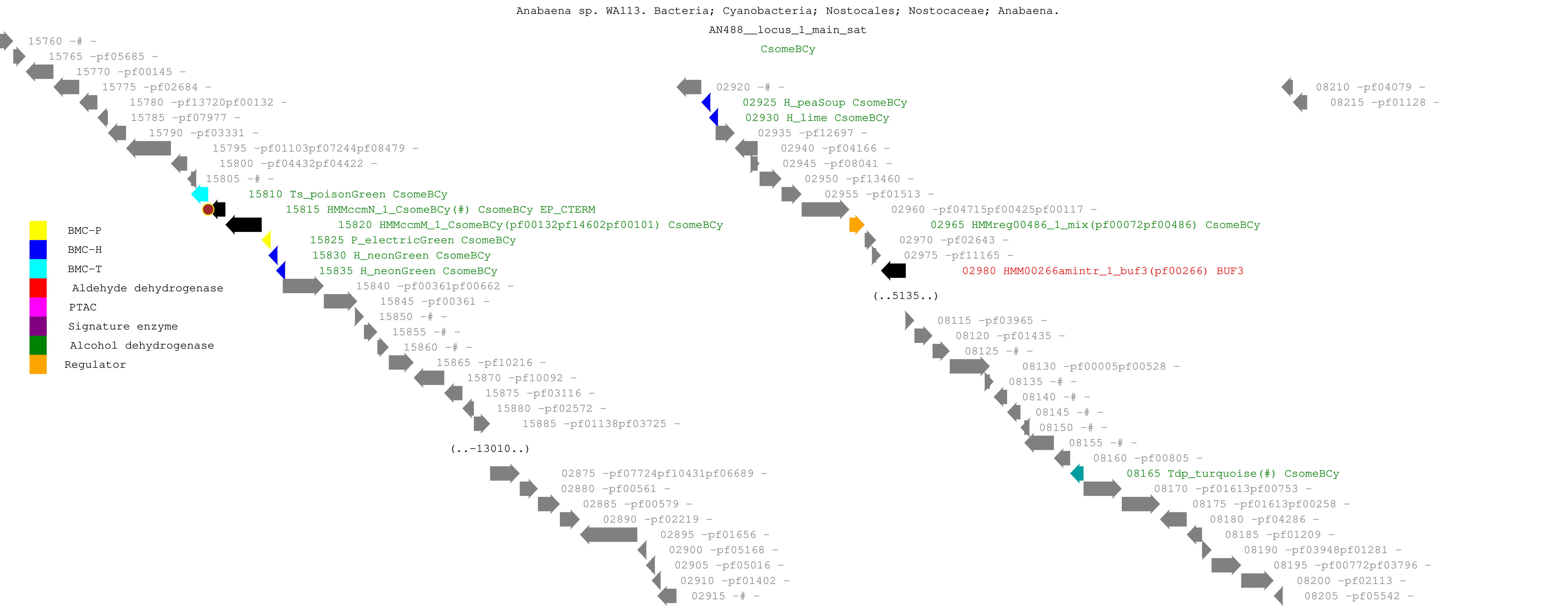
Anabaena sp. WA113. Bacteria; Cyanobacteria; Nostocales; Nostocaceae; Anabaena.
AN488__locus_1_main_sat
CsomeBCy
15760 -# -
15765 -pf05685 -
15770 -pf00145 -
15775 -pf02684 -
15780 -pf13720pf00132 -
15785 -pf07977 -
15790 -pf03331 -
15795 -pf01103pf07244pf08479 -
15800 -pf04432pf04422 -
15805 -# -
15810 Ts_poisonGreen CsomeBCy
15815 HMMccmN_1_CsomeBCy(#) CsomeBCy EP_CTERM
15820 HMMccmM_1_CsomeBCy(pf00132pf14602pf00101) CsomeBCy
15825 P_electricGreen CsomeBCy
15830 H_neonGreen CsomeBCy
15835 H_neonGreen CsomeBCy
15840 -pf00361pf00662 -
15845 -pf00361 -
15850 -# -
15855 -# -
15860 -# -
15865 -pf10216 -
15870 -pf10092 -
15875 -pf03116 -
15880 -pf02572 -
15885 -pf01138pf03725 -
(..-13010..)
02875 -pf07724pf10431pf06689 -
02880 -pf00561 -
02885 -pf00579 -
02890 -pf02219 -
02895 -pf01656 -
02900 -pf05168 -
02905 -pf05016 -
02910 -pf01402 -
02915 -# -
BMC-P
BMC-H
BMC-T
Aldehyde dehydrogenase
PTAC
Signature enzyme
Alcohol dehydrogenase
Regulator
02920 -# -
02925 H_peaSoup CsomeBCy
02930 H_lime CsomeBCy
02935 -pf12697 -
02940 -pf04166 -
02945 -pf08041 -
02950 -pf13460 -
02955 -pf01513 -
02960 -pf04715pf00425pf00117 -
02965 HMMreg00486_1_mix(pf00072pf00486) CsomeBCy
02970 -pf02643 -
02975 -pf11165 -
02980 HMM00266amintr_1_buf3(pf00266) BUF3
(..5135..)
08115 -pf03965 -
08120 -pf01435 -
08125 -# -
08130 -pf00005pf00528 -
08135 -# -
08140 -# -
08145 -# -
08150 -# -
08155 -# -
08160 -pf00805 -
08165 Tdp_turquoise(#) CsomeBCy
08170 -pf01613pf00753 -
08175 -pf01613pf00258 -
08180 -pf04286 -
08185 -pf01209 -
08190 -pf03948pf01281 -
08195 -pf00772pf03796 -
08200 -pf02113 -
08205 -pf05542 -
08210 -pf04079 -
08215 -pf01128 -

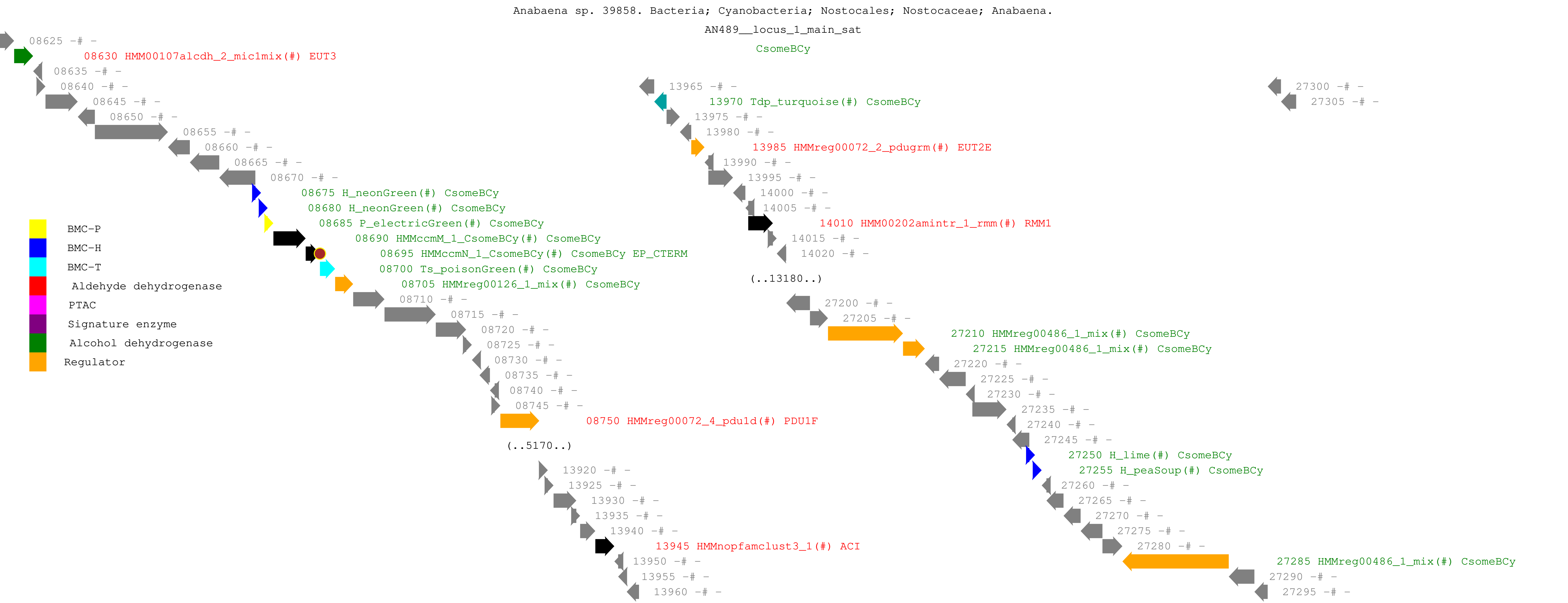
Anabaena sp. 39858. Bacteria; Cyanobacteria; Nostocales; Nostocaceae; Anabaena.
AN489__locus_1_main_sat
CsomeBCy
08625 -# -
08630 HMM00107alcdh_2_mic1mix(#) EUT3
08635 -# -
08640 -# -
08645 -# -
08650 -# -
08655 -# -
08660 -# -
08665 -# -
08670 -# -
08675 H_neonGreen(#) CsomeBCy
08680 H_neonGreen(#) CsomeBCy
08685 P_electricGreen(#) CsomeBCy
08690 HMMccmM_1_CsomeBCy(#) CsomeBCy
08695 HMMccmN_1_CsomeBCy(#) CsomeBCy EP_CTERM
08700 Ts_poisonGreen(#) CsomeBCy
08705 HMMreg00126_1_mix(#) CsomeBCy
08710 -# -
08715 -# -
08720 -# -
08725 -# -
08730 -# -
08735 -# -
08740 -# -
08745 -# -
08750 HMMreg00072_4_pdu1d(#) PDU1F
(..5170..)
13920 -# -
13925 -# -
13930 -# -
13935 -# -
13940 -# -
13945 HMMnopfamclust3_1(#) ACI
13950 -# -
13955 -# -
13960 -# -
13965 -# -
13970 Tdp_turquoise(#) CsomeBCy
13975 -# -
13980 -# -
13985 HMMreg00072_2_pdugrm(#) EUT2E
13990 -# -
13995 -# -
14000 -# -
14005 -# -
14010 HMM00202amintr_1_rmm(#) RMM1
14015 -# -
14020 -# -
(..13180..)
27200 -# -
27205 -# -
27210 HMMreg00486_1_mix(#) CsomeBCy
27215 HMMreg00486_1_mix(#) CsomeBCy
27220 -# -
27225 -# -
27230 -# -
27235 -# -
27240 -# -
27245 -# -
27250 H_lime(#) CsomeBCy
27255 H_peaSoup(#) CsomeBCy
27260 -# -
27265 -# -
27270 -# -
27275 -# -
27280 -# -
27285 HMMreg00486_1_mix(#) CsomeBCy
27290 -# -
27295 -# -
27300 -# -
27305 -# -
BMC-P
BMC-H
BMC-T
Aldehyde dehydrogenase
PTAC
Signature enzyme
Alcohol dehydrogenase
Regulator

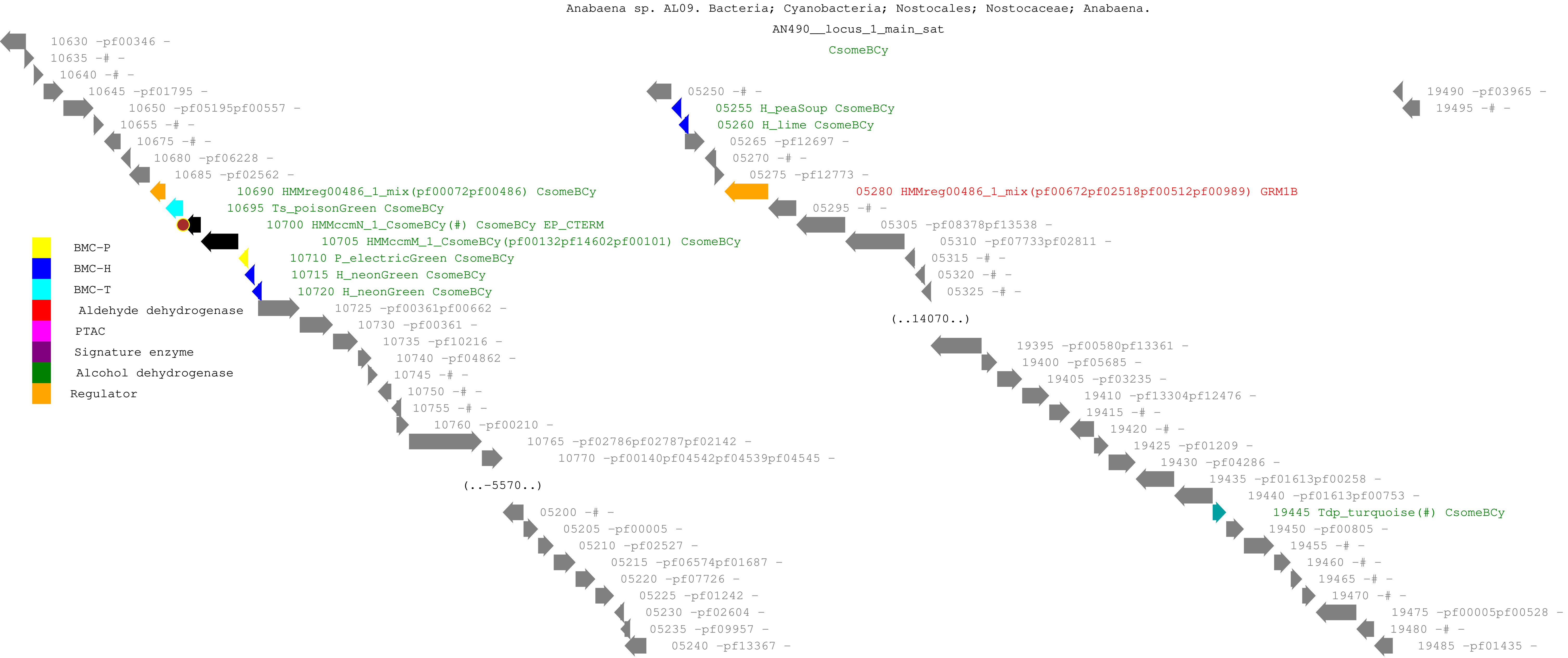
Anabaena sp. AL09. Bacteria; Cyanobacteria; Nostocales; Nostocaceae; Anabaena.
AN490__locus_1_main_sat
CsomeBCy
10630 -pf00346 -
10635 -# -
10640 -# -
10645 -pf01795 -
10650 -pf05195pf00557 -
10655 -# -
10675 -# -
10680 -pf06228 -
10685 -pf02562 -
10690 HMMreg00486_1_mix(pf00072pf00486) CsomeBCy
10695 Ts_poisonGreen CsomeBCy
10700 HMMccmN_1_CsomeBCy(#) CsomeBCy EP_CTERM
10705 HMMccmM_1_CsomeBCy(pf00132pf14602pf00101) CsomeBCy
10710 P_electricGreen CsomeBCy
10715 H_neonGreen CsomeBCy
10720 H_neonGreen CsomeBCy
10725 -pf00361pf00662 -
10730 -pf00361 -
10735 -pf10216 -
10740 -pf04862 -
10745 -# -
10750 -# -
10755 -# -
10760 -pf00210 -
10765 -pf02786pf02787pf02142 -
10770 -pf00140pf04542pf04539pf04545 -
(..-5570..)
05200 -# -
05205 -pf00005 -
05210 -pf02527 -
05215 -pf06574pf01687 -
05220 -pf07726 -
05225 -pf01242 -
05230 -pf02604 -
05235 -pf09957 -
05240 -pf13367 -
05250 -# -
05255 H_peaSoup CsomeBCy
05260 H_lime CsomeBCy
05265 -pf12697 -
05270 -# -
05275 -pf12773 -
05280 HMMreg00486_1_mix(pf00672pf02518pf00512pf00989) GRM1B
05295 -# -
05305 -pf08378pf13538 -
05310 -pf07733pf02811 -
05315 -# -
05320 -# -
05325 -# -
(..14070..)
19395 -pf00580pf13361 -
19400 -pf05685 -
19405 -pf03235 -
19410 -pf13304pf12476 -
19415 -# -
19420 -# -
19425 -pf01209 -
19430 -pf04286 -
19435 -pf01613pf00258 -
19440 -pf01613pf00753 -
19445 Tdp_turquoise(#) CsomeBCy
19450 -pf00805 -
19455 -# -
19460 -# -
19465 -# -
19470 -# -
19475 -pf00005pf00528 -
19480 -# -
19485 -pf01435 -
19490 -pf03965 -
19495 -# -
BMC-P
BMC-H
BMC-T
Aldehyde dehydrogenase
PTAC
Signature enzyme
Alcohol dehydrogenase
Regulator

Anabaena sp. 90. Bacteria; Cyanobacteria; Nostocales; Nostocaceae; Anabaena.

ANA_C_locus_1_main_sat

CsomeBCy

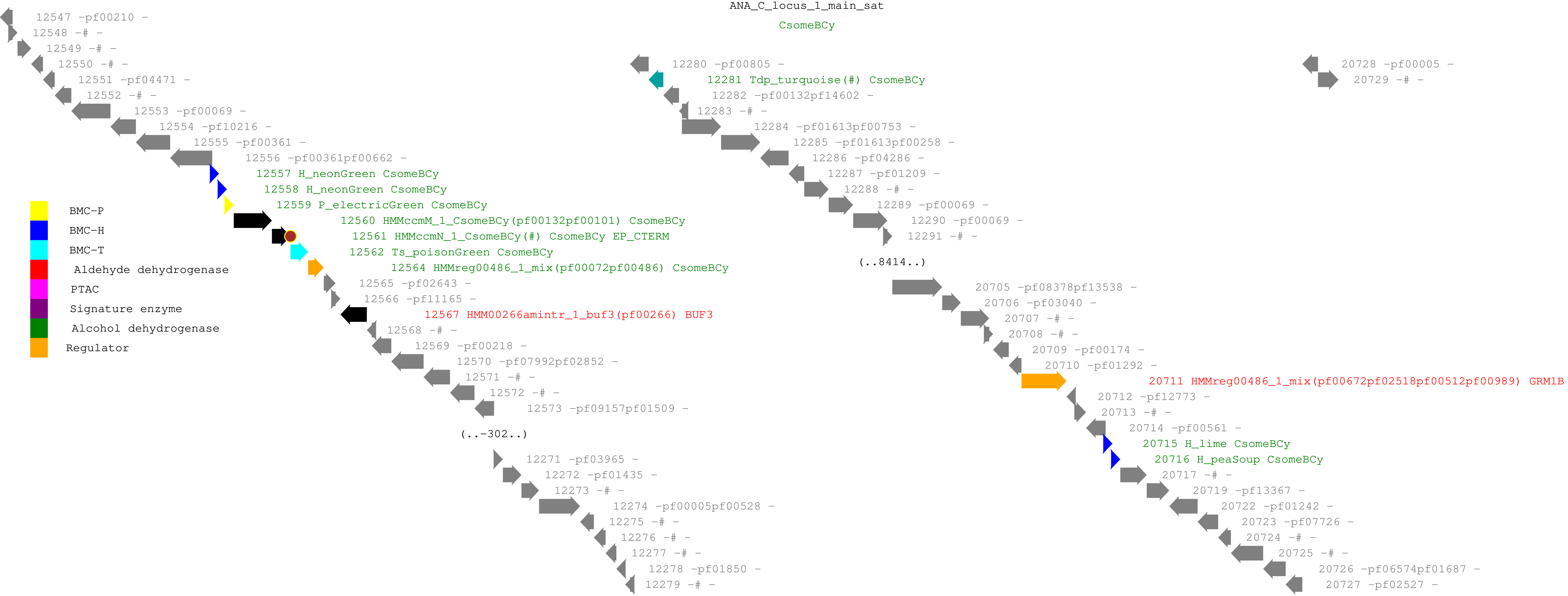

Anabaena cylindrica (strain ATCC 27899 / PCC 7122). Bacteria; Cyanobacteria; Nostocales; Nostocaceae; Anabaena.

Anacy__locus_1_main_sat

CsomeBCy

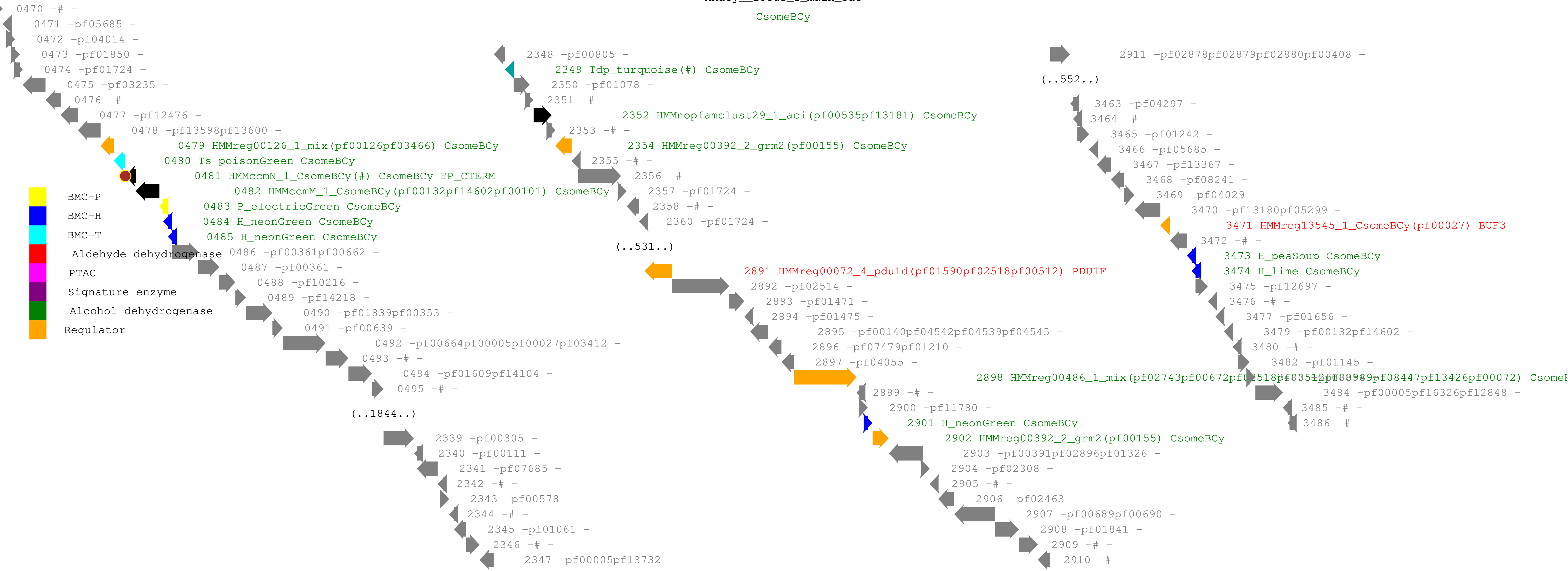

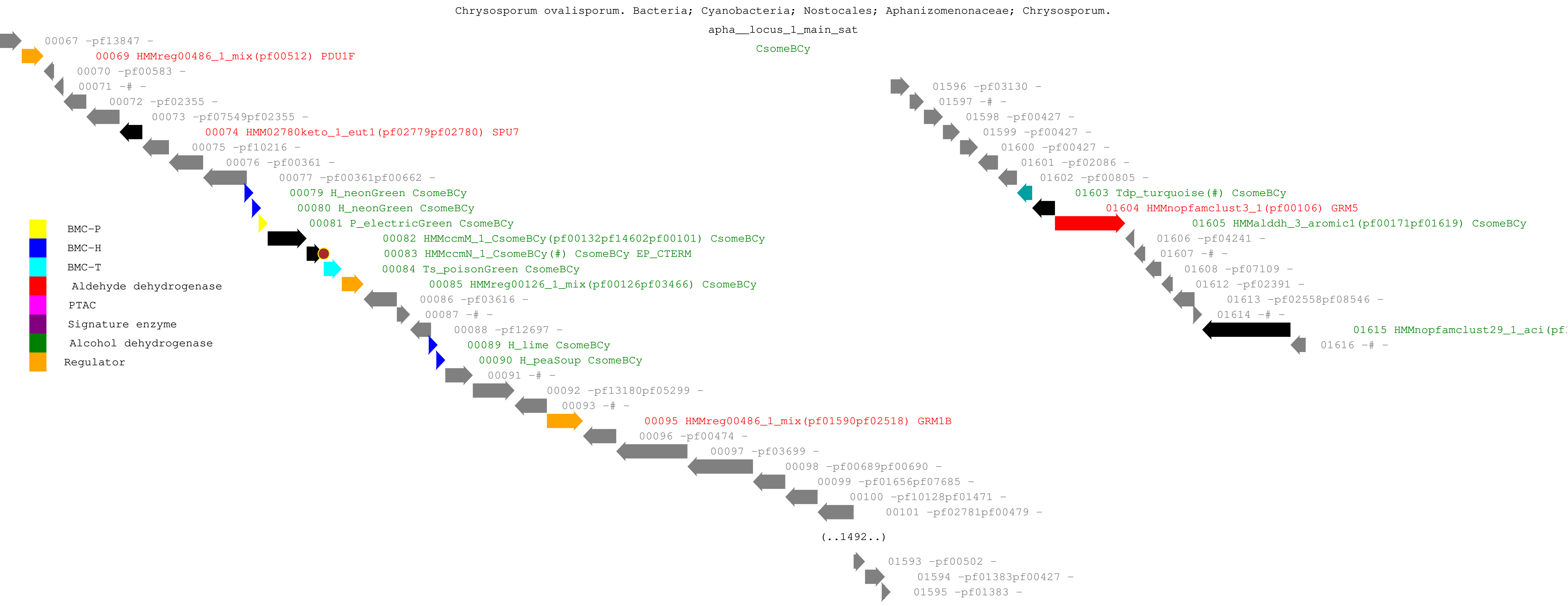
Chrysosporum ovalisporum. Bacteria; Cyanobacteria; Nostocales; Aphanizomenonaceae; Chrysosporum.
apha__locus_1_main_sat
CsomeBCy
00067 -pf13847 -
00069 HMMreg00486_1_mix(pf00512) PDU1F
00070 -pf00583 -
00071 -# -
00072 -pf02355 -
00073 -pf07549pf02355 -
00074 HMM02780keto_1_eut1(pf02779pf02780) SPU7
00075 -pf10216 -
00076 -pf00361 -
00077 -pf00361pf00662 -
00079 H_neonGreen CsomeBCy
00080 H_neonGreen CsomeBCy
00081 P_electricGreen CsomeBCy
00082 HMMccmM_1_CsomeBCy(pf00132pf14602pf00101) CsomeBCy
00083 HMMccmN_1_CsomeBCy(#) CsomeBCy EP_CTERM
00084 Ts_poisonGreen CsomeBCy
00085 HMMreg00126_1_mix(pf00126pf03466) CsomeBCy
00086 -pf03616 -
00087 -# -
00088 -pf12697 -
00089 H_lime CsomeBCy
00090 H_peaSoup CsomeBCy
00091 -# -
00092 -pf13180pf05299 -
00093 -# -
00095 HMMreg00486_1_mix(pf01590pf02518) GRM1B
00096 -pf00474 -
00097 -pf03699 -
00098 -pf00689pf00690 -
00099 -pf01656pf07685 -
00100 -pf10128pf01471 -
00101 -pf02781pf00479 -
(..1492..)
01593 -pf00502 -
01594 -pf01383pf00427 -
01595 -pf01383 -
01596 -pf03130 -
01597 -# -
01598 -pf00427 -
01599 -pf00427 -
01600 -pf00427 -
01601 -pf02086 -
01602 -pf00805 -
01603 Tdp_turquoise(#) CsomeBCy
01604 HMMnopfamclust3_1(pf00106) GRM5
01605 HMMalddh_3_aromic1(pf00171pf01619) CsomeBCy
01606 -pf04241 -
01607 -# -
01608 -pf07109 -
01612 -pf02391 -
01613 -pf02558pf08546 -
01614 -# -
01615 HMMnopfamclust29_1_aci(pf
01616 -# -
BMC-P
BMC-H
BMC-T
Aldehyde dehydrogenase
PTAC
Signature enzyme
Alcohol dehydrogenase
Regulator

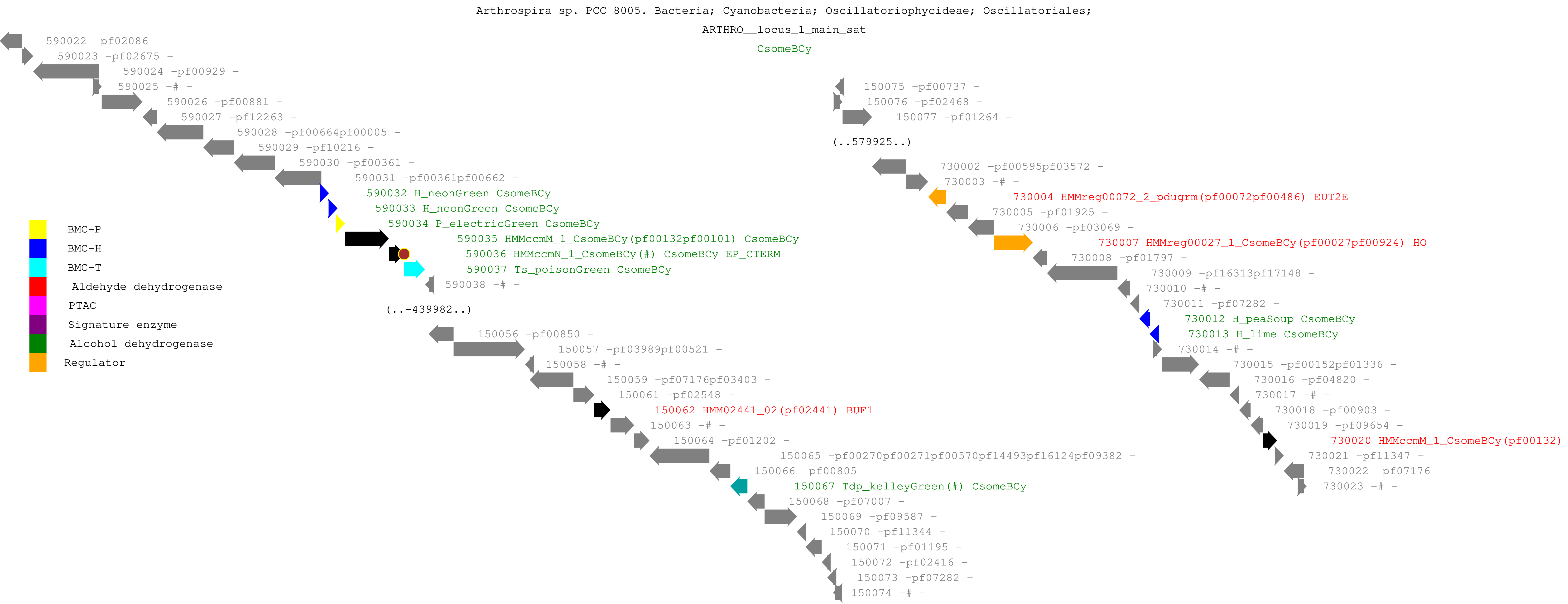

Arthrospira sp. PCC 8005. Bacteria; Cyanobacteria; Oscillatoriophycideae; Oscillatoriales;
ARTHRO__locus_1_main_sat
CsomeBCy
590022 -pf02086 -
590023 -pf02675 -
590024 -pf00929 -
590025 -# -
590026 -pf00881 -
590027 -pf12263 -
590028 -pf00664pf00005 -
590029 -pf10216 -
590030 -pf00361 -
590031 -pf00361pf00662 -
590032 H_neonGreen CsomeBCy
590033 H_neonGreen CsomeBCy
590034 P_electricGreen CsomeBCy
590035 HMMccmM_1_CsomeBCy(pf00132pf00101) CsomeBCy
590036 HMMccmN_1_CsomeBCy(#) CsomeBCy EP_CTERM
590037 Ts_poisonGreen CsomeBCy
590038 -# -
(..-439982..)
150056 -pf00850 -
150057 -pf03989pf00521 -
150058 -# -
150059 -pf07176pf03403 -
150061 -pf02548 -
150062 HMM02441_02(pf02441) BUF1
150063 -# -
150064 -pf01202 -
150065 -pf00270pf00271pf00570pf14493pf16124pf09382 -
150066 -pf00805 -
150067 Tdp_kelleyGreen(#) CsomeBCy
150068 -pf07007 -
150069 -pf09587 -
150070 -pf11344 -
150071 -pf01195 -
150072 -pf02416 -
150073 -pf07282 -
150074 -# -
150075 -pf00737 -
150076 -pf02468 -
150077 -pf01264 -
(..579925..)
730002 -pf00595pf03572 -
730003 -# -
730004 HMMreg00072_2_pdugrm(pf00072pf00486) EUT2E
730005 -pf01925 -
730006 -pf03069 -
730007 HMMreg00027_1_CsomeBCy(pf00027pf00924) HO
730008 -pf01797 -
730009 -pf16313pf17148 -
730010 -# -
730011 -pf07282 -
730012 H_peaSoup CsomeBCy
730013 H_lime CsomeBCy
730014 -# -
730015 -pf00152pf01336 -
730016 -pf04820 -
730017 -# -
730018 -pf00903 -
730019 -pf09654 -
730020 HMMccmM_1_CsomeBCy(pf00132)
730021 -pf11347 -
730022 -pf07176 -
730023 -# -
BMC-P
BMC-H
BMC-T
Aldehyde dehydrogenase
PTAC
Signature enzyme
Alcohol dehydrogenase
Regulator

Aphanothece sacrum FPU1. Bacteria; Cyanobacteria; Oscillatoriophycideae; Chroococcales;

AsFPU1__locus_1_main_sat

CsomeBCy

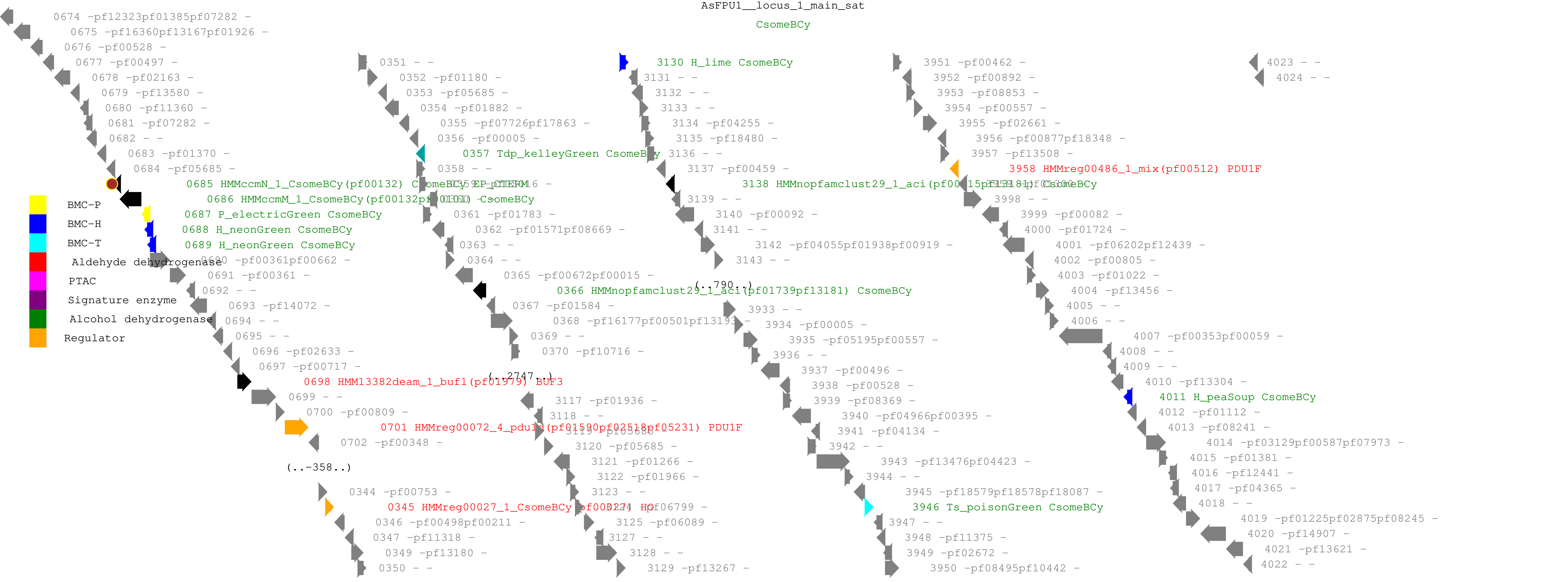

Cylindrospermopsis sp. CR12. Bacteria; Cyanobacteria; Nostocales; Aphanizomenonaceae;

ASL19__locus_1_main_sat

CsomeBCy

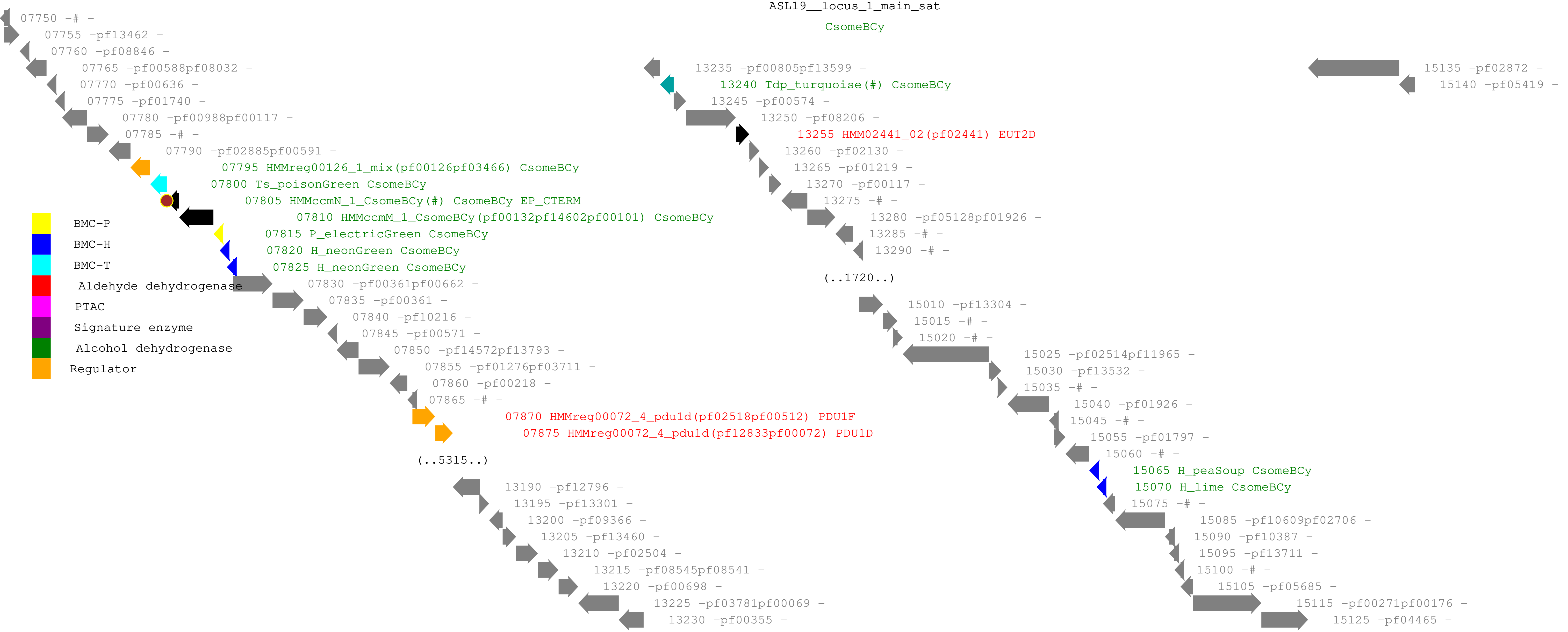

Oscillatoriales cyanobacterium CG2_30_40_61. Bacteria; Cyanobacteria; Oscillatoriophycideae; Oscillatoriales;

AUK43__locus_1_main_sat

CsomeBCy

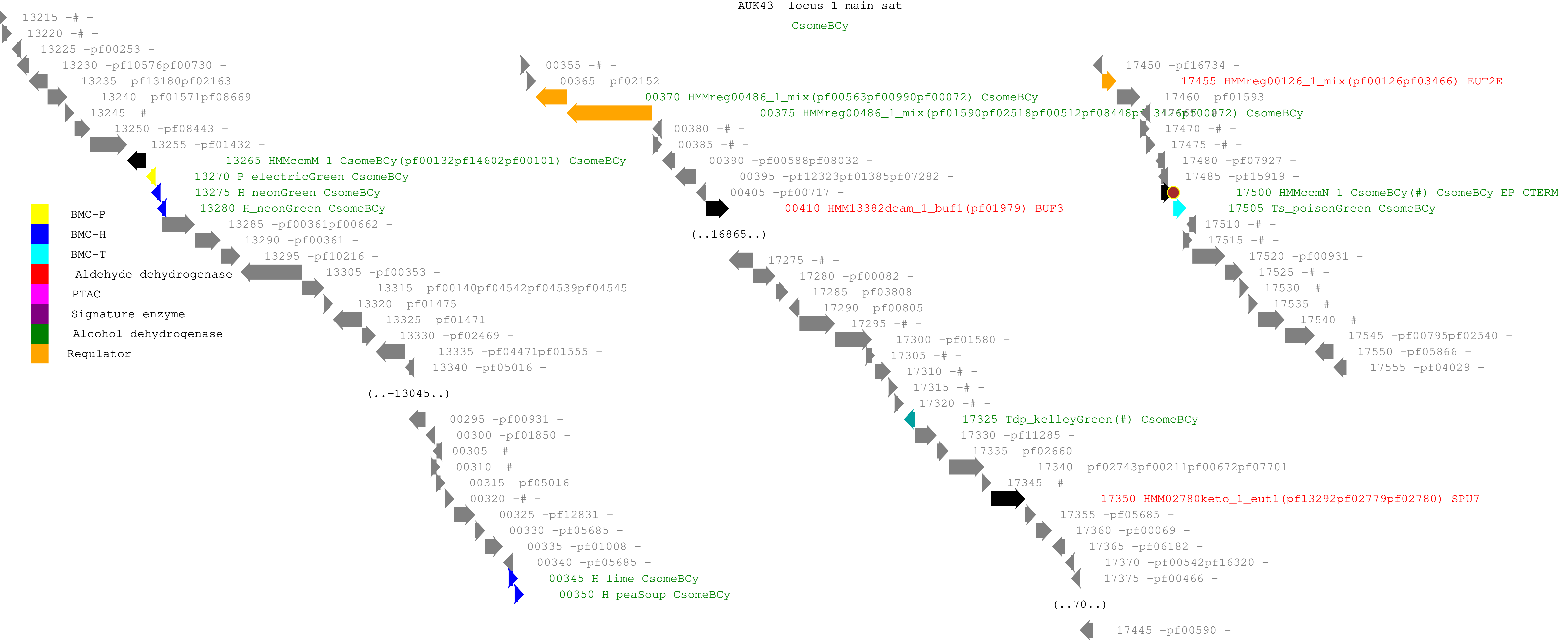

Oscillatoriales cyanobacterium CG2_30_44_21. Bacteria; Cyanobacteria; Oscillatoriophycideae; Oscillatoriales;

AUK48__locus_1_main_sat

CsomeBCy

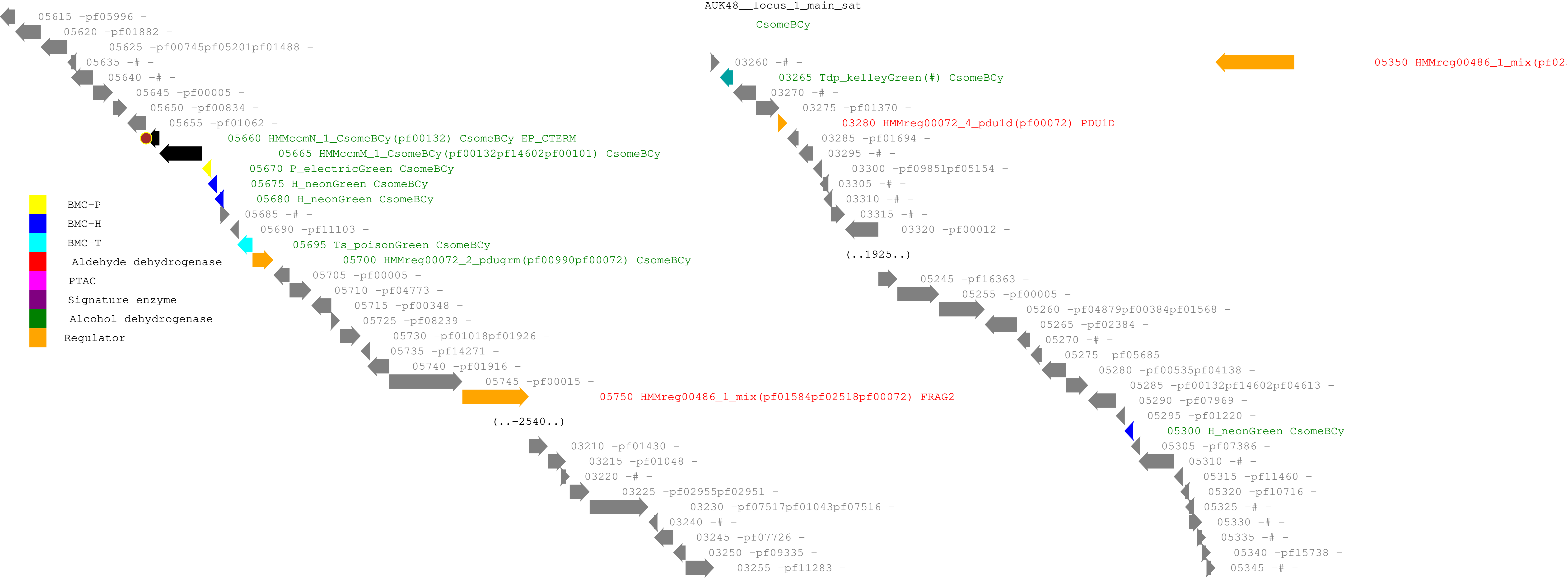

Anabaena variabilis (strain ATCC 29413 / PCC 7937). Bacteria; Cyanobacteria; Nostocales; Nostocaceae; Anabaena.

Ava__locus_1_main_sat

CsomeBCy

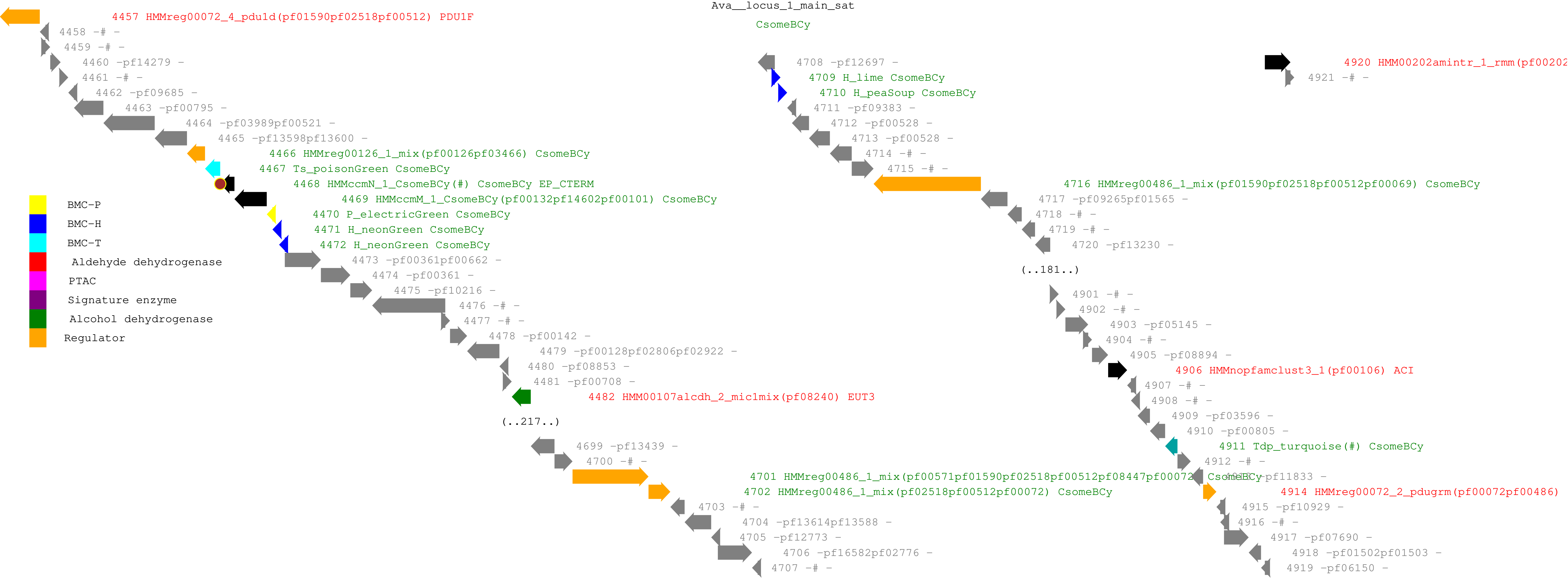

Synechococcus sp. PCC 7003. Bacteria; Cyanobacteria; Synechococcales; Synechococcaceae;

AWQ21__locus_1_main_sat

CsomeBCy

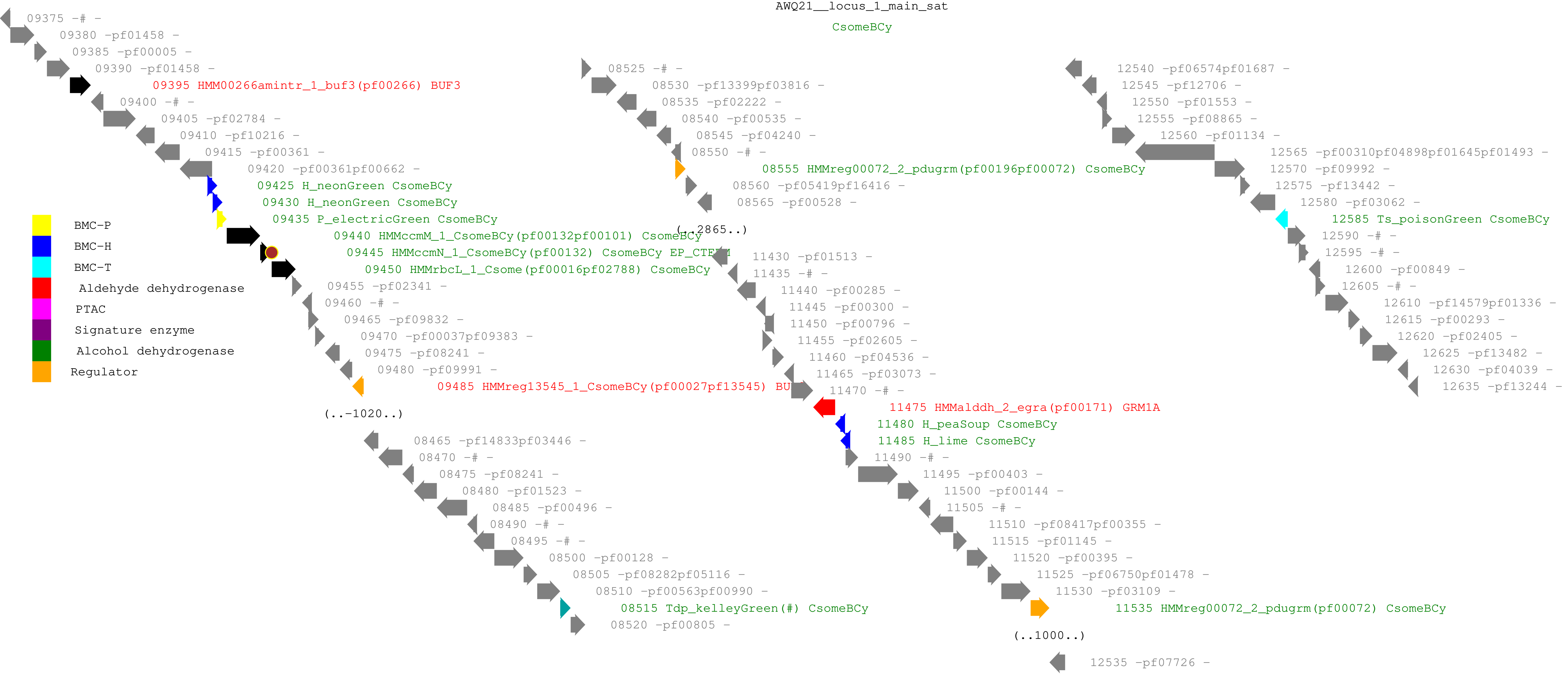

Synechococcus sp. PCC 7117. Bacteria; Cyanobacteria; Synechococcales; Synechococcaceae;

AWQ22__locus_1_main_sat

CsomeBCy

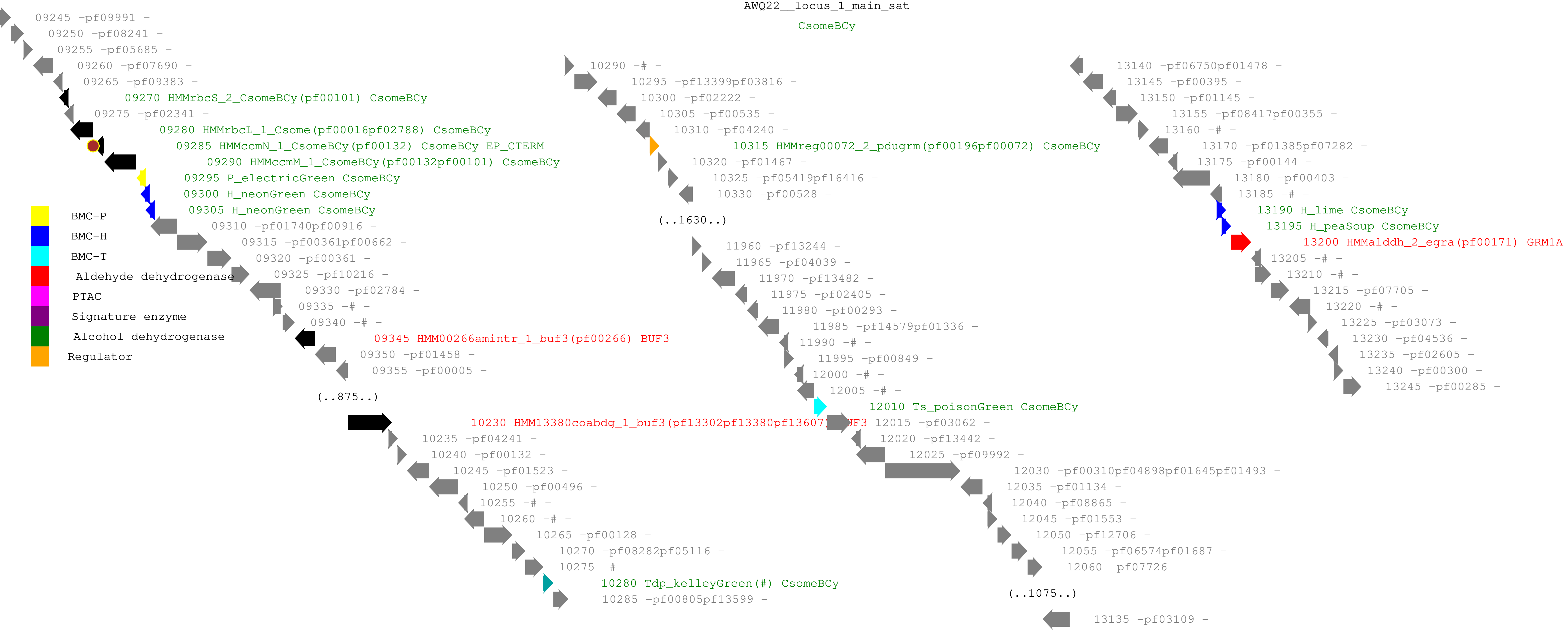

Phormidium willei BDU 130791. Bacteria; Cyanobacteria; Oscillatoriophycideae; Oscillatoriales;

AY600__locus_1_main_sat

CsomeBCy

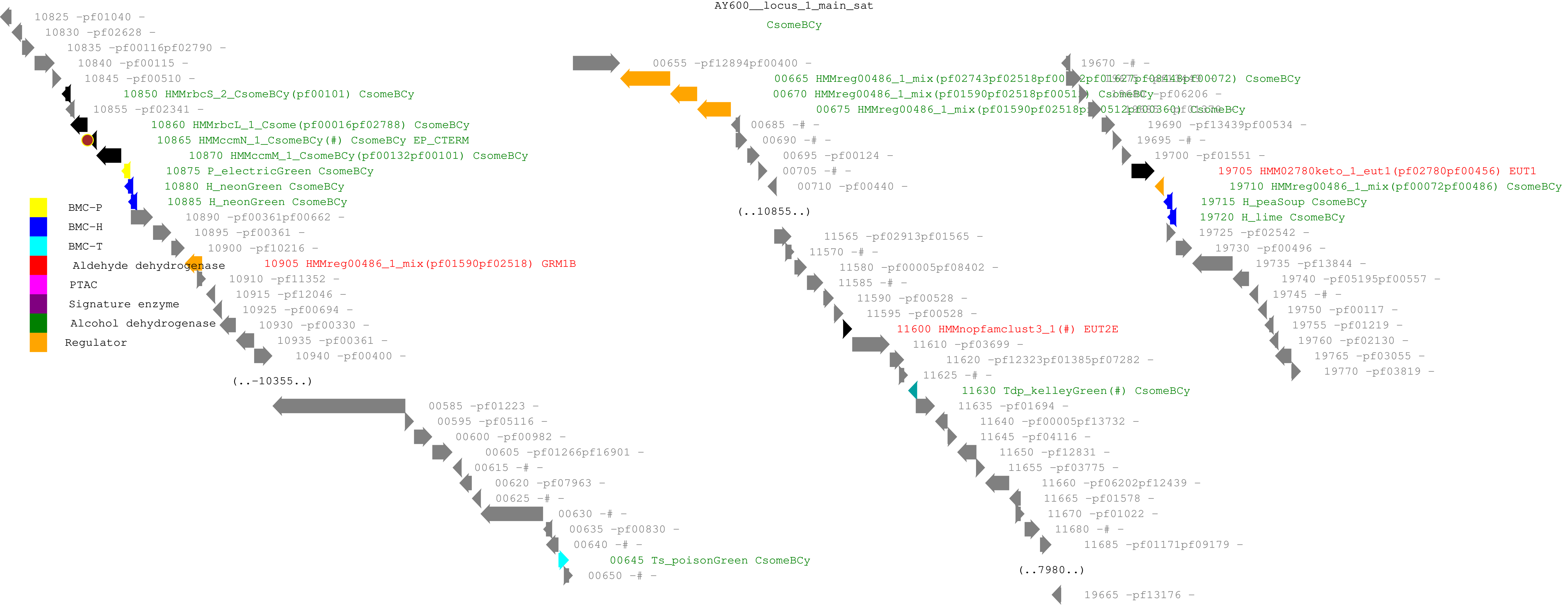

Alkalinema sp. CACIAM 70d. Bacteria; Cyanobacteria; Synechococcales; Pseudanabaenaceae;

B0A82__locus_1_main_sat

CsomeBCy

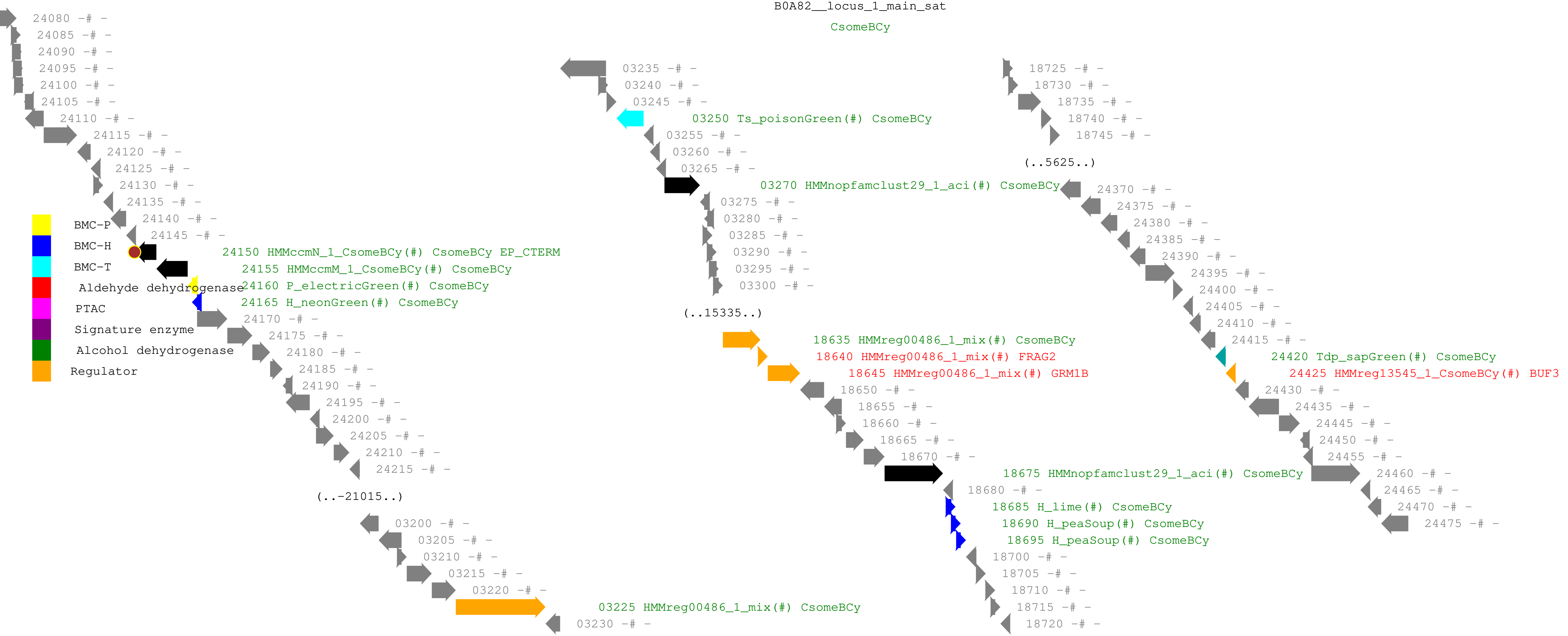

Chroococcidiopsis sp. TS-821. Bacteria; Cyanobacteria; Chroococcidiopsidales;

B1A85__locus_1_main_sat

CsomeBCy

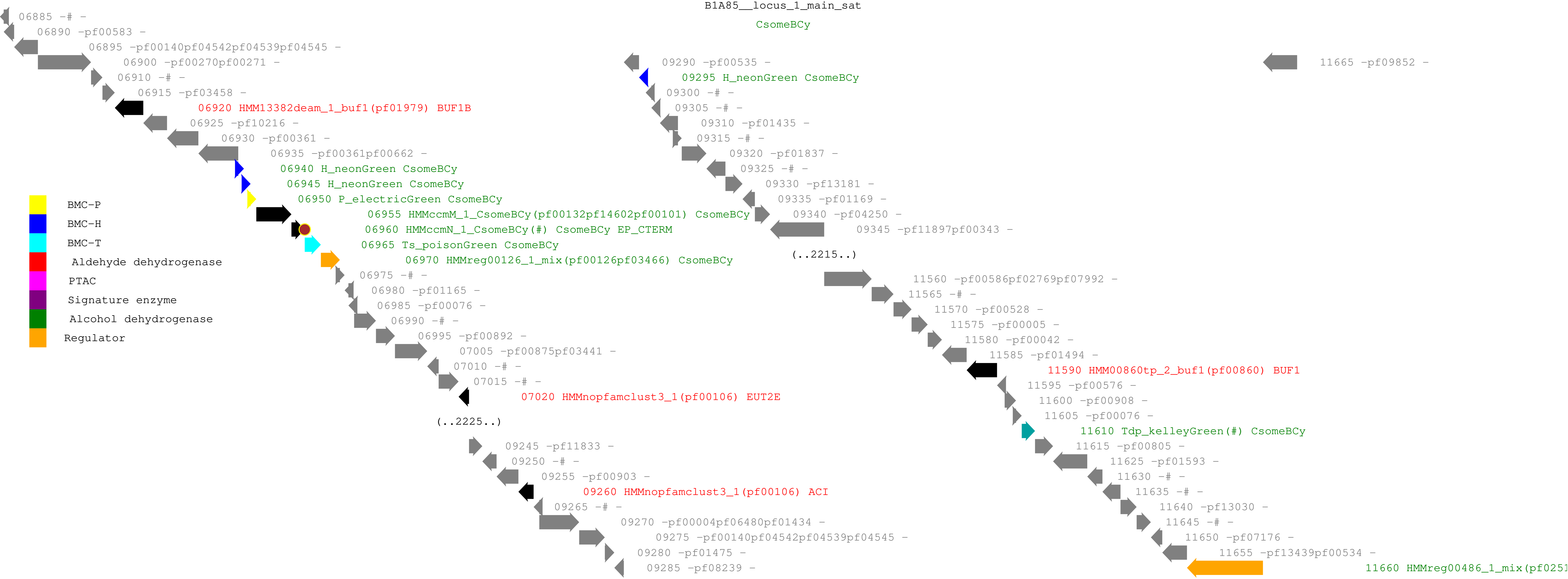

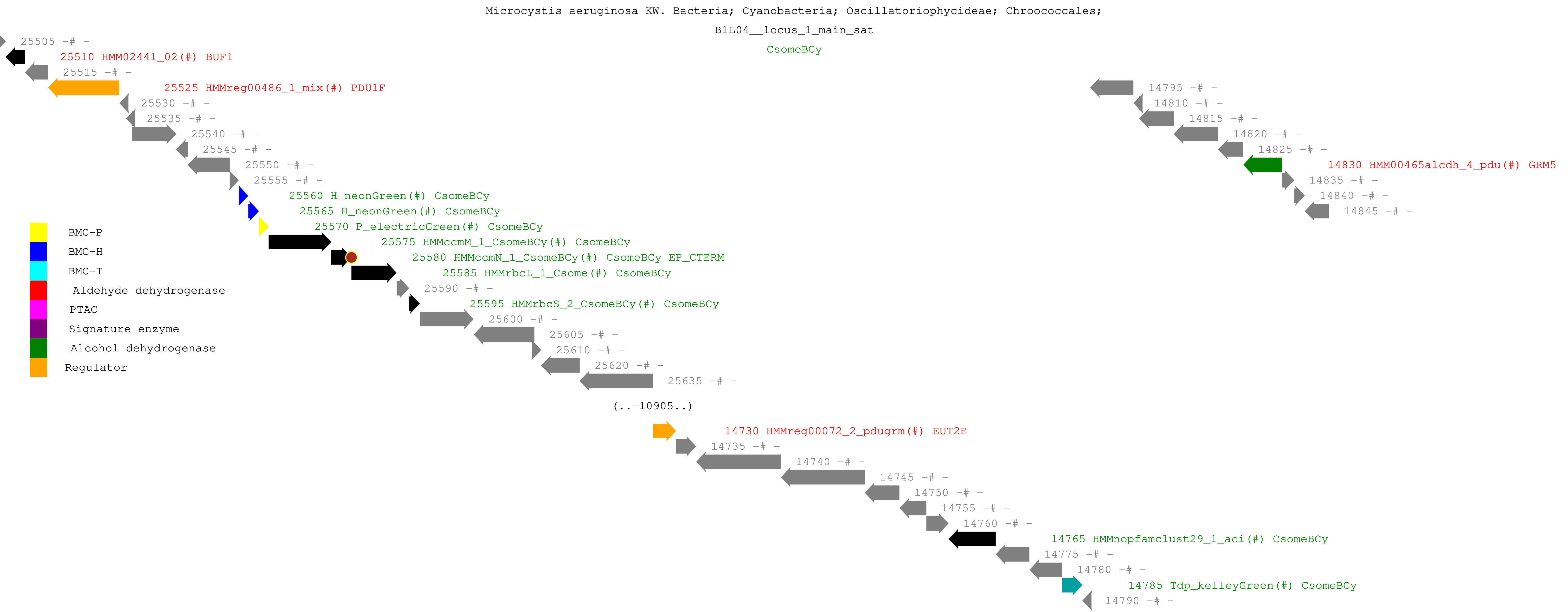
Microcystis aeruginosa KW. Bacteria; Cyanobacteria; Oscillatoriophycideae; Chroococcales;
B1L04__locus_1_main_sat
CsomeBCy
25505 -# -
25510 HMM02441_02(#) BUF1
25515 -# -
25525 HMMreg00486_1_mix(#) PDU1F
25530 -# -
25535 -# -
25540 -# -
25545 -# -
25550 -# -
25555 -# -
25560 H_neonGreen(#) CsomeBCy
25565 H_neonGreen(#) CsomeBCy
25570 P_electricGreen(#) CsomeBCy
25575 HMMccmM_1_CsomeBCy(#) CsomeBCy
25580 HMMccmN_1_CsomeBCy(#) CsomeBCy EP_CTERM
25585 HMMrbcL_1_Csome(#) CsomeBCy
25590 -# -
25595 HMMrbcS_2_CsomeBCy(#) CsomeBCy
25600 -# -
25605 -# -
25610 -# -
25620 -# -
25635 -# -
(..-10905..)
14730 HMMreg00072_2_pdugrm(#) EUT2E
14735 -# -
14740 -# -
14745 -# -
14750 -# -
14755 -# -
14760 -# -
14765 HMMnopfamclust29_1_aci(#) CsomeBCy
14775 -# -
14780 -# -
14785 Tdp_kelleyGreen(#) CsomeBCy
14790 -# -
14795 -# -
14810 -# -
14815 -# -
14820 -# -
14825 -# -
14830 HMM00465alcdh_4_pdu(#) GRM5
14835 -# -
14840 -# -
14845 -# -
BMC-P
BMC-H
BMC-T
Aldehyde dehydrogenase
PTAC
Signature enzyme
Alcohol dehydrogenase
Regulator

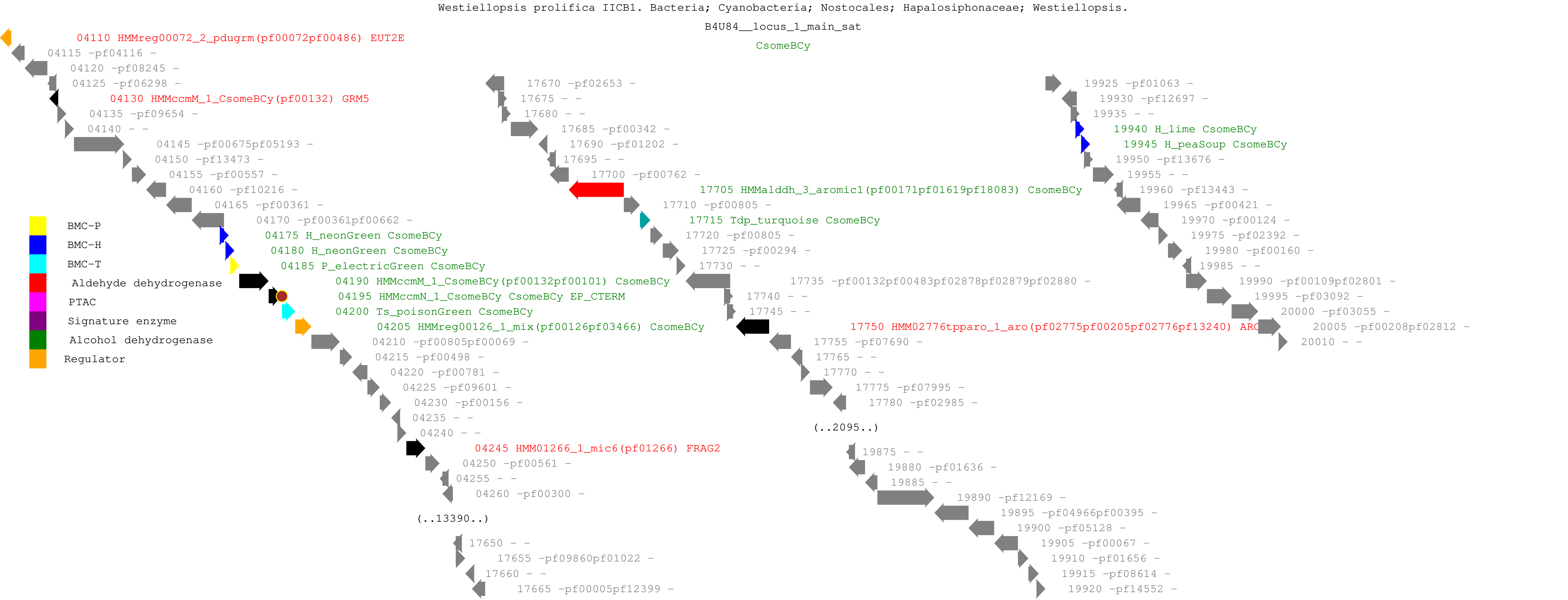
Westiellopsis prolifica IICB1. Bacteria; Cyanobacteria; Nostocales; Hapalosiphonaceae; Westiellopsis.
B4U84__locus_1_main_sat
CsomeBCy
04110 HMMreg00072_2_pdugrm(pf00072pf00486) EUT2E
04115 -pf04116 -
04120 -pf08245 -
04125 -pf06298 -
04130 HMMccmM_1_CsomeBCy(pf00132) GRM5
04135 -pf09654 -
04140 - -
04145 -pf00675pf05193 -
04150 -pf13473 -
04155 -pf00557 -
04160 -pf10216 -
04165 -pf00361 -
04170 -pf00361pf00662 -
04175 H_neonGreen CsomeBCy
04180 H_neonGreen CsomeBCy
04185 P_electricGreen CsomeBCy
04190 HMMccmM_1_CsomeBCy(pf00132pf00101) CsomeBCy
04195 HMMccmN_1_CsomeBCy CsomeBCy EP_CTERM
04200 Ts_poisonGreen CsomeBCy
04205 HMMreg00126_1_mix(pf00126pf03466) CsomeBCy
04210 -pf00805pf00069 -
04215 -pf00498 -
04220 -pf00781 -
04225 -pf09601 -
04230 -pf00156 -
04235 - -
04240 - -
04245 HMM01266_1_mic6(pf01266) FRAG2
04250 -pf00561 -
04255 - -
04260 -pf00300 -
(..13390..)
17650 - -
17655 -pf09860pf01022 -
17660 - -
17665 -pf00005pf12399 -
BMC-P
BMC-H
BMC-T
Aldehyde dehydrogenase
PTAC
Signature enzyme
Alcohol dehydrogenase
Regulator
17670 -pf02653 -
17675 - -
17680 - -
17685 -pf00342 -
17690 -pf01202 -
17695 - -
17700 -pf00762 -
17705 HMMaldh_3_aromic1(pf00171pf01619pf18083) CsomeBCy
17710 -pf00805 -
17715 Tdp_turquoise CsomeBCy
17720 -pf00805 -
17725 -pf00294 -
17730 - -
17735 -pf00132pf00483pf02878pf02879pf02880 -
17740 - -
17745 - -
17750 HMM02776tpparo_1_aro(pf02775pf00205pf02776pf13240) ARO
17755 -pf07690 -
17765 - -
17770 - -
17775 -pf07995 -
17780 -pf02985 -
(..2095..)
19875 - -
19880 -pf01636 -
19885 - -
19890 -pf12169 -
19895 -pf04966pf00395 -
19900 -pf05128 -
19905 -pf00067 -
19910 -pf01656 -
19915 -pf08614 -
19920 -pf14552 -
19925 -pf01063 -
19930 -pf12697 -
19935 - -
19940 H_lime CsomeBCy
19945 H_peaSoup CsomeBCy
19950 -pf13676 -
19955 - -
19960 -pf13443 -
19965 -pf00421 -
19970 -pf00124 -
19975 -pf02392 -
19980 -pf00160 -
19985 - -
19990 -pf00109pf02801 -
19995 -pf03092 -
20000 -pf03055 -
20005 -pf00208pf02812 -
20010 - -

Microcystis sp. MC19. Bacteria; Cyanobacteria; Oscillatoriophycideae; Chroococcales;

B5D77__locus_1_main_sat

CsomeBCy

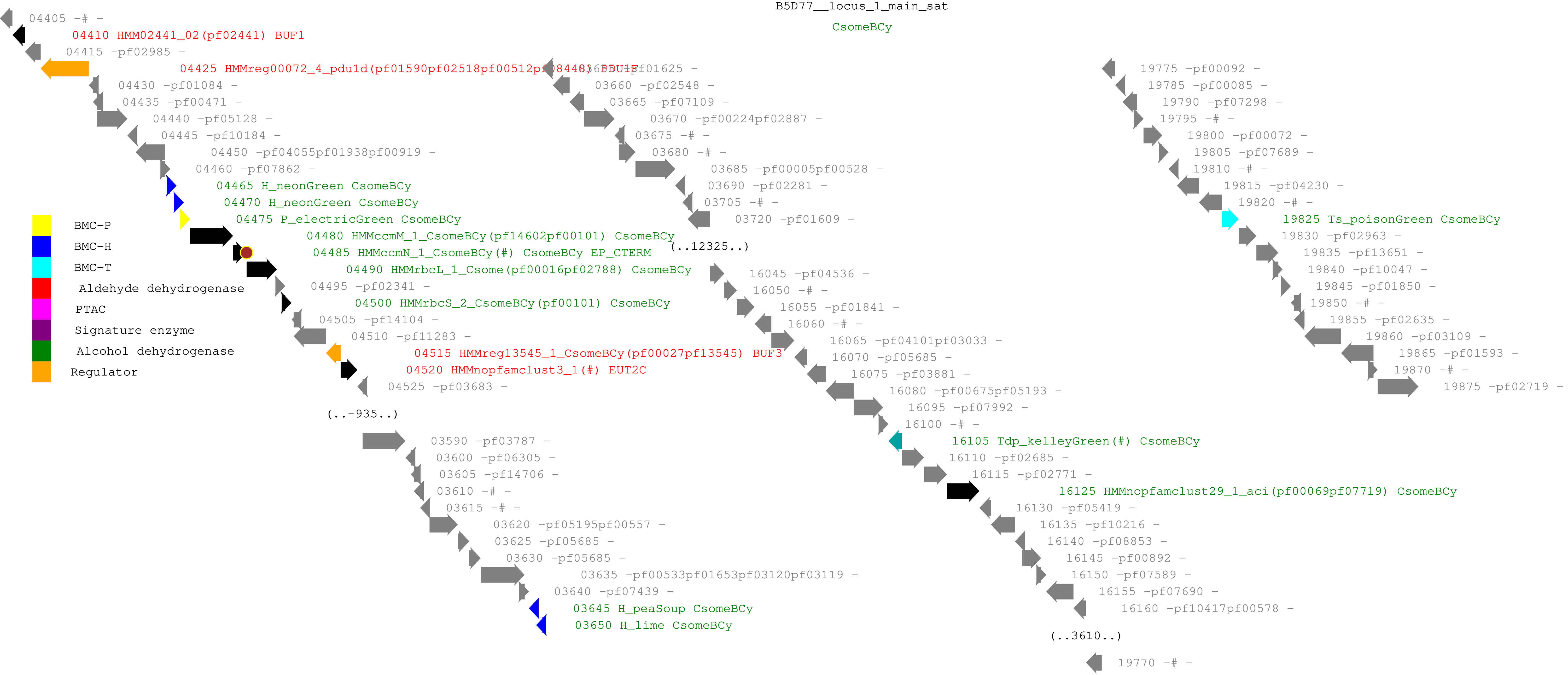

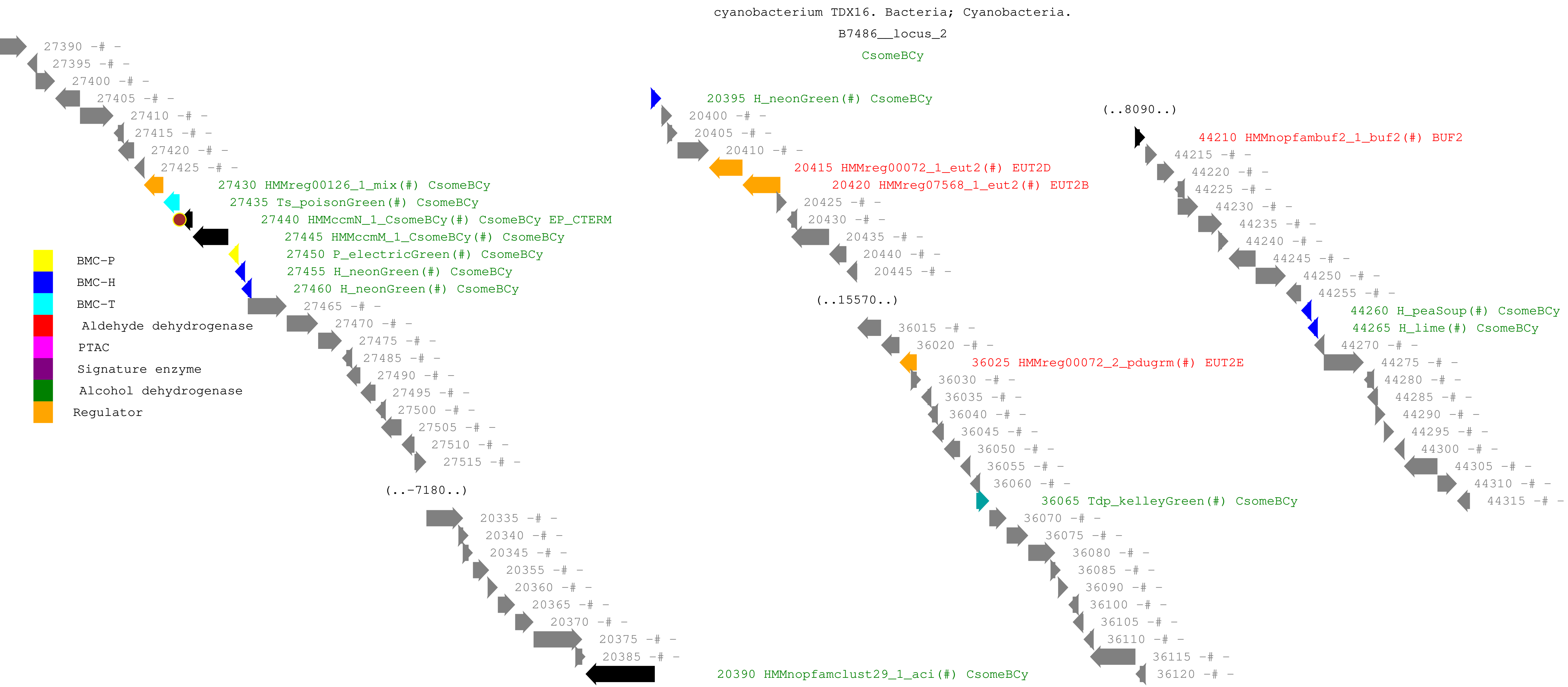

cyanobacterium TDX16. Bacteria; Cyanobacteria.
B7486__locus_2
CsomeBCy
27390 -# -
27395 -# -
27400 -# -
27405 -# -
27410 -# -
27415 -# -
27420 -# -
27425 -# -
27430 HMMreg00126_1_mix(#) CsomeBCy
27435 Ts_poisonGreen(#) CsomeBCy
27440 HMMccmN_1_CsomeBCy(#) CsomeBCy EP_CTERM
27445 HMMccmM_1_CsomeBCy(#) CsomeBCy
27450 P_electricGreen(#) CsomeBCy
27455 H_neonGreen(#) CsomeBCy
27460 H_neonGreen(#) CsomeBCy
27465 -# -
27470 -# -
27475 -# -
27485 -# -
27490 -# -
27495 -# -
27500 -# -
27505 -# -
27510 -# -
27515 -# -
(..-7180..)
20335 -# -
20340 -# -
20345 -# -
20355 -# -
20360 -# -
20365 -# -
20370 -# -
20375 -# -
20385 -# -
20390 HMMnopfamclust29_1_aci(#) CsomeBCy
20395 H_neonGreen(#) CsomeBCy
20400 -# -
20405 -# -
20410 -# -
20415 HMMreg00072_1_eut2(#) EUT2D
20420 HMMreg07568_1_eut2(#) EUT2B
20425 -# -
20430 -# -
20435 -# -
20440 -# -
20445 -# -
(..15570..)
36015 -# -
36020 -# -
36025 HMMreg00072_2_pdugrm(#) EUT2E
36030 -# -
36035 -# -
36040 -# -
36045 -# -
36050 -# -
36055 -# -
36060 -# -
36065 Tdp_kelleyGreen(#) CsomeBCy
36070 -# -
36075 -# -
36080 -# -
36085 -# -
36090 -# -
36100 -# -
36105 -# -
36110 -# -
36115 -# -
36120 -# -
(..8090..)
44210 HMMnopfambuf2_1_buf2(#) BUF2
44215 -# -
44220 -# -
44225 -# -
44230 -# -
44235 -# -
44240 -# -
44245 -# -
44250 -# -
44255 -# -
44260 H_peaSoup(#) CsomeBCy
44265 H_lime(#) CsomeBCy
44270 -# -
44275 -# -
44280 -# -
44285 -# -
44290 -# -
44295 -# -
44300 -# -
44305 -# -
44310 -# -
44315 -# -
BMC-P
BMC-H
BMC-T
Aldehyde dehydrogenase
PTAC
Signature enzyme
Alcohol dehydrogenase
Regulator

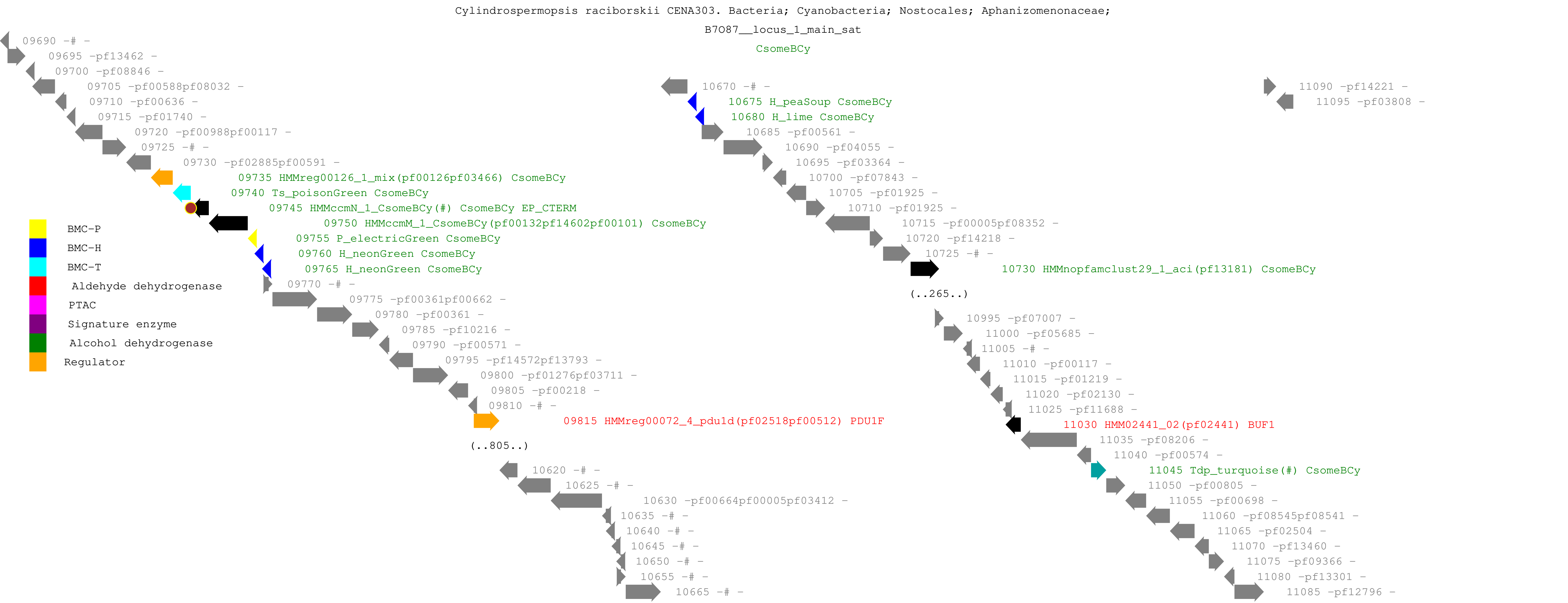

Cylindrospermopsis raciborskii CENA303. Bacteria; Cyanobacteria; Nostocales; Aphanizomenonaceae;
B7087__locus_1_main_sat
CsomeBCy
09690 -# -
09695 -pf13462 -
09700 -pf08846 -
09705 -pf00588pf08032 -
09710 -pf00636 -
09715 -pf01740 -
09720 -pf00988pf00117 -
09725 -# -
09730 -pf02885pf00591 -
09735 HMMreg00126_1_mix(pf00126pf03466) CsomeBCy
09740 Ts_poisonGreen CsomeBCy
09745 HMMccmN_1_CsomeBCy(#) CsomeBCy EP_CTERM
09750 HMMccmM_1_CsomeBCy(pf00132pf14602pf00101) CsomeBCy
09755 P_electricGreen CsomeBCy
09760 H_neonGreen CsomeBCy
09765 H_neonGreen CsomeBCy
09770 -# -
09775 -pf00361pf00662 -
09780 -pf00361 -
09785 -pf10216 -
09790 -pf00571 -
09795 -pf14572pf13793 -
09800 -pf01276pf03711 -
09805 -pf00218 -
09810 -# -
09815 HMMreg00072_4_pdu1d(pf02518pf00512) PDU1F
(..805..)
10620 -# -
10625 -# -
10630 -pf00664pf00005pf03412 -
10635 -# -
10640 -# -
10645 -# -
10650 -# -
10655 -# -
10665 -# -
10670 -# -
10675 H_peaSoup CsomeBCy
10680 H_lime CsomeBCy
10685 -pf00561 -
10690 -pf04055 -
10695 -pf03364 -
10700 -pf07843 -
10705 -pf01925 -
10710 -pf01925 -
10715 -pf00005pf08352 -
10720 -pf14218 -
10725 -# -
10730 HMMnopfamclust29_1_aci(pf13181) CsomeBCy
(..265..)
10995 -pf07007 -
11000 -pf05685 -
11005 -# -
11010 -pf00117 -
11015 -pf01219 -
11020 -pf02130 -
11025 -pf11688 -
11030 HMM02441_02(pf02441) BUF1
11035 -pf08206 -
11040 -pf00574 -
11045 Tdp_turquoise(#) CsomeBCy
11050 -pf00805 -
11055 -pf00698 -
11060 -pf08545pf08541 -
11065 -pf02504 -
11070 -pf13460 -
11075 -pf09366 -
11080 -pf13301 -
11085 -pf12796 -
11090 -pf14221 -
11095 -pf03808 -
BMC-P
BMC-H
BMC-T
Aldehyde dehydrogenase
PTAC
Signature enzyme
Alcohol dehydrogenase
Regulator

Pseudanabaena sp. SR411. Bacteria; Cyanobacteria; Synechococcales; Pseudanabaenaceae;

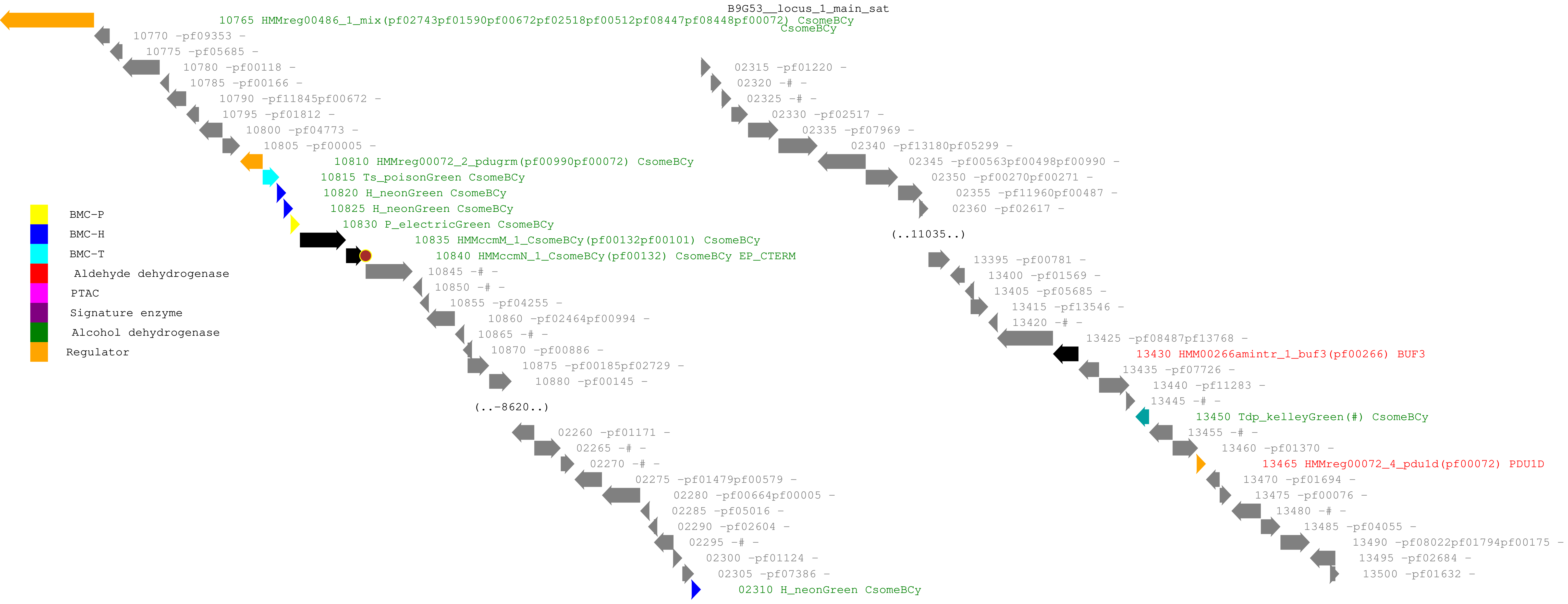

Arthrospira sp. 09.13F. Bacteria; Cyanobacteria; Oscillatoriophycideae; Oscillatoriales;

B9S53__locus_1_main_sat

CsomeBCy

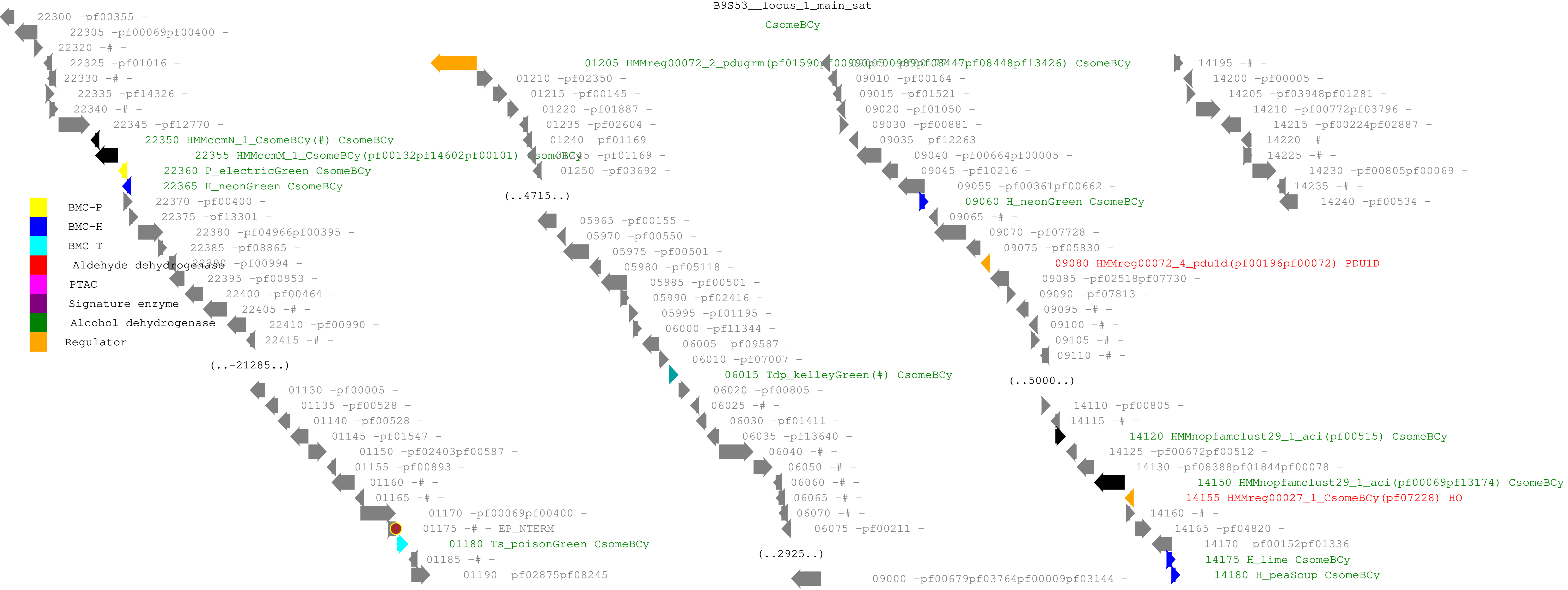

Mastigocoleus testarum BC008. Bacteria; Cyanobacteria; Nostocales; Hapalosiphonaceae; Mastigocoleus.

BC008__locus_1_main_sat

CsomeBCy

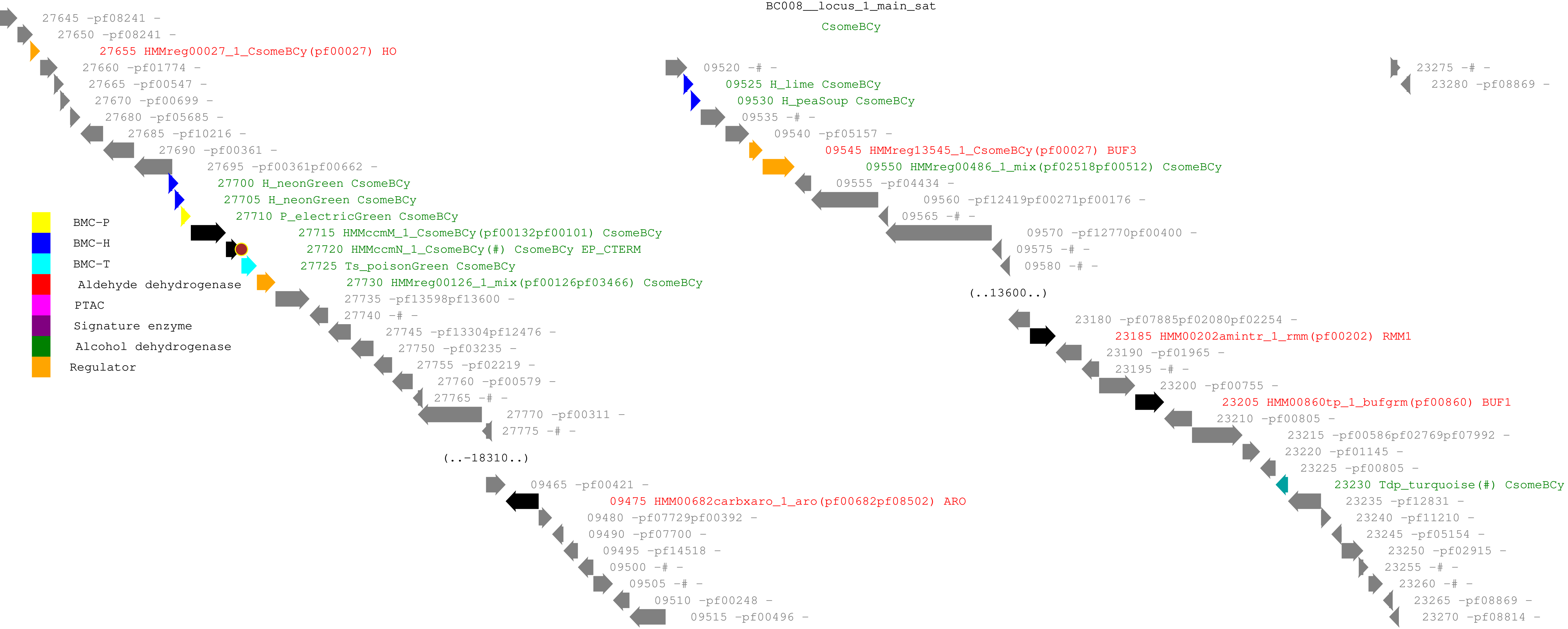

Nostoc sp. MBR 210. Bacteria; Cyanobacteria; Nostocales; Nostocaceae; Nostoc.

BCD64__locus_1_main_sat

CsomeBCy

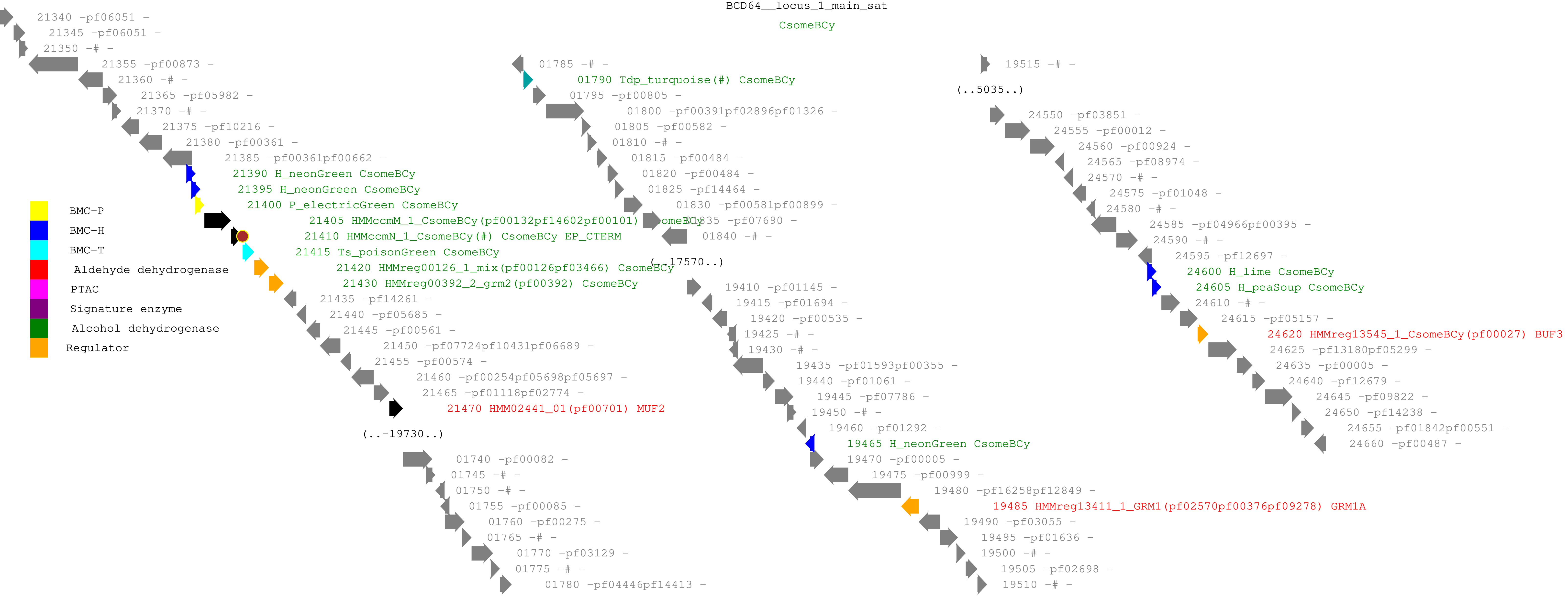

Oscillatoriales cyanobacterium USR001. Bacteria; Cyanobacteria; Oscillatoriophycideae; Oscillatoriales;

BCD67__locus_1_main_sat

CsomeBCy

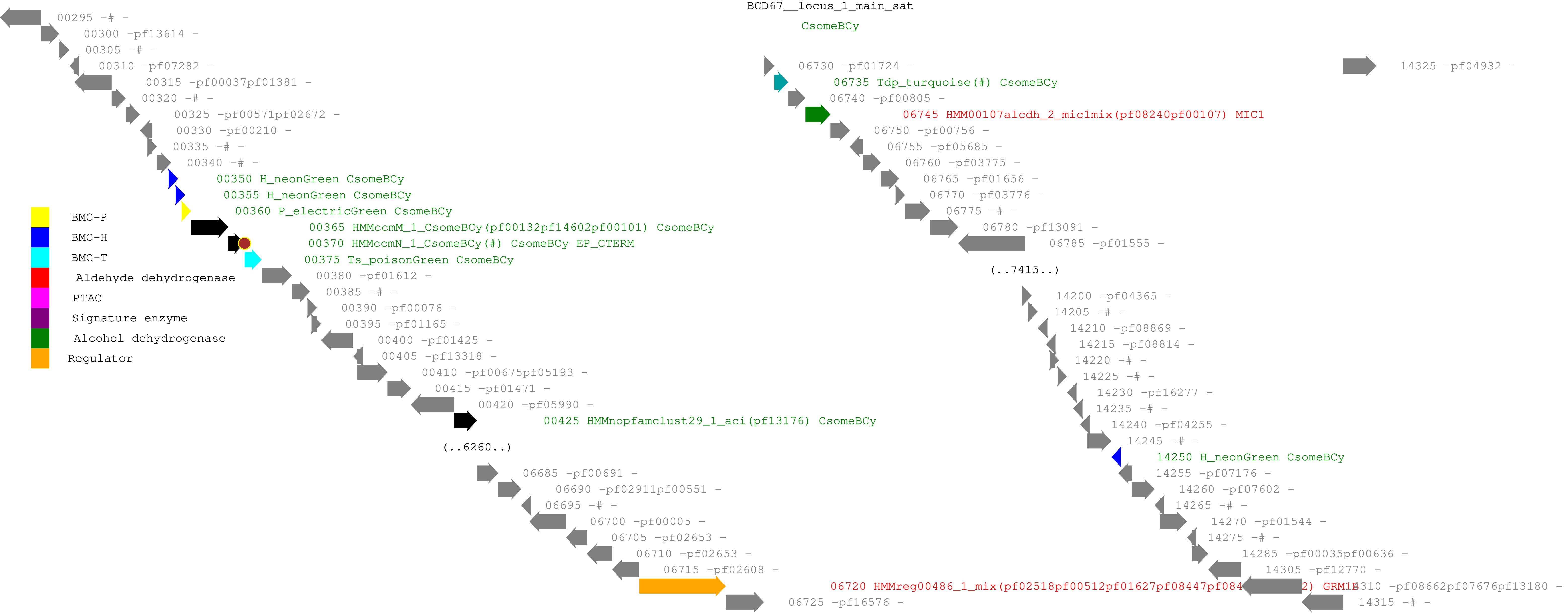

Limnothrix sp. P13C2. Bacteria; Cyanobacteria; Synechococcales; Pseudanabaenaceae;

BCR12__locus_1_main_sat

CsomeBCy

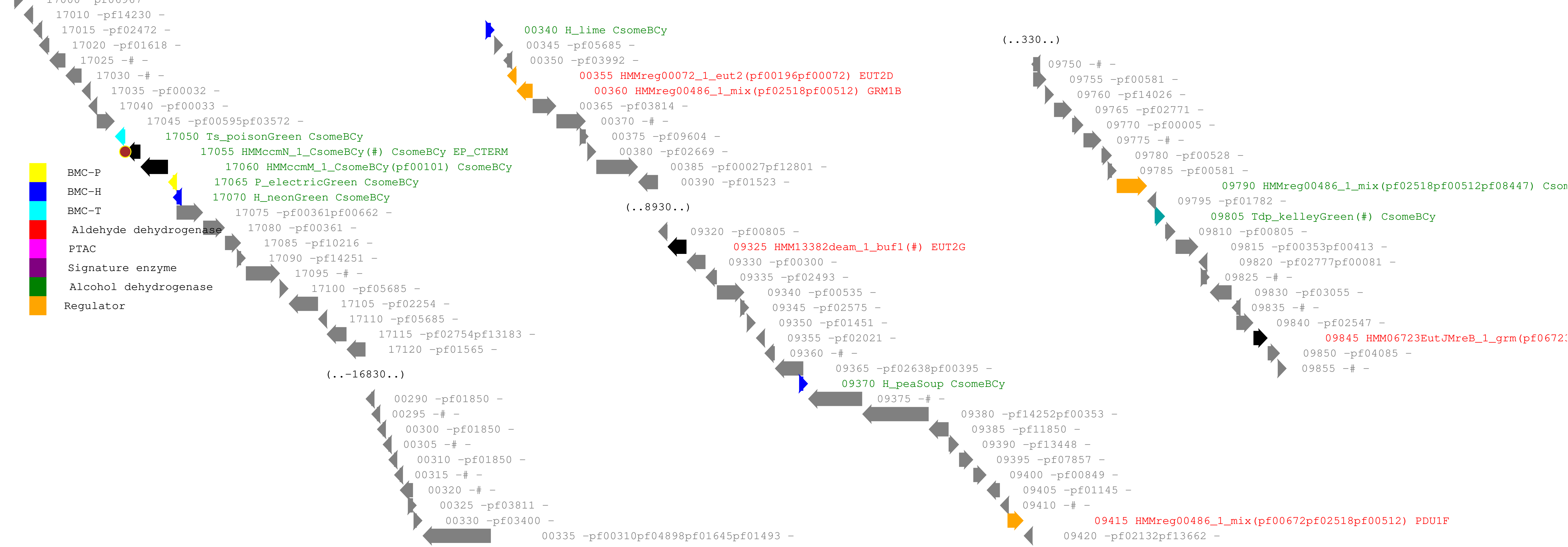

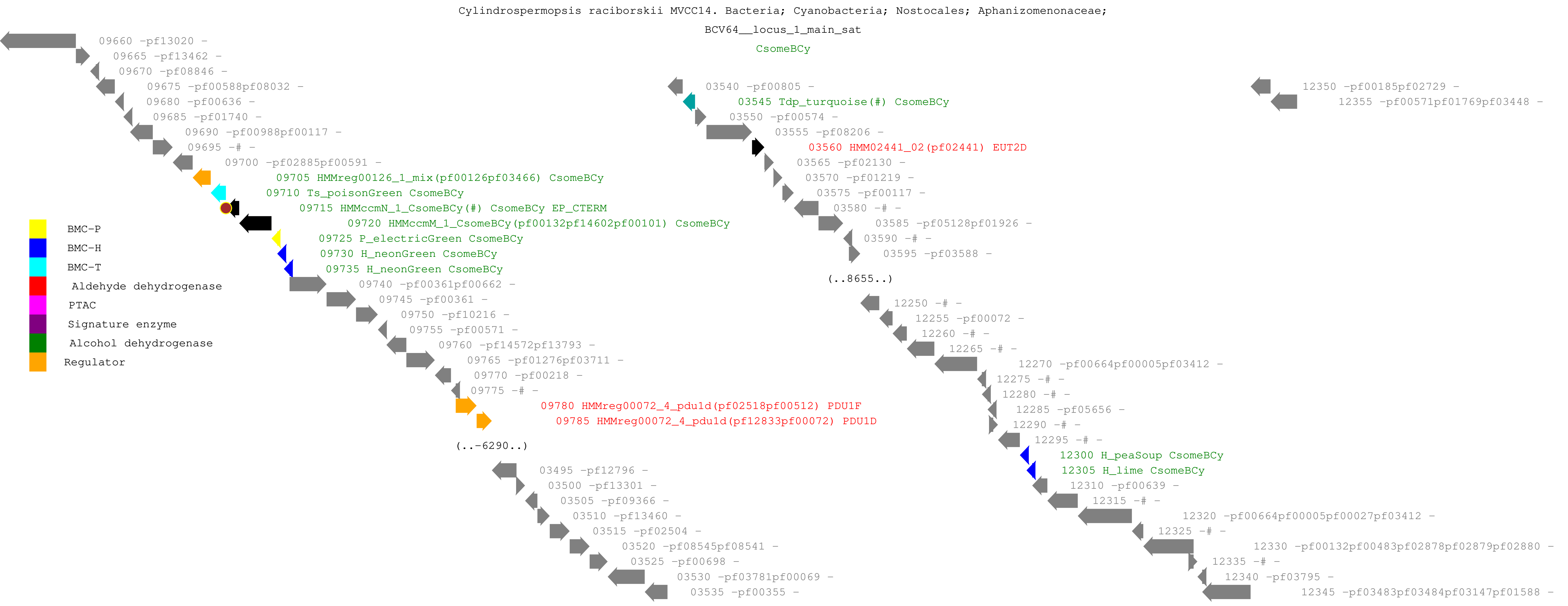
Cylindrospermopsis raciborskii MVCC14. Bacteria; Cyanobacteria; Nostocales; Aphanizomenonaceae;
BCV64__locus_1_main_sat
CsomeBCy
09660 -pf13020 -
09665 -pf13462 -
09670 -pf08846 -
09675 -pf00588pf08032 -
09680 -pf00636 -
09685 -pf01740 -
09690 -pf00988pf00117 -
09695 -# -
09700 -pf02885pf00591 -
09705 HMMreg00126_1_mix(pf00126pf03466) CsomeBCy
09710 Ts_poisonGreen CsomeBCy
09715 HMMccmN_1_CsomeBCy(#) CsomeBCy EP_CTERM
09720 HMMccmM_1_CsomeBCy(pf00132pf14602pf00101) CsomeBCy
09725 P_electricGreen CsomeBCy
09730 H_neonGreen CsomeBCy
09735 H_neonGreen CsomeBCy
09740 -pf00361pf00662 -
09745 -pf00361 -
09750 -pf10216 -
09755 -pf00571 -
09760 -pf14572pf13793 -
09765 -pf01276pf03711 -
09770 -pf00218 -
09775 -# -
09780 HMMreg00072_4_pdu1d(pf02518pf00512) PDU1F
09785 HMMreg00072_4_pdu1d(pf12833pf00072) PDU1D
(..-6290..)
03495 -pf12796 -
03500 -pf13301 -
03505 -pf09366 -
03510 -pf13460 -
03515 -pf02504 -
03520 -pf08545pf08541 -
03525 -pf00698 -
03530 -pf03781pf00069 -
03535 -pf00355 -
03540 -pf00805 -
03545 Tdp_turquoise(#) CsomeBCy
03550 -pf00574 -
03555 -pf08206 -
03560 HMM02441_02(pf02441) EUT2D
03565 -pf02130 -
03570 -pf01219 -
03575 -pf00117 -
03580 -# -
03585 -pf05128pf01926 -
03590 -# -
03595 -pf03588 -
(..8655..)
12250 -# -
12255 -pf00072 -
12260 -# -
12265 -# -
12270 -pf00664pf00005pf03412 -
12275 -# -
12280 -# -
12285 -pf05656 -
12290 -# -
12295 -# -
12300 H_peaSoup CsomeBCy
12305 H_lime CsomeBCy
12310 -pf00639 -
12315 -# -
12320 -pf00664pf00005pf00027pf03412 -
12325 -# -
12330 -pf00132pf00483pf02878pf02879pf02880 -
12335 -# -
12340 -pf03795 -
12345 -pf03483pf03484pf03147pf01588 -
12350 -pf00185pf02729 -
12355 -pf00571pf01769pf03448 -
BMC-P
BMC-H
BMC-T
Aldehyde dehydrogenase
PTAC
Signature enzyme
Alcohol dehydrogenase
Regulator

Halothece sp. (strain PCC 7418) (Synechococcus sp. (strain PCC 7418)). Bacteria; Cyanobacteria; Oscillatoriophycideae; Chroococcales;

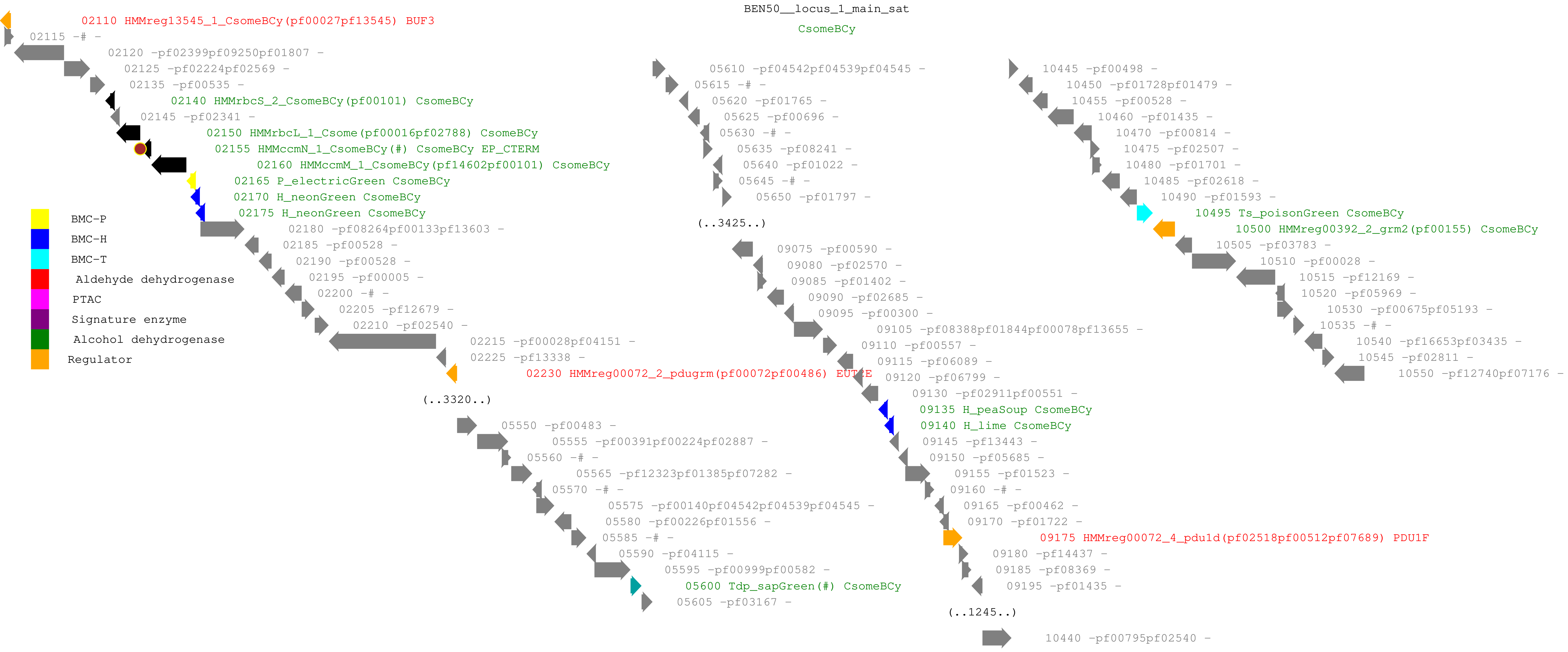

Bacillus subtilis BEST7613. Bacteria; Firmicutes; Bacilli; Bacillales; Bacillaceae; Bacillus.

BEST7613__locus_1_main_sat

CsomeBCy

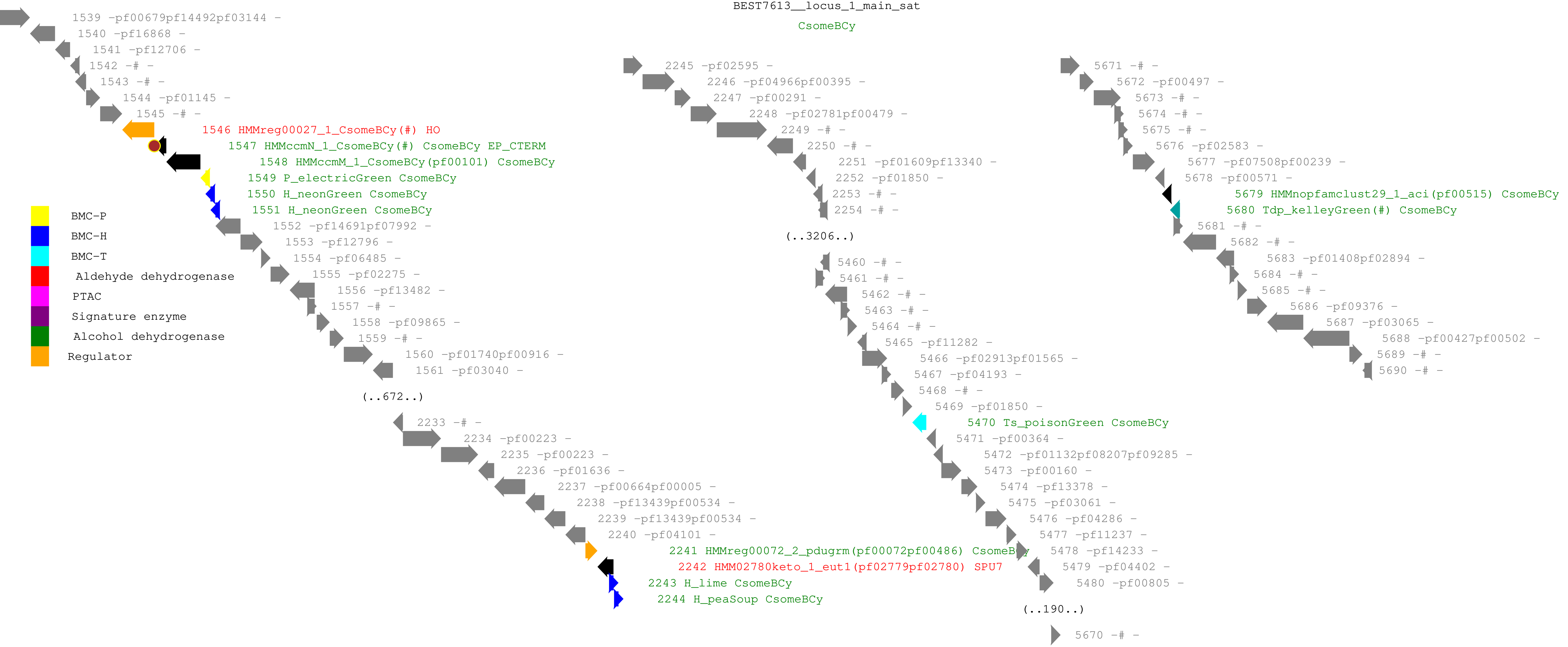

Microcystis aeruginosa NIES-98. Bacteria; Cyanobacteria; Oscillatoriophycideae; Chroococcales;
BFG60__locus_1_main_sat
CsomeBCy

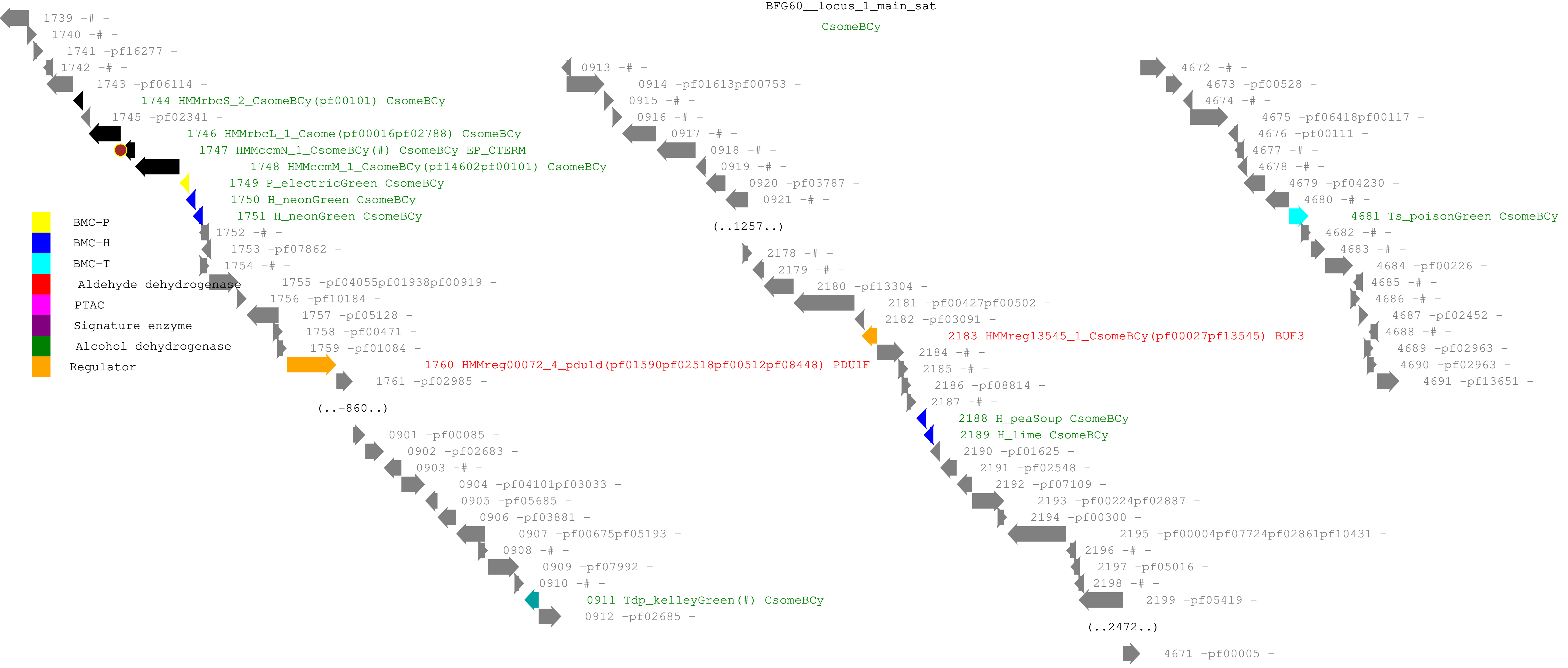

Microcystis aeruginosa PCC 7806SL. Bacteria; Cyanobacteria; Oscillatoriophycideae; Chroococcales;

BH695__locus_1_main_sat

CsomeBCy

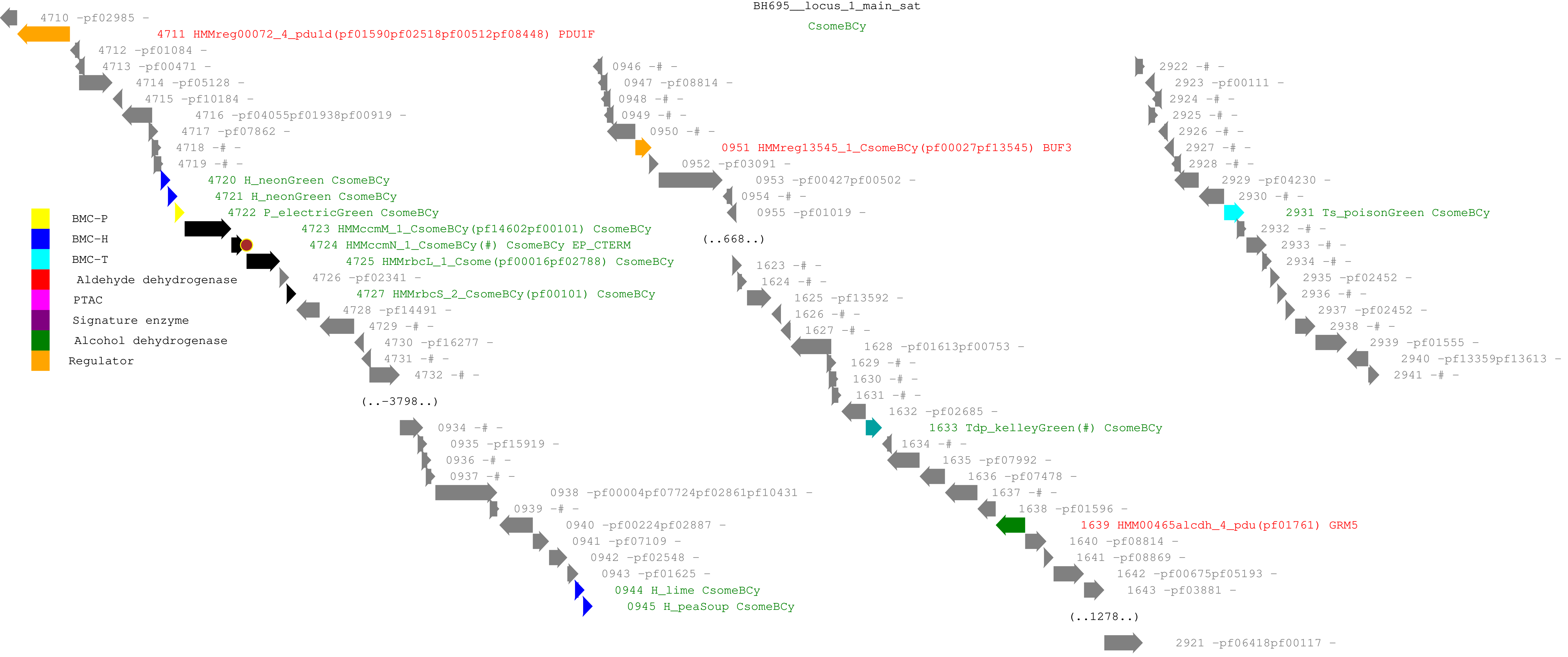

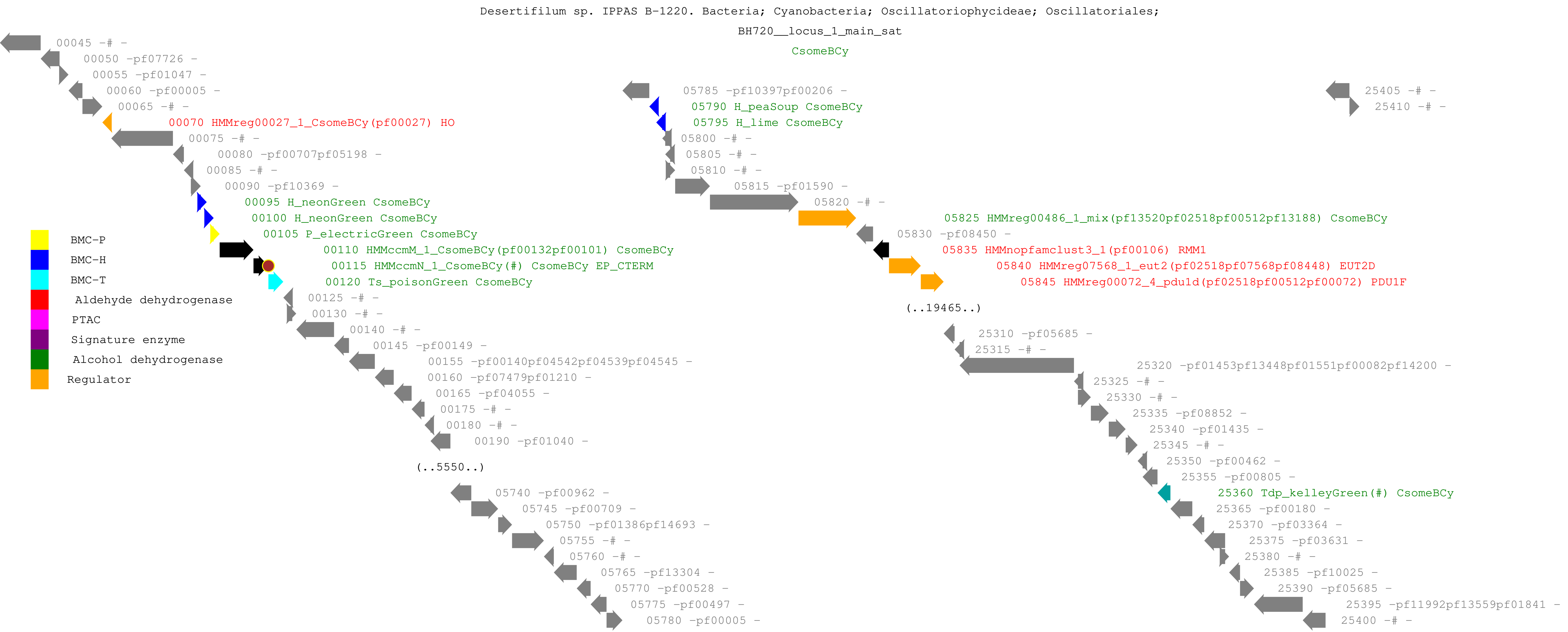
Desertifilum sp. IPPAS B-1220. Bacteria; Cyanobacteria; Oscillatoriophycideae; Oscillatoriales;
BH720__locus_1_main_sat
CsomeBCy
00045 -# -
00050 -pf07726 -
00055 -pf01047 -
00060 -pf00005 -
00065 -# -
00070 HMMreg00027_1_CsomeBCy(pf00027) HO
00075 -# -
00080 -pf00707pf05198 -
00085 -# -
00090 -pf10369 -
00095 H_neonGreen CsomeBCy
00100 H_neonGreen CsomeBCy
00105 P_electricGreen CsomeBCy
00110 HMMccmM_1_CsomeBCy(pf00132pf00101) CsomeBCy
00115 HMMccmN_1_CsomeBCy(#) CsomeBCy EP_CTERM
00120 Ts_poisonGreen CsomeBCy
00125 -# -
00130 -# -
00140 -# -
00145 -pf00149 -
00155 -pf00140pf04542pf04539pf04545 -
00160 -pf07479pf01210 -
00165 -pf04055 -
00175 -# -
00180 -# -
00190 -pf01040 -
(..5550..)
05740 -pf00962 -
05745 -pf00709 -
05750 -pf01386pf14693 -
05755 -# -
05760 -# -
05765 -pf13304 -
05770 -pf00528 -
05775 -pf00497 -
05780 -pf00005 -
05785 -pf10397pf00206 -
05790 H_peaSoup CsomeBCy
05795 H_lime CsomeBCy
05800 -# -
05805 -# -
05810 -# -
05815 -pf01590 -
05820 -# -
05825 HMMreg00486_1_mix(pf13520pf02518pf00512pf13188) CsomeBCy
05830 -pf08450 -
05835 HMMnopfamclust3_1(pf00106) RMM1
05840 HMMreg07568_1_eut2(pf02518pf07568pf08448) EUT2D
05845 HMMreg00072_4_pdu1d(pf02518pf00512pf00072) PDU1F
(..19465..)
25310 -pf05685 -
25315 -# -
25320 -pf01453pf13448pf01551pf00082pf14200 -
25325 -# -
25330 -# -
25335 -pf08852 -
25340 -pf01435 -
25345 -# -
25350 -pf00462 -
25355 -pf00805 -
25360 Tdp_kelleyGreen(#) CsomeBCy
25365 -pf00180 -
25370 -pf03364 -
25375 -pf03631 -
25380 -# -
25385 -pf10025 -
25390 -pf05685 -
25395 -pf11992pf13559pf01841 -
25400 -# -
25405 -# -
25410 -# -
BMC-P
BMC-H
BMC-T
Aldehyde dehydrogenase
PTAC
Signature enzyme
Alcohol dehydrogenase
Regulator

Roseofilum reptotaenium AO1-A. Bacteria; Cyanobacteria; Oscillatoriophycideae; Oscillatoriales;

BI308__locus_1_main_sat

CsomeBCy

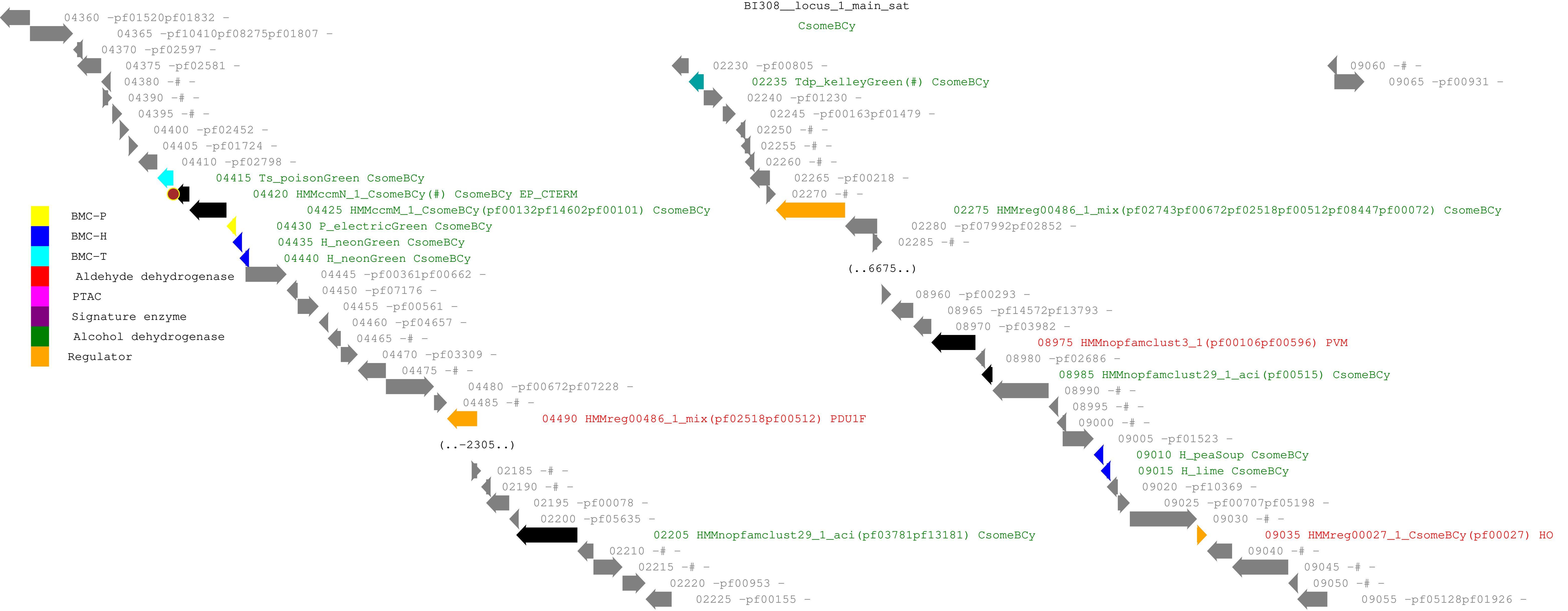

Limnothrix sp. CACIAM 69d. Bacteria; Cyanobacteria; Synechococcales; Pseudanabaenaceae; Limnothrix;

BJG00__locus_1_main_sat

CsomeBCy

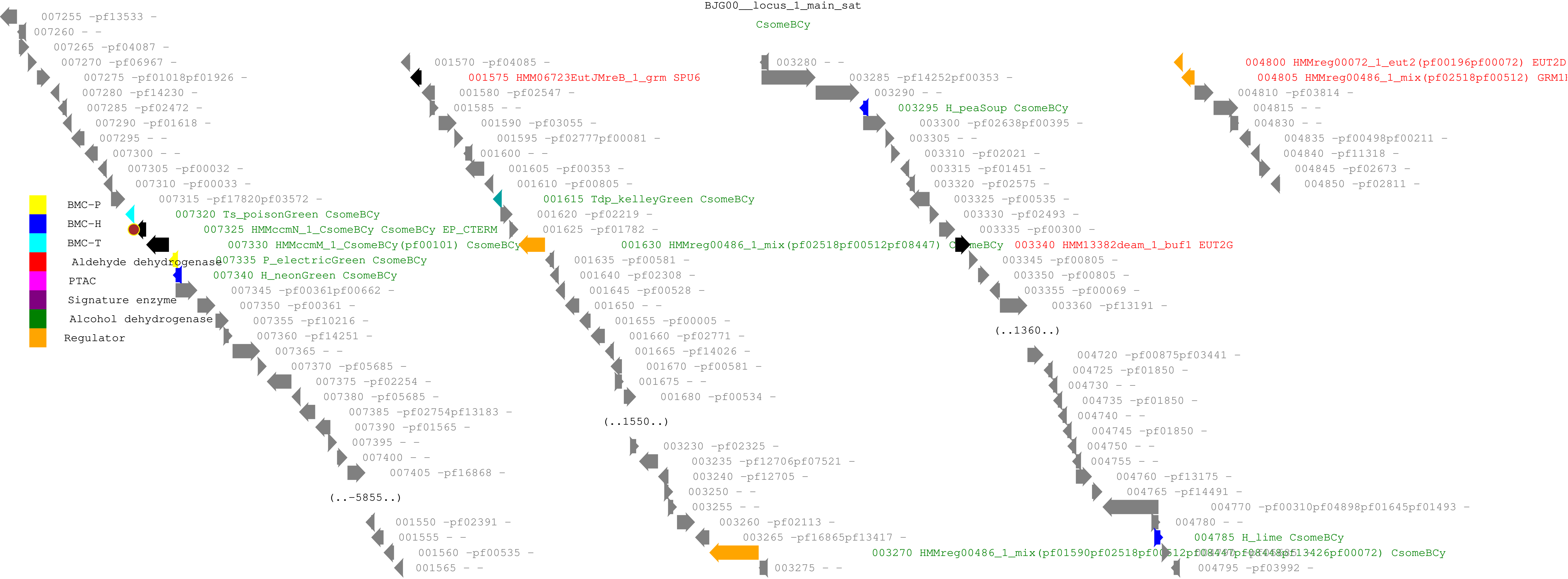

Moorea producens PAL-8-15-08-1. Bacteria; Cyanobacteria; Oscillatoriophycideae; Oscillatoriales;

BJP34__locus_1_main_sat

CsomeBCy

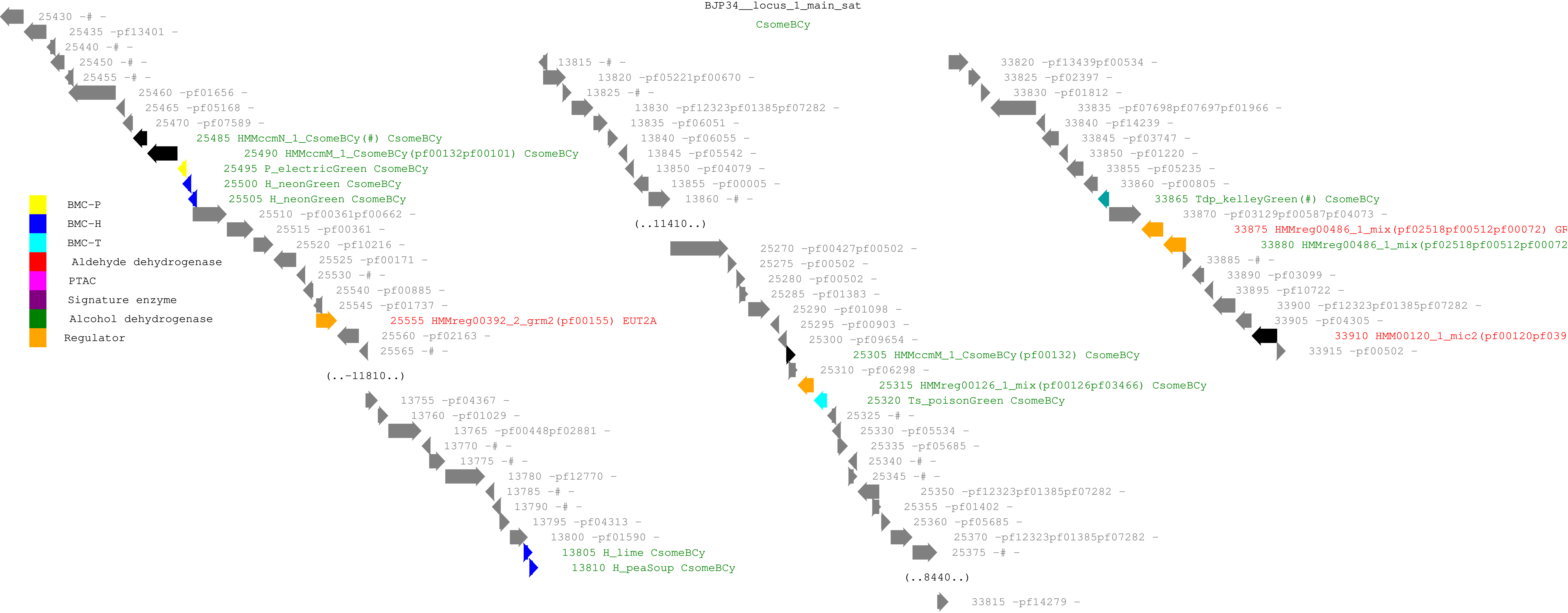

Moorea producens JHB. Bacteria; Cyanobacteria; Oscillatoriophycideae; Oscillatoriales;

BJP36__locus_1_main_sat

CsomeBCy

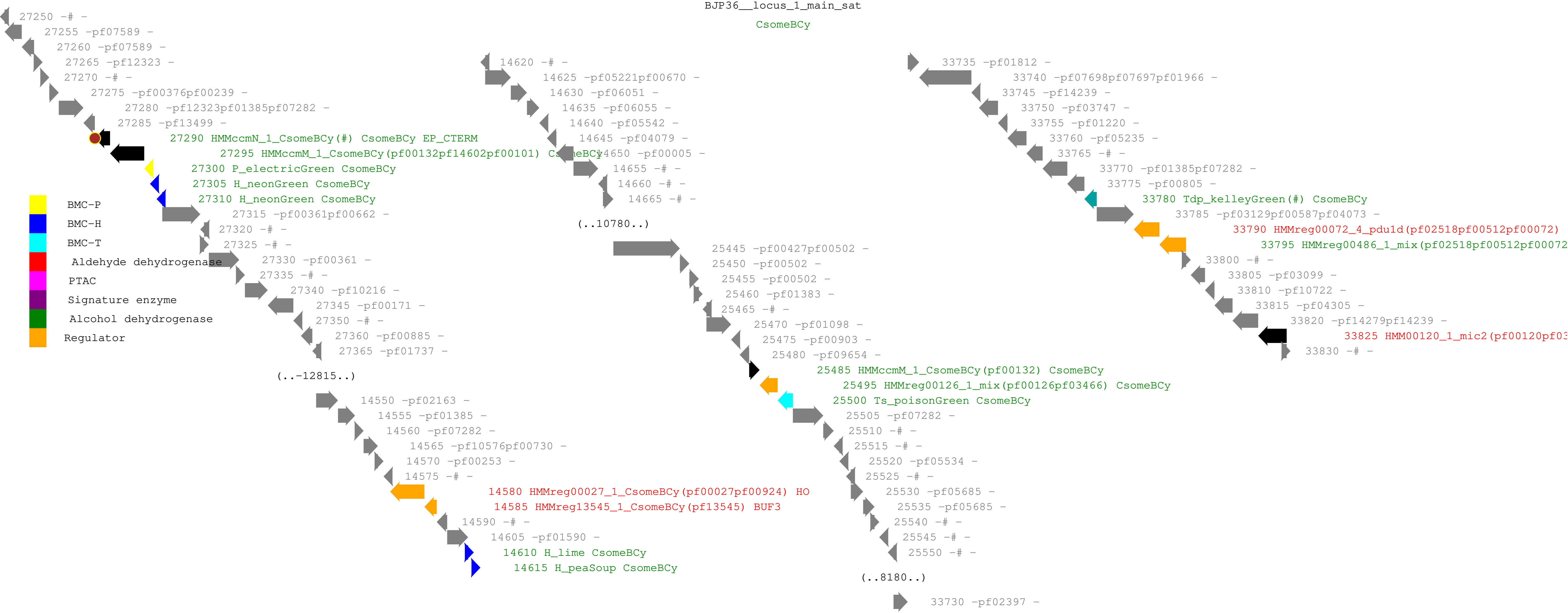

Moorea bouillonii PNG. Bacteria; Cyanobacteria; Oscillatoriophycideae; Oscillatoriales;

BJP37__locus_1_main_sat

CsomeBCy

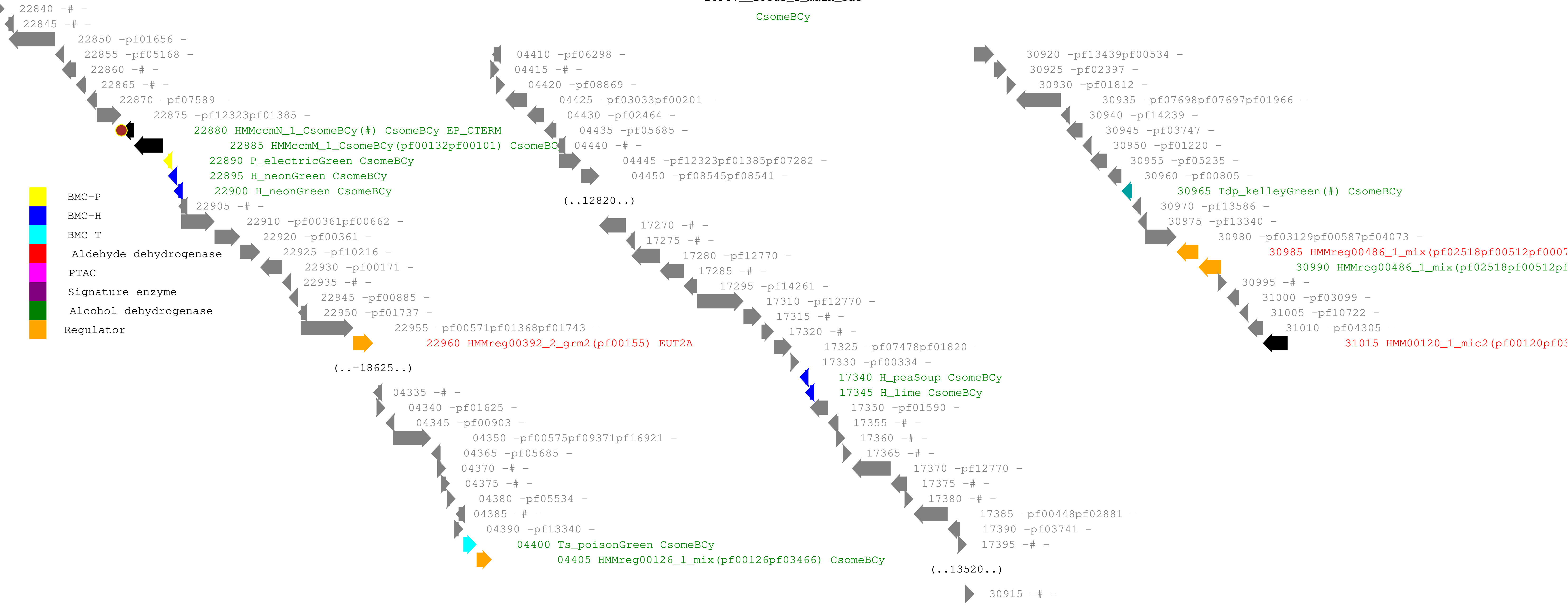

Mastigocladus laminosus UU774. Bacteria; Cyanobacteria; Nostocales; Hapalosiphonaceae; Mastigocladus.

BLD44__locus_1_main_sat

CsomeBCy

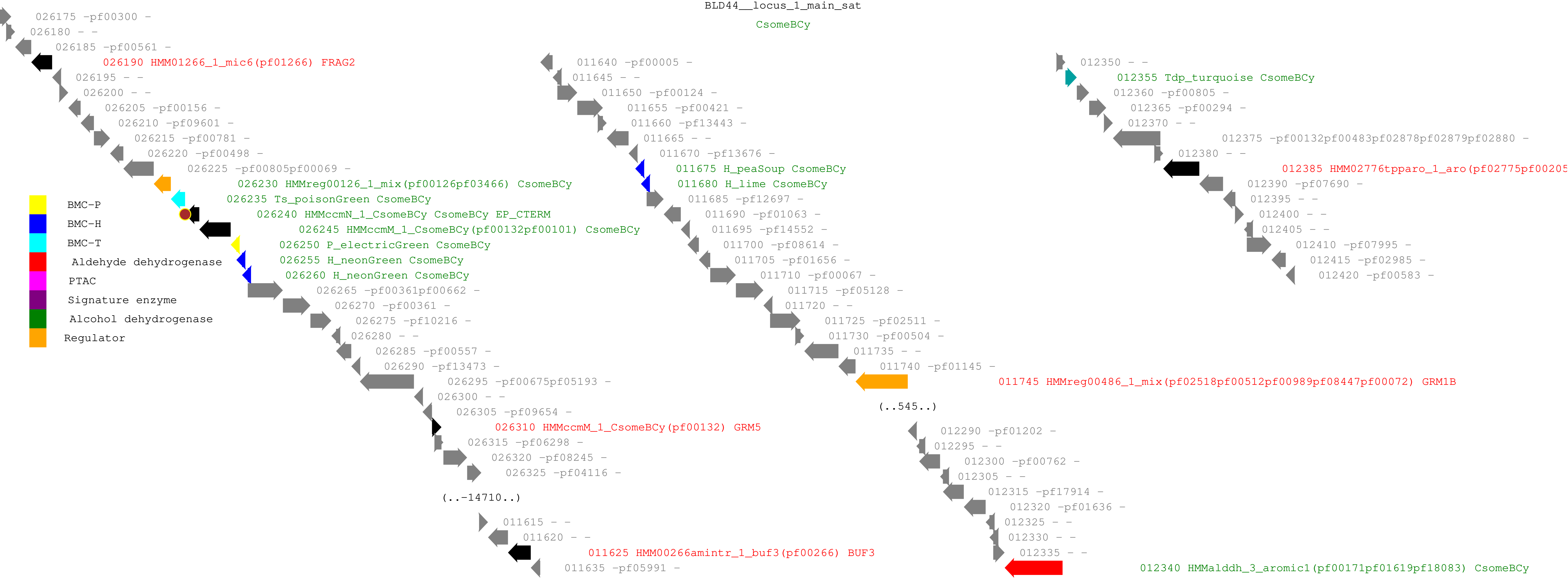

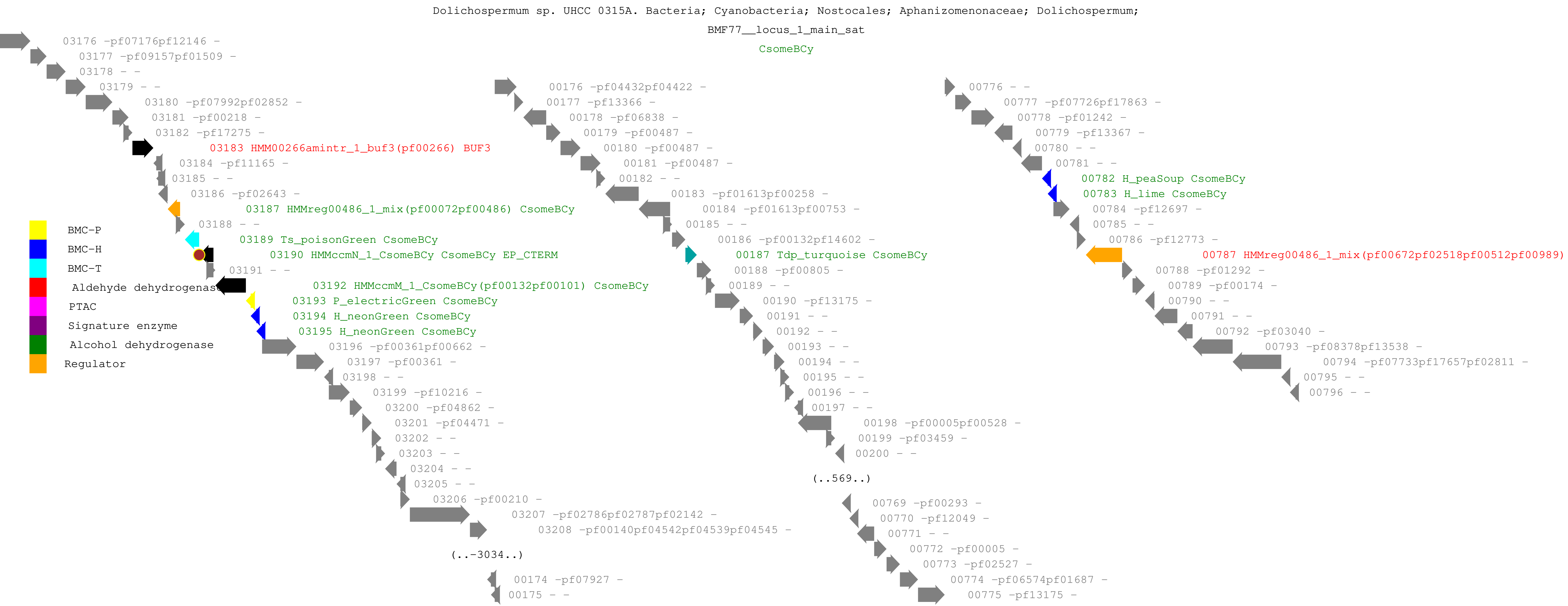
Dolichospermum sp. UHCC 0315A. Bacteria; Cyanobacteria; Nostocales; Aphanizomenonaceae; Dolichospermum;
BMF77__locus_1_main_sat
CsomeBCy
03176 -pf07176pf12146 -
03177 -pf09157pf01509 -
03178 - -
03179 - -
03180 -pf07992pf02852 -
03181 -pf00218 -
03182 -pf17275 -
03183 HMM00266amintr_1_buf3(pf00266) BUF3
03184 -pf11165 -
03185 - -
03186 -pf02643 -
03187 HMMreg00486_1_mix(pf00072pf00486) CsomeBCy
03188 - -
03189 Ts_poisonGreen CsomeBCy
03190 HMMccmN_1_CsomeBCy CsomeBCy EP_CTERM
03191 - -
03192 HMMccmM_1_CsomeBCy(pf00132pf00101) CsomeBCy
03193 P_electricGreen CsomeBCy
03194 H_neonGreen CsomeBCy
03195 H_neonGreen CsomeBCy
03196 -pf00361pf00662 -
03197 -pf00361 -
03198 - -
03199 -pf10216 -
03200 -pf04862 -
03201 -pf04471 -
03202 - -
03203 - -
03204 - -
03205 - -
03206 -pf00210 -
03207 -pf02786pf02787pf02142 -
03208 -pf00140pf04542pf04539pf04545 -
(..-3034..)
00174 -pf07927 -
00175 - -
00176 -pf04432pf04422 -
00177 -pf13366 -
00178 -pf06838 -
00179 -pf00487 -
00180 -pf00487 -
00181 -pf00487 -
00182 - -
00183 -pf01613pf00258 -
00184 -pf01613pf00753 -
00185 - -
00186 -pf00132pf14602 -
00187 Tdp_turquoise CsomeBCy
00188 -pf00805 -
00189 - -
00190 -pf13175 -
00191 - -
00192 - -
00193 - -
00194 - -
00195 - -
00196 - -
00197 - -
00198 -pf00005pf00528 -
00199 -pf03459 -
00200 - -
(..569..)
00769 -pf00293 -
00770 -pf12049 -
00771 - -
00772 -pf00005 -
00773 -pf02527 -
00774 -pf06574pf01687 -
00775 -pf13175 -
00776 - -
00777 -pf07726pf17863 -
00778 -pf01242 -
00779 -pf13367 -
00780 - -
00781 - -
00782 H_peaSoup CsomeBCy
00783 H_lime CsomeBCy
00784 -pf12697 -
00785 - -
00786 -pf12773 -
00787 HMMreg00486_1_mix(pf00672pf02518pf00512pf00989)
00788 -pf01292 -
00789 -pf00174 -
00790 - -
00791 - -
00792 -pf03040 -
00793 -pf08378pf13538 -
00794 -pf07733pf17657pf02811 -
00795 - -
00796 - -
BMC-P
BMC-H
BMC-T
Aldehyde dehydrogenase
PTAC
Signature enzyme
Alcohol dehydrogenase
Regulator

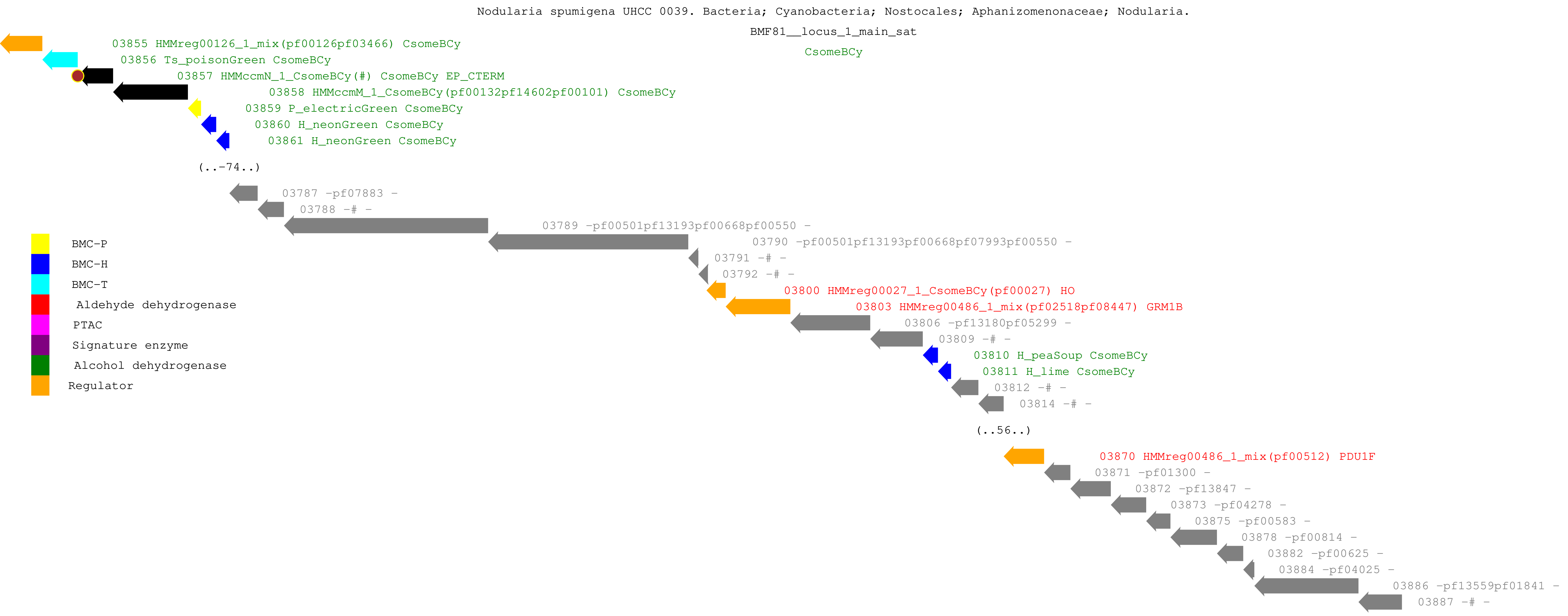
Nodularia spumigena UHCC 0039. Bacteria; Cyanobacteria; Nostocales; Aphanizomenonaceae; Nodularia.
BMF81__locus_1_main_sat
CsomeBCy
03855 HMMreg00126_1_mix(pf00126pf03466) CsomeBCy
03856 Ts_poisonGreen CsomeBCy
03857 HMMccmN_1_CsomeBCy(#) CsomeBCy EP_CTERM
03858 HMMccmM_1_CsomeBCy(pf00132pf14602pf00101) CsomeBCy
03859 P_electricGreen CsomeBCy
03860 H_neonGreen CsomeBCy
03861 H_neonGreen CsomeBCy
(..-74..)
03787 -pf07883 -
03788 -# -
03789 -pf00501pf13193pf00668pf00550 -
03790 -pf00501pf13193pf00668pf07993pf00550 -
03791 -# -
03792 -# -
03800 HMMreg00027_1_CsomeBCy(pf00027) HO
03803 HMMreg00486_1_mix(pf02518pf08447) GRM1B
03806 -pf13180pf05299 -
03809 -# -
03810 H_peaSoup CsomeBCy
03811 H_lime CsomeBCy
03812 -# -
03814 -# -
(..56..)
03870 HMMreg00486_1_mix(pf00512) PDU1F
03871 -pf01300 -
03872 -pf13847 -
03873 -pf04278 -
03875 -pf00583 -
03878 -pf00814 -
03882 -pf00625 -
03884 -pf04025 -
03886 -pf13559pf01841 -
03887 -# -
BMC-P
BMC-H
BMC-T
Aldehyde dehydrogenase
PTAC
Signature enzyme
Alcohol dehydrogenase
Regulator

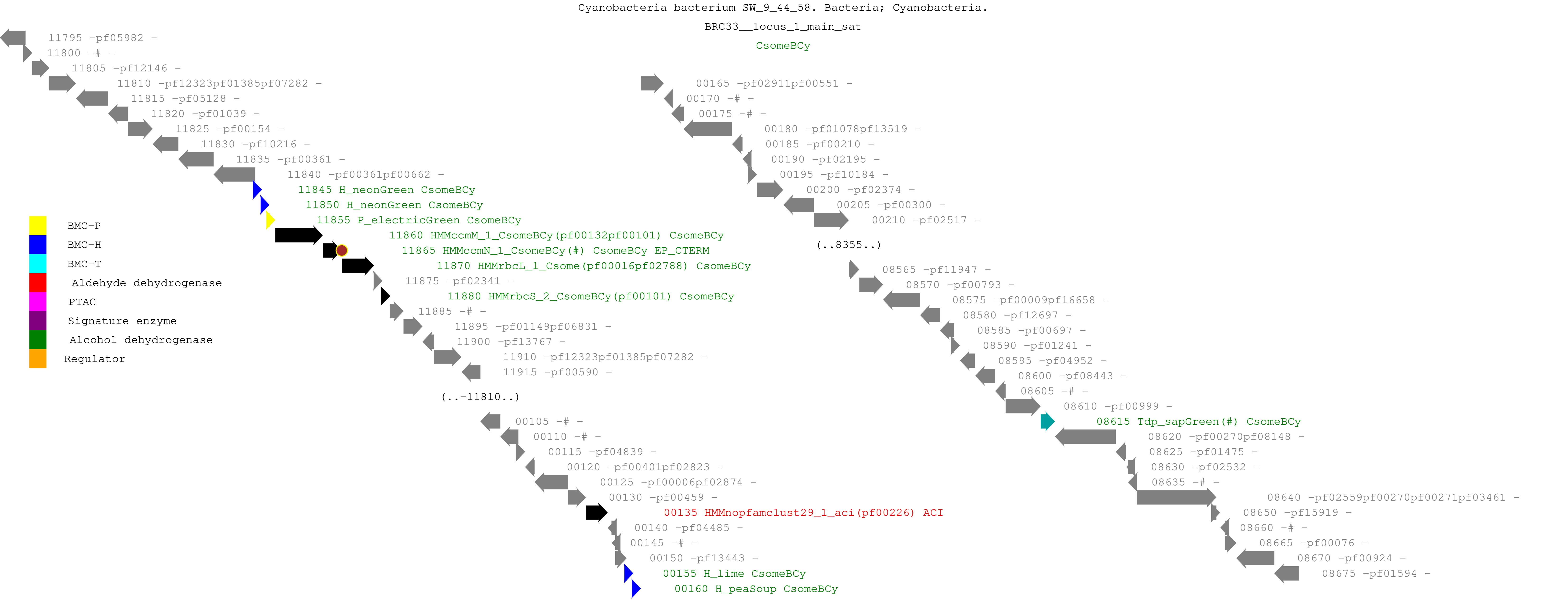
Cyanobacteria bacterium SW_9_44_58. Bacteria; Cyanobacteria.
BRC33__locus_1_main_sat
CsomeBCy
11795 -pf05982 -
11800 -# -
11805 -pf12146 -
11810 -pf12323pf01385pf07282 -
11815 -pf05128 -
11820 -pf01039 -
11825 -pf00154 -
11830 -pf10216 -
11835 -pf00361 -
11840 -pf00361pf00662 -
11845 H_neonGreen CsomeBCy
11850 H_neonGreen CsomeBCy
11855 P_electricGreen CsomeBCy
11860 HMMccmM_1_CsomeBCy(pf00132pf00101) CsomeBCy
11865 HMMccmN_1_CsomeBCy(#) CsomeBCy EP_CTERM
11870 HMMrbcL_1_Csome(pf00016pf02788) CsomeBCy
11875 -pf02341 -
11880 HMMrbcS_2_CsomeBCy(pf00101) CsomeBCy
11885 -# -
11895 -pf01149pf06831 -
11900 -pf13767 -
11910 -pf12323pf01385pf07282 -
11915 -pf00590 -
(..-11810..)
00105 -# -
00110 -# -
00115 -pf04839 -
00120 -pf00401pf02823 -
00125 -pf00006pf02874 -
00130 -pf00459 -
00135 HMMnopfamclust29_1_aci(pf00226) ACI
00140 -pf04485 -
00145 -# -
00150 -pf13443 -
00155 H_lime CsomeBCy
00160 H_peaSoup CsomeBCy
BMC-P
BMC-H
BMC-T
Aldehyde dehydrogenase
PTAC
Signature enzyme
Alcohol dehydrogenase
Regulator
00165 -pf02911pf00551 -
00170 -# -
00175 -# -
00180 -pf01078pf13519 -
00185 -pf00210 -
00190 -pf02195 -
00195 -pf10184 -
00200 -pf02374 -
00205 -pf00300 -
00210 -pf02517 -
(..8355..)
08565 -pf11947 -
08570 -pf00793 -
08575 -pf00009pf16658 -
08580 -pf12697 -
08585 -pf00697 -
08590 -pf01241 -
08595 -pf04952 -
08600 -pf08443 -
08605 -# -
08610 -pf00999 -
08615 Tdp_sapGreen(#) CsomeBCy
08620 -pf00270pf08148 -
08625 -pf01475 -
08630 -pf02532 -
08635 -# -
08640 -pf02559pf00270pf00271pf03461 -
08650 -pf15919 -
08660 -# -
08665 -pf00076 -
08670 -pf00924 -
08675 -pf01594 -

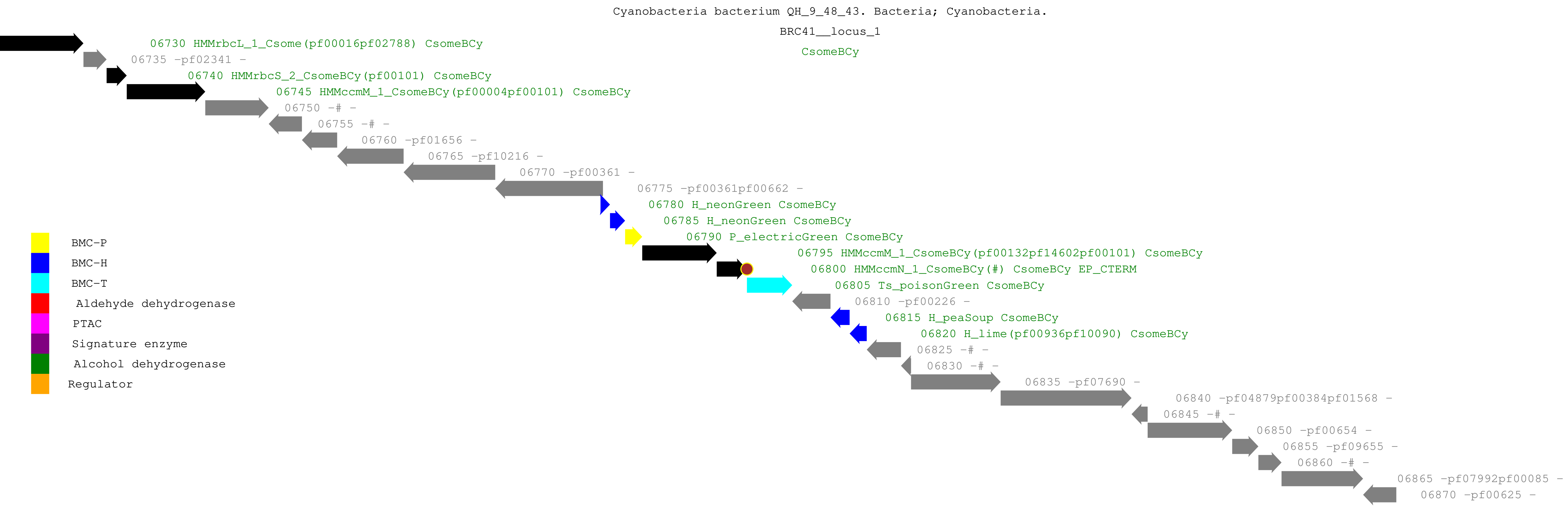
Cyanobacteria bacterium QH_9_48_43. Bacteria; Cyanobacteria.
BRC41__locus_1
CsomeBCy
06730 HMMrbcL_1_Csome(pf00016pf02788) CsomeBCy
06735 -pf02341 -
06740 HMMrbcS_2_CsomeBCy(pf00101) CsomeBCy
06745 HMMccmM_1_CsomeBCy(pf00004pf00101) CsomeBCy
06750 -# -
06755 -# -
06760 -pf01656 -
06765 -pf10216 -
06770 -pf00361 -
06775 -pf00361pf00662 -
06780 H_neonGreen CsomeBCy
06785 H_neonGreen CsomeBCy
06790 P_electricGreen CsomeBCy
06795 HMMccmM_1_CsomeBCy(pf00132pf14602pf00101) CsomeBCy
06800 HMMccmN_1_CsomeBCy(#) CsomeBCy EP_CTERM
06805 Ts_poisonGreen CsomeBCy
06810 -pf00226 -
06815 H_peaSoup CsomeBCy
06820 H_lime(pf00936pf10090) CsomeBCy
06825 -# -
06830 -# -
06835 -pf07690 -
06840 -pf04879pf00384pf01568 -
06845 -# -
06850 -pf00654 -
06855 -pf09655 -
06860 -# -
06865 -pf07992pf00085 -
06870 -pf00625 -
BMC-P
BMC-H
BMC-T
Aldehyde dehydrogenase
PTAC
Signature enzyme
Alcohol dehydrogenase
Regulator

Synechococcus lividus PCC 6715. Bacteria; Cyanobacteria; Synechococcales; Synechococcaceae;

BRW62__locus_1_main_sat

CsomeBCy

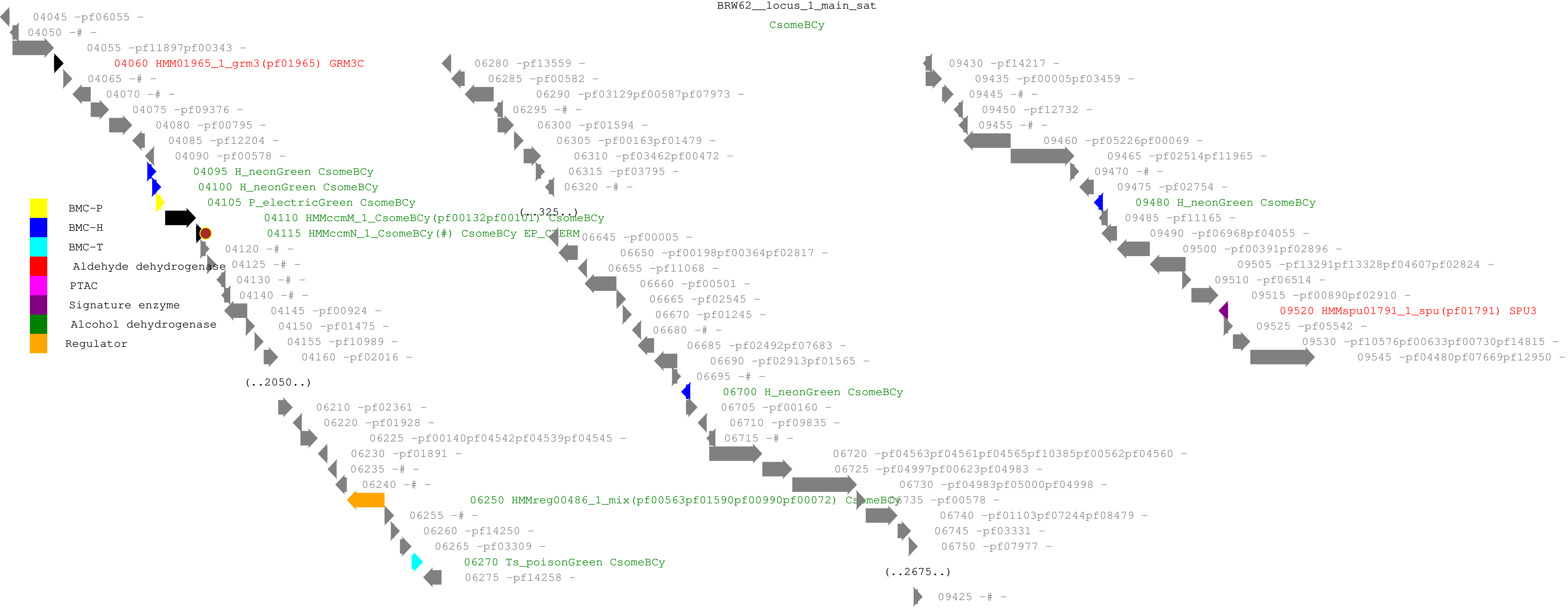

Oscillatoriales cyanobacterium 'hensonii'. Bacteria; Cyanobacteria; Oscillatoriophycideae; Oscillatoriales;

BST81__locus_1_main_sat

CsomeBCy

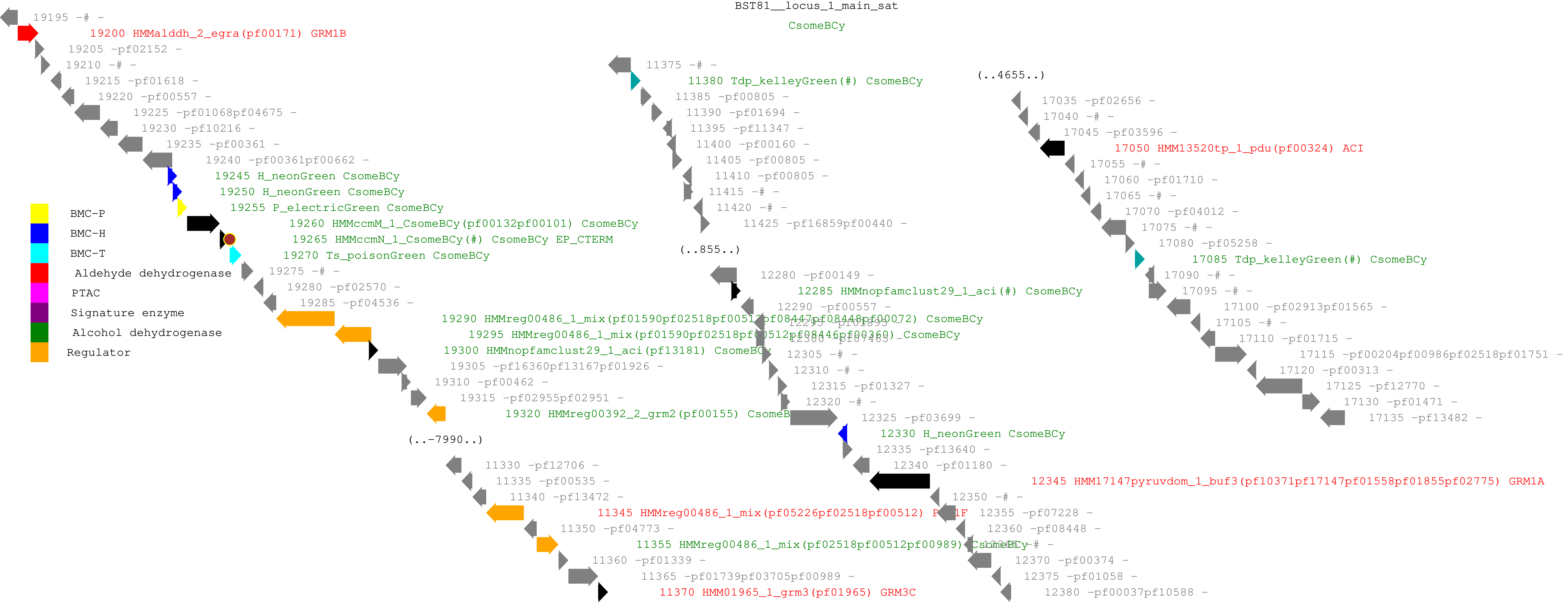

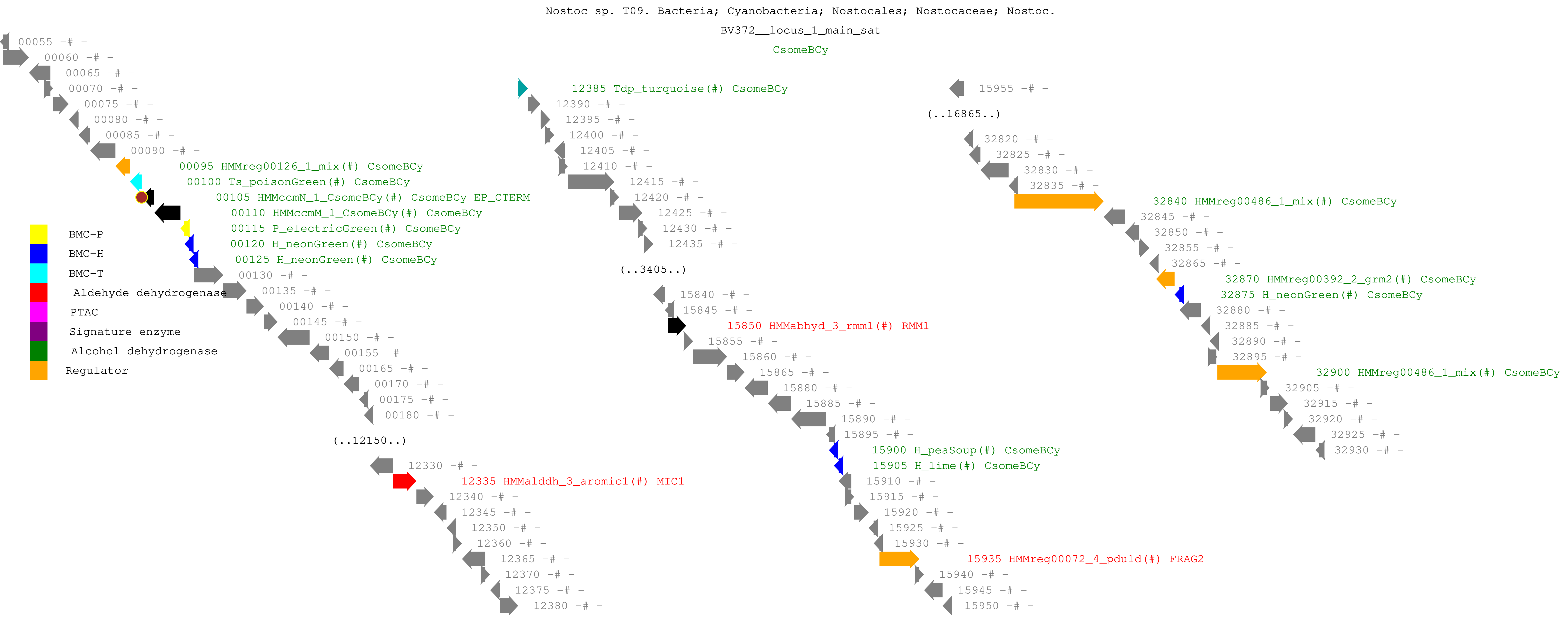

Nostoc sp. T09. Bacteria; Cyanobacteria; Nostocales; Nostocaceae; Nostoc.
BV372__locus_1_main_sat
CsomeBCy
00055 -# -
00060 -# -
00065 -# -
00070 -# -
00075 -# -
00080 -# -
00085 -# -
00090 -# -
00095 HMMreg00126_1_mix(#) CsomeBCy
00100 Ts_poisonGreen(#) CsomeBCy
00105 HMMccmN_1_CsomeBCy(#) CsomeBCy EP_CTERM
00110 HMMccmM_1_CsomeBCy(#) CsomeBCy
00115 P_electricGreen(#) CsomeBCy
00120 H_neonGreen(#) CsomeBCy
00125 H_neonGreen(#) CsomeBCy
00130 -# -
00135 -# -
00140 -# -
00145 -# -
00150 -# -
00155 -# -
00165 -# -
00170 -# -
00175 -# -
00180 -# -
(..12150..)
12330 -# -
12335 HMMaldh_3_aromic1(#) MIC1
12340 -# -
12345 -# -
12350 -# -
12360 -# -
12365 -# -
12370 -# -
12375 -# -
12380 -# -
BMC-P
BMC-H
BMC-T
Aldehyde dehydrogenase
PTAC
Signature enzyme
Alcohol dehydrogenase
Regulator
12385 Tdp_turquoise(#) CsomeBCy
12390 -# -
12395 -# -
12400 -# -
12405 -# -
12410 -# -
12415 -# -
12420 -# -
12425 -# -
12430 -# -
12435 -# -
(..3405..)
15840 -# -
15845 -# -
15850 HMMabhyd_3_rmm1(#) RMM1
15855 -# -
15860 -# -
15865 -# -
15880 -# -
15885 -# -
15890 -# -
15895 -# -
15900 H_peaSoup(#) CsomeBCy
15905 H_lime(#) CsomeBCy
15910 -# -
15915 -# -
15920 -# -
15925 -# -
15930 -# -
15935 HMMreg00072_4_pdu1d(#) FRAG2
15940 -# -
15945 -# -
15950 -# -
15955 -# -
(..16865..)
32820 -# -
32825 -# -
32830 -# -
32835 -# -
32840 HMMreg00486_1_mix(#) CsomeBCy
32845 -# -
32850 -# -
32855 -# -
32865 -# -
32870 HMMreg00392_2_grm2(#) CsomeBCy
32875 H_neonGreen(#) CsomeBCy
32880 -# -
32885 -# -
32890 -# -
32895 -# -
32900 HMMreg00486_1_mix(#) CsomeBCy
32905 -# -
32915 -# -
32920 -# -
32925 -# -
32930 -# -

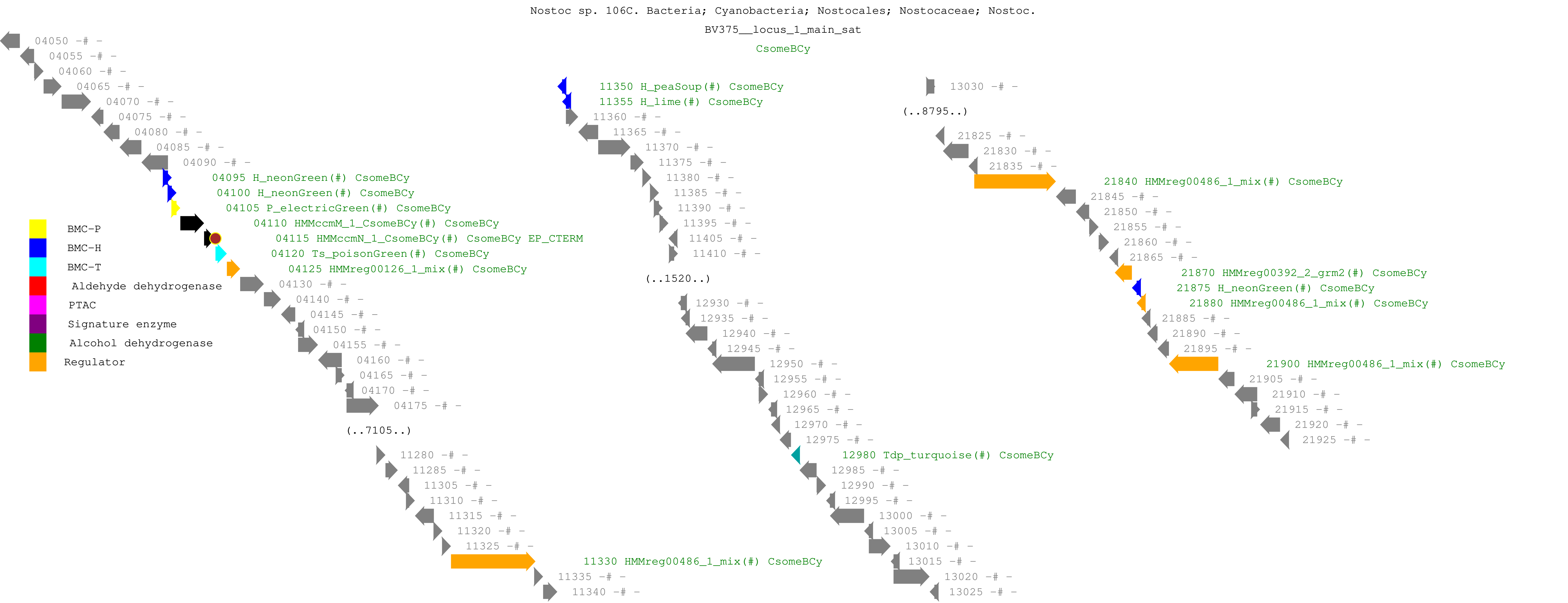

Nostoc sp. 106C. Bacteria; Cyanobacteria; Nostocales; Nostocaceae; Nostoc.
BV375__locus_1_main_sat
CsomeBCy
04050 -# -
04055 -# -
04060 -# -
04065 -# -
04070 -# -
04075 -# -
04080 -# -
04085 -# -
04090 -# -
04095 H_neonGreen(#) CsomeBCy
04100 H_neonGreen(#) CsomeBCy
04105 P_electricGreen(#) CsomeBCy
04110 HMMccmM_1_CsomeBCy(#) CsomeBCy
04115 HMMccmN_1_CsomeBCy(#) CsomeBCy EP_CTERM
04120 Ts_poisonGreen(#) CsomeBCy
04125 HMMreg00126_1_mix(#) CsomeBCy
04130 -# -
04140 -# -
04145 -# -
04150 -# -
04155 -# -
04160 -# -
04165 -# -
04170 -# -
04175 -# -
(..7105..)
11280 -# -
11285 -# -
11305 -# -
11310 -# -
11315 -# -
11320 -# -
11325 -# -
11330 HMMreg00486_1_mix(#) CsomeBCy
11335 -# -
11340 -# -
11350 H_peaSoup(#) CsomeBCy
11355 H_lime(#) CsomeBCy
11360 -# -
11365 -# -
11370 -# -
11375 -# -
11380 -# -
11385 -# -
11390 -# -
11395 -# -
11405 -# -
11410 -# -
(..1520..)
12930 -# -
12935 -# -
12940 -# -
12945 -# -
12950 -# -
12955 -# -
12960 -# -
12965 -# -
12970 -# -
12975 -# -
12980 Tdp_turquoise(#) CsomeBCy
12985 -# -
12990 -# -
12995 -# -
13000 -# -
13005 -# -
13010 -# -
13015 -# -
13020 -# -
13025 -# -
13030 -# -
(..8795..)
21825 -# -
21830 -# -
21835 -# -
21840 HMMreg00486_1_mix(#) CsomeBCy
21845 -# -
21850 -# -
21855 -# -
21860 -# -
21865 -# -
21870 HMMreg00392_2_grm2(#) CsomeBCy
21875 H_neonGreen(#) CsomeBCy
21880 HMMreg00486_1_mix(#) CsomeBCy
21885 -# -
21890 -# -
21895 -# -
21900 HMMreg00486_1_mix(#) CsomeBCy
21905 -# -
21910 -# -
21915 -# -
21920 -# -
21925 -# -
BMC-P
BMC-H
BMC-T
Aldehyde dehydrogenase
PTAC
Signature enzyme
Alcohol dehydrogenase
Regulator

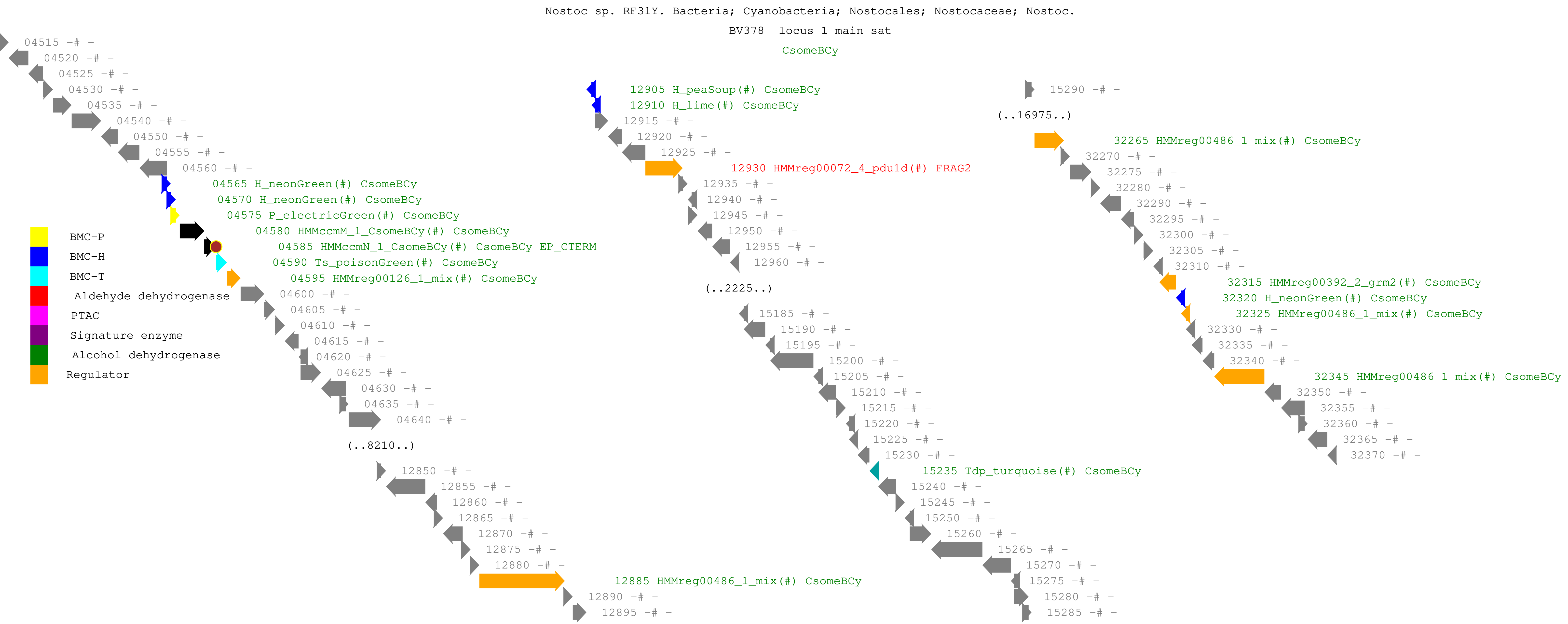
Nostoc sp. RF31Y. Bacteria; Cyanobacteria; Nostocales; Nostocaceae; Nostoc.
BV378__locus_1_main_sat
CsomeBCy
04515 -# -
04520 -# -
04525 -# -
04530 -# -
04535 -# -
04540 -# -
04550 -# -
04555 -# -
04560 -# -
04565 H_neonGreen(#) CsomeBCy
04570 H_neonGreen(#) CsomeBCy
04575 P_electricGreen(#) CsomeBCy
04580 HMMccmM_1_CsomeBCy(#) CsomeBCy
04585 HMMccmN_1_CsomeBCy(#) CsomeBCy EP_CTERM
04590 Ts_poisonGreen(#) CsomeBCy
04595 HMMreg00126_1_mix(#) CsomeBCy
04600 -# -
04605 -# -
04610 -# -
04615 -# -
04620 -# -
04625 -# -
04630 -# -
04635 -# -
04640 -# -
(..8210..)
12850 -# -
12855 -# -
12860 -# -
12865 -# -
12870 -# -
12875 -# -
12880 -# -
12885 HMMreg00486_1_mix(#) CsomeBCy
12890 -# -
12895 -# -
BMC-P
BMC-H
BMC-T
Aldehyde dehydrogenase
PTAC
Signature enzyme
Alcohol dehydrogenase
Regulator
12905 H_peaSoup(#) CsomeBCy
12910 H_lime(#) CsomeBCy
12915 -# -
12920 -# -
12925 -# -
12930 HMMreg00072_4_pdu1d(#) FRAG2
12935 -# -
12940 -# -
12945 -# -
12950 -# -
12955 -# -
12960 -# -
(..2225..)
15185 -# -
15190 -# -
15195 -# -
15200 -# -
15205 -# -
15210 -# -
15215 -# -
15220 -# -
15225 -# -
15230 -# -
15235 Tdp_turquoise(#) CsomeBCy
15240 -# -
15245 -# -
15250 -# -
15260 -# -
15265 -# -
15270 -# -
15275 -# -
15280 -# -
15285 -# -
15290 -# -
(..16975..)
32265 HMMreg00486_1_mix(#) CsomeBCy
32270 -# -
32275 -# -
32280 -# -
32290 -# -
32295 -# -
32300 -# -
32305 -# -
32310 -# -
32315 HMMreg00392_2_grm2(#) CsomeBCy
32320 H_neonGreen(#) CsomeBCy
32325 HMMreg00486_1_mix(#) CsomeBCy
32330 -# -
32335 -# -
32340 -# -
32345 HMMreg00486_1_mix(#) CsomeBCy
32350 -# -
32355 -# -
32360 -# -
32365 -# -
32370 -# -

Nostocales cyanobacterium HT-58-2. Bacteria; Cyanobacteria; Nostocales.

BZZ01__locus_1_main_sat

CsomeBCy

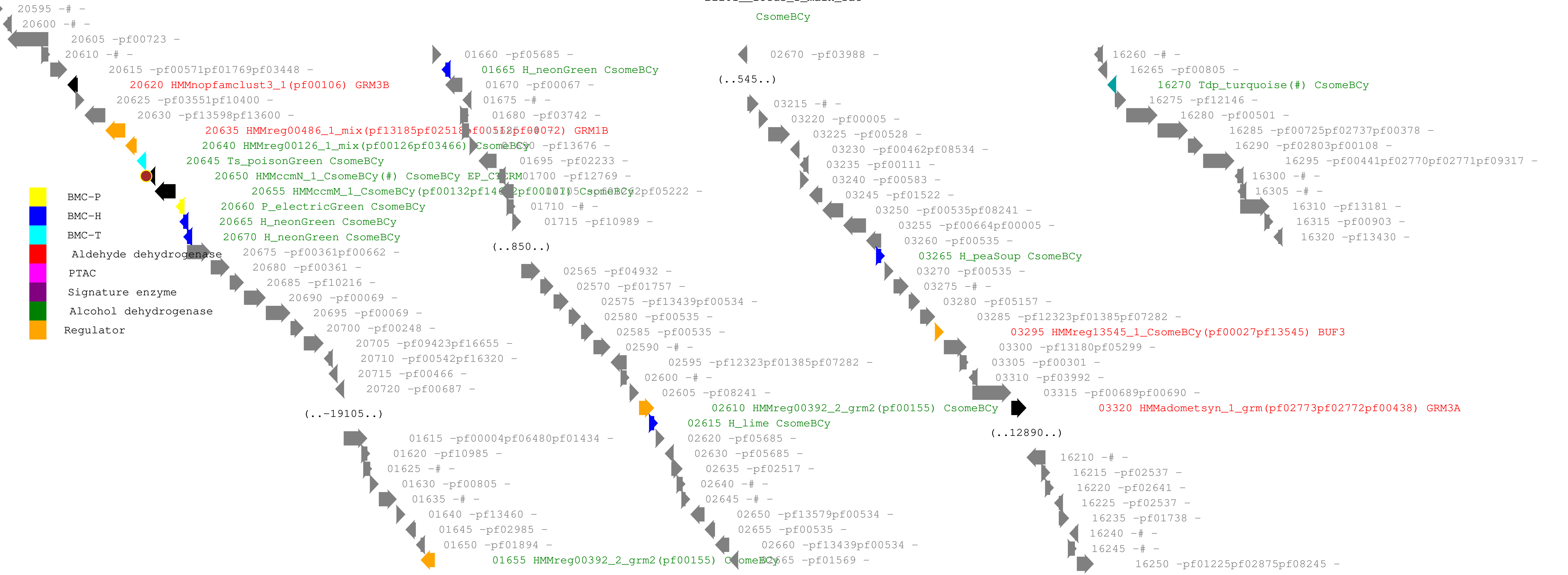

Acaryochloris sp. RCC1774. Bacteria; Cyanobacteria; Synechococcales; Acaryochloridaceae;

C1752__locus_1_main_sat

CsomeBCy

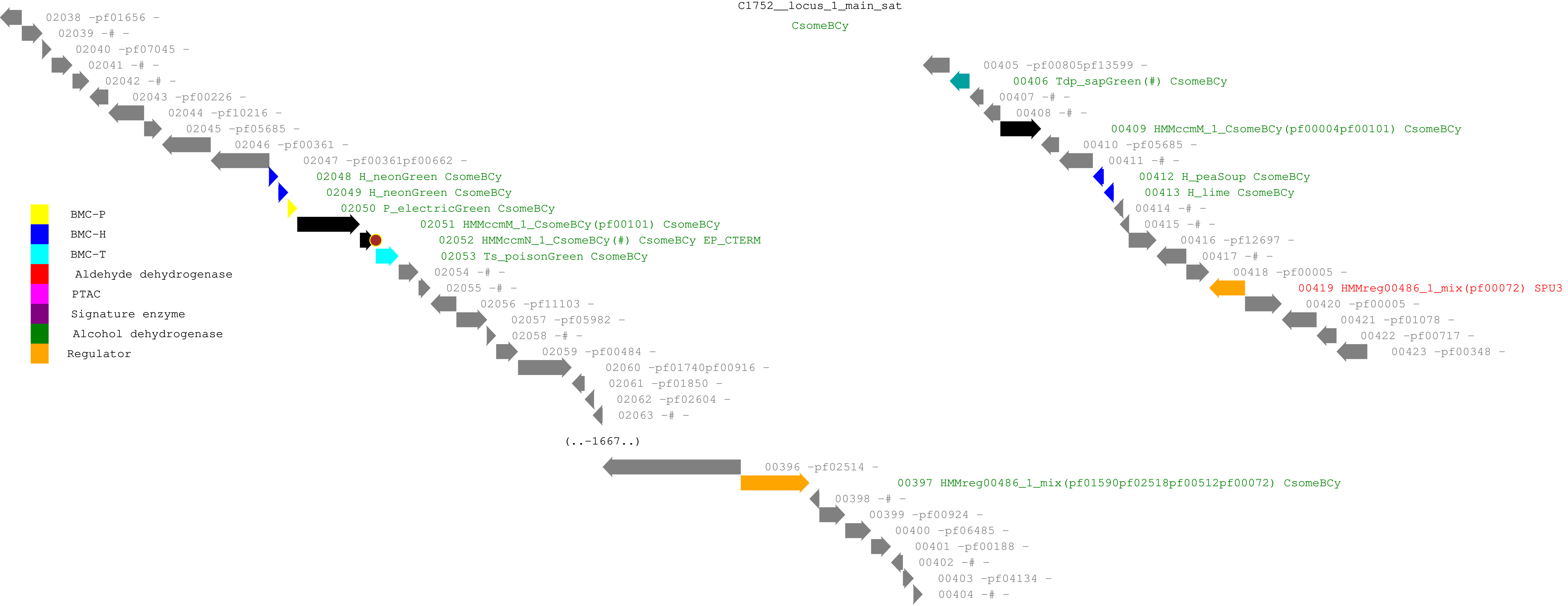

Hapalosiphonaceae cyanobacterium JJU2. Bacteria; Cyanobacteria; Nostocales; Hapalosiphonaceae.

C6Y22__locus_1_main_sat

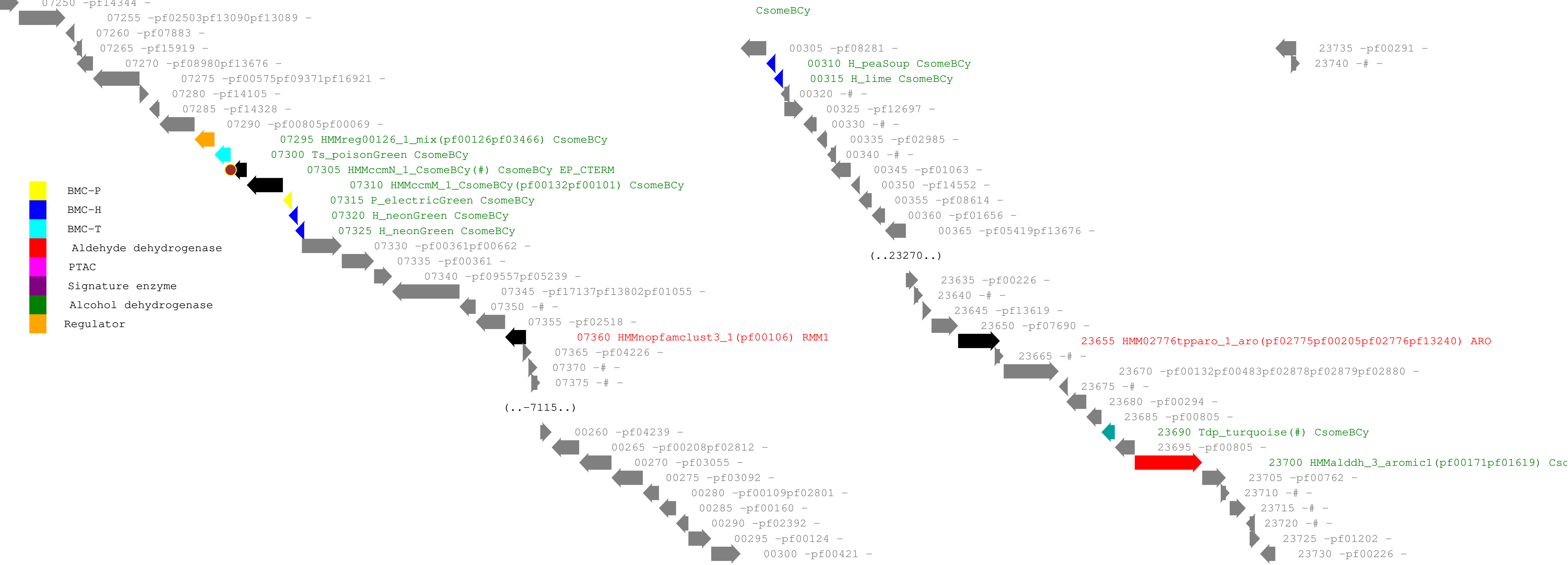

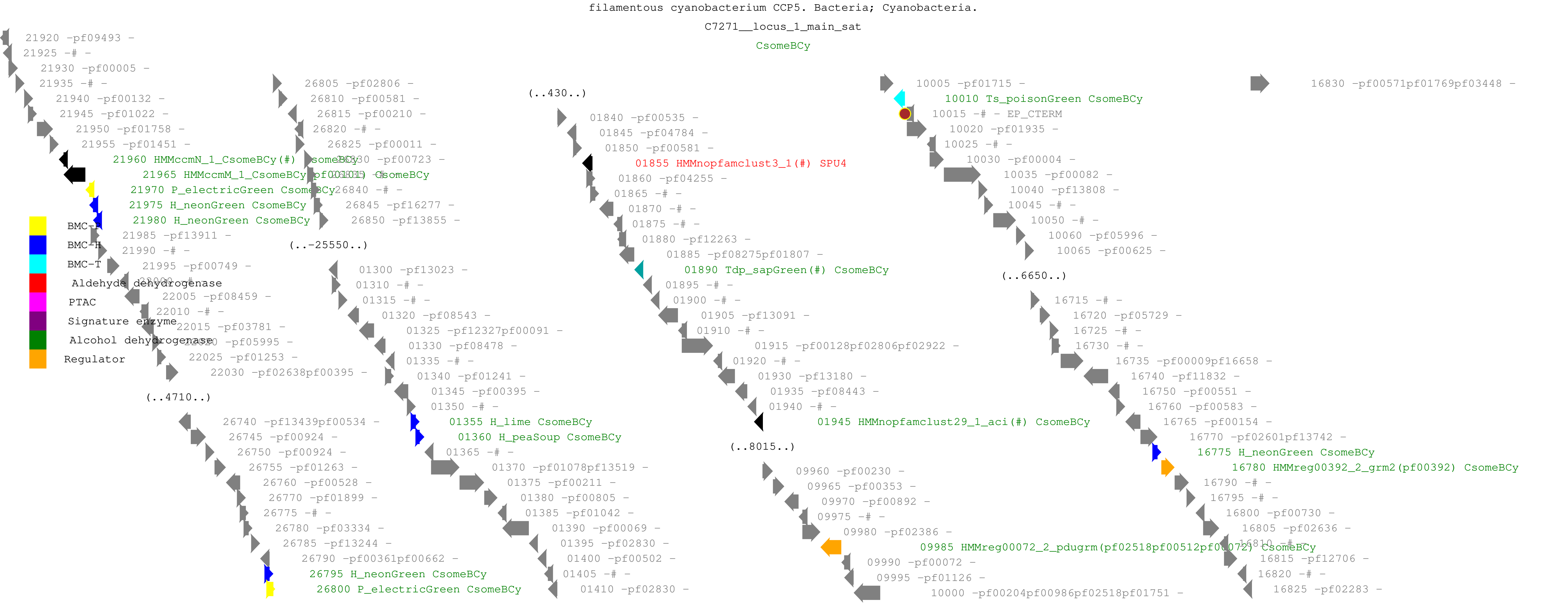

filamentous cyanobacterium CCP5. Bacteria; Cyanobacteria.
C7271__locus_1_main_sat
CsomeBCy
BMC-P
BMC-H
BMC-T
Aldehyde dehydrogenase
PTAC
Signature enzyme
Alcohol dehydrogenase
Regulator
21920 -pf09493 -
21925 -# -
21930 -pf00005 -
21935 -# -
21940 -pf00132 -
21945 -pf01022 -
21950 -pf01758 -
21955 -pf01451 -
21960 HMMccmN_1_CsomeBCy(#) CsomeBCy
21965 HMMccmM_1_CsomeBCy(pf03319) CsomeBCy
21970 P_electricGreen CsomeBCy
21975 H_neonGreen CsomeBCy
21980 H_neonGreen CsomeBCy
21985 -pf13911 -
21990 -# -
21995 -pf00749 -
22000 -# -
22005 -pf08459 -
22010 -# -
22015 -pf03781 -
22020 -pf05995 -
22025 -pf01253 -
22030 -pf02638pf00395 -
(..4710..)
26740 -pf13439pf00534 -
26745 -pf00924 -
26750 -pf00924 -
26755 -pf01263 -
26760 -pf00528 -
26770 -pf01899 -
26775 -# -
26780 -pf03334 -
26785 -pf13244 -
26790 -pf00361pf00662 -
26795 H_neonGreen CsomeBCy
26800 P_electricGreen CsomeBCy
26805 -pf02806 -
26810 -pf00581 -
26815 -pf00210 -
26820 -# -
26825 -pf00011 -
26830 -pf00723 -
26835 -# -
26840 -# -
26845 -pf16277 -
26850 -pf13855 -
(..-25550..)
01300 -pf13023 -
01310 -# -
01315 -# -
01320 -pf08543 -
01325 -pf12327pf00091 -
01330 -pf08478 -
01335 -# -
01340 -pf01241 -
01345 -pf00395 -
01350 -# -
01355 H_lime CsomeBCy
01360 H_peaSoup CsomeBCy
01365 -# -
01370 -pf01078pf13519 -
01375 -pf00211 -
01380 -pf00805 -
01385 -pf01042 -
01390 -pf00069 -
01395 -pf02830 -
01400 -pf00502 -
01405 -# -
01410 -pf02830 -
(..430..)
01840 -pf00535 -
01845 -pf04784 -
01850 -pf00581 -
01855 HMMnopfamclust3_1(#) SPU4
01860 -pf04255 -
01865 -# -
01870 -# -
01875 -# -
01880 -pf12263 -
01885 -pf08275pf01807 -
01890 Tdp_sapGreen(#) CsomeBCy
01895 -# -
01900 -# -
01905 -pf13091 -
01910 -# -
01915 -pf00128pf02806pf02922 -
01920 -# -
01930 -pf13180 -
01935 -pf08443 -
01940 -# -
01945 HMMnopfamclust29_1_aci(#) CsomeBCy
(..8015..)
09960 -pf00230 -
09965 -pf00353 -
09970 -pf00892 -
09975 -# -
09980 -pf02386 -
09985 HMMreg00072_2_pdugrm(pf02518pf00512pf00072) CsomeBCy
09990 -pf00072 -
09995 -pf01126 -
10000 -pf00204pf00986pf02518pf01751 -
10005 -pf01715 -
10010 Ts_poisonGreen CsomeBCy
10015 -# - EP_CTERM
10020 -pf01935 -
10025 -# -
10030 -pf00004 -
10035 -pf00082 -
10040 -pf13808 -
10045 -# -
10050 -# -
10060 -pf05996 -
10065 -pf00625 -
(..6650..)
16715 -# -
16720 -pf05729 -
16725 -# -
16730 -# -
16735 -pf00009pf16658 -
16740 -pf11832 -
16750 -pf00551 -
16760 -pf00583 -
16765 -pf00154 -
16770 -pf02601pf13742 -
16775 H_neonGreen CsomeBCy
16780 HMMreg00392_2_grm2(pf00392) CsomeBCy
16790 -# -
16795 -# -
16800 -pf00730 -
16805 -pf02636 -
16810 -# -
16815 -pf12706 -
16820 -# -
16825 -pf02283 -
16830 -pf00571pf01769pf03448 -

filamentous cyanobacterium CCT1. Bacteria; Cyanobacteria.

C7293__locus_1_main_sat

CsomeBCy

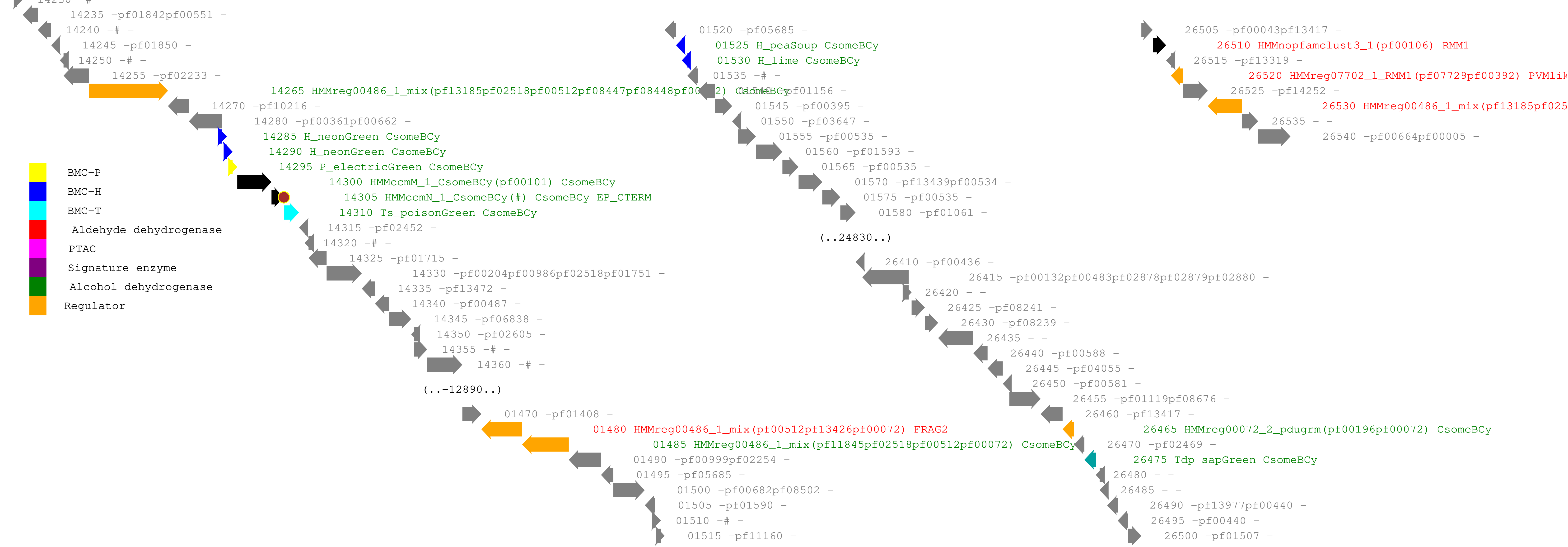

Microcystis aeruginosa DIANCHI905. Bacteria; Cyanobacteria; Oscillatoriophycideae; Chroococcales;

C789__locus_1_main_sat

CsomeBCy

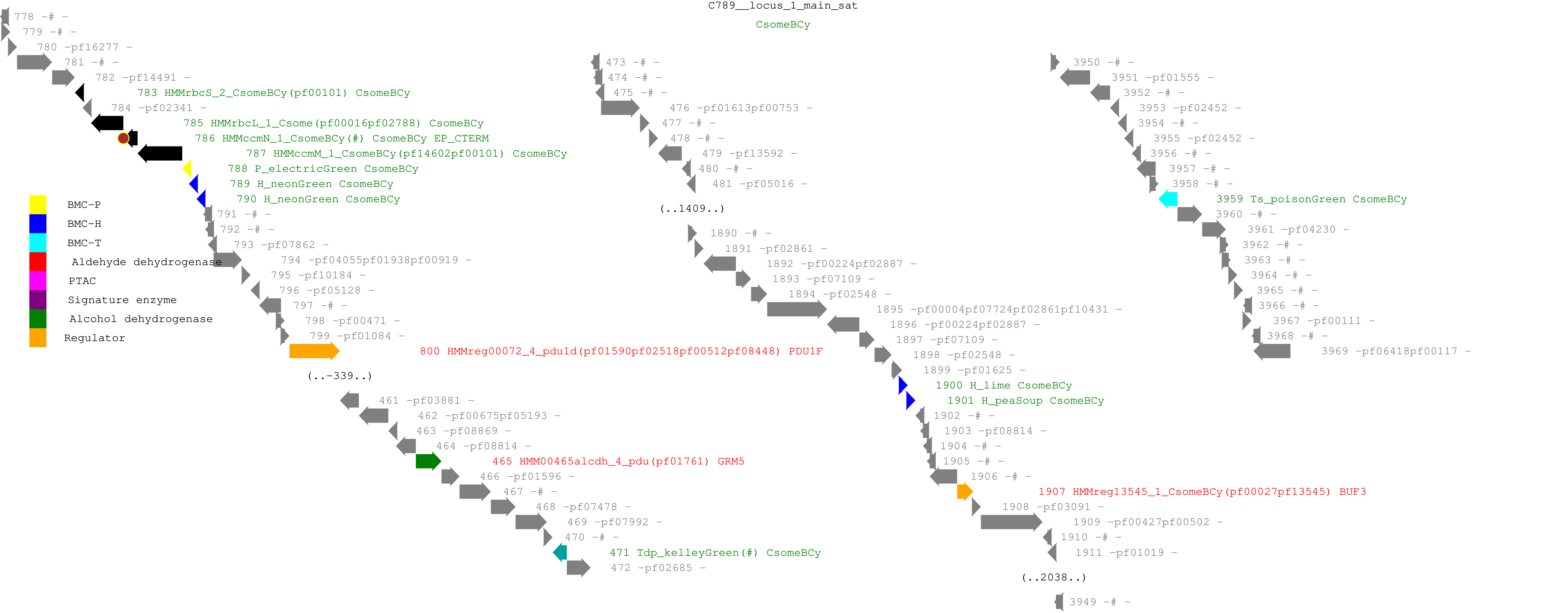

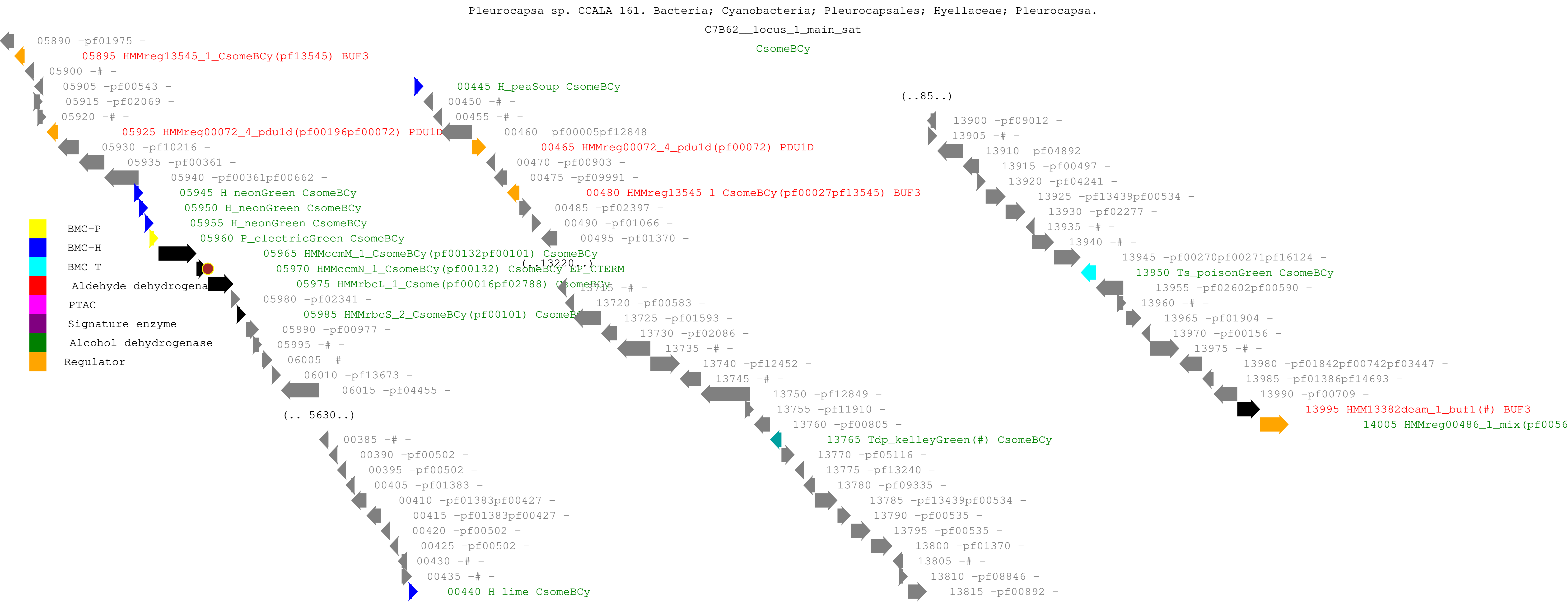

Pleurocapsa sp. CCALA 161. Bacteria; Cyanobacteria; Pleurocapsales; Hyellaceae; Pleurocapsa.
C7B62__locus_1_main_sat
CsomeBCy
05890 -pf01975 -
05895 HMMreg13545_1_CsomeBCy(pf13545) BUF3
05900 -# -
05905 -pf00543 -
05915 -pf02069 -
05920 -# -
05925 HMMreg00072_4_pdu1d(pf00196pf00072) PDU1D
05930 -pf10216 -
05935 -pf00361 -
05940 -pf00361pf00662 -
05945 H_neonGreen CsomeBCy
05950 H_neonGreen CsomeBCy
05955 H_neonGreen CsomeBCy
05960 P_electricGreen CsomeBCy
05965 HMMccmM_1_CsomeBCy(pf00132pf00101) CsomeBCy
05970 HMMccmN_1_CsomeBCy(pf00132) CsomeBCy EP_CTERM
05975 HMMrbcL_1_Csome(pf00016pf02788) CsomeBCy
05980 -pf02341 -
05985 HMMrbcS_2_CsomeBCy(pf00101) CsomeB
05990 -pf00977 -
05995 -# -
06005 -# -
06010 -pf13673 -
06015 -pf04455 -
(..-5630..)
00385 -# -
00390 -pf00502 -
00395 -pf00502 -
00405 -pf01383 -
00410 -pf01383pf00427 -
00415 -pf01383pf00427 -
00420 -pf00502 -
00425 -pf00502 -
00430 -# -
00435 -# -
00440 H_lime CsomeBCy
00445 H_peaSoup CsomeBCy
00450 -# -
00455 -# -
00460 -pf00005pf12848 -
00465 HMMreg00072_4_pdu1d(pf00072) PDU1D
00470 -pf00903 -
00475 -pf09991 -
00480 HMMreg13545_1_CsomeBCy(pf00027pf13545) BUF3
00485 -pf02397 -
00490 -pf01066 -
00495 -pf01370 -
(..13220..)
13715 -# -
13720 -pf00583 -
13725 -pf01593 -
13730 -pf02086 -
13735 -# -
13740 -pf12452 -
13745 -# -
13750 -pf12849 -
13755 -pf11910 -
13760 -pf00805 -
13765 Tdp_kelleyGreen(#) CsomeBCy
13770 -pf05116 -
13775 -pf13240 -
13780 -pf09335 -
13785 -pf13439pf00534 -
13790 -pf00535 -
13795 -pf00535 -
13800 -pf01370 -
13805 -# -
13810 -pf08846 -
13815 -pf00892 -
(..85..)
13900 -pf09012 -
13905 -# -
13910 -pf04892 -
13915 -pf00497 -
13920 -pf04241 -
13925 -pf13439pf00534 -
13930 -pf02277 -
13935 -# -
13940 -# -
13945 -pf00270pf00271pf16124 -
13950 Ts_poisonGreen CsomeBCy
13955 -pf02602pf00590 -
13960 -# -
13965 -pf01904 -
13970 -pf00156 -
13975 -# -
13980 -pf01842pf00742pf03447 -
13985 -pf01386pf14693 -
13990 -pf00709 -
13995 HMM13382deam_1_buf1(#) BUF3
14005 HMMreg00486_1_mix(pf0056
BMC-P
BMC-H
BMC-T
Aldehyde dehydrogena
PTAC
Signature enzyme
Alcohol dehydrogenase
Regulator

Merismopedia glauca CCAP 1448/3. Bacteria; Cyanobacteria; Synechococcales; Merismopediaceae;

C7B64__locus_1_main_sat

CsomeBCy

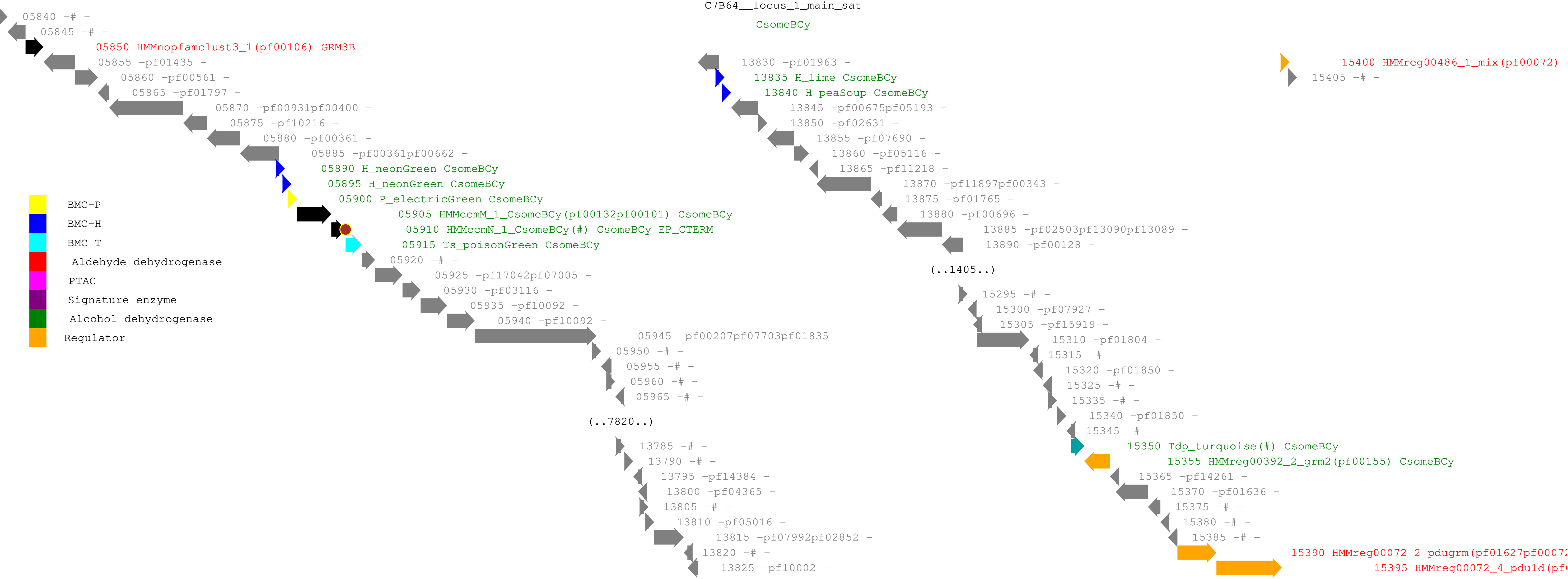

Phormidesmis priestleyi ULC007. Bacteria; Cyanobacteria; Synechococcales; Leptolyngbyaceae;

C7B65__locus_1_main_sat

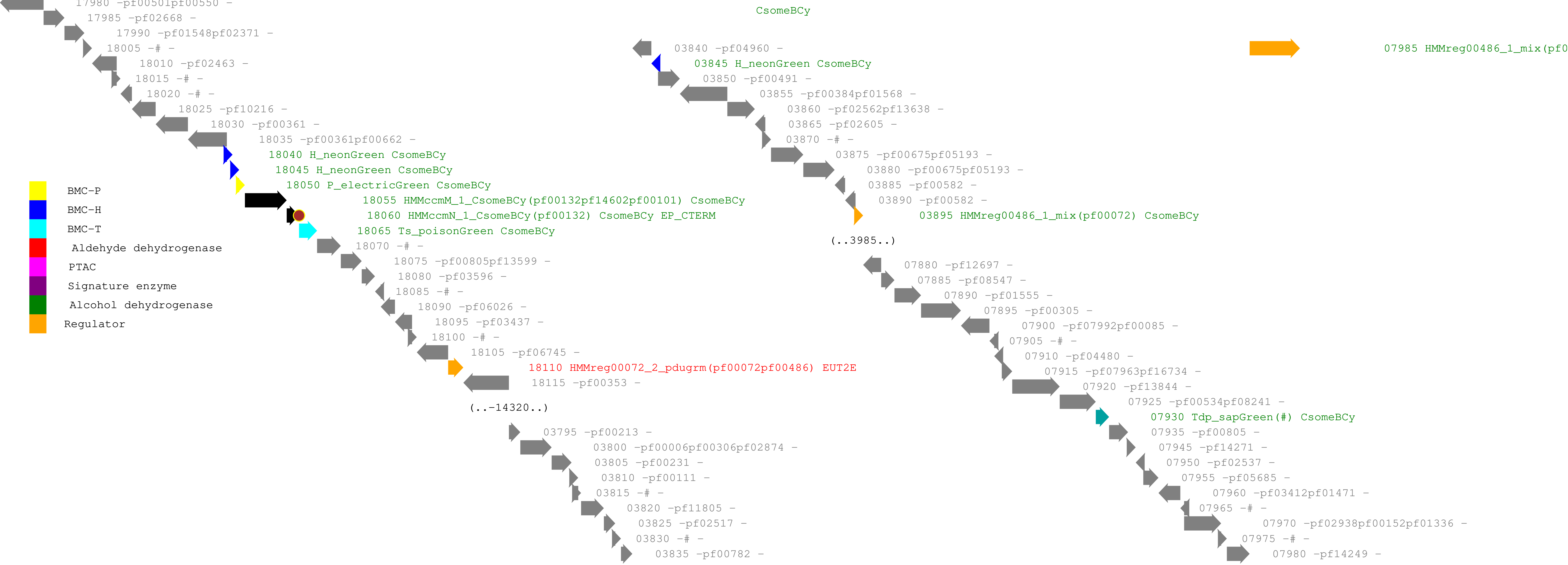

filamentous cyanobacterium Phorm 6. Bacteria; Cyanobacteria.

C7B67__locus_1_main_sat

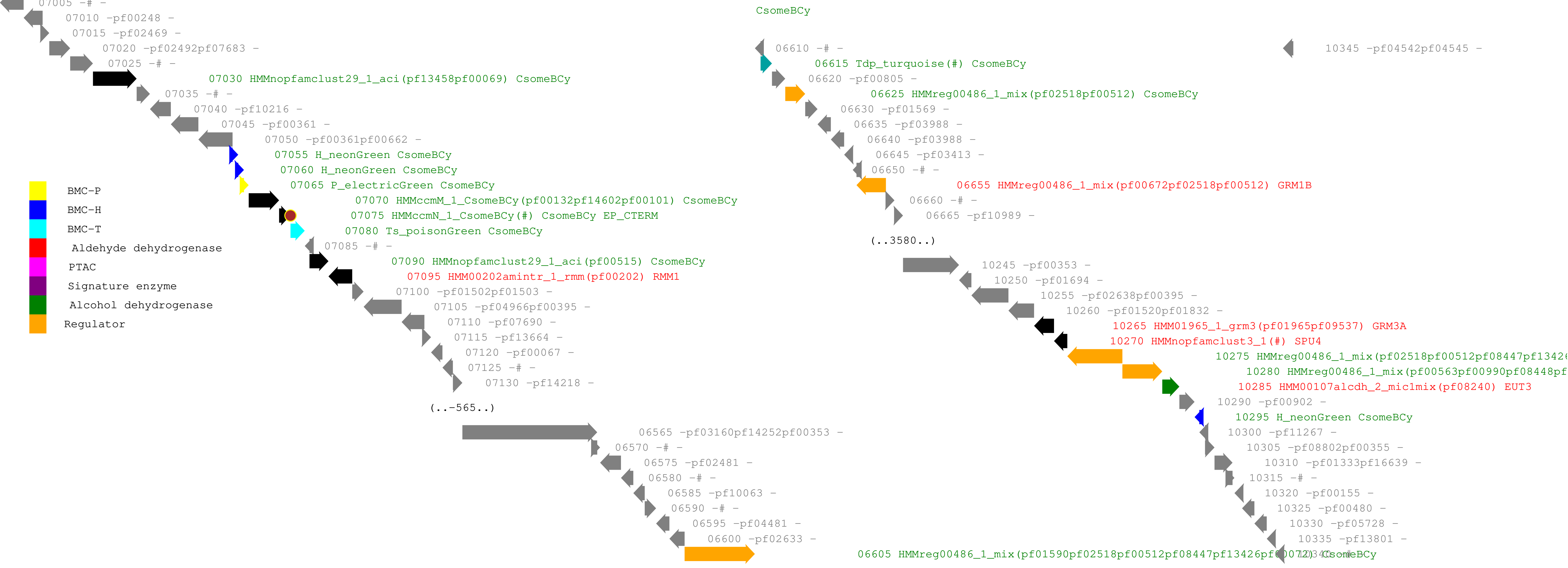

filamentous cyanobacterium Phorm 46. Bacteria; Cyanobacteria.

C7B69__locus_1_main_sat

CsomeBCy

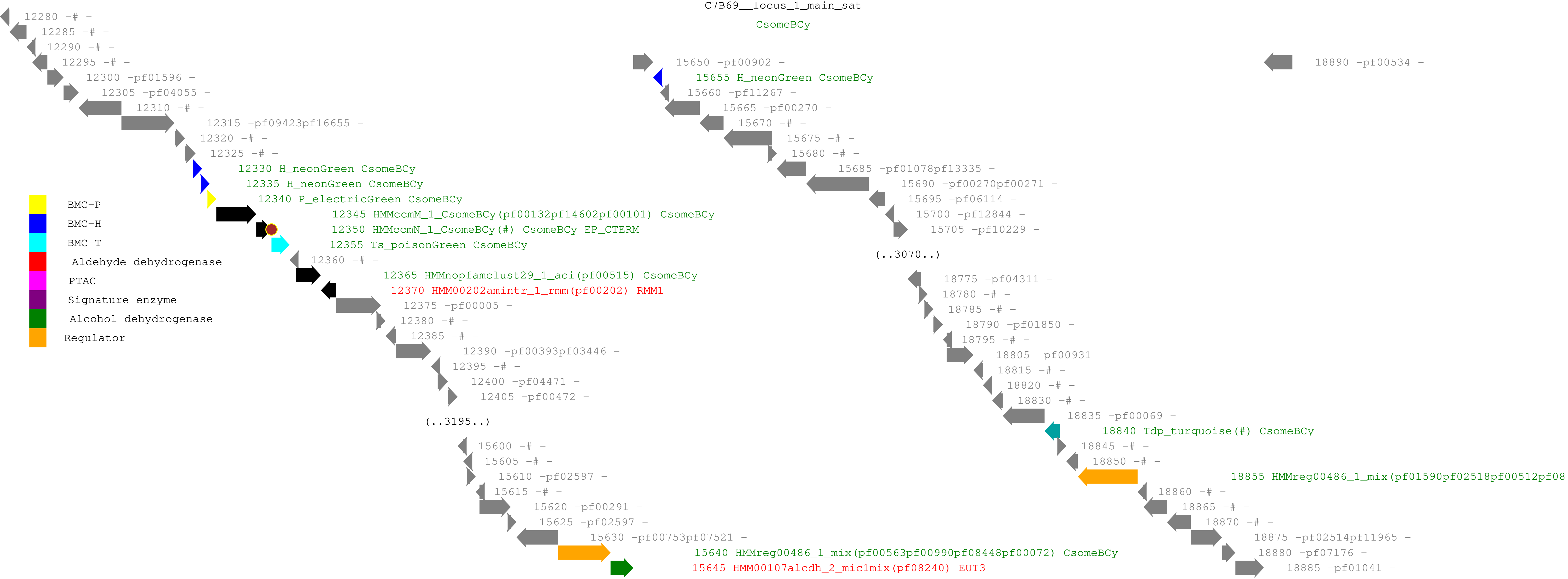

Chlorogloea sp. CCALA 695. Bacteria; Cyanobacteria; Oscillatoriophycideae; Chroococcales;

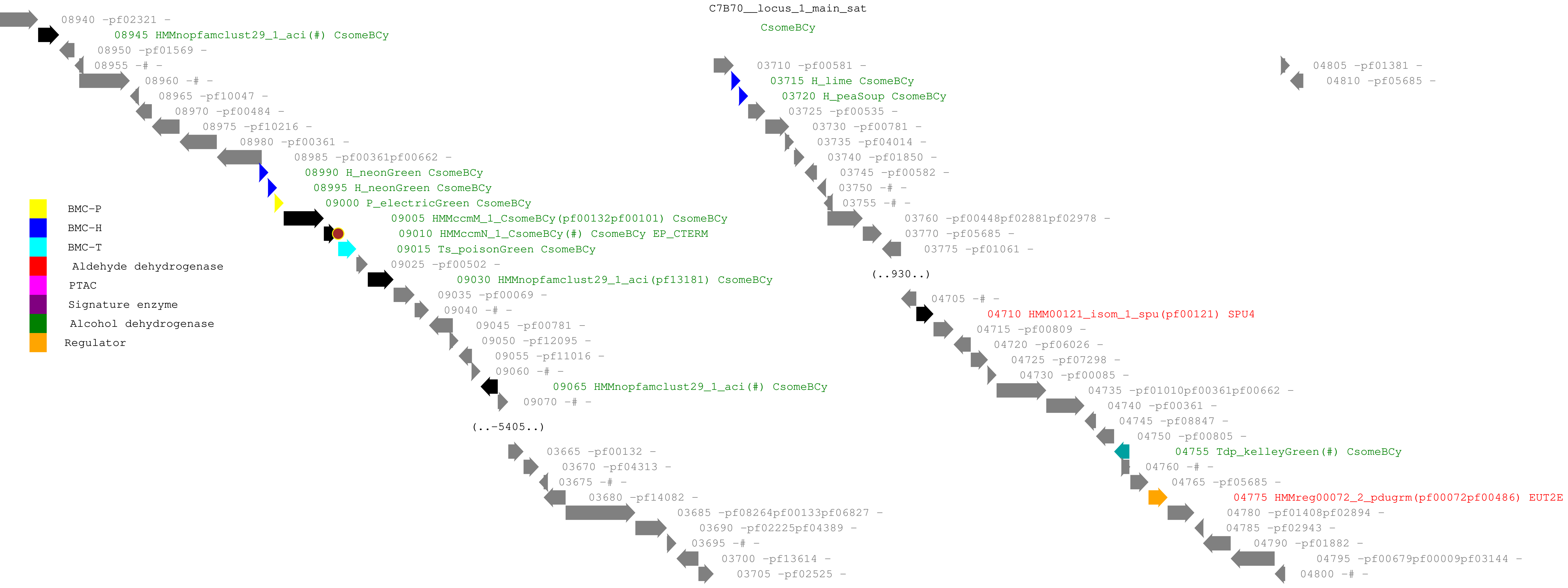

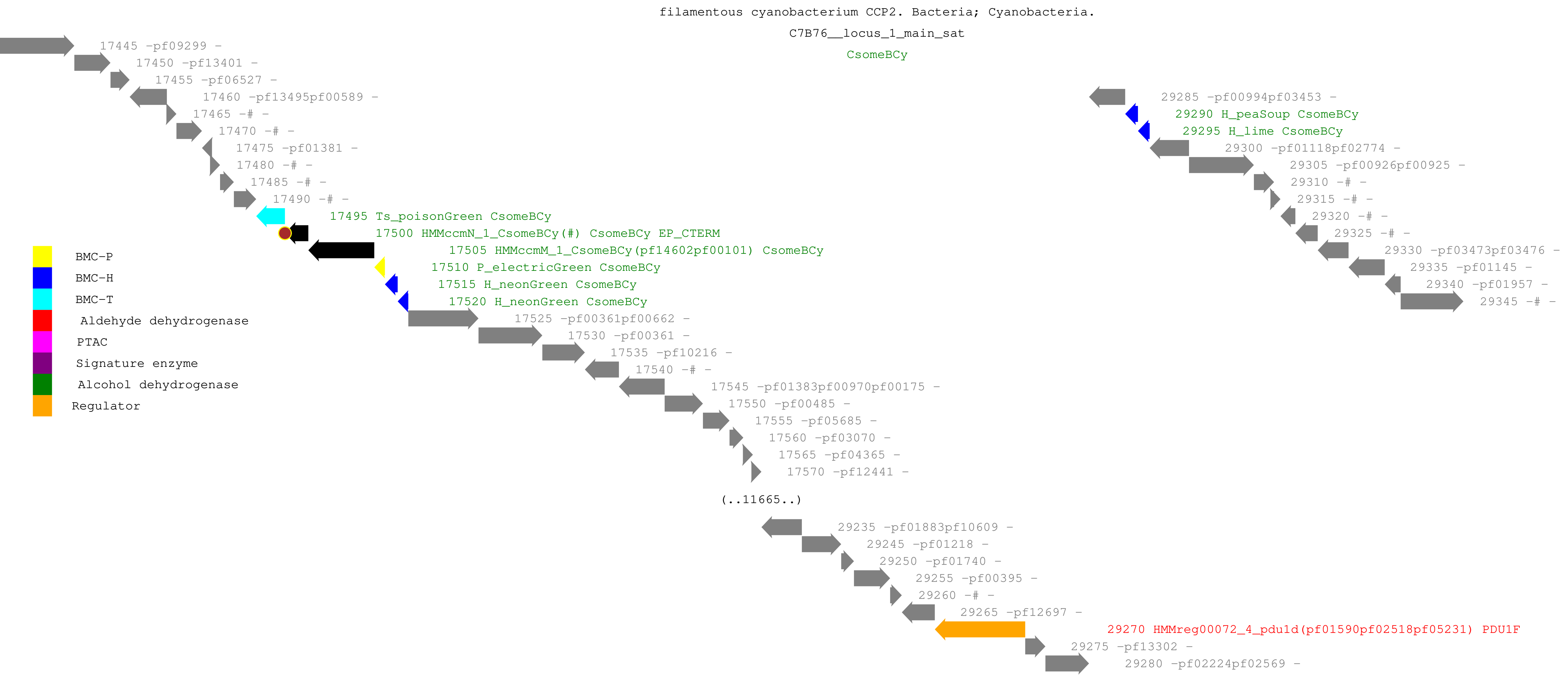
filamentous cyanobacterium CCP2. Bacteria; Cyanobacteria.
C7B76__locus_1_main_sat
CsomeBCy
17445 -pf09299 -
17450 -pf13401 -
17455 -pf06527 -
17460 -pf13495pf00589 -
17465 -# -
17470 -# -
17475 -pf01381 -
17480 -# -
17485 -# -
17490 -# -
17495 Ts_poisonGreen CsomeBCy
17500 HMMccmN_1_CsomeBCy(#) CsomeBCy EP_CTERM
17505 HMMccmM_1_CsomeBCy(pf14602pf00101) CsomeBCy
17510 P_electricGreen CsomeBCy
17515 H_neonGreen CsomeBCy
17520 H_neonGreen CsomeBCy
17525 -pf00361pf00662 -
17530 -pf00361 -
17535 -pf10216 -
17540 -# -
17545 -pf01383pf00970pf00175 -
17550 -pf00485 -
17555 -pf05685 -
17560 -pf03070 -
17565 -pf04365 -
17570 -pf12441 -
(..11665..)
29235 -pf01883pf10609 -
29245 -pf01218 -
29250 -pf01740 -
29255 -pf00395 -
29260 -# -
29265 -pf12697 -
29270 HMMreg00072_4_pdu1d(pf01590pf02518pf05231) PDU1F
29275 -pf13302 -
29280 -pf02224pf02569 -
29285 -pf00994pf03453 -
29290 H_peaSoup CsomeBCy
29295 H_lime CsomeBCy
29300 -pf01118pf02774 -
29305 -pf00926pf00925 -
29310 -# -
29315 -# -
29320 -# -
29325 -# -
29330 -pf03473pf03476 -
29335 -pf01145 -
29340 -pf01957 -
29345 -# -
BMC-P
BMC-H
BMC-T
Aldehyde dehydrogenase
PTAC
Signature enzyme
Alcohol dehydrogenase
Regulator

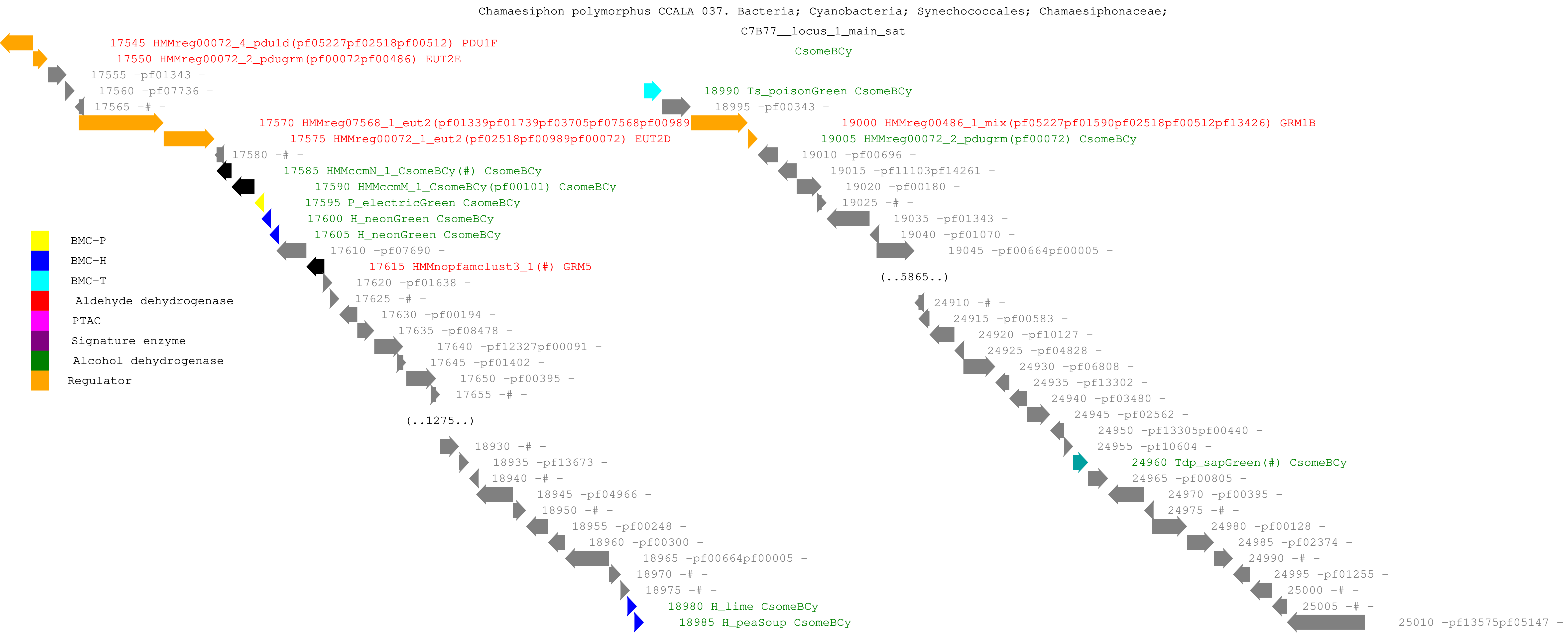
Chamaesiphon polymorphus CCALA 037. Bacteria; Cyanobacteria; Synechococcales; Chamaesiphonaceae;
C7B77__locus_1_main_sat
CsomeBCy
17545 HMMreg00072_4_pdu1d(pf05227pf02518pf00512) PDU1F
17550 HMMreg00072_2_pdugrm(pf00072pf00486) EUT2E
17555 -pf01343 -
17560 -pf07736 -
17565 -# -
17570 HMMreg07568_1_eut2(pf01339pf01739pf03705pf07568pf00989
17575 HMMreg00072_1_eut2(pf02518pf00989pf00072) EUT2D
17580 -# -
17585 HMMccmN_1_CsomeBCy(#) CsomeBCy
17590 HMMccmM_1_CsomeBCy(pf00101) CsomeBCy
17595 P_electricGreen CsomeBCy
17600 H_neonGreen CsomeBCy
17605 H_neonGreen CsomeBCy
17610 -pf07690 -
17615 HMMnopfamclust3_1(#) GRM5
17620 -pf01638 -
17625 -# -
17630 -pf00194 -
17635 -pf08478 -
17640 -pf12327pf00091 -
17645 -pf01402 -
17650 -pf00395 -
17655 -# -
(..1275..)
18930 -# -
18935 -pf13673 -
18940 -# -
18945 -pf04966 -
18950 -# -
18955 -pf00248 -
18960 -pf00300 -
18965 -pf00664pf00005 -
18970 -# -
18975 -# -
18980 H_lime CsomeBCy
18985 H_peaSoup CsomeBCy
18990 Ts_poisonGreen CsomeBCy
18995 -pf00343 -
19000 HMMreg00486_1_mix(pf05227pf01590pf02518pf00512pf13426) GRM1B
19005 HMMreg00072_2_pdugrm(pf00072) CsomeBCy
19010 -pf00696 -
19015 -pf11103pf14261 -
19020 -pf00180 -
19025 -# -
19035 -pf01343 -
19040 -pf01070 -
19045 -pf00664pf00005 -
(..5865..)
24910 -# -
24915 -pf00583 -
24920 -pf10127 -
24925 -pf04828 -
24930 -pf06808 -
24935 -pf13302 -
24940 -pf03480 -
24945 -pf02562 -
24950 -pf13305pf00440 -
24955 -pf10604 -
24960 Tdp_sapGreen(#) CsomeBCy
24965 -pf00805 -
24970 -pf00395 -
24975 -# -
24980 -pf00128 -
24985 -pf02374 -
24990 -# -
24995 -pf01255 -
25000 -# -
25005 -# -
25010 -pf13575pf05147 -
BMC-P
BMC-H
BMC-T
Aldehyde dehydrogenase
PTAC
Signature enzyme
Alcohol dehydrogenase
Regulator

Chroococcidiopsis cubana CCALA 043. Bacteria; Cyanobacteria; Chroococcidiopsidales;

C7B79__locus_1_main_sat

CsomeBCy

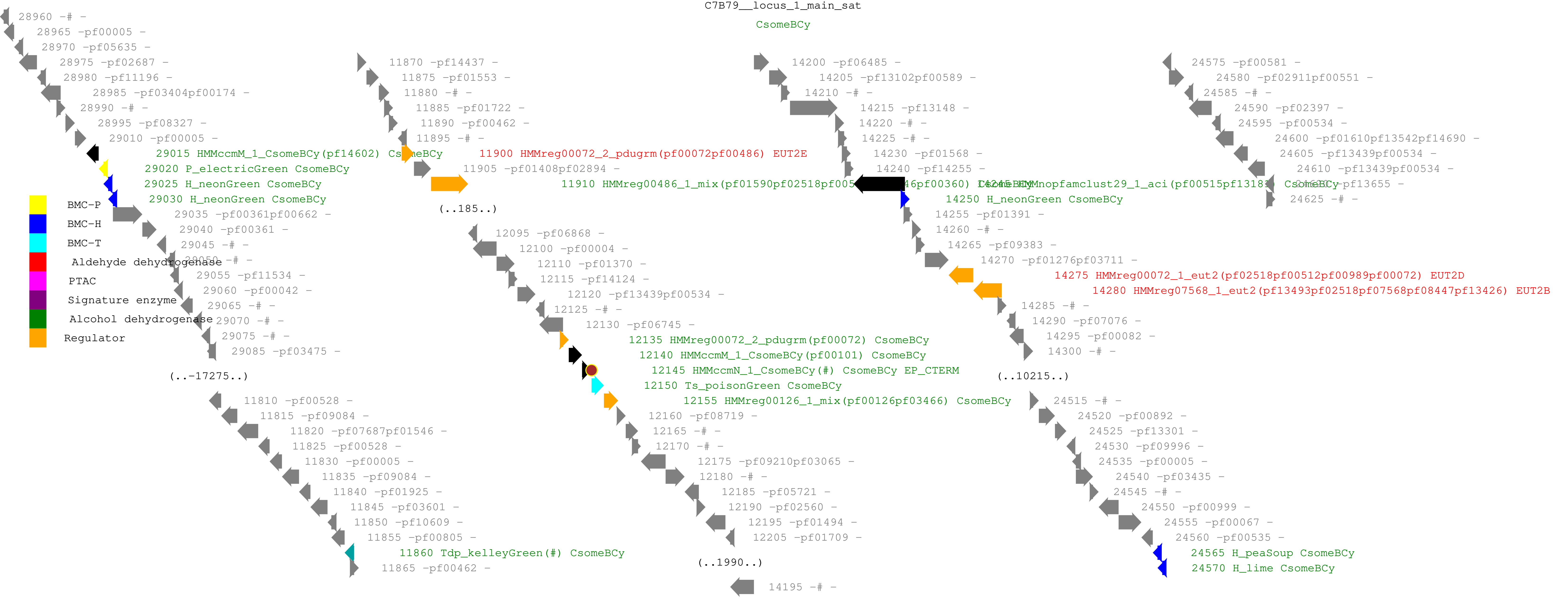

Cyanosarcina cf. burmensis CCALA 770. Bacteria; Cyanobacteria; Oscillatoriophycideae; Chroococcales;

C7B80__locus_1_main_sat

CsomeBCy

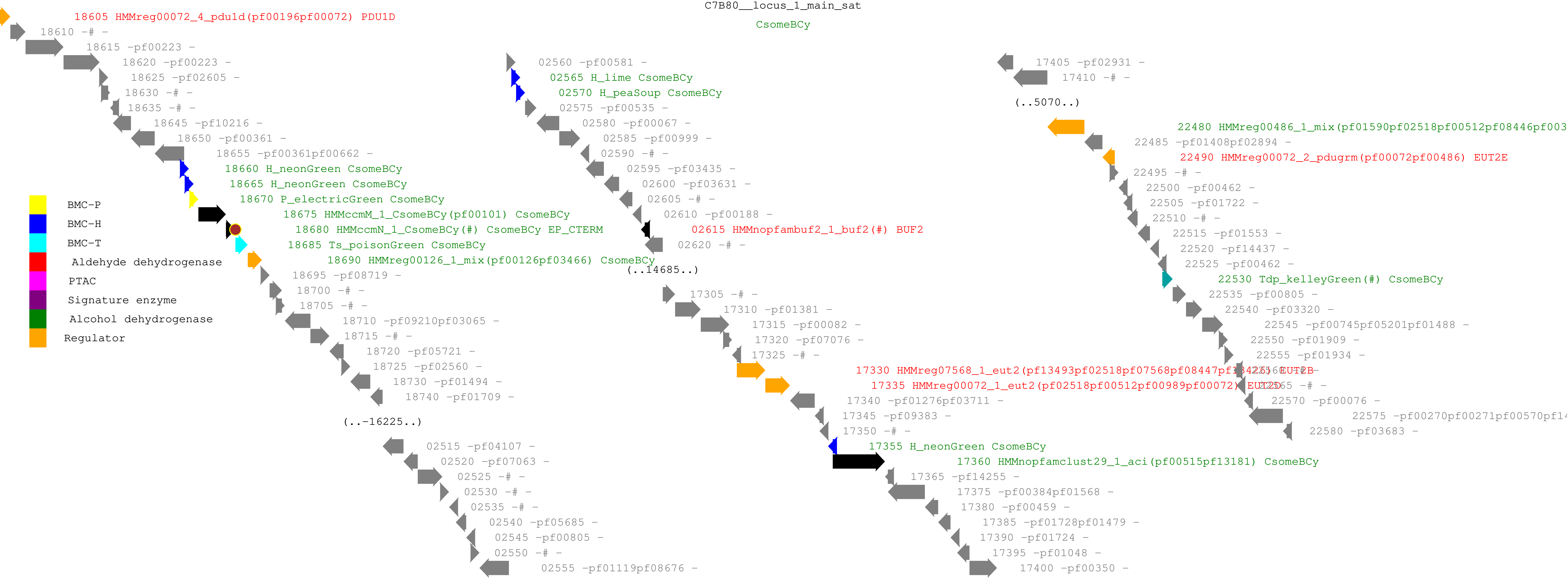

Leptolyngbya frigida ULC18. Bacteria; Cyanobacteria; Synechococcales; Leptolyngbyaceae;

C7B82__locus_1_main_sat

CsomeBCy

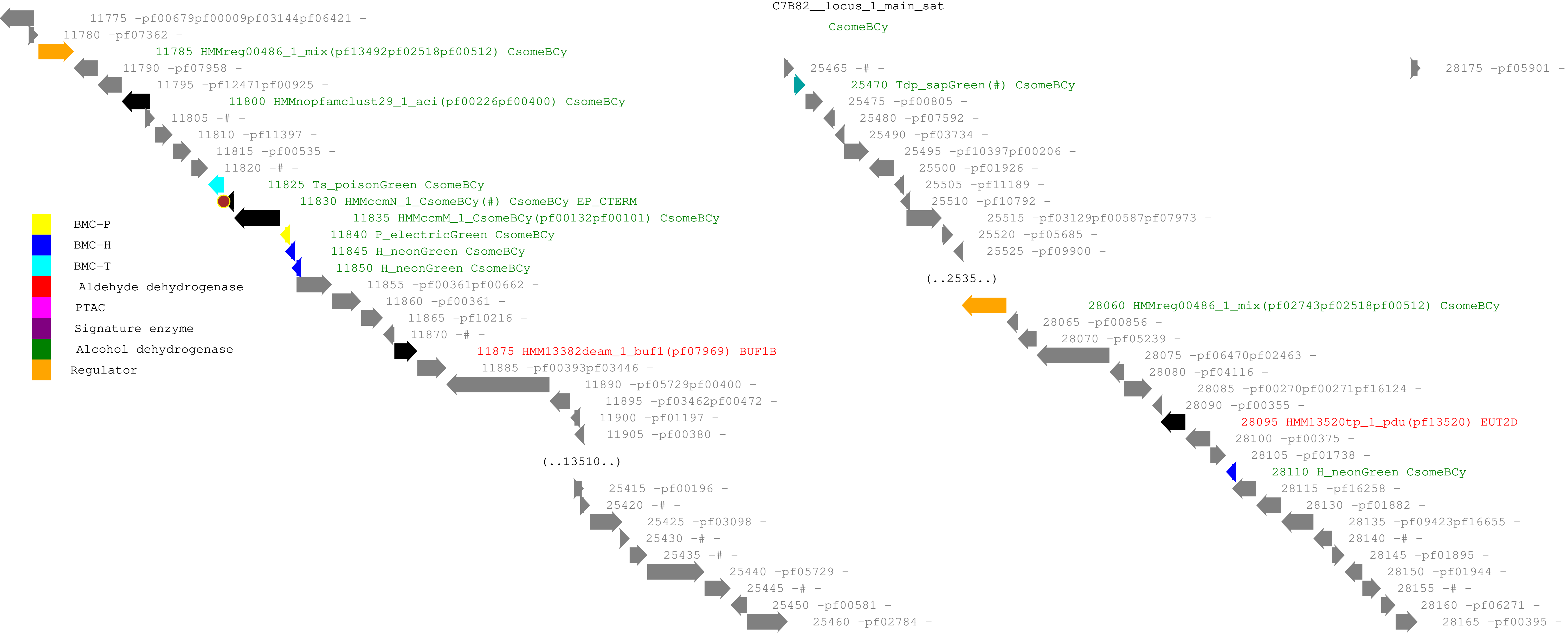

Aphanothece hegewaldii CCALA 016. Bacteria; Cyanobacteria; Oscillatoriophycideae; Chroococcales;

C7H19__locus_1_main_sat

CsomeBCy

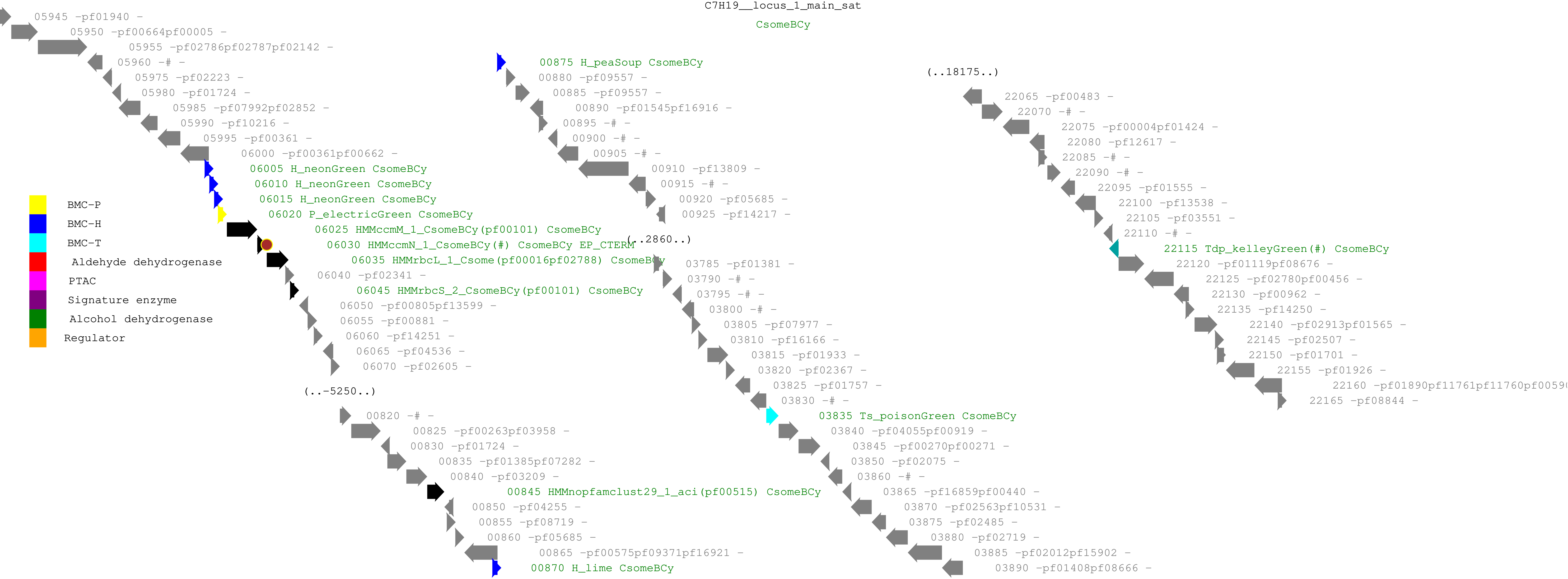

Chroococcidiopsis sp. CCALA 051. Bacteria; Cyanobacteria; Chroococcidiopsidales;

C7Y66__locus_1_main_sat

CsomeBCy

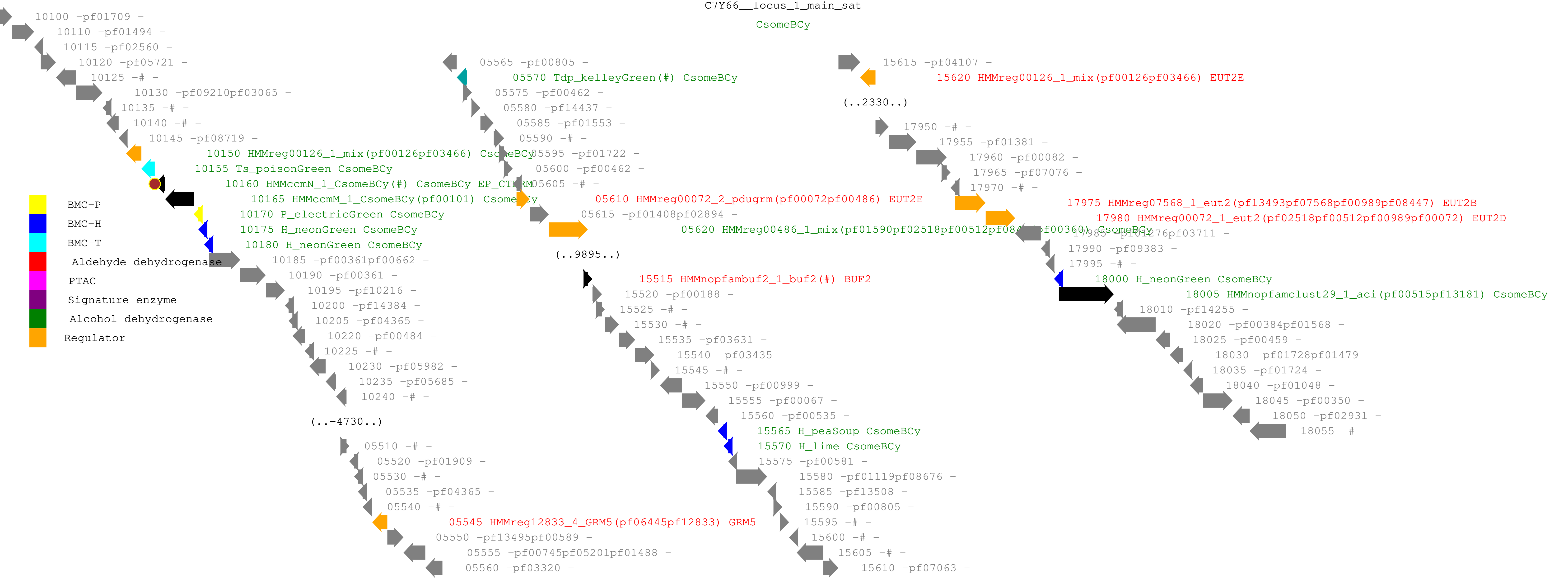

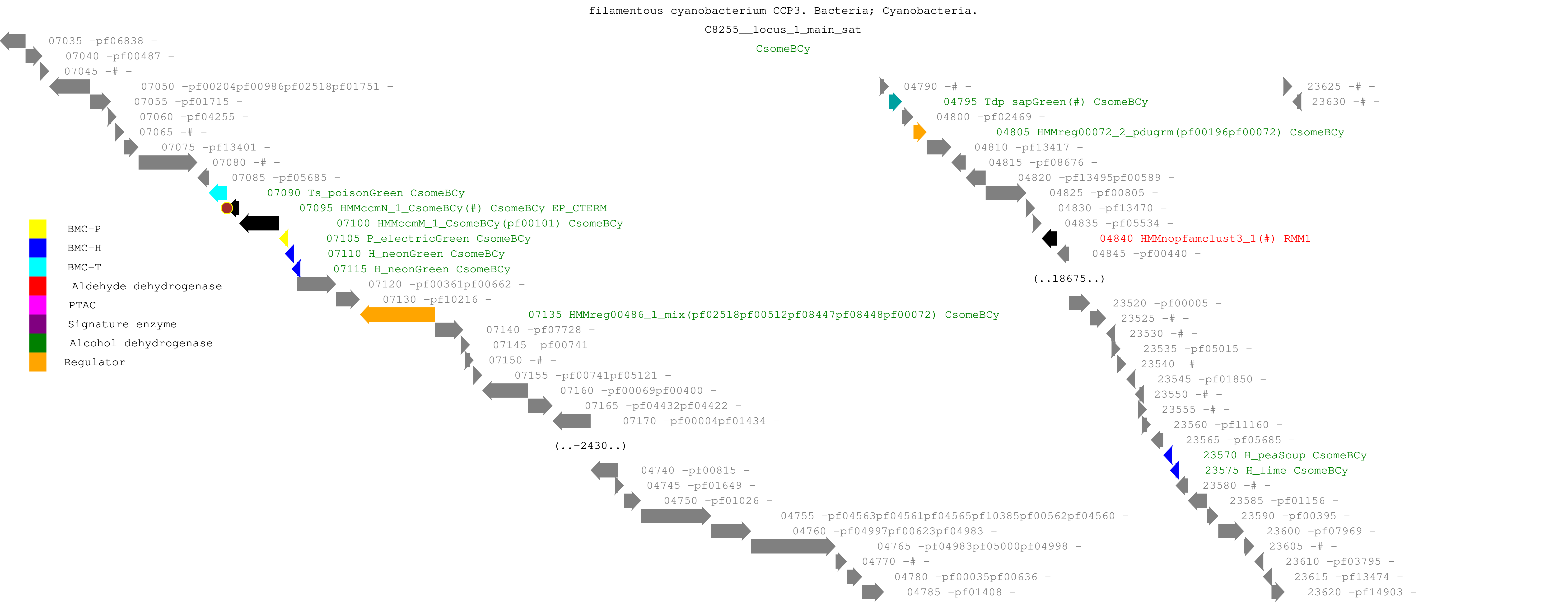
filamentous cyanobacterium CCP3. Bacteria; Cyanobacteria.
C8255__locus_1_main_sat
CsomeBCy
07035 -pf06838 -
07040 -pf00487 -
07045 -# -
07050 -pf00204pf00986pf02518pf01751 -
07055 -pf01715 -
07060 -pf04255 -
07065 -# -
07075 -pf13401 -
07080 -# -
07085 -pf05685 -
07090 Ts_poisonGreen CsomeBCy
07095 HMMccmN_1_CsomeBCy(#) CsomeBCy EP_CTERM
07100 HMMccmM_1_CsomeBCy(pf00101) CsomeBCy
07105 P_electricGreen CsomeBCy
07110 H_neonGreen CsomeBCy
07115 H_neonGreen CsomeBCy
07120 -pf00361pf00662 -
07130 -pf10216 -
07135 HMMreg00486_1_mix(pf02518pf00512pf08447pf08448pf00072) CsomeBCy
07140 -pf07728 -
07145 -pf00741 -
07150 -# -
07155 -pf00741pf05121 -
07160 -pf00069pf00400 -
07165 -pf04432pf04422 -
07170 -pf00004pf01434 -
(..-2430..)
04740 -pf00815 -
04745 -pf01649 -
04750 -pf01026 -
04755 -pf04563pf04561pf04565pf10385pf00562pf04560 -
04760 -pf04997pf00623pf04983 -
04765 -pf04983pf05000pf04998 -
04770 -# -
04780 -pf00035pf00636 -
04785 -pf01408 -
04790 -# -
04795 Tdp_sapGreen(#) CsomeBCy
04800 -pf02469 -
04805 HMMreg00072_2_pdugrm(pf00196pf00072) CsomeBCy
04810 -pf13417 -
04815 -pf08676 -
04820 -pf13495pf00589 -
04825 -pf00805 -
04830 -pf13470 -
04835 -pf05534 -
04840 HMMnopfamclust3_1(#) RMM1
04845 -pf00440 -
(..18675..)
23520 -pf00005 -
23525 -# -
23530 -# -
23535 -pf05015 -
23540 -# -
23545 -pf01850 -
23550 -# -
23555 -# -
23560 -pf11160 -
23565 -pf05685 -
23570 H_peaSoup CsomeBCy
23575 H_lime CsomeBCy
23580 -# -
23585 -pf01156 -
23590 -pf00395 -
23600 -pf07969 -
23605 -# -
23610 -pf03795 -
23615 -pf13474 -
23620 -pf14903 -
23625 -# -
23630 -# -
BMC-P
BMC-H
BMC-T
Aldehyde dehydrogenase
PTAC
Signature enzyme
Alcohol dehydrogenase
Regulator

filamentous cyanobacterium CCP4. Bacteria; Cyanobacteria.

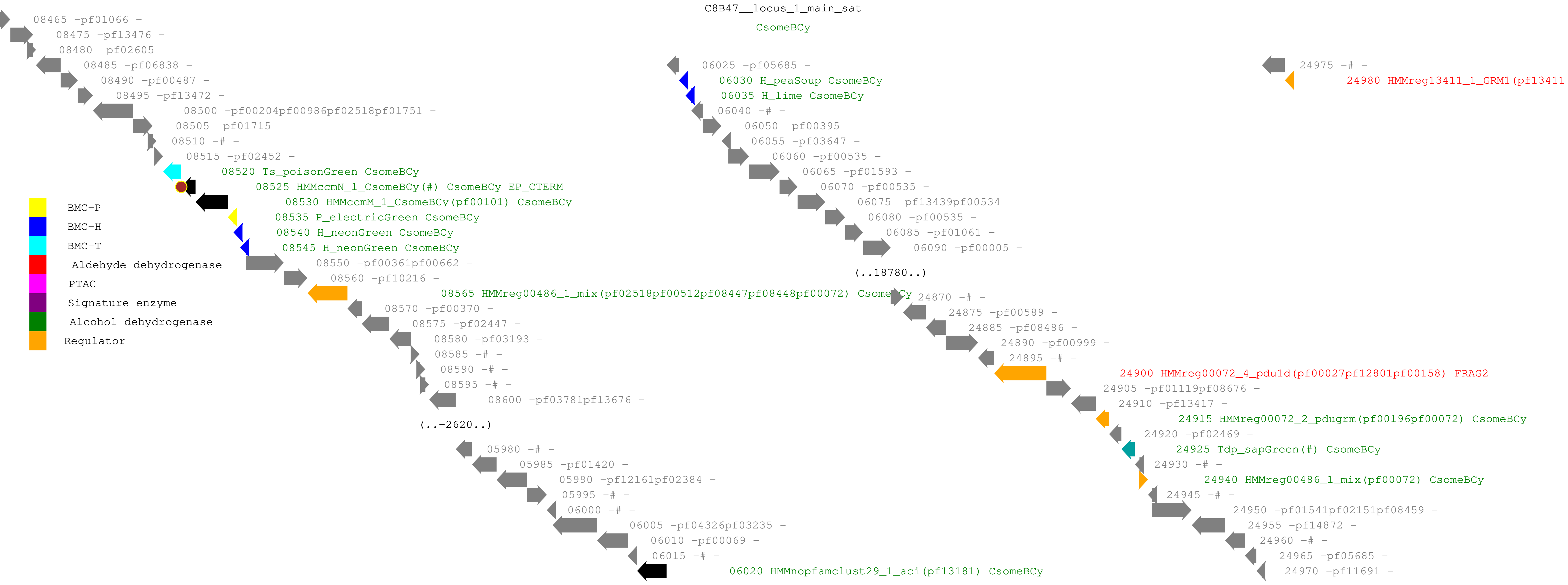

Calothrix sp. PCC 6303. Bacteria; Cyanobacteria; Nostocales; Rivulariaceae; Calothrix.

Cal6303__locus_1_main_sat

CsomeBCy

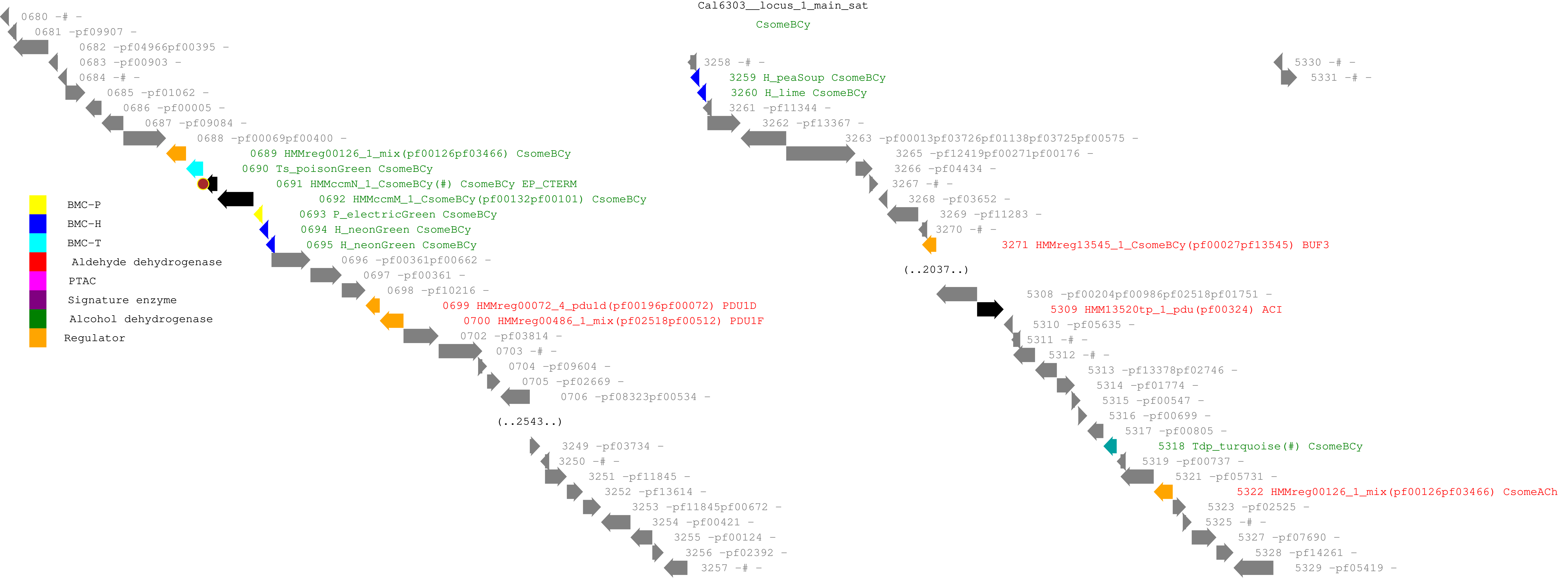

Calothrix sp. PCC 7507. Bacteria; Cyanobacteria; Nostocales; Rivulariaceae; Calothrix.

Cal7507__locus_1_main_sat

CsomeBCy

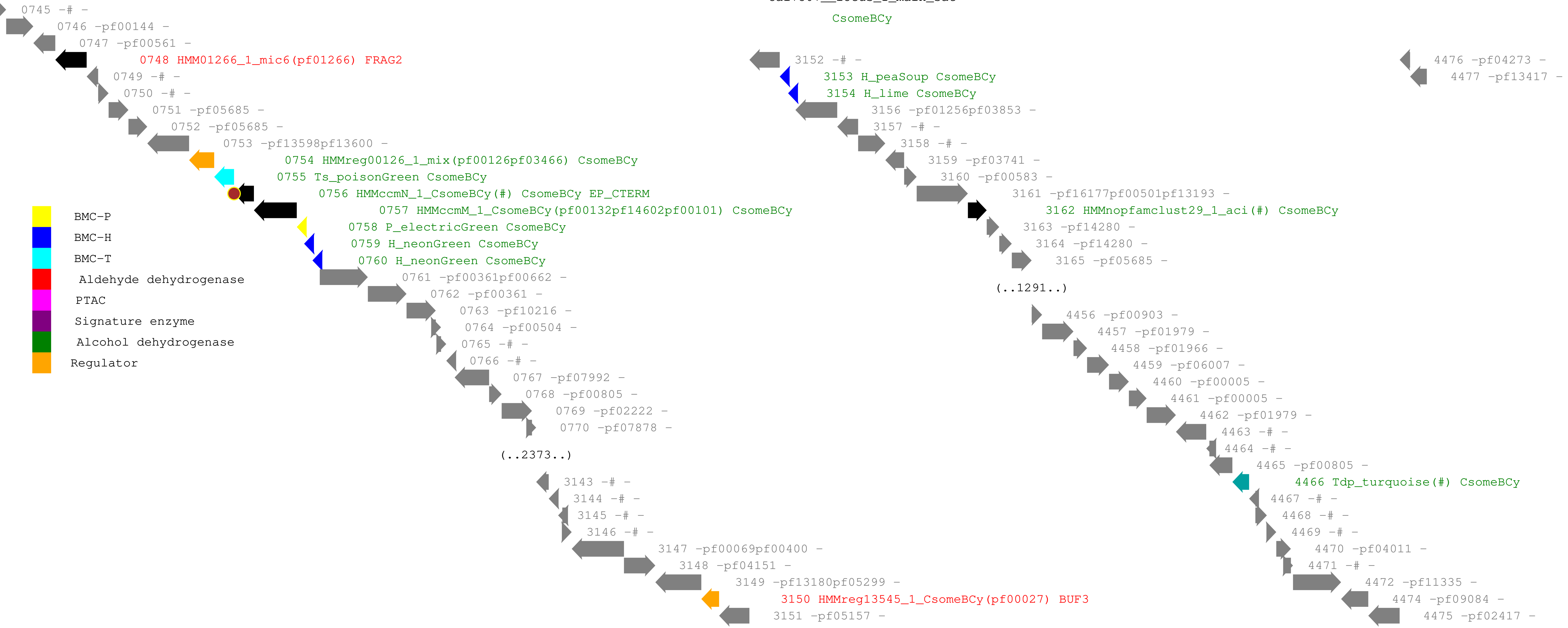

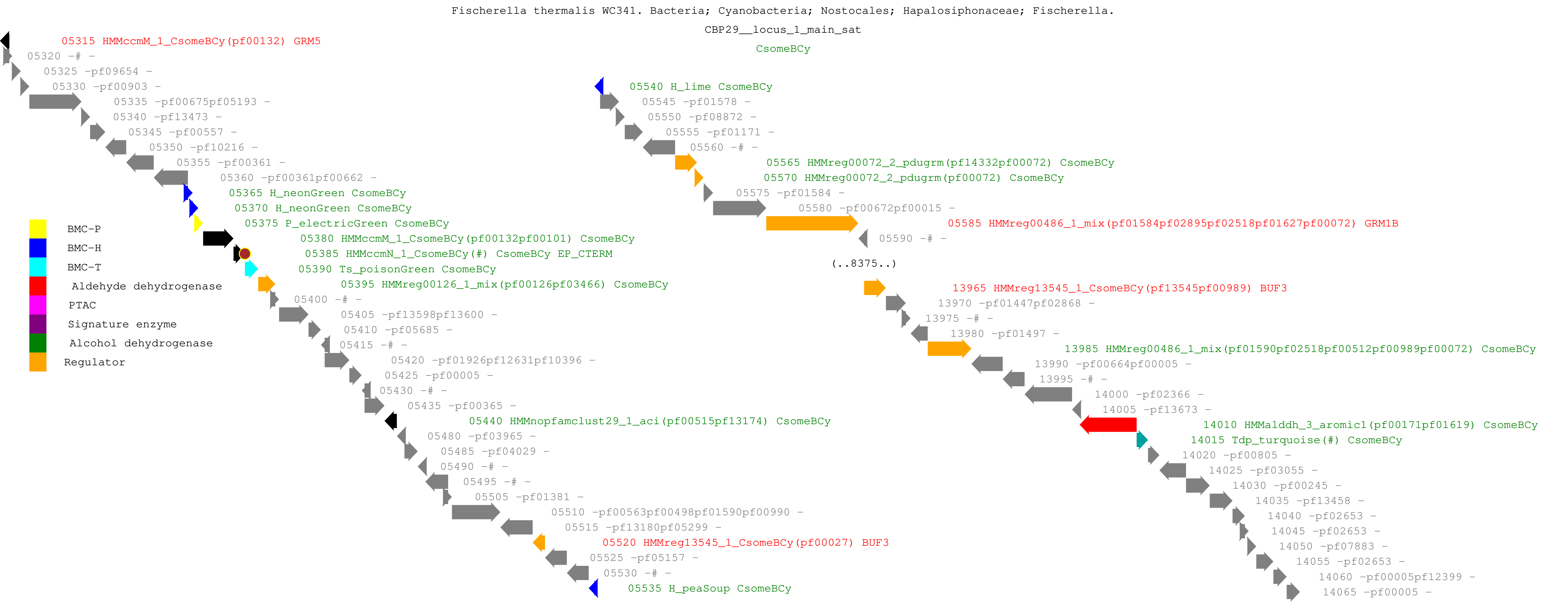
Fischerella thermalis WC341. Bacteria; Cyanobacteria; Nostocales; Hapalosiphonaceae; Fischerella.
CBP29__locus_1_main_sat
CsomeBCy
05315 HMMccmM_1_CsomeBCy(pf00132) GRM5
05320 -# -
05325 -pf09654 -
05330 -pf00903 -
05335 -pf00675pf05193 -
05340 -pf13473 -
05345 -pf00557 -
05350 -pf10216 -
05355 -pf00361 -
05360 -pf00361pf00662 -
05365 H_neonGreen CsomeBCy
05370 H_neonGreen CsomeBCy
05375 P_electricGreen CsomeBCy
05380 HMMccmM_1_CsomeBCy(pf00132pf00101) CsomeBCy
05385 HMMccmN_1_CsomeBCy(#) CsomeBCy EP_CTERM
05390 Ts_poisonGreen CsomeBCy
05395 HMMreg00126_1_mix(pf00126pf03466) CsomeBCy
05400 -# -
05405 -pf13598pf13600 -
05410 -pf05685 -
05415 -# -
05420 -pf01926pf12631pf10396 -
05425 -pf00005 -
05430 -# -
05435 -pf00365 -
05440 HMMnopfamclust29_1_aci(pf00515pf13174) CsomeBCy
05480 -pf03965 -
05485 -pf04029 -
05490 -# -
05495 -# -
05505 -pf01381 -
05510 -pf00563pf00498pf01590pf00990 -
05515 -pf13180pf05299 -
05520 HMMreg13545_1_CsomeBCy(pf00027) BUF3
05525 -pf05157 -
05530 -# -
05535 H_peaSoup CsomeBCy
BMC-P
BMC-H
BMC-T
Aldehyde dehydrogenase
PTAC
Signature enzyme
Alcohol dehydrogenase
Regulator
05540 H_lime CsomeBCy
05545 -pf01578 -
05550 -pf08872 -
05555 -pf01171 -
05560 -# -
05565 HMMreg00072_2_pdugrm(pf14332pf00072) CsomeBCy
05570 HMMreg00072_2_pdugrm(pf00072) CsomeBCy
05575 -pf01584 -
05580 -pf00672pf00015 -
05585 HMMreg00486_1_mix(pf01584pf02895pf02518pf01627pf00072) GRM1B
05590 -# -
(..8375..)
13965 HMMreg13545_1_CsomeBCy(pf13545pf00989) BUF3
13970 -pf01447pf02868 -
13975 -# -
13980 -pf01497 -
13985 HMMreg00486_1_mix(pf01590pf02518pf00512pf00989pf00072) CsomeBCy
13990 -pf00664pf00005 -
13995 -# -
14000 -pf02366 -
14005 -pf13673 -
14010 HMMaldh_3_aromic1(pf00171pf01619) CsomeBCy
14015 Tdp_turquoise(#) CsomeBCy
14020 -pf00805 -
14025 -pf03055 -
14030 -pf00245 -
14035 -pf13458 -
14040 -pf02653 -
14045 -pf02653 -
14050 -pf07883 -
14055 -pf02653 -
14060 -pf00005pf12399 -
14065 -pf00005 -

Cyanothece sp. (strain ATCC 51142). Bacteria; Cyanobacteria; Oscillatoriophycideae; Oscillatoriales;

cce__locus_1_main_sat

CsomeBCy

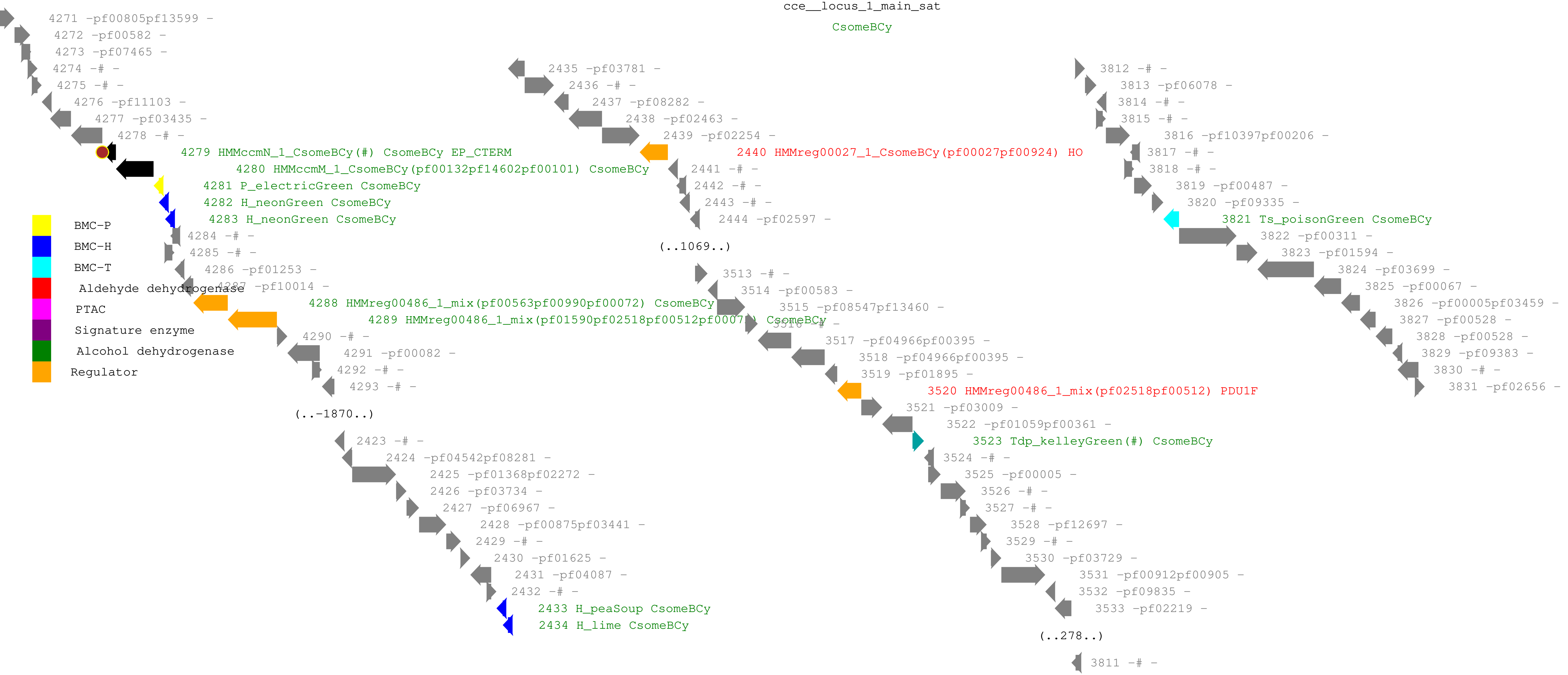

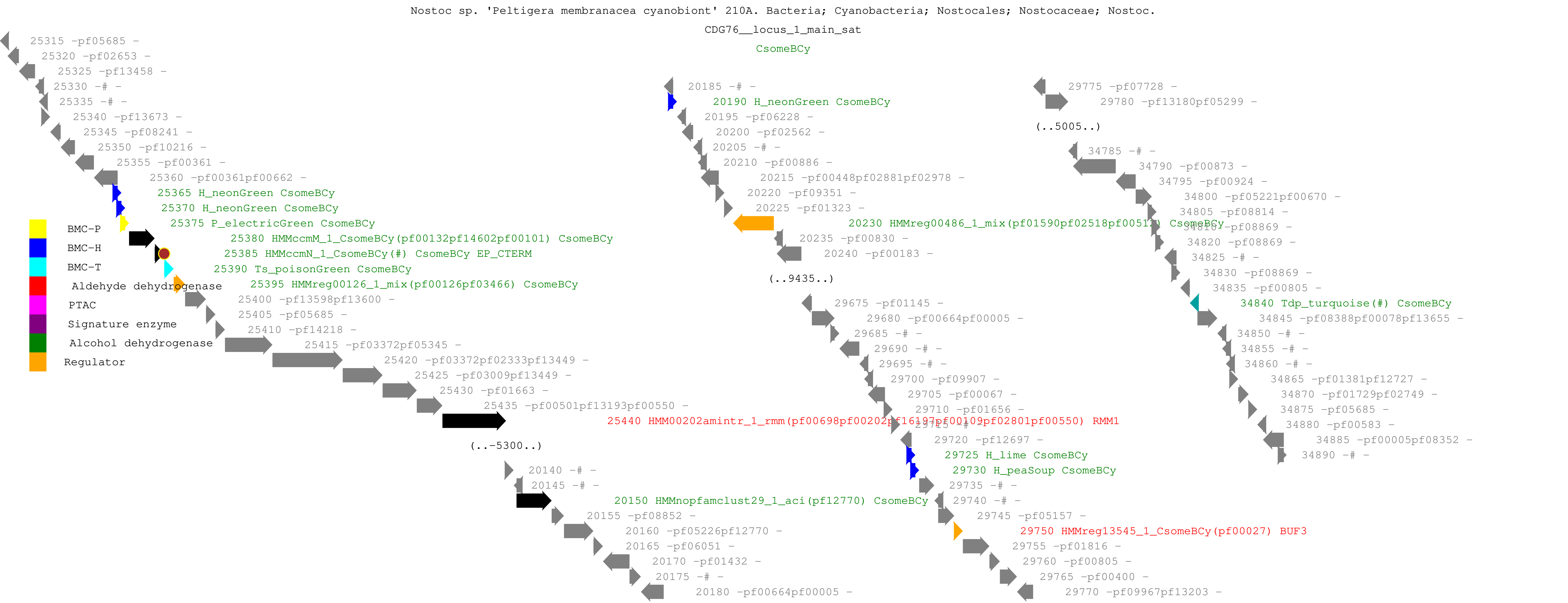
Nostoc sp. 'Peltigera membranacea cyanobiont' 210A. Bacteria; Cyanobacteria; Nostocales; Nostocaceae; Nostoc.
CDG76__locus_1_main_sat
CsomeBCy
BMC-P
BMC-H
BMC-T
Aldehyde dehydrogenase
PTAC
Signature enzyme
Alcohol dehydrogenase
Regulator
25315 -pf05685 -
25320 -pf02653 -
25325 -pf13458 -
25330 -# -
25335 -# -
25340 -pf13673 -
25345 -pf08241 -
25350 -pf10216 -
25355 -pf00361 -
25360 -pf00361pf00662 -
25365 H_neonGreen CsomeBCy
25370 H_neonGreen CsomeBCy
25375 P_electricGreen CsomeBCy
25380 HMMccmM_1_CsomeBCy(pf00132pf14602pf00101) CsomeBCy
25385 HMMccmN_1_CsomeBCy(#) CsomeBCy EP_CTERM
25390 Ts_poisonGreen CsomeBCy
25395 HMMreg00126_1_mix(pf00126pf03466) CsomeBCy
25400 -pf13598pf13600 -
25405 -pf05685 -
25410 -pf14218 -
25415 -pf03372pf05345 -
25420 -pf03372pf02333pf13449 -
25425 -pf03009pf13449 -
25430 -pf01663 -
25435 -pf00501pf13193pf00550 -
25440 HMM00202amintr_1_rmm(pf00698pf00202pf16197pf00109pf02801pf00550) RMM1
(..-5300..)
20140 -# -
20145 -# -
20150 HMMnopfamclust29_1_aci(pf12770) CsomeBCy
20155 -pf08852 -
20160 -pf05226pf12770 -
20165 -pf06051 -
20170 -pf01432 -
20175 -# -
20180 -pf00664pf00005 -
20185 -# -
20190 H_neonGreen CsomeBCy
20195 -pf06228 -
20200 -pf02562 -
20205 -# -
20210 -pf00886 -
20215 -pf00448pf02881pf02978 -
20220 -pf09351 -
20225 -pf01323 -
20230 HMMreg00486_1_mix(pf01590pf02518pf0051[illegible]) CsomeBCy
20235 -pf00830 -
20240 -pf00183 -
(..9435..)
29675 -pf01145 -
29680 -pf00664pf00005 -
29685 -# -
29690 -# -
29695 -# -
29700 -pf09907 -
29705 -pf00067 -
29710 -pf01656 -
29715 -# -
29720 -pf12697 -
29725 H_lime CsomeBCy
29730 H_peaSoup CsomeBCy
29735 -# -
29740 -# -
29745 -pf05157 -
29750 HMMreg13545_1_CsomeBCy(pf00027) BUF3
29755 -pf01816 -
29760 -pf00805 -
29765 -pf00400 -
29770 -pf09967pf13203 -
29775 -pf07728 -
29780 -pf13180pf05299 -
(..5005..)
34785 -# -
34790 -pf00873 -
34795 -pf00924 -
34800 -pf05221pf00670 -
34805 -pf08814 -
34810 -pf08869 -
34820 -pf08869 -
34825 -# -
34830 -pf08869 -
34835 -pf00805 -
34840 Tdp_turquoise(#) CsomeBCy
34845 -pf08388pf00078pf13655 -
34850 -# -
34855 -# -
34860 -# -
34865 -pf01381pf12727 -
34870 -pf01729pf02749 -
34875 -pf05685 -
34880 -pf00583 -
34885 -pf00005pf08352 -
34890 -# -

Nostoc sp. 'Peltigera membranacea cyanobiont' 213. Bacteria; Cyanobacteria; Nostocales; Nostocaceae; Nostoc.

CDG77__locus_1_main_sat

CsomeBCy

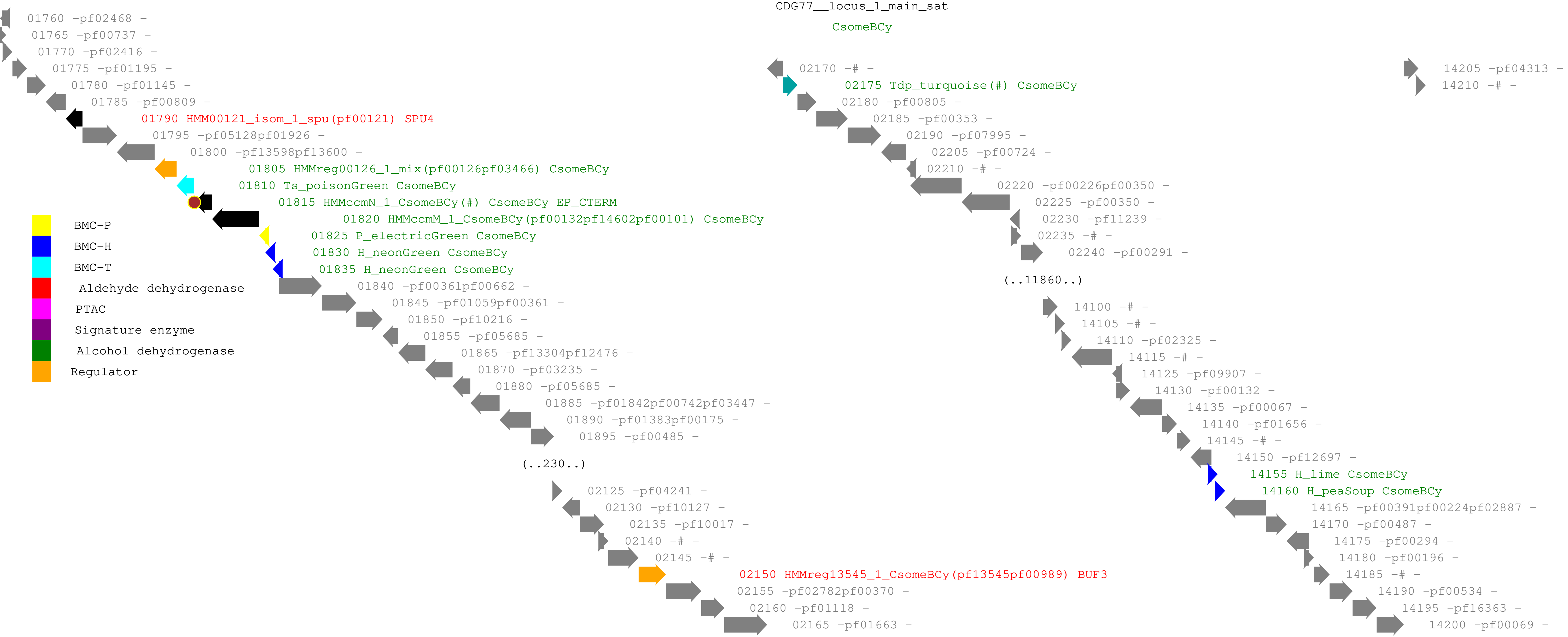

Nostoc sp. 'Peltigera membranacea cyanobiont' 232. Bacteria; Cyanobacteria; Nostocales; Nostocaceae; Nostoc.

CDG79__locus_1_main_sat

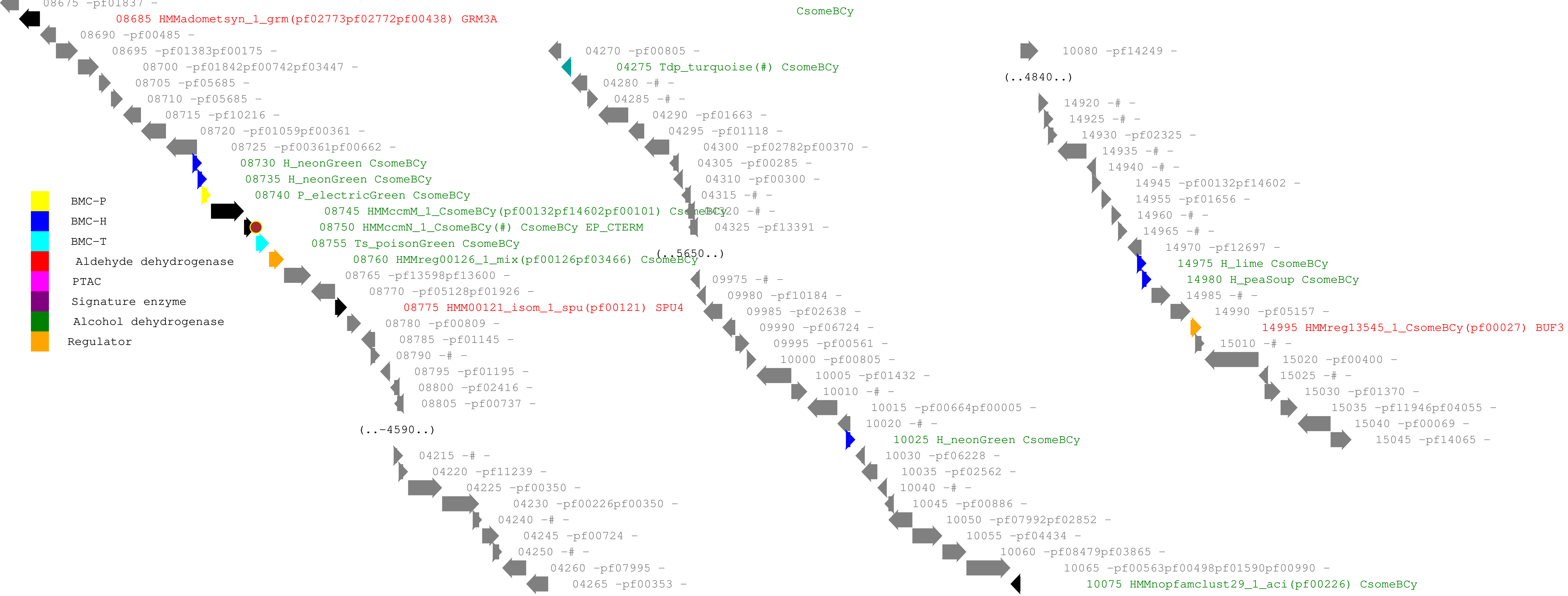

Fischerella thermalis CCMEE 5201. Bacteria; Cyanobacteria; Nostocales; Hapalosiphonaceae; Fischerella.

CEN39__locus_1_main_sat

CsomeBCy

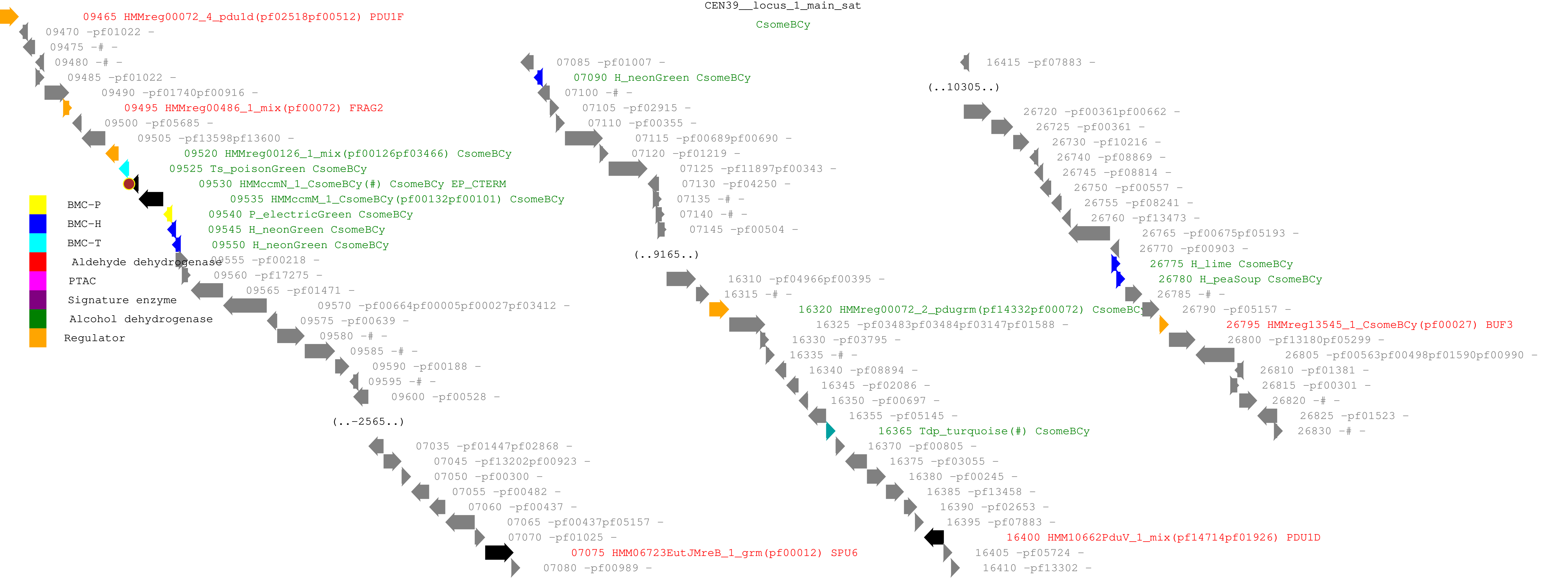

Fischerella thermalis CCMEE 5205. Bacteria; Cyanobacteria; Nostocales; Hapalosiphonaceae; Fischerella.

CEN40__locus_1_main_sat

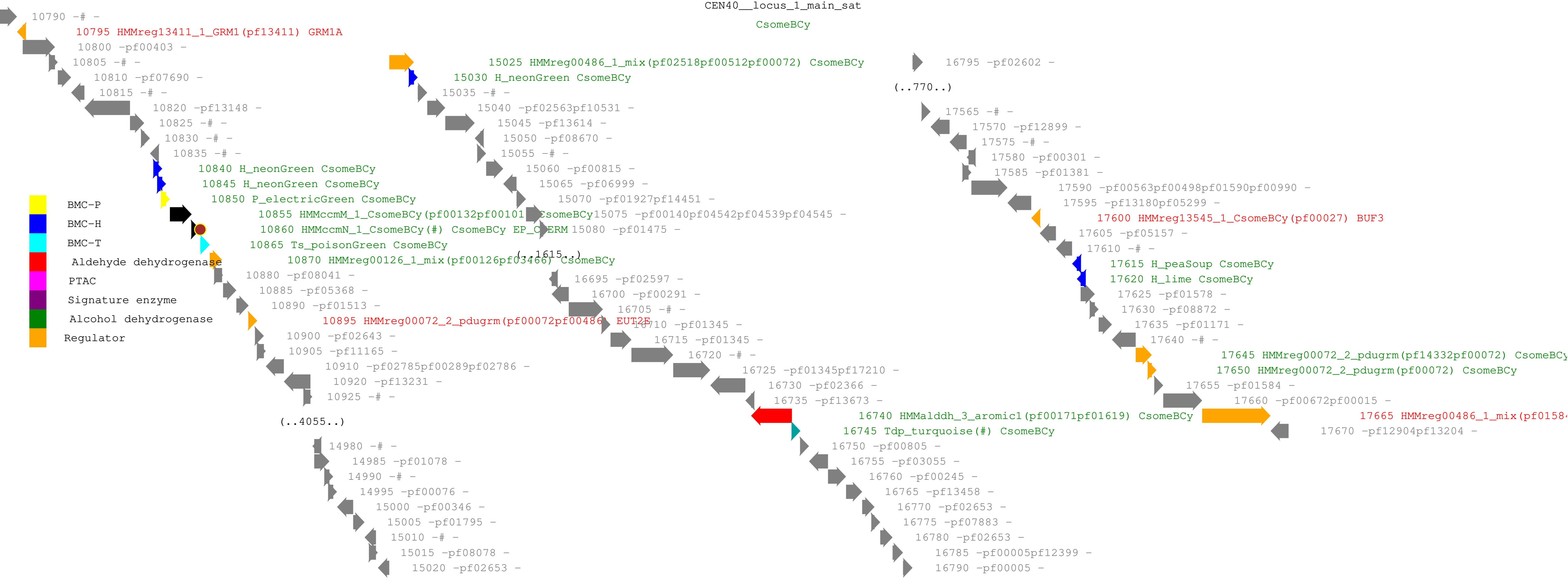

Fischerella thermalis CCMEE 5330. Bacteria; Cyanobacteria; Nostocales; Hapalosiphonaceae; Fischerella.

CEN41__locus_1_main_sat

CsomeBCy

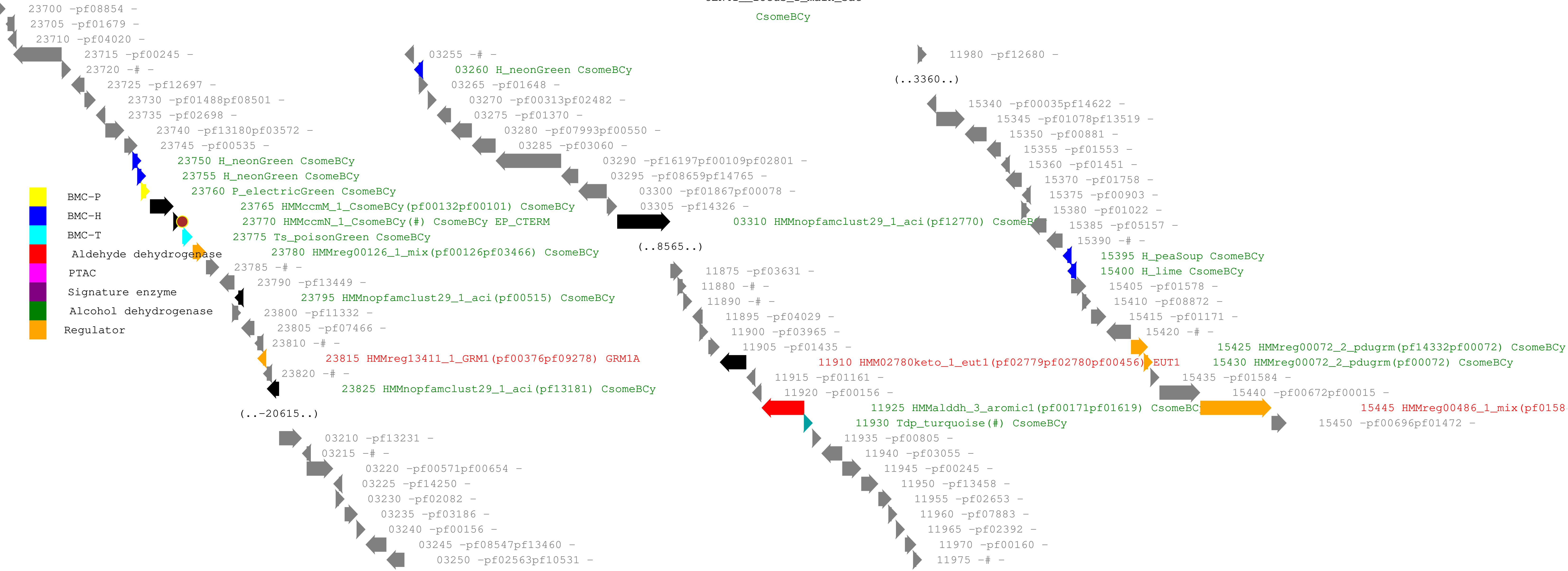

Fischerella muscicola CCMEE 5323. Bacteria; Cyanobacteria; Nostocales; Hapalosiphonaceae; Fischerella.

CEN44__locus_1_main_sat

CsomeBCy

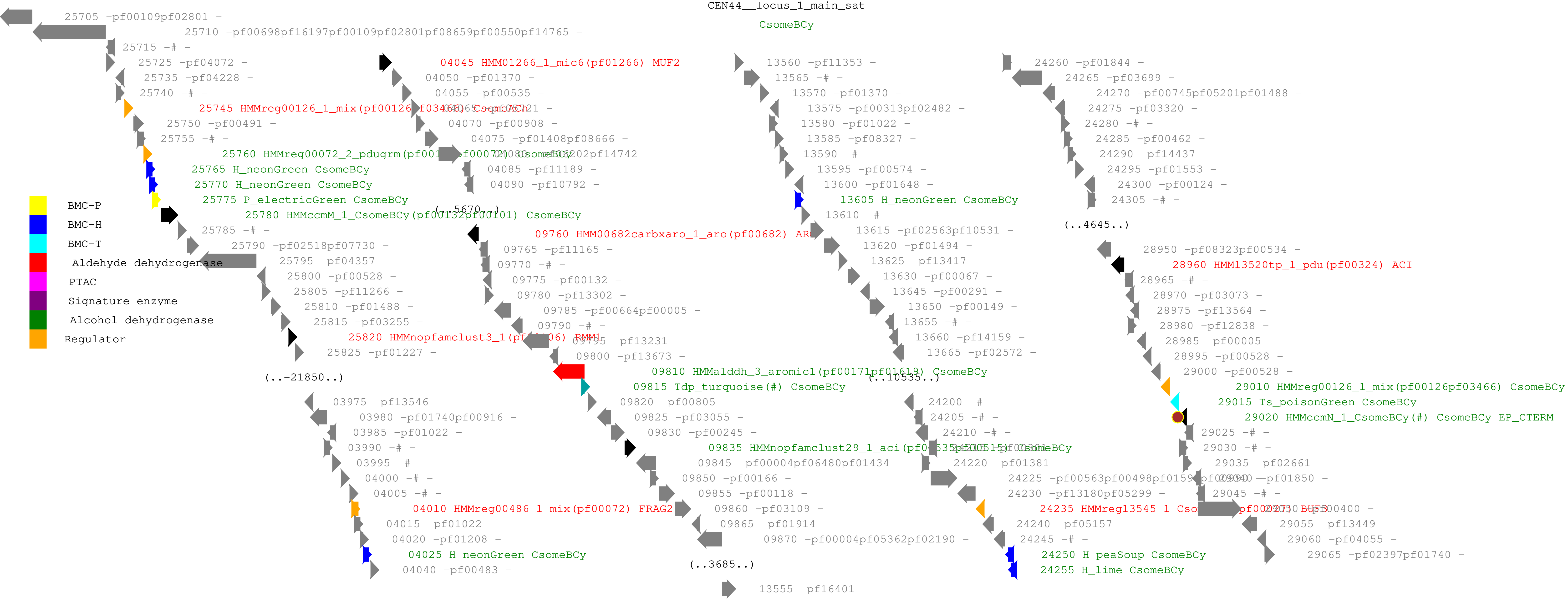

Fischerella thermalis CCMEE 5318. Bacteria; Cyanobacteria; Nostocales; Hapalosiphonaceae; Fischerella.

CEN46__locus_1_main_sat

CsomeBCy

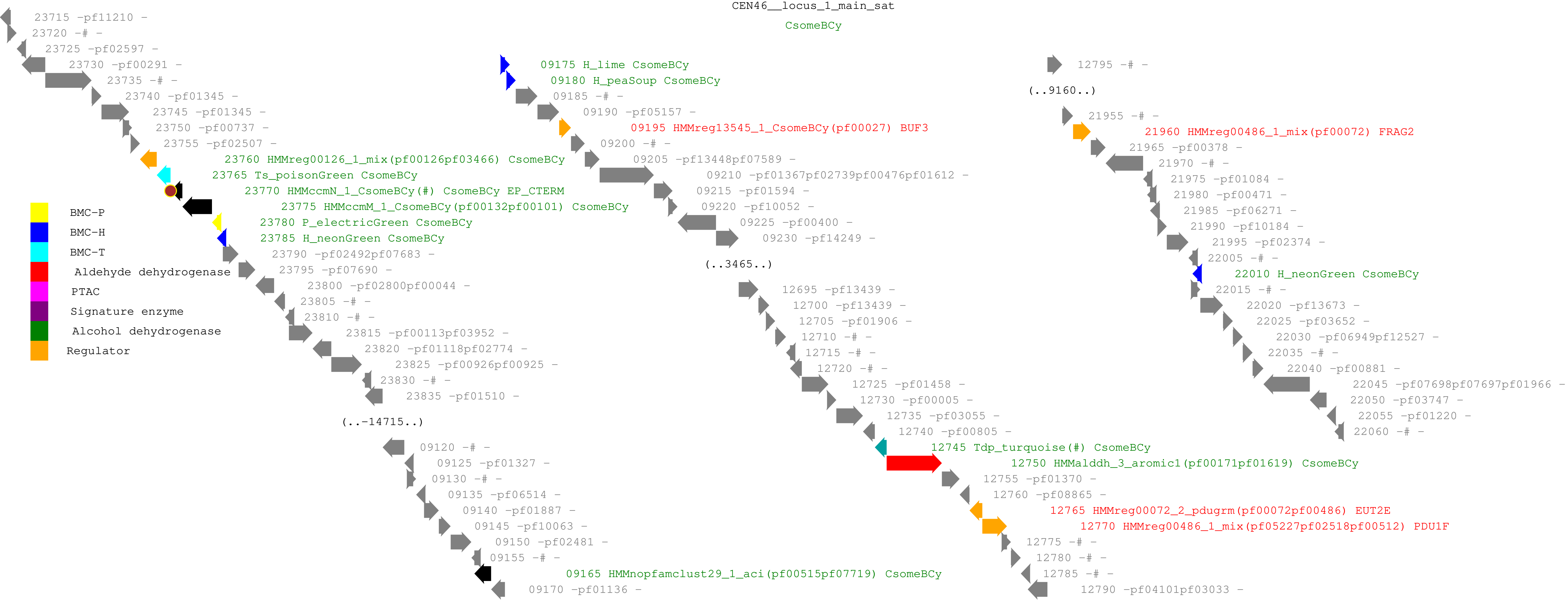

Fischerella thermalis CCMEE 5319. Bacteria; Cyanobacteria; Nostocales; Hapalosiphonaceae; Fischerella.

CEN47__locus_1_main_sat

CsomeBCy

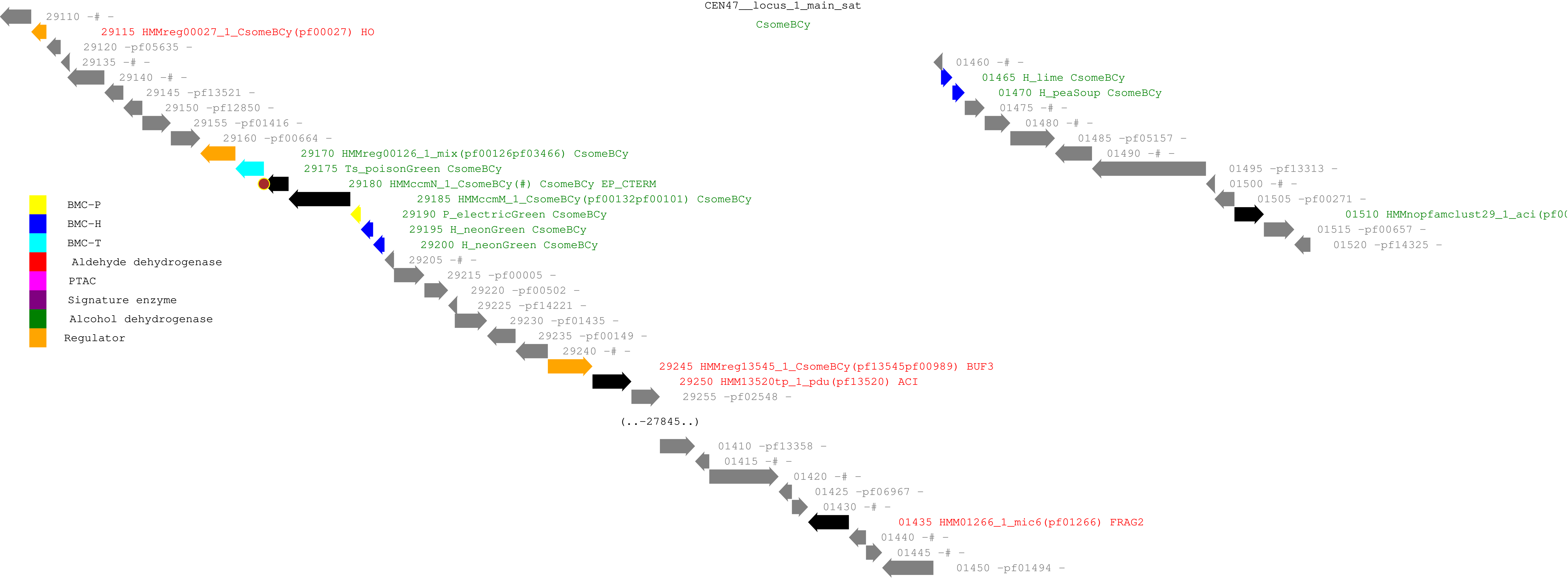

Fischerella thermalis CCMEE 5273. Bacteria; Cyanobacteria; Nostocales; Hapalosiphonaceae; Fischerella.

CEN49__locus_1_main_sat

CsomeBCy

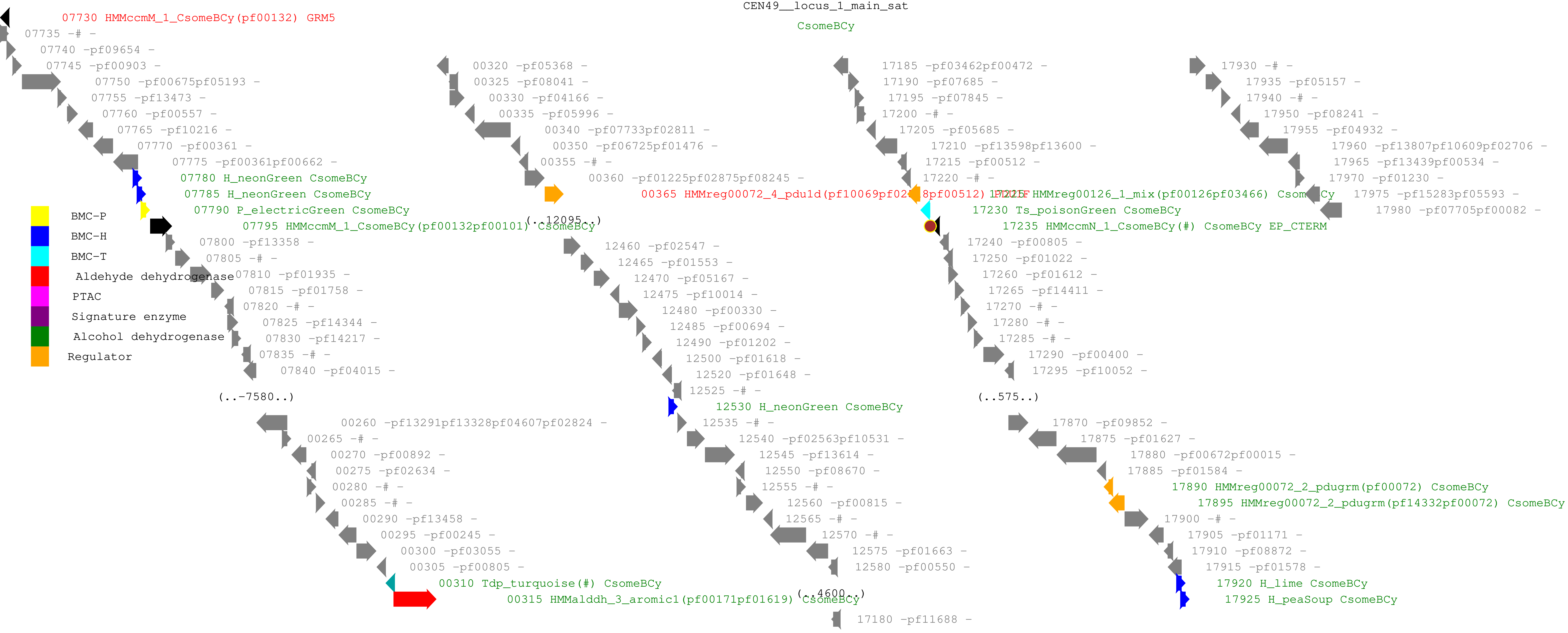

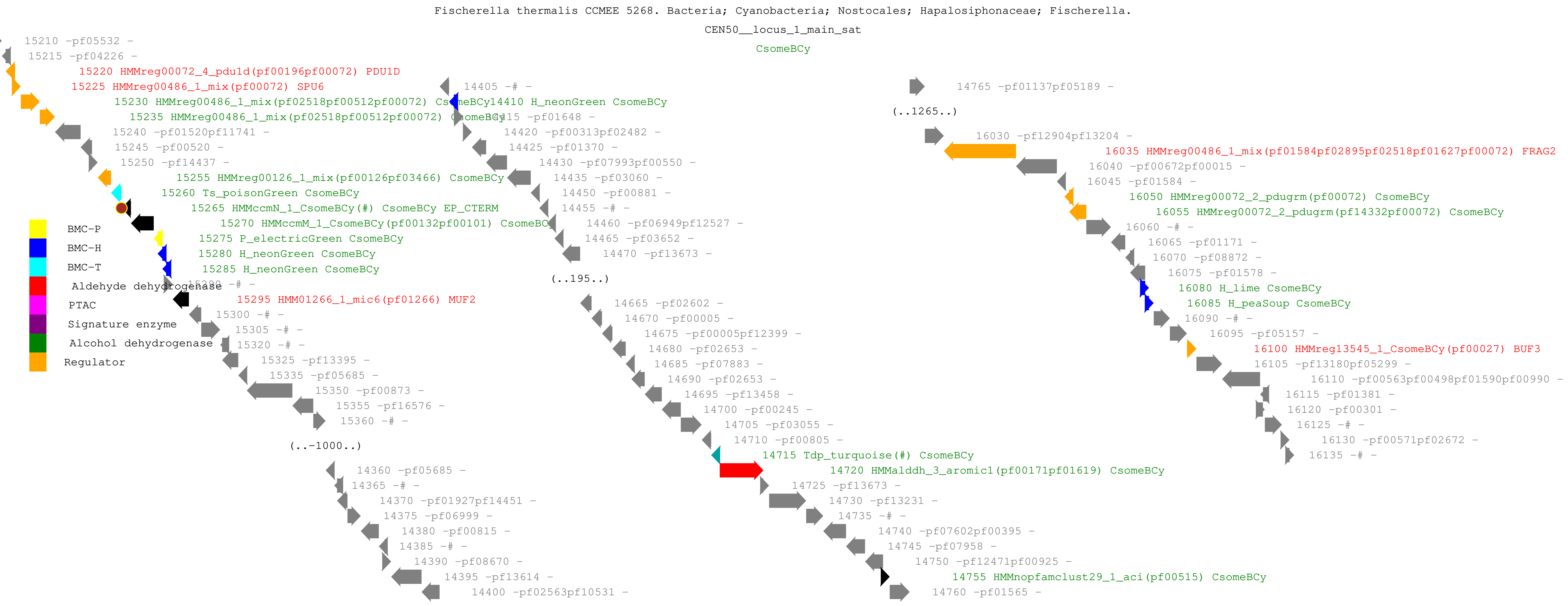

Fischerella thermalis CCMEE 5268. Bacteria; Cyanobacteria; Nostocales; Hapalosiphonaceae; Fischerella.
CEN50__locus_1_main_sat
CsomeBCy
15210 -pf05532 -
15215 -pf04226 -
15220 HMMreg00072_4_pdu1d(pf00196pf00072) PDU1D
15225 HMMreg00486_1_mix(pf00072) SPU6
15230 HMMreg00486_1_mix(pf02518pf00512pf00072) CsomeBCy
15235 HMMreg00486_1_mix(pf02518pf00512pf00072) CsomeBCy
15240 -pf01520pf11741 -
15245 -pf00520 -
15250 -pf14437 -
15255 HMMreg00126_1_mix(pf00126pf03466) CsomeBCy
15260 Ts_poisonGreen CsomeBCy
15265 HMMccmN_1_CsomeBCy(#) CsomeBCy EP_CTERM
15270 HMMccmM_1_CsomeBCy(pf00132pf00101) CsomeBCy
15275 P_electricGreen CsomeBCy
15280 H_neonGreen CsomeBCy
15285 H_neonGreen CsomeBCy
15290 -# -
15295 HMM01266_1_mic6(pf01266) MUF2
15300 -# -
15305 -# -
15320 -# -
15325 -pf13395 -
15335 -pf05685 -
15350 -pf00873 -
15355 -pf16576 -
15360 -# -
(..-1000..)
14360 -pf05685 -
14365 -# -
14370 -pf01927pf14451 -
14375 -pf06999 -
14380 -pf00815 -
14385 -# -
14390 -pf08670 -
14395 -pf13614 -
14400 -pf02563pf10531 -
14405 -# -
14410 H_neonGreen CsomeBCy
14415 -pf01648 -
14420 -pf00313pf02482 -
14425 -pf01370 -
14430 -pf07993pf00550 -
14435 -pf03060 -
14450 -pf00881 -
14455 -# -
14460 -pf06949pf12527 -
14465 -pf03652 -
14470 -pf13673 -
(..195..)
14665 -pf02602 -
14670 -pf00005 -
14675 -pf00005pf12399 -
14680 -pf02653 -
14685 -pf07883 -
14690 -pf02653 -
14695 -pf13458 -
14700 -pf00245 -
14705 -pf03055 -
14710 -pf00805 -
14715 Tdp_turquoise(#) CsomeBCy
14720 HMMalddh_3_aromic1(pf00171pf01619) CsomeBCy
14725 -pf13673 -
14730 -pf13231 -
14735 -# -
14740 -pf07602pf00395 -
14745 -pf07958 -
14750 -pf12471pf00925 -
14755 HMMnopfamclust29_1_aci(pf00515) CsomeBCy
14760 -pf01565 -
14765 -pf01137pf05189 -
(..1265..)
16030 -pf12904pf13204 -
16035 HMMreg00486_1_mix(pf01584pf02895pf02518pf01627pf00072) FRAG2
16040 -pf00672pf00015 -
16045 -pf01584 -
16050 HMMreg00072_2_pdugrm(pf00072) CsomeBCy
16055 HMMreg00072_2_pdugrm(pf14332pf00072) CsomeBCy
16060 -# -
16065 -pf01171 -
16070 -pf08872 -
16075 -pf01578 -
16080 H_lime CsomeBCy
16085 H_peaSoup CsomeBCy
16090 -# -
16095 -pf05157 -
16100 HMMreg13545_1_CsomeBCy(pf00027) BUF3
16105 -pf13180pf05299 -
16110 -pf00563pf00498pf01590pf00990 -
16115 -pf01381 -
16120 -pf00301 -
16125 -# -
16130 -pf00571pf02672 -
16135 -# -
BMC-P
BMC-H
BMC-T
Aldehyde dehydrogenase
PTAC
Signature enzyme
Alcohol dehydrogenase
Regulator

Cylindrospermopsis raciborskii S05. Bacteria; Cyanobacteria; Nostocales; Aphanizomenonaceae;

CEP08__locus_1_main_sat

CsomeBCy

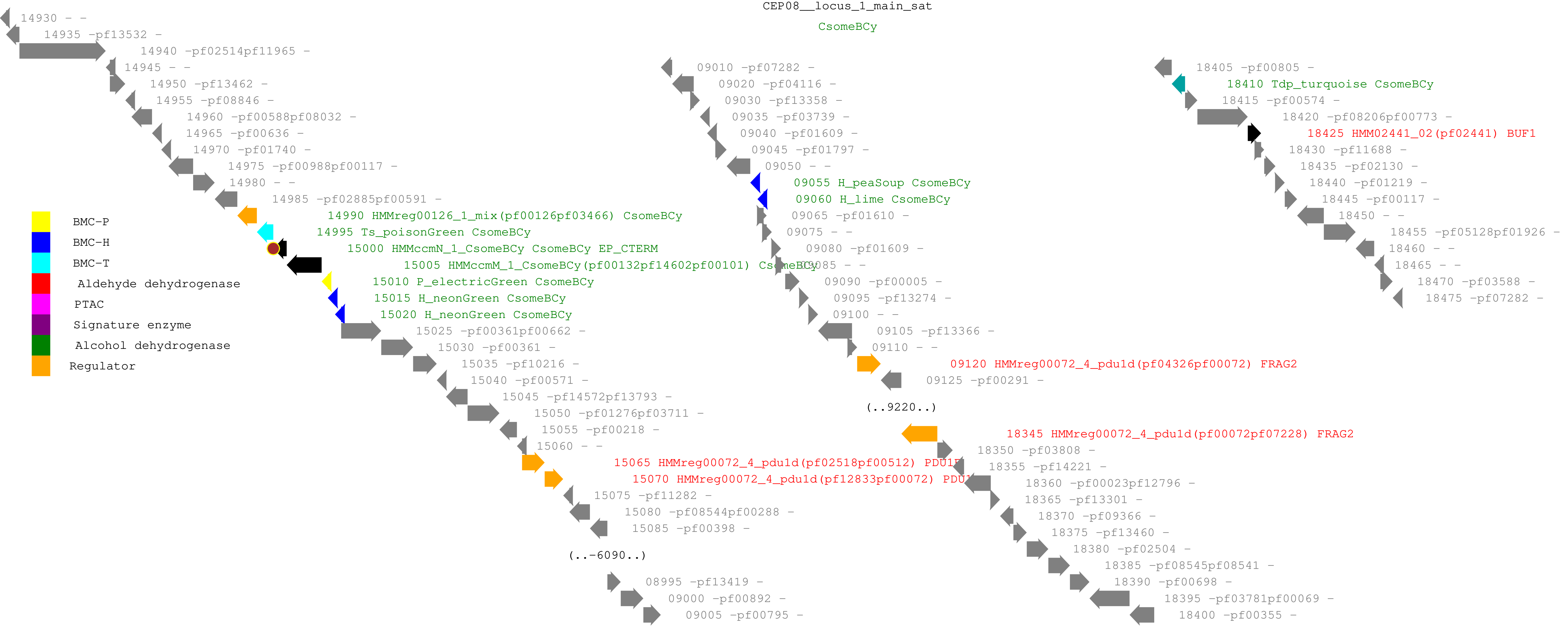

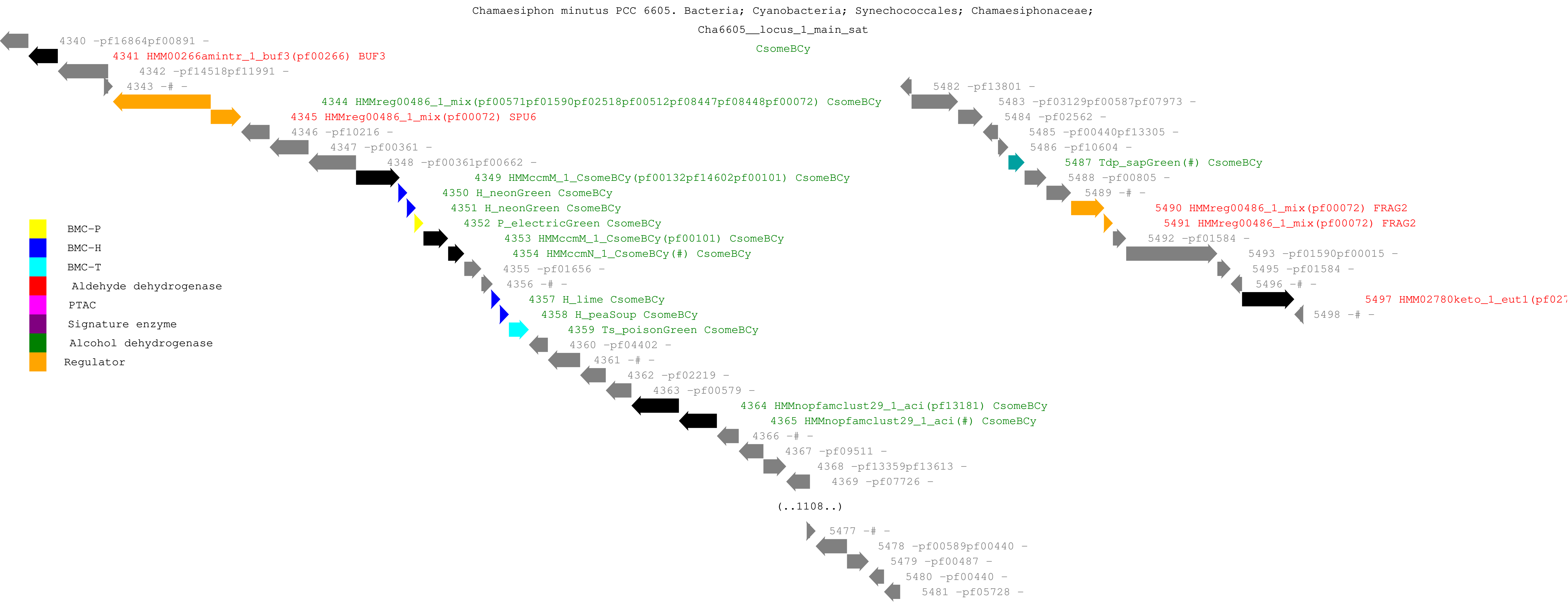
Chamaesiphon minutus PCC 6605. Bacteria; Cyanobacteria; Synechococcales; Chamaesiphonaceae;
Cha6605__locus_1_main_sat
CsomeBCy
4340 -pf16864pf00891 -
4341 HMM00266amintr_1_buf3(pf00266) BUF3
4342 -pf14518pf11991 -
4343 -# -
4344 HMMreg00486_1_mix(pf00571pf01590pf02518pf00512pf08447pf08448pf00072) CsomeBCy
4345 HMMreg00486_1_mix(pf00072) SPU6
4346 -pf10216 -
4347 -pf00361 -
4348 -pf00361pf00662 -
4349 HMMccmM_1_CsomeBCy(pf00132pf14602pf00101) CsomeBCy
4350 H_neonGreen CsomeBCy
4351 H_neonGreen CsomeBCy
4352 P_electricGreen CsomeBCy
4353 HMMccmM_1_CsomeBCy(pf00101) CsomeBCy
4354 HMMccmN_1_CsomeBCy(#) CsomeBCy
4355 -pf01656 -
4356 -# -
4357 H_lime CsomeBCy
4358 H_peaSoup CsomeBCy
4359 Ts_poisonGreen CsomeBCy
4360 -pf04402 -
4361 -# -
4362 -pf02219 -
4363 -pf00579 -
4364 HMMnopfamclust29_1_aci(pf13181) CsomeBCy
4365 HMMnopfamclust29_1_aci(#) CsomeBCy
4366 -# -
4367 -pf09511 -
4368 -pf13359pf13613 -
4369 -pf07726 -
(..1108..)
5477 -# -
5478 -pf00589pf00440 -
5479 -pf00487 -
5480 -pf00440 -
5481 -pf05728 -
5482 -pf13801 -
5483 -pf03129pf00587pf07973 -
5484 -pf02562 -
5485 -pf00440pf13305 -
5486 -pf10604 -
5487 Tdp_sapGreen(#) CsomeBCy
5488 -pf00805 -
5489 -# -
5490 HMMreg00486_1_mix(pf00072) FRAG2
5491 HMMreg00486_1_mix(pf00072) FRAG2
5492 -pf01584 -
5493 -pf01590pf00015 -
5495 -pf01584 -
5496 -# -
5497 HMM02780keto_1_eut1(pf027
5498 -# -
BMC-P
BMC-H
BMC-T
Aldehyde dehydrogenase
PTAC
Signature enzyme
Alcohol dehydrogenase
Regulator

Chroococcidiopsis thermalis PCC 7203. Bacteria; Cyanobacteria; Chroococcidiopsidales;

Chro__locus_1_main_sat

CsomeBCy

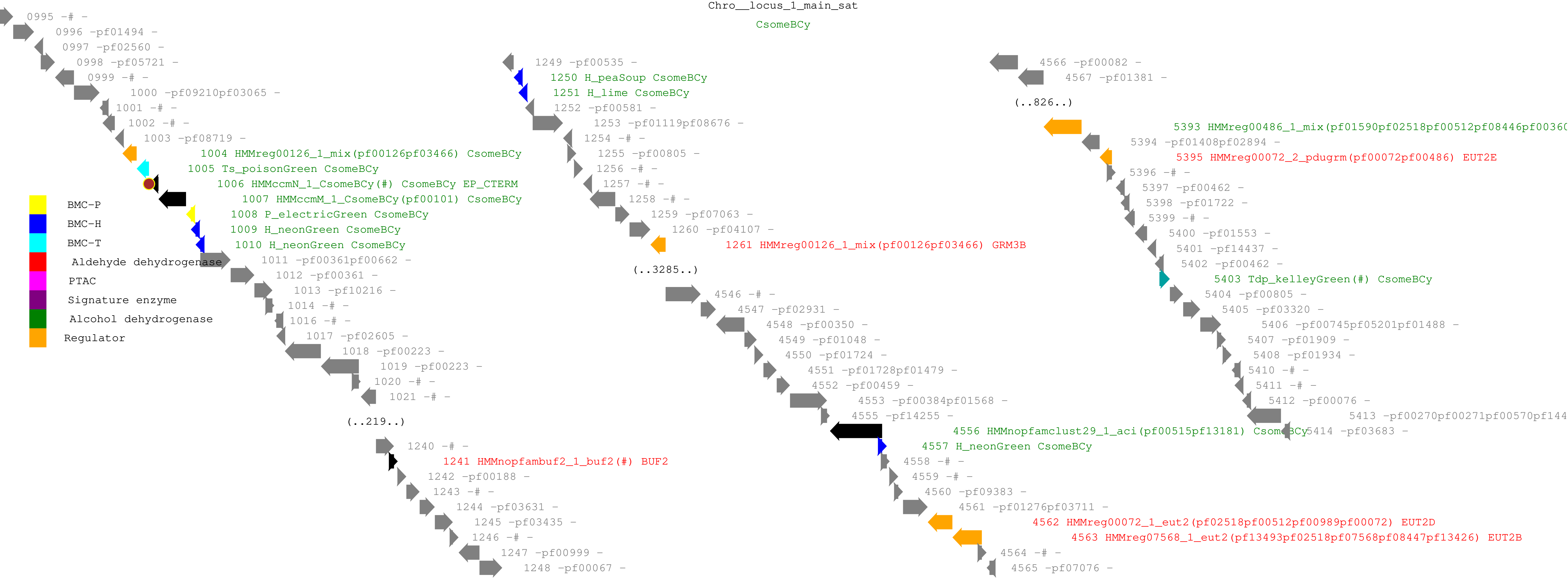

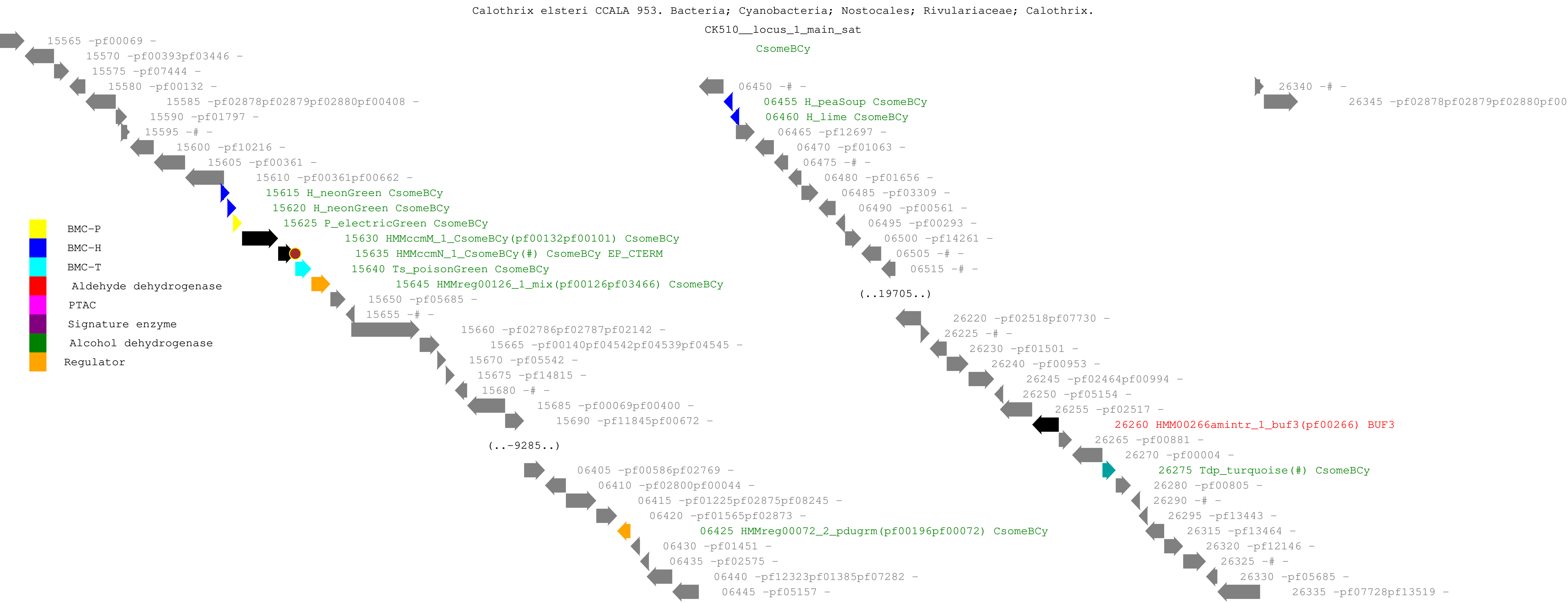
Calothrix elsteri CCALA 953. Bacteria; Cyanobacteria; Nostocales; Rivulariaceae; Calothrix.
CK510__locus_1_main_sat
CsomeBCy
15565 -pf00069 -
15570 -pf00393pf03446 -
15575 -pf07444 -
15580 -pf00132 -
15585 -pf02878pf02879pf02880pf00408 -
15590 -pf01797 -
15595 -# -
15600 -pf10216 -
15605 -pf00361 -
15610 -pf00361pf00662 -
15615 H_neonGreen CsomeBCy
15620 H_neonGreen CsomeBCy
15625 P_electricGreen CsomeBCy
15630 HMMccmM_1_CsomeBCy(pf00132pf00101) CsomeBCy
15635 HMMccmN_1_CsomeBCy(#) CsomeBCy EP_CTERM
15640 Ts_poisonGreen CsomeBCy
15645 HMMreg00126_1_mix(pf00126pf03466) CsomeBCy
15650 -pf05685 -
15655 -# -
15660 -pf02786pf02787pf02142 -
15665 -pf00140pf04542pf04539pf04545 -
15670 -pf05542 -
15675 -pf14815 -
15680 -# -
15685 -pf00069pf00400 -
15690 -pf11845pf00672 -
(..-9285..)
06405 -pf00586pf02769 -
06410 -pf02800pf00044 -
06415 -pf01225pf02875pf08245 -
06420 -pf01565pf02873 -
06425 HMMreg00072_2_pdugrm(pf00196pf00072) CsomeBCy
06430 -pf01451 -
06435 -pf02575 -
06440 -pf12323pf01385pf07282 -
06445 -pf05157 -
06450 -# -
06455 H_peaSoup CsomeBCy
06460 H_lime CsomeBCy
06465 -pf12697 -
06470 -pf01063 -
06475 -# -
06480 -pf01656 -
06485 -pf03309 -
06490 -pf00561 -
06495 -pf00293 -
06500 -pf14261 -
06505 -# -
06515 -# -
(..19705..)
26220 -pf02518pf07730 -
26225 -# -
26230 -pf01501 -
26240 -pf00953 -
26245 -pf02464pf00994 -
26250 -pf05154 -
26255 -pf02517 -
26260 HMM00266amintr_1_buf3(pf00266) BUF3
26265 -pf00881 -
26270 -pf00004 -
26275 Tdp_turquoise(#) CsomeBCy
26280 -pf00805 -
26290 -# -
26295 -pf13443 -
26315 -pf13464 -
26320 -pf12146 -
26325 -# -
26330 -pf05685 -
26335 -pf07728pf13519 -
26340 -# -
26345 -pf02878pf02879pf02880pf00
BMC-P
BMC-H
BMC-T
Aldehyde dehydrogenase
PTAC
Signature enzyme
Alcohol dehydrogenase
Regulator

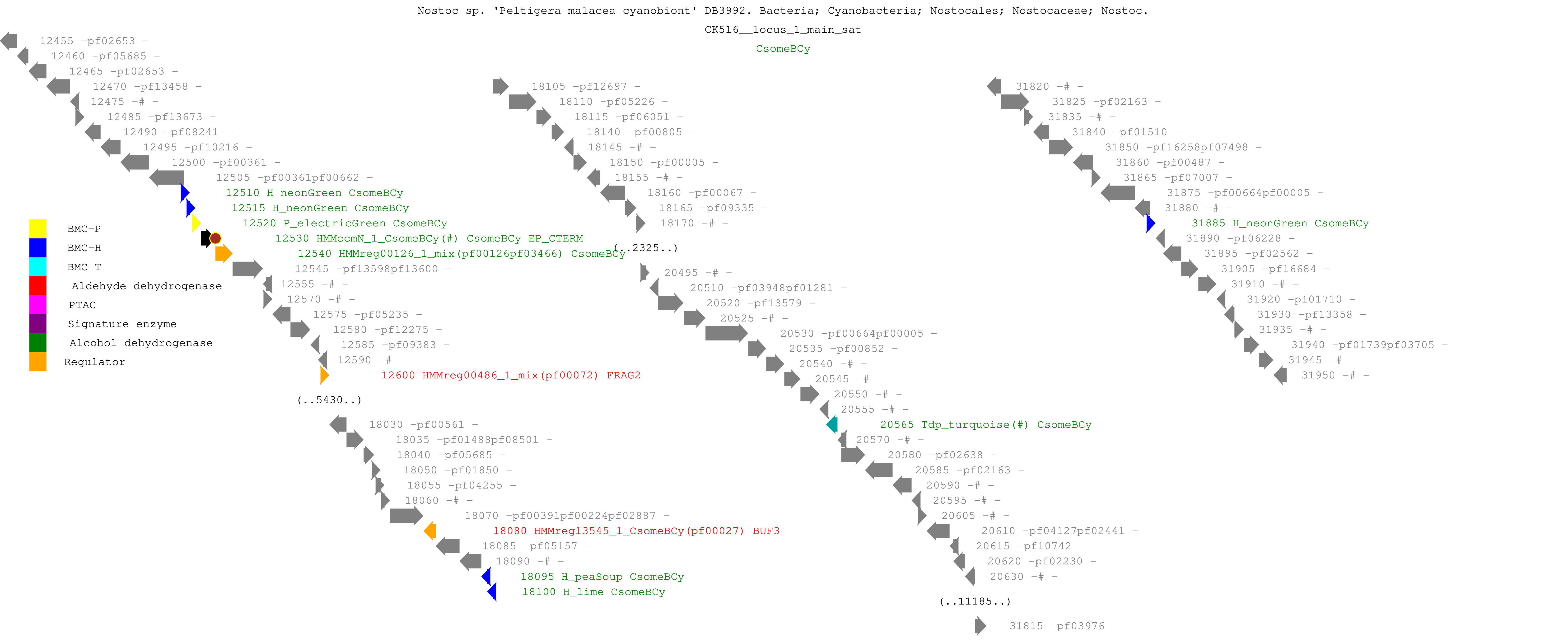
Nostoc sp. 'Peltigera malacea cyanobiont' DB3992. Bacteria; Cyanobacteria; Nostocales; Nostocaceae; Nostoc.
CK516__locus_1_main_sat
CsomeBCy
BMC-P
BMC-H
BMC-T
Aldehyde dehydrogenase
PTAC
Signature enzyme
Alcohol dehydrogenase
Regulator
12455 -pf02653 -
12460 -pf05685 -
12465 -pf02653 -
12470 -pf13458 -
12475 -# -
12485 -pf13673 -
12490 -pf08241 -
12495 -pf10216 -
12500 -pf00361 -
12505 -pf00361pf00662 -
12510 H_neonGreen CsomeBCy
12515 H_neonGreen CsomeBCy
12520 P_electricGreen CsomeBCy
12530 HMMccmN_1_CsomeBCy(#) CsomeBCy EP_CTERM
12540 HMMreg00126_1_mix(pf00126pf03466) CsomeBCy
12545 -pf13598pf13600 -
12555 -# -
12570 -# -
12575 -pf05235 -
12580 -pf12275 -
12585 -pf09383 -
12590 -# -
12600 HMMreg00486_1_mix(pf00072) FRAG2
(..5430..)
18030 -pf00561 -
18035 -pf01488pf08501 -
18040 -pf05685 -
18050 -pf01850 -
18055 -pf04255 -
18060 -# -
18070 -pf00391pf00224pf02887 -
18080 HMMreg13545_1_CsomeBCy(pf00027) BUF3
18085 -pf05157 -
18090 -# -
18095 H_peaSoup CsomeBCy
18100 H_lime CsomeBCy
18105 -pf12697 -
18110 -pf05226 -
18115 -pf06051 -
18140 -pf00805 -
18145 -# -
18150 -pf00005 -
18155 -# -
18160 -pf00067 -
18165 -pf09335 -
18170 -# -
(..2325..)
20495 -# -
20510 -pf03948pf01281 -
20520 -pf13579 -
20525 -# -
20530 -pf00664pf00005 -
20535 -pf00852 -
20540 -# -
20545 -# -
20550 -# -
20555 -# -
20565 Tdp_turquoise(#) CsomeBCy
20570 -# -
20580 -pf02638 -
20585 -pf02163 -
20590 -# -
20595 -# -
20605 -# -
20610 -pf04127pf02441 -
20615 -pf10742 -
20620 -pf02230 -
20630 -# -
(..11185..)
31815 -pf03976 -
31820 -# -
31825 -pf02163 -
31835 -# -
31840 -pf01510 -
31850 -pf16258pf07498 -
31860 -pf00487 -
31865 -pf07007 -
31875 -pf00664pf00005 -
31880 -# -
31885 H_neonGreen CsomeBCy
31890 -pf06228 -
31895 -pf02562 -
31905 -pf16684 -
31910 -# -
31920 -pf01710 -
31930 -pf13358 -
31935 -# -
31940 -pf01739pf03705 -
31945 -# -
31950 -# -

Nostoc sp. CENA543. Bacteria; Cyanobacteria; Nostocales; Nostocaceae; Nostoc.

CLI64__locus_1_main_sat

CsomeBCy

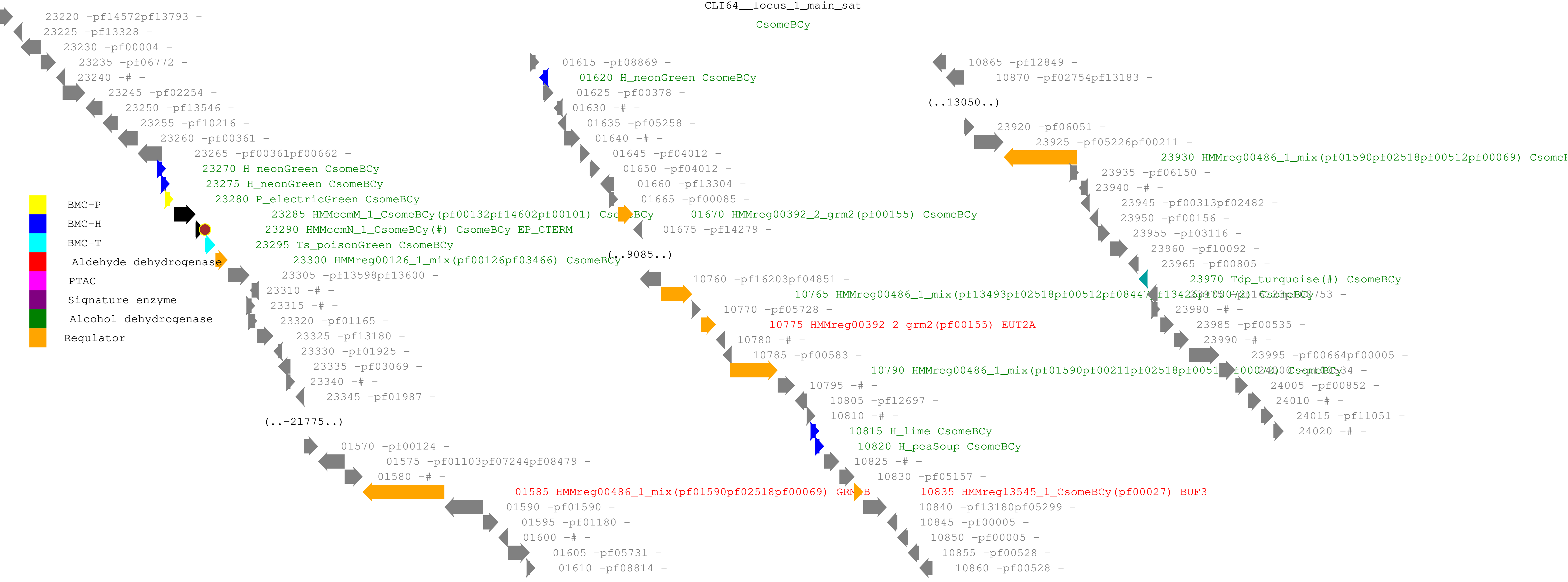

Nostoc flagelliforme CCNUN1. Bacteria; Cyanobacteria; Nostocales; Nostocaceae; Nostoc.

C0091__locus_1_main_sat

CsomeBCy

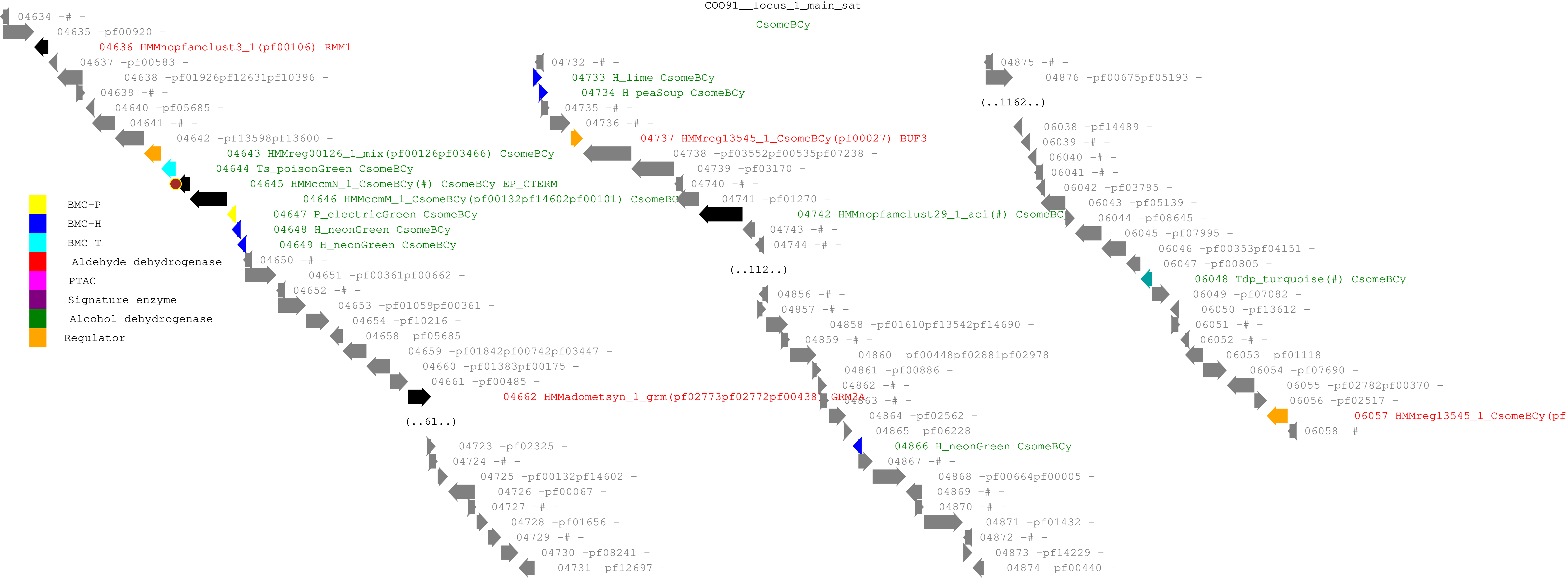

Tychonema bourrellyi FEM_GT703. Bacteria; Cyanobacteria; Oscillatoriophycideae; Oscillatoriales;

CP500__locus_1_main_sat

CsomeBCy

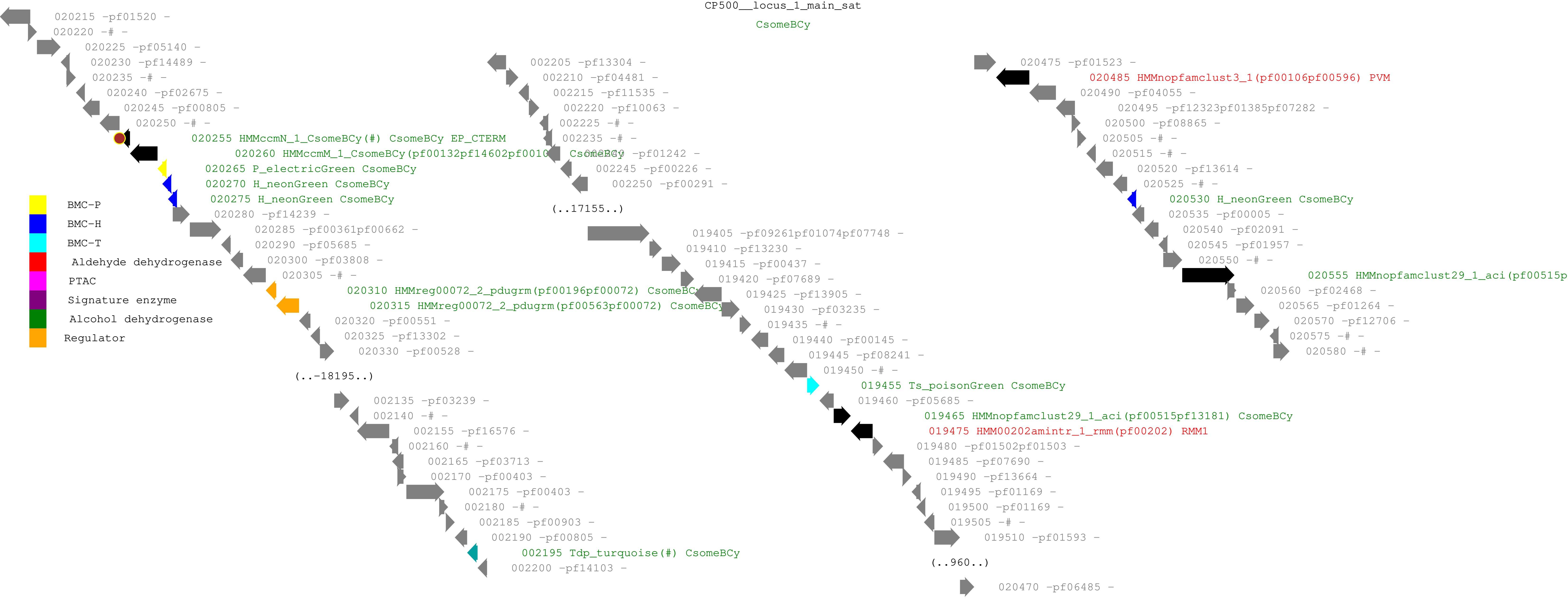

Cylindrospermopsis raciborskii CS-505. Bacteria; Cyanobacteria; Nostocales; Aphanizomenonaceae;

CRC__locus_1_main_sat

CsomeBCy

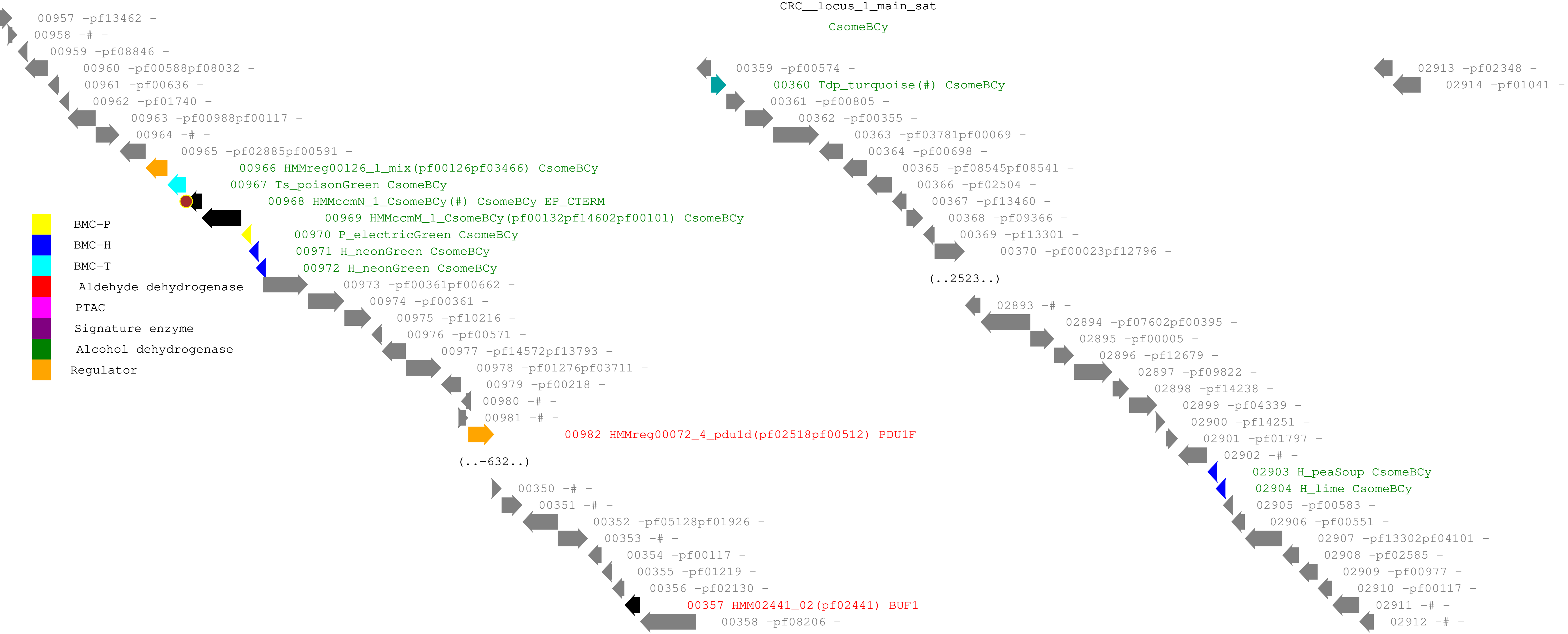

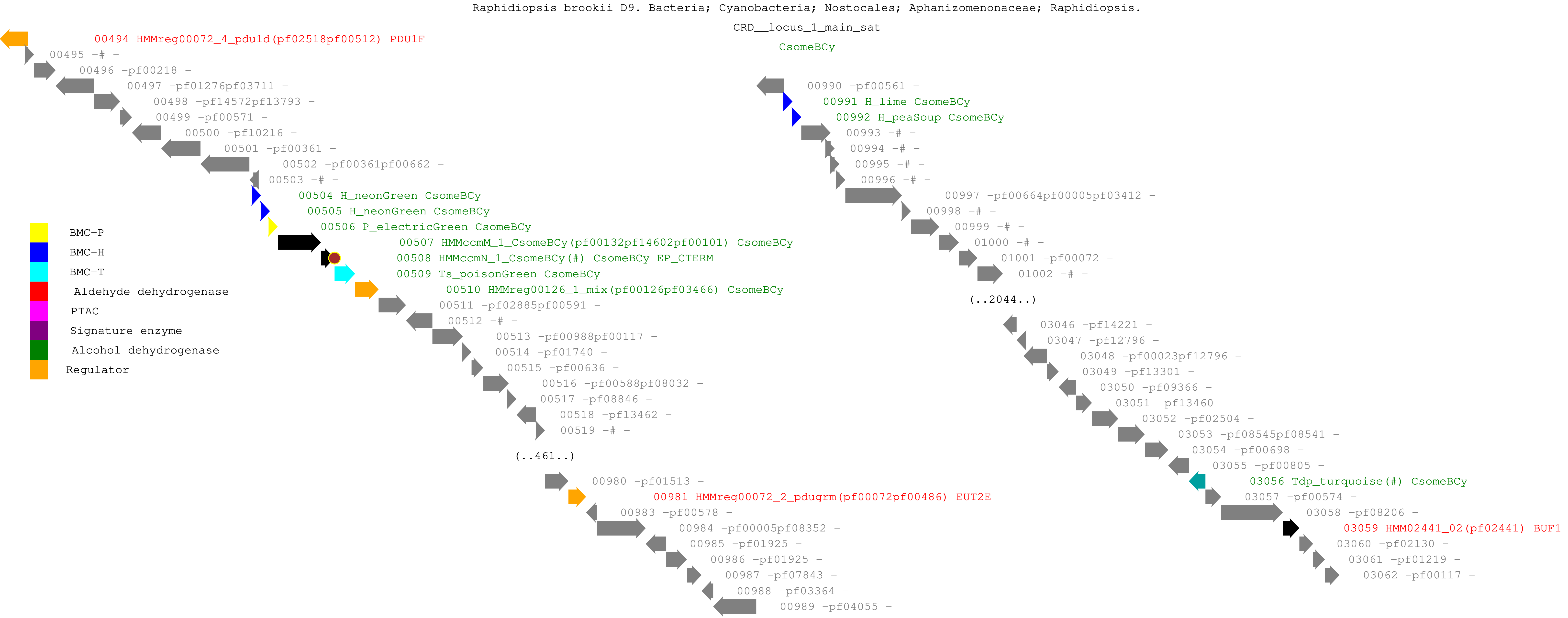
Raphidiopsis brookii D9. Bacteria; Cyanobacteria; Nostocales; Aphanizomenonaceae; Raphidiopsis.
CRD__locus_1_main_sat
CsomeBCy
00494 HMMreg00072_4_pdu1d(pf02518pf00512) PDU1F
00495 -# -
00496 -pf00218 -
00497 -pf01276pf03711 -
00498 -pf14572pf13793 -
00499 -pf00571 -
00500 -pf10216 -
00501 -pf00361 -
00502 -pf00361pf00662 -
00503 -# -
00504 H_neonGreen CsomeBCy
00505 H_neonGreen CsomeBCy
00506 P_electricGreen CsomeBCy
00507 HMMccmM_1_CsomeBCy(pf00132pf14602pf00101) CsomeBCy
00508 HMMccmN_1_CsomeBCy(#) CsomeBCy EP_CTERM
00509 Ts_poisonGreen CsomeBCy
00510 HMMreg00126_1_mix(pf00126pf03466) CsomeBCy
00511 -pf02885pf00591 -
00512 -# -
00513 -pf00988pf00117 -
00514 -pf01740 -
00515 -pf00636 -
00516 -pf00588pf08032 -
00517 -pf08846 -
00518 -pf13462 -
00519 -# -
(..461..)
00980 -pf01513 -
00981 HMMreg00072_2_pdugrm(pf00072pf00486) EUT2E
00983 -pf00578 -
00984 -pf00005pf08352 -
00985 -pf01925 -
00986 -pf01925 -
00987 -pf07843 -
00988 -pf03364 -
00989 -pf04055 -
00990 -pf00561 -
00991 H_lime CsomeBCy
00992 H_peaSoup CsomeBCy
00993 -# -
00994 -# -
00995 -# -
00996 -# -
00997 -pf00664pf00005pf03412 -
00998 -# -
00999 -# -
01000 -# -
01001 -pf00072 -
01002 -# -
(..2044..)
03046 -pf14221 -
03047 -pf12796 -
03048 -pf00023pf12796 -
03049 -pf13301 -
03050 -pf09366 -
03051 -pf13460 -
03052 -pf02504 -
03053 -pf08545pf08541 -
03054 -pf00698 -
03055 -pf00805 -
03056 Tdp_turquoise(#) CsomeBCy
03057 -pf00574 -
03058 -pf08206 -
03059 HMM02441_02(pf02441) BUF1
03060 -pf02130 -
03061 -pf01219 -
03062 -pf00117 -
BMC-P
BMC-H
BMC-T
Aldehyde dehydrogenase
PTAC
Signature enzyme
Alcohol dehydrogenase
Regulator

Crinalium epipsammum PCC 9333. Bacteria; Cyanobacteria; Oscillatoriophycideae; Oscillatoriales;

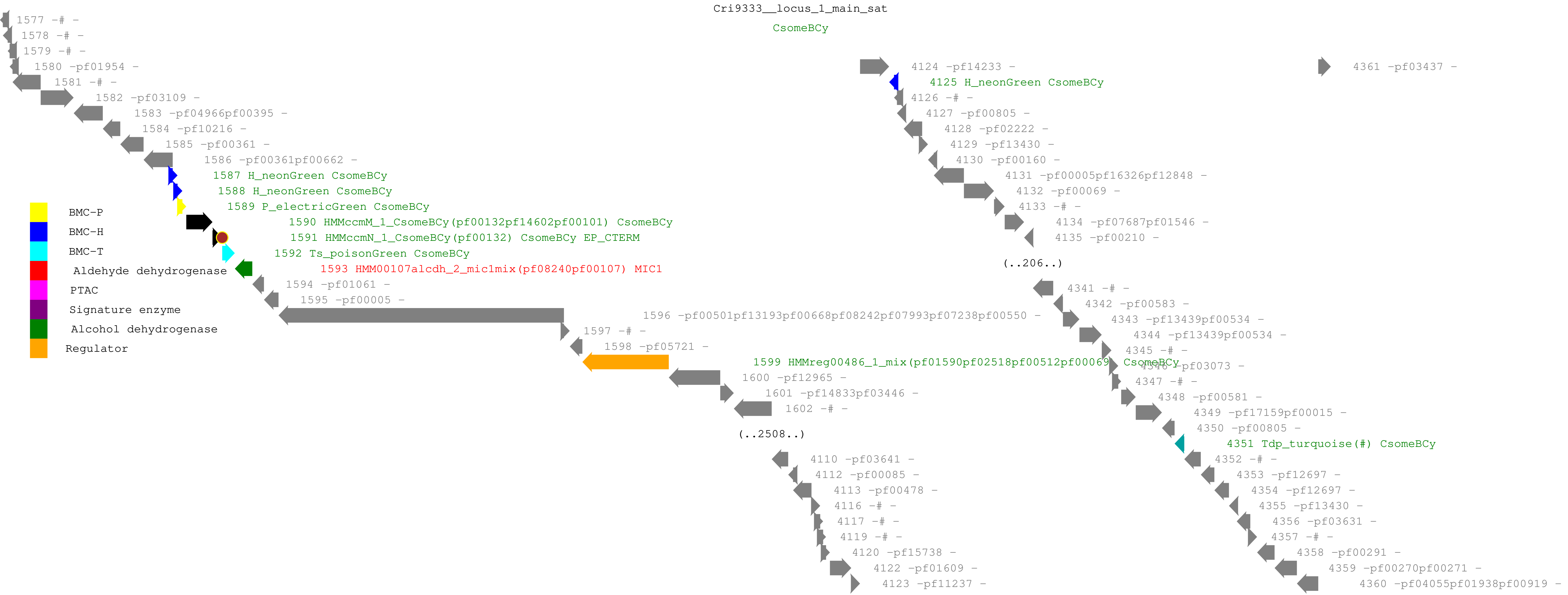

Chroococcales cyanobacterium IPPAS B-1203. Bacteria; Cyanobacteria; Oscillatoriophycideae; Chroococcales.

CSQ79__locus_1_main_sat

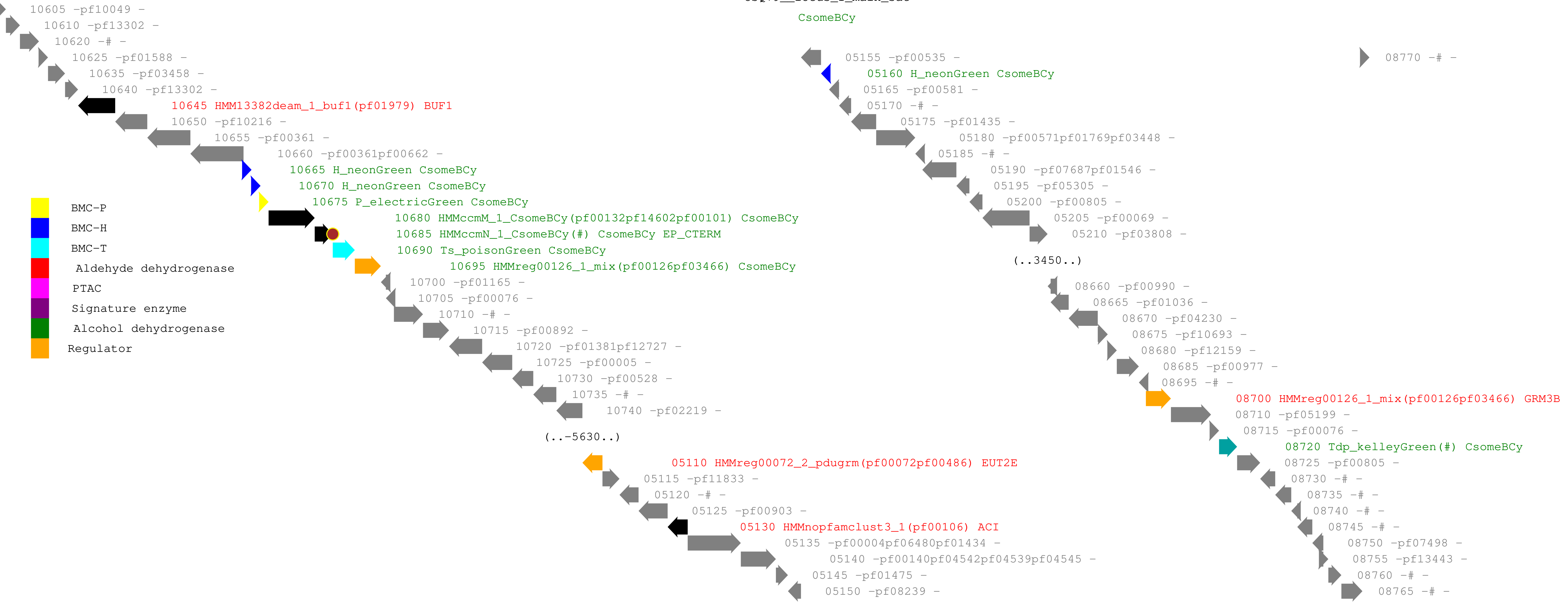

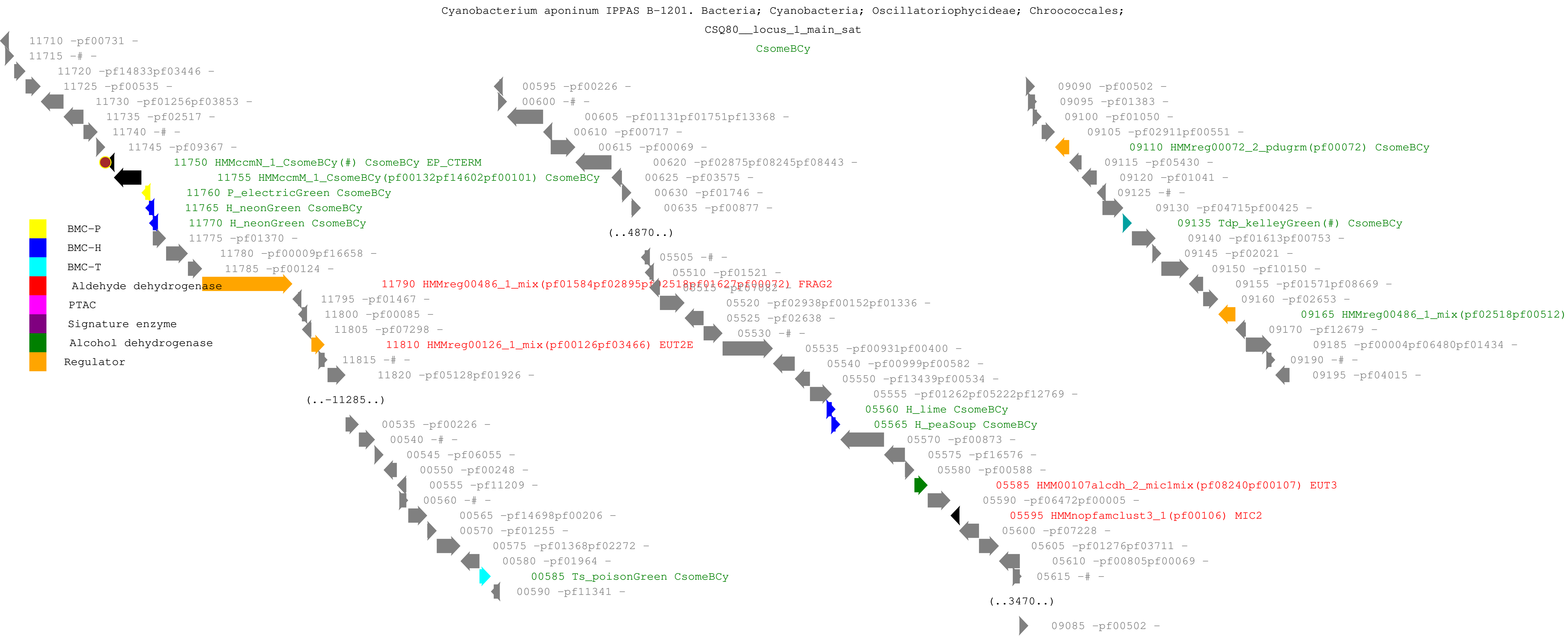
Cyanobacterium aponinum IPPAS B-1201. Bacteria; Cyanobacteria; Oscillatoriophycideae; Chroococcales;
CSQ80__locus_1_main_sat
CsomeBCy
11710 -pf00731 -
11715 -# -
11720 -pf14833pf03446 -
11725 -pf00535 -
11730 -pf01256pf03853 -
11735 -pf02517 -
11740 -# -
11745 -pf09367 -
11750 HMMccmN_1_CsomeBCy(#) CsomeBCy EP_CTERM
11755 HMMccmM_1_CsomeBCy(pf00132pf14602pf00101) CsomeBCy
11760 P_electricGreen CsomeBCy
11765 H_neonGreen CsomeBCy
11770 H_neonGreen CsomeBCy
11775 -pf01370 -
11780 -pf00009pf16658 -
11785 -pf00124 -
11790 HMMreg00486_1_mix(pf01584pf02895pf02518pf01627pf00072) FRAG2
11795 -pf01467 -
11800 -pf00085 -
11805 -pf07298 -
11810 HMMreg00126_1_mix(pf00126pf03466) EUT2E
11815 -# -
11820 -pf05128pf01926 -
(..-11285..)
00535 -pf00226 -
00540 -# -
00545 -pf06055 -
00550 -pf00248 -
00555 -pf11209 -
00560 -# -
00565 -pf14698pf00206 -
00570 -pf01255 -
00575 -pf01368pf02272 -
00580 -pf01964 -
00585 Ts_poisonGreen CsomeBCy
00590 -pf11341 -
BMC-P
BMC-H
BMC-T
Aldehyde dehydrogenase
PTAC
Signature enzyme
Alcohol dehydrogenase
Regulator
00595 -pf00226 -
00600 -# -
00605 -pf01131pf01751pf13368 -
00610 -pf00717 -
00615 -pf00069 -
00620 -pf02875pf08245pf08443 -
00625 -pf03575 -
00630 -pf01746 -
00635 -pf00877 -
(..4870..)
05505 -# -
05510 -pf01521 -
05515 -pf07682 -
05520 -pf02938pf00152pf01336 -
05525 -pf02638 -
05530 -# -
05535 -pf00931pf00400 -
05540 -pf00999pf00582 -
05550 -pf13439pf00534 -
05555 -pf01262pf05222pf12769 -
05560 H_lime CsomeBCy
05565 H_peaSoup CsomeBCy
05570 -pf00873 -
05575 -pf16576 -
05580 -pf00588 -
05585 HMM00107alcdh_2_mic1mix(pf08240pf00107) EUT3
05590 -pf06472pf00005 -
05595 HMMnopfamclust3_1(pf00106) MIC2
05600 -pf07228 -
05605 -pf01276pf03711 -
05610 -pf00805pf00069 -
05615 -# -
(..3470..)
09085 -pf00502 -
09090 -pf00502 -
09095 -pf01383 -
09100 -pf01050 -
09105 -pf02911pf00551 -
09110 HMMreg00072_2_pdugrm(pf00072) CsomeBCy
09115 -pf05430 -
09120 -pf01041 -
09125 -# -
09130 -pf04715pf00425 -
09135 Tdp_kelleyGreen(#) CsomeBCy
09140 -pf01613pf00753 -
09145 -pf02021 -
09150 -pf10150 -
09155 -pf01571pf08669 -
09160 -pf02653 -
09165 HMMreg00486_1_mix(pf02518pf00512)
09170 -pf12679 -
09185 -pf00004pf06480pf01434 -
09190 -# -
09195 -pf04015 -

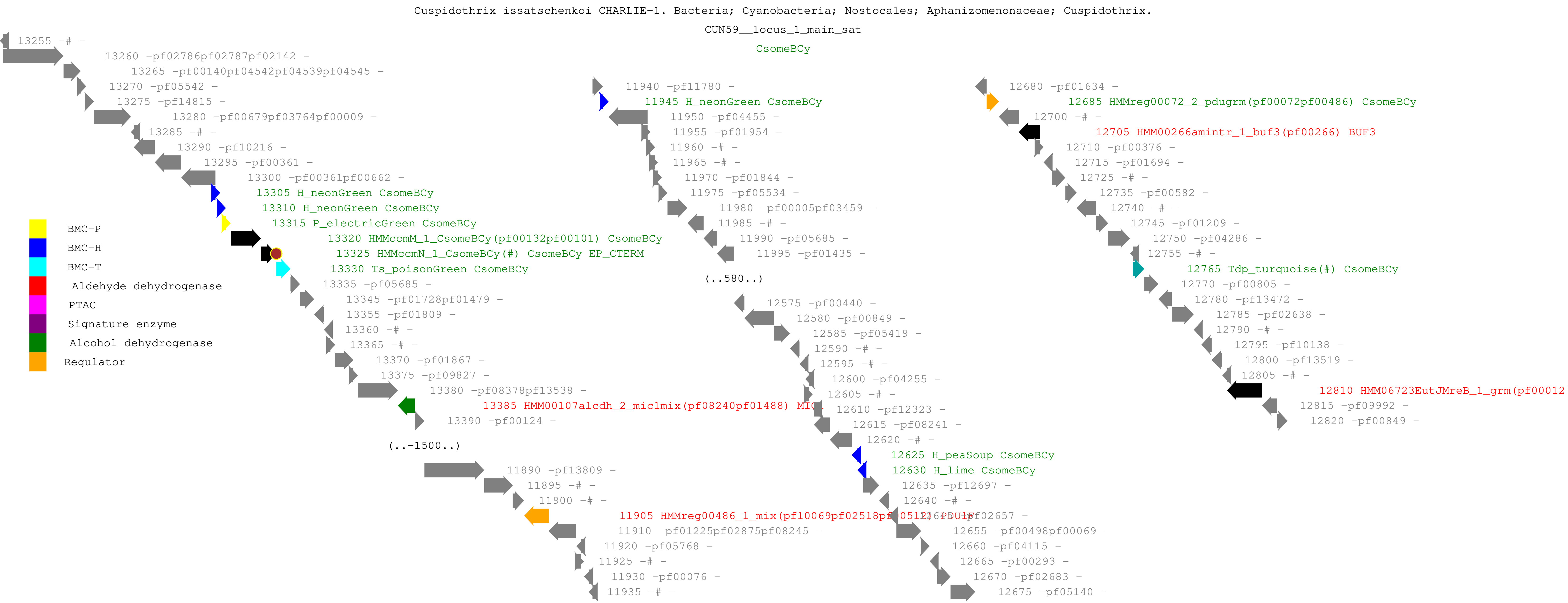

Cuspidothrix issatschenkoi CHARLIE-1. Bacteria; Cyanobacteria; Nostocales; Aphanizomenonaceae; Cuspidothrix.
CUN59__locus_1_main_sat
CsomeBCy
BMC-P
BMC-H
BMC-T
Aldehyde dehydrogenase
PTAC
Signature enzyme
Alcohol dehydrogenase
Regulator
13255 -# -
13260 -pf02786pf02787pf02142 -
13265 -pf00140pf04542pf04539pf04545 -
13270 -pf05542 -
13275 -pf14815 -
13280 -pf00679pf03764pf00009 -
13285 -# -
13290 -pf10216 -
13295 -pf00361 -
13300 -pf00361pf00662 -
13305 H_neonGreen CsomeBCy
13310 H_neonGreen CsomeBCy
13315 P_electricGreen CsomeBCy
13320 HMMccmM_1_CsomeBCy(pf00132pf00101) CsomeBCy
13325 HMMccmN_1_CsomeBCy(#) CsomeBCy EP_CTERM
13330 Ts_poisonGreen CsomeBCy
13335 -pf05685 -
13345 -pf01728pf01479 -
13355 -pf01809 -
13360 -# -
13365 -# -
13370 -pf01867 -
13375 -pf09827 -
13380 -pf08378pf13538 -
13385 HMM00107alcdh_2_mic1mix(pf08240pf01488) MIC
13390 -pf00124 -
(..-1500..)
11890 -pf13809 -
11895 -# -
11900 -# -
11905 HMMreg00486_1_mix(pf10069pf02518pf00512) PDU1
11910 -pf01225pf02875pf08245 -
11920 -pf05768 -
11925 -# -
11930 -pf00076 -
11935 -# -
11940 -pf11780 -
11945 H_neonGreen CsomeBCy
11950 -pf04455 -
11955 -pf01954 -
11960 -# -
11965 -# -
11970 -pf01844 -
11975 -pf05534 -
11980 -pf00005pf03459 -
11985 -# -
11990 -pf05685 -
11995 -pf01435 -
(..580..)
12575 -pf00440 -
12580 -pf00849 -
12585 -pf05419 -
12590 -# -
12595 -# -
12600 -pf04255 -
12605 -# -
12610 -pf12323 -
12615 -pf08241 -
12620 -# -
12625 H_peaSoup CsomeBCy
12630 H_lime CsomeBCy
12635 -pf12697 -
12640 -# -
pf02657 -
12655 -pf00498pf00069 -
12660 -pf04115 -
12665 -pf00293 -
12670 -pf02683 -
12675 -pf05140 -
12680 -pf01634 -
12685 HMMreg00072_2_pdugrm(pf00072pf00486) CsomeBCy
12700 -# -
12705 HMM00266amintr_1_buf3(pf00266) BUF3
12710 -pf00376 -
12715 -pf01694 -
12725 -# -
12735 -pf00582 -
12740 -# -
12745 -pf01209 -
12750 -pf04286 -
12755 -# -
12765 Tdp_turquoise(#) CsomeBCy
12770 -pf00805 -
12780 -pf13472 -
12785 -pf02638 -
12790 -# -
12795 -pf10138 -
12800 -pf13519 -
12805 -# -
12810 HMM06723EutJMreB_1_grm(pf00012
12815 -pf09992 -
12820 -pf00849 -

Cyanobacteria bacterium M5B4. Bacteria; Cyanobacteria.

CV045__locus_fragmented

CsomeBCy

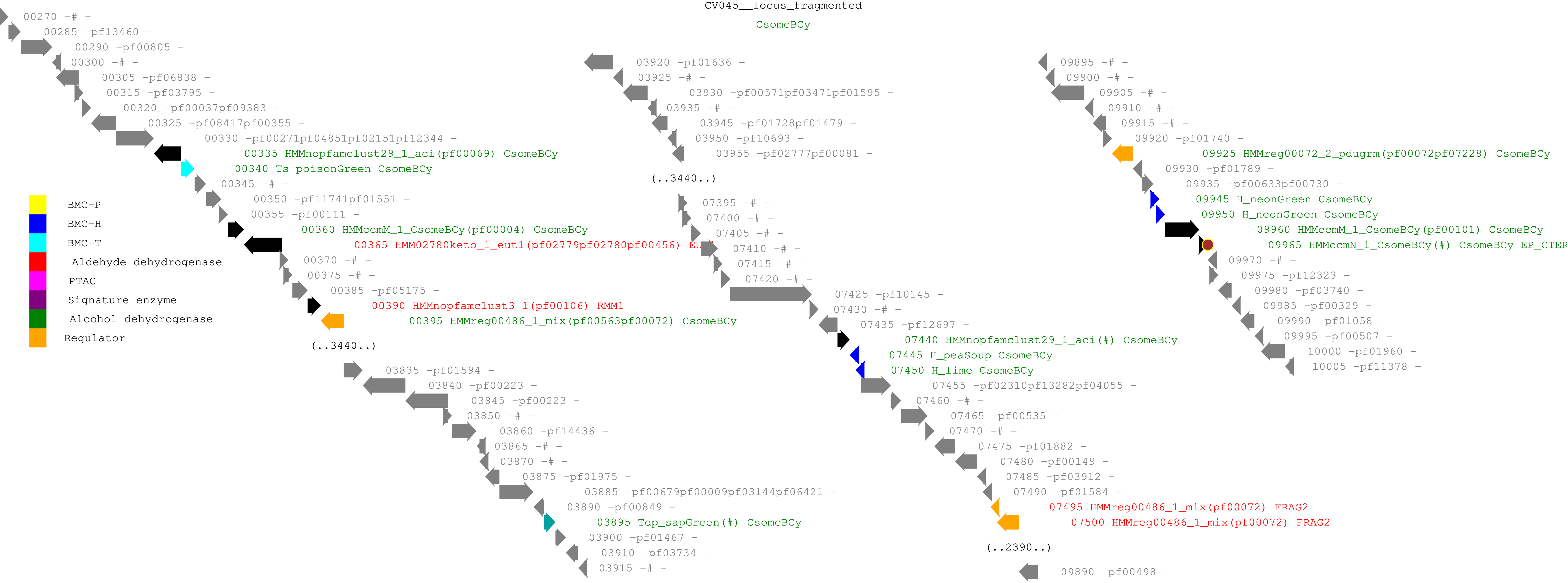

Crocosphaera watsonii WH 8501. Bacteria; Cyanobacteria; Oscillatoriophycideae; Chroococcales;

CwatDRAFT__locus_1_main_sat

CsomeBCy

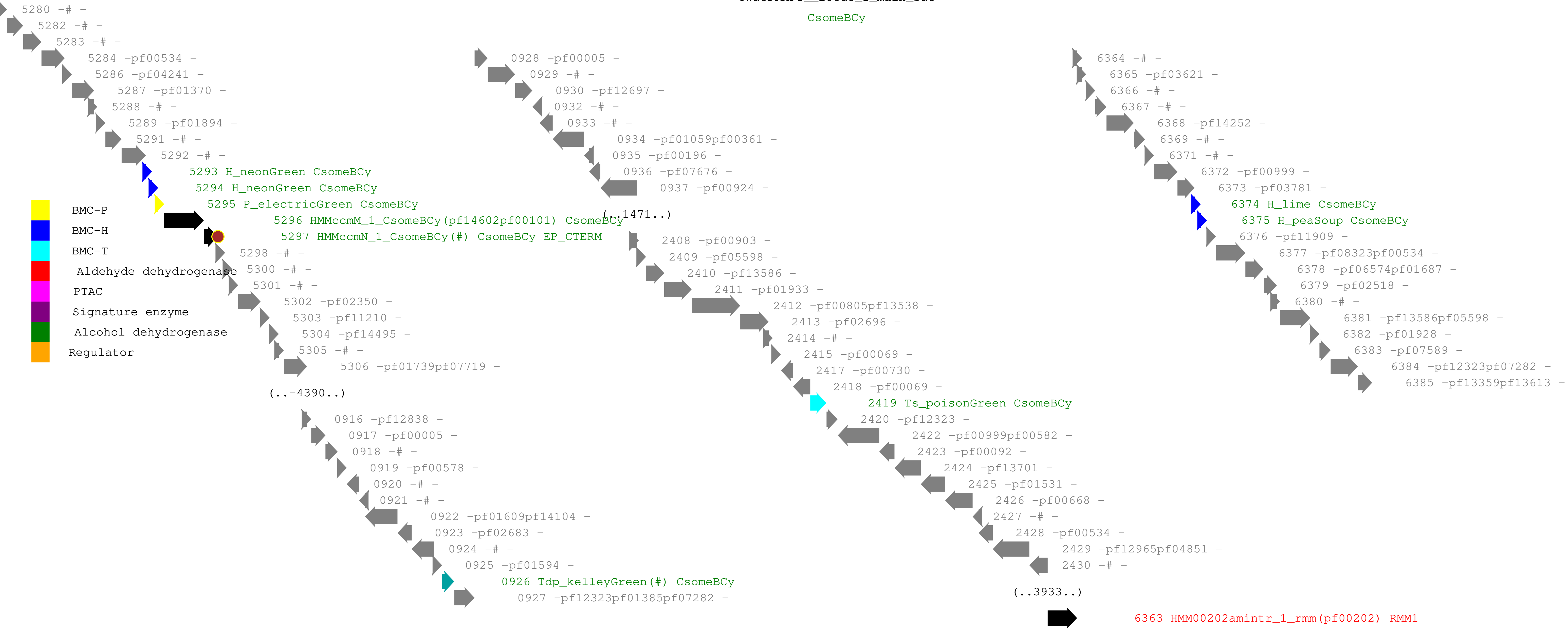

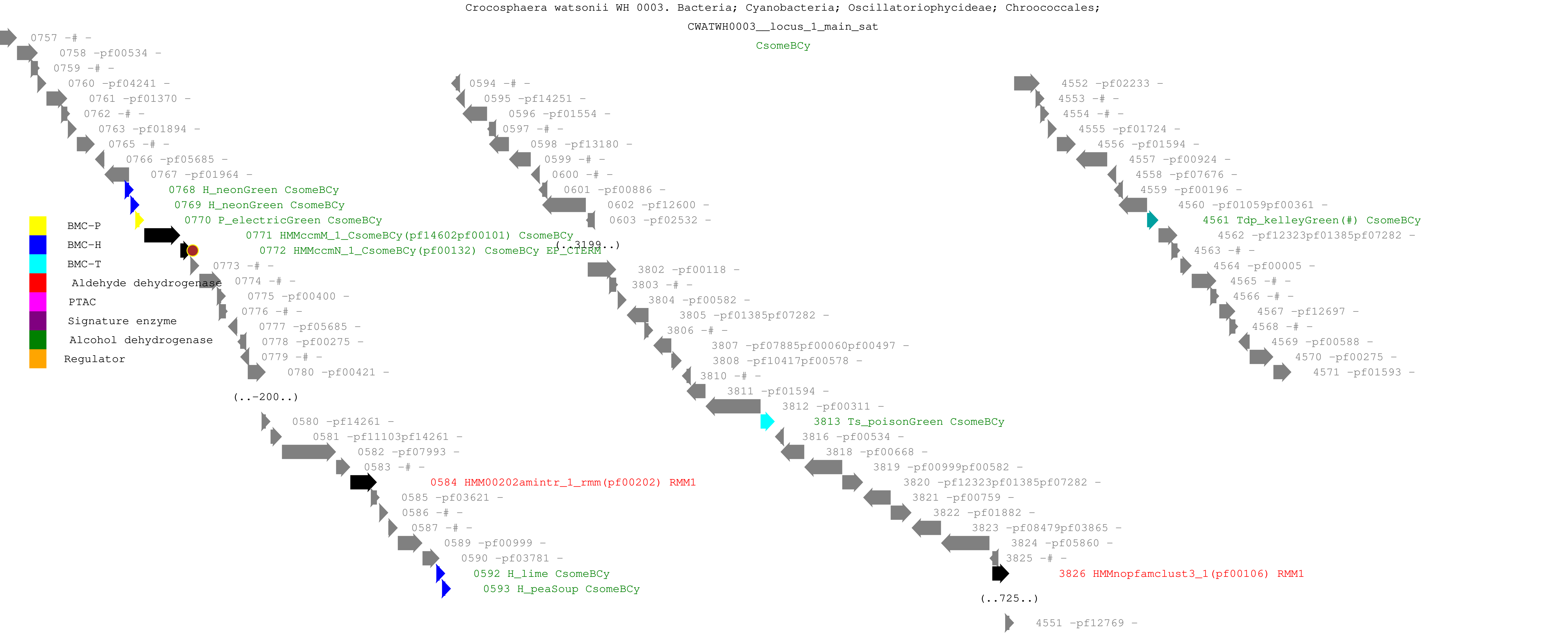
Crocosphaera watsonii WH 0003. Bacteria; Cyanobacteria; Oscillatoriophycideae; Chroococcales;
CWATWH0003__locus_1_main_sat
CsomeBCy
BMC-P
BMC-H
BMC-T
Aldehyde dehydrogenase
PTAC
Signature enzyme
Alcohol dehydrogenase
Regulator
0757 -# -
0758 -pf00534 -
0759 -# -
0760 -pf04241 -
0761 -pf01370 -
0762 -# -
0763 -pf01894 -
0765 -# -
0766 -pf05685 -
0767 -pf01964 -
0768 H_neonGreen CsomeBCy
0769 H_neonGreen CsomeBCy
0770 P_electricGreen CsomeBCy
0771 HMMccmM_1_CsomeBCy(pf14602pf00101) CsomeBCy
0772 HMMccmN_1_CsomeBCy(pf00132) CsomeBCy EP_CTERM
0773 -# -
0774 -# -
0775 -pf00400 -
0776 -# -
0777 -pf05685 -
0778 -pf00275 -
0779 -# -
0780 -pf00421 -
(..-200..)
0580 -pf14261 -
0581 -pf11103pf14261 -
0582 -pf07993 -
0583 -# -
0584 HMM00202amintr_1_rmm(pf00202) RMM1
0585 -pf03621 -
0586 -# -
0587 -# -
0589 -pf00999 -
0590 -pf03781 -
0592 H_lime CsomeBCy
0593 H_peaSoup CsomeBCy
0594 -# -
0595 -pf14251 -
0596 -pf01554 -
0597 -# -
0598 -pf13180 -
0599 -# -
0600 -# -
0601 -pf00886 -
0602 -pf12600 -
0603 -pf02532 -
(..3199..)
3802 -pf00118 -
3803 -# -
3804 -pf00582 -
3805 -pf01385pf07282 -
3806 -# -
3807 -pf07885pf00060pf00497 -
3808 -pf10417pf00578 -
3810 -# -
3811 -pf01594 -
3812 -pf00311 -
3813 Ts_poisonGreen CsomeBCy
3816 -pf00534 -
3818 -pf00668 -
3819 -pf00999pf00582 -
3820 -pf12323pf01385pf07282 -
3821 -pf00759 -
3822 -pf01882 -
3823 -pf08479pf03865 -
3824 -pf05860 -
3825 -# -
3826 HMMnopfamclust3_1(pf00106) RMM1
(..725..)
4551 -pf12769 -
4552 -pf02233 -
4553 -# -
4554 -# -
4555 -pf01724 -
4556 -pf01594 -
4557 -pf00924 -
4558 -pf07676 -
4559 -pf00196 -
4560 -pf01059pf00361 -
4561 Tdp_kelleyGreen(#) CsomeBCy
4562 -pf12323pf01385pf07282 -
4563 -# -
4564 -pf00005 -
4565 -# -
4566 -# -
4567 -pf12697 -
4568 -# -
4569 -pf00588 -
4570 -pf00275 -
4571 -pf01593 -

Crocosphaera watsonii WH 0005. Bacteria; Cyanobacteria; Oscillatoriophycideae; Chroococcales;

CWATWH0005__locus_fragmented

CsomeBCy

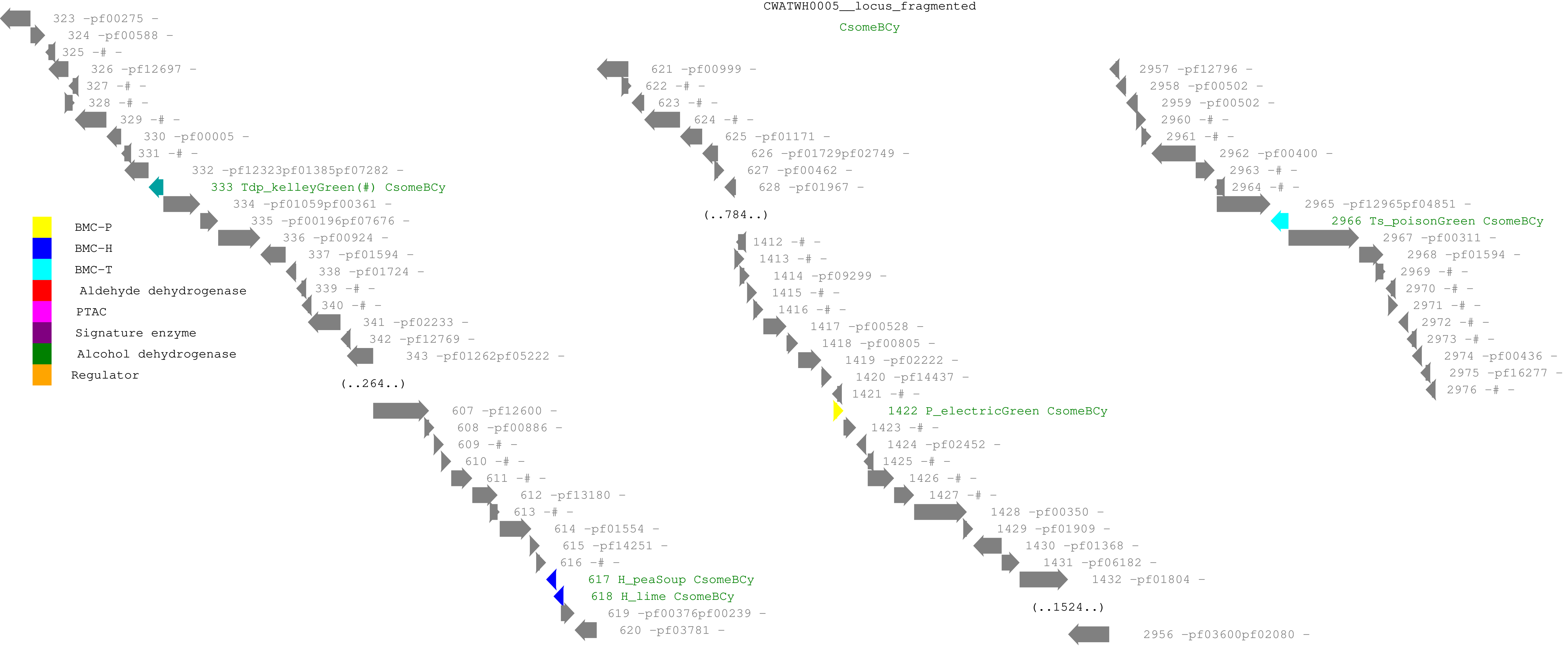

Crocosphaera watsonii WH 0401. Bacteria; Cyanobacteria; Oscillatoriophycideae; Chroococcales;

CWATWH0401__locus_1_main_sat

CsomeBCy

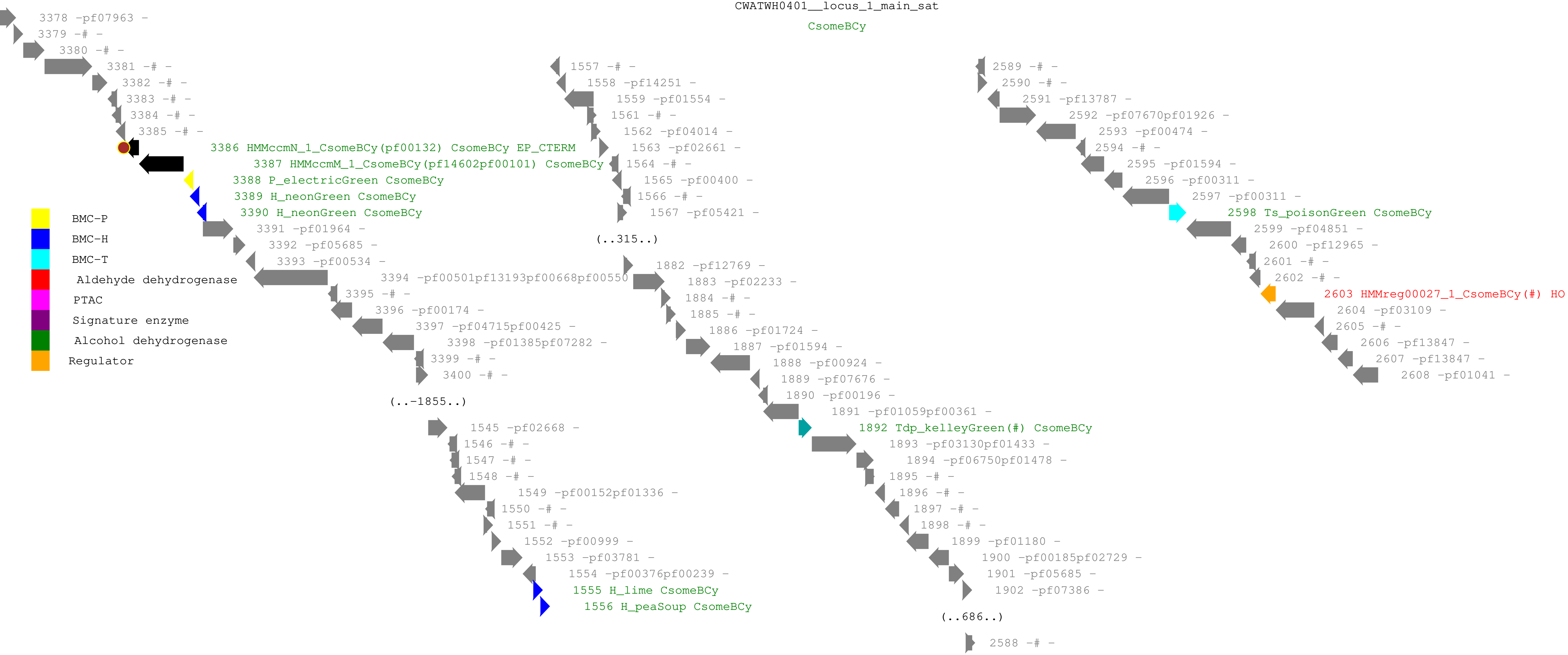

Crocosphaera watsonii WH 0402. Bacteria; Cyanobacteria; Oscillatoriophycideae; Chroococcales;

CWATWH0402__locus_1_main_sat

CsomeBCy

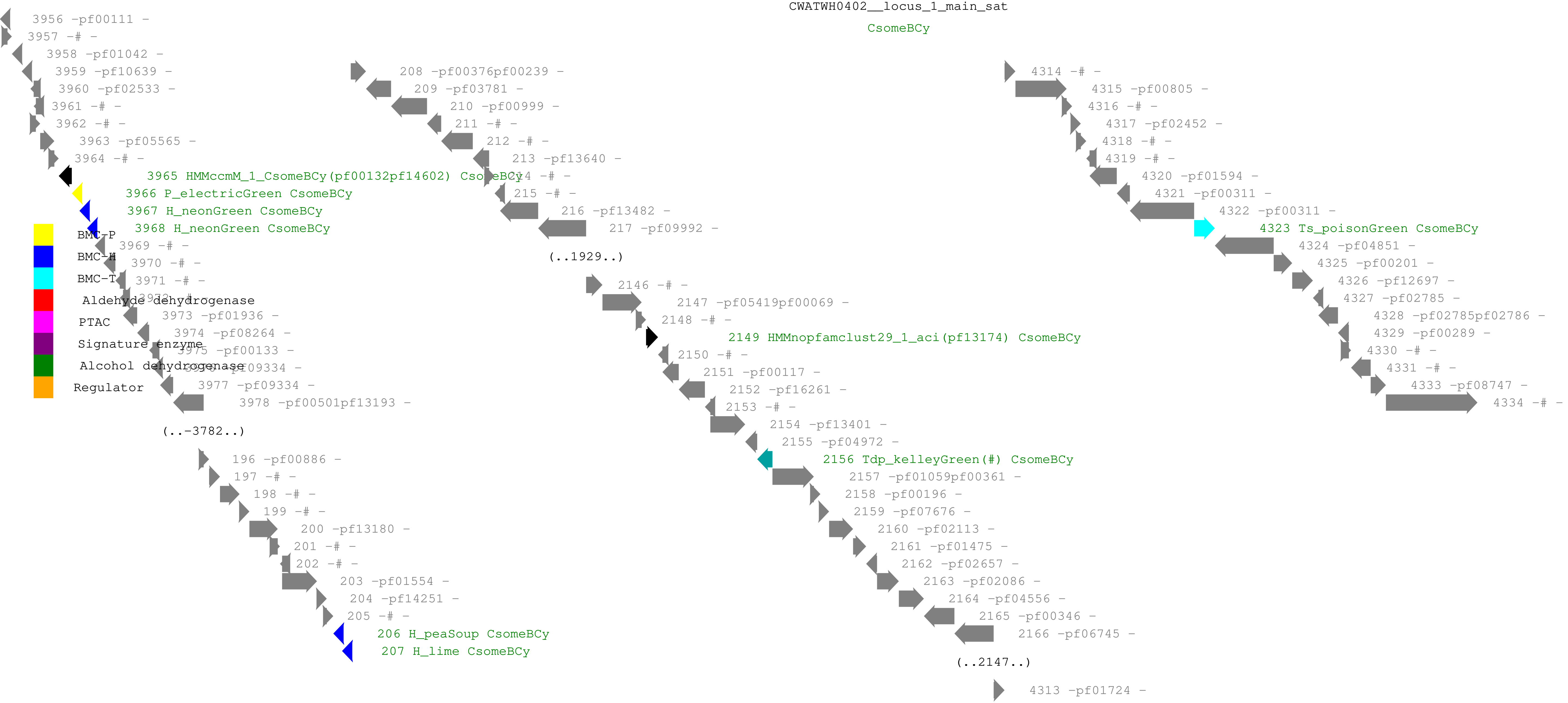

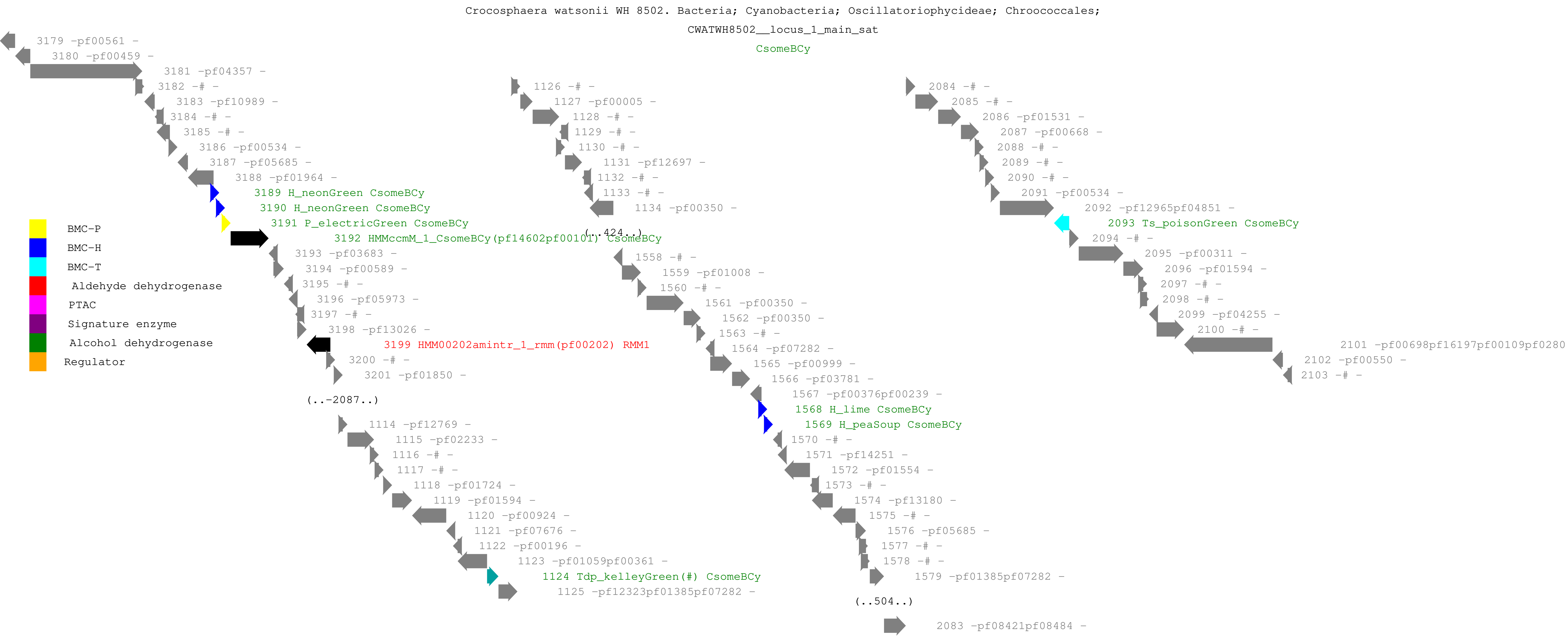

Crocosphaera watsonii WH 8502. Bacteria; Cyanobacteria; Oscillatoriophycideae; Chroococcales;
CWATWH8502__locus_1_main_sat
CsomeBCy
BMC-P
BMC-H
BMC-T
Aldehyde dehydrogenase
PTAC
Signature enzyme
Alcohol dehydrogenase
Regulator
3179 -pf00561 -
3180 -pf00459 -
3181 -pf04357 -
3182 -# -
3183 -pf10989 -
3184 -# -
3185 -# -
3186 -pf00534 -
3187 -pf05685 -
3188 -pf01964 -
3189 H_neonGreen CsomeBCy
3190 H_neonGreen CsomeBCy
3191 P_electricGreen CsomeBCy
3192 HMMccmM_1_CsomeBCy(pf14602pf00101) CsomeBCy
3193 -pf03683 -
3194 -pf00589 -
3195 -# -
3196 -pf05973 -
3197 -# -
3198 -pf13026 -
3199 HMM00202amintr_1_rmm(pf00202) RMM1
3200 -# -
3201 -pf01850 -
(..-2087..)
1114 -pf12769 -
1115 -pf02233 -
1116 -# -
1117 -# -
1118 -pf01724 -
1119 -pf01594 -
1120 -pf00924 -
1121 -pf07676 -
1122 -pf00196 -
1123 -pf01059pf00361 -
1124 Tdp_kelleyGreen(#) CsomeBCy
1125 -pf12323pf01385pf07282 -
1126 -# -
1127 -pf00005 -
1128 -# -
1129 -# -
1130 -# -
1131 -pf12697 -
1132 -# -
1133 -# -
1134 -pf00350 -
(..424..)
1558 -# -
1559 -pf01008 -
1560 -# -
1561 -pf00350 -
1562 -pf00350 -
1563 -# -
1564 -pf07282 -
1565 -pf00999 -
1566 -pf03781 -
1567 -pf00376pf00239 -
1568 H_lime CsomeBCy
1569 H_peaSoup CsomeBCy
1570 -# -
1571 -pf14251 -
1572 -pf01554 -
1573 -# -
1574 -pf13180 -
1575 -# -
1576 -pf05685 -
1577 -# -
1578 -# -
1579 -pf01385pf07282 -
(..504..)
2083 -pf08421pf08484 -
2084 -# -
2085 -# -
2086 -pf01531 -
2087 -pf00668 -
2088 -# -
2089 -# -
2090 -# -
2091 -pf00534 -
2092 -pf12965pf04851 -
2093 Ts_poisonGreen CsomeBCy
2094 -# -
2095 -pf00311 -
2096 -pf01594 -
2097 -# -
2098 -# -
2099 -pf04255 -
2100 -# -
2101 -pf00698pf16197pf00109pf0280
2102 -pf00550 -
2103 -# -

Cyanothece sp. CCY0110. Bacteria; Cyanobacteria; Oscillatoriophycideae; Oscillatoriales;

CY0110__locus_1_main_sat

CsomeBCy

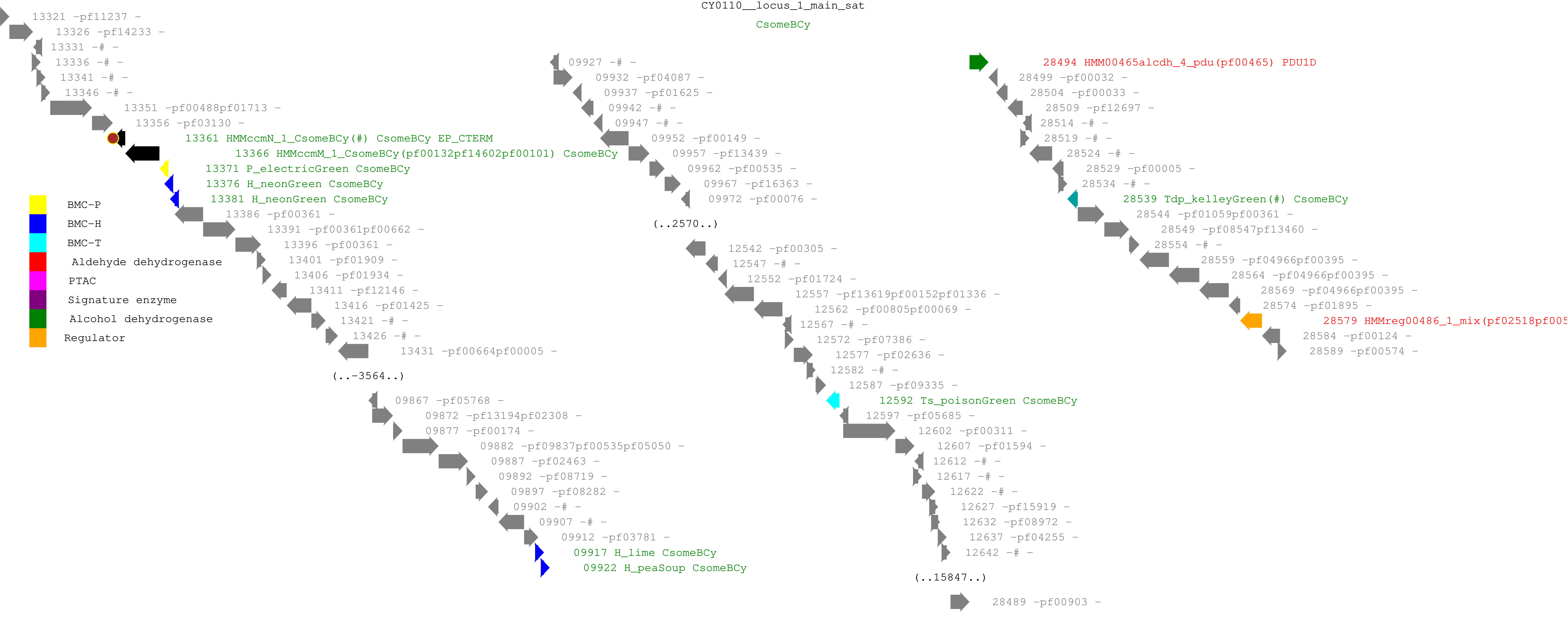

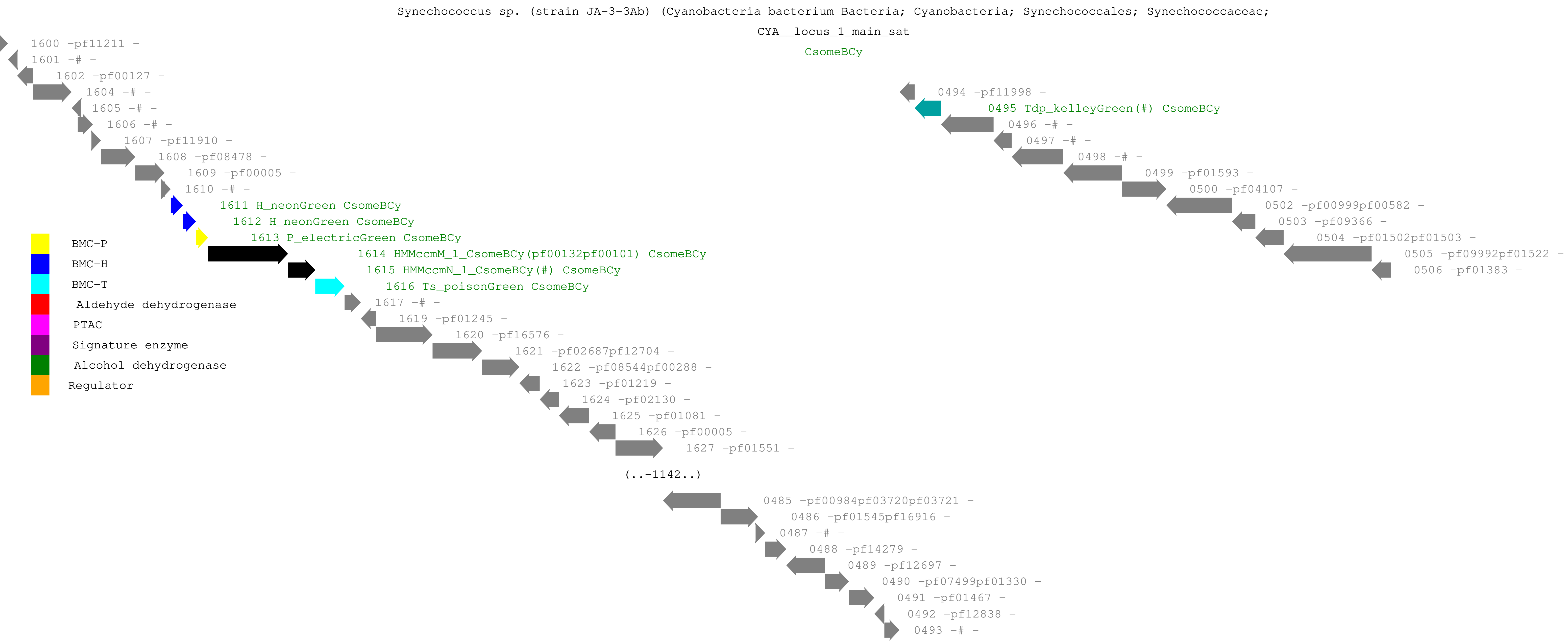
Synechococcus sp. (strain JA-3-3Ab) (Cyanobacteria bacterium Bacteria; Cyanobacteria; Synechococcales; Synechococcaceae;
CYA__locus_1_main_sat
CsomeBCy
1600 -pf11211 -
1601 -# -
1602 -pf00127 -
1604 -# -
1605 -# -
1606 -# -
1607 -pf11910 -
1608 -pf08478 -
1609 -pf00005 -
1610 -# -
1611 H_neonGreen CsomeBCy
1612 H_neonGreen CsomeBCy
1613 P_electricGreen CsomeBCy
1614 HMMccmM_1_CsomeBCy(pf00132pf00101) CsomeBCy
1615 HMMccmN_1_CsomeBCy(#) CsomeBCy
1616 Ts_poisonGreen CsomeBCy
1617 -# -
1619 -pf01245 -
1620 -pf16576 -
1621 -pf02687pf12704 -
1622 -pf08544pf00288 -
1623 -pf01219 -
1624 -pf02130 -
1625 -pf01081 -
1626 -pf00005 -
1627 -pf01551 -
(..-1142..)
0485 -pf00984pf03720pf03721 -
0486 -pf01545pf16916 -
0487 -# -
0488 -pf14279 -
0489 -pf12697 -
0490 -pf07499pf01330 -
0491 -pf01467 -
0492 -pf12838 -
0493 -# -
0494 -pf11998 -
0495 Tdp_kelleyGreen(#) CsomeBCy
0496 -# -
0497 -# -
0498 -# -
0499 -pf01593 -
0500 -pf04107 -
0502 -pf00999pf00582 -
0503 -pf09366 -
0504 -pf01502pf01503 -
0505 -pf09992pf01522 -
0506 -pf01383 -
BMC-P
BMC-H
BMC-T
Aldehyde dehydrogenase
PTAC
Signature enzyme
Alcohol dehydrogenase
Regulator

Cyanobacterium aponinum (strain PCC 10605). Bacteria; Cyanobacteria; Oscillatoriophycideae; Chroococcales;

Cyan10605__locus_1_main_sat

CsomeBCy

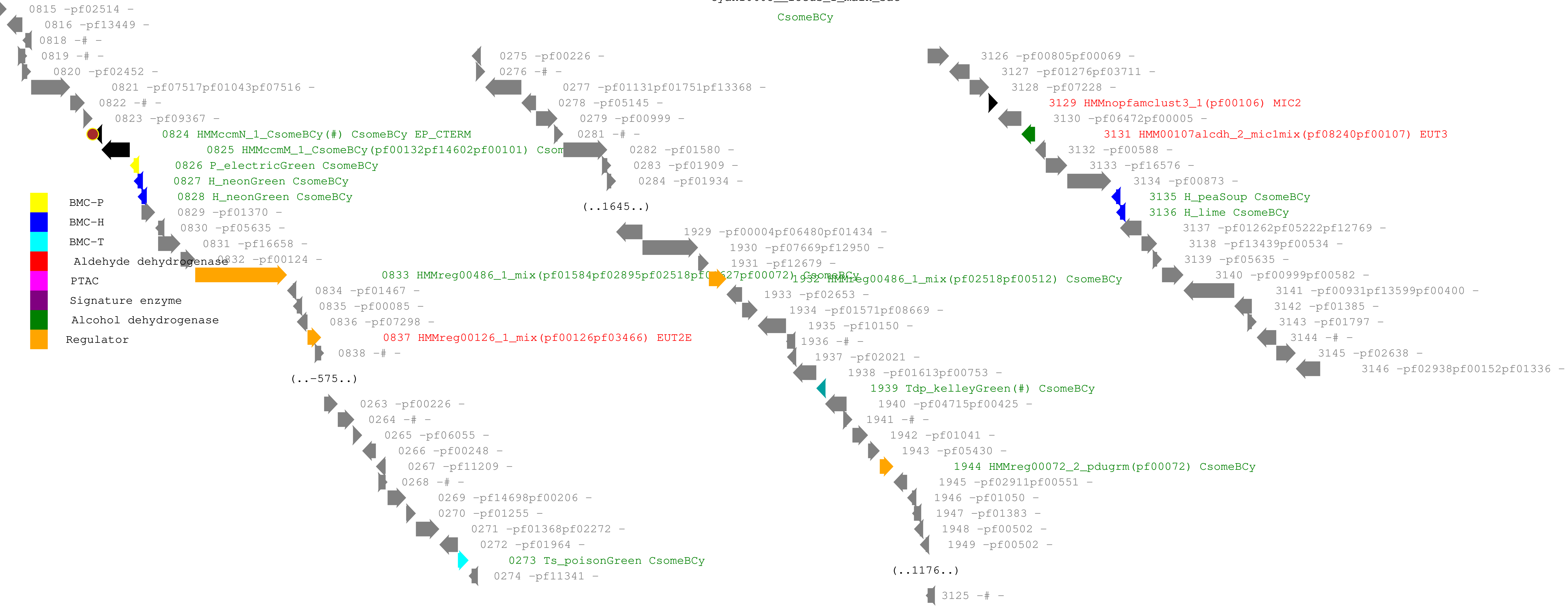

Cyanothece sp. (strain PCC 7425 / ATCC 29141). Bacteria; Cyanobacteria; Oscillatoriophycideae; Oscillatoriales;

Cyan7425__locus_1_main_sat

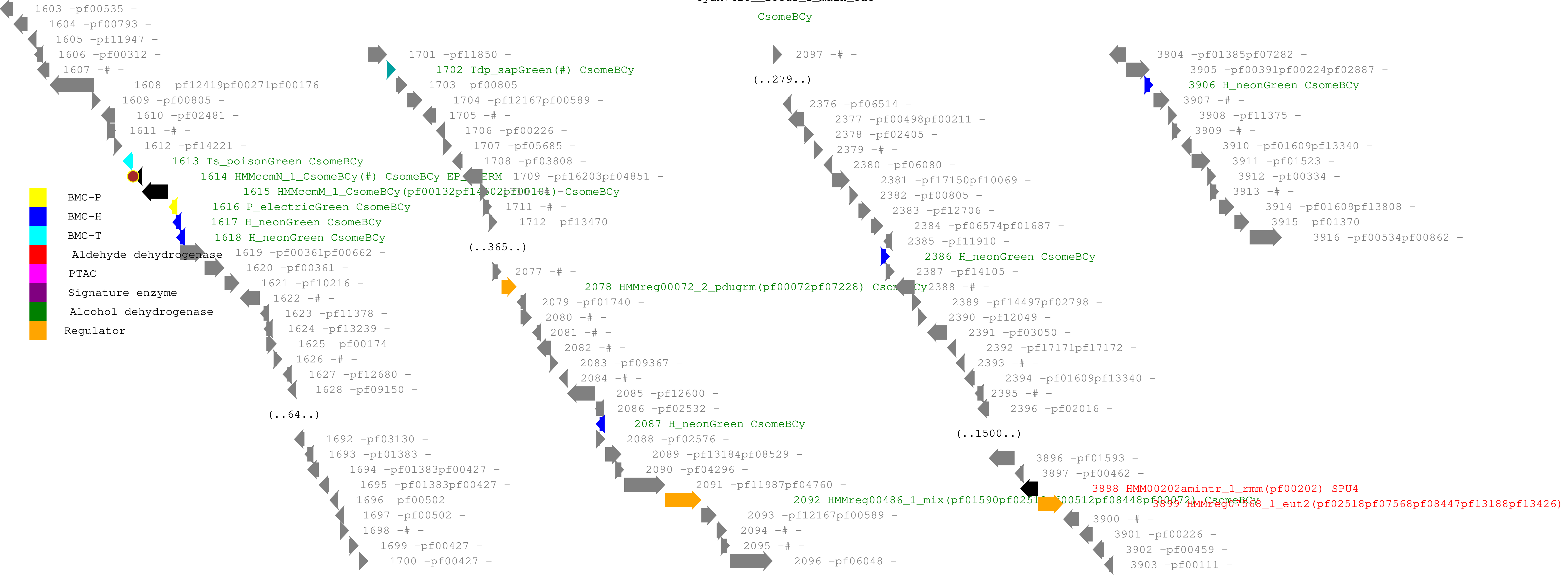

Cyanothece sp. (strain PCC 7822). Bacteria; Cyanobacteria; Oscillatoriophycideae; Oscillatoriales;

Cyan7822__locus_1_main_sat

CsomeBCy

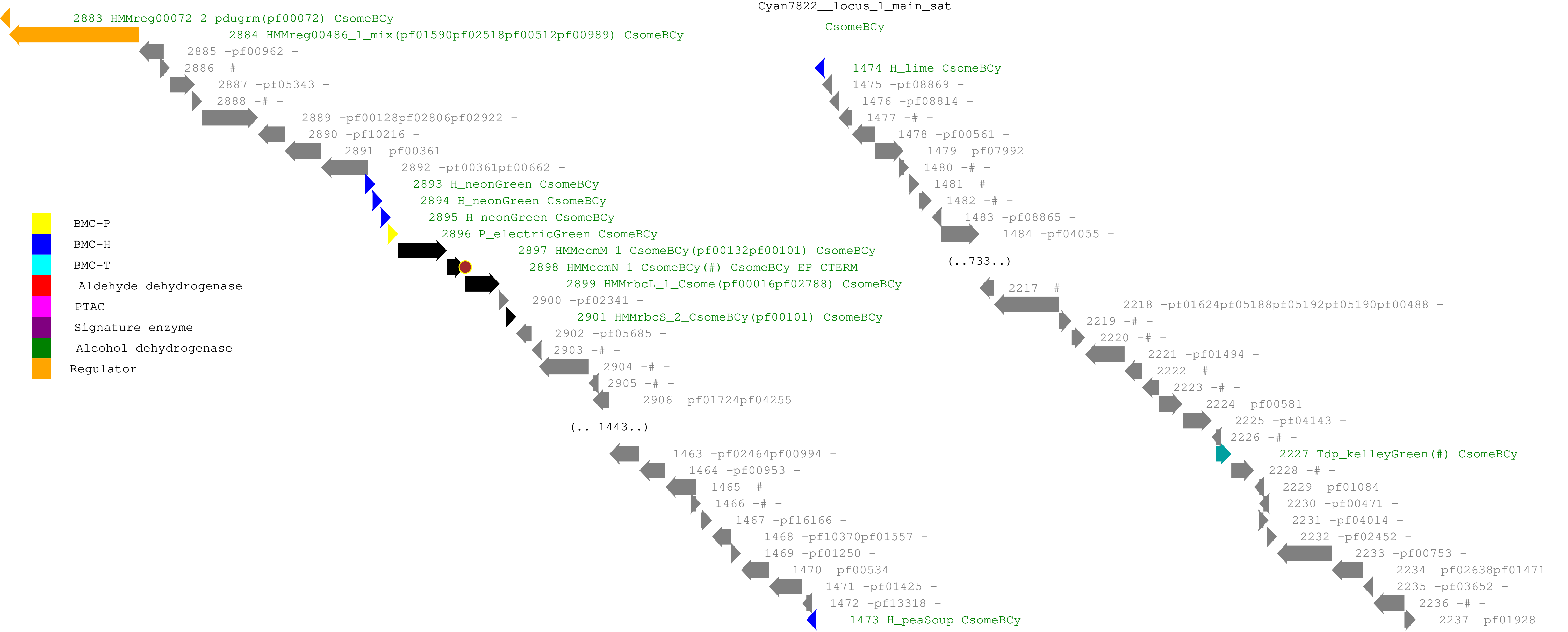

Cyanobacterium stanieri (strain ATCC 29140 / PCC 7202). Bacteria; Cyanobacteria; Oscillatoriophycideae; Chroococcales;

Cyast__locus_1_main_sat

CsomeBCy

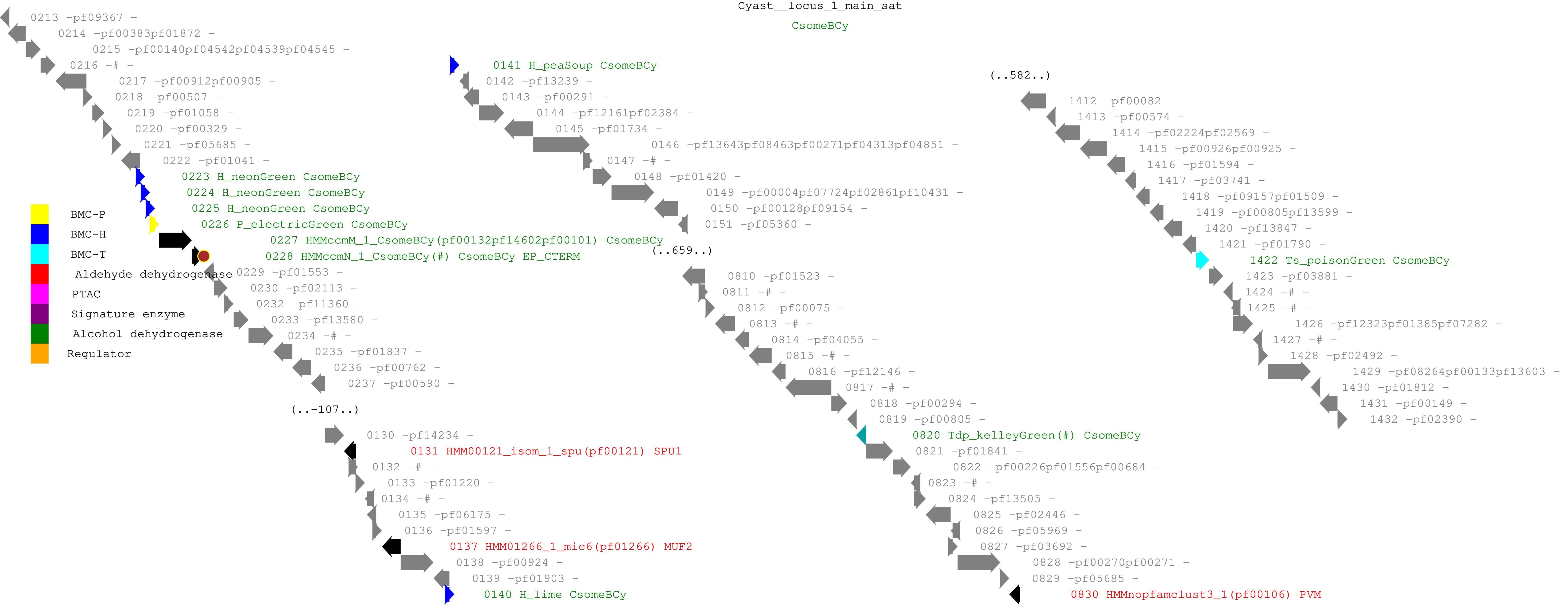

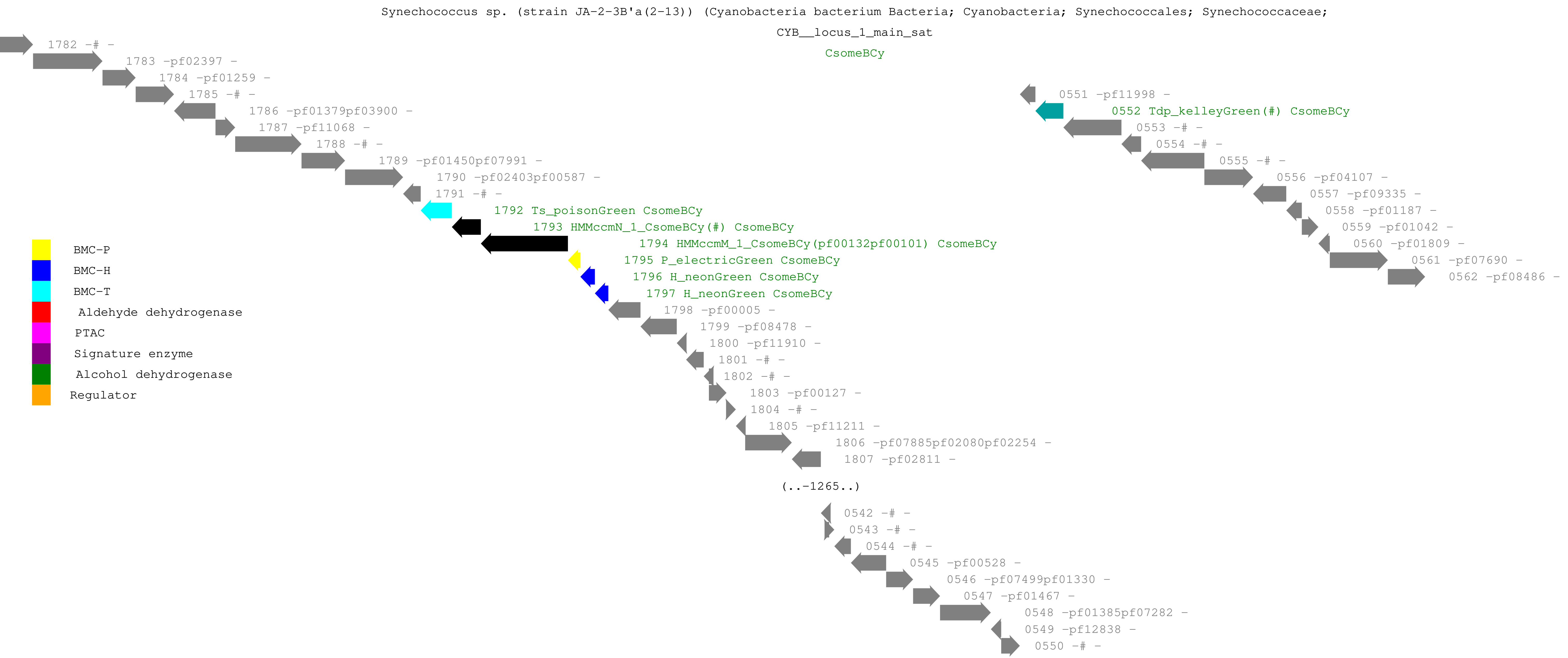

Synechococcus sp. (strain JA-2-3B'a(2-13)) (Cyanobacteria bacterium Bacteria; Cyanobacteria; Synechococcales; Synechococcaceae;
CYB__locus_1_main_sat
CsomeBCy
1782 -# -
1783 -pf02397 -
1784 -pf01259 -
1785 -# -
1786 -pf01379pf03900 -
1787 -pf11068 -
1788 -# -
1789 -pf01450pf07991 -
1790 -pf02403pf00587 -
1791 -# -
1792 Ts_poisonGreen CsomeBCy
1793 HMMccmN_1_CsomeBCy(#) CsomeBCy
1794 HMMccmM_1_CsomeBCy(pf00132pf00101) CsomeBCy
1795 P_electricGreen CsomeBCy
1796 H_neonGreen CsomeBCy
1797 H_neonGreen CsomeBCy
1798 -pf00005 -
1799 -pf08478 -
1800 -pf11910 -
1801 -# -
1802 -# -
1803 -pf00127 -
1804 -# -
1805 -pf11211 -
1806 -pf07885pf02080pf02254 -
1807 -pf02811 -
(..-1265..)
0542 -# -
0543 -# -
0544 -# -
0545 -pf00528 -
0546 -pf07499pf01330 -
0547 -pf01467 -
0548 -pf01385pf07282 -
0549 -pf12838 -
0550 -# -
0551 -pf11998 -
0552 Tdp_kelleyGreen(#) CsomeBCy
0553 -# -
0554 -# -
0555 -# -
0556 -pf04107 -
0557 -pf09335 -
0558 -pf01187 -
0559 -pf01042 -
0560 -pf01809 -
0561 -pf07690 -
0562 -pf08486 -
BMC-P
BMC-H
BMC-T
Aldehyde dehydrogenase
PTAC
Signature enzyme
Alcohol dehydrogenase
Regulator

Cylindrospermum stagnale PCC 7417. Bacteria; Cyanobacteria; Nostocales; Nostocaceae; Cylindrospermum.

Cylst__locus_1_main_sat

CsomeBCy

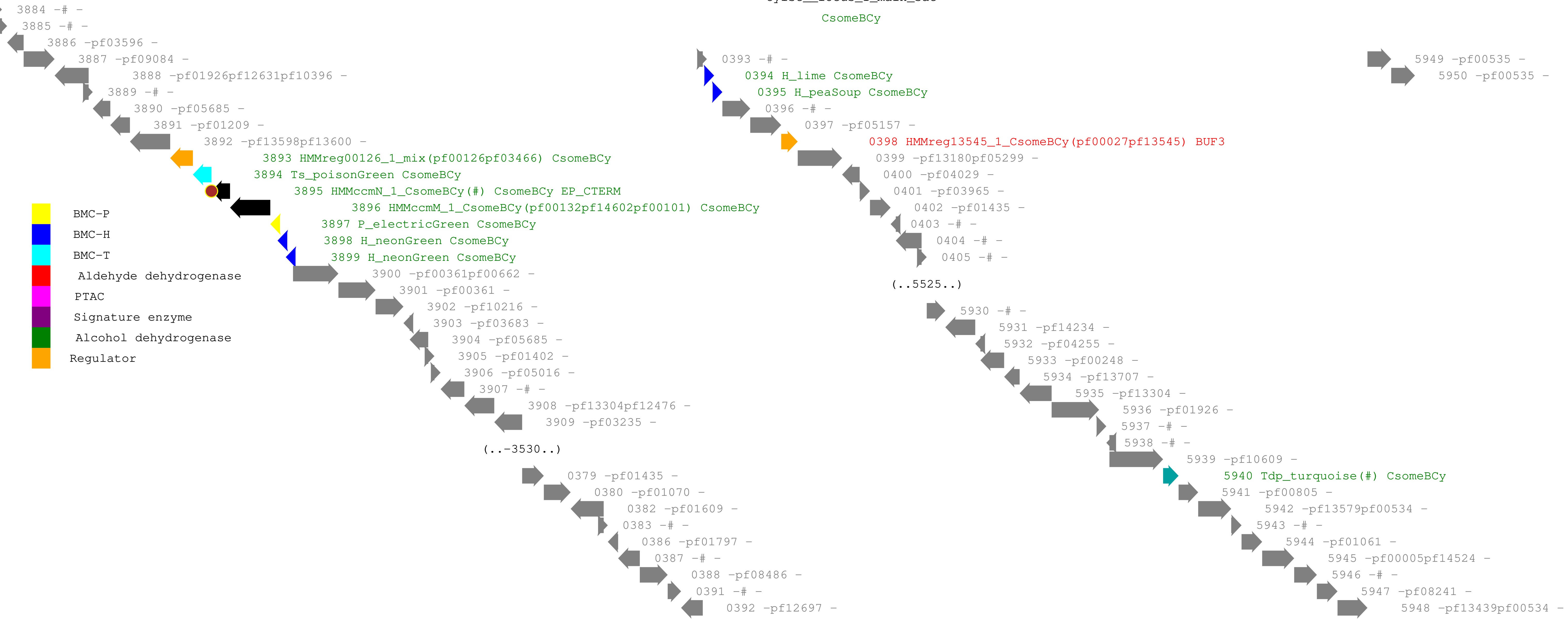

Synechocystis sp. (strain PCC 6714) (Aphanocapsa sp. (strain PCC Bacteria; Cyanobacteria; Synechococcales; Merismopediaceae;

D082__locus_1_main_sat

CsomeBCy

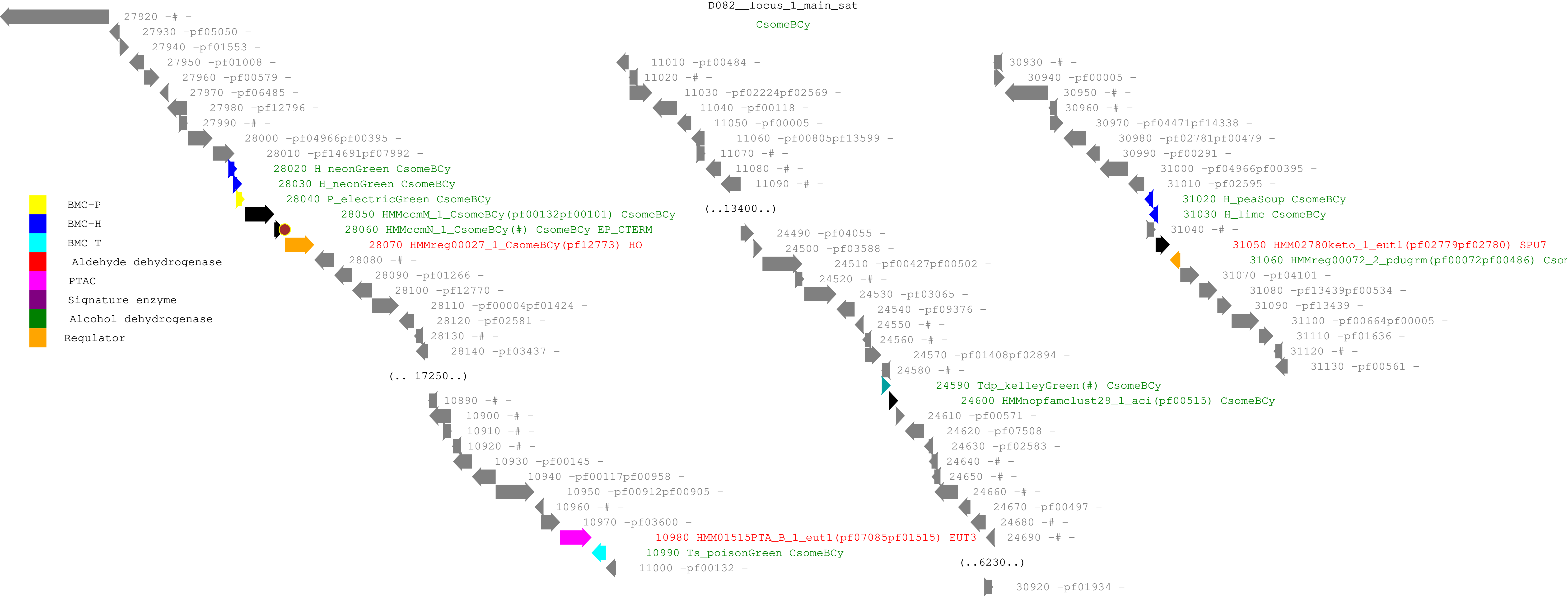

Okeania hirsuta. Bacteria; Cyanobacteria; Oscillatoriophycideae; Oscillatoriales;

D5R40__locus_1_main_sat

CsomeBCy

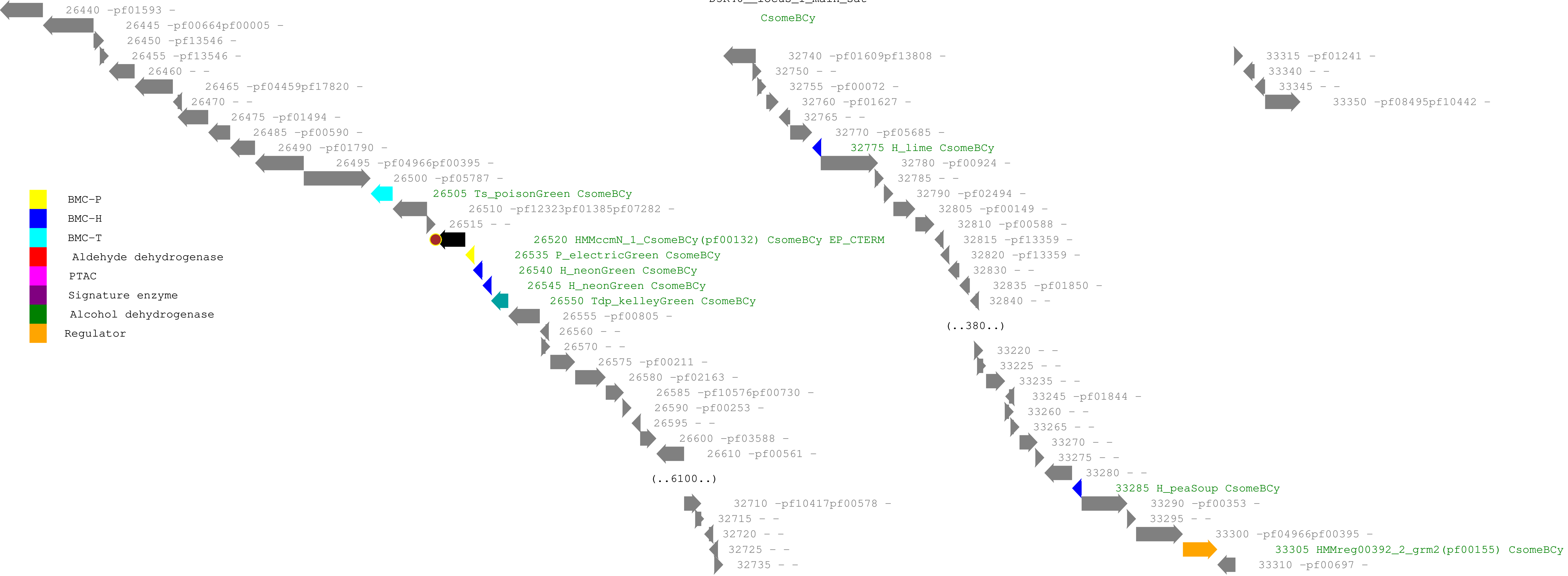

Cyanobacteria bacterium J003. Bacteria; Cyanobacteria.

D6676__locus_1_main_sat

CsomeBCy

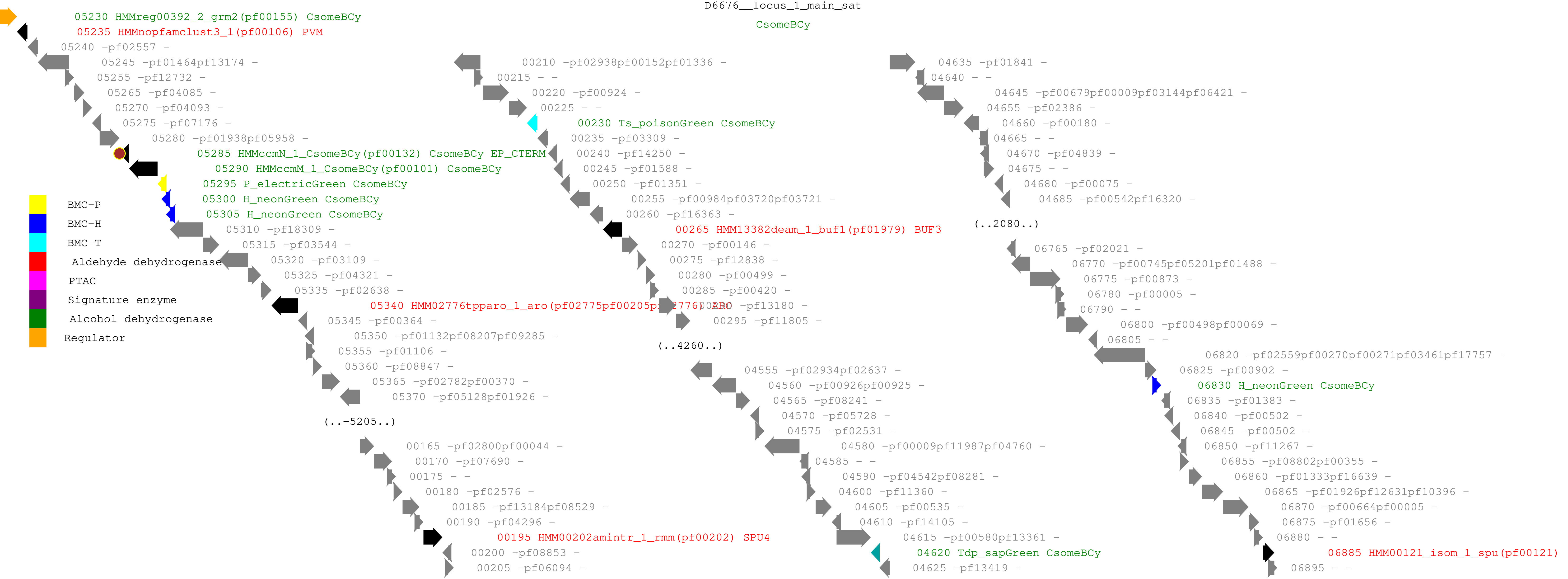

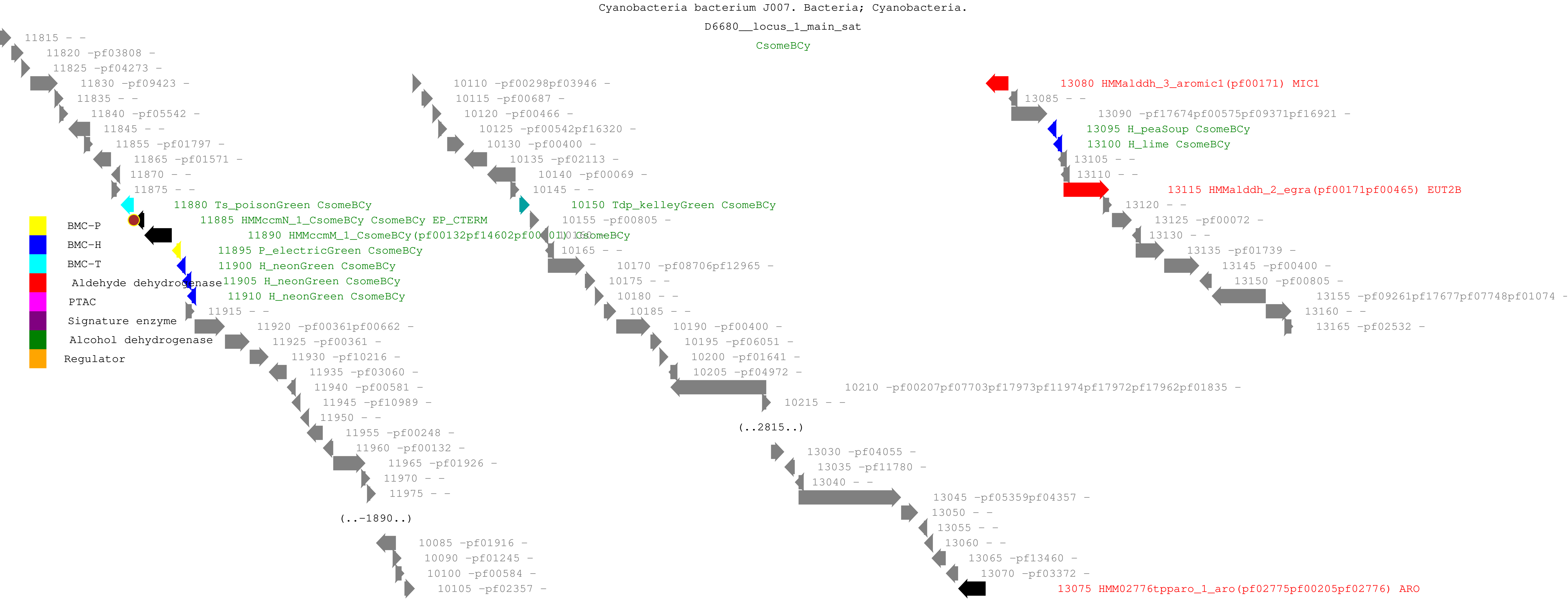
Cyanobacteria bacterium J007. Bacteria; Cyanobacteria.
D6680__locus_1_main_sat
CsomeBCy
11815 - -
11820 -pf03808 -
11825 -pf04273 -
11830 -pf09423 -
11835 - -
11840 -pf05542 -
11845 - -
11855 -pf01797 -
11865 -pf01571 -
11870 - -
11875 - -
11880 Ts_poisonGreen CsomeBCy
11885 HMMccmN_1_CsomeBCy CsomeBCy EP_CTERM
11890 HMMccmM_1_CsomeBCy(pf00132pf14602pf00101) CsomeBCy
11895 P_electricGreen CsomeBCy
11900 H_neonGreen CsomeBCy
11905 H_neonGreen CsomeBCy
11910 H_neonGreen CsomeBCy
11915 - -
11920 -pf00361pf00662 -
11925 -pf00361 -
11930 -pf10216 -
11935 -pf03060 -
11940 -pf00581 -
11945 -pf10989 -
11950 - -
11955 -pf00248 -
11960 -pf00132 -
11965 -pf01926 -
11970 - -
11975 - -
(..-1890..)
10085 -pf01916 -
10090 -pf01245 -
10100 -pf00584 -
10105 -pf02357 -
BMC-P
BMC-H
BMC-T
Aldehyde dehydrogenase
PTAC
Signature enzyme
Alcohol dehydrogenase
Regulator
10110 -pf00298pf03946 -
10115 -pf00687 -
10120 -pf00466 -
10125 -pf00542pf16320 -
10130 -pf00400 -
10135 -pf02113 -
10140 -pf00069 -
10145 - -
10150 Tdp_kelleyGreen CsomeBCy
10155 -pf00805 -
10160
10165 - -
10170 -pf08706pf12965 -
10175 - -
10180 - -
10185 - -
10190 -pf00400 -
10195 -pf06051 -
10200 -pf01641 -
10205 -pf04972 -
10210 -pf00207pf07703pf17973pf11974pf17972pf17962pf01835 -
10215 - -
(..2815..)
13030 -pf04055 -
13035 -pf11780 -
13040 - -
13045 -pf05359pf04357 -
13050 - -
13055 - -
13060 - -
13065 -pf13460 -
13070 -pf03372 -
13075 HMM02776tpparo_1_aro(pf02775pf00205pf02776) ARO
13080 HMMalddh_3_aromic1(pf00171) MIC1
13085 - -
13090 -pf17674pf00575pf09371pf16921 -
13095 H_peaSoup CsomeBCy
13100 H_lime CsomeBCy
13105 - -
13110 - -
13115 HMMaldddh_2_egra(pf00171pf00465) EUT2B
13120 - -
13125 -pf00072 -
13130 - -
13135 -pf01739 -
13145 -pf00400 -
13150 -pf00805 -
13155 -pf09261pf17677pf07748pf01074 -
13160 - -
13165 -pf02532 -

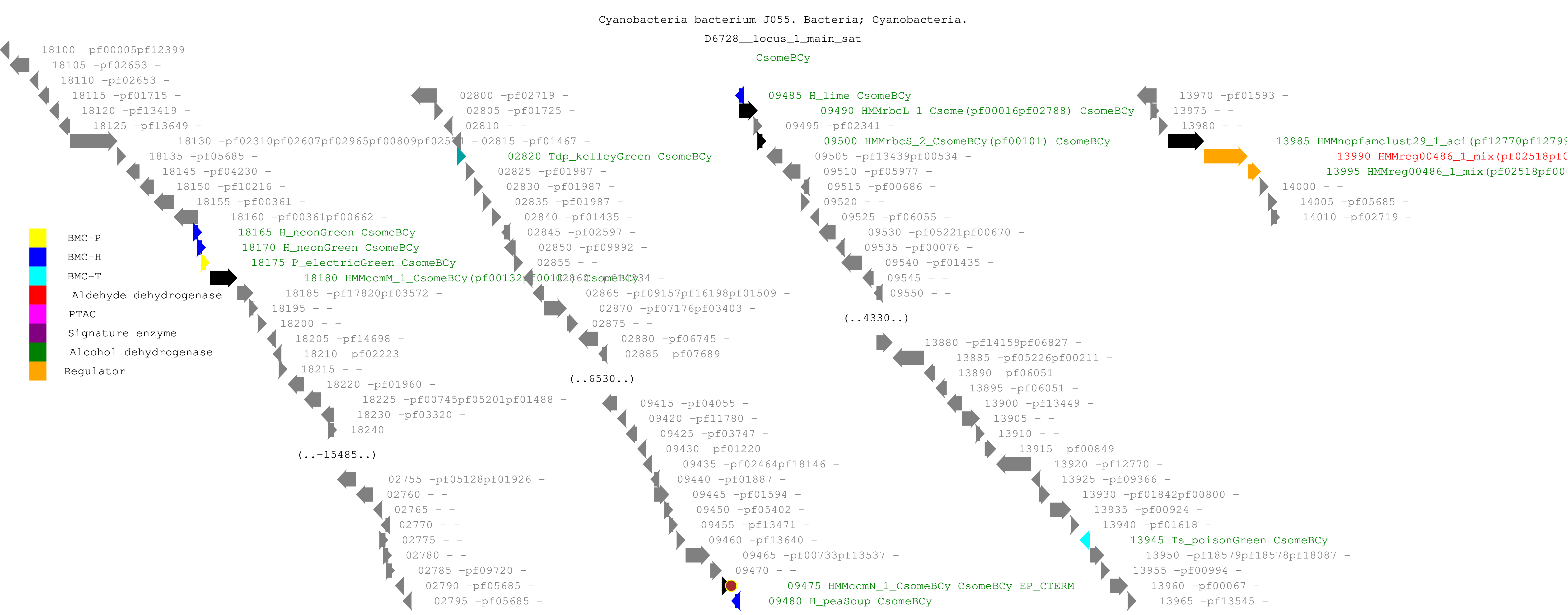

Cyanobacteria bacterium J055. Bacteria; Cyanobacteria.
D6728__locus_1_main_sat
CsomeBCy
BMC-P
BMC-H
BMC-T
Aldehyde dehydrogenase
PTAC
Signature enzyme
Alcohol dehydrogenase
Regulator
18100 -pf00005pf12399 -
18105 -pf02653 -
18110 -pf02653 -
18115 -pf01715 -
18120 -pf13419 -
18125 -pf13649 -
18130 -pf02310pf02607pf02965pf00809pf025
18135 -pf05685 -
18145 -pf04230 -
18150 -pf10216 -
18155 -pf00361 -
18160 -pf00361pf00662 -
18165 H_neonGreen CsomeBCy
18170 H_neonGreen CsomeBCy
18175 P_electricGreen CsomeBCy
18180 HMMccmM_1_CsomeBCy(pf00132pf00101) CsomeBCy
18185 -pf17820pf03572 -
18195 - -
18200 - -
18205 -pf14698 -
18210 -pf02223 -
18215 - -
18220 -pf01960 -
18225 -pf00745pf05201pf01488 -
18230 -pf03320 -
18240 - -
(..-15485..)
02755 -pf05128pf01926 -
02760 - -
02765 - -
02770 - -
02775 - -
02780 - -
02785 -pf09720 -
02790 -pf05685 -
02795 -pf05685 -
02800 -pf02719 -
02805 -pf01725 -
02810 - -
02815 -pf01467 -
02820 Tdp_kelleyGreen CsomeBCy
02825 -pf01987 -
02830 -pf01987 -
02835 -pf01987 -
02840 -pf01435 -
02845 -pf02597 -
02850 -pf09992 -
02855 - -
02860 -pf13434 -
02865 -pf09157pf16198pf01509 -
02870 -pf07176pf03403 -
02875 - -
02880 -pf06745 -
02885 -pf07689 -
(..6530..)
09415 -pf04055 -
09420 -pf11780 -
09425 -pf03747 -
09430 -pf01220 -
09435 -pf02464pf18146 -
09440 -pf01887 -
09445 -pf01594 -
09450 -pf05402 -
09455 -pf13471 -
09460 -pf13640 -
09465 -pf00733pf13537 -
09470 - -
09475 HMMccmN_1_CsomeBCy CsomeBCy EP_CTERM
09480 H_peaSoup CsomeBCy
09485 H_lime CsomeBCy
09490 HMMrbcL_1_Csome(pf00016pf02788) CsomeBCy
09495 -pf02341 -
09500 HMMrbcS_2_CsomeBCy(pf00101) CsomeBCy
09505 -pf13439pf00534 -
09510 -pf05977 -
09515 -pf00686 -
09520 - -
09525 -pf06055 -
09530 -pf05221pf00670 -
09535 -pf00076 -
09540 -pf01435 -
09545 - -
09550 - -
(..4330..)
13880 -pf14159pf06827 -
13885 -pf05226pf00211 -
13890 -pf06051 -
13895 -pf06051 -
13900 -pf13449 -
13905 - -
13910 - -
13915 -pf00849 -
13920 -pf12770 -
13925 -pf09366 -
13930 -pf01842pf00800 -
13935 -pf00924 -
13940 -pf01618 -
13945 Ts_poisonGreen CsomeBCy
13950 -pf18579pf18578pf18087 -
13955 -pf00994 -
13960 -pf00067 -
13965 -pf13545 -
13970 -pf01593 -
13975 - -
13980 - -
13985 HMMnopfamclust29_1_aci(pf12770pf1279
13990 HMMreg00486_1_mix(pf02518pf0
13995 HMMreg00486_1_mix(pf02518pf00
14000 - -
14005 -pf05685 -
14010 -pf02719 -

Cyanobacteria bacterium J069. Bacteria; Cyanobacteria.

D6742__locus_1_main_sat

CsomeBCy

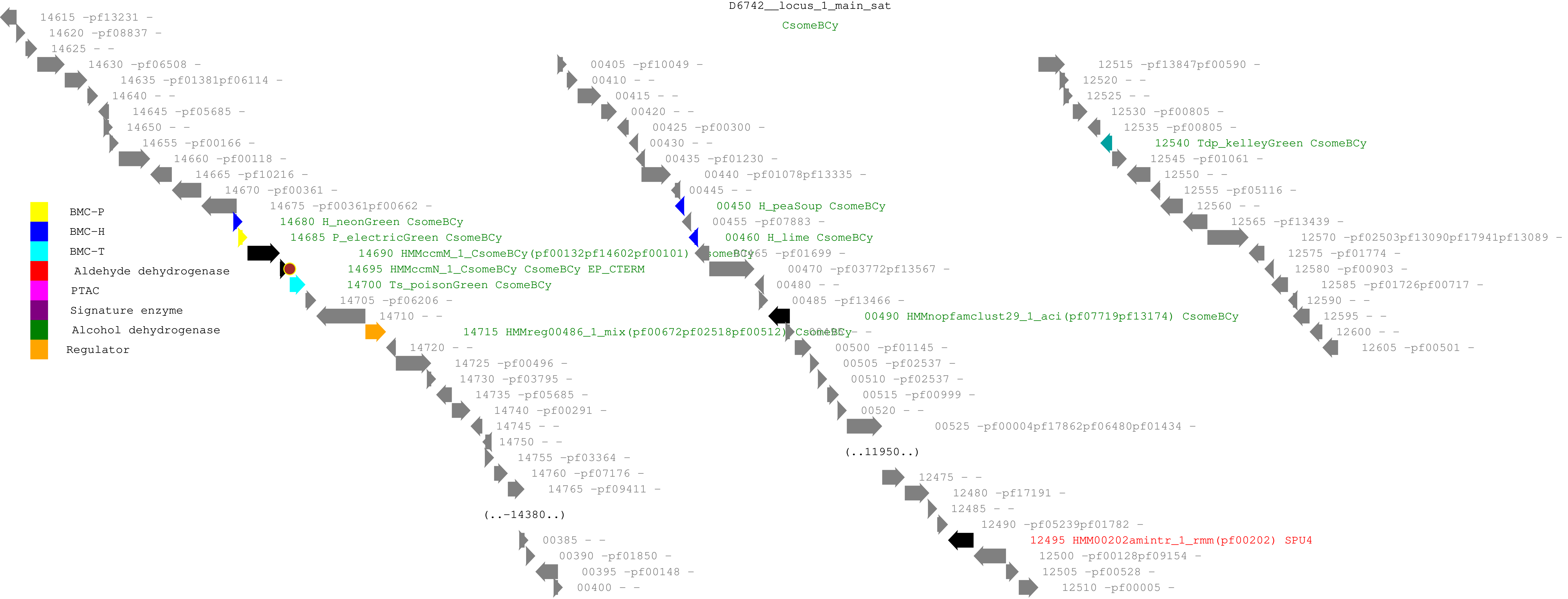

Cyanobacteria bacterium J083. Bacteria; Cyanobacteria.

D6756__locus_1_main_sat

CsomeBCy

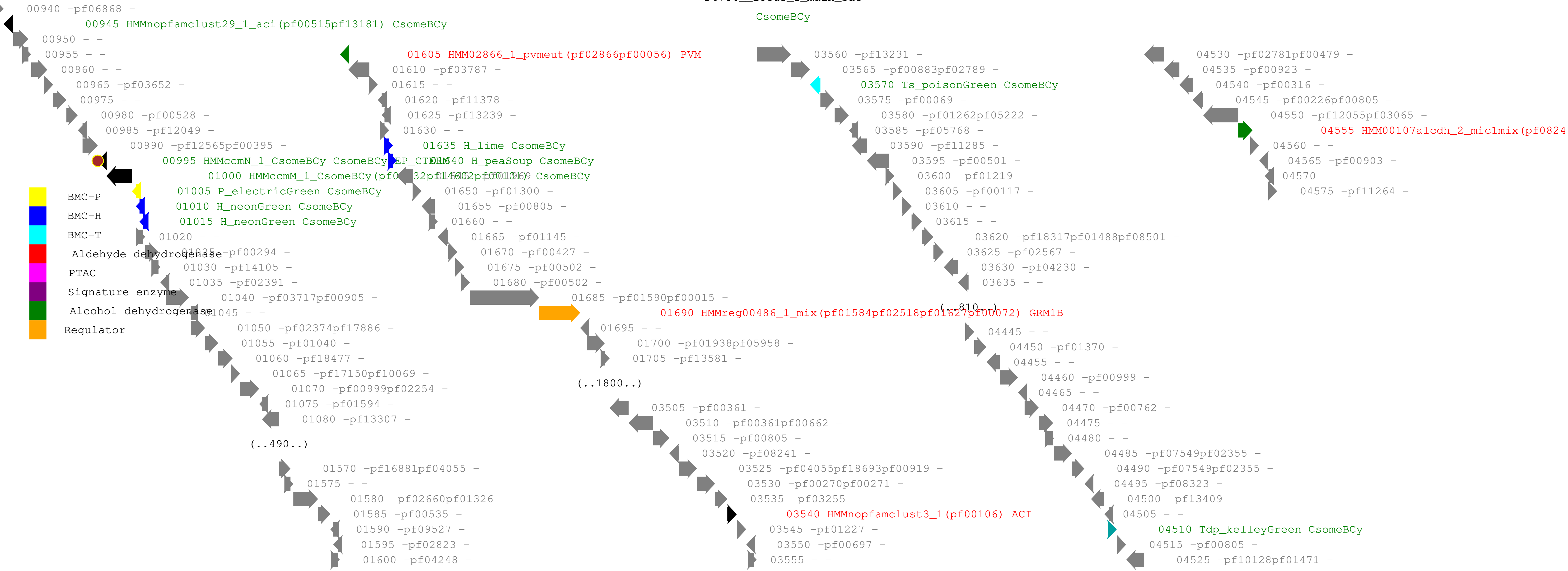

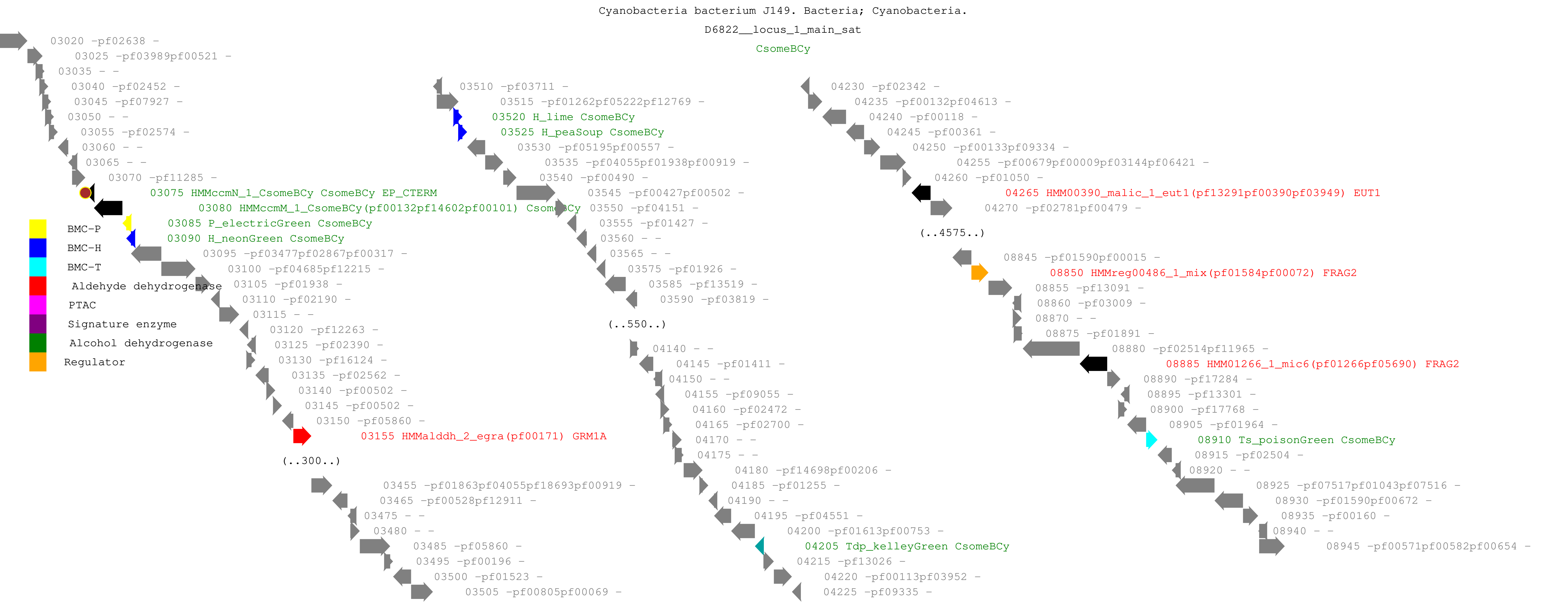

Cyanobacteria bacterium J149. Bacteria; Cyanobacteria.
D6822__locus_1_main_sat
CsomeBCy
03020 -pf02638 -
03025 -pf03989pf00521 -
03035 - -
03040 -pf02452 -
03045 -pf07927 -
03050 - -
03055 -pf02574 -
03060 - -
03065 - -
03070 -pf11285 -
03075 HMMccmN_1_CsomeBCy CsomeBCy EP_CTERM
03080 HMMccmM_1_CsomeBCy(pf00132pf14602pf00101) CsomeBCy
03085 P_electricGreen CsomeBCy
03090 H_neonGreen CsomeBCy
03095 -pf03477pf02867pf00317 -
03100 -pf04685pf12215 -
03105 -pf01938 -
03110 -pf02190 -
03115 - -
03120 -pf12263 -
03125 -pf02390 -
03130 -pf16124 -
03135 -pf02562 -
03140 -pf00502 -
03145 -pf00502 -
03150 -pf05860 -
03155 HMMalddh_2_egra(pf00171) GRM1A
(..300..)
03455 -pf01863pf04055pf18693pf00919 -
03465 -pf00528pf12911 -
03475 - -
03480 - -
03485 -pf05860 -
03495 -pf00196 -
03500 -pf01523 -
03505 -pf00805pf00069 -
BMC-P
BMC-H
BMC-T
Aldehyde dehydrogenase
PTAC
Signature enzyme
Alcohol dehydrogenase
Regulator
03510 -pf03711 -
03515 -pf01262pf05222pf12769 -
03520 H_lime CsomeBCy
03525 H_peaSoup CsomeBCy
03530 -pf05195pf00557 -
03535 -pf04055pf01938pf00919 -
03540 -pf00490 -
03545 -pf00427pf00502 -
03550 -pf04151 -
03555 -pf01427 -
03560 - -
03565 - -
03575 -pf01926 -
03585 -pf13519 -
03590 -pf03819 -
(..550..)
04140 - -
04145 -pf01411 -
04150 - -
04155 -pf09055 -
04160 -pf02472 -
04165 -pf02700 -
04170 - -
04175 - -
04180 -pf14698pf00206 -
04185 -pf01255 -
04190 - -
04195 -pf04551 -
04200 -pf01613pf00753 -
04205 Tdp_kelleyGreen CsomeBCy
04215 -pf13026 -
04220 -pf00113pf03952 -
04225 -pf09335 -
04230 -pf02342 -
04235 -pf00132pf04613 -
04240 -pf00118 -
04245 -pf00361 -
04250 -pf00133pf09334 -
04255 -pf00679pf00009pf03144pf06421 -
04260 -pf01050 -
04265 HMM00390_malic_1_eut1(pf13291pf00390pf03949) EUT1
04270 -pf02781pf00479 -
(..4575..)
08845 -pf01590pf00015 -
08850 HMMreg00486_1_mix(pf01584pf00072) FRAG2
08855 -pf13091 -
08860 -pf03009 -
08870 - -
08875 -pf01891 -
08880 -pf02514pf11965 -
08885 HMM01266_1_mic6(pf01266pf05690) FRAG2
08890 -pf17284 -
08895 -pf13301 -
08900 -pf17768 -
08905 -pf01964 -
08910 Ts_poisonGreen CsomeBCy
08915 -pf02504 -
08920 - -
08925 -pf07517pf01043pf07516 -
08930 -pf01590pf00672 -
08935 -pf00160 -
08940 - -
08945 -pf00571pf00582pf00654 -

Tolypothrix bouteillei VB521301. Bacteria; Cyanobacteria; Nostocales; Tolypothrichaceae; Tolypothrix.

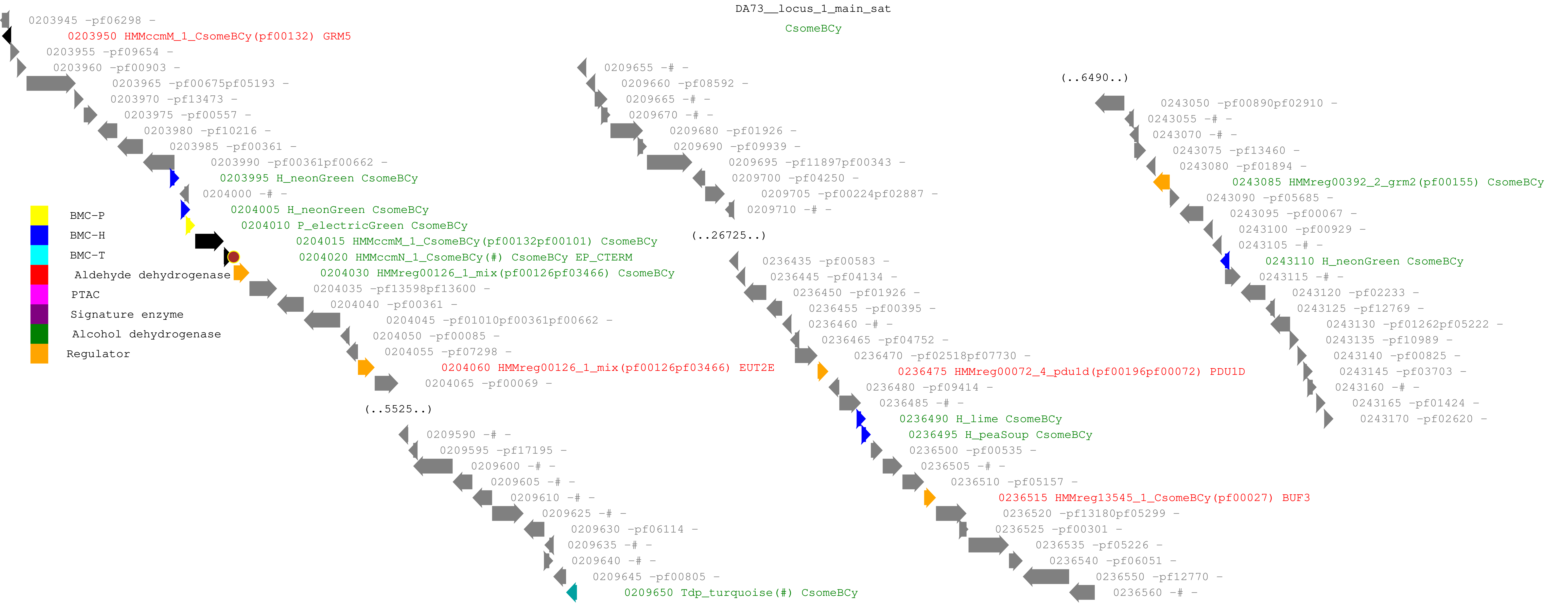

Dactylococcopsis salina PCC 8305. Bacteria; Cyanobacteria; Synechococcales; Synechococcaceae;

Dacsa__locus_1_main_sat

CsomeBCy

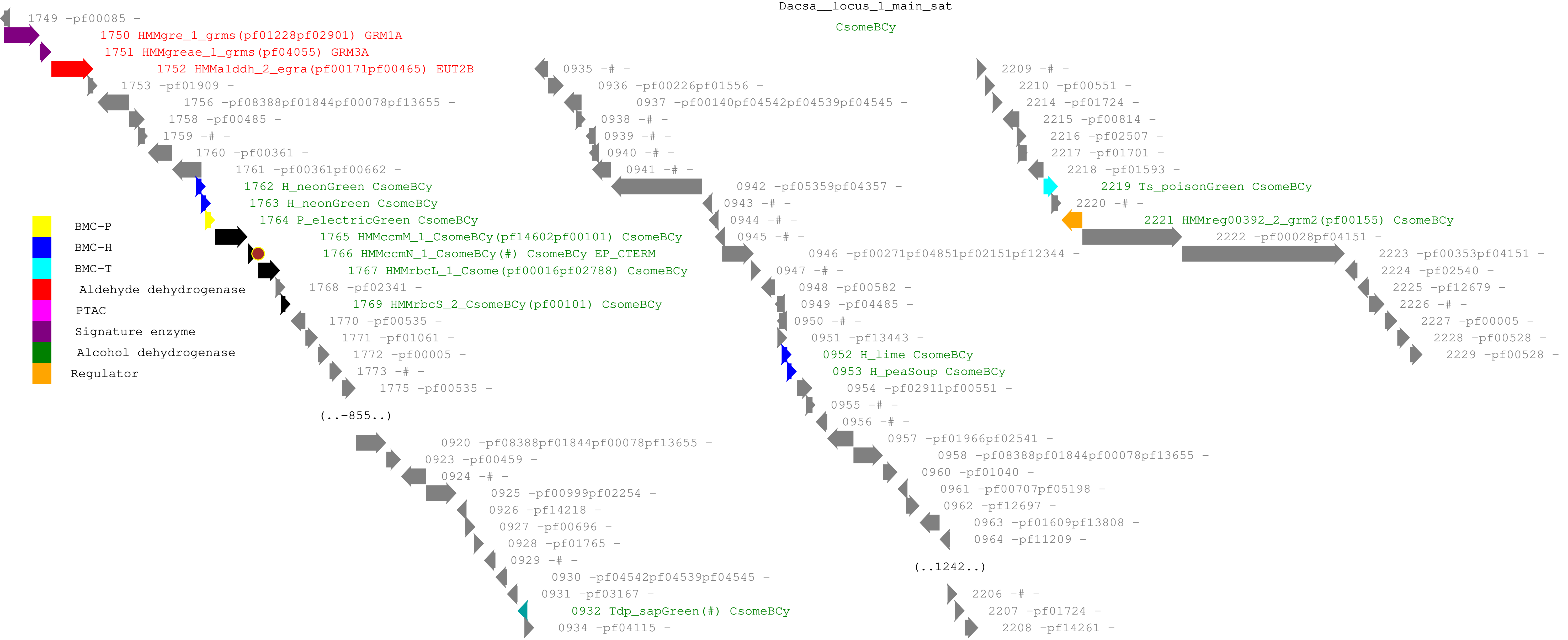

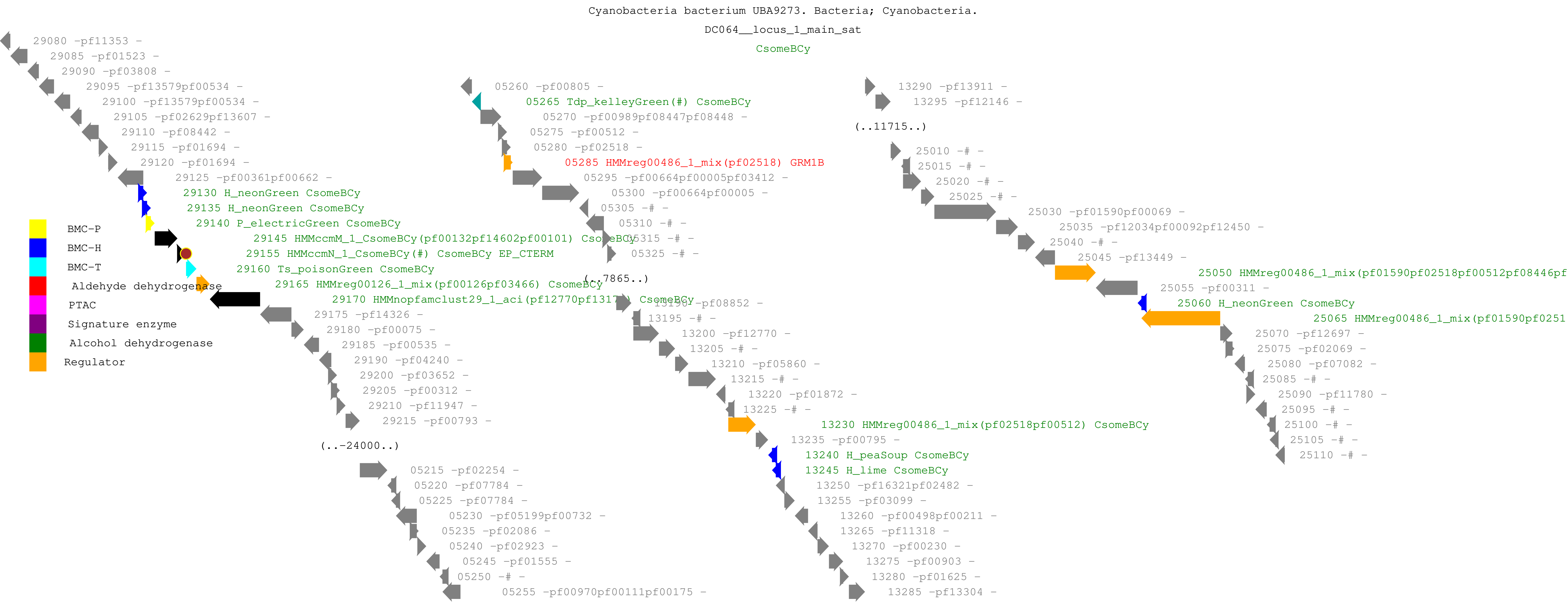
Cyanobacteria bacterium UBA9273. Bacteria; Cyanobacteria.
DC064__locus_1_main_sat
CsomeBCy
BMC-P
BMC-H
BMC-T
Aldehyde dehydrogenase
PTAC
Signature enzyme
Alcohol dehydrogenase
Regulator
29080 -pf11353 -
29085 -pf01523 -
29090 -pf03808 -
29095 -pf13579pf00534 -
29100 -pf13579pf00534 -
29105 -pf02629pf13607 -
29110 -pf08442 -
29115 -pf01694 -
29120 -pf01694 -
29125 -pf00361pf00662 -
29130 H_neonGreen CsomeBCy
29135 H_neonGreen CsomeBCy
29140 P_electricGreen CsomeBCy
29145 HMMccmM_1_CsomeBCy(pf00132pf14602pf00101) CsomeBCy
29155 HMMccmN_1_CsomeBCy(#) CsomeBCy EP_CTERM
29160 Ts_poisonGreen CsomeBCy
29165 HMMreg00126_1_mix(pf00126pf03466) CsomeBCy
29170 HMMnopfamclust29_1_aci(pf12770pf1317..) CsomeBCy
29175 -pf14326 -
29180 -pf00075 -
29185 -pf00535 -
29190 -pf04240 -
29200 -pf03652 -
29205 -pf00312 -
29210 -pf11947 -
29215 -pf00793 -
(..-24000..)
05215 -pf02254 -
05220 -pf07784 -
05225 -pf07784 -
05230 -pf05199pf00732 -
05235 -pf02086 -
05240 -pf02923 -
05245 -pf01555 -
05250 -# -
05255 -pf00970pf00111pf00175 -
05260 -pf00805 -
05265 Tdp_kelleyGreen(#) CsomeBCy
05270 -pf00989pf08447pf08448 -
05275 -pf00512 -
05280 -pf02518 -
05285 HMMreg00486_1_mix(pf02518) GRM1B
05295 -pf00664pf00005pf03412 -
05300 -pf00664pf00005 -
05305 -# -
05310 -# -
05315 -# -
05325 -# -
(..7865..)
13190 -pf08852 -
13195 -# -
13200 -pf12770 -
13205 -# -
13210 -pf05860 -
13215 -# -
13220 -pf01872 -
13225 -# -
13230 HMMreg00486_1_mix(pf02518pf00512) CsomeBCy
13235 -pf00795 -
13240 H_peaSoup CsomeBCy
13245 H_lime CsomeBCy
13250 -pf16321pf02482 -
13255 -pf03099 -
13260 -pf00498pf00211 -
13265 -pf11318 -
13270 -pf00230 -
13275 -pf00903 -
13280 -pf01625 -
13285 -pf13304 -
13290 -pf13911 -
13295 -pf12146 -
(..11715..)
25010 -# -
25015 -# -
25020 -# -
25025 -# -
25030 -pf01590pf00069 -
25035 -pf12034pf00092pf12450 -
25040 -# -
25045 -pf13449 -
25050 HMMreg00486_1_mix(pf01590pf02518pf00512pf08446pf
25055 -pf00311 -
25060 H_neonGreen CsomeBCy
25065 HMMreg00486_1_mix(pf01590pf0251
25070 -pf12697 -
25075 -pf02069 -
25080 -pf07082 -
25085 -# -
25090 -pf11780 -
25095 -# -
25100 -# -
25105 -# -
25110 -# -

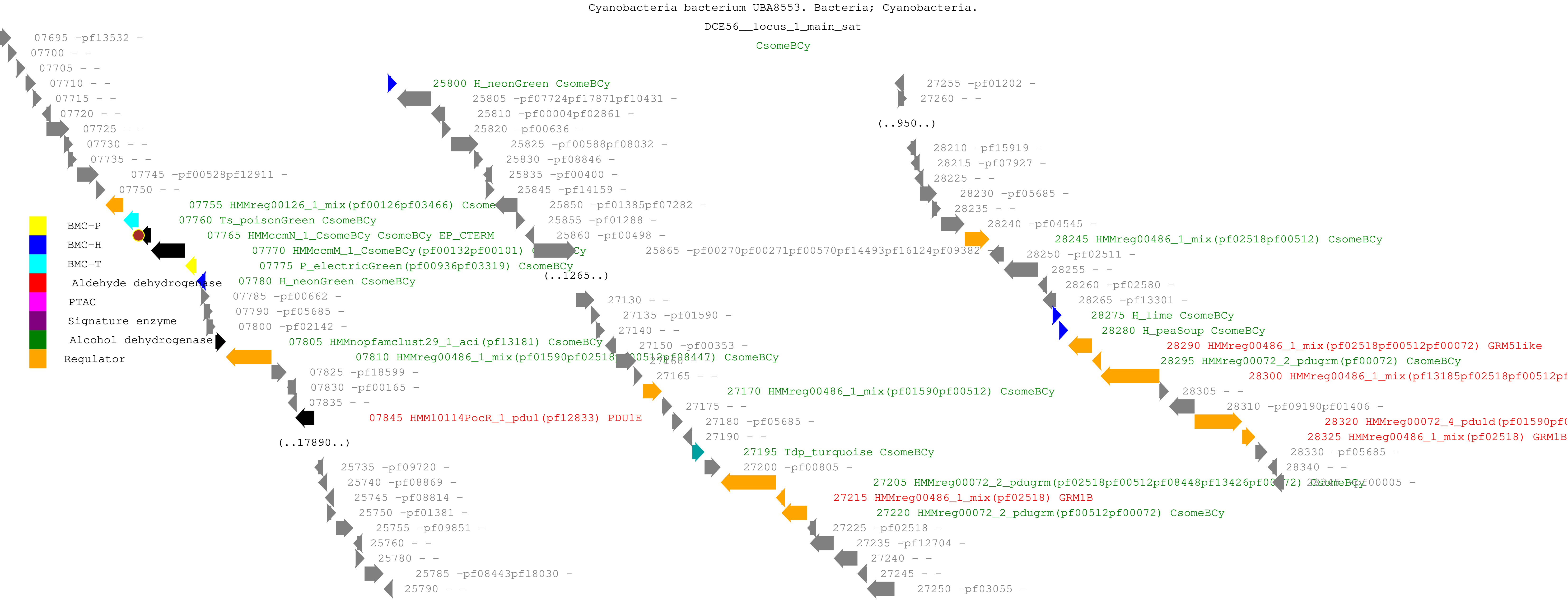
Cyanobacteria bacterium UBA8553. Bacteria; Cyanobacteria.
DCE56__locus_1_main_sat
CsomeBCy
BMC-P
BMC-H
BMC-T
Aldehyde dehydrogenase
PTAC
Signature enzyme
Alcohol dehydrogenase
Regulator
07695 -pf13532 -
07700 - -
07705 - -
07710 - -
07715 - -
07720 - -
07725 - -
07730 - -
07735 - -
07745 -pf00528pf12911 -
07750 - -
07755 HMMreg00126_1_mix(pf00126pf03466) Csome
07760 Ts_poisonGreen CsomeBCy
07765 HMMccmN_1_CsomeBCy CsomeBCy EP_CTERM
07770 HMMccmM_1_CsomeBCy(pf00132pf00101)
07775 P_electricGreen(pf00936pf03319) CsomeBCy
07780 H_neonGreen CsomeBCy
07785 -pf00662 -
07790 -pf05685 -
07800 -pf02142 -
07805 HMMnopfamclust29_1_aci(pf13181) CsomeBCy
07810 HMMreg00486_1_mix(pf01590pf02518pf00512pf08447) CsomeBCy
07825 -pf18599 -
07830 -pf00165 -
07835 - -
07845 HMM10114PocR_1_pdu1(pf12833) PDU1E
(..17890..)
25735 -pf09720 -
25740 -pf08869 -
25745 -pf08814 -
25750 -pf01381 -
25755 -pf09851 -
25760 - -
25780 - -
25785 -pf08443pf18030 -
25790 - -
25800 H_neonGreen CsomeBCy
25805 -pf07724pf17871pf10431 -
25810 -pf00004pf02861 -
25820 -pf00636 -
25825 -pf00588pf08032 -
25830 -pf08846 -
25835 -pf00400 -
25845 -pf14159 -
25850 -pf01385pf07282 -
25855 -pf01288 -
25860 -pf00498 -
25865 -pf00270pf00271pf00570pf14493pf16124pf09382 -
(..1265..)
27130 - -
27135 -pf01590 -
27140 - -
27150 -pf00353 -
27160
27165 - -
27170 HMMreg00486_1_mix(pf01590pf00512) CsomeBCy
27175 - -
27180 -pf05685 -
27190 - -
27195 Tdp_turquoise CsomeBCy
27200 -pf00805 -
27205 HMMreg00072_2_pdugrm(pf02518pf00512pf08448pf13426pf00072) CsomeBCy
27215 HMMreg00486_1_mix(pf02518) GRM1B
27220 HMMreg00072_2_pdugrm(pf00512pf00072) CsomeBCy
27225 -pf02518 -
27235 -pf12704 -
27240 - -
27245 - -
27250 -pf03055 -
27255 -pf01202 -
27260 - -
(..950..)
28210 -pf15919 -
28215 -pf07927 -
28225 - -
28230 -pf05685 -
28235 - -
28240 -pf04545 -
28245 HMMreg00486_1_mix(pf02518pf00512) CsomeBCy
28250 -pf02511 -
28255 - -
28260 -pf02580 -
28265 -pf13301 -
28275 H_lime CsomeBCy
28280 H_peaSoup CsomeBCy
28290 HMMreg00486_1_mix(pf02518pf00512pf00072) GRM5like
28295 HMMreg00072_2_pdugrm(pf00072) CsomeBCy
28300 HMMreg00486_1_mix(pf13185pf02518pf00512pf
28305 - -
28310 -pf09190pf01406 -
28320 HMMreg00072_4_pdu1d(pf01590pf
28325 HMMreg00486_1_mix(pf02518) GRM1B
28330 -pf05685 -
28340 - -
28345 -pf00005 -

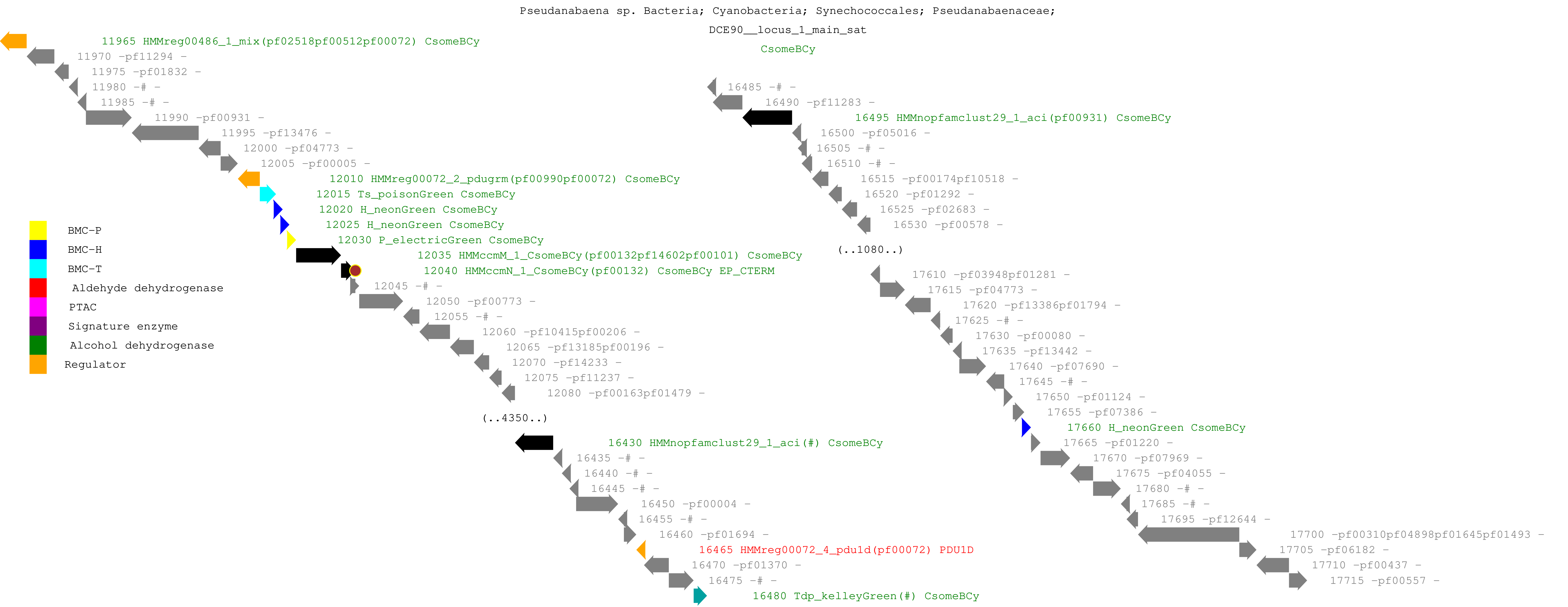

Pseudanabaena sp. Bacteria; Cyanobacteria; Synechococcales; Pseudanabaenaceae;
DCE90__locus_1_main_sat
CsomeBCy
11965 HMMreg00486_1_mix(pf02518pf00512pf00072) CsomeBCy
11970 -pf11294 -
11975 -pf01832 -
11980 -# -
11985 -# -
11990 -pf00931 -
11995 -pf13476 -
12000 -pf04773 -
12005 -pf00005 -
12010 HMMreg00072_2_pdugrm(pf00990pf00072) CsomeBCy
12015 Ts_poisonGreen CsomeBCy
12020 H_neonGreen CsomeBCy
12025 H_neonGreen CsomeBCy
12030 P_electricGreen CsomeBCy
12035 HMMccmM_1_CsomeBCy(pf00132pf14602pf00101) CsomeBCy
12040 HMMccmN_1_CsomeBCy(pf00132) CsomeBCy EP_CTERM
12045 -# -
12050 -pf00773 -
12055 -# -
12060 -pf10415pf00206 -
12065 -pf13185pf00196 -
12070 -pf14233 -
12075 -pf11237 -
12080 -pf00163pf01479 -
(..4350..)
16430 HMMnopfamclust29_1_aci(#) CsomeBCy
16435 -# -
16440 -# -
16445 -# -
16450 -pf00004 -
16455 -# -
16460 -pf01694 -
16465 HMMreg00072_4_pdu1d(pf00072) PDU1D
16470 -pf01370 -
16475 -# -
16480 Tdp_kelleyGreen(#) CsomeBCy
16485 -# -
16490 -pf11283 -
16495 HMMnopfamclust29_1_aci(pf00931) CsomeBCy
16500 -pf05016 -
16505 -# -
16510 -# -
16515 -pf00174pf10518 -
16520 -pf01292 -
16525 -pf02683 -
16530 -pf00578 -
(..1080..)
17610 -pf03948pf01281 -
17615 -pf04773 -
17620 -pf13386pf01794 -
17625 -# -
17630 -pf00080 -
17635 -pf13442 -
17640 -pf07690 -
17645 -# -
17650 -pf01124 -
17655 -pf07386 -
17660 H_neonGreen CsomeBCy
17665 -pf01220 -
17670 -pf07969 -
17675 -pf04055 -
17680 -# -
17685 -# -
17695 -pf12644 -
17700 -pf00310pf04898pf01645pf01493 -
17705 -pf06182 -
17710 -pf00437 -
17715 -pf00557 -
BMC-P
BMC-H
BMC-T
Aldehyde dehydrogenase
PTAC
Signature enzyme
Alcohol dehydrogenase
Regulator

Snowella sp. Bacteria; Cyanobacteria; Synechococcales; Coelosphaeriaceae; Snowella.

DCF12__locus_1_main_sat

CsomeBCy

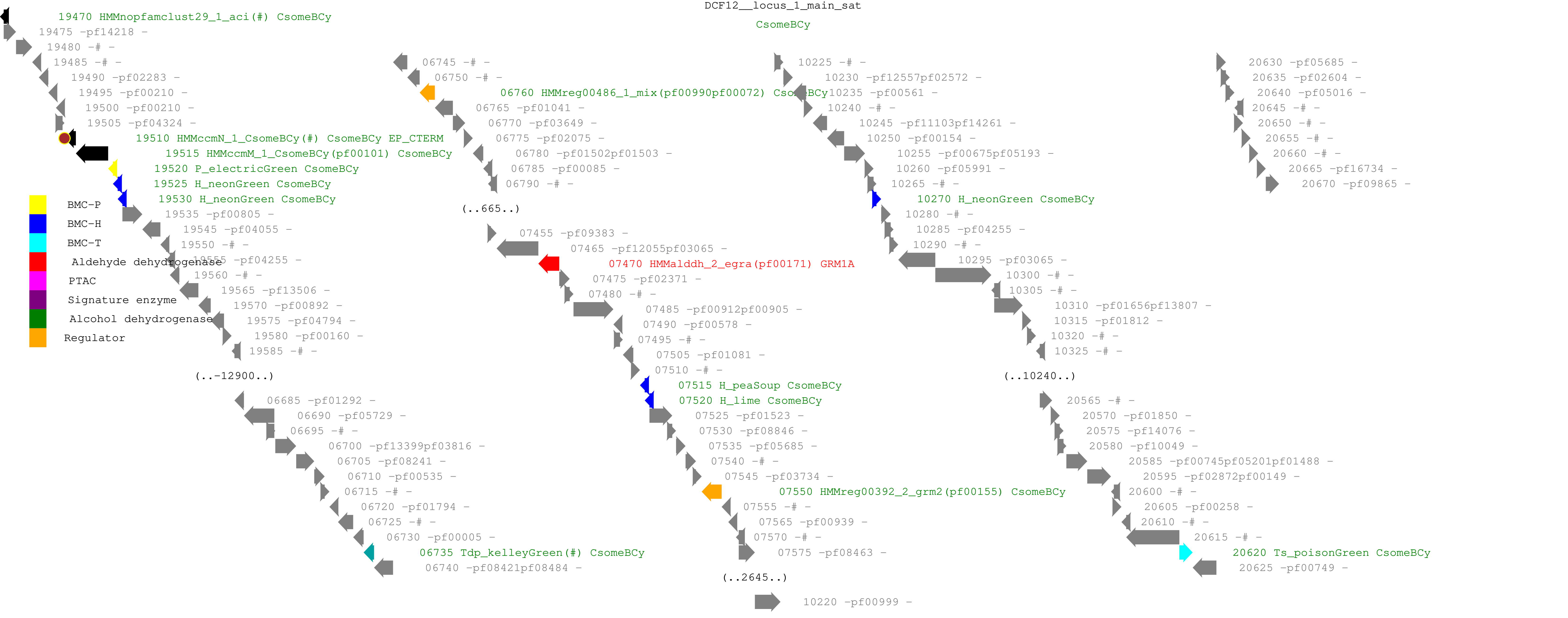

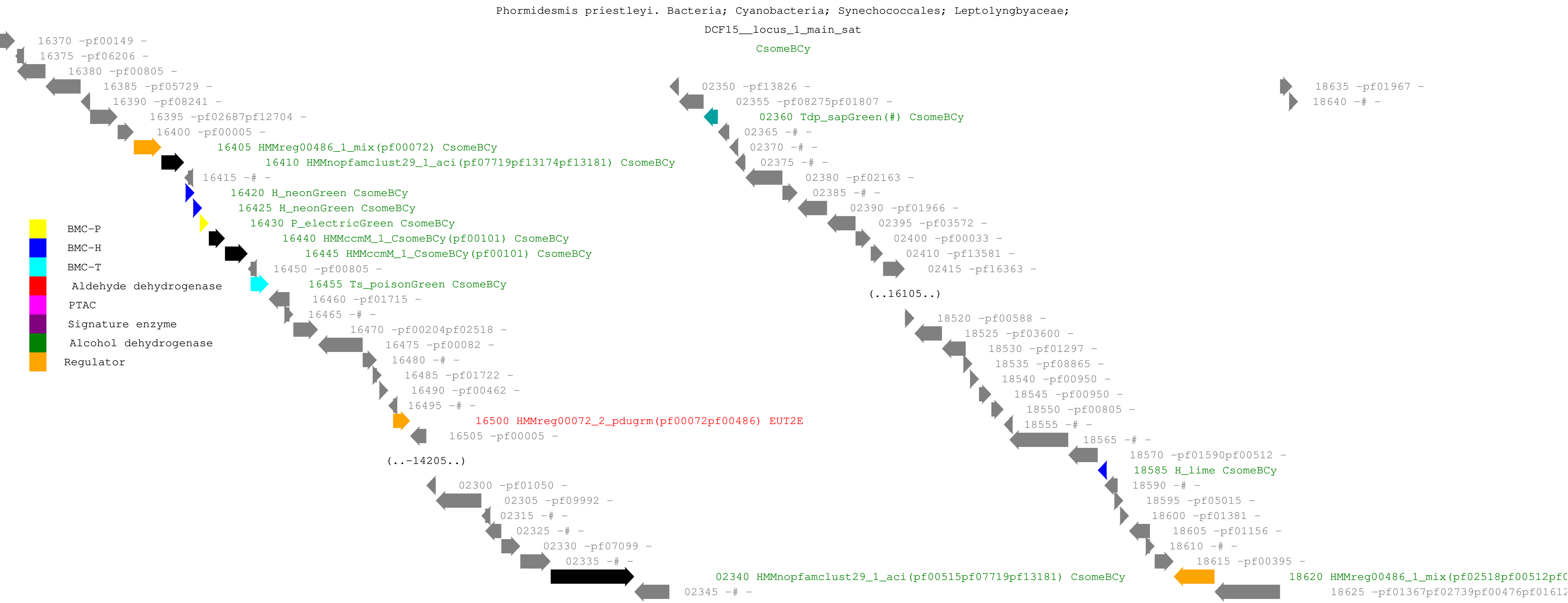

Phormidesmis priestleyi. Bacteria; Cyanobacteria; Synechococcales; Leptolyngbyaceae;
DCF15__locus_1_main_sat
CsomeBCy
16370 -pf00149 -
16375 -pf06206 -
16380 -pf00805 -
16385 -pf05729 -
16390 -pf08241 -
16395 -pf02687pf12704 -
16400 -pf00005 -
16405 HMMreg00486_1_mix(pf00072) CsomeBCy
16410 HMMnopfamclust29_1_aci(pf07719pf13174pf13181) CsomeBCy
16415 -# -
16420 H_neonGreen CsomeBCy
16425 H_neonGreen CsomeBCy
16430 P_electricGreen CsomeBCy
16440 HMMccmM_1_CsomeBCy(pf00101) CsomeBCy
16445 HMMccmM_1_CsomeBCy(pf00101) CsomeBCy
16450 -pf00805 -
16455 Ts_poisonGreen CsomeBCy
16460 -pf01715 -
16465 -# -
16470 -pf00204pf02518 -
16475 -pf00082 -
16480 -# -
16485 -pf01722 -
16490 -pf00462 -
16495 -# -
16500 HMMreg00072_2_pdugrm(pf00072pf00486) EUT2E
16505 -pf00005 -
(..-14205..)
02300 -pf01050 -
02305 -pf09992 -
02315 -# -
02325 -# -
02330 -pf07099 -
02335 -# -
02340 HMMnopfamclust29_1_aci(pf00515pf07719pf13181) CsomeBCy
02345 -# -
02350 -pf13826 -
02355 -pf08275pf01807 -
02360 Tdp_sapGreen(#) CsomeBCy
02365 -# -
02370 -# -
02375 -# -
02380 -pf02163 -
02385 -# -
02390 -pf01966 -
02395 -pf03572 -
02400 -pf00033 -
02410 -pf13581 -
02415 -pf16363 -
(..16105..)
18520 -pf00588 -
18525 -pf03600 -
18530 -pf01297 -
18535 -pf08865 -
18540 -pf00950 -
18545 -pf00950 -
18550 -pf00805 -
18555 -# -
18565 -# -
18570 -pf01590pf00512 -
18585 H_lime CsomeBCy
18590 -# -
18595 -pf05015 -
18600 -pf01381 -
18605 -pf01156 -
18610 -# -
18615 -pf00395 -
18620 HMMreg00486_1_mix(pf02518pf00512pf0
18625 -pf01367pf02739pf00476pf0161
18635 -pf01967 -
18640 -# -
BMC-P
BMC-H
BMC-T
Aldehyde dehydrogenase
PTAC
Signature enzyme
Alcohol dehydrogenase
Regulator

Leptolyngbya antarctica. Bacteria; Cyanobacteria; Synechococcales; Leptolyngbyaceae;

DCF17__locus_1_main_sat

CsomeBCy

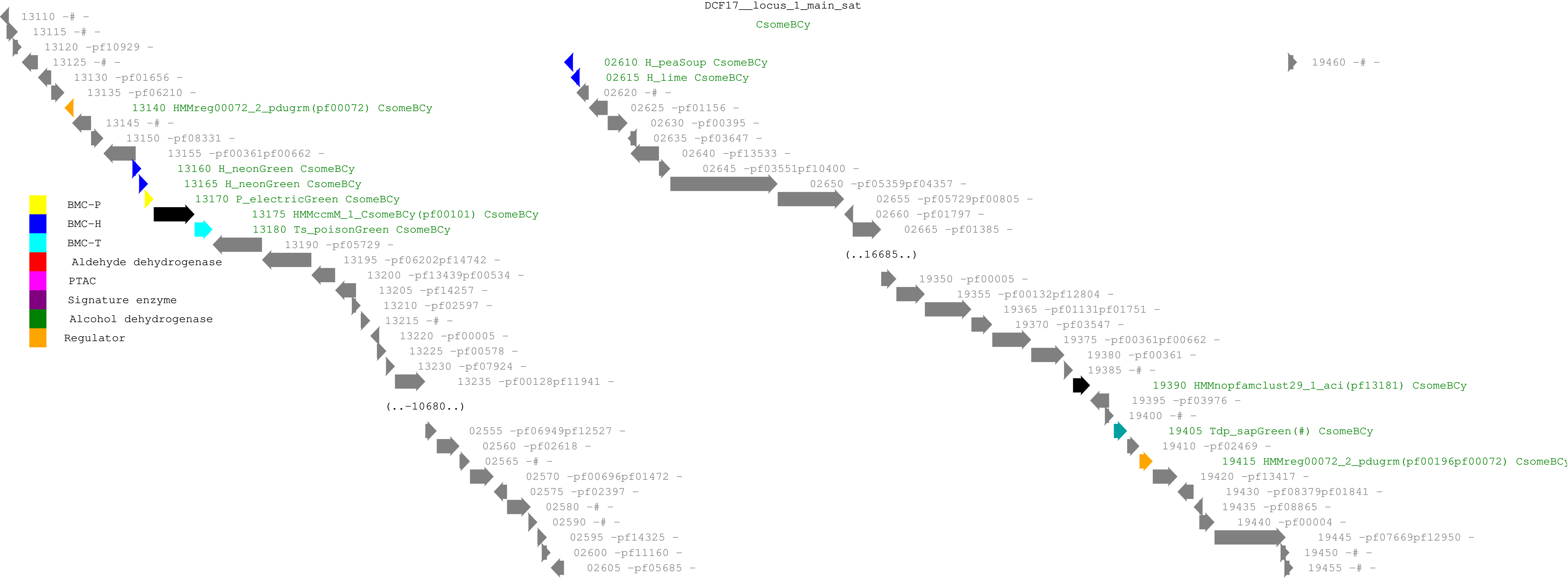

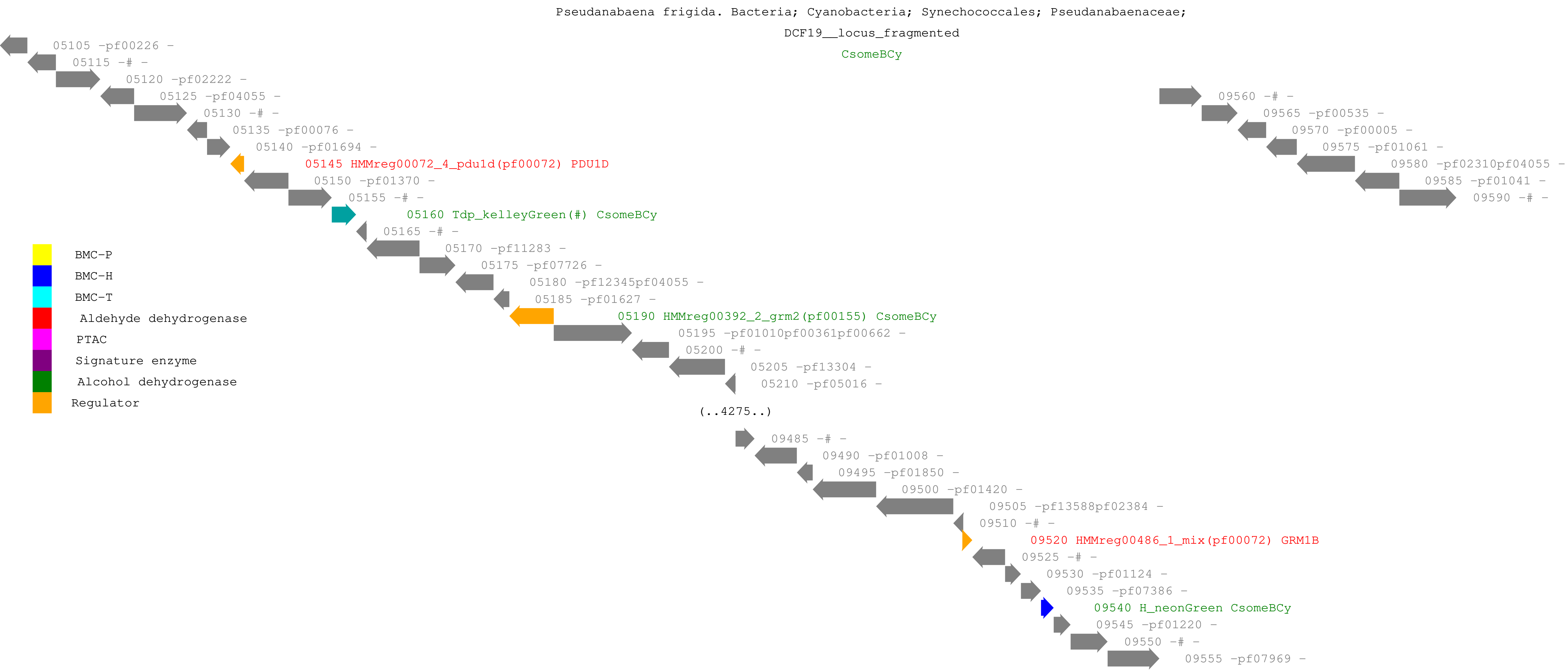
Pseudanabaena frigida. Bacteria; Cyanobacteria; Synechococcales; Pseudanabaenaceae;
DCF19__locus_fragmented
CsomeBCy
05105 -pf00226 -
05115 -# -
05120 -pf02222 -
05125 -pf04055 -
05130 -# -
05135 -pf00076 -
05140 -pf01694 -
05145 HMMreg00072_4_pdu1d(pf00072) PDU1D
05150 -pf01370 -
05155 -# -
05160 Tdp_kelleyGreen(#) CsomeBCy
05165 -# -
05170 -pf11283 -
05175 -pf07726 -
05180 -pf12345pf04055 -
05185 -pf01627 -
05190 HMMreg00392_2_grm2(pf00155) CsomeBCy
05195 -pf01010pf00361pf00662 -
05200 -# -
05205 -pf13304 -
05210 -pf05016 -
(..4275..)
09485 -# -
09490 -pf01008 -
09495 -pf01850 -
09500 -pf01420 -
09505 -pf13588pf02384 -
09510 -# -
09520 HMMreg00486_1_mix(pf00072) GRM1B
09525 -# -
09530 -pf01124 -
09535 -pf07386 -
09540 H_neonGreen CsomeBCy
09545 -pf01220 -
09550 -# -
09555 -pf07969 -
09560 -# -
09565 -pf00535 -
09570 -pf00005 -
09575 -pf01061 -
09580 -pf02310pf04055 -
09585 -pf01041 -
09590 -# -
BMC-P
BMC-H
BMC-T
Aldehyde dehydrogenase
PTAC
Signature enzyme
Alcohol dehydrogenase
Regulator

Pseudanabaena sp. Bacteria; Cyanobacteria; Synechococcales; Pseudanabaenaceae;

DCF20__locus_1_main_sat

CsomeBCy

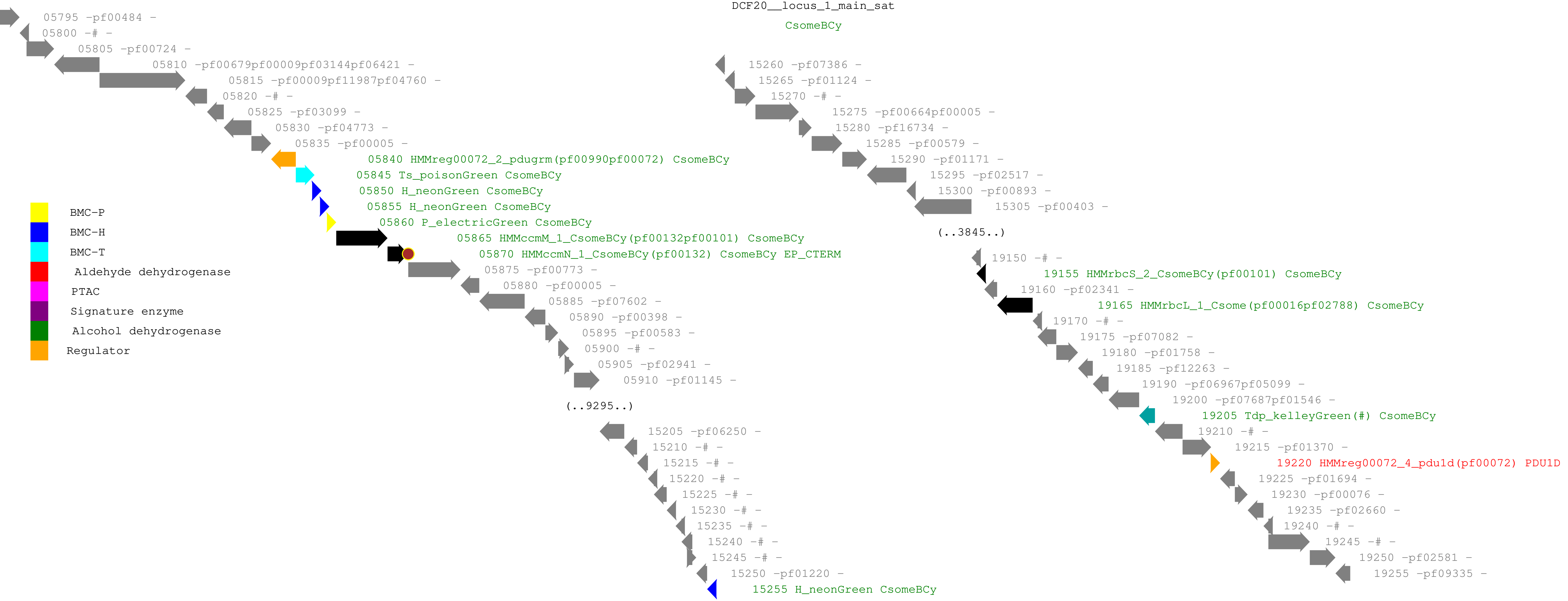

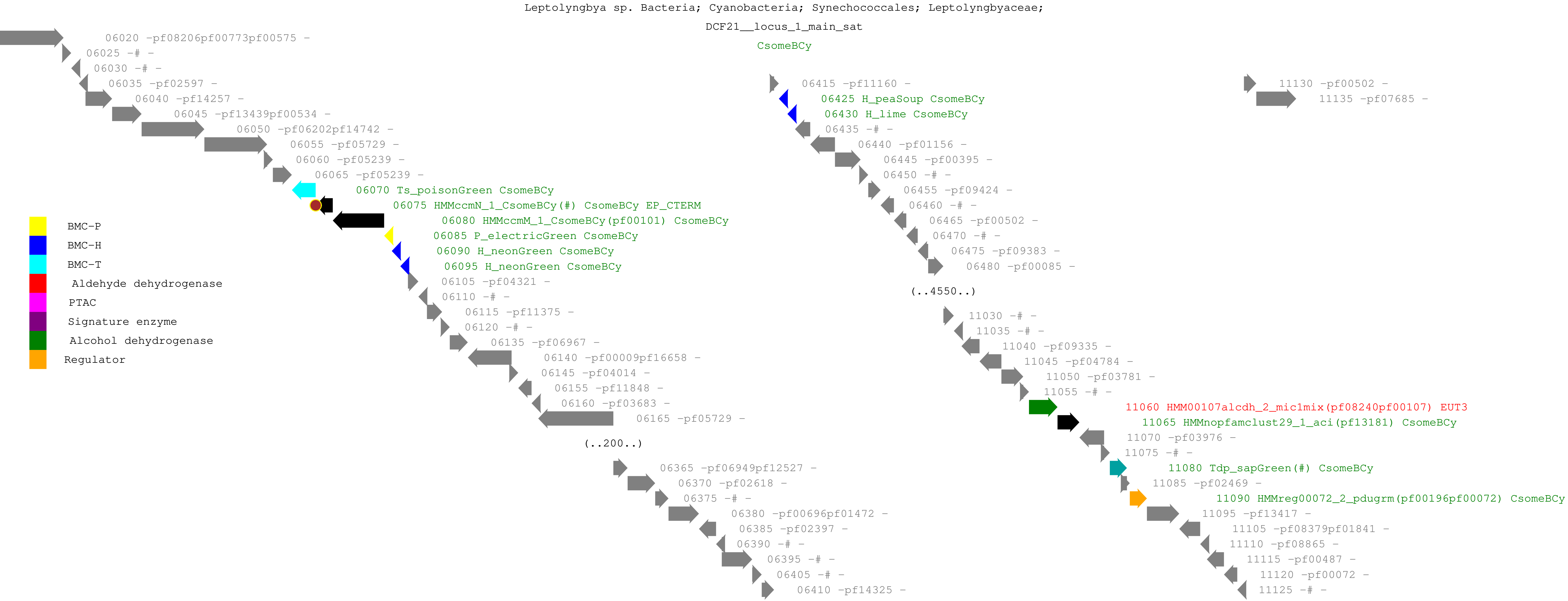
Leptolyngbya sp. Bacteria; Cyanobacteria; Synechococcales; Leptolyngbyaceae;
DCF21__locus_1_main_sat
CsomeBCy
06020 -pf08206pf00773pf00575 -
06025 -# -
06030 -# -
06035 -pf02597 -
06040 -pf14257 -
06045 -pf13439pf00534 -
06050 -pf06202pf14742 -
06055 -pf05729 -
06060 -pf05239 -
06065 -pf05239 -
06070 Ts_poisonGreen CsomeBCy
06075 HMMccmN_1_CsomeBCy(#) CsomeBCy EP_CTERM
06080 HMMccmM_1_CsomeBCy(pf00101) CsomeBCy
06085 P_electricGreen CsomeBCy
06090 H_neonGreen CsomeBCy
06095 H_neonGreen CsomeBCy
06105 -pf04321 -
06110 -# -
06115 -pf11375 -
06120 -# -
06135 -pf06967 -
06140 -pf00009pf16658 -
06145 -pf04014 -
06155 -pf11848 -
06160 -pf03683 -
06165 -pf05729 -
(..200..)
06365 -pf06949pf12527 -
06370 -pf02618 -
06375 -# -
06380 -pf00696pf01472 -
06385 -pf02397 -
06390 -# -
06395 -# -
06405 -# -
06410 -pf14325 -
06415 -pf11160 -
06425 H_peaSoup CsomeBCy
06430 H_lime CsomeBCy
06435 -# -
06440 -pf01156 -
06445 -pf00395 -
06450 -# -
06455 -pf09424 -
06460 -# -
06465 -pf00502 -
06470 -# -
06475 -pf09383 -
06480 -pf00085 -
(..4550..)
11030 -# -
11035 -# -
11040 -pf09335 -
11045 -pf04784 -
11050 -pf03781 -
11055 -# -
11060 HMM00107alcdh_2_mic1mix(pf08240pf00107) EUT3
11065 HMMnopfamclust29_1_aci(pf13181) CsomeBCy
11070 -pf03976 -
11075 -# -
11080 Tdp_sapGreen(#) CsomeBCy
11085 -pf02469 -
11090 HMMreg00072_2_pdugrm(pf00196pf00072) CsomeBCy
11095 -pf13417 -
11105 -pf08379pf01841 -
11110 -pf08865 -
11115 -pf00487 -
11120 -pf00072 -
11125 -# -
11130 -pf00502 -
11135 -pf07685 -
BMC-P
BMC-H
BMC-T
Aldehyde dehydrogenase
PTAC
Signature enzyme
Alcohol dehydrogenase
Regulator

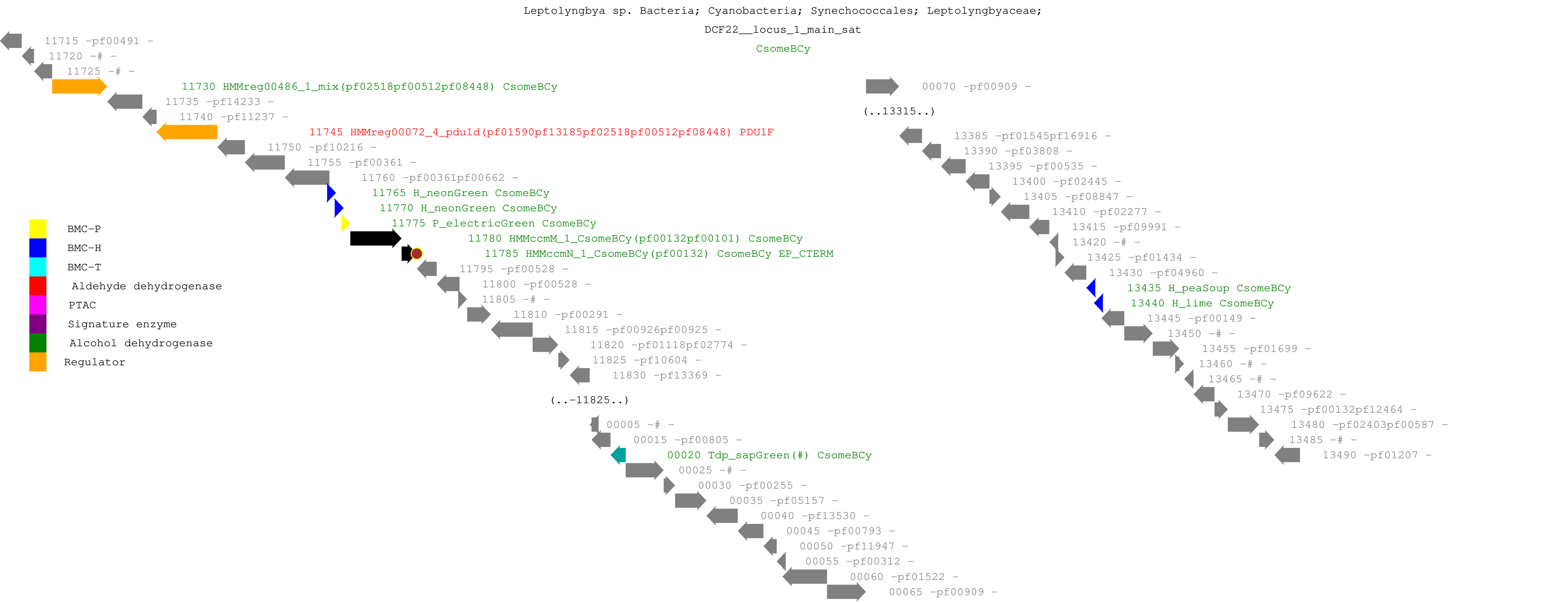

Leptolyngbya sp. Bacteria; Cyanobacteria; Synechococcales; Leptolyngbyaceae;
DCF22__locus_1_main_sat
CsomeBCy
11715 -pf00491 -
11720 -# -
11725 -# -
11730 HMMreg00486_1_mix(pf02518pf00512pf08448) CsomeBCy
11735 -pf14233 -
11740 -pf11237 -
11745 HMMreg00072_4_pdu1d(pf01590pf13185pf02518pf00512pf08448) PDU1F
11750 -pf10216 -
11755 -pf00361 -
11760 -pf00361pf00662 -
11765 H_neonGreen CsomeBCy
11770 H_neonGreen CsomeBCy
11775 P_electricGreen CsomeBCy
11780 HMMccmM_1_CsomeBCy(pf00132pf00101) CsomeBCy
11785 HMMccmN_1_CsomeBCy(pf00132) CsomeBCy EP_CTERM
11795 -pf00528 -
11800 -pf00528 -
11805 -# -
11810 -pf00291 -
11815 -pf00926pf00925 -
11820 -pf01118pf02774 -
11825 -pf10604 -
11830 -pf13369 -
(..-11825..)
00005 -# -
00015 -pf00805 -
00020 Tdp_sapGreen(#) CsomeBCy
00025 -# -
00030 -pf00255 -
00035 -pf05157 -
00040 -pf13530 -
00045 -pf00793 -
00050 -pf11947 -
00055 -pf00312 -
00060 -pf01522 -
00065 -pf00909 -
00070 -pf00909 -
(..13315..)
13385 -pf01545pf16916 -
13390 -pf03808 -
13395 -pf00535 -
13400 -pf02445 -
13405 -pf08847 -
13410 -pf02277 -
13415 -pf09991 -
13420 -# -
13425 -pf01434 -
13430 -pf04960 -
13435 H_peaSoup CsomeBCy
13440 H_lime CsomeBCy
13445 -pf00149 -
13450 -# -
13455 -pf01699 -
13460 -# -
13465 -# -
13470 -pf09622 -
13475 -pf00132pf12464 -
13480 -pf02403pf00587 -
13485 -# -
13490 -pf01207 -
BMC-P
BMC-H
BMC-T
Aldehyde dehydrogenase
PTAC
Signature enzyme
Alcohol dehydrogenase
Regulator

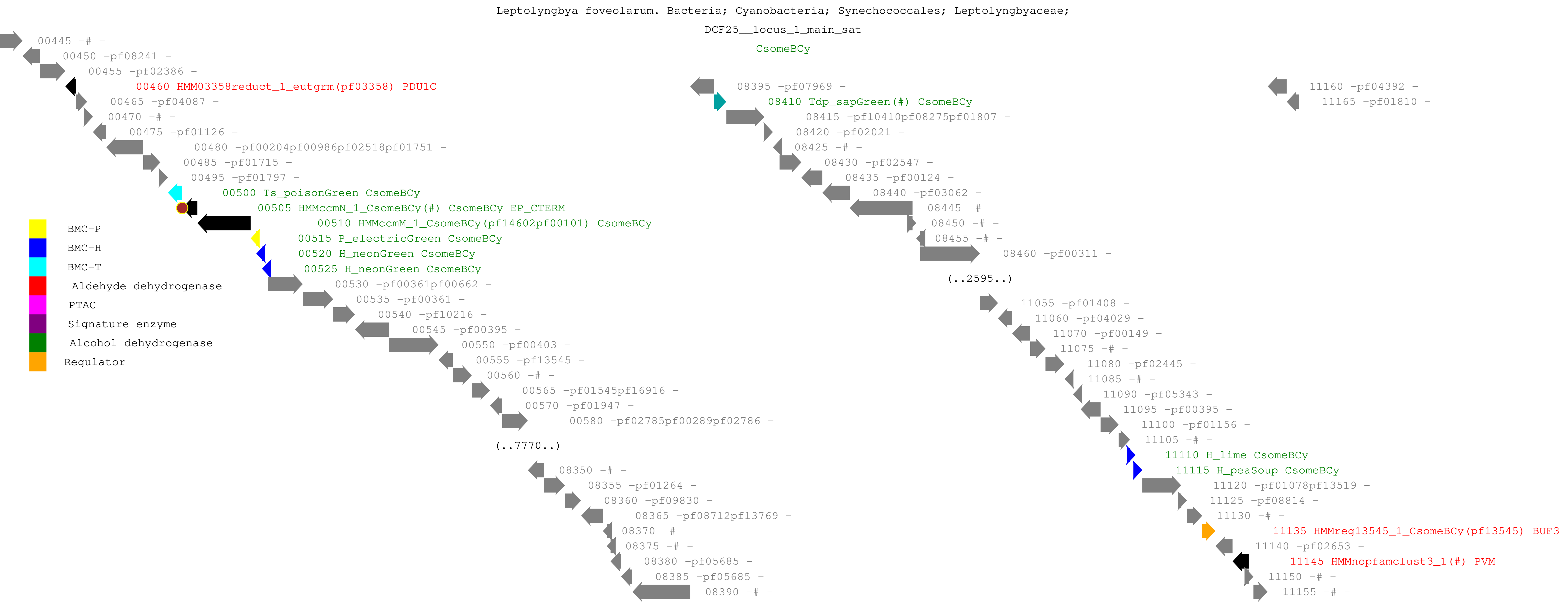
Leptolyngbya foveolarum. Bacteria; Cyanobacteria; Synechococcales; Leptolyngbyaceae;
DCF25__locus_1_main_sat
CsomeBCy
00445 -# -
00450 -pf08241 -
00455 -pf02386 -
00460 HMM03358reduct_1_eutgrm(pf03358) PDU1C
00465 -pf04087 -
00470 -# -
00475 -pf01126 -
00480 -pf00204pf00986pf02518pf01751 -
00485 -pf01715 -
00495 -pf01797 -
00500 Ts_poisonGreen CsomeBCy
00505 HMMccmN_1_CsomeBCy(#) CsomeBCy EP_CTERM
00510 HMMccmM_1_CsomeBCy(pf14602pf00101) CsomeBCy
00515 P_electricGreen CsomeBCy
00520 H_neonGreen CsomeBCy
00525 H_neonGreen CsomeBCy
00530 -pf00361pf00662 -
00535 -pf00361 -
00540 -pf10216 -
00545 -pf00395 -
00550 -pf00403 -
00555 -pf13545 -
00560 -# -
00565 -pf01545pf16916 -
00570 -pf01947 -
00580 -pf02785pf00289pf02786 -
(..7770..)
08350 -# -
08355 -pf01264 -
08360 -pf09830 -
08365 -pf08712pf13769 -
08370 -# -
08375 -# -
08380 -pf05685 -
08385 -pf05685 -
08390 -# -
08395 -pf07969 -
08410 Tdp_sapGreen(#) CsomeBCy
08415 -pf10410pf08275pf01807 -
08420 -pf02021 -
08425 -# -
08430 -pf02547 -
08435 -pf00124 -
08440 -pf03062 -
08445 -# -
08450 -# -
08455 -# -
08460 -pf00311 -
(..2595..)
11055 -pf01408 -
11060 -pf04029 -
11070 -pf00149 -
11075 -# -
11080 -pf02445 -
11085 -# -
11090 -pf05343 -
11095 -pf00395 -
11100 -pf01156 -
11105 -# -
11110 H_lime CsomeBCy
11115 H_peaSoup CsomeBCy
11120 -pf01078pf13519 -
11125 -pf08814 -
11130 -# -
11135 HMMreg13545_1_CsomeBCy(pf13545) BUF3
11140 -pf02653 -
11145 HMMnopfamclust3_1(#) PVM
11150 -# -
11155 -# -
11160 -pf04392 -
11165 -pf01810 -
BMC-P
BMC-H
BMC-T
Aldehyde dehydrogenase
PTAC
Signature enzyme
Alcohol dehydrogenase
Regulator

Leptolyngbya sp. Bacteria; Cyanobacteria; Synechococcales; Leptolyngbyaceae;

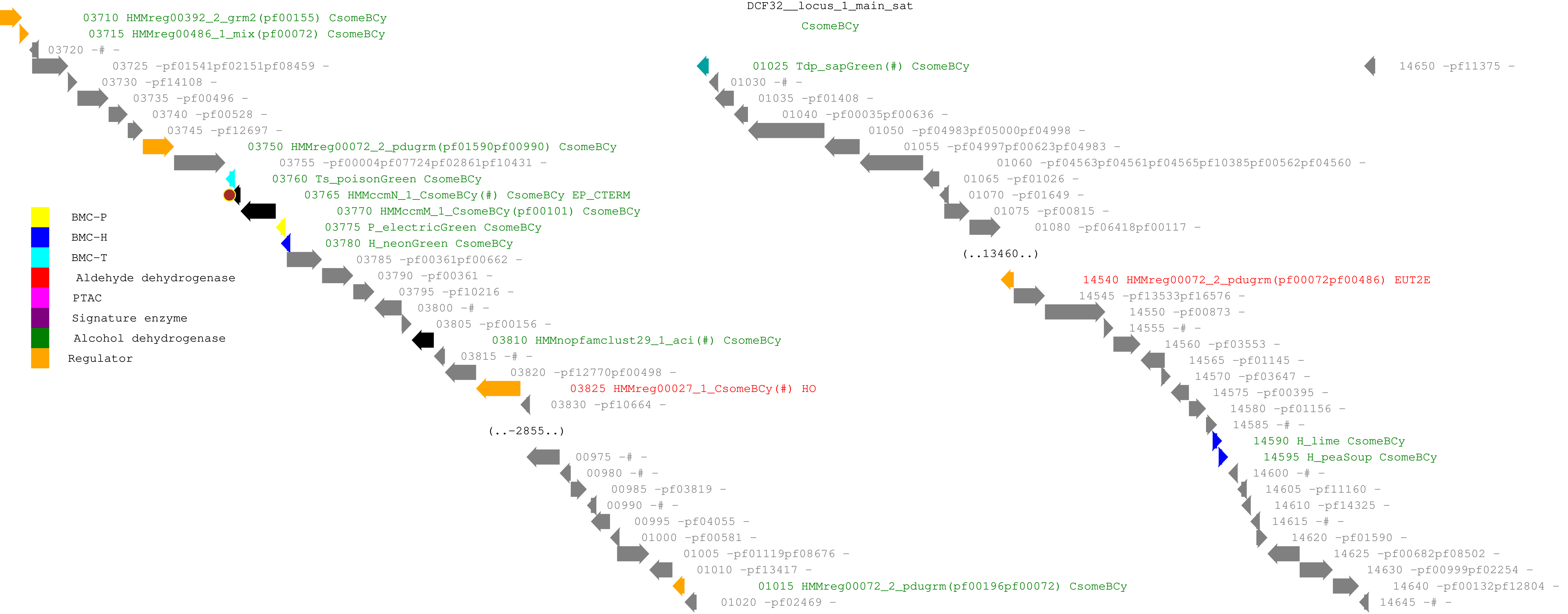

Cyanothece sp. UBA12306. Bacteria; Cyanobacteria; Oscillatoriophycideae; Oscillatoriales;

DCF68__locus_1_main_sat

CsomeBCy

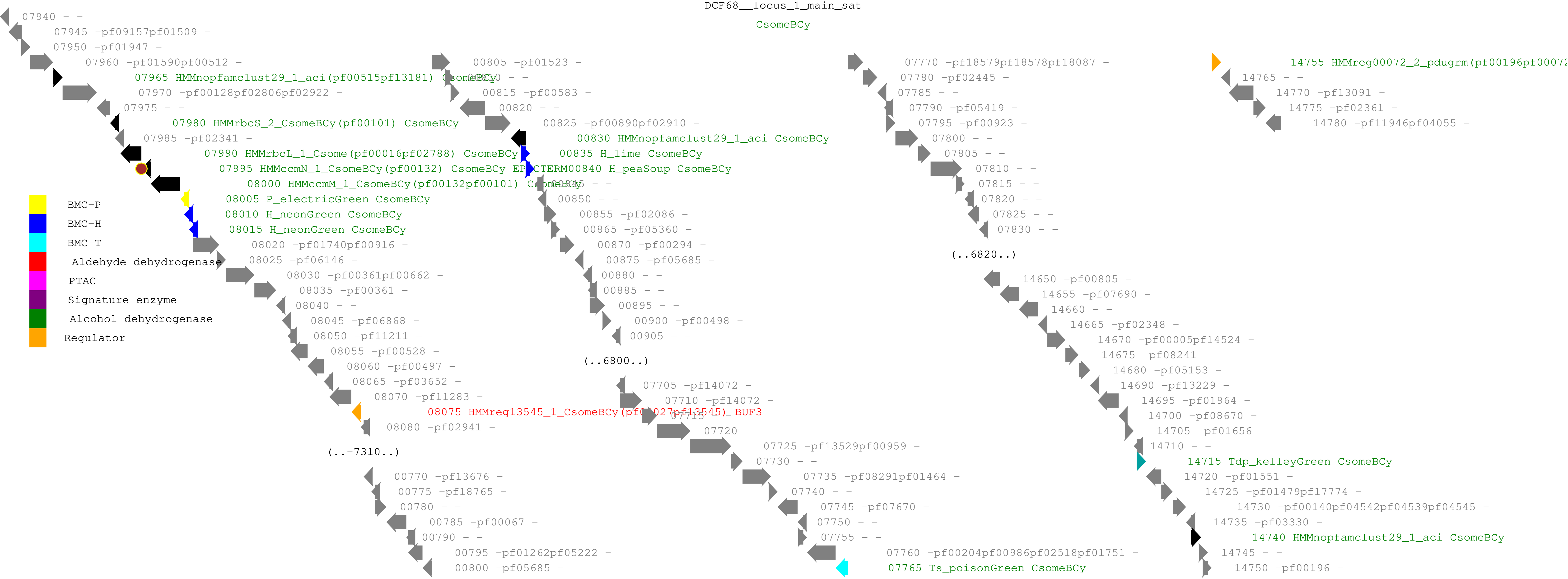

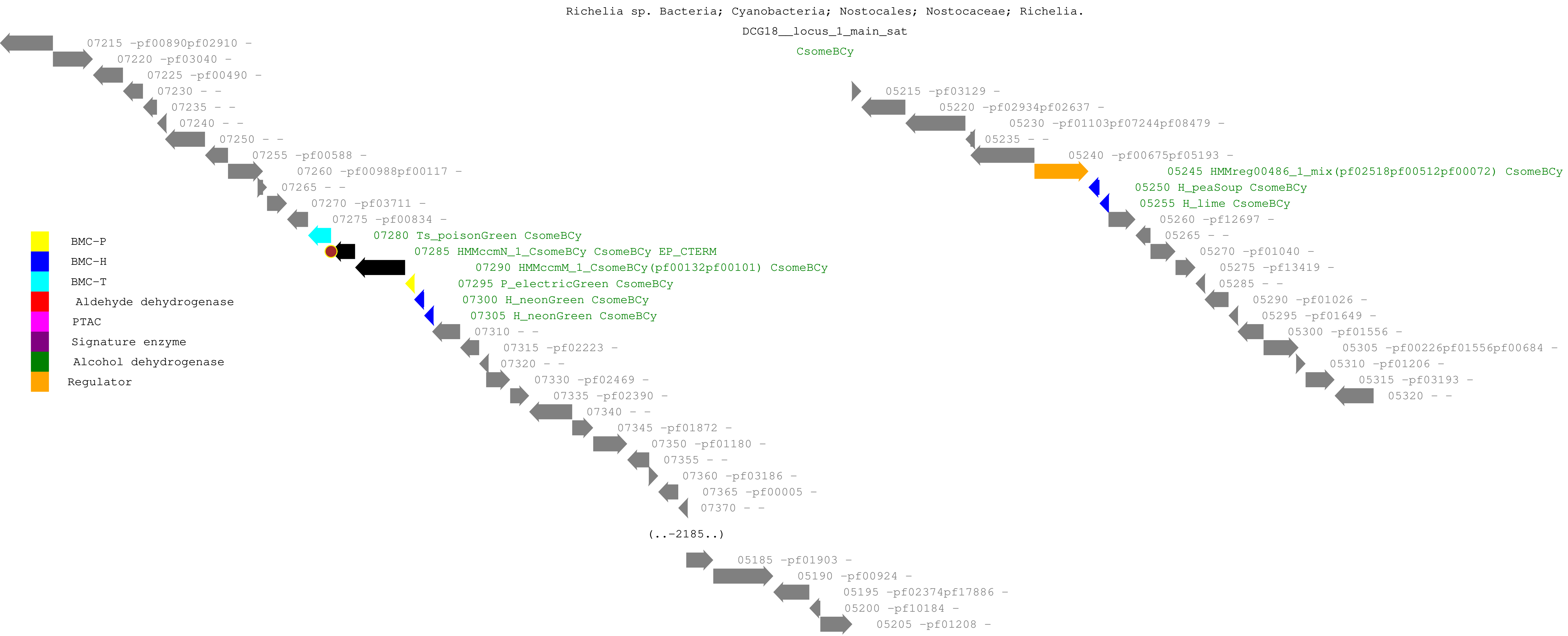
Richelia sp. Bacteria; Cyanobacteria; Nostocales; Nostocaceae; Richelia.
DCG18__locus_1_main_sat
CsomeBCy
07215 -pf00890pf02910 -
07220 -pf03040 -
07225 -pf00490 -
07230 - -
07235 - -
07240 - -
07250 - -
07255 -pf00588 -
07260 -pf00988pf00117 -
07265 - -
07270 -pf03711 -
07275 -pf00834 -
07280 Ts_poisonGreen CsomeBCy
07285 HMMccmN_1_CsomeBCy CsomeBCy EP_CTERM
07290 HMMccmM_1_CsomeBCy(pf00132pf00101) CsomeBCy
07295 P_electricGreen CsomeBCy
07300 H_neonGreen CsomeBCy
07305 H_neonGreen CsomeBCy
07310 - -
07315 -pf02223 -
07320 - -
07330 -pf02469 -
07335 -pf02390 -
07340 - -
07345 -pf01872 -
07350 -pf01180 -
07355 - -
07360 -pf03186 -
07365 -pf00005 -
07370 - -
(..-2185..)
05185 -pf01903 -
05190 -pf00924 -
05195 -pf02374pf17886 -
05200 -pf10184 -
05205 -pf01208 -
05215 -pf03129 -
05220 -pf02934pf02637 -
05230 -pf01103pf07244pf08479 -
05235 - -
05240 -pf00675pf05193 -
05245 HMMreg00486_1_mix(pf02518pf00512pf00072) CsomeBCy
05250 H_peaSoup CsomeBCy
05255 H_lime CsomeBCy
05260 -pf12697 -
05265 - -
05270 -pf01040 -
05275 -pf13419 -
05285 - -
05290 -pf01026 -
05295 -pf01649 -
05300 -pf01556 -
05305 -pf00226pf01556pf00684 -
05310 -pf01206 -
05315 -pf03193 -
05320 - -
BMC-P
BMC-H
BMC-T
Aldehyde dehydrogenase
PTAC
Signature enzyme
Alcohol dehydrogenase
Regulator

Cyanobacteria bacterium UBA8543. Bacteria; Cyanobacteria.

DCP31__locus_fragmented

CsomeBCy

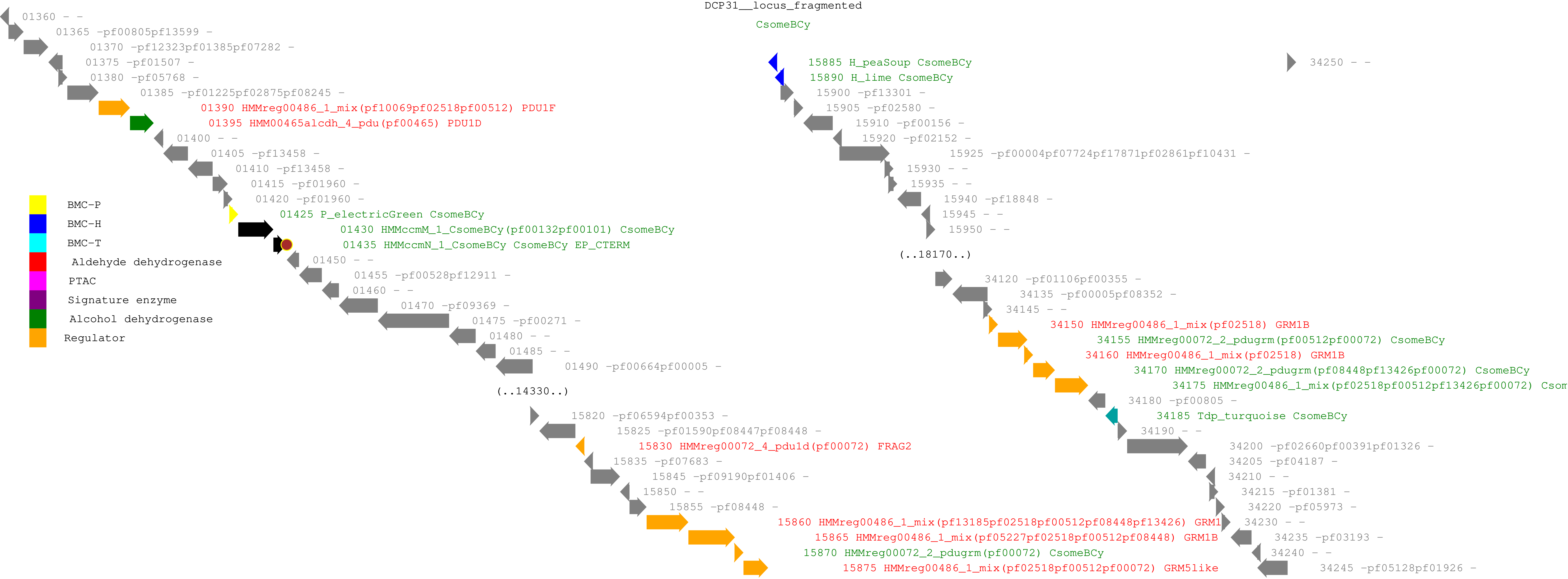

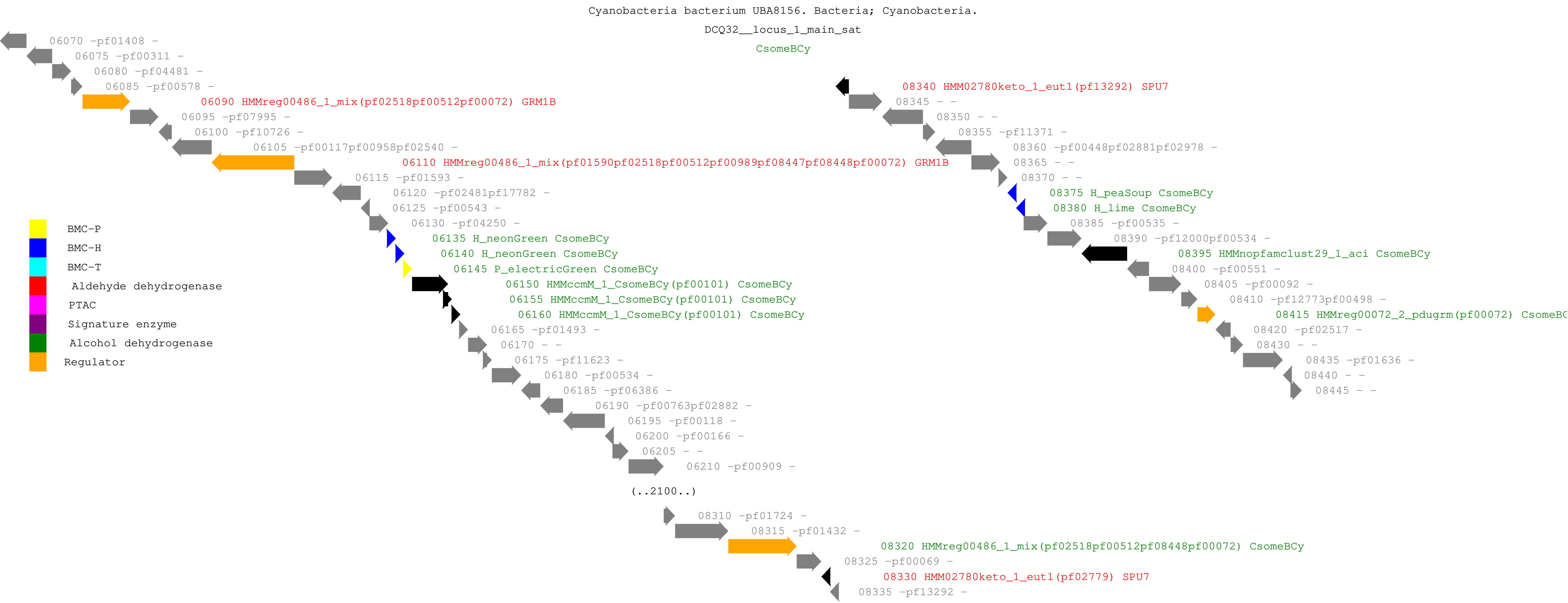

Cyanobacteria bacterium UBA8156. Bacteria; Cyanobacteria.
DCQ32__locus_1_main_sat
CsomeBCy
06070 -pf01408 -
06075 -pf00311 -
06080 -pf04481 -
06085 -pf00578 -
06090 HMMreg00486_1_mix(pf02518pf00512pf00072) GRM1B
06095 -pf07995 -
06100 -pf10726 -
06105 -pf00117pf00958pf02540 -
06110 HMMreg00486_1_mix(pf01590pf02518pf00512pf00989pf08447pf08448pf00072) GRM1B
06115 -pf01593 -
06120 -pf02481pf17782 -
06125 -pf00543 -
06130 -pf04250 -
06135 H_neonGreen CsomeBCy
06140 H_neonGreen CsomeBCy
06145 P_electricGreen CsomeBCy
06150 HMMccmM_1_CsomeBCy(pf00101) CsomeBCy
06155 HMMccmM_1_CsomeBCy(pf00101) CsomeBCy
06160 HMMccmM_1_CsomeBCy(pf00101) CsomeBCy
06165 -pf01493 -
06170 - -
06175 -pf11623 -
06180 -pf00534 -
06185 -pf06386 -
06190 -pf00763pf02882 -
06195 -pf00118 -
06200 -pf00166 -
06205 - -
06210 -pf00909 -
(..2100..)
08310 -pf01724 -
08315 -pf01432 -
08320 HMMreg00486_1_mix(pf02518pf00512pf08448pf00072) CsomeBCy
08325 -pf00069 -
08330 HMM02780keto_1_eut1(pf02779) SPU7
08335 -pf13292 -
08340 HMM02780keto_1_eut1(pf13292) SPU7
08345 - -
08350 - -
08355 -pf11371 -
08360 -pf00448pf02881pf02978 -
08365 - -
08370 - -
08375 H_peaSoup CsomeBCy
08380 H_lime CsomeBCy
08385 -pf00535 -
08390 -pf12000pf00534 -
08395 HMMnopfamclust29_1_aci CsomeBCy
08400 -pf00551 -
08405 -pf00092 -
08410 -pf12773pf00498 -
08415 HMMreg00072_2_pdugrm(pf00072) CsomeBC
08420 -pf02517 -
08430 - -
08435 -pf01636 -
08440 - -
08445 - -
BMC-P
BMC-H
BMC-T
Aldehyde dehydrogenase
PTAC
Signature enzyme
Alcohol dehydrogenase
Regulator

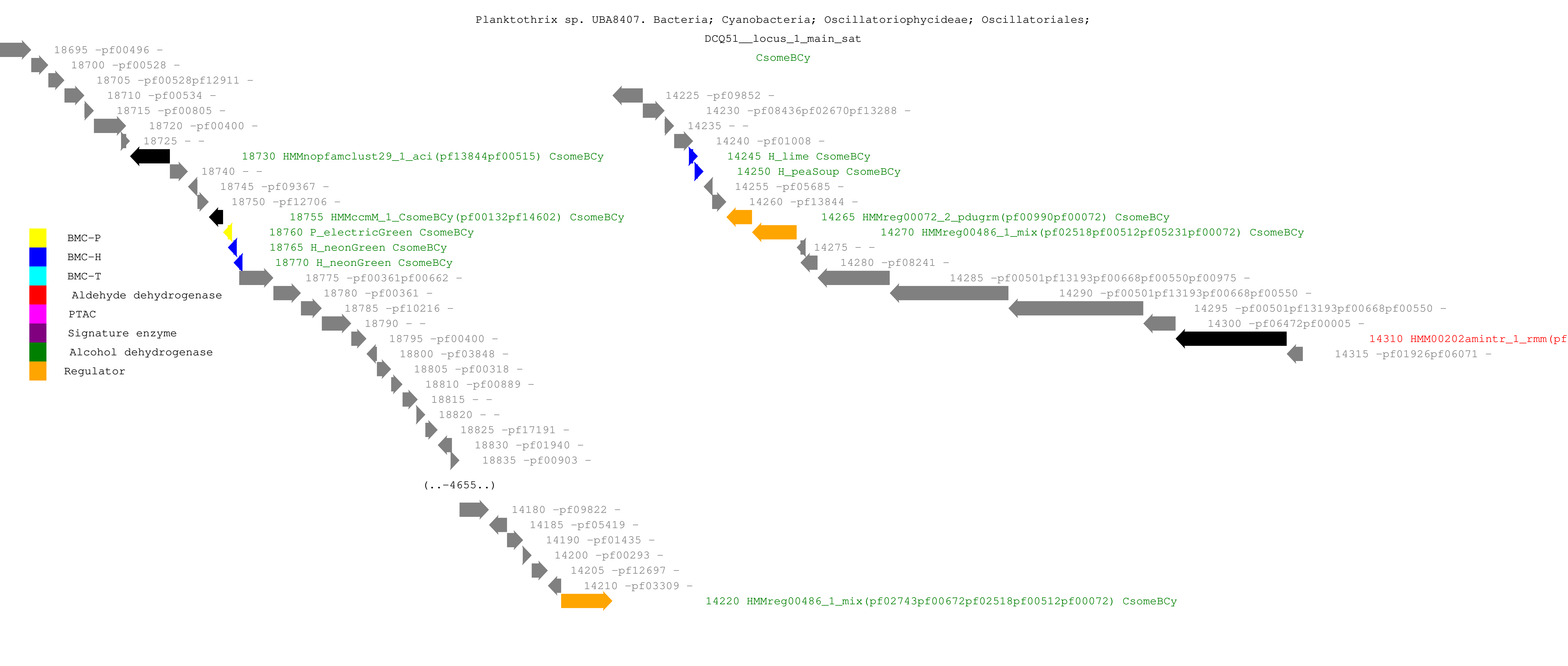
Planktothrix sp. UBA8407. Bacteria; Cyanobacteria; Oscillatoriophycideae; Oscillatoriales;
DCQ51__locus_1_main_sat
CsomeBCy
18695 -pf00496 -
18700 -pf00528 -
18705 -pf00528pf12911 -
18710 -pf00534 -
18715 -pf00805 -
18720 -pf00400 -
18725 - -
18730 HMMnopfamclust29_1_aci(pf13844pf00515) CsomeBCy
18740 - -
18745 -pf09367 -
18750 -pf12706 -
18755 HMMccmM_1_CsomeBCy(pf00132pf14602) CsomeBCy
18760 P_electricGreen CsomeBCy
18765 H_neonGreen CsomeBCy
18770 H_neonGreen CsomeBCy
18775 -pf00361pf00662 -
18780 -pf00361 -
18785 -pf10216 -
18790 - -
18795 -pf00400 -
18800 -pf03848 -
18805 -pf00318 -
18810 -pf00889 -
18815 - -
18820 - -
18825 -pf17191 -
18830 -pf01940 -
18835 -pf00903 -
(..-4655..)
14180 -pf09822 -
14185 -pf05419 -
14190 -pf01435 -
14200 -pf00293 -
14205 -pf12697 -
14210 -pf03309 -
14220 HMMreg00486_1_mix(pf02743pf00672pf02518pf00512pf00072) CsomeBCy
14225 -pf09852 -
14230 -pf08436pf02670pf13288 -
14235 - -
14240 -pf01008 -
14245 H_lime CsomeBCy
14250 H_peaSoup CsomeBCy
14255 -pf05685 -
14260 -pf13844 -
14265 HMMreg00072_2_pdugrm(pf00990pf00072) CsomeBCy
14270 HMMreg00486_1_mix(pf02518pf00512pf05231pf00072) CsomeBCy
14275 - -
14280 -pf08241 -
14285 -pf00501pf13193pf00668pf00550pf00975 -
14290 -pf00501pf13193pf00668pf00550 -
14295 -pf00501pf13193pf00668pf00550 -
14300 -pf06472pf00005 -
14310 HMM00202amintr_1_rmm(pf
14315 -pf01926pf06071 -
BMC-P
BMC-H
BMC-T
Aldehyde dehydrogenase
PTAC
Signature enzyme
Alcohol dehydrogenase
Regulator

Planktothrix sp. UBA8402. Bacteria; Cyanobacteria; Oscillatoriophycideae; Oscillatoriales;

DCQ63__locus_fragmented

CsomeBCy

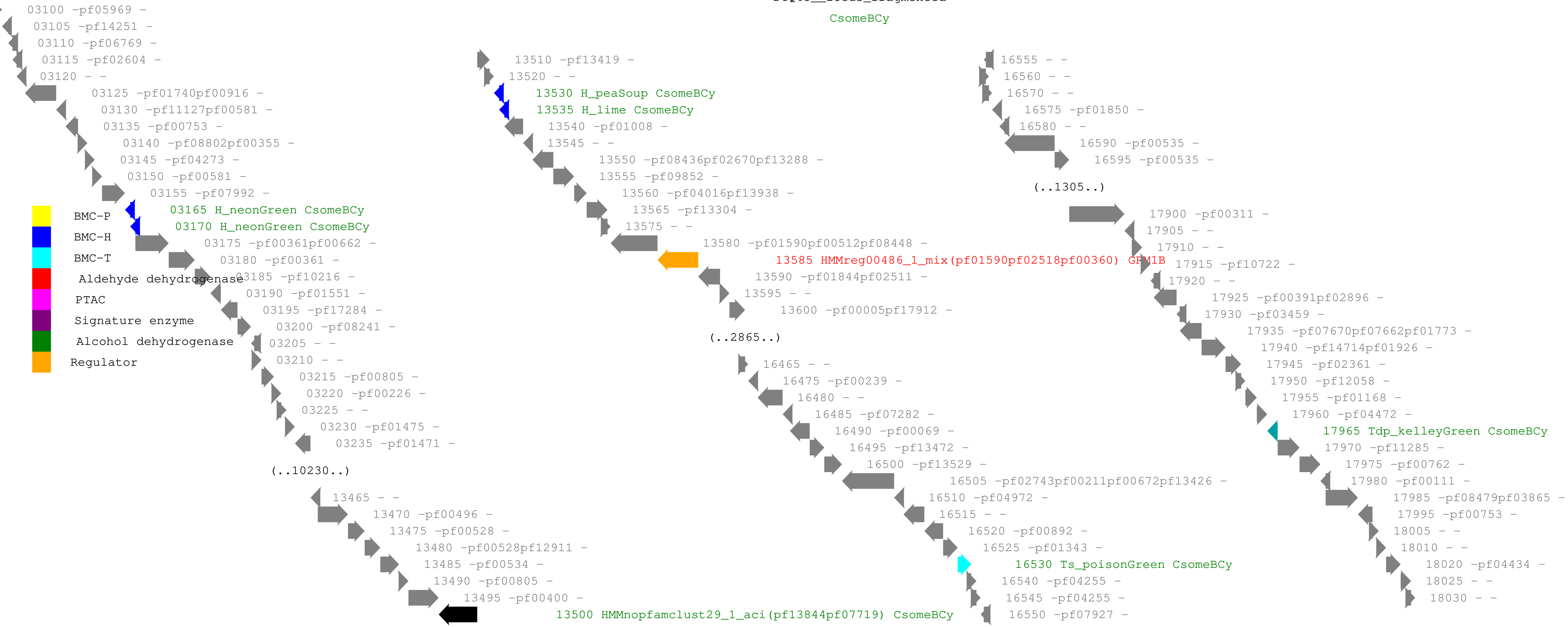

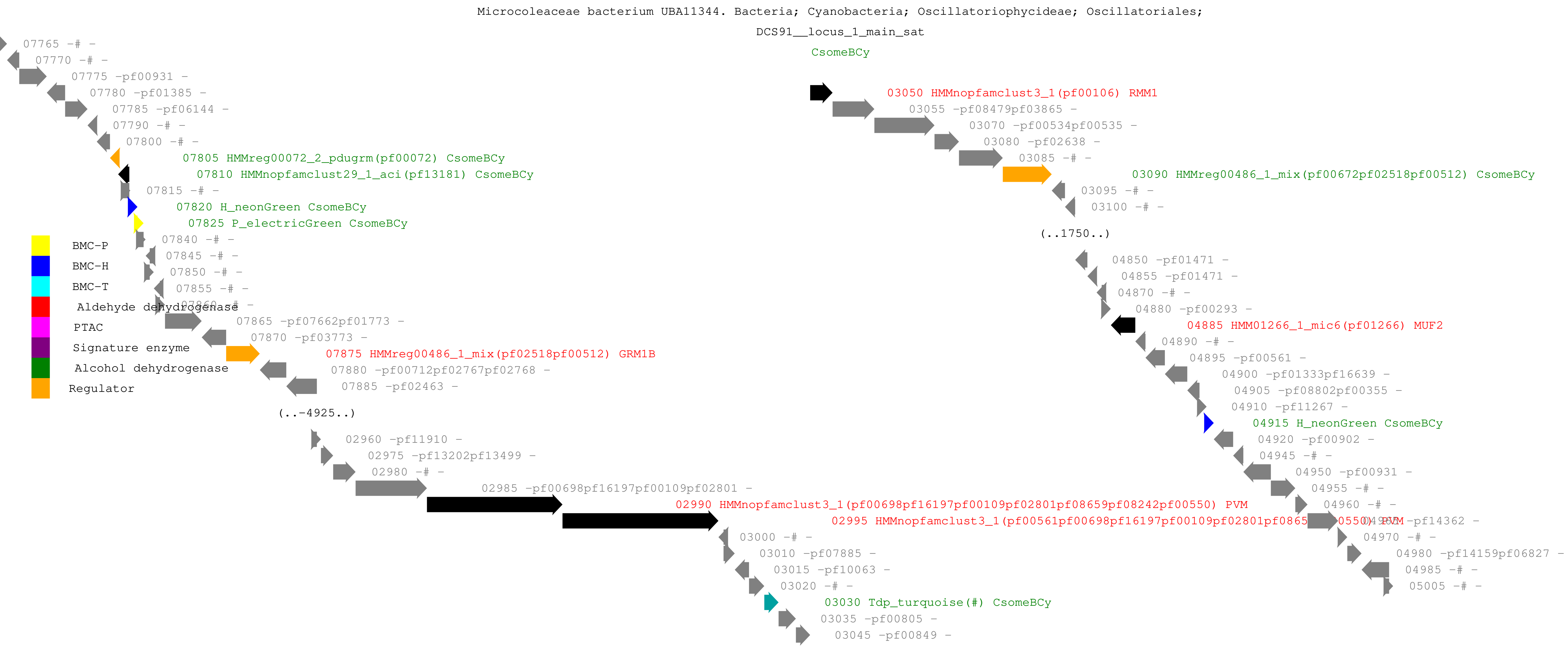
Microcoleaceae bacterium UBA11344. Bacteria; Cyanobacteria; Oscillatoriophycideae; Oscillatoriales;
DCS91__locus_1_main_sat
CsomeBCy
07765 -# -
07770 -# -
07775 -pf00931 -
07780 -pf01385 -
07785 -pf06144 -
07790 -# -
07800 -# -
07805 HMMreg00072_2_pdugrm(pf00072) CsomeBCy
07810 HMMnopfamclust29_1_aci(pf13181) CsomeBCy
07815 -# -
07820 H_neonGreen CsomeBCy
07825 P_electricGreen CsomeBCy
07840 -# -
07845 -# -
07850 -# -
07855 -# -
07865 -pf07662pf01773 -
07870 -pf03773 -
07875 HMMreg00486_1_mix(pf02518pf00512) GRM1B
07880 -pf00712pf02767pf02768 -
07885 -pf02463 -
(..-4925..)
02960 -pf11910 -
02975 -pf13202pf13499 -
02980 -# -
02985 -pf00698pf16197pf00109pf02801 -
02990 HMMnopfamclust3_1(pf00698pf16197pf00109pf02801pf08659pf08242pf00550) PVM
02995 HMMnopfamclust3_1(pf00561pf00698pf16197pf00109pf02801pf0865 0550) PVM
03000 -# -
03010 -pf07885 -
03015 -pf10063 -
03020 -# -
03030 Tdp_turquoise(#) CsomeBCy
03035 -pf00805 -
03045 -pf00849 -
03050 HMMnopfamclust3_1(pf00106) RMM1
03055 -pf08479pf03865 -
03070 -pf00534pf00535 -
03080 -pf02638 -
03085 -# -
03090 HMMreg00486_1_mix(pf00672pf02518pf00512) CsomeBCy
03095 -# -
03100 -# -
(..1750..)
04850 -pf01471 -
04855 -pf01471 -
04870 -# -
04880 -pf00293 -
04885 HMM01266_1_mic6(pf01266) MUF2
04890 -# -
04895 -pf00561 -
04900 -pf01333pf16639 -
04905 -pf08802pf00355 -
04910 -pf11267 -
04915 H_neonGreen CsomeBCy
04920 -pf00902 -
04945 -# -
04950 -pf00931 -
04955 -# -
04960 -# -
-pf14362 -
04970 -# -
04980 -pf14159pf06827 -
04985 -# -
05005 -# -
BMC-P
BMC-H
BMC-T
Aldehyde dehydrogenase
PTAC
Signature enzyme
Alcohol dehydrogenase
Regulator

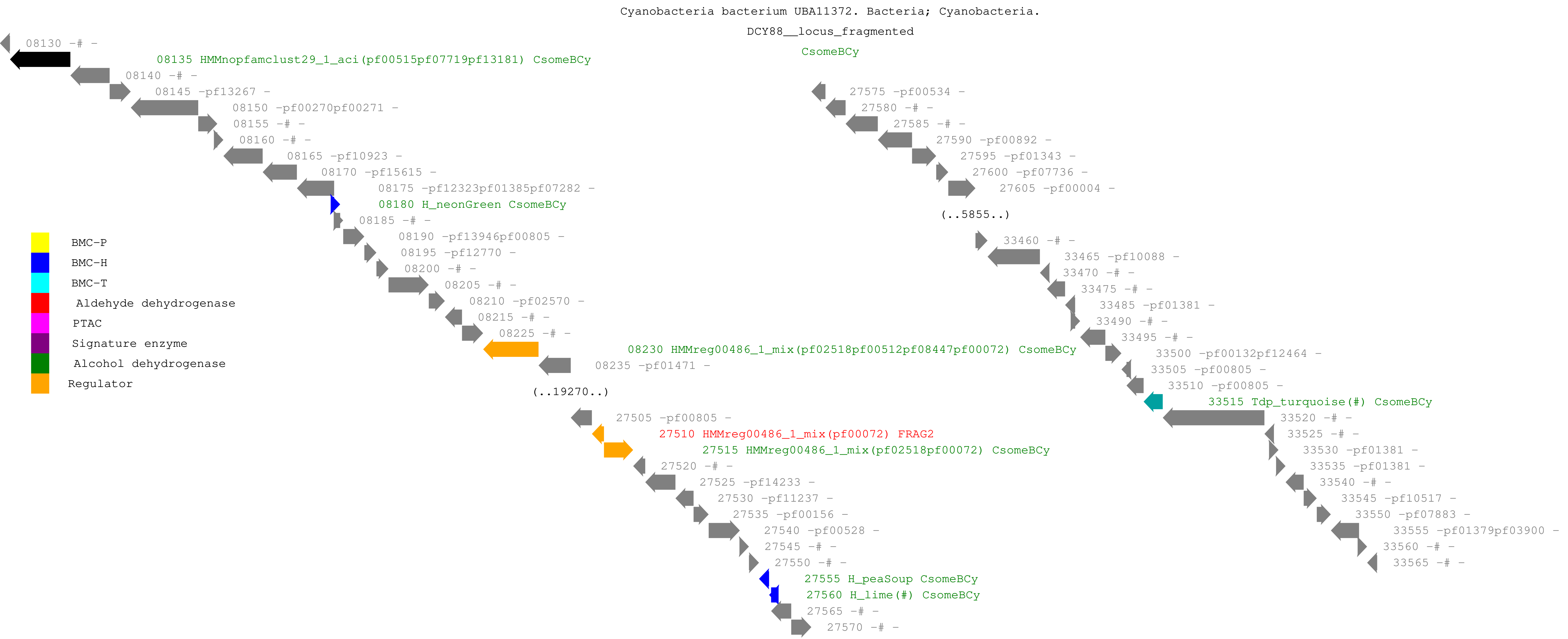

Cyanobacteria bacterium UBA11372. Bacteria; Cyanobacteria.
DCY88__locus_fragmented
CsomeBCy
08130 -# -
08135 HMMnopfamclust29_1_aci(pf00515pf07719pf13181) CsomeBCy
08140 -# -
08145 -pf13267 -
08150 -pf00270pf00271 -
08155 -# -
08160 -# -
08165 -pf10923 -
08170 -pf15615 -
08175 -pf12323pf01385pf07282 -
08180 H_neonGreen CsomeBCy
08185 -# -
08190 -pf13946pf00805 -
08195 -pf12770 -
08200 -# -
08205 -# -
08210 -pf02570 -
08215 -# -
08225 -# -
08230 HMMreg00486_1_mix(pf02518pf00512pf08447pf00072) CsomeBCy
08235 -pf01471 -
(..19270..)
27505 -pf00805 -
27510 HMMreg00486_1_mix(pf00072) FRAG2
27515 HMMreg00486_1_mix(pf02518pf00072) CsomeBCy
27520 -# -
27525 -pf14233 -
27530 -pf11237 -
27535 -pf00156 -
27540 -pf00528 -
27545 -# -
27550 -# -
27555 H_peaSoup CsomeBCy
27560 H_lime(#) CsomeBCy
27565 -# -
27570 -# -
27575 -pf00534 -
27580 -# -
27585 -# -
27590 -pf00892 -
27595 -pf01343 -
27600 -pf07736 -
27605 -pf00004 -
(..5855..)
33460 -# -
33465 -pf10088 -
33470 -# -
33475 -# -
33485 -pf01381 -
33490 -# -
33495 -# -
33500 -pf00132pf12464 -
33505 -pf00805 -
33510 -pf00805 -
33515 Tdp_turquoise(#) CsomeBCy
33520 -# -
33525 -# -
33530 -pf01381 -
33535 -pf01381 -
33540 -# -
33545 -pf10517 -
33550 -pf07883 -
33555 -pf01379pf03900 -
33560 -# -
33565 -# -
BMC-P
BMC-H
BMC-T
Aldehyde dehydrogenase
PTAC
Signature enzyme
Alcohol dehydrogenase
Regulator

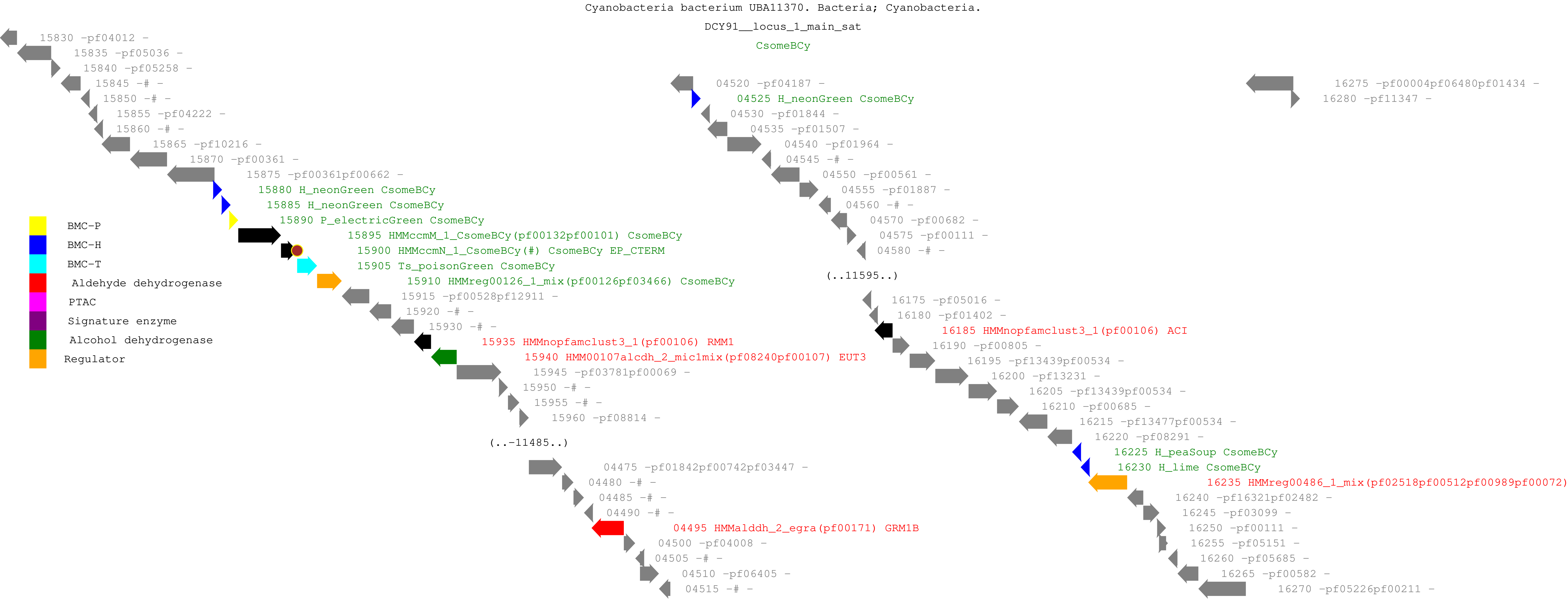
Cyanobacteria bacterium UBA11370. Bacteria; Cyanobacteria.
DCY91__locus_1_main_sat
CsomeBCy
15830 -pf04012 -
15835 -pf05036 -
15840 -pf05258 -
15845 -# -
15850 -# -
15855 -pf04222 -
15860 -# -
15865 -pf10216 -
15870 -pf00361 -
15875 -pf00361pf00662 -
15880 H_neonGreen CsomeBCy
15885 H_neonGreen CsomeBCy
15890 P_electricGreen CsomeBCy
15895 HMMccmM_1_CsomeBCy(pf00132pf00101) CsomeBCy
15900 HMMccmN_1_CsomeBCy(#) CsomeBCy EP_CTERM
15905 Ts_poisonGreen CsomeBCy
15910 HMMreg00126_1_mix(pf00126pf03466) CsomeBCy
15915 -pf00528pf12911 -
15920 -# -
15930 -# -
15935 HMMnopfamclust3_1(pf00106) RMM1
15940 HMM00107alcdh_2_mic1mix(pf08240pf00107) EUT3
15945 -pf03781pf00069 -
15950 -# -
15955 -# -
15960 -pf08814 -
(..-11485..)
04475 -pf01842pf00742pf03447 -
04480 -# -
04485 -# -
04490 -# -
04495 HMMalddh_2_egra(pf00171) GRM1B
04500 -pf04008 -
04505 -# -
04510 -pf06405 -
04515 -# -
04520 -pf04187 -
04525 H_neonGreen CsomeBCy
04530 -pf01844 -
04535 -pf01507 -
04540 -pf01964 -
04545 -# -
04550 -pf00561 -
04555 -pf01887 -
04560 -# -
04570 -pf00682 -
04575 -pf00111 -
04580 -# -
(..11595..)
16175 -pf05016 -
16180 -pf01402 -
16185 HMMnopfamclust3_1(pf00106) ACI
16190 -pf00805 -
16195 -pf13439pf00534 -
16200 -pf13231 -
16205 -pf13439pf00534 -
16210 -pf00685 -
16215 -pf13477pf00534 -
16220 -pf08291 -
16225 H_peaSoup CsomeBCy
16230 H_lime CsomeBCy
16235 HMMreg00486_1_mix(pf02518pf00512pf00989pf00072)
16240 -pf16321pf02482 -
16245 -pf03099 -
16250 -pf00111 -
16255 -pf05151 -
16260 -pf05685 -
16265 -pf00582 -
16270 -pf05226pf00211 -
16275 -pf00004pf06480pf01434 -
16280 -pf11347 -
BMC-P
BMC-H
BMC-T
Aldehyde dehydrogenase
PTAC
Signature enzyme
Alcohol dehydrogenase
Regulator

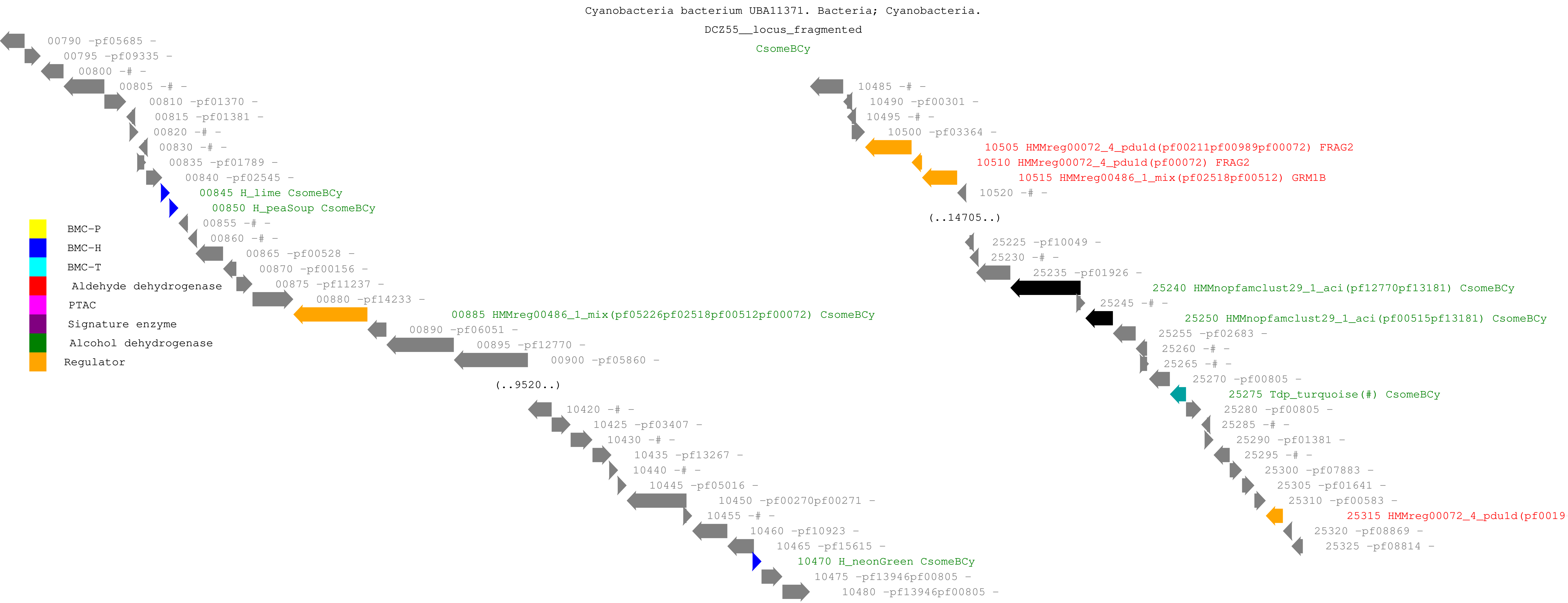

Cyanobacteria bacterium UBA11371. Bacteria; Cyanobacteria.
DCZ55__locus_fragmented
CsomeBCy
00790 -pf05685 -
00795 -pf09335 -
00800 -# -
00805 -# -
00810 -pf01370 -
00815 -pf01381 -
00820 -# -
00830 -# -
00835 -pf01789 -
00840 -pf02545 -
00845 H_lime CsomeBCy
00850 H_peaSoup CsomeBCy
00855 -# -
00860 -# -
00865 -pf00528 -
00870 -pf00156 -
00875 -pf11237 -
00880 -pf14233 -
00885 HMMreg00486_1_mix(pf05226pf02518pf00512pf00072) CsomeBCy
00890 -pf06051 -
00895 -pf12770 -
00900 -pf05860 -
(..9520..)
10420 -# -
10425 -pf03407 -
10430 -# -
10435 -pf13267 -
10440 -# -
10445 -pf05016 -
10450 -pf00270pf00271 -
10455 -# -
10460 -pf10923 -
10465 -pf15615 -
10470 H_neonGreen CsomeBCy
10475 -pf13946pf00805 -
10480 -pf13946pf00805 -
10485 -# -
10490 -pf00301 -
10495 -# -
10500 -pf03364 -
10505 HMMreg00072_4_pdu1d(pf00211pf00989pf00072) FRAG2
10510 HMMreg00072_4_pdu1d(pf00072) FRAG2
10515 HMMreg00486_1_mix(pf02518pf00512) GRM1B
10520 -# -
(..14705..)
25225 -pf10049 -
25230 -# -
25235 -pf01926 -
25240 HMMnopfamclust29_1_aci(pf12770pf13181) CsomeBCy
25245 -# -
25250 HMMnopfamclust29_1_aci(pf00515pf13181) CsomeBCy
25255 -pf02683 -
25260 -# -
25265 -# -
25270 -pf00805 -
25275 Tdp_turquoise(#) CsomeBCy
25280 -pf00805 -
25285 -# -
25290 -pf01381 -
25295 -# -
25300 -pf07883 -
25305 -pf01641 -
25310 -pf00583 -
25315 HMMreg00072_4_pdu1d(pf0019
25320 -pf08869 -
25325 -pf08814 -
BMC-P
BMC-H
BMC-T
Aldehyde dehydrogenase
PTAC
Signature enzyme
Alcohol dehydrogenase
Regulator

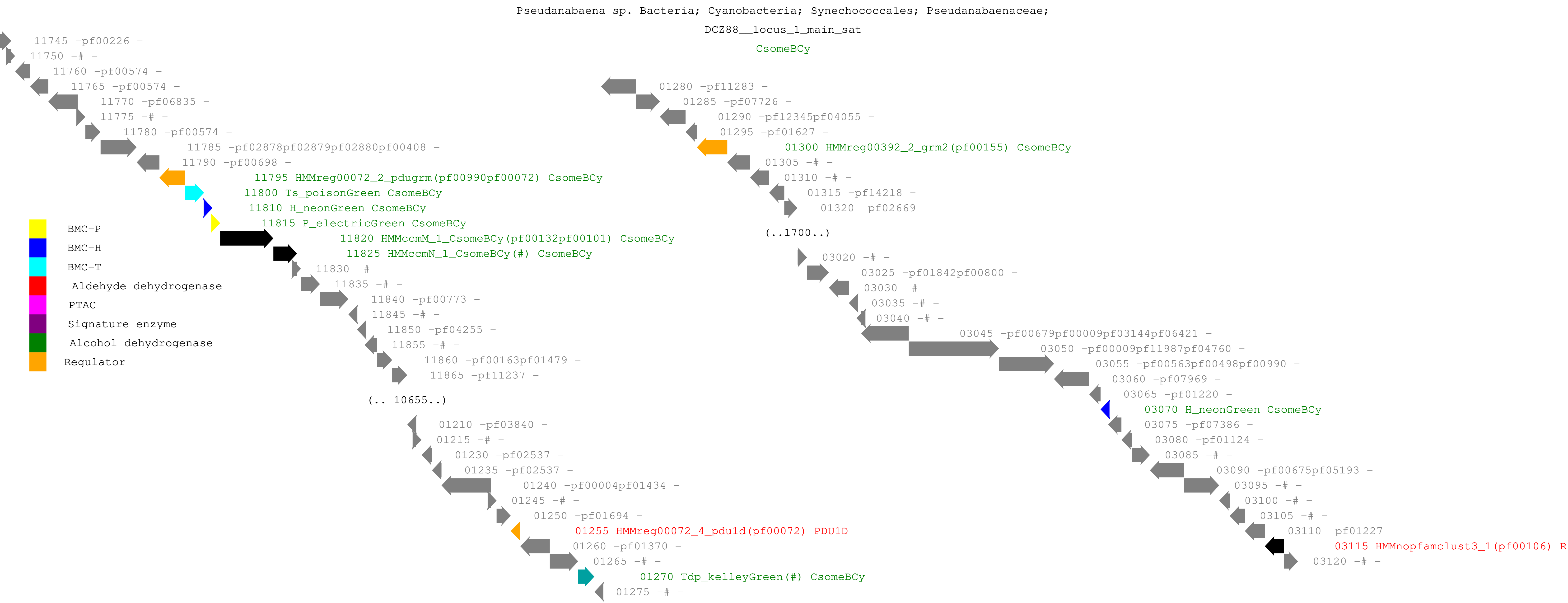
Pseudanabaena sp. Bacteria; Cyanobacteria; Synechococcales; Pseudanabaenaceae;
DCZ88__locus_1_main_sat
CsomeBCy
11745 -pf00226 -
11750 -# -
11760 -pf00574 -
11765 -pf00574 -
11770 -pf06835 -
11775 -# -
11780 -pf00574 -
11785 -pf02878pf02879pf02880pf00408 -
11790 -pf00698 -
11795 HMMreg00072_2_pdugrm(pf00990pf00072) CsomeBCy
11800 Ts_poisonGreen CsomeBCy
11810 H_neonGreen CsomeBCy
11815 P_electricGreen CsomeBCy
11820 HMMccmM_1_CsomeBCy(pf00132pf00101) CsomeBCy
11825 HMMccmN_1_CsomeBCy(#) CsomeBCy
11830 -# -
11835 -# -
11840 -pf00773 -
11845 -# -
11850 -pf04255 -
11855 -# -
11860 -pf00163pf01479 -
11865 -pf11237 -
(..-10655..)
01210 -pf03840 -
01215 -# -
01230 -pf02537 -
01235 -pf02537 -
01240 -pf00004pf01434 -
01245 -# -
01250 -pf01694 -
01255 HMMreg00072_4_pdu1d(pf00072) PDU1D
01260 -pf01370 -
01265 -# -
01270 Tdp_kelleyGreen(#) CsomeBCy
01275 -# -
01280 -pf11283 -
01285 -pf07726 -
01290 -pf12345pf04055 -
01295 -pf01627 -
01300 HMMreg00392_2_grm2(pf00155) CsomeBCy
01305 -# -
01310 -# -
01315 -pf14218 -
01320 -pf02669 -
(..1700..)
03020 -# -
03025 -pf01842pf00800 -
03030 -# -
03035 -# -
03040 -# -
03045 -pf00679pf00009pf03144pf06421 -
03050 -pf00009pf11987pf04760 -
03055 -pf00563pf00498pf00990 -
03060 -pf07969 -
03065 -pf01220 -
03070 H_neonGreen CsomeBCy
03075 -pf07386 -
03080 -pf01124 -
03085 -# -
03090 -pf00675pf05193 -
03095 -# -
03100 -# -
03105 -# -
03110 -pf01227 -
03115 HMMnopfamclust3_1(pf00106) R
03120 -# -
BMC-P
BMC-H
BMC-T
Aldehyde dehydrogenase
PTAC
Signature enzyme
Alcohol dehydrogenase
Regulator

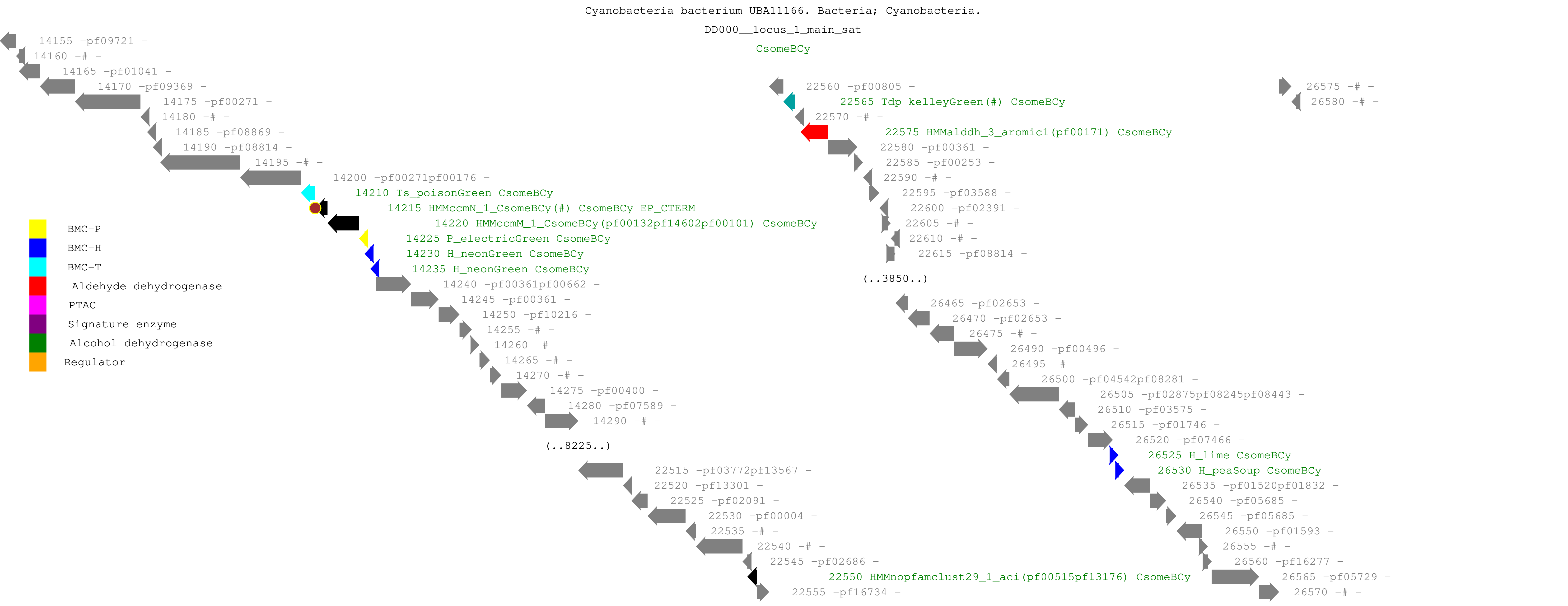
Cyanobacteria bacterium UBA11166. Bacteria; Cyanobacteria.
DD000__locus_1_main_sat
CsomeBCy
14155 -pf09721 -
14160 -# -
14165 -pf01041 -
14170 -pf09369 -
14175 -pf00271 -
14180 -# -
14185 -pf08869 -
14190 -pf08814 -
14195 -# -
14200 -pf00271pf00176 -
14210 Ts_poisonGreen CsomeBCy
14215 HMMccmN_1_CsomeBCy(#) CsomeBCy EP_CTERM
14220 HMMccmM_1_CsomeBCy(pf00132pf14602pf00101) CsomeBCy
14225 P_electricGreen CsomeBCy
14230 H_neonGreen CsomeBCy
14235 H_neonGreen CsomeBCy
14240 -pf00361pf00662 -
14245 -pf00361 -
14250 -pf10216 -
14255 -# -
14260 -# -
14265 -# -
14270 -# -
14275 -pf00400 -
14280 -pf07589 -
14290 -# -
(..8225..)
22515 -pf03772pf13567 -
22520 -pf13301 -
22525 -pf02091 -
22530 -pf00004 -
22535 -# -
22540 -# -
22545 -pf02686 -
22550 HMMnopfamclust29_1_aci(pf00515pf13176) CsomeBCy
22555 -pf16734 -
22560 -pf00805 -
22565 Tdp_kelleyGreen(#) CsomeBCy
22570 -# -
22575 HMMalddh_3_aromic1(pf00171) CsomeBCy
22580 -pf00361 -
22585 -pf00253 -
22590 -# -
22595 -pf03588 -
22600 -pf02391 -
22605 -# -
22610 -# -
22615 -pf08814 -
(..3850..)
26465 -pf02653 -
26470 -pf02653 -
26475 -# -
26490 -pf00496 -
26495 -# -
26500 -pf04542pf08281 -
26505 -pf02875pf08245pf08443 -
26510 -pf03575 -
26515 -pf01746 -
26520 -pf07466 -
26525 H_lime CsomeBCy
26530 H_peaSoup CsomeBCy
26535 -pf01520pf01832 -
26540 -pf05685 -
26545 -pf05685 -
26550 -pf01593 -
26555 -# -
26560 -pf16277 -
26565 -pf05729 -
26570 -# -
26575 -# -
26580 -# -
BMC-P
BMC-H
BMC-T
Aldehyde dehydrogenase
PTAC
Signature enzyme
Alcohol dehydrogenase
Regulator

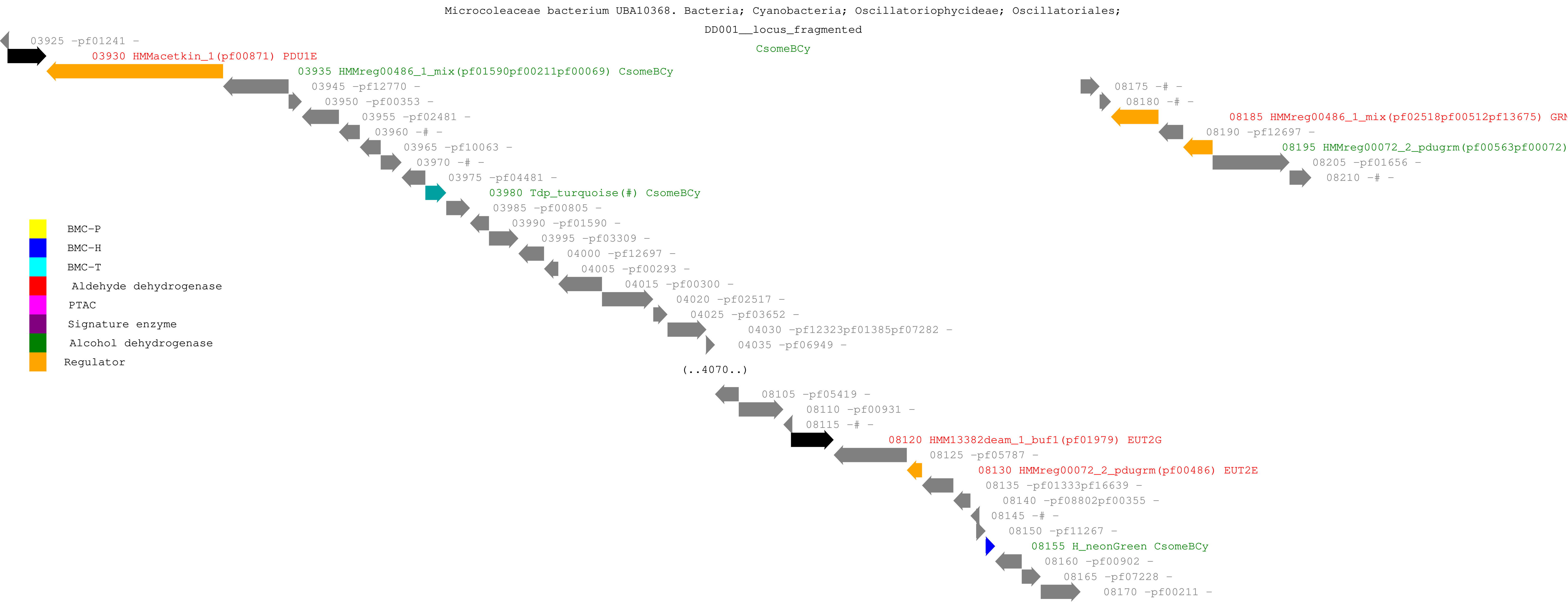
Microcoleaceae bacterium UBA10368. Bacteria; Cyanobacteria; Oscillatoriophycideae; Oscillatoriales;
DD001__locus_fragmented
CsomeBCy
03925 -pf01241 -
03930 HMMacetkin_1(pf00871) PDU1E
03935 HMMreg00486_1_mix(pf01590pf00211pf00069) CsomeBCy
03945 -pf12770 -
03950 -pf00353 -
03955 -pf02481 -
03960 -# -
03965 -pf10063 -
03970 -# -
03975 -pf04481 -
03980 Tdp_turquoise(#) CsomeBCy
03985 -pf00805 -
03990 -pf01590 -
03995 -pf03309 -
04000 -pf12697 -
04005 -pf00293 -
04015 -pf00300 -
04020 -pf02517 -
04025 -pf03652 -
04030 -pf12323pf01385pf07282 -
04035 -pf06949 -
(..4070..)
08105 -pf05419 -
08110 -pf00931 -
08115 -# -
08120 HMM13382deam_1_buf1(pf01979) EUT2G
08125 -pf05787 -
08130 HMMreg00072_2_pdugrm(pf00486) EUT2E
08135 -pf01333pf16639 -
08140 -pf08802pf00355 -
08145 -# -
08150 -pf11267 -
08155 H_neonGreen CsomeBCy
08160 -pf00902 -
08165 -pf07228 -
08170 -pf00211 -
08175 -# -
08180 -# -
08185 HMMreg00486_1_mix(pf02518pf00512pf13675) GRM
08190 -pf12697 -
08195 HMMreg00072_2_pdugrm(pf00563pf00072)
08205 -pf01656 -
08210 -# -
BMC-P
BMC-H
BMC-T
Aldehyde dehydrogenase
PTAC
Signature enzyme
Alcohol dehydrogenase
Regulator

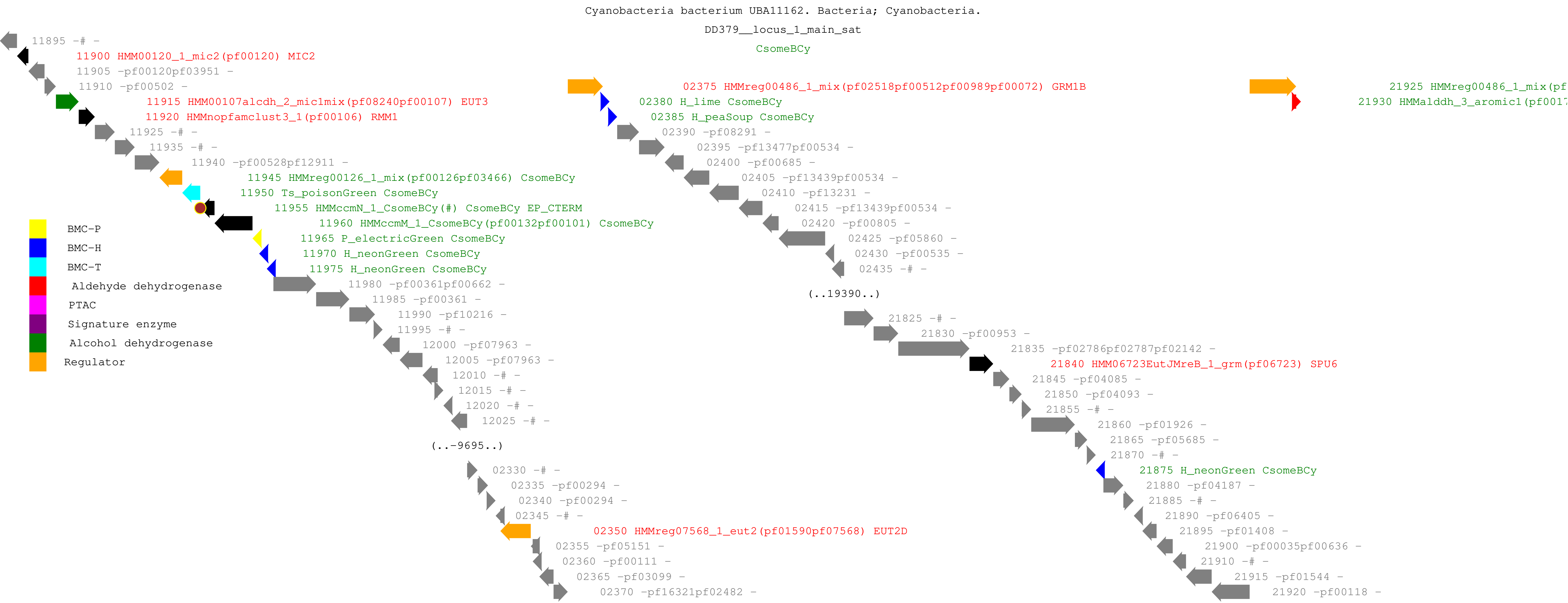

Cyanobacteria bacterium UBA11162. Bacteria; Cyanobacteria.
DD379__locus_1_main_sat
CsomeBCy
11895 -# -
11900 HMM00120_1_mic2(pf00120) MIC2
11905 -pf00120pf03951 -
11910 -pf00502 -
11915 HMM00107alcdh_2_mic1mix(pf08240pf00107) EUT3
11920 HMMnopfamclust3_1(pf00106) RMM1
11925 -# -
11935 -# -
11940 -pf00528pf12911 -
11945 HMMreg00126_1_mix(pf00126pf03466) CsomeBCy
11950 Ts_poisonGreen CsomeBCy
11955 HMMccmN_1_CsomeBCy(#) CsomeBCy EP_CTERM
11960 HMMccmM_1_CsomeBCy(pf00132pf00101) CsomeBCy
11965 P_electricGreen CsomeBCy
11970 H_neonGreen CsomeBCy
11975 H_neonGreen CsomeBCy
11980 -pf00361pf00662 -
11985 -pf00361 -
11990 -pf10216 -
11995 -# -
12000 -pf07963 -
12005 -pf07963 -
12010 -# -
12015 -# -
12020 -# -
12025 -# -
(..-9695..)
02330 -# -
02335 -pf00294 -
02340 -pf00294 -
02345 -# -
02350 HMMreg07568_1_eut2(pf01590pf07568) EUT2D
02355 -pf05151 -
02360 -pf00111 -
02365 -pf03099 -
02370 -pf16321pf02482 -
02375 HMMreg00486_1_mix(pf02518pf00512pf00989pf00072) GRM1B
02380 H_lime CsomeBCy
02385 H_peaSoup CsomeBCy
02390 -pf08291 -
02395 -pf13477pf00534 -
02400 -pf00685 -
02405 -pf13439pf00534 -
02410 -pf13231 -
02415 -pf13439pf00534 -
02420 -pf00805 -
02425 -pf05860 -
02430 -pf00535 -
02435 -# -
(..19390..)
21825 -# -
21830 -pf00953 -
21835 -pf02786pf02787pf02142 -
21840 HMM06723EutJMreB_1_grm(pf06723) SPU6
21845 -pf04085 -
21850 -pf04093 -
21855 -# -
21860 -pf01926 -
21865 -pf05685 -
21870 -# -
21875 H_neonGreen CsomeBCy
21880 -pf04187 -
21885 -# -
21890 -pf06405 -
21895 -pf01408 -
21900 -pf00035pf00636 -
21910 -# -
21915 -pf01544 -
21920 -pf00118 -
21925 HMMreg00486_1_mix(pf
21930 HMMalddh_3_aromic1(pf0017
BMC-P
BMC-H
BMC-T
Aldehyde dehydrogenase
PTAC
Signature enzyme
Alcohol dehydrogenase
Regulator

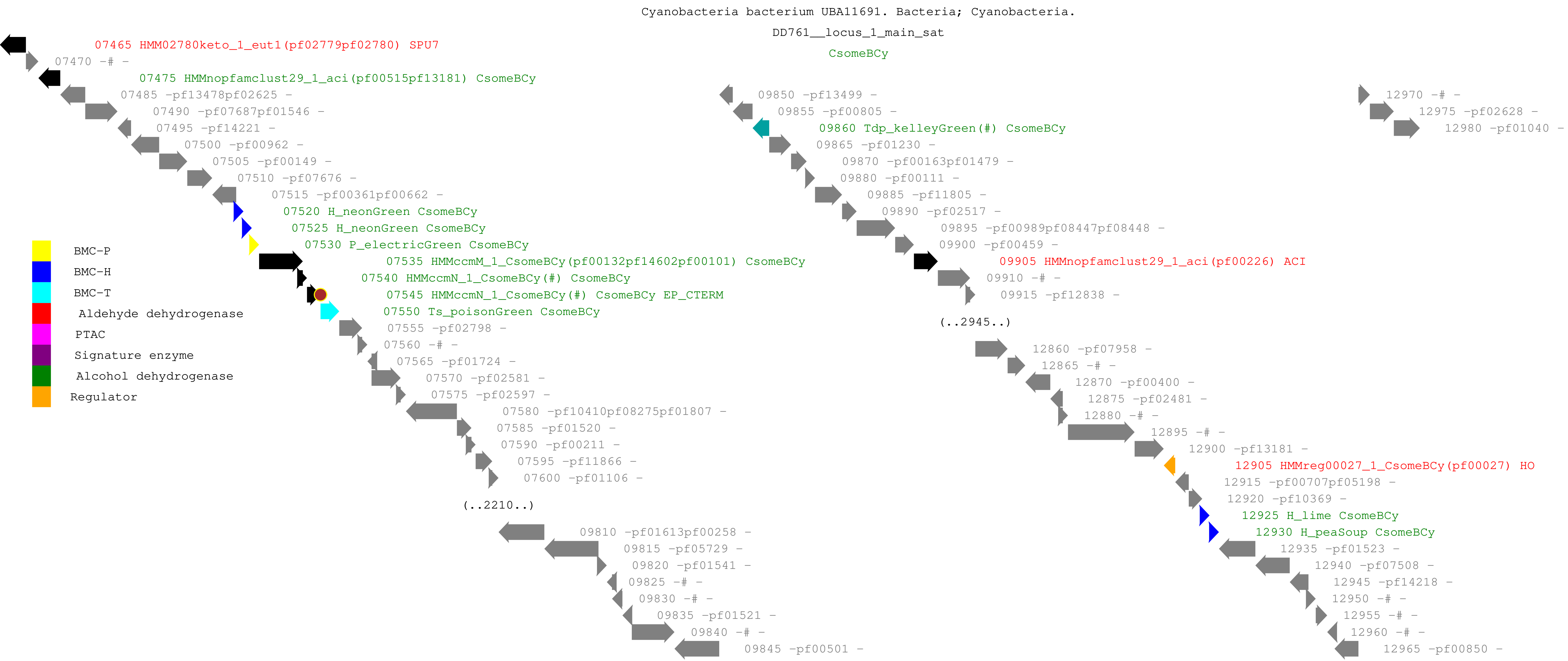

Cyanobacteria bacterium UBA11691. Bacteria; Cyanobacteria.
DD761__locus_1_main_sat
CsomeBCy
07465 HMM02780keto_1_eut1(pf02779pf02780) SPU7
07470 -# -
07475 HMMnopfamclust29_1_aci(pf00515pf13181) CsomeBCy
07485 -pf13478pf02625 -
07490 -pf07687pf01546 -
07495 -pf14221 -
07500 -pf00962 -
07505 -pf00149 -
07510 -pf07676 -
07515 -pf00361pf00662 -
07520 H_neonGreen CsomeBCy
07525 H_neonGreen CsomeBCy
07530 P_electricGreen CsomeBCy
07535 HMMccmM_1_CsomeBCy(pf00132pf14602pf00101) CsomeBCy
07540 HMMccmN_1_CsomeBCy(#) CsomeBCy
07545 HMMccmN_1_CsomeBCy(#) CsomeBCy EP_CTERM
07550 Ts_poisonGreen CsomeBCy
07555 -pf02798 -
07560 -# -
07565 -pf01724 -
07570 -pf02581 -
07575 -pf02597 -
07580 -pf10410pf08275pf01807 -
07585 -pf01520 -
07590 -pf00211 -
07595 -pf11866 -
07600 -pf01106 -
(..2210..)
09810 -pf01613pf00258 -
09815 -pf05729 -
09820 -pf01541 -
09825 -# -
09830 -# -
09835 -pf01521 -
09840 -# -
09845 -pf00501 -
BMC-P
BMC-H
BMC-T
Aldehyde dehydrogenase
PTAC
Signature enzyme
Alcohol dehydrogenase
Regulator
09850 -pf13499 -
09855 -pf00805 -
09860 Tdp_kelleyGreen(#) CsomeBCy
09865 -pf01230 -
09870 -pf00163pf01479 -
09880 -pf00111 -
09885 -pf11805 -
09890 -pf02517 -
09895 -pf00989pf08447pf08448 -
09900 -pf00459 -
09905 HMMnopfamclust29_1_aci(pf00226) ACI
09910 -# -
09915 -pf12838 -
(..2945..)
12860 -pf07958 -
12865 -# -
12870 -pf00400 -
12875 -pf02481 -
12880 -# -
12895 -# -
12900 -pf13181 -
12905 HMMreg00027_1_CsomeBCy(pf00027) HO
12915 -pf00707pf05198 -
12920 -pf10369 -
12925 H_lime CsomeBCy
12930 H_peaSoup CsomeBCy
12935 -pf01523 -
12940 -pf07508 -
12945 -pf14218 -
12950 -# -
12955 -# -
12960 -# -
12965 -pf00850 -
12970 -# -
12975 -pf02628 -
12980 -pf01040 -

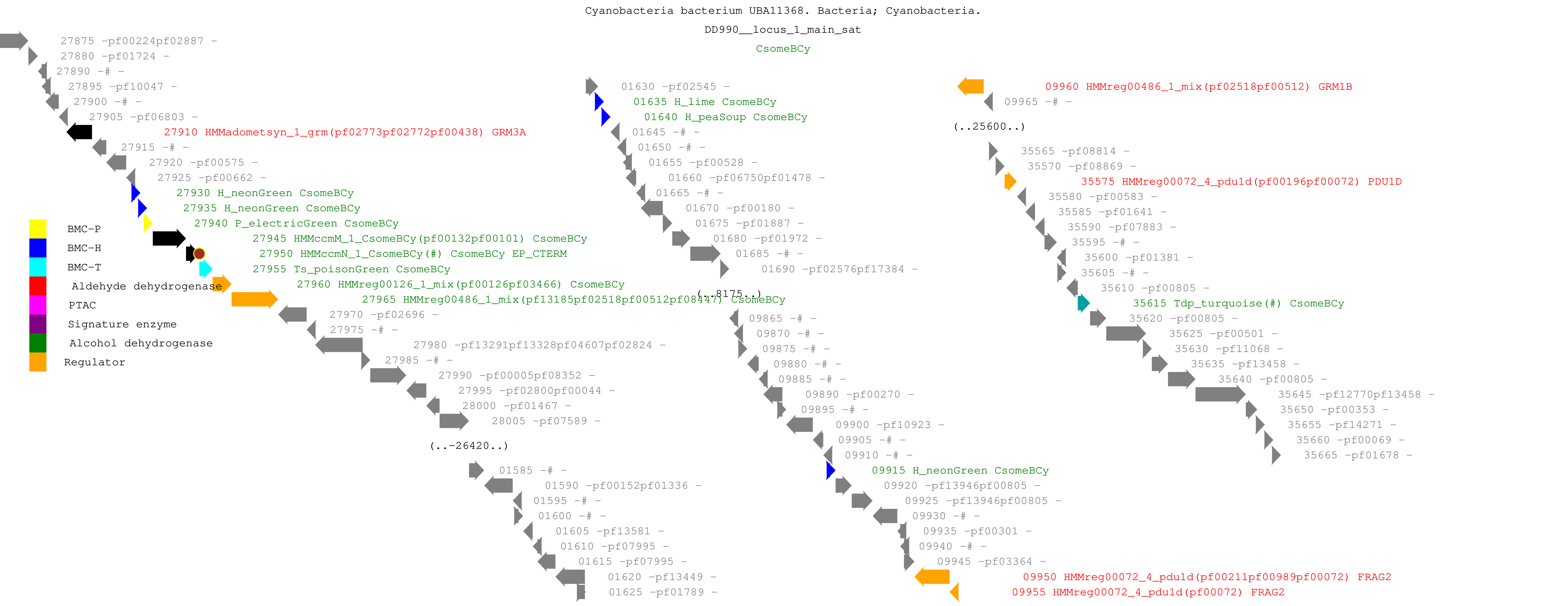

Cyanobacteria bacterium UBA11368. Bacteria; Cyanobacteria.
DD990__locus_1_main_sat
CsomeBCy
27875 -pf00224pf02887 -
27880 -pf01724 -
27890 -# -
27895 -pf10047 -
27900 -# -
27905 -pf06803 -
27910 HMMadometsyn_1_grm(pf02773pf02772pf00438) GRM3A
27915 -# -
27920 -pf00575 -
27925 -pf00662 -
27930 H_neonGreen CsomeBCy
27935 H_neonGreen CsomeBCy
27940 P_electricGreen CsomeBCy
27945 HMMccmM_1_CsomeBCy(pf00132pf00101) CsomeBCy
27950 HMMccmN_1_CsomeBCy(#) CsomeBCy EP_CTERM
27955 Ts_poisonGreen CsomeBCy
27960 HMMreg00126_1_mix(pf00126pf03466) CsomeBCy
27965 HMMreg00486_1_mix(pf13185pf02518pf00512pf08447) CsomeBCy
27970 -pf02696 -
27975 -# -
27980 -pf13291pf13328pf04607pf02824 -
27985 -# -
27990 -pf00005pf08352 -
27995 -pf02800pf00044 -
28000 -pf01467 -
28005 -pf07589 -
(..-26420..)
01585 -# -
01590 -pf00152pf01336 -
01595 -# -
01600 -# -
01605 -pf13581 -
01610 -pf07995 -
01615 -pf07995 -
01620 -pf13449 -
01625 -pf01789 -
01630 -pf02545 -
01635 H_lime CsomeBCy
01640 H_peaSoup CsomeBCy
01645 -# -
01650 -# -
01655 -pf00528 -
01660 -pf06750pf01478 -
01665 -# -
01670 -pf00180 -
01675 -pf01887 -
01680 -pf01972 -
01685 -# -
01690 -pf02576pf17384 -
(..8175..)
09865 -# -
09870 -# -
09875 -# -
09880 -# -
09885 -# -
09890 -pf00270 -
09895 -# -
09900 -pf10923 -
09905 -# -
09910 -# -
09915 H_neonGreen CsomeBCy
09920 -pf13946pf00805 -
09925 -pf13946pf00805 -
09930 -# -
09935 -pf00301 -
09940 -# -
09945 -pf03364 -
09950 HMMreg00072_4_pdu1d(pf00211pf00989pf00072) FRAG2
09955 HMMreg00072_4_pdu1d(pf00072) FRAG2
09960 HMMreg00486_1_mix(pf02518pf00512) GRM1B
09965 -# -
(..25600..)
35565 -pf08814 -
35570 -pf08869 -
35575 HMMreg00072_4_pdu1d(pf00196pf00072) PDU1D
35580 -pf00583 -
35585 -pf01641 -
35590 -pf07883 -
35595 -# -
35600 -pf01381 -
35605 -# -
35610 -pf00805 -
35615 Tdp_turquoise(#) CsomeBCy
35620 -pf00805 -
35625 -pf00501 -
35630 -pf11068 -
35635 -pf13458 -
35640 -pf00805 -
35645 -pf12770pf13458 -
35650 -pf00353 -
35655 -pf14271 -
35660 -pf00069 -
35665 -pf01678 -
BMC-P
BMC-H
BMC-T
Aldehyde dehydrogenase
PTAC
Signature enzyme
Alcohol dehydrogenase
Regulator

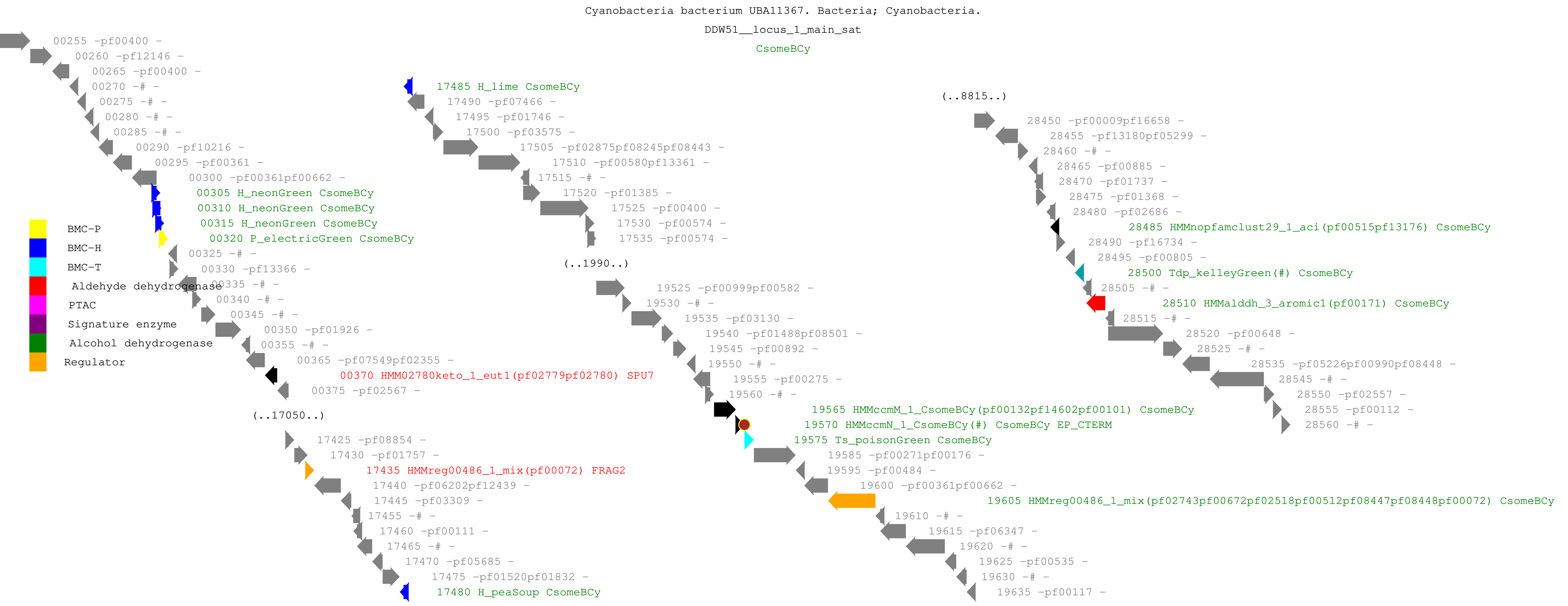

Cyanobacteria bacterium UBA11367. Bacteria; Cyanobacteria.
DDW51__locus_1_main_sat
CsomeBCy
00255 -pf00400 -
00260 -pf12146 -
00265 -pf00400 -
00270 -# -
00275 -# -
00280 -# -
00285 -# -
00290 -pf10216 -
00295 -pf00361 -
00300 -pf00361pf00662 -
00305 H_neonGreen CsomeBCy
00310 H_neonGreen CsomeBCy
00315 H_neonGreen CsomeBCy
00320 P_electricGreen CsomeBCy
00325 -# -
00330 -pf13366 -
00335 -# -
00340 -# -
00345 -# -
00350 -pf01926 -
00355 -# -
00365 -pf07549pf02355 -
00370 HMM02780keto_1_eut1(pf02779pf02780) SPU7
00375 -pf02567 -
(..17050..)
17425 -pf08854 -
17430 -pf01757 -
17435 HMMreg00486_1_mix(pf00072) FRAG2
17440 -pf06202pf12439 -
17445 -pf03309 -
17455 -# -
17460 -pf00111 -
17465 -# -
17470 -pf05685 -
17475 -pf01520pf01832 -
17480 H_peaSoup CsomeBCy
17485 H_lime CsomeBCy
17490 -pf07466 -
17495 -pf01746 -
17500 -pf03575 -
17505 -pf02875pf08245pf08443 -
17510 -pf00580pf13361 -
17515 -# -
17520 -pf01385 -
17525 -pf00400 -
17530 -pf00574 -
17535 -pf00574 -
(..1990..)
19525 -pf00999pf00582 -
19530 -# -
19535 -pf03130 -
19540 -pf01488pf08501 -
19545 -pf00892 -
19550 -# -
19555 -pf00275 -
19560 -# -
19565 HMMccmM_1_CsomeBCy(pf00132pf14602pf00101) CsomeBCy
19570 HMMccmN_1_CsomeBCy(#) CsomeBCy EP_CTERM
19575 Ts_poisonGreen CsomeBCy
19585 -pf00271pf00176 -
19595 -pf00484 -
19600 -pf00361pf00662 -
19605 HMMreg00486_1_mix(pf02743pf00672pf02518pf00512pf08447pf08448pf00072) CsomeBCy
19610 -# -
19615 -pf06347 -
19620 -# -
19625 -pf00535 -
19630 -# -
19635 -pf00117 -
(..8815..)
28450 -pf00009pf16658 -
28455 -pf13180pf05299 -
28460 -# -
28465 -pf00885 -
28470 -pf01737 -
28475 -pf01368 -
28480 -pf02686 -
28485 HMMnopfamclust29_1_aci(pf00515pf13176) CsomeBCy
28490 -pf16734 -
28495 -pf00805 -
28500 Tdp_kelleyGreen(#) CsomeBCy
28505 -# -
28510 HMMalddh_3_aromic1(pf00171) CsomeBCy
28515 -# -
28520 -pf00648 -
28525 -# -
28535 -pf05226pf00990pf08448 -
28545 -# -
28550 -pf02557 -
28555 -pf00112 -
28560 -# -
BMC-P
BMC-H
BMC-T
Aldehyde dehydrogenase
PTAC
Signature enzyme
Alcohol dehydrogenase
Regulator

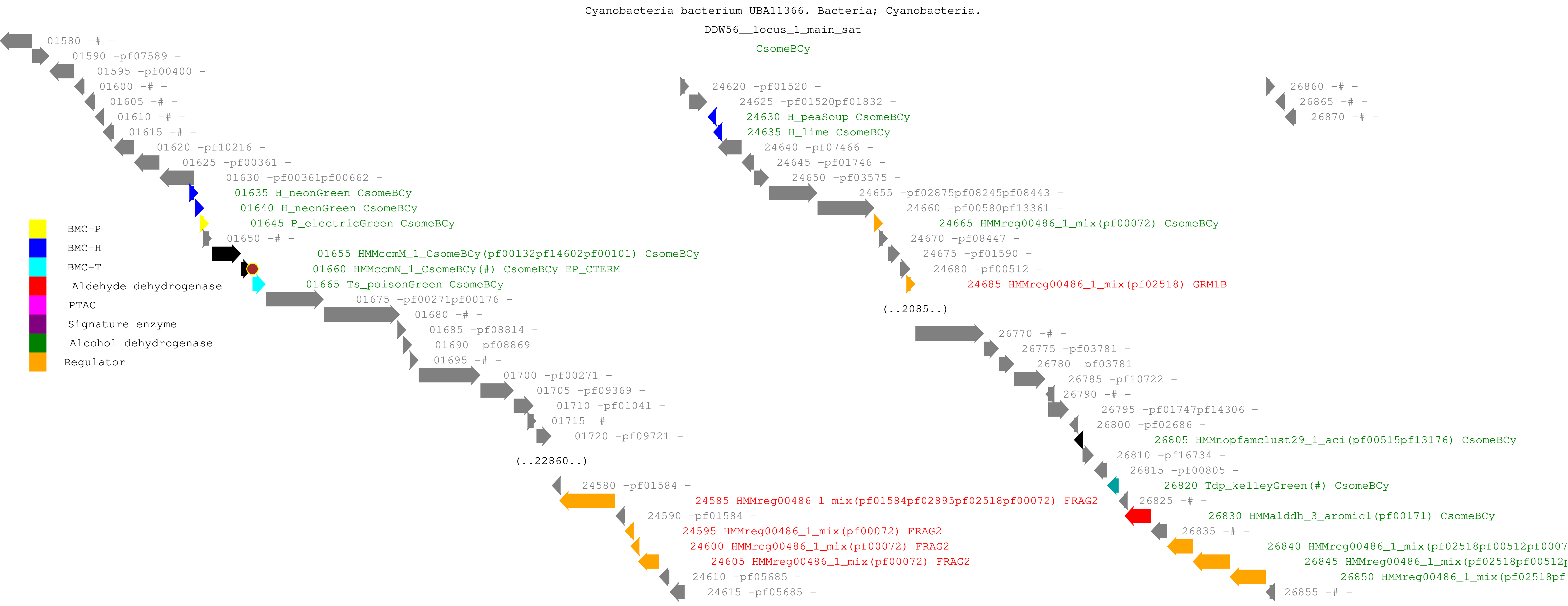

Cyanobacteria bacterium UBA11366. Bacteria; Cyanobacteria.
DDW56__locus_1_main_sat
CsomeBCy
01580 -# -
01590 -pf07589 -
01595 -pf00400 -
01600 -# -
01605 -# -
01610 -# -
01615 -# -
01620 -pf10216 -
01625 -pf00361 -
01630 -pf00361pf00662 -
01635 H_neonGreen CsomeBCy
01640 H_neonGreen CsomeBCy
01645 P_electricGreen CsomeBCy
01650 -# -
01655 HMMccmM_1_CsomeBCy(pf00132pf14602pf00101) CsomeBCy
01660 HMMccmN_1_CsomeBCy(#) CsomeBCy EP_CTERM
01665 Ts_poisonGreen CsomeBCy
01675 -pf00271pf00176 -
01680 -# -
01685 -pf08814 -
01690 -pf08869 -
01695 -# -
01700 -pf00271 -
01705 -pf09369 -
01710 -pf01041 -
01715 -# -
01720 -pf09721 -
(..22860..)
24580 -pf01584 -
24585 HMMreg00486_1_mix(pf01584pf02895pf02518pf00072) FRAG2
24590 -pf01584 -
24595 HMMreg00486_1_mix(pf00072) FRAG2
24600 HMMreg00486_1_mix(pf00072) FRAG2
24605 HMMreg00486_1_mix(pf00072) FRAG2
24610 -pf05685 -
24615 -pf05685 -
BMC-P
BMC-H
BMC-T
Aldehyde dehydrogenase
PTAC
Signature enzyme
Alcohol dehydrogenase
Regulator
24620 -pf01520 -
24625 -pf01520pf01832 -
24630 H_peaSoup CsomeBCy
24635 H_lime CsomeBCy
24640 -pf07466 -
24645 -pf01746 -
24650 -pf03575 -
24655 -pf02875pf08245pf08443 -
24660 -pf00580pf13361 -
24665 HMMreg00486_1_mix(pf00072) CsomeBCy
24670 -pf08447 -
24675 -pf01590 -
24680 -pf00512 -
24685 HMMreg00486_1_mix(pf02518) GRM1B
(..2085..)
26770 -# -
26775 -pf03781 -
26780 -pf03781 -
26785 -pf10722 -
26790 -# -
26795 -pf01747pf14306 -
26800 -pf02686 -
26805 HMMnopfamclust29_1_aci(pf00515pf13176) CsomeBCy
26810 -pf16734 -
26815 -pf00805 -
26820 Tdp_kelleyGreen(#) CsomeBCy
26825 -# -
26830 HMMalddh_3_aromic1(pf00171) CsomeBCy
26835 -# -
26840 HMMreg00486_1_mix(pf02518pf00512pf0007
26845 HMMreg00486_1_mix(pf02518pf00512p
26850 HMMreg00486_1_mix(pf02518pf
26855 -# -
26860 -# -
26865 -# -
26870 -# -

Cyanobacteria bacterium UBA11369. Bacteria; Cyanobacteria.

DDW76__locus_1_main_sat

CsomeBCy

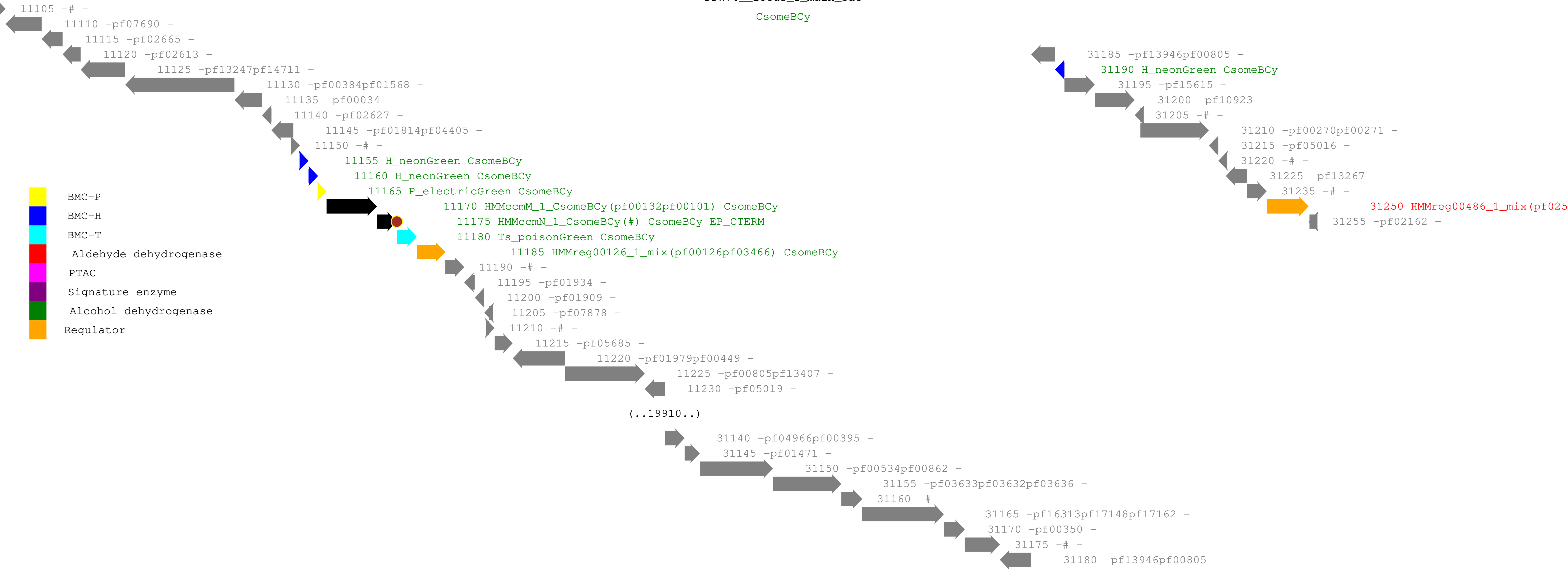

Planktothrix sp. UBA10369. Bacteria; Cyanobacteria; Oscillatoriophycideae; Oscillatoriales;

DDZ60__locus_1_main_sat

CsomeBCy

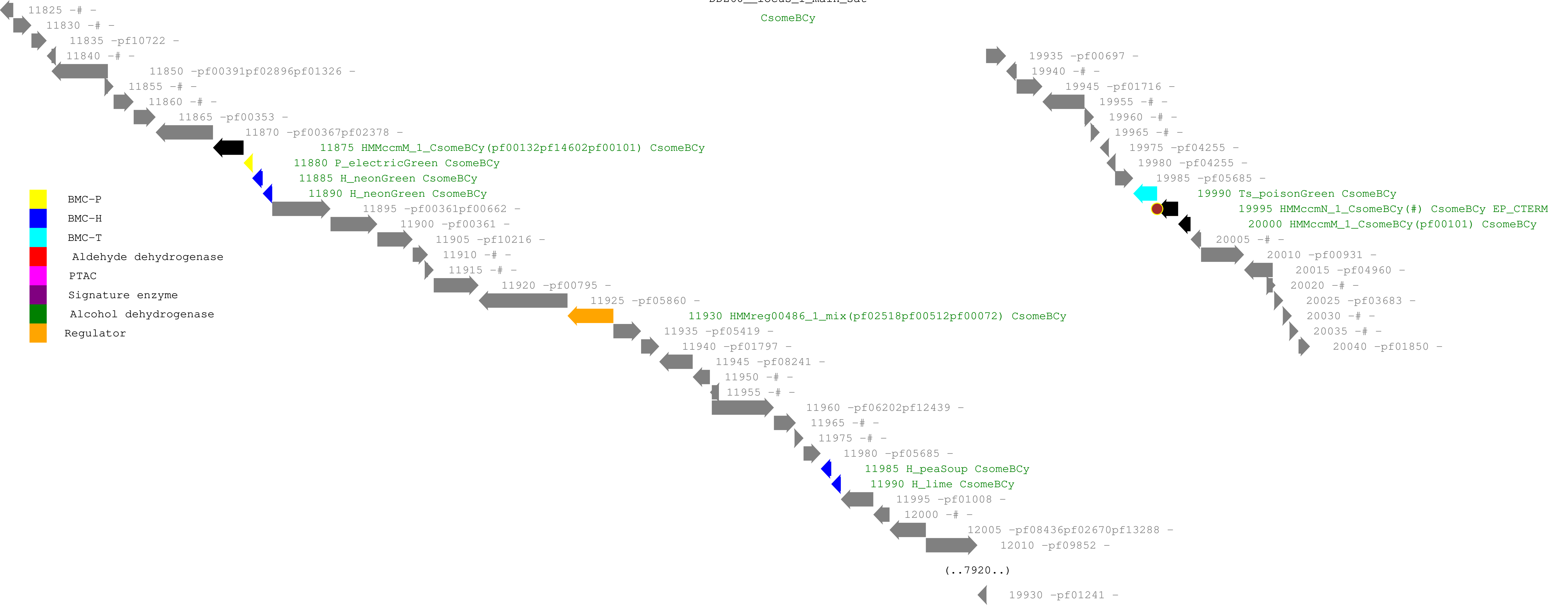

Cyanobacteria bacterium UBA8803. Bacteria; Cyanobacteria.

DDZ80__locus_1_main_sat

CsomeBCy

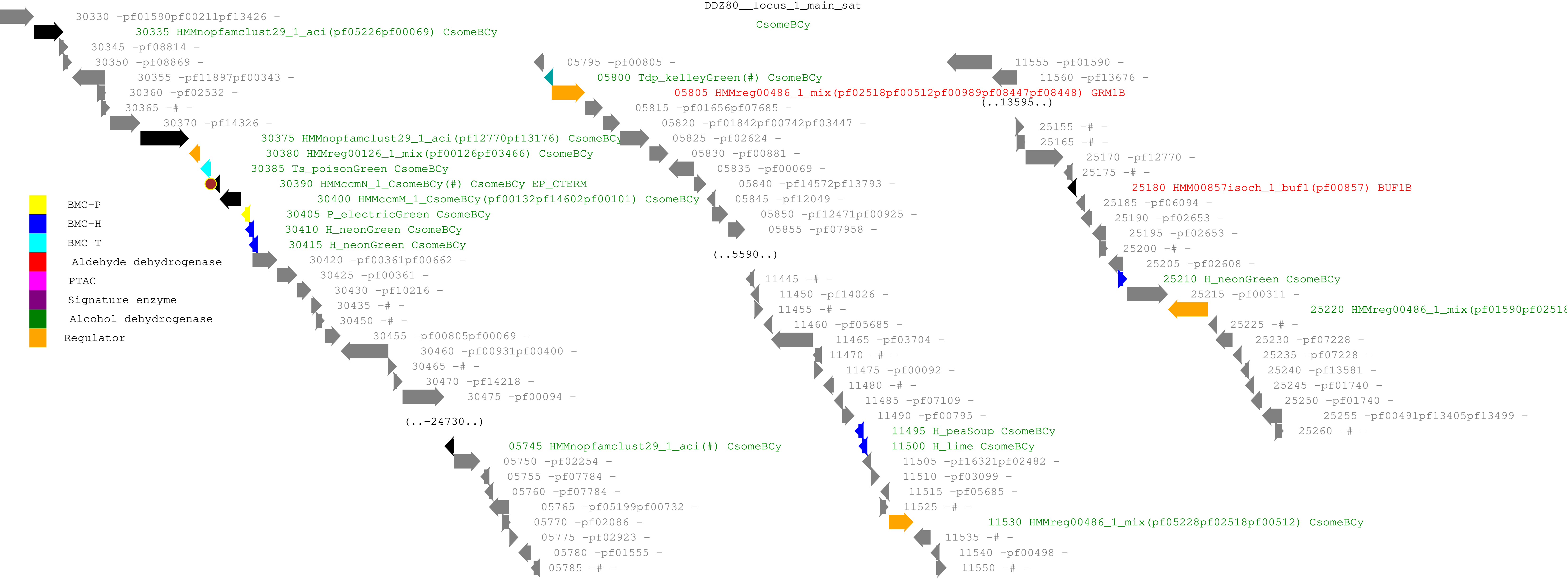

Cyanobacteria bacterium UBA11159. Bacteria; Cyanobacteria.

DEA78__locus_1_main_sat

CsomeBCy

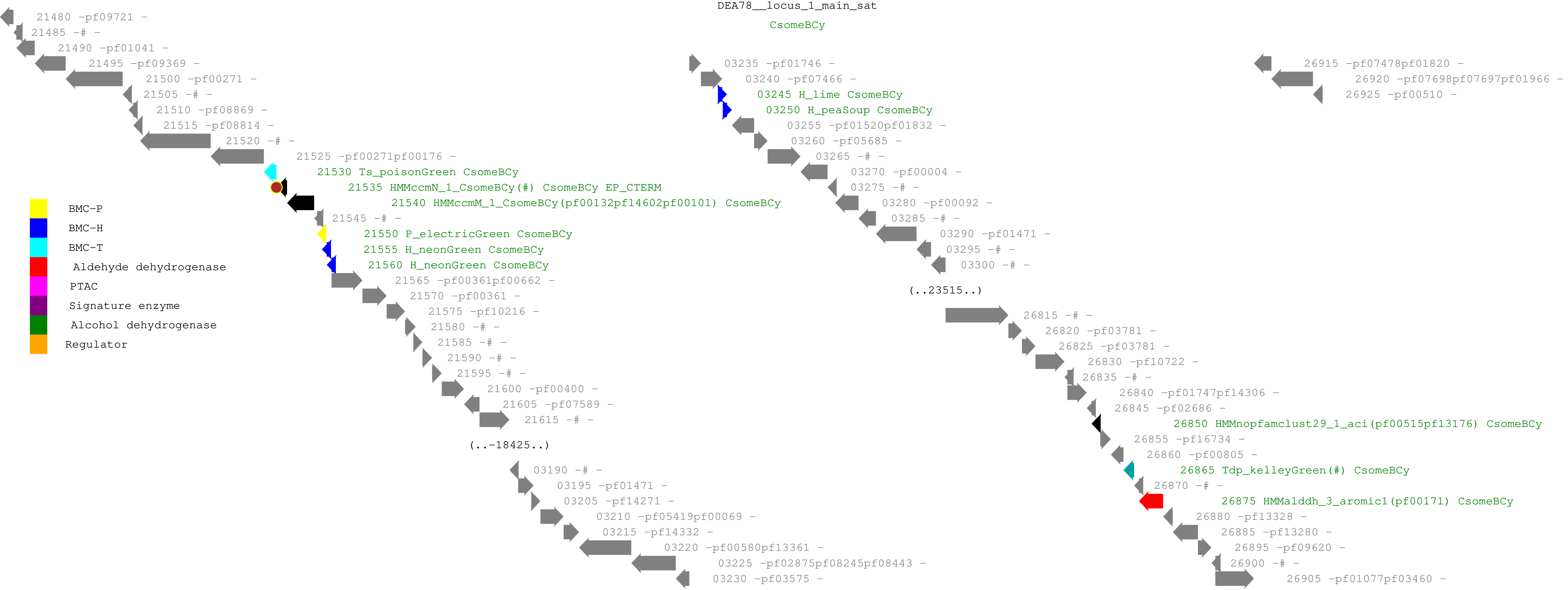

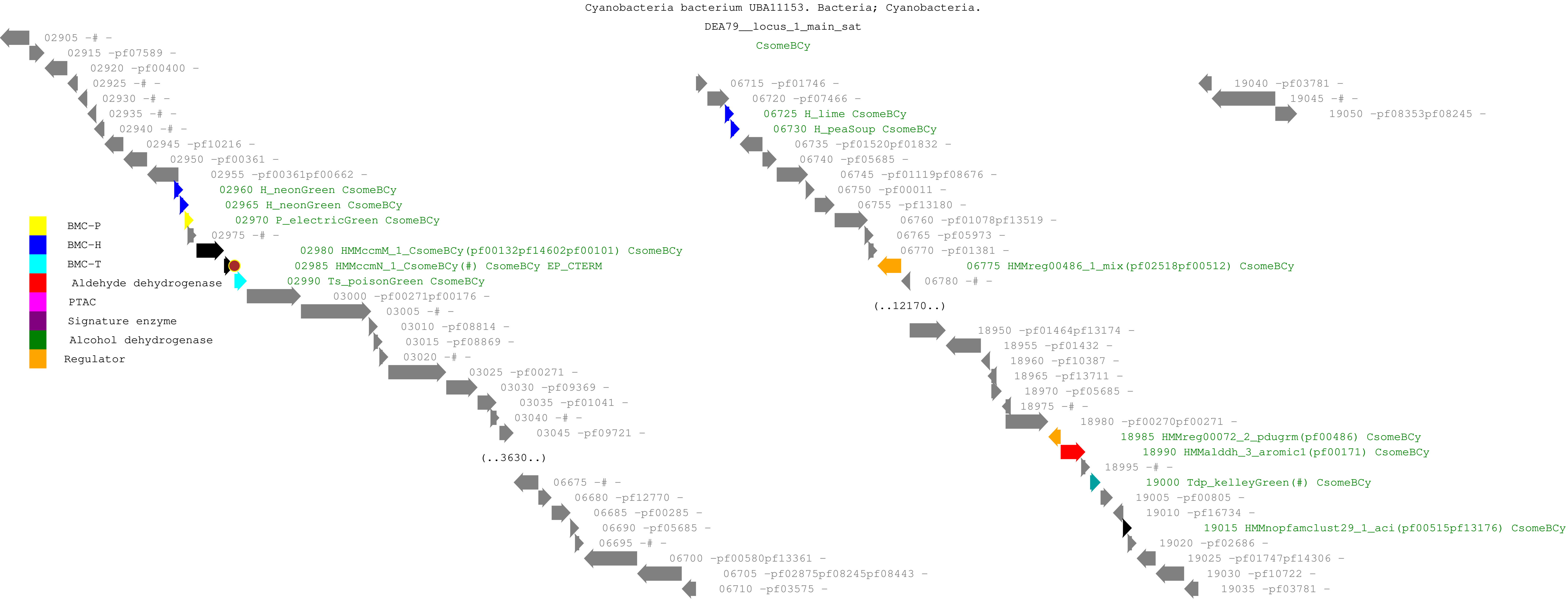
Cyanobacteria bacterium UBA11153. Bacteria; Cyanobacteria.
DEA79__locus_1_main_sat
CsomeBCy
02905 -# -
02915 -pf07589 -
02920 -pf00400 -
02925 -# -
02930 -# -
02935 -# -
02940 -# -
02945 -pf10216 -
02950 -pf00361 -
02955 -pf00361pf00662 -
02960 H_neonGreen CsomeBCy
02965 H_neonGreen CsomeBCy
02970 P_electricGreen CsomeBCy
02975 -# -
02980 HMMccmM_1_CsomeBCy(pf00132pf14602pf00101) CsomeBCy
02985 HMMccmN_1_CsomeBCy(#) CsomeBCy EP_CTERM
02990 Ts_poisonGreen CsomeBCy
03000 -pf00271pf00176 -
03005 -# -
03010 -pf08814 -
03015 -pf08869 -
03020 -# -
03025 -pf00271 -
03030 -pf09369 -
03035 -pf01041 -
03040 -# -
03045 -pf09721 -
(..3630..)
06675 -# -
06680 -pf12770 -
06685 -pf00285 -
06690 -pf05685 -
06695 -# -
06700 -pf00580pf13361 -
06705 -pf02875pf08245pf08443 -
06710 -pf03575 -
06715 -pf01746 -
06720 -pf07466 -
06725 H_lime CsomeBCy
06730 H_peaSoup CsomeBCy
06735 -pf01520pf01832 -
06740 -pf05685 -
06745 -pf01119pf08676 -
06750 -pf00011 -
06755 -pf13180 -
06760 -pf01078pf13519 -
06765 -pf05973 -
06770 -pf01381 -
06775 HMMreg00486_1_mix(pf02518pf00512) CsomeBCy
06780 -# -
(..12170..)
18950 -pf01464pf13174 -
18955 -pf01432 -
18960 -pf10387 -
18965 -pf13711 -
18970 -pf05685 -
18975 -# -
18980 -pf00270pf00271 -
18985 HMMreg00072_2_pdugrm(pf00486) CsomeBCy
18990 HMMalddh_3_aromic1(pf00171) CsomeBCy
18995 -# -
19000 Tdp_kelleyGreen(#) CsomeBCy
19005 -pf00805 -
19010 -pf16734 -
19015 HMMnopfamclust29_1_aci(pf00515pf13176) CsomeBCy
19020 -pf02686 -
19025 -pf01747pf14306 -
19030 -pf10722 -
19035 -pf03781 -
19040 -pf03781 -
19045 -# -
19050 -pf08353pf08245 -
BMC-P
BMC-H
BMC-T
Aldehyde dehydrogenase
PTAC
Signature enzyme
Alcohol dehydrogenase
Regulator

Oscillatoriales bacterium UBA8482. Bacteria; Cyanobacteria; Oscillatoriophycideae; Oscillatoriales;

DEF27__locus_fragmented

CsomeBCy

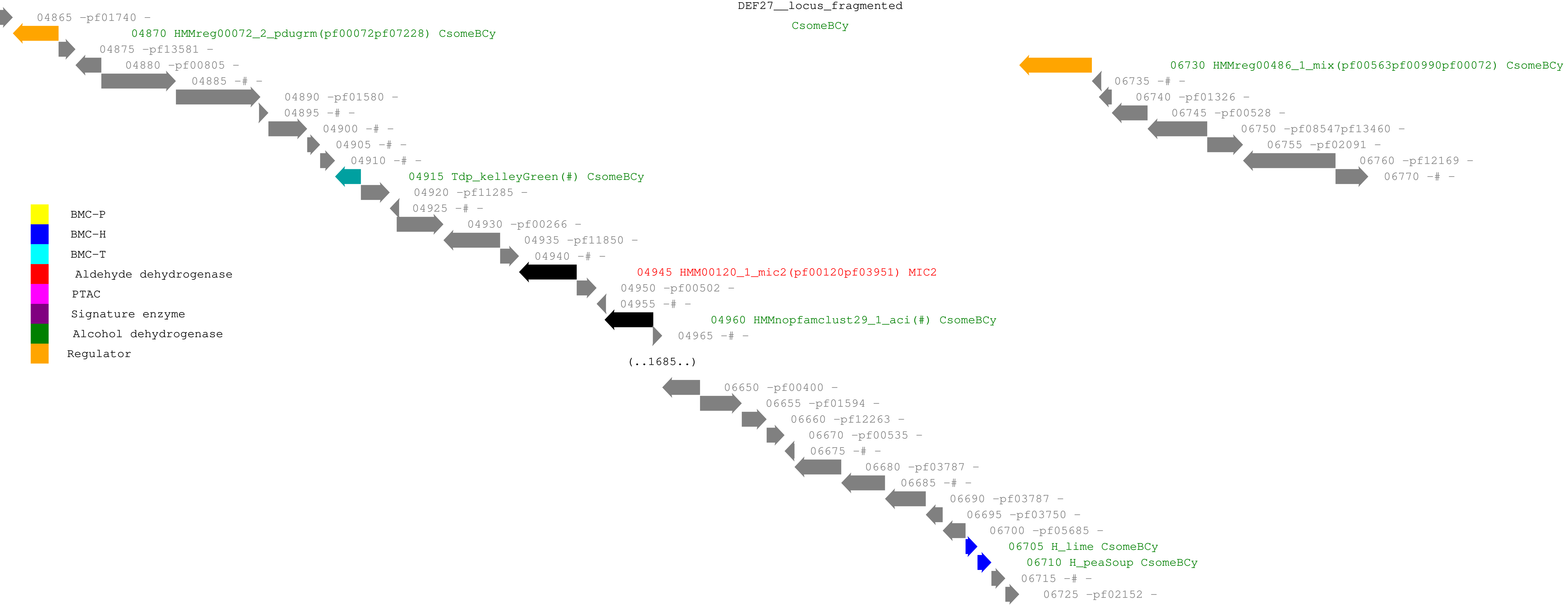

Nostoc sp. UBA8866. Bacteria; Cyanobacteria; Nostocales; Nostocaceae; Nostoc.

DEF48__locus_1_main_sat

CsomeBCy

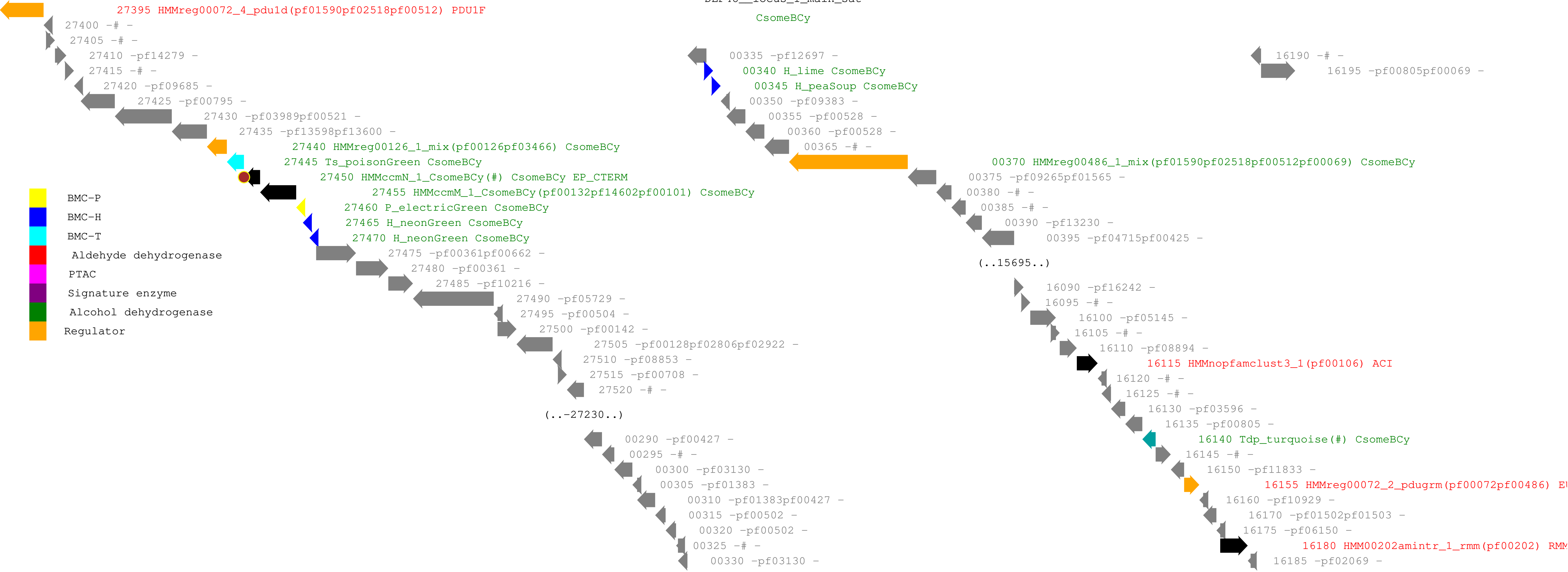

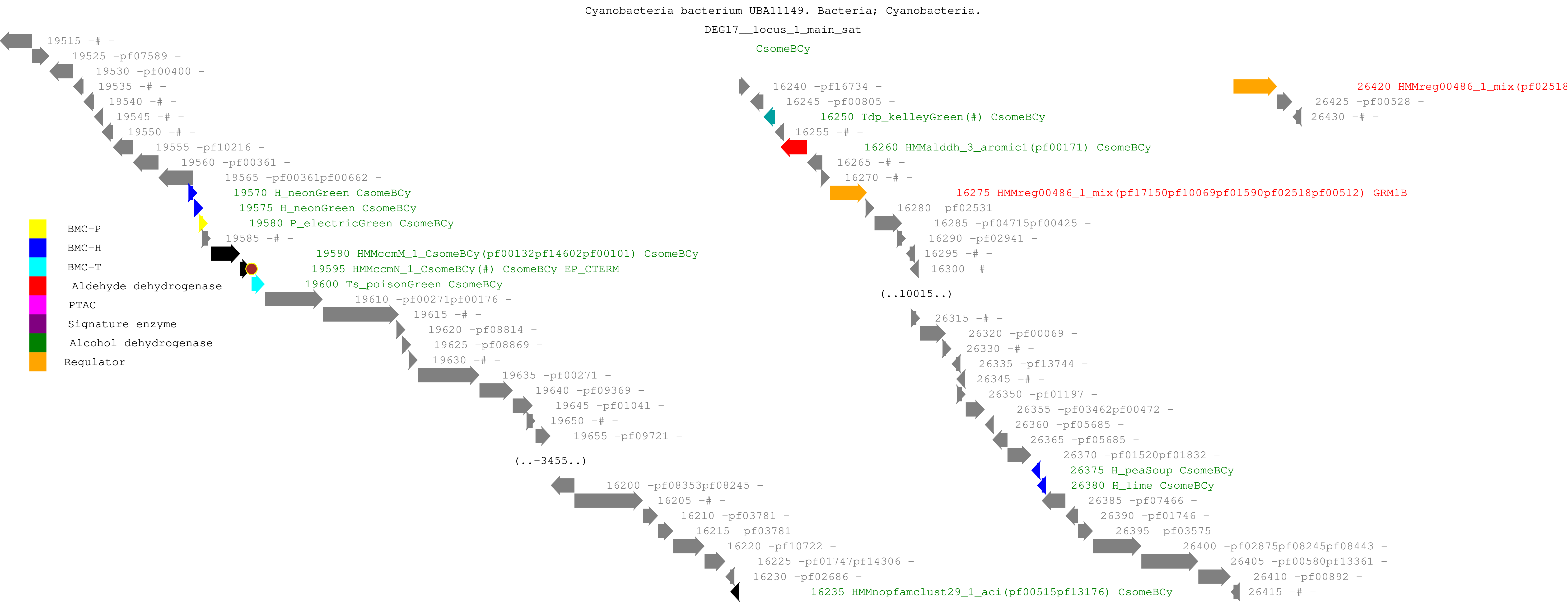
Cyanobacteria bacterium UBA11149. Bacteria; Cyanobacteria.
DEG17__locus_1_main_sat
CsomeBCy
19515 -# -
19525 -pf07589 -
19530 -pf00400 -
19535 -# -
19540 -# -
19545 -# -
19550 -# -
19555 -pf10216 -
19560 -pf00361 -
19565 -pf00361pf00662 -
19570 H_neonGreen CsomeBCy
19575 H_neonGreen CsomeBCy
19580 P_electricGreen CsomeBCy
19585 -# -
19590 HMMccmM_1_CsomeBCy(pf00132pf14602pf00101) CsomeBCy
19595 HMMccmN_1_CsomeBCy(#) CsomeBCy EP_CTERM
19600 Ts_poisonGreen CsomeBCy
19610 -pf00271pf00176 -
19615 -# -
19620 -pf08814 -
19625 -pf08869 -
19630 -# -
19635 -pf00271 -
19640 -pf09369 -
19645 -pf01041 -
19650 -# -
19655 -pf09721 -
(..-3455..)
16200 -pf08353pf08245 -
16205 -# -
16210 -pf03781 -
16215 -pf03781 -
16220 -pf10722 -
16225 -pf01747pf14306 -
16230 -pf02686 -
16235 HMMnopfamclust29_1_aci(pf00515pf13176) CsomeBCy
BMC-P
BMC-H
BMC-T
Aldehyde dehydrogenase
PTAC
Signature enzyme
Alcohol dehydrogenase
Regulator
16240 -pf16734 -
16245 -pf00805 -
16250 Tdp_kelleyGreen(#) CsomeBCy
16255 -# -
16260 HMMaldh_3_aromic1(pf00171) CsomeBCy
16265 -# -
16270 -# -
16275 HMMreg00486_1_mix(pf17150pf10069pf01590pf02518pf00512) GRM1B
16280 -pf02531 -
16285 -pf04715pf00425 -
16290 -pf02941 -
16295 -# -
16300 -# -
(..10015..)
26315 -# -
26320 -pf00069 -
26330 -# -
26335 -pf13744 -
26345 -# -
26350 -pf01197 -
26355 -pf03462pf00472 -
26360 -pf05685 -
26365 -pf05685 -
26370 -pf01520pf01832 -
26375 H_peaSoup CsomeBCy
26380 H_lime CsomeBCy
26385 -pf07466 -
26390 -pf01746 -
26395 -pf03575 -
26400 -pf02875pf08245pf08443 -
26405 -pf00580pf13361 -
26410 -pf00892 -
26415 -# -
26420 HMMreg00486_1_mix(pf02518
26425 -pf00528 -
26430 -# -

Cyanobacteria bacterium UBA11148. Bacteria; Cyanobacteria.

DEG47__locus_1_main_sat

CsomeBCy

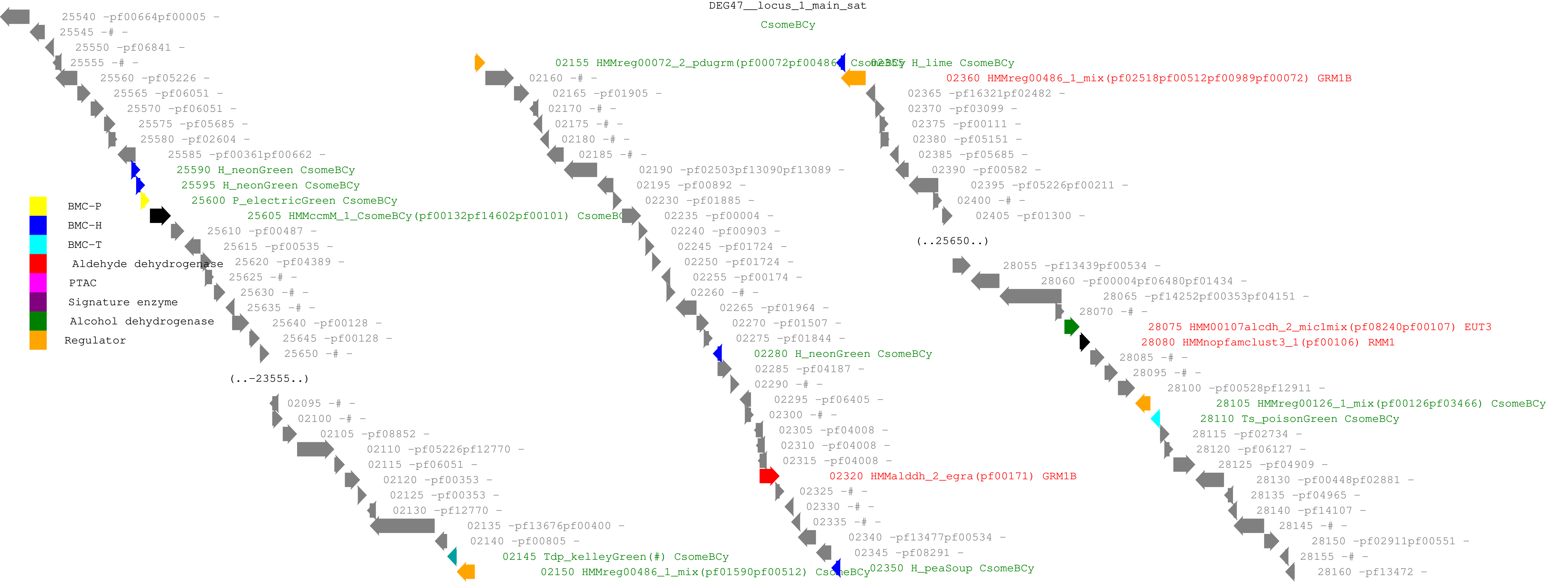

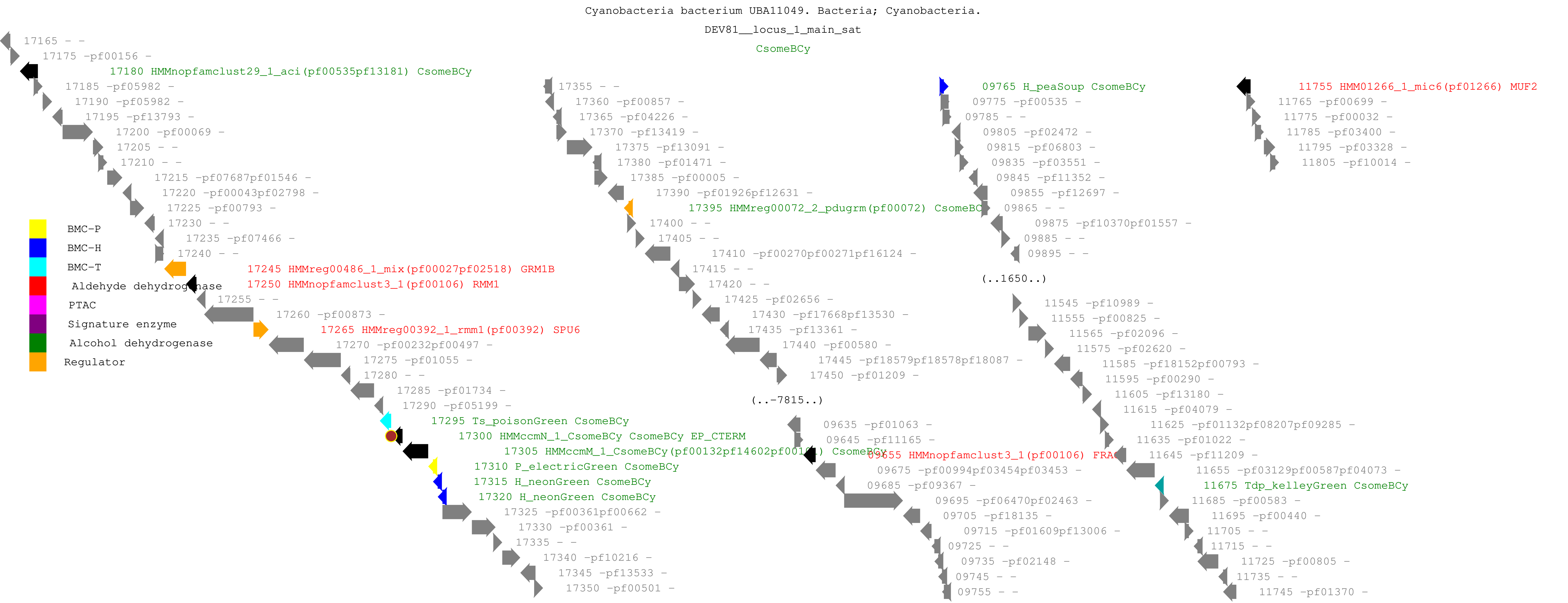

Cyanobacteria bacterium UBA11049. Bacteria; Cyanobacteria.
DEV81__locus_1_main_sat
CsomeBCy
BMC-P
BMC-H
BMC-T
Aldehyde dehydrogenase
PTAC
Signature enzyme
Alcohol dehydrogenase
Regulator
17165 - -
17175 -pf00156 -
17180 HMMnopfamclust29_1_aci(pf00535pf13181) CsomeBCy
17185 -pf05982 -
17190 -pf05982 -
17195 -pf13793 -
17200 -pf00069 -
17205 - -
17210 - -
17215 -pf07687pf01546 -
17220 -pf00043pf02798 -
17225 -pf00793 -
17230 - -
17235 -pf07466 -
17240 - -
17245 HMMreg00486_1_mix(pf00027pf02518) GRM1B
17250 HMMnopfamclust3_1(pf00106) RMM1
17255 - -
17260 -pf00873 -
17265 HMMreg00392_1_rmm1(pf00392) SPU6
17270 -pf00232pf00497 -
17275 -pf01055 -
17280 - -
17285 -pf01734 -
17290 -pf05199 -
17295 Ts_poisonGreen CsomeBCy
17300 HMMccmN_1_CsomeBCy CsomeBCy EP_CTERM
17305 HMMccmM_1_CsomeBCy(pf00132pf14602pf00101) CsomeBCy
17310 P_electricGreen CsomeBCy
17315 H_neonGreen CsomeBCy
17320 H_neonGreen CsomeBCy
17325 -pf00361pf00662 -
17330 -pf00361 -
17335 - -
17340 -pf10216 -
17345 -pf13533 -
17350 -pf00501 -
17355 - -
17360 -pf00857 -
17365 -pf04226 -
17370 -pf13419 -
17375 -pf13091 -
17380 -pf01471 -
17385 -pf00005 -
17390 -pf01926pf12631 -
17395 HMMreg00072_2_pdugrm(pf00072) CsomeBCy
17400 - -
17405 - -
17410 -pf00270pf00271pf16124 -
17415 - -
17420 - -
17425 -pf02656 -
17430 -pf17668pf13530 -
17435 -pf13361 -
17440 -pf00580 -
17445 -pf18579pf18578pf18087 -
17450 -pf01209 -
(..-7815..)
09635 -pf01063 -
09645 -pf11165 -
09655 HMMnopfamclust3_1(pf00106) FRA
09675 -pf00994pf03454pf03453 -
09685 -pf09367 -
09695 -pf06470pf02463 -
09705 -pf18135 -
09715 -pf01609pf13006 -
09725 - -
09735 -pf02148 -
09745 - -
09755 - -
09765 H_peaSoup CsomeBCy
09775 -pf00535 -
09785 - -
09805 -pf02472 -
09815 -pf06803 -
09835 -pf03551 -
09845 -pf11352 -
09855 -pf12697 -
09865 - -
09875 -pf10370pf01557 -
09885 - -
09895 - -
(..1650..)
11545 -pf10989 -
11555 -pf00825 -
11565 -pf02096 -
11575 -pf02620 -
11585 -pf18152pf00793 -
11595 -pf00290 -
11605 -pf13180 -
11615 -pf04079 -
11625 -pf01132pf08207pf09285 -
11635 -pf01022 -
11645 -pf11209 -
11655 -pf03129pf00587pf04073 -
11675 Tdp_kelleyGreen CsomeBCy
11685 -pf00583 -
11695 -pf00440 -
11705 - -
11715 - -
11725 -pf00805 -
11735 - -
11745 -pf01370 -
11755 HMM01266_1_mic6(pf01266) MUF2
11765 -pf00699 -
11775 -pf00032 -
11785 -pf03400 -
11795 -pf03328 -
11805 -pf10014 -

Microcoleaceae bacterium UBA9251. Bacteria; Cyanobacteria; Oscillatoriophycideae; Oscillatoriales;

DGO89__locus_fragmented

CsomeBCy

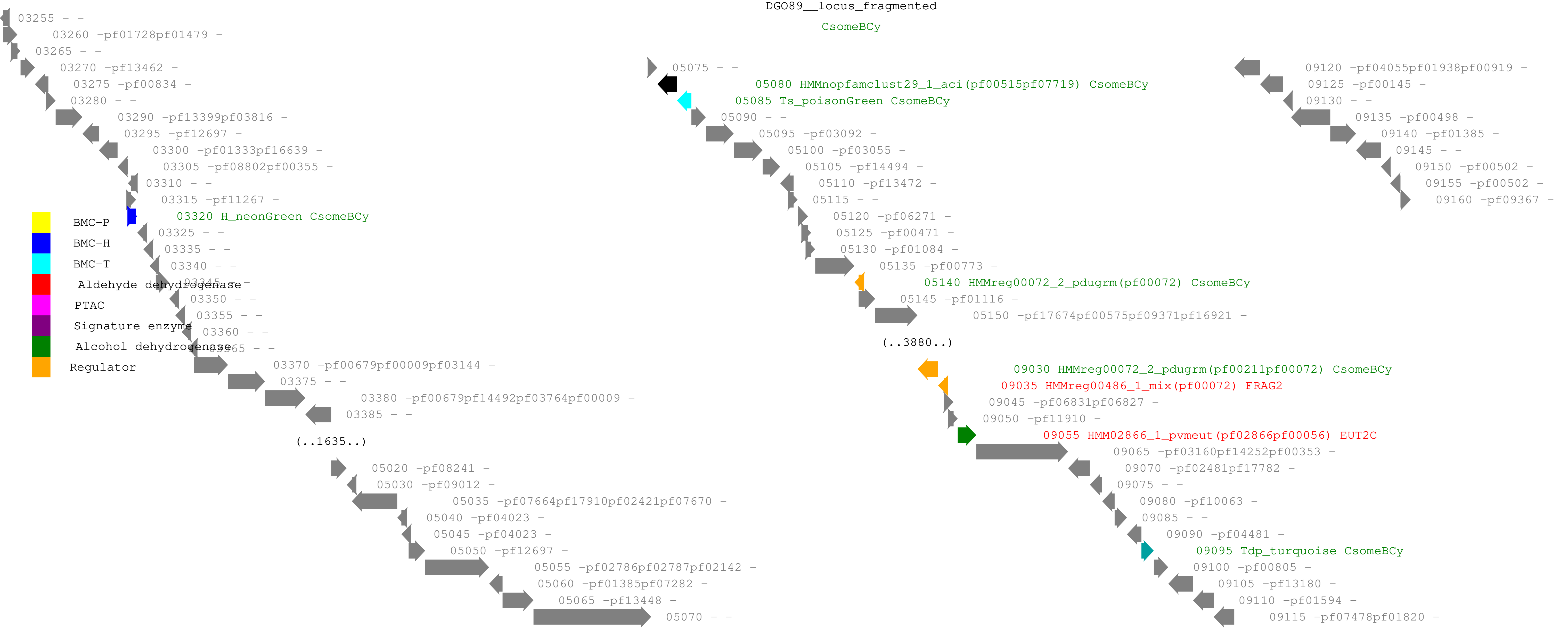

Anabaena sp. UBA12330. Bacteria; Cyanobacteria; Nostocales; Nostocaceae; Anabaena;

DIU28__locus_1_main_sat

CsomeBCy

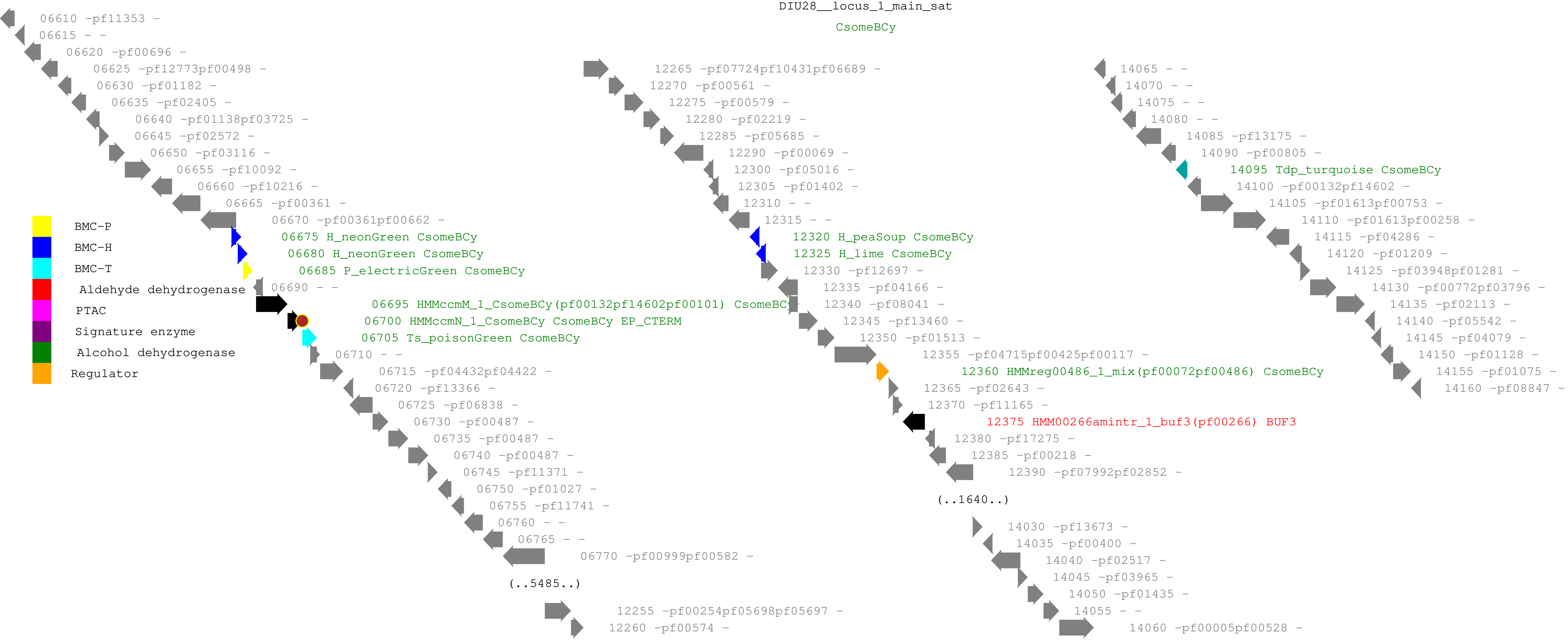

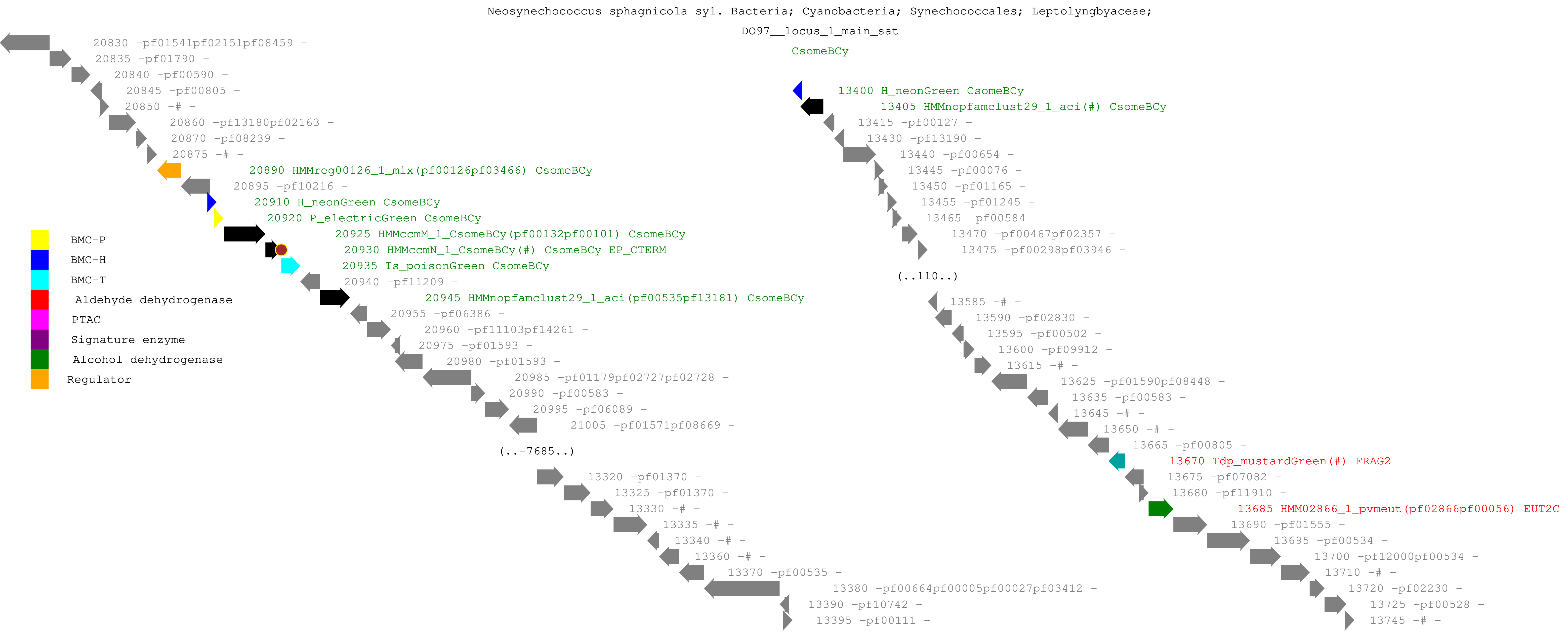

Neosynechococcus sphagnicola sy1. Bacteria; Cyanobacteria; Synechococcales; Leptolyngbyaceae;
DO97__locus_1_main_sat
CsomeBCy
20830 -pf01541pf02151pf08459 -
20835 -pf01790 -
20840 -pf00590 -
20845 -pf00805 -
20850 -# -
20860 -pf13180pf02163 -
20870 -pf08239 -
20875 -# -
20890 HMMreg00126_1_mix(pf00126pf03466) CsomeBCy
20895 -pf10216 -
20910 H_neonGreen CsomeBCy
20920 P_electricGreen CsomeBCy
20925 HMMccmM_1_CsomeBCy(pf00132pf00101) CsomeBCy
20930 HMMccmN_1_CsomeBCy(#) CsomeBCy EP_CTERM
20935 Ts_poisonGreen CsomeBCy
20940 -pf11209 -
20945 HMMnopfamclust29_1_aci(pf00535pf13181) CsomeBCy
20955 -pf06386 -
20960 -pf11103pf14261 -
20975 -pf01593 -
20980 -pf01593 -
20985 -pf01179pf02727pf02728 -
20990 -pf00583 -
20995 -pf06089 -
21005 -pf01571pf08669 -
(..-7685..)
13320 -pf01370 -
13325 -pf01370 -
13330 -# -
13335 -# -
13340 -# -
13360 -# -
13370 -pf00535 -
13380 -pf00664pf00005pf00027pf03412 -
13390 -pf10742 -
13395 -pf00111 -
BMC-P
BMC-H
BMC-T
Aldehyde dehydrogenase
PTAC
Signature enzyme
Alcohol dehydrogenase
Regulator
13400 H_neonGreen CsomeBCy
13405 HMMnopfamclust29_1_aci(#) CsomeBCy
13415 -pf00127 -
13430 -pf13190 -
13440 -pf00654 -
13445 -pf00076 -
13450 -pf01165 -
13455 -pf01245 -
13465 -pf00584 -
13470 -pf00467pf02357 -
13475 -pf00298pf03946 -
(..110..)
13585 -# -
13590 -pf02830 -
13595 -pf00502 -
13600 -pf09912 -
13615 -# -
13625 -pf01590pf08448 -
13635 -pf00583 -
13645 -# -
13650 -# -
13665 -pf00805 -
13670 Tdp_mustardGreen(#) FRAG2
13675 -pf07082 -
13680 -pf11910 -
13685 HMM02866_1_pvmeut(pf02866pf00056) EUT2C
13690 -pf01555 -
13695 -pf00534 -
13700 -pf12000pf00534 -
13710 -# -
13720 -pf02230 -
13725 -pf00528 -
13745 -# -

Synechococcus elongatus PCC 11801. Bacteria; Cyanobacteria; Synechococcales; Synechococcaceae; Synechococcus.

DOP62__locus_1_main_sat

CsomeBCy

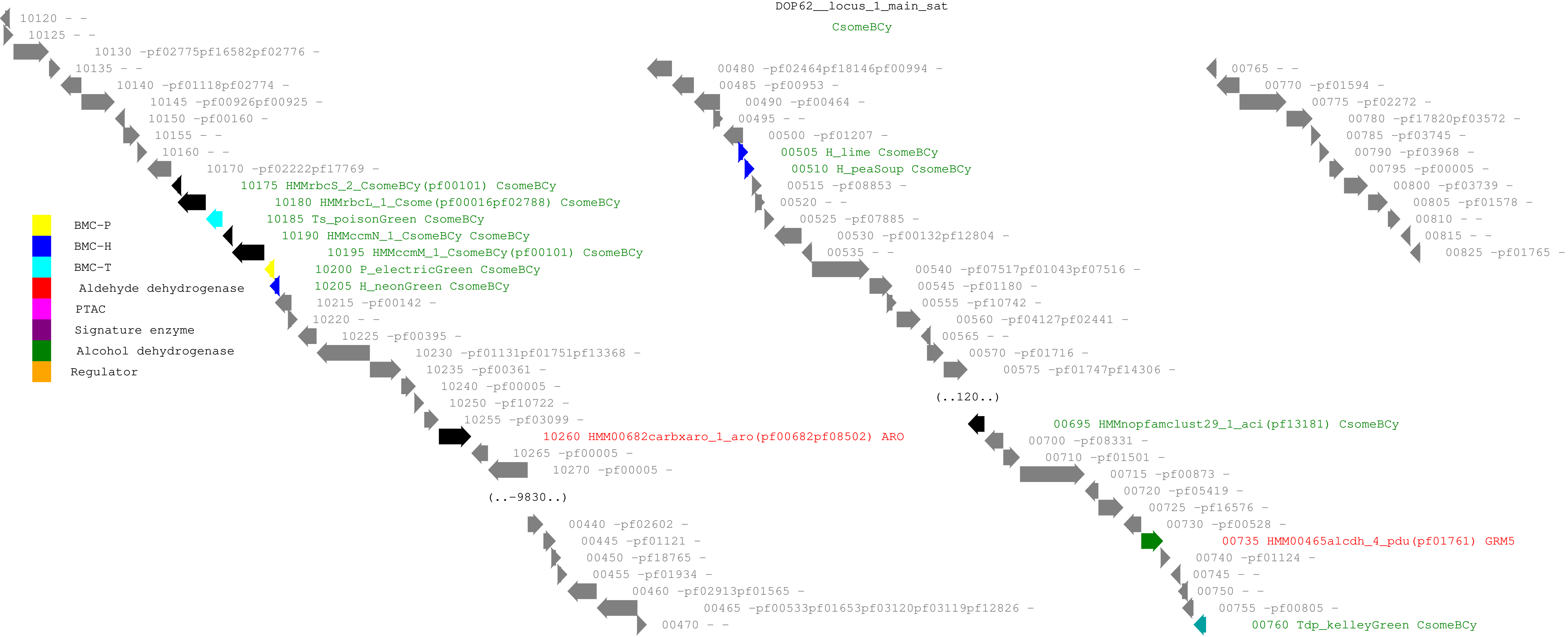

Calothrix desertica PCC 7102. Bacteria; Cyanobacteria; Nostocales; Calotrichaceae; Calothrix.

DSM106972__locus_1_main_sat

CsomeBCy

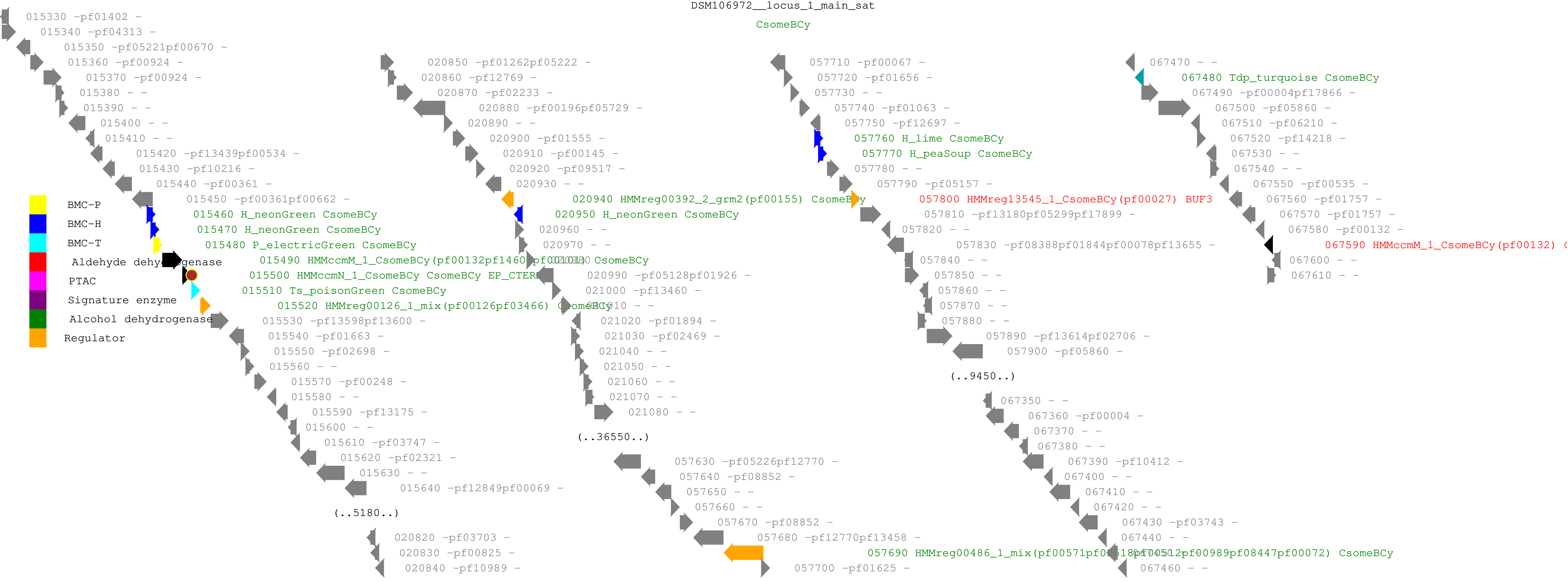

Trichormus variabilis SAG 1403-4b. Bacteria; Cyanobacteria; Nostocales; Nostocaceae; Trichormus.

DSM107003__locus_1_main_sat

CsomeBCy

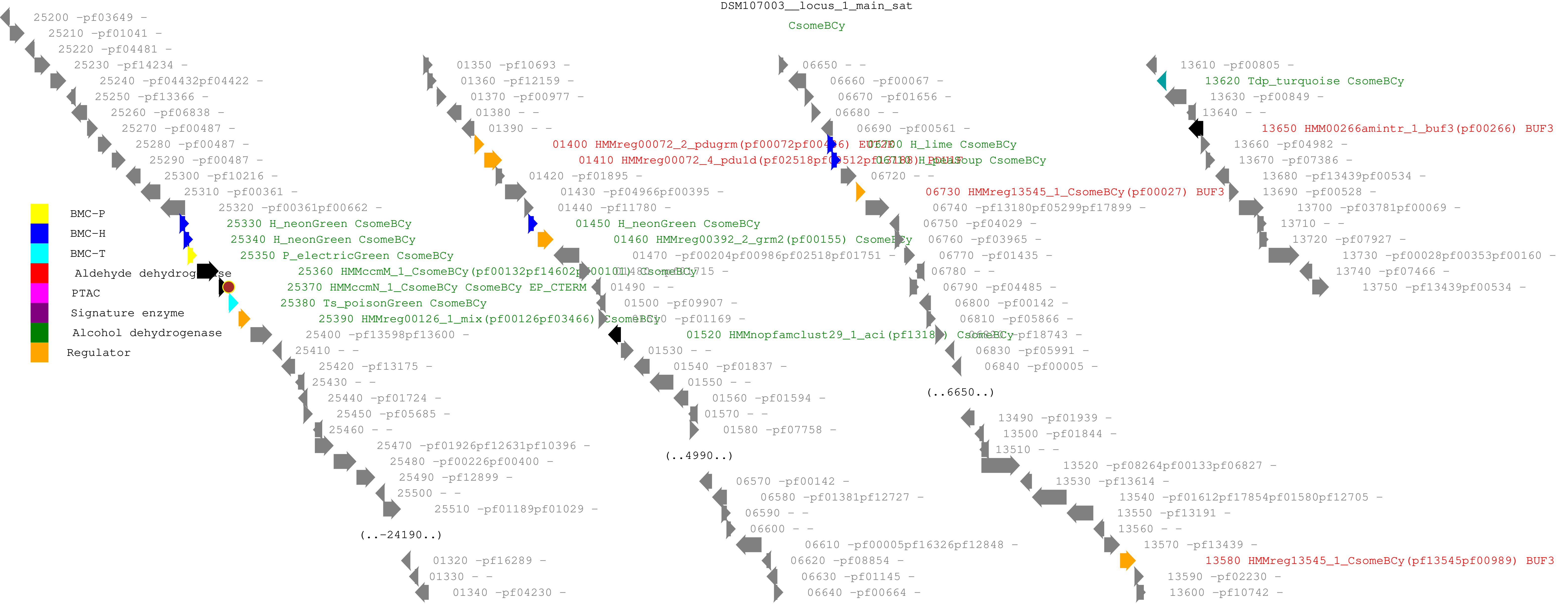

Chroococcidiopsis cubana SAG 39.79. Bacteria; Cyanobacteria; Chroococcidiopsidales; Chroococcidiopsidaceae;

DSM107010__locus_1_main_sat

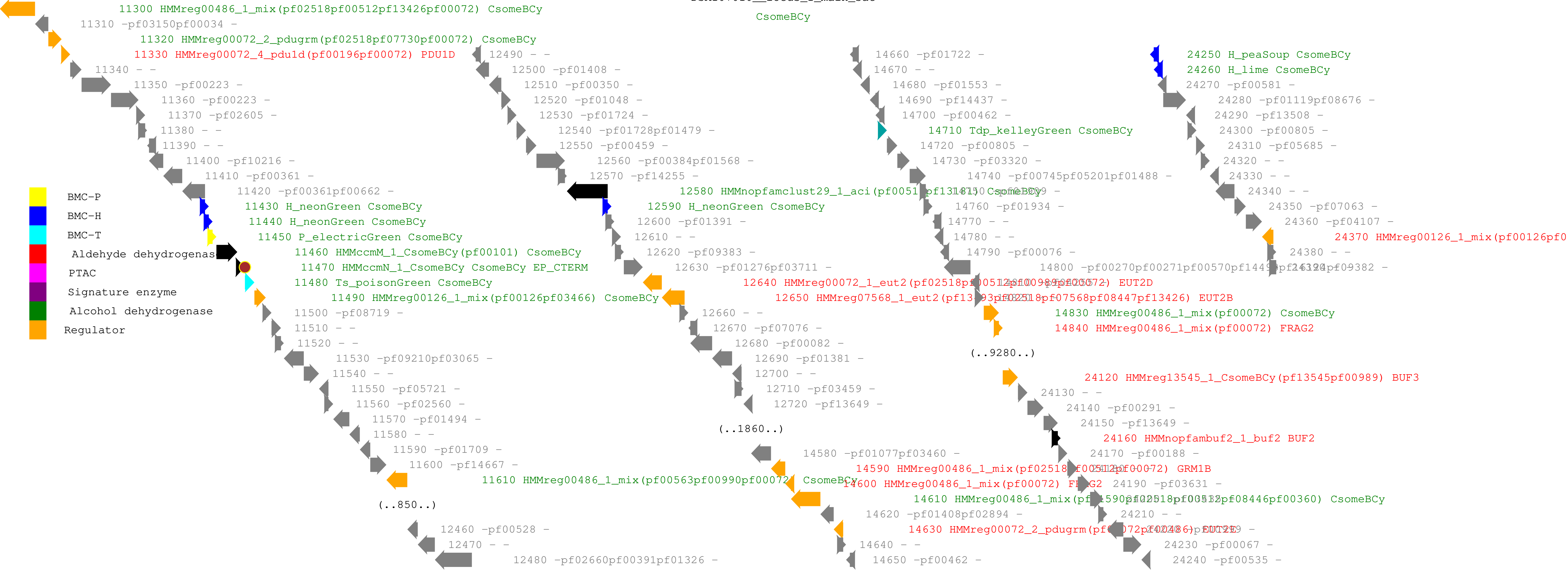

Microcystis wesenbergii TW10. Bacteria; Cyanobacteria; Oscillatoriophycideae; Chroococcales;

DWQ51__locus_1_main_sat

CsomeBCy

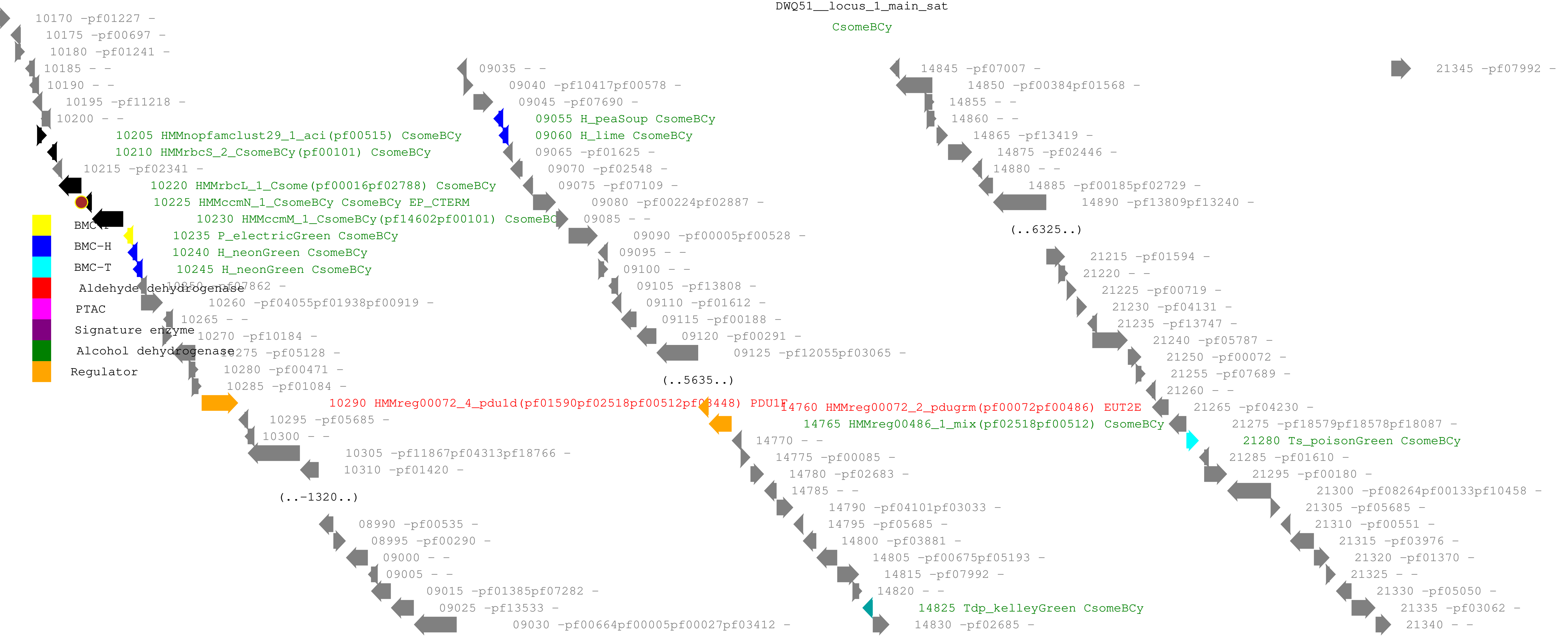

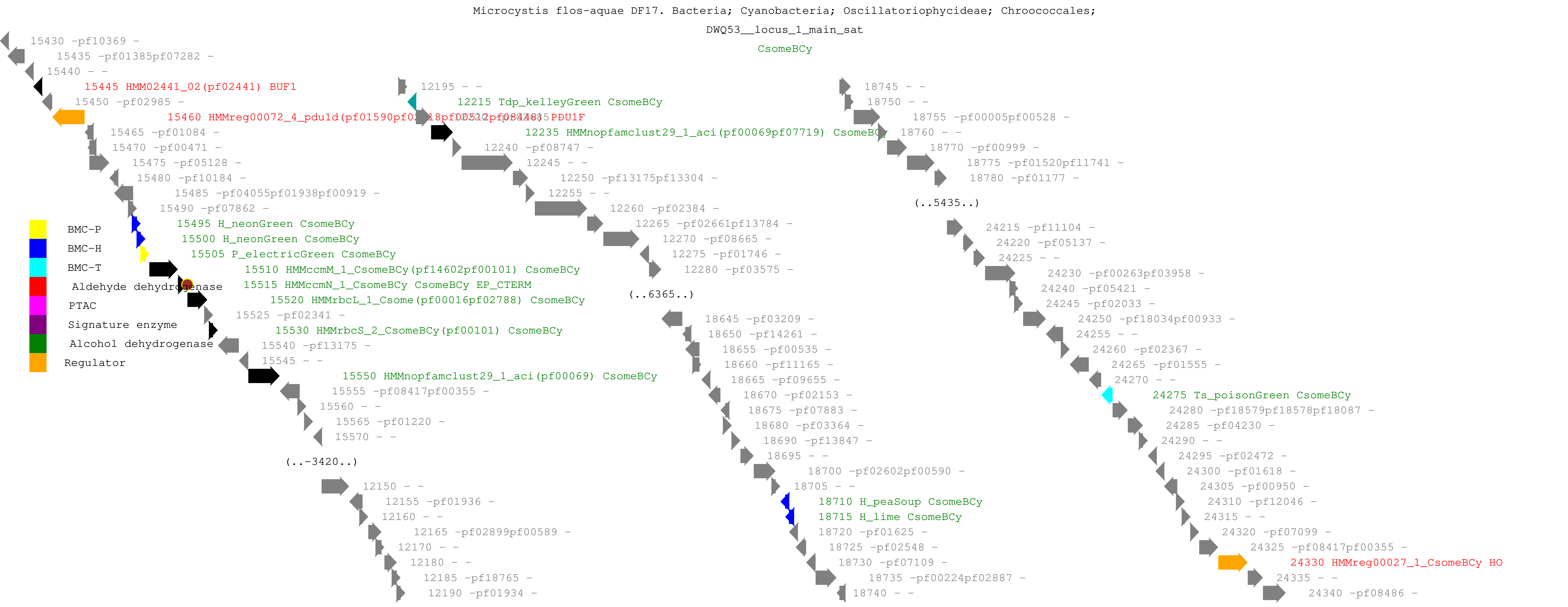
Microcystis flos-aquae DF17. Bacteria; Cyanobacteria; Oscillatoriophycideae; Chroococcales;
DWQ53__locus_1_main_sat
CsomeBCy
15430 -pf10369 -
15435 -pf01385pf07282 -
15440 - -
15445 HMM02441_02(pf02441) BUF1
15450 -pf02985 -
15460 HMMreg00072_4_pdu1d(pf01590pf0[illegible]18pf02512pf08448) PDU1F
15465 -pf01084 -
15470 -pf00471 -
15475 -pf05128 -
15480 -pf10184 -
15485 -pf04055pf01938pf00919 -
15490 -pf07862 -
15495 H_neonGreen CsomeBCy
15500 H_neonGreen CsomeBCy
15505 P_electricGreen CsomeBCy
15510 HMMccmM_1_CsomeBCy(pf14602pf00101) CsomeBCy
15515 HMMccmN_1_CsomeBCy CsomeBCy EP_CTERM
15520 HMMrbcL_1_Csome(pf00016pf02788) CsomeBCy
15525 -pf02341 -
15530 HMMrbcS_2_CsomeBCy(pf00101) CsomeBCy
15540 -pf13175 -
15545 - -
15550 HMMnopfamclust29_1_aci(pf00069) CsomeBCy
15555 -pf08417pf00355 -
15560 - -
15565 -pf01220 -
15570 - -
(..-3420..)
12150 - -
12155 -pf01936 -
12160 - -
12165 -pf02899pf00589 -
12170 - -
12180 - -
12185 -pf18765 -
12190 -pf01934 -
12195 - -
12215 Tdp_kelleyGreen CsomeBCy
12235 HMMnopfamclust29_1_aci(pf00069pf07719) CsomeBCy
12240 -pf08747 -
12245 - -
12250 -pf13175pf13304 -
12255 - -
12260 -pf02384 -
12265 -pf02661pf13784 -
12270 -pf08665 -
12275 -pf01746 -
12280 -pf03575 -
(..6365..)
18645 -pf03209 -
18650 -pf14261 -
18655 -pf00535 -
18660 -pf11165 -
18665 -pf09655 -
18670 -pf02153 -
18675 -pf07883 -
18680 -pf03364 -
18690 -pf13847 -
18695 - -
18700 -pf02602pf00590 -
18705 - -
18710 H_peaSoup CsomeBCy
18715 H_lime CsomeBCy
18720 -pf01625 -
18725 -pf02548 -
18730 -pf07109 -
18735 -pf00224pf02887 -
18740 - -
18745 - -
18750 - -
18755 -pf00005pf00528 -
18760 - -
18770 -pf00999 -
18775 -pf01520pf11741 -
18780 -pf01177 -
(..5435..)
24215 -pf11104 -
24220 -pf05137 -
24225 - -
24230 -pf00263pf03958 -
24240 -pf05421 -
24245 -pf02033 -
24250 -pf18034pf00933 -
24255 - -
24260 -pf02367 -
24265 -pf01555 -
24270 - -
24275 Ts_poisonGreen CsomeBCy
24280 -pf18579pf18578pf18087 -
24285 -pf04230 -
24290 - -
24295 -pf02472 -
24300 -pf01618 -
24305 -pf00950 -
24310 -pf12046 -
24315 - -
24320 -pf07099 -
24325 -pf08417pf00355 -
24330 HMMreg00027_1_CsomeBCy HO
24335 - -
24340 -pf08486 -
BMC-P
BMC-H
BMC-T
Aldehyde dehydrogenase
PTAC
Signature enzyme
Alcohol dehydrogenase
Regulator

Microcystis flos-aquae TF09. Bacteria; Cyanobacteria; Oscillatoriophycideae; Chroococcales;

DWQ54__locus_1_main_sat

CsomeBCy

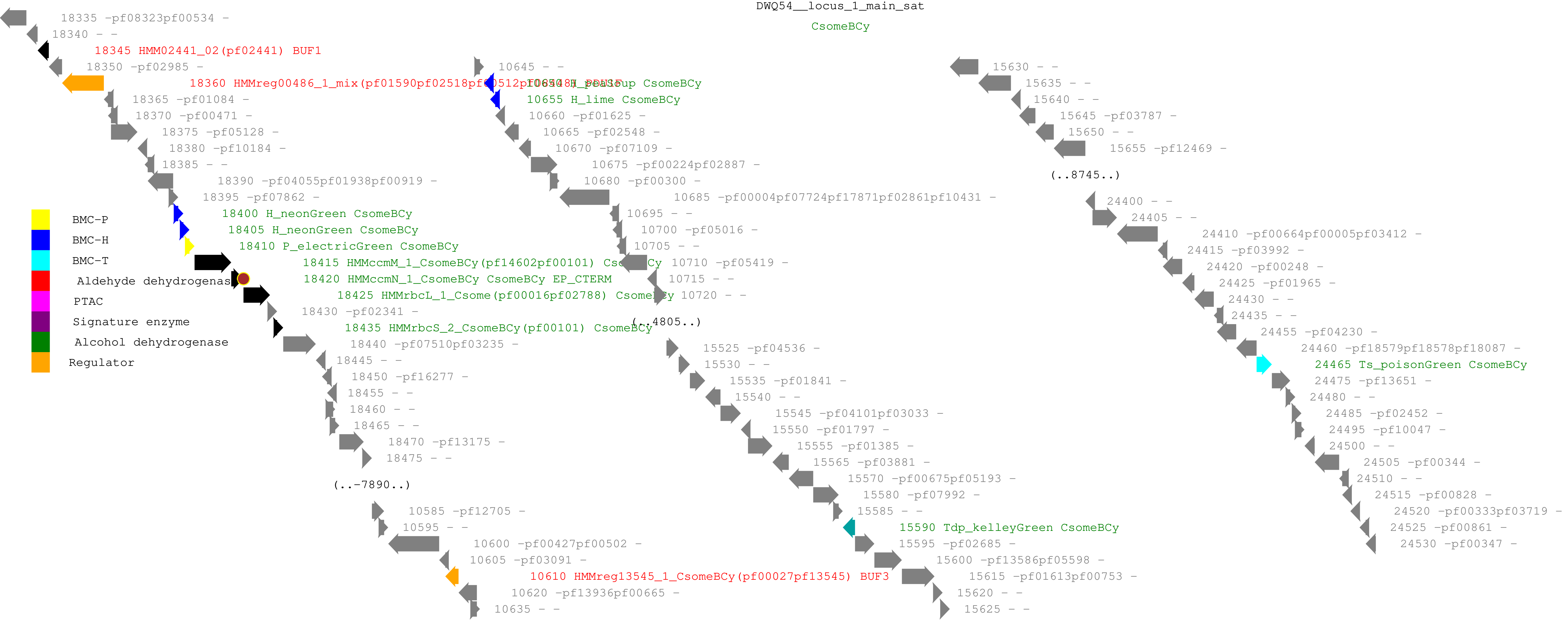

Microcystis aeruginosa DA14. Bacteria; Cyanobacteria; Oscillatoriophycideae; Chroococcales;

DWQ56__locus_1_main_sat

CsomeBCy

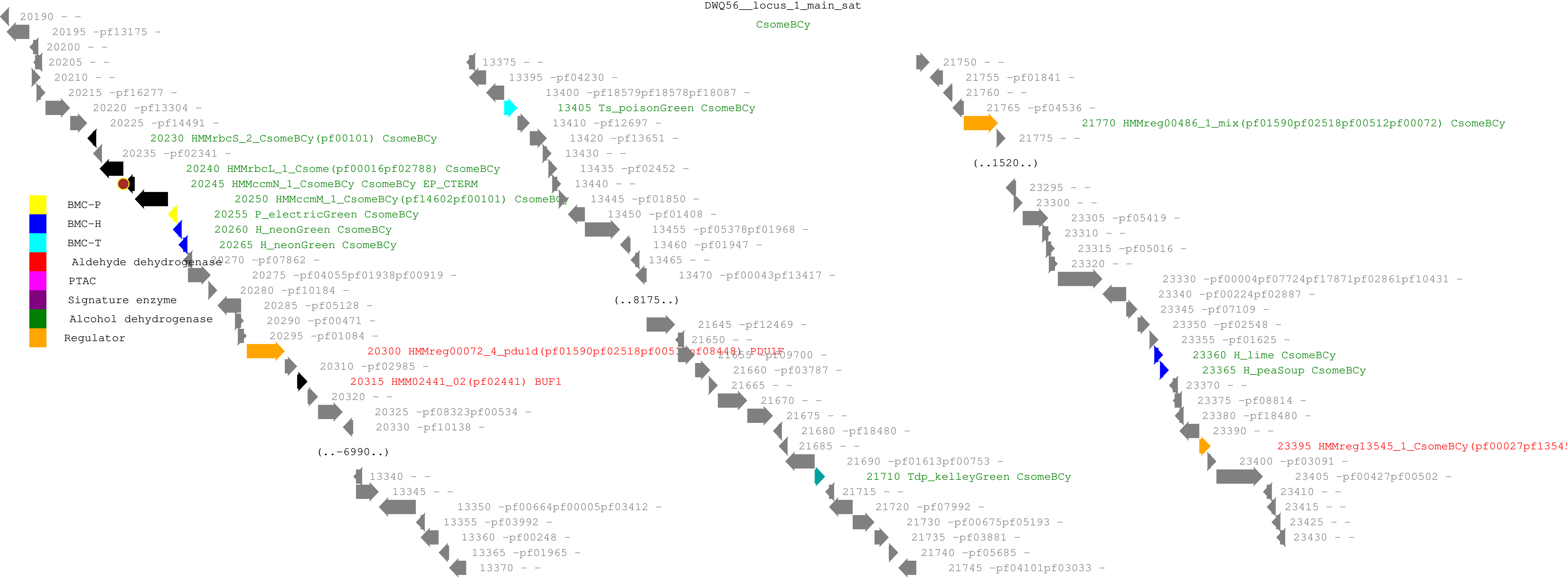

Microcystis aeruginosa TA09. Bacteria; Cyanobacteria; Oscillatoriophycideae; Chroococcales;

DWQ58__locus_1_main_sat

CsomeBCy

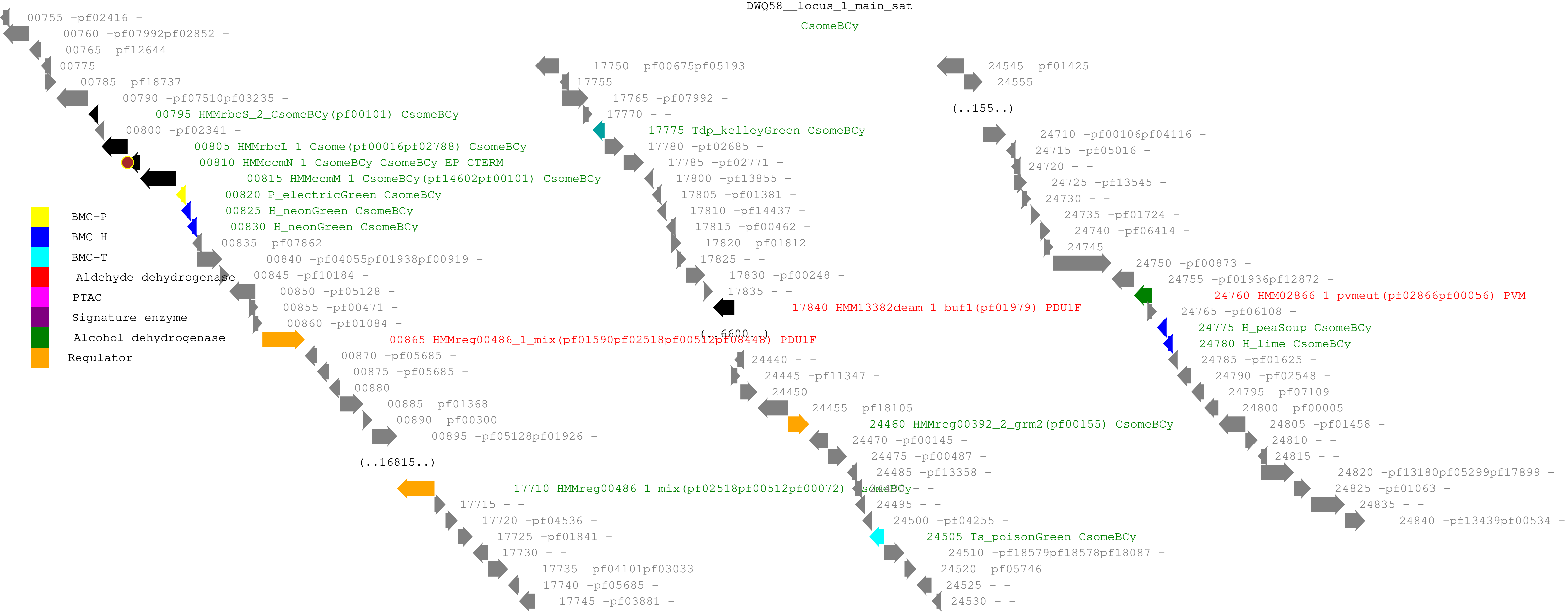

Halomicronema excentricum str. Lakshadweep. Bacteria; Cyanobacteria; Synechococcales; Prochlorotrichaceae;

DYY88__locus_1_main_sat

CsomeBCy

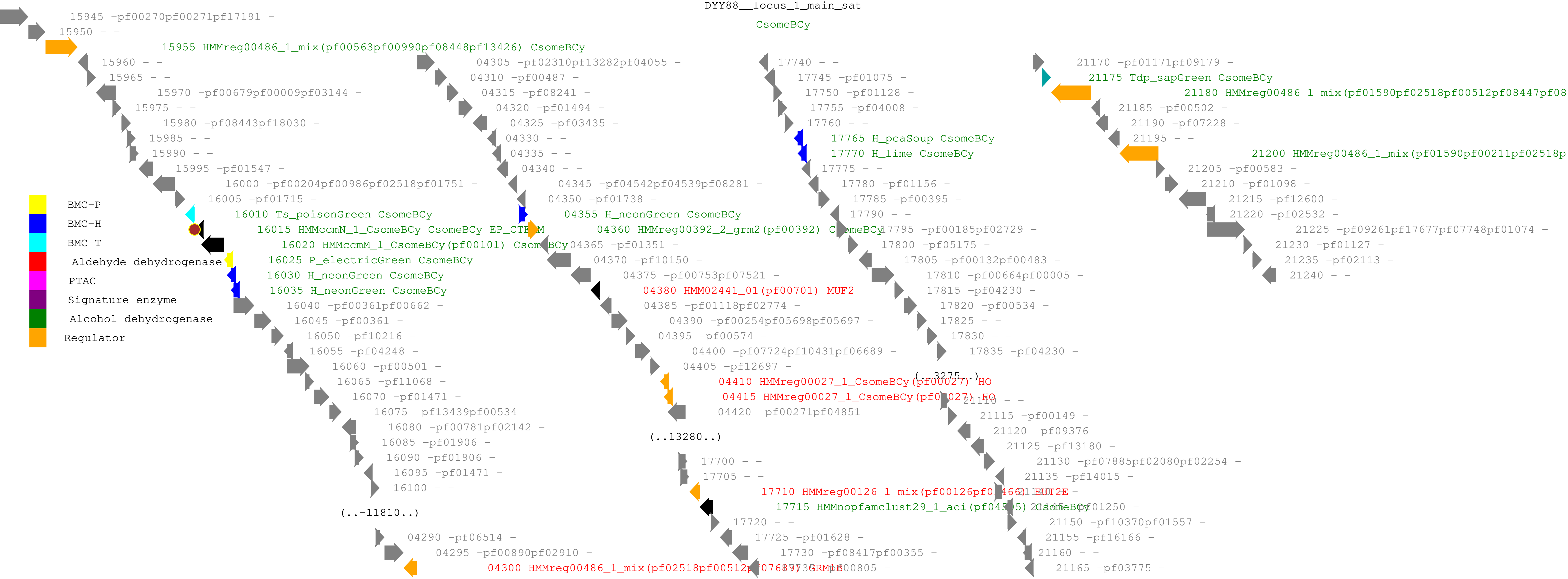

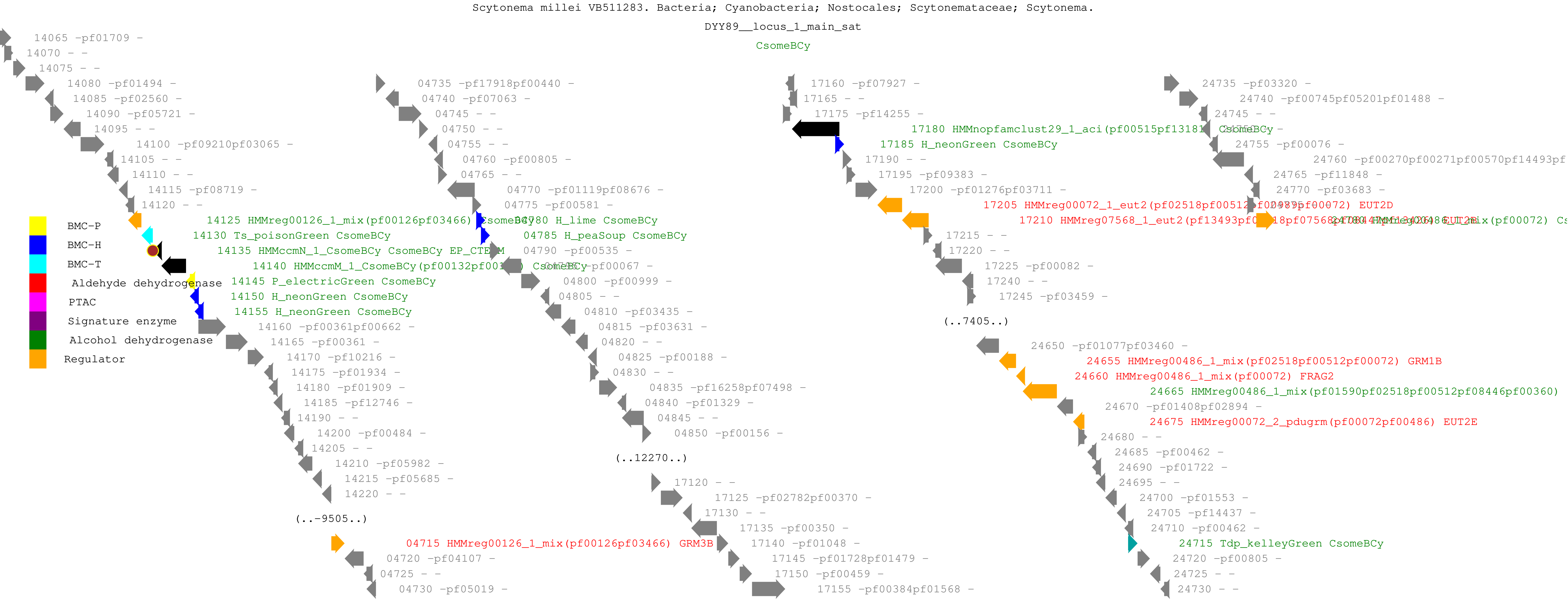

Scytonema millei VB511283. Bacteria; Cyanobacteria; Nostocales; Scytonemataceae; Scytonema.
DYY89__locus_1_main_sat
CsomeBCy
BMC-P
BMC-H
BMC-T
Aldehyde dehydrogenase
PTAC
Signature enzyme
Alcohol dehydrogenase
Regulator
14065 -pf01709 -
14070 - -
14075 - -
14080 -pf01494 -
14085 -pf02560 -
14090 -pf05721 -
14095 - -
14100 -pf09210pf03065 -
14105 - -
14110 - -
14115 -pf08719 -
14120 - -
14125 HMMreg00126_1_mix(pf00126pf03466) CsomeBCy
14130 Ts_poisonGreen CsomeBCy
14135 HMMccmN_1_CsomeBCy CsomeBCy EP_CTE_M
14140 HMMccmM_1_CsomeBCy(pf00132pf001
14145 P_electricGreen CsomeBCy
14150 H_neonGreen CsomeBCy
14155 H_neonGreen CsomeBCy
14160 -pf00361pf00662 -
14165 -pf00361 -
14170 -pf10216 -
14175 -pf01934 -
14180 -pf01909 -
14185 -pf12746 -
14190 - -
14200 -pf00484 -
14205 - -
14210 -pf05982 -
14215 -pf05685 -
14220 - -
(..-9505..)
04715 HMMreg00126_1_mix(pf00126pf03466) GRM3B
04720 -pf04107 -
04725 - -
04730 -pf05019 -
04735 -pf17918pf00440 -
04740 -pf07063 -
04745 - -
04750 - -
04755 - -
04760 -pf00805 -
04765 - -
04770 -pf01119pf08676 -
04775 -pf00581 -
04780 H_lime CsomeBCy
04785 H_peaSoup CsomeBCy
04790 -pf00535 -
) CsomeBCy
-pf00067 -
04800 -pf00999 -
04805 - -
04810 -pf03435 -
04815 -pf03631 -
04820 - -
04825 -pf00188 -
04830 - -
04835 -pf16258pf07498 -
04840 -pf01329 -
04845 - -
04850 -pf00156 -
(..12270..)
17120 - -
17125 -pf02782pf00370 -
17130 - -
17135 -pf00350 -
17140 -pf01048 -
17145 -pf01728pf01479 -
17150 -pf00459 -
17155 -pf00384pf01568 -
17160 -pf07927 -
17165 - -
17175 -pf14255 -
17180 HMMnopfamclust29_1_aci(pf00515pf13181
17185 H_neonGreen CsomeBCy
17190 - -
17195 -pf09383 -
17200 -pf01276pf03711 -
17205 HMMreg00072_1_eut2(pf02518pf00512
pf00072) EUT2D
17210 HMMreg07568_1_eut2(pf13493pf0
8pf07568
17215 - -
17220 - -
17225 -pf00082 -
17240 - -
17245 -pf03459 -
(..7405..)
24650 -pf01077pf03460 -
24655 HMMreg00486_1_mix(pf02518pf00512pf00072) GRM1B
24660 HMMreg00486_1_mix(pf00072) FRAG2
24665 HMMreg00486_1_mix(pf01590pf02518pf00512pf08446pf00360)
24670 -pf01408pf02894 -
24675 HMMreg00072_2_pdugrm(pf00072pf00486) EUT2E
24680 - -
24685 -pf00462 -
24690 -pf01722 -
24695 - -
24700 -pf01553 -
24705 -pf14437 -
24710 -pf00462 -
24715 Tdp_kelleyGreen CsomeBCy
24720 -pf00805 -
24725 - -
24730 - -
24735 -pf03320 -
24740 -pf00745pf05201pf01488 -
24745 - -
CsomeBCy
24755 -pf00076 -
24760 -pf00270pf00271pf00570pf14493pf
24765 -pf11848 -
24770 -pf03683 -
EUT2E
mix(pf00072) Cs

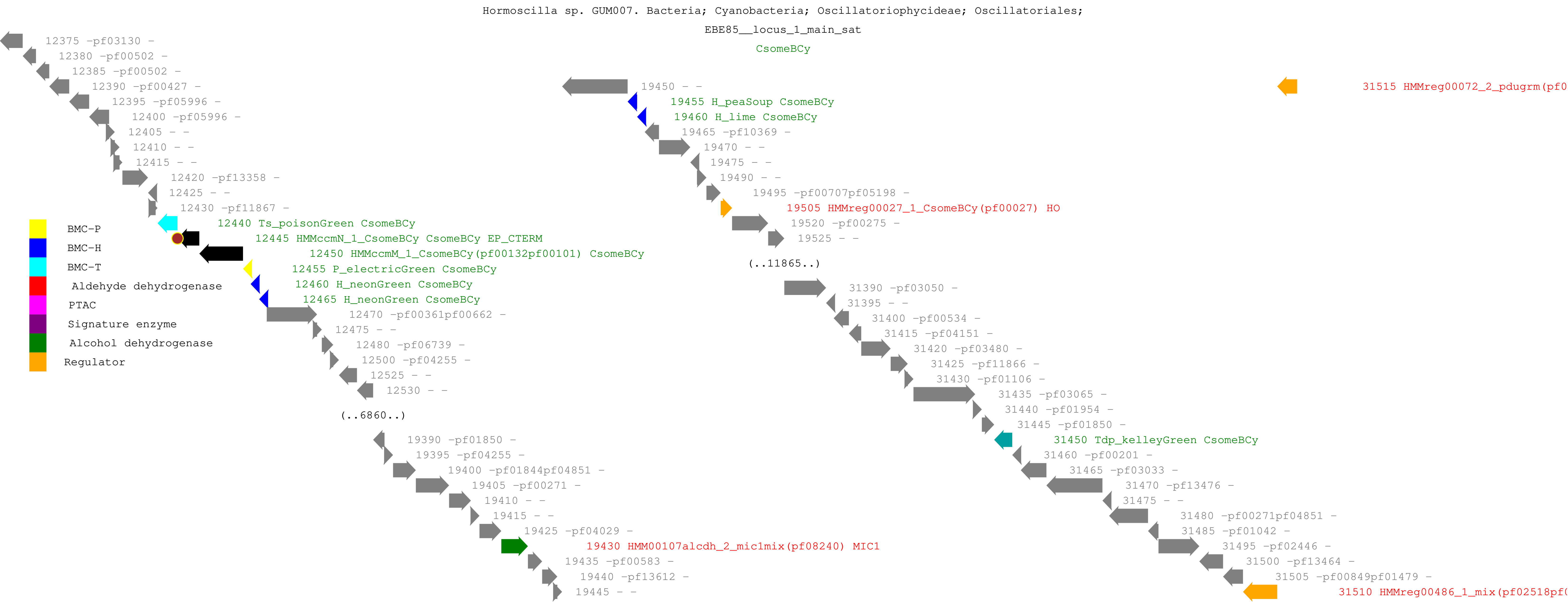
Hormoscilla sp. GUM007. Bacteria; Cyanobacteria; Oscillatoriophycideae; Oscillatoriales;
EBE85__locus_1_main_sat
CsomeBCy
12375 -pf03130 -
12380 -pf00502 -
12385 -pf00502 -
12390 -pf00427 -
12395 -pf05996 -
12400 -pf05996 -
12405 - -
12410 - -
12415 - -
12420 -pf13358 -
12425 - -
12430 -pf11867 -
12440 Ts_poisonGreen CsomeBCy
12445 HMMccmN_1_CsomeBCy CsomeBCy EP_CTERM
12450 HMMccmM_1_CsomeBCy(pf00132pf00101) CsomeBCy
12455 P_electricGreen CsomeBCy
12460 H_neonGreen CsomeBCy
12465 H_neonGreen CsomeBCy
12470 -pf00361pf00662 -
12475 - -
12480 -pf06739 -
12500 -pf04255 -
12525 - -
12530 - -
(..6860..)
19390 -pf01850 -
19395 -pf04255 -
19400 -pf01844pf04851 -
19405 -pf00271 -
19410 - -
19415 - -
19425 -pf04029 -
19430 HMM00107alcdh_2_mic1mix(pf08240) MIC1
19435 -pf00583 -
19440 -pf13612 -
19445 - -
19450 - -
19455 H_peaSoup CsomeBCy
19460 H_lime CsomeBCy
19465 -pf10369 -
19470 - -
19475 - -
19490 - -
19495 -pf00707pf05198 -
19505 HMMreg00027_1_CsomeBCy(pf00027) HO
19520 -pf00275 -
19525 - -
(..11865..)
31390 -pf03050 -
31395 - -
31400 -pf00534 -
31415 -pf04151 -
31420 -pf03480 -
31425 -pf11866 -
31430 -pf01106 -
31435 -pf03065 -
31440 -pf01954 -
31445 -pf01850 -
31450 Tdp_kelleyGreen CsomeBCy
31460 -pf00201 -
31465 -pf03033 -
31470 -pf13476 -
31475 - -
31480 -pf00271pf04851 -
31485 -pf01042 -
31495 -pf02446 -
31500 -pf13464 -
31505 -pf00849pf01479 -
31510 HMMreg00486_1_mix(pf02518pf
31515 HMMreg00072_2_pdugrm(pf0
BMC-P
BMC-H
BMC-T
Aldehyde dehydrogenase
PTAC
Signature enzyme
Alcohol dehydrogenase
Regulator

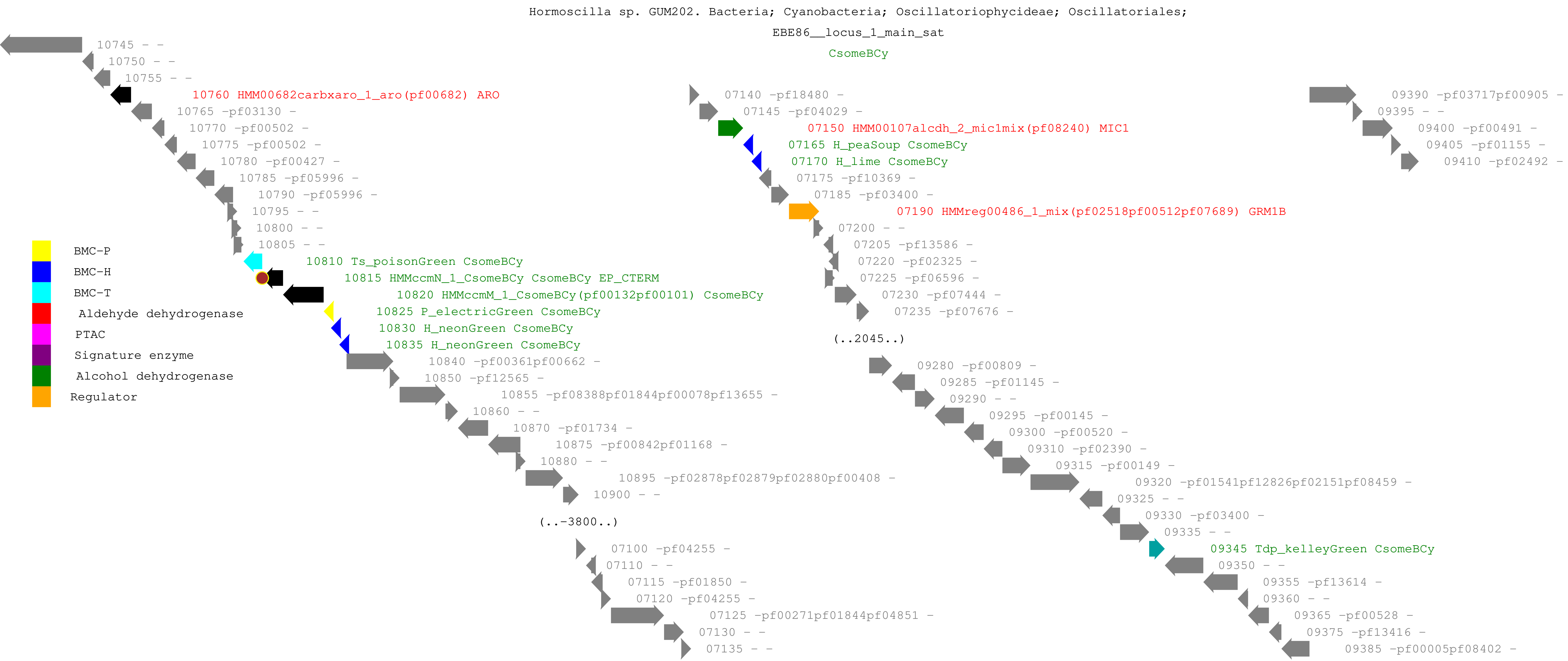
Hormoscilla sp. GUM202. Bacteria; Cyanobacteria; Oscillatoriophycideae; Oscillatoriales;
EBE86__locus_1_main_sat
CsomeBCy
10745 - -
10750 - -
10755 - -
10760 HMM00682carbxaro_1_aro(pf00682) ARO
10765 -pf03130 -
10770 -pf00502 -
10775 -pf00502 -
10780 -pf00427 -
10785 -pf05996 -
10790 -pf05996 -
10795 - -
10800 - -
10805 - -
10810 Ts_poisonGreen CsomeBCy
10815 HMMccmN_1_CsomeBCy CsomeBCy EP_CTERM
10820 HMMccmM_1_CsomeBCy(pf00132pf00101) CsomeBCy
10825 P_electricGreen CsomeBCy
10830 H_neonGreen CsomeBCy
10835 H_neonGreen CsomeBCy
10840 -pf00361pf00662 -
10850 -pf12565 -
10855 -pf08388pf01844pf00078pf13655 -
10860 - -
10870 -pf01734 -
10875 -pf00842pf01168 -
10880 - -
10895 -pf02878pf02879pf02880pf00408 -
10900 - -
(..-3800..)
07100 -pf04255 -
07110 - -
07115 -pf01850 -
07120 -pf04255 -
07125 -pf00271pf01844pf04851 -
07130 - -
07135 - -
07140 -pf18480 -
07145 -pf04029 -
07150 HMM00107alcdh_2_mic1mix(pf08240) MIC1
07165 H_peaSoup CsomeBCy
07170 H_lime CsomeBCy
07175 -pf10369 -
07185 -pf03400 -
07190 HMMreg00486_1_mix(pf02518pf00512pf07689) GRM1B
07200 - -
07205 -pf13586 -
07220 -pf02325 -
07225 -pf06596 -
07230 -pf07444 -
07235 -pf07676 -
(..2045..)
09280 -pf00809 -
09285 -pf01145 -
09290 - -
09295 -pf00145 -
09300 -pf00520 -
09310 -pf02390 -
09315 -pf00149 -
09320 -pf01541pf12826pf02151pf08459 -
09325 - -
09330 -pf03400 -
09335 - -
09345 Tdp_kelleyGreen CsomeBCy
09350 - -
09355 -pf13614 -
09360 - -
09365 -pf00528 -
09375 -pf13416 -
09385 -pf00005pf08402 -
09390 -pf03717pf00905 -
09395 - -
09400 -pf00491 -
09405 -pf01155 -
09410 -pf02492 -
BMC-P
BMC-H
BMC-T
Aldehyde dehydrogenase
PTAC
Signature enzyme
Alcohol dehydrogenase
Regulator

Microcystis aeruginosa FACHB-524. Bacteria; Cyanobacteria; Oscillatoriophycideae; Chroococcales;

ED562__locus_1_main_sat

CsomeBCy

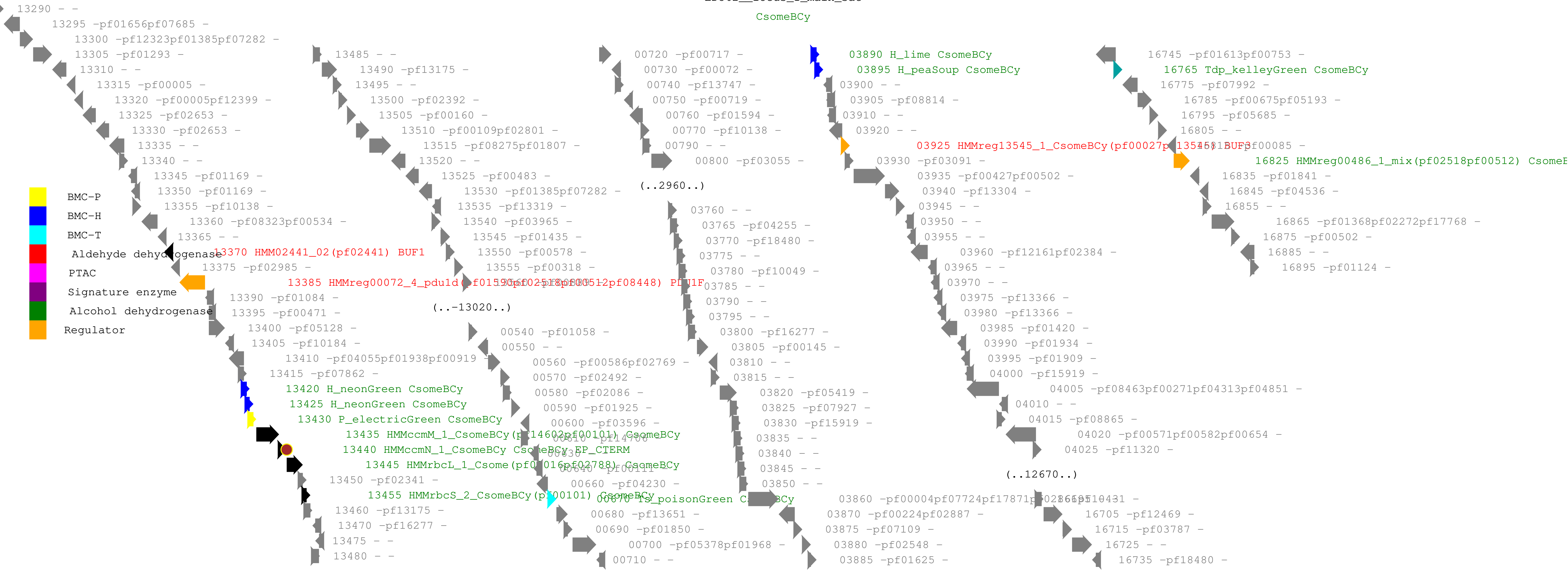

Leptolyngbya sp. IPPAS B-1204. Bacteria; Cyanobacteria; Synechococcales; Leptolyngbyaceae; Leptolyngbya.

EDM05__locus_1_main_sat

CsomeBCy

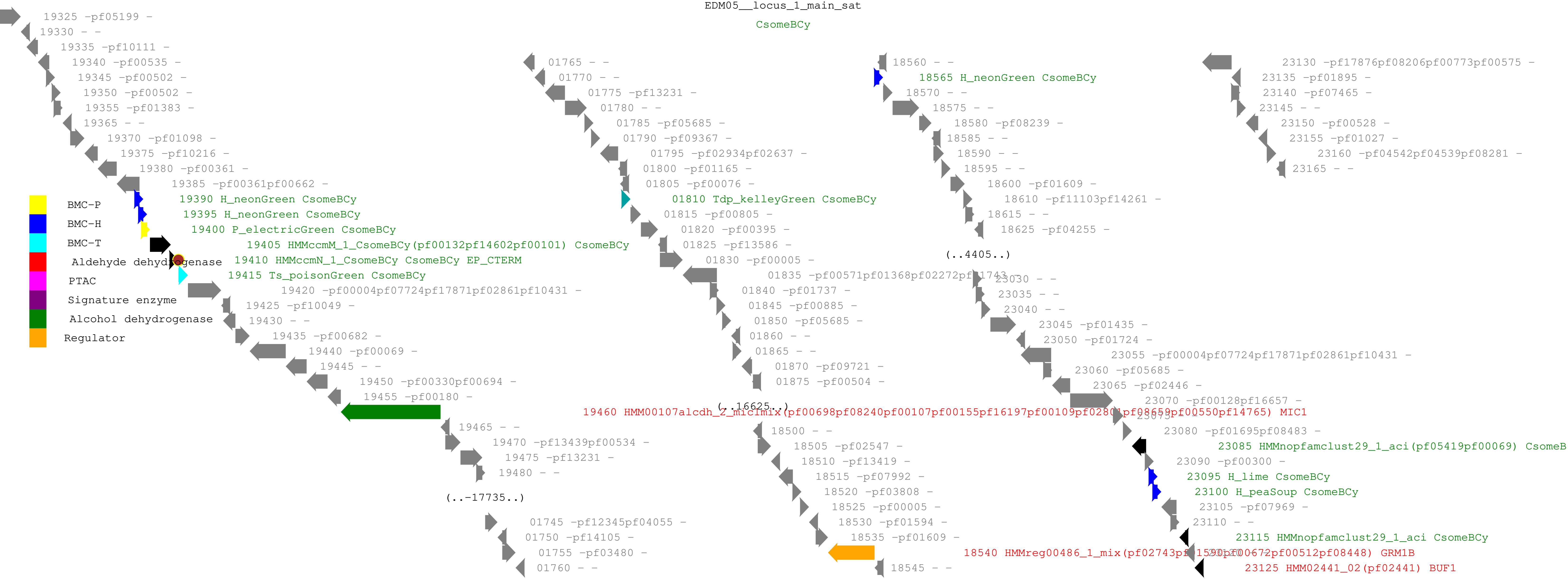

Chroococcales cyanobacterium metabat2.561. Bacteria; Cyanobacteria; Oscillatoriophycideae; Chroococcales.

EHM73__locus_fragmented

CsomeBCy

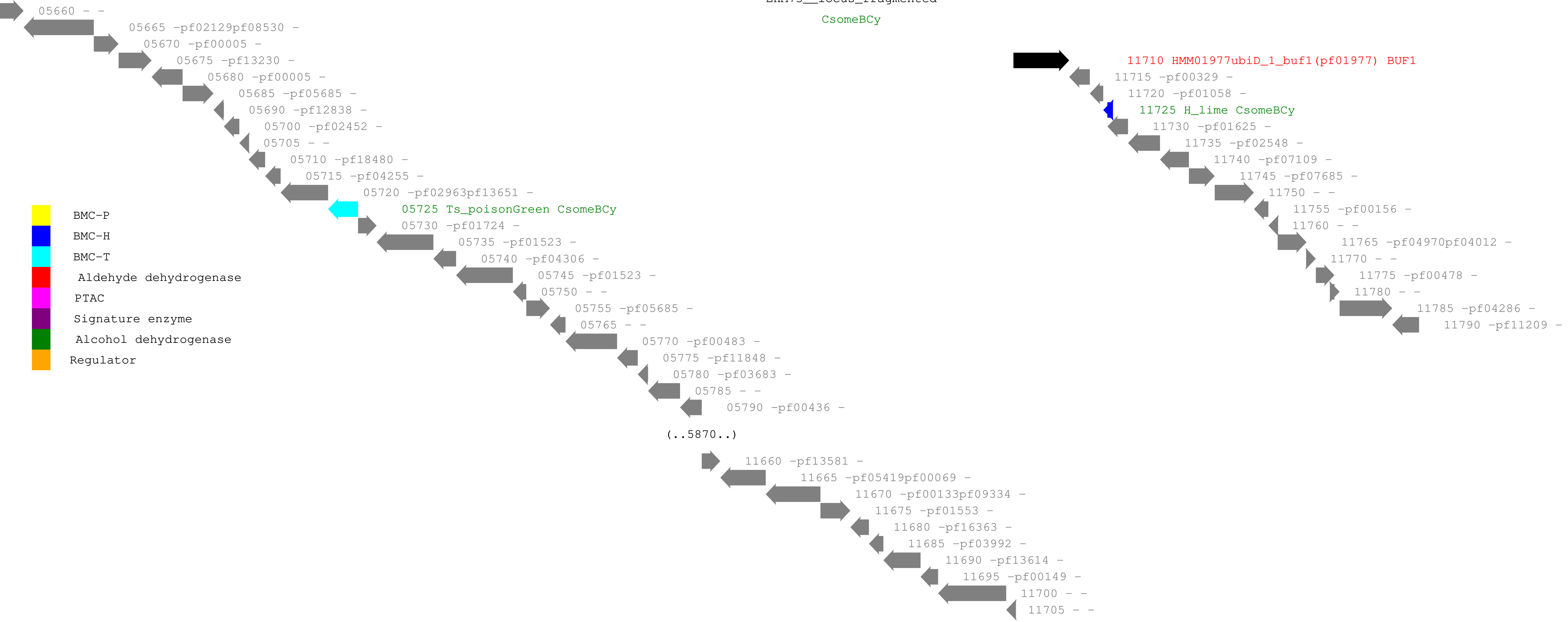

Microcystis flos-aquae Mf_WU_F_19750830_S460. Bacteria; Cyanobacteria; Oscillatoriophycideae; Chroococcales;

EWV40__locus_1_main_sat

CsomeBCy

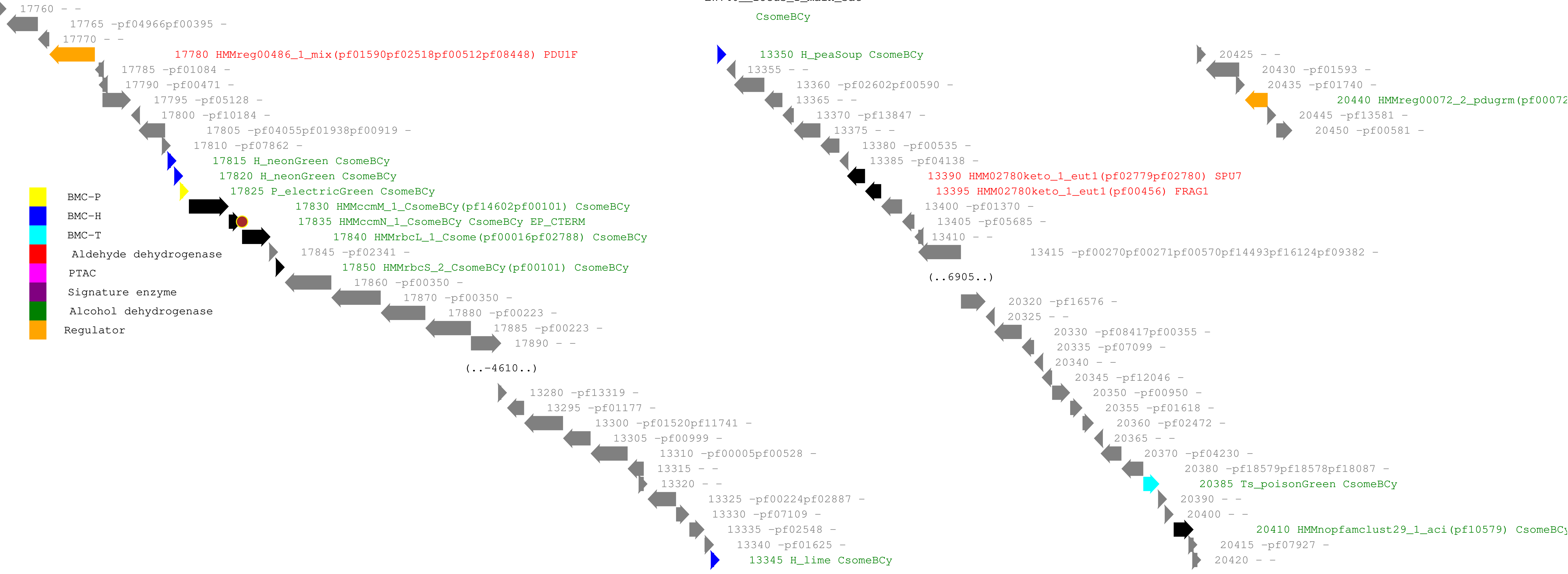

Microcystis flos-aquae Mf_QC_C_20070823_S10D. Bacteria; Cyanobacteria; Oscillatoriophycideae; Chroococcales;

EWV45__locus_1_main_sat

CsomeBCy

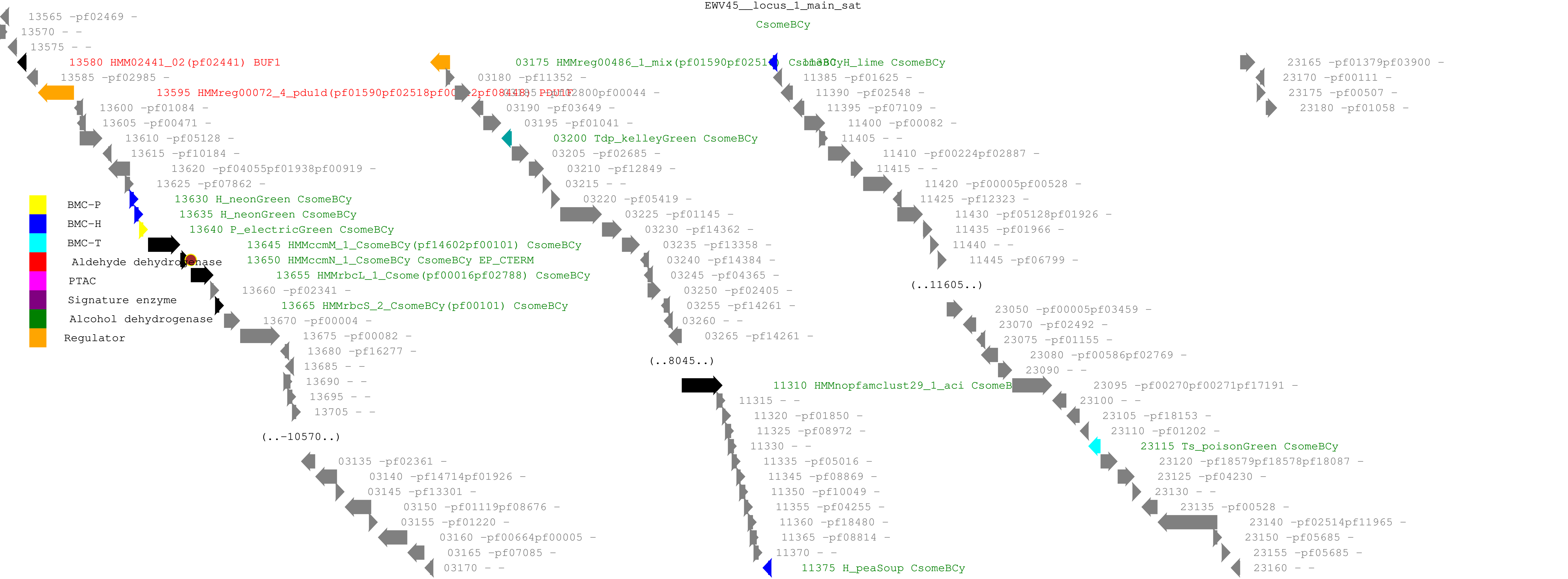

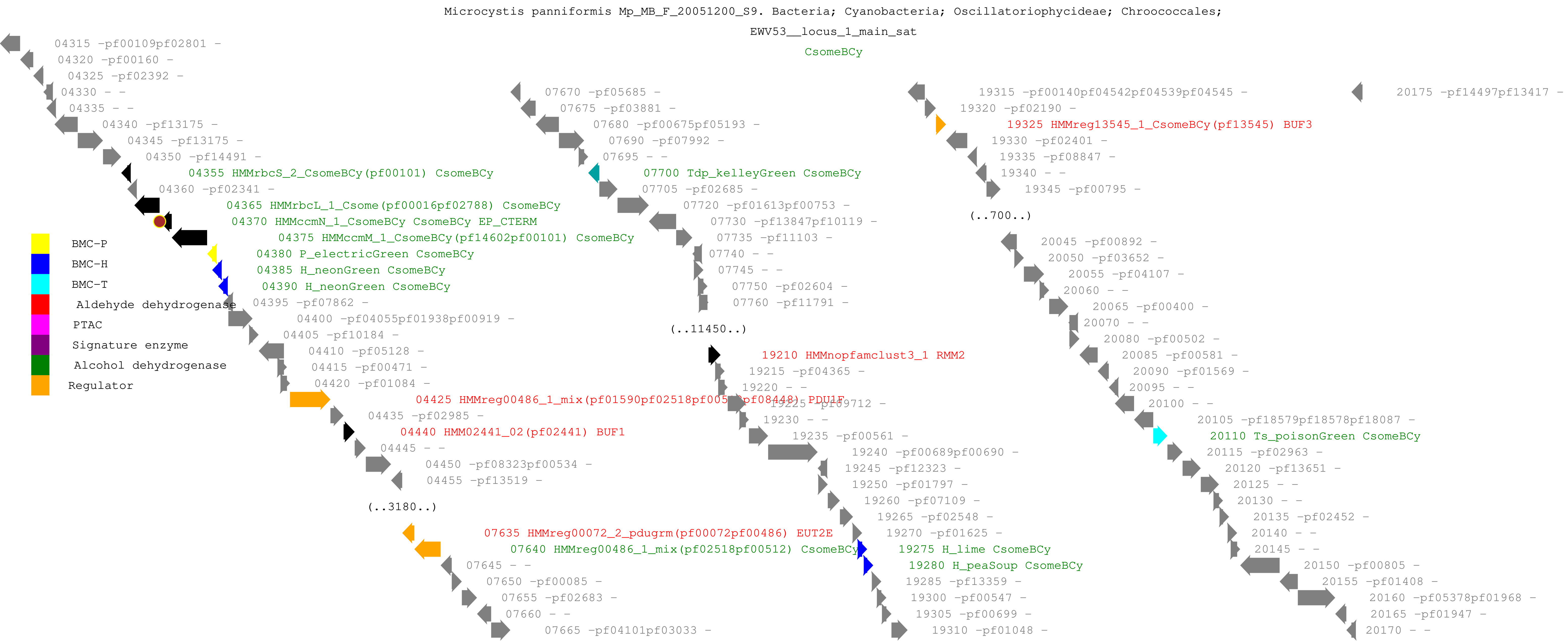
Microcystis panniformis Mp_MB_F_20051200_S9. Bacteria; Cyanobacteria; Oscillatoriophycideae; Chroococcales;
EWV53__locus_1_main_sat
CsomeBCy
04315 -pf00109pf02801 -
04320 -pf00160 -
04325 -pf02392 -
04330 - -
04335 - -
04340 -pf13175 -
04345 -pf13175 -
04350 -pf14491 -
04355 HMMrbcS_2_CsomeBCy(pf00101) CsomeBCy
04360 -pf02341 -
04365 HMMrbcL_1_Csome(pf00016pf02788) CsomeBCy
04370 HMMccmN_1_CsomeBCy CsomeBCy EP_CTERM
04375 HMMccmM_1_CsomeBCy(pf14602pf00101) CsomeBCy
04380 P_electricGreen CsomeBCy
04385 H_neonGreen CsomeBCy
04390 H_neonGreen CsomeBCy
04395 -pf07862 -
04400 -pf04055pf01938pf00919 -
04405 -pf10184 -
04410 -pf05128 -
04415 -pf00471 -
04420 -pf01084 -
04425 HMMreg00486_1_mix(pf01590pf02518pf005 pf08448) PDU1F
04435 -pf02985 -
04440 HMM02441_02(pf02441) BUF1
04445 - -
04450 -pf08323pf00534 -
04455 -pf13519 -
(..3180..)
07635 HMMreg00072_2_pdugrm(pf00072pf00486) EUT2E
07640 HMMreg00486_1_mix(pf02518pf00512) CsomeBCy
07645 - -
07650 -pf00085 -
07655 -pf02683 -
07660 - -
07665 -pf04101pf03033 -
07670 -pf05685 -
07675 -pf03881 -
07680 -pf00675pf05193 -
07690 -pf07992 -
07695 - -
07700 Tdp_kelleyGreen CsomeBCy
07705 -pf02685 -
07720 -pf01613pf00753 -
07730 -pf13847pf10119 -
07735 -pf11103 -
07740 - -
07745 - -
07750 -pf02604 -
07760 -pf11791 -
(..11450..)
19210 HMMnopfamclust3_1 RMM2
19215 -pf04365 -
19220 - -
19225 -pf09712 -
19230 - -
19235 -pf00561 -
19240 -pf00689pf00690 -
19245 -pf12323 -
19250 -pf01797 -
19260 -pf07109 -
19265 -pf02548 -
19270 -pf01625 -
19275 H_lime CsomeBCy
19280 H_peaSoup CsomeBCy
19285 -pf13359 -
19300 -pf00547 -
19305 -pf00699 -
19310 -pf01048 -
19315 -pf00140pf04542pf04539pf04545 -
19320 -pf02190 -
19325 HMMreg13545_1_CsomeBCy(pf13545) BUF3
19330 -pf02401 -
19335 -pf08847 -
19340 - -
19345 -pf00795 -
(..700..)
20045 -pf00892 -
20050 -pf03652 -
20055 -pf04107 -
20060 - -
20065 -pf00400 -
20070 - -
20080 -pf00502 -
20085 -pf00581 -
20090 -pf01569 -
20095 - -
20100 - -
20105 -pf18579pf18578pf18087 -
20110 Ts_poisonGreen CsomeBCy
20115 -pf02963 -
20120 -pf13651 -
20125 - -
20130 - -
20135 -pf02452 -
20140 - -
20145 - -
20150 -pf00805 -
20155 -pf01408 -
20160 -pf05378pf01968 -
20165 -pf01947 -
20170 - -
20175 -pf14497pf13417 -
BMC-P
BMC-H
BMC-T
Aldehyde dehydrogenase
PTAC
Signature enzyme
Alcohol dehydrogenase
Regulator

Microcystis novacekii Mn_MB_F_20050700_S1D. Bacteria; Cyanobacteria; Oscillatoriophycideae; Chroococcales;

EWV54__locus_1_main_sat

CsomeBCy

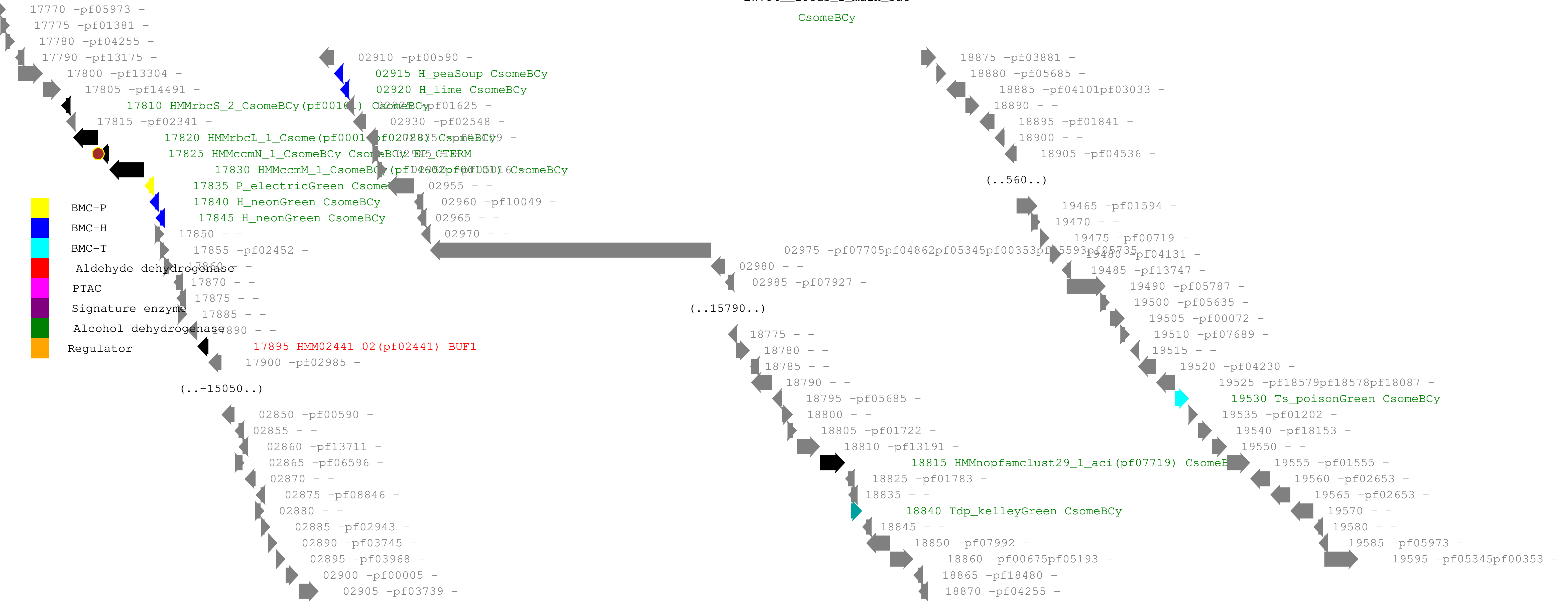

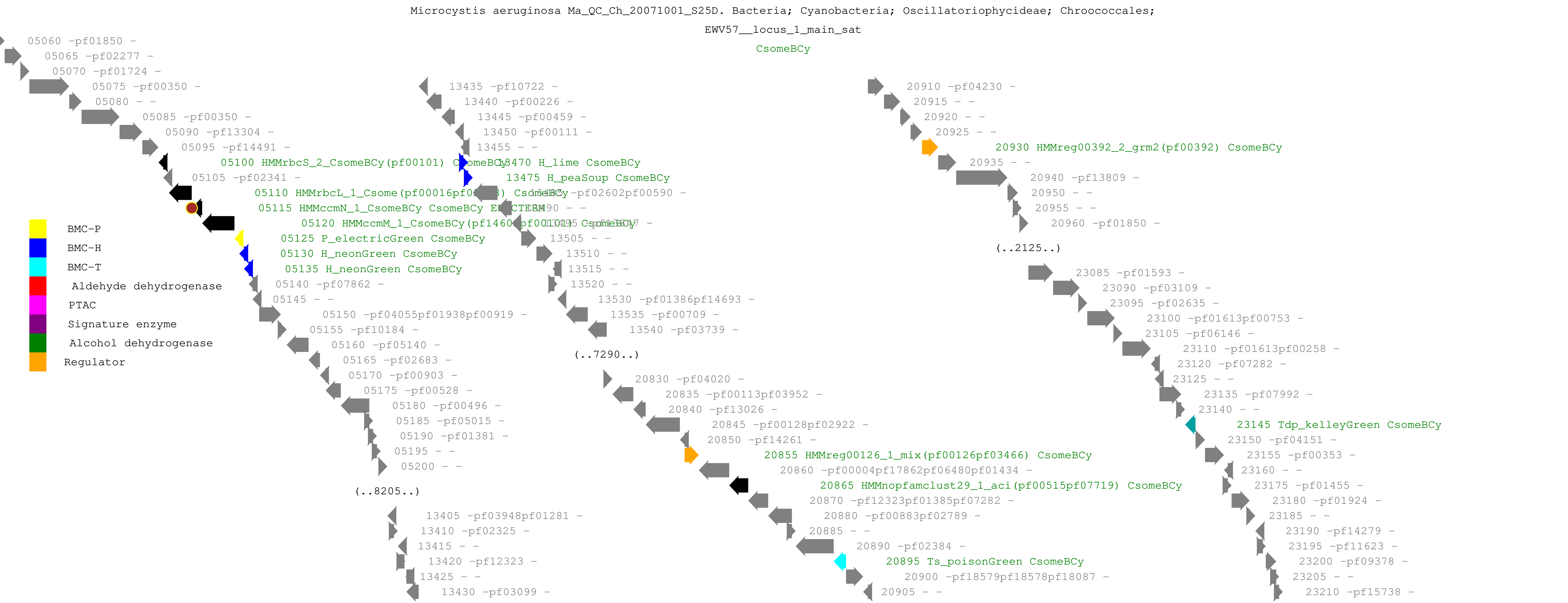
Microcystis aeruginosa Ma_QC_Ch_20071001_S25D. Bacteria; Cyanobacteria; Oscillatoriophycideae; Chroococcales;
EWV57__locus_1_main_sat
CsomeBCy
BMC-P
BMC-H
BMC-T
Aldehyde dehydrogenase
PTAC
Signature enzyme
Alcohol dehydrogenase
Regulator
05060 -pf01850 -
05065 -pf02277 -
05070 -pf01724 -
05075 -pf00350 -
05080 - -
05085 -pf00350 -
05090 -pf13304 -
05095 -pf14491 -
05100 HMMrbcS_2_CsomeBCy(pf00101) CsomeBCy
05105 -pf02341 -
05110 HMMrbcL_1_Csome(pf00016pf0[illegible]) CsomeBCy
05115 HMMccmN_1_CsomeBCy CsomeBCy EPCTERM
05120 HMMccmM_1_CsomeBCy(pf1460[illegible]pf00132) CsomeBCy
05125 P_electricGreen CsomeBCy
05130 H_neonGreen CsomeBCy
05135 H_neonGreen CsomeBCy
05140 -pf07862 -
05145 - -
05150 -pf04055pf01938pf00919 -
05155 -pf10184 -
05160 -pf05140 -
05165 -pf02683 -
05170 -pf00903 -
05175 -pf00528 -
05180 -pf00496 -
05185 -pf05015 -
05190 -pf01381 -
05195 - -
05200 - -
(..8205..)
13405 -pf03948pf01281 -
13410 -pf02325 -
13415 - -
13420 -pf12323 -
13425 - -
13430 -pf03099 -
13435 -pf10722 -
13440 -pf00226 -
13445 -pf00459 -
13450 -pf00111 -
13455 - -
13470 H_lime CsomeBCy
13475 H_peaSoup CsomeBCy
13480 -pf02602pf00590 -
13490 - -
13495 -pf13847 -
13505 - -
13510 - -
13515 - -
13520 - -
13530 -pf01386pf14693 -
13535 -pf00709 -
13540 -pf03739 -
(..7290..)
20830 -pf04020 -
20835 -pf00113pf03952 -
20840 -pf13026 -
20845 -pf00128pf02922 -
20850 -pf14261 -
20855 HMMreg00126_1_mix(pf00126pf03466) CsomeBCy
20860 -pf00004pf17862pf06480pf01434 -
20865 HMMnopfamclust29_1_aci(pf00515pf07719) CsomeBCy
20870 -pf12323pf01385pf07282 -
20880 -pf00883pf02789 -
20885 - -
20890 -pf02384 -
20895 Ts_poisonGreen CsomeBCy
20900 -pf18579pf18578pf18087 -
20905 - -
20910 -pf04230 -
20915 - -
20920 - -
20925 - -
20930 HMMreg00392_2_grm2(pf00392) CsomeBCy
20935 - -
20940 -pf13809 -
20950 - -
20955 - -
20960 -pf01850 -
(..2125..)
23085 -pf01593 -
23090 -pf03109 -
23095 -pf02635 -
23100 -pf01613pf00753 -
23105 -pf06146 -
23110 -pf01613pf00258 -
23120 -pf07282 -
23125 - -
23135 -pf07992 -
23140 - -
23145 Tdp_kelleyGreen CsomeBCy
23150 -pf04151 -
23155 -pf00353 -
23160 - -
23175 -pf01455 -
23180 -pf01924 -
23185 - -
23190 -pf14279 -
23195 -pf11623 -
23200 -pf09378 -
23205 - -
23210 -pf15738 -

Microcystis sp. Msp_OC_L_20101000_S702. Bacteria; Cyanobacteria; Oscillatoriophycideae; Chroococcales;

EWV60__locus_1_main_sat

CsomeBCy

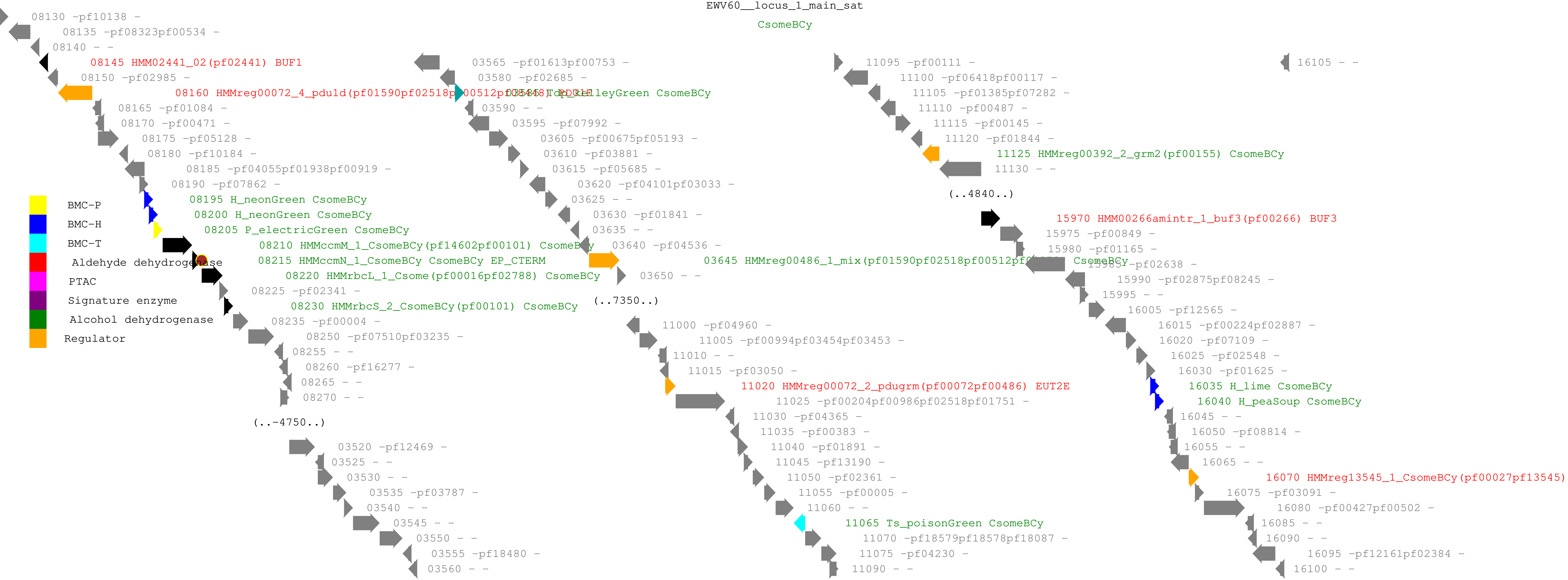

Microcystis aeruginosa Ma_OC_LR_19540900_S633. Bacteria; Cyanobacteria; Oscillatoriophycideae; Chroococcales;

EWV62__locus_1_main_sat

CsomeBCy

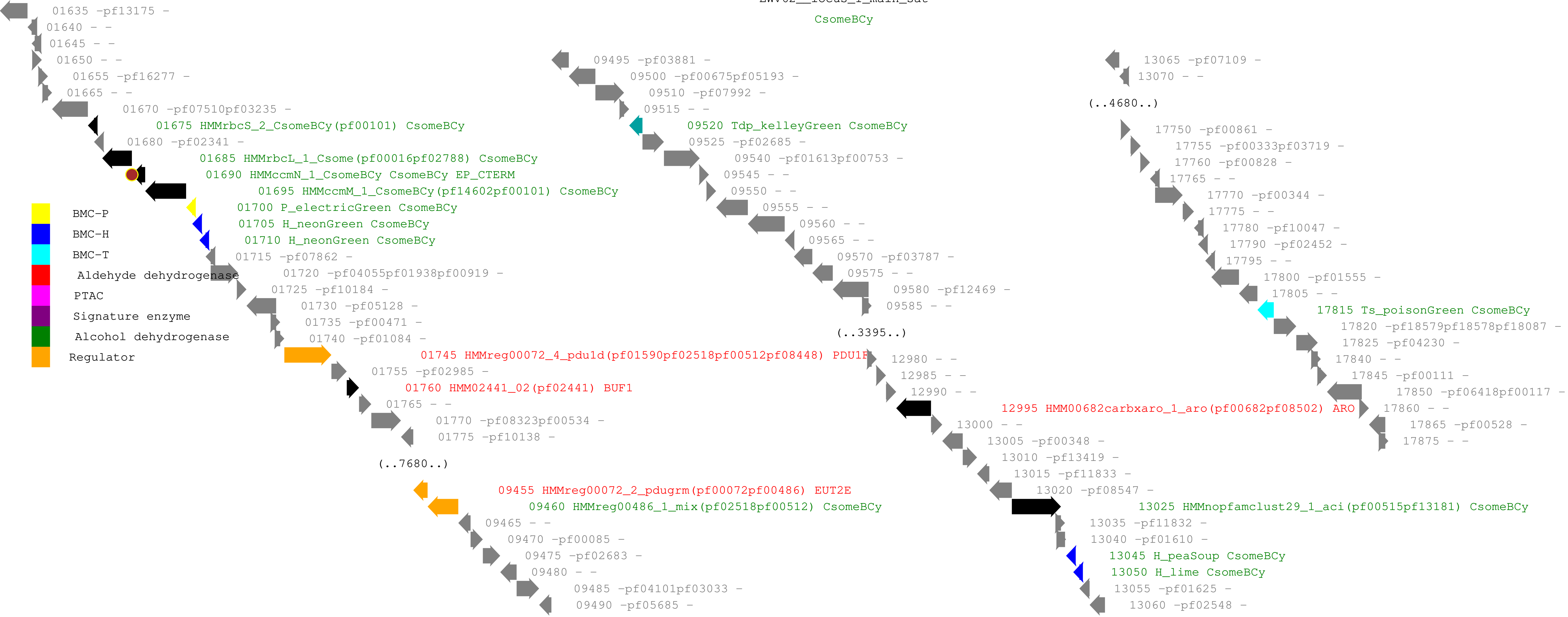

Microcystis aeruginosa Ma_OC_H_19870700_S124. Bacteria; Cyanobacteria; Oscillatoriophycideae; Chroococcales;

EWV63__locus_1_main_sat

CsomeBCy

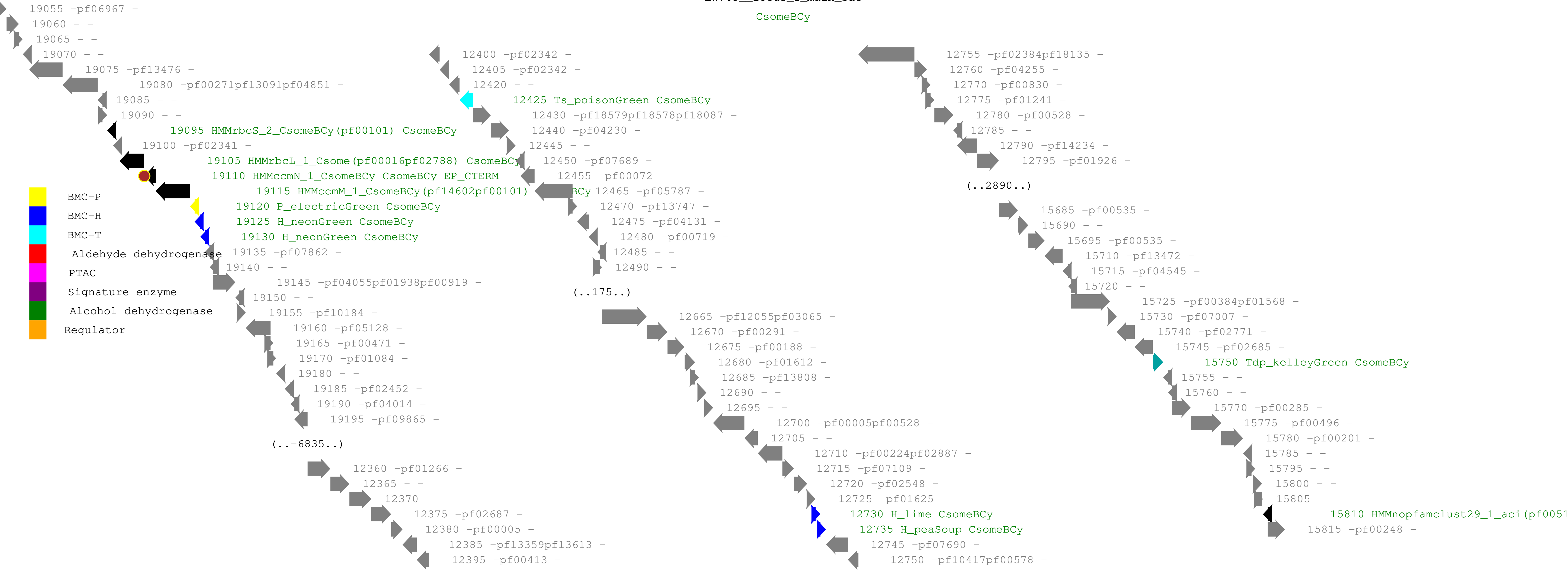

Microcystis sp. M_OC_Ca_00000000_C217Col. Bacteria; Cyanobacteria; Oscillatoriophycideae; Chroococcales;

EWV66__locus_1_main_sat

CsomeBCy

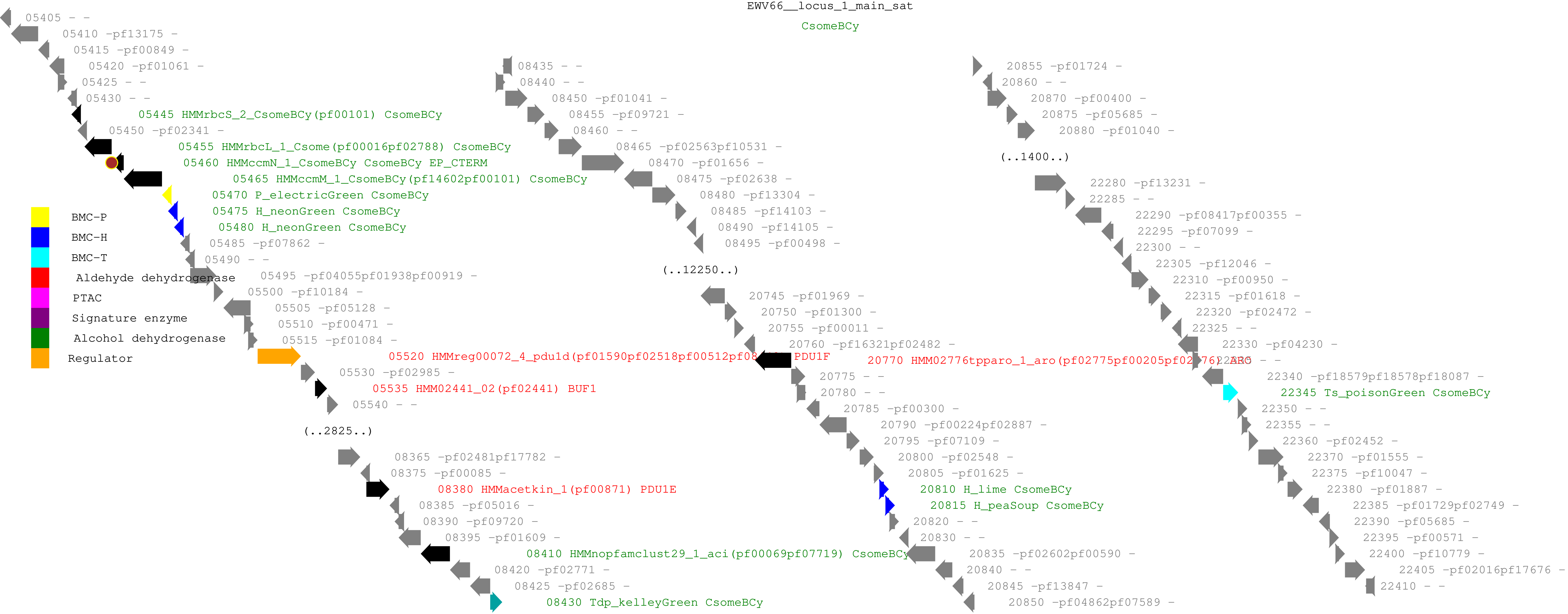

Microcystis sp. M_QC_C_20170808_M9Col. Bacteria; Cyanobacteria; Oscillatoriophycideae; Chroococcales;

EWV68__locus_1_main_sat

CsomeBCy

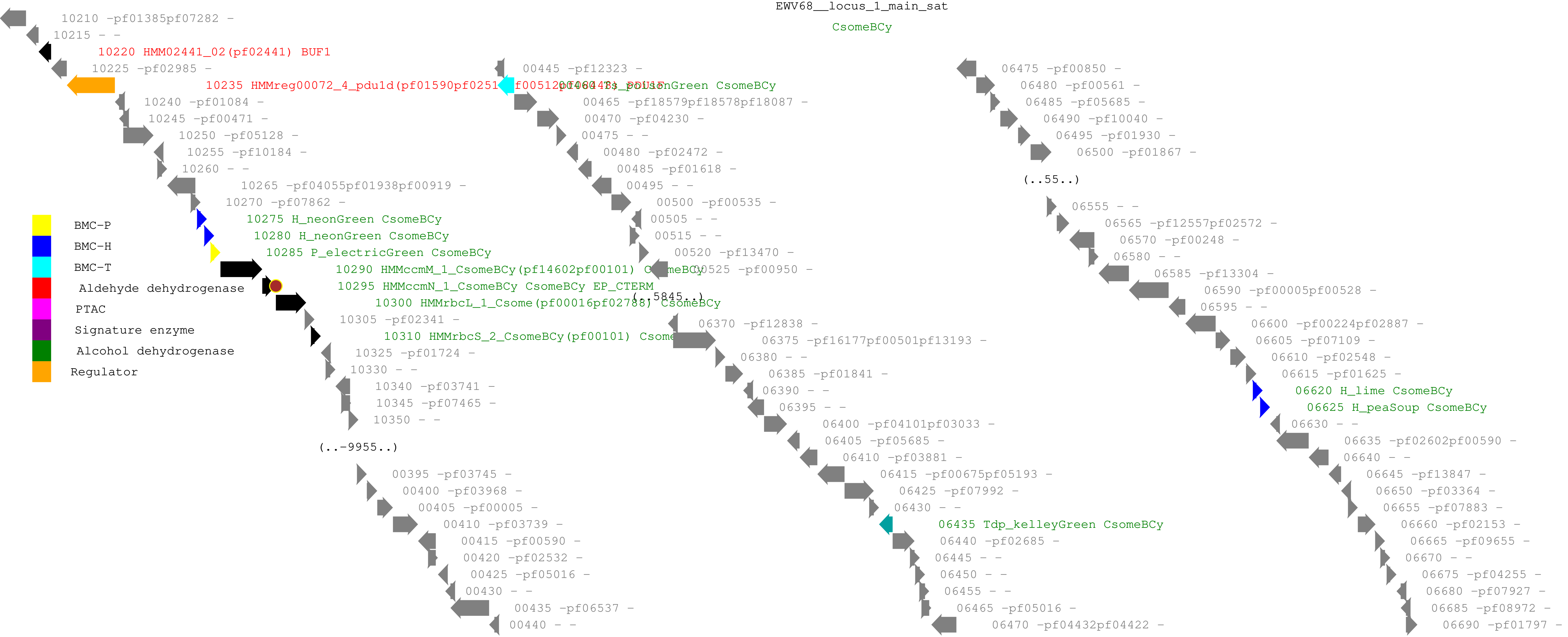

Microcystis wesenbergii Mw_QC_S_20081001_S30D. Bacteria; Cyanobacteria; Oscillatoriophycideae; Chroococcales;

EWV75__locus_1_main_sat

CsomeBCy

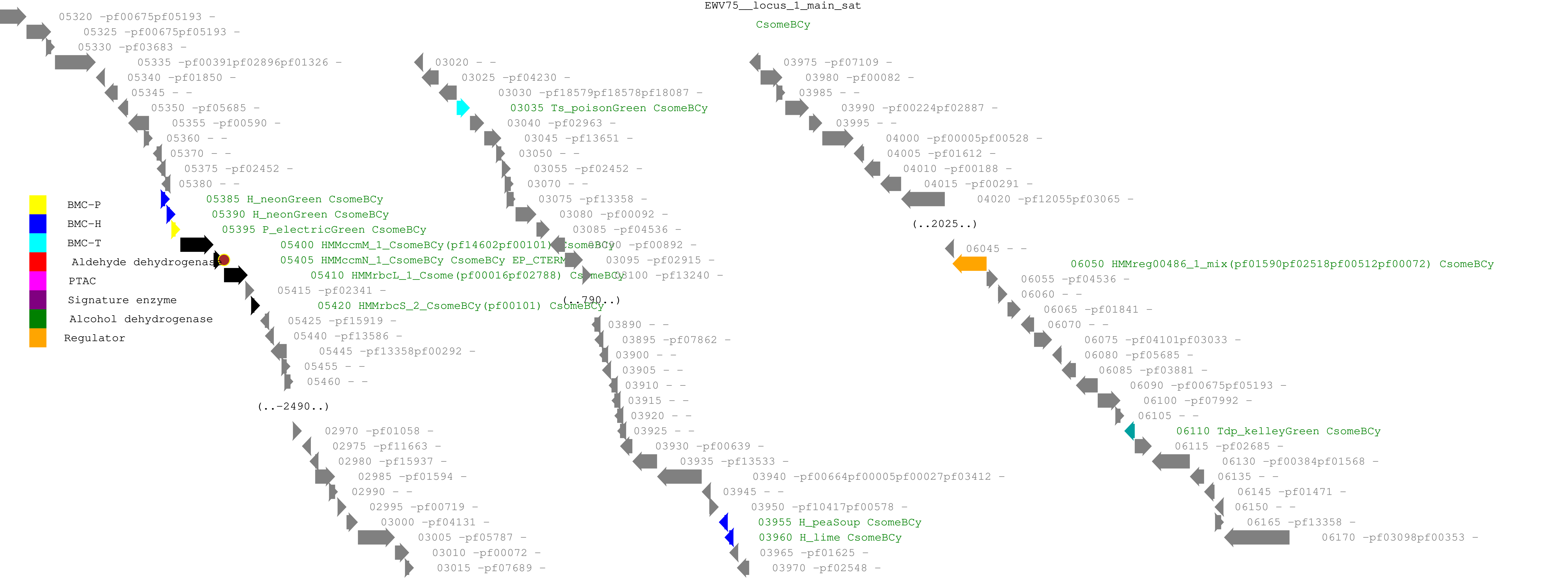

Microcystis viridis Mv_BB_P_19951000_S68D. Bacteria; Cyanobacteria; Oscillatoriophycideae; Chroococcales;

EWV77__locus_1_main_sat

CsomeBCy

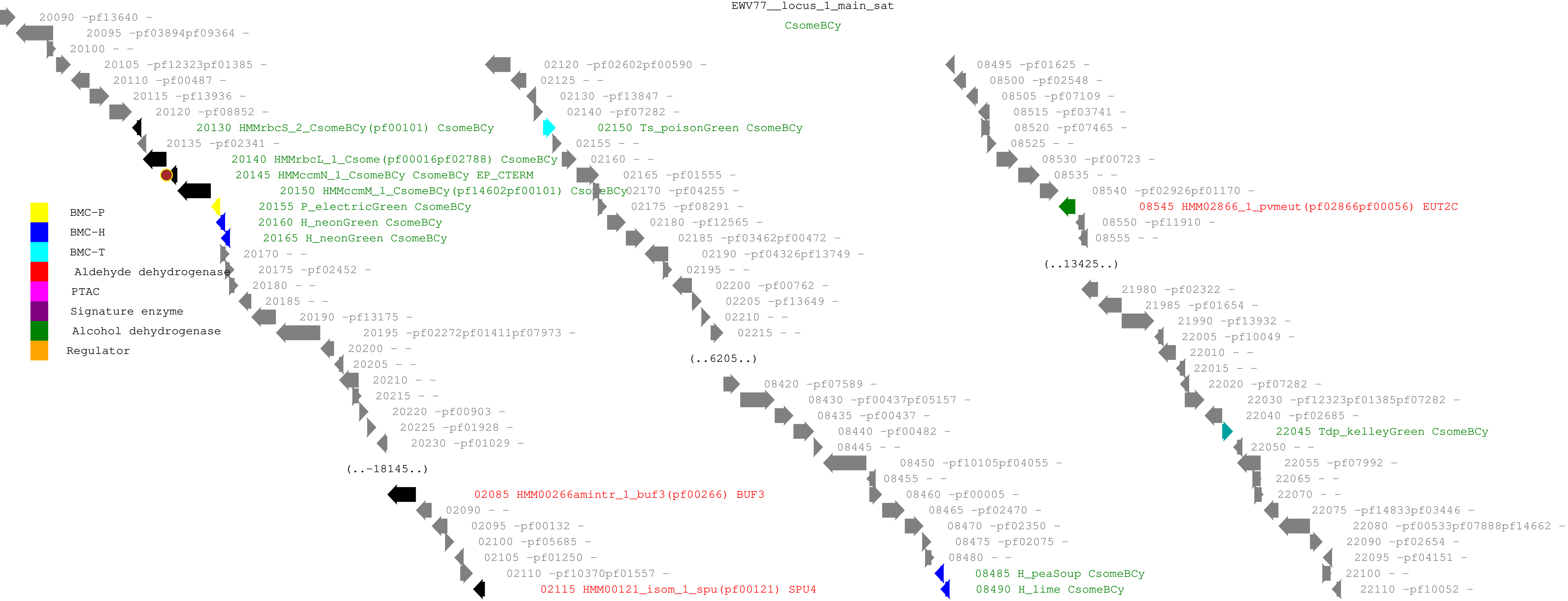

Microcystis aeruginosa Ma_MB_F_20061100_S20D. Bacteria; Cyanobacteria; Oscillatoriophycideae; Chroococcales;

EWV78__locus_1_main_sat

CsomeBCy

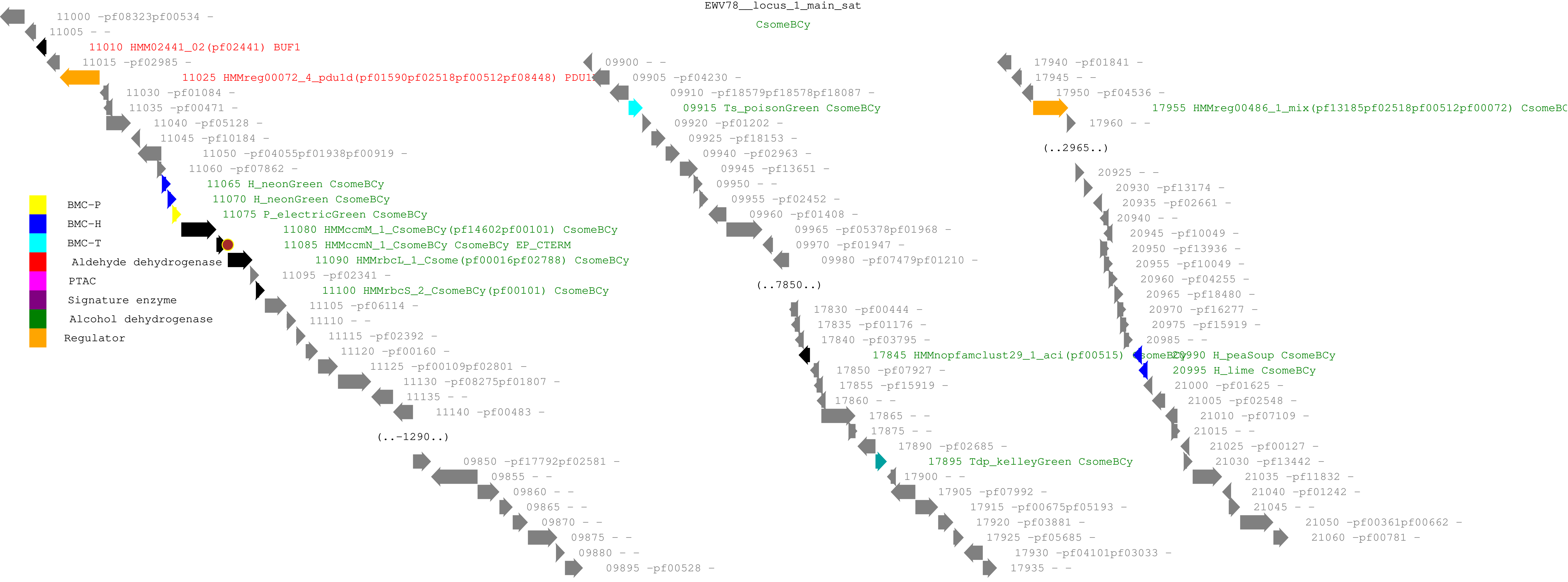

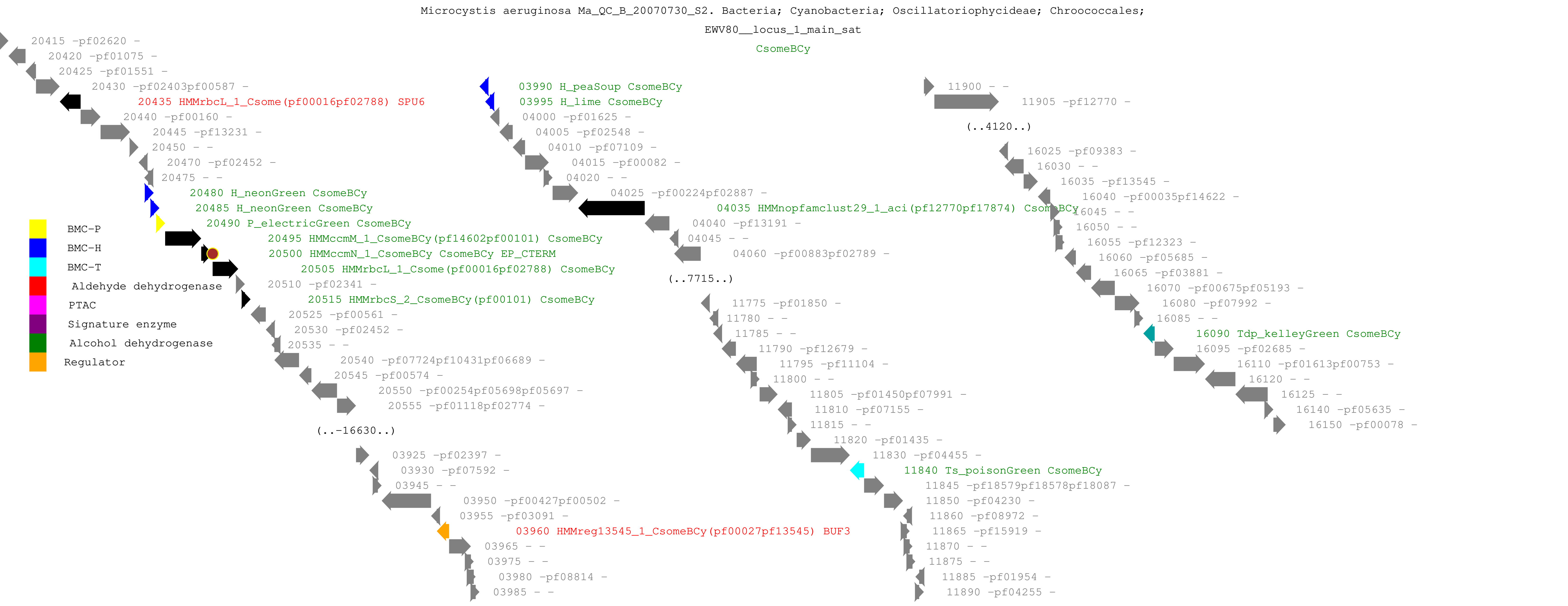
Microcystis aeruginosa Ma_QC_B_20070730_S2. Bacteria; Cyanobacteria; Oscillatoriophycideae; Chroococcales;
EWV80__locus_1_main_sat
CsomeBCy
20415 -pf02620 -
20420 -pf01075 -
20425 -pf01551 -
20430 -pf02403pf00587 -
20435 HMMrbcL_1_Csome(pf00016pf02788) SPU6
20440 -pf00160 -
20445 -pf13231 -
20450 - -
20470 -pf02452 -
20475 - -
20480 H_neonGreen CsomeBCy
20485 H_neonGreen CsomeBCy
20490 P_electricGreen CsomeBCy
20495 HMMccmM_1_CsomeBCy(pf14602pf00101) CsomeBCy
20500 HMMccmN_1_CsomeBCy CsomeBCy EP_CTERM
20505 HMMrbcL_1_Csome(pf00016pf02788) CsomeBCy
20510 -pf02341 -
20515 HMMrbcS_2_CsomeBCy(pf00101) CsomeBCy
20525 -pf00561 -
20530 -pf02452 -
20535 - -
20540 -pf07724pf10431pf06689 -
20545 -pf00574 -
20550 -pf00254pf05698pf05697 -
20555 -pf01118pf02774 -
(..-16630..)
03925 -pf02397 -
03930 -pf07592 -
03945 - -
03950 -pf00427pf00502 -
03955 -pf03091 -
03960 HMMreg13545_1_CsomeBCy(pf00027pf13545) BUF3
03965 - -
03975 - -
03980 -pf08814 -
03985 - -
BMC-P
BMC-H
BMC-T
Aldehyde dehydrogenase
PTAC
Signature enzyme
Alcohol dehydrogenase
Regulator
03990 H_peaSoup CsomeBCy
03995 H_lime CsomeBCy
04000 -pf01625 -
04005 -pf02548 -
04010 -pf07109 -
04015 -pf00082 -
04020 - -
04025 -pf00224pf02887 -
04035 HMMnopfamclust29_1_aci(pf12770pf17874) CsomeBCy
04040 -pf13191 -
04045 - -
04060 -pf00883pf02789 -
(..7715..)
11775 -pf01850 -
11780 - -
11785 - -
11790 -pf12679 -
11795 -pf11104 -
11800 - -
11805 -pf01450pf07991 -
11810 -pf07155 -
11815 - -
11820 -pf01435 -
11830 -pf04455 -
11840 Ts_poisonGreen CsomeBCy
11845 -pf18579pf18578pf18087 -
11850 -pf04230 -
11860 -pf08972 -
11865 -pf15919 -
11870 - -
11875 - -
11885 -pf01954 -
11890 -pf04255 -
11900 - -
11905 -pf12770 -
(..4120..)
16025 -pf09383 -
16030 - -
16035 -pf13545 -
16040 -pf00035pf14622 -
16045 - -
16050 - -
16055 -pf12323 -
16060 -pf05685 -
16065 -pf03881 -
16070 -pf00675pf05193 -
16080 -pf07992 -
16085 - -
16090 Tdp_kelleyGreen CsomeBCy
16095 -pf02685 -
16110 -pf01613pf00753 -
16120 - -
16125 - -
16140 -pf05635 -
16150 -pf00078 -

Microcystis aeruginosa Ma_SC_T_19800800_S464. Bacteria; Cyanobacteria; Oscillatoriophycideae; Chroococcales;

EWV81__locus_1_main_sat

CsomeBCy

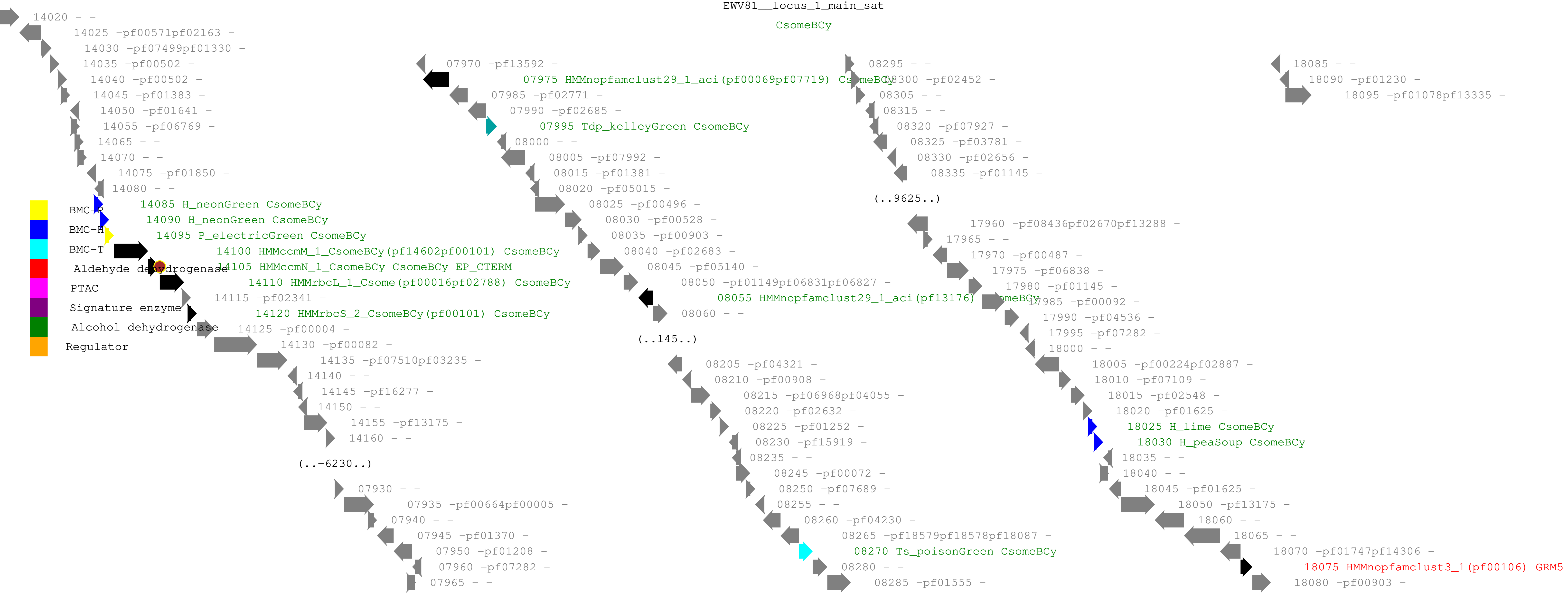

Microcystis aeruginosa Ma_AC_P_19900807_S299. Bacteria; Cyanobacteria; Oscillatoriophycideae; Chroococcales;

EWV82__locus_1_main_sat

CsomeBCy

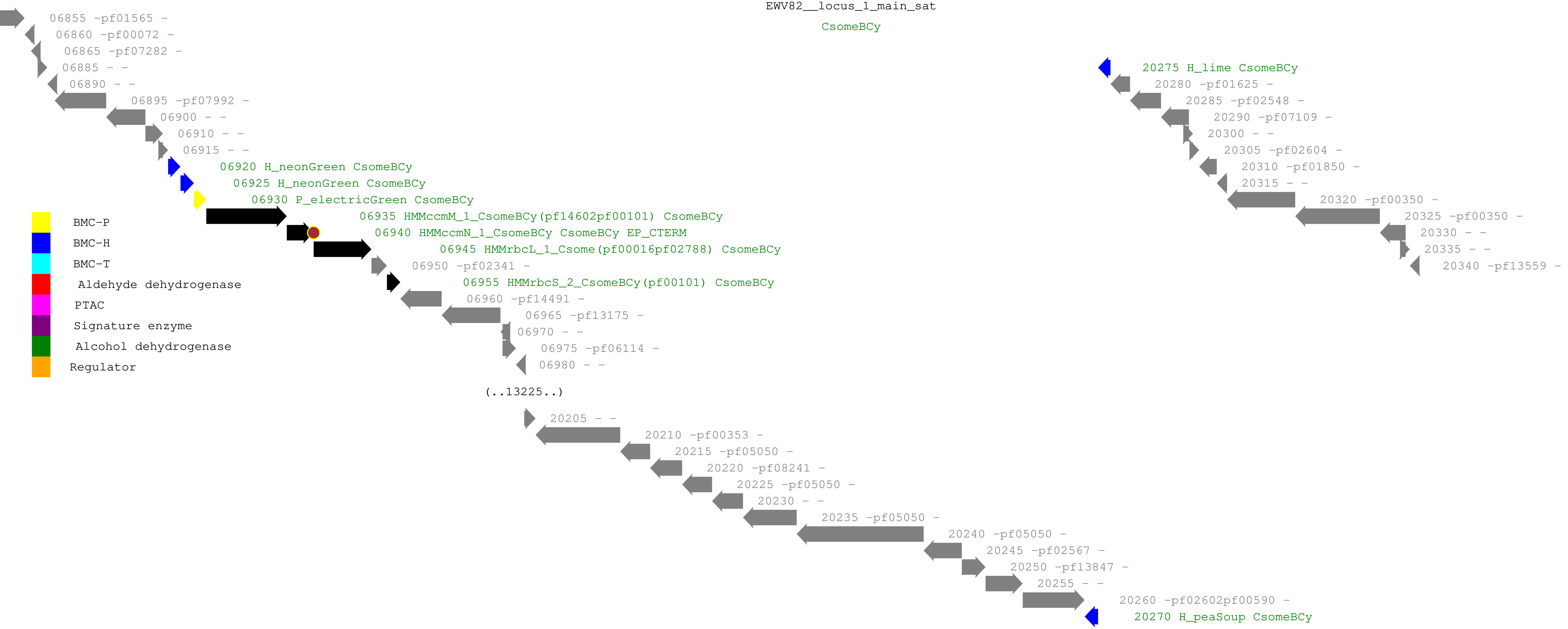

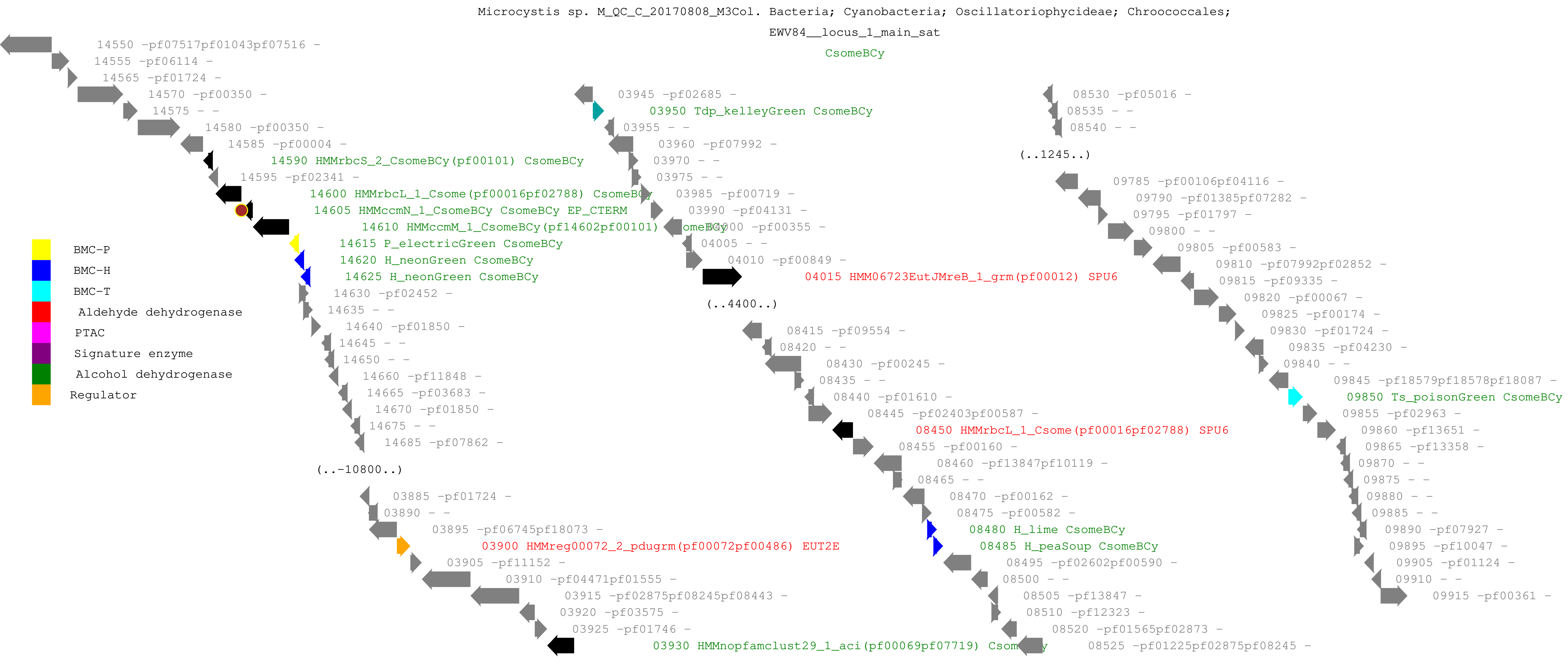
Microcystis sp. M_QC_C_20170808_M3Col. Bacteria; Cyanobacteria; Oscillatoriophycideae; Chroococcales;
EWV84__locus_1_main_sat
CsomeBCy
14550 -pf07517pf01043pf07516 -
14555 -pf06114 -
14565 -pf01724 -
14570 -pf00350 -
14575 - -
14580 -pf00350 -
14585 -pf00004 -
14590 HMMrbcS_2_CsomeBCy(pf00101) CsomeBCy
14595 -pf02341 -
14600 HMMrbcL_1_Csome(pf00016pf02788) CsomeBCy
14605 HMMccmN_1_CsomeBCy CsomeBCy EP_CTERM
14610 HMMccmM_1_CsomeBCy(pf14602pf00101) CsomeBCy
14615 P_electricGreen CsomeBCy
14620 H_neonGreen CsomeBCy
14625 H_neonGreen CsomeBCy
14630 -pf02452 -
14635 - -
14640 -pf01850 -
14645 - -
14650 - -
14660 -pf11848 -
14665 -pf03683 -
14670 -pf01850 -
14675 - -
14685 -pf07862 -
(..-10800..)
03885 -pf01724 -
03890 - -
03895 -pf06745pf18073 -
03900 HMMreg00072_2_pdugrm(pf00072pf00486) EUT2E
03905 -pf11152 -
03910 -pf04471pf01555 -
03915 -pf02875pf08245pf08443 -
03920 -pf03575 -
03925 -pf01746 -
03930 HMMnopfamclust29_1_aci(pf00069pf07719) CsomeBCy
BMC-P
BMC-H
BMC-T
Aldehyde dehydrogenase
PTAC
Signature enzyme
Alcohol dehydrogenase
Regulator
03945 -pf02685 -
03950 Tdp_kelleyGreen CsomeBCy
03955 - -
03960 -pf07992 -
03970 - -
03975 - -
03985 -pf00719 -
03990 -pf04131 -
04000 -pf00355 -
04005 - -
04010 -pf00849 -
04015 HMM06723EutJMreB_1_grm(pf00012) SPU6
(..4400..)
08415 -pf09554 -
08420 - -
08430 -pf00245 -
08435 - -
08440 -pf01610 -
08445 -pf02403pf00587 -
08450 HMMrbcL_1_Csome(pf00016pf02788) SPU6
08455 -pf00160 -
08460 -pf13847pf10119 -
08465 - -
08470 -pf00162 -
08475 -pf00582 -
08480 H_lime CsomeBCy
08485 H_peaSoup CsomeBCy
08495 -pf02602pf00590 -
08500 - -
08505 -pf13847 -
08510 -pf12323 -
08520 -pf01565pf02873 -
08525 -pf01225pf02875pf08245 -
08530 -pf05016 -
08535 - -
08540 - -
(..1245..)
09785 -pf00106pf04116 -
09790 -pf01385pf07282 -
09795 -pf01797 -
09800 - -
09805 -pf00583 -
09810 -pf07992pf02852 -
09815 -pf09335 -
09820 -pf00067 -
09825 -pf00174 -
09830 -pf01724 -
09835 -pf04230 -
09840 - -
09845 -pf18579pf18578pf18087 -
09850 Ts_poisonGreen CsomeBCy
09855 -pf02963 -
09860 -pf13651 -
09865 -pf13358 -
09870 - -
09875 - -
09880 - -
09885 - -
09890 -pf07927 -
09895 -pf10047 -
09905 -pf01124 -
09910 - -
09915 -pf00361 -

Microcystis aeruginosa Ma_QC_C_20070703_M131. Bacteria; Cyanobacteria; Oscillatoriophycideae; Chroococcales;

EWV85__locus_1_main_sat

CsomeBCy

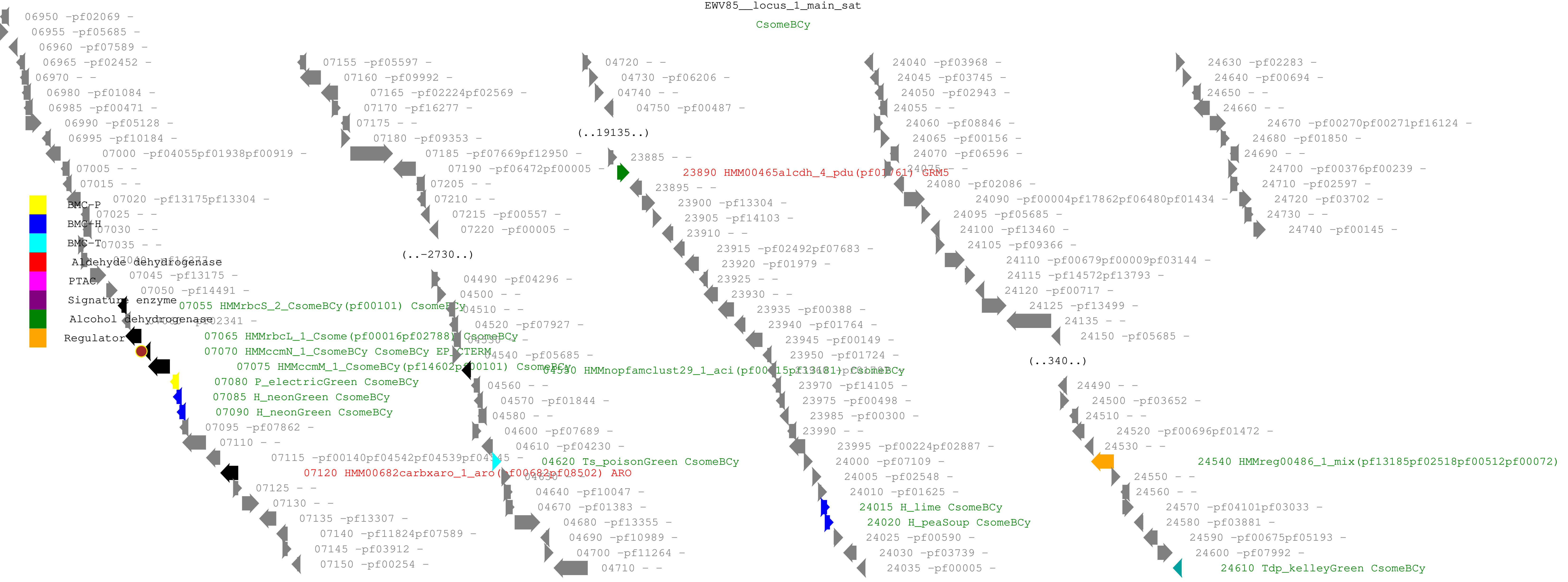

Microcystis wesenbergii Mw_MB_S_20031200_S109D. Bacteria; Cyanobacteria; Oscillatoriophycideae; Chroococcales;

EWV88__locus_1_main_sat

CsomeBCy

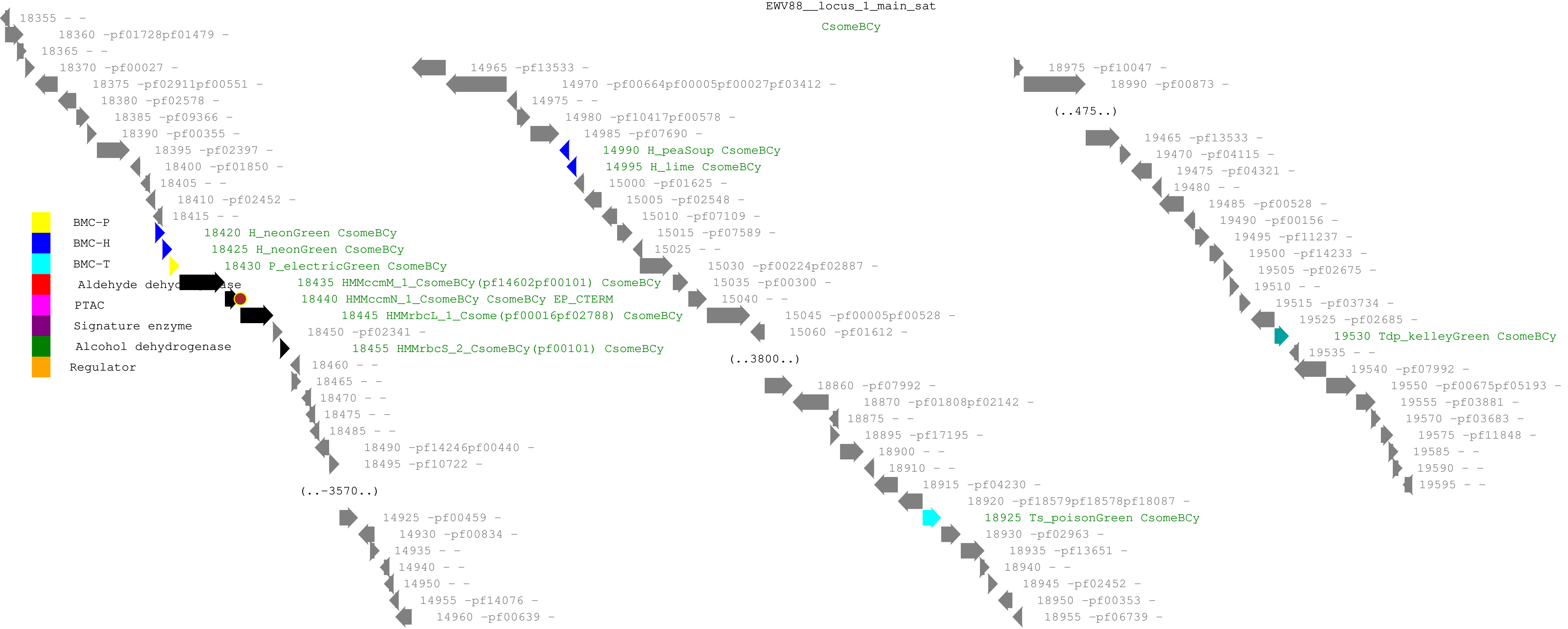

Microcystis aeruginosa Ma_QC_Ca_00000000_S207. Bacteria; Cyanobacteria; Oscillatoriophycideae; Chroococcales;

EWV91__locus_1_main_sat

CsomeBCy

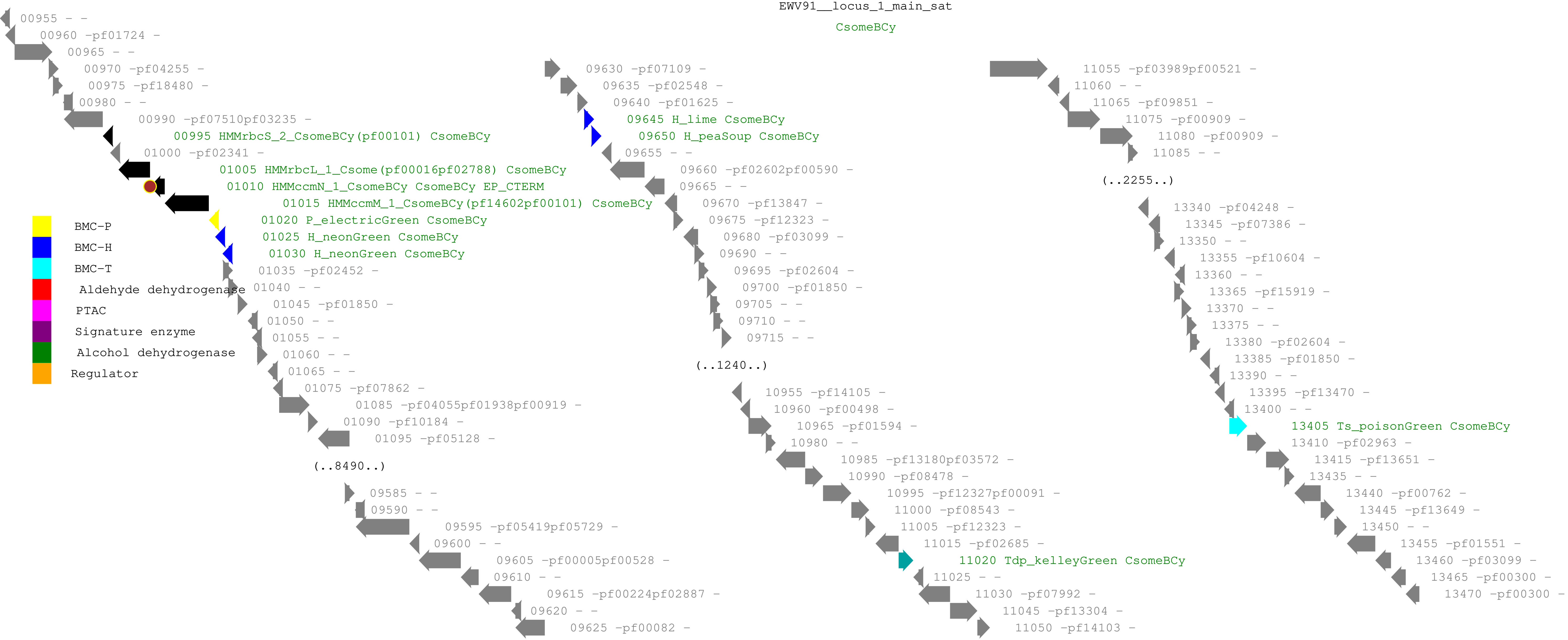

Microcystis aeruginosa Ma_MB_S_20031200_S102. Bacteria; Cyanobacteria; Oscillatoriophycideae; Chroococcales;

EWV92__locus_1_main_sat

CsomeBCy

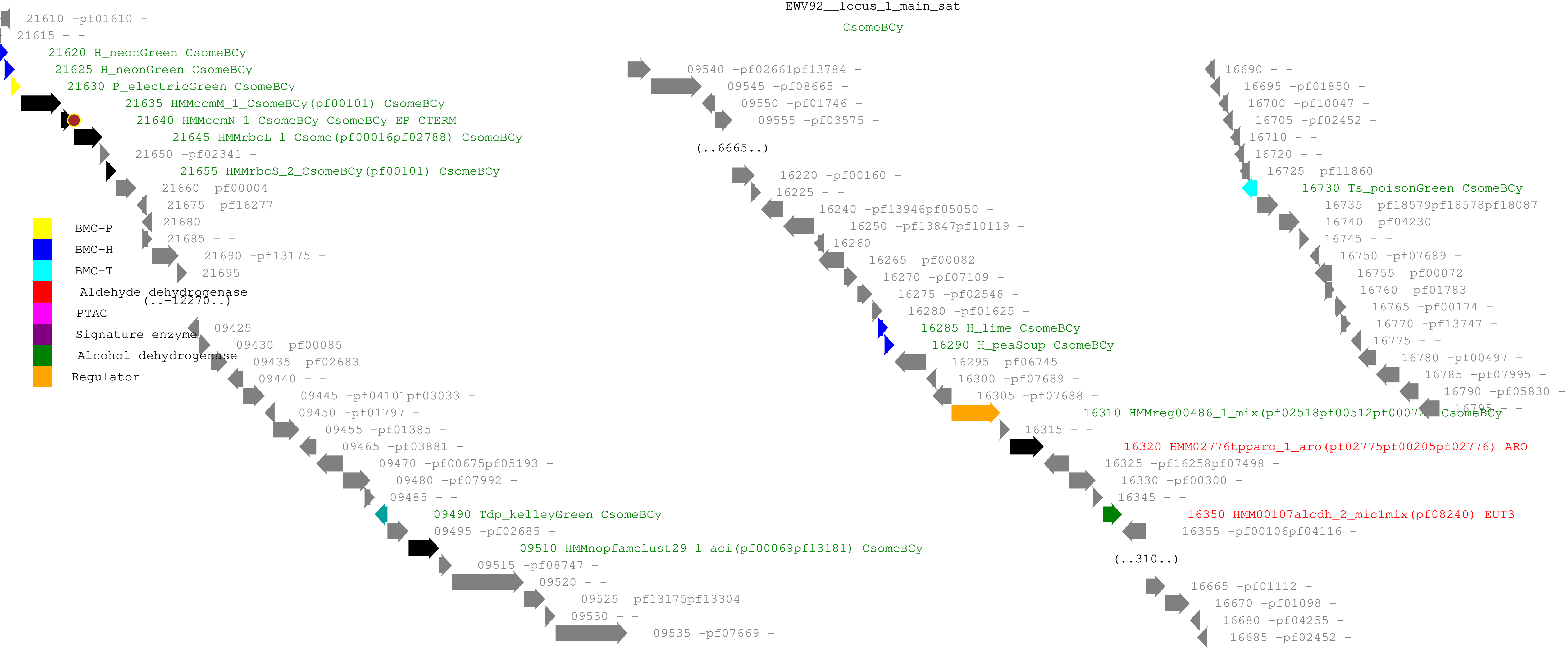

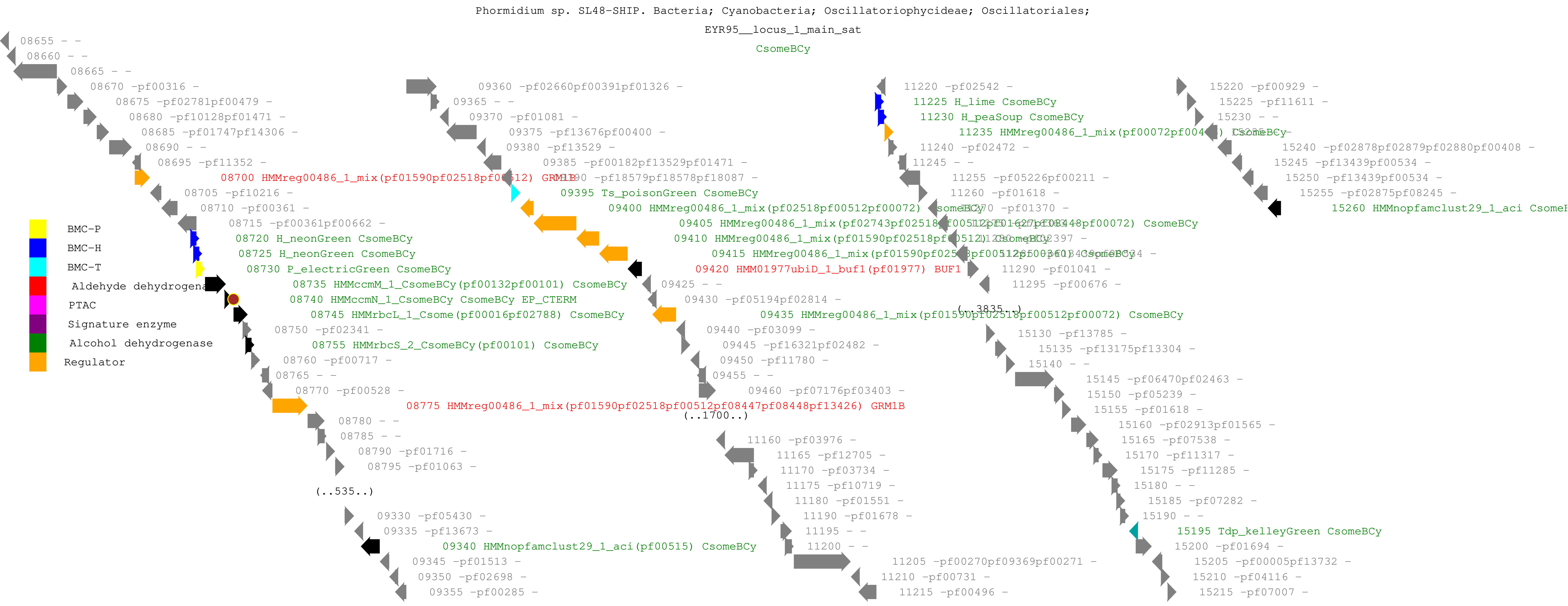
Phormidium sp. SL48-SHIP. Bacteria; Cyanobacteria; Oscillatoriophycideae; Oscillatoriales;
EYR95__locus_1_main_sat
CsomeBCy
BMC-P
BMC-H
BMC-T
Aldehyde dehydrogenase
PTAC
Signature enzyme
Alcohol dehydrogenase
Regulator
08655 - -
08660 - -
08665 - -
08670 -pf00316 -
08675 -pf02781pf00479 -
08680 -pf10128pf01471 -
08685 -pf01747pf14306 -
08690 - -
08695 -pf11352 -
08700 HMMreg00486_1_mix(pf01590pf02518pf00512) GRM1B
08705 -pf10216 -
08710 -pf00361 -
08715 -pf00361pf00662 -
08720 H_neonGreen CsomeBCy
08725 H_neonGreen CsomeBCy
08730 P_electricGreen CsomeBCy
08735 HMMccmM_1_CsomeBCy(pf00132pf00101) CsomeBCy
08740 HMMccmN_1_CsomeBCy CsomeBCy EP_CTERM
08745 HMMrbcL_1_Csome(pf00016pf02788) CsomeBCy
08750 -pf02341 -
08755 HMMrbcS_2_CsomeBCy(pf00101) CsomeBCy
08760 -pf00717 -
08765 - -
08770 -pf00528 -
08775 HMMreg00486_1_mix(pf01590pf02518pf00512pf08447pf08448pf13426) GRM1B
08780 - -
08785 - -
08790 -pf01716 -
08795 -pf01063 -
(..535..)
09330 -pf05430 -
09335 -pf13673 -
09340 HMMnopfamclust29_1_aci(pf00515) CsomeBCy
09345 -pf01513 -
09350 -pf02698 -
09355 -pf00285 -
09360 -pf02660pf00391pf01326 -
09365 - -
09370 -pf01081 -
09375 -pf13676pf00400 -
09380 -pf13529 -
09385 -pf00182pf13529pf01471 -
09390 -pf18579pf18578pf18087 -
09395 Ts_poisonGreen CsomeBCy
09400 HMMreg00486_1_mix(pf02518pf00512pf00072) CsomeBCy
09405 HMMreg00486_1_mix(pf02743pf02518pf00512pf01627pf08448pf00072) CsomeBCy
09410 HMMreg00486_1_mix(pf01590pf02518pf00512) CsomeBCy
09415 HMMreg00486_1_mix(pf01590pf02518pf00512pf00360) CsomeBCy
09420 HMM01977ubiD_1_buf1(pf01977) BUF1
09425 - -
09430 -pf05194pf02814 -
09435 HMMreg00486_1_mix(pf01590pf02518pf00512pf00072) CsomeBCy
09440 -pf03099 -
09445 -pf16321pf02482 -
09450 -pf11780 -
09455 - -
09460 -pf07176pf03403 -
(..1700..)
11160 -pf03976 -
11165 -pf12705 -
11170 -pf03734 -
11175 -pf10719 -
11180 -pf01551 -
11190 -pf01678 -
11195 - -
11200 - -
11205 -pf00270pf09369pf00271 -
11210 -pf00731 -
11215 -pf00496 -
11220 -pf02542 -
11225 H_lime CsomeBCy
11230 H_peaSoup CsomeBCy
11235 HMMreg00486_1_mix(pf00072pf04[illegible]) CsomeBCy
11240 -pf02472 -
11245 - -
11255 -pf05226pf00211 -
11260 -pf01618 -
11270 -pf01370 -
11290 -pf01041 -
11295 -pf00676 -
(..3835..)
15130 -pf13785 -
15135 -pf13175pf13304 -
15140 - -
15145 -pf06470pf02463 -
15150 -pf05239 -
15155 -pf01618 -
15160 -pf02913pf01565 -
15165 -pf07538 -
15170 -pf11317 -
15175 -pf11285 -
15180 - -
15185 -pf07282 -
15190 - -
15195 Tdp_kelleyGreen CsomeBCy
15200 -pf01694 -
15205 -pf00005pf13732 -
15210 -pf04116 -
15215 -pf07007 -
15220 -pf00929 -
15225 -pf11611 -
15230 - -
15235 - -
15240 -pf02878pf02879pf02880pf00408 -
15245 -pf13439pf00534 -
15250 -pf13439pf00534 -
15255 -pf02875pf08245 -
15260 HMMnopfamclust29_1_aci CsomeB

Microcystis aeruginosa EAWAG127a. Bacteria; Cyanobacteria; Oscillatoriophycideae; Chroococcales;

EZJ55__locus_1_main_sat

CsomeBCy

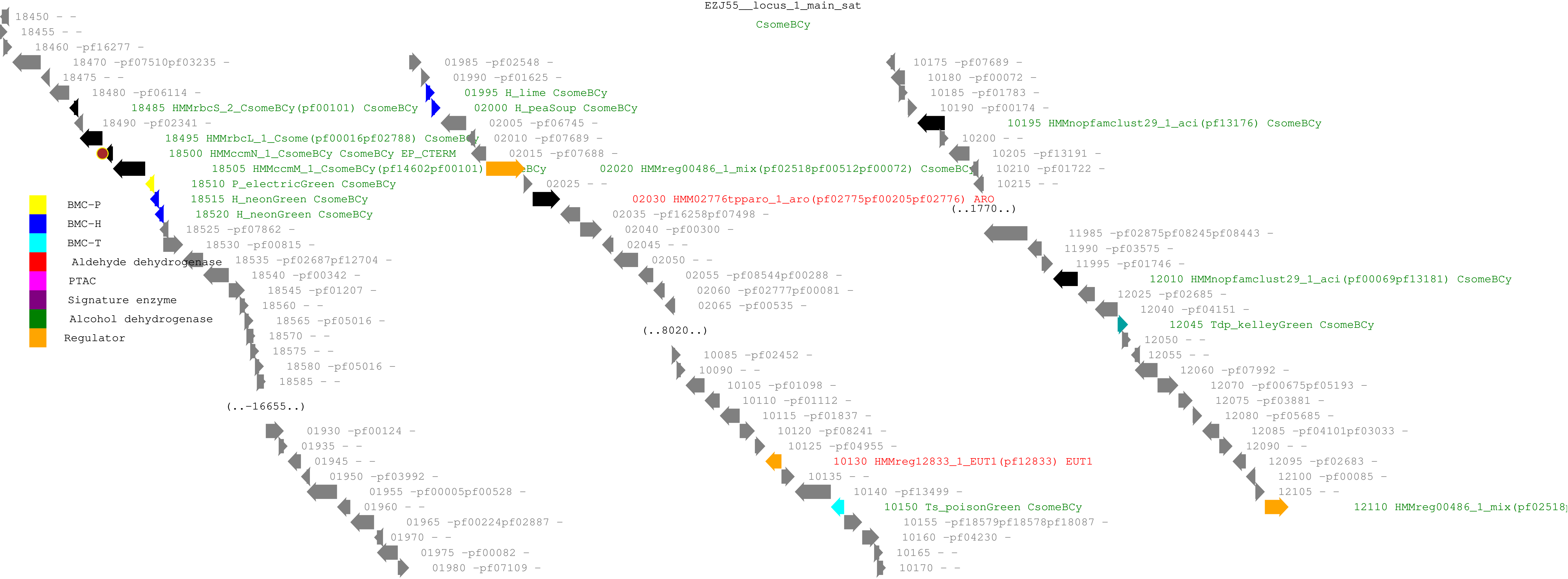

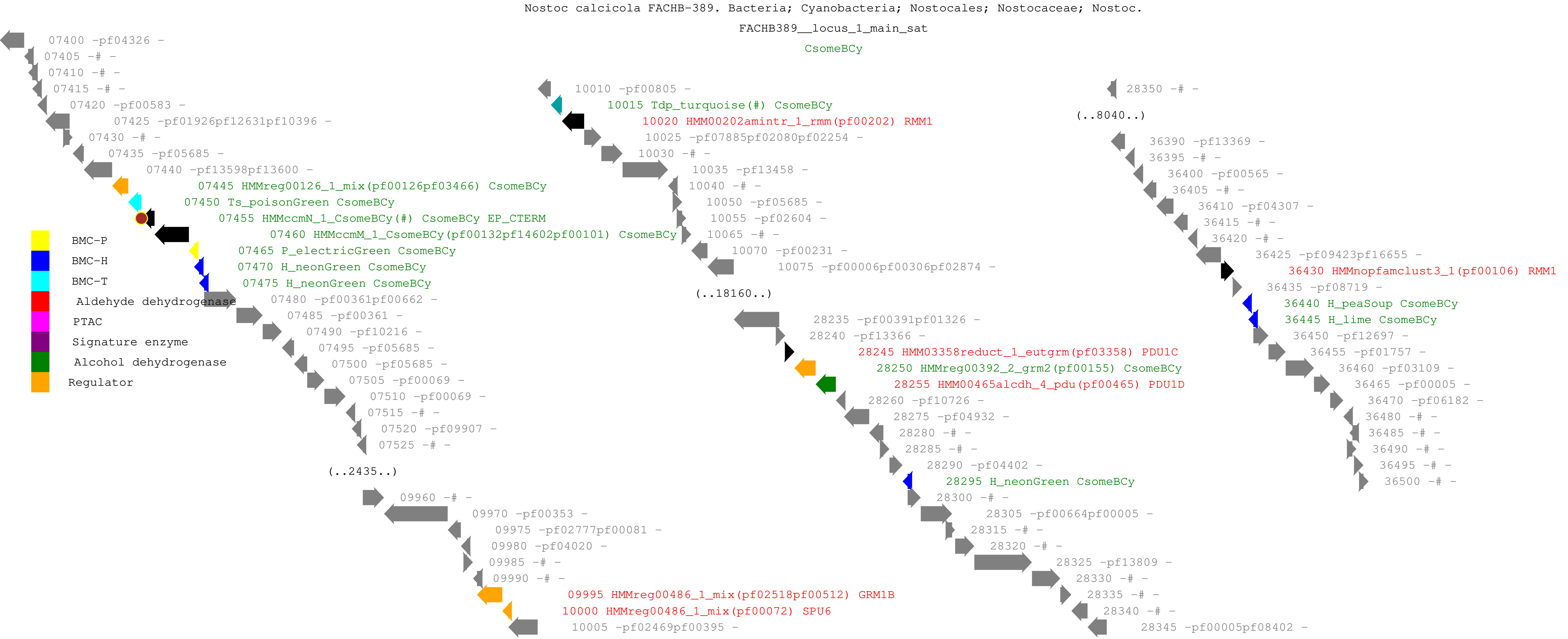

Nostoc calcicola FACHB-389. Bacteria; Cyanobacteria; Nostocales; Nostocaceae; Nostoc.
FACHB389__locus_1_main_sat
CsomeBCy
07400 -pf04326 -
07405 -# -
07410 -# -
07415 -# -
07420 -pf00583 -
07425 -pf01926pf12631pf10396 -
07430 -# -
07435 -pf05685 -
07440 -pf13598pf13600 -
07445 HMMreg00126_1_mix(pf00126pf03466) CsomeBCy
07450 Ts_poisonGreen CsomeBCy
07455 HMMccmN_1_CsomeBCy(#) CsomeBCy EP_CTERM
07460 HMMccmM_1_CsomeBCy(pf00132pf14602pf00101) CsomeBCy
07465 P_electricGreen CsomeBCy
07470 H_neonGreen CsomeBCy
07475 H_neonGreen CsomeBCy
07480 -pf00361pf00662 -
07485 -pf00361 -
07490 -pf10216 -
07495 -pf05685 -
07500 -pf05685 -
07505 -pf00069 -
07510 -pf00069 -
07515 -# -
07520 -pf09907 -
07525 -# -
(..2435..)
09960 -# -
09970 -pf00353 -
09975 -pf02777pf00081 -
09980 -pf04020 -
09985 -# -
09990 -# -
09995 HMMreg00486_1_mix(pf02518pf00512) GRM1B
10000 HMMreg00486_1_mix(pf00072) SPU6
10005 -pf02469pf00395 -
10010 -pf00805 -
10015 Tdp_turquoise(#) CsomeBCy
10020 HMM00202amintr_1_rmm(pf00202) RMM1
10025 -pf07885pf02080pf02254 -
10030 -# -
10035 -pf13458 -
10040 -# -
10050 -pf05685 -
10055 -pf02604 -
10065 -# -
10070 -pf00231 -
10075 -pf00006pf00306pf02874 -
(..18160..)
28235 -pf00391pf01326 -
28240 -pf13366 -
28245 HMM03358reduct_1_eutgrm(pf03358) PDU1C
28250 HMMreg00392_2_grm2(pf00155) CsomeBCy
28255 HMM00465alcdh_4_pdu(pf00465) PDU1D
28260 -pf10726 -
28275 -pf04932 -
28280 -# -
28285 -# -
28290 -pf04402 -
28295 H_neonGreen CsomeBCy
28300 -# -
28305 -pf00664pf00005 -
28315 -# -
28320 -# -
28325 -pf13809 -
28330 -# -
28335 -# -
28340 -# -
28345 -pf00005pf08402 -
28350 -# -
(..8040..)
36390 -pf13369 -
36395 -# -
36400 -pf00565 -
36405 -# -
36410 -pf04307 -
36415 -# -
36420 -# -
36425 -pf09423pf16655 -
36430 HMMnopfamclust3_1(pf00106) RMM1
36435 -pf08719 -
36440 H_peaSoup CsomeBCy
36445 H_lime CsomeBCy
36450 -pf12697 -
36455 -pf01757 -
36460 -pf03109 -
36465 -pf00005 -
36470 -pf06182 -
36480 -# -
36485 -# -
36490 -# -
36495 -# -
36500 -# -
BMC-P
BMC-H
BMC-T
Aldehyde dehydrogenase
PTAC
Signature enzyme
Alcohol dehydrogenase
Regulator

Tolypothrix sp. PCC 7601. Bacteria; Cyanobacteria; Nostocales; Tolypothrichaceae; Tolypothrix.

FDUTEX481__locus_1_main_sat

CsomeBCy

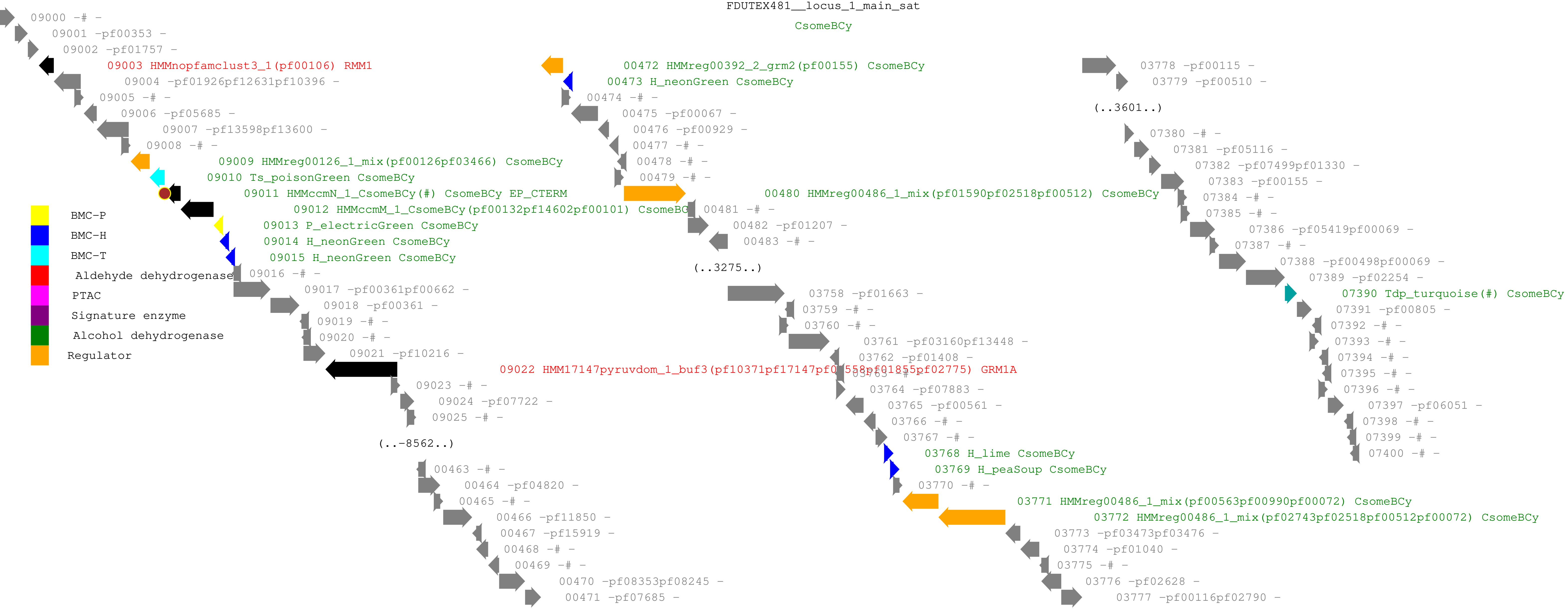

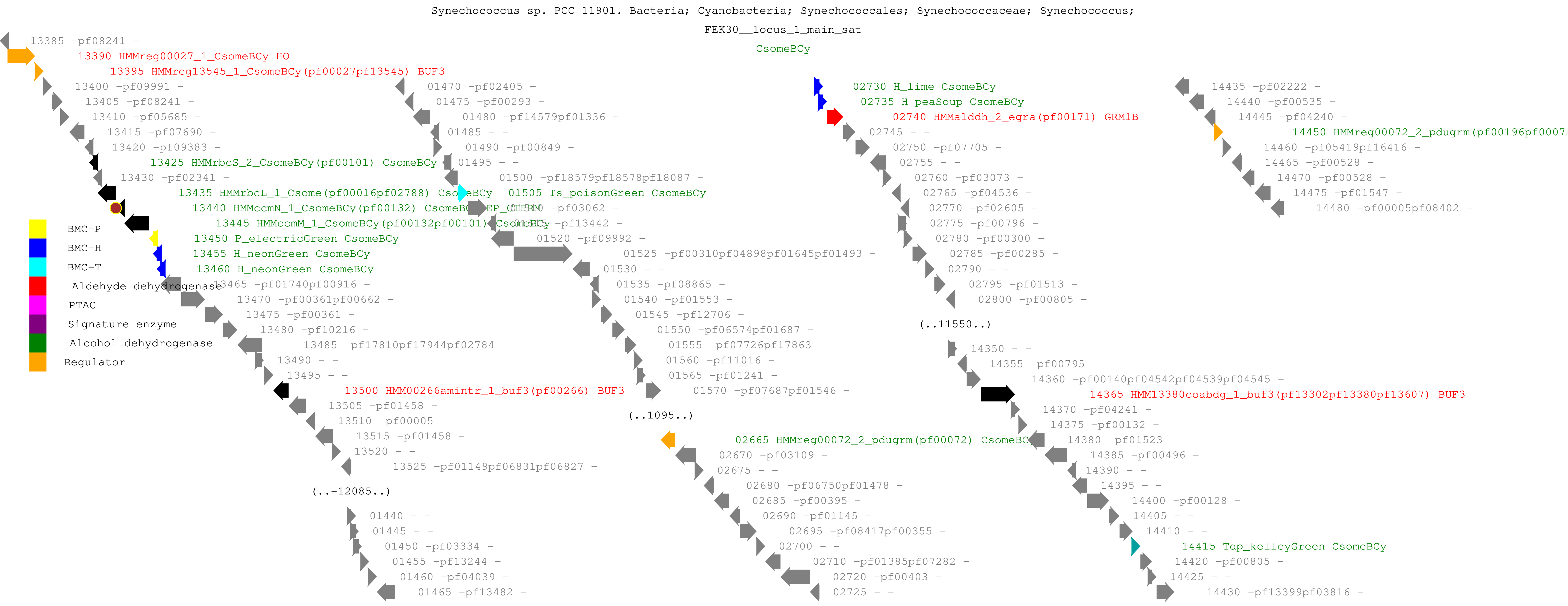

Synechococcus sp. PCC 11901. Bacteria; Cyanobacteria; Synechococcales; Synechococcaceae; Synechococcus;
FEK30__locus_1_main_sat
CsomeBCy
13385 -pf08241 -
13390 HMMreg00027_1_CsomeBCy HO
13395 HMMreg13545_1_CsomeBCy(pf00027pf13545) BUF3
13400 -pf09991 -
13405 -pf08241 -
13410 -pf05685 -
13415 -pf07690 -
13420 -pf09383 -
13425 HMMrbcS_2_CsomeBCy(pf00101) CsomeBCy
13430 -pf02341 -
13435 HMMrbcL_1_Csome(pf00016pf02788) CsomeBCy
13440 HMMccmN_1_CsomeBCy(pf00132) CsomeBCy
13445 HMMccmM_1_CsomeBCy(pf00132pf00101) CsomeBCy
13450 P_electricGreen CsomeBCy
13455 H_neonGreen CsomeBCy
13460 H_neonGreen CsomeBCy
13465 -pf01740pf00916 -
13470 -pf00361pf00662 -
13475 -pf00361 -
13480 -pf10216 -
13485 -pf17810pf17944pf02784 -
13490 - -
13495 - -
13500 HMM00266amintr_1_buf3(pf00266) BUF3
13505 -pf01458 -
13510 -pf00005 -
13515 -pf01458 -
13520 - -
13525 -pf01149pf06831pf06827 -
(..-12085..)
01440 - -
01445 - -
01450 -pf03334 -
01455 -pf13244 -
01460 -pf04039 -
01465 -pf13482 -
BMC-P
BMC-H
BMC-T
Aldehyde dehydrogenase
PTAC
Signature enzyme
Alcohol dehydrogenase
Regulator
01470 -pf02405 -
01475 -pf00293 -
01480 -pf14579pf01336 -
01485 - -
01490 -pf00849 -
01495 - -
01500 -pf18579pf18578pf18087 -
01505 Ts_poisonGreen CsomeBCy
EP_CTERM
-pf03062 -
-pf13442 -
01520 -pf09992 -
01525 -pf00310pf04898pf01645pf01493 -
01530 - -
01535 -pf08865 -
01540 -pf01553 -
01545 -pf12706 -
01550 -pf06574pf01687 -
01555 -pf07726pf17863 -
01560 -pf11016 -
01565 -pf01241 -
01570 -pf07687pf01546 -
(..1095..)
02665 HMMreg00072_2_pdugrm(pf00072) CsomeBCy
02670 -pf03109 -
02675 - -
02680 -pf06750pf01478 -
02685 -pf00395 -
02690 -pf01145 -
02695 -pf08417pf00355 -
02700 - -
02710 -pf01385pf07282 -
02720 -pf00403 -
02725 - -
02730 H_lime CsomeBCy
02735 H_peaSoup CsomeBCy
02740 HMMalddh_2_egra(pf00171) GRM1B
02745 - -
02750 -pf07705 -
02755 - -
02760 -pf03073 -
02765 -pf04536 -
02770 -pf02605 -
02775 -pf00796 -
02780 -pf00300 -
02785 -pf00285 -
02790 - -
02795 -pf01513 -
02800 -pf00805 -
(..11550..)
14350 - -
14355 -pf00795 -
14360 -pf00140pf04542pf04539pf04545 -
14365 HMM13380coabdg_1_buf3(pf13302pf13380pf13607) BUF3
14370 -pf04241 -
14375 -pf00132 -
14380 -pf01523 -
14385 -pf00496 -
14390 - -
14395 - -
14400 -pf00128 -
14405 - -
14410 - -
14415 Tdp_kelleyGreen CsomeBCy
14420 -pf00805 -
14425 - -
14430 -pf13399pf03816 -
14435 -pf02222 -
14440 -pf00535 -
14445 -pf04240 -
14450 HMMreg00072_2_pdugrm(pf00196pf0007
14460 -pf05419pf16416 -
14465 -pf00528 -
14470 -pf00528 -
14475 -pf01547 -
14480 -pf00005pf08402 -

Thermosynechococcus sp. CL-1. Bacteria; Cyanobacteria; Synechococcales; Synechococcaceae;

FFX45__locus_1_main_sat

CsomeBCy

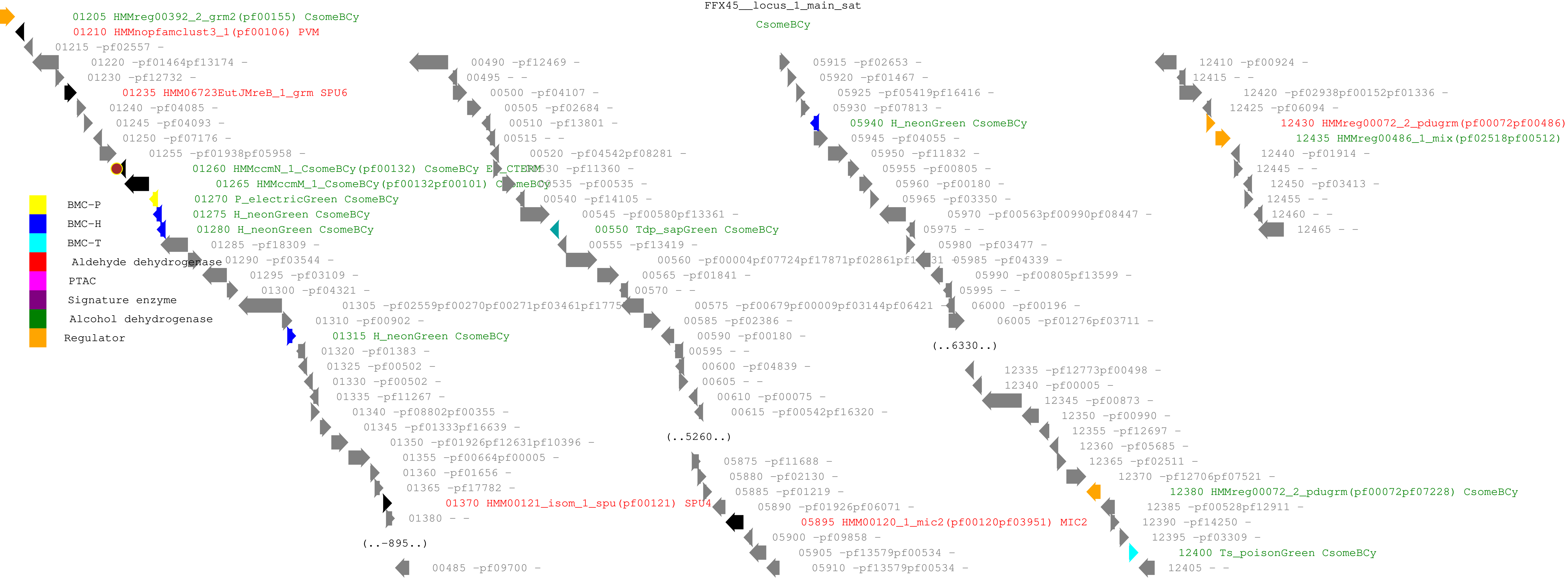

Fischerella sp. NIES-3754. Bacteria; Cyanobacteria; Nostocales; Hapalosiphonaceae; Fischerella.

FIS3754__locus_1_main_sat

CsomeBCy

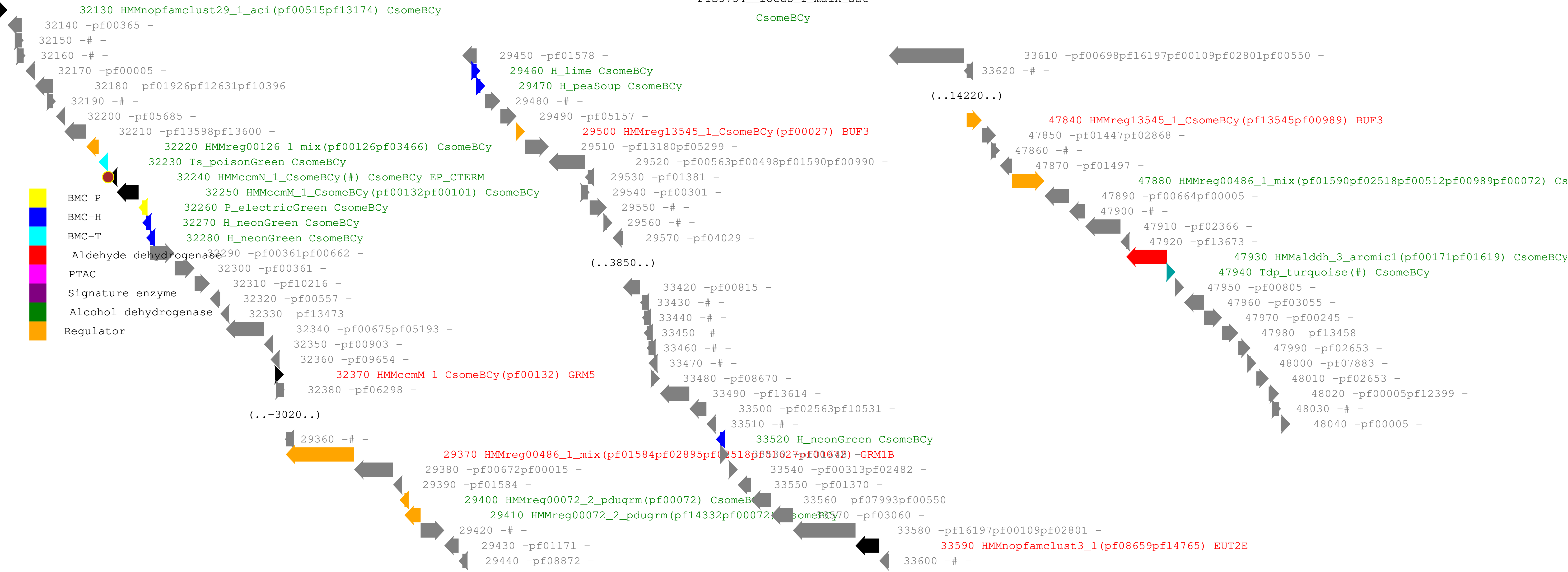

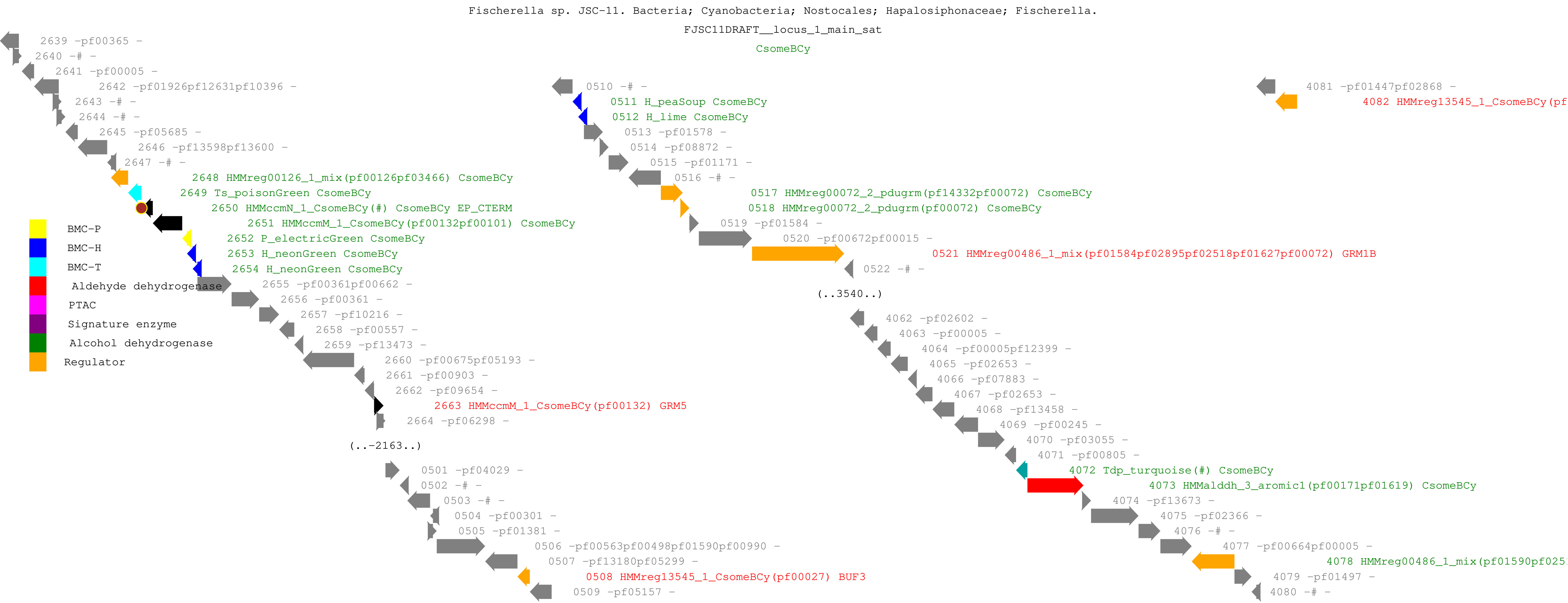

Fischerella sp. JSC-11. Bacteria; Cyanobacteria; Nostocales; Hapalosiphonaceae; Fischerella.
FJSC11DRAFT__locus_1_main_sat
CsomeBCy
2639 -pf00365 -
2640 -# -
2641 -pf00005 -
2642 -pf01926pf12631pf10396 -
2643 -# -
2644 -# -
2645 -pf05685 -
2646 -pf13598pf13600 -
2647 -# -
2648 HMMreg00126_1_mix(pf00126pf03466) CsomeBCy
2649 Ts_poisonGreen CsomeBCy
2650 HMMccmN_1_CsomeBCy(#) CsomeBCy EP_CTERM
2651 HMMccmM_1_CsomeBCy(pf00132pf00101) CsomeBCy
2652 P_electricGreen CsomeBCy
2653 H_neonGreen CsomeBCy
2654 H_neonGreen CsomeBCy
2655 -pf00361pf00662 -
2656 -pf00361 -
2657 -pf10216 -
2658 -pf00557 -
2659 -pf13473 -
2660 -pf00675pf05193 -
2661 -pf00903 -
2662 -pf09654 -
2663 HMMccmM_1_CsomeBCy(pf00132) GRM5
2664 -pf06298 -
(..-2163..)
0501 -pf04029 -
0502 -# -
0503 -# -
0504 -pf00301 -
0505 -pf01381 -
0506 -pf00563pf00498pf01590pf00990 -
0507 -pf13180pf05299 -
0508 HMMreg13545_1_CsomeBCy(pf00027) BUF3
0509 -pf05157 -
BMC-P
BMC-H
BMC-T
Aldehyde dehydrogenase
PTAC
Signature enzyme
Alcohol dehydrogenase
Regulator
0510 -# -
0511 H_peaSoup CsomeBCy
0512 H_lime CsomeBCy
0513 -pf01578 -
0514 -pf08872 -
0515 -pf01171 -
0516 -# -
0517 HMMreg00072_2_pdugrm(pf14332pf00072) CsomeBCy
0518 HMMreg00072_2_pdugrm(pf00072) CsomeBCy
0519 -pf01584 -
0520 -pf00672pf00015 -
0521 HMMreg00486_1_mix(pf01584pf02895pf02518pf01627pf00072) GRM1B
0522 -# -
(..3540..)
4062 -pf02602 -
4063 -pf00005 -
4064 -pf00005pf12399 -
4065 -pf02653 -
4066 -pf07883 -
4067 -pf02653 -
4068 -pf13458 -
4069 -pf00245 -
4070 -pf03055 -
4071 -pf00805 -
4072 Tdp_turquoise(#) CsomeBCy
4073 HMMalddh_3_aromic1(pf00171pf01619) CsomeBCy
4074 -pf13673 -
4075 -pf02366 -
4076 -# -
4077 -pf00664pf00005 -
4078 HMMreg00486_1_mix(pf01590pf025
4079 -pf01497 -
4080 -# -
4081 -pf01447pf02868 -
4082 HMMreg13545_1_CsomeBCy(pf

Euhalothece natronophila Z-M001. Bacteria; Cyanobacteria; Oscillatoriophycideae; Chroococcales;

FRE64__locus_1_main_sat

CsomeBCy

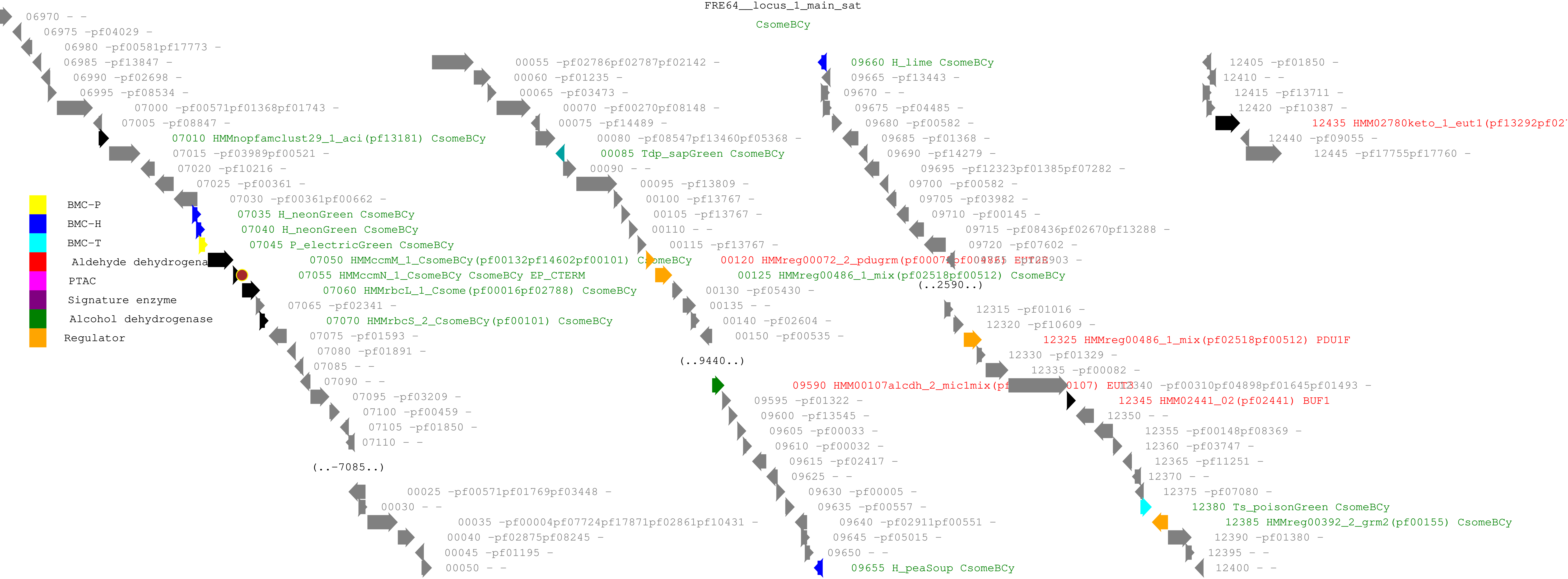

Microcystis aeruginosa KLA2. Bacteria; Cyanobacteria; Oscillatoriophycideae; Chroococcales;

FXO09__locus_1_main_sat

CsomeBCy

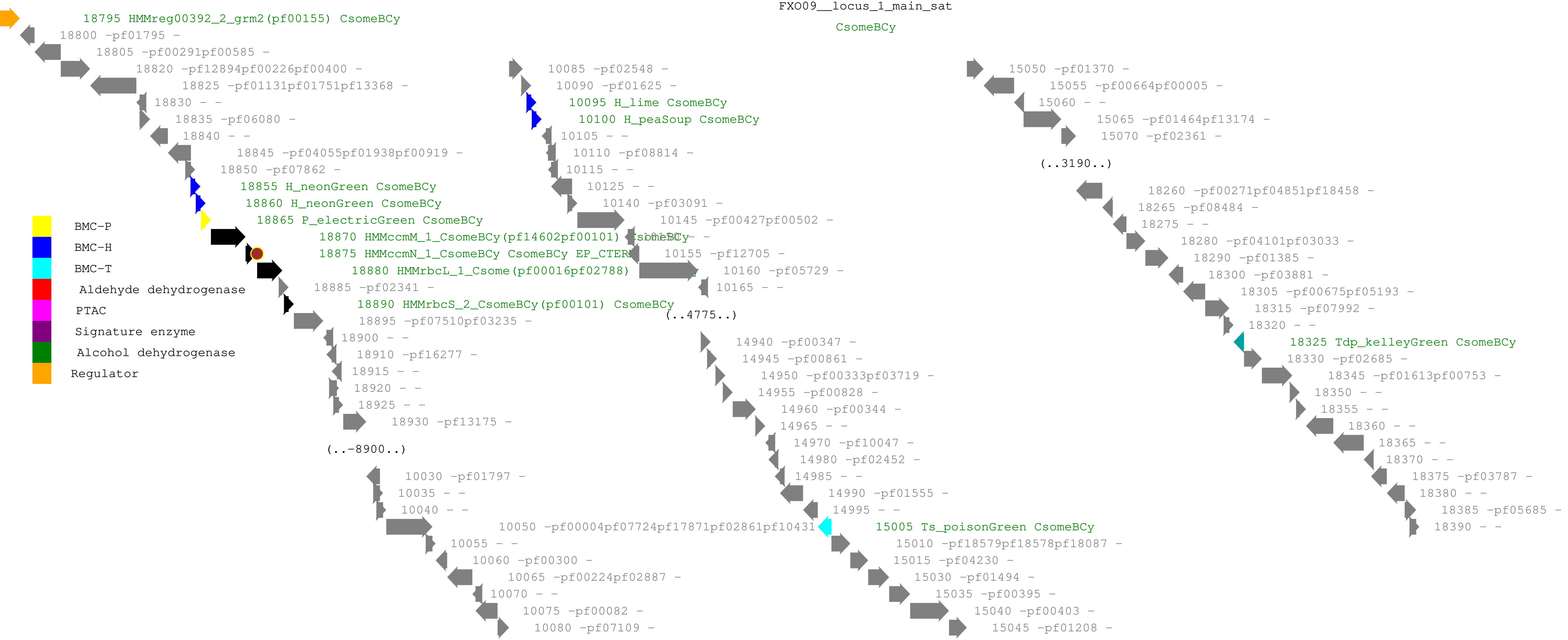

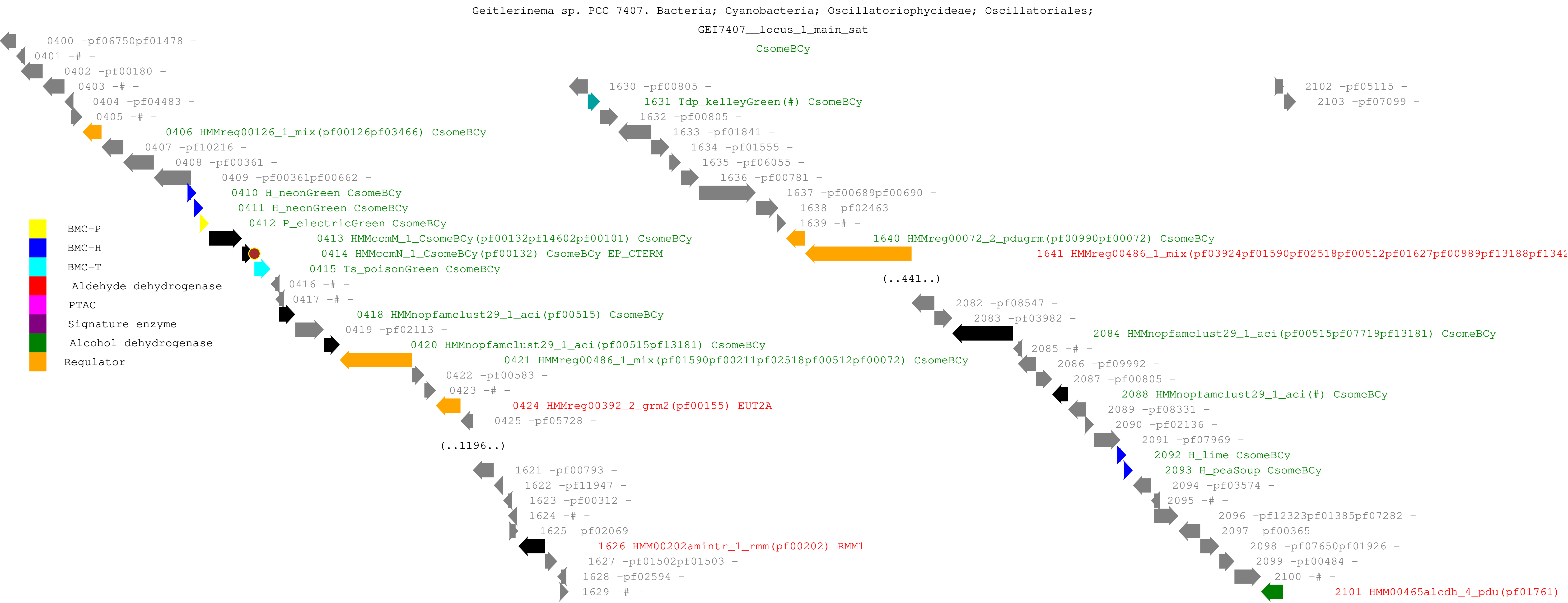

Geitlerinema sp. PCC 7407. Bacteria; Cyanobacteria; Oscillatoriophycideae; Oscillatoriales;
GEI7407__locus_1_main_sat
CsomeBCy
0400 -pf06750pf01478 -
0401 -# -
0402 -pf00180 -
0403 -# -
0404 -pf04483 -
0405 -# -
0406 HMMreg00126_1_mix(pf00126pf03466) CsomeBCy
0407 -pf10216 -
0408 -pf00361 -
0409 -pf00361pf00662 -
0410 H_neonGreen CsomeBCy
0411 H_neonGreen CsomeBCy
0412 P_electricGreen CsomeBCy
0413 HMMccmM_1_CsomeBCy(pf00132pf14602pf00101) CsomeBCy
0414 HMMccmN_1_CsomeBCy(pf00132) CsomeBCy EP_CTERM
0415 Ts_poisonGreen CsomeBCy
0416 -# -
0417 -# -
0418 HMMnopfamclust29_1_aci(pf00515) CsomeBCy
0419 -pf02113 -
0420 HMMnopfamclust29_1_aci(pf00515pf13181) CsomeBCy
0421 HMMreg00486_1_mix(pf01590pf00211pf02518pf00512pf00072) CsomeBCy
0422 -pf00583 -
0423 -# -
0424 HMMreg00392_2_grm2(pf00155) EUT2A
0425 -pf05728 -
(..1196..)
1621 -pf00793 -
1622 -pf11947 -
1623 -pf00312 -
1624 -# -
1625 -pf02069 -
1626 HMM00202amintr_1_rmm(pf00202) RMM1
1627 -pf01502pf01503 -
1628 -pf02594 -
1629 -# -
1630 -pf00805 -
1631 Tdp_kelleyGreen(#) CsomeBCy
1632 -pf00805 -
1633 -pf01841 -
1634 -pf01555 -
1635 -pf06055 -
1636 -pf00781 -
1637 -pf00689pf00690 -
1638 -pf02463 -
1639 -# -
1640 HMMreg00072_2_pdugrm(pf00990pf00072) CsomeBCy
1641 HMMreg00486_1_mix(pf03924pf01590pf02518pf00512pf01627pf00989pf13188pf1342
(..441..)
2082 -pf08547 -
2083 -pf03982 -
2084 HMMnopfamclust29_1_aci(pf00515pf07719pf13181) CsomeBCy
2085 -# -
2086 -pf09992 -
2087 -pf00805 -
2088 HMMnopfamclust29_1_aci(#) CsomeBCy
2089 -pf08331 -
2090 -pf02136 -
2091 -pf07969 -
2092 H_lime CsomeBCy
2093 H_peaSoup CsomeBCy
2094 -pf03574 -
2095 -# -
2096 -pf12323pf01385pf07282 -
2097 -pf00365 -
2098 -pf07650pf01926 -
2099 -pf00484 -
2100 -# -
2101 HMM00465alcdh_4_pdu(pf01761)
2102 -pf05115 -
2103 -pf07099 -
BMC-P
BMC-H
BMC-T
Aldehyde dehydrogenase
PTAC
Signature enzyme
Alcohol dehydrogenase
Regulator

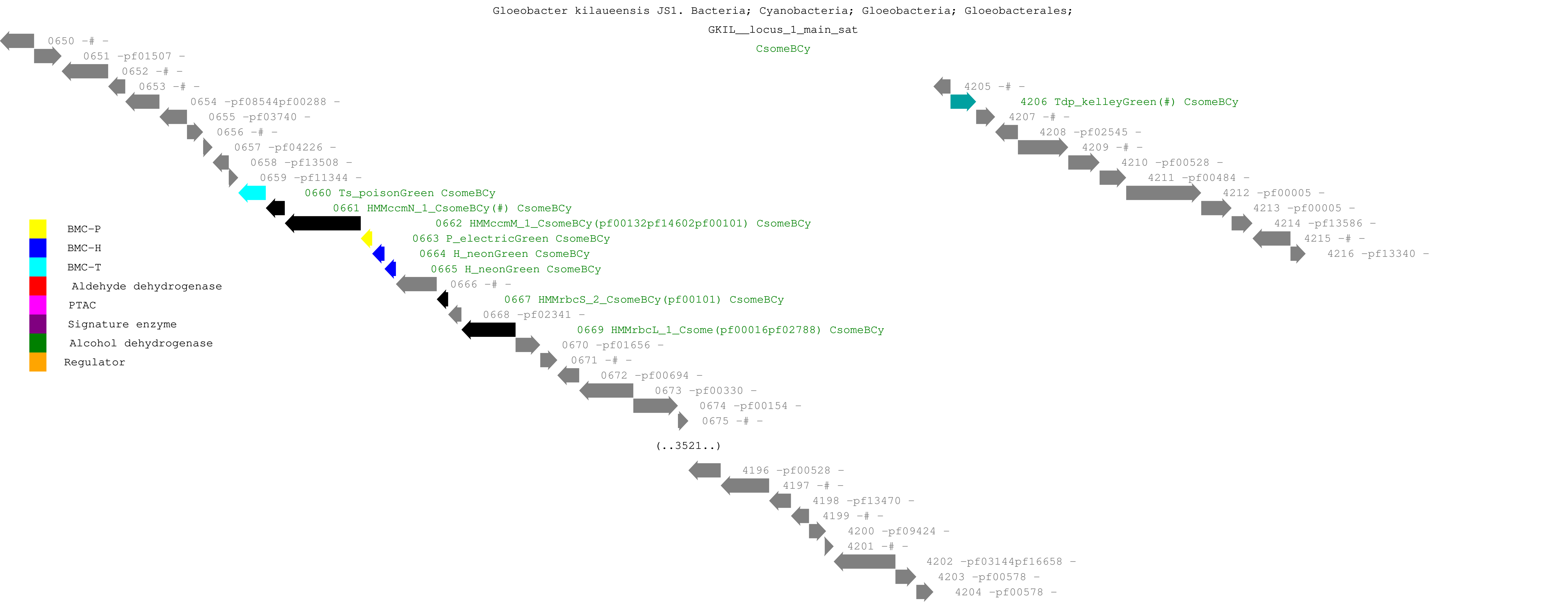
Gloeobacter kilaueensis JS1. Bacteria; Cyanobacteria; Gloeobacteria; Gloeobacterales;
GKIL__locus_1_main_sat
CsomeBCy
0650 -# -
0651 -pf01507 -
0652 -# -
0653 -# -
0654 -pf08544pf00288 -
0655 -pf03740 -
0656 -# -
0657 -pf04226 -
0658 -pf13508 -
0659 -pf11344 -
0660 Ts_poisonGreen CsomeBCy
0661 HMMccmN_1_CsomeBCy(#) CsomeBCy
0662 HMMccmM_1_CsomeBCy(pf00132pf14602pf00101) CsomeBCy
0663 P_electricGreen CsomeBCy
0664 H_neonGreen CsomeBCy
0665 H_neonGreen CsomeBCy
0666 -# -
0667 HMMrbcS_2_CsomeBCy(pf00101) CsomeBCy
0668 -pf02341 -
0669 HMMrbcL_1_Csome(pf00016pf02788) CsomeBCy
0670 -pf01656 -
0671 -# -
0672 -pf00694 -
0673 -pf00330 -
0674 -pf00154 -
0675 -# -
(..3521..)
4196 -pf00528 -
4197 -# -
4198 -pf13470 -
4199 -# -
4200 -pf09424 -
4201 -# -
4202 -pf03144pf16658 -
4203 -pf00578 -
4204 -pf00578 -
4205 -# -
4206 Tdp_kelleyGreen(#) CsomeBCy
4207 -# -
4208 -pf02545 -
4209 -# -
4210 -pf00528 -
4211 -pf00484 -
4212 -pf00005 -
4213 -pf00005 -
4214 -pf13586 -
4215 -# -
4216 -pf13340 -
BMC-P
BMC-H
BMC-T
Aldehyde dehydrogenase
PTAC
Signature enzyme
Alcohol dehydrogenase
Regulator

Gloeomargarita lithophora Alchichica-D10. Bacteria; Cyanobacteria; Gloeomargaritales; Gloeomargaritaceae;

GlitD10__locus_1_main_sat

CsomeBCy

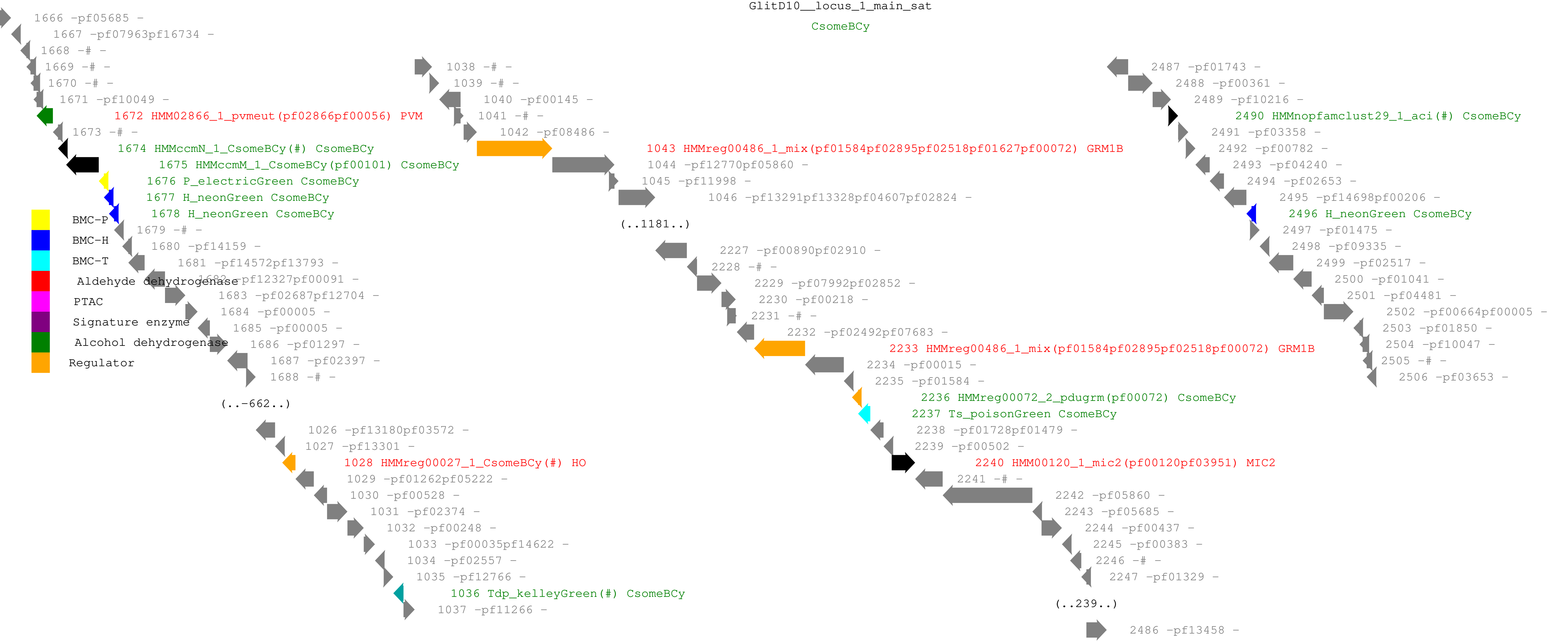

Gloeocapsa sp. PCC 73106. Bacteria; Cyanobacteria; Oscillatoriophycideae; Chroococcales;

GLO73106DRAFT__locus_1_main_sat

CsomeBCy

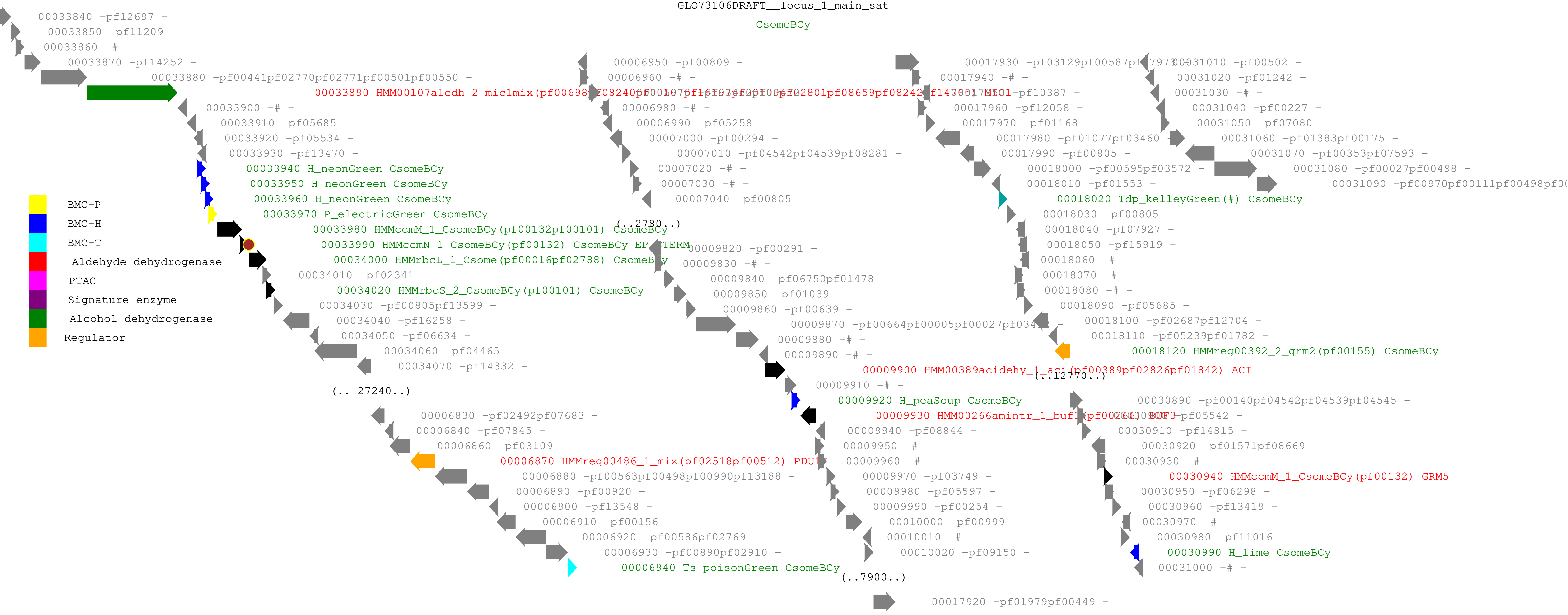

Gloeocapsa sp. PCC 7428. Bacteria; Cyanobacteria; Oscillatoriophycideae; Chroococcales;

Glo7428__locus_1_main_sat

CsomeBCy

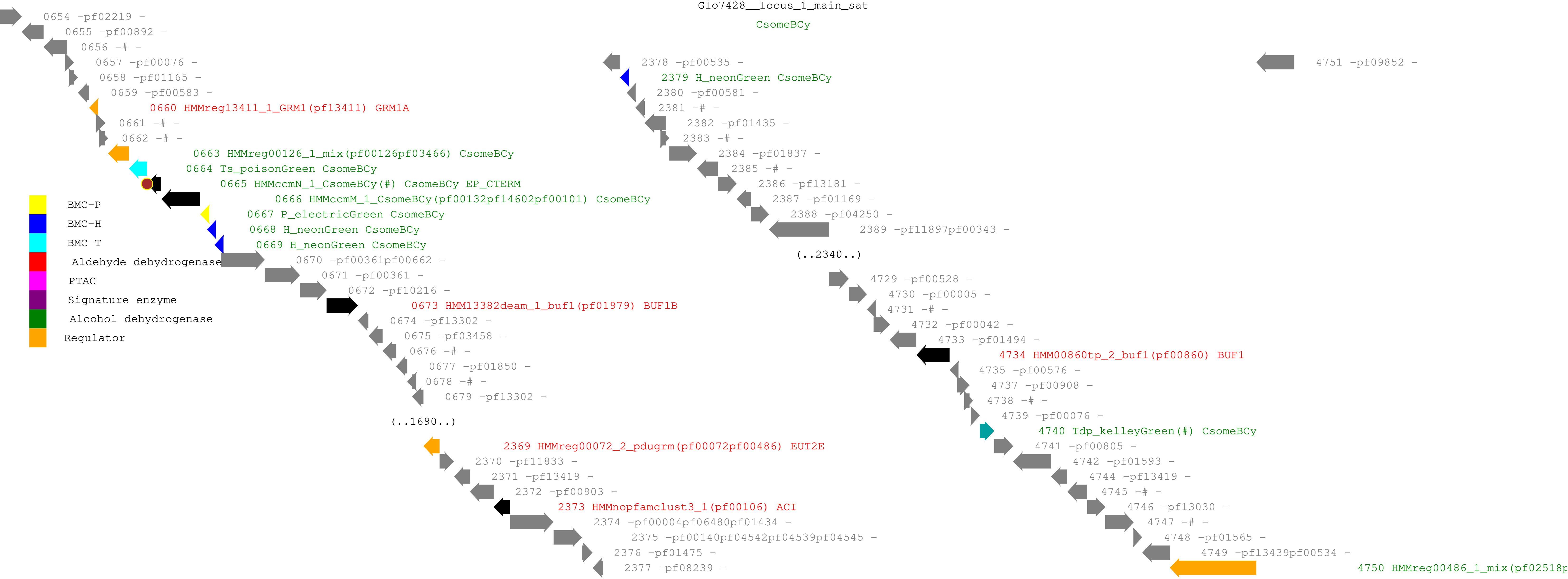

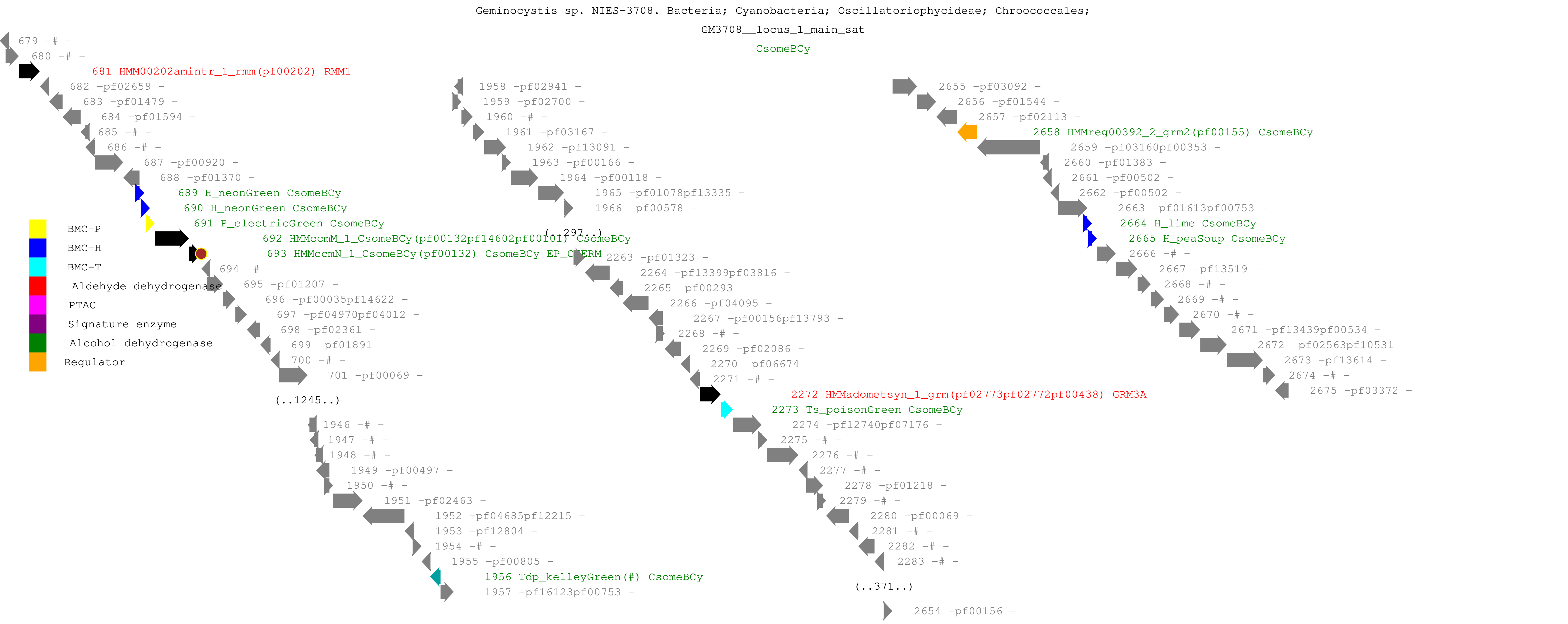

Geminocystis sp. NIES-3708. Bacteria; Cyanobacteria; Oscillatoriophycideae; Chroococcales;
GM3708__locus_1_main_sat
CsomeBCy
679 -# -
680 -# -
681 HMM00202amintr_1_rmm(pf00202) RMM1
682 -pf02659 -
683 -pf01479 -
684 -pf01594 -
685 -# -
686 -# -
687 -pf00920 -
688 -pf01370 -
689 H_neonGreen CsomeBCy
690 H_neonGreen CsomeBCy
691 P_electricGreen CsomeBCy
692 HMMccmM_1_CsomeBCy(pf00132pf14602pf00101) CsomeBCy
693 HMMccmN_1_CsomeBCy(pf00132) CsomeBCy EP_CTERM
694 -# -
695 -pf01207 -
696 -pf00035pf14622 -
697 -pf04970pf04012 -
698 -pf02361 -
699 -pf01891 -
700 -# -
701 -pf00069 -
(..1245..)
1946 -# -
1947 -# -
1948 -# -
1949 -pf00497 -
1950 -# -
1951 -pf02463 -
1952 -pf04685pf12215 -
1953 -pf12804 -
1954 -# -
1955 -pf00805 -
1956 Tdp_kelleyGreen(#) CsomeBCy
1957 -pf16123pf00753 -
1958 -pf02941 -
1959 -pf02700 -
1960 -# -
1961 -pf03167 -
1962 -pf13091 -
1963 -pf00166 -
1964 -pf00118 -
1965 -pf01078pf13335 -
1966 -pf00578 -
(..297..)
2263 -pf01323 -
2264 -pf13399pf03816 -
2265 -pf00293 -
2266 -pf04095 -
2267 -pf00156pf13793 -
2268 -# -
2269 -pf02086 -
2270 -pf06674 -
2271 -# -
2272 HMMadometsyn_1_grm(pf02773pf02772pf00438) GRM3A
2273 Ts_poisonGreen CsomeBCy
2274 -pf12740pf07176 -
2275 -# -
2276 -# -
2277 -# -
2278 -pf01218 -
2279 -# -
2280 -pf00069 -
2281 -# -
2282 -# -
2283 -# -
(..371..)
2654 -pf00156 -
2655 -pf03092 -
2656 -pf01544 -
2657 -pf02113 -
2658 HMMreg00392_2_grm2(pf00155) CsomeBCy
2659 -pf03160pf00353 -
2660 -pf01383 -
2661 -pf00502 -
2662 -pf00502 -
2663 -pf01613pf00753 -
2664 H_lime CsomeBCy
2665 H_peaSoup CsomeBCy
2666 -# -
2667 -pf13519 -
2668 -# -
2669 -# -
2670 -# -
2671 -pf13439pf00534 -
2672 -pf02563pf10531 -
2673 -pf13614 -
2674 -# -
2675 -pf03372 -
BMC-P
BMC-H
BMC-T
Aldehyde dehydrogenase
PTAC
Signature enzyme
Alcohol dehydrogenase
Regulator

Geminocystis sp. NIES-3709. Bacteria; Cyanobacteria; Oscillatoriophycideae; Chroococcales;

GM3709__locus_1_main_sat

CsomeBCy

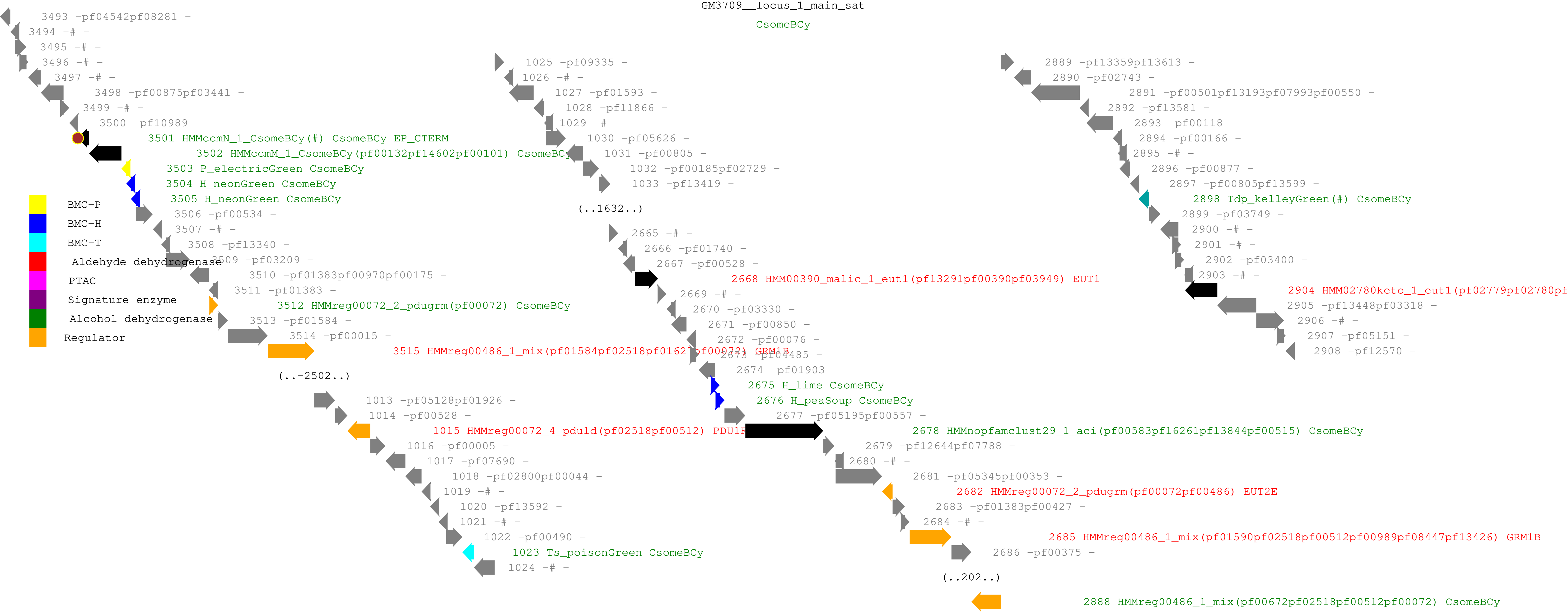

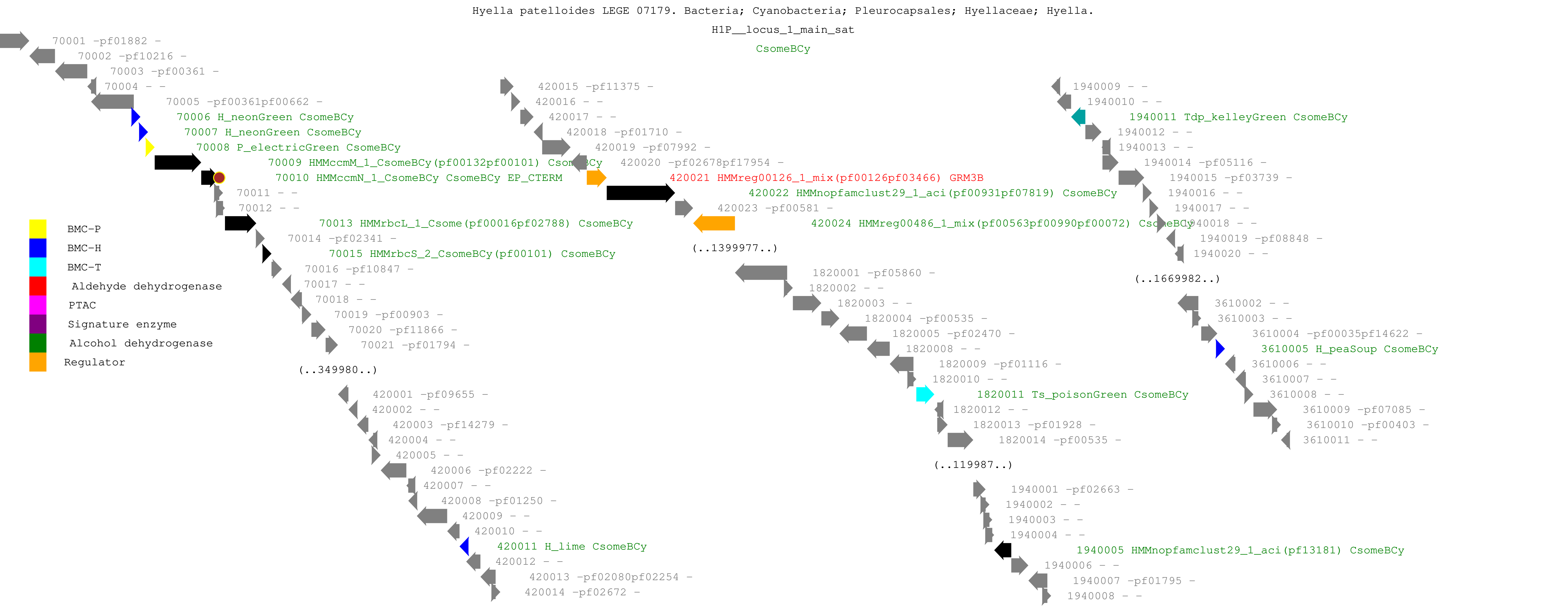
Hyella patelloides LEGE 07179. Bacteria; Cyanobacteria; Pleurocapsales; Hyellaceae; Hyella.
H1P__locus_1_main_sat
CsomeBCy
70001 -pf01882 -
70002 -pf10216 -
70003 -pf00361 -
70004 - -
70005 -pf00361pf00662 -
70006 H_neonGreen CsomeBCy
70007 H_neonGreen CsomeBCy
70008 P_electricGreen CsomeBCy
70009 HMMccmM_1_CsomeBCy(pf00132pf00101)
70010 HMMccmN_1_CsomeBCy CsomeBCy EP_CTERM
70011 - -
70012 - -
70013 HMMrbcL_1_Csome(pf00016pf02788) CsomeBCy
70014 -pf02341 -
70015 HMMrbcS_2_CsomeBCy(pf00101) CsomeBCy
70016 -pf10847 -
70017 - -
70018 - -
70019 -pf00903 -
70020 -pf11866 -
70021 -pf01794 -
(..349980..)
420001 -pf09655 -
420002 - -
420003 -pf14279 -
420004 - -
420005 - -
420006 -pf02222 -
420007 - -
420008 -pf01250 -
420009 - -
420010 - -
420011 H_lime CsomeBCy
420012 - -
420013 -pf02080pf02254 -
420014 -pf02672 -
420015 -pf11375 -
420016 - -
420017 - -
420018 -pf01710 -
420019 -pf07992 -
420020 -pf02678pf17954 -
420021 HMMreg00126_1_mix(pf00126pf03466) GRM3B
420022 HMMnopfamclust29_1_aci(pf00931pf07819) CsomeBCy
420023 -pf00581 -
420024 HMMreg00486_1_mix(pf00563pf00990pf00072)
(..1399977..)
1820001 -pf05860 -
1820002 - -
1820003 - -
1820004 -pf00535 -
1820005 -pf02470 -
1820008 - -
1820009 -pf01116 -
1820010 - -
1820011 Ts_poisonGreen CsomeBCy
1820012 - -
1820013 -pf01928 -
1820014 -pf00535 -
(..119987..)
1940001 -pf02663 -
1940002 - -
1940003 - -
1940004 - -
1940005 HMMnopfamclust29_1_aci(pf13181) CsomeBCy
1940006 - -
1940007 -pf01795 -
1940008 - -
1940009 - -
1940010 - -
1940011 Tdp_kelleyGreen CsomeBCy
1940012 - -
1940013 - -
1940014 -pf05116 -
1940015 -pf03739 -
1940016 - -
1940017 - -
1940018 - -
1940019 -pf08848 -
1940020 - -
(..1669982..)
3610002 - -
3610003 - -
3610004 -pf00035pf14622 -
3610005 H_peaSoup CsomeBCy
3610006 - -
3610007 - -
3610008 - -
3610009 -pf07085 -
3610010 -pf00403 -
3610011 - -
BMC-P
BMC-H
BMC-T
Aldehyde dehydrogenase
PTAC
Signature enzyme
Alcohol dehydrogenase
Regulator

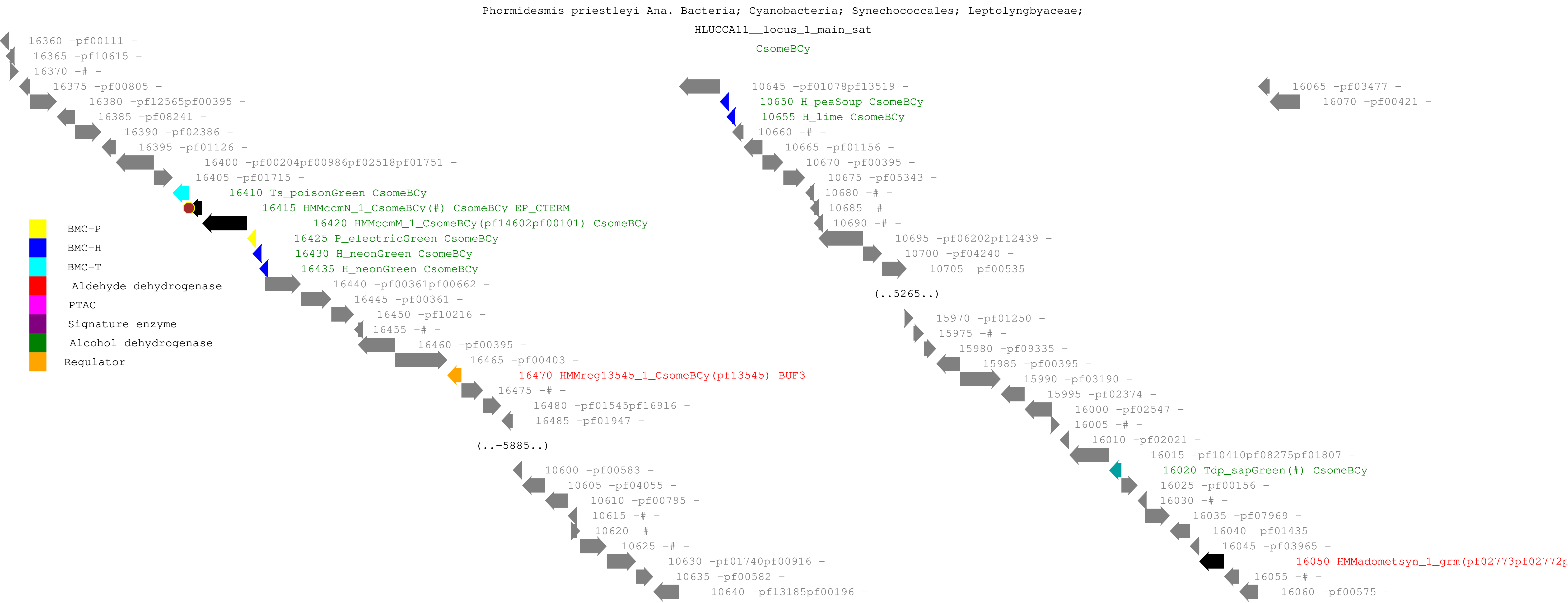

Phormidesmis priestleyi Ana. Bacteria; Cyanobacteria; Synechococcales; Leptolyngbyaceae;
HLUCCA11__locus_1_main_sat
CsomeBCy
16360 -pf00111 -
16365 -pf10615 -
16370 -# -
16375 -pf00805 -
16380 -pf12565pf00395 -
16385 -pf08241 -
16390 -pf02386 -
16395 -pf01126 -
16400 -pf00204pf00986pf02518pf01751 -
16405 -pf01715 -
16410 Ts_poisonGreen CsomeBCy
16415 HMMccmN_1_CsomeBCy(#) CsomeBCy EP_CTERM
16420 HMMccmM_1_CsomeBCy(pf14602pf00101) CsomeBCy
16425 P_electricGreen CsomeBCy
16430 H_neonGreen CsomeBCy
16435 H_neonGreen CsomeBCy
16440 -pf00361pf00662 -
16445 -pf00361 -
16450 -pf10216 -
16455 -# -
16460 -pf00395 -
16465 -pf00403 -
16470 HMMreg13545_1_CsomeBCy(pf13545) BUF3
16475 -# -
16480 -pf01545pf16916 -
16485 -pf01947 -
(..-5885..)
10600 -pf00583 -
10605 -pf04055 -
10610 -pf00795 -
10615 -# -
10620 -# -
10625 -# -
10630 -pf01740pf00916 -
10635 -pf00582 -
10640 -pf13185pf00196 -
BMC-P
BMC-H
BMC-T
Aldehyde dehydrogenase
PTAC
Signature enzyme
Alcohol dehydrogenase
Regulator
10645 -pf01078pf13519 -
10650 H_peaSoup CsomeBCy
10655 H_lime CsomeBCy
10660 -# -
10665 -pf01156 -
10670 -pf00395 -
10675 -pf05343 -
10680 -# -
10685 -# -
10690 -# -
10695 -pf06202pf12439 -
10700 -pf04240 -
10705 -pf00535 -
(..5265..)
15970 -pf01250 -
15975 -# -
15980 -pf09335 -
15985 -pf00395 -
15990 -pf03190 -
15995 -pf02374 -
16000 -pf02547 -
16005 -# -
16010 -pf02021 -
16015 -pf10410pf08275pf01807 -
16020 Tdp_sapGreen(#) CsomeBCy
16025 -pf00156 -
16030 -# -
16035 -pf07969 -
16040 -pf01435 -
16045 -pf03965 -
16050 HMMadometsyn_1_grm(pf02773pf02772p
16055 -# -
16060 -pf00575 -
16065 -pf03477 -
16070 -pf00421 -

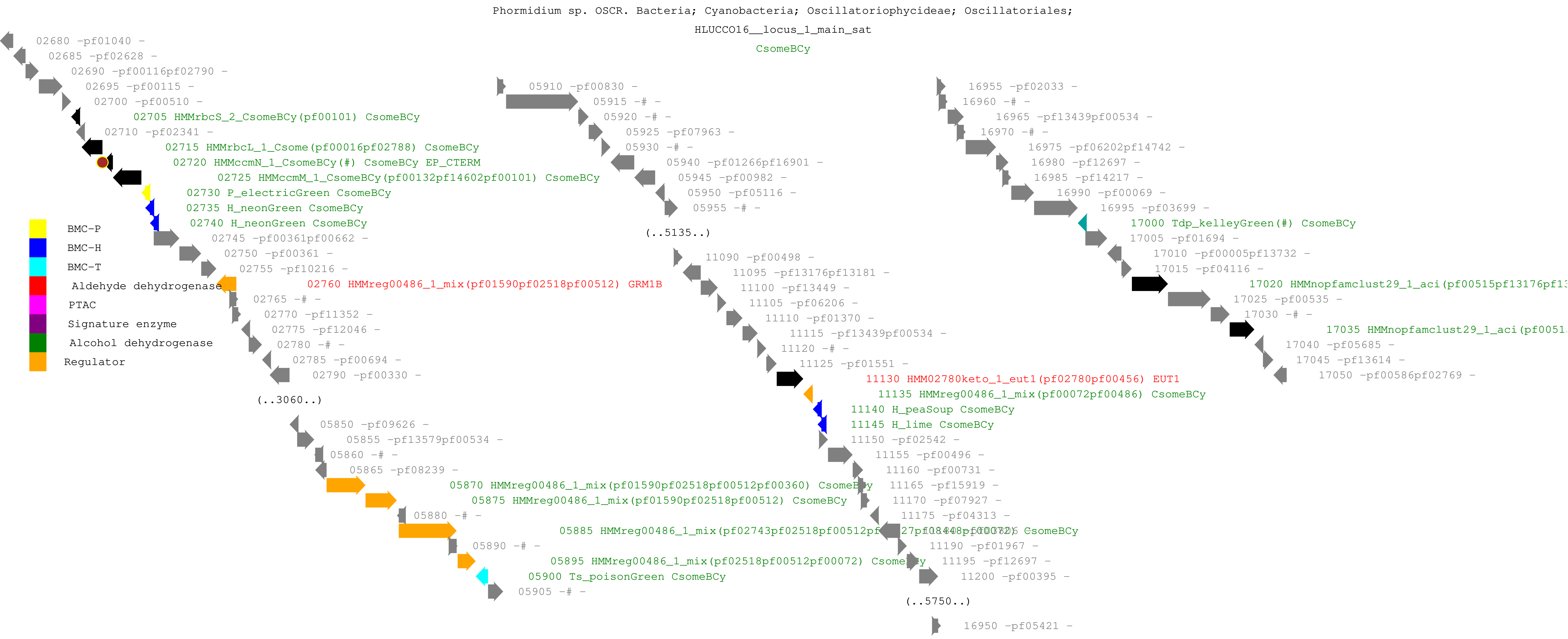

Phormidium sp. OSCR. Bacteria; Cyanobacteria; Oscillatoriophycideae; Oscillatoriales;
HLUCCO16__locus_1_main_sat
CsomeBCy
02680 -pf01040 -
02685 -pf02628 -
02690 -pf00116pf02790 -
02695 -pf00115 -
02700 -pf00510 -
02705 HMMrbcS_2_CsomeBCy(pf00101) CsomeBCy
02710 -pf02341 -
02715 HMMrbcL_1_Csome(pf00016pf02788) CsomeBCy
02720 HMMccmN_1_CsomeBCy(#) CsomeBCy EP_CTERM
02725 HMMccmM_1_CsomeBCy(pf00132pf14602pf00101) CsomeBCy
02730 P_electricGreen CsomeBCy
02735 H_neonGreen CsomeBCy
02740 H_neonGreen CsomeBCy
02745 -pf00361pf00662 -
02750 -pf00361 -
02755 -pf10216 -
02760 HMMreg00486_1_mix(pf01590pf02518pf00512) GRM1B
02765 -# -
02770 -pf11352 -
02775 -pf12046 -
02780 -# -
02785 -pf00694 -
02790 -pf00330 -
(..3060..)
05850 -pf09626 -
05855 -pf13579pf00534 -
05860 -# -
05865 -pf08239 -
05870 HMMreg00486_1_mix(pf01590pf02518pf00512pf00360) CsomeBCy
05875 HMMreg00486_1_mix(pf01590pf02518pf00512) CsomeBCy
05880 -# -
05885 HMMreg00486_1_mix(pf02743pf02518pf00512pf
05890 -# -
05895 HMMreg00486_1_mix(pf02518pf00512pf00072) CsomeBCy
05900 Ts_poisonGreen CsomeBCy
05905 -# -
05910 -pf00830 -
05915 -# -
05920 -# -
05925 -pf07963 -
05930 -# -
05940 -pf01266pf16901 -
05945 -pf00982 -
05950 -pf05116 -
05955 -# -
(..5135..)
11090 -pf00498 -
11095 -pf13176pf13181 -
11100 -pf13449 -
11105 -pf06206 -
11110 -pf01370 -
11115 -pf13439pf00534 -
11120 -# -
11125 -pf01551 -
11130 HMM02780keto_1_eut1(pf02780pf00456) EUT1
11135 HMMreg00486_1_mix(pf00072pf00486) CsomeBCy
11140 H_peaSoup CsomeBCy
11145 H_lime CsomeBCy
11150 -pf02542 -
11155 -pf00496 -
11160 -pf00731 -
11165 -pf15919 -
11170 -pf07927 -
11175 -pf04313 -
11190 -pf01967 -
11195 -pf12697 -
11200 -pf00395 -
(..5750..)
16950 -pf05421 -
16955 -pf02033 -
16960 -# -
16965 -pf13439pf00534 -
16970 -# -
16975 -pf06202pf14742 -
16980 -pf12697 -
16985 -pf14217 -
16990 -pf00069 -
16995 -pf03699 -
17000 Tdp_kelleyGreen(#) CsomeBCy
17005 -pf01694 -
17010 -pf00005pf13732 -
17015 -pf04116 -
17020 HMMnopfamclust29_1_aci(pf00515pf13176pf1
17025 -pf00535 -
17030 -# -
17035 HMMnopfamclust29_1_aci(pf0051
17040 -pf05685 -
17045 -pf13614 -
17050 -pf00586pf02769 -
BMC-P
BMC-H
BMC-T
Aldehyde dehydrogenase
PTAC
Signature enzyme
Alcohol dehydrogenase
Regulator

Calothrix sp. 336/3. Bacteria; Cyanobacteria; Nostocales; Rivulariaceae; Calothrix.

IJ00__locus_1_main_sat

CsomeBCy

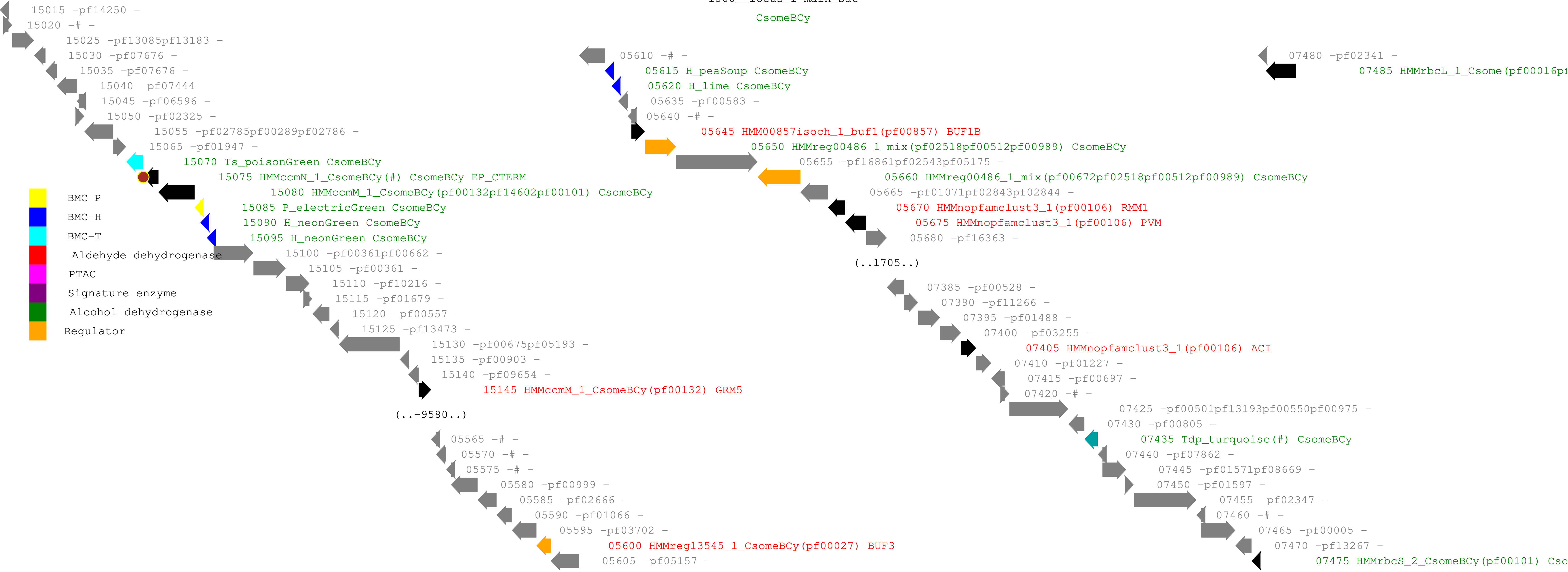

Microcystis aeruginosa PCC 7806. Bacteria; Cyanobacteria; Oscillatoriophycideae; Chroococcales;

IPF__locus_fragmented

CsomeBCy

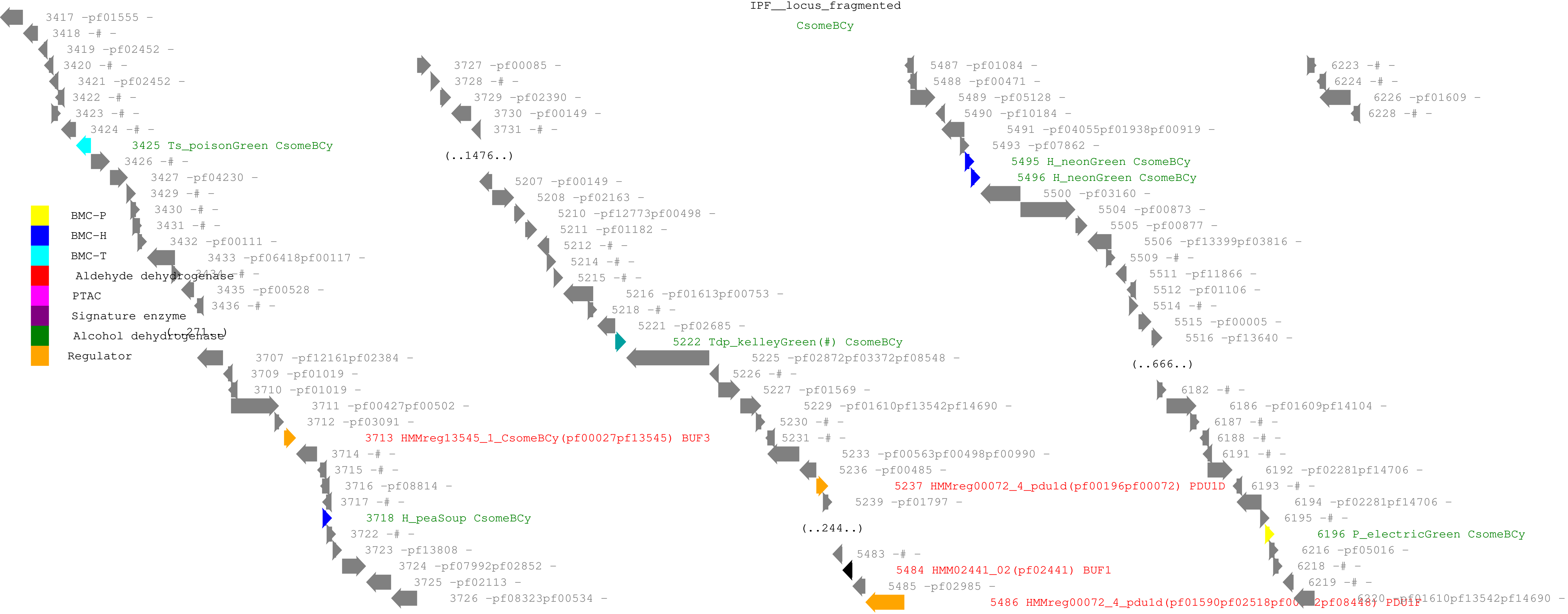

Rubidibacter lacunae KORDI 51-2. Bacteria; Cyanobacteria; Oscillatoriophycideae; Chroococcales;

KR51__locus_1_main_sat

CsomeBCy

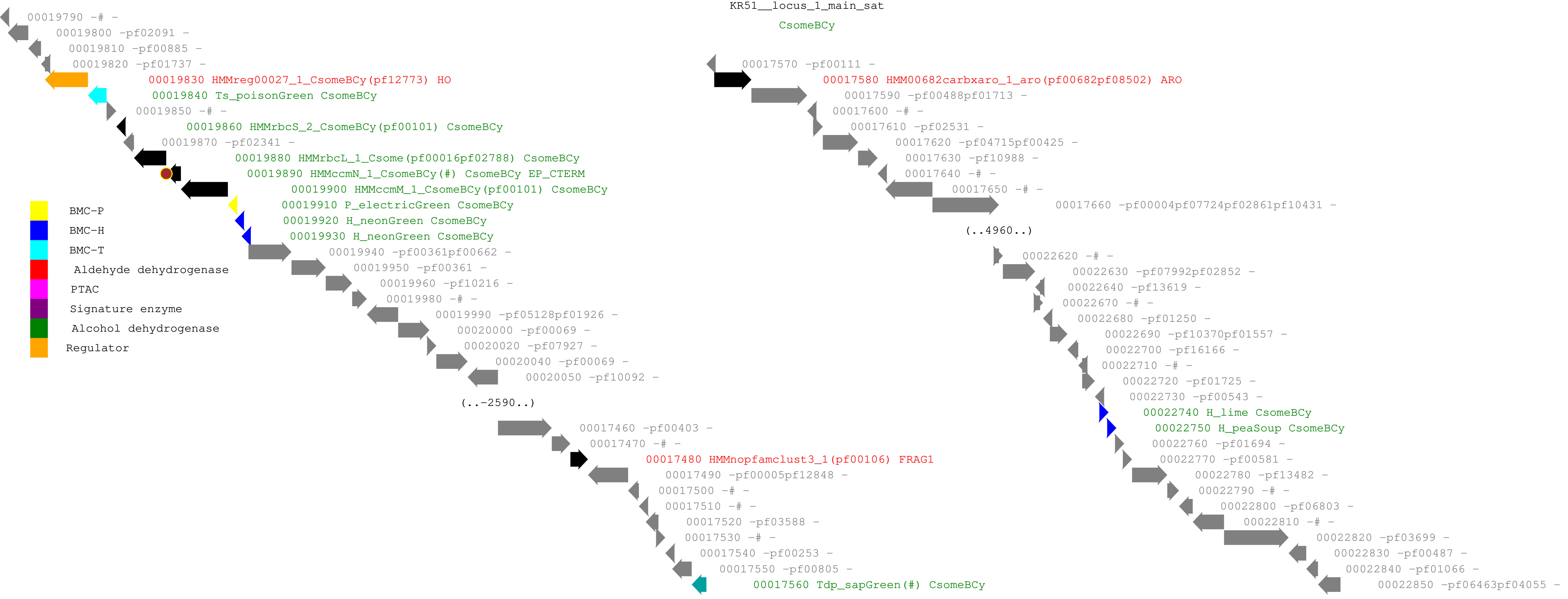

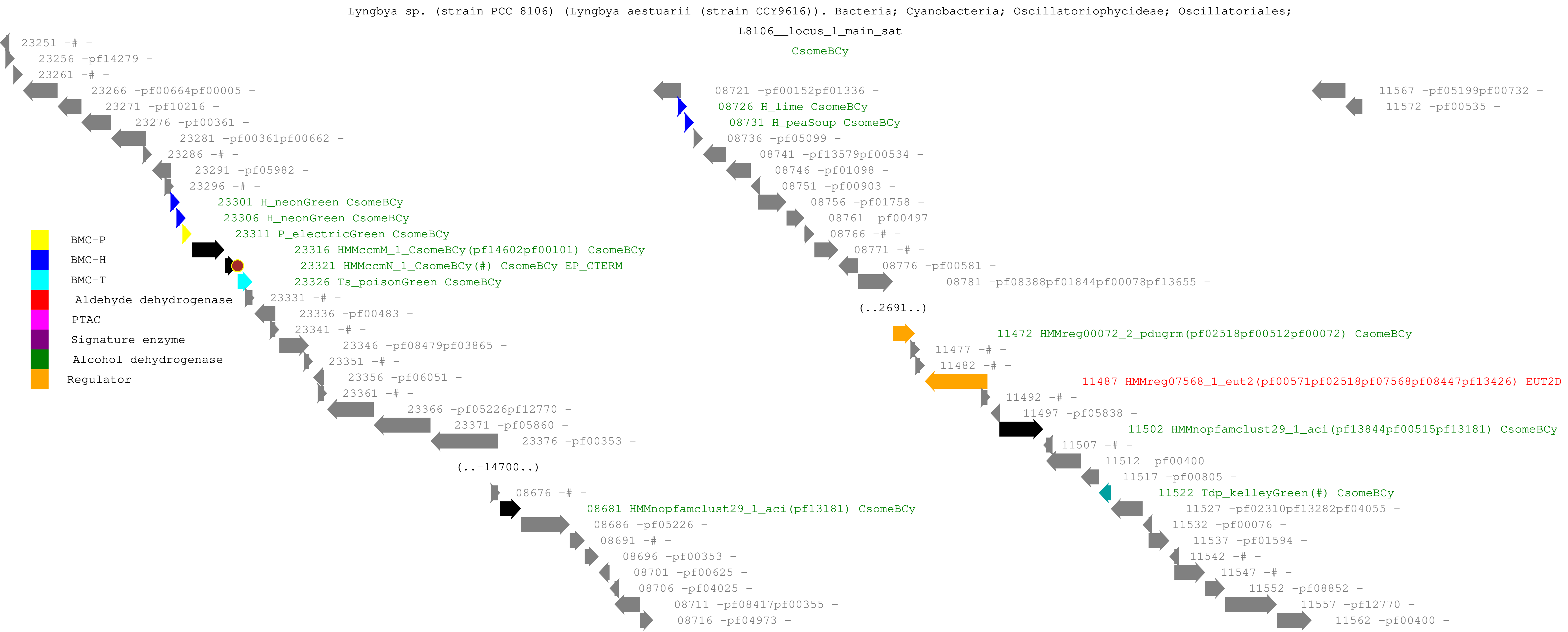
Lyngbya sp. (strain PCC 8106) (Lyngbya aestuarii (strain CCY9616)). Bacteria; Cyanobacteria; Oscillatoriophycideae; Oscillatoriales;
L8106__locus_1_main_sat
CsomeBCy
23251 -# -
23256 -pf14279 -
23261 -# -
23266 -pf00664pf00005 -
23271 -pf10216 -
23276 -pf00361 -
23281 -pf00361pf00662 -
23286 -# -
23291 -pf05982 -
23296 -# -
23301 H_neonGreen CsomeBCy
23306 H_neonGreen CsomeBCy
23311 P_electricGreen CsomeBCy
23316 HMMccmM_1_CsomeBCy(pf14602pf00101) CsomeBCy
23321 HMMccmN_1_CsomeBCy(#) CsomeBCy EP_CTERM
23326 Ts_poisonGreen CsomeBCy
23331 -# -
23336 -pf00483 -
23341 -# -
23346 -pf08479pf03865 -
23351 -# -
23356 -pf06051 -
23361 -# -
23366 -pf05226pf12770 -
23371 -pf05860 -
23376 -pf00353 -
(..-14700..)
08676 -# -
08681 HMMnopfamclust29_1_aci(pf13181) CsomeBCy
08686 -pf05226 -
08691 -# -
08696 -pf00353 -
08701 -pf00625 -
08706 -pf04025 -
08711 -pf08417pf00355 -
08716 -pf04973 -
08721 -pf00152pf01336 -
08726 H_lime CsomeBCy
08731 H_peaSoup CsomeBCy
08736 -pf05099 -
08741 -pf13579pf00534 -
08746 -pf01098 -
08751 -pf00903 -
08756 -pf01758 -
08761 -pf00497 -
08766 -# -
08771 -# -
08776 -pf00581 -
08781 -pf08388pf01844pf00078pf13655 -
(..2691..)
11472 HMMreg00072_2_pdugrm(pf02518pf00512pf00072) CsomeBCy
11477 -# -
11482 -# -
11487 HMMreg07568_1_eut2(pf00571pf02518pf07568pf08447pf13426) EUT2D
11492 -# -
11497 -pf05838 -
11502 HMMnopfamclust29_1_aci(pf13844pf00515pf13181) CsomeBCy
11507 -# -
11512 -pf00400 -
11517 -pf00805 -
11522 Tdp_kelleyGreen(#) CsomeBCy
11527 -pf02310pf13282pf04055 -
11532 -pf00076 -
11537 -pf01594 -
11542 -# -
11547 -# -
11552 -pf08852 -
11557 -pf12770 -
11562 -pf00400 -
11567 -pf05199pf00732 -
11572 -pf00535 -
BMC-P
BMC-H
BMC-T
Aldehyde dehydrogenase
PTAC
Signature enzyme
Alcohol dehydrogenase
Regulator

Leptolyngbya sp. NIES-3755. Bacteria; Cyanobacteria; Synechococcales; Leptolyngbyaceae;

LEP3755__locus_1_main_sat

CsomeBCy

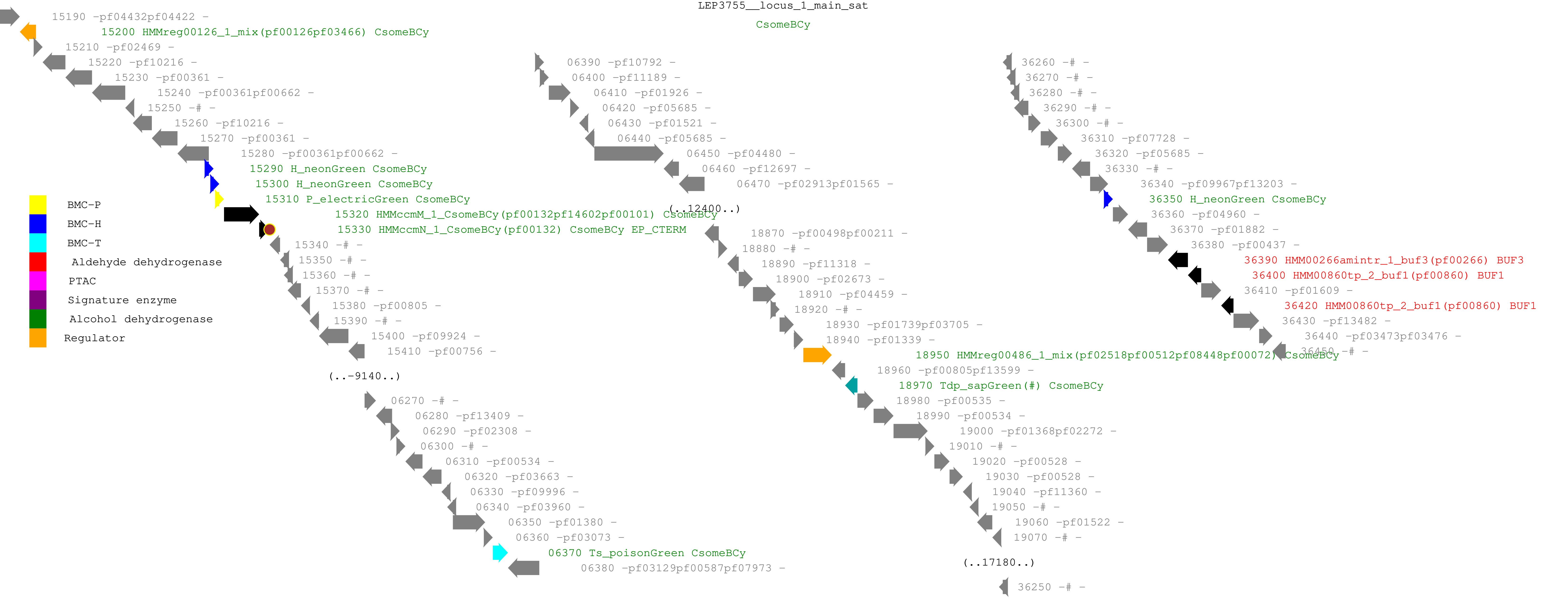

Leptolyngbya sp. PCC 7375. Bacteria; Cyanobacteria; Synechococcales; Leptolyngbyaceae;

Lepto7375DRAFT__locus_1_main_sat

CsomeBCy

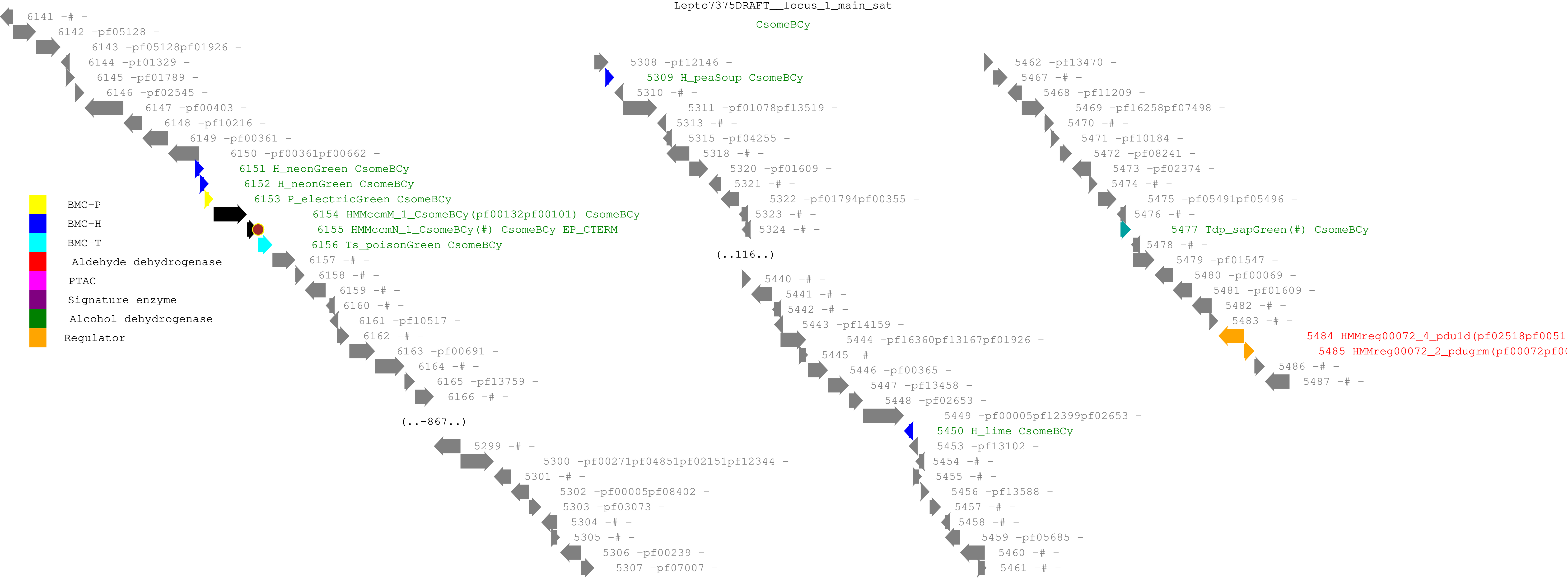

Leptolyngbya sp. PCC 7376. Bacteria; Cyanobacteria; Synechococcales; Leptolyngbyaceae;

Lepto7376__locus_1_main_sat

CsomeBCy

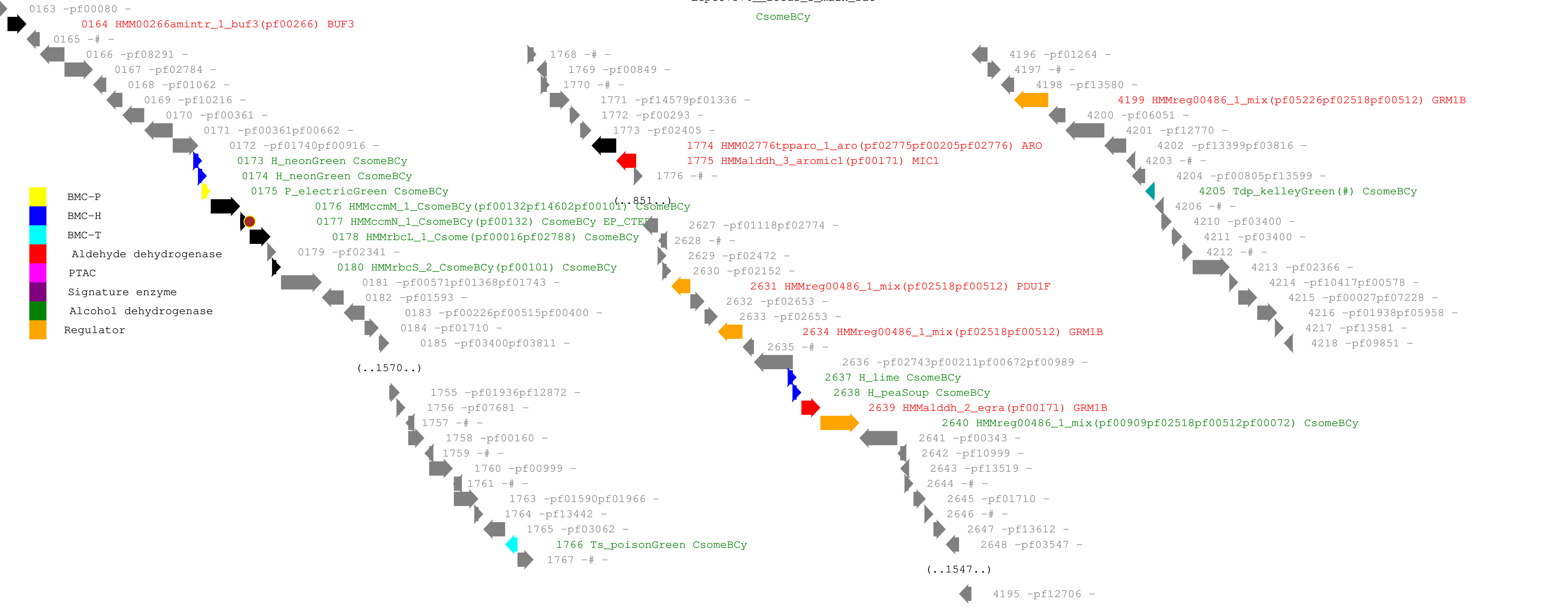

Moorea producens 3L. Bacteria; Cyanobacteria; Oscillatoriophycideae; Oscillatoriales;

LYNGBM3L__locus_1_main_sat

CsomeBCy

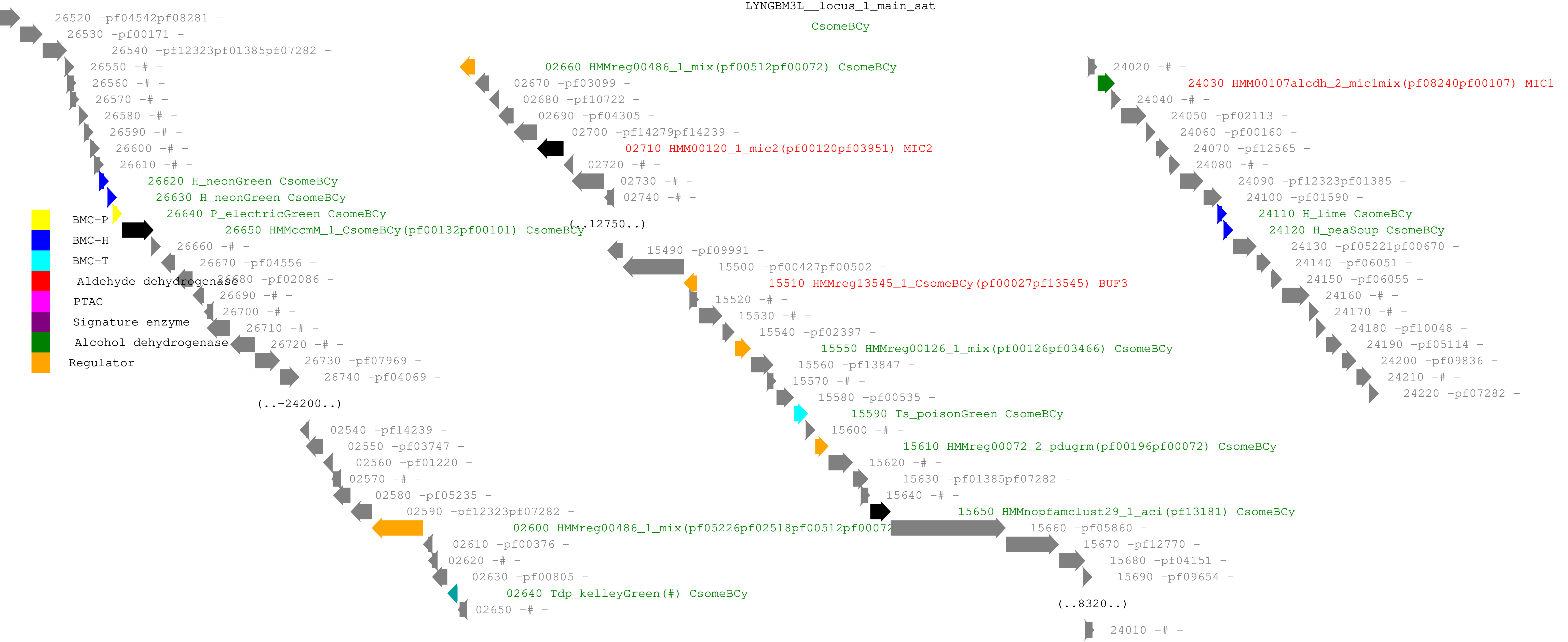

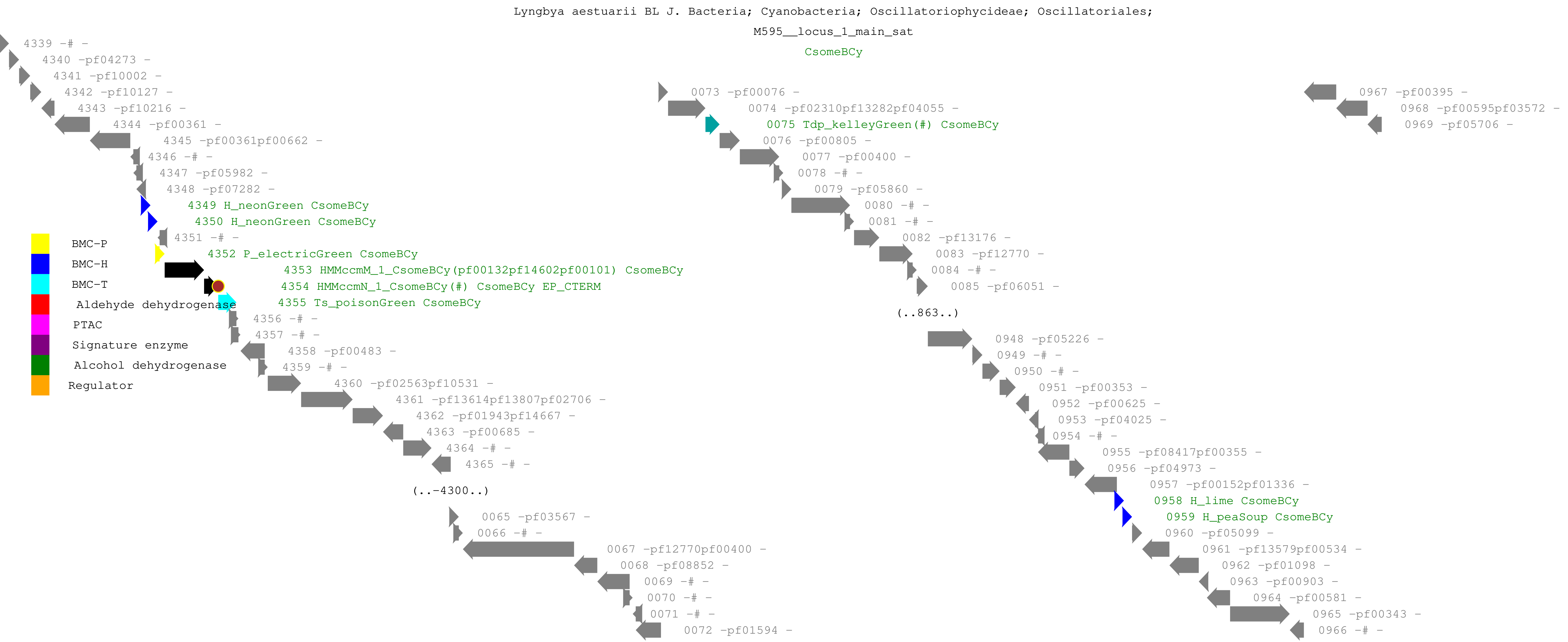
Lyngbya aestuarii BL J. Bacteria; Cyanobacteria; Oscillatoriophycideae; Oscillatoriales;
M595__locus_1_main_sat
CsomeBCy
BMC-P
BMC-H
BMC-T
Aldehyde dehydrogenase
PTAC
Signature enzyme
Alcohol dehydrogenase
Regulator
4339 -# -
4340 -pf04273 -
4341 -pf10002 -
4342 -pf10127 -
4343 -pf10216 -
4344 -pf00361 -
4345 -pf00361pf00662 -
4346 -# -
4347 -pf05982 -
4348 -pf07282 -
4349 H_neonGreen CsomeBCy
4350 H_neonGreen CsomeBCy
4351 -# -
4352 P_electricGreen CsomeBCy
4353 HMMccmM_1_CsomeBCy(pf00132pf14602pf00101) CsomeBCy
4354 HMMccmN_1_CsomeBCy(#) CsomeBCy EP_CTERM
4355 Ts_poisonGreen CsomeBCy
4356 -# -
4357 -# -
4358 -pf00483 -
4359 -# -
4360 -pf02563pf10531 -
4361 -pf13614pf13807pf02706 -
4362 -pf01943pf14667 -
4363 -pf00685 -
4364 -# -
4365 -# -
(..-4300..)
0065 -pf03567 -
0066 -# -
0067 -pf12770pf00400 -
0068 -pf08852 -
0069 -# -
0070 -# -
0071 -# -
0072 -pf01594 -
0073 -pf00076 -
0074 -pf02310pf13282pf04055 -
0075 Tdp_kelleyGreen(#) CsomeBCy
0076 -pf00805 -
0077 -pf00400 -
0078 -# -
0079 -pf05860 -
0080 -# -
0081 -# -
0082 -pf13176 -
0083 -pf12770 -
0084 -# -
0085 -pf06051 -
(..863..)
0948 -pf05226 -
0949 -# -
0950 -# -
0951 -pf00353 -
0952 -pf00625 -
0953 -pf04025 -
0954 -# -
0955 -pf08417pf00355 -
0956 -pf04973 -
0957 -pf00152pf01336 -
0958 H_lime CsomeBCy
0959 H_peaSoup CsomeBCy
0960 -pf05099 -
0961 -pf13579pf00534 -
0962 -pf01098 -
0963 -pf00903 -
0964 -pf00581 -
0965 -pf00343 -
0966 -# -
0967 -pf00395 -
0968 -pf00595pf03572 -
0969 -pf05706 -

Microcystis aeruginosa 11-30S32. Bacteria; Cyanobacteria; Oscillatoriophycideae; Chroococcales;

MAE30S32__locus_1_main_sat

CsomeBCy

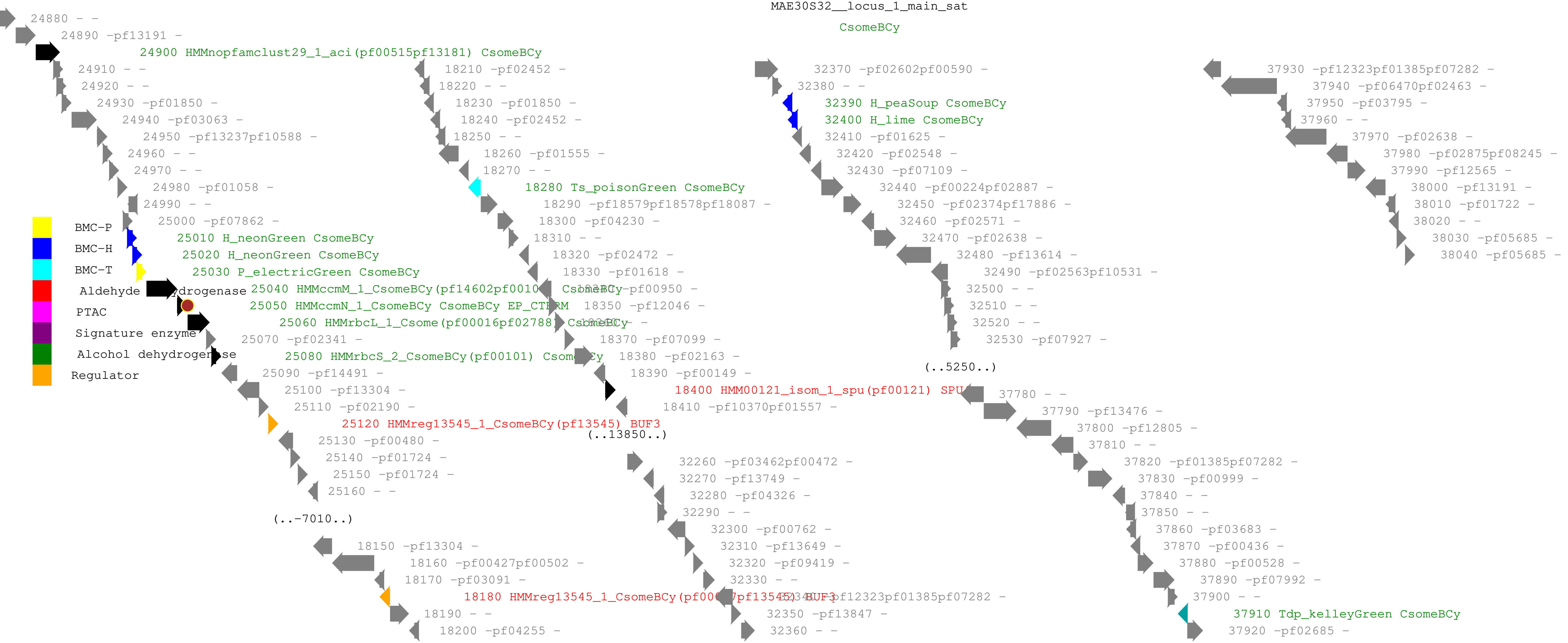

Microcystis aeruginosa (strain NIES-843). Bacteria; Cyanobacteria; Oscillatoriophycideae; Chroococcales;

MAE__locus_1_main_sat

CsomeBCy

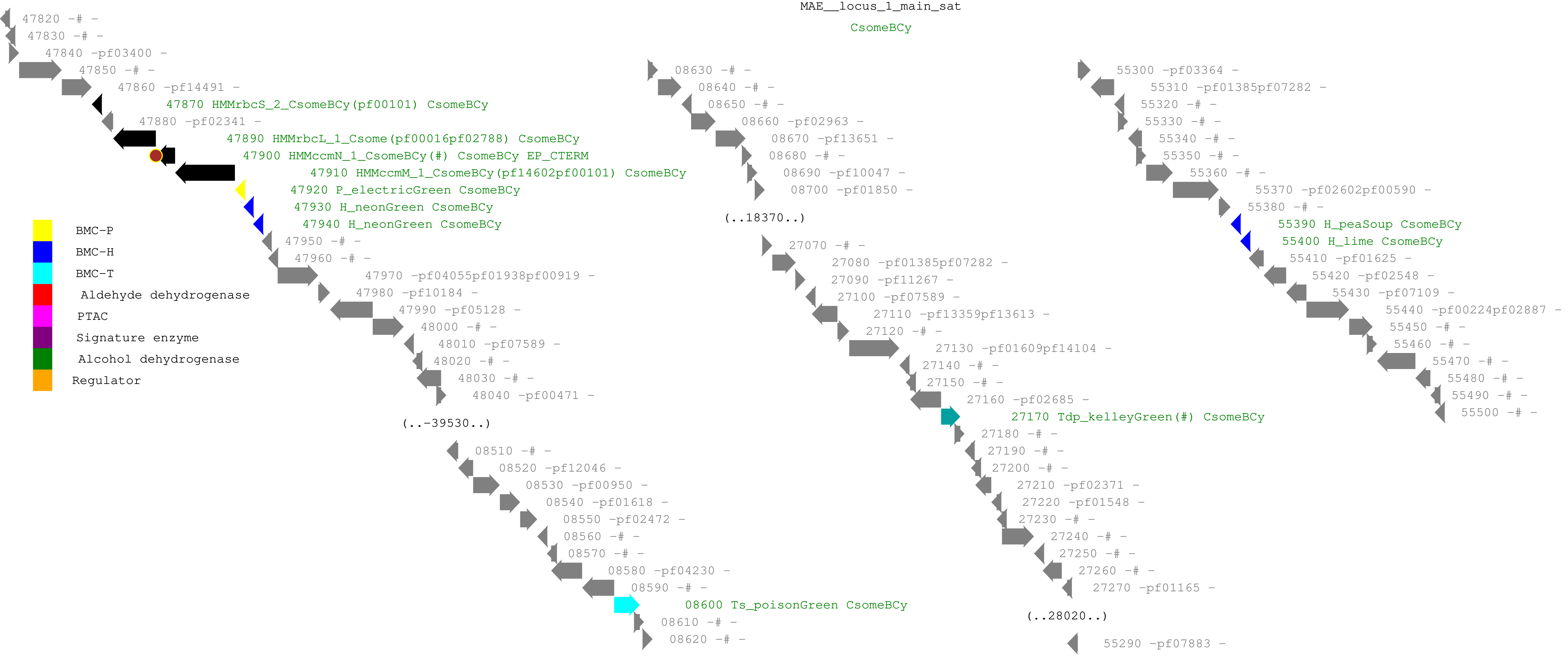

Microcystis aeruginosa SPC777. Bacteria; Cyanobacteria; Oscillatoriophycideae; Chroococcales;

MAESPC__locus_1_main_sat

CsomeBCy

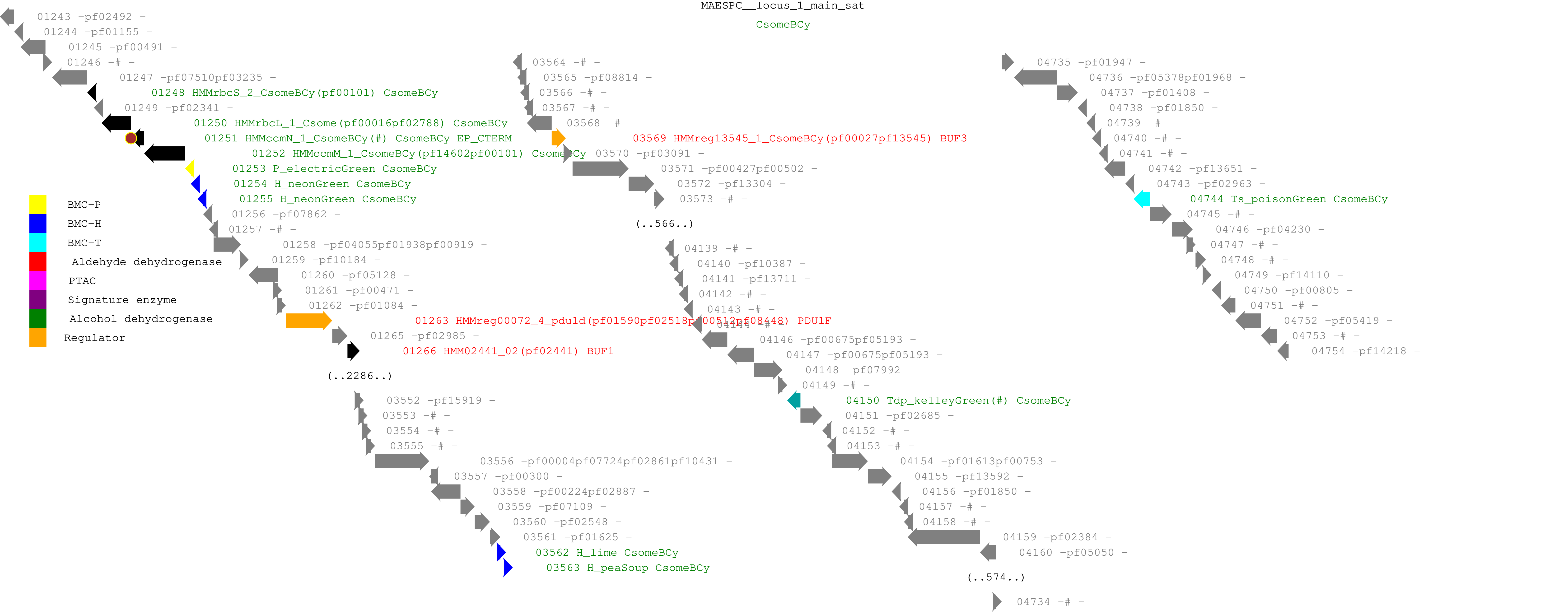

Coleofasciculus chthonoplastes PCC 7420. Bacteria; Cyanobacteria; Oscillatoriophycideae; Oscillatoriales;

MC7420__locus_1_main_sat

CsomeBCy

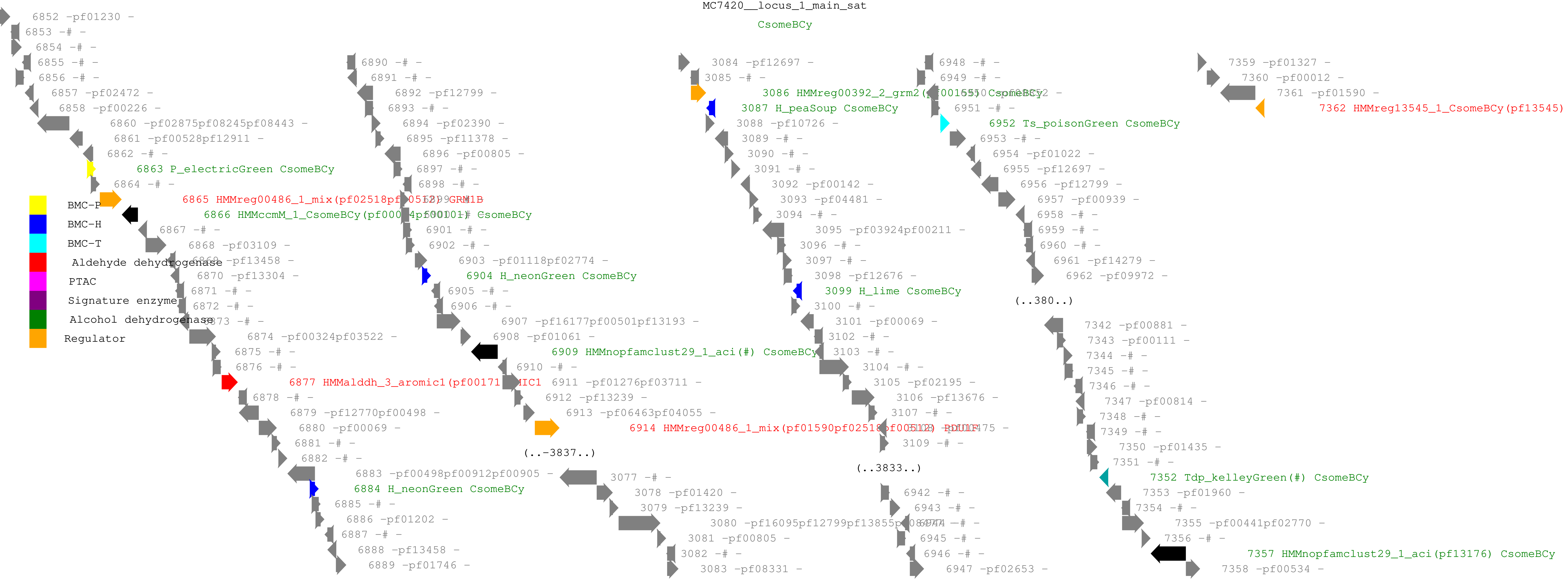

Microcystis aeruginosa NIES-4285. Bacteria; Cyanobacteria; Oscillatoriophycideae; Chroococcales;

MiAbB__locus_1_main_sat

CsomeBCy

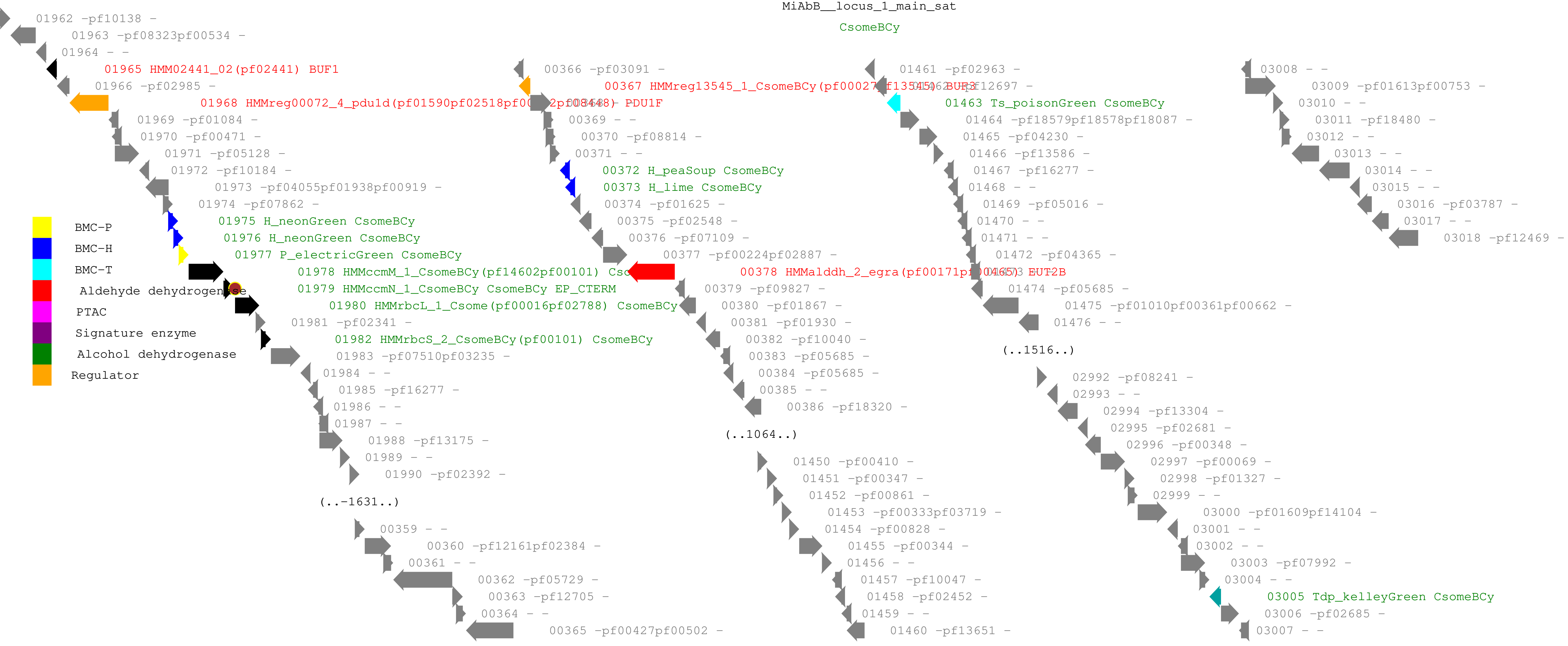

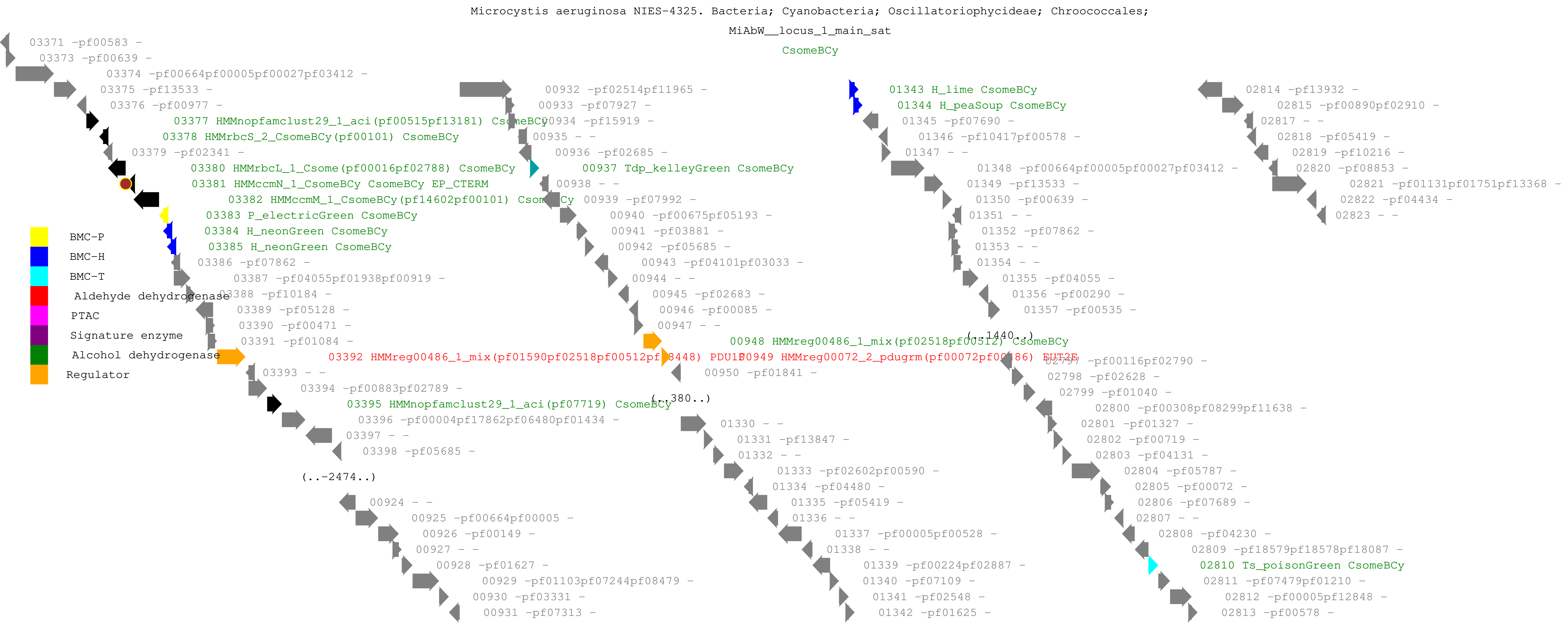
Microcystis aeruginosa NIES-4325. Bacteria; Cyanobacteria; Oscillatoriophycideae; Chroococcales;
MiAbW__locus_1_main_sat
CsomeBCy
03371 -pf00583 -
03373 -pf00639 -
03374 -pf00664pf00005pf00027pf03412 -
03375 -pf13533 -
03376 -pf00977 -
03377 HMMnopfamclust29_1_aci(pf00515pf13181) CsomeBCy
03378 HMMrbcS_2_CsomeBCy(pf00101) CsomeBCy
03379 -pf02341 -
03380 HMMrbcL_1_Csome(pf00016pf02788) CsomeBCy
03381 HMMccmN_1_CsomeBCy CsomeBCy EP_CTERM
03382 HMMccmM_1_CsomeBCy(pf14602pf00101) CsomeBCy
03383 P_electricGreen CsomeBCy
03384 H_neonGreen CsomeBCy
03385 H_neonGreen CsomeBCy
03386 -pf07862 -
03387 -pf04055pf01938pf00919 -
03388 -pf10184 -
03389 -pf05128 -
03390 -pf00471 -
03391 -pf01084 -
03392 HMMreg00486_1_mix(pf01590pf02518pf00512pf03448) PDU1
03393 - -
03394 -pf00883pf02789 -
03395 HMMnopfamclust29_1_aci(pf07719) CsomeBCy
03396 -pf00004pf17862pf06480pf01434 -
03397 - -
03398 -pf05685 -
(..-2474..)
00924 - -
00925 -pf00664pf00005 -
00926 -pf00149 -
00927 - -
00928 -pf01627 -
00929 -pf01103pf07244pf08479 -
00930 -pf03331 -
00931 -pf07313 -
00932 -pf02514pf11965 -
00933 -pf07927 -
00934 -pf15919 -
00935 - -
00936 -pf02685 -
00937 Tdp_kelleyGreen CsomeBCy
00938 - -
00939 -pf07992 -
00940 -pf00675pf05193 -
00941 -pf03881 -
00942 -pf05685 -
00943 -pf04101pf03033 -
00944 - -
00945 -pf02683 -
00946 -pf00085 -
00947 - -
00948 HMMreg00486_1_mix(pf02518pf00512) CsomeBCy
00949 HMMreg00072_2_pdugrm(pf00072pf00486) EUT2E
00950 -pf01841 -
(..380..)
01330 - -
01331 -pf13847 -
01332 - -
01333 -pf02602pf00590 -
01334 -pf04480 -
01335 -pf05419 -
01336 - -
01337 -pf00005pf00528 -
01338 - -
01339 -pf00224pf02887 -
01340 -pf07109 -
01341 -pf02548 -
01342 -pf01625 -
01343 H_lime CsomeBCy
01344 H_peaSoup CsomeBCy
01345 -pf07690 -
01346 -pf10417pf00578 -
01347 - -
01348 -pf00664pf00005pf00027pf03412 -
01349 -pf13533 -
01350 -pf00639 -
01351 - -
01352 -pf07862 -
01353 - -
01354 - -
01355 -pf04055 -
01356 -pf00290 -
01357 -pf00535 -
(..1440..)
02797 -pf00116pf02790 -
02798 -pf02628 -
02799 -pf01040 -
02800 -pf00308pf08299pf11638 -
02801 -pf01327 -
02802 -pf00719 -
02803 -pf04131 -
02804 -pf05787 -
02805 -pf00072 -
02806 -pf07689 -
02807 - -
02808 -pf04230 -
02809 -pf18579pf18578pf18087 -
02810 Ts_poisonGreen CsomeBCy
02811 -pf07479pf01210 -
02812 -pf00005pf12848 -
02813 -pf00578 -
02814 -pf13932 -
02815 -pf00890pf02910 -
02817 - -
02818 -pf05419 -
02819 -pf10216 -
02820 -pf08853 -
02821 -pf01131pf01751pf13368 -
02822 -pf04434 -
02823 - -
BMC-P
BMC-H
BMC-T
Aldehyde dehydrogenase
PTAC
Signature enzyme
Alcohol dehydrogenase
Regulator

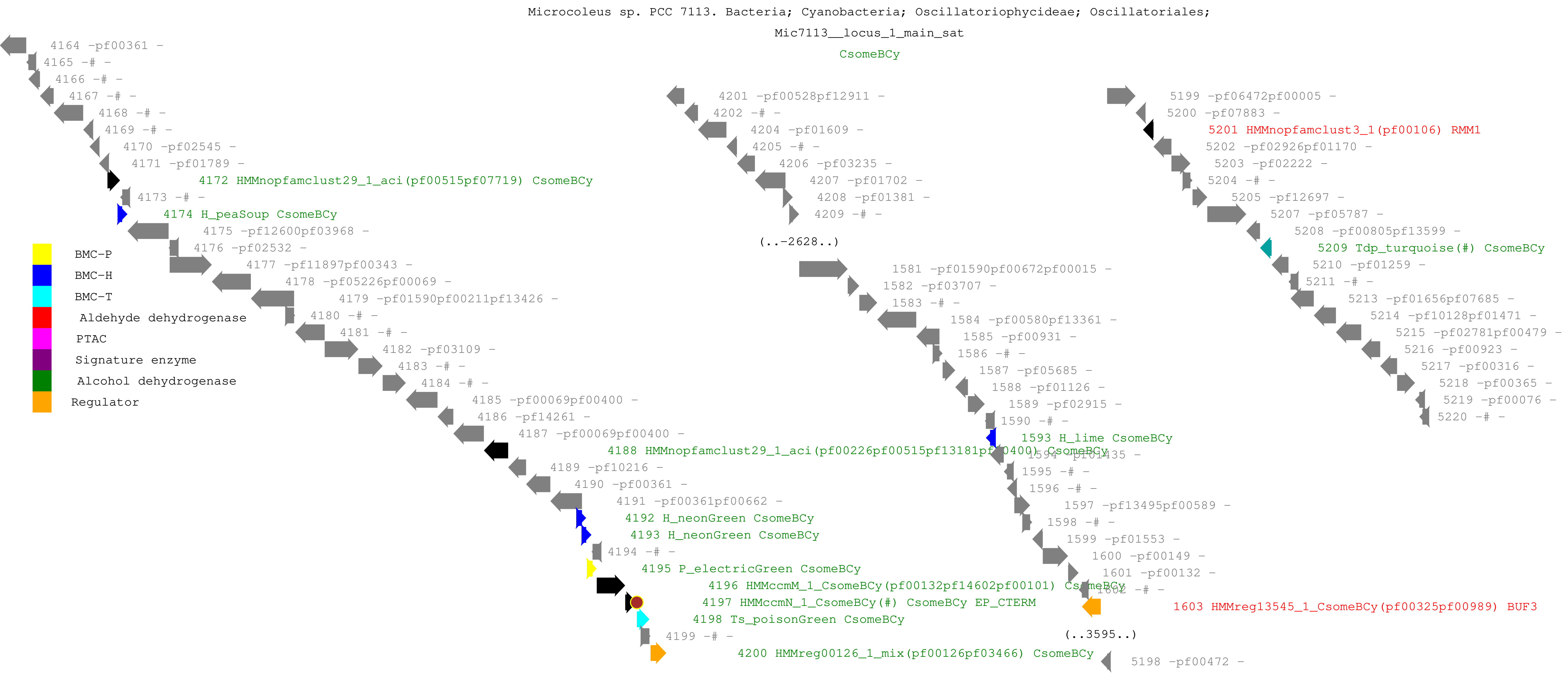
Microcoleus sp. PCC 7113. Bacteria; Cyanobacteria; Oscillatoriophycideae; Oscillatoriales;
Mic7113__locus_1_main_sat
CsomeBCy
4164 -pf00361 -
4165 -# -
4166 -# -
4167 -# -
4168 -# -
4169 -# -
4170 -pf02545 -
4171 -pf01789 -
4172 HMMnopfamclust29_1_aci(pf00515pf07719) CsomeBCy
4173 -# -
4174 H_peaSoup CsomeBCy
4175 -pf12600pf03968 -
4176 -pf02532 -
4177 -pf11897pf00343 -
4178 -pf05226pf00069 -
4179 -pf01590pf00211pf13426 -
4180 -# -
4181 -# -
4182 -pf03109 -
4183 -# -
4184 -# -
4185 -pf00069pf00400 -
4186 -pf14261 -
4187 -pf00069pf00400 -
4188 HMMnopfamclust29_1_aci(pf00226pf00515pf13181pf 0400) CsomeBCy
4189 -pf10216 -
4190 -pf00361 -
4191 -pf00361pf00662 -
4192 H_neonGreen CsomeBCy
4193 H_neonGreen CsomeBCy
4194 -# -
4195 P_electricGreen CsomeBCy
4196 HMMccmM_1_CsomeBCy(pf00132pf14602pf00101) CsomeBCy
4197 HMMccmN_1_CsomeBCy(#) CsomeBCy EP_CTERM
4198 Ts_poisonGreen CsomeBCy
4199 -# -
4200 HMMreg00126_1_mix(pf00126pf03466) CsomeBCy
BMC-P
BMC-H
BMC-T
Aldehyde dehydrogenase
PTAC
Signature enzyme
Alcohol dehydrogenase
Regulator
4201 -pf00528pf12911 -
4202 -# -
4204 -pf01609 -
4205 -# -
4206 -pf03235 -
4207 -pf01702 -
4208 -pf01381 -
4209 -# -
(..-2628..)
1581 -pf01590pf00672pf00015 -
1582 -pf03707 -
1583 -# -
1584 -pf00580pf13361 -
1585 -pf00931 -
1586 -# -
1587 -pf05685 -
1588 -pf01126 -
1589 -pf02915 -
1590 -# -
1593 H_lime CsomeBCy
1594 -pf01435 -
1595 -# -
1596 -# -
1597 -pf13495pf00589 -
1598 -# -
1599 -pf01553 -
1600 -pf00149 -
1601 -pf00132 -
1602 -# -
1603 HMMreg13545_1_CsomeBCy(pf00325pf00989) BUF3
(..3595..)
5198 -pf00472 -
5199 -pf06472pf00005 -
5200 -pf07883 -
5201 HMMnopfamclust3_1(pf00106) RMM1
5202 -pf02926pf01170 -
5203 -pf02222 -
5204 -# -
5205 -pf12697 -
5207 -pf05787 -
5208 -pf00805pf13599 -
5209 Tdp_turquoise(#) CsomeBCy
5210 -pf01259 -
5211 -# -
5213 -pf01656pf07685 -
5214 -pf10128pf01471 -
5215 -pf02781pf00479 -
5216 -pf00923 -
5217 -pf00316 -
5218 -pf00365 -
5219 -pf00076 -
5220 -# -

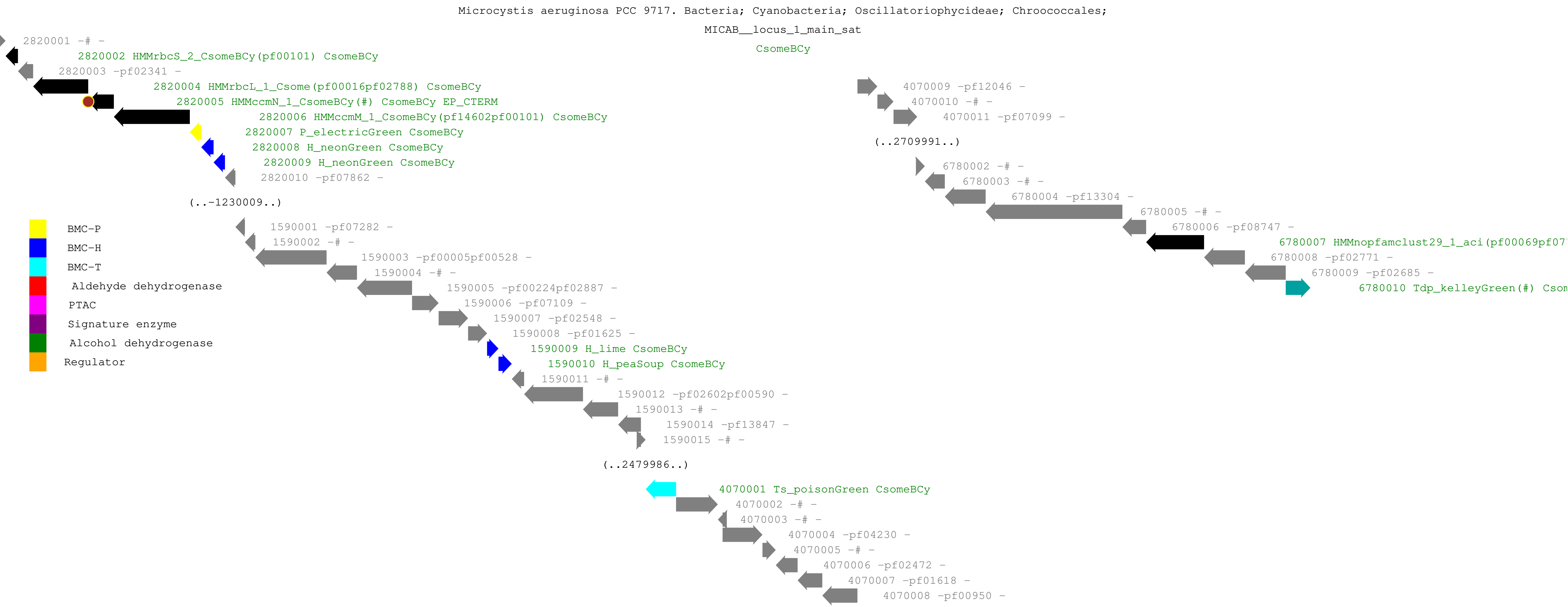
Microcystis aeruginosa PCC 9717. Bacteria; Cyanobacteria; Oscillatoriophycideae; Chroococcales;
MICAB__locus_1_main_sat
CsomeBCy
2820001 -# -
2820002 HMMrbcS_2_CsomeBCy(pf00101) CsomeBCy
2820003 -pf02341 -
2820004 HMMrbcL_1_Csome(pf00016pf02788) CsomeBCy
2820005 HMMccmN_1_CsomeBCy(#) CsomeBCy EP_CTERM
2820006 HMMccmM_1_CsomeBCy(pf14602pf00101) CsomeBCy
2820007 P_electricGreen CsomeBCy
2820008 H_neonGreen CsomeBCy
2820009 H_neonGreen CsomeBCy
2820010 -pf07862 -
(..-1230009..)
BMC-P
BMC-H
BMC-T
Aldehyde dehydrogenase
PTAC
Signature enzyme
Alcohol dehydrogenase
Regulator
1590001 -pf07282 -
1590002 -# -
1590003 -pf00005pf00528 -
1590004 -# -
1590005 -pf00224pf02887 -
1590006 -pf07109 -
1590007 -pf02548 -
1590008 -pf01625 -
1590009 H_lime CsomeBCy
1590010 H_peaSoup CsomeBCy
1590011 -# -
1590012 -pf02602pf00590 -
1590013 -# -
1590014 -pf13847 -
1590015 -# -
(..2479986..)
4070001 Ts_poisonGreen CsomeBCy
4070002 -# -
4070003 -# -
4070004 -pf04230 -
4070005 -# -
4070006 -pf02472 -
4070007 -pf01618 -
4070008 -pf00950 -
4070009 -pf12046 -
4070010 -# -
4070011 -pf07099 -
(..2709991..)
6780002 -# -
6780003 -# -
6780004 -pf13304 -
6780005 -# -
6780006 -pf08747 -
6780007 HMMnopfamclust29_1_aci(pf00069pf07
6780008 -pf02771 -
6780009 -pf02685 -
6780010 Tdp_kelleyGreen(#) Csom

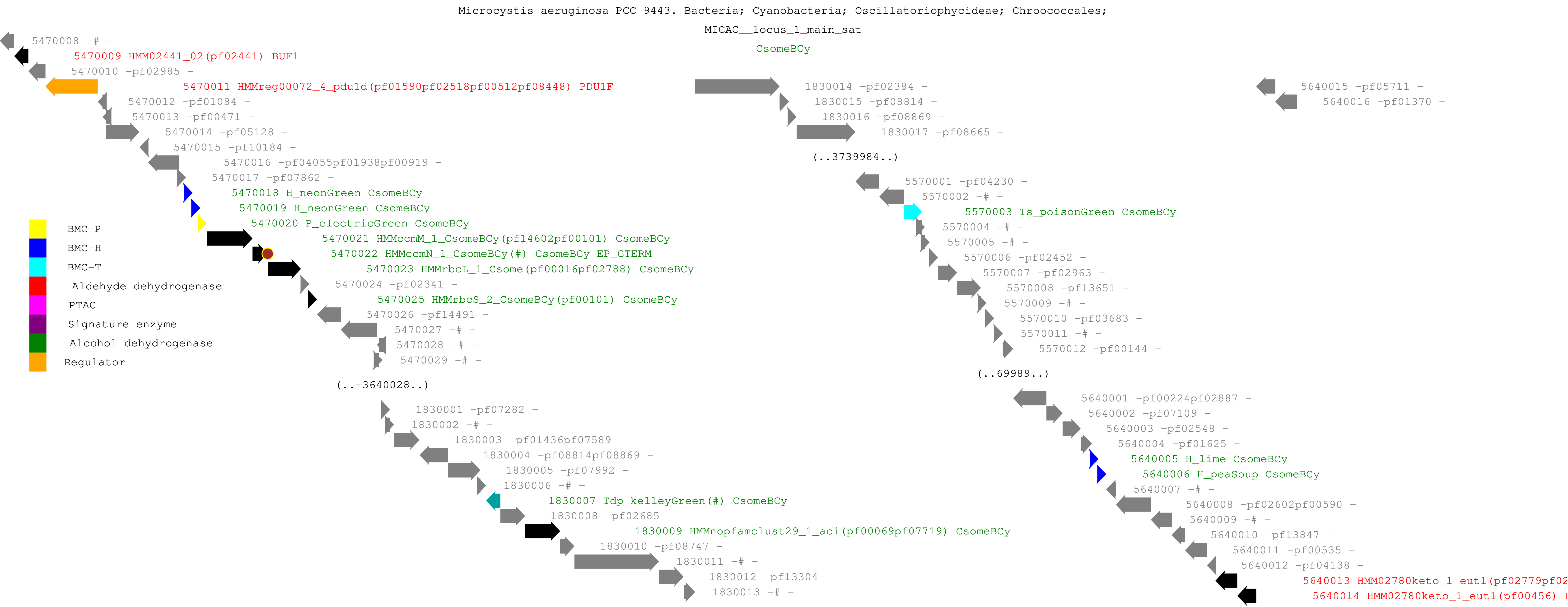

Microcystis aeruginosa PCC 9443. Bacteria; Cyanobacteria; Oscillatoriophycideae; Chroococcales;
MICAC__locus_1_main_sat
CsomeBCy
5470008 -# -
5470009 HMM02441_02(pf02441) BUF1
5470010 -pf02985 -
5470011 HMMreg00072_4_pdu1d(pf01590pf02518pf00512pf08448) PDU1F
5470012 -pf01084 -
5470013 -pf00471 -
5470014 -pf05128 -
5470015 -pf10184 -
5470016 -pf04055pf01938pf00919 -
5470017 -pf07862 -
5470018 H_neonGreen CsomeBCy
5470019 H_neonGreen CsomeBCy
5470020 P_electricGreen CsomeBCy
5470021 HMMccmM_1_CsomeBCy(pf14602pf00101) CsomeBCy
5470022 HMMccmN_1_CsomeBCy(#) CsomeBCy EP_CTERM
5470023 HMMrbcL_1_Csome(pf00016pf02788) CsomeBCy
5470024 -pf02341 -
5470025 HMMrbcS_2_CsomeBCy(pf00101) CsomeBCy
5470026 -pf14491 -
5470027 -# -
5470028 -# -
5470029 -# -
(..-3640028..)
1830001 -pf07282 -
1830002 -# -
1830003 -pf01436pf07589 -
1830004 -pf08814pf08869 -
1830005 -pf07992 -
1830006 -# -
1830007 Tdp_kelleyGreen(#) CsomeBCy
1830008 -pf02685 -
1830009 HMMnopfamclust29_1_aci(pf00069pf07719) CsomeBCy
1830010 -pf08747 -
1830011 -# -
1830012 -pf13304 -
1830013 -# -
1830014 -pf02384 -
1830015 -pf08814 -
1830016 -pf08869 -
1830017 -pf08665 -
(..3739984..)
5570001 -pf04230 -
5570002 -# -
5570003 Ts_poisonGreen CsomeBCy
5570004 -# -
5570005 -# -
5570006 -pf02452 -
5570007 -pf02963 -
5570008 -pf13651 -
5570009 -# -
5570010 -pf03683 -
5570011 -# -
5570012 -pf00144 -
(..69989..)
5640001 -pf00224pf02887 -
5640002 -pf07109 -
5640003 -pf02548 -
5640004 -pf01625 -
5640005 H_lime CsomeBCy
5640006 H_peaSoup CsomeBCy
5640007 -# -
5640008 -pf02602pf00590 -
5640009 -# -
5640010 -pf13847 -
5640011 -pf00535 -
5640012 -pf04138 -
5640013 HMM02780keto_1_eut1(pf02779pf02
5640014 HMM02780keto_1_eut1(pf00456)
5640015 -pf05711 -
5640016 -pf01370 -
BMC-P
BMC-H
BMC-T
Aldehyde dehydrogenase
PTAC
Signature enzyme
Alcohol dehydrogenase
Regulator

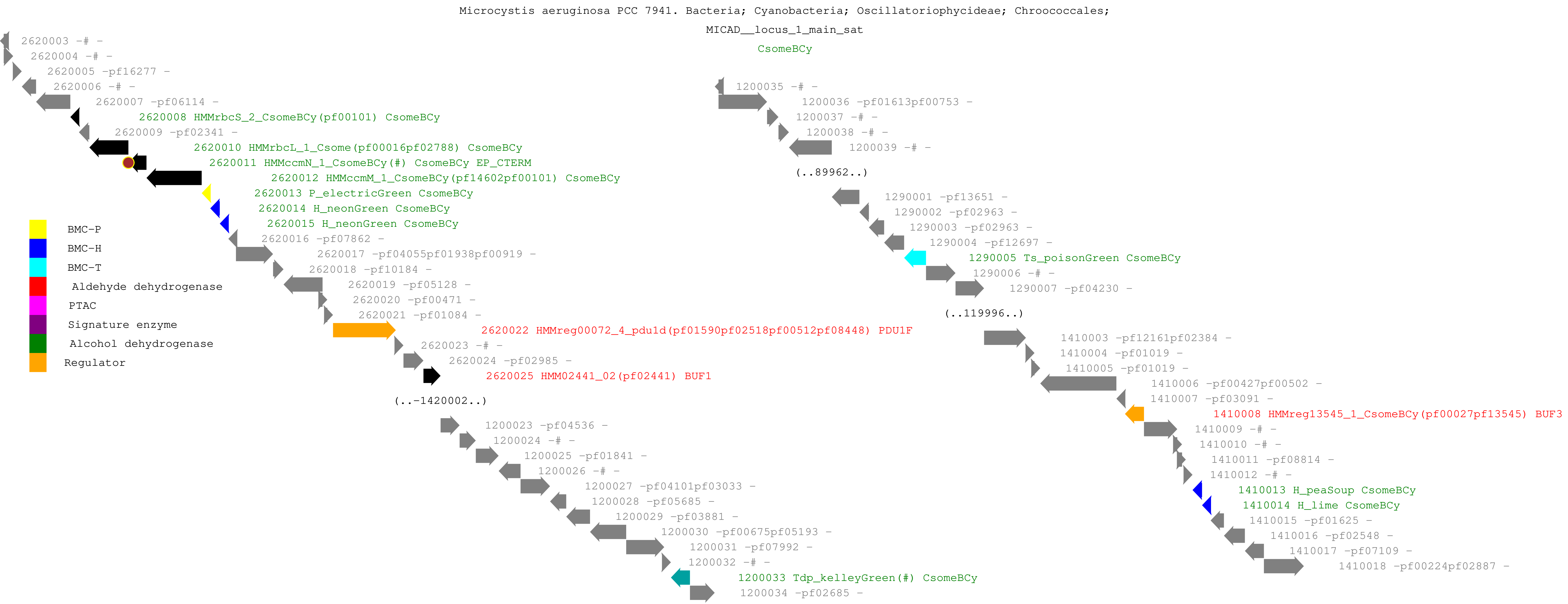
Microcystis aeruginosa PCC 7941. Bacteria; Cyanobacteria; Oscillatoriophycideae; Chroococcales;
MICAD__locus_1_main_sat
CsomeBCy
2620003 -# -
2620004 -# -
2620005 -pf16277 -
2620006 -# -
2620007 -pf06114 -
2620008 HMMrbcS_2_CsomeBCy(pf00101) CsomeBCy
2620009 -pf02341 -
2620010 HMMrbcL_1_Csome(pf00016pf02788) CsomeBCy
2620011 HMMccmN_1_CsomeBCy(#) CsomeBCy EP_CTERM
2620012 HMMccmM_1_CsomeBCy(pf14602pf00101) CsomeBCy
2620013 P_electricGreen CsomeBCy
2620014 H_neonGreen CsomeBCy
2620015 H_neonGreen CsomeBCy
2620016 -pf07862 -
2620017 -pf04055pf01938pf00919 -
2620018 -pf10184 -
2620019 -pf05128 -
2620020 -pf00471 -
2620021 -pf01084 -
2620022 HMMreg00072_4_pdu1d(pf01590pf02518pf00512pf08448) PDU1F
2620023 -# -
2620024 -pf02985 -
2620025 HMM02441_02(pf02441) BUF1
(..-1420002..)
1200023 -pf04536 -
1200024 -# -
1200025 -pf01841 -
1200026 -# -
1200027 -pf04101pf03033 -
1200028 -pf05685 -
1200029 -pf03881 -
1200030 -pf00675pf05193 -
1200031 -pf07992 -
1200032 -# -
1200033 Tdp_kelleyGreen(#) CsomeBCy
1200034 -pf02685 -
1200035 -# -
1200036 -pf01613pf00753 -
1200037 -# -
1200038 -# -
1200039 -# -
(..89962..)
1290001 -pf13651 -
1290002 -pf02963 -
1290003 -pf02963 -
1290004 -pf12697 -
1290005 Ts_poisonGreen CsomeBCy
1290006 -# -
1290007 -pf04230 -
(..119996..)
1410003 -pf12161pf02384 -
1410004 -pf01019 -
1410005 -pf01019 -
1410006 -pf00427pf00502 -
1410007 -pf03091 -
1410008 HMMreg13545_1_CsomeBCy(pf00027pf13545) BUF3
1410009 -# -
1410010 -# -
1410011 -pf08814 -
1410012 -# -
1410013 H_peaSoup CsomeBCy
1410014 H_lime CsomeBCy
1410015 -pf01625 -
1410016 -pf02548 -
1410017 -pf07109 -
1410018 -pf00224pf02887 -
BMC-P
BMC-H
BMC-T
Aldehyde dehydrogenase
PTAC
Signature enzyme
Alcohol dehydrogenase
Regulator

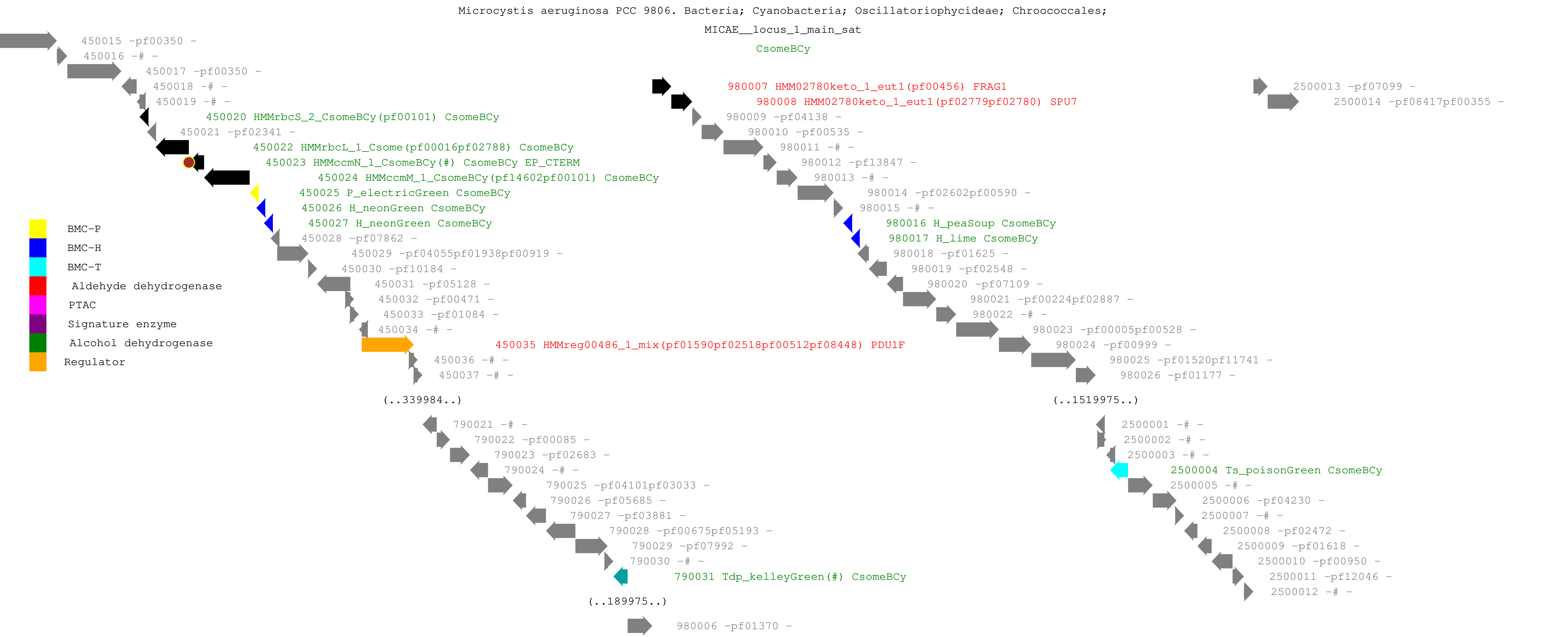

Microcystis aeruginosa PCC 9806. Bacteria; Cyanobacteria; Oscillatoriophycideae; Chroococcales;
MICAE__locus_1_main_sat
CsomeBCy
450015 -pf00350 -
450016 -# -
450017 -pf00350 -
450018 -# -
450019 -# -
450020 HMMrbcS_2_CsomeBCy(pf00101) CsomeBCy
450021 -pf02341 -
450022 HMMrbcL_1_Csome(pf00016pf02788) CsomeBCy
450023 HMMccmN_1_CsomeBCy(#) CsomeBCy EP_CTERM
450024 HMMccmM_1_CsomeBCy(pf14602pf00101) CsomeBCy
450025 P_electricGreen CsomeBCy
450026 H_neonGreen CsomeBCy
450027 H_neonGreen CsomeBCy
450028 -pf07862 -
450029 -pf04055pf01938pf00919 -
450030 -pf10184 -
450031 -pf05128 -
450032 -pf00471 -
450033 -pf01084 -
450034 -# -
450035 HMMreg00486_1_mix(pf01590pf02518pf00512pf08448) PDU1F
450036 -# -
450037 -# -
(..339984..)
790021 -# -
790022 -pf00085 -
790023 -pf02683 -
790024 -# -
790025 -pf04101pf03033 -
790026 -pf05685 -
790027 -pf03881 -
790028 -pf00675pf05193 -
790029 -pf07992 -
790030 -# -
790031 Tdp_kelleyGreen(#) CsomeBCy
(..189975..)
980006 -pf01370 -
980007 HMM02780keto_1_eut1(pf00456) FRAG1
980008 HMM02780keto_1_eut1(pf02779pf02780) SPU7
980009 -pf04138 -
980010 -pf00535 -
980011 -# -
980012 -pf13847 -
980013 -# -
980014 -pf02602pf00590 -
980015 -# -
980016 H_peaSoup CsomeBCy
980017 H_lime CsomeBCy
980018 -pf01625 -
980019 -pf02548 -
980020 -pf07109 -
980021 -pf00224pf02887 -
980022 -# -
980023 -pf00005pf00528 -
980024 -pf00999 -
980025 -pf01520pf11741 -
980026 -pf01177 -
(..1519975..)
2500001 -# -
2500002 -# -
2500003 -# -
2500004 Ts_poisonGreen CsomeBCy
2500005 -# -
2500006 -pf04230 -
2500007 -# -
2500008 -pf02472 -
2500009 -pf01618 -
2500010 -pf00950 -
2500011 -pf12046 -
2500012 -# -
2500013 -pf07099 -
2500014 -pf08417pf00355 -
BMC-P
BMC-H
BMC-T
Aldehyde dehydrogenase
PTAC
Signature enzyme
Alcohol dehydrogenase
Regulator

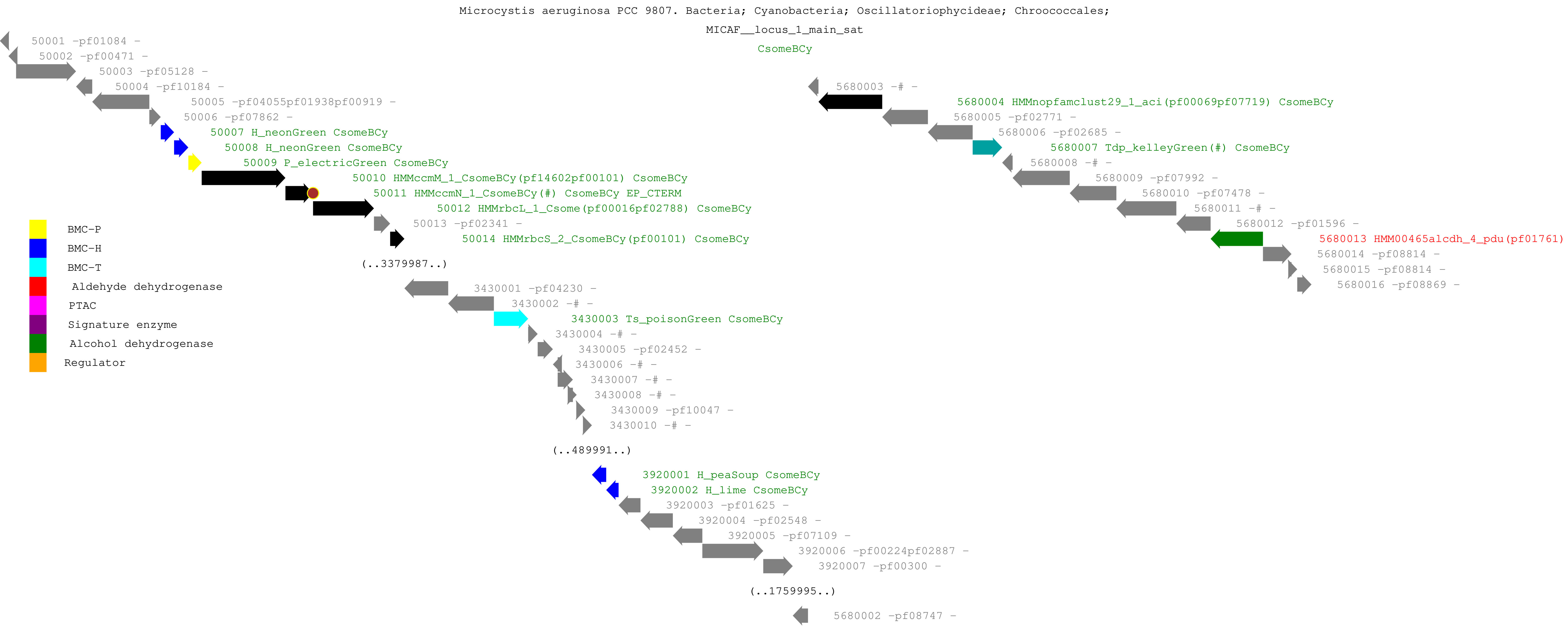
Microcystis aeruginosa PCC 9807. Bacteria; Cyanobacteria; Oscillatoriophycideae; Chroococcales;
MICAF__locus_1_main_sat
CsomeBCy
50001 -pf01084 -
50002 -pf00471 -
50003 -pf05128 -
50004 -pf10184 -
50005 -pf04055pf01938pf00919 -
50006 -pf07862 -
50007 H_neonGreen CsomeBCy
50008 H_neonGreen CsomeBCy
50009 P_electricGreen CsomeBCy
50010 HMMccmM_1_CsomeBCy(pf14602pf00101) CsomeBCy
50011 HMMccmN_1_CsomeBCy(#) CsomeBCy EP_CTERM
50012 HMMrbcL_1_Csome(pf00016pf02788) CsomeBCy
50013 -pf02341 -
50014 HMMrbcS_2_CsomeBCy(pf00101) CsomeBCy
(..3379987..)
3430001 -pf04230 -
3430002 -# -
3430003 Ts_poisonGreen CsomeBCy
3430004 -# -
3430005 -pf02452 -
3430006 -# -
3430007 -# -
3430008 -# -
3430009 -pf10047 -
3430010 -# -
(..489991..)
3920001 H_peaSoup CsomeBCy
3920002 H_lime CsomeBCy
3920003 -pf01625 -
3920004 -pf02548 -
3920005 -pf07109 -
3920006 -pf00224pf02887 -
3920007 -pf00300 -
(..1759995..)
5680002 -pf08747 -
5680003 -# -
5680004 HMMnopfamclust29_1_aci(pf00069pf07719) CsomeBCy
5680005 -pf02771 -
5680006 -pf02685 -
5680007 Tdp_kelleyGreen(#) CsomeBCy
5680008 -# -
5680009 -pf07992 -
5680010 -pf07478 -
5680011 -# -
5680012 -pf01596 -
5680013 HMM00465alcdh_4_pdu(pf01761)
5680014 -pf08814 -
5680015 -pf08814 -
5680016 -pf08869 -
BMC-P
BMC-H
BMC-T
Aldehyde dehydrogenase
PTAC
Signature enzyme
Alcohol dehydrogenase
Regulator

Microcystis aeruginosa PCC 9808. Bacteria; Cyanobacteria; Oscillatoriophycideae; Chroococcales;

MICAG__locus_1_main_sat

CsomeBCy

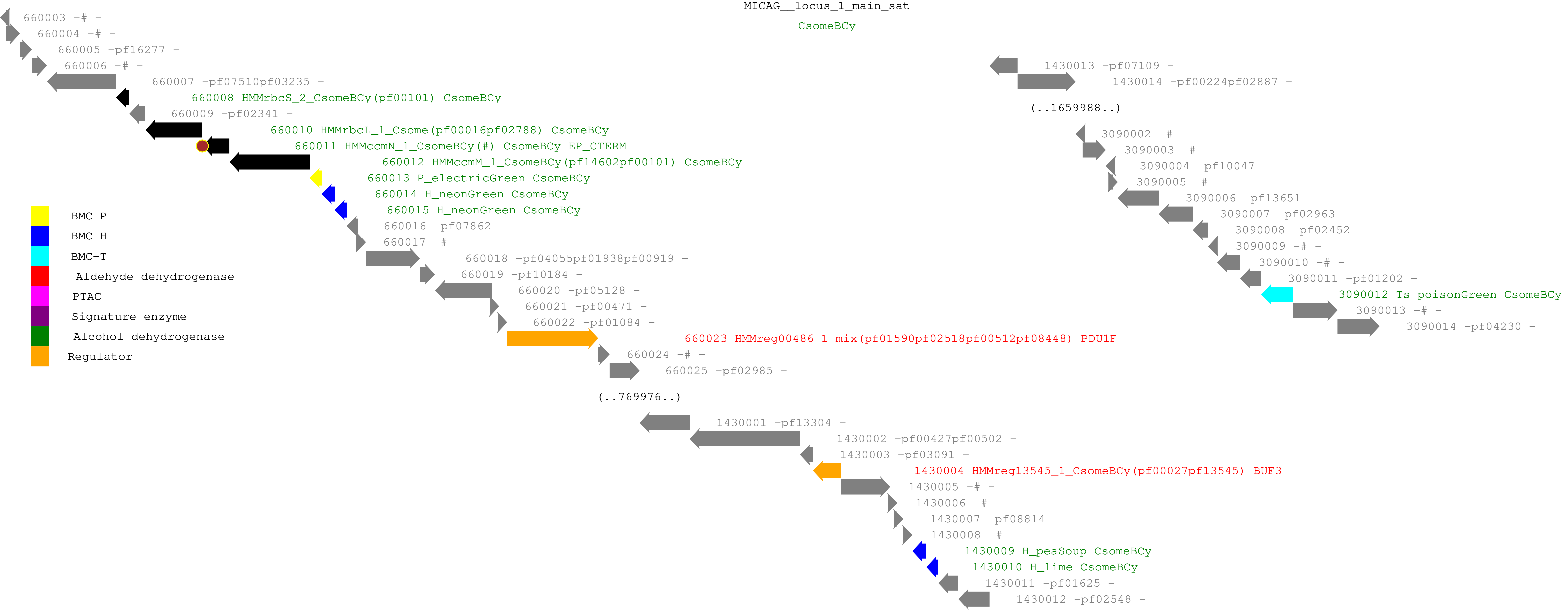

Microcystis aeruginosa PCC 9809. Bacteria; Cyanobacteria; Oscillatoriophycideae; Chroococcales;

MICAH__locus_1_main_sat

CsomeBCy

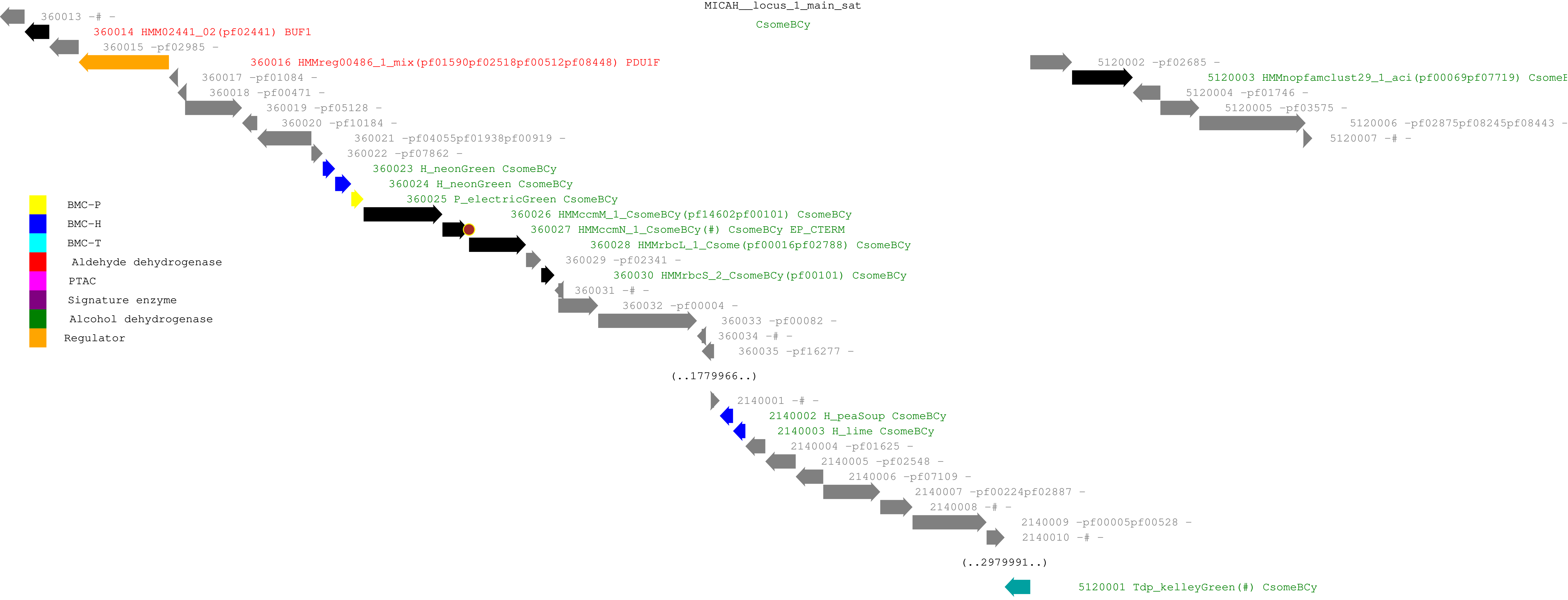

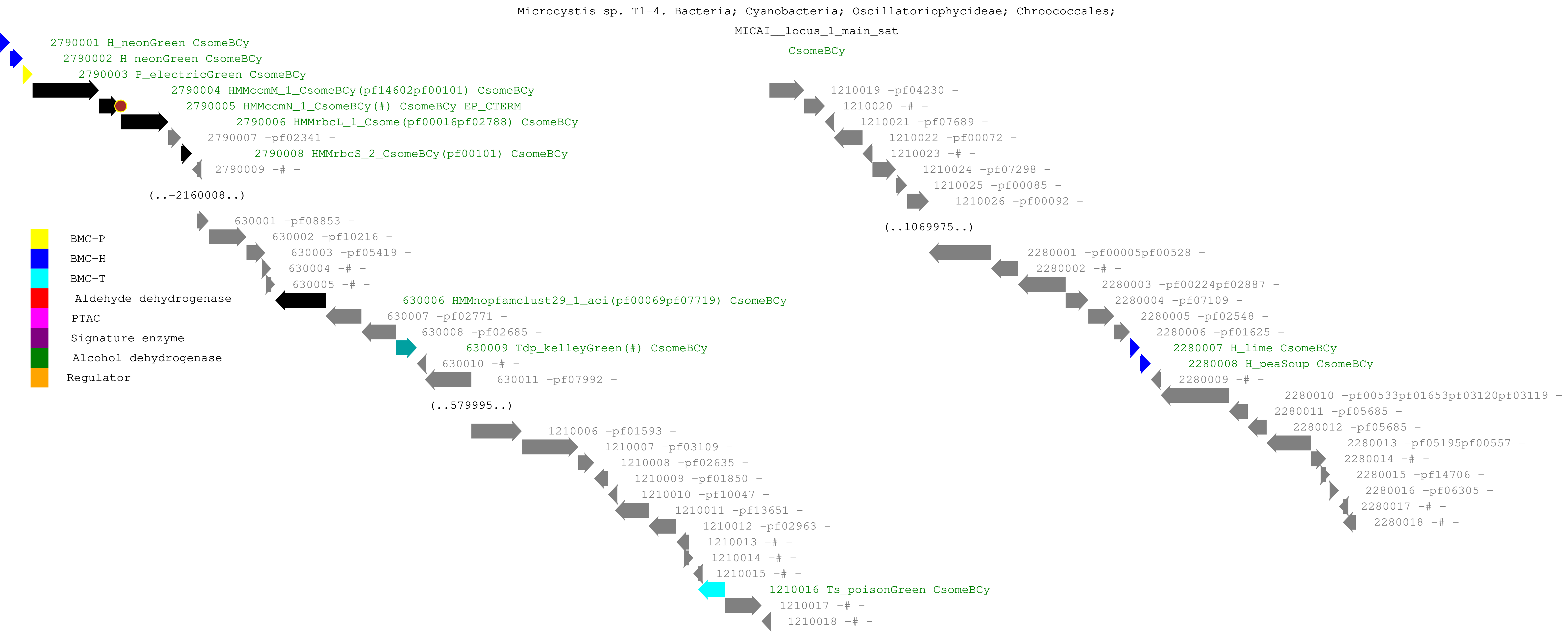

Microcystis sp. T1-4. Bacteria; Cyanobacteria; Oscillatoriophycideae; Chroococcales;
MICAI__locus_1_main_sat
CsomeBCy
2790001 H_neonGreen CsomeBCy
2790002 H_neonGreen CsomeBCy
2790003 P_electricGreen CsomeBCy
2790004 HMMccmM_1_CsomeBCy(pf14602pf00101) CsomeBCy
2790005 HMMccmN_1_CsomeBCy(#) CsomeBCy EP_CTERM
2790006 HMMrbcL_1_Csome(pf00016pf02788) CsomeBCy
2790007 -pf02341 -
2790008 HMMrbcS_2_CsomeBCy(pf00101) CsomeBCy
2790009 -# -
(..-2160008..)
630001 -pf08853 -
630002 -pf10216 -
630003 -pf05419 -
630004 -# -
630005 -# -
630006 HMMnopfamclust29_1_aci(pf00069pf07719) CsomeBCy
630007 -pf02771 -
630008 -pf02685 -
630009 Tdp_kelleyGreen(#) CsomeBCy
630010 -# -
630011 -pf07992 -
(..579995..)
1210006 -pf01593 -
1210007 -pf03109 -
1210008 -pf02635 -
1210009 -pf01850 -
1210010 -pf10047 -
1210011 -pf13651 -
1210012 -pf02963 -
1210013 -# -
1210014 -# -
1210015 -# -
1210016 Ts_poisonGreen CsomeBCy
1210017 -# -
1210018 -# -
1210019 -pf04230 -
1210020 -# -
1210021 -pf07689 -
1210022 -pf00072 -
1210023 -# -
1210024 -pf07298 -
1210025 -pf00085 -
1210026 -pf00092 -
(..1069975..)
2280001 -pf00005pf00528 -
2280002 -# -
2280003 -pf00224pf02887 -
2280004 -pf07109 -
2280005 -pf02548 -
2280006 -pf01625 -
2280007 H_lime CsomeBCy
2280008 H_peaSoup CsomeBCy
2280009 -# -
2280010 -pf00533pf01653pf03120pf03119 -
2280011 -pf05685 -
2280012 -pf05685 -
2280013 -pf05195pf00557 -
2280014 -# -
2280015 -pf14706 -
2280016 -pf06305 -
2280017 -# -
2280018 -# -
BMC-P
BMC-H
BMC-T
Aldehyde dehydrogenase
PTAC
Signature enzyme
Alcohol dehydrogenase
Regulator

Microcystis aeruginosa PCC 9701. Bacteria; Cyanobacteria; Oscillatoriophycideae; Chroococcales;

MICAK__locus_1_main_sat

CsomeBCy

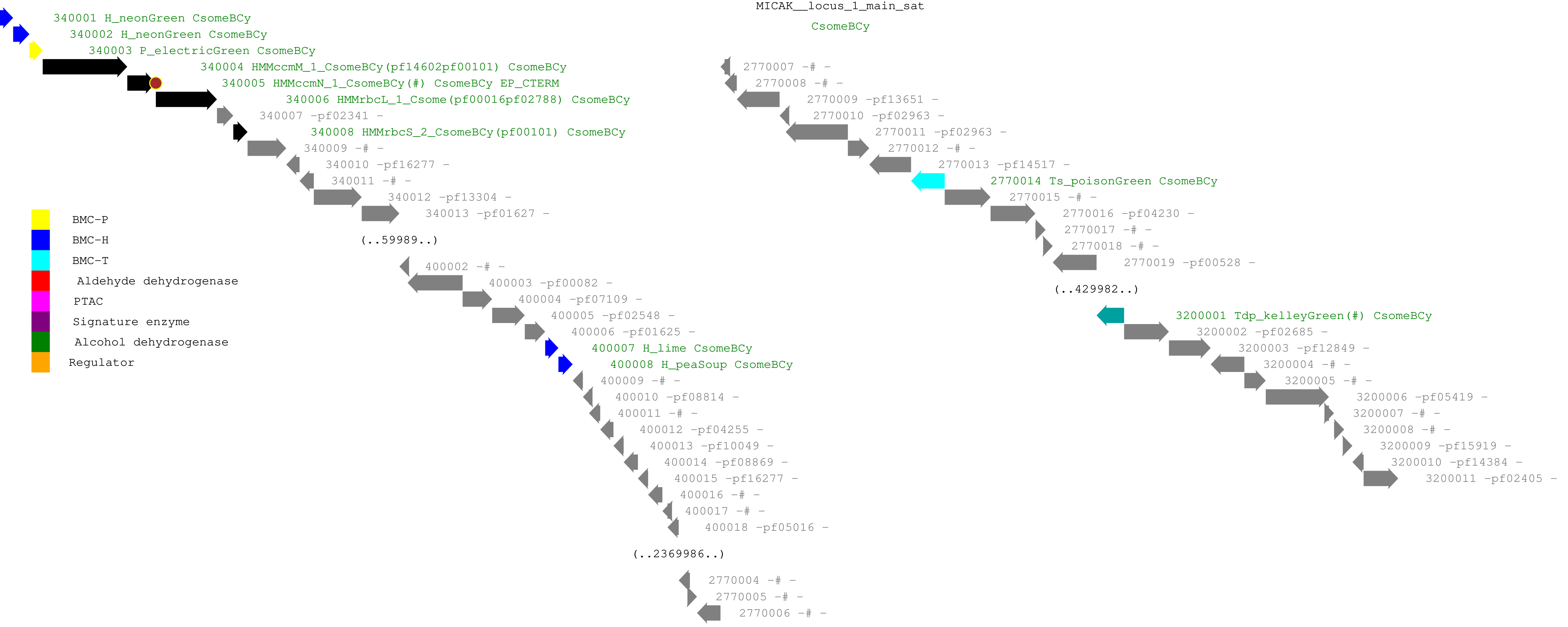

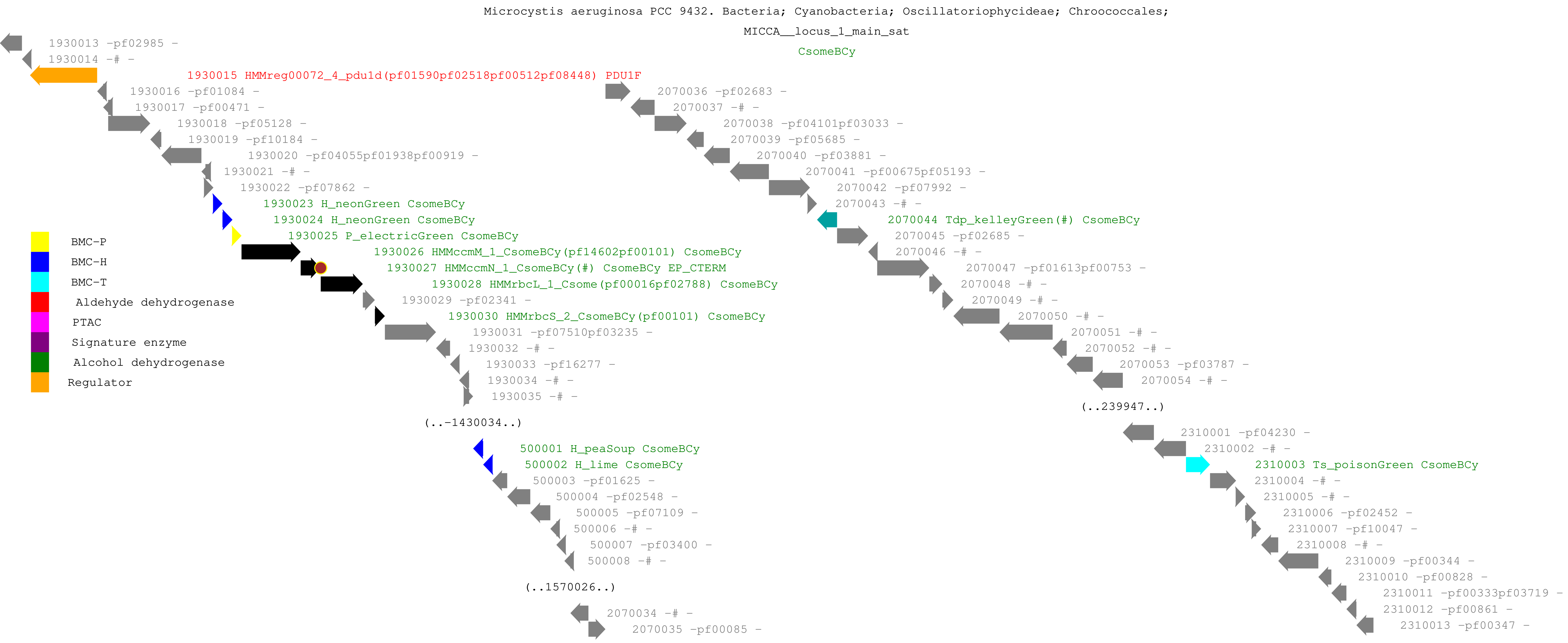

Microcystis aeruginosa PCC 9432. Bacteria; Cyanobacteria; Oscillatoriophycideae; Chroococcales;
MICCA__locus_1_main_sat
CsomeBCy
1930013 -pf02985 -
1930014 -# -
1930015 HMMreg00072_4_pdu1d(pf01590pf02518pf00512pf08448) PDU1F
1930016 -pf01084 -
1930017 -pf00471 -
1930018 -pf05128 -
1930019 -pf10184 -
1930020 -pf04055pf01938pf00919 -
1930021 -# -
1930022 -pf07862 -
1930023 H_neonGreen CsomeBCy
1930024 H_neonGreen CsomeBCy
1930025 P_electricGreen CsomeBCy
1930026 HMMccmM_1_CsomeBCy(pf14602pf00101) CsomeBCy
1930027 HMMccmN_1_CsomeBCy(#) CsomeBCy EP_CTERM
1930028 HMMrbcL_1_Csome(pf00016pf02788) CsomeBCy
1930029 -pf02341 -
1930030 HMMrbcS_2_CsomeBCy(pf00101) CsomeBCy
1930031 -pf07510pf03235 -
1930032 -# -
1930033 -pf16277 -
1930034 -# -
1930035 -# -
(..-1430034..)
500001 H_peaSoup CsomeBCy
500002 H_lime CsomeBCy
500003 -pf01625 -
500004 -pf02548 -
500005 -pf07109 -
500006 -# -
500007 -pf03400 -
500008 -# -
(..1570026..)
2070034 -# -
2070035 -pf00085 -
2070036 -pf02683 -
2070037 -# -
2070038 -pf04101pf03033 -
2070039 -pf05685 -
2070040 -pf03881 -
2070041 -pf00675pf05193 -
2070042 -pf07992 -
2070043 -# -
2070044 Tdp_kelleyGreen(#) CsomeBCy
2070045 -pf02685 -
2070046 -# -
2070047 -pf01613pf00753 -
2070048 -# -
2070049 -# -
2070050 -# -
2070051 -# -
2070052 -# -
2070053 -pf03787 -
2070054 -# -
(..239947..)
2310001 -pf04230 -
2310002 -# -
2310003 Ts_poisonGreen CsomeBCy
2310004 -# -
2310005 -# -
2310006 -pf02452 -
2310007 -pf10047 -
2310008 -# -
2310009 -pf00344 -
2310010 -pf00828 -
2310011 -pf00333pf03719 -
2310012 -pf00861 -
2310013 -pf00347 -
BMC-P
BMC-H
BMC-T
Aldehyde dehydrogenase
PTAC
Signature enzyme
Alcohol dehydrogenase
Regulator

Microcoleus vaginatus FGP-2. Bacteria; Cyanobacteria; Oscillatoriophycideae; Oscillatoriales;

MicvaDRAFT__locus_1_main_sat

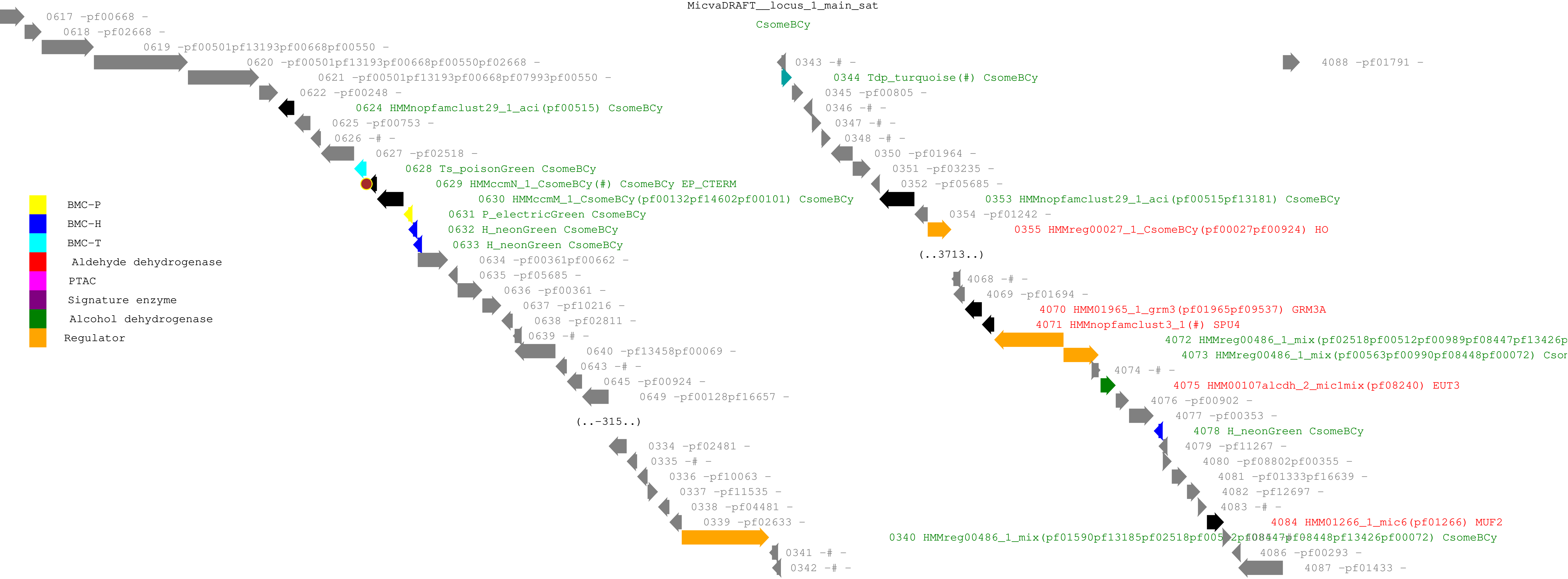

Microcystis aeruginosa NIES-2520. Bacteria; Cyanobacteria; Oscillatoriophycideae; Chroococcales;

MiTe__locus_1_main_sat

CsomeBCy

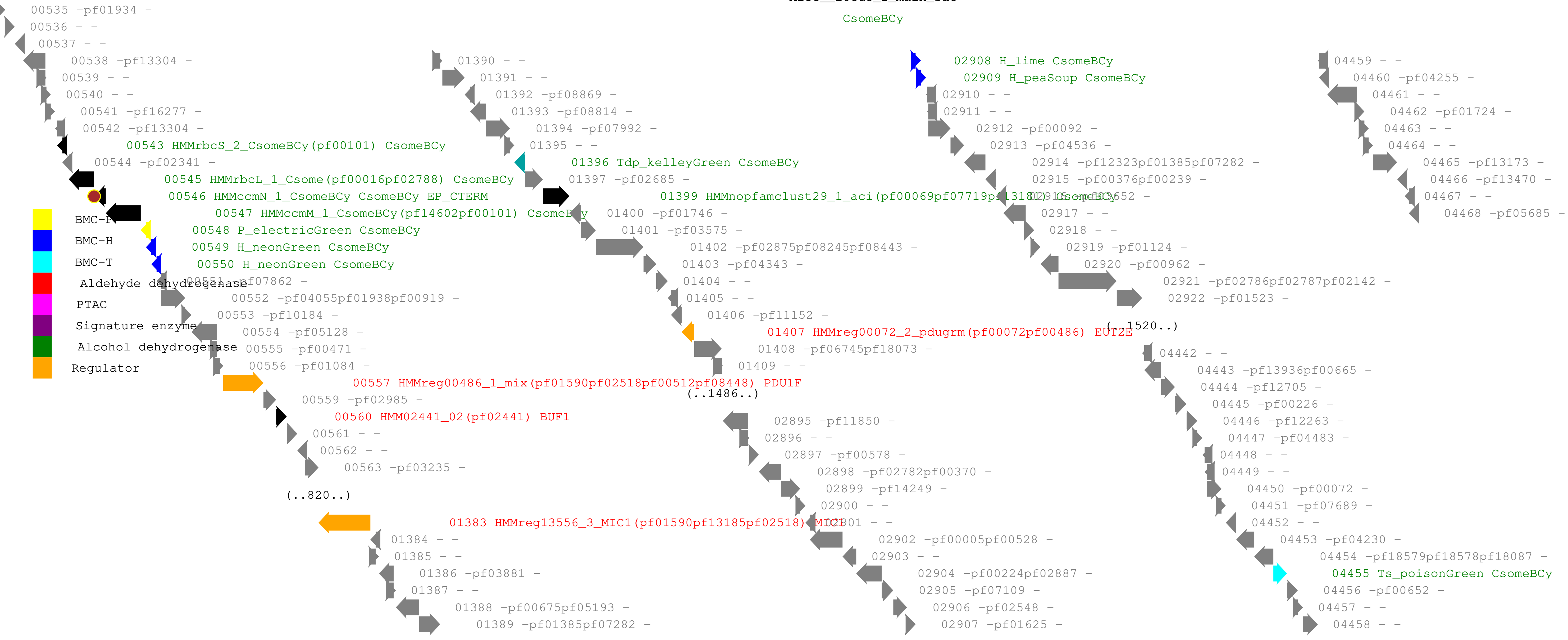

Microcystis aeruginosa NIES-2521. Bacteria; Cyanobacteria; Oscillatoriophycideae; Chroococcales;

MiTs__locus_1_main_sat

CsomeBCy

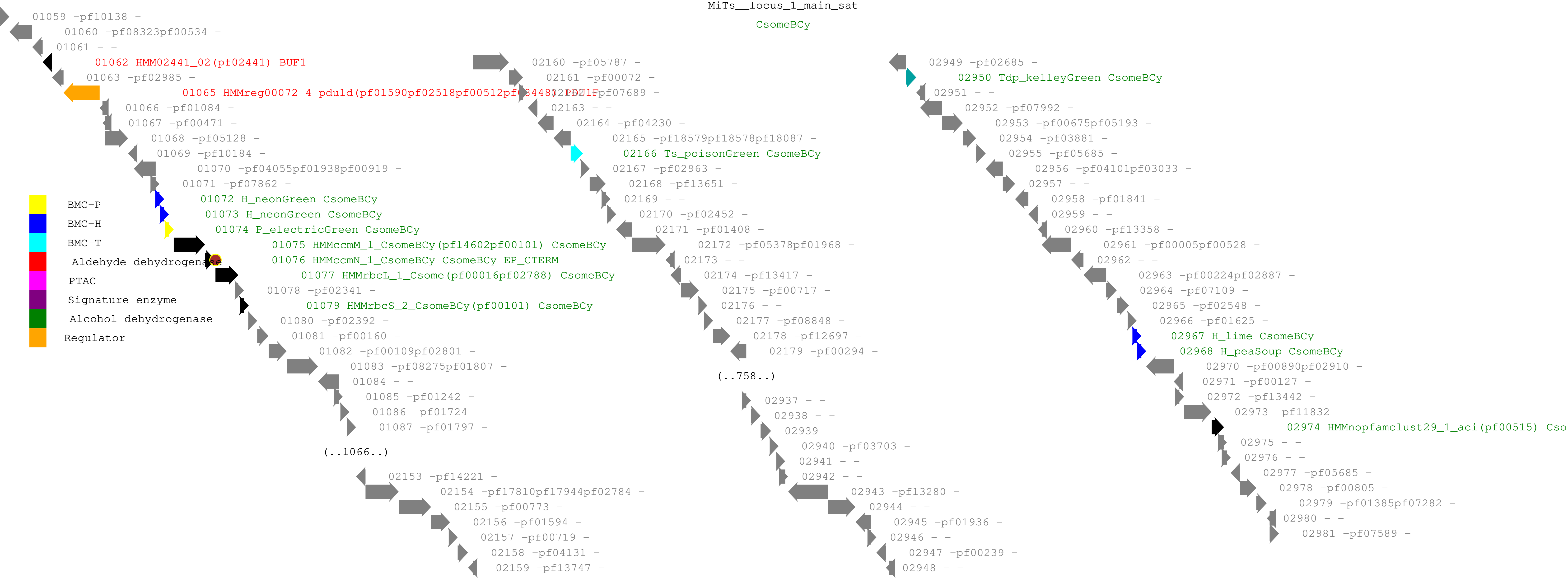

Microcystis aeruginosa NIES-2519. Bacteria; Cyanobacteria; Oscillatoriophycideae; Chroococcales;

MiYa__locus_1_main_sat

CsomeBCy

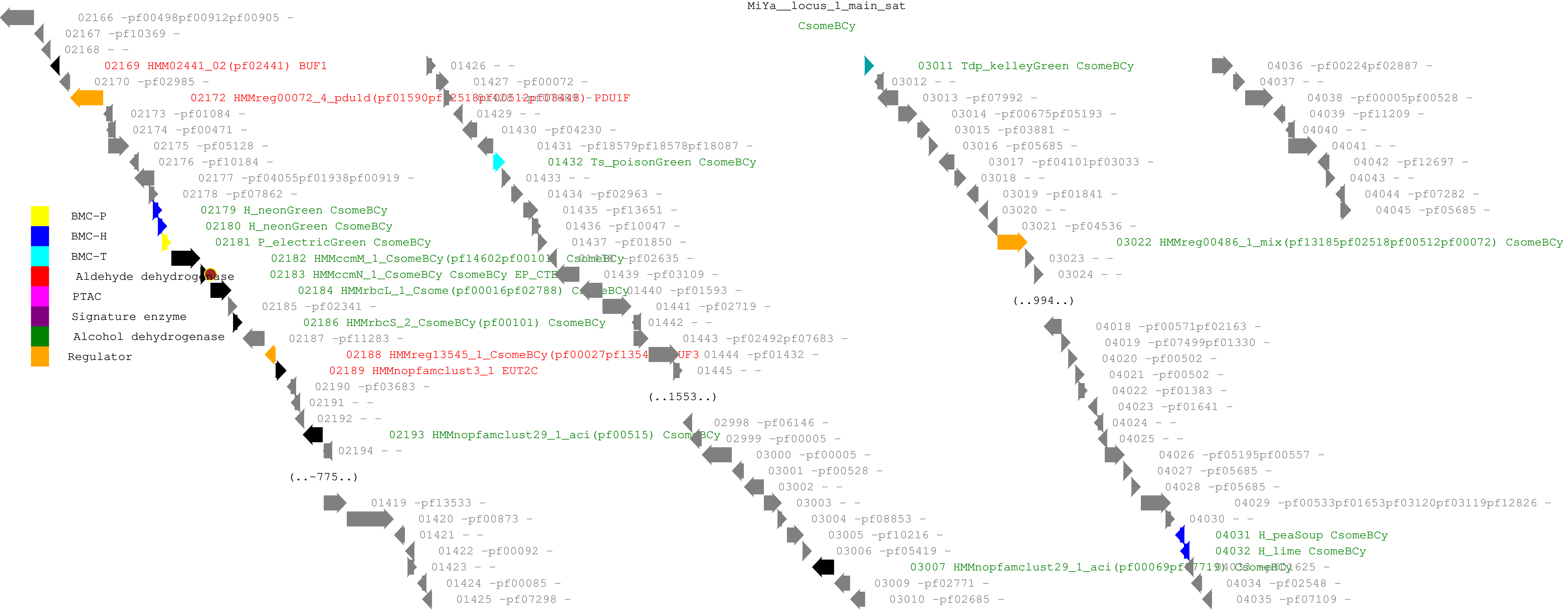

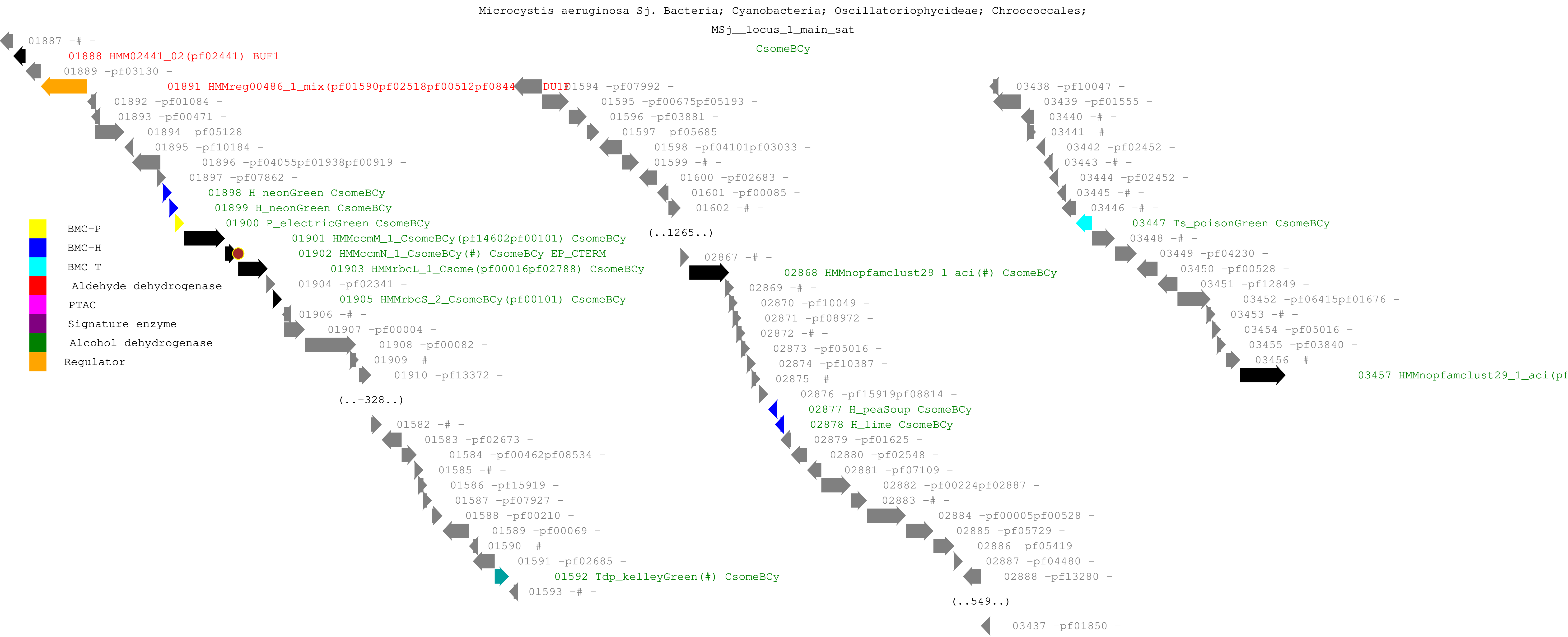
Microcystis aeruginosa Sj. Bacteria; Cyanobacteria; Oscillatoriophycideae; Chroococcales;
MSj__locus_1_main_sat
CsomeBCy
01887 -# -
01888 HMM02441_02(pf02441) BUF1
01889 -pf03130 -
01891 HMMreg00486_1_mix(pf01590pf02518pf00512pf0844 DU1F
01892 -pf01084 -
01893 -pf00471 -
01894 -pf05128 -
01895 -pf10184 -
01896 -pf04055pf01938pf00919 -
01897 -pf07862 -
01898 H_neonGreen CsomeBCy
01899 H_neonGreen CsomeBCy
01900 P_electricGreen CsomeBCy
01901 HMMccmM_1_CsomeBCy(pf14602pf00101) CsomeBCy
01902 HMMccmN_1_CsomeBCy(#) CsomeBCy EP_CTERM
01903 HMMrbcL_1_Csome(pf00016pf02788) CsomeBCy
01904 -pf02341 -
01905 HMMrbcS_2_CsomeBCy(pf00101) CsomeBCy
01906 -# -
01907 -pf00004 -
01908 -pf00082 -
01909 -# -
01910 -pf13372 -
(..-328..)
01582 -# -
01583 -pf02673 -
01584 -pf00462pf08534 -
01585 -# -
01586 -pf15919 -
01587 -pf07927 -
01588 -pf00210 -
01589 -pf00069 -
01590 -# -
01591 -pf02685 -
01592 Tdp_kelleyGreen(#) CsomeBCy
01593 -# -
01594 -pf07992 -
01595 -pf00675pf05193 -
01596 -pf03881 -
01597 -pf05685 -
01598 -pf04101pf03033 -
01599 -# -
01600 -pf02683 -
01601 -pf00085 -
01602 -# -
(..1265..)
02867 -# -
02868 HMMnopfamclust29_1_aci(#) CsomeBCy
02869 -# -
02870 -pf10049 -
02871 -pf08972 -
02872 -# -
02873 -pf05016 -
02874 -pf10387 -
02875 -# -
02876 -pf15919pf08814 -
02877 H_peaSoup CsomeBCy
02878 H_lime CsomeBCy
02879 -pf01625 -
02880 -pf02548 -
02881 -pf07109 -
02882 -pf00224pf02887 -
02883 -# -
02884 -pf00005pf00528 -
02885 -pf05729 -
02886 -pf05419 -
02887 -pf04480 -
02888 -pf13280 -
(..549..)
03437 -pf01850 -
03438 -pf10047 -
03439 -pf01555 -
03440 -# -
03441 -# -
03442 -pf02452 -
03443 -# -
03444 -pf02452 -
03445 -# -
03446 -# -
03447 Ts_poisonGreen CsomeBCy
03448 -# -
03449 -pf04230 -
03450 -pf00528 -
03451 -pf12849 -
03452 -pf06415pf01676 -
03453 -# -
03454 -pf05016 -
03455 -pf03840 -
03456 -# -
03457 HMMnopfamclust29_1_aci(pf
BMC-P
BMC-H
BMC-T
Aldehyde dehydrogenase
PTAC
Signature enzyme
Alcohol dehydrogenase
Regulator

Microcystis aeruginosa NIES-1211. Bacteria; Cyanobacteria; Oscillatoriophycideae; Chroococcales;
MTo__locus_1_main_sat
CsomeBCy

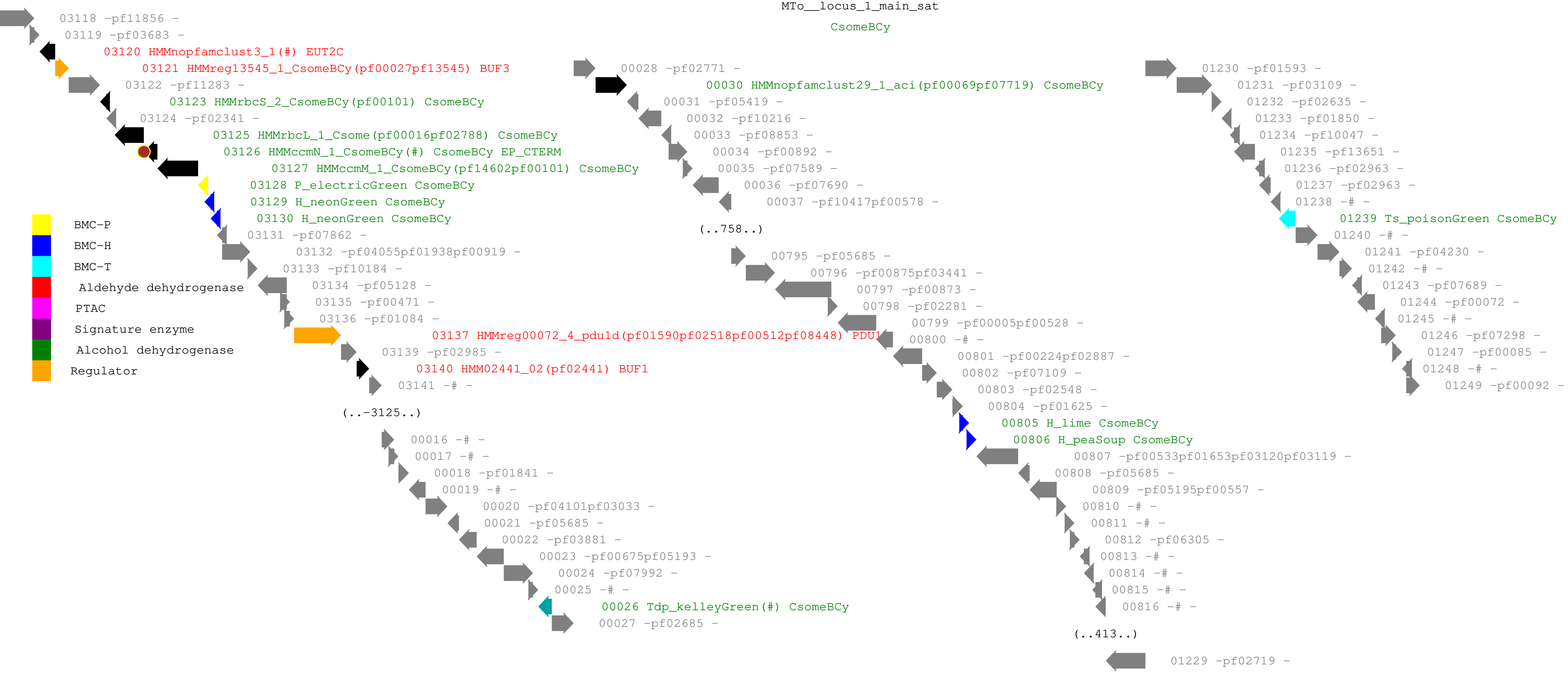

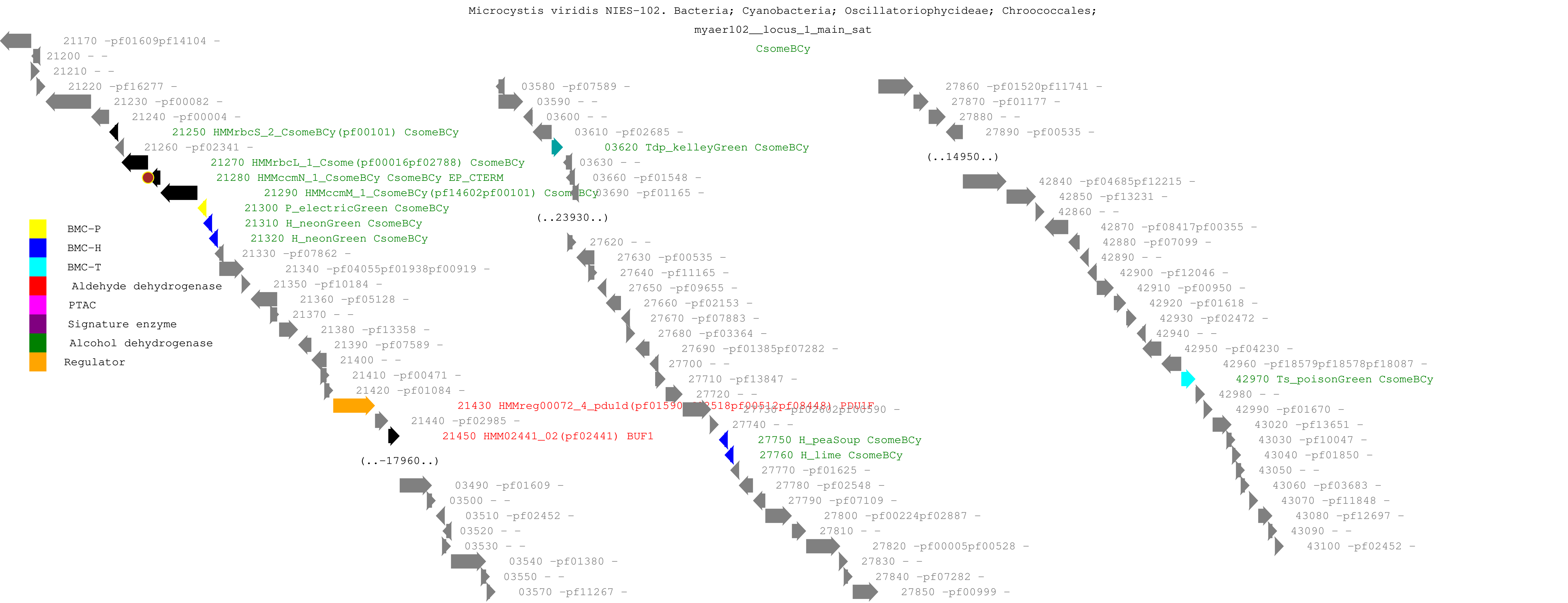

Microcystis viridis NIES-102. Bacteria; Cyanobacteria; Oscillatoriophycideae; Chroococcales;
myaer102__locus_1_main_sat
CsomeBCy
21170 -pf01609pf14104 -
21200 - -
21210 - -
21220 -pf16277 -
21230 -pf00082 -
21240 -pf00004 -
21250 HMMrbcS_2_CsomeBCy(pf00101) CsomeBCy
21260 -pf02341 -
21270 HMMrbcL_1_Csome(pf00016pf02788) CsomeBCy
21280 HMMccmN_1_CsomeBCy CsomeBCy EP_CTERM
21290 HMMccmM_1_CsomeBCy(pf14602pf00101) CsomeBCy
21300 P_electricGreen CsomeBCy
21310 H_neonGreen CsomeBCy
21320 H_neonGreen CsomeBCy
21330 -pf07862 -
21340 -pf04055pf01938pf00919 -
21350 -pf10184 -
21360 -pf05128 -
21370 - -
21380 -pf13358 -
21390 -pf07589 -
21400 - -
21410 -pf00471 -
21420 -pf01084 -
21430 HMMreg00072_4_pdu1d(pf01590 ...518pf00512pf08448) PDU1F
21440 -pf02985 -
21450 HMM02441_02(pf02441) BUF1
(..-17960..)
03490 -pf01609 -
03500 - -
03510 -pf02452 -
03520 - -
03530 - -
03540 -pf01380 -
03550 - -
03570 -pf11267 -
03580 -pf07589 -
03590 - -
03600 - -
03610 -pf02685 -
03620 Tdp_kelleyGreen CsomeBCy
03630 - -
03660 -pf01548 -
03690 -pf01165 -
(..23930..)
27620 - -
27630 -pf00535 -
27640 -pf11165 -
27650 -pf09655 -
27660 -pf02153 -
27670 -pf07883 -
27680 -pf03364 -
27690 -pf01385pf07282 -
27700 - -
27710 -pf13847 -
27720 - -
27730 -pf02602pf00590 -
27740 - -
27750 H_peaSoup CsomeBCy
27760 H_lime CsomeBCy
27770 -pf01625 -
27780 -pf02548 -
27790 -pf07109 -
27800 -pf00224pf02887 -
27810 - -
27820 -pf00005pf00528 -
27830 - -
27840 -pf07282 -
27850 -pf00999 -
27860 -pf01520pf11741 -
27870 -pf01177 -
27880 - -
27890 -pf00535 -
(..14950..)
42840 -pf04685pf12215 -
42850 -pf13231 -
42860 - -
42870 -pf08417pf00355 -
42880 -pf07099 -
42890 - -
42900 -pf12046 -
42910 -pf00950 -
42920 -pf01618 -
42930 -pf02472 -
42940 - -
42950 -pf04230 -
42960 -pf18579pf18578pf18087 -
42970 Ts_poisonGreen CsomeBCy
42980 - -
42990 -pf01670 -
43020 -pf13651 -
43030 -pf10047 -
43040 -pf01850 -
43050 - -
43060 -pf03683 -
43070 -pf11848 -
43080 -pf12697 -
43090 - -
43100 -pf02452 -
BMC-P
BMC-H
BMC-T
Aldehyde dehydrogenase
PTAC
Signature enzyme
Alcohol dehydrogenase
Regulator

Microcystis aeruginosa NIES-87. Bacteria; Cyanobacteria; Oscillatoriophycideae; Chroococcales;

myaer87__locus_1_main_sat

CsomeBCy

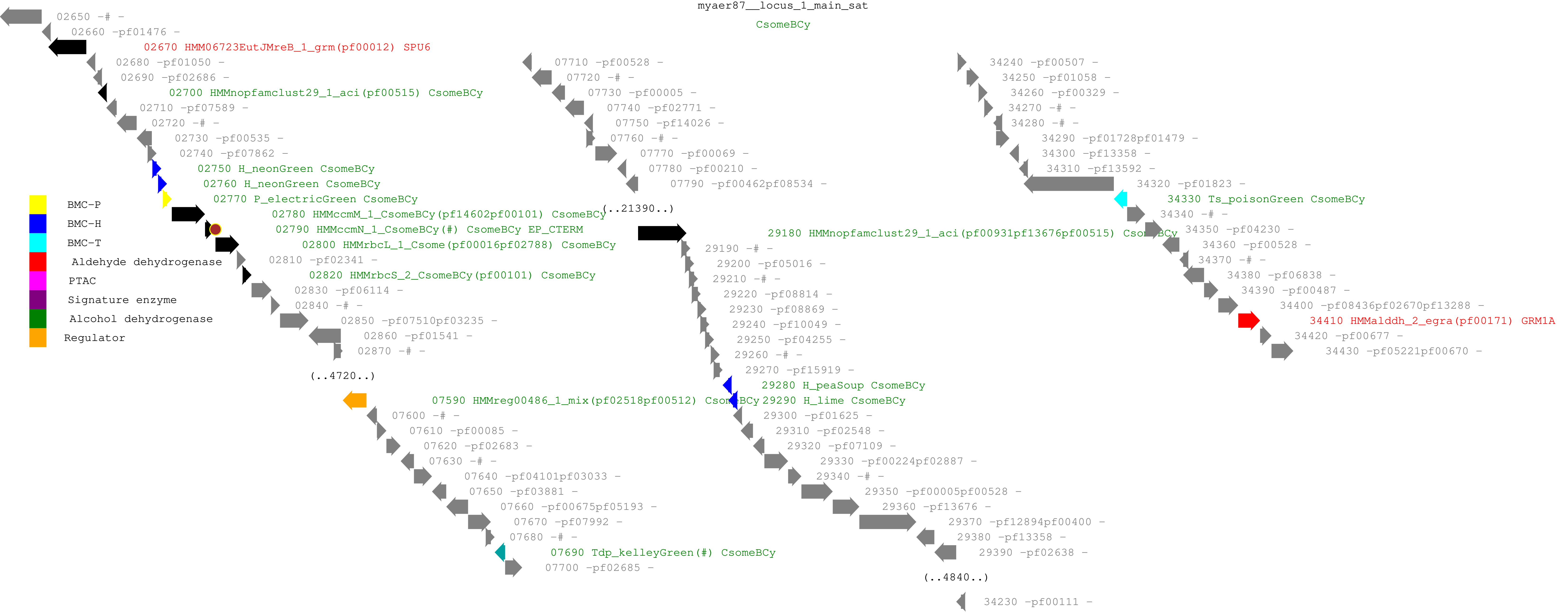

Microcystis aeruginosa NIES-2549. Bacteria; Cyanobacteria; Oscillatoriophycideae; Chroococcales;

MYAER__locus_1_main_sat

CsomeBCy

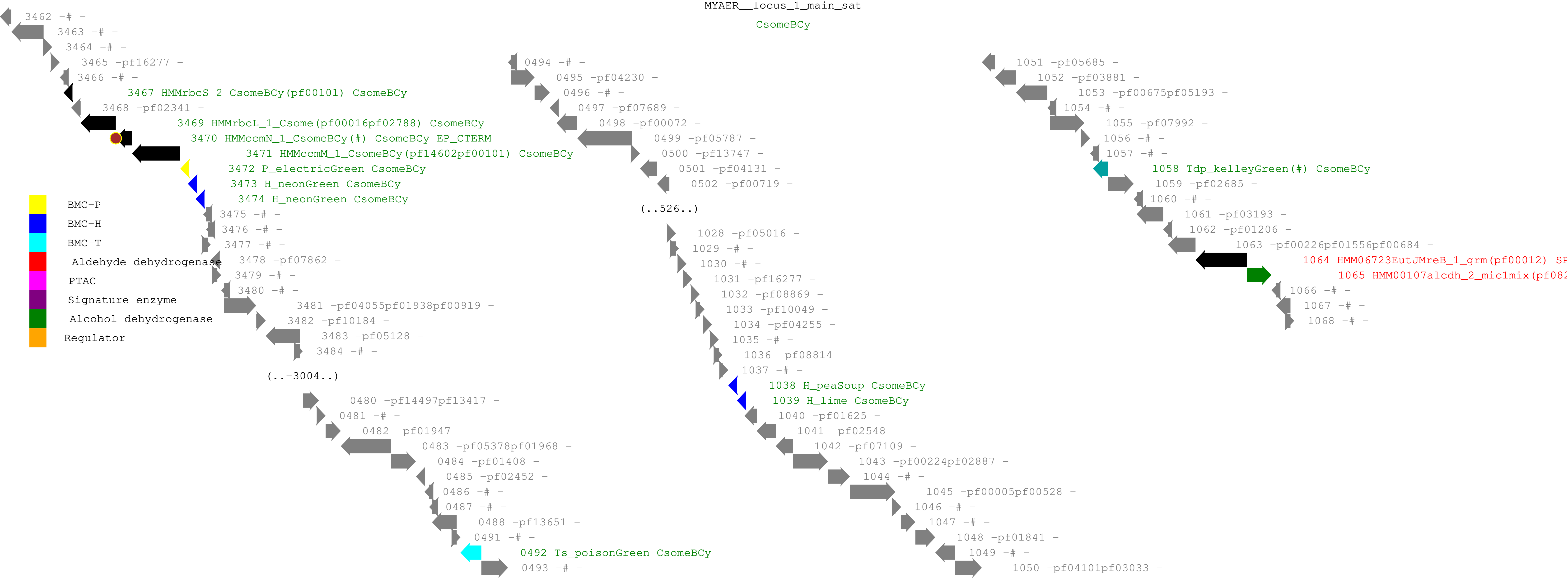

Microcystis sp. 0824. Bacteria; Cyanobacteria; Oscillatoriophycideae; Chroococcales;

N0824__locus_1_main_sat

CsomeBCy

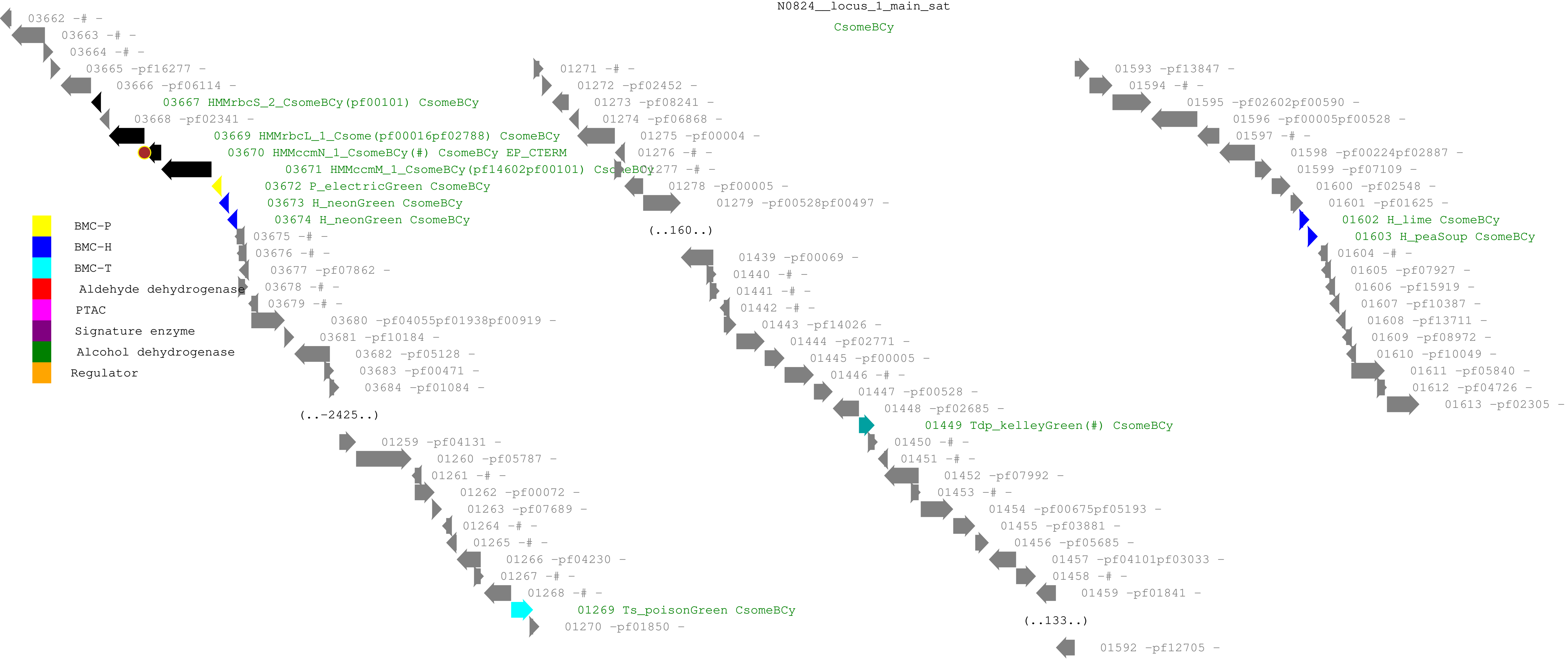

Microcystis aeruginosa NIES-44. Bacteria; Cyanobacteria; Oscillatoriophycideae; Chroococcales;

N44__locus_1_main_sat

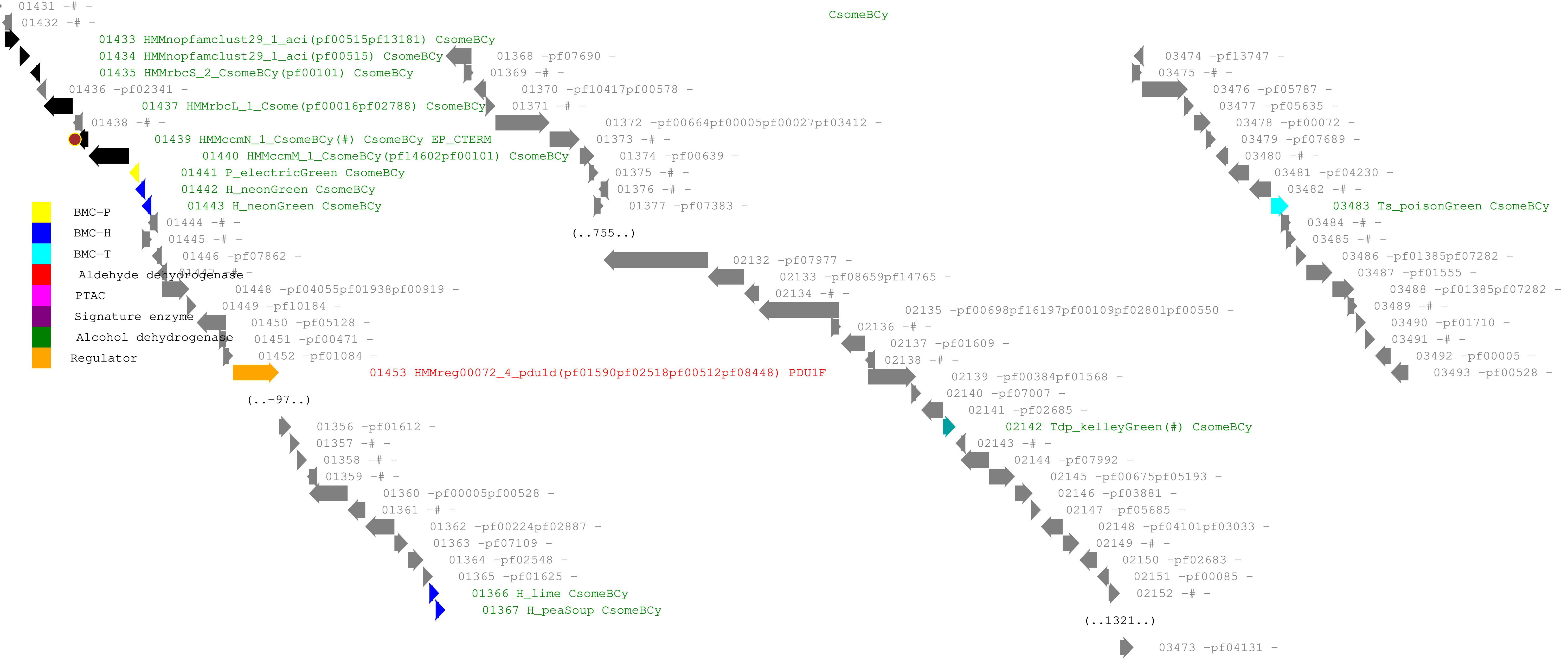

Leptolyngbya sp. Heron Island J. Bacteria; Cyanobacteria; Synechococcales; Leptolyngbyaceae;

N836__locus_1_main_sat

CsomeBCy

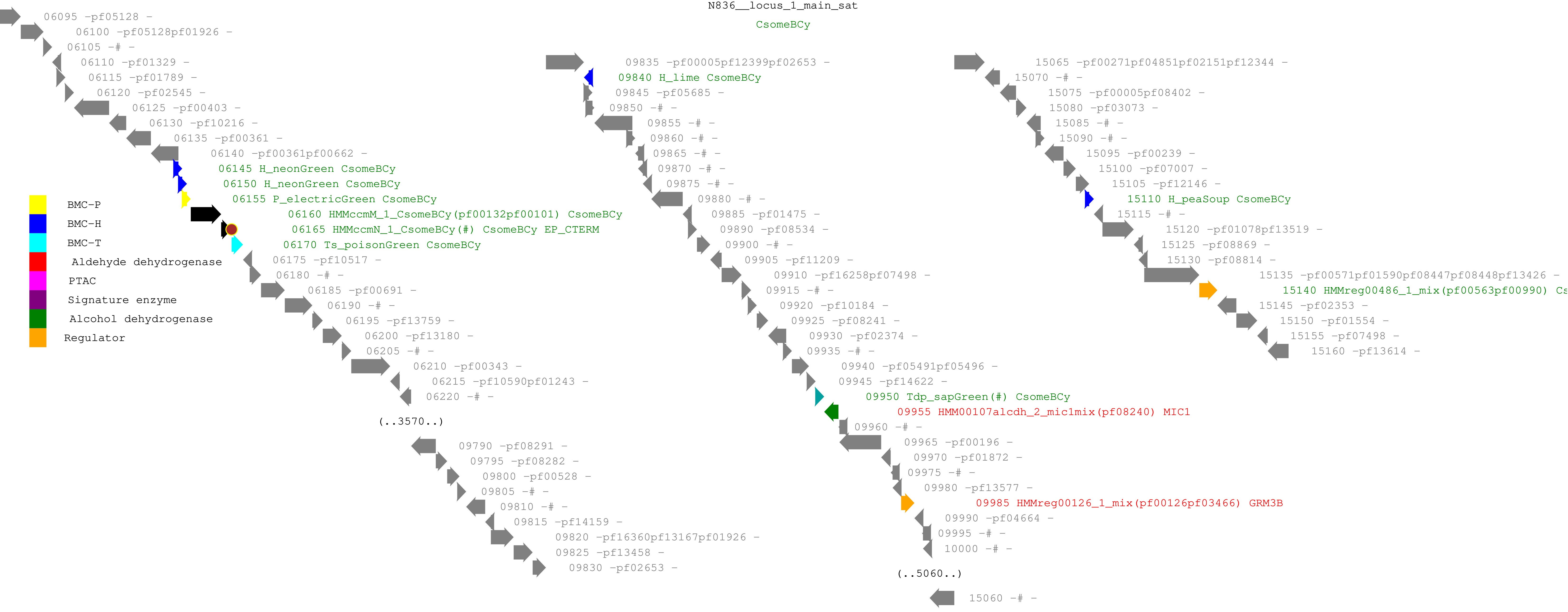

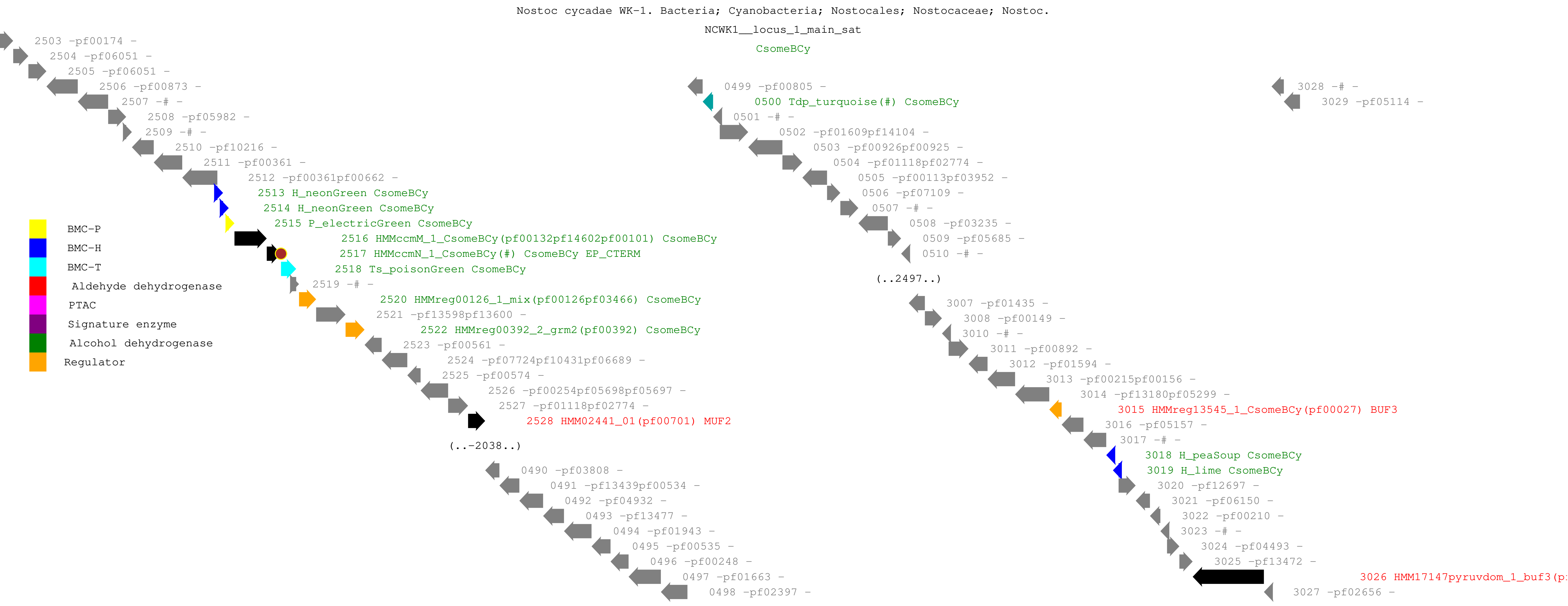
Nostoc cycadae WK-1. Bacteria; Cyanobacteria; Nostocales; Nostocaceae; Nostoc.
NCWK1__locus_1_main_sat
CsomeBCy
2503 -pf00174 -
2504 -pf06051 -
2505 -pf06051 -
2506 -pf00873 -
2507 -# -
2508 -pf05982 -
2509 -# -
2510 -pf10216 -
2511 -pf00361 -
2512 -pf00361pf00662 -
2513 H_neonGreen CsomeBCy
2514 H_neonGreen CsomeBCy
2515 P_electricGreen CsomeBCy
2516 HMMccmM_1_CsomeBCy(pf00132pf14602pf00101) CsomeBCy
2517 HMMccmN_1_CsomeBCy(#) CsomeBCy EP_CTERM
2518 Ts_poisonGreen CsomeBCy
2519 -# -
2520 HMMreg00126_1_mix(pf00126pf03466) CsomeBCy
2521 -pf13598pf13600 -
2522 HMMreg00392_2_grm2(pf00392) CsomeBCy
2523 -pf00561 -
2524 -pf07724pf10431pf06689 -
2525 -pf00574 -
2526 -pf00254pf05698pf05697 -
2527 -pf01118pf02774 -
2528 HMM02441_01(pf00701) MUF2
(..-2038..)
0490 -pf03808 -
0491 -pf13439pf00534 -
0492 -pf04932 -
0493 -pf13477 -
0494 -pf01943 -
0495 -pf00535 -
0496 -pf00248 -
0497 -pf01663 -
0498 -pf02397 -
BMC-P
BMC-H
BMC-T
Aldehyde dehydrogenase
PTAC
Signature enzyme
Alcohol dehydrogenase
Regulator
0499 -pf00805 -
0500 Tdp_turquoise(#) CsomeBCy
0501 -# -
0502 -pf01609pf14104 -
0503 -pf00926pf00925 -
0504 -pf01118pf02774 -
0505 -pf00113pf03952 -
0506 -pf07109 -
0507 -# -
0508 -pf03235 -
0509 -pf05685 -
0510 -# -
(..2497..)
3007 -pf01435 -
3008 -pf00149 -
3010 -# -
3011 -pf00892 -
3012 -pf01594 -
3013 -pf00215pf00156 -
3014 -pf13180pf05299 -
3015 HMMreg13545_1_CsomeBCy(pf00027) BUF3
3016 -pf05157 -
3017 -# -
3018 H_peaSoup CsomeBCy
3019 H_lime CsomeBCy
3020 -pf12697 -
3021 -pf06150 -
3022 -pf00210 -
3023 -# -
3024 -pf04493 -
3025 -pf13472 -
3026 HMM17147pyruvdom_1_buf3(p
3027 -pf02656 -
3028 -# -
3029 -pf05114 -

Chroogloeocystis siderophila 5.2 s.c.1. Bacteria; Cyanobacteria; Oscillatoriophycideae; Chroococcales;

NIES1031__locus_1_main_sat

CsomeBCy

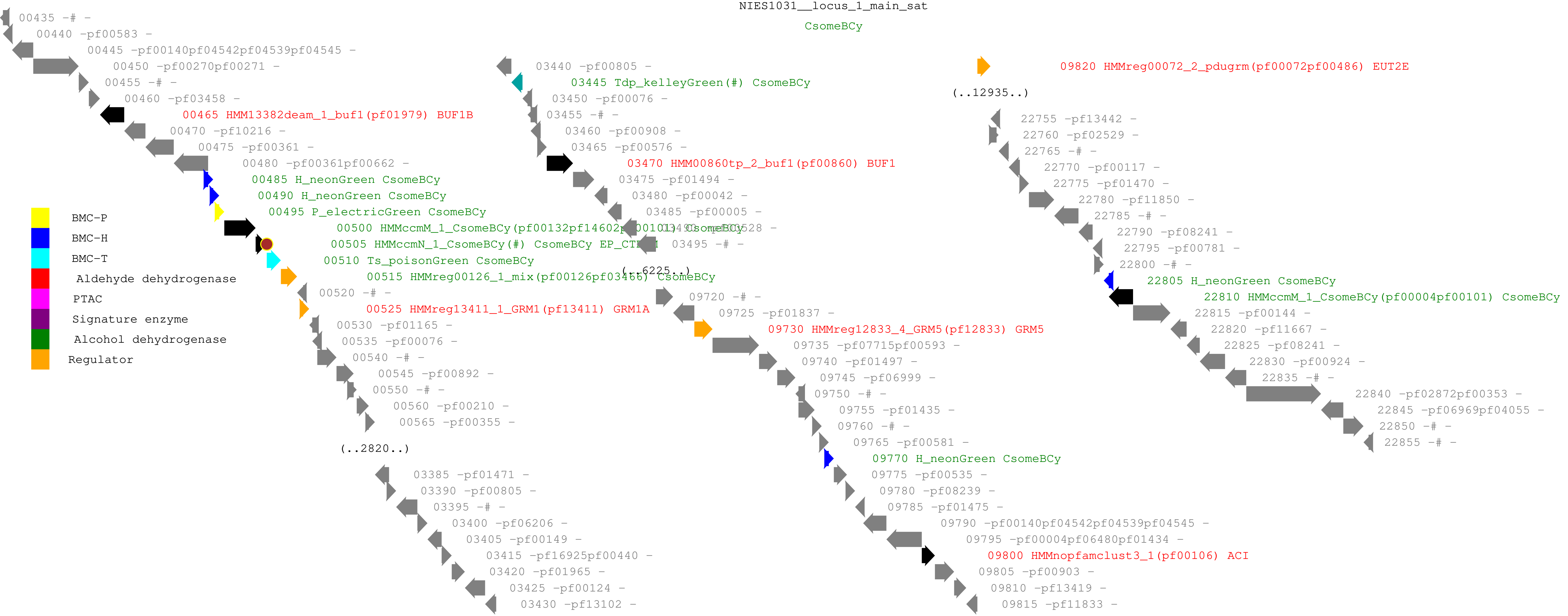

Anabaena cylindrica (strain ATCC 27899 / PCC 7122). Bacteria; Cyanobacteria; Nostocales; Nostocaceae; Anabaena.

NIES19__locus_1_main_sat

CsomeBCy

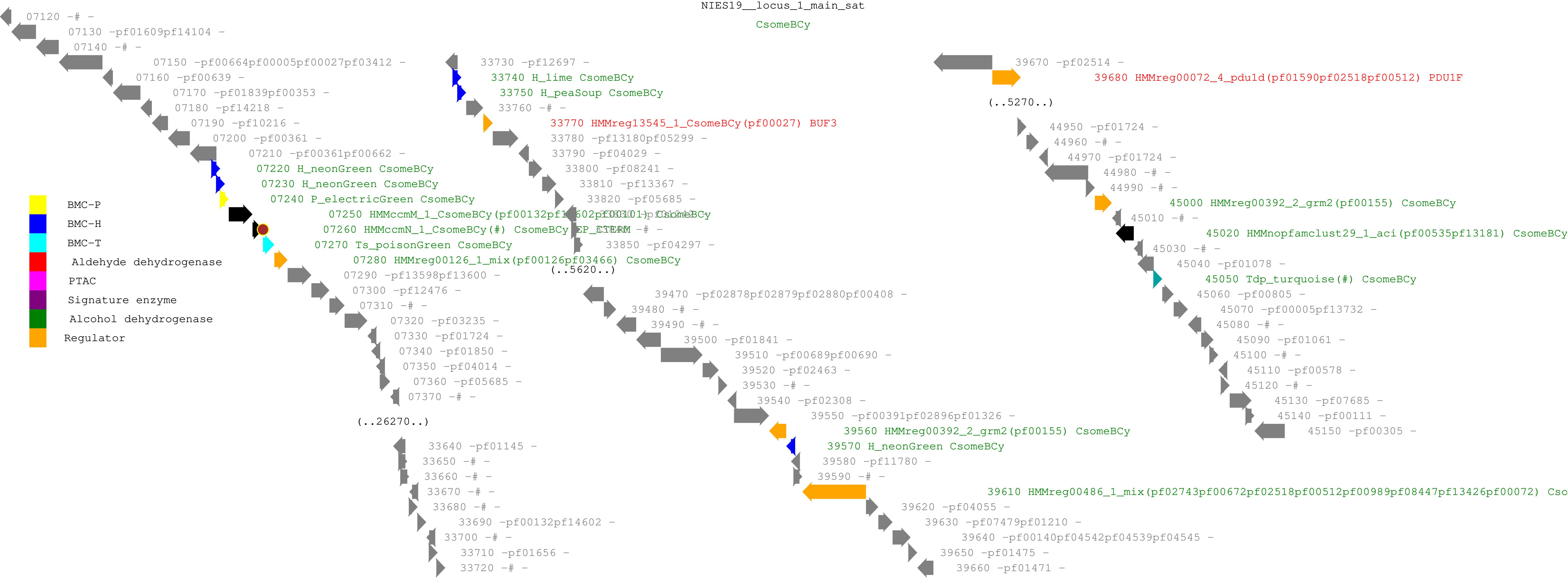

Planktothrix agardhii NIES-204. Bacteria; Cyanobacteria; Oscillatoriophycideae; Oscillatoriales;

NIES204__locus_1_main_sat

CsomeBCy

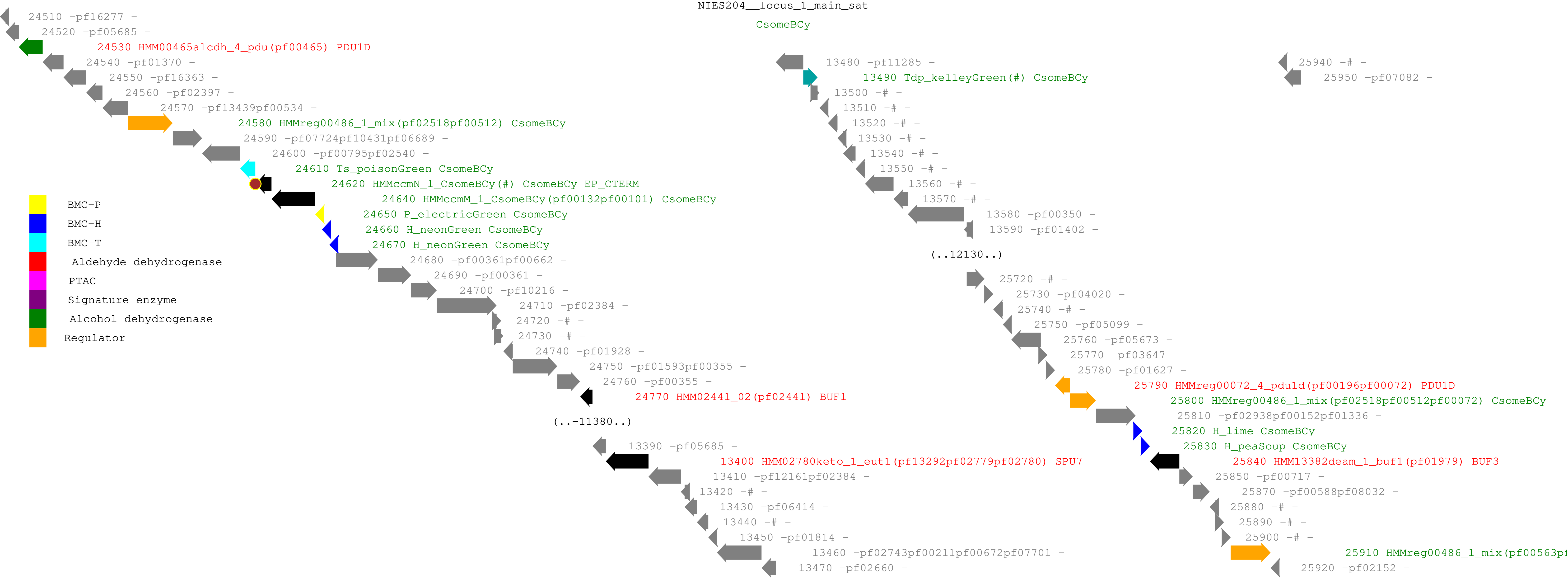

Limnothrix rosea IAM M-220. Bacteria; Cyanobacteria; Synechococcales; Pseudanabaenaceae;

NIES208__locus_1_main_sat

CsomeBCy

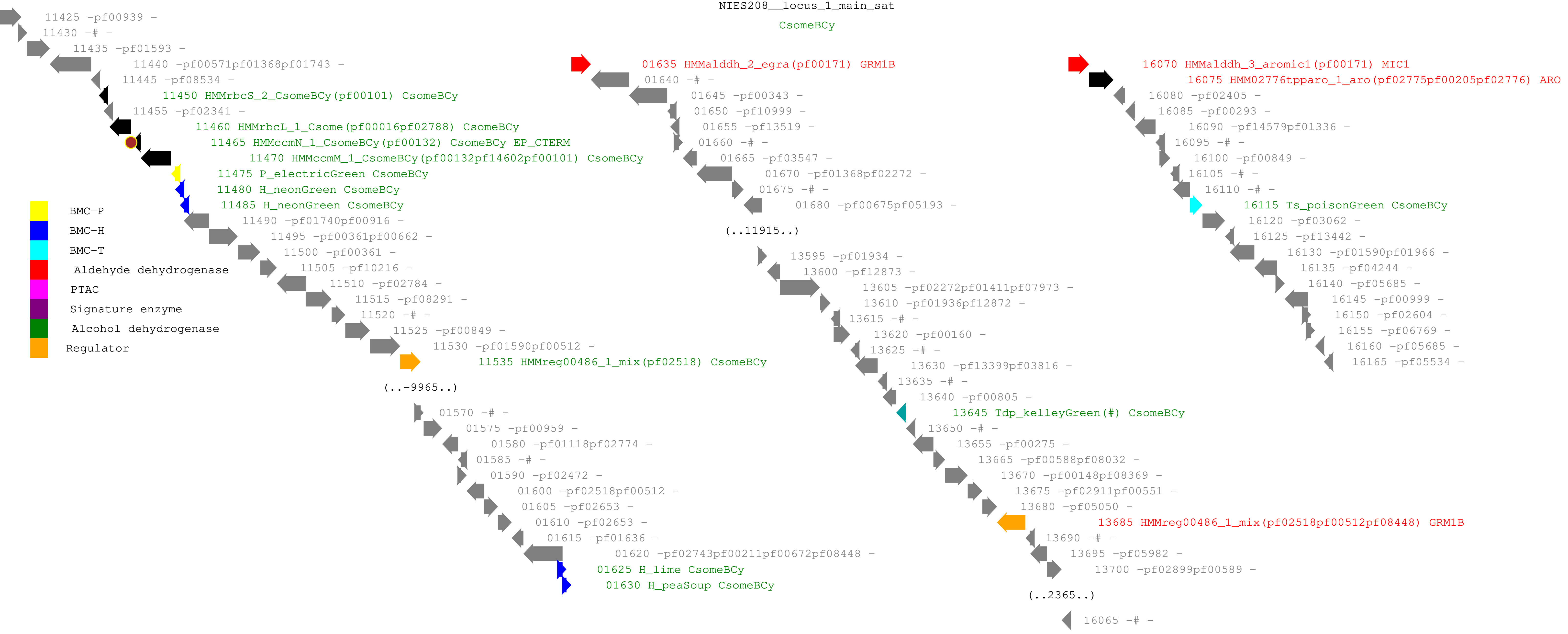

Calothrix sp. NIES-2098. Bacteria; Cyanobacteria; Nostocales; Rivulariaceae; Calothrix.

NIES2098__locus_1_main_sat

CsomeBCy

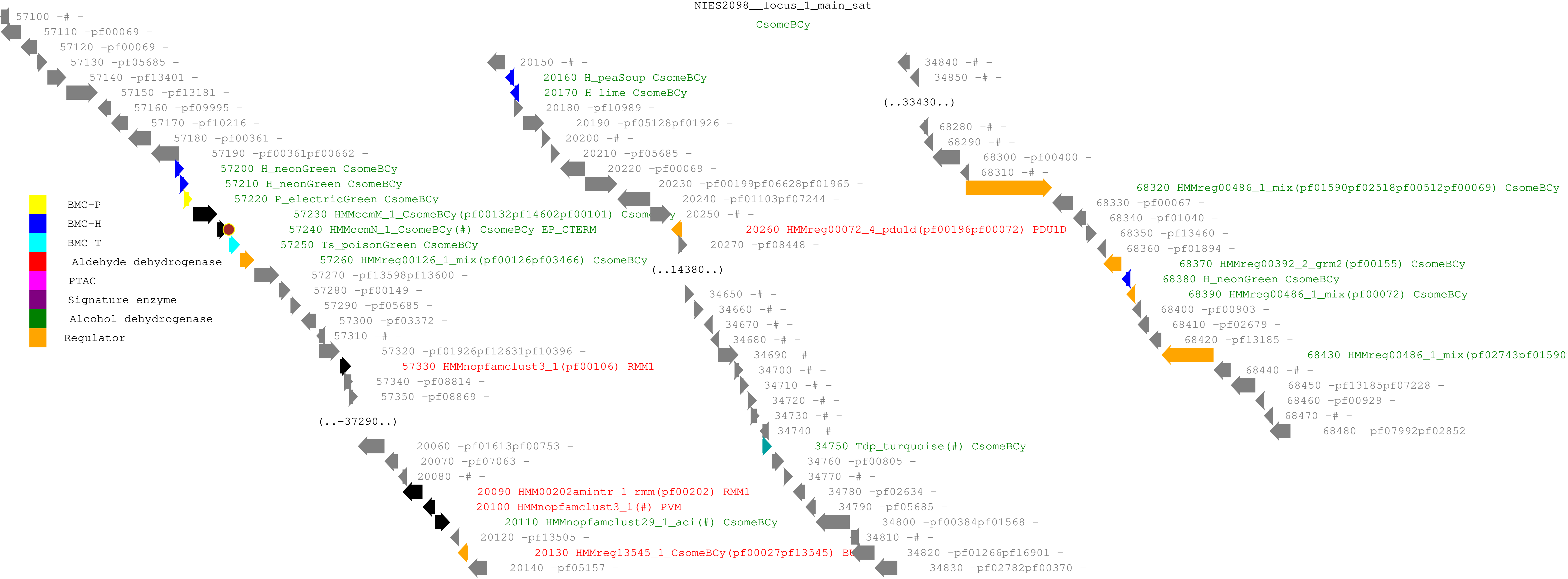

Calothrix sp. NIES-2100. Bacteria; Cyanobacteria; Nostocales; Rivulariaceae; Calothrix.

NIES2100__locus_1_main_sat

CsomeBCy

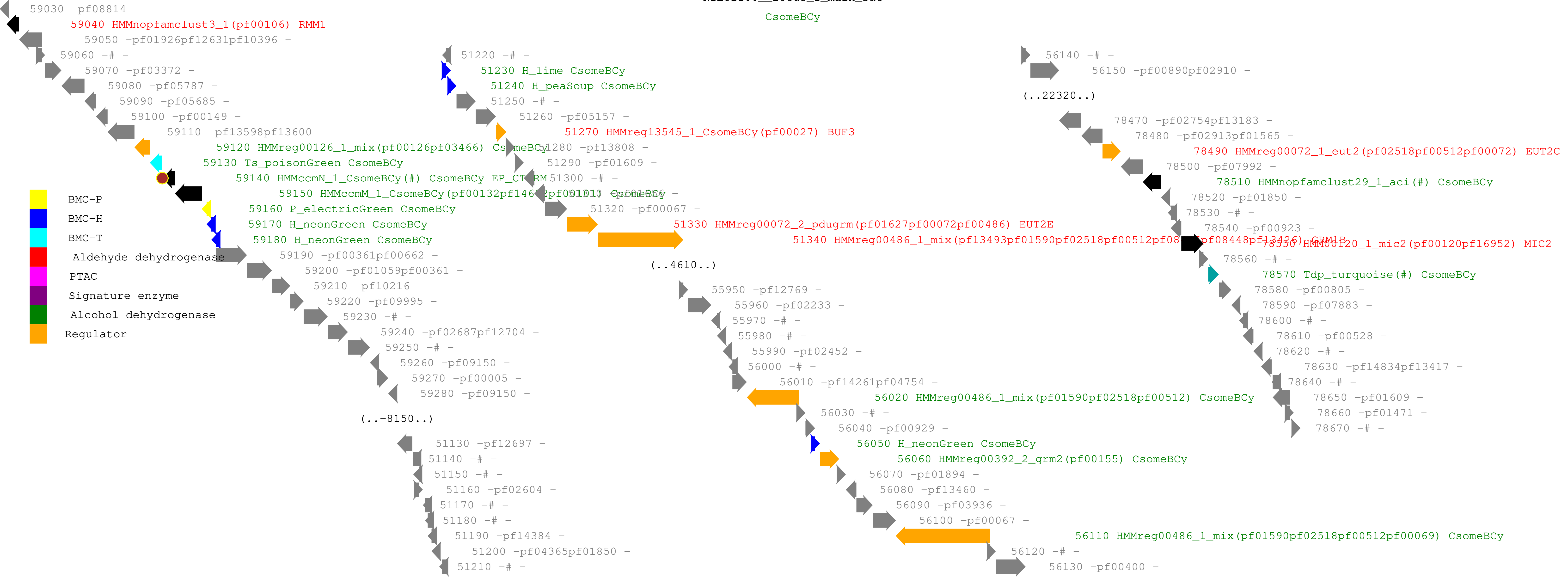

Calothrix sp. HK-06. Bacteria; Cyanobacteria; Nostocales; Rivulariaceae; Calothrix.

NIES2101__locus_1_main_sat

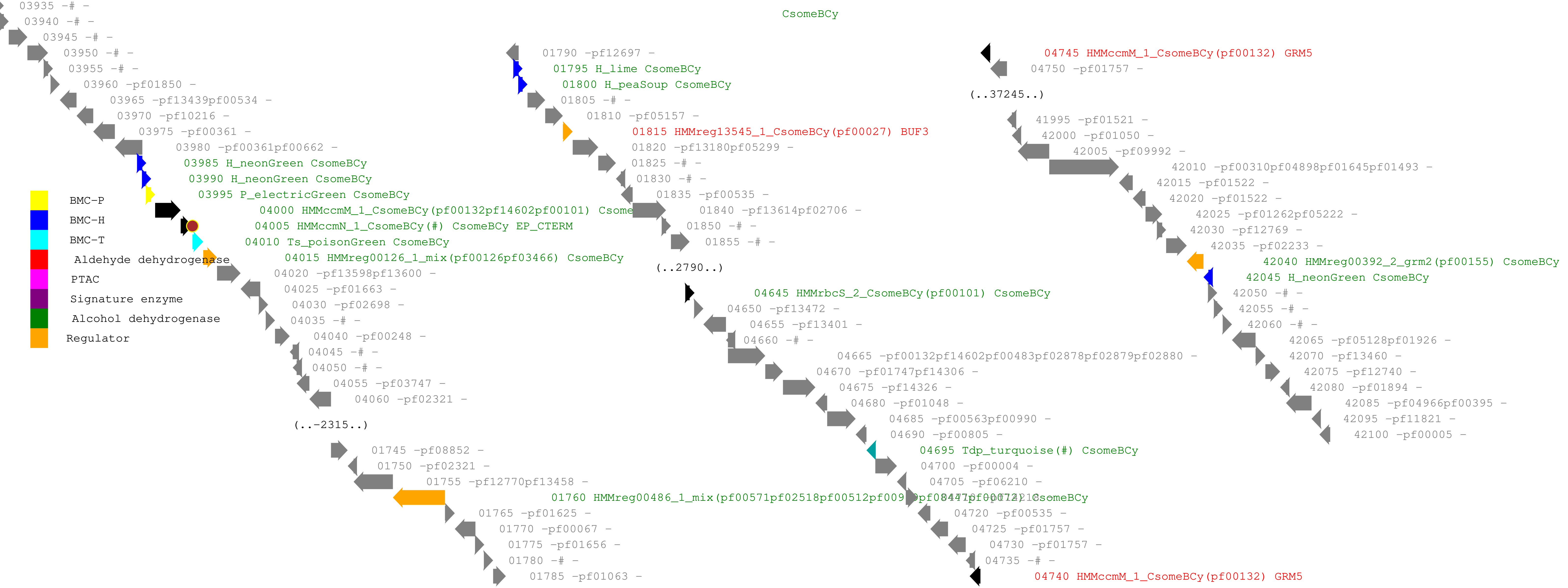

Leptolyngbya sp. NIES-2104. Bacteria; Cyanobacteria; Synechococcales; Leptolyngbyaceae;

NIES2104__locus_1_main_sat

CsomeBCy

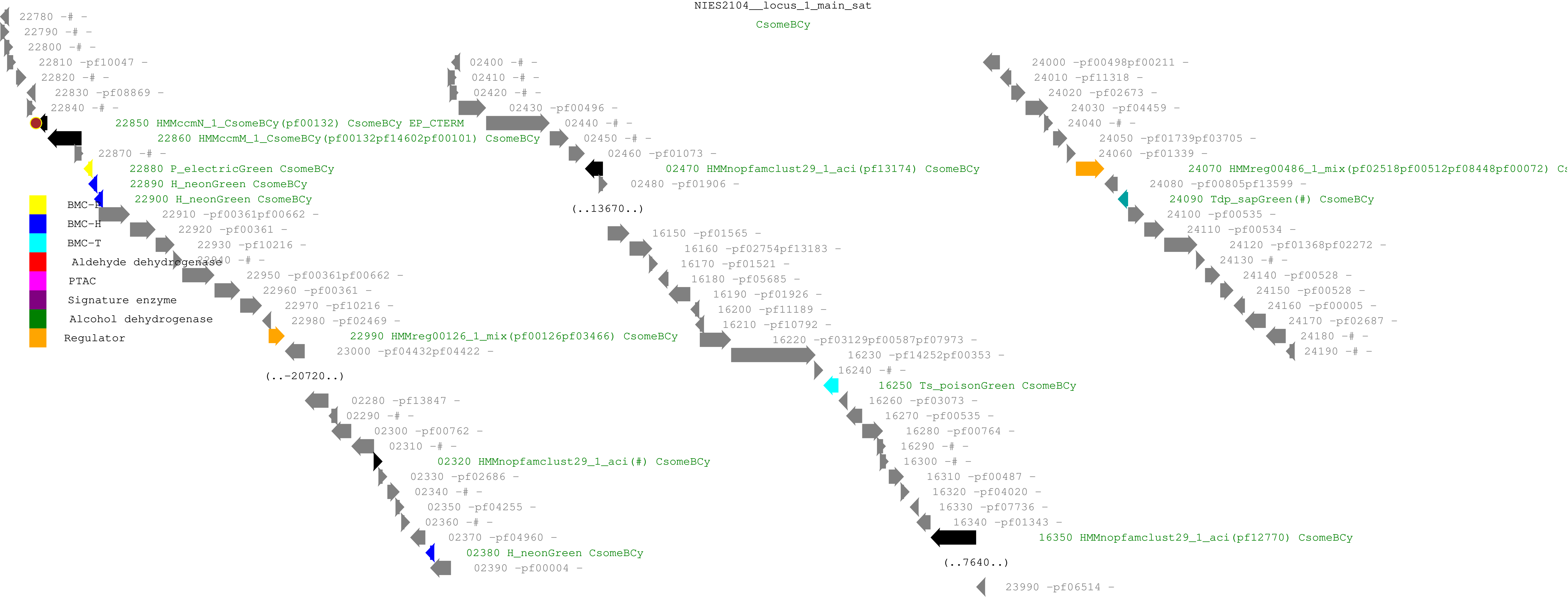

Nostoc carneum NIES-2107. Bacteria; Cyanobacteria; Nostocales; Nostocaceae; Nostoc.

NIES2107__locus_1_main_sat

CsomeBCy

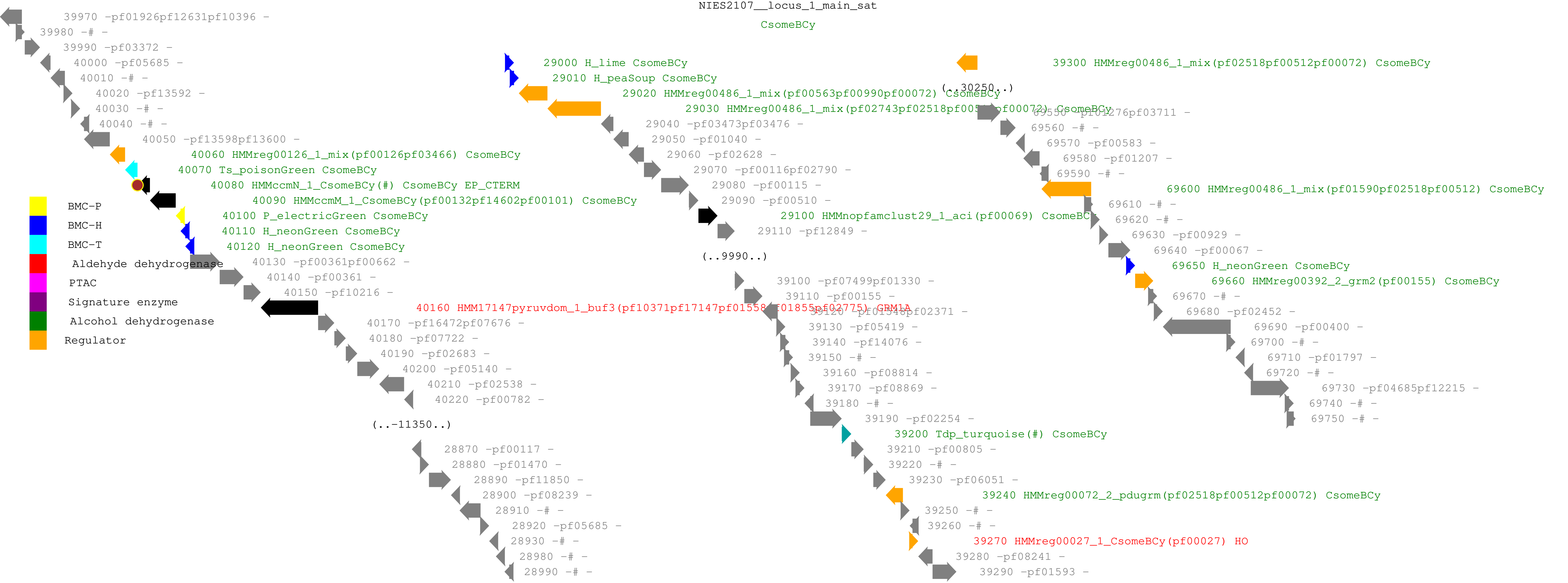

Nostoc sp. HK-01. Bacteria; Cyanobacteria; Nostocales; Nostocaceae; Nostoc.

NIES2109__locus_1_main_sat

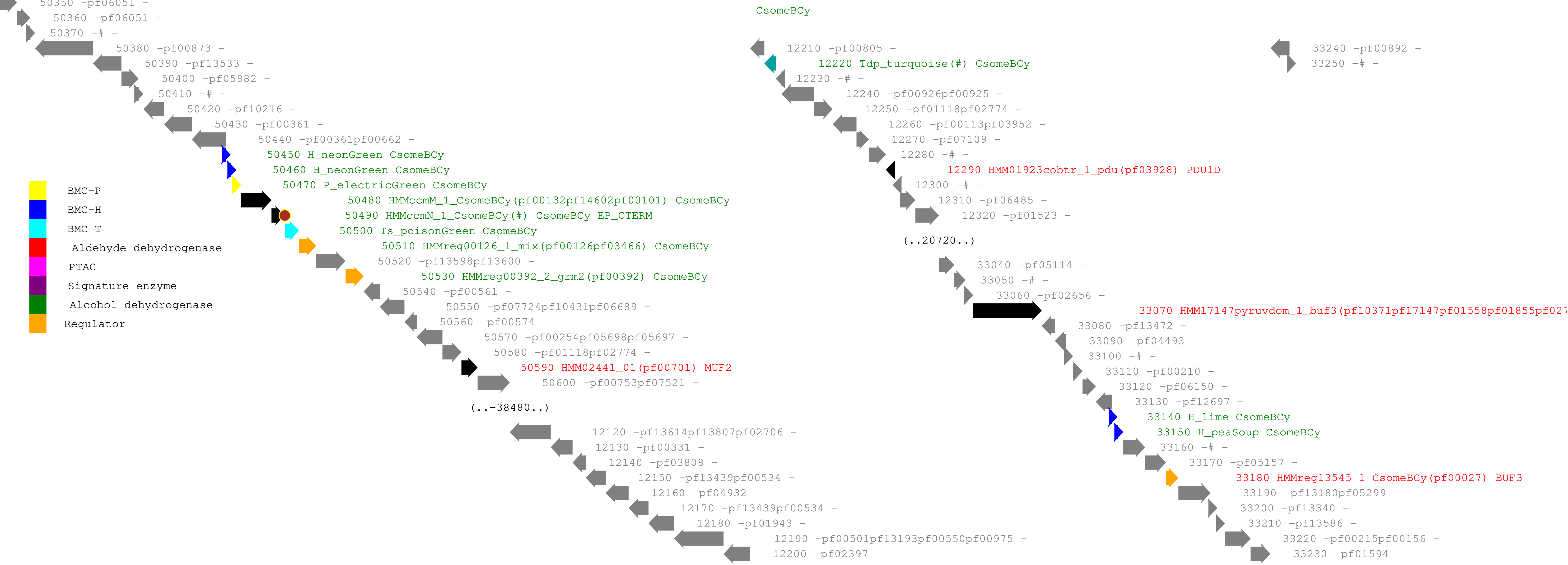

Nostoc sp. NIES-2111. Bacteria; Cyanobacteria; Nostocales; Nostocaceae; Nostoc.

NIES2111__locus_1_main_sat

CsomeBCy

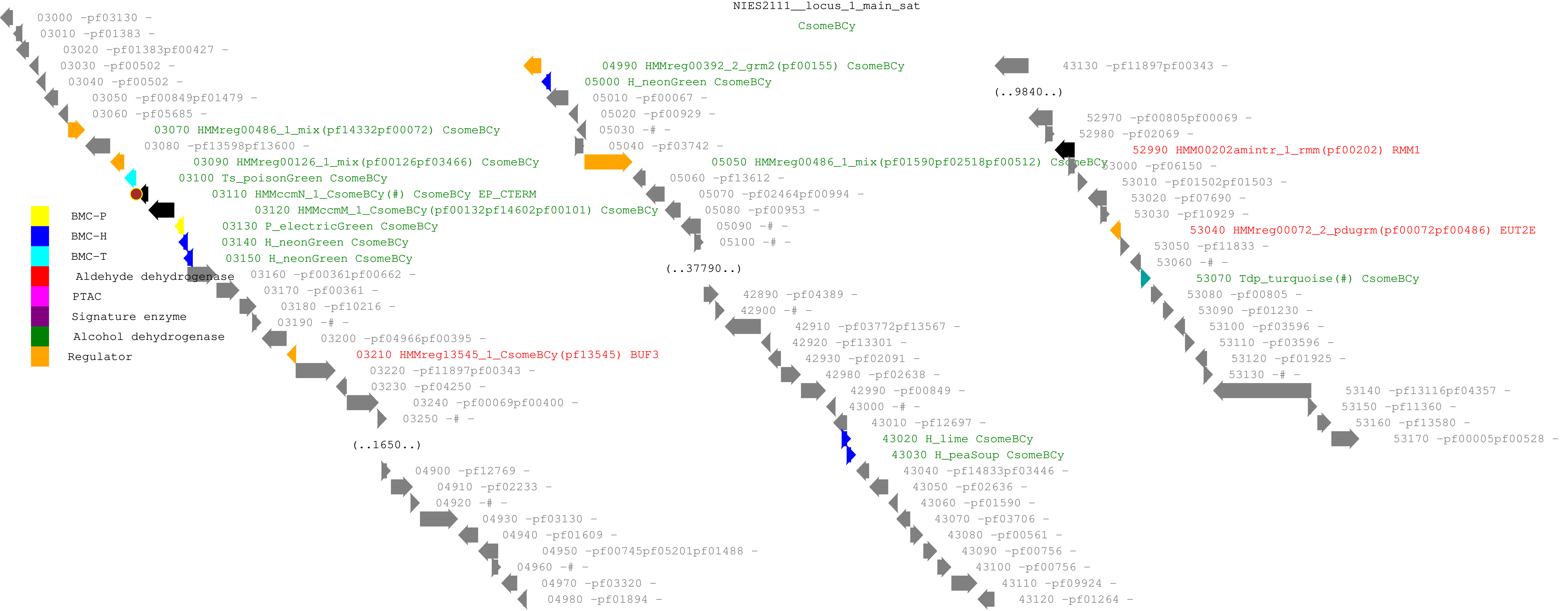

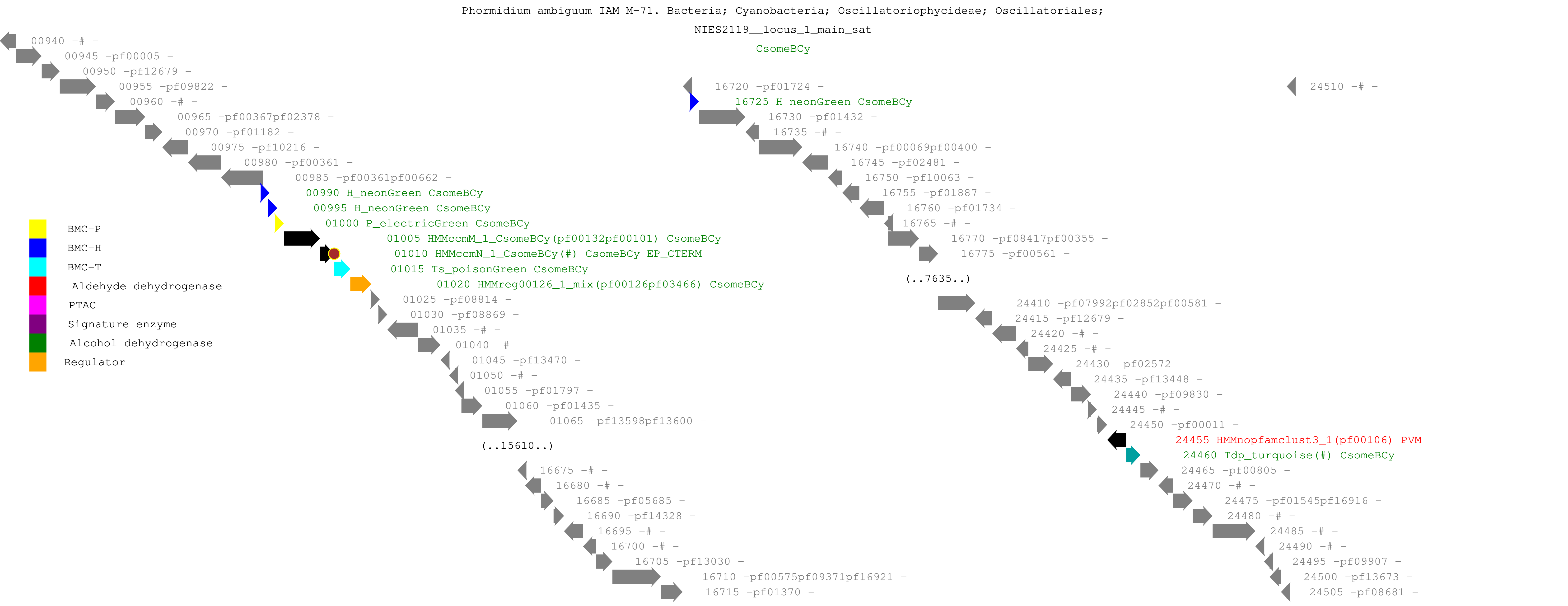

Phormidium ambiguum IAM M-71. Bacteria; Cyanobacteria; Oscillatoriophycideae; Oscillatoriales;
NIES2119__locus_1_main_sat
CsomeBCy
00940 -# -
00945 -pf00005 -
00950 -pf12679 -
00955 -pf09822 -
00960 -# -
00965 -pf00367pf02378 -
00970 -pf01182 -
00975 -pf10216 -
00980 -pf00361 -
00985 -pf00361pf00662 -
00990 H_neonGreen CsomeBCy
00995 H_neonGreen CsomeBCy
01000 P_electricGreen CsomeBCy
01005 HMMccmM_1_CsomeBCy(pf00132pf00101) CsomeBCy
01010 HMMccmN_1_CsomeBCy(#) CsomeBCy EP_CTERM
01015 Ts_poisonGreen CsomeBCy
01020 HMMreg00126_1_mix(pf00126pf03466) CsomeBCy
01025 -pf08814 -
01030 -pf08869 -
01035 -# -
01040 -# -
01045 -pf13470 -
01050 -# -
01055 -pf01797 -
01060 -pf01435 -
01065 -pf13598pf13600 -
(..15610..)
16675 -# -
16680 -# -
16685 -pf05685 -
16690 -pf14328 -
16695 -# -
16700 -# -
16705 -pf13030 -
16710 -pf00575pf09371pf16921 -
16715 -pf01370 -
16720 -pf01724 -
16725 H_neonGreen CsomeBCy
16730 -pf01432 -
16735 -# -
16740 -pf00069pf00400 -
16745 -pf02481 -
16750 -pf10063 -
16755 -pf01887 -
16760 -pf01734 -
16765 -# -
16770 -pf08417pf00355 -
16775 -pf00561 -
(..7635..)
24410 -pf07992pf02852pf00581 -
24415 -pf12679 -
24420 -# -
24425 -# -
24430 -pf02572 -
24435 -pf13448 -
24440 -pf09830 -
24445 -# -
24450 -pf00011 -
24455 HMMnopfamclust3_1(pf00106) PVM
24460 Tdp_turquoise(#) CsomeBCy
24465 -pf00805 -
24470 -# -
24475 -pf01545pf16916 -
24480 -# -
24485 -# -
24490 -# -
24495 -pf09907 -
24500 -pf13673 -
24505 -pf08681 -
24510 -# -
BMC-P
BMC-H
BMC-T
Aldehyde dehydrogenase
PTAC
Signature enzyme
Alcohol dehydrogenase
Regulator

Scytonema sp. HK-05. Bacteria; Cyanobacteria; Nostocales; Scytonemataceae; Scytonema.

NIES2130__locus_1_main_sat

CsomeBCy

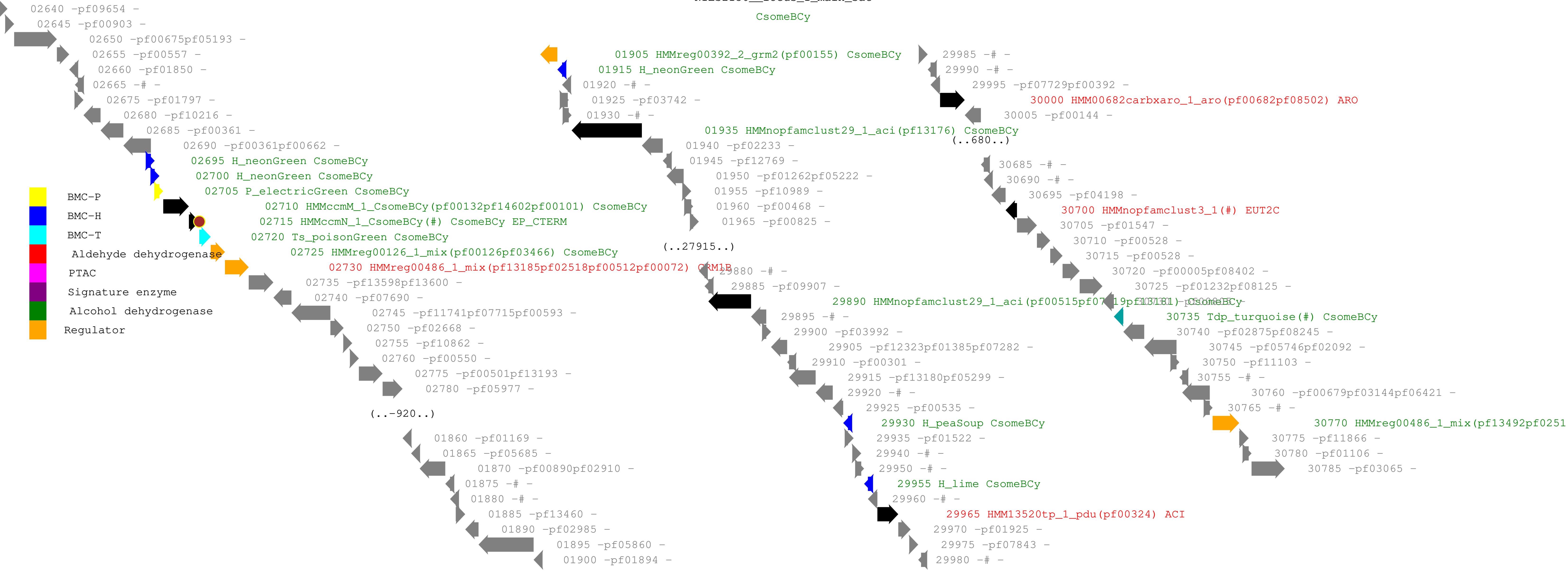

Leptolyngbya boryana NIES-2135. Bacteria; Cyanobacteria; Synechococcales; Leptolyngbyaceae;

NIES2135__locus_1_main_sat

CsomeBCy

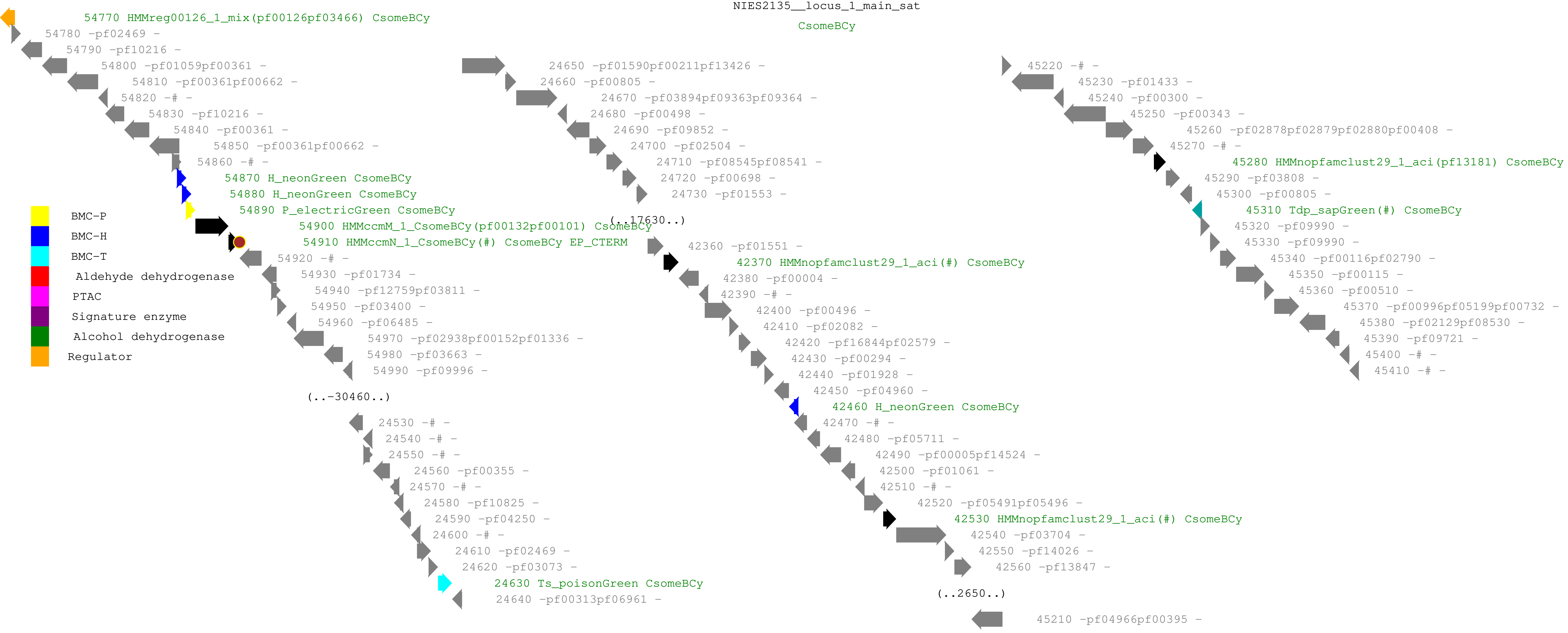

Anabaenopsis circularis NIES-21. Bacteria; Cyanobacteria; Nostocales; Aphanizomenonaceae; Anabaenopsis.

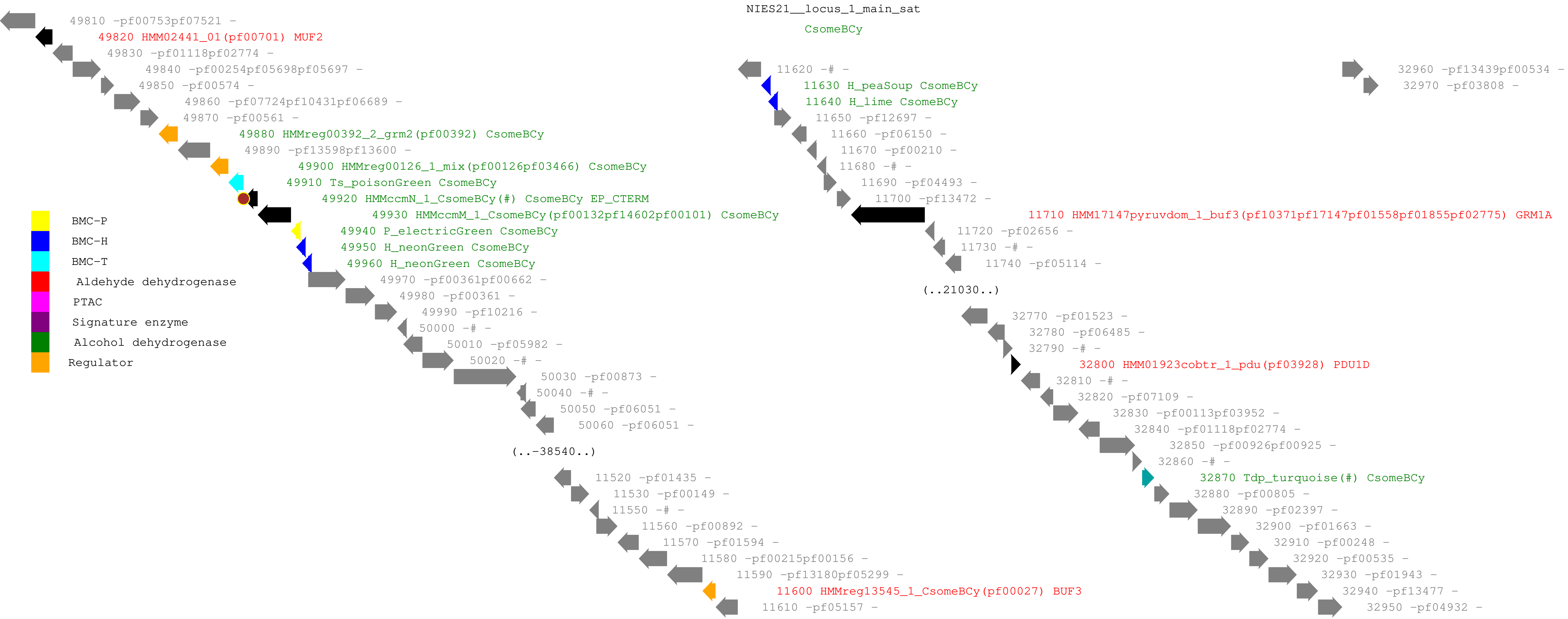

Calothrix brevissima NIES-22. Bacteria; Cyanobacteria; Nostocales; Rivulariaceae; Calothrix.

NIES22__locus_1_main_sat

CsomeBCy

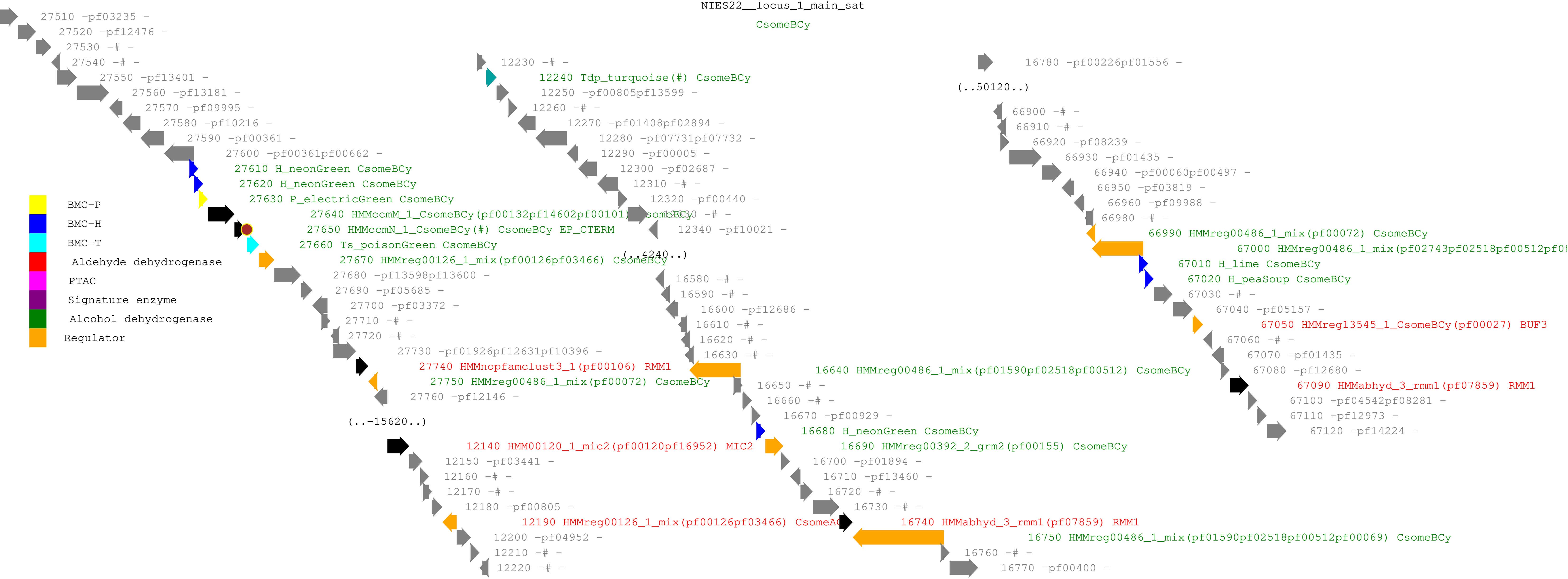

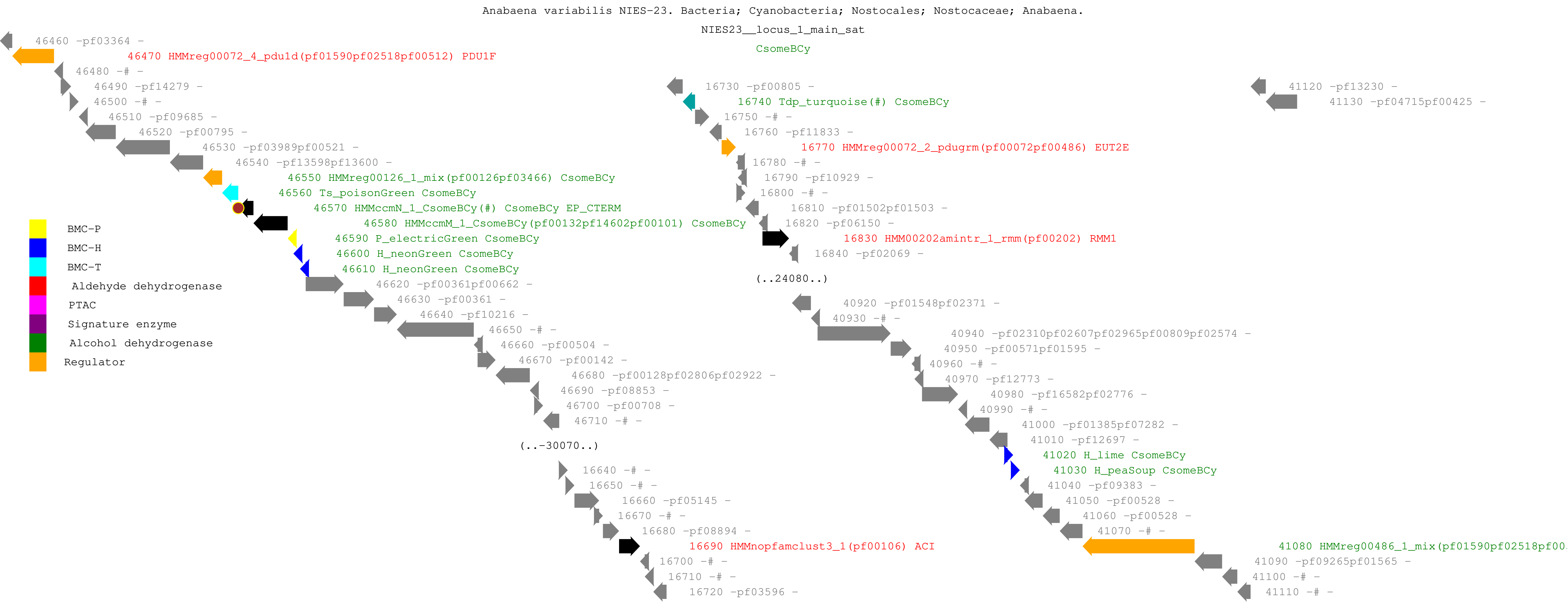
Anabaena variabilis NIES-23. Bacteria; Cyanobacteria; Nostocales; Nostocaceae; Anabaena.
NIES23__locus_1_main_sat
CsomeBCy
46460 -pf03364 -
46470 HMMreg00072_4_pdu1d(pf01590pf02518pf00512) PDU1F
46480 -# -
46490 -pf14279 -
46500 -# -
46510 -pf09685 -
46520 -pf00795 -
46530 -pf03989pf00521 -
46540 -pf13598pf13600 -
46550 HMMreg00126_1_mix(pf00126pf03466) CsomeBCy
46560 Ts_poisonGreen CsomeBCy
46570 HMMccmN_1_CsomeBCy(#) CsomeBCy EP_CTERM
46580 HMMccmM_1_CsomeBCy(pf00132pf14602pf00101) CsomeBCy
46590 P_electricGreen CsomeBCy
46600 H_neonGreen CsomeBCy
46610 H_neonGreen CsomeBCy
46620 -pf00361pf00662 -
46630 -pf00361 -
46640 -pf10216 -
46650 -# -
46660 -pf00504 -
46670 -pf00142 -
46680 -pf00128pf02806pf02922 -
46690 -pf08853 -
46700 -pf00708 -
46710 -# -
(..-30070..)
16640 -# -
16650 -# -
16660 -pf05145 -
16670 -# -
16680 -pf08894 -
16690 HMMnopfamclust3_1(pf00106) ACI
16700 -# -
16710 -# -
16720 -pf03596 -
16730 -pf00805 -
16740 Tdp_turquoise(#) CsomeBCy
16750 -# -
16760 -pf11833 -
16770 HMMreg00072_2_pdugrm(pf00072pf00486) EUT2E
16780 -# -
16790 -pf10929 -
16800 -# -
16810 -pf01502pf01503 -
16820 -pf06150 -
16830 HMM00202amintr_1_rmm(pf00202) RMM1
16840 -pf02069 -
(..24080..)
40920 -pf01548pf02371 -
40930 -# -
40940 -pf02310pf02607pf02965pf00809pf02574 -
40950 -pf00571pf01595 -
40960 -# -
40970 -pf12773 -
40980 -pf16582pf02776 -
40990 -# -
41000 -pf01385pf07282 -
41010 -pf12697 -
41020 H_lime CsomeBCy
41030 H_peaSoup CsomeBCy
41040 -pf09383 -
41050 -pf00528 -
41060 -pf00528 -
41070 -# -
41080 HMMreg00486_1_mix(pf01590pf02518pf00
41090 -pf09265pf01565 -
41100 -# -
41110 -# -
41120 -pf13230 -
41130 -pf04715pf00425 -
BMC-P
BMC-H
BMC-T
Aldehyde dehydrogenase
PTAC
Signature enzyme
Alcohol dehydrogenase
Regulator

Nostoc linckia NIES-25. Bacteria; Cyanobacteria; Nostocales; Nostocaceae; Nostoc.

NIES25__locus_1_main_sat

CsomeBCy

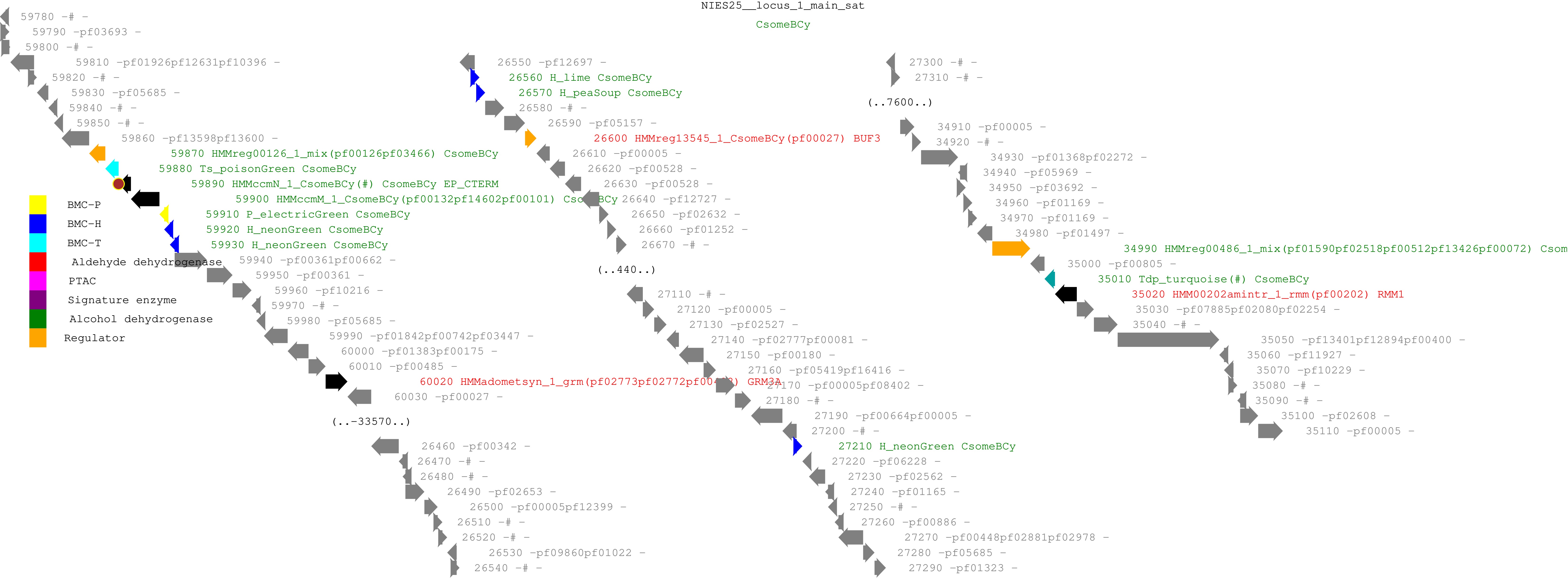

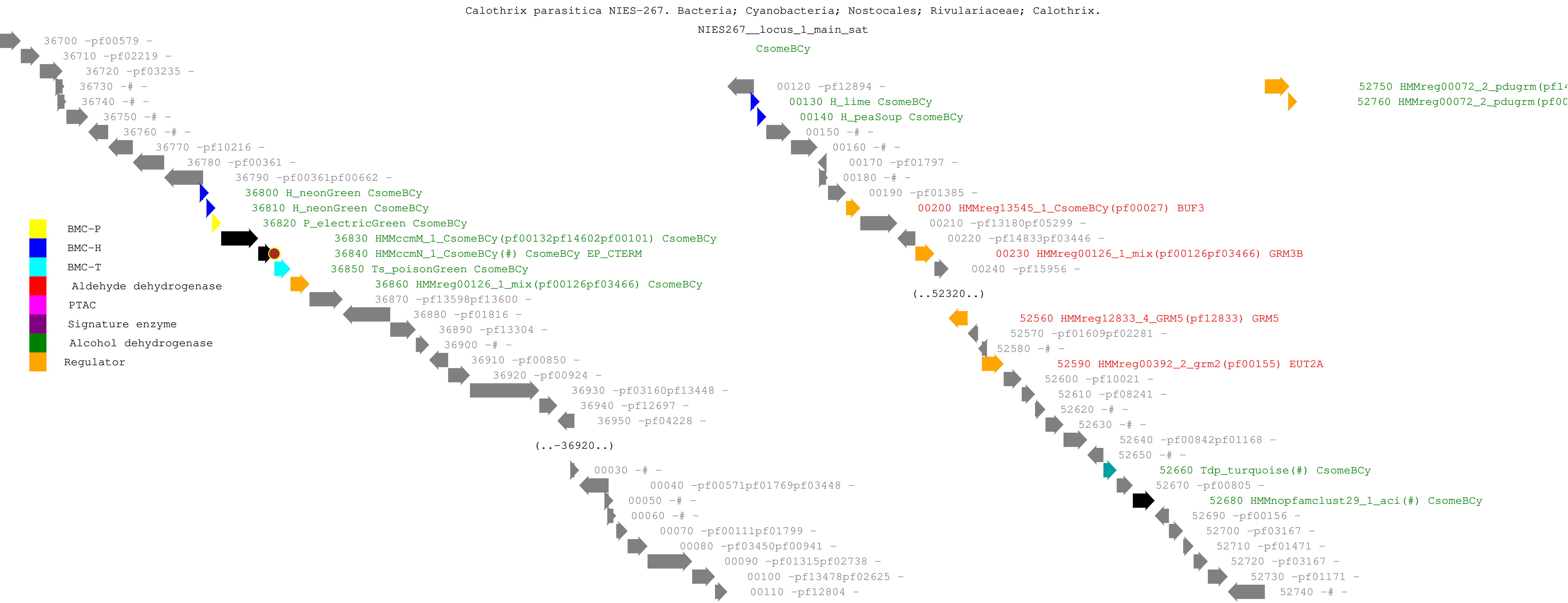

Calothrix parasitica NIES-267. Bacteria; Cyanobacteria; Nostocales; Rivulariaceae; Calothrix.
NIES267__locus_1_main_sat
CsomeBCy
36700 -pf00579 -
36710 -pf02219 -
36720 -pf03235 -
36730 -# -
36740 -# -
36750 -# -
36760 -# -
36770 -pf10216 -
36780 -pf00361 -
36790 -pf00361pf00662 -
36800 H_neonGreen CsomeBCy
36810 H_neonGreen CsomeBCy
36820 P_electricGreen CsomeBCy
36830 HMMccmM_1_CsomeBCy(pf00132pf14602pf00101) CsomeBCy
36840 HMMccmN_1_CsomeBCy(#) CsomeBCy EP_CTERM
36850 Ts_poisonGreen CsomeBCy
36860 HMMreg00126_1_mix(pf00126pf03466) CsomeBCy
36870 -pf13598pf13600 -
36880 -pf01816 -
36890 -pf13304 -
36900 -# -
36910 -pf00850 -
36920 -pf00924 -
36930 -pf03160pf13448 -
36940 -pf12697 -
36950 -pf04228 -
(..-36920..)
00030 -# -
00040 -pf00571pf01769pf03448 -
00050 -# -
00060 -# -
00070 -pf00111pf01799 -
00080 -pf03450pf00941 -
00090 -pf01315pf02738 -
00100 -pf13478pf02625 -
00110 -pf12804 -
00120 -pf12894 -
00130 H_lime CsomeBCy
00140 H_peaSoup CsomeBCy
00150 -# -
00160 -# -
00170 -pf01797 -
00180 -# -
00190 -pf01385 -
00200 HMMreg13545_1_CsomeBCy(pf00027) BUF3
00210 -pf13180pf05299 -
00220 -pf14833pf03446 -
00230 HMMreg00126_1_mix(pf00126pf03466) GRM3B
00240 -pf15956 -
(..52320..)
52560 HMMreg12833_4_GRM5(pf12833) GRM5
52570 -pf01609pf02281 -
52580 -# -
52590 HMMreg00392_2_grm2(pf00155) EUT2A
52600 -pf10021 -
52610 -pf08241 -
52620 -# -
52630 -# -
52640 -pf00842pf01168 -
52650 -# -
52660 Tdp_turquoise(#) CsomeBCy
52670 -pf00805 -
52680 HMMnopfamclust29_1_aci(#) CsomeBCy
52690 -pf00156 -
52700 -pf03167 -
52710 -pf01471 -
52720 -pf03167 -
52730 -pf01171 -
52740 -# -
52750 HMMreg00072_2_pdugrm(pf1
52760 HMMreg00072_2_pdugrm(pf0
BMC-P
BMC-H
BMC-T
Aldehyde dehydrogenase
PTAC
Signature enzyme
Alcohol dehydrogenase
Regulator

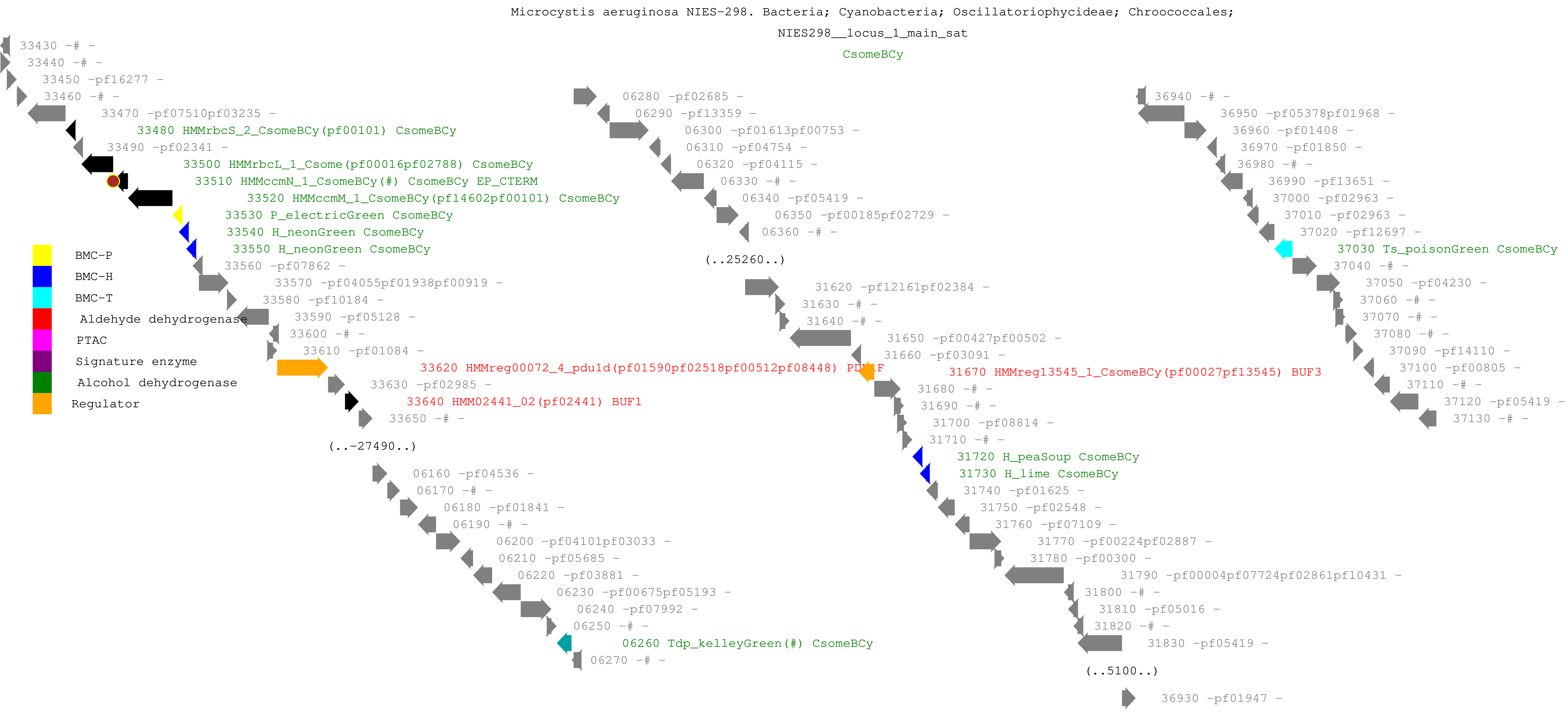
Microcystis aeruginosa NIES-298. Bacteria; Cyanobacteria; Oscillatoriophycideae; Chroococcales;
NIES298__locus_1_main_sat
CsomeBCy
33430 -# -
33440 -# -
33450 -pf16277 -
33460 -# -
33470 -pf07510pf03235 -
33480 HMMrbcS_2_CsomeBCy(pf00101) CsomeBCy
33490 -pf02341 -
33500 HMMrbcL_1_Csome(pf00016pf02788) CsomeBCy
33510 HMMccmN_1_CsomeBCy(#) CsomeBCy EP_CTERM
33520 HMMccmM_1_CsomeBCy(pf14602pf00101) CsomeBCy
33530 P_electricGreen CsomeBCy
33540 H_neonGreen CsomeBCy
33550 H_neonGreen CsomeBCy
33560 -pf07862 -
33570 -pf04055pf01938pf00919 -
33580 -pf10184 -
33590 -pf05128 -
33600 -# -
33610 -pf01084 -
33620 HMMreg00072_4_pdu1d(pf01590pf02518pf00512pf08448) PDUEF
33630 -pf02985 -
33640 HMM02441_02(pf02441) BUF1
33650 -# -
(..-27490..)
06160 -pf04536 -
06170 -# -
06180 -pf01841 -
06190 -# -
06200 -pf04101pf03033 -
06210 -pf05685 -
06220 -pf03881 -
06230 -pf00675pf05193 -
06240 -pf07992 -
06250 -# -
06260 Tdp_kelleyGreen(#) CsomeBCy
06270 -# -
06280 -pf02685 -
06290 -pf13359 -
06300 -pf01613pf00753 -
06310 -pf04754 -
06320 -pf04115 -
06330 -# -
06340 -pf05419 -
06350 -pf00185pf02729 -
06360 -# -
(..25260..)
31620 -pf12161pf02384 -
31630 -# -
31640 -# -
31650 -pf00427pf00502 -
31660 -pf03091 -
31670 HMMreg13545_1_CsomeBCy(pf00027pf13545) BUF3
31680 -# -
31690 -# -
31700 -pf08814 -
31710 -# -
31720 H_peaSoup CsomeBCy
31730 H_lime CsomeBCy
31740 -pf01625 -
31750 -pf02548 -
31760 -pf07109 -
31770 -pf00224pf02887 -
31780 -pf00300 -
31790 -pf00004pf07724pf02861pf10431 -
31800 -# -
31810 -pf05016 -
31820 -# -
31830 -pf05419 -
(..5100..)
36930 -pf01947 -
36940 -# -
36950 -pf05378pf01968 -
36960 -pf01408 -
36970 -pf01850 -
36980 -# -
36990 -pf13651 -
37000 -pf02963 -
37010 -pf02963 -
37020 -pf12697 -
37030 Ts_poisonGreen CsomeBCy
37040 -# -
37050 -pf04230 -
37060 -# -
37070 -# -
37080 -# -
37090 -pf14110 -
37100 -pf00805 -
37110 -# -
37120 -pf05419 -
37130 -# -
BMC-P
BMC-H
BMC-T
Aldehyde dehydrogenase
PTAC
Signature enzyme
Alcohol dehydrogenase
Regulator

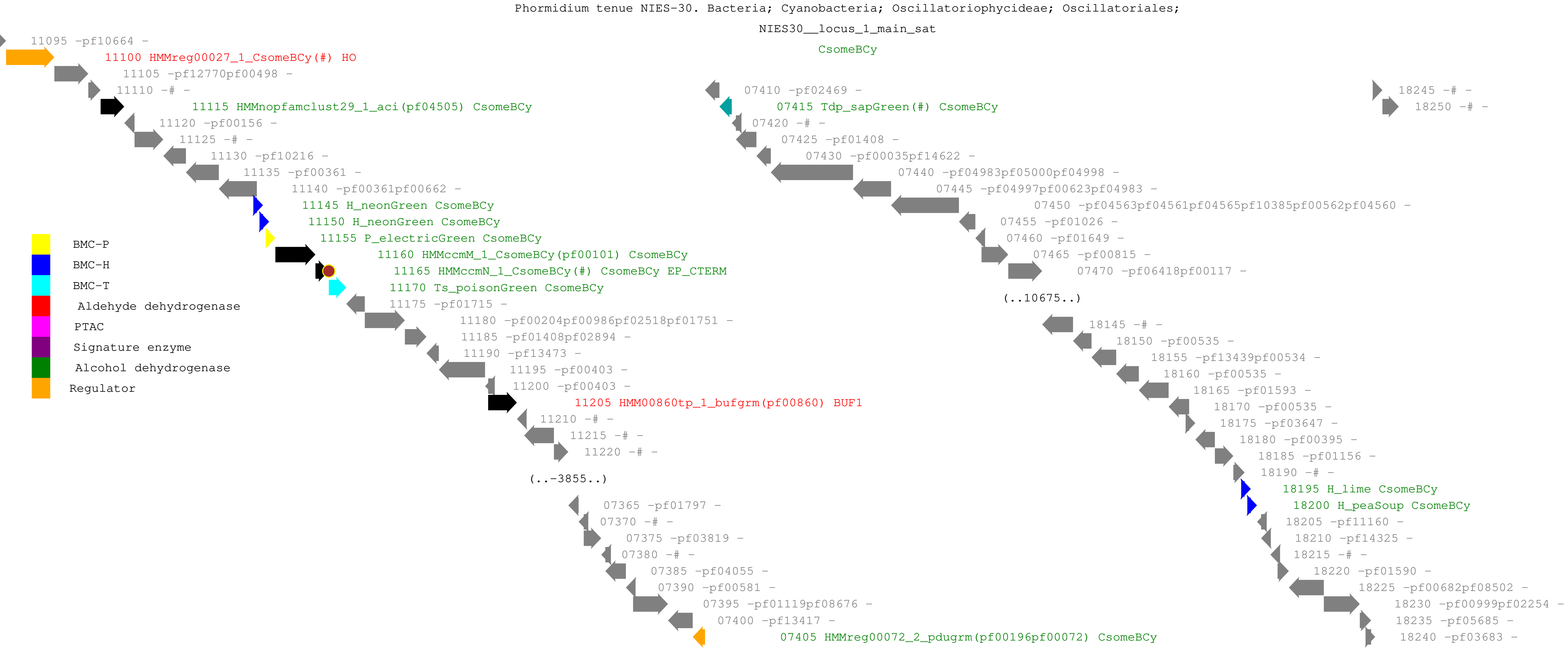
Phormidium tenue NIES-30. Bacteria; Cyanobacteria; Oscillatoriophycideae; Oscillatoriales;
NIES30__locus_1_main_sat
CsomeBCy
11095 -pf10664 -
11100 HMMreg00027_1_CsomeBCy(#) HO
11105 -pf12770pf00498 -
11110 -# -
11115 HMMnopfamclust29_1_aci(pf04505) CsomeBCy
11120 -pf00156 -
11125 -# -
11130 -pf10216 -
11135 -pf00361 -
11140 -pf00361pf00662 -
11145 H_neonGreen CsomeBCy
11150 H_neonGreen CsomeBCy
11155 P_electricGreen CsomeBCy
11160 HMMccmM_1_CsomeBCy(pf00101) CsomeBCy
11165 HMMccmN_1_CsomeBCy(#) CsomeBCy EP_CTERM
11170 Ts_poisonGreen CsomeBCy
11175 -pf01715 -
11180 -pf00204pf00986pf02518pf01751 -
11185 -pf01408pf02894 -
11190 -pf13473 -
11195 -pf00403 -
11200 -pf00403 -
11205 HMM00860tp_1_bufgrm(pf00860) BUF1
11210 -# -
11215 -# -
11220 -# -
(..-3855..)
07365 -pf01797 -
07370 -# -
07375 -pf03819 -
07380 -# -
07385 -pf04055 -
07390 -pf00581 -
07395 -pf01119pf08676 -
07400 -pf13417 -
07405 HMMreg00072_2_pdugrm(pf00196pf00072) CsomeBCy
07410 -pf02469 -
07415 Tdp_sapGreen(#) CsomeBCy
07420 -# -
07425 -pf01408 -
07430 -pf00035pf14622 -
07440 -pf04983pf05000pf04998 -
07445 -pf04997pf00623pf04983 -
07450 -pf04563pf04561pf04565pf10385pf00562pf04560 -
07455 -pf01026 -
07460 -pf01649 -
07465 -pf00815 -
07470 -pf06418pf00117 -
(..10675..)
18145 -# -
18150 -pf00535 -
18155 -pf13439pf00534 -
18160 -pf00535 -
18165 -pf01593 -
18170 -pf00535 -
18175 -pf03647 -
18180 -pf00395 -
18185 -pf01156 -
18190 -# -
18195 H_lime CsomeBCy
18200 H_peaSoup CsomeBCy
18205 -pf11160 -
18210 -pf14325 -
18215 -# -
18220 -pf01590 -
18225 -pf00682pf08502 -
18230 -pf00999pf02254 -
18235 -pf05685 -
18240 -pf03683 -
18245 -# -
18250 -# -
BMC-P
BMC-H
BMC-T
Aldehyde dehydrogenase
PTAC
Signature enzyme
Alcohol dehydrogenase
Regulator

Fremyella diplosiphon NIES-3275. Bacteria; Cyanobacteria; Nostocales; Rivulariaceae; Microchaete.

NIES3275__locus_1_main_sat

CsomeBCy

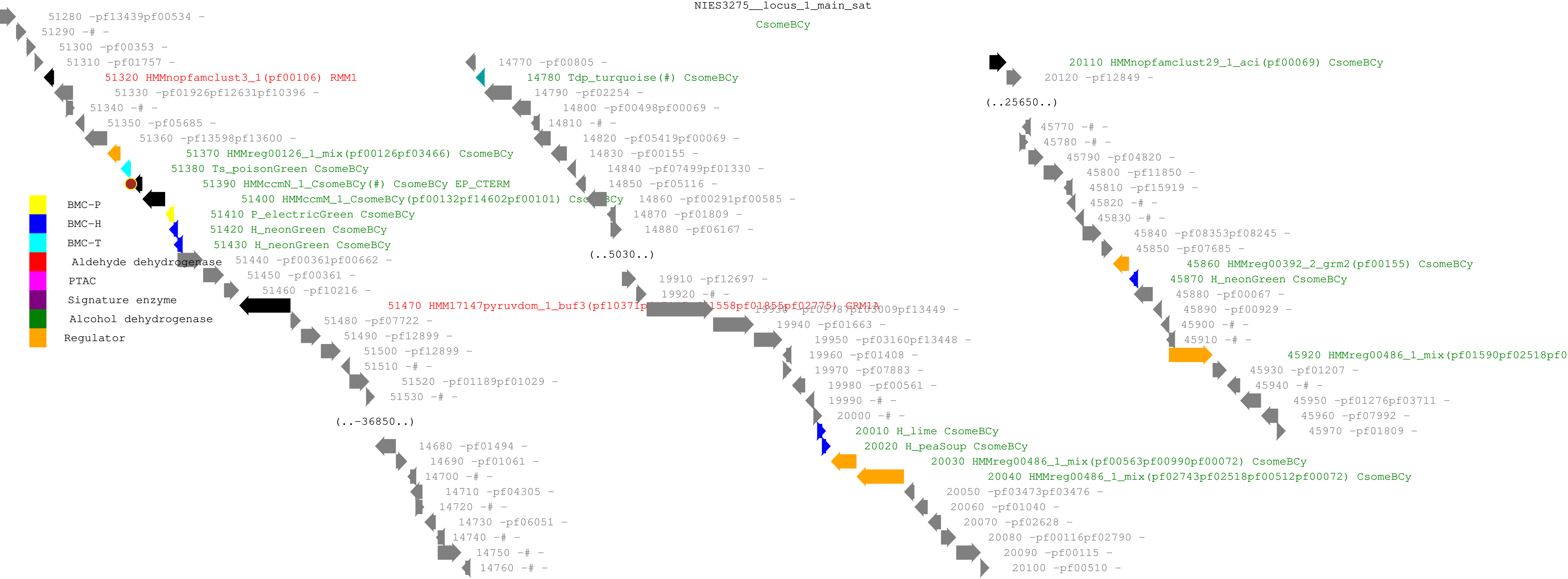

Nodularia sp. NIES-3585. Bacteria; Cyanobacteria; Nostocales; Aphanizomenonaceae; Nodularia.

NIES3585__locus_1_main_sat

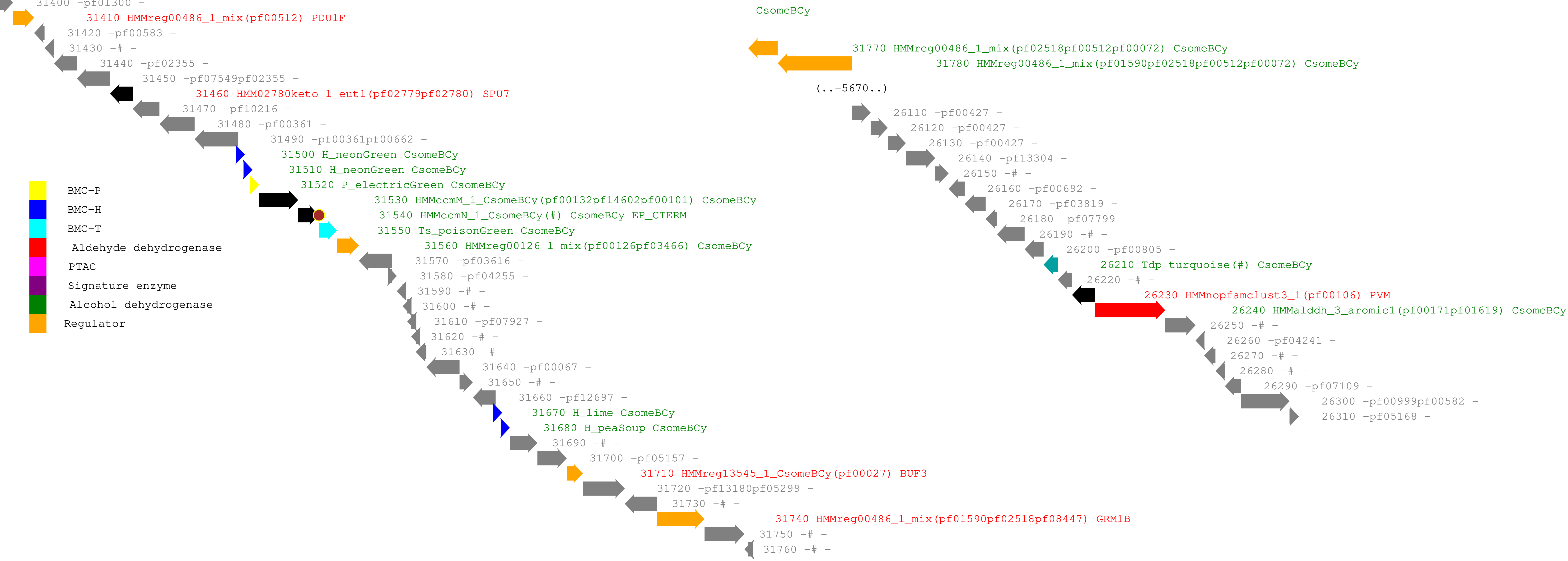

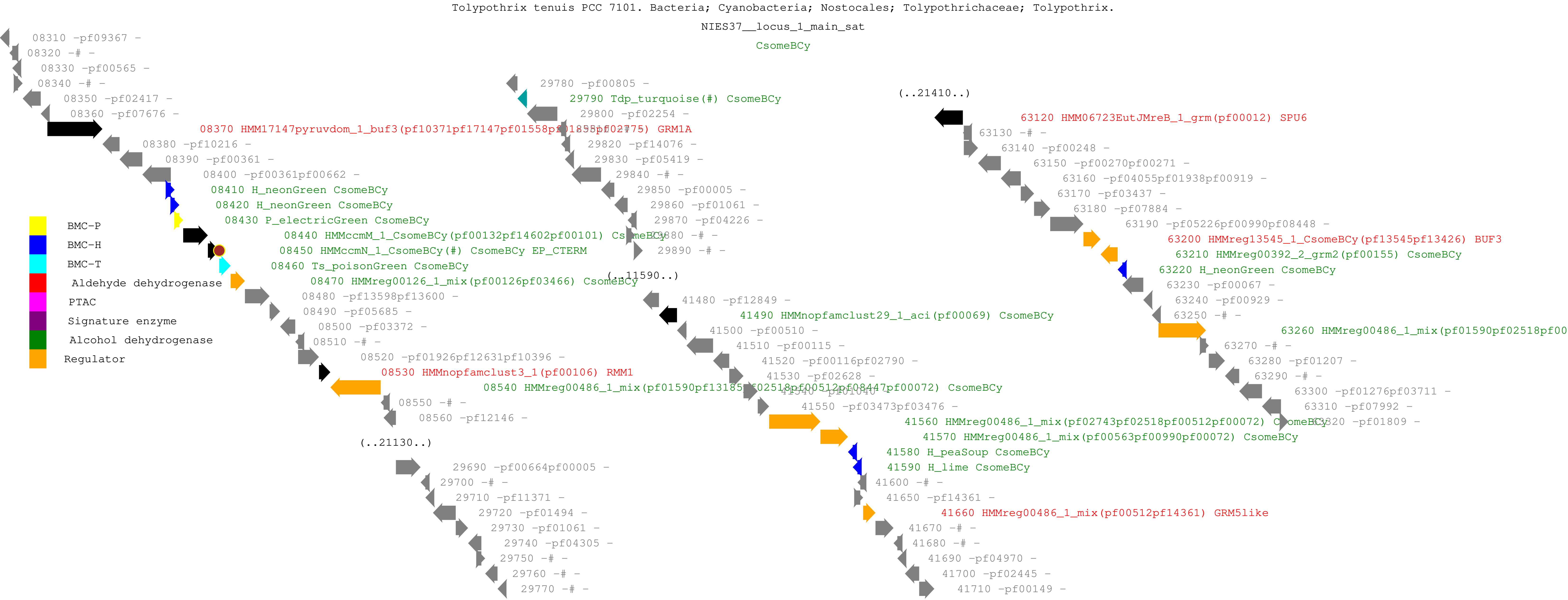

Tolypothrix tenuis PCC 7101. Bacteria; Cyanobacteria; Nostocales; Tolypothrichaceae; Tolypothrix.
NIES37__locus_1_main_sat
CsomeBCy
BMC-P
BMC-H
BMC-T
Aldehyde dehydrogenase
PTAC
Signature enzyme
Alcohol dehydrogenase
Regulator
08310 -pf09367 -
08320 -# -
08330 -pf00565 -
08340 -# -
08350 -pf02417 -
08360 -pf07676 -
08370 HMM17147pyruvdom_1_buf3(pf10371pf17147pf01558pf01855pf02775) GRM1A
08380 -pf10216 -
08390 -pf00361 -
08400 -pf00361pf00662 -
08410 H_neonGreen CsomeBCy
08420 H_neonGreen CsomeBCy
08430 P_electricGreen CsomeBCy
08440 HMMccmM_1_CsomeBCy(pf00132pf14602pf00101) CsomeBCy
08450 HMMccmN_1_CsomeBCy(#) CsomeBCy EP_CTERM
08460 Ts_poisonGreen CsomeBCy
08470 HMMreg00126_1_mix(pf00126pf03466) CsomeBCy
08480 -pf13598pf13600 -
08490 -pf05685 -
08500 -pf03372 -
08510 -# -
08520 -pf01926pf12631pf10396 -
08530 HMMnopfamclust3_1(pf00106) RMM1
08540 HMMreg00486_1_mix(pf01590pf13185pf02518pf00512pf08447pf00072) CsomeBCy
08550 -# -
08560 -pf12146 -
(..21130..)
29690 -pf00664pf00005 -
29700 -# -
29710 -pf11371 -
29720 -pf01494 -
29730 -pf01061 -
29740 -pf04305 -
29750 -# -
29760 -# -
29770 -# -
29780 -pf00805 -
29790 Tdp_turquoise(#) CsomeBCy
29800 -pf02254 -
29820 -pf14076 -
29830 -pf05419 -
29840 -# -
29850 -pf00005 -
29860 -pf01061 -
29870 -pf04226 -
29880 -# -
29890 -# -
(..11590..)
41480 -pf12849 -
41490 HMMnopfamclust29_1_aci(pf00069) CsomeBCy
41500 -pf00510 -
41510 -pf00115 -
41520 -pf00116pf02790 -
41530 -pf02628 -
41540 -pf01040 -
41550 -pf03473pf03476 -
41560 HMMreg00486_1_mix(pf02743pf02518pf00512pf00072) CsomeBCy
41570 HMMreg00486_1_mix(pf00563pf00990pf00072) CsomeBCy
41580 H_peaSoup CsomeBCy
41590 H_lime CsomeBCy
41600 -# -
41650 -pf14361 -
41660 HMMreg00486_1_mix(pf00512pf14361) GRM5like
41670 -# -
41680 -# -
41690 -pf04970 -
41700 -pf02445 -
41710 -pf00149 -
(..21410..)
63120 HMM06723EutJMreB_1_grm(pf00012) SPU6
63130 -# -
63140 -pf00248 -
63150 -pf00270pf00271 -
63160 -pf04055pf01938pf00919 -
63170 -pf03437 -
63180 -pf07884 -
63190 -pf05226pf00990pf08448 -
63200 HMMreg13545_1_CsomeBCy(pf13545pf13426) BUF3
63210 HMMreg00392_2_grm2(pf00155) CsomeBCy
63220 H_neonGreen CsomeBCy
63230 -pf00067 -
63240 -pf00929 -
63250 -# -
63260 HMMreg00486_1_mix(pf01590pf02518pf00
63270 -# -
63280 -pf01207 -
63290 -# -
63300 -pf01276pf03711 -
63310 -pf07992 -
63320 -pf01809 -

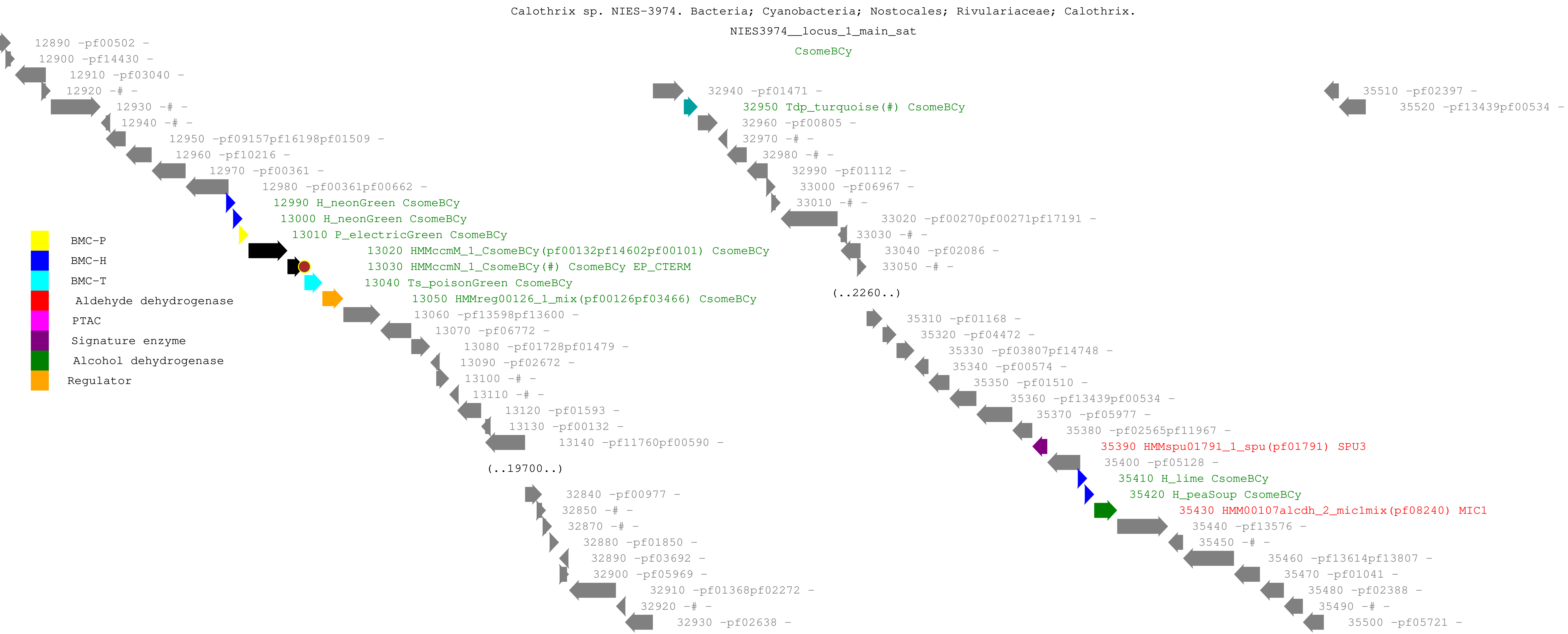

Calothrix sp. NIES-3974. Bacteria; Cyanobacteria; Nostocales; Rivulariaceae; Calothrix.
NIES3974__locus_1_main_sat
CsomeBCy
12890 -pf00502 -
12900 -pf14430 -
12910 -pf03040 -
12920 -# -
12930 -# -
12940 -# -
12950 -pf09157pf16198pf01509 -
12960 -pf10216 -
12970 -pf00361 -
12980 -pf00361pf00662 -
12990 H_neonGreen CsomeBCy
13000 H_neonGreen CsomeBCy
13010 P_electricGreen CsomeBCy
13020 HMMccmM_1_CsomeBCy(pf00132pf14602pf00101) CsomeBCy
13030 HMMccmN_1_CsomeBCy(#) CsomeBCy EP_CTERM
13040 Ts_poisonGreen CsomeBCy
13050 HMMreg00126_1_mix(pf00126pf03466) CsomeBCy
13060 -pf13598pf13600 -
13070 -pf06772 -
13080 -pf01728pf01479 -
13090 -pf02672 -
13100 -# -
13110 -# -
13120 -pf01593 -
13130 -pf00132 -
13140 -pf11760pf00590 -
(..19700..)
32840 -pf00977 -
32850 -# -
32870 -# -
32880 -pf01850 -
32890 -pf03692 -
32900 -pf05969 -
32910 -pf01368pf02272 -
32920 -# -
32930 -pf02638 -
32940 -pf01471 -
32950 Tdp_turquoise(#) CsomeBCy
32960 -pf00805 -
32970 -# -
32980 -# -
32990 -pf01112 -
33000 -pf06967 -
33010 -# -
33020 -pf00270pf00271pf17191 -
33030 -# -
33040 -pf02086 -
33050 -# -
(..2260..)
35310 -pf01168 -
35320 -pf04472 -
35330 -pf03807pf14748 -
35340 -pf00574 -
35350 -pf01510 -
35360 -pf13439pf00534 -
35370 -pf05977 -
35380 -pf02565pf11967 -
35390 HMMspu01791_1_spu(pf01791) SPU3
35400 -pf05128 -
35410 H_lime CsomeBCy
35420 H_peaSoup CsomeBCy
35430 HMM00107alcdh_2_mic1mix(pf08240) MIC1
35440 -pf13576 -
35450 -# -
35460 -pf13614pf13807 -
35470 -pf01041 -
35480 -pf02388 -
35490 -# -
35500 -pf05721 -
35510 -pf02397 -
35520 -pf13439pf00534 -
BMC-P
BMC-H
BMC-T
Aldehyde dehydrogenase
PTAC
Signature enzyme
Alcohol dehydrogenase
Regulator

Arthrospira platensis (strain NIES-39 / IAM M-135) (Spirulina Bacteria; Cyanobacteria; Oscillatoriophycideae; Oscillatoriales;

NIES39_K_locus_1

CsomeBCy

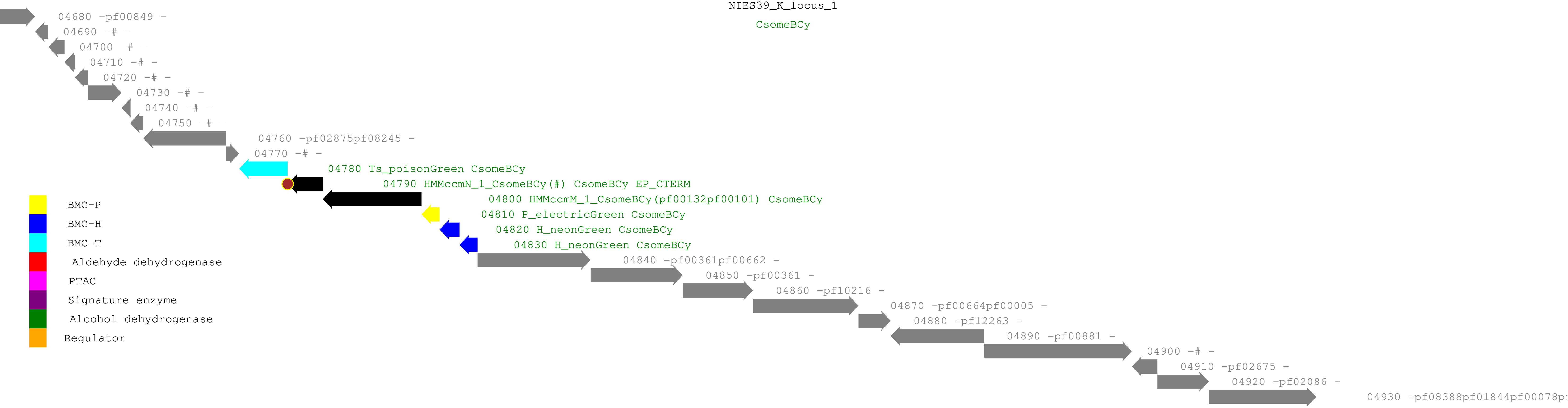

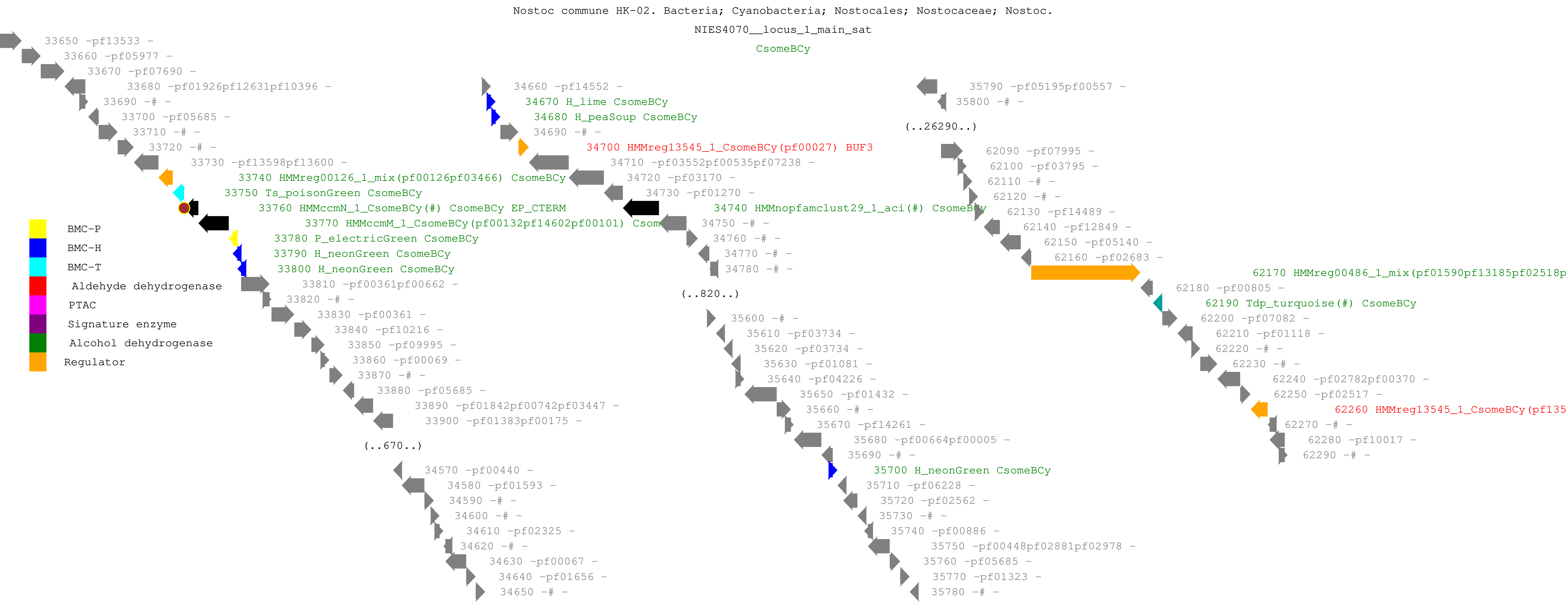
Nostoc commune HK-02. Bacteria; Cyanobacteria; Nostocales; Nostocaceae; Nostoc.
NIES4070__locus_1_main_sat
CsomeBCy
33650 -pf13533 -
33660 -pf05977 -
33670 -pf07690 -
33680 -pf01926pf12631pf10396 -
33690 -# -
33700 -pf05685 -
33710 -# -
33720 -# -
33730 -pf13598pf13600 -
33740 HMMreg00126_1_mix(pf00126pf03466) CsomeBCy
33750 Ts_poisonGreen CsomeBCy
33760 HMMccmN_1_CsomeBCy(#) CsomeBCy EP_CTERM
33770 HMMccmM_1_CsomeBCy(pf00132pf14602pf00101) Csom
33780 P_electricGreen CsomeBCy
33790 H_neonGreen CsomeBCy
33800 H_neonGreen CsomeBCy
33810 -pf00361pf00662 -
33820 -# -
33830 -pf00361 -
33840 -pf10216 -
33850 -pf09995 -
33860 -pf00069 -
33870 -# -
33880 -pf05685 -
33890 -pf01842pf00742pf03447 -
33900 -pf01383pf00175 -
(..670..)
34570 -pf00440 -
34580 -pf01593 -
34590 -# -
34600 -# -
34610 -pf02325 -
34620 -# -
34630 -pf00067 -
34640 -pf01656 -
34650 -# -
34660 -pf14552 -
34670 H_lime CsomeBCy
34680 H_peaSoup CsomeBCy
34690 -# -
34700 HMMreg13545_1_CsomeBCy(pf00027) BUF3
34710 -pf03552pf00535pf07238 -
34720 -pf03170 -
34730 -pf01270 -
34740 HMMnopfamclust29_1_aci(#) CsomeB
34750 -# -
34760 -# -
34770 -# -
34780 -# -
(..820..)
35600 -# -
35610 -pf03734 -
35620 -pf03734 -
35630 -pf01081 -
35640 -pf04226 -
35650 -pf01432 -
35660 -# -
35670 -pf14261 -
35680 -pf00664pf00005 -
35690 -# -
35700 H_neonGreen CsomeBCy
35710 -pf06228 -
35720 -pf02562 -
35730 -# -
35740 -pf00886 -
35750 -pf00448pf02881pf02978 -
35760 -pf05685 -
35770 -pf01323 -
35780 -# -
35790 -pf05195pf00557 -
35800 -# -
(..26290..)
62090 -pf07995 -
62100 -pf03795 -
62110 -# -
62120 -# -
62130 -pf14489 -
62140 -pf12849 -
62150 -pf05140 -
62160 -pf02683 -
62170 HMMreg00486_1_mix(pf01590pf13185pf02518p
62180 -pf00805 -
62190 Tdp_turquoise(#) CsomeBCy
62200 -pf07082 -
62210 -pf01118 -
62220 -# -
62230 -# -
62240 -pf02782pf00370 -
62250 -pf02517 -
62260 HMMreg13545_1_CsomeBCy(pf135
62270 -# -
62280 -pf10017 -
62290 -# -
BMC-P
BMC-H
BMC-T
Aldehyde dehydrogenase
PTAC
Signature enzyme
Alcohol dehydrogenase
Regulator

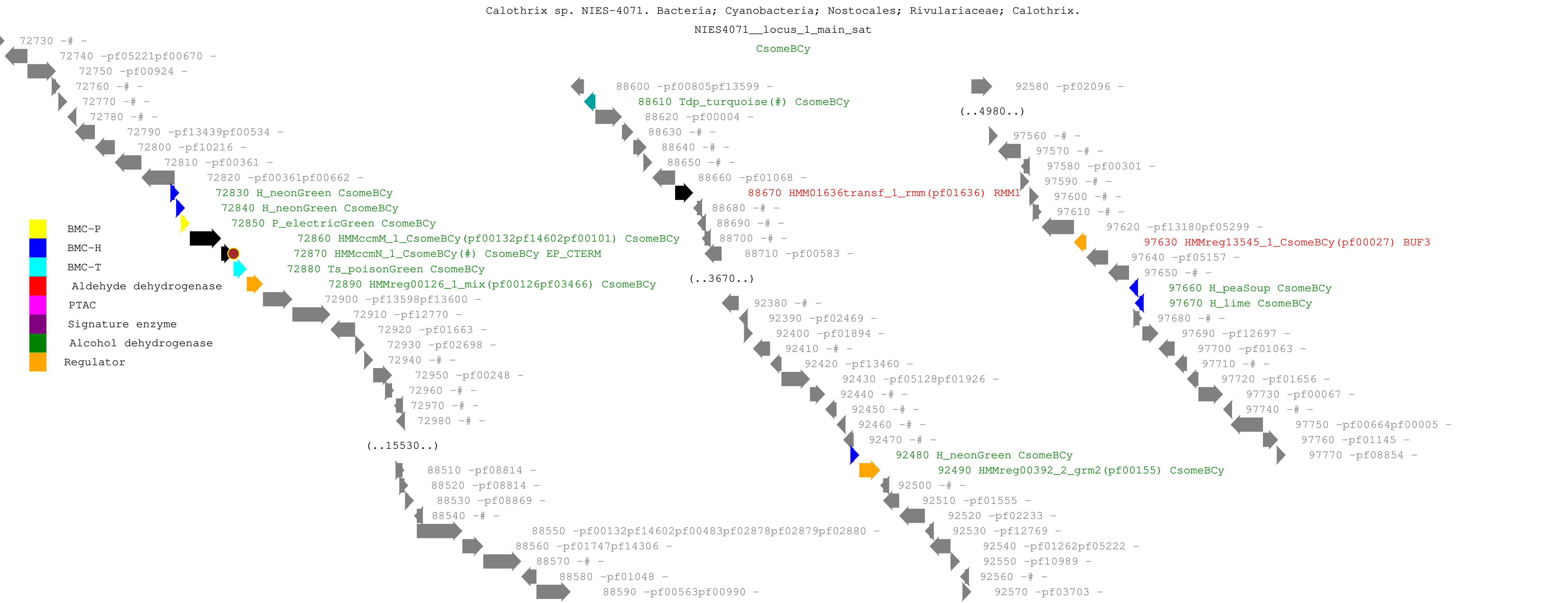
Calothrix sp. NIES-4071. Bacteria; Cyanobacteria; Nostocales; Rivulariaceae; Calothrix.
NIES4071__locus_1_main_sat
CsomeBCy
72730 -# -
72740 -pf05221pf00670 -
72750 -pf00924 -
72760 -# -
72770 -# -
72780 -# -
72790 -pf13439pf00534 -
72800 -pf10216 -
72810 -pf00361 -
72820 -pf00361pf00662 -
72830 H_neonGreen CsomeBCy
72840 H_neonGreen CsomeBCy
72850 P_electricGreen CsomeBCy
72860 HMMccmM_1_CsomeBCy(pf00132pf14602pf00101) CsomeBCy
72870 HMMccmN_1_CsomeBCy(#) CsomeBCy EP_CTERM
72880 Ts_poisonGreen CsomeBCy
72890 HMMreg00126_1_mix(pf00126pf03466) CsomeBCy
72900 -pf13598pf13600 -
72910 -pf12770 -
72920 -pf01663 -
72930 -pf02698 -
72940 -# -
72950 -pf00248 -
72960 -# -
72970 -# -
72980 -# -
(..15530..)
88510 -pf08814 -
88520 -pf08814 -
88530 -pf08869 -
88540 -# -
88550 -pf00132pf14602pf00483pf02878pf02879pf02880 -
88560 -pf01747pf14306 -
88570 -# -
88580 -pf01048 -
88590 -pf00563pf00990 -
BMC-P
BMC-H
BMC-T
Aldehyde dehydrogenase
PTAC
Signature enzyme
Alcohol dehydrogenase
Regulator
88600 -pf00805pf13599 -
88610 Tdp_turquoise(#) CsomeBCy
88620 -pf00004 -
88630 -# -
88640 -# -
88650 -# -
88660 -pf01068 -
88670 HMM01636transf_1_rmm(pf01636) RMM1
88680 -# -
88690 -# -
88700 -# -
88710 -pf00583 -
(..3670..)
92380 -# -
92390 -pf02469 -
92400 -pf01894 -
92410 -# -
92420 -pf13460 -
92430 -pf05128pf01926 -
92440 -# -
92450 -# -
92460 -# -
92470 -# -
92480 H_neonGreen CsomeBCy
92490 HMMreg00392_2_grm2(pf00155) CsomeBCy
92500 -# -
92510 -pf01555 -
92520 -pf02233 -
92530 -pf12769 -
92540 -pf01262pf05222 -
92550 -pf10989 -
92560 -# -
92570 -pf03703 -
92580 -pf02096 -
(..4980..)
97560 -# -
97570 -# -
97580 -pf00301 -
97590 -# -
97600 -# -
97610 -# -
97620 -pf13180pf05299 -
97630 HMMreg13545_1_CsomeBCy(pf00027) BUF3
97640 -pf05157 -
97650 -# -
97660 H_peaSoup CsomeBCy
97670 H_lime CsomeBCy
97680 -# -
97690 -pf12697 -
97700 -pf01063 -
97710 -# -
97720 -pf01656 -
97730 -pf00067 -
97740 -# -
97750 -pf00664pf00005 -
97760 -pf01145 -
97770 -pf08854 -

Nostoc commune NIES-4072. Bacteria; Cyanobacteria; Nostocales; Nostocaceae; Nostoc.

NIES4072__locus_1_main_sat

CsomeBCy

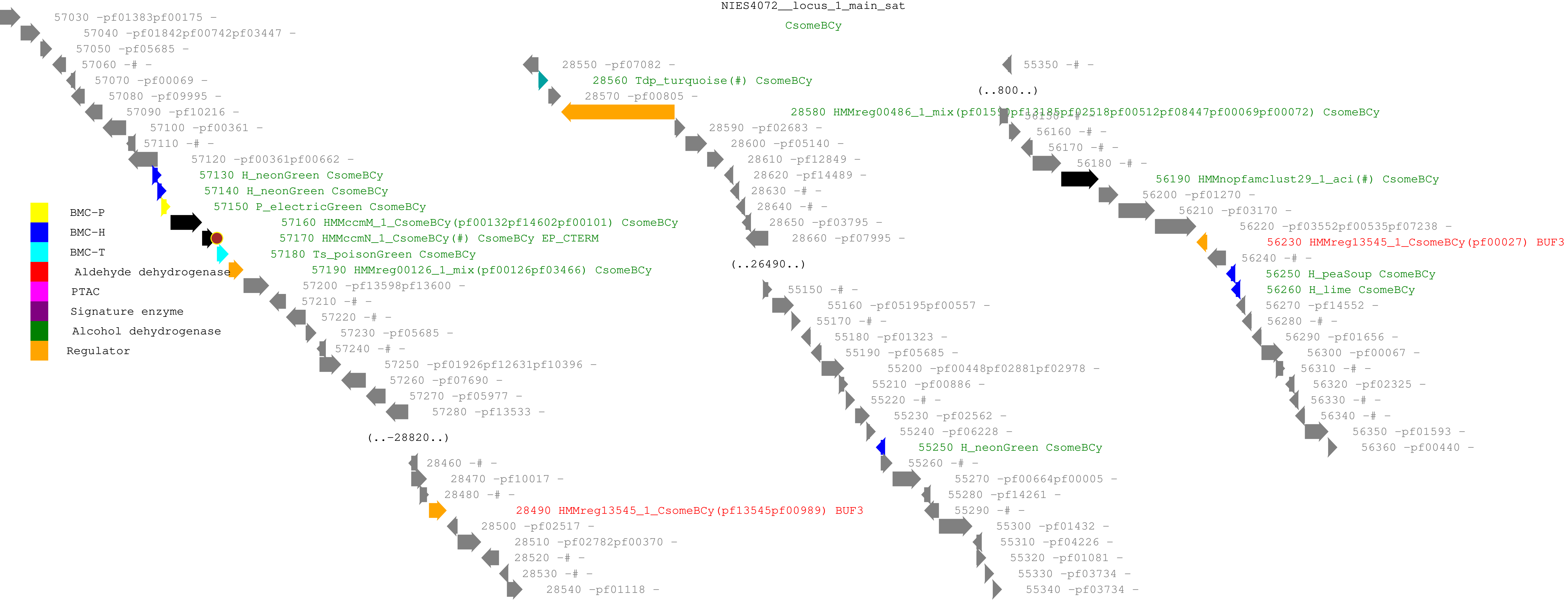

Scytonema sp. NIES-4073. Bacteria; Cyanobacteria; Nostocales; Scytonemataceae; Scytonema.

NIES4073__locus_1_main_sat

CsomeBCy

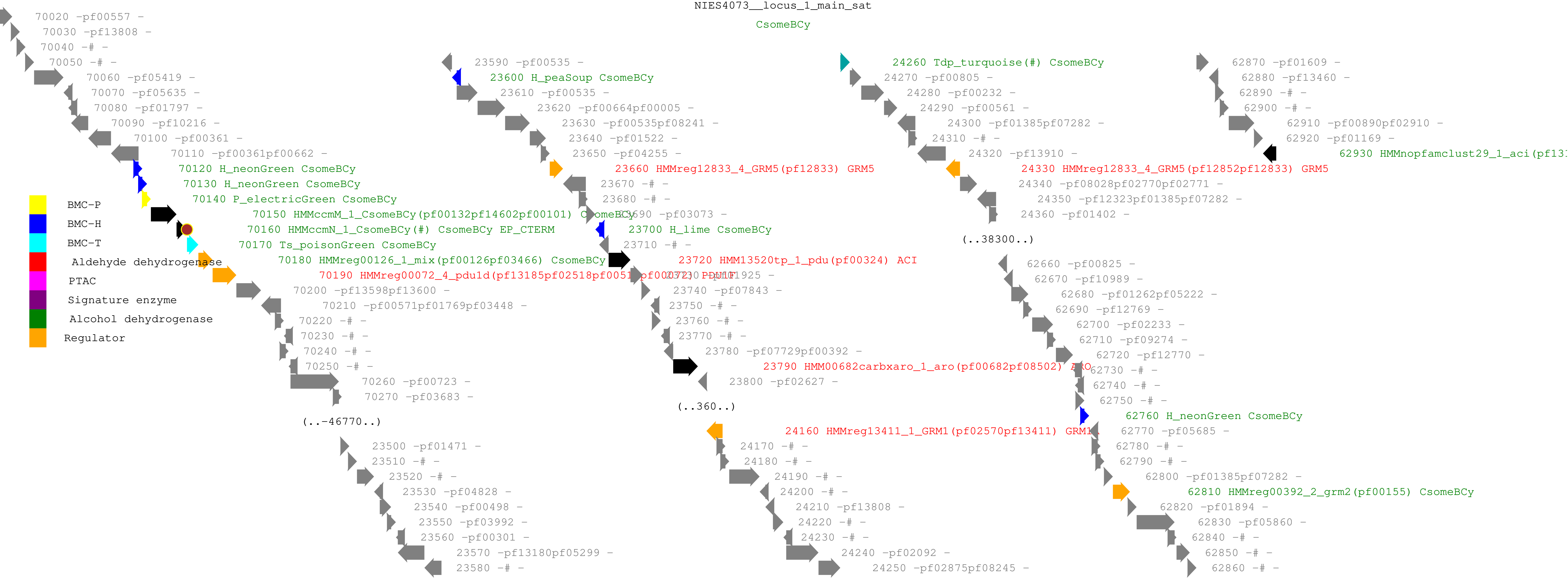

Cylindrospermum sp. NIES-4074. Bacteria; Cyanobacteria; Nostocales; Nostocaceae; Cylindrospermum.

NIES4074__locus_1_main_sat

CsomeBCy

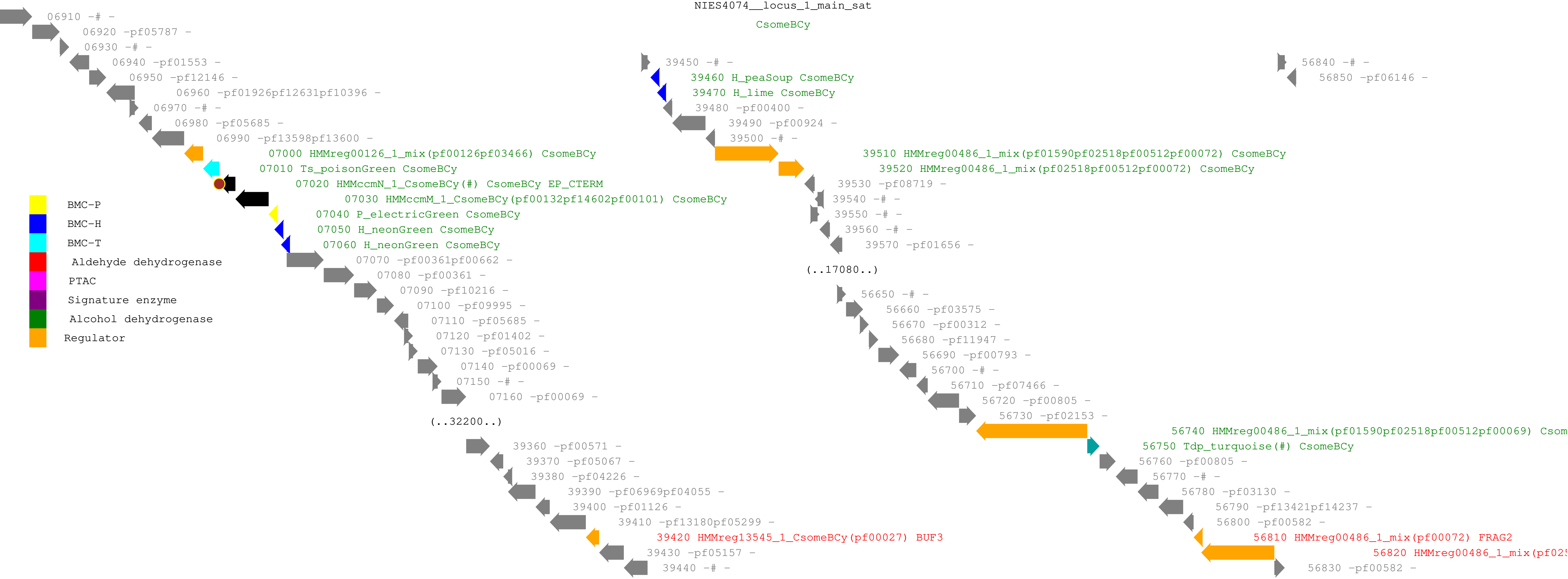

Tolypothrix sp. NIES-4075. Bacteria; Cyanobacteria; Nostocales; Tolypothrichaceae; Tolypothrix.

NIES4075__locus_1_main_sat

CsomeBCy

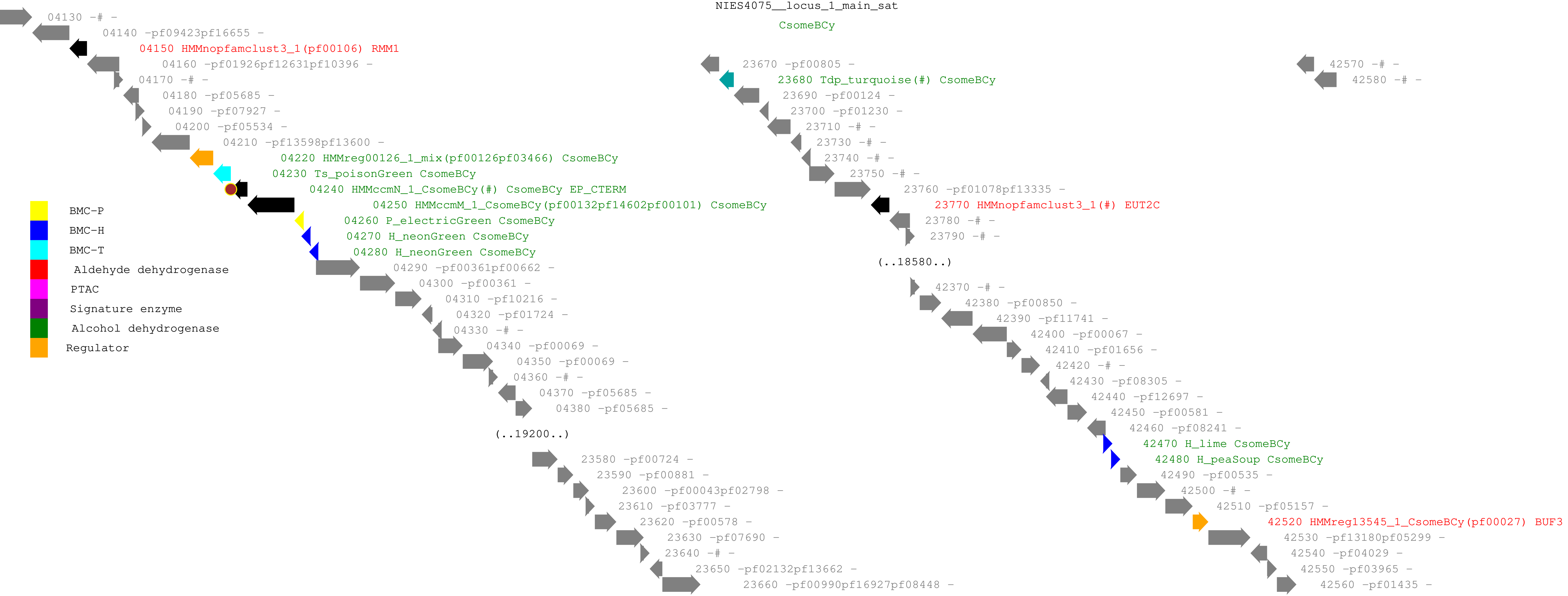

Calothrix sp. NIES-4101. Bacteria; Cyanobacteria; Nostocales; Rivulariaceae; Calothrix.

NIES4101__locus_1_main_sat

CsomeBCy

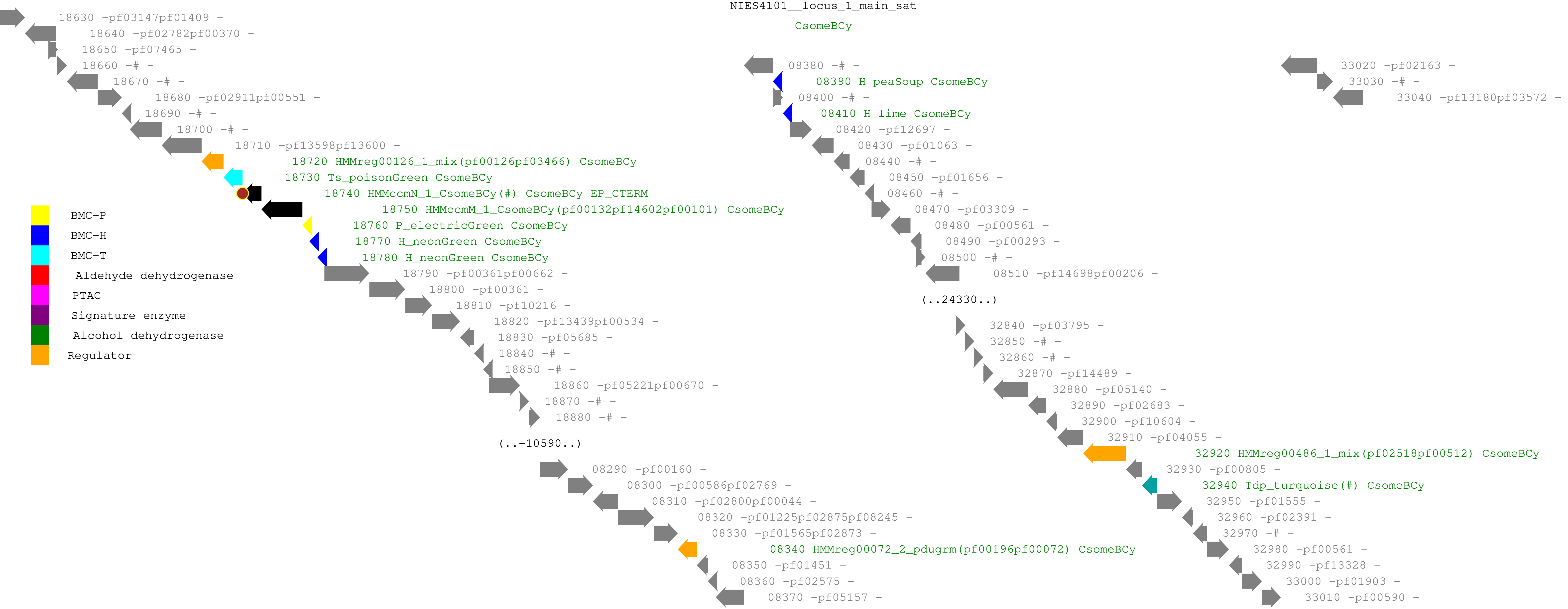

Chondrocystis sp. NIES-4102. Bacteria; Cyanobacteria; Oscillatoriophycideae; Chroococcales;

NIES4102__locus_1_main_sat

CsomeBCy

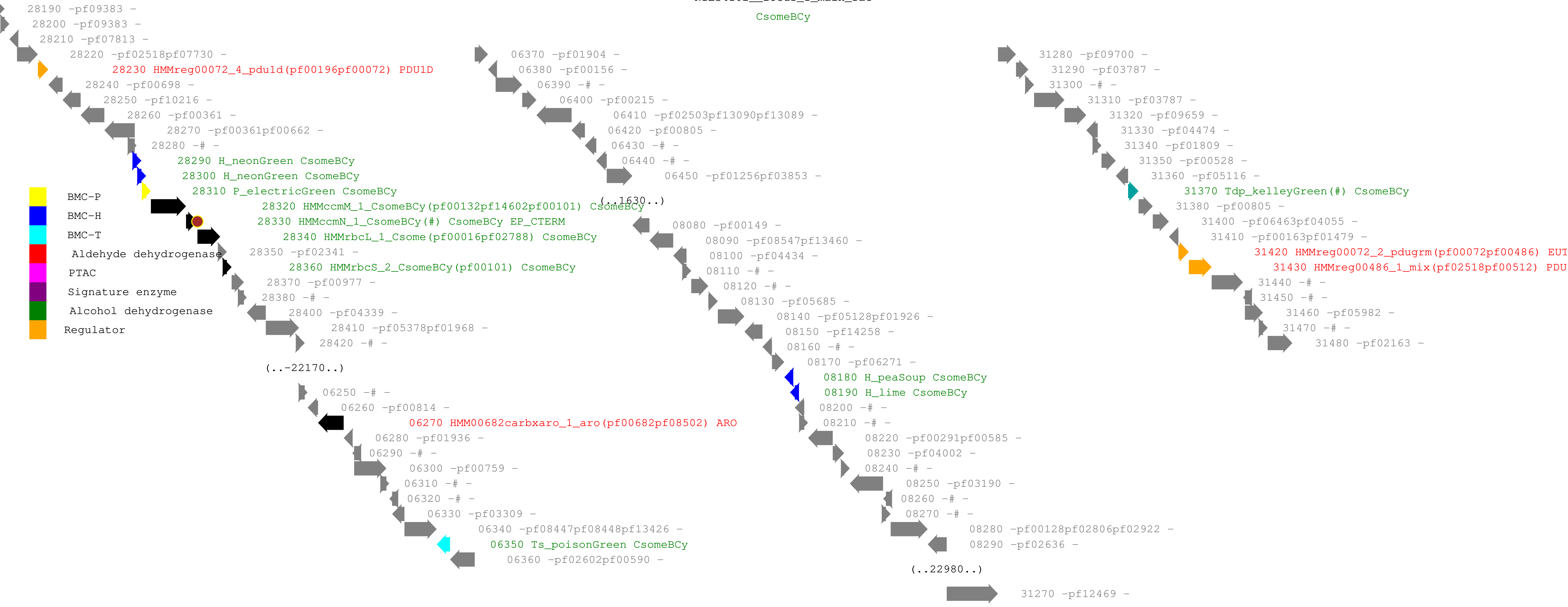

Nostoc sp. NIES-4103. Bacteria; Cyanobacteria; Nostocales; Nostocaceae; Nostoc.

NIES4103__locus_1_main_sat

CsomeBCy

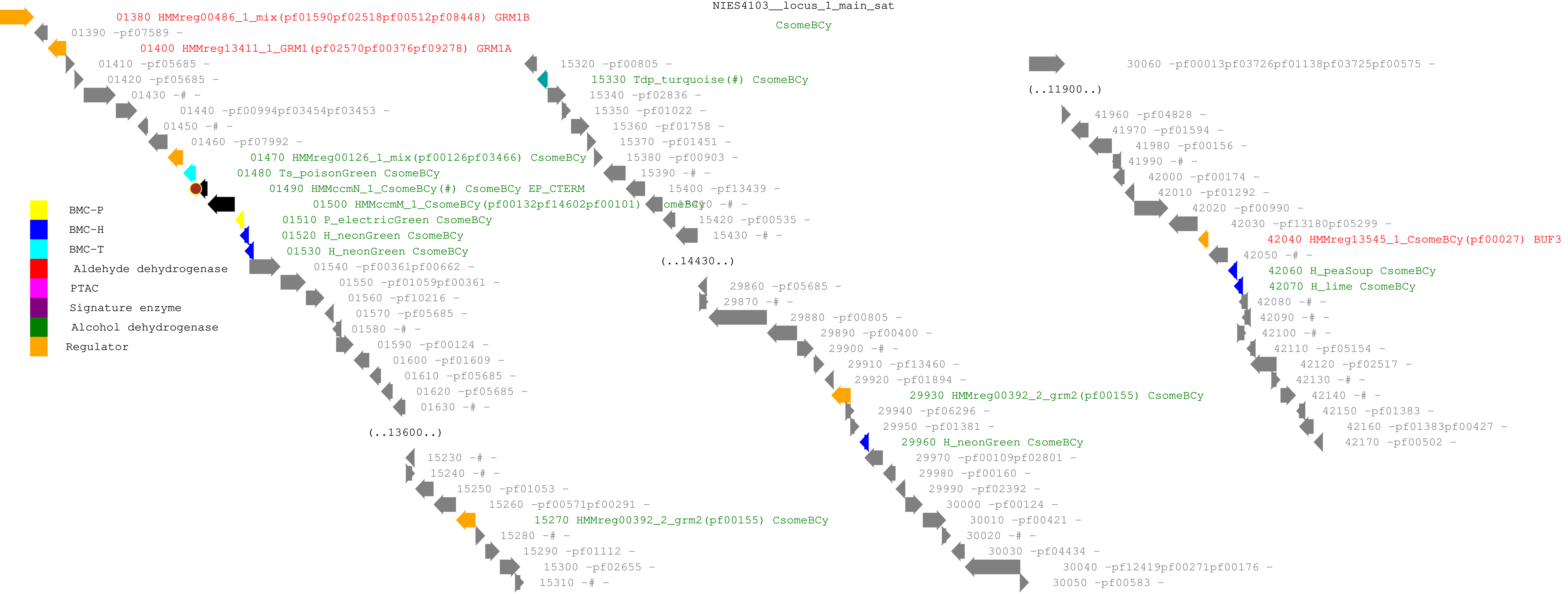

Calothrix sp. NIES-4105. Bacteria; Cyanobacteria; Nostocales; Rivulariaceae; Calothrix.

NIES4105__locus_1_main_sat

CsomeBCy

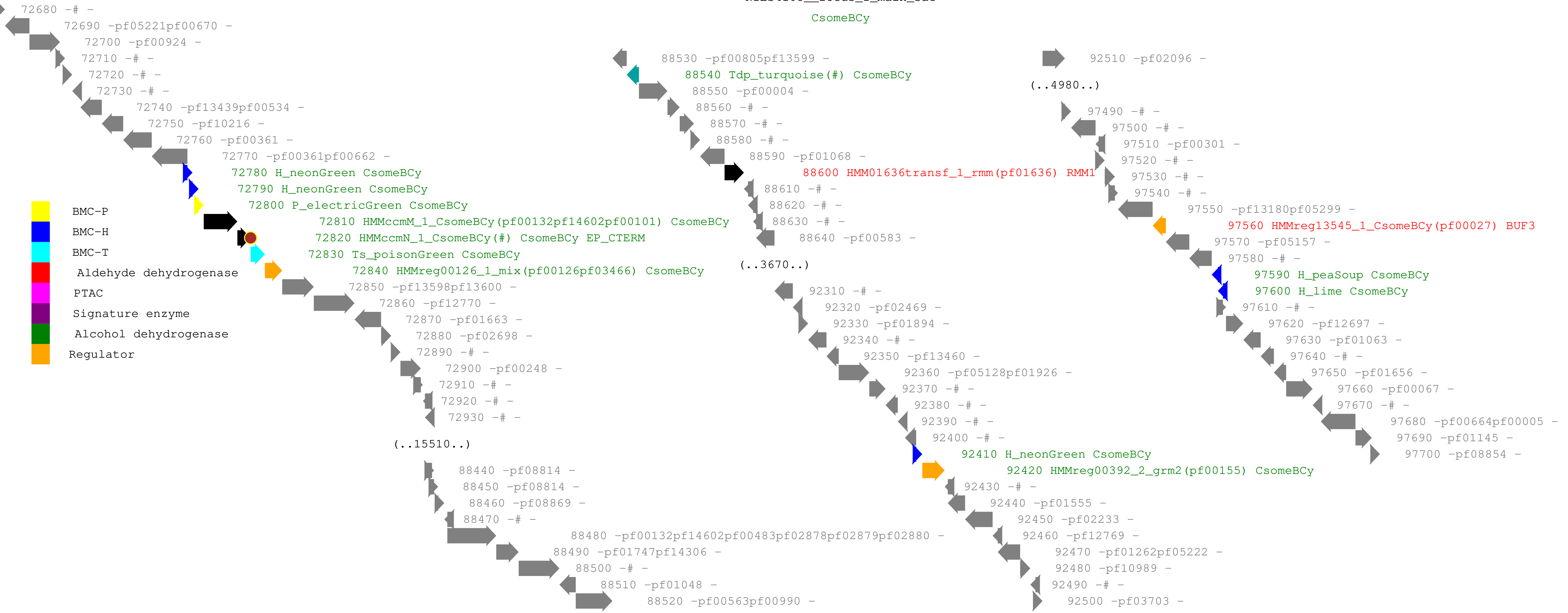

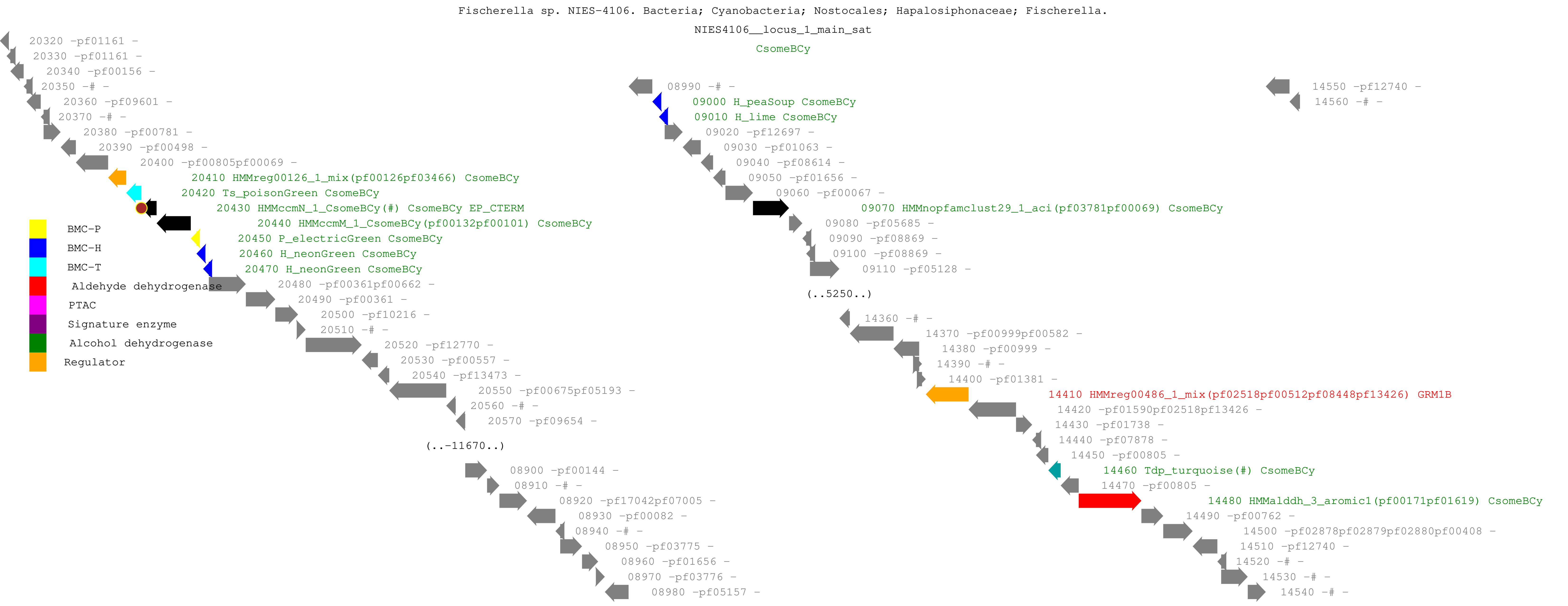
Fischerella sp. NIES-4106. Bacteria; Cyanobacteria; Nostocales; Hapalosiphonaceae; Fischerella.
NIES4106__locus_1_main_sat
CsomeBCy
20320 -pf01161 -
20330 -pf01161 -
20340 -pf00156 -
20350 -# -
20360 -pf09601 -
20370 -# -
20380 -pf00781 -
20390 -pf00498 -
20400 -pf00805pf00069 -
20410 HMMreg00126_1_mix(pf00126pf03466) CsomeBCy
20420 Ts_poisonGreen CsomeBCy
20430 HMMccmN_1_CsomeBCy(#) CsomeBCy EP_CTERM
20440 HMMccmM_1_CsomeBCy(pf00132pf00101) CsomeBCy
20450 P_electricGreen CsomeBCy
20460 H_neonGreen CsomeBCy
20470 H_neonGreen CsomeBCy
20480 -pf00361pf00662 -
20490 -pf00361 -
20500 -pf10216 -
20510 -# -
20520 -pf12770 -
20530 -pf00557 -
20540 -pf13473 -
20550 -pf00675pf05193 -
20560 -# -
20570 -pf09654 -
(..-11670..)
08900 -pf00144 -
08910 -# -
08920 -pf17042pf07005 -
08930 -pf00082 -
08940 -# -
08950 -pf03775 -
08960 -pf01656 -
08970 -pf03776 -
08980 -pf05157 -
BMC-P
BMC-H
BMC-T
Aldehyde dehydrogenase
PTAC
Signature enzyme
Alcohol dehydrogenase
Regulator
08990 -# -
09000 H_peaSoup CsomeBCy
09010 H_lime CsomeBCy
09020 -pf12697 -
09030 -pf01063 -
09040 -pf08614 -
09050 -pf01656 -
09060 -pf00067 -
09070 HMMnopfamclust29_1_aci(pf03781pf00069) CsomeBCy
09080 -pf05685 -
09090 -pf08869 -
09100 -pf08869 -
09110 -pf05128 -
(..5250..)
14360 -# -
14370 -pf00999pf00582 -
14380 -pf00999 -
14390 -# -
14400 -pf01381 -
14410 HMMreg00486_1_mix(pf02518pf00512pf08448pf13426) GRM1B
14420 -pf01590pf02518pf13426 -
14430 -pf01738 -
14440 -pf07878 -
14450 -pf00805 -
14460 Tdp_turquoise(#) CsomeBCy
14470 -pf00805 -
14480 HMMaldh_3_aromic1(pf00171pf01619) CsomeBCy
14490 -pf00762 -
14500 -pf02878pf02879pf02880pf00408 -
14510 -pf12740 -
14520 -# -
14530 -# -
14540 -# -
14550 -pf12740 -
14560 -# -

Aulosira laxa NIES-50. Bacteria; Cyanobacteria; Nostocales; Fortieaceae; Aulosira.

NIES50__locus_1_main_sat

CsomeBCy

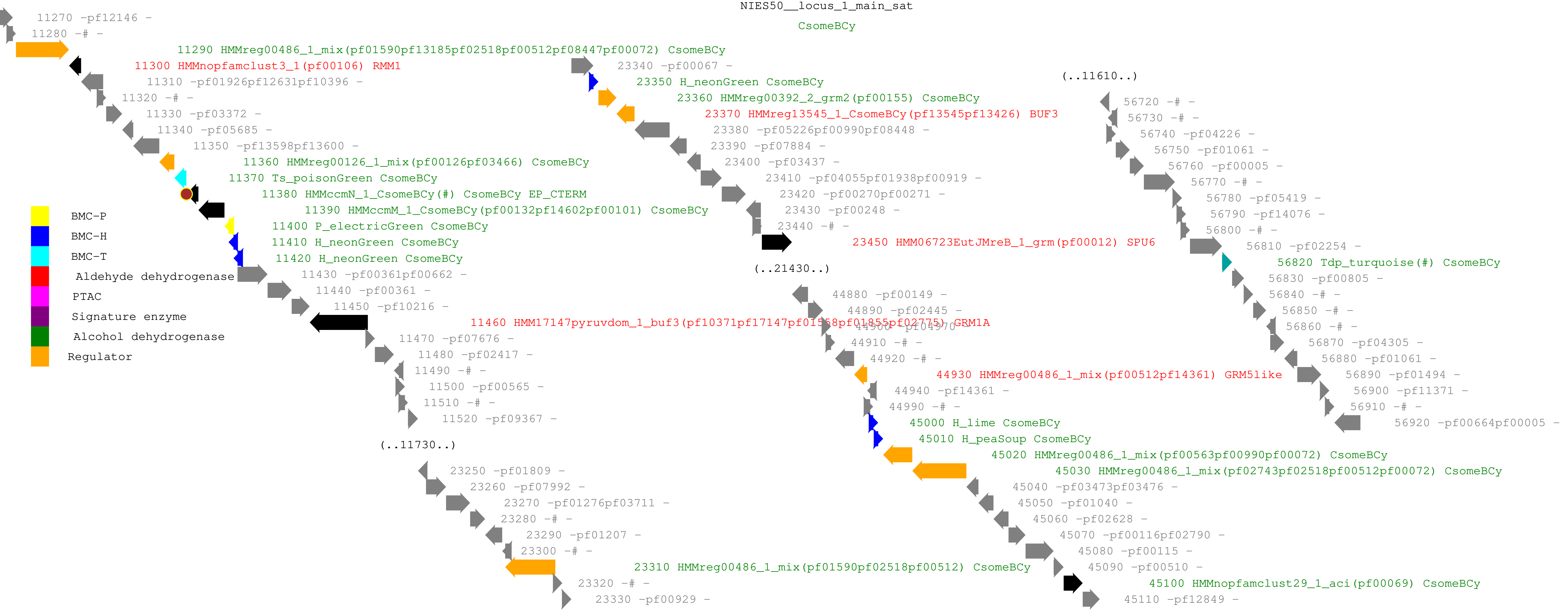

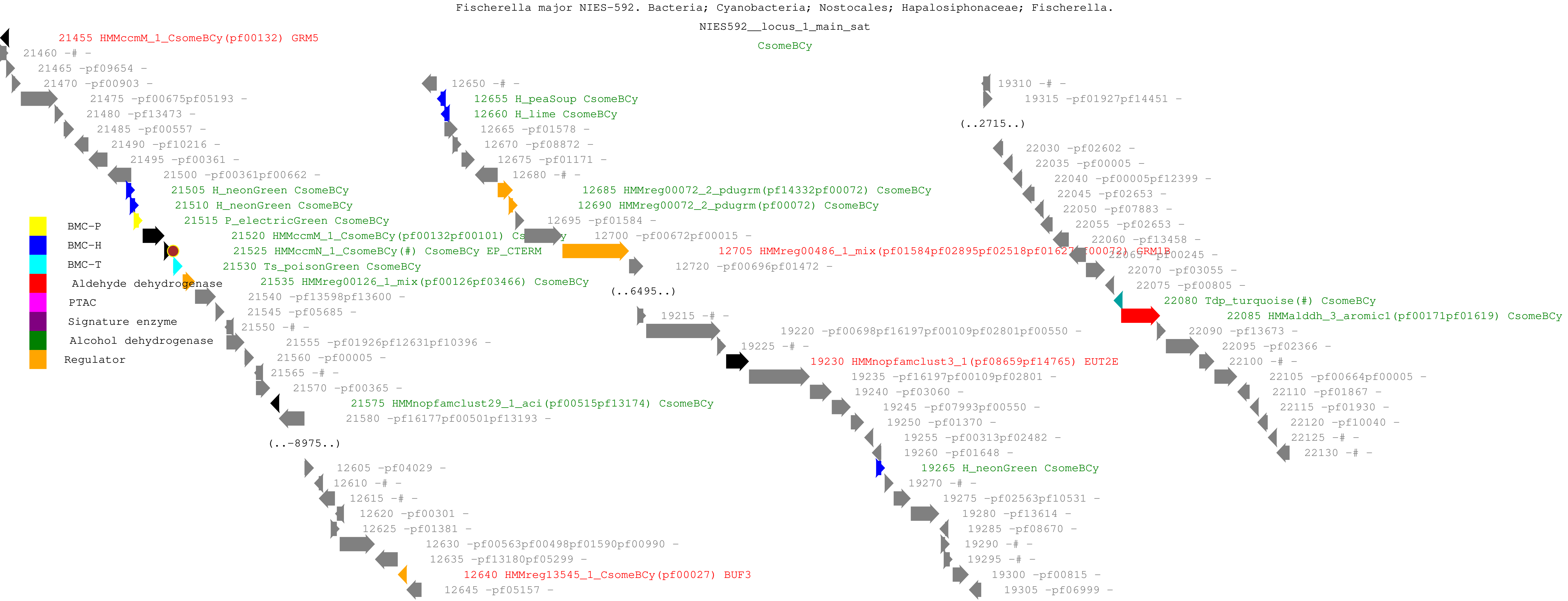

Fischerella major NIES-592. Bacteria; Cyanobacteria; Nostocales; Hapalosiphonaceae; Fischerella.
NIES592__locus_1_main_sat
CsomeBCy
21455 HMMccmM_1_CsomeBCy(pf00132) GRM5
21460 -# -
21465 -pf09654 -
21470 -pf00903 -
21475 -pf00675pf05193 -
21480 -pf13473 -
21485 -pf00557 -
21490 -pf10216 -
21495 -pf00361 -
21500 -pf00361pf00662 -
21505 H_neonGreen CsomeBCy
21510 H_neonGreen CsomeBCy
21515 P_electricGreen CsomeBCy
21520 HMMccmM_1_CsomeBCy(pf00132pf00101) CsomeBCy
21525 HMMccmN_1_CsomeBCy(#) CsomeBCy EP_CTERM
21530 Ts_poisonGreen CsomeBCy
21535 HMMreg00126_1_mix(pf00126pf03466) CsomeBCy
21540 -pf13598pf13600 -
21545 -pf05685 -
21550 -# -
21555 -pf01926pf12631pf10396 -
21560 -pf00005 -
21565 -# -
21570 -pf00365 -
21575 HMMnopfamclust29_1_aci(pf00515pf13174) CsomeBCy
21580 -pf16177pf00501pf13193 -
(..-8975..)
12605 -pf04029 -
12610 -# -
12615 -# -
12620 -pf00301 -
12625 -pf01381 -
12630 -pf00563pf00498pf01590pf00990 -
12635 -pf13180pf05299 -
12640 HMMreg13545_1_CsomeBCy(pf00027) BUF3
12645 -pf05157 -
12650 -# -
12655 H_peaSoup CsomeBCy
12660 H_lime CsomeBCy
12665 -pf01578 -
12670 -pf08872 -
12675 -pf01171 -
12680 -# -
12685 HMMreg00072_2_pdugrm(pf14332pf00072) CsomeBCy
12690 HMMreg00072_2_pdugrm(pf00072) CsomeBCy
12695 -pf01584 -
12700 -pf00672pf00015 -
12705 HMMreg00486_1_mix(pf01584pf02895pf02518pf01627pf00072) GRM1B
12720 -pf00696pf01472 -
(..6495..)
19215 -# -
19220 -pf00698pf16197pf00109pf02801pf00550 -
19225 -# -
19230 HMMnopfamclust3_1(pf08659pf14765) EUT2E
19235 -pf16197pf00109pf02801 -
19240 -pf03060 -
19245 -pf07993pf00550 -
19250 -pf01370 -
19255 -pf00313pf02482 -
19260 -pf01648 -
19265 H_neonGreen CsomeBCy
19270 -# -
19275 -pf02563pf10531 -
19280 -pf13614 -
19285 -pf08670 -
19290 -# -
19295 -# -
19300 -pf00815 -
19305 -pf06999 -
19310 -# -
19315 -pf01927pf14451 -
(..2715..)
22030 -pf02602 -
22035 -pf00005 -
22040 -pf00005pf12399 -
22045 -pf02653 -
22050 -pf07883 -
22055 -pf02653 -
22060 -pf13458 -
22065 -pf00245 -
22070 -pf03055 -
22075 -pf00805 -
22080 Tdp_turquoise(#) CsomeBCy
22085 HMMalddh_3_aromic1(pf00171pf01619) CsomeBCy
22090 -pf13673 -
22095 -pf02366 -
22100 -# -
22105 -pf00664pf00005 -
22110 -pf01867 -
22115 -pf01930 -
22120 -pf10040 -
22125 -# -
22130 -# -
BMC-P
BMC-H
BMC-T
Aldehyde dehydrogenase
PTAC
Signature enzyme
Alcohol dehydrogenase
Regulator

Hydrococcus rivularis NIES-593. Bacteria; Cyanobacteria; Pleurocapsales; Hydrococcaceae; Hydrococcus.

NIES593__locus_1_main_sat

CsomeBCy

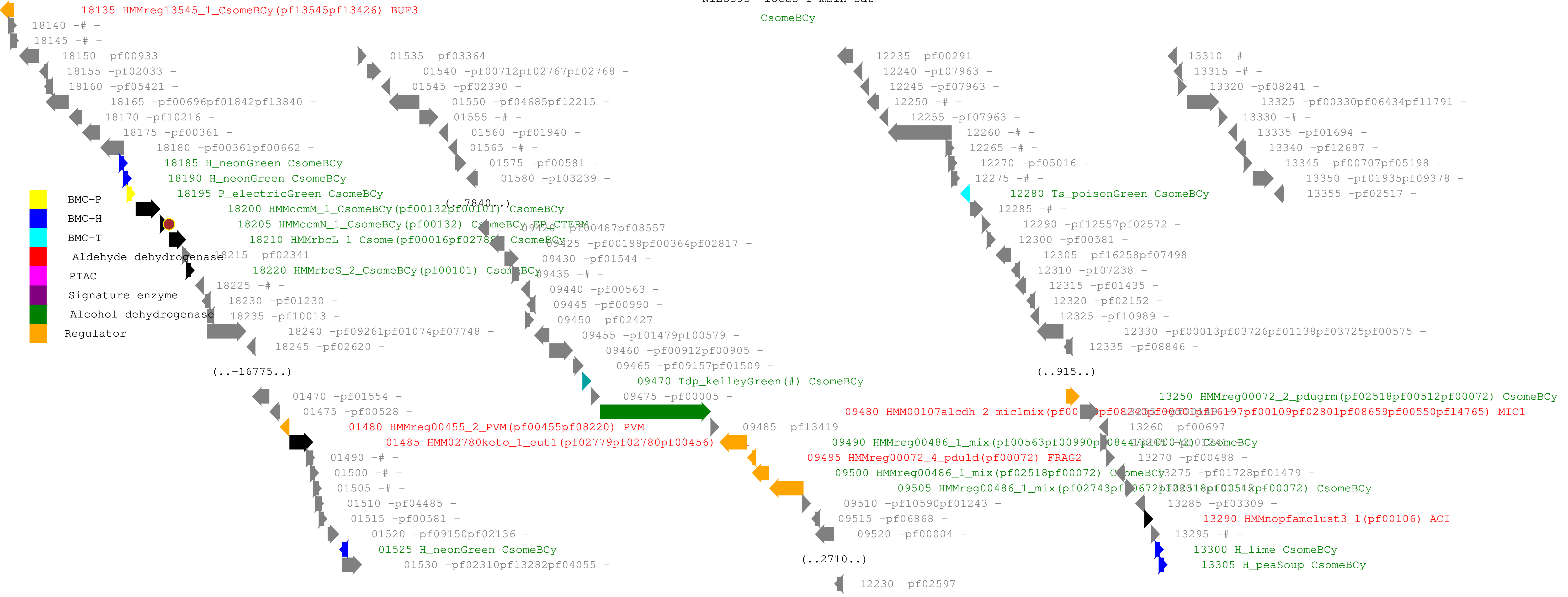

Sphaerospermopsis kisseleviana NIES-73. Bacteria; Cyanobacteria; Nostocales; Aphanizomenonaceae;

NIES73__locus_1_main_sat

CsomeBCy

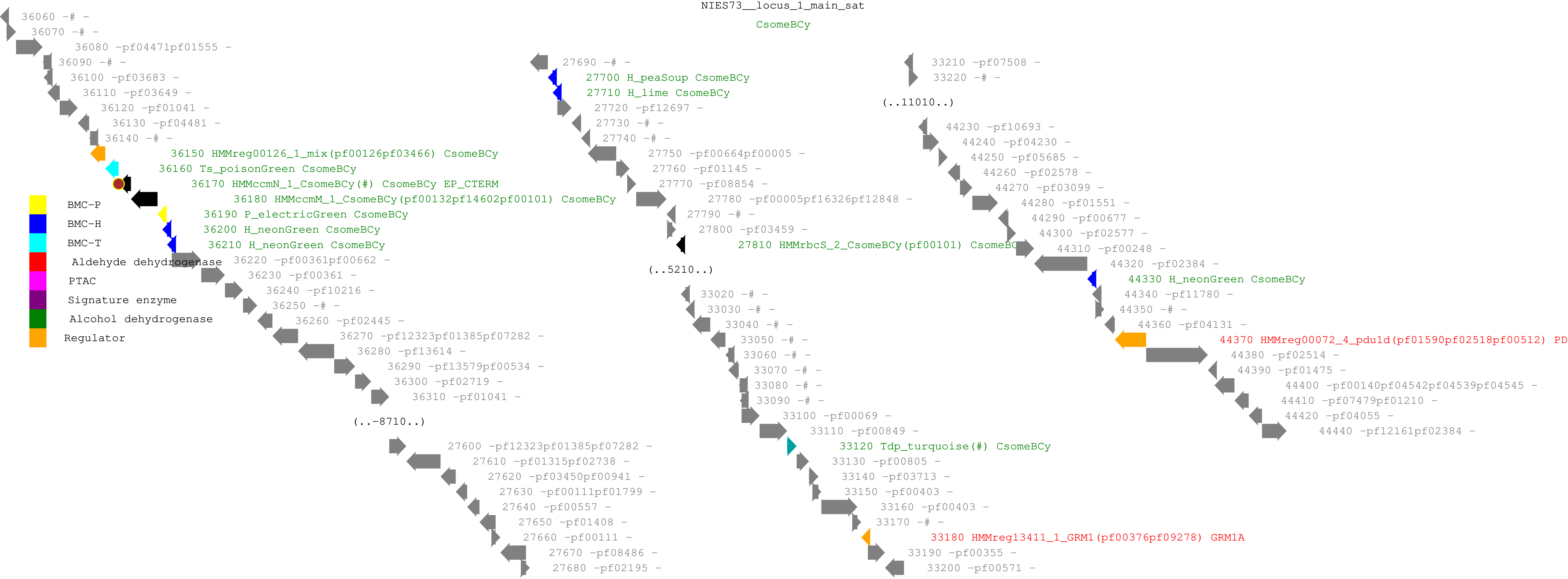

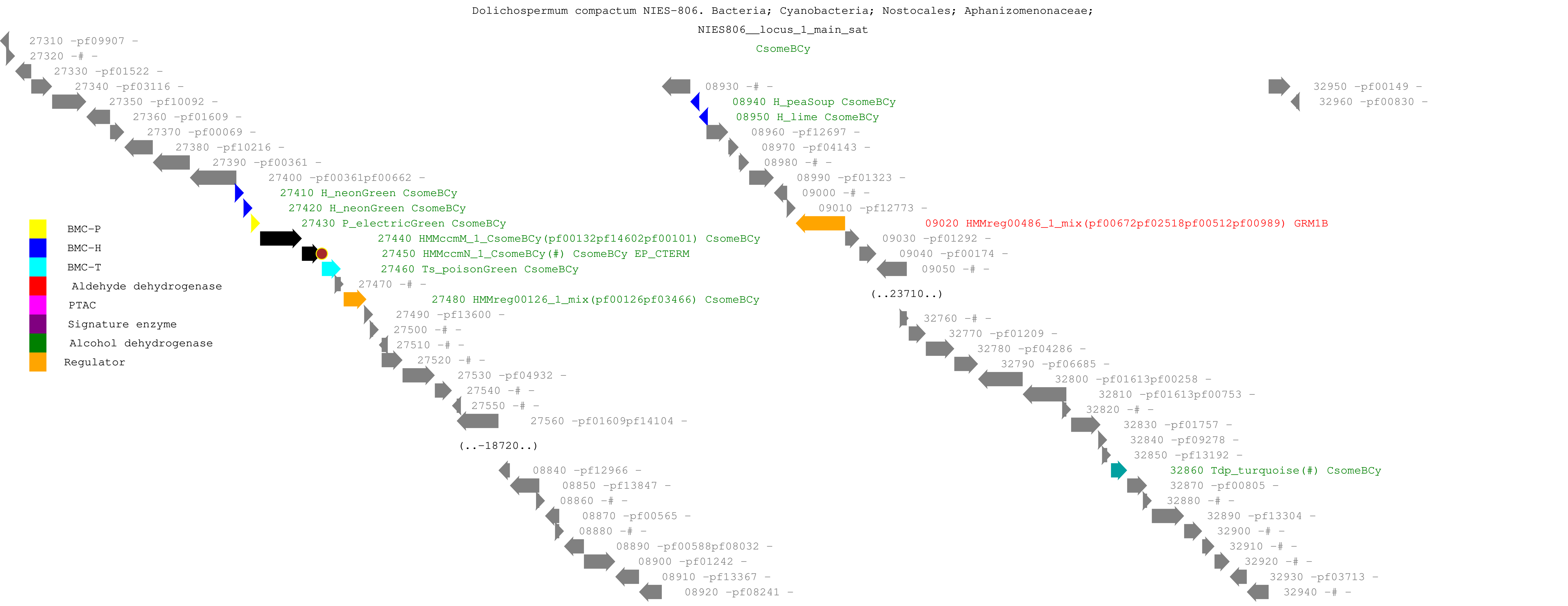

Dolichospermum compactum NIES-806. Bacteria; Cyanobacteria; Nostocales; Aphanizomenonaceae;
NIES806__locus_1_main_sat
CsomeBCy
27310 -pf09907 -
27320 -# -
27330 -pf01522 -
27340 -pf03116 -
27350 -pf10092 -
27360 -pf01609 -
27370 -pf00069 -
27380 -pf10216 -
27390 -pf00361 -
27400 -pf00361pf00662 -
27410 H_neonGreen CsomeBCy
27420 H_neonGreen CsomeBCy
27430 P_electricGreen CsomeBCy
27440 HMMccmM_1_CsomeBCy(pf00132pf14602pf00101) CsomeBCy
27450 HMMccmN_1_CsomeBCy(#) CsomeBCy EP_CTERM
27460 Ts_poisonGreen CsomeBCy
27470 -# -
27480 HMMreg00126_1_mix(pf00126pf03466) CsomeBCy
27490 -pf13600 -
27500 -# -
27510 -# -
27520 -# -
27530 -pf04932 -
27540 -# -
27550 -# -
27560 -pf01609pf14104 -
(..-18720..)
08840 -pf12966 -
08850 -pf13847 -
08860 -# -
08870 -pf00565 -
08880 -# -
08890 -pf00588pf08032 -
08900 -pf01242 -
08910 -pf13367 -
08920 -pf08241 -
08930 -# -
08940 H_peaSoup CsomeBCy
08950 H_lime CsomeBCy
08960 -pf12697 -
08970 -pf04143 -
08980 -# -
08990 -pf01323 -
09000 -# -
09010 -pf12773 -
09020 HMMreg00486_1_mix(pf00672pf02518pf00512pf00989) GRM1B
09030 -pf01292 -
09040 -pf00174 -
09050 -# -
(..23710..)
32760 -# -
32770 -pf01209 -
32780 -pf04286 -
32790 -pf06685 -
32800 -pf01613pf00258 -
32810 -pf01613pf00753 -
32820 -# -
32830 -pf01757 -
32840 -pf09278 -
32850 -pf13192 -
32860 Tdp_turquoise(#) CsomeBCy
32870 -pf00805 -
32880 -# -
32890 -pf13304 -
32900 -# -
32910 -# -
32920 -# -
32930 -pf03713 -
32940 -# -
32950 -pf00149 -
32960 -pf00830 -
BMC-P
BMC-H
BMC-T
Aldehyde dehydrogenase
PTAC
Signature enzyme
Alcohol dehydrogenase
Regulator

Dolichospermum planctonicum. Bacteria; Cyanobacteria; Nostocales; Aphanizomenonaceae; Dolichospermum.

NIES80__locus_1_main_sat

CsomeBCy

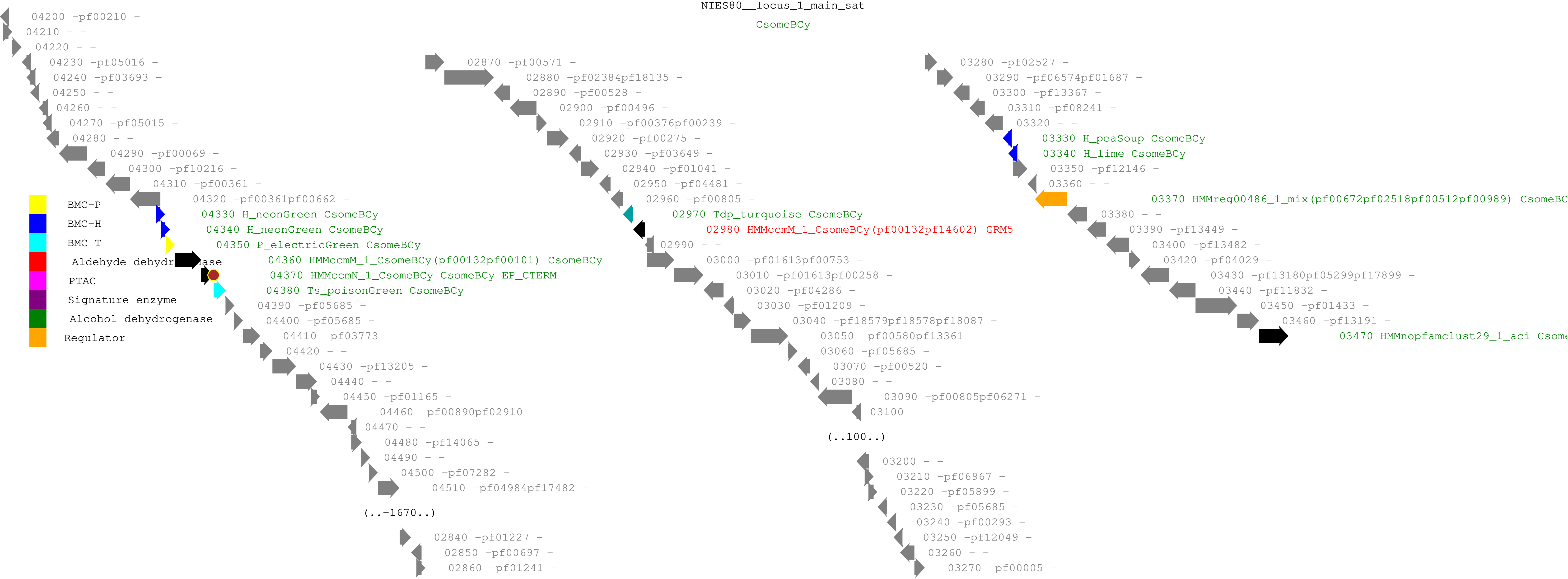

Raphidiopsis curvata NIES-932. Bacteria; Cyanobacteria; Nostocales; Aphanizomenonaceae; Raphidiopsis.

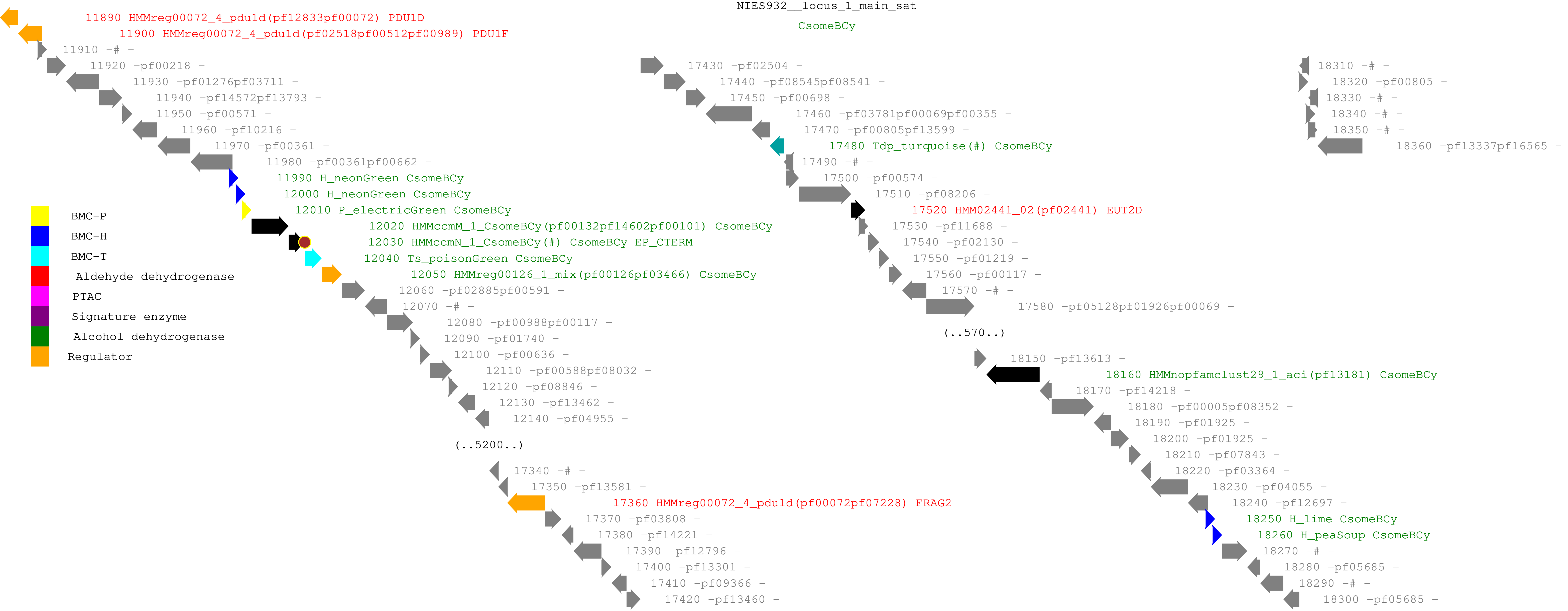

Synechococcus sp. NIES-970. Bacteria; Cyanobacteria; Synechococcales; Synechococcaceae;

NIES970__locus_1_main_sat

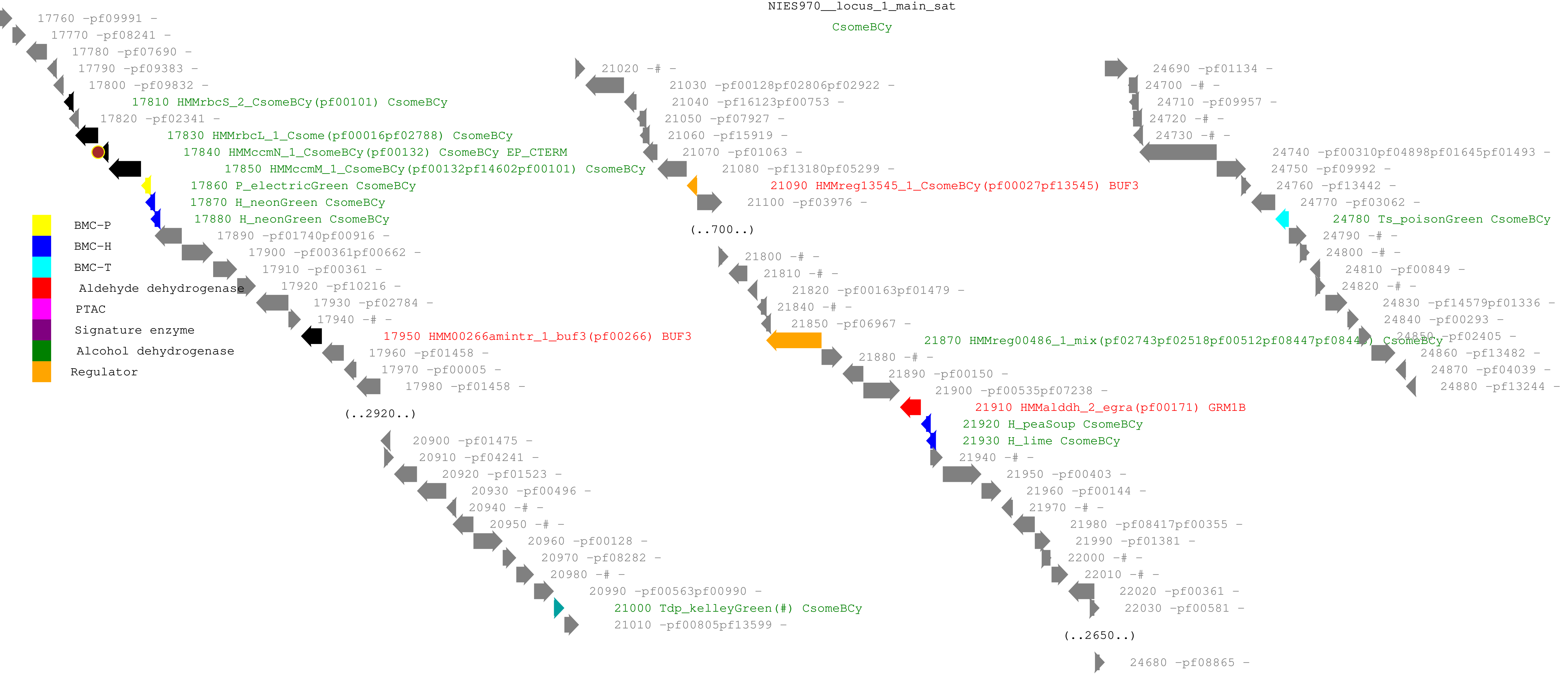

Thermosynechococcus sp. NK55a. Bacteria; Cyanobacteria; Synechococcales; Synechococcaceae;

NK55__locus_1_main_sat

CsomeBCy

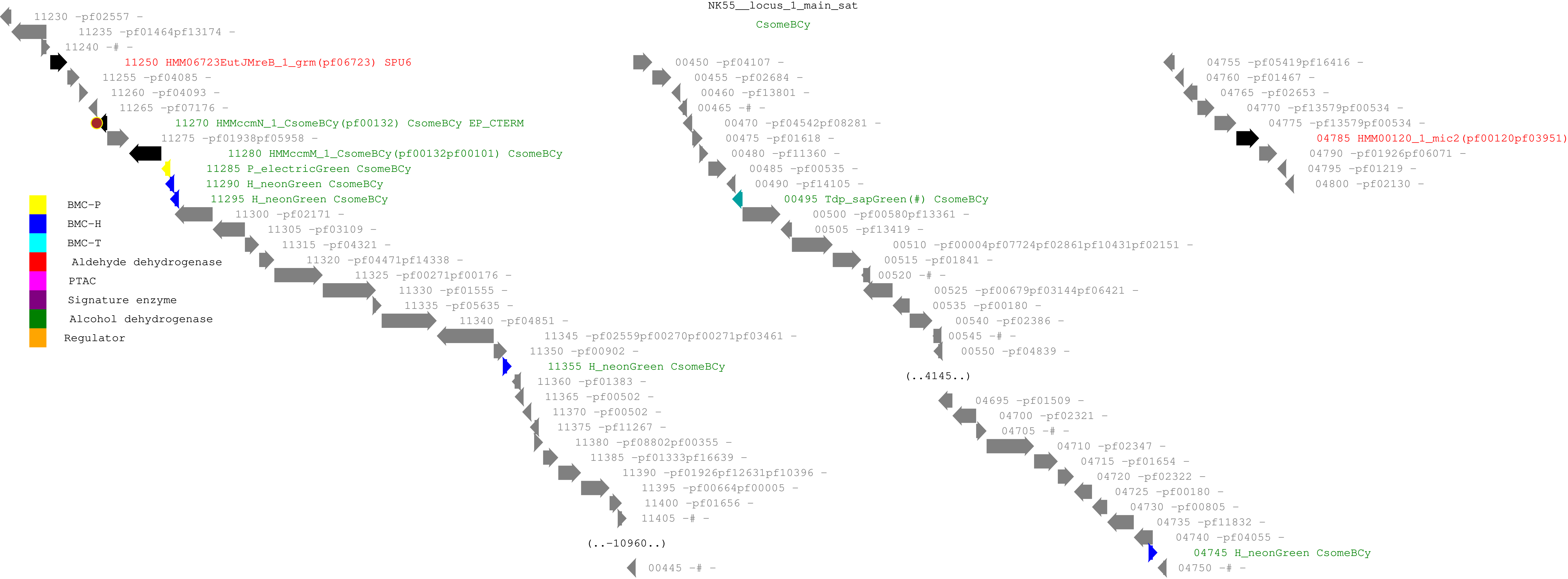

Nostoc sp. 'Lobaria pulmonaria (5183) cyanobiont'. Bacteria; Cyanobacteria; Nostocales; Nostocaceae; Nostoc.

NLP__locus_1_main_sat

CsomeBCy

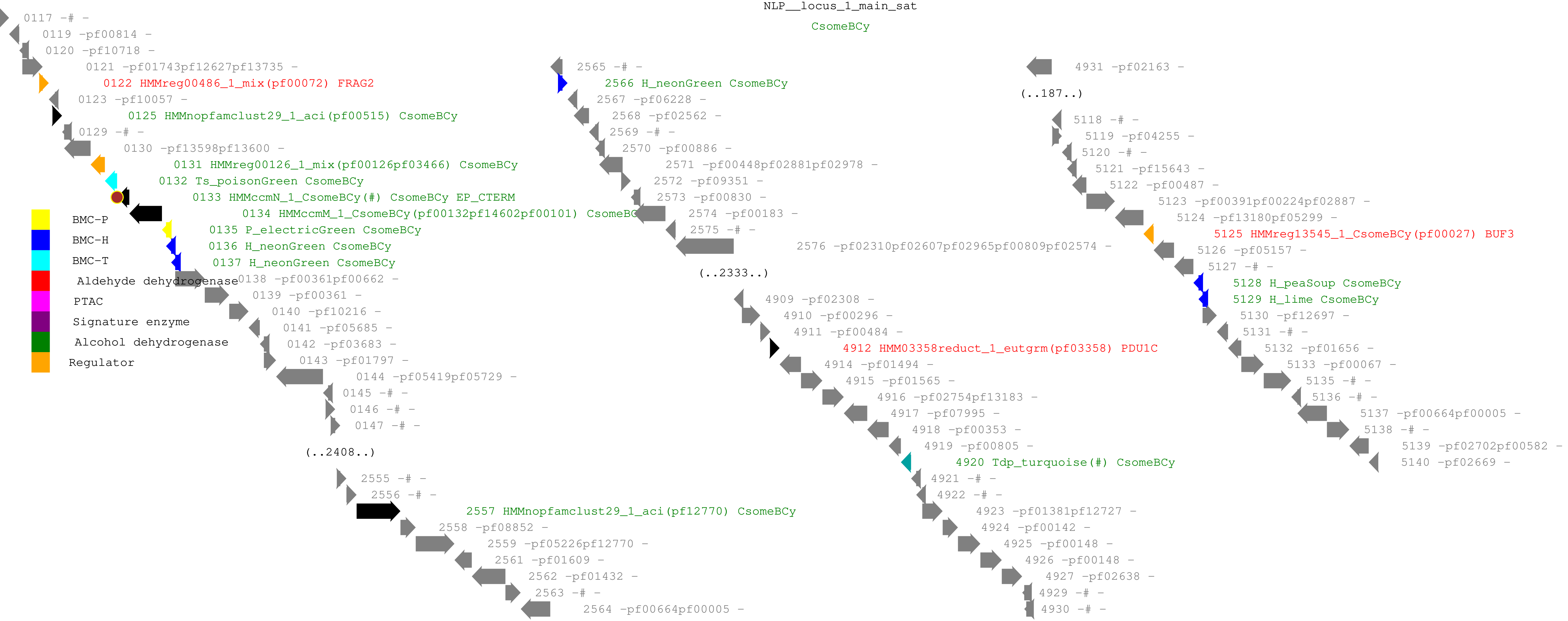

Nostoc sp. NIES-3756. Bacteria; Cyanobacteria; Nostocales; Nostocaceae; Nostoc.

NOS3756__locus_1_main_sat

CsomeBCy

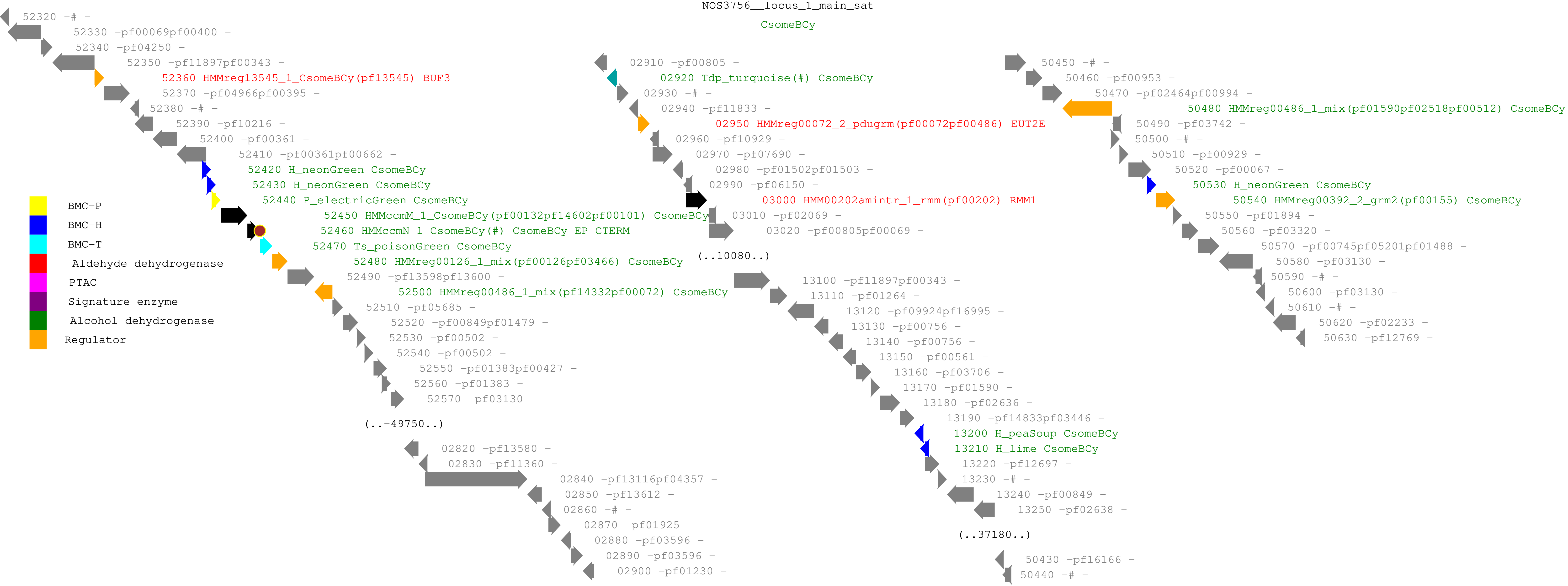

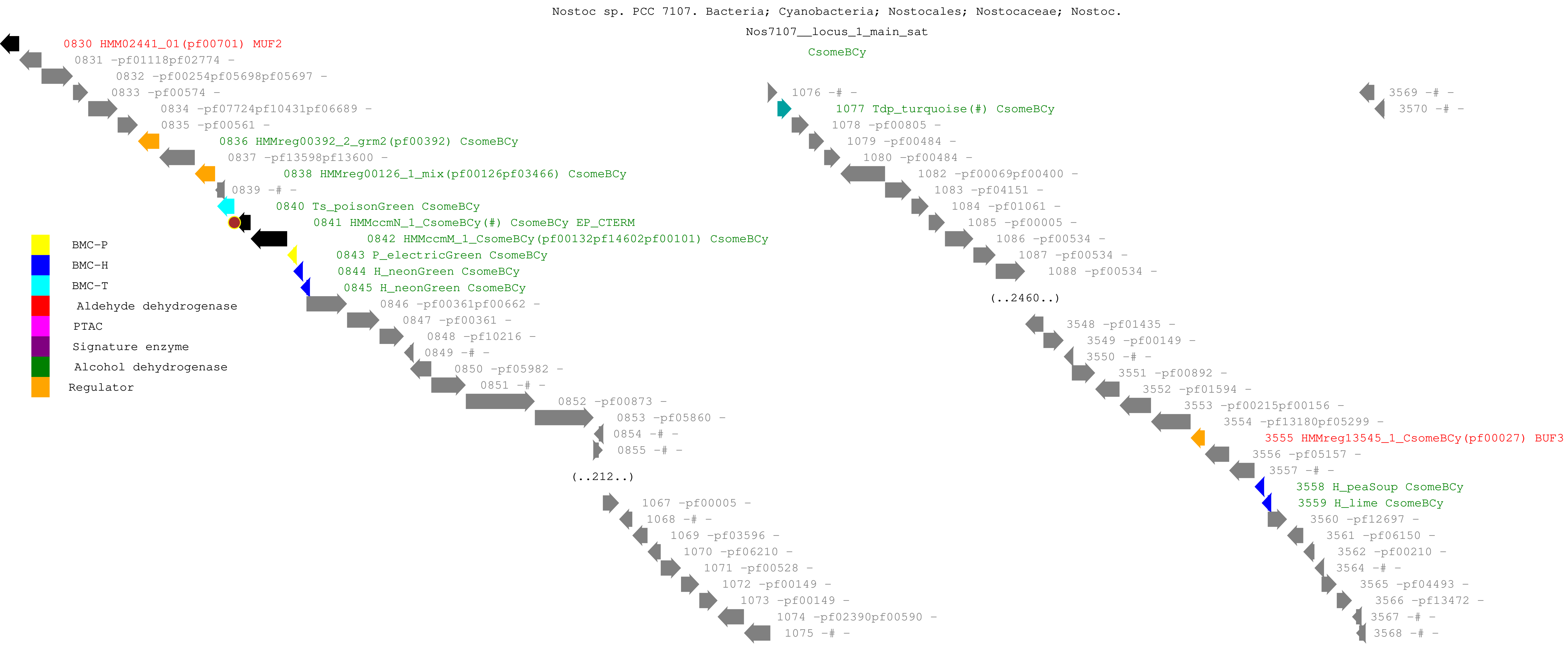

Nostoc sp. PCC 7107. Bacteria; Cyanobacteria; Nostocales; Nostocaceae; Nostoc.
Nos7107__locus_1_main_sat
CsomeBCy
0830 HMM02441_01(pf00701) MUF2
0831 -pf01118pf02774 -
0832 -pf00254pf05698pf05697 -
0833 -pf00574 -
0834 -pf07724pf10431pf06689 -
0835 -pf00561 -
0836 HMMreg00392_2_grm2(pf00392) CsomeBCy
0837 -pf13598pf13600 -
0838 HMMreg00126_1_mix(pf00126pf03466) CsomeBCy
0839 -# -
0840 Ts_poisonGreen CsomeBCy
0841 HMMccmN_1_CsomeBCy(#) CsomeBCy EP_CTERM
0842 HMMccmM_1_CsomeBCy(pf00132pf14602pf00101) CsomeBCy
0843 P_electricGreen CsomeBCy
0844 H_neonGreen CsomeBCy
0845 H_neonGreen CsomeBCy
0846 -pf00361pf00662 -
0847 -pf00361 -
0848 -pf10216 -
0849 -# -
0850 -pf05982 -
0851 -# -
0852 -pf00873 -
0853 -pf05860 -
0854 -# -
0855 -# -
(..212..)
1067 -pf00005 -
1068 -# -
1069 -pf03596 -
1070 -pf06210 -
1071 -pf00528 -
1072 -pf00149 -
1073 -pf00149 -
1074 -pf02390pf00590 -
1075 -# -
1076 -# -
1077 Tdp_turquoise(#) CsomeBCy
1078 -pf00805 -
1079 -pf00484 -
1080 -pf00484 -
1082 -pf00069pf00400 -
1083 -pf04151 -
1084 -pf01061 -
1085 -pf00005 -
1086 -pf00534 -
1087 -pf00534 -
1088 -pf00534 -
(..2460..)
3548 -pf01435 -
3549 -pf00149 -
3550 -# -
3551 -pf00892 -
3552 -pf01594 -
3553 -pf00215pf00156 -
3554 -pf13180pf05299 -
3555 HMMreg13545_1_CsomeBCy(pf00027) BUF3
3556 -pf05157 -
3557 -# -
3558 H_peaSoup CsomeBCy
3559 H_lime CsomeBCy
3560 -pf12697 -
3561 -pf06150 -
3562 -pf00210 -
3564 -# -
3565 -pf04493 -
3566 -pf13472 -
3567 -# -
3568 -# -
3569 -# -
3570 -# -
BMC-P
BMC-H
BMC-T
Aldehyde dehydrogenase
PTAC
Signature enzyme
Alcohol dehydrogenase
Regulator

Nostoc sp. (strain ATCC 29411 / PCC 7524). Bacteria; Cyanobacteria; Nostocales; Nostocaceae; Nostoc.

Nos7524__locus_1_main_sat

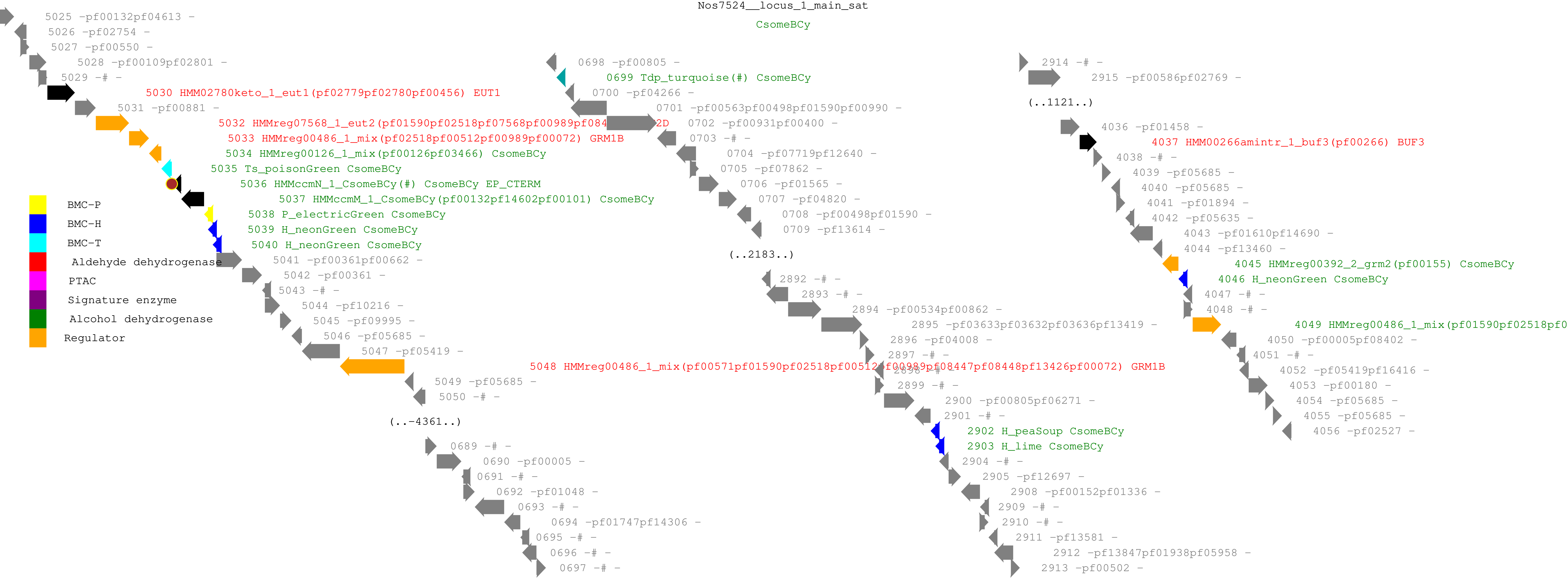

Nostoc sp. 'Peltigera membranacea cyanobiont N6'. Bacteria; Cyanobacteria; Nostocales; Nostocaceae; Nostoc.

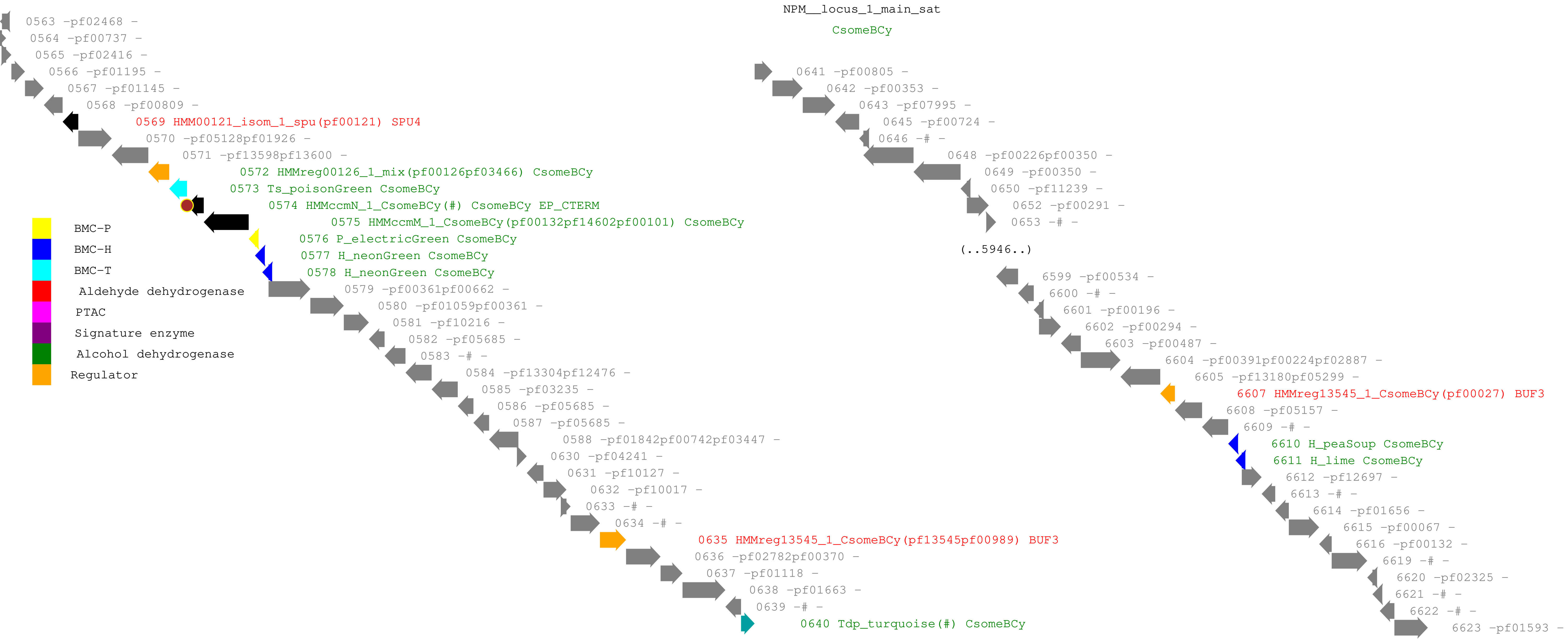

Nostoc punctiforme (strain ATCC 29133 / PCC 73102). Bacteria; Cyanobacteria; Nostocales; Nostocaceae; Nostoc.

Npun_F_locus_1_main_sat

CsomeBCy

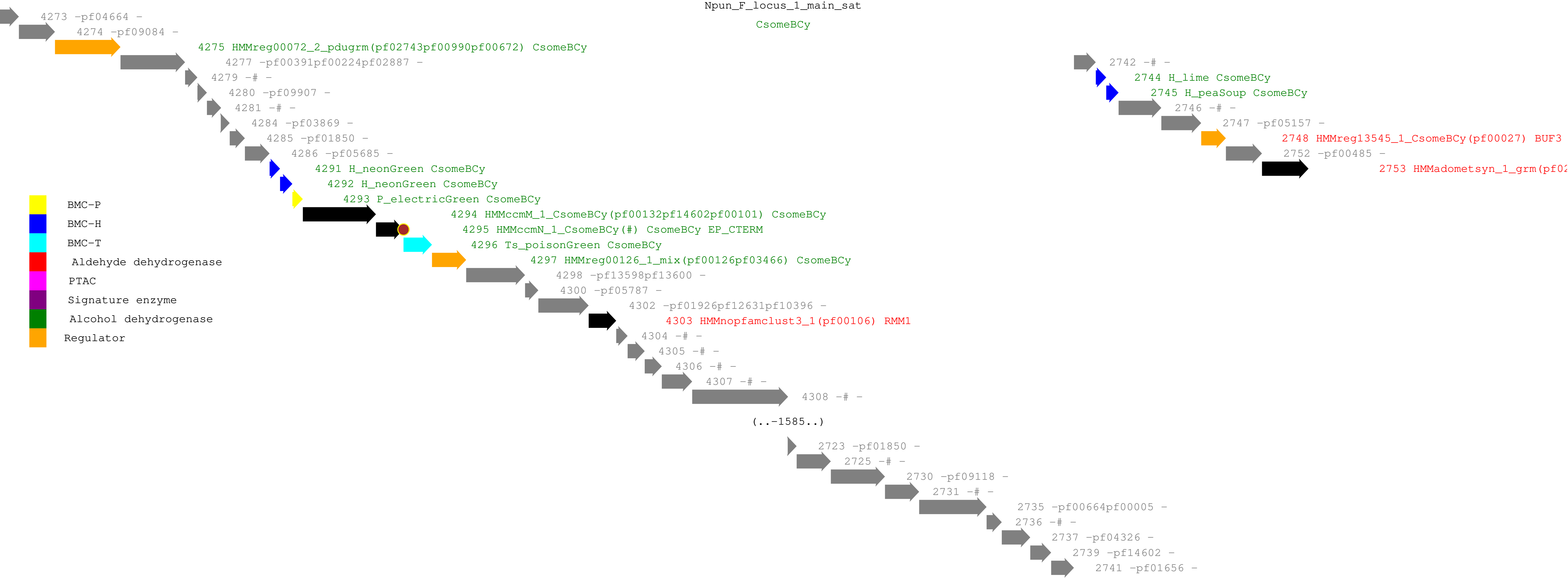

Nodularia spumigena CCY9414. Bacteria; Cyanobacteria; Nostocales; Aphanizomenonaceae; Nodularia.

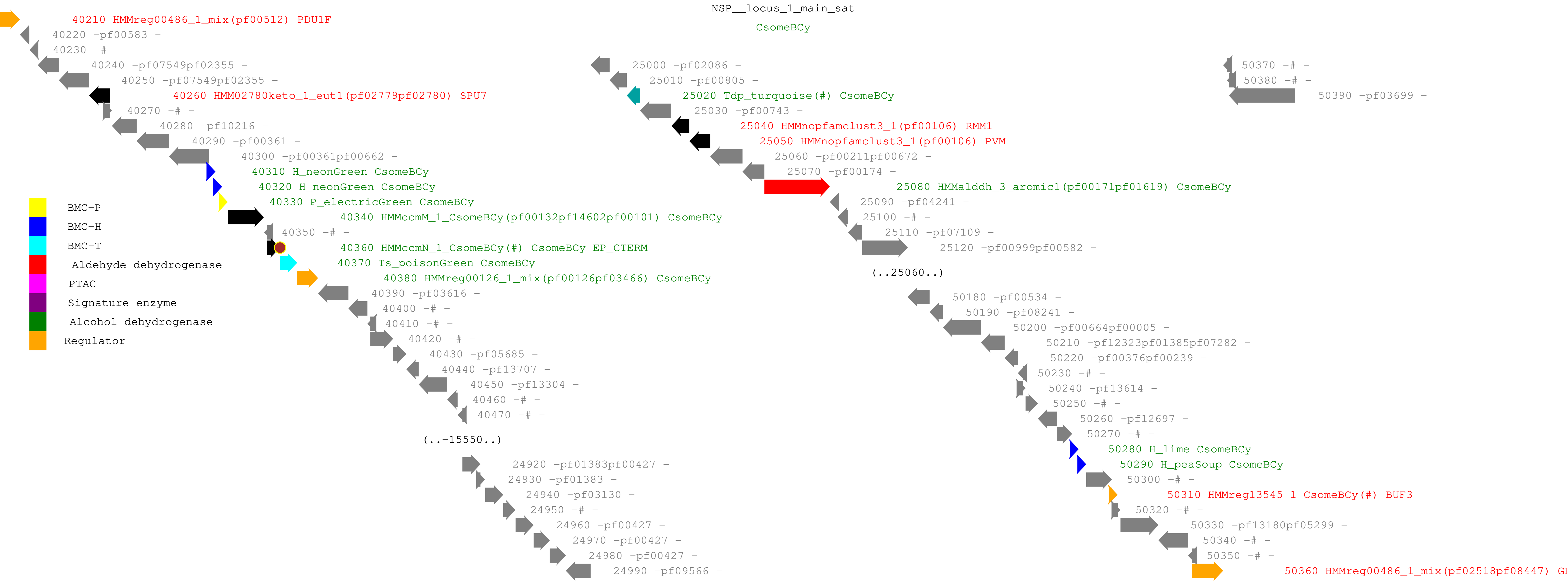

Microcystis aeruginosa TAIHU98. Bacteria; Cyanobacteria; Oscillatoriophycideae; Chroococcales;

O53__locus_fragmented

CsomeBCy

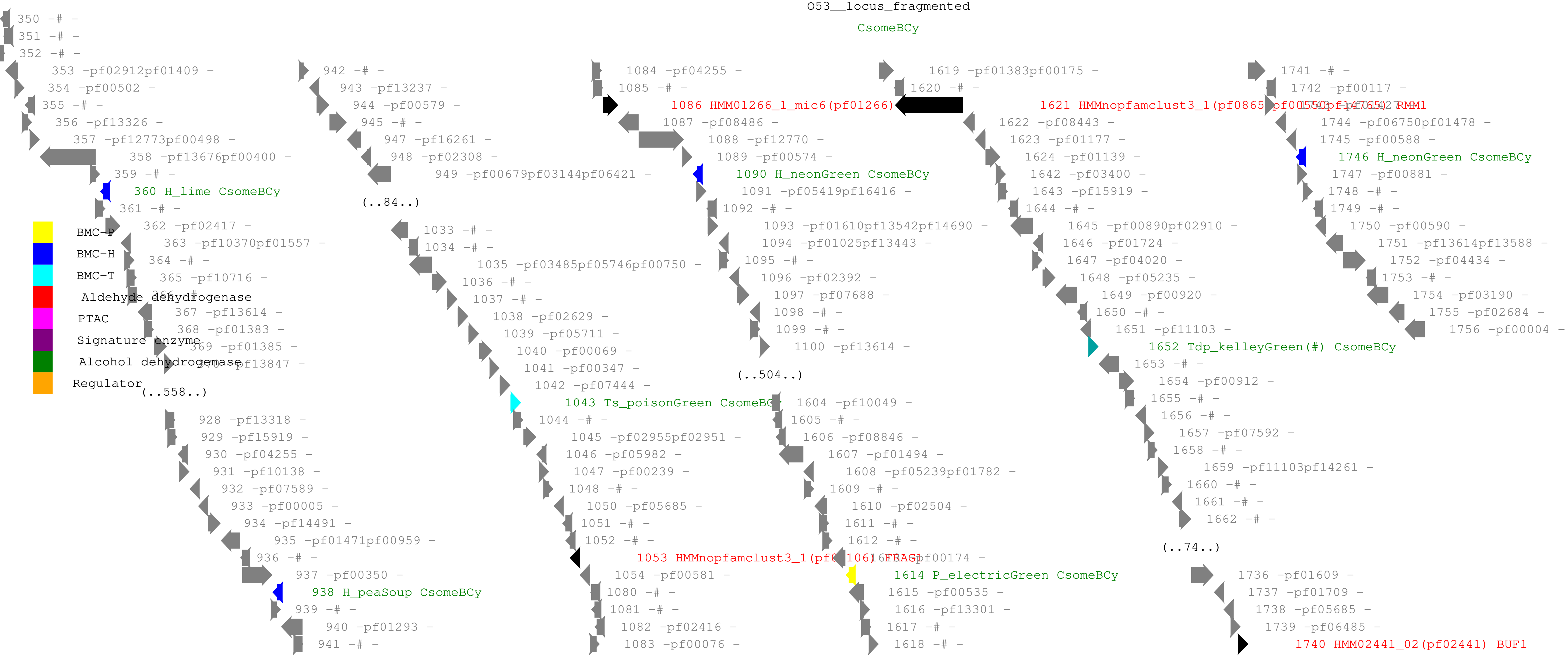

Leptolyngbya sp. O-77. Bacteria; Cyanobacteria; Synechococcales; Leptolyngbyaceae;

O77CONTIG1__locus_1_main_sat

CsomeBCy

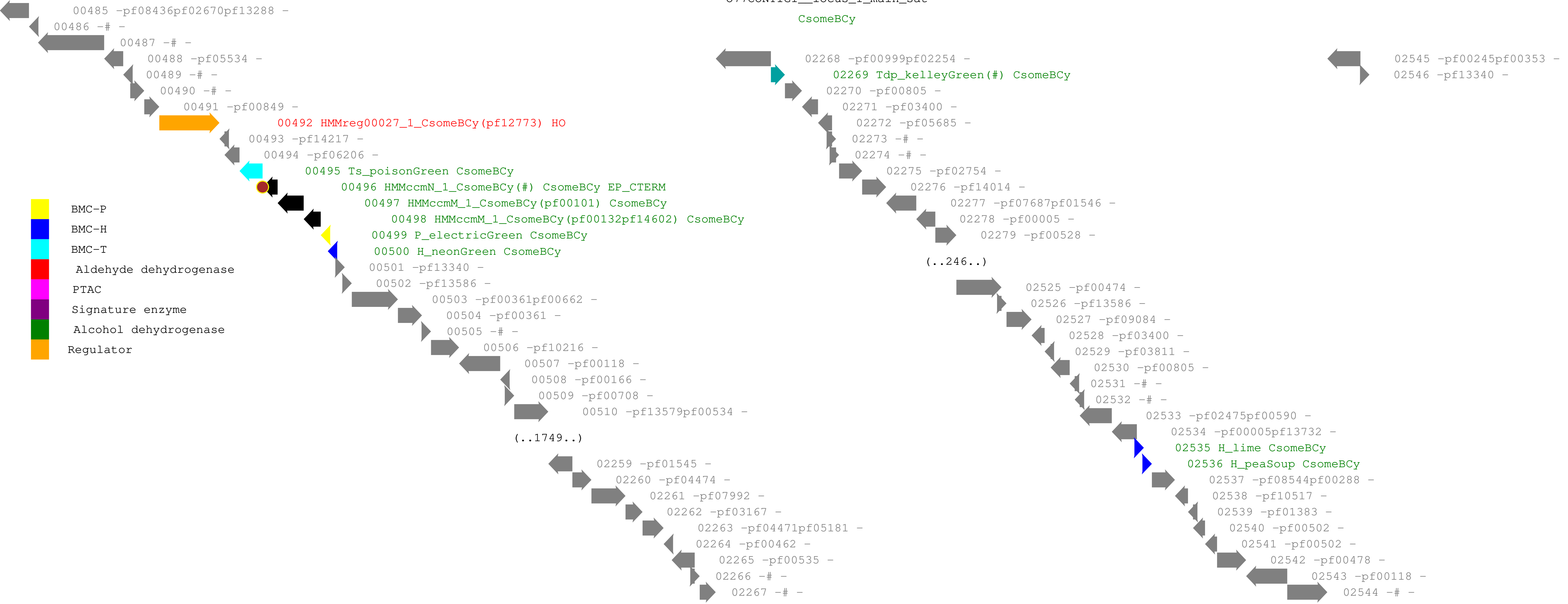

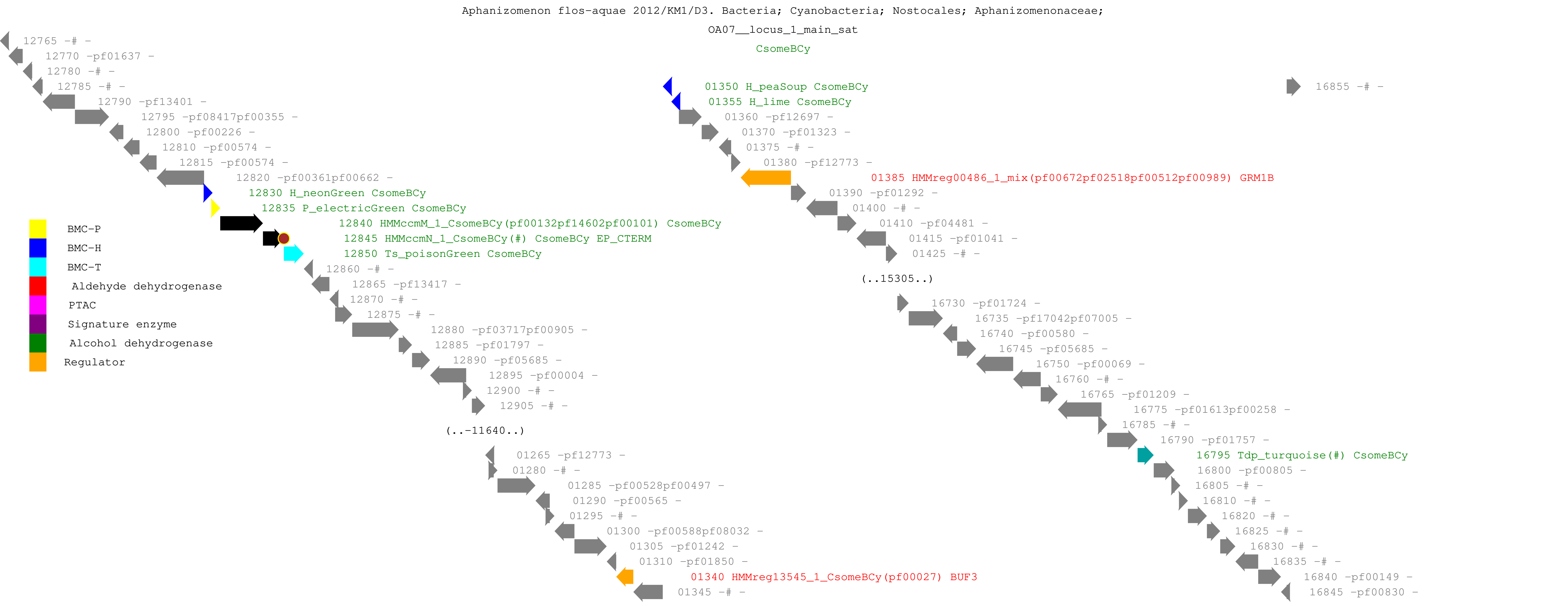
Aphanizomenon flos-aquae 2012/KM1/D3. Bacteria; Cyanobacteria; Nostocales; Aphanizomenonaceae;
OA07__locus_1_main_sat
CsomeBCy
12765 -# -
12770 -pf01637 -
12780 -# -
12785 -# -
12790 -pf13401 -
12795 -pf08417pf00355 -
12800 -pf00226 -
12810 -pf00574 -
12815 -pf00574 -
12820 -pf00361pf00662 -
12830 H_neonGreen CsomeBCy
12835 P_electricGreen CsomeBCy
12840 HMMccmM_1_CsomeBCy(pf00132pf14602pf00101) CsomeBCy
12845 HMMccmN_1_CsomeBCy(#) CsomeBCy EP_CTERM
12850 Ts_poisonGreen CsomeBCy
12860 -# -
12865 -pf13417 -
12870 -# -
12875 -# -
12880 -pf03717pf00905 -
12885 -pf01797 -
12890 -pf05685 -
12895 -pf00004 -
12900 -# -
12905 -# -
(..-11640..)
01265 -pf12773 -
01280 -# -
01285 -pf00528pf00497 -
01290 -pf00565 -
01295 -# -
01300 -pf00588pf08032 -
01305 -pf01242 -
01310 -pf01850 -
01340 HMMreg13545_1_CsomeBCy(pf00027) BUF3
01345 -# -
01350 H_peaSoup CsomeBCy
01355 H_lime CsomeBCy
01360 -pf12697 -
01370 -pf01323 -
01375 -# -
01380 -pf12773 -
01385 HMMreg00486_1_mix(pf00672pf02518pf00512pf00989) GRM1B
01390 -pf01292 -
01400 -# -
01410 -pf04481 -
01415 -pf01041 -
01425 -# -
(..15305..)
16730 -pf01724 -
16735 -pf17042pf07005 -
16740 -pf00580 -
16745 -pf05685 -
16750 -pf00069 -
16760 -# -
16765 -pf01209 -
16775 -pf01613pf00258 -
16785 -# -
16790 -pf01757 -
16795 Tdp_turquoise(#) CsomeBCy
16800 -pf00805 -
16805 -# -
16810 -# -
16820 -# -
16825 -# -
16830 -# -
16835 -# -
16840 -pf00149 -
16845 -pf00830 -
16855 -# -
BMC-P
BMC-H
BMC-T
Aldehyde dehydrogenase
PTAC
Signature enzyme
Alcohol dehydrogenase
Regulator

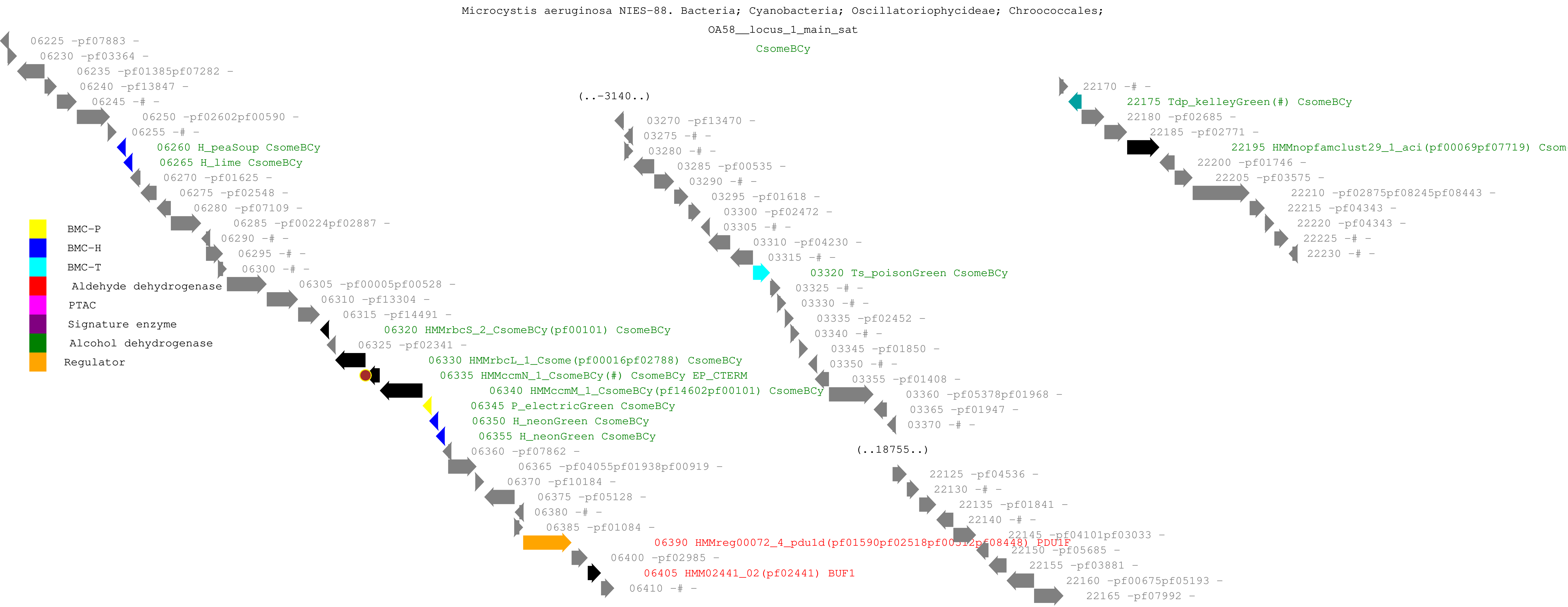
Microcystis aeruginosa NIES-88. Bacteria; Cyanobacteria; Oscillatoriophycideae; Chroococcales;
OA58__locus_1_main_sat
CsomeBCy
BMC-P
BMC-H
BMC-T
Aldehyde dehydrogenase
PTAC
Signature enzyme
Alcohol dehydrogenase
Regulator
06225 -pf07883 -
06230 -pf03364 -
06235 -pf01385pf07282 -
06240 -pf13847 -
06245 -# -
06250 -pf02602pf00590 -
06255 -# -
06260 H_peaSoup CsomeBCy
06265 H_lime CsomeBCy
06270 -pf01625 -
06275 -pf02548 -
06280 -pf07109 -
06285 -pf00224pf02887 -
06290 -# -
06295 -# -
06300 -# -
06305 -pf00005pf00528 -
06310 -pf13304 -
06315 -pf14491 -
06320 HMMrbcS_2_CsomeBCy(pf00101) CsomeBCy
06325 -pf02341 -
06330 HMMrbcL_1_Csome(pf00016pf02788) CsomeBCy
06335 HMMccmN_1_CsomeBCy(#) CsomeBCy EP_CTERM
06340 HMMccmM_1_CsomeBCy(pf14602pf00101) CsomeBCy
06345 P_electricGreen CsomeBCy
06350 H_neonGreen CsomeBCy
06355 H_neonGreen CsomeBCy
06360 -pf07862 -
06365 -pf04055pf01938pf00919 -
06370 -pf10184 -
06375 -pf05128 -
06380 -# -
06385 -pf01084 -
06390 HMMreg00072_4_pdu1d(pf01590pf02518pf00512pf08448) PDU1F
06400 -pf02985 -
06405 HMM02441_02(pf02441) BUF1
06410 -# -
(..-3140..)
03270 -pf13470 -
03275 -# -
03280 -# -
03285 -pf00535 -
03290 -# -
03295 -pf01618 -
03300 -pf02472 -
03305 -# -
03310 -pf04230 -
03315 -# -
03320 Ts_poisonGreen CsomeBCy
03325 -# -
03330 -# -
03335 -pf02452 -
03340 -# -
03345 -pf01850 -
03350 -# -
03355 -pf01408 -
03360 -pf05378pf01968 -
03365 -pf01947 -
03370 -# -
(..18755..)
22125 -pf04536 -
22130 -# -
22135 -pf01841 -
22140 -# -
22145 -pf04101pf03033 -
22150 -pf05685 -
22155 -pf03881 -
22160 -pf00675pf05193 -
22165 -pf07992 -
22170 -# -
22175 Tdp_kelleyGreen(#) CsomeBCy
22180 -pf02685 -
22185 -pf02771 -
22195 HMMnopfamclust29_1_aci(pf00069pf07719) Csom
22200 -pf01746 -
22205 -pf03575 -
22210 -pf02875pf08245pf08443 -
22215 -pf04343 -
22220 -pf04343 -
22225 -# -
22230 -# -

Oscillatoria nigro-viridis PCC 7112. Bacteria; Cyanobacteria; Oscillatoriophycideae; Oscillatoriales;

Osc7112__locus_1_main_sat

CsomeBCy

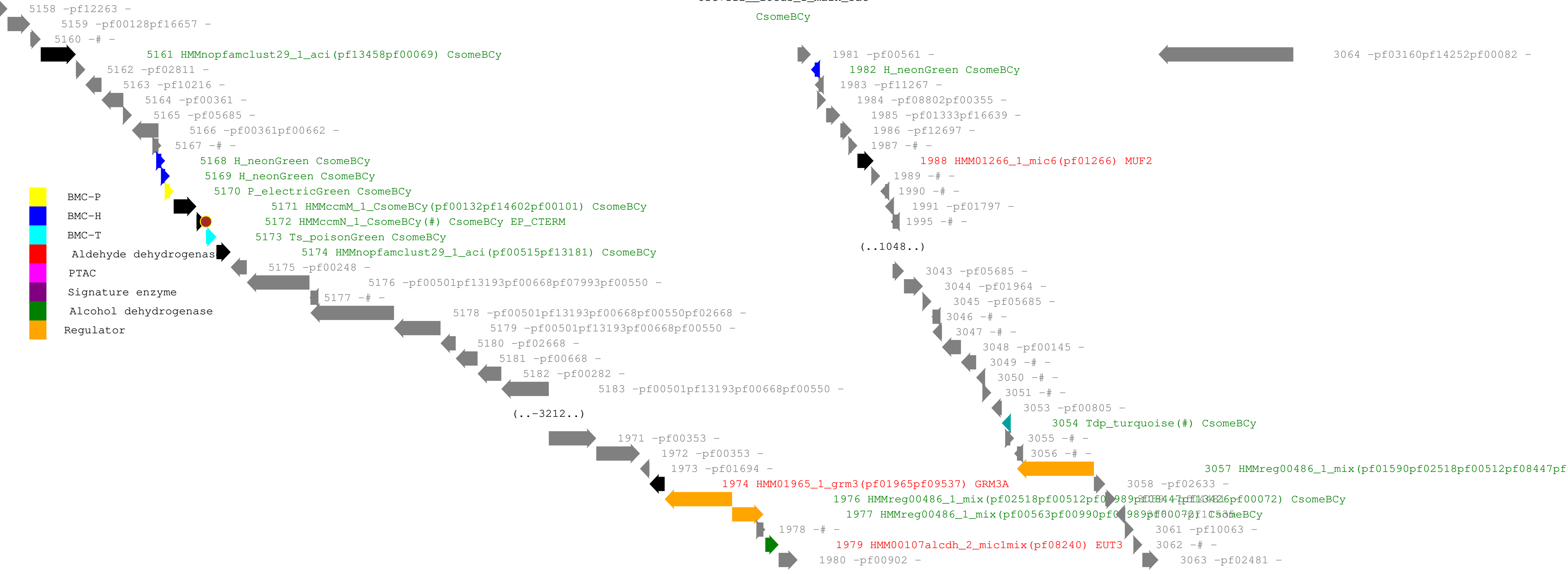

Oscillatoriales cyanobacterium JSC-12. Bacteria; Cyanobacteria; Oscillatoriophycideae; Oscillatoriales;

OsccyDRAFT__locus_1_main_sat

CsomeBCy

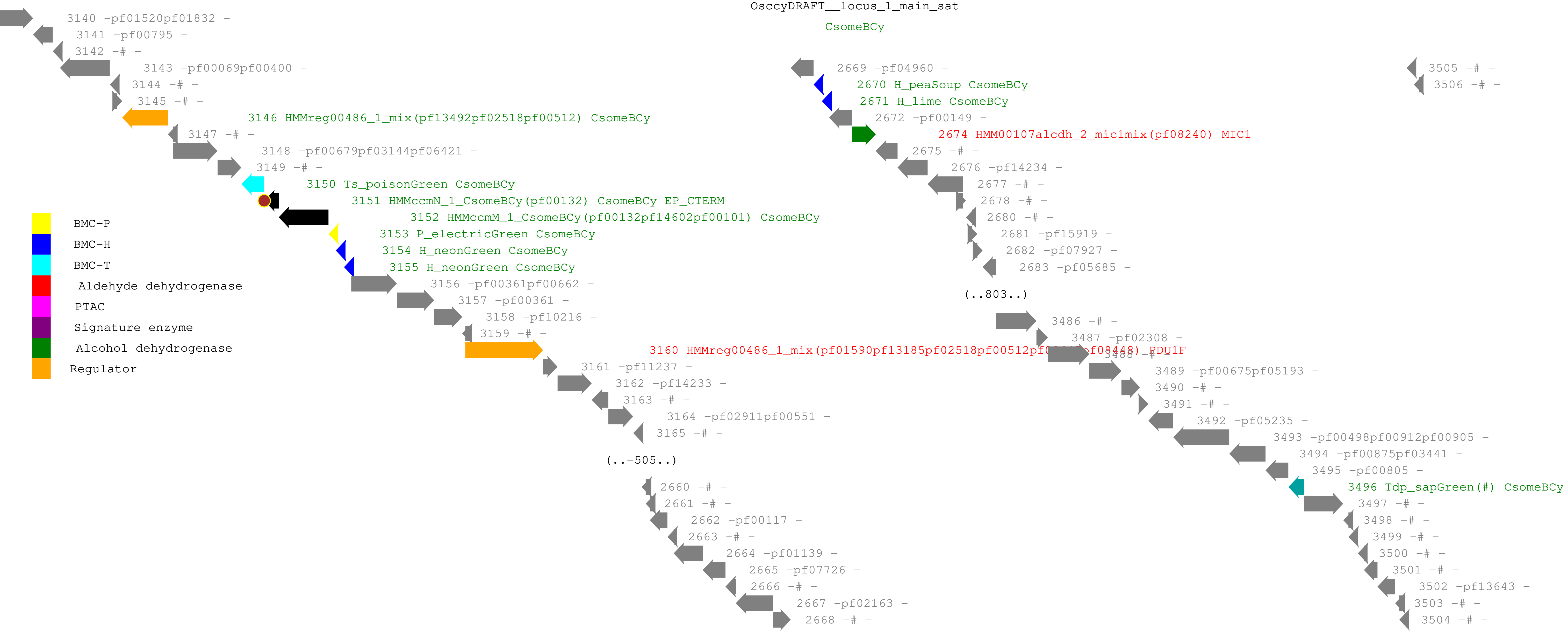

Oscillatoria acuminata PCC 6304. Bacteria; Cyanobacteria; Oscillatoriophycideae; Oscillatoriales;

Oscil6304__locus_1_main_sat

CsomeBCy

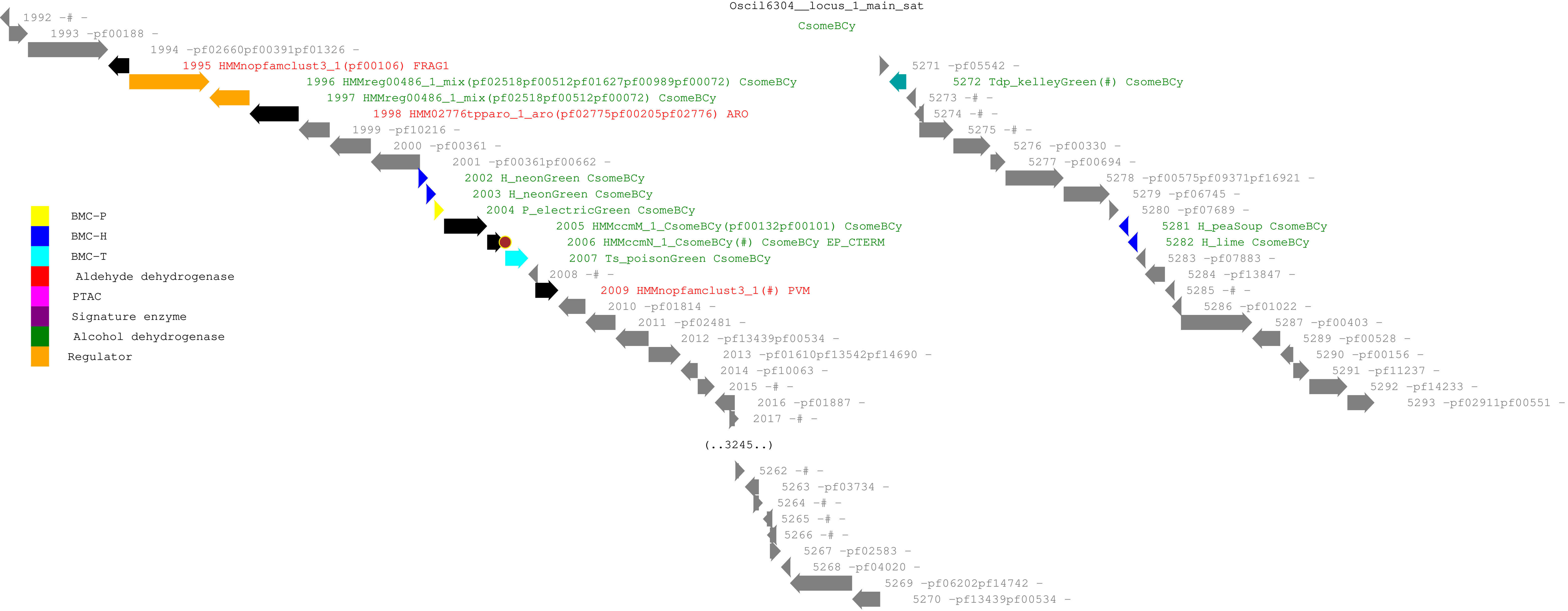

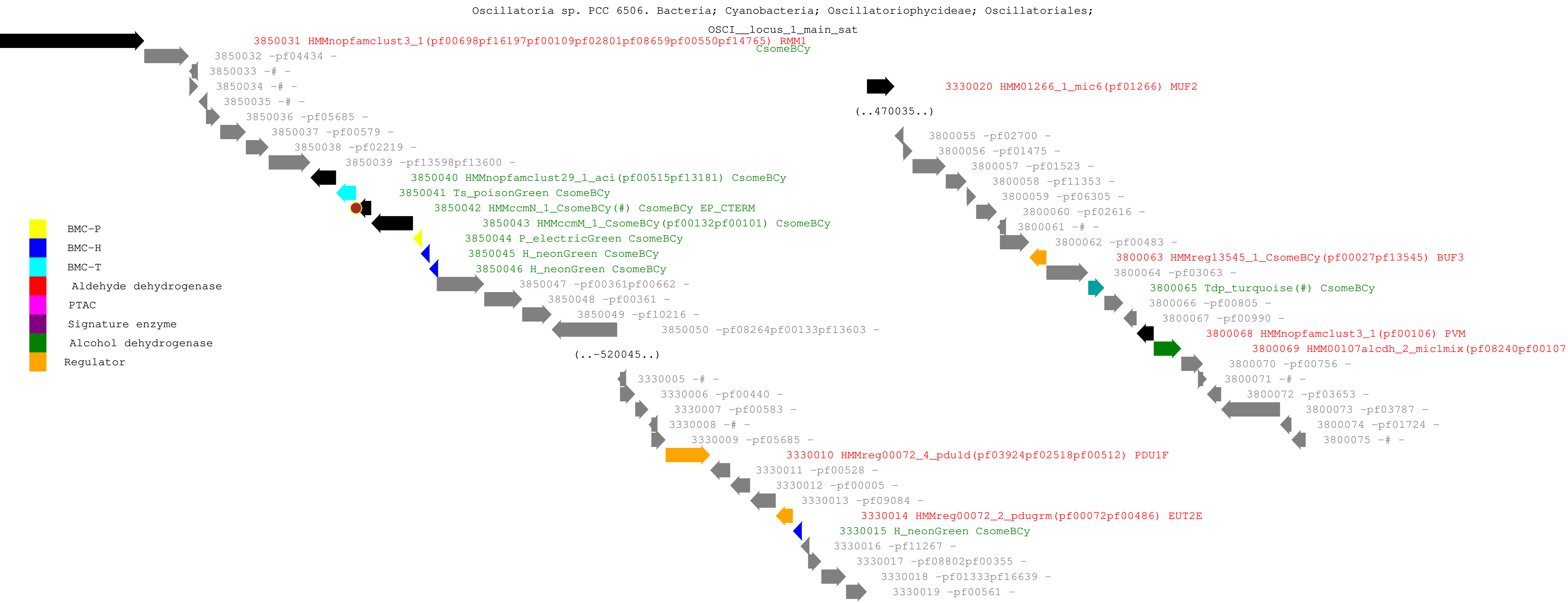
Oscillatoria sp. PCC 6506. Bacteria; Cyanobacteria; Oscillatoriophycideae; Oscillatoriales;
OSCI__locus_1_main_sat
3850031 HMMnopfamclust3_1(pf00698pf16197pf00109pf02801pf08659pf00550pf14765) RMM1
CsomeBCy
3850032 -pf04434 -
3850033 -# -
3850034 -# -
3850035 -# -
3850036 -pf05685 -
3850037 -pf00579 -
3850038 -pf02219 -
3850039 -pf13598pf13600 -
3850040 HMMnopfamclust29_1_aci(pf00515pf13181) CsomeBCy
3850041 Ts_poisonGreen CsomeBCy
3850042 HMMccmN_1_CsomeBCy(#) CsomeBCy EP_CTERM
3850043 HMMccmM_1_CsomeBCy(pf00132pf00101) CsomeBCy
3850044 P_electricGreen CsomeBCy
3850045 H_neonGreen CsomeBCy
3850046 H_neonGreen CsomeBCy
3850047 -pf00361pf00662 -
3850048 -pf00361 -
3850049 -pf10216 -
3850050 -pf08264pf00133pf13603 -
(..-520045..)
3330005 -# -
3330006 -pf00440 -
3330007 -pf00583 -
3330008 -# -
3330009 -pf05685 -
3330010 HMMreg00072_4_pdu1d(pf03924pf02518pf00512) PDU1F
3330011 -pf00528 -
3330012 -pf00005 -
3330013 -pf09084 -
3330014 HMMreg00072_2_pdugrm(pf00072pf00486) EUT2E
3330015 H_neonGreen CsomeBCy
3330016 -pf11267 -
3330017 -pf08802pf00355 -
3330018 -pf01333pf16639 -
3330019 -pf00561 -
3330020 HMM01266_1_mic6(pf01266) MUF2
(..470035..)
3800055 -pf02700 -
3800056 -pf01475 -
3800057 -pf01523 -
3800058 -pf11353 -
3800059 -pf06305 -
3800060 -pf02616 -
3800061 -# -
3800062 -pf00483 -
3800063 HMMreg13545_1_CsomeBCy(pf00027pf13545) BUF3
3800064 -pf03063 -
3800065 Tdp_turquoise(#) CsomeBCy
3800066 -pf00805 -
3800067 -pf00990 -
3800068 HMMnopfamclust3_1(pf00106) PVM
3800069 HMM00107alcdh_2_mic1mix(pf08240pf00107
3800070 -pf00756 -
3800071 -# -
3800072 -pf03653 -
3800073 -pf03787 -
3800074 -pf01724 -
3800075 -# -
BMC-P
BMC-H
BMC-T
Aldehyde dehydrogenase
PTAC
Signature enzyme
Alcohol dehydrogenase
Regulator

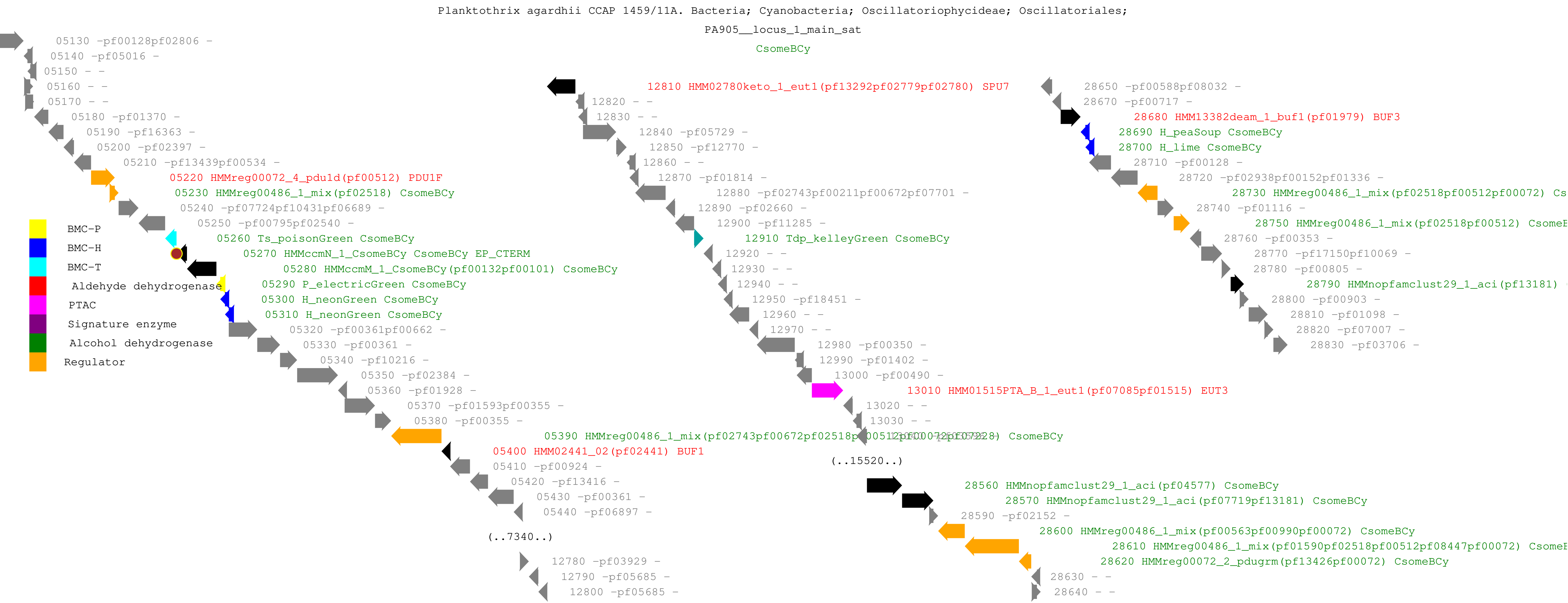
Planktothrix agardhii CCAP 1459/11A. Bacteria; Cyanobacteria; Oscillatoriophycideae; Oscillatoriales;
PA905__locus_1_main_sat
CsomeBCy
BMC-P
BMC-H
BMC-T
Aldehyde dehydrogenase
PTAC
Signature enzyme
Alcohol dehydrogenase
Regulator
05130 -pf00128pf02806 -
05140 -pf05016 -
05150 - -
05160 - -
05170 - -
05180 -pf01370 -
05190 -pf16363 -
05200 -pf02397 -
05210 -pf13439pf00534 -
05220 HMMreg00072_4_pdu1d(pf00512) PDU1F
05230 HMMreg00486_1_mix(pf02518) CsomeBCy
05240 -pf07724pf10431pf06689 -
05250 -pf00795pf02540 -
05260 Ts_poisonGreen CsomeBCy
05270 HMMccmN_1_CsomeBCy CsomeBCy EP_CTERM
05280 HMMccmM_1_CsomeBCy(pf00132pf00101) CsomeBCy
05290 P_electricGreen CsomeBCy
05300 H_neonGreen CsomeBCy
05310 H_neonGreen CsomeBCy
05320 -pf00361pf00662 -
05330 -pf00361 -
05340 -pf10216 -
05350 -pf02384 -
05360 -pf01928 -
05370 -pf01593pf00355 -
05380 -pf00355 -
05390 HMMreg00486_1_mix(pf02743pf00672pf02518pf00512pf00072) CsomeBCy
05400 HMM02441_02(pf02441) BUF1
05410 -pf00924 -
05420 -pf13416 -
05430 -pf00361 -
05440 -pf06897 -
(..7340..)
12780 -pf03929 -
12790 -pf05685 -
12800 -pf05685 -
12810 HMM02780keto_1_eut1(pf13292pf02779pf02780) SPU7
12820 - -
12830 - -
12840 -pf05729 -
12850 -pf12770 -
12860 - -
12870 -pf01814 -
12880 -pf02743pf00211pf00672pf07701 -
12890 -pf02660 -
12900 -pf11285 -
12910 Tdp_kelleyGreen CsomeBCy
12920 - -
12930 - -
12940 - -
12950 -pf18451 -
12960 - -
12970 - -
12980 -pf00350 -
12990 -pf01402 -
13000 -pf00490 -
13010 HMM01515PTA_B_1_eut1(pf07085pf01515) EUT3
13020 - -
13030 - -
(..15520..)
28560 HMMnopfamclust29_1_aci(pf04577) CsomeBCy
28570 HMMnopfamclust29_1_aci(pf07719pf13181) CsomeBCy
28590 -pf02152 -
28600 HMMreg00486_1_mix(pf00563pf00990pf00072) CsomeBCy
28610 HMMreg00486_1_mix(pf01590pf02518pf00512pf08447pf00072) CsomeE
28620 HMMreg00072_2_pdugrm(pf13426pf00072) CsomeBCy
28630 - -
28640 - -
28650 -pf00588pf08032 -
28670 -pf00717 -
28680 HMM13382deam_1_buf1(pf01979) BUF3
28690 H_peaSoup CsomeBCy
28700 H_lime CsomeBCy
28710 -pf00128 -
28720 -pf02938pf00152pf01336 -
28730 HMMreg00486_1_mix(pf02518pf00512pf00072) Cs
28740 -pf01116 -
28750 HMMreg00486_1_mix(pf02518pf00512) CsomeE
28760 -pf00353 -
28770 -pf17150pf10069 -
28780 -pf00805 -
28790 HMMnopfamclust29_1_aci(pf13181)
28800 -pf00903 -
28810 -pf01098 -
28820 -pf07007 -
28830 -pf03706 -

Chlorogloeopsis fritschii PCC 6912. Bacteria; Cyanobacteria; Nostocales; Chlorogloeopsidaceae; Chlorogloeopsis.

PCC6912__locus_1_main_sat

CsomeBCy

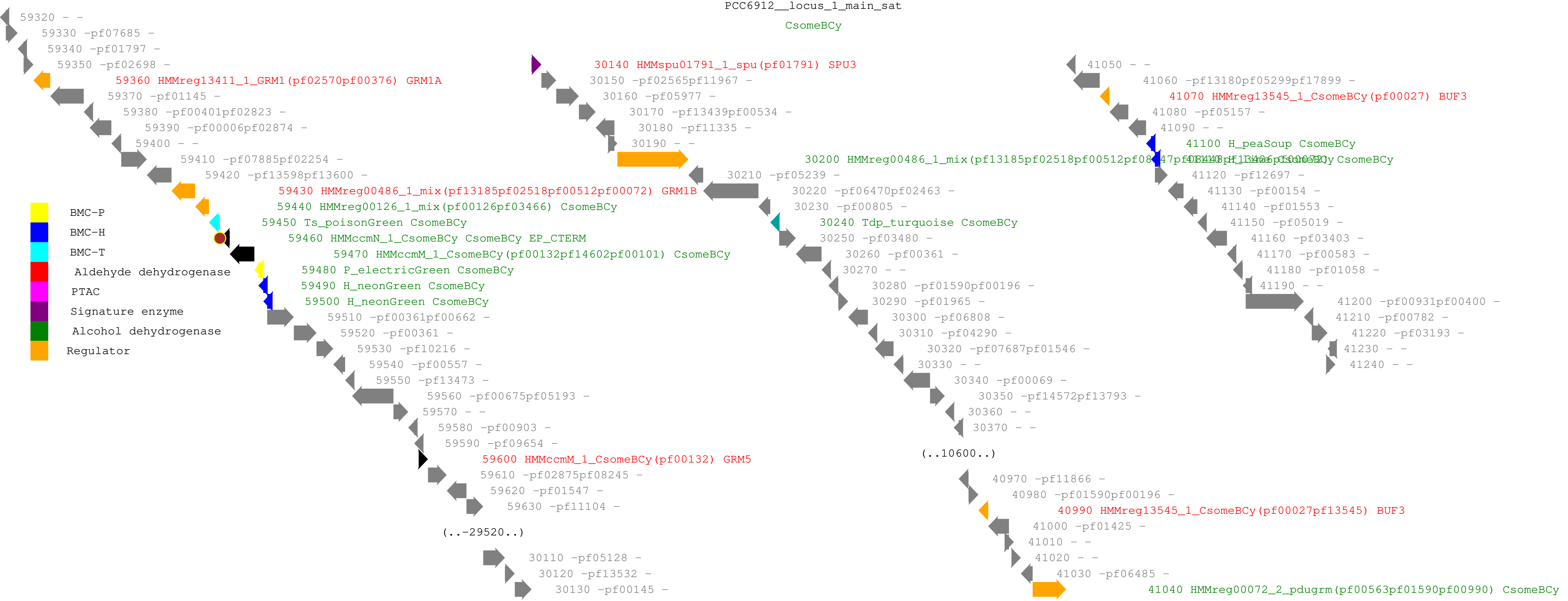

Halothece sp. (strain PCC 7418) (Synechococcus sp. (strain PCC 7418)). Bacteria; Cyanobacteria; Oscillatoriophycideae; Chroococcales;

PCC7418__locus_1_main_sat

CsomeBCy

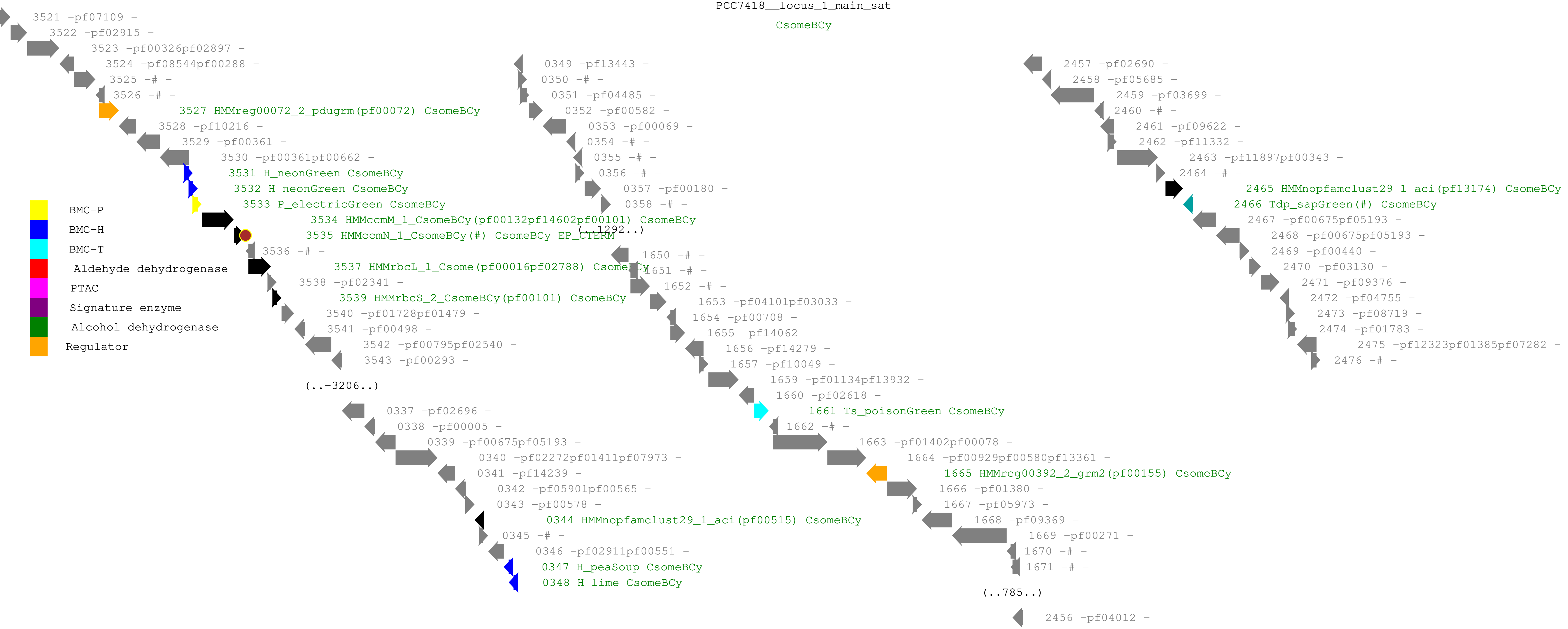

Cyanothece sp. (strain PCC 7424) (Synechococcus sp. (strain ATCC Bacteria; Cyanobacteria; Oscillatoriophycideae; Oscillatoriales;

PCC7424__locus_1_main_sat

CsomeBCy

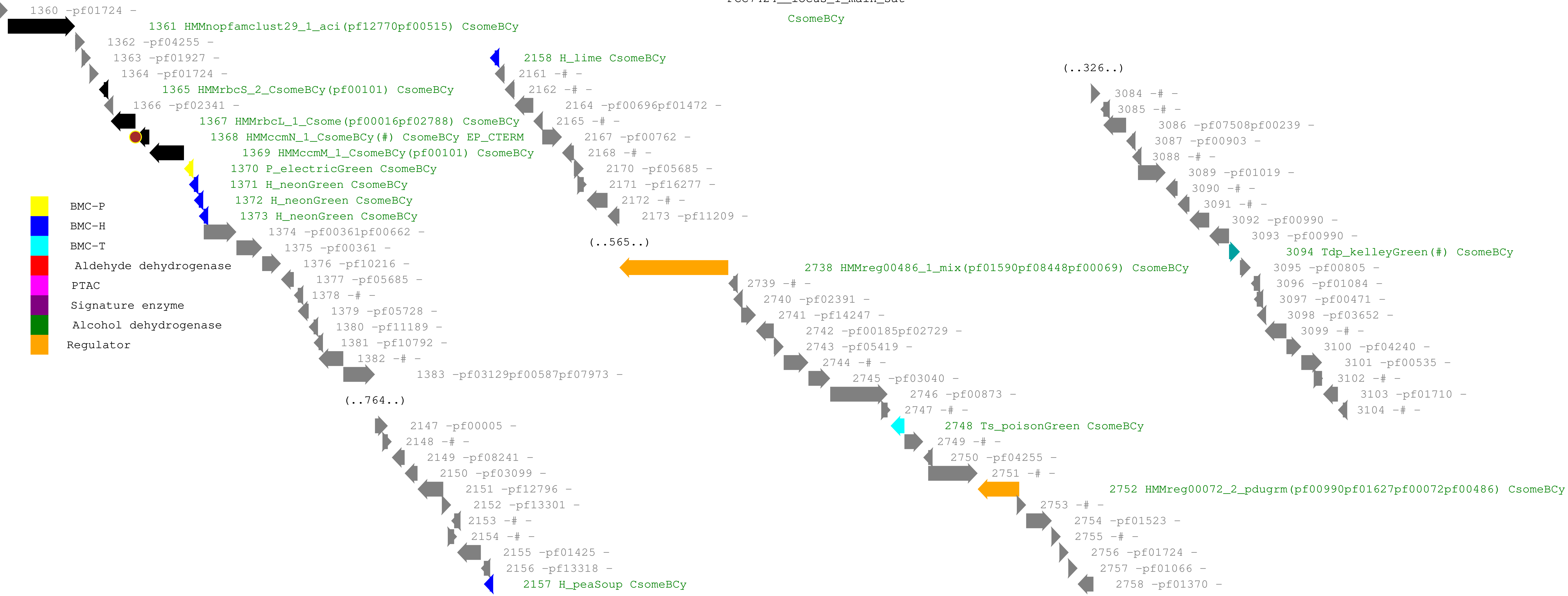

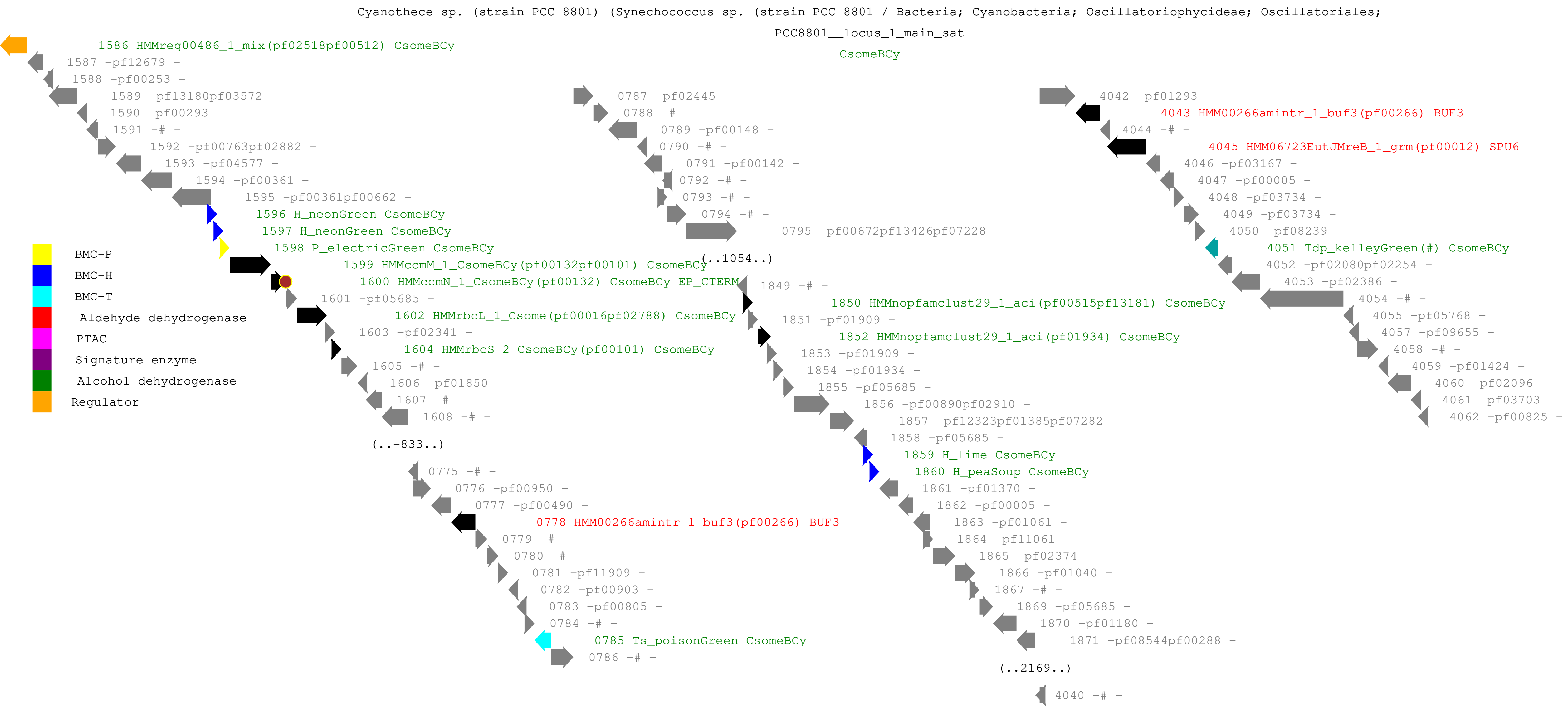
Cyanothece sp. (strain PCC 8801) (Synechococcus sp. (strain PCC 8801 / Bacteria; Cyanobacteria; Oscillatoriophycideae; Oscillatoriales;
PCC8801__locus_1_main_sat
CsomeBCy
1586 HMMreg00486_1_mix(pf02518pf00512) CsomeBCy
1587 -pf12679 -
1588 -pf00253 -
1589 -pf13180pf03572 -
1590 -pf00293 -
1591 -# -
1592 -pf00763pf02882 -
1593 -pf04577 -
1594 -pf00361 -
1595 -pf00361pf00662 -
1596 H_neonGreen CsomeBCy
1597 H_neonGreen CsomeBCy
1598 P_electricGreen CsomeBCy
1599 HMMccmM_1_CsomeBCy(pf00132pf00101) CsomeBCy
1600 HMMccmN_1_CsomeBCy(pf00132) CsomeBCy EP_CTERM
1601 -pf05685 -
1602 HMMrbcL_1_Csome(pf00016pf02788) CsomeBCy
1603 -pf02341 -
1604 HMMrbcS_2_CsomeBCy(pf00101) CsomeBCy
1605 -# -
1606 -pf01850 -
1607 -# -
1608 -# -
(..-833..)
0775 -# -
0776 -pf00950 -
0777 -pf00490 -
0778 HMM00266amintr_1_buf3(pf00266) BUF3
0779 -# -
0780 -# -
0781 -pf11909 -
0782 -pf00903 -
0783 -pf00805 -
0784 -# -
0785 Ts_poisonGreen CsomeBCy
0786 -# -
0787 -pf02445 -
0788 -# -
0789 -pf00148 -
0790 -# -
0791 -pf00142 -
0792 -# -
0793 -# -
0794 -# -
0795 -pf00672pf13426pf07228 -
(..1054..)
1849 -# -
1850 HMMnopfamclust29_1_aci(pf00515pf13181) CsomeBCy
1851 -pf01909 -
1852 HMMnopfamclust29_1_aci(pf01934) CsomeBCy
1853 -pf01909 -
1854 -pf01934 -
1855 -pf05685 -
1856 -pf00890pf02910 -
1857 -pf12323pf01385pf07282 -
1858 -pf05685 -
1859 H_lime CsomeBCy
1860 H_peaSoup CsomeBCy
1861 -pf01370 -
1862 -pf00005 -
1863 -pf01061 -
1864 -pf11061 -
1865 -pf02374 -
1866 -pf01040 -
1867 -# -
1869 -pf05685 -
1870 -pf01180 -
1871 -pf08544pf00288 -
(..2169..)
4040 -# -
4042 -pf01293 -
4043 HMM00266amintr_1_buf3(pf00266) BUF3
4044 -# -
4045 HMM06723EutJMreB_1_grm(pf00012) SPU6
4046 -pf03167 -
4047 -pf00005 -
4048 -pf03734 -
4049 -pf03734 -
4050 -pf08239 -
4051 Tdp_kelleyGreen(#) CsomeBCy
4052 -pf02080pf02254 -
4053 -pf02386 -
4054 -# -
4055 -pf05768 -
4057 -pf09655 -
4058 -# -
4059 -pf01424 -
4060 -pf02096 -
4061 -pf03703 -
4062 -pf00825 -
BMC-P
BMC-H
BMC-T
Aldehyde dehydrogenase
PTAC
Signature enzyme
Alcohol dehydrogenase
Regulator

Hassallia byssoidea VB512170. Bacteria; Cyanobacteria; Nostocales; Tolypothrichaceae; Hassallia.

PI95__locus_1_main_sat

CsomeBCy

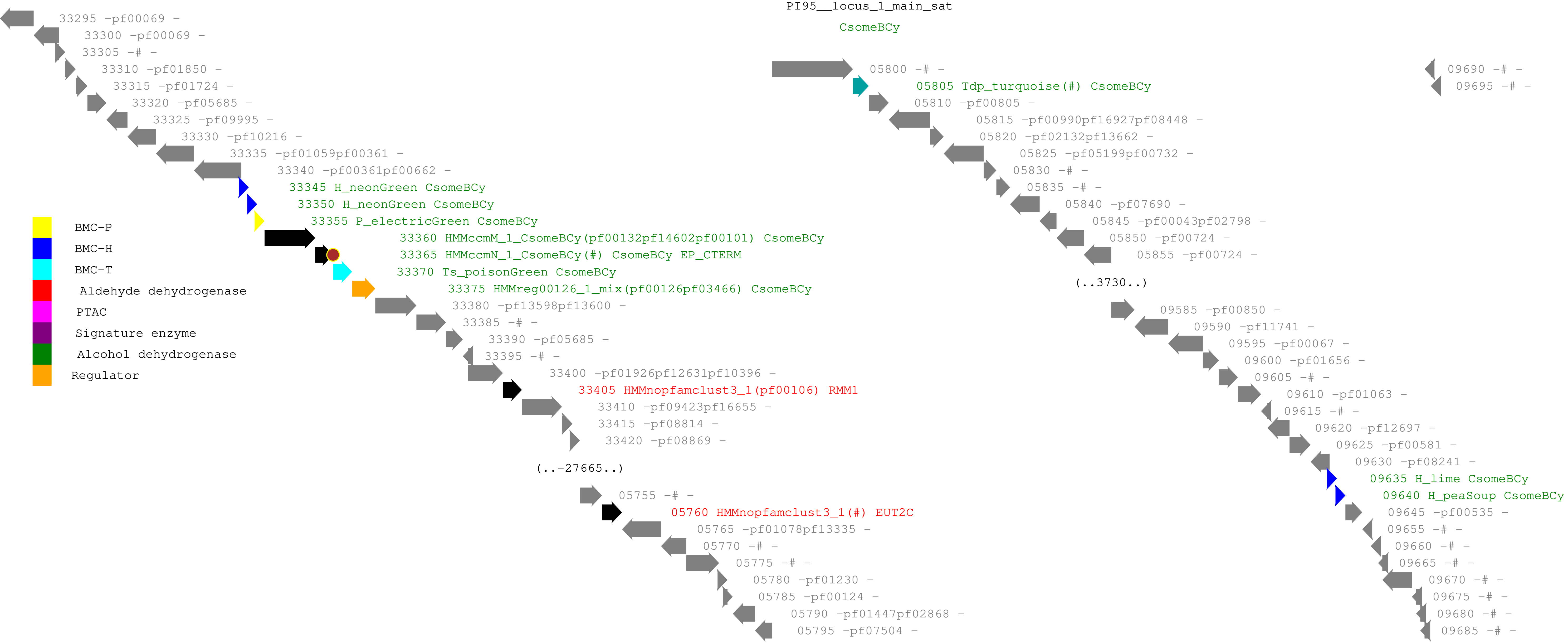

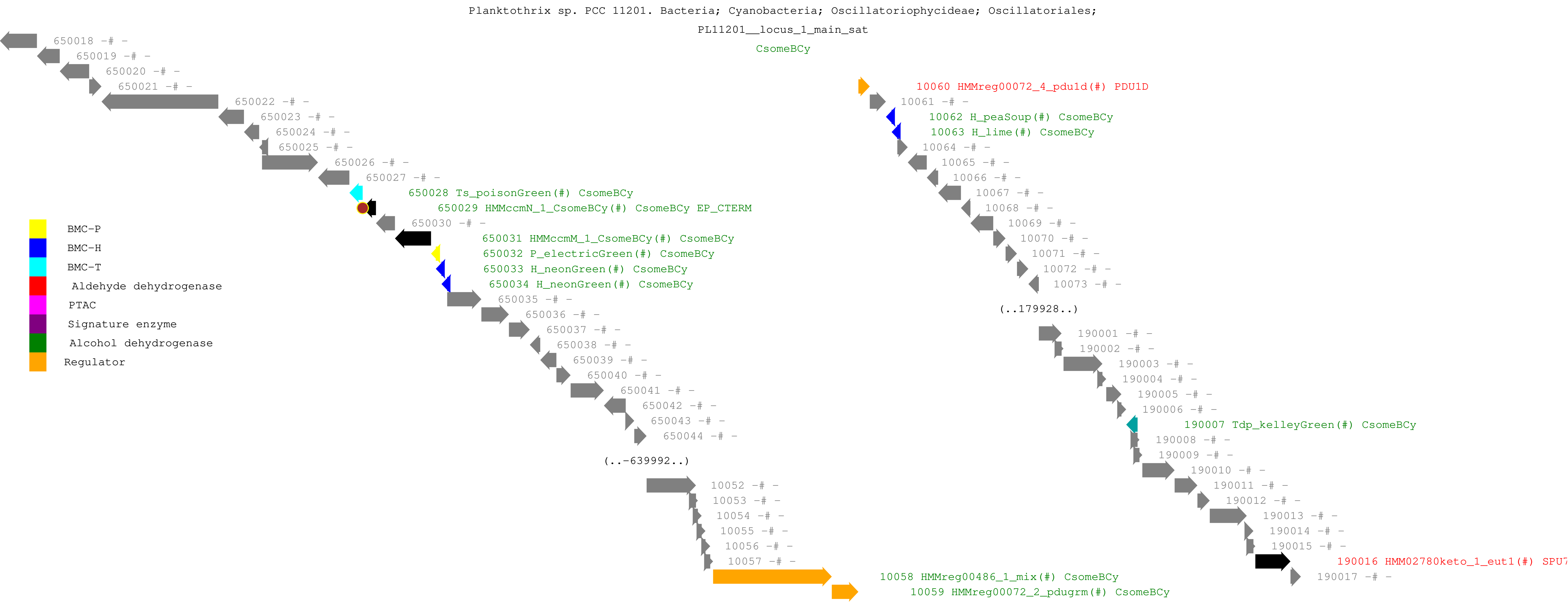

Planktothrix sp. PCC 11201. Bacteria; Cyanobacteria; Oscillatoriophycideae; Oscillatoriales;
PL11201__locus_1_main_sat
CsomeBCy
650018 -# -
650019 -# -
650020 -# -
650021 -# -
650022 -# -
650023 -# -
650024 -# -
650025 -# -
650026 -# -
650027 -# -
650028 Ts_poisonGreen(#) CsomeBCy
650029 HMMccmN_1_CsomeBCy(#) CsomeBCy EP_CTERM
650030 -# -
650031 HMMccmM_1_CsomeBCy(#) CsomeBCy
650032 P_electricGreen(#) CsomeBCy
650033 H_neonGreen(#) CsomeBCy
650034 H_neonGreen(#) CsomeBCy
650035 -# -
650036 -# -
650037 -# -
650038 -# -
650039 -# -
650040 -# -
650041 -# -
650042 -# -
650043 -# -
650044 -# -
(..-639992..)
10052 -# -
10053 -# -
10054 -# -
10055 -# -
10056 -# -
10057 -# -
10058 HMMreg00486_1_mix(#) CsomeBCy
10059 HMMreg00072_2_pdugrm(#) CsomeBCy
10060 HMMreg00072_4_pdu1d(#) PDU1D
10061 -# -
10062 H_peaSoup(#) CsomeBCy
10063 H_lime(#) CsomeBCy
10064 -# -
10065 -# -
10066 -# -
10067 -# -
10068 -# -
10069 -# -
10070 -# -
10071 -# -
10072 -# -
10073 -# -
(..179928..)
190001 -# -
190002 -# -
190003 -# -
190004 -# -
190005 -# -
190006 -# -
190007 Tdp_kelleyGreen(#) CsomeBCy
190008 -# -
190009 -# -
190010 -# -
190011 -# -
190012 -# -
190013 -# -
190014 -# -
190015 -# -
190016 HMM02780keto_1_eut1(#) SPU7
190017 -# -
BMC-P
BMC-H
BMC-T
Aldehyde dehydrogenase
PTAC
Signature enzyme
Alcohol dehydrogenase
Regulator

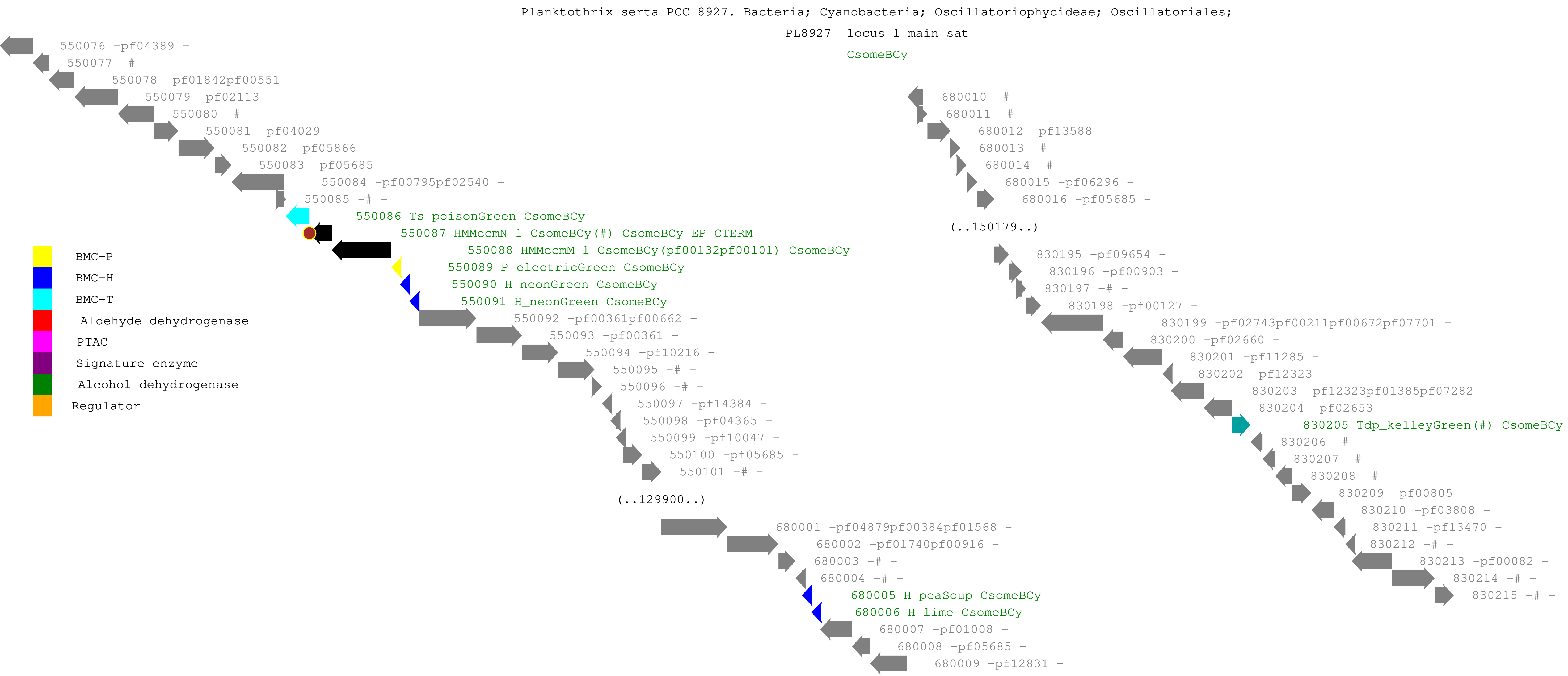
Planktothrix serta PCC 8927. Bacteria; Cyanobacteria; Oscillatoriophycideae; Oscillatoriales;
PL8927__locus_1_main_sat
CsomeBCy
550076 -pf04389 -
550077 -# -
550078 -pf01842pf00551 -
550079 -pf02113 -
550080 -# -
550081 -pf04029 -
550082 -pf05866 -
550083 -pf05685 -
550084 -pf00795pf02540 -
550085 -# -
550086 Ts_poisonGreen CsomeBCy
550087 HMMccmN_1_CsomeBCy(#) CsomeBCy EP_CTERM
550088 HMMccmM_1_CsomeBCy(pf00132pf00101) CsomeBCy
550089 P_electricGreen CsomeBCy
550090 H_neonGreen CsomeBCy
550091 H_neonGreen CsomeBCy
550092 -pf00361pf00662 -
550093 -pf00361 -
550094 -pf10216 -
550095 -# -
550096 -# -
550097 -pf14384 -
550098 -pf04365 -
550099 -pf10047 -
550100 -pf05685 -
550101 -# -
(..129900..)
680001 -pf04879pf00384pf01568 -
680002 -pf01740pf00916 -
680003 -# -
680004 -# -
680005 H_peaSoup CsomeBCy
680006 H_lime CsomeBCy
680007 -pf01008 -
680008 -pf05685 -
680009 -pf12831 -
680010 -# -
680011 -# -
680012 -pf13588 -
680013 -# -
680014 -# -
680015 -pf06296 -
680016 -pf05685 -
(..150179..)
830195 -pf09654 -
830196 -pf00903 -
830197 -# -
830198 -pf00127 -
830199 -pf02743pf00211pf00672pf07701 -
830200 -pf02660 -
830201 -pf11285 -
830202 -pf12323 -
830203 -pf12323pf01385pf07282 -
830204 -pf02653 -
830205 Tdp_kelleyGreen(#) CsomeBCy
830206 -# -
830207 -# -
830208 -# -
830209 -pf00805 -
830210 -pf03808 -
830211 -pf13470 -
830212 -# -
830213 -pf00082 -
830214 -# -
830215 -# -
BMC-P
BMC-H
BMC-T
Aldehyde dehydrogenase
PTAC
Signature enzyme
Alcohol dehydrogenase
Regulator

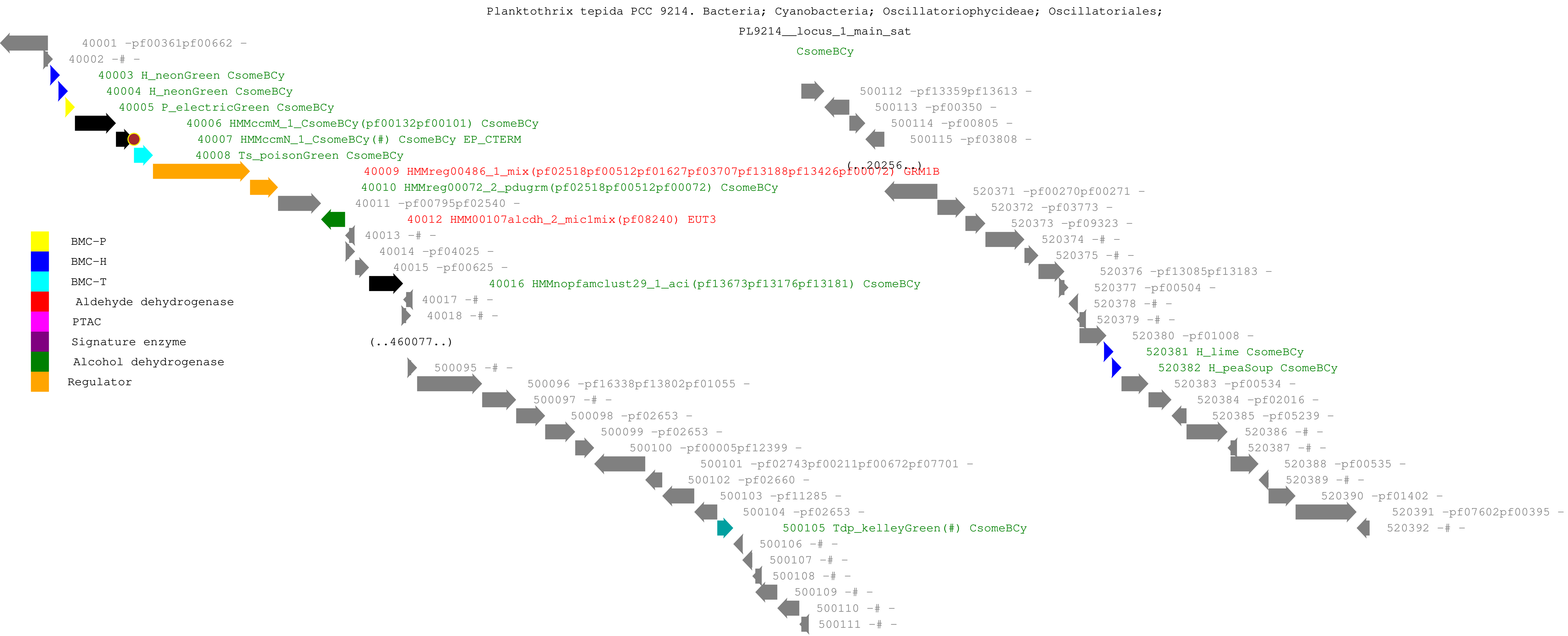
Planktothrix tepida PCC 9214. Bacteria; Cyanobacteria; Oscillatoriophycideae; Oscillatoriales;
PL9214__locus_1_main_sat
CsomeBCy
40001 -pf00361pf00662 -
40002 -# -
40003 H_neonGreen CsomeBCy
40004 H_neonGreen CsomeBCy
40005 P_electricGreen CsomeBCy
40006 HMMccmM_1_CsomeBCy(pf00132pf00101) CsomeBCy
40007 HMMccmN_1_CsomeBCy(#) CsomeBCy EP_CTERM
40008 Ts_poisonGreen CsomeBCy
40009 HMMreg00486_1_mix(pf02518pf00512pf01627pf03707pf13188pf13426pf00072) GRM1B
40010 HMMreg00072_2_pdugrm(pf02518pf00512pf00072) CsomeBCy
40011 -pf00795pf02540 -
40012 HMM00107alcdh_2_mic1mix(pf08240) EUT3
40013 -# -
40014 -pf04025 -
40015 -pf00625 -
40016 HMMnopfamclust29_1_aci(pf13673pf13176pf13181) CsomeBCy
40017 -# -
40018 -# -
(..460077..)
500095 -# -
500096 -pf16338pf13802pf01055 -
500097 -# -
500098 -pf02653 -
500099 -pf02653 -
500100 -pf00005pf12399 -
500101 -pf02743pf00211pf00672pf07701 -
500102 -pf02660 -
500103 -pf11285 -
500104 -pf02653 -
500105 Tdp_kelleyGreen(#) CsomeBCy
500106 -# -
500107 -# -
500108 -# -
500109 -# -
500110 -# -
500111 -# -
500112 -pf13359pf13613 -
500113 -pf00350 -
500114 -pf00805 -
500115 -pf03808 -
(..20256..)
520371 -pf00270pf00271 -
520372 -pf03773 -
520373 -pf09323 -
520374 -# -
520375 -# -
520376 -pf13085pf13183 -
520377 -pf00504 -
520378 -# -
520379 -# -
520380 -pf01008 -
520381 H_lime CsomeBCy
520382 H_peaSoup CsomeBCy
520383 -pf00534 -
520384 -pf02016 -
520385 -pf05239 -
520386 -# -
520387 -# -
520388 -pf00535 -
520389 -# -
520390 -pf01402 -
520391 -pf07602pf00395 -
520392 -# -
BMC-P
BMC-H
BMC-T
Aldehyde dehydrogenase
PTAC
Signature enzyme
Alcohol dehydrogenase
Regulator

Planktothrix paucivesiculata PCC 9631. Bacteria; Cyanobacteria; Oscillatoriophycideae; Oscillatoriales;

PL9631__locus_1_main_sat

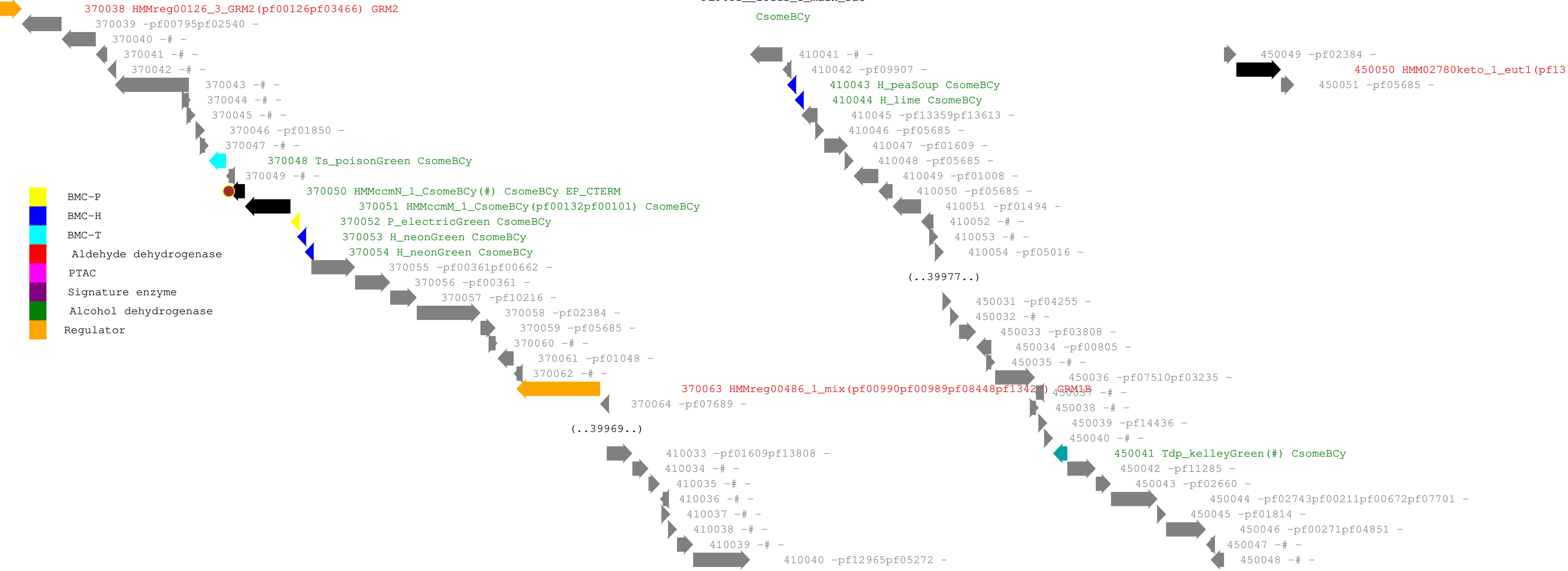

Planktothrix agardhii (Oscillatoria agardhii). Bacteria; Cyanobacteria; Oscillatoriophycideae; Oscillatoriales;

PLAM__locus_1_main_sat

CsomeBCy

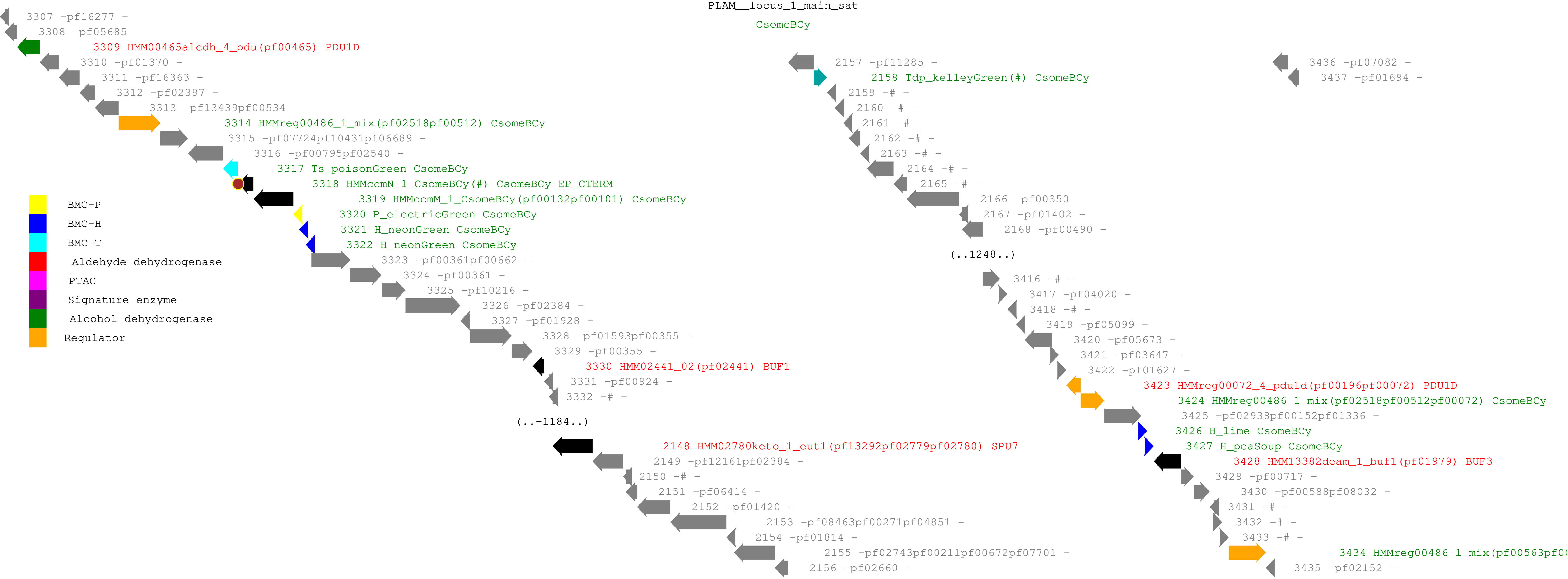

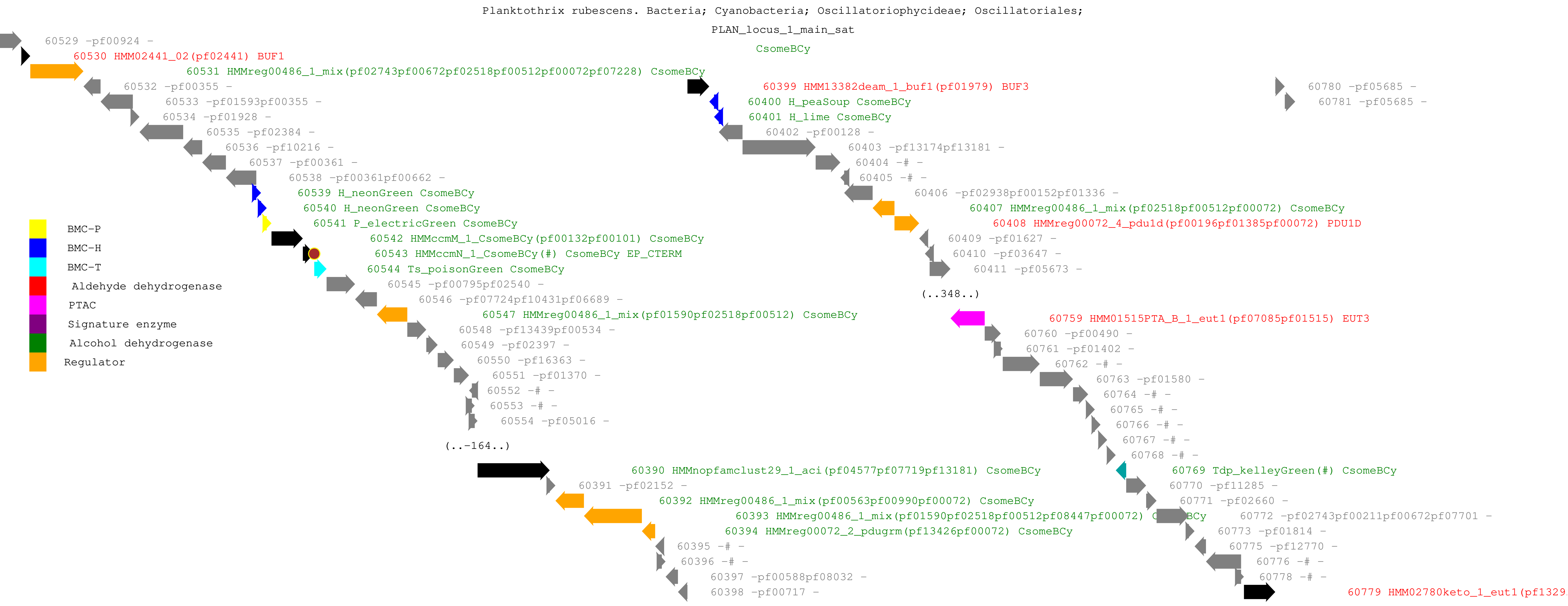

Planktothrix rubescens. Bacteria; Cyanobacteria; Oscillatoriophycideae; Oscillatoriales;
PLAN_locus_1_main_sat
CsomeBCy
60529 -pf00924 -
60530 HMM02441_02(pf02441) BUF1
60531 HMMreg00486_1_mix(pf02743pf00672pf02518pf00512pf00072pf07228) CsomeBCy
60532 -pf00355 -
60533 -pf01593pf00355 -
60534 -pf01928 -
60535 -pf02384 -
60536 -pf10216 -
60537 -pf00361 -
60538 -pf00361pf00662 -
60539 H_neonGreen CsomeBCy
60540 H_neonGreen CsomeBCy
60541 P_electricGreen CsomeBCy
60542 HMMccmM_1_CsomeBCy(pf00132pf00101) CsomeBCy
60543 HMMccmN_1_CsomeBCy(#) CsomeBCy EP_CTERM
60544 Ts_poisonGreen CsomeBCy
60545 -pf00795pf02540 -
60546 -pf07724pf10431pf06689 -
60547 HMMreg00486_1_mix(pf01590pf02518pf00512) CsomeBCy
60548 -pf13439pf00534 -
60549 -pf02397 -
60550 -pf16363 -
60551 -pf01370 -
60552 -# -
60553 -# -
60554 -pf05016 -
(..-164..)
60390 HMMnopfamclust29_1_aci(pf04577pf07719pf13181) CsomeBCy
60391 -pf02152 -
60392 HMMreg00486_1_mix(pf00563pf00990pf00072) CsomeBCy
60393 HMMreg00486_1_mix(pf01590pf02518pf00512pf08447pf00072) CsomeBCy
60394 HMMreg00072_2_pdugrm(pf13426pf00072) CsomeBCy
60395 -# -
60396 -# -
60397 -pf00588pf08032 -
60398 -pf00717 -
60399 HMM13382deam_1_buf1(pf01979) BUF3
60400 H_peaSoup CsomeBCy
60401 H_lime CsomeBCy
60402 -pf00128 -
60403 -pf13174pf13181 -
60404 -# -
60405 -# -
60406 -pf02938pf00152pf01336 -
60407 HMMreg00486_1_mix(pf02518pf00512pf00072) CsomeBCy
60408 HMMreg00072_4_pdu1d(pf00196pf01385pf00072) PDU1D
60409 -pf01627 -
60410 -pf03647 -
60411 -pf05673 -
(..348..)
60759 HMM01515PTA_B_1_eut1(pf07085pf01515) EUT3
60760 -pf00490 -
60761 -pf01402 -
60762 -# -
60763 -pf01580 -
60764 -# -
60765 -# -
60766 -# -
60767 -# -
60768 -# -
60769 Tdp_kelleyGreen(#) CsomeBCy
60770 -pf11285 -
60771 -pf02660 -
60772 -pf02743pf00211pf00672pf07701 -
60773 -pf01814 -
60775 -pf12770 -
60776 -# -
60778 -# -
60779 HMM02780keto_1_eut1(pf1329
60780 -pf05685 -
60781 -pf05685 -
BMC-P
BMC-H
BMC-T
Aldehyde dehydrogenase
PTAC
Signature enzyme
Alcohol dehydrogenase
Regulator

Pleurocapsa sp. PCC 7327. Bacteria; Cyanobacteria; Pleurocapsales; Hyellaceae; Pleurocapsa.

Ple7327__locus_1_main_sat

CsomeBCy

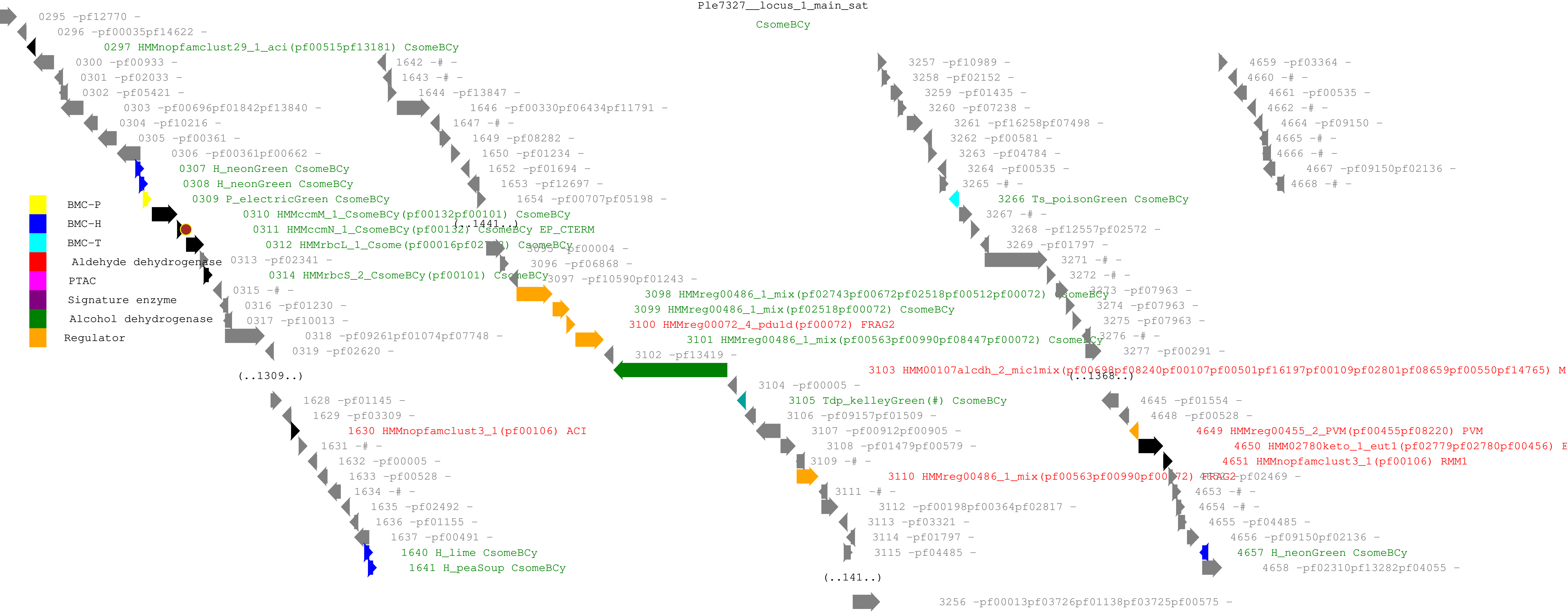

Prochlorothrix hollandica PCC 9006 = CALU 1027. Bacteria; Cyanobacteria; Synechococcales; Pseudanabaenaceae;

PROH__locus_1_main_sat

CsomeBCy

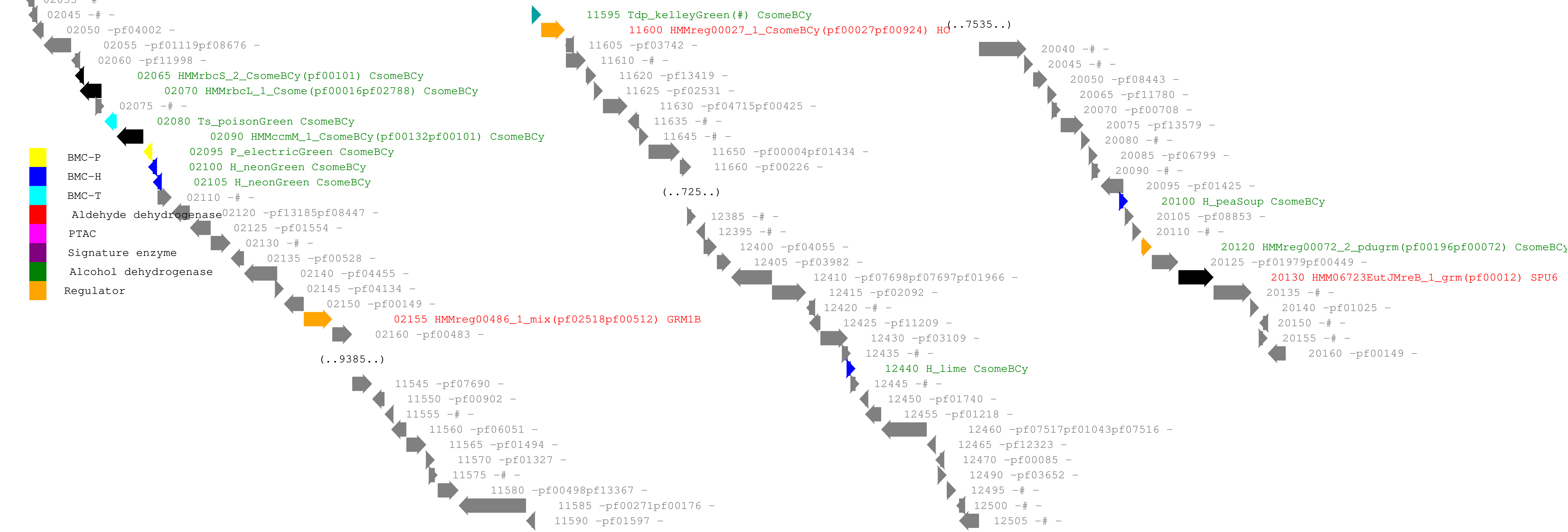

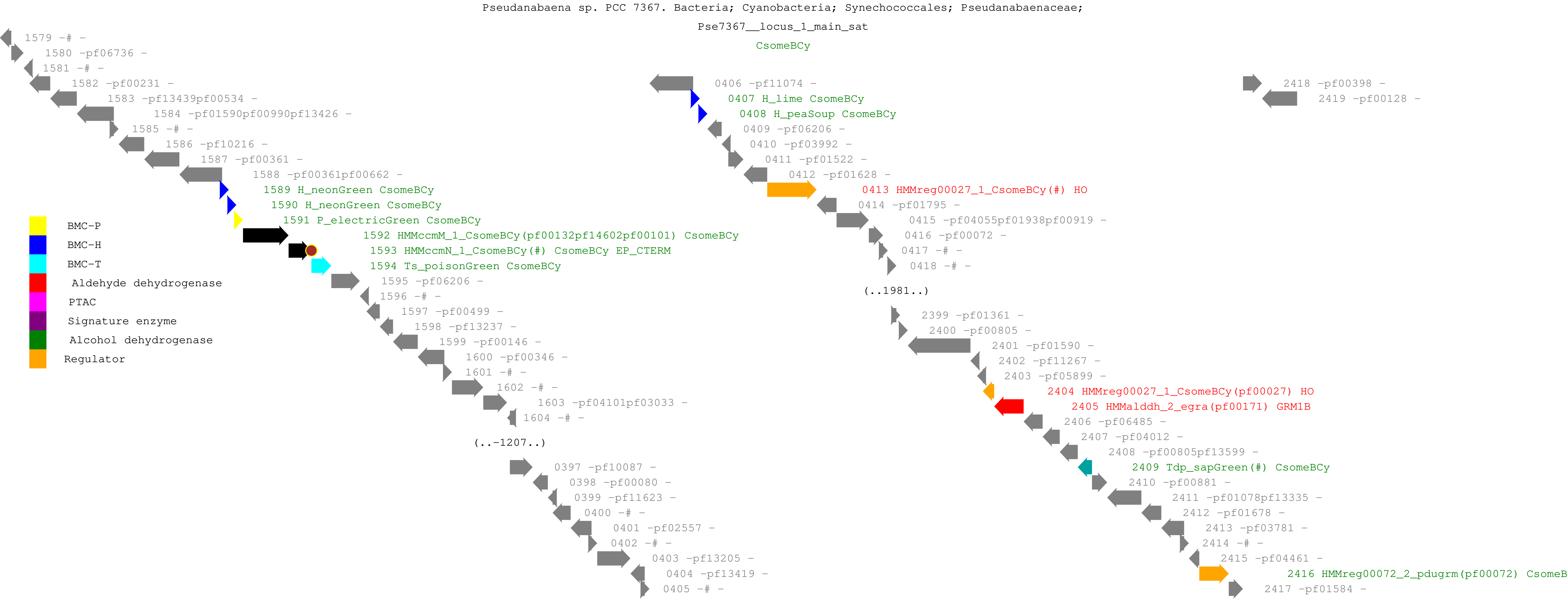

Pseudanabaena sp. PCC 7367. Bacteria; Cyanobacteria; Synechococcales; Pseudanabaenaceae;
Pse7367__locus_1_main_sat
CsomeBCy
1579 -# -
1580 -pf06736 -
1581 -# -
1582 -pf00231 -
1583 -pf13439pf00534 -
1584 -pf01590pf00990pf13426 -
1585 -# -
1586 -pf10216 -
1587 -pf00361 -
1588 -pf00361pf00662 -
1589 H_neonGreen CsomeBCy
1590 H_neonGreen CsomeBCy
1591 P_electricGreen CsomeBCy
1592 HMMccmM_1_CsomeBCy(pf00132pf14602pf00101) CsomeBCy
1593 HMMccmN_1_CsomeBCy(#) CsomeBCy EP_CTERM
1594 Ts_poisonGreen CsomeBCy
1595 -pf06206 -
1596 -# -
1597 -pf00499 -
1598 -pf13237 -
1599 -pf00146 -
1600 -pf00346 -
1601 -# -
1602 -# -
1603 -pf04101pf03033 -
1604 -# -
(..-1207..)
0397 -pf10087 -
0398 -pf00080 -
0399 -pf11623 -
0400 -# -
0401 -pf02557 -
0402 -# -
0403 -pf13205 -
0404 -pf13419 -
0405 -# -
BMC-P
BMC-H
BMC-T
Aldehyde dehydrogenase
PTAC
Signature enzyme
Alcohol dehydrogenase
Regulator
0406 -pf11074 -
0407 H_lime CsomeBCy
0408 H_peaSoup CsomeBCy
0409 -pf06206 -
0410 -pf03992 -
0411 -pf01522 -
0412 -pf01628 -
0413 HMMreg00027_1_CsomeBCy(#) HO
0414 -pf01795 -
0415 -pf04055pf01938pf00919 -
0416 -pf00072 -
0417 -# -
0418 -# -
(..1981..)
2399 -pf01361 -
2400 -pf00805 -
2401 -pf01590 -
2402 -pf11267 -
2403 -pf05899 -
2404 HMMreg00027_1_CsomeBCy(pf00027) HO
2405 HMMaldh_2_egra(pf00171) GRM1B
2406 -pf06485 -
2407 -pf04012 -
2408 -pf00805pf13599 -
2409 Tdp_sapGreen(#) CsomeBCy
2410 -pf00881 -
2411 -pf01078pf13335 -
2412 -pf01678 -
2413 -pf03781 -
2414 -# -
2415 -pf04461 -
2416 HMMreg00072_2_pdugrm(pf00072) CsomeB
2417 -pf01584 -
2418 -pf00398 -
2419 -pf00128 -

Pseudanabaena biceps PCC 7429. Bacteria; Cyanobacteria; Synechococcales; Pseudanabaenaceae;

Pse7429DRAFT__locus_1_main_sat

CsomeBCy

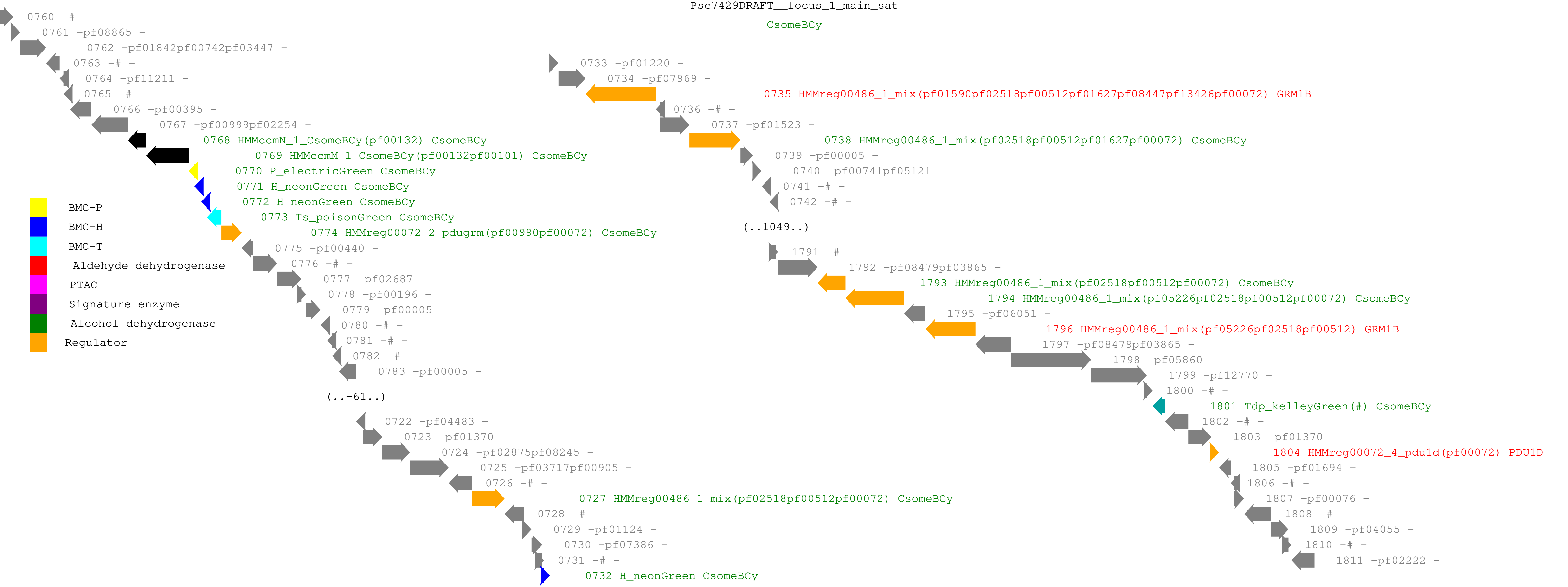

Scytonema millei VB511283. Bacteria; Cyanobacteria; Nostocales; Scytonemataceae; Scytonema.

QH73__locus_1_main_sat

CsomeBCy

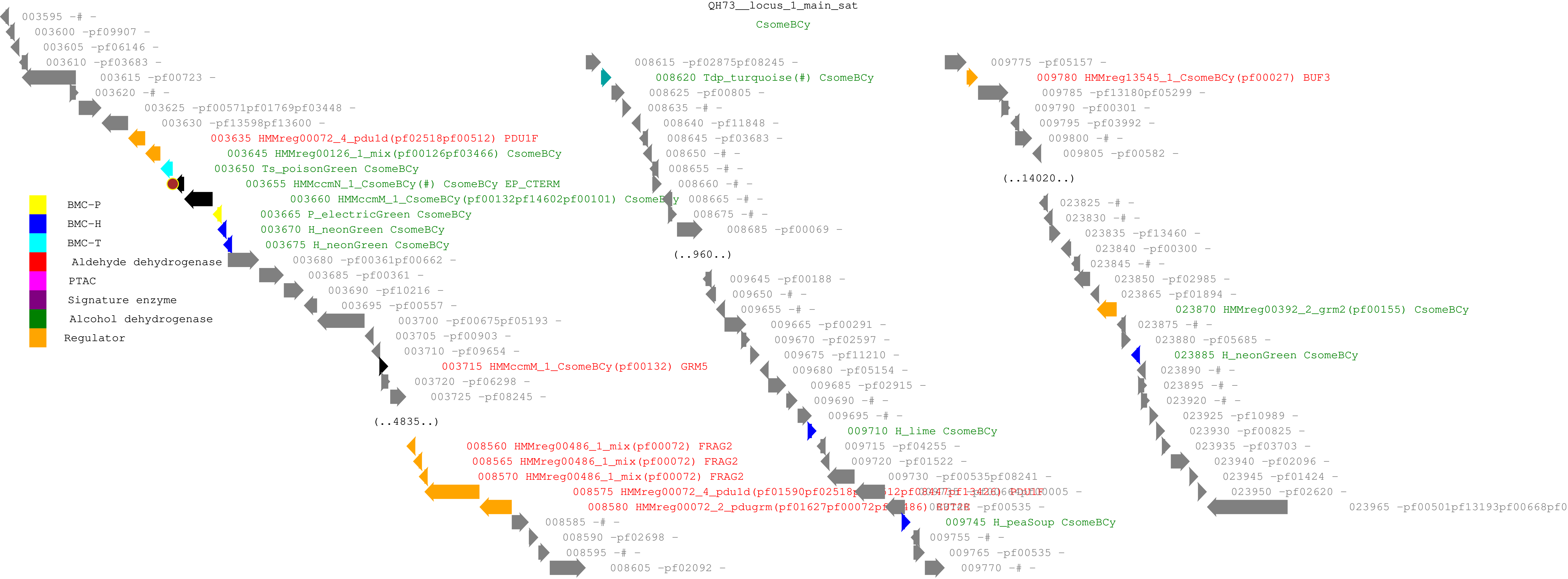

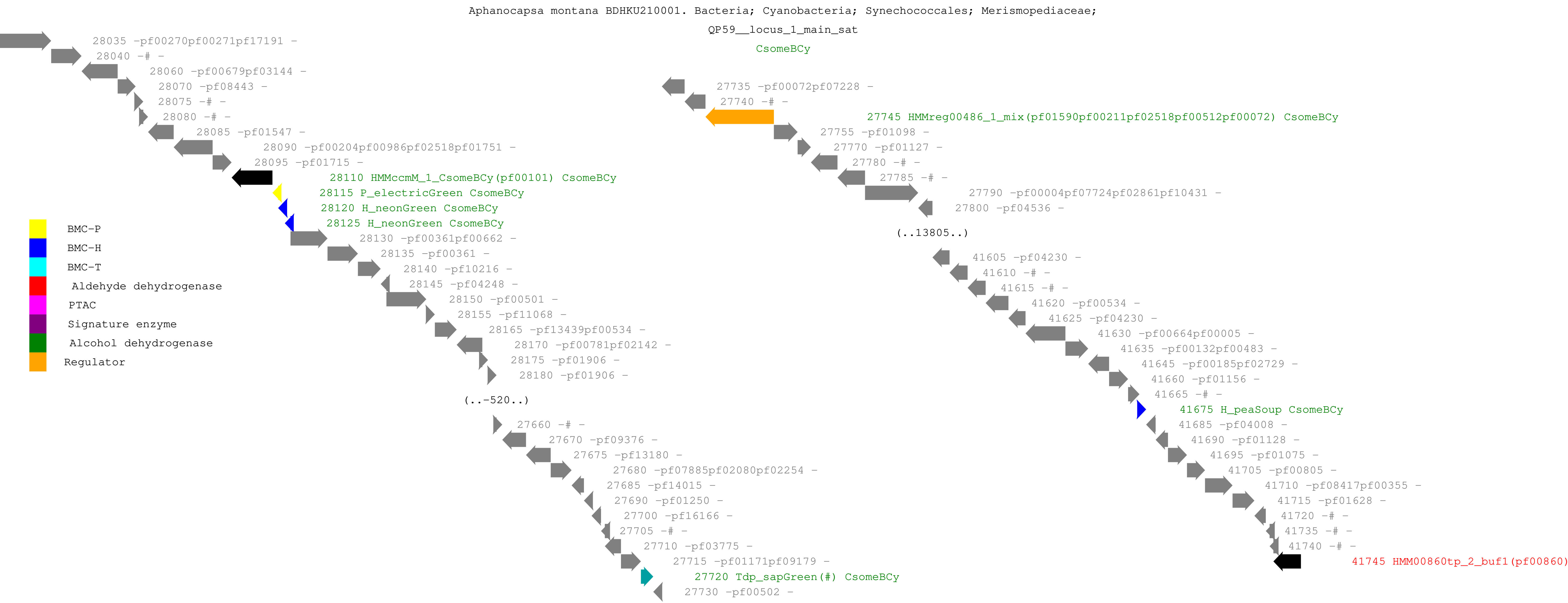
Aphanocapsa montana BDHKU210001. Bacteria; Cyanobacteria; Synechococcales; Merismopediaceae;
QP59__locus_1_main_sat
CsomeBCy
28035 -pf00270pf00271pf17191 -
28040 -# -
28060 -pf00679pf03144 -
28070 -pf08443 -
28075 -# -
28080 -# -
28085 -pf01547 -
28090 -pf00204pf00986pf02518pf01751 -
28095 -pf01715 -
28110 HMMccmM_1_CsomeBCy(pf00101) CsomeBCy
28115 P_electricGreen CsomeBCy
28120 H_neonGreen CsomeBCy
28125 H_neonGreen CsomeBCy
28130 -pf00361pf00662 -
28135 -pf00361 -
28140 -pf10216 -
28145 -pf04248 -
28150 -pf00501 -
28155 -pf11068 -
28165 -pf13439pf00534 -
28170 -pf00781pf02142 -
28175 -pf01906 -
28180 -pf01906 -
(..-520..)
27660 -# -
27670 -pf09376 -
27675 -pf13180 -
27680 -pf07885pf02080pf02254 -
27685 -pf14015 -
27690 -pf01250 -
27700 -pf16166 -
27705 -# -
27710 -pf03775 -
27715 -pf01171pf09179 -
27720 Tdp_sapGreen(#) CsomeBCy
27730 -pf00502 -
27735 -pf00072pf07228 -
27740 -# -
27745 HMMreg00486_1_mix(pf01590pf00211pf02518pf00512pf00072) CsomeBCy
27755 -pf01098 -
27770 -pf01127 -
27780 -# -
27785 -# -
27790 -pf00004pf07724pf02861pf10431 -
27800 -pf04536 -
(..13805..)
41605 -pf04230 -
41610 -# -
41615 -# -
41620 -pf00534 -
41625 -pf04230 -
41630 -pf00664pf00005 -
41635 -pf00132pf00483 -
41645 -pf00185pf02729 -
41660 -pf01156 -
41665 -# -
41675 H_peaSoup CsomeBCy
41685 -pf04008 -
41690 -pf01128 -
41695 -pf01075 -
41705 -pf00805 -
41710 -pf08417pf00355 -
41715 -pf01628 -
41720 -# -
41735 -# -
41740 -# -
41745 HMM00860tp_2_buf1(pf00860)
BMC-P
BMC-H
BMC-T
Aldehyde dehydrogenase
PTAC
Signature enzyme
Alcohol dehydrogenase
Regulator

Lyngbya confervoides BDU141951. Bacteria; Cyanobacteria; Oscillatoriophycideae; Oscillatoriales;
QQ91__locus_1_main_sat
CsomeBCy

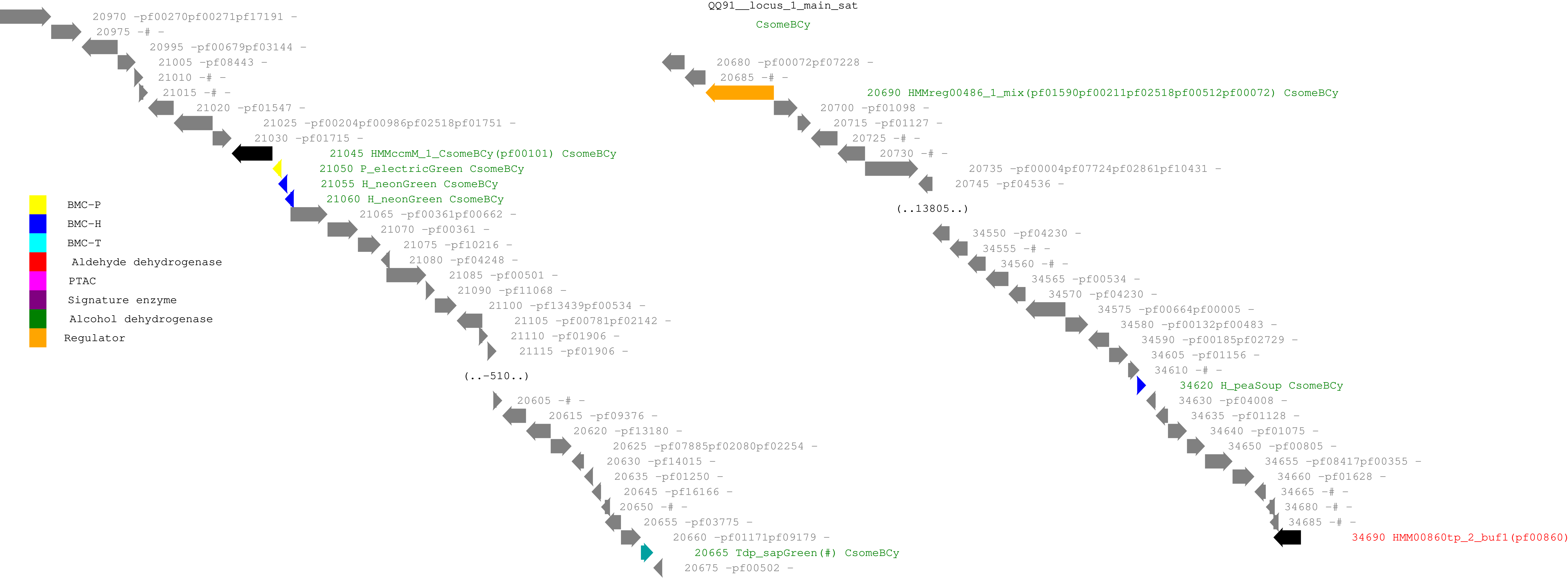

Richelia intracellularis HH01. Bacteria; Cyanobacteria; Nostocales; Nostocaceae; Richelia.

RINTHH__locus_1_main_sat

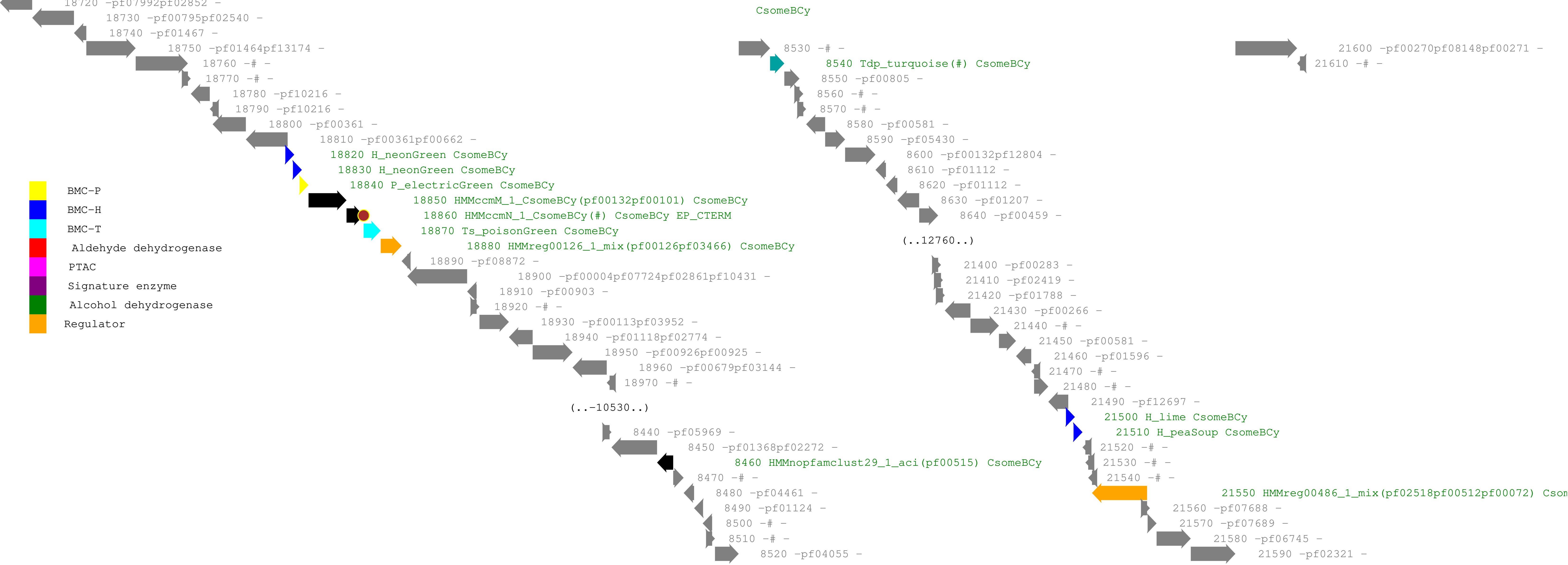

Richelia intracellularis HM01. Bacteria; Cyanobacteria; Nostocales; Nostocaceae; Richelia.

RINTHM__locus_fragmented

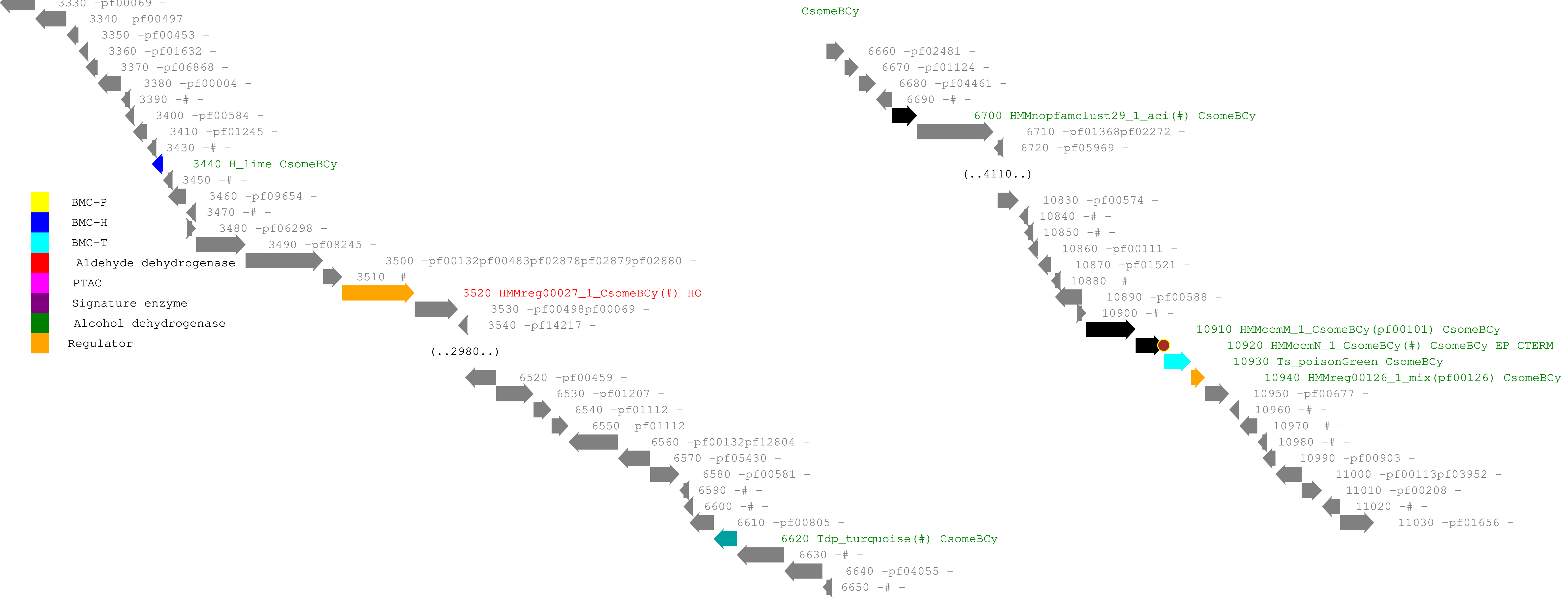

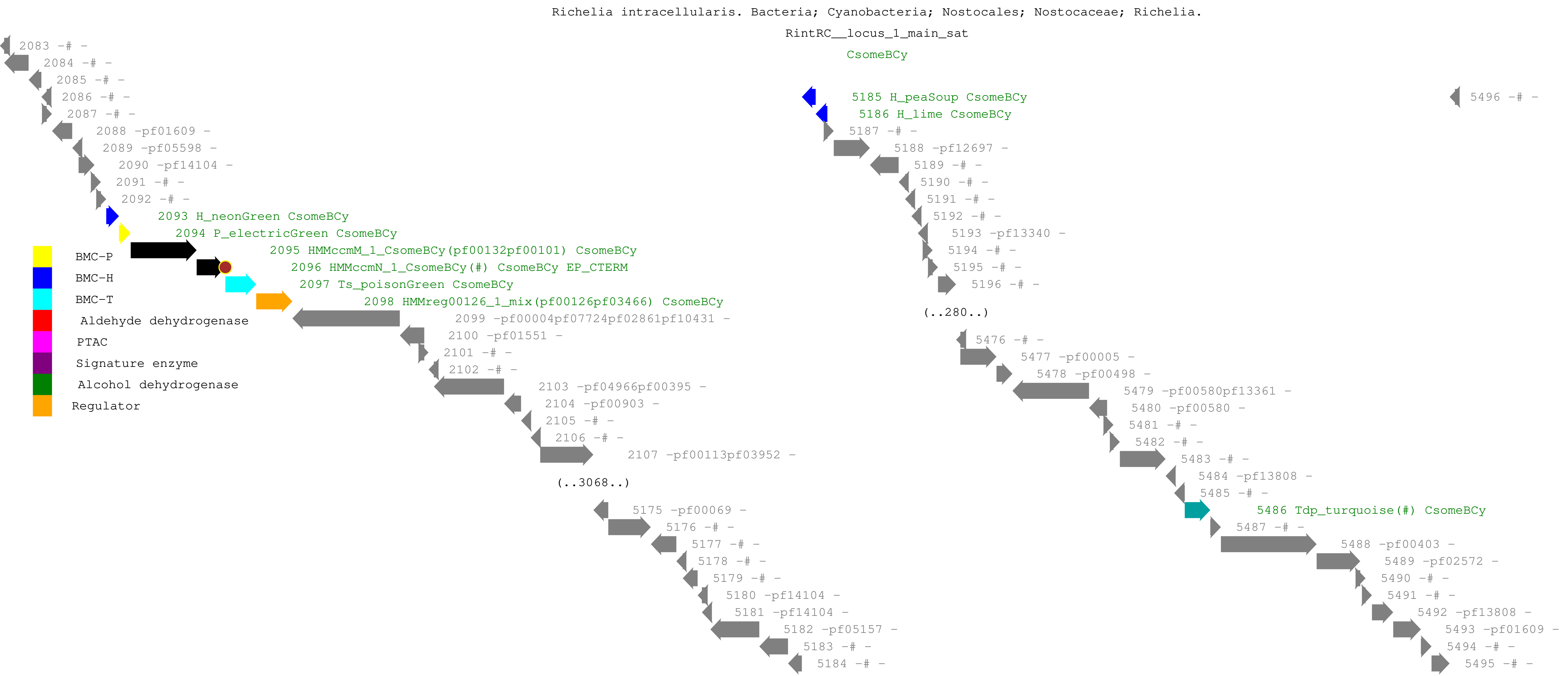
Richelia intracellularis. Bacteria; Cyanobacteria; Nostocales; Nostocaceae; Richelia.
RintRC__locus_1_main_sat
CsomeBCy
2083 -# -
2084 -# -
2085 -# -
2086 -# -
2087 -# -
2088 -pf01609 -
2089 -pf05598 -
2090 -pf14104 -
2091 -# -
2092 -# -
2093 H_neonGreen CsomeBCy
2094 P_electricGreen CsomeBCy
2095 HMMccmM_1_CsomeBCy(pf00132pf00101) CsomeBCy
2096 HMMccmN_1_CsomeBCy(#) CsomeBCy EP_CTERM
2097 Ts_poisonGreen CsomeBCy
2098 HMMreg00126_1_mix(pf00126pf03466) CsomeBCy
2099 -pf00004pf07724pf02861pf10431 -
2100 -pf01551 -
2101 -# -
2102 -# -
2103 -pf04966pf00395 -
2104 -pf00903 -
2105 -# -
2106 -# -
2107 -pf00113pf03952 -
(..3068..)
5175 -pf00069 -
5176 -# -
5177 -# -
5178 -# -
5179 -# -
5180 -pf14104 -
5181 -pf14104 -
5182 -pf05157 -
5183 -# -
5184 -# -
5185 H_peaSoup CsomeBCy
5186 H_lime CsomeBCy
5187 -# -
5188 -pf12697 -
5189 -# -
5190 -# -
5191 -# -
5192 -# -
5193 -pf13340 -
5194 -# -
5195 -# -
5196 -# -
(..280..)
5476 -# -
5477 -pf00005 -
5478 -pf00498 -
5479 -pf00580pf13361 -
5480 -pf00580 -
5481 -# -
5482 -# -
5483 -# -
5484 -pf13808 -
5485 -# -
5486 Tdp_turquoise(#) CsomeBCy
5487 -# -
5488 -pf00403 -
5489 -pf02572 -
5490 -# -
5491 -# -
5492 -pf13808 -
5493 -pf01609 -
5494 -# -
5495 -# -
5496 -# -
BMC-P
BMC-H
BMC-T
Aldehyde dehydrogenase
PTAC
Signature enzyme
Alcohol dehydrogenase
Regulator

Rivularia sp. PCC 7116. Bacteria; Cyanobacteria; Nostocales; Rivulariaceae; Rivularia.

Riv7116__locus_1_main_sat

CsomeBCy

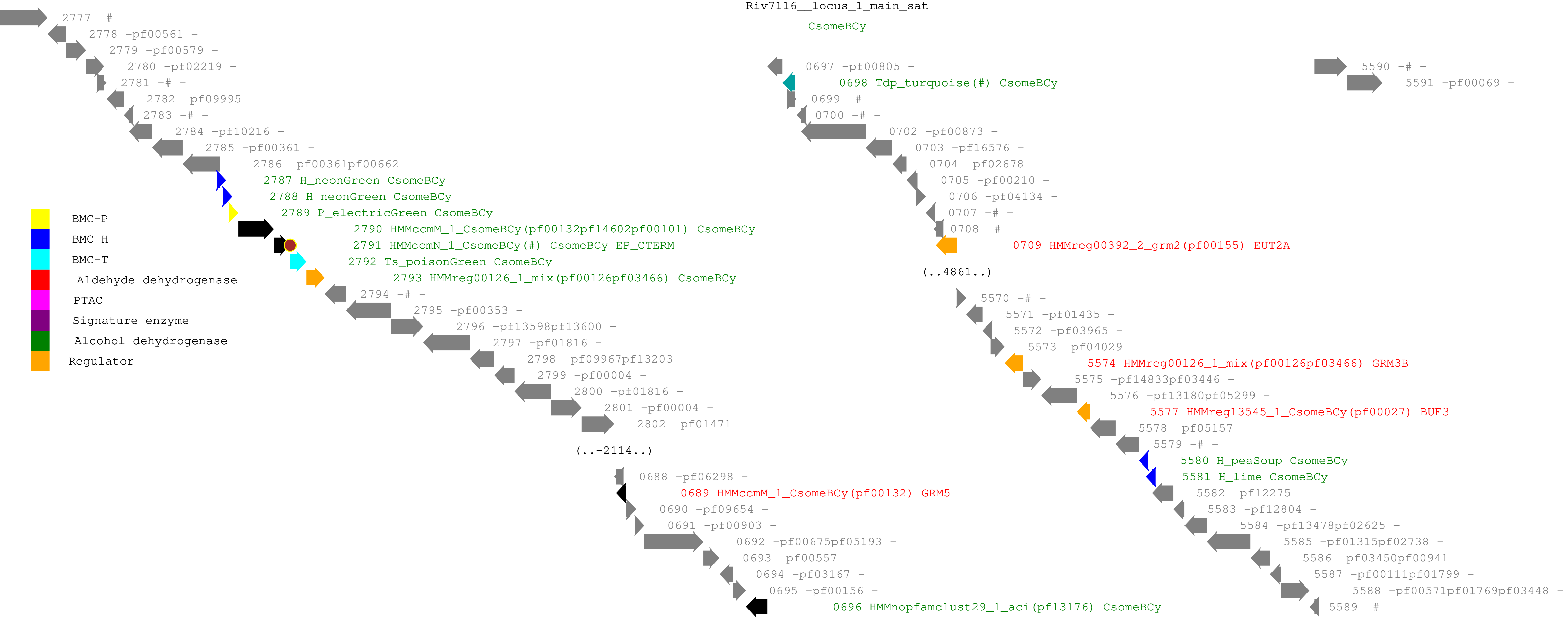

Scytonema sp. HK-05. Bacteria; Cyanobacteria; Nostocales; Scytonemataceae; Scytonema.

SAMD00079811__locus_1_main_sat

CsomeBCy

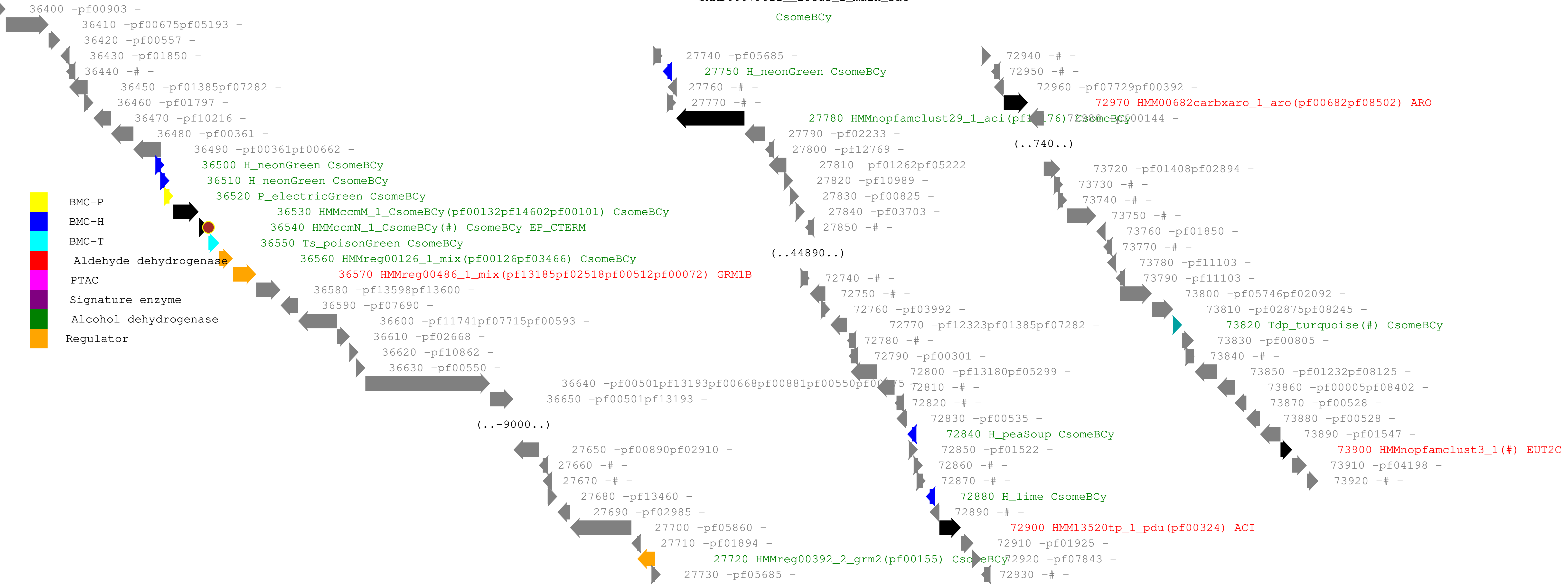

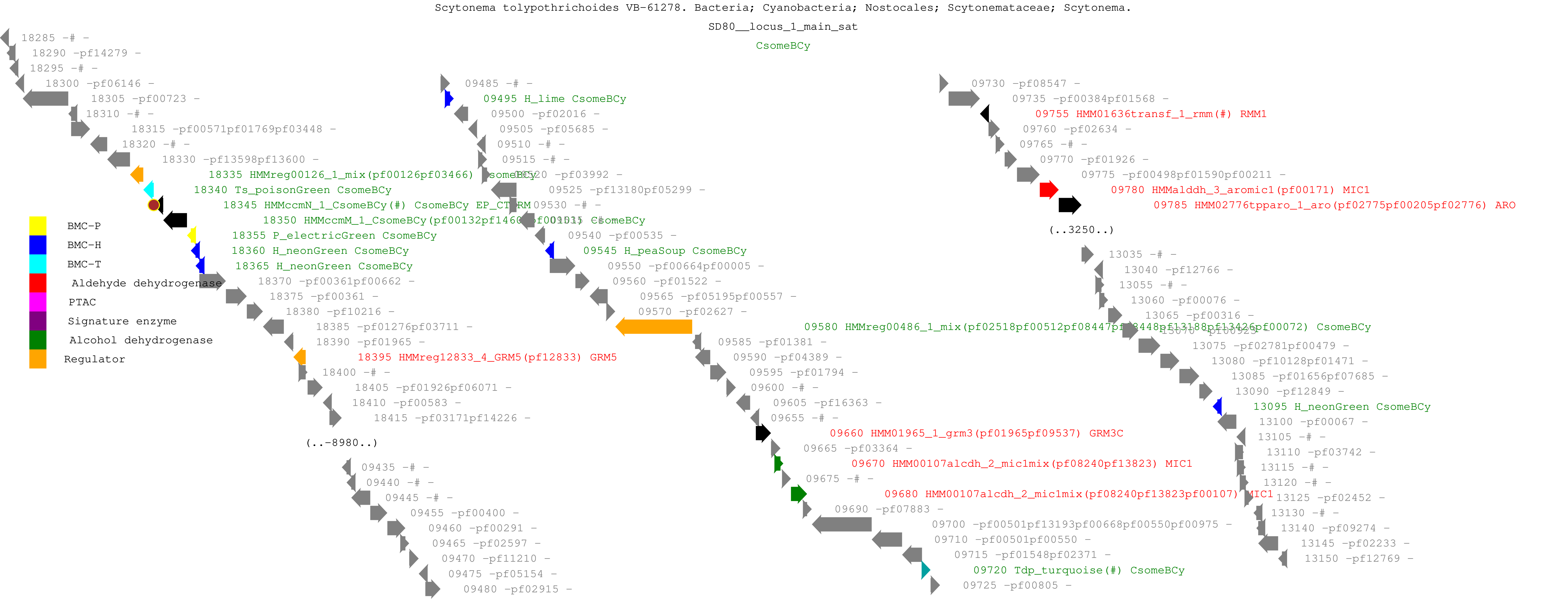
Scytonema tolypothrichoides VB-61278. Bacteria; Cyanobacteria; Nostocales; Scytonemataceae; Scytonema.
SD80__locus_1_main_sat
CsomeBCy
18285 -# -
18290 -pf14279 -
18295 -# -
18300 -pf06146 -
18305 -pf00723 -
18310 -# -
18315 -pf00571pf01769pf03448 -
18320 -# -
18330 -pf13598pf13600 -
18335 HMMreg00126_1_mix(pf00126pf03466) CsomeBCy
18340 Ts_poisonGreen CsomeBCy
18345 HMMccmN_1_CsomeBCy(#) CsomeBCy EP_CTERM
18350 HMMccmM_1_CsomeBCy(pf00132pf14602pf00101) CsomeBCy
18355 P_electricGreen CsomeBCy
18360 H_neonGreen CsomeBCy
18365 H_neonGreen CsomeBCy
18370 -pf00361pf00662 -
18375 -pf00361 -
18380 -pf10216 -
18385 -pf01276pf03711 -
18390 -pf01965 -
18395 HMMreg12833_4_GRM5(pf12833) GRM5
18400 -# -
18405 -pf01926pf06071 -
18410 -pf00583 -
18415 -pf03171pf14226 -
(..-8980..)
09435 -# -
09440 -# -
09445 -# -
09455 -pf00400 -
09460 -pf00291 -
09465 -pf02597 -
09470 -pf11210 -
09475 -pf05154 -
09480 -pf02915 -
09485 -# -
09495 H_lime CsomeBCy
09500 -pf02016 -
09505 -pf05685 -
09510 -# -
09515 -# -
09520 -pf03992 -
09525 -pf13180pf05299 -
09530 -# -
09535 CsomeBCy
09540 -pf00535 -
09545 H_peaSoup CsomeBCy
09550 -pf00664pf00005 -
09560 -pf01522 -
09565 -pf05195pf00557 -
09570 -pf02627 -
09580 HMMreg00486_1_mix(pf02518pf00512pf08447pf08448pf13188pf13426pf00072) CsomeBCy
09585 -pf01381 -
09590 -pf04389 -
09595 -pf01794 -
09600 -# -
09605 -pf16363 -
09655 -# -
09660 HMM01965_1_grm3(pf01965pf09537) GRM3C
09665 -pf03364 -
09670 HMM00107alcdh_2_mic1mix(pf08240pf13823) MIC1
09675 -# -
09680 HMM00107alcdh_2_mic1mix(pf08240pf13823pf00107) MIC1
09690 -pf07883 -
09700 -pf00501pf13193pf00668pf00550pf00975 -
09710 -pf00501pf00550 -
09715 -pf01548pf02371 -
09720 Tdp_turquoise(#) CsomeBCy
09725 -pf00805 -
09730 -pf08547 -
09735 -pf00384pf01568 -
09755 HMM01636transf_1_rmm(#) RMM1
09760 -pf02634 -
09765 -# -
09770 -pf01926 -
09775 -pf00498pf01590pf00211 -
09780 HMMaldh_3_aromic1(pf00171) MIC1
09785 HMM02776tpparo_1_aro(pf02775pf00205pf02776) ARO
(..3250..)
13035 -# -
13040 -pf12766 -
13055 -# -
13060 -pf00076 -
13065 -pf00316 -
13070 -pf00923 -
13075 -pf02781pf00479 -
13080 -pf10128pf01471 -
13085 -pf01656pf07685 -
13090 -pf12849 -
13095 H_neonGreen CsomeBCy
13100 -pf00067 -
13105 -# -
13110 -pf03742 -
13115 -# -
13120 -# -
13125 -pf02452 -
13130 -# -
13140 -pf09274 -
13145 -pf02233 -
13150 -pf12769 -
BMC-P
BMC-H
BMC-T
Aldehyde dehydrogenase
PTAC
Signature enzyme
Alcohol dehydrogenase
Regulator

Tolypothrix campylonemoides VB511288. Bacteria; Cyanobacteria; Nostocales; Tolypothrichaceae; Tolypothrix.

SD81__locus_1_main_sat

CsomeBCy

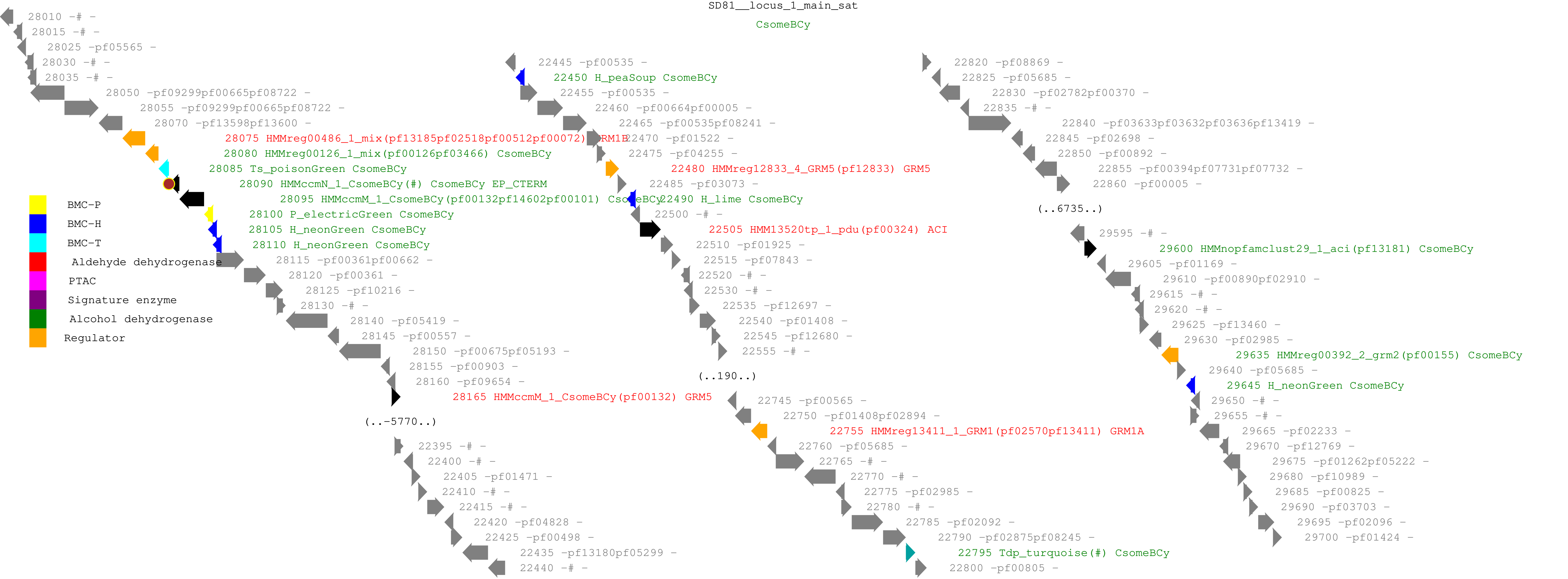

Synechocystis sp. (strain PCC 6803 / Kazusa). Bacteria; Cyanobacteria; Synechococcales; Merismopediaceae;

slr_locus_fragmented

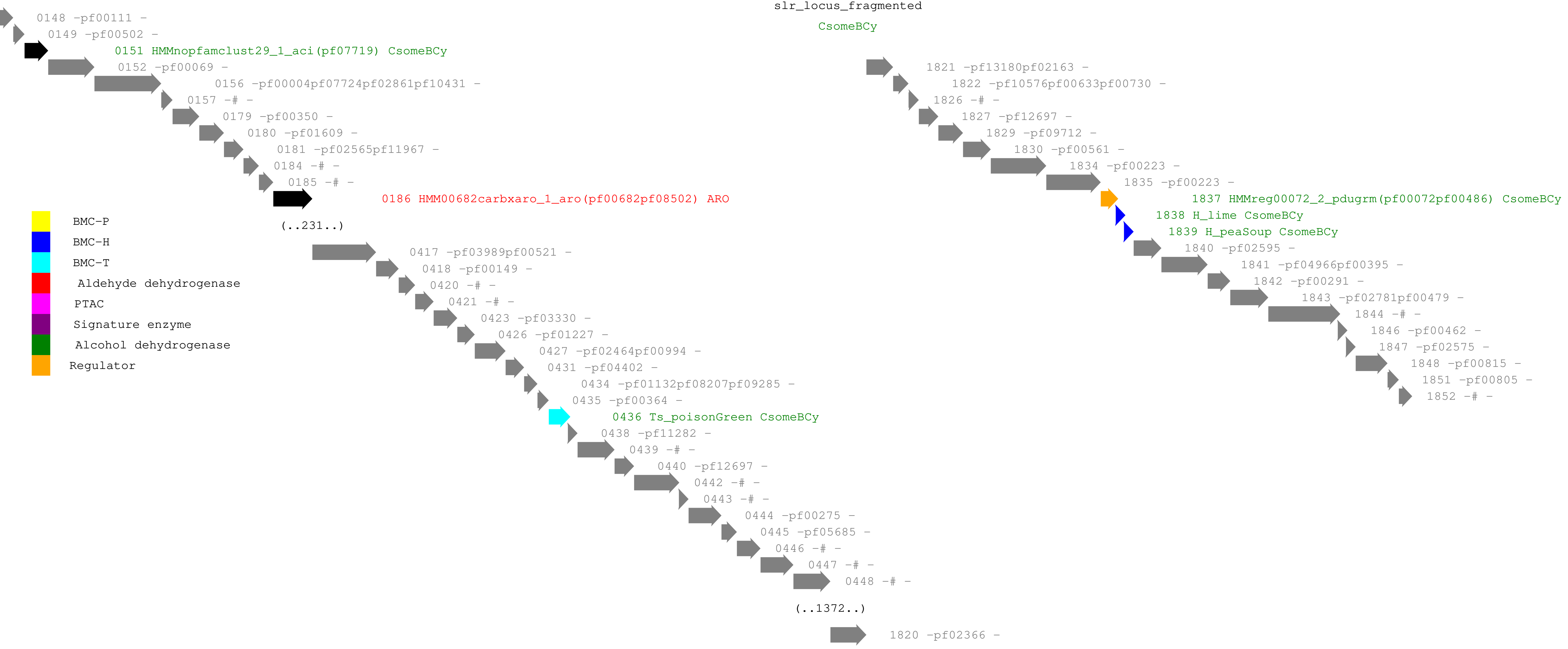

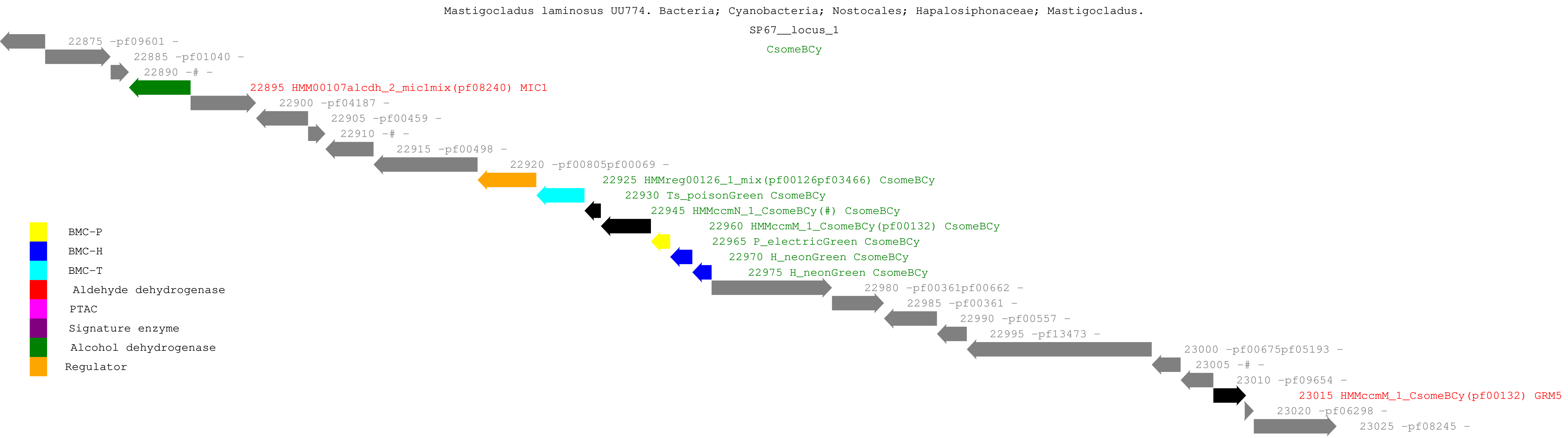
Mastigocladus laminosus UU774. Bacteria; Cyanobacteria; Nostocales; Hapalosiphonaceae; Mastigocladus.
SP67__locus_1
CsomeBCy
22875 -pf09601 -
22885 -pf01040 -
22890 -# -
22895 HMM00107alcdh_2_mic1mix(pf08240) MIC1
22900 -pf04187 -
22905 -pf00459 -
22910 -# -
22915 -pf00498 -
22920 -pf00805pf00069 -
22925 HMMreg00126_1_mix(pf00126pf03466) CsomeBCy
22930 Ts_poisonGreen CsomeBCy
22945 HMMccmN_1_CsomeBCy(#) CsomeBCy
22960 HMMccmM_1_CsomeBCy(pf00132) CsomeBCy
22965 P_electricGreen CsomeBCy
22970 H_neonGreen CsomeBCy
22975 H_neonGreen CsomeBCy
22980 -pf00361pf00662 -
22985 -pf00361 -
22990 -pf00557 -
22995 -pf13473 -
23000 -pf00675pf05193 -
23005 -# -
23010 -pf09654 -
23015 HMMccmM_1_CsomeBCy(pf00132) GRM5
23020 -pf06298 -
23025 -pf08245 -
BMC-P
BMC-H
BMC-T
Aldehyde dehydrogenase
PTAC
Signature enzyme
Alcohol dehydrogenase
Regulator

Arthrospira platensis C1. Bacteria; Cyanobacteria; Oscillatoriophycideae; Oscillatoriales;
SPLC1_S_locus_1_main_sat
CsomeBCy

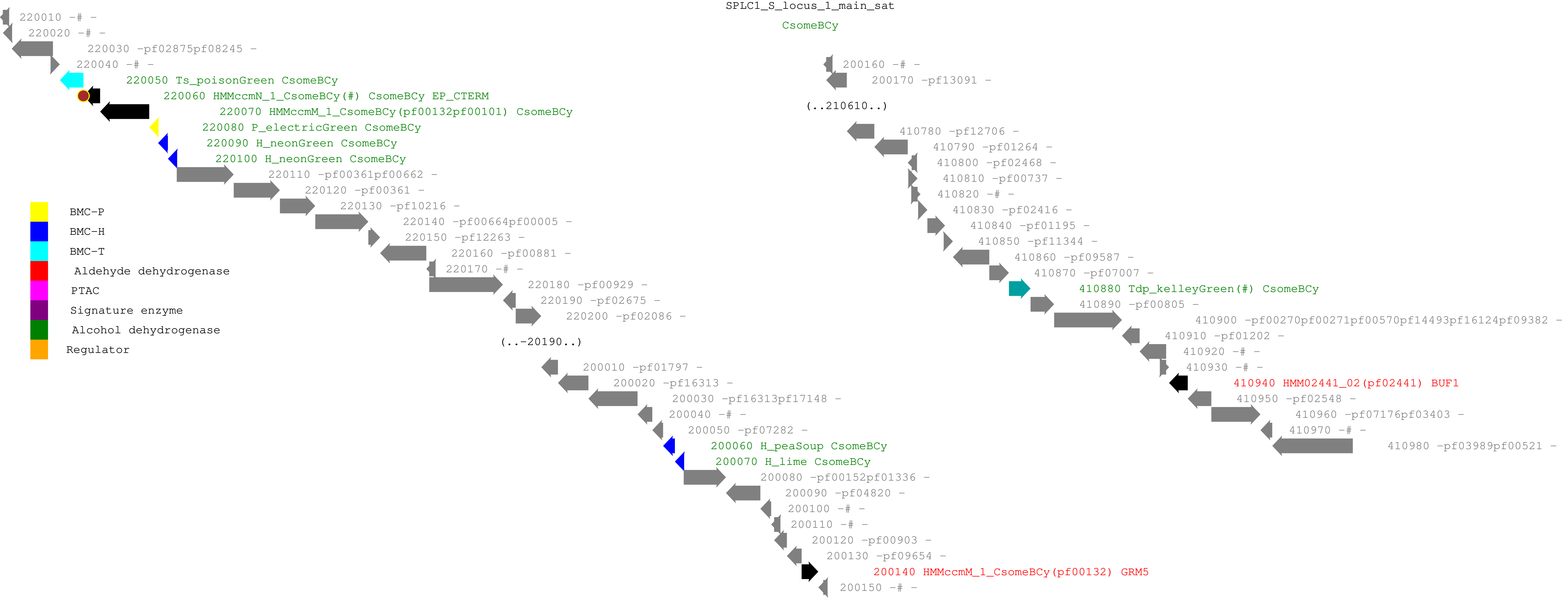

Sphaerospermopsis reniformis. Bacteria; Cyanobacteria; Nostocales; Aphanizomenonaceae; Sphaerospermopsis.

SR1949__locus_1_main_sat

CsomeBCy

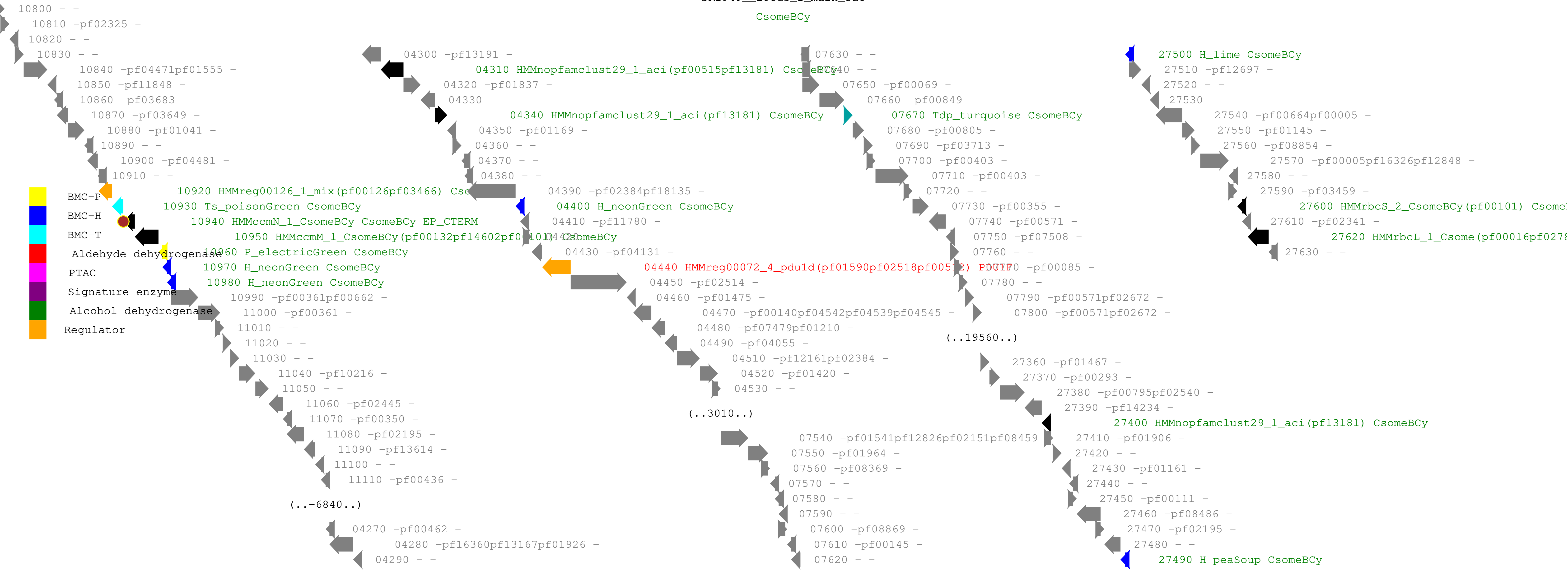

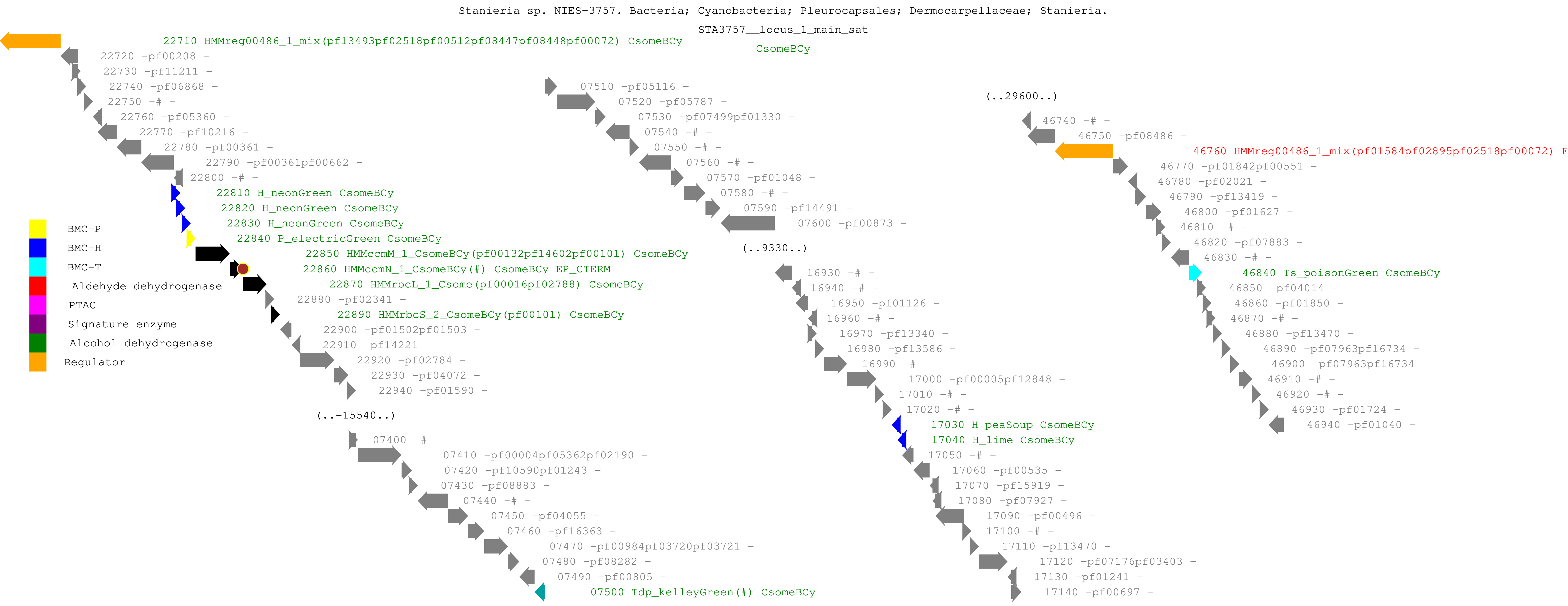

Stanieria sp. NIES-3757. Bacteria; Cyanobacteria; Pleurocapsales; Dermocarpellaceae; Stanieria.
STA3757__locus_1_main_sat
CsomeBCy
22710 HMMreg00486_1_mix(pf13493pf02518pf00512pf08447pf08448pf00072) CsomeBCy
22720 -pf00208 -
22730 -pf11211 -
22740 -pf06868 -
22750 -# -
22760 -pf05360 -
22770 -pf10216 -
22780 -pf00361 -
22790 -pf00361pf00662 -
22800 -# -
22810 H_neonGreen CsomeBCy
22820 H_neonGreen CsomeBCy
22830 H_neonGreen CsomeBCy
22840 P_electricGreen CsomeBCy
22850 HMMccmM_1_CsomeBCy(pf00132pf14602pf00101) CsomeBCy
22860 HMMccmN_1_CsomeBCy(#) CsomeBCy EP_CTERM
22870 HMMrbcL_1_Csome(pf00016pf02788) CsomeBCy
22880 -pf02341 -
22890 HMMrbcS_2_CsomeBCy(pf00101) CsomeBCy
22900 -pf01502pf01503 -
22910 -pf14221 -
22920 -pf02784 -
22930 -pf04072 -
22940 -pf01590 -
(..-15540..)
07400 -# -
07410 -pf00004pf05362pf02190 -
07420 -pf10590pf01243 -
07430 -pf08883 -
07440 -# -
07450 -pf04055 -
07460 -pf16363 -
07470 -pf00984pf03720pf03721 -
07480 -pf08282 -
07490 -pf00805 -
07500 Tdp_kelleyGreen(#) CsomeBCy
07510 -pf05116 -
07520 -pf05787 -
07530 -pf07499pf01330 -
07540 -# -
07550 -# -
07560 -# -
07570 -pf01048 -
07580 -# -
07590 -pf14491 -
07600 -pf00873 -
(..9330..)
16930 -# -
16940 -# -
16950 -pf01126 -
16960 -# -
16970 -pf13340 -
16980 -pf13586 -
16990 -# -
17000 -pf00005pf12848 -
17010 -# -
17020 -# -
17030 H_peaSoup CsomeBCy
17040 H_lime CsomeBCy
17050 -# -
17060 -pf00535 -
17070 -pf15919 -
17080 -pf07927 -
17090 -pf00496 -
17100 -# -
17110 -pf13470 -
17120 -pf07176pf03403 -
17130 -pf01241 -
17140 -pf00697 -
(..29600..)
46740 -# -
46750 -pf08486 -
46760 HMMreg00486_1_mix(pf01584pf02895pf02518pf00072) F
46770 -pf01842pf00551 -
46780 -pf02021 -
46790 -pf13419 -
46800 -pf01627 -
46810 -# -
46820 -pf07883 -
46830 -# -
46840 Ts_poisonGreen CsomeBCy
46850 -pf04014 -
46860 -pf01850 -
46870 -# -
46880 -pf13470 -
46890 -pf07963pf16734 -
46900 -pf07963pf16734 -
46910 -# -
46920 -# -
46930 -pf01724 -
46940 -pf01040 -
BMC-P
BMC-H
BMC-T
Aldehyde dehydrogenase
PTAC
Signature enzyme
Alcohol dehydrogenase
Regulator

Stanieria cyanosphaera (strain ATCC 29371 / PCC 7437). Bacteria; Cyanobacteria; Pleurocapsales; Dermocarpellaceae; Stanieria.

Sta7437__locus_1_main_sat

CsomeBCy

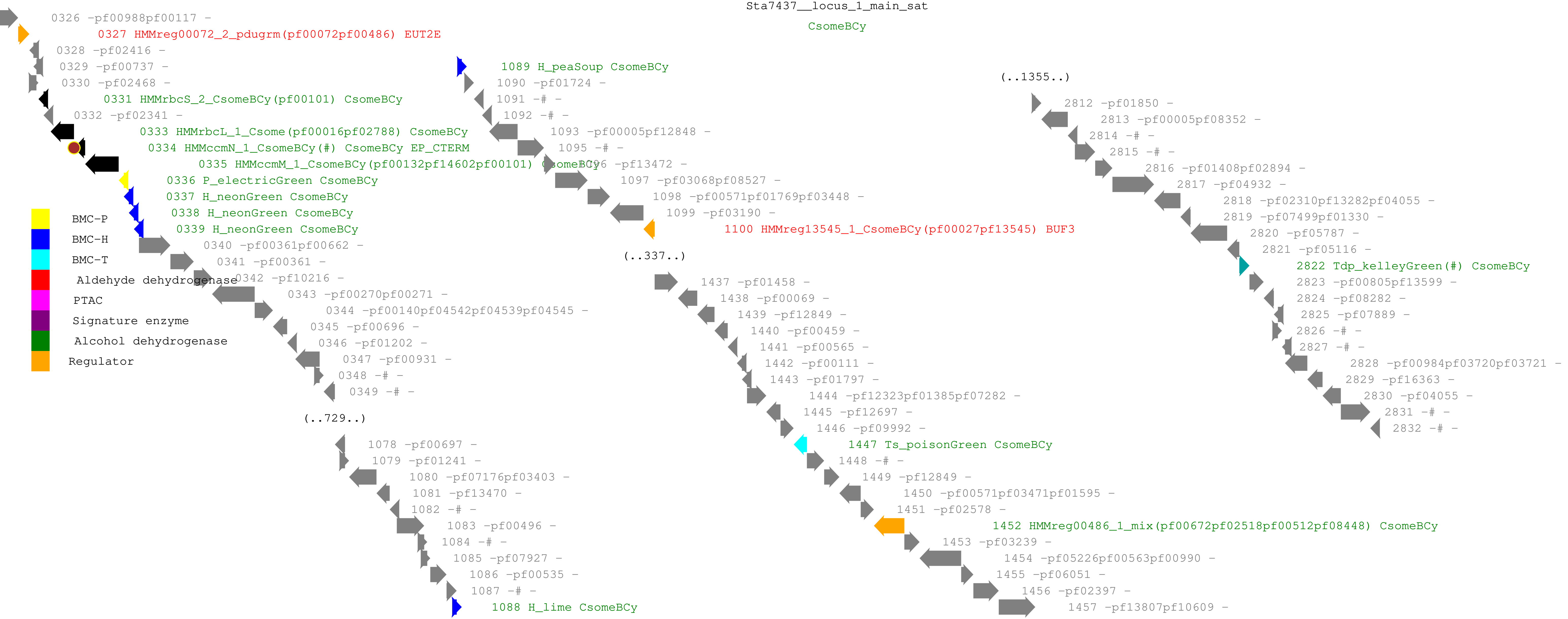

Synechococcus sp. 60AY4M2. Bacteria; Cyanobacteria; Synechococcales; Synechococcaceae;
SYN60AY4M2__locus_1_main_sat
CsomeBCy

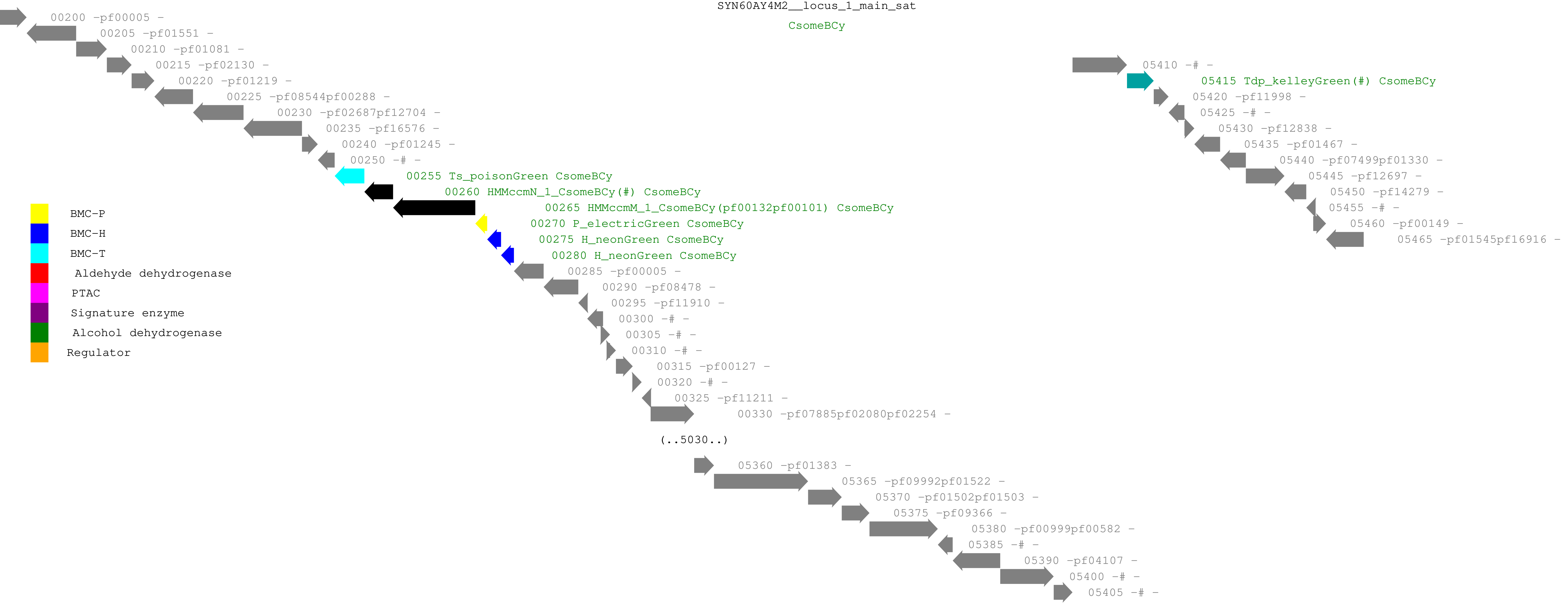

Synechococcus sp. (strain ATCC 27167 / PCC 6312). Bacteria; Cyanobacteria; Synechococcales; Synechococcaceae;

Syn6312__locus_1_main_sat

CsomeBCy

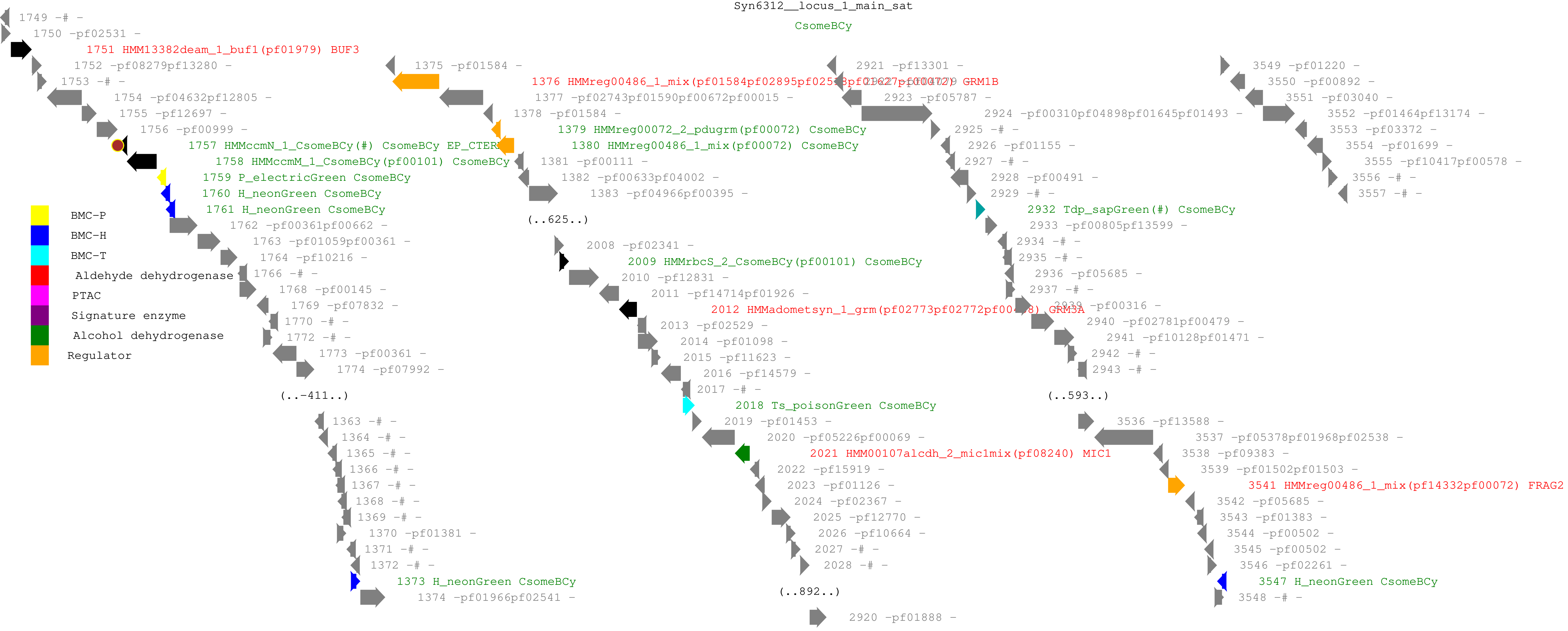

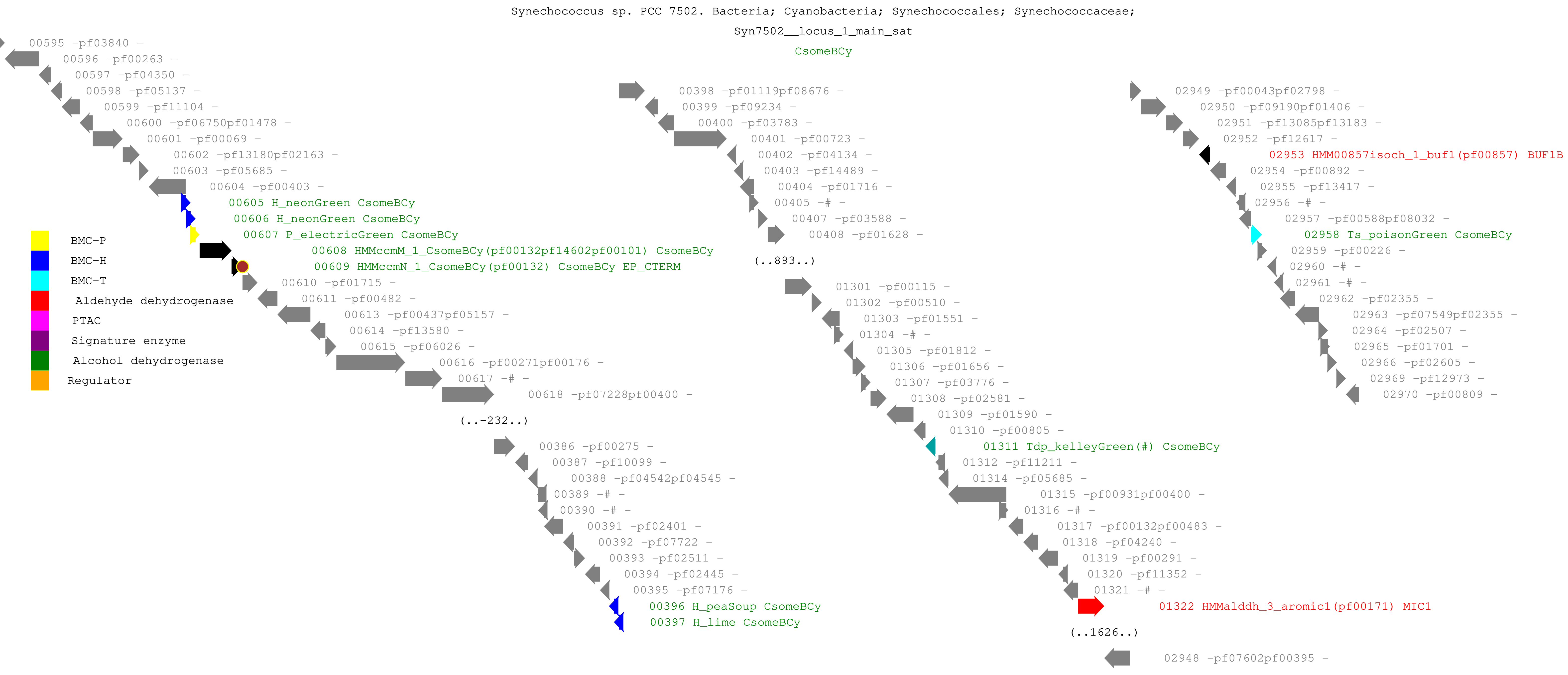
Synechococcus sp. PCC 7502. Bacteria; Cyanobacteria; Synechococcales; Synechococcaceae;
Syn7502__locus_1_main_sat
CsomeBCy
00595 -pf03840 -
00596 -pf00263 -
00597 -pf04350 -
00598 -pf05137 -
00599 -pf11104 -
00600 -pf06750pf01478 -
00601 -pf00069 -
00602 -pf13180pf02163 -
00603 -pf05685 -
00604 -pf00403 -
00605 H_neonGreen CsomeBCy
00606 H_neonGreen CsomeBCy
00607 P_electricGreen CsomeBCy
00608 HMMccmM_1_CsomeBCy(pf00132pf14602pf00101) CsomeBCy
00609 HMMccmN_1_CsomeBCy(pf00132) CsomeBCy EP_CTERM
00610 -pf01715 -
00611 -pf00482 -
00613 -pf00437pf05157 -
00614 -pf13580 -
00615 -pf06026 -
00616 -pf00271pf00176 -
00617 -# -
00618 -pf07228pf00400 -
(..-232..)
00386 -pf00275 -
00387 -pf10099 -
00388 -pf04542pf04545 -
00389 -# -
00390 -# -
00391 -pf02401 -
00392 -pf07722 -
00393 -pf02511 -
00394 -pf02445 -
00395 -pf07176 -
00396 H_peaSoup CsomeBCy
00397 H_lime CsomeBCy
00398 -pf01119pf08676 -
00399 -pf09234 -
00400 -pf03783 -
00401 -pf00723 -
00402 -pf04134 -
00403 -pf14489 -
00404 -pf01716 -
00405 -# -
00407 -pf03588 -
00408 -pf01628 -
(..893..)
01301 -pf00115 -
01302 -pf00510 -
01303 -pf01551 -
01304 -# -
01305 -pf01812 -
01306 -pf01656 -
01307 -pf03776 -
01308 -pf02581 -
01309 -pf01590 -
01310 -pf00805 -
01311 Tdp_kelleyGreen(#) CsomeBCy
01312 -pf11211 -
01314 -pf05685 -
01315 -pf00931pf00400 -
01316 -# -
01317 -pf00132pf00483 -
01318 -pf04240 -
01319 -pf00291 -
01320 -pf11352 -
01321 -# -
01322 HMMaldh_3_aromic1(pf00171) MIC1
(..1626..)
02948 -pf07602pf00395 -
02949 -pf00043pf02798 -
02950 -pf09190pf01406 -
02951 -pf13085pf13183 -
02952 -pf12617 -
02953 HMM00857isoch_1_buf1(pf00857) BUF1B
02954 -pf00892 -
02955 -pf13417 -
02956 -# -
02957 -pf00588pf08032 -
02958 Ts_poisonGreen CsomeBCy
02959 -pf00226 -
02960 -# -
02961 -# -
02962 -pf02355 -
02963 -pf07549pf02355 -
02964 -pf02507 -
02965 -pf01701 -
02966 -pf02605 -
02969 -pf12973 -
02970 -pf00809 -
BMC-P
BMC-H
BMC-T
Aldehyde dehydrogenase
PTAC
Signature enzyme
Alcohol dehydrogenase
Regulator

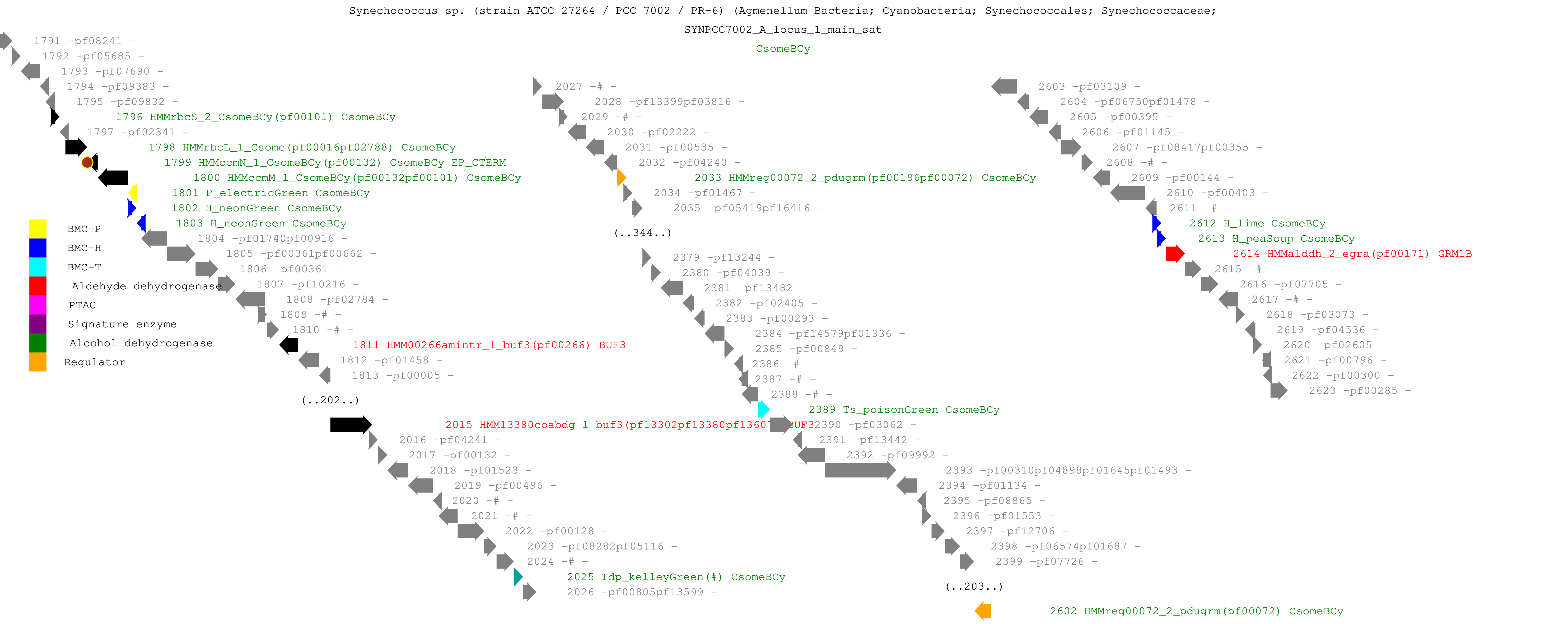

Synechococcus sp. (strain ATCC 27264 / PCC 7002 / PR-6) (Agmenellum Bacteria; Cyanobacteria; Synechococcales; Synechococcaceae;
SYNPCC7002_A_locus_1_main_sat
CsomeBCy
1791 -pf08241 -
1792 -pf05685 -
1793 -pf07690 -
1794 -pf09383 -
1795 -pf09832 -
1796 HMMrbcS_2_CsomeBCy(pf00101) CsomeBCy
1797 -pf02341 -
1798 HMMrbcL_1_Csome(pf00016pf02788) CsomeBCy
1799 HMMccmN_1_CsomeBCy(pf00132) CsomeBCy EP_CTERM
1800 HMMccmM_1_CsomeBCy(pf00132pf00101) CsomeBCy
1801 P_electricGreen CsomeBCy
1802 H_neonGreen CsomeBCy
1803 H_neonGreen CsomeBCy
1804 -pf01740pf00916 -
1805 -pf00361pf00662 -
1806 -pf00361 -
1807 -pf10216 -
1808 -pf02784 -
1809 -# -
1810 -# -
1811 HMM00266amintr_1_buf3(pf00266) BUF3
1812 -pf01458 -
1813 -pf00005 -
(..202..)
2015 HMM13380coabdg_1_buf3(pf13302pf13380pf13607) BUF3
2016 -pf04241 -
2017 -pf00132 -
2018 -pf01523 -
2019 -pf00496 -
2020 -# -
2021 -# -
2022 -pf00128 -
2023 -pf08282pf05116 -
2024 -# -
2025 Tdp_kelleyGreen(#) CsomeBCy
2026 -pf00805pf13599 -
2027 -# -
2028 -pf13399pf03816 -
2029 -# -
2030 -pf02222 -
2031 -pf00535 -
2032 -pf04240 -
2033 HMMreg00072_2_pdugrm(pf00196pf00072) CsomeBCy
2034 -pf01467 -
2035 -pf05419pf16416 -
(..344..)
2379 -pf13244 -
2380 -pf04039 -
2381 -pf13482 -
2382 -pf02405 -
2383 -pf00293 -
2384 -pf14579pf01336 -
2385 -pf00849 -
2386 -# -
2387 -# -
2388 -# -
2389 Ts_poisonGreen CsomeBCy
2390 -pf03062 -
2391 -pf13442 -
2392 -pf09992 -
2393 -pf00310pf04898pf01645pf01493 -
2394 -pf01134 -
2395 -pf08865 -
2396 -pf01553 -
2397 -pf12706 -
2398 -pf06574pf01687 -
2399 -pf07726 -
(..203..)
2602 HMMreg00072_2_pdugrm(pf00072) CsomeBCy
2603 -pf03109 -
2604 -pf06750pf01478 -
2605 -pf00395 -
2606 -pf01145 -
2607 -pf08417pf00355 -
2608 -# -
2609 -pf00144 -
2610 -pf00403 -
2611 -# -
2612 H_lime CsomeBCy
2613 H_peaSoup CsomeBCy
2614 HMMalddh_2_egra(pf00171) GRM1B
2615 -# -
2616 -pf07705 -
2617 -# -
2618 -pf03073 -
2619 -pf04536 -
2620 -pf02605 -
2621 -pf00796 -
2622 -pf00300 -
2623 -pf00285 -
BMC-P
BMC-H
BMC-T
Aldehyde dehydrogenase
PTAC
Signature enzyme
Alcohol dehydrogenase
Regulator

Synechococcus elongatus (strain PCC 7942) (Anacystis nidulans R2). Bacteria; Cyanobacteria; Synechococcales; Synechococcaceae;

Synpcc7942__locus_1_main_sat

CsomeBCy

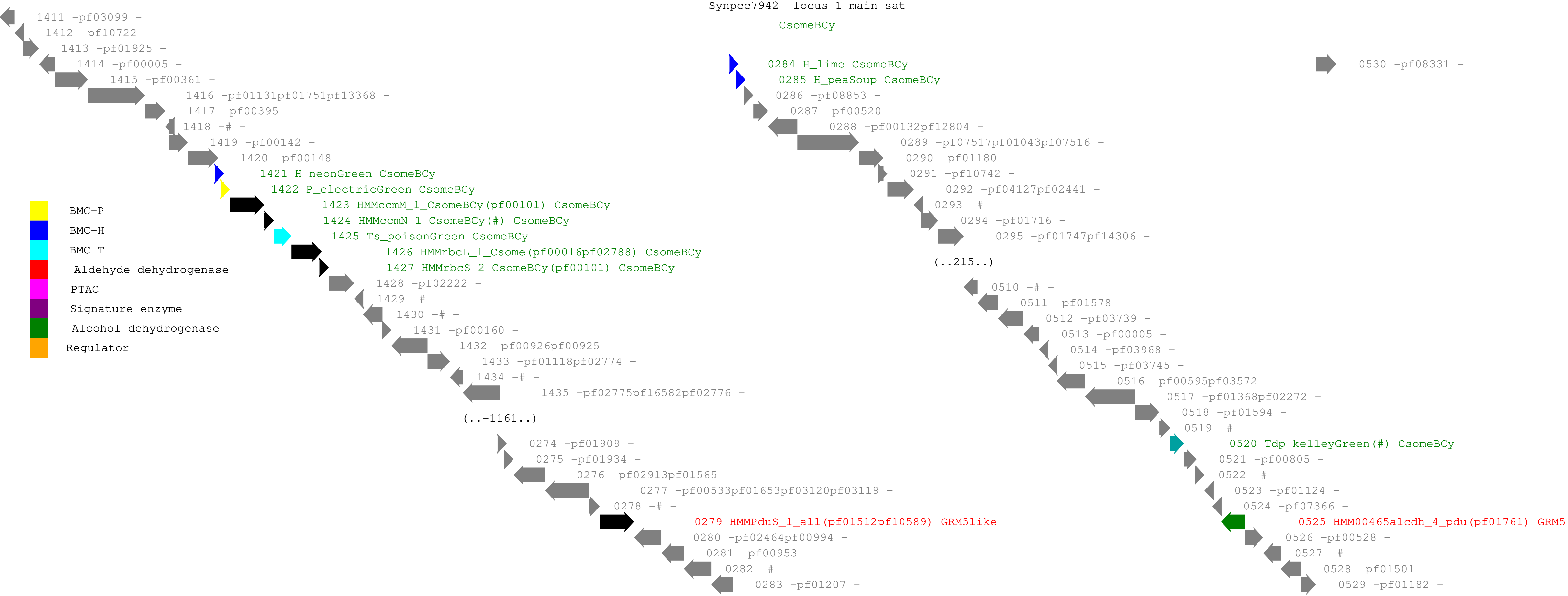

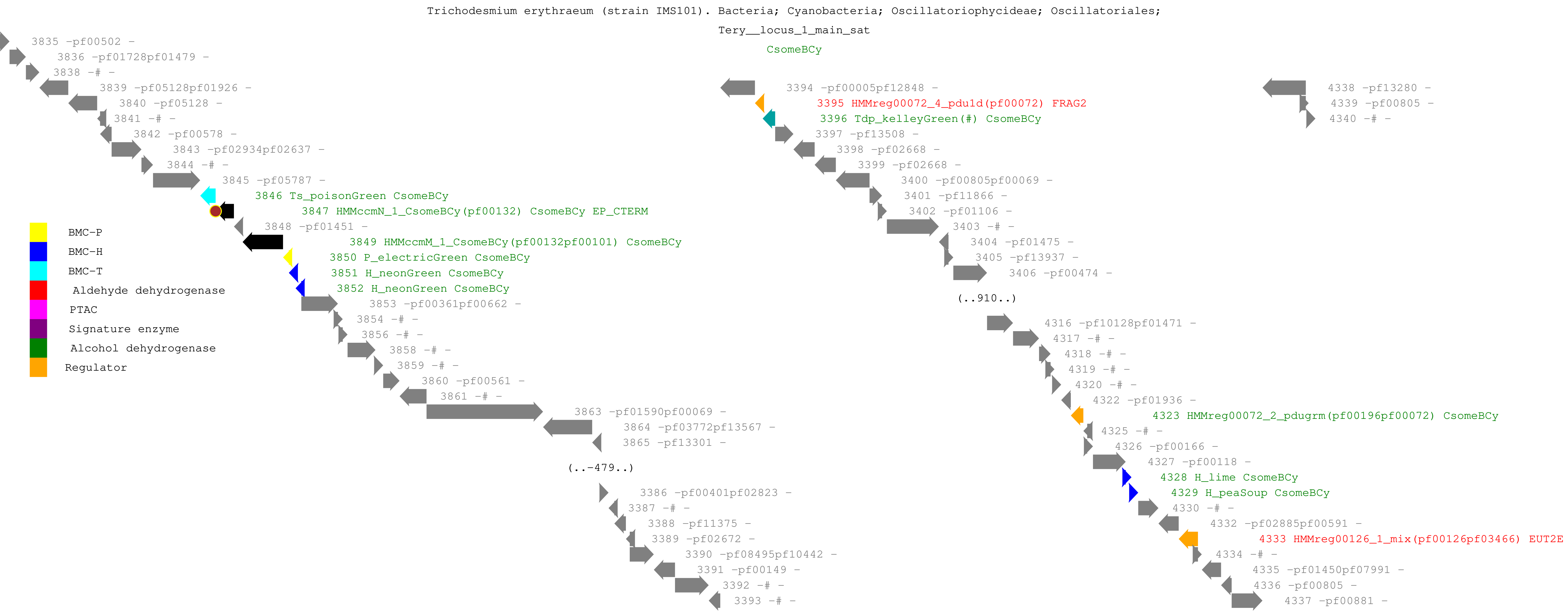
Trichodesmium erythraeum (strain IMS101). Bacteria; Cyanobacteria; Oscillatoriophycideae; Oscillatoriales;
Tery__locus_1_main_sat
CsomeBCy
3835 -pf00502 -
3836 -pf01728pf01479 -
3838 -# -
3839 -pf05128pf01926 -
3840 -pf05128 -
3841 -# -
3842 -pf00578 -
3843 -pf02934pf02637 -
3844 -# -
3845 -pf05787 -
3846 Ts_poisonGreen CsomeBCy
3847 HMMccmN_1_CsomeBCy(pf00132) CsomeBCy EP_CTERM
3848 -pf01451 -
3849 HMMccmM_1_CsomeBCy(pf00132pf00101) CsomeBCy
3850 P_electricGreen CsomeBCy
3851 H_neonGreen CsomeBCy
3852 H_neonGreen CsomeBCy
3853 -pf00361pf00662 -
3854 -# -
3856 -# -
3858 -# -
3859 -# -
3860 -pf00561 -
3861 -# -
3863 -pf01590pf00069 -
3864 -pf03772pf13567 -
3865 -pf13301 -
(..-479..)
3386 -pf00401pf02823 -
3387 -# -
3388 -pf11375 -
3389 -pf02672 -
3390 -pf08495pf10442 -
3391 -pf00149 -
3392 -# -
3393 -# -
3394 -pf00005pf12848 -
3395 HMMreg00072_4_pduld(pf00072) FRAG2
3396 Tdp_kelleyGreen(#) CsomeBCy
3397 -pf13508 -
3398 -pf02668 -
3399 -pf02668 -
3400 -pf00805pf00069 -
3401 -pf11866 -
3402 -pf01106 -
3403 -# -
3404 -pf01475 -
3405 -pf13937 -
3406 -pf00474 -
(..910..)
4316 -pf10128pf01471 -
4317 -# -
4318 -# -
4319 -# -
4320 -# -
4322 -pf01936 -
4323 HMMreg00072_2_pdugrm(pf00196pf00072) CsomeBCy
4325 -# -
4326 -pf00166 -
4327 -pf00118 -
4328 H_lime CsomeBCy
4329 H_peaSoup CsomeBCy
4330 -# -
4332 -pf02885pf00591 -
4333 HMMreg00126_1_mix(pf00126pf03466) EUT2E
4334 -# -
4335 -pf01450pf07991 -
4336 -pf00805 -
4337 -pf00881 -
4338 -pf13280 -
4339 -pf00805 -
4340 -# -
BMC-P
BMC-H
BMC-T
Aldehyde dehydrogenase
PTAC
Signature enzyme
Alcohol dehydrogenase
Regulator

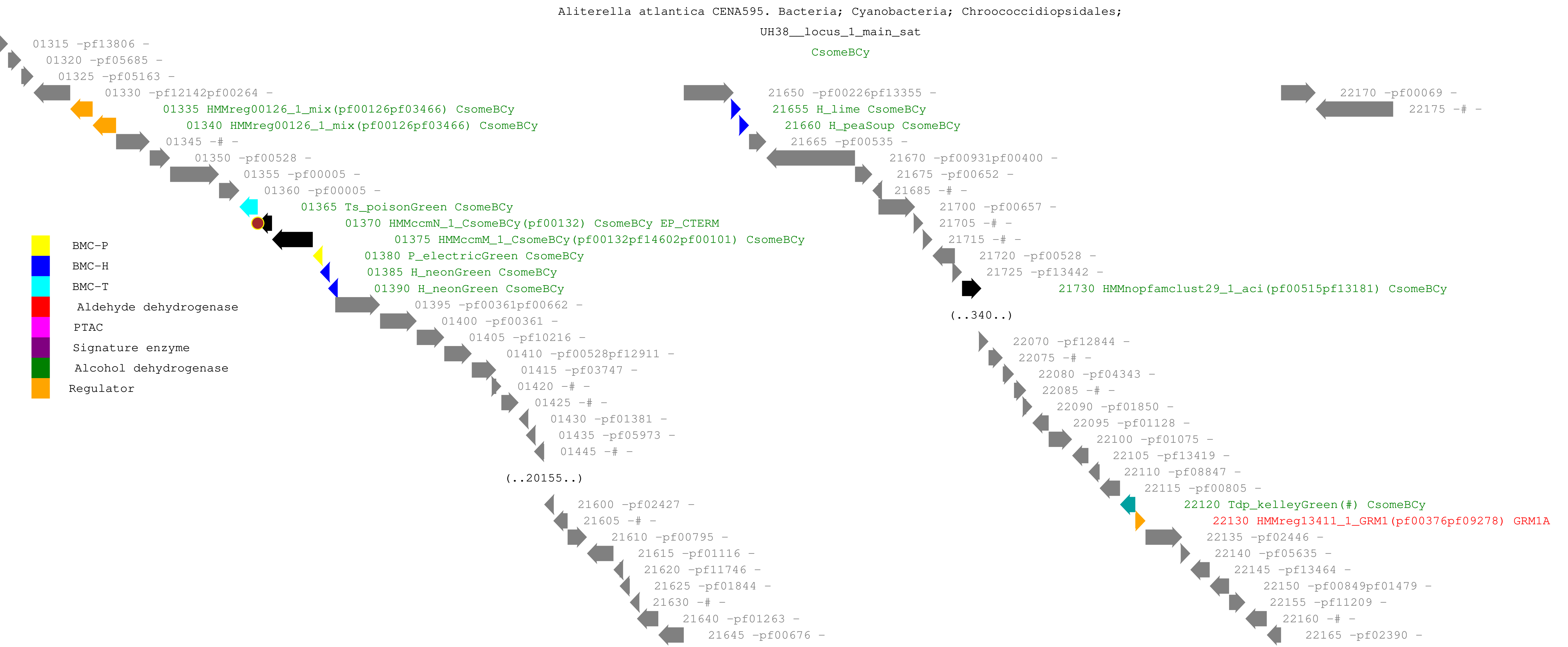

Aliterella atlantica CENA595. Bacteria; Cyanobacteria; Chroococcidiopsidales;
UH38__locus_1_main_sat
CsomeBCy
01315 -pf13806 -
01320 -pf05685 -
01325 -pf05163 -
01330 -pf12142pf00264 -
01335 HMMreg00126_1_mix(pf00126pf03466) CsomeBCy
01340 HMMreg00126_1_mix(pf00126pf03466) CsomeBCy
01345 -# -
01350 -pf00528 -
01355 -pf00005 -
01360 -pf00005 -
01365 Ts_poisonGreen CsomeBCy
01370 HMMccmN_1_CsomeBCy(pf00132) CsomeBCy EP_CTERM
01375 HMMccmM_1_CsomeBCy(pf00132pf14602pf00101) CsomeBCy
01380 P_electricGreen CsomeBCy
01385 H_neonGreen CsomeBCy
01390 H_neonGreen CsomeBCy
01395 -pf00361pf00662 -
01400 -pf00361 -
01405 -pf10216 -
01410 -pf00528pf12911 -
01415 -pf03747 -
01420 -# -
01425 -# -
01430 -pf01381 -
01435 -pf05973 -
01445 -# -
(..20155..)
21600 -pf02427 -
21605 -# -
21610 -pf00795 -
21615 -pf01116 -
21620 -pf11746 -
21625 -pf01844 -
21630 -# -
21640 -pf01263 -
21645 -pf00676 -
21650 -pf00226pf13355 -
21655 H_lime CsomeBCy
21660 H_peaSoup CsomeBCy
21665 -pf00535 -
21670 -pf00931pf00400 -
21675 -pf00652 -
21685 -# -
21700 -pf00657 -
21705 -# -
21715 -# -
21720 -pf00528 -
21725 -pf13442 -
21730 HMMnopfamclust29_1_aci(pf00515pf13181) CsomeBCy
(..340..)
22070 -pf12844 -
22075 -# -
22080 -pf04343 -
22085 -# -
22090 -pf01850 -
22095 -pf01128 -
22100 -pf01075 -
22105 -pf13419 -
22110 -pf08847 -
22115 -pf00805 -
22120 Tdp_kelleyGreen(#) CsomeBCy
22130 HMMreg13411_1_GRM1(pf00376pf09278) GRM1A
22135 -pf02446 -
22140 -pf05635 -
22145 -pf13464 -
22150 -pf00849pf01479 -
22155 -pf11209 -
22160 -# -
22165 -pf02390 -
22170 -pf00069 -
22175 -# -
BMC-P
BMC-H
BMC-T
Aldehyde dehydrogenase
PTAC
Signature enzyme
Alcohol dehydrogenase
Regulator

Nostoc linckia z16. Bacteria; Cyanobacteria; Nostocales; Nostocaceae; Nostoc.

VF13__locus_1_main_sat

CsomeBCy

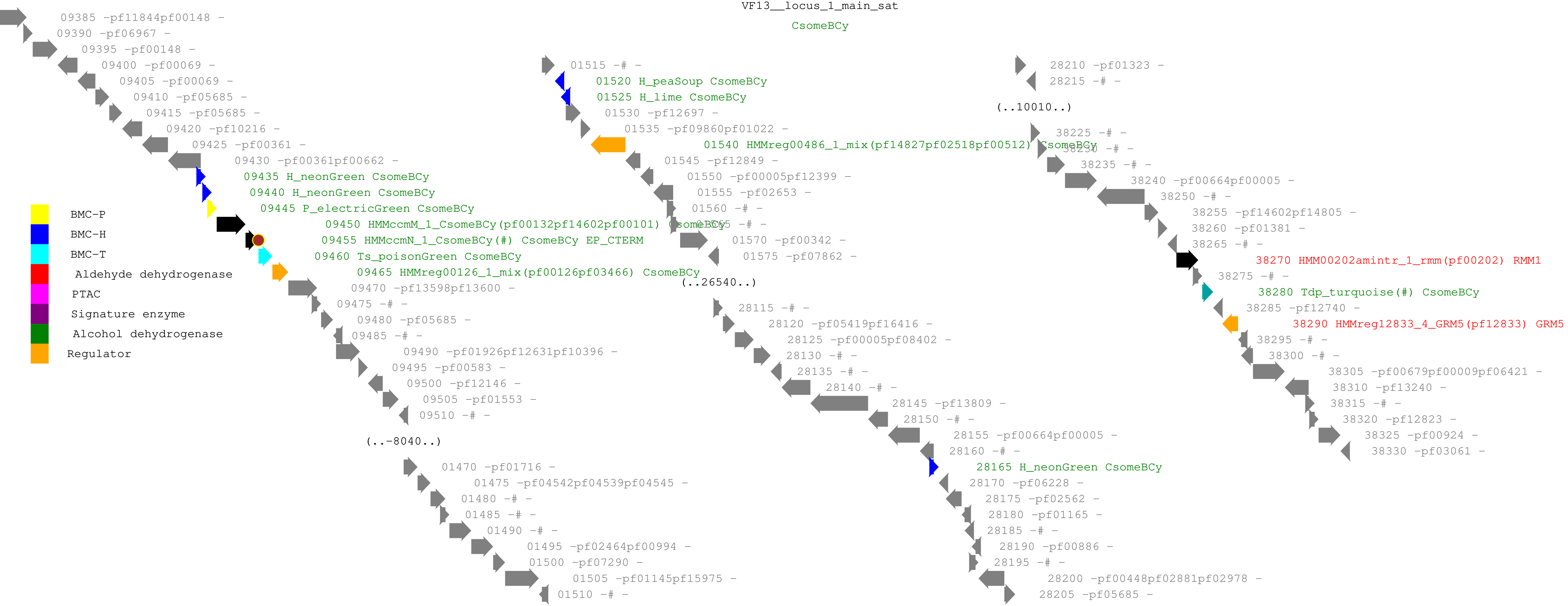

Microcystis panniformis FACHB-1757. Bacteria; Cyanobacteria; Oscillatoriophycideae; Chroococcales;

VL20__locus_1_main_sat

CsomeBCy

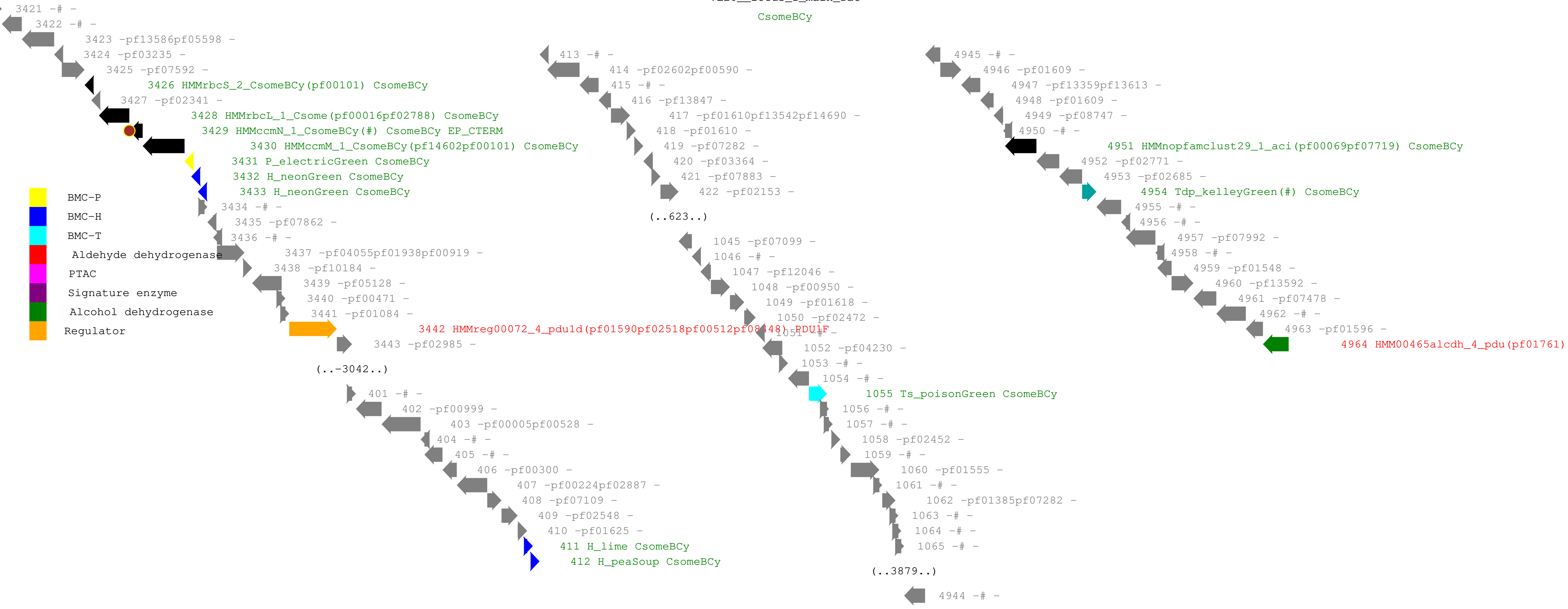

Scytonema hofmannii PCC 7110. Bacteria; Cyanobacteria; Nostocales; Scytonemataceae; Scytonema.

WA1__locus_1_main_sat

CsomeBCy

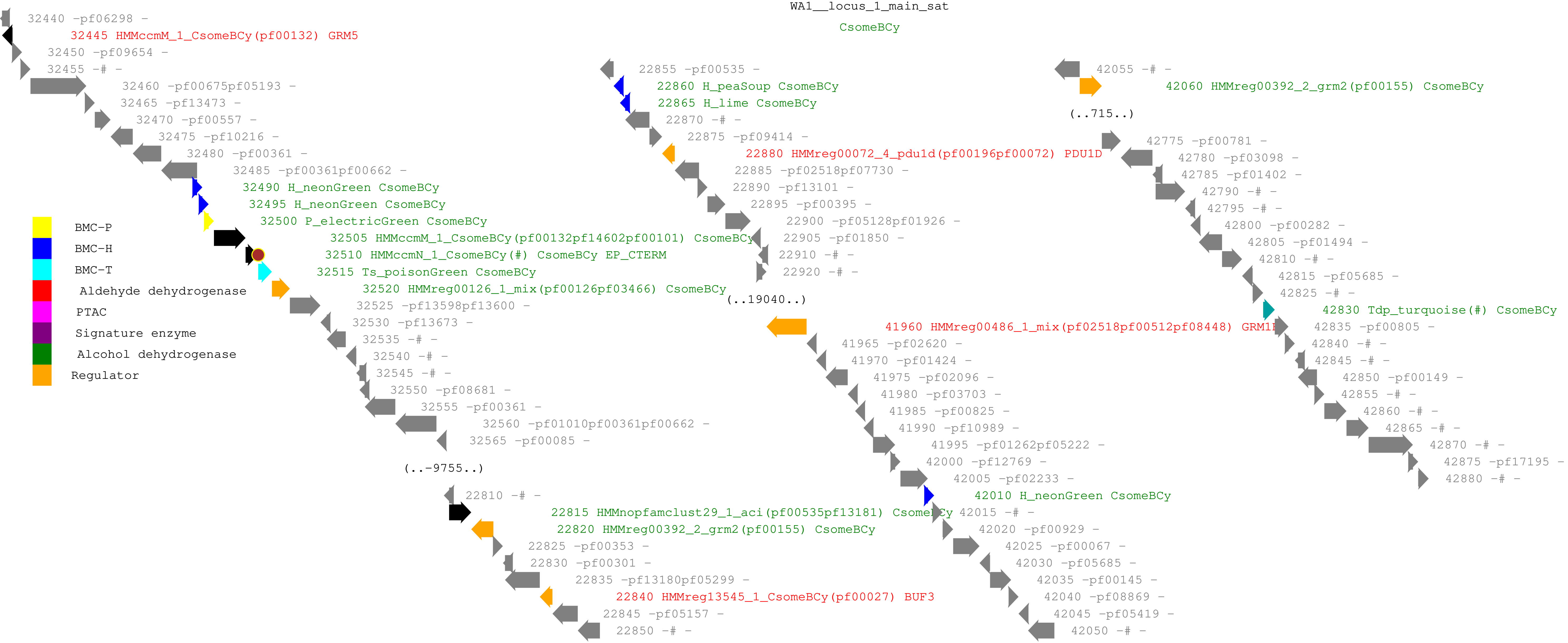

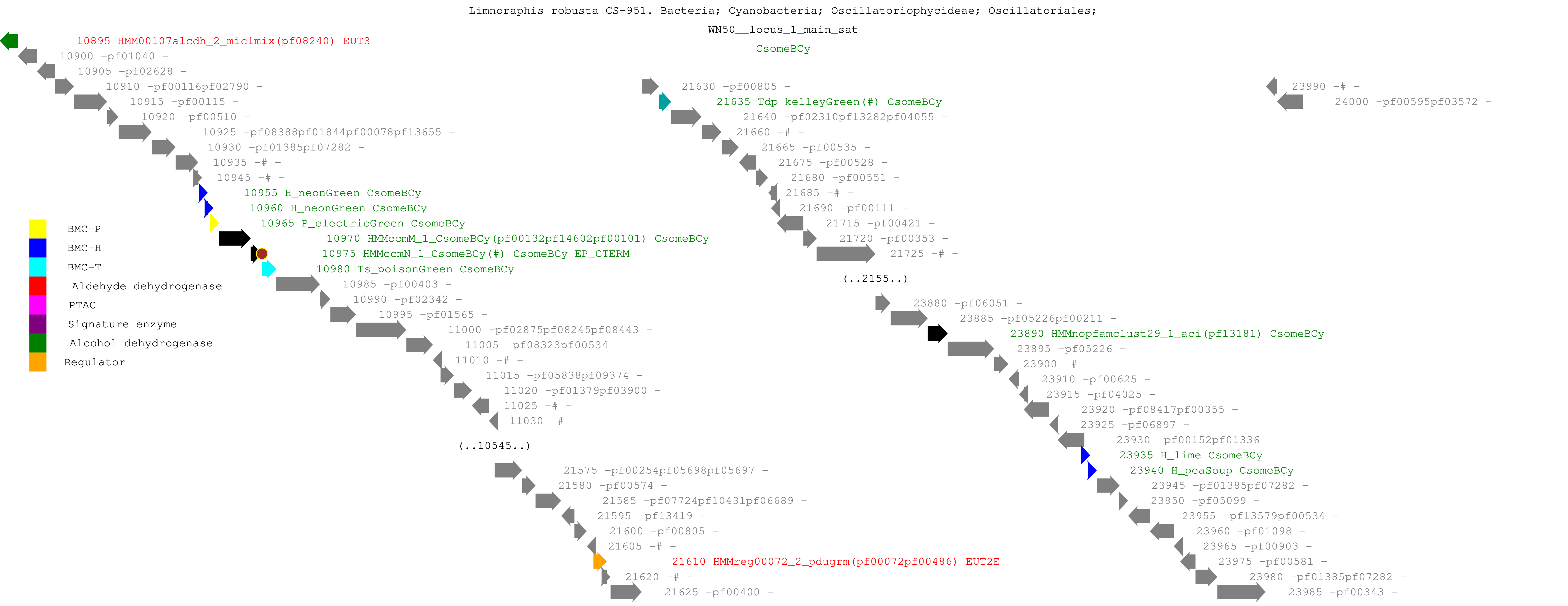
Limnoraphis robusta CS-951. Bacteria; Cyanobacteria; Oscillatoriophycideae; Oscillatoriales;
WN50__locus_1_main_sat
CsomeBCy
10895 HMM00107alcdh_2_mic1mix(pf08240) EUT3
10900 -pf01040 -
10905 -pf02628 -
10910 -pf00116pf02790 -
10915 -pf00115 -
10920 -pf00510 -
10925 -pf08388pf01844pf00078pf13655 -
10930 -pf01385pf07282 -
10935 -# -
10945 -# -
10955 H_neonGreen CsomeBCy
10960 H_neonGreen CsomeBCy
10965 P_electricGreen CsomeBCy
10970 HMMccmM_1_CsomeBCy(pf00132pf14602pf00101) CsomeBCy
10975 HMMccmN_1_CsomeBCy(#) CsomeBCy EP_CTERM
10980 Ts_poisonGreen CsomeBCy
10985 -pf00403 -
10990 -pf02342 -
10995 -pf01565 -
11000 -pf02875pf08245pf08443 -
11005 -pf08323pf00534 -
11010 -# -
11015 -pf05838pf09374 -
11020 -pf01379pf03900 -
11025 -# -
11030 -# -
(..10545..)
21575 -pf00254pf05698pf05697 -
21580 -pf00574 -
21585 -pf07724pf10431pf06689 -
21595 -pf13419 -
21600 -pf00805 -
21605 -# -
21610 HMMreg00072_2_pdugrm(pf00072pf00486) EUT2E
21620 -# -
21625 -pf00400 -
21630 -pf00805 -
21635 Tdp_kelleyGreen(#) CsomeBCy
21640 -pf02310pf13282pf04055 -
21660 -# -
21665 -pf00535 -
21675 -pf00528 -
21680 -pf00551 -
21685 -# -
21690 -pf00111 -
21715 -pf00421 -
21720 -pf00353 -
21725 -# -
(..2155..)
23880 -pf06051 -
23885 -pf05226pf00211 -
23890 HMMnopfamclust29_1_aci(pf13181) CsomeBCy
23895 -pf05226 -
23900 -# -
23910 -pf00625 -
23915 -pf04025 -
23920 -pf08417pf00355 -
23925 -pf06897 -
23930 -pf00152pf01336 -
23935 H_lime CsomeBCy
23940 H_peaSoup CsomeBCy
23945 -pf01385pf07282 -
23950 -pf05099 -
23955 -pf13579pf00534 -
23960 -pf01098 -
23965 -pf00903 -
23975 -pf00581 -
23980 -pf01385pf07282 -
23985 -pf00343 -
23990 -# -
24000 -pf00595pf03572 -
BMC-P
BMC-H
BMC-T
Aldehyde dehydrogenase
PTAC
Signature enzyme
Alcohol dehydrogenase
Regulator

Xenococcus sp. PCC 7305. Bacteria; Cyanobacteria; Pleurocapsales; Xenococcaceae; Xenococcus.

Xen7305DRAFT__locus_1_main_sat

CsomeBCy

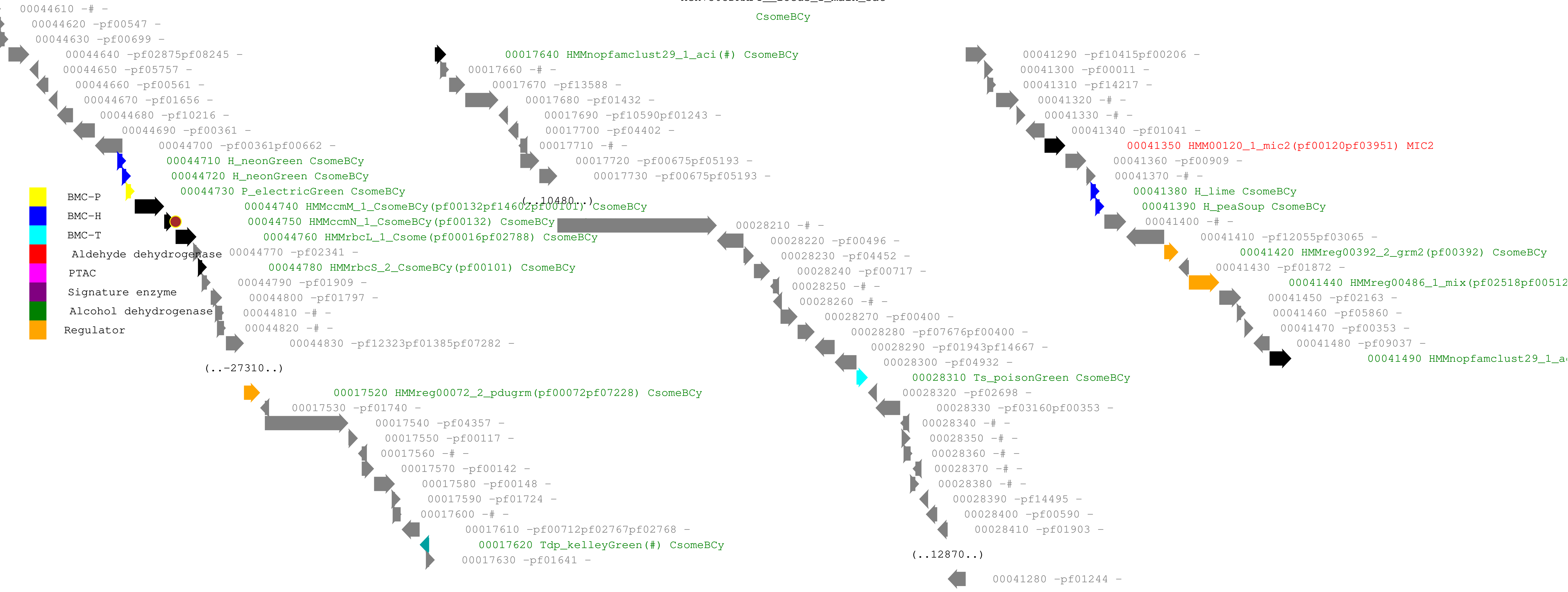

Halomicronema hongdechloris C2206. Bacteria; Cyanobacteria; Synechococcales; Leptolyngbyaceae;

XM38__locus_1_main_sat

CsomeBCy

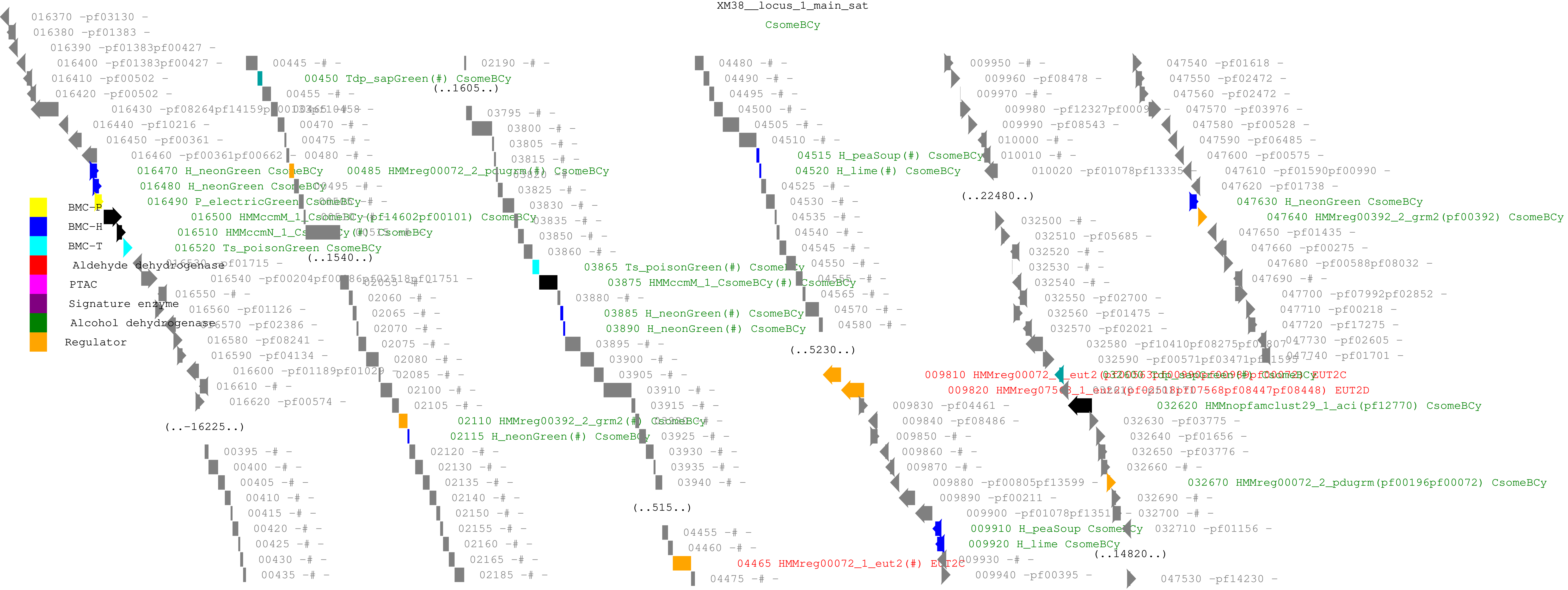

Clostridium kluyveri. Bacteria; Firmicutes; Clostridia; Clostridiales; Clostridiaceae;

BS101__locus_1_main_sat

ETU

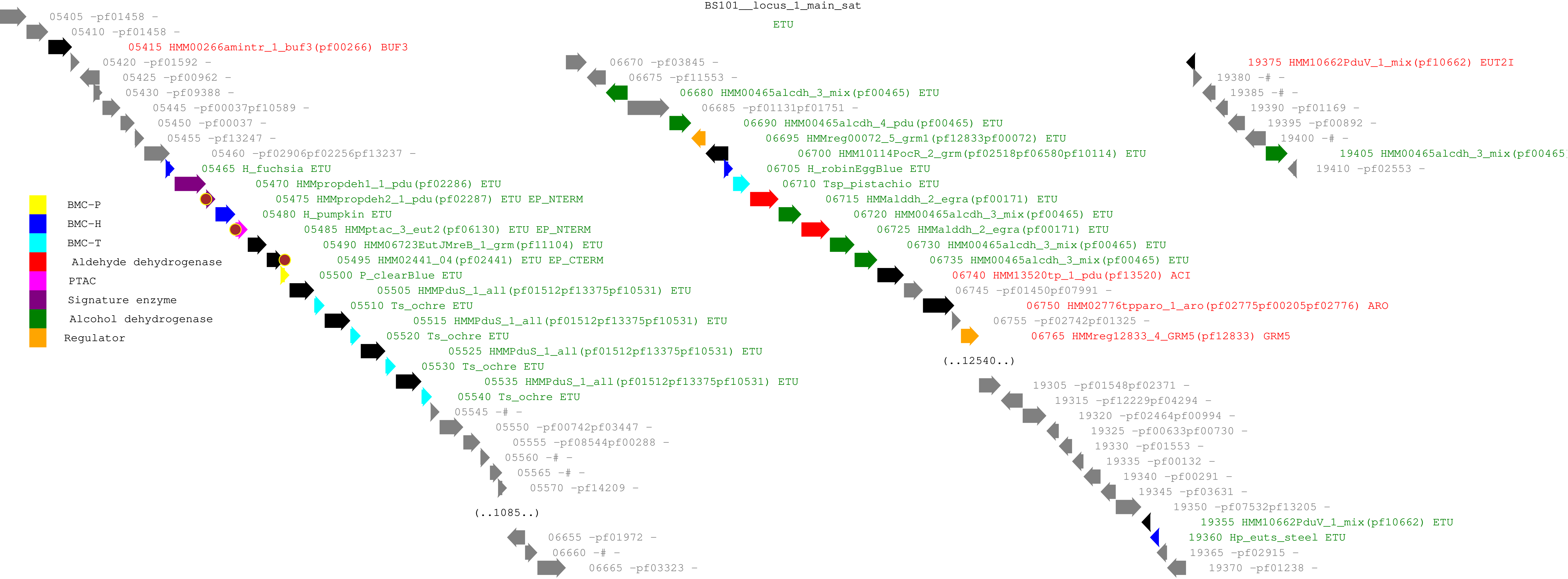

Clostridium kluyveri (strain ATCC 8527 / DSM 555 / NCIMB 10680). Bacteria; Firmicutes; Clostridia; Clostridiales; Clostridiaceae;

CKL__locus_1_main_sat

ETU

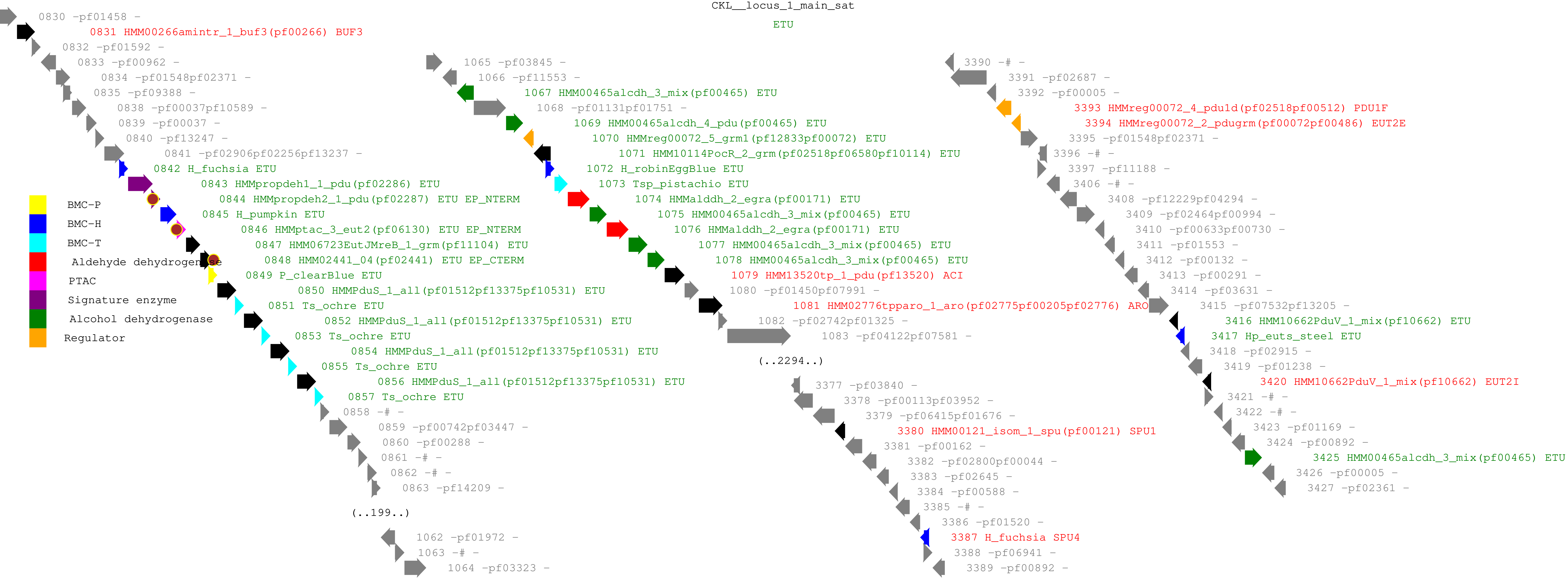

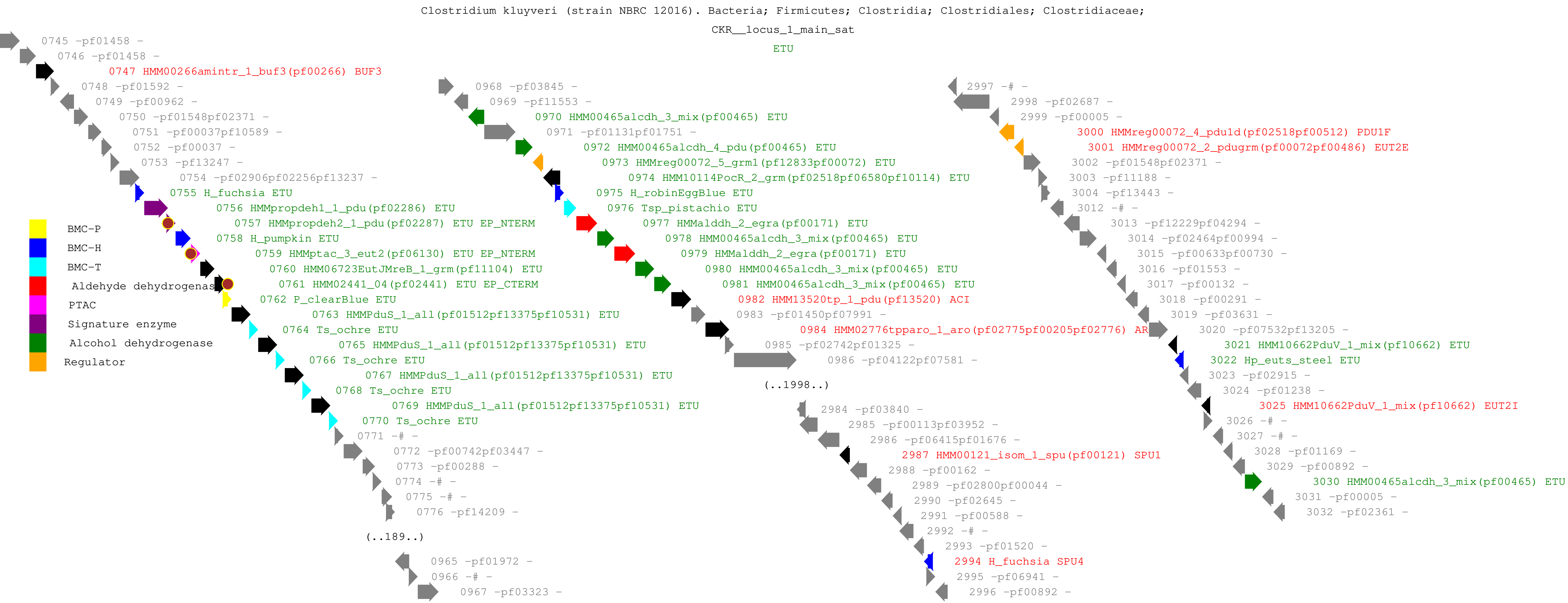
Clostridium kluyveri (strain NBRC 12016). Bacteria; Firmicutes; Clostridia; Clostridiales; Clostridiaceae;
CKR__locus_1_main_sat
ETU
0745 -pf01458 -
0746 -pf01458 -
0747 HMM00266amintr_1_buf3(pf00266) BUF3
0748 -pf01592 -
0749 -pf00962 -
0750 -pf01548pf02371 -
0751 -pf00037pf10589 -
0752 -pf00037 -
0753 -pf13247 -
0754 -pf02906pf02256pf13237 -
0755 H_fuchsia ETU
0756 HMMpropdeh1_1_pdu(pf02286) ETU
0757 HMMpropdeh2_1_pdu(pf02287) ETU EP_NTERM
0758 H_pumpkin ETU
0759 HMMptac_3_eut2(pf06130) ETU EP_NTERM
0760 HMM06723EutJMreB_1_grm(pf11104) ETU
0761 HMM02441_04(pf02441) ETU EP_CTERM
0762 P_clearBlue ETU
0763 HMMPduS_1_all(pf01512pf13375pf10531) ETU
0764 Ts_ochre ETU
0765 HMMPduS_1_all(pf01512pf13375pf10531) ETU
0766 Ts_ochre ETU
0767 HMMPduS_1_all(pf01512pf13375pf10531) ETU
0768 Ts_ochre ETU
0769 HMMPduS_1_all(pf01512pf13375pf10531) ETU
0770 Ts_ochre ETU
0771 -# -
0772 -pf00742pf03447 -
0773 -pf00288 -
0774 -# -
0775 -# -
0776 -pf14209 -
(..189..)
0965 -pf01972 -
0966 -# -
0967 -pf03323 -
BMC-P
BMC-H
BMC-T
Aldehyde dehydrogenas
PTAC
Signature enzyme
Alcohol dehydrogenase
Regulator
0968 -pf03845 -
0969 -pf11553 -
0970 HMM00465alcdh_3_mix(pf00465) ETU
0971 -pf01131pf01751 -
0972 HMM00465alcdh_4_pdu(pf00465) ETU
0973 HMMreg00072_5_grm1(pf12833pf00072) ETU
0974 HMM10114PocR_2_grm(pf02518pf06580pf10114) ETU
0975 H_robinEggBlue ETU
0976 Tsp_pistachio ETU
0977 HMMalddh_2_egra(pf00171) ETU
0978 HMM00465alcdh_3_mix(pf00465) ETU
0979 HMMalddh_2_egra(pf00171) ETU
0980 HMM00465alcdh_3_mix(pf00465) ETU
0981 HMM00465alcdh_3_mix(pf00465) ETU
0982 HMM13520tp_1_pdu(pf13520) ACI
0983 -pf01450pf07991 -
0984 HMM02776tpparo_1_aro(pf02775pf00205pf02776) AR
0985 -pf02742pf01325 -
0986 -pf04122pf07581 -
(..1998..)
2984 -pf03840 -
2985 -pf00113pf03952 -
2986 -pf06415pf01676 -
2987 HMM00121_isom_1_spu(pf00121) SPU1
2988 -pf00162 -
2989 -pf02800pf00044 -
2990 -pf02645 -
2991 -pf00588 -
2992 -# -
2993 -pf01520 -
2994 H_fuchsia SPU4
2995 -pf06941 -
2996 -pf00892 -
2997 -# -
2998 -pf02687 -
2999 -pf00005 -
3000 HMMreg00072_4_pdu1d(pf02518pf00512) PDU1F
3001 HMMreg00072_2_pdugrm(pf00072pf00486) EUT2E
3002 -pf01548pf02371 -
3003 -pf11188 -
3004 -pf13443 -
3012 -# -
3013 -pf12229pf04294 -
3014 -pf02464pf00994 -
3015 -pf00633pf00730 -
3016 -pf01553 -
3017 -pf00132 -
3018 -pf00291 -
3019 -pf03631 -
3020 -pf07532pf13205 -
3021 HMM10662PduV_1_mix(pf10662) ETU
3022 Hp_euts_steel ETU
3023 -pf02915 -
3024 -pf01238 -
3025 HMM10662PduV_1_mix(pf10662) EUT2I
3026 -# -
3027 -# -
3028 -pf01169 -
3029 -pf00892 -
3030 HMM00465alcdh_3_mix(pf00465) ETU
3031 -pf00005 -
3032 -pf02361 -

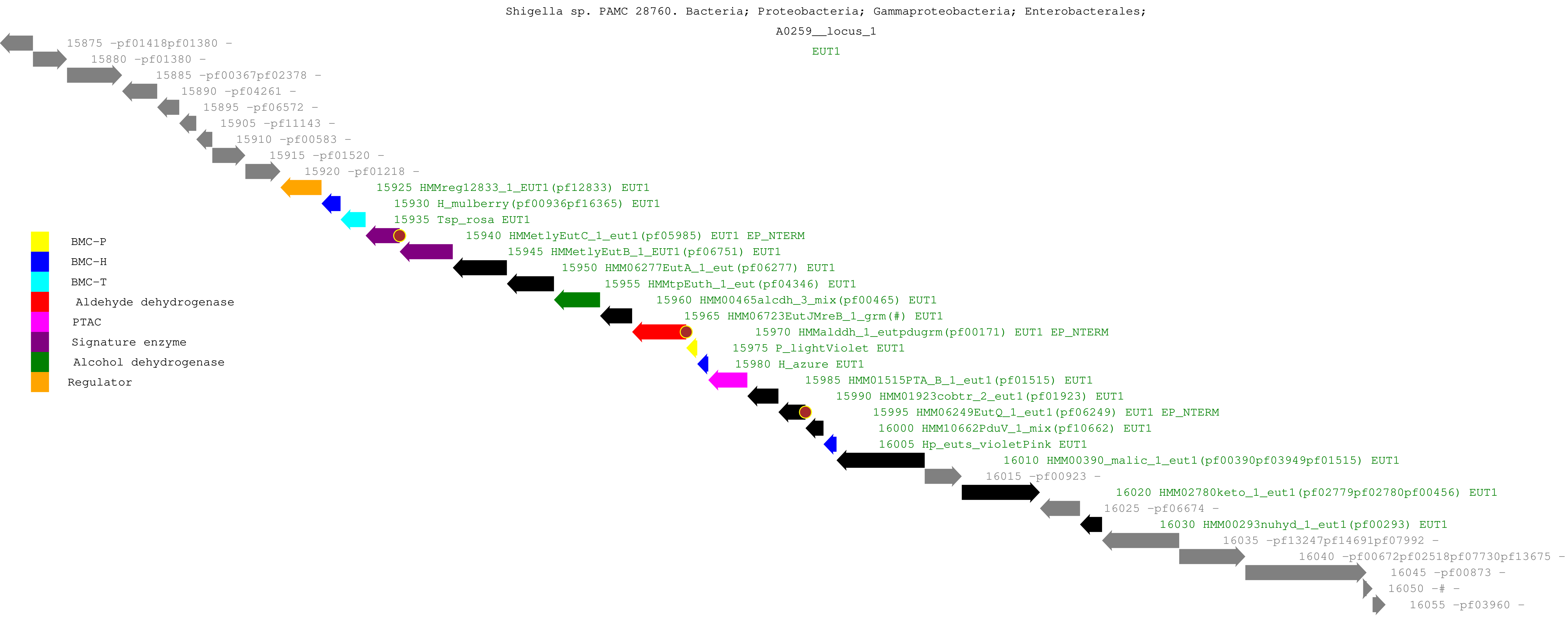
Shigella sp. PAMC 28760. Bacteria; Proteobacteria; Gammaproteobacteria; Enterobacterales;
A0259__locus_1
EUT1
15875 -pf01418pf01380 -
15880 -pf01380 -
15885 -pf00367pf02378 -
15890 -pf04261 -
15895 -pf06572 -
15905 -pf11143 -
15910 -pf00583 -
15915 -pf01520 -
15920 -pf01218 -
15925 HMMreg12833_1_EUT1(pf12833) EUT1
15930 H_mulberry(pf00936pf16365) EUT1
15935 Tsp_rosa EUT1
15940 HMMetlyEutC_1_eut1(pf05985) EUT1 EP_NTERM
15945 HMMetlyEutB_1_EUT1(pf06751) EUT1
15950 HMM06277EutA_1_eut(pf06277) EUT1
15955 HMMtpEuth_1_eut(pf04346) EUT1
15960 HMM00465alcdh_3_mix(pf00465) EUT1
15965 HMM06723EutJMreB_1_grm(#) EUT1
15970 HMMalddh_1_eutpdugrm(pf00171) EUT1 EP_NTERM
15975 P_lightViolet EUT1
15980 H_azure EUT1
15985 HMM01515PTA_B_1_eut1(pf01515) EUT1
15990 HMM01923cobtr_2_eut1(pf01923) EUT1
15995 HMM06249EutQ_1_eut1(pf06249) EUT1 EP_NTERM
16000 HMM10662PduV_1_mix(pf10662) EUT1
16005 Hp_euts_violetPink EUT1
16010 HMM00390_malic_1_eut1(pf00390pf03949pf01515) EUT1
16015 -pf00923 -
16020 HMM02780keto_1_eut1(pf02779pf02780pf00456) EUT1
16025 -pf06674 -
16030 HMM00293nuhyd_1_eut1(pf00293) EUT1
16035 -pf13247pf14691pf07992 -
16040 -pf00672pf02518pf07730pf13675 -
16045 -pf00873 -
16050 -# -
16055 -pf03960 -
BMC-P
BMC-H
BMC-T
Aldehyde dehydrogenase
PTAC
Signature enzyme
Alcohol dehydrogenase
Regulator

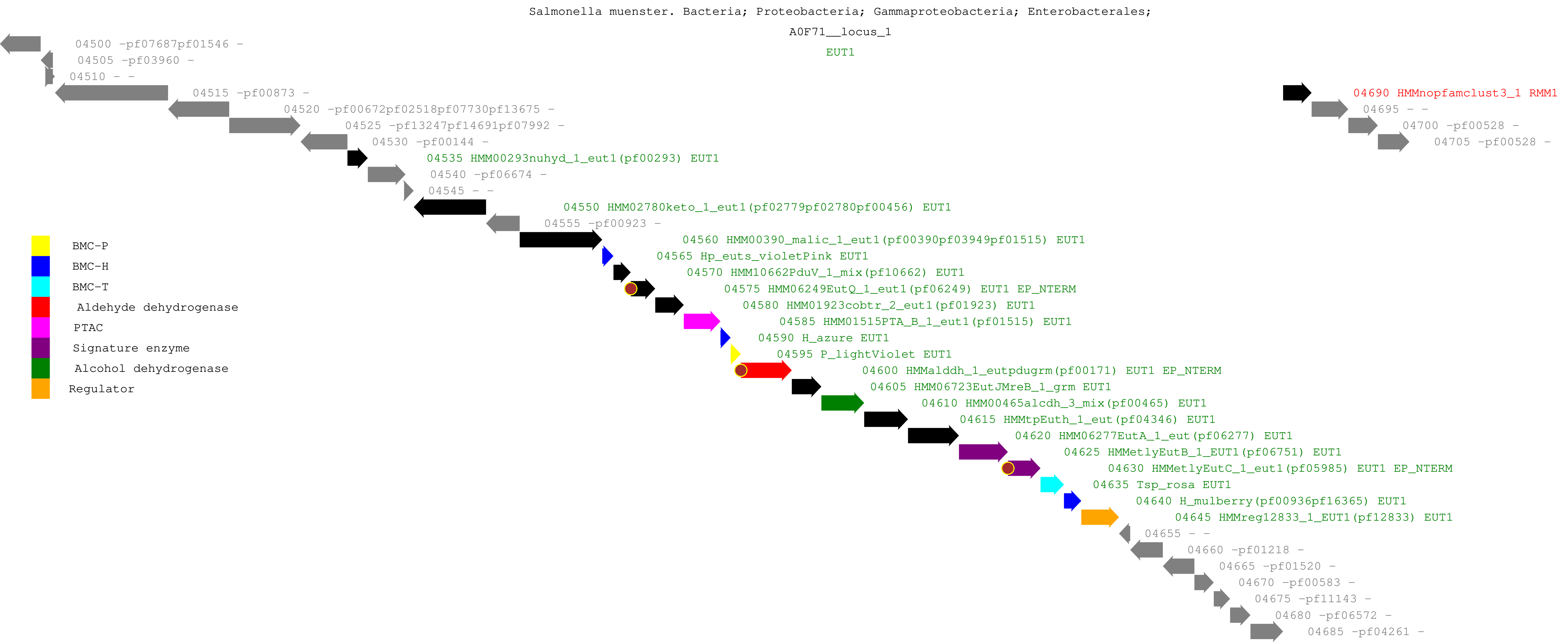

Salmonella muenster. Bacteria; Proteobacteria; Gammaproteobacteria; Enterobacterales;
A0F71__locus_1
EUT1
04500 -pf07687pf01546 -
04505 -pf03960 -
04510 - -
04515 -pf00873 -
04520 -pf00672pf02518pf07730pf13675 -
04525 -pf13247pf14691pf07992 -
04530 -pf00144 -
04535 HMM00293nuhyd_1_eut1(pf00293) EUT1
04540 -pf06674 -
04545 - -
04550 HMM02780keto_1_eut1(pf02779pf02780pf00456) EUT1
04555 -pf00923 -
04560 HMM00390_malic_1_eut1(pf00390pf03949pf01515) EUT1
04565 Hp_euts_violetPink EUT1
04570 HMM10662PduV_1_mix(pf10662) EUT1
04575 HMM06249EutQ_1_eut1(pf06249) EUT1 EP_NTERM
04580 HMM01923cobtr_2_eut1(pf01923) EUT1
04585 HMM01515PTA_B_1_eut1(pf01515) EUT1
04590 H_azure EUT1
04595 P_lightViolet EUT1
04600 HMMaldh_1_eutpdugrm(pf00171) EUT1 EP_NTERM
04605 HMM06723EutJMreB_1_grm EUT1
04610 HMM00465alcdh_3_mix(pf00465) EUT1
04615 HMMtpEuth_1_eut(pf04346) EUT1
04620 HMM06277EutA_1_eut(pf06277) EUT1
04625 HMMetlyEutB_1_EUT1(pf06751) EUT1
04630 HMMetlyEutC_1_eut1(pf05985) EUT1 EP_NTERM
04635 Tsp_rosa EUT1
04640 H_mulberry(pf00936pf16365) EUT1
04645 HMMreg12833_1_EUT1(pf12833) EUT1
04655 - -
04660 -pf01218 -
04665 -pf01520 -
04670 -pf00583 -
04675 -pf11143 -
04680 -pf06572 -
04685 -pf04261 -
04690 HMMnopfamclust3_1 RMM1
04695 - -
04700 -pf00528 -
04705 -pf00528 -
BMC-P
BMC-H
BMC-T
Aldehyde dehydrogenase
PTAC
Signature enzyme
Alcohol dehydrogenase
Regulator

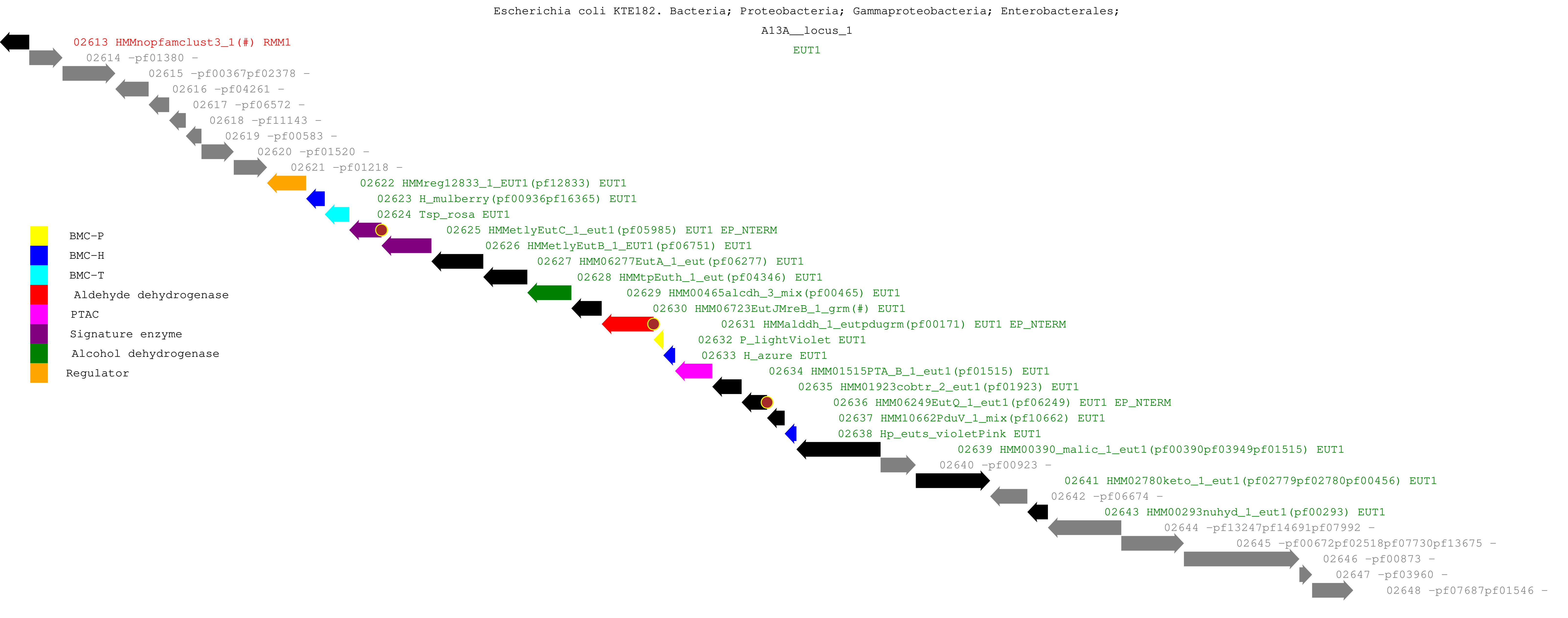
Escherichia coli KTE182. Bacteria; Proteobacteria; Gammaproteobacteria; Enterobacterales;
A13A__locus_1
EUT1
02613 HMMnopfamclust3_1(#) RMM1
02614 -pf01380 -
02615 -pf00367pf02378 -
02616 -pf04261 -
02617 -pf06572 -
02618 -pf11143 -
02619 -pf00583 -
02620 -pf01520 -
02621 -pf01218 -
02622 HMMreg12833_1_EUT1(pf12833) EUT1
02623 H_mulberry(pf00936pf16365) EUT1
02624 Tsp_rosa EUT1
02625 HMMetlyEutC_1_eut1(pf05985) EUT1 EP_NTERM
02626 HMMetlyEutB_1_EUT1(pf06751) EUT1
02627 HMM06277EutA_1_eut(pf06277) EUT1
02628 HMMtpEuth_1_eut(pf04346) EUT1
02629 HMM00465alcdh_3_mix(pf00465) EUT1
02630 HMM06723EutJMreB_1_grm(#) EUT1
02631 HMMalddh_1_eutpdugrm(pf00171) EUT1 EP_NTERM
02632 P_lightViolet EUT1
02633 H_azure EUT1
02634 HMM01515PTA_B_1_eut1(pf01515) EUT1
02635 HMM01923cobtr_2_eut1(pf01923) EUT1
02636 HMM06249EutQ_1_eut1(pf06249) EUT1 EP_NTERM
02637 HMM10662PduV_1_mix(pf10662) EUT1
02638 Hp_euts_violetPink EUT1
02639 HMM00390_malic_1_eut1(pf00390pf03949pf01515) EUT1
02640 -pf00923 -
02641 HMM02780keto_1_eut1(pf02779pf02780pf00456) EUT1
02642 -pf06674 -
02643 HMM00293nuhyd_1_eut1(pf00293) EUT1
02644 -pf13247pf14691pf07992 -
02645 -pf00672pf02518pf07730pf13675 -
02646 -pf00873 -
02647 -pf03960 -
02648 -pf07687pf01546 -
BMC-P
BMC-H
BMC-T
Aldehyde dehydrogenase
PTAC
Signature enzyme
Alcohol dehydrogenase
Regulator

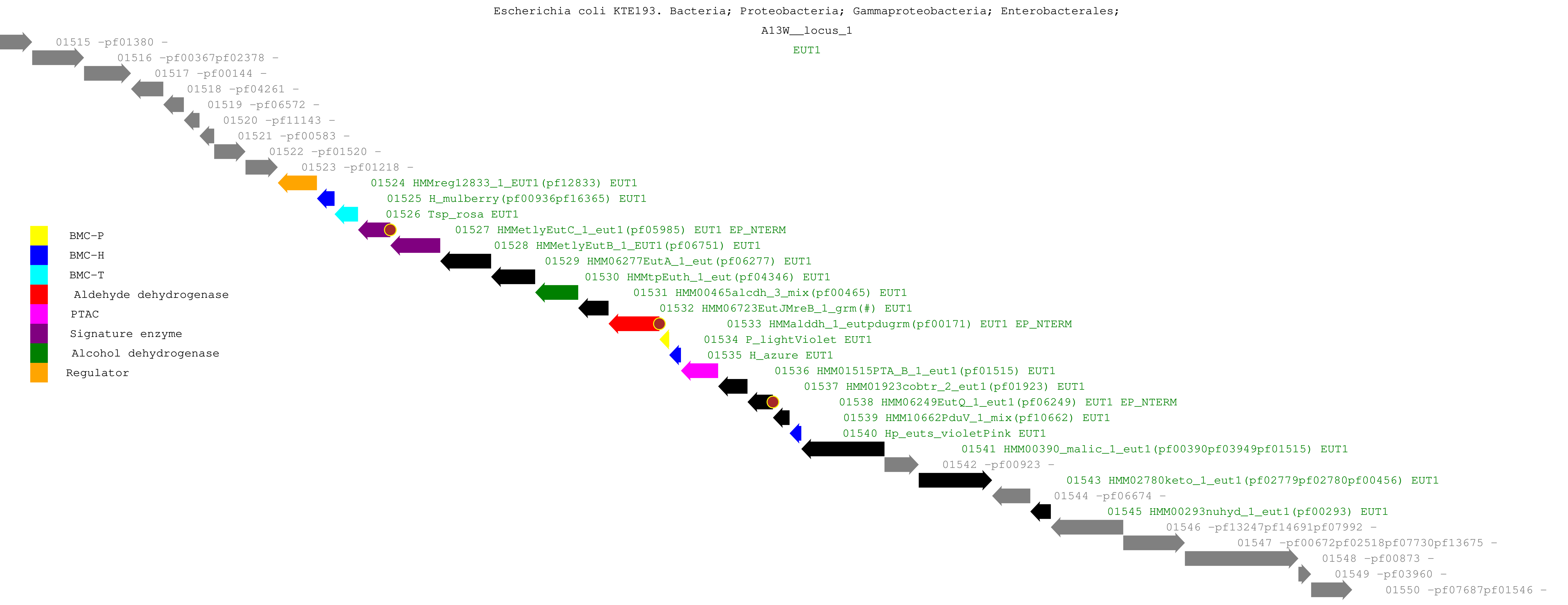

Escherichia coli KTE193. Bacteria; Proteobacteria; Gammaproteobacteria; Enterobacterales;
A13W__locus_1
EUT1
01515 -pf01380 -
01516 -pf00367pf02378 -
01517 -pf00144 -
01518 -pf04261 -
01519 -pf06572 -
01520 -pf11143 -
01521 -pf00583 -
01522 -pf01520 -
01523 -pf01218 -
01524 HMMreg12833_1_EUT1(pf12833) EUT1
01525 H_mulberry(pf00936pf16365) EUT1
01526 Tsp_rosa EUT1
01527 HMMetlyEutC_1_eut1(pf05985) EUT1 EP_NTERM
01528 HMMetlyEutB_1_EUT1(pf06751) EUT1
01529 HMM06277EutA_1_eut(pf06277) EUT1
01530 HMMtpEuth_1_eut(pf04346) EUT1
01531 HMM00465alcdh_3_mix(pf00465) EUT1
01532 HMM06723EutJMreB_1_grm(#) EUT1
01533 HMMalddh_1_eutpdugrm(pf00171) EUT1 EP_NTERM
01534 P_lightViolet EUT1
01535 H_azure EUT1
01536 HMM01515PTA_B_1_eut1(pf01515) EUT1
01537 HMM01923cobtr_2_eut1(pf01923) EUT1
01538 HMM06249EutQ_1_eut1(pf06249) EUT1 EP_NTERM
01539 HMM10662PduV_1_mix(pf10662) EUT1
01540 Hp_euts_violetPink EUT1
01541 HMM00390_malic_1_eut1(pf00390pf03949pf01515) EUT1
01542 -pf00923 -
01543 HMM02780keto_1_eut1(pf02779pf02780pf00456) EUT1
01544 -pf06674 -
01545 HMM00293nuhyd_1_eut1(pf00293) EUT1
01546 -pf13247pf14691pf07992 -
01547 -pf00672pf02518pf07730pf13675 -
01548 -pf00873 -
01549 -pf03960 -
01550 -pf07687pf01546 -
BMC-P
BMC-H
BMC-T
Aldehyde dehydrogenase
PTAC
Signature enzyme
Alcohol dehydrogenase
Regulator

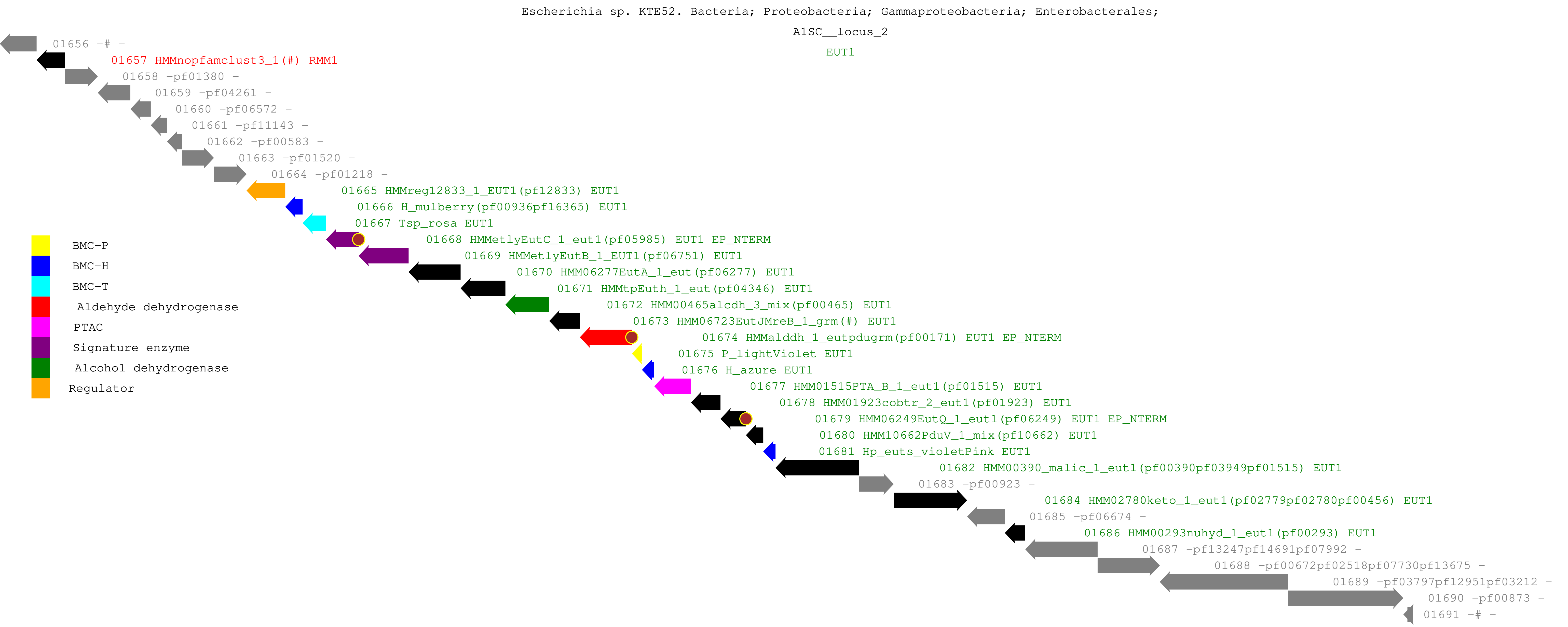
Escherichia sp. KTE52. Bacteria; Proteobacteria; Gammaproteobacteria; Enterobacterales;
A1SC__locus_2
EUT1
01656 -# -
01657 HMMnopfamclust3_1(#) RMM1
01658 -pf01380 -
01659 -pf04261 -
01660 -pf06572 -
01661 -pf11143 -
01662 -pf00583 -
01663 -pf01520 -
01664 -pf01218 -
01665 HMMreg12833_1_EUT1(pf12833) EUT1
01666 H_mulberry(pf00936pf16365) EUT1
01667 Tsp_rosa EUT1
01668 HMMetlyEutC_1_eut1(pf05985) EUT1 EP_NTERM
01669 HMMetlyEutB_1_EUT1(pf06751) EUT1
01670 HMM06277EutA_1_eut(pf06277) EUT1
01671 HMMtpEuth_1_eut(pf04346) EUT1
01672 HMM00465alcdh_3_mix(pf00465) EUT1
01673 HMM06723EutJMreB_1_grm(#) EUT1
01674 HMMalddh_1_eutpdugrm(pf00171) EUT1 EP_NTERM
01675 P_lightViolet EUT1
01676 H_azure EUT1
01677 HMM01515PTA_B_1_eut1(pf01515) EUT1
01678 HMM01923cobtr_2_eut1(pf01923) EUT1
01679 HMM06249EutQ_1_eut1(pf06249) EUT1 EP_NTERM
01680 HMM10662PduV_1_mix(pf10662) EUT1
01681 Hp_euts_violetPink EUT1
01682 HMM00390_malic_1_eut1(pf00390pf03949pf01515) EUT1
01683 -pf00923 -
01684 HMM02780keto_1_eut1(pf02779pf02780pf00456) EUT1
01685 -pf06674 -
01686 HMM00293nuhyd_1_eut1(pf00293) EUT1
01687 -pf13247pf14691pf07992 -
01688 -pf00672pf02518pf07730pf13675 -
01689 -pf03797pf12951pf03212 -
01690 -pf00873 -
01691 -# -
BMC-P
BMC-H
BMC-T
Aldehyde dehydrogenase
PTAC
Signature enzyme
Alcohol dehydrogenase
Regulator

Escherichia coli KTE64. Bacteria; Proteobacteria; Gammaproteobacteria; Enterobacterales;

A1U1__locus_1

EUT1

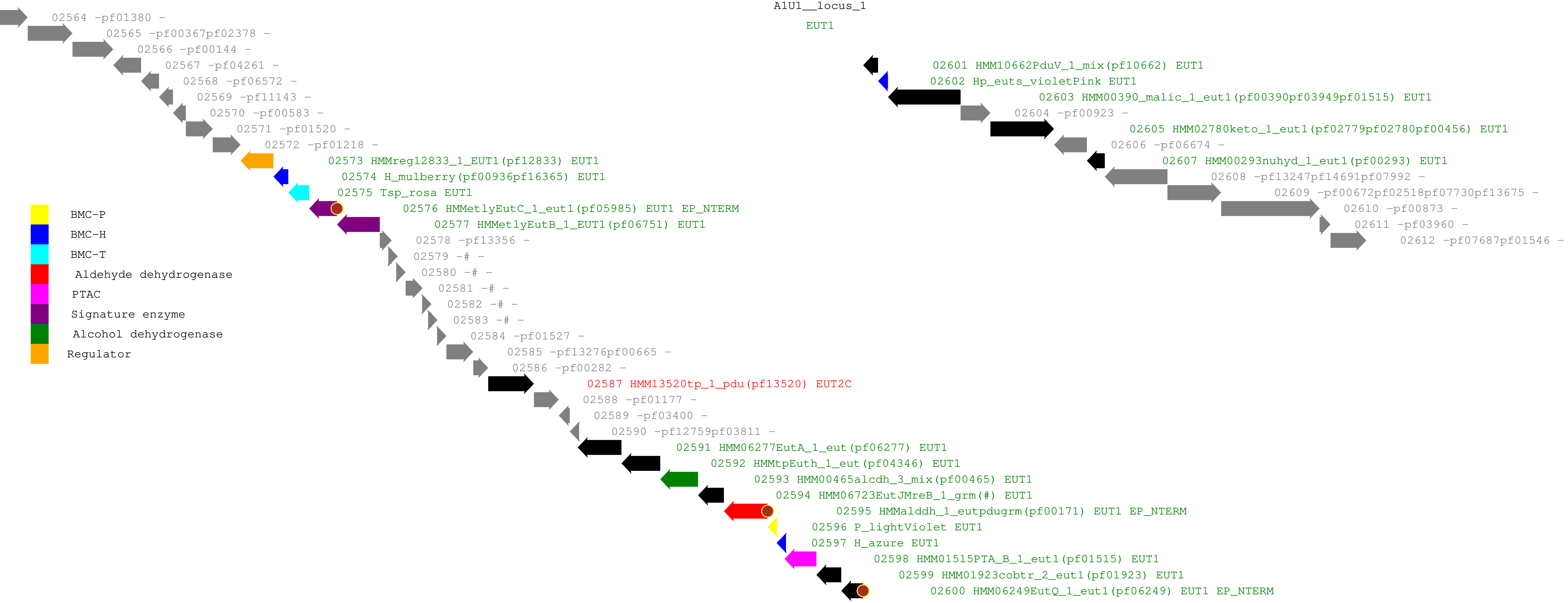

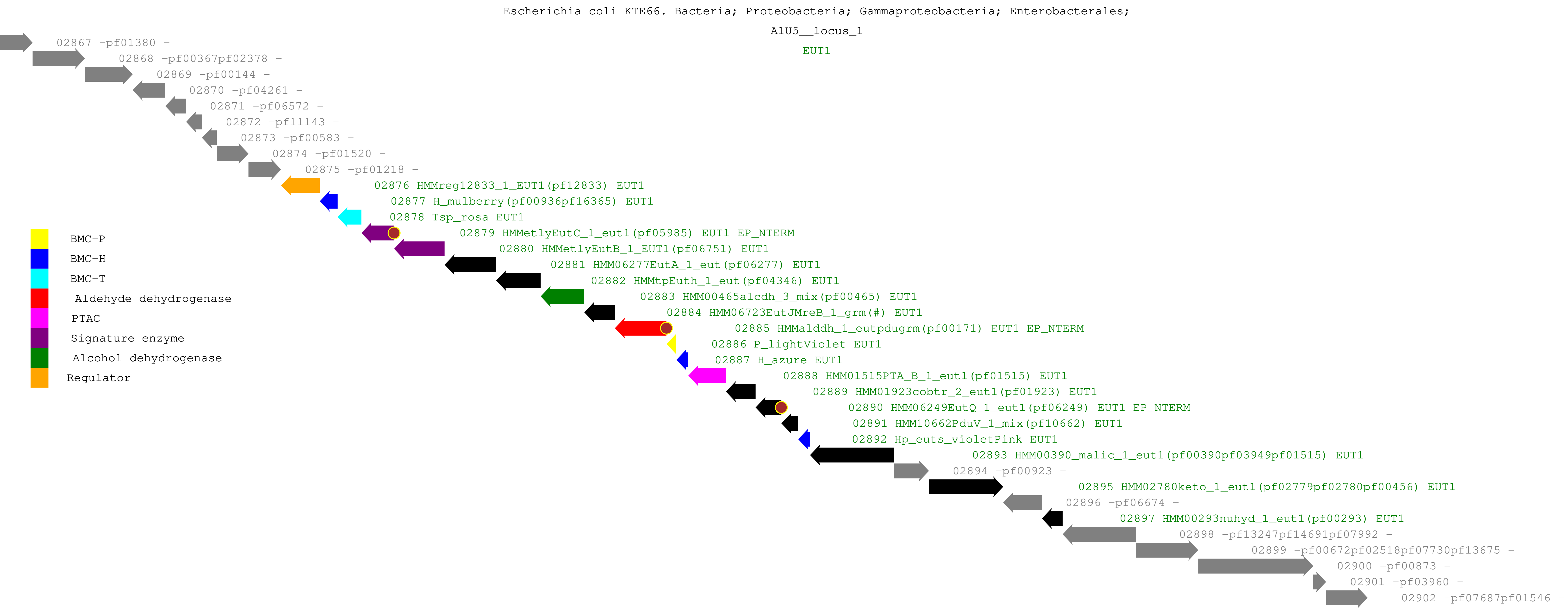
Escherichia coli KTE66. Bacteria; Proteobacteria; Gammaproteobacteria; Enterobacterales;
A1U5__locus_1
EUT1
02867 -pf01380 -
02868 -pf00367pf02378 -
02869 -pf00144 -
02870 -pf04261 -
02871 -pf06572 -
02872 -pf11143 -
02873 -pf00583 -
02874 -pf01520 -
02875 -pf01218 -
02876 HMMreg12833_1_EUT1(pf12833) EUT1
02877 H_mulberry(pf00936pf16365) EUT1
02878 Tsp_rosa EUT1
02879 HMMetlyEutC_1_eut1(pf05985) EUT1 EP_NTERM
02880 HMMetlyEutB_1_EUT1(pf06751) EUT1
02881 HMM06277EutA_1_eut(pf06277) EUT1
02882 HMMtpEuth_1_eut(pf04346) EUT1
02883 HMM00465alcdh_3_mix(pf00465) EUT1
02884 HMM06723EutJMreB_1_grm(#) EUT1
02885 HMMalddh_1_eutpdugrm(pf00171) EUT1 EP_NTERM
02886 P_lightViolet EUT1
02887 H_azure EUT1
02888 HMM01515PTA_B_1_eut1(pf01515) EUT1
02889 HMM01923cobtr_2_eut1(pf01923) EUT1
02890 HMM06249EutQ_1_eut1(pf06249) EUT1 EP_NTERM
02891 HMM10662PduV_1_mix(pf10662) EUT1
02892 Hp_euts_violetPink EUT1
02893 HMM00390_malic_1_eut1(pf00390pf03949pf01515) EUT1
02894 -pf00923 -
02895 HMM02780keto_1_eut1(pf02779pf02780pf00456) EUT1
02896 -pf06674 -
02897 HMM00293nuhyd_1_eut1(pf00293) EUT1
02898 -pf13247pf14691pf07992 -
02899 -pf00672pf02518pf07730pf13675 -
02900 -pf00873 -
02901 -pf03960 -
02902 -pf07687pf01546 -
BMC-P
BMC-H
BMC-T
Aldehyde dehydrogenase
PTAC
Signature enzyme
Alcohol dehydrogenase
Regulator

Escherichia coli KTE73. Bacteria; Proteobacteria; Gammaproteobacteria; Enterobacterales;

A1UI__locus_1

EUT1

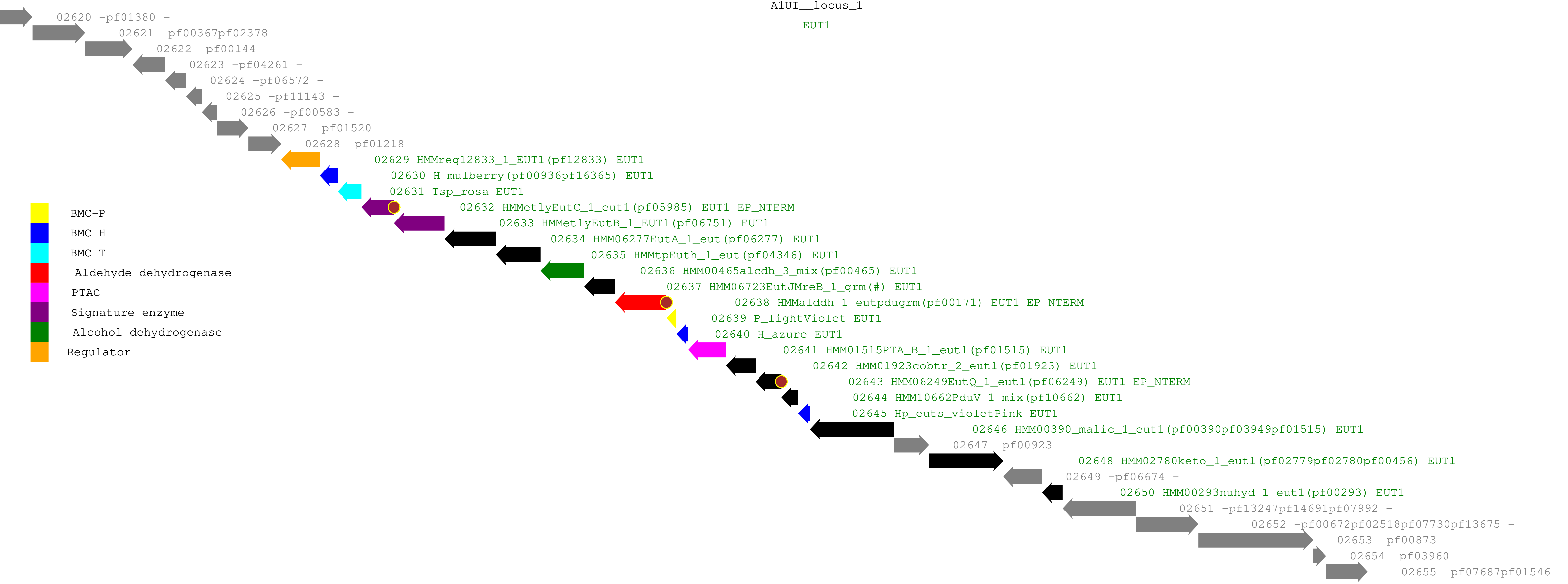

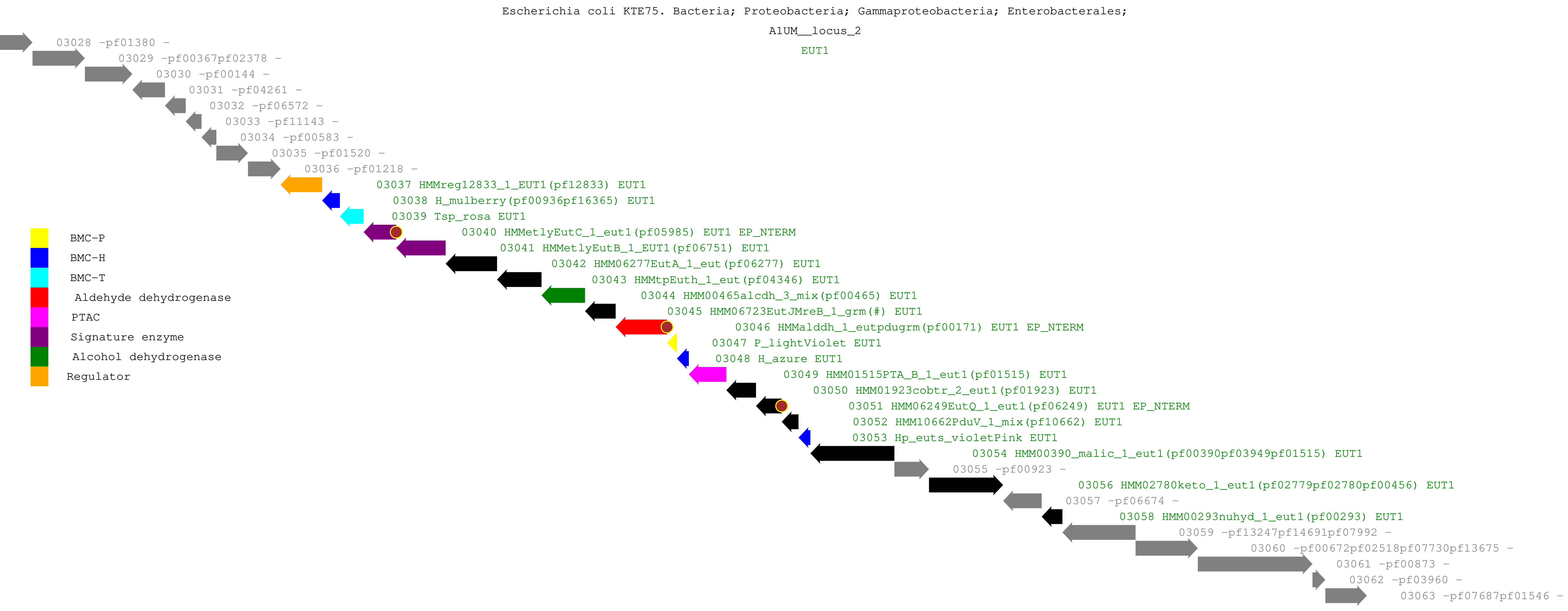
Escherichia coli KTE75. Bacteria; Proteobacteria; Gammaproteobacteria; Enterobacterales;
A1UM__locus_2
EUT1
03028 -pf01380 -
03029 -pf00367pf02378 -
03030 -pf00144 -
03031 -pf04261 -
03032 -pf06572 -
03033 -pf11143 -
03034 -pf00583 -
03035 -pf01520 -
03036 -pf01218 -
03037 HMMreg12833_1_EUT1(pf12833) EUT1
03038 H_mulberry(pf00936pf16365) EUT1
03039 Tsp_rosa EUT1
03040 HMMetlyEutC_1_eut1(pf05985) EUT1 EP_NTERM
03041 HMMetlyEutB_1_EUT1(pf06751) EUT1
03042 HMM06277EutA_1_eut(pf06277) EUT1
03043 HMMtpEuth_1_eut(pf04346) EUT1
03044 HMM00465alcdh_3_mix(pf00465) EUT1
03045 HMM06723EutJMreB_1_grm(#) EUT1
03046 HMMalddh_1_eutpdugrm(pf00171) EUT1 EP_NTERM
03047 P_lightViolet EUT1
03048 H_azure EUT1
03049 HMM01515PTA_B_1_eut1(pf01515) EUT1
03050 HMM01923cobtr_2_eut1(pf01923) EUT1
03051 HMM06249EutQ_1_eut1(pf06249) EUT1 EP_NTERM
03052 HMM10662PduV_1_mix(pf10662) EUT1
03053 Hp_euts_violetPink EUT1
03054 HMM00390_malic_1_eut1(pf00390pf03949pf01515) EUT1
03055 -pf00923 -
03056 HMM02780keto_1_eut1(pf02779pf02780pf00456) EUT1
03057 -pf06674 -
03058 HMM00293nuhyd_1_eut1(pf00293) EUT1
03059 -pf13247pf14691pf07992 -
03060 -pf00672pf02518pf07730pf13675 -
03061 -pf00873 -
03062 -pf03960 -
03063 -pf07687pf01546 -
BMC-P
BMC-H
BMC-T
Aldehyde dehydrogenase
PTAC
Signature enzyme
Alcohol dehydrogenase
Regulator

Escherichia coli KTE76. Bacteria; Proteobacteria; Gammaproteobacteria; Enterobacterales;

A1UO__locus_1

EUT1

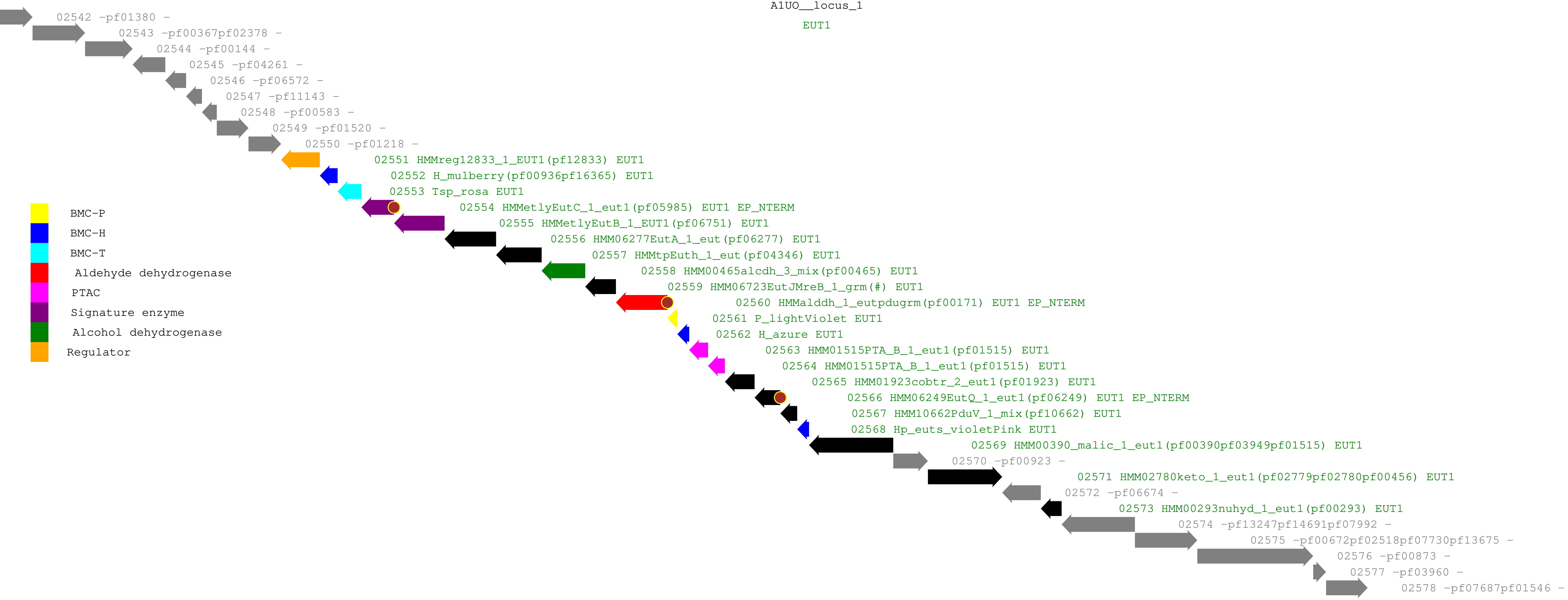

Escherichia sp. KTE96. Bacteria; Proteobacteria; Gammaproteobacteria; Enterobacterales;

A1WG__locus_2

EUT1

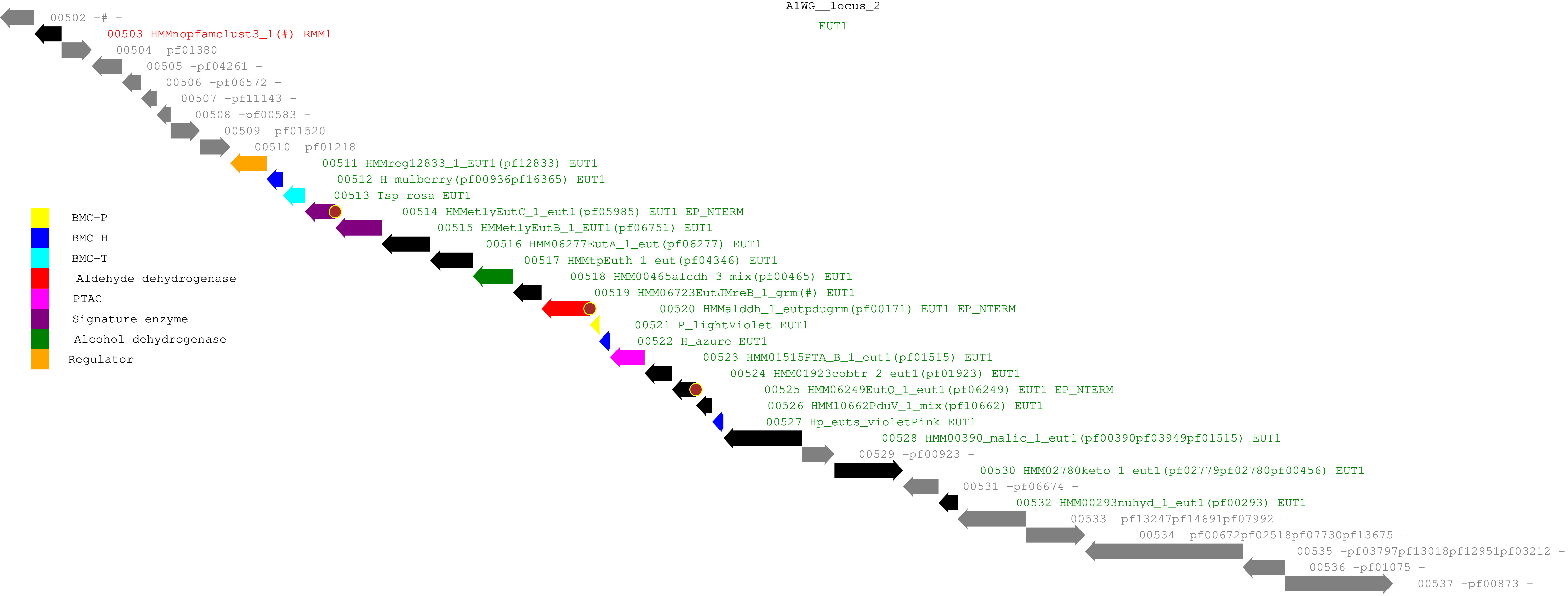

Escherichia coli KTE100. Bacteria; Proteobacteria; Gammaproteobacteria; Enterobacterales;

A1WK__locus_1

EUT1

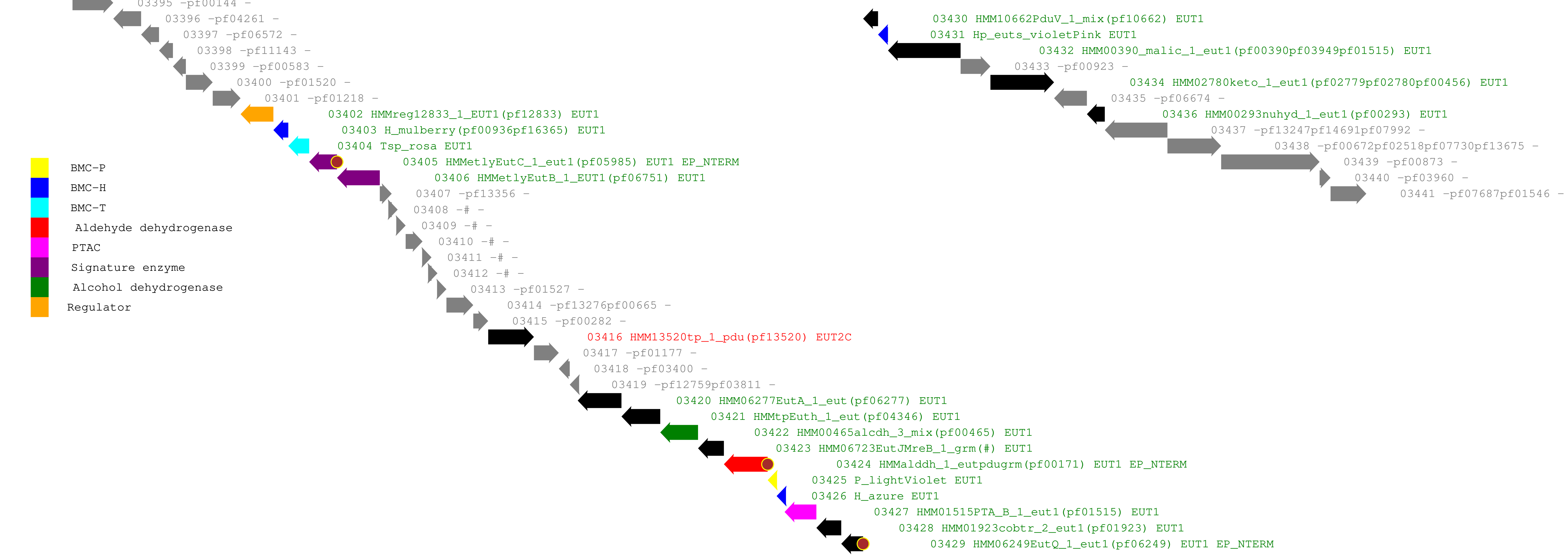

Escherichia coli KTE107. Bacteria; Proteobacteria; Gammaproteobacteria; Enterobacterales;

A1WS__locus_1

EUT1

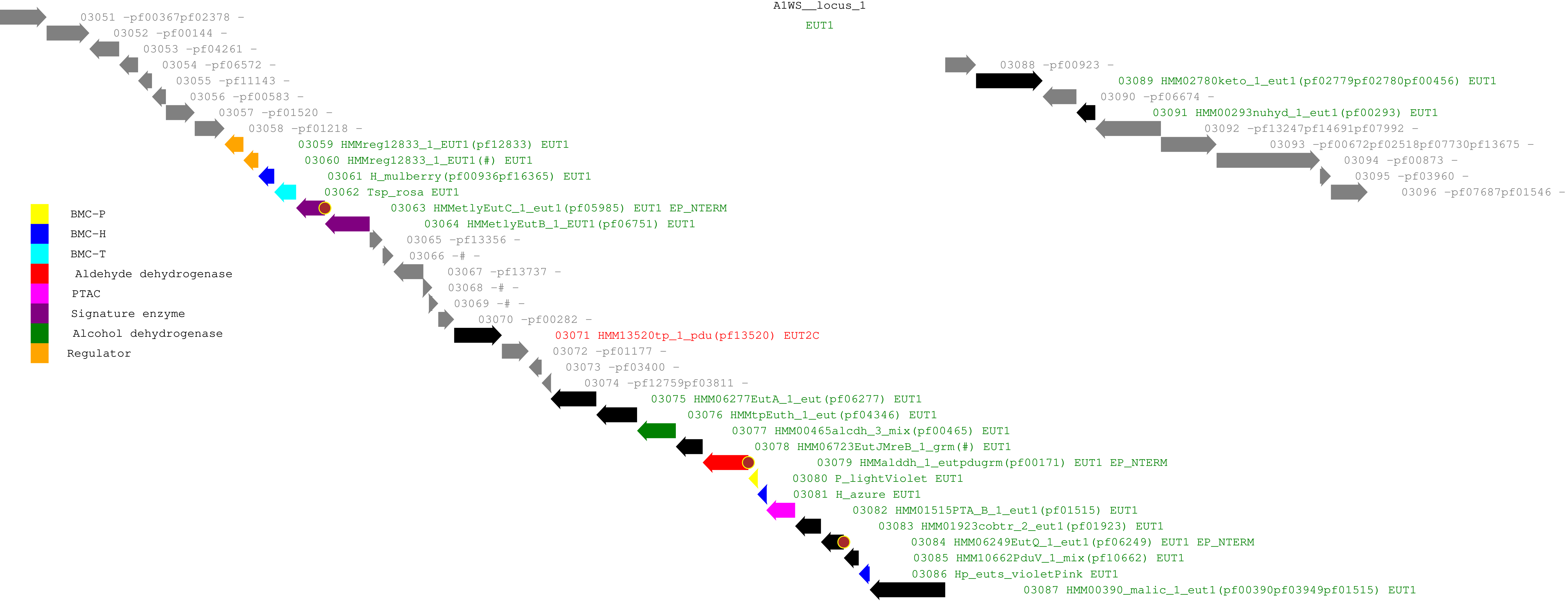

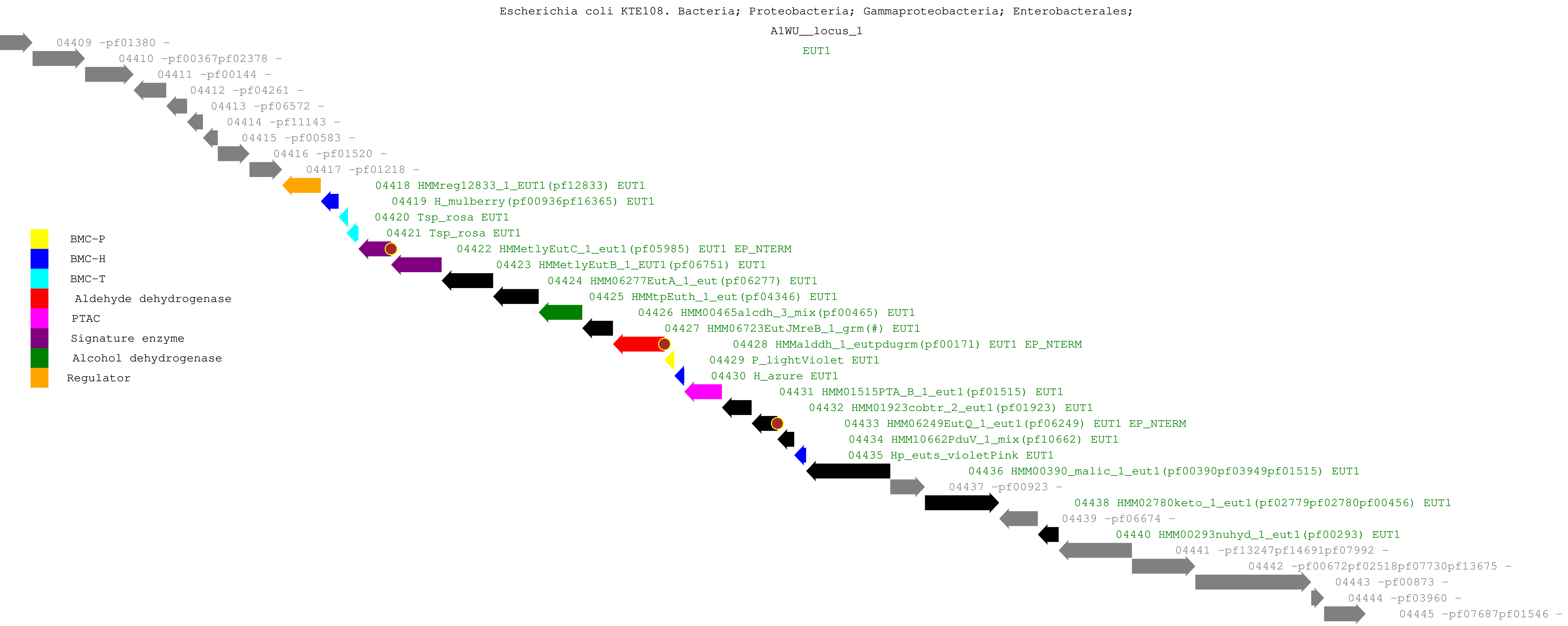
Escherichia coli KTE108. Bacteria; Proteobacteria; Gammaproteobacteria; Enterobacterales;
A1WU__locus_1
EUT1
04409 -pf01380 -
04410 -pf00367pf02378 -
04411 -pf00144 -
04412 -pf04261 -
04413 -pf06572 -
04414 -pf11143 -
04415 -pf00583 -
04416 -pf01520 -
04417 -pf01218 -
04418 HMMreg12833_1_EUT1(pf12833) EUT1
04419 H_mulberry(pf00936pf16365) EUT1
04420 Tsp_rosa EUT1
04421 Tsp_rosa EUT1
04422 HMMetlyEutC_1_eut1(pf05985) EUT1 EP_NTERM
04423 HMMetlyEutB_1_EUT1(pf06751) EUT1
04424 HMM06277EutA_1_eut(pf06277) EUT1
04425 HMMtpEuth_1_eut(pf04346) EUT1
04426 HMM00465alcdh_3_mix(pf00465) EUT1
04427 HMM06723EutJMreB_1_grm(#) EUT1
04428 HMMalddh_1_eutpdugrm(pf00171) EUT1 EP_NTERM
04429 P_lightViolet EUT1
04430 H_azure EUT1
04431 HMM01515PTA_B_1_eut1(pf01515) EUT1
04432 HMM01923cobtr_2_eut1(pf01923) EUT1
04433 HMM06249EutQ_1_eut1(pf06249) EUT1 EP_NTERM
04434 HMM10662PduV_1_mix(pf10662) EUT1
04435 Hp_euts_violetPink EUT1
04436 HMM00390_malic_1_eut1(pf00390pf03949pf01515) EUT1
04437 -pf00923 -
04438 HMM02780keto_1_eut1(pf02779pf02780pf00456) EUT1
04439 -pf06674 -
04440 HMM00293nuhyd_1_eut1(pf00293) EUT1
04441 -pf13247pf14691pf07992 -
04442 -pf00672pf02518pf07730pf13675 -
04443 -pf00873 -
04444 -pf03960 -
04445 -pf07687pf01546 -
BMC-P
BMC-H
BMC-T
Aldehyde dehydrogenase
PTAC
Signature enzyme
Alcohol dehydrogenase
Regulator

Klebsiella michiganensis E718. Bacteria; Proteobacteria; Gammaproteobacteria; Enterobacterales;

A225__locus_2

EUT1

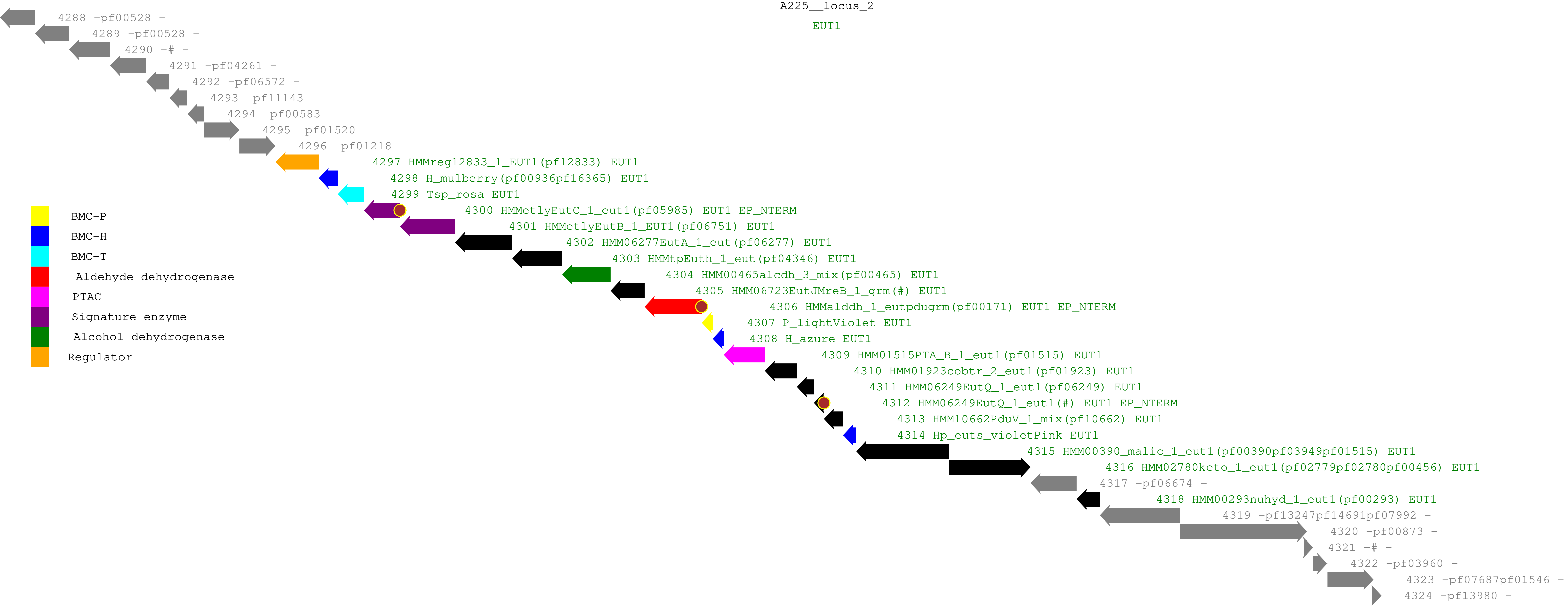

Burkholderiales bacterium RIFOXYC12_FULL_60_6. Bacteria; Proteobacteria; Betaproteobacteria; Burkholderiales.

A2496__locus_1_main_sat

EUT1

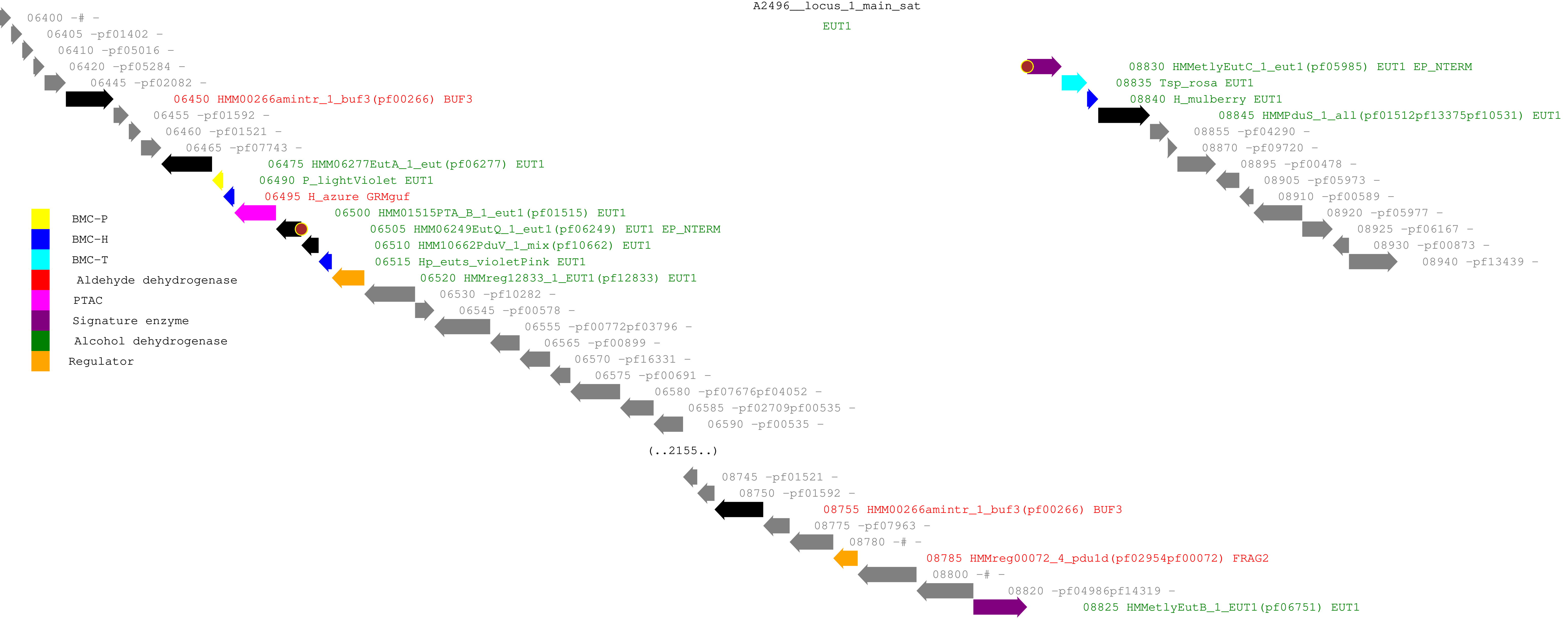

Burkholderiales bacterium RIFOXYD12_FULL_59_19. Bacteria; Proteobacteria; Betaproteobacteria; Burkholderiales.

A2503__locus_1

EUT1

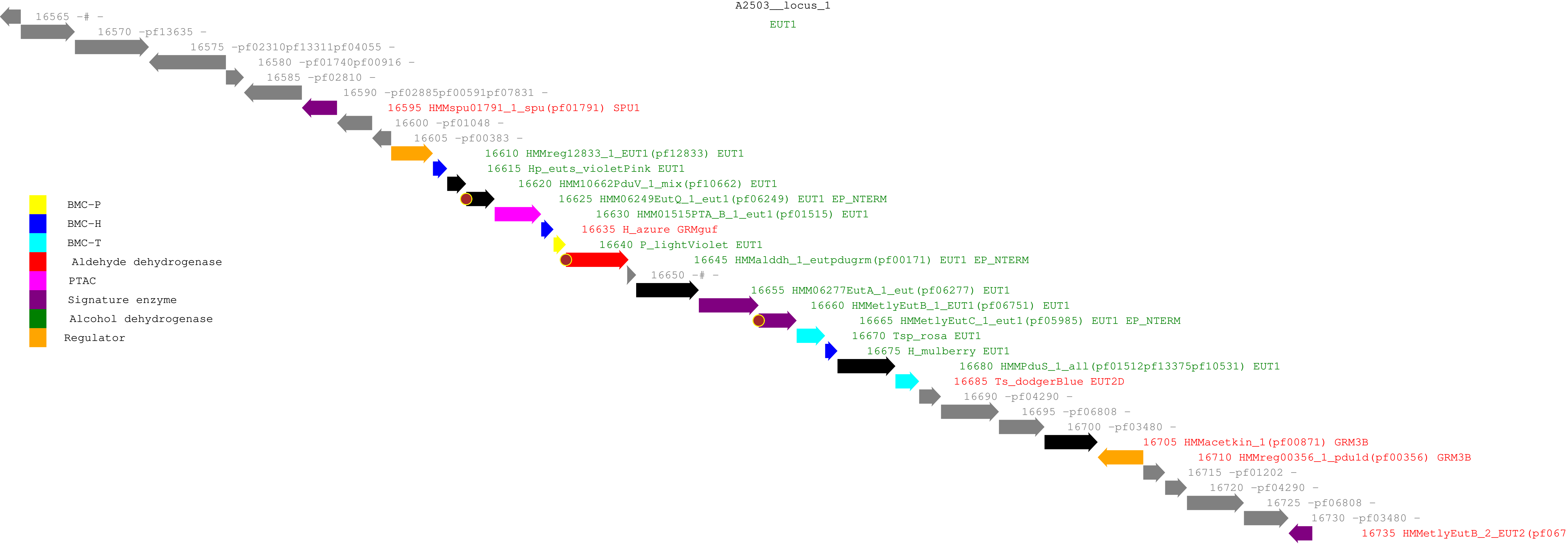

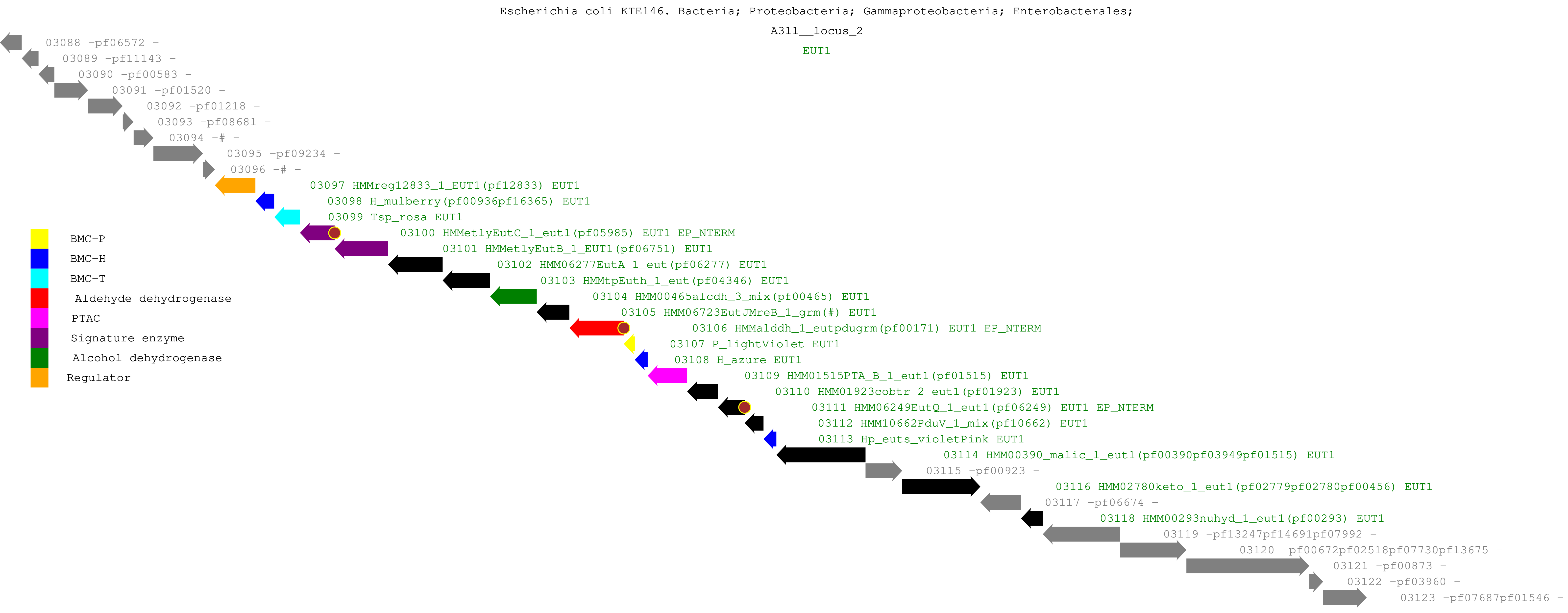

Escherichia coli KTE146. Bacteria; Proteobacteria; Gammaproteobacteria; Enterobacterales;
A311__locus_2
EUT1
03088 -pf06572 -
03089 -pf11143 -
03090 -pf00583 -
03091 -pf01520 -
03092 -pf01218 -
03093 -pf08681 -
03094 -# -
03095 -pf09234 -
03096 -# -
03097 HMMreg12833_1_EUT1(pf12833) EUT1
03098 H_mulberry(pf00936pf16365) EUT1
03099 Tsp_rosa EUT1
03100 HMMetlyEutC_1_eut1(pf05985) EUT1 EP_NTERM
03101 HMMetlyEutB_1_EUT1(pf06751) EUT1
03102 HMM06277EutA_1_eut(pf06277) EUT1
03103 HMMtpEuth_1_eut(pf04346) EUT1
03104 HMM00465alcdh_3_mix(pf00465) EUT1
03105 HMM06723EutJMreB_1_grm(#) EUT1
03106 HMMalddh_1_eutpdugrm(pf00171) EUT1 EP_NTERM
03107 P_lightViolet EUT1
03108 H_azure EUT1
03109 HMM01515PTA_B_1_eut1(pf01515) EUT1
03110 HMM01923cobtr_2_eut1(pf01923) EUT1
03111 HMM06249EutQ_1_eut1(pf06249) EUT1 EP_NTERM
03112 HMM10662PduV_1_mix(pf10662) EUT1
03113 Hp_euts_violetPink EUT1
03114 HMM00390_malic_1_eut1(pf00390pf03949pf01515) EUT1
03115 -pf00923 -
03116 HMM02780keto_1_eut1(pf02779pf02780pf00456) EUT1
03117 -pf06674 -
03118 HMM00293nuhyd_1_eut1(pf00293) EUT1
03119 -pf13247pf14691pf07992 -
03120 -pf00672pf02518pf07730pf13675 -
03121 -pf00873 -
03122 -pf03960 -
03123 -pf07687pf01546 -
BMC-P
BMC-H
BMC-T
Aldehyde dehydrogenase
PTAC
Signature enzyme
Alcohol dehydrogenase
Regulator

Escherichia sp. KTE159. Bacteria; Proteobacteria; Gammaproteobacteria; Enterobacterales;

A31E__locus_2

EUT1

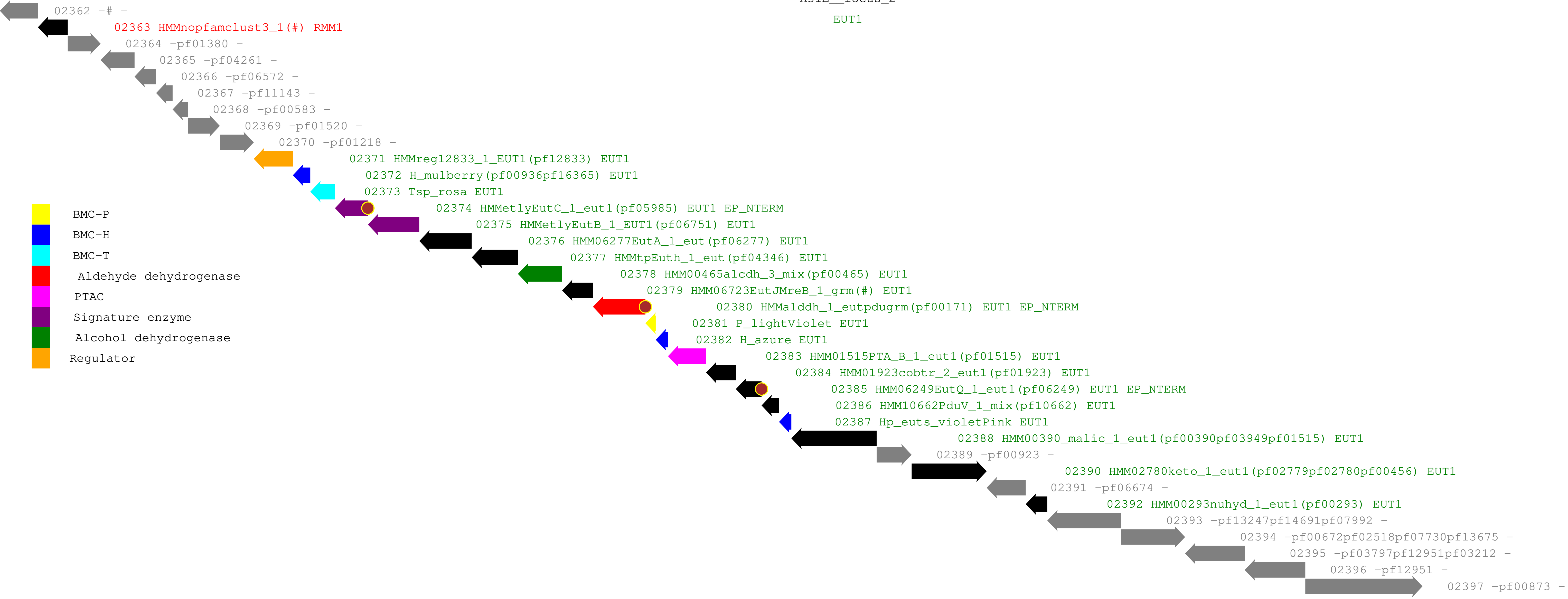

Salmonella paratyphi A. Bacteria; Proteobacteria; Gammaproteobacteria; Enterobacterales;

A3456__locus_1

EUT1

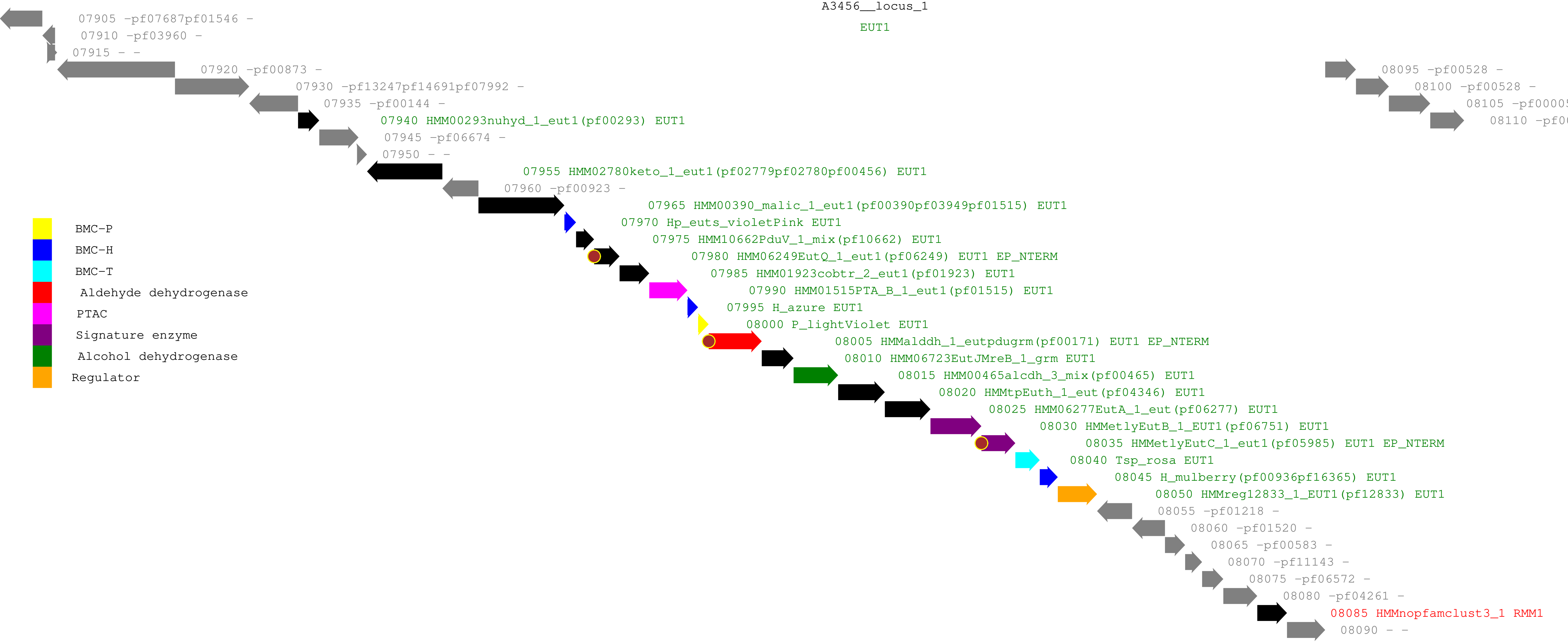

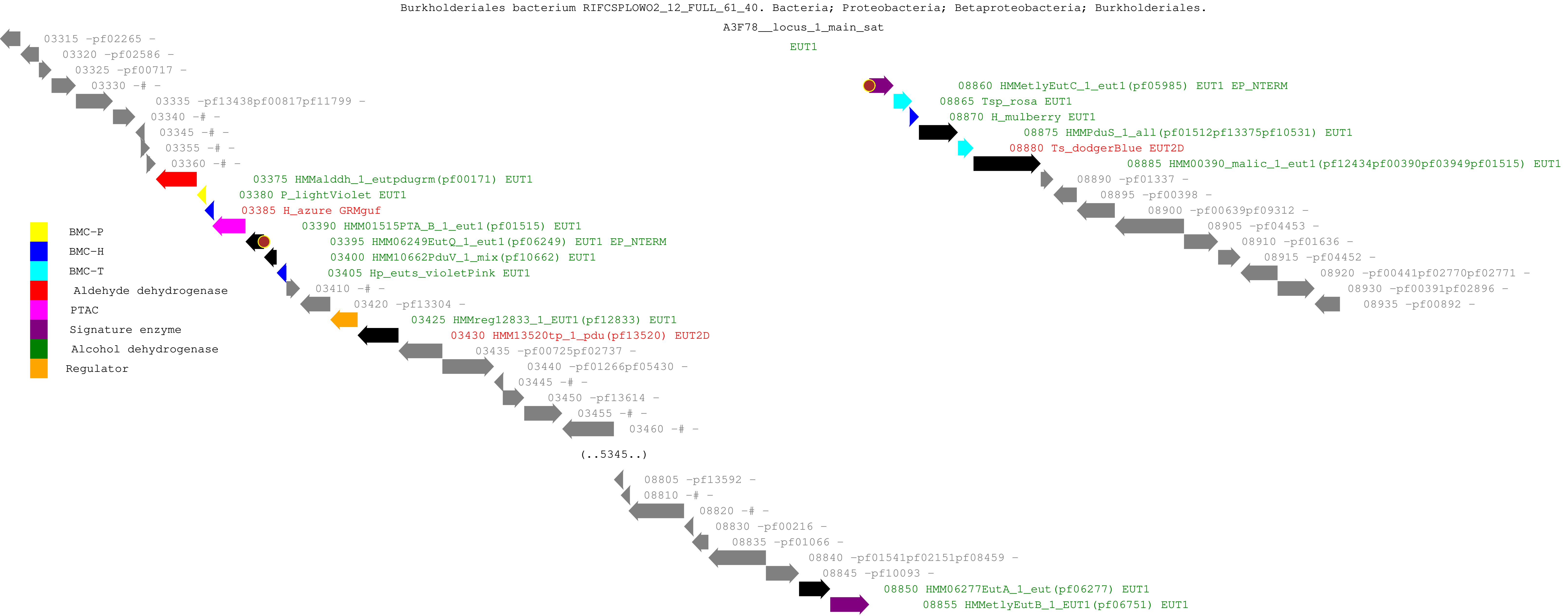
Burkholderiales bacterium RIFCSPLOWO2_12_FULL_61_40. Bacteria; Proteobacteria; Betaproteobacteria; Burkholderiales.
A3F78__locus_1_main_sat
EUT1
03315 -pf02265 -
03320 -pf02586 -
03325 -pf00717 -
03330 -# -
03335 -pf13438pf00817pf11799 -
03340 -# -
03345 -# -
03355 -# -
03360 -# -
03375 HMMalddh_1_eutpdugrm(pf00171) EUT1
03380 P_lightViolet EUT1
03385 H_azure GRMguf
03390 HMM01515PTA_B_1_eut1(pf01515) EUT1
03395 HMM06249EutQ_1_eut1(pf06249) EUT1 EP_NTERM
03400 HMM10662PduV_1_mix(pf10662) EUT1
03405 Hp_euts_violetPink EUT1
03410 -# -
03420 -pf13304 -
03425 HMMreg12833_1_EUT1(pf12833) EUT1
03430 HMM13520tp_1_pdu(pf13520) EUT2D
03435 -pf00725pf02737 -
03440 -pf01266pf05430 -
03445 -# -
03450 -pf13614 -
03455 -# -
03460 -# -
(..5345..)
08805 -pf13592 -
08810 -# -
08820 -# -
08830 -pf00216 -
08835 -pf01066 -
08840 -pf01541pf02151pf08459 -
08845 -pf10093 -
08850 HMM06277EutA_1_eut(pf06277) EUT1
08855 HMMetlyEutB_1_EUT1(pf06751) EUT1
08860 HMMetlyEutC_1_eut1(pf05985) EUT1 EP_NTERM
08865 Tsp_rosa EUT1
08870 H_mulberry EUT1
08875 HMMPduS_1_all(pf01512pf13375pf10531) EUT1
08880 Ts_dodgerBlue EUT2D
08885 HMM00390_malic_1_eut1(pf12434pf00390pf03949pf01515) EUT1
08890 -pf01337 -
08895 -pf00398 -
08900 -pf00639pf09312 -
08905 -pf04453 -
08910 -pf01636 -
08915 -pf04452 -
08920 -pf00441pf02770pf02771 -
08930 -pf00391pf02896 -
08935 -pf00892 -
BMC-P
BMC-H
BMC-T
Aldehyde dehydrogenase
PTAC
Signature enzyme
Alcohol dehydrogenase
Regulator

Salmonella enterica I. Bacteria; Proteobacteria; Gammaproteobacteria; Enterobacterales;

A3T25__locus_1

EUT1

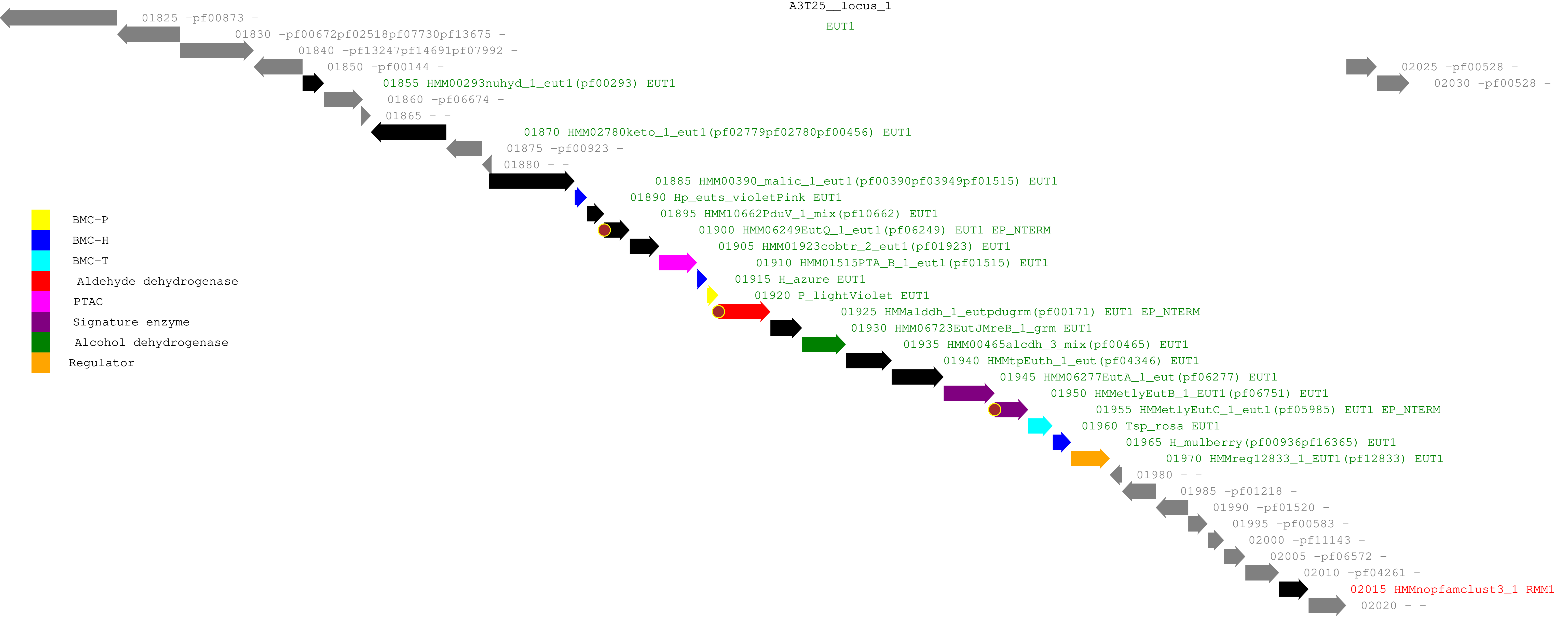

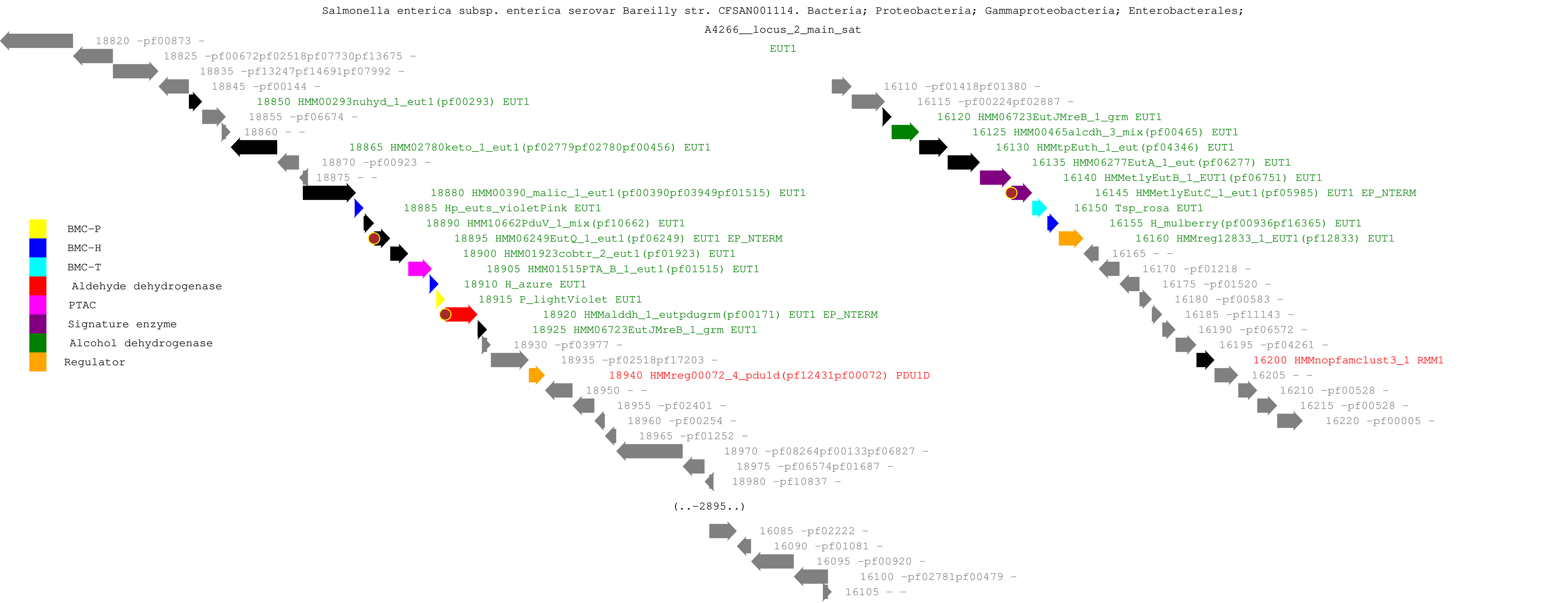
Salmonella enterica subsp. enterica serovar Bareilly str. CFSAN001114. Bacteria; Proteobacteria; Gammaproteobacteria; Enterobacterales;
A4266__locus_2_main_sat
EUT1
18820 -pf00873 -
18825 -pf00672pf02518pf07730pf13675 -
18835 -pf13247pf14691pf07992 -
18845 -pf00144 -
18850 HMM00293nuhyd_1_eut1(pf00293) EUT1
18855 -pf06674 -
18860 - -
18865 HMM02780keto_1_eut1(pf02779pf02780pf00456) EUT1
18870 -pf00923 -
18875 - -
18880 HMM00390_malic_1_eut1(pf00390pf03949pf01515) EUT1
18885 Hp_euts_violetPink EUT1
18890 HMM10662PduV_1_mix(pf10662) EUT1
18895 HMM06249EutQ_1_eut1(pf06249) EUT1 EP_NTERM
18900 HMM01923cobtr_2_eut1(pf01923) EUT1
18905 HMM01515PTA_B_1_eut1(pf01515) EUT1
18910 H_azure EUT1
18915 P_lightViolet EUT1
18920 HMMalddh_1_eutpdugrm(pf00171) EUT1 EP_NTERM
18925 HMM06723EutJMreB_1_grm EUT1
18930 -pf03977 -
18935 -pf02518pf17203 -
18940 HMMreg00072_4_pdu1d(pf12431pf00072) PDU1D
18950 - -
18955 -pf02401 -
18960 -pf00254 -
18965 -pf01252 -
18970 -pf08264pf00133pf06827 -
18975 -pf06574pf01687 -
18980 -pf10837 -
(..-2895..)
16085 -pf02222 -
16090 -pf01081 -
16095 -pf00920 -
16100 -pf02781pf00479 -
16105 - -
16110 -pf01418pf01380 -
16115 -pf00224pf02887 -
16120 HMM06723EutJMreB_1_grm EUT1
16125 HMM00465alcdh_3_mix(pf00465) EUT1
16130 HMMtpEuth_1_eut(pf04346) EUT1
16135 HMM06277EutA_1_eut(pf06277) EUT1
16140 HMMetlyEutB_1_EUT1(pf06751) EUT1
16145 HMMetlyEutC_1_eut1(pf05985) EUT1 EP_NTERM
16150 Tsp_rosa EUT1
16155 H_mulberry(pf00936pf16365) EUT1
16160 HMMreg12833_1_EUT1(pf12833) EUT1
16165 - -
16170 -pf01218 -
16175 -pf01520 -
16180 -pf00583 -
16185 -pf11143 -
16190 -pf06572 -
16195 -pf04261 -
16200 HMMnopfamclust3_1 RMM1
16205 - -
16210 -pf00528 -
16215 -pf00528 -
16220 -pf00005 -
BMC-P
BMC-H
BMC-T
Aldehyde dehydrogenase
PTAC
Signature enzyme
Alcohol dehydrogenase
Regulator

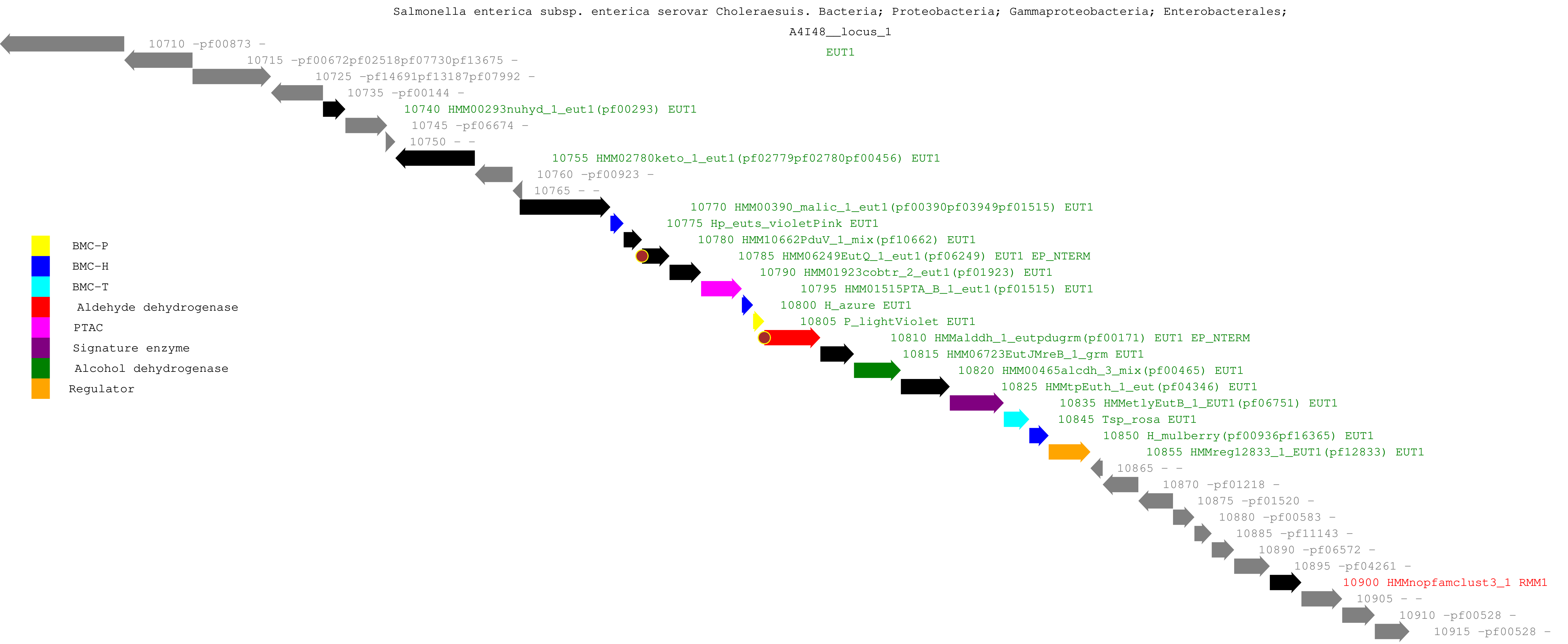
Salmonella enterica subsp. enterica serovar Choleraesuis. Bacteria; Proteobacteria; Gammaproteobacteria; Enterobacterales;
A4I48__locus_1
EUT1
10710 -pf00873 -
10715 -pf00672pf02518pf07730pf13675 -
10725 -pf14691pf13187pf07992 -
10735 -pf00144 -
10740 HMM00293nuhyd_1_eut1(pf00293) EUT1
10745 -pf06674 -
10750 - -
10755 HMM02780keto_1_eut1(pf02779pf02780pf00456) EUT1
10760 -pf00923 -
10765 - -
10770 HMM00390_malic_1_eut1(pf00390pf03949pf01515) EUT1
10775 Hp_euts_violetPink EUT1
10780 HMM10662PduV_1_mix(pf10662) EUT1
10785 HMM06249EutQ_1_eut1(pf06249) EUT1 EP_NTERM
10790 HMM01923cobtr_2_eut1(pf01923) EUT1
10795 HMM01515PTA_B_1_eut1(pf01515) EUT1
10800 H_azure EUT1
10805 P_lightViolet EUT1
10810 HMMalddh_1_eutpdugrm(pf00171) EUT1 EP_NTERM
10815 HMM06723EutJMreB_1_grm EUT1
10820 HMM00465alcdh_3_mix(pf00465) EUT1
10825 HMMtpEuth_1_eut(pf04346) EUT1
10835 HMMetlyEutB_1_EUT1(pf06751) EUT1
10845 Tsp_rosa EUT1
10850 H_mulberry(pf00936pf16365) EUT1
10855 HMMreg12833_1_EUT1(pf12833) EUT1
10865 - -
10870 -pf01218 -
10875 -pf01520 -
10880 -pf00583 -
10885 -pf11143 -
10890 -pf06572 -
10895 -pf04261 -
10900 HMMnopfamclust3_1 RMM1
10905 - -
10910 -pf00528 -
10915 -pf00528 -
BMC-P
BMC-H
BMC-T
Aldehyde dehydrogenase
PTAC
Signature enzyme
Alcohol dehydrogenase
Regulator

Salmonella enterica subsp. enterica serovar Panama. Bacteria; Proteobacteria; Gammaproteobacteria; Enterobacterales;

A4I59__locus_1

EUT1

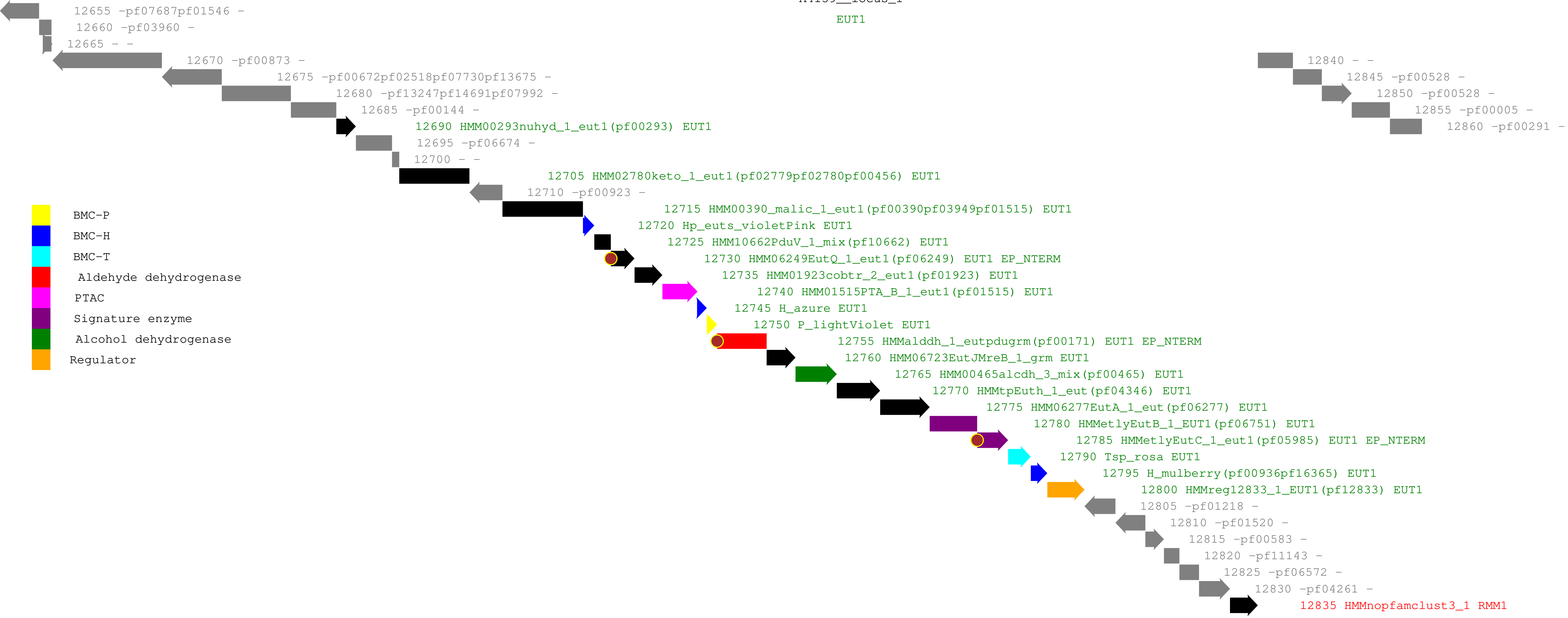

Salmonella enterica subsp. enterica serovar Richmond. Bacteria; Proteobacteria; Gammaproteobacteria; Enterobacterales;

A4I74__locus_1

EUT1

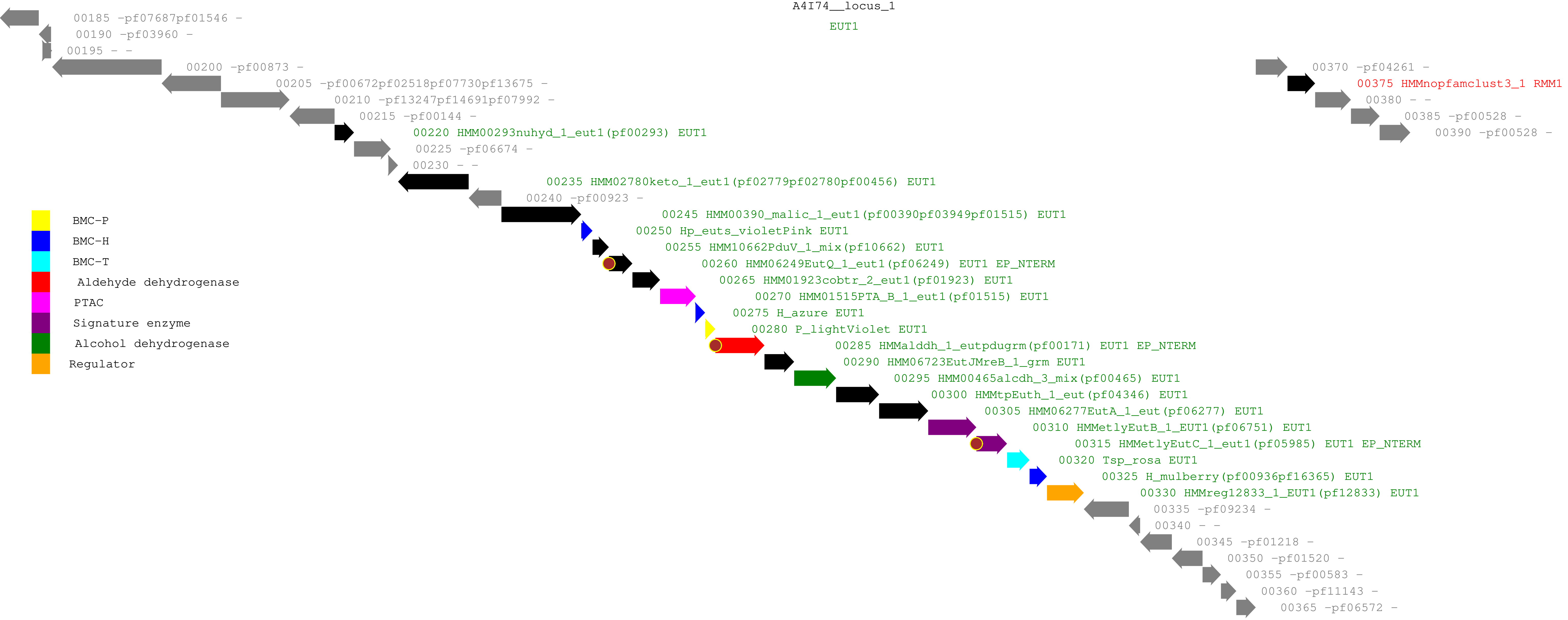

Salmonella enterica subsp. enterica serovar Kedougou. Bacteria; Proteobacteria; Gammaproteobacteria; Enterobacterales;

A4I75__locus_2

EUT1

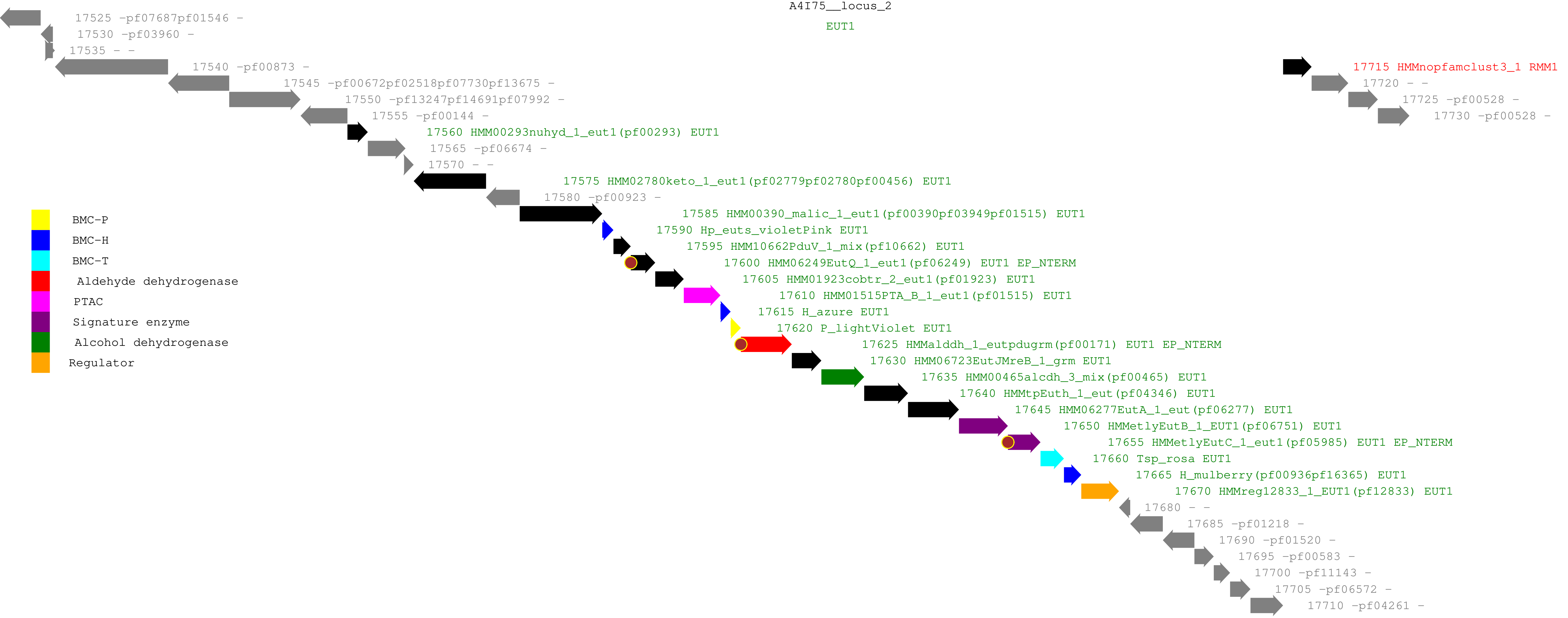

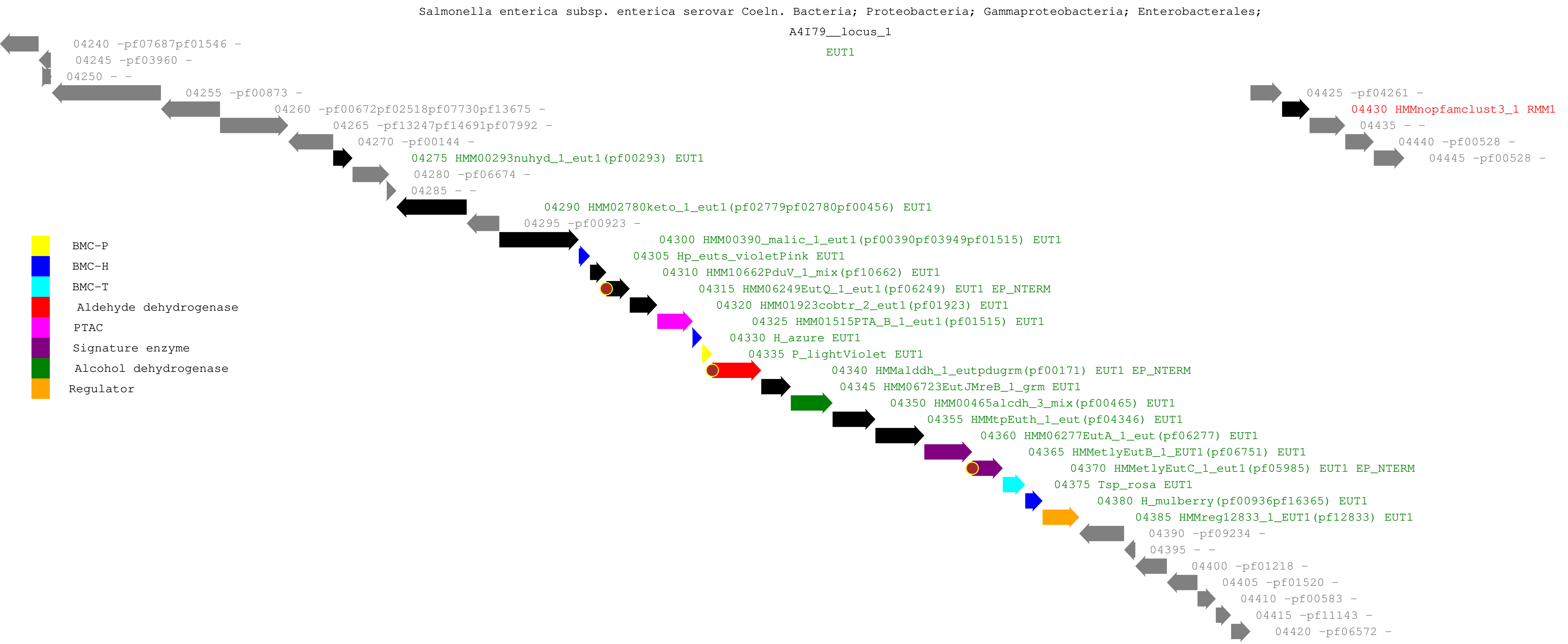

Salmonella enterica subsp. enterica serovar Coeln. Bacteria; Proteobacteria; Gammaproteobacteria; Enterobacterales;
A4I79__locus_1
EUT1
04240 -pf07687pf01546 -
04245 -pf03960 -
04250 - -
04255 -pf00873 -
04260 -pf00672pf02518pf07730pf13675 -
04265 -pf13247pf14691pf07992 -
04270 -pf00144 -
04275 HMM00293nuhyd_1_eut1(pf00293) EUT1
04280 -pf06674 -
04285 - -
04290 HMM02780keto_1_eut1(pf02779pf02780pf00456) EUT1
04295 -pf00923 -
04300 HMM00390_malic_1_eut1(pf00390pf03949pf01515) EUT1
04305 Hp_euts_violetPink EUT1
04310 HMM10662PduV_1_mix(pf10662) EUT1
04315 HMM06249EutQ_1_eut1(pf06249) EUT1 EP_NTERM
04320 HMM01923cobtr_2_eut1(pf01923) EUT1
04325 HMM01515PTA_B_1_eut1(pf01515) EUT1
04330 H_azure EUT1
04335 P_lightViolet EUT1
04340 HMMalddh_1_eutpdugrm(pf00171) EUT1 EP_NTERM
04345 HMM06723EutJMreB_1_grm EUT1
04350 HMM00465alcdh_3_mix(pf00465) EUT1
04355 HMMtpEuth_1_eut(pf04346) EUT1
04360 HMM06277EutA_1_eut(pf06277) EUT1
04365 HMMetlyEutB_1_EUT1(pf06751) EUT1
04370 HMMetlyEutC_1_eut1(pf05985) EUT1 EP_NTERM
04375 Tsp_rosa EUT1
04380 H_mulberry(pf00936pf16365) EUT1
04385 HMMreg12833_1_EUT1(pf12833) EUT1
04390 -pf09234 -
04395 - -
04400 -pf01218 -
04405 -pf01520 -
04410 -pf00583 -
04415 -pf11143 -
04420 -pf06572 -
04425 -pf04261 -
04430 HMMnopfamclust3_1 RMM1
04435 - -
04440 -pf00528 -
04445 -pf00528 -
BMC-P
BMC-H
BMC-T
Aldehyde dehydrogenase
PTAC
Signature enzyme
Alcohol dehydrogenase
Regulator

Salmonella oranienberg. Bacteria; Proteobacteria; Gammaproteobacteria; Enterobacterales;

A4I87__locus_1

EUT1

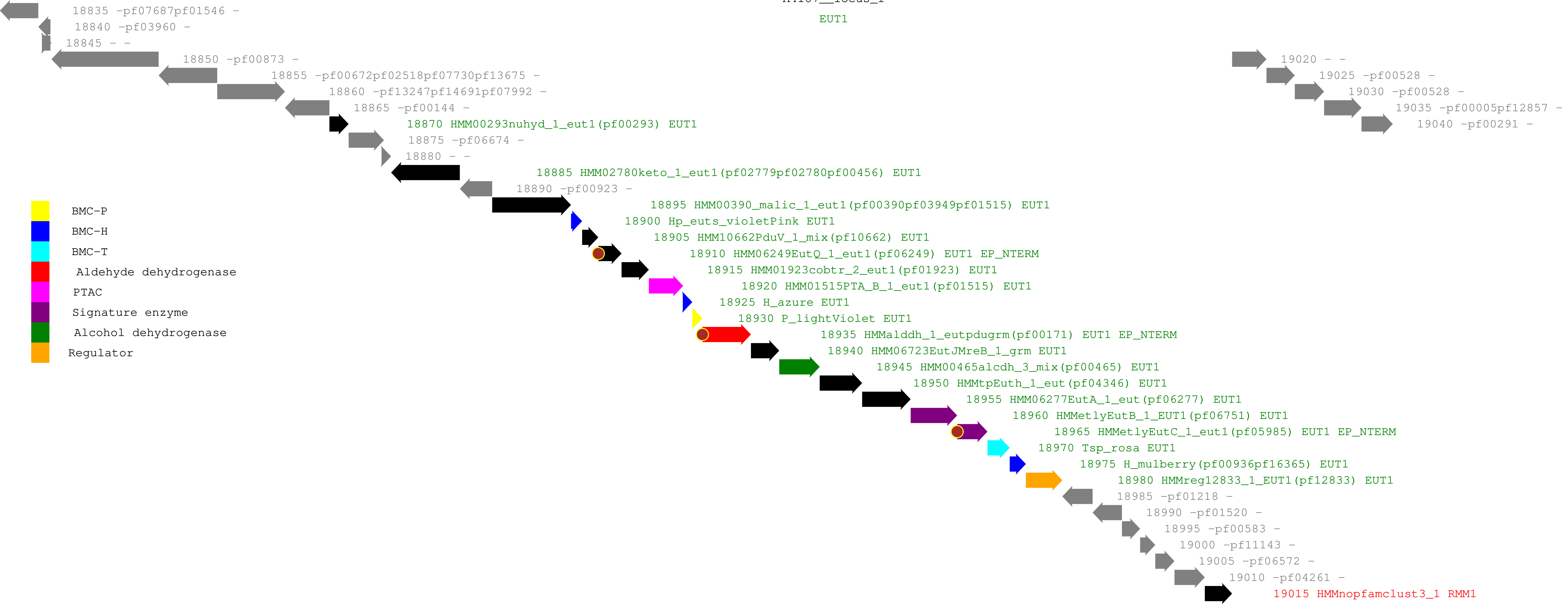

Salmonella enterica subsp. enterica serovar London. Bacteria; Proteobacteria; Gammaproteobacteria; Enterobacterales;

A4I94__locus_2

EUT1

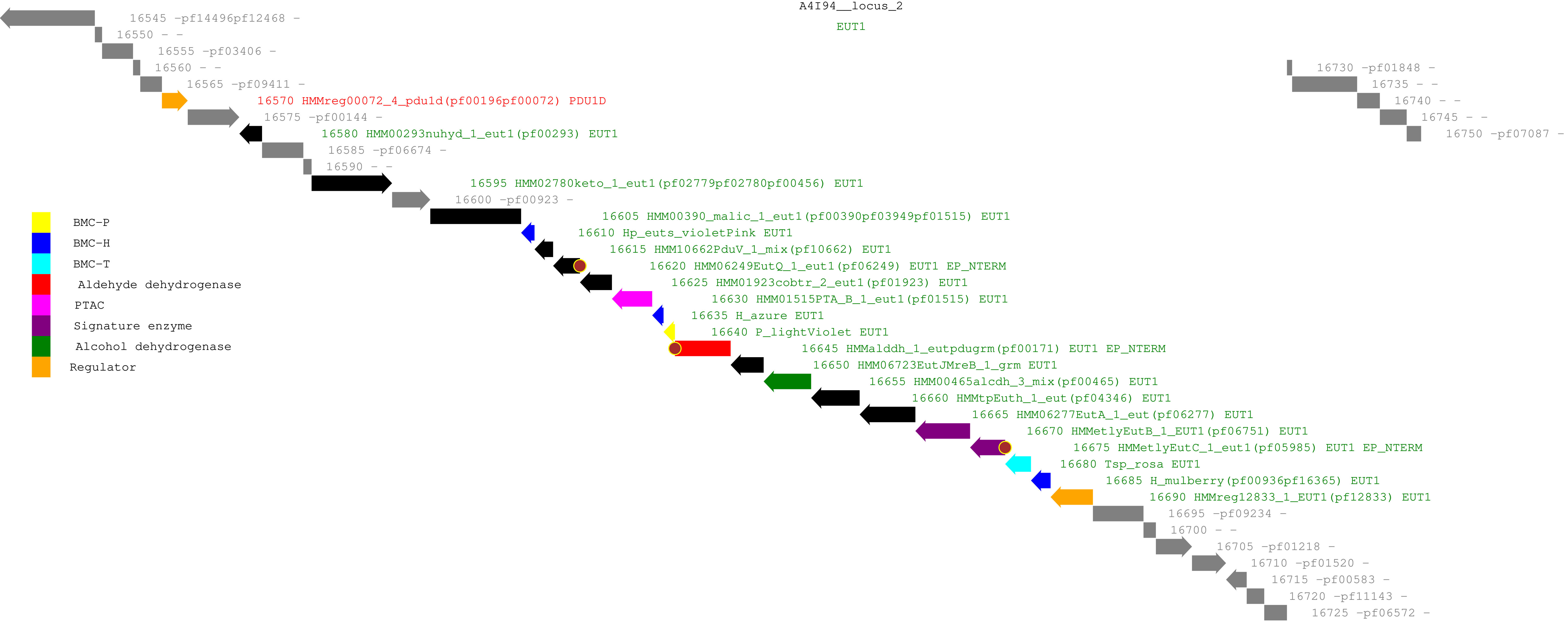

Salmonella enterica subsp. enterica serovar Sandiego. Bacteria; Proteobacteria; Gammaproteobacteria; Enterobacterales;

A4J53__locus_2

EUT1

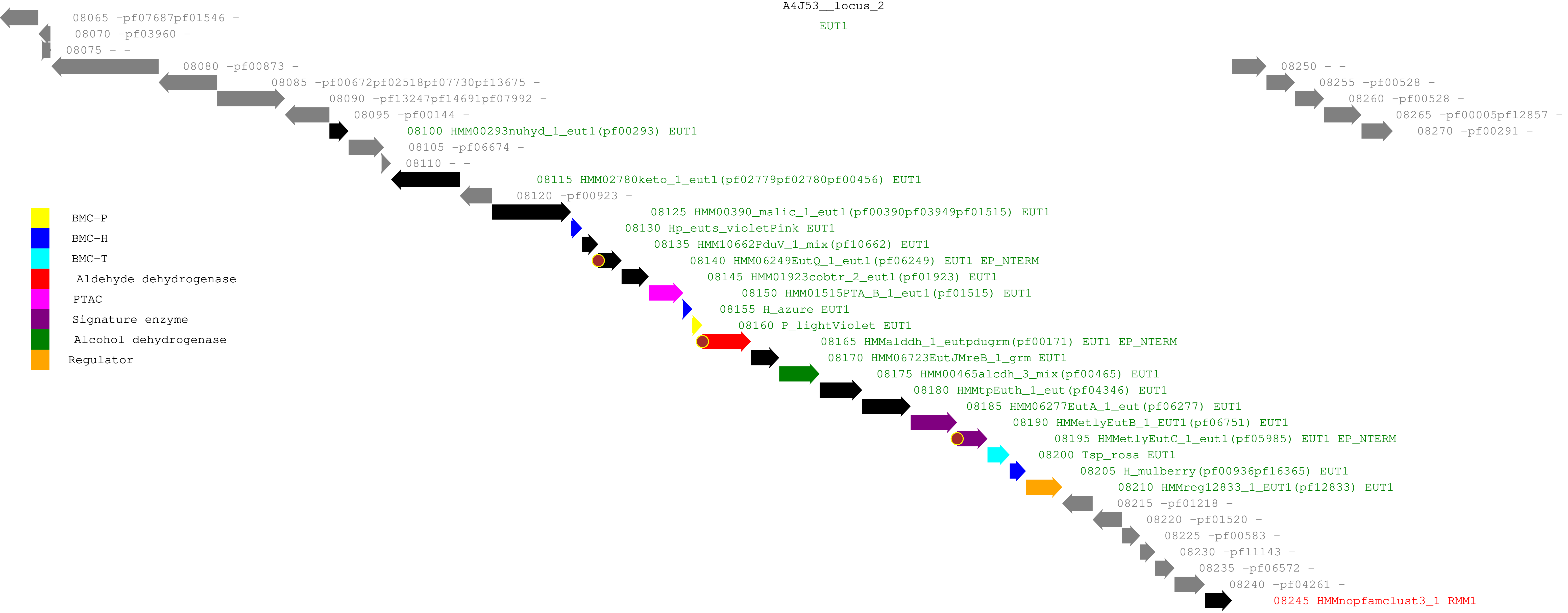

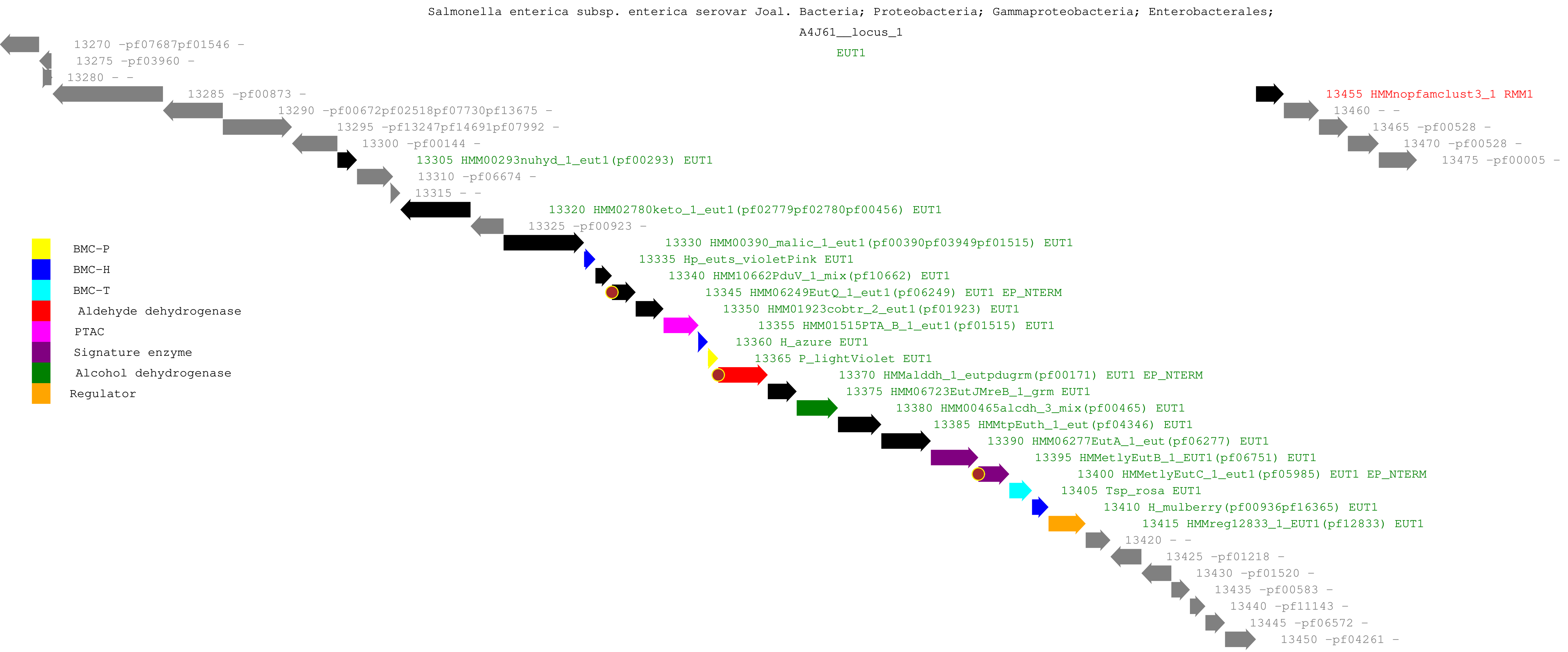
Salmonella enterica subsp. enterica serovar Joal. Bacteria; Proteobacteria; Gammaproteobacteria; Enterobacterales;
A4J61__locus_1
EUT1
13270 -pf07687pf01546 -
13275 -pf03960 -
13280 - -
13285 -pf00873 -
13290 -pf00672pf02518pf07730pf13675 -
13295 -pf13247pf14691pf07992 -
13300 -pf00144 -
13305 HMM00293nuhyd_1_eut1(pf00293) EUT1
13310 -pf06674 -
13315 - -
13320 HMM02780keto_1_eut1(pf02779pf02780pf00456) EUT1
13325 -pf00923 -
13330 HMM00390_malic_1_eut1(pf00390pf03949pf01515) EUT1
13335 Hp_euts_violetPink EUT1
13340 HMM10662PduV_1_mix(pf10662) EUT1
13345 HMM06249EutQ_1_eut1(pf06249) EUT1 EP_NTERM
13350 HMM01923cobtr_2_eut1(pf01923) EUT1
13355 HMM01515PTA_B_1_eut1(pf01515) EUT1
13360 H_azure EUT1
13365 P_lightViolet EUT1
13370 HMMalddh_1_eutpdugrm(pf00171) EUT1 EP_NTERM
13375 HMM06723EutJMreB_1_grm EUT1
13380 HMM00465alcdh_3_mix(pf00465) EUT1
13385 HMMtpEuth_1_eut(pf04346) EUT1
13390 HMM06277EutA_1_eut(pf06277) EUT1
13395 HMMetlyEutB_1_EUT1(pf06751) EUT1
13400 HMMetlyEutC_1_eut1(pf05985) EUT1 EP_NTERM
13405 Tsp_rosa EUT1
13410 H_mulberry(pf00936pf16365) EUT1
13415 HMMreg12833_1_EUT1(pf12833) EUT1
13420 - -
13425 -pf01218 -
13430 -pf01520 -
13435 -pf00583 -
13440 -pf11143 -
13445 -pf06572 -
13450 -pf04261 -
13455 HMMnopfamclust3_1 RMM1
13460 - -
13465 -pf00528 -
13470 -pf00528 -
13475 -pf00005 -
BMC-P
BMC-H
BMC-T
Aldehyde dehydrogenase
PTAC
Signature enzyme
Alcohol dehydrogenase
Regulator

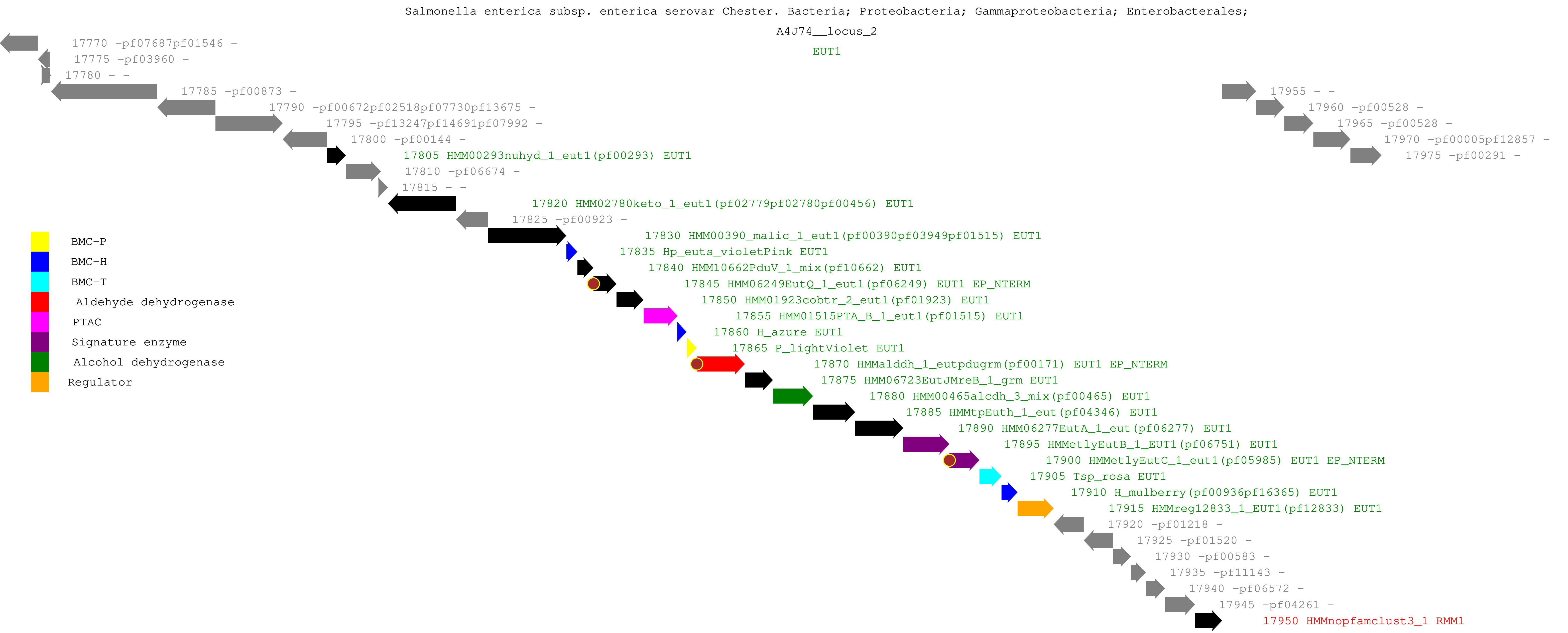

Salmonella enterica subsp. enterica serovar Chester. Bacteria; Proteobacteria; Gammaproteobacteria; Enterobacterales;
A4J74__locus_2
EUT1
17770 -pf07687pf01546 -
17775 -pf03960 -
17780 - -
17785 -pf00873 -
17790 -pf00672pf02518pf07730pf13675 -
17795 -pf13247pf14691pf07992 -
17800 -pf00144 -
17805 HMM00293nuhyd_1_eut1(pf00293) EUT1
17810 -pf06674 -
17815 - -
17820 HMM02780keto_1_eut1(pf02779pf02780pf00456) EUT1
17825 -pf00923 -
17830 HMM00390_malic_1_eut1(pf00390pf03949pf01515) EUT1
17835 Hp_euts_violetPink EUT1
17840 HMM10662PduV_1_mix(pf10662) EUT1
17845 HMM06249EutQ_1_eut1(pf06249) EUT1 EP_NTERM
17850 HMM01923cobtr_2_eut1(pf01923) EUT1
17855 HMM01515PTA_B_1_eut1(pf01515) EUT1
17860 H_azure EUT1
17865 P_lightViolet EUT1
17870 HMMaldh_1_eutpdugrm(pf00171) EUT1 EP_NTERM
17875 HMM06723EutJMreB_1_grm EUT1
17880 HMM00465alcdh_3_mix(pf00465) EUT1
17885 HMMtpEuth_1_eut(pf04346) EUT1
17890 HMM06277EutA_1_eut(pf06277) EUT1
17895 HMMetlyEutB_1_EUT1(pf06751) EUT1
17900 HMMetlyEutC_1_eut1(pf05985) EUT1 EP_NTERM
17905 Tsp_rosa EUT1
17910 H_mulberry(pf00936pf16365) EUT1
17915 HMMreg12833_1_EUT1(pf12833) EUT1
17920 -pf01218 -
17925 -pf01520 -
17930 -pf00583 -
17935 -pf11143 -
17940 -pf06572 -
17945 -pf04261 -
17950 HMMnopfamclust3_1 RMM1
17955 - -
17960 -pf00528 -
17965 -pf00528 -
17970 -pf00005pf12857 -
17975 -pf00291 -
BMC-P
BMC-H
BMC-T
Aldehyde dehydrogenase
PTAC
Signature enzyme
Alcohol dehydrogenase
Regulator

Salmonella enterica subsp. enterica serovar Meleagridis. Bacteria; Proteobacteria; Gammaproteobacteria; Enterobacterales;

A4K05__locus_2

EUT1

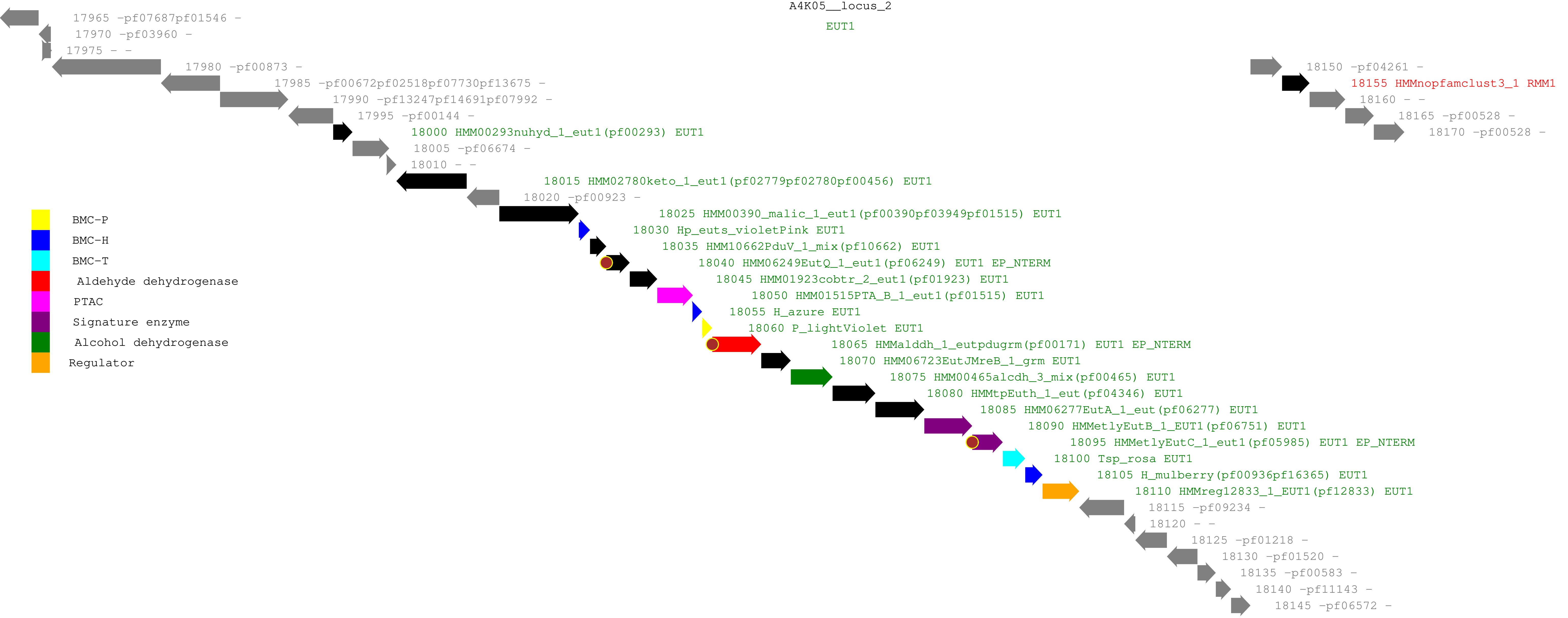

Salmonella enterica subsp. enterica serovar Bakau. Bacteria; Proteobacteria; Gammaproteobacteria; Enterobacterales;

A4K08__locus_2

EUT1

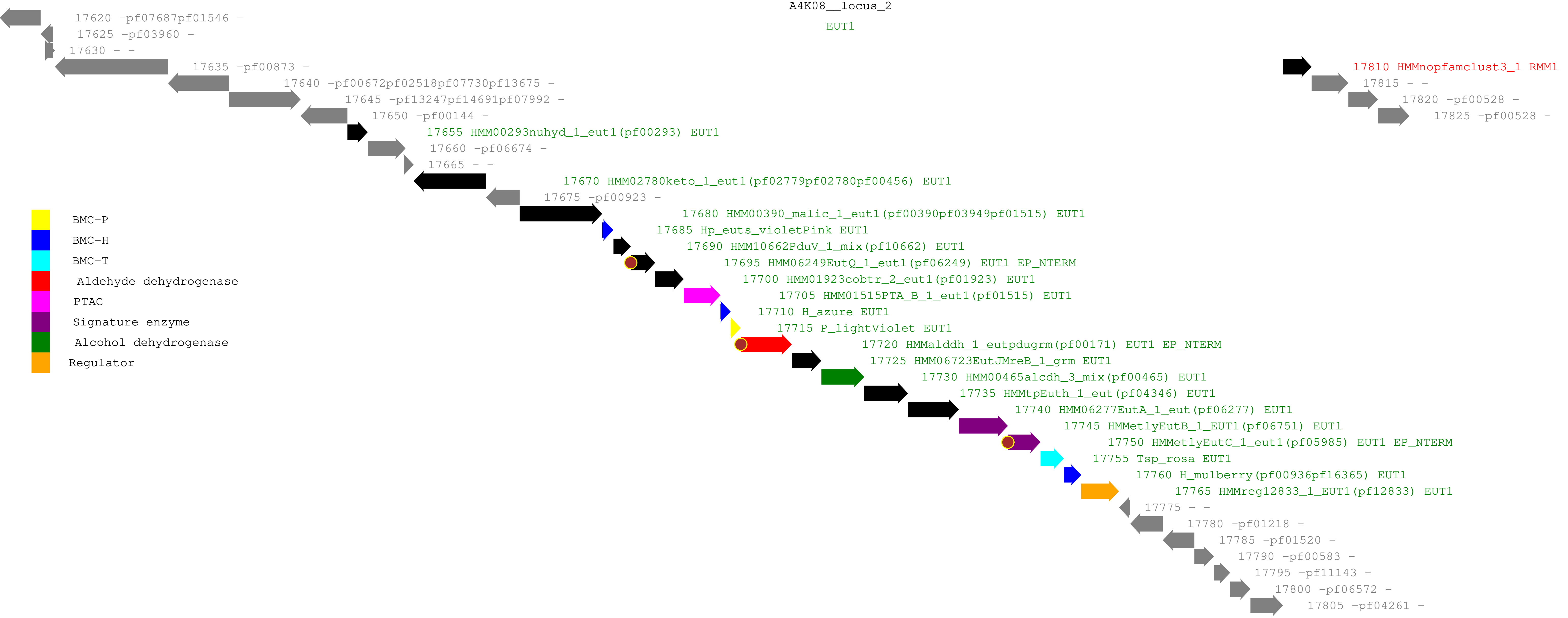

Salmonella enterica subsp. enterica serovar Kingabwa. Bacteria; Proteobacteria; Gammaproteobacteria; Enterobacterales;

A4K20__locus_2

EUT1

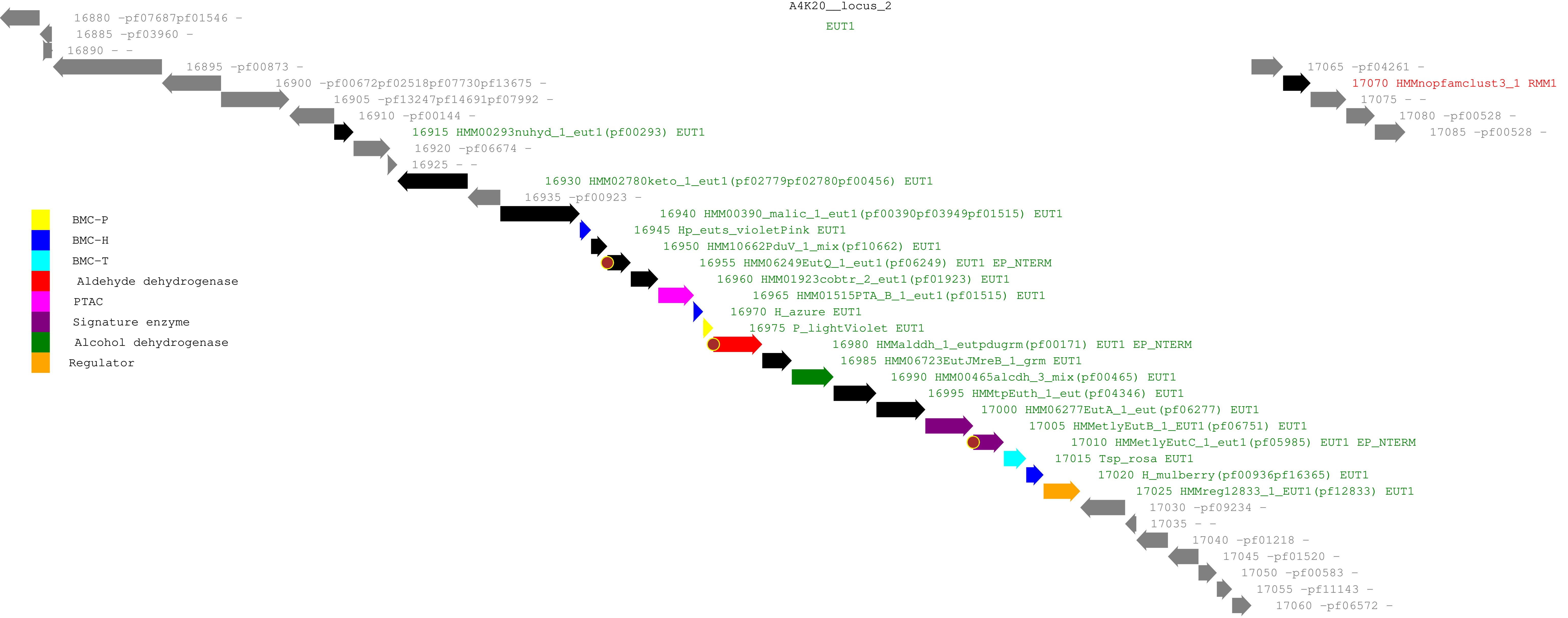

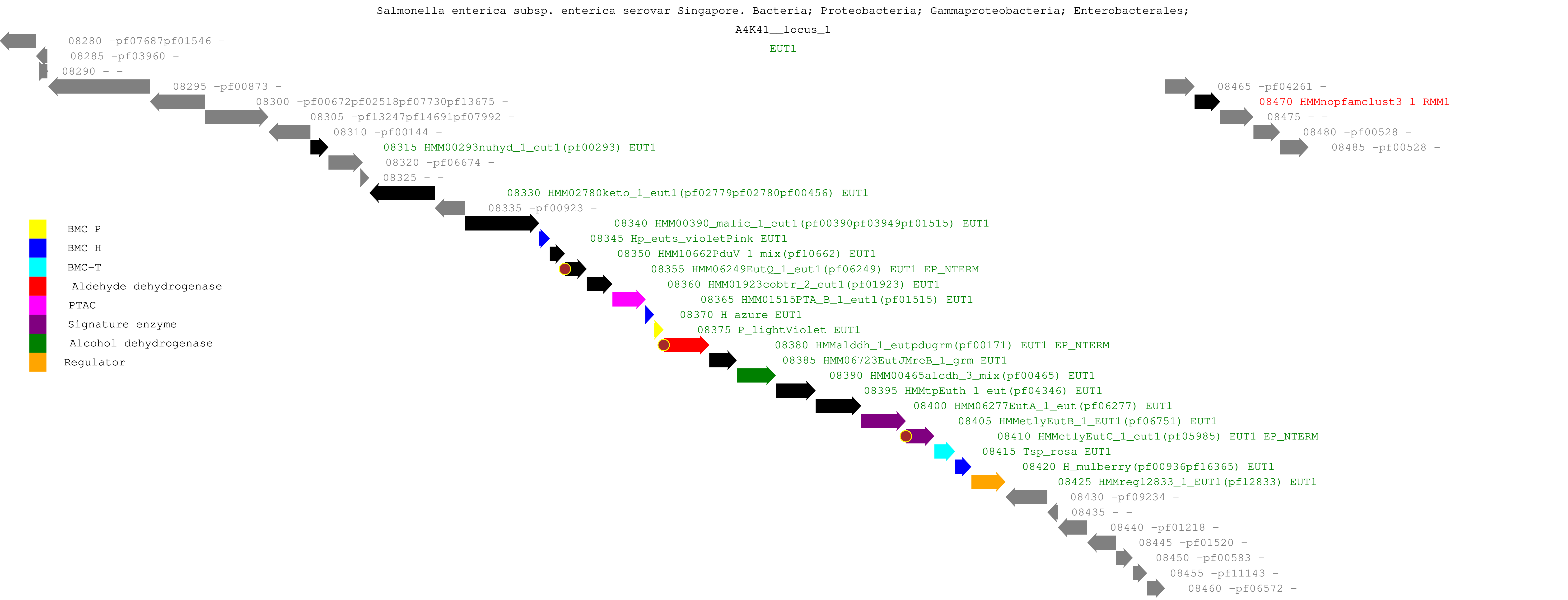

Salmonella enterica subsp. enterica serovar Singapore. Bacteria; Proteobacteria; Gammaproteobacteria; Enterobacterales;
A4K41__locus_1
EUT1
08280 -pf07687pf01546 -
08285 -pf03960 -
08290 - -
08295 -pf00873 -
08300 -pf00672pf02518pf07730pf13675 -
08305 -pf13247pf14691pf07992 -
08310 -pf00144 -
08315 HMM00293nuhyd_1_eut1(pf00293) EUT1
08320 -pf06674 -
08325 - -
08330 HMM02780keto_1_eut1(pf02779pf02780pf00456) EUT1
08335 -pf00923 -
08340 HMM00390_malic_1_eut1(pf00390pf03949pf01515) EUT1
08345 Hp_euts_violetPink EUT1
08350 HMM10662PduV_1_mix(pf10662) EUT1
08355 HMM06249EutQ_1_eut1(pf06249) EUT1 EP_NTERM
08360 HMM01923cobtr_2_eut1(pf01923) EUT1
08365 HMM01515PTA_B_1_eut1(pf01515) EUT1
08370 H_azure EUT1
08375 P_lightViolet EUT1
08380 HMMalddh_1_eutpdugrm(pf00171) EUT1 EP_NTERM
08385 HMM06723EutJMreB_1_grm EUT1
08390 HMM00465alcdh_3_mix(pf00465) EUT1
08395 HMMtpEuth_1_eut(pf04346) EUT1
08400 HMM06277EutA_1_eut(pf06277) EUT1
08405 HMMetlyEutB_1_EUT1(pf06751) EUT1
08410 HMMetlyEutC_1_eut1(pf05985) EUT1 EP_NTERM
08415 Tsp_rosa EUT1
08420 H_mulberry(pf00936pf16365) EUT1
08425 HMMreg12833_1_EUT1(pf12833) EUT1
08430 -pf09234 -
08435 - -
08440 -pf01218 -
08445 -pf01520 -
08450 -pf00583 -
08455 -pf11143 -
08460 -pf06572 -
08465 -pf04261 -
08470 HMMnopfamclust3_1 RMM1
08475 - -
08480 -pf00528 -
08485 -pf00528 -
BMC-P
BMC-H
BMC-T
Aldehyde dehydrogenase
PTAC
Signature enzyme
Alcohol dehydrogenase
Regulator

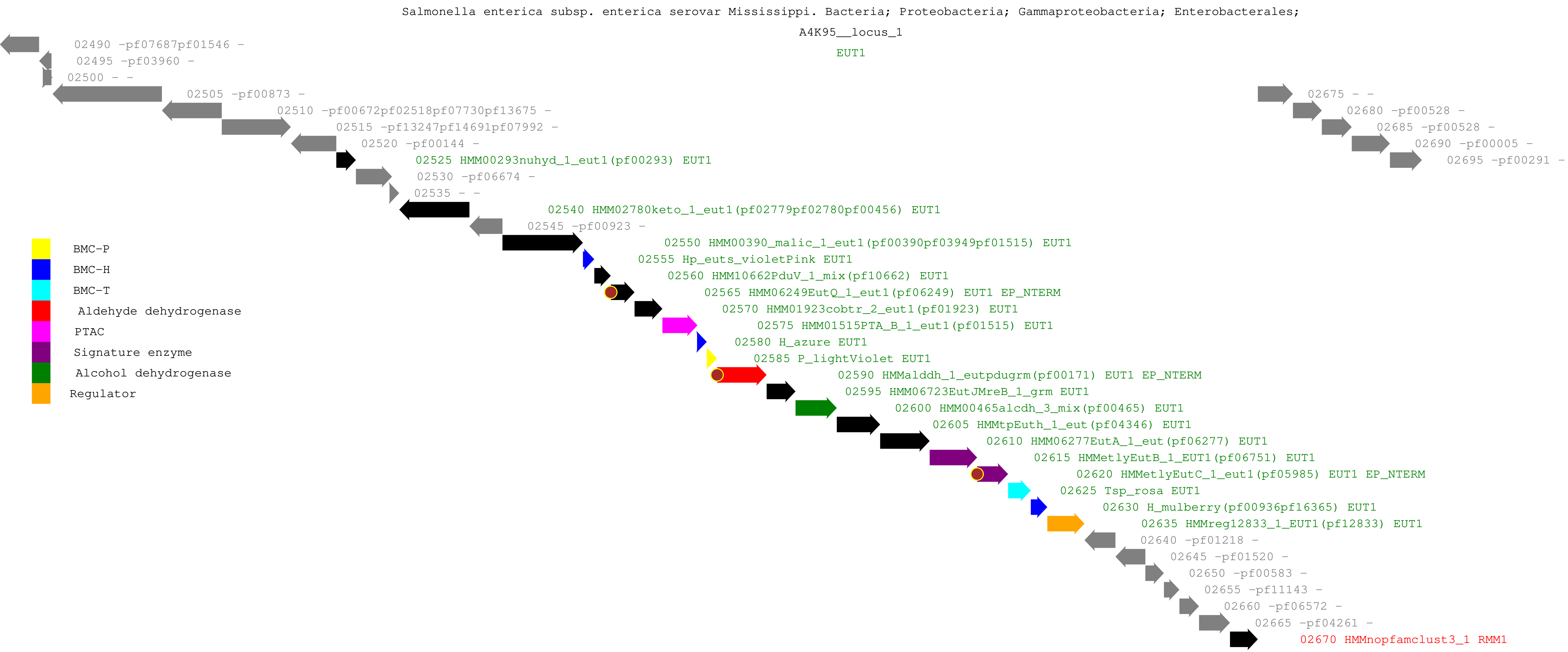
Salmonella enterica subsp. enterica serovar Mississippi. Bacteria; Proteobacteria; Gammaproteobacteria; Enterobacterales;
A4K95__locus_1
EUT1
02490 -pf07687pf01546 -
02495 -pf03960 -
02500 - -
02505 -pf00873 -
02510 -pf00672pf02518pf07730pf13675 -
02515 -pf13247pf14691pf07992 -
02520 -pf00144 -
02525 HMM00293nuhyd_1_eut1(pf00293) EUT1
02530 -pf06674 -
02535 - -
02540 HMM02780keto_1_eut1(pf02779pf02780pf00456) EUT1
02545 -pf00923 -
02550 HMM00390_malic_1_eut1(pf00390pf03949pf01515) EUT1
02555 Hp_euts_violetPink EUT1
02560 HMM10662PduV_1_mix(pf10662) EUT1
02565 HMM06249EutQ_1_eut1(pf06249) EUT1 EP_NTERM
02570 HMM01923cobtr_2_eut1(pf01923) EUT1
02575 HMM01515PTA_B_1_eut1(pf01515) EUT1
02580 H_azure EUT1
02585 P_lightViolet EUT1
02590 HMMaldh_1_eutpdugrm(pf00171) EUT1 EP_NTERM
02595 HMM06723EutJMreB_1_grm EUT1
02600 HMM00465alcdh_3_mix(pf00465) EUT1
02605 HMMtpEuth_1_eut(pf04346) EUT1
02610 HMM06277EutA_1_eut(pf06277) EUT1
02615 HMMetlyEutB_1_EUT1(pf06751) EUT1
02620 HMMetlyEutC_1_eut1(pf05985) EUT1 EP_NTERM
02625 Tsp_rosa EUT1
02630 H_mulberry(pf00936pf16365) EUT1
02635 HMMreg12833_1_EUT1(pf12833) EUT1
02640 -pf01218 -
02645 -pf01520 -
02650 -pf00583 -
02655 -pf11143 -
02660 -pf06572 -
02665 -pf04261 -
02670 HMMnopfamclust3_1 RMM1
02675 - -
02680 -pf00528 -
02685 -pf00528 -
02690 -pf00005 -
02695 -pf00291 -
BMC-P
BMC-H
BMC-T
Aldehyde dehydrogenase
PTAC
Signature enzyme
Alcohol dehydrogenase
Regulator

Salmonella dublin. Bacteria; Proteobacteria; Gammaproteobacteria; Enterobacterales;

A4L74__locus_2

EUT1

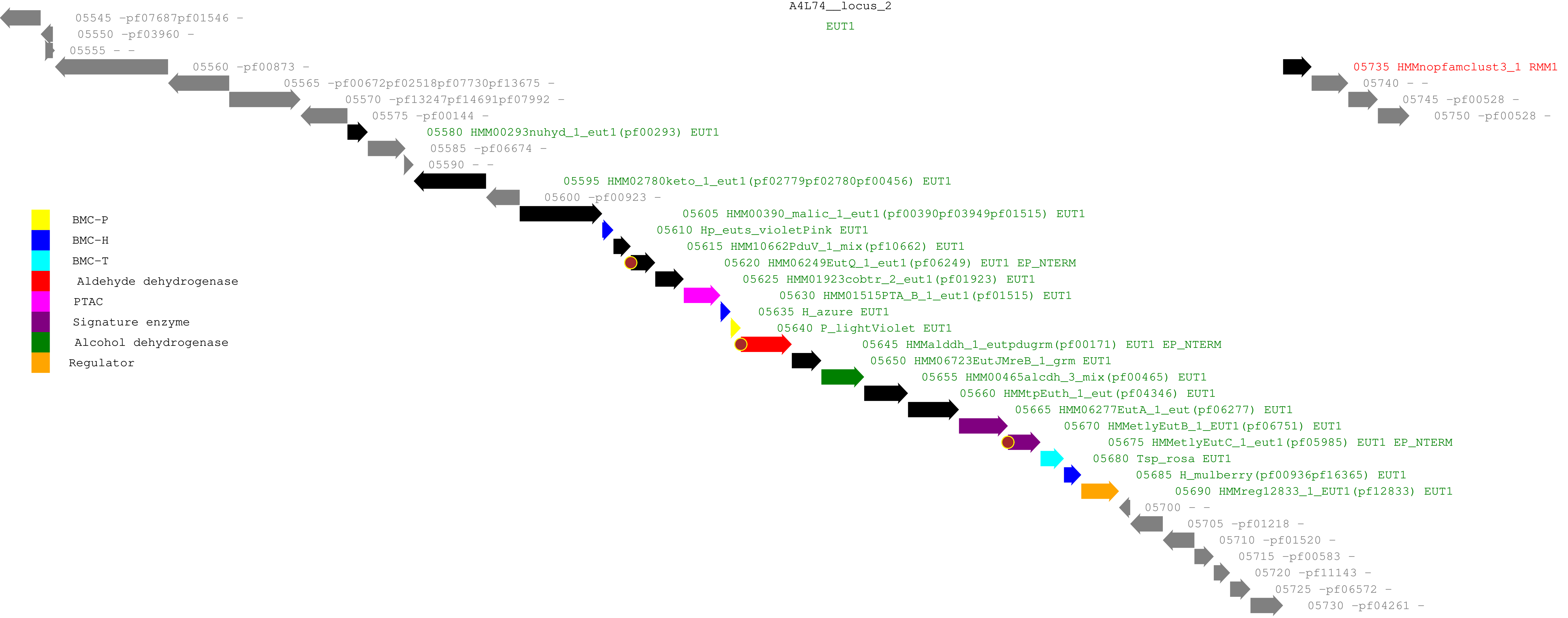

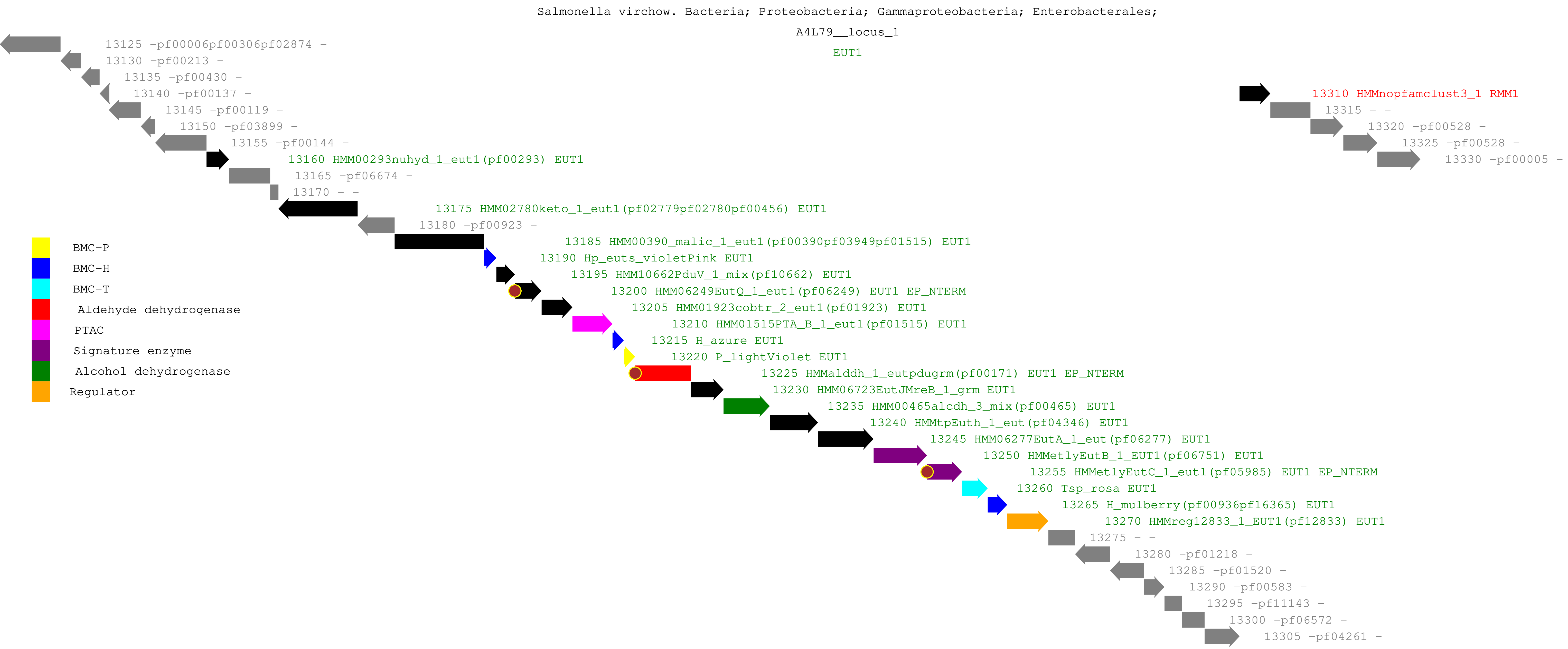
Salmonella virchow. Bacteria; Proteobacteria; Gammaproteobacteria; Enterobacterales;
A4L79__locus_1
EUT1
13125 -pf00006pf00306pf02874 -
13130 -pf00213 -
13135 -pf00430 -
13140 -pf00137 -
13145 -pf00119 -
13150 -pf03899 -
13155 -pf00144 -
13160 HMM00293nuhyd_1_eut1(pf00293) EUT1
13165 -pf06674 -
13170 - -
13175 HMM02780keto_1_eut1(pf02779pf02780pf00456) EUT1
13180 -pf00923 -
13185 HMM00390_malic_1_eut1(pf00390pf03949pf01515) EUT1
13190 Hp_euts_violetPink EUT1
13195 HMM10662PduV_1_mix(pf10662) EUT1
13200 HMM06249EutQ_1_eut1(pf06249) EUT1 EP_NTERM
13205 HMM01923cobtr_2_eut1(pf01923) EUT1
13210 HMM01515PTA_B_1_eut1(pf01515) EUT1
13215 H_azure EUT1
13220 P_lightViolet EUT1
13225 HMMaldh_1_eutpdugrm(pf00171) EUT1 EP_NTERM
13230 HMM06723EutJMreB_1_grm EUT1
13235 HMM00465alcdh_3_mix(pf00465) EUT1
13240 HMMtpEuth_1_eut(pf04346) EUT1
13245 HMM06277EutA_1_eut(pf06277) EUT1
13250 HMMetlyEutB_1_EUT1(pf06751) EUT1
13255 HMMetlyEutC_1_eut1(pf05985) EUT1 EP_NTERM
13260 Tsp_rosa EUT1
13265 H_mulberry(pf00936pf16365) EUT1
13270 HMMreg12833_1_EUT1(pf12833) EUT1
13275 - -
13280 -pf01218 -
13285 -pf01520 -
13290 -pf00583 -
13295 -pf11143 -
13300 -pf06572 -
13305 -pf04261 -
13310 HMMnopfamclust3_1 RMM1
13315 - -
13320 -pf00528 -
13325 -pf00528 -
13330 -pf00005 -
BMC-P
BMC-H
BMC-T
Aldehyde dehydrogenase
PTAC
Signature enzyme
Alcohol dehydrogenase
Regulator

Salmonella enterica subsp. enterica serovar Chester. Bacteria; Proteobacteria; Gammaproteobacteria; Enterobacterales;

A4M00__locus_2

EUT1

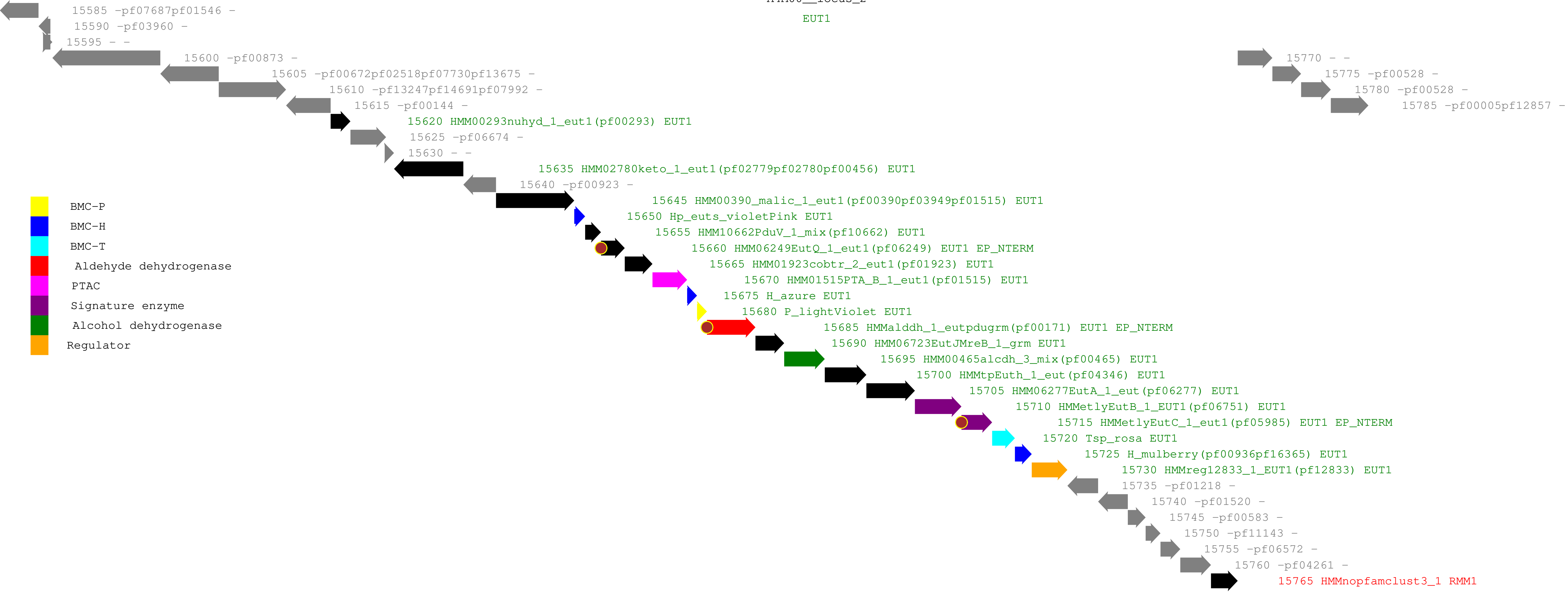

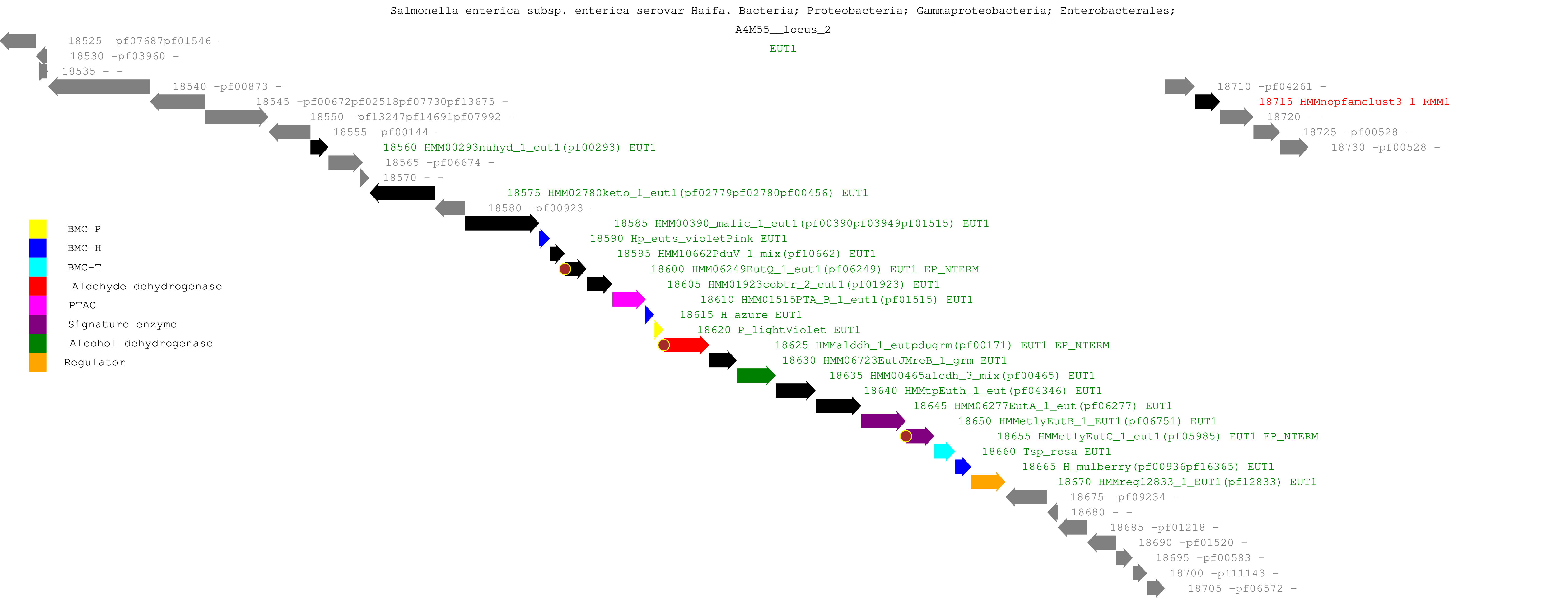

Salmonella enterica subsp. enterica serovar Haifa. Bacteria; Proteobacteria; Gammaproteobacteria; Enterobacterales;
A4M55__locus_2
EUT1
18525 -pf07687pf01546 -
18530 -pf03960 -
18535 - -
18540 -pf00873 -
18545 -pf00672pf02518pf07730pf13675 -
18550 -pf13247pf14691pf07992 -
18555 -pf00144 -
18560 HMM00293nuhyd_1_eut1(pf00293) EUT1
18565 -pf06674 -
18570 - -
18575 HMM02780keto_1_eut1(pf02779pf02780pf00456) EUT1
18580 -pf00923 -
18585 HMM00390_malic_1_eut1(pf00390pf03949pf01515) EUT1
18590 Hp_euts_violetPink EUT1
18595 HMM10662PduV_1_mix(pf10662) EUT1
18600 HMM06249EutQ_1_eut1(pf06249) EUT1 EP_NTERM
18605 HMM01923cobtr_2_eut1(pf01923) EUT1
18610 HMM01515PTA_B_1_eut1(pf01515) EUT1
18615 H_azure EUT1
18620 P_lightViolet EUT1
18625 HMMalddh_1_eutpdugrm(pf00171) EUT1 EP_NTERM
18630 HMM06723EutJMreB_1_grm EUT1
18635 HMM00465alcdh_3_mix(pf00465) EUT1
18640 HMMtpEuth_1_eut(pf04346) EUT1
18645 HMM06277EutA_1_eut(pf06277) EUT1
18650 HMMetlyEutB_1_EUT1(pf06751) EUT1
18655 HMMetlyEutC_1_eut1(pf05985) EUT1 EP_NTERM
18660 Tsp_rosa EUT1
18665 H_mulberry(pf00936pf16365) EUT1
18670 HMMreg12833_1_EUT1(pf12833) EUT1
18675 -pf09234 -
18680 - -
18685 -pf01218 -
18690 -pf01520 -
18695 -pf00583 -
18700 -pf11143 -
18705 -pf06572 -
18710 -pf04261 -
18715 HMMnopfamclust3_1 RMM1
18720 - -
18725 -pf00528 -
18730 -pf00528 -
BMC-P
BMC-H
BMC-T
Aldehyde dehydrogenase
PTAC
Signature enzyme
Alcohol dehydrogenase
Regulator

Salmonella enterica subsp. enterica serovar Braenderup. Bacteria; Proteobacteria; Gammaproteobacteria; Enterobacterales;

A4N09__locus_2

EUT1

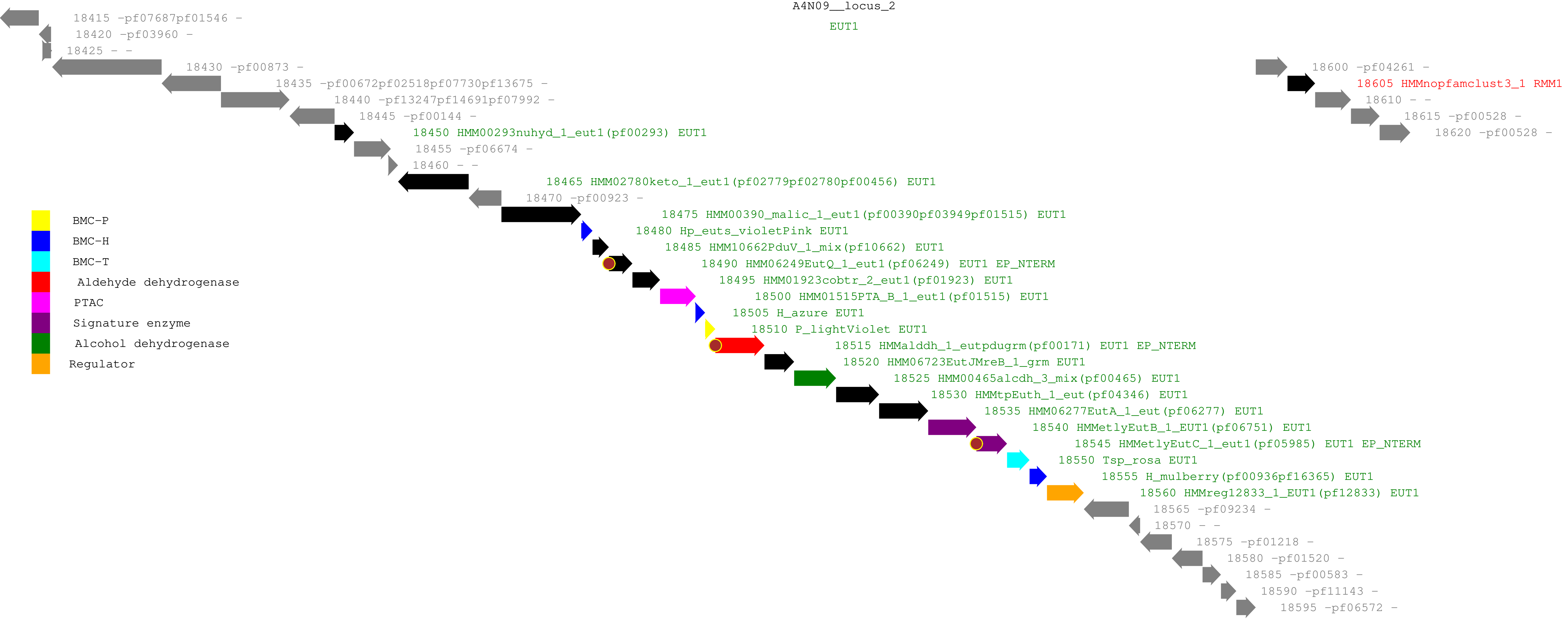

Salmonella enterica subsp. enterica serovar Isangi. Bacteria; Proteobacteria; Gammaproteobacteria; Enterobacterales;

A4N97__locus_1

EUT1

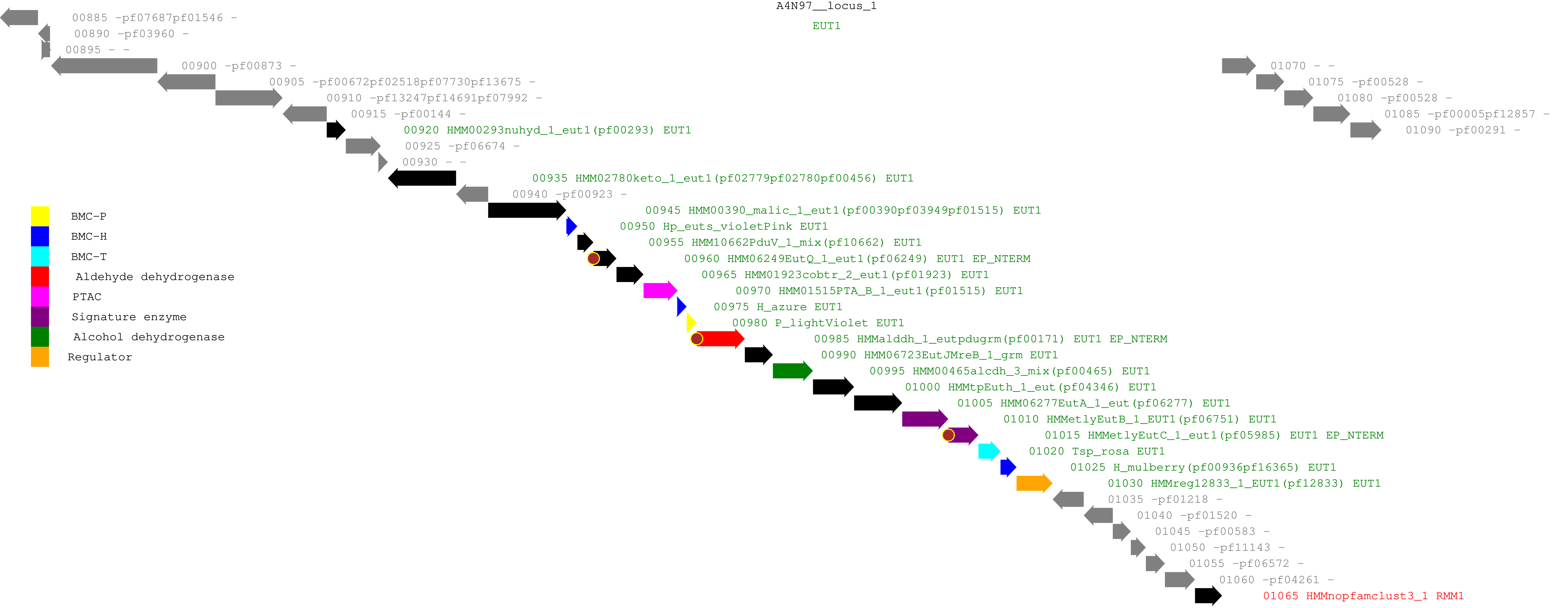

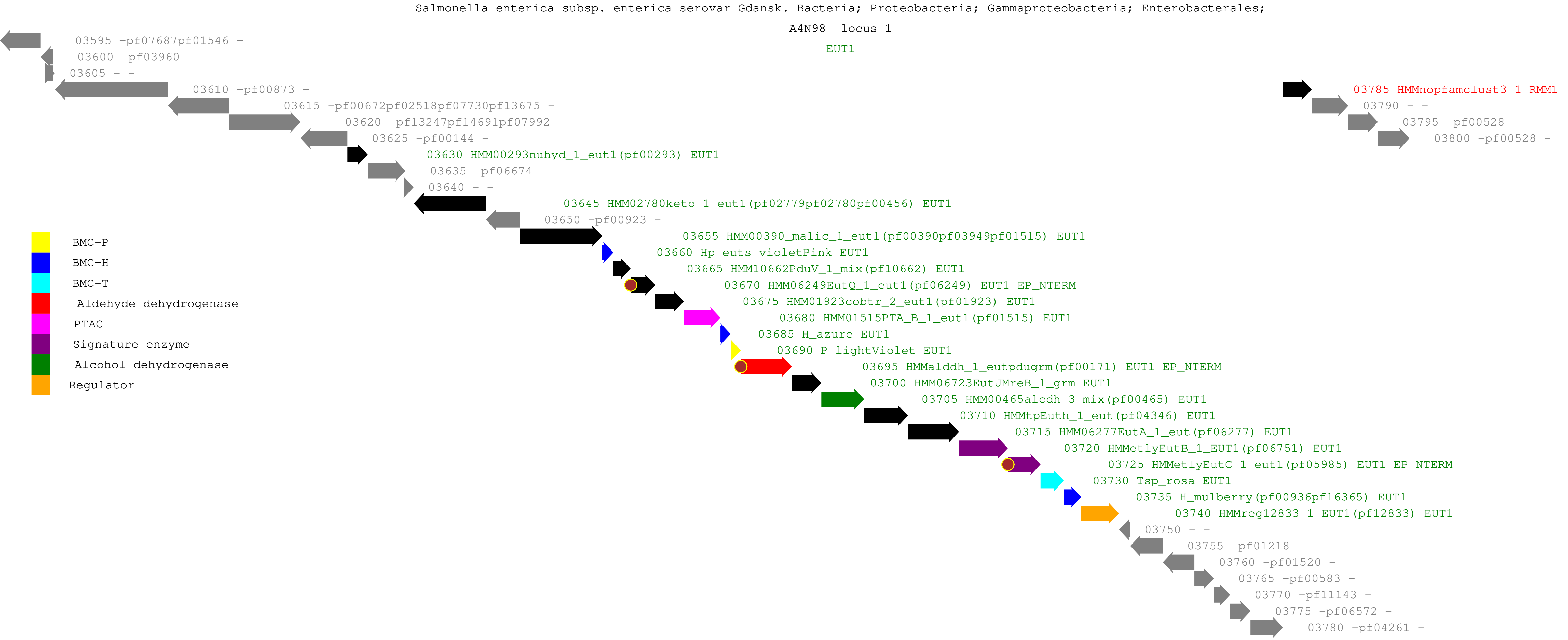

Salmonella enterica subsp. enterica serovar Gdansk. Bacteria; Proteobacteria; Gammaproteobacteria; Enterobacterales;
A4N98__locus_1
EUT1
03595 -pf07687pf01546 -
03600 -pf03960 -
03605 - -
03610 -pf00873 -
03615 -pf00672pf02518pf07730pf13675 -
03620 -pf13247pf14691pf07992 -
03625 -pf00144 -
03630 HMM00293nuhyd_1_eut1(pf00293) EUT1
03635 -pf06674 -
03640 - -
03645 HMM02780keto_1_eut1(pf02779pf02780pf00456) EUT1
03650 -pf00923 -
03655 HMM00390_malic_1_eut1(pf00390pf03949pf01515) EUT1
03660 Hp_euts_violetPink EUT1
03665 HMM10662PduV_1_mix(pf10662) EUT1
03670 HMM06249EutQ_1_eut1(pf06249) EUT1 EP_NTERM
03675 HMM01923cobtr_2_eut1(pf01923) EUT1
03680 HMM01515PTA_B_1_eut1(pf01515) EUT1
03685 H_azure EUT1
03690 P_lightViolet EUT1
03695 HMMalddh_1_eutpdugrm(pf00171) EUT1 EP_NTERM
03700 HMM06723EutJMreB_1_grm EUT1
03705 HMM00465alcdh_3_mix(pf00465) EUT1
03710 HMMtpEuth_1_eut(pf04346) EUT1
03715 HMM06277EutA_1_eut(pf06277) EUT1
03720 HMMetlyEutB_1_EUT1(pf06751) EUT1
03725 HMMetlyEutC_1_eut1(pf05985) EUT1 EP_NTERM
03730 Tsp_rosa EUT1
03735 H_mulberry(pf00936pf16365) EUT1
03740 HMMreg12833_1_EUT1(pf12833) EUT1
03750 - -
03755 -pf01218 -
03760 -pf01520 -
03765 -pf00583 -
03770 -pf11143 -
03775 -pf06572 -
03780 -pf04261 -
03785 HMMnopfamclust3_1 RMM1
03790 - -
03795 -pf00528 -
03800 -pf00528 -
BMC-P
BMC-H
BMC-T
Aldehyde dehydrogenase
PTAC
Signature enzyme
Alcohol dehydrogenase
Regulator

Salmonella enterica I. Bacteria; Proteobacteria; Gammaproteobacteria; Enterobacterales;

A4023__locus_2

EUT1

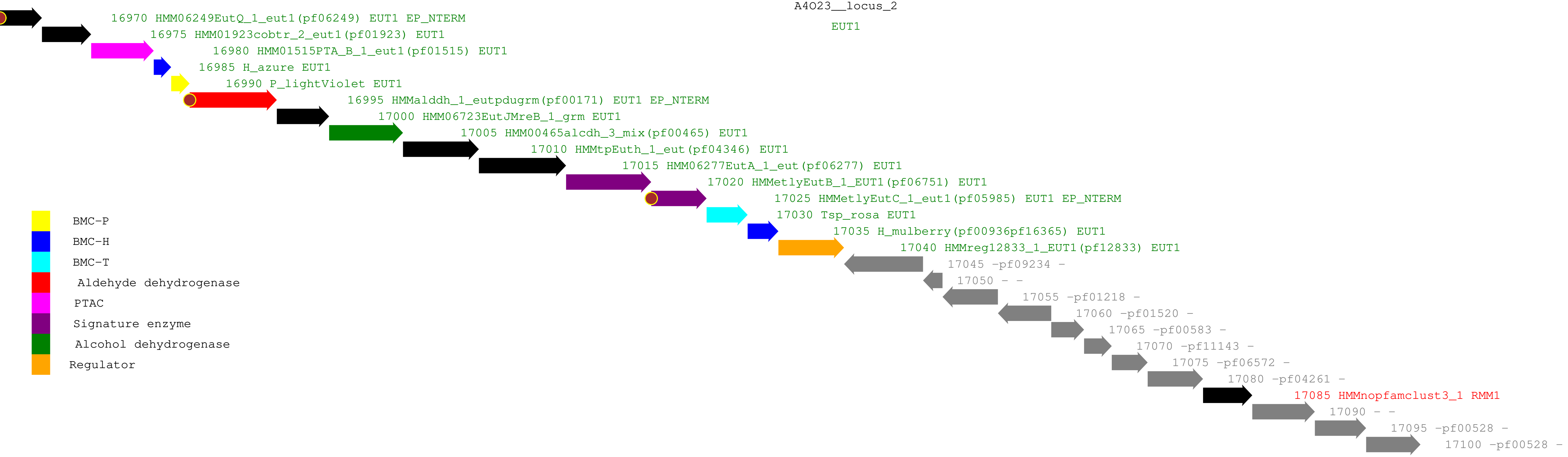

Salmonella rubislaw. Bacteria; Proteobacteria; Gammaproteobacteria; Enterobacterales;

A4T11__locus_1

EUT1

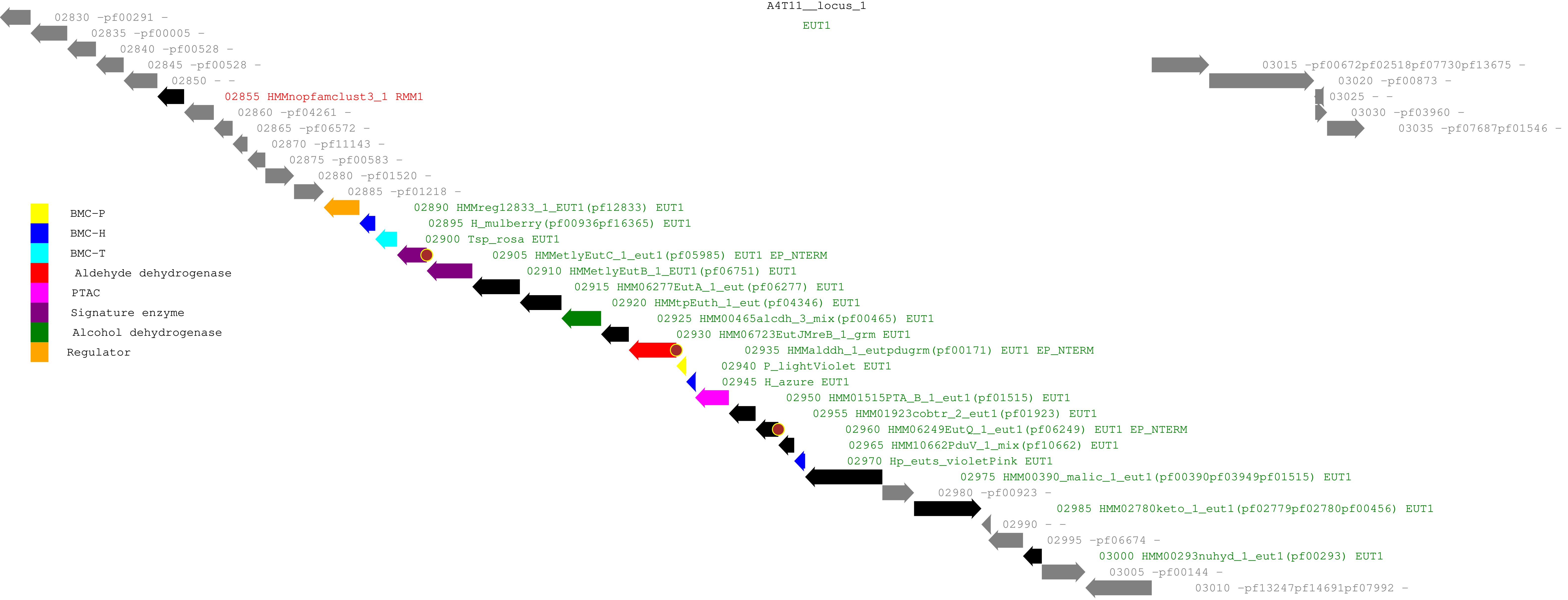

Escherichia coli. Bacteria; Proteobacteria; Gammaproteobacteria; Enterobacterales;

A4T40__locus_1

EUT1

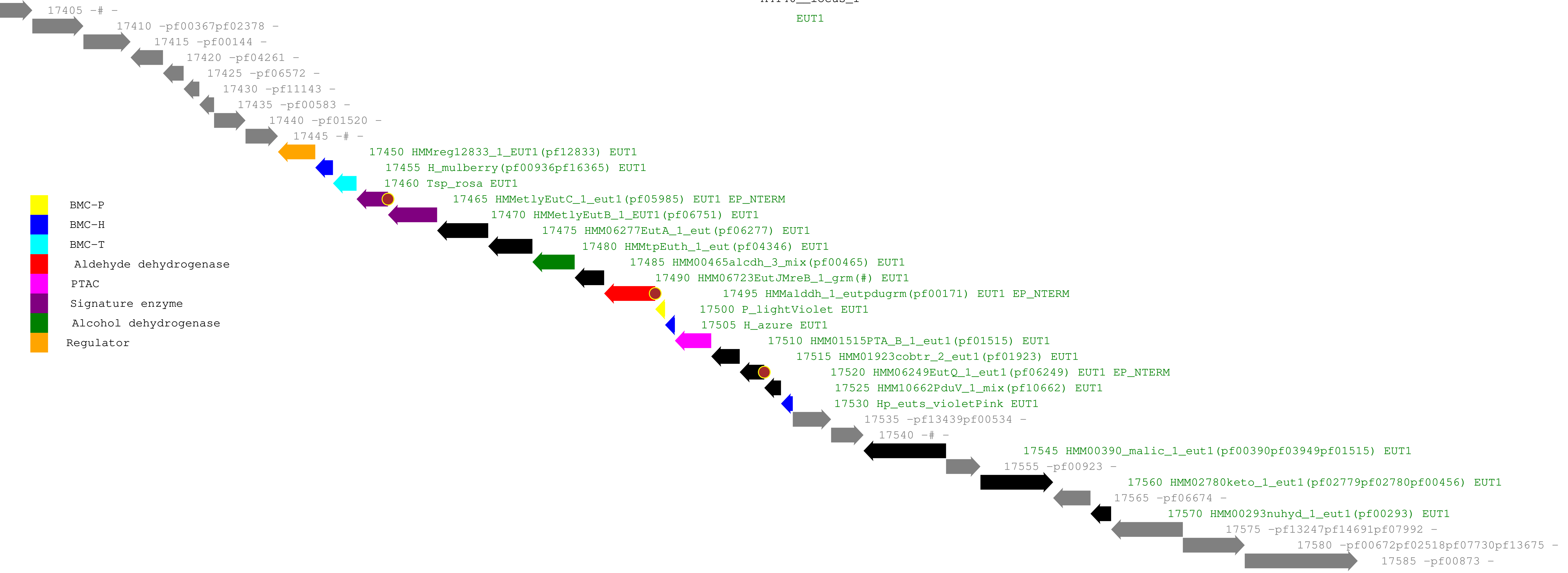

Escherichia coli. Bacteria; Proteobacteria; Gammaproteobacteria; Enterobacterales;

A4T50__locus_1

EUT1

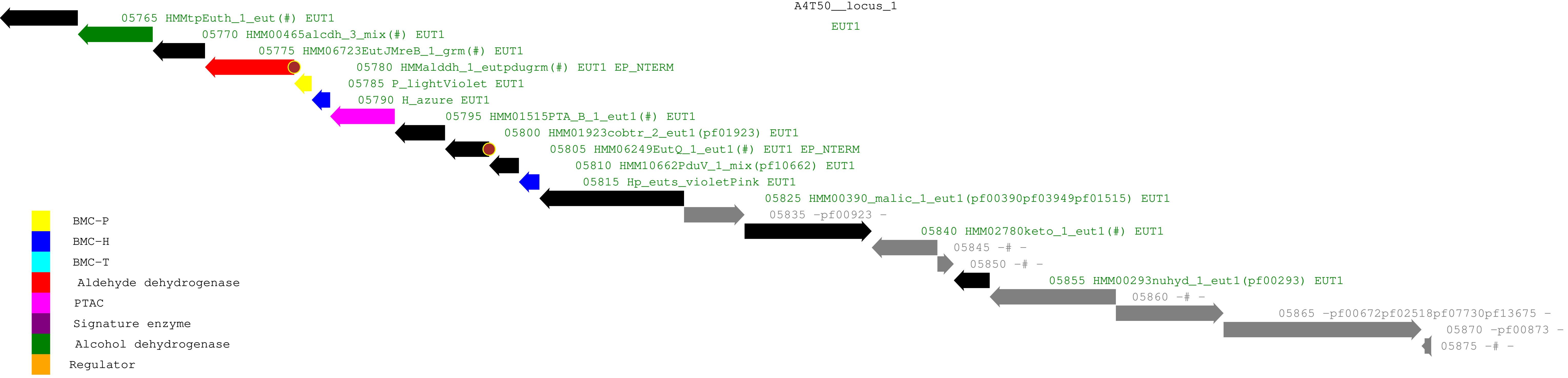

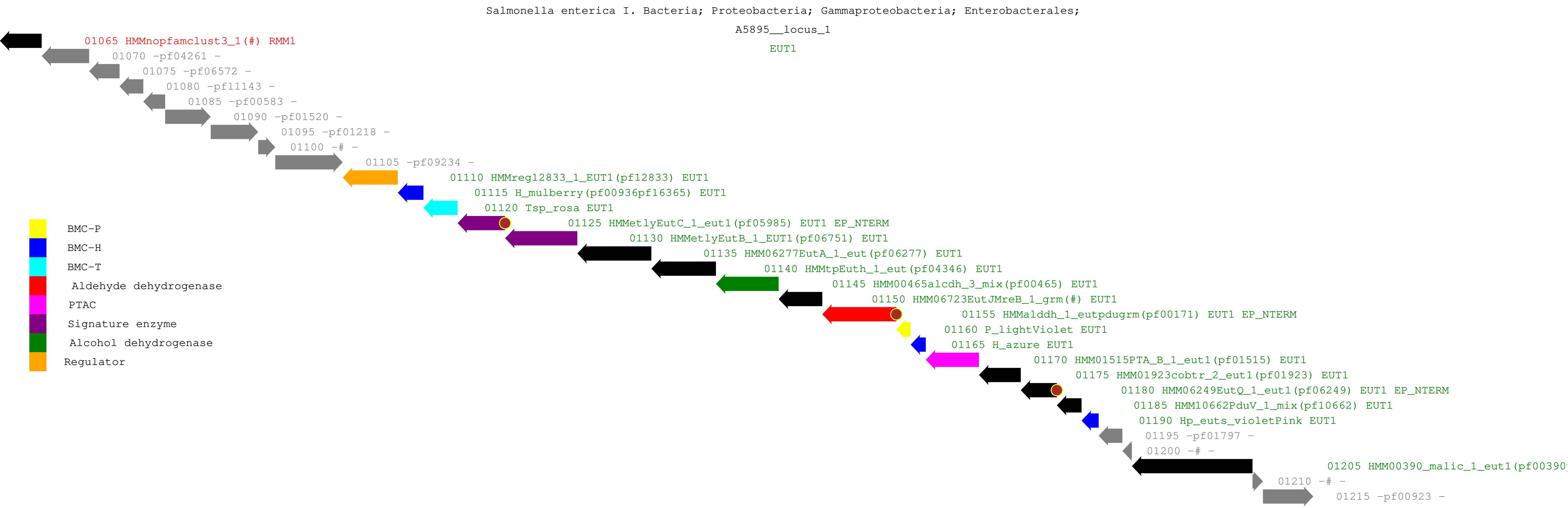

Salmonella enterica I. Bacteria; Proteobacteria; Gammaproteobacteria; Enterobacterales;
A5895__locus_1
EUT1
01065 HMMnopfamclust3_1(#) RMM1
01070 -pf04261 -
01075 -pf06572 -
01080 -pf11143 -
01085 -pf00583 -
01090 -pf01520 -
01095 -pf01218 -
01100 -# -
01105 -pf09234 -
01110 HMMreg12833_1_EUT1(pf12833) EUT1
01115 H_mulberry(pf00936pf16365) EUT1
01120 Tsp_rosa EUT1
01125 HMMetlyEutC_1_eut1(pf05985) EUT1 EP_NTERM
01130 HMMetlyEutB_1_EUT1(pf06751) EUT1
01135 HMM06277EutA_1_eut(pf06277) EUT1
01140 HMMtpEuth_1_eut(pf04346) EUT1
01145 HMM00465alcdh_3_mix(pf00465) EUT1
01150 HMM06723EutJMreB_1_grm(#) EUT1
01155 HMMalddh_1_eutpdugrm(pf00171) EUT1 EP_NTERM
01160 P_lightViolet EUT1
01165 H_azure EUT1
01170 HMM01515PTA_B_1_eut1(pf01515) EUT1
01175 HMM01923cobtr_2_eut1(pf01923) EUT1
01180 HMM06249EutQ_1_eut1(pf06249) EUT1 EP_NTERM
01185 HMM10662PduV_1_mix(pf10662) EUT1
01190 Hp_euts_violetPink EUT1
01195 -pf01797 -
01200 -# -
01205 HMM00390_malic_1_eut1(pf00390
01210 -# -
01215 -pf00923 -
BMC-P
BMC-H
BMC-T
Aldehyde dehydrogenase
PTAC
Signature enzyme
Alcohol dehydrogenase
Regulator

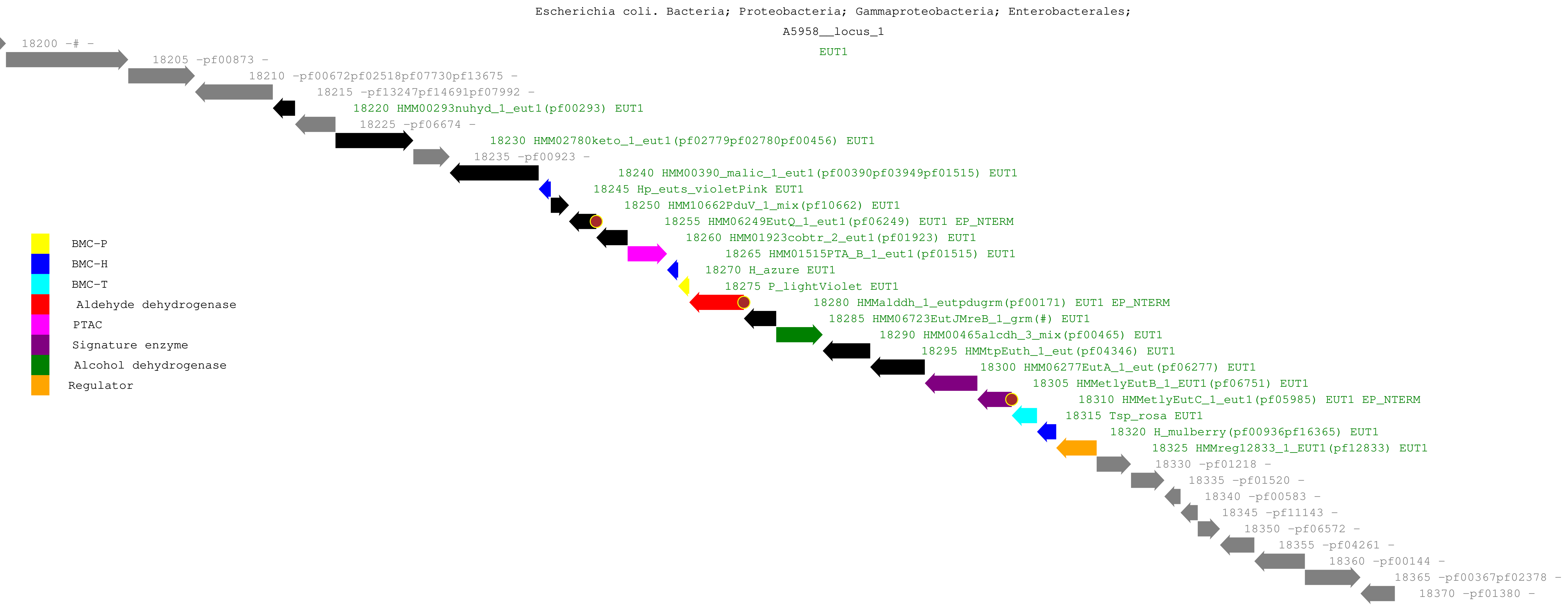
Escherichia coli. Bacteria; Proteobacteria; Gammaproteobacteria; Enterobacterales;
A5958__locus_1
EUT1
18200 -# -
18205 -pf00873 -
18210 -pf00672pf02518pf07730pf13675 -
18215 -pf13247pf14691pf07992 -
18220 HMM00293nuhyd_1_eut1(pf00293) EUT1
18225 -pf06674 -
18230 HMM02780keto_1_eut1(pf02779pf02780pf00456) EUT1
18235 -pf00923 -
18240 HMM00390_malic_1_eut1(pf00390pf03949pf01515) EUT1
18245 Hp_euts_violetPink EUT1
18250 HMM10662PduV_1_mix(pf10662) EUT1
18255 HMM06249EutQ_1_eut1(pf06249) EUT1 EP_NTERM
18260 HMM01923cobtr_2_eut1(pf01923) EUT1
18265 HMM01515PTA_B_1_eut1(pf01515) EUT1
18270 H_azure EUT1
18275 P_lightViolet EUT1
18280 HMMalddh_1_eutpdugrm(pf00171) EUT1 EP_NTERM
18285 HMM06723EutJMreB_1_grm(#) EUT1
18290 HMM00465alcdh_3_mix(pf00465) EUT1
18295 HMMtpEuth_1_eut(pf04346) EUT1
18300 HMM06277EutA_1_eut(pf06277) EUT1
18305 HMMetlyEutB_1_EUT1(pf06751) EUT1
18310 HMMetlyEutC_1_eut1(pf05985) EUT1 EP_NTERM
18315 Tsp_rosa EUT1
18320 H_mulberry(pf00936pf16365) EUT1
18325 HMMreg12833_1_EUT1(pf12833) EUT1
18330 -pf01218 -
18335 -pf01520 -
18340 -pf00583 -
18345 -pf11143 -
18350 -pf06572 -
18355 -pf04261 -
18360 -pf00144 -
18365 -pf00367pf02378 -
18370 -pf01380 -
BMC-P
BMC-H
BMC-T
Aldehyde dehydrogenase
PTAC
Signature enzyme
Alcohol dehydrogenase
Regulator

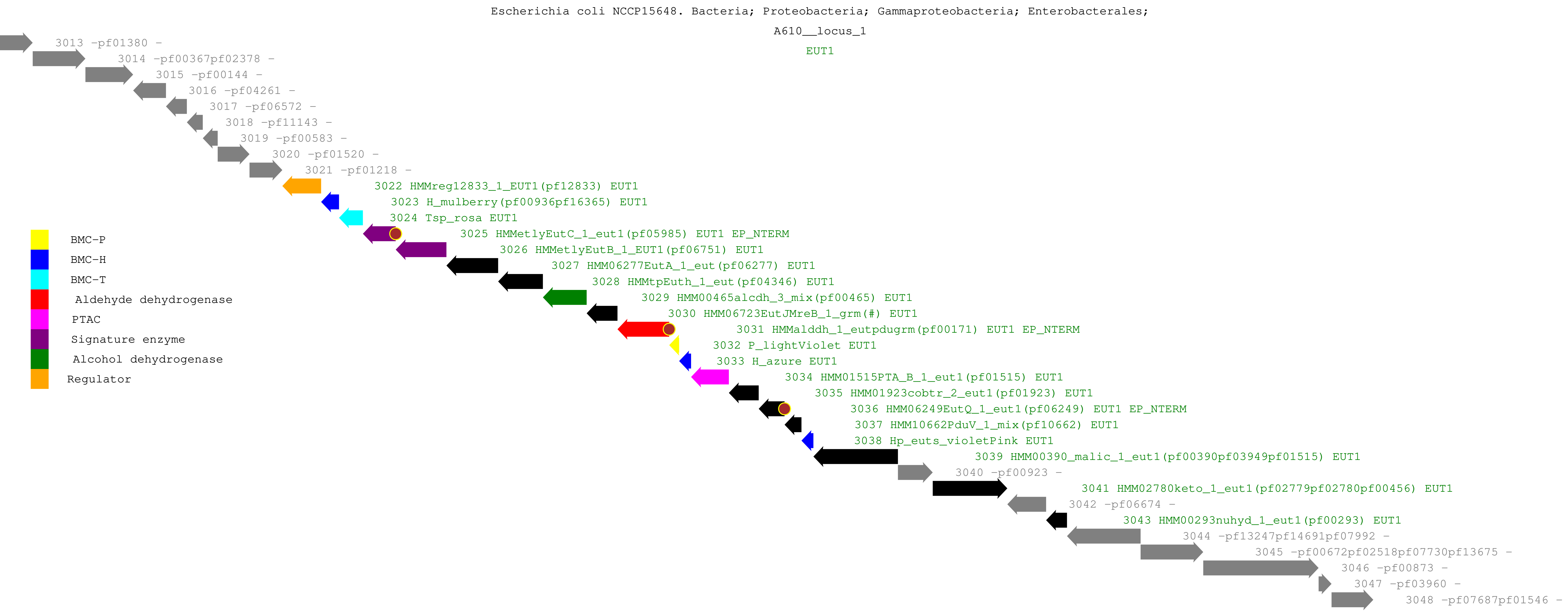

Escherichia coli NCCP15648. Bacteria; Proteobacteria; Gammaproteobacteria; Enterobacterales;
A610__locus_1
EUT1
3013 -pf01380 -
3014 -pf00367pf02378 -
3015 -pf00144 -
3016 -pf04261 -
3017 -pf06572 -
3018 -pf11143 -
3019 -pf00583 -
3020 -pf01520 -
3021 -pf01218 -
3022 HMMreg12833_1_EUT1(pf12833) EUT1
3023 H_mulberry(pf00936pf16365) EUT1
3024 Tsp_rosa EUT1
3025 HMMetlyEutC_1_eut1(pf05985) EUT1 EP_NTERM
3026 HMMetlyEutB_1_EUT1(pf06751) EUT1
3027 HMM06277EutA_1_eut(pf06277) EUT1
3028 HMMtpEuth_1_eut(pf04346) EUT1
3029 HMM00465alcdh_3_mix(pf00465) EUT1
3030 HMM06723EutJMreB_1_grm(#) EUT1
3031 HMMalddh_1_eutpdugrm(pf00171) EUT1 EP_NTERM
3032 P_lightViolet EUT1
3033 H_azure EUT1
3034 HMM01515PTA_B_1_eut1(pf01515) EUT1
3035 HMM01923cobtr_2_eut1(pf01923) EUT1
3036 HMM06249EutQ_1_eut1(pf06249) EUT1 EP_NTERM
3037 HMM10662PduV_1_mix(pf10662) EUT1
3038 Hp_euts_violetPink EUT1
3039 HMM00390_malic_1_eut1(pf00390pf03949pf01515) EUT1
3040 -pf00923 -
3041 HMM02780keto_1_eut1(pf02779pf02780pf00456) EUT1
3042 -pf06674 -
3043 HMM00293nuhyd_1_eut1(pf00293) EUT1
3044 -pf13247pf14691pf07992 -
3045 -pf00672pf02518pf07730pf13675 -
3046 -pf00873 -
3047 -pf03960 -
3048 -pf07687pf01546 -
BMC-P
BMC-H
BMC-T
Aldehyde dehydrogenase
PTAC
Signature enzyme
Alcohol dehydrogenase
Regulator

Salmonella enterica subsp. enterica serovar Cubana str. 76814. Bacteria; Proteobacteria; Gammaproteobacteria; Enterobacterales;

A628__locus_1

EUT1

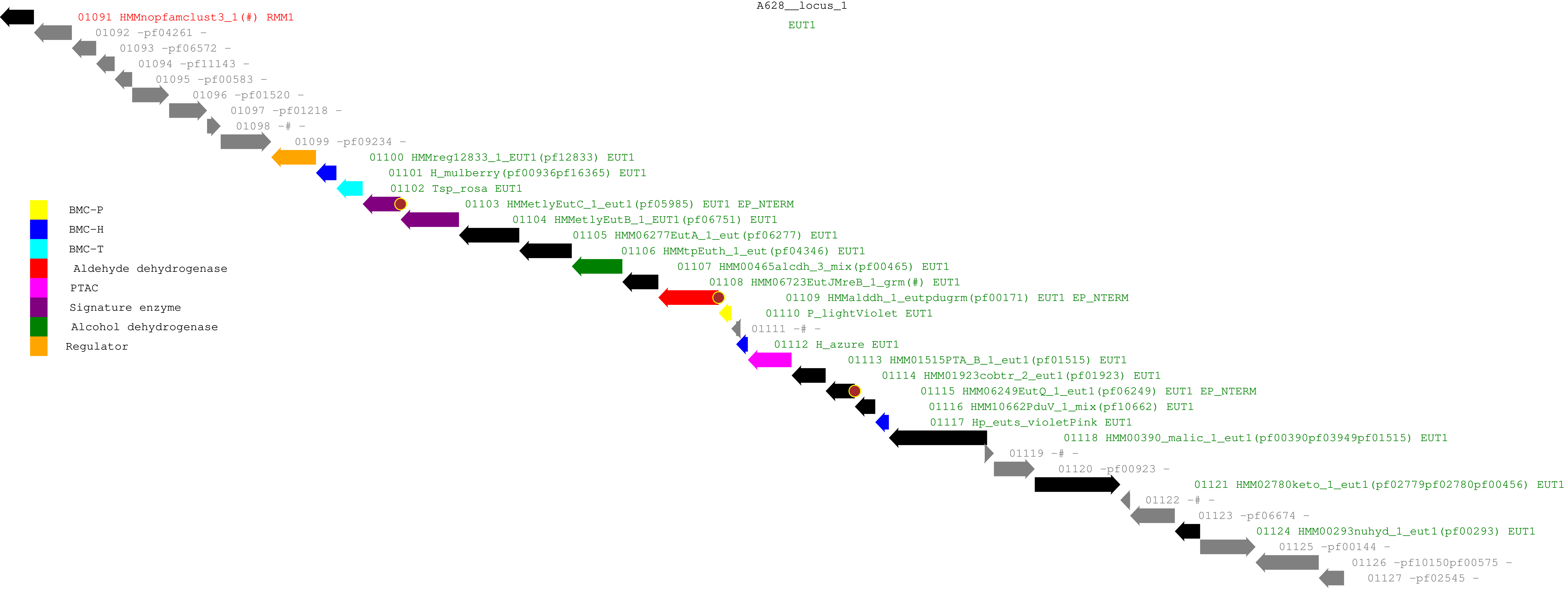

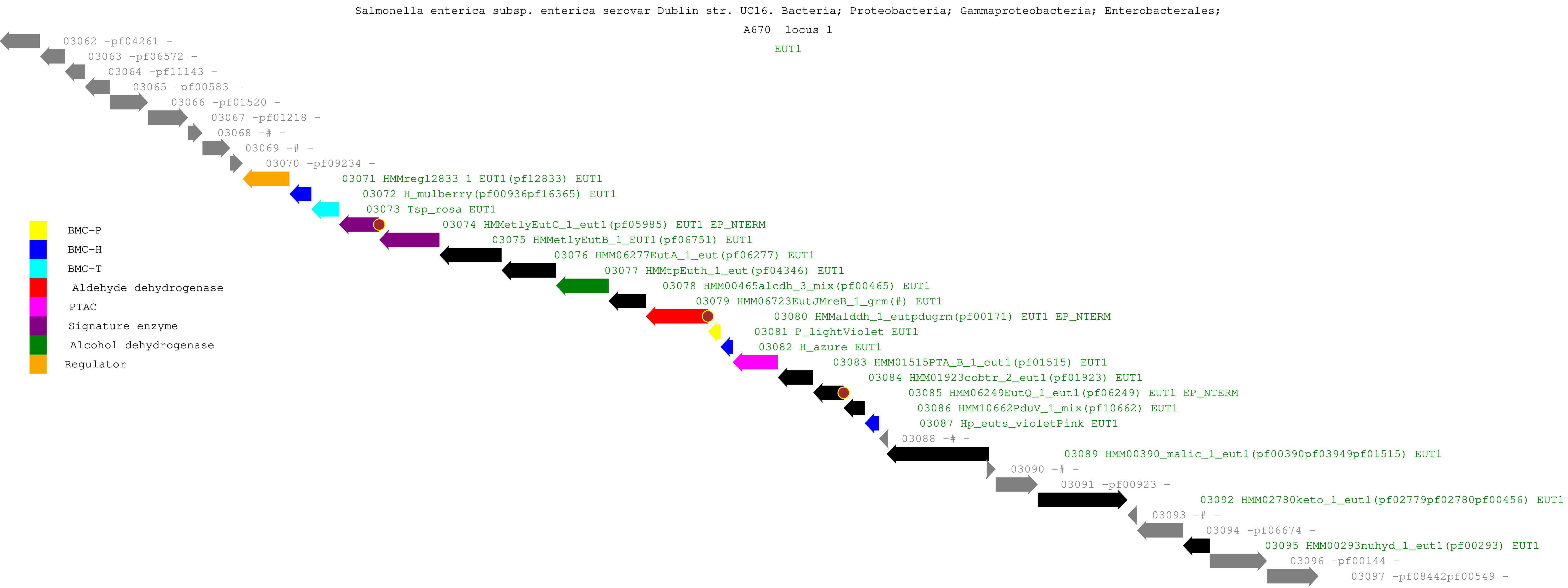
Salmonella enterica subsp. enterica serovar Dublin str. UC16. Bacteria; Proteobacteria; Gammaproteobacteria; Enterobacterales;
A670__locus_1
EUT1
03062 -pf04261 -
03063 -pf06572 -
03064 -pf11143 -
03065 -pf00583 -
03066 -pf01520 -
03067 -pf01218 -
03068 -# -
03069 -# -
03070 -pf09234 -
03071 HMMreg12833_1_EUT1(pf12833) EUT1
03072 H_mulberry(pf00936pf16365) EUT1
03073 Tsp_rosa EUT1
03074 HMMetlyEutC_1_eut1(pf05985) EUT1 EP_NTERM
03075 HMMetlyEutB_1_EUT1(pf06751) EUT1
03076 HMM06277EutA_1_eut(pf06277) EUT1
03077 HMMtpEuth_1_eut(pf04346) EUT1
03078 HMM00465alcdh_3_mix(pf00465) EUT1
03079 HMM06723EutJMreB_1_grm(#) EUT1
03080 HMMaldddh_1_eutpdugrm(pf00171) EUT1 EP_NTERM
03081 P_lightViolet EUT1
03082 H_azure EUT1
03083 HMM01515PTA_B_1_eut1(pf01515) EUT1
03084 HMM01923cobtr_2_eut1(pf01923) EUT1
03085 HMM06249EutQ_1_eut1(pf06249) EUT1 EP_NTERM
03086 HMM10662PduV_1_mix(pf10662) EUT1
03087 Hp_euts_violetPink EUT1
03088 -# -
03089 HMM00390_malic_1_eut1(pf00390pf03949pf01515) EUT1
03090 -# -
03091 -pf00923 -
03092 HMM02780keto_1_eut1(pf02779pf02780pf00456) EUT1
03093 -# -
03094 -pf06674 -
03095 HMM00293nuhyd_1_eut1(pf00293) EUT1
03096 -pf00144 -
03097 -pf08442pf00549 -
BMC-P
BMC-H
BMC-T
Aldehyde dehydrogenase
PTAC
Signature enzyme
Alcohol dehydrogenase
Regulator

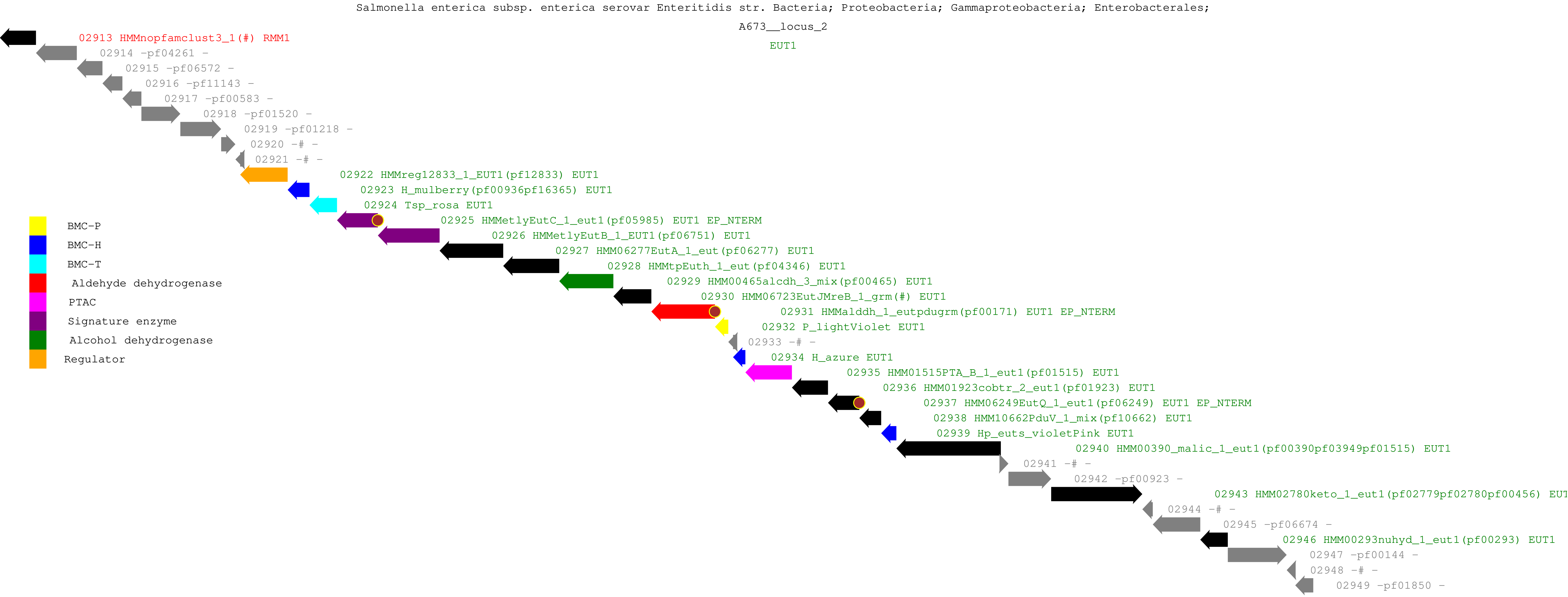

Salmonella enterica subsp. enterica serovar Enteritidis str. Bacteria; Proteobacteria; Gammaproteobacteria; Enterobacterales;
A673__locus_2
EUT1
02913 HMMnopfamclust3_1(#) RMM1
02914 -pf04261 -
02915 -pf06572 -
02916 -pf11143 -
02917 -pf00583 -
02918 -pf01520 -
02919 -pf01218 -
02920 -# -
02921 -# -
02922 HMMreg12833_1_EUT1(pf12833) EUT1
02923 H_mulberry(pf00936pf16365) EUT1
02924 Tsp_rosa EUT1
02925 HMMetlyEutC_1_eut1(pf05985) EUT1 EP_NTERM
02926 HMMetlyEutB_1_EUT1(pf06751) EUT1
02927 HMM06277EutA_1_eut(pf06277) EUT1
02928 HMMtpEuth_1_eut(pf04346) EUT1
02929 HMM00465alcdh_3_mix(pf00465) EUT1
02930 HMM06723EutJMreB_1_grm(#) EUT1
02931 HMMalddh_1_eutpdugrm(pf00171) EUT1 EP_NTERM
02932 P_lightViolet EUT1
02933 -# -
02934 H_azure EUT1
02935 HMM01515PTA_B_1_eut1(pf01515) EUT1
02936 HMM01923cobtr_2_eut1(pf01923) EUT1
02937 HMM06249EutQ_1_eut1(pf06249) EUT1 EP_NTERM
02938 HMM10662PduV_1_mix(pf10662) EUT1
02939 Hp_euts_violetPink EUT1
02940 HMM00390_malic_1_eut1(pf00390pf03949pf01515) EUT1
02941 -# -
02942 -pf00923 -
02943 HMM02780keto_1_eut1(pf02779pf02780pf00456) EUT
02944 -# -
02945 -pf06674 -
02946 HMM00293nuhyd_1_eut1(pf00293) EUT1
02947 -pf00144 -
02948 -# -
02949 -pf01850 -
BMC-P
BMC-H
BMC-T
Aldehyde dehydrogenase
PTAC
Signature enzyme
Alcohol dehydrogenase
Regulator

Klebsiella michiganensis. Bacteria; Proteobacteria; Gammaproteobacteria; Enterobacterales;

A6A30__locus_2

EUT1

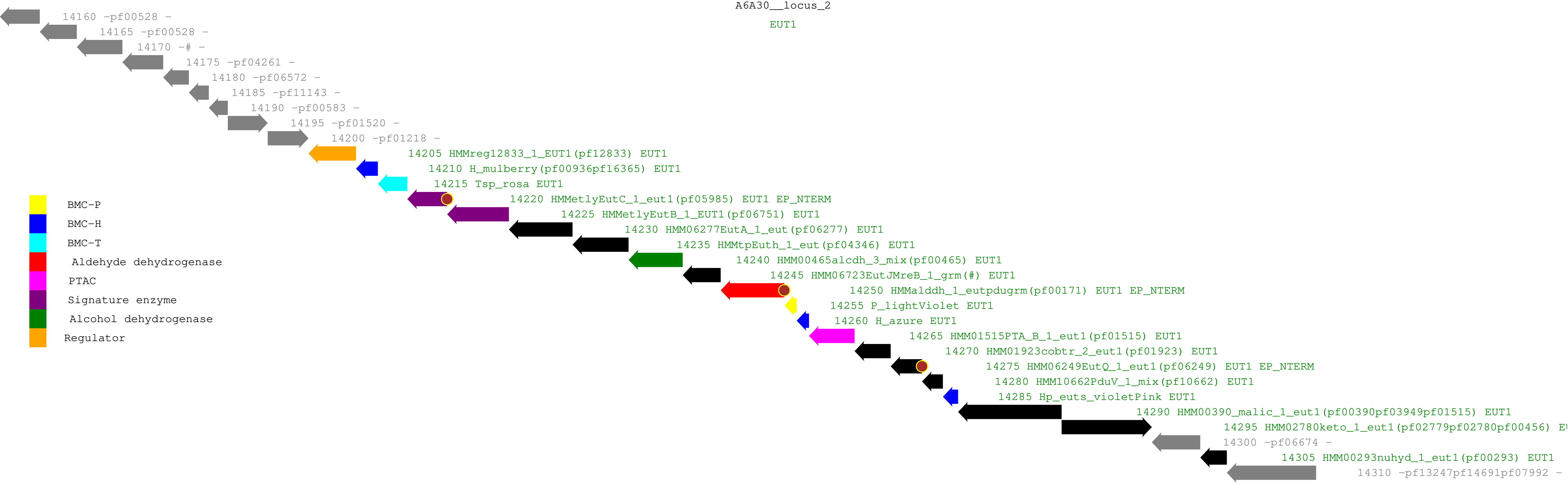

Salmonella enterica subsp. enterica serovar Rissen. Bacteria; Proteobacteria; Gammaproteobacteria; Enterobacterales;

A6S79__locus_1

EUT1

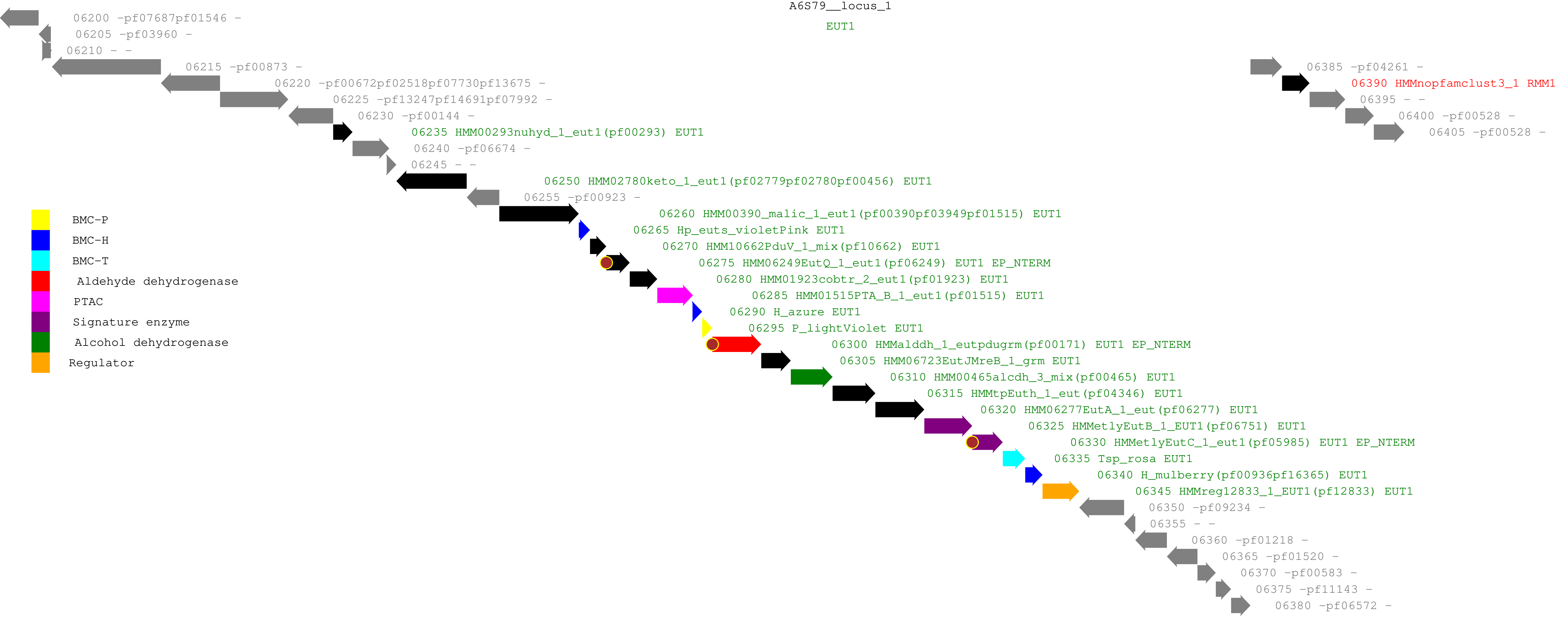

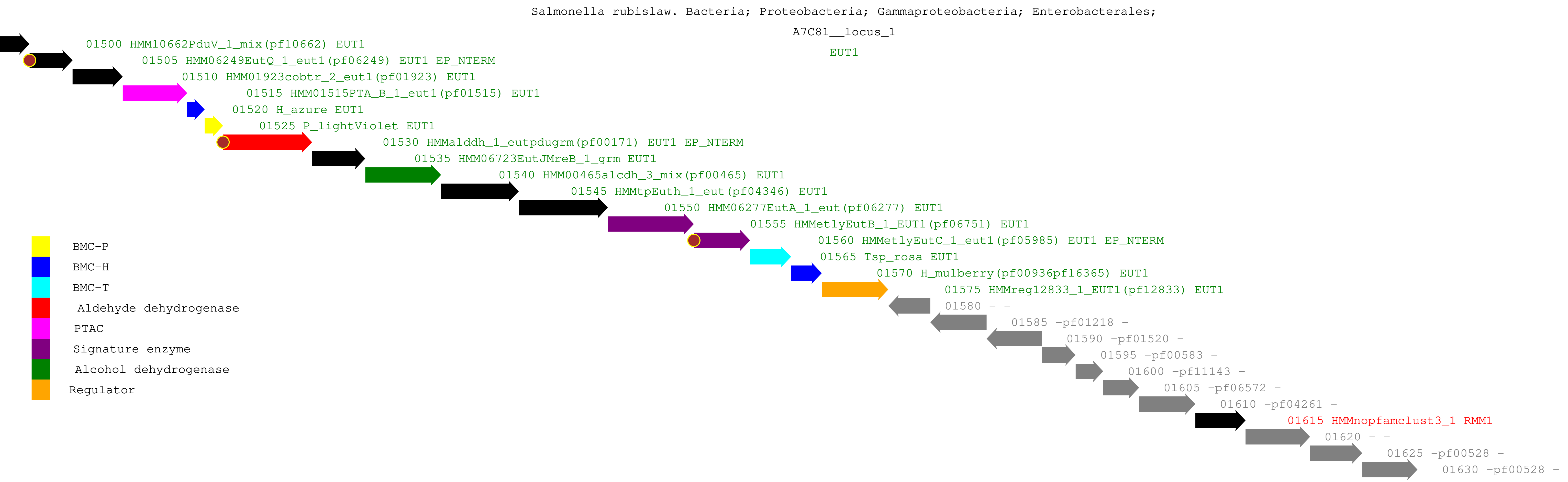
Salmonella rubislaw. Bacteria; Proteobacteria; Gammaproteobacteria; Enterobacterales;
A7C81__locus_1
EUT1
01500 HMM10662PduV_1_mix(pf10662) EUT1
01505 HMM06249EutQ_1_eut1(pf06249) EUT1 EP_NTERM
01510 HMM01923cobtr_2_eut1(pf01923) EUT1
01515 HMM01515PTA_B_1_eut1(pf01515) EUT1
01520 H_azure EUT1
01525 P_lightViolet EUT1
01530 HMMalddh_1_eutpdugrm(pf00171) EUT1 EP_NTERM
01535 HMM06723EutJMreB_1_grm EUT1
01540 HMM00465alcdh_3_mix(pf00465) EUT1
01545 HMMtpEuth_1_eut(pf04346) EUT1
01550 HMM06277EutA_1_eut(pf06277) EUT1
01555 HMMetlyEutB_1_EUT1(pf06751) EUT1
01560 HMMetlyEutC_1_eut1(pf05985) EUT1 EP_NTERM
01565 Tsp_rosa EUT1
01570 H_mulberry(pf00936pf16365) EUT1
01575 HMMreg12833_1_EUT1(pf12833) EUT1
01580 - -
01585 -pf01218 -
01590 -pf01520 -
01595 -pf00583 -
01600 -pf11143 -
01605 -pf06572 -
01610 -pf04261 -
01615 HMMnopfamclust3_1 RMM1
01620 - -
01625 -pf00528 -
01630 -pf00528 -
BMC-P
BMC-H
BMC-T
Aldehyde dehydrogenase
PTAC
Signature enzyme
Alcohol dehydrogenase
Regulator

Salmonella enterica (Salmonella choleraesuis). Bacteria; Proteobacteria; Gammaproteobacteria; Enterobacterales;

A7D45__locus_2

EUT1

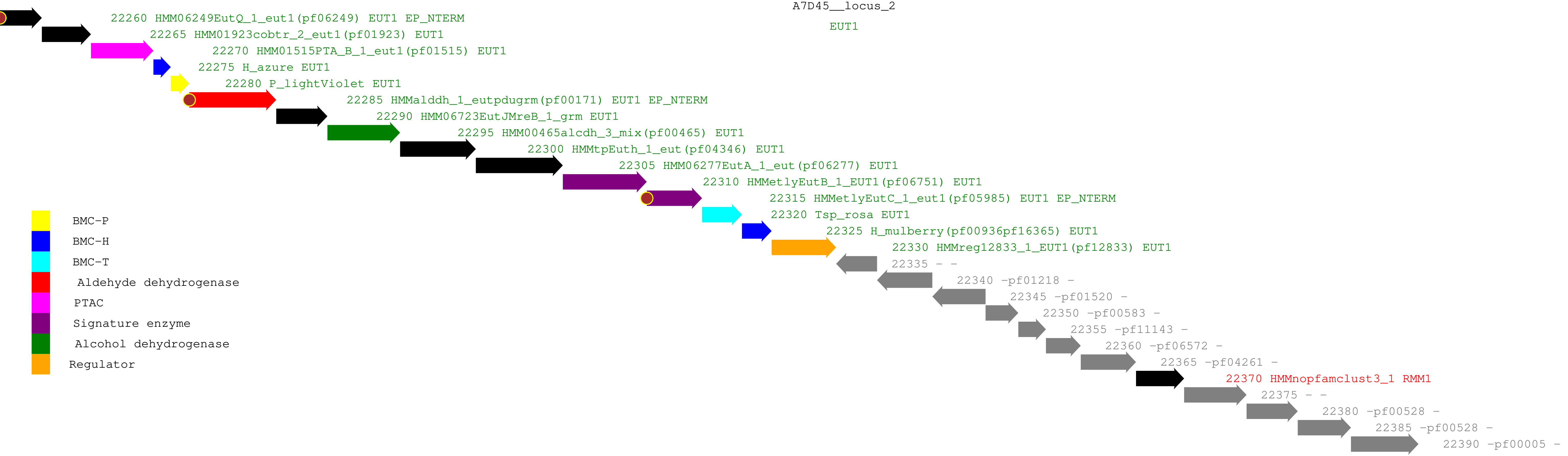

Salmonella enterica (Salmonella choleraesuis). Bacteria; Proteobacteria; Gammaproteobacteria; Enterobacterales;

A7K84__locus_2

EUT1

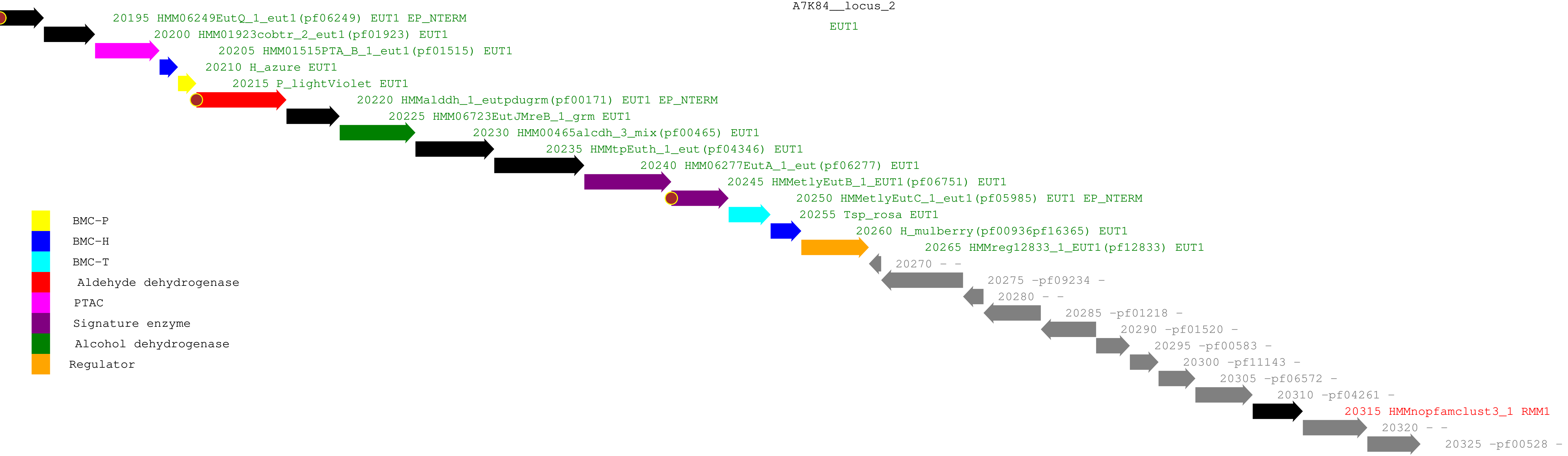

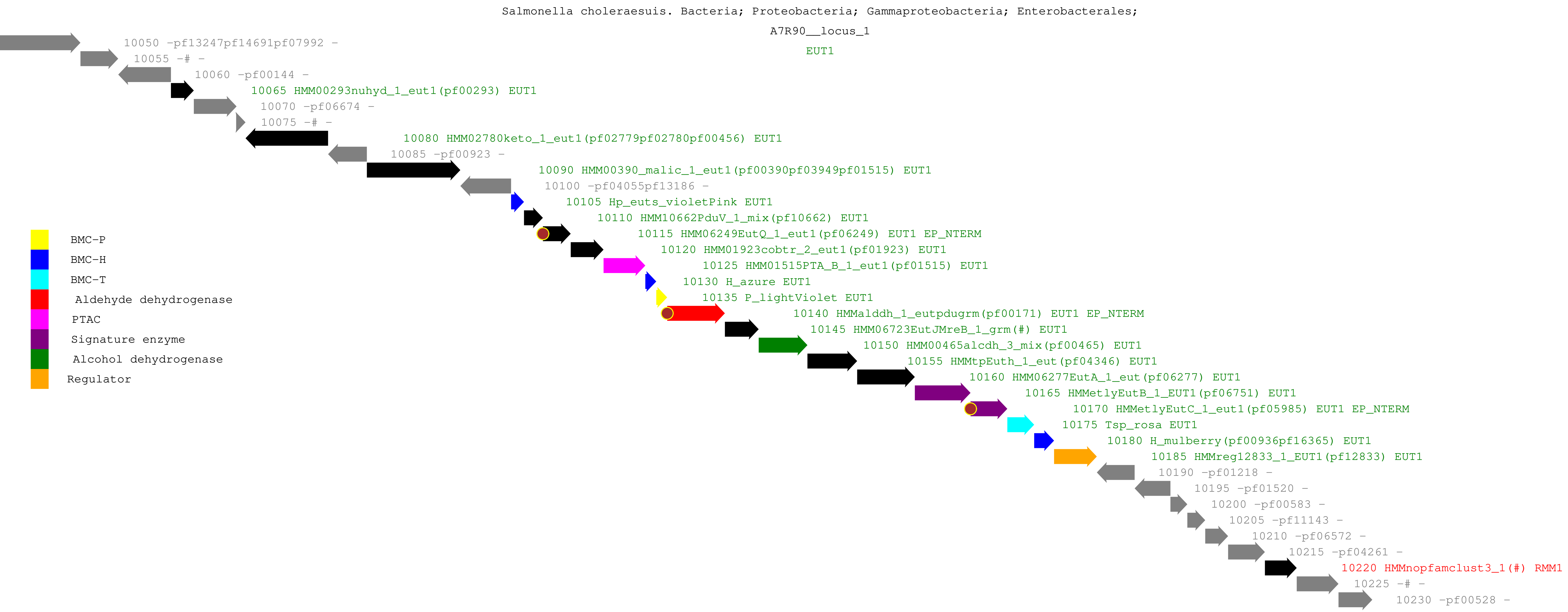
Salmonella choleraesuis. Bacteria; Proteobacteria; Gammaproteobacteria; Enterobacterales;
A7R90__locus_1
EUT1
10050 -pf13247pf14691pf07992 -
10055 -# -
10060 -pf00144 -
10065 HMM00293nuhyd_1_eut1(pf00293) EUT1
10070 -pf06674 -
10075 -# -
10080 HMM02780keto_1_eut1(pf02779pf02780pf00456) EUT1
10085 -pf00923 -
10090 HMM00390_malic_1_eut1(pf00390pf03949pf01515) EUT1
10100 -pf04055pf13186 -
10105 Hp_euts_violetPink EUT1
10110 HMM10662PduV_1_mix(pf10662) EUT1
10115 HMM06249EutQ_1_eut1(pf06249) EUT1 EP_NTERM
10120 HMM01923cobtr_2_eut1(pf01923) EUT1
10125 HMM01515PTA_B_1_eut1(pf01515) EUT1
10130 H_azure EUT1
10135 P_lightViolet EUT1
10140 HMMalddh_1_eutpdugrm(pf00171) EUT1 EP_NTERM
10145 HMM06723EutJMreB_1_grm(#) EUT1
10150 HMM00465alcdh_3_mix(pf00465) EUT1
10155 HMMtpEuth_1_eut(pf04346) EUT1
10160 HMM06277EutA_1_eut(pf06277) EUT1
10165 HMMetlyEutB_1_EUT1(pf06751) EUT1
10170 HMMetlyEutC_1_eut1(pf05985) EUT1 EP_NTERM
10175 Tsp_rosa EUT1
10180 H_mulberry(pf00936pf16365) EUT1
10185 HMMreg12833_1_EUT1(pf12833) EUT1
10190 -pf01218 -
10195 -pf01520 -
10200 -pf00583 -
10205 -pf11143 -
10210 -pf06572 -
10215 -pf04261 -
10220 HMMnopfamclust3_1(#) RMM1
10225 -# -
10230 -pf00528 -
BMC-P
BMC-H
BMC-T
Aldehyde dehydrogenase
PTAC
Signature enzyme
Alcohol dehydrogenase
Regulator

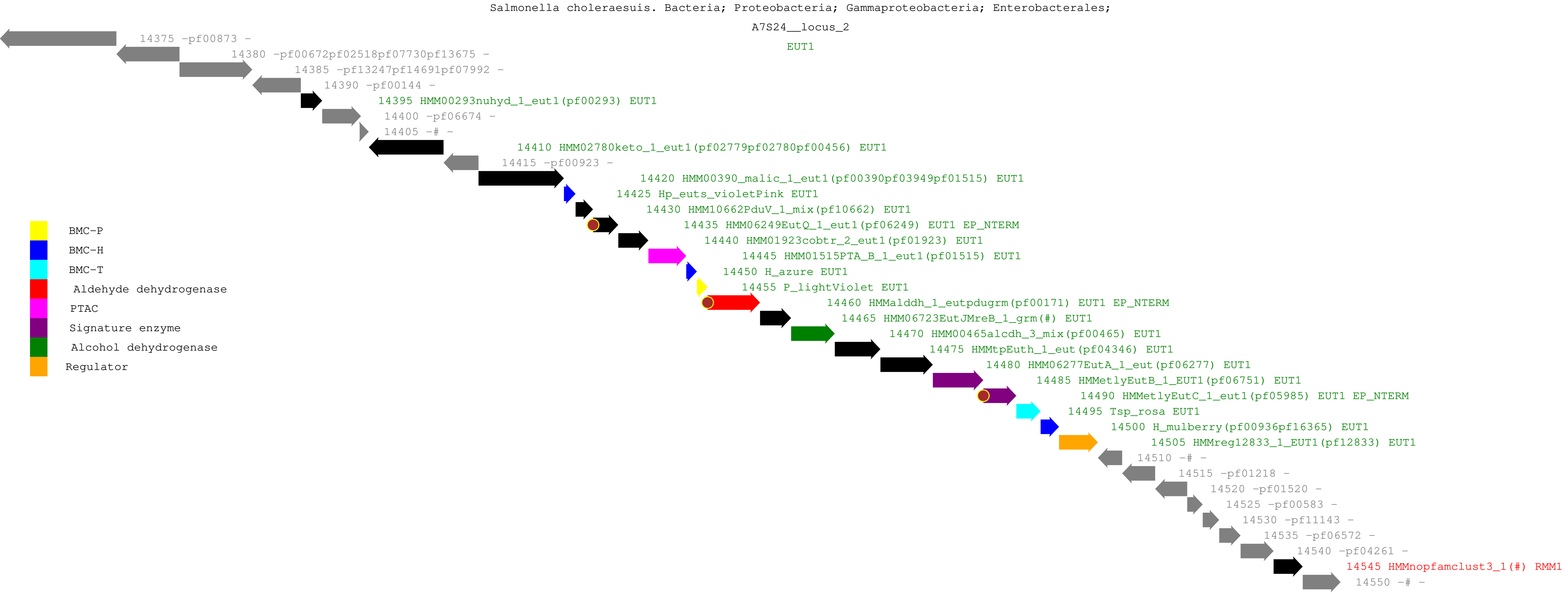
Salmonella choleraesuis. Bacteria; Proteobacteria; Gammaproteobacteria; Enterobacterales;
A7S24__locus_2
EUT1
14375 -pf00873 -
14380 -pf00672pf02518pf07730pf13675 -
14385 -pf13247pf14691pf07992 -
14390 -pf00144 -
14395 HMM00293nuhyd_1_eut1(pf00293) EUT1
14400 -pf06674 -
14405 -# -
14410 HMM02780keto_1_eut1(pf02779pf02780pf00456) EUT1
14415 -pf00923 -
14420 HMM00390_malic_1_eut1(pf00390pf03949pf01515) EUT1
14425 Hp_euts_violetPink EUT1
14430 HMM10662PduV_1_mix(pf10662) EUT1
14435 HMM06249EutQ_1_eut1(pf06249) EUT1 EP_NTERM
14440 HMM01923cobtr_2_eut1(pf01923) EUT1
14445 HMM01515PTA_B_1_eut1(pf01515) EUT1
14450 H_azure EUT1
14455 P_lightViolet EUT1
14460 HMMalddh_1_eutpdugrm(pf00171) EUT1 EP_NTERM
14465 HMM06723EutJMreB_1_grm(#) EUT1
14470 HMM00465alcdh_3_mix(pf00465) EUT1
14475 HMMtpEuth_1_eut(pf04346) EUT1
14480 HMM06277EutA_1_eut(pf06277) EUT1
14485 HMMetlyEutB_1_EUT1(pf06751) EUT1
14490 HMMetlyEutC_1_eut1(pf05985) EUT1 EP_NTERM
14495 Tsp_rosa EUT1
14500 H_mulberry(pf00936pf16365) EUT1
14505 HMMreg12833_1_EUT1(pf12833) EUT1
14510 -# -
14515 -pf01218 -
14520 -pf01520 -
14525 -pf00583 -
14530 -pf11143 -
14535 -pf06572 -
14540 -pf04261 -
14545 HMMnopfamclust3_1(#) RMM1
14550 -# -
BMC-P
BMC-H
BMC-T
Aldehyde dehydrogenase
PTAC
Signature enzyme
Alcohol dehydrogenase
Regulator

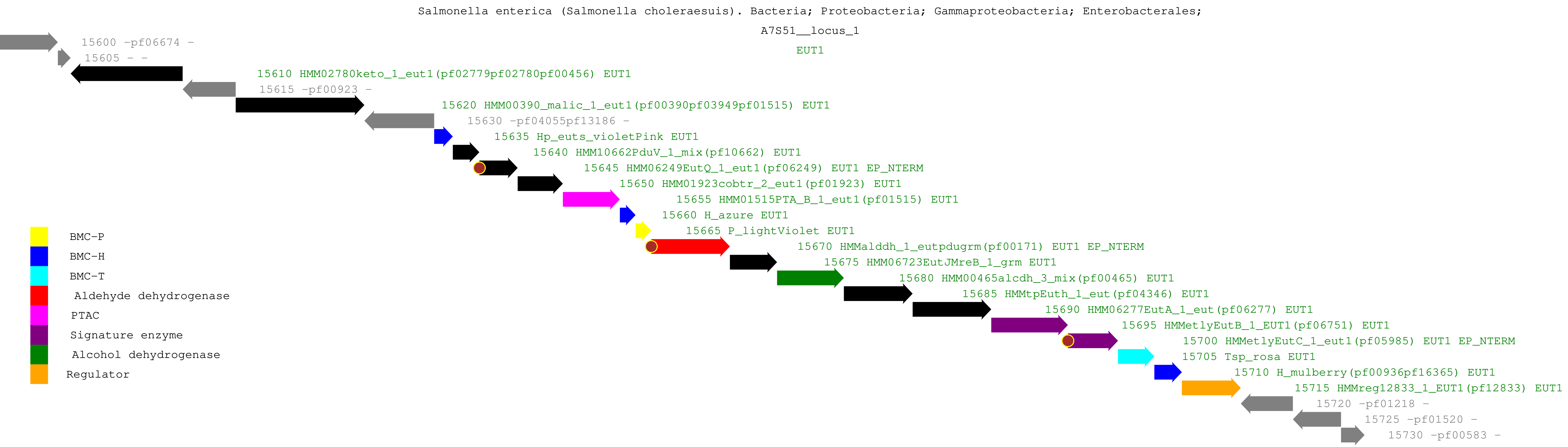
Salmonella enterica (Salmonella choleraesuis). Bacteria; Proteobacteria; Gammaproteobacteria; Enterobacterales;
A7S51__locus_1
EUT1
15600 -pf06674 -
15605 - -
15610 HMM02780keto_1_eut1(pf02779pf02780pf00456) EUT1
15615 -pf00923 -
15620 HMM00390_malic_1_eut1(pf00390pf03949pf01515) EUT1
15630 -pf04055pf13186 -
15635 Hp_euts_violetPink EUT1
15640 HMM10662PduV_1_mix(pf10662) EUT1
15645 HMM06249EutQ_1_eut1(pf06249) EUT1 EP_NTERM
15650 HMM01923cobtr_2_eut1(pf01923) EUT1
15655 HMM01515PTA_B_1_eut1(pf01515) EUT1
15660 H_azure EUT1
15665 P_lightViolet EUT1
15670 HMMalddh_1_eutpdugrm(pf00171) EUT1 EP_NTERM
15675 HMM06723EutJMreB_1_grm EUT1
15680 HMM00465alcdh_3_mix(pf00465) EUT1
15685 HMMtpEuth_1_eut(pf04346) EUT1
15690 HMM06277EutA_1_eut(pf06277) EUT1
15695 HMMetlyEutB_1_EUT1(pf06751) EUT1
15700 HMMetlyEutC_1_eut1(pf05985) EUT1 EP_NTERM
15705 Tsp_rosa EUT1
15710 H_mulberry(pf00936pf16365) EUT1
15715 HMMreg12833_1_EUT1(pf12833) EUT1
15720 -pf01218 -
15725 -pf01520 -
15730 -pf00583 -
BMC-P
BMC-H
BMC-T
Aldehyde dehydrogenase
PTAC
Signature enzyme
Alcohol dehydrogenase
Regulator

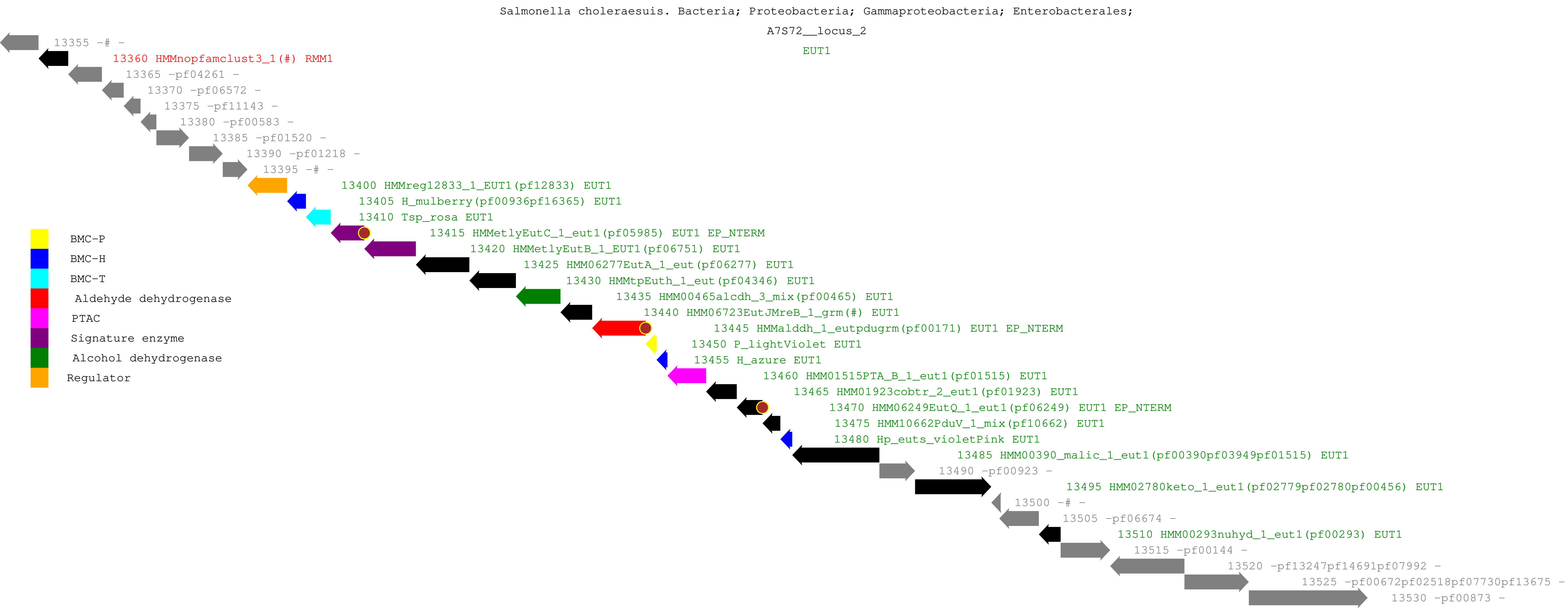
Salmonella choleraesuis. Bacteria; Proteobacteria; Gammaproteobacteria; Enterobacterales;
A7S72__locus_2
EUT1
13355 -# -
13360 HMMnopfamclust3_1(#) RMM1
13365 -pf04261 -
13370 -pf06572 -
13375 -pf11143 -
13380 -pf00583 -
13385 -pf01520 -
13390 -pf01218 -
13395 -# -
13400 HMMreg12833_1_EUT1(pf12833) EUT1
13405 H_mulberry(pf00936pf16365) EUT1
13410 Tsp_rosa EUT1
13415 HMMetlyEutC_1_eut1(pf05985) EUT1 EP_NTERM
13420 HMMetlyEutB_1_EUT1(pf06751) EUT1
13425 HMM06277EutA_1_eut(pf06277) EUT1
13430 HMMtpEuth_1_eut(pf04346) EUT1
13435 HMM00465alcdh_3_mix(pf00465) EUT1
13440 HMM06723EutJMreB_1_grm(#) EUT1
13445 HMMalddh_1_eutpdugrm(pf00171) EUT1 EP_NTERM
13450 P_lightViolet EUT1
13455 H_azure EUT1
13460 HMM01515PTA_B_1_eut1(pf01515) EUT1
13465 HMM01923cobtr_2_eut1(pf01923) EUT1
13470 HMM06249EutQ_1_eut1(pf06249) EUT1 EP_NTERM
13475 HMM10662PduV_1_mix(pf10662) EUT1
13480 Hp_euts_violetPink EUT1
13485 HMM00390_malic_1_eut1(pf00390pf03949pf01515) EUT1
13490 -pf00923 -
13495 HMM02780keto_1_eut1(pf02779pf02780pf00456) EUT1
13500 -# -
13505 -pf06674 -
13510 HMM00293nuhyd_1_eut1(pf00293) EUT1
13515 -pf00144 -
13520 -pf13247pf14691pf07992 -
13525 -pf00672pf02518pf07730pf13675 -
13530 -pf00873 -
BMC-P
BMC-H
BMC-T
Aldehyde dehydrogenase
PTAC
Signature enzyme
Alcohol dehydrogenase
Regulator

Salmonella enterica subsp. enterica serovar Saintpaul. Bacteria; Proteobacteria; Gammaproteobacteria; Enterobacterales;

A7T00__locus_1

EUT1

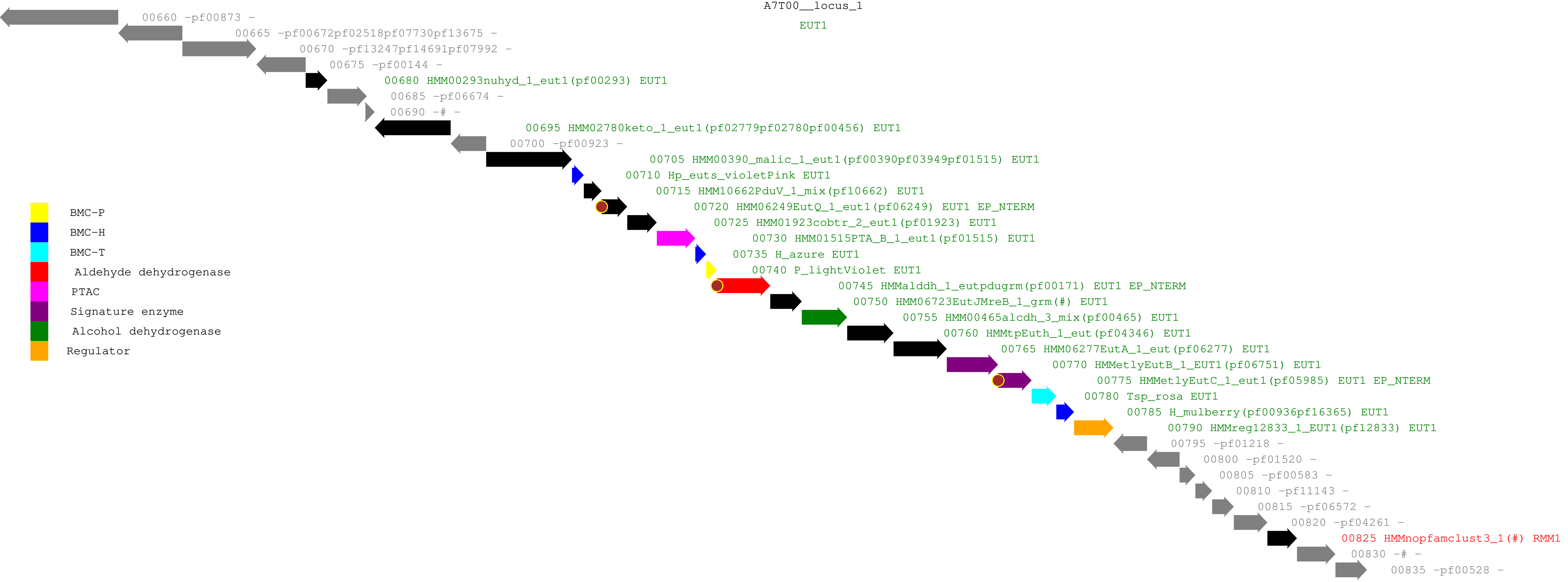

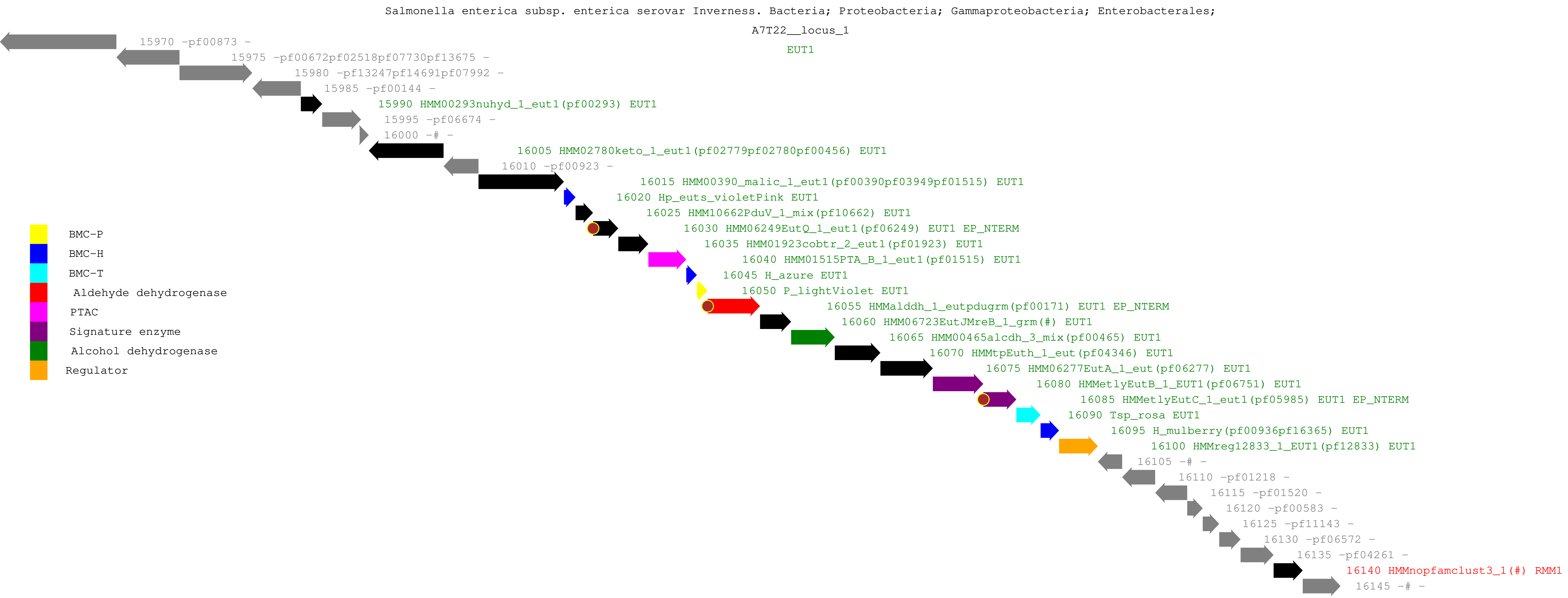

Salmonella enterica subsp. enterica serovar Inverness. Bacteria; Proteobacteria; Gammaproteobacteria; Enterobacterales;
A7T22__locus_1
EUT1
15970 -pf00873 -
15975 -pf00672pf02518pf07730pf13675 -
15980 -pf13247pf14691pf07992 -
15985 -pf00144 -
15990 HMM00293nuhyd_1_eut1(pf00293) EUT1
15995 -pf06674 -
16000 -# -
16005 HMM02780keto_1_eut1(pf02779pf02780pf00456) EUT1
16010 -pf00923 -
16015 HMM00390_malic_1_eut1(pf00390pf03949pf01515) EUT1
16020 Hp_euts_violetPink EUT1
16025 HMM10662PduV_1_mix(pf10662) EUT1
16030 HMM06249EutQ_1_eut1(pf06249) EUT1 EP_NTERM
16035 HMM01923cobtr_2_eut1(pf01923) EUT1
16040 HMM01515PTA_B_1_eut1(pf01515) EUT1
16045 H_azure EUT1
16050 P_lightViolet EUT1
16055 HMMalddh_1_eutpdugrm(pf00171) EUT1 EP_NTERM
16060 HMM06723EutJMreB_1_grm(#) EUT1
16065 HMM00465alcdh_3_mix(pf00465) EUT1
16070 HMMtpEuth_1_eut(pf04346) EUT1
16075 HMM06277EutA_1_eut(pf06277) EUT1
16080 HMMetlyEutB_1_EUT1(pf06751) EUT1
16085 HMMetlyEutC_1_eut1(pf05985) EUT1 EP_NTERM
16090 Tsp_rosa EUT1
16095 H_mulberry(pf00936pf16365) EUT1
16100 HMMreg12833_1_EUT1(pf12833) EUT1
16105 -# -
16110 -pf01218 -
16115 -pf01520 -
16120 -pf00583 -
16125 -pf11143 -
16130 -pf06572 -
16135 -pf04261 -
16140 HMMnopfamclust3_1(#) RMM1
16145 -# -
BMC-P
BMC-H
BMC-T
Aldehyde dehydrogenase
PTAC
Signature enzyme
Alcohol dehydrogenase
Regulator

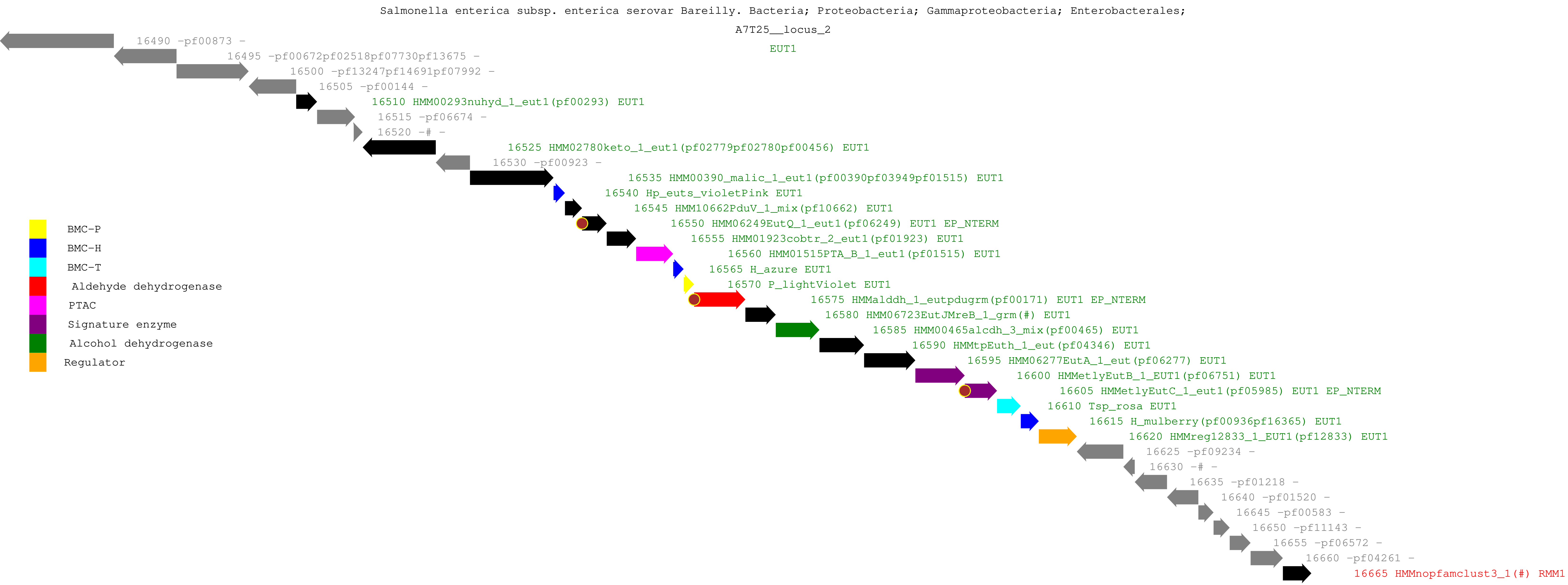
Salmonella enterica subsp. enterica serovar Bareilly. Bacteria; Proteobacteria; Gammaproteobacteria; Enterobacterales;
A7T25__locus_2
EUT1
16490 -pf00873 -
16495 -pf00672pf02518pf07730pf13675 -
16500 -pf13247pf14691pf07992 -
16505 -pf00144 -
16510 HMM00293nuhyd_1_eut1(pf00293) EUT1
16515 -pf06674 -
16520 -# -
16525 HMM02780keto_1_eut1(pf02779pf02780pf00456) EUT1
16530 -pf00923 -
16535 HMM00390_malic_1_eut1(pf00390pf03949pf01515) EUT1
16540 Hp_euts_violetPink EUT1
16545 HMM10662PduV_1_mix(pf10662) EUT1
16550 HMM06249EutQ_1_eut1(pf06249) EUT1 EP_NTERM
16555 HMM01923cobtr_2_eut1(pf01923) EUT1
16560 HMM01515PTA_B_1_eut1(pf01515) EUT1
16565 H_azure EUT1
16570 P_lightViolet EUT1
16575 HMMalddh_1_eutpdugrm(pf00171) EUT1 EP_NTERM
16580 HMM06723EutJMreB_1_grm(#) EUT1
16585 HMM00465alcdh_3_mix(pf00465) EUT1
16590 HMMtpEuth_1_eut(pf04346) EUT1
16595 HMM06277EutA_1_eut(pf06277) EUT1
16600 HMMetlyEutB_1_EUT1(pf06751) EUT1
16605 HMMetlyEutC_1_eut1(pf05985) EUT1 EP_NTERM
16610 Tsp_rosa EUT1
16615 H_mulberry(pf00936pf16365) EUT1
16620 HMMreg12833_1_EUT1(pf12833) EUT1
16625 -pf09234 -
16630 -# -
16635 -pf01218 -
16640 -pf01520 -
16645 -pf00583 -
16650 -pf11143 -
16655 -pf06572 -
16660 -pf04261 -
16665 HMMnopfamclust3_1(#) RMM1
BMC-P
BMC-H
BMC-T
Aldehyde dehydrogenase
PTAC
Signature enzyme
Alcohol dehydrogenase
Regulator

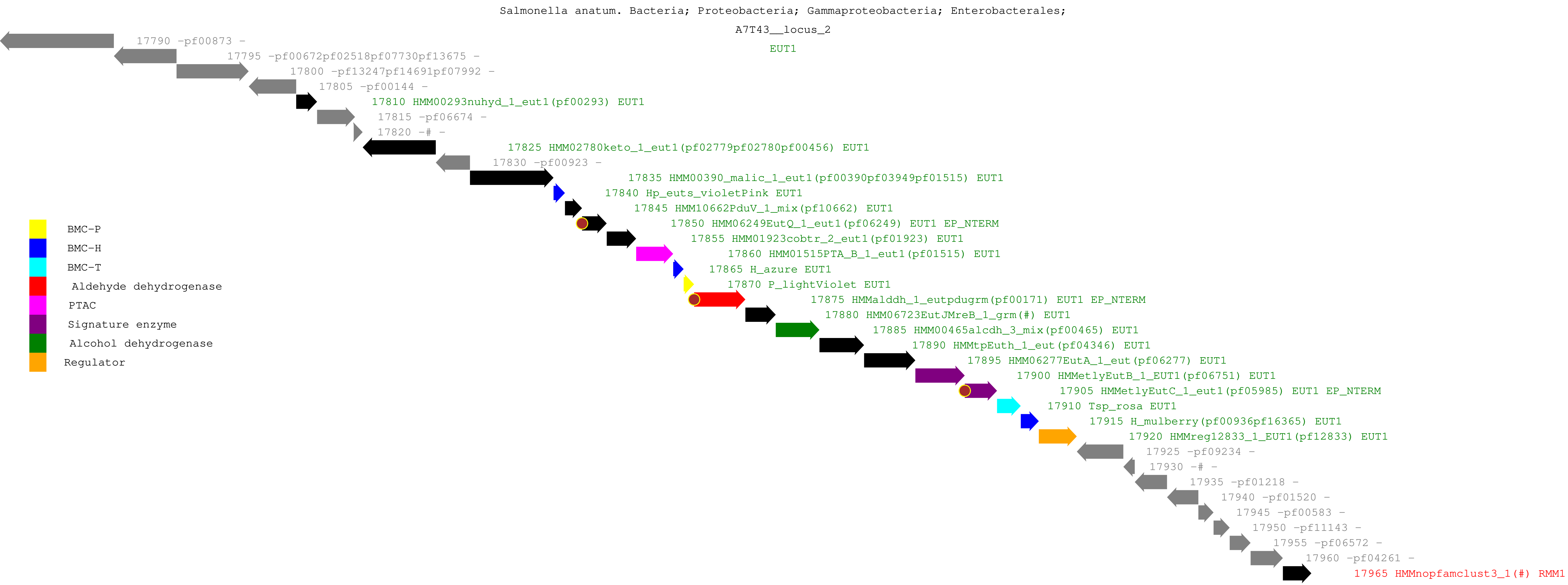

Salmonella anatum. Bacteria; Proteobacteria; Gammaproteobacteria; Enterobacterales;
A7T43__locus_2
EUT1
17790 -pf00873 -
17795 -pf00672pf02518pf07730pf13675 -
17800 -pf13247pf14691pf07992 -
17805 -pf00144 -
17810 HMM00293nuhyd_1_eut1(pf00293) EUT1
17815 -pf06674 -
17820 -# -
17825 HMM02780keto_1_eut1(pf02779pf02780pf00456) EUT1
17830 -pf00923 -
17835 HMM00390_malic_1_eut1(pf00390pf03949pf01515) EUT1
17840 Hp_euts_violetPink EUT1
17845 HMM10662PduV_1_mix(pf10662) EUT1
17850 HMM06249EutQ_1_eut1(pf06249) EUT1 EP_NTERM
17855 HMM01923cobtr_2_eut1(pf01923) EUT1
17860 HMM01515PTA_B_1_eut1(pf01515) EUT1
17865 H_azure EUT1
17870 P_lightViolet EUT1
17875 HMMalddh_1_eutpdugrm(pf00171) EUT1 EP_NTERM
17880 HMM06723EutJMreB_1_grm(#) EUT1
17885 HMM00465alcdh_3_mix(pf00465) EUT1
17890 HMMtpEuth_1_eut(pf04346) EUT1
17895 HMM06277EutA_1_eut(pf06277) EUT1
17900 HMMetlyEutB_1_EUT1(pf06751) EUT1
17905 HMMetlyEutC_1_eut1(pf05985) EUT1 EP_NTERM
17910 Tsp_rosa EUT1
17915 H_mulberry(pf00936pf16365) EUT1
17920 HMMreg12833_1_EUT1(pf12833) EUT1
17925 -pf09234 -
17930 -# -
17935 -pf01218 -
17940 -pf01520 -
17945 -pf00583 -
17950 -pf11143 -
17955 -pf06572 -
17960 -pf04261 -
17965 HMMnopfamclust3_1(#) RMM1
BMC-P
BMC-H
BMC-T
Aldehyde dehydrogenase
PTAC
Signature enzyme
Alcohol dehydrogenase
Regulator

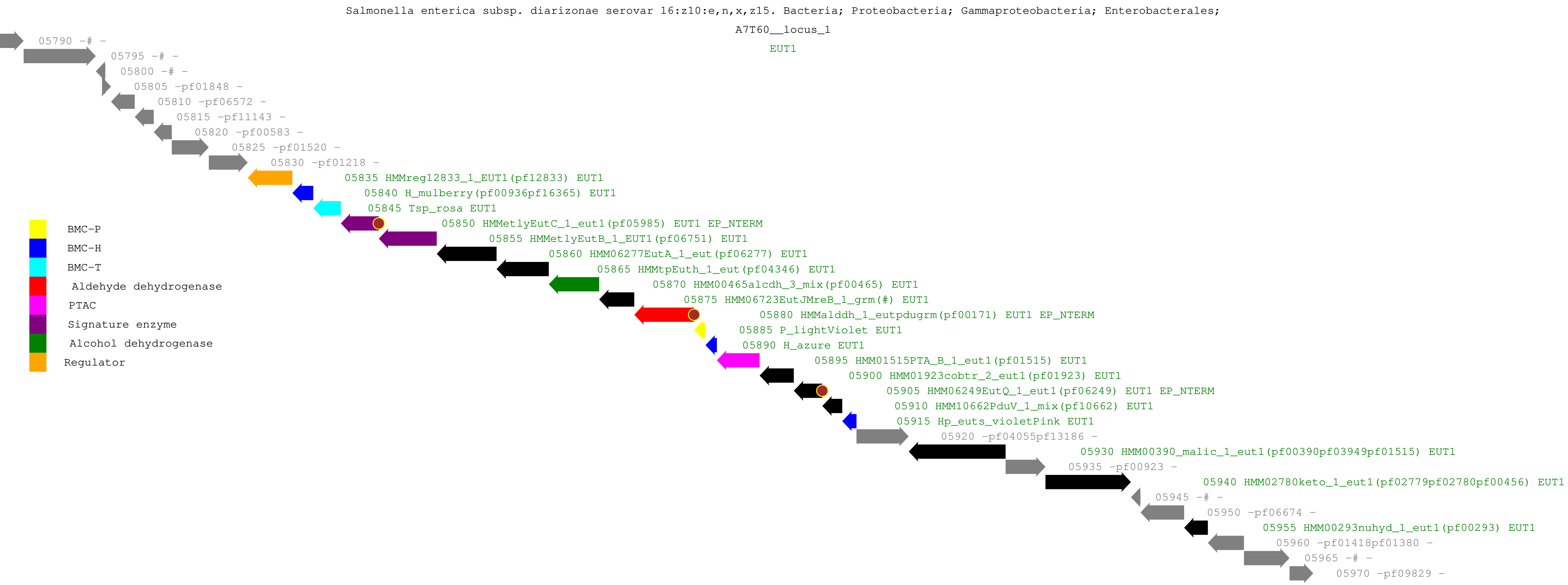

Salmonella enterica subsp. diarizonae serovar 16:z10:e,n,x,z15. Bacteria; Proteobacteria; Gammaproteobacteria; Enterobacterales;
A7T60__locus_1
EUT1
05790 -# -
05795 -# -
05800 -# -
05805 -pf01848 -
05810 -pf06572 -
05815 -pf11143 -
05820 -pf00583 -
05825 -pf01520 -
05830 -pf01218 -
05835 HMMreg12833_1_EUT1(pf12833) EUT1
05840 H_mulberry(pf00936pf16365) EUT1
05845 Tsp_rosa EUT1
05850 HMMetlyEutC_1_eut1(pf05985) EUT1 EP_NTERM
05855 HMMetlyEutB_1_EUT1(pf06751) EUT1
05860 HMM06277EutA_1_eut(pf06277) EUT1
05865 HMMtpEuth_1_eut(pf04346) EUT1
05870 HMM00465alcdh_3_mix(pf00465) EUT1
05875 HMM06723EutJMreB_1_grm(#) EUT1
05880 HMMalddh_1_eutpdugrm(pf00171) EUT1 EP_NTERM
05885 P_lightViolet EUT1
05890 H_azure EUT1
05895 HMM01515PTA_B_1_eut1(pf01515) EUT1
05900 HMM01923cobtr_2_eut1(pf01923) EUT1
05905 HMM06249EutQ_1_eut1(pf06249) EUT1 EP_NTERM
05910 HMM10662PduV_1_mix(pf10662) EUT1
05915 Hp_euts_violetPink EUT1
05920 -pf04055pf13186 -
05930 HMM00390_malic_1_eut1(pf00390pf03949pf01515) EUT1
05935 -pf00923 -
05940 HMM02780keto_1_eut1(pf02779pf02780pf00456) EUT1
05945 -# -
05950 -pf06674 -
05955 HMM00293nuhyd_1_eut1(pf00293) EUT1
05960 -pf01418pf01380 -
05965 -# -
05970 -pf09829 -
BMC-P
BMC-H
BMC-T
Aldehyde dehydrogenase
PTAC
Signature enzyme
Alcohol dehydrogenase
Regulator

Salmonella enterica subsp. enterica serovar Montevideo str. 609458-2. Bacteria; Proteobacteria; Gammaproteobacteria; Enterobacterales;

A7W92__locus_2

EUT1

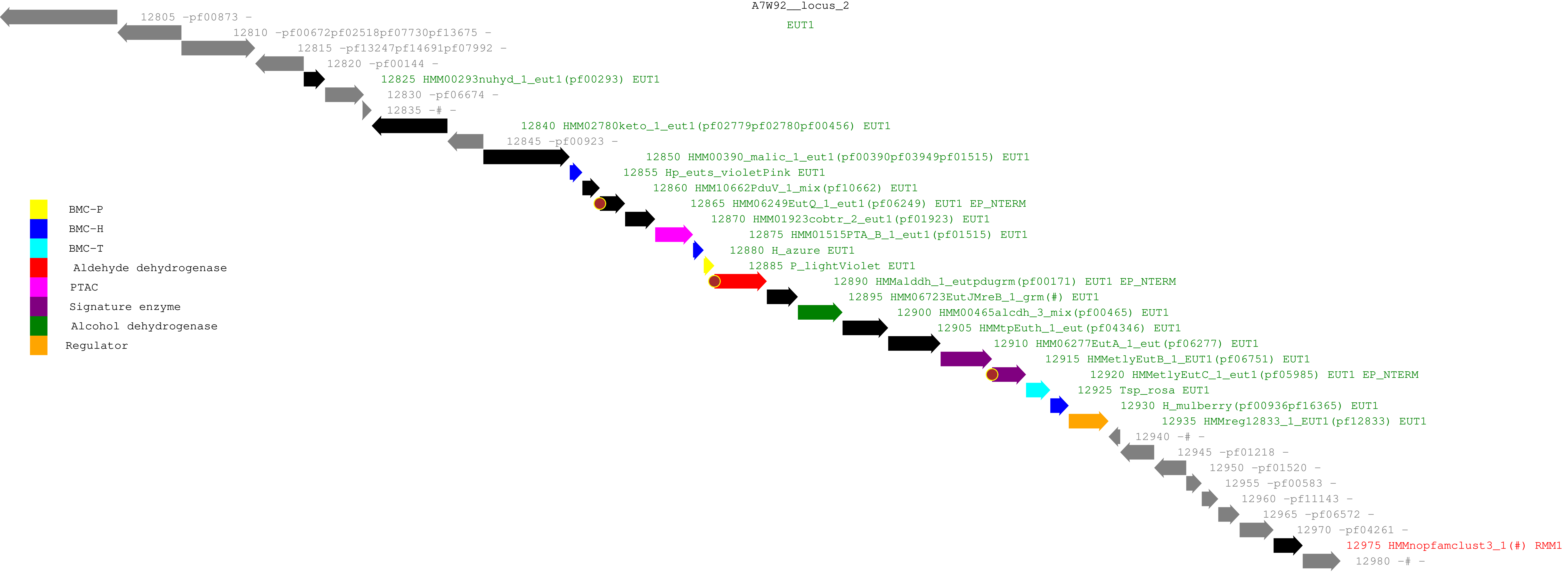

Escherichia coli. Bacteria; Proteobacteria; Gammaproteobacteria; Enterobacterales;

A8C65__locus_1

EUT1

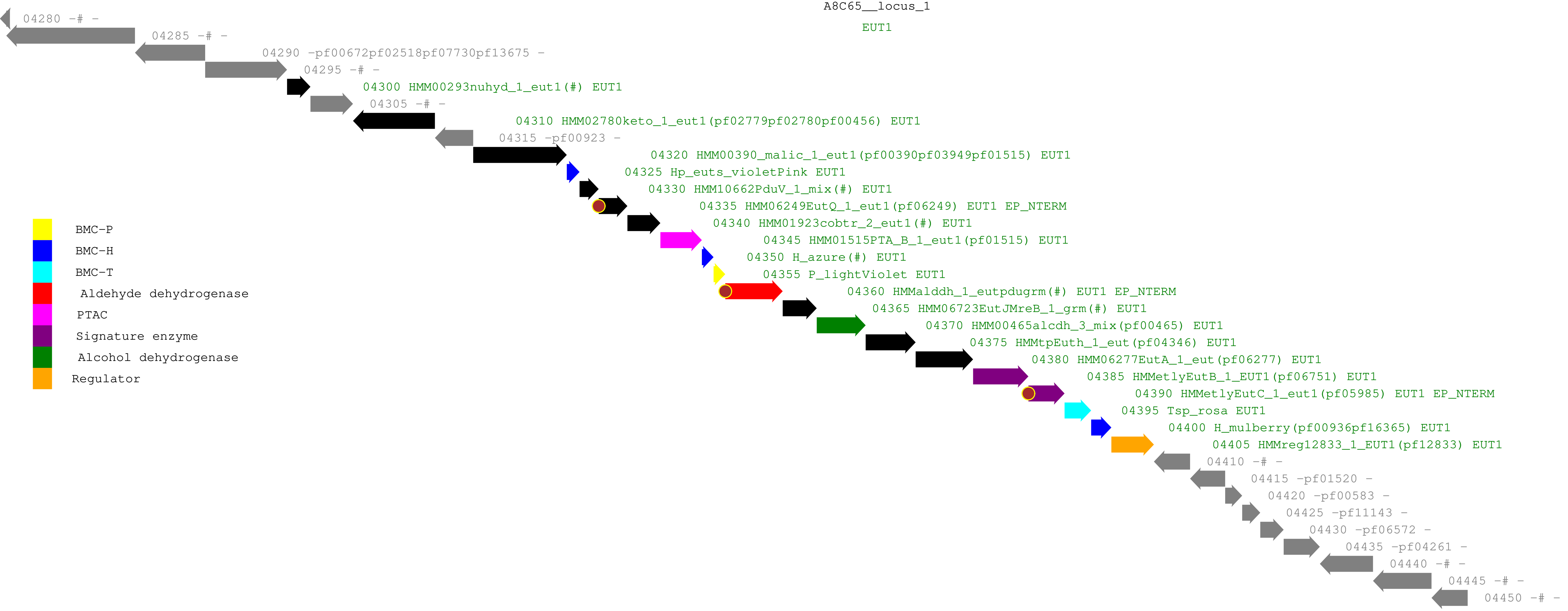

Escherichia coli. Bacteria; Proteobacteria; Gammaproteobacteria; Enterobacterales;

A8G17__locus_1

EUT1

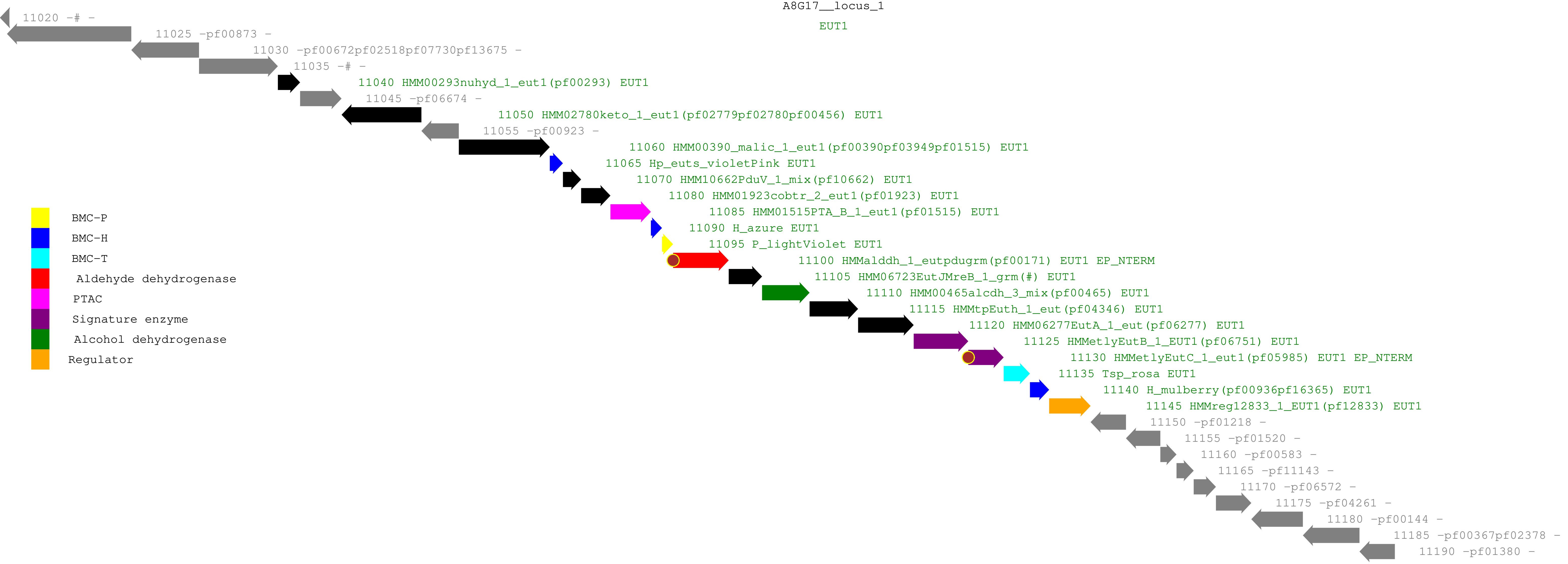

Escherichia coli. Bacteria; Proteobacteria; Gammaproteobacteria; Enterobacterales;

A8M42__locus_1

EUT1

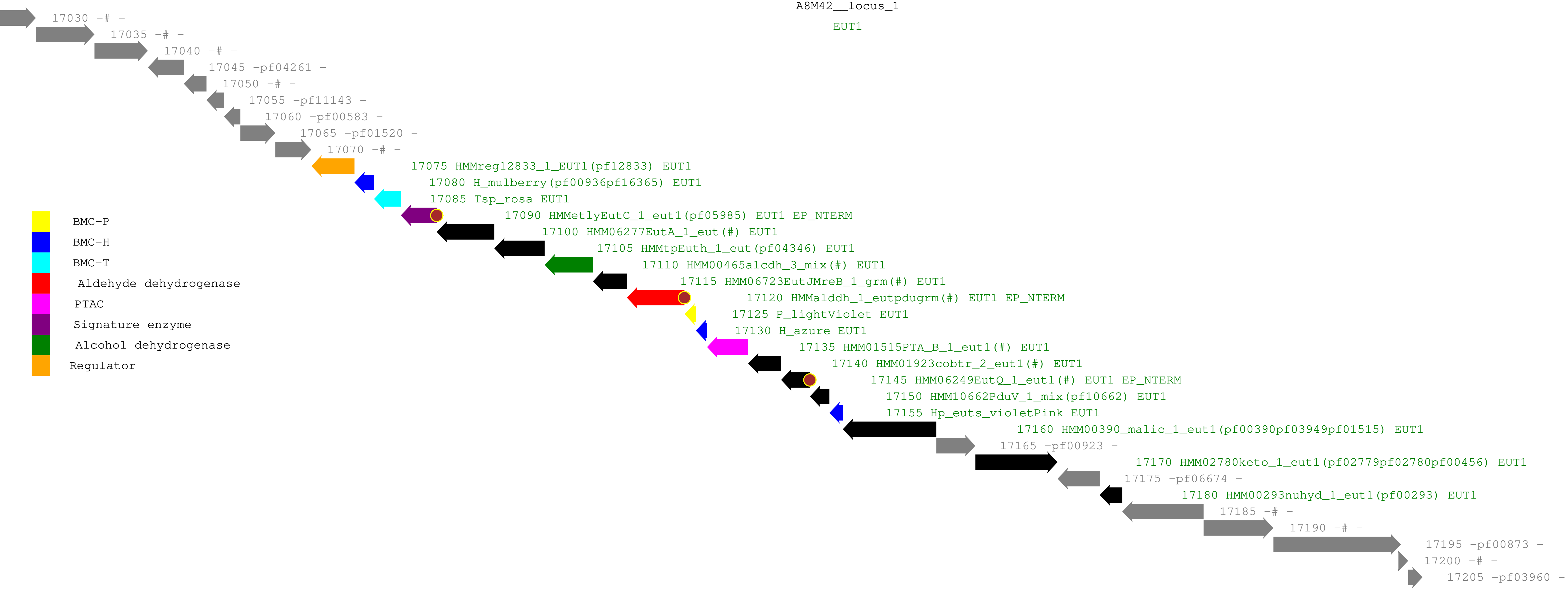

Escherichia coli. Bacteria; Proteobacteria; Gammaproteobacteria; Enterobacterales;

A8M44__locus_1

EUT1

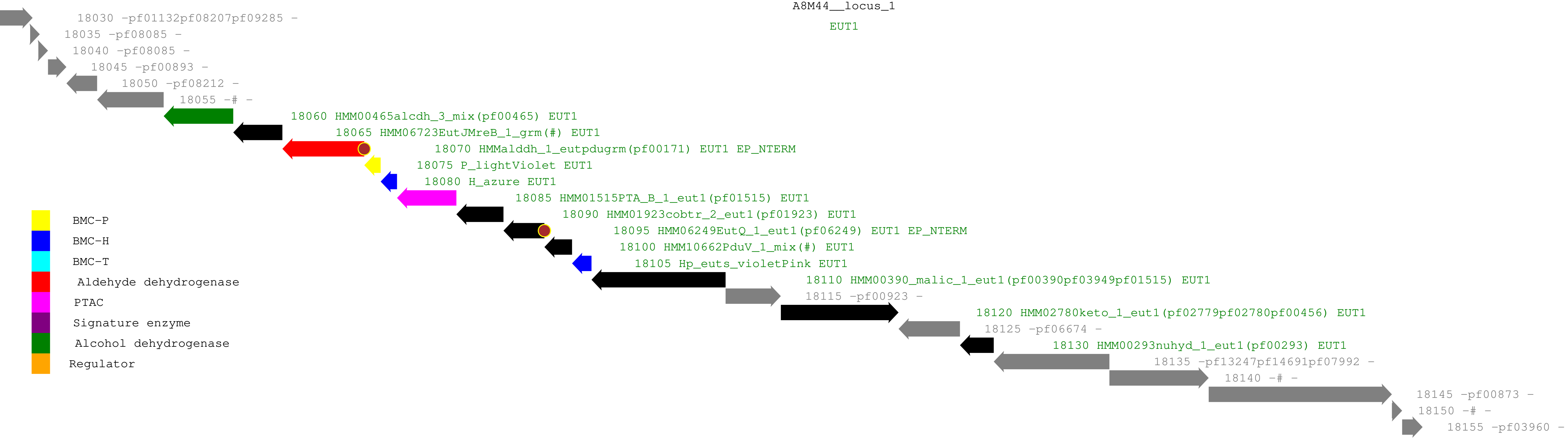

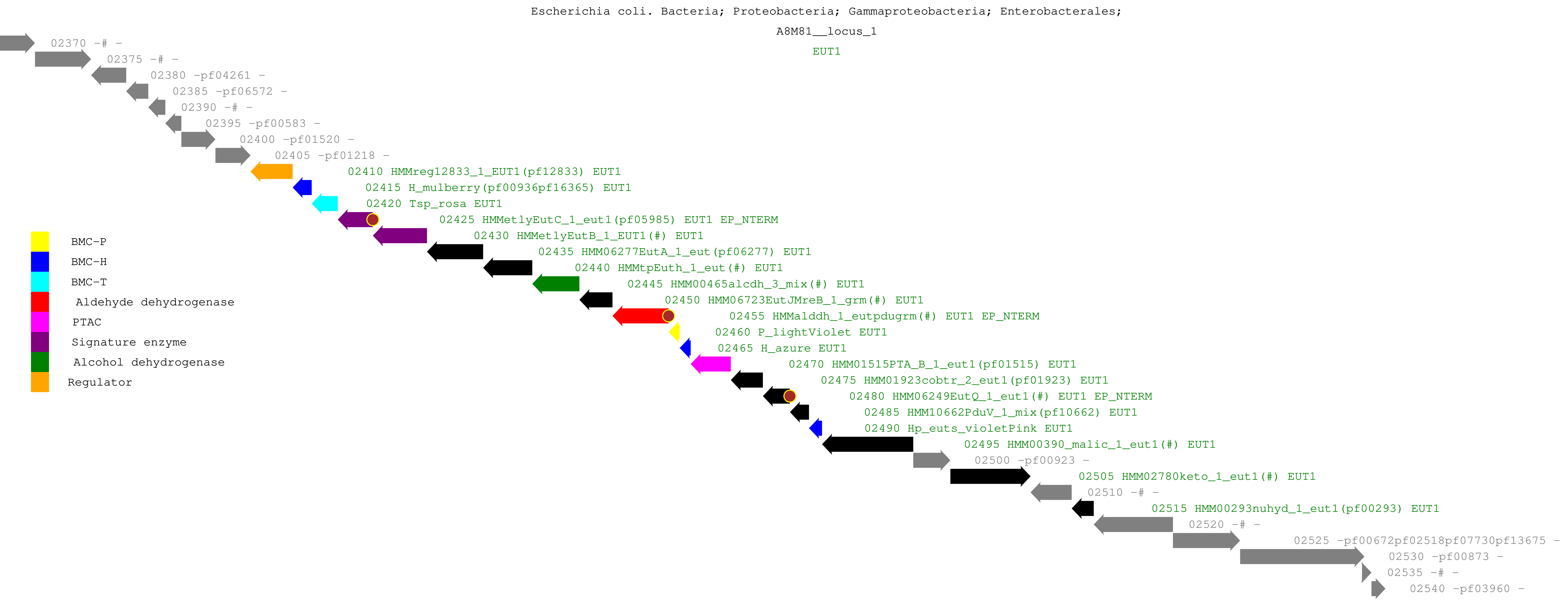
Escherichia coli. Bacteria; Proteobacteria; Gammaproteobacteria; Enterobacterales;
A8M81__locus_1
EUT1
02370 -# -
02375 -# -
02380 -pf04261 -
02385 -pf06572 -
02390 -# -
02395 -pf00583 -
02400 -pf01520 -
02405 -pf01218 -
02410 HMMreg12833_1_EUT1(pf12833) EUT1
02415 H_mulberry(pf00936pf16365) EUT1
02420 Tsp_rosa EUT1
02425 HMMetlyEutC_1_eut1(pf05985) EUT1 EP_NTERM
02430 HMMetlyEutB_1_EUT1(#) EUT1
02435 HMM06277EutA_1_eut(pf06277) EUT1
02440 HMMtpEuth_1_eut(#) EUT1
02445 HMM00465alcdh_3_mix(#) EUT1
02450 HMM06723EutJMreB_1_grm(#) EUT1
02455 HMMalddh_1_eutpdugrm(#) EUT1 EP_NTERM
02460 P_lightViolet EUT1
02465 H_azure EUT1
02470 HMM01515PTA_B_1_eut1(pf01515) EUT1
02475 HMM01923cobtr_2_eut1(pf01923) EUT1
02480 HMM06249EutQ_1_eut1(#) EUT1 EP_NTERM
02485 HMM10662PduV_1_mix(pf10662) EUT1
02490 Hp_euts_violetPink EUT1
02495 HMM00390_malic_1_eut1(#) EUT1
02500 -pf00923 -
02505 HMM02780keto_1_eut1(#) EUT1
02510 -# -
02515 HMM00293nuhyd_1_eut1(pf00293) EUT1
02520 -# -
02525 -pf00672pf02518pf07730pf13675 -
02530 -pf00873 -
02535 -# -
02540 -pf03960 -
BMC-P
BMC-H
BMC-T
Aldehyde dehydrogenase
PTAC
Signature enzyme
Alcohol dehydrogenase
Regulator

Salmonella enterica subsp. enterica serovar Napoli. Bacteria; Proteobacteria; Gammaproteobacteria; Enterobacterales;

A8N98__locus_2

EUT1

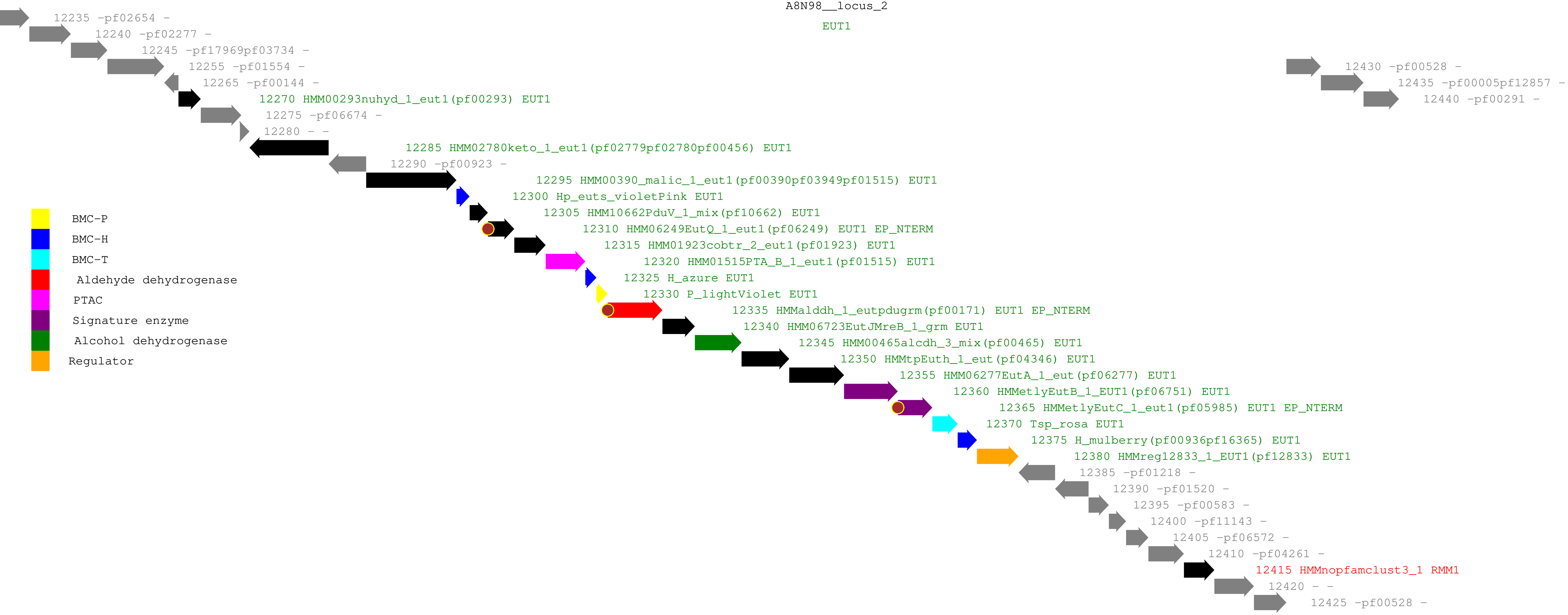

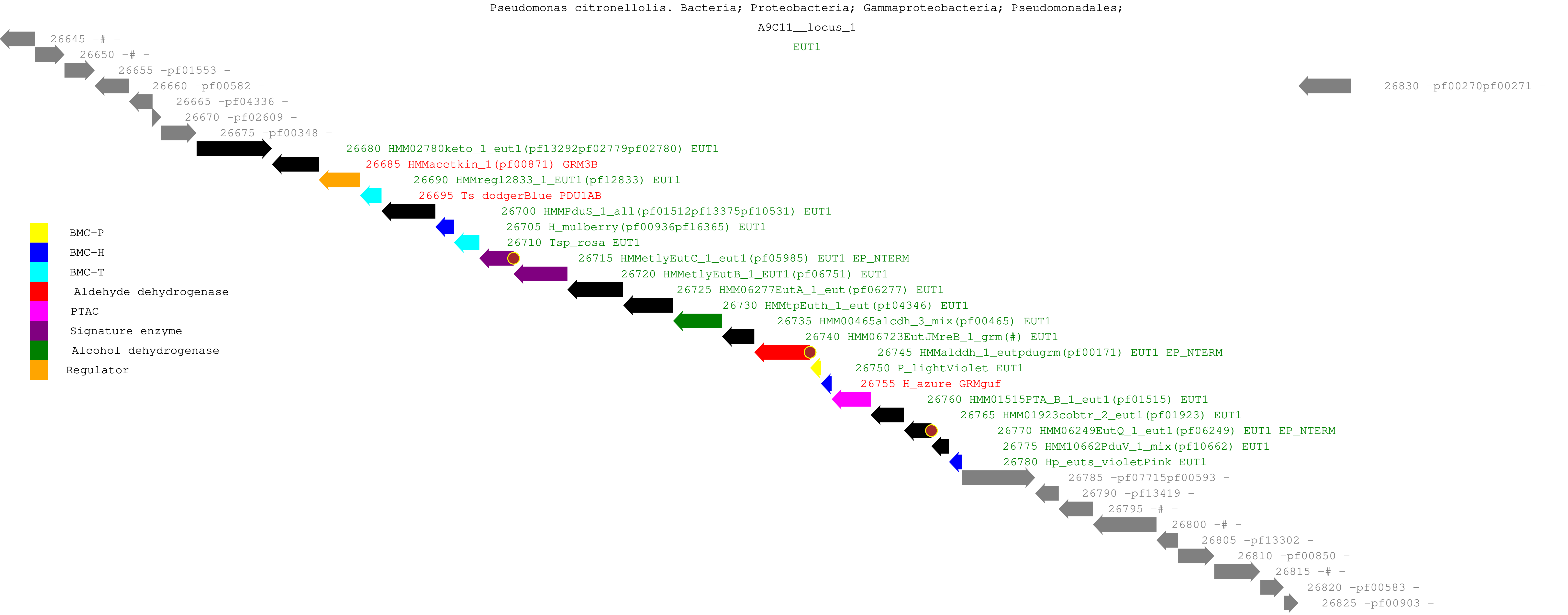

Pseudomonas citronellolis. Bacteria; Proteobacteria; Gammaproteobacteria; Pseudomonadales;
A9C11__locus_1
EUT1
26645 -# -
26650 -# -
26655 -pf01553 -
26660 -pf00582 -
26665 -pf04336 -
26670 -pf02609 -
26675 -pf00348 -
26680 HMM02780keto_1_eut1(pf13292pf02779pf02780) EUT1
26685 HMMacetkin_1(pf00871) GRM3B
26690 HMMreg12833_1_EUT1(pf12833) EUT1
26695 Ts_dodgerBlue PDU1AB
26700 HMMPduS_1_all(pf01512pf13375pf10531) EUT1
26705 H_mulberry(pf00936pf16365) EUT1
26710 Tsp_rosa EUT1
26715 HMMetlyEutC_1_eut1(pf05985) EUT1 EP_NTERM
26720 HMMetlyEutB_1_EUT1(pf06751) EUT1
26725 HMM06277EutA_1_eut(pf06277) EUT1
26730 HMMtpEuth_1_eut(pf04346) EUT1
26735 HMM00465alcdh_3_mix(pf00465) EUT1
26740 HMM06723EutJMreB_1_grm(#) EUT1
26745 HMMaldh_1_eutpdugrm(pf00171) EUT1 EP_NTERM
26750 P_lightViolet EUT1
26755 H_azure GRMguf
26760 HMM01515PTA_B_1_eut1(pf01515) EUT1
26765 HMM01923cobtr_2_eut1(pf01923) EUT1
26770 HMM06249EutQ_1_eut1(pf06249) EUT1 EP_NTERM
26775 HMM10662PduV_1_mix(pf10662) EUT1
26780 Hp_euts_violetPink EUT1
26785 -pf07715pf00593 -
26790 -pf13419 -
26795 -# -
26800 -# -
26805 -pf13302 -
26810 -pf00850 -
26815 -# -
26820 -pf00583 -
26825 -pf00903 -
26830 -pf00270pf00271 -
BMC-P
BMC-H
BMC-T
Aldehyde dehydrogenase
PTAC
Signature enzyme
Alcohol dehydrogenase
Regulator

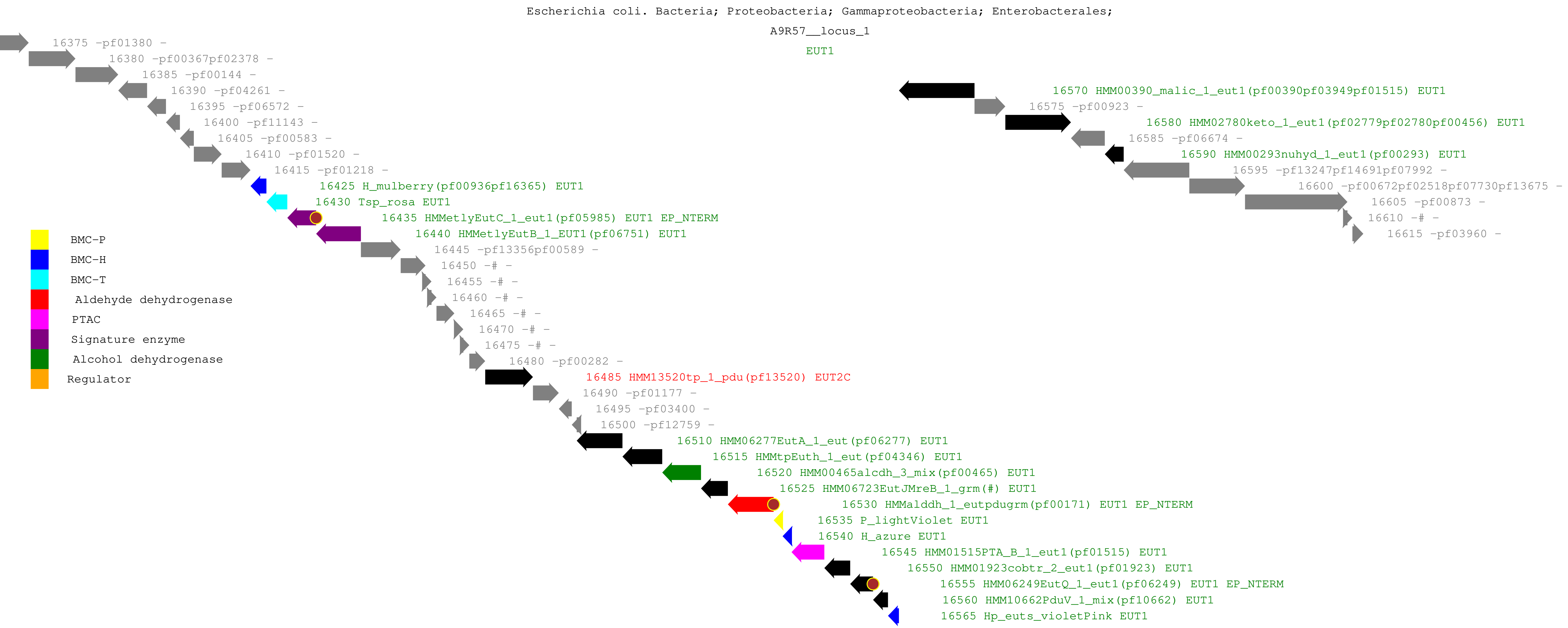

Escherichia coli. Bacteria; Proteobacteria; Gammaproteobacteria; Enterobacterales;
A9R57__locus_1
EUT1
16375 -pf01380 -
16380 -pf00367pf02378 -
16385 -pf00144 -
16390 -pf04261 -
16395 -pf06572 -
16400 -pf11143 -
16405 -pf00583 -
16410 -pf01520 -
16415 -pf01218 -
16425 H_mulberry(pf00936pf16365) EUT1
16430 Tsp_rosa EUT1
16435 HMMetlyEutC_1_eut1(pf05985) EUT1 EP_NTERM
16440 HMMetlyEutB_1_EUT1(pf06751) EUT1
16445 -pf13356pf00589 -
16450 -# -
16455 -# -
16460 -# -
16465 -# -
16470 -# -
16475 -# -
16480 -pf00282 -
16485 HMM13520tp_1_pdu(pf13520) EUT2C
16490 -pf01177 -
16495 -pf03400 -
16500 -pf12759 -
16510 HMM06277EutA_1_eut(pf06277) EUT1
16515 HMMtpEuth_1_eut(pf04346) EUT1
16520 HMM00465alcdh_3_mix(pf00465) EUT1
16525 HMM06723EutJMreB_1_grm(#) EUT1
16530 HMMaldh_1_eutpdugrm(pf00171) EUT1 EP_NTERM
16535 P_lightViolet EUT1
16540 H_azure EUT1
16545 HMM01515PTA_B_1_eut1(pf01515) EUT1
16550 HMM01923cobtr_2_eut1(pf01923) EUT1
16555 HMM06249EutQ_1_eut1(pf06249) EUT1 EP_NTERM
16560 HMM10662PduV_1_mix(pf10662) EUT1
16565 Hp_euts_violetPink EUT1
16570 HMM00390_malic_1_eut1(pf00390pf03949pf01515) EUT1
16575 -pf00923 -
16580 HMM02780keto_1_eut1(pf02779pf02780pf00456) EUT1
16585 -pf06674 -
16590 HMM00293nuhyd_1_eut1(pf00293) EUT1
16595 -pf13247pf14691pf07992 -
16600 -pf00672pf02518pf07730pf13675 -
16605 -pf00873 -
16610 -# -
16615 -pf03960 -
BMC-P
BMC-H
BMC-T
Aldehyde dehydrogenase
PTAC
Signature enzyme
Alcohol dehydrogenase
Regulator

Escherichia coli. Bacteria; Proteobacteria; Gammaproteobacteria; Enterobacterales;

A9X62__locus_1

EUT1

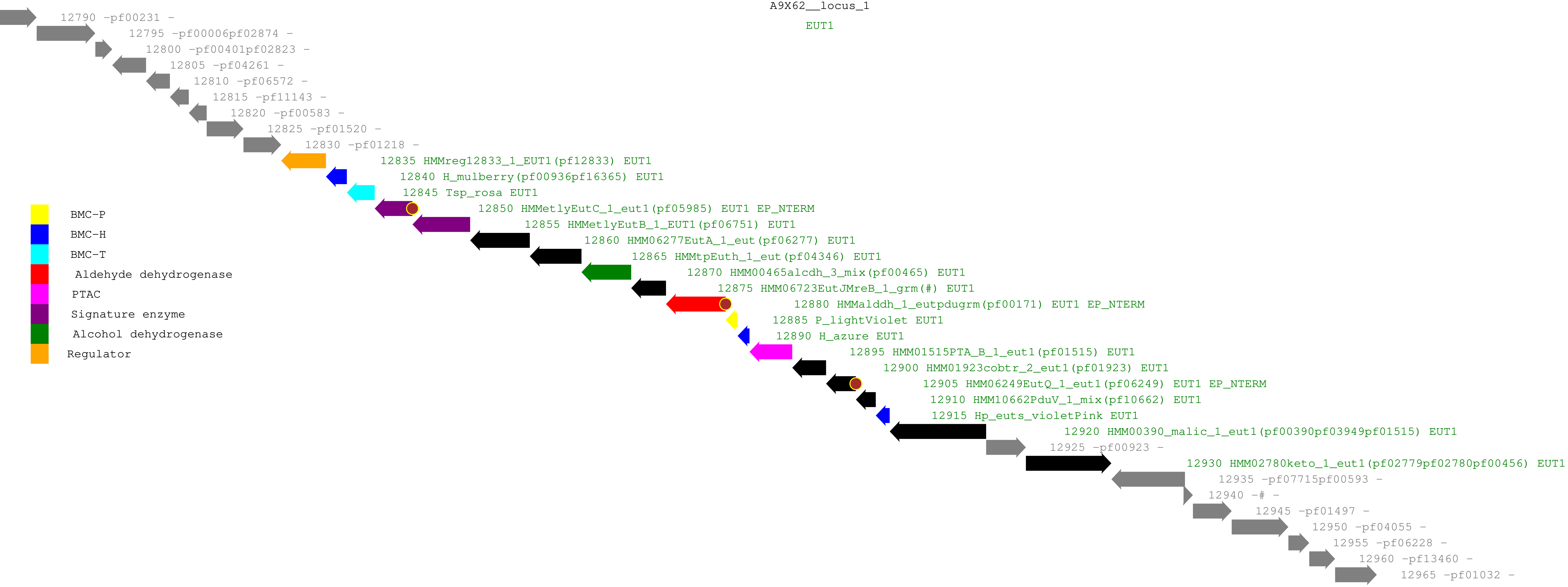

Salmonella thompson. Bacteria; Proteobacteria; Gammaproteobacteria; Enterobacterales;

AA556__locus_2

EUT1

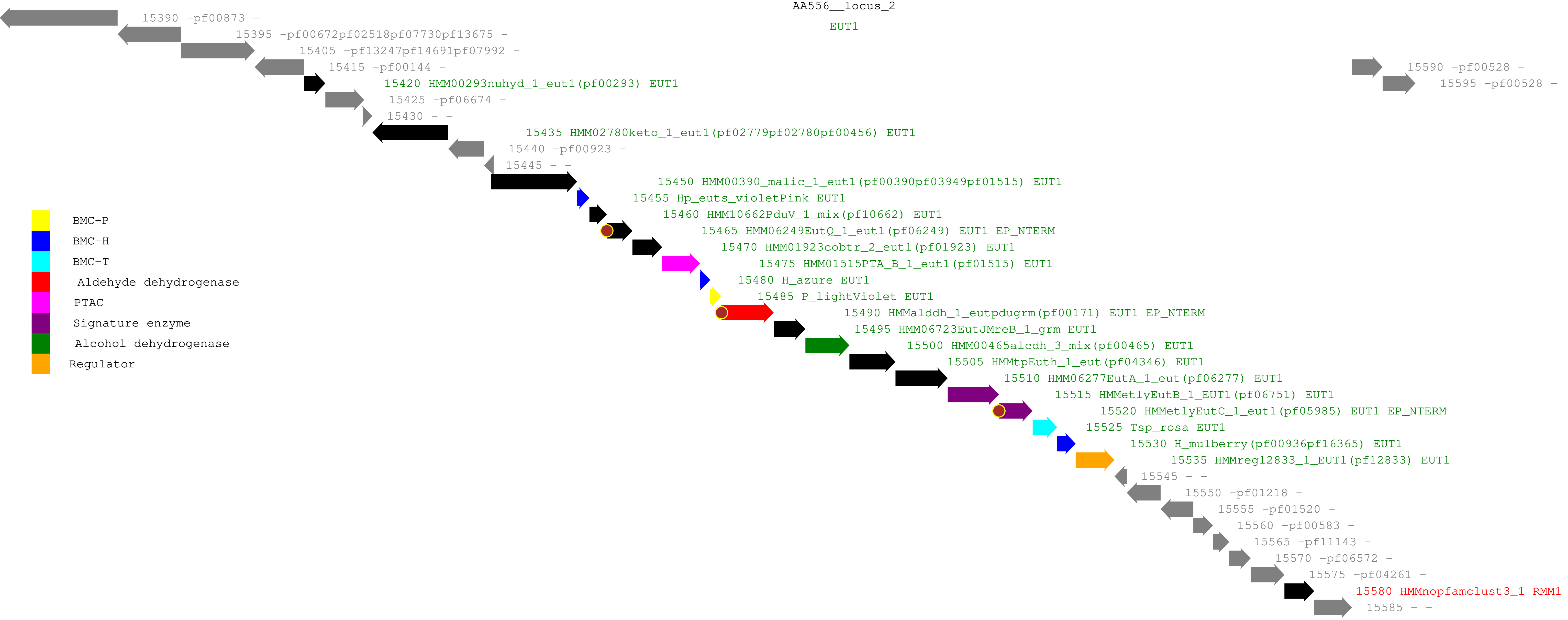

Salmonella enteritidis. Bacteria; Proteobacteria; Gammaproteobacteria; Enterobacterales;

AA689__locus_2

EUT1

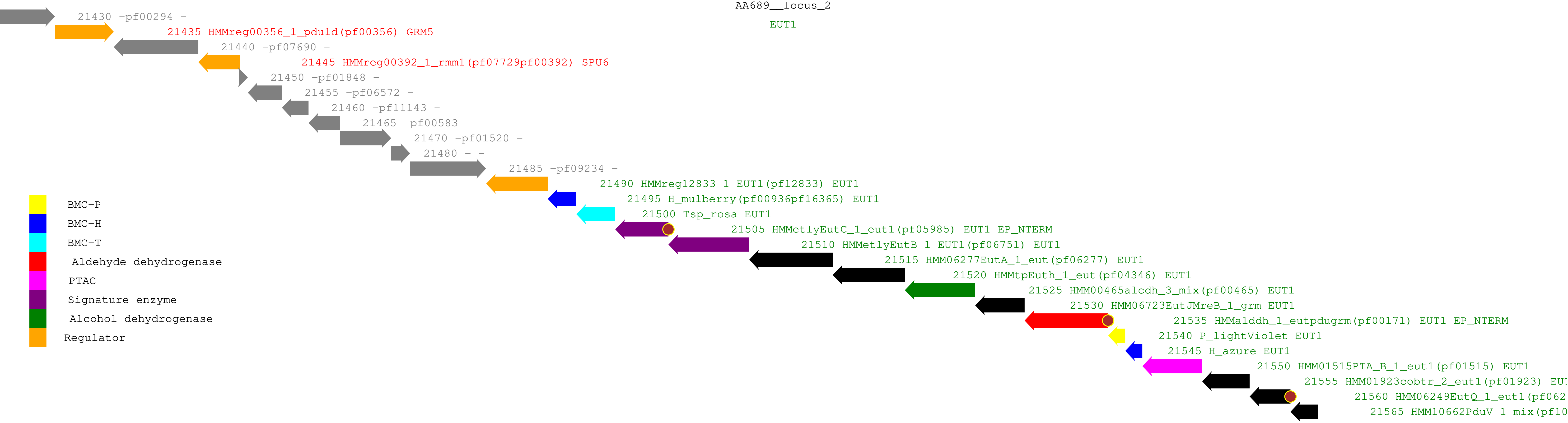

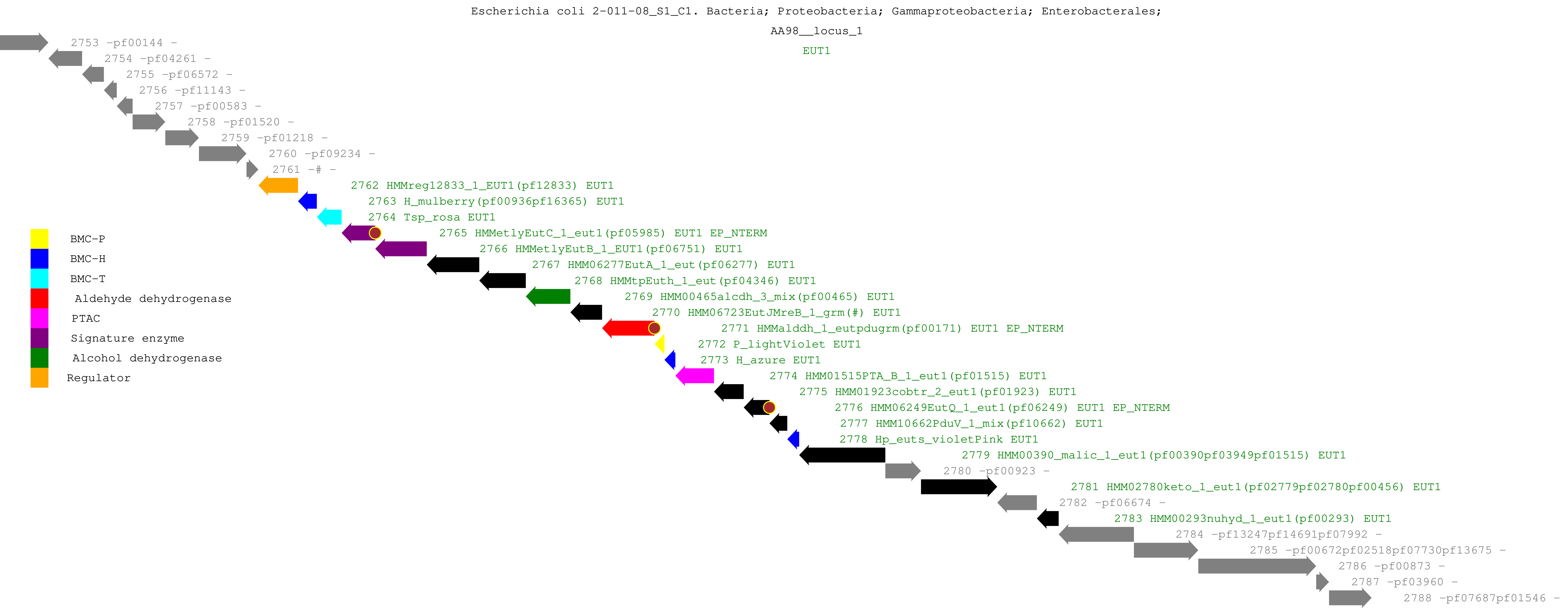

Escherichia coli 2-011-08_S1_C1. Bacteria; Proteobacteria; Gammaproteobacteria; Enterobacterales;
AA98__locus_1
EUT1
2753 -pf00144 -
2754 -pf04261 -
2755 -pf06572 -
2756 -pf11143 -
2757 -pf00583 -
2758 -pf01520 -
2759 -pf01218 -
2760 -pf09234 -
2761 -# -
2762 HMMreg12833_1_EUT1(pf12833) EUT1
2763 H_mulberry(pf00936pf16365) EUT1
2764 Tsp_rosa EUT1
2765 HMMetlyEutC_1_eut1(pf05985) EUT1 EP_NTERM
2766 HMMetlyEutB_1_EUT1(pf06751) EUT1
2767 HMM06277EutA_1_eut(pf06277) EUT1
2768 HMMtpEuth_1_eut(pf04346) EUT1
2769 HMM00465alcdh_3_mix(pf00465) EUT1
2770 HMM06723EutJMreB_1_grm(#) EUT1
2771 HMMaldh_1_eutpdugrm(pf00171) EUT1 EP_NTERM
2772 P_lightViolet EUT1
2773 H_azure EUT1
2774 HMM01515PTA_B_1_eut1(pf01515) EUT1
2775 HMM01923cobtr_2_eut1(pf01923) EUT1
2776 HMM06249EutQ_1_eut1(pf06249) EUT1 EP_NTERM
2777 HMM10662PduV_1_mix(pf10662) EUT1
2778 Hp_euts_violetPink EUT1
2779 HMM00390_malic_1_eut1(pf00390pf03949pf01515) EUT1
2780 -pf00923 -
2781 HMM02780keto_1_eut1(pf02779pf02780pf00456) EUT1
2782 -pf06674 -
2783 HMM00293nuhyd_1_eut1(pf00293) EUT1
2784 -pf13247pf14691pf07992 -
2785 -pf00672pf02518pf07730pf13675 -
2786 -pf00873 -
2787 -pf03960 -
2788 -pf07687pf01546 -
BMC-P
BMC-H
BMC-T
Aldehyde dehydrogenase
PTAC
Signature enzyme
Alcohol dehydrogenase
Regulator

Salmonella newport. Bacteria; Proteobacteria; Gammaproteobacteria; Enterobacterales;

AAJ88__locus_2_main_sat

EUT1

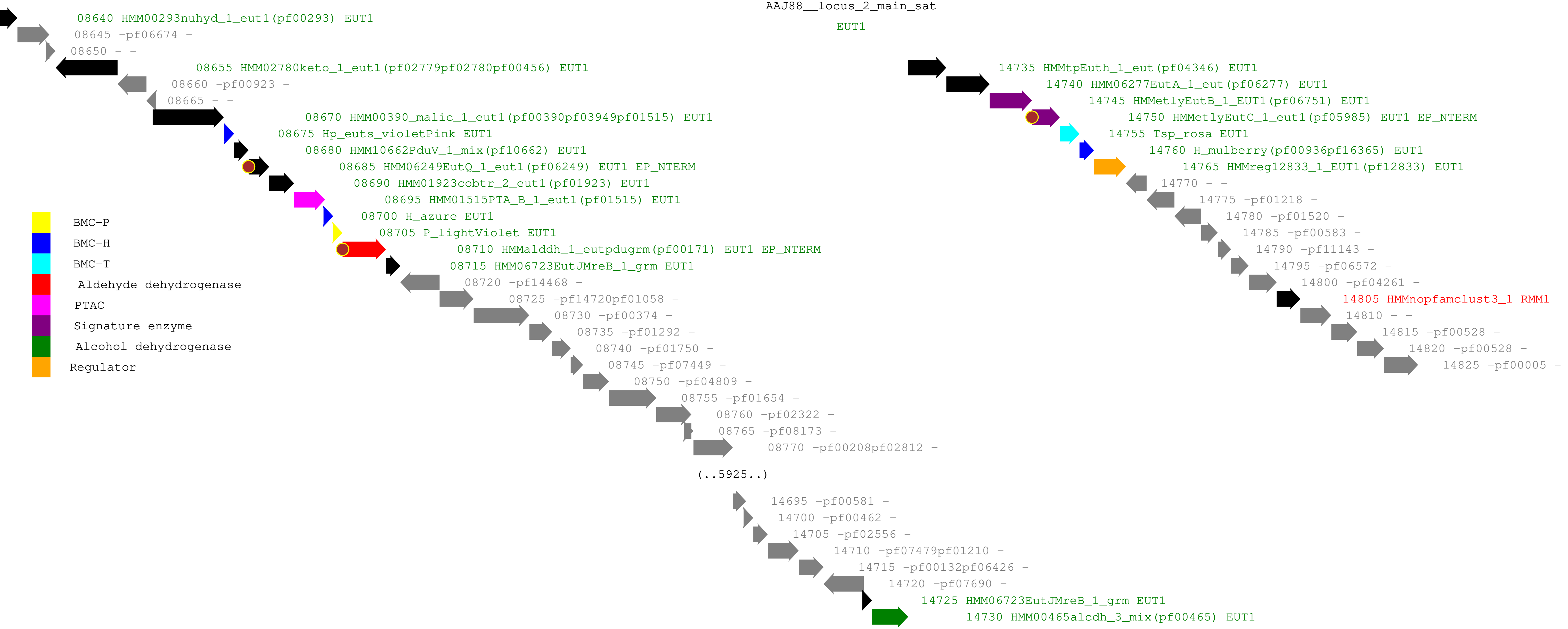

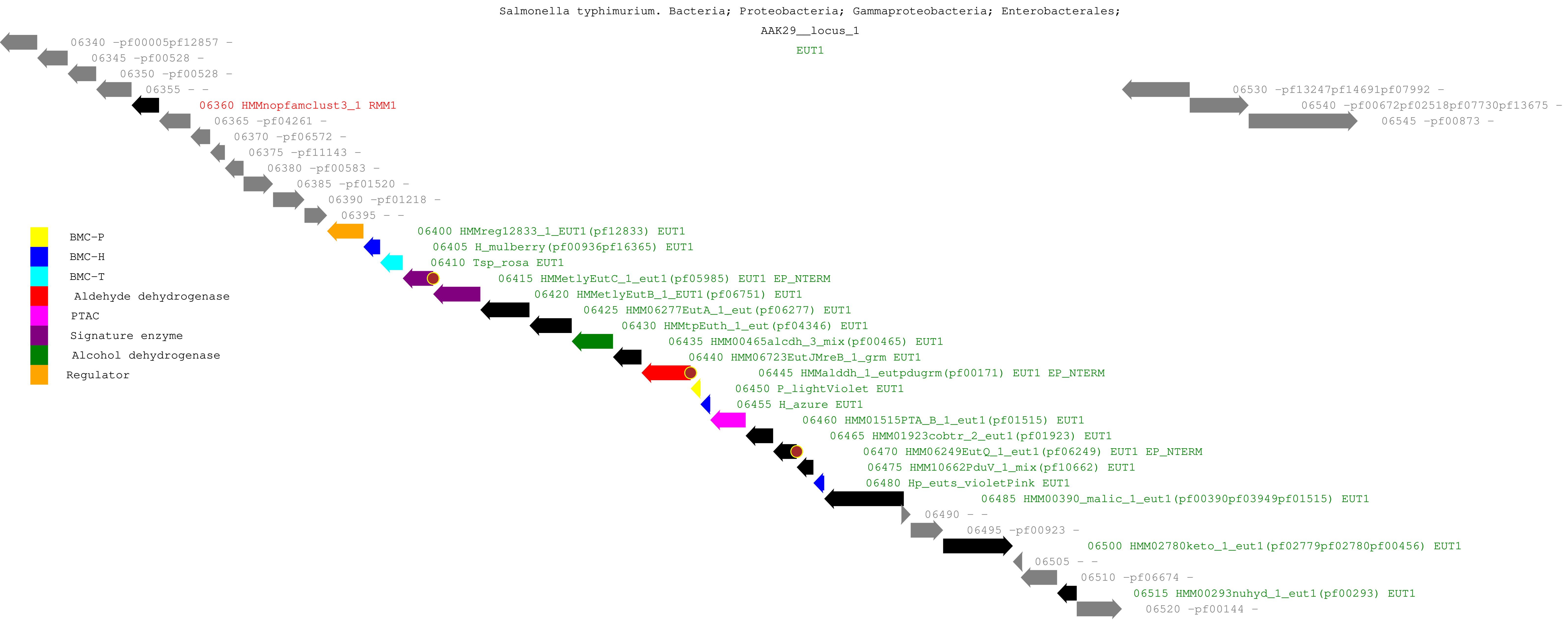

Salmonella typhimurium. Bacteria; Proteobacteria; Gammaproteobacteria; Enterobacterales;
AAK29__locus_1
EUT1
06340 -pf00005pf12857 -
06345 -pf00528 -
06350 -pf00528 -
06355 - -
06360 HMMnopfamclust3_1 RMM1
06365 -pf04261 -
06370 -pf06572 -
06375 -pf11143 -
06380 -pf00583 -
06385 -pf01520 -
06390 -pf01218 -
06395 - -
06400 HMMreg12833_1_EUT1(pf12833) EUT1
06405 H_mulberry(pf00936pf16365) EUT1
06410 Tsp_rosa EUT1
06415 HMMetlyEutC_1_eut1(pf05985) EUT1 EP_NTERM
06420 HMMetlyEutB_1_EUT1(pf06751) EUT1
06425 HMM06277EutA_1_eut(pf06277) EUT1
06430 HMMtpEuth_1_eut(pf04346) EUT1
06435 HMM00465alcdh_3_mix(pf00465) EUT1
06440 HMM06723EutJMreB_1_grm EUT1
06445 HMMalddh_1_eutpdugrm(pf00171) EUT1 EP_NTERM
06450 P_lightViolet EUT1
06455 H_azure EUT1
06460 HMM01515PTA_B_1_eut1(pf01515) EUT1
06465 HMM01923cobtr_2_eut1(pf01923) EUT1
06470 HMM06249EutQ_1_eut1(pf06249) EUT1 EP_NTERM
06475 HMM10662PduV_1_mix(pf10662) EUT1
06480 Hp_euts_violetPink EUT1
06485 HMM00390_malic_1_eut1(pf00390pf03949pf01515) EUT1
06490 - -
06495 -pf00923 -
06500 HMM02780keto_1_eut1(pf02779pf02780pf00456) EUT1
06505 - -
06510 -pf06674 -
06515 HMM00293nuhyd_1_eut1(pf00293) EUT1
06520 -pf00144 -
06530 -pf13247pf14691pf07992 -
06540 -pf00672pf02518pf07730pf13675 -
06545 -pf00873 -
BMC-P
BMC-H
BMC-T
Aldehyde dehydrogenase
PTAC
Signature enzyme
Alcohol dehydrogenase
Regulator

Salmonella enterica I. Bacteria; Proteobacteria; Gammaproteobacteria; Enterobacterales;

AAL03__locus_1

EUT1

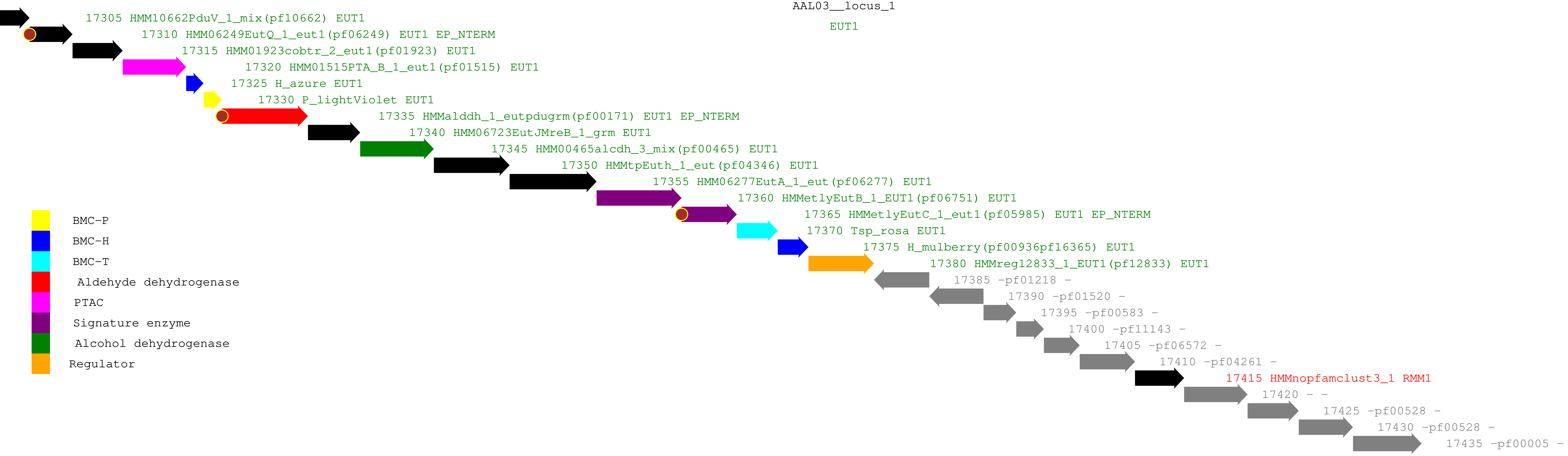

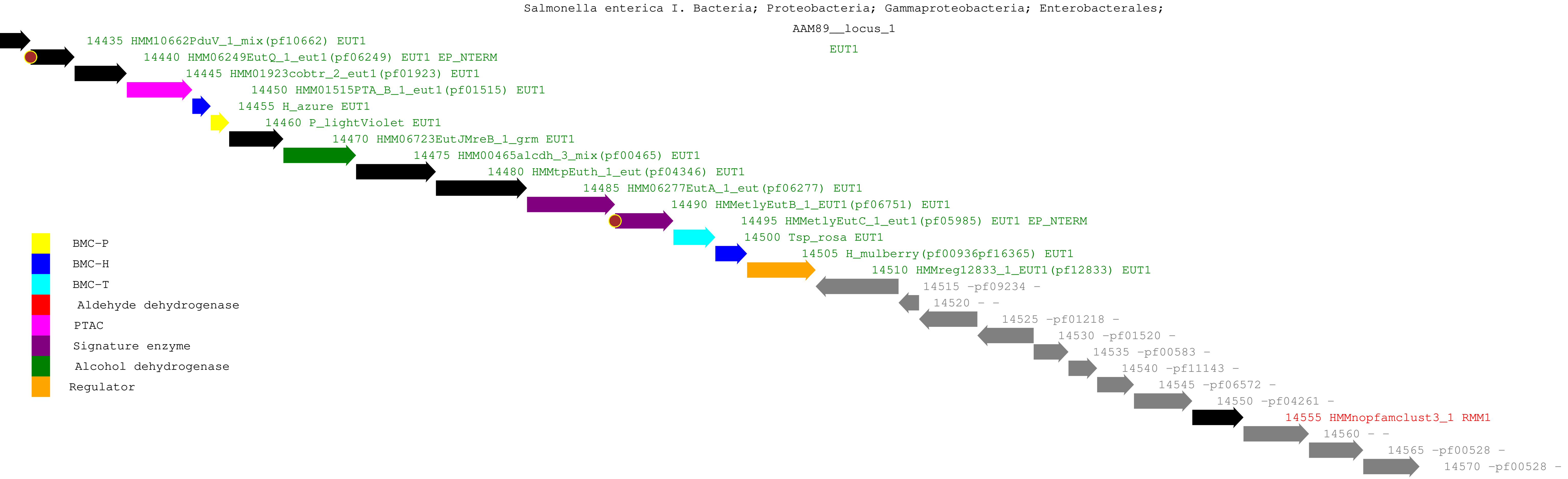

Salmonella enterica I. Bacteria; Proteobacteria; Gammaproteobacteria; Enterobacterales;
AAM89__locus_1
EUT1
14435 HMM10662PduV_1_mix(pf10662) EUT1
14440 HMM06249EutQ_1_eut1(pf06249) EUT1 EP_NTERM
14445 HMM01923cobtr_2_eut1(pf01923) EUT1
14450 HMM01515PTA_B_1_eut1(pf01515) EUT1
14455 H_azure EUT1
14460 P_lightViolet EUT1
14470 HMM06723EutJMreB_1_grm EUT1
14475 HMM00465alcdh_3_mix(pf00465) EUT1
14480 HMMtpEuth_1_eut(pf04346) EUT1
14485 HMM06277EutA_1_eut(pf06277) EUT1
14490 HMMetlyEutB_1_EUT1(pf06751) EUT1
14495 HMMetlyEutC_1_eut1(pf05985) EUT1 EP_NTERM
14500 Tsp_rosa EUT1
14505 H_mulberry(pf00936pf16365) EUT1
14510 HMMreg12833_1_EUT1(pf12833) EUT1
14515 -pf09234 -
14520 - -
14525 -pf01218 -
14530 -pf01520 -
14535 -pf00583 -
14540 -pf11143 -
14545 -pf06572 -
14550 -pf04261 -
14555 HMMnopfamclust3_1 RMM1
14560 - -
14565 -pf00528 -
14570 -pf00528 -
BMC-P
BMC-H
BMC-T
Aldehyde dehydrogenase
PTAC
Signature enzyme
Alcohol dehydrogenase
Regulator

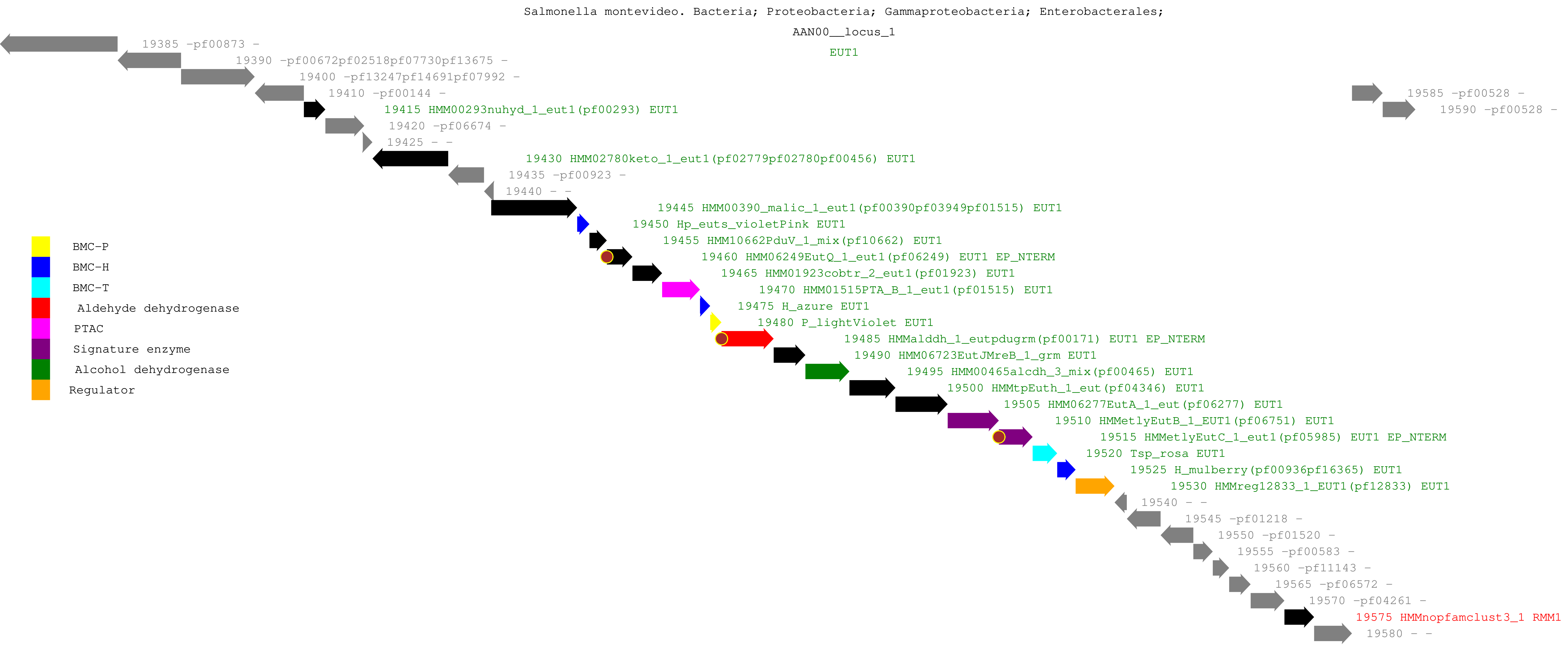
Salmonella montevideo. Bacteria; Proteobacteria; Gammaproteobacteria; Enterobacterales;
AAN00__locus_1
EUT1
19385 -pf00873 -
19390 -pf00672pf02518pf07730pf13675 -
19400 -pf13247pf14691pf07992 -
19410 -pf00144 -
19415 HMM00293nuhyd_1_eut1(pf00293) EUT1
19420 -pf06674 -
19425 - -
19430 HMM02780keto_1_eut1(pf02779pf02780pf00456) EUT1
19435 -pf00923 -
19440 - -
19445 HMM00390_malic_1_eut1(pf00390pf03949pf01515) EUT1
19450 Hp_euts_violetPink EUT1
19455 HMM10662PduV_1_mix(pf10662) EUT1
19460 HMM06249EutQ_1_eut1(pf06249) EUT1 EP_NTERM
19465 HMM01923cobtr_2_eut1(pf01923) EUT1
19470 HMM01515PTA_B_1_eut1(pf01515) EUT1
19475 H_azure EUT1
19480 P_lightViolet EUT1
19485 HMMaldh_1_eutpdugrm(pf00171) EUT1 EP_NTERM
19490 HMM06723EutJMreB_1_grm EUT1
19495 HMM00465alcdh_3_mix(pf00465) EUT1
19500 HMMtpEuth_1_eut(pf04346) EUT1
19505 HMM06277EutA_1_eut(pf06277) EUT1
19510 HMMetlyEutB_1_EUT1(pf06751) EUT1
19515 HMMetlyEutC_1_eut1(pf05985) EUT1 EP_NTERM
19520 Tsp_rosa EUT1
19525 H_mulberry(pf00936pf16365) EUT1
19530 HMMreg12833_1_EUT1(pf12833) EUT1
19540 - -
19545 -pf01218 -
19550 -pf01520 -
19555 -pf00583 -
19560 -pf11143 -
19565 -pf06572 -
19570 -pf04261 -
19575 HMMnopfamclust3_1 RMM1
19580 - -
19585 -pf00528 -
19590 -pf00528 -
BMC-P
BMC-H
BMC-T
Aldehyde dehydrogenase
PTAC
Signature enzyme
Alcohol dehydrogenase
Regulator

Salmonella derby. Bacteria; Proteobacteria; Gammaproteobacteria; Enterobacterales;

AAN63__locus_2

EUT1

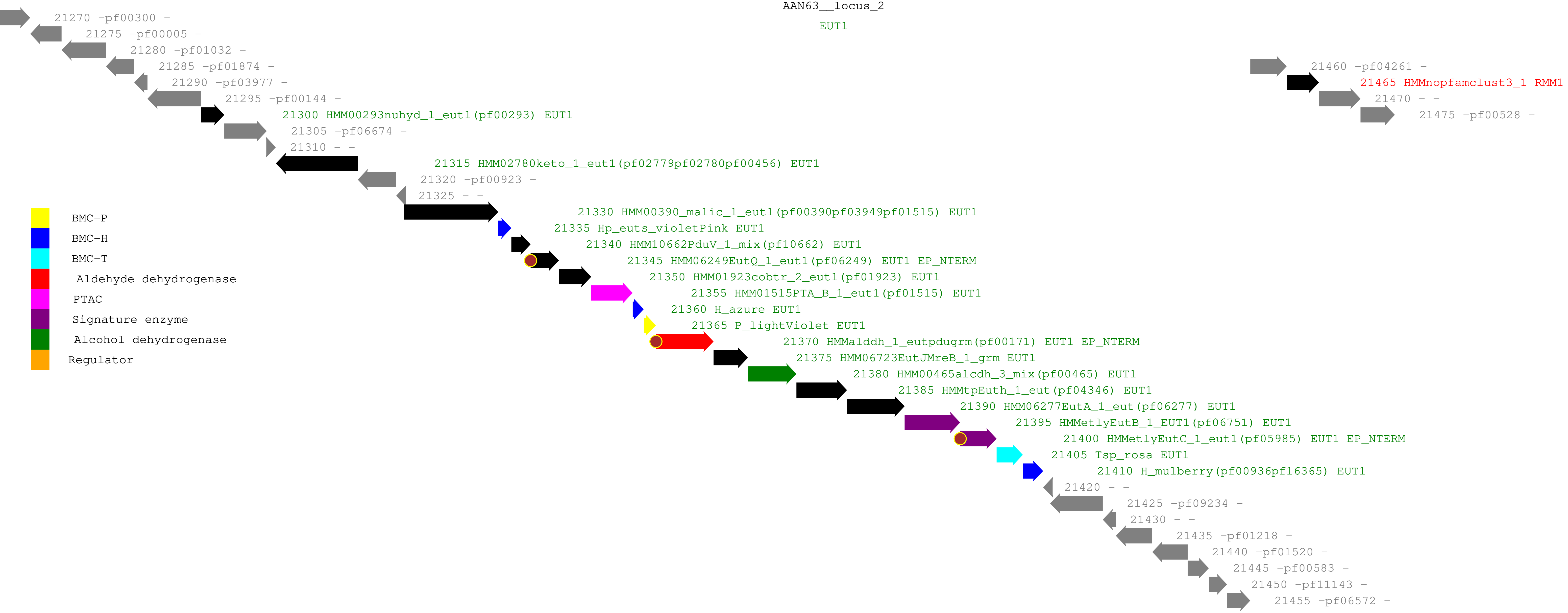

Salmonella typhimurium. Bacteria; Proteobacteria; Gammaproteobacteria; Enterobacterales;

AAP89__locus_1

EUT1

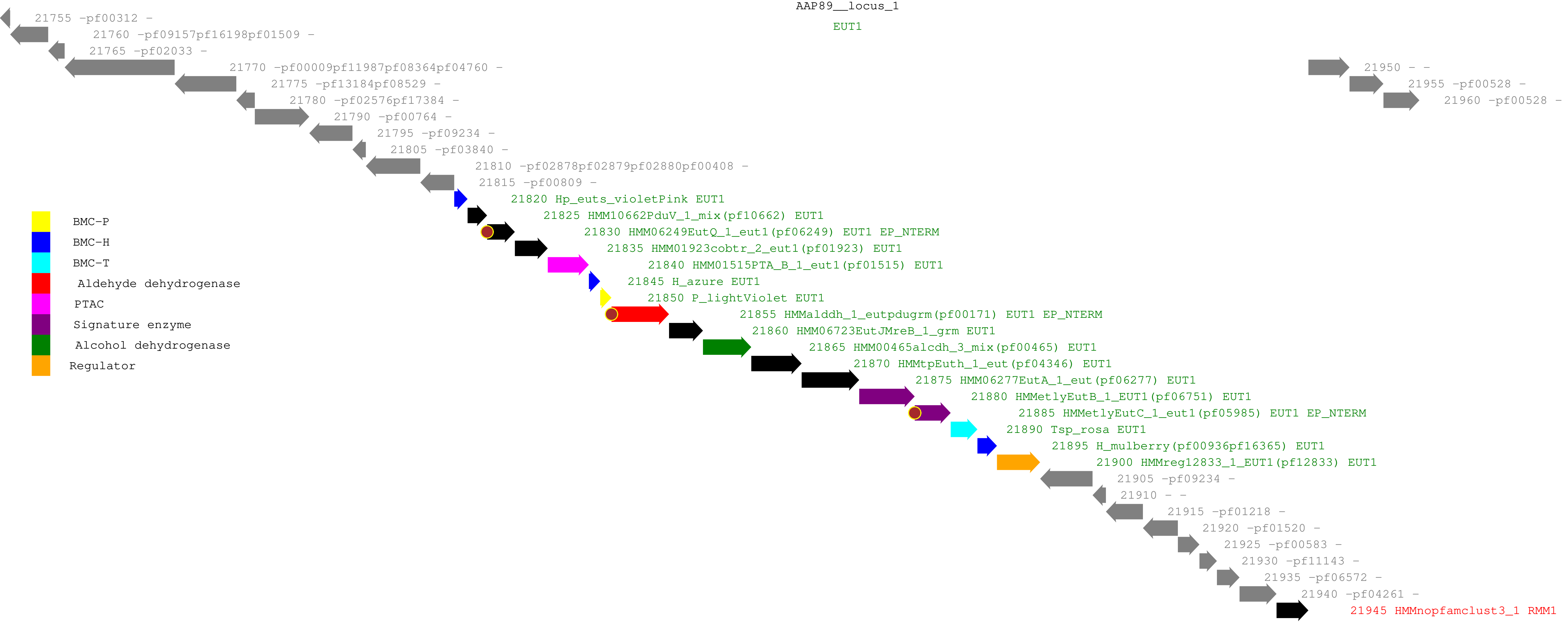

Escherichia coli. Bacteria; Proteobacteria; Gammaproteobacteria; Enterobacterales;

AAW06__locus_2

EUT1

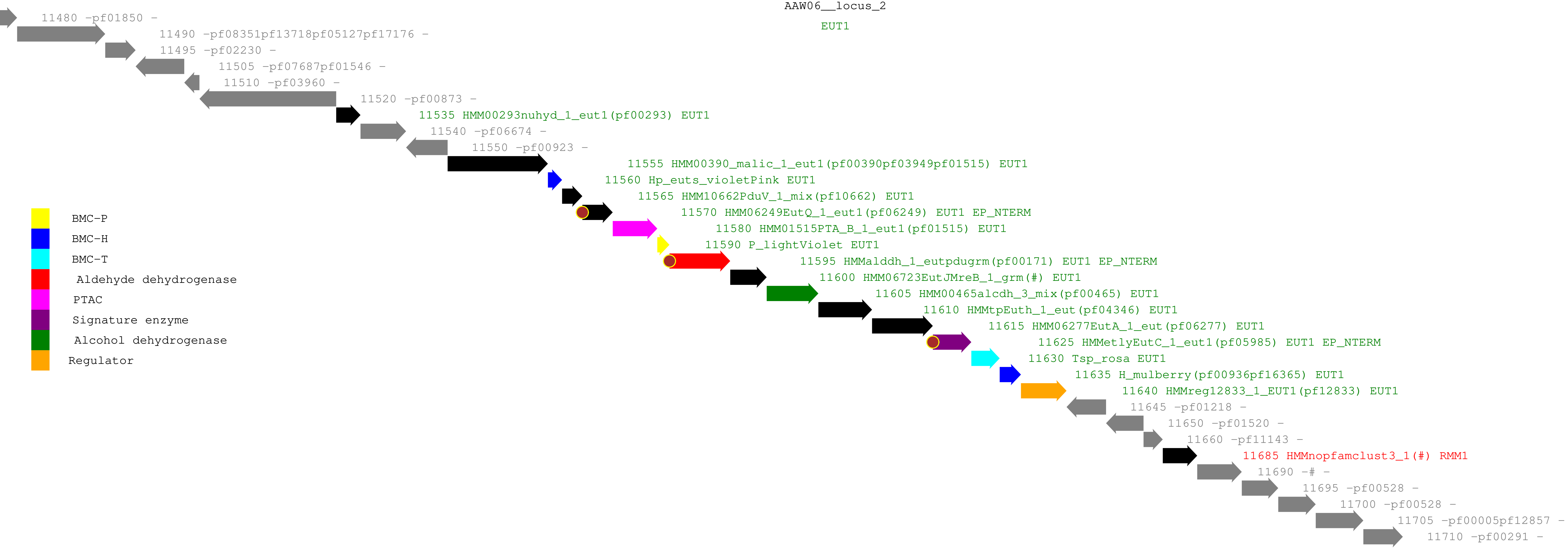

Sedimenticola thiotaurini. Bacteria; Proteobacteria; Gammaproteobacteria; Sedimenticola.

AAY24__locus_1

EUT1

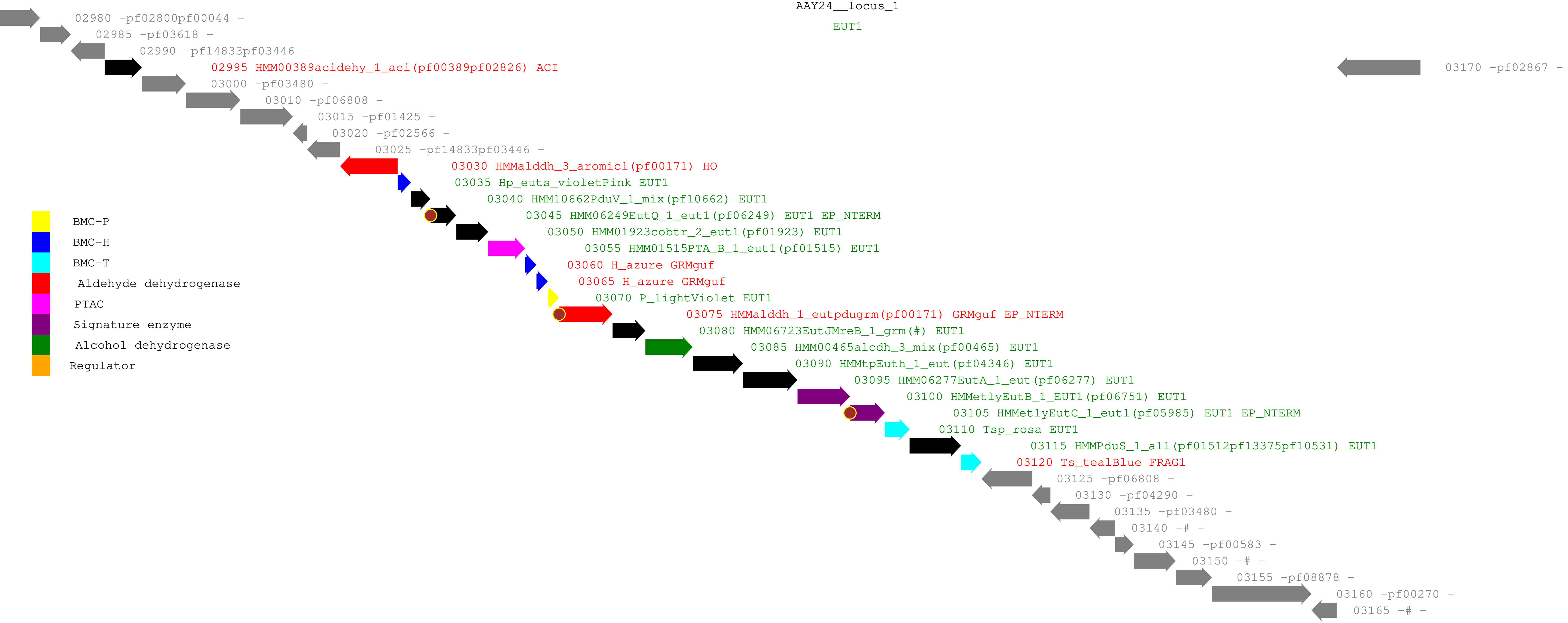

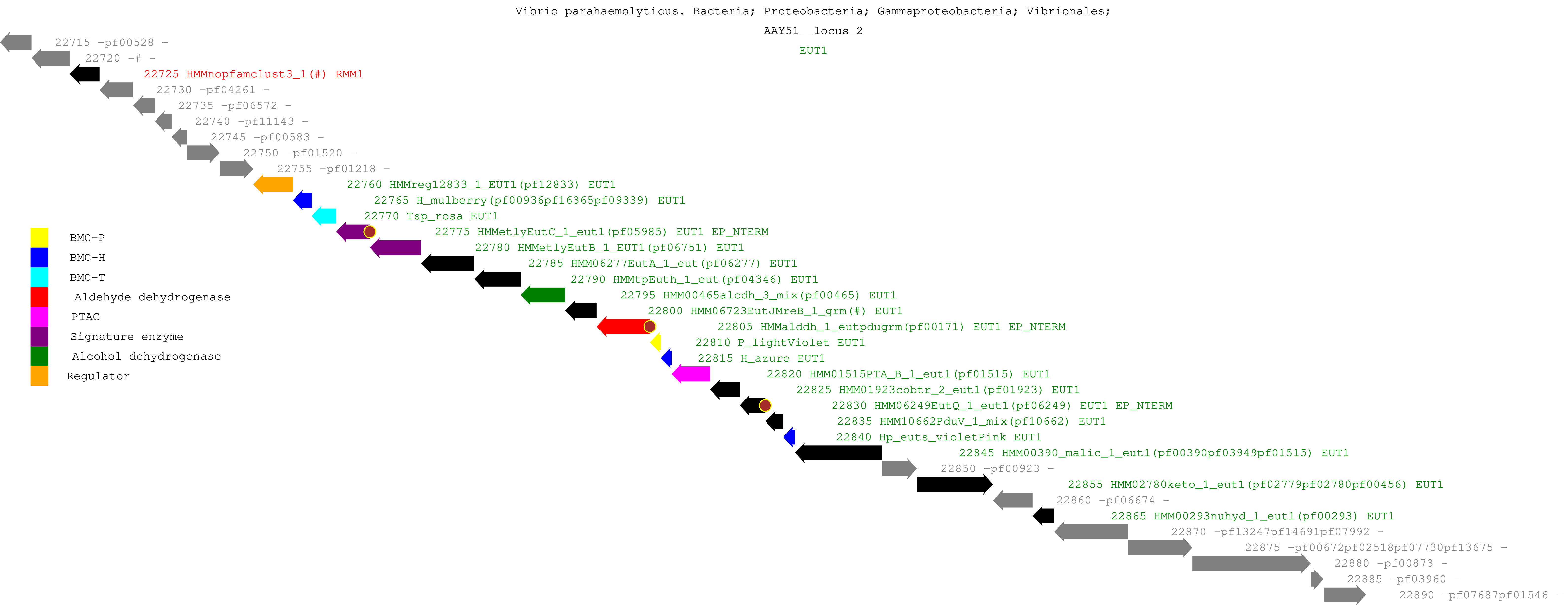

Vibrio parahaemolyticus. Bacteria; Proteobacteria; Gammaproteobacteria; Vibrionales;
AAY51__locus_2
EUT1
22715 -pf00528 -
22720 -# -
22725 HMMnopfamclust3_1(#) RMM1
22730 -pf04261 -
22735 -pf06572 -
22740 -pf11143 -
22745 -pf00583 -
22750 -pf01520 -
22755 -pf01218 -
22760 HMMreg12833_1_EUT1(pf12833) EUT1
22765 H_mulberry(pf00936pf16365pf09339) EUT1
22770 Tsp_rosa EUT1
22775 HMMetlyEutC_1_eut1(pf05985) EUT1 EP_NTERM
22780 HMMetlyEutB_1_EUT1(pf06751) EUT1
22785 HMM06277EutA_1_eut(pf06277) EUT1
22790 HMMtpEuth_1_eut(pf04346) EUT1
22795 HMM00465alcdh_3_mix(pf00465) EUT1
22800 HMM06723EutJMreB_1_grm(#) EUT1
22805 HMMalddh_1_eutpdugrm(pf00171) EUT1 EP_NTERM
22810 P_lightViolet EUT1
22815 H_azure EUT1
22820 HMM01515PTA_B_1_eut1(pf01515) EUT1
22825 HMM01923cobtr_2_eut1(pf01923) EUT1
22830 HMM06249EutQ_1_eut1(pf06249) EUT1 EP_NTERM
22835 HMM10662PduV_1_mix(pf10662) EUT1
22840 Hp_euts_violetPink EUT1
22845 HMM00390_malic_1_eut1(pf00390pf03949pf01515) EUT1
22850 -pf00923 -
22855 HMM02780keto_1_eut1(pf02779pf02780pf00456) EUT1
22860 -pf06674 -
22865 HMM00293nuhyd_1_eut1(pf00293) EUT1
22870 -pf13247pf14691pf07992 -
22875 -pf00672pf02518pf07730pf13675 -
22880 -pf00873 -
22885 -pf03960 -
22890 -pf07687pf01546 -
BMC-P
BMC-H
BMC-T
Aldehyde dehydrogenase
PTAC
Signature enzyme
Alcohol dehydrogenase
Regulator

Escherichia coli 5-366-08_S1_C1. Bacteria; Proteobacteria; Gammaproteobacteria; Enterobacterales;

AB08__locus_1

EUT1

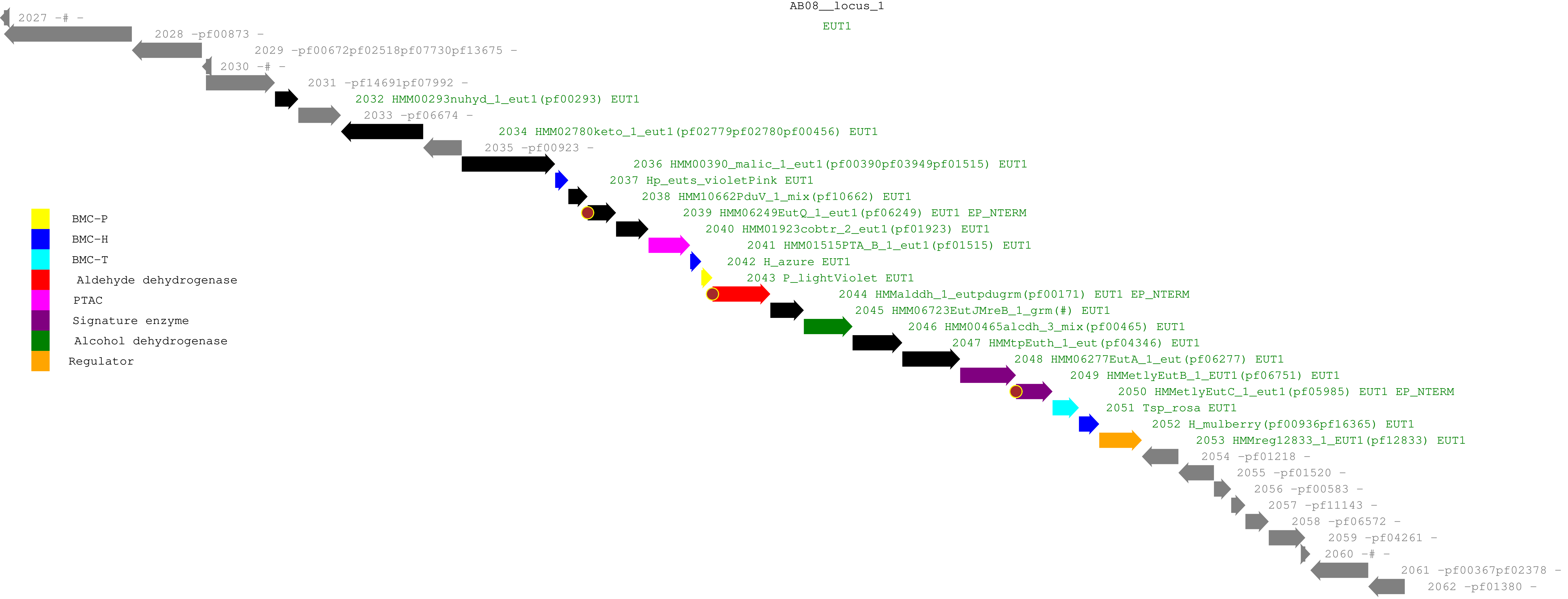

Klebsiella oxytoca. Bacteria; Proteobacteria; Gammaproteobacteria; Enterobacterales;

AB185__locus_2

EUT1

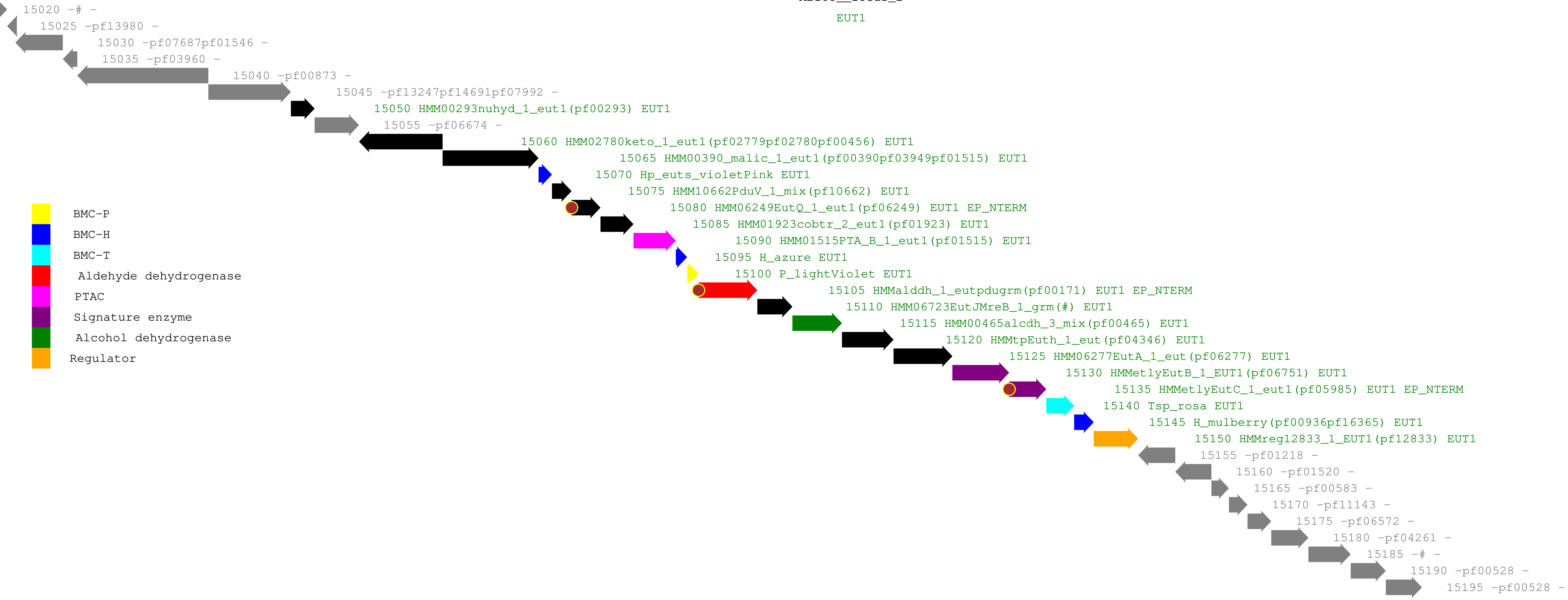

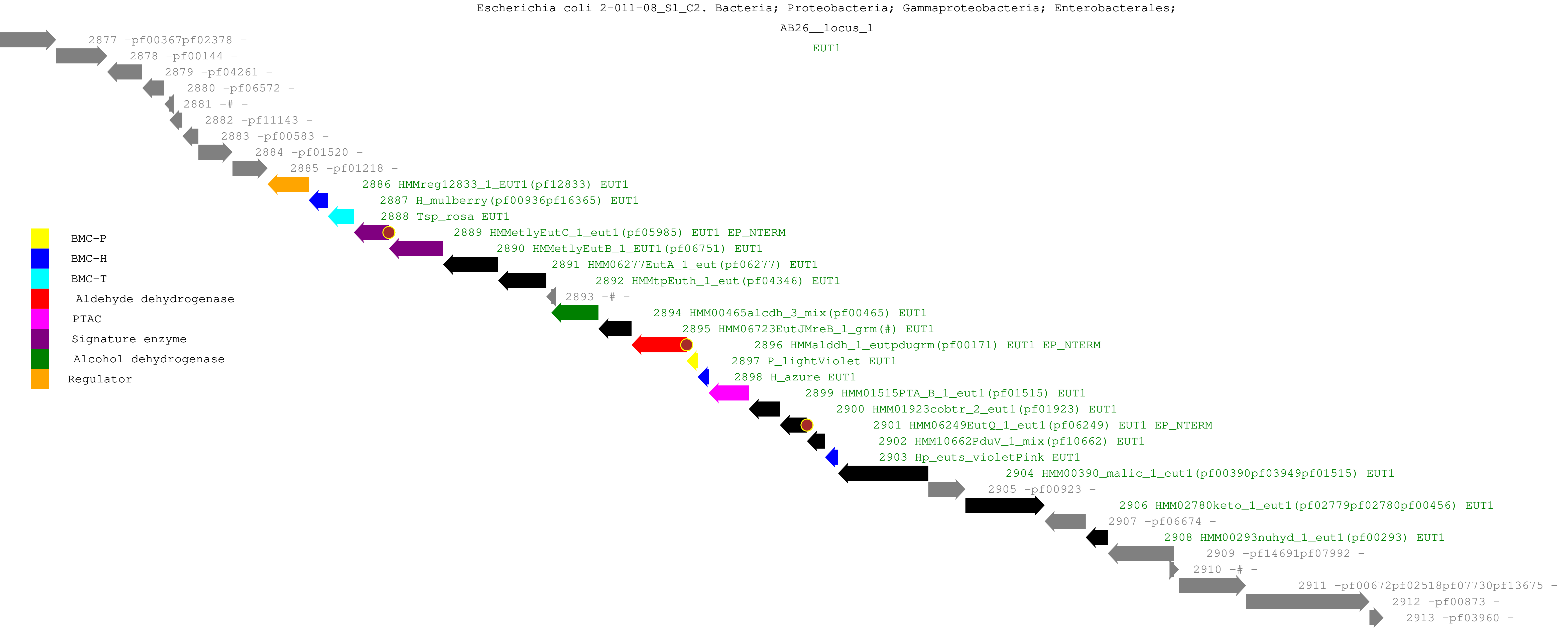

Escherichia coli 2-011-08_S1_C2. Bacteria; Proteobacteria; Gammaproteobacteria; Enterobacterales;
AB26__locus_1
EUT1
2877 -pf00367pf02378 -
2878 -pf00144 -
2879 -pf04261 -
2880 -pf06572 -
2881 -# -
2882 -pf11143 -
2883 -pf00583 -
2884 -pf01520 -
2885 -pf01218 -
2886 HMMreg12833_1_EUT1(pf12833) EUT1
2887 H_mulberry(pf00936pf16365) EUT1
2888 Tsp_rosa EUT1
2889 HMMetlyEutC_1_eut1(pf05985) EUT1 EP_NTERM
2890 HMMetlyEutB_1_EUT1(pf06751) EUT1
2891 HMM06277EutA_1_eut(pf06277) EUT1
2892 HMMtpEuth_1_eut(pf04346) EUT1
2893 -# -
2894 HMM00465alcdh_3_mix(pf00465) EUT1
2895 HMM06723EutJMreB_1_grm(#) EUT1
2896 HMMalddh_1_eutpdugrm(pf00171) EUT1 EP_NTERM
2897 P_lightViolet EUT1
2898 H_azure EUT1
2899 HMM01515PTA_B_1_eut1(pf01515) EUT1
2900 HMM01923cobtr_2_eut1(pf01923) EUT1
2901 HMM06249EutQ_1_eut1(pf06249) EUT1 EP_NTERM
2902 HMM10662PduV_1_mix(pf10662) EUT1
2903 Hp_euts_violetPink EUT1
2904 HMM00390_malic_1_eut1(pf00390pf03949pf01515) EUT1
2905 -pf00923 -
2906 HMM02780keto_1_eut1(pf02779pf02780pf00456) EUT1
2907 -pf06674 -
2908 HMM00293nuhyd_1_eut1(pf00293) EUT1
2909 -pf14691pf07992 -
2910 -# -
2911 -pf00672pf02518pf07730pf13675 -
2912 -pf00873 -
2913 -pf03960 -
BMC-P
BMC-H
BMC-T
Aldehyde dehydrogenase
PTAC
Signature enzyme
Alcohol dehydrogenase
Regulator

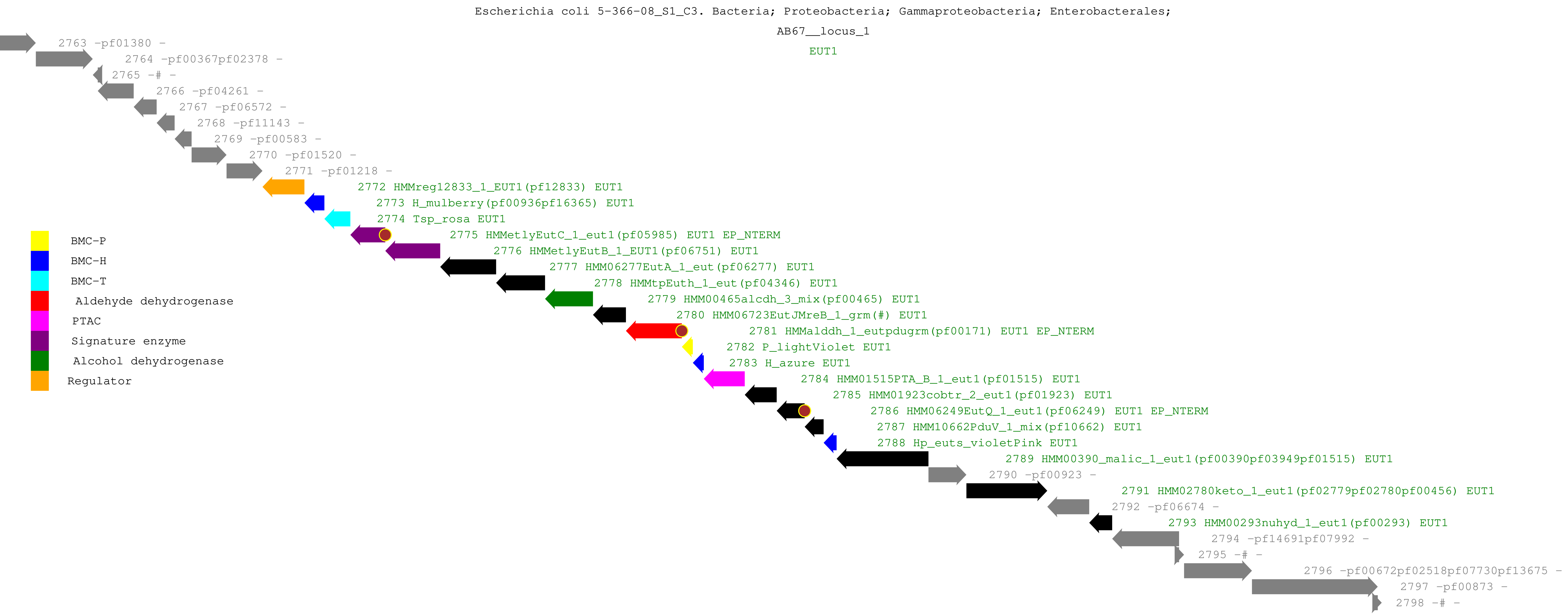

Escherichia coli 5-366-08_S1_C3. Bacteria; Proteobacteria; Gammaproteobacteria; Enterobacterales;
AB67__locus_1
EUT1
2763 -pf01380 -
2764 -pf00367pf02378 -
2765 -# -
2766 -pf04261 -
2767 -pf06572 -
2768 -pf11143 -
2769 -pf00583 -
2770 -pf01520 -
2771 -pf01218 -
2772 HMMreg12833_1_EUT1(pf12833) EUT1
2773 H_mulberry(pf00936pf16365) EUT1
2774 Tsp_rosa EUT1
2775 HMMetlyEutC_1_eut1(pf05985) EUT1 EP_NTERM
2776 HMMetlyEutB_1_EUT1(pf06751) EUT1
2777 HMM06277EutA_1_eut(pf06277) EUT1
2778 HMMtpEuth_1_eut(pf04346) EUT1
2779 HMM00465alcdh_3_mix(pf00465) EUT1
2780 HMM06723EutJMreB_1_grm(#) EUT1
2781 HMMalddh_1_eutpdugrm(pf00171) EUT1 EP_NTERM
2782 P_lightViolet EUT1
2783 H_azure EUT1
2784 HMM01515PTA_B_1_eut1(pf01515) EUT1
2785 HMM01923cobtr_2_eut1(pf01923) EUT1
2786 HMM06249EutQ_1_eut1(pf06249) EUT1 EP_NTERM
2787 HMM10662PduV_1_mix(pf10662) EUT1
2788 Hp_euts_violetPink EUT1
2789 HMM00390_malic_1_eut1(pf00390pf03949pf01515) EUT1
2790 -pf00923 -
2791 HMM02780keto_1_eut1(pf02779pf02780pf00456) EUT1
2792 -pf06674 -
2793 HMM00293nuhyd_1_eut1(pf00293) EUT1
2794 -pf14691pf07992 -
2795 -# -
2796 -pf00672pf02518pf07730pf13675 -
2797 -pf00873 -
2798 -# -
BMC-P
BMC-H
BMC-T
Aldehyde dehydrogenase
PTAC
Signature enzyme
Alcohol dehydrogenase
Regulator

Escherichia coli 6-537-08_S1_C3. Bacteria; Proteobacteria; Gammaproteobacteria; Enterobacterales;

AB81__locus_1

EUT1

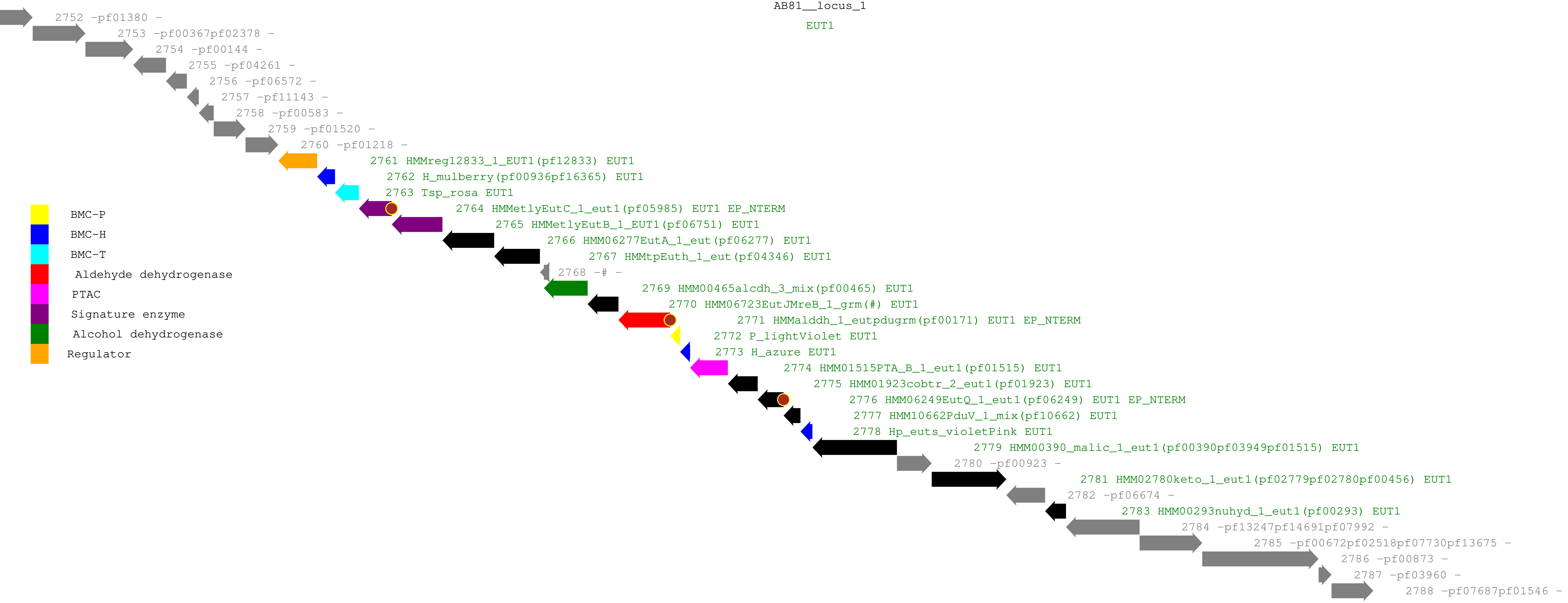

Escherichia coli 1-110-08_S3_C1. Bacteria; Proteobacteria; Gammaproteobacteria; Enterobacterales;

AB97__locus_1

EUT1

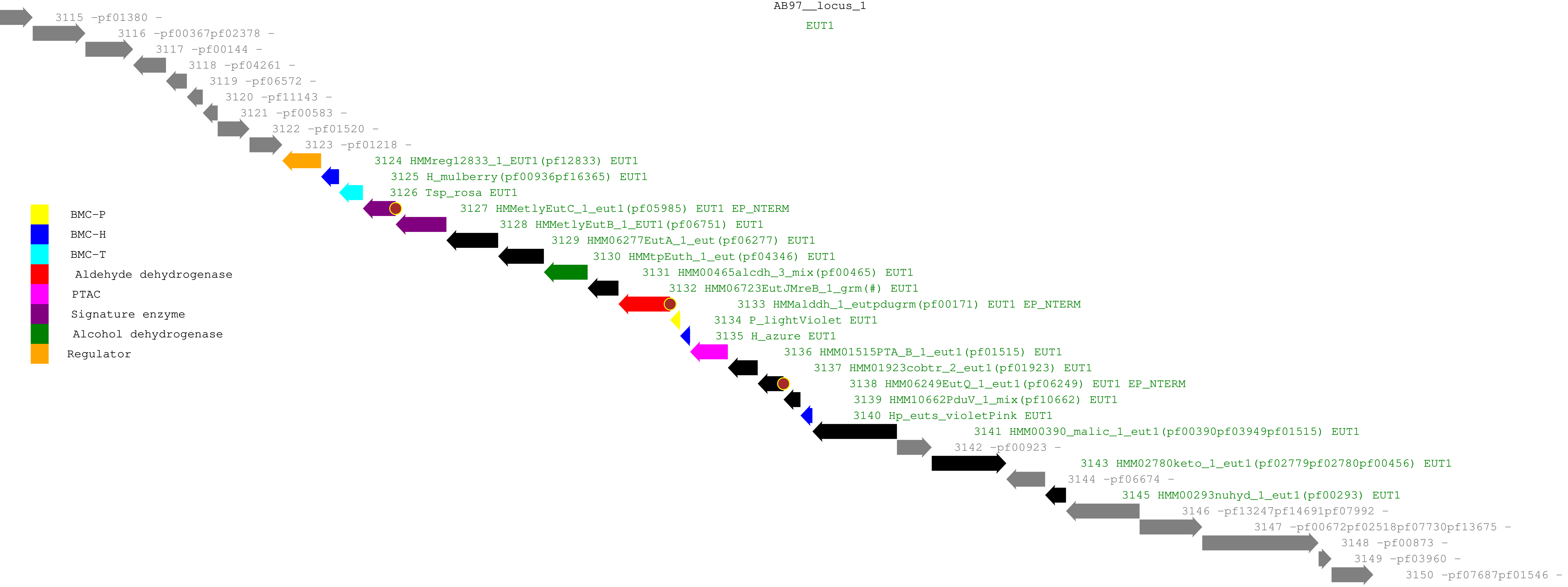

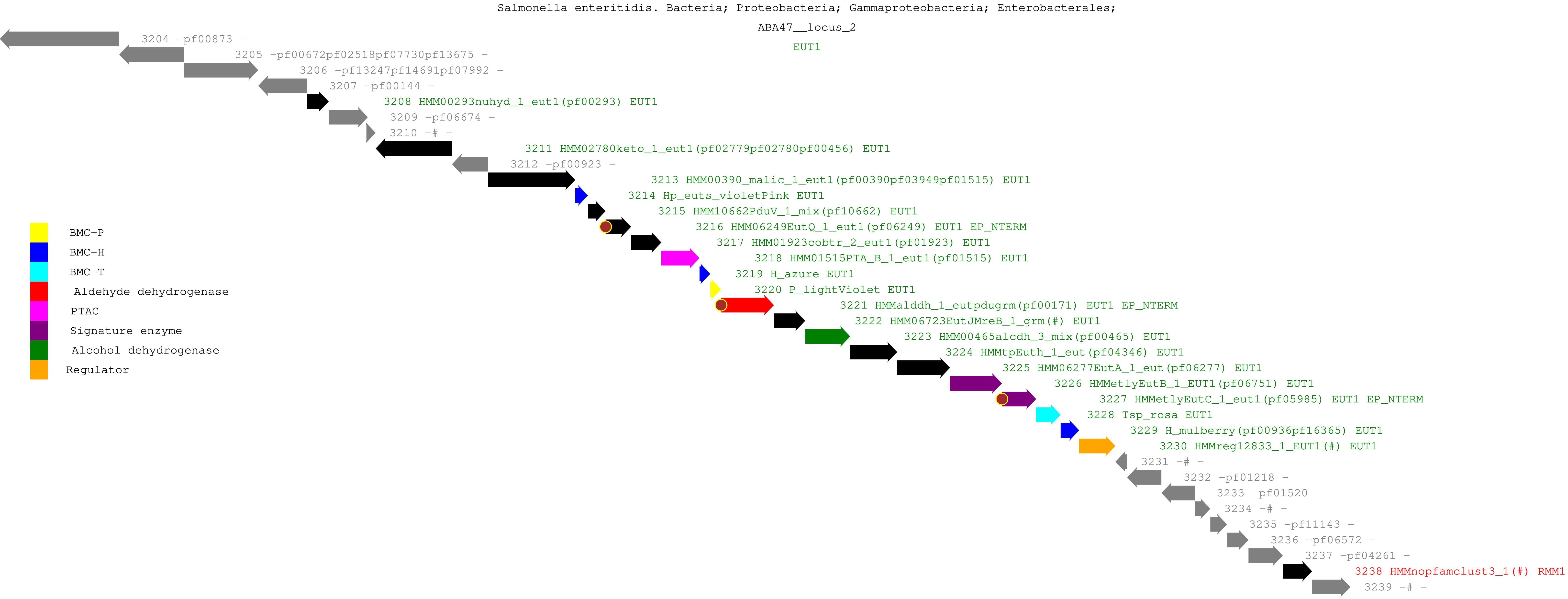

Salmonella enteritidis. Bacteria; Proteobacteria; Gammaproteobacteria; Enterobacterales;
ABA47__locus_2
EUT1
3204 -pf00873 -
3205 -pf00672pf02518pf07730pf13675 -
3206 -pf13247pf14691pf07992 -
3207 -pf00144 -
3208 HMM00293nuhyd_1_eut1(pf00293) EUT1
3209 -pf06674 -
3210 -# -
3211 HMM02780keto_1_eut1(pf02779pf02780pf00456) EUT1
3212 -pf00923 -
3213 HMM00390_malic_1_eut1(pf00390pf03949pf01515) EUT1
3214 Hp_euts_violetPink EUT1
3215 HMM10662PduV_1_mix(pf10662) EUT1
3216 HMM06249EutQ_1_eut1(pf06249) EUT1 EP_NTERM
3217 HMM01923cobtr_2_eut1(pf01923) EUT1
3218 HMM01515PTA_B_1_eut1(pf01515) EUT1
3219 H_azure EUT1
3220 P_lightViolet EUT1
3221 HMMalddh_1_eutpdugrm(pf00171) EUT1 EP_NTERM
3222 HMM06723EutJMreB_1_grm(#) EUT1
3223 HMM00465alcdh_3_mix(pf00465) EUT1
3224 HMMtpEuth_1_eut(pf04346) EUT1
3225 HMM06277EutA_1_eut(pf06277) EUT1
3226 HMMetlyEutB_1_EUT1(pf06751) EUT1
3227 HMMetlyEutC_1_eut1(pf05985) EUT1 EP_NTERM
3228 Tsp_rosa EUT1
3229 H_mulberry(pf00936pf16365) EUT1
3230 HMMreg12833_1_EUT1(#) EUT1
3231 -# -
3232 -pf01218 -
3233 -pf01520 -
3234 -# -
3235 -pf11143 -
3236 -pf06572 -
3237 -pf04261 -
3238 HMMnopfamclust3_1(#) RMM1
3239 -# -
BMC-P
BMC-H
BMC-T
Aldehyde dehydrogenase
PTAC
Signature enzyme
Alcohol dehydrogenase
Regulator

Shigella boydii. Bacteria; Proteobacteria; Gammaproteobacteria; Enterobacterales;

ABE81__locus_1

EUT1

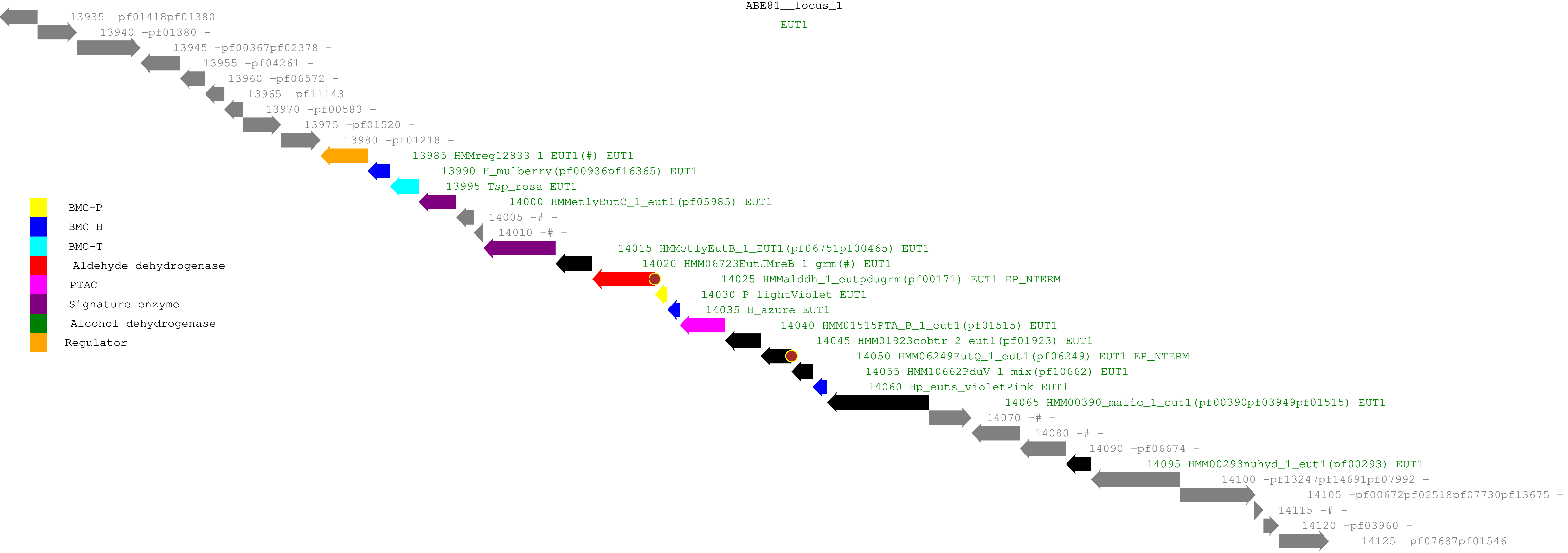

Salmonella enterica subsp. enterica serovar Mbandaka. Bacteria; Proteobacteria; Gammaproteobacteria; Enterobacterales;

ABJ60__locus_2_main_sat

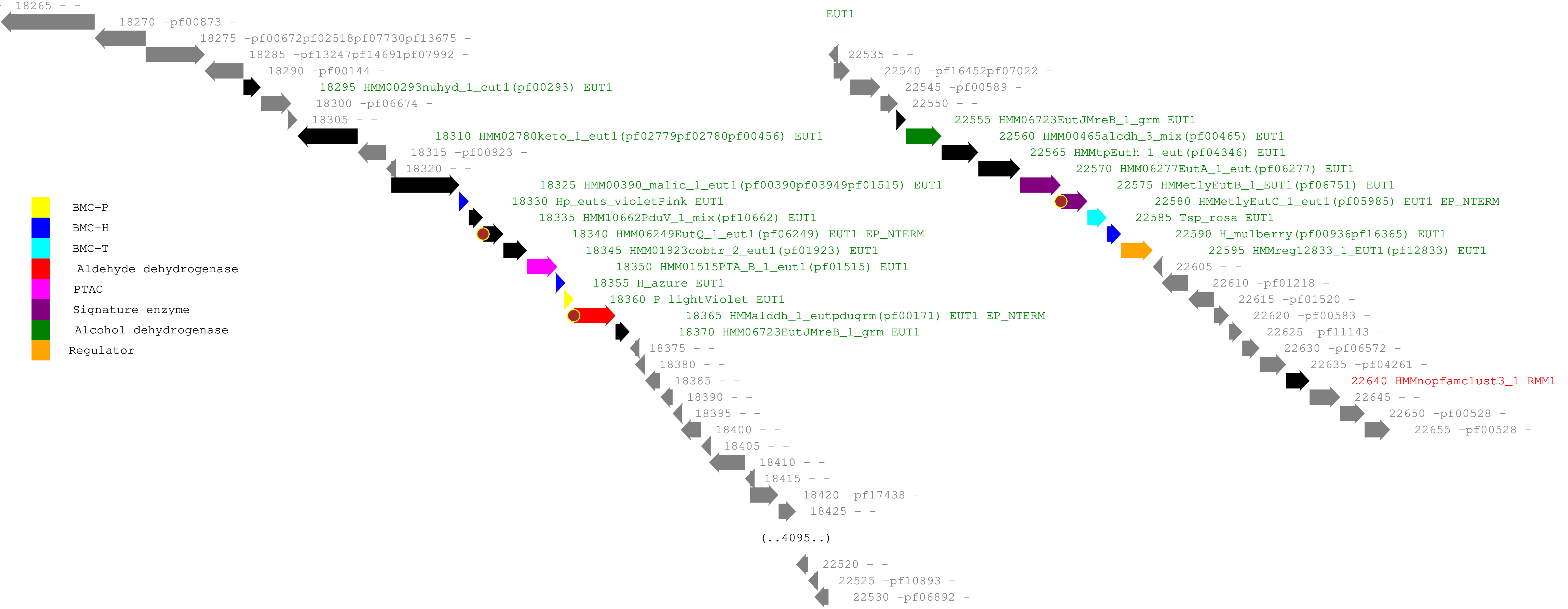

Salmonella senftenberg. Bacteria; Proteobacteria; Gammaproteobacteria; Enterobacterales;

ABM45__locus_2_main_sat

EUT1

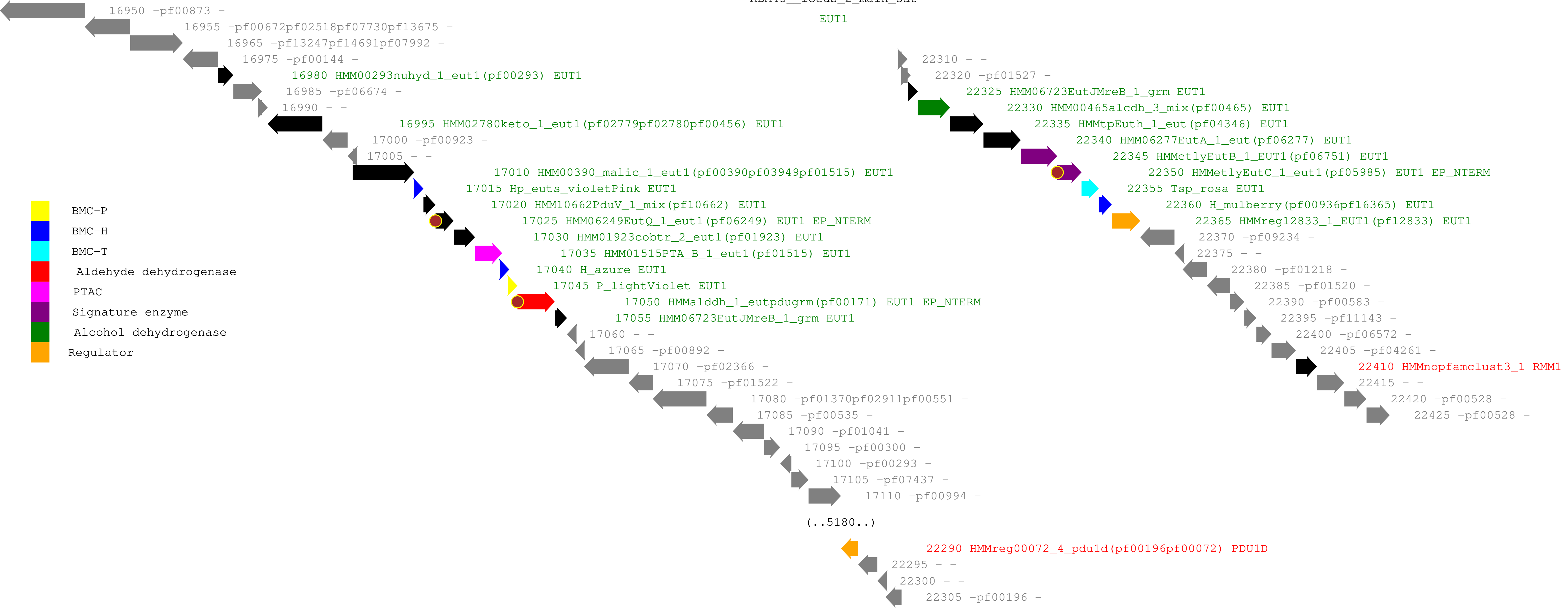

Salmonella typhi. Bacteria; Proteobacteria; Gammaproteobacteria; Enterobacterales;

ABP05__locus_1_main_sat

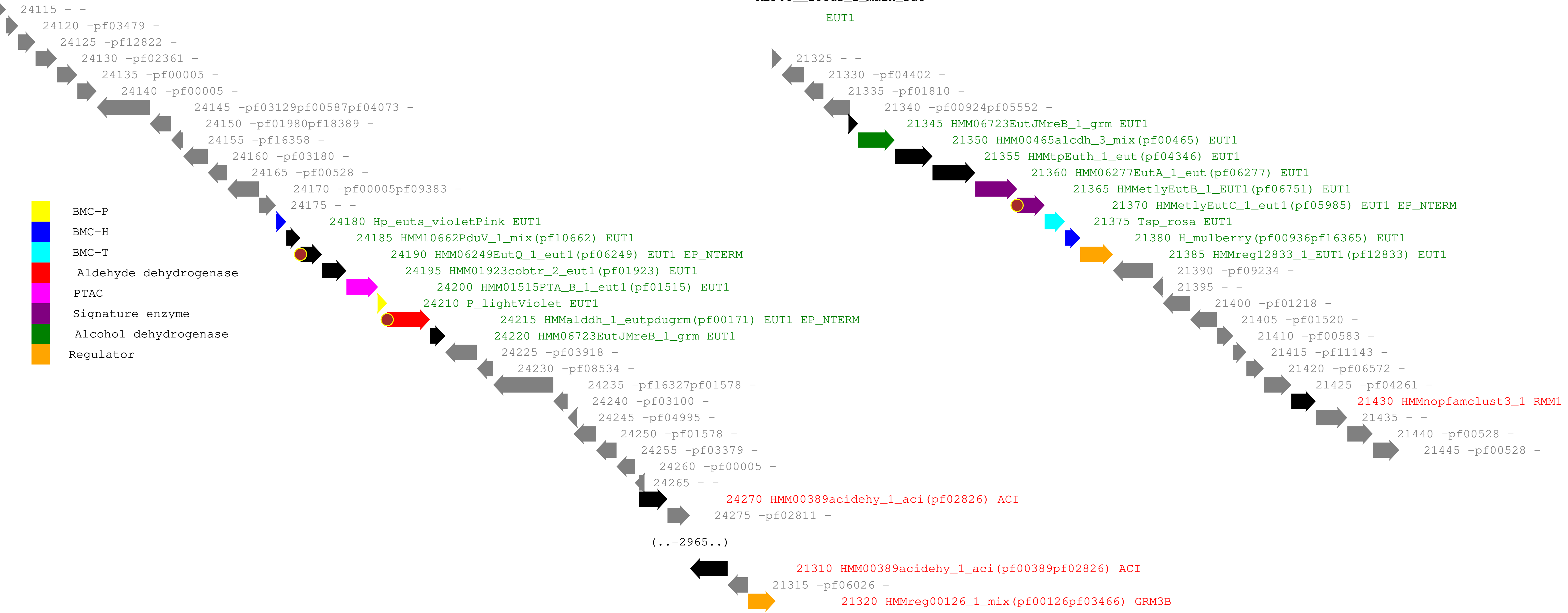

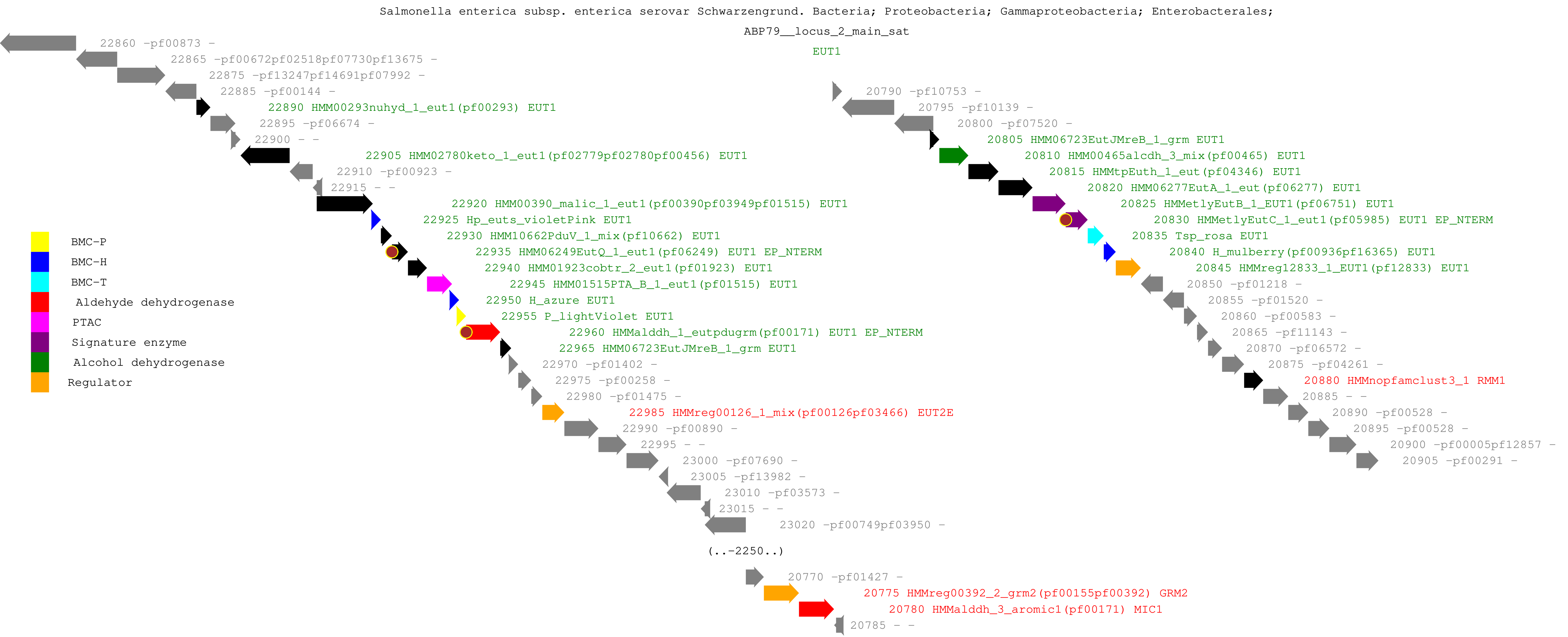
Salmonella enterica subsp. enterica serovar Schwarzengrund. Bacteria; Proteobacteria; Gammaproteobacteria; Enterobacterales;
ABP79__locus_2_main_sat
EUT1
22860 -pf00873 -
22865 -pf00672pf02518pf07730pf13675 -
22875 -pf13247pf14691pf07992 -
22885 -pf00144 -
22890 HMM00293nuhyd_1_eut1(pf00293) EUT1
22895 -pf06674 -
22900 - -
22905 HMM02780keto_1_eut1(pf02779pf02780pf00456) EUT1
22910 -pf00923 -
22915 - -
22920 HMM00390_malic_1_eut1(pf00390pf03949pf01515) EUT1
22925 Hp_euts_violetPink EUT1
22930 HMM10662PduV_1_mix(pf10662) EUT1
22935 HMM06249EutQ_1_eut1(pf06249) EUT1 EP_NTERM
22940 HMM01923cobtr_2_eut1(pf01923) EUT1
22945 HMM01515PTA_B_1_eut1(pf01515) EUT1
22950 H_azure EUT1
22955 P_lightViolet EUT1
22960 HMMaldh_1_eutpdugrm(pf00171) EUT1 EP_NTERM
22965 HMM06723EutJMreB_1_grm EUT1
22970 -pf01402 -
22975 -pf00258 -
22980 -pf01475 -
22985 HMMreg00126_1_mix(pf00126pf03466) EUT2E
22990 -pf00890 -
22995 - -
23000 -pf07690 -
23005 -pf13982 -
23010 -pf03573 -
23015 - -
23020 -pf00749pf03950 -
(..-2250..)
20770 -pf01427 -
20775 HMMreg00392_2_grm2(pf00155pf00392) GRM2
20780 HMMaldh_3_aromic1(pf00171) MIC1
20785 - -
20790 -pf10753 -
20795 -pf10139 -
20800 -pf07520 -
20805 HMM06723EutJMreB_1_grm EUT1
20810 HMM00465alcdh_3_mix(pf00465) EUT1
20815 HMMtpEuth_1_eut(pf04346) EUT1
20820 HMM06277EutA_1_eut(pf06277) EUT1
20825 HMMetlyEutB_1_EUT1(pf06751) EUT1
20830 HMMetlyEutC_1_eut1(pf05985) EUT1 EP_NTERM
20835 Tsp_rosa EUT1
20840 H_mulberry(pf00936pf16365) EUT1
20845 HMMreg12833_1_EUT1(pf12833) EUT1
20850 -pf01218 -
20855 -pf01520 -
20860 -pf00583 -
20865 -pf11143 -
20870 -pf06572 -
20875 -pf04261 -
20880 HMMnopfamclust3_1 RMM1
20885 - -
20890 -pf00528 -
20895 -pf00528 -
20900 -pf00005pf12857 -
20905 -pf00291 -
BMC-P
BMC-H
BMC-T
Aldehyde dehydrogenase
PTAC
Signature enzyme
Alcohol dehydrogenase
Regulator

Enterobacter sp. GN02600. Bacteria; Proteobacteria; Gammaproteobacteria; Enterobacterales;

ABR34__locus_2

EUT1

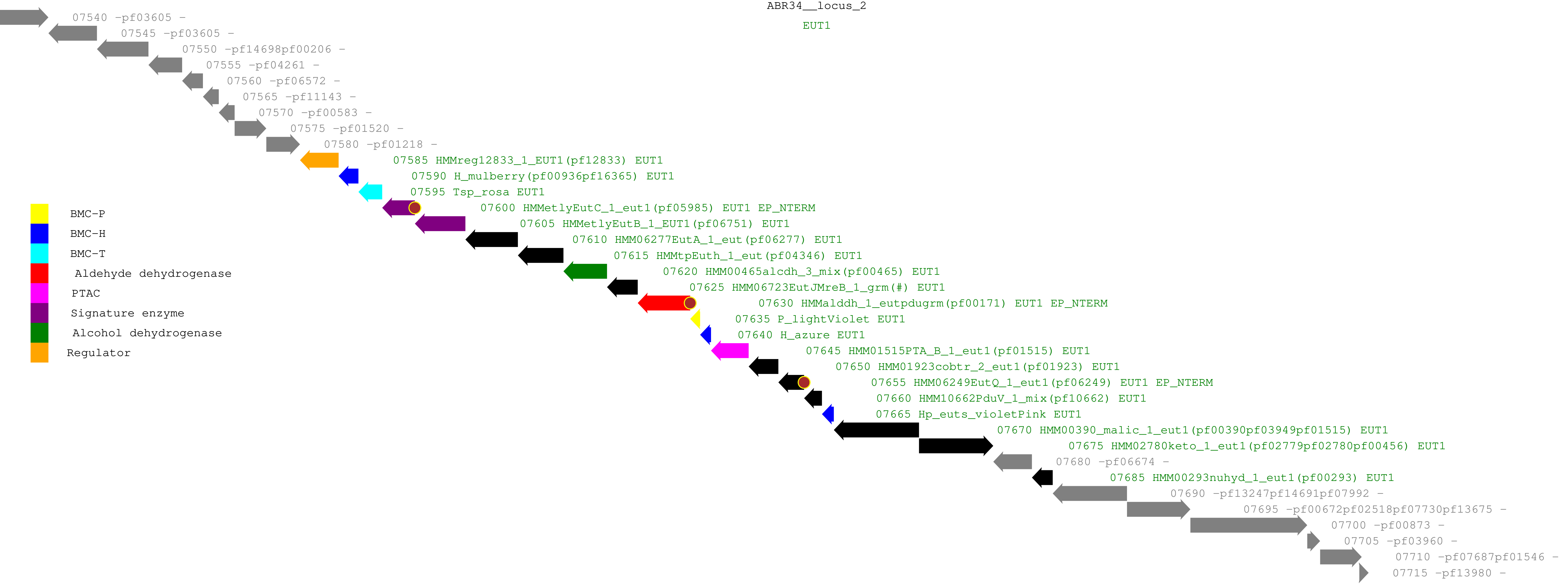

Photobacterium aquae. Bacteria; Proteobacteria; Gammaproteobacteria; Vibrionales;

ABT56__locus_1

EUT1

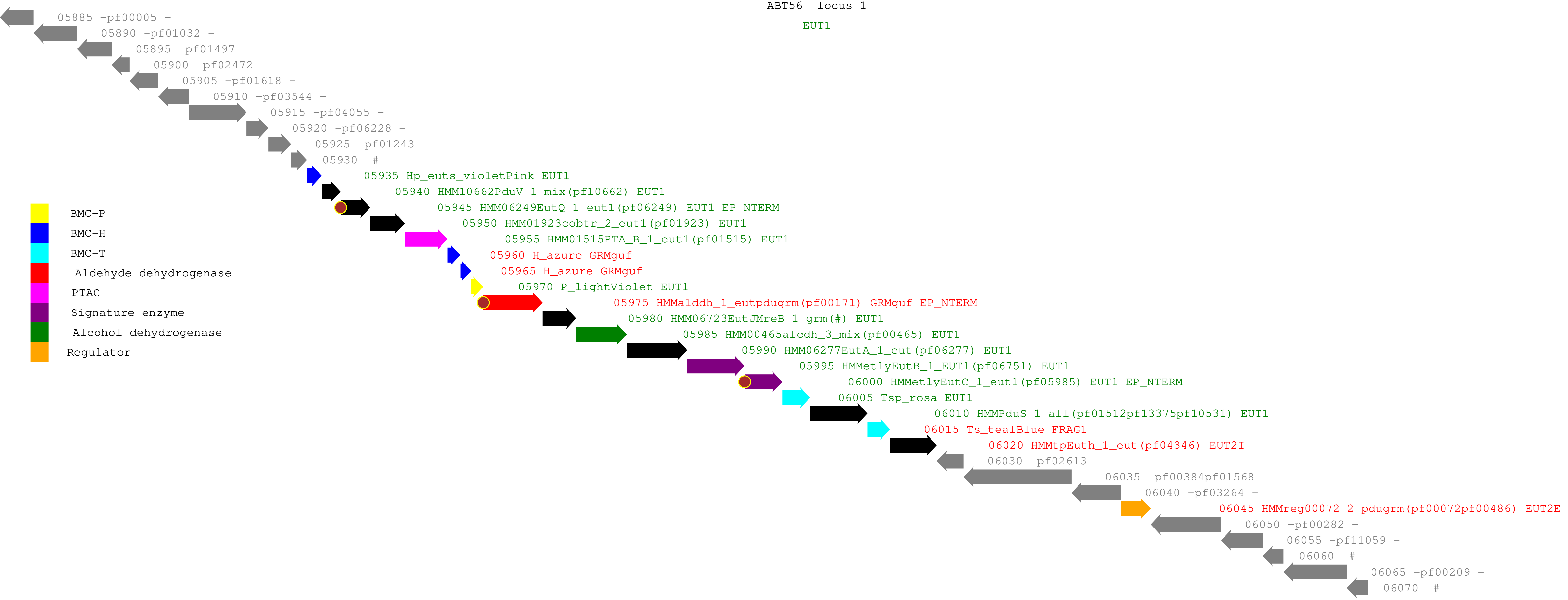

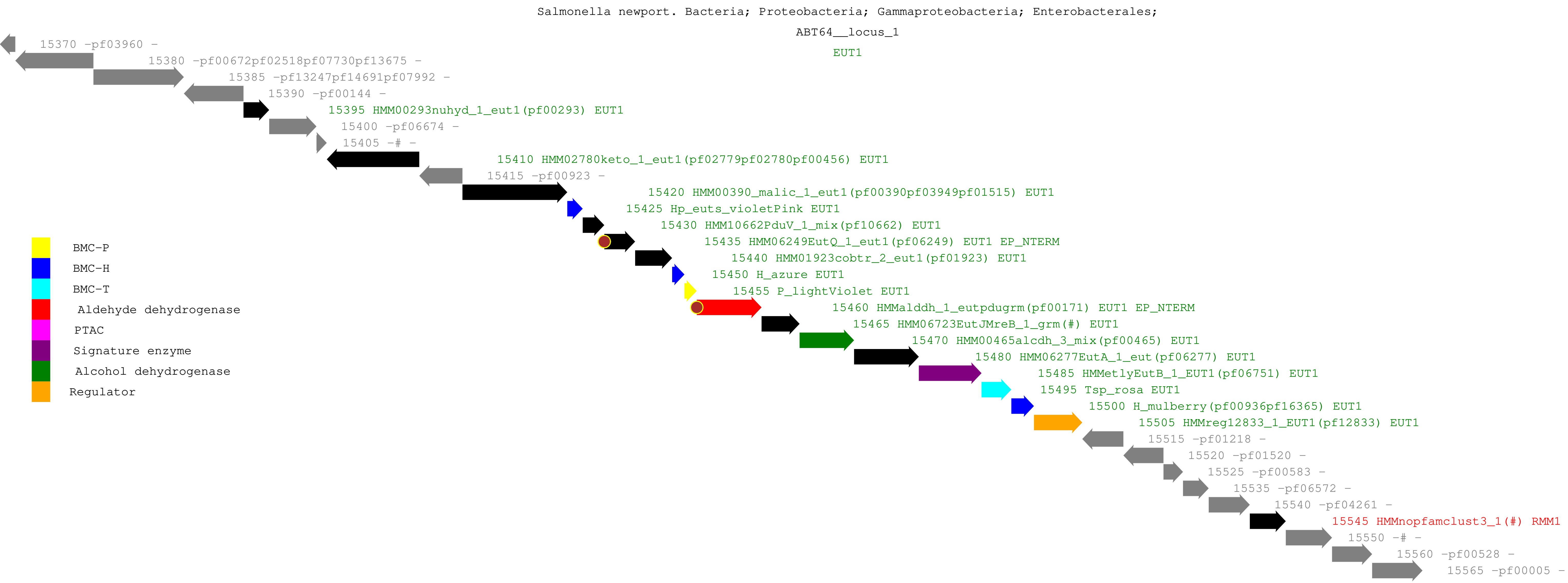
Salmonella newport. Bacteria; Proteobacteria; Gammaproteobacteria; Enterobacterales;
ABT64__locus_1
EUT1
15370 -pf03960 -
15380 -pf00672pf02518pf07730pf13675 -
15385 -pf13247pf14691pf07992 -
15390 -pf00144 -
15395 HMM00293nuhyd_1_eut1(pf00293) EUT1
15400 -pf06674 -
15405 -# -
15410 HMM02780keto_1_eut1(pf02779pf02780pf00456) EUT1
15415 -pf00923 -
15420 HMM00390_malic_1_eut1(pf00390pf03949pf01515) EUT1
15425 Hp_euts_violetPink EUT1
15430 HMM10662PduV_1_mix(pf10662) EUT1
15435 HMM06249EutQ_1_eut1(pf06249) EUT1 EP_NTERM
15440 HMM01923cobtr_2_eut1(pf01923) EUT1
15450 H_azure EUT1
15455 P_lightViolet EUT1
15460 HMMalddh_1_eutpdugrm(pf00171) EUT1 EP_NTERM
15465 HMM06723EutJMreB_1_grm(#) EUT1
15470 HMM00465alcdh_3_mix(pf00465) EUT1
15480 HMM06277EutA_1_eut(pf06277) EUT1
15485 HMMetlyEutB_1_EUT1(pf06751) EUT1
15495 Tsp_rosa EUT1
15500 H_mulberry(pf00936pf16365) EUT1
15505 HMMreg12833_1_EUT1(pf12833) EUT1
15515 -pf01218 -
15520 -pf01520 -
15525 -pf00583 -
15535 -pf06572 -
15540 -pf04261 -
15545 HMMnopfamclust3_1(#) RMM1
15550 -# -
15560 -pf00528 -
15565 -pf00005 -
BMC-P
BMC-H
BMC-T
Aldehyde dehydrogenase
PTAC
Signature enzyme
Alcohol dehydrogenase
Regulator

Pluralibacter gergoviae (Enterobacter gergoviae). Bacteria; Proteobacteria; Gammaproteobacteria; Enterobacterales;

ABW06__locus_1

EUT1

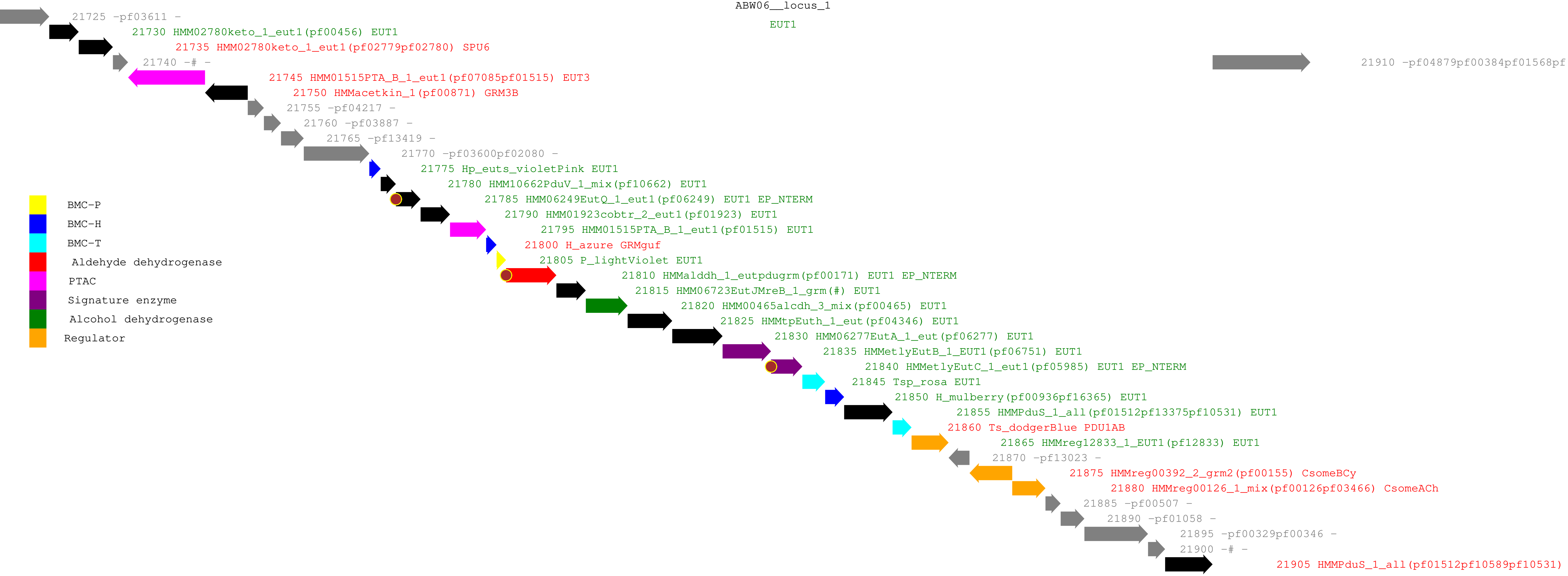

Escherichia coli. Bacteria; Proteobacteria; Gammaproteobacteria; Enterobacterales;

AC067__locus_1

EUT1

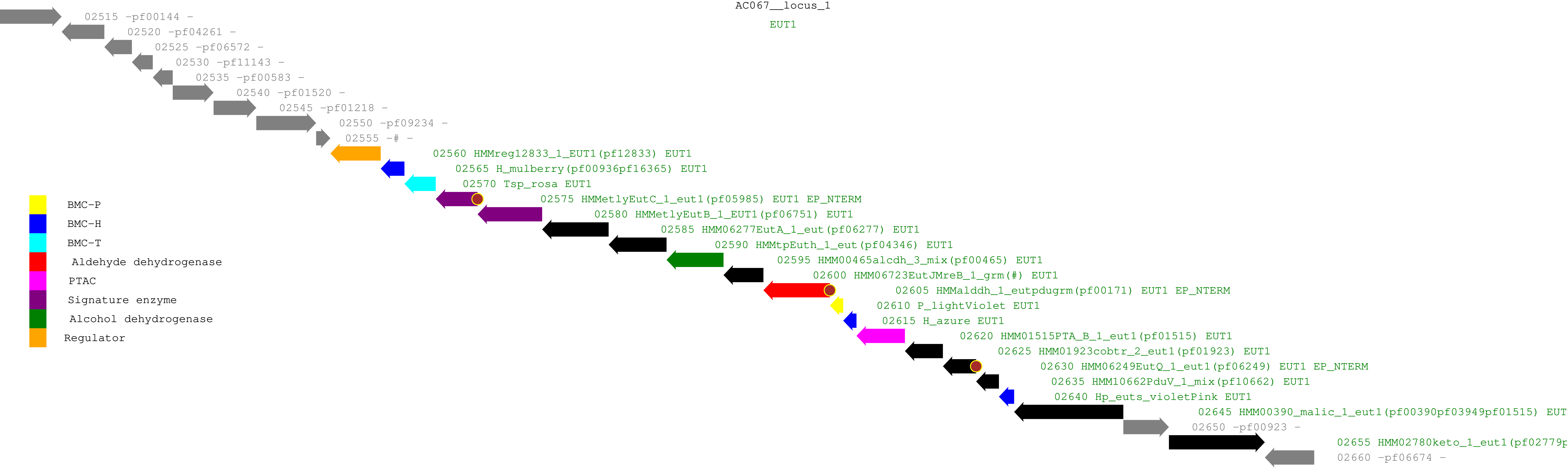

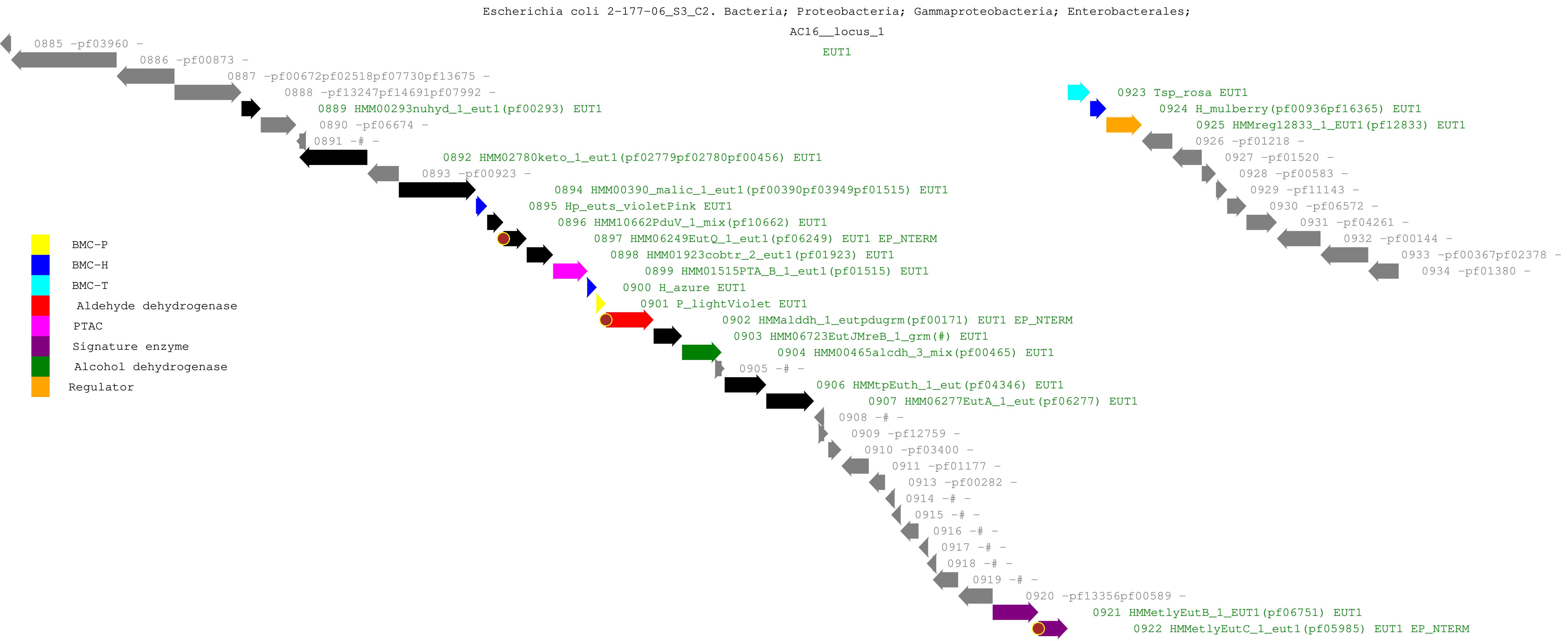
Escherichia coli 2-177-06_S3_C2. Bacteria; Proteobacteria; Gammaproteobacteria; Enterobacterales;
AC16__locus_1
EUT1
0885 -pf03960 -
0886 -pf00873 -
0887 -pf00672pf02518pf07730pf13675 -
0888 -pf13247pf14691pf07992 -
0889 HMM00293nuhyd_1_eut1(pf00293) EUT1
0890 -pf06674 -
0891 -# -
0892 HMM02780keto_1_eut1(pf02779pf02780pf00456) EUT1
0893 -pf00923 -
0894 HMM00390_malic_1_eut1(pf00390pf03949pf01515) EUT1
0895 Hp_euts_violetPink EUT1
0896 HMM10662PduV_1_mix(pf10662) EUT1
0897 HMM06249EutQ_1_eut1(pf06249) EUT1 EP_NTERM
0898 HMM01923cobtr_2_eut1(pf01923) EUT1
0899 HMM01515PTA_B_1_eut1(pf01515) EUT1
0900 H_azure EUT1
0901 P_lightViolet EUT1
0902 HMMaldh_1_eutpdugrm(pf00171) EUT1 EP_NTERM
0903 HMM06723EutJMreB_1_grm(#) EUT1
0904 HMM00465alcdh_3_mix(pf00465) EUT1
0905 -# -
0906 HMMtpEuth_1_eut(pf04346) EUT1
0907 HMM06277EutA_1_eut(pf06277) EUT1
0908 -# -
0909 -pf12759 -
0910 -pf03400 -
0911 -pf01177 -
0913 -pf00282 -
0914 -# -
0915 -# -
0916 -# -
0917 -# -
0918 -# -
0919 -# -
0920 -pf13356pf00589 -
0921 HMMetlyEutB_1_EUT1(pf06751) EUT1
0922 HMMetlyEutC_1_eut1(pf05985) EUT1 EP_NTERM
0923 Tsp_rosa EUT1
0924 H_mulberry(pf00936pf16365) EUT1
0925 HMMreg12833_1_EUT1(pf12833) EUT1
0926 -pf01218 -
0927 -pf01520 -
0928 -pf00583 -
0929 -pf11143 -
0930 -pf06572 -
0931 -pf04261 -
0932 -pf00144 -
0933 -pf00367pf02378 -
0934 -pf01380 -
BMC-P
BMC-H
BMC-T
Aldehyde dehydrogenase
PTAC
Signature enzyme
Alcohol dehydrogenase
Regulator

Salmonella enterica subsp. enterica serovar Brandenburg. Bacteria; Proteobacteria; Gammaproteobacteria; Enterobacterales;

AC218__locus_2

EUT1

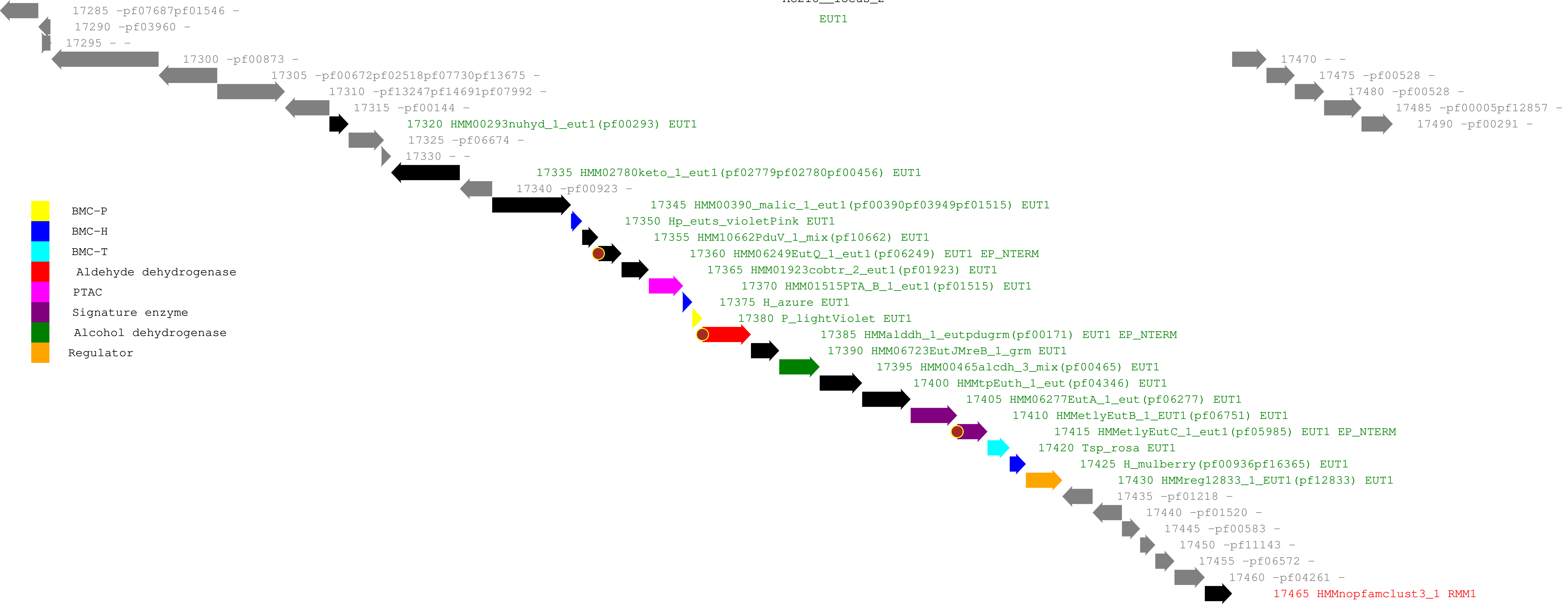

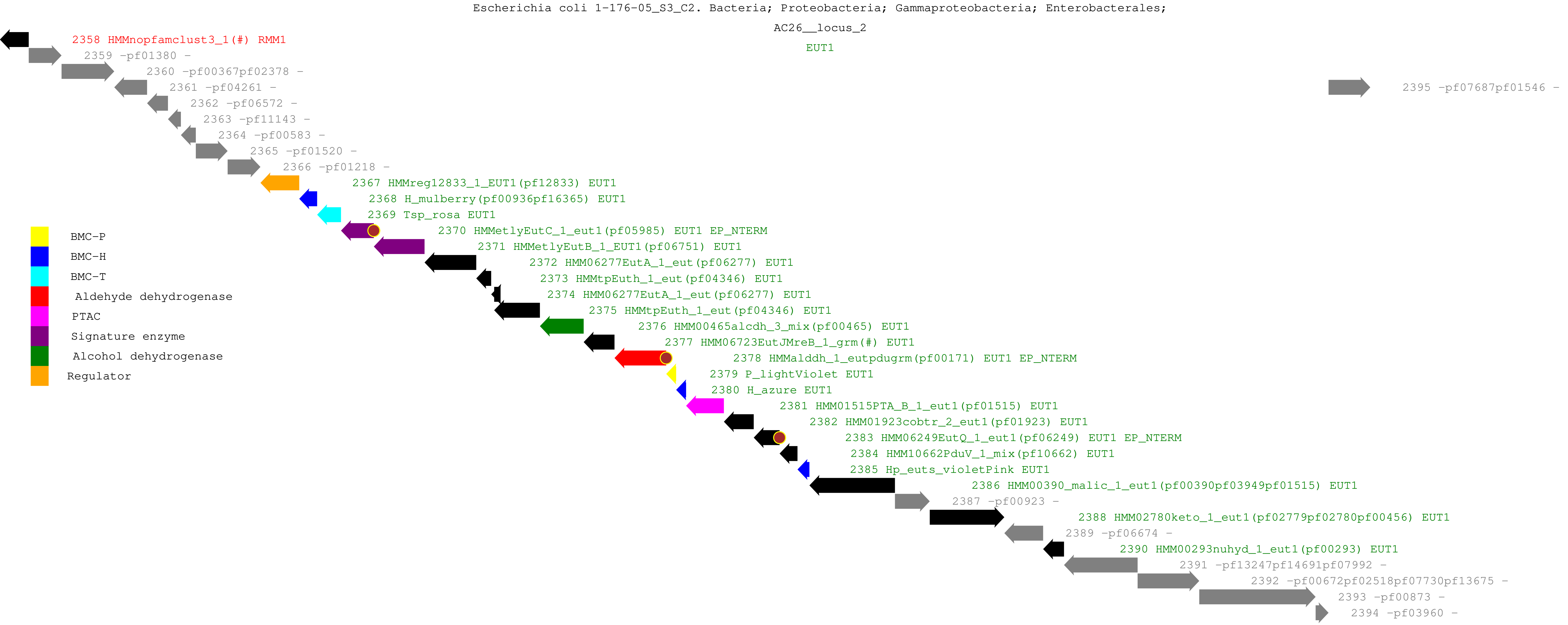
Escherichia coli 1-176-05_S3_C2. Bacteria; Proteobacteria; Gammaproteobacteria; Enterobacterales;
AC26__locus_2
EUT1
2358 HMMnopfamclust3_1(#) RMM1
2359 -pf01380 -
2360 -pf00367pf02378 -
2361 -pf04261 -
2362 -pf06572 -
2363 -pf11143 -
2364 -pf00583 -
2365 -pf01520 -
2366 -pf01218 -
2367 HMMreg12833_1_EUT1(pf12833) EUT1
2368 H_mulberry(pf00936pf16365) EUT1
2369 Tsp_rosa EUT1
2370 HMMetlyEutC_1_eut1(pf05985) EUT1 EP_NTERM
2371 HMMetlyEutB_1_EUT1(pf06751) EUT1
2372 HMM06277EutA_1_eut(pf06277) EUT1
2373 HMMtpEuth_1_eut(pf04346) EUT1
2374 HMM06277EutA_1_eut(pf06277) EUT1
2375 HMMtpEuth_1_eut(pf04346) EUT1
2376 HMM00465alcdh_3_mix(pf00465) EUT1
2377 HMM06723EutJMreB_1_grm(#) EUT1
2378 HMMalddh_1_eutpdugrm(pf00171) EUT1 EP_NTERM
2379 P_lightViolet EUT1
2380 H_azure EUT1
2381 HMM01515PTA_B_1_eut1(pf01515) EUT1
2382 HMM01923cobtr_2_eut1(pf01923) EUT1
2383 HMM06249EutQ_1_eut1(pf06249) EUT1 EP_NTERM
2384 HMM10662PduV_1_mix(pf10662) EUT1
2385 Hp_euts_violetPink EUT1
2386 HMM00390_malic_1_eut1(pf00390pf03949pf01515) EUT1
2387 -pf00923 -
2388 HMM02780keto_1_eut1(pf02779pf02780pf00456) EUT1
2389 -pf06674 -
2390 HMM00293nuhyd_1_eut1(pf00293) EUT1
2391 -pf13247pf14691pf07992 -
2392 -pf00672pf02518pf07730pf13675 -
2393 -pf00873 -
2394 -pf03960 -
2395 -pf07687pf01546 -
BMC-P
BMC-H
BMC-T
Aldehyde dehydrogenase
PTAC
Signature enzyme
Alcohol dehydrogenase
Regulator

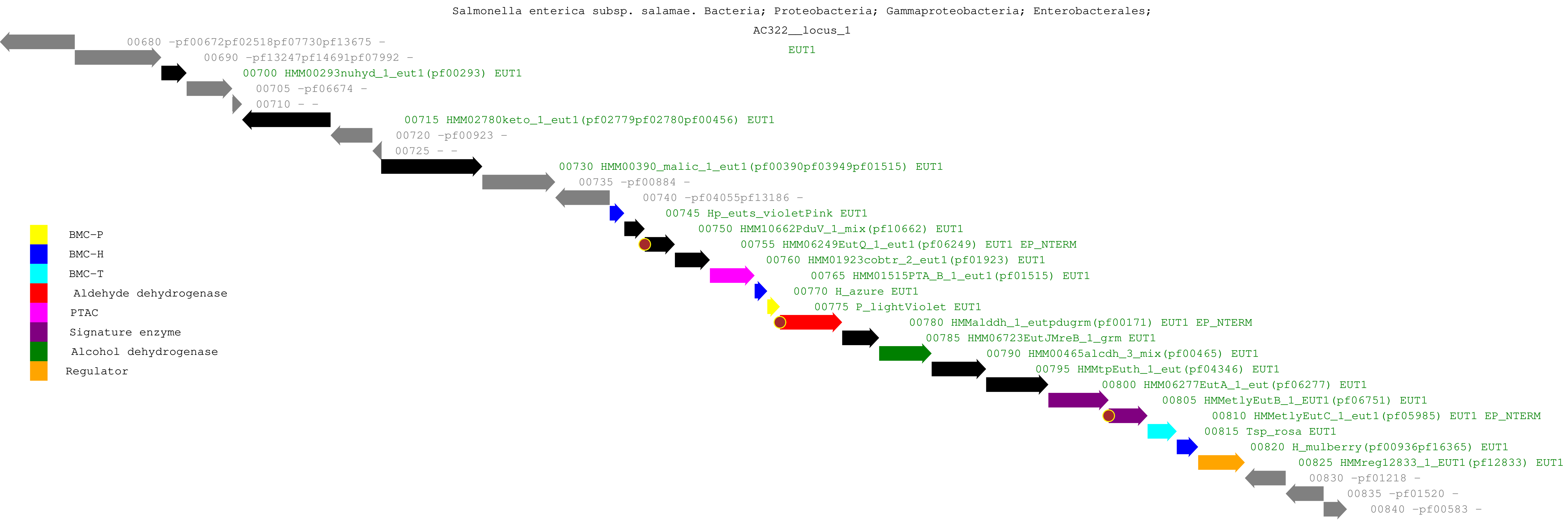
Salmonella enterica subsp. salamae. Bacteria; Proteobacteria; Gammaproteobacteria; Enterobacterales;
AC322__locus_1
EUT1
00680 -pf00672pf02518pf07730pf13675 -
00690 -pf13247pf14691pf07992 -
00700 HMM00293nuhyd_1_eut1(pf00293) EUT1
00705 -pf06674 -
00710 - -
00715 HMM02780keto_1_eut1(pf02779pf02780pf00456) EUT1
00720 -pf00923 -
00725 - -
00730 HMM00390_malic_1_eut1(pf00390pf03949pf01515) EUT1
00735 -pf00884 -
00740 -pf04055pf13186 -
00745 Hp_euts_violetPink EUT1
00750 HMM10662PduV_1_mix(pf10662) EUT1
00755 HMM06249EutQ_1_eut1(pf06249) EUT1 EP_NTERM
00760 HMM01923cobtr_2_eut1(pf01923) EUT1
00765 HMM01515PTA_B_1_eut1(pf01515) EUT1
00770 H_azure EUT1
00775 P_lightViolet EUT1
00780 HMMaldh_1_eutpdugrm(pf00171) EUT1 EP_NTERM
00785 HMM06723EutJMreB_1_grm EUT1
00790 HMM00465alcdh_3_mix(pf00465) EUT1
00795 HMMtpEuth_1_eut(pf04346) EUT1
00800 HMM06277EutA_1_eut(pf06277) EUT1
00805 HMMetlyEutB_1_EUT1(pf06751) EUT1
00810 HMMetlyEutC_1_eut1(pf05985) EUT1 EP_NTERM
00815 Tsp_rosa EUT1
00820 H_mulberry(pf00936pf16365) EUT1
00825 HMMreg12833_1_EUT1(pf12833) EUT1
00830 -pf01218 -
00835 -pf01520 -
00840 -pf00583 -
BMC-P
BMC-H
BMC-T
Aldehyde dehydrogenase
PTAC
Signature enzyme
Alcohol dehydrogenase
Regulator

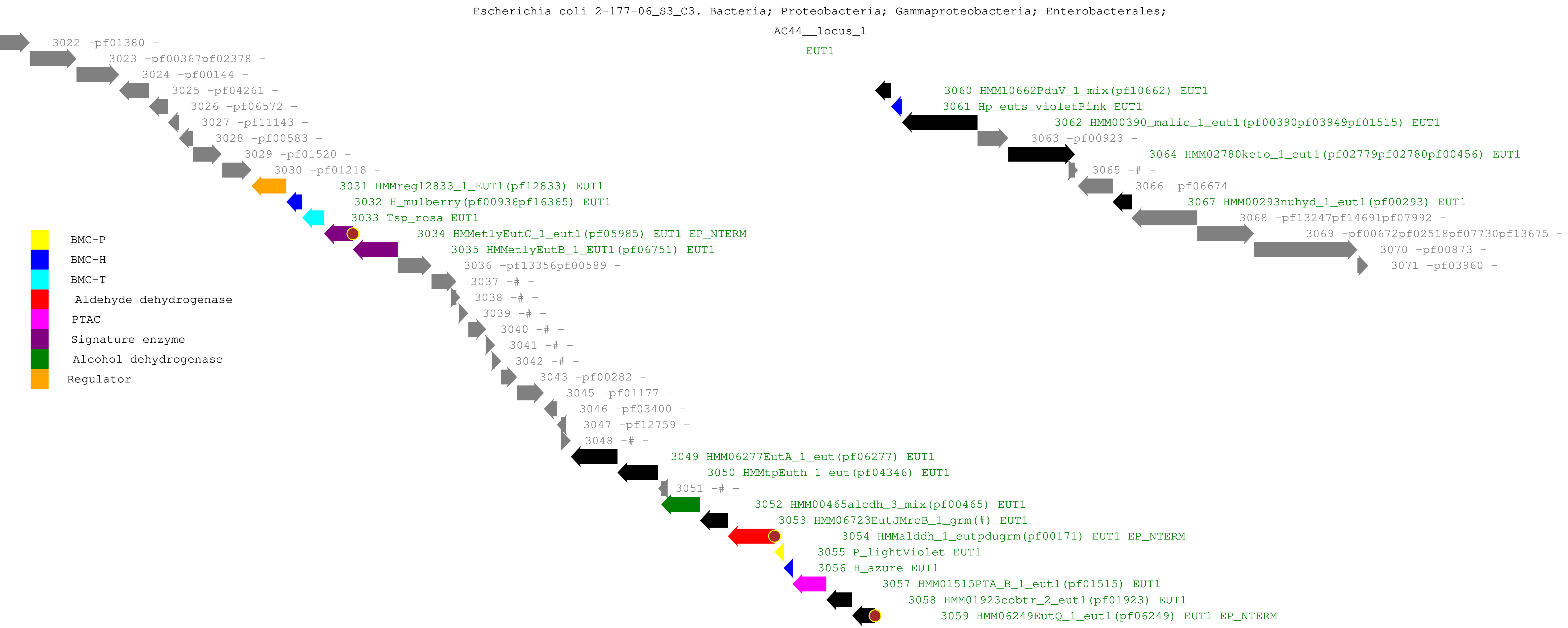
Escherichia coli 2-177-06_S3_C3. Bacteria; Proteobacteria; Gammaproteobacteria; Enterobacterales;
AC44__locus_1
EUT1
3022 -pf01380 -
3023 -pf00367pf02378 -
3024 -pf00144 -
3025 -pf04261 -
3026 -pf06572 -
3027 -pf11143 -
3028 -pf00583 -
3029 -pf01520 -
3030 -pf01218 -
3031 HMMreg12833_1_EUT1(pf12833) EUT1
3032 H_mulberry(pf00936pf16365) EUT1
3033 Tsp_rosa EUT1
3034 HMMetlyEutC_1_eut1(pf05985) EUT1 EP_NTERM
3035 HMMetlyEutB_1_EUT1(pf06751) EUT1
3036 -pf13356pf00589 -
3037 -# -
3038 -# -
3039 -# -
3040 -# -
3041 -# -
3042 -# -
3043 -pf00282 -
3045 -pf01177 -
3046 -pf03400 -
3047 -pf12759 -
3048 -# -
3049 HMM06277EutA_1_eut(pf06277) EUT1
3050 HMMtpEuth_1_eut(pf04346) EUT1
3051 -# -
3052 HMM00465alcdh_3_mix(pf00465) EUT1
3053 HMM06723EutJMreB_1_grm(#) EUT1
3054 HMMalddh_1_eutpdugrm(pf00171) EUT1 EP_NTERM
3055 P_lightViolet EUT1
3056 H_azure EUT1
3057 HMM01515PTA_B_1_eut1(pf01515) EUT1
3058 HMM01923cobtr_2_eut1(pf01923) EUT1
3059 HMM06249EutQ_1_eut1(pf06249) EUT1 EP_NTERM
3060 HMM10662PduV_1_mix(pf10662) EUT1
3061 Hp_euts_violetPink EUT1
3062 HMM00390_malic_1_eut1(pf00390pf03949pf01515) EUT1
3063 -pf00923 -
3064 HMM02780keto_1_eut1(pf02779pf02780pf00456) EUT1
3065 -# -
3066 -pf06674 -
3067 HMM00293nuhyd_1_eut1(pf00293) EUT1
3068 -pf13247pf14691pf07992 -
3069 -pf00672pf02518pf07730pf13675 -
3070 -pf00873 -
3071 -pf03960 -
BMC-P
BMC-H
BMC-T
Aldehyde dehydrogenase
PTAC
Signature enzyme
Alcohol dehydrogenase
Regulator

Escherichia coli 2-210-07_S3_C3. Bacteria; Proteobacteria; Gammaproteobacteria; Enterobacterales;

AC45__locus_1

EUT1

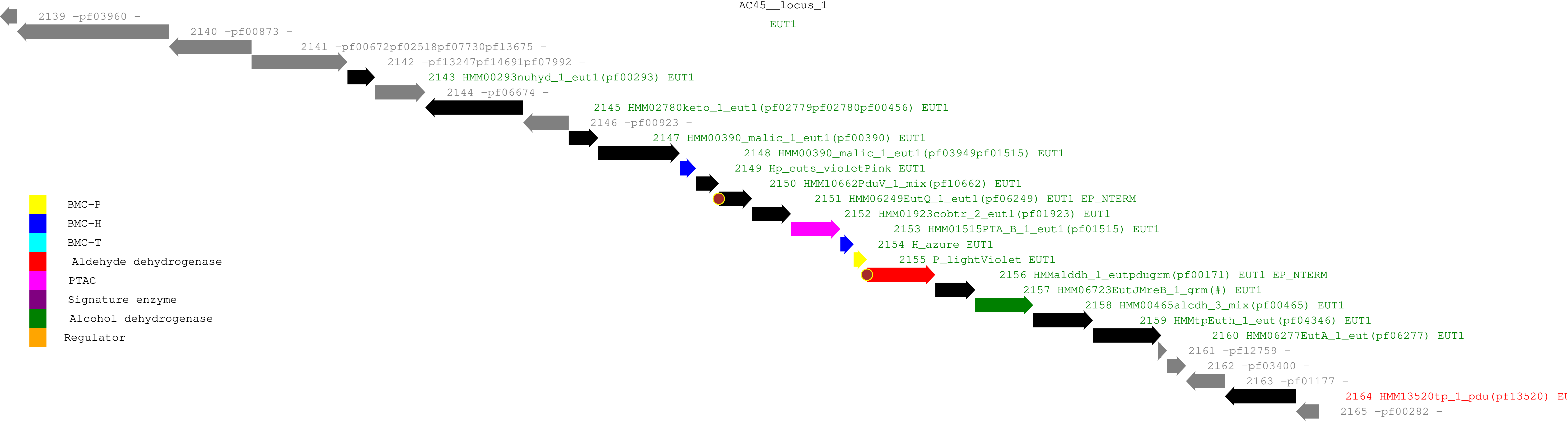

Escherichia coli 1-182-04_S3_C3. Bacteria; Proteobacteria; Gammaproteobacteria; Enterobacterales;

AC56__locus_1

EUT1

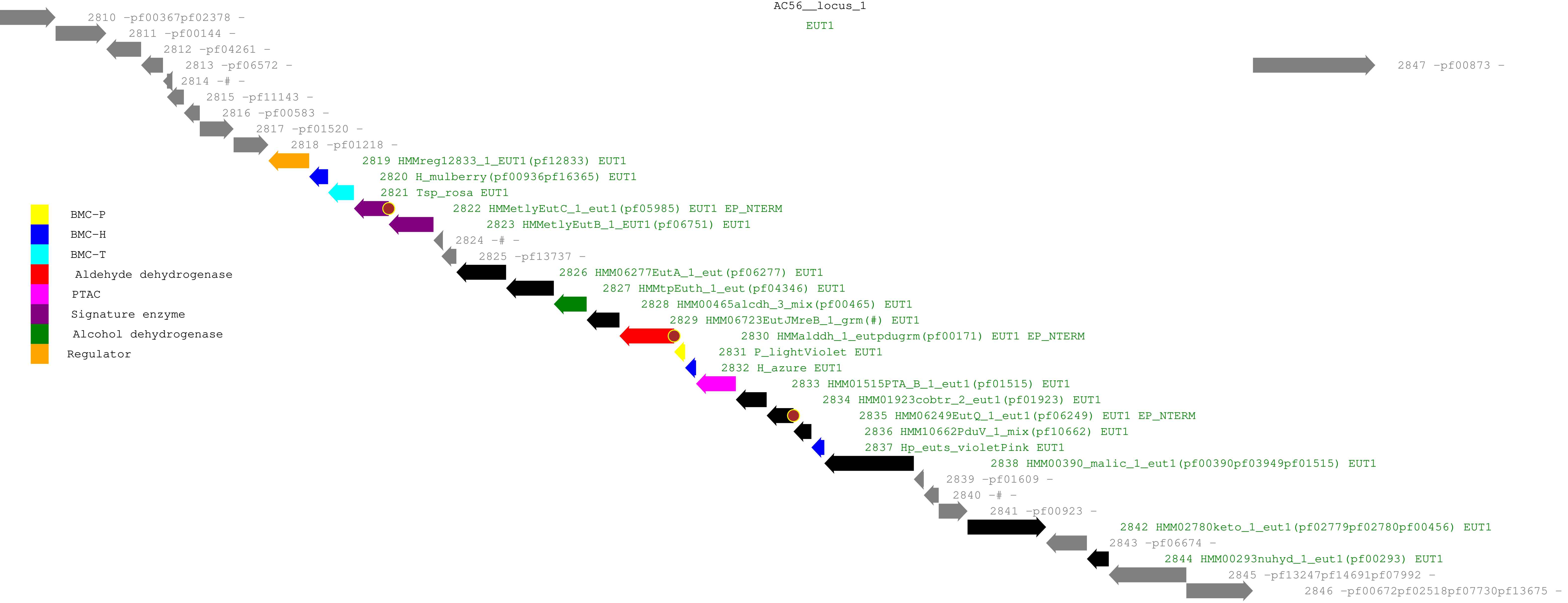

Escherichia coli 1-392-07_S4_C1. Bacteria; Proteobacteria; Gammaproteobacteria; Enterobacterales;

AC84__locus_1

EUT1

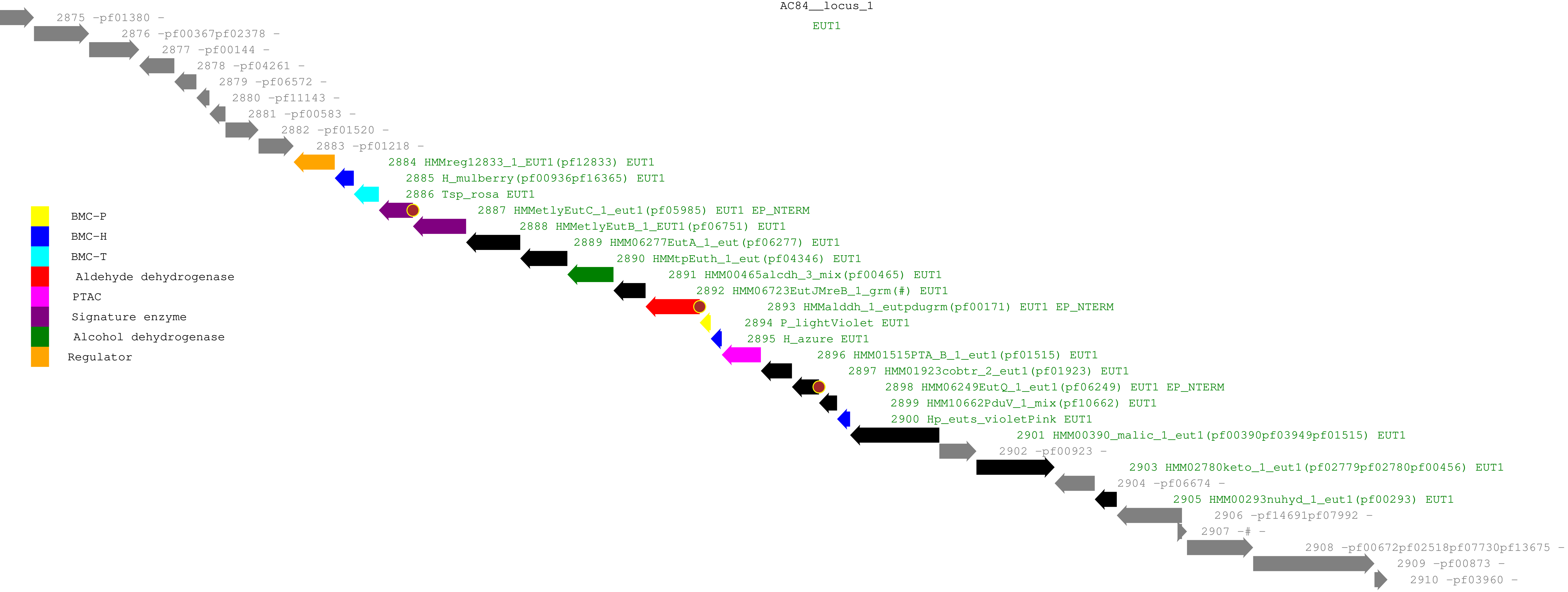

Salmonella senftenberg. Bacteria; Proteobacteria; Gammaproteobacteria; Enterobacterales;

AC863__locus_1

EUT1

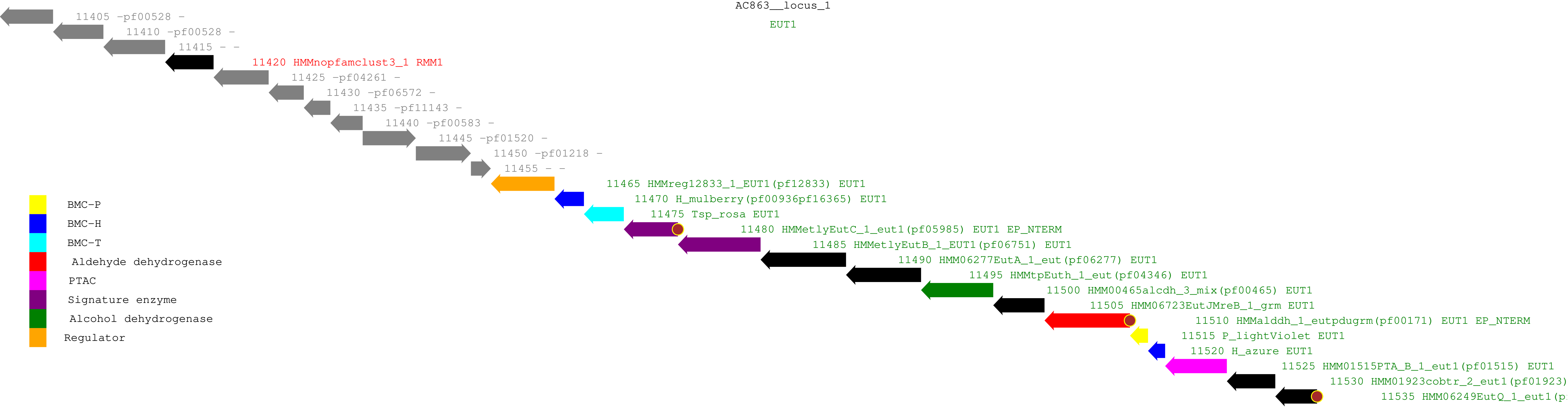

Escherichia coli 3-267-03_S4_C1. Bacteria; Proteobacteria; Gammaproteobacteria; Enterobacterales;

AC88__locus_1

EUT1

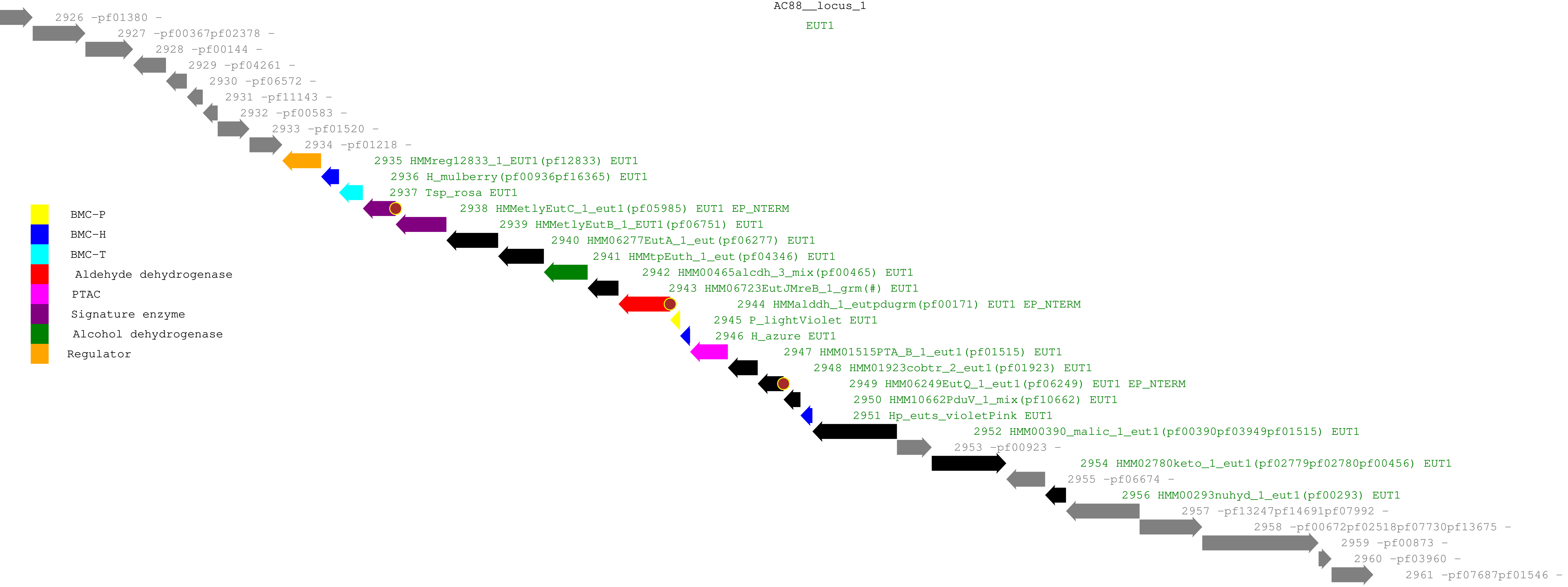

Escherichia coli 2-005-03_S4_C2. Bacteria; Proteobacteria; Gammaproteobacteria; Enterobacterales;

AC93__locus_1

EUT1

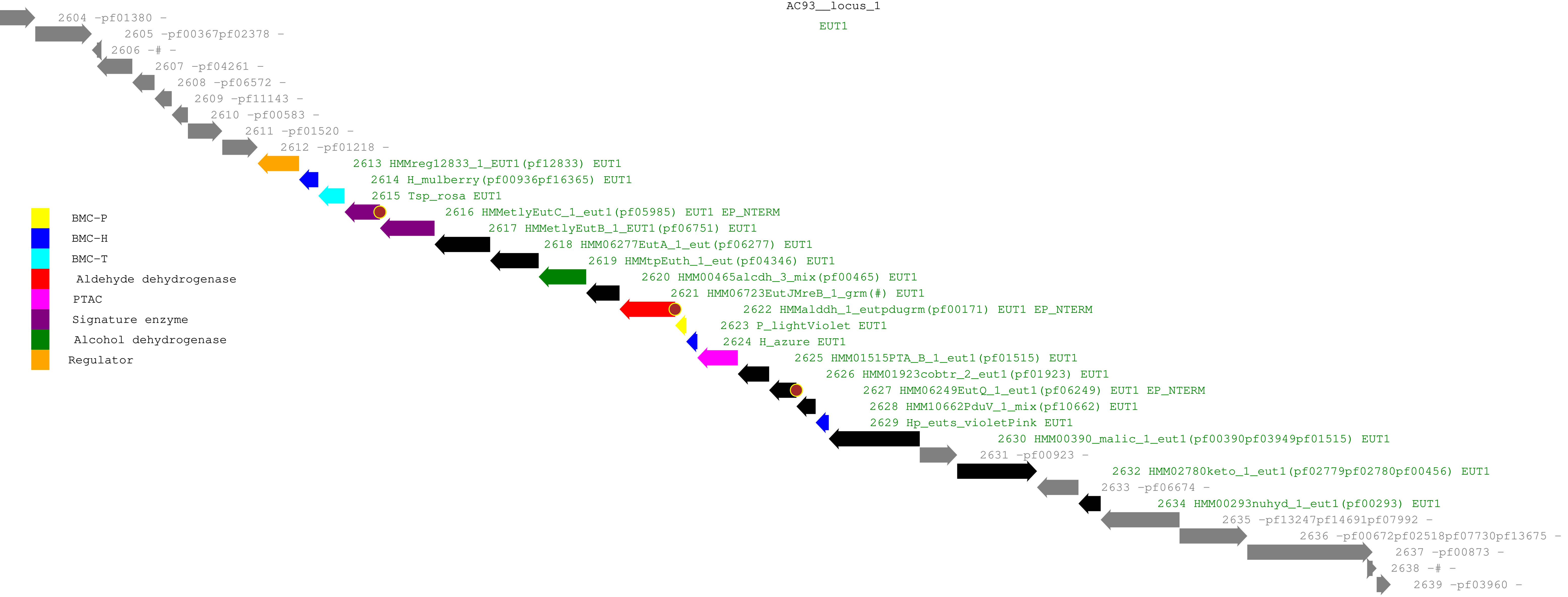

Escherichia coli. Bacteria; Proteobacteria; Gammaproteobacteria; Enterobacterales;

ACN002__locus_1

EUT1

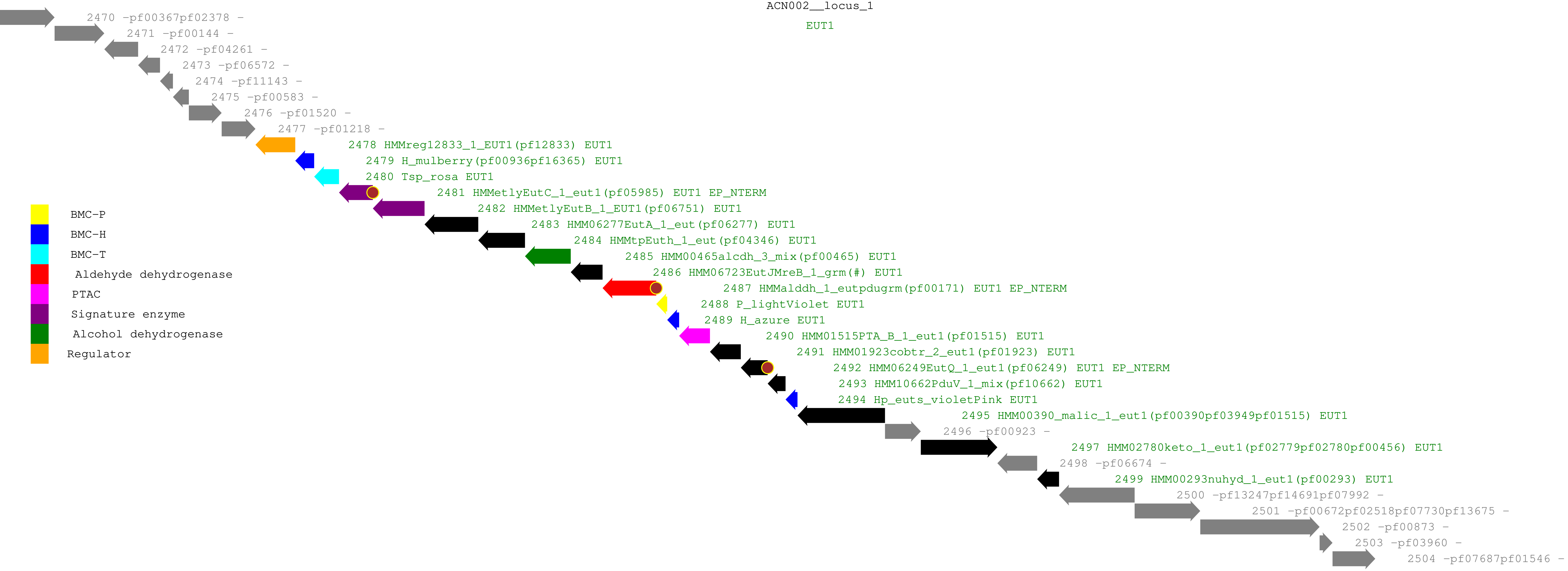

Escherichia coli. Bacteria; Proteobacteria; Gammaproteobacteria; Enterobacterales;

ACU57__locus_1

EUT1

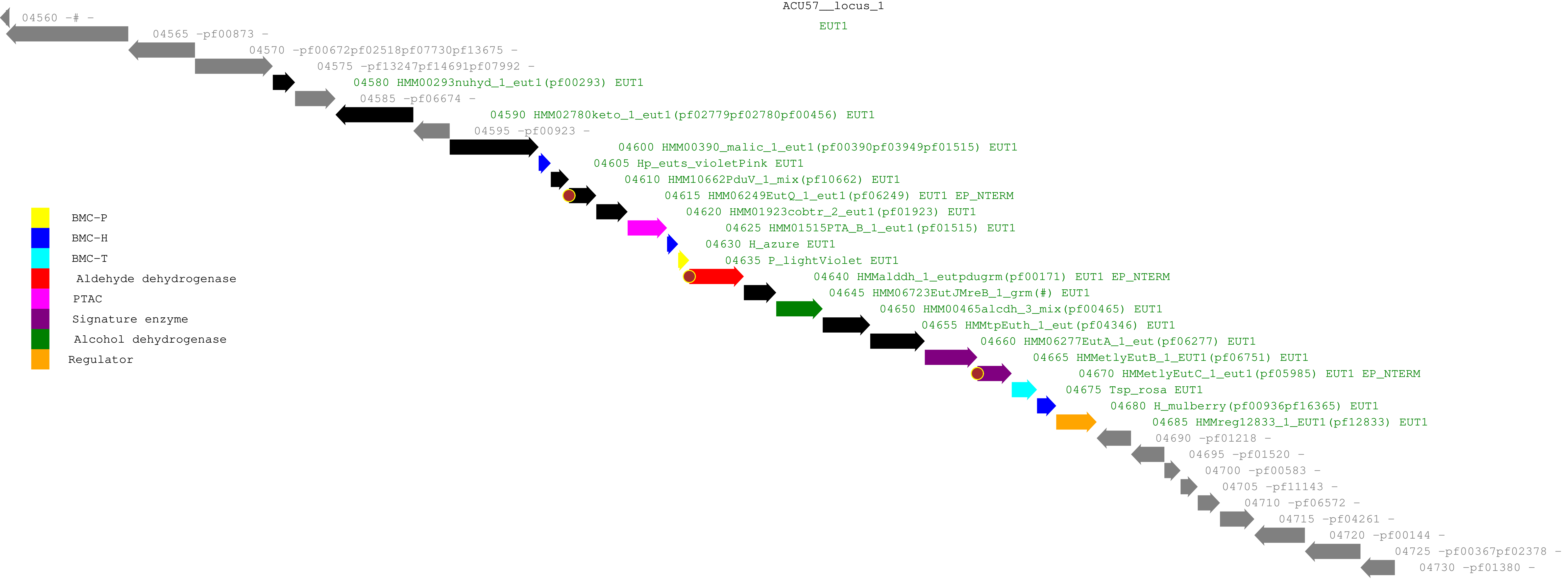

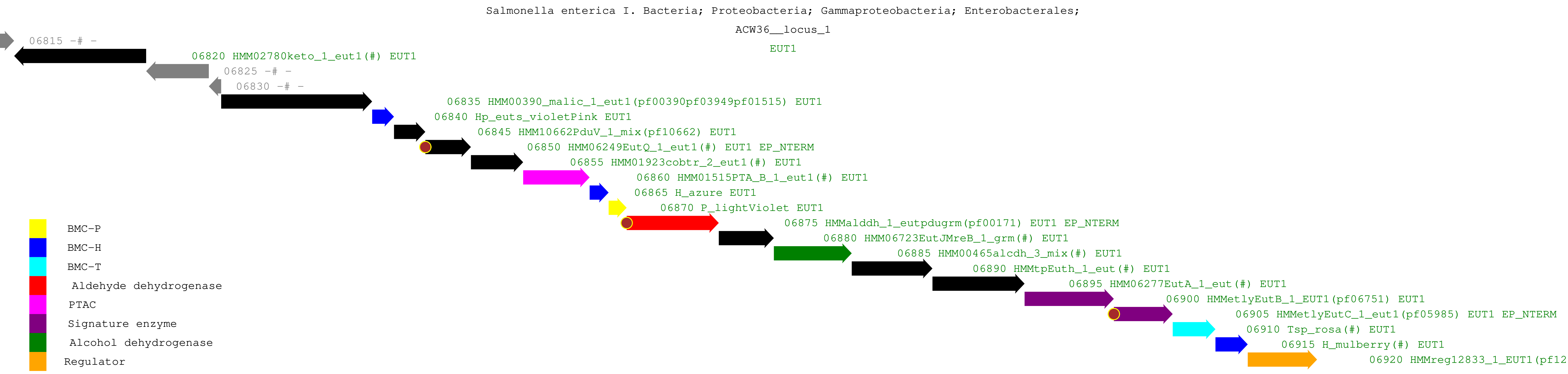
Salmonella enterica I. Bacteria; Proteobacteria; Gammaproteobacteria; Enterobacterales;
ACW36__locus_1
EUT1
06815 -# -
06820 HMM02780keto_1_eut1(#) EUT1
06825 -# -
06830 -# -
06835 HMM00390_malic_1_eut1(pf00390pf03949pf01515) EUT1
06840 Hp_euts_violetPink EUT1
06845 HMM10662PduV_1_mix(pf10662) EUT1
06850 HMM06249EutQ_1_eut1(#) EUT1 EP_NTERM
06855 HMM01923cobtr_2_eut1(#) EUT1
06860 HMM01515PTA_B_1_eut1(#) EUT1
06865 H_azure EUT1
06870 P_lightViolet EUT1
06875 HMMalddh_1_eutpdugrm(pf00171) EUT1 EP_NTERM
06880 HMM06723EutJMreB_1_grm(#) EUT1
06885 HMM00465alcdh_3_mix(#) EUT1
06890 HMMtpEuth_1_eut(#) EUT1
06895 HMM06277EutA_1_eut(#) EUT1
06900 HMMetlyEutB_1_EUT1(pf06751) EUT1
06905 HMMetlyEutC_1_eut1(pf05985) EUT1 EP_NTERM
06910 Tsp_rosa(#) EUT1
06915 H_mulberry(#) EUT1
06920 HMMreg12833_1_EUT1(pf12
BMC-P
BMC-H
BMC-T
Aldehyde dehydrogenase
PTAC
Signature enzyme
Alcohol dehydrogenase
Regulator

Salmonella enterica I. Bacteria; Proteobacteria; Gammaproteobacteria; Enterobacterales;

ACW51__locus_1

EUT1

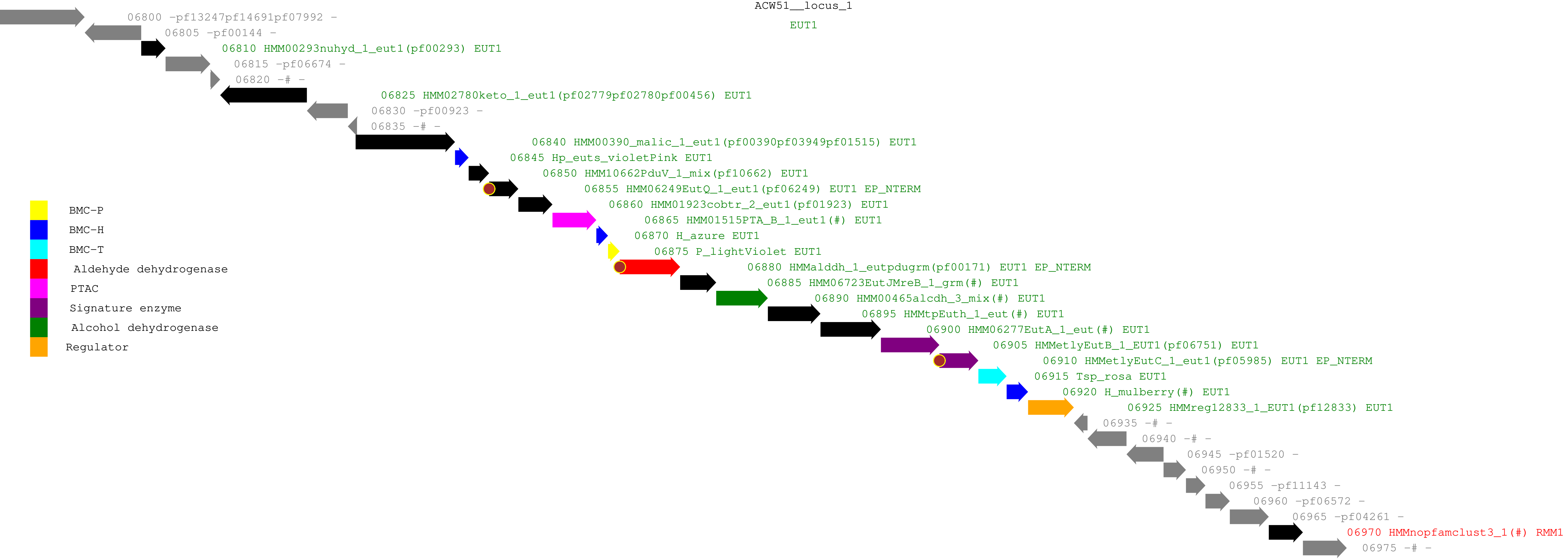

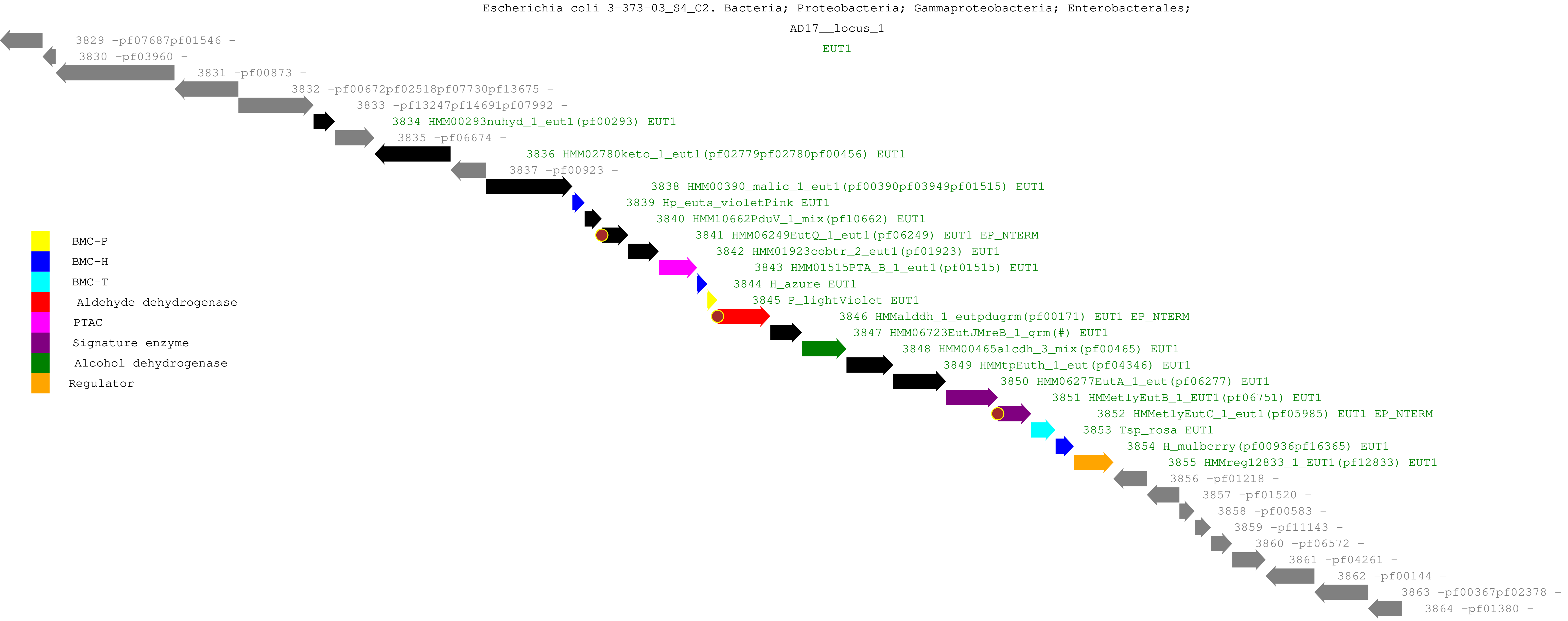
Escherichia coli 3-373-03_S4_C2. Bacteria; Proteobacteria; Gammaproteobacteria; Enterobacterales;
AD17__locus_1
EUT1
3829 -pf07687pf01546 -
3830 -pf03960 -
3831 -pf00873 -
3832 -pf00672pf02518pf07730pf13675 -
3833 -pf13247pf14691pf07992 -
3834 HMM00293nuhyd_1_eut1(pf00293) EUT1
3835 -pf06674 -
3836 HMM02780keto_1_eut1(pf02779pf02780pf00456) EUT1
3837 -pf00923 -
3838 HMM00390_malic_1_eut1(pf00390pf03949pf01515) EUT1
3839 Hp_euts_violetPink EUT1
3840 HMM10662PduV_1_mix(pf10662) EUT1
3841 HMM06249EutQ_1_eut1(pf06249) EUT1 EP_NTERM
3842 HMM01923cobtr_2_eut1(pf01923) EUT1
3843 HMM01515PTA_B_1_eut1(pf01515) EUT1
3844 H_azure EUT1
3845 P_lightViolet EUT1
3846 HMMalddh_1_eutpdugrm(pf00171) EUT1 EP_NTERM
3847 HMM06723EutJMreB_1_grm(#) EUT1
3848 HMM00465alcdh_3_mix(pf00465) EUT1
3849 HMMtpEuth_1_eut(pf04346) EUT1
3850 HMM06277EutA_1_eut(pf06277) EUT1
3851 HMMetlyEutB_1_EUT1(pf06751) EUT1
3852 HMMetlyEutC_1_eut1(pf05985) EUT1 EP_NTERM
3853 Tsp_rosa EUT1
3854 H_mulberry(pf00936pf16365) EUT1
3855 HMMreg12833_1_EUT1(pf12833) EUT1
3856 -pf01218 -
3857 -pf01520 -
3858 -pf00583 -
3859 -pf11143 -
3860 -pf06572 -
3861 -pf04261 -
3862 -pf00144 -
3863 -pf00367pf02378 -
3864 -pf01380 -
BMC-P
BMC-H
BMC-T
Aldehyde dehydrogenase
PTAC
Signature enzyme
Alcohol dehydrogenase
Regulator

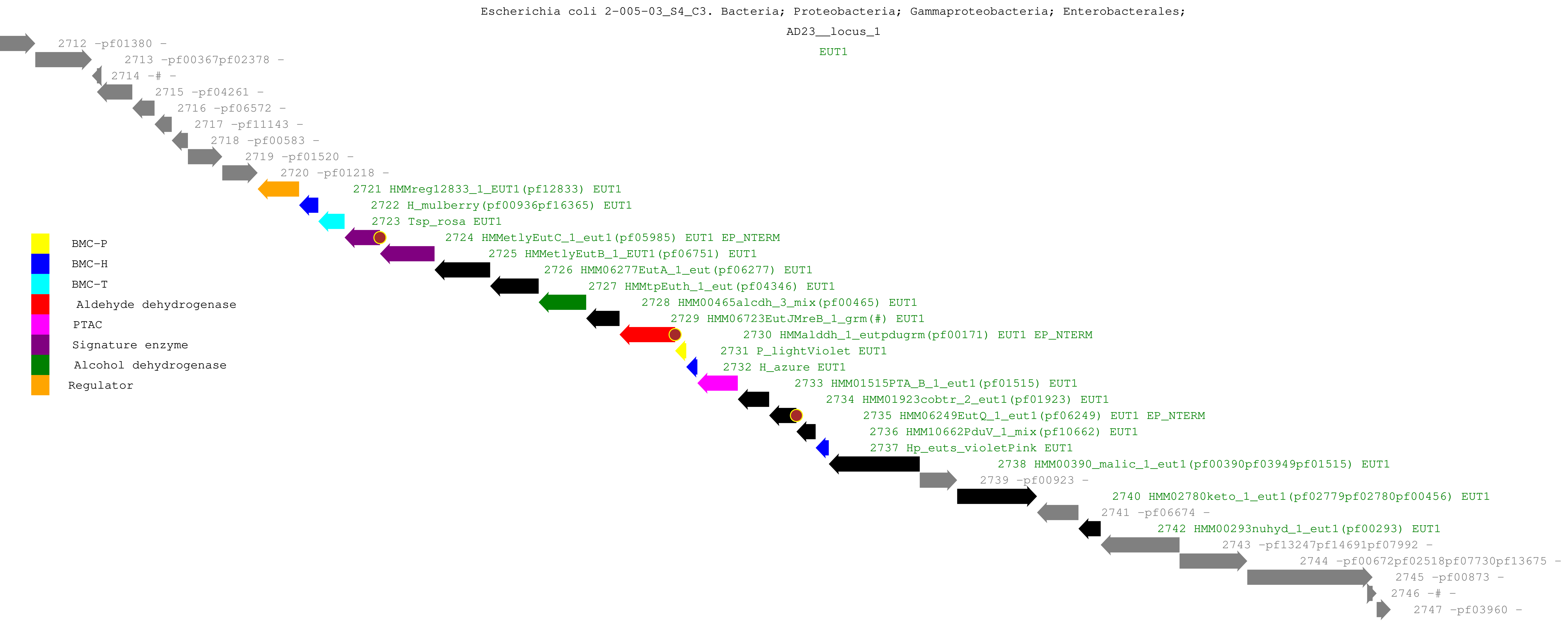

Escherichia coli 2-005-03_S4_C3. Bacteria; Proteobacteria; Gammaproteobacteria; Enterobacterales;
AD23__locus_1
EUT1
2712 -pf01380 -
2713 -pf00367pf02378 -
2714 -# -
2715 -pf04261 -
2716 -pf06572 -
2717 -pf11143 -
2718 -pf00583 -
2719 -pf01520 -
2720 -pf01218 -
2721 HMMreg12833_1_EUT1(pf12833) EUT1
2722 H_mulberry(pf00936pf16365) EUT1
2723 Tsp_rosa EUT1
2724 HMMetlyEutC_1_eut1(pf05985) EUT1 EP_NTERM
2725 HMMetlyEutB_1_EUT1(pf06751) EUT1
2726 HMM06277EutA_1_eut(pf06277) EUT1
2727 HMMtpEuth_1_eut(pf04346) EUT1
2728 HMM00465alcdh_3_mix(pf00465) EUT1
2729 HMM06723EutJMreB_1_grm(#) EUT1
2730 HMMalddh_1_eutpdugrm(pf00171) EUT1 EP_NTERM
2731 P_lightViolet EUT1
2732 H_azure EUT1
2733 HMM01515PTA_B_1_eut1(pf01515) EUT1
2734 HMM01923cobtr_2_eut1(pf01923) EUT1
2735 HMM06249EutQ_1_eut1(pf06249) EUT1 EP_NTERM
2736 HMM10662PduV_1_mix(pf10662) EUT1
2737 Hp_euts_violetPink EUT1
2738 HMM00390_malic_1_eut1(pf00390pf03949pf01515) EUT1
2739 -pf00923 -
2740 HMM02780keto_1_eut1(pf02779pf02780pf00456) EUT1
2741 -pf06674 -
2742 HMM00293nuhyd_1_eut1(pf00293) EUT1
2743 -pf13247pf14691pf07992 -
2744 -pf00672pf02518pf07730pf13675 -
2745 -pf00873 -
2746 -# -
2747 -pf03960 -
BMC-P
BMC-H
BMC-T
Aldehyde dehydrogenase
PTAC
Signature enzyme
Alcohol dehydrogenase
Regulator

Escherichia coli 2-427-07_S4_C3. Bacteria; Proteobacteria; Gammaproteobacteria; Enterobacterales;

AD31__locus_1

EUT1

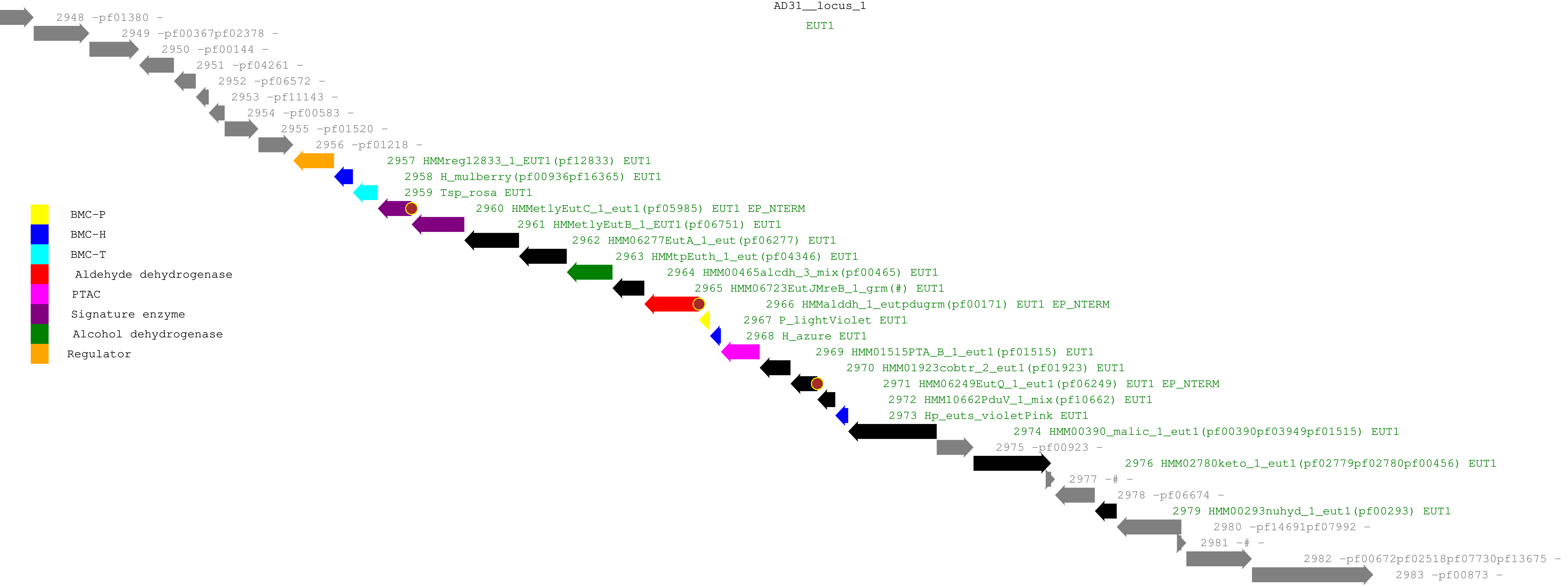

Escherichia coli 1-392-07_S4_C3. Bacteria; Proteobacteria; Gammaproteobacteria; Enterobacterales;

AD40__locus_1

EUT1

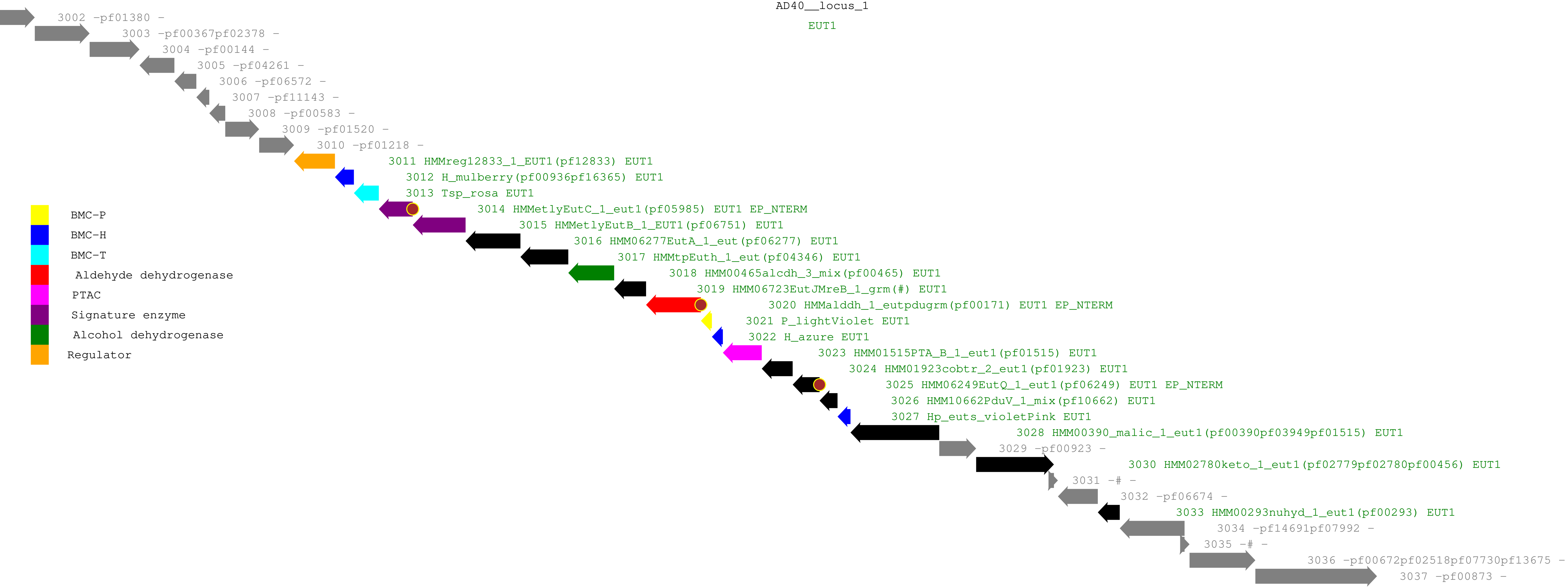

Salmonella enterica I. Bacteria; Proteobacteria; Gammaproteobacteria; Enterobacterales;

ADQ53__locus_1

EUT1

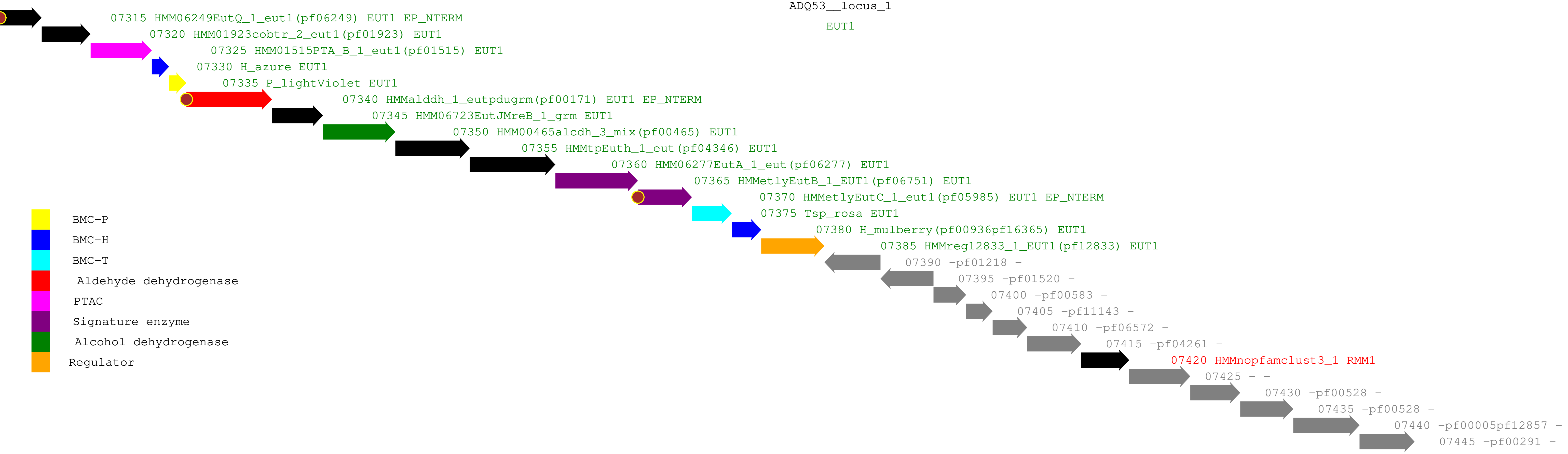

Salmonella enterica (Salmonella choleraesuis). Bacteria; Proteobacteria; Gammaproteobacteria; Enterobacterales;

ADQ66__locus_2

EUT1

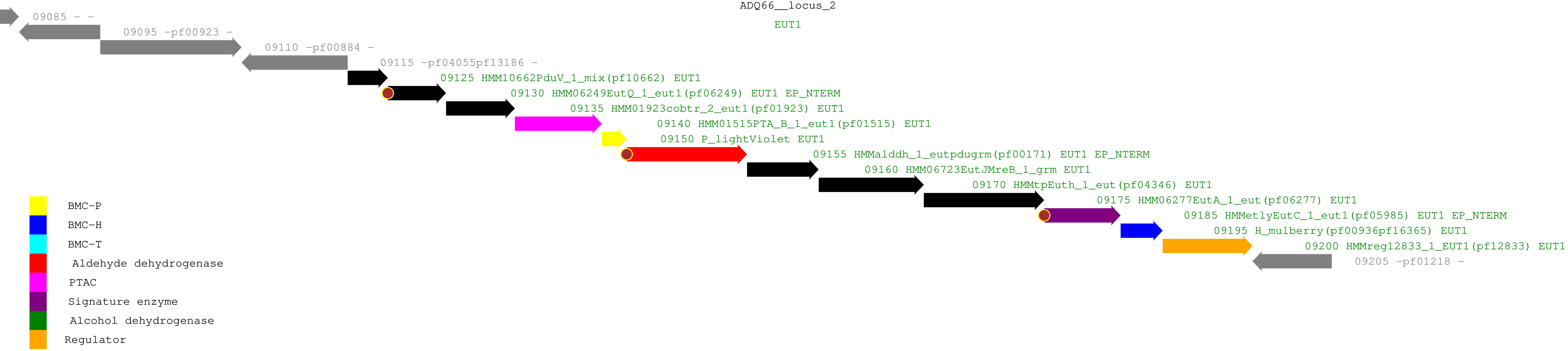

Escherichia coli UCI 65. Bacteria; Proteobacteria; Gammaproteobacteria; Enterobacterales;

AE24__locus_1

EUT1

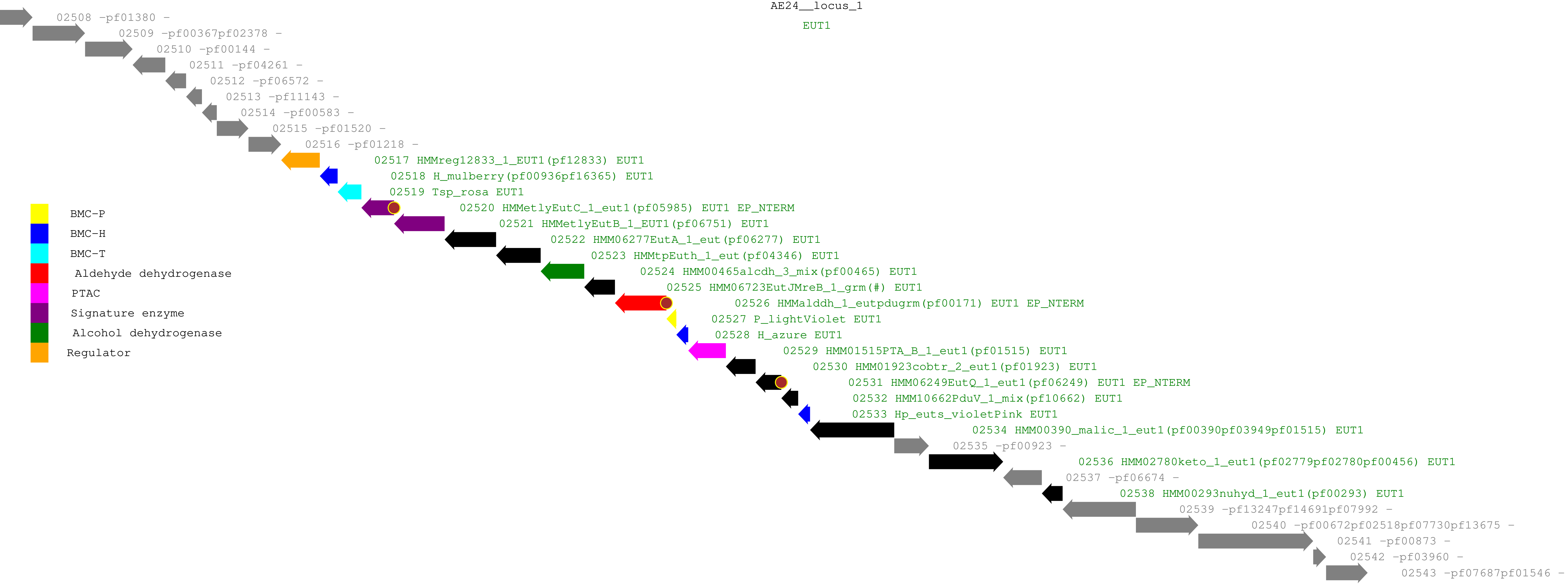

Salmonella enterica subsp. enterica serovar Tennessee. Bacteria; Proteobacteria; Gammaproteobacteria; Enterobacterales;

AE390__locus_1

EUT1

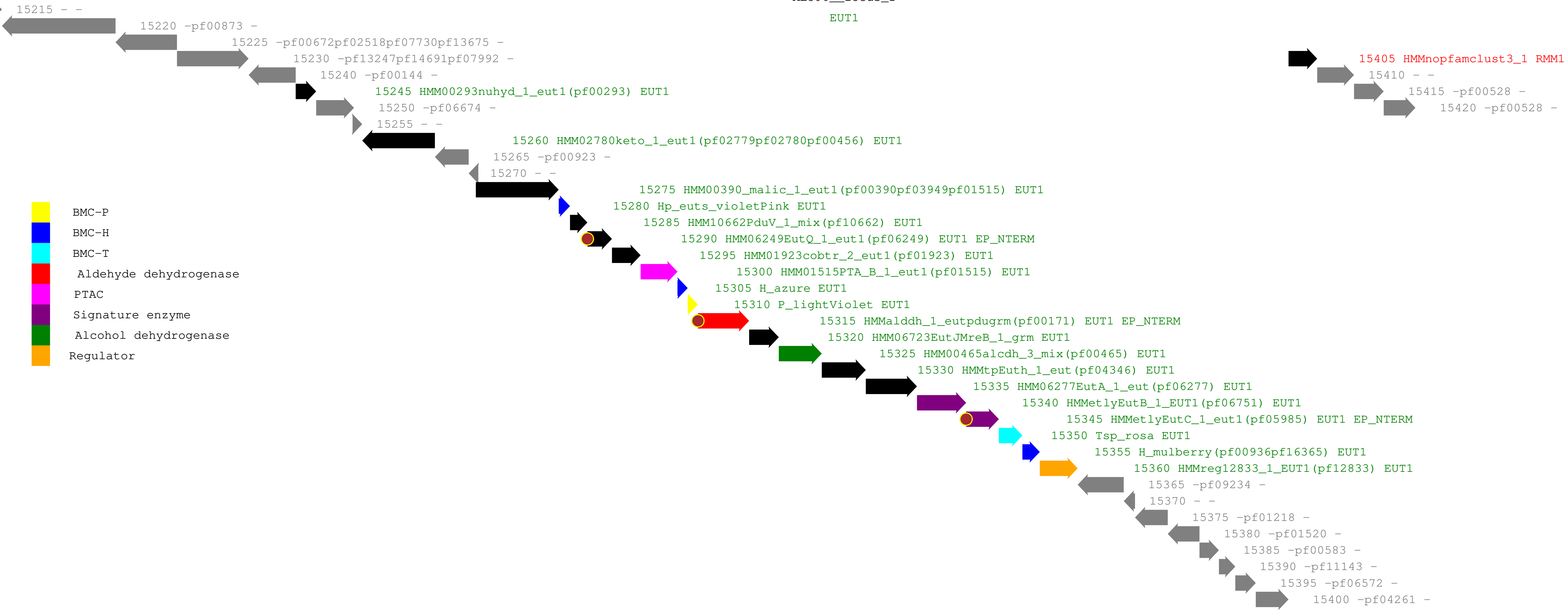

Salmonella enterica subsp. enterica serovar Heidelberg. Bacteria; Proteobacteria; Gammaproteobacteria; Enterobacterales;

AEV26__locus_1

EUT1

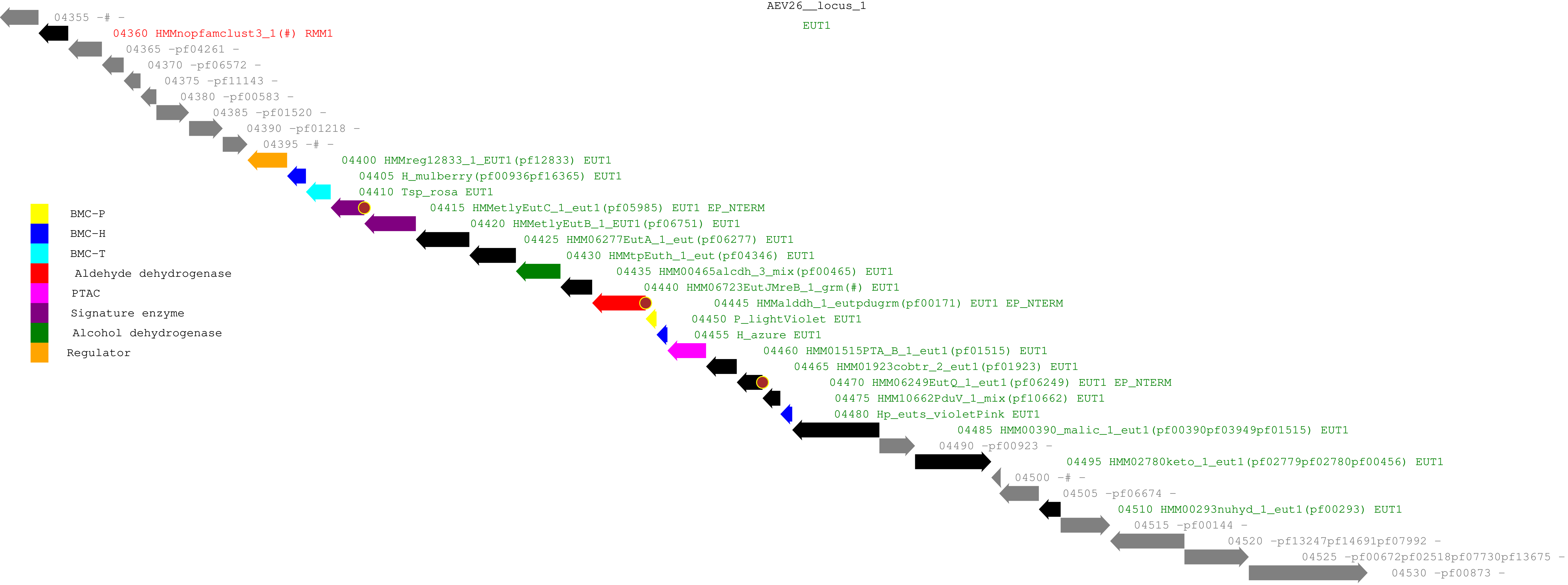

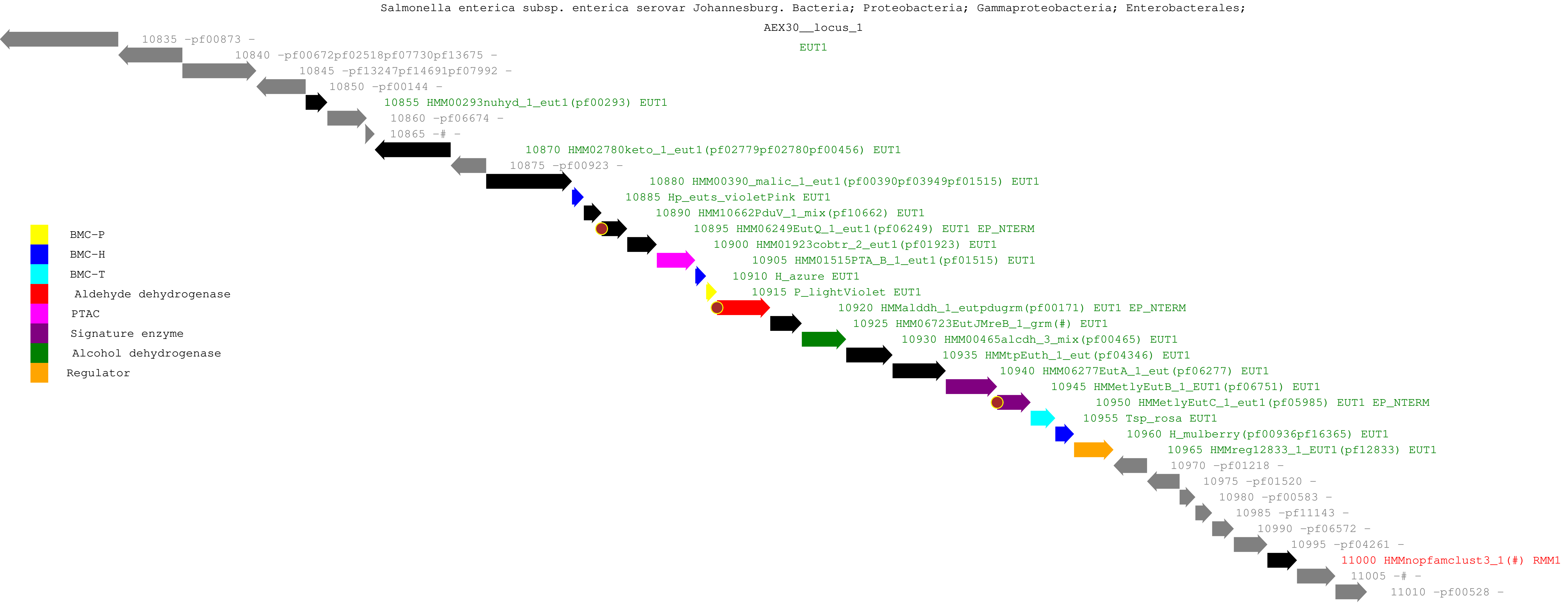
Salmonella enterica subsp. enterica serovar Johannesburg. Bacteria; Proteobacteria; Gammaproteobacteria; Enterobacterales;
AEX30__locus_1
EUT1
10835 -pf00873 -
10840 -pf00672pf02518pf07730pf13675 -
10845 -pf13247pf14691pf07992 -
10850 -pf00144 -
10855 HMM00293nuhyd_1_eut1(pf00293) EUT1
10860 -pf06674 -
10865 -# -
10870 HMM02780keto_1_eut1(pf02779pf02780pf00456) EUT1
10875 -pf00923 -
10880 HMM00390_malic_1_eut1(pf00390pf03949pf01515) EUT1
10885 Hp_euts_violetPink EUT1
10890 HMM10662PduV_1_mix(pf10662) EUT1
10895 HMM06249EutQ_1_eut1(pf06249) EUT1 EP_NTERM
10900 HMM01923cobtr_2_eut1(pf01923) EUT1
10905 HMM01515PTA_B_1_eut1(pf01515) EUT1
10910 H_azure EUT1
10915 P_lightViolet EUT1
10920 HMMalddh_1_eutpdugrm(pf00171) EUT1 EP_NTERM
10925 HMM06723EutJMreB_1_grm(#) EUT1
10930 HMM00465alcdh_3_mix(pf00465) EUT1
10935 HMMtpEuth_1_eut(pf04346) EUT1
10940 HMM06277EutA_1_eut(pf06277) EUT1
10945 HMMetlyEutB_1_EUT1(pf06751) EUT1
10950 HMMetlyEutC_1_eut1(pf05985) EUT1 EP_NTERM
10955 Tsp_rosa EUT1
10960 H_mulberry(pf00936pf16365) EUT1
10965 HMMreg12833_1_EUT1(pf12833) EUT1
10970 -pf01218 -
10975 -pf01520 -
10980 -pf00583 -
10985 -pf11143 -
10990 -pf06572 -
10995 -pf04261 -
11000 HMMnopfamclust3_1(#) RMM1
11005 -# -
11010 -pf00528 -
BMC-P
BMC-H
BMC-T
Aldehyde dehydrogenase
PTAC
Signature enzyme
Alcohol dehydrogenase
Regulator

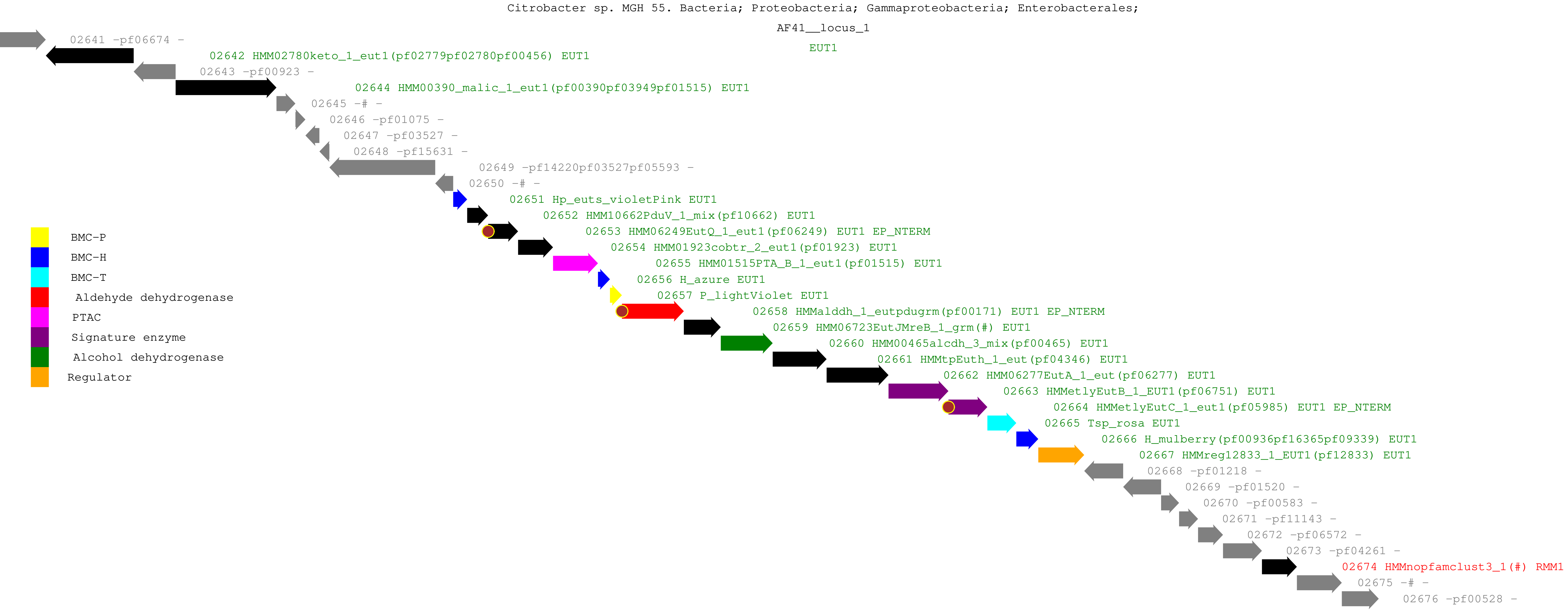
Citrobacter sp. MGH 55. Bacteria; Proteobacteria; Gammaproteobacteria; Enterobacterales;
AF41__locus_1
EUT1
02641 -pf06674 -
02642 HMM02780keto_1_eut1(pf02779pf02780pf00456) EUT1
02643 -pf00923 -
02644 HMM00390_malic_1_eut1(pf00390pf03949pf01515) EUT1
02645 -# -
02646 -pf01075 -
02647 -pf03527 -
02648 -pf15631 -
02649 -pf14220pf03527pf05593 -
02650 -# -
02651 Hp_euts_violetPink EUT1
02652 HMM10662PduV_1_mix(pf10662) EUT1
02653 HMM06249EutQ_1_eut1(pf06249) EUT1 EP_NTERM
02654 HMM01923cobtr_2_eut1(pf01923) EUT1
02655 HMM01515PTA_B_1_eut1(pf01515) EUT1
02656 H_azure EUT1
02657 P_lightViolet EUT1
02658 HMMalddh_1_eutpdugrm(pf00171) EUT1 EP_NTERM
02659 HMM06723EutJMreB_1_grm(#) EUT1
02660 HMM00465alcdh_3_mix(pf00465) EUT1
02661 HMMtpEuth_1_eut(pf04346) EUT1
02662 HMM06277EutA_1_eut(pf06277) EUT1
02663 HMMetlyEutB_1_EUT1(pf06751) EUT1
02664 HMMetlyEutC_1_eut1(pf05985) EUT1 EP_NTERM
02665 Tsp_rosa EUT1
02666 H_mulberry(pf00936pf16365pf09339) EUT1
02667 HMMreg12833_1_EUT1(pf12833) EUT1
02668 -pf01218 -
02669 -pf01520 -
02670 -pf00583 -
02671 -pf11143 -
02672 -pf06572 -
02673 -pf04261 -
02674 HMMnopfamclust3_1(#) RMM1
02675 -# -
02676 -pf00528 -
BMC-P
BMC-H
BMC-T
Aldehyde dehydrogenase
PTAC
Signature enzyme
Alcohol dehydrogenase
Regulator

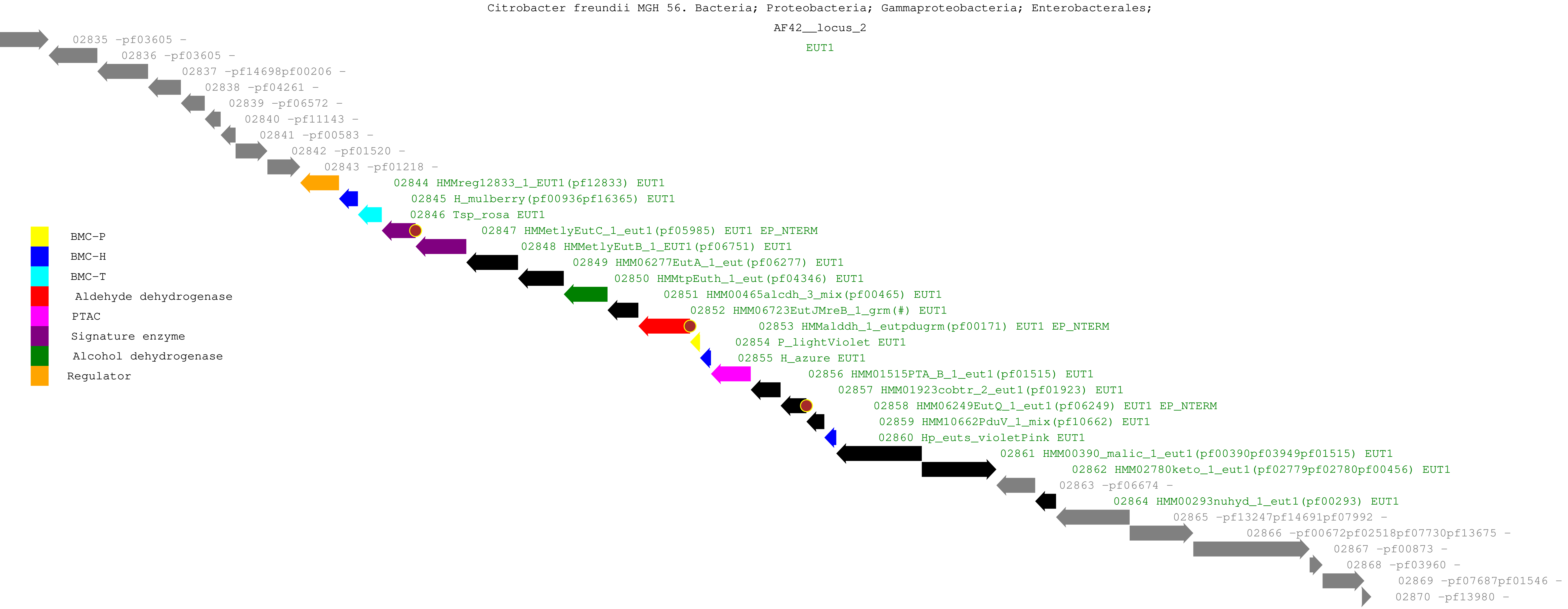

Citrobacter freundii MGH 56. Bacteria; Proteobacteria; Gammaproteobacteria; Enterobacterales;
AF42__locus_2
EUT1
02835 -pf03605 -
02836 -pf03605 -
02837 -pf14698pf00206 -
02838 -pf04261 -
02839 -pf06572 -
02840 -pf11143 -
02841 -pf00583 -
02842 -pf01520 -
02843 -pf01218 -
02844 HMMreg12833_1_EUT1(pf12833) EUT1
02845 H_mulberry(pf00936pf16365) EUT1
02846 Tsp_rosa EUT1
02847 HMMetlyEutC_1_eut1(pf05985) EUT1 EP_NTERM
02848 HMMetlyEutB_1_EUT1(pf06751) EUT1
02849 HMM06277EutA_1_eut(pf06277) EUT1
02850 HMMtpEuth_1_eut(pf04346) EUT1
02851 HMM00465alcdh_3_mix(pf00465) EUT1
02852 HMM06723EutJMreB_1_grm(#) EUT1
02853 HMMalddh_1_eutpdugrm(pf00171) EUT1 EP_NTERM
02854 P_lightViolet EUT1
02855 H_azure EUT1
02856 HMM01515PTA_B_1_eut1(pf01515) EUT1
02857 HMM01923cobtr_2_eut1(pf01923) EUT1
02858 HMM06249EutQ_1_eut1(pf06249) EUT1 EP_NTERM
02859 HMM10662PduV_1_mix(pf10662) EUT1
02860 Hp_euts_violetPink EUT1
02861 HMM00390_malic_1_eut1(pf00390pf03949pf01515) EUT1
02862 HMM02780keto_1_eut1(pf02779pf02780pf00456) EUT1
02863 -pf06674 -
02864 HMM00293nuhyd_1_eut1(pf00293) EUT1
02865 -pf13247pf14691pf07992 -
02866 -pf00672pf02518pf07730pf13675 -
02867 -pf00873 -
02868 -pf03960 -
02869 -pf07687pf01546 -
02870 -pf13980 -
BMC-P
BMC-H
BMC-T
Aldehyde dehydrogenase
PTAC
Signature enzyme
Alcohol dehydrogenase
Regulator

Salmonella enterica I. Bacteria; Proteobacteria; Gammaproteobacteria; Enterobacterales;

AG53__locus_1

EUT1

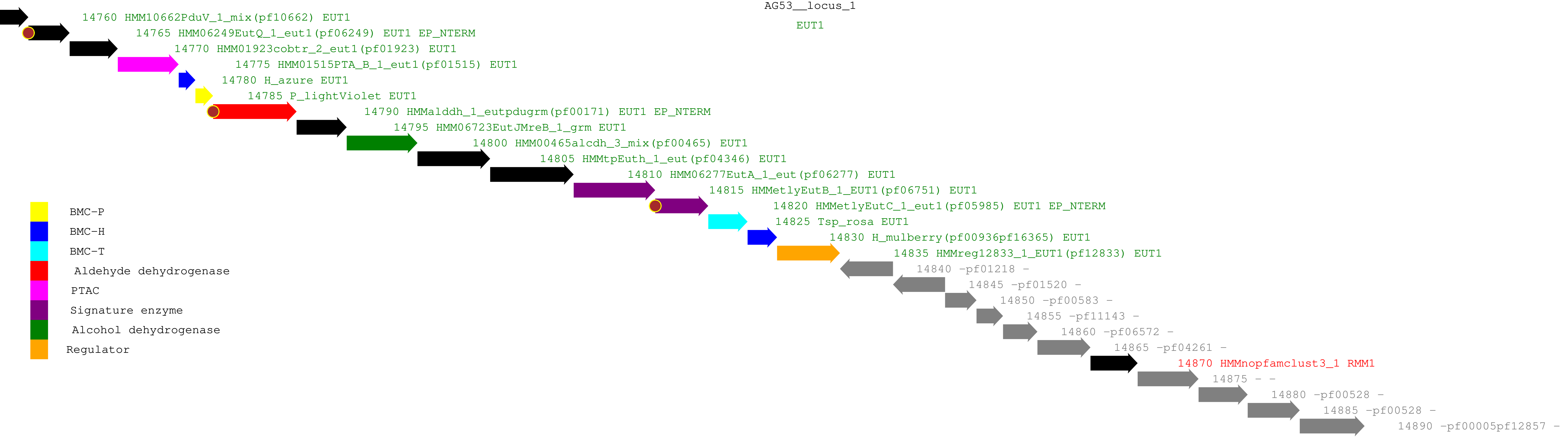

Klebsiella pneumoniae. Bacteria; Proteobacteria; Gammaproteobacteria; Enterobacterales;

AGG09__locus_2

EUT1

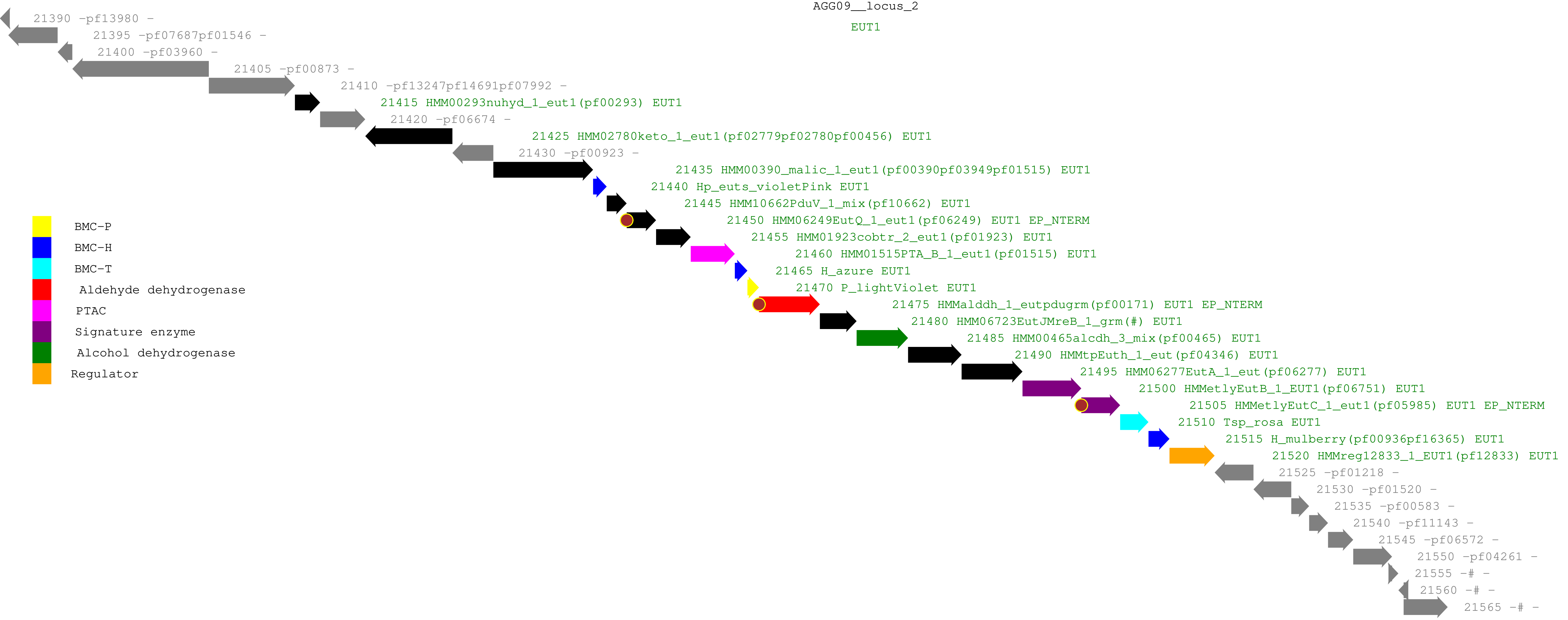

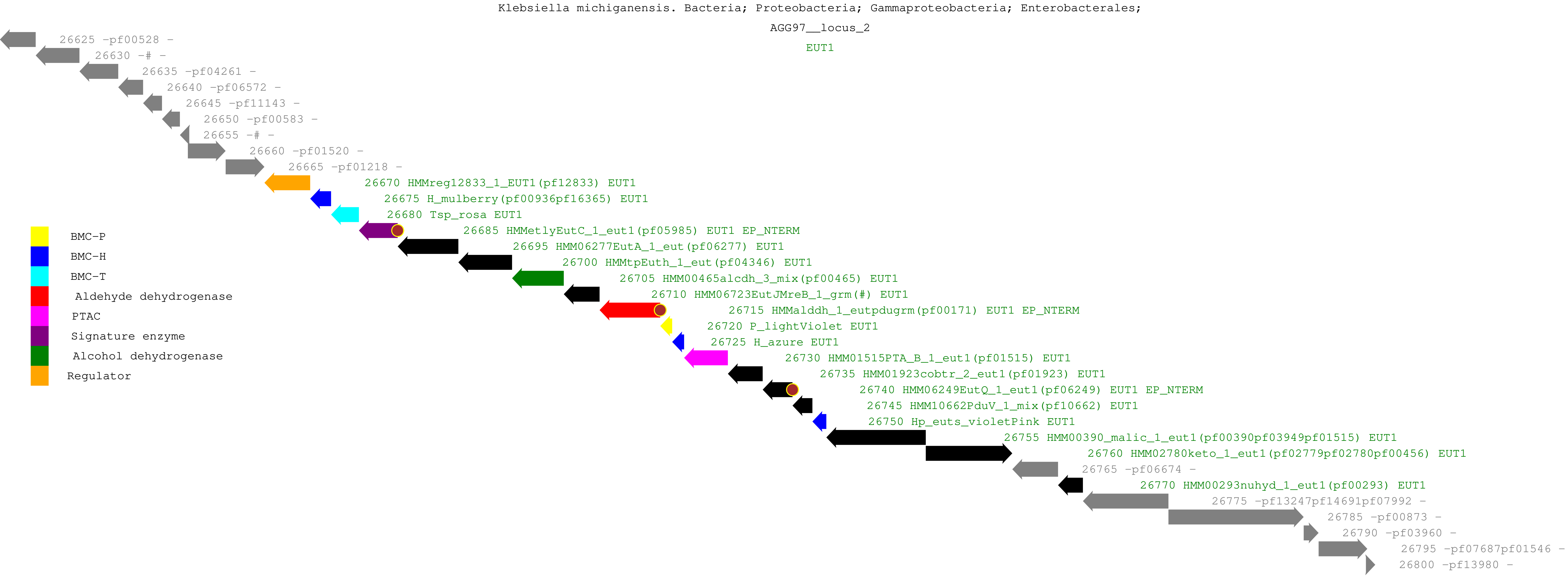
Klebsiella michiganensis. Bacteria; Proteobacteria; Gammaproteobacteria; Enterobacterales;
AGG97__locus_2
EUT1
26625 -pf00528 -
26630 -# -
26635 -pf04261 -
26640 -pf06572 -
26645 -pf11143 -
26650 -pf00583 -
26655 -# -
26660 -pf01520 -
26665 -pf01218 -
26670 HMMreg12833_1_EUT1(pf12833) EUT1
26675 H_mulberry(pf00936pf16365) EUT1
26680 Tsp_rosa EUT1
26685 HMMetlyEutC_1_eut1(pf05985) EUT1 EP_NTERM
26695 HMM06277EutA_1_eut(pf06277) EUT1
26700 HMMtpEuth_1_eut(pf04346) EUT1
26705 HMM00465alcdh_3_mix(pf00465) EUT1
26710 HMM06723EutJMreB_1_grm(#) EUT1
26715 HMMalddh_1_eutpdugrm(pf00171) EUT1 EP_NTERM
26720 P_lightViolet EUT1
26725 H_azure EUT1
26730 HMM01515PTA_B_1_eut1(pf01515) EUT1
26735 HMM01923cobtr_2_eut1(pf01923) EUT1
26740 HMM06249EutQ_1_eut1(pf06249) EUT1 EP_NTERM
26745 HMM10662PduV_1_mix(pf10662) EUT1
26750 Hp_euts_violetPink EUT1
26755 HMM00390_malic_1_eut1(pf00390pf03949pf01515) EUT1
26760 HMM02780keto_1_eut1(pf02779pf02780pf00456) EUT1
26765 -pf06674 -
26770 HMM00293nuhyd_1_eut1(pf00293) EUT1
26775 -pf13247pf14691pf07992 -
26785 -pf00873 -
26790 -pf03960 -
26795 -pf07687pf01546 -
26800 -pf13980 -
BMC-P
BMC-H
BMC-T
Aldehyde dehydrogenase
PTAC
Signature enzyme
Alcohol dehydrogenase
Regulator

Salmonella enterica subsp. enterica serovar Kentucky. Bacteria; Proteobacteria; Gammaproteobacteria; Enterobacterales;

AH329__locus_2

EUT1

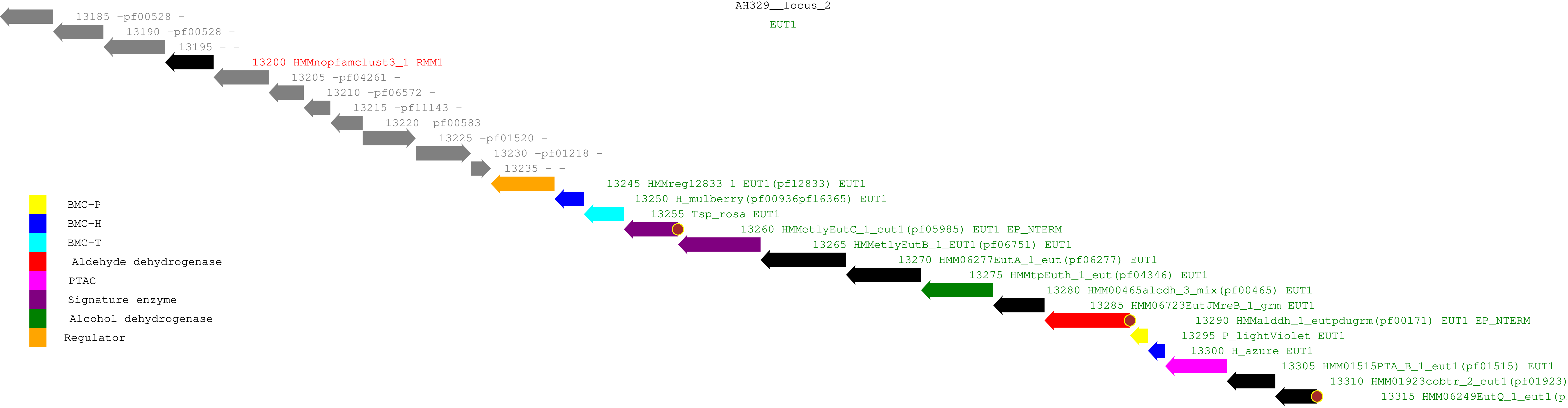

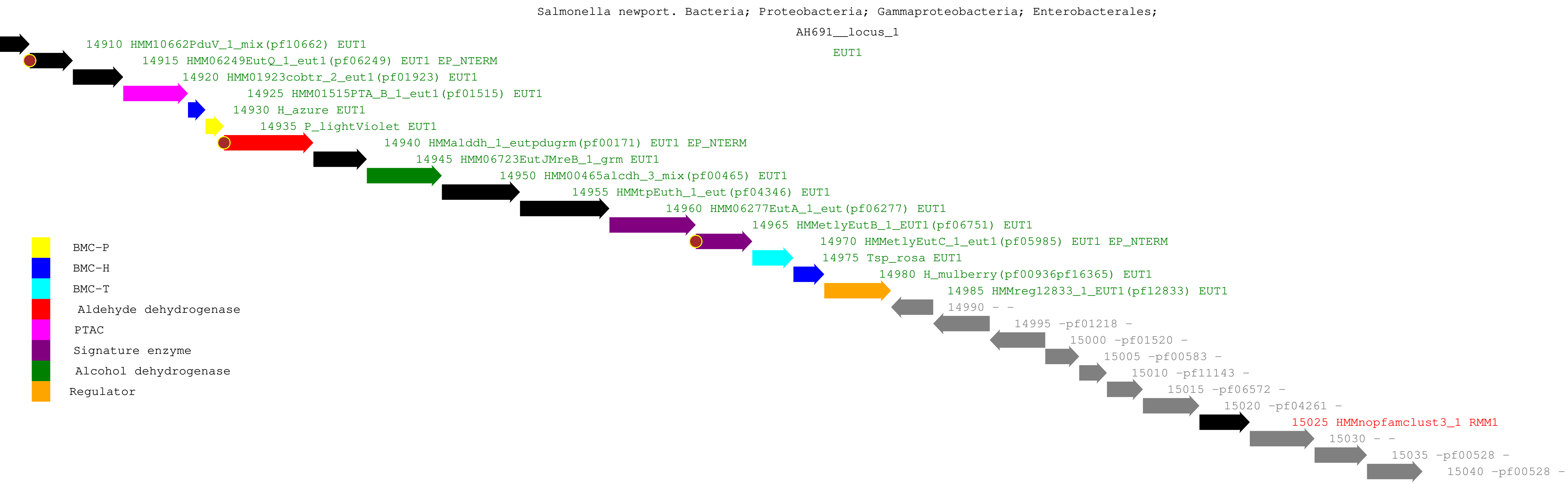
Salmonella newport. Bacteria; Proteobacteria; Gammaproteobacteria; Enterobacterales;
AH691__locus_1
EUT1
14910 HMM10662PduV_1_mix(pf10662) EUT1
14915 HMM06249EutQ_1_eut1(pf06249) EUT1 EP_NTERM
14920 HMM01923cobtr_2_eut1(pf01923) EUT1
14925 HMM01515PTA_B_1_eut1(pf01515) EUT1
14930 H_azure EUT1
14935 P_lightViolet EUT1
14940 HMMalddh_1_eutpdugrm(pf00171) EUT1 EP_NTERM
14945 HMM06723EutJMreB_1_grm EUT1
14950 HMM00465alcdh_3_mix(pf00465) EUT1
14955 HMMtpEuth_1_eut(pf04346) EUT1
14960 HMM06277EutA_1_eut(pf06277) EUT1
14965 HMMetlyEutB_1_EUT1(pf06751) EUT1
14970 HMMetlyEutC_1_eut1(pf05985) EUT1 EP_NTERM
14975 Tsp_rosa EUT1
14980 H_mulberry(pf00936pf16365) EUT1
14985 HMMreg12833_1_EUT1(pf12833) EUT1
14990 - -
14995 -pf01218 -
15000 -pf01520 -
15005 -pf00583 -
15010 -pf11143 -
15015 -pf06572 -
15020 -pf04261 -
15025 HMMnopfamclust3_1 RMM1
15030 - -
15035 -pf00528 -
15040 -pf00528 -
BMC-P
BMC-H
BMC-T
Aldehyde dehydrogenase
PTAC
Signature enzyme
Alcohol dehydrogenase
Regulator

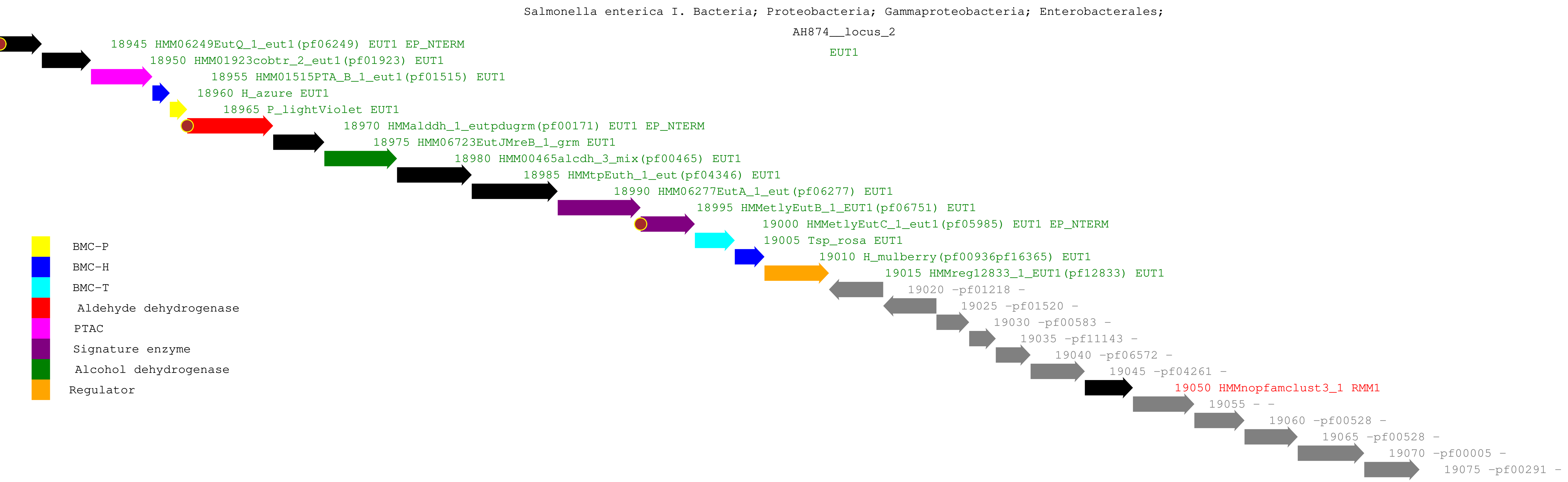

Salmonella enterica I. Bacteria; Proteobacteria; Gammaproteobacteria; Enterobacterales;
AH874__locus_2
EUT1
18945 HMM06249EutQ_1_eut1(pf06249) EUT1 EP_NTERM
18950 HMM01923cobtr_2_eut1(pf01923) EUT1
18955 HMM01515PTA_B_1_eut1(pf01515) EUT1
18960 H_azure EUT1
18965 P_lightViolet EUT1
18970 HMMalddh_1_eutpdugrm(pf00171) EUT1 EP_NTERM
18975 HMM06723EutJMreB_1_grm EUT1
18980 HMM00465alcdh_3_mix(pf00465) EUT1
18985 HMMtpEuth_1_eut(pf04346) EUT1
18990 HMM06277EutA_1_eut(pf06277) EUT1
18995 HMMetlyEutB_1_EUT1(pf06751) EUT1
19000 HMMetlyEutC_1_eut1(pf05985) EUT1 EP_NTERM
19005 Tsp_rosa EUT1
19010 H_mulberry(pf00936pf16365) EUT1
19015 HMMreg12833_1_EUT1(pf12833) EUT1
19020 -pf01218 -
19025 -pf01520 -
19030 -pf00583 -
19035 -pf11143 -
19040 -pf06572 -
19045 -pf04261 -
19050 HMMnopfamclust3_1 RMM1
19055 - -
19060 -pf00528 -
19065 -pf00528 -
19070 -pf00005 -
19075 -pf00291 -
BMC-P
BMC-H
BMC-T
Aldehyde dehydrogenase
PTAC
Signature enzyme
Alcohol dehydrogenase
Regulator

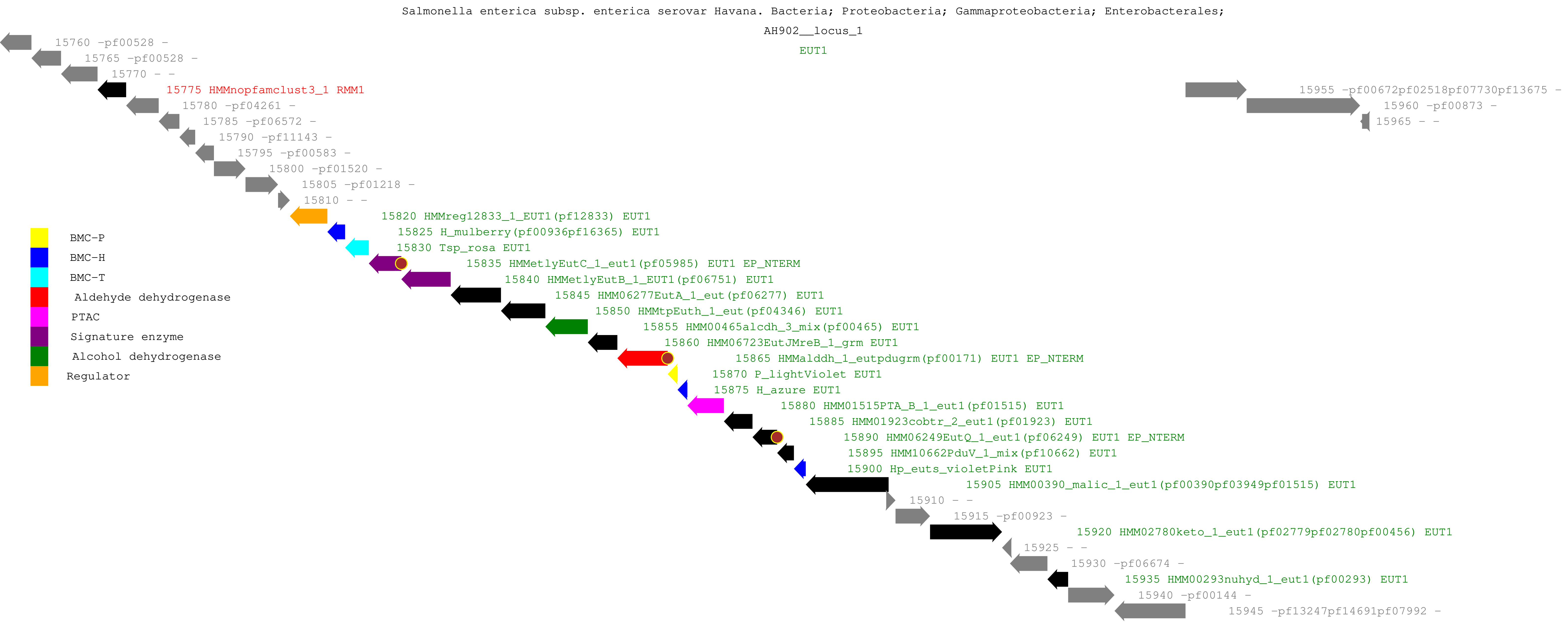

Salmonella enterica subsp. enterica serovar Havana. Bacteria; Proteobacteria; Gammaproteobacteria; Enterobacterales;
AH902__locus_1
EUT1
15760 -pf00528 -
15765 -pf00528 -
15770 - -
15775 HMMnopfamclust3_1 RMM1
15780 -pf04261 -
15785 -pf06572 -
15790 -pf11143 -
15795 -pf00583 -
15800 -pf01520 -
15805 -pf01218 -
15810 - -
15820 HMMreg12833_1_EUT1(pf12833) EUT1
15825 H_mulberry(pf00936pf16365) EUT1
15830 Tsp_rosa EUT1
15835 HMMetlyEutC_1_eut1(pf05985) EUT1 EP_NTERM
15840 HMMetlyEutB_1_EUT1(pf06751) EUT1
15845 HMM06277EutA_1_eut(pf06277) EUT1
15850 HMMtpEuth_1_eut(pf04346) EUT1
15855 HMM00465alcdh_3_mix(pf00465) EUT1
15860 HMM06723EutJMreB_1_grm EUT1
15865 HMMalddh_1_eutpdugrm(pf00171) EUT1 EP_NTERM
15870 P_lightViolet EUT1
15875 H_azure EUT1
15880 HMM01515PTA_B_1_eut1(pf01515) EUT1
15885 HMM01923cobtr_2_eut1(pf01923) EUT1
15890 HMM06249EutQ_1_eut1(pf06249) EUT1 EP_NTERM
15895 HMM10662PduV_1_mix(pf10662) EUT1
15900 Hp_euts_violetPink EUT1
15905 HMM00390_malic_1_eut1(pf00390pf03949pf01515) EUT1
15910 - -
15915 -pf00923 -
15920 HMM02780keto_1_eut1(pf02779pf02780pf00456) EUT1
15925 - -
15930 -pf06674 -
15935 HMM00293nuhyd_1_eut1(pf00293) EUT1
15940 -pf00144 -
15945 -pf13247pf14691pf07992 -
15955 -pf00672pf02518pf07730pf13675 -
15960 -pf00873 -
15965 - -
BMC-P
BMC-H
BMC-T
Aldehyde dehydrogenase
PTAC
Signature enzyme
Alcohol dehydrogenase
Regulator

Salmonella enterica subsp. enterica serovar Paratyphi B. Bacteria; Proteobacteria; Gammaproteobacteria; Enterobacterales;

AHS25__locus_2_main_sat

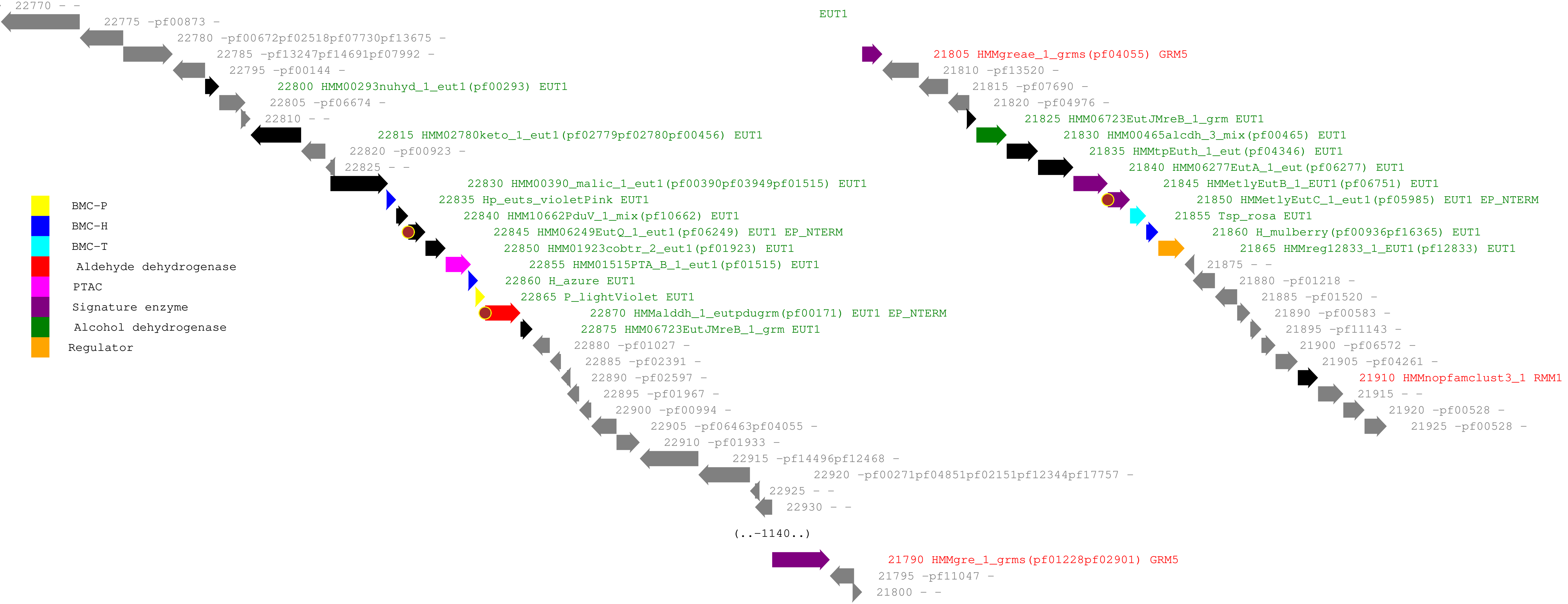

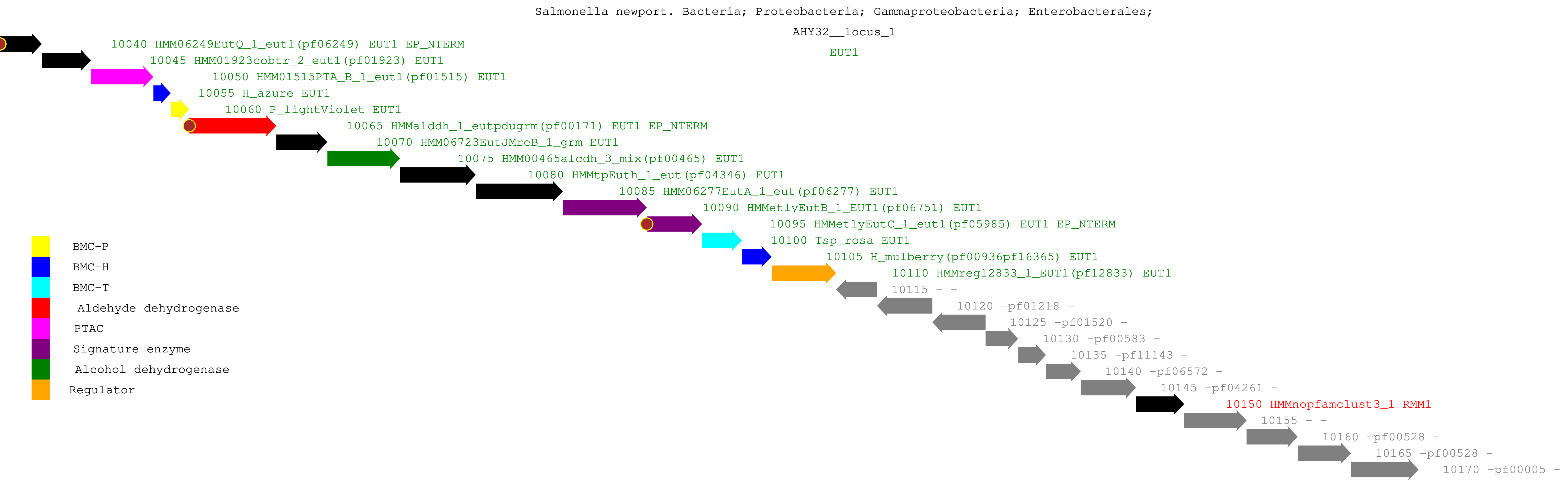

Salmonella newport. Bacteria; Proteobacteria; Gammaproteobacteria; Enterobacterales;
AHY32__locus_1
EUT1
10040 HMM06249EutQ_1_eut1(pf06249) EUT1 EP_NTERM
10045 HMM01923cobtr_2_eut1(pf01923) EUT1
10050 HMM01515PTA_B_1_eut1(pf01515) EUT1
10055 H_azure EUT1
10060 P_lightViolet EUT1
10065 HMMalddh_1_eutpdugrm(pf00171) EUT1 EP_NTERM
10070 HMM06723EutJMreB_1_grm EUT1
10075 HMM00465alcdh_3_mix(pf00465) EUT1
10080 HMMtpEuth_1_eut(pf04346) EUT1
10085 HMM06277EutA_1_eut(pf06277) EUT1
10090 HMMetlyEutB_1_EUT1(pf06751) EUT1
10095 HMMetlyEutC_1_eut1(pf05985) EUT1 EP_NTERM
10100 Tsp_rosa EUT1
10105 H_mulberry(pf00936pf16365) EUT1
10110 HMMreg12833_1_EUT1(pf12833) EUT1
10115 - -
10120 -pf01218 -
10125 -pf01520 -
10130 -pf00583 -
10135 -pf11143 -
10140 -pf06572 -
10145 -pf04261 -
10150 HMMnopfamclust3_1 RMM1
10155 - -
10160 -pf00528 -
10165 -pf00528 -
10170 -pf00005 -
BMC-P
BMC-H
BMC-T
Aldehyde dehydrogenase
PTAC
Signature enzyme
Alcohol dehydrogenase
Regulator

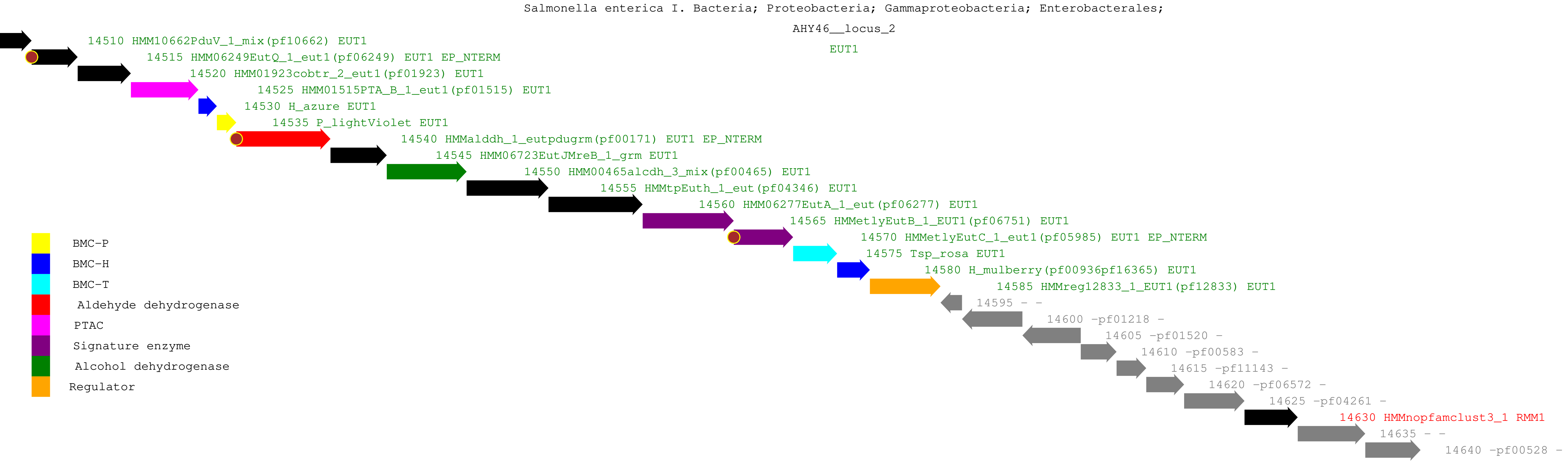

Salmonella enterica I. Bacteria; Proteobacteria; Gammaproteobacteria; Enterobacterales;
AHY46__locus_2
EUT1
14510 HMM10662PduV_1_mix(pf10662) EUT1
14515 HMM06249EutQ_1_eut1(pf06249) EUT1 EP_NTERM
14520 HMM01923cobtr_2_eut1(pf01923) EUT1
14525 HMM01515PTA_B_1_eut1(pf01515) EUT1
14530 H_azure EUT1
14535 P_lightViolet EUT1
14540 HMMalddh_1_eutpdugrm(pf00171) EUT1 EP_NTERM
14545 HMM06723EutJMreB_1_grm EUT1
14550 HMM00465alcdh_3_mix(pf00465) EUT1
14555 HMMtpEuth_1_eut(pf04346) EUT1
14560 HMM06277EutA_1_eut(pf06277) EUT1
14565 HMMetlyEutB_1_EUT1(pf06751) EUT1
14570 HMMetlyEutC_1_eut1(pf05985) EUT1 EP_NTERM
14575 Tsp_rosa EUT1
14580 H_mulberry(pf00936pf16365) EUT1
14585 HMMreg12833_1_EUT1(pf12833) EUT1
14595 - -
14600 -pf01218 -
14605 -pf01520 -
14610 -pf00583 -
14615 -pf11143 -
14620 -pf06572 -
14625 -pf04261 -
14630 HMMnopfamclust3_1 RMM1
14635 - -
14640 -pf00528 -
BMC-P
BMC-H
BMC-T
Aldehyde dehydrogenase
PTAC
Signature enzyme
Alcohol dehydrogenase
Regulator

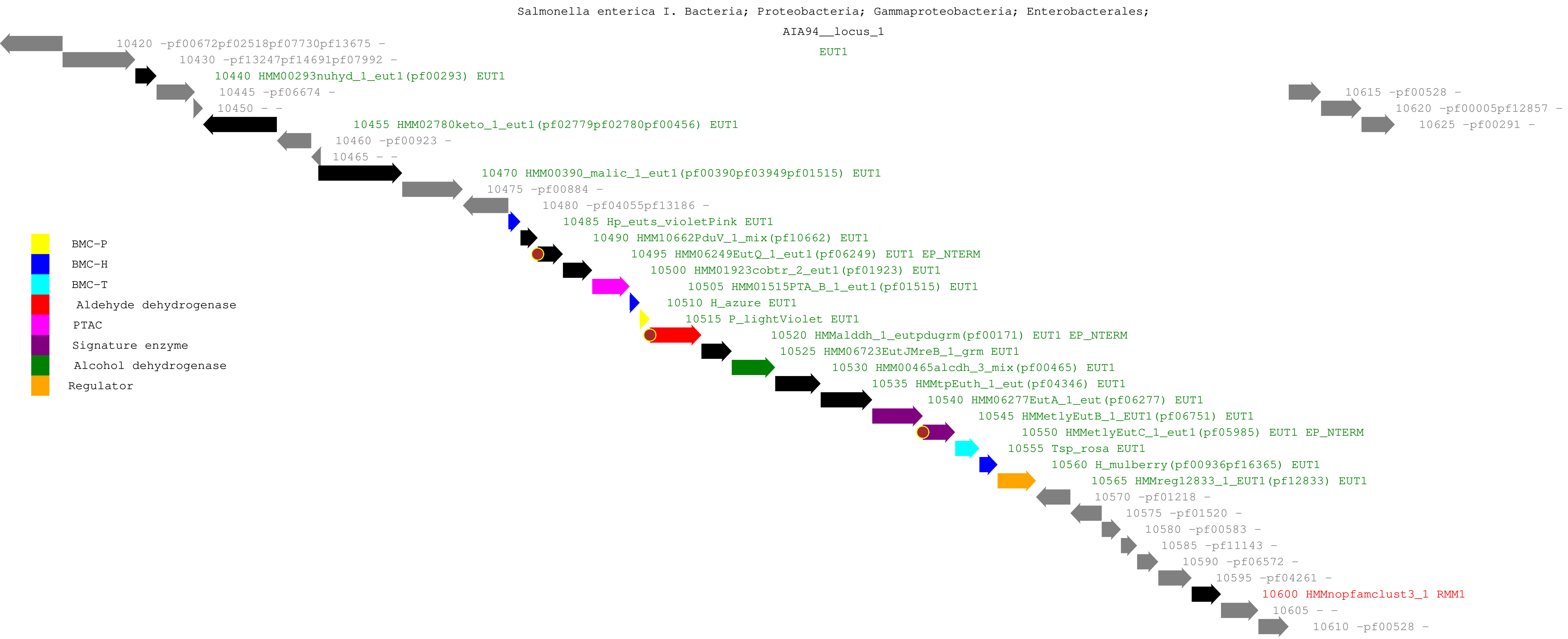
Salmonella enterica I. Bacteria; Proteobacteria; Gammaproteobacteria; Enterobacterales;
AIA94__locus_1
EUT1
10420 -pf00672pf02518pf07730pf13675 -
10430 -pf13247pf14691pf07992 -
10440 HMM00293nuhyd_1_eut1(pf00293) EUT1
10445 -pf06674 -
10450 - -
10455 HMM02780keto_1_eut1(pf02779pf02780pf00456) EUT1
10460 -pf00923 -
10465 - -
10470 HMM00390_malic_1_eut1(pf00390pf03949pf01515) EUT1
10475 -pf00884 -
10480 -pf04055pf13186 -
10485 Hp_euts_violetPink EUT1
10490 HMM10662PduV_1_mix(pf10662) EUT1
10495 HMM06249EutQ_1_eut1(pf06249) EUT1 EP_NTERM
10500 HMM01923cobtr_2_eut1(pf01923) EUT1
10505 HMM01515PTA_B_1_eut1(pf01515) EUT1
10510 H_azure EUT1
10515 P_lightViolet EUT1
10520 HMMalddh_1_eutpdugrm(pf00171) EUT1 EP_NTERM
10525 HMM06723EutJMreB_1_grm EUT1
10530 HMM00465alcdh_3_mix(pf00465) EUT1
10535 HMMtpEuth_1_eut(pf04346) EUT1
10540 HMM06277EutA_1_eut(pf06277) EUT1
10545 HMMetlyEutB_1_EUT1(pf06751) EUT1
10550 HMMetlyEutC_1_eut1(pf05985) EUT1 EP_NTERM
10555 Tsp_rosa EUT1
10560 H_mulberry(pf00936pf16365) EUT1
10565 HMMreg12833_1_EUT1(pf12833) EUT1
10570 -pf01218 -
10575 -pf01520 -
10580 -pf00583 -
10585 -pf11143 -
10590 -pf06572 -
10595 -pf04261 -
10600 HMMnopfamclust3_1 RMM1
10605 - -
10610 -pf00528 -
10615 -pf00528 -
10620 -pf00005pf12857 -
10625 -pf00291 -
BMC-P
BMC-H
BMC-T
Aldehyde dehydrogenase
PTAC
Signature enzyme
Alcohol dehydrogenase
Regulator

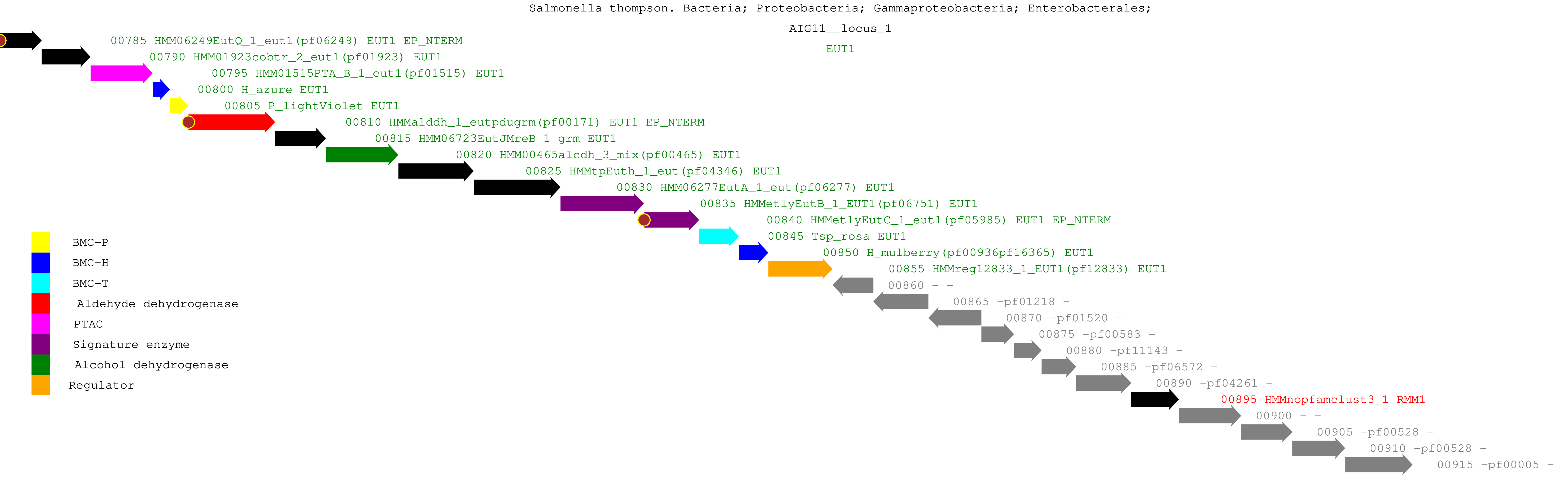

Salmonella thompson. Bacteria; Proteobacteria; Gammaproteobacteria; Enterobacterales;
AIG11__locus_1
EUT1
00785 HMM06249EutQ_1_eut1(pf06249) EUT1 EP_NTERM
00790 HMM01923cobtr_2_eut1(pf01923) EUT1
00795 HMM01515PTA_B_1_eut1(pf01515) EUT1
00800 H_azure EUT1
00805 P_lightViolet EUT1
00810 HMMalddh_1_eutpdugrm(pf00171) EUT1 EP_NTERM
00815 HMM06723EutJMreB_1_grm EUT1
00820 HMM00465alcdh_3_mix(pf00465) EUT1
00825 HMMtpEuth_1_eut(pf04346) EUT1
00830 HMM06277EutA_1_eut(pf06277) EUT1
00835 HMMetlyEutB_1_EUT1(pf06751) EUT1
00840 HMMetlyEutC_1_eut1(pf05985) EUT1 EP_NTERM
00845 Tsp_rosa EUT1
00850 H_mulberry(pf00936pf16365) EUT1
00855 HMMreg12833_1_EUT1(pf12833) EUT1
00860 - -
00865 -pf01218 -
00870 -pf01520 -
00875 -pf00583 -
00880 -pf11143 -
00885 -pf06572 -
00890 -pf04261 -
00895 HMMnopfamclust3_1 RMM1
00900 - -
00905 -pf00528 -
00910 -pf00528 -
00915 -pf00005 -
BMC-P
BMC-H
BMC-T
Aldehyde dehydrogenase
PTAC
Signature enzyme
Alcohol dehydrogenase
Regulator

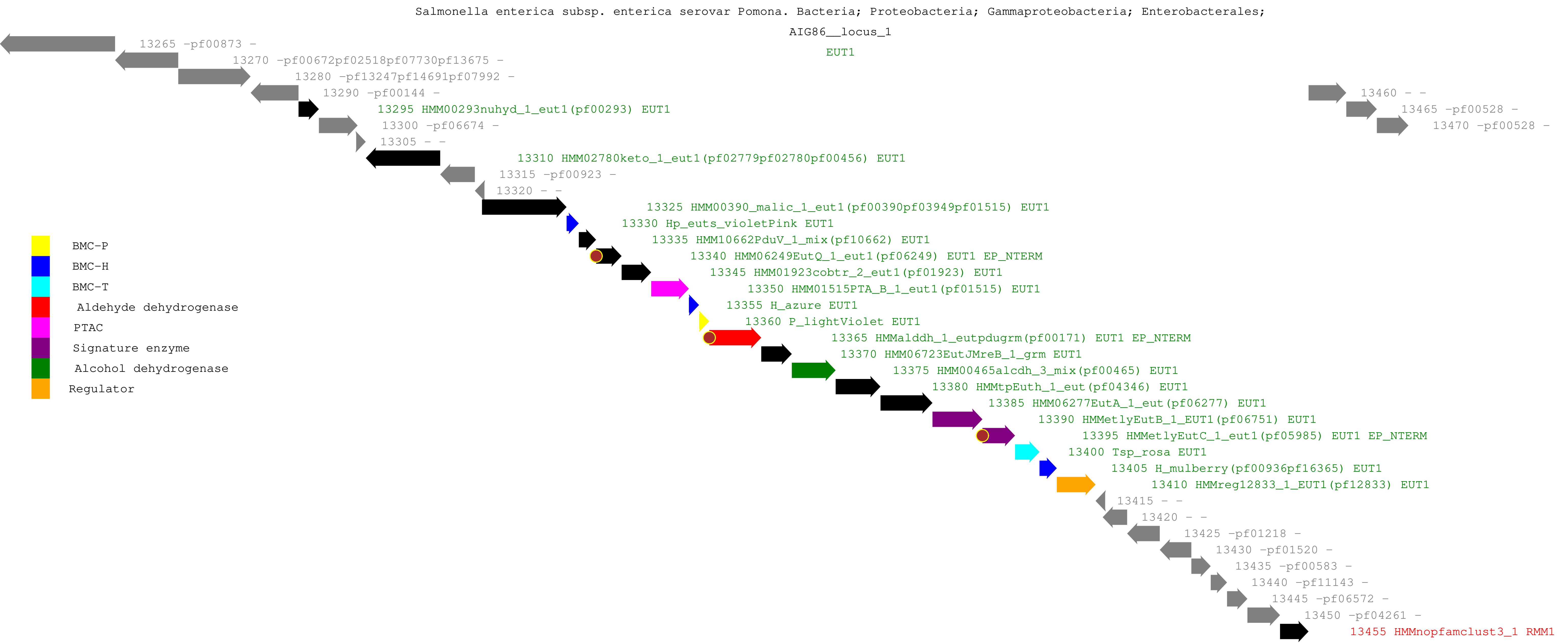
Salmonella enterica subsp. enterica serovar Pomona. Bacteria; Proteobacteria; Gammaproteobacteria; Enterobacterales;
AIG86__locus_1
EUT1
13265 -pf00873 -
13270 -pf00672pf02518pf07730pf13675 -
13280 -pf13247pf14691pf07992 -
13290 -pf00144 -
13295 HMM00293nuhyd_1_eut1(pf00293) EUT1
13300 -pf06674 -
13305 - -
13310 HMM02780keto_1_eut1(pf02779pf02780pf00456) EUT1
13315 -pf00923 -
13320 - -
13325 HMM00390_malic_1_eut1(pf00390pf03949pf01515) EUT1
13330 Hp_euts_violetPink EUT1
13335 HMM10662PduV_1_mix(pf10662) EUT1
13340 HMM06249EutQ_1_eut1(pf06249) EUT1 EP_NTERM
13345 HMM01923cobtr_2_eut1(pf01923) EUT1
13350 HMM01515PTA_B_1_eut1(pf01515) EUT1
13355 H_azure EUT1
13360 P_lightViolet EUT1
13365 HMMaldh_1_eutpdugrm(pf00171) EUT1 EP_NTERM
13370 HMM06723EutJMreB_1_grm EUT1
13375 HMM00465alcdh_3_mix(pf00465) EUT1
13380 HMMtpEuth_1_eut(pf04346) EUT1
13385 HMM06277EutA_1_eut(pf06277) EUT1
13390 HMMetlyEutB_1_EUT1(pf06751) EUT1
13395 HMMetlyEutC_1_eut1(pf05985) EUT1 EP_NTERM
13400 Tsp_rosa EUT1
13405 H_mulberry(pf00936pf16365) EUT1
13410 HMMreg12833_1_EUT1(pf12833) EUT1
13415 - -
13420 - -
13425 -pf01218 -
13430 -pf01520 -
13435 -pf00583 -
13440 -pf11143 -
13445 -pf06572 -
13450 -pf04261 -
13455 HMMnopfamclust3_1 RMM1
13460 - -
13465 -pf00528 -
13470 -pf00528 -
BMC-P
BMC-H
BMC-T
Aldehyde dehydrogenase
PTAC
Signature enzyme
Alcohol dehydrogenase
Regulator

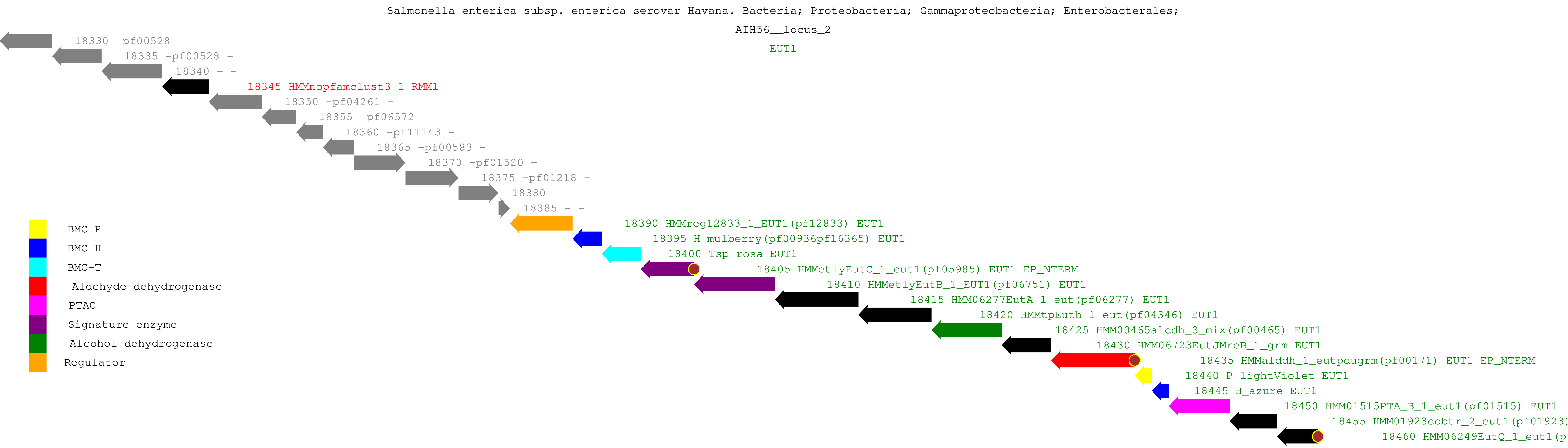

Salmonella enterica subsp. enterica serovar Havana. Bacteria; Proteobacteria; Gammaproteobacteria; Enterobacterales;
AIH56__locus_2
EUT1
18330 -pf00528 -
18335 -pf00528 -
18340 - -
18345 HMMnopfamclust3_1 RMM1
18350 -pf04261 -
18355 -pf06572 -
18360 -pf11143 -
18365 -pf00583 -
18370 -pf01520 -
18375 -pf01218 -
18380 - -
18385 - -
18390 HMMreg12833_1_EUT1(pf12833) EUT1
18395 H_mulberry(pf00936pf16365) EUT1
18400 Tsp_rosa EUT1
18405 HMMetlyEutC_1_eut1(pf05985) EUT1 EP_NTERM
18410 HMMetlyEutB_1_EUT1(pf06751) EUT1
18415 HMM06277EutA_1_eut(pf06277) EUT1
18420 HMMtpEuth_1_eut(pf04346) EUT1
18425 HMM00465alcdh_3_mix(pf00465) EUT1
18430 HMM06723EutJMreB_1_grm EUT1
18435 HMMalddh_1_eutpdugrm(pf00171) EUT1 EP_NTERM
18440 P_lightViolet EUT1
18445 H_azure EUT1
18450 HMM01515PTA_B_1_eut1(pf01515) EUT1
18455 HMM01923cobtr_2_eut1(pf01923)
18460 HMM06249EutQ_1_eut1(p
BMC-P
BMC-H
BMC-T
Aldehyde dehydrogenase
PTAC
Signature enzyme
Alcohol dehydrogenase
Regulator

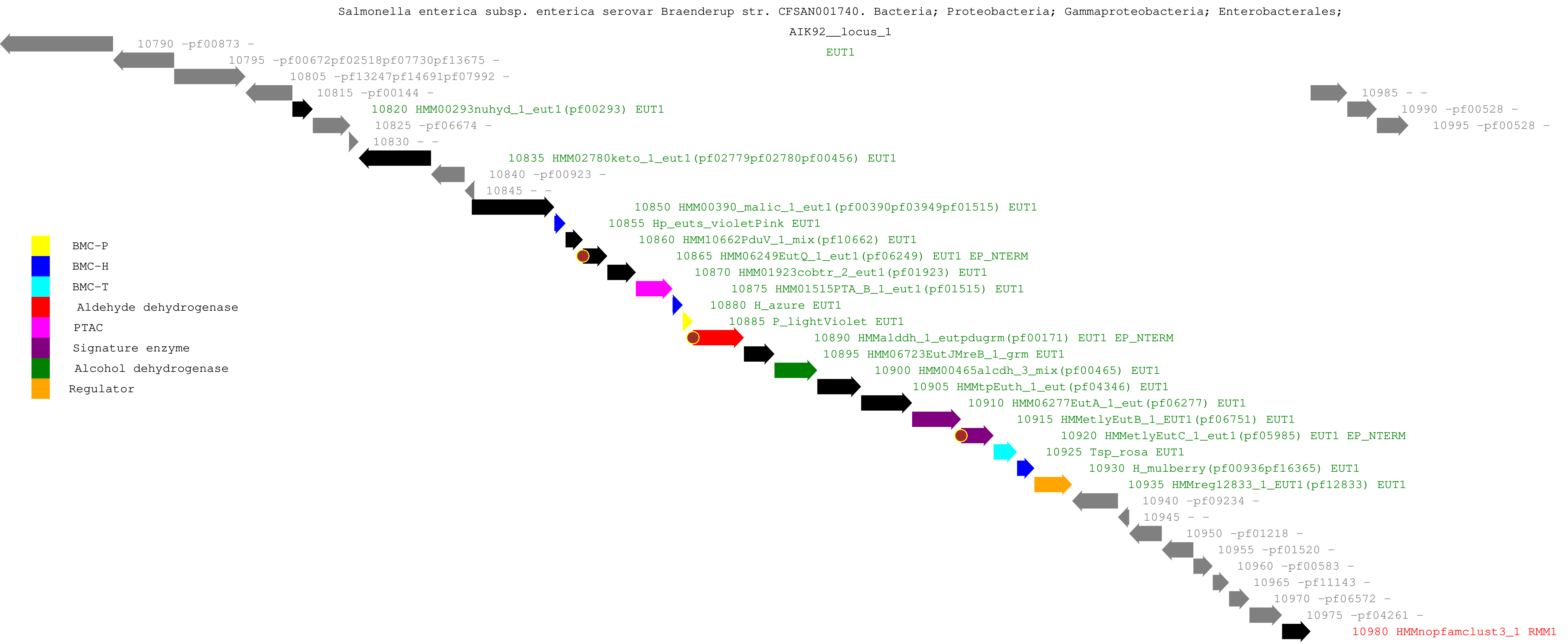
Salmonella enterica subsp. enterica serovar Braenderup str. CFSAN001740. Bacteria; Proteobacteria; Gammaproteobacteria; Enterobacterales;
AIK92__locus_1
EUT1
10790 -pf00873 -
10795 -pf00672pf02518pf07730pf13675 -
10805 -pf13247pf14691pf07992 -
10815 -pf00144 -
10820 HMM00293nuhyd_1_eut1(pf00293) EUT1
10825 -pf06674 -
10830 - -
10835 HMM02780keto_1_eut1(pf02779pf02780pf00456) EUT1
10840 -pf00923 -
10845 - -
10850 HMM00390_malic_1_eut1(pf00390pf03949pf01515) EUT1
10855 Hp_euts_violetPink EUT1
10860 HMM10662PduV_1_mix(pf10662) EUT1
10865 HMM06249EutQ_1_eut1(pf06249) EUT1 EP_NTERM
10870 HMM01923cobtr_2_eut1(pf01923) EUT1
10875 HMM01515PTA_B_1_eut1(pf01515) EUT1
10880 H_azure EUT1
10885 P_lightViolet EUT1
10890 HMMalddh_1_eutpdugrm(pf00171) EUT1 EP_NTERM
10895 HMM06723EutJMreB_1_grm EUT1
10900 HMM00465alcdh_3_mix(pf00465) EUT1
10905 HMMtpEuth_1_eut(pf04346) EUT1
10910 HMM06277EutA_1_eut(pf06277) EUT1
10915 HMMetlyEutB_1_EUT1(pf06751) EUT1
10920 HMMetlyEutC_1_eut1(pf05985) EUT1 EP_NTERM
10925 Tsp_rosa EUT1
10930 H_mulberry(pf00936pf16365) EUT1
10935 HMMreg12833_1_EUT1(pf12833) EUT1
10940 -pf09234 -
10945 - -
10950 -pf01218 -
10955 -pf01520 -
10960 -pf00583 -
10965 -pf11143 -
10970 -pf06572 -
10975 -pf04261 -
10980 HMMnopfamclust3_1 RMM1
10985 - -
10990 -pf00528 -
10995 -pf00528 -
BMC-P
BMC-H
BMC-T
Aldehyde dehydrogenase
PTAC
Signature enzyme
Alcohol dehydrogenase
Regulator

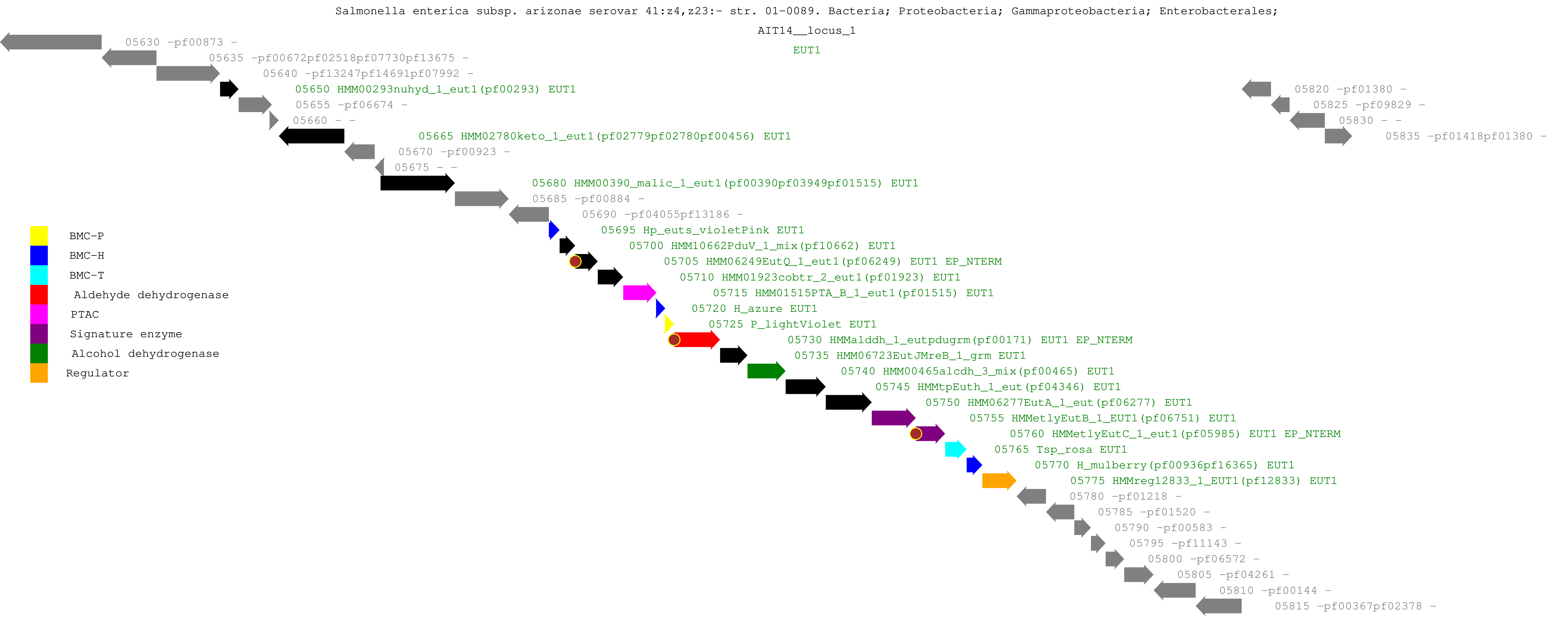
Salmonella enterica subsp. arizonae serovar 41:z4,z23:- str. 01-0089. Bacteria; Proteobacteria; Gammaproteobacteria; Enterobacterales;
AIT14__locus_1
EUT1
05630 -pf00873 -
05635 -pf00672pf02518pf07730pf13675 -
05640 -pf13247pf14691pf07992 -
05650 HMM00293nuhyd_1_eut1(pf00293) EUT1
05655 -pf06674 -
05660 - -
05665 HMM02780keto_1_eut1(pf02779pf02780pf00456) EUT1
05670 -pf00923 -
05675 - -
05680 HMM00390_malic_1_eut1(pf00390pf03949pf01515) EUT1
05685 -pf00884 -
05690 -pf04055pf13186 -
05695 Hp_euts_violetPink EUT1
05700 HMM10662PduV_1_mix(pf10662) EUT1
05705 HMM06249EutQ_1_eut1(pf06249) EUT1 EP_NTERM
05710 HMM01923cobtr_2_eut1(pf01923) EUT1
05715 HMM01515PTA_B_1_eut1(pf01515) EUT1
05720 H_azure EUT1
05725 P_lightViolet EUT1
05730 HMMaldh_1_eutpdugrm(pf00171) EUT1 EP_NTERM
05735 HMM06723EutJMreB_1_grm EUT1
05740 HMM00465alcdh_3_mix(pf00465) EUT1
05745 HMMtpEuth_1_eut(pf04346) EUT1
05750 HMM06277EutA_1_eut(pf06277) EUT1
05755 HMMetlyEutB_1_EUT1(pf06751) EUT1
05760 HMMetlyEutC_1_eut1(pf05985) EUT1 EP_NTERM
05765 Tsp_rosa EUT1
05770 H_mulberry(pf00936pf16365) EUT1
05775 HMMreg12833_1_EUT1(pf12833) EUT1
05780 -pf01218 -
05785 -pf01520 -
05790 -pf00583 -
05795 -pf11143 -
05800 -pf06572 -
05805 -pf04261 -
05810 -pf00144 -
05815 -pf00367pf02378 -
05820 -pf01380 -
05825 -pf09829 -
05830 - -
05835 -pf01418pf01380 -
BMC-P
BMC-H
BMC-T
Aldehyde dehydrogenase
PTAC
Signature enzyme
Alcohol dehydrogenase
Regulator

Escherichia coli. Bacteria; Proteobacteria; Gammaproteobacteria; Enterobacterales;

AJ318__locus_1

EUT1

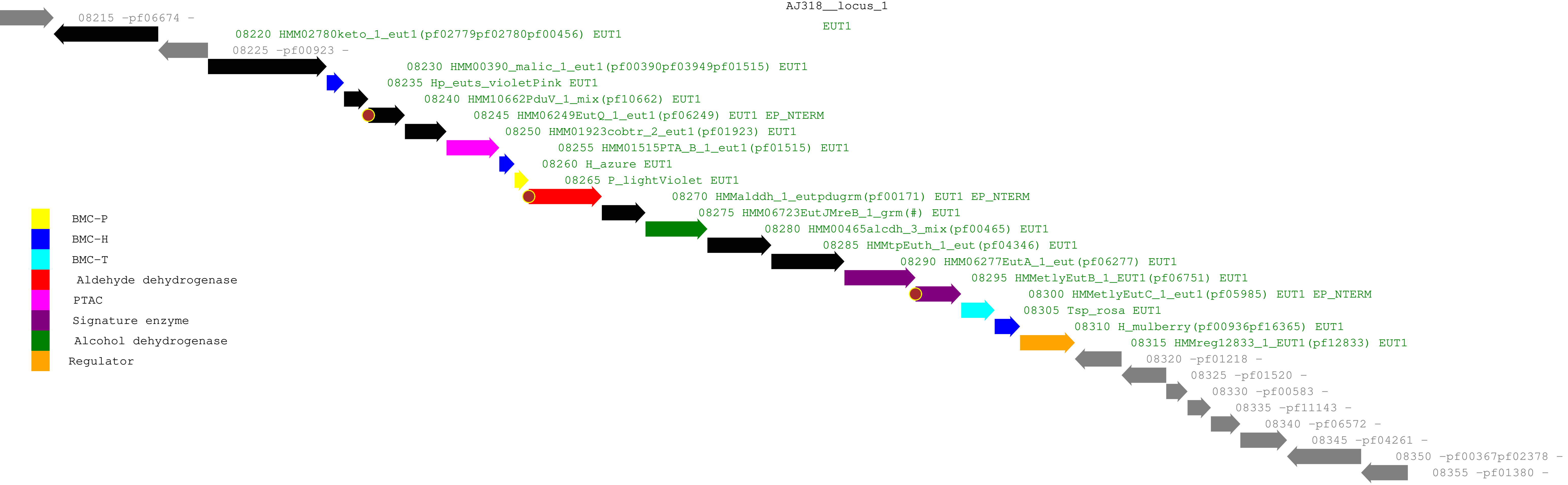

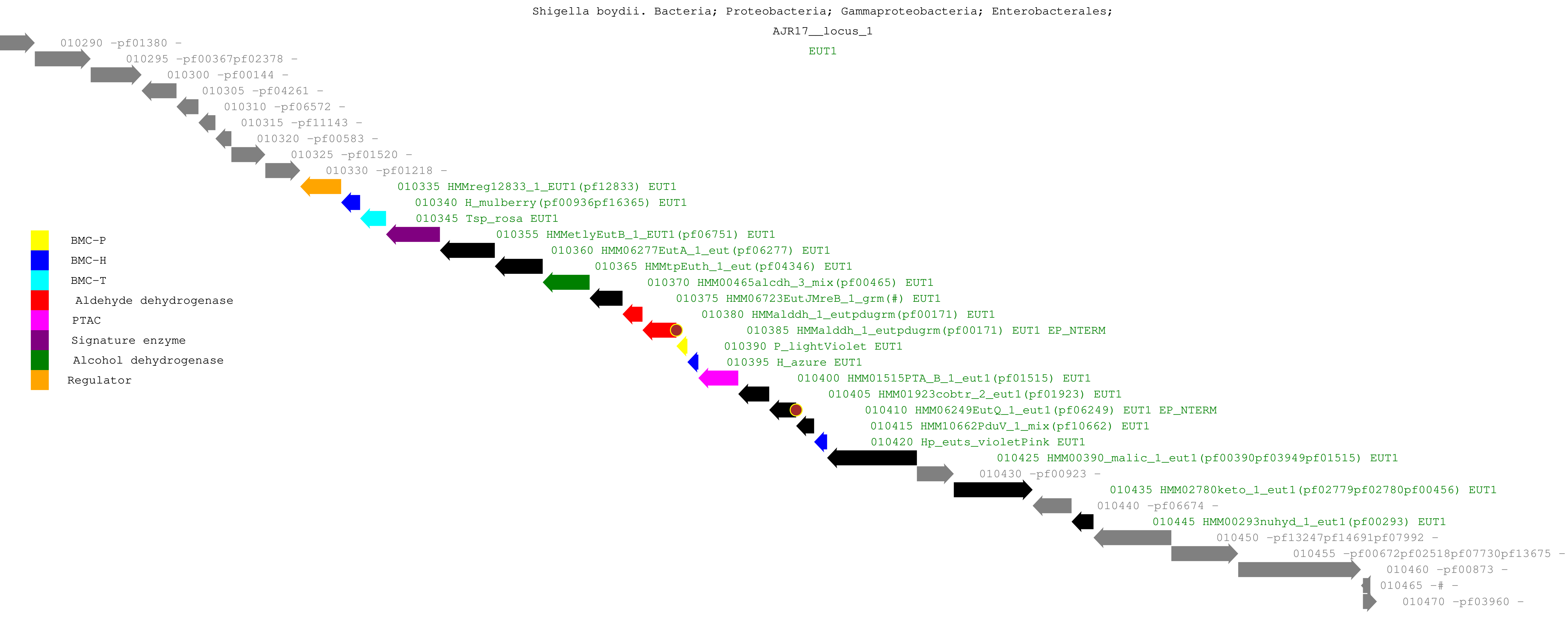

Shigella boydii. Bacteria; Proteobacteria; Gammaproteobacteria; Enterobacterales;
AJR17__locus_1
EUT1
010290 -pf01380 -
010295 -pf00367pf02378 -
010300 -pf00144 -
010305 -pf04261 -
010310 -pf06572 -
010315 -pf11143 -
010320 -pf00583 -
010325 -pf01520 -
010330 -pf01218 -
010335 HMMreg12833_1_EUT1(pf12833) EUT1
010340 H_mulberry(pf00936pf16365) EUT1
010345 Tsp_rosa EUT1
010355 HMMetlyEutB_1_EUT1(pf06751) EUT1
010360 HMM06277EutA_1_eut(pf06277) EUT1
010365 HMMtpEuth_1_eut(pf04346) EUT1
010370 HMM00465alcdh_3_mix(pf00465) EUT1
010375 HMM06723EutJMreB_1_grm(#) EUT1
010380 HMMalddh_1_eutpdugrm(pf00171) EUT1
010385 HMMalddh_1_eutpdugrm(pf00171) EUT1 EP_NTERM
010390 P_lightViolet EUT1
010395 H_azure EUT1
010400 HMM01515PTA_B_1_eut1(pf01515) EUT1
010405 HMM01923cobtr_2_eut1(pf01923) EUT1
010410 HMM06249EutQ_1_eut1(pf06249) EUT1 EP_NTERM
010415 HMM10662PduV_1_mix(pf10662) EUT1
010420 Hp_euts_violetPink EUT1
010425 HMM00390_malic_1_eut1(pf00390pf03949pf01515) EUT1
010430 -pf00923 -
010435 HMM02780keto_1_eut1(pf02779pf02780pf00456) EUT1
010440 -pf06674 -
010445 HMM00293nuhyd_1_eut1(pf00293) EUT1
010450 -pf13247pf14691pf07992 -
010455 -pf00672pf02518pf07730pf13675 -
010460 -pf00873 -
010465 -# -
010470 -pf03960 -
BMC-P
BMC-H
BMC-T
Aldehyde dehydrogenase
PTAC
Signature enzyme
Alcohol dehydrogenase
Regulator

Shigella boydii. Bacteria; Proteobacteria; Gammaproteobacteria; Enterobacterales;

AJR18__locus_1_main_sat

EUT1

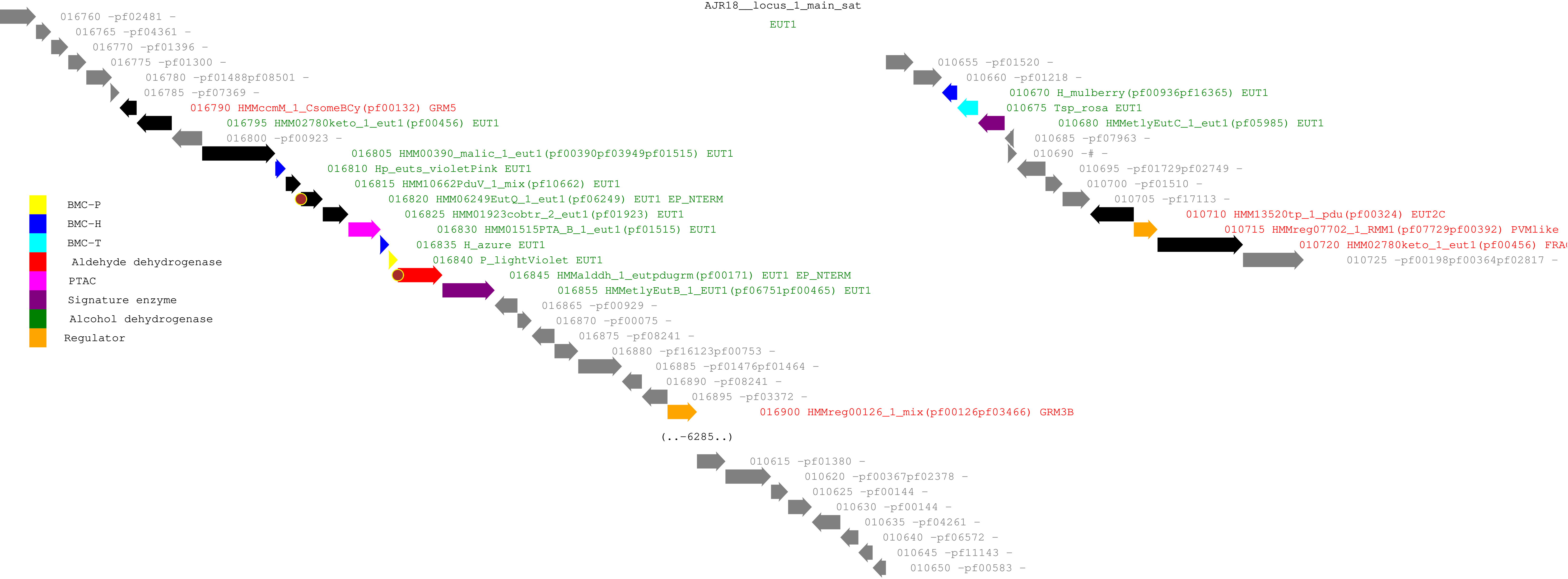

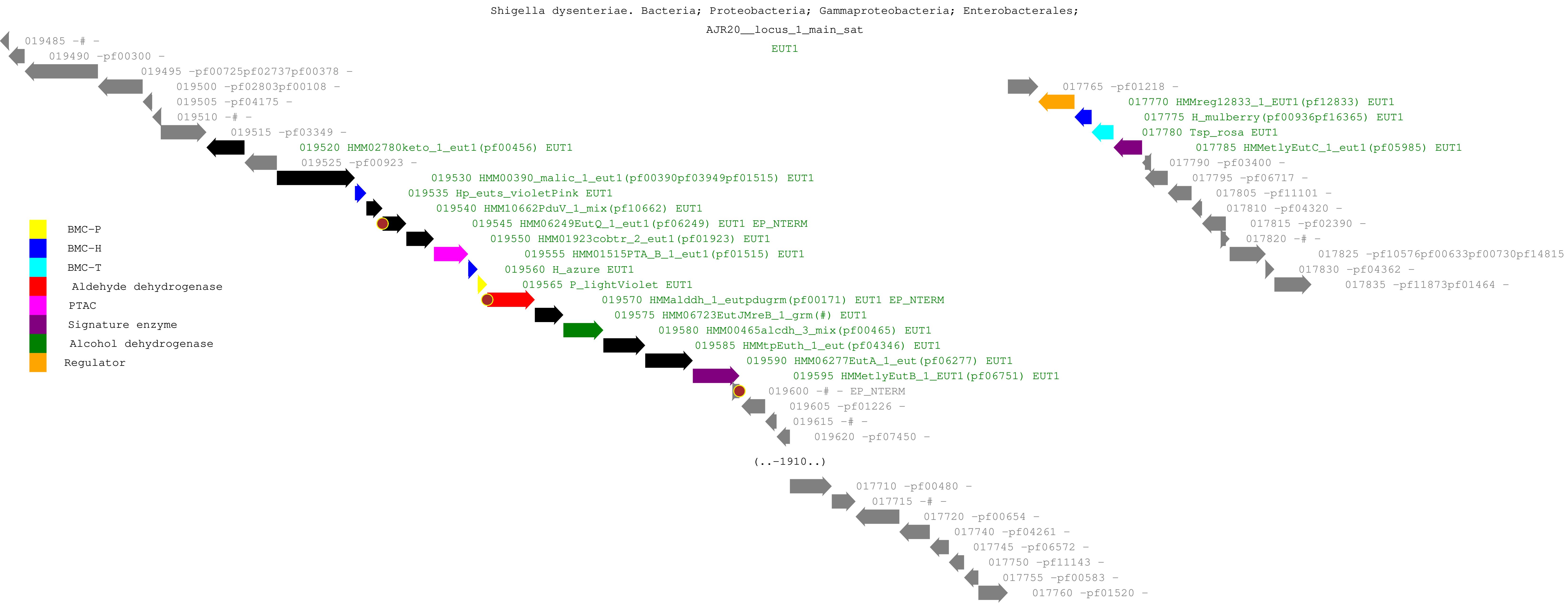

Shigella dysenteriae. Bacteria; Proteobacteria; Gammaproteobacteria; Enterobacterales;
AJR20__locus_1_main_sat
EUT1
019485 -# -
019490 -pf00300 -
019495 -pf00725pf02737pf00378 -
019500 -pf02803pf00108 -
019505 -pf04175 -
019510 -# -
019515 -pf03349 -
019520 HMM02780keto_1_eut1(pf00456) EUT1
019525 -pf00923 -
019530 HMM00390_malic_1_eut1(pf00390pf03949pf01515) EUT1
019535 Hp_euts_violetPink EUT1
019540 HMM10662PduV_1_mix(pf10662) EUT1
019545 HMM06249EutQ_1_eut1(pf06249) EUT1 EP_NTERM
019550 HMM01923cobtr_2_eut1(pf01923) EUT1
019555 HMM01515PTA_B_1_eut1(pf01515) EUT1
019560 H_azure EUT1
019565 P_lightViolet EUT1
019570 HMMalddh_1_eutpdugrm(pf00171) EUT1 EP_NTERM
019575 HMM06723EutJMreB_1_grm(#) EUT1
019580 HMM00465alcdh_3_mix(pf00465) EUT1
019585 HMMtpEuth_1_eut(pf04346) EUT1
019590 HMM06277EutA_1_eut(pf06277) EUT1
019595 HMMetlyEutB_1_EUT1(pf06751) EUT1
019600 -# - EP_NTERM
019605 -pf01226 -
019615 -# -
019620 -pf07450 -
(..-1910..)
017710 -pf00480 -
017715 -# -
017720 -pf00654 -
017740 -pf04261 -
017745 -pf06572 -
017750 -pf11143 -
017755 -pf00583 -
017760 -pf01520 -
017765 -pf01218 -
017770 HMMreg12833_1_EUT1(pf12833) EUT1
017775 H_mulberry(pf00936pf16365) EUT1
017780 Tsp_rosa EUT1
017785 HMMetlyEutC_1_eut1(pf05985) EUT1
017790 -pf03400 -
017795 -pf06717 -
017805 -pf11101 -
017810 -pf04320 -
017815 -pf02390 -
017820 -# -
017825 -pf10576pf00633pf00730pf14815
017830 -pf04362 -
017835 -pf11873pf01464 -
BMC-P
BMC-H
BMC-T
Aldehyde dehydrogenase
PTAC
Signature enzyme
Alcohol dehydrogenase
Regulator

Shigella dysenteriae. Bacteria; Proteobacteria; Gammaproteobacteria; Enterobacterales;

AJR21__locus_1

EUT1

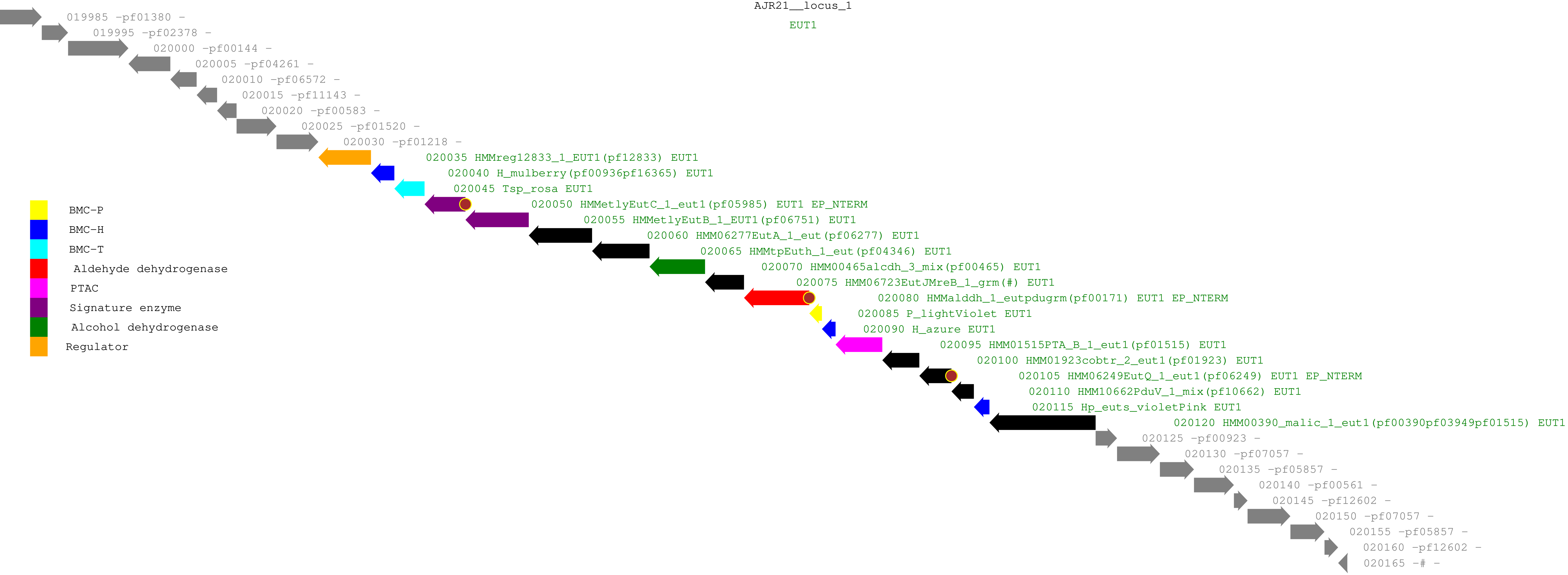

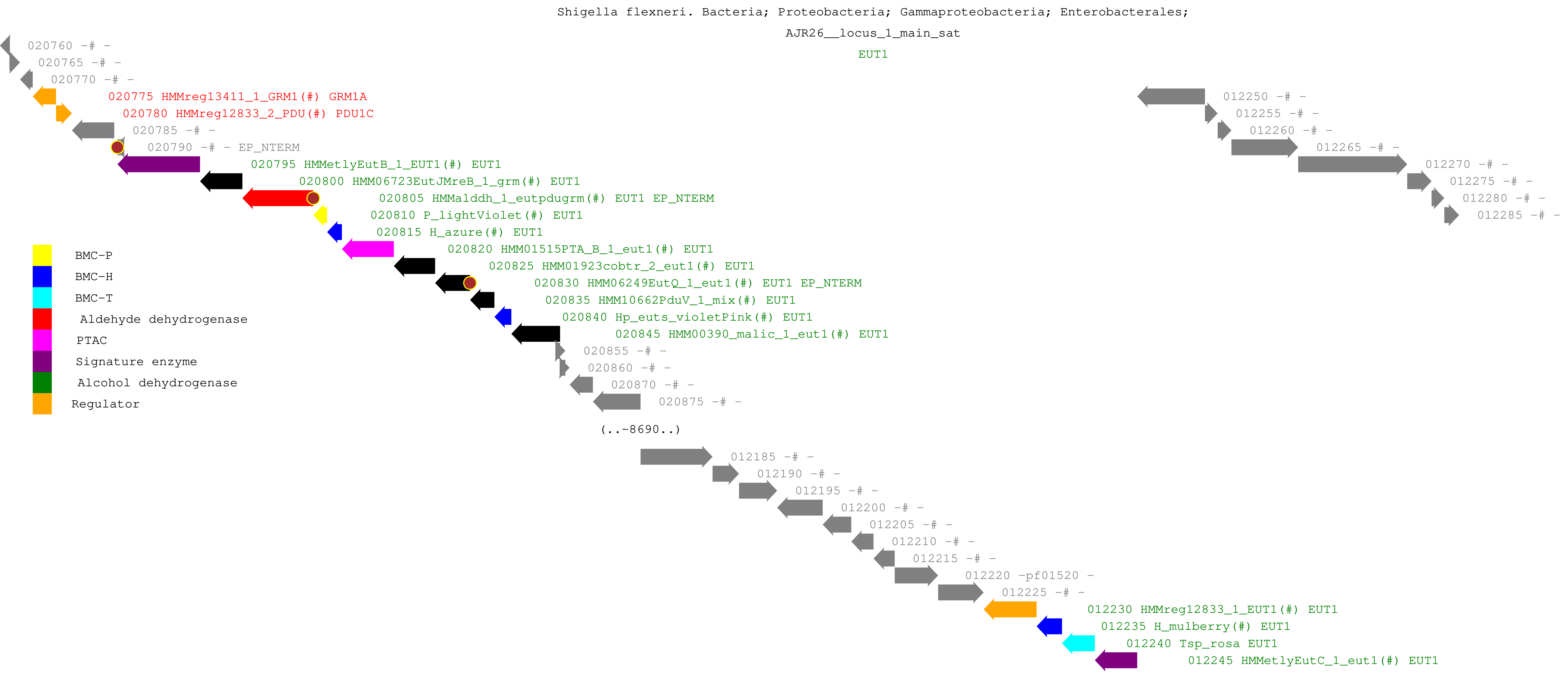
Shigella flexneri. Bacteria; Proteobacteria; Gammaproteobacteria; Enterobacterales;
AJR26__locus_1_main_sat
EUT1
020760 -# -
020765 -# -
020770 -# -
020775 HMMreg13411_1_GRM1(#) GRM1A
020780 HMMreg12833_2_PDU(#) PDU1C
020785 -# -
020790 -# - EP_NTERM
020795 HMMetlyEutB_1_EUT1(#) EUT1
020800 HMM06723EutJMreB_1_grm(#) EUT1
020805 HMMalddh_1_eutpdugrm(#) EUT1 EP_NTERM
020810 P_lightViolet(#) EUT1
020815 H_azure(#) EUT1
020820 HMM01515PTA_B_1_eut1(#) EUT1
020825 HMM01923cobtr_2_eut1(#) EUT1
020830 HMM06249EutQ_1_eut1(#) EUT1 EP_NTERM
020835 HMM10662PduV_1_mix(#) EUT1
020840 Hp_euts_violetPink(#) EUT1
020845 HMM00390_malic_1_eut1(#) EUT1
020855 -# -
020860 -# -
020870 -# -
020875 -# -
(..-8690..)
012185 -# -
012190 -# -
012195 -# -
012200 -# -
012205 -# -
012210 -# -
012215 -# -
012220 -pf01520 -
012225 -# -
012230 HMMreg12833_1_EUT1(#) EUT1
012235 H_mulberry(#) EUT1
012240 Tsp_rosa EUT1
012245 HMMetlyEutC_1_eut1(#) EUT1
012250 -# -
012255 -# -
012260 -# -
012265 -# -
012270 -# -
012275 -# -
012280 -# -
012285 -# -
BMC-P
BMC-H
BMC-T
Aldehyde dehydrogenase
PTAC
Signature enzyme
Alcohol dehydrogenase
Regulator

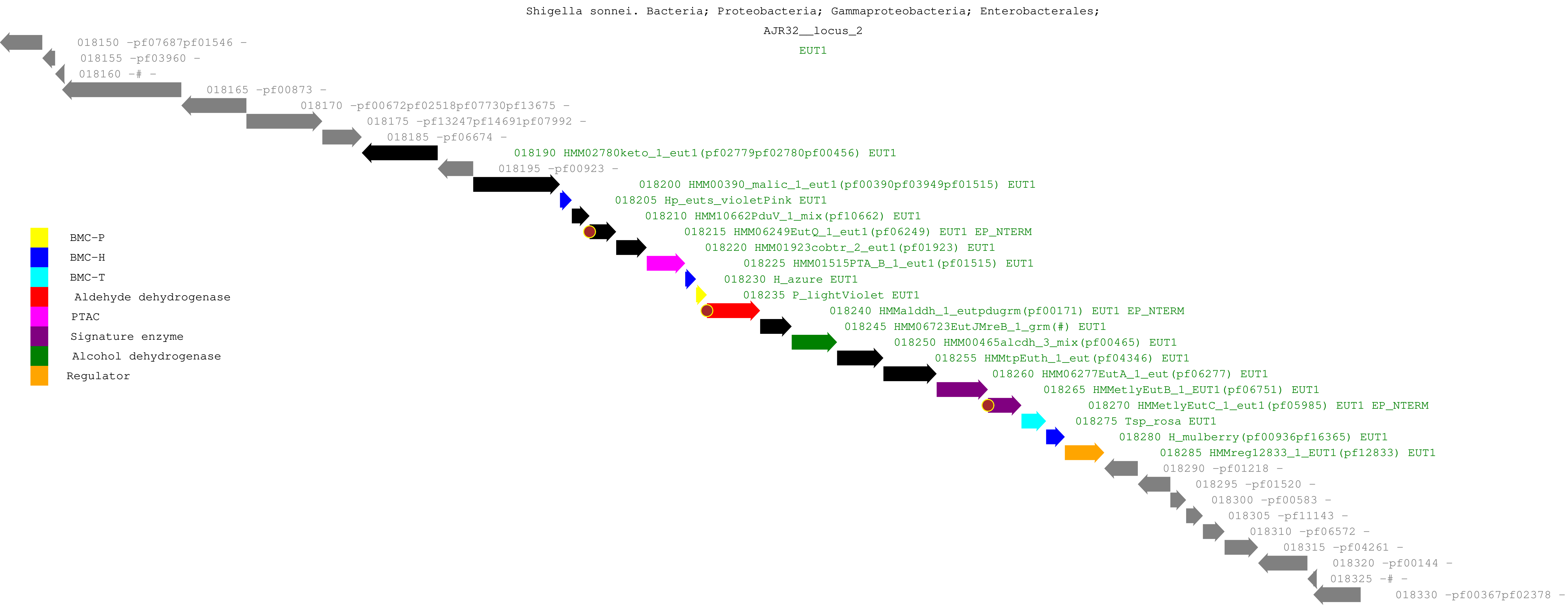

Shigella sonnei. Bacteria; Proteobacteria; Gammaproteobacteria; Enterobacterales;
AJR32__locus_2
EUT1
018150 -pf07687pf01546 -
018155 -pf03960 -
018160 -# -
018165 -pf00873 -
018170 -pf00672pf02518pf07730pf13675 -
018175 -pf13247pf14691pf07992 -
018185 -pf06674 -
018190 HMM02780keto_1_eut1(pf02779pf02780pf00456) EUT1
018195 -pf00923 -
018200 HMM00390_malic_1_eut1(pf00390pf03949pf01515) EUT1
018205 Hp_euts_violetPink EUT1
018210 HMM10662PduV_1_mix(pf10662) EUT1
018215 HMM06249EutQ_1_eut1(pf06249) EUT1 EP_NTERM
018220 HMM01923cobtr_2_eut1(pf01923) EUT1
018225 HMM01515PTA_B_1_eut1(pf01515) EUT1
018230 H_azure EUT1
018235 P_lightViolet EUT1
018240 HMMalddh_1_eutpdugrm(pf00171) EUT1 EP_NTERM
018245 HMM06723EutJMreB_1_grm(#) EUT1
018250 HMM00465alcdh_3_mix(pf00465) EUT1
018255 HMMtpEuth_1_eut(pf04346) EUT1
018260 HMM06277EutA_1_eut(pf06277) EUT1
018265 HMMetlyEutB_1_EUT1(pf06751) EUT1
018270 HMMetlyEutC_1_eut1(pf05985) EUT1 EP_NTERM
018275 Tsp_rosa EUT1
018280 H_mulberry(pf00936pf16365) EUT1
018285 HMMreg12833_1_EUT1(pf12833) EUT1
018290 -pf01218 -
018295 -pf01520 -
018300 -pf00583 -
018305 -pf11143 -
018310 -pf06572 -
018315 -pf04261 -
018320 -pf00144 -
018325 -# -
018330 -pf00367pf02378 -
BMC-P
BMC-H
BMC-T
Aldehyde dehydrogenase
PTAC
Signature enzyme
Alcohol dehydrogenase
Regulator

Escherichia coli. Bacteria; Proteobacteria; Gammaproteobacteria; Enterobacterales;

AKG99__locus_1

EUT1

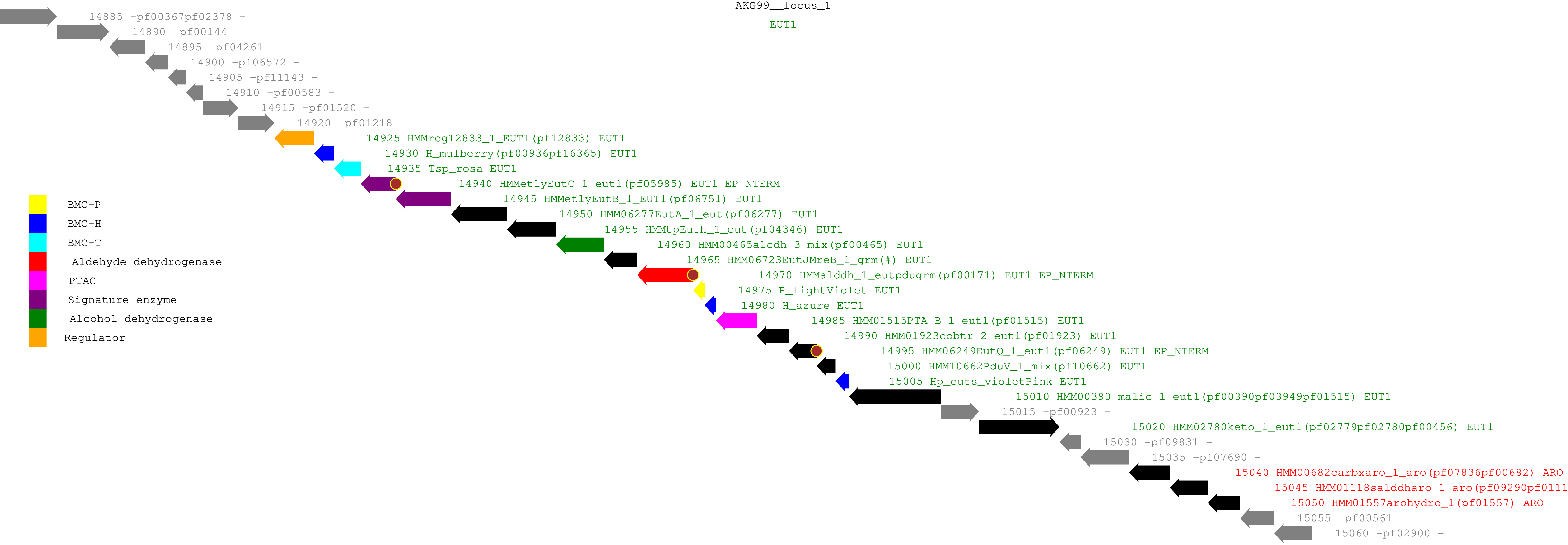

Escherichia coli. Bacteria; Proteobacteria; Gammaproteobacteria; Enterobacterales;

AKN40__locus_1

EUT1

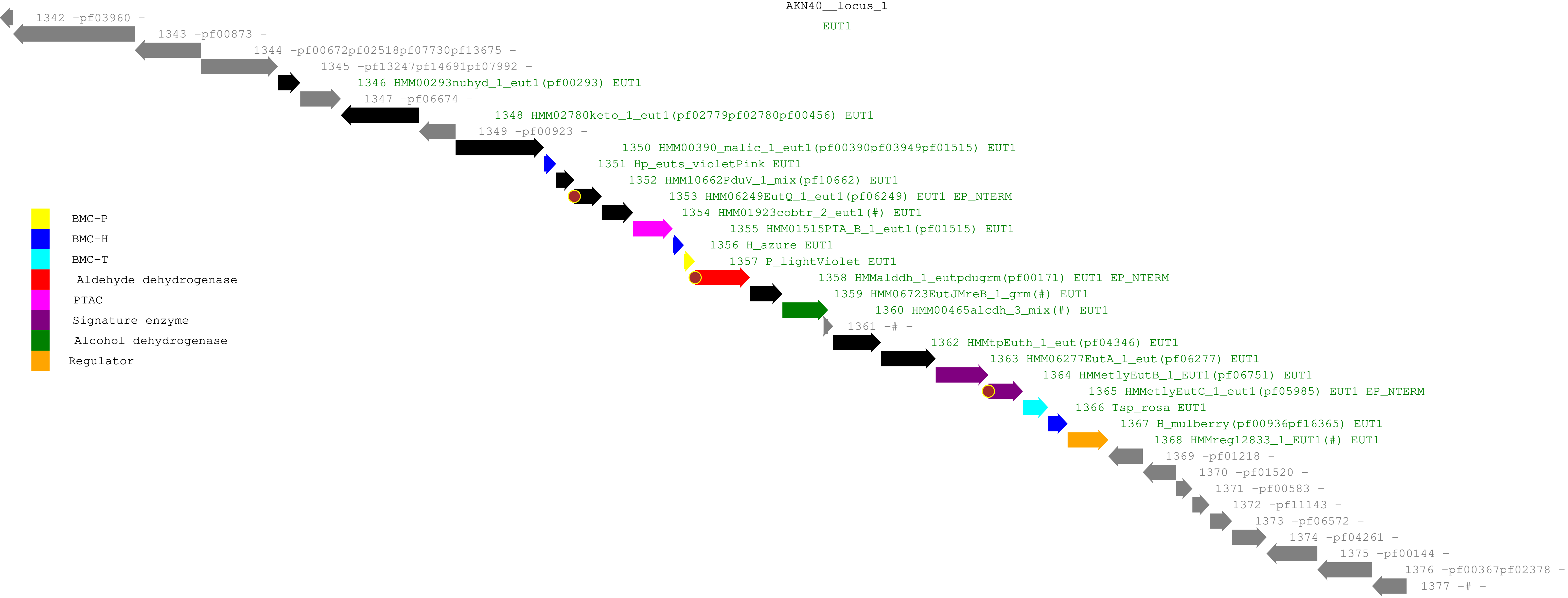

Citrobacter amalonaticus. Bacteria; Proteobacteria; Gammaproteobacteria; Enterobacterales;

AL012__locus_1

EUT1

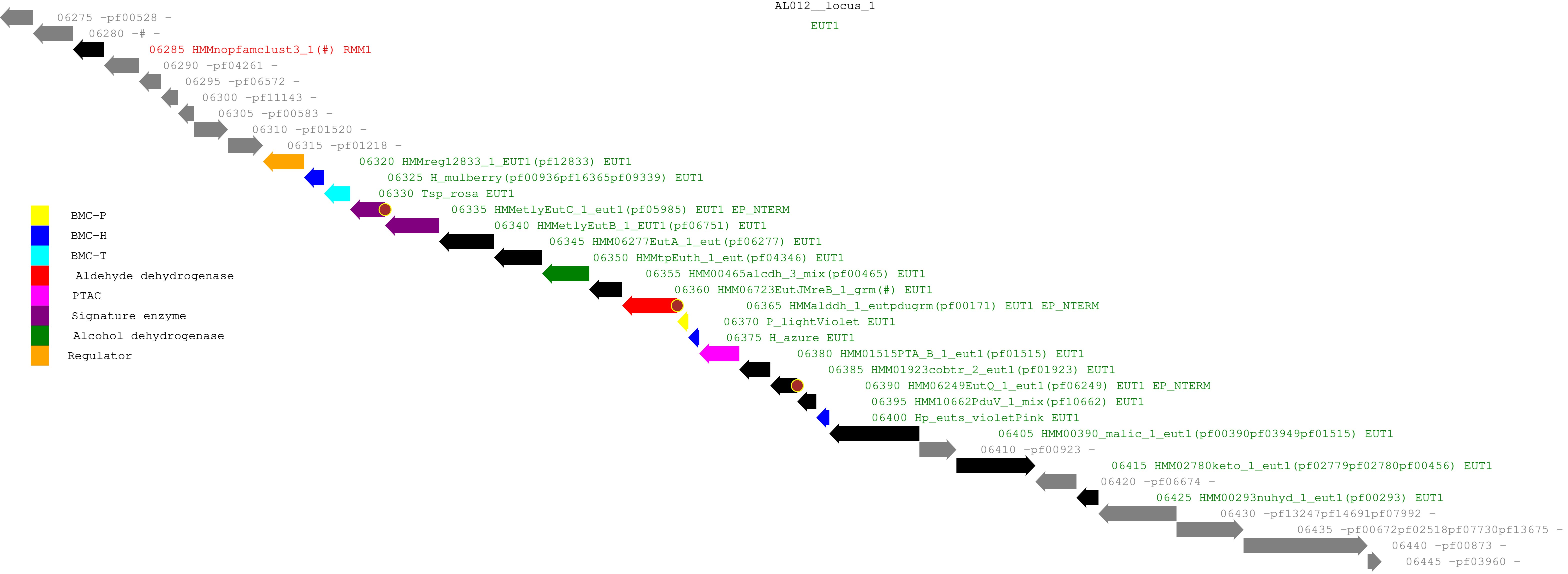

Citrobacter amalonaticus. Bacteria; Proteobacteria; Gammaproteobacteria; Enterobacterales;

AL479__locus_2

EUT1

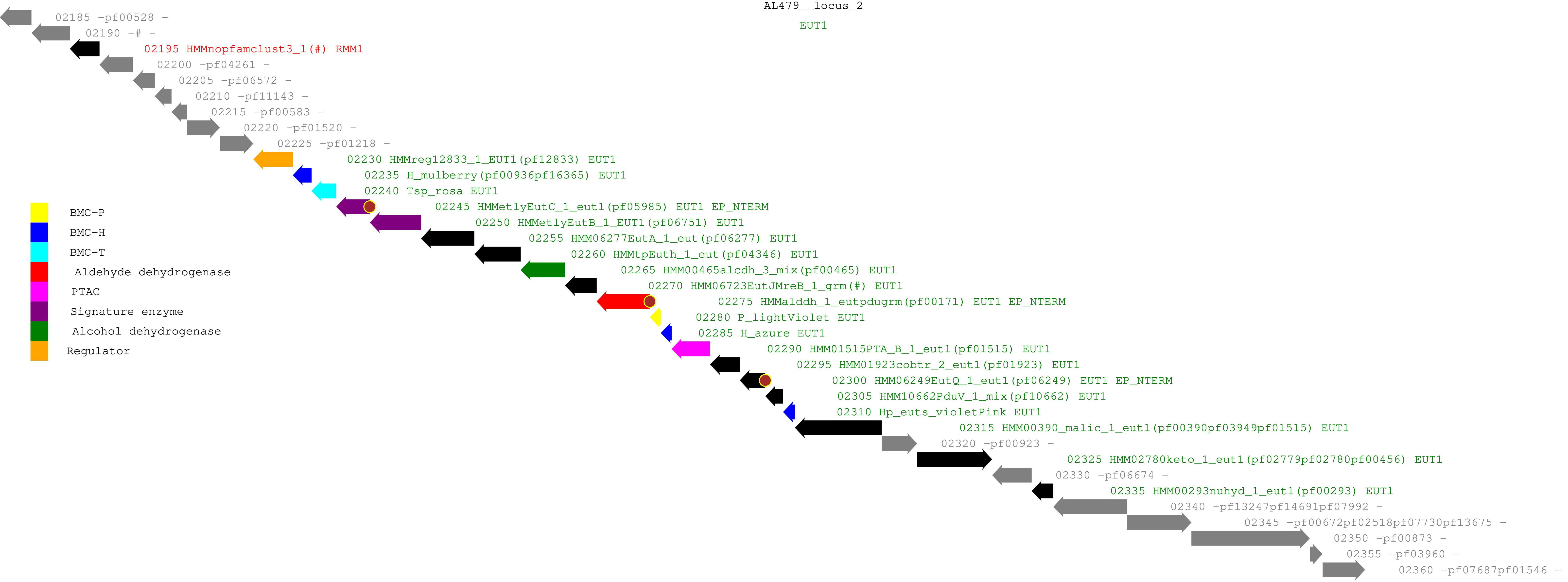

Escherichia fergusonii. Bacteria; Proteobacteria; Gammaproteobacteria; Enterobacterales;

AL530__locus_1

EUT1

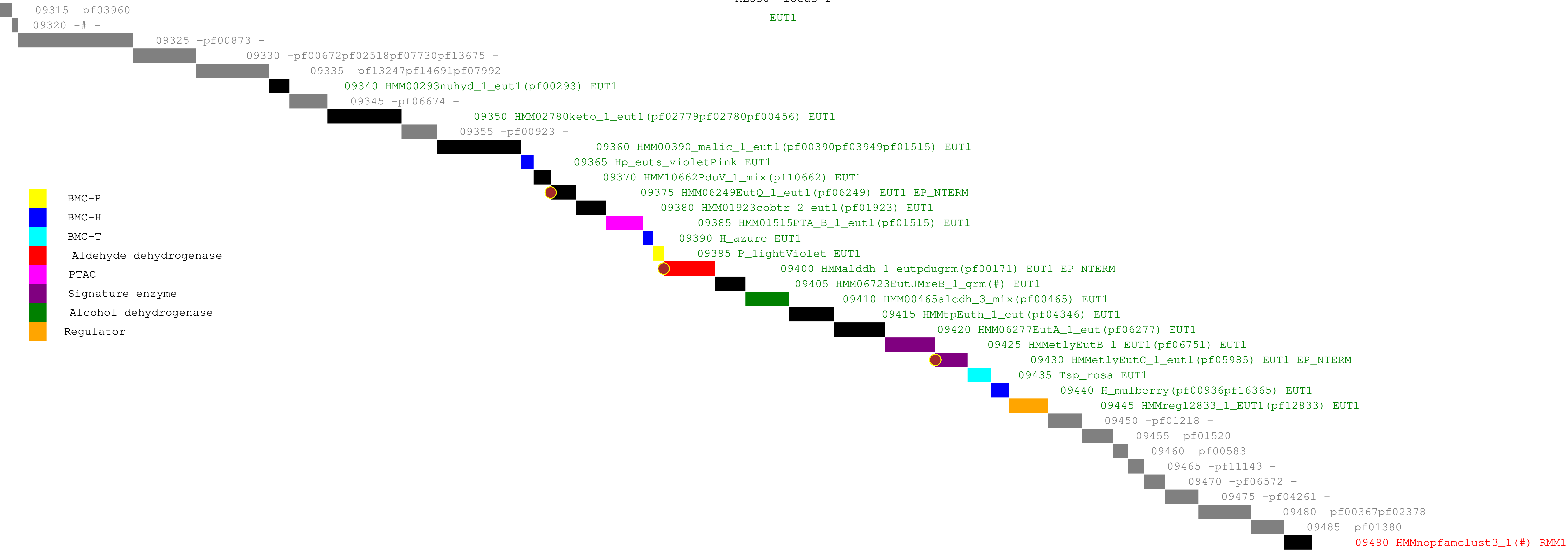

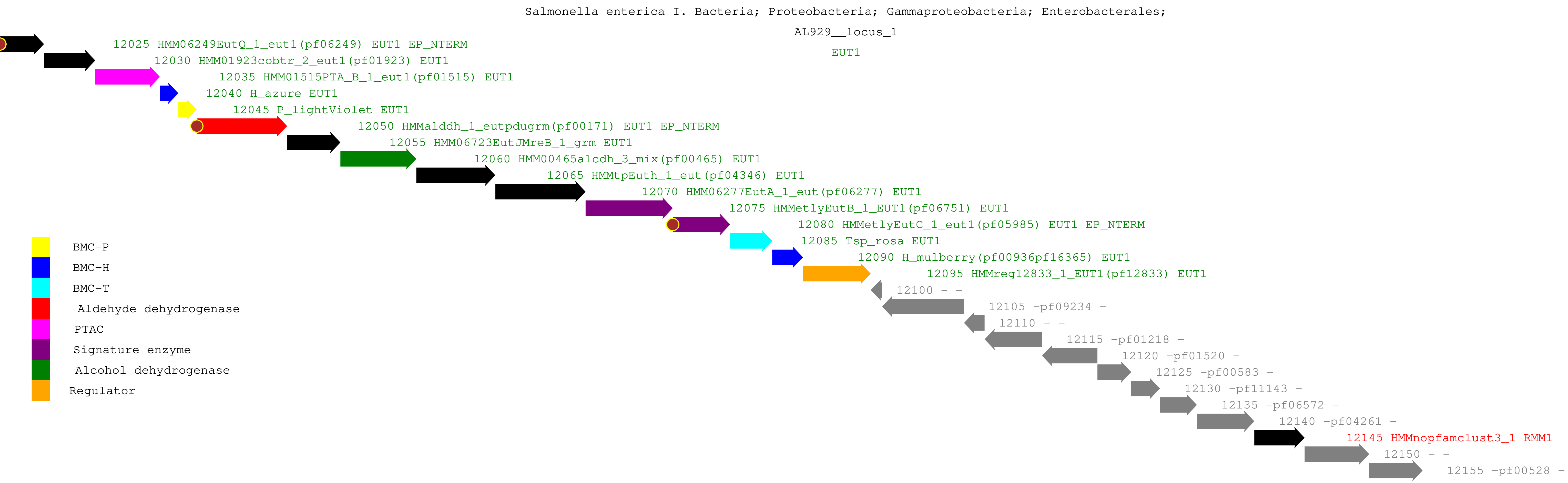

Salmonella enterica I. Bacteria; Proteobacteria; Gammaproteobacteria; Enterobacterales;
AL929__locus_1
EUT1
12025 HMM06249EutQ_1_eut1(pf06249) EUT1 EP_NTERM
12030 HMM01923cobtr_2_eut1(pf01923) EUT1
12035 HMM01515PTA_B_1_eut1(pf01515) EUT1
12040 H_azure EUT1
12045 P_lightViolet EUT1
12050 HMMalddh_1_eutpdugrm(pf00171) EUT1 EP_NTERM
12055 HMM06723EutJMreB_1_grm EUT1
12060 HMM00465alcdh_3_mix(pf00465) EUT1
12065 HMMtpEuth_1_eut(pf04346) EUT1
12070 HMM06277EutA_1_eut(pf06277) EUT1
12075 HMMetlyEutB_1_EUT1(pf06751) EUT1
12080 HMMetlyEutC_1_eut1(pf05985) EUT1 EP_NTERM
12085 Tsp_rosa EUT1
12090 H_mulberry(pf00936pf16365) EUT1
12095 HMMreg12833_1_EUT1(pf12833) EUT1
12100 - -
12105 -pf09234 -
12110 - -
12115 -pf01218 -
12120 -pf01520 -
12125 -pf00583 -
12130 -pf11143 -
12135 -pf06572 -
12140 -pf04261 -
12145 HMMnopfamclust3_1 RMM1
12150 - -
12155 -pf00528 -
BMC-P
BMC-H
BMC-T
Aldehyde dehydrogenase
PTAC
Signature enzyme
Alcohol dehydrogenase
Regulator

Salmonella infantis. Bacteria; Proteobacteria; Gammaproteobacteria; Enterobacterales;

AL960__locus_1

EUT1

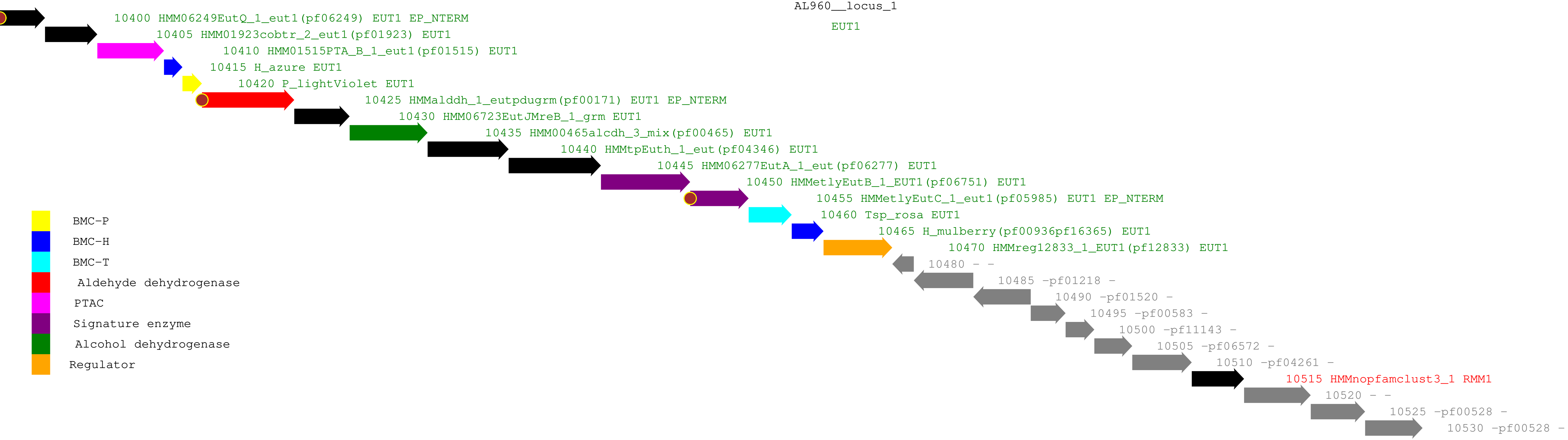

Salmonella enterica subsp. enterica serovar Pomona. Bacteria; Proteobacteria; Gammaproteobacteria; Enterobacterales;

AL980__locus_2

EUT1

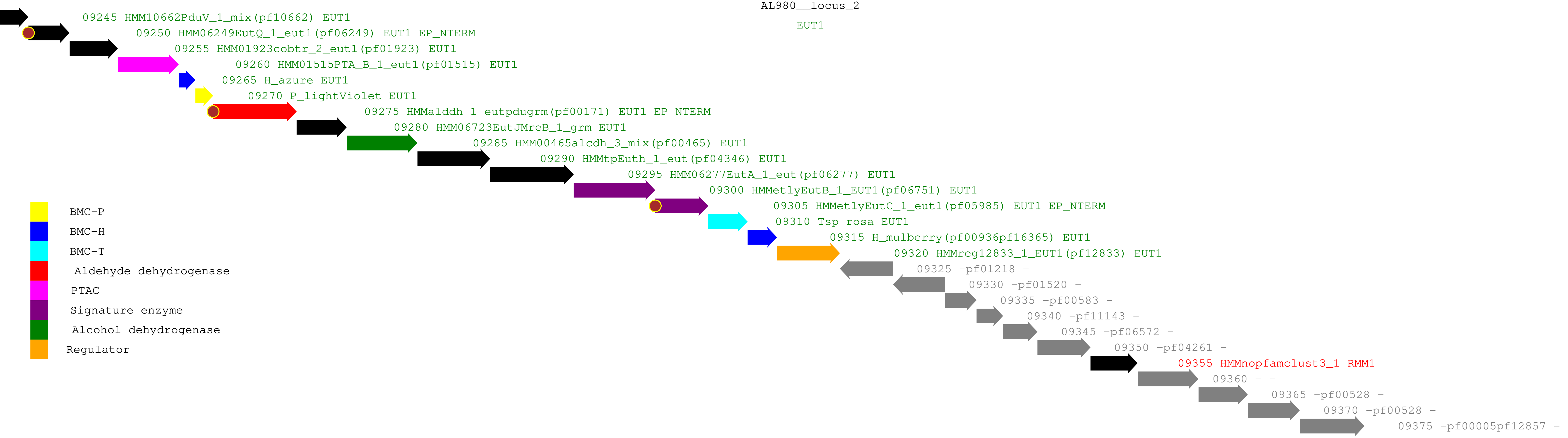

Salmonella typhimurium. Bacteria; Proteobacteria; Gammaproteobacteria; Enterobacterales;

AM358__locus_1

EUT1

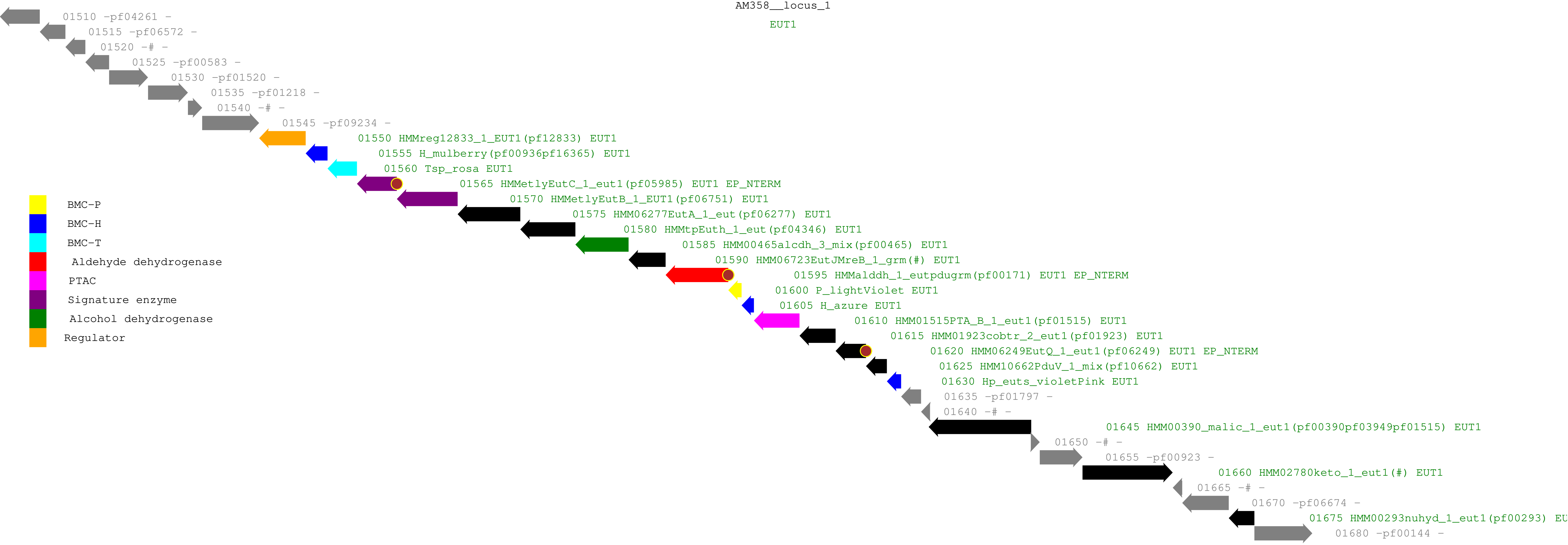

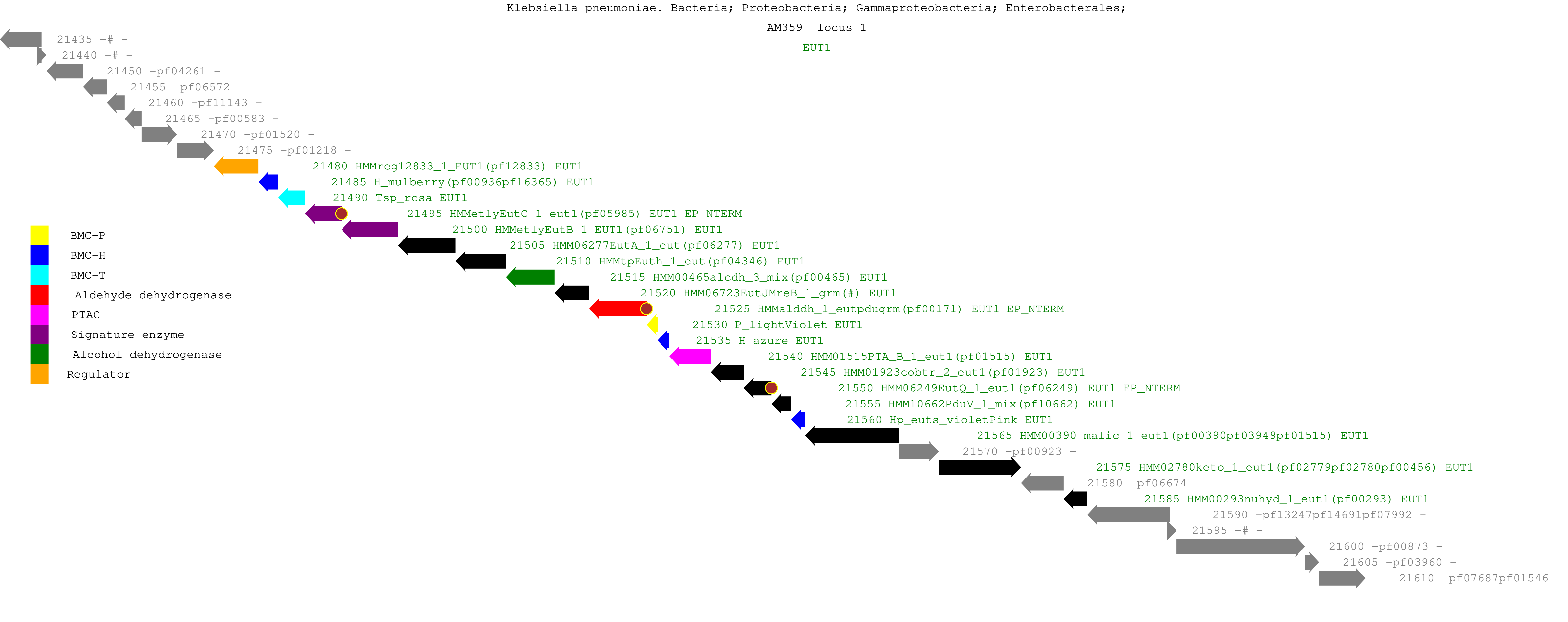
Klebsiella pneumoniae. Bacteria; Proteobacteria; Gammaproteobacteria; Enterobacterales;
AM359__locus_1
EUT1
21435 -# -
21440 -# -
21450 -pf04261 -
21455 -pf06572 -
21460 -pf11143 -
21465 -pf00583 -
21470 -pf01520 -
21475 -pf01218 -
21480 HMMreg12833_1_EUT1(pf12833) EUT1
21485 H_mulberry(pf00936pf16365) EUT1
21490 Tsp_rosa EUT1
21495 HMMetlyEutC_1_eut1(pf05985) EUT1 EP_NTERM
21500 HMMetlyEutB_1_EUT1(pf06751) EUT1
21505 HMM06277EutA_1_eut(pf06277) EUT1
21510 HMMtpEuth_1_eut(pf04346) EUT1
21515 HMM00465alcdh_3_mix(pf00465) EUT1
21520 HMM06723EutJMreB_1_grm(#) EUT1
21525 HMMalddh_1_eutpdugrm(pf00171) EUT1 EP_NTERM
21530 P_lightViolet EUT1
21535 H_azure EUT1
21540 HMM01515PTA_B_1_eut1(pf01515) EUT1
21545 HMM01923cobtr_2_eut1(pf01923) EUT1
21550 HMM06249EutQ_1_eut1(pf06249) EUT1 EP_NTERM
21555 HMM10662PduV_1_mix(pf10662) EUT1
21560 Hp_euts_violetPink EUT1
21565 HMM00390_malic_1_eut1(pf00390pf03949pf01515) EUT1
21570 -pf00923 -
21575 HMM02780keto_1_eut1(pf02779pf02780pf00456) EUT1
21580 -pf06674 -
21585 HMM00293nuhyd_1_eut1(pf00293) EUT1
21590 -pf13247pf14691pf07992 -
21595 -# -
21600 -pf00873 -
21605 -pf03960 -
21610 -pf07687pf01546 -
BMC-P
BMC-H
BMC-T
Aldehyde dehydrogenase
PTAC
Signature enzyme
Alcohol dehydrogenase
Regulator

Morganella morganii (Proteus morganii). Bacteria; Proteobacteria; Gammaproteobacteria; Enterobacterales;

AM380__locus_1

EUT1

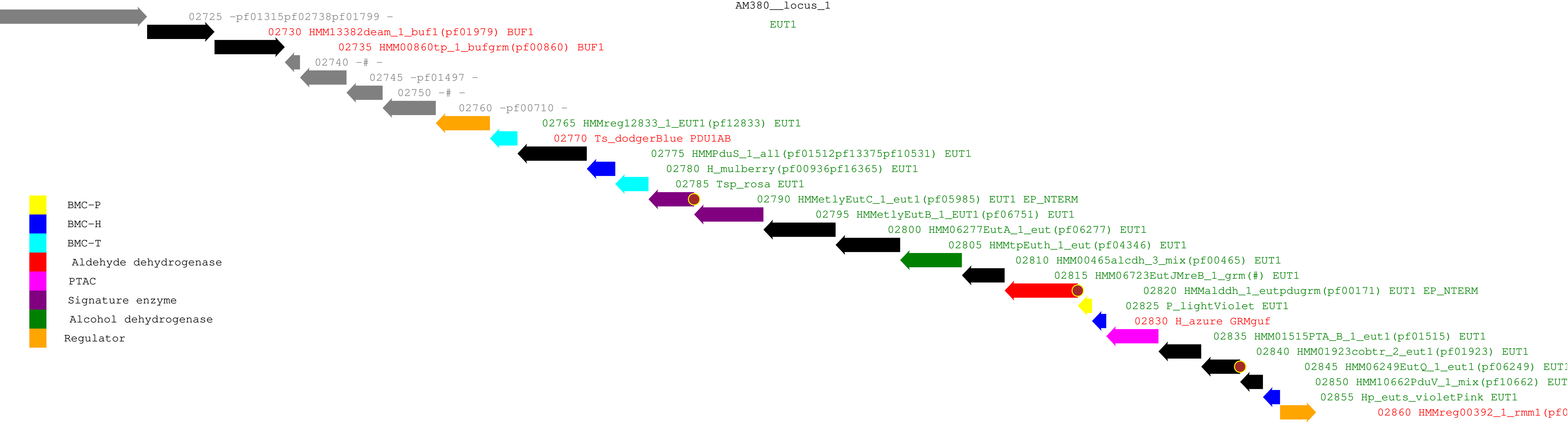

Klebsiella pneumoniae. Bacteria; Proteobacteria; Gammaproteobacteria; Enterobacterales;

AM390__locus_1

EUT1

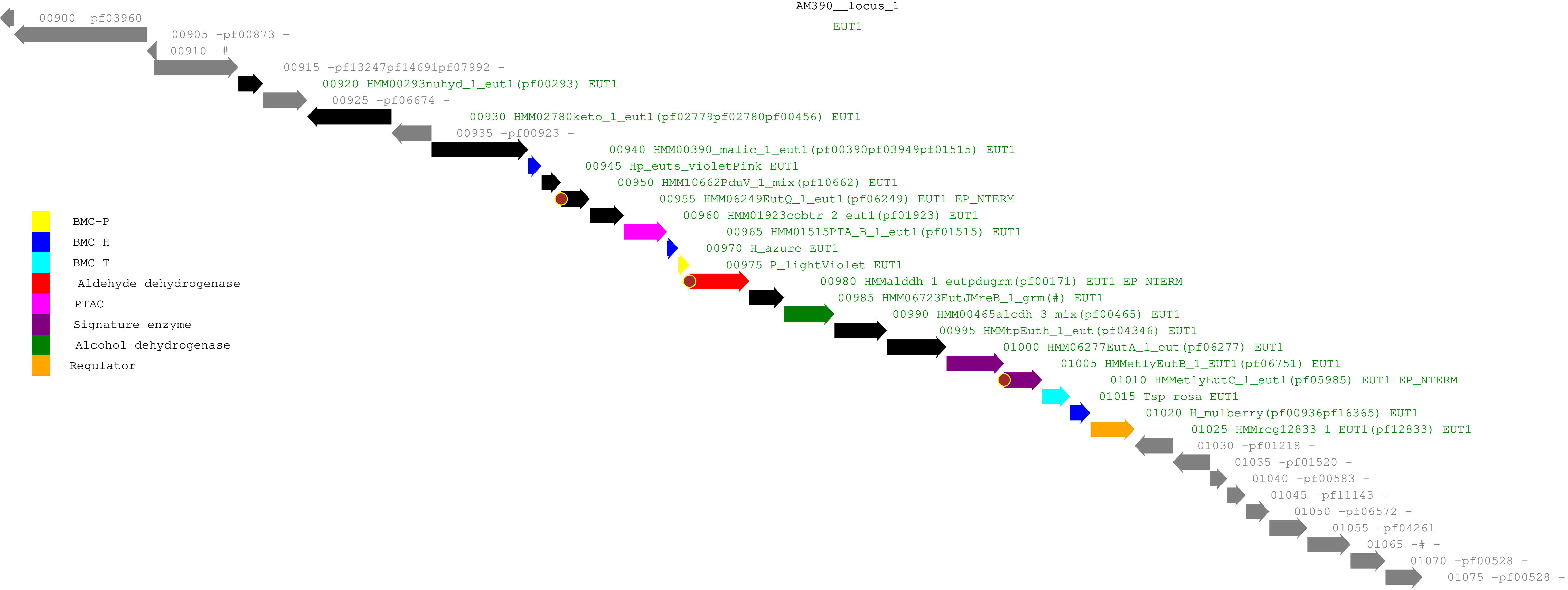

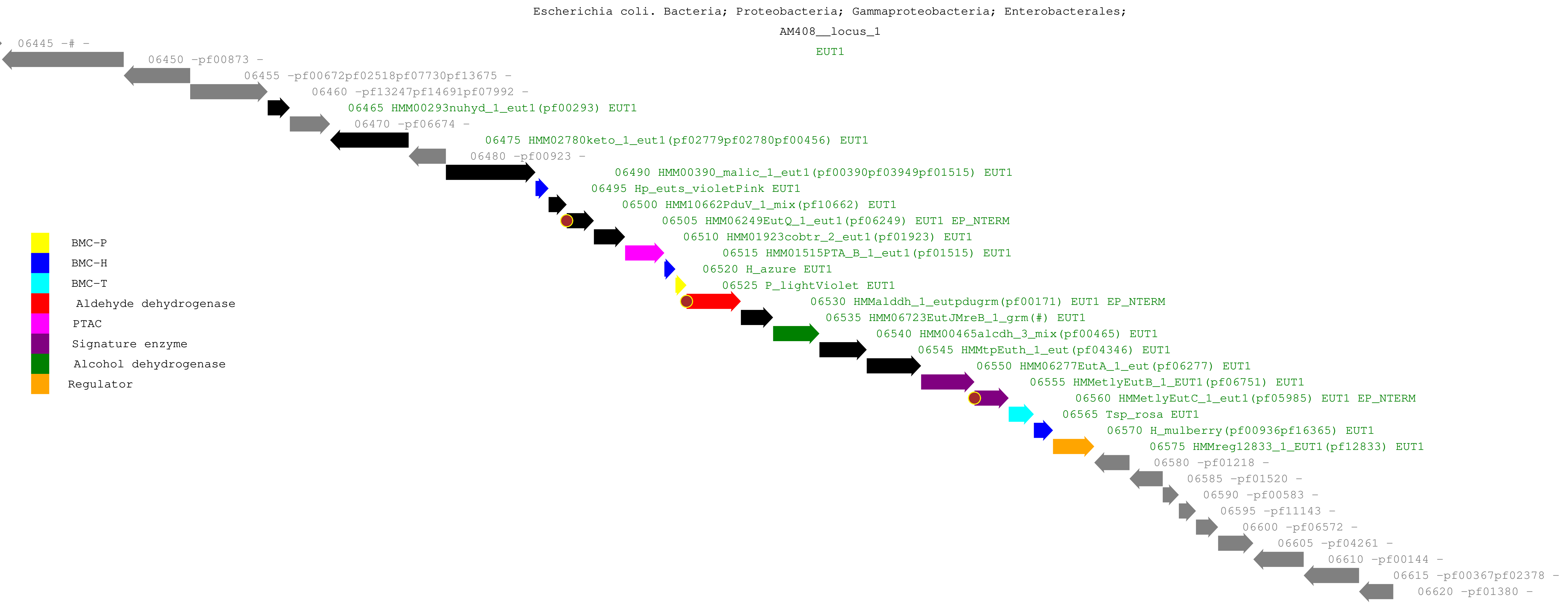

Escherichia coli. Bacteria; Proteobacteria; Gammaproteobacteria; Enterobacterales;
AM408__locus_1
EUT1
06445 -# -
06450 -pf00873 -
06455 -pf00672pf02518pf07730pf13675 -
06460 -pf13247pf14691pf07992 -
06465 HMM00293nuhyd_1_eut1(pf00293) EUT1
06470 -pf06674 -
06475 HMM02780keto_1_eut1(pf02779pf02780pf00456) EUT1
06480 -pf00923 -
06490 HMM00390_malic_1_eut1(pf00390pf03949pf01515) EUT1
06495 Hp_euts_violetPink EUT1
06500 HMM10662PduV_1_mix(pf10662) EUT1
06505 HMM06249EutQ_1_eut1(pf06249) EUT1 EP_NTERM
06510 HMM01923cobtr_2_eut1(pf01923) EUT1
06515 HMM01515PTA_B_1_eut1(pf01515) EUT1
06520 H_azure EUT1
06525 P_lightViolet EUT1
06530 HMMalddh_1_eutpdugrm(pf00171) EUT1 EP_NTERM
06535 HMM06723EutJMreB_1_grm(#) EUT1
06540 HMM00465alcdh_3_mix(pf00465) EUT1
06545 HMMtpEuth_1_eut(pf04346) EUT1
06550 HMM06277EutA_1_eut(pf06277) EUT1
06555 HMMetlyEutB_1_EUT1(pf06751) EUT1
06560 HMMetlyEutC_1_eut1(pf05985) EUT1 EP_NTERM
06565 Tsp_rosa EUT1
06570 H_mulberry(pf00936pf16365) EUT1
06575 HMMreg12833_1_EUT1(pf12833) EUT1
06580 -pf01218 -
06585 -pf01520 -
06590 -pf00583 -
06595 -pf11143 -
06600 -pf06572 -
06605 -pf04261 -
06610 -pf00144 -
06615 -pf00367pf02378 -
06620 -pf01380 -
BMC-P
BMC-H
BMC-T
Aldehyde dehydrogenase
PTAC
Signature enzyme
Alcohol dehydrogenase
Regulator

Klebsiella pneumoniae. Bacteria; Proteobacteria; Gammaproteobacteria; Enterobacterales;

AM447__locus_2

EUT1

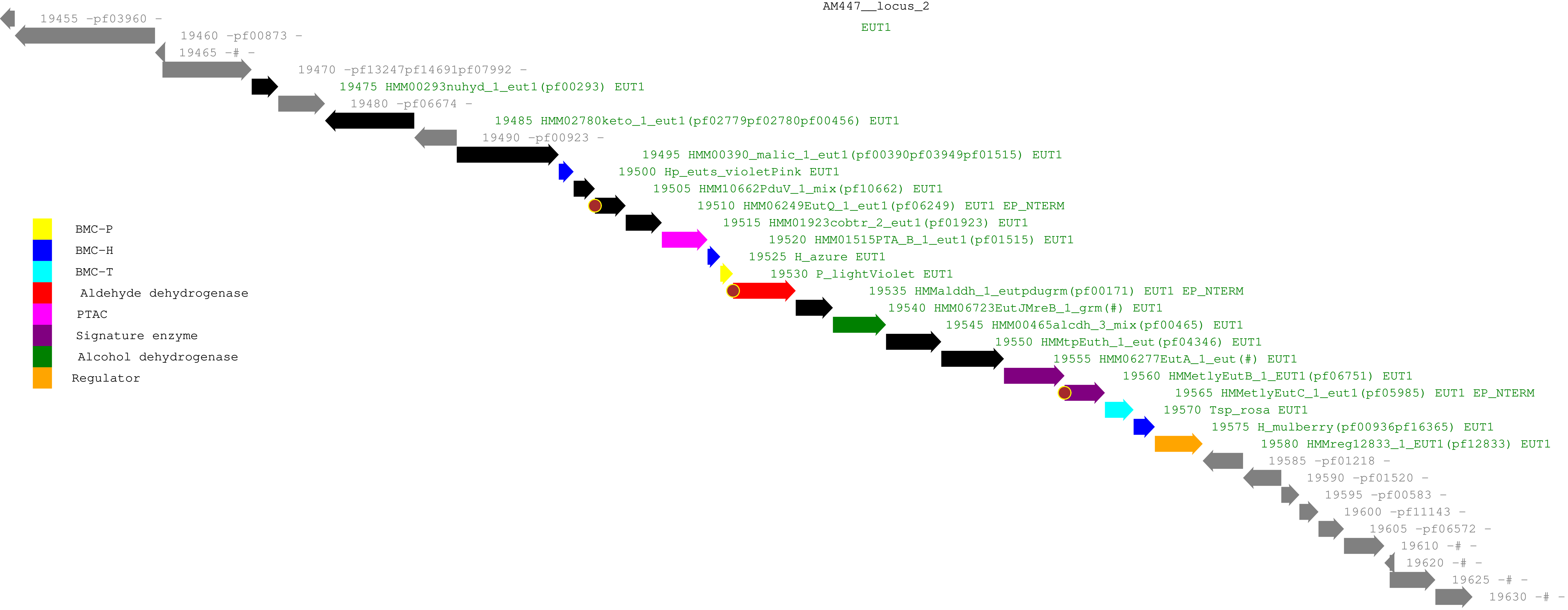

Salmonella enterica subsp. enterica serovar Brandenburg. Bacteria; Proteobacteria; Gammaproteobacteria; Enterobacterales;

AMA33__locus_1

EUT1

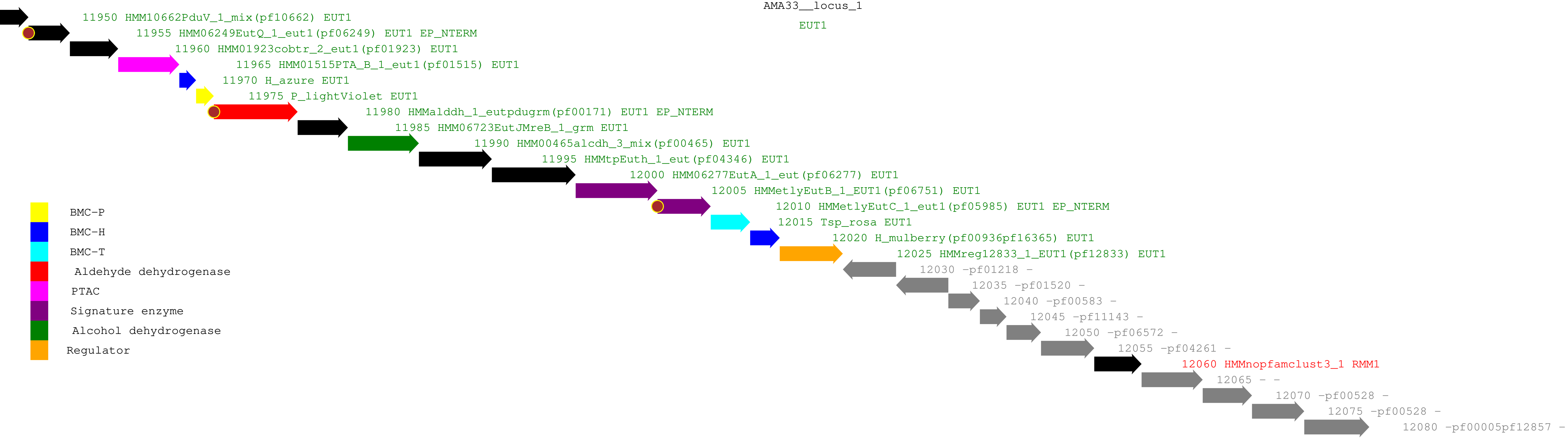

Escherichia coli. Bacteria; Proteobacteria; Gammaproteobacteria; Enterobacterales;

AML35__locus_1

EUT1

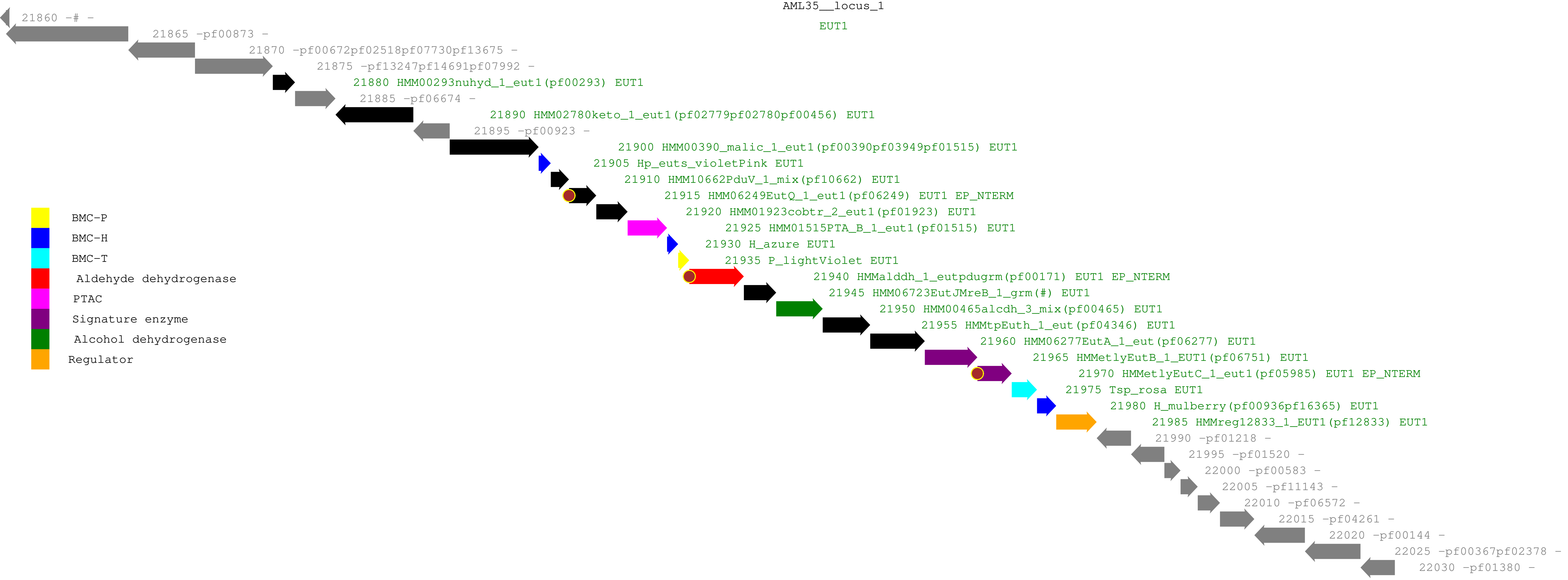

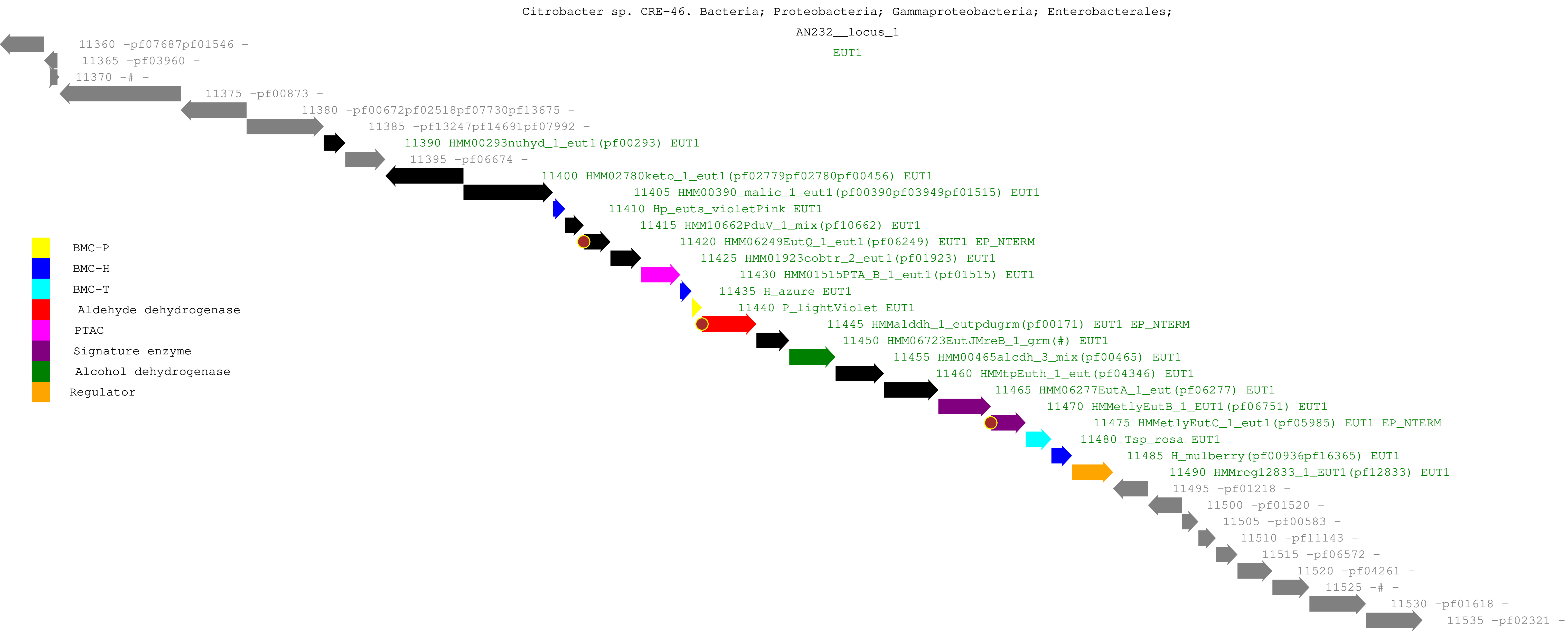
Citrobacter sp. CRE-46. Bacteria; Proteobacteria; Gammaproteobacteria; Enterobacterales;
AN232__locus_1
EUT1
11360 -pf07687pf01546 -
11365 -pf03960 -
11370 -# -
11375 -pf00873 -
11380 -pf00672pf02518pf07730pf13675 -
11385 -pf13247pf14691pf07992 -
11390 HMM00293nuhyd_1_eut1(pf00293) EUT1
11395 -pf06674 -
11400 HMM02780keto_1_eut1(pf02779pf02780pf00456) EUT1
11405 HMM00390_malic_1_eut1(pf00390pf03949pf01515) EUT1
11410 Hp_euts_violetPink EUT1
11415 HMM10662PduV_1_mix(pf10662) EUT1
11420 HMM06249EutQ_1_eut1(pf06249) EUT1 EP_NTERM
11425 HMM01923cobtr_2_eut1(pf01923) EUT1
11430 HMM01515PTA_B_1_eut1(pf01515) EUT1
11435 H_azure EUT1
11440 P_lightViolet EUT1
11445 HMMalddh_1_eutpdugrm(pf00171) EUT1 EP_NTERM
11450 HMM06723EutJMreB_1_grm(#) EUT1
11455 HMM00465alcdh_3_mix(pf00465) EUT1
11460 HMMtpEuth_1_eut(pf04346) EUT1
11465 HMM06277EutA_1_eut(pf06277) EUT1
11470 HMMetlyEutB_1_EUT1(pf06751) EUT1
11475 HMMetlyEutC_1_eut1(pf05985) EUT1 EP_NTERM
11480 Tsp_rosa EUT1
11485 H_mulberry(pf00936pf16365) EUT1
11490 HMMreg12833_1_EUT1(pf12833) EUT1
11495 -pf01218 -
11500 -pf01520 -
11505 -pf00583 -
11510 -pf11143 -
11515 -pf06572 -
11520 -pf04261 -
11525 -# -
11530 -pf01618 -
11535 -pf02321 -
BMC-P
BMC-H
BMC-T
Aldehyde dehydrogenase
PTAC
Signature enzyme
Alcohol dehydrogenase
Regulator

Citrobacter freundii. Bacteria; Proteobacteria; Gammaproteobacteria; Enterobacterales;

AN672__locus_1

EUT1

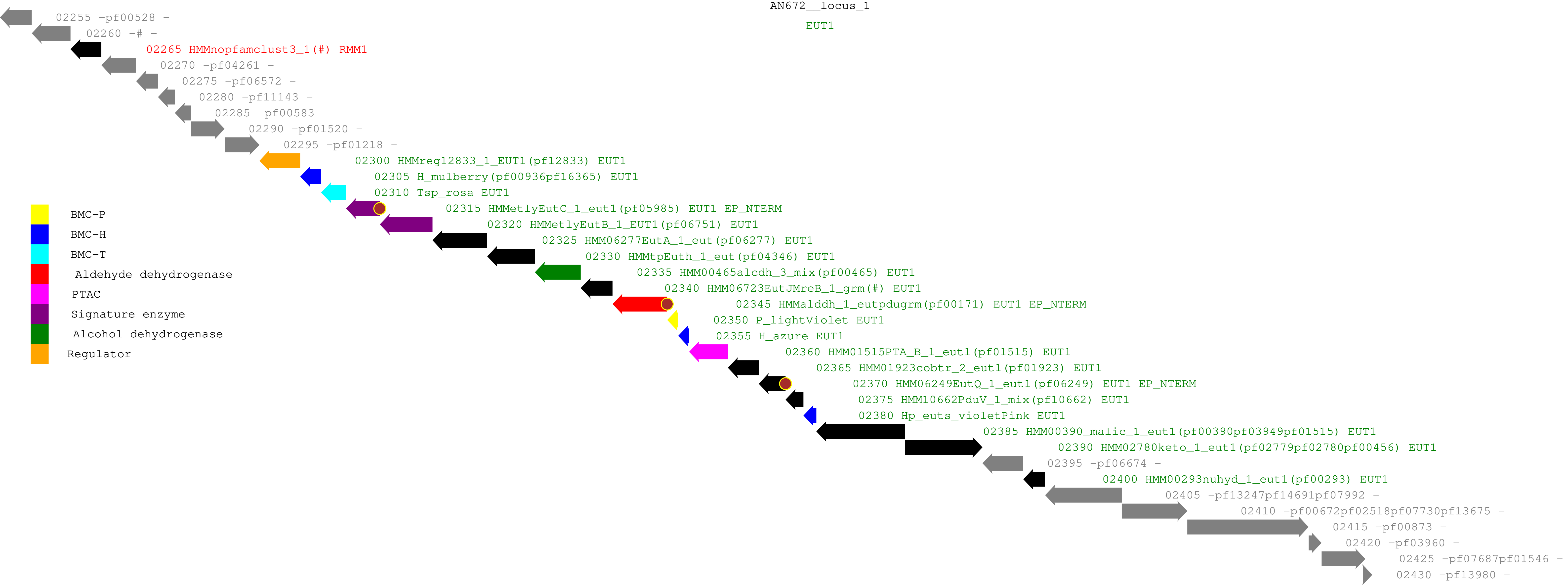

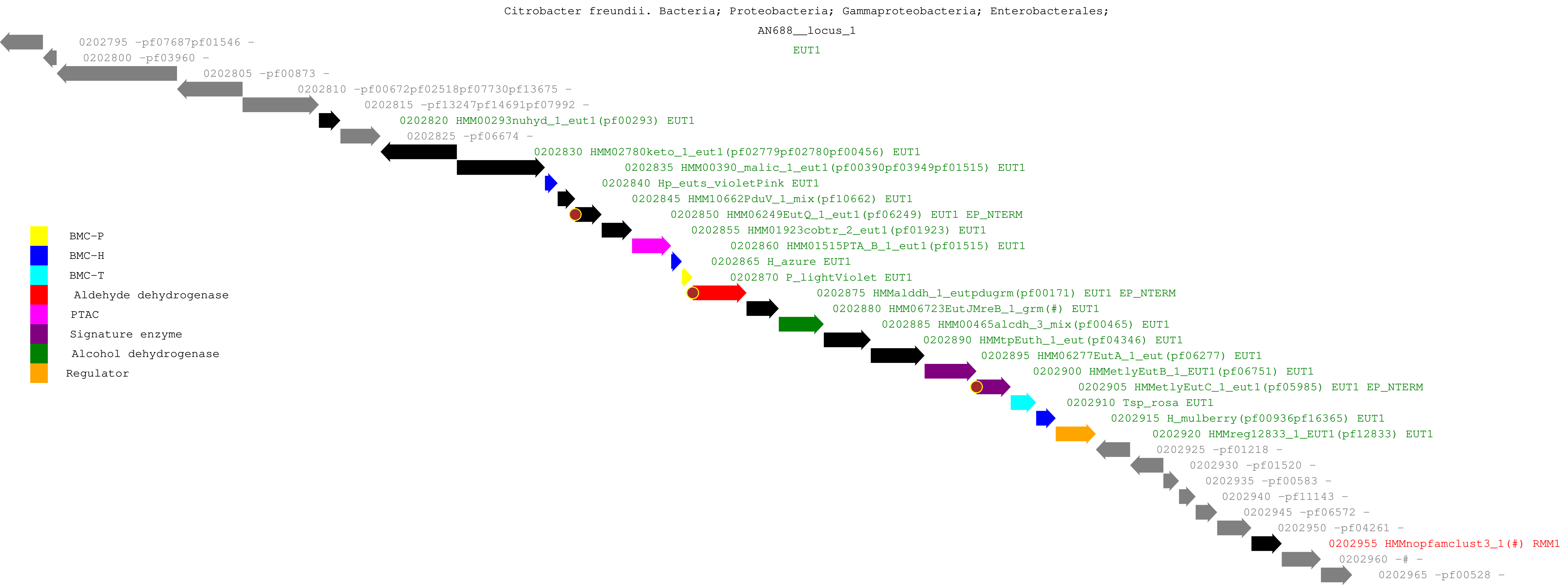
Citrobacter freundii. Bacteria; Proteobacteria; Gammaproteobacteria; Enterobacterales;
AN688__locus_1
EUT1
0202795 -pf07687pf01546 -
0202800 -pf03960 -
0202805 -pf00873 -
0202810 -pf00672pf02518pf07730pf13675 -
0202815 -pf13247pf14691pf07992 -
0202820 HMM00293nuhyd_1_eut1(pf00293) EUT1
0202825 -pf06674 -
0202830 HMM02780keto_1_eut1(pf02779pf02780pf00456) EUT1
0202835 HMM00390_malic_1_eut1(pf00390pf03949pf01515) EUT1
0202840 Hp_euts_violetPink EUT1
0202845 HMM10662PduV_1_mix(pf10662) EUT1
0202850 HMM06249EutQ_1_eut1(pf06249) EUT1 EP_NTERM
0202855 HMM01923cobtr_2_eut1(pf01923) EUT1
0202860 HMM01515PTA_B_1_eut1(pf01515) EUT1
0202865 H_azure EUT1
0202870 P_lightViolet EUT1
0202875 HMMalddh_1_eutpdugrm(pf00171) EUT1 EP_NTERM
0202880 HMM06723EutJMreB_1_grm(#) EUT1
0202885 HMM00465alcdh_3_mix(pf00465) EUT1
0202890 HMMtpEuth_1_eut(pf04346) EUT1
0202895 HMM06277EutA_1_eut(pf06277) EUT1
0202900 HMMetlyEutB_1_EUT1(pf06751) EUT1
0202905 HMMetlyEutC_1_eut1(pf05985) EUT1 EP_NTERM
0202910 Tsp_rosa EUT1
0202915 H_mulberry(pf00936pf16365) EUT1
0202920 HMMreg12833_1_EUT1(pf12833) EUT1
0202925 -pf01218 -
0202930 -pf01520 -
0202935 -pf00583 -
0202940 -pf11143 -
0202945 -pf06572 -
0202950 -pf04261 -
0202955 HMMnopfamclust3_1(#) RMM1
0202960 -# -
0202965 -pf00528 -
BMC-P
BMC-H
BMC-T
Aldehyde dehydrogenase
PTAC
Signature enzyme
Alcohol dehydrogenase
Regulator

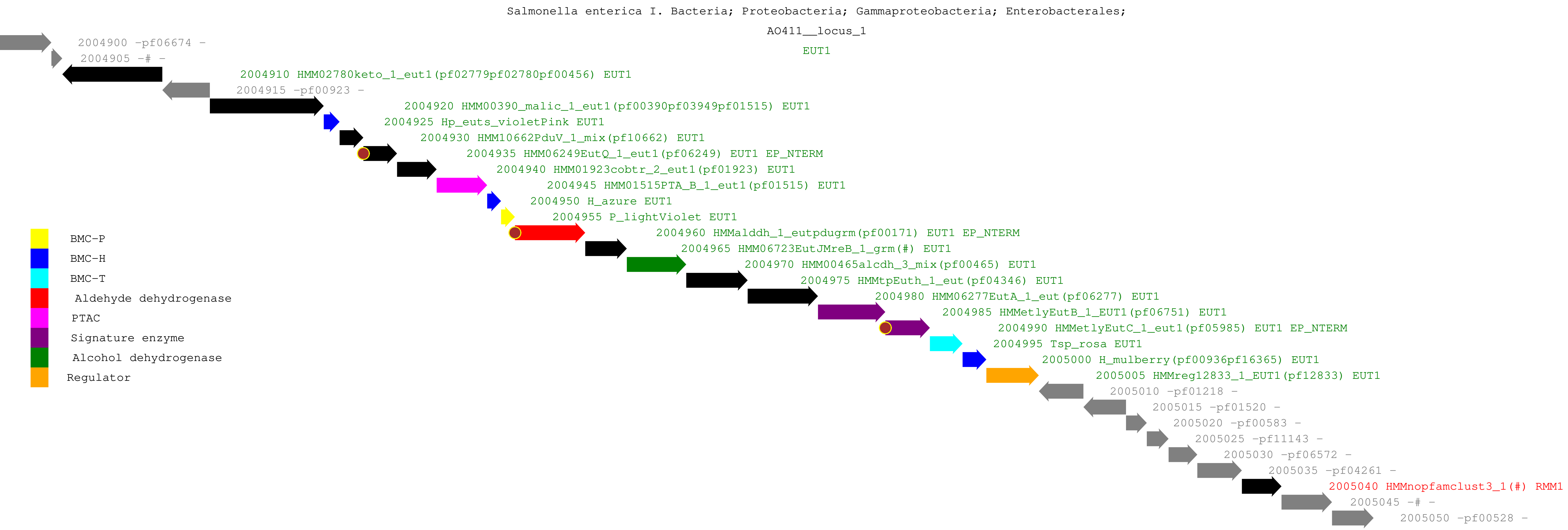
Salmonella enterica I. Bacteria; Proteobacteria; Gammaproteobacteria; Enterobacterales;
A0411__locus_1
EUT1
2004900 -pf06674 -
2004905 -# -
2004910 HMM02780keto_1_eut1(pf02779pf02780pf00456) EUT1
2004915 -pf00923 -
2004920 HMM00390_malic_1_eut1(pf00390pf03949pf01515) EUT1
2004925 Hp_euts_violetPink EUT1
2004930 HMM10662PduV_1_mix(pf10662) EUT1
2004935 HMM06249EutQ_1_eut1(pf06249) EUT1 EP_NTERM
2004940 HMM01923cobtr_2_eut1(pf01923) EUT1
2004945 HMM01515PTA_B_1_eut1(pf01515) EUT1
2004950 H_azure EUT1
2004955 P_lightViolet EUT1
2004960 HMMalddh_1_eutpdugrm(pf00171) EUT1 EP_NTERM
2004965 HMM06723EutJMreB_1_grm(#) EUT1
2004970 HMM00465alcdh_3_mix(pf00465) EUT1
2004975 HMMtpEuth_1_eut(pf04346) EUT1
2004980 HMM06277EutA_1_eut(pf06277) EUT1
2004985 HMMetlyEutB_1_EUT1(pf06751) EUT1
2004990 HMMetlyEutC_1_eut1(pf05985) EUT1 EP_NTERM
2004995 Tsp_rosa EUT1
2005000 H_mulberry(pf00936pf16365) EUT1
2005005 HMMreg12833_1_EUT1(pf12833) EUT1
2005010 -pf01218 -
2005015 -pf01520 -
2005020 -pf00583 -
2005025 -pf11143 -
2005030 -pf06572 -
2005035 -pf04261 -
2005040 HMMnopfamclust3_1(#) RMM1
2005045 -# -
2005050 -pf00528 -
BMC-P
BMC-H
BMC-T
Aldehyde dehydrogenase
PTAC
Signature enzyme
Alcohol dehydrogenase
Regulator

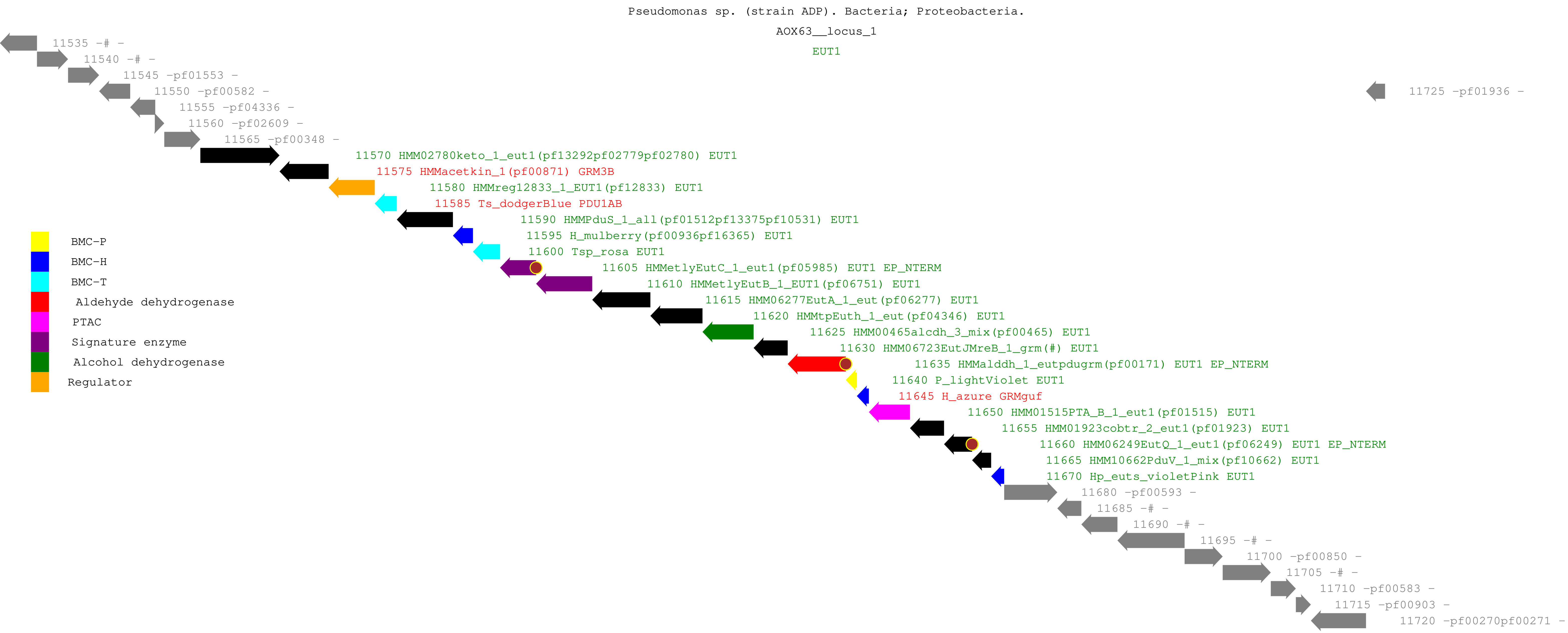
Pseudomonas sp. (strain ADP). Bacteria; Proteobacteria.
AOX63__locus_1
EUT1
11535 -# -
11540 -# -
11545 -pf01553 -
11550 -pf00582 -
11555 -pf04336 -
11560 -pf02609 -
11565 -pf00348 -
11570 HMM02780keto_1_eut1(pf13292pf02779pf02780) EUT1
11575 HMMacetkin_1(pf00871) GRM3B
11580 HMMreg12833_1_EUT1(pf12833) EUT1
11585 Ts_dodgerBlue PDU1AB
11590 HMMPduS_1_all(pf01512pf13375pf10531) EUT1
11595 H_mulberry(pf00936pf16365) EUT1
11600 Tsp_rosa EUT1
11605 HMMetlyEutC_1_eut1(pf05985) EUT1 EP_NTERM
11610 HMMetlyEutB_1_EUT1(pf06751) EUT1
11615 HMM06277EutA_1_eut(pf06277) EUT1
11620 HMMtpEuth_1_eut(pf04346) EUT1
11625 HMM00465alcdh_3_mix(pf00465) EUT1
11630 HMM06723EutJMreB_1_grm(#) EUT1
11635 HMMalddh_1_eutpdugrm(pf00171) EUT1 EP_NTERM
11640 P_lightViolet EUT1
11645 H_azure GRMguf
11650 HMM01515PTA_B_1_eut1(pf01515) EUT1
11655 HMM01923cobtr_2_eut1(pf01923) EUT1
11660 HMM06249EutQ_1_eut1(pf06249) EUT1 EP_NTERM
11665 HMM10662PduV_1_mix(pf10662) EUT1
11670 Hp_euts_violetPink EUT1
11680 -pf00593 -
11685 -# -
11690 -# -
11695 -# -
11700 -pf00850 -
11705 -# -
11710 -pf00583 -
11715 -pf00903 -
11720 -pf00270pf00271 -
11725 -pf01936 -
BMC-P
BMC-H
BMC-T
Aldehyde dehydrogenase
PTAC
Signature enzyme
Alcohol dehydrogenase
Regulator

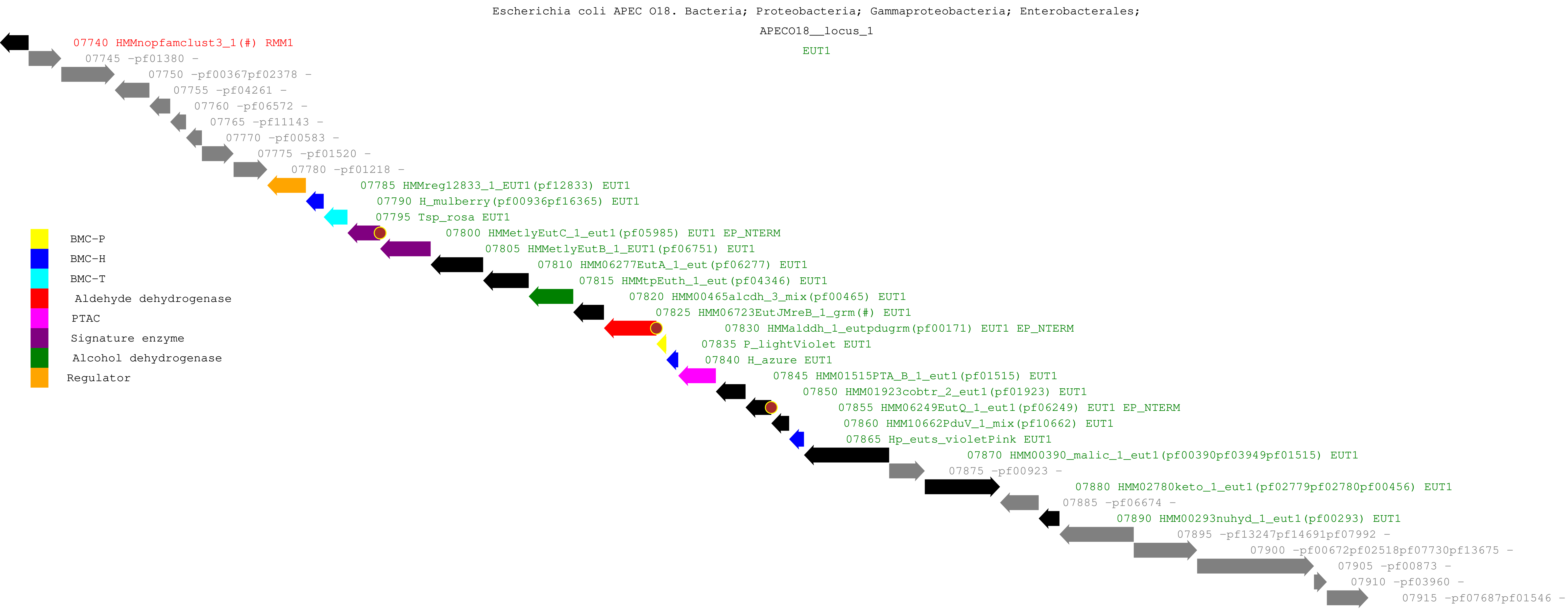
Escherichia coli APEC O18. Bacteria; Proteobacteria; Gammaproteobacteria; Enterobacterales;
APECO18__locus_1
EUT1
07740 HMMnopfamclust3_1(#) RMM1
07745 -pf01380 -
07750 -pf00367pf02378 -
07755 -pf04261 -
07760 -pf06572 -
07765 -pf11143 -
07770 -pf00583 -
07775 -pf01520 -
07780 -pf01218 -
07785 HMMreg12833_1_EUT1(pf12833) EUT1
07790 H_mulberry(pf00936pf16365) EUT1
07795 Tsp_rosa EUT1
07800 HMMetlyEutC_1_eut1(pf05985) EUT1 EP_NTERM
07805 HMMetlyEutB_1_EUT1(pf06751) EUT1
07810 HMM06277EutA_1_eut(pf06277) EUT1
07815 HMMtpEuth_1_eut(pf04346) EUT1
07820 HMM00465alcdh_3_mix(pf00465) EUT1
07825 HMM06723EutJMreB_1_grm(#) EUT1
07830 HMMalddh_1_eutpdugrm(pf00171) EUT1 EP_NTERM
07835 P_lightViolet EUT1
07840 H_azure EUT1
07845 HMM01515PTA_B_1_eut1(pf01515) EUT1
07850 HMM01923cobtr_2_eut1(pf01923) EUT1
07855 HMM06249EutQ_1_eut1(pf06249) EUT1 EP_NTERM
07860 HMM10662PduV_1_mix(pf10662) EUT1
07865 Hp_euts_violetPink EUT1
07870 HMM00390_malic_1_eut1(pf00390pf03949pf01515) EUT1
07875 -pf00923 -
07880 HMM02780keto_1_eut1(pf02779pf02780pf00456) EUT1
07885 -pf06674 -
07890 HMM00293nuhyd_1_eut1(pf00293) EUT1
07895 -pf13247pf14691pf07992 -
07900 -pf00672pf02518pf07730pf13675 -
07905 -pf00873 -
07910 -pf03960 -
07915 -pf07687pf01546 -
BMC-P
BMC-H
BMC-T
Aldehyde dehydrogenase
PTAC
Signature enzyme
Alcohol dehydrogenase
Regulator

Escherichia coli O1:K1 / APEC. Bacteria; Proteobacteria; Gammaproteobacteria; Enterobacterales;

APECO1__locus_2

EUT1

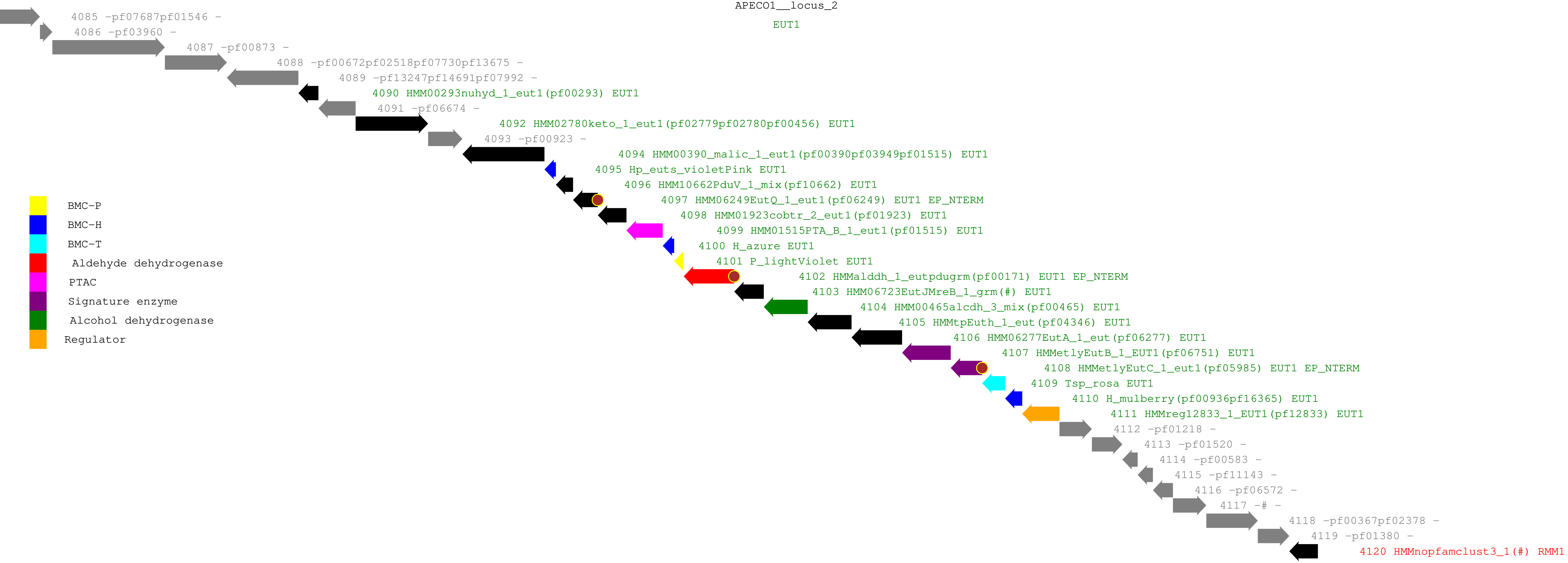

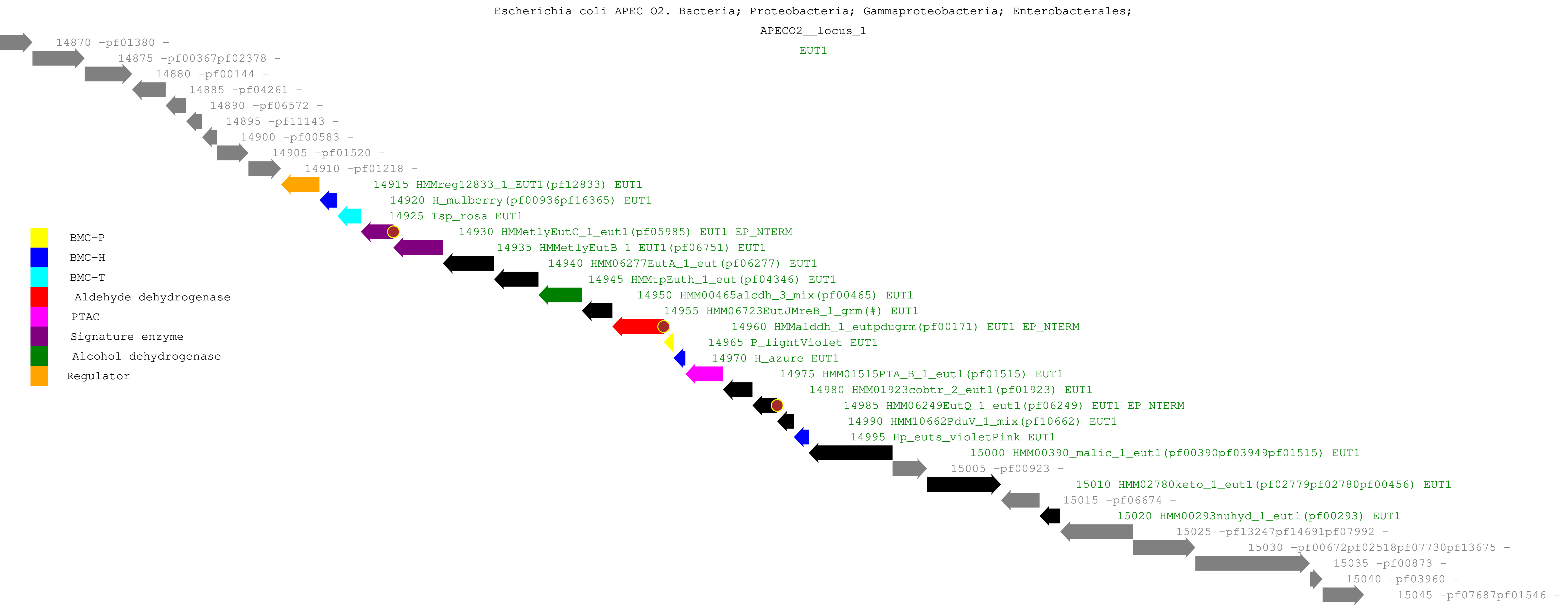
Escherichia coli APEC O2. Bacteria; Proteobacteria; Gammaproteobacteria; Enterobacterales;
APECO2__locus_1
EUT1
14870 -pf01380 -
14875 -pf00367pf02378 -
14880 -pf00144 -
14885 -pf04261 -
14890 -pf06572 -
14895 -pf11143 -
14900 -pf00583 -
14905 -pf01520 -
14910 -pf01218 -
14915 HMMreg12833_1_EUT1(pf12833) EUT1
14920 H_mulberry(pf00936pf16365) EUT1
14925 Tsp_rosa EUT1
14930 HMMetlyEutC_1_eut1(pf05985) EUT1 EP_NTERM
14935 HMMetlyEutB_1_EUT1(pf06751) EUT1
14940 HMM06277EutA_1_eut(pf06277) EUT1
14945 HMMtpEuth_1_eut(pf04346) EUT1
14950 HMM00465alcdh_3_mix(pf00465) EUT1
14955 HMM06723EutJMreB_1_grm(#) EUT1
14960 HMMalddh_1_eutpdugrm(pf00171) EUT1 EP_NTERM
14965 P_lightViolet EUT1
14970 H_azure EUT1
14975 HMM01515PTA_B_1_eut1(pf01515) EUT1
14980 HMM01923cobtr_2_eut1(pf01923) EUT1
14985 HMM06249EutQ_1_eut1(pf06249) EUT1 EP_NTERM
14990 HMM10662PduV_1_mix(pf10662) EUT1
14995 Hp_euts_violetPink EUT1
15000 HMM00390_malic_1_eut1(pf00390pf03949pf01515) EUT1
15005 -pf00923 -
15010 HMM02780keto_1_eut1(pf02779pf02780pf00456) EUT1
15015 -pf06674 -
15020 HMM00293nuhyd_1_eut1(pf00293) EUT1
15025 -pf13247pf14691pf07992 -
15030 -pf00672pf02518pf07730pf13675 -
15035 -pf00873 -
15040 -pf03960 -
15045 -pf07687pf01546 -
BMC-P
BMC-H
BMC-T
Aldehyde dehydrogenase
PTAC
Signature enzyme
Alcohol dehydrogenase
Regulator

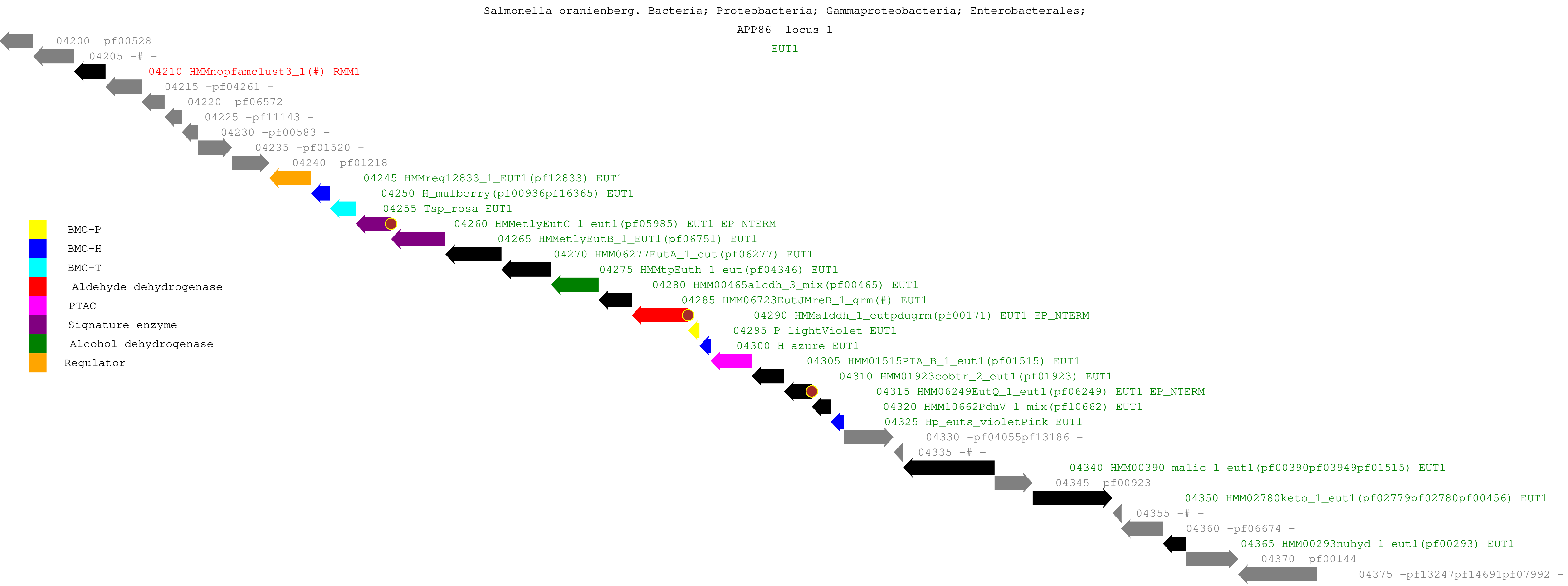
Salmonella oranienberg. Bacteria; Proteobacteria; Gammaproteobacteria; Enterobacterales;
APP86__locus_1
EUT1
04200 -pf00528 -
04205 -# -
04210 HMMnopfamclust3_1(#) RMM1
04215 -pf04261 -
04220 -pf06572 -
04225 -pf11143 -
04230 -pf00583 -
04235 -pf01520 -
04240 -pf01218 -
04245 HMMreg12833_1_EUT1(pf12833) EUT1
04250 H_mulberry(pf00936pf16365) EUT1
04255 Tsp_rosa EUT1
04260 HMMetlyEutC_1_eut1(pf05985) EUT1 EP_NTERM
04265 HMMetlyEutB_1_EUT1(pf06751) EUT1
04270 HMM06277EutA_1_eut(pf06277) EUT1
04275 HMMtpEuth_1_eut(pf04346) EUT1
04280 HMM00465alcdh_3_mix(pf00465) EUT1
04285 HMM06723EutJMreB_1_grm(#) EUT1
04290 HMMalddh_1_eutpdugrm(pf00171) EUT1 EP_NTERM
04295 P_lightViolet EUT1
04300 H_azure EUT1
04305 HMM01515PTA_B_1_eut1(pf01515) EUT1
04310 HMM01923cobtr_2_eut1(pf01923) EUT1
04315 HMM06249EutQ_1_eut1(pf06249) EUT1 EP_NTERM
04320 HMM10662PduV_1_mix(pf10662) EUT1
04325 Hp_euts_violetPink EUT1
04330 -pf04055pf13186 -
04335 -# -
04340 HMM00390_malic_1_eut1(pf00390pf03949pf01515) EUT1
04345 -pf00923 -
04350 HMM02780keto_1_eut1(pf02779pf02780pf00456) EUT1
04355 -# -
04360 -pf06674 -
04365 HMM00293nuhyd_1_eut1(pf00293) EUT1
04370 -pf00144 -
04375 -pf13247pf14691pf07992 -
BMC-P
BMC-H
BMC-T
Aldehyde dehydrogenase
PTAC
Signature enzyme
Alcohol dehydrogenase
Regulator

Escherichia coli. Bacteria; Proteobacteria; Gammaproteobacteria; Enterobacterales;

APT94__locus_1

EUT1

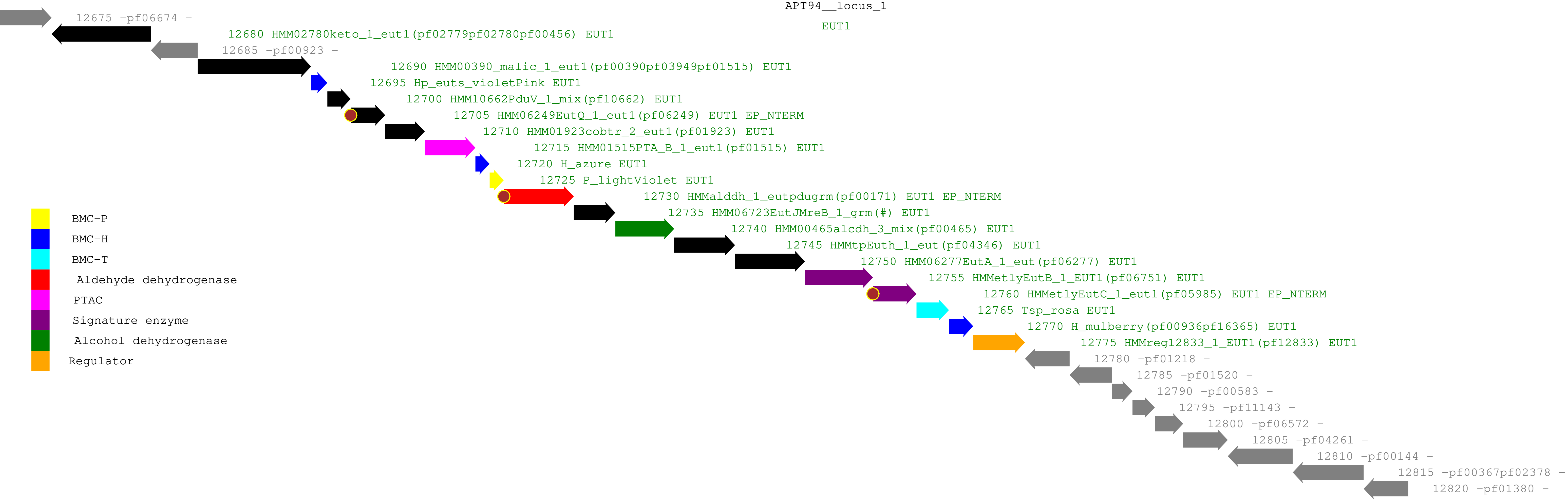

Citrobacter sp. 50677481. Bacteria; Proteobacteria; Gammaproteobacteria; Enterobacterales;

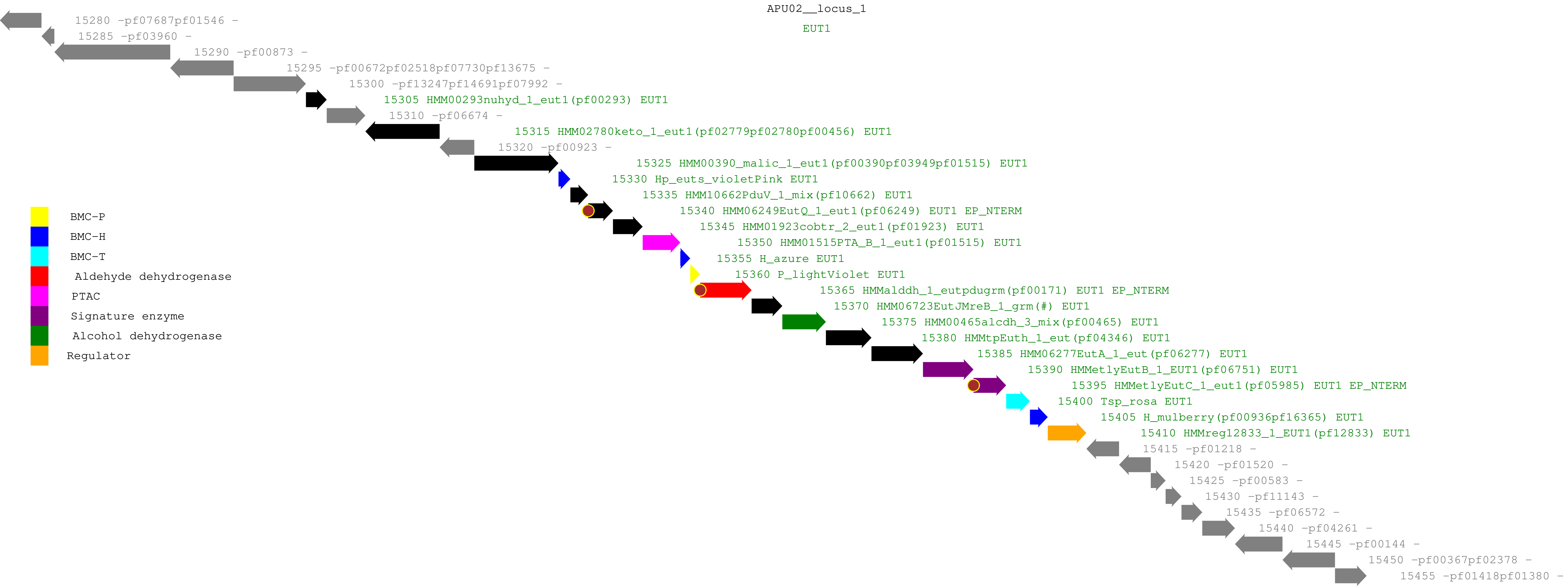

Escherichia coli. Bacteria; Proteobacteria; Gammaproteobacteria; Enterobacterales;

APU18__locus_1

EUT1

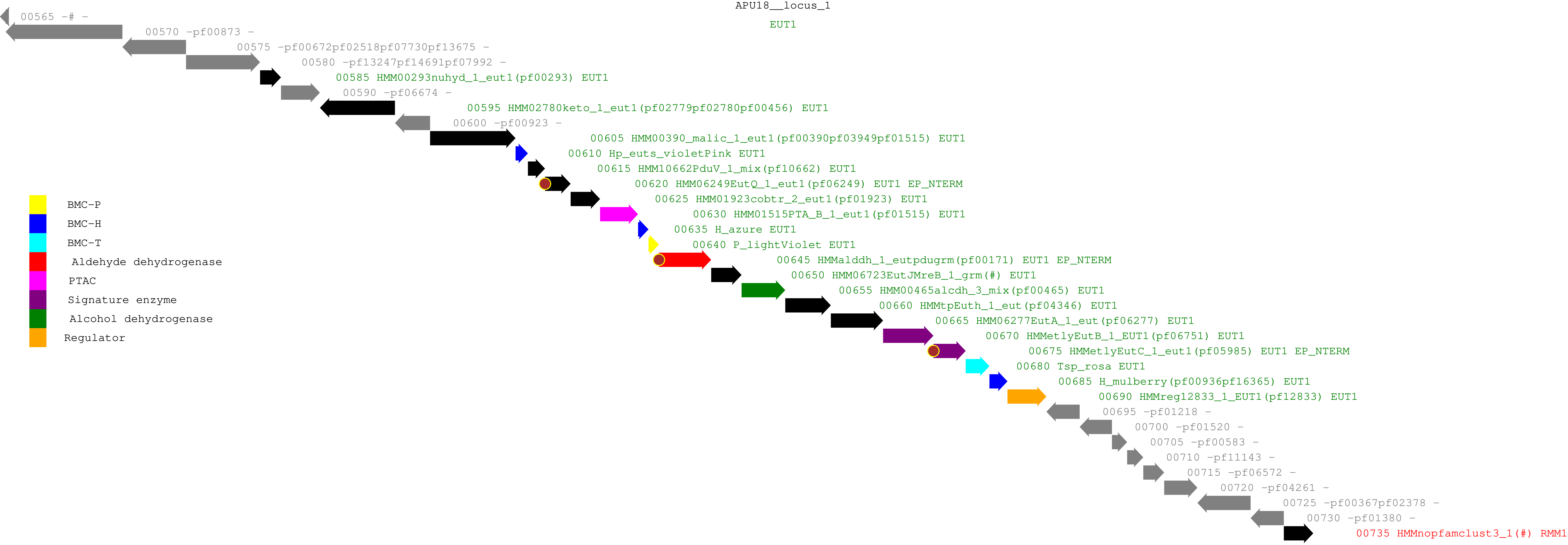

Salmonella enterica subsp. enterica serovar 4,12:d:-. Bacteria; Proteobacteria; Gammaproteobacteria; Enterobacterales;

AR931__locus_1

EUT1

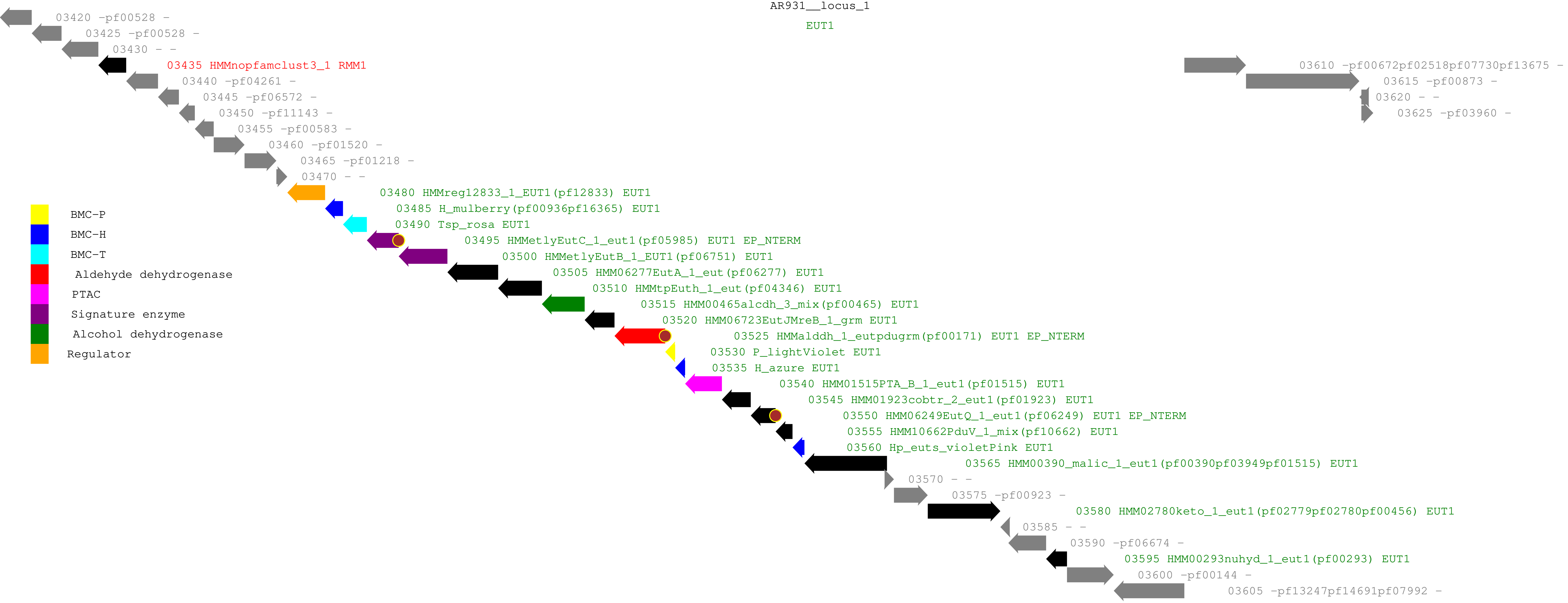

Escherichia coli. Bacteria; Proteobacteria; Gammaproteobacteria; Enterobacterales;

ARC77__locus_1

EUT1

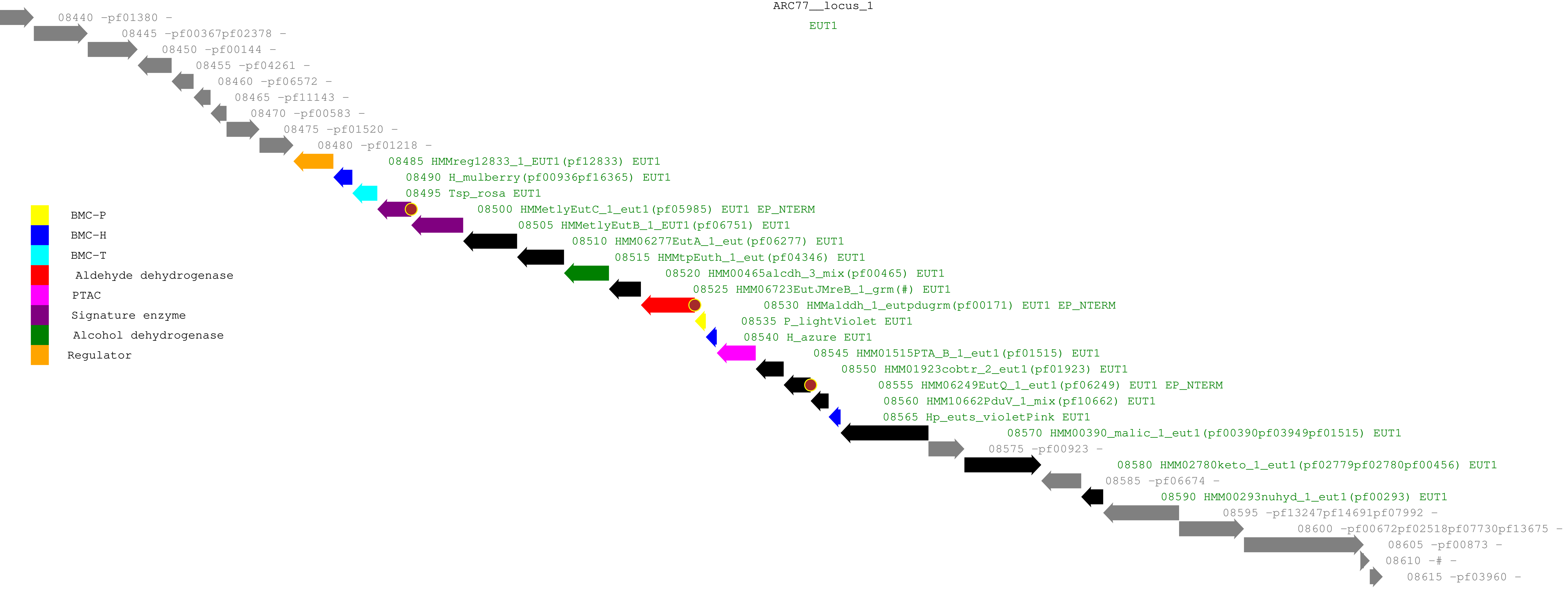

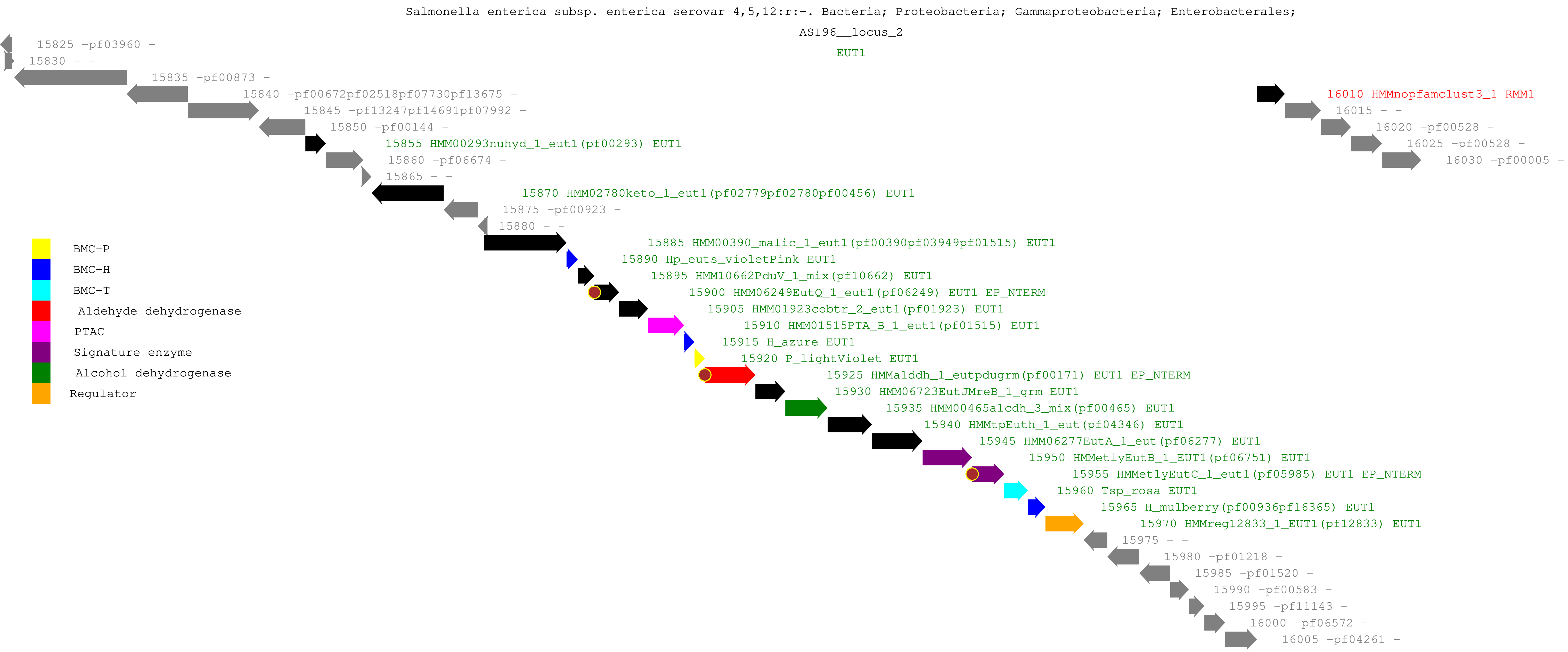
Salmonella enterica subsp. enterica serovar 4,5,12:r:-. Bacteria; Proteobacteria; Gammaproteobacteria; Enterobacterales;
ASI96__locus_2
EUT1
15825 -pf03960 -
15830 - -
15835 -pf00873 -
15840 -pf00672pf02518pf07730pf13675 -
15845 -pf13247pf14691pf07992 -
15850 -pf00144 -
15855 HMM00293nuhyd_1_eut1(pf00293) EUT1
15860 -pf06674 -
15865 - -
15870 HMM02780keto_1_eut1(pf02779pf02780pf00456) EUT1
15875 -pf00923 -
15880 - -
15885 HMM00390_malic_1_eut1(pf00390pf03949pf01515) EUT1
15890 Hp_euts_violetPink EUT1
15895 HMM10662PduV_1_mix(pf10662) EUT1
15900 HMM06249EutQ_1_eut1(pf06249) EUT1 EP_NTERM
15905 HMM01923cobtr_2_eut1(pf01923) EUT1
15910 HMM01515PTA_B_1_eut1(pf01515) EUT1
15915 H_azure EUT1
15920 P_lightViolet EUT1
15925 HMMaldh_1_eutpdugrm(pf00171) EUT1 EP_NTERM
15930 HMM06723EutJMreB_1_grm EUT1
15935 HMM00465alcdh_3_mix(pf00465) EUT1
15940 HMMtpEuth_1_eut(pf04346) EUT1
15945 HMM06277EutA_1_eut(pf06277) EUT1
15950 HMMetlyEutB_1_EUT1(pf06751) EUT1
15955 HMMetlyEutC_1_eut1(pf05985) EUT1 EP_NTERM
15960 Tsp_rosa EUT1
15965 H_mulberry(pf00936pf16365) EUT1
15970 HMMreg12833_1_EUT1(pf12833) EUT1
15975 - -
15980 -pf01218 -
15985 -pf01520 -
15990 -pf00583 -
15995 -pf11143 -
16000 -pf06572 -
16005 -pf04261 -
16010 HMMnopfamclust3_1 RMM1
16015 - -
16020 -pf00528 -
16025 -pf00528 -
16030 -pf00005 -
BMC-P
BMC-H
BMC-T
Aldehyde dehydrogenase
PTAC
Signature enzyme
Alcohol dehydrogenase
Regulator

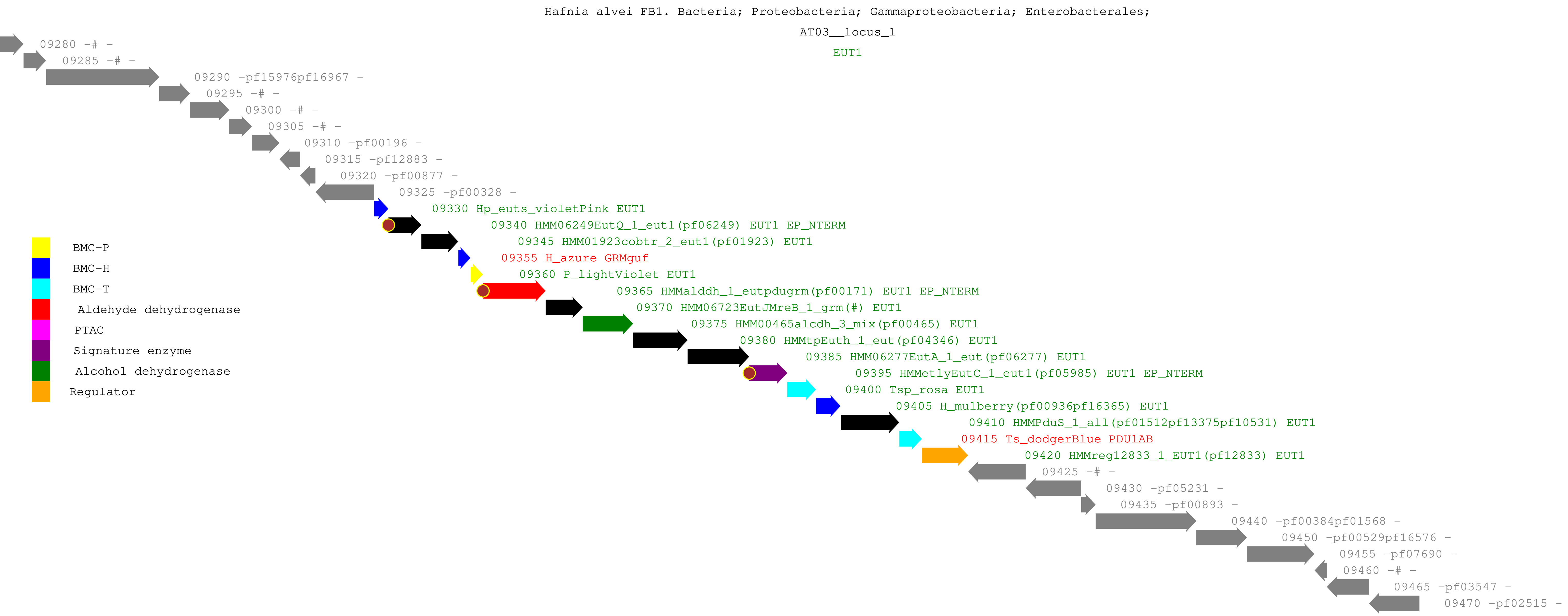
Hafnia alvei FB1. Bacteria; Proteobacteria; Gammaproteobacteria; Enterobacterales;
AT03__locus_1
EUT1
09280 -# -
09285 -# -
09290 -pf15976pf16967 -
09295 -# -
09300 -# -
09305 -# -
09310 -pf00196 -
09315 -pf12883 -
09320 -pf00877 -
09325 -pf00328 -
09330 Hp_euts_violetPink EUT1
09340 HMM06249EutQ_1_eut1(pf06249) EUT1 EP_NTERM
09345 HMM01923cobtr_2_eut1(pf01923) EUT1
09355 H_azure GRMguf
09360 P_lightViolet EUT1
09365 HMMaldh_1_eutpdugrm(pf00171) EUT1 EP_NTERM
09370 HMM06723EutJMreB_1_grm(#) EUT1
09375 HMM00465alcdh_3_mix(pf00465) EUT1
09380 HMMtpEuth_1_eut(pf04346) EUT1
09385 HMM06277EutA_1_eut(pf06277) EUT1
09395 HMMetlyEutC_1_eut1(pf05985) EUT1 EP_NTERM
09400 Tsp_rosa EUT1
09405 H_mulberry(pf00936pf16365) EUT1
09410 HMMPduS_1_all(pf01512pf13375pf10531) EUT1
09415 Ts_dodgerBlue PDU1AB
09420 HMMreg12833_1_EUT1(pf12833) EUT1
09425 -# -
09430 -pf05231 -
09435 -pf00893 -
09440 -pf00384pf01568 -
09450 -pf00529pf16576 -
09455 -pf07690 -
09460 -# -
09465 -pf03547 -
09470 -pf02515 -
BMC-P
BMC-H
BMC-T
Aldehyde dehydrogenase
PTAC
Signature enzyme
Alcohol dehydrogenase
Regulator

Marinobacter sp. LV10R520-4. Bacteria; Proteobacteria; Gammaproteobacteria; Alteromonadales;

ATG98__locus_1

EUT1

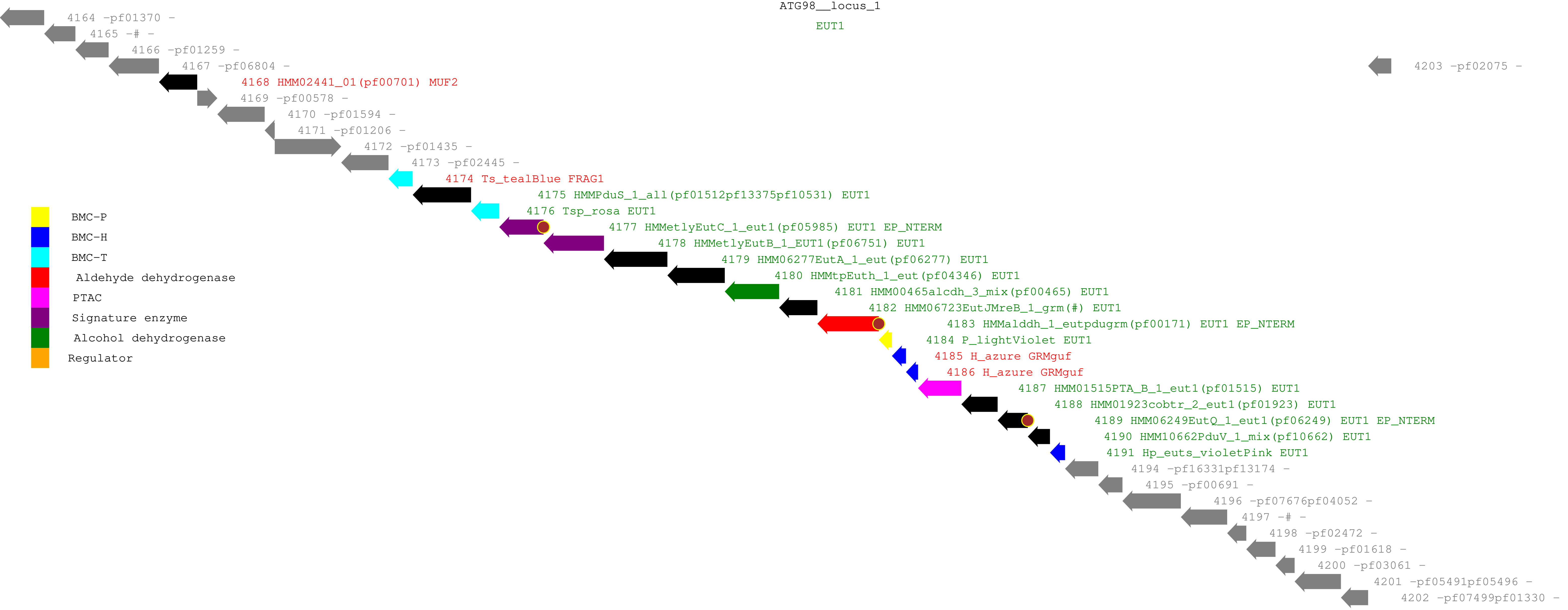

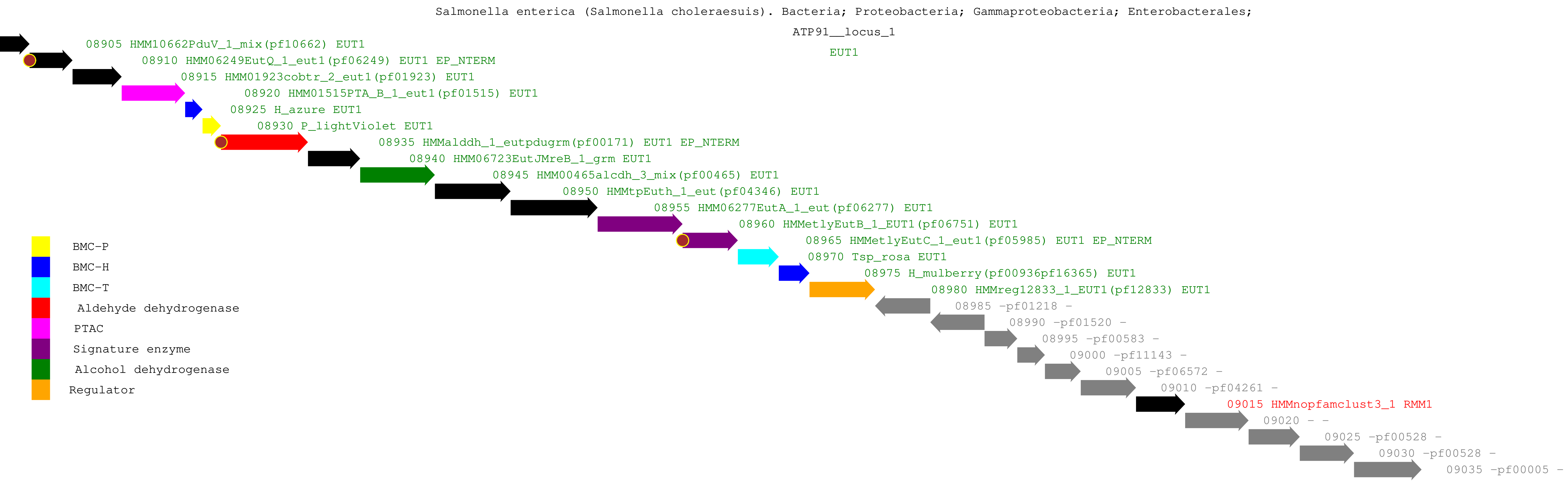

Salmonella enterica (Salmonella choleraesuis). Bacteria; Proteobacteria; Gammaproteobacteria; Enterobacterales;
ATP91__locus_1
EUT1
08905 HMM10662PduV_1_mix(pf10662) EUT1
08910 HMM06249EutQ_1_eut1(pf06249) EUT1 EP_NTERM
08915 HMM01923cobtr_2_eut1(pf01923) EUT1
08920 HMM01515PTA_B_1_eut1(pf01515) EUT1
08925 H_azure EUT1
08930 P_lightViolet EUT1
08935 HMMaldh_1_eutpdugrm(pf00171) EUT1 EP_NTERM
08940 HMM06723EutJMreB_1_grm EUT1
08945 HMM00465alcdh_3_mix(pf00465) EUT1
08950 HMMtpEuth_1_eut(pf04346) EUT1
08955 HMM06277EutA_1_eut(pf06277) EUT1
08960 HMMetlyEutB_1_EUT1(pf06751) EUT1
08965 HMMetlyEutC_1_eut1(pf05985) EUT1 EP_NTERM
08970 Tsp_rosa EUT1
08975 H_mulberry(pf00936pf16365) EUT1
08980 HMMreg12833_1_EUT1(pf12833) EUT1
08985 -pf01218 -
08990 -pf01520 -
08995 -pf00583 -
09000 -pf11143 -
09005 -pf06572 -
09010 -pf04261 -
09015 HMMnopfamclust3_1 RMM1
09020 - -
09025 -pf00528 -
09030 -pf00528 -
09035 -pf00005 -
BMC-P
BMC-H
BMC-T
Aldehyde dehydrogenase
PTAC
Signature enzyme
Alcohol dehydrogenase
Regulator

Salmonella typhimurium (strain ATCC 68169 / UK-1). Bacteria; Proteobacteria; Gammaproteobacteria; Enterobacterales;

AU596__locus_2

EUT1

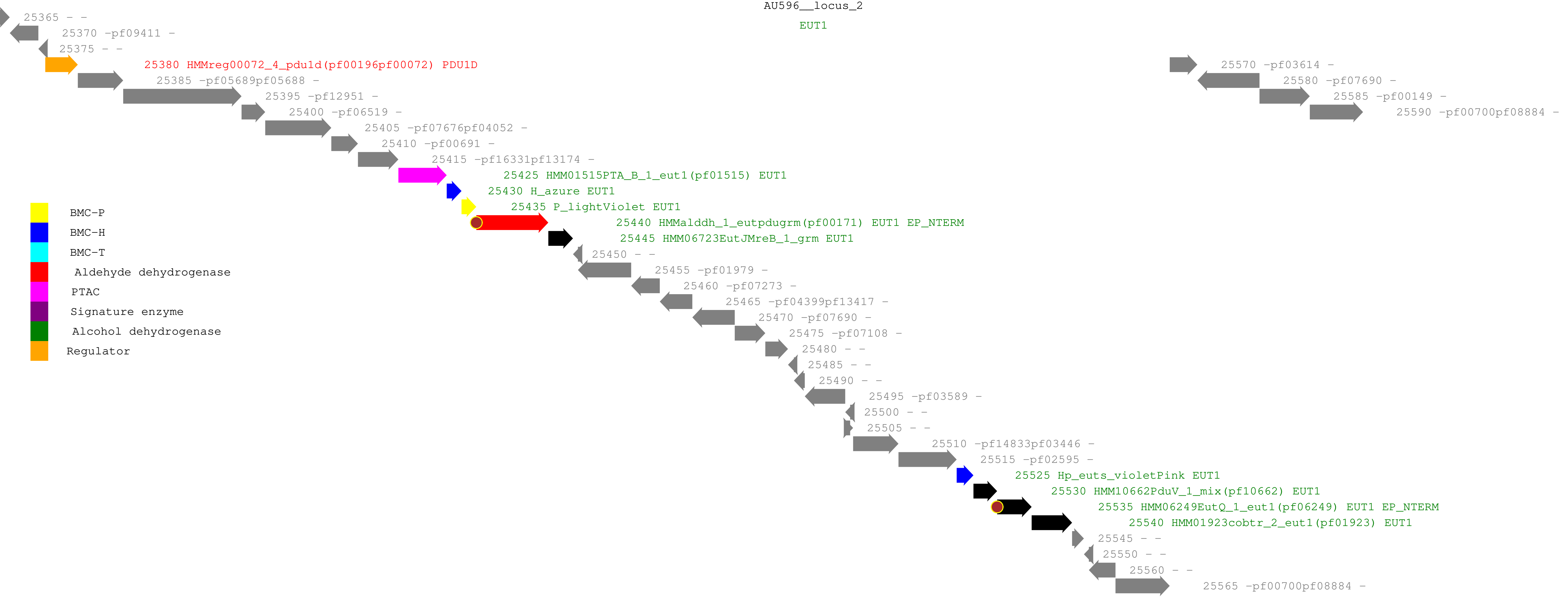

Salmonella enterica subsp. enterica serovar Sekondi. Bacteria; Proteobacteria; Gammaproteobacteria; Enterobacterales;

AU626__locus_3_main_sat

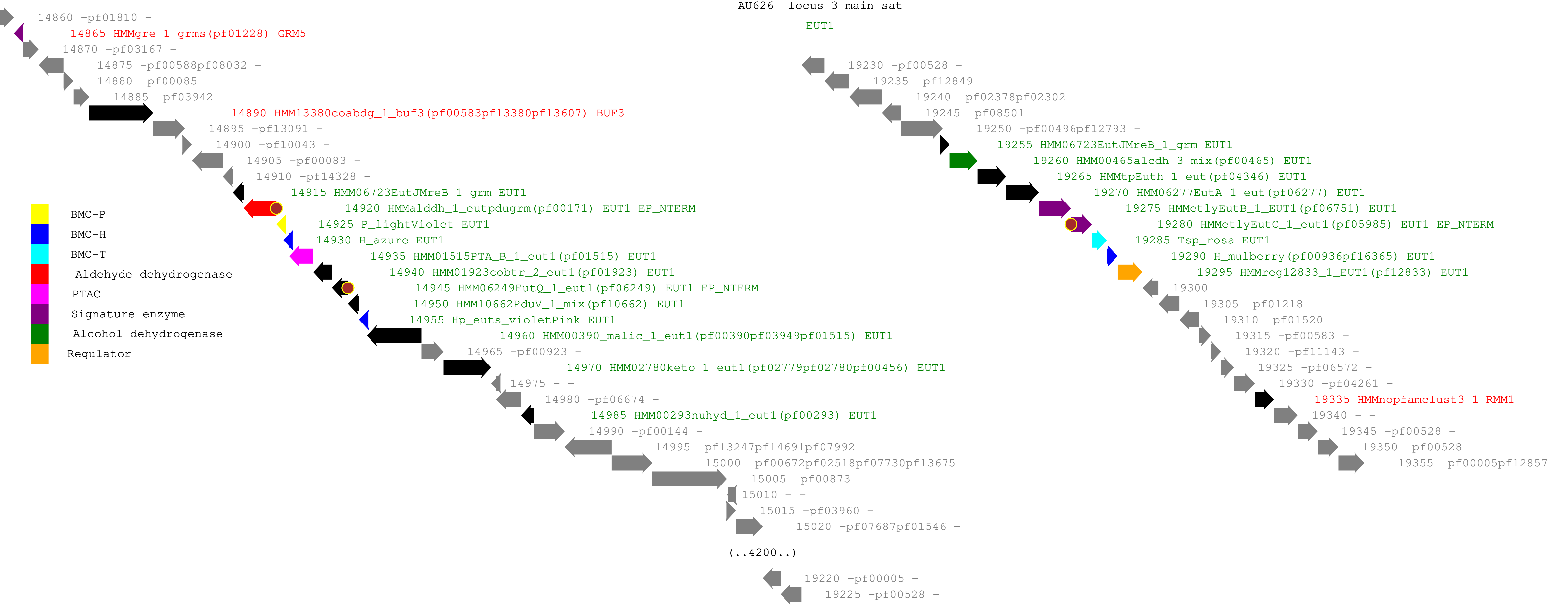

Salmonella enterica subsp. enterica serovar Ridge. Bacteria; Proteobacteria; Gammaproteobacteria; Enterobacterales;

AU648__locus_2

EUT1

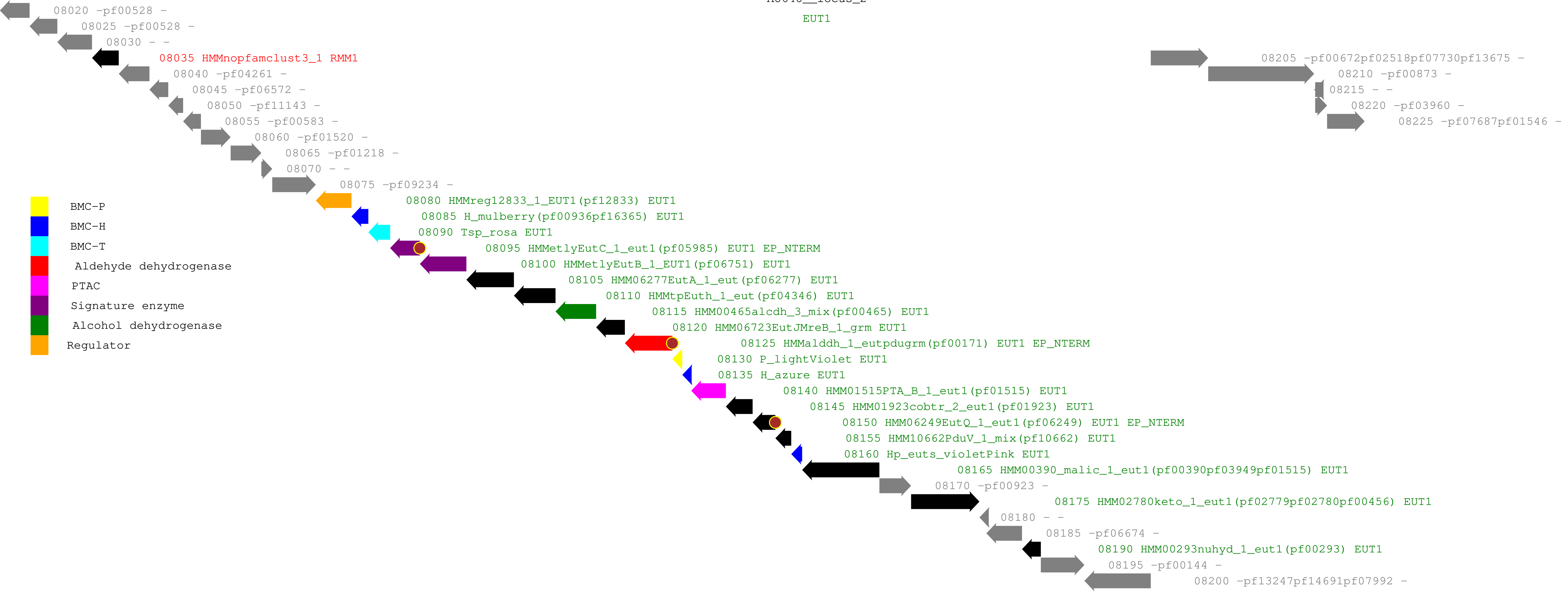

Salmonella abony. Bacteria; Proteobacteria; Gammaproteobacteria; Enterobacterales;

AU743__locus_2_main_sat

EUT1

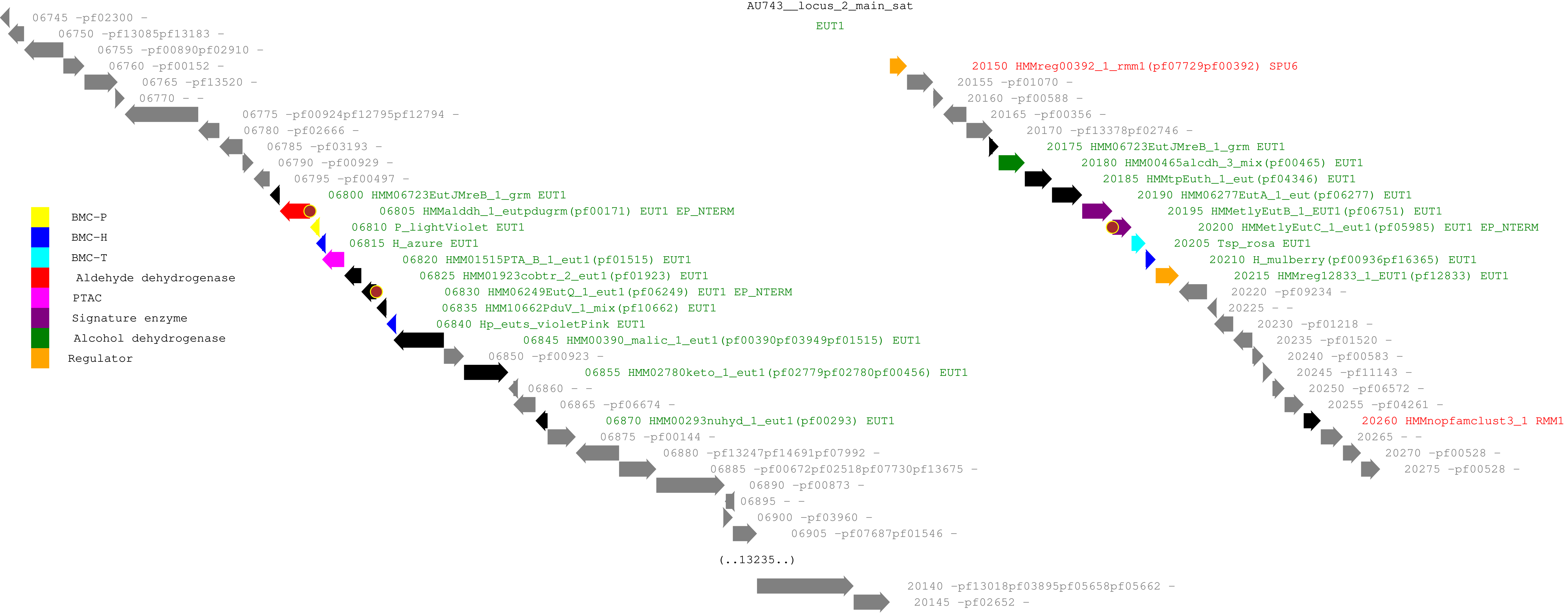

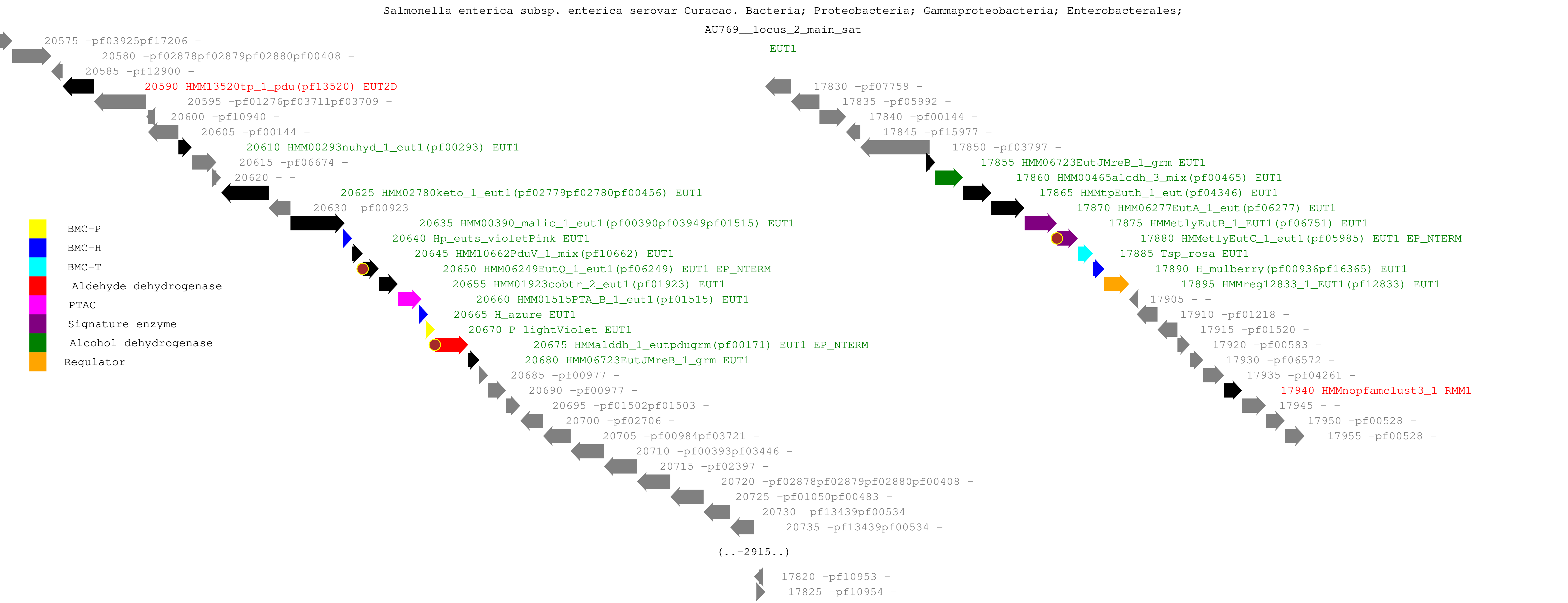

Salmonella enterica subsp. enterica serovar Curacao. Bacteria; Proteobacteria; Gammaproteobacteria; Enterobacterales;
AU769__locus_2_main_sat
EUT1
20575 -pf03925pf17206 -
20580 -pf02878pf02879pf02880pf00408 -
20585 -pf12900 -
20590 HMM13520tp_1_pdu(pf13520) EUT2D
20595 -pf01276pf03711pf03709 -
20600 -pf10940 -
20605 -pf00144 -
20610 HMM00293nuhyd_1_eut1(pf00293) EUT1
20615 -pf06674 -
20620 - -
20625 HMM02780keto_1_eut1(pf02779pf02780pf00456) EUT1
20630 -pf00923 -
20635 HMM00390_malic_1_eut1(pf00390pf03949pf01515) EUT1
20640 Hp_euts_violetPink EUT1
20645 HMM10662PduV_1_mix(pf10662) EUT1
20650 HMM06249EutQ_1_eut1(pf06249) EUT1 EP_NTERM
20655 HMM01923cobtr_2_eut1(pf01923) EUT1
20660 HMM01515PTA_B_1_eut1(pf01515) EUT1
20665 H_azure EUT1
20670 P_lightViolet EUT1
20675 HMMaldh_1_eutpdugrm(pf00171) EUT1 EP_NTERM
20680 HMM06723EutJMreB_1_grm EUT1
20685 -pf00977 -
20690 -pf00977 -
20695 -pf01502pf01503 -
20700 -pf02706 -
20705 -pf00984pf03721 -
20710 -pf00393pf03446 -
20715 -pf02397 -
20720 -pf02878pf02879pf02880pf00408 -
20725 -pf01050pf00483 -
20730 -pf13439pf00534 -
20735 -pf13439pf00534 -
(..-2915..)
17820 -pf10953 -
17825 -pf10954 -
17830 -pf07759 -
17835 -pf05992 -
17840 -pf00144 -
17845 -pf15977 -
17850 -pf03797 -
17855 HMM06723EutJMreB_1_grm EUT1
17860 HMM00465alcdh_3_mix(pf00465) EUT1
17865 HMMtpEuth_1_eut(pf04346) EUT1
17870 HMM06277EutA_1_eut(pf06277) EUT1
17875 HMMetlyEutB_1_EUT1(pf06751) EUT1
17880 HMMetlyEutC_1_eut1(pf05985) EUT1 EP_NTERM
17885 Tsp_rosa EUT1
17890 H_mulberry(pf00936pf16365) EUT1
17895 HMMreg12833_1_EUT1(pf12833) EUT1
17905 - -
17910 -pf01218 -
17915 -pf01520 -
17920 -pf00583 -
17930 -pf06572 -
17935 -pf04261 -
17940 HMMnopfamclust3_1 RMM1
17945 - -
17950 -pf00528 -
17955 -pf00528 -
BMC-P
BMC-H
BMC-T
Aldehyde dehydrogenase
PTAC
Signature enzyme
Alcohol dehydrogenase
Regulator

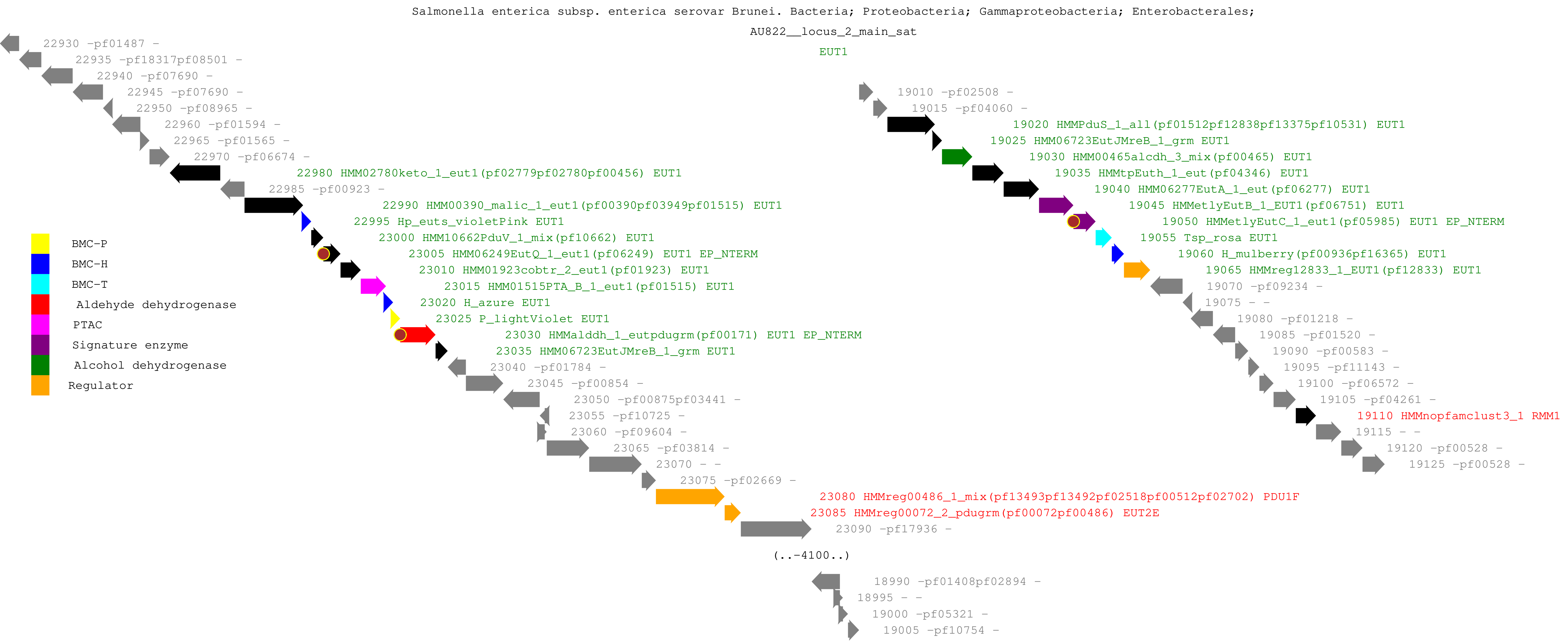
Salmonella enterica subsp. enterica serovar Brunei. Bacteria; Proteobacteria; Gammaproteobacteria; Enterobacterales;
AU822__locus_2_main_sat
EUT1
22930 -pf01487 -
22935 -pf18317pf08501 -
22940 -pf07690 -
22945 -pf07690 -
22950 -pf08965 -
22960 -pf01594 -
22965 -pf01565 -
22970 -pf06674 -
22980 HMM02780keto_1_eut1(pf02779pf02780pf00456) EUT1
22985 -pf00923 -
22990 HMM00390_malic_1_eut1(pf00390pf03949pf01515) EUT1
22995 Hp_euts_violetPink EUT1
23000 HMM10662PduV_1_mix(pf10662) EUT1
23005 HMM06249EutQ_1_eut1(pf06249) EUT1 EP_NTERM
23010 HMM01923cobtr_2_eut1(pf01923) EUT1
23015 HMM01515PTA_B_1_eut1(pf01515) EUT1
23020 H_azure EUT1
23025 P_lightViolet EUT1
23030 HMMalddh_1_eutpdugrm(pf00171) EUT1 EP_NTERM
23035 HMM06723EutJMreB_1_grm EUT1
23040 -pf01784 -
23045 -pf00854 -
23050 -pf00875pf03441 -
23055 -pf10725 -
23060 -pf09604 -
23065 -pf03814 -
23070 - -
23075 -pf02669 -
23080 HMMreg00486_1_mix(pf13493pf13492pf02518pf00512pf02702) PDU1F
23085 HMMreg00072_2_pdugrm(pf00072pf00486) EUT2E
23090 -pf17936 -
(..-4100..)
18990 -pf01408pf02894 -
18995 - -
19000 -pf05321 -
19005 -pf10754 -
19010 -pf02508 -
19015 -pf04060 -
19020 HMMPduS_1_all(pf01512pf12838pf13375pf10531) EUT1
19025 HMM06723EutJMreB_1_grm EUT1
19030 HMM00465alcdh_3_mix(pf00465) EUT1
19035 HMMtpEuth_1_eut(pf04346) EUT1
19040 HMM06277EutA_1_eut(pf06277) EUT1
19045 HMMetlyEutB_1_EUT1(pf06751) EUT1
19050 HMMetlyEutC_1_eut1(pf05985) EUT1 EP_NTERM
19055 Tsp_rosa EUT1
19060 H_mulberry(pf00936pf16365) EUT1
19065 HMMreg12833_1_EUT1(pf12833) EUT1
19070 -pf09234 -
19075 - -
19080 -pf01218 -
19085 -pf01520 -
19090 -pf00583 -
19095 -pf11143 -
19100 -pf06572 -
19105 -pf04261 -
19110 HMMnopfamclust3_1 RMM1
19115 - -
19120 -pf00528 -
19125 -pf00528 -
BMC-P
BMC-H
BMC-T
Aldehyde dehydrogenase
PTAC
Signature enzyme
Alcohol dehydrogenase
Regulator

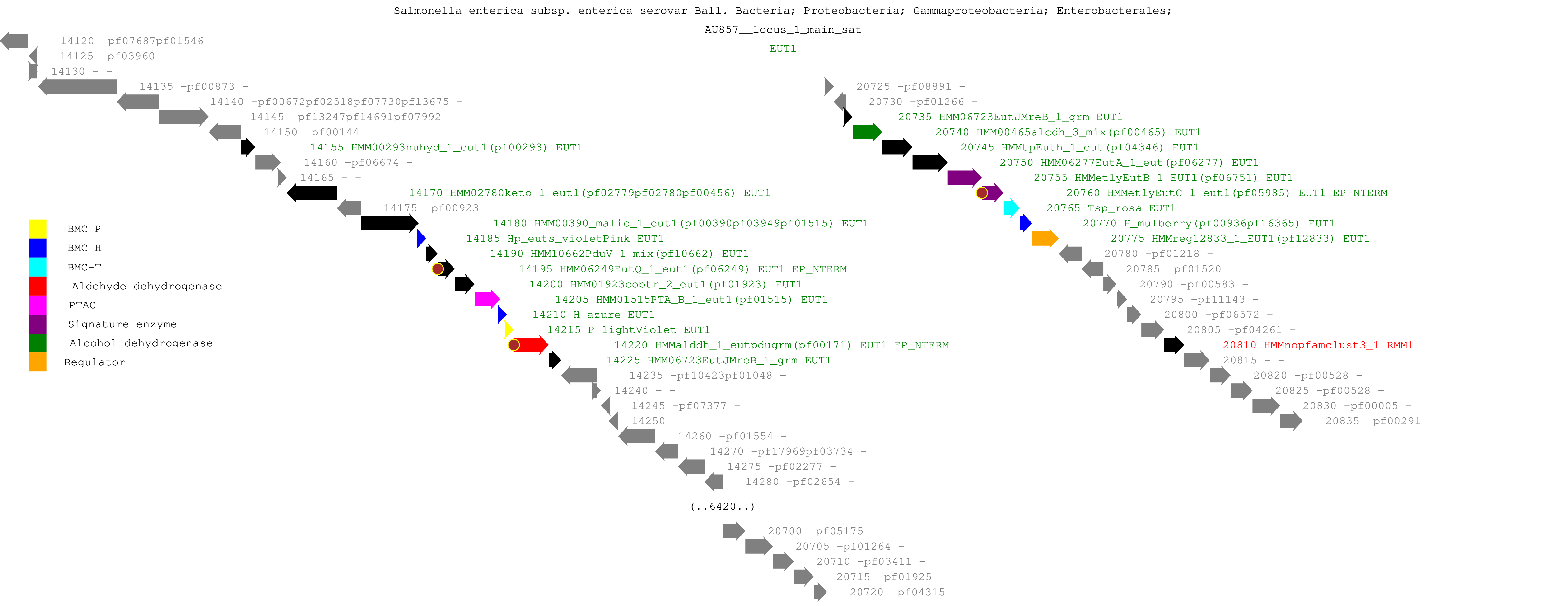
Salmonella enterica subsp. enterica serovar Ball. Bacteria; Proteobacteria; Gammaproteobacteria; Enterobacterales;
AU857__locus_1_main_sat
EUT1
14120 -pf07687pf01546 -
14125 -pf03960 -
14130 - -
14135 -pf00873 -
14140 -pf00672pf02518pf07730pf13675 -
14145 -pf13247pf14691pf07992 -
14150 -pf00144 -
14155 HMM00293nuhyd_1_eut1(pf00293) EUT1
14160 -pf06674 -
14165 - -
14170 HMM02780keto_1_eut1(pf02779pf02780pf00456) EUT1
14175 -pf00923 -
14180 HMM00390_malic_1_eut1(pf00390pf03949pf01515) EUT1
14185 Hp_euts_violetPink EUT1
14190 HMM10662PduV_1_mix(pf10662) EUT1
14195 HMM06249EutQ_1_eut1(pf06249) EUT1 EP_NTERM
14200 HMM01923cobtr_2_eut1(pf01923) EUT1
14205 HMM01515PTA_B_1_eut1(pf01515) EUT1
14210 H_azure EUT1
14215 P_lightViolet EUT1
14220 HMMalddh_1_eutpdugrm(pf00171) EUT1 EP_NTERM
14225 HMM06723EutJMreB_1_grm EUT1
14235 -pf10423pf01048 -
14240 - -
14245 -pf07377 -
14250 - -
14260 -pf01554 -
14270 -pf17969pf03734 -
14275 -pf02277 -
14280 -pf02654 -
(..6420..)
20700 -pf05175 -
20705 -pf01264 -
20710 -pf03411 -
20715 -pf01925 -
20720 -pf04315 -
20725 -pf08891 -
20730 -pf01266 -
20735 HMM06723EutJMreB_1_grm EUT1
20740 HMM00465alcdh_3_mix(pf00465) EUT1
20745 HMMtpEuth_1_eut(pf04346) EUT1
20750 HMM06277EutA_1_eut(pf06277) EUT1
20755 HMMetlyEutB_1_EUT1(pf06751) EUT1
20760 HMMetlyEutC_1_eut1(pf05985) EUT1 EP_NTERM
20765 Tsp_rosa EUT1
20770 H_mulberry(pf00936pf16365) EUT1
20775 HMMreg12833_1_EUT1(pf12833) EUT1
20780 -pf01218 -
20785 -pf01520 -
20790 -pf00583 -
20795 -pf11143 -
20800 -pf06572 -
20805 -pf04261 -
20810 HMMnopfamclust3_1 RMM1
20815 - -
20820 -pf00528 -
20825 -pf00528 -
20830 -pf00005 -
20835 -pf00291 -
BMC-P
BMC-H
BMC-T
Aldehyde dehydrogenase
PTAC
Signature enzyme
Alcohol dehydrogenase
Regulator

Salmonella enterica subsp. enterica serovar Ibadan. Bacteria; Proteobacteria; Gammaproteobacteria; Enterobacterales;

AU867__locus_2

EUT1

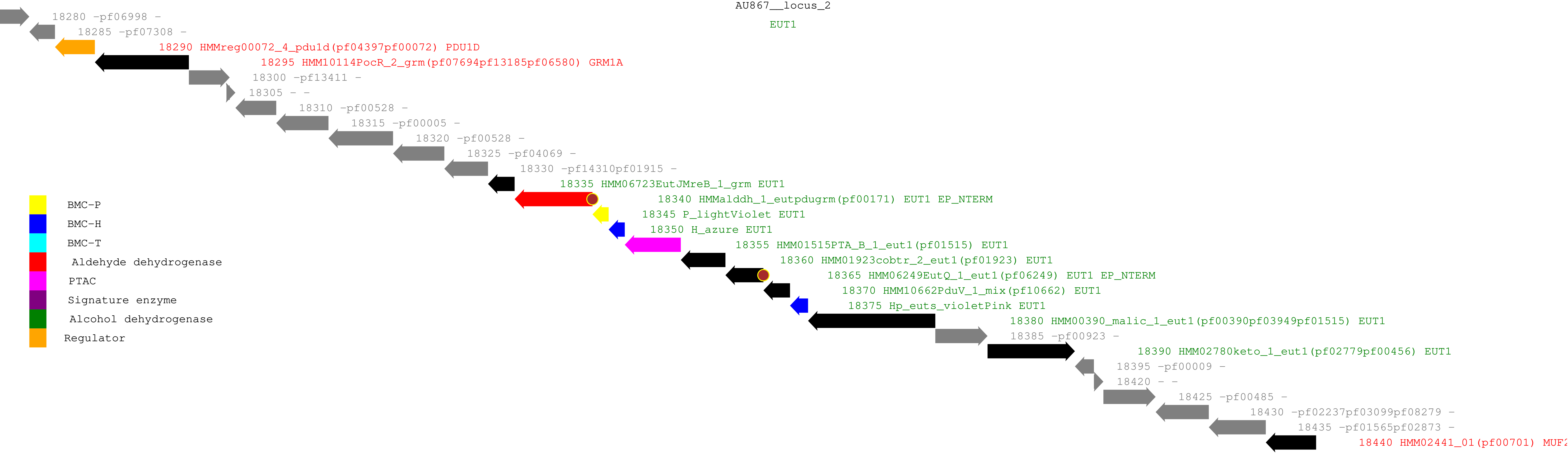

Salmonella enterica subsp. enterica serovar Kottbus. Bacteria; Proteobacteria; Gammaproteobacteria; Enterobacterales;

AU884__locus_2

EUT1

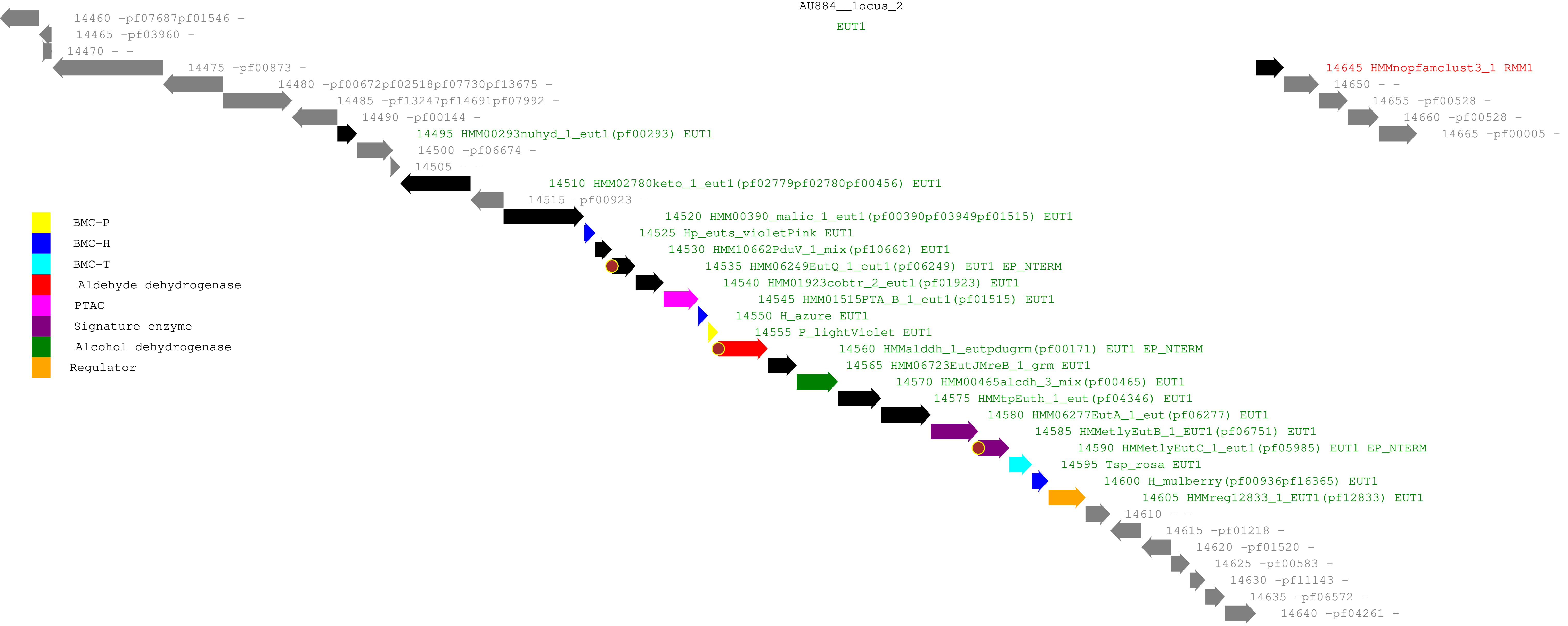

Salmonella enterica subsp. enterica serovar Ago. Bacteria; Proteobacteria; Gammaproteobacteria; Enterobacterales;

AU897__locus_2_main_sat

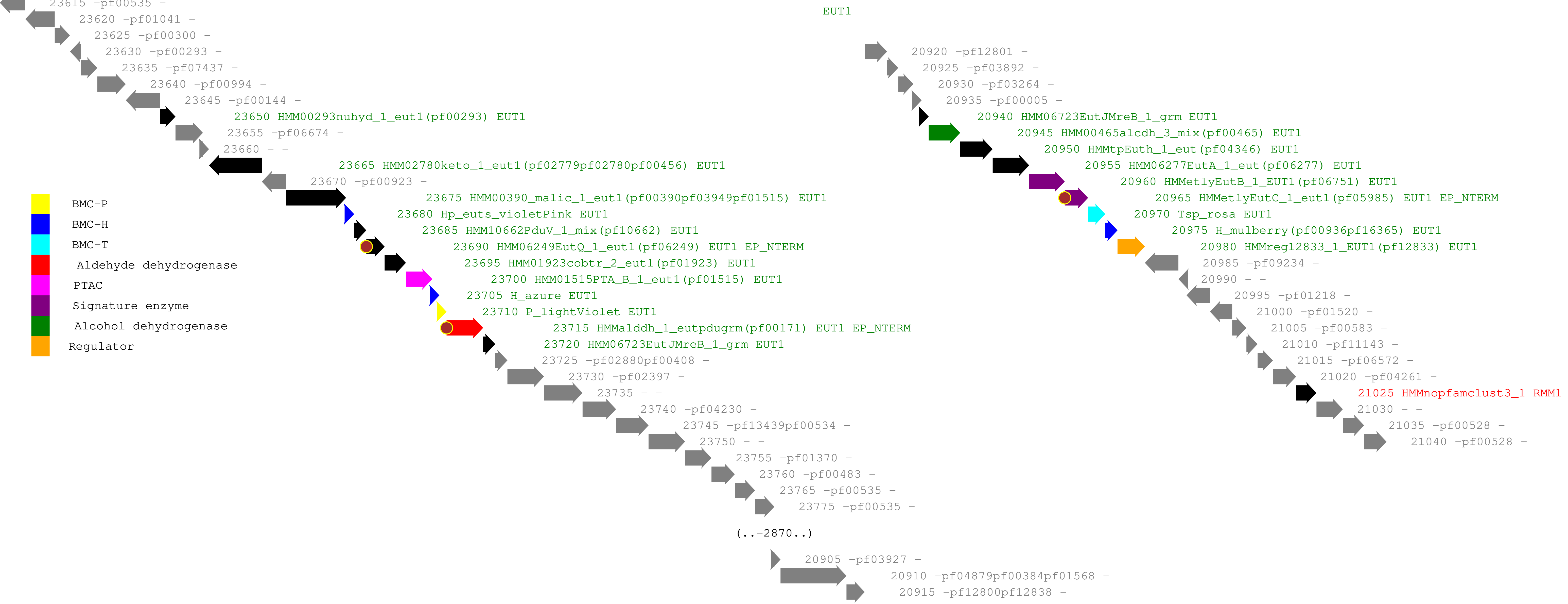

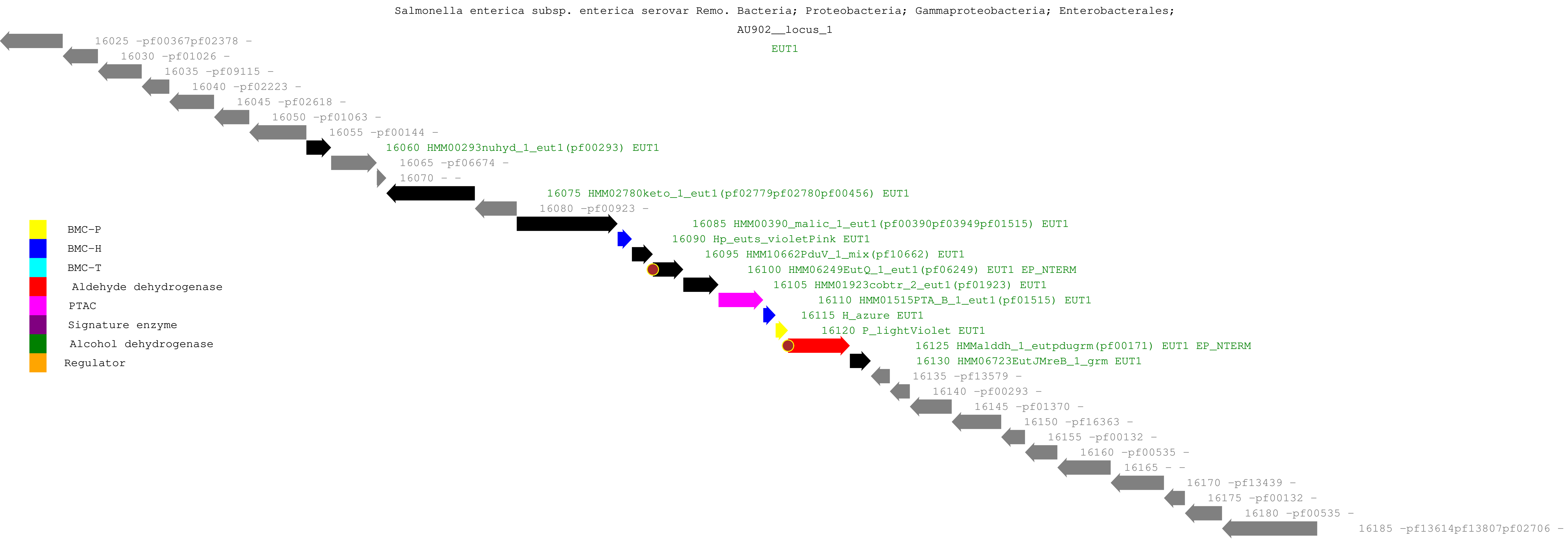
Salmonella enterica subsp. enterica serovar Remo. Bacteria; Proteobacteria; Gammaproteobacteria; Enterobacterales;
AU902__locus_1
EUT1
16025 -pf00367pf02378 -
16030 -pf01026 -
16035 -pf09115 -
16040 -pf02223 -
16045 -pf02618 -
16050 -pf01063 -
16055 -pf00144 -
16060 HMM00293nuhyd_1_eut1(pf00293) EUT1
16065 -pf06674 -
16070 - -
16075 HMM02780keto_1_eut1(pf02779pf02780pf00456) EUT1
16080 -pf00923 -
16085 HMM00390_malic_1_eut1(pf00390pf03949pf01515) EUT1
16090 Hp_euts_violetPink EUT1
16095 HMM10662PduV_1_mix(pf10662) EUT1
16100 HMM06249EutQ_1_eut1(pf06249) EUT1 EP_NTERM
16105 HMM01923cobtr_2_eut1(pf01923) EUT1
16110 HMM01515PTA_B_1_eut1(pf01515) EUT1
16115 H_azure EUT1
16120 P_lightViolet EUT1
16125 HMMalddh_1_eutpdugrm(pf00171) EUT1 EP_NTERM
16130 HMM06723EutJMreB_1_grm EUT1
16135 -pf13579 -
16140 -pf00293 -
16145 -pf01370 -
16150 -pf16363 -
16155 -pf00132 -
16160 -pf00535 -
16165 - -
16170 -pf13439 -
16175 -pf00132 -
16180 -pf00535 -
16185 -pf13614pf13807pf02706 -
BMC-P
BMC-H
BMC-T
Aldehyde dehydrogenase
PTAC
Signature enzyme
Alcohol dehydrogenase
Regulator

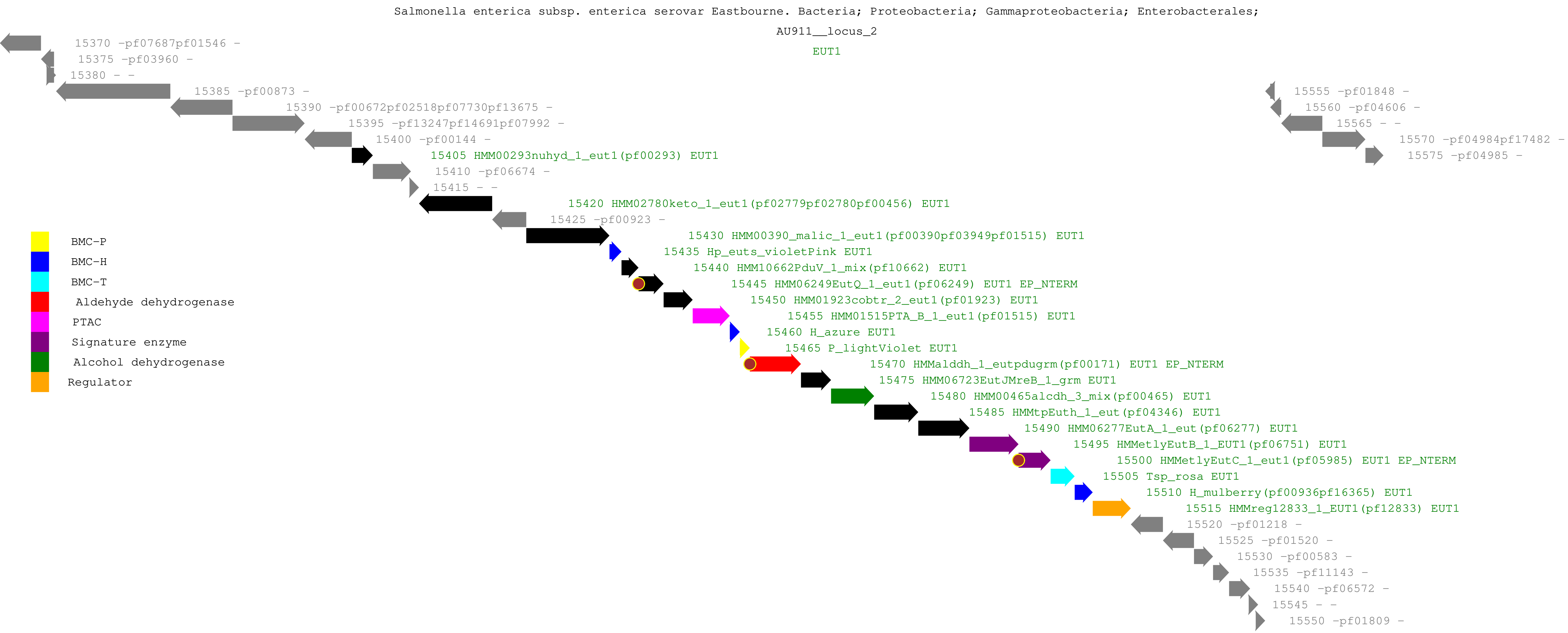
Salmonella enterica subsp. enterica serovar Eastbourne. Bacteria; Proteobacteria; Gammaproteobacteria; Enterobacterales;
AU911__locus_2
EUT1
15370 -pf07687pf01546 -
15375 -pf03960 -
15380 - -
15385 -pf00873 -
15390 -pf00672pf02518pf07730pf13675 -
15395 -pf13247pf14691pf07992 -
15400 -pf00144 -
15405 HMM00293nuhyd_1_eut1(pf00293) EUT1
15410 -pf06674 -
15415 - -
15420 HMM02780keto_1_eut1(pf02779pf02780pf00456) EUT1
15425 -pf00923 -
15430 HMM00390_malic_1_eut1(pf00390pf03949pf01515) EUT1
15435 Hp_euts_violetPink EUT1
15440 HMM10662PduV_1_mix(pf10662) EUT1
15445 HMM06249EutQ_1_eut1(pf06249) EUT1 EP_NTERM
15450 HMM01923cobtr_2_eut1(pf01923) EUT1
15455 HMM01515PTA_B_1_eut1(pf01515) EUT1
15460 H_azure EUT1
15465 P_lightViolet EUT1
15470 HMMalddh_1_eutpdugrm(pf00171) EUT1 EP_NTERM
15475 HMM06723EutJMreB_1_grm EUT1
15480 HMM00465alcdh_3_mix(pf00465) EUT1
15485 HMMtpEuth_1_eut(pf04346) EUT1
15490 HMM06277EutA_1_eut(pf06277) EUT1
15495 HMMetlyEutB_1_EUT1(pf06751) EUT1
15500 HMMetlyEutC_1_eut1(pf05985) EUT1 EP_NTERM
15505 Tsp_rosa EUT1
15510 H_mulberry(pf00936pf16365) EUT1
15515 HMMreg12833_1_EUT1(pf12833) EUT1
15520 -pf01218 -
15525 -pf01520 -
15530 -pf00583 -
15535 -pf11143 -
15540 -pf06572 -
15545 - -
15550 -pf01809 -
15555 -pf01848 -
15560 -pf04606 -
15565 - -
15570 -pf04984pf17482 -
15575 -pf04985 -
BMC-P
BMC-H
BMC-T
Aldehyde dehydrogenase
PTAC
Signature enzyme
Alcohol dehydrogenase
Regulator

Salmonella enterica subsp. enterica serovar Takoradi. Bacteria; Proteobacteria; Gammaproteobacteria; Enterobacterales;

AU931__locus_1

EUT1

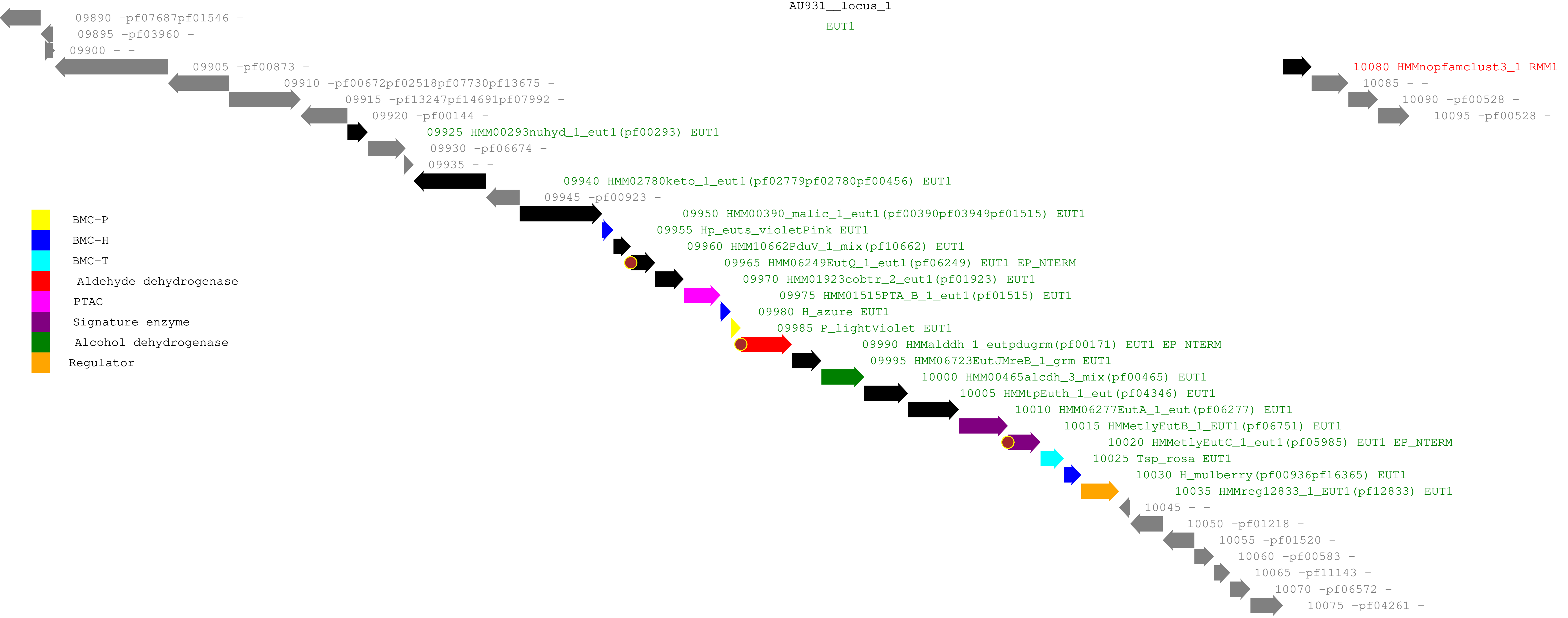

Salmonella enterica subsp. enterica serovar Telelkebir. Bacteria; Proteobacteria; Gammaproteobacteria; Enterobacterales;

AU975__locus_2_main_sat

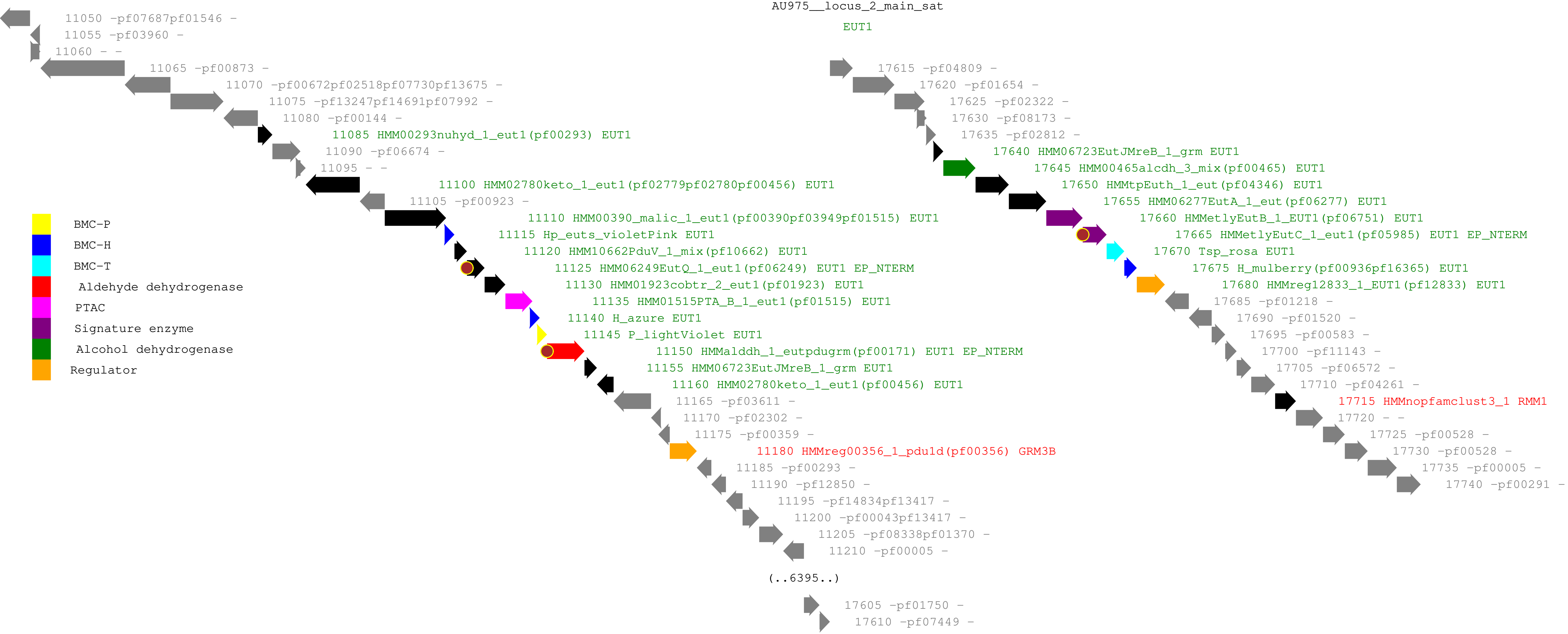

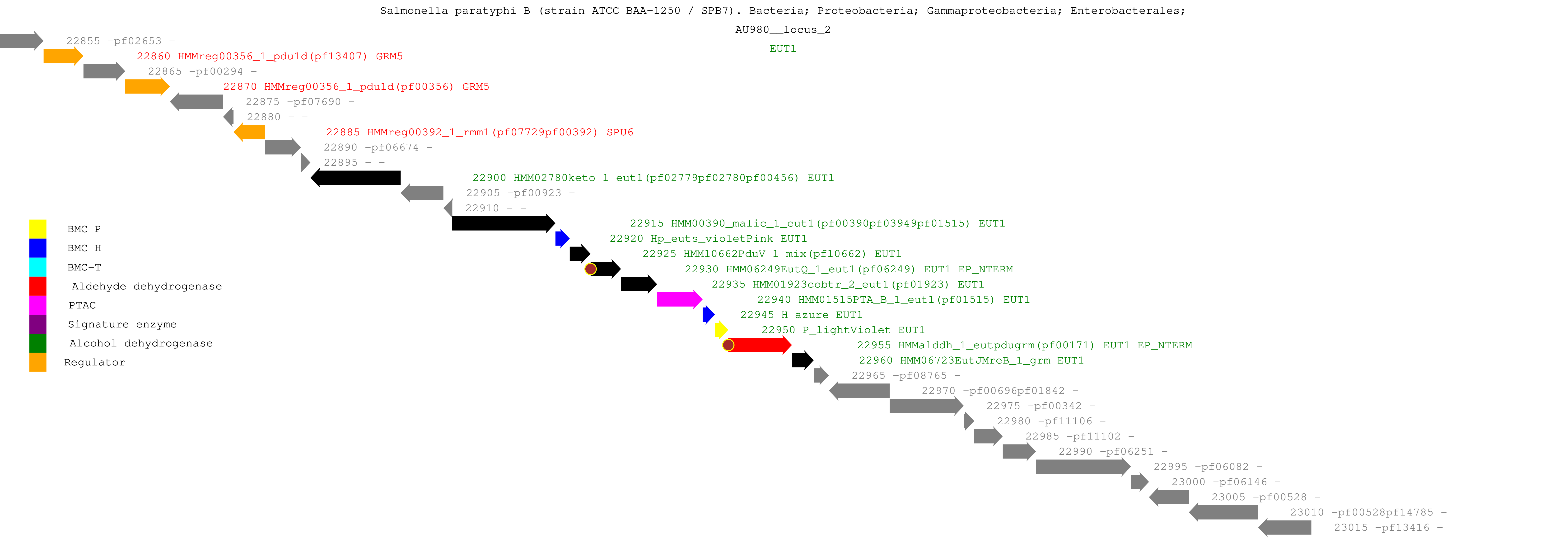

Salmonella paratyphi B (strain ATCC BAA-1250 / SPB7). Bacteria; Proteobacteria; Gammaproteobacteria; Enterobacterales;
AU980__locus_2
EUT1
22855 -pf02653 -
22860 HMMreg00356_1_pdu1d(pf13407) GRM5
22865 -pf00294 -
22870 HMMreg00356_1_pdu1d(pf00356) GRM5
22875 -pf07690 -
22880 - -
22885 HMMreg00392_1_rmm1(pf07729pf00392) SPU6
22890 -pf06674 -
22895 - -
22900 HMM02780keto_1_eut1(pf02779pf02780pf00456) EUT1
22905 -pf00923 -
22910 - -
22915 HMM00390_malic_1_eut1(pf00390pf03949pf01515) EUT1
22920 Hp_euts_violetPink EUT1
22925 HMM10662PduV_1_mix(pf10662) EUT1
22930 HMM06249EutQ_1_eut1(pf06249) EUT1 EP_NTERM
22935 HMM01923cobtr_2_eut1(pf01923) EUT1
22940 HMM01515PTA_B_1_eut1(pf01515) EUT1
22945 H_azure EUT1
22950 P_lightViolet EUT1
22955 HMMalddh_1_eutpdugrm(pf00171) EUT1 EP_NTERM
22960 HMM06723EutJMreB_1_grm EUT1
22965 -pf08765 -
22970 -pf00696pf01842 -
22975 -pf00342 -
22980 -pf11106 -
22985 -pf11102 -
22990 -pf06251 -
22995 -pf06082 -
23000 -pf06146 -
23005 -pf00528 -
23010 -pf00528pf14785 -
23015 -pf13416 -
BMC-P
BMC-H
BMC-T
Aldehyde dehydrogenase
PTAC
Signature enzyme
Alcohol dehydrogenase
Regulator

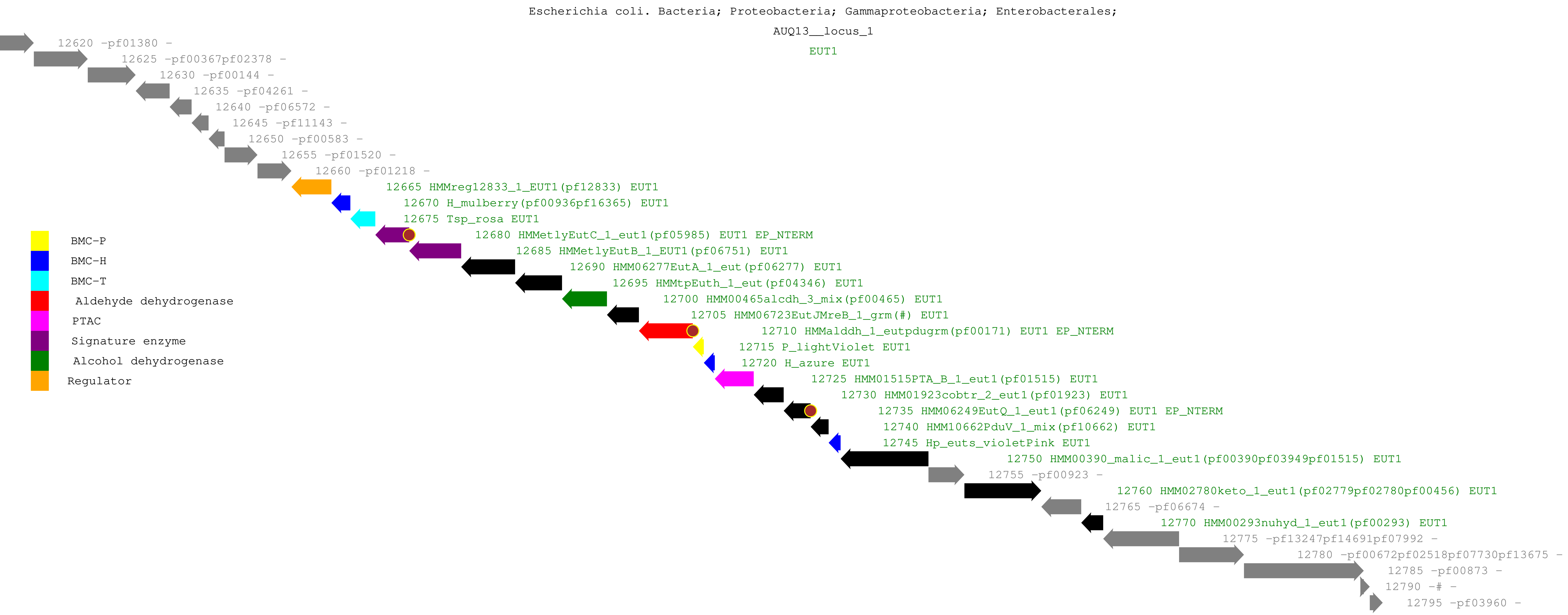

Escherichia coli. Bacteria; Proteobacteria; Gammaproteobacteria; Enterobacterales;
AUQ13__locus_1
EUT1
12620 -pf01380 -
12625 -pf00367pf02378 -
12630 -pf00144 -
12635 -pf04261 -
12640 -pf06572 -
12645 -pf11143 -
12650 -pf00583 -
12655 -pf01520 -
12660 -pf01218 -
12665 HMMreg12833_1_EUT1(pf12833) EUT1
12670 H_mulberry(pf00936pf16365) EUT1
12675 Tsp_rosa EUT1
12680 HMMetlyEutC_1_eut1(pf05985) EUT1 EP_NTERM
12685 HMMetlyEutB_1_EUT1(pf06751) EUT1
12690 HMM06277EutA_1_eut(pf06277) EUT1
12695 HMMtpEuth_1_eut(pf04346) EUT1
12700 HMM00465alcdh_3_mix(pf00465) EUT1
12705 HMM06723EutJMreB_1_grm(#) EUT1
12710 HMMalddh_1_eutpdugrm(pf00171) EUT1 EP_NTERM
12715 P_lightViolet EUT1
12720 H_azure EUT1
12725 HMM01515PTA_B_1_eut1(pf01515) EUT1
12730 HMM01923cobtr_2_eut1(pf01923) EUT1
12735 HMM06249EutQ_1_eut1(pf06249) EUT1 EP_NTERM
12740 HMM10662PduV_1_mix(pf10662) EUT1
12745 Hp_euts_violetPink EUT1
12750 HMM00390_malic_1_eut1(pf00390pf03949pf01515) EUT1
12755 -pf00923 -
12760 HMM02780keto_1_eut1(pf02779pf02780pf00456) EUT1
12765 -pf06674 -
12770 HMM00293nuhyd_1_eut1(pf00293) EUT1
12775 -pf13247pf14691pf07992 -
12780 -pf00672pf02518pf07730pf13675 -
12785 -pf00873 -
12790 -# -
12795 -pf03960 -
BMC-P
BMC-H
BMC-T
Aldehyde dehydrogenase
PTAC
Signature enzyme
Alcohol dehydrogenase
Regulator

Salmonella enterica subsp. enterica serovar Senftenberg str. Bacteria; Proteobacteria; Gammaproteobacteria; Enterobacterales;

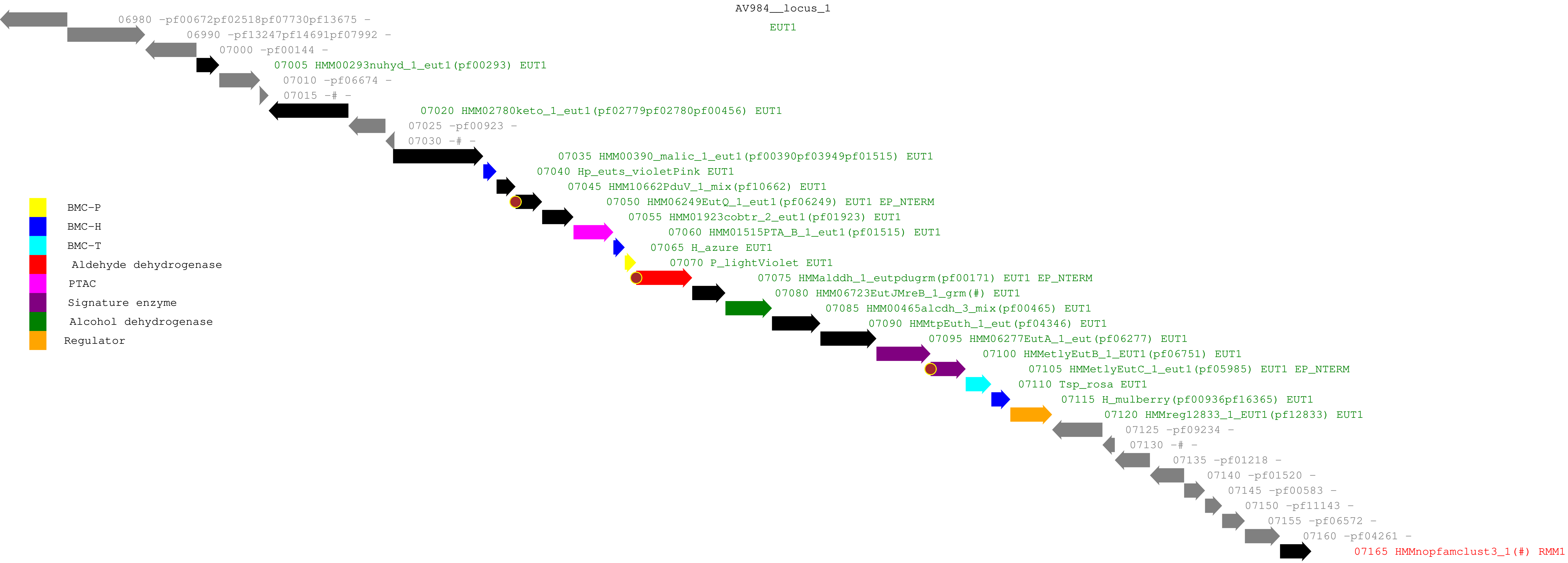

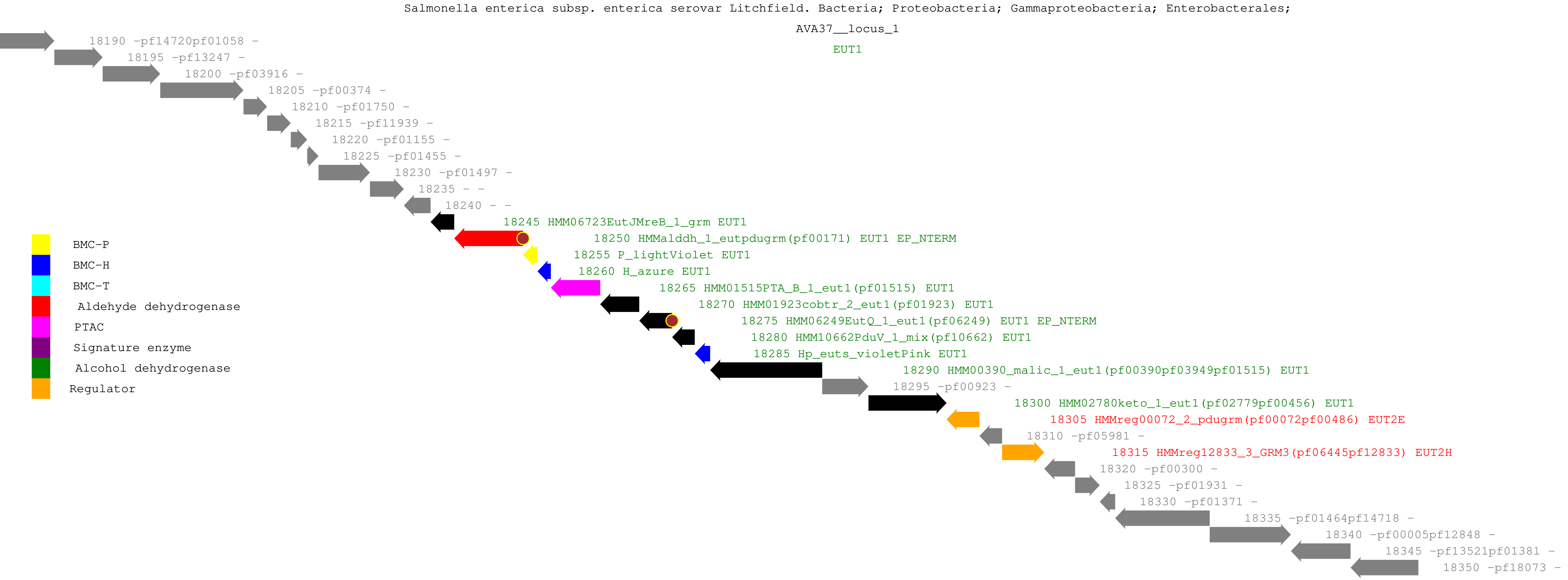
Salmonella enterica subsp. enterica serovar Litchfield. Bacteria; Proteobacteria; Gammaproteobacteria; Enterobacterales;
AVA37__locus_1
EUT1
18190 -pf14720pf01058 -
18195 -pf13247 -
18200 -pf03916 -
18205 -pf00374 -
18210 -pf01750 -
18215 -pf11939 -
18220 -pf01155 -
18225 -pf01455 -
18230 -pf01497 -
18235 - -
18240 - -
18245 HMM06723EutJMreB_1_grm EUT1
18250 HMMalddh_1_eutpdugrm(pf00171) EUT1 EP_NTERM
18255 P_lightViolet EUT1
18260 H_azure EUT1
18265 HMM01515PTA_B_1_eut1(pf01515) EUT1
18270 HMM01923cobtr_2_eut1(pf01923) EUT1
18275 HMM06249EutQ_1_eut1(pf06249) EUT1 EP_NTERM
18280 HMM10662PduV_1_mix(pf10662) EUT1
18285 Hp_euts_violetPink EUT1
18290 HMM00390_malic_1_eut1(pf00390pf03949pf01515) EUT1
18295 -pf00923 -
18300 HMM02780keto_1_eut1(pf02779pf00456) EUT1
18305 HMMreg00072_2_pdugrm(pf00072pf00486) EUT2E
18310 -pf05981 -
18315 HMMreg12833_3_GRM3(pf06445pf12833) EUT2H
18320 -pf00300 -
18325 -pf01931 -
18330 -pf01371 -
18335 -pf01464pf14718 -
18340 -pf00005pf12848 -
18345 -pf13521pf01381 -
18350 -pf18073 -
BMC-P
BMC-H
BMC-T
Aldehyde dehydrogenase
PTAC
Signature enzyme
Alcohol dehydrogenase
Regulator

Salmonella enterica subsp. enterica serovar Bredeney. Bacteria; Proteobacteria; Gammaproteobacteria; Enterobacterales;

AVA51__locus_1

EUT1

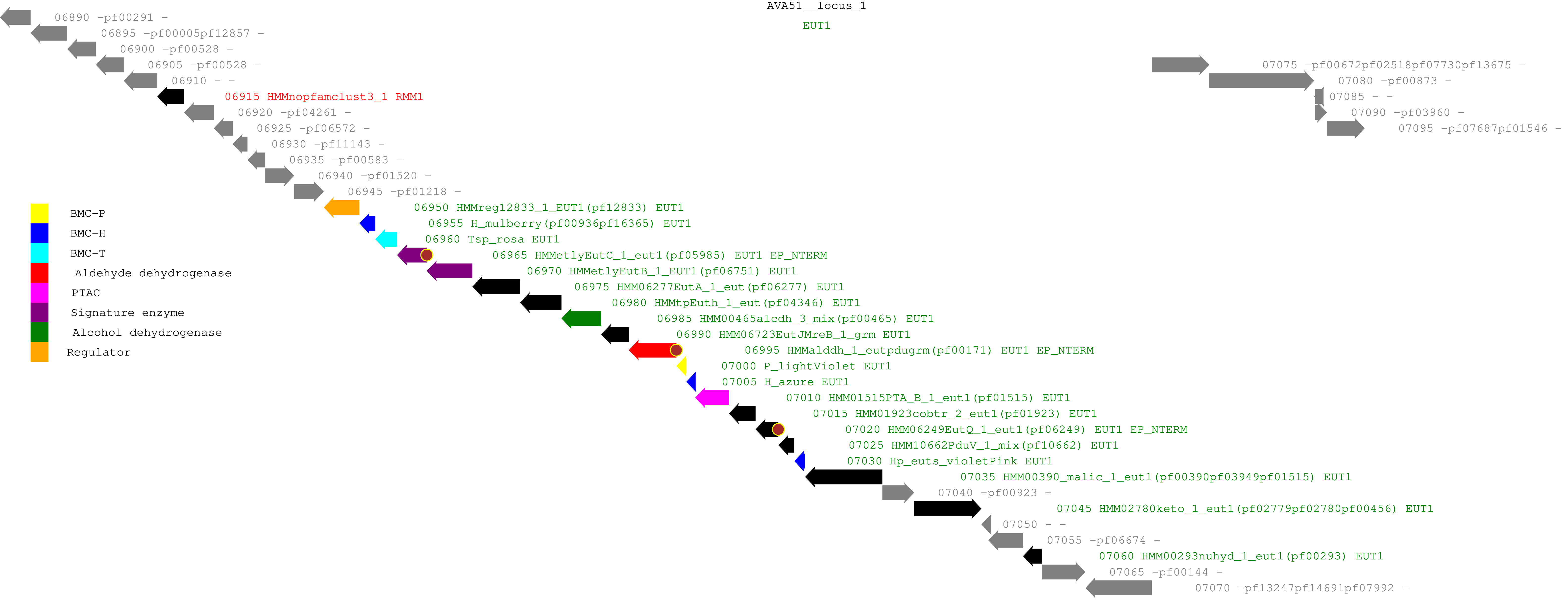

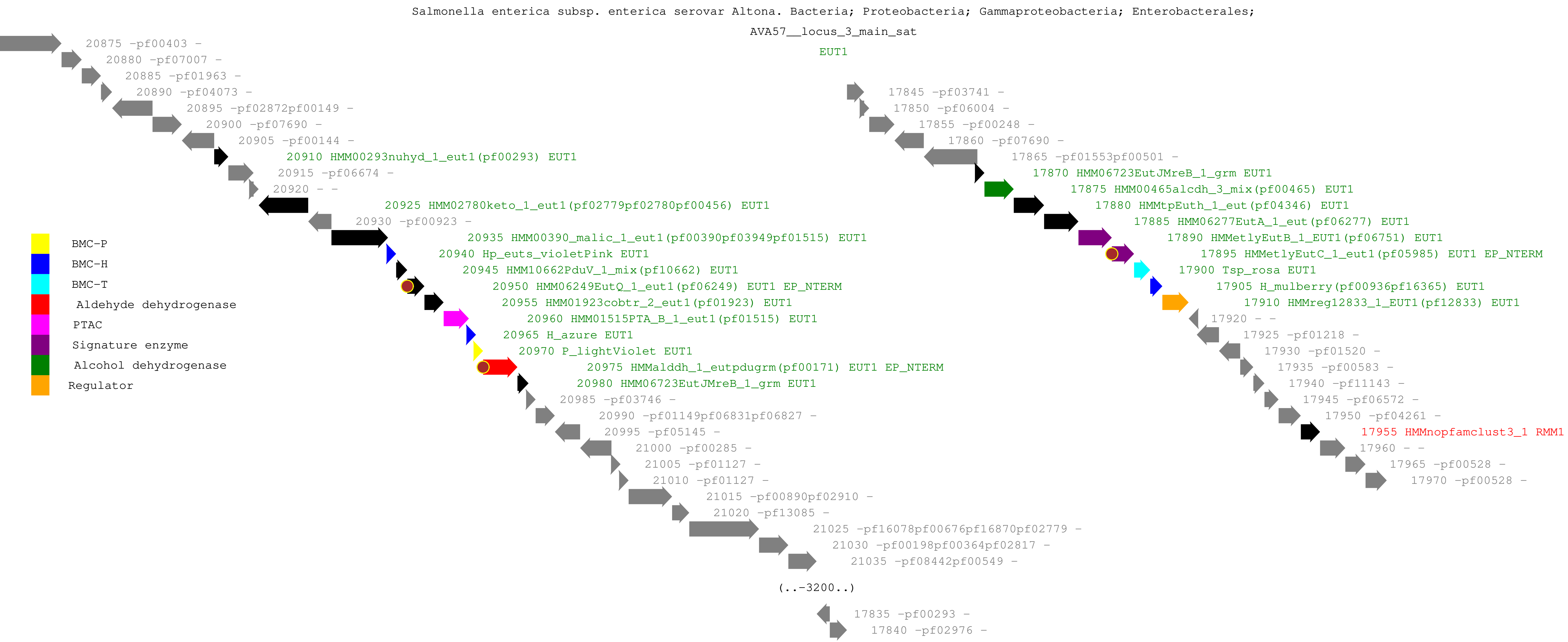
Salmonella enterica subsp. enterica serovar Altona. Bacteria; Proteobacteria; Gammaproteobacteria; Enterobacterales;
AVA57__locus_3_main_sat
EUT1
20875 -pf00403 -
20880 -pf07007 -
20885 -pf01963 -
20890 -pf04073 -
20895 -pf02872pf00149 -
20900 -pf07690 -
20905 -pf00144 -
20910 HMM00293nuhyd_1_eut1(pf00293) EUT1
20915 -pf06674 -
20920 - -
20925 HMM02780keto_1_eut1(pf02779pf02780pf00456) EUT1
20930 -pf00923 -
20935 HMM00390_malic_1_eut1(pf00390pf03949pf01515) EUT1
20940 Hp_euts_violetPink EUT1
20945 HMM10662PduV_1_mix(pf10662) EUT1
20950 HMM06249EutQ_1_eut1(pf06249) EUT1 EP_NTERM
20955 HMM01923cobtr_2_eut1(pf01923) EUT1
20960 HMM01515PTA_B_1_eut1(pf01515) EUT1
20965 H_azure EUT1
20970 P_lightViolet EUT1
20975 HMMaldh_1_eutpdugrm(pf00171) EUT1 EP_NTERM
20980 HMM06723EutJMreB_1_grm EUT1
20985 -pf03746 -
20990 -pf01149pf06831pf06827 -
20995 -pf05145 -
21000 -pf00285 -
21005 -pf01127 -
21010 -pf01127 -
21015 -pf00890pf02910 -
21020 -pf13085 -
21025 -pf16078pf00676pf16870pf02779 -
21030 -pf00198pf00364pf02817 -
21035 -pf08442pf00549 -
(..-3200..)
17835 -pf00293 -
17840 -pf02976 -
17845 -pf03741 -
17850 -pf06004 -
17855 -pf00248 -
17860 -pf07690 -
17865 -pf01553pf00501 -
17870 HMM06723EutJMreB_1_grm EUT1
17875 HMM00465alcdh_3_mix(pf00465) EUT1
17880 HMMtpEuth_1_eut(pf04346) EUT1
17885 HMM06277EutA_1_eut(pf06277) EUT1
17890 HMMetlyEutB_1_EUT1(pf06751) EUT1
17895 HMMetlyEutC_1_eut1(pf05985) EUT1 EP_NTERM
17900 Tsp_rosa EUT1
17905 H_mulberry(pf00936pf16365) EUT1
17910 HMMreg12833_1_EUT1(pf12833) EUT1
17920 - -
17925 -pf01218 -
17930 -pf01520 -
17935 -pf00583 -
17940 -pf11143 -
17945 -pf06572 -
17950 -pf04261 -
17955 HMMnopfamclust3_1 RMM1
17960 - -
17965 -pf00528 -
17970 -pf00528 -
BMC-P
BMC-H
BMC-T
Aldehyde dehydrogenase
PTAC
Signature enzyme
Alcohol dehydrogenase
Regulator

Salmonella enterica subsp. enterica serovar Nima. Bacteria; Proteobacteria; Gammaproteobacteria; Enterobacterales;

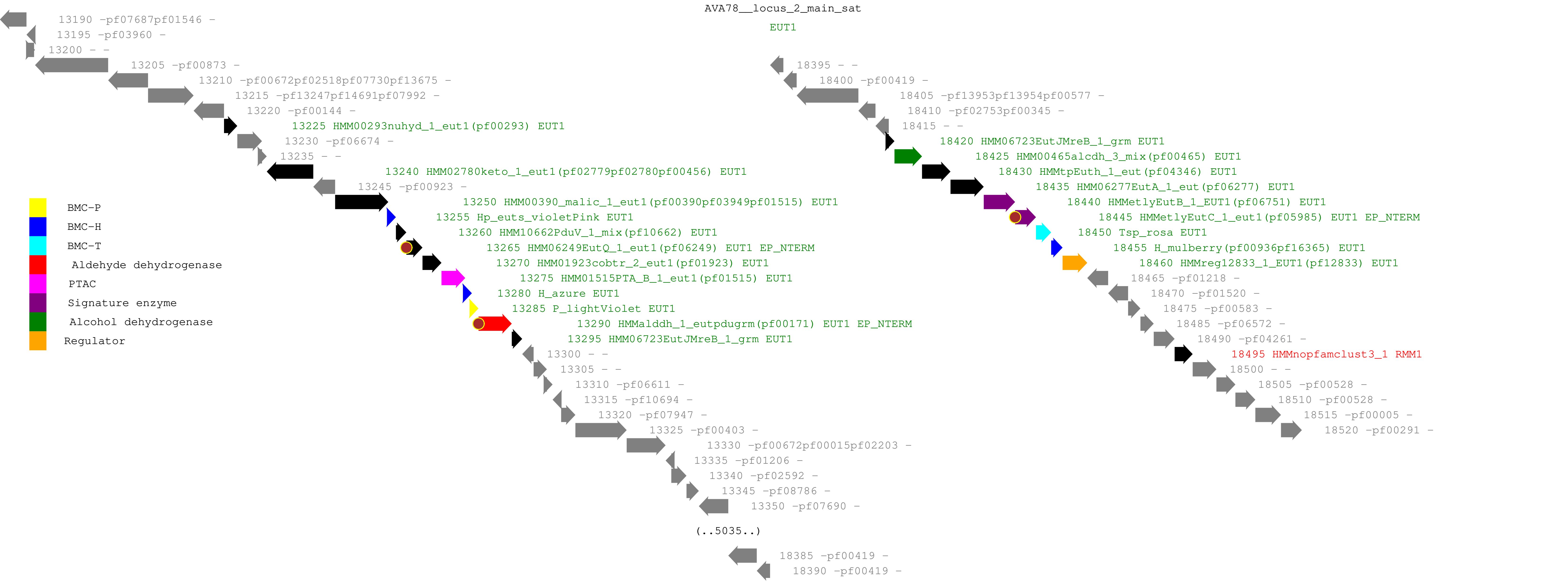

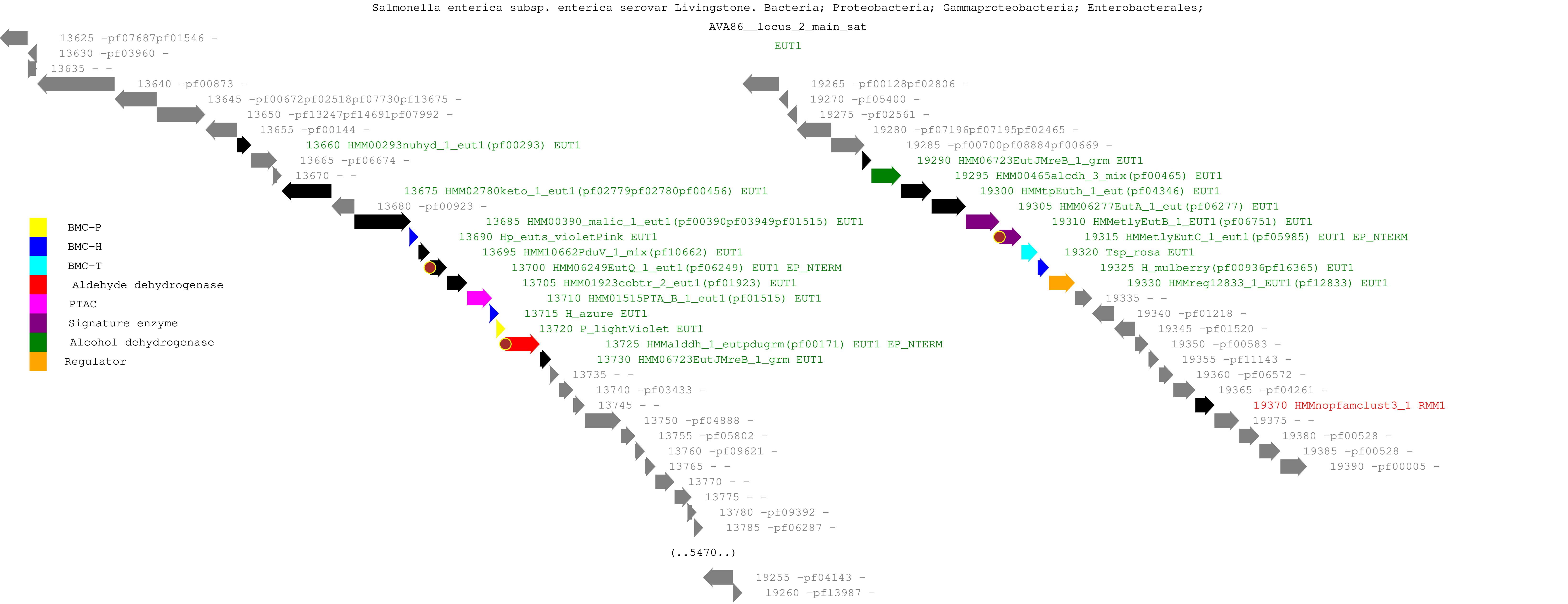

Salmonella enterica subsp. enterica serovar Livingstone. Bacteria; Proteobacteria; Gammaproteobacteria; Enterobacterales;
AVA86__locus_2_main_sat
EUT1
13625 -pf07687pf01546 -
13630 -pf03960 -
13635 - -
13640 -pf00873 -
13645 -pf00672pf02518pf07730pf13675 -
13650 -pf13247pf14691pf07992 -
13655 -pf00144 -
13660 HMM00293nuhyd_1_eut1(pf00293) EUT1
13665 -pf06674 -
13670 - -
13675 HMM02780keto_1_eut1(pf02779pf02780pf00456) EUT1
13680 -pf00923 -
13685 HMM00390_malic_1_eut1(pf00390pf03949pf01515) EUT1
13690 Hp_euts_violetPink EUT1
13695 HMM10662PduV_1_mix(pf10662) EUT1
13700 HMM06249EutQ_1_eut1(pf06249) EUT1 EP_NTERM
13705 HMM01923cobtr_2_eut1(pf01923) EUT1
13710 HMM01515PTA_B_1_eut1(pf01515) EUT1
13715 H_azure EUT1
13720 P_lightViolet EUT1
13725 HMMalddh_1_eutpdugrm(pf00171) EUT1 EP_NTERM
13730 HMM06723EutJMreB_1_grm EUT1
13735 - -
13740 -pf03433 -
13745 - -
13750 -pf04888 -
13755 -pf05802 -
13760 -pf09621 -
13765 - -
13770 - -
13775 - -
13780 -pf09392 -
13785 -pf06287 -
(..5470..)
19255 -pf04143 -
19260 -pf13987 -
19265 -pf00128pf02806 -
19270 -pf05400 -
19275 -pf02561 -
19280 -pf07196pf07195pf02465 -
19285 -pf00700pf08884pf00669 -
19290 HMM06723EutJMreB_1_grm EUT1
19295 HMM00465alcdh_3_mix(pf00465) EUT1
19300 HMMtpEuth_1_eut(pf04346) EUT1
19305 HMM06277EutA_1_eut(pf06277) EUT1
19310 HMMetlyEutB_1_EUT1(pf06751) EUT1
19315 HMMetlyEutC_1_eut1(pf05985) EUT1 EP_NTERM
19320 Tsp_rosa EUT1
19325 H_mulberry(pf00936pf16365) EUT1
19330 HMMreg12833_1_EUT1(pf12833) EUT1
19335 - -
19340 -pf01218 -
19345 -pf01520 -
19350 -pf00583 -
19355 -pf11143 -
19360 -pf06572 -
19365 -pf04261 -
19370 HMMnopfamclust3_1 RMM1
19375 - -
19380 -pf00528 -
19385 -pf00528 -
19390 -pf00005 -
BMC-P
BMC-H
BMC-T
Aldehyde dehydrogenase
PTAC
Signature enzyme
Alcohol dehydrogenase
Regulator

Salmonella enterica subsp. enterica serovar Hvittingfoss. Bacteria; Proteobacteria; Gammaproteobacteria; Enterobacterales;

AVB75__locus_2_main_sat

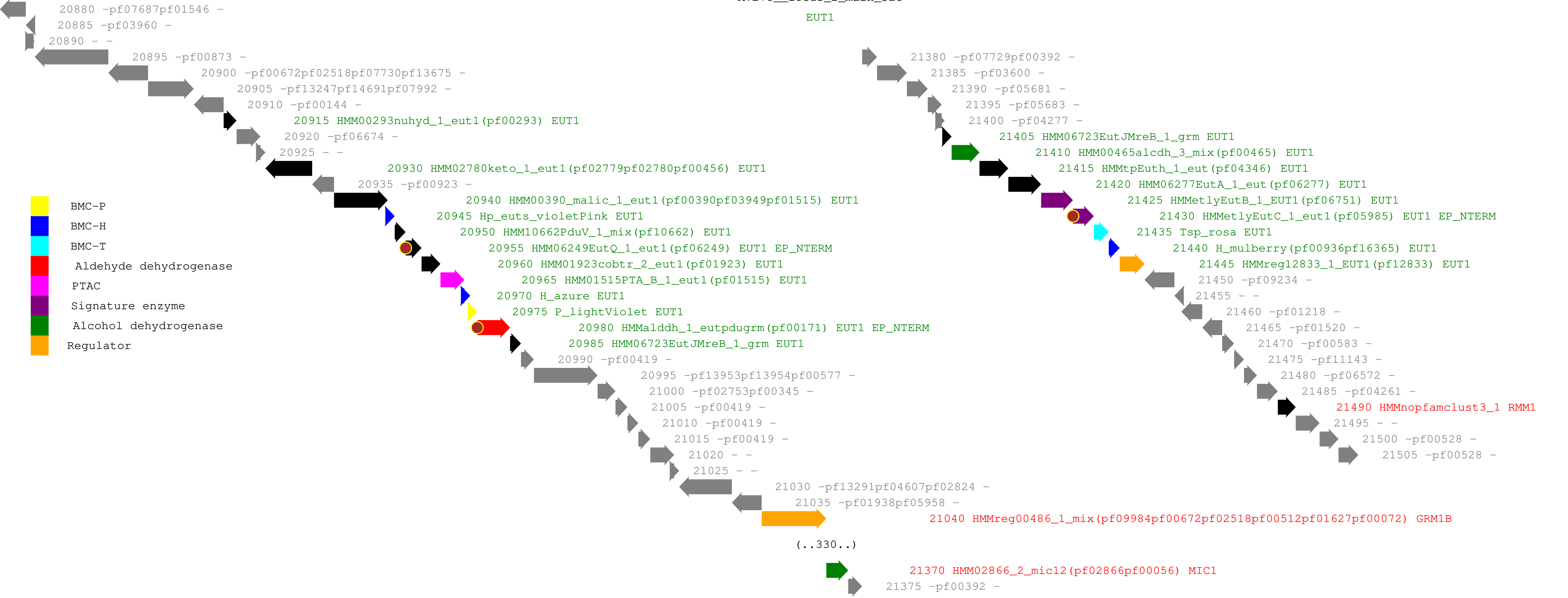

Salmonella enterica subsp. enterica serovar Vitkin. Bacteria; Proteobacteria; Gammaproteobacteria; Enterobacterales;

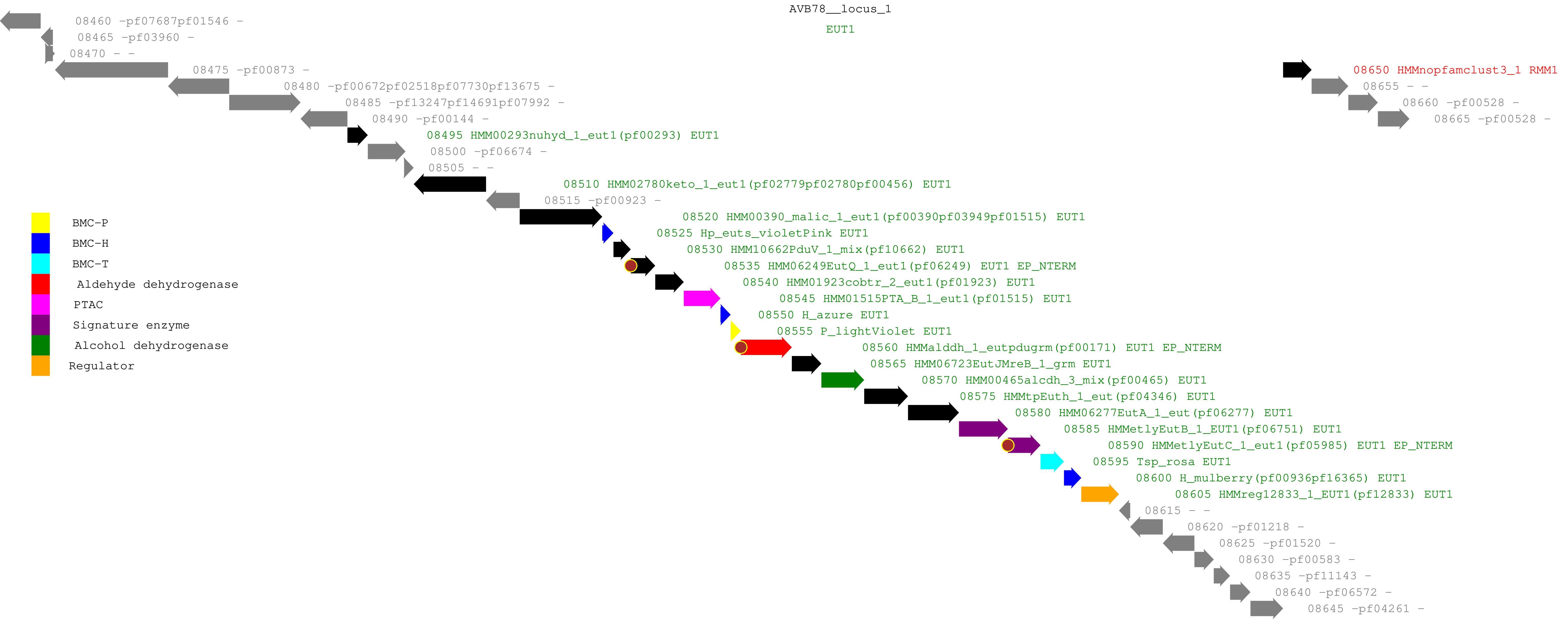

Salmonella enterica subsp. enterica serovar Ajiobo. Bacteria; Proteobacteria; Gammaproteobacteria; Enterobacterales;

AVB95__locus_2

EUT1

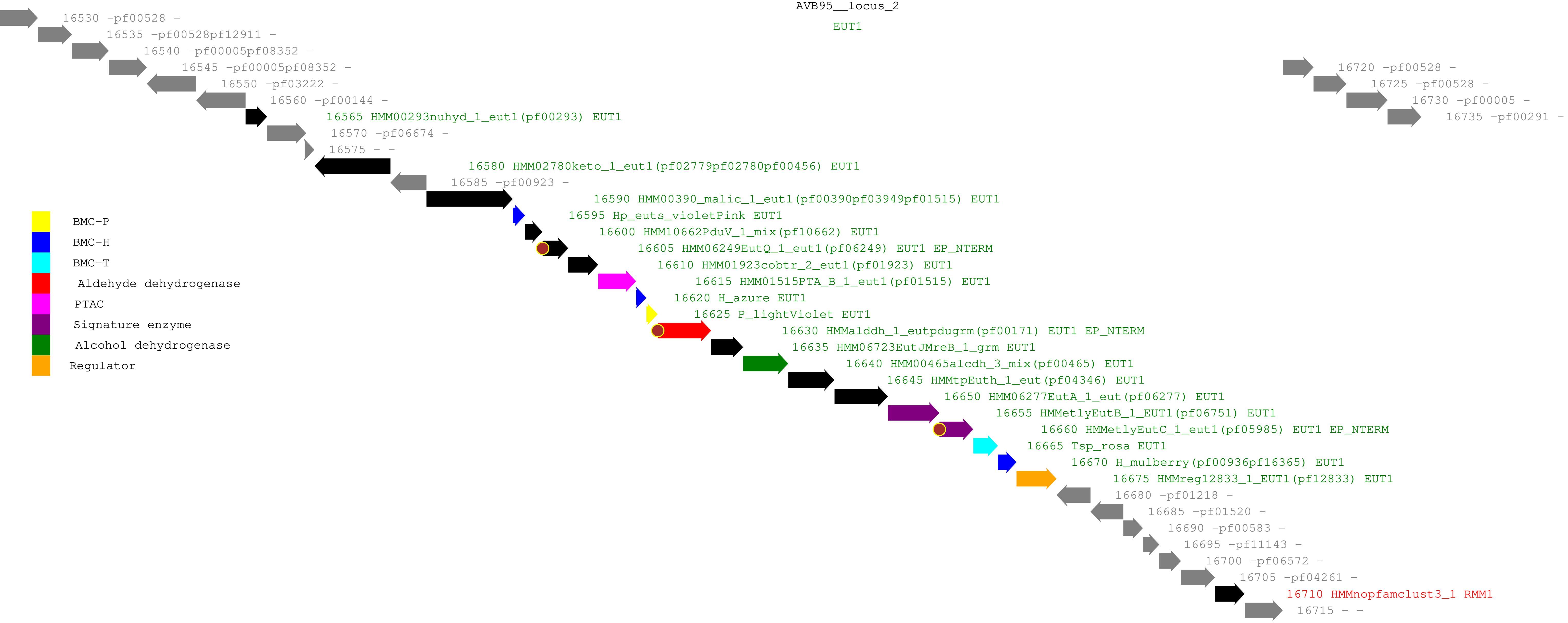

Salmonella enterica subsp. enterica serovar Kambole. Bacteria; Proteobacteria; Gammaproteobacteria; Enterobacterales;

AVC51__locus_1_main_sat

EUT1

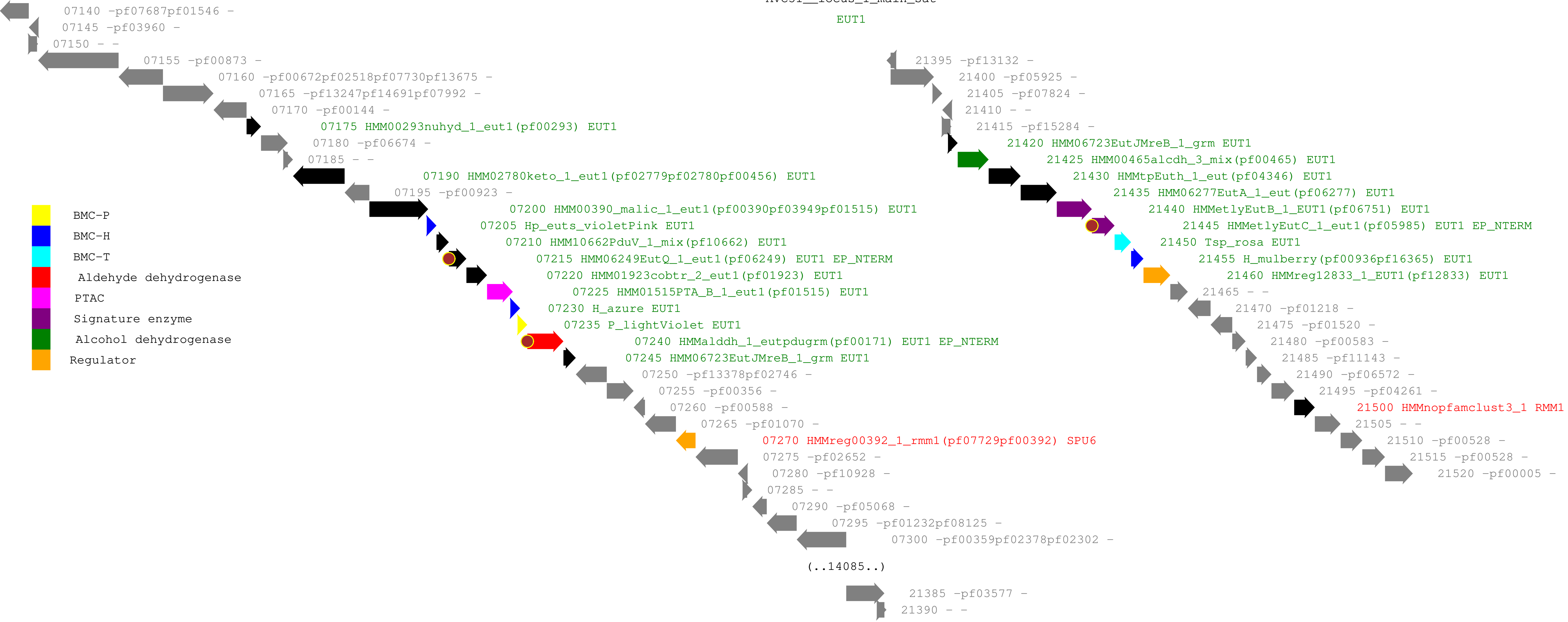

Salmonella enterica subsp. enterica serovar Indiana. Bacteria; Proteobacteria; Gammaproteobacteria; Enterobacterales;

AVC75__locus_1

EUT1

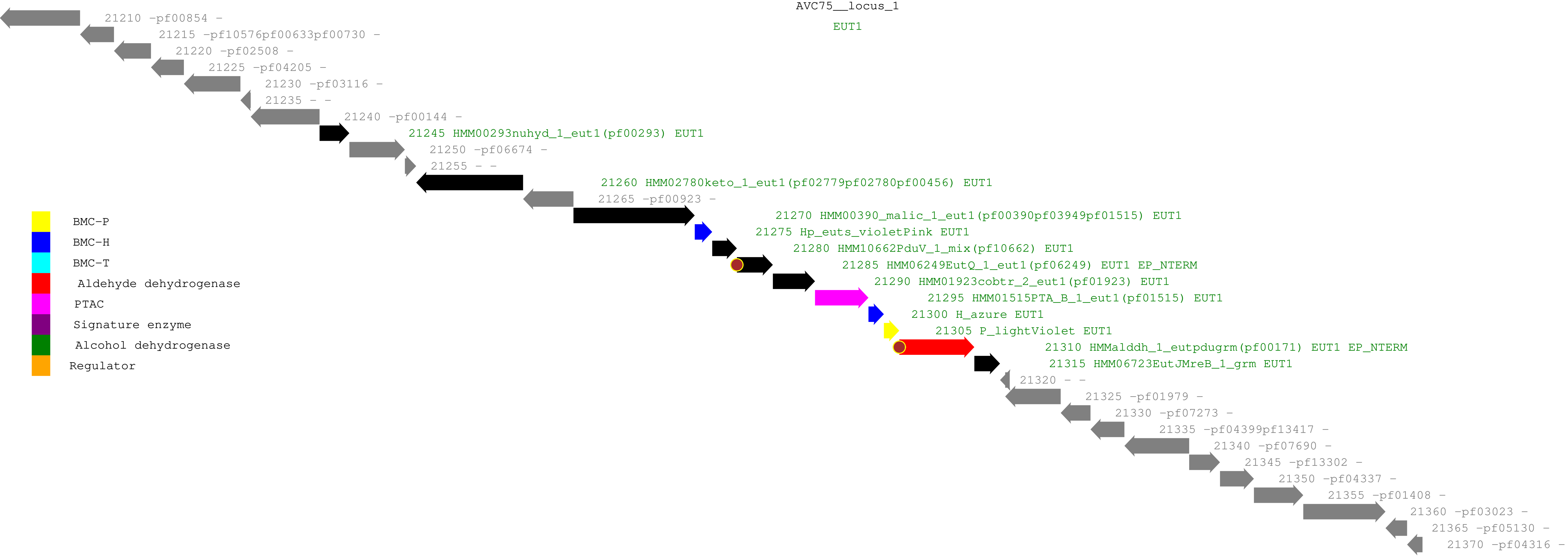

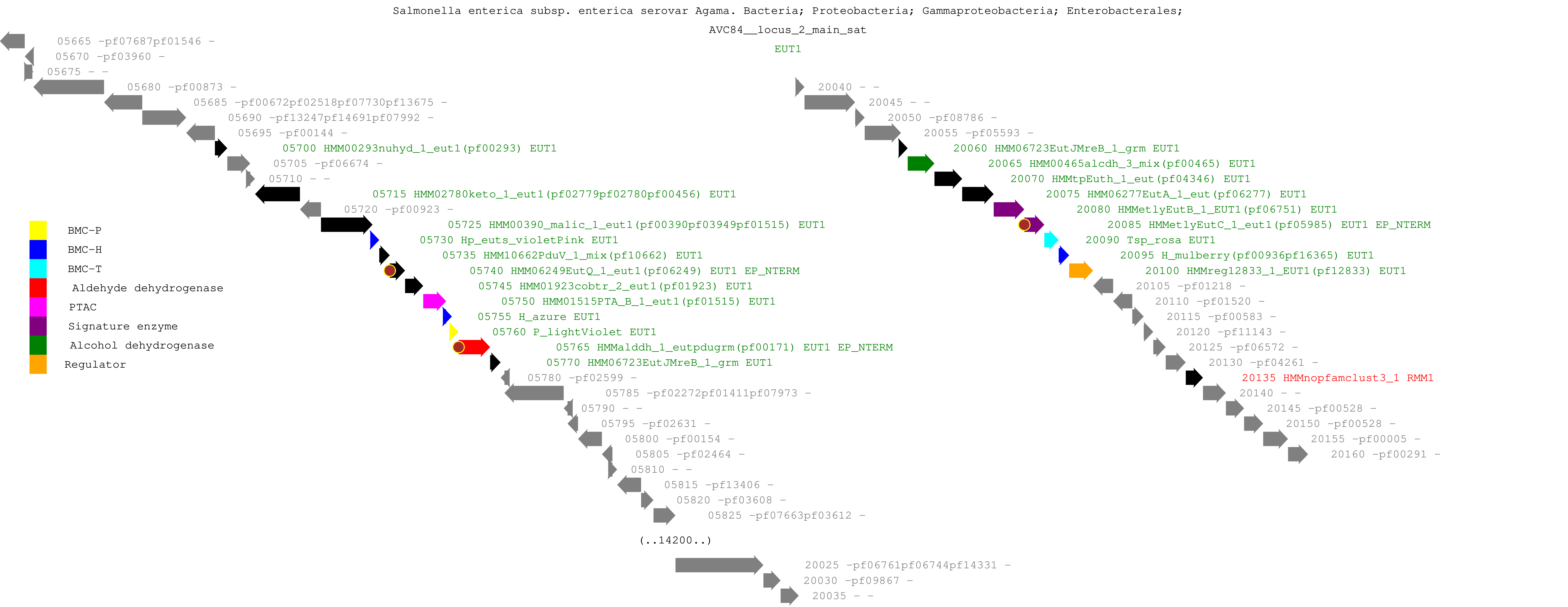
Salmonella enterica subsp. enterica serovar Agama. Bacteria; Proteobacteria; Gammaproteobacteria; Enterobacterales;
AVC84__locus_2_main_sat
EUT1
05665 -pf07687pf01546 -
05670 -pf03960 -
05675 - -
05680 -pf00873 -
05685 -pf00672pf02518pf07730pf13675 -
05690 -pf13247pf14691pf07992 -
05695 -pf00144 -
05700 HMM00293nuhyd_1_eut1(pf00293) EUT1
05705 -pf06674 -
05710 - -
05715 HMM02780keto_1_eut1(pf02779pf02780pf00456) EUT1
05720 -pf00923 -
05725 HMM00390_malic_1_eut1(pf00390pf03949pf01515) EUT1
05730 Hp_euts_violetPink EUT1
05735 HMM10662PduV_1_mix(pf10662) EUT1
05740 HMM06249EutQ_1_eut1(pf06249) EUT1 EP_NTERM
05745 HMM01923cobtr_2_eut1(pf01923) EUT1
05750 HMM01515PTA_B_1_eut1(pf01515) EUT1
05755 H_azure EUT1
05760 P_lightViolet EUT1
05765 HMMalddh_1_eutpdugrm(pf00171) EUT1 EP_NTERM
05770 HMM06723EutJMreB_1_grm EUT1
05780 -pf02599 -
05785 -pf02272pf01411pf07973 -
05790 - -
05795 -pf02631 -
05800 -pf00154 -
05805 -pf02464 -
05810 - -
05815 -pf13406 -
05820 -pf03608 -
05825 -pf07663pf03612 -
(..14200..)
20025 -pf06761pf06744pf14331 -
20030 -pf09867 -
20035 - -
20040 - -
20045 - -
20050 -pf08786 -
20055 -pf05593 -
20060 HMM06723EutJMreB_1_grm EUT1
20065 HMM00465alcdh_3_mix(pf00465) EUT1
20070 HMMtpEuth_1_eut(pf04346) EUT1
20075 HMM06277EutA_1_eut(pf06277) EUT1
20080 HMMetlyEutB_1_EUT1(pf06751) EUT1
20085 HMMetlyEutC_1_eut1(pf05985) EUT1 EP_NTERM
20090 Tsp_rosa EUT1
20095 H_mulberry(pf00936pf16365) EUT1
20100 HMMreg12833_1_EUT1(pf12833) EUT1
20105 -pf01218 -
20110 -pf01520 -
20115 -pf00583 -
20120 -pf11143 -
20125 -pf06572 -
20130 -pf04261 -
20135 HMMnopfamclust3_1 RMM1
20140 - -
20145 -pf00528 -
20150 -pf00528 -
20155 -pf00005 -
20160 -pf00291 -
BMC-P
BMC-H
BMC-T
Aldehyde dehydrogenase
PTAC
Signature enzyme
Alcohol dehydrogenase
Regulator

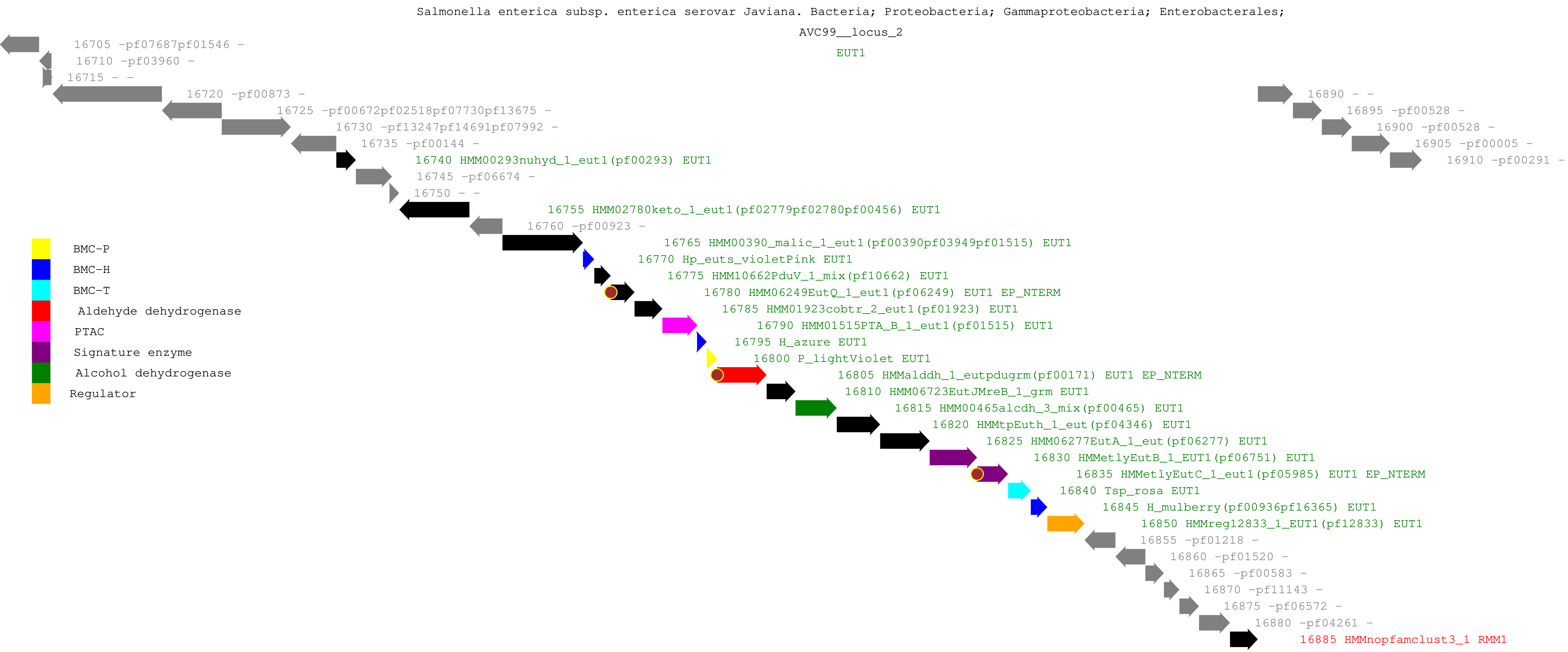

Salmonella enterica subsp. enterica serovar Javiana. Bacteria; Proteobacteria; Gammaproteobacteria; Enterobacterales;
AVC99__locus_2
EUT1
16705 -pf07687pf01546 -
16710 -pf03960 -
16715 - -
16720 -pf00873 -
16725 -pf00672pf02518pf07730pf13675 -
16730 -pf13247pf14691pf07992 -
16735 -pf00144 -
16740 HMM00293nuhyd_1_eut1(pf00293) EUT1
16745 -pf06674 -
16750 - -
16755 HMM02780keto_1_eut1(pf02779pf02780pf00456) EUT1
16760 -pf00923 -
16765 HMM00390_malic_1_eut1(pf00390pf03949pf01515) EUT1
16770 Hp_euts_violetPink EUT1
16775 HMM10662PduV_1_mix(pf10662) EUT1
16780 HMM06249EutQ_1_eut1(pf06249) EUT1 EP_NTERM
16785 HMM01923cobtr_2_eut1(pf01923) EUT1
16790 HMM01515PTA_B_1_eut1(pf01515) EUT1
16795 H_azure EUT1
16800 P_lightViolet EUT1
16805 HMMalddh_1_eutpdugrm(pf00171) EUT1 EP_NTERM
16810 HMM06723EutJMreB_1_grm EUT1
16815 HMM00465alcdh_3_mix(pf00465) EUT1
16820 HMMtpEuth_1_eut(pf04346) EUT1
16825 HMM06277EutA_1_eut(pf06277) EUT1
16830 HMMetlyEutB_1_EUT1(pf06751) EUT1
16835 HMMetlyEutC_1_eut1(pf05985) EUT1 EP_NTERM
16840 Tsp_rosa EUT1
16845 H_mulberry(pf00936pf16365) EUT1
16850 HMMreg12833_1_EUT1(pf12833) EUT1
16855 -pf01218 -
16860 -pf01520 -
16865 -pf00583 -
16870 -pf11143 -
16875 -pf06572 -
16880 -pf04261 -
16885 HMMnopfamclust3_1 RMM1
16890 - -
16895 -pf00528 -
16900 -pf00528 -
16905 -pf00005 -
16910 -pf00291 -
BMC-P
BMC-H
BMC-T
Aldehyde dehydrogenase
PTAC
Signature enzyme
Alcohol dehydrogenase
Regulator

Salmonella enterica subsp. enterica serovar Alachua. Bacteria; Proteobacteria; Gammaproteobacteria; Enterobacterales;

AVD57__locus_2

EUT1

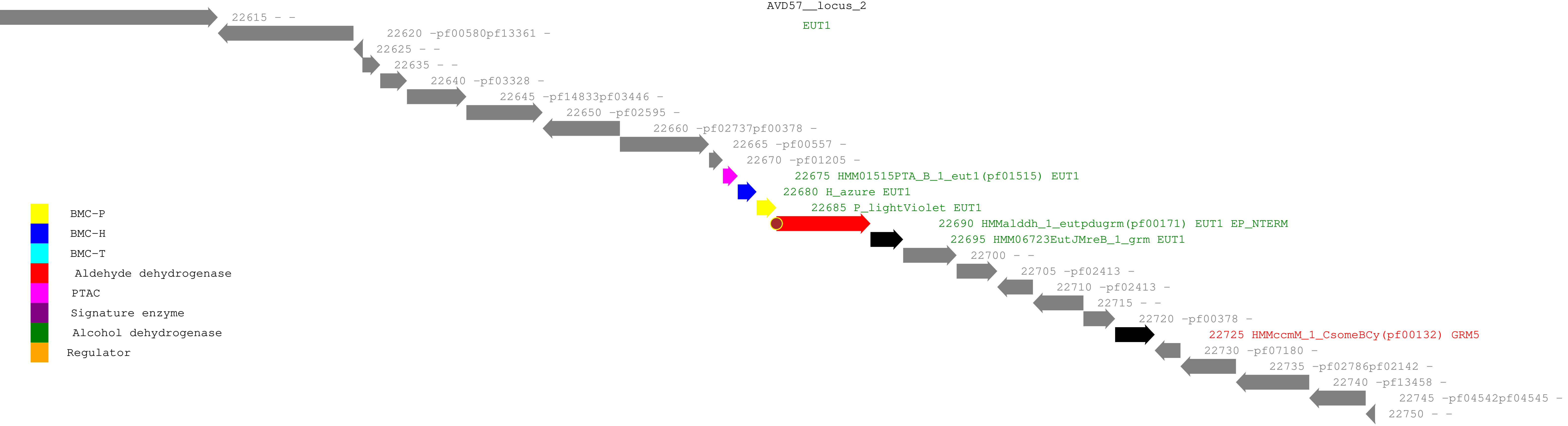

Salmonella enterica subsp. enterica serovar Agbeni. Bacteria; Proteobacteria; Gammaproteobacteria; Enterobacterales;

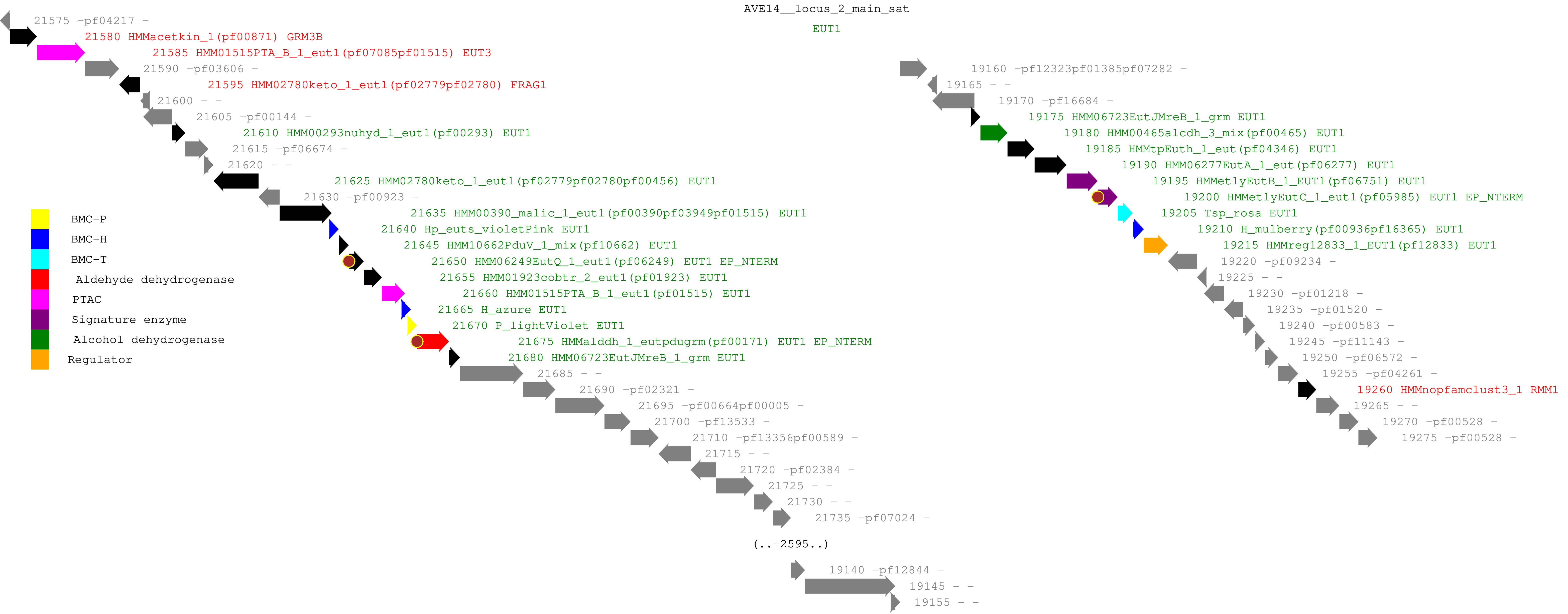

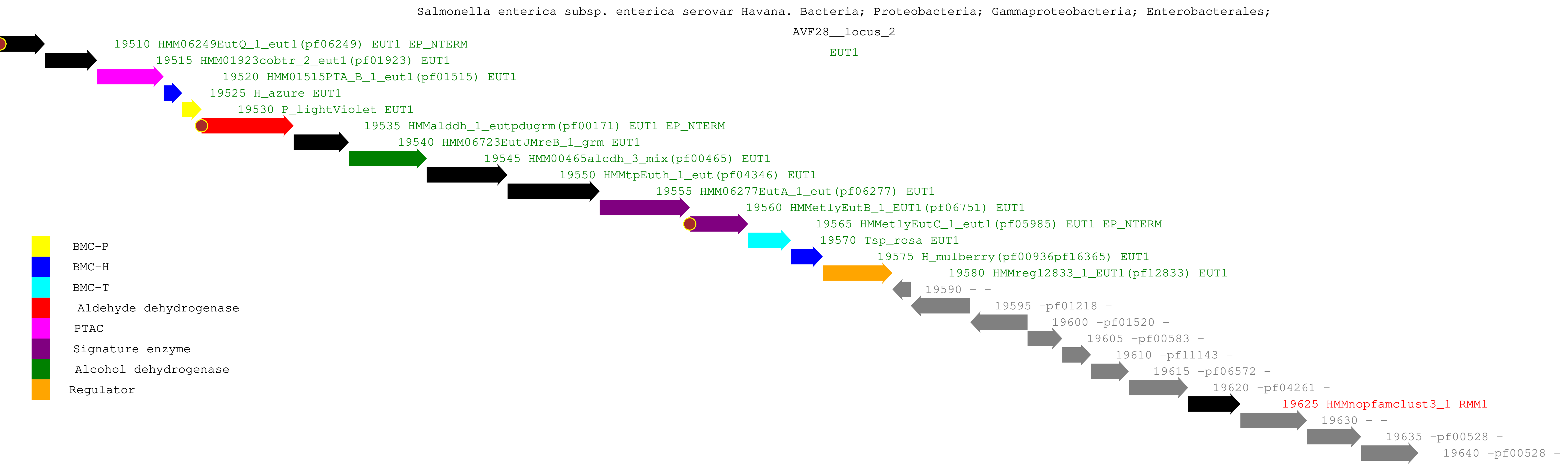

Salmonella enterica subsp. enterica serovar Havana. Bacteria; Proteobacteria; Gammaproteobacteria; Enterobacterales;
AVF28__locus_2
EUT1
19510 HMM06249EutQ_1_eut1(pf06249) EUT1 EP_NTERM
19515 HMM01923cobtr_2_eut1(pf01923) EUT1
19520 HMM01515PTA_B_1_eut1(pf01515) EUT1
19525 H_azure EUT1
19530 P_lightViolet EUT1
19535 HMMaldh_1_eutpdugrm(pf00171) EUT1 EP_NTERM
19540 HMM06723EutJMreB_1_grm EUT1
19545 HMM00465alcdh_3_mix(pf00465) EUT1
19550 HMMtpEuth_1_eut(pf04346) EUT1
19555 HMM06277EutA_1_eut(pf06277) EUT1
19560 HMMetlyEutB_1_EUT1(pf06751) EUT1
19565 HMMetlyEutC_1_eut1(pf05985) EUT1 EP_NTERM
19570 Tsp_rosa EUT1
19575 H_mulberry(pf00936pf16365) EUT1
19580 HMMreg12833_1_EUT1(pf12833) EUT1
19590 - -
19595 -pf01218 -
19600 -pf01520 -
19605 -pf00583 -
19610 -pf11143 -
19615 -pf06572 -
19620 -pf04261 -
19625 HMMnopfamclust3_1 RMM1
19630 - -
19635 -pf00528 -
19640 -pf00528 -
BMC-P
BMC-H
BMC-T
Aldehyde dehydrogenase
PTAC
Signature enzyme
Alcohol dehydrogenase
Regulator

Salmonella enterica subsp. enterica serovar Mikawasima. Bacteria; Proteobacteria; Gammaproteobacteria; Enterobacterales;

AVF35__locus_2_main_sat

EUT1

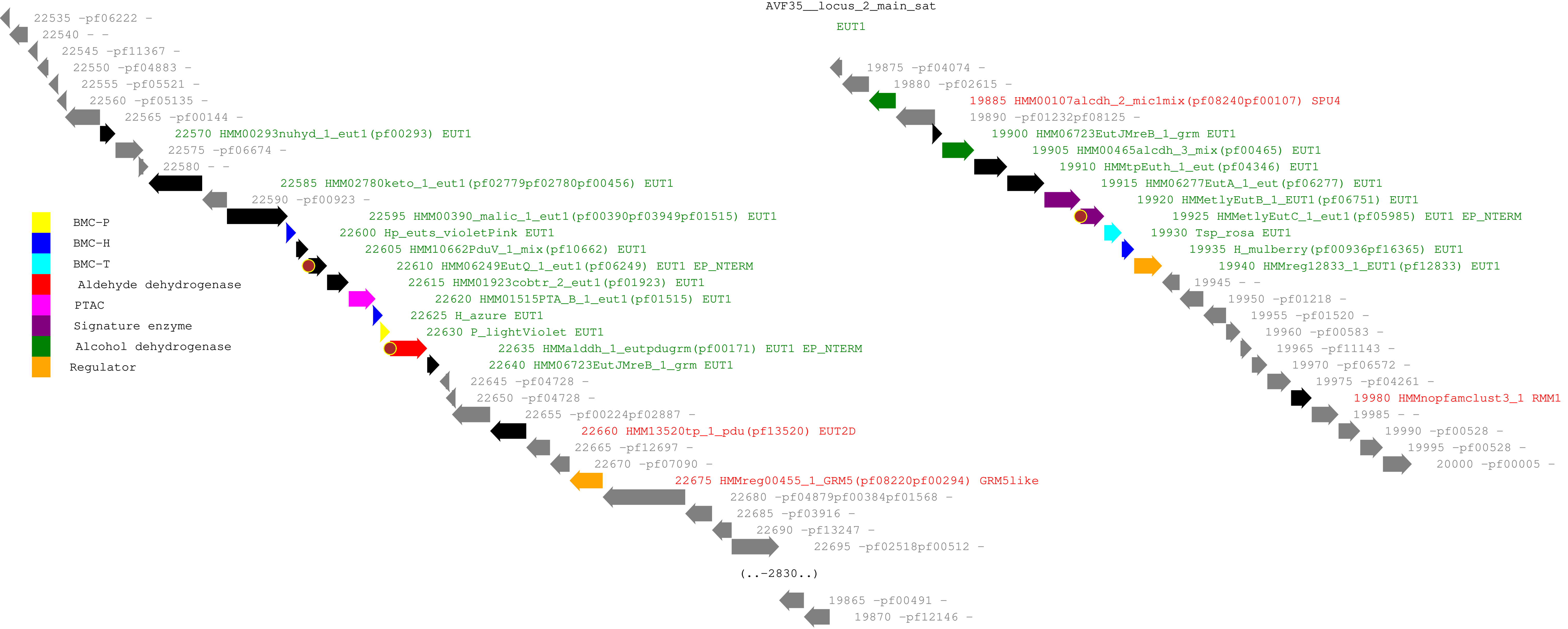

Salmonella enterica subsp. enterica serovar Kottbus. Bacteria; Proteobacteria; Gammaproteobacteria; Enterobacterales;

AVF53__locus_2_main_sat

EUT1

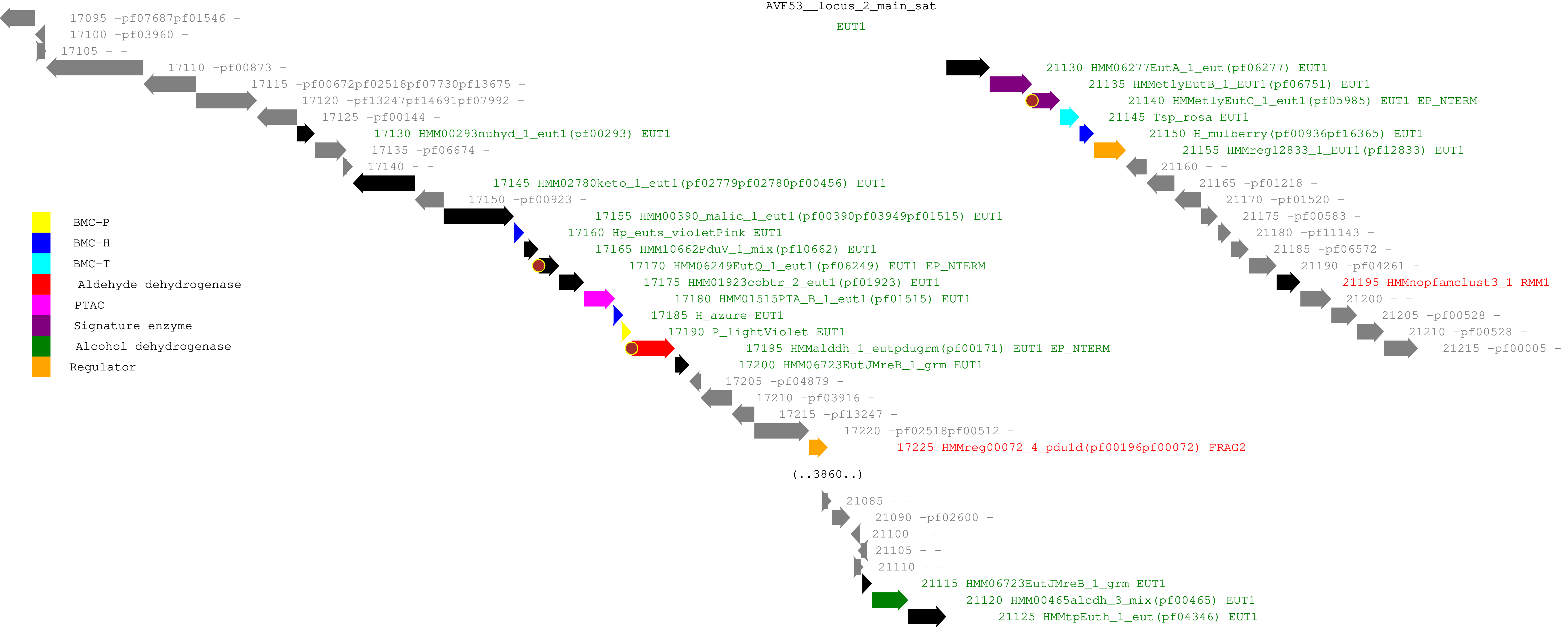

Salmonella enterica subsp. enterica serovar Vitkin. Bacteria; Proteobacteria; Gammaproteobacteria; Enterobacterales;

AVG84__locus_2

EUT1

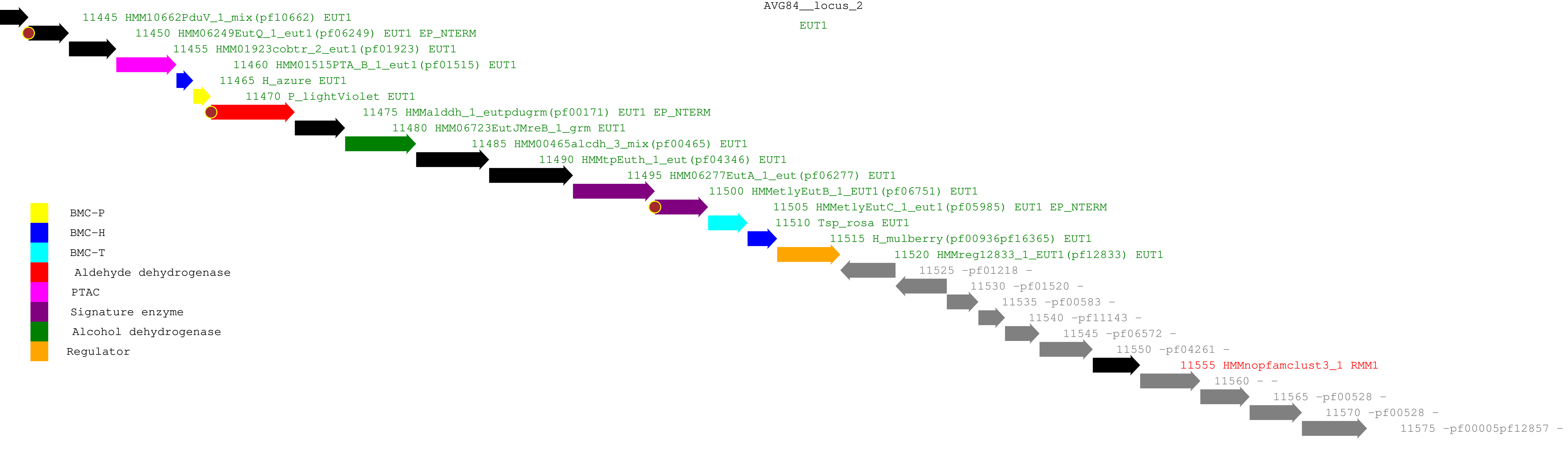

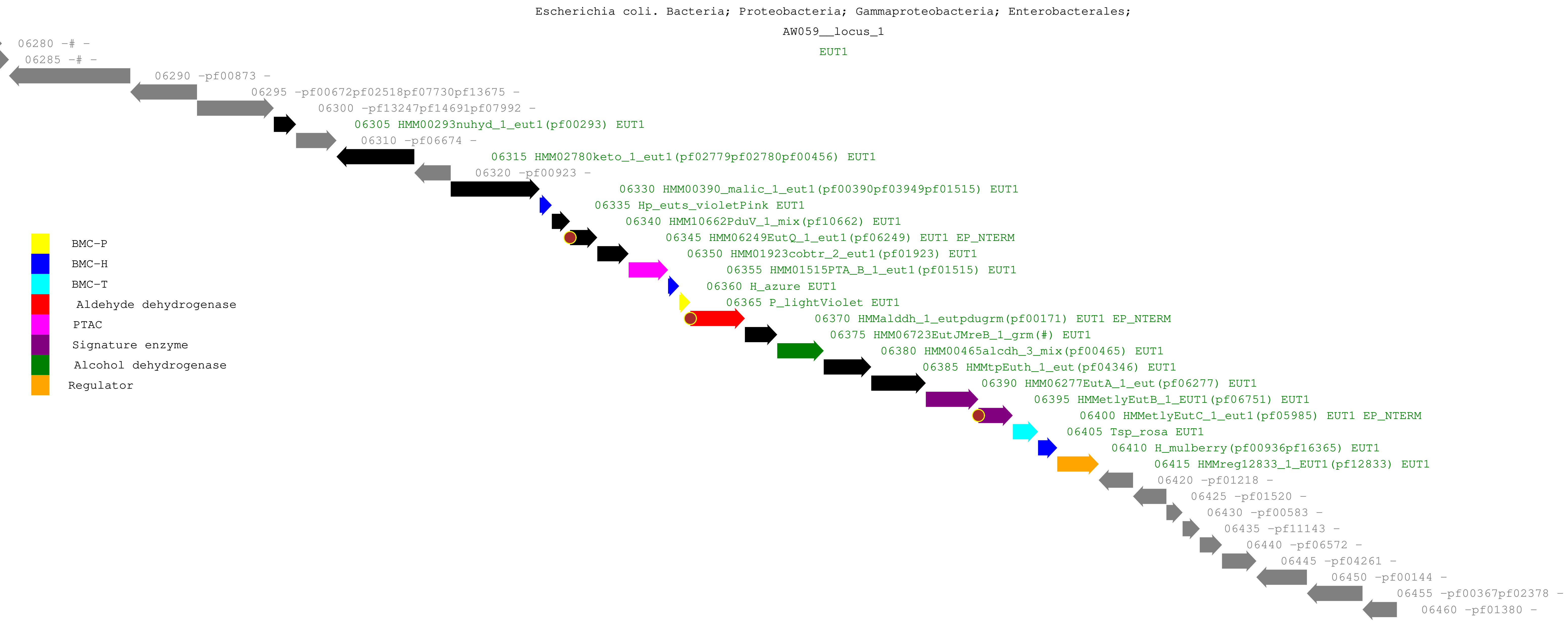

Escherichia coli. Bacteria; Proteobacteria; Gammaproteobacteria; Enterobacterales;
AW059__locus_1
EUT1
06280 -# -
06285 -# -
06290 -pf00873 -
06295 -pf00672pf02518pf07730pf13675 -
06300 -pf13247pf14691pf07992 -
06305 HMM00293nuhyd_1_eut1(pf00293) EUT1
06310 -pf06674 -
06315 HMM02780keto_1_eut1(pf02779pf02780pf00456) EUT1
06320 -pf00923 -
06330 HMM00390_malic_1_eut1(pf00390pf03949pf01515) EUT1
06335 Hp_euts_violetPink EUT1
06340 HMM10662PduV_1_mix(pf10662) EUT1
06345 HMM06249EutQ_1_eut1(pf06249) EUT1 EP_NTERM
06350 HMM01923cobtr_2_eut1(pf01923) EUT1
06355 HMM01515PTA_B_1_eut1(pf01515) EUT1
06360 H_azure EUT1
06365 P_lightViolet EUT1
06370 HMMalddh_1_eutpdugrm(pf00171) EUT1 EP_NTERM
06375 HMM06723EutJMreB_1_grm(#) EUT1
06380 HMM00465alcdh_3_mix(pf00465) EUT1
06385 HMMtpEuth_1_eut(pf04346) EUT1
06390 HMM06277EutA_1_eut(pf06277) EUT1
06395 HMMetlyEutB_1_EUT1(pf06751) EUT1
06400 HMMetlyEutC_1_eut1(pf05985) EUT1 EP_NTERM
06405 Tsp_rosa EUT1
06410 H_mulberry(pf00936pf16365) EUT1
06415 HMMreg12833_1_EUT1(pf12833) EUT1
06420 -pf01218 -
06425 -pf01520 -
06430 -pf00583 -
06435 -pf11143 -
06440 -pf06572 -
06445 -pf04261 -
06450 -pf00144 -
06455 -pf00367pf02378 -
06460 -pf01380 -
BMC-P
BMC-H
BMC-T
Aldehyde dehydrogenase
PTAC
Signature enzyme
Alcohol dehydrogenase
Regulator

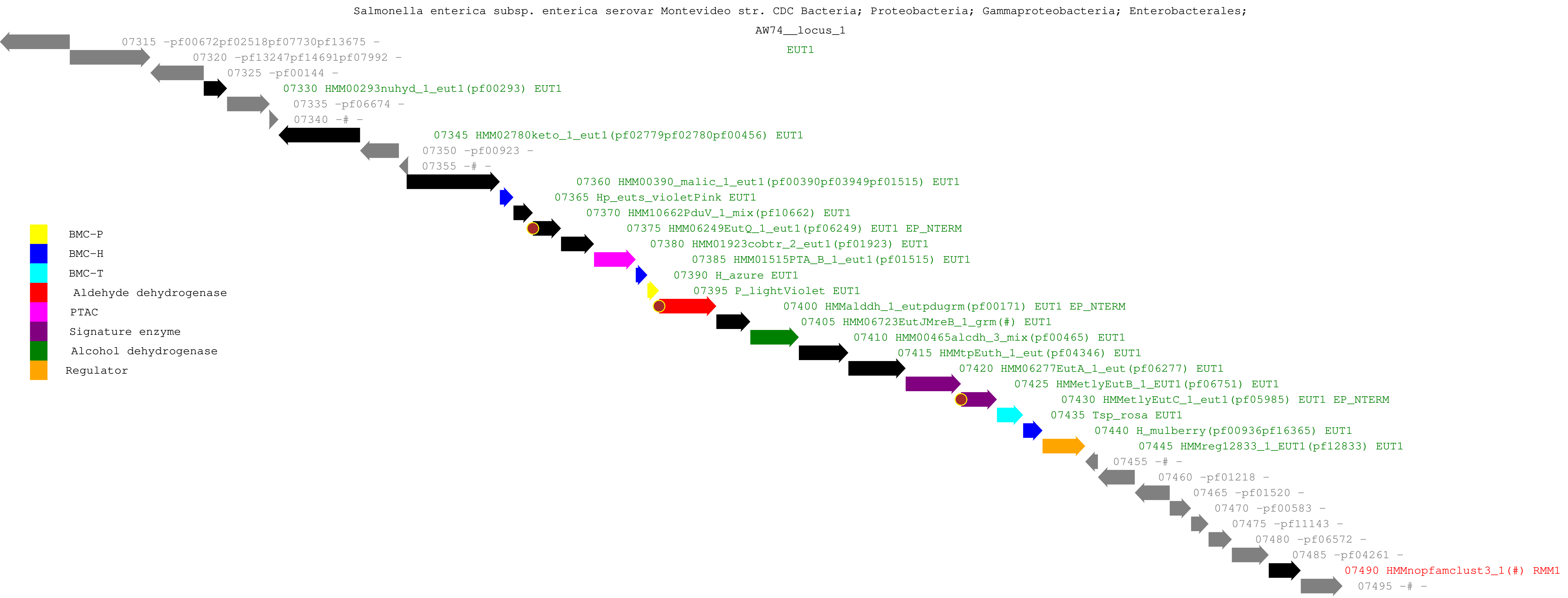
Salmonella enterica subsp. enterica serovar Montevideo str. CDC Bacteria; Proteobacteria; Gammaproteobacteria; Enterobacterales;
AW74__locus_1
EUT1
07315 -pf00672pf02518pf07730pf13675 -
07320 -pf13247pf14691pf07992 -
07325 -pf00144 -
07330 HMM00293nuhyd_1_eut1(pf00293) EUT1
07335 -pf06674 -
07340 -# -
07345 HMM02780keto_1_eut1(pf02779pf02780pf00456) EUT1
07350 -pf00923 -
07355 -# -
07360 HMM00390_malic_1_eut1(pf00390pf03949pf01515) EUT1
07365 Hp_euts_violetPink EUT1
07370 HMM10662PduV_1_mix(pf10662) EUT1
07375 HMM06249EutQ_1_eut1(pf06249) EUT1 EP_NTERM
07380 HMM01923cobtr_2_eut1(pf01923) EUT1
07385 HMM01515PTA_B_1_eut1(pf01515) EUT1
07390 H_azure EUT1
07395 P_lightViolet EUT1
07400 HMMaldh_1_eutpdugrm(pf00171) EUT1 EP_NTERM
07405 HMM06723EutJMreB_1_grm(#) EUT1
07410 HMM00465alcdh_3_mix(pf00465) EUT1
07415 HMMtpEuth_1_eut(pf04346) EUT1
07420 HMM06277EutA_1_eut(pf06277) EUT1
07425 HMMetlyEutB_1_EUT1(pf06751) EUT1
07430 HMMetlyEutC_1_eut1(pf05985) EUT1 EP_NTERM
07435 Tsp_rosa EUT1
07440 H_mulberry(pf00936pf16365) EUT1
07445 HMMreg12833_1_EUT1(pf12833) EUT1
07455 -# -
07460 -pf01218 -
07465 -pf01520 -
07470 -pf00583 -
07475 -pf11143 -
07480 -pf06572 -
07485 -pf04261 -
07490 HMMnopfamclust3_1(#) RMM1
07495 -# -
BMC-P
BMC-H
BMC-T
Aldehyde dehydrogenase
PTAC
Signature enzyme
Alcohol dehydrogenase
Regulator

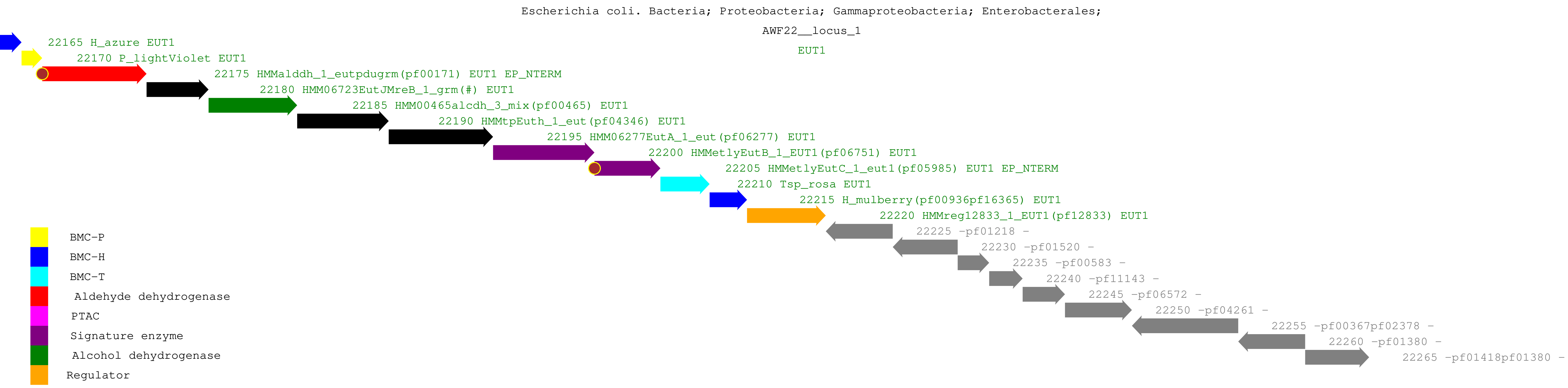

Escherichia coli. Bacteria; Proteobacteria; Gammaproteobacteria; Enterobacterales;
AWF22__locus_1
EUT1
22165 H_azure EUT1
22170 P_lightViolet EUT1
22175 HMMalddh_1_eutpdugrm(pf00171) EUT1 EP_NTERM
22180 HMM06723EutJMreB_1_grm(#) EUT1
22185 HMM00465alcdh_3_mix(pf00465) EUT1
22190 HMMtpEuth_1_eut(pf04346) EUT1
22195 HMM06277EutA_1_eut(pf06277) EUT1
22200 HMMetlyEutB_1_EUT1(pf06751) EUT1
22205 HMMetlyEutC_1_eut1(pf05985) EUT1 EP_NTERM
22210 Tsp_rosa EUT1
22215 H_mulberry(pf00936pf16365) EUT1
22220 HMMreg12833_1_EUT1(pf12833) EUT1
22225 -pf01218 -
22230 -pf01520 -
22235 -pf00583 -
22240 -pf11143 -
22245 -pf06572 -
22250 -pf04261 -
22255 -pf00367pf02378 -
22260 -pf01380 -
22265 -pf01418pf01380 -
BMC-P
BMC-H
BMC-T
Aldehyde dehydrogenase
PTAC
Signature enzyme
Alcohol dehydrogenase
Regulator

Escherichia coli. Bacteria; Proteobacteria; Gammaproteobacteria; Enterobacterales;

AWG90__locus_2

EUT1

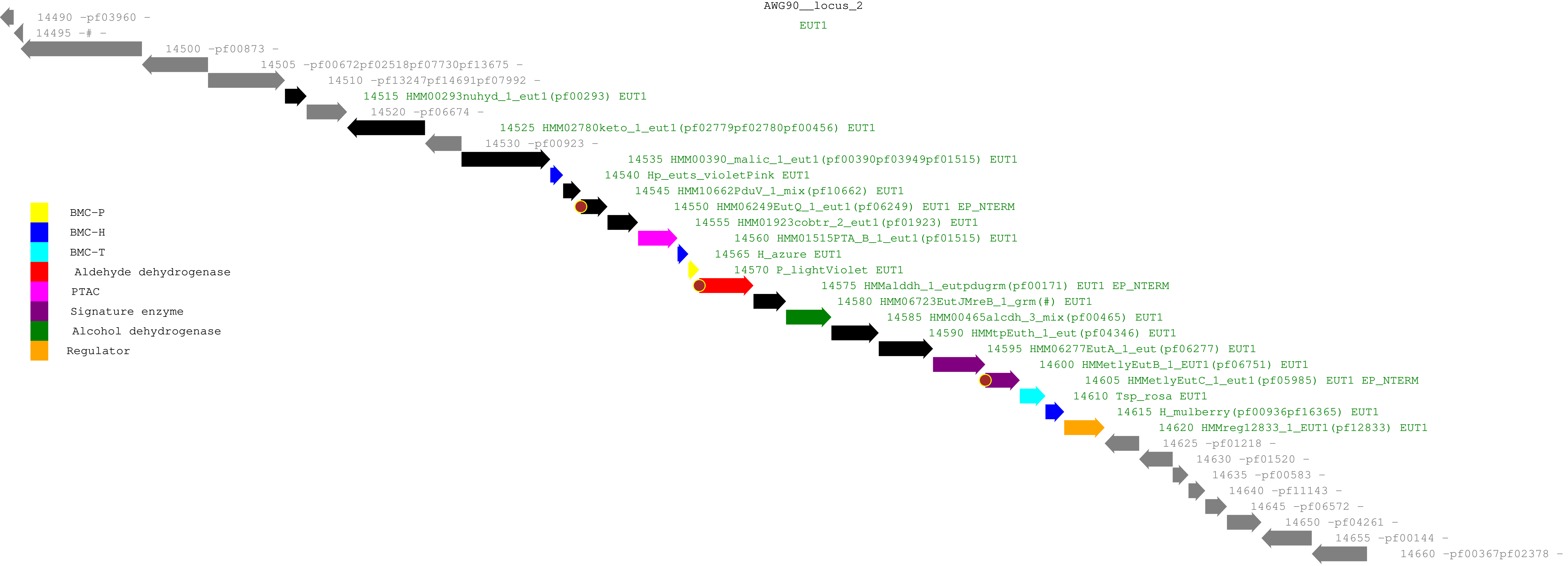

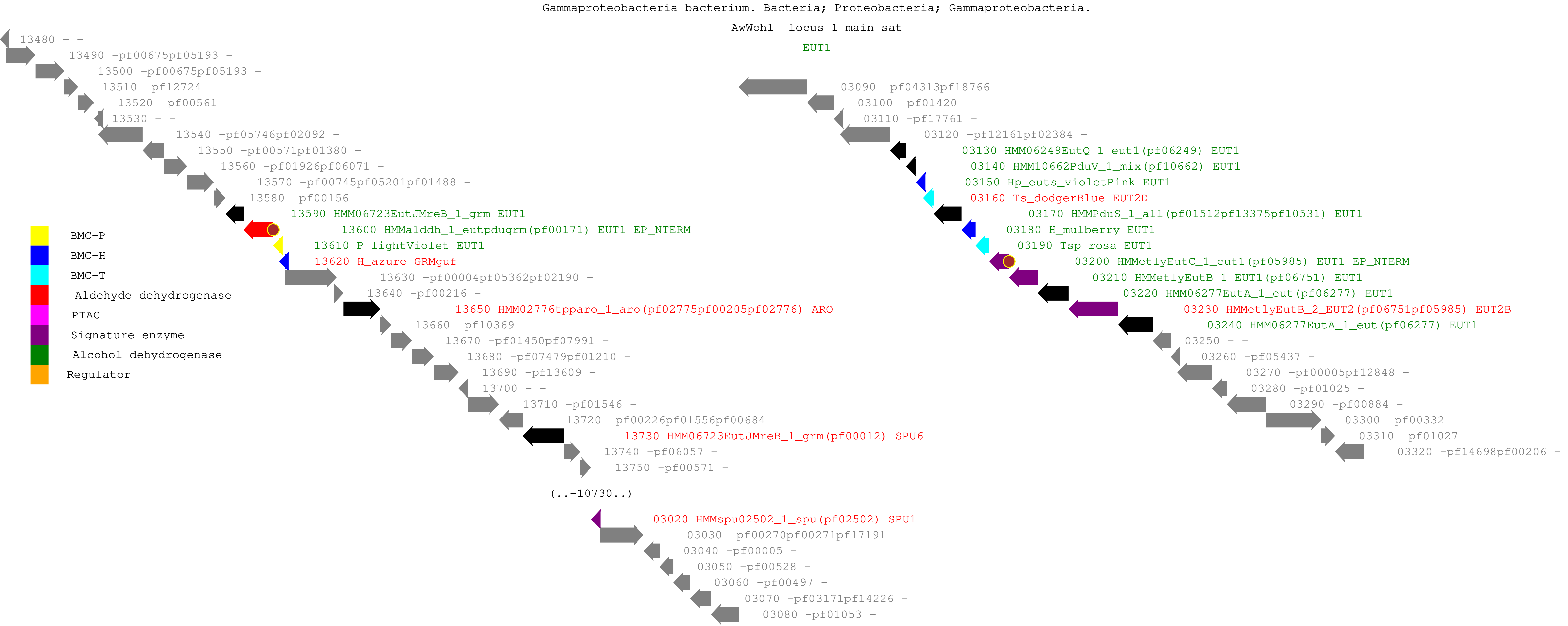

Gammaproteobacteria bacterium. Bacteria; Proteobacteria; Gammaproteobacteria.
AwWohl__locus_1_main_sat
EUT1
13480 - -
13490 -pf00675pf05193 -
13500 -pf00675pf05193 -
13510 -pf12724 -
13520 -pf00561 -
13530 - -
13540 -pf05746pf02092 -
13550 -pf00571pf01380 -
13560 -pf01926pf06071 -
13570 -pf00745pf05201pf01488 -
13580 -pf00156 -
13590 HMM06723EutJMreB_1_grm EUT1
13600 HMMalddh_1_eutpdugrm(pf00171) EUT1 EP_NTERM
13610 P_lightViolet EUT1
13620 H_azure GRMguf
13630 -pf00004pf05362pf02190 -
13640 -pf00216 -
13650 HMM02776tpparo_1_aro(pf02775pf00205pf02776) ARO
13660 -pf10369 -
13670 -pf01450pf07991 -
13680 -pf07479pf01210 -
13690 -pf13609 -
13700 - -
13710 -pf01546 -
13720 -pf00226pf01556pf00684 -
13730 HMM06723EutJMreB_1_grm(pf00012) SPU6
13740 -pf06057 -
13750 -pf00571 -
(..-10730..)
03020 HMMspu02502_1_spu(pf02502) SPU1
03030 -pf00270pf00271pf17191 -
03040 -pf00005 -
03050 -pf00528 -
03060 -pf00497 -
03070 -pf03171pf14226 -
03080 -pf01053 -
BMC-P
BMC-H
BMC-T
Aldehyde dehydrogenase
PTAC
Signature enzyme
Alcohol dehydrogenase
Regulator
03090 -pf04313pf18766 -
03100 -pf01420 -
03110 -pf17761 -
03120 -pf12161pf02384 -
03130 HMM06249EutQ_1_eut1(pf06249) EUT1
03140 HMM10662PduV_1_mix(pf10662) EUT1
03150 Hp_euts_violetPink EUT1
03160 Ts_dodgerBlue EUT2D
03170 HMMPduS_1_all(pf01512pf13375pf10531) EUT1
03180 H_mulberry EUT1
03190 Tsp_rosa EUT1
03200 HMMetlyEutC_1_eut1(pf05985) EUT1 EP_NTERM
03210 HMMetlyEutB_1_EUT1(pf06751) EUT1
03220 HMM06277EutA_1_eut(pf06277) EUT1
03230 HMMetlyEutB_2_EUT2(pf06751pf05985) EUT2B
03240 HMM06277EutA_1_eut(pf06277) EUT1
03250 - -
03260 -pf05437 -
03270 -pf00005pf12848 -
03280 -pf01025 -
03290 -pf00884 -
03300 -pf00332 -
03310 -pf01027 -
03320 -pf14698pf00206 -

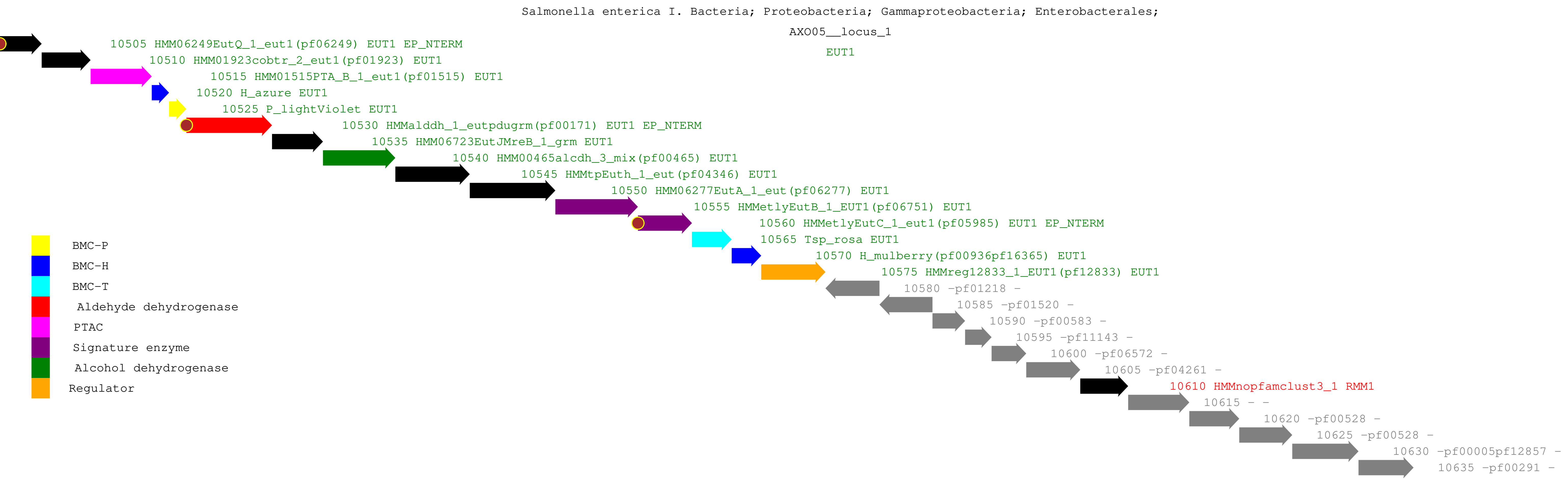
Salmonella enterica I. Bacteria; Proteobacteria; Gammaproteobacteria; Enterobacterales;
AXO05__locus_1
EUT1
10505 HMM06249EutQ_1_eut1(pf06249) EUT1 EP_NTERM
10510 HMM01923cobtr_2_eut1(pf01923) EUT1
10515 HMM01515PTA_B_1_eut1(pf01515) EUT1
10520 H_azure EUT1
10525 P_lightViolet EUT1
10530 HMMalddh_1_eutpdugrm(pf00171) EUT1 EP_NTERM
10535 HMM06723EutJMreB_1_grm EUT1
10540 HMM00465alcdh_3_mix(pf00465) EUT1
10545 HMMtpEuth_1_eut(pf04346) EUT1
10550 HMM06277EutA_1_eut(pf06277) EUT1
10555 HMMetlyEutB_1_EUT1(pf06751) EUT1
10560 HMMetlyEutC_1_eut1(pf05985) EUT1 EP_NTERM
10565 Tsp_rosa EUT1
10570 H_mulberry(pf00936pf16365) EUT1
10575 HMMreg12833_1_EUT1(pf12833) EUT1
10580 -pf01218 -
10585 -pf01520 -
10590 -pf00583 -
10595 -pf11143 -
10600 -pf06572 -
10605 -pf04261 -
10610 HMMnopfamclust3_1 RMM1
10615 - -
10620 -pf00528 -
10625 -pf00528 -
10630 -pf00005pf12857 -
10635 -pf00291 -
BMC-P
BMC-H
BMC-T
Aldehyde dehydrogenase
PTAC
Signature enzyme
Alcohol dehydrogenase
Regulator

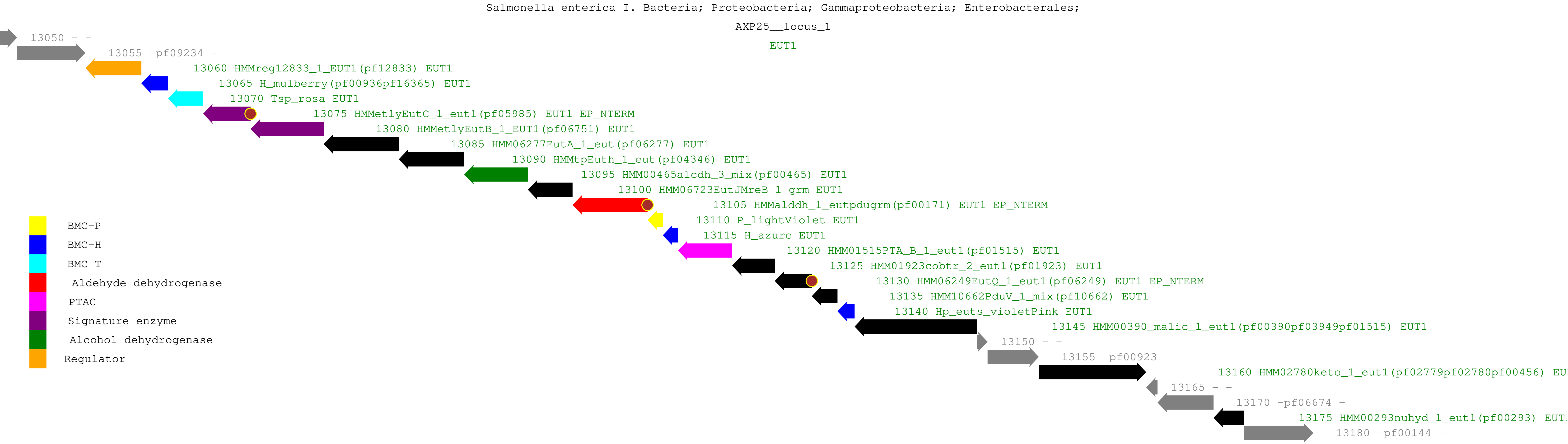

Salmonella enterica I. Bacteria; Proteobacteria; Gammaproteobacteria; Enterobacterales;
AXP25__locus_1
EUT1
13050 - -
13055 -pf09234 -
13060 HMMreg12833_1_EUT1(pf12833) EUT1
13065 H_mulberry(pf00936pf16365) EUT1
13070 Tsp_rosa EUT1
13075 HMMetlyEutC_1_eut1(pf05985) EUT1 EP_NTERM
13080 HMMetlyEutB_1_EUT1(pf06751) EUT1
13085 HMM06277EutA_1_eut(pf06277) EUT1
13090 HMMtpEuth_1_eut(pf04346) EUT1
13095 HMM00465alcdh_3_mix(pf00465) EUT1
13100 HMM06723EutJMreB_1_grm EUT1
13105 HMMalddh_1_eutpdugrm(pf00171) EUT1 EP_NTERM
13110 P_lightViolet EUT1
13115 H_azure EUT1
13120 HMM01515PTA_B_1_eut1(pf01515) EUT1
13125 HMM01923cobtr_2_eut1(pf01923) EUT1
13130 HMM06249EutQ_1_eut1(pf06249) EUT1 EP_NTERM
13135 HMM10662PduV_1_mix(pf10662) EUT1
13140 Hp_euts_violetPink EUT1
13145 HMM00390_malic_1_eut1(pf00390pf03949pf01515) EUT1
13150 - -
13155 -pf00923 -
13160 HMM02780keto_1_eut1(pf02779pf02780pf00456) EU
13165 - -
13170 -pf06674 -
13175 HMM00293nuhyd_1_eut1(pf00293) EUT
13180 -pf00144 -
BMC-P
BMC-H
BMC-T
Aldehyde dehydrogenase
PTAC
Signature enzyme
Alcohol dehydrogenase
Regulator

Salmonella montevideo. Bacteria; Proteobacteria; Gammaproteobacteria; Enterobacterales;

AXU21__locus_1

EUT1

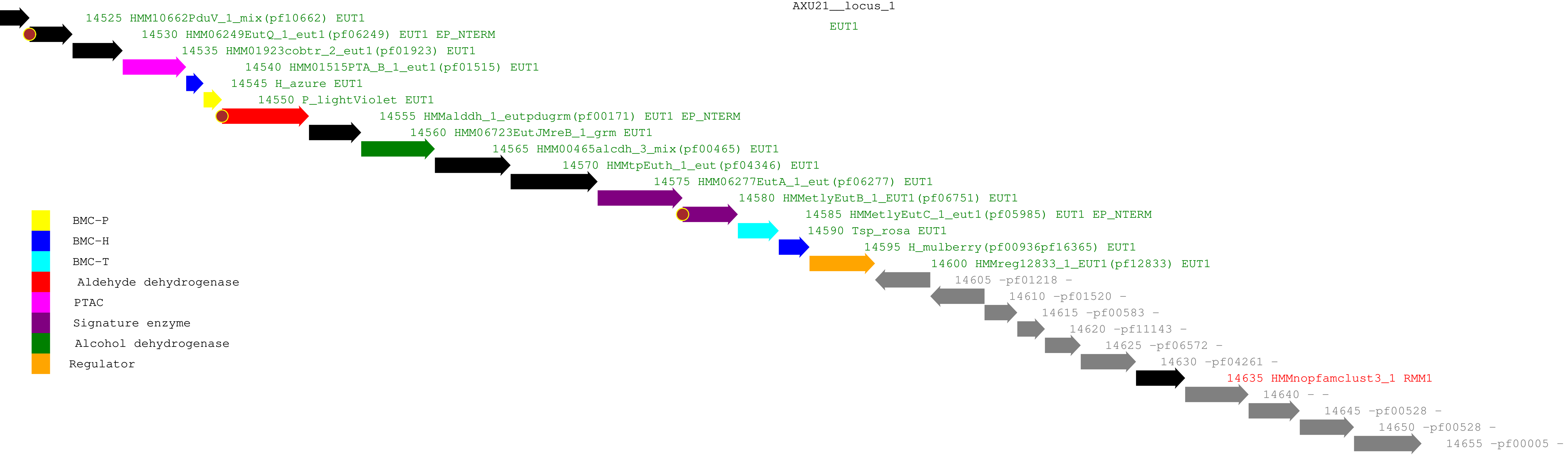

Morganella psychrotolerans. Bacteria; Proteobacteria; Gammaproteobacteria; Enterobacterales;

AYY16__locus_1

EUT1

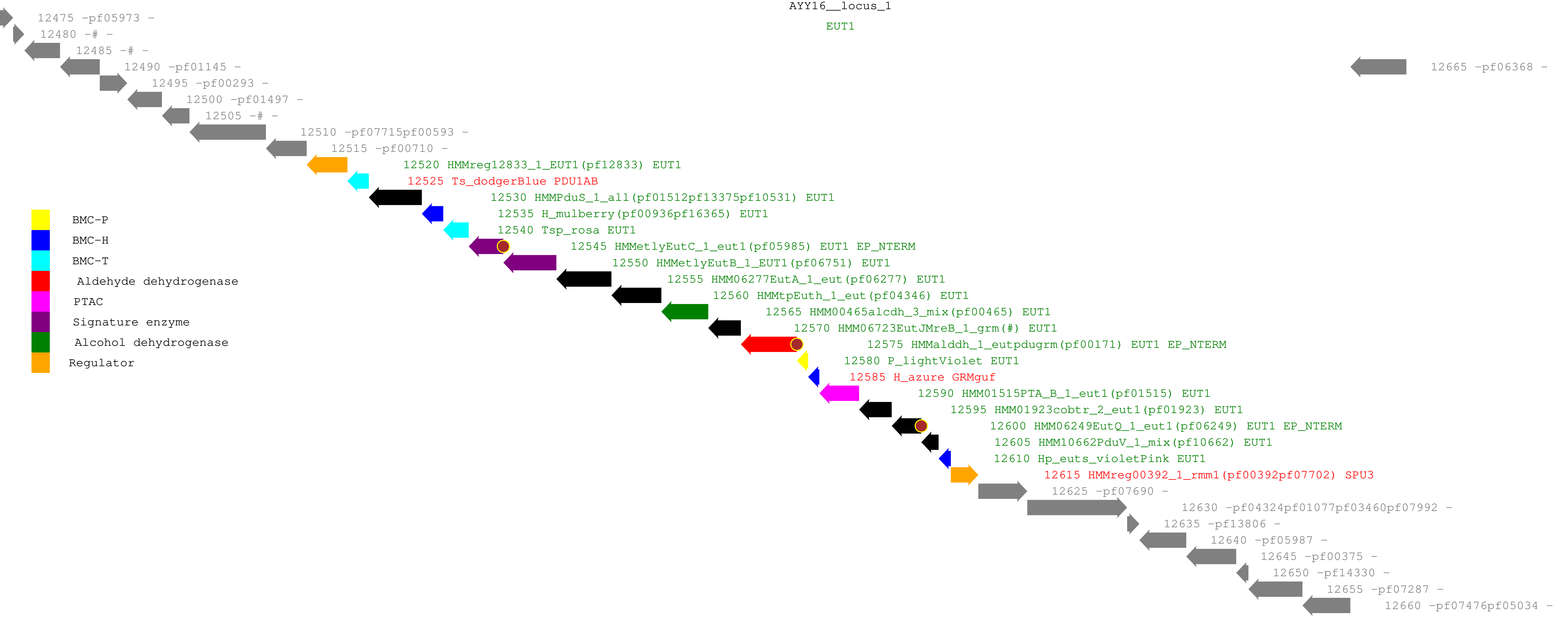

Morganella psychrotolerans. Bacteria; Proteobacteria; Gammaproteobacteria; Enterobacterales;

AYY17__locus_1

EUT1

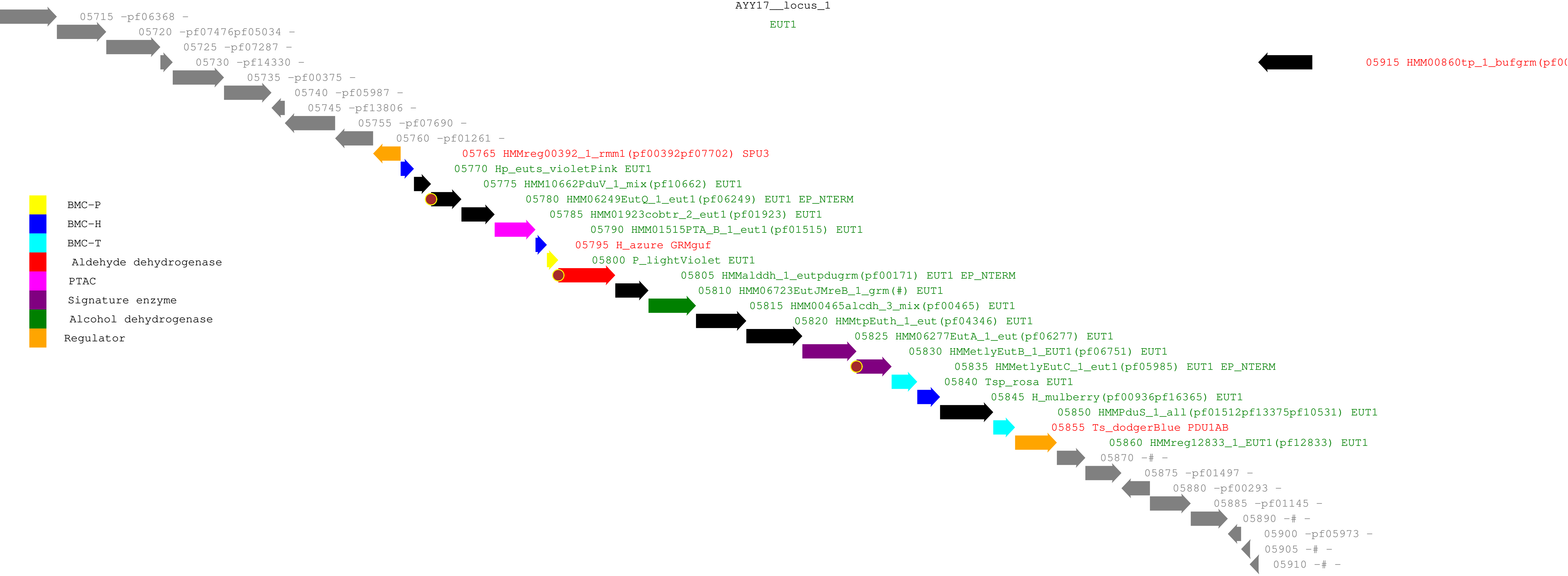

Morganella psychrotolerans. Bacteria; Proteobacteria; Gammaproteobacteria; Enterobacterales;

AYY18__locus_1

EUT1

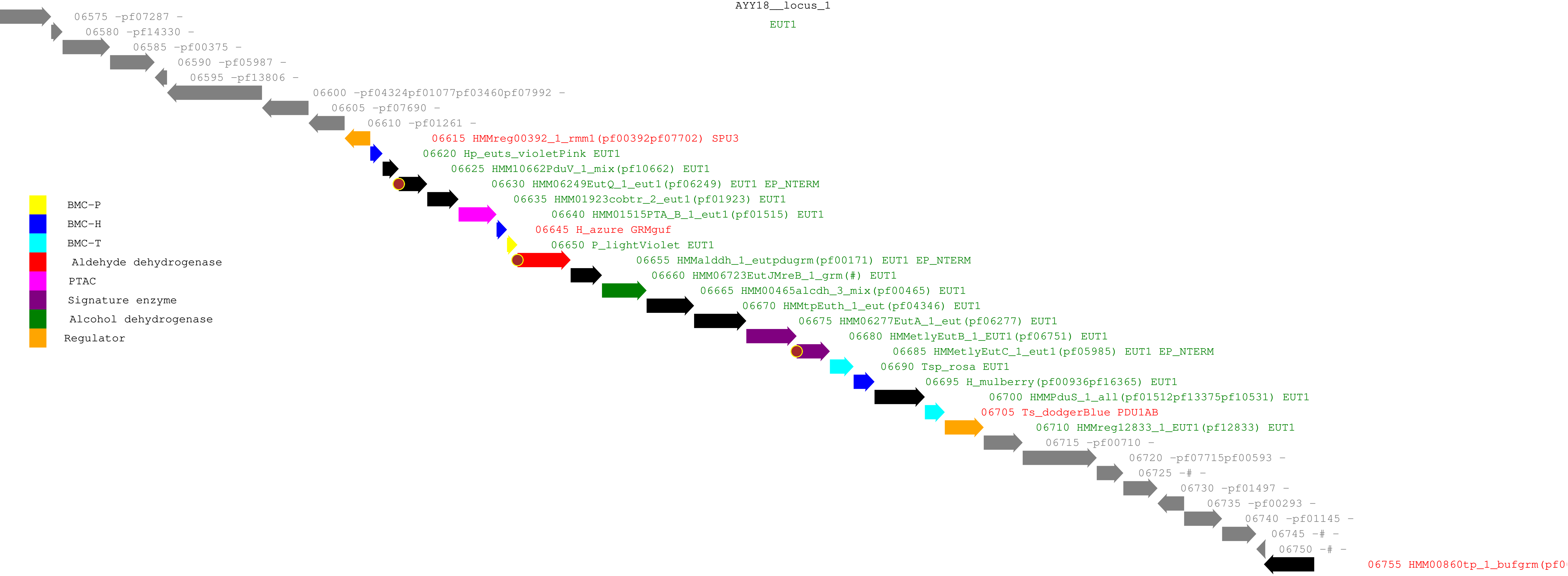

Citrobacter freundii. Bacteria; Proteobacteria; Gammaproteobacteria; Enterobacterales;

AZ002__locus_1

EUT1

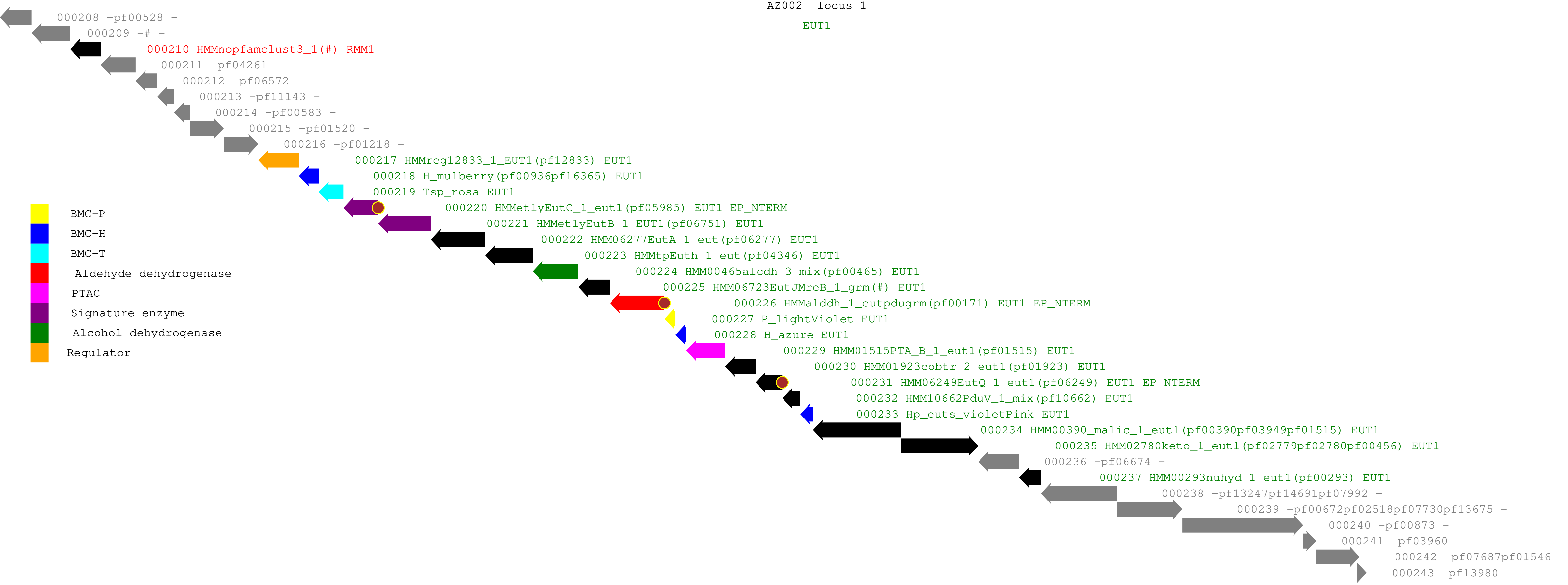

Citrobacter freundii. Bacteria; Proteobacteria; Gammaproteobacteria; Enterobacterales;

AZ006__locus_1

EUT1

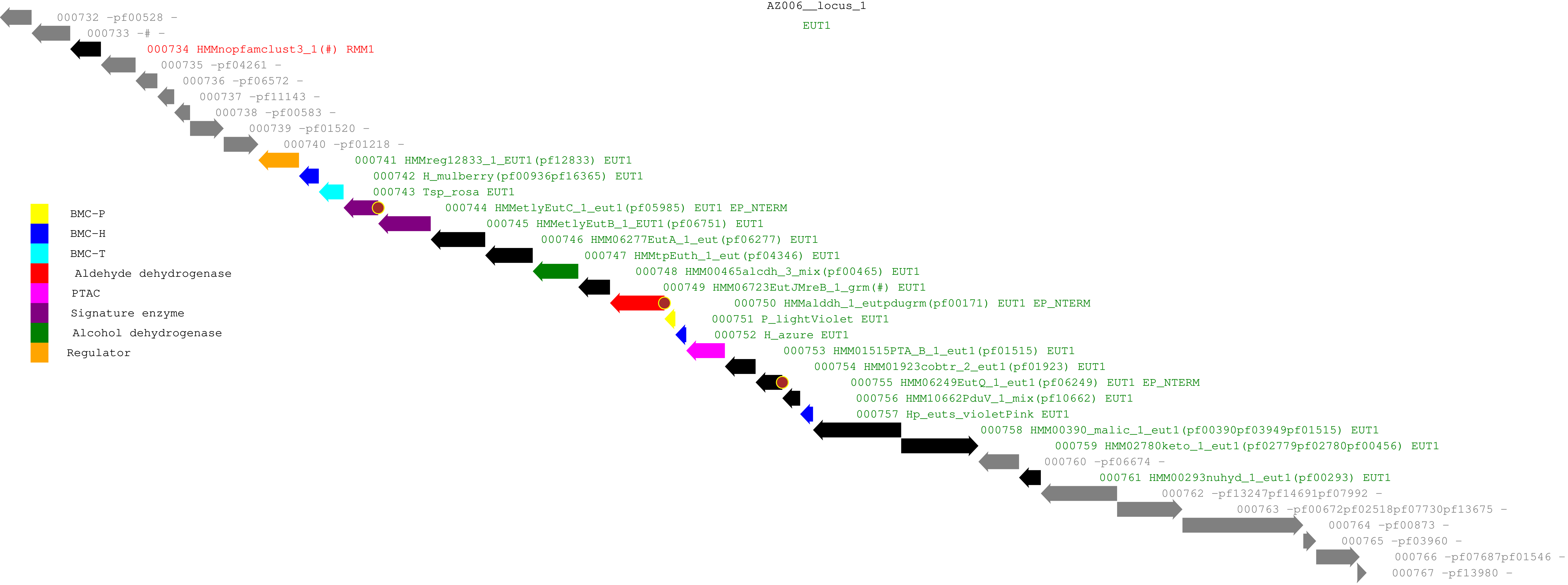

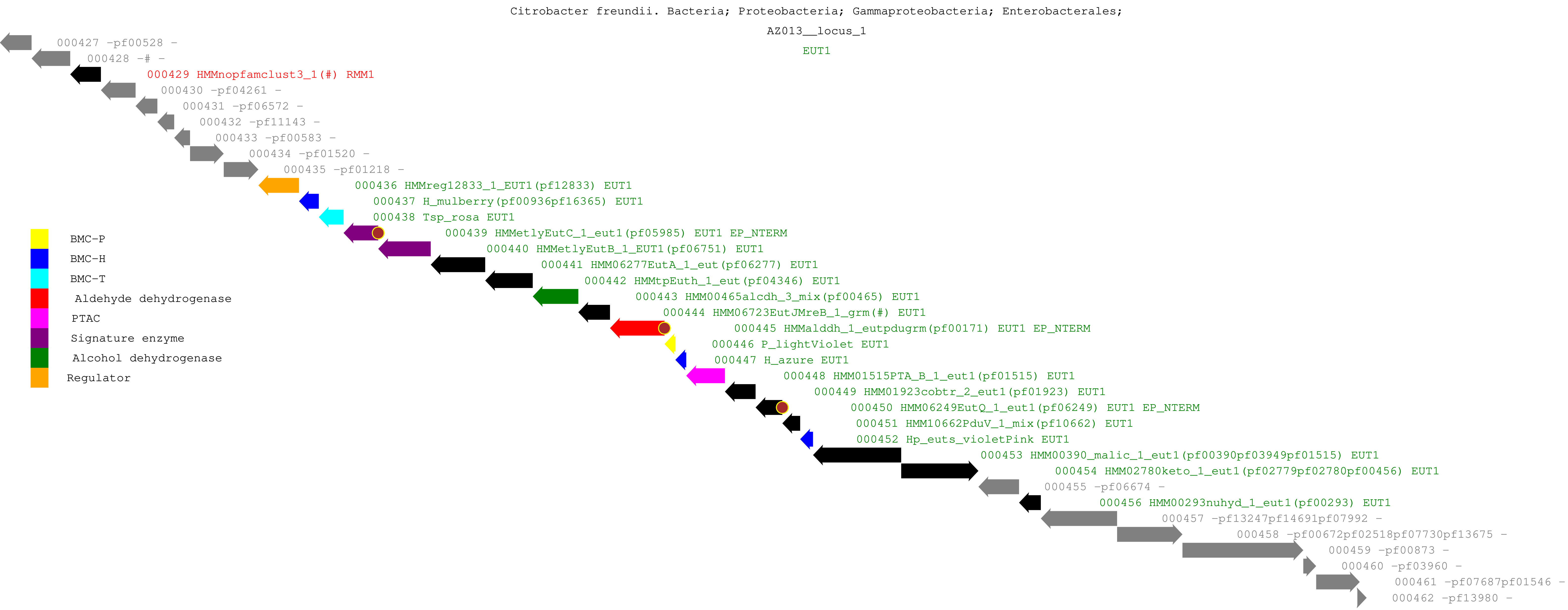
Citrobacter freundii. Bacteria; Proteobacteria; Gammaproteobacteria; Enterobacterales;
AZ013__locus_1
EUT1
000427 -pf00528 -
000428 -# -
000429 HMMnopfamclust3_1(#) RMM1
000430 -pf04261 -
000431 -pf06572 -
000432 -pf11143 -
000433 -pf00583 -
000434 -pf01520 -
000435 -pf01218 -
000436 HMMreg12833_1_EUT1(pf12833) EUT1
000437 H_mulberry(pf00936pf16365) EUT1
000438 Tsp_rosa EUT1
000439 HMMetlyEutC_1_eut1(pf05985) EUT1 EP_NTERM
000440 HMMetlyEutB_1_EUT1(pf06751) EUT1
000441 HMM06277EutA_1_eut(pf06277) EUT1
000442 HMMtpEuth_1_eut(pf04346) EUT1
000443 HMM00465alcdh_3_mix(pf00465) EUT1
000444 HMM06723EutJMreB_1_grm(#) EUT1
000445 HMMalddh_1_eutpdugrm(pf00171) EUT1 EP_NTERM
000446 P_lightViolet EUT1
000447 H_azure EUT1
000448 HMM01515PTA_B_1_eut1(pf01515) EUT1
000449 HMM01923cobtr_2_eut1(pf01923) EUT1
000450 HMM06249EutQ_1_eut1(pf06249) EUT1 EP_NTERM
000451 HMM10662PduV_1_mix(pf10662) EUT1
000452 Hp_euts_violetPink EUT1
000453 HMM00390_malic_1_eut1(pf00390pf03949pf01515) EUT1
000454 HMM02780keto_1_eut1(pf02779pf02780pf00456) EUT1
000455 -pf06674 -
000456 HMM00293nuhyd_1_eut1(pf00293) EUT1
000457 -pf13247pf14691pf07992 -
000458 -pf00672pf02518pf07730pf13675 -
000459 -pf00873 -
000460 -pf03960 -
000461 -pf07687pf01546 -
000462 -pf13980 -
BMC-P
BMC-H
BMC-T
Aldehyde dehydrogenase
PTAC
Signature enzyme
Alcohol dehydrogenase
Regulator

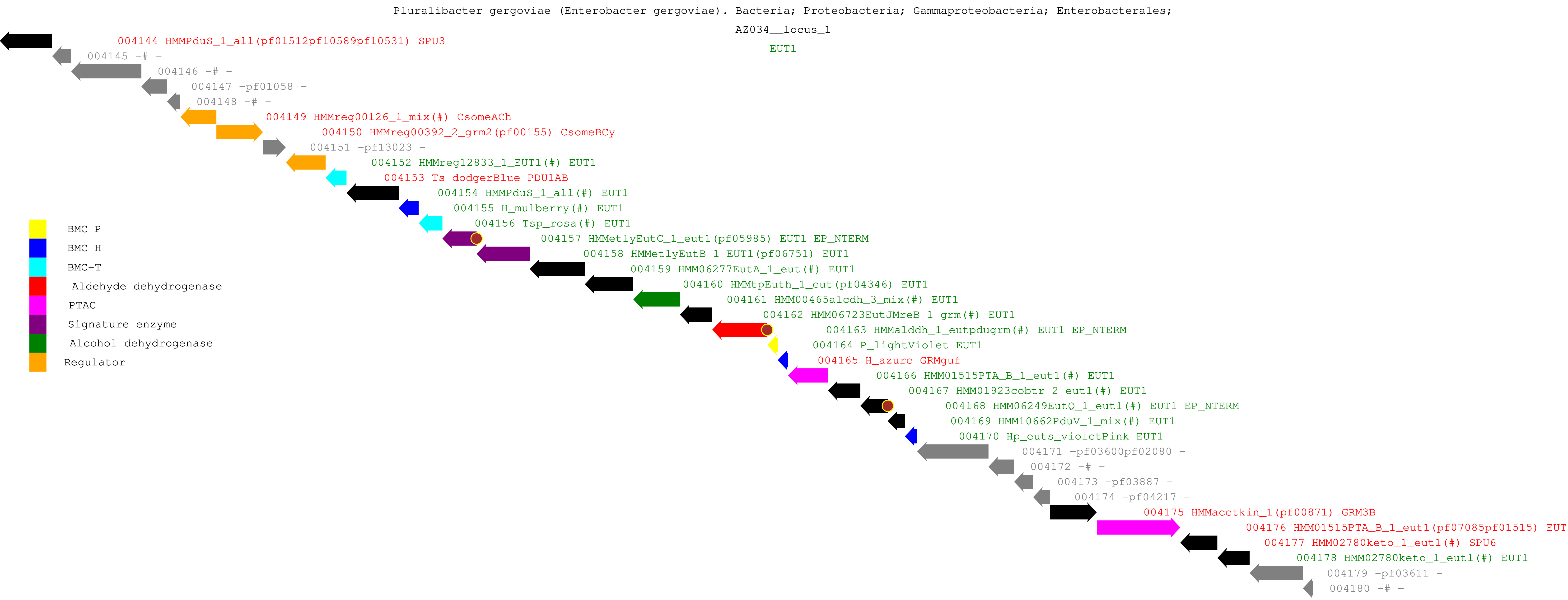

Pluralibacter gergoviae (Enterobacter gergoviae). Bacteria; Proteobacteria; Gammaproteobacteria; Enterobacterales;
AZ034__locus_1
EUT1
004144 HMMPduS_1_all(pf01512pf10589pf10531) SPU3
004145 -# -
004146 -# -
004147 -pf01058 -
004148 -# -
004149 HMMreg00126_1_mix(#) CsomeACh
004150 HMMreg00392_2_grm2(pf00155) CsomeBCy
004151 -pf13023 -
004152 HMMreg12833_1_EUT1(#) EUT1
004153 Ts_dodgerBlue PDU1AB
004154 HMMPduS_1_all(#) EUT1
004155 H_mulberry(#) EUT1
004156 Tsp_rosa(#) EUT1
004157 HMMetlyEutC_1_eut1(pf05985) EUT1 EP_NTERM
004158 HMMetlyEutB_1_EUT1(pf06751) EUT1
004159 HMM06277EutA_1_eut(#) EUT1
004160 HMMtpEuth_1_eut(pf04346) EUT1
004161 HMM00465alcdh_3_mix(#) EUT1
004162 HMM06723EutJMreB_1_grm(#) EUT1
004163 HMMaldh_1_eutpdugrm(#) EUT1 EP_NTERM
004164 P_lightViolet EUT1
004165 H_azure GRMguf
004166 HMM01515PTA_B_1_eut1(#) EUT1
004167 HMM01923cobtr_2_eut1(#) EUT1
004168 HMM06249EutQ_1_eut1(#) EUT1 EP_NTERM
004169 HMM10662PduV_1_mix(#) EUT1
004170 Hp_euts_violetPink EUT1
004171 -pf03600pf02080 -
004172 -# -
004173 -pf03887 -
004174 -pf04217 -
004175 HMMacetkin_1(pf00871) GRM3B
004176 HMM01515PTA_B_1_eut1(pf07085pf01515) EUT
004177 HMM02780keto_1_eut1(#) SPU6
004178 HMM02780keto_1_eut1(#) EUT1
004179 -pf03611 -
004180 -# -
BMC-P
BMC-H
BMC-T
Aldehyde dehydrogenase
PTAC
Signature enzyme
Alcohol dehydrogenase
Regulator

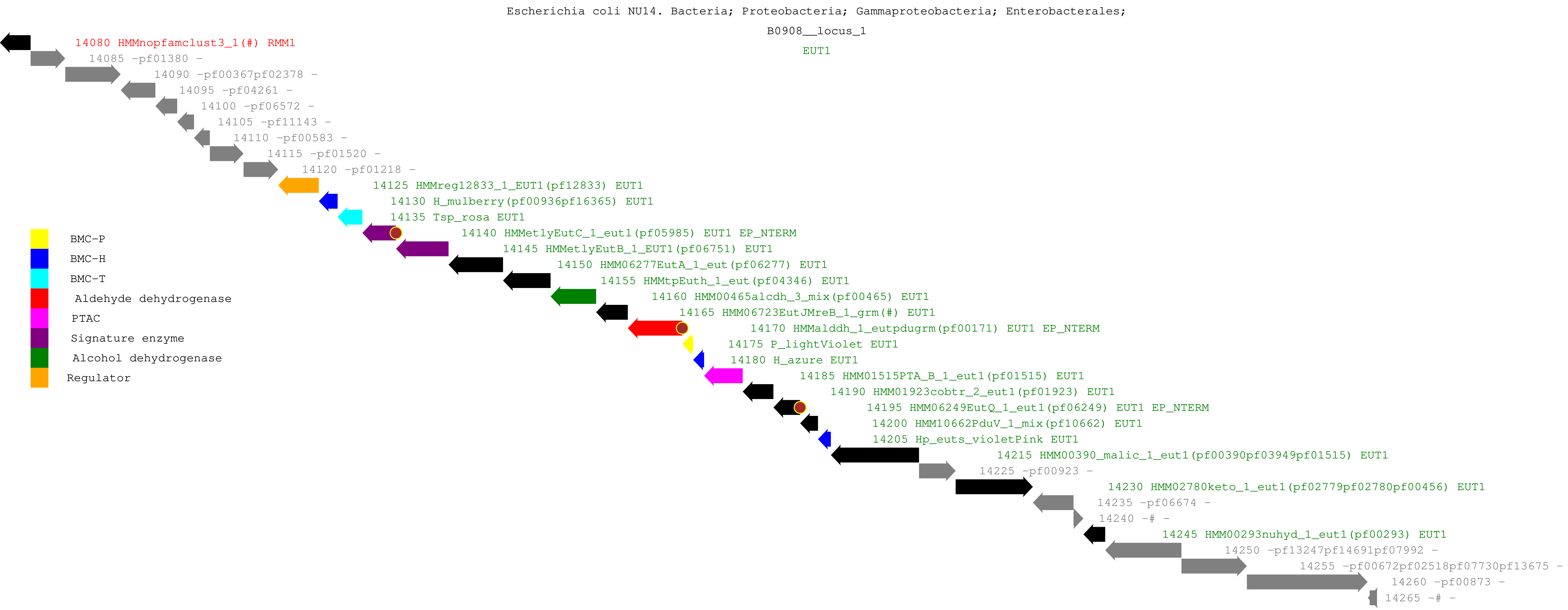

Escherichia coli NU14. Bacteria; Proteobacteria; Gammaproteobacteria; Enterobacterales;
B0908__locus_1
EUT1
14080 HMMnopfamclust3_1(#) RMM1
14085 -pf01380 -
14090 -pf00367pf02378 -
14095 -pf04261 -
14100 -pf06572 -
14105 -pf11143 -
14110 -pf00583 -
14115 -pf01520 -
14120 -pf01218 -
14125 HMMreg12833_1_EUT1(pf12833) EUT1
14130 H_mulberry(pf00936pf16365) EUT1
14135 Tsp_rosa EUT1
14140 HMMetlyEutC_1_eut1(pf05985) EUT1 EP_NTERM
14145 HMMetlyEutB_1_EUT1(pf06751) EUT1
14150 HMM06277EutA_1_eut(pf06277) EUT1
14155 HMMtpEuth_1_eut(pf04346) EUT1
14160 HMM00465alcdh_3_mix(pf00465) EUT1
14165 HMM06723EutJMreB_1_grm(#) EUT1
14170 HMMalddh_1_eutpdugrm(pf00171) EUT1 EP_NTERM
14175 P_lightViolet EUT1
14180 H_azure EUT1
14185 HMM01515PTA_B_1_eut1(pf01515) EUT1
14190 HMM01923cobtr_2_eut1(pf01923) EUT1
14195 HMM06249EutQ_1_eut1(pf06249) EUT1 EP_NTERM
14200 HMM10662PduV_1_mix(pf10662) EUT1
14205 Hp_euts_violetPink EUT1
14215 HMM00390_malic_1_eut1(pf00390pf03949pf01515) EUT1
14225 -pf00923 -
14230 HMM02780keto_1_eut1(pf02779pf02780pf00456) EUT1
14235 -pf06674 -
14240 -# -
14245 HMM00293nuhyd_1_eut1(pf00293) EUT1
14250 -pf13247pf14691pf07992 -
14255 -pf00672pf02518pf07730pf13675 -
14260 -pf00873 -
14265 -# -
BMC-P
BMC-H
BMC-T
Aldehyde dehydrogenase
PTAC
Signature enzyme
Alcohol dehydrogenase
Regulator

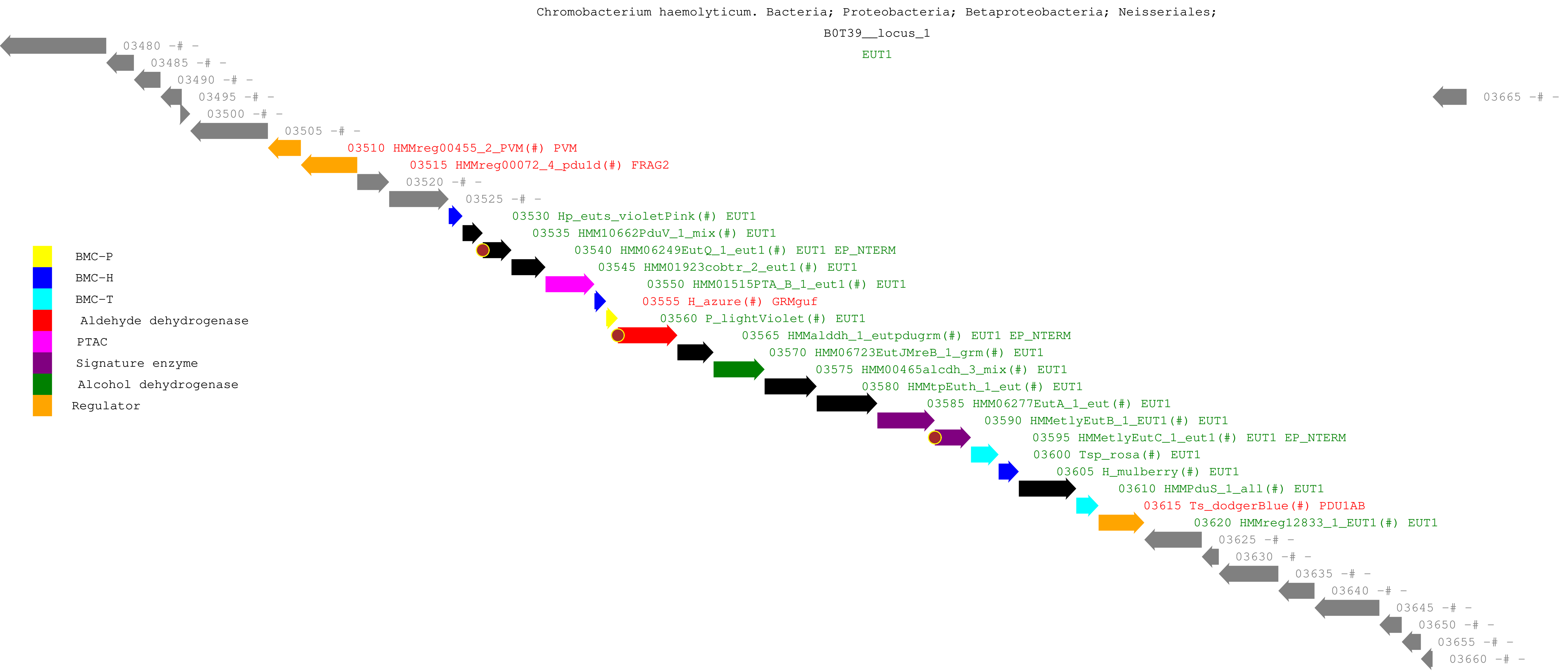
Chromobacterium haemolyticum. Bacteria; Proteobacteria; Betaproteobacteria; Neisseriales;
B0T39__locus_1
EUT1
03480 -# -
03485 -# -
03490 -# -
03495 -# -
03500 -# -
03505 -# -
03510 HMMreg00455_2_PVM(#) PVM
03515 HMMreg00072_4_pdu1d(#) FRAG2
03520 -# -
03525 -# -
03530 Hp_euts_violetPink(#) EUT1
03535 HMM10662PduV_1_mix(#) EUT1
03540 HMM06249EutQ_1_eut1(#) EUT1 EP_NTERM
03545 HMM01923cobtr_2_eut1(#) EUT1
03550 HMM01515PTA_B_1_eut1(#) EUT1
03555 H_azure(#) GRMguf
03560 P_lightViolet(#) EUT1
03565 HMMalddh_1_eutpdugrm(#) EUT1 EP_NTERM
03570 HMM06723EutJMreB_1_grm(#) EUT1
03575 HMM00465alcdh_3_mix(#) EUT1
03580 HMMtpEuth_1_eut(#) EUT1
03585 HMM06277EutA_1_eut(#) EUT1
03590 HMMetlyEutB_1_EUT1(#) EUT1
03595 HMMetlyEutC_1_eut1(#) EUT1 EP_NTERM
03600 Tsp_rosa(#) EUT1
03605 H_mulberry(#) EUT1
03610 HMMPduS_1_all(#) EUT1
03615 Ts_dodgerBlue(#) PDU1AB
03620 HMMreg12833_1_EUT1(#) EUT1
03625 -# -
03630 -# -
03635 -# -
03640 -# -
03645 -# -
03650 -# -
03655 -# -
03660 -# -
03665 -# -
BMC-P
BMC-H
BMC-T
Aldehyde dehydrogenase
PTAC
Signature enzyme
Alcohol dehydrogenase
Regulator

Chromobacterium haemolyticum. Bacteria; Proteobacteria; Betaproteobacteria; Neisseriales;

B0T40__locus_1

EUT1

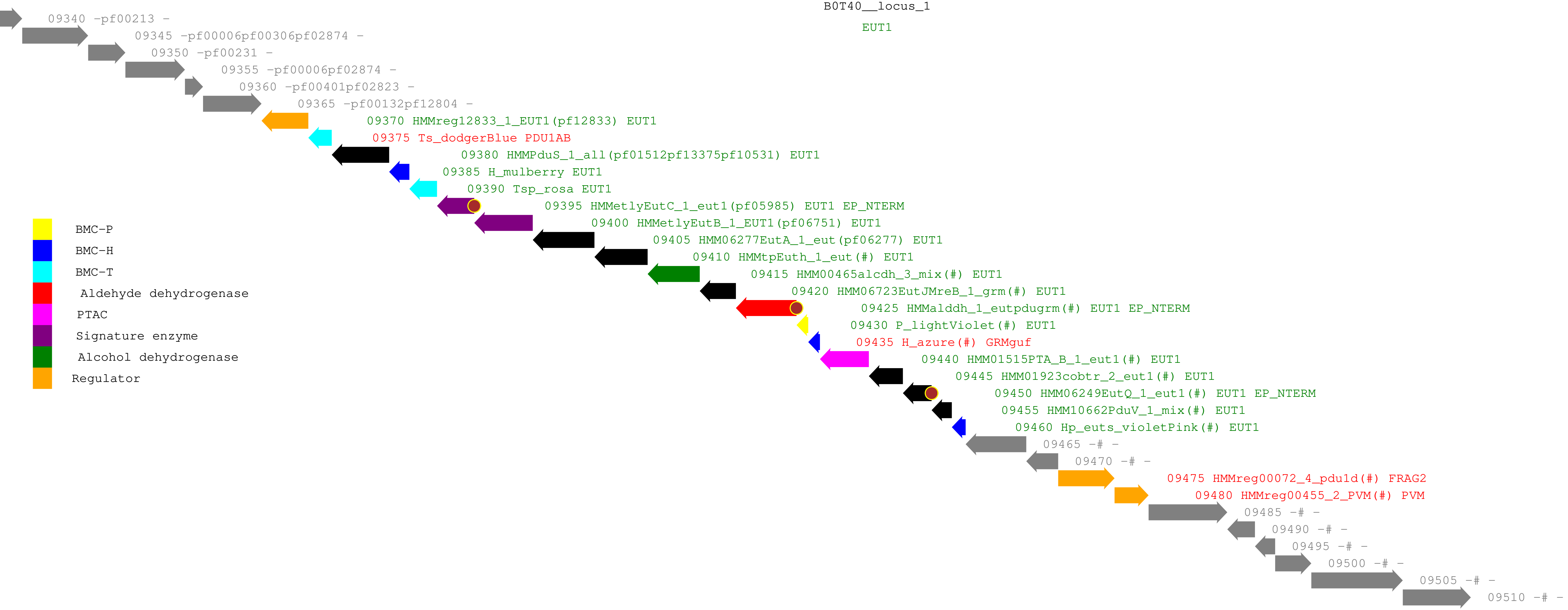

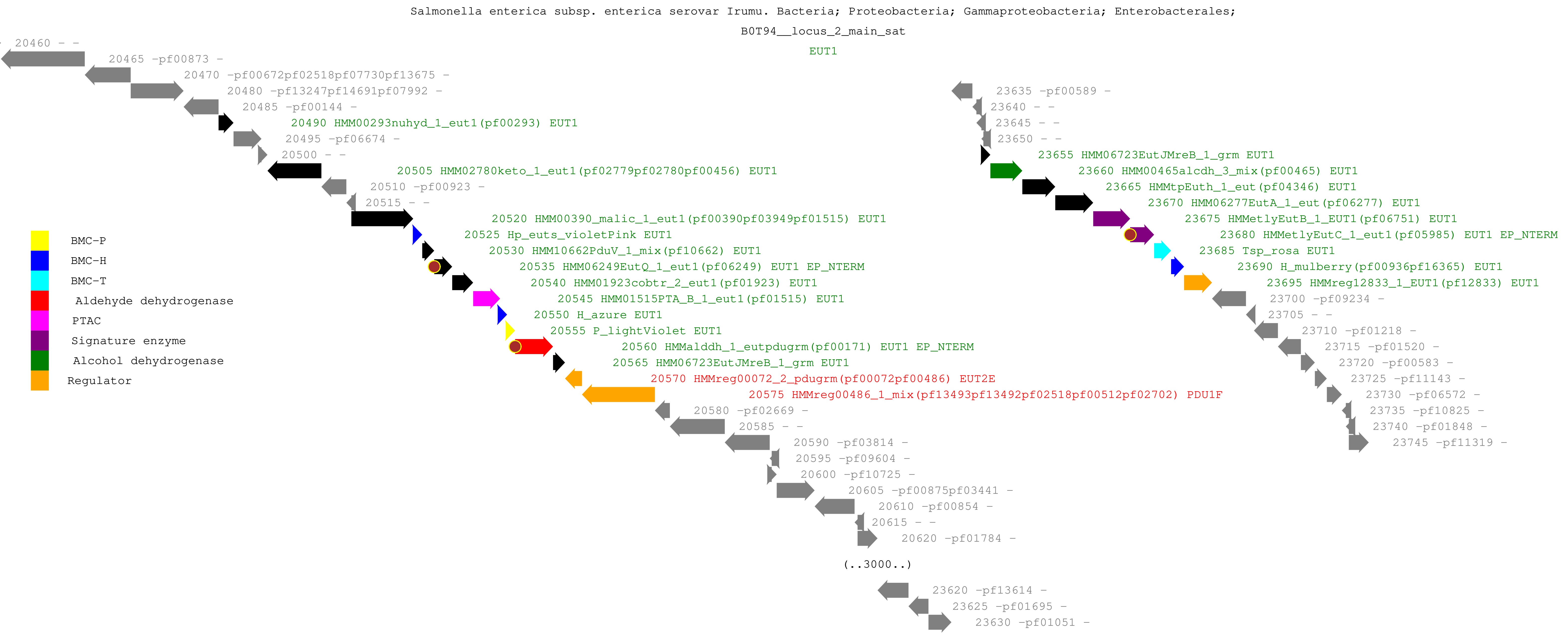

Salmonella enterica subsp. enterica serovar Irumu. Bacteria; Proteobacteria; Gammaproteobacteria; Enterobacterales;
B0T94__locus_2_main_sat
EUT1
20460 - -
20465 -pf00873 -
20470 -pf00672pf02518pf07730pf13675 -
20480 -pf13247pf14691pf07992 -
20485 -pf00144 -
20490 HMM00293nuhyd_1_eut1(pf00293) EUT1
20495 -pf06674 -
20500 - -
20505 HMM02780keto_1_eut1(pf02779pf02780pf00456) EUT1
20510 -pf00923 -
20515 - -
20520 HMM00390_malic_1_eut1(pf00390pf03949pf01515) EUT1
20525 Hp_euts_violetPink EUT1
20530 HMM10662PduV_1_mix(pf10662) EUT1
20535 HMM06249EutQ_1_eut1(pf06249) EUT1 EP_NTERM
20540 HMM01923cobtr_2_eut1(pf01923) EUT1
20545 HMM01515PTA_B_1_eut1(pf01515) EUT1
20550 H_azure EUT1
20555 P_lightViolet EUT1
20560 HMMalddh_1_eutpdugrm(pf00171) EUT1 EP_NTERM
20565 HMM06723EutJMreB_1_grm EUT1
20570 HMMreg00072_2_pdugrm(pf00072pf00486) EUT2E
20575 HMMreg00486_1_mix(pf13493pf13492pf02518pf00512pf02702) PDU1F
20580 -pf02669 -
20585 - -
20590 -pf03814 -
20595 -pf09604 -
20600 -pf10725 -
20605 -pf00875pf03441 -
20610 -pf00854 -
20615 - -
20620 -pf01784 -
(..3000..)
23620 -pf13614 -
23625 -pf01695 -
23630 -pf01051 -
23635 -pf00589 -
23640 - -
23645 - -
23650 - -
23655 HMM06723EutJMreB_1_grm EUT1
23660 HMM00465alcdh_3_mix(pf00465) EUT1
23665 HMMtpEuth_1_eut(pf04346) EUT1
23670 HMM06277EutA_1_eut(pf06277) EUT1
23675 HMMetlyEutB_1_EUT1(pf06751) EUT1
23680 HMMetlyEutC_1_eut1(pf05985) EUT1 EP_NTERM
23685 Tsp_rosa EUT1
23690 H_mulberry(pf00936pf16365) EUT1
23695 HMMreg12833_1_EUT1(pf12833) EUT1
23700 -pf09234 -
23705 - -
23710 -pf01218 -
23715 -pf01520 -
23720 -pf00583 -
23725 -pf11143 -
23730 -pf06572 -
23735 -pf10825 -
23740 -pf01848 -
23745 -pf11319 -
BMC-P
BMC-H
BMC-T
Aldehyde dehydrogenase
PTAC
Signature enzyme
Alcohol dehydrogenase
Regulator

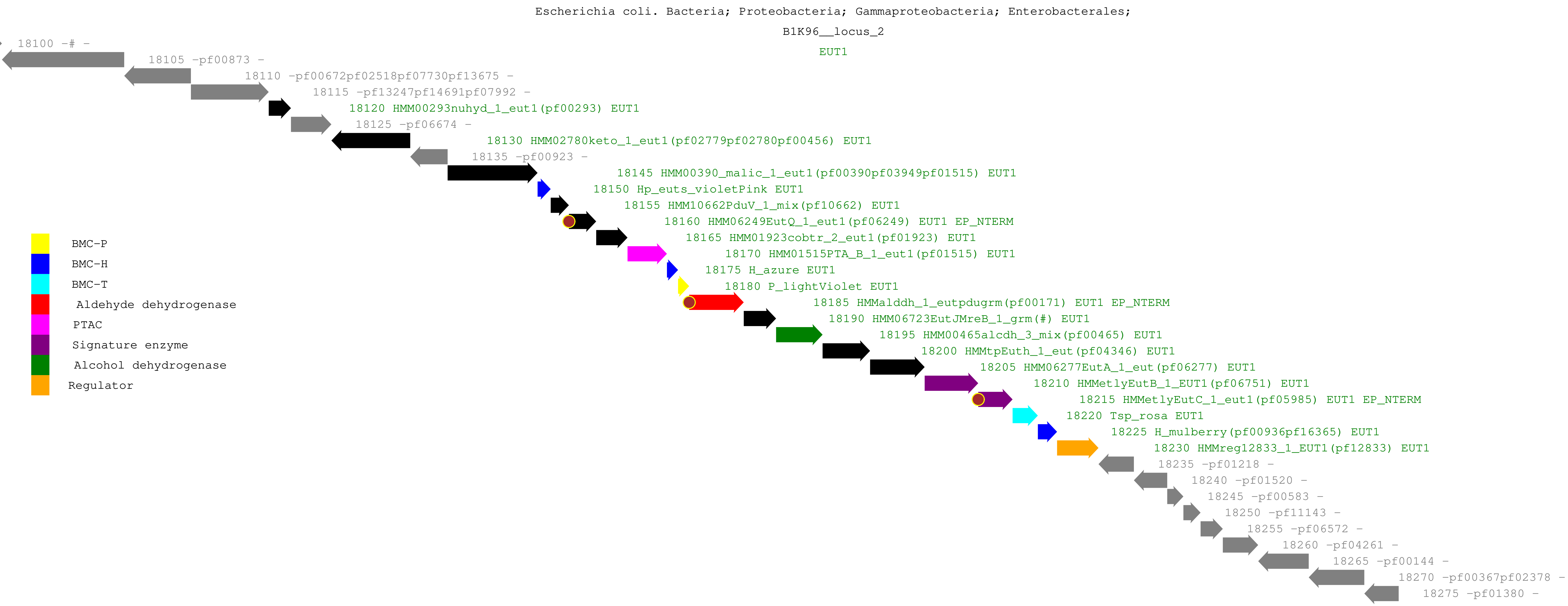
Escherichia coli. Bacteria; Proteobacteria; Gammaproteobacteria; Enterobacterales;
B1K96__locus_2
EUT1
18100 -# -
18105 -pf00873 -
18110 -pf00672pf02518pf07730pf13675 -
18115 -pf13247pf14691pf07992 -
18120 HMM00293nuhyd_1_eut1(pf00293) EUT1
18125 -pf06674 -
18130 HMM02780keto_1_eut1(pf02779pf02780pf00456) EUT1
18135 -pf00923 -
18145 HMM00390_malic_1_eut1(pf00390pf03949pf01515) EUT1
18150 Hp_euts_violetPink EUT1
18155 HMM10662PduV_1_mix(pf10662) EUT1
18160 HMM06249EutQ_1_eut1(pf06249) EUT1 EP_NTERM
18165 HMM01923cobtr_2_eut1(pf01923) EUT1
18170 HMM01515PTA_B_1_eut1(pf01515) EUT1
18175 H_azure EUT1
18180 P_lightViolet EUT1
18185 HMMalddh_1_eutpdugrm(pf00171) EUT1 EP_NTERM
18190 HMM06723EutJMreB_1_grm(#) EUT1
18195 HMM00465alcdh_3_mix(pf00465) EUT1
18200 HMMtpEuth_1_eut(pf04346) EUT1
18205 HMM06277EutA_1_eut(pf06277) EUT1
18210 HMMetlyEutB_1_EUT1(pf06751) EUT1
18215 HMMetlyEutC_1_eut1(pf05985) EUT1 EP_NTERM
18220 Tsp_rosa EUT1
18225 H_mulberry(pf00936pf16365) EUT1
18230 HMMreg12833_1_EUT1(pf12833) EUT1
18235 -pf01218 -
18240 -pf01520 -
18245 -pf00583 -
18250 -pf11143 -
18255 -pf06572 -
18260 -pf04261 -
18265 -pf00144 -
18270 -pf00367pf02378 -
18275 -pf01380 -
BMC-P
BMC-H
BMC-T
Aldehyde dehydrogenase
PTAC
Signature enzyme
Alcohol dehydrogenase
Regulator

Escherichia coli M8. Bacteria; Proteobacteria; Gammaproteobacteria; Enterobacterales;

B2H83__locus_2

EUT1

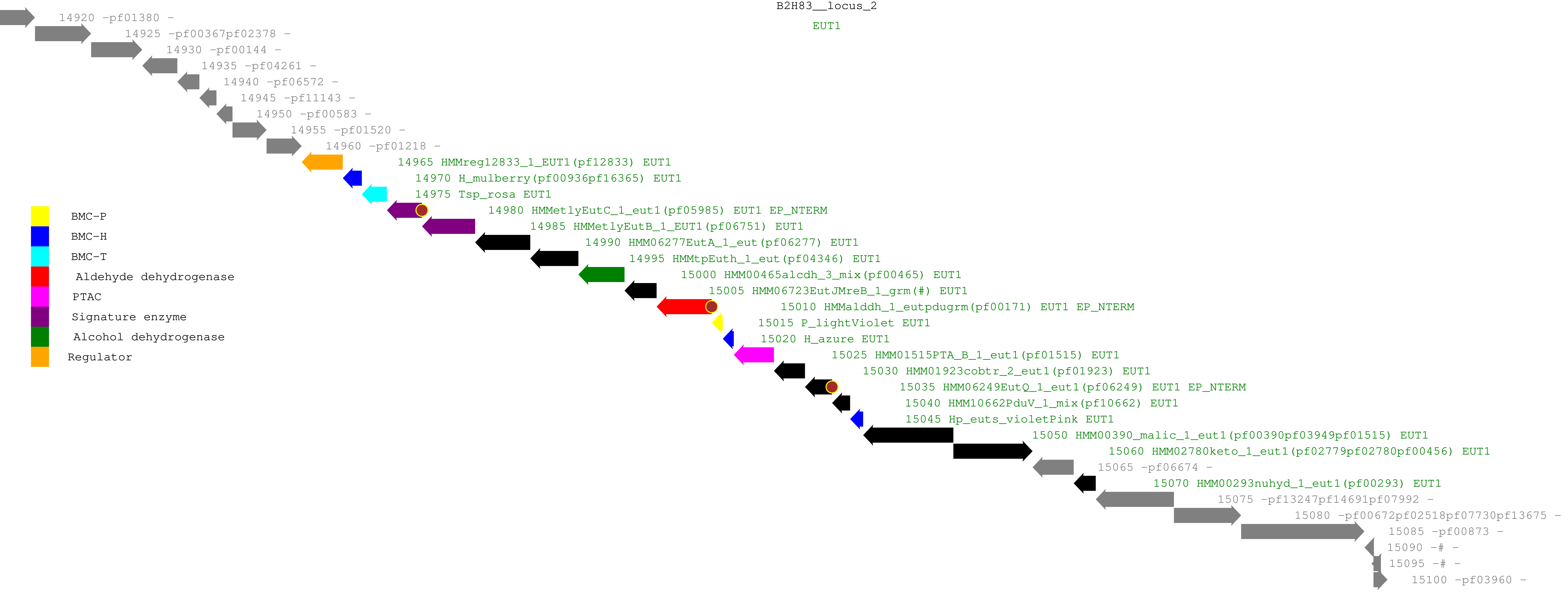

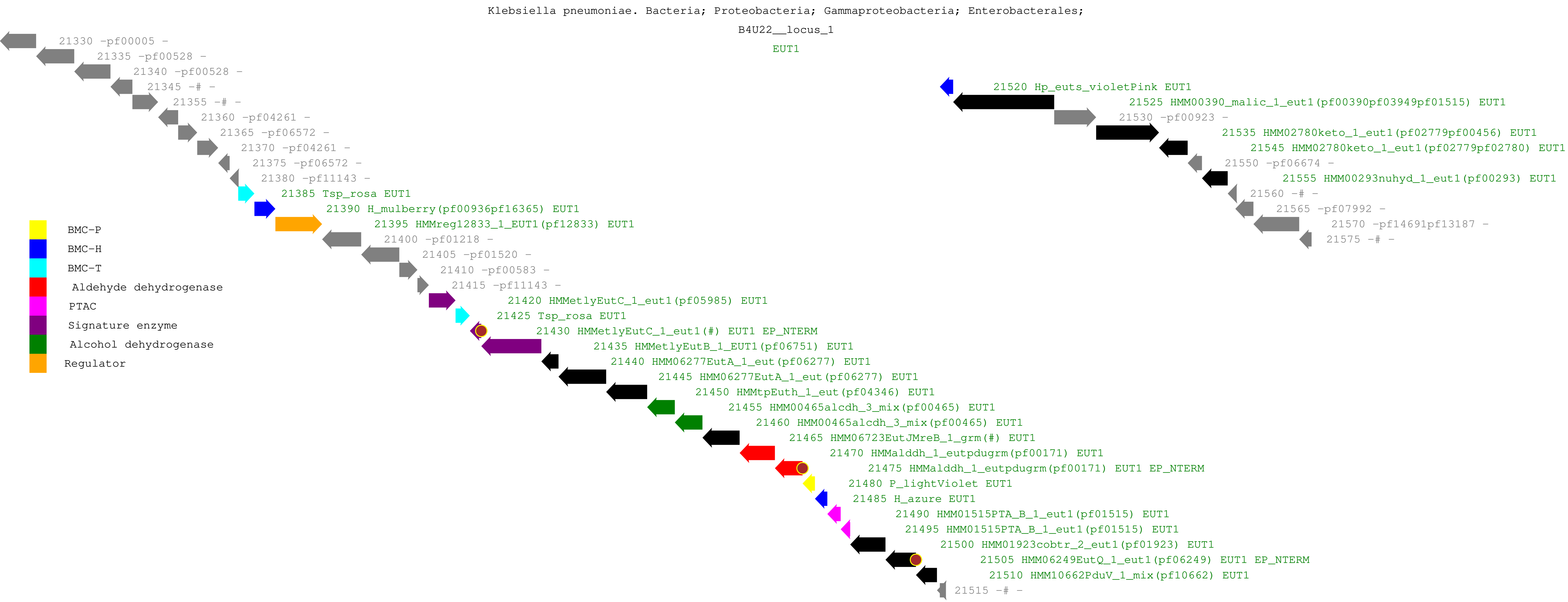
Klebsiella pneumoniae. Bacteria; Proteobacteria; Gammaproteobacteria; Enterobacterales;
B4U22__locus_1
EUT1
21330 -pf00005 -
21335 -pf00528 -
21340 -pf00528 -
21345 -# -
21355 -# -
21360 -pf04261 -
21365 -pf06572 -
21370 -pf04261 -
21375 -pf06572 -
21380 -pf11143 -
21385 Tsp_rosa EUT1
21390 H_mulberry(pf00936pf16365) EUT1
21395 HMMreg12833_1_EUT1(pf12833) EUT1
21400 -pf01218 -
21405 -pf01520 -
21410 -pf00583 -
21415 -pf11143 -
21420 HMMetlyEutC_1_eut1(pf05985) EUT1
21425 Tsp_rosa EUT1
21430 HMMetlyEutC_1_eut1(#) EUT1 EP_NTERM
21435 HMMetlyEutB_1_EUT1(pf06751) EUT1
21440 HMM06277EutA_1_eut(pf06277) EUT1
21445 HMM06277EutA_1_eut(pf06277) EUT1
21450 HMMtpEuth_1_eut(pf04346) EUT1
21455 HMM00465alcdh_3_mix(pf00465) EUT1
21460 HMM00465alcdh_3_mix(pf00465) EUT1
21465 HMM06723EutJMreB_1_grm(#) EUT1
21470 HMMalddh_1_eutpdugrm(pf00171) EUT1
21475 HMMalddh_1_eutpdugrm(pf00171) EUT1 EP_NTERM
21480 P_lightViolet EUT1
21485 H_azure EUT1
21490 HMM01515PTA_B_1_eut1(pf01515) EUT1
21495 HMM01515PTA_B_1_eut1(pf01515) EUT1
21500 HMM01923cobtr_2_eut1(pf01923) EUT1
21505 HMM06249EutQ_1_eut1(pf06249) EUT1 EP_NTERM
21510 HMM10662PduV_1_mix(pf10662) EUT1
21515 -# -
21520 Hp_euts_violetPink EUT1
21525 HMM00390_malic_1_eut1(pf00390pf03949pf01515) EUT1
21530 -pf00923 -
21535 HMM02780keto_1_eut1(pf02779pf00456) EUT1
21545 HMM02780keto_1_eut1(pf02779pf02780) EUT1
21550 -pf06674 -
21555 HMM00293nuhyd_1_eut1(pf00293) EUT1
21560 -# -
21565 -pf07992 -
21570 -pf14691pf13187 -
21575 -# -
BMC-P
BMC-H
BMC-T
Aldehyde dehydrogenase
PTAC
Signature enzyme
Alcohol dehydrogenase
Regulator

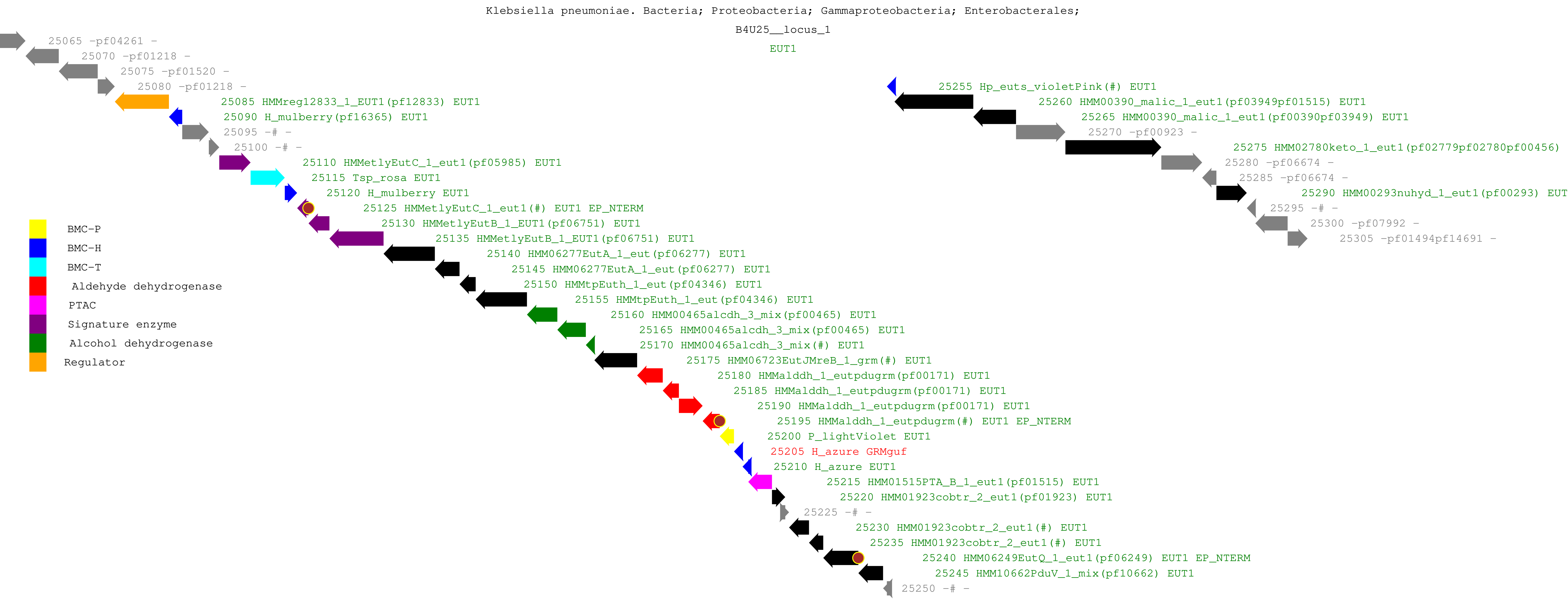

Klebsiella pneumoniae. Bacteria; Proteobacteria; Gammaproteobacteria; Enterobacterales;
B4U25__locus_1
EUT1
25065 -pf04261 -
25070 -pf01218 -
25075 -pf01520 -
25080 -pf01218 -
25085 HMMreg12833_1_EUT1(pf12833) EUT1
25090 H_mulberry(pf16365) EUT1
25095 -# -
25100 -# -
25110 HMMetlyEutC_1_eut1(pf05985) EUT1
25115 Tsp_rosa EUT1
25120 H_mulberry EUT1
25125 HMMetlyEutC_1_eut1(#) EUT1 EP_NTERM
25130 HMMetlyEutB_1_EUT1(pf06751) EUT1
25135 HMMetlyEutB_1_EUT1(pf06751) EUT1
25140 HMM06277EutA_1_eut(pf06277) EUT1
25145 HMM06277EutA_1_eut(pf06277) EUT1
25150 HMMtpEuth_1_eut(pf04346) EUT1
25155 HMMtpEuth_1_eut(pf04346) EUT1
25160 HMM00465alcdh_3_mix(pf00465) EUT1
25165 HMM00465alcdh_3_mix(pf00465) EUT1
25170 HMM00465alcdh_3_mix(#) EUT1
25175 HMM06723EutJMreB_1_grm(#) EUT1
25180 HMMalddh_1_eutpdugrm(pf00171) EUT1
25185 HMMalddh_1_eutpdugrm(pf00171) EUT1
25190 HMMalddh_1_eutpdugrm(pf00171) EUT1
25195 HMMalddh_1_eutpdugrm(#) EUT1 EP_NTERM
25200 P_lightViolet EUT1
25205 H_azure GRMguf
25210 H_azure EUT1
25215 HMM01515PTA_B_1_eut1(pf01515) EUT1
25220 HMM01923cobtr_2_eut1(pf01923) EUT1
25225 -# -
25230 HMM01923cobtr_2_eut1(#) EUT1
25235 HMM01923cobtr_2_eut1(#) EUT1
25240 HMM06249EutQ_1_eut1(pf06249) EUT1 EP_NTERM
25245 HMM10662PduV_1_mix(pf10662) EUT1
25250 -# -
25255 Hp_euts_violetPink(#) EUT1
25260 HMM00390_malic_1_eut1(pf03949pf01515) EUT1
25265 HMM00390_malic_1_eut1(pf00390pf03949) EUT1
25270 -pf00923 -
25275 HMM02780keto_1_eut1(pf02779pf02780pf00456)
25280 -pf06674 -
25285 -pf06674 -
25290 HMM00293nuhyd_1_eut1(pf00293) EUT
25295 -# -
25300 -pf07992 -
25305 -pf01494pf14691 -
BMC-P
BMC-H
BMC-T
Aldehyde dehydrogenase
PTAC
Signature enzyme
Alcohol dehydrogenase
Regulator

Klebsiella pneumoniae. Bacteria; Proteobacteria; Gammaproteobacteria; Enterobacterales;

B4U27__locus_1

EUT1

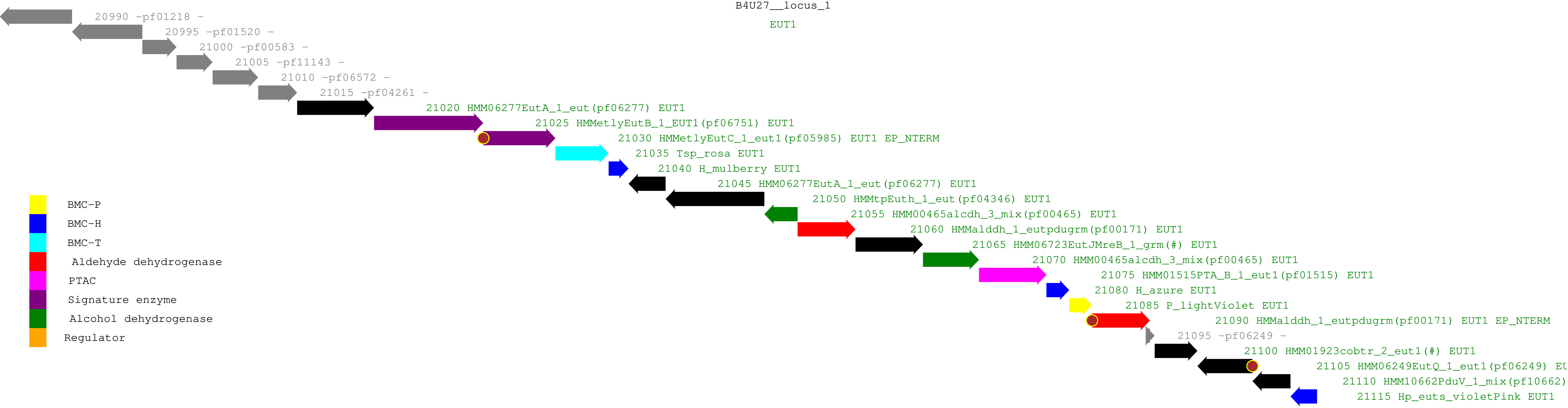

Klebsiella pneumoniae. Bacteria; Proteobacteria; Gammaproteobacteria; Enterobacterales;

B4U30__locus_1

EUT1

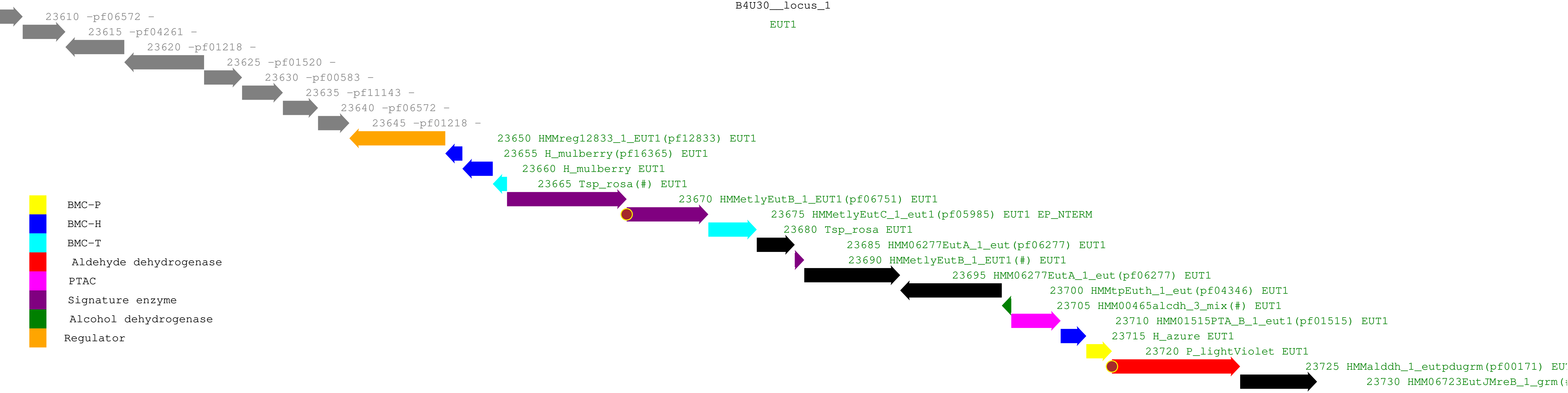

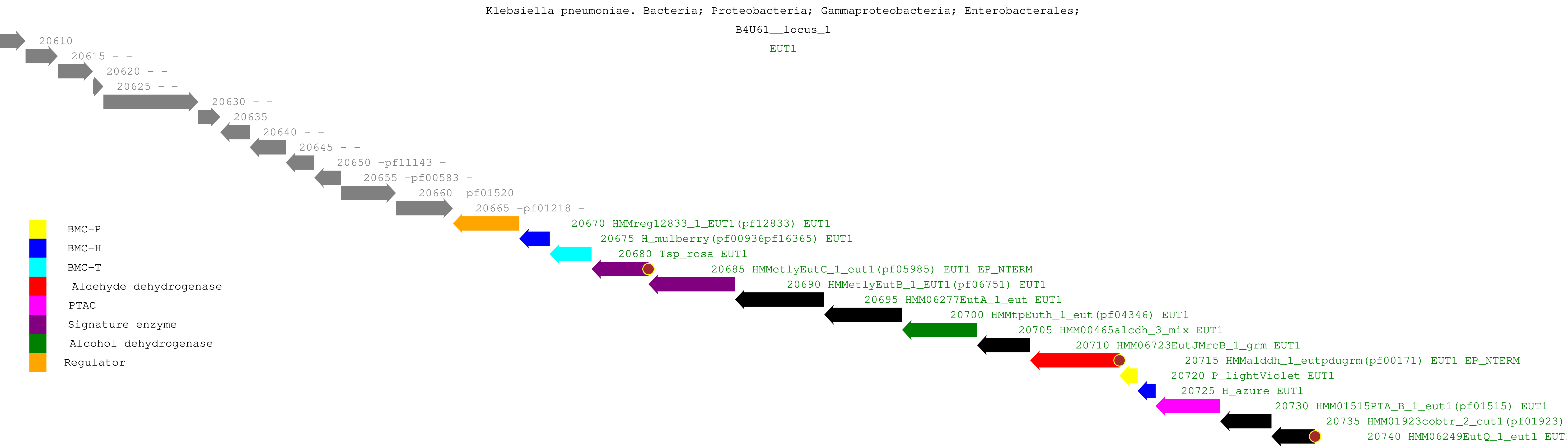
Klebsiella pneumoniae. Bacteria; Proteobacteria; Gammaproteobacteria; Enterobacterales;
B4U61__locus_1
EUT1
20610 - -
20615 - -
20620 - -
20625 - -
20630 - -
20635 - -
20640 - -
20645 - -
20650 -pf11143 -
20655 -pf00583 -
20660 -pf01520 -
20665 -pf01218 -
20670 HMMreg12833_1_EUT1(pf12833) EUT1
20675 H_mulberry(pf00936pf16365) EUT1
20680 Tsp_rosa EUT1
20685 HMMetlyEutC_1_eut1(pf05985) EUT1 EP_NTERM
20690 HMMetlyEutB_1_EUT1(pf06751) EUT1
20695 HMM06277EutA_1_eut EUT1
20700 HMMtpEuth_1_eut(pf04346) EUT1
20705 HMM00465alcdh_3_mix EUT1
20710 HMM06723EutJMreB_1_grm EUT1
20715 HMMalddh_1_eutpdugrm(pf00171) EUT1 EP_NTERM
20720 P_lightViolet EUT1
20725 H_azure EUT1
20730 HMM01515PTA_B_1_eut1(pf01515) EUT1
20735 HMM01923cobtr_2_eut1(pf01923)
20740 HMM06249EutQ_1_eut1 EUT
BMC-P
BMC-H
BMC-T
Aldehyde dehydrogenase
PTAC
Signature enzyme
Alcohol dehydrogenase
Regulator

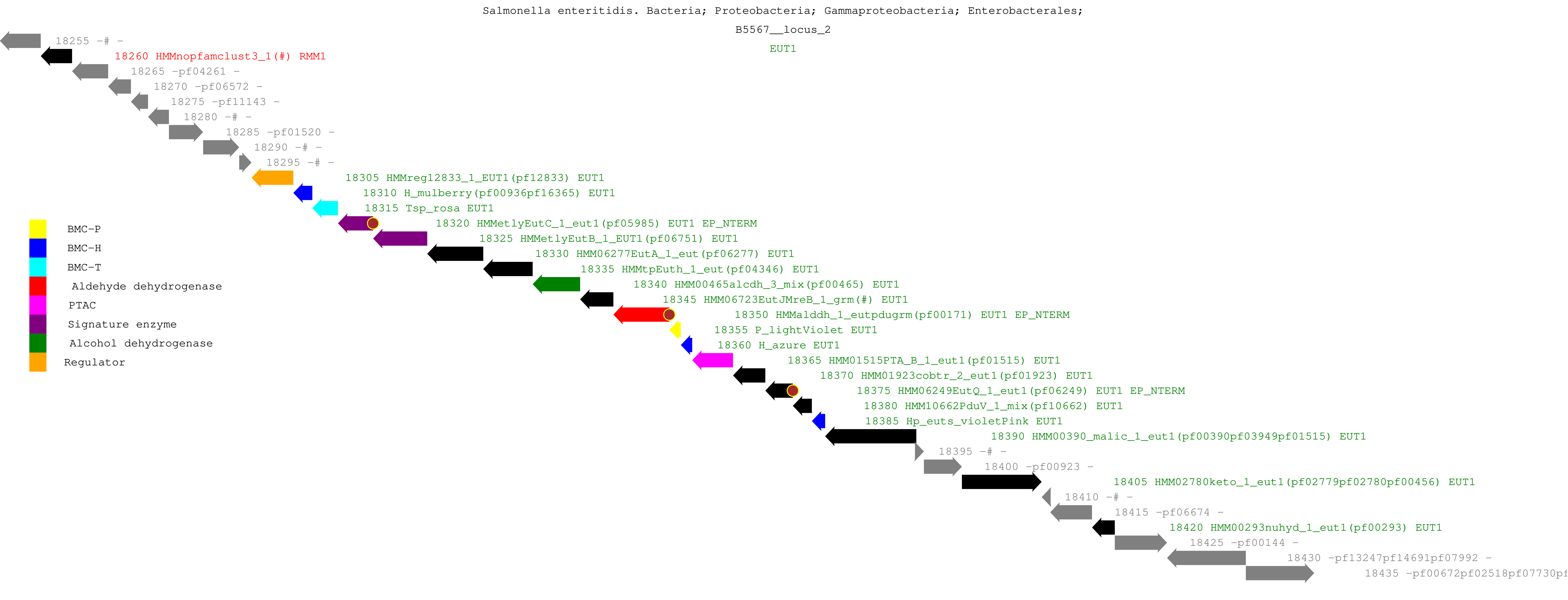

Salmonella enteritidis. Bacteria; Proteobacteria; Gammaproteobacteria; Enterobacterales;
B5567__locus_2
EUT1
18255 -# -
18260 HMMnopfamclust3_1(#) RMM1
18265 -pf04261 -
18270 -pf06572 -
18275 -pf11143 -
18280 -# -
18285 -pf01520 -
18290 -# -
18295 -# -
18305 HMMreg12833_1_EUT1(pf12833) EUT1
18310 H_mulberry(pf00936pf16365) EUT1
18315 Tsp_rosa EUT1
18320 HMMetlyEutC_1_eut1(pf05985) EUT1 EP_NTERM
18325 HMMetlyEutB_1_EUT1(pf06751) EUT1
18330 HMM06277EutA_1_eut(pf06277) EUT1
18335 HMMtpEuth_1_eut(pf04346) EUT1
18340 HMM00465alcdh_3_mix(pf00465) EUT1
18345 HMM06723EutJMreB_1_grm(#) EUT1
18350 HMMalddh_1_eutpdugrm(pf00171) EUT1 EP_NTERM
18355 P_lightViolet EUT1
18360 H_azure EUT1
18365 HMM01515PTA_B_1_eut1(pf01515) EUT1
18370 HMM01923cobtr_2_eut1(pf01923) EUT1
18375 HMM06249EutQ_1_eut1(pf06249) EUT1 EP_NTERM
18380 HMM10662PduV_1_mix(pf10662) EUT1
18385 Hp_euts_violetPink EUT1
18390 HMM00390_malic_1_eut1(pf00390pf03949pf01515) EUT1
18395 -# -
18400 -pf00923 -
18405 HMM02780keto_1_eut1(pf02779pf02780pf00456) EUT1
18410 -# -
18415 -pf06674 -
18420 HMM00293nuhyd_1_eut1(pf00293) EUT1
18425 -pf00144 -
18430 -pf13247pf14691pf07992 -
18435 -pf00672pf02518pf07730pf
BMC-P
BMC-H
BMC-T
Aldehyde dehydrogenase
PTAC
Signature enzyme
Alcohol dehydrogenase
Regulator

Morganella morganii (Proteus morganii). Bacteria; Proteobacteria; Gammaproteobacteria; Enterobacterales;

B5724__locus_1

EUT1

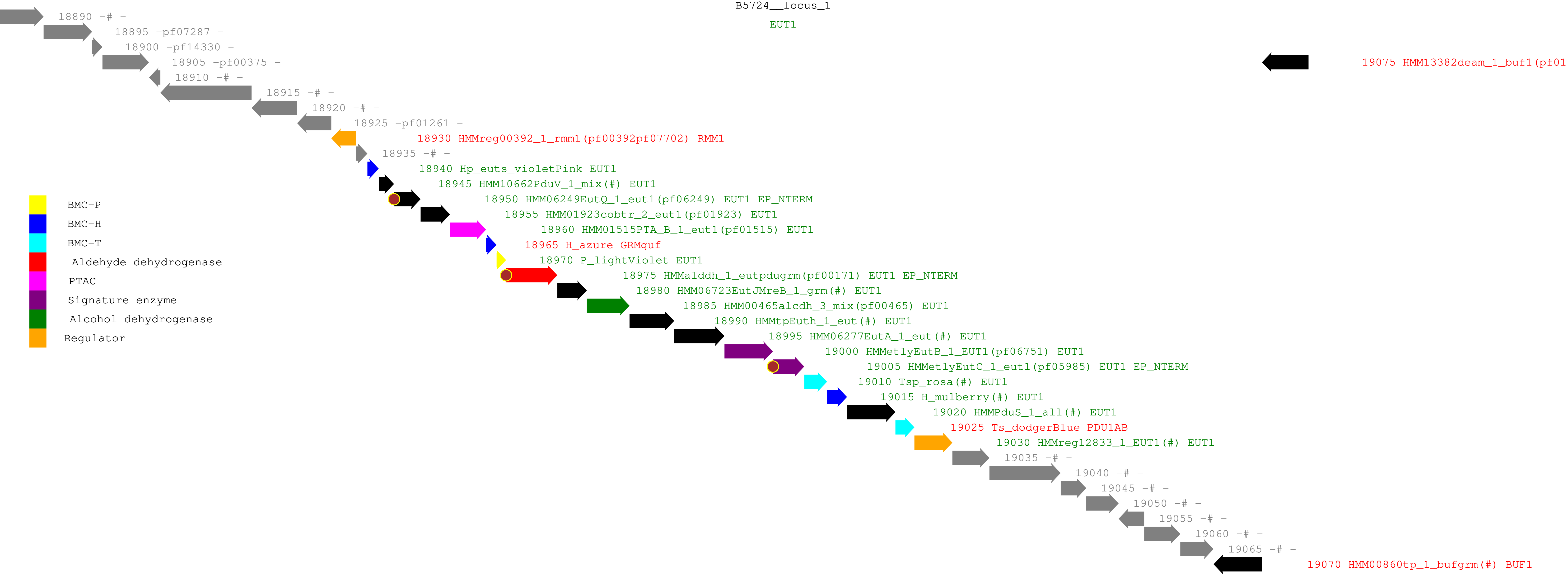

Klebsiella pneumoniae. Bacteria; Proteobacteria; Gammaproteobacteria; Enterobacterales;

B5G58__locus_2

EUT1

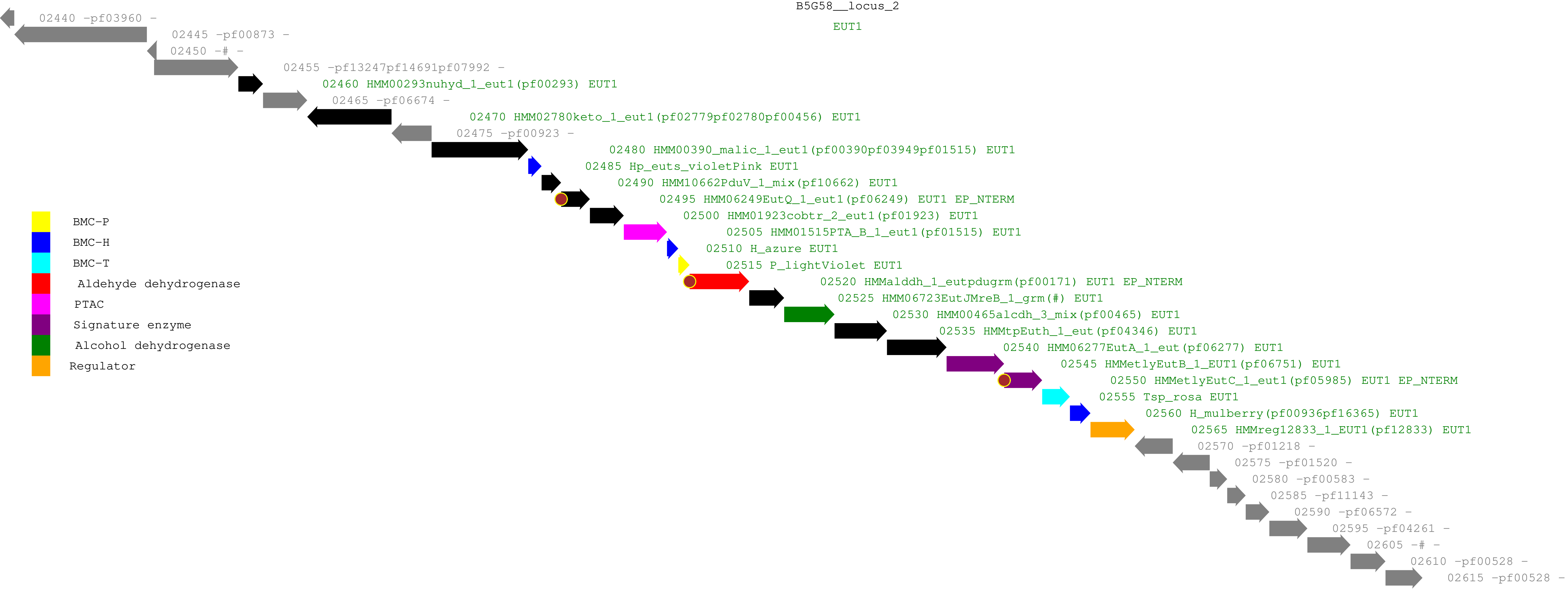

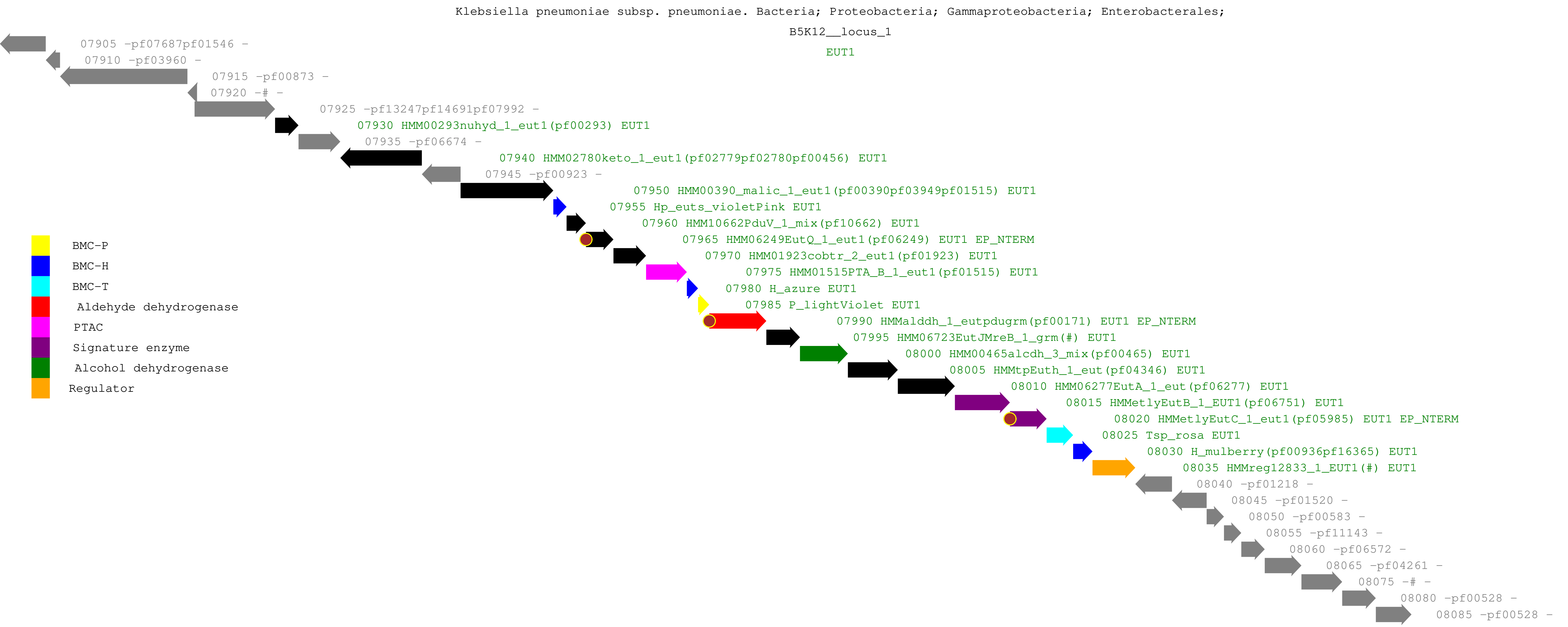
Klebsiella pneumoniae subsp. pneumoniae. Bacteria; Proteobacteria; Gammaproteobacteria; Enterobacterales;
B5K12__locus_1
EUT1
07905 -pf07687pf01546 -
07910 -pf03960 -
07915 -pf00873 -
07920 -# -
07925 -pf13247pf14691pf07992 -
07930 HMM00293nuhyd_1_eut1(pf00293) EUT1
07935 -pf06674 -
07940 HMM02780keto_1_eut1(pf02779pf02780pf00456) EUT1
07945 -pf00923 -
07950 HMM00390_malic_1_eut1(pf00390pf03949pf01515) EUT1
07955 Hp_euts_violetPink EUT1
07960 HMM10662PduV_1_mix(pf10662) EUT1
07965 HMM06249EutQ_1_eut1(pf06249) EUT1 EP_NTERM
07970 HMM01923cobtr_2_eut1(pf01923) EUT1
07975 HMM01515PTA_B_1_eut1(pf01515) EUT1
07980 H_azure EUT1
07985 P_lightViolet EUT1
07990 HMMalddh_1_eutpdugrm(pf00171) EUT1 EP_NTERM
07995 HMM06723EutJMreB_1_grm(#) EUT1
08000 HMM00465alcdh_3_mix(pf00465) EUT1
08005 HMMtpEuth_1_eut(pf04346) EUT1
08010 HMM06277EutA_1_eut(pf06277) EUT1
08015 HMMetlyEutB_1_EUT1(pf06751) EUT1
08020 HMMetlyEutC_1_eut1(pf05985) EUT1 EP_NTERM
08025 Tsp_rosa EUT1
08030 H_mulberry(pf00936pf16365) EUT1
08035 HMMreg12833_1_EUT1(#) EUT1
08040 -pf01218 -
08045 -pf01520 -
08050 -pf00583 -
08055 -pf11143 -
08060 -pf06572 -
08065 -pf04261 -
08075 -# -
08080 -pf00528 -
08085 -pf00528 -
BMC-P
BMC-H
BMC-T
Aldehyde dehydrogenase
PTAC
Signature enzyme
Alcohol dehydrogenase
Regulator

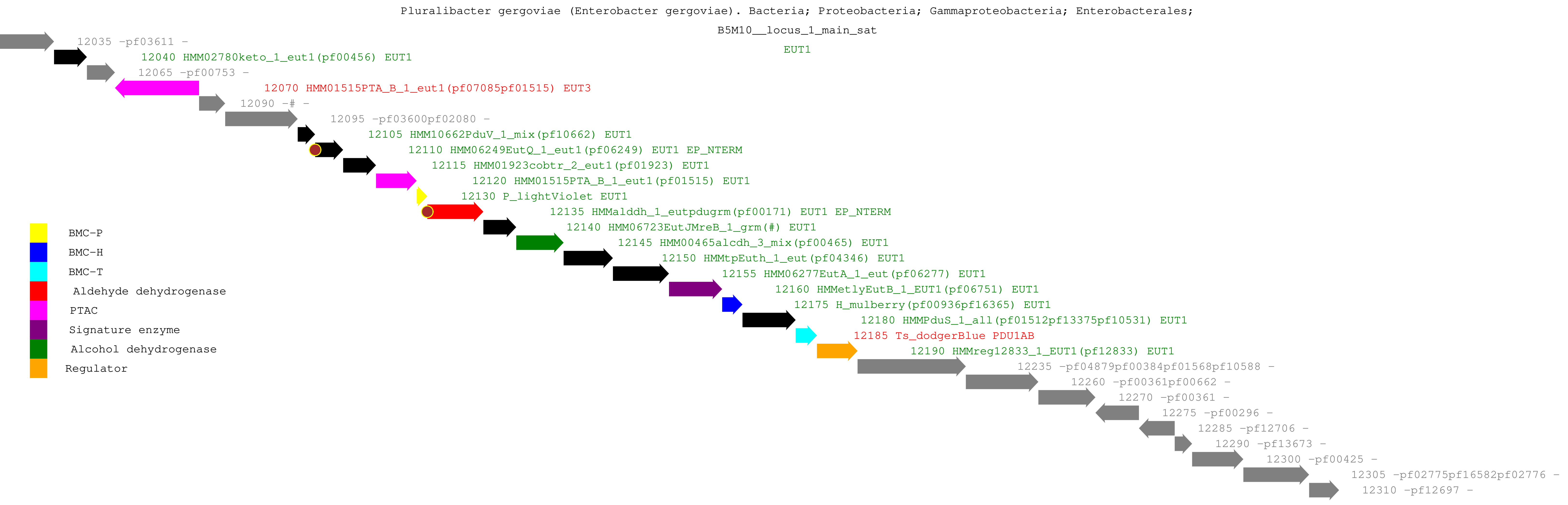
Pluralibacter gergoviae (Enterobacter gergoviae). Bacteria; Proteobacteria; Gammaproteobacteria; Enterobacterales;
B5M10__locus_1_main_sat
EUT1
12035 -pf03611 -
12040 HMM02780keto_1_eut1(pf00456) EUT1
12065 -pf00753 -
12070 HMM01515PTA_B_1_eut1(pf07085pf01515) EUT3
12090 -# -
12095 -pf03600pf02080 -
12105 HMM10662PduV_1_mix(pf10662) EUT1
12110 HMM06249EutQ_1_eut1(pf06249) EUT1 EP_NTERM
12115 HMM01923cobtr_2_eut1(pf01923) EUT1
12120 HMM01515PTA_B_1_eut1(pf01515) EUT1
12130 P_lightViolet EUT1
12135 HMMalddh_1_eutpdugrm(pf00171) EUT1 EP_NTERM
12140 HMM06723EutJMreB_1_grm(#) EUT1
12145 HMM00465alcdh_3_mix(pf00465) EUT1
12150 HMMtpEuth_1_eut(pf04346) EUT1
12155 HMM06277EutA_1_eut(pf06277) EUT1
12160 HMMetlyEutB_1_EUT1(pf06751) EUT1
12175 H_mulberry(pf00936pf16365) EUT1
12180 HMMPduS_1_all(pf01512pf13375pf10531) EUT1
12185 Ts_dodgerBlue PDU1AB
12190 HMMreg12833_1_EUT1(pf12833) EUT1
12235 -pf04879pf00384pf01568pf10588 -
12260 -pf00361pf00662 -
12270 -pf00361 -
12275 -pf00296 -
12285 -pf12706 -
12290 -pf13673 -
12300 -pf00425 -
12305 -pf02775pf16582pf02776 -
12310 -pf12697 -
BMC-P
BMC-H
BMC-T
Aldehyde dehydrogenase
PTAC
Signature enzyme
Alcohol dehydrogenase
Regulator

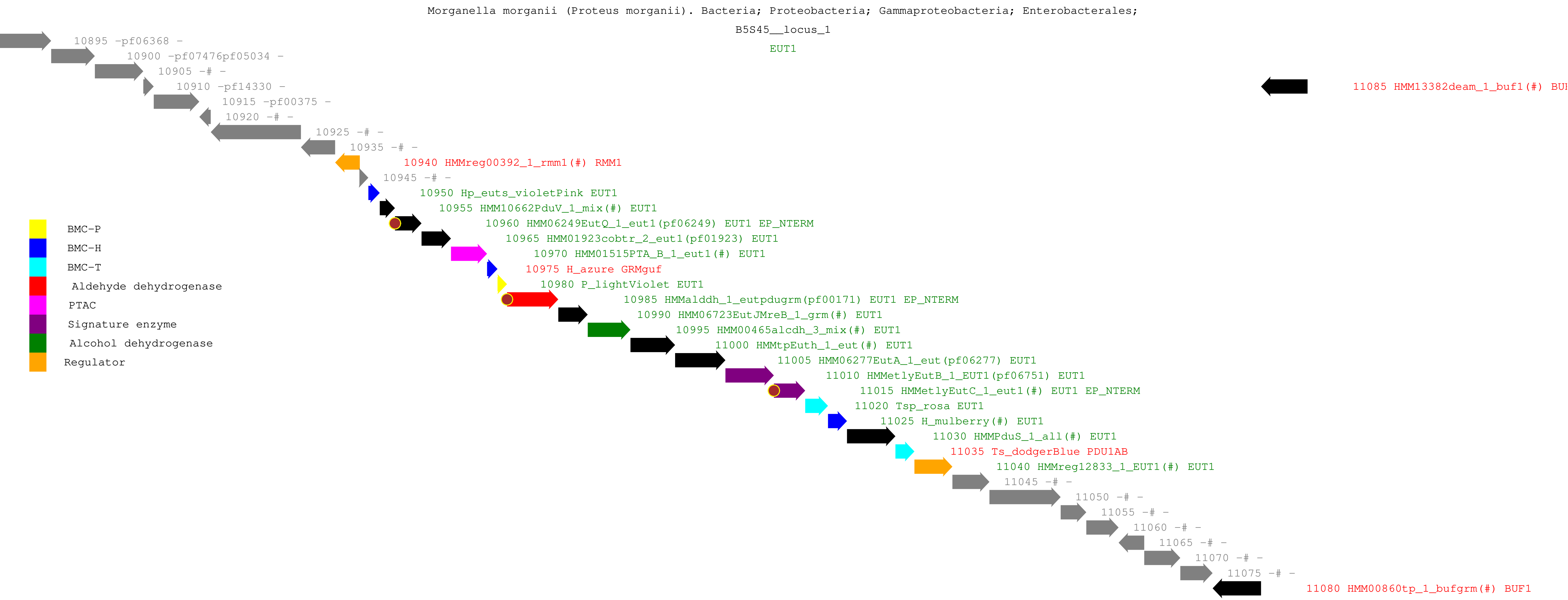

Morganella morganii (Proteus morganii). Bacteria; Proteobacteria; Gammaproteobacteria; Enterobacterales;
B5S45__locus_1
EUT1
10895 -pf06368 -
10900 -pf07476pf05034 -
10905 -# -
10910 -pf14330 -
10915 -pf00375 -
10920 -# -
10925 -# -
10935 -# -
10940 HMMreg00392_1_rmm1(#) RMM1
10945 -# -
10950 Hp_euts_violetPink EUT1
10955 HMM10662PduV_1_mix(#) EUT1
10960 HMM06249EutQ_1_eut1(pf06249) EUT1 EP_NTERM
10965 HMM01923cobtr_2_eut1(pf01923) EUT1
10970 HMM01515PTA_B_1_eut1(#) EUT1
10975 H_azure GRMguf
10980 P_lightViolet EUT1
10985 HMMalddh_1_eutpdugrm(pf00171) EUT1 EP_NTERM
10990 HMM06723EutJMreB_1_grm(#) EUT1
10995 HMM00465alcdh_3_mix(#) EUT1
11000 HMMtpEuth_1_eut(#) EUT1
11005 HMM06277EutA_1_eut(pf06277) EUT1
11010 HMMetlyEutB_1_EUT1(pf06751) EUT1
11015 HMMetlyEutC_1_eut1(#) EUT1 EP_NTERM
11020 Tsp_rosa EUT1
11025 H_mulberry(#) EUT1
11030 HMMPduS_1_all(#) EUT1
11035 Ts_dodgerBlue PDU1AB
11040 HMMreg12833_1_EUT1(#) EUT1
11045 -# -
11050 -# -
11055 -# -
11060 -# -
11065 -# -
11070 -# -
11075 -# -
11080 HMM00860tp_1_bufgrm(#) BUF1
11085 HMM13382deam_1_buf1(#) BUI
BMC-P
BMC-H
BMC-T
Aldehyde dehydrogenase
PTAC
Signature enzyme
Alcohol dehydrogenase
Regulator

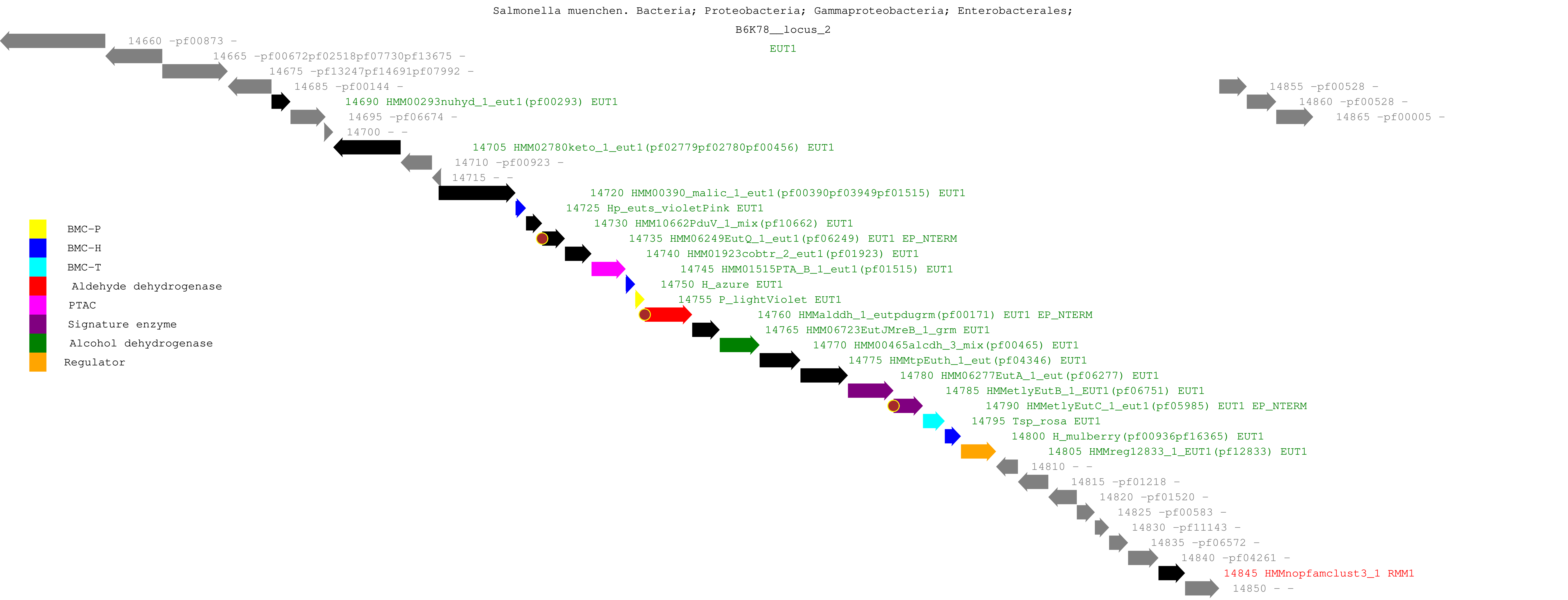

Salmonella muenchen. Bacteria; Proteobacteria; Gammaproteobacteria; Enterobacterales;
B6K78__locus_2
EUT1
14660 -pf00873 -
14665 -pf00672pf02518pf07730pf13675 -
14675 -pf13247pf14691pf07992 -
14685 -pf00144 -
14690 HMM00293nuhyd_1_eut1(pf00293) EUT1
14695 -pf06674 -
14700 - -
14705 HMM02780keto_1_eut1(pf02779pf02780pf00456) EUT1
14710 -pf00923 -
14715 - -
14720 HMM00390_malic_1_eut1(pf00390pf03949pf01515) EUT1
14725 Hp_euts_violetPink EUT1
14730 HMM10662PduV_1_mix(pf10662) EUT1
14735 HMM06249EutQ_1_eut1(pf06249) EUT1 EP_NTERM
14740 HMM01923cobtr_2_eut1(pf01923) EUT1
14745 HMM01515PTA_B_1_eut1(pf01515) EUT1
14750 H_azure EUT1
14755 P_lightViolet EUT1
14760 HMMalddh_1_eutpdugrm(pf00171) EUT1 EP_NTERM
14765 HMM06723EutJMreB_1_grm EUT1
14770 HMM00465alcdh_3_mix(pf00465) EUT1
14775 HMMtpEuth_1_eut(pf04346) EUT1
14780 HMM06277EutA_1_eut(pf06277) EUT1
14785 HMMetlyEutB_1_EUT1(pf06751) EUT1
14790 HMMetlyEutC_1_eut1(pf05985) EUT1 EP_NTERM
14795 Tsp_rosa EUT1
14800 H_mulberry(pf00936pf16365) EUT1
14805 HMMreg12833_1_EUT1(pf12833) EUT1
14810 - -
14815 -pf01218 -
14820 -pf01520 -
14825 -pf00583 -
14830 -pf11143 -
14835 -pf06572 -
14840 -pf04261 -
14845 HMMnopfamclust3_1 RMM1
14850 - -
14855 -pf00528 -
14860 -pf00528 -
14865 -pf00005 -
BMC-P
BMC-H
BMC-T
Aldehyde dehydrogenase
PTAC
Signature enzyme
Alcohol dehydrogenase
Regulator

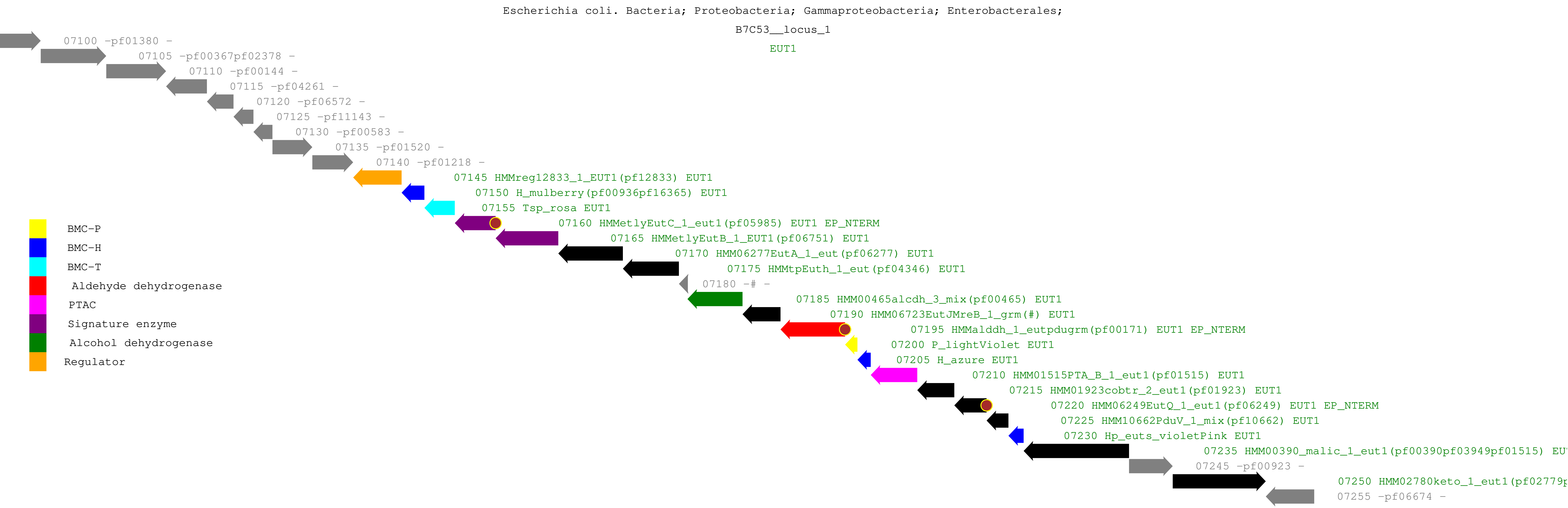

Escherichia coli. Bacteria; Proteobacteria; Gammaproteobacteria; Enterobacterales;
B7C53__locus_1
EUT1
07100 -pf01380 -
07105 -pf00367pf02378 -
07110 -pf00144 -
07115 -pf04261 -
07120 -pf06572 -
07125 -pf11143 -
07130 -pf00583 -
07135 -pf01520 -
07140 -pf01218 -
07145 HMMreg12833_1_EUT1(pf12833) EUT1
07150 H_mulberry(pf00936pf16365) EUT1
07155 Tsp_rosa EUT1
07160 HMMetlyEutC_1_eut1(pf05985) EUT1 EP_NTERM
07165 HMMetlyEutB_1_EUT1(pf06751) EUT1
07170 HMM06277EutA_1_eut(pf06277) EUT1
07175 HMMtpEuth_1_eut(pf04346) EUT1
07180 -# -
07185 HMM00465alcdh_3_mix(pf00465) EUT1
07190 HMM06723EutJMreB_1_grm(#) EUT1
07195 HMMaldh_1_eutpdugrm(pf00171) EUT1 EP_NTERM
07200 P_lightViolet EUT1
07205 H_azure EUT1
07210 HMM01515PTA_B_1_eut1(pf01515) EUT1
07215 HMM01923cobtr_2_eut1(pf01923) EUT1
07220 HMM06249EutQ_1_eut1(pf06249) EUT1 EP_NTERM
07225 HMM10662PduV_1_mix(pf10662) EUT1
07230 Hp_euts_violetPink EUT1
07235 HMM00390_malic_1_eut1(pf00390pf03949pf01515) EU
07245 -pf00923 -
07250 HMM02780keto_1_eut1(pf02779p
07255 -pf06674 -
BMC-P
BMC-H
BMC-T
Aldehyde dehydrogenase
PTAC
Signature enzyme
Alcohol dehydrogenase
Regulator

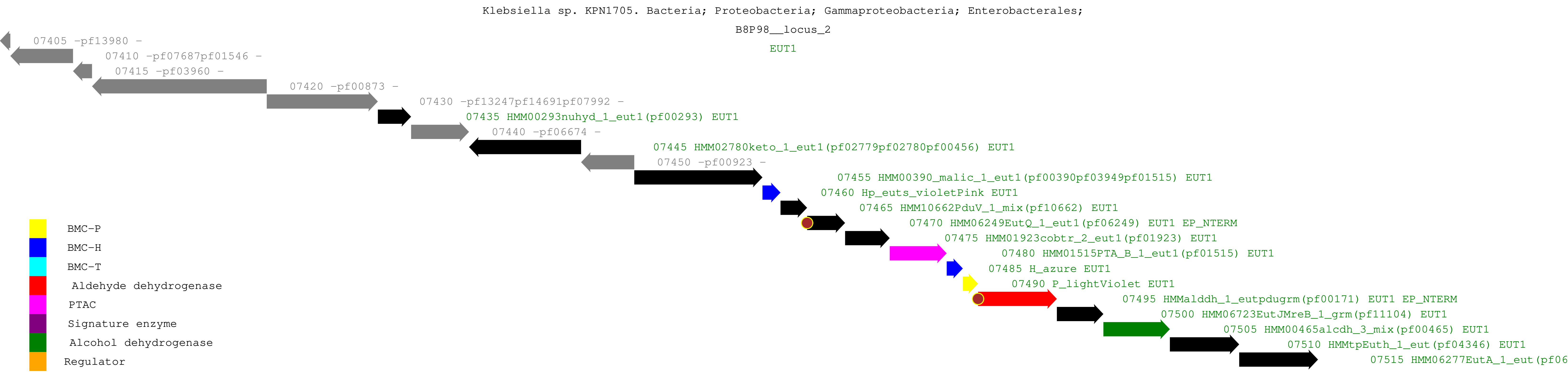
Klebsiella sp. KPN1705. Bacteria; Proteobacteria; Gammaproteobacteria; Enterobacterales;
B8P98__locus_2
EUT1
07405 -pf13980 -
07410 -pf07687pf01546 -
07415 -pf03960 -
07420 -pf00873 -
07430 -pf13247pf14691pf07992 -
07435 HMM00293nuhyd_1_eut1(pf00293) EUT1
07440 -pf06674 -
07445 HMM02780keto_1_eut1(pf02779pf02780pf00456) EUT1
07450 -pf00923 -
07455 HMM00390_malic_1_eut1(pf00390pf03949pf01515) EUT1
07460 Hp_euts_violetPink EUT1
07465 HMM10662PduV_1_mix(pf10662) EUT1
07470 HMM06249EutQ_1_eut1(pf06249) EUT1 EP_NTERM
07475 HMM01923cobtr_2_eut1(pf01923) EUT1
07480 HMM01515PTA_B_1_eut1(pf01515) EUT1
07485 H_azure EUT1
07490 P_lightViolet EUT1
07495 HMMalddh_1_eutpdugrm(pf00171) EUT1 EP_NTERM
07500 HMM06723EutJMreB_1_grm(pf11104) EUT1
07505 HMM00465alcdh_3_mix(pf00465) EUT1
07510 HMMtpEuth_1_eut(pf04346) EUT1
07515 HMM06277EutA_1_eut(pf06
BMC-P
BMC-H
BMC-T
Aldehyde dehydrogenase
PTAC
Signature enzyme
Alcohol dehydrogenase
Regulator

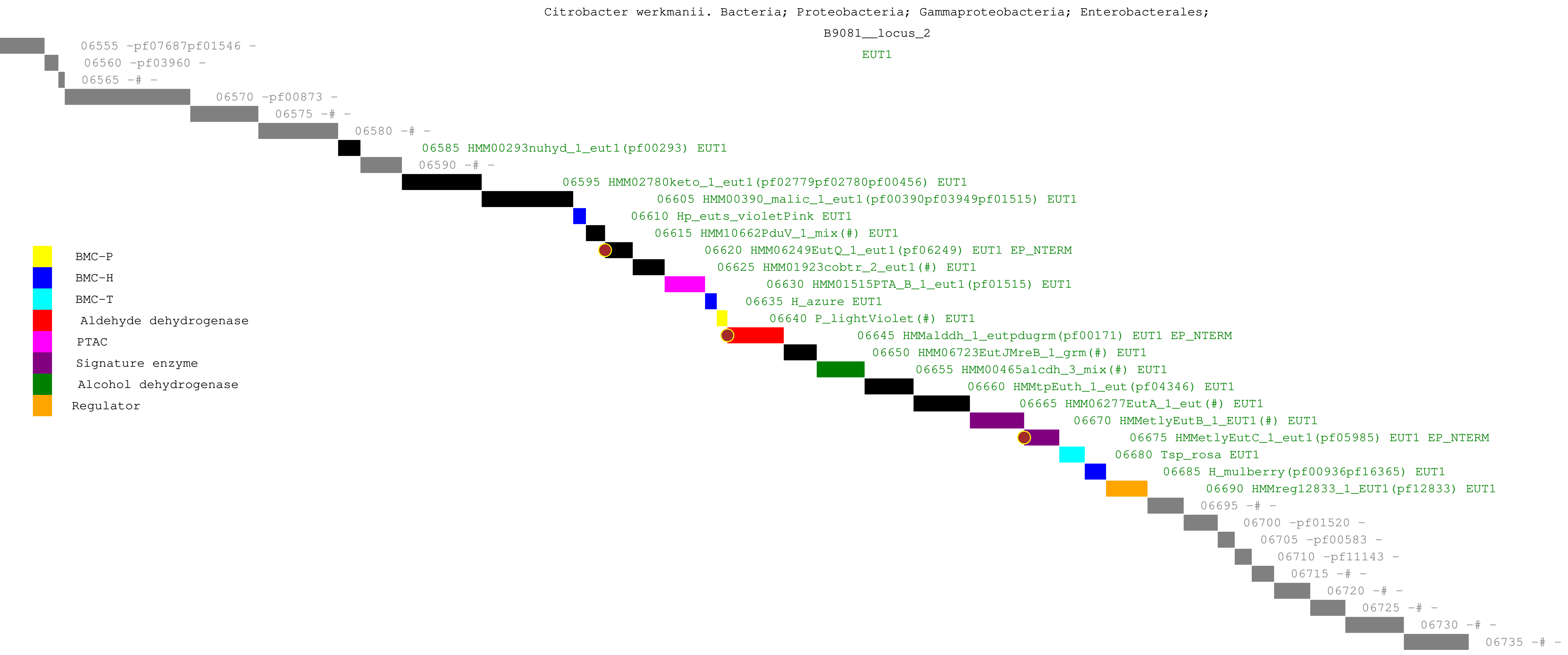
Citrobacter werkmanii. Bacteria; Proteobacteria; Gammaproteobacteria; Enterobacterales;
B9081__locus_2
EUT1
06555 -pf07687pf01546 -
06560 -pf03960 -
06565 -# -
06570 -pf00873 -
06575 -# -
06580 -# -
06585 HMM00293nuhyd_1_eut1(pf00293) EUT1
06590 -# -
06595 HMM02780keto_1_eut1(pf02779pf02780pf00456) EUT1
06605 HMM00390_malic_1_eut1(pf00390pf03949pf01515) EUT1
06610 Hp_euts_violetPink EUT1
06615 HMM10662PduV_1_mix(#) EUT1
06620 HMM06249EutQ_1_eut1(pf06249) EUT1 EP_NTERM
06625 HMM01923cobtr_2_eut1(#) EUT1
06630 HMM01515PTA_B_1_eut1(pf01515) EUT1
06635 H_azure EUT1
06640 P_lightViolet(#) EUT1
06645 HMMalddh_1_eutpdugrm(pf00171) EUT1 EP_NTERM
06650 HMM06723EutJMreB_1_grm(#) EUT1
06655 HMM00465alcdh_3_mix(#) EUT1
06660 HMMtpEuth_1_eut(pf04346) EUT1
06665 HMM06277EutA_1_eut(#) EUT1
06670 HMMetlyEutB_1_EUT1(#) EUT1
06675 HMMetlyEutC_1_eut1(pf05985) EUT1 EP_NTERM
06680 Tsp_rosa EUT1
06685 H_mulberry(pf00936pf16365) EUT1
06690 HMMreg12833_1_EUT1(pf12833) EUT1
06695 -# -
06700 -pf01520 -
06705 -pf00583 -
06710 -pf11143 -
06715 -# -
06720 -# -
06725 -# -
06730 -# -
06735 -# -
BMC-P
BMC-H
BMC-T
Aldehyde dehydrogenase
PTAC
Signature enzyme
Alcohol dehydrogenase
Regulator

Citrobacter sp. L55. Bacteria; Proteobacteria; Gammaproteobacteria; Enterobacterales;

B9P82__locus_2

EUT1

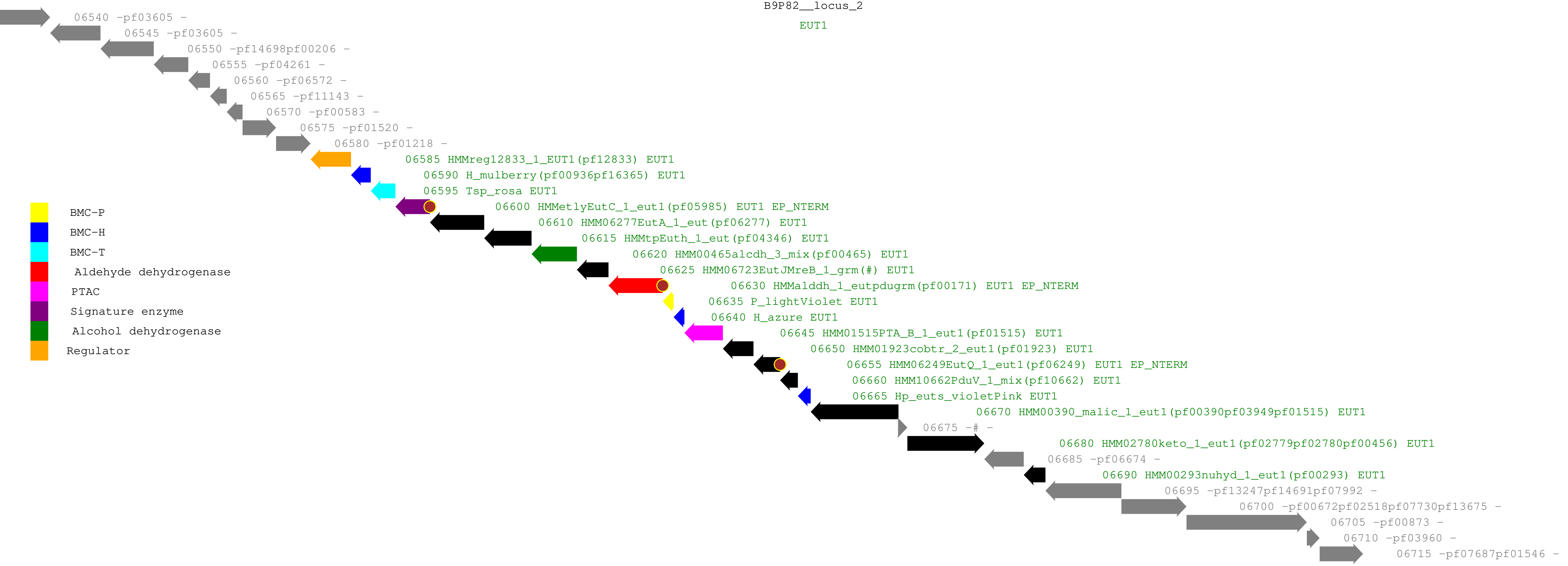

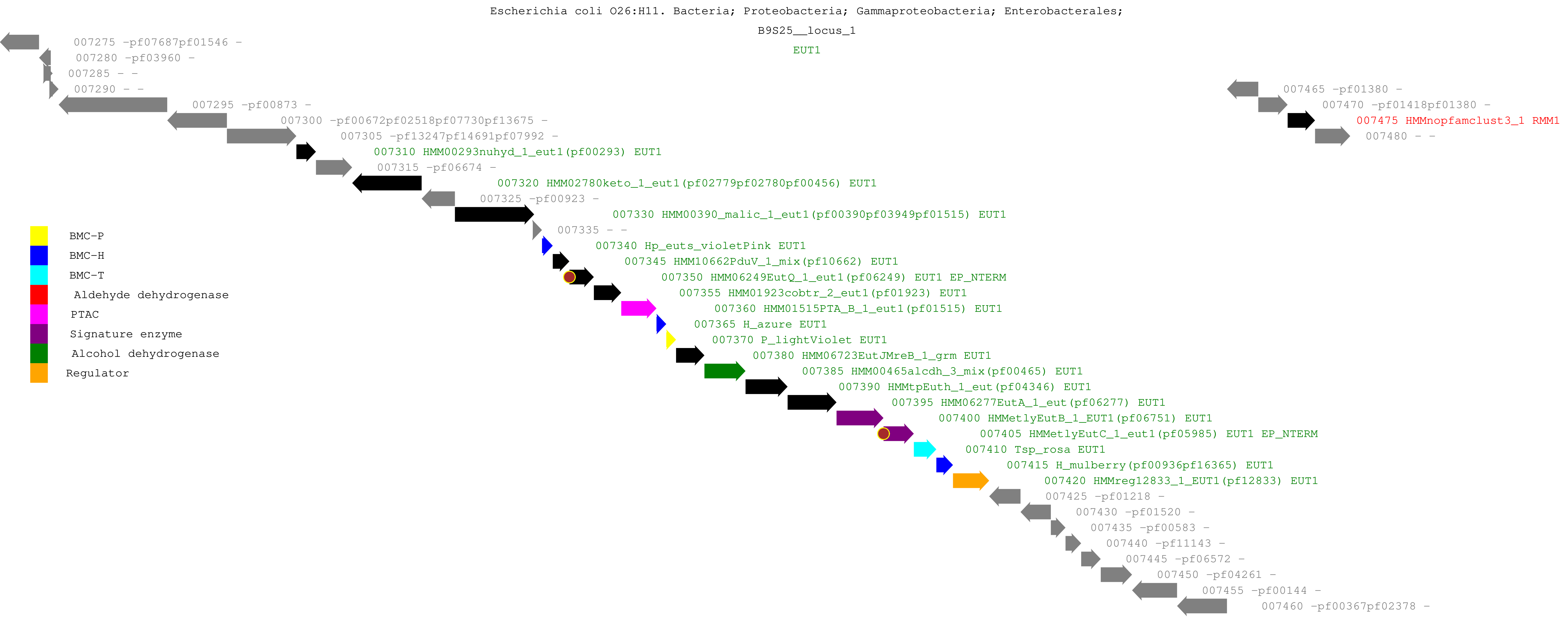

Escherichia coli O26:H11. Bacteria; Proteobacteria; Gammaproteobacteria; Enterobacterales;
B9S25__locus_1
EUT1
007275 -pf07687pf01546 -
007280 -pf03960 -
007285 - -
007290 - -
007295 -pf00873 -
007300 -pf00672pf02518pf07730pf13675 -
007305 -pf13247pf14691pf07992 -
007310 HMM00293nuhyd_1_eut1(pf00293) EUT1
007315 -pf06674 -
007320 HMM02780keto_1_eut1(pf02779pf02780pf00456) EUT1
007325 -pf00923 -
007330 HMM00390_malic_1_eut1(pf00390pf03949pf01515) EUT1
007335 - -
007340 Hp_euts_violetPink EUT1
007345 HMM10662PduV_1_mix(pf10662) EUT1
007350 HMM06249EutQ_1_eut1(pf06249) EUT1 EP_NTERM
007355 HMM01923cobtr_2_eut1(pf01923) EUT1
007360 HMM01515PTA_B_1_eut1(pf01515) EUT1
007365 H_azure EUT1
007370 P_lightViolet EUT1
007380 HMM06723EutJMreB_1_grm EUT1
007385 HMM00465alcdh_3_mix(pf00465) EUT1
007390 HMMtpEuth_1_eut(pf04346) EUT1
007395 HMM06277EutA_1_eut(pf06277) EUT1
007400 HMMetlyEutB_1_EUT1(pf06751) EUT1
007405 HMMetlyEutC_1_eut1(pf05985) EUT1 EP_NTERM
007410 Tsp_rosa EUT1
007415 H_mulberry(pf00936pf16365) EUT1
007420 HMMreg12833_1_EUT1(pf12833) EUT1
007425 -pf01218 -
007430 -pf01520 -
007435 -pf00583 -
007440 -pf11143 -
007445 -pf06572 -
007450 -pf04261 -
007455 -pf00144 -
007460 -pf00367pf02378 -
007465 -pf01380 -
007470 -pf01418pf01380 -
007475 HMMnopfamclust3_1 RMM1
007480 - -
BMC-P
BMC-H
BMC-T
Aldehyde dehydrogenase
PTAC
Signature enzyme
Alcohol dehydrogenase
Regulator

Escherichia coli O121:H19. Bacteria; Proteobacteria; Gammaproteobacteria; Enterobacterales;

B9X35__locus_1

EUT1

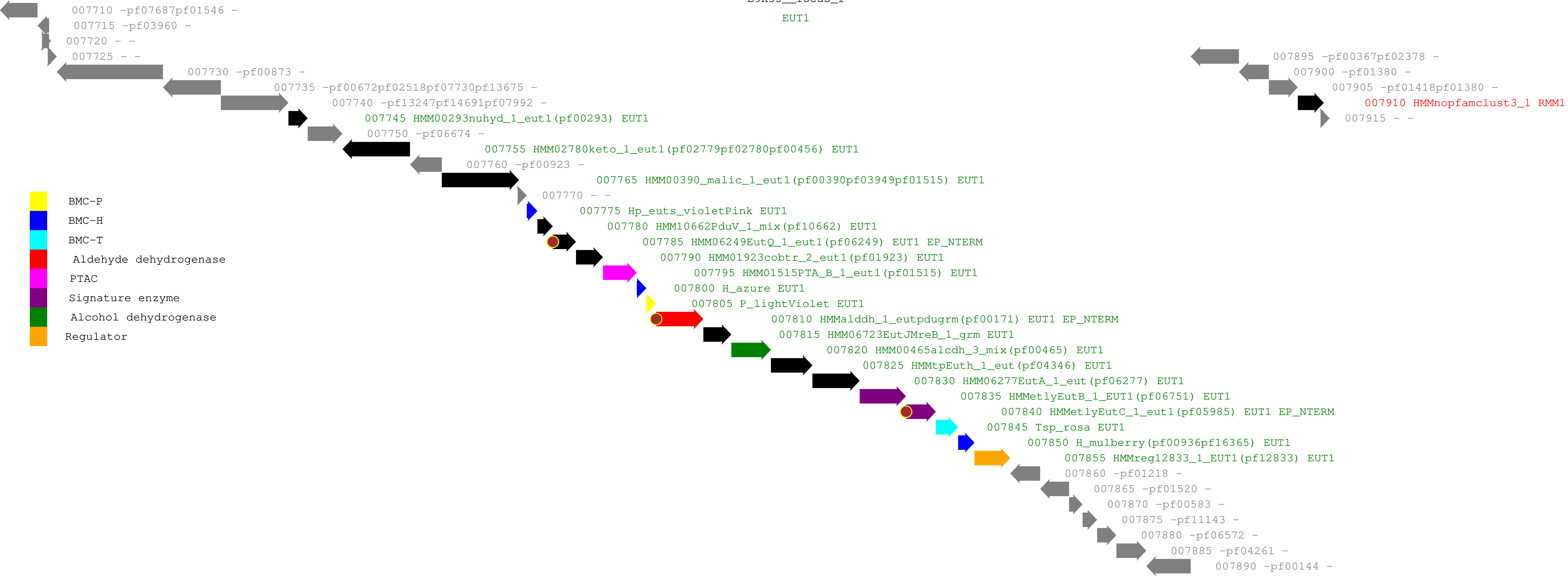

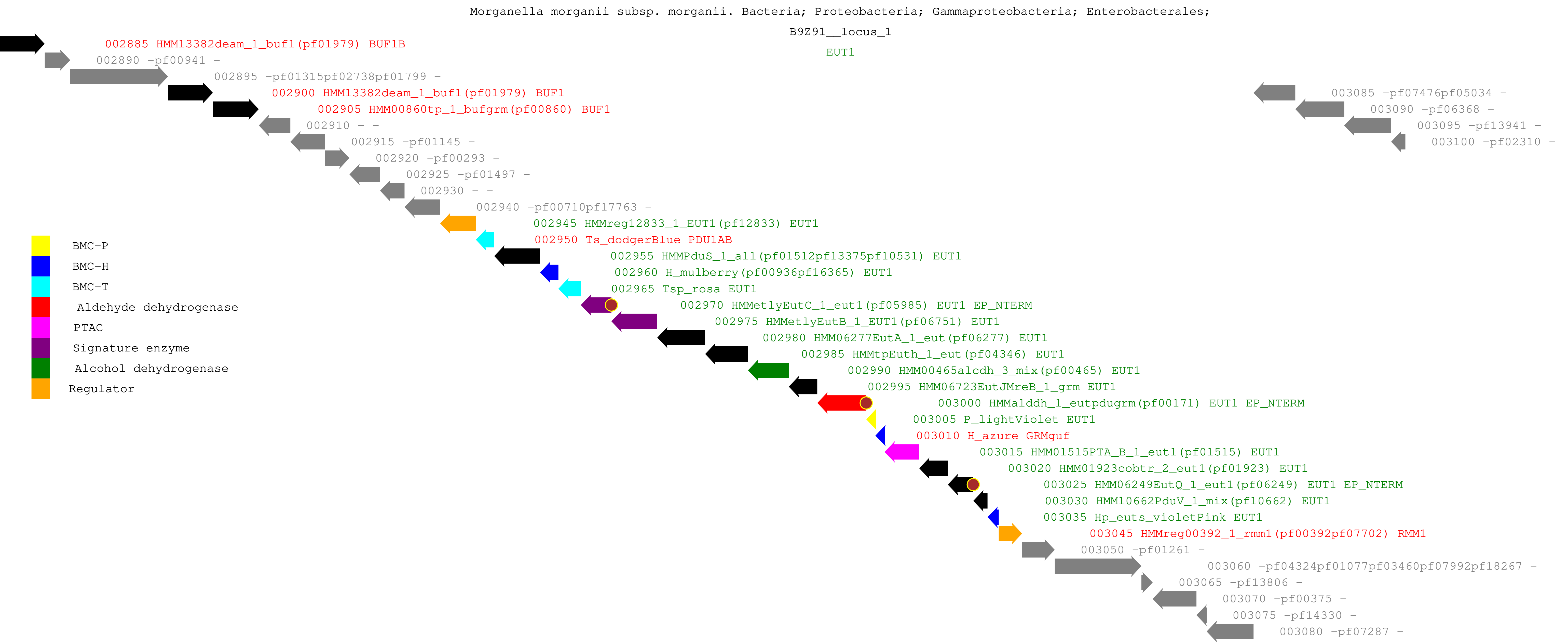
Morganella morganii subsp. morganii. Bacteria; Proteobacteria; Gammaproteobacteria; Enterobacterales;
B9Z91__locus_1
EUT1
002885 HMM13382deam_1_buf1(pf01979) BUF1B
002890 -pf00941 -
002895 -pf01315pf02738pf01799 -
002900 HMM13382deam_1_buf1(pf01979) BUF1
002905 HMM00860tp_1_bufgrm(pf00860) BUF1
002910 - -
002915 -pf01145 -
002920 -pf00293 -
002925 -pf01497 -
002930 - -
002940 -pf00710pf17763 -
002945 HMMreg12833_1_EUT1(pf12833) EUT1
002950 Ts_dodgerBlue PDU1AB
002955 HMMPduS_1_all(pf01512pf13375pf10531) EUT1
002960 H_mulberry(pf00936pf16365) EUT1
002965 Tsp_rosa EUT1
002970 HMMetlyEutC_1_eut1(pf05985) EUT1 EP_NTERM
002975 HMMetlyEutB_1_EUT1(pf06751) EUT1
002980 HMM06277EutA_1_eut(pf06277) EUT1
002985 HMMtpEuth_1_eut(pf04346) EUT1
002990 HMM00465alcdh_3_mix(pf00465) EUT1
002995 HMM06723EutJMreB_1_grm EUT1
003000 HMMalddh_1_eutpdugrm(pf00171) EUT1 EP_NTERM
003005 P_lightViolet EUT1
003010 H_azure GRMguf
003015 HMM01515PTA_B_1_eut1(pf01515) EUT1
003020 HMM01923cobtr_2_eut1(pf01923) EUT1
003025 HMM06249EutQ_1_eut1(pf06249) EUT1 EP_NTERM
003030 HMM10662PduV_1_mix(pf10662) EUT1
003035 Hp_euts_violetPink EUT1
003045 HMMreg00392_1_rmm1(pf00392pf07702) RMM1
003050 -pf01261 -
003060 -pf04324pf01077pf03460pf07992pf18267 -
003065 -pf13806 -
003070 -pf00375 -
003075 -pf14330 -
003080 -pf07287 -
003085 -pf07476pf05034 -
003090 -pf06368 -
003095 -pf13941 -
003100 -pf02310 -
BMC-P
BMC-H
BMC-T
Aldehyde dehydrogenase
PTAC
Signature enzyme
Alcohol dehydrogenase
Regulator

Salmonella choleraesuis. Bacteria; Proteobacteria; Gammaproteobacteria; Enterobacterales;

BA753__locus_2

EUT1

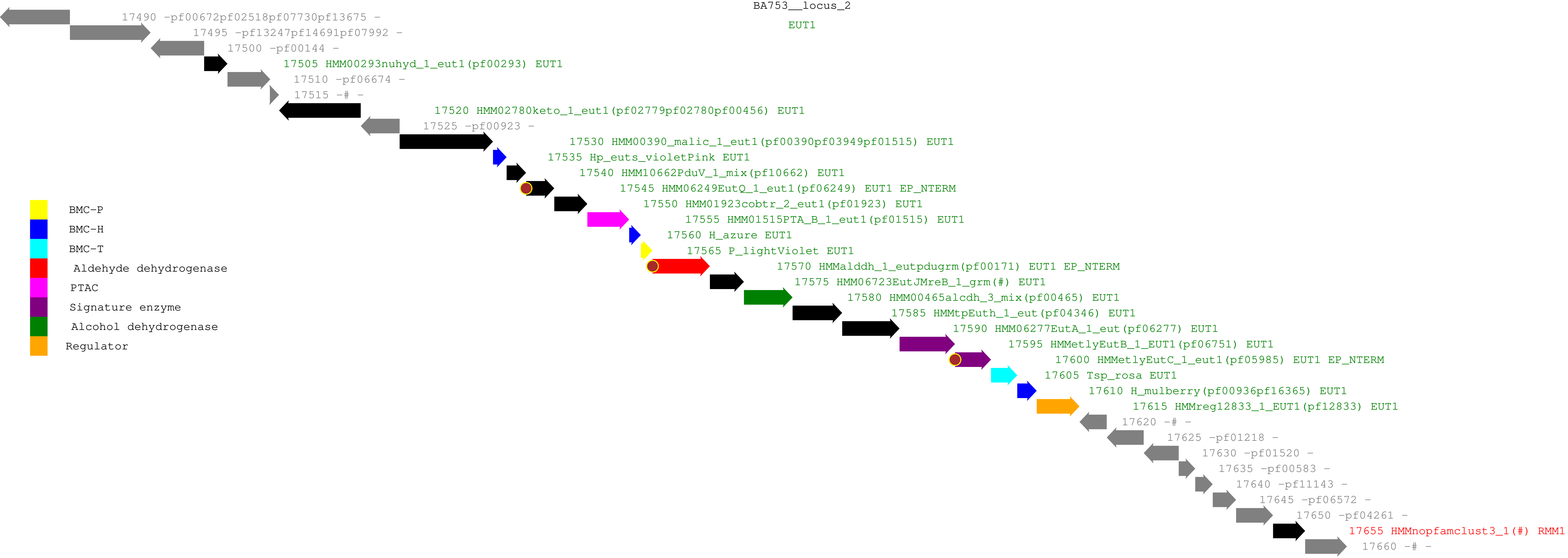

Pseudomonas sp. EGD-AKN5. Bacteria; Proteobacteria; Gammaproteobacteria; Pseudomonadales;

BAE52__locus_1

EUT1

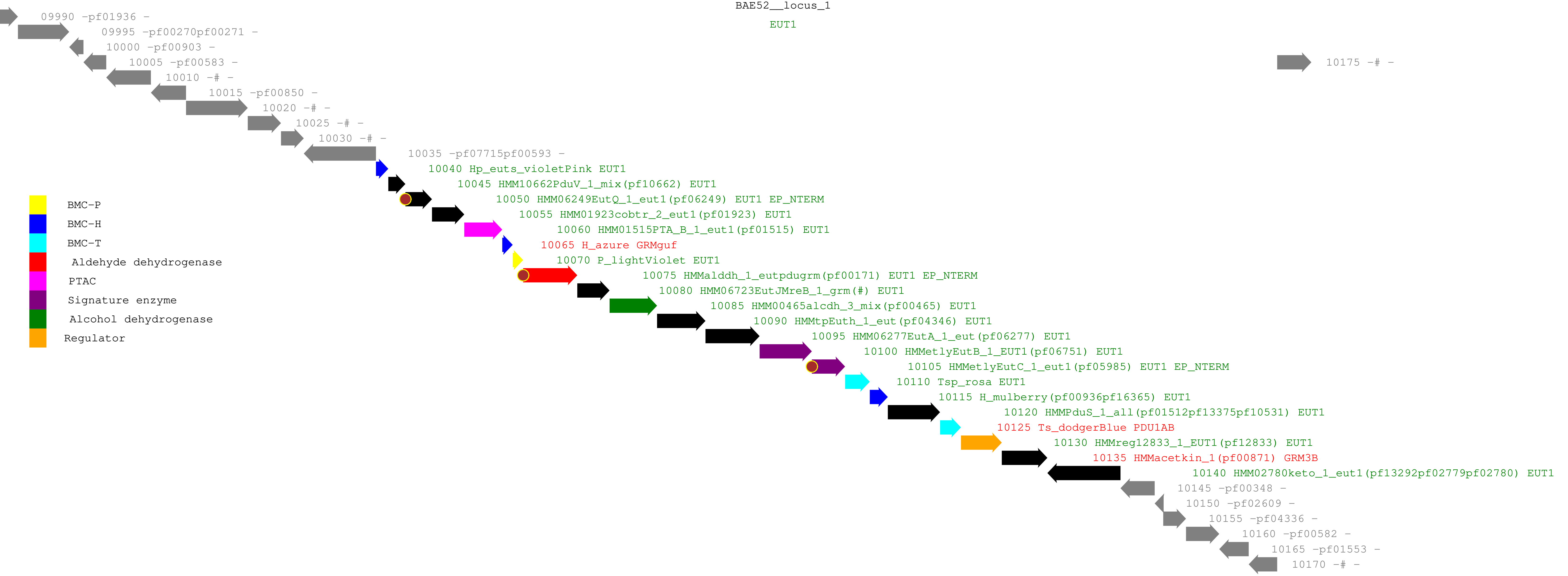

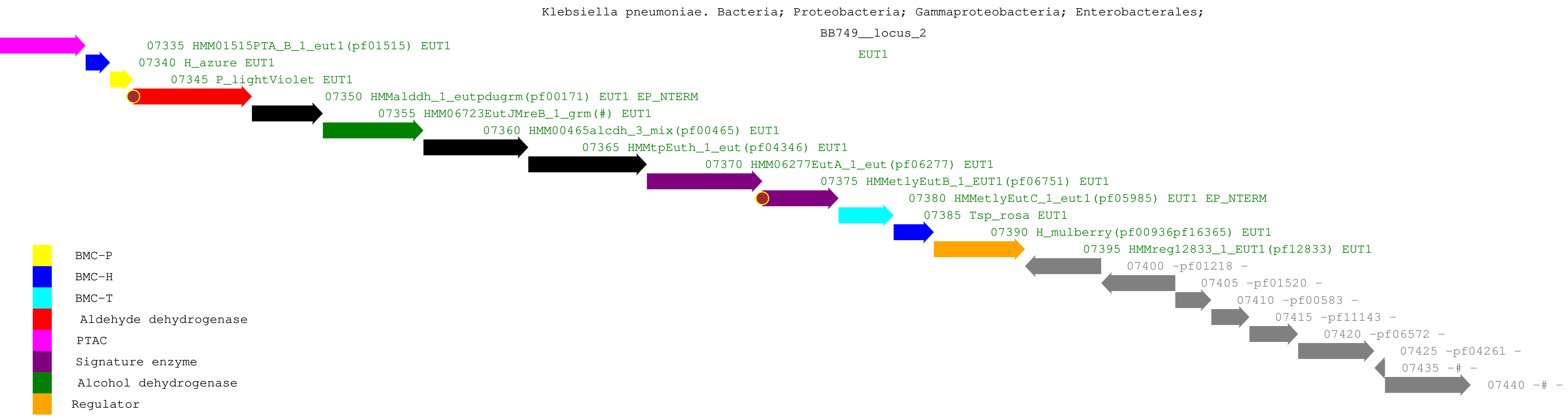

Klebsiella pneumoniae. Bacteria; Proteobacteria; Gammaproteobacteria; Enterobacterales;
BB749__locus_2
EUT1
07335 HMM01515PTA_B_1_eut1(pf01515) EUT1
07340 H_azure EUT1
07345 P_lightViolet EUT1
07350 HMMalddh_1_eutpdugrm(pf00171) EUT1 EP_NTERM
07355 HMM06723EutJMreB_1_grm(#) EUT1
07360 HMM00465alcdh_3_mix(pf00465) EUT1
07365 HMMtpEuth_1_eut(pf04346) EUT1
07370 HMM06277EutA_1_eut(pf06277) EUT1
07375 HMMetlyEutB_1_EUT1(pf06751) EUT1
07380 HMMetlyEutC_1_eut1(pf05985) EUT1 EP_NTERM
07385 Tsp_rosa EUT1
07390 H_mulberry(pf00936pf16365) EUT1
07395 HMMreg12833_1_EUT1(pf12833) EUT1
07400 -pf01218 -
07405 -pf01520 -
07410 -pf00583 -
07415 -pf11143 -
07420 -pf06572 -
07425 -pf04261 -
07435 -# -
07440 -# -
BMC-P
BMC-H
BMC-T
Aldehyde dehydrogenase
PTAC
Signature enzyme
Alcohol dehydrogenase
Regulator

Pluralibacter gergoviae (Enterobacter gergoviae). Bacteria; Proteobacteria; Gammaproteobacteria; Enterobacterales;

BB778__locus_1

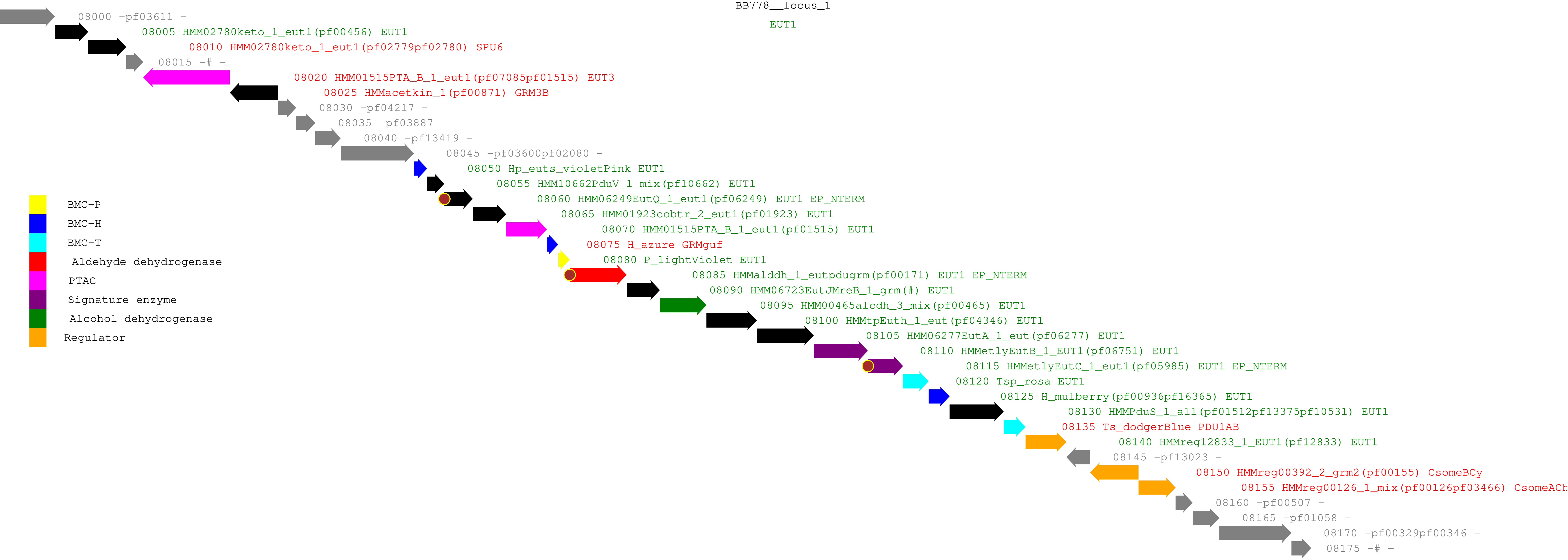

Salmonella enterica subsp. enterica serovar Javiana str. CFSAN000904. Bacteria; Proteobacteria; Gammaproteobacteria; Enterobacterales;

BBD49__locus_2

EUT1

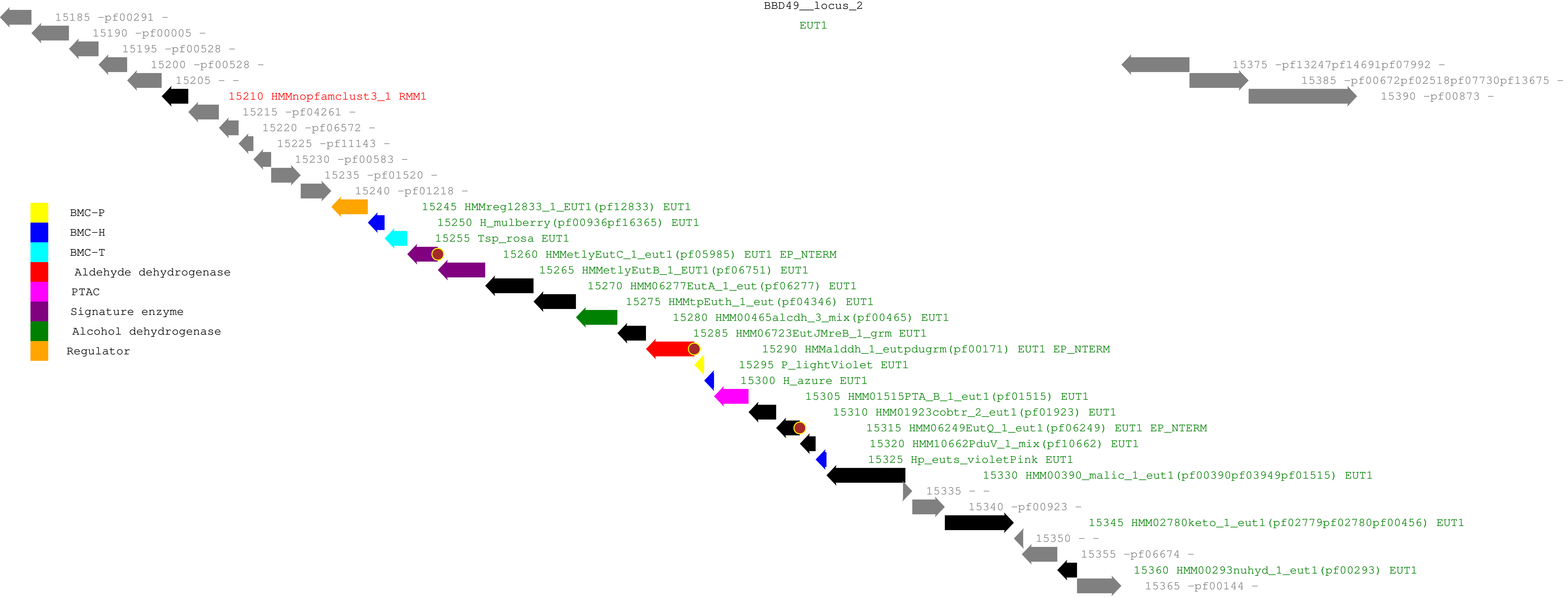

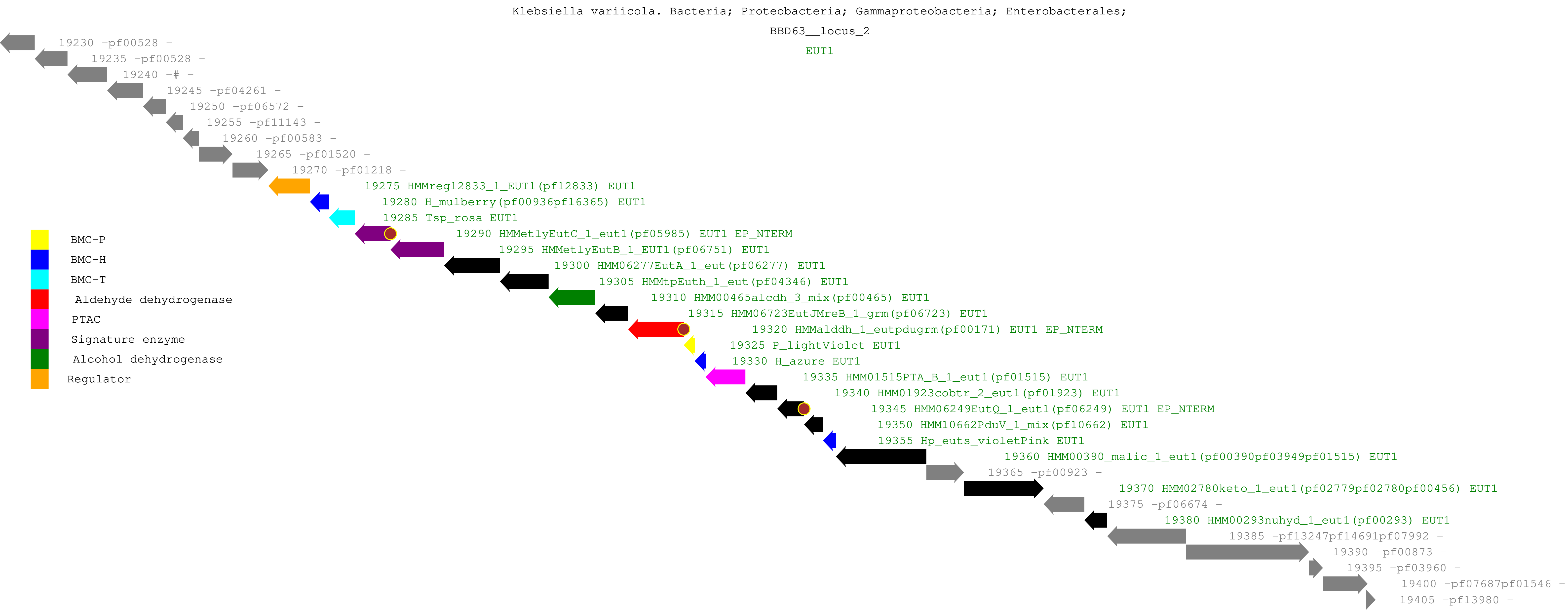
Klebsiella variicola. Bacteria; Proteobacteria; Gammaproteobacteria; Enterobacterales;
BBD63__locus_2
EUT1
19230 -pf00528 -
19235 -pf00528 -
19240 -# -
19245 -pf04261 -
19250 -pf06572 -
19255 -pf11143 -
19260 -pf00583 -
19265 -pf01520 -
19270 -pf01218 -
19275 HMMreg12833_1_EUT1(pf12833) EUT1
19280 H_mulberry(pf00936pf16365) EUT1
19285 Tsp_rosa EUT1
19290 HMMetlyEutC_1_eut1(pf05985) EUT1 EP_NTERM
19295 HMMetlyEutB_1_EUT1(pf06751) EUT1
19300 HMM06277EutA_1_eut(pf06277) EUT1
19305 HMMtpEuth_1_eut(pf04346) EUT1
19310 HMM00465alcdh_3_mix(pf00465) EUT1
19315 HMM06723EutJMreB_1_grm(pf06723) EUT1
19320 HMMalddh_1_eutpdugrm(pf00171) EUT1 EP_NTERM
19325 P_lightViolet EUT1
19330 H_azure EUT1
19335 HMM01515PTA_B_1_eut1(pf01515) EUT1
19340 HMM01923cobtr_2_eut1(pf01923) EUT1
19345 HMM06249EutQ_1_eut1(pf06249) EUT1 EP_NTERM
19350 HMM10662PduV_1_mix(pf10662) EUT1
19355 Hp_euts_violetPink EUT1
19360 HMM00390_malic_1_eut1(pf00390pf03949pf01515) EUT1
19365 -pf00923 -
19370 HMM02780keto_1_eut1(pf02779pf02780pf00456) EUT1
19375 -pf06674 -
19380 HMM00293nuhyd_1_eut1(pf00293) EUT1
19385 -pf13247pf14691pf07992 -
19390 -pf00873 -
19395 -pf03960 -
19400 -pf07687pf01546 -
19405 -pf13980 -
BMC-P
BMC-H
BMC-T
Aldehyde dehydrogenase
PTAC
Signature enzyme
Alcohol dehydrogenase
Regulator

Salmonella enteritidis. Bacteria; Proteobacteria; Gammaproteobacteria; Enterobacterales;

BCA92__locus_1

EUT1

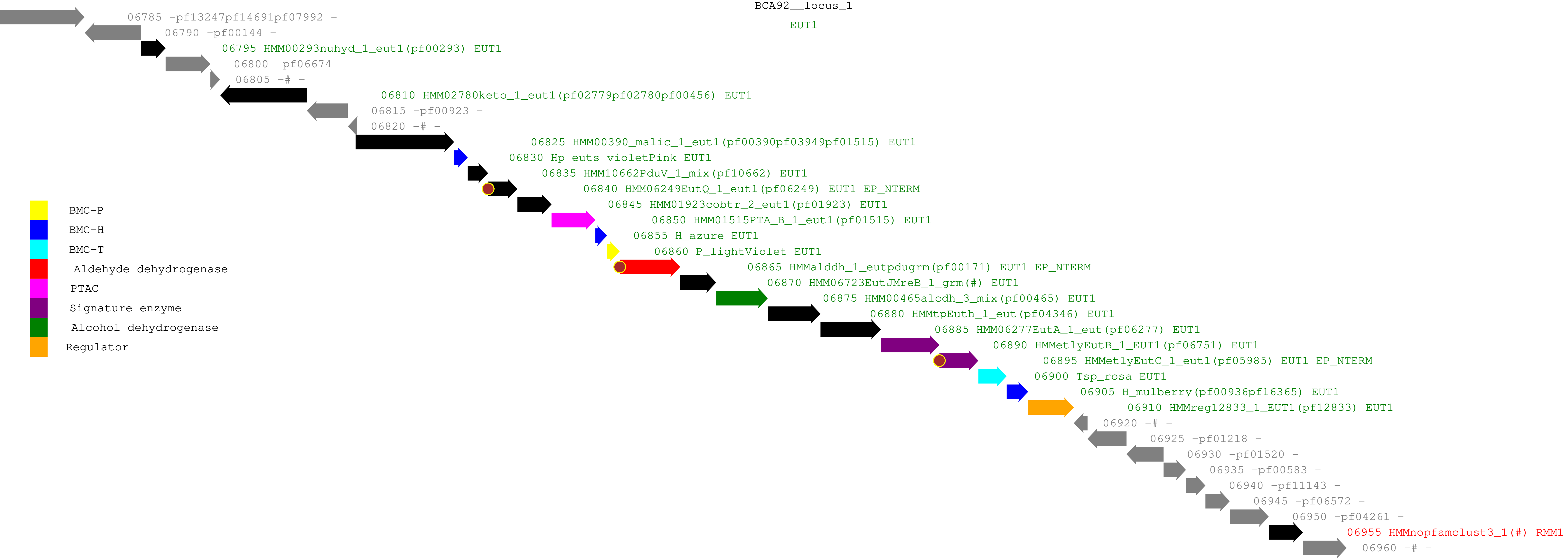

Klebsiella pneumoniae. Bacteria; Proteobacteria; Gammaproteobacteria; Enterobacterales;

BCB67__locus_2

EUT1

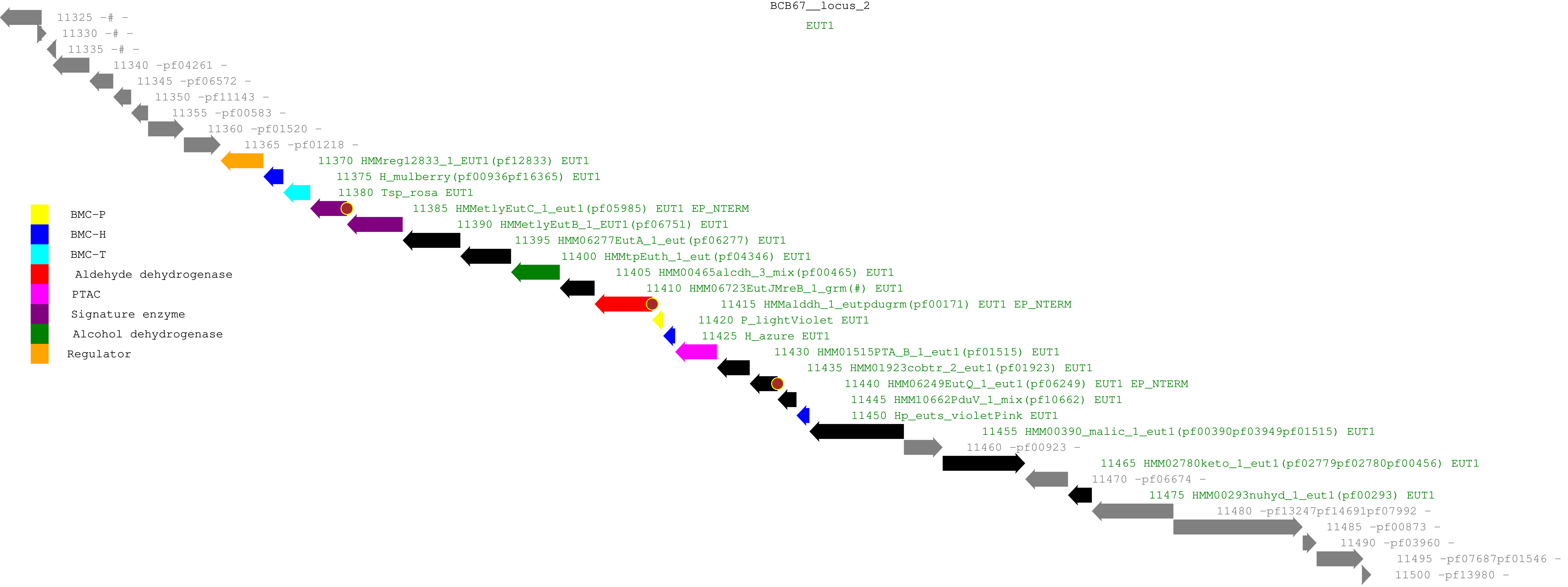

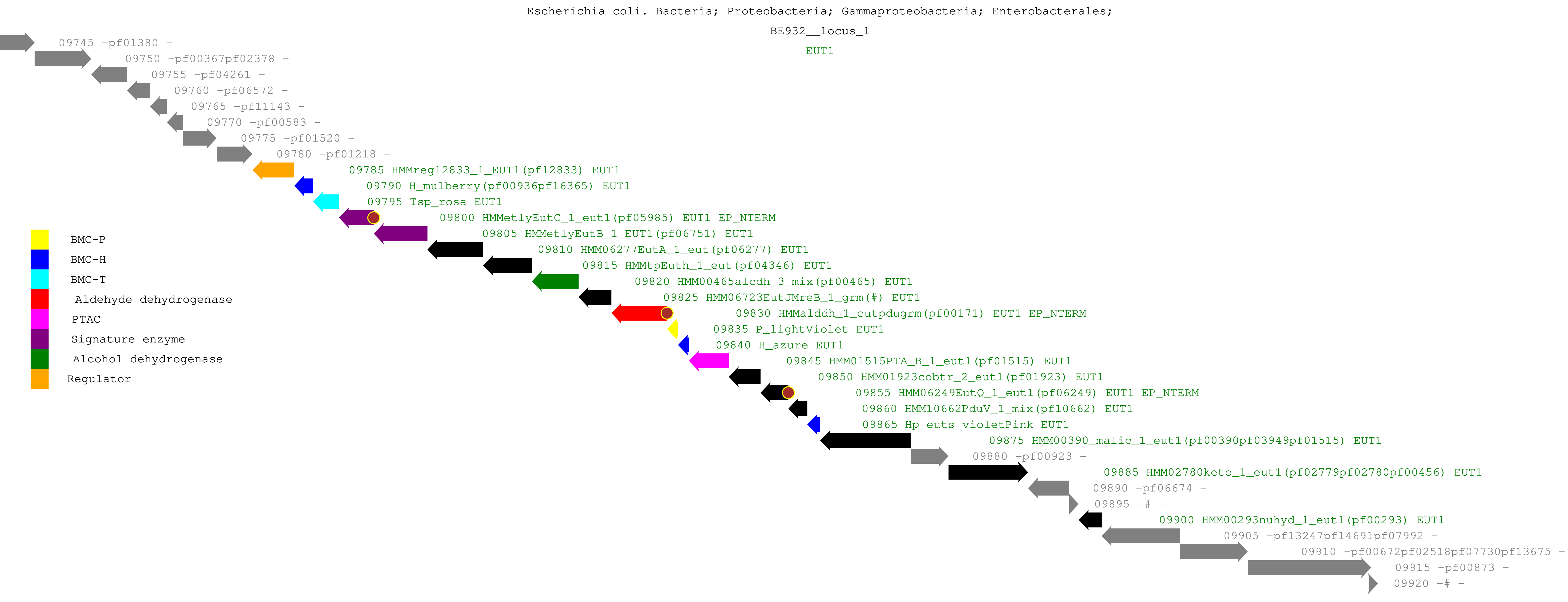

Escherichia coli. Bacteria; Proteobacteria; Gammaproteobacteria; Enterobacterales;
BE932__locus_1
EUT1
09745 -pf01380 -
09750 -pf00367pf02378 -
09755 -pf04261 -
09760 -pf06572 -
09765 -pf11143 -
09770 -pf00583 -
09775 -pf01520 -
09780 -pf01218 -
09785 HMMreg12833_1_EUT1(pf12833) EUT1
09790 H_mulberry(pf00936pf16365) EUT1
09795 Tsp_rosa EUT1
09800 HMMetlyEutC_1_eut1(pf05985) EUT1 EP_NTERM
09805 HMMetlyEutB_1_EUT1(pf06751) EUT1
09810 HMM06277EutA_1_eut(pf06277) EUT1
09815 HMMtpEuth_1_eut(pf04346) EUT1
09820 HMM00465alcdh_3_mix(pf00465) EUT1
09825 HMM06723EutJMreB_1_grm(#) EUT1
09830 HMMalddh_1_eutpdugrm(pf00171) EUT1 EP_NTERM
09835 P_lightViolet EUT1
09840 H_azure EUT1
09845 HMM01515PTA_B_1_eut1(pf01515) EUT1
09850 HMM01923cobtr_2_eut1(pf01923) EUT1
09855 HMM06249EutQ_1_eut1(pf06249) EUT1 EP_NTERM
09860 HMM10662PduV_1_mix(pf10662) EUT1
09865 Hp_euts_violetPink EUT1
09875 HMM00390_malic_1_eut1(pf00390pf03949pf01515) EUT1
09880 -pf00923 -
09885 HMM02780keto_1_eut1(pf02779pf02780pf00456) EUT1
09890 -pf06674 -
09895 -# -
09900 HMM00293nuhyd_1_eut1(pf00293) EUT1
09905 -pf13247pf14691pf07992 -
09910 -pf00672pf02518pf07730pf13675 -
09915 -pf00873 -
09920 -# -
BMC-P
BMC-H
BMC-T
Aldehyde dehydrogenase
PTAC
Signature enzyme
Alcohol dehydrogenase
Regulator

Escherichia coli. Bacteria; Proteobacteria; Gammaproteobacteria; Enterobacterales;

BE963__locus_1

EUT1

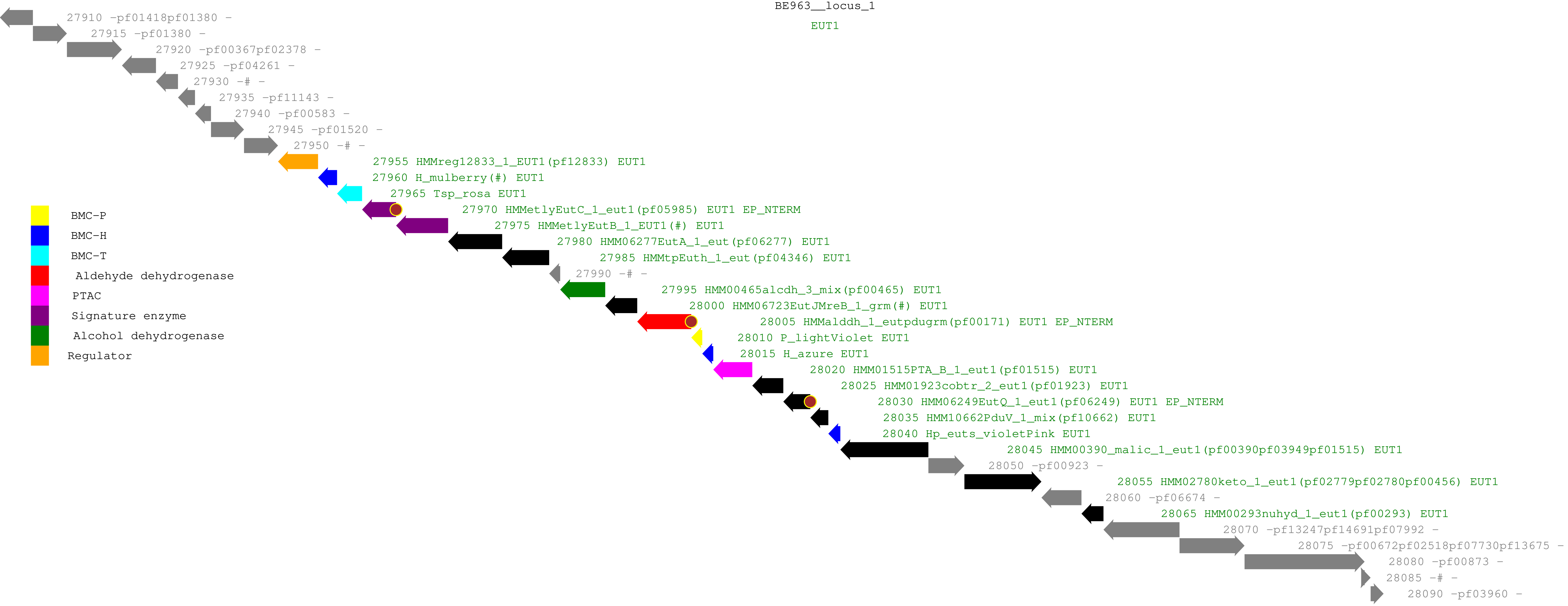

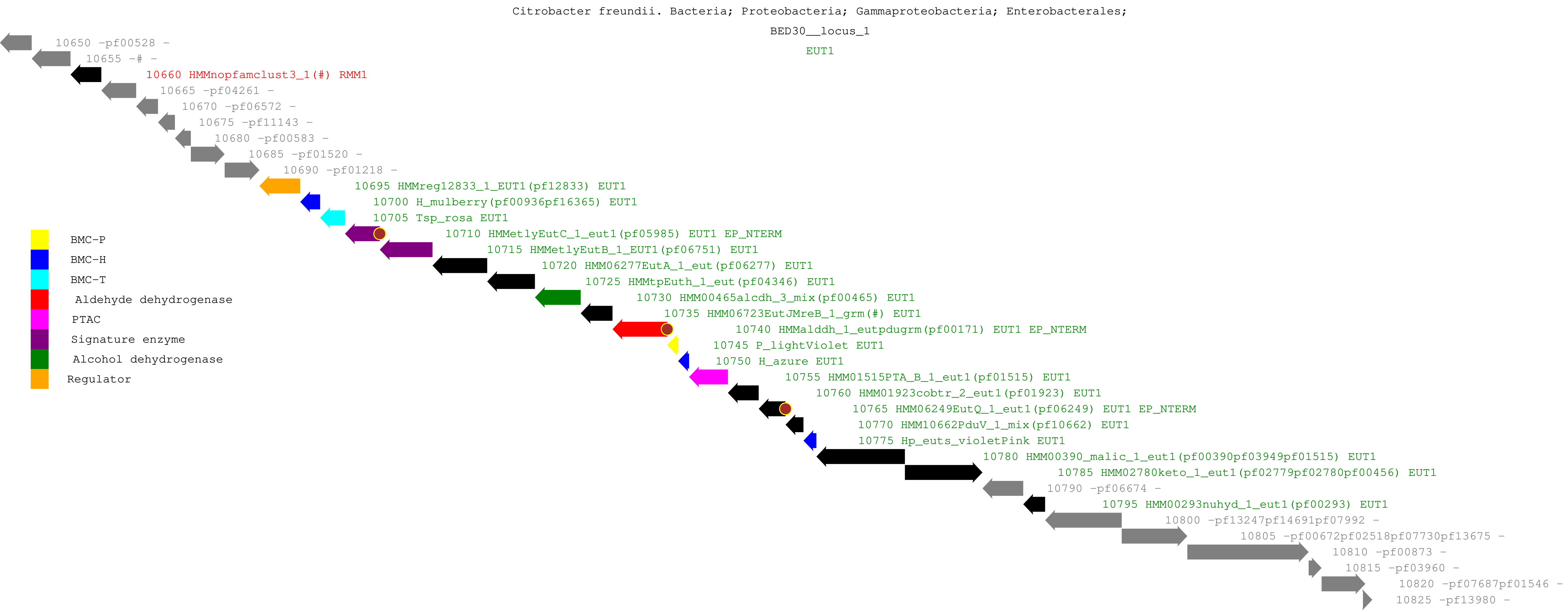

Citrobacter freundii. Bacteria; Proteobacteria; Gammaproteobacteria; Enterobacterales;
BED30__locus_1
EUT1
10650 -pf00528 -
10655 -# -
10660 HMMnopfamclust3_1(#) RMM1
10665 -pf04261 -
10670 -pf06572 -
10675 -pf11143 -
10680 -pf00583 -
10685 -pf01520 -
10690 -pf01218 -
10695 HMMreg12833_1_EUT1(pf12833) EUT1
10700 H_mulberry(pf00936pf16365) EUT1
10705 Tsp_rosa EUT1
10710 HMMetlyEutC_1_eut1(pf05985) EUT1 EP_NTERM
10715 HMMetlyEutB_1_EUT1(pf06751) EUT1
10720 HMM06277EutA_1_eut(pf06277) EUT1
10725 HMMtpEuth_1_eut(pf04346) EUT1
10730 HMM00465alcdh_3_mix(pf00465) EUT1
10735 HMM06723EutJMreB_1_grm(#) EUT1
10740 HMMaldh_1_eutpdugrm(pf00171) EUT1 EP_NTERM
10745 P_lightViolet EUT1
10750 H_azure EUT1
10755 HMM01515PTA_B_1_eut1(pf01515) EUT1
10760 HMM01923cobtr_2_eut1(pf01923) EUT1
10765 HMM06249EutQ_1_eut1(pf06249) EUT1 EP_NTERM
10770 HMM10662PduV_1_mix(pf10662) EUT1
10775 Hp_euts_violetPink EUT1
10780 HMM00390_malic_1_eut1(pf00390pf03949pf01515) EUT1
10785 HMM02780keto_1_eut1(pf02779pf02780pf00456) EUT1
10790 -pf06674 -
10795 HMM00293nuhyd_1_eut1(pf00293) EUT1
10800 -pf13247pf14691pf07992 -
10805 -pf00672pf02518pf07730pf13675 -
10810 -pf00873 -
10815 -pf03960 -
10820 -pf07687pf01546 -
10825 -pf13980 -
BMC-P
BMC-H
BMC-T
Aldehyde dehydrogenase
PTAC
Signature enzyme
Alcohol dehydrogenase
Regulator

Citrobacter freundii. Bacteria; Proteobacteria; Gammaproteobacteria; Enterobacterales;

BED45__locus_2

EUT1

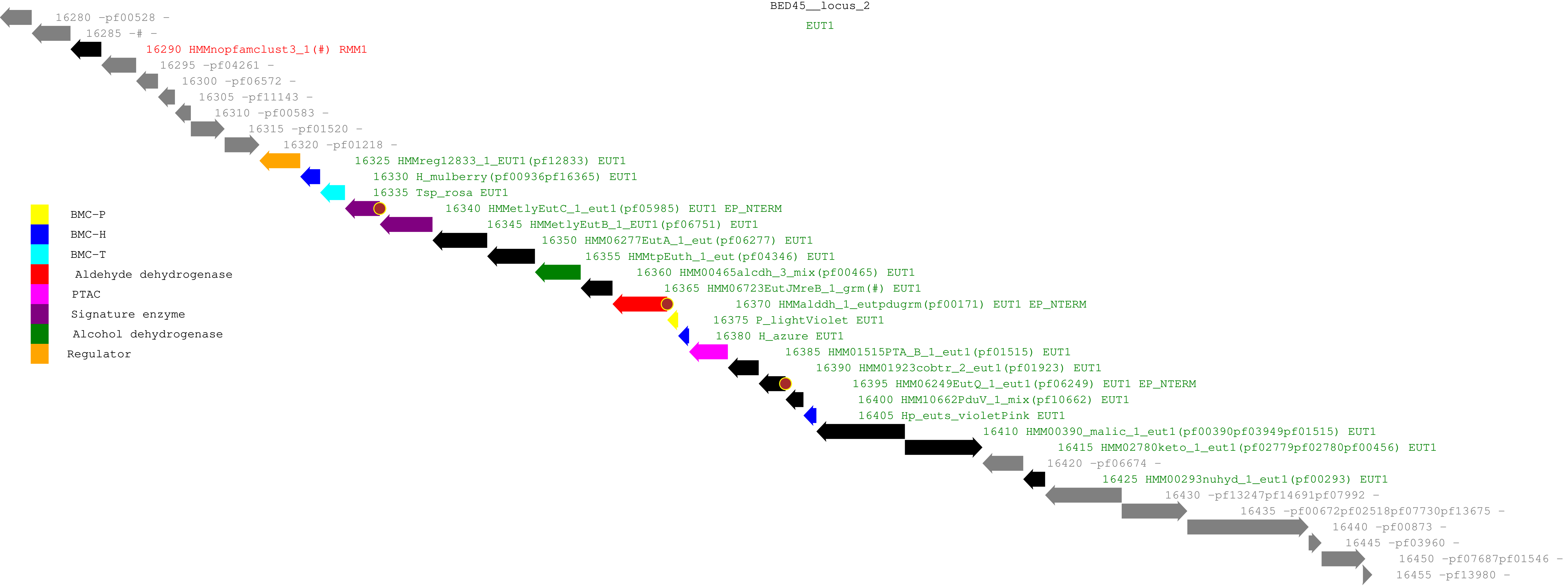

Salmonella enterica subsp. enterica serovar Grumpensis. Bacteria; Proteobacteria; Gammaproteobacteria; Enterobacterales;

BEL51__locus_1

EUT1

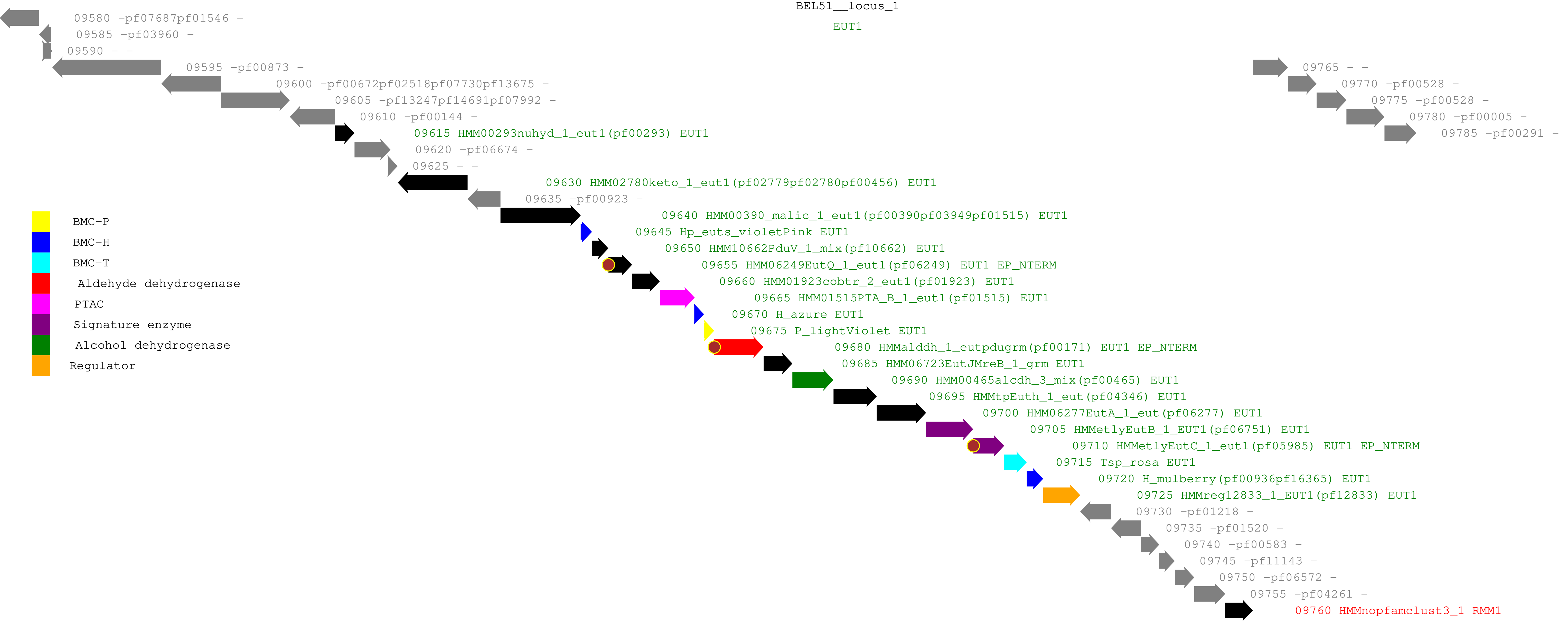

Escherichia sp. MOD1-EC5451. Bacteria; Proteobacteria; Gammaproteobacteria; Enterobacterales;

BEM40__locus_1

EUT1

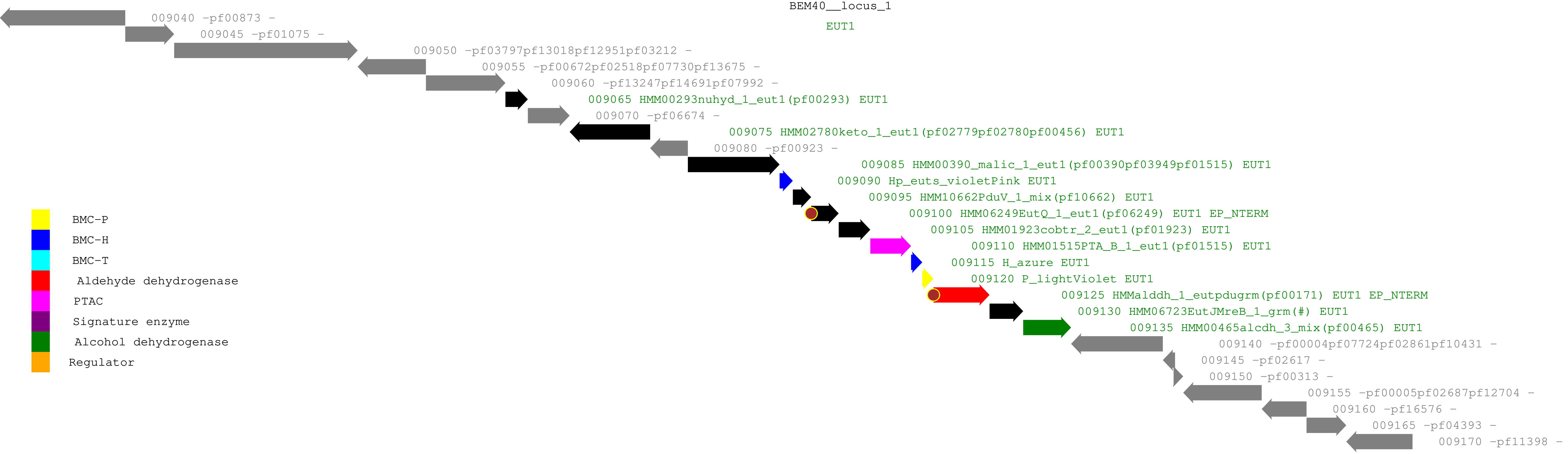

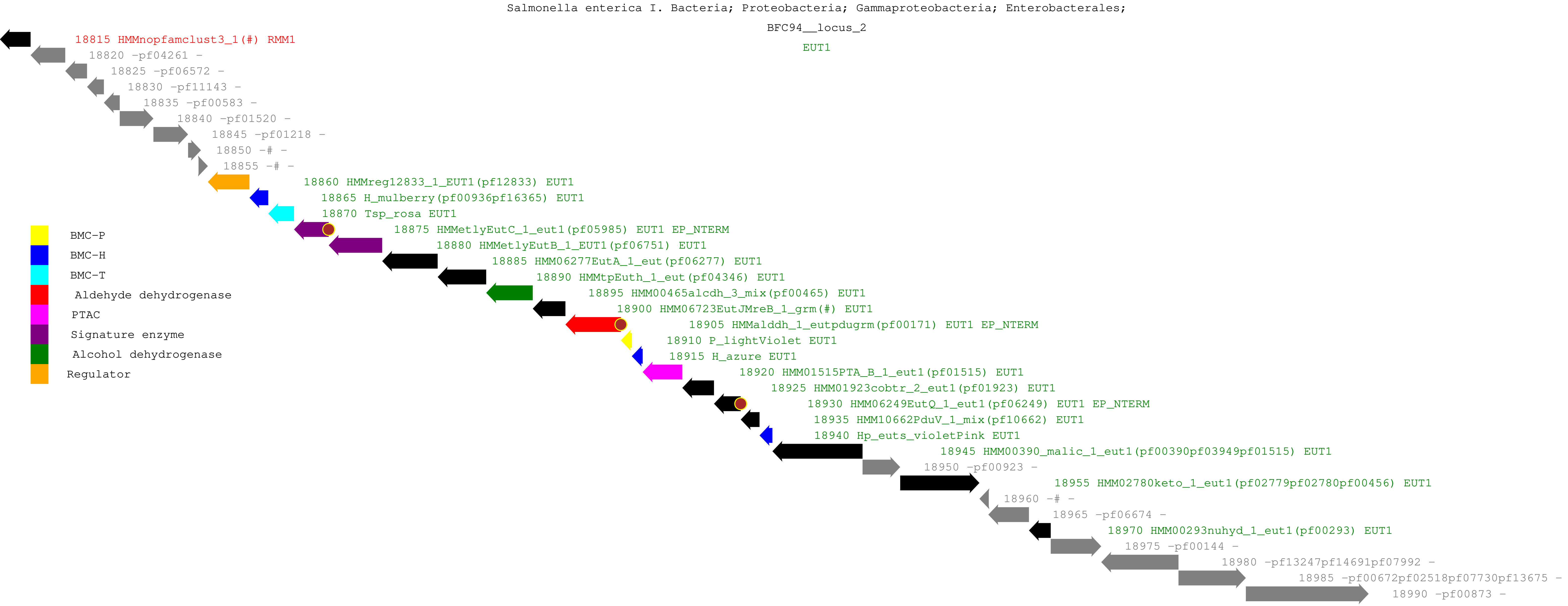
Salmonella enterica I. Bacteria; Proteobacteria; Gammaproteobacteria; Enterobacterales;
BFC94__locus_2
EUT1
18815 HMMnopfamclust3_1(#) RMM1
18820 -pf04261 -
18825 -pf06572 -
18830 -pf11143 -
18835 -pf00583 -
18840 -pf01520 -
18845 -pf01218 -
18850 -# -
18855 -# -
18860 HMMreg12833_1_EUT1(pf12833) EUT1
18865 H_mulberry(pf00936pf16365) EUT1
18870 Tsp_rosa EUT1
18875 HMMetlyEutC_1_eut1(pf05985) EUT1 EP_NTERM
18880 HMMetlyEutB_1_EUT1(pf06751) EUT1
18885 HMM06277EutA_1_eut(pf06277) EUT1
18890 HMMtpEuth_1_eut(pf04346) EUT1
18895 HMM00465alcdh_3_mix(pf00465) EUT1
18900 HMM06723EutJMreB_1_grm(#) EUT1
18905 HMMalddh_1_eutpdugrm(pf00171) EUT1 EP_NTERM
18910 P_lightViolet EUT1
18915 H_azure EUT1
18920 HMM01515PTA_B_1_eut1(pf01515) EUT1
18925 HMM01923cobtr_2_eut1(pf01923) EUT1
18930 HMM06249EutQ_1_eut1(pf06249) EUT1 EP_NTERM
18935 HMM10662PduV_1_mix(pf10662) EUT1
18940 Hp_euts_violetPink EUT1
18945 HMM00390_malic_1_eut1(pf00390pf03949pf01515) EUT1
18950 -pf00923 -
18955 HMM02780keto_1_eut1(pf02779pf02780pf00456) EUT1
18960 -# -
18965 -pf06674 -
18970 HMM00293nuhyd_1_eut1(pf00293) EUT1
18975 -pf00144 -
18980 -pf13247pf14691pf07992 -
18985 -pf00672pf02518pf07730pf13675 -
18990 -pf00873 -
BMC-P
BMC-H
BMC-T
Aldehyde dehydrogenase
PTAC
Signature enzyme
Alcohol dehydrogenase
Regulator

Shigella sp. FC2928. Bacteria; Proteobacteria; Gammaproteobacteria; Enterobacterales;

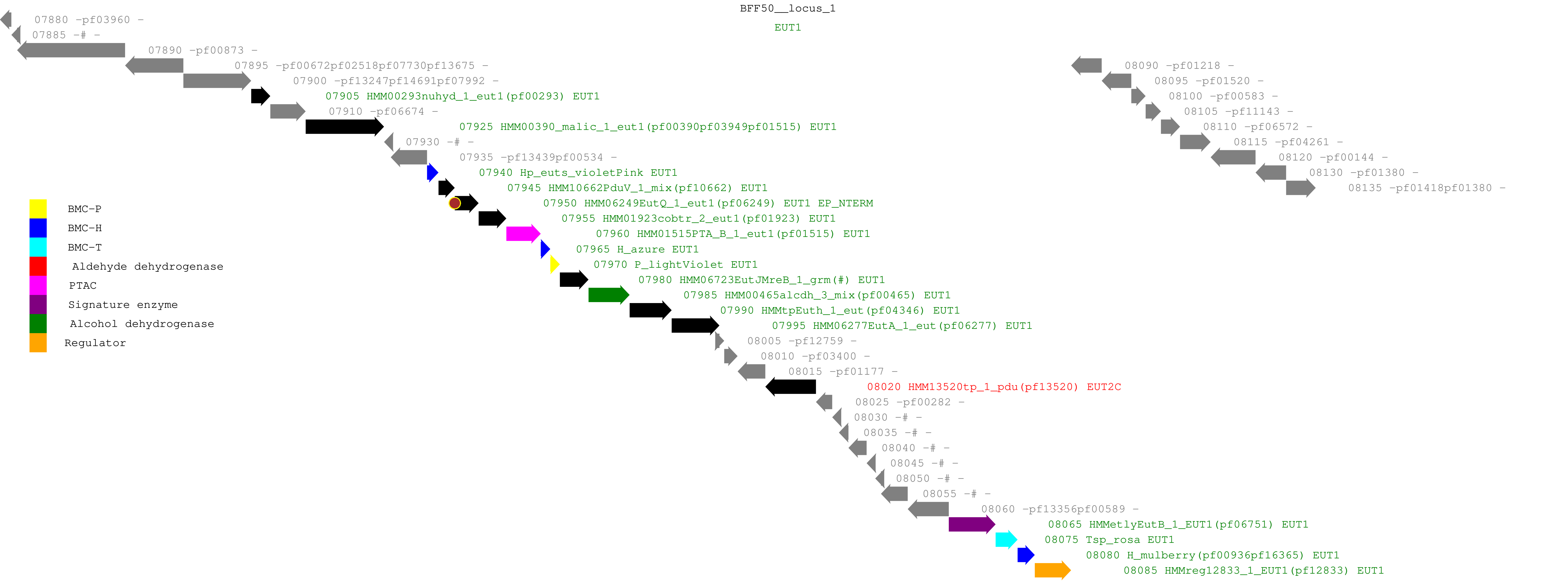

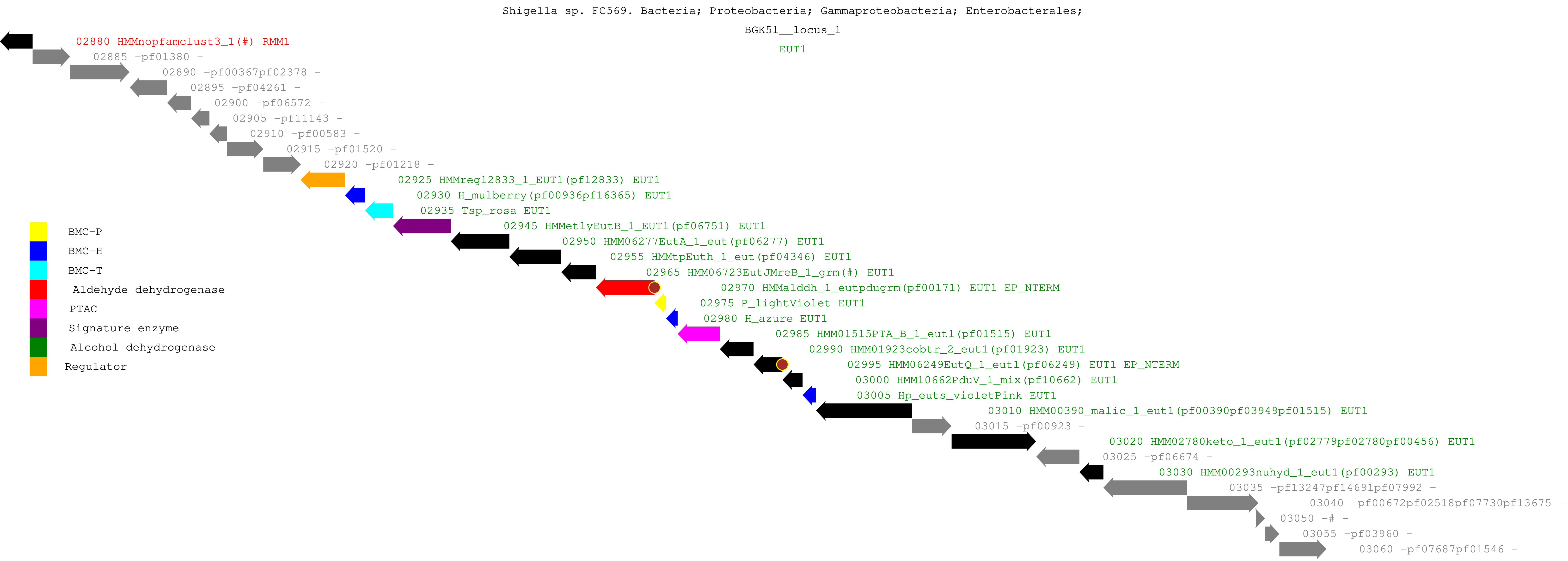
Shigella sp. FC569. Bacteria; Proteobacteria; Gammaproteobacteria; Enterobacterales;
BGK51__locus_1
EUT1
02880 HMMnopfamclust3_1(#) RMM1
02885 -pf01380 -
02890 -pf00367pf02378 -
02895 -pf04261 -
02900 -pf06572 -
02905 -pf11143 -
02910 -pf00583 -
02915 -pf01520 -
02920 -pf01218 -
02925 HMMreg12833_1_EUT1(pf12833) EUT1
02930 H_mulberry(pf00936pf16365) EUT1
02935 Tsp_rosa EUT1
02945 HMMetlyEutB_1_EUT1(pf06751) EUT1
02950 HMM06277EutA_1_eut(pf06277) EUT1
02955 HMMtpEuth_1_eut(pf04346) EUT1
02965 HMM06723EutJMreB_1_grm(#) EUT1
02970 HMMalddh_1_eutpdugrm(pf00171) EUT1 EP_NTERM
02975 P_lightViolet EUT1
02980 H_azure EUT1
02985 HMM01515PTA_B_1_eut1(pf01515) EUT1
02990 HMM01923cobtr_2_eut1(pf01923) EUT1
02995 HMM06249EutQ_1_eut1(pf06249) EUT1 EP_NTERM
03000 HMM10662PduV_1_mix(pf10662) EUT1
03005 Hp_euts_violetPink EUT1
03010 HMM00390_malic_1_eut1(pf00390pf03949pf01515) EUT1
03015 -pf00923 -
03020 HMM02780keto_1_eut1(pf02779pf02780pf00456) EUT1
03025 -pf06674 -
03030 HMM00293nuhyd_1_eut1(pf00293) EUT1
03035 -pf13247pf14691pf07992 -
03040 -pf00672pf02518pf07730pf13675 -
03050 -# -
03055 -pf03960 -
03060 -pf07687pf01546 -
BMC-P
BMC-H
BMC-T
Aldehyde dehydrogenase
PTAC
Signature enzyme
Alcohol dehydrogenase
Regulator

Salmonella enterica (Salmonella choleraesuis). Bacteria; Proteobacteria; Gammaproteobacteria; Enterobacterales;

BH006__locus_1

EUT1

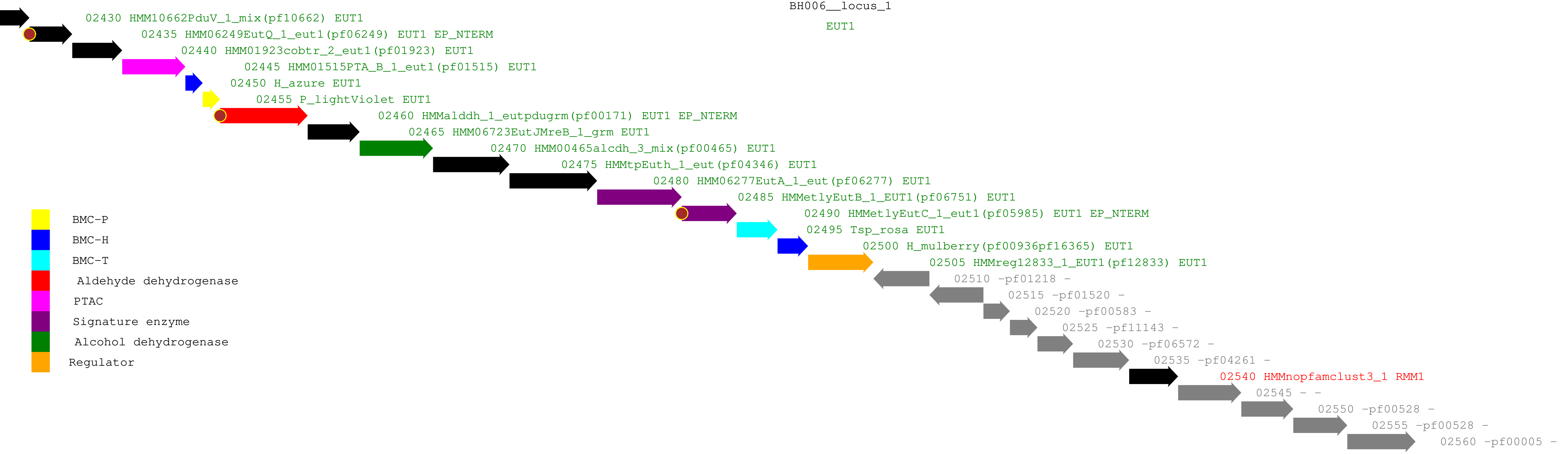

Escherichia coli. Bacteria; Proteobacteria; Gammaproteobacteria; Enterobacterales;

BIQ87__locus_1

EUT1

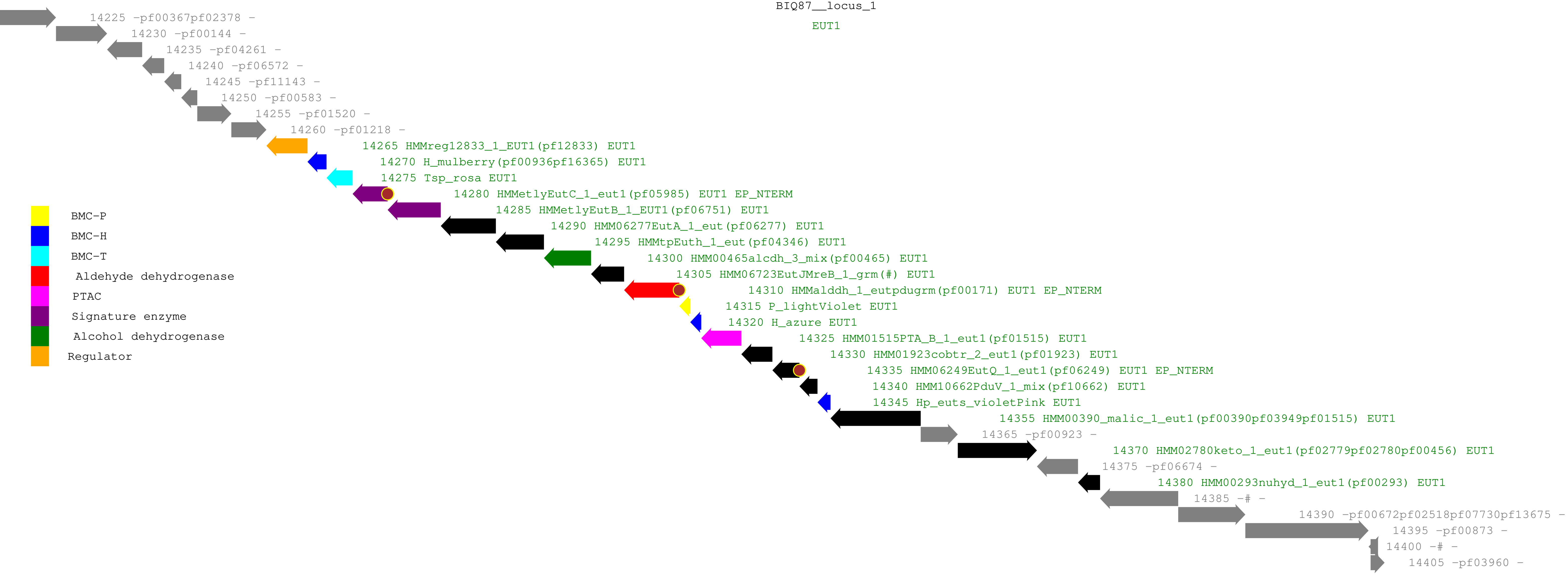

Klebsiella sp. LTGPAF-6F. Bacteria; Proteobacteria; Gammaproteobacteria; Enterobacterales;

BJF97__locus_2

EUT1

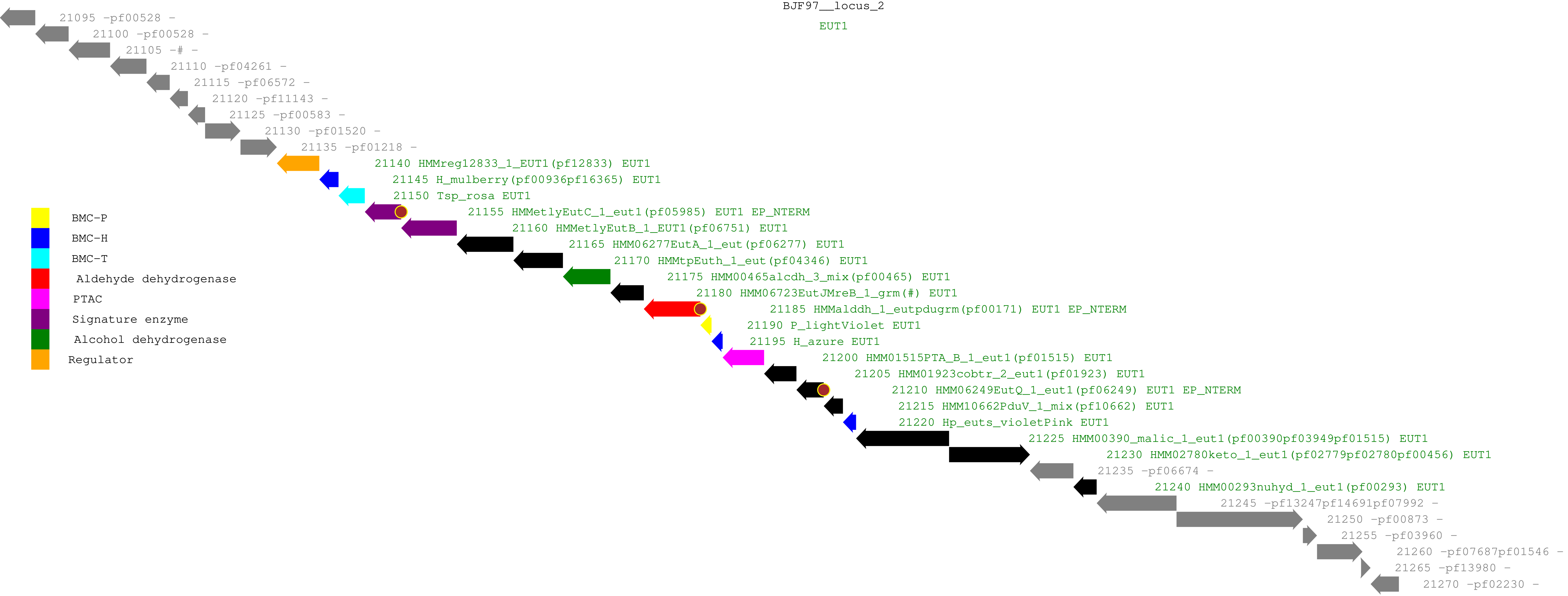

Escherichia coli. Bacteria; Proteobacteria; Gammaproteobacteria; Enterobacterales;

BK373__locus_1

EUT1

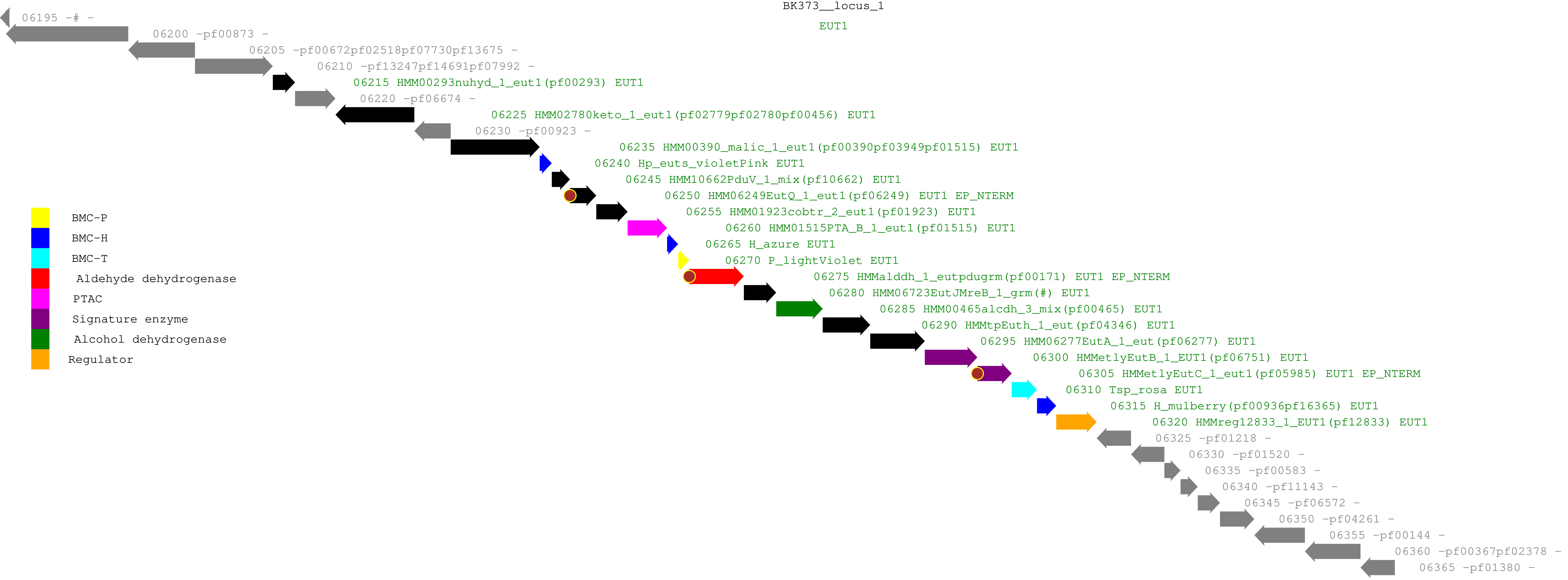

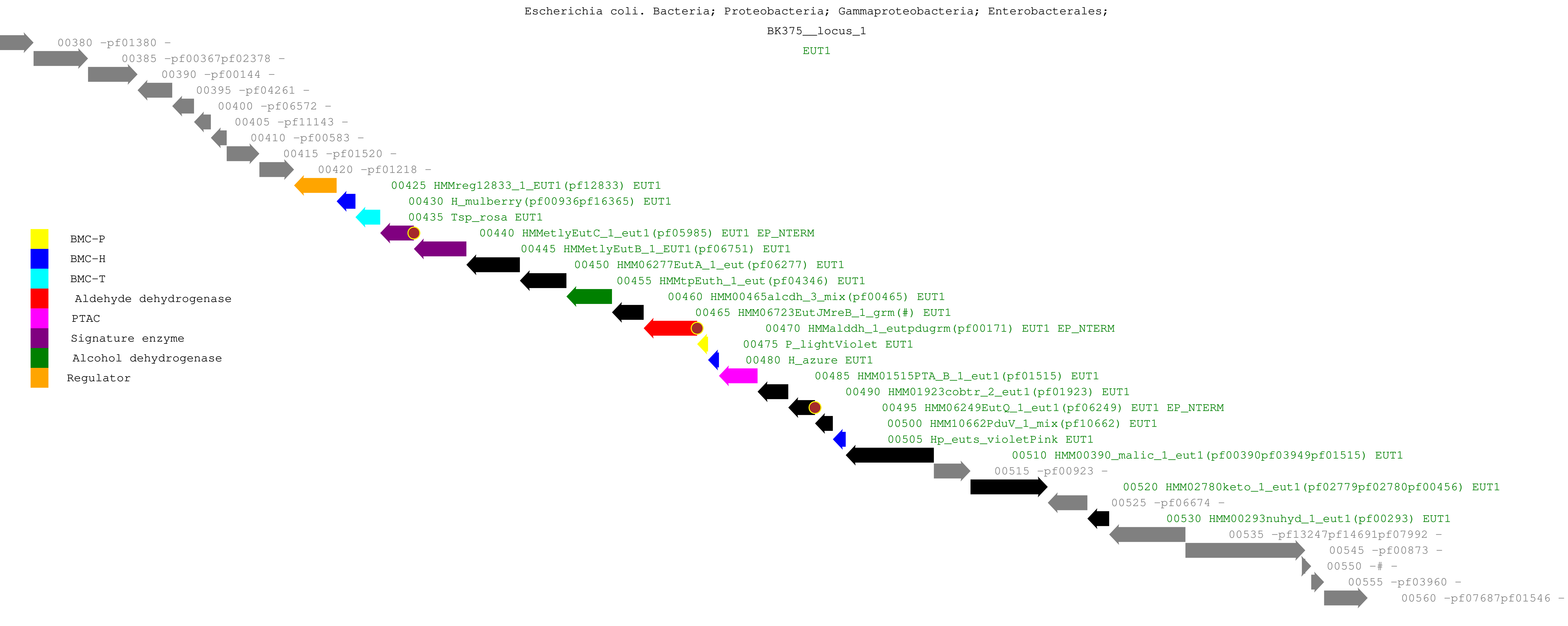

Escherichia coli. Bacteria; Proteobacteria; Gammaproteobacteria; Enterobacterales;
BK375__locus_1
EUT1
00380 -pf01380 -
00385 -pf00367pf02378 -
00390 -pf00144 -
00395 -pf04261 -
00400 -pf06572 -
00405 -pf11143 -
00410 -pf00583 -
00415 -pf01520 -
00420 -pf01218 -
00425 HMMreg12833_1_EUT1(pf12833) EUT1
00430 H_mulberry(pf00936pf16365) EUT1
00435 Tsp_rosa EUT1
00440 HMMetlyEutC_1_eut1(pf05985) EUT1 EP_NTERM
00445 HMMetlyEutB_1_EUT1(pf06751) EUT1
00450 HMM06277EutA_1_eut(pf06277) EUT1
00455 HMMtpEuth_1_eut(pf04346) EUT1
00460 HMM00465alcdh_3_mix(pf00465) EUT1
00465 HMM06723EutJMreB_1_grm(#) EUT1
00470 HMMalddh_1_eutpdugrm(pf00171) EUT1 EP_NTERM
00475 P_lightViolet EUT1
00480 H_azure EUT1
00485 HMM01515PTA_B_1_eut1(pf01515) EUT1
00490 HMM01923cobtr_2_eut1(pf01923) EUT1
00495 HMM06249EutQ_1_eut1(pf06249) EUT1 EP_NTERM
00500 HMM10662PduV_1_mix(pf10662) EUT1
00505 Hp_euts_violetPink EUT1
00510 HMM00390_malic_1_eut1(pf00390pf03949pf01515) EUT1
00515 -pf00923 -
00520 HMM02780keto_1_eut1(pf02779pf02780pf00456) EUT1
00525 -pf06674 -
00530 HMM00293nuhyd_1_eut1(pf00293) EUT1
00535 -pf13247pf14691pf07992 -
00545 -pf00873 -
00550 -# -
00555 -pf03960 -
00560 -pf07687pf01546 -
BMC-P
BMC-H
BMC-T
Aldehyde dehydrogenase
PTAC
Signature enzyme
Alcohol dehydrogenase
Regulator

Escherichia coli. Bacteria; Proteobacteria; Gammaproteobacteria; Enterobacterales;

BK383__locus_1

EUT1

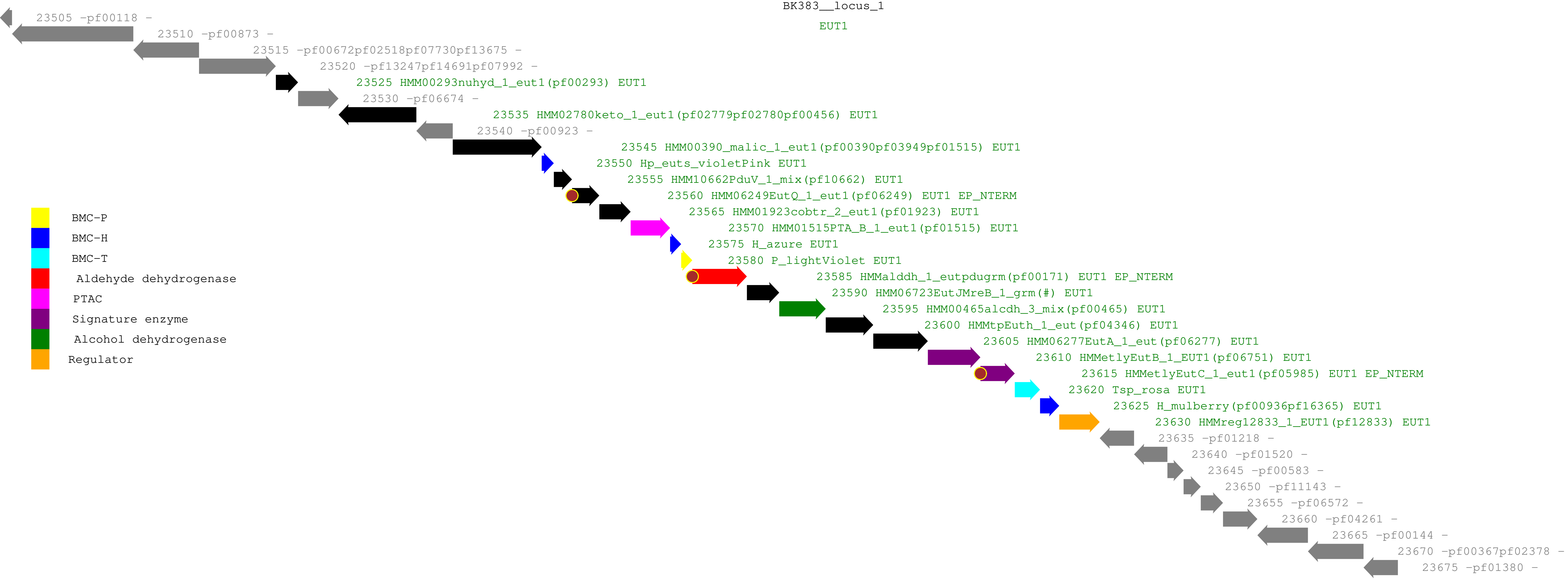

Escherichia coli. Bacteria; Proteobacteria; Gammaproteobacteria; Enterobacterales;

BK400__locus_1

EUT1

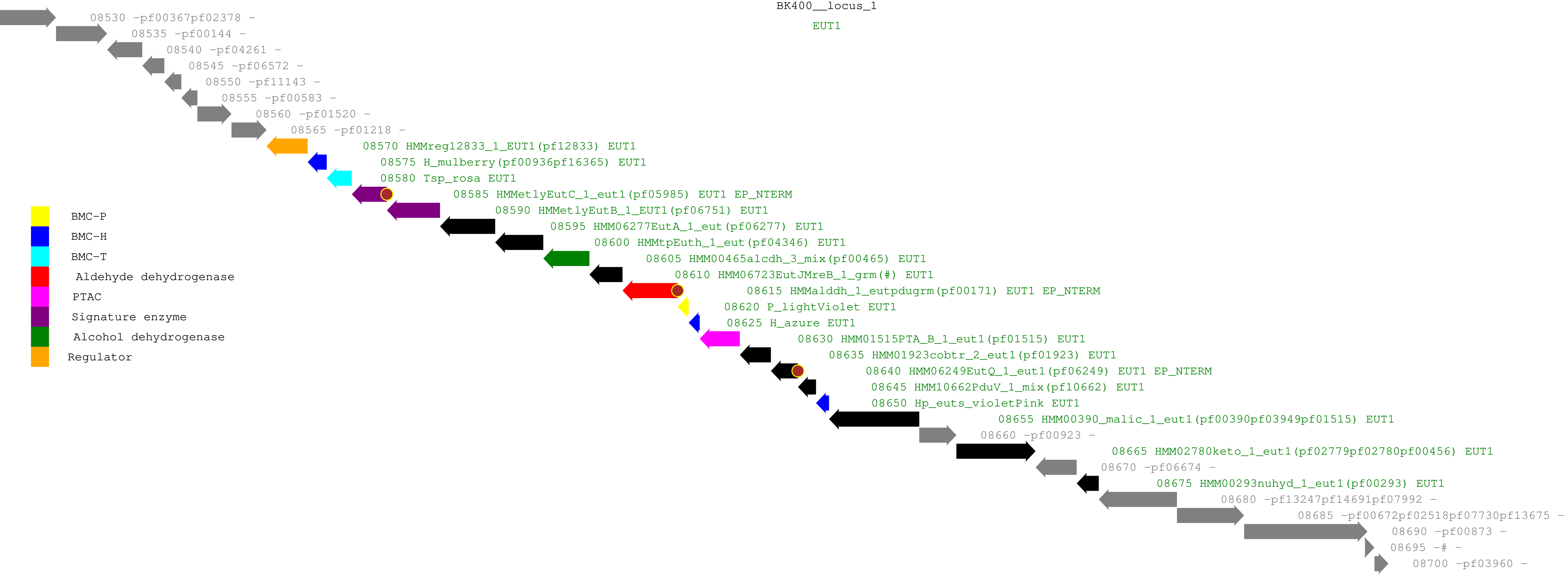

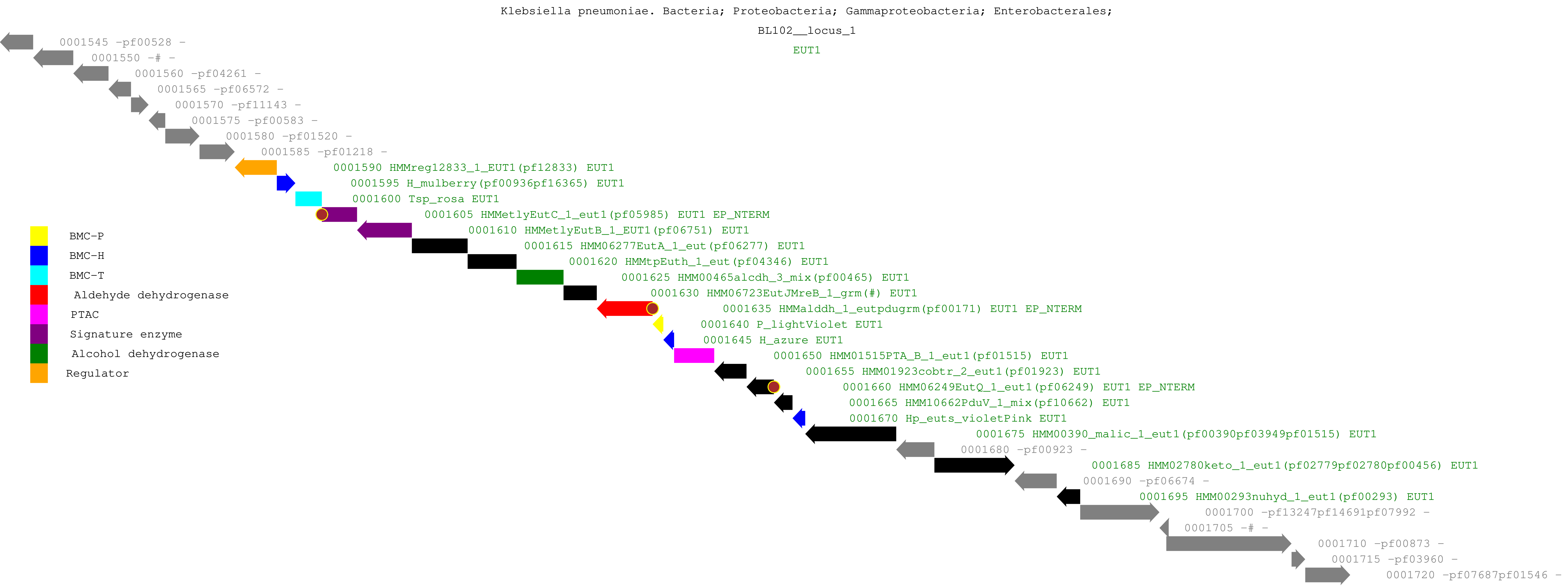

Klebsiella pneumoniae. Bacteria; Proteobacteria; Gammaproteobacteria; Enterobacterales;
BL102__locus_1
EUT1
0001545 -pf00528 -
0001550 -# -
0001560 -pf04261 -
0001565 -pf06572 -
0001570 -pf11143 -
0001575 -pf00583 -
0001580 -pf01520 -
0001585 -pf01218 -
0001590 HMMreg12833_1_EUT1(pf12833) EUT1
0001595 H_mulberry(pf00936pf16365) EUT1
0001600 Tsp_rosa EUT1
0001605 HMMetlyEutC_1_eut1(pf05985) EUT1 EP_NTERM
0001610 HMMetlyEutB_1_EUT1(pf06751) EUT1
0001615 HMM06277EutA_1_eut(pf06277) EUT1
0001620 HMMtpEuth_1_eut(pf04346) EUT1
0001625 HMM00465alcdh_3_mix(pf00465) EUT1
0001630 HMM06723EutJMreB_1_grm(#) EUT1
0001635 HMMaldдh_1_eutpdugrm(pf00171) EUT1 EP_NTERM
0001640 P_lightViolet EUT1
0001645 H_azure EUT1
0001650 HMM01515PTA_B_1_eut1(pf01515) EUT1
0001655 HMM01923cobtr_2_eut1(pf01923) EUT1
0001660 HMM06249EutQ_1_eut1(pf06249) EUT1 EP_NTERM
0001665 HMM10662PduV_1_mix(pf10662) EUT1
0001670 Hp_euts_violetPink EUT1
0001675 HMM00390_malic_1_eut1(pf00390pf03949pf01515) EUT1
0001680 -pf00923 -
0001685 HMM02780keto_1_eut1(pf02779pf02780pf00456) EUT1
0001690 -pf06674 -
0001695 HMM00293nuhyd_1_eut1(pf00293) EUT1
0001700 -pf13247pf14691pf07992 -
0001705 -# -
0001710 -pf00873 -
0001715 -pf03960 -
0001720 -pf07687pf01546 -
BMC-P
BMC-H
BMC-T
Aldehyde dehydrogenase
PTAC
Signature enzyme
Alcohol dehydrogenase
Regulator

Klebsiella sp. PO552. Bacteria; Proteobacteria; Gammaproteobacteria; Enterobacterales;

BMD99__locus_2

EUT1

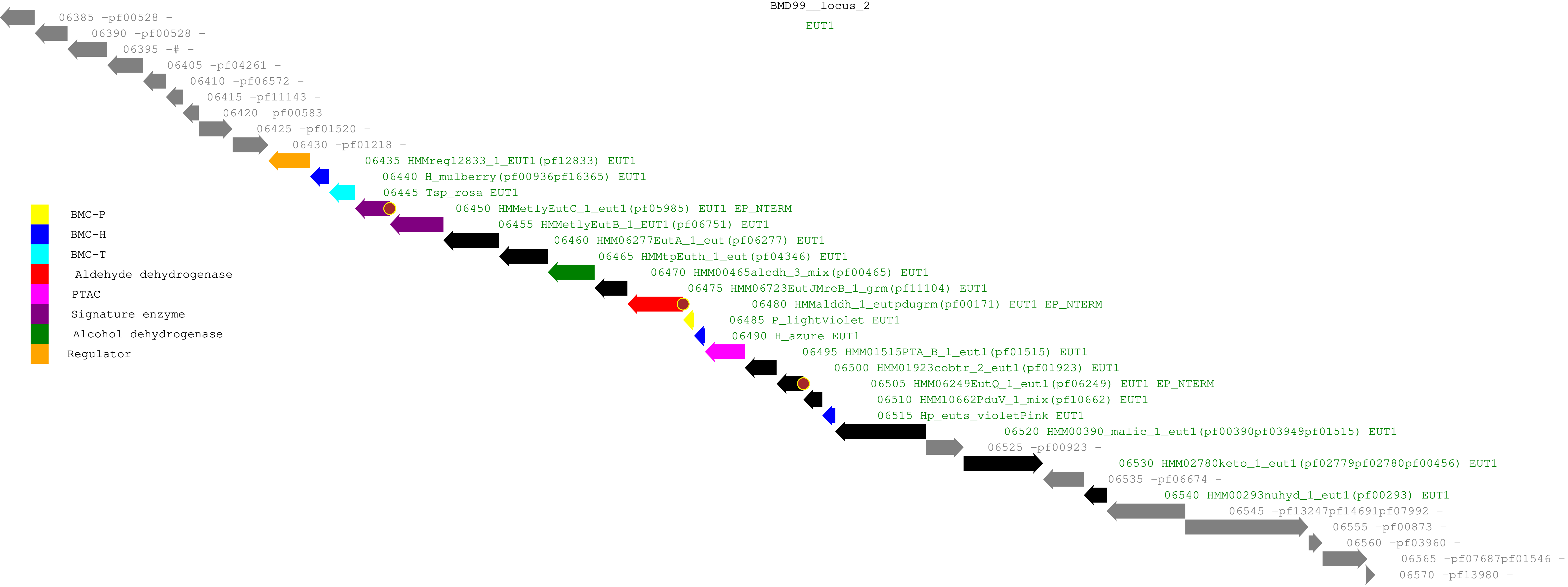

Hafnia alvei. Bacteria; Proteobacteria; Gammaproteobacteria; Enterobacterales;

BN1044__locus_1

EUT1

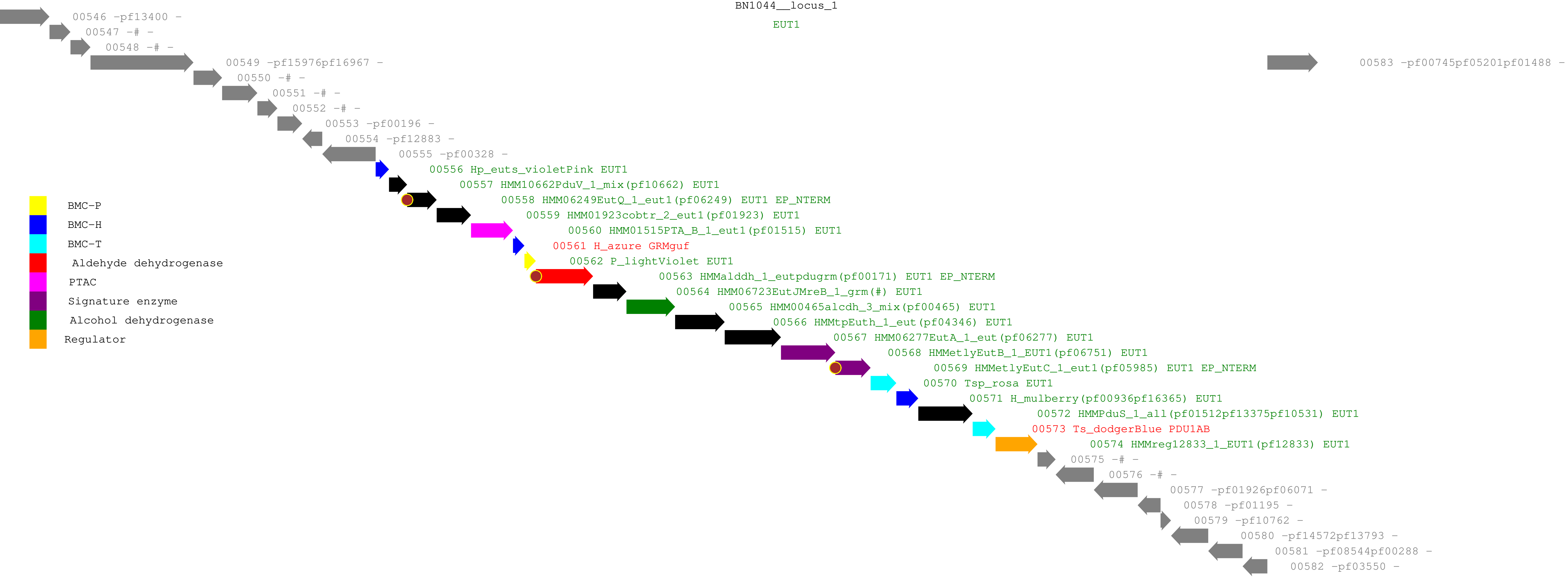

Citrobacter koseri (Citrobacter diversus). Bacteria; Proteobacteria; Gammaproteobacteria; Enterobacterales;

BN1086__locus_1

EUT1

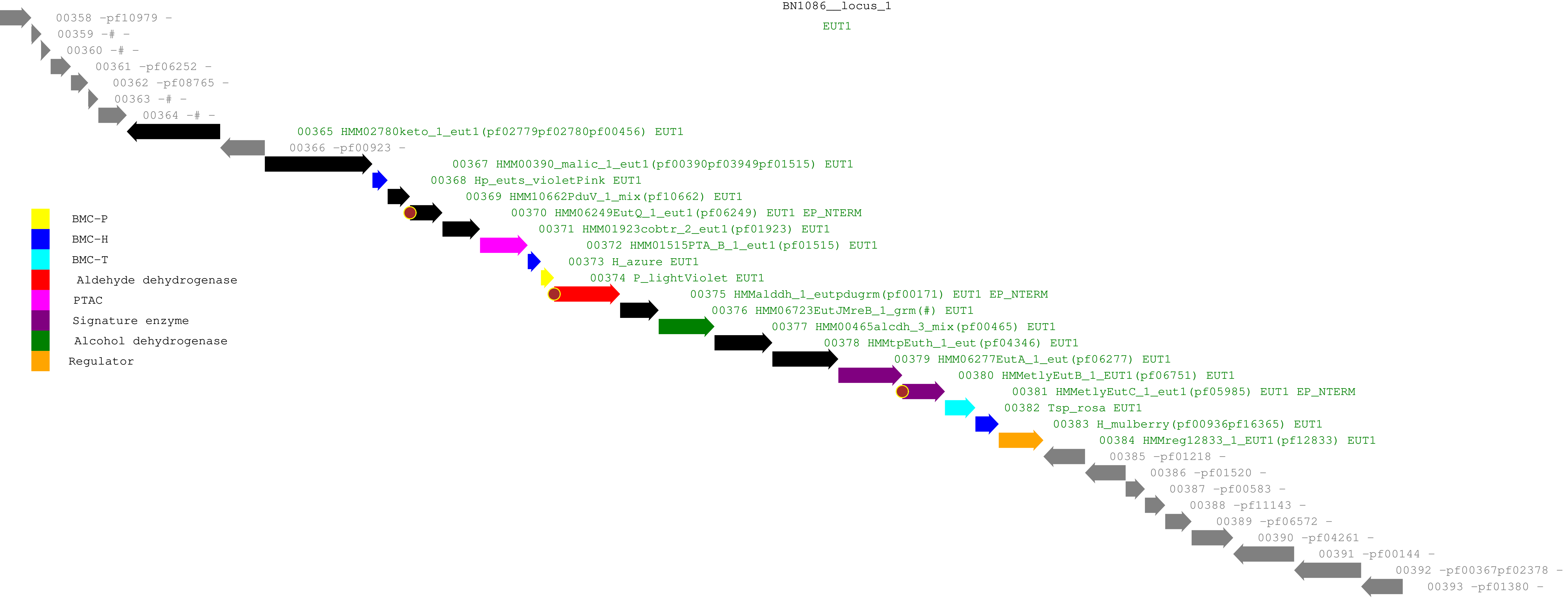

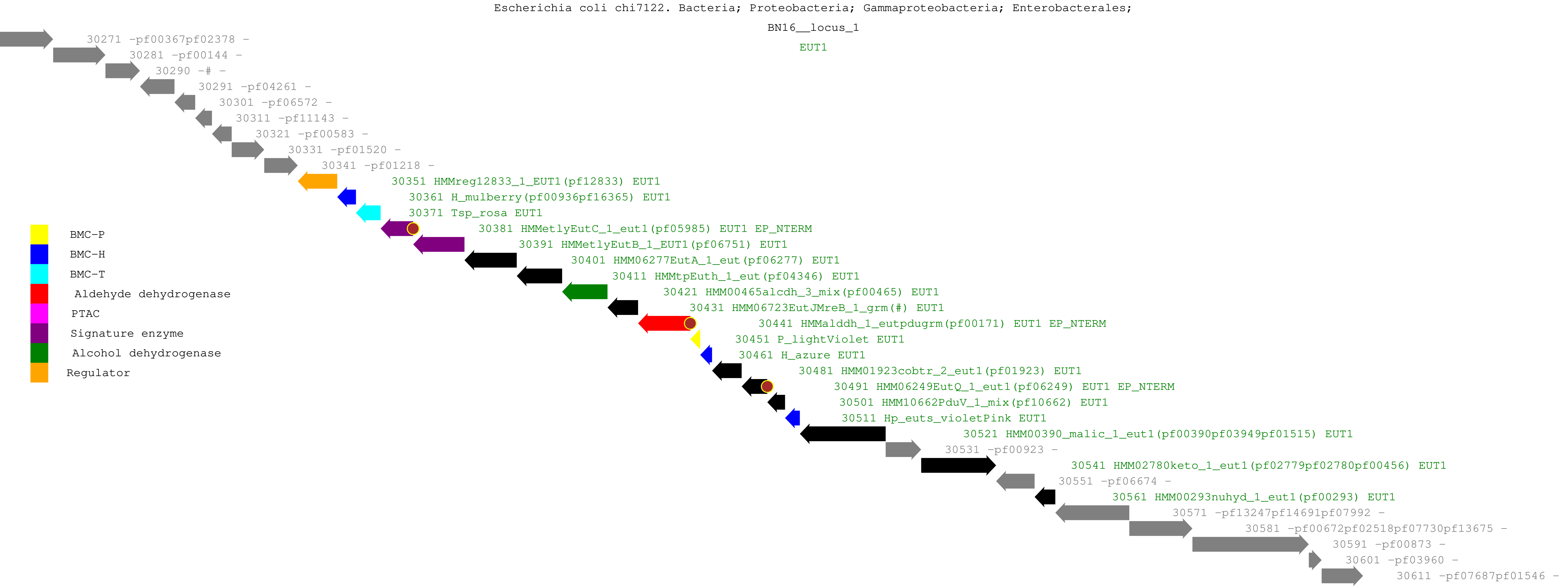

Escherichia coli chi7122. Bacteria; Proteobacteria; Gammaproteobacteria; Enterobacterales;
BN16__locus_1
EUT1
30271 -pf00367pf02378 -
30281 -pf00144 -
30290 -# -
30291 -pf04261 -
30301 -pf06572 -
30311 -pf11143 -
30321 -pf00583 -
30331 -pf01520 -
30341 -pf01218 -
30351 HMMreg12833_1_EUT1(pf12833) EUT1
30361 H_mulberry(pf00936pf16365) EUT1
30371 Tsp_rosa EUT1
30381 HMMetlyEutC_1_eut1(pf05985) EUT1 EP_NTERM
30391 HMMetlyEutB_1_EUT1(pf06751) EUT1
30401 HMM06277EutA_1_eut(pf06277) EUT1
30411 HMMtpEuth_1_eut(pf04346) EUT1
30421 HMM00465alcdh_3_mix(pf00465) EUT1
30431 HMM06723EutJMreB_1_grm(#) EUT1
30441 HMMalddh_1_eutpdugrm(pf00171) EUT1 EP_NTERM
30451 P_lightViolet EUT1
30461 H_azure EUT1
30481 HMM01923cobtr_2_eut1(pf01923) EUT1
30491 HMM06249EutQ_1_eut1(pf06249) EUT1 EP_NTERM
30501 HMM10662PduV_1_mix(pf10662) EUT1
30511 Hp_euts_violetPink EUT1
30521 HMM00390_malic_1_eut1(pf00390pf03949pf01515) EUT1
30531 -pf00923 -
30541 HMM02780keto_1_eut1(pf02779pf02780pf00456) EUT1
30551 -pf06674 -
30561 HMM00293nuhyd_1_eut1(pf00293) EUT1
30571 -pf13247pf14691pf07992 -
30581 -pf00672pf02518pf07730pf13675 -
30591 -pf00873 -
30601 -pf03960 -
30611 -pf07687pf01546 -
BMC-P
BMC-H
BMC-T
Aldehyde dehydrogenase
PTAC
Signature enzyme
Alcohol dehydrogenase
Regulator

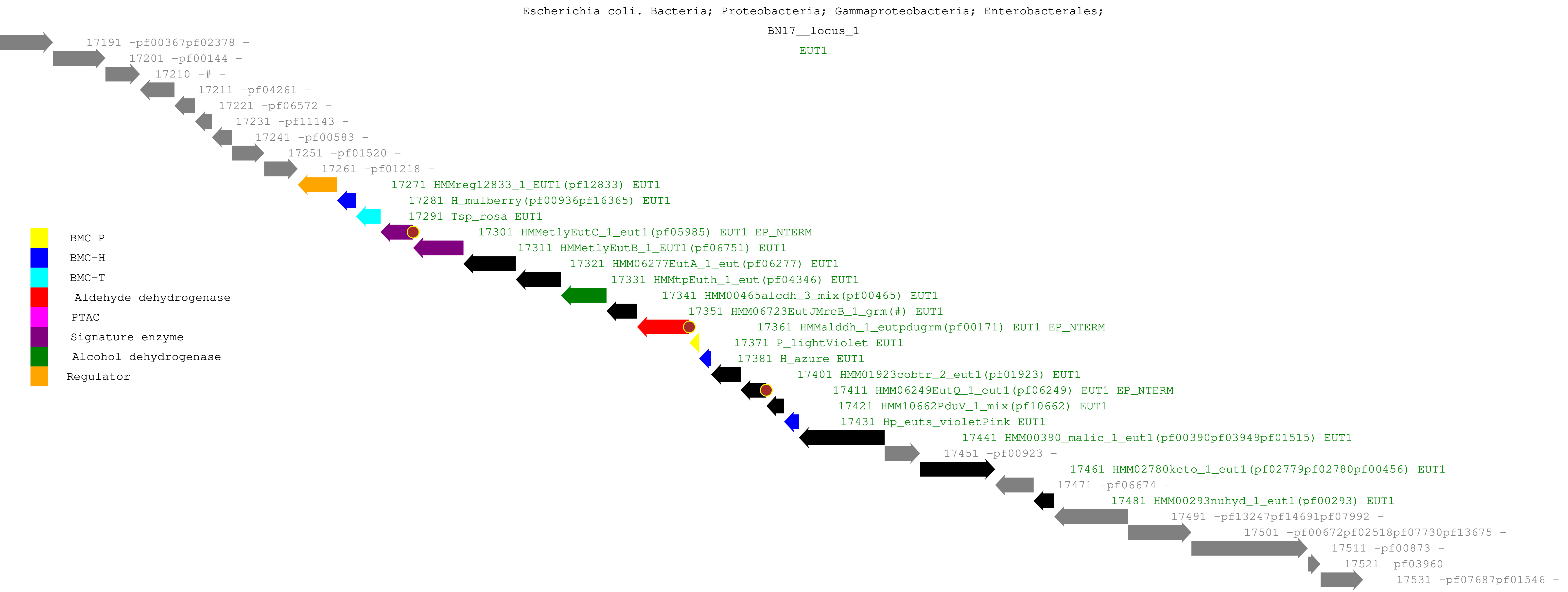
Escherichia coli. Bacteria; Proteobacteria; Gammaproteobacteria; Enterobacterales;
BN17__locus_1
EUT1
17191 -pf00367pf02378 -
17201 -pf00144 -
17210 -# -
17211 -pf04261 -
17221 -pf06572 -
17231 -pf11143 -
17241 -pf00583 -
17251 -pf01520 -
17261 -pf01218 -
17271 HMMreg12833_1_EUT1(pf12833) EUT1
17281 H_mulberry(pf00936pf16365) EUT1
17291 Tsp_rosa EUT1
17301 HMMetlyEutC_1_eut1(pf05985) EUT1 EP_NTERM
17311 HMMetlyEutB_1_EUT1(pf06751) EUT1
17321 HMM06277EutA_1_eut(pf06277) EUT1
17331 HMMtpEuth_1_eut(pf04346) EUT1
17341 HMM00465alcdh_3_mix(pf00465) EUT1
17351 HMM06723EutJMreB_1_grm(#) EUT1
17361 HMMalddh_1_eutpdugrm(pf00171) EUT1 EP_NTERM
17371 P_lightViolet EUT1
17381 H_azure EUT1
17401 HMM01923cobtr_2_eut1(pf01923) EUT1
17411 HMM06249EutQ_1_eut1(pf06249) EUT1 EP_NTERM
17421 HMM10662PduV_1_mix(pf10662) EUT1
17431 Hp_euts_violetPink EUT1
17441 HMM00390_malic_1_eut1(pf00390pf03949pf01515) EUT1
17451 -pf00923 -
17461 HMM02780keto_1_eut1(pf02779pf02780pf00456) EUT1
17471 -pf06674 -
17481 HMM00293nuhyd_1_eut1(pf00293) EUT1
17491 -pf13247pf14691pf07992 -
17501 -pf00672pf02518pf07730pf13675 -
17511 -pf00873 -
17521 -pf03960 -
17531 -pf07687pf01546 -
BMC-P
BMC-H
BMC-T
Aldehyde dehydrogenase
PTAC
Signature enzyme
Alcohol dehydrogenase
Regulator

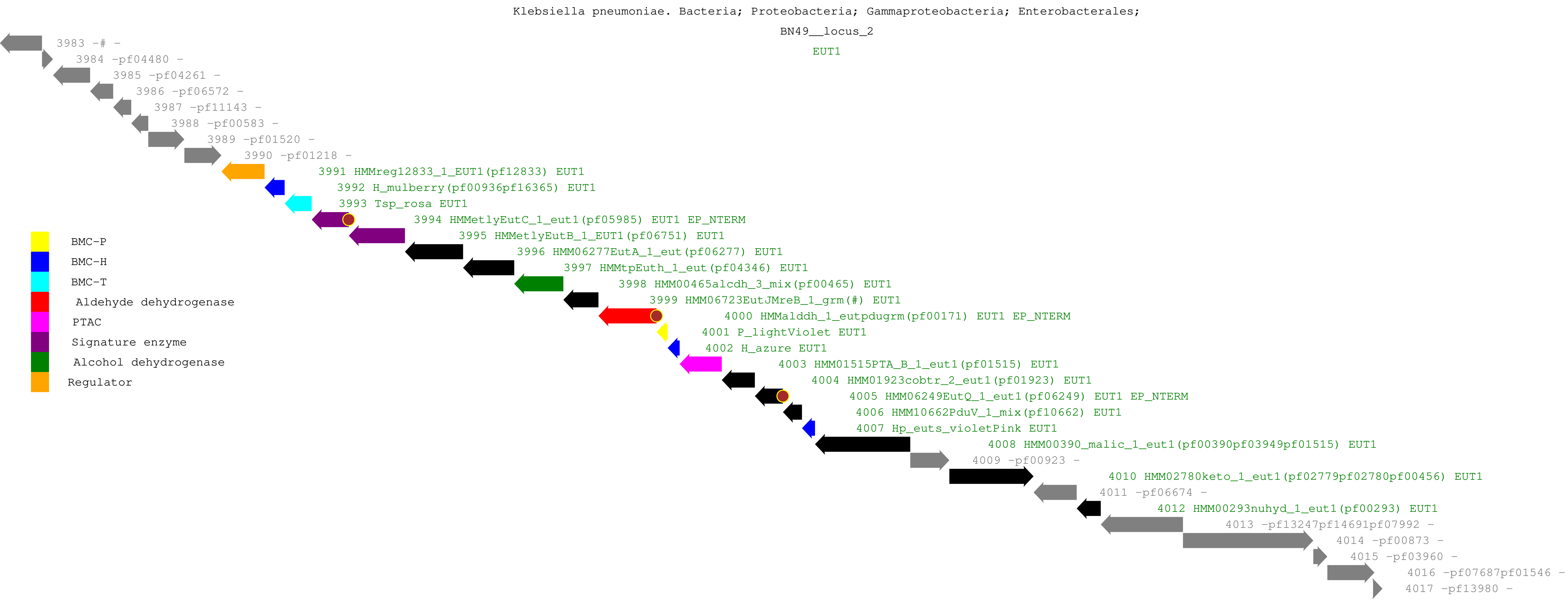
Klebsiella pneumoniae. Bacteria; Proteobacteria; Gammaproteobacteria; Enterobacterales;
BN49__locus_2
EUT1
3983 -# -
3984 -pf04480 -
3985 -pf04261 -
3986 -pf06572 -
3987 -pf11143 -
3988 -pf00583 -
3989 -pf01520 -
3990 -pf01218 -
3991 HMMreg12833_1_EUT1(pf12833) EUT1
3992 H_mulberry(pf00936pf16365) EUT1
3993 Tsp_rosa EUT1
3994 HMMetlyEutC_1_eut1(pf05985) EUT1 EP_NTERM
3995 HMMetlyEutB_1_EUT1(pf06751) EUT1
3996 HMM06277EutA_1_eut(pf06277) EUT1
3997 HMMtpEuth_1_eut(pf04346) EUT1
3998 HMM00465alcdh_3_mix(pf00465) EUT1
3999 HMM06723EutJMreB_1_grm(#) EUT1
4000 HMMalddh_1_eutpdugrm(pf00171) EUT1 EP_NTERM
4001 P_lightViolet EUT1
4002 H_azure EUT1
4003 HMM01515PTA_B_1_eut1(pf01515) EUT1
4004 HMM01923cobtr_2_eut1(pf01923) EUT1
4005 HMM06249EutQ_1_eut1(pf06249) EUT1 EP_NTERM
4006 HMM10662PduV_1_mix(pf10662) EUT1
4007 Hp_euts_violetPink EUT1
4008 HMM00390_malic_1_eut1(pf00390pf03949pf01515) EUT1
4009 -pf00923 -
4010 HMM02780keto_1_eut1(pf02779pf02780pf00456) EUT1
4011 -pf06674 -
4012 HMM00293nuhyd_1_eut1(pf00293) EUT1
4013 -pf13247pf14691pf07992 -
4014 -pf00873 -
4015 -pf03960 -
4016 -pf07687pf01546 -
4017 -pf13980 -
BMC-P
BMC-H
BMC-T
Aldehyde dehydrogenase
PTAC
Signature enzyme
Alcohol dehydrogenase
Regulator

Escherichia coli O127:H6. Bacteria; Proteobacteria; Gammaproteobacteria; Enterobacterales;

BQ9544__locus_2

EUT1

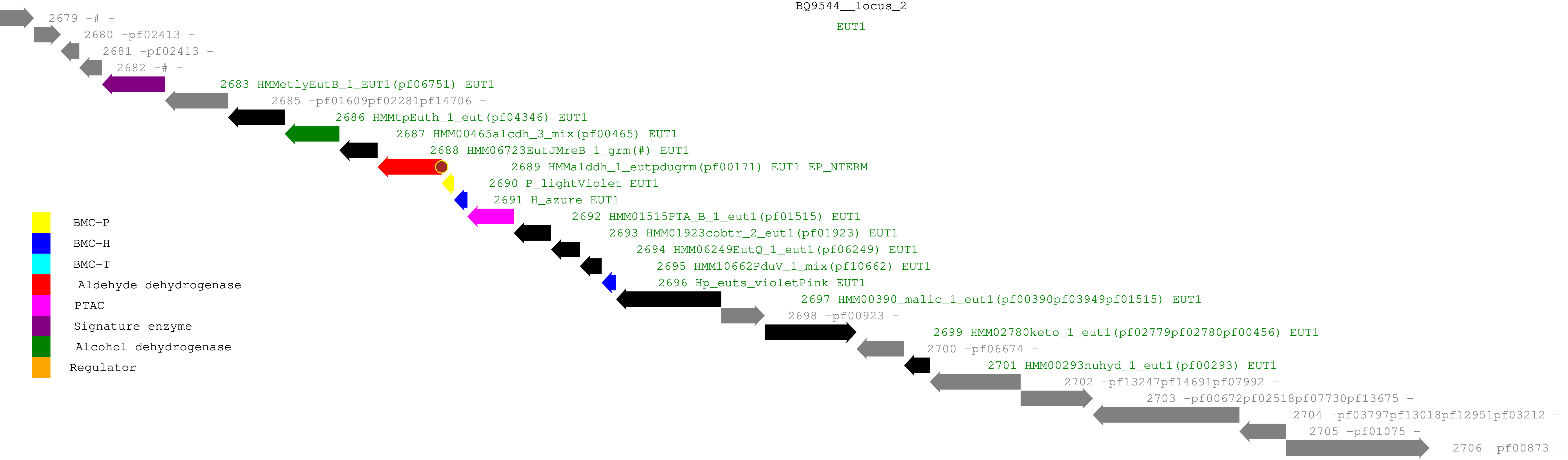

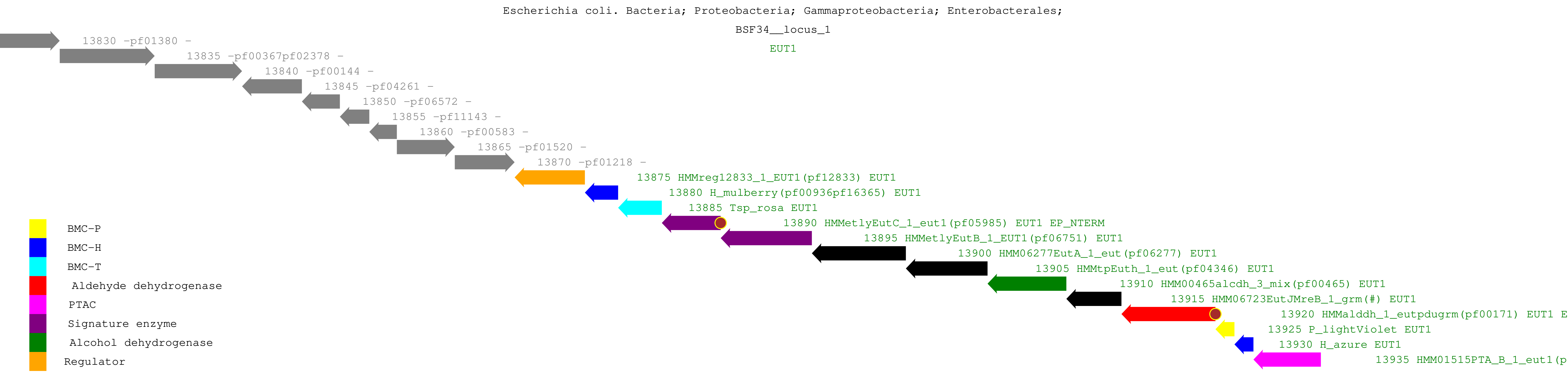

Escherichia coli. Bacteria; Proteobacteria; Gammaproteobacteria; Enterobacterales;
BSF34__locus_1
EUT1
13830 -pf01380 -
13835 -pf00367pf02378 -
13840 -pf00144 -
13845 -pf04261 -
13850 -pf06572 -
13855 -pf11143 -
13860 -pf00583 -
13865 -pf01520 -
13870 -pf01218 -
13875 HMMreg12833_1_EUT1(pf12833) EUT1
13880 H_mulberry(pf00936pf16365) EUT1
13885 Tsp_rosa EUT1
13890 HMMetlyEutC_1_eut1(pf05985) EUT1 EP_NTERM
13895 HMMetlyEutB_1_EUT1(pf06751) EUT1
13900 HMM06277EutA_1_eut(pf06277) EUT1
13905 HMMtpEuth_1_eut(pf04346) EUT1
13910 HMM00465alcdh_3_mix(pf00465) EUT1
13915 HMM06723EutJMreB_1_grm(#) EUT1
13920 HMMaldh_1_eutpdugrm(pf00171) EUT1 E
13925 P_lightViolet EUT1
13930 H_azure EUT1
13935 HMM01515PTA_B_1_eut1(p
BMC-P
BMC-H
BMC-T
Aldehyde dehydrogenase
PTAC
Signature enzyme
Alcohol dehydrogenase
Regulator

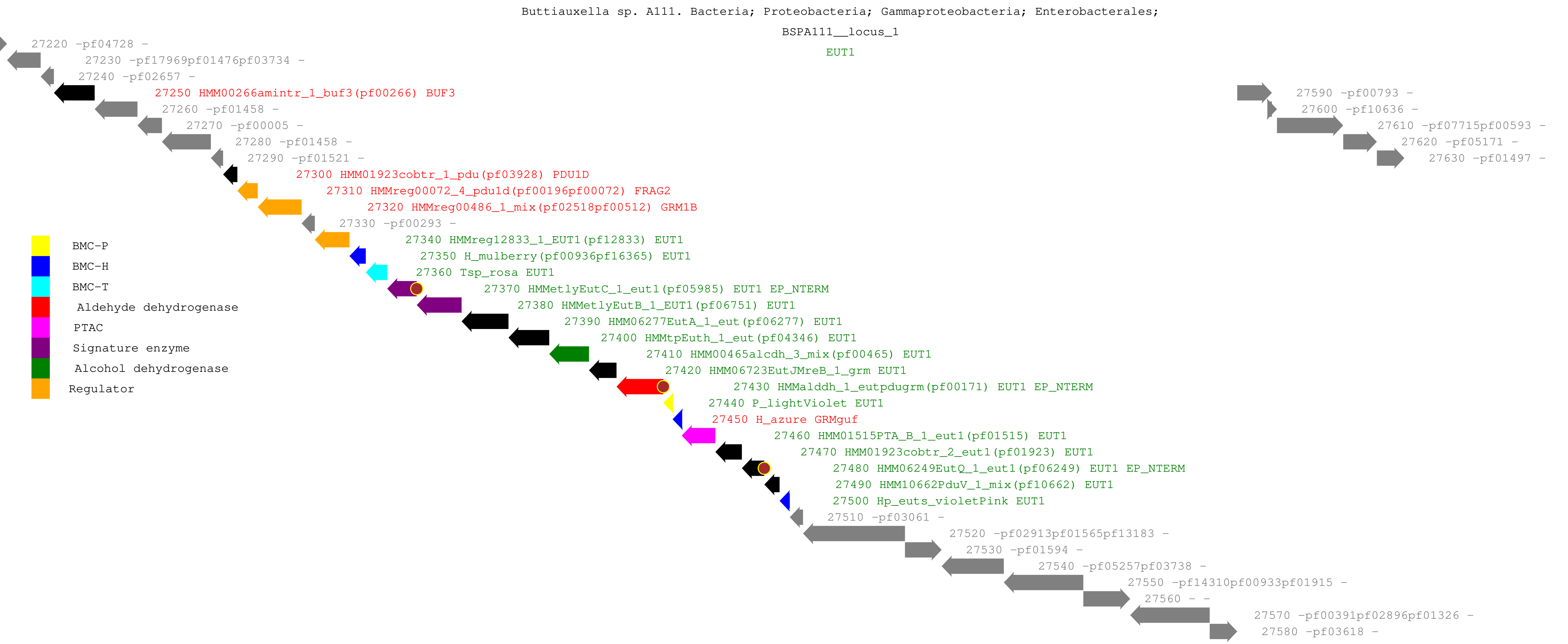

Buttiauxella sp. A111. Bacteria; Proteobacteria; Gammaproteobacteria; Enterobacterales;
BSPA111__locus_1
EUT1
27220 -pf04728 -
27230 -pf17969pf01476pf03734 -
27240 -pf02657 -
27250 HMM00266amintr_1_buf3(pf00266) BUF3
27260 -pf01458 -
27270 -pf00005 -
27280 -pf01458 -
27290 -pf01521 -
27300 HMM01923cobtr_1_pdu(pf03928) PDU1D
27310 HMMreg00072_4_pdu1d(pf00196pf00072) FRAG2
27320 HMMreg00486_1_mix(pf02518pf00512) GRM1B
27330 -pf00293 -
27340 HMMreg12833_1_EUT1(pf12833) EUT1
27350 H_mulberry(pf00936pf16365) EUT1
27360 Tsp_rosa EUT1
27370 HMMetlyEutC_1_eut1(pf05985) EUT1 EP_NTERM
27380 HMMetlyEutB_1_EUT1(pf06751) EUT1
27390 HMM06277EutA_1_eut(pf06277) EUT1
27400 HMMtpEuth_1_eut(pf04346) EUT1
27410 HMM00465alcdh_3_mix(pf00465) EUT1
27420 HMM06723EutJMreB_1_grm EUT1
27430 HMMalddh_1_eutpdugrm(pf00171) EUT1 EP_NTERM
27440 P_lightViolet EUT1
27450 H_azure GRMguf
27460 HMM01515PTA_B_1_eut1(pf01515) EUT1
27470 HMM01923cobtr_2_eut1(pf01923) EUT1
27480 HMM06249EutQ_1_eut1(pf06249) EUT1 EP_NTERM
27490 HMM10662PduV_1_mix(pf10662) EUT1
27500 Hp_euts_violetPink EUT1
27510 -pf03061 -
27520 -pf02913pf01565pf13183 -
27530 -pf01594 -
27540 -pf05257pf03738 -
27550 -pf14310pf00933pf01915 -
27560 - -
27570 -pf00391pf02896pf01326 -
27580 -pf03618 -
27590 -pf00793 -
27600 -pf10636 -
27610 -pf07715pf00593 -
27620 -pf05171 -
27630 -pf01497 -
BMC-P
BMC-H
BMC-T
Aldehyde dehydrogenase
PTAC
Signature enzyme
Alcohol dehydrogenase
Regulator

Salmonella enteritidis. Bacteria; Proteobacteria; Gammaproteobacteria; Enterobacterales;

BTN74__locus_2

EUT1

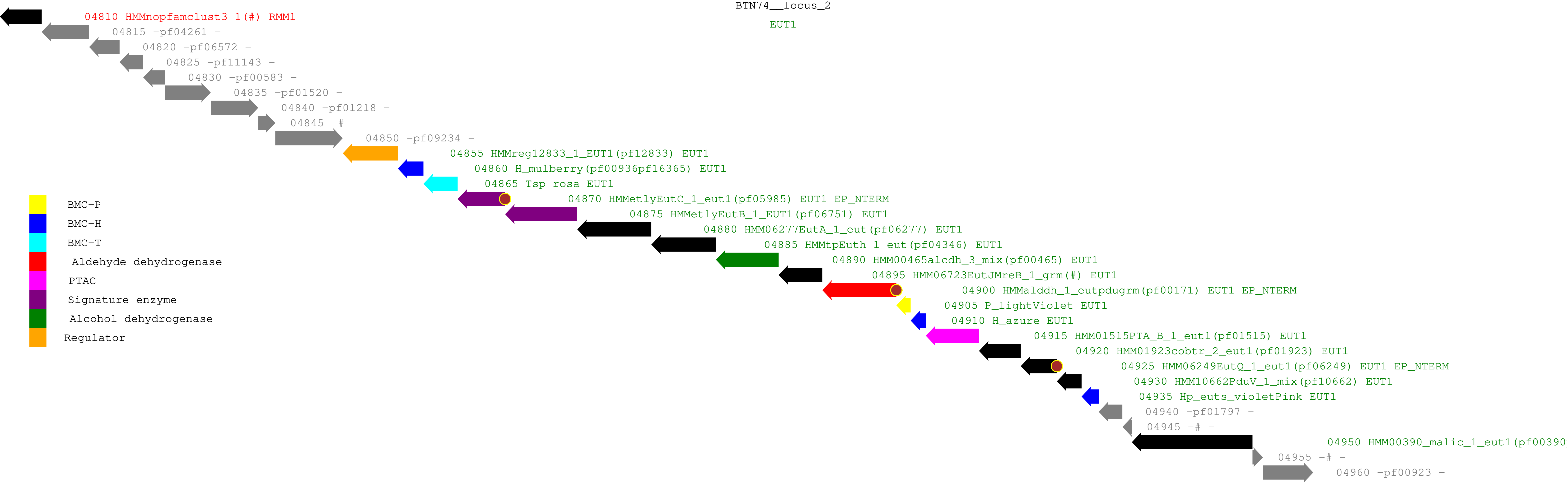

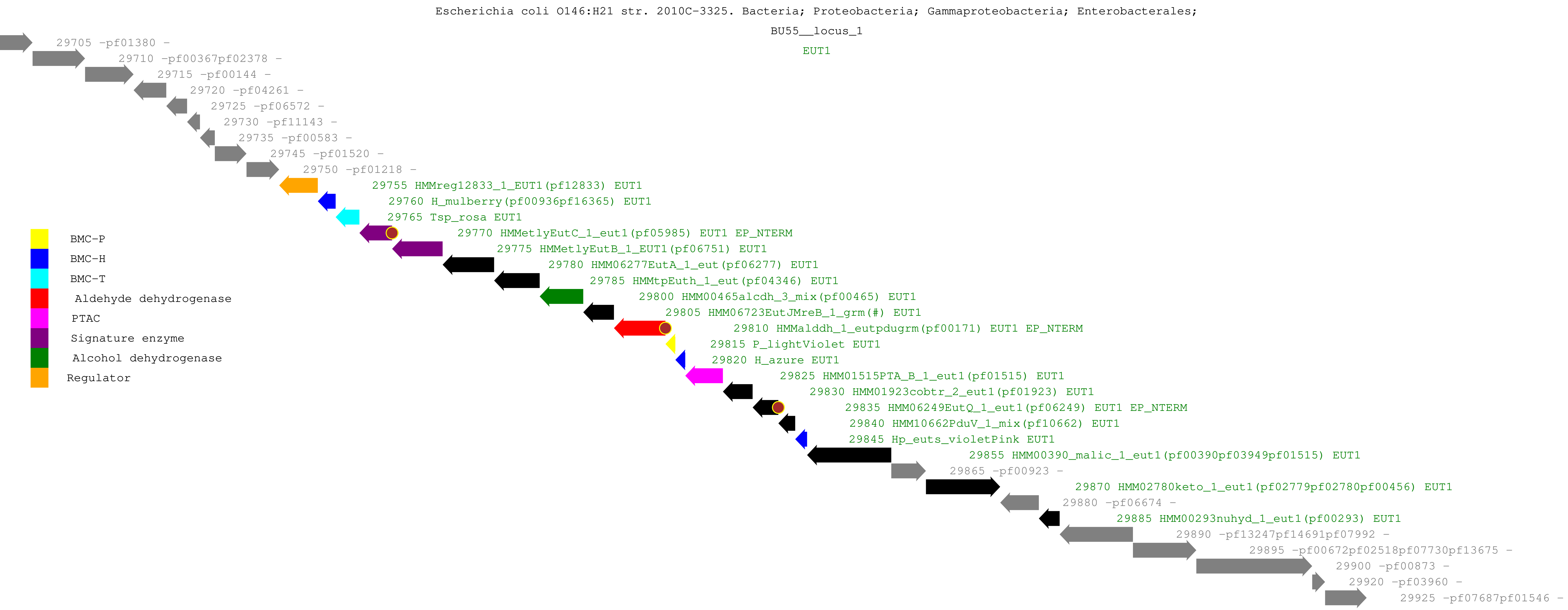

Escherichia coli O146:H21 str. 2010C-3325. Bacteria; Proteobacteria; Gammaproteobacteria; Enterobacterales;
BU55__locus_1
EUT1
29705 -pf01380 -
29710 -pf00367pf02378 -
29715 -pf00144 -
29720 -pf04261 -
29725 -pf06572 -
29730 -pf11143 -
29735 -pf00583 -
29745 -pf01520 -
29750 -pf01218 -
29755 HMMreg12833_1_EUT1(pf12833) EUT1
29760 H_mulberry(pf00936pf16365) EUT1
29765 Tsp_rosa EUT1
29770 HMMetlyEutC_1_eut1(pf05985) EUT1 EP_NTERM
29775 HMMetlyEutB_1_EUT1(pf06751) EUT1
29780 HMM06277EutA_1_eut(pf06277) EUT1
29785 HMMtpEuth_1_eut(pf04346) EUT1
29800 HMM00465alcdh_3_mix(pf00465) EUT1
29805 HMM06723EutJMreB_1_grm(#) EUT1
29810 HMMalddh_1_eutpdugrm(pf00171) EUT1 EP_NTERM
29815 P_lightViolet EUT1
29820 H_azure EUT1
29825 HMM01515PTA_B_1_eut1(pf01515) EUT1
29830 HMM01923cobtr_2_eut1(pf01923) EUT1
29835 HMM06249EutQ_1_eut1(pf06249) EUT1 EP_NTERM
29840 HMM10662PduV_1_mix(pf10662) EUT1
29845 Hp_euts_violetPink EUT1
29855 HMM00390_malic_1_eut1(pf00390pf03949pf01515) EUT1
29865 -pf00923 -
29870 HMM02780keto_1_eut1(pf02779pf02780pf00456) EUT1
29880 -pf06674 -
29885 HMM00293nuhyd_1_eut1(pf00293) EUT1
29890 -pf13247pf14691pf07992 -
29895 -pf00672pf02518pf07730pf13675 -
29900 -pf00873 -
29920 -pf03960 -
29925 -pf07687pf01546 -
BMC-P
BMC-H
BMC-T
Aldehyde dehydrogenase
PTAC
Signature enzyme
Alcohol dehydrogenase
Regulator

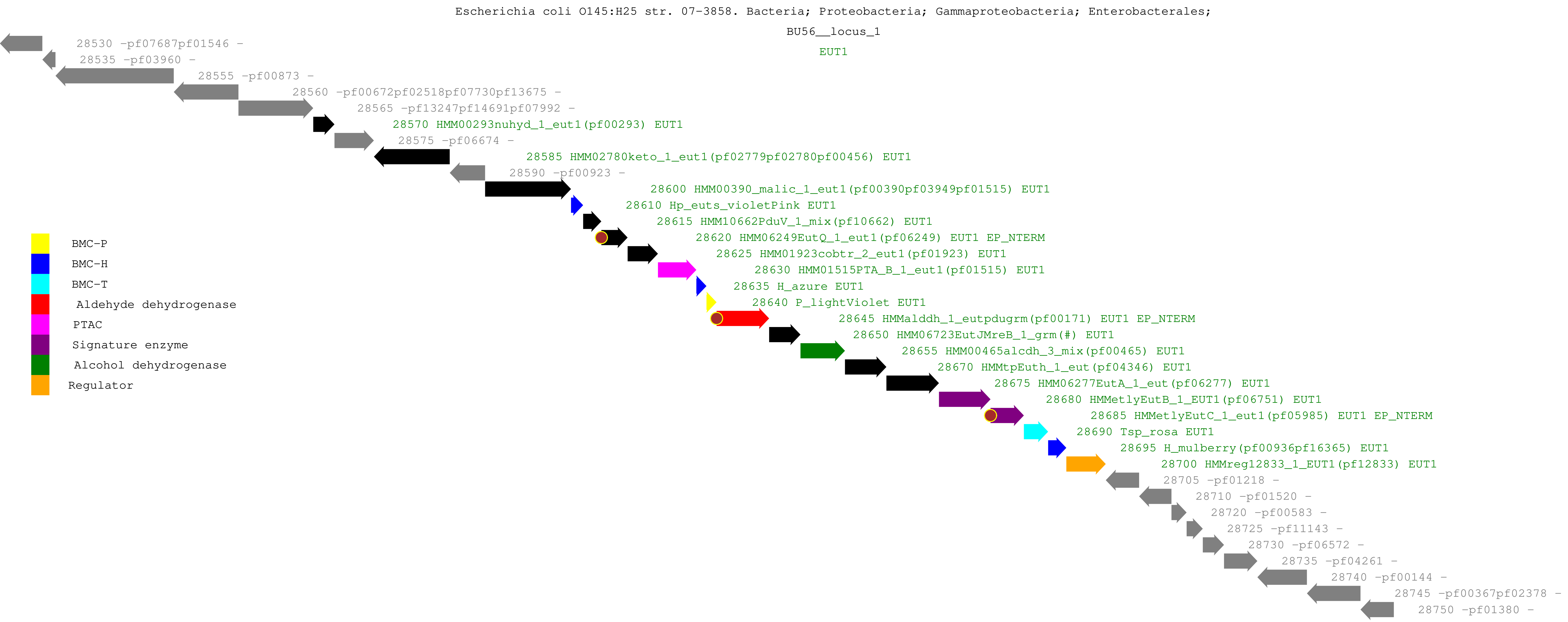

Escherichia coli O145:H25 str. 07-3858. Bacteria; Proteobacteria; Gammaproteobacteria; Enterobacterales;
BU56__locus_1
EUT1
28530 -pf07687pf01546 -
28535 -pf03960 -
28555 -pf00873 -
28560 -pf00672pf02518pf07730pf13675 -
28565 -pf13247pf14691pf07992 -
28570 HMM00293nuhyd_1_eut1(pf00293) EUT1
28575 -pf06674 -
28585 HMM02780keto_1_eut1(pf02779pf02780pf00456) EUT1
28590 -pf00923 -
28600 HMM00390_malic_1_eut1(pf00390pf03949pf01515) EUT1
28610 Hp_euts_violetPink EUT1
28615 HMM10662PduV_1_mix(pf10662) EUT1
28620 HMM06249EutQ_1_eut1(pf06249) EUT1 EP_NTERM
28625 HMM01923cobtr_2_eut1(pf01923) EUT1
28630 HMM01515PTA_B_1_eut1(pf01515) EUT1
28635 H_azure EUT1
28640 P_lightViolet EUT1
28645 HMMalddh_1_eutpdugrm(pf00171) EUT1 EP_NTERM
28650 HMM06723EutJMreB_1_grm(#) EUT1
28655 HMM00465alcdh_3_mix(pf00465) EUT1
28670 HMMtpEuth_1_eut(pf04346) EUT1
28675 HMM06277EutA_1_eut(pf06277) EUT1
28680 HMMetlyEutB_1_EUT1(pf06751) EUT1
28685 HMMetlyEutC_1_eut1(pf05985) EUT1 EP_NTERM
28690 Tsp_rosa EUT1
28695 H_mulberry(pf00936pf16365) EUT1
28700 HMMreg12833_1_EUT1(pf12833) EUT1
28705 -pf01218 -
28710 -pf01520 -
28720 -pf00583 -
28725 -pf11143 -
28730 -pf06572 -
28735 -pf04261 -
28740 -pf00144 -
28745 -pf00367pf02378 -
28750 -pf01380 -
BMC-P
BMC-H
BMC-T
Aldehyde dehydrogenase
PTAC
Signature enzyme
Alcohol dehydrogenase
Regulator

Escherichia coli O128:H2 str. 2011C-3317. Bacteria; Proteobacteria; Gammaproteobacteria; Enterobacterales;

BU64__locus_1

EUT1

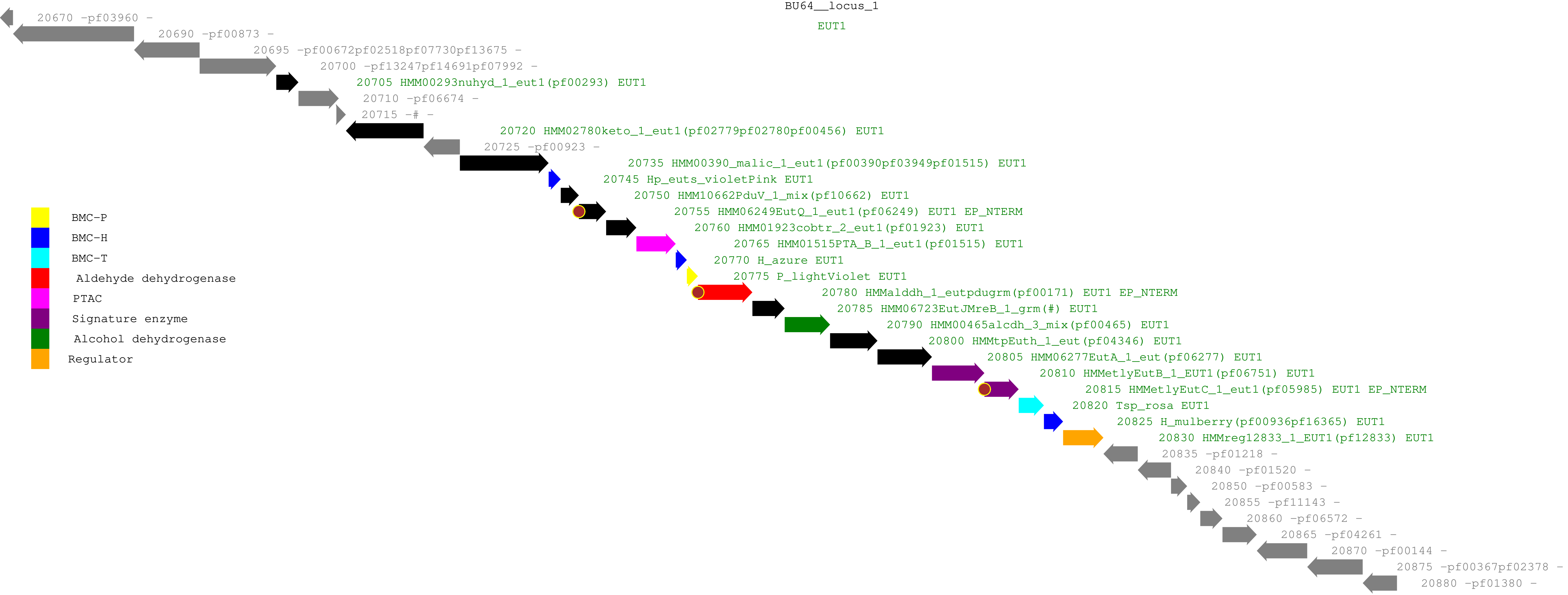

Klebsiella pneumoniae subsp. pneumoniae. Bacteria; Proteobacteria; Gammaproteobacteria; Enterobacterales;

BU813__locus_1

EUT1

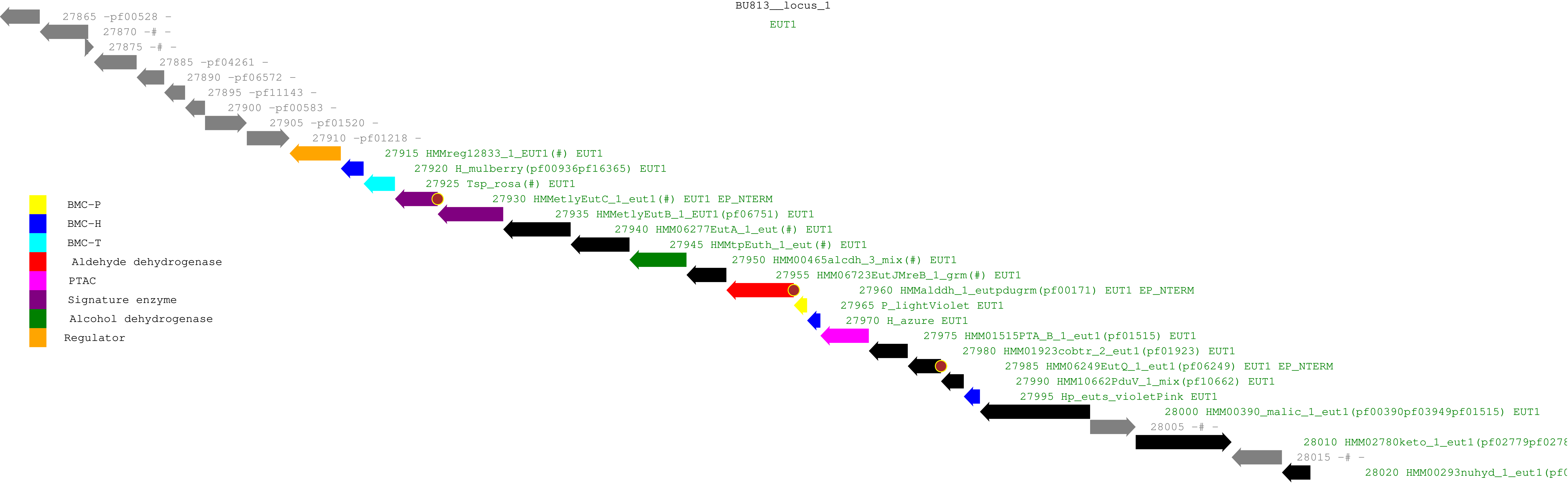

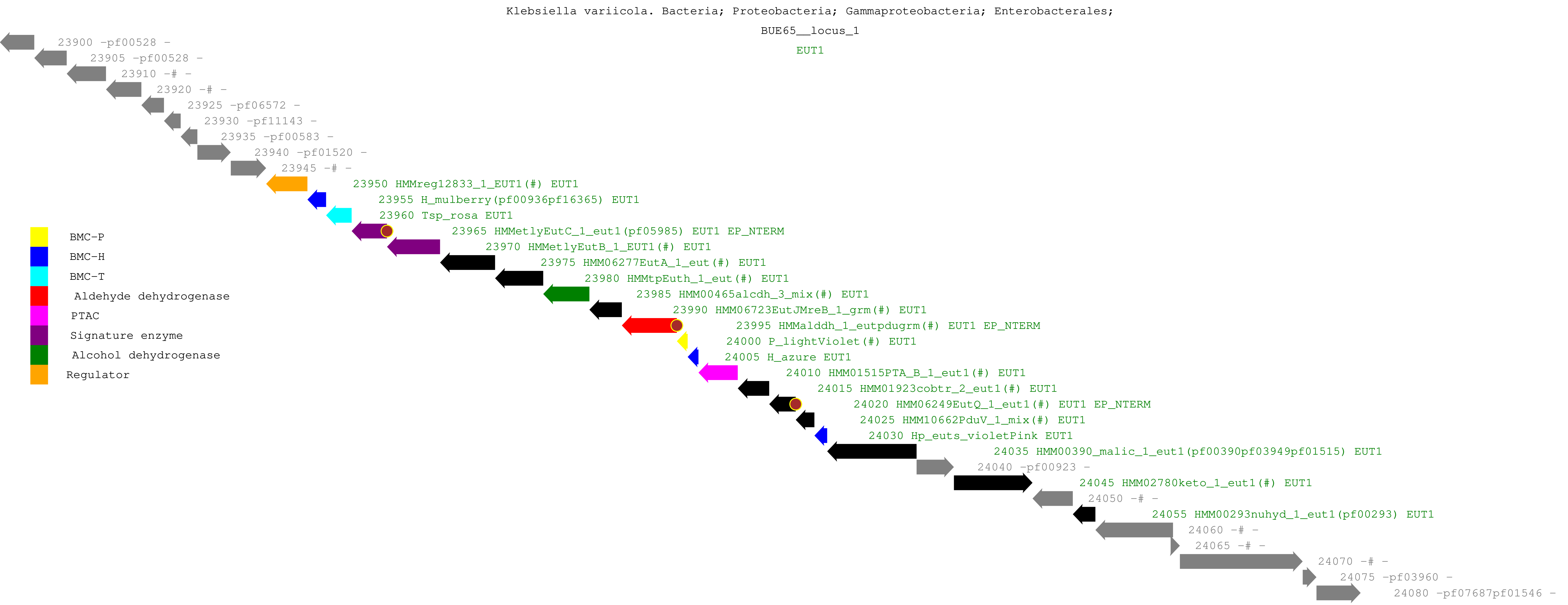

Klebsiella variicola. Bacteria; Proteobacteria; Gammaproteobacteria; Enterobacterales;
BUE65__locus_1
EUT1
23900 -pf00528 -
23905 -pf00528 -
23910 -# -
23920 -# -
23925 -pf06572 -
23930 -pf11143 -
23935 -pf00583 -
23940 -pf01520 -
23945 -# -
23950 HMMreg12833_1_EUT1(#) EUT1
23955 H_mulberry(pf00936pf16365) EUT1
23960 Tsp_rosa EUT1
23965 HMMetlyEutC_1_eut1(pf05985) EUT1 EP_NTERM
23970 HMMetlyEutB_1_EUT1(#) EUT1
23975 HMM06277EutA_1_eut(#) EUT1
23980 HMMtpEuth_1_eut(#) EUT1
23985 HMM00465alcdh_3_mix(#) EUT1
23990 HMM06723EutJMreB_1_grm(#) EUT1
23995 HMMalddh_1_eutpdugrm(#) EUT1 EP_NTERM
24000 P_lightViolet(#) EUT1
24005 H_azure EUT1
24010 HMM01515PTA_B_1_eut1(#) EUT1
24015 HMM01923cobtr_2_eut1(#) EUT1
24020 HMM06249EutQ_1_eut1(#) EUT1 EP_NTERM
24025 HMM10662PduV_1_mix(#) EUT1
24030 Hp_euts_violetPink EUT1
24035 HMM00390_malic_1_eut1(pf00390pf03949pf01515) EUT1
24040 -pf00923 -
24045 HMM02780keto_1_eut1(#) EUT1
24050 -# -
24055 HMM00293nuhyd_1_eut1(pf00293) EUT1
24060 -# -
24065 -# -
24070 -# -
24075 -pf03960 -
24080 -pf07687pf01546 -
BMC-P
BMC-H
BMC-T
Aldehyde dehydrogenase
PTAC
Signature enzyme
Alcohol dehydrogenase
Regulator

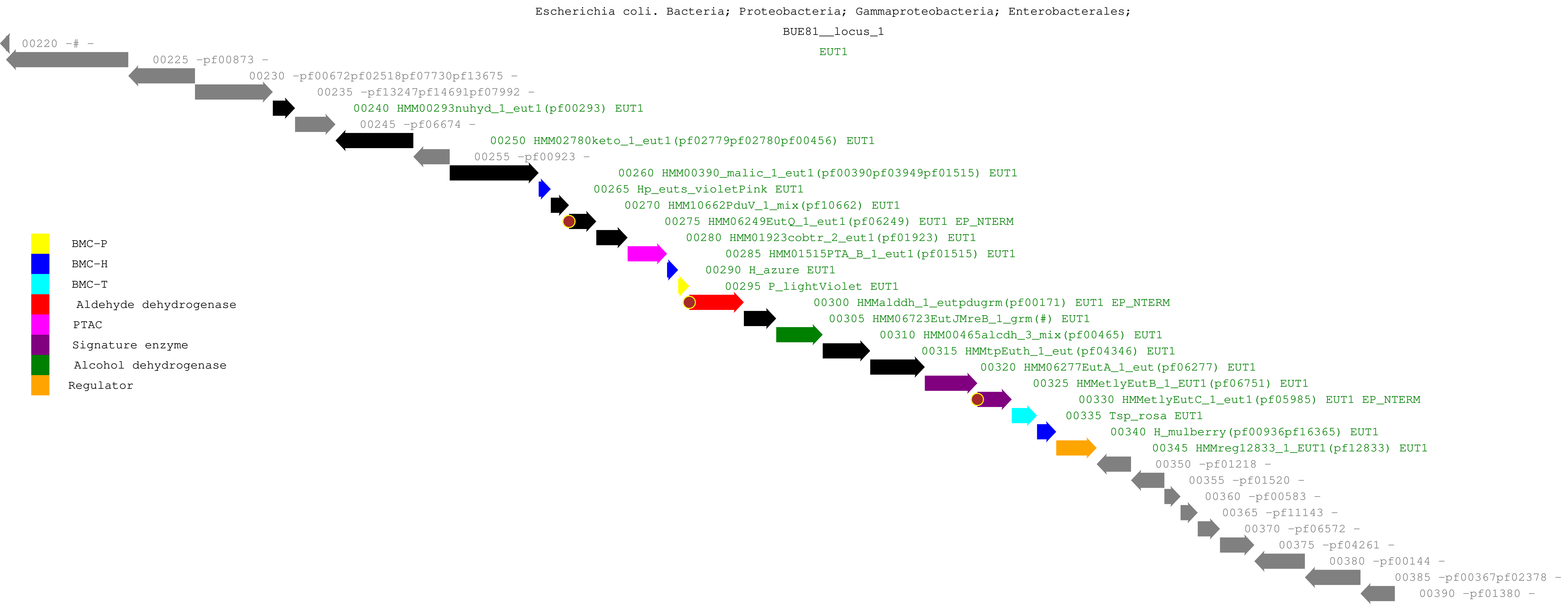

Escherichia coli. Bacteria; Proteobacteria; Gammaproteobacteria; Enterobacterales;
BUE81__locus_1
EUT1
00220 -# -
00225 -pf00873 -
00230 -pf00672pf02518pf07730pf13675 -
00235 -pf13247pf14691pf07992 -
00240 HMM00293nuhyd_1_eut1(pf00293) EUT1
00245 -pf06674 -
00250 HMM02780keto_1_eut1(pf02779pf02780pf00456) EUT1
00255 -pf00923 -
00260 HMM00390_malic_1_eut1(pf00390pf03949pf01515) EUT1
00265 Hp_euts_violetPink EUT1
00270 HMM10662PduV_1_mix(pf10662) EUT1
00275 HMM06249EutQ_1_eut1(pf06249) EUT1 EP_NTERM
00280 HMM01923cobtr_2_eut1(pf01923) EUT1
00285 HMM01515PTA_B_1_eut1(pf01515) EUT1
00290 H_azure EUT1
00295 P_lightViolet EUT1
00300 HMMaldddh_1_eutpdugrm(pf00171) EUT1 EP_NTERM
00305 HMM06723EutJMreB_1_grm(#) EUT1
00310 HMM00465alcdh_3_mix(pf00465) EUT1
00315 HMMtpEuth_1_eut(pf04346) EUT1
00320 HMM06277EutA_1_eut(pf06277) EUT1
00325 HMMetlyEutB_1_EUT1(pf06751) EUT1
00330 HMMetlyEutC_1_eut1(pf05985) EUT1 EP_NTERM
00335 Tsp_rosa EUT1
00340 H_mulberry(pf00936pf16365) EUT1
00345 HMMreg12833_1_EUT1(pf12833) EUT1
00350 -pf01218 -
00355 -pf01520 -
00360 -pf00583 -
00365 -pf11143 -
00370 -pf06572 -
00375 -pf04261 -
00380 -pf00144 -
00385 -pf00367pf02378 -
00390 -pf01380 -
BMC-P
BMC-H
BMC-T
Aldehyde dehydrogenase
PTAC
Signature enzyme
Alcohol dehydrogenase
Regulator

Salmonella enterica subsp. diarizonae serovar 60:r:e,n,x,z15. Bacteria; Proteobacteria; Gammaproteobacteria; Enterobacterales;

BUJ13__locus_1

EUT1

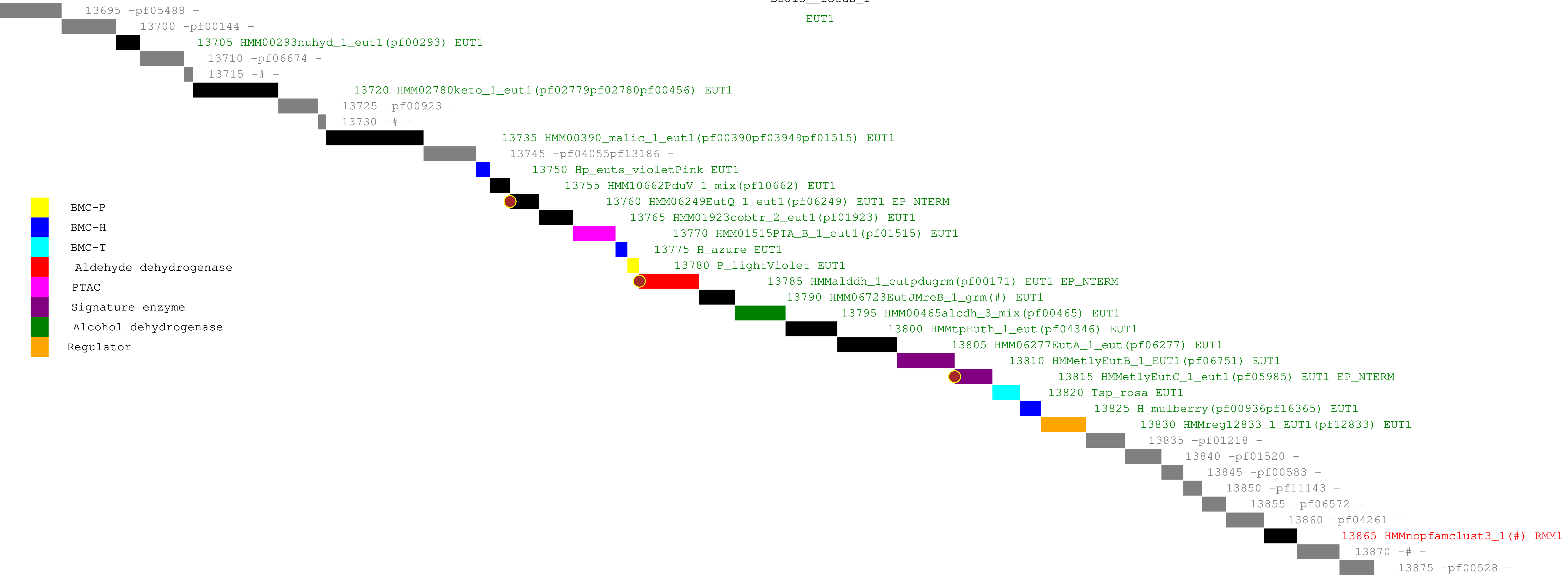

Salmonella rubislaw. Bacteria; Proteobacteria; Gammaproteobacteria; Enterobacterales;

BUJ14__locus_1

EUT1

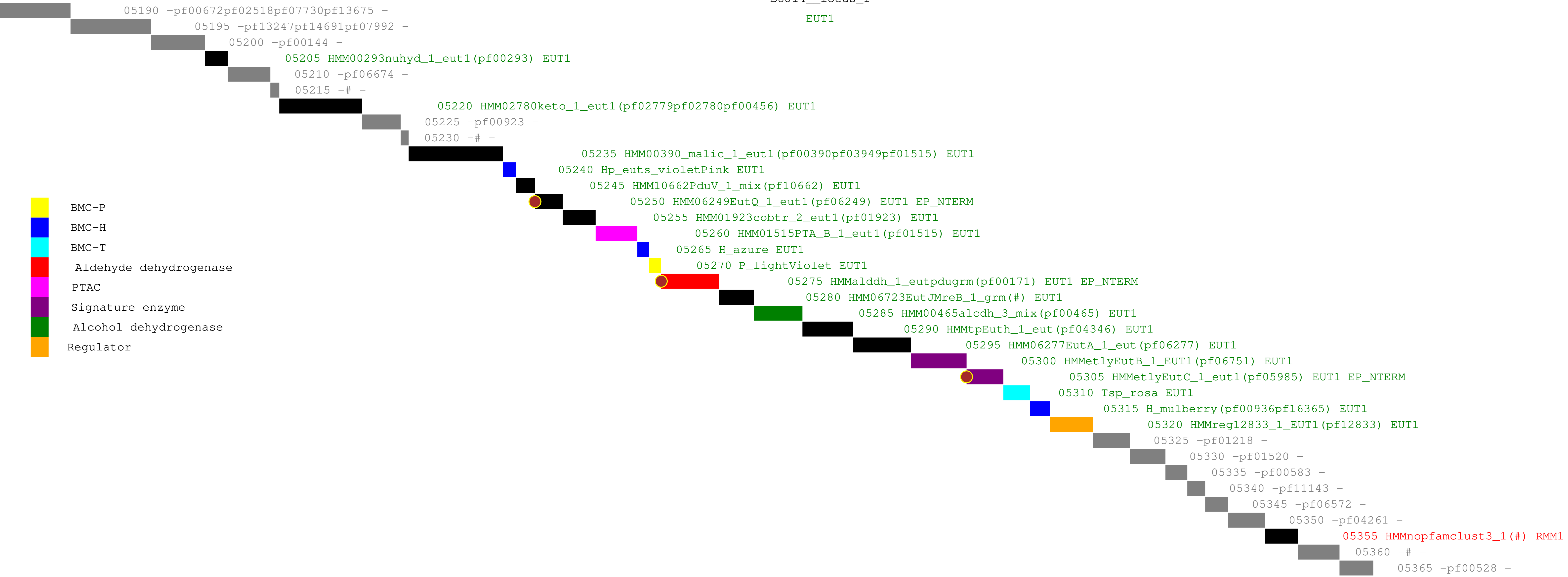

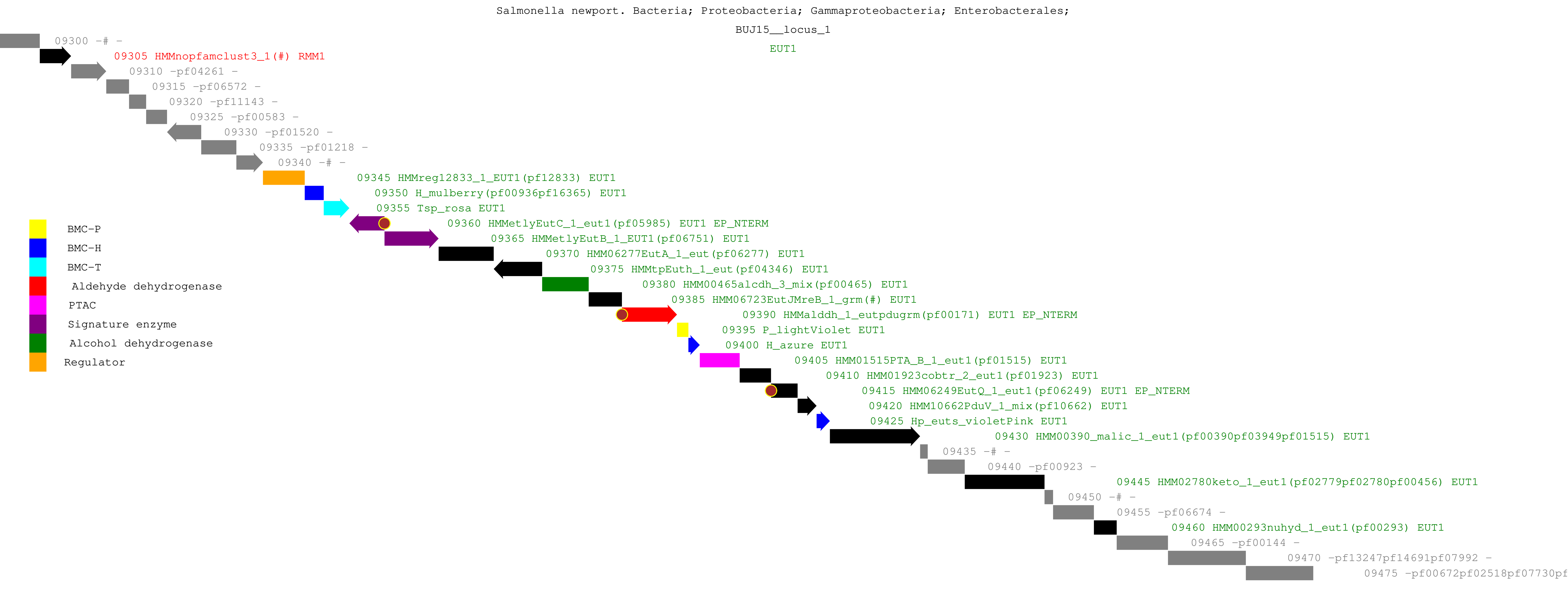
Salmonella newport. Bacteria; Proteobacteria; Gammaproteobacteria; Enterobacterales;
BUJ15__locus_1
EUT1
09300 -# -
09305 HMMnopfamclust3_1(#) RMM1
09310 -pf04261 -
09315 -pf06572 -
09320 -pf11143 -
09325 -pf00583 -
09330 -pf01520 -
09335 -pf01218 -
09340 -# -
09345 HMMreg12833_1_EUT1(pf12833) EUT1
09350 H_mulberry(pf00936pf16365) EUT1
09355 Tsp_rosa EUT1
09360 HMMetlyEutC_1_eut1(pf05985) EUT1 EP_NTERM
09365 HMMetlyEutB_1_EUT1(pf06751) EUT1
09370 HMM06277EutA_1_eut(pf06277) EUT1
09375 HMMtpEuth_1_eut(pf04346) EUT1
09380 HMM00465alcdh_3_mix(pf00465) EUT1
09385 HMM06723EutJMreB_1_grm(#) EUT1
09390 HMMalddh_1_eutpdugrm(pf00171) EUT1 EP_NTERM
09395 P_lightViolet EUT1
09400 H_azure EUT1
09405 HMM01515PTA_B_1_eut1(pf01515) EUT1
09410 HMM01923cobtr_2_eut1(pf01923) EUT1
09415 HMM06249EutQ_1_eut1(pf06249) EUT1 EP_NTERM
09420 HMM10662PduV_1_mix(pf10662) EUT1
09425 Hp_euts_violetPink EUT1
09430 HMM00390_malic_1_eut1(pf00390pf03949pf01515) EUT1
09435 -# -
09440 -pf00923 -
09445 HMM02780keto_1_eut1(pf02779pf02780pf00456) EUT1
09450 -# -
09455 -pf06674 -
09460 HMM00293nuhyd_1_eut1(pf00293) EUT1
09465 -pf00144 -
09470 -pf13247pf14691pf07992 -
09475 -pf00672pf02518pf07730pf
BMC-P
BMC-H
BMC-T
Aldehyde dehydrogenase
PTAC
Signature enzyme
Alcohol dehydrogenase
Regulator

Salmonella choleraesuis. Bacteria; Proteobacteria; Gammaproteobacteria; Enterobacterales;

BUJ19__locus_1

EUT1

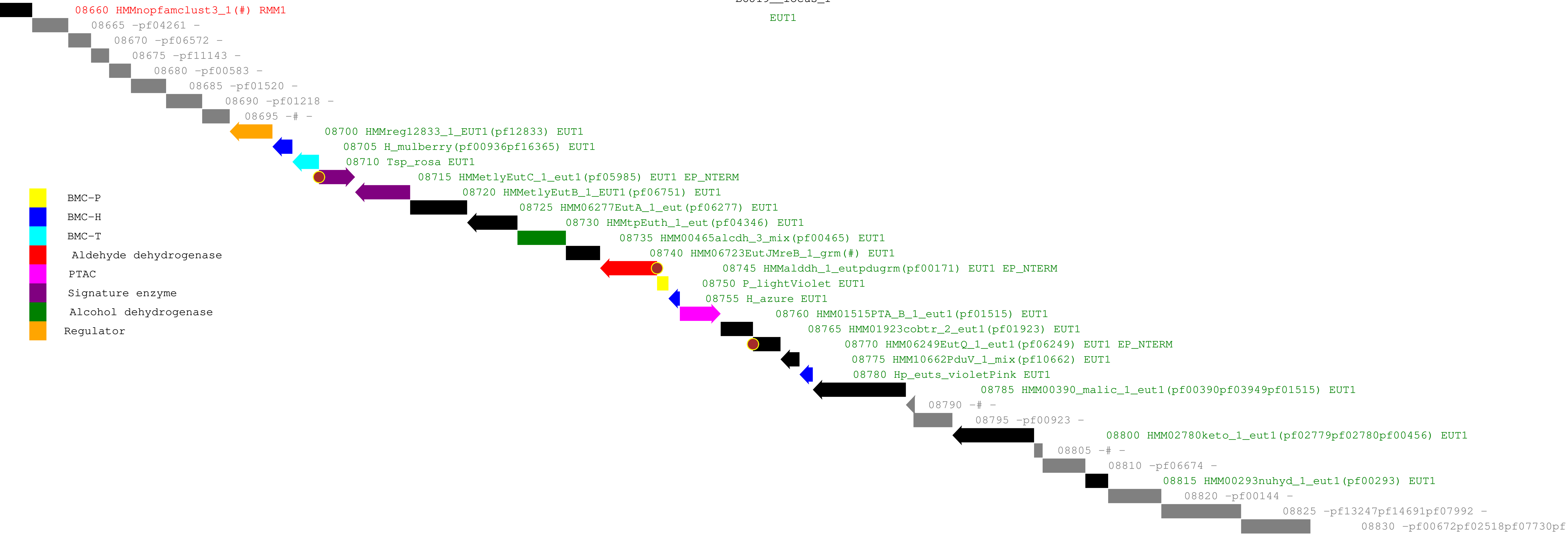

Salmonella newport. Bacteria; Proteobacteria; Gammaproteobacteria; Enterobacterales;

BUJ24__locus_1

EUT1

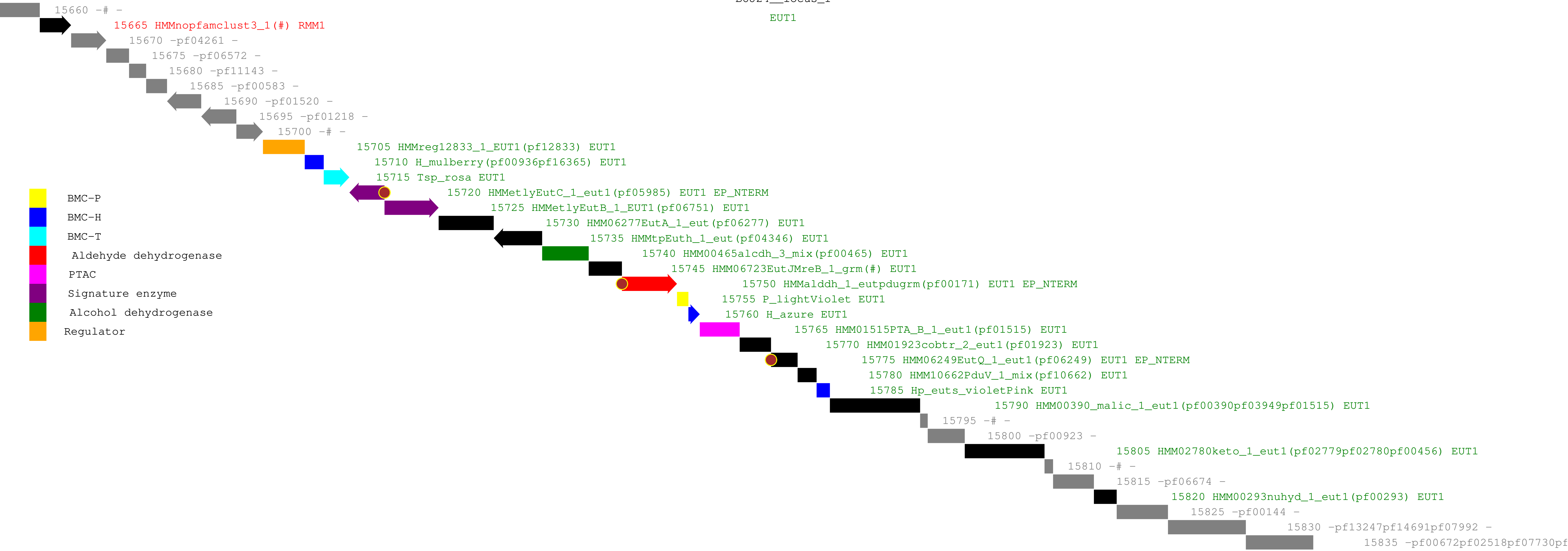

Salmonella enterica subsp. enterica serovar Bardo. Bacteria; Proteobacteria; Gammaproteobacteria; Enterobacterales;

BUJ26__locus_1

EUT1

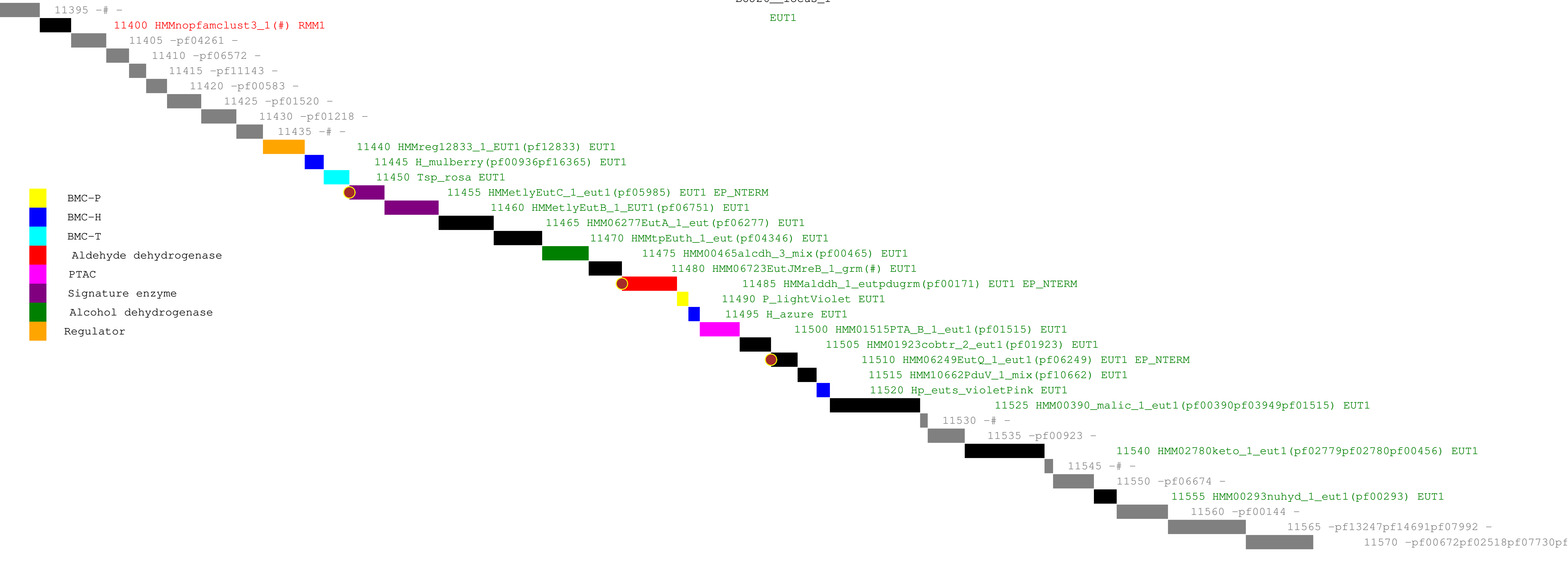

Salmonella enterica subsp. enterica serovar Hartford. Bacteria; Proteobacteria; Gammaproteobacteria; Enterobacterales;

BUJ27__locus_1

EUT1

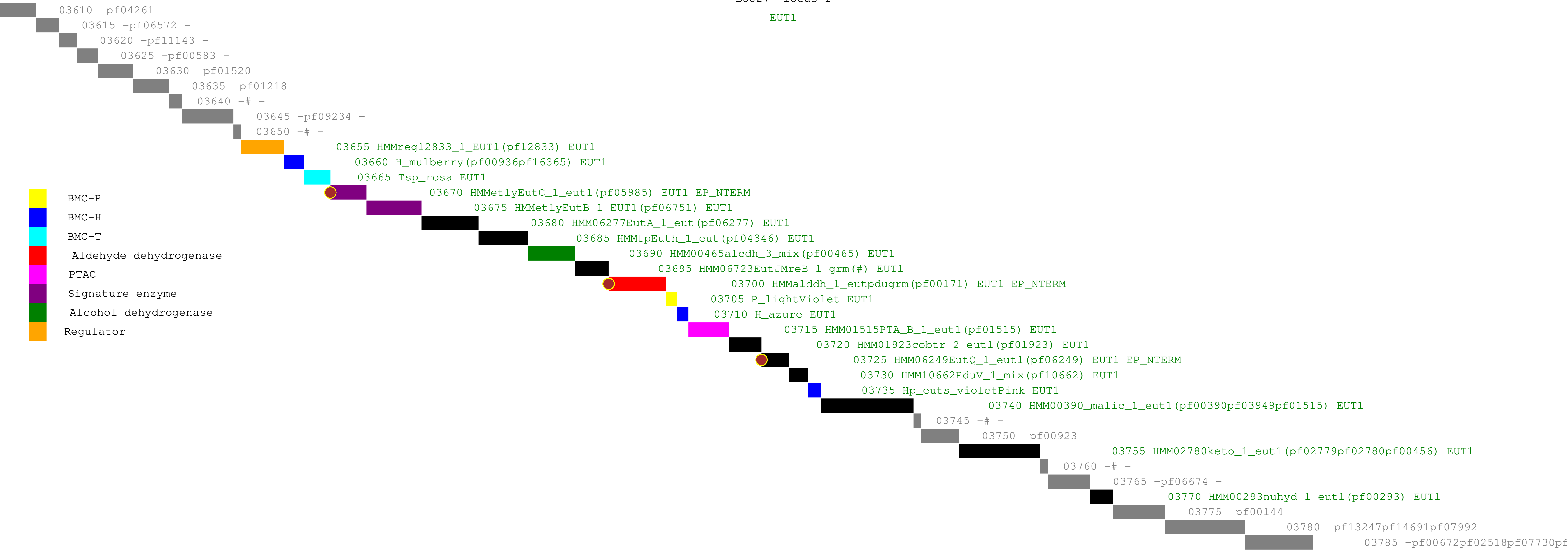

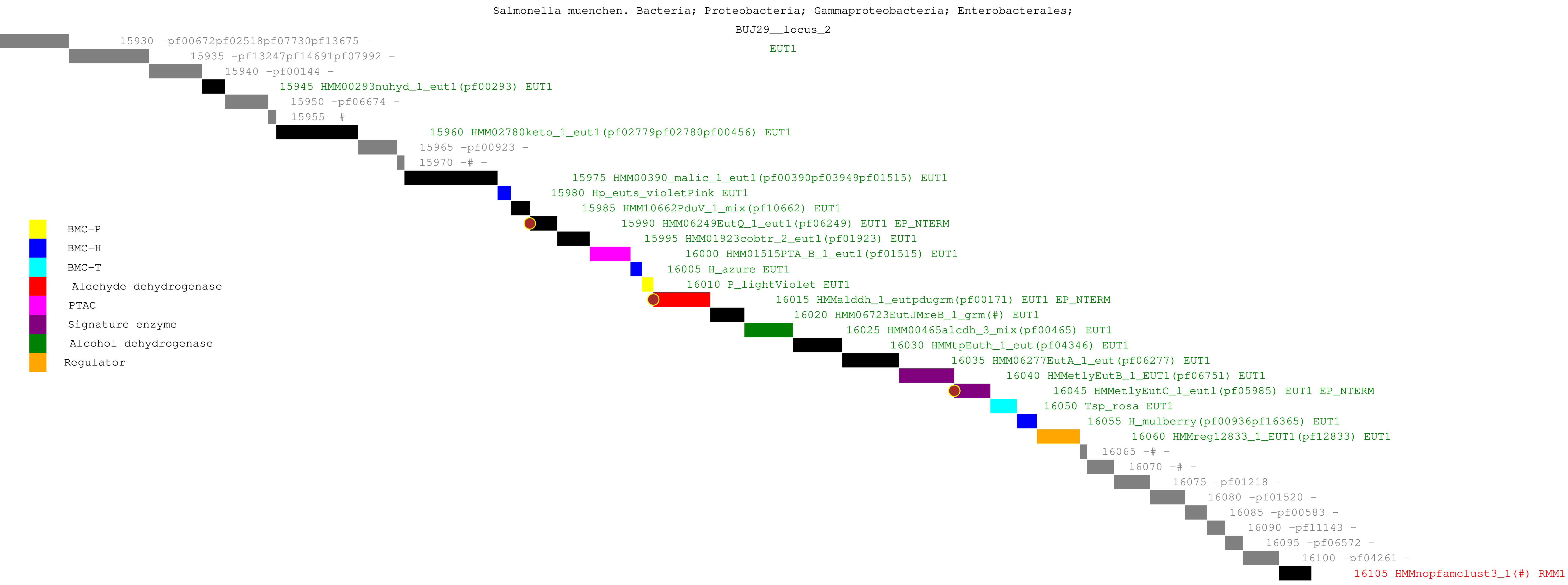
Salmonella muenchen. Bacteria; Proteobacteria; Gammaproteobacteria; Enterobacterales;
BUJ29__locus_2
EUT1
15930 -pf00672pf02518pf07730pf13675 -
15935 -pf13247pf14691pf07992 -
15940 -pf00144 -
15945 HMM00293nuhyd_1_eut1(pf00293) EUT1
15950 -pf06674 -
15955 -# -
15960 HMM02780keto_1_eut1(pf02779pf02780pf00456) EUT1
15965 -pf00923 -
15970 -# -
15975 HMM00390_malic_1_eut1(pf00390pf03949pf01515) EUT1
15980 Hp_euts_violetPink EUT1
15985 HMM10662PduV_1_mix(pf10662) EUT1
15990 HMM06249EutQ_1_eut1(pf06249) EUT1 EP_NTERM
15995 HMM01923cobtr_2_eut1(pf01923) EUT1
16000 HMM01515PTA_B_1_eut1(pf01515) EUT1
16005 H_azure EUT1
16010 P_lightViolet EUT1
16015 HMMalddh_1_eutpdugrm(pf00171) EUT1 EP_NTERM
16020 HMM06723EutJMreB_1_grm(#) EUT1
16025 HMM00465alcdh_3_mix(pf00465) EUT1
16030 HMMtpEuth_1_eut(pf04346) EUT1
16035 HMM06277EutA_1_eut(pf06277) EUT1
16040 HMMetlyEutB_1_EUT1(pf06751) EUT1
16045 HMMetlyEutC_1_eut1(pf05985) EUT1 EP_NTERM
16050 Tsp_rosa EUT1
16055 H_mulberry(pf00936pf16365) EUT1
16060 HMMreg12833_1_EUT1(pf12833) EUT1
16065 -# -
16070 -# -
16075 -pf01218 -
16080 -pf01520 -
16085 -pf00583 -
16090 -pf11143 -
16095 -pf06572 -
16100 -pf04261 -
16105 HMMnopfamclust3_1(#) RMM1
BMC-P
BMC-H
BMC-T
Aldehyde dehydrogenase
PTAC
Signature enzyme
Alcohol dehydrogenase
Regulator

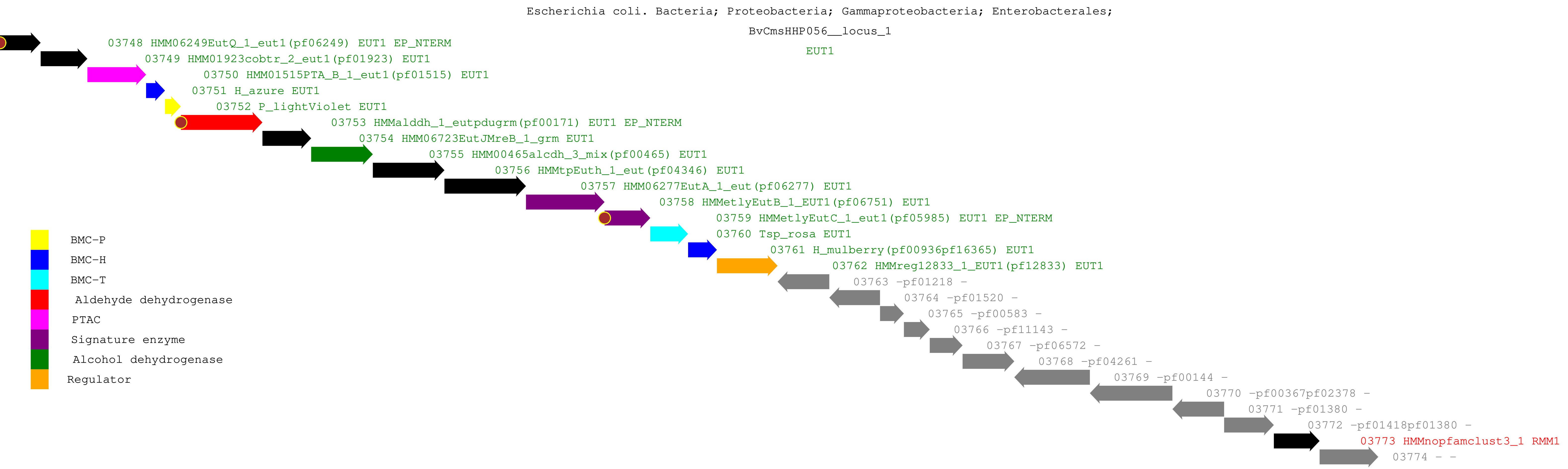
Escherichia coli. Bacteria; Proteobacteria; Gammaproteobacteria; Enterobacterales;
BvCmsHHP056__locus_1
EUT1
03748 HMM06249EutQ_1_eut1(pf06249) EUT1 EP_NTERM
03749 HMM01923cobtr_2_eut1(pf01923) EUT1
03750 HMM01515PTA_B_1_eut1(pf01515) EUT1
03751 H_azure EUT1
03752 P_lightViolet EUT1
03753 HMMaldh_1_eutpdugrm(pf00171) EUT1 EP_NTERM
03754 HMM06723EutJMreB_1_grm EUT1
03755 HMM00465alcdh_3_mix(pf00465) EUT1
03756 HMMtpEuth_1_eut(pf04346) EUT1
03757 HMM06277EutA_1_eut(pf06277) EUT1
03758 HMMetlyEutB_1_EUT1(pf06751) EUT1
03759 HMMetlyEutC_1_eut1(pf05985) EUT1 EP_NTERM
03760 Tsp_rosa EUT1
03761 H_mulberry(pf00936pf16365) EUT1
03762 HMMreg12833_1_EUT1(pf12833) EUT1
03763 -pf01218 -
03764 -pf01520 -
03765 -pf00583 -
03766 -pf11143 -
03767 -pf06572 -
03768 -pf04261 -
03769 -pf00144 -
03770 -pf00367pf02378 -
03771 -pf01380 -
03772 -pf01418pf01380 -
03773 HMMnopfamclust3_1 RMM1
03774 - -
BMC-P
BMC-H
BMC-T
Aldehyde dehydrogenase
PTAC
Signature enzyme
Alcohol dehydrogenase
Regulator

Escherichia coli. Bacteria; Proteobacteria; Gammaproteobacteria; Enterobacterales;

BvCmsKSP026__locus_1

EUT1

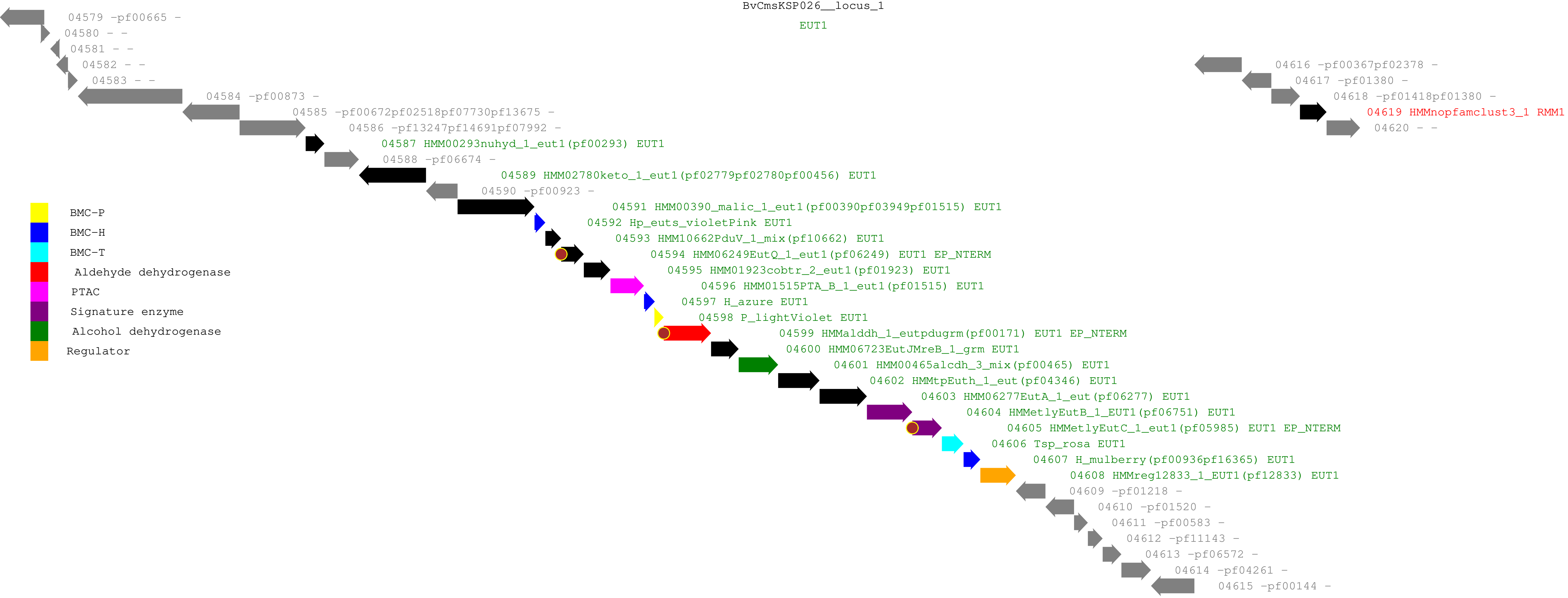

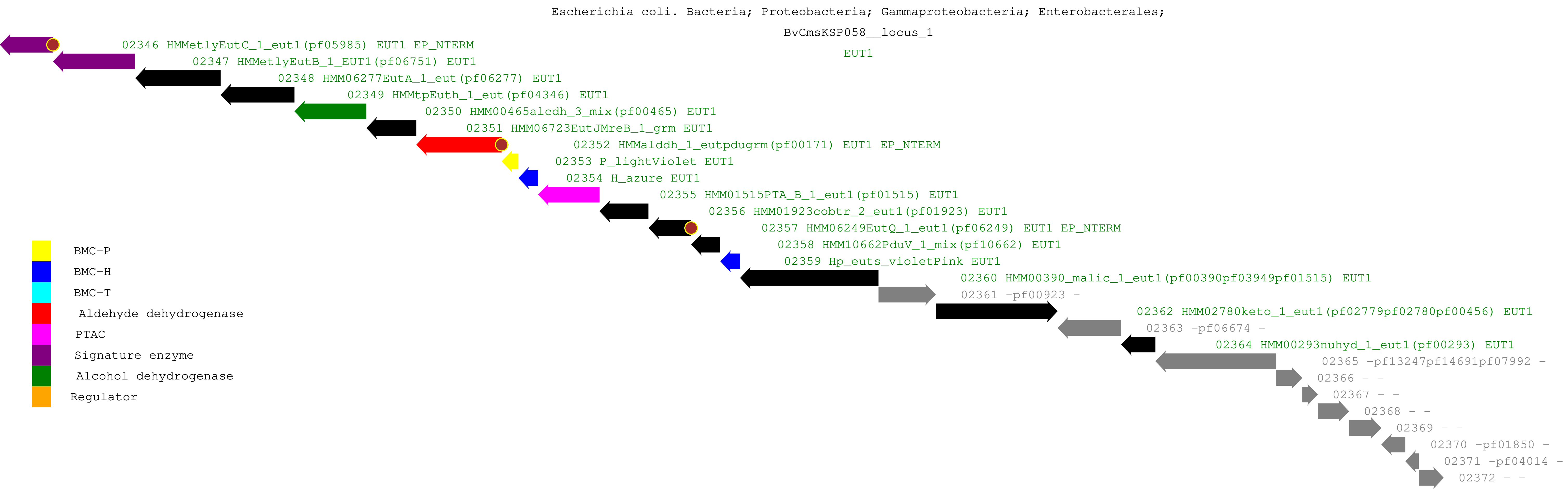
Escherichia coli. Bacteria; Proteobacteria; Gammaproteobacteria; Enterobacterales;
BvCmsKSP058__locus_1
EUT1
02346 HMMetlyEutC_1_eut1(pf05985) EUT1 EP_NTERM
02347 HMMetlyEutB_1_EUT1(pf06751) EUT1
02348 HMM06277EutA_1_eut(pf06277) EUT1
02349 HMMtpEuth_1_eut(pf04346) EUT1
02350 HMM00465alcdh_3_mix(pf00465) EUT1
02351 HMM06723EutJMreB_1_grm EUT1
02352 HMMalddh_1_eutpdugrm(pf00171) EUT1 EP_NTERM
02353 P_lightViolet EUT1
02354 H_azure EUT1
02355 HMM01515PTA_B_1_eut1(pf01515) EUT1
02356 HMM01923cobtr_2_eut1(pf01923) EUT1
02357 HMM06249EutQ_1_eut1(pf06249) EUT1 EP_NTERM
02358 HMM10662PduV_1_mix(pf10662) EUT1
02359 Hp_euts_violetPink EUT1
02360 HMM00390_malic_1_eut1(pf00390pf03949pf01515) EUT1
02361 -pf00923 -
02362 HMM02780keto_1_eut1(pf02779pf02780pf00456) EUT1
02363 -pf06674 -
02364 HMM00293nuhyd_1_eut1(pf00293) EUT1
02365 -pf13247pf14691pf07992 -
02366 - -
02367 - -
02368 - -
02369 - -
02370 -pf01850 -
02371 -pf04014 -
02372 - -
BMC-P
BMC-H
BMC-T
Aldehyde dehydrogenase
PTAC
Signature enzyme
Alcohol dehydrogenase
Regulator

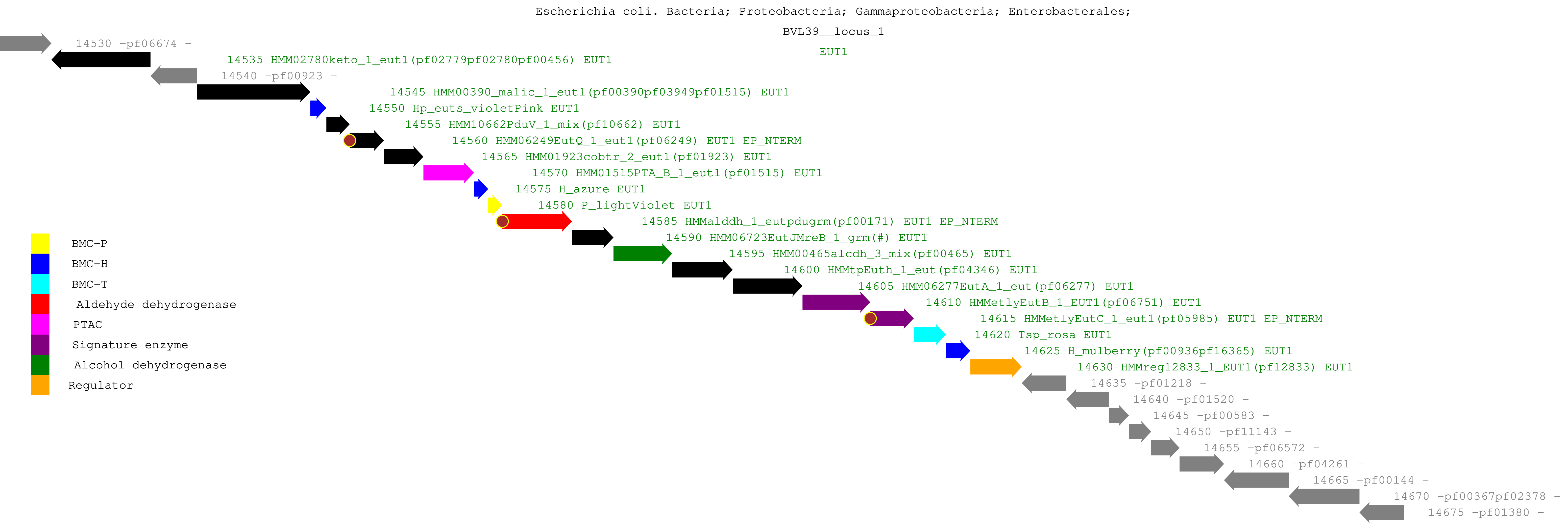

Escherichia coli. Bacteria; Proteobacteria; Gammaproteobacteria; Enterobacterales;
BVL39__locus_1
EUT1
14530 -pf06674 -
14535 HMM02780keto_1_eut1(pf02779pf02780pf00456) EUT1
14540 -pf00923 -
14545 HMM00390_malic_1_eut1(pf00390pf03949pf01515) EUT1
14550 Hp_euts_violetPink EUT1
14555 HMM10662PduV_1_mix(pf10662) EUT1
14560 HMM06249EutQ_1_eut1(pf06249) EUT1 EP_NTERM
14565 HMM01923cobtr_2_eut1(pf01923) EUT1
14570 HMM01515PTA_B_1_eut1(pf01515) EUT1
14575 H_azure EUT1
14580 P_lightViolet EUT1
14585 HMMalddh_1_eutpdugrm(pf00171) EUT1 EP_NTERM
14590 HMM06723EutJMreB_1_grm(#) EUT1
14595 HMM00465alcdh_3_mix(pf00465) EUT1
14600 HMMtpEuth_1_eut(pf04346) EUT1
14605 HMM06277EutA_1_eut(pf06277) EUT1
14610 HMMetlyEutB_1_EUT1(pf06751) EUT1
14615 HMMetlyEutC_1_eut1(pf05985) EUT1 EP_NTERM
14620 Tsp_rosa EUT1
14625 H_mulberry(pf00936pf16365) EUT1
14630 HMMreg12833_1_EUT1(pf12833) EUT1
14635 -pf01218 -
14640 -pf01520 -
14645 -pf00583 -
14650 -pf11143 -
14655 -pf06572 -
14660 -pf04261 -
14665 -pf00144 -
14670 -pf00367pf02378 -
14675 -pf01380 -
BMC-P
BMC-H
BMC-T
Aldehyde dehydrogenase
PTAC
Signature enzyme
Alcohol dehydrogenase
Regulator

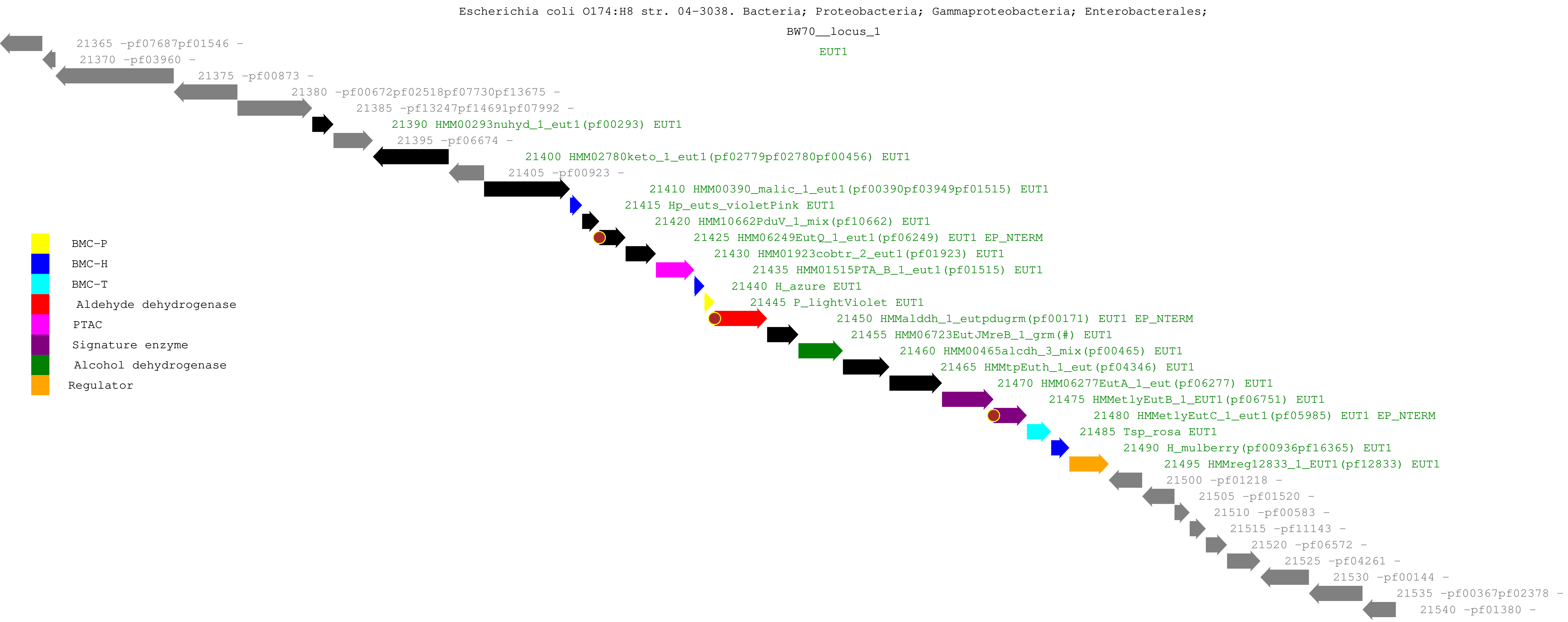
Escherichia coli O174:H8 str. 04-3038. Bacteria; Proteobacteria; Gammaproteobacteria; Enterobacterales;
BW70__locus_1
EUT1
21365 -pf07687pf01546 -
21370 -pf03960 -
21375 -pf00873 -
21380 -pf00672pf02518pf07730pf13675 -
21385 -pf13247pf14691pf07992 -
21390 HMM00293nuhyd_1_eut1(pf00293) EUT1
21395 -pf06674 -
21400 HMM02780keto_1_eut1(pf02779pf02780pf00456) EUT1
21405 -pf00923 -
21410 HMM00390_malic_1_eut1(pf00390pf03949pf01515) EUT1
21415 Hp_euts_violetPink EUT1
21420 HMM10662PduV_1_mix(pf10662) EUT1
21425 HMM06249EutQ_1_eut1(pf06249) EUT1 EP_NTERM
21430 HMM01923cobtr_2_eut1(pf01923) EUT1
21435 HMM01515PTA_B_1_eut1(pf01515) EUT1
21440 H_azure EUT1
21445 P_lightViolet EUT1
21450 HMMalddh_1_eutpdugrm(pf00171) EUT1 EP_NTERM
21455 HMM06723EutJMreB_1_grm(#) EUT1
21460 HMM00465alcdh_3_mix(pf00465) EUT1
21465 HMMtpEuth_1_eut(pf04346) EUT1
21470 HMM06277EutA_1_eut(pf06277) EUT1
21475 HMMetlyEutB_1_EUT1(pf06751) EUT1
21480 HMMetlyEutC_1_eut1(pf05985) EUT1 EP_NTERM
21485 Tsp_rosa EUT1
21490 H_mulberry(pf00936pf16365) EUT1
21495 HMMreg12833_1_EUT1(pf12833) EUT1
21500 -pf01218 -
21505 -pf01520 -
21510 -pf00583 -
21515 -pf11143 -
21520 -pf06572 -
21525 -pf04261 -
21530 -pf00144 -
21535 -pf00367pf02378 -
21540 -pf01380 -
BMC-P
BMC-H
BMC-T
Aldehyde dehydrogenase
PTAC
Signature enzyme
Alcohol dehydrogenase
Regulator

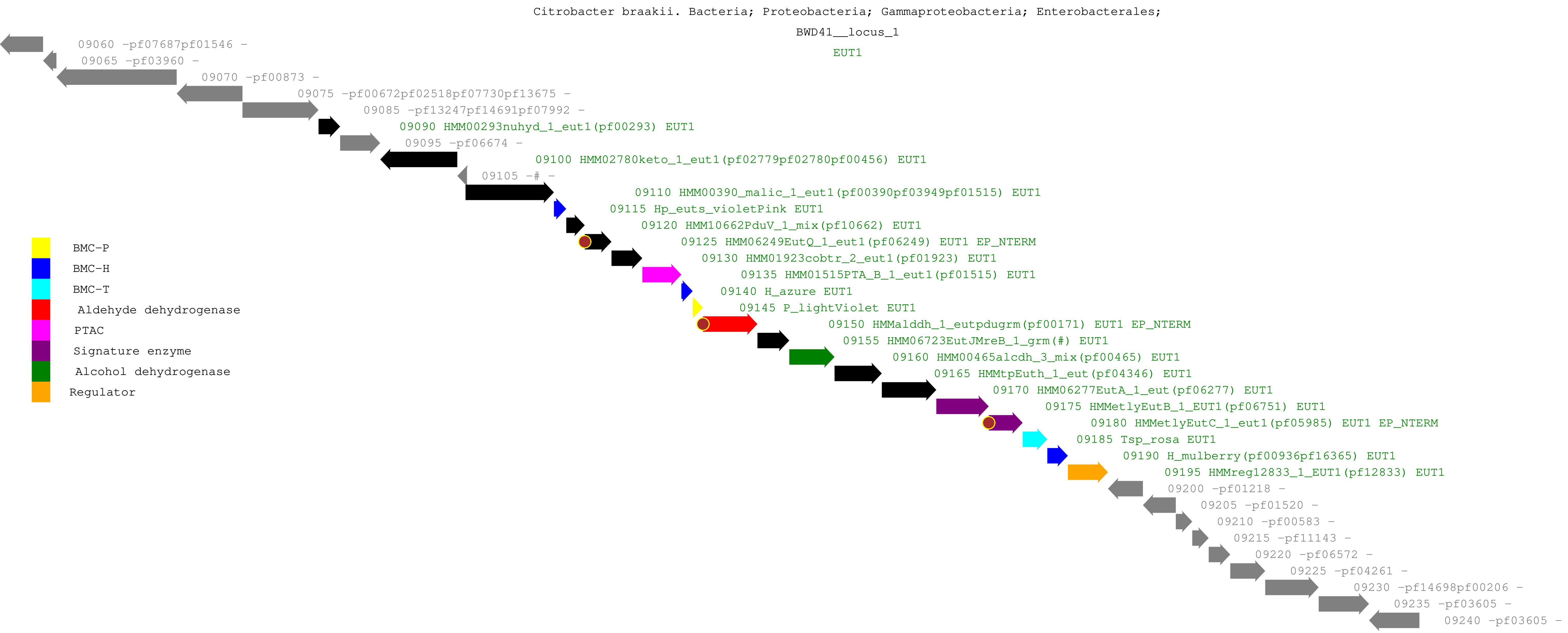
Citrobacter braakii. Bacteria; Proteobacteria; Gammaproteobacteria; Enterobacterales;
BWD41__locus_1
EUT1
09060 -pf07687pf01546 -
09065 -pf03960 -
09070 -pf00873 -
09075 -pf00672pf02518pf07730pf13675 -
09085 -pf13247pf14691pf07992 -
09090 HMM00293nuhyd_1_eut1(pf00293) EUT1
09095 -pf06674 -
09100 HMM02780keto_1_eut1(pf02779pf02780pf00456) EUT1
09105 -# -
09110 HMM00390_malic_1_eut1(pf00390pf03949pf01515) EUT1
09115 Hp_euts_violetPink EUT1
09120 HMM10662PduV_1_mix(pf10662) EUT1
09125 HMM06249EutQ_1_eut1(pf06249) EUT1 EP_NTERM
09130 HMM01923cobtr_2_eut1(pf01923) EUT1
09135 HMM01515PTA_B_1_eut1(pf01515) EUT1
09140 H_azure EUT1
09145 P_lightViolet EUT1
09150 HMMalddh_1_eutpdugrm(pf00171) EUT1 EP_NTERM
09155 HMM06723EutJMreB_1_grm(#) EUT1
09160 HMM00465alcdh_3_mix(pf00465) EUT1
09165 HMMtpEuth_1_eut(pf04346) EUT1
09170 HMM06277EutA_1_eut(pf06277) EUT1
09175 HMMetlyEutB_1_EUT1(pf06751) EUT1
09180 HMMetlyEutC_1_eut1(pf05985) EUT1 EP_NTERM
09185 Tsp_rosa EUT1
09190 H_mulberry(pf00936pf16365) EUT1
09195 HMMreg12833_1_EUT1(pf12833) EUT1
09200 -pf01218 -
09205 -pf01520 -
09210 -pf00583 -
09215 -pf11143 -
09220 -pf06572 -
09225 -pf04261 -
09230 -pf14698pf00206 -
09235 -pf03605 -
09240 -pf03605 -
BMC-P
BMC-H
BMC-T
Aldehyde dehydrogenase
PTAC
Signature enzyme
Alcohol dehydrogenase
Regulator

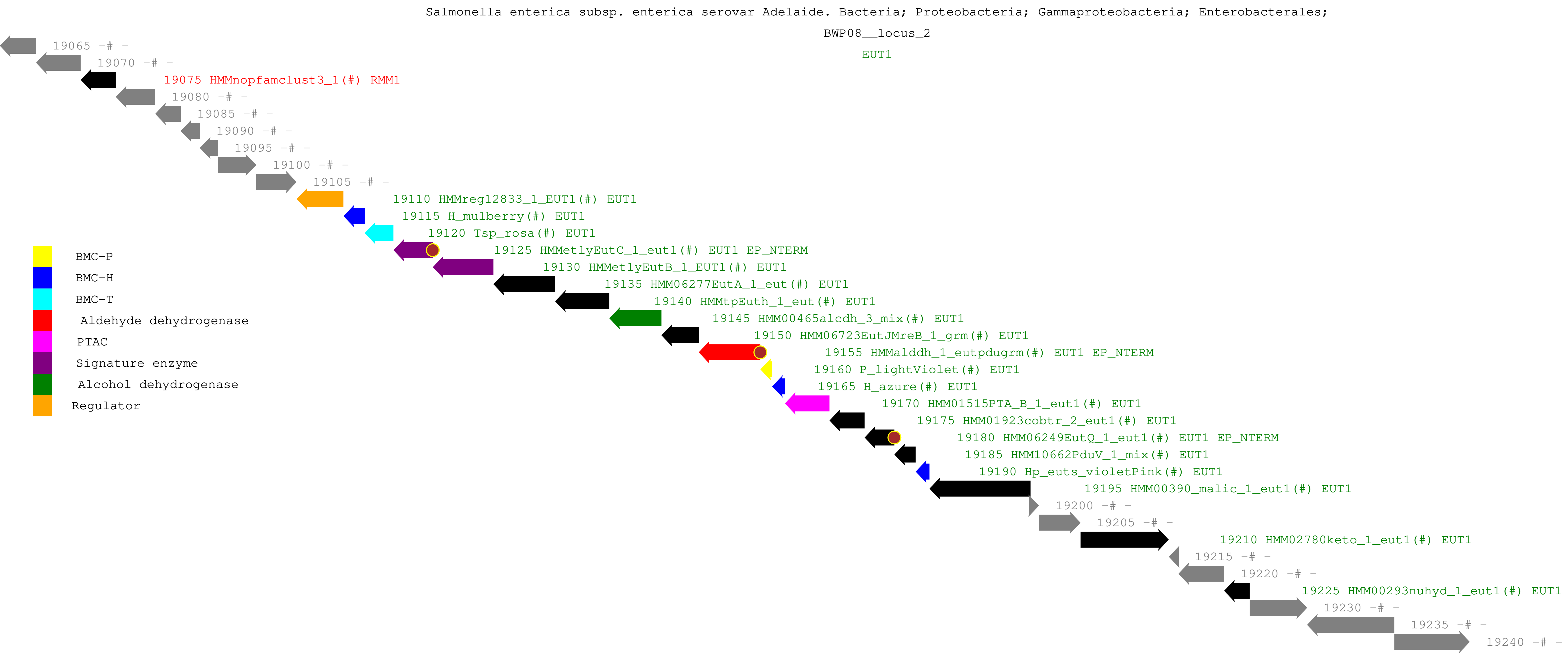

Salmonella enterica subsp. enterica serovar Adelaide. Bacteria; Proteobacteria; Gammaproteobacteria; Enterobacterales;
BWP08__locus_2
EUT1
19065 -# -
19070 -# -
19075 HMMnopfamclust3_1(#) RMM1
19080 -# -
19085 -# -
19090 -# -
19095 -# -
19100 -# -
19105 -# -
19110 HMMreg12833_1_EUT1(#) EUT1
19115 H_mulberry(#) EUT1
19120 Tsp_rosa(#) EUT1
19125 HMMetlyEutC_1_eut1(#) EUT1 EP_NTERM
19130 HMMetlyEutB_1_EUT1(#) EUT1
19135 HMM06277EutA_1_eut(#) EUT1
19140 HMMtpEuth_1_eut(#) EUT1
19145 HMM00465alcdh_3_mix(#) EUT1
19150 HMM06723EutJMreB_1_grm(#) EUT1
19155 HMMalddh_1_eutpdugrm(#) EUT1 EP_NTERM
19160 P_lightViolet(#) EUT1
19165 H_azure(#) EUT1
19170 HMM01515PTA_B_1_eut1(#) EUT1
19175 HMM01923cobtr_2_eut1(#) EUT1
19180 HMM06249EutQ_1_eut1(#) EUT1 EP_NTERM
19185 HMM10662PduV_1_mix(#) EUT1
19190 Hp_euts_violetPink(#) EUT1
19195 HMM00390_malic_1_eut1(#) EUT1
19200 -# -
19205 -# -
19210 HMM02780keto_1_eut1(#) EUT1
19215 -# -
19220 -# -
19225 HMM00293nuhyd_1_eut1(#) EUT1
19230 -# -
19235 -# -
19240 -# -
BMC-P
BMC-H
BMC-T
Aldehyde dehydrogenase
PTAC
Signature enzyme
Alcohol dehydrogenase
Regulator

Salmonella enterica subsp. enterica serovar Worthington. Bacteria; Proteobacteria; Gammaproteobacteria; Enterobacterales;

BWP16__locus_1

EUT1

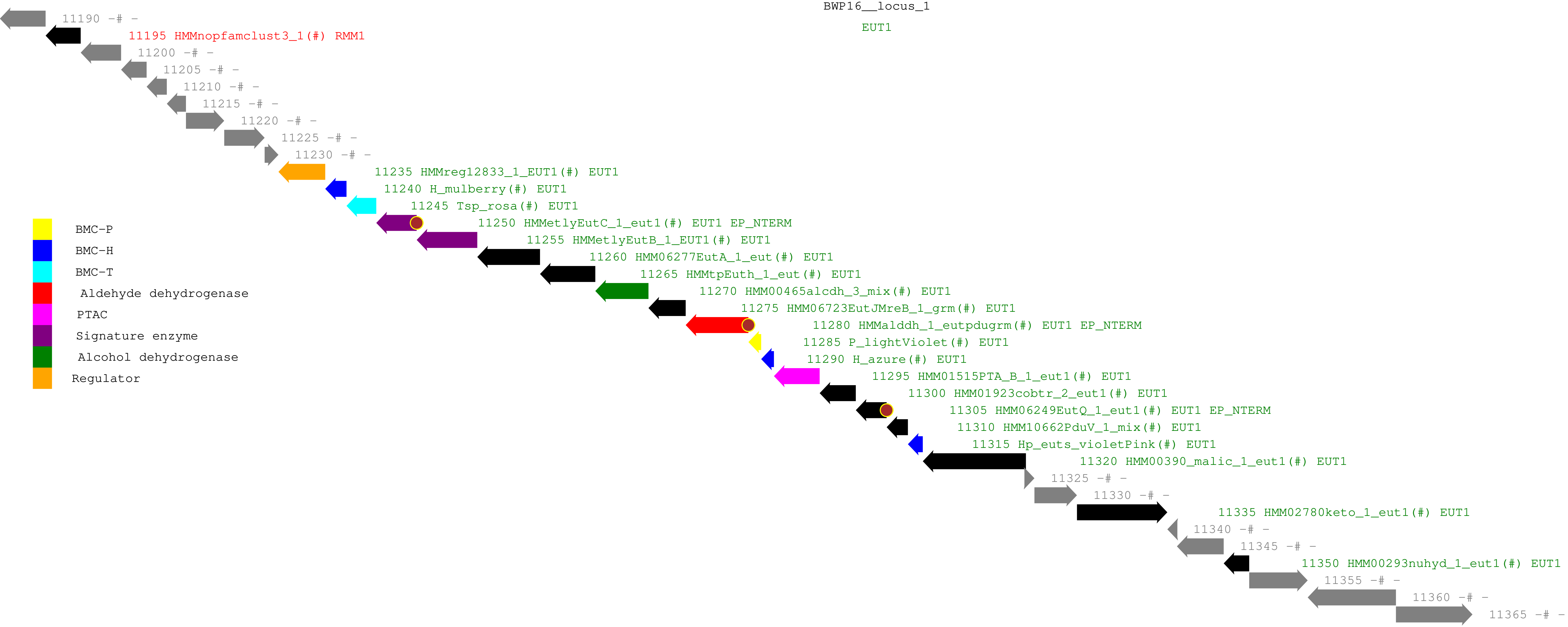

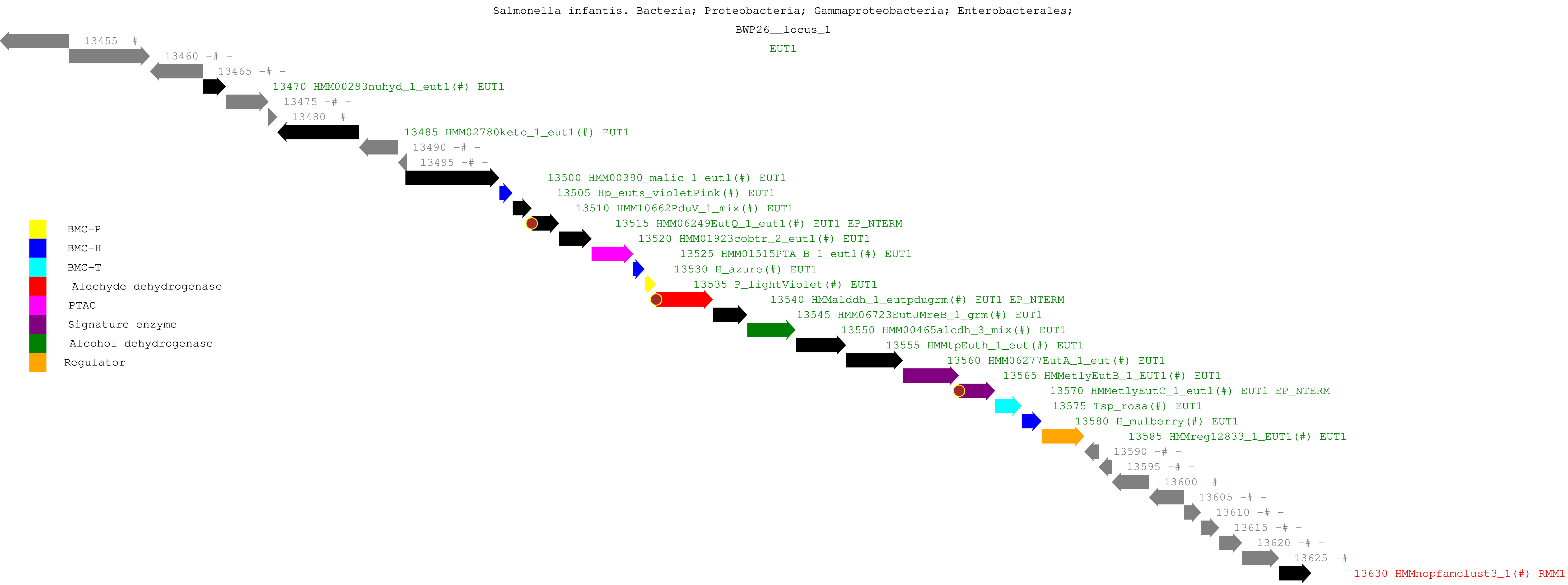
Salmonella infantis. Bacteria; Proteobacteria; Gammaproteobacteria; Enterobacterales;
BWP26__locus_1
EUT1
13455 -# -
13460 -# -
13465 -# -
13470 HMM00293nuhyd_1_eut1(#) EUT1
13475 -# -
13480 -# -
13485 HMM02780keto_1_eut1(#) EUT1
13490 -# -
13495 -# -
13500 HMM00390_malic_1_eut1(#) EUT1
13505 Hp_euts_violetPink(#) EUT1
13510 HMM10662PduV_1_mix(#) EUT1
13515 HMM06249EutQ_1_eut1(#) EUT1 EP_NTERM
13520 HMM01923cobtr_2_eut1(#) EUT1
13525 HMM01515PTA_B_1_eut1(#) EUT1
13530 H_azure(#) EUT1
13535 P_lightViolet(#) EUT1
13540 HMMaldh_1_eutpdugrm(#) EUT1 EP_NTERM
13545 HMM06723EutJMreB_1_grm(#) EUT1
13550 HMM00465alcdh_3_mix(#) EUT1
13555 HMMtpEuth_1_eut(#) EUT1
13560 HMM06277EutA_1_eut(#) EUT1
13565 HMMetlyEutB_1_EUT1(#) EUT1
13570 HMMetlyEutC_1_eut1(#) EUT1 EP_NTERM
13575 Tsp_rosa(#) EUT1
13580 H_mulberry(#) EUT1
13585 HMMreg12833_1_EUT1(#) EUT1
13590 -# -
13595 -# -
13600 -# -
13605 -# -
13610 -# -
13615 -# -
13620 -# -
13625 -# -
13630 HMMnopfamclust3_1(#) RMM1
BMC-P
BMC-H
BMC-T
Aldehyde dehydrogenase
PTAC
Signature enzyme
Alcohol dehydrogenase
Regulator

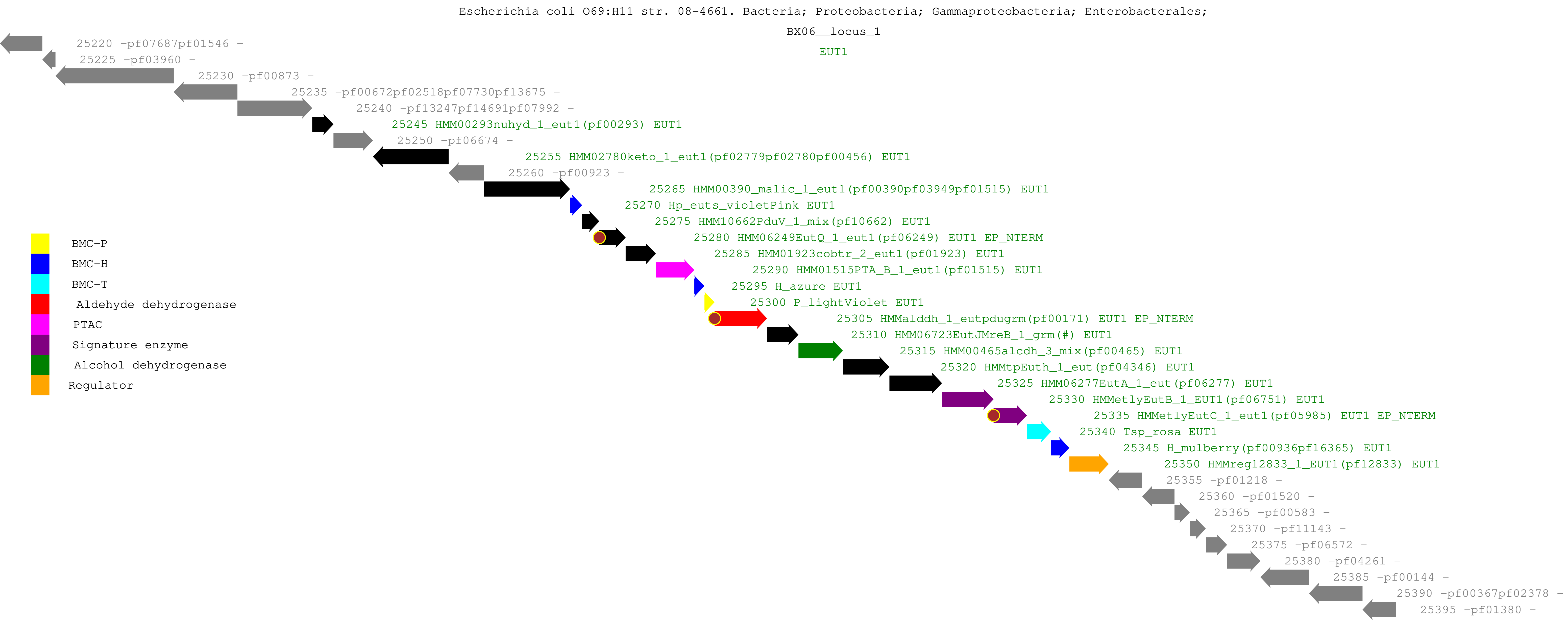
Escherichia coli O69:H11 str. 08-4661. Bacteria; Proteobacteria; Gammaproteobacteria; Enterobacterales;
BX06__locus_1
EUT1
25220 -pf07687pf01546 -
25225 -pf03960 -
25230 -pf00873 -
25235 -pf00672pf02518pf07730pf13675 -
25240 -pf13247pf14691pf07992 -
25245 HMM00293nuhyd_1_eut1(pf00293) EUT1
25250 -pf06674 -
25255 HMM02780keto_1_eut1(pf02779pf02780pf00456) EUT1
25260 -pf00923 -
25265 HMM00390_malic_1_eut1(pf00390pf03949pf01515) EUT1
25270 Hp_euts_violetPink EUT1
25275 HMM10662PduV_1_mix(pf10662) EUT1
25280 HMM06249EutQ_1_eut1(pf06249) EUT1 EP_NTERM
25285 HMM01923cobtr_2_eut1(pf01923) EUT1
25290 HMM01515PTA_B_1_eut1(pf01515) EUT1
25295 H_azure EUT1
25300 P_lightViolet EUT1
25305 HMMalddh_1_eutpdugrm(pf00171) EUT1 EP_NTERM
25310 HMM06723EutJMreB_1_grm(#) EUT1
25315 HMM00465alcdh_3_mix(pf00465) EUT1
25320 HMMtpEuth_1_eut(pf04346) EUT1
25325 HMM06277EutA_1_eut(pf06277) EUT1
25330 HMMetlyEutB_1_EUT1(pf06751) EUT1
25335 HMMetlyEutC_1_eut1(pf05985) EUT1 EP_NTERM
25340 Tsp_rosa EUT1
25345 H_mulberry(pf00936pf16365) EUT1
25350 HMMreg12833_1_EUT1(pf12833) EUT1
25355 -pf01218 -
25360 -pf01520 -
25365 -pf00583 -
25370 -pf11143 -
25375 -pf06572 -
25380 -pf04261 -
25385 -pf00144 -
25390 -pf00367pf02378 -
25395 -pf01380 -
BMC-P
BMC-H
BMC-T
Aldehyde dehydrogenase
PTAC
Signature enzyme
Alcohol dehydrogenase
Regulator

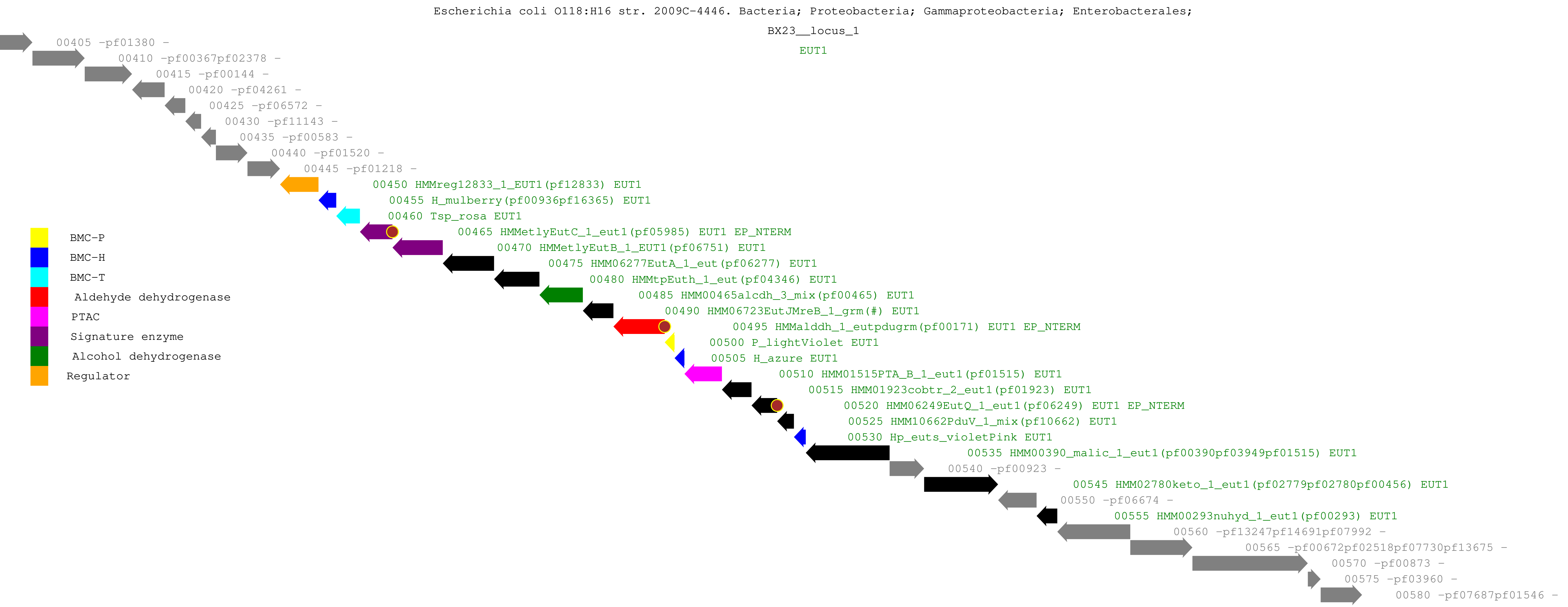
Escherichia coli O118:H16 str. 2009C-4446. Bacteria; Proteobacteria; Gammaproteobacteria; Enterobacterales;
BX23__locus_1
EUT1
00405 -pf01380 -
00410 -pf00367pf02378 -
00415 -pf00144 -
00420 -pf04261 -
00425 -pf06572 -
00430 -pf11143 -
00435 -pf00583 -
00440 -pf01520 -
00445 -pf01218 -
00450 HMMreg12833_1_EUT1(pf12833) EUT1
00455 H_mulberry(pf00936pf16365) EUT1
00460 Tsp_rosa EUT1
00465 HMMetlyEutC_1_eut1(pf05985) EUT1 EP_NTERM
00470 HMMetlyEutB_1_EUT1(pf06751) EUT1
00475 HMM06277EutA_1_eut(pf06277) EUT1
00480 HMMtpEuth_1_eut(pf04346) EUT1
00485 HMM00465alcdh_3_mix(pf00465) EUT1
00490 HMM06723EutJMreB_1_grm(#) EUT1
00495 HMMalddh_1_eutpdugrm(pf00171) EUT1 EP_NTERM
00500 P_lightViolet EUT1
00505 H_azure EUT1
00510 HMM01515PTA_B_1_eut1(pf01515) EUT1
00515 HMM01923cobtr_2_eut1(pf01923) EUT1
00520 HMM06249EutQ_1_eut1(pf06249) EUT1 EP_NTERM
00525 HMM10662PduV_1_mix(pf10662) EUT1
00530 Hp_euts_violetPink EUT1
00535 HMM00390_malic_1_eut1(pf00390pf03949pf01515) EUT1
00540 -pf00923 -
00545 HMM02780keto_1_eut1(pf02779pf02780pf00456) EUT1
00550 -pf06674 -
00555 HMM00293nuhyd_1_eut1(pf00293) EUT1
00560 -pf13247pf14691pf07992 -
00565 -pf00672pf02518pf07730pf13675 -
00570 -pf00873 -
00575 -pf03960 -
00580 -pf07687pf01546 -
BMC-P
BMC-H
BMC-T
Aldehyde dehydrogenase
PTAC
Signature enzyme
Alcohol dehydrogenase
Regulator

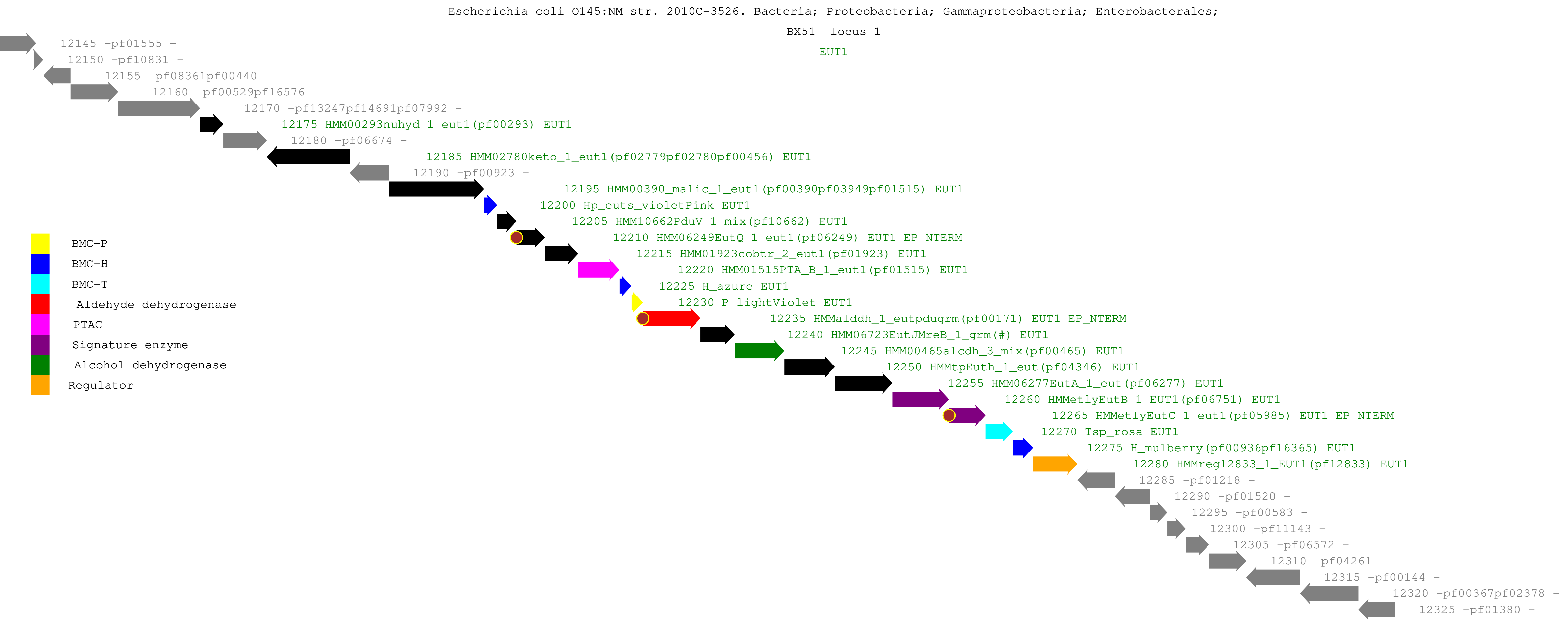
Escherichia coli O145:NM str. 2010C-3526. Bacteria; Proteobacteria; Gammaproteobacteria; Enterobacterales;
BX51__locus_1
EUT1
12145 -pf01555 -
12150 -pf10831 -
12155 -pf08361pf00440 -
12160 -pf00529pf16576 -
12170 -pf13247pf14691pf07992 -
12175 HMM00293nuhyd_1_eut1(pf00293) EUT1
12180 -pf06674 -
12185 HMM02780keto_1_eut1(pf02779pf02780pf00456) EUT1
12190 -pf00923 -
12195 HMM00390_malic_1_eut1(pf00390pf03949pf01515) EUT1
12200 Hp_euts_violetPink EUT1
12205 HMM10662PduV_1_mix(pf10662) EUT1
12210 HMM06249EutQ_1_eut1(pf06249) EUT1 EP_NTERM
12215 HMM01923cobtr_2_eut1(pf01923) EUT1
12220 HMM01515PTA_B_1_eut1(pf01515) EUT1
12225 H_azure EUT1
12230 P_lightViolet EUT1
12235 HMMalddh_1_eutpdugrm(pf00171) EUT1 EP_NTERM
12240 HMM06723EutJMreB_1_grm(#) EUT1
12245 HMM00465alcdh_3_mix(pf00465) EUT1
12250 HMMtpEuth_1_eut(pf04346) EUT1
12255 HMM06277EutA_1_eut(pf06277) EUT1
12260 HMMetlyEutB_1_EUT1(pf06751) EUT1
12265 HMMetlyEutC_1_eut1(pf05985) EUT1 EP_NTERM
12270 Tsp_rosa EUT1
12275 H_mulberry(pf00936pf16365) EUT1
12280 HMMreg12833_1_EUT1(pf12833) EUT1
12285 -pf01218 -
12290 -pf01520 -
12295 -pf00583 -
12300 -pf11143 -
12305 -pf06572 -
12310 -pf04261 -
12315 -pf00144 -
12320 -pf00367pf02378 -
12325 -pf01380 -
BMC-P
BMC-H
BMC-T
Aldehyde dehydrogenase
PTAC
Signature enzyme
Alcohol dehydrogenase
Regulator

Escherichia coli O121:H19 str. 2010C-3609. Bacteria; Proteobacteria; Gammaproteobacteria; Enterobacterales;

BX52__locus_1

EUT1

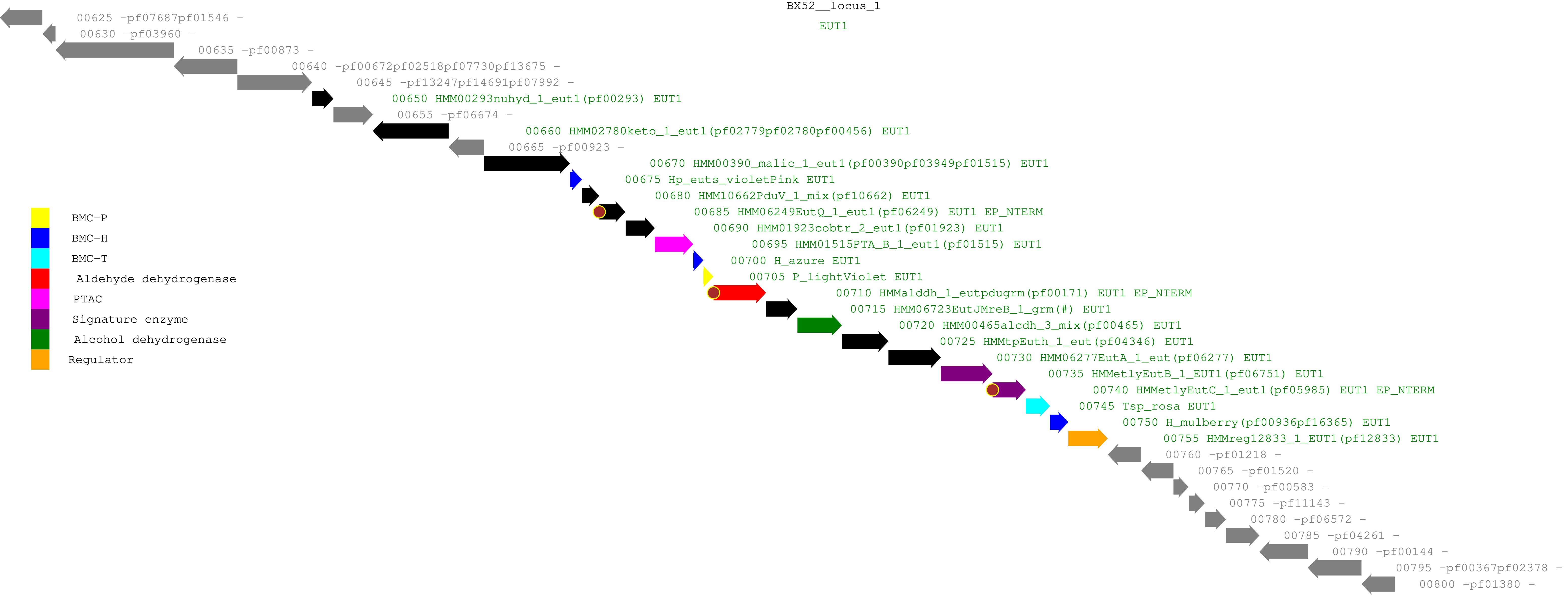

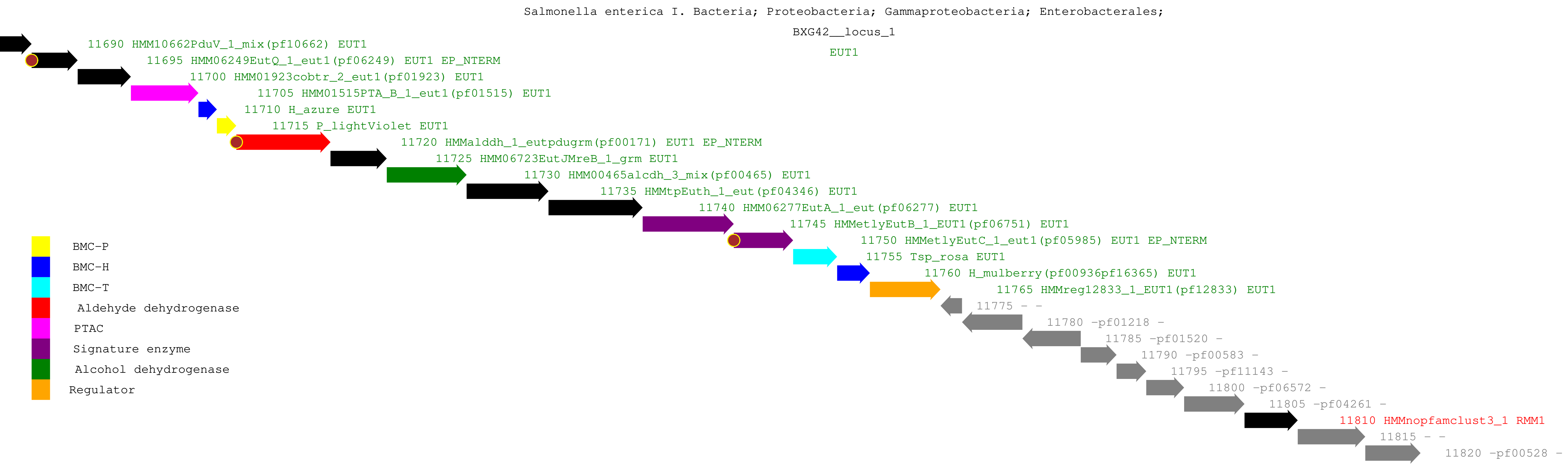

Salmonella enterica I. Bacteria; Proteobacteria; Gammaproteobacteria; Enterobacterales;
BXG42__locus_1
EUT1
11690 HMM10662PduV_1_mix(pf10662) EUT1
11695 HMM06249EutQ_1_eut1(pf06249) EUT1 EP_NTERM
11700 HMM01923cobtr_2_eut1(pf01923) EUT1
11705 HMM01515PTA_B_1_eut1(pf01515) EUT1
11710 H_azure EUT1
11715 P_lightViolet EUT1
11720 HMMalddh_1_eutpdugrm(pf00171) EUT1 EP_NTERM
11725 HMM06723EutJMreB_1_grm EUT1
11730 HMM00465alcdh_3_mix(pf00465) EUT1
11735 HMMtpEuth_1_eut(pf04346) EUT1
11740 HMM06277EutA_1_eut(pf06277) EUT1
11745 HMMetlyEutB_1_EUT1(pf06751) EUT1
11750 HMMetlyEutC_1_eut1(pf05985) EUT1 EP_NTERM
11755 Tsp_rosa EUT1
11760 H_mulberry(pf00936pf16365) EUT1
11765 HMMreg12833_1_EUT1(pf12833) EUT1
11775 - -
11780 -pf01218 -
11785 -pf01520 -
11790 -pf00583 -
11795 -pf11143 -
11800 -pf06572 -
11805 -pf04261 -
11810 HMMnopfamclust3_1 RMM1
11815 - -
11820 -pf00528 -
BMC-P
BMC-H
BMC-T
Aldehyde dehydrogenase
PTAC
Signature enzyme
Alcohol dehydrogenase
Regulator

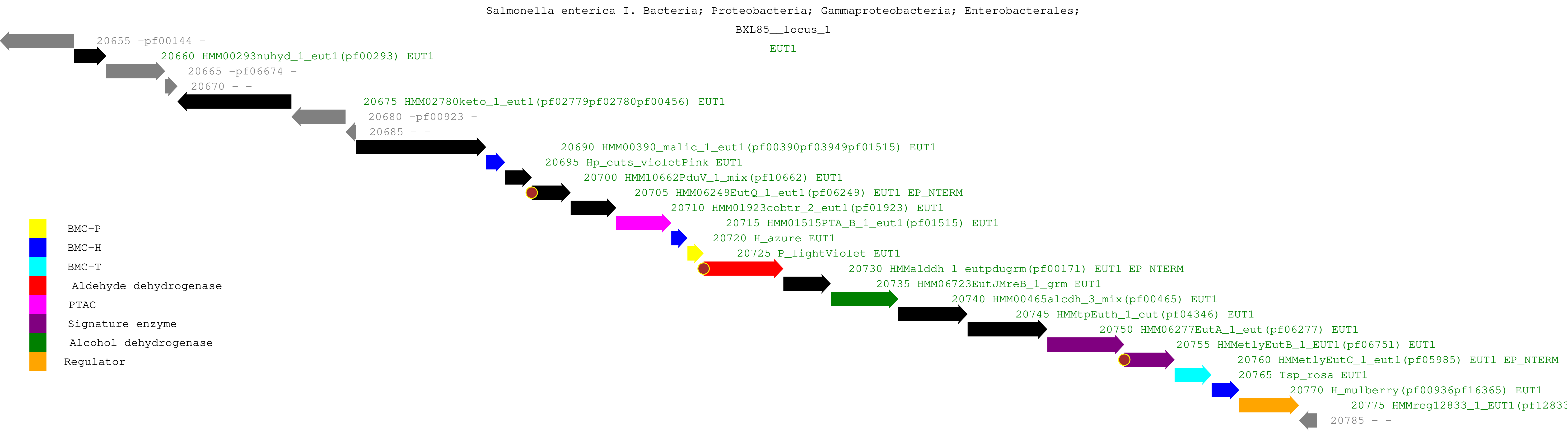
Salmonella enterica I. Bacteria; Proteobacteria; Gammaproteobacteria; Enterobacterales;
BXL85__locus_1
EUT1
20655 -pf00144 -
20660 HMM00293nuhyd_1_eut1(pf00293) EUT1
20665 -pf06674 -
20670 - -
20675 HMM02780keto_1_eut1(pf02779pf02780pf00456) EUT1
20680 -pf00923 -
20685 - -
20690 HMM00390_malic_1_eut1(pf00390pf03949pf01515) EUT1
20695 Hp_euts_violetPink EUT1
20700 HMM10662PduV_1_mix(pf10662) EUT1
20705 HMM06249EutQ_1_eut1(pf06249) EUT1 EP_NTERM
20710 HMM01923cobtr_2_eut1(pf01923) EUT1
20715 HMM01515PTA_B_1_eut1(pf01515) EUT1
20720 H_azure EUT1
20725 P_lightViolet EUT1
20730 HMMalddh_1_eutpdugrm(pf00171) EUT1 EP_NTERM
20735 HMM06723EutJMreB_1_grm EUT1
20740 HMM00465alcdh_3_mix(pf00465) EUT1
20745 HMMtpEuth_1_eut(pf04346) EUT1
20750 HMM06277EutA_1_eut(pf06277) EUT1
20755 HMMetlyEutB_1_EUT1(pf06751) EUT1
20760 HMMetlyEutC_1_eut1(pf05985) EUT1 EP_NTERM
20765 Tsp_rosa EUT1
20770 H_mulberry(pf00936pf16365) EUT1
20775 HMMreg12833_1_EUT1(pf12833
20785 - -
BMC-P
BMC-H
BMC-T
Aldehyde dehydrogenase
PTAC
Signature enzyme
Alcohol dehydrogenase
Regulator

Salmonella enterica subsp. enterica serovar Java. Bacteria; Proteobacteria; Gammaproteobacteria; Enterobacterales;

BXN20__locus_2

EUT1

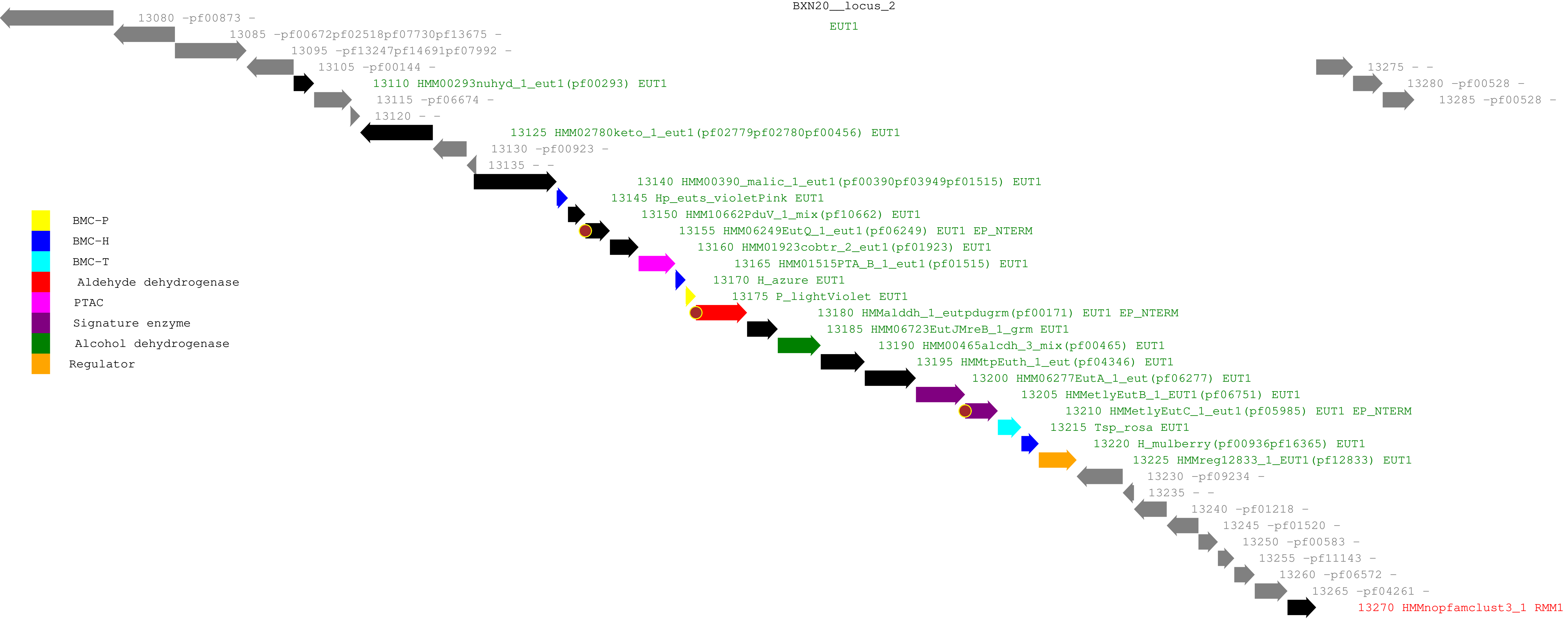

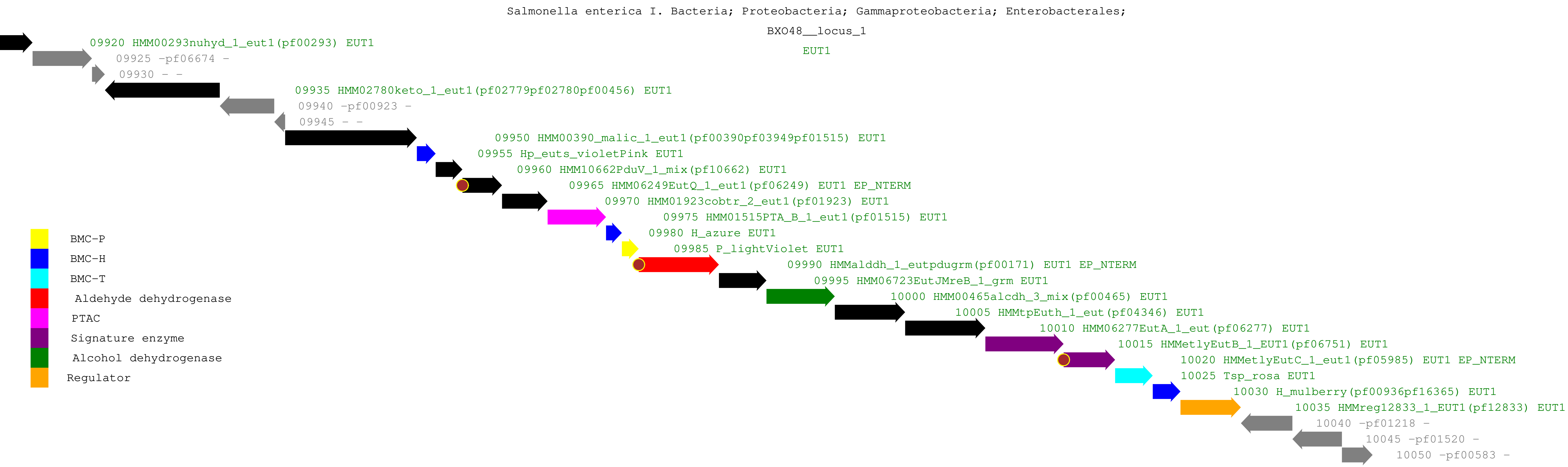
Salmonella enterica I. Bacteria; Proteobacteria; Gammaproteobacteria; Enterobacterales;
BX048__locus_1
EUT1
09920 HMM00293nuhyd_1_eut1(pf00293) EUT1
09925 -pf06674 -
09930 - -
09935 HMM02780keto_1_eut1(pf02779pf02780pf00456) EUT1
09940 -pf00923 -
09945 - -
09950 HMM00390_malic_1_eut1(pf00390pf03949pf01515) EUT1
09955 Hp_euts_violetPink EUT1
09960 HMM10662PduV_1_mix(pf10662) EUT1
09965 HMM06249EutQ_1_eut1(pf06249) EUT1 EP_NTERM
09970 HMM01923cobtr_2_eut1(pf01923) EUT1
09975 HMM01515PTA_B_1_eut1(pf01515) EUT1
09980 H_azure EUT1
09985 P_lightViolet EUT1
09990 HMMalddh_1_eutpdugrm(pf00171) EUT1 EP_NTERM
09995 HMM06723EutJMreB_1_grm EUT1
10000 HMM00465alcdh_3_mix(pf00465) EUT1
10005 HMMtpEuth_1_eut(pf04346) EUT1
10010 HMM06277EutA_1_eut(pf06277) EUT1
10015 HMMetlyEutB_1_EUT1(pf06751) EUT1
10020 HMMetlyEutC_1_eut1(pf05985) EUT1 EP_NTERM
10025 Tsp_rosa EUT1
10030 H_mulberry(pf00936pf16365) EUT1
10035 HMMreg12833_1_EUT1(pf12833) EUT1
10040 -pf01218 -
10045 -pf01520 -
10050 -pf00583 -
BMC-P
BMC-H
BMC-T
Aldehyde dehydrogenase
PTAC
Signature enzyme
Alcohol dehydrogenase
Regulator

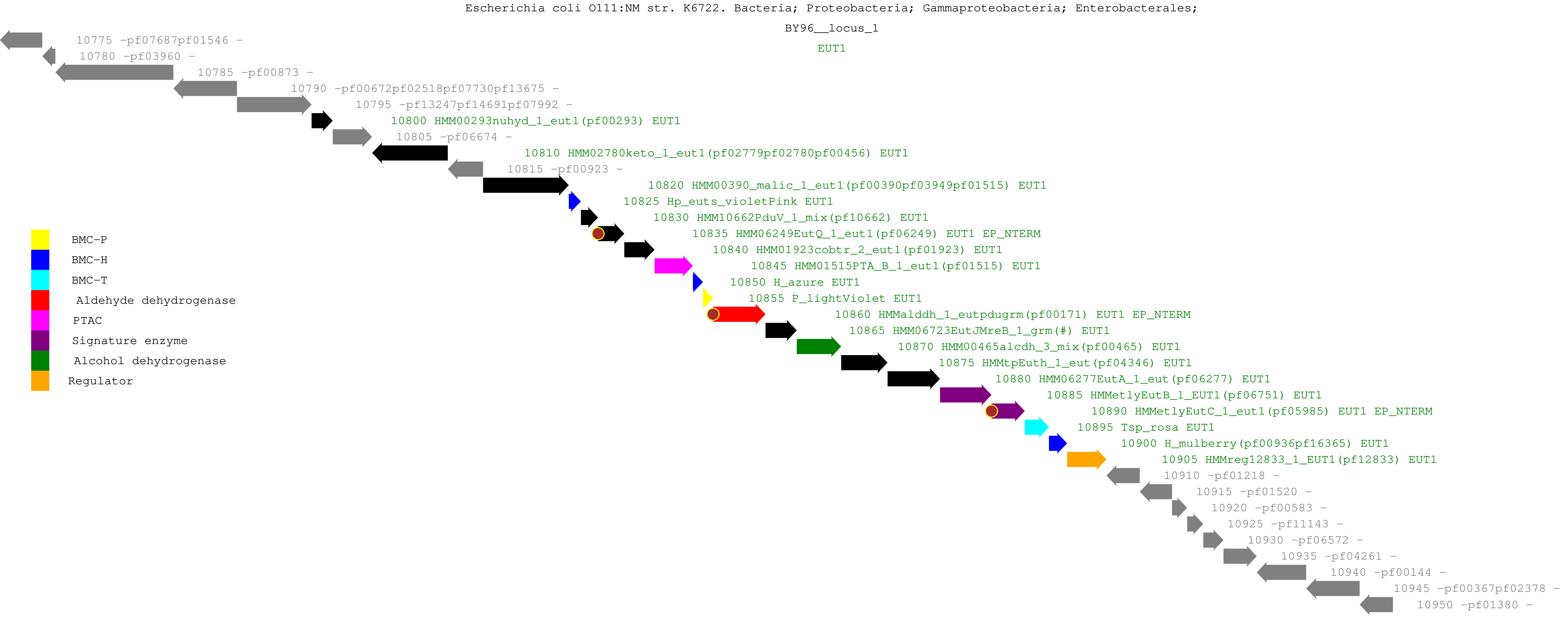
Escherichia coli O111:NM str. K6722. Bacteria; Proteobacteria; Gammaproteobacteria; Enterobacterales;
BY96__locus_1
EUT1
10775 -pf07687pf01546 -
10780 -pf03960 -
10785 -pf00873 -
10790 -pf00672pf02518pf07730pf13675 -
10795 -pf13247pf14691pf07992 -
10800 HMM00293nuhyd_1_eut1(pf00293) EUT1
10805 -pf06674 -
10810 HMM02780keto_1_eut1(pf02779pf02780pf00456) EUT1
10815 -pf00923 -
10820 HMM00390_malic_1_eut1(pf00390pf03949pf01515) EUT1
10825 Hp_euts_violetPink EUT1
10830 HMM10662PduV_1_mix(pf10662) EUT1
10835 HMM06249EutQ_1_eut1(pf06249) EUT1 EP_NTERM
10840 HMM01923cobtr_2_eut1(pf01923) EUT1
10845 HMM01515PTA_B_1_eut1(pf01515) EUT1
10850 H_azure EUT1
10855 P_lightViolet EUT1
10860 HMMalddh_1_eutpdugrm(pf00171) EUT1 EP_NTERM
10865 HMM06723EutJMreB_1_grm(#) EUT1
10870 HMM00465alcdh_3_mix(pf00465) EUT1
10875 HMMtpEuth_1_eut(pf04346) EUT1
10880 HMM06277EutA_1_eut(pf06277) EUT1
10885 HMMetlyEutB_1_EUT1(pf06751) EUT1
10890 HMMetlyEutC_1_eut1(pf05985) EUT1 EP_NTERM
10895 Tsp_rosa EUT1
10900 H_mulberry(pf00936pf16365) EUT1
10905 HMMreg12833_1_EUT1(pf12833) EUT1
10910 -pf01218 -
10915 -pf01520 -
10920 -pf00583 -
10925 -pf11143 -
10930 -pf06572 -
10935 -pf04261 -
10940 -pf00144 -
10945 -pf00367pf02378 -
10950 -pf01380 -
BMC-P
BMC-H
BMC-T
Aldehyde dehydrogenase
PTAC
Signature enzyme
Alcohol dehydrogenase
Regulator

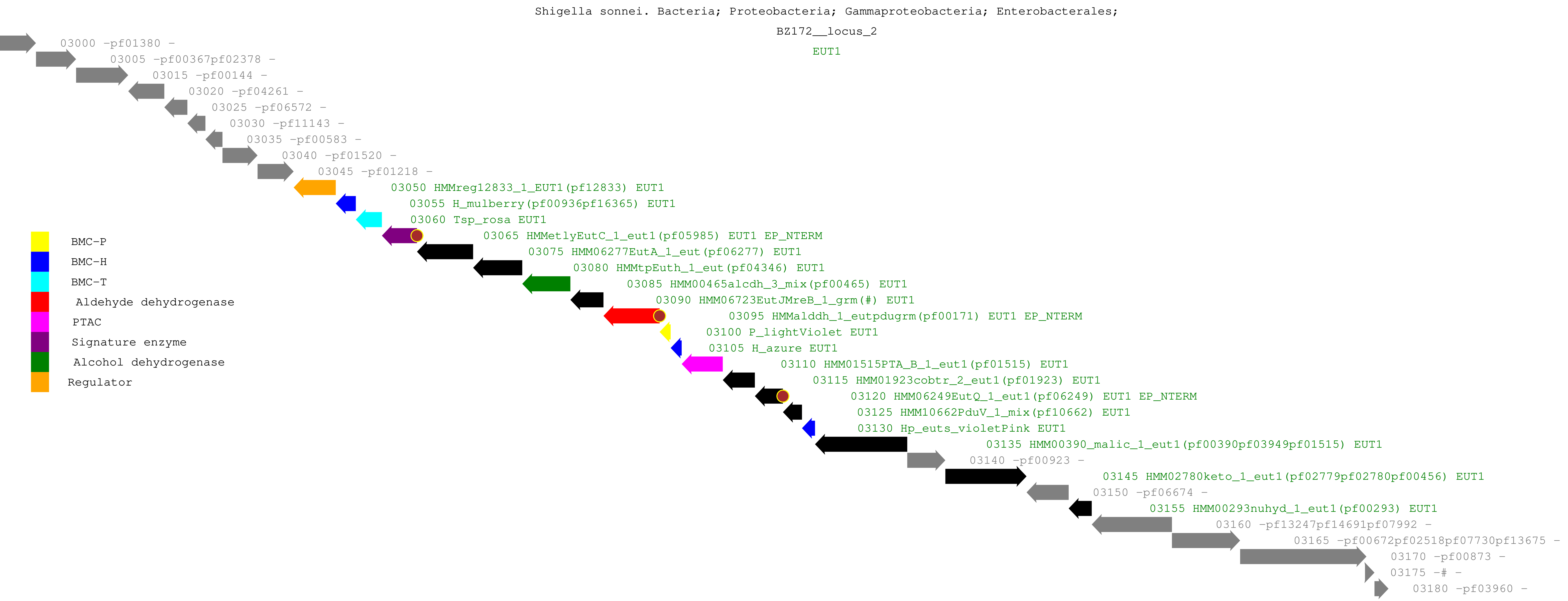
Shigella sonnei. Bacteria; Proteobacteria; Gammaproteobacteria; Enterobacterales;
BZ172__locus_2
EUT1
03000 -pf01380 -
03005 -pf00367pf02378 -
03015 -pf00144 -
03020 -pf04261 -
03025 -pf06572 -
03030 -pf11143 -
03035 -pf00583 -
03040 -pf01520 -
03045 -pf01218 -
03050 HMMreg12833_1_EUT1(pf12833) EUT1
03055 H_mulberry(pf00936pf16365) EUT1
03060 Tsp_rosa EUT1
03065 HMMetlyEutC_1_eut1(pf05985) EUT1 EP_NTERM
03075 HMM06277EutA_1_eut(pf06277) EUT1
03080 HMMtpEuth_1_eut(pf04346) EUT1
03085 HMM00465alcdh_3_mix(pf00465) EUT1
03090 HMM06723EutJMreB_1_grm(#) EUT1
03095 HMMalddh_1_eutpdugrm(pf00171) EUT1 EP_NTERM
03100 P_lightViolet EUT1
03105 H_azure EUT1
03110 HMM01515PTA_B_1_eut1(pf01515) EUT1
03115 HMM01923cobtr_2_eut1(pf01923) EUT1
03120 HMM06249EutQ_1_eut1(pf06249) EUT1 EP_NTERM
03125 HMM10662PduV_1_mix(pf10662) EUT1
03130 Hp_euts_violetPink EUT1
03135 HMM00390_malic_1_eut1(pf00390pf03949pf01515) EUT1
03140 -pf00923 -
03145 HMM02780keto_1_eut1(pf02779pf02780pf00456) EUT1
03150 -pf06674 -
03155 HMM00293nuhyd_1_eut1(pf00293) EUT1
03160 -pf13247pf14691pf07992 -
03165 -pf00672pf02518pf07730pf13675 -
03170 -pf00873 -
03175 -# -
03180 -pf03960 -
BMC-P
BMC-H
BMC-T
Aldehyde dehydrogenase
PTAC
Signature enzyme
Alcohol dehydrogenase
Regulator

Citrobacter sp. A316. Bacteria; Proteobacteria; Gammaproteobacteria; Enterobacterales;

BZK41__locus_1

EUT1

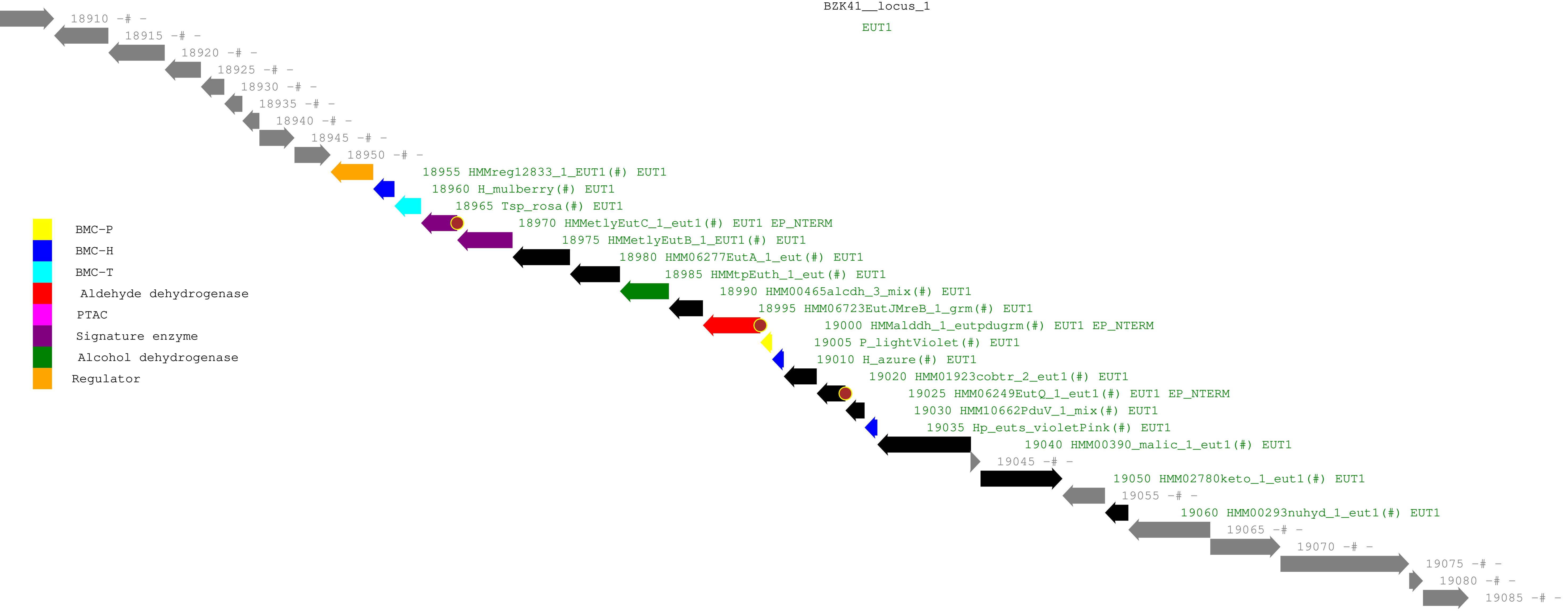

Citrobacter braakii. Bacteria; Proteobacteria; Gammaproteobacteria; Enterobacterales;

BZK42__locus_2

EUT1

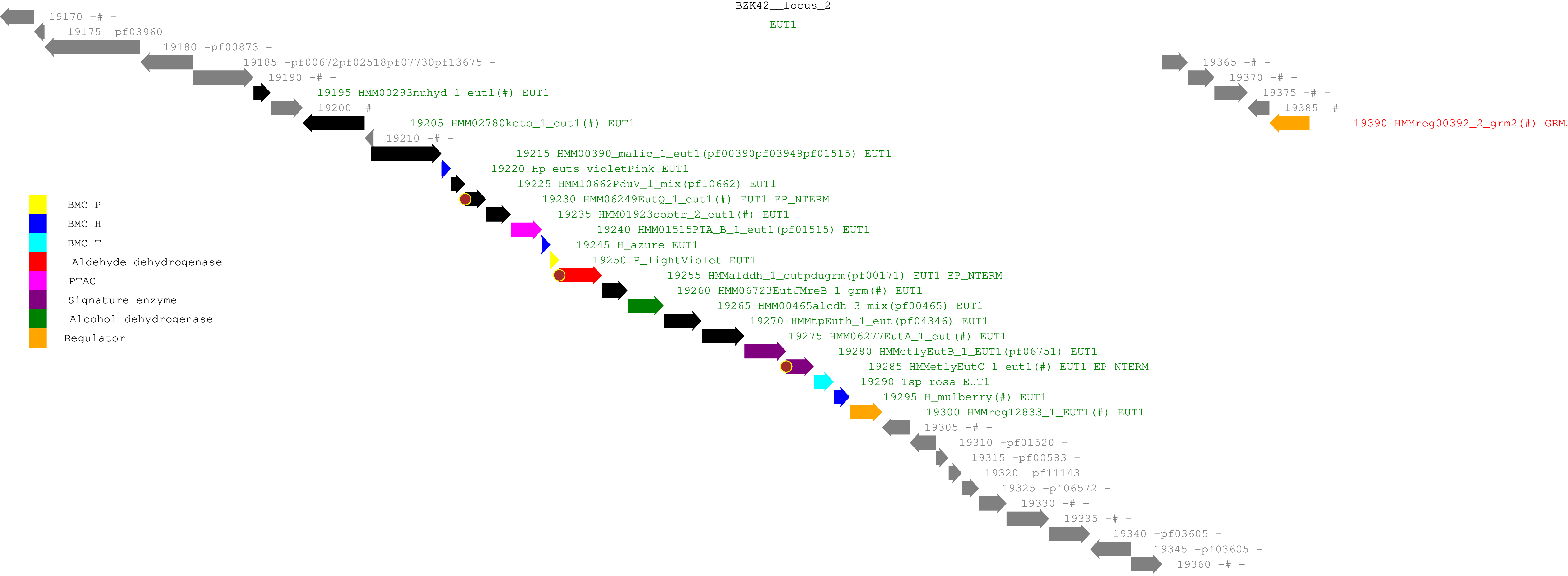

Escherichia coli. Bacteria; Proteobacteria; Gammaproteobacteria; Enterobacterales;

BZL69__locus_1

EUT1

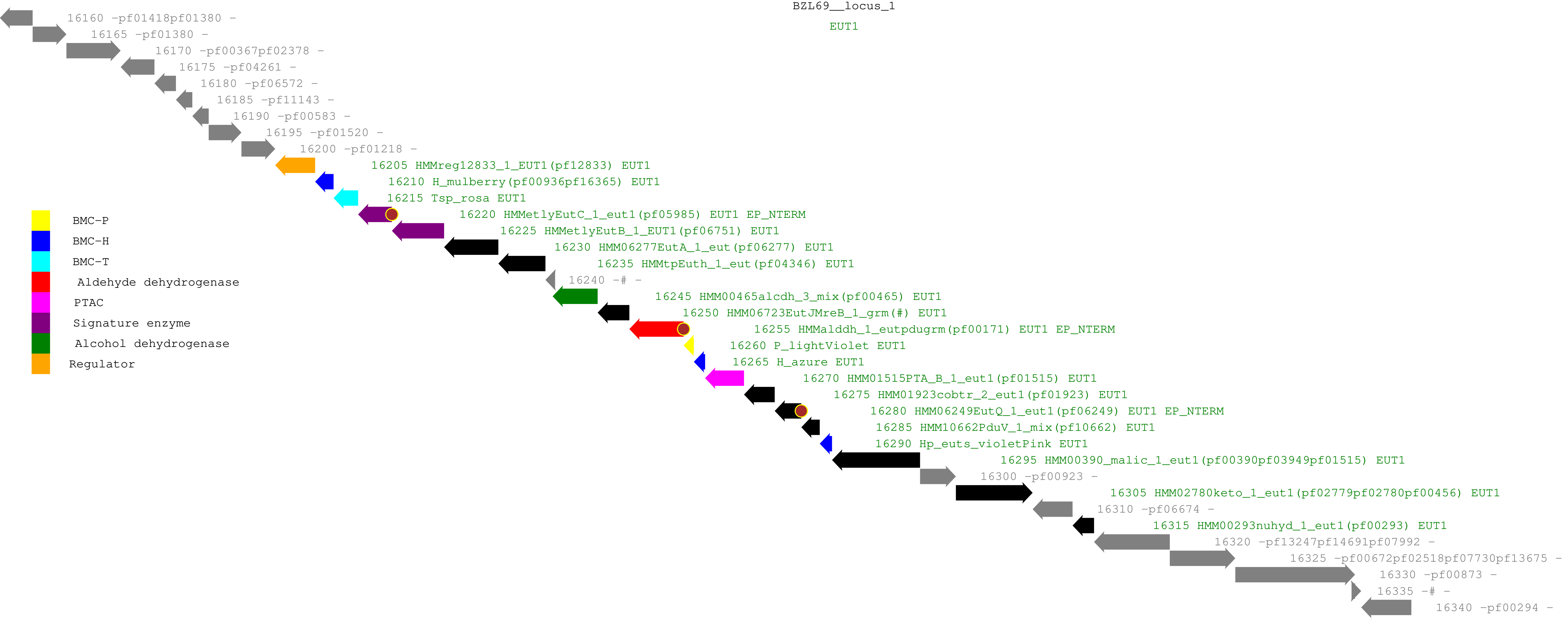

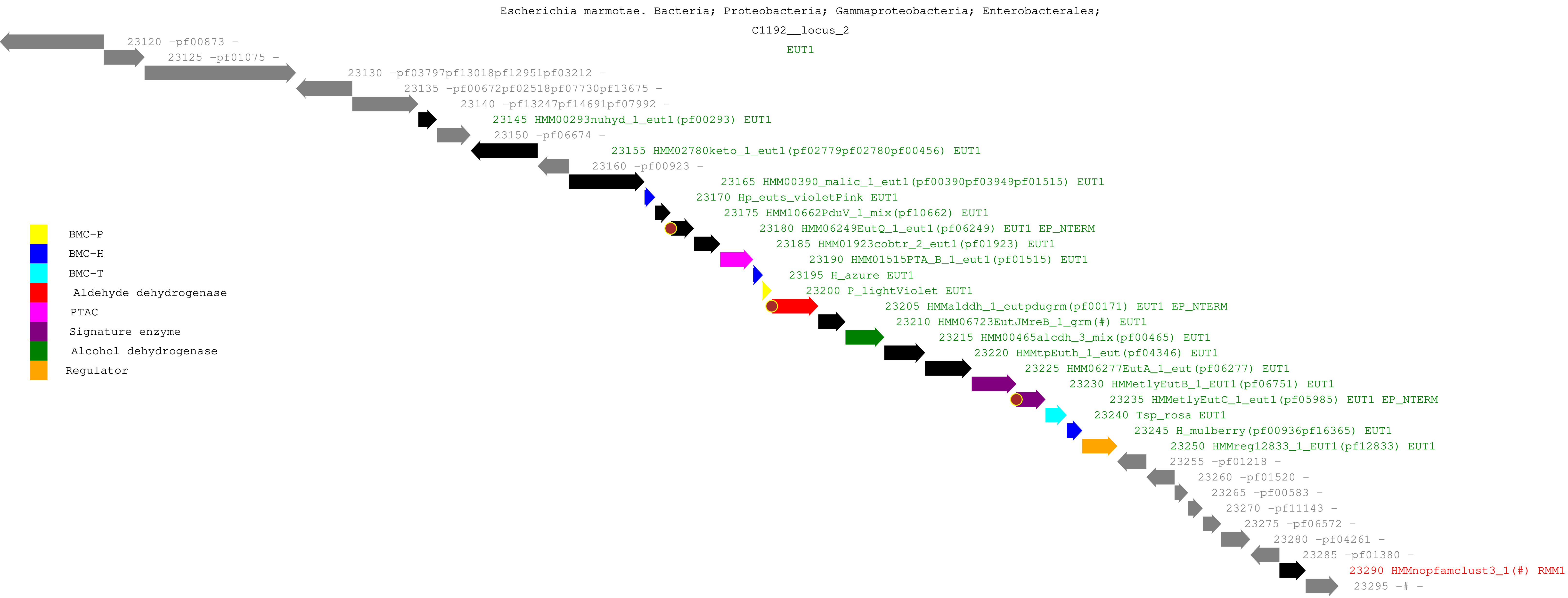
Escherichia marmotae. Bacteria; Proteobacteria; Gammaproteobacteria; Enterobacterales;
C1192__locus_2
EUT1
23120 -pf00873 -
23125 -pf01075 -
23130 -pf03797pf13018pf12951pf03212 -
23135 -pf00672pf02518pf07730pf13675 -
23140 -pf13247pf14691pf07992 -
23145 HMM00293nuhyd_1_eut1(pf00293) EUT1
23150 -pf06674 -
23155 HMM02780keto_1_eut1(pf02779pf02780pf00456) EUT1
23160 -pf00923 -
23165 HMM00390_malic_1_eut1(pf00390pf03949pf01515) EUT1
23170 Hp_euts_violetPink EUT1
23175 HMM10662PduV_1_mix(pf10662) EUT1
23180 HMM06249EutQ_1_eut1(pf06249) EUT1 EP_NTERM
23185 HMM01923cobtr_2_eut1(pf01923) EUT1
23190 HMM01515PTA_B_1_eut1(pf01515) EUT1
23195 H_azure EUT1
23200 P_lightViolet EUT1
23205 HMMalddh_1_eutpdugrm(pf00171) EUT1 EP_NTERM
23210 HMM06723EutJMreB_1_grm(#) EUT1
23215 HMM00465alcdh_3_mix(pf00465) EUT1
23220 HMMtpEuth_1_eut(pf04346) EUT1
23225 HMM06277EutA_1_eut(pf06277) EUT1
23230 HMMetlyEutB_1_EUT1(pf06751) EUT1
23235 HMMetlyEutC_1_eut1(pf05985) EUT1 EP_NTERM
23240 Tsp_rosa EUT1
23245 H_mulberry(pf00936pf16365) EUT1
23250 HMMreg12833_1_EUT1(pf12833) EUT1
23255 -pf01218 -
23260 -pf01520 -
23265 -pf00583 -
23270 -pf11143 -
23275 -pf06572 -
23280 -pf04261 -
23285 -pf01380 -
23290 HMMnopfamclust3_1(#) RMM1
23295 -# -
BMC-P
BMC-H
BMC-T
Aldehyde dehydrogenase
PTAC
Signature enzyme
Alcohol dehydrogenase
Regulator

Klebsiella pneumoniae. Bacteria; Proteobacteria; Gammaproteobacteria; Enterobacterales;

C1459__locus_1

EUT1

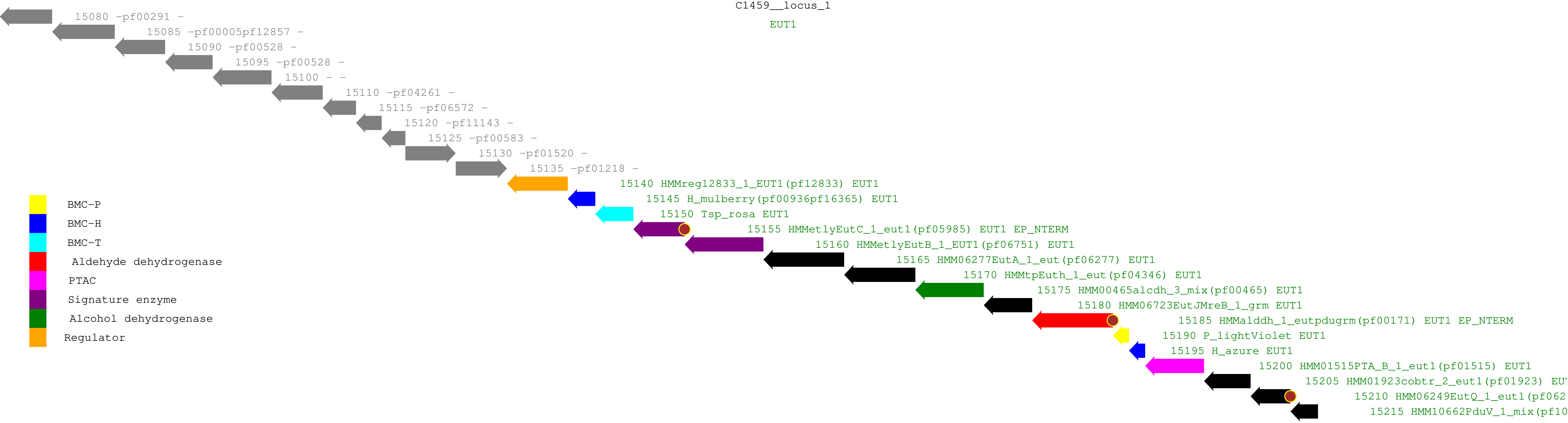

Escherichia coli. Bacteria; Proteobacteria; Gammaproteobacteria; Enterobacterales;

C1I39__locus_2

EUT1

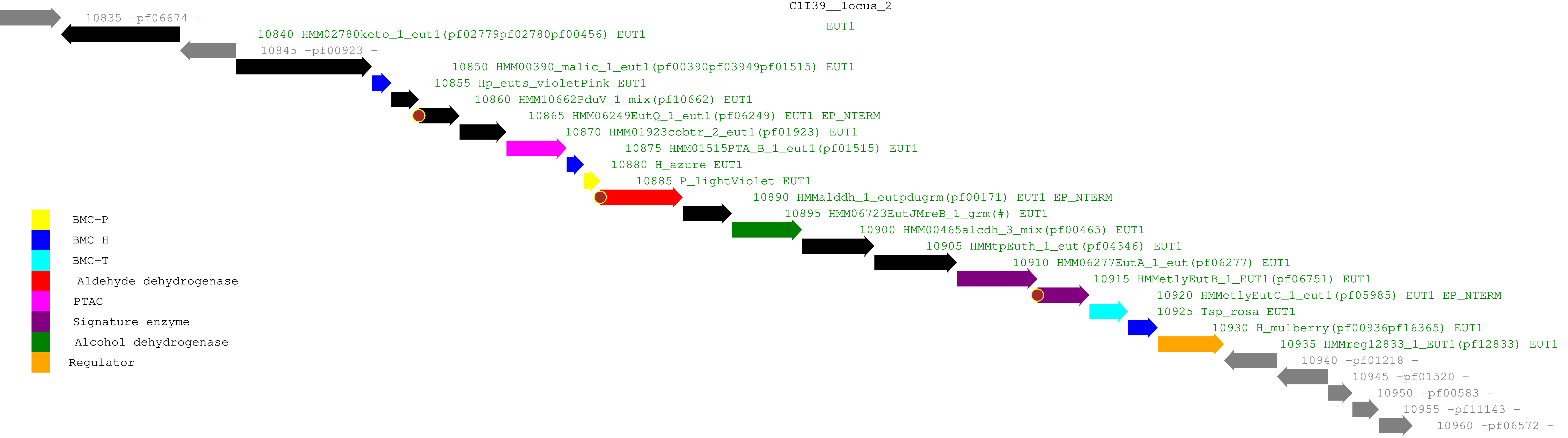

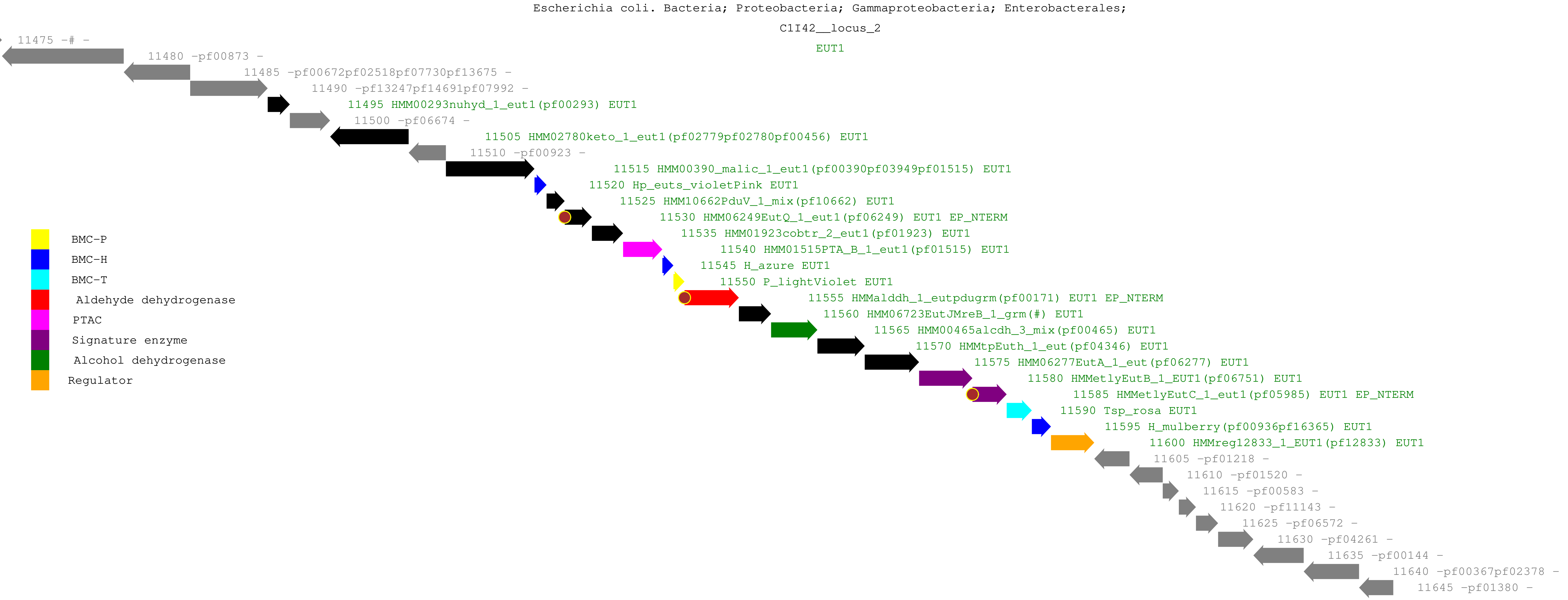

Escherichia coli. Bacteria; Proteobacteria; Gammaproteobacteria; Enterobacterales;
C1I42__locus_2
EUT1
11475 -# -
11480 -pf00873 -
11485 -pf00672pf02518pf07730pf13675 -
11490 -pf13247pf14691pf07992 -
11495 HMM00293nuhyd_1_eut1(pf00293) EUT1
11500 -pf06674 -
11505 HMM02780keto_1_eut1(pf02779pf02780pf00456) EUT1
11510 -pf00923 -
11515 HMM00390_malic_1_eut1(pf00390pf03949pf01515) EUT1
11520 Hp_euts_violetPink EUT1
11525 HMM10662PduV_1_mix(pf10662) EUT1
11530 HMM06249EutQ_1_eut1(pf06249) EUT1 EP_NTERM
11535 HMM01923cobtr_2_eut1(pf01923) EUT1
11540 HMM01515PTA_B_1_eut1(pf01515) EUT1
11545 H_azure EUT1
11550 P_lightViolet EUT1
11555 HMMalddh_1_eutpdugrm(pf00171) EUT1 EP_NTERM
11560 HMM06723EutJMreB_1_grm(#) EUT1
11565 HMM00465alcdh_3_mix(pf00465) EUT1
11570 HMMtpEuth_1_eut(pf04346) EUT1
11575 HMM06277EutA_1_eut(pf06277) EUT1
11580 HMMetlyEutB_1_EUT1(pf06751) EUT1
11585 HMMetlyEutC_1_eut1(pf05985) EUT1 EP_NTERM
11590 Tsp_rosa EUT1
11595 H_mulberry(pf00936pf16365) EUT1
11600 HMMreg12833_1_EUT1(pf12833) EUT1
11605 -pf01218 -
11610 -pf01520 -
11615 -pf00583 -
11620 -pf11143 -
11625 -pf06572 -
11630 -pf04261 -
11635 -pf00144 -
11640 -pf00367pf02378 -
11645 -pf01380 -
BMC-P
BMC-H
BMC-T
Aldehyde dehydrogenase
PTAC
Signature enzyme
Alcohol dehydrogenase
Regulator

Salmonella gallinarum. Bacteria; Proteobacteria; Gammaproteobacteria; Enterobacterales;

C2296__locus_1

EUT1

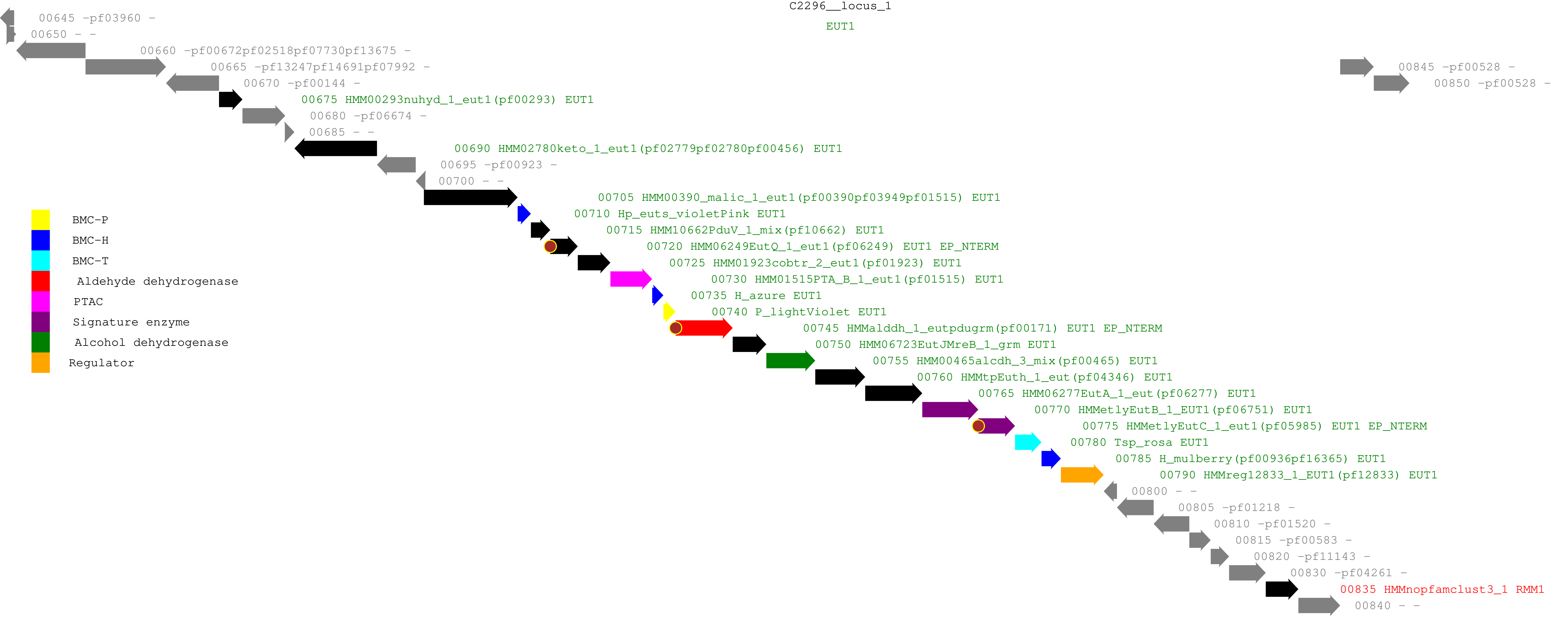

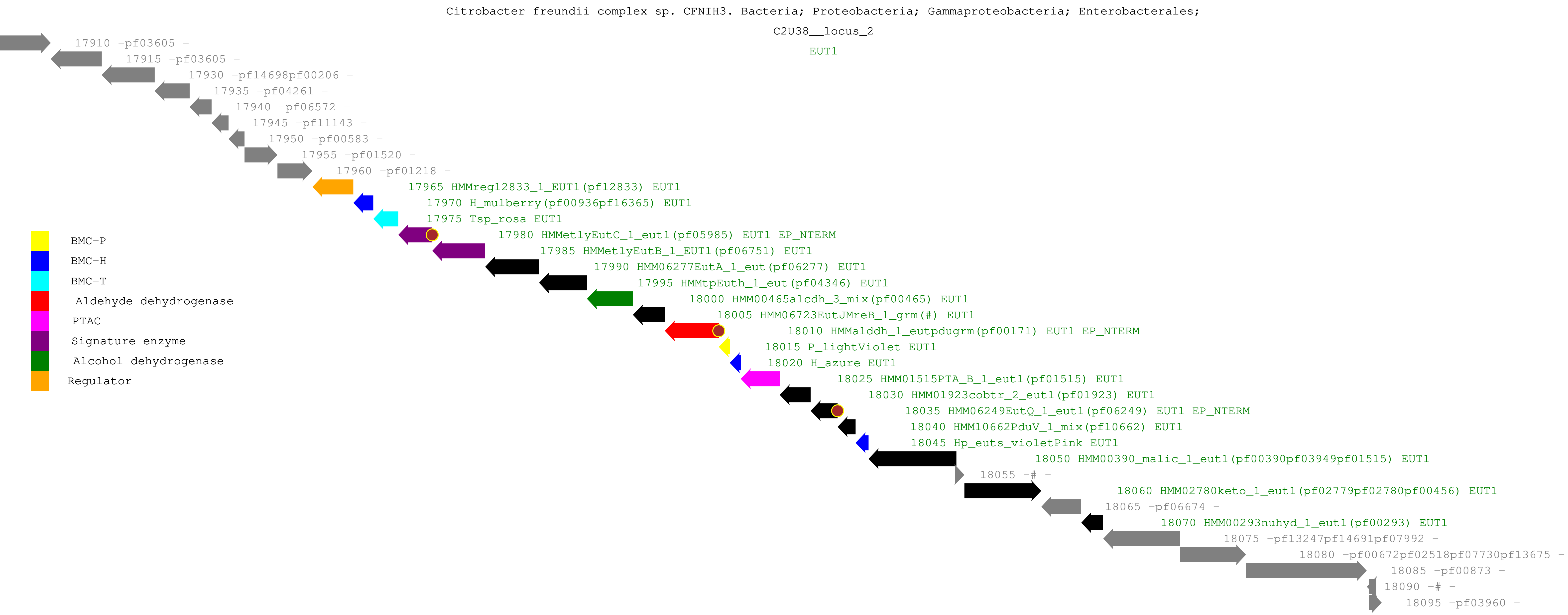
Citrobacter freundii complex sp. CFNIH3. Bacteria; Proteobacteria; Gammaproteobacteria; Enterobacterales;
C2U38__locus_2
EUT1
17910 -pf03605 -
17915 -pf03605 -
17930 -pf14698pf00206 -
17935 -pf04261 -
17940 -pf06572 -
17945 -pf11143 -
17950 -pf00583 -
17955 -pf01520 -
17960 -pf01218 -
17965 HMMreg12833_1_EUT1(pf12833) EUT1
17970 H_mulberry(pf00936pf16365) EUT1
17975 Tsp_rosa EUT1
17980 HMMetlyEutC_1_eut1(pf05985) EUT1 EP_NTERM
17985 HMMetlyEutB_1_EUT1(pf06751) EUT1
17990 HMM06277EutA_1_eut(pf06277) EUT1
17995 HMMtpEuth_1_eut(pf04346) EUT1
18000 HMM00465alcdh_3_mix(pf00465) EUT1
18005 HMM06723EutJMreB_1_grm(#) EUT1
18010 HMMalddh_1_eutpdugrm(pf00171) EUT1 EP_NTERM
18015 P_lightViolet EUT1
18020 H_azure EUT1
18025 HMM01515PTA_B_1_eut1(pf01515) EUT1
18030 HMM01923cobtr_2_eut1(pf01923) EUT1
18035 HMM06249EutQ_1_eut1(pf06249) EUT1 EP_NTERM
18040 HMM10662PduV_1_mix(pf10662) EUT1
18045 Hp_euts_violetPink EUT1
18050 HMM00390_malic_1_eut1(pf00390pf03949pf01515) EUT1
18055 -# -
18060 HMM02780keto_1_eut1(pf02779pf02780pf00456) EUT1
18065 -pf06674 -
18070 HMM00293nuhyd_1_eut1(pf00293) EUT1
18075 -pf13247pf14691pf07992 -
18080 -pf00672pf02518pf07730pf13675 -
18085 -pf00873 -
18090 -# -
18095 -pf03960 -
BMC-P
BMC-H
BMC-T
Aldehyde dehydrogenase
PTAC
Signature enzyme
Alcohol dehydrogenase
Regulator

Citrobacter freundii complex sp. CFNIH4. Bacteria; Proteobacteria; Gammaproteobacteria; Enterobacterales;

C2U41__locus_2

EUT1

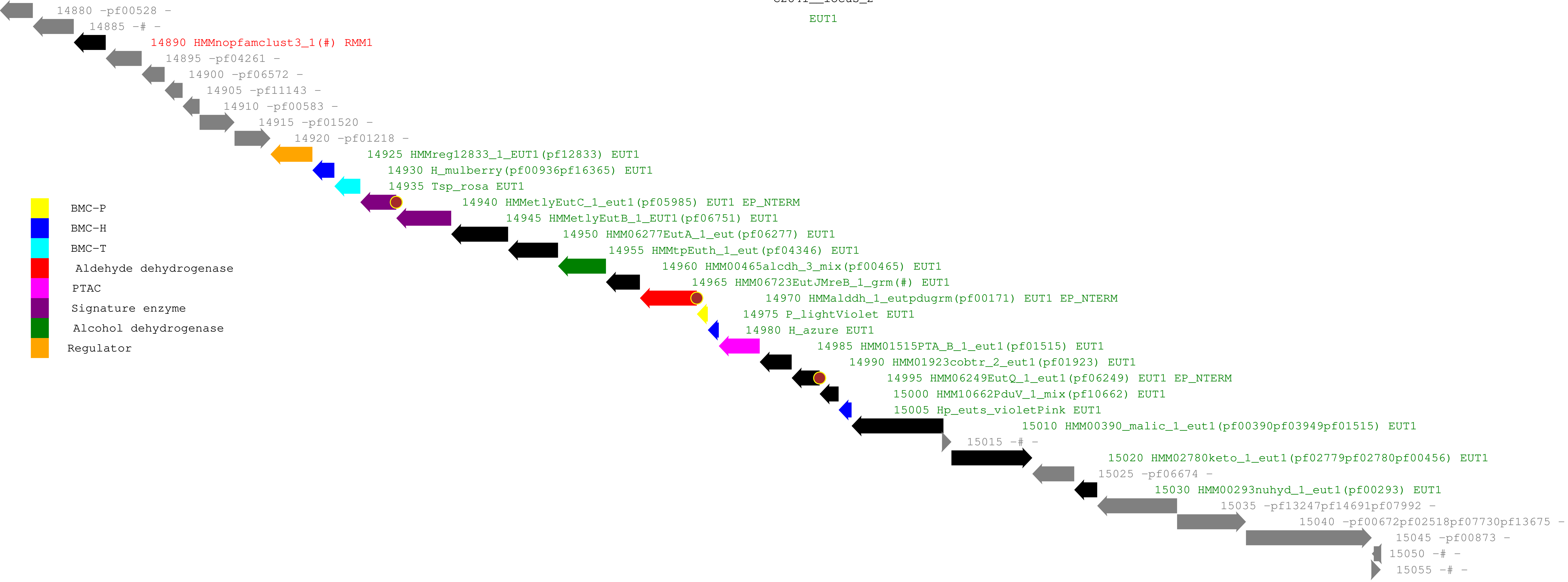

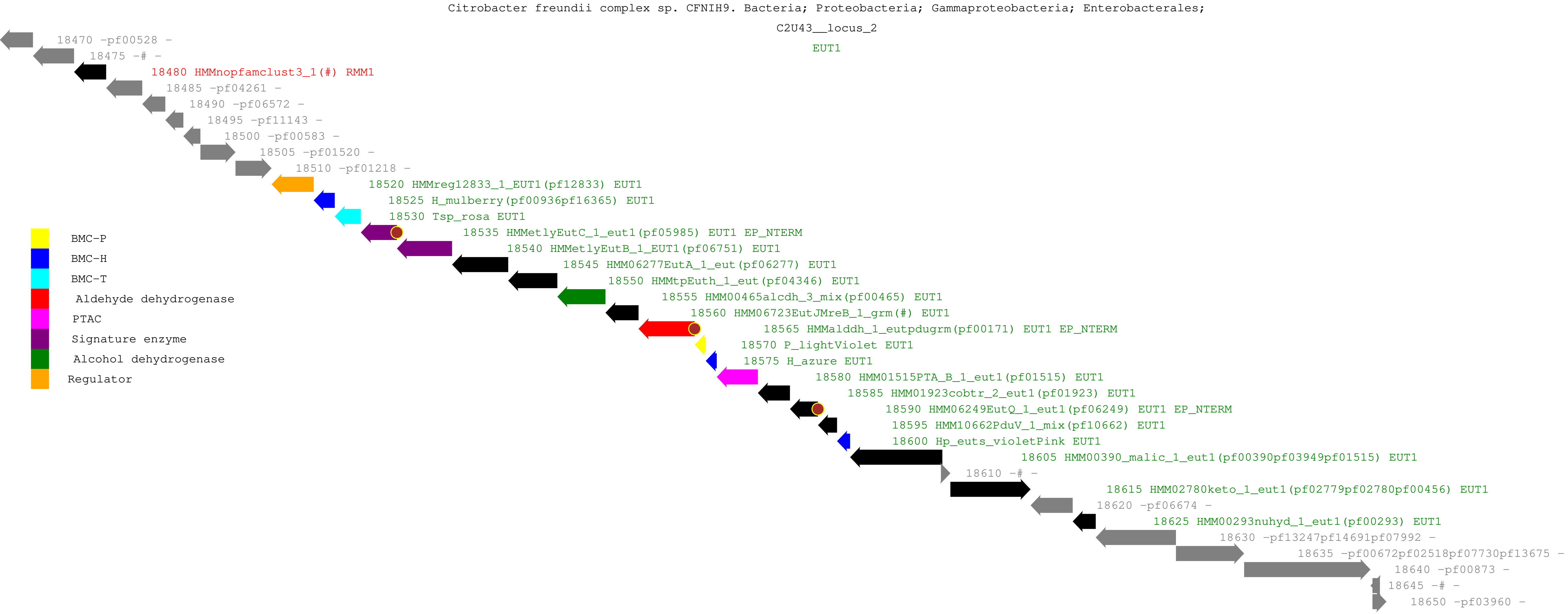

Citrobacter freundii complex sp. CFNIH9. Bacteria; Proteobacteria; Gammaproteobacteria; Enterobacterales;
C2U43__locus_2
EUT1
18470 -pf00528 -
18475 -# -
18480 HMMnopfamclust3_1(#) RMM1
18485 -pf04261 -
18490 -pf06572 -
18495 -pf11143 -
18500 -pf00583 -
18505 -pf01520 -
18510 -pf01218 -
18520 HMMreg12833_1_EUT1(pf12833) EUT1
18525 H_mulberry(pf00936pf16365) EUT1
18530 Tsp_rosa EUT1
18535 HMMetlyEutC_1_eut1(pf05985) EUT1 EP_NTERM
18540 HMMetlyEutB_1_EUT1(pf06751) EUT1
18545 HMM06277EutA_1_eut(pf06277) EUT1
18550 HMMtpEuth_1_eut(pf04346) EUT1
18555 HMM00465alcdh_3_mix(pf00465) EUT1
18560 HMM06723EutJMreB_1_grm(#) EUT1
18565 HMMalddh_1_eutpdugrm(pf00171) EUT1 EP_NTERM
18570 P_lightViolet EUT1
18575 H_azure EUT1
18580 HMM01515PTA_B_1_eut1(pf01515) EUT1
18585 HMM01923cobtr_2_eut1(pf01923) EUT1
18590 HMM06249EutQ_1_eut1(pf06249) EUT1 EP_NTERM
18595 HMM10662PduV_1_mix(pf10662) EUT1
18600 Hp_euts_violetPink EUT1
18605 HMM00390_malic_1_eut1(pf00390pf03949pf01515) EUT1
18610 -# -
18615 HMM02780keto_1_eut1(pf02779pf02780pf00456) EUT1
18620 -pf06674 -
18625 HMM00293nuhyd_1_eut1(pf00293) EUT1
18630 -pf13247pf14691pf07992 -
18635 -pf00672pf02518pf07730pf13675 -
18640 -pf00873 -
18645 -# -
18650 -pf03960 -
BMC-P
BMC-H
BMC-T
Aldehyde dehydrogenase
PTAC
Signature enzyme
Alcohol dehydrogenase
Regulator

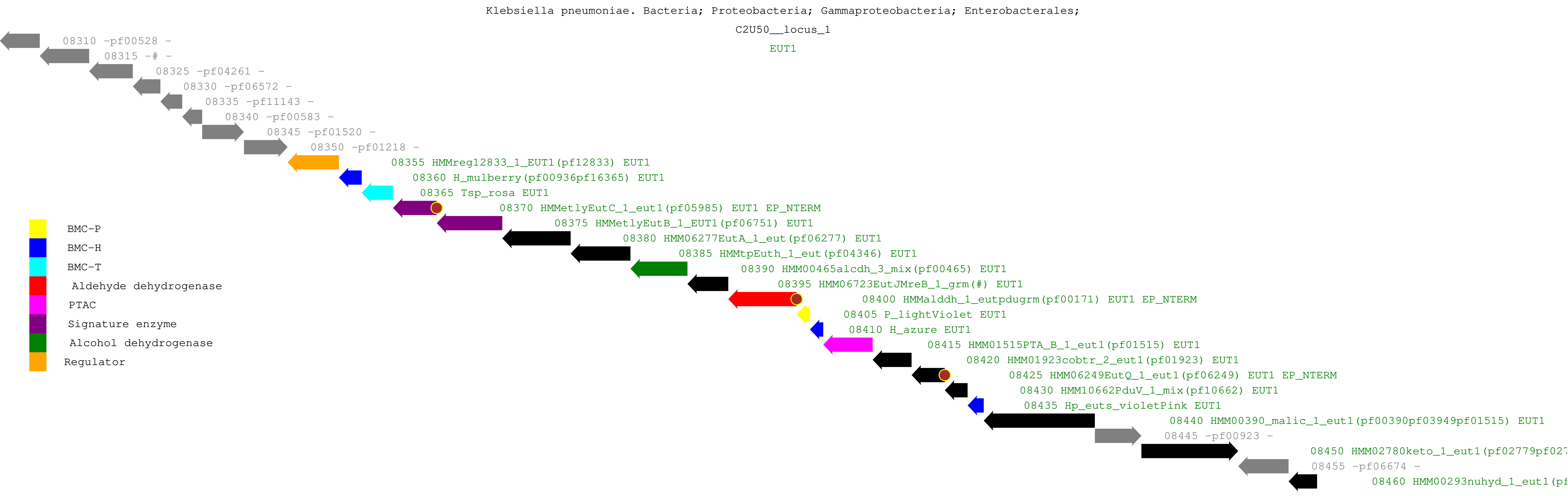
Klebsiella pneumoniae. Bacteria; Proteobacteria; Gammaproteobacteria; Enterobacterales;
C2U50__locus_1
EUT1
08310 -pf00528 -
08315 -# -
08325 -pf04261 -
08330 -pf06572 -
08335 -pf11143 -
08340 -pf00583 -
08345 -pf01520 -
08350 -pf01218 -
08355 HMMreg12833_1_EUT1(pf12833) EUT1
08360 H_mulberry(pf00936pf16365) EUT1
08365 Tsp_rosa EUT1
08370 HMMetlyEutC_1_eut1(pf05985) EUT1 EP_NTERM
08375 HMMetlyEutB_1_EUT1(pf06751) EUT1
08380 HMM06277EutA_1_eut(pf06277) EUT1
08385 HMMtpEuth_1_eut(pf04346) EUT1
08390 HMM00465alcdh_3_mix(pf00465) EUT1
08395 HMM06723EutJMreB_1_grm(#) EUT1
08400 HMMalddh_1_eutpdugrm(pf00171) EUT1 EP_NTERM
08405 P_lightViolet EUT1
08410 H_azure EUT1
08415 HMM01515PTA_B_1_eut1(pf01515) EUT1
08420 HMM01923cobtr_2_eut1(pf01923) EUT1
08425 HMM06249EutQ_1_eut1(pf06249) EUT1 EP_NTERM
08430 HMM10662PduV_1_mix(pf10662) EUT1
08435 Hp_euts_violetPink EUT1
08440 HMM00390_malic_1_eut1(pf00390pf03949pf01515) EUT1
08445 -pf00923 -
08450 HMM02780keto_1_eut1(pf02779pf027
08455 -pf06674 -
08460 HMM00293nuhyd_1_eut1(pf
BMC-P
BMC-H
BMC-T
Aldehyde dehydrogenase
PTAC
Signature enzyme
Alcohol dehydrogenase
Regulator

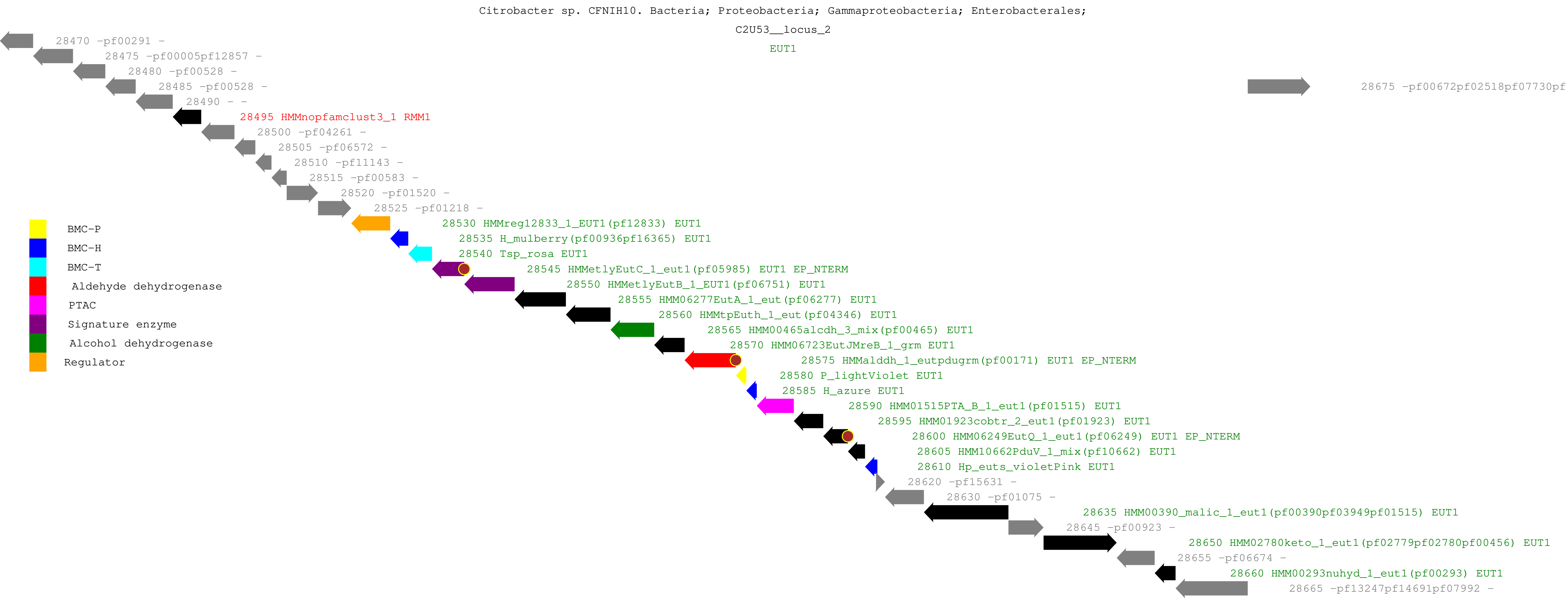
Citrobacter sp. CFNIH10. Bacteria; Proteobacteria; Gammaproteobacteria; Enterobacterales;
C2U53__locus_2
EUT1
28470 -pf00291 -
28475 -pf00005pf12857 -
28480 -pf00528 -
28485 -pf00528 -
28490 - -
28495 HMMnopfamclust3_1 RMM1
28500 -pf04261 -
28505 -pf06572 -
28510 -pf11143 -
28515 -pf00583 -
28520 -pf01520 -
28525 -pf01218 -
28530 HMMreg12833_1_EUT1(pf12833) EUT1
28535 H_mulberry(pf00936pf16365) EUT1
28540 Tsp_rosa EUT1
28545 HMMetlyEutC_1_eut1(pf05985) EUT1 EP_NTERM
28550 HMMetlyEutB_1_EUT1(pf06751) EUT1
28555 HMM06277EutA_1_eut(pf06277) EUT1
28560 HMMtpEuth_1_eut(pf04346) EUT1
28565 HMM00465alcdh_3_mix(pf00465) EUT1
28570 HMM06723EutJMreB_1_grm EUT1
28575 HMMalddh_1_eutpdugrm(pf00171) EUT1 EP_NTERM
28580 P_lightViolet EUT1
28585 H_azure EUT1
28590 HMM01515PTA_B_1_eut1(pf01515) EUT1
28595 HMM01923cobtr_2_eut1(pf01923) EUT1
28600 HMM06249EutQ_1_eut1(pf06249) EUT1 EP_NTERM
28605 HMM10662PduV_1_mix(pf10662) EUT1
28610 Hp_euts_violetPink EUT1
28620 -pf15631 -
28630 -pf01075 -
28635 HMM00390_malic_1_eut1(pf00390pf03949pf01515) EUT1
28645 -pf00923 -
28650 HMM02780keto_1_eut1(pf02779pf02780pf00456) EUT1
28655 -pf06674 -
28660 HMM00293nuhyd_1_eut1(pf00293) EUT1
28665 -pf13247pf14691pf07992 -
28675 -pf00672pf02518pf07730pf
BMC-P
BMC-H
BMC-T
Aldehyde dehydrogenase
PTAC
Signature enzyme
Alcohol dehydrogenase
Regulator

Citrobacter amalonaticus. Bacteria; Proteobacteria; Gammaproteobacteria; Enterobacterales;

C3430__locus_2

EUT1

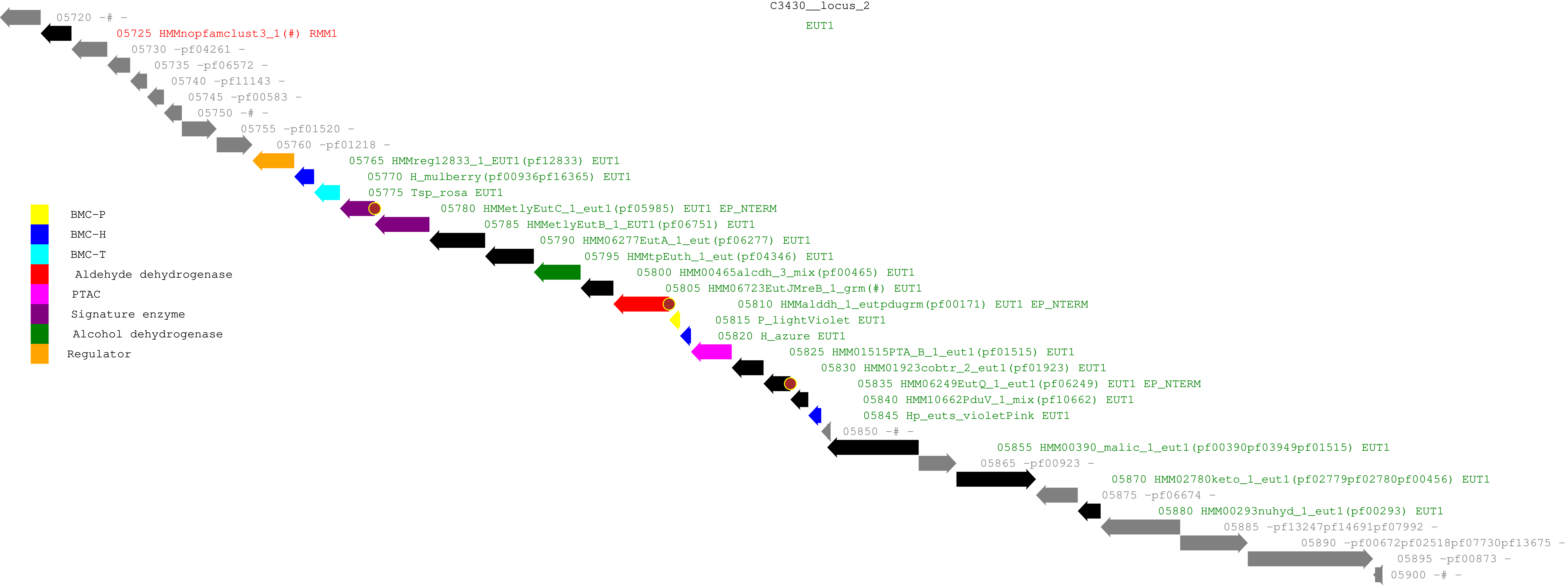

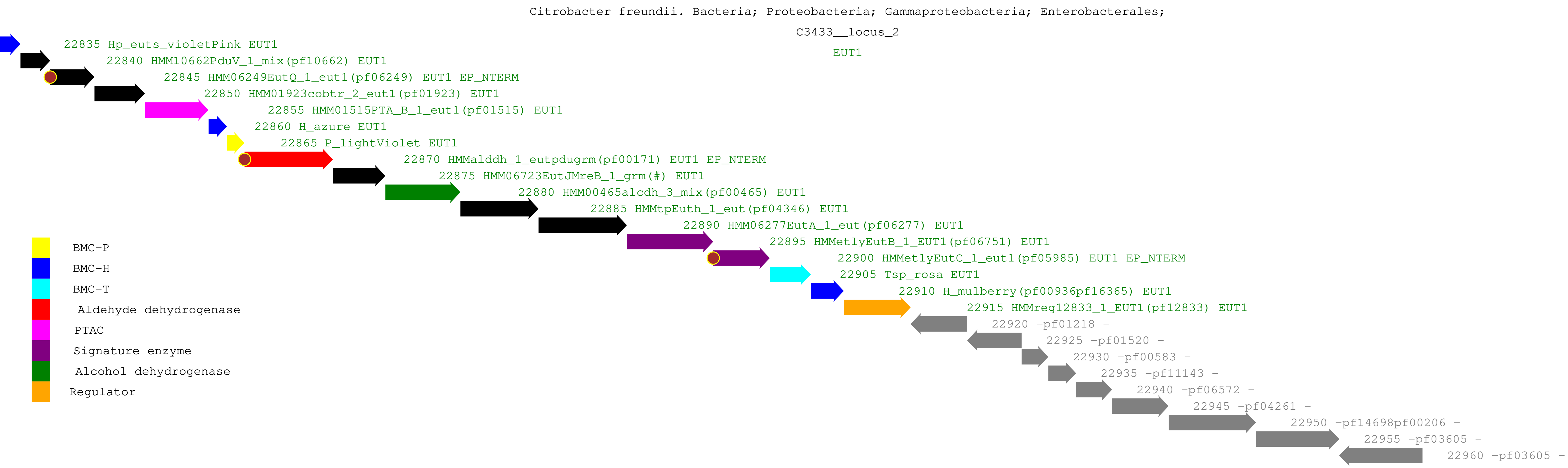
Citrobacter freundii. Bacteria; Proteobacteria; Gammaproteobacteria; Enterobacterales;
C3433__locus_2
EUT1
22835 Hp_euts_violetPink EUT1
22840 HMM10662PduV_1_mix(pf10662) EUT1
22845 HMM06249EutQ_1_eut1(pf06249) EUT1 EP_NTERM
22850 HMM01923cobtr_2_eut1(pf01923) EUT1
22855 HMM01515PTA_B_1_eut1(pf01515) EUT1
22860 H_azure EUT1
22865 P_lightViolet EUT1
22870 HMMalddh_1_eutpdugrm(pf00171) EUT1 EP_NTERM
22875 HMM06723EutJMreB_1_grm(#) EUT1
22880 HMM00465alcdh_3_mix(pf00465) EUT1
22885 HMMtpEuth_1_eut(pf04346) EUT1
22890 HMM06277EutA_1_eut(pf06277) EUT1
22895 HMMetlyEutB_1_EUT1(pf06751) EUT1
22900 HMMetlyEutC_1_eut1(pf05985) EUT1 EP_NTERM
22905 Tsp_rosa EUT1
22910 H_mulberry(pf00936pf16365) EUT1
22915 HMMreg12833_1_EUT1(pf12833) EUT1
22920 -pf01218 -
22925 -pf01520 -
22930 -pf00583 -
22935 -pf11143 -
22940 -pf06572 -
22945 -pf04261 -
22950 -pf14698pf00206 -
22955 -pf03605 -
22960 -pf03605 -
BMC-P
BMC-H
BMC-T
Aldehyde dehydrogenase
PTAC
Signature enzyme
Alcohol dehydrogenase
Regulator

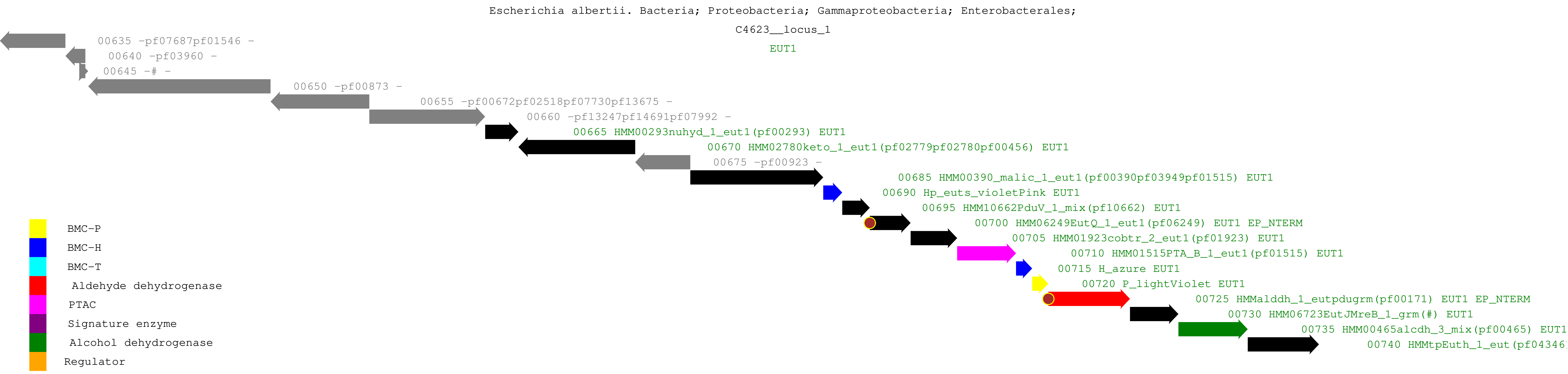

Escherichia albertii. Bacteria; Proteobacteria; Gammaproteobacteria; Enterobacterales;
C4623__locus_1
EUT1
00635 -pf07687pf01546 -
00640 -pf03960 -
00645 -# -
00650 -pf00873 -
00655 -pf00672pf02518pf07730pf13675 -
00660 -pf13247pf14691pf07992 -
00665 HMM00293nuhyd_1_eut1(pf00293) EUT1
00670 HMM02780keto_1_eut1(pf02779pf02780pf00456) EUT1
00675 -pf00923 -
00685 HMM00390_malic_1_eut1(pf00390pf03949pf01515) EUT1
00690 Hp_euts_violetPink EUT1
00695 HMM10662PduV_1_mix(pf10662) EUT1
00700 HMM06249EutQ_1_eut1(pf06249) EUT1 EP_NTERM
00705 HMM01923cobtr_2_eut1(pf01923) EUT1
00710 HMM01515PTA_B_1_eut1(pf01515) EUT1
00715 H_azure EUT1
00720 P_lightViolet EUT1
00725 HMMaldh_1_eutpdugrm(pf00171) EUT1 EP_NTERM
00730 HMM06723EutJMreB_1_grm(#) EUT1
00735 HMM00465alcdh_3_mix(pf00465) EUT1
00740 HMMtpEuth_1_eut(pf04346)
BMC-P
BMC-H
BMC-T
Aldehyde dehydrogenase
PTAC
Signature enzyme
Alcohol dehydrogenase
Regulator

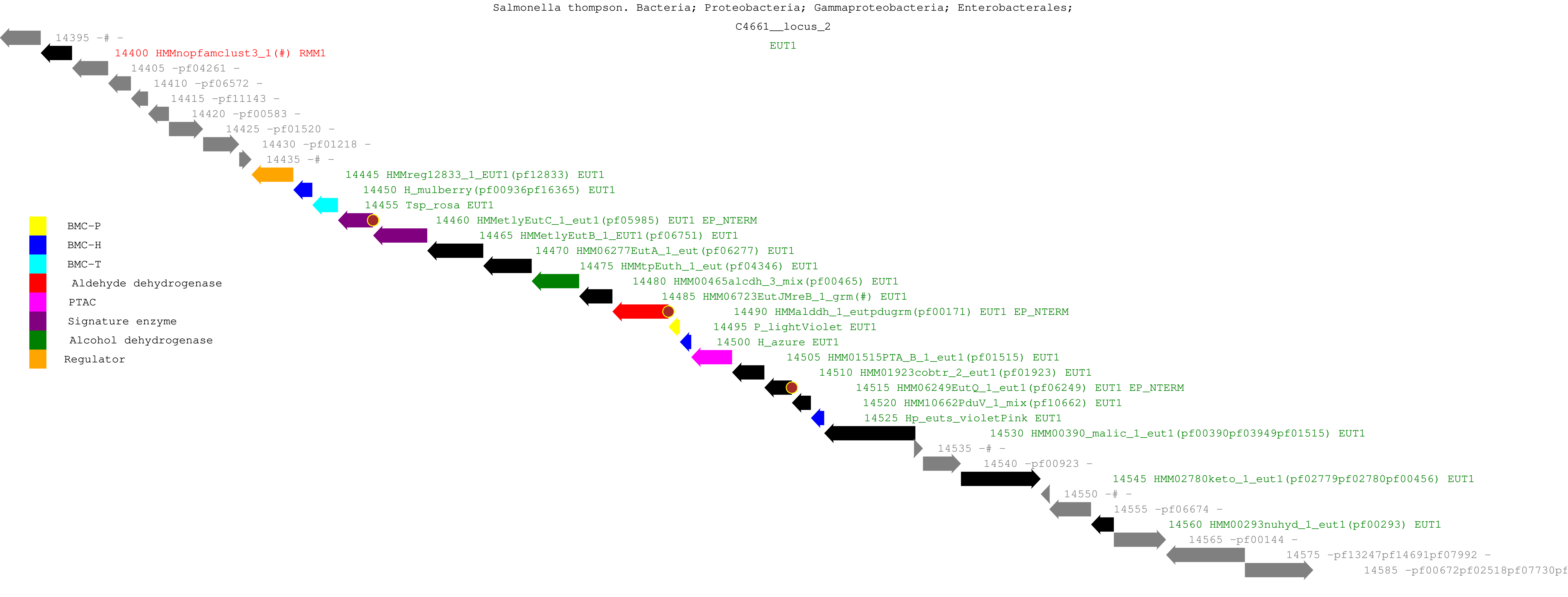

Salmonella thompson. Bacteria; Proteobacteria; Gammaproteobacteria; Enterobacterales;
C4661__locus_2
EUT1
14395 -# -
14400 HMMnopfamclust3_1(#) RMM1
14405 -pf04261 -
14410 -pf06572 -
14415 -pf11143 -
14420 -pf00583 -
14425 -pf01520 -
14430 -pf01218 -
14435 -# -
14445 HMMreg12833_1_EUT1(pf12833) EUT1
14450 H_mulberry(pf00936pf16365) EUT1
14455 Tsp_rosa EUT1
14460 HMMetlyEutC_1_eut1(pf05985) EUT1 EP_NTERM
14465 HMMetlyEutB_1_EUT1(pf06751) EUT1
14470 HMM06277EutA_1_eut(pf06277) EUT1
14475 HMMtpEuth_1_eut(pf04346) EUT1
14480 HMM00465alcdh_3_mix(pf00465) EUT1
14485 HMM06723EutJMreB_1_grm(#) EUT1
14490 HMMalddh_1_eutpdugrm(pf00171) EUT1 EP_NTERM
14495 P_lightViolet EUT1
14500 H_azure EUT1
14505 HMM01515PTA_B_1_eut1(pf01515) EUT1
14510 HMM01923cobtr_2_eut1(pf01923) EUT1
14515 HMM06249EutQ_1_eut1(pf06249) EUT1 EP_NTERM
14520 HMM10662PduV_1_mix(pf10662) EUT1
14525 Hp_euts_violetPink EUT1
14530 HMM00390_malic_1_eut1(pf00390pf03949pf01515) EUT1
14535 -# -
14540 -pf00923 -
14545 HMM02780keto_1_eut1(pf02779pf02780pf00456) EUT1
14550 -# -
14555 -pf06674 -
14560 HMM00293nuhyd_1_eut1(pf00293) EUT1
14565 -pf00144 -
14575 -pf13247pf14691pf07992 -
14585 -pf00672pf02518pf07730pf
BMC-P
BMC-H
BMC-T
Aldehyde dehydrogenase
PTAC
Signature enzyme
Alcohol dehydrogenase
Regulator

Salmonella enterica subsp. enterica serovar Bareilly. Bacteria; Proteobacteria; Gammaproteobacteria; Enterobacterales;

C4680__locus_1

EUT1

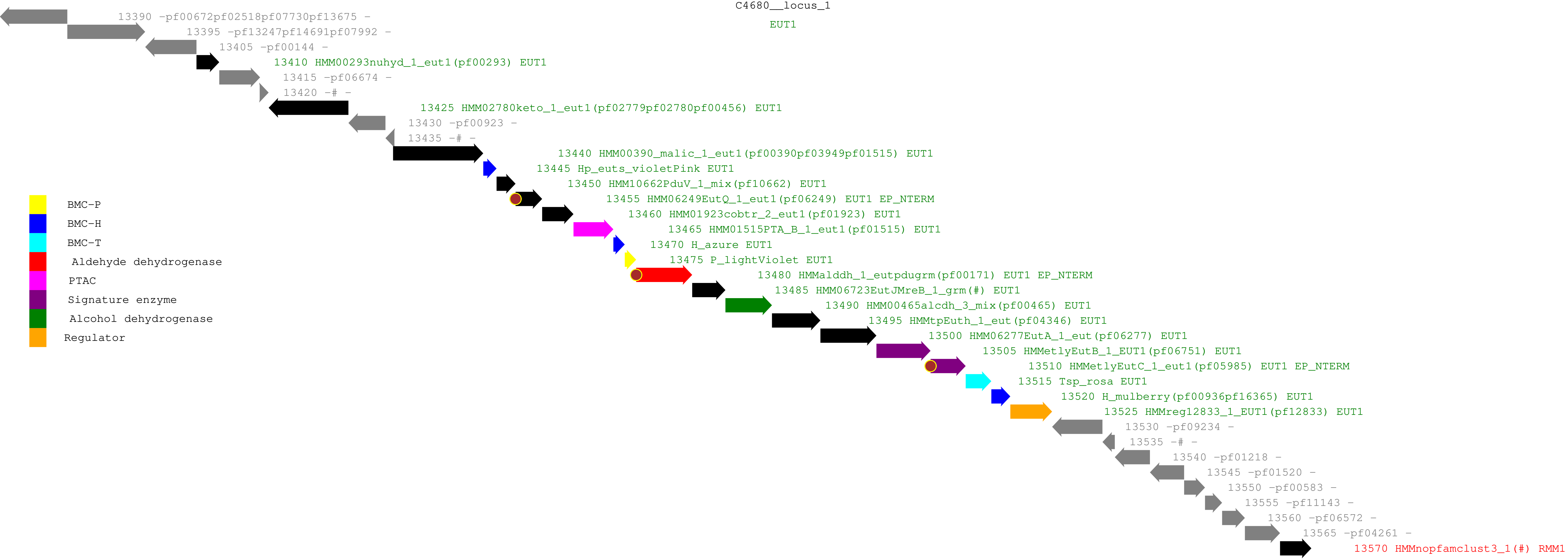

Salmonella enterica subsp. enterica serovar Cerro. Bacteria; Proteobacteria; Gammaproteobacteria; Enterobacterales;

C4780__locus_1

EUT1

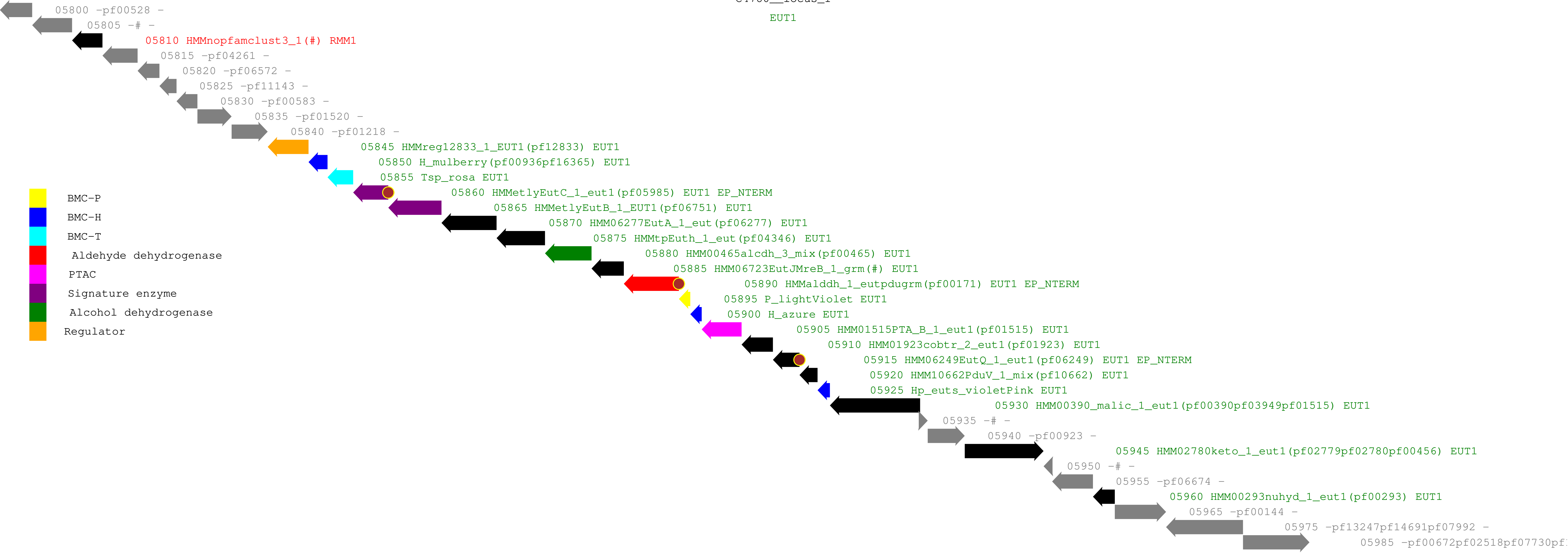

Salmonella enterica subsp. enterica serovar Agona. Bacteria; Proteobacteria; Gammaproteobacteria; Enterobacterales;

C4792__locus_1

EUT1

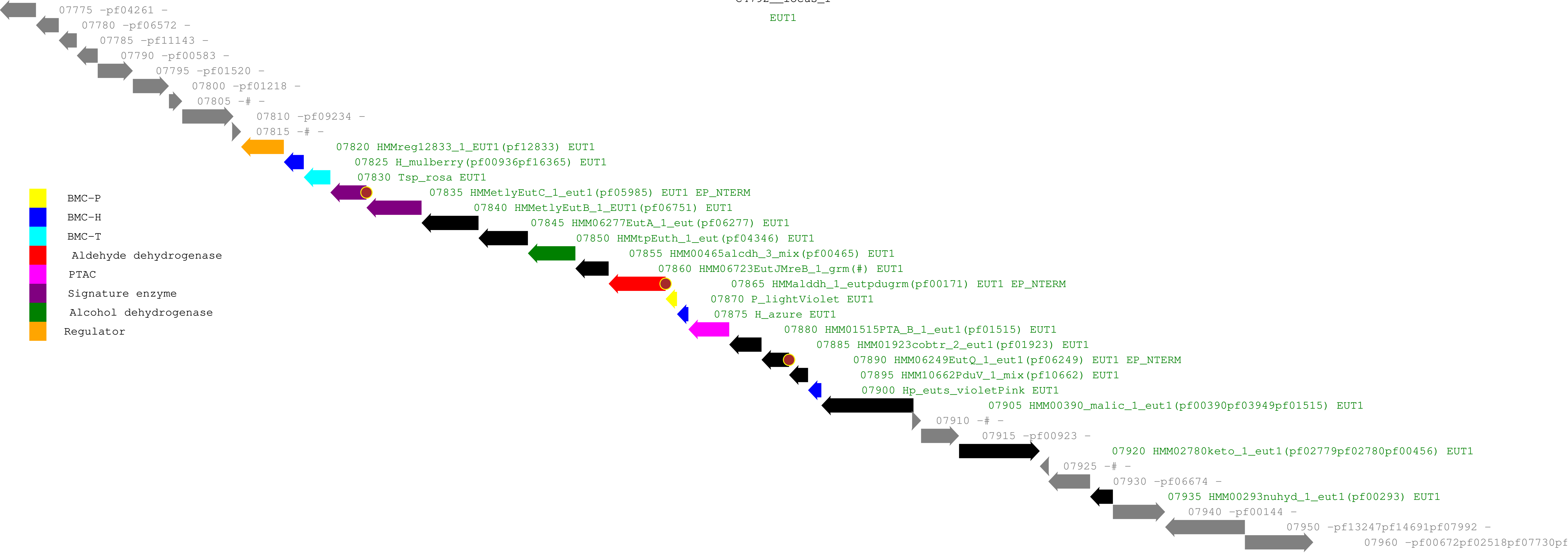

Salmonella choleraesuis. Bacteria; Proteobacteria; Gammaproteobacteria; Enterobacterales;

C4801__locus_1

EUT1

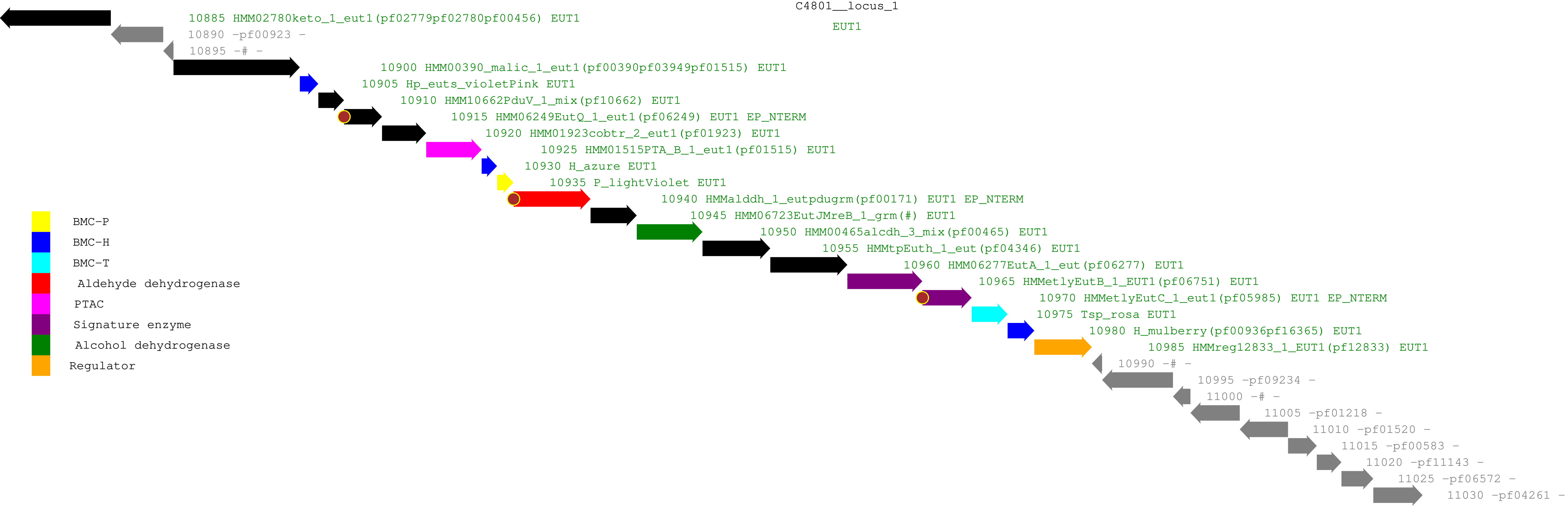

Salmonella enterica subsp. arizonae serovar 51:g,z51:-. Bacteria; Proteobacteria; Gammaproteobacteria; Enterobacterales;

C4803__locus_2

EUT1

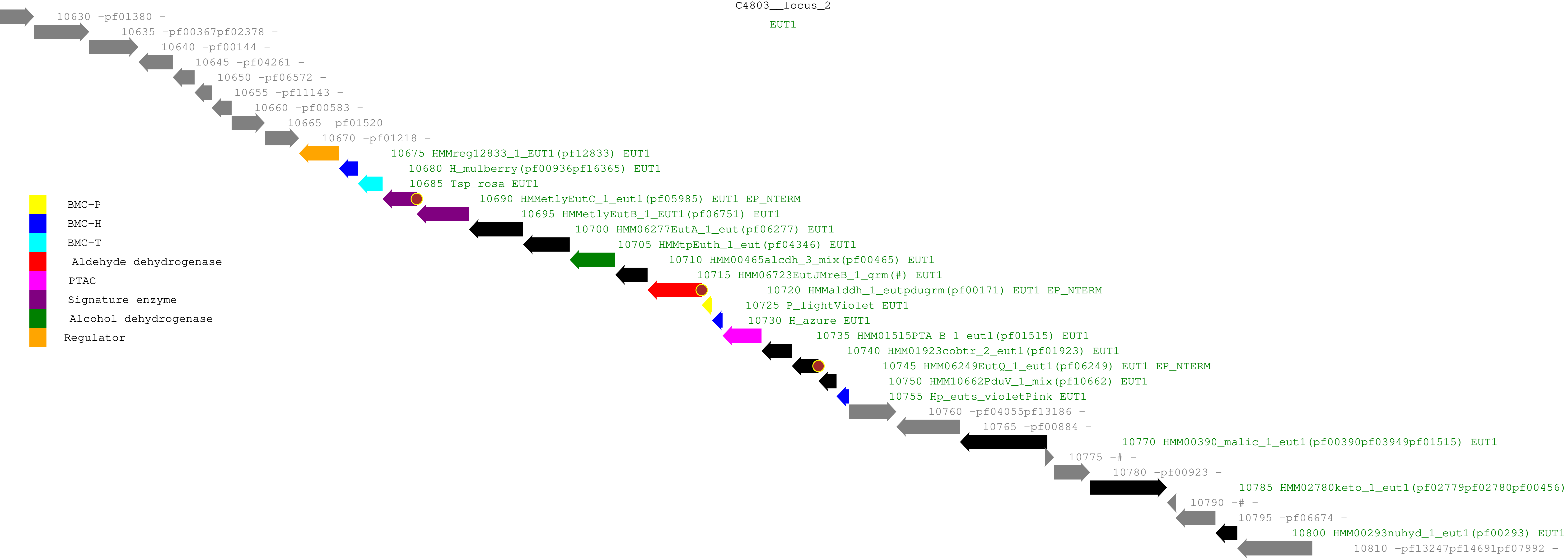

Salmonella enterica subsp. enterica serovar Give. Bacteria; Proteobacteria; Gammaproteobacteria; Enterobacterales;

C4832__locus_2

EUT1

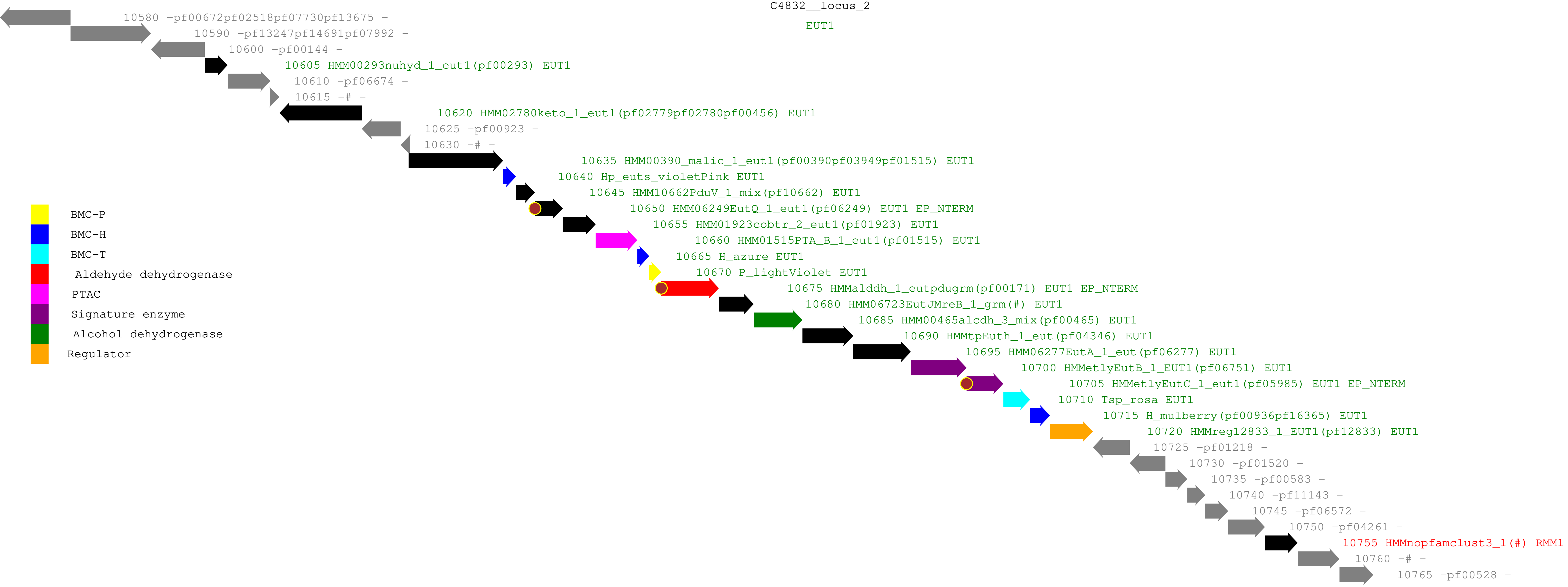

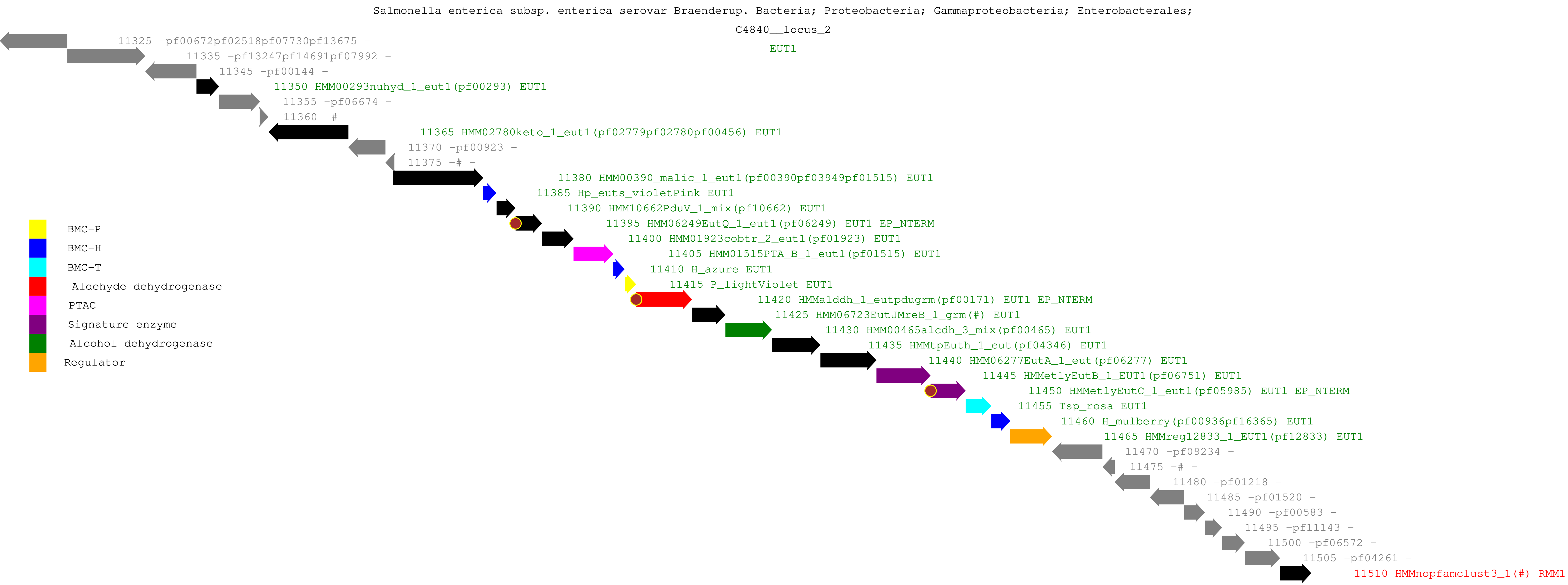

Salmonella enterica subsp. enterica serovar Braenderup. Bacteria; Proteobacteria; Gammaproteobacteria; Enterobacterales;
C4840__locus_2
EUT1
11325 -pf00672pf02518pf07730pf13675 -
11335 -pf13247pf14691pf07992 -
11345 -pf00144 -
11350 HMM00293nuhyd_1_eut1(pf00293) EUT1
11355 -pf06674 -
11360 -# -
11365 HMM02780keto_1_eut1(pf02779pf02780pf00456) EUT1
11370 -pf00923 -
11375 -# -
11380 HMM00390_malic_1_eut1(pf00390pf03949pf01515) EUT1
11385 Hp_euts_violetPink EUT1
11390 HMM10662PduV_1_mix(pf10662) EUT1
11395 HMM06249EutQ_1_eut1(pf06249) EUT1 EP_NTERM
11400 HMM01923cobtr_2_eut1(pf01923) EUT1
11405 HMM01515PTA_B_1_eut1(pf01515) EUT1
11410 H_azure EUT1
11415 P_lightViolet EUT1
11420 HMMalddh_1_eutpdugrm(pf00171) EUT1 EP_NTERM
11425 HMM06723EutJMreB_1_grm(#) EUT1
11430 HMM00465alcdh_3_mix(pf00465) EUT1
11435 HMMtpEuth_1_eut(pf04346) EUT1
11440 HMM06277EutA_1_eut(pf06277) EUT1
11445 HMMetlyEutB_1_EUT1(pf06751) EUT1
11450 HMMetlyEutC_1_eut1(pf05985) EUT1 EP_NTERM
11455 Tsp_rosa EUT1
11460 H_mulberry(pf00936pf16365) EUT1
11465 HMMreg12833_1_EUT1(pf12833) EUT1
11470 -pf09234 -
11475 -# -
11480 -pf01218 -
11485 -pf01520 -
11490 -pf00583 -
11495 -pf11143 -
11500 -pf06572 -
11505 -pf04261 -
11510 HMMnopfamclust3_1(#) RMM1
BMC-P
BMC-H
BMC-T
Aldehyde dehydrogenase
PTAC
Signature enzyme
Alcohol dehydrogenase
Regulator

Salmonella enterica subsp. enterica serovar Javiana. Bacteria; Proteobacteria; Gammaproteobacteria; Enterobacterales;

C4843__locus_1

EUT1

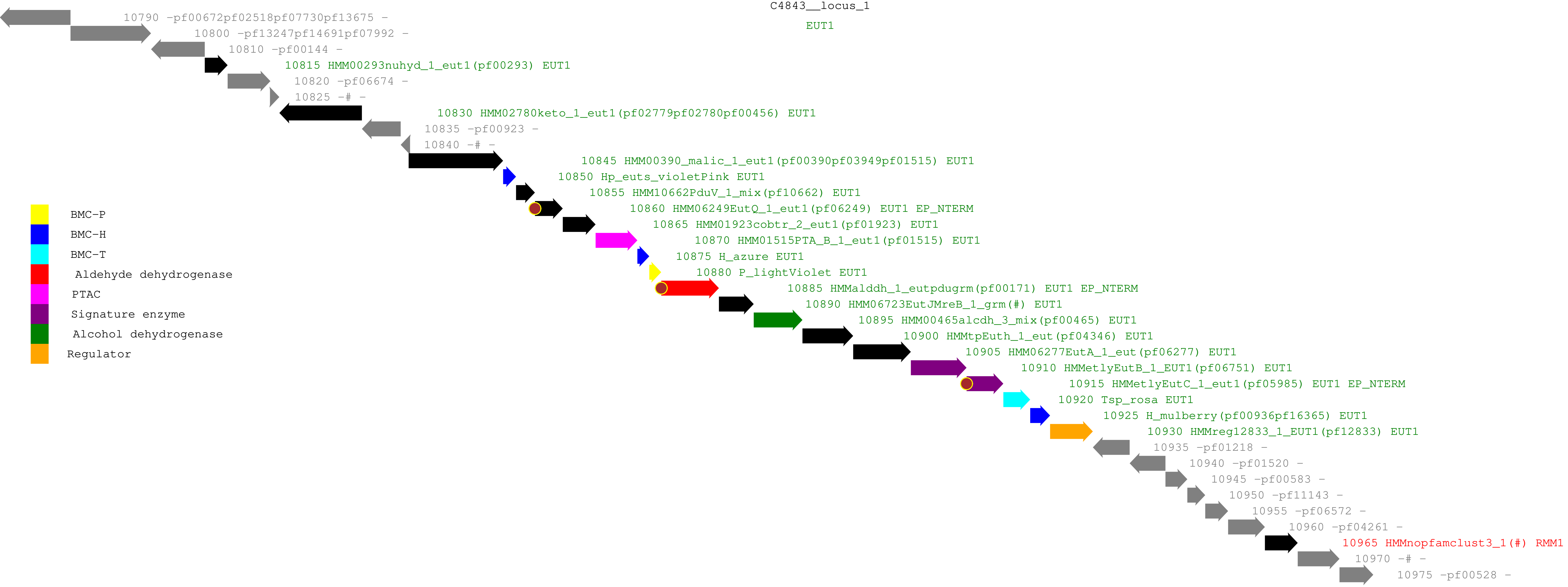

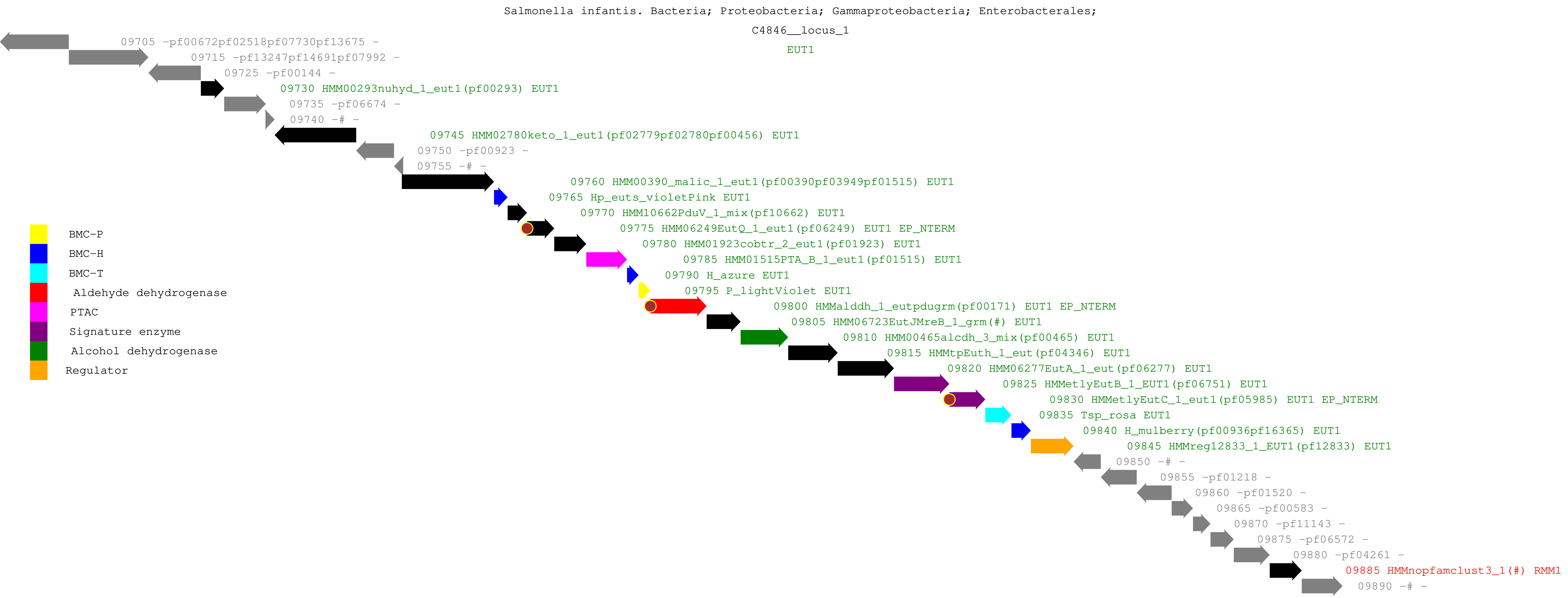

Salmonella infantis. Bacteria; Proteobacteria; Gammaproteobacteria; Enterobacterales;
C4846__locus_1
EUT1
09705 -pf00672pf02518pf07730pf13675 -
09715 -pf13247pf14691pf07992 -
09725 -pf00144 -
09730 HMM00293nuhyd_1_eut1(pf00293) EUT1
09735 -pf06674 -
09740 -# -
09745 HMM02780keto_1_eut1(pf02779pf02780pf00456) EUT1
09750 -pf00923 -
09755 -# -
09760 HMM00390_malic_1_eut1(pf00390pf03949pf01515) EUT1
09765 Hp_euts_violetPink EUT1
09770 HMM10662PduV_1_mix(pf10662) EUT1
09775 HMM06249EutQ_1_eut1(pf06249) EUT1 EP_NTERM
09780 HMM01923cobtr_2_eut1(pf01923) EUT1
09785 HMM01515PTA_B_1_eut1(pf01515) EUT1
09790 H_azure EUT1
09795 P_lightViolet EUT1
09800 HMMaldh_1_eutpdugrm(pf00171) EUT1 EP_NTERM
09805 HMM06723EutJMreB_1_grm(#) EUT1
09810 HMM00465alcdh_3_mix(pf00465) EUT1
09815 HMMtpEuth_1_eut(pf04346) EUT1
09820 HMM06277EutA_1_eut(pf06277) EUT1
09825 HMMetlyEutB_1_EUT1(pf06751) EUT1
09830 HMMetlyEutC_1_eut1(pf05985) EUT1 EP_NTERM
09835 Tsp_rosa EUT1
09840 H_mulberry(pf00936pf16365) EUT1
09845 HMMreg12833_1_EUT1(pf12833) EUT1
09850 -# -
09855 -pf01218 -
09860 -pf01520 -
09865 -pf00583 -
09870 -pf11143 -
09875 -pf06572 -
09880 -pf04261 -
09885 HMMnopfamclust3_1(#) RMM1
09890 -# -
BMC-P
BMC-H
BMC-T
Aldehyde dehydrogenase
PTAC
Signature enzyme
Alcohol dehydrogenase
Regulator

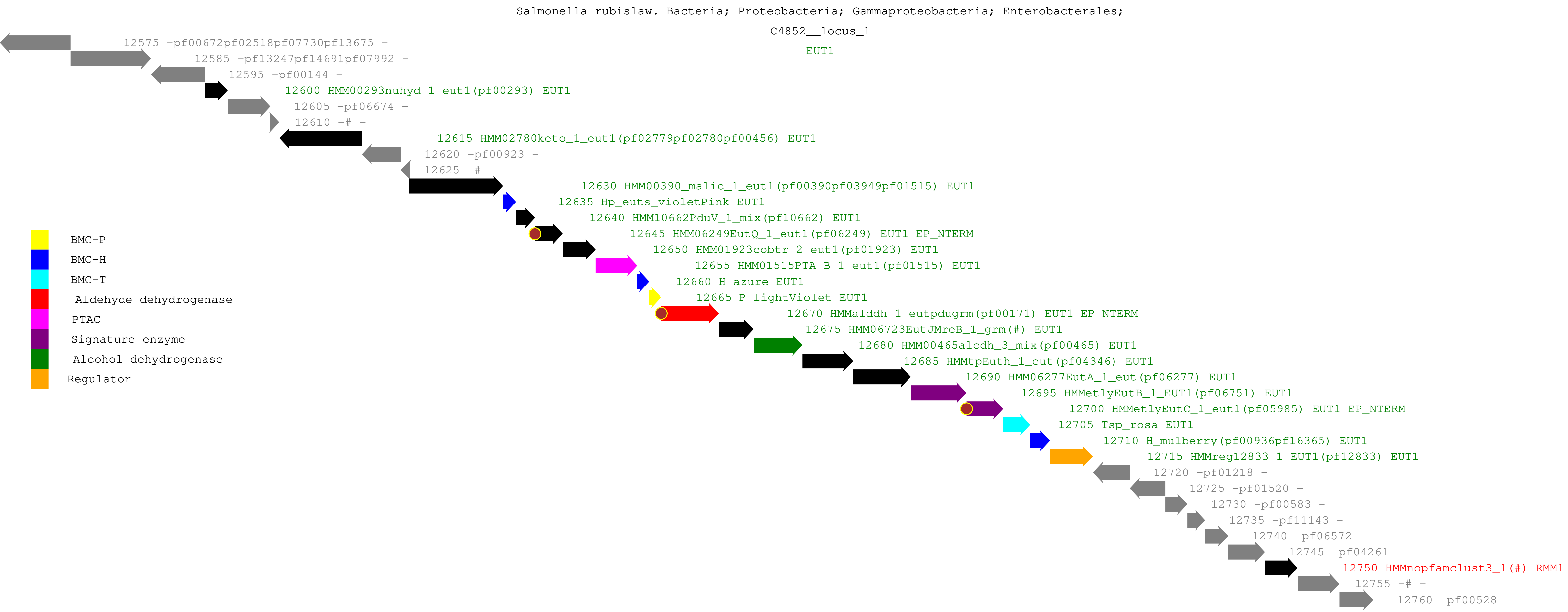
Salmonella rubislaw. Bacteria; Proteobacteria; Gammaproteobacteria; Enterobacterales;
C4852__locus_1
EUT1
12575 -pf00672pf02518pf07730pf13675 -
12585 -pf13247pf14691pf07992 -
12595 -pf00144 -
12600 HMM00293nuhyd_1_eut1(pf00293) EUT1
12605 -pf06674 -
12610 -# -
12615 HMM02780keto_1_eut1(pf02779pf02780pf00456) EUT1
12620 -pf00923 -
12625 -# -
12630 HMM00390_malic_1_eut1(pf00390pf03949pf01515) EUT1
12635 Hp_euts_violetPink EUT1
12640 HMM10662PduV_1_mix(pf10662) EUT1
12645 HMM06249EutQ_1_eut1(pf06249) EUT1 EP_NTERM
12650 HMM01923cobtr_2_eut1(pf01923) EUT1
12655 HMM01515PTA_B_1_eut1(pf01515) EUT1
12660 H_azure EUT1
12665 P_lightViolet EUT1
12670 HMMaldh_1_eutpdugrm(pf00171) EUT1 EP_NTERM
12675 HMM06723EutJMreB_1_grm(#) EUT1
12680 HMM00465alcdh_3_mix(pf00465) EUT1
12685 HMMtpEuth_1_eut(pf04346) EUT1
12690 HMM06277EutA_1_eut(pf06277) EUT1
12695 HMMetlyEutB_1_EUT1(pf06751) EUT1
12700 HMMetlyEutC_1_eut1(pf05985) EUT1 EP_NTERM
12705 Tsp_rosa EUT1
12710 H_mulberry(pf00936pf16365) EUT1
12715 HMMreg12833_1_EUT1(pf12833) EUT1
12720 -pf01218 -
12725 -pf01520 -
12730 -pf00583 -
12735 -pf11143 -
12740 -pf06572 -
12745 -pf04261 -
12750 HMMnopfamclust3_1(#) RMM1
12755 -# -
12760 -pf00528 -
BMC-P
BMC-H
BMC-T
Aldehyde dehydrogenase
PTAC
Signature enzyme
Alcohol dehydrogenase
Regulator

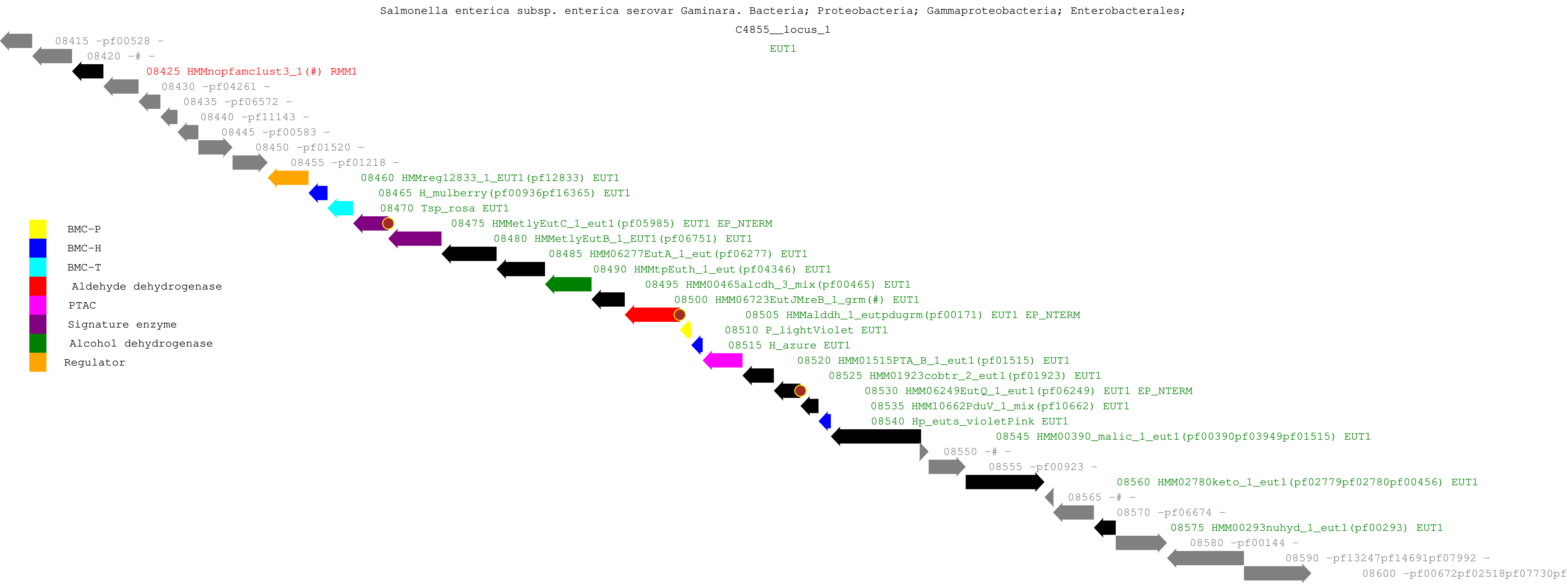
Salmonella enterica subsp. enterica serovar Gaminara. Bacteria; Proteobacteria; Gammaproteobacteria; Enterobacterales;
C4855__locus_1
EUT1
08415 -pf00528 -
08420 -# -
08425 HMMnopfamclust3_1(#) RMM1
08430 -pf04261 -
08435 -pf06572 -
08440 -pf11143 -
08445 -pf00583 -
08450 -pf01520 -
08455 -pf01218 -
08460 HMMreg12833_1_EUT1(pf12833) EUT1
08465 H_mulberry(pf00936pf16365) EUT1
08470 Tsp_rosa EUT1
08475 HMMetlyEutC_1_eut1(pf05985) EUT1 EP_NTERM
08480 HMMetlyEutB_1_EUT1(pf06751) EUT1
08485 HMM06277EutA_1_eut(pf06277) EUT1
08490 HMMtpEuth_1_eut(pf04346) EUT1
08495 HMM00465alcdh_3_mix(pf00465) EUT1
08500 HMM06723EutJMreB_1_grm(#) EUT1
08505 HMMalddh_1_eutpdugrm(pf00171) EUT1 EP_NTERM
08510 P_lightViolet EUT1
08515 H_azure EUT1
08520 HMM01515PTA_B_1_eut1(pf01515) EUT1
08525 HMM01923cobtr_2_eut1(pf01923) EUT1
08530 HMM06249EutQ_1_eut1(pf06249) EUT1 EP_NTERM
08535 HMM10662PduV_1_mix(pf10662) EUT1
08540 Hp_euts_violetPink EUT1
08545 HMM00390_malic_1_eut1(pf00390pf03949pf01515) EUT1
08550 -# -
08555 -pf00923 -
08560 HMM02780keto_1_eut1(pf02779pf02780pf00456) EUT1
08565 -# -
08570 -pf06674 -
08575 HMM00293nuhyd_1_eut1(pf00293) EUT1
08580 -pf00144 -
08590 -pf13247pf14691pf07992 -
08600 -pf00672pf02518pf07730pf
BMC-P
BMC-H
BMC-T
Aldehyde dehydrogenase
PTAC
Signature enzyme
Alcohol dehydrogenase
Regulator

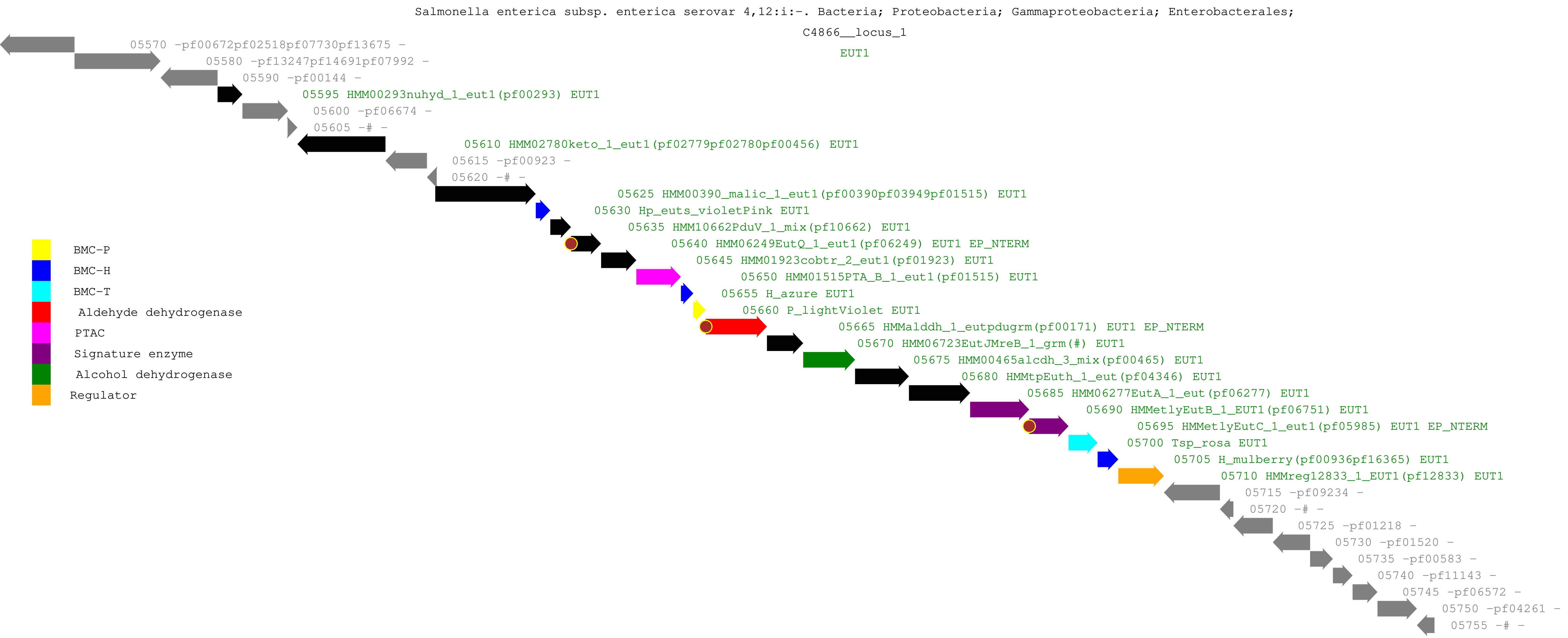

Salmonella enterica subsp. enterica serovar 4,12:i:-. Bacteria; Proteobacteria; Gammaproteobacteria; Enterobacterales;
C4866__locus_1
EUT1
05570 -pf00672pf02518pf07730pf13675 -
05580 -pf13247pf14691pf07992 -
05590 -pf00144 -
05595 HMM00293nuhyd_1_eut1(pf00293) EUT1
05600 -pf06674 -
05605 -# -
05610 HMM02780keto_1_eut1(pf02779pf02780pf00456) EUT1
05615 -pf00923 -
05620 -# -
05625 HMM00390_malic_1_eut1(pf00390pf03949pf01515) EUT1
05630 Hp_euts_violetPink EUT1
05635 HMM10662PduV_1_mix(pf10662) EUT1
05640 HMM06249EutQ_1_eut1(pf06249) EUT1 EP_NTERM
05645 HMM01923cobtr_2_eut1(pf01923) EUT1
05650 HMM01515PTA_B_1_eut1(pf01515) EUT1
05655 H_azure EUT1
05660 P_lightViolet EUT1
05665 HMMaldh_1_eutpdugrm(pf00171) EUT1 EP_NTERM
05670 HMM06723EutJMreB_1_grm(#) EUT1
05675 HMM00465alcdh_3_mix(pf00465) EUT1
05680 HMMtpEuth_1_eut(pf04346) EUT1
05685 HMM06277EutA_1_eut(pf06277) EUT1
05690 HMMetlyEutB_1_EUT1(pf06751) EUT1
05695 HMMetlyEutC_1_eut1(pf05985) EUT1 EP_NTERM
05700 Tsp_rosa EUT1
05705 H_mulberry(pf00936pf16365) EUT1
05710 HMMreg12833_1_EUT1(pf12833) EUT1
05715 -pf09234 -
05720 -# -
05725 -pf01218 -
05730 -pf01520 -
05735 -pf00583 -
05740 -pf11143 -
05745 -pf06572 -
05750 -pf04261 -
05755 -# -
BMC-P
BMC-H
BMC-T
Aldehyde dehydrogenase
PTAC
Signature enzyme
Alcohol dehydrogenase
Regulator

Salmonella anatum. Bacteria; Proteobacteria; Gammaproteobacteria; Enterobacterales;

C4876__locus_1

EUT1

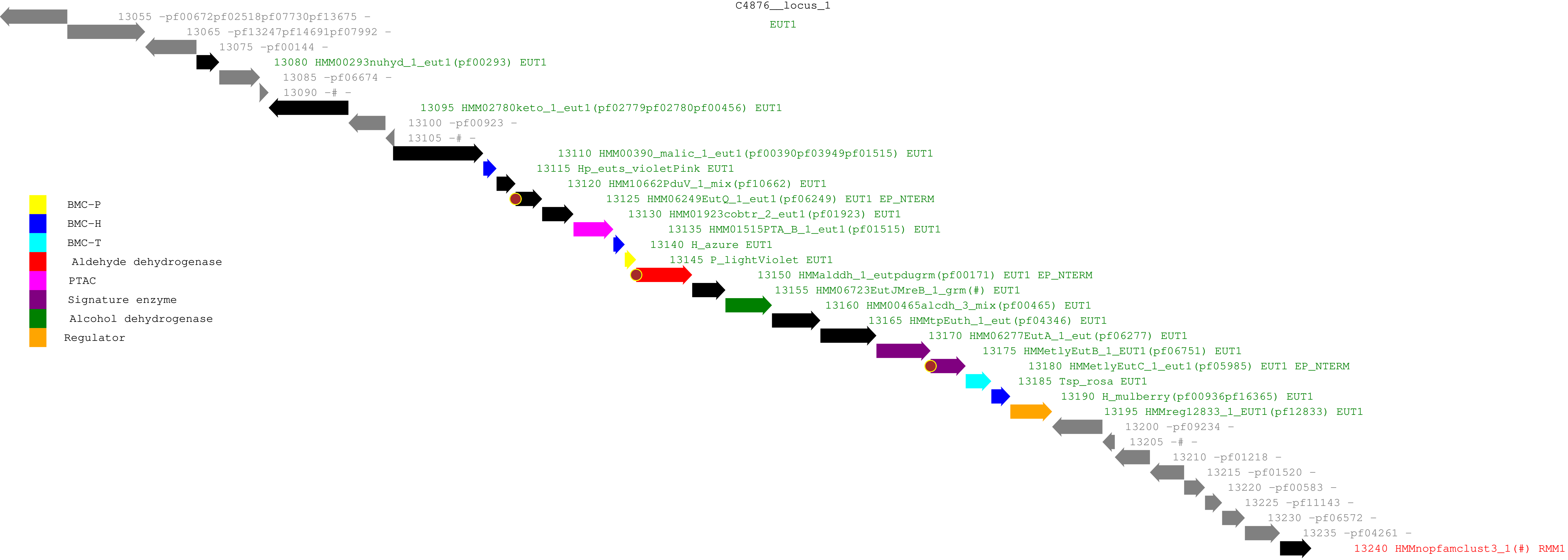

Salmonella enterica subsp. enterica serovar Kentucky. Bacteria; Proteobacteria; Gammaproteobacteria; Enterobacterales;

C4878__locus_2

EUT1

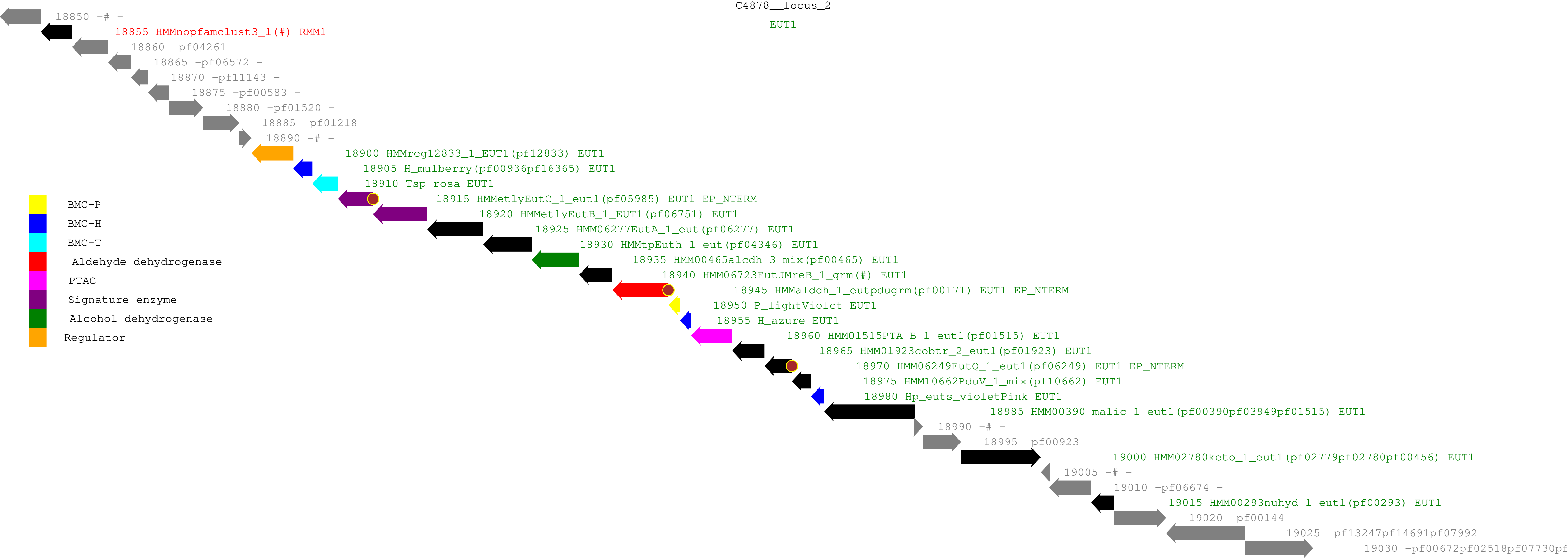

Escherichia marmotae. Bacteria; Proteobacteria; Gammaproteobacteria; Enterobacterales;

C4A13__locus_1_main_sat

EUT1

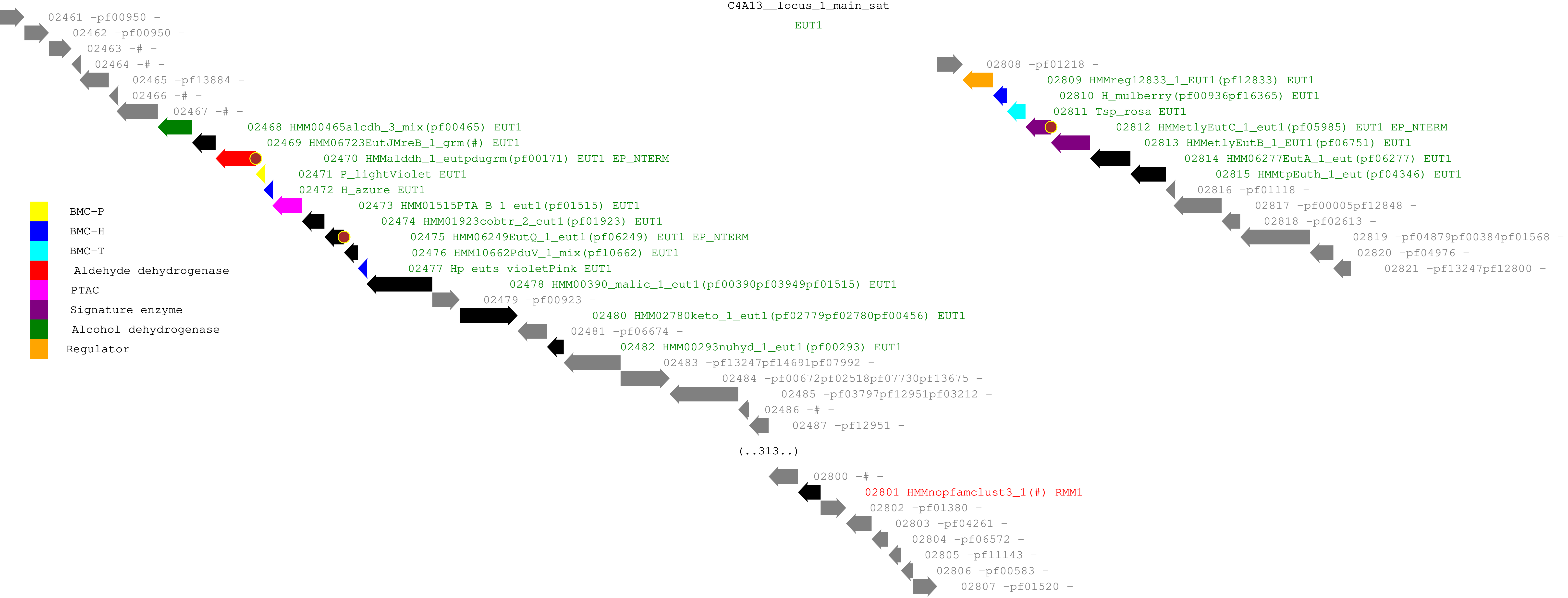

Salmonella enterica subsp. enterica serovar Teko. Bacteria; Proteobacteria; Gammaproteobacteria; Enterobacterales;

C4V46__locus_2

EUT1

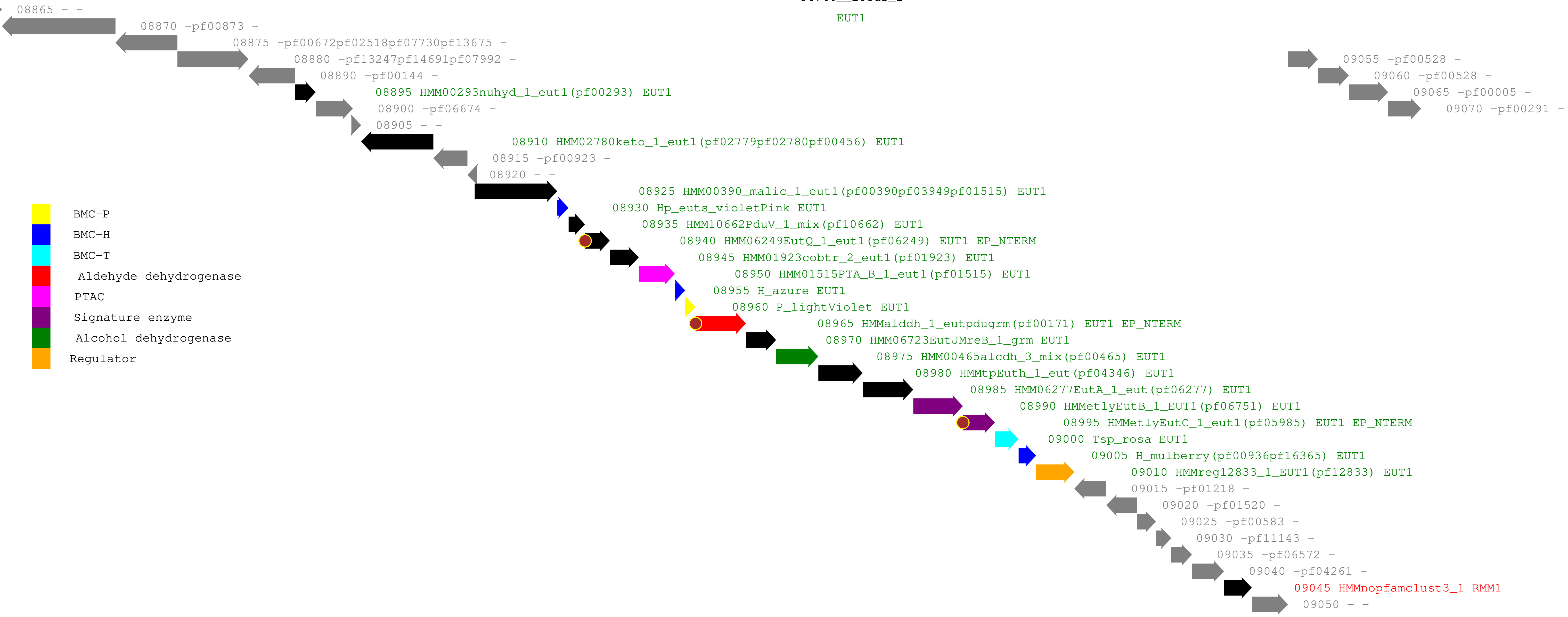

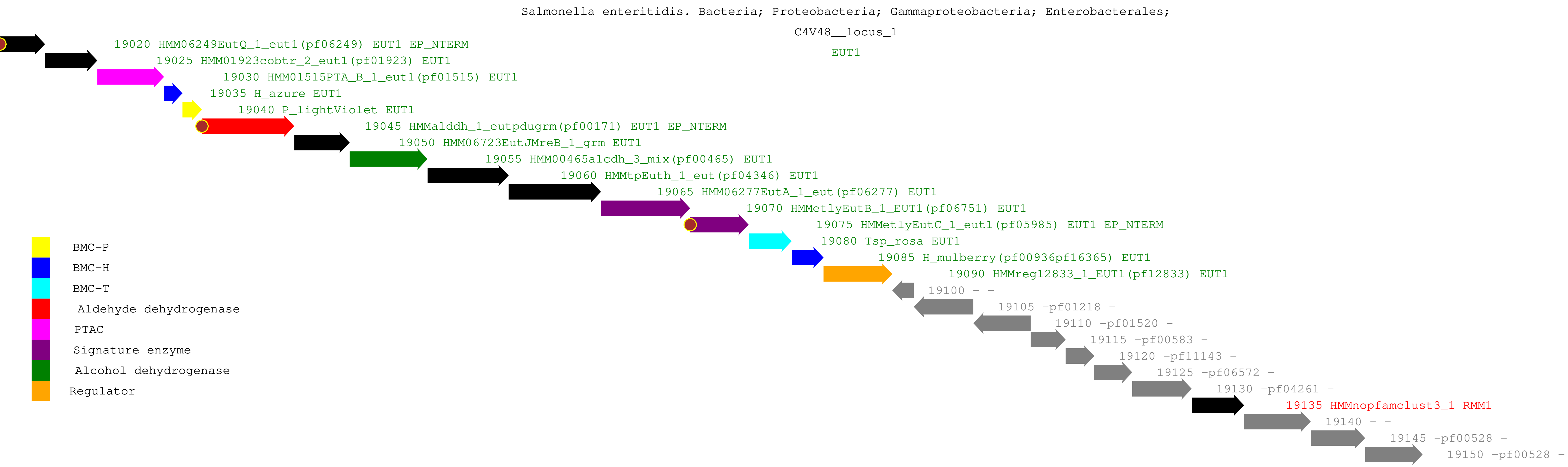
Salmonella enteritidis. Bacteria; Proteobacteria; Gammaproteobacteria; Enterobacterales;
C4V48__locus_1
EUT1
19020 HMM06249EutQ_1_eut1(pf06249) EUT1 EP_NTERM
19025 HMM01923cobtr_2_eut1(pf01923) EUT1
19030 HMM01515PTA_B_1_eut1(pf01515) EUT1
19035 H_azure EUT1
19040 P_lightViolet EUT1
19045 HMMalddh_1_eutpdugrm(pf00171) EUT1 EP_NTERM
19050 HMM06723EutJMreB_1_grm EUT1
19055 HMM00465alcdh_3_mix(pf00465) EUT1
19060 HMMtpEuth_1_eut(pf04346) EUT1
19065 HMM06277EutA_1_eut(pf06277) EUT1
19070 HMMetlyEutB_1_EUT1(pf06751) EUT1
19075 HMMetlyEutC_1_eut1(pf05985) EUT1 EP_NTERM
19080 Tsp_rosa EUT1
19085 H_mulberry(pf00936pf16365) EUT1
19090 HMMreg12833_1_EUT1(pf12833) EUT1
19100 - -
19105 -pf01218 -
19110 -pf01520 -
19115 -pf00583 -
19120 -pf11143 -
19125 -pf06572 -
19130 -pf04261 -
19135 HMMnopfamclust3_1 RMM1
19140 - -
19145 -pf00528 -
19150 -pf00528 -
BMC-P
BMC-H
BMC-T
Aldehyde dehydrogenase
PTAC
Signature enzyme
Alcohol dehydrogenase
Regulator

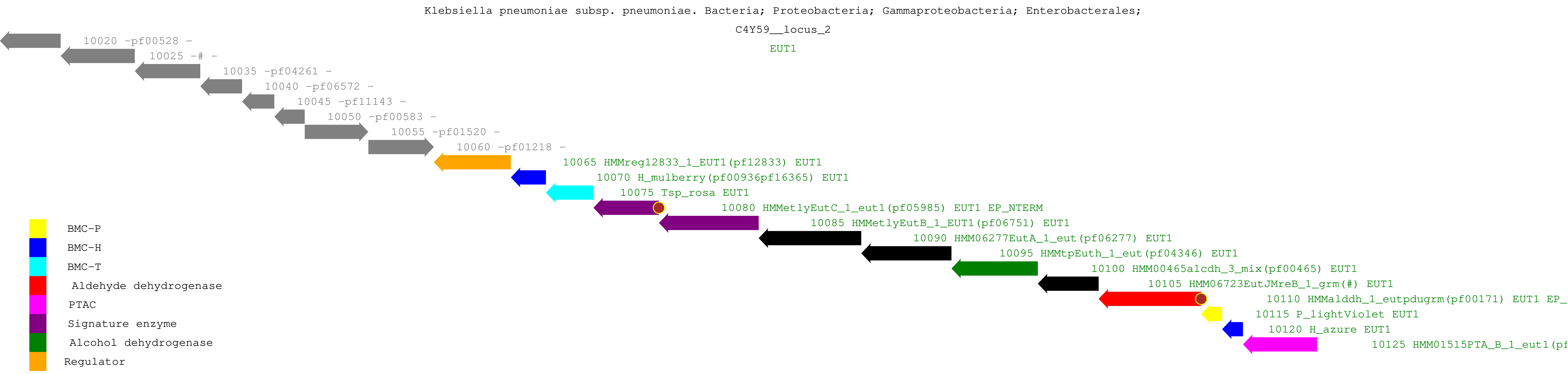

Klebsiella pneumoniae subsp. pneumoniae. Bacteria; Proteobacteria; Gammaproteobacteria; Enterobacterales;
C4Y59__locus_2
EUT1
10020 -pf00528 -
10025 -# -
10035 -pf04261 -
10040 -pf06572 -
10045 -pf11143 -
10050 -pf00583 -
10055 -pf01520 -
10060 -pf01218 -
10065 HMMreg12833_1_EUT1(pf12833) EUT1
10070 H_mulberry(pf00936pf16365) EUT1
10075 Tsp_rosa EUT1
10080 HMMetlyEutC_1_eut1(pf05985) EUT1 EP_NTERM
10085 HMMetlyEutB_1_EUT1(pf06751) EUT1
10090 HMM06277EutA_1_eut(pf06277) EUT1
10095 HMMtpEuth_1_eut(pf04346) EUT1
10100 HMM00465alcdh_3_mix(pf00465) EUT1
10105 HMM06723EutJMreB_1_grm(#) EUT1
10110 HMMaldh_1_eutpdugrm(pf00171) EUT1 EP_
10115 P_lightViolet EUT1
10120 H_azure EUT1
10125 HMM01515PTA_B_1_eut1(pf
BMC-P
BMC-H
BMC-T
Aldehyde dehydrogenase
PTAC
Signature enzyme
Alcohol dehydrogenase
Regulator

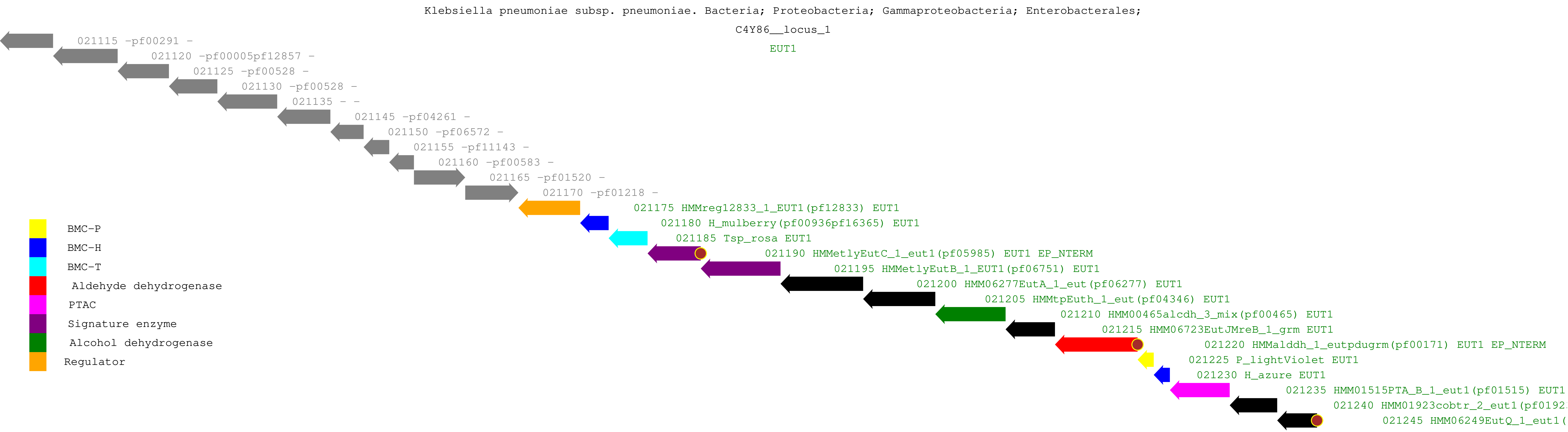

Klebsiella pneumoniae subsp. pneumoniae. Bacteria; Proteobacteria; Gammaproteobacteria; Enterobacterales;
C4Y86__locus_1
EUT1
021115 -pf00291 -
021120 -pf00005pf12857 -
021125 -pf00528 -
021130 -pf00528 -
021135 - -
021145 -pf04261 -
021150 -pf06572 -
021155 -pf11143 -
021160 -pf00583 -
021165 -pf01520 -
021170 -pf01218 -
021175 HMMreg12833_1_EUT1(pf12833) EUT1
021180 H_mulberry(pf00936pf16365) EUT1
021185 Tsp_rosa EUT1
021190 HMMetlyEutC_1_eut1(pf05985) EUT1 EP_NTERM
021195 HMMetlyEutB_1_EUT1(pf06751) EUT1
021200 HMM06277EutA_1_eut(pf06277) EUT1
021205 HMMtpEuth_1_eut(pf04346) EUT1
021210 HMM00465alcdh_3_mix(pf00465) EUT1
021215 HMM06723EutJMreB_1_grm EUT1
021220 HMMaldh_1_eutpdugrm(pf00171) EUT1 EP_NTERM
021225 P_lightViolet EUT1
021230 H_azure EUT1
021235 HMM01515PTA_B_1_eut1(pf01515) EUT1
021240 HMM01923cobtr_2_eut1(pf0192
021245 HMM06249EutQ_1_eut1(
BMC-P
BMC-H
BMC-T
Aldehyde dehydrogenase
PTAC
Signature enzyme
Alcohol dehydrogenase
Regulator

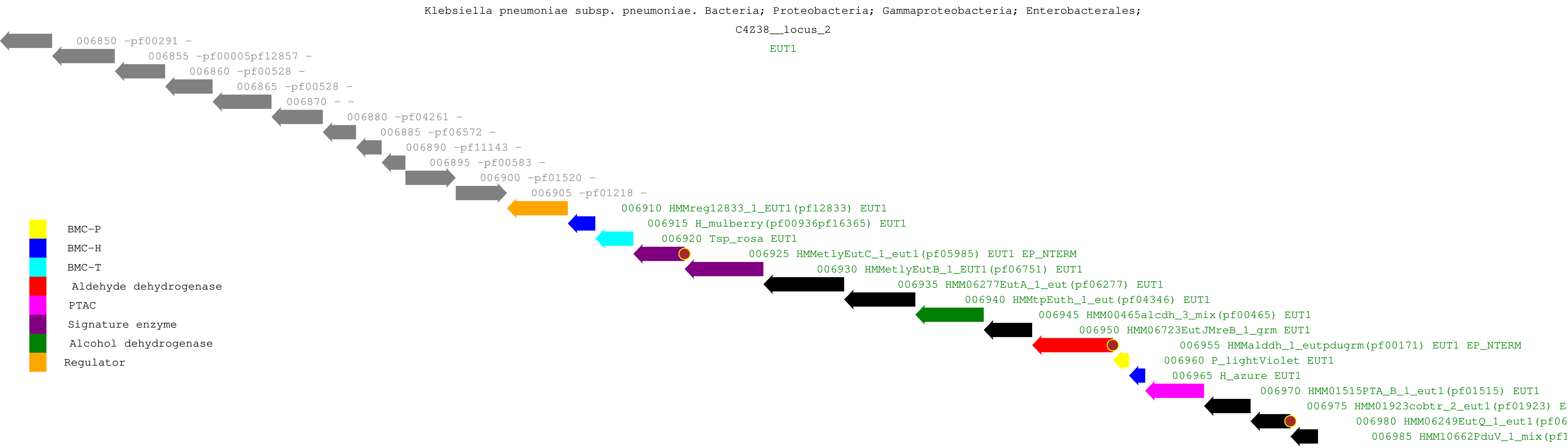
Klebsiella pneumoniae subsp. pneumoniae. Bacteria; Proteobacteria; Gammaproteobacteria; Enterobacterales;
C4Z38__locus_2
EUT1
006850 -pf00291 -
006855 -pf00005pf12857 -
006860 -pf00528 -
006865 -pf00528 -
006870 - -
006880 -pf04261 -
006885 -pf06572 -
006890 -pf11143 -
006895 -pf00583 -
006900 -pf01520 -
006905 -pf01218 -
006910 HMMreg12833_1_EUT1(pf12833) EUT1
006915 H_mulberry(pf00936pf16365) EUT1
006920 Tsp_rosa EUT1
006925 HMMetlyEutC_1_eut1(pf05985) EUT1 EP_NTERM
006930 HMMetlyEutB_1_EUT1(pf06751) EUT1
006935 HMM06277EutA_1_eut(pf06277) EUT1
006940 HMMtpEuth_1_eut(pf04346) EUT1
006945 HMM00465alcdh_3_mix(pf00465) EUT1
006950 HMM06723EutJMreB_1_grm EUT1
006955 HMMaldh_1_eutpdugrm(pf00171) EUT1 EP_NTERM
006960 P_lightViolet EUT1
006965 H_azure EUT1
006970 HMM01515PTA_B_1_eut1(pf01515) EUT1
006975 HMM01923cobtr_2_eut1(pf01923) E
006980 HMM06249EutQ_1_eut1(pf06
006985 HMM10662PduV_1_mix(pf1
BMC-P
BMC-H
BMC-T
Aldehyde dehydrogenase
PTAC
Signature enzyme
Alcohol dehydrogenase
Regulator

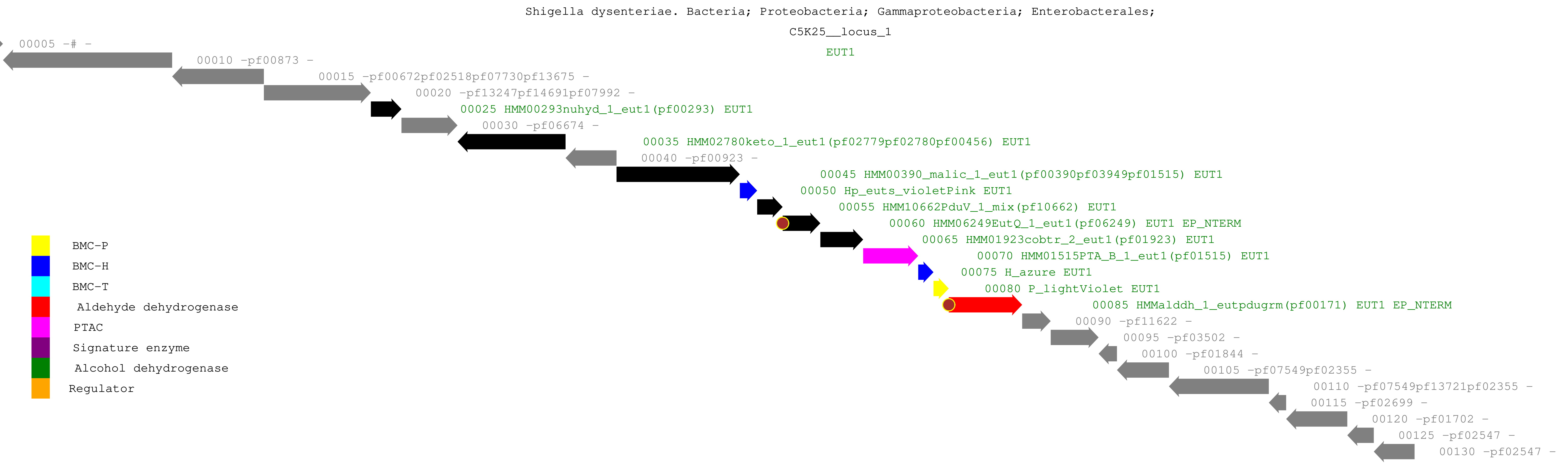
Shigella dysenteriae. Bacteria; Proteobacteria; Gammaproteobacteria; Enterobacterales;
C5K25__locus_1
EUT1
00005 -# -
00010 -pf00873 -
00015 -pf00672pf02518pf07730pf13675 -
00020 -pf13247pf14691pf07992 -
00025 HMM00293nuhyd_1_eut1(pf00293) EUT1
00030 -pf06674 -
00035 HMM02780keto_1_eut1(pf02779pf02780pf00456) EUT1
00040 -pf00923 -
00045 HMM00390_malic_1_eut1(pf00390pf03949pf01515) EUT1
00050 Hp_euts_violetPink EUT1
00055 HMM10662PduV_1_mix(pf10662) EUT1
00060 HMM06249EutQ_1_eut1(pf06249) EUT1 EP_NTERM
00065 HMM01923cobtr_2_eut1(pf01923) EUT1
00070 HMM01515PTA_B_1_eut1(pf01515) EUT1
00075 H_azure EUT1
00080 P_lightViolet EUT1
00085 HMMalddh_1_eutpdugrm(pf00171) EUT1 EP_NTERM
00090 -pf11622 -
00095 -pf03502 -
00100 -pf01844 -
00105 -pf07549pf02355 -
00110 -pf07549pf13721pf02355 -
00115 -pf02699 -
00120 -pf01702 -
00125 -pf02547 -
00130 -pf02547 -
BMC-P
BMC-H
BMC-T
Aldehyde dehydrogenase
PTAC
Signature enzyme
Alcohol dehydrogenase
Regulator

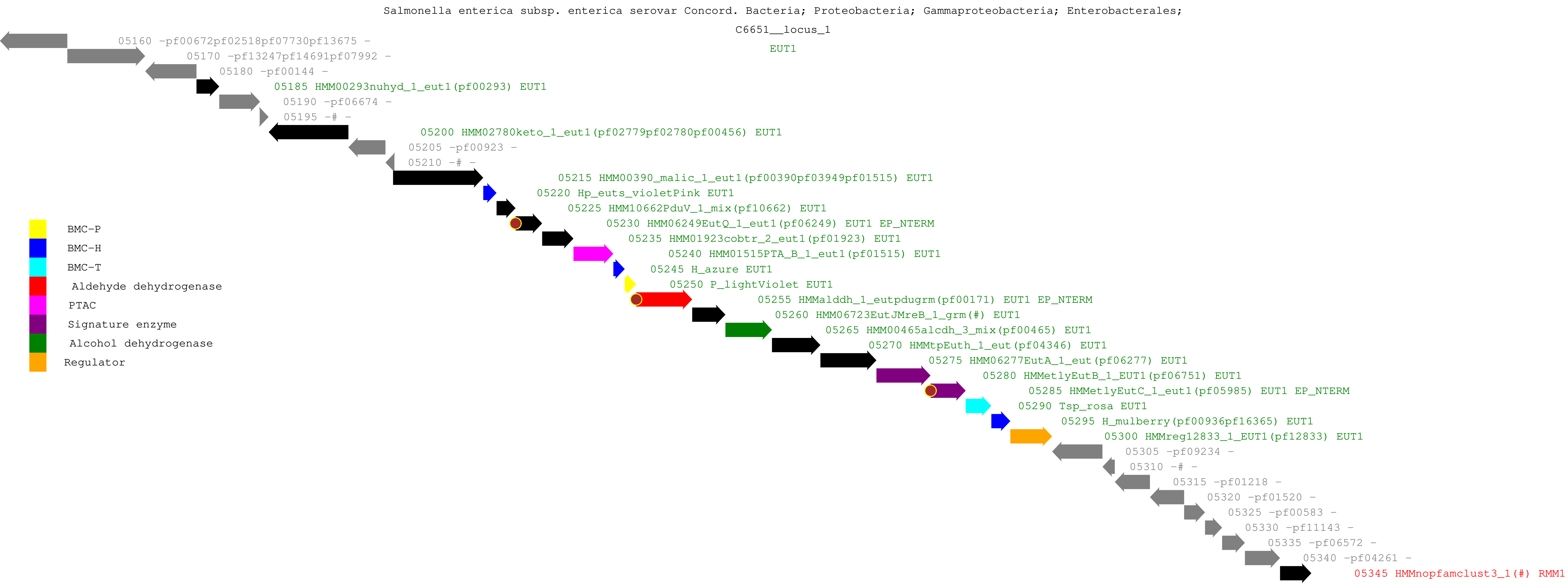
Salmonella enterica subsp. enterica serovar Concord. Bacteria; Proteobacteria; Gammaproteobacteria; Enterobacterales;
C6651__locus_1
EUT1
05160 -pf00672pf02518pf07730pf13675 -
05170 -pf13247pf14691pf07992 -
05180 -pf00144 -
05185 HMM00293nuhyd_1_eut1(pf00293) EUT1
05190 -pf06674 -
05195 -# -
05200 HMM02780keto_1_eut1(pf02779pf02780pf00456) EUT1
05205 -pf00923 -
05210 -# -
05215 HMM00390_malic_1_eut1(pf00390pf03949pf01515) EUT1
05220 Hp_euts_violetPink EUT1
05225 HMM10662PduV_1_mix(pf10662) EUT1
05230 HMM06249EutQ_1_eut1(pf06249) EUT1 EP_NTERM
05235 HMM01923cobtr_2_eut1(pf01923) EUT1
05240 HMM01515PTA_B_1_eut1(pf01515) EUT1
05245 H_azure EUT1
05250 P_lightViolet EUT1
05255 HMMalddh_1_eutpdugrm(pf00171) EUT1 EP_NTERM
05260 HMM06723EutJMreB_1_grm(#) EUT1
05265 HMM00465alcdh_3_mix(pf00465) EUT1
05270 HMMtpEuth_1_eut(pf04346) EUT1
05275 HMM06277EutA_1_eut(pf06277) EUT1
05280 HMMetlyEutB_1_EUT1(pf06751) EUT1
05285 HMMetlyEutC_1_eut1(pf05985) EUT1 EP_NTERM
05290 Tsp_rosa EUT1
05295 H_mulberry(pf00936pf16365) EUT1
05300 HMMreg12833_1_EUT1(pf12833) EUT1
05305 -pf09234 -
05310 -# -
05315 -pf01218 -
05320 -pf01520 -
05325 -pf00583 -
05330 -pf11143 -
05335 -pf06572 -
05340 -pf04261 -
05345 HMMnopfamclust3_1(#) RMM1
BMC-P
BMC-H
BMC-T
Aldehyde dehydrogenase
PTAC
Signature enzyme
Alcohol dehydrogenase
Regulator

Thauera linaloolentis 47Lol = DSM 12138. Bacteria; Proteobacteria; Betaproteobacteria; Rhodocyclales;

C666__locus_1_main_sat

EUT1

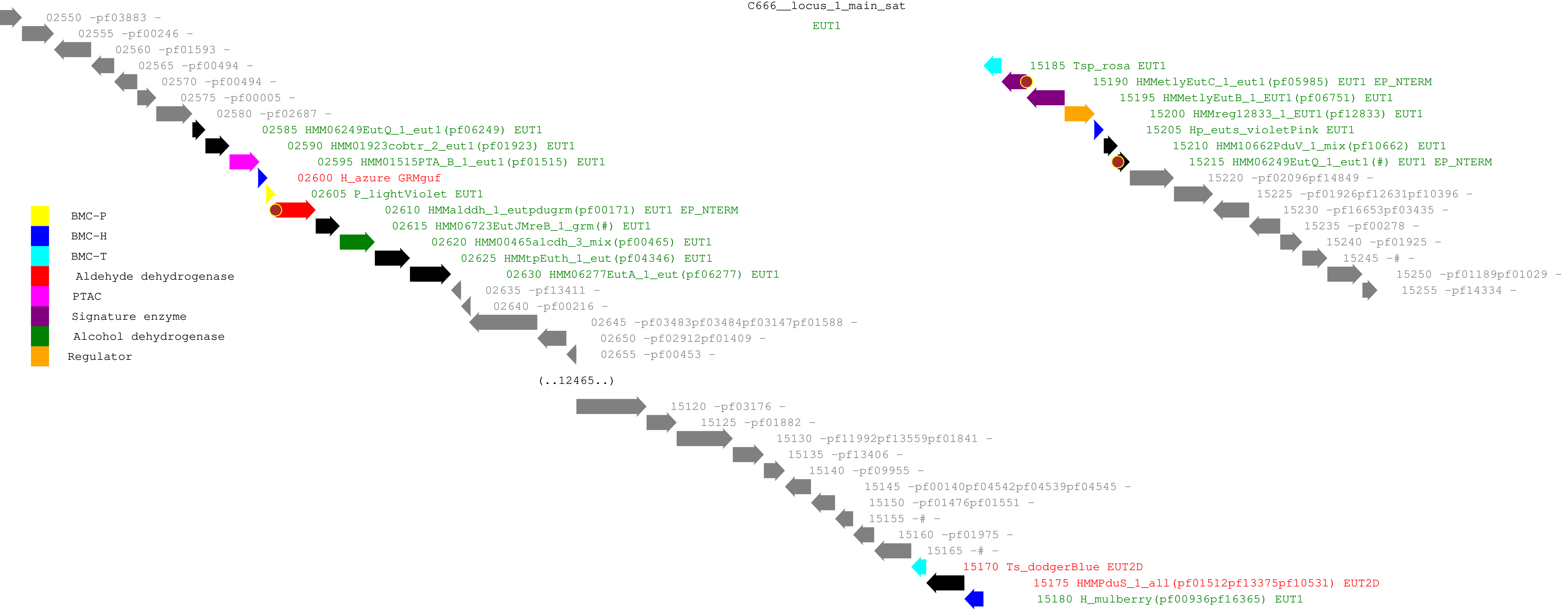

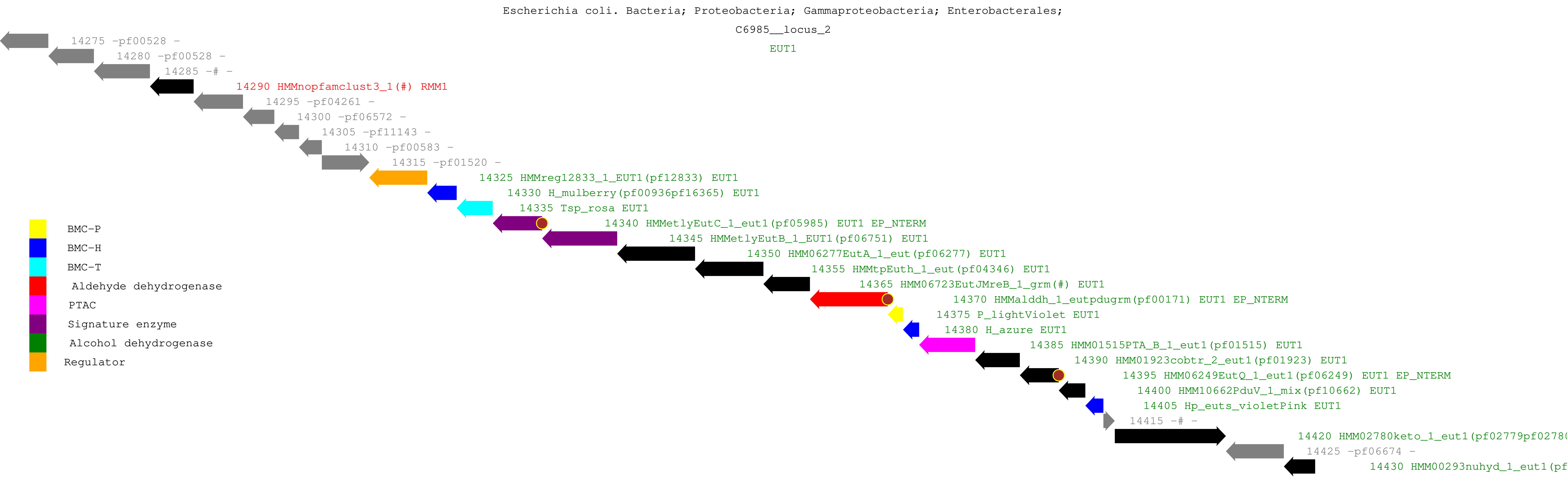

Escherichia coli. Bacteria; Proteobacteria; Gammaproteobacteria; Enterobacterales;
C6985__locus_2
EUT1
14275 -pf00528 -
14280 -pf00528 -
14285 -# -
14290 HMMnopfamclust3_1(#) RMM1
14295 -pf04261 -
14300 -pf06572 -
14305 -pf11143 -
14310 -pf00583 -
14315 -pf01520 -
14325 HMMreg12833_1_EUT1(pf12833) EUT1
14330 H_mulberry(pf00936pf16365) EUT1
14335 Tsp_rosa EUT1
14340 HMMetlyEutC_1_eut1(pf05985) EUT1 EP_NTERM
14345 HMMetlyEutB_1_EUT1(pf06751) EUT1
14350 HMM06277EutA_1_eut(pf06277) EUT1
14355 HMMtpEuth_1_eut(pf04346) EUT1
14365 HMM06723EutJMreB_1_grm(#) EUT1
14370 HMMalddh_1_eutpdugrm(pf00171) EUT1 EP_NTERM
14375 P_lightViolet EUT1
14380 H_azure EUT1
14385 HMM01515PTA_B_1_eut1(pf01515) EUT1
14390 HMM01923cobtr_2_eut1(pf01923) EUT1
14395 HMM06249EutQ_1_eut1(pf06249) EUT1 EP_NTERM
14400 HMM10662PduV_1_mix(pf10662) EUT1
14405 Hp_euts_violetPink EUT1
14415 -# -
14420 HMM02780keto_1_eut1(pf02779pf0278
14425 -pf06674 -
14430 HMM00293nuhyd_1_eut1(pf
BMC-P
BMC-H
BMC-T
Aldehyde dehydrogenase
PTAC
Signature enzyme
Alcohol dehydrogenase
Regulator

Escherichia coli. Bacteria; Proteobacteria; Gammaproteobacteria; Enterobacterales;

C6B13__locus_1

EUT1

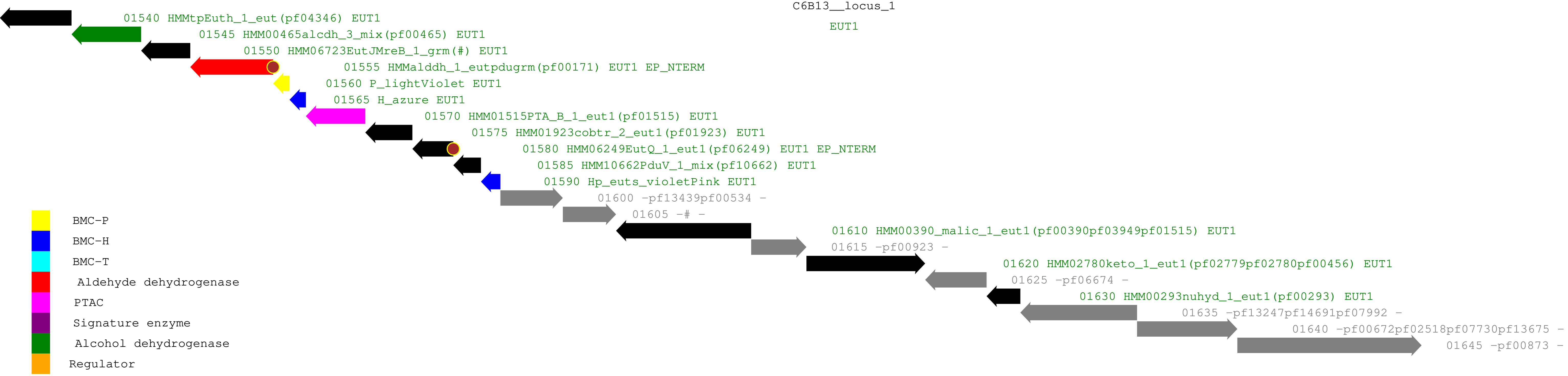

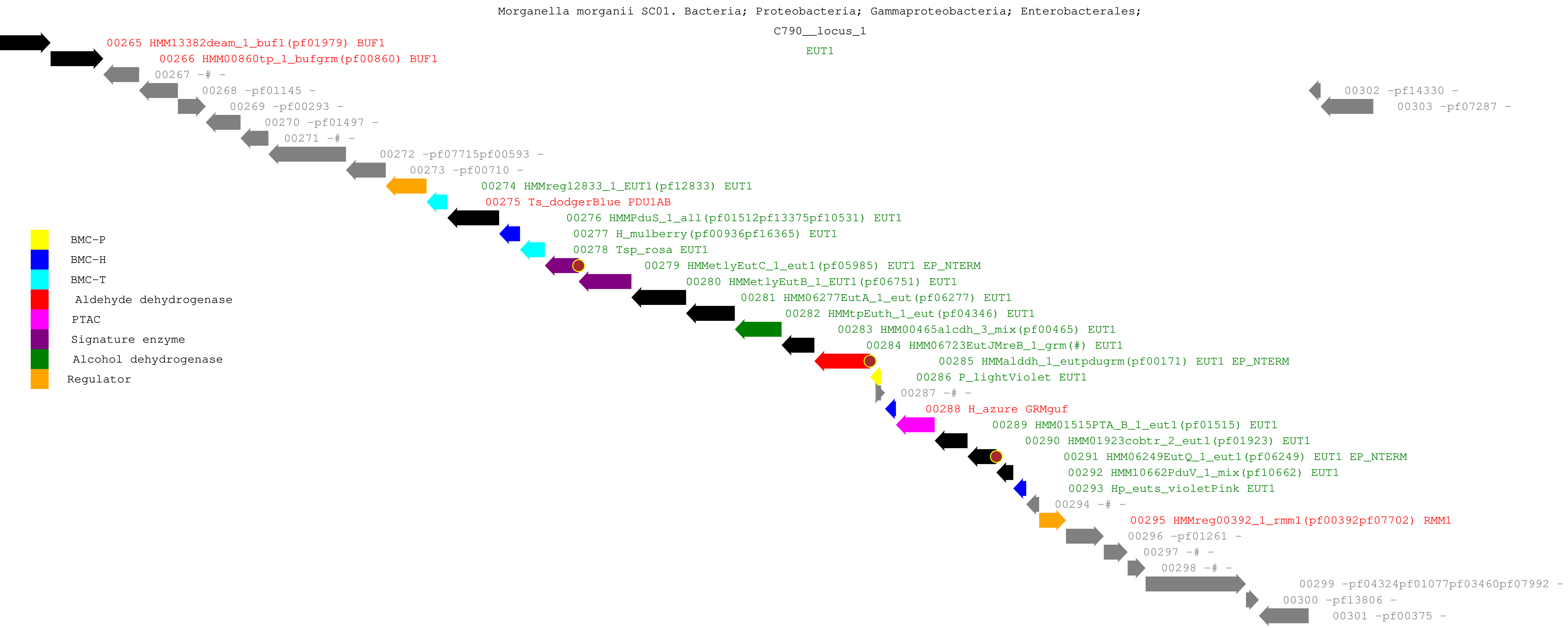
Morganella morganii SC01. Bacteria; Proteobacteria; Gammaproteobacteria; Enterobacterales;
C790__locus_1
EUT1
00265 HMM13382deam_1_buf1(pf01979) BUF1
00266 HMM00860tp_1_bufgrm(pf00860) BUF1
00267 -# -
00268 -pf01145 -
00269 -pf00293 -
00270 -pf01497 -
00271 -# -
00272 -pf07715pf00593 -
00273 -pf00710 -
00274 HMMreg12833_1_EUT1(pf12833) EUT1
00275 Ts_dodgerBlue PDU1AB
00276 HMMPduS_1_all(pf01512pf13375pf10531) EUT1
00277 H_mulberry(pf00936pf16365) EUT1
00278 Tsp_rosa EUT1
00279 HMMetlyEutC_1_eut1(pf05985) EUT1 EP_NTERM
00280 HMMetlyEutB_1_EUT1(pf06751) EUT1
00281 HMM06277EutA_1_eut(pf06277) EUT1
00282 HMMtpEuth_1_eut(pf04346) EUT1
00283 HMM00465alcdh_3_mix(pf00465) EUT1
00284 HMM06723EutJMreB_1_grm(#) EUT1
00285 HMMalddh_1_eutpdugrm(pf00171) EUT1 EP_NTERM
00286 P_lightViolet EUT1
00287 -# -
00288 H_azure GRMguf
00289 HMM01515PTA_B_1_eut1(pf01515) EUT1
00290 HMM01923cobtr_2_eut1(pf01923) EUT1
00291 HMM06249EutQ_1_eut1(pf06249) EUT1 EP_NTERM
00292 HMM10662PduV_1_mix(pf10662) EUT1
00293 Hp_euts_violetPink EUT1
00294 -# -
00295 HMMreg00392_1_rmm1(pf00392pf07702) RMM1
00296 -pf01261 -
00297 -# -
00298 -# -
00299 -pf04324pf01077pf03460pf07992 -
00300 -pf13806 -
00301 -pf00375 -
00302 -pf14330 -
00303 -pf07287 -
BMC-P
BMC-H
BMC-T
Aldehyde dehydrogenase
PTAC
Signature enzyme
Alcohol dehydrogenase
Regulator

Escherichia sp. 4726-5. Bacteria; Proteobacteria; Gammaproteobacteria; Enterobacterales;

C7B04__locus_2

EUT1

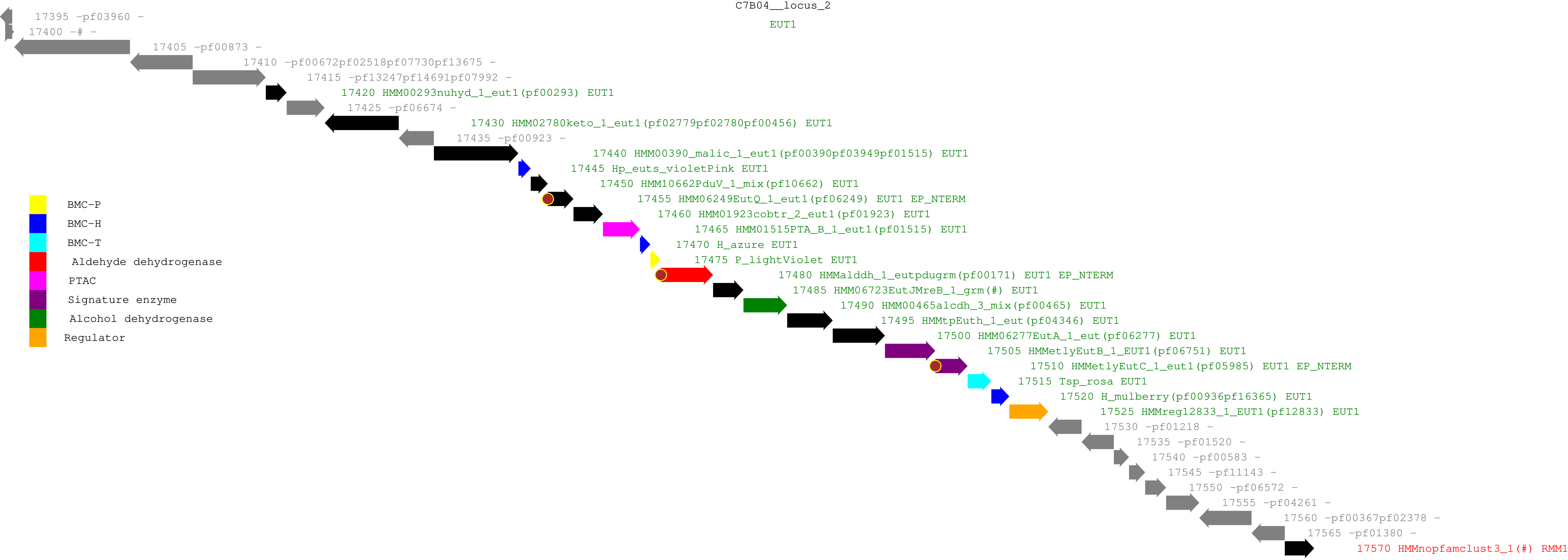

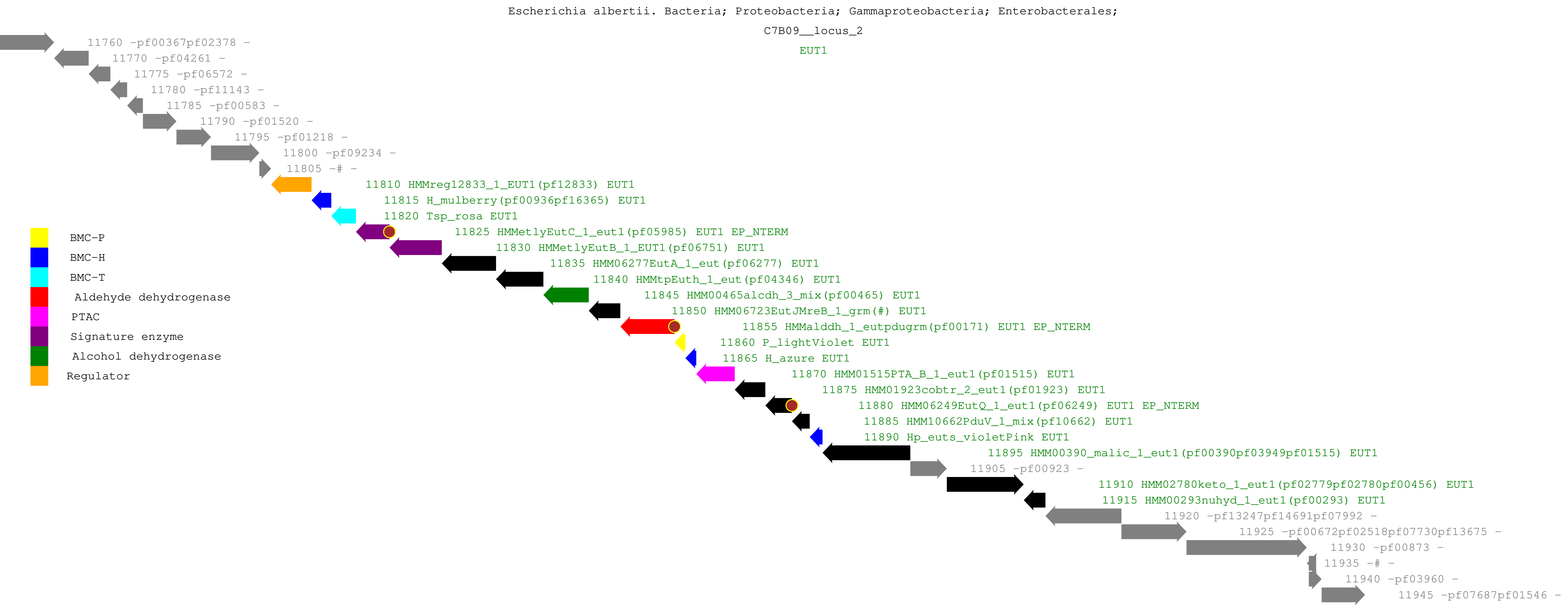

Escherichia albertii. Bacteria; Proteobacteria; Gammaproteobacteria; Enterobacterales;
C7B09__locus_2
EUT1
11760 -pf00367pf02378 -
11770 -pf04261 -
11775 -pf06572 -
11780 -pf11143 -
11785 -pf00583 -
11790 -pf01520 -
11795 -pf01218 -
11800 -pf09234 -
11805 -# -
11810 HMMreg12833_1_EUT1(pf12833) EUT1
11815 H_mulberry(pf00936pf16365) EUT1
11820 Tsp_rosa EUT1
11825 HMMetlyEutC_1_eut1(pf05985) EUT1 EP_NTERM
11830 HMMetlyEutB_1_EUT1(pf06751) EUT1
11835 HMM06277EutA_1_eut(pf06277) EUT1
11840 HMMtpEuth_1_eut(pf04346) EUT1
11845 HMM00465alcdh_3_mix(pf00465) EUT1
11850 HMM06723EutJMreB_1_grm(#) EUT1
11855 HMMalddh_1_eutpdugrm(pf00171) EUT1 EP_NTERM
11860 P_lightViolet EUT1
11865 H_azure EUT1
11870 HMM01515PTA_B_1_eut1(pf01515) EUT1
11875 HMM01923cobtr_2_eut1(pf01923) EUT1
11880 HMM06249EutQ_1_eut1(pf06249) EUT1 EP_NTERM
11885 HMM10662PduV_1_mix(pf10662) EUT1
11890 Hp_euts_violetPink EUT1
11895 HMM00390_malic_1_eut1(pf00390pf03949pf01515) EUT1
11905 -pf00923 -
11910 HMM02780keto_1_eut1(pf02779pf02780pf00456) EUT1
11915 HMM00293nuhyd_1_eut1(pf00293) EUT1
11920 -pf13247pf14691pf07992 -
11925 -pf00672pf02518pf07730pf13675 -
11930 -pf00873 -
11935 -# -
11940 -pf03960 -
11945 -pf07687pf01546 -
BMC-P
BMC-H
BMC-T
Aldehyde dehydrogenase
PTAC
Signature enzyme
Alcohol dehydrogenase
Regulator

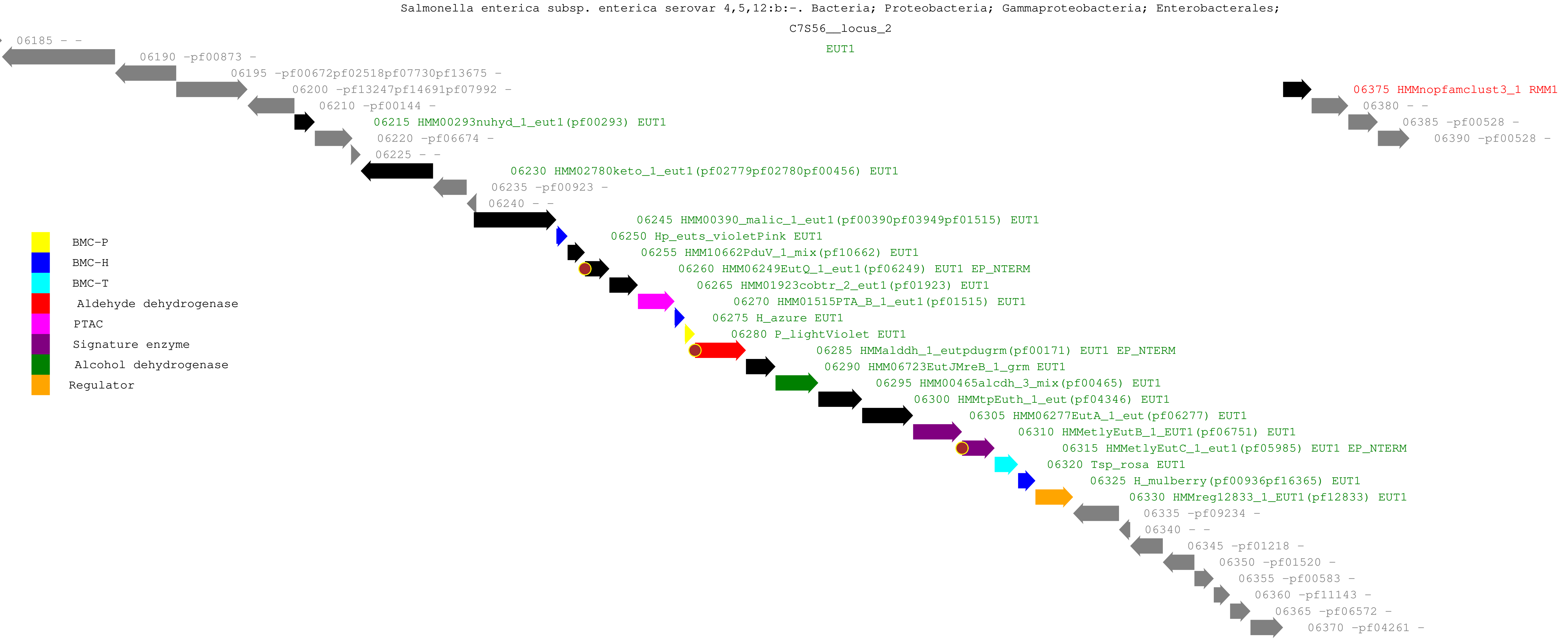
Salmonella enterica subsp. enterica serovar 4,5,12:b:-. Bacteria; Proteobacteria; Gammaproteobacteria; Enterobacterales;
C7S56__locus_2
EUT1
06185 - -
06190 -pf00873 -
06195 -pf00672pf02518pf07730pf13675 -
06200 -pf13247pf14691pf07992 -
06210 -pf00144 -
06215 HMM00293nuhyd_1_eut1(pf00293) EUT1
06220 -pf06674 -
06225 - -
06230 HMM02780keto_1_eut1(pf02779pf02780pf00456) EUT1
06235 -pf00923 -
06240 - -
06245 HMM00390_malic_1_eut1(pf00390pf03949pf01515) EUT1
06250 Hp_euts_violetPink EUT1
06255 HMM10662PduV_1_mix(pf10662) EUT1
06260 HMM06249EutQ_1_eut1(pf06249) EUT1 EP_NTERM
06265 HMM01923cobtr_2_eut1(pf01923) EUT1
06270 HMM01515PTA_B_1_eut1(pf01515) EUT1
06275 H_azure EUT1
06280 P_lightViolet EUT1
06285 HMMaldh_1_eutpdugrm(pf00171) EUT1 EP_NTERM
06290 HMM06723EutJMreB_1_grm EUT1
06295 HMM00465alcdh_3_mix(pf00465) EUT1
06300 HMMtpEuth_1_eut(pf04346) EUT1
06305 HMM06277EutA_1_eut(pf06277) EUT1
06310 HMMetlyEutB_1_EUT1(pf06751) EUT1
06315 HMMetlyEutC_1_eut1(pf05985) EUT1 EP_NTERM
06320 Tsp_rosa EUT1
06325 H_mulberry(pf00936pf16365) EUT1
06330 HMMreg12833_1_EUT1(pf12833) EUT1
06335 -pf09234 -
06340 - -
06345 -pf01218 -
06350 -pf01520 -
06355 -pf00583 -
06360 -pf11143 -
06365 -pf06572 -
06370 -pf04261 -
06375 HMMnopfamclust3_1 RMM1
06380 - -
06385 -pf00528 -
06390 -pf00528 -
BMC-P
BMC-H
BMC-T
Aldehyde dehydrogenase
PTAC
Signature enzyme
Alcohol dehydrogenase
Regulator

Salmonella enterica subsp. enterica serovar Javiana. Bacteria; Proteobacteria; Gammaproteobacteria; Enterobacterales;

C7S57__locus_1

EUT1

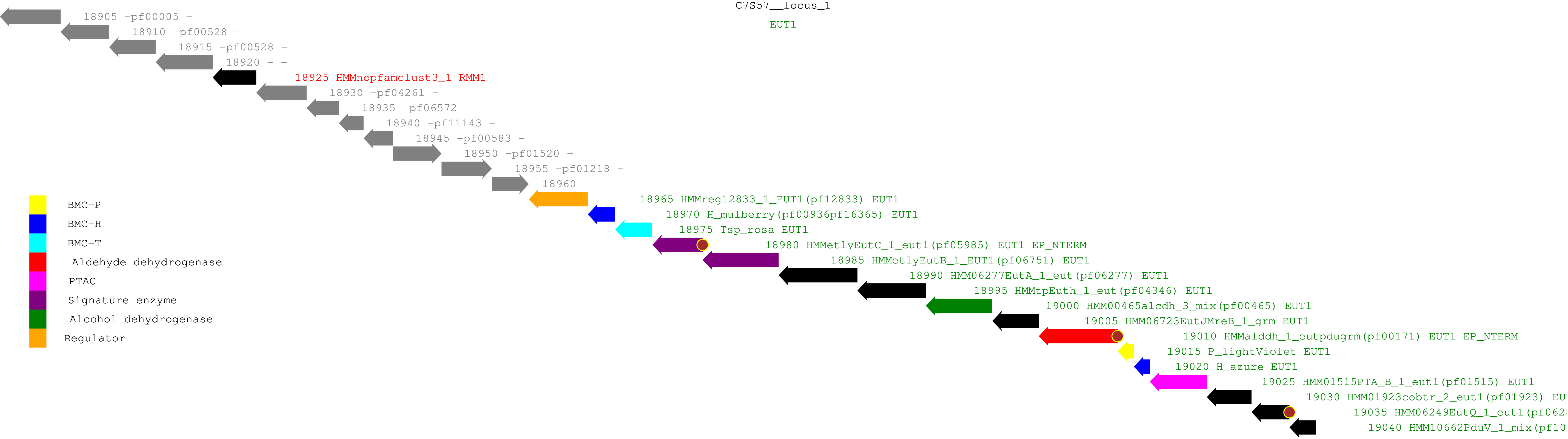

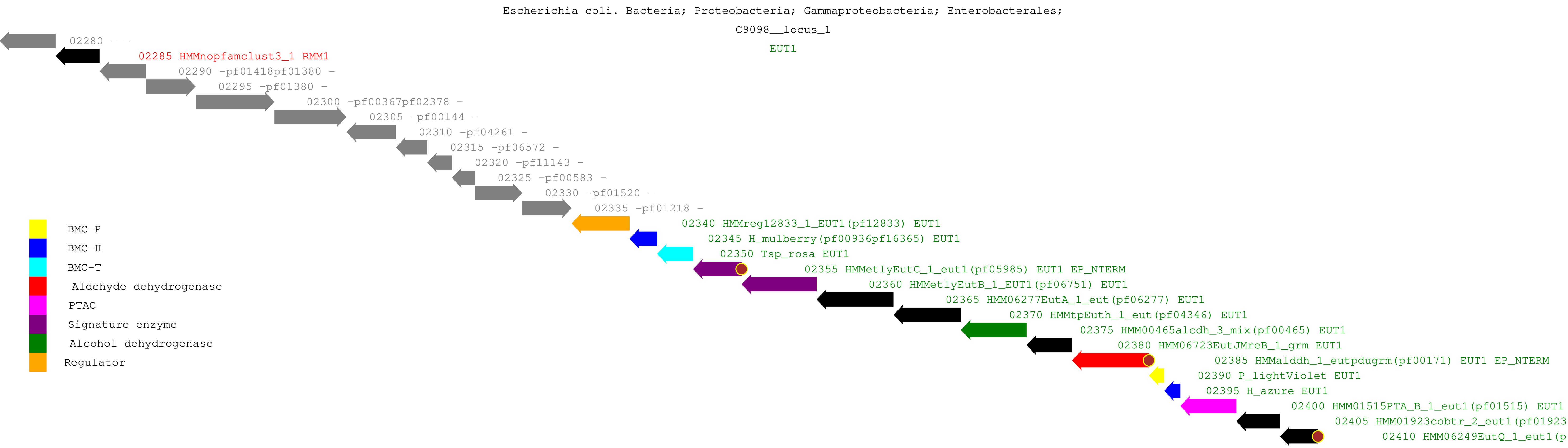

Escherichia coli. Bacteria; Proteobacteria; Gammaproteobacteria; Enterobacterales;
C9098__locus_1
EUT1
02280 - -
02285 HMMnopfamclust3_1 RMM1
02290 -pf01418pf01380 -
02295 -pf01380 -
02300 -pf00367pf02378 -
02305 -pf00144 -
02310 -pf04261 -
02315 -pf06572 -
02320 -pf11143 -
02325 -pf00583 -
02330 -pf01520 -
02335 -pf01218 -
02340 HMMreg12833_1_EUT1(pf12833) EUT1
02345 H_mulberry(pf00936pf16365) EUT1
02350 Tsp_rosa EUT1
02355 HMMetlyEutC_1_eut1(pf05985) EUT1 EP_NTERM
02360 HMMetlyEutB_1_EUT1(pf06751) EUT1
02365 HMM06277EutA_1_eut(pf06277) EUT1
02370 HMMtpEuth_1_eut(pf04346) EUT1
02375 HMM00465alcdh_3_mix(pf00465) EUT1
02380 HMM06723EutJMreB_1_grm EUT1
02385 HMMaldd h_1_eutpdugrm(pf00171) EUT1 EP_NTERM
02390 P_lightViolet EUT1
02395 H_azure EUT1
02400 HMM01515PTA_B_1_eut1(pf01515) EUT1
02405 HMM01923cobtr_2_eut1(pf01923
02410 HMM06249EutQ_1_eut1(p
BMC-P
BMC-H
BMC-T
Aldehyde dehydrogenase
PTAC
Signature enzyme
Alcohol dehydrogenase
Regulator

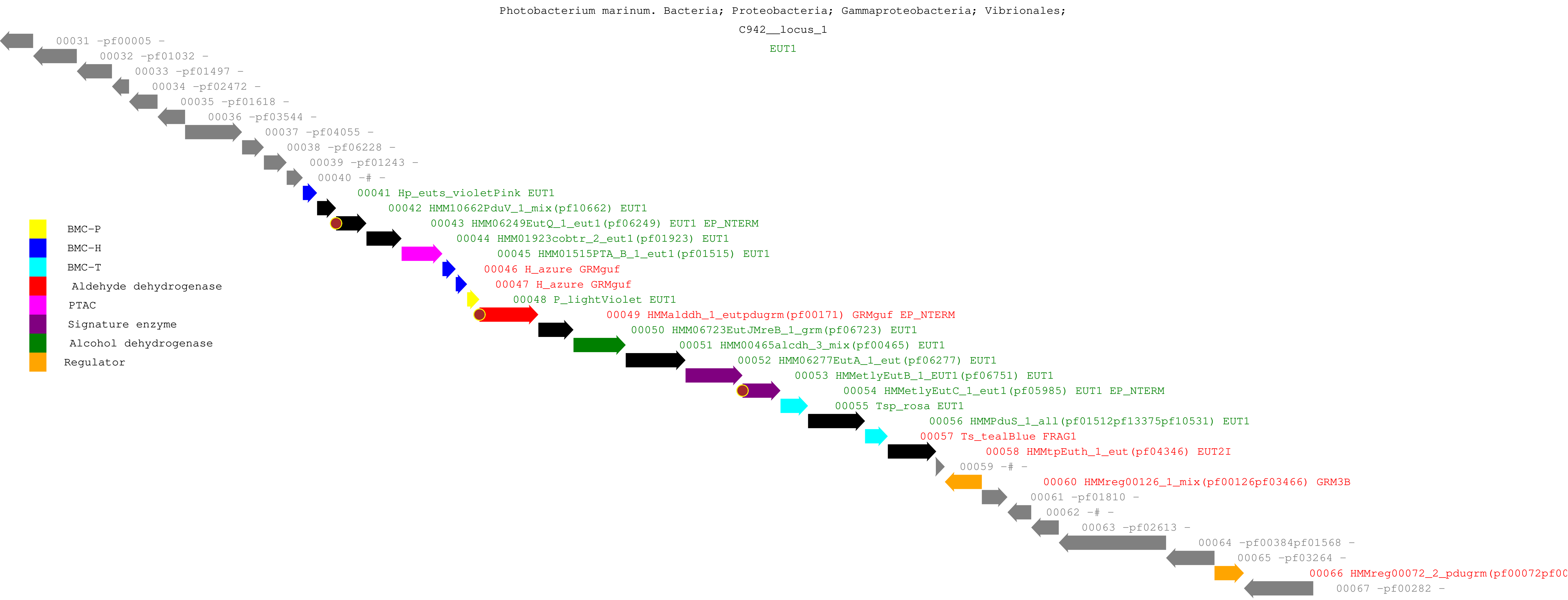
Photobacterium marinum. Bacteria; Proteobacteria; Gammaproteobacteria; Vibrionales;
C942__locus_1
EUT1
00031 -pf00005 -
00032 -pf01032 -
00033 -pf01497 -
00034 -pf02472 -
00035 -pf01618 -
00036 -pf03544 -
00037 -pf04055 -
00038 -pf06228 -
00039 -pf01243 -
00040 -# -
00041 Hp_euts_violetPink EUT1
00042 HMM10662PduV_1_mix(pf10662) EUT1
00043 HMM06249EutQ_1_eut1(pf06249) EUT1 EP_NTERM
00044 HMM01923cobtr_2_eut1(pf01923) EUT1
00045 HMM01515PTA_B_1_eut1(pf01515) EUT1
00046 H_azure GRMguf
00047 H_azure GRMguf
00048 P_lightViolet EUT1
00049 HMMalddh_1_eutpdugrm(pf00171) GRMguf EP_NTERM
00050 HMM06723EutJMreB_1_grm(pf06723) EUT1
00051 HMM00465alcdh_3_mix(pf00465) EUT1
00052 HMM06277EutA_1_eut(pf06277) EUT1
00053 HMMetlyEutB_1_EUT1(pf06751) EUT1
00054 HMMetlyEutC_1_eut1(pf05985) EUT1 EP_NTERM
00055 Tsp_rosa EUT1
00056 HMMPduS_1_all(pf01512pf13375pf10531) EUT1
00057 Ts_tealBlue FRAG1
00058 HMMtpEuth_1_eut(pf04346) EUT2I
00059 -# -
00060 HMMreg00126_1_mix(pf00126pf03466) GRM3B
00061 -pf01810 -
00062 -# -
00063 -pf02613 -
00064 -pf00384pf01568 -
00065 -pf03264 -
00066 HMMreg00072_2_pdugrm(pf00072pf00
00067 -pf00282 -
BMC-P
BMC-H
BMC-T
Aldehyde dehydrogenase
PTAC
Signature enzyme
Alcohol dehydrogenase
Regulator

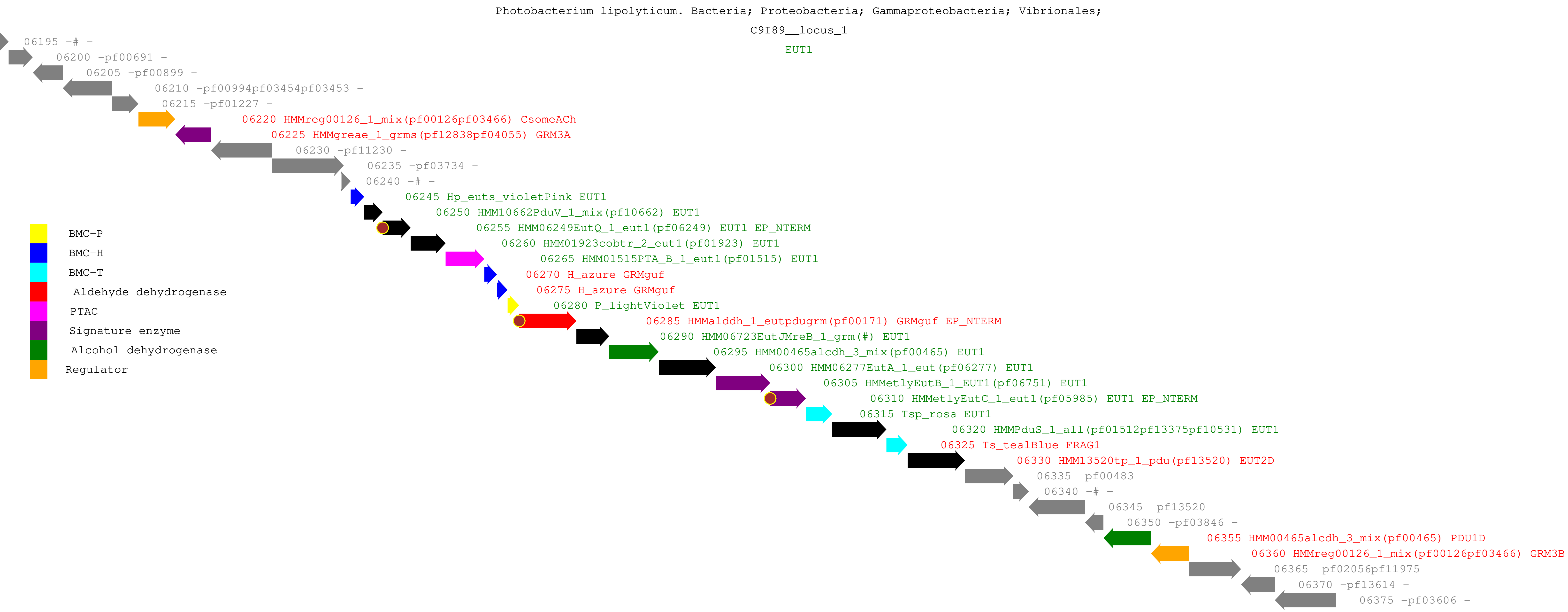

Photobacterium lipolyticum. Bacteria; Proteobacteria; Gammaproteobacteria; Vibrionales;
C9I89__locus_1
EUT1
06195 -# -
06200 -pf00691 -
06205 -pf00899 -
06210 -pf00994pf03454pf03453 -
06215 -pf01227 -
06220 HMMreg00126_1_mix(pf00126pf03466) CsomeACh
06225 HMMgreae_1_grms(pf12838pf04055) GRM3A
06230 -pf11230 -
06235 -pf03734 -
06240 -# -
06245 Hp_euts_violetPink EUT1
06250 HMM10662PduV_1_mix(pf10662) EUT1
06255 HMM06249EutQ_1_eut1(pf06249) EUT1 EP_NTERM
06260 HMM01923cobtr_2_eut1(pf01923) EUT1
06265 HMM01515PTA_B_1_eut1(pf01515) EUT1
06270 H_azure GRMguf
06275 H_azure GRMguf
06280 P_lightViolet EUT1
06285 HMMaldh_1_eutpdugrm(pf00171) GRMguf EP_NTERM
06290 HMM06723EutJMreB_1_grm(#) EUT1
06295 HMM00465alcdh_3_mix(pf00465) EUT1
06300 HMM06277EutA_1_eut(pf06277) EUT1
06305 HMMetlyEutB_1_EUT1(pf06751) EUT1
06310 HMMetlyEutC_1_eut1(pf05985) EUT1 EP_NTERM
06315 Tsp_rosa EUT1
06320 HMMPduS_1_all(pf01512pf13375pf10531) EUT1
06325 Ts_tealBlue FRAG1
06330 HMM13520tp_1_pdu(pf13520) EUT2D
06335 -pf00483 -
06340 -# -
06345 -pf13520 -
06350 -pf03846 -
06355 HMM00465alcdh_3_mix(pf00465) PDU1D
06360 HMMreg00126_1_mix(pf00126pf03466) GRM3B
06365 -pf02056pf11975 -
06370 -pf13614 -
06375 -pf03606 -
BMC-P
BMC-H
BMC-T
Aldehyde dehydrogenase
PTAC
Signature enzyme
Alcohol dehydrogenase
Regulator

Photobacterium lutimaris. Bacteria; Proteobacteria; Gammaproteobacteria; Vibrionales;

C9I99__locus_1

EUT1

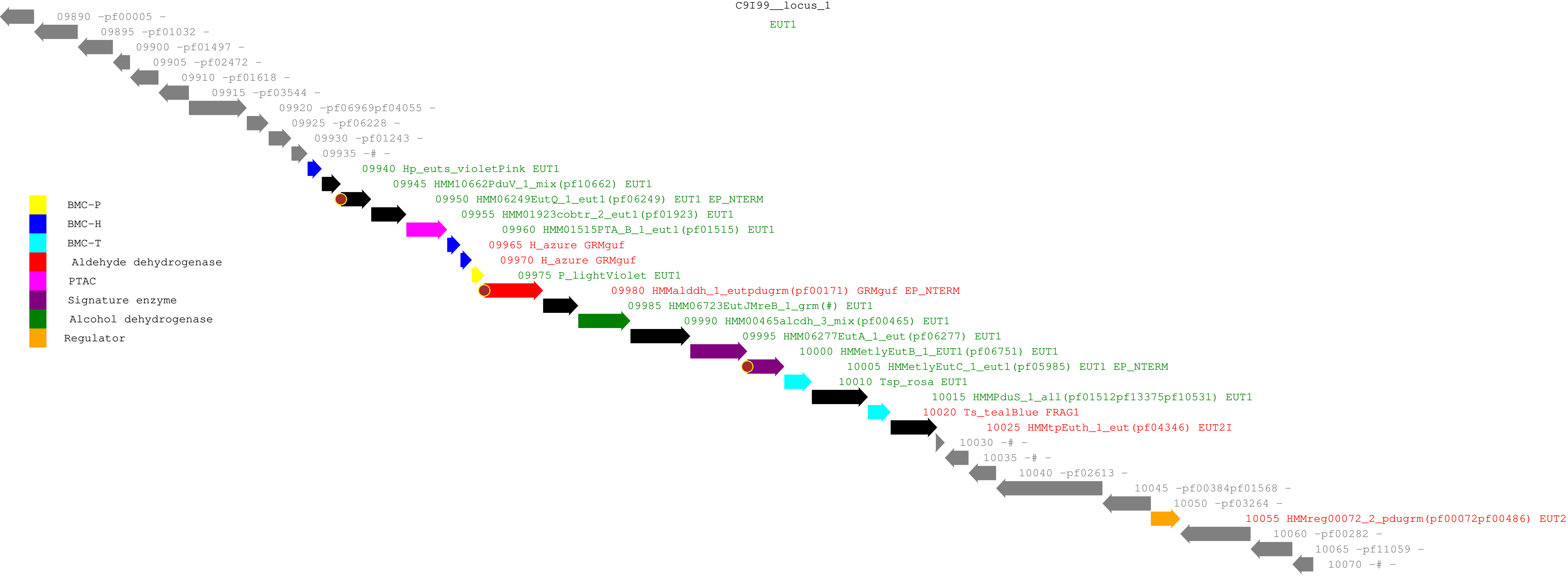

Photobacterium indicum. Bacteria; Proteobacteria; Gammaproteobacteria; Vibrionales;

C9J47__locus_1

EUT1

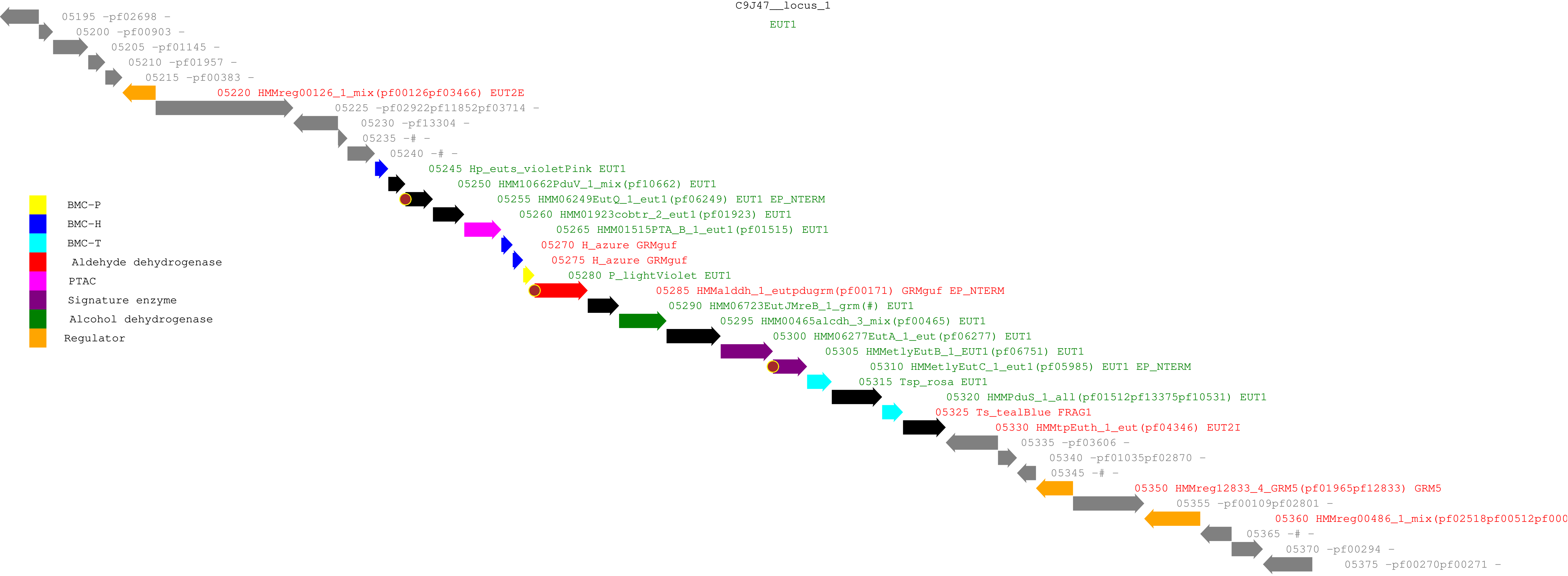

Salmonella enterica I. Bacteria; Proteobacteria; Gammaproteobacteria; Enterobacterales;

C9P23__locus_1

EUT1

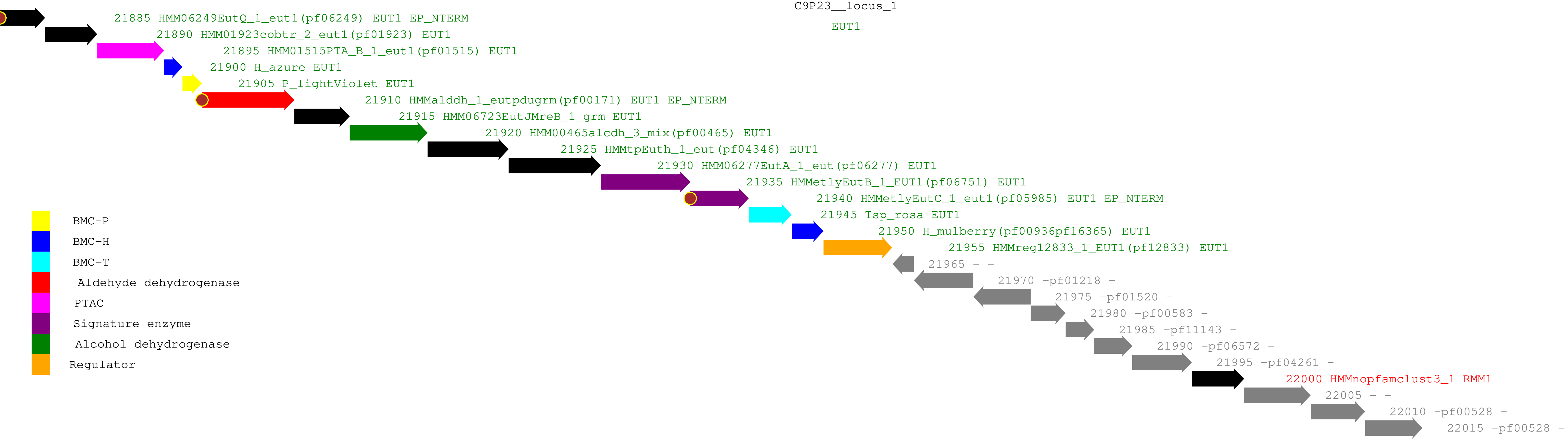

Salmonella enteritidis. Bacteria; Proteobacteria; Gammaproteobacteria; Enterobacterales;

CA285__locus_2

EUT1

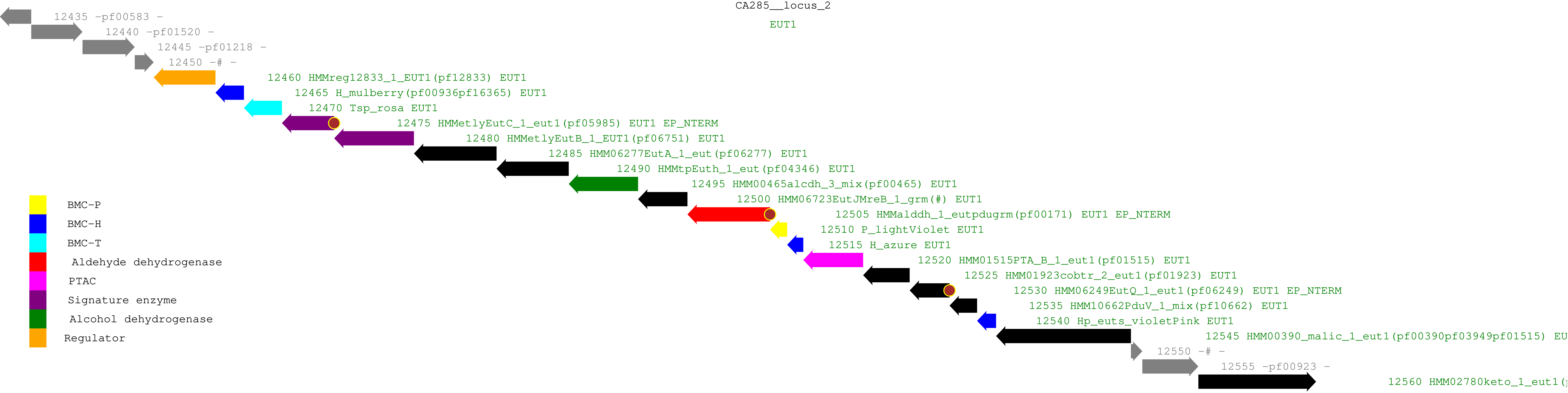

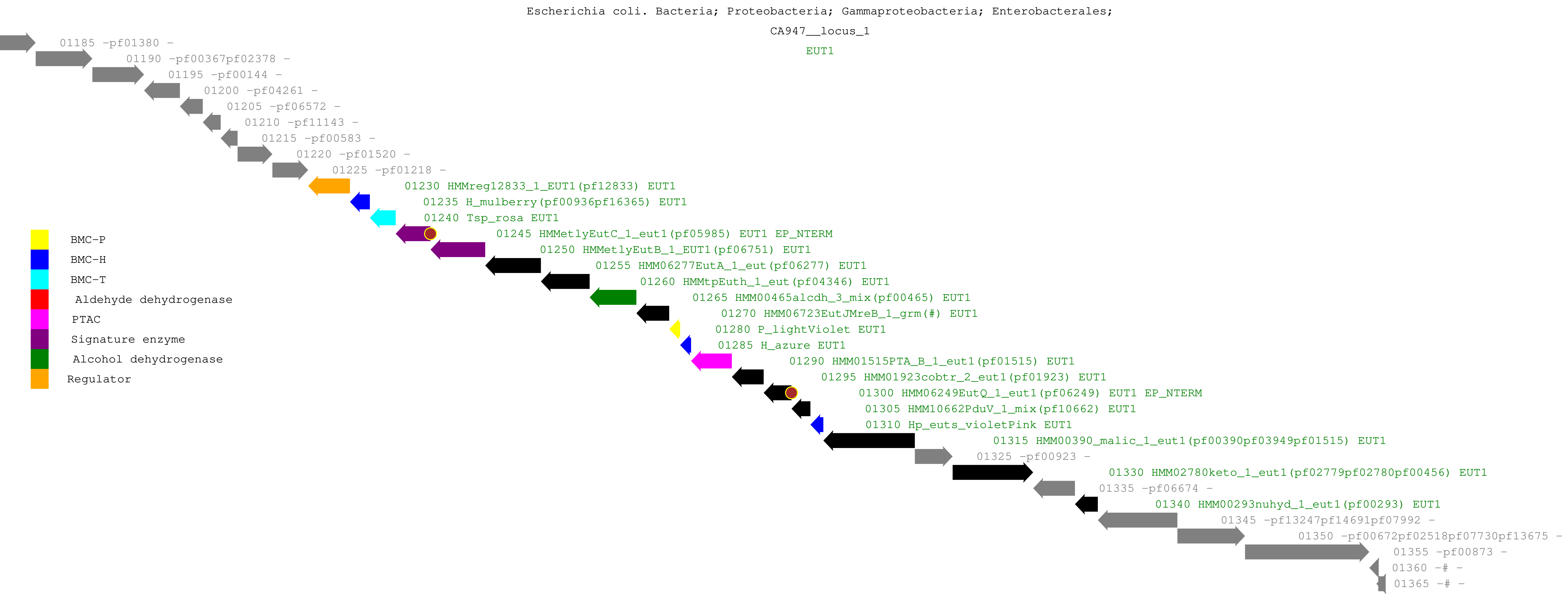
Escherichia coli. Bacteria; Proteobacteria; Gammaproteobacteria; Enterobacterales;
CA947__locus_1
EUT1
01185 -pf01380 -
01190 -pf00367pf02378 -
01195 -pf00144 -
01200 -pf04261 -
01205 -pf06572 -
01210 -pf11143 -
01215 -pf00583 -
01220 -pf01520 -
01225 -pf01218 -
01230 HMMreg12833_1_EUT1(pf12833) EUT1
01235 H_mulberry(pf00936pf16365) EUT1
01240 Tsp_rosa EUT1
01245 HMMetlyEutC_1_eut1(pf05985) EUT1 EP_NTERM
01250 HMMetlyEutB_1_EUT1(pf06751) EUT1
01255 HMM06277EutA_1_eut(pf06277) EUT1
01260 HMMtpEuth_1_eut(pf04346) EUT1
01265 HMM00465alcdh_3_mix(pf00465) EUT1
01270 HMM06723EutJMreB_1_grm(#) EUT1
01280 P_lightViolet EUT1
01285 H_azure EUT1
01290 HMM01515PTA_B_1_eut1(pf01515) EUT1
01295 HMM01923cobtr_2_eut1(pf01923) EUT1
01300 HMM06249EutQ_1_eut1(pf06249) EUT1 EP_NTERM
01305 HMM10662PduV_1_mix(pf10662) EUT1
01310 Hp_euts_violetPink EUT1
01315 HMM00390_malic_1_eut1(pf00390pf03949pf01515) EUT1
01325 -pf00923 -
01330 HMM02780keto_1_eut1(pf02779pf02780pf00456) EUT1
01335 -pf06674 -
01340 HMM00293nuhyd_1_eut1(pf00293) EUT1
01345 -pf13247pf14691pf07992 -
01350 -pf00672pf02518pf07730pf13675 -
01355 -pf00873 -
01360 -# -
01365 -# -
BMC-P
BMC-H
BMC-T
Aldehyde dehydrogenase
PTAC
Signature enzyme
Alcohol dehydrogenase
Regulator

Salmonella enterica subsp. enterica serovar Paratyphi B str. SARA61. Bacteria; Proteobacteria; Gammaproteobacteria; Enterobacterales;

CAH10__locus_1

EUT1

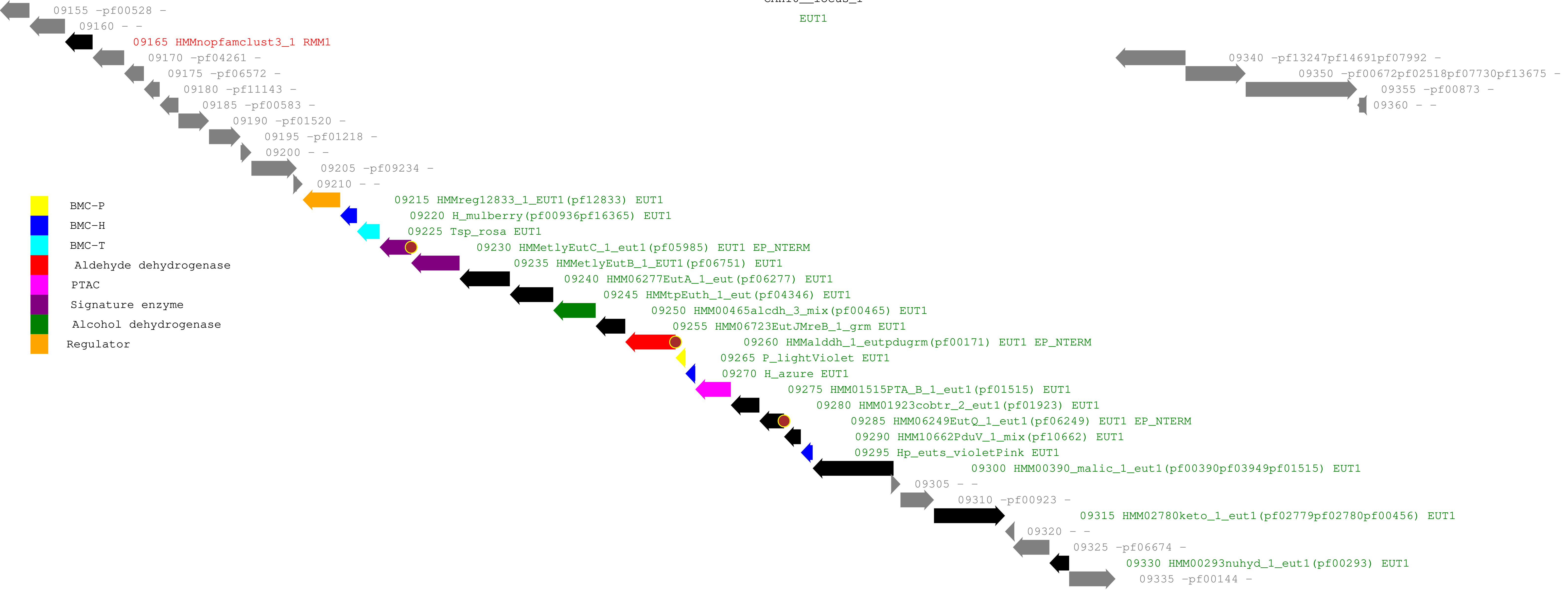

Salmonella enterica subsp. enterica serovar Typhimurium str. CFSAN000648. Bacteria; Proteobacteria; Gammaproteobacteria; Enterobacterales;

CAI81__locus_2

EUT1

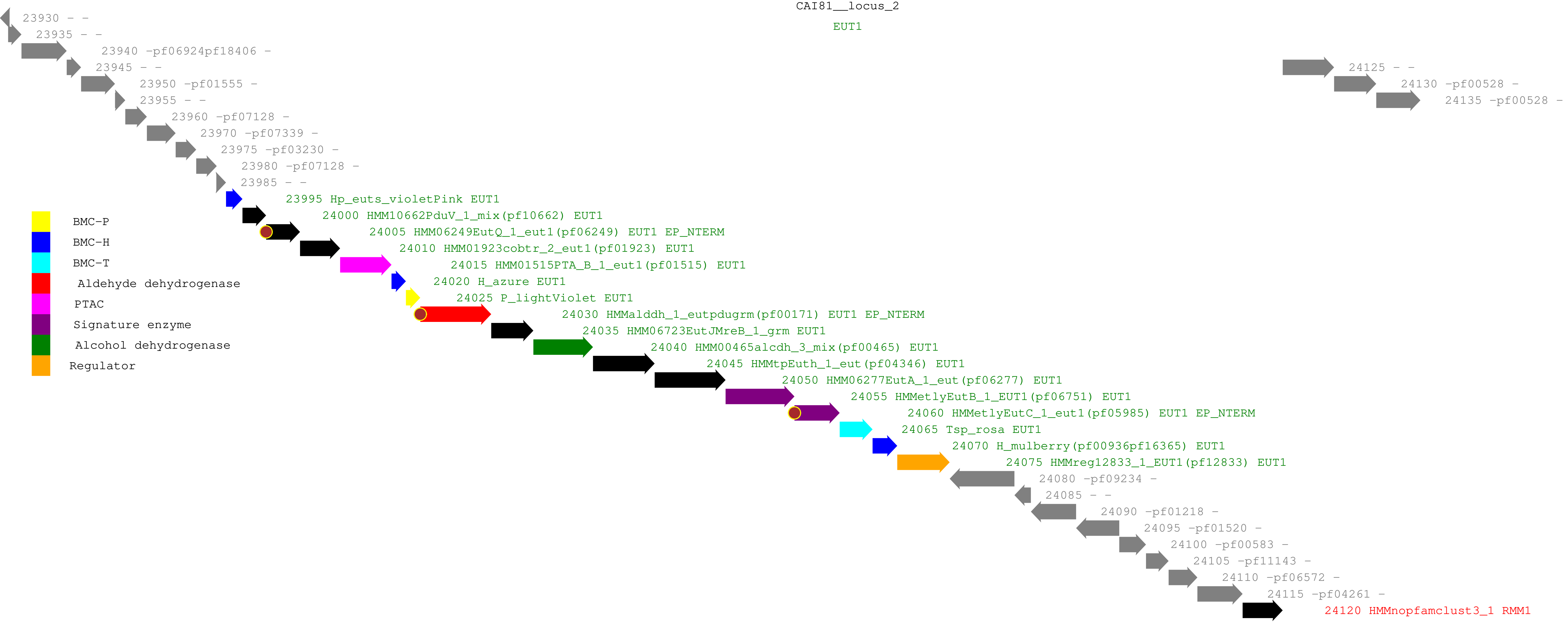

Escherichia coli O157:H7. Bacteria; Proteobacteria; Gammaproteobacteria; Enterobacterales;

CAM50__locus_1

EUT1

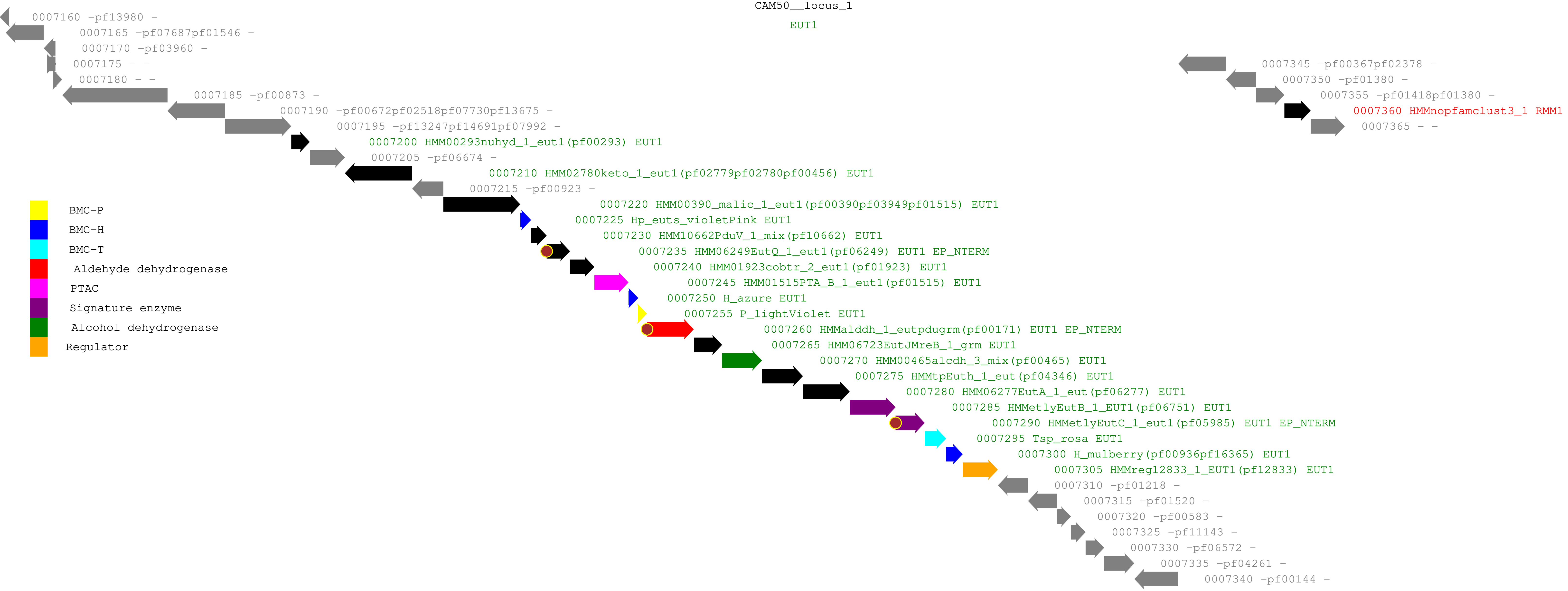

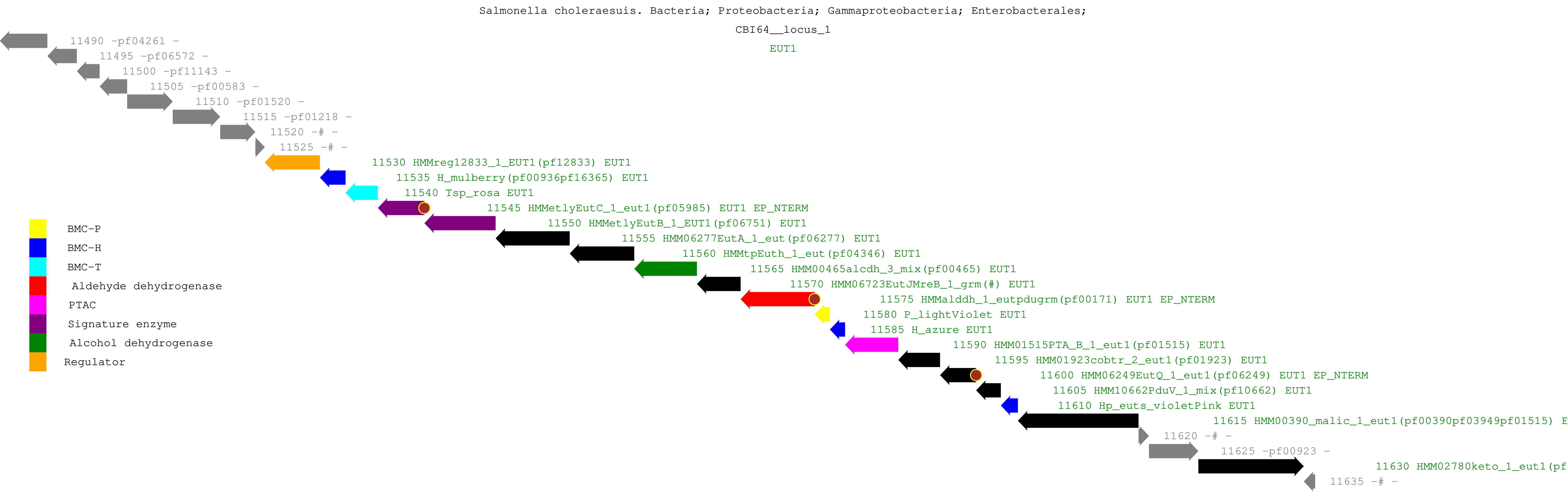
Salmonella choleraesuis. Bacteria; Proteobacteria; Gammaproteobacteria; Enterobacterales;
CBI64__locus_1
EUT1
11490 -pf04261 -
11495 -pf06572 -
11500 -pf11143 -
11505 -pf00583 -
11510 -pf01520 -
11515 -pf01218 -
11520 -# -
11525 -# -
11530 HMMreg12833_1_EUT1(pf12833) EUT1
11535 H_mulberry(pf00936pf16365) EUT1
11540 Tsp_rosa EUT1
11545 HMMetlyEutC_1_eut1(pf05985) EUT1 EP_NTERM
11550 HMMetlyEutB_1_EUT1(pf06751) EUT1
11555 HMM06277EutA_1_eut(pf06277) EUT1
11560 HMMtpEuth_1_eut(pf04346) EUT1
11565 HMM00465alcdh_3_mix(pf00465) EUT1
11570 HMM06723EutJMreB_1_grm(#) EUT1
11575 HMMalddh_1_eutpdugrm(pf00171) EUT1 EP_NTERM
11580 P_lightViolet EUT1
11585 H_azure EUT1
11590 HMM01515PTA_B_1_eut1(pf01515) EUT1
11595 HMM01923cobtr_2_eut1(pf01923) EUT1
11600 HMM06249EutQ_1_eut1(pf06249) EUT1 EP_NTERM
11605 HMM10662PduV_1_mix(pf10662) EUT1
11610 Hp_euts_violetPink EUT1
11615 HMM00390_malic_1_eut1(pf00390pf03949pf01515) E
11620 -# -
11625 -pf00923 -
11630 HMM02780keto_1_eut1(pf
11635 -# -
BMC-P
BMC-H
BMC-T
Aldehyde dehydrogenase
PTAC
Signature enzyme
Alcohol dehydrogenase
Regulator

Shigella flexneri. Bacteria; Proteobacteria; Gammaproteobacteria; Enterobacterales;

CBL21__locus_1

EUT1

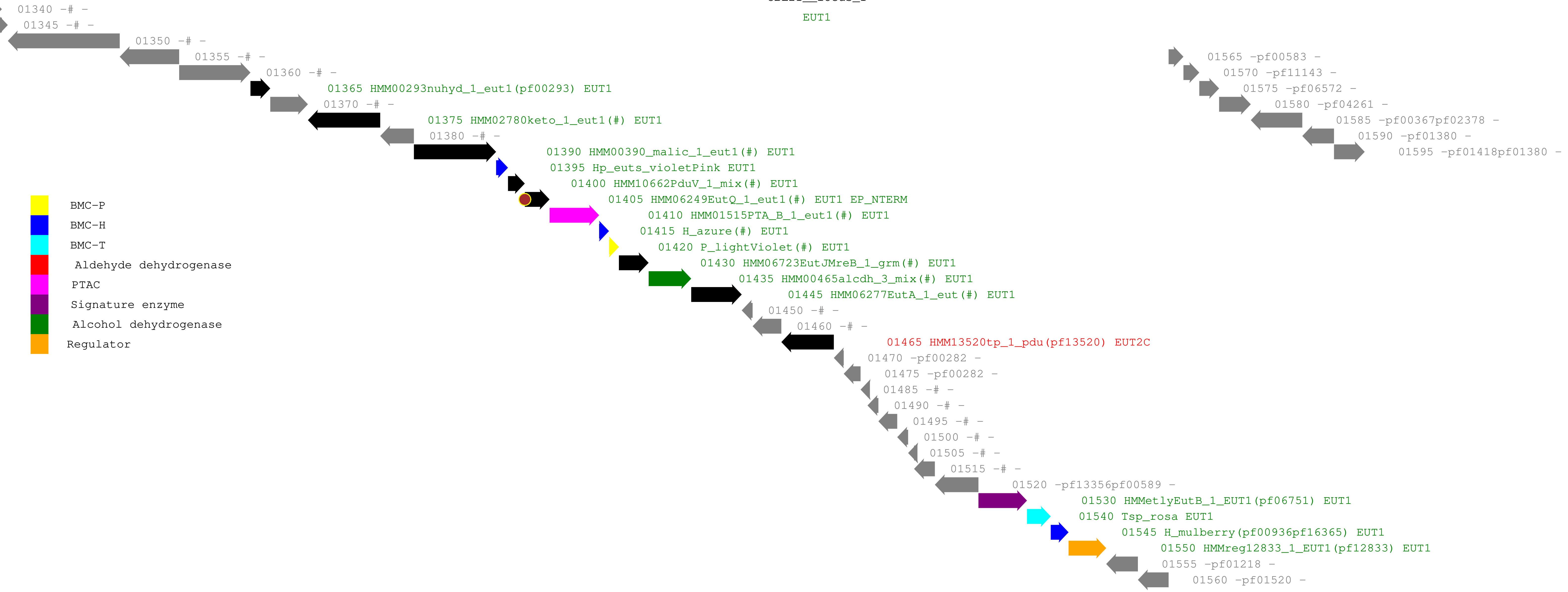

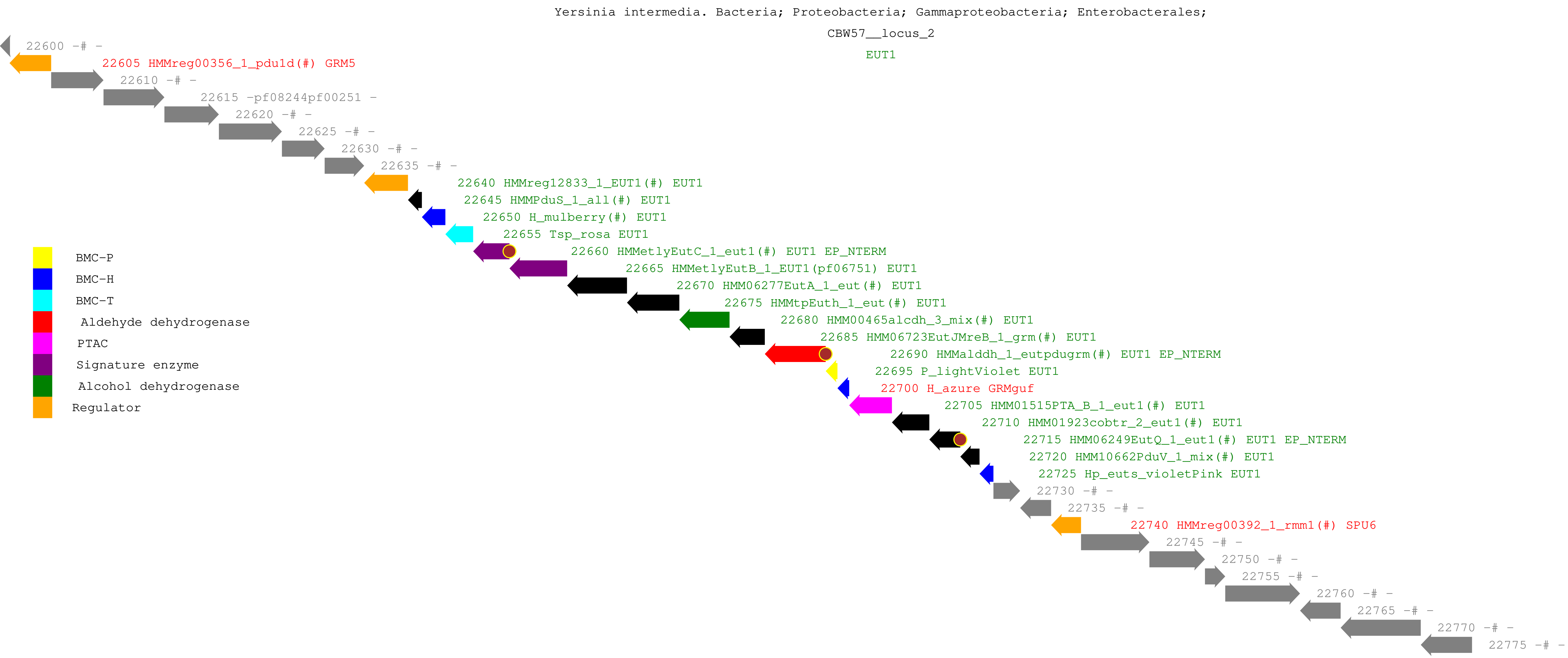
Yersinia intermedia. Bacteria; Proteobacteria; Gammaproteobacteria; Enterobacterales;
CBW57__locus_2
EUT1
22600 -# -
22605 HMMreg00356_1_pdu1d(#) GRM5
22610 -# -
22615 -pf08244pf00251 -
22620 -# -
22625 -# -
22630 -# -
22635 -# -
22640 HMMreg12833_1_EUT1(#) EUT1
22645 HMMPduS_1_all(#) EUT1
22650 H_mulberry(#) EUT1
22655 Tsp_rosa EUT1
22660 HMMetlyEutC_1_eut1(#) EUT1 EP_NTERM
22665 HMMetlyEutB_1_EUT1(pf06751) EUT1
22670 HMM06277EutA_1_eut(#) EUT1
22675 HMMtpEuth_1_eut(#) EUT1
22680 HMM00465alcdh_3_mix(#) EUT1
22685 HMM06723EutJMreB_1_grm(#) EUT1
22690 HMMalddh_1_eutpdugrm(#) EUT1 EP_NTERM
22695 P_lightViolet EUT1
22700 H_azure GRMguf
22705 HMM01515PTA_B_1_eut1(#) EUT1
22710 HMM01923cobtr_2_eut1(#) EUT1
22715 HMM06249EutQ_1_eut1(#) EUT1 EP_NTERM
22720 HMM10662PduV_1_mix(#) EUT1
22725 Hp_euts_violetPink EUT1
22730 -# -
22735 -# -
22740 HMMreg00392_1_rmm1(#) SPU6
22745 -# -
22750 -# -
22755 -# -
22760 -# -
22765 -# -
22770 -# -
22775 -# -
BMC-P
BMC-H
BMC-T
Aldehyde dehydrogenase
PTAC
Signature enzyme
Alcohol dehydrogenase
Regulator

Escherichia coli O145:NM. Bacteria; Proteobacteria; Gammaproteobacteria; Enterobacterales;

CCU01__locus_1

EUT1

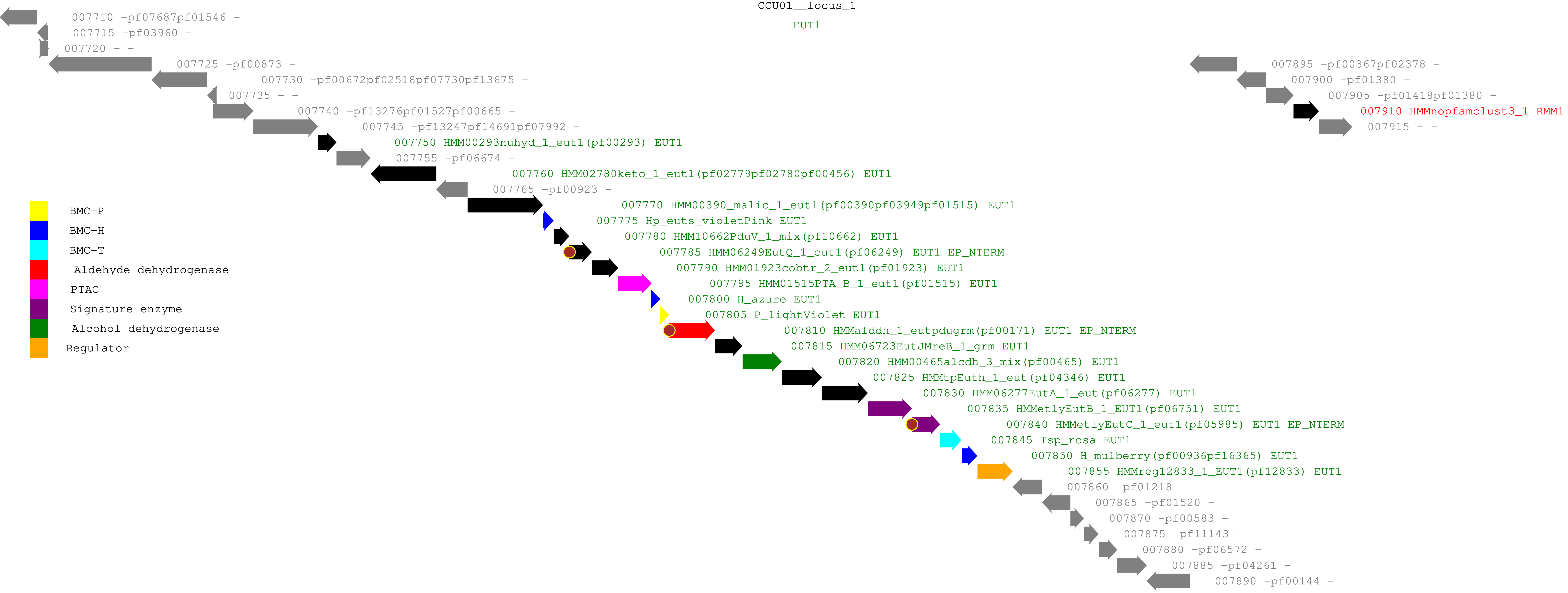

Escherichia coli O103:H2. Bacteria; Proteobacteria; Gammaproteobacteria; Enterobacterales;

CCU04__locus_1

EUT1

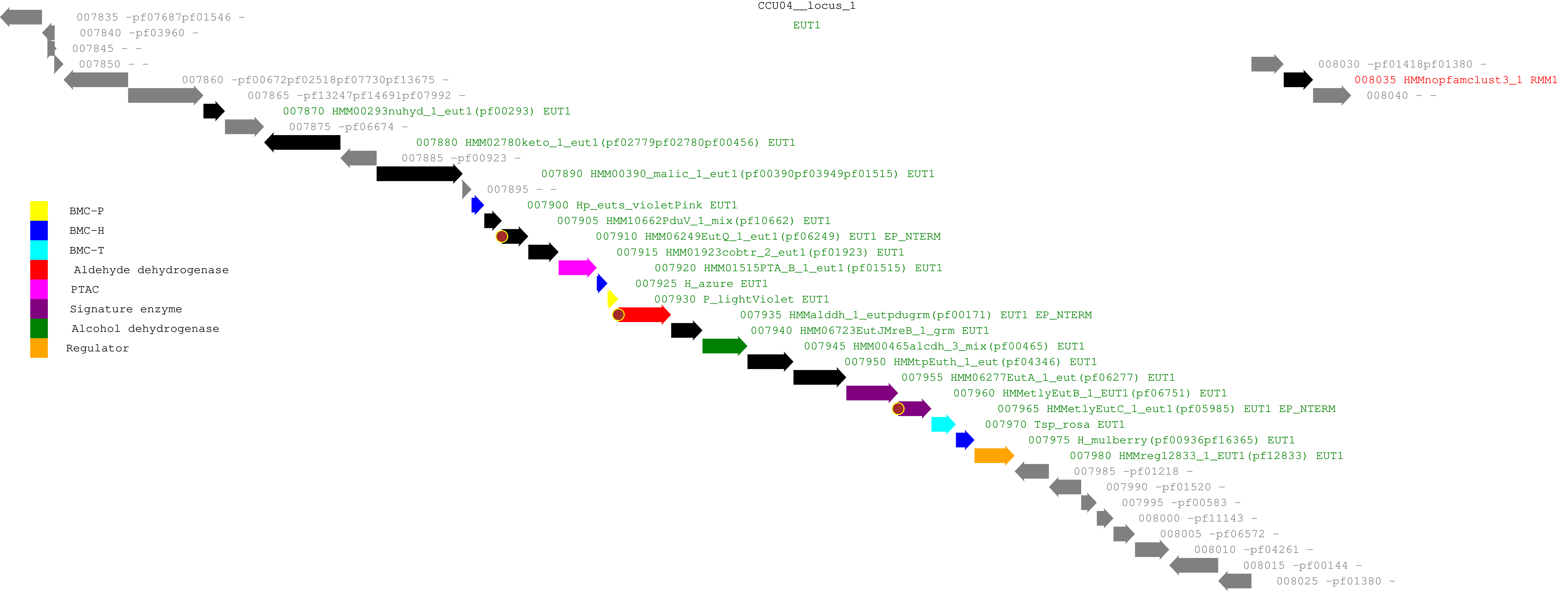

Escherichia coli. Bacteria; Proteobacteria; Gammaproteobacteria; Enterobacterales;

CCZ11__locus_1

EUT1

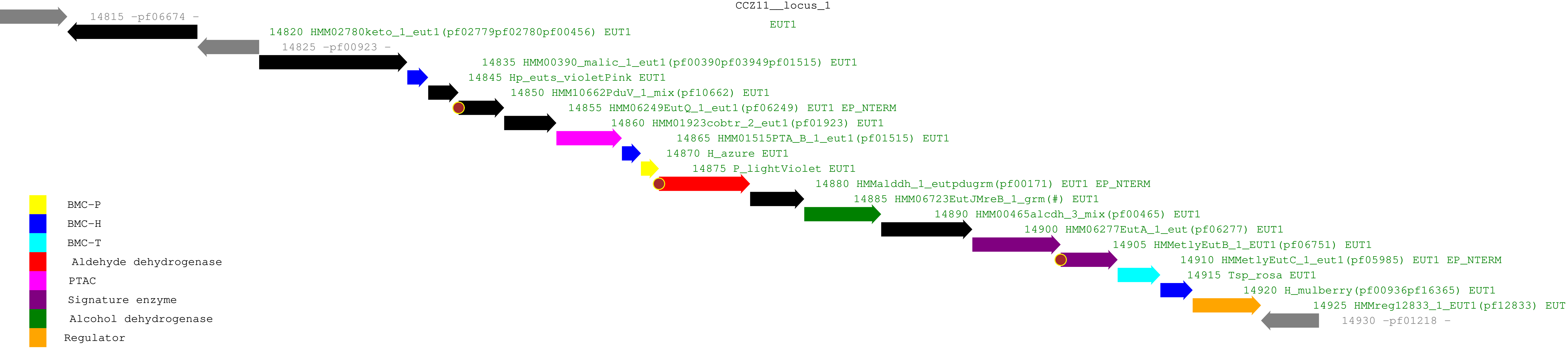

Citrobacter youngae. Bacteria; Proteobacteria; Gammaproteobacteria; Enterobacterales;

CD187__locus_1

EUT1

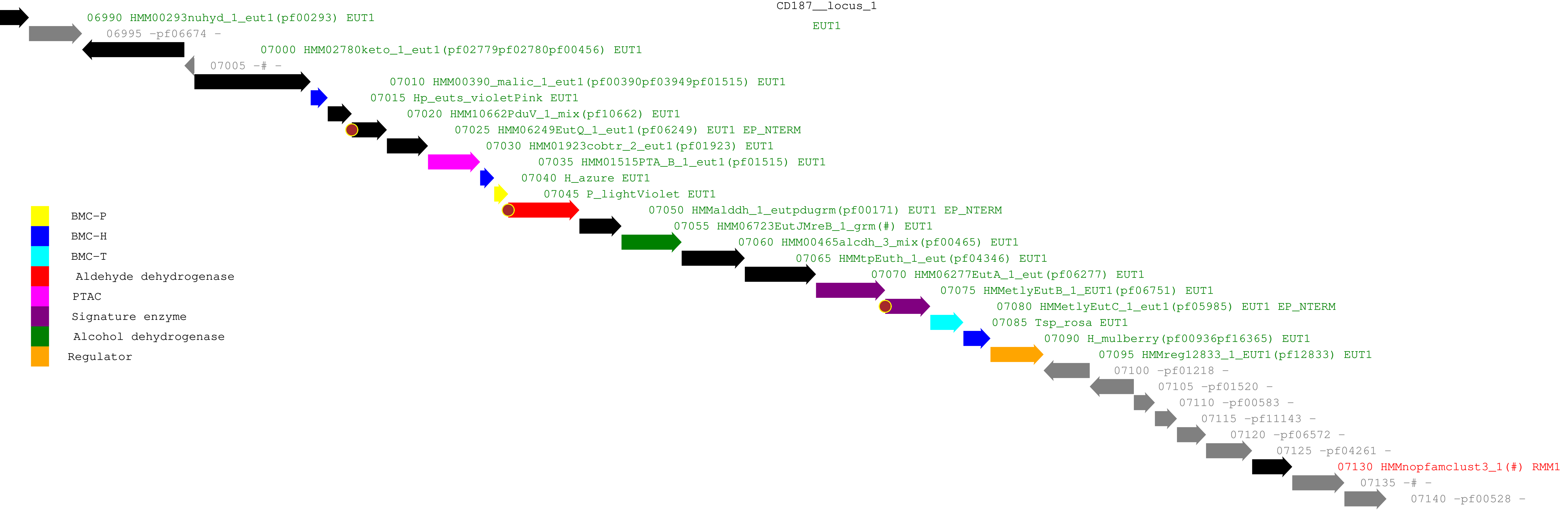

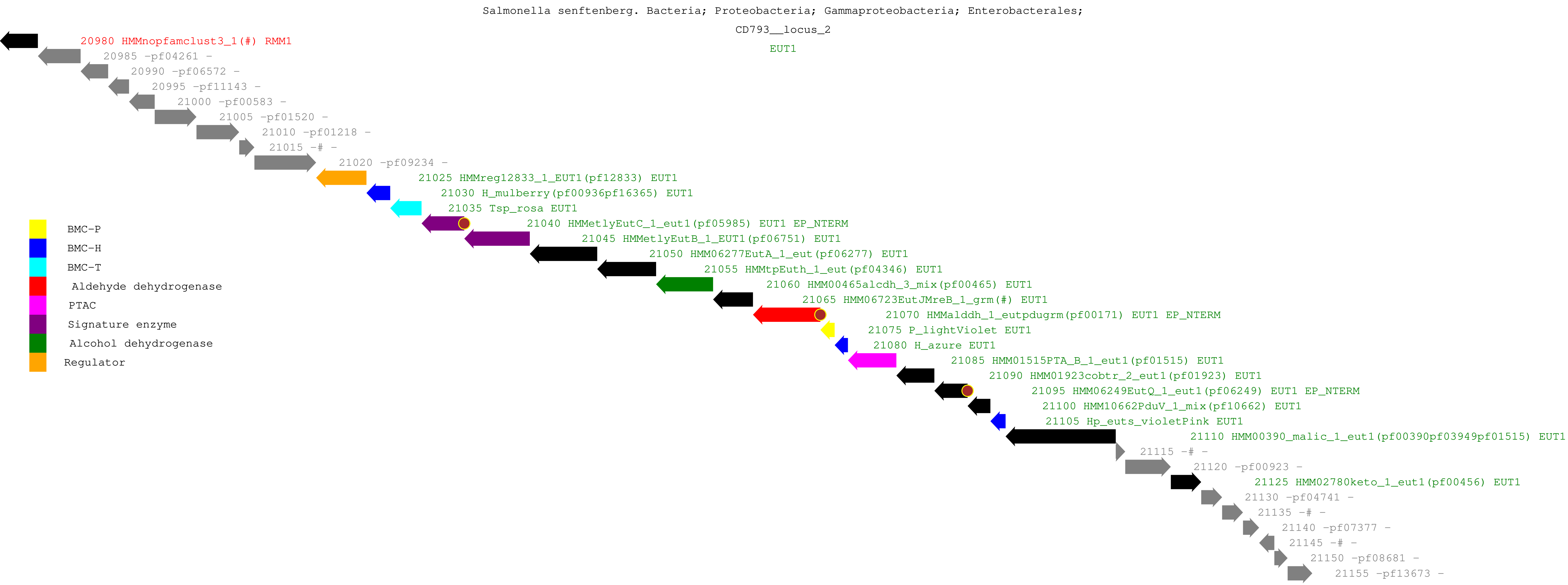
Salmonella senftenberg. Bacteria; Proteobacteria; Gammaproteobacteria; Enterobacterales;
CD793__locus_2
EUT1
20980 HMMnopfamclust3_1(#) RMM1
20985 -pf04261 -
20990 -pf06572 -
20995 -pf11143 -
21000 -pf00583 -
21005 -pf01520 -
21010 -pf01218 -
21015 -# -
21020 -pf09234 -
21025 HMMreg12833_1_EUT1(pf12833) EUT1
21030 H_mulberry(pf00936pf16365) EUT1
21035 Tsp_rosa EUT1
21040 HMMetlyEutC_1_eut1(pf05985) EUT1 EP_NTERM
21045 HMMetlyEutB_1_EUT1(pf06751) EUT1
21050 HMM06277EutA_1_eut(pf06277) EUT1
21055 HMMtpEuth_1_eut(pf04346) EUT1
21060 HMM00465alcdh_3_mix(pf00465) EUT1
21065 HMM06723EutJMreB_1_grm(#) EUT1
21070 HMMalddh_1_eutpdugrm(pf00171) EUT1 EP_NTERM
21075 P_lightViolet EUT1
21080 H_azure EUT1
21085 HMM01515PTA_B_1_eut1(pf01515) EUT1
21090 HMM01923cobtr_2_eut1(pf01923) EUT1
21095 HMM06249EutQ_1_eut1(pf06249) EUT1 EP_NTERM
21100 HMM10662PduV_1_mix(pf10662) EUT1
21105 Hp_euts_violetPink EUT1
21110 HMM00390_malic_1_eut1(pf00390pf03949pf01515) EUT1
21115 -# -
21120 -pf00923 -
21125 HMM02780keto_1_eut1(pf00456) EUT1
21130 -pf04741 -
21135 -# -
21140 -pf07377 -
21145 -# -
21150 -pf08681 -
21155 -pf13673 -
BMC-P
BMC-H
BMC-T
Aldehyde dehydrogenase
PTAC
Signature enzyme
Alcohol dehydrogenase
Regulator

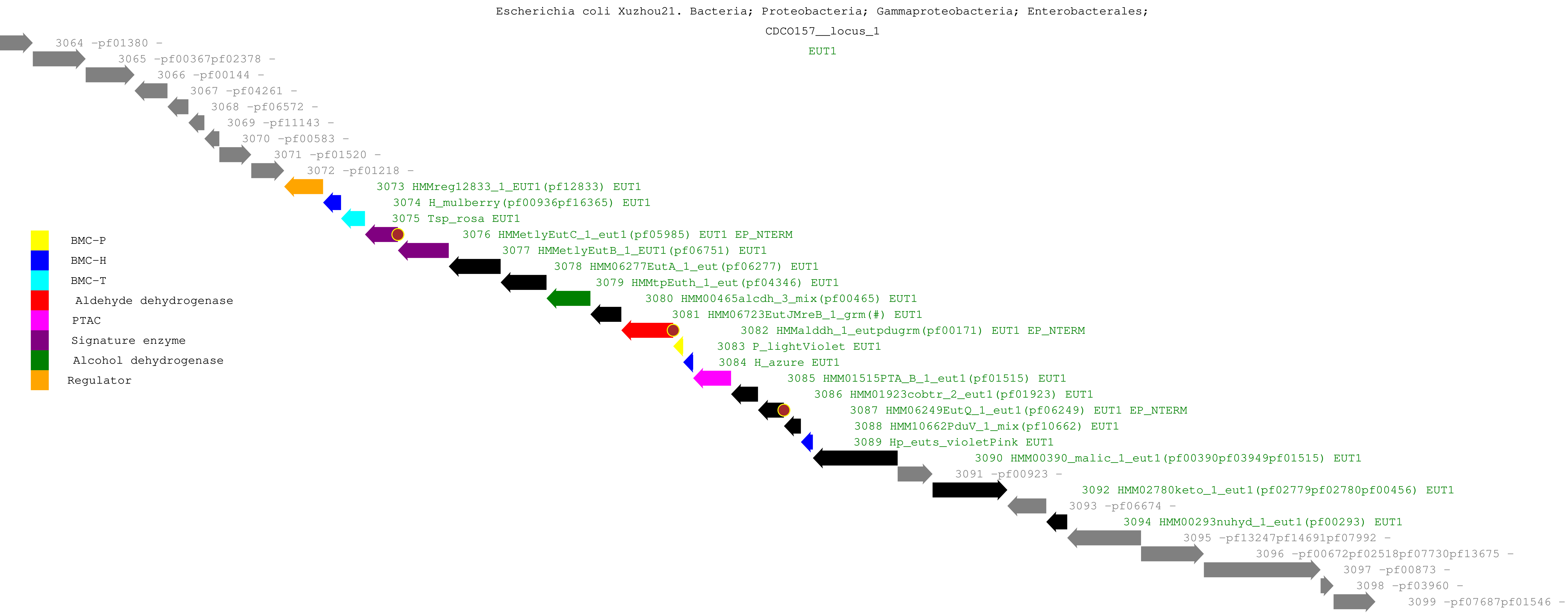

Escherichia coli Xuzhou21. Bacteria; Proteobacteria; Gammaproteobacteria; Enterobacterales;
CDCO157__locus_1
EUT1
3064 -pf01380 -
3065 -pf00367pf02378 -
3066 -pf00144 -
3067 -pf04261 -
3068 -pf06572 -
3069 -pf11143 -
3070 -pf00583 -
3071 -pf01520 -
3072 -pf01218 -
3073 HMMreg12833_1_EUT1(pf12833) EUT1
3074 H_mulberry(pf00936pf16365) EUT1
3075 Tsp_rosa EUT1
3076 HMMetlyEutC_1_eut1(pf05985) EUT1 EP_NTERM
3077 HMMetlyEutB_1_EUT1(pf06751) EUT1
3078 HMM06277EutA_1_eut(pf06277) EUT1
3079 HMMtpEuth_1_eut(pf04346) EUT1
3080 HMM00465alcdh_3_mix(pf00465) EUT1
3081 HMM06723EutJMreB_1_grm(#) EUT1
3082 HMMaldh_1_eutpdugrm(pf00171) EUT1 EP_NTERM
3083 P_lightViolet EUT1
3084 H_azure EUT1
3085 HMM01515PTA_B_1_eut1(pf01515) EUT1
3086 HMM01923cobtr_2_eut1(pf01923) EUT1
3087 HMM06249EutQ_1_eut1(pf06249) EUT1 EP_NTERM
3088 HMM10662PduV_1_mix(pf10662) EUT1
3089 Hp_euts_violetPink EUT1
3090 HMM00390_malic_1_eut1(pf00390pf03949pf01515) EUT1
3091 -pf00923 -
3092 HMM02780keto_1_eut1(pf02779pf02780pf00456) EUT1
3093 -pf06674 -
3094 HMM00293nuhyd_1_eut1(pf00293) EUT1
3095 -pf13247pf14691pf07992 -
3096 -pf00672pf02518pf07730pf13675 -
3097 -pf00873 -
3098 -pf03960 -
3099 -pf07687pf01546 -
BMC-P
BMC-H
BMC-T
Aldehyde dehydrogenase
PTAC
Signature enzyme
Alcohol dehydrogenase
Regulator

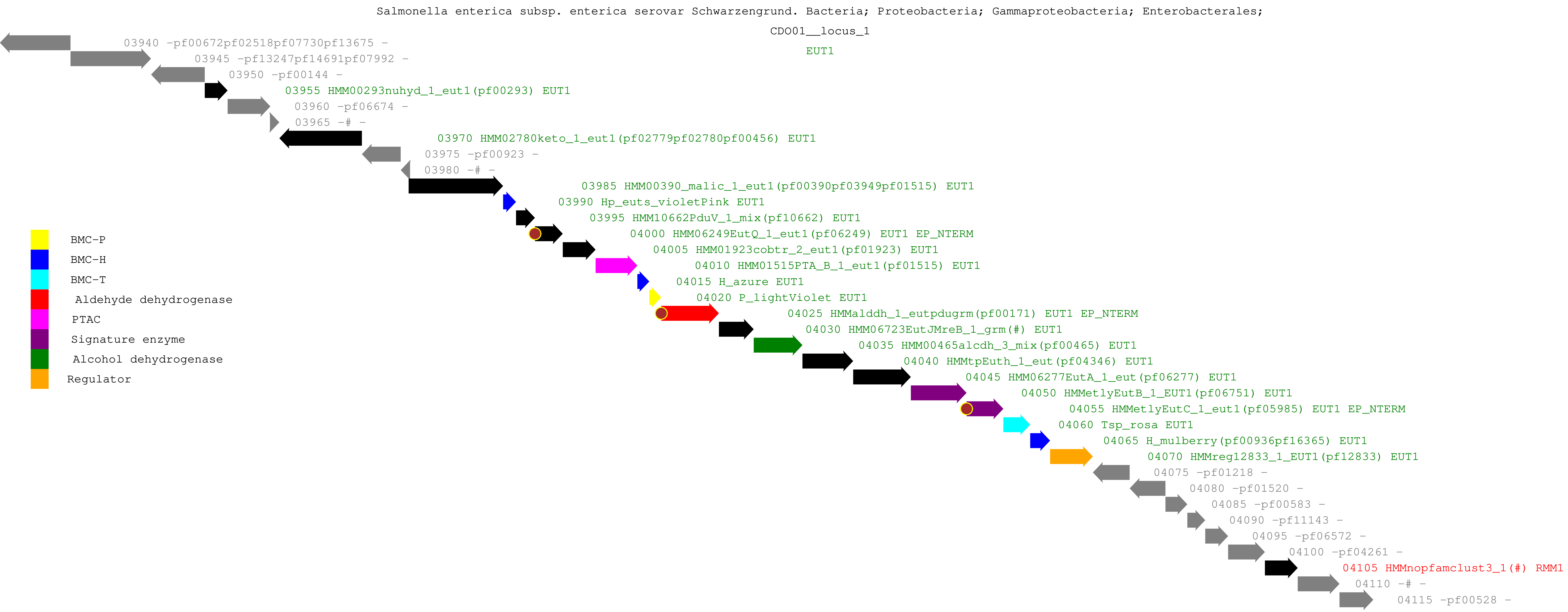
Salmonella enterica subsp. enterica serovar Schwarzengrund. Bacteria; Proteobacteria; Gammaproteobacteria; Enterobacterales;
CDO01__locus_1
EUT1
03940 -pf00672pf02518pf07730pf13675 -
03945 -pf13247pf14691pf07992 -
03950 -pf00144 -
03955 HMM00293nuhyd_1_eut1(pf00293) EUT1
03960 -pf06674 -
03965 -# -
03970 HMM02780keto_1_eut1(pf02779pf02780pf00456) EUT1
03975 -pf00923 -
03980 -# -
03985 HMM00390_malic_1_eut1(pf00390pf03949pf01515) EUT1
03990 Hp_euts_violetPink EUT1
03995 HMM10662PduV_1_mix(pf10662) EUT1
04000 HMM06249EutQ_1_eut1(pf06249) EUT1 EP_NTERM
04005 HMM01923cobtr_2_eut1(pf01923) EUT1
04010 HMM01515PTA_B_1_eut1(pf01515) EUT1
04015 H_azure EUT1
04020 P_lightViolet EUT1
04025 HMMaldh_1_eutpdugrm(pf00171) EUT1 EP_NTERM
04030 HMM06723EutJMreB_1_grm(#) EUT1
04035 HMM00465alcdh_3_mix(pf00465) EUT1
04040 HMMtpEuth_1_eut(pf04346) EUT1
04045 HMM06277EutA_1_eut(pf06277) EUT1
04050 HMMetlyEutB_1_EUT1(pf06751) EUT1
04055 HMMetlyEutC_1_eut1(pf05985) EUT1 EP_NTERM
04060 Tsp_rosa EUT1
04065 H_mulberry(pf00936pf16365) EUT1
04070 HMMreg12833_1_EUT1(pf12833) EUT1
04075 -pf01218 -
04080 -pf01520 -
04085 -pf00583 -
04090 -pf11143 -
04095 -pf06572 -
04100 -pf04261 -
04105 HMMnopfamclust3_1(#) RMM1
04110 -# -
04115 -pf00528 -
BMC-P
BMC-H
BMC-T
Aldehyde dehydrogenase
PTAC
Signature enzyme
Alcohol dehydrogenase
Regulator

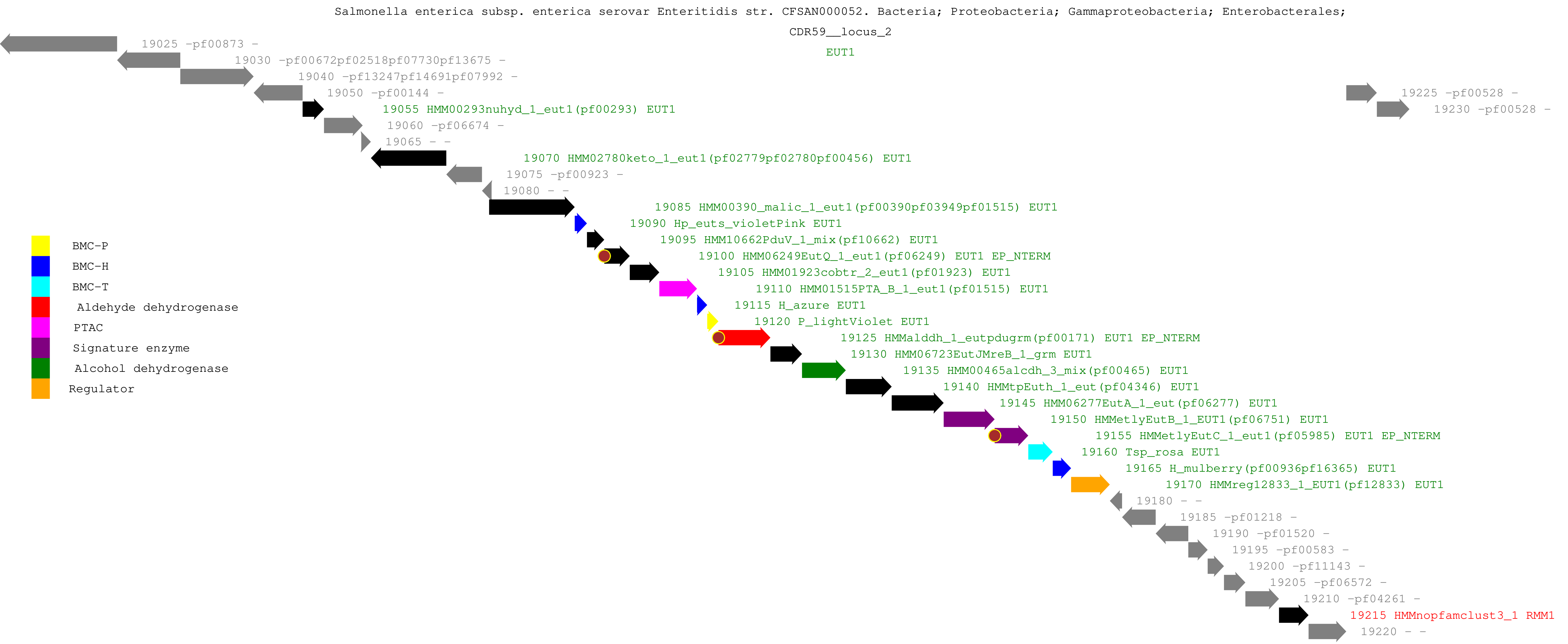
Salmonella enterica subsp. enterica serovar Enteritidis str. CFSAN000052. Bacteria; Proteobacteria; Gammaproteobacteria; Enterobacterales;
CDR59__locus_2
EUT1
19025 -pf00873 -
19030 -pf00672pf02518pf07730pf13675 -
19040 -pf13247pf14691pf07992 -
19050 -pf00144 -
19055 HMM00293nuhyd_1_eut1(pf00293) EUT1
19060 -pf06674 -
19065 - -
19070 HMM02780keto_1_eut1(pf02779pf02780pf00456) EUT1
19075 -pf00923 -
19080 - -
19085 HMM00390_malic_1_eut1(pf00390pf03949pf01515) EUT1
19090 Hp_euts_violetPink EUT1
19095 HMM10662PduV_1_mix(pf10662) EUT1
19100 HMM06249EutQ_1_eut1(pf06249) EUT1 EP_NTERM
19105 HMM01923cobtr_2_eut1(pf01923) EUT1
19110 HMM01515PTA_B_1_eut1(pf01515) EUT1
19115 H_azure EUT1
19120 P_lightViolet EUT1
19125 HMMalddh_1_eutpdugrm(pf00171) EUT1 EP_NTERM
19130 HMM06723EutJMreB_1_grm EUT1
19135 HMM00465alcdh_3_mix(pf00465) EUT1
19140 HMMtpEuth_1_eut(pf04346) EUT1
19145 HMM06277EutA_1_eut(pf06277) EUT1
19150 HMMetlyEutB_1_EUT1(pf06751) EUT1
19155 HMMetlyEutC_1_eut1(pf05985) EUT1 EP_NTERM
19160 Tsp_rosa EUT1
19165 H_mulberry(pf00936pf16365) EUT1
19170 HMMreg12833_1_EUT1(pf12833) EUT1
19180 - -
19185 -pf01218 -
19190 -pf01520 -
19195 -pf00583 -
19200 -pf11143 -
19205 -pf06572 -
19210 -pf04261 -
19215 HMMnopfamclust3_1 RMM1
19220 - -
19225 -pf00528 -
19230 -pf00528 -
BMC-P
BMC-H
BMC-T
Aldehyde dehydrogenase
PTAC
Signature enzyme
Alcohol dehydrogenase
Regulator

Escherichia coli O7:K1 str. CE10. Bacteria; Proteobacteria; Gammaproteobacteria; Enterobacterales;

CE10__locus_1

EUT1

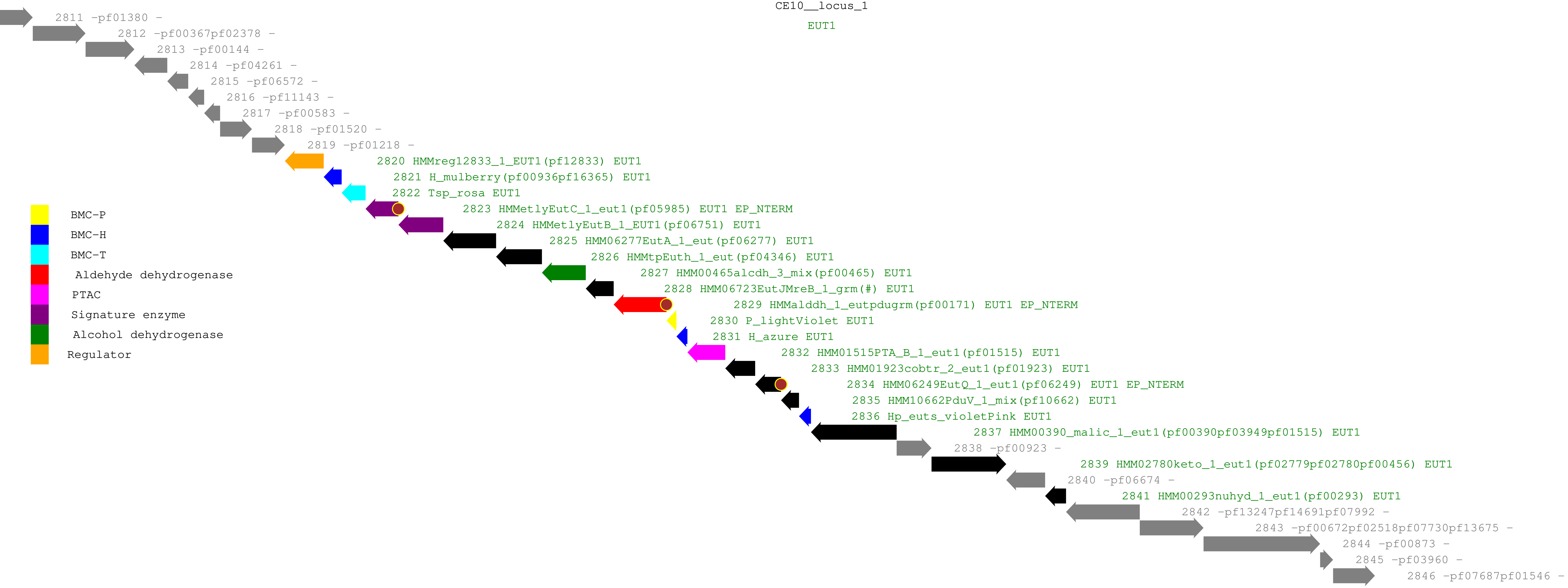

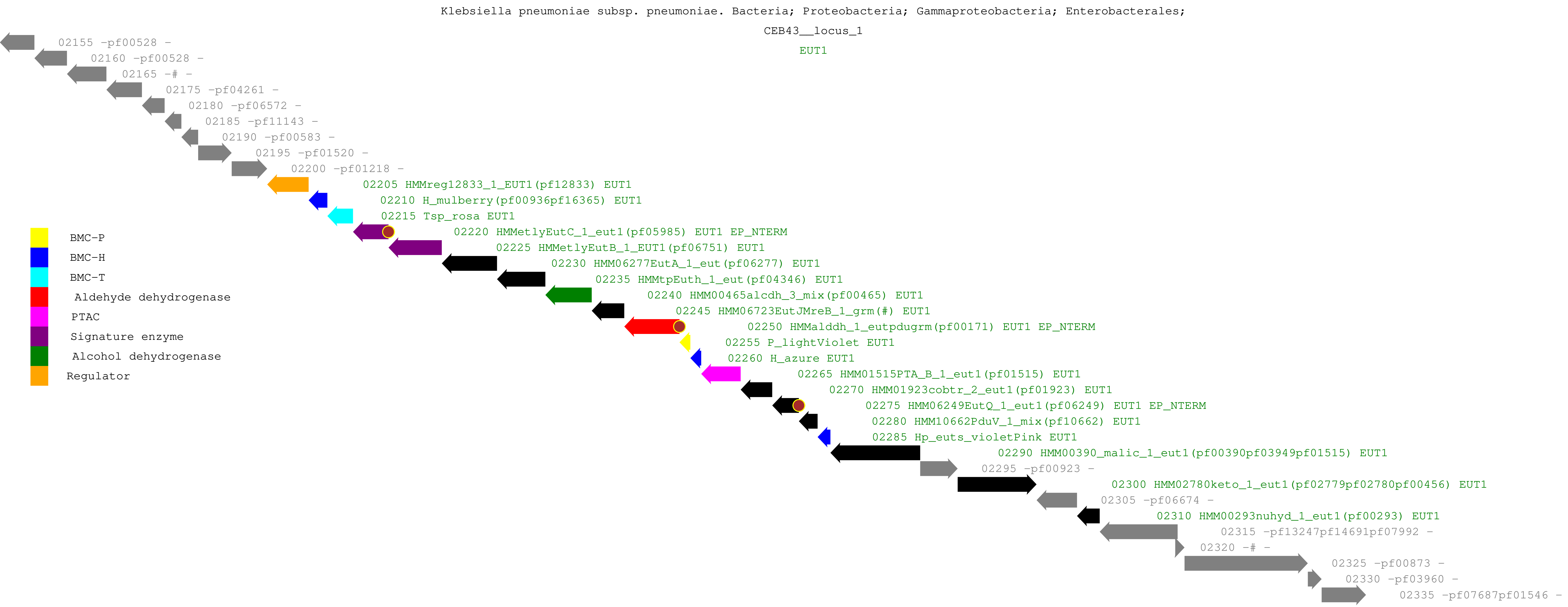
Klebsiella pneumoniae subsp. pneumoniae. Bacteria; Proteobacteria; Gammaproteobacteria; Enterobacterales;
CEB43__locus_1
EUT1
02155 -pf00528 -
02160 -pf00528 -
02165 -# -
02175 -pf04261 -
02180 -pf06572 -
02185 -pf11143 -
02190 -pf00583 -
02195 -pf01520 -
02200 -pf01218 -
02205 HMMreg12833_1_EUT1(pf12833) EUT1
02210 H_mulberry(pf00936pf16365) EUT1
02215 Tsp_rosa EUT1
02220 HMMetlyEutC_1_eut1(pf05985) EUT1 EP_NTERM
02225 HMMetlyEutB_1_EUT1(pf06751) EUT1
02230 HMM06277EutA_1_eut(pf06277) EUT1
02235 HMMtpEuth_1_eut(pf04346) EUT1
02240 HMM00465alcdh_3_mix(pf00465) EUT1
02245 HMM06723EutJMreB_1_grm(#) EUT1
02250 HMMalddh_1_eutpdugrm(pf00171) EUT1 EP_NTERM
02255 P_lightViolet EUT1
02260 H_azure EUT1
02265 HMM01515PTA_B_1_eut1(pf01515) EUT1
02270 HMM01923cobtr_2_eut1(pf01923) EUT1
02275 HMM06249EutQ_1_eut1(pf06249) EUT1 EP_NTERM
02280 HMM10662PduV_1_mix(pf10662) EUT1
02285 Hp_euts_violetPink EUT1
02290 HMM00390_malic_1_eut1(pf00390pf03949pf01515) EUT1
02295 -pf00923 -
02300 HMM02780keto_1_eut1(pf02779pf02780pf00456) EUT1
02305 -pf06674 -
02310 HMM00293nuhyd_1_eut1(pf00293) EUT1
02315 -pf13247pf14691pf07992 -
02320 -# -
02325 -pf00873 -
02330 -pf03960 -
02335 -pf07687pf01546 -
BMC-P
BMC-H
BMC-T
Aldehyde dehydrogenase
PTAC
Signature enzyme
Alcohol dehydrogenase
Regulator

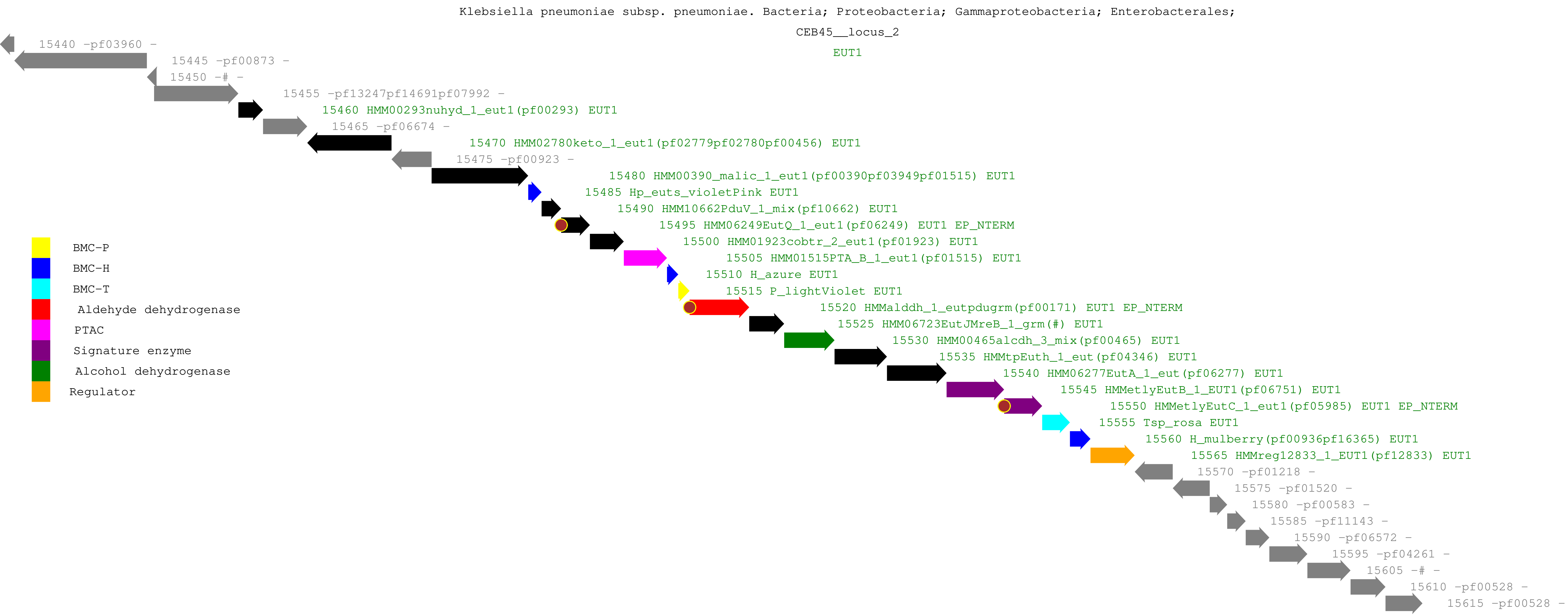
Klebsiella pneumoniae subsp. pneumoniae. Bacteria; Proteobacteria; Gammaproteobacteria; Enterobacterales;
CEB45__locus_2
EUT1
15440 -pf03960 -
15445 -pf00873 -
15450 -# -
15455 -pf13247pf14691pf07992 -
15460 HMM00293nuhyd_1_eut1(pf00293) EUT1
15465 -pf06674 -
15470 HMM02780keto_1_eut1(pf02779pf02780pf00456) EUT1
15475 -pf00923 -
15480 HMM00390_malic_1_eut1(pf00390pf03949pf01515) EUT1
15485 Hp_euts_violetPink EUT1
15490 HMM10662PduV_1_mix(pf10662) EUT1
15495 HMM06249EutQ_1_eut1(pf06249) EUT1 EP_NTERM
15500 HMM01923cobtr_2_eut1(pf01923) EUT1
15505 HMM01515PTA_B_1_eut1(pf01515) EUT1
15510 H_azure EUT1
15515 P_lightViolet EUT1
15520 HMMalddh_1_eutpdugrm(pf00171) EUT1 EP_NTERM
15525 HMM06723EutJMreB_1_grm(#) EUT1
15530 HMM00465alcdh_3_mix(pf00465) EUT1
15535 HMMtpEuth_1_eut(pf04346) EUT1
15540 HMM06277EutA_1_eut(pf06277) EUT1
15545 HMMetlyEutB_1_EUT1(pf06751) EUT1
15550 HMMetlyEutC_1_eut1(pf05985) EUT1 EP_NTERM
15555 Tsp_rosa EUT1
15560 H_mulberry(pf00936pf16365) EUT1
15565 HMMreg12833_1_EUT1(pf12833) EUT1
15570 -pf01218 -
15575 -pf01520 -
15580 -pf00583 -
15585 -pf11143 -
15590 -pf06572 -
15595 -pf04261 -
15605 -# -
15610 -pf00528 -
15615 -pf00528 -
BMC-P
BMC-H
BMC-T
Aldehyde dehydrogenase
PTAC
Signature enzyme
Alcohol dehydrogenase
Regulator

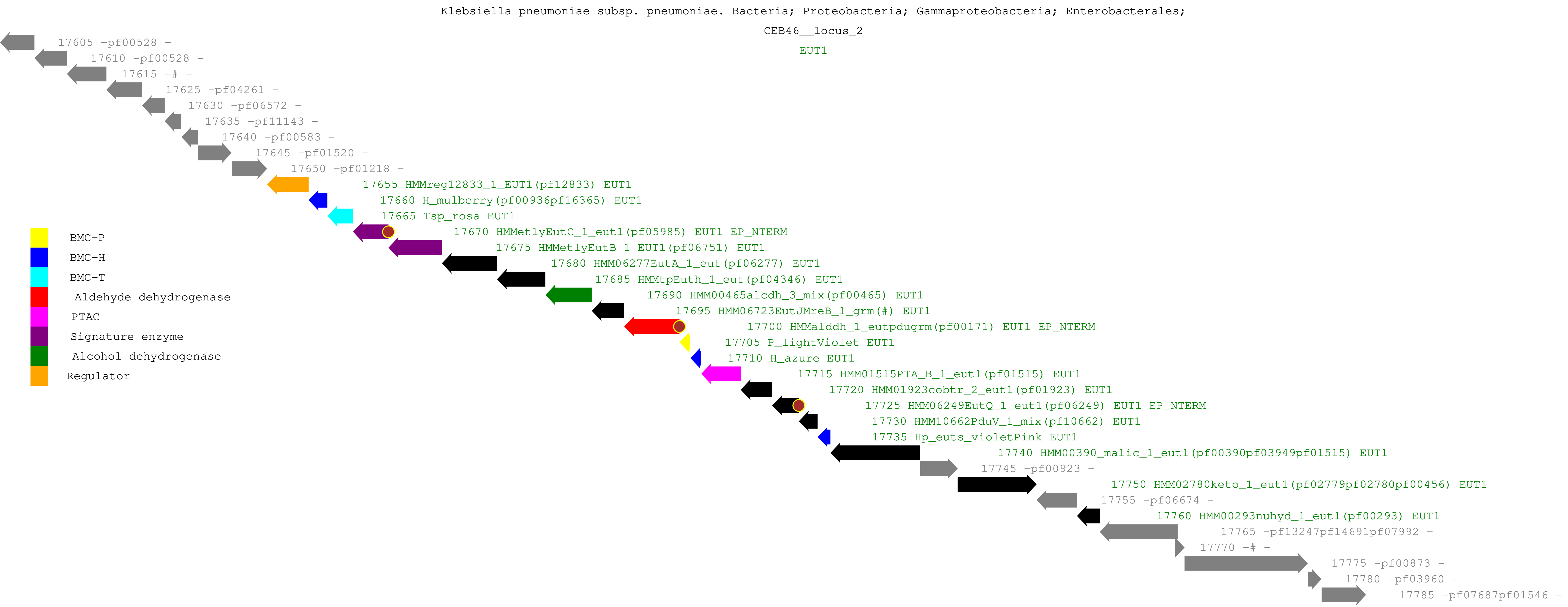

Klebsiella pneumoniae subsp. pneumoniae. Bacteria; Proteobacteria; Gammaproteobacteria; Enterobacterales;
CEB46__locus_2
EUT1
17605 -pf00528 -
17610 -pf00528 -
17615 -# -
17625 -pf04261 -
17630 -pf06572 -
17635 -pf11143 -
17640 -pf00583 -
17645 -pf01520 -
17650 -pf01218 -
17655 HMMreg12833_1_EUT1(pf12833) EUT1
17660 H_mulberry(pf00936pf16365) EUT1
17665 Tsp_rosa EUT1
17670 HMMetlyEutC_1_eut1(pf05985) EUT1 EP_NTERM
17675 HMMetlyEutB_1_EUT1(pf06751) EUT1
17680 HMM06277EutA_1_eut(pf06277) EUT1
17685 HMMtpEuth_1_eut(pf04346) EUT1
17690 HMM00465alcdh_3_mix(pf00465) EUT1
17695 HMM06723EutJMreB_1_grm(#) EUT1
17700 HMMaldh_1_eutpdugrm(pf00171) EUT1 EP_NTERM
17705 P_lightViolet EUT1
17710 H_azure EUT1
17715 HMM01515PTA_B_1_eut1(pf01515) EUT1
17720 HMM01923cobtr_2_eut1(pf01923) EUT1
17725 HMM06249EutQ_1_eut1(pf06249) EUT1 EP_NTERM
17730 HMM10662PduV_1_mix(pf10662) EUT1
17735 Hp_euts_violetPink EUT1
17740 HMM00390_malic_1_eut1(pf00390pf03949pf01515) EUT1
17745 -pf00923 -
17750 HMM02780keto_1_eut1(pf02779pf02780pf00456) EUT1
17755 -pf06674 -
17760 HMM00293nuhyd_1_eut1(pf00293) EUT1
17765 -pf13247pf14691pf07992 -
17770 -# -
17775 -pf00873 -
17780 -pf03960 -
17785 -pf07687pf01546 -
BMC-P
BMC-H
BMC-T
Aldehyde dehydrogenase
PTAC
Signature enzyme
Alcohol dehydrogenase
Regulator

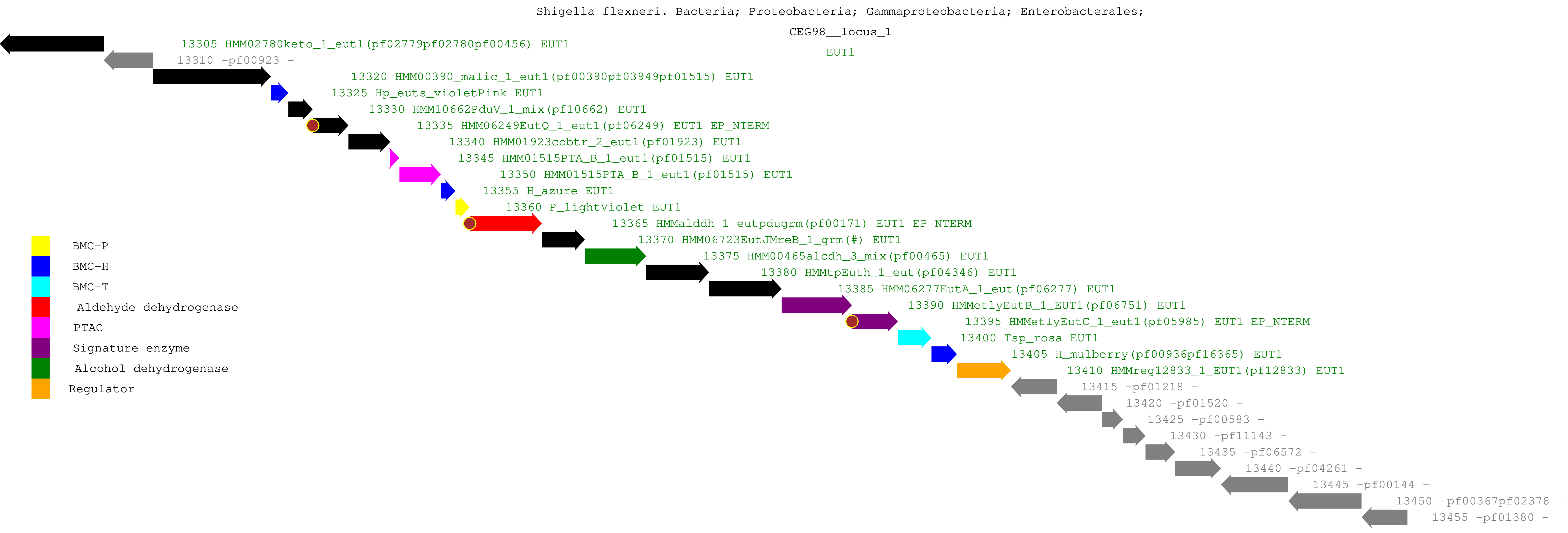

Shigella flexneri. Bacteria; Proteobacteria; Gammaproteobacteria; Enterobacterales;
CEG98__locus_1
EUT1
13305 HMM02780keto_1_eut1(pf02779pf02780pf00456) EUT1
13310 -pf00923 -
13320 HMM00390_malic_1_eut1(pf00390pf03949pf01515) EUT1
13325 Hp_euts_violetPink EUT1
13330 HMM10662PduV_1_mix(pf10662) EUT1
13335 HMM06249EutQ_1_eut1(pf06249) EUT1 EP_NTERM
13340 HMM01923cobtr_2_eut1(pf01923) EUT1
13345 HMM01515PTA_B_1_eut1(pf01515) EUT1
13350 HMM01515PTA_B_1_eut1(pf01515) EUT1
13355 H_azure EUT1
13360 P_lightViolet EUT1
13365 HMMalddh_1_eutpdugrm(pf00171) EUT1 EP_NTERM
13370 HMM06723EutJMreB_1_grm(#) EUT1
13375 HMM00465alcdh_3_mix(pf00465) EUT1
13380 HMMtpEuth_1_eut(pf04346) EUT1
13385 HMM06277EutA_1_eut(pf06277) EUT1
13390 HMMetlyEutB_1_EUT1(pf06751) EUT1
13395 HMMetlyEutC_1_eut1(pf05985) EUT1 EP_NTERM
13400 Tsp_rosa EUT1
13405 H_mulberry(pf00936pf16365) EUT1
13410 HMMreg12833_1_EUT1(pf12833) EUT1
13415 -pf01218 -
13420 -pf01520 -
13425 -pf00583 -
13430 -pf11143 -
13435 -pf06572 -
13440 -pf04261 -
13445 -pf00144 -
13450 -pf00367pf02378 -
13455 -pf01380 -
BMC-P
BMC-H
BMC-T
Aldehyde dehydrogenase
PTAC
Signature enzyme
Alcohol dehydrogenase
Regulator

Xenophilus sp. AP218F. Bacteria; Proteobacteria; Betaproteobacteria; Burkholderiales;

CEK28__locus_1

EUT1

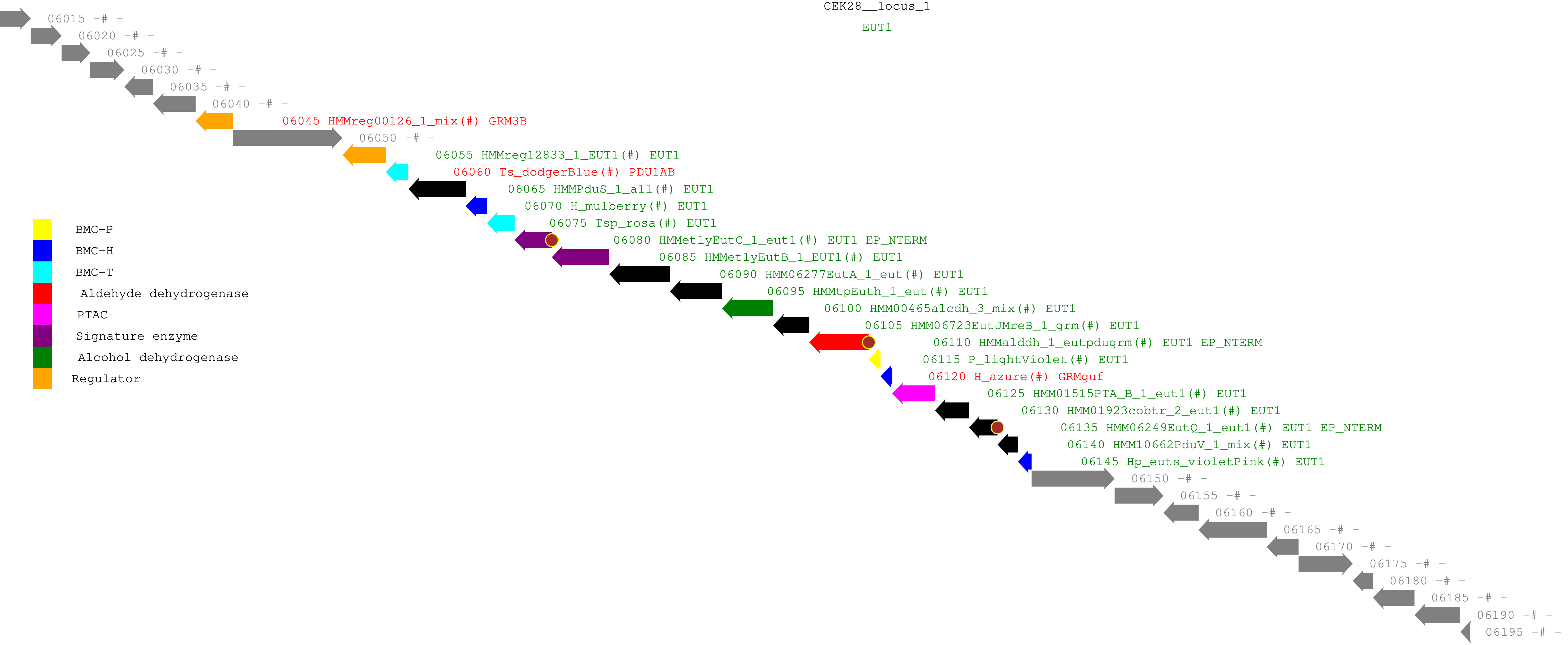

Escherichia coli O157. Bacteria; Proteobacteria; Gammaproteobacteria; Enterobacterales;

CEP72__locus_1

EUT1

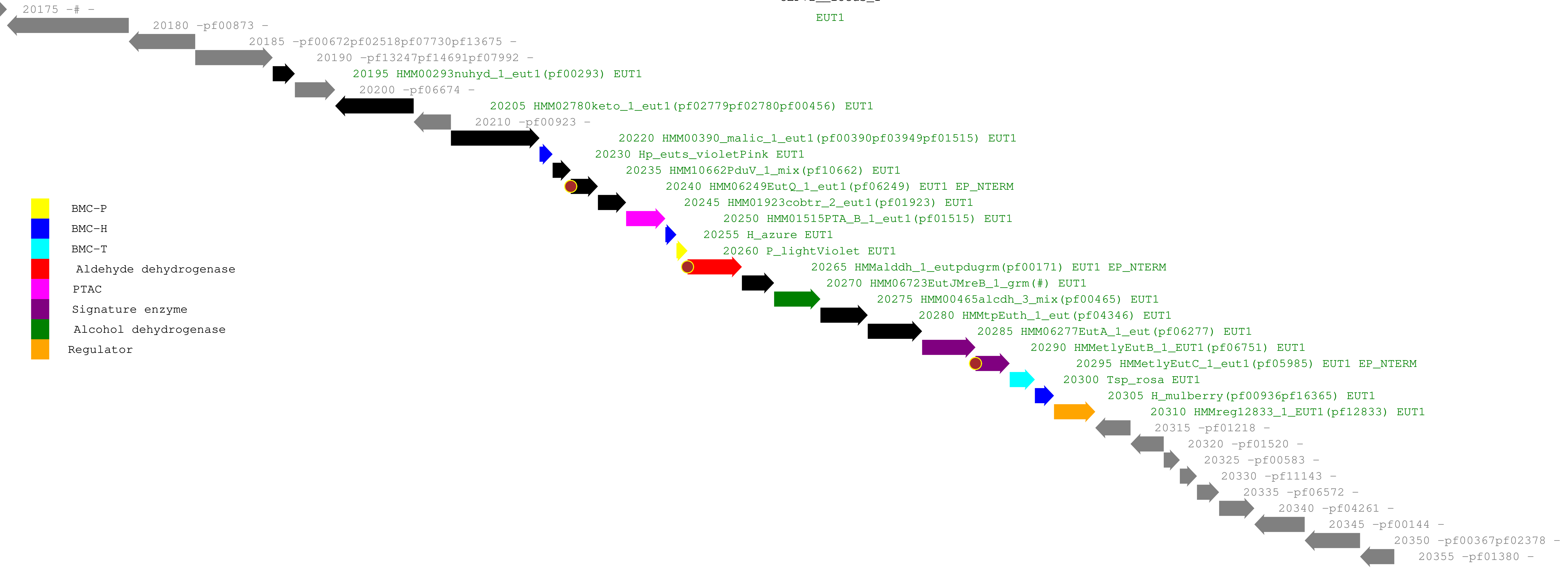

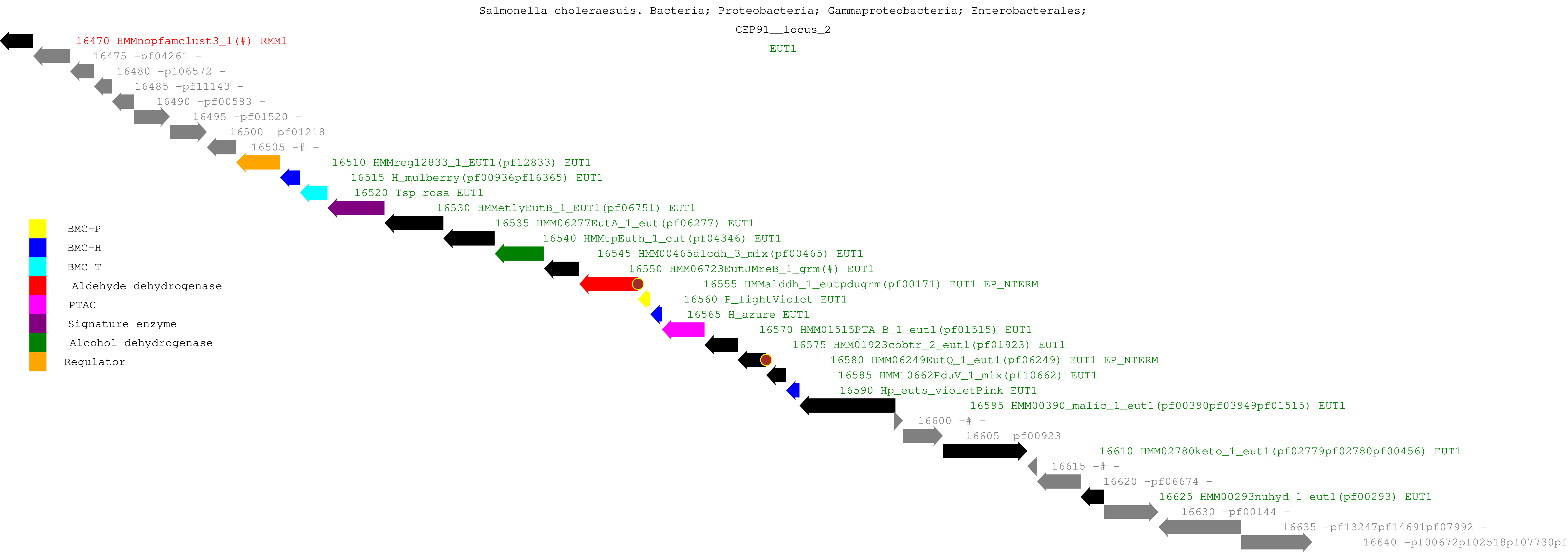

Salmonella choleraesuis. Bacteria; Proteobacteria; Gammaproteobacteria; Enterobacterales;
CEP91__locus_2
EUT1
16470 HMMnopfamclust3_1(#) RMM1
16475 -pf04261 -
16480 -pf06572 -
16485 -pf11143 -
16490 -pf00583 -
16495 -pf01520 -
16500 -pf01218 -
16505 -# -
16510 HMMreg12833_1_EUT1(pf12833) EUT1
16515 H_mulberry(pf00936pf16365) EUT1
16520 Tsp_rosa EUT1
16530 HMMetlyEutB_1_EUT1(pf06751) EUT1
16535 HMM06277EutA_1_eut(pf06277) EUT1
16540 HMMtpEuth_1_eut(pf04346) EUT1
16545 HMM00465alcdh_3_mix(pf00465) EUT1
16550 HMM06723EutJMreB_1_grm(#) EUT1
16555 HMMalddh_1_eutpdugrm(pf00171) EUT1 EP_NTERM
16560 P_lightViolet EUT1
16565 H_azure EUT1
16570 HMM01515PTA_B_1_eut1(pf01515) EUT1
16575 HMM01923cobtr_2_eut1(pf01923) EUT1
16580 HMM06249EutQ_1_eut1(pf06249) EUT1 EP_NTERM
16585 HMM10662PduV_1_mix(pf10662) EUT1
16590 Hp_euts_violetPink EUT1
16595 HMM00390_malic_1_eut1(pf00390pf03949pf01515) EUT1
16600 -# -
16605 -pf00923 -
16610 HMM02780keto_1_eut1(pf02779pf02780pf00456) EUT1
16615 -# -
16620 -pf06674 -
16625 HMM00293nuhyd_1_eut1(pf00293) EUT1
16630 -pf00144 -
16635 -pf13247pf14691pf07992 -
16640 -pf00672pf02518pf07730pf
BMC-P
BMC-H
BMC-T
Aldehyde dehydrogenase
PTAC
Signature enzyme
Alcohol dehydrogenase
Regulator

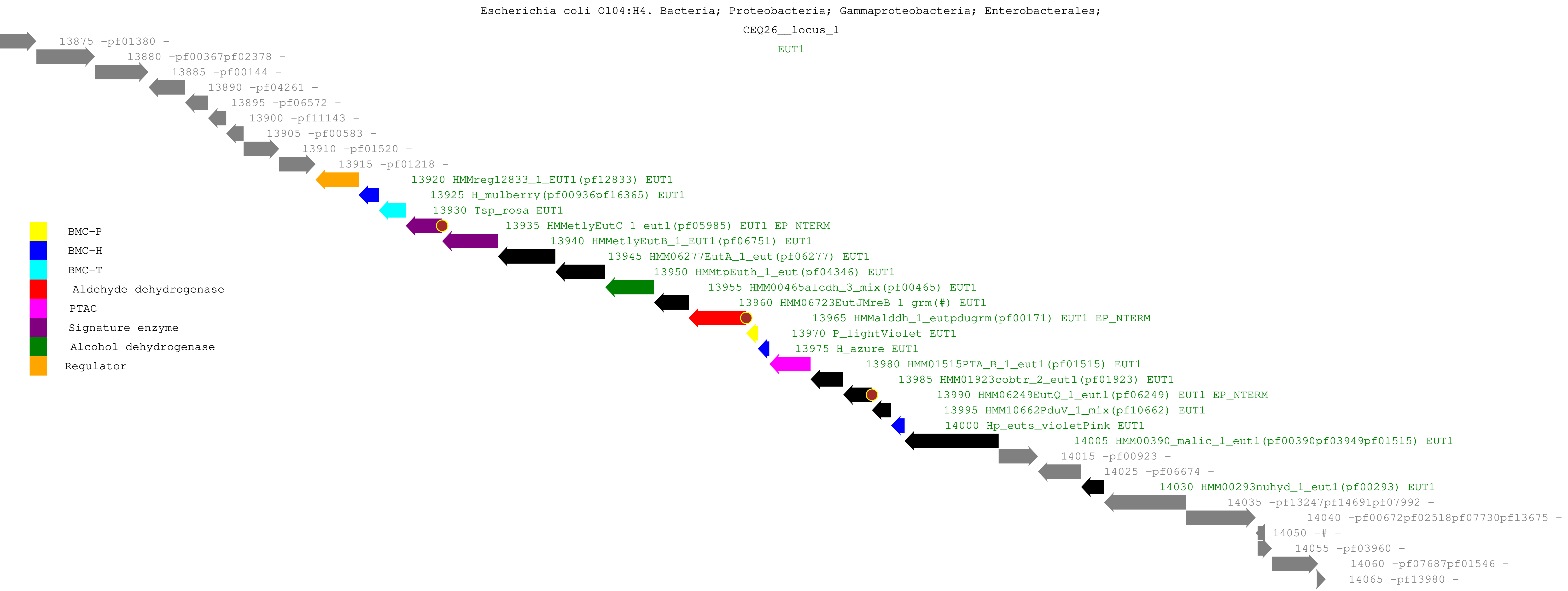
Escherichia coli O104:H4. Bacteria; Proteobacteria; Gammaproteobacteria; Enterobacterales;
CEQ26__locus_1
EUT1
13875 -pf01380 -
13880 -pf00367pf02378 -
13885 -pf00144 -
13890 -pf04261 -
13895 -pf06572 -
13900 -pf11143 -
13905 -pf00583 -
13910 -pf01520 -
13915 -pf01218 -
13920 HMMreg12833_1_EUT1(pf12833) EUT1
13925 H_mulberry(pf00936pf16365) EUT1
13930 Tsp_rosa EUT1
13935 HMMetlyEutC_1_eut1(pf05985) EUT1 EP_NTERM
13940 HMMetlyEutB_1_EUT1(pf06751) EUT1
13945 HMM06277EutA_1_eut(pf06277) EUT1
13950 HMMtpEuth_1_eut(pf04346) EUT1
13955 HMM00465alcdh_3_mix(pf00465) EUT1
13960 HMM06723EutJMreB_1_grm(#) EUT1
13965 HMMalddh_1_eutpdugrm(pf00171) EUT1 EP_NTERM
13970 P_lightViolet EUT1
13975 H_azure EUT1
13980 HMM01515PTA_B_1_eut1(pf01515) EUT1
13985 HMM01923cobtr_2_eut1(pf01923) EUT1
13990 HMM06249EutQ_1_eut1(pf06249) EUT1 EP_NTERM
13995 HMM10662PduV_1_mix(pf10662) EUT1
14000 Hp_euts_violetPink EUT1
14005 HMM00390_malic_1_eut1(pf00390pf03949pf01515) EUT1
14015 -pf00923 -
14025 -pf06674 -
14030 HMM00293nuhyd_1_eut1(pf00293) EUT1
14035 -pf13247pf14691pf07992 -
14040 -pf00672pf02518pf07730pf13675 -
14050 -# -
14055 -pf03960 -
14060 -pf07687pf01546 -
14065 -pf13980 -
BMC-P
BMC-H
BMC-T
Aldehyde dehydrogenase
PTAC
Signature enzyme
Alcohol dehydrogenase
Regulator

Hafnia alvei. Bacteria; Proteobacteria; Gammaproteobacteria; Enterobacterales;

CEQ28__locus_1

EUT1

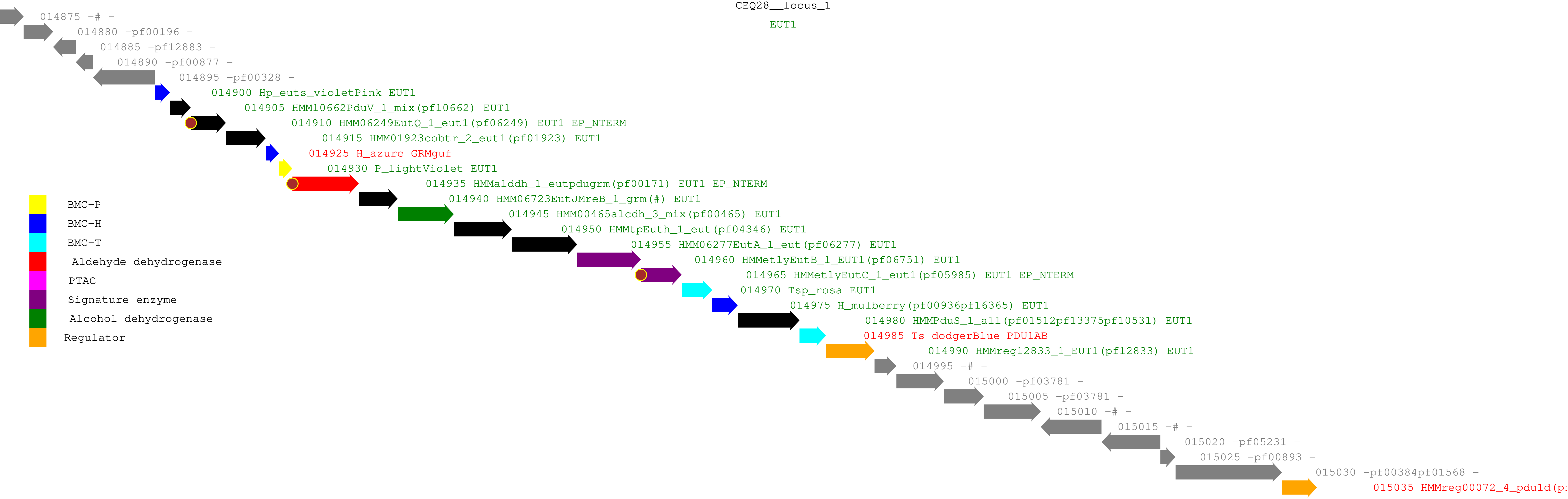

Salmonella enterica subsp. enterica serovar Bredeney. Bacteria; Proteobacteria; Gammaproteobacteria; Enterobacterales;

CF29__locus_2

EUT1

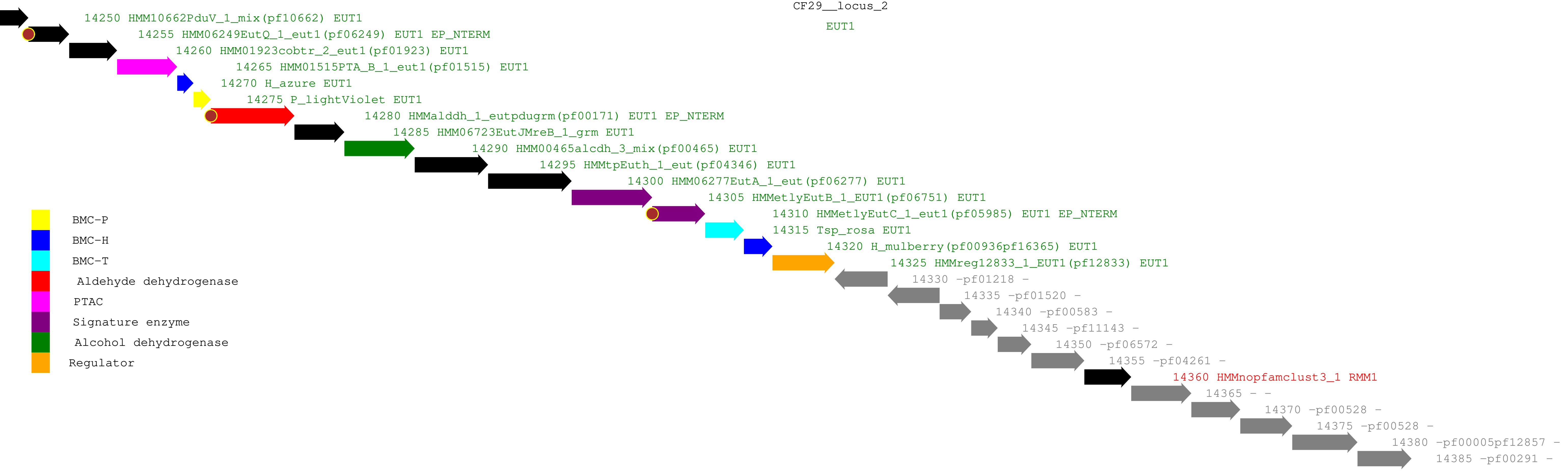

Citrobacter freundii CFNIH1. Bacteria; Proteobacteria; Gammaproteobacteria; Enterobacterales;

CFNIH1__locus_2

EUT1

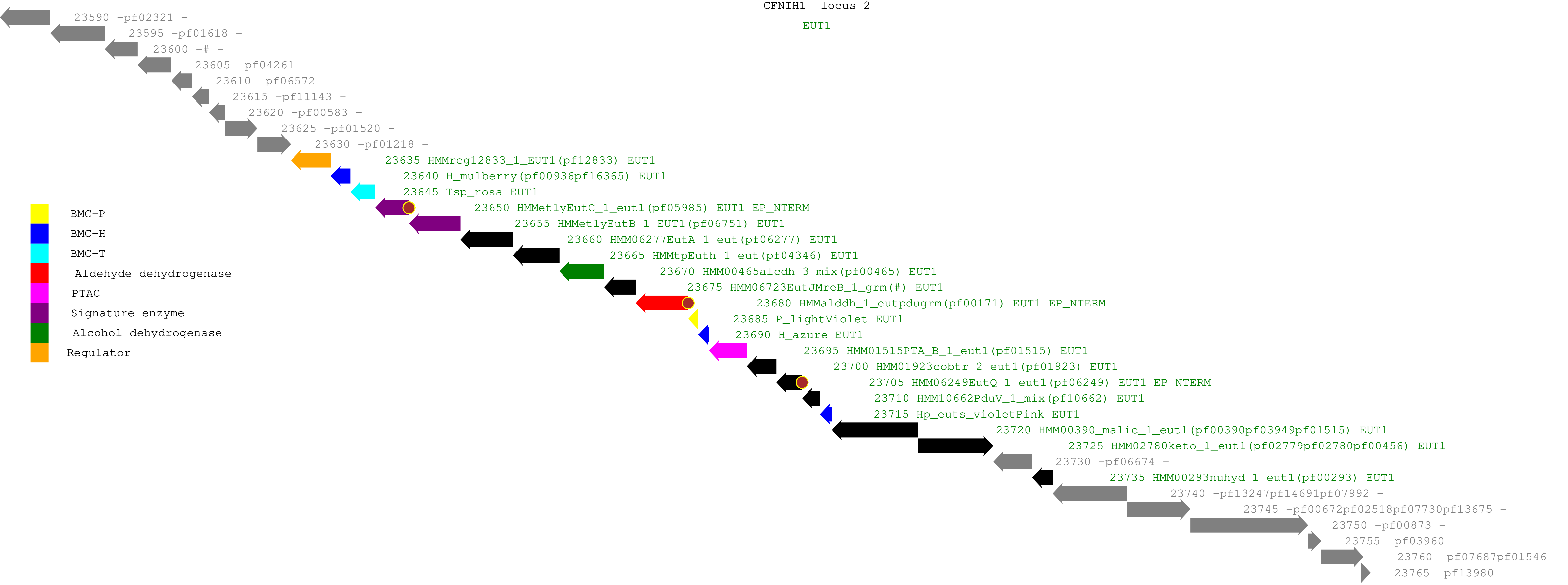

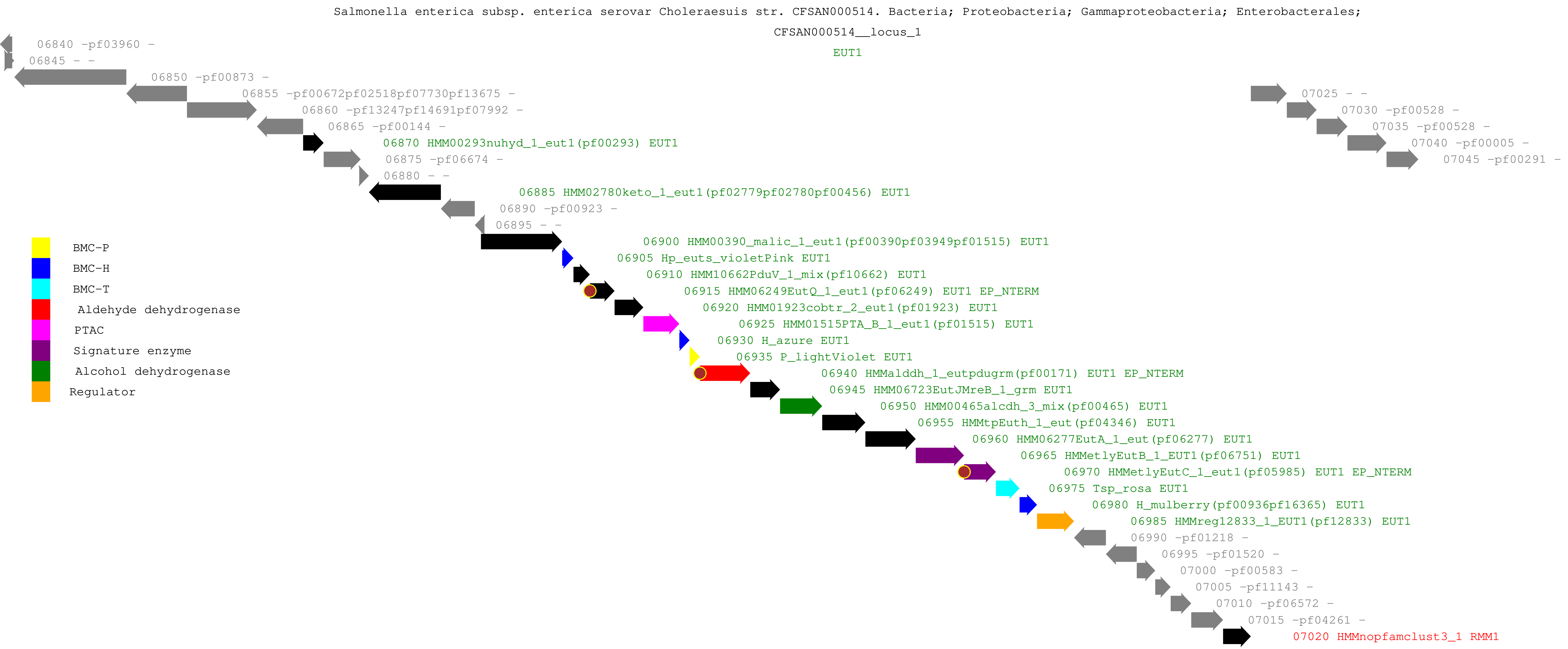

Salmonella enterica subsp. enterica serovar Choleraesuis str. CFSAN000514. Bacteria; Proteobacteria; Gammaproteobacteria; Enterobacterales;
CFSAN000514__locus_1
EUT1
06840 -pf03960 -
06845 - -
06850 -pf00873 -
06855 -pf00672pf02518pf07730pf13675 -
06860 -pf13247pf14691pf07992 -
06865 -pf00144 -
06870 HMM00293nuhyd_1_eut1(pf00293) EUT1
06875 -pf06674 -
06880 - -
06885 HMM02780keto_1_eut1(pf02779pf02780pf00456) EUT1
06890 -pf00923 -
06895 - -
06900 HMM00390_malic_1_eut1(pf00390pf03949pf01515) EUT1
06905 Hp_euts_violetPink EUT1
06910 HMM10662PduV_1_mix(pf10662) EUT1
06915 HMM06249EutQ_1_eut1(pf06249) EUT1 EP_NTERM
06920 HMM01923cobtr_2_eut1(pf01923) EUT1
06925 HMM01515PTA_B_1_eut1(pf01515) EUT1
06930 H_azure EUT1
06935 P_lightViolet EUT1
06940 HMMalddh_1_eutpdugrm(pf00171) EUT1 EP_NTERM
06945 HMM06723EutJMreB_1_grm EUT1
06950 HMM00465alcdh_3_mix(pf00465) EUT1
06955 HMMtpEuth_1_eut(pf04346) EUT1
06960 HMM06277EutA_1_eut(pf06277) EUT1
06965 HMMetlyEutB_1_EUT1(pf06751) EUT1
06970 HMMetlyEutC_1_eut1(pf05985) EUT1 EP_NTERM
06975 Tsp_rosa EUT1
06980 H_mulberry(pf00936pf16365) EUT1
06985 HMMreg12833_1_EUT1(pf12833) EUT1
06990 -pf01218 -
06995 -pf01520 -
07000 -pf00583 -
07005 -pf11143 -
07010 -pf06572 -
07015 -pf04261 -
07020 HMMnopfamclust3_1 RMM1
07025 - -
07030 -pf00528 -
07035 -pf00528 -
07040 -pf00005 -
07045 -pf00291 -
BMC-P
BMC-H
BMC-T
Aldehyde dehydrogenase
PTAC
Signature enzyme
Alcohol dehydrogenase
Regulator

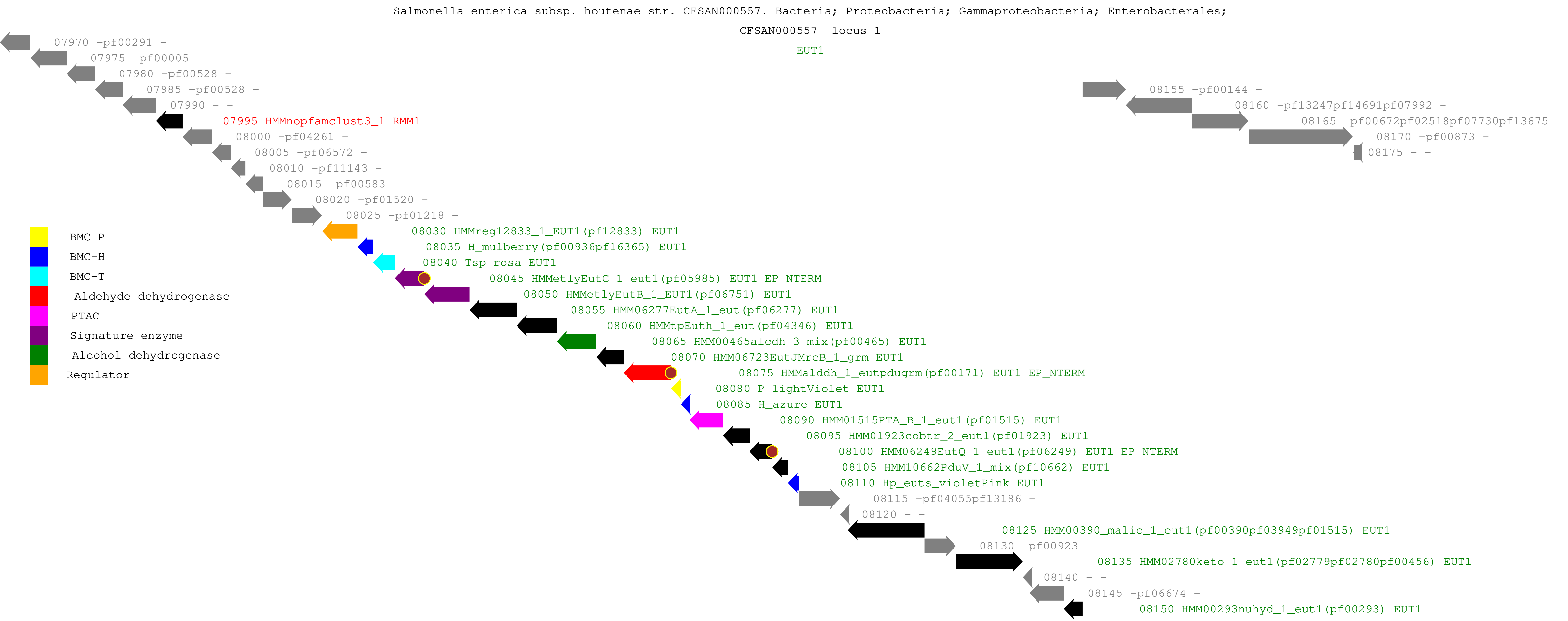

Salmonella enterica subsp. houtenae str. CFSAN000557. Bacteria; Proteobacteria; Gammaproteobacteria; Enterobacterales;
CFSAN000557__locus_1
EUT1
07970 -pf00291 -
07975 -pf00005 -
07980 -pf00528 -
07985 -pf00528 -
07990 - -
07995 HMMnopfamclust3_1 RMM1
08000 -pf04261 -
08005 -pf06572 -
08010 -pf11143 -
08015 -pf00583 -
08020 -pf01520 -
08025 -pf01218 -
08030 HMMreg12833_1_EUT1(pf12833) EUT1
08035 H_mulberry(pf00936pf16365) EUT1
08040 Tsp_rosa EUT1
08045 HMMetlyEutC_1_eut1(pf05985) EUT1 EP_NTERM
08050 HMMetlyEutB_1_EUT1(pf06751) EUT1
08055 HMM06277EutA_1_eut(pf06277) EUT1
08060 HMMtpEuth_1_eut(pf04346) EUT1
08065 HMM00465alcdh_3_mix(pf00465) EUT1
08070 HMM06723EutJMreB_1_grm EUT1
08075 HMMalddh_1_eutpdugrm(pf00171) EUT1 EP_NTERM
08080 P_lightViolet EUT1
08085 H_azure EUT1
08090 HMM01515PTA_B_1_eut1(pf01515) EUT1
08095 HMM01923cobtr_2_eut1(pf01923) EUT1
08100 HMM06249EutQ_1_eut1(pf06249) EUT1 EP_NTERM
08105 HMM10662PduV_1_mix(pf10662) EUT1
08110 Hp_euts_violetPink EUT1
08115 -pf04055pf13186 -
08120 - -
08125 HMM00390_malic_1_eut1(pf00390pf03949pf01515) EUT1
08130 -pf00923 -
08135 HMM02780keto_1_eut1(pf02779pf02780pf00456) EUT1
08140 - -
08145 -pf06674 -
08150 HMM00293nuhyd_1_eut1(pf00293) EUT1
08155 -pf00144 -
08160 -pf13247pf14691pf07992 -
08165 -pf00672pf02518pf07730pf13675 -
08170 -pf00873 -
08175 - -
BMC-P
BMC-H
BMC-T
Aldehyde dehydrogenase
PTAC
Signature enzyme
Alcohol dehydrogenase
Regulator

Salmonella enterica subsp. salamae str. CFSAN000559. Bacteria; Proteobacteria; Gammaproteobacteria; Enterobacterales;

CFSAN000559__locus_1

EUT1

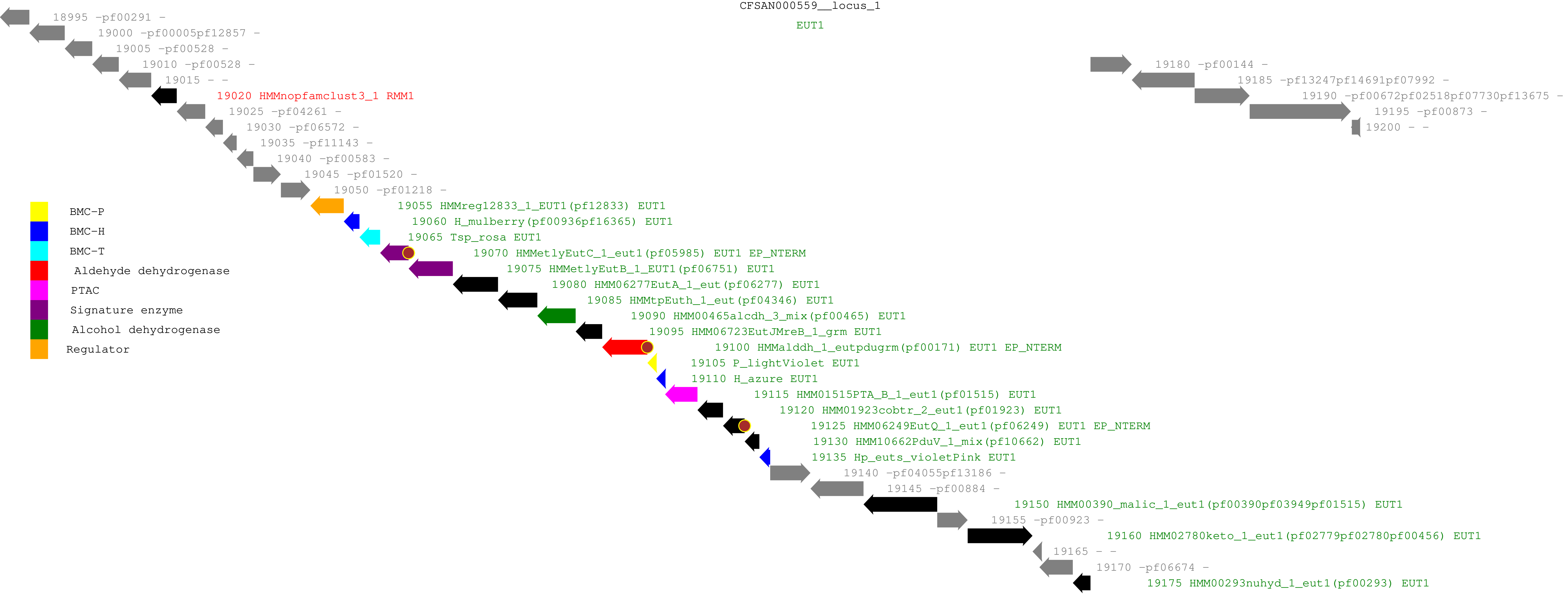

Salmonella enterica subsp. arizonae str. CFSAN000560. Bacteria; Proteobacteria; Gammaproteobacteria; Enterobacterales;

CFSAN000560__locus_1

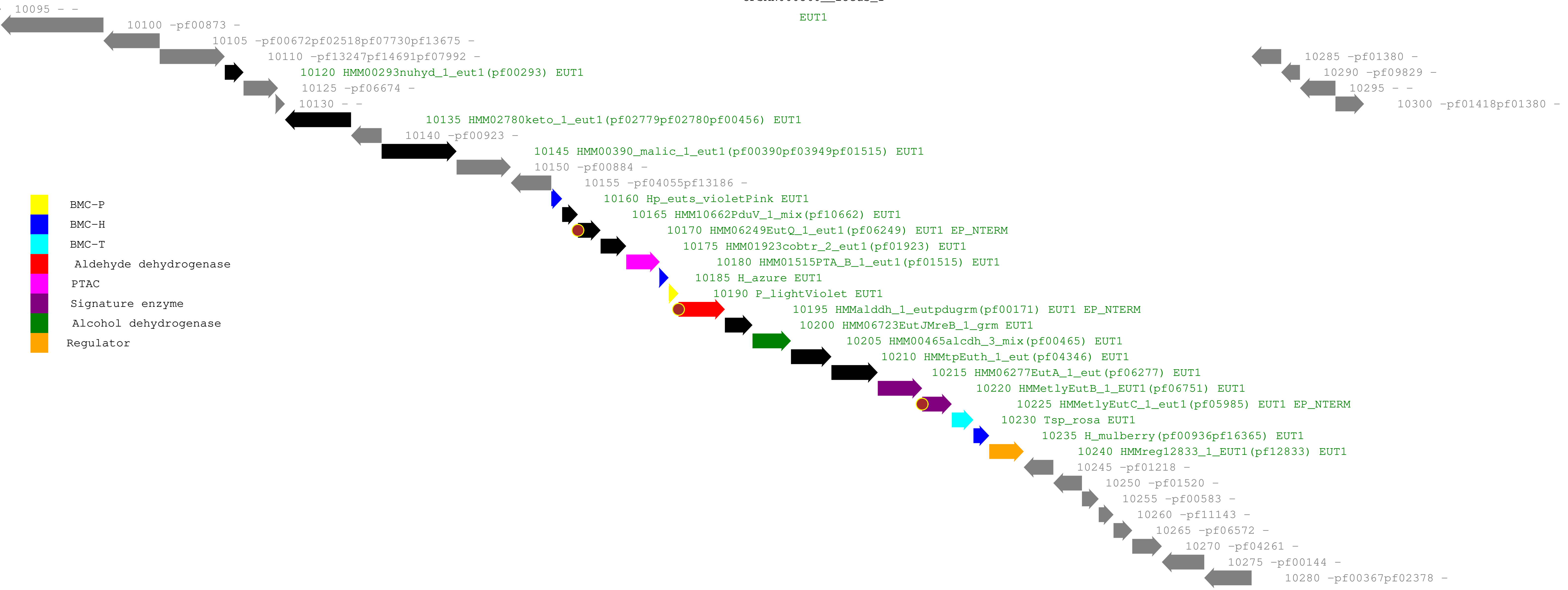

Salmonella enterica subsp. enterica serovar Gallinarum str. CFSAN000571. Bacteria; Proteobacteria; Gammaproteobacteria; Enterobacterales;

CFSAN000571__locus_2

EUT1

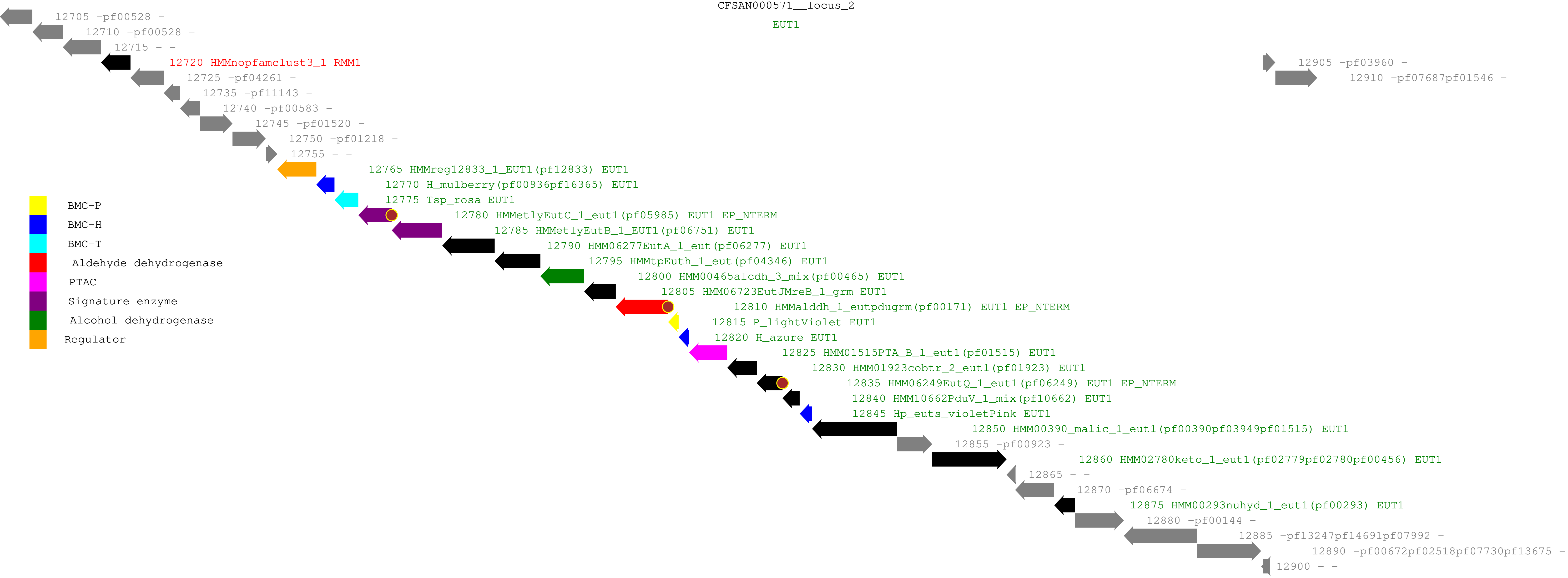

Salmonella enterica subsp. enterica serovar Muenchen str. CFSAN000593. Bacteria; Proteobacteria; Gammaproteobacteria; Enterobacterales;

CFSAN000593__locus_2

EUT1

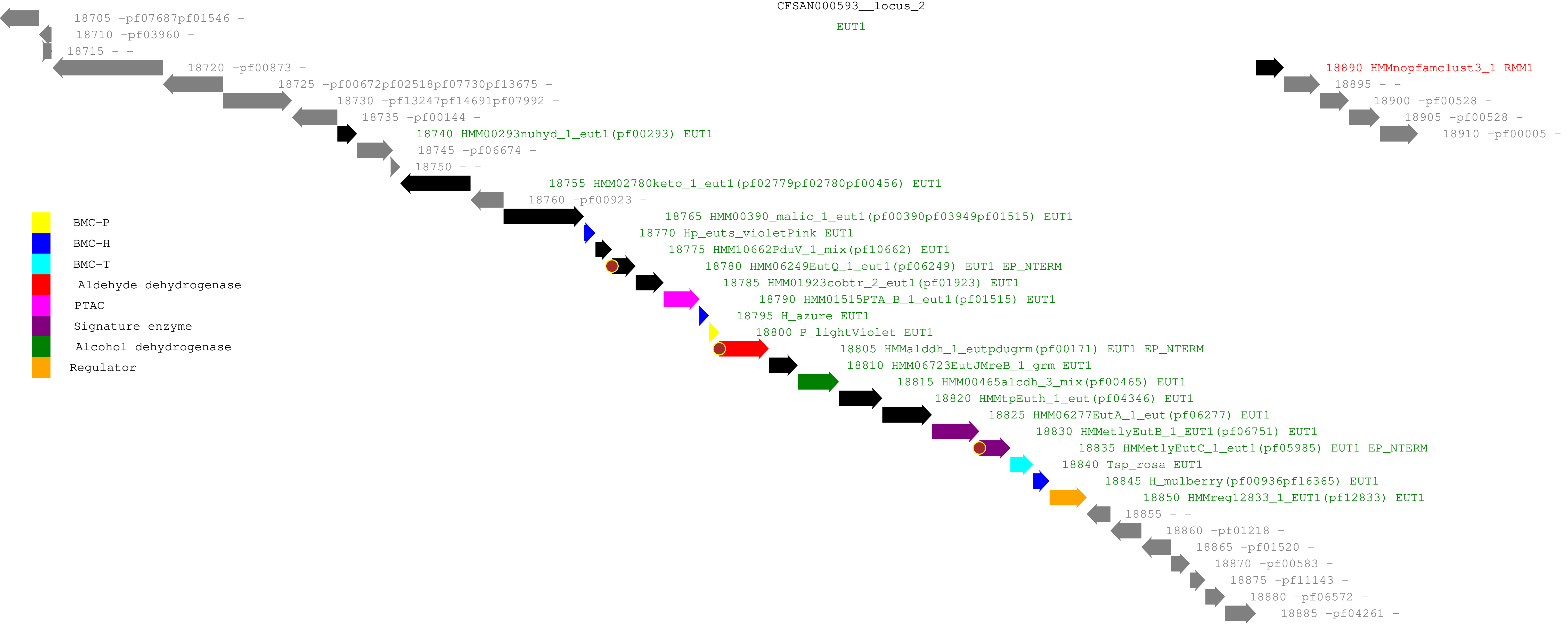

Salmonella enterica subsp. enterica serovar Muenchen str. CFSAN000596. Bacteria; Proteobacteria; Gammaproteobacteria; Enterobacterales;

CFSAN000596__locus_1

EUT1

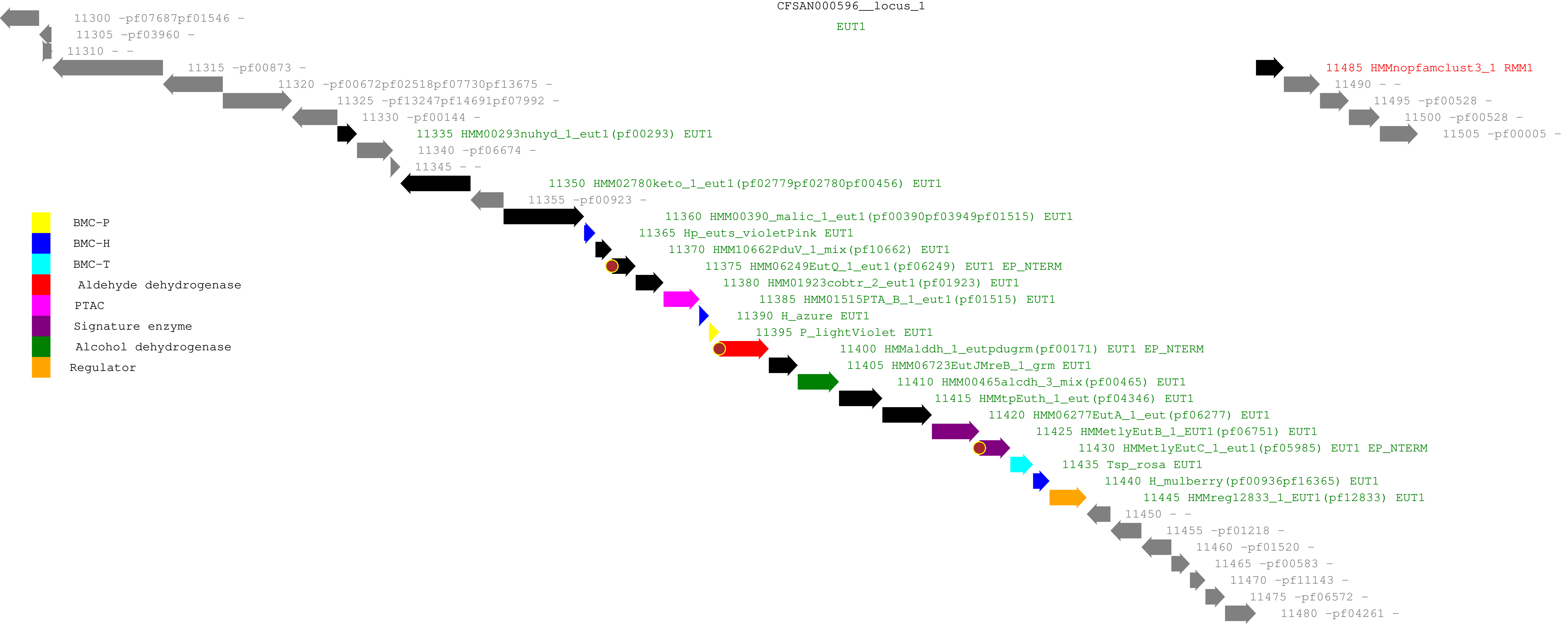

Salmonella enterica subsp. enterica serovar Panama str. CFSAN000600. Bacteria; Proteobacteria; Gammaproteobacteria; Enterobacterales;

CFSAN000600__locus_2

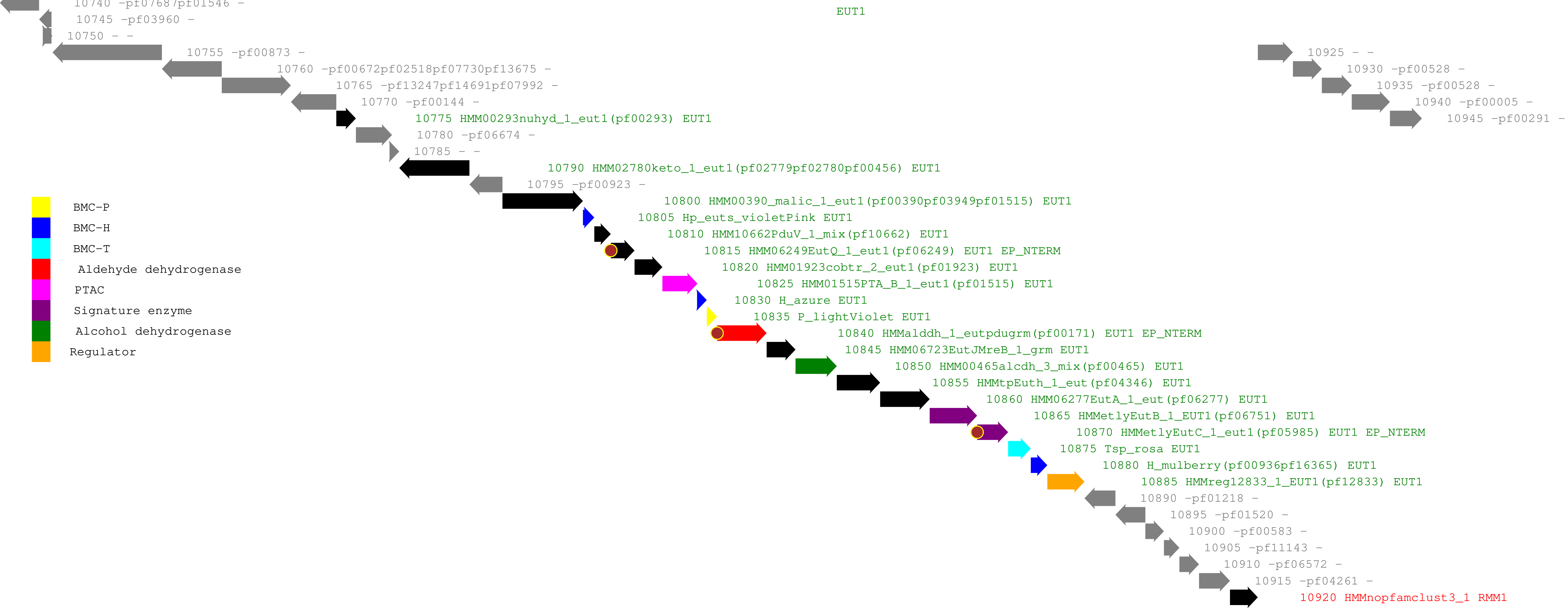

Salmonella enterica subsp. enterica serovar Saintpaul str. CFSAN000618. Bacteria; Proteobacteria; Gammaproteobacteria; Enterobacterales;

CFSAN000618__locus_1

EUT1

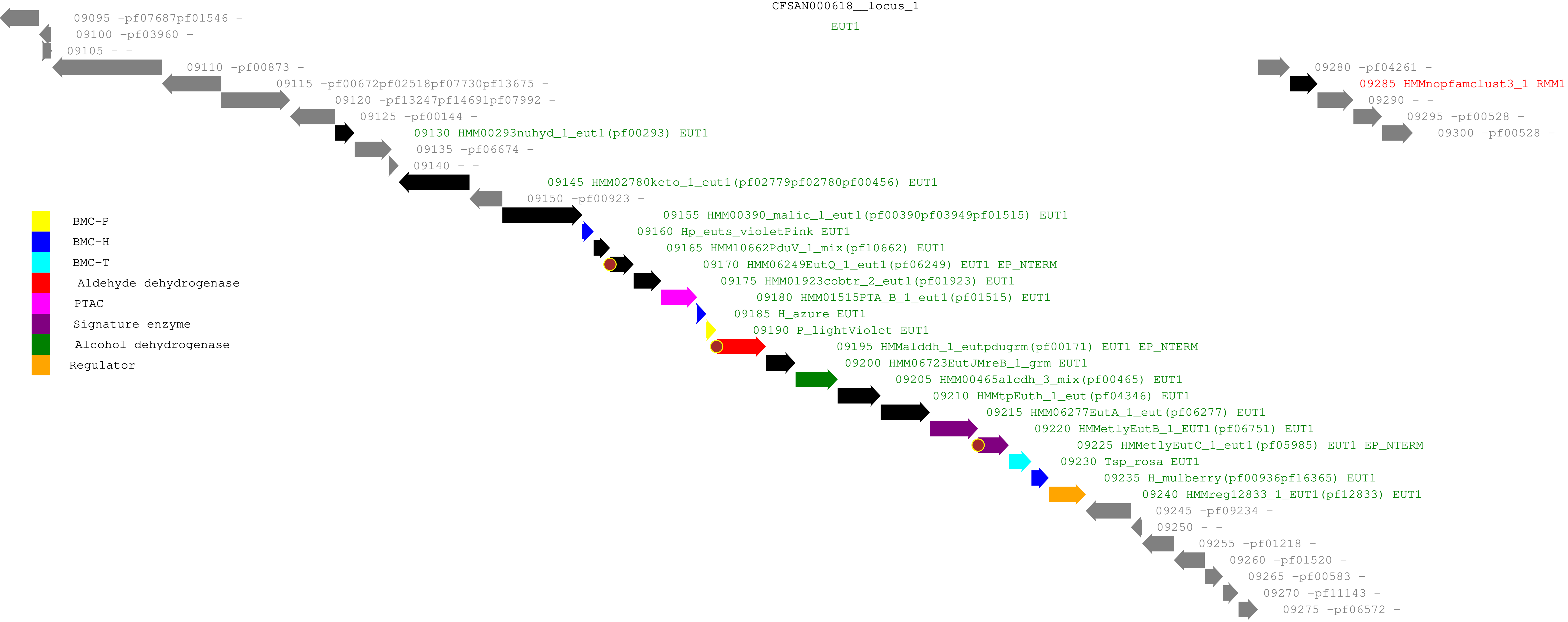

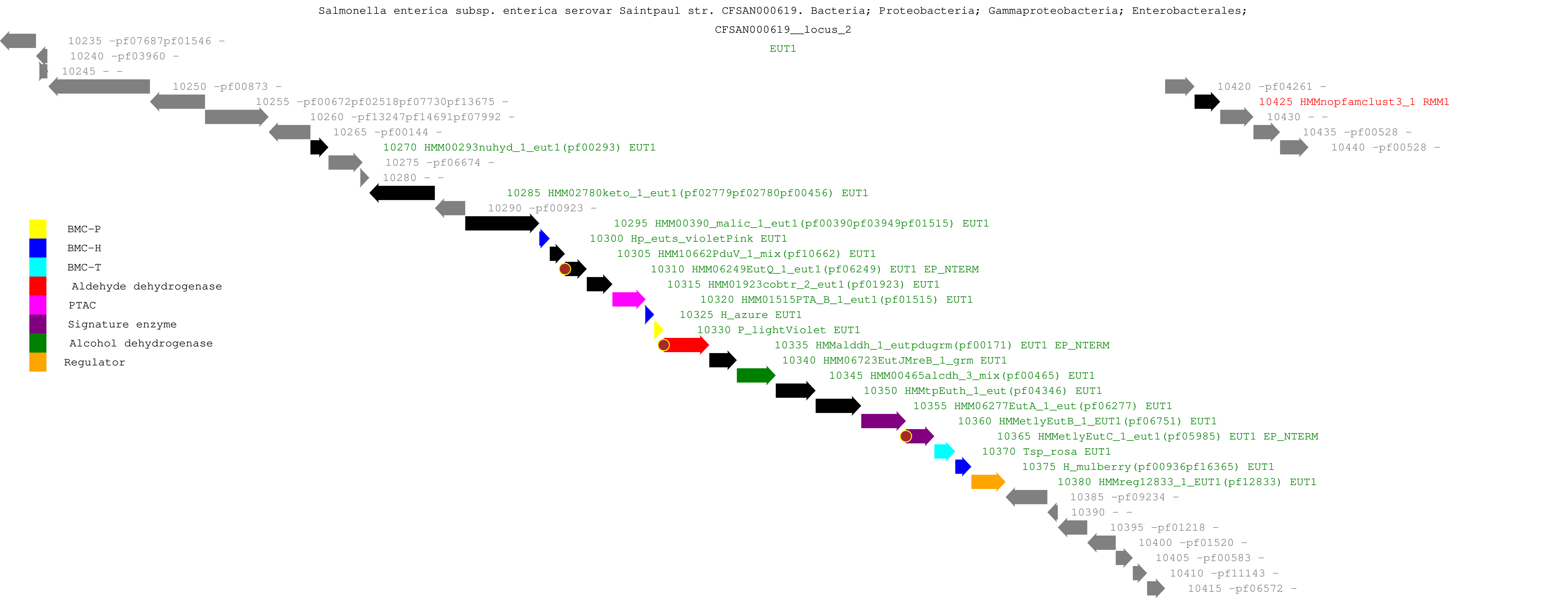

Salmonella enterica subsp. enterica serovar Saintpaul str. CFSAN000619. Bacteria; Proteobacteria; Gammaproteobacteria; Enterobacterales;
CFSAN000619__locus_2
EUT1
10235 -pf07687pf01546 -
10240 -pf03960 -
10245 - -
10250 -pf00873 -
10255 -pf00672pf02518pf07730pf13675 -
10260 -pf13247pf14691pf07992 -
10265 -pf00144 -
10270 HMM00293nuhyd_1_eut1(pf00293) EUT1
10275 -pf06674 -
10280 - -
10285 HMM02780keto_1_eut1(pf02779pf02780pf00456) EUT1
10290 -pf00923 -
10295 HMM00390_malic_1_eut1(pf00390pf03949pf01515) EUT1
10300 Hp_euts_violetPink EUT1
10305 HMM10662PduV_1_mix(pf10662) EUT1
10310 HMM06249EutQ_1_eut1(pf06249) EUT1 EP_NTERM
10315 HMM01923cobtr_2_eut1(pf01923) EUT1
10320 HMM01515PTA_B_1_eut1(pf01515) EUT1
10325 H_azure EUT1
10330 P_lightViolet EUT1
10335 HMMalddh_1_eutpdugrm(pf00171) EUT1 EP_NTERM
10340 HMM06723EutJMreB_1_grm EUT1
10345 HMM00465alcdh_3_mix(pf00465) EUT1
10350 HMMtpEuth_1_eut(pf04346) EUT1
10355 HMM06277EutA_1_eut(pf06277) EUT1
10360 HMMetlyEutB_1_EUT1(pf06751) EUT1
10365 HMMetlyEutC_1_eut1(pf05985) EUT1 EP_NTERM
10370 Tsp_rosa EUT1
10375 H_mulberry(pf00936pf16365) EUT1
10380 HMMreg12833_1_EUT1(pf12833) EUT1
10385 -pf09234 -
10390 - -
10395 -pf01218 -
10400 -pf01520 -
10405 -pf00583 -
10410 -pf11143 -
10415 -pf06572 -
10420 -pf04261 -
10425 HMMnopfamclust3_1 RMM1
10430 - -
10435 -pf00528 -
10440 -pf00528 -
BMC-P
BMC-H
BMC-T
Aldehyde dehydrogenase
PTAC
Signature enzyme
Alcohol dehydrogenase
Regulator

Salmonella enterica subsp. enterica serovar Stanley str. CFSAN000623. Bacteria; Proteobacteria; Gammaproteobacteria; Enterobacterales;

CFSAN000623__locus_2

EUT1

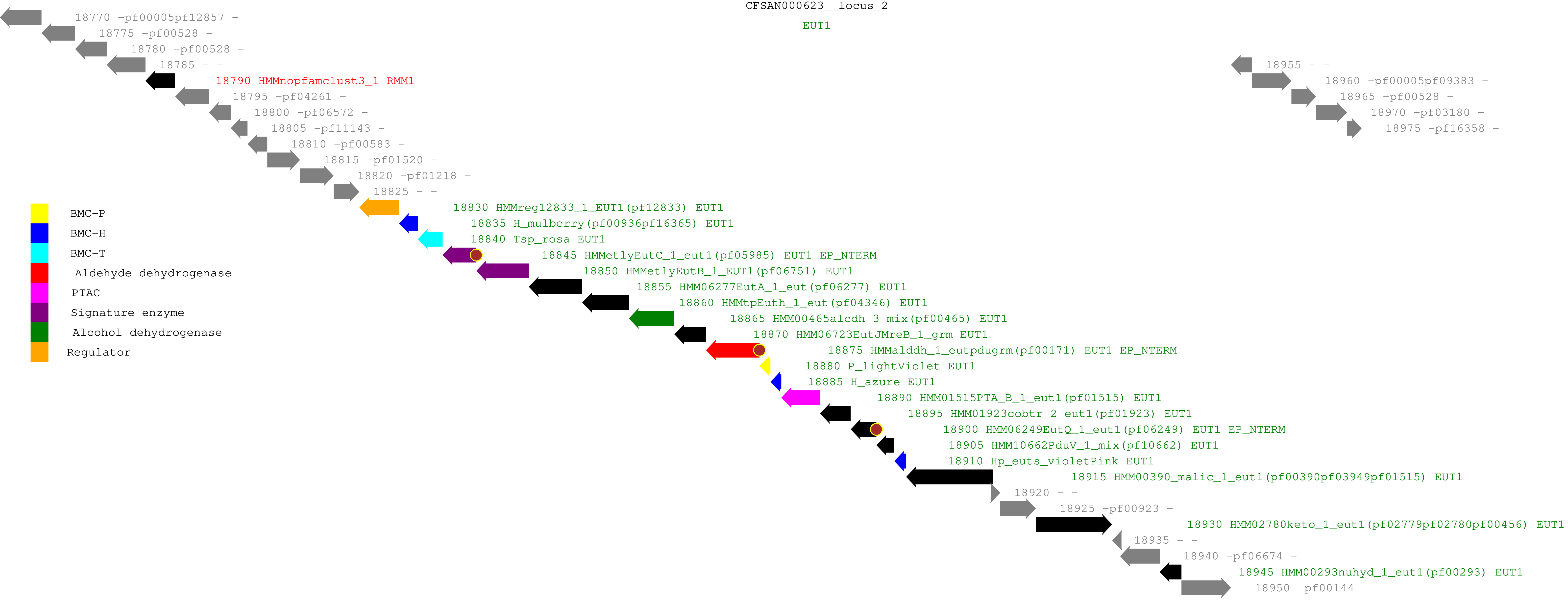

Salmonella enterica subsp. enterica serovar Thompson str. CFSAN000625. Bacteria; Proteobacteria; Gammaproteobacteria; Enterobacterales;

CFSAN000625__locus_2

EUT1

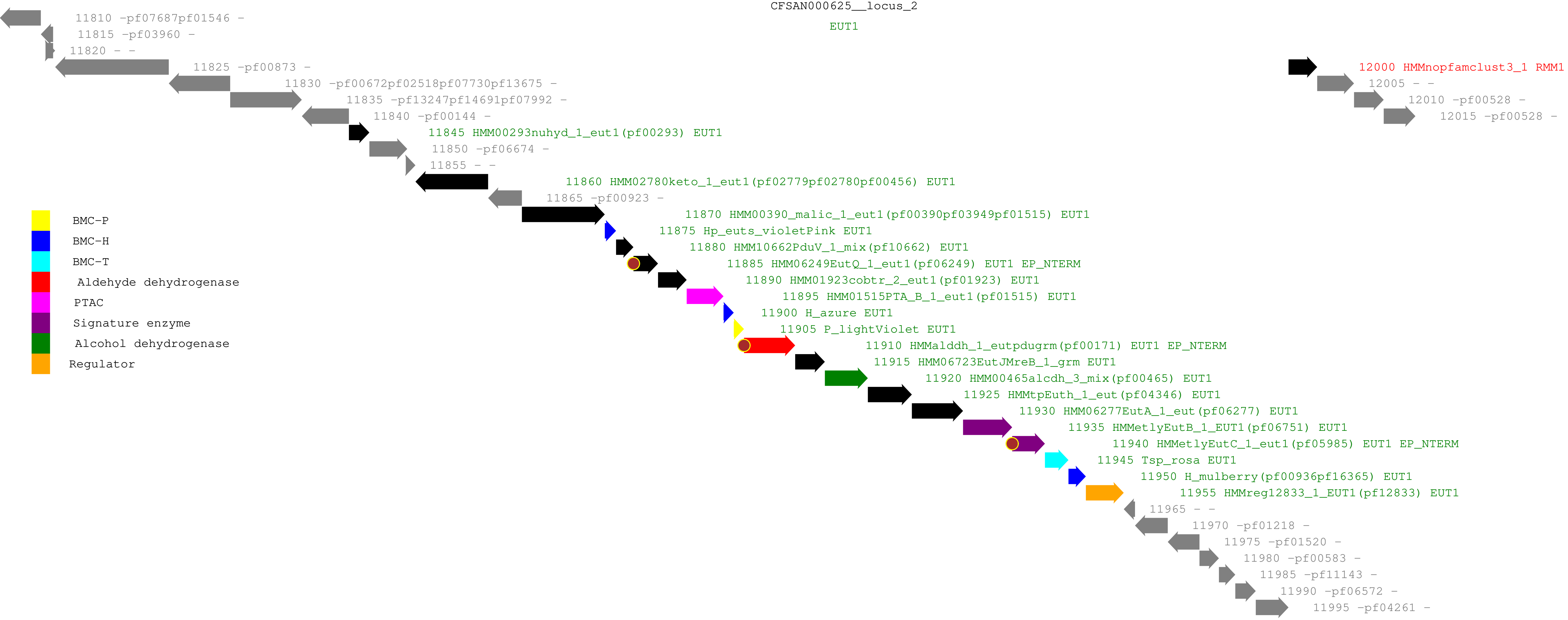

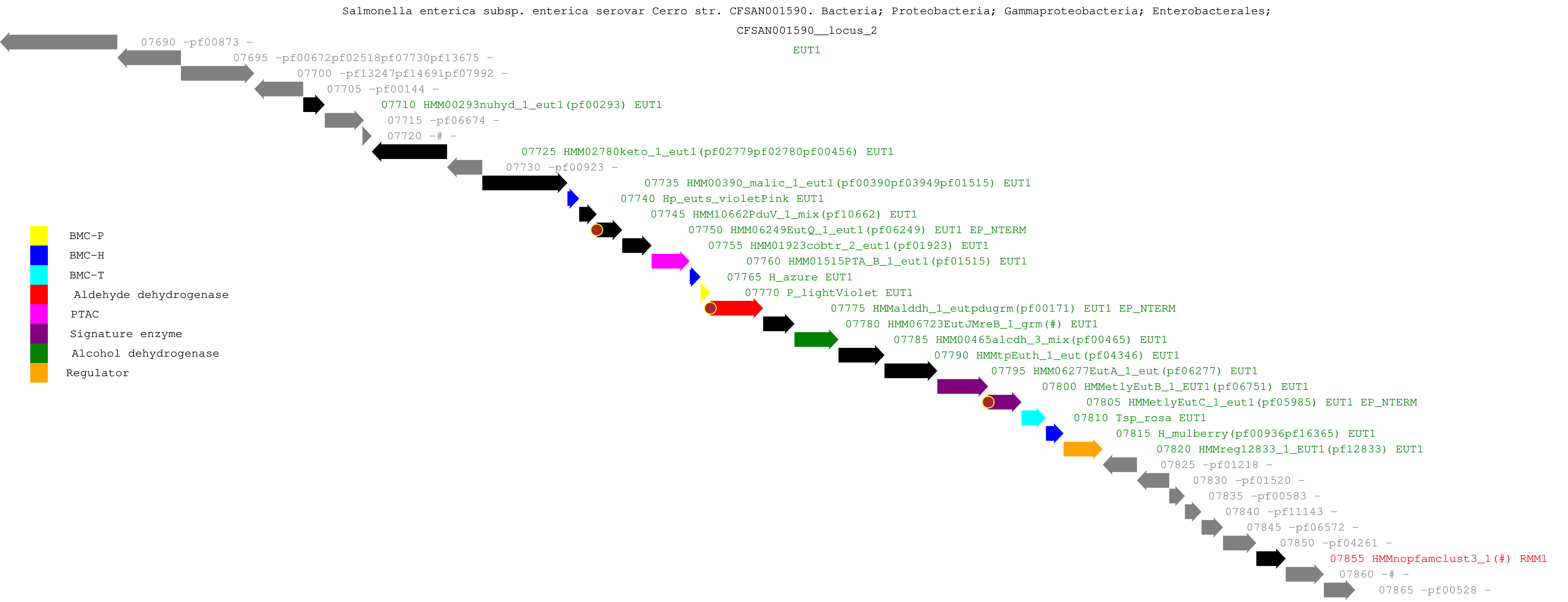
Salmonella enterica subsp. enterica serovar Cerro str. CFSAN001590. Bacteria; Proteobacteria; Gammaproteobacteria; Enterobacterales;
CFSAN001590__locus_2
EUT1
07690 -pf00873 -
07695 -pf00672pf02518pf07730pf13675 -
07700 -pf13247pf14691pf07992 -
07705 -pf00144 -
07710 HMM00293nuhyd_1_eut1(pf00293) EUT1
07715 -pf06674 -
07720 -# -
07725 HMM02780keto_1_eut1(pf02779pf02780pf00456) EUT1
07730 -pf00923 -
07735 HMM00390_malic_1_eut1(pf00390pf03949pf01515) EUT1
07740 Hp_euts_violetPink EUT1
07745 HMM10662PduV_1_mix(pf10662) EUT1
07750 HMM06249EutQ_1_eut1(pf06249) EUT1 EP_NTERM
07755 HMM01923cobtr_2_eut1(pf01923) EUT1
07760 HMM01515PTA_B_1_eut1(pf01515) EUT1
07765 H_azure EUT1
07770 P_lightViolet EUT1
07775 HMMalddh_1_eutpdugrm(pf00171) EUT1 EP_NTERM
07780 HMM06723EutJMreB_1_grm(#) EUT1
07785 HMM00465alcdh_3_mix(pf00465) EUT1
07790 HMMtpEuth_1_eut(pf04346) EUT1
07795 HMM06277EutA_1_eut(pf06277) EUT1
07800 HMMetlyEutB_1_EUT1(pf06751) EUT1
07805 HMMetlyEutC_1_eut1(pf05985) EUT1 EP_NTERM
07810 Tsp_rosa EUT1
07815 H_mulberry(pf00936pf16365) EUT1
07820 HMMreg12833_1_EUT1(pf12833) EUT1
07825 -pf01218 -
07830 -pf01520 -
07835 -pf00583 -
07840 -pf11143 -
07845 -pf06572 -
07850 -pf04261 -
07855 HMMnopfamclust3_1(#) RMM1
07860 -# -
07865 -pf00528 -
BMC-P
BMC-H
BMC-T
Aldehyde dehydrogenase
PTAC
Signature enzyme
Alcohol dehydrogenase
Regulator

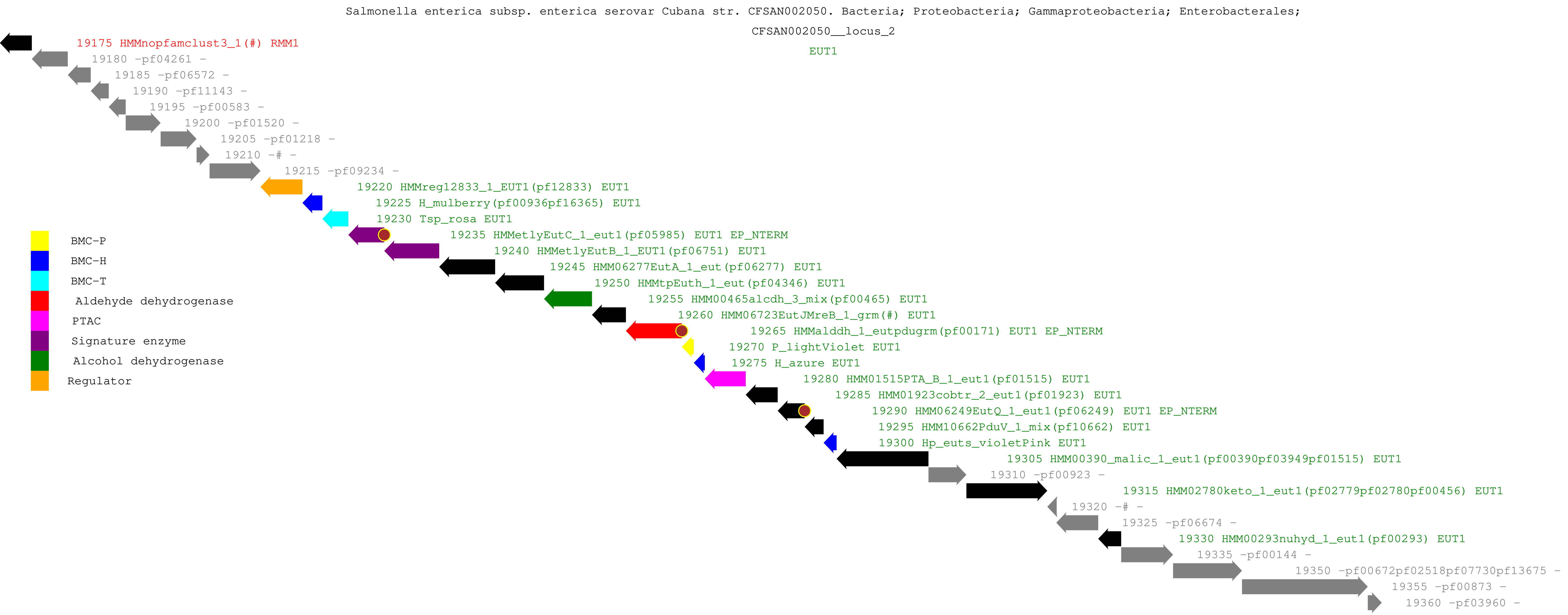

Salmonella enterica subsp. enterica serovar Cubana str. CFSAN002050. Bacteria; Proteobacteria; Gammaproteobacteria; Enterobacterales;
CFSAN002050__locus_2
EUT1
19175 HMMnopfamclust3_1(#) RMM1
19180 -pf04261 -
19185 -pf06572 -
19190 -pf11143 -
19195 -pf00583 -
19200 -pf01520 -
19205 -pf01218 -
19210 -# -
19215 -pf09234 -
19220 HMMreg12833_1_EUT1(pf12833) EUT1
19225 H_mulberry(pf00936pf16365) EUT1
19230 Tsp_rosa EUT1
19235 HMMetlyEutC_1_eut1(pf05985) EUT1 EP_NTERM
19240 HMMetlyEutB_1_EUT1(pf06751) EUT1
19245 HMM06277EutA_1_eut(pf06277) EUT1
19250 HMMtpEuth_1_eut(pf04346) EUT1
19255 HMM00465alcdh_3_mix(pf00465) EUT1
19260 HMM06723EutJMreB_1_grm(#) EUT1
19265 HMMalddh_1_eutpdugrm(pf00171) EUT1 EP_NTERM
19270 P_lightViolet EUT1
19275 H_azure EUT1
19280 HMM01515PTA_B_1_eut1(pf01515) EUT1
19285 HMM01923cobtr_2_eut1(pf01923) EUT1
19290 HMM06249EutQ_1_eut1(pf06249) EUT1 EP_NTERM
19295 HMM10662PduV_1_mix(pf10662) EUT1
19300 Hp_euts_violetPink EUT1
19305 HMM00390_malic_1_eut1(pf00390pf03949pf01515) EUT1
19310 -pf00923 -
19315 HMM02780keto_1_eut1(pf02779pf02780pf00456) EUT1
19320 -# -
19325 -pf06674 -
19330 HMM00293nuhyd_1_eut1(pf00293) EUT1
19335 -pf00144 -
19350 -pf00672pf02518pf07730pf13675 -
19355 -pf00873 -
19360 -pf03960 -
BMC-P
BMC-H
BMC-T
Aldehyde dehydrogenase
PTAC
Signature enzyme
Alcohol dehydrogenase
Regulator

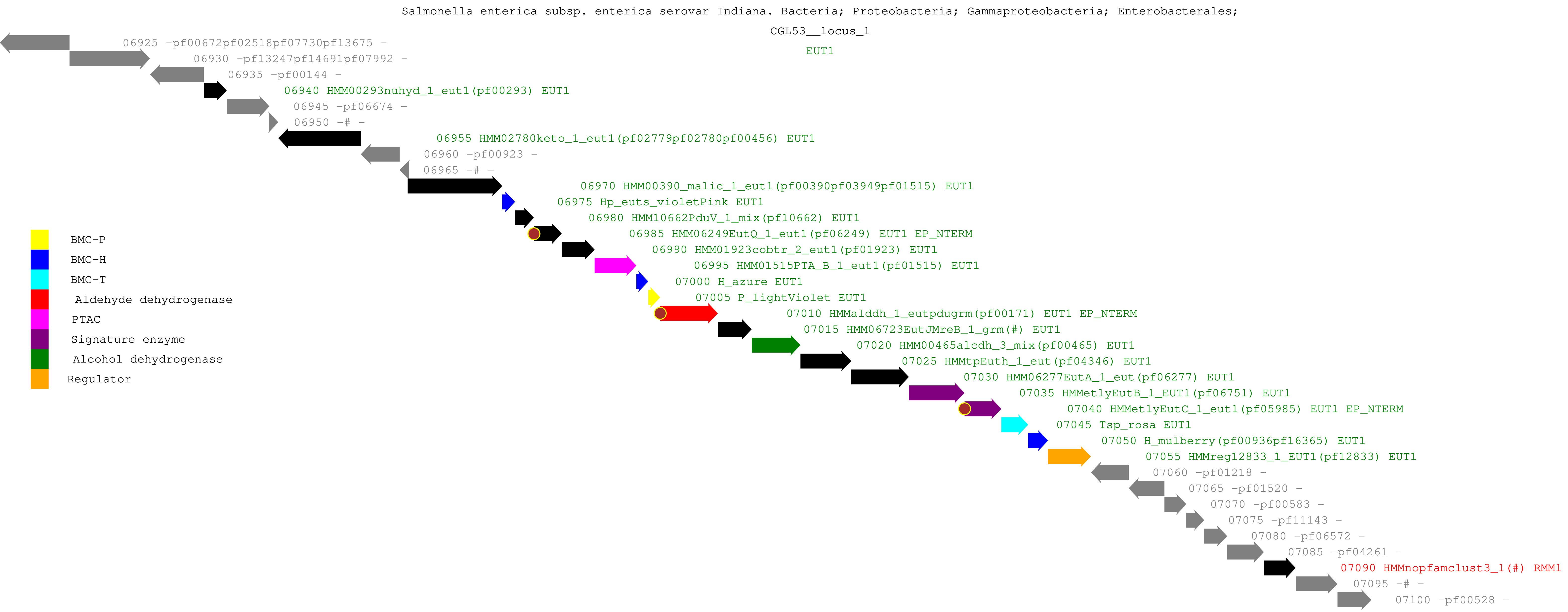
Salmonella enterica subsp. enterica serovar Indiana. Bacteria; Proteobacteria; Gammaproteobacteria; Enterobacterales;
CGL53__locus_1
EUT1
06925 -pf00672pf02518pf07730pf13675 -
06930 -pf13247pf14691pf07992 -
06935 -pf00144 -
06940 HMM00293nuhyd_1_eut1(pf00293) EUT1
06945 -pf06674 -
06950 -# -
06955 HMM02780keto_1_eut1(pf02779pf02780pf00456) EUT1
06960 -pf00923 -
06965 -# -
06970 HMM00390_malic_1_eut1(pf00390pf03949pf01515) EUT1
06975 Hp_euts_violetPink EUT1
06980 HMM10662PduV_1_mix(pf10662) EUT1
06985 HMM06249EutQ_1_eut1(pf06249) EUT1 EP_NTERM
06990 HMM01923cobtr_2_eut1(pf01923) EUT1
06995 HMM01515PTA_B_1_eut1(pf01515) EUT1
07000 H_azure EUT1
07005 P_lightViolet EUT1
07010 HMMaldh_1_eutpdugrm(pf00171) EUT1 EP_NTERM
07015 HMM06723EutJMreB_1_grm(#) EUT1
07020 HMM00465alcdh_3_mix(pf00465) EUT1
07025 HMMtpEuth_1_eut(pf04346) EUT1
07030 HMM06277EutA_1_eut(pf06277) EUT1
07035 HMMetlyEutB_1_EUT1(pf06751) EUT1
07040 HMMetlyEutC_1_eut1(pf05985) EUT1 EP_NTERM
07045 Tsp_rosa EUT1
07050 H_mulberry(pf00936pf16365) EUT1
07055 HMMreg12833_1_EUT1(pf12833) EUT1
07060 -pf01218 -
07065 -pf01520 -
07070 -pf00583 -
07075 -pf11143 -
07080 -pf06572 -
07085 -pf04261 -
07090 HMMnopfamclust3_1(#) RMM1
07095 -# -
07100 -pf00528 -
BMC-P
BMC-H
BMC-T
Aldehyde dehydrogenase
PTAC
Signature enzyme
Alcohol dehydrogenase
Regulator

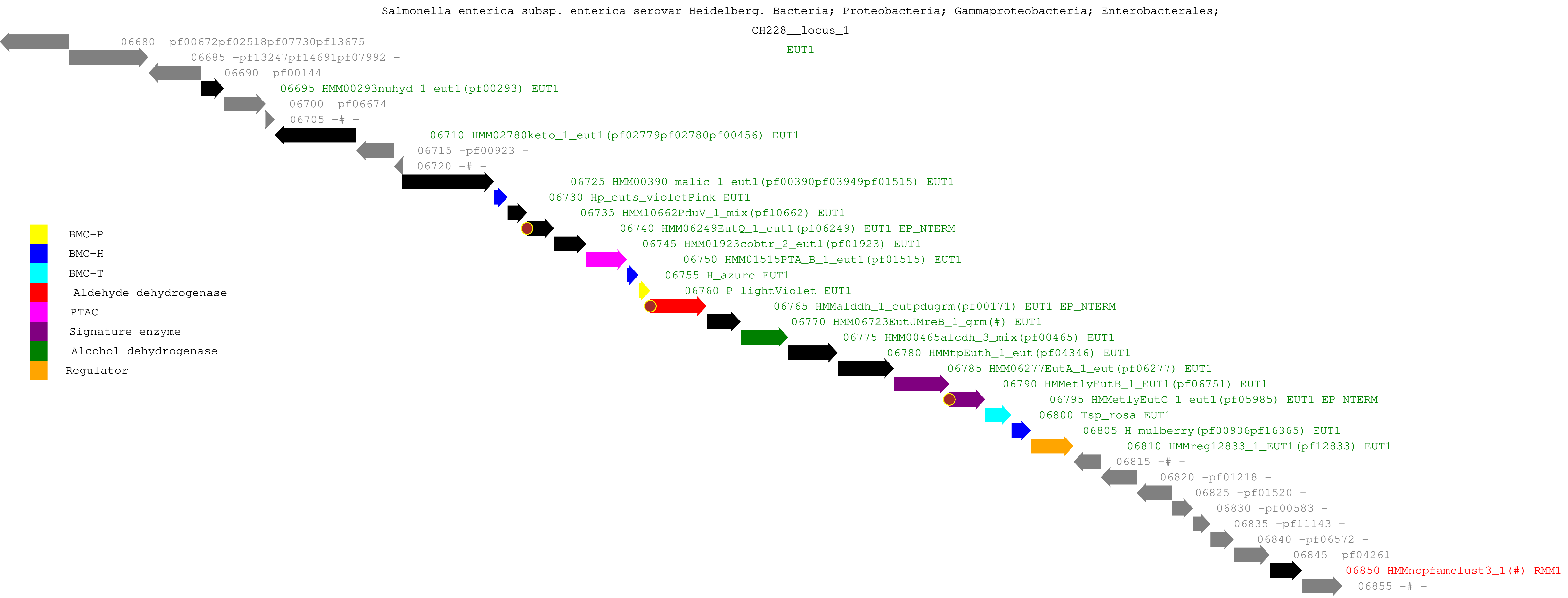

Salmonella enterica subsp. enterica serovar Heidelberg. Bacteria; Proteobacteria; Gammaproteobacteria; Enterobacterales;
CH228__locus_1
EUT1
06680 -pf00672pf02518pf07730pf13675 -
06685 -pf13247pf14691pf07992 -
06690 -pf00144 -
06695 HMM00293nuhyd_1_eut1(pf00293) EUT1
06700 -pf06674 -
06705 -# -
06710 HMM02780keto_1_eut1(pf02779pf02780pf00456) EUT1
06715 -pf00923 -
06720 -# -
06725 HMM00390_malic_1_eut1(pf00390pf03949pf01515) EUT1
06730 Hp_euts_violetPink EUT1
06735 HMM10662PduV_1_mix(pf10662) EUT1
06740 HMM06249EutQ_1_eut1(pf06249) EUT1 EP_NTERM
06745 HMM01923cobtr_2_eut1(pf01923) EUT1
06750 HMM01515PTA_B_1_eut1(pf01515) EUT1
06755 H_azure EUT1
06760 P_lightViolet EUT1
06765 HMMalddh_1_eutpdugrm(pf00171) EUT1 EP_NTERM
06770 HMM06723EutJMreB_1_grm(#) EUT1
06775 HMM00465alcdh_3_mix(pf00465) EUT1
06780 HMMtpEuth_1_eut(pf04346) EUT1
06785 HMM06277EutA_1_eut(pf06277) EUT1
06790 HMMetlyEutB_1_EUT1(pf06751) EUT1
06795 HMMetlyEutC_1_eut1(pf05985) EUT1 EP_NTERM
06800 Tsp_rosa EUT1
06805 H_mulberry(pf00936pf16365) EUT1
06810 HMMreg12833_1_EUT1(pf12833) EUT1
06815 -# -
06820 -pf01218 -
06825 -pf01520 -
06830 -pf00583 -
06835 -pf11143 -
06840 -pf06572 -
06845 -pf04261 -
06850 HMMnopfamclust3_1(#) RMM1
06855 -# -
BMC-P
BMC-H
BMC-T
Aldehyde dehydrogenase
PTAC
Signature enzyme
Alcohol dehydrogenase
Regulator

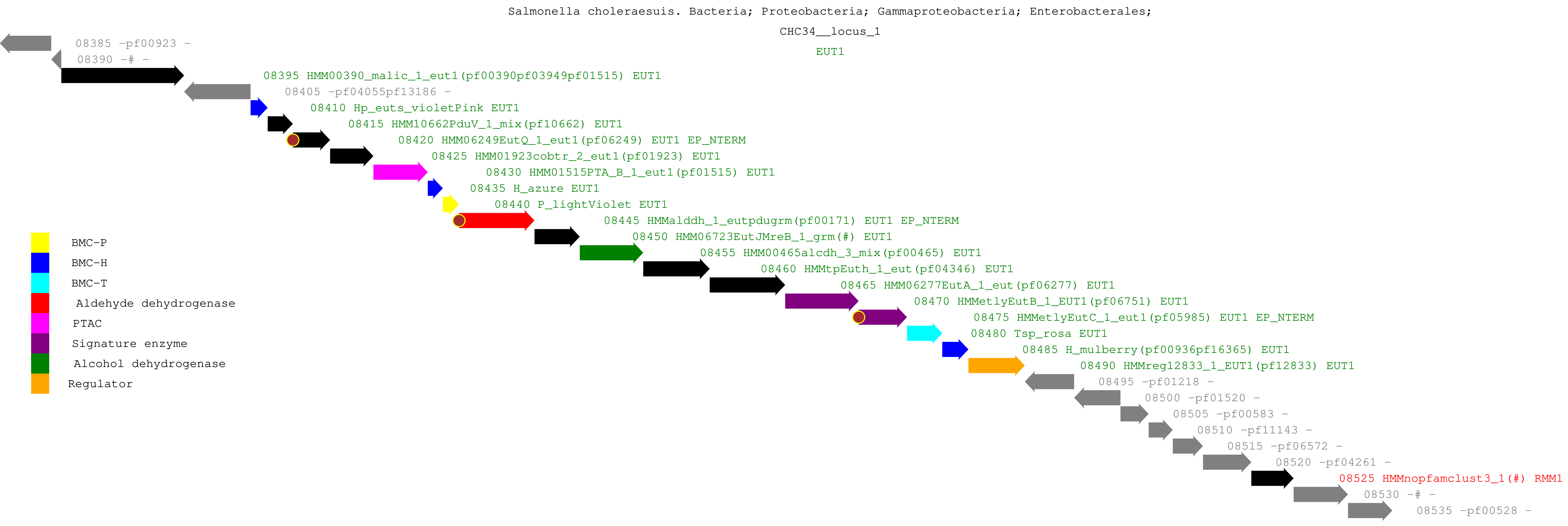

Salmonella choleraesuis. Bacteria; Proteobacteria; Gammaproteobacteria; Enterobacterales;
CHC34__locus_1
EUT1
08385 -pf00923 -
08390 -# -
08395 HMM00390_malic_1_eut1(pf00390pf03949pf01515) EUT1
08405 -pf04055pf13186 -
08410 Hp_euts_violetPink EUT1
08415 HMM10662PduV_1_mix(pf10662) EUT1
08420 HMM06249EutQ_1_eut1(pf06249) EUT1 EP_NTERM
08425 HMM01923cobtr_2_eut1(pf01923) EUT1
08430 HMM01515PTA_B_1_eut1(pf01515) EUT1
08435 H_azure EUT1
08440 P_lightViolet EUT1
08445 HMMalddh_1_eutpdugrm(pf00171) EUT1 EP_NTERM
08450 HMM06723EutJMreB_1_grm(#) EUT1
08455 HMM00465alcdh_3_mix(pf00465) EUT1
08460 HMMtpEuth_1_eut(pf04346) EUT1
08465 HMM06277EutA_1_eut(pf06277) EUT1
08470 HMMetlyEutB_1_EUT1(pf06751) EUT1
08475 HMMetlyEutC_1_eut1(pf05985) EUT1 EP_NTERM
08480 Tsp_rosa EUT1
08485 H_mulberry(pf00936pf16365) EUT1
08490 HMMreg12833_1_EUT1(pf12833) EUT1
08495 -pf01218 -
08500 -pf01520 -
08505 -pf00583 -
08510 -pf11143 -
08515 -pf06572 -
08520 -pf04261 -
08525 HMMnopfamclust3_1(#) RMM1
08530 -# -
08535 -pf00528 -
BMC-P
BMC-H
BMC-T
Aldehyde dehydrogenase
PTAC
Signature enzyme
Alcohol dehydrogenase
Regulator

Salmonella choleraesuis. Bacteria; Proteobacteria; Gammaproteobacteria; Enterobacterales;

CHD23__locus_1

EUT1

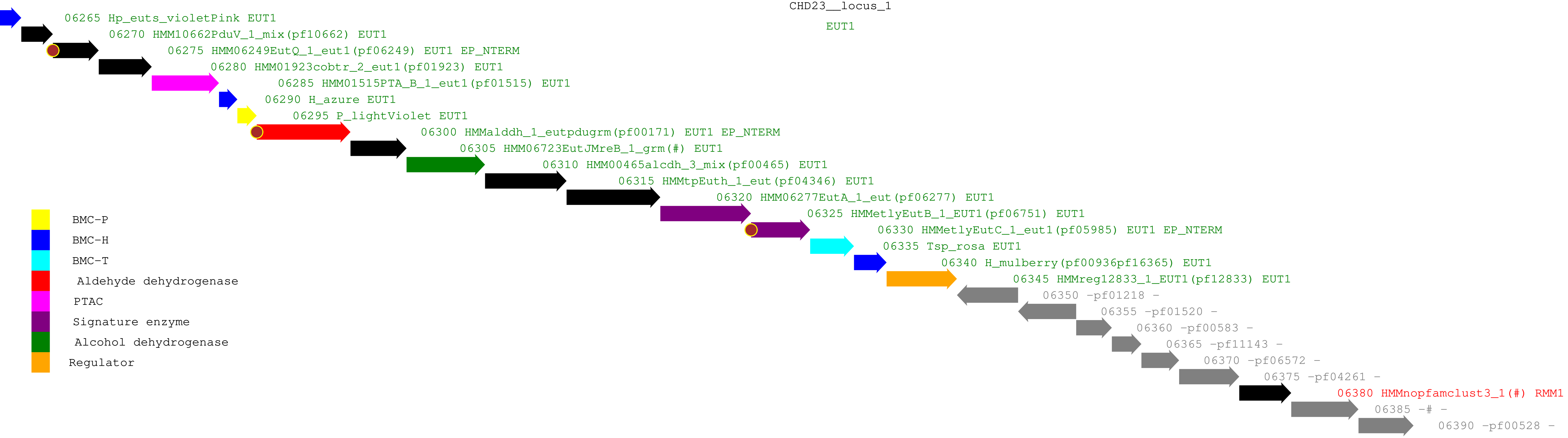

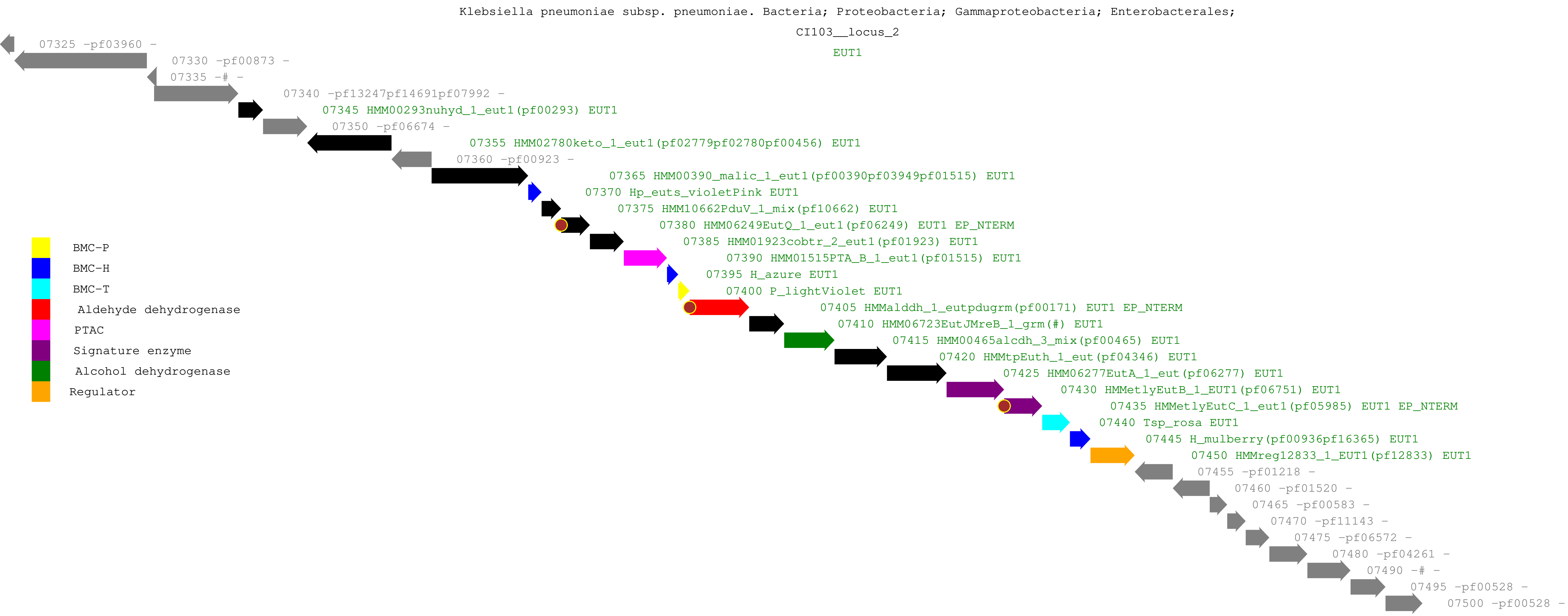

Klebsiella pneumoniae subsp. pneumoniae. Bacteria; Proteobacteria; Gammaproteobacteria; Enterobacterales;
CI103__locus_2
EUT1
07325 -pf03960 -
07330 -pf00873 -
07335 -# -
07340 -pf13247pf14691pf07992 -
07345 HMM00293nuhyd_1_eut1(pf00293) EUT1
07350 -pf06674 -
07355 HMM02780keto_1_eut1(pf02779pf02780pf00456) EUT1
07360 -pf00923 -
07365 HMM00390_malic_1_eut1(pf00390pf03949pf01515) EUT1
07370 Hp_euts_violetPink EUT1
07375 HMM10662PduV_1_mix(pf10662) EUT1
07380 HMM06249EutQ_1_eut1(pf06249) EUT1 EP_NTERM
07385 HMM01923cobtr_2_eut1(pf01923) EUT1
07390 HMM01515PTA_B_1_eut1(pf01515) EUT1
07395 H_azure EUT1
07400 P_lightViolet EUT1
07405 HMMalddh_1_eutpdugrm(pf00171) EUT1 EP_NTERM
07410 HMM06723EutJMreB_1_grm(#) EUT1
07415 HMM00465alcdh_3_mix(pf00465) EUT1
07420 HMMtpEuth_1_eut(pf04346) EUT1
07425 HMM06277EutA_1_eut(pf06277) EUT1
07430 HMMetlyEutB_1_EUT1(pf06751) EUT1
07435 HMMetlyEutC_1_eut1(pf05985) EUT1 EP_NTERM
07440 Tsp_rosa EUT1
07445 H_mulberry(pf00936pf16365) EUT1
07450 HMMreg12833_1_EUT1(pf12833) EUT1
07455 -pf01218 -
07460 -pf01520 -
07465 -pf00583 -
07470 -pf11143 -
07475 -pf06572 -
07480 -pf04261 -
07490 -# -
07495 -pf00528 -
07500 -pf00528 -
BMC-P
BMC-H
BMC-T
Aldehyde dehydrogenase
PTAC
Signature enzyme
Alcohol dehydrogenase
Regulator

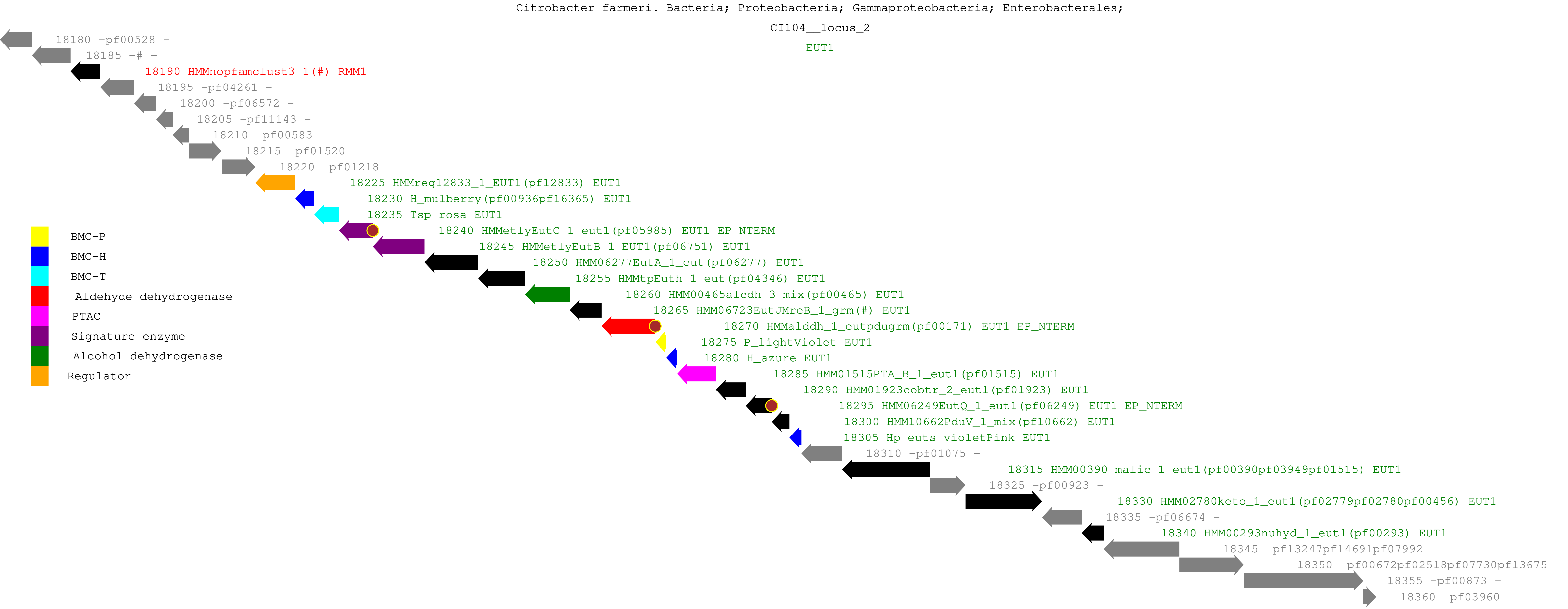
Citrobacter farmeri. Bacteria; Proteobacteria; Gammaproteobacteria; Enterobacterales;
CI104__locus_2
EUT1
18180 -pf00528 -
18185 -# -
18190 HMMnopfamclust3_1(#) RMM1
18195 -pf04261 -
18200 -pf06572 -
18205 -pf11143 -
18210 -pf00583 -
18215 -pf01520 -
18220 -pf01218 -
18225 HMMreg12833_1_EUT1(pf12833) EUT1
18230 H_mulberry(pf00936pf16365) EUT1
18235 Tsp_rosa EUT1
18240 HMMetlyEutC_1_eut1(pf05985) EUT1 EP_NTERM
18245 HMMetlyEutB_1_EUT1(pf06751) EUT1
18250 HMM06277EutA_1_eut(pf06277) EUT1
18255 HMMtpEuth_1_eut(pf04346) EUT1
18260 HMM00465alcdh_3_mix(pf00465) EUT1
18265 HMM06723EutJMreB_1_grm(#) EUT1
18270 HMMalddh_1_eutpdugrm(pf00171) EUT1 EP_NTERM
18275 P_lightViolet EUT1
18280 H_azure EUT1
18285 HMM01515PTA_B_1_eut1(pf01515) EUT1
18290 HMM01923cobtr_2_eut1(pf01923) EUT1
18295 HMM06249EutQ_1_eut1(pf06249) EUT1 EP_NTERM
18300 HMM10662PduV_1_mix(pf10662) EUT1
18305 Hp_euts_violetPink EUT1
18310 -pf01075 -
18315 HMM00390_malic_1_eut1(pf00390pf03949pf01515) EUT1
18325 -pf00923 -
18330 HMM02780keto_1_eut1(pf02779pf02780pf00456) EUT1
18335 -pf06674 -
18340 HMM00293nuhyd_1_eut1(pf00293) EUT1
18345 -pf13247pf14691pf07992 -
18350 -pf00672pf02518pf07730pf13675 -
18355 -pf00873 -
18360 -pf03960 -
BMC-P
BMC-H
BMC-T
Aldehyde dehydrogenase
PTAC
Signature enzyme
Alcohol dehydrogenase
Regulator

Shigella sonnei. Bacteria; Proteobacteria; Gammaproteobacteria; Enterobacterales;

CI632__locus_2

EUT1

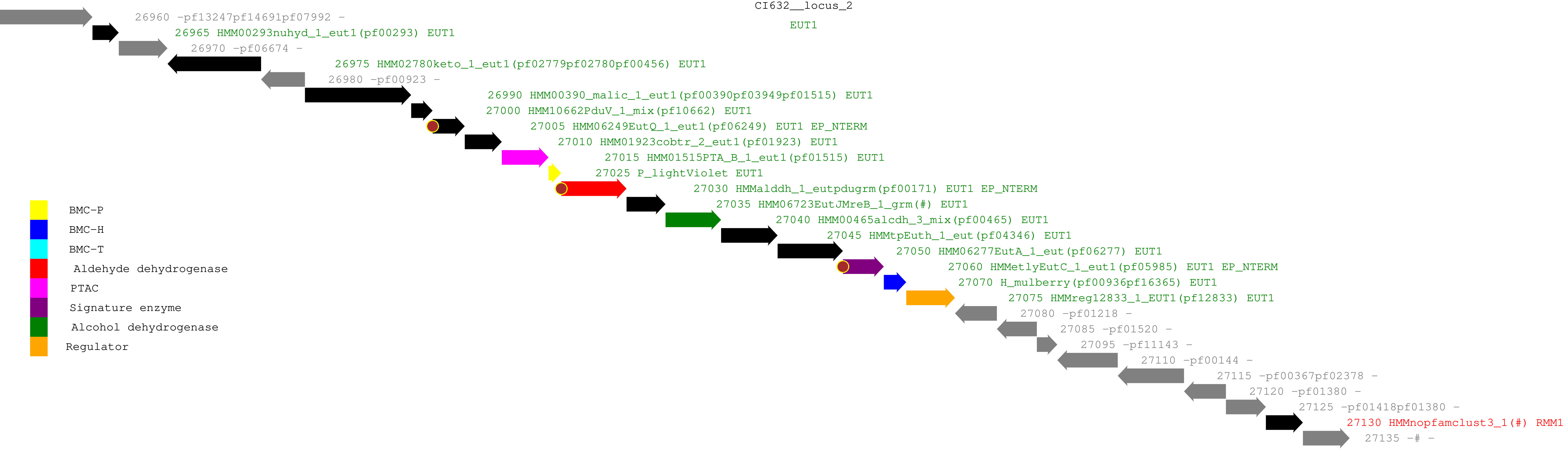

Shigella sonnei. Bacteria; Proteobacteria; Gammaproteobacteria; Enterobacterales;

CI641__locus_2

EUT1

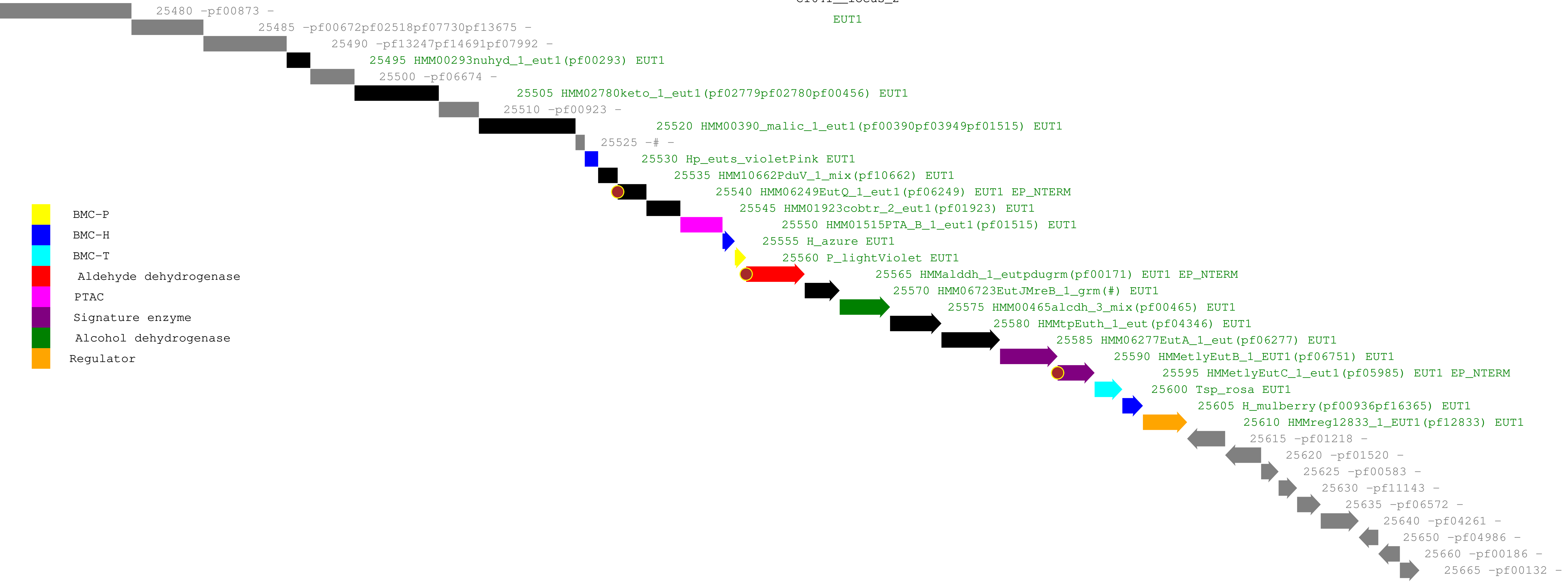

Klebsiella pneumoniae subsp. pneumoniae. Bacteria; Proteobacteria; Gammaproteobacteria; Enterobacterales;

CI682__locus_1

EUT1

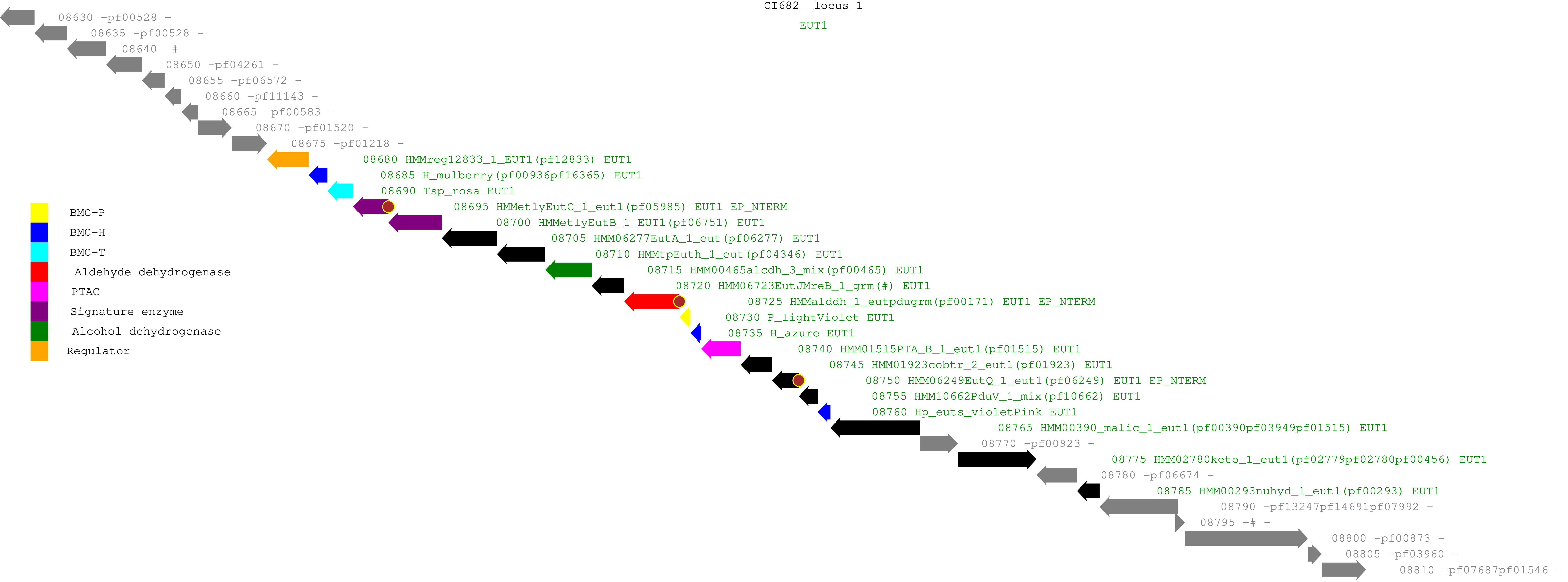

Klebsiella pneumoniae subsp. pneumoniae. Bacteria; Proteobacteria; Gammaproteobacteria; Enterobacterales;

CI699__locus_1

EUT1

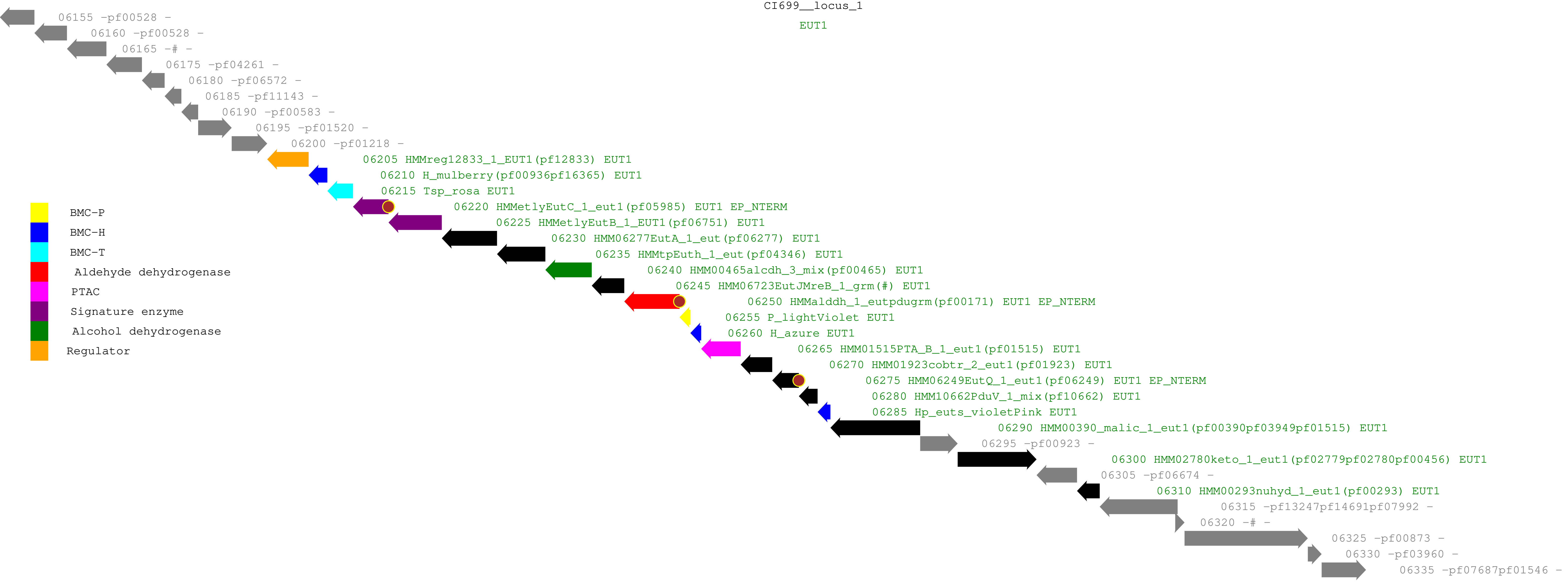

Shigella boydii. Bacteria; Proteobacteria; Gammaproteobacteria; Enterobacterales;

CI719__locus_1

EUT1

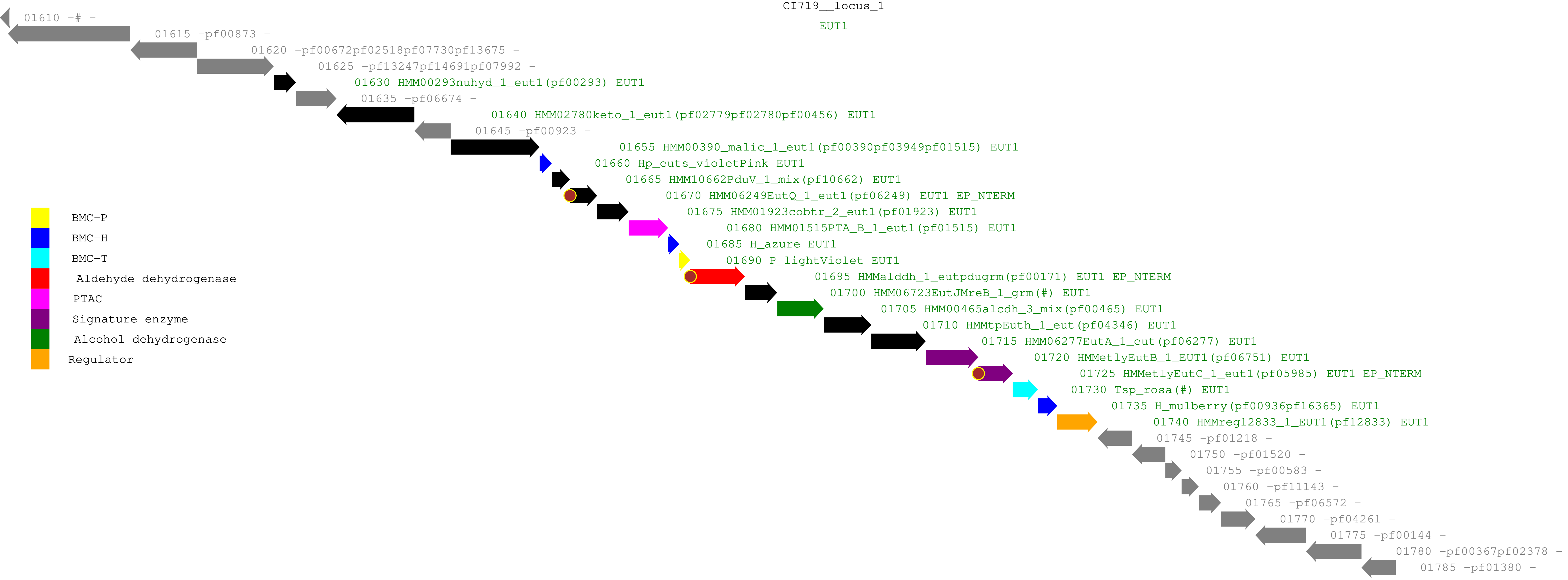

Shigella boydii. Bacteria; Proteobacteria; Gammaproteobacteria; Enterobacterales;

CI736__locus_1

EUT1

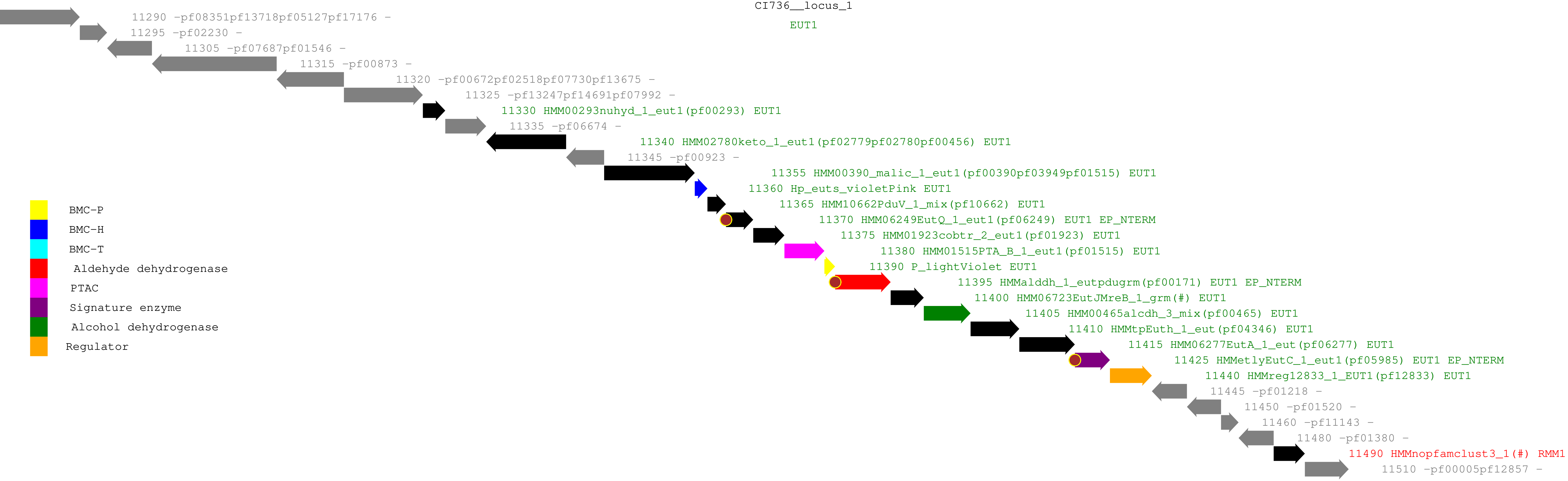

Klebsiella pneumoniae subsp. pneumoniae. Bacteria; Proteobacteria; Gammaproteobacteria; Enterobacterales;

CI751__locus_2

EUT1

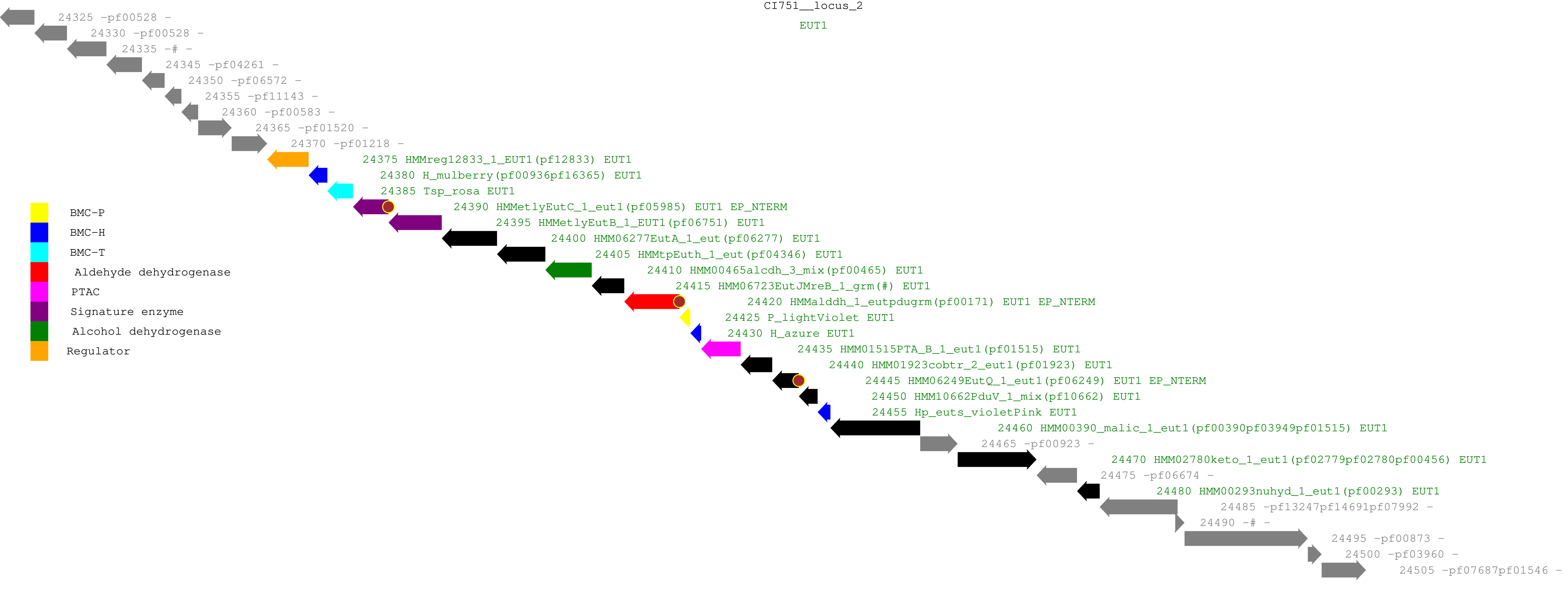

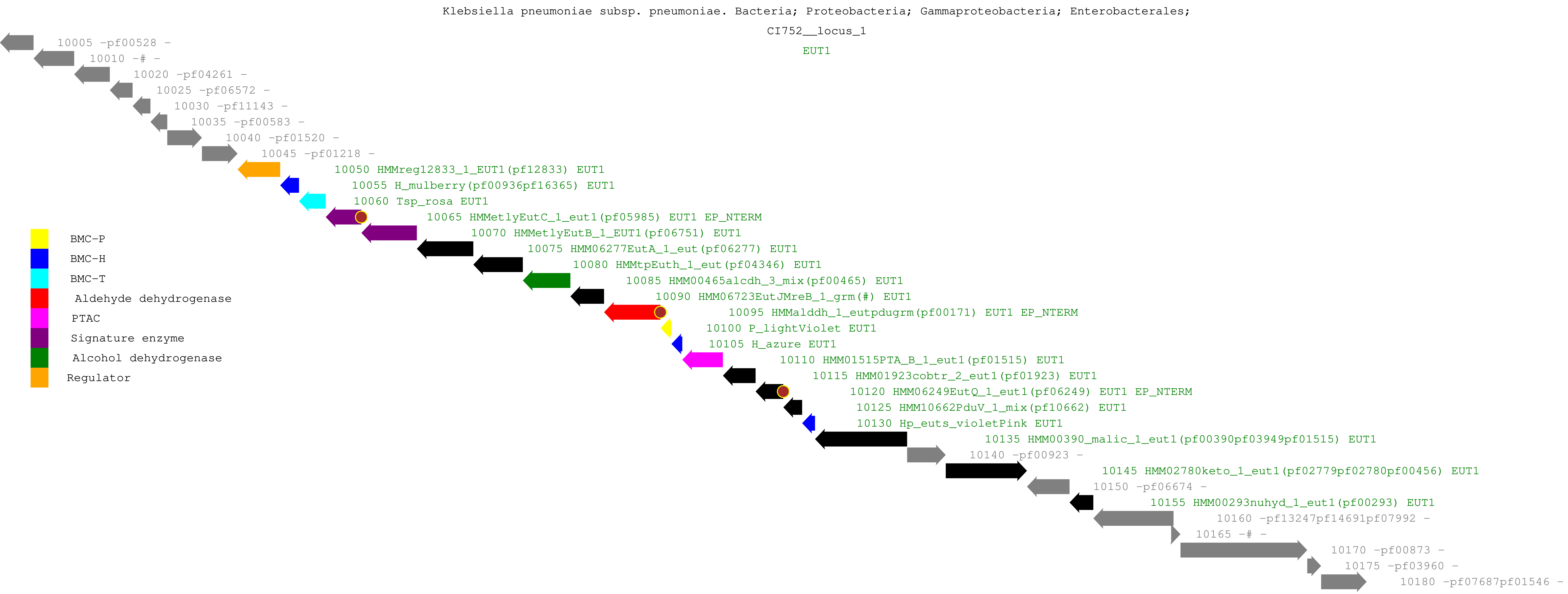

Klebsiella pneumoniae subsp. pneumoniae. Bacteria; Proteobacteria; Gammaproteobacteria; Enterobacterales;
CI752__locus_1
EUT1
10005 -pf00528 -
10010 -# -
10020 -pf04261 -
10025 -pf06572 -
10030 -pf11143 -
10035 -pf00583 -
10040 -pf01520 -
10045 -pf01218 -
10050 HMMreg12833_1_EUT1(pf12833) EUT1
10055 H_mulberry(pf00936pf16365) EUT1
10060 Tsp_rosa EUT1
10065 HMMetlyEutC_1_eut1(pf05985) EUT1 EP_NTERM
10070 HMMetlyEutB_1_EUT1(pf06751) EUT1
10075 HMM06277EutA_1_eut(pf06277) EUT1
10080 HMMtpEuth_1_eut(pf04346) EUT1
10085 HMM00465alcdh_3_mix(pf00465) EUT1
10090 HMM06723EutJMreB_1_grm(#) EUT1
10095 HMMalddh_1_eutpdugrm(pf00171) EUT1 EP_NTERM
10100 P_lightViolet EUT1
10105 H_azure EUT1
10110 HMM01515PTA_B_1_eut1(pf01515) EUT1
10115 HMM01923cobtr_2_eut1(pf01923) EUT1
10120 HMM06249EutQ_1_eut1(pf06249) EUT1 EP_NTERM
10125 HMM10662PduV_1_mix(pf10662) EUT1
10130 Hp_euts_violetPink EUT1
10135 HMM00390_malic_1_eut1(pf00390pf03949pf01515) EUT1
10140 -pf00923 -
10145 HMM02780keto_1_eut1(pf02779pf02780pf00456) EUT1
10150 -pf06674 -
10155 HMM00293nuhyd_1_eut1(pf00293) EUT1
10160 -pf13247pf14691pf07992 -
10165 -# -
10170 -pf00873 -
10175 -pf03960 -
10180 -pf07687pf01546 -
BMC-P
BMC-H
BMC-T
Aldehyde dehydrogenase
PTAC
Signature enzyme
Alcohol dehydrogenase
Regulator

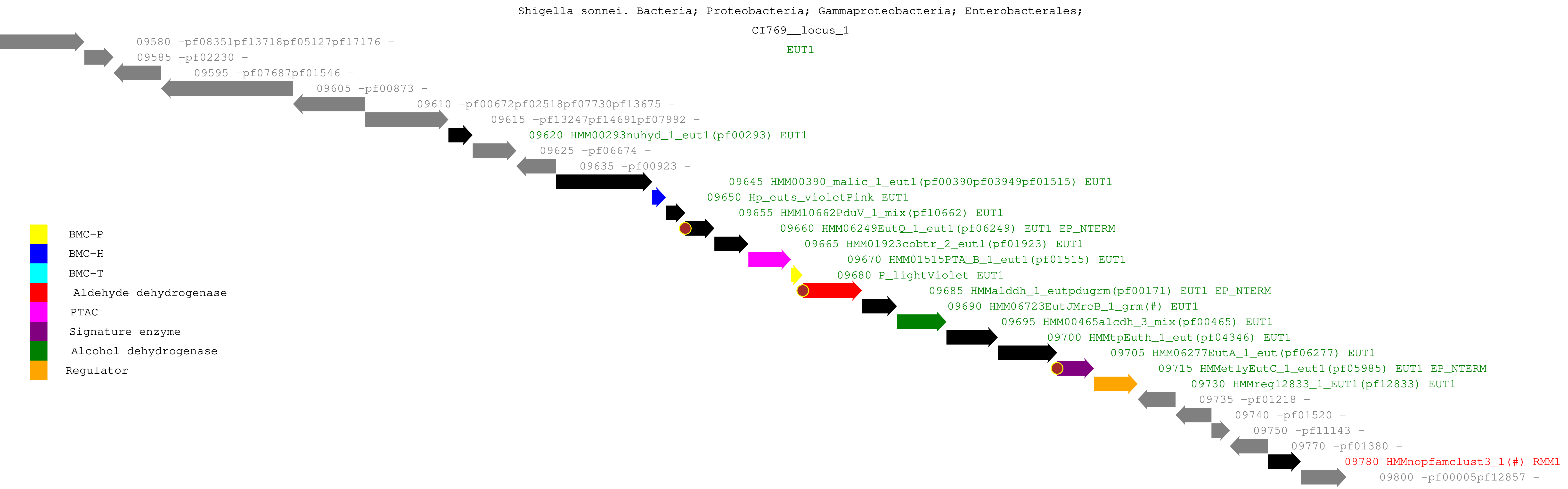

Shigella sonnei. Bacteria; Proteobacteria; Gammaproteobacteria; Enterobacterales;
CI769__locus_1
EUT1
09580 -pf08351pf13718pf05127pf17176 -
09585 -pf02230 -
09595 -pf07687pf01546 -
09605 -pf00873 -
09610 -pf00672pf02518pf07730pf13675 -
09615 -pf13247pf14691pf07992 -
09620 HMM00293nuhyd_1_eut1(pf00293) EUT1
09625 -pf06674 -
09635 -pf00923 -
09645 HMM00390_malic_1_eut1(pf00390pf03949pf01515) EUT1
09650 Hp_euts_violetPink EUT1
09655 HMM10662PduV_1_mix(pf10662) EUT1
09660 HMM06249EutQ_1_eut1(pf06249) EUT1 EP_NTERM
09665 HMM01923cobtr_2_eut1(pf01923) EUT1
09670 HMM01515PTA_B_1_eut1(pf01515) EUT1
09680 P_lightViolet EUT1
09685 HMMalddh_1_eutpdugrm(pf00171) EUT1 EP_NTERM
09690 HMM06723EutJMreB_1_grm(#) EUT1
09695 HMM00465alcdh_3_mix(pf00465) EUT1
09700 HMMtpEuth_1_eut(pf04346) EUT1
09705 HMM06277EutA_1_eut(pf06277) EUT1
09715 HMMetlyEutC_1_eut1(pf05985) EUT1 EP_NTERM
09730 HMMreg12833_1_EUT1(pf12833) EUT1
09735 -pf01218 -
09740 -pf01520 -
09750 -pf11143 -
09770 -pf01380 -
09780 HMMnopfamclust3_1(#) RMM1
09800 -pf00005pf12857 -
BMC-P
BMC-H
BMC-T
Aldehyde dehydrogenase
PTAC
Signature enzyme
Alcohol dehydrogenase
Regulator

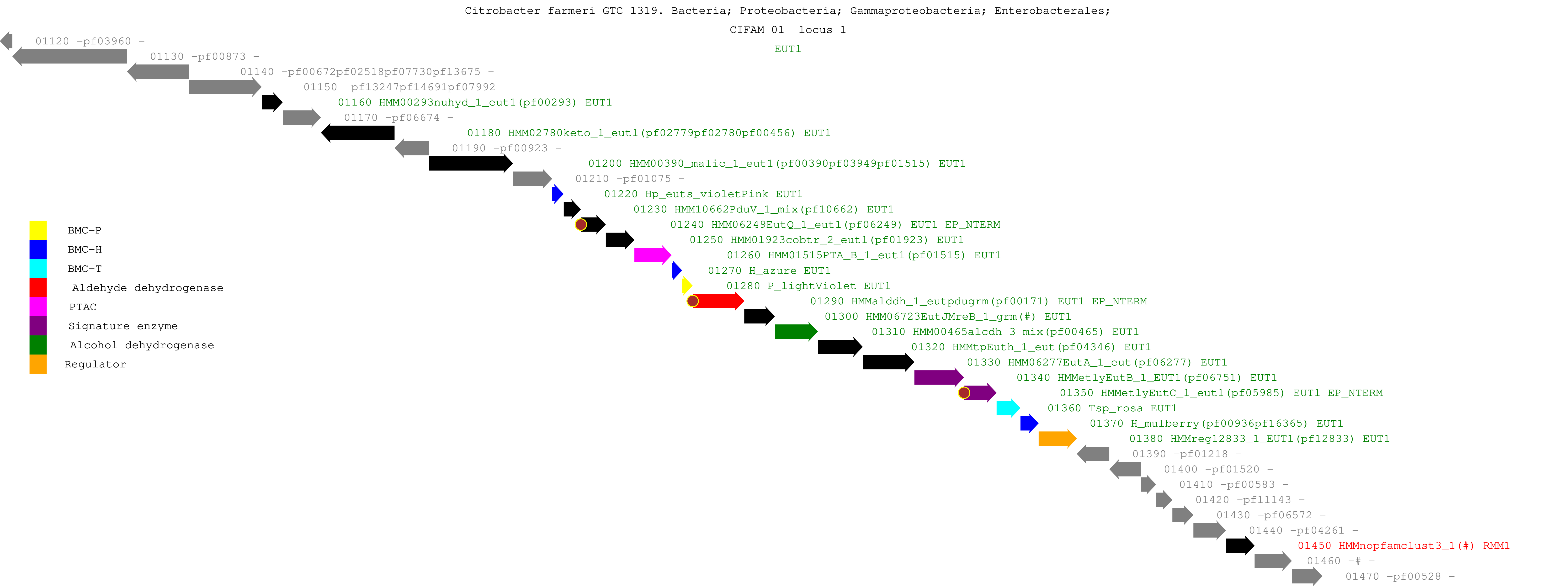

Citrobacter farmeri GTC 1319. Bacteria; Proteobacteria; Gammaproteobacteria; Enterobacterales;
CIFAM_01__locus_1
EUT1
01120 -pf03960 -
01130 -pf00873 -
01140 -pf00672pf02518pf07730pf13675 -
01150 -pf13247pf14691pf07992 -
01160 HMM00293nuhyd_1_eut1(pf00293) EUT1
01170 -pf06674 -
01180 HMM02780keto_1_eut1(pf02779pf02780pf00456) EUT1
01190 -pf00923 -
01200 HMM00390_malic_1_eut1(pf00390pf03949pf01515) EUT1
01210 -pf01075 -
01220 Hp_euts_violetPink EUT1
01230 HMM10662PduV_1_mix(pf10662) EUT1
01240 HMM06249EutQ_1_eut1(pf06249) EUT1 EP_NTERM
01250 HMM01923cobtr_2_eut1(pf01923) EUT1
01260 HMM01515PTA_B_1_eut1(pf01515) EUT1
01270 H_azure EUT1
01280 P_lightViolet EUT1
01290 HMMaldh_1_eutpdugrm(pf00171) EUT1 EP_NTERM
01300 HMM06723EutJMreB_1_grm(#) EUT1
01310 HMM00465alcdh_3_mix(pf00465) EUT1
01320 HMMtpEuth_1_eut(pf04346) EUT1
01330 HMM06277EutA_1_eut(pf06277) EUT1
01340 HMMetlyEutB_1_EUT1(pf06751) EUT1
01350 HMMetlyEutC_1_eut1(pf05985) EUT1 EP_NTERM
01360 Tsp_rosa EUT1
01370 H_mulberry(pf00936pf16365) EUT1
01380 HMMreg12833_1_EUT1(pf12833) EUT1
01390 -pf01218 -
01400 -pf01520 -
01410 -pf00583 -
01420 -pf11143 -
01430 -pf06572 -
01440 -pf04261 -
01450 HMMnopfamclust3_1(#) RMM1
01460 -# -
01470 -pf00528 -
BMC-P
BMC-H
BMC-T
Aldehyde dehydrogenase
PTAC
Signature enzyme
Alcohol dehydrogenase
Regulator

Citrobacter youngae ATCC 29220. Bacteria; Proteobacteria; Gammaproteobacteria; Enterobacterales;

CIT292__locus_1

EUT1

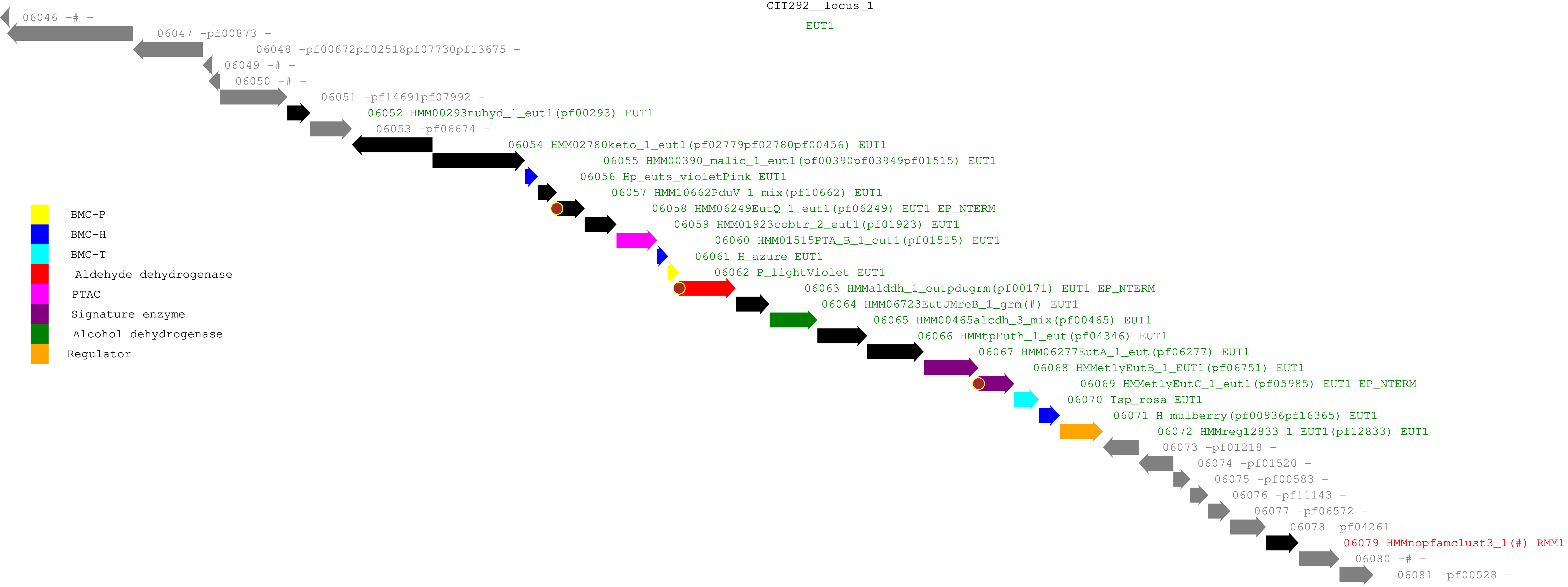

Citrobacter sp. 86. Bacteria; Proteobacteria; Gammaproteobacteria; Enterobacterales;

CITRO86__locus_1

EUT1

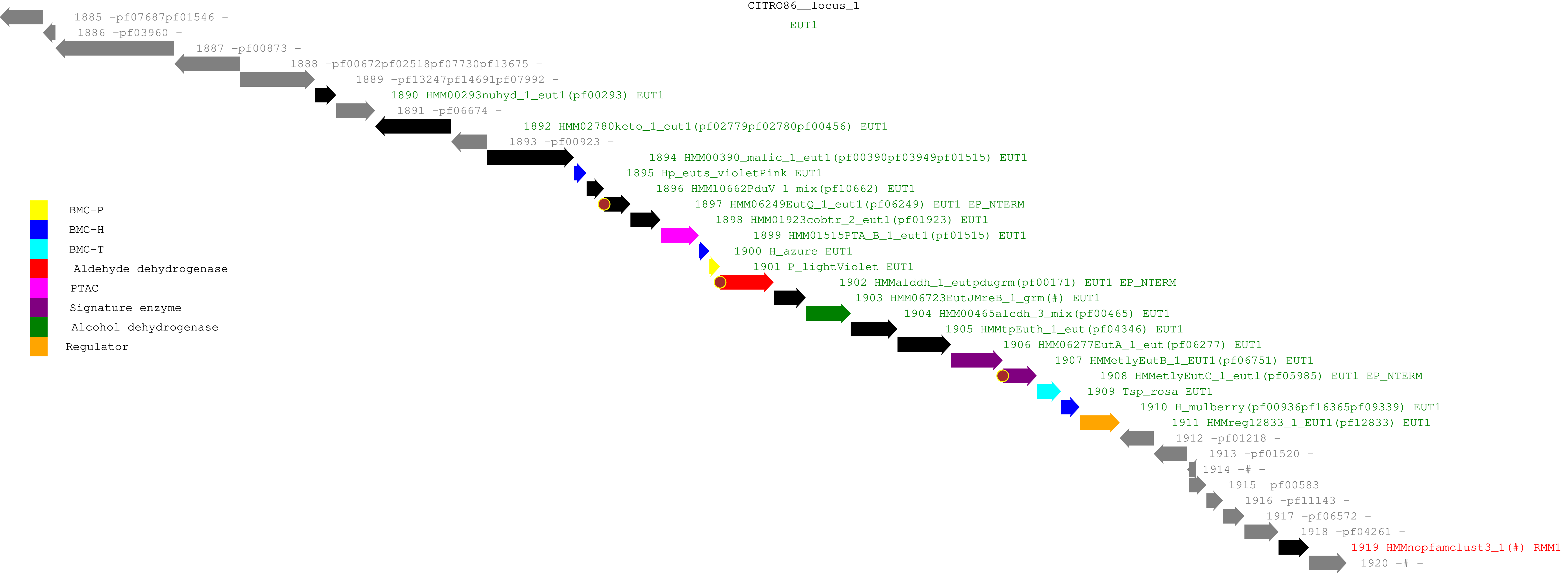

Citrobacter sp. 92. Bacteria; Proteobacteria; Gammaproteobacteria; Enterobacterales;

CITRO92__locus_2

EUT1

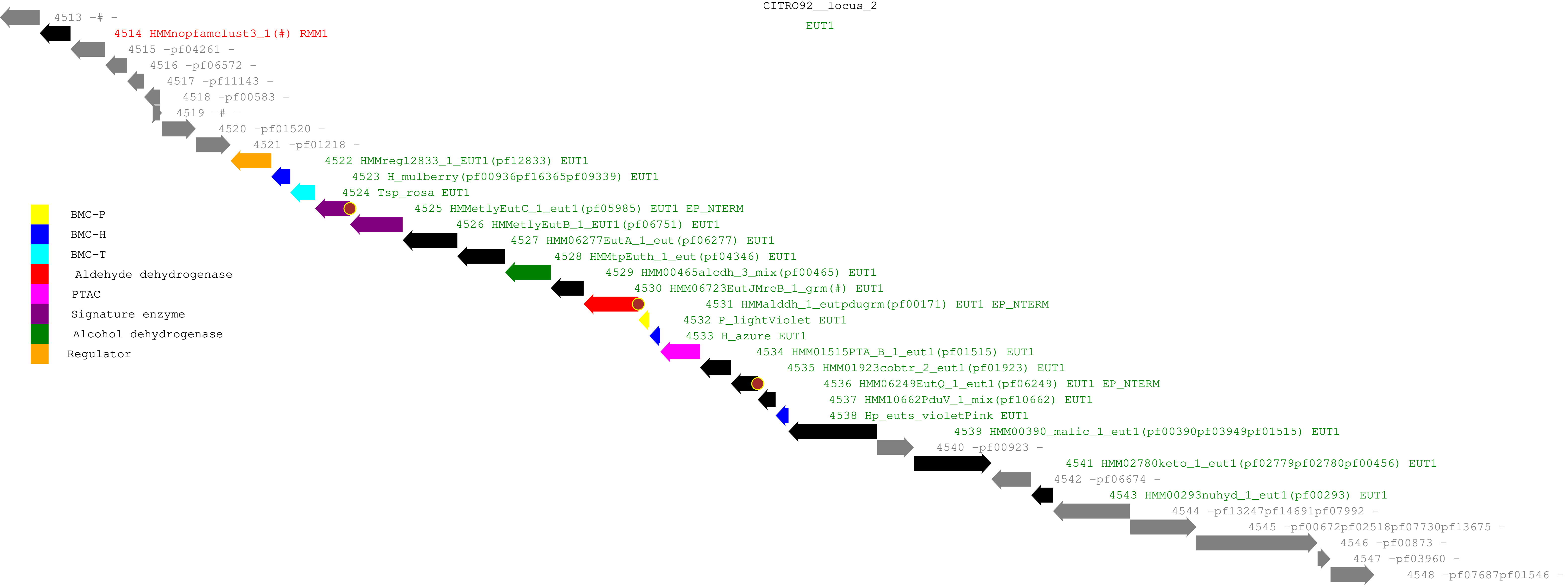

Citrobacter werkmanii NBRC 105721. Bacteria; Proteobacteria; Gammaproteobacteria; Enterobacterales;

CIWKM_02__locus_1

EUT1

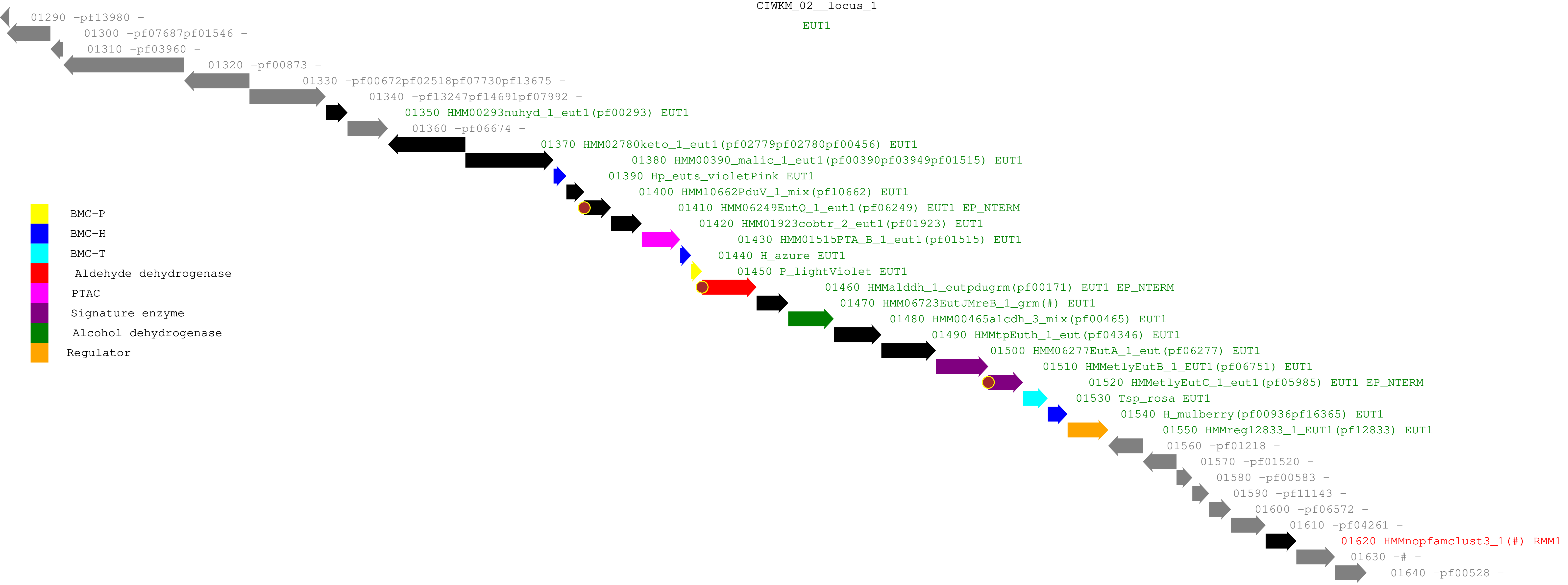

Salmonella enterica subsp. enterica serovar Schwarzengrund. Bacteria; Proteobacteria; Gammaproteobacteria; Enterobacterales;

CJS39__locus_2

EUT1

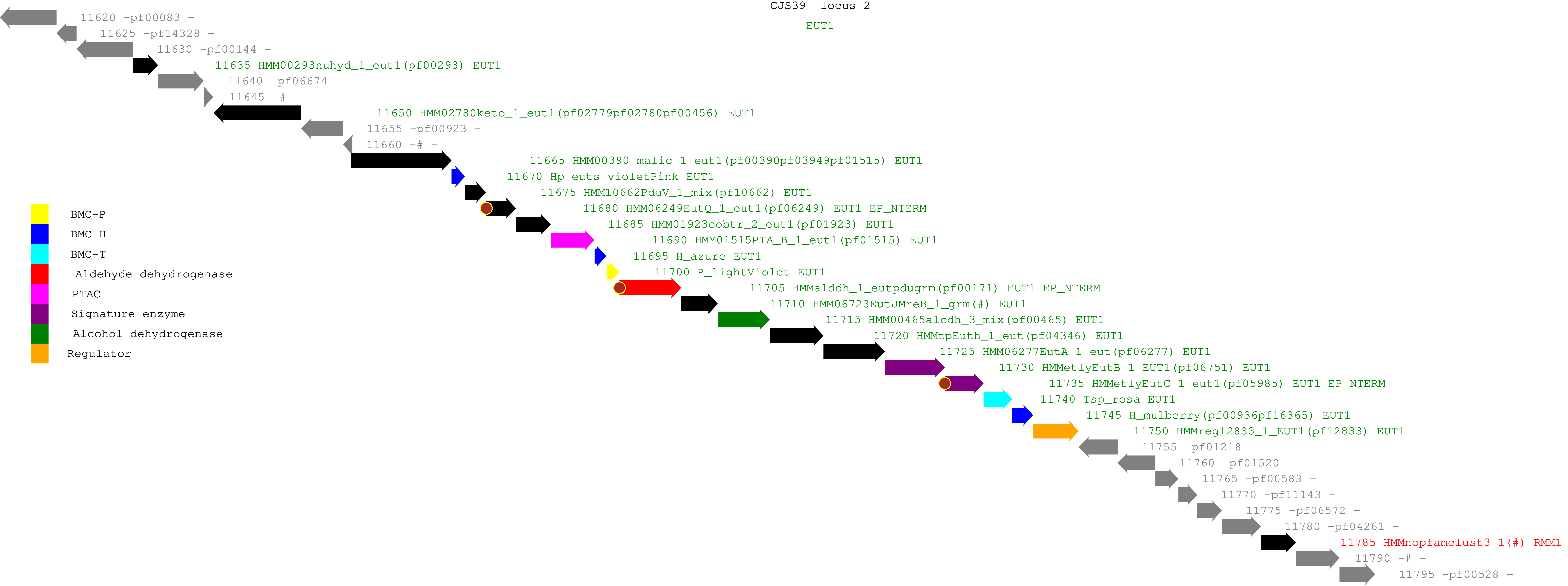

Salmonella enterica subsp. enterica serovar Kentucky. Bacteria; Proteobacteria; Gammaproteobacteria; Enterobacterales;

CJS41__locus_2

EUT1

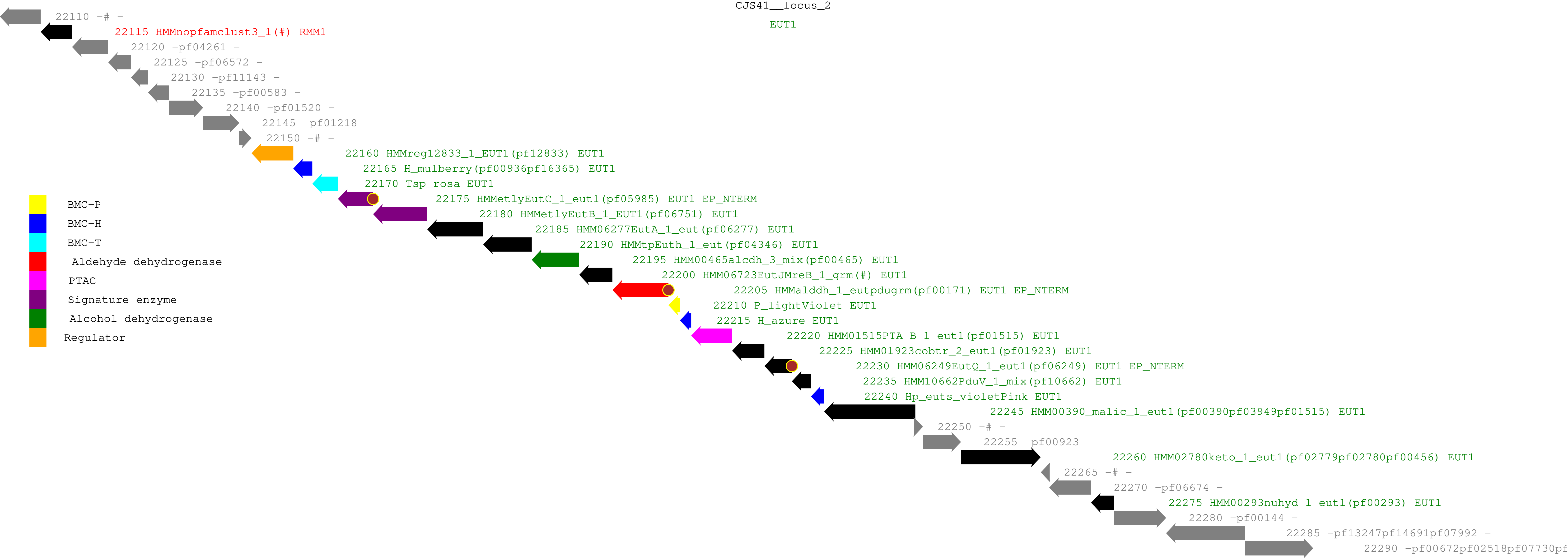

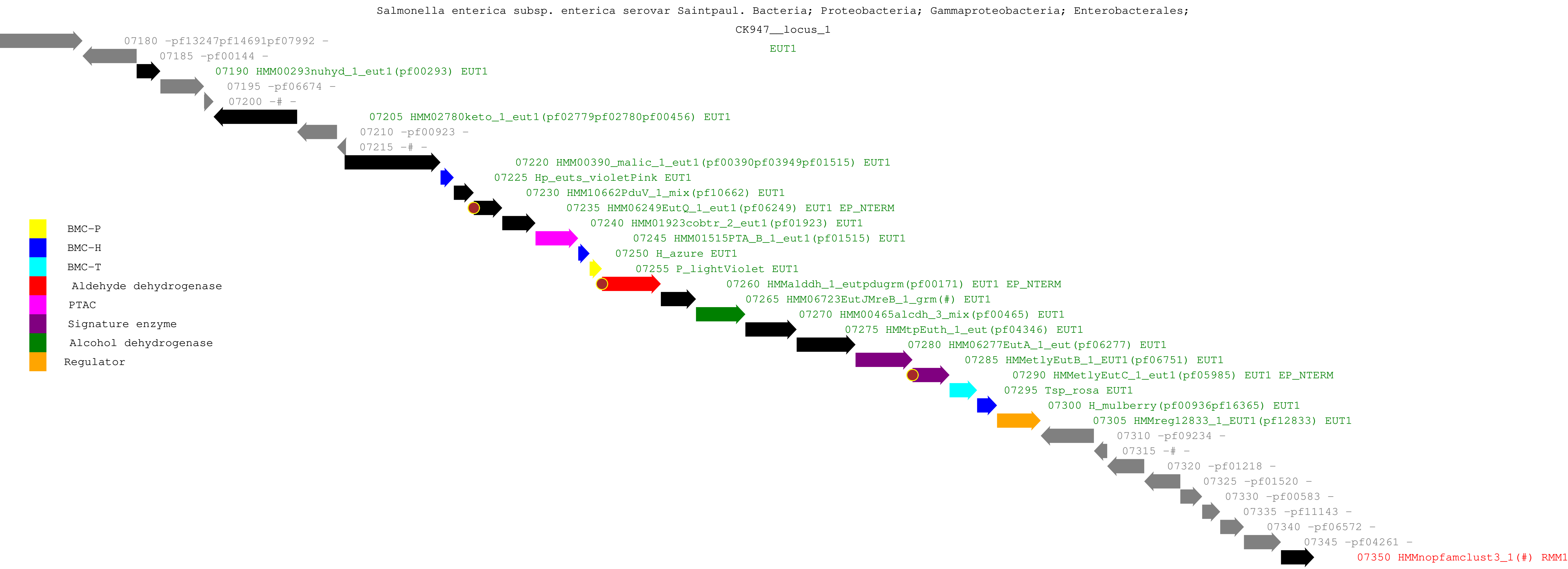
Salmonella enterica subsp. enterica serovar Saintpaul. Bacteria; Proteobacteria; Gammaproteobacteria; Enterobacterales;
CK947__locus_1
EUT1
07180 -pf13247pf14691pf07992 -
07185 -pf00144 -
07190 HMM00293nuhyd_1_eut1(pf00293) EUT1
07195 -pf06674 -
07200 -# -
07205 HMM02780keto_1_eut1(pf02779pf02780pf00456) EUT1
07210 -pf00923 -
07215 -# -
07220 HMM00390_malic_1_eut1(pf00390pf03949pf01515) EUT1
07225 Hp_euts_violetPink EUT1
07230 HMM10662PduV_1_mix(pf10662) EUT1
07235 HMM06249EutQ_1_eut1(pf06249) EUT1 EP_NTERM
07240 HMM01923cobtr_2_eut1(pf01923) EUT1
07245 HMM01515PTA_B_1_eut1(pf01515) EUT1
07250 H_azure EUT1
07255 P_lightViolet EUT1
07260 HMMalddh_1_eutpdugrm(pf00171) EUT1 EP_NTERM
07265 HMM06723EutJMreB_1_grm(#) EUT1
07270 HMM00465alcdh_3_mix(pf00465) EUT1
07275 HMMtpEuth_1_eut(pf04346) EUT1
07280 HMM06277EutA_1_eut(pf06277) EUT1
07285 HMMetlyEutB_1_EUT1(pf06751) EUT1
07290 HMMetlyEutC_1_eut1(pf05985) EUT1 EP_NTERM
07295 Tsp_rosa EUT1
07300 H_mulberry(pf00936pf16365) EUT1
07305 HMMreg12833_1_EUT1(pf12833) EUT1
07310 -pf09234 -
07315 -# -
07320 -pf01218 -
07325 -pf01520 -
07330 -pf00583 -
07335 -pf11143 -
07340 -pf06572 -
07345 -pf04261 -
07350 HMMnopfamclust3_1(#) RMM1
BMC-P
BMC-H
BMC-T
Aldehyde dehydrogenase
PTAC
Signature enzyme
Alcohol dehydrogenase
Regulator

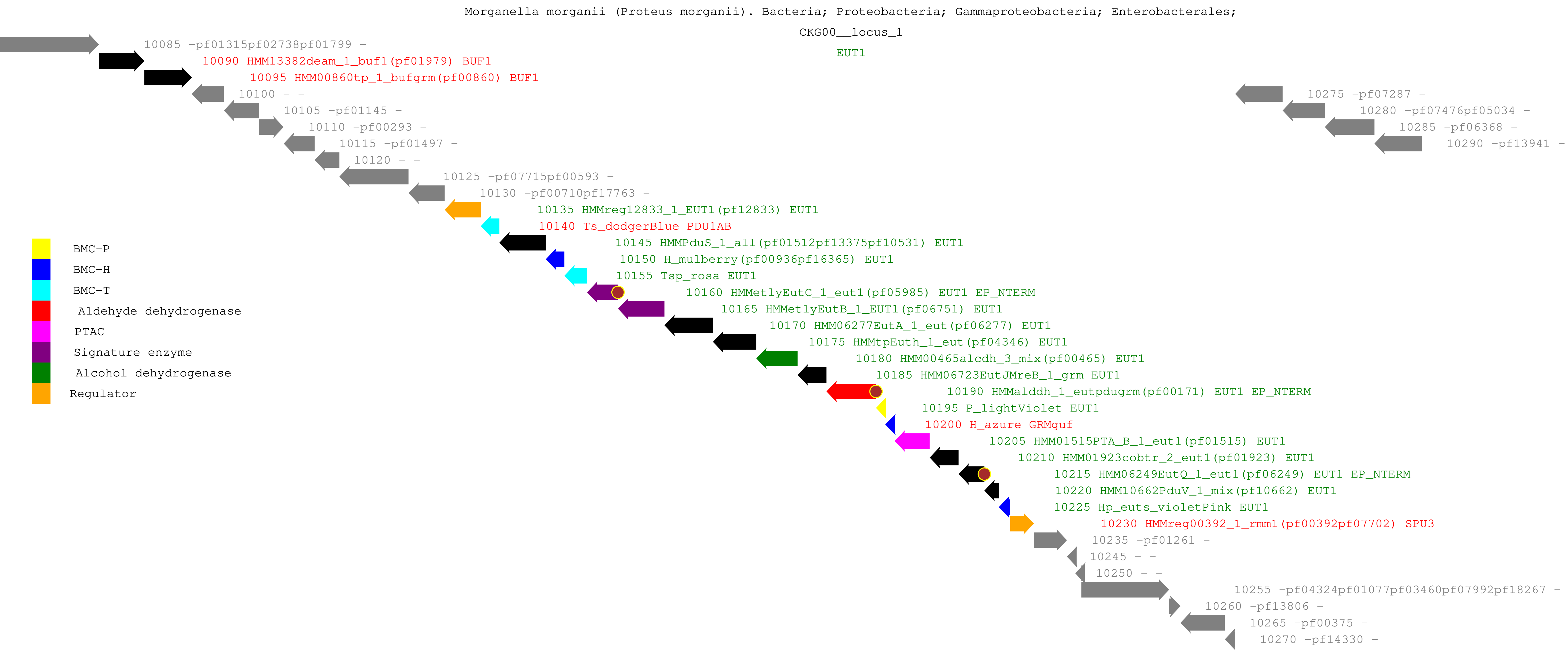
Morganella morganii (Proteus morganii). Bacteria; Proteobacteria; Gammaproteobacteria; Enterobacterales;
CKG00__locus_1
EUT1
10085 -pf01315pf02738pf01799 -
10090 HMM13382deam_1_buf1(pf01979) BUF1
10095 HMM00860tp_1_bufgrm(pf00860) BUF1
10100 - -
10105 -pf01145 -
10110 -pf00293 -
10115 -pf01497 -
10120 - -
10125 -pf07715pf00593 -
10130 -pf00710pf17763 -
10135 HMMreg12833_1_EUT1(pf12833) EUT1
10140 Ts_dodgerBlue PDU1AB
10145 HMMPduS_1_all(pf01512pf13375pf10531) EUT1
10150 H_mulberry(pf00936pf16365) EUT1
10155 Tsp_rosa EUT1
10160 HMMetlyEutC_1_eut1(pf05985) EUT1 EP_NTERM
10165 HMMetlyEutB_1_EUT1(pf06751) EUT1
10170 HMM06277EutA_1_eut(pf06277) EUT1
10175 HMMtpEuth_1_eut(pf04346) EUT1
10180 HMM00465alcdh_3_mix(pf00465) EUT1
10185 HMM06723EutJMreB_1_grm EUT1
10190 HMMalddh_1_eutpdugrm(pf00171) EUT1 EP_NTERM
10195 P_lightViolet EUT1
10200 H_azure GRMguf
10205 HMM01515PTA_B_1_eut1(pf01515) EUT1
10210 HMM01923cobtr_2_eut1(pf01923) EUT1
10215 HMM06249EutQ_1_eut1(pf06249) EUT1 EP_NTERM
10220 HMM10662PduV_1_mix(pf10662) EUT1
10225 Hp_euts_violetPink EUT1
10230 HMMreg00392_1_rmm1(pf00392pf07702) SPU3
10235 -pf01261 -
10245 - -
10250 - -
10255 -pf04324pf01077pf03460pf07992pf18267 -
10260 -pf13806 -
10265 -pf00375 -
10270 -pf14330 -
10275 -pf07287 -
10280 -pf07476pf05034 -
10285 -pf06368 -
10290 -pf13941 -
BMC-P
BMC-H
BMC-T
Aldehyde dehydrogenase
PTAC
Signature enzyme
Alcohol dehydrogenase
Regulator

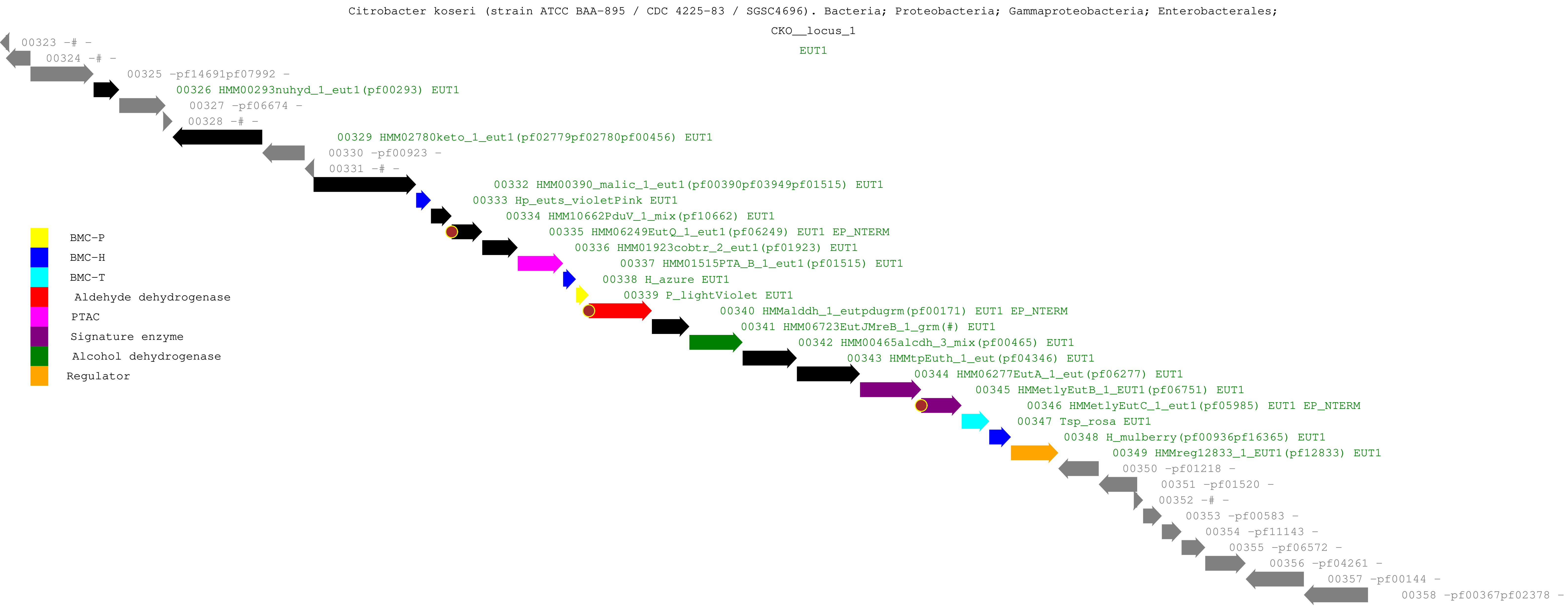
Citrobacter koseri (strain ATCC BAA-895 / CDC 4225-83 / SGSC4696). Bacteria; Proteobacteria; Gammaproteobacteria; Enterobacterales;
CKO__locus_1
EUT1
00323 -# -
00324 -# -
00325 -pf14691pf07992 -
00326 HMM00293nuhyd_1_eut1(pf00293) EUT1
00327 -pf06674 -
00328 -# -
00329 HMM02780keto_1_eut1(pf02779pf02780pf00456) EUT1
00330 -pf00923 -
00331 -# -
00332 HMM00390_malic_1_eut1(pf00390pf03949pf01515) EUT1
00333 Hp_euts_violetPink EUT1
00334 HMM10662PduV_1_mix(pf10662) EUT1
00335 HMM06249EutQ_1_eut1(pf06249) EUT1 EP_NTERM
00336 HMM01923cobtr_2_eut1(pf01923) EUT1
00337 HMM01515PTA_B_1_eut1(pf01515) EUT1
00338 H_azure EUT1
00339 P_lightViolet EUT1
00340 HMMalddh_1_eutpdugrm(pf00171) EUT1 EP_NTERM
00341 HMM06723EutJMreB_1_grm(#) EUT1
00342 HMM00465alcdh_3_mix(pf00465) EUT1
00343 HMMtpEuth_1_eut(pf04346) EUT1
00344 HMM06277EutA_1_eut(pf06277) EUT1
00345 HMMetlyEutB_1_EUT1(pf06751) EUT1
00346 HMMetlyEutC_1_eut1(pf05985) EUT1 EP_NTERM
00347 Tsp_rosa EUT1
00348 H_mulberry(pf00936pf16365) EUT1
00349 HMMreg12833_1_EUT1(pf12833) EUT1
00350 -pf01218 -
00351 -pf01520 -
00352 -# -
00353 -pf00583 -
00354 -pf11143 -
00355 -pf06572 -
00356 -pf04261 -
00357 -pf00144 -
00358 -pf00367pf02378 -
BMC-P
BMC-H
BMC-T
Aldehyde dehydrogenase
PTAC
Signature enzyme
Alcohol dehydrogenase
Regulator

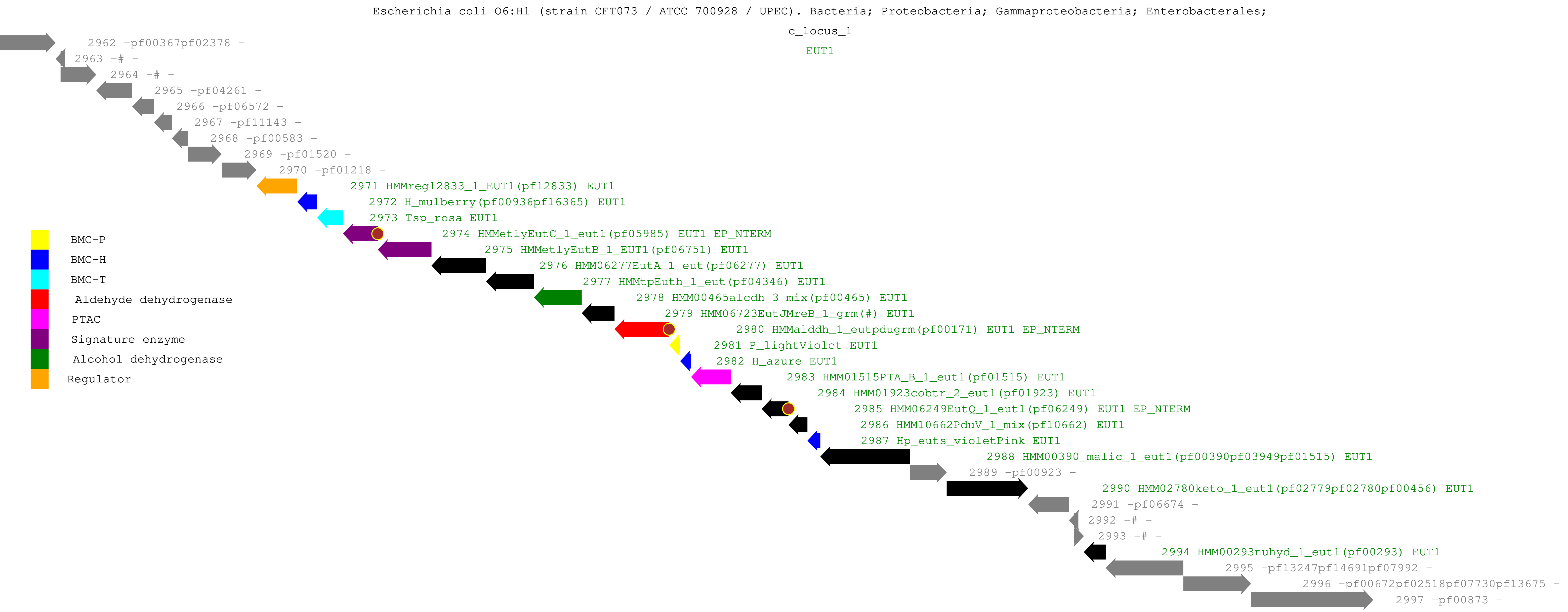
Escherichia coli O6:H1 (strain CFT073 / ATCC 700928 / UPEC). Bacteria; Proteobacteria; Gammaproteobacteria; Enterobacterales;
c_locus_1
EUT1
2962 -pf00367pf02378 -
2963 -# -
2964 -# -
2965 -pf04261 -
2966 -pf06572 -
2967 -pf11143 -
2968 -pf00583 -
2969 -pf01520 -
2970 -pf01218 -
2971 HMMreg12833_1_EUT1(pf12833) EUT1
2972 H_mulberry(pf00936pf16365) EUT1
2973 Tsp_rosa EUT1
2974 HMMetlyEutC_1_eut1(pf05985) EUT1 EP_NTERM
2975 HMMetlyEutB_1_EUT1(pf06751) EUT1
2976 HMM06277EutA_1_eut(pf06277) EUT1
2977 HMMtpEuth_1_eut(pf04346) EUT1
2978 HMM00465alcdh_3_mix(pf00465) EUT1
2979 HMM06723EutJMreB_1_grm(#) EUT1
2980 HMMaldh_1_eutpdugrm(pf00171) EUT1 EP_NTERM
2981 P_lightViolet EUT1
2982 H_azure EUT1
2983 HMM01515PTA_B_1_eut1(pf01515) EUT1
2984 HMM01923cobtr_2_eut1(pf01923) EUT1
2985 HMM06249EutQ_1_eut1(pf06249) EUT1 EP_NTERM
2986 HMM10662PduV_1_mix(pf10662) EUT1
2987 Hp_euts_violetPink EUT1
2988 HMM00390_malic_1_eut1(pf00390pf03949pf01515) EUT1
2989 -pf00923 -
2990 HMM02780keto_1_eut1(pf02779pf02780pf00456) EUT1
2991 -pf06674 -
2992 -# -
2993 -# -
2994 HMM00293nuhyd_1_eut1(pf00293) EUT1
2995 -pf13247pf14691pf07992 -
2996 -pf00672pf02518pf07730pf13675 -
2997 -pf00873 -
BMC-P
BMC-H
BMC-T
Aldehyde dehydrogenase
PTAC
Signature enzyme
Alcohol dehydrogenase
Regulator

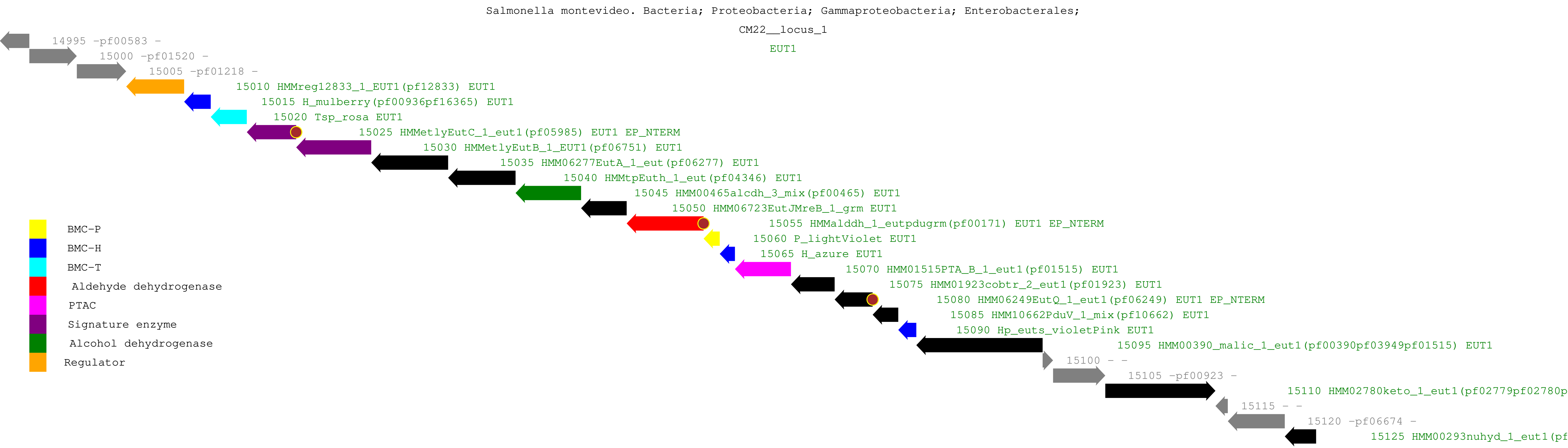

Salmonella montevideo. Bacteria; Proteobacteria; Gammaproteobacteria; Enterobacterales;
CM22__locus_1
EUT1
14995 -pf00583 -
15000 -pf01520 -
15005 -pf01218 -
15010 HMMreg12833_1_EUT1(pf12833) EUT1
15015 H_mulberry(pf00936pf16365) EUT1
15020 Tsp_rosa EUT1
15025 HMMetlyEutC_1_eut1(pf05985) EUT1 EP_NTERM
15030 HMMetlyEutB_1_EUT1(pf06751) EUT1
15035 HMM06277EutA_1_eut(pf06277) EUT1
15040 HMMtpEuth_1_eut(pf04346) EUT1
15045 HMM00465alcdh_3_mix(pf00465) EUT1
15050 HMM06723EutJMreB_1_grm EUT1
15055 HMMaldh_1_eutpdugrm(pf00171) EUT1 EP_NTERM
15060 P_lightViolet EUT1
15065 H_azure EUT1
15070 HMM01515PTA_B_1_eut1(pf01515) EUT1
15075 HMM01923cobtr_2_eut1(pf01923) EUT1
15080 HMM06249EutQ_1_eut1(pf06249) EUT1 EP_NTERM
15085 HMM10662PduV_1_mix(pf10662) EUT1
15090 Hp_euts_violetPink EUT1
15095 HMM00390_malic_1_eut1(pf00390pf03949pf01515) EUT1
15100 - -
15105 -pf00923 -
15110 HMM02780keto_1_eut1(pf02779pf02780p
15115 - -
15120 -pf06674 -
15125 HMM00293nuhyd_1_eut1(pf
BMC-P
BMC-H
BMC-T
Aldehyde dehydrogenase
PTAC
Signature enzyme
Alcohol dehydrogenase
Regulator

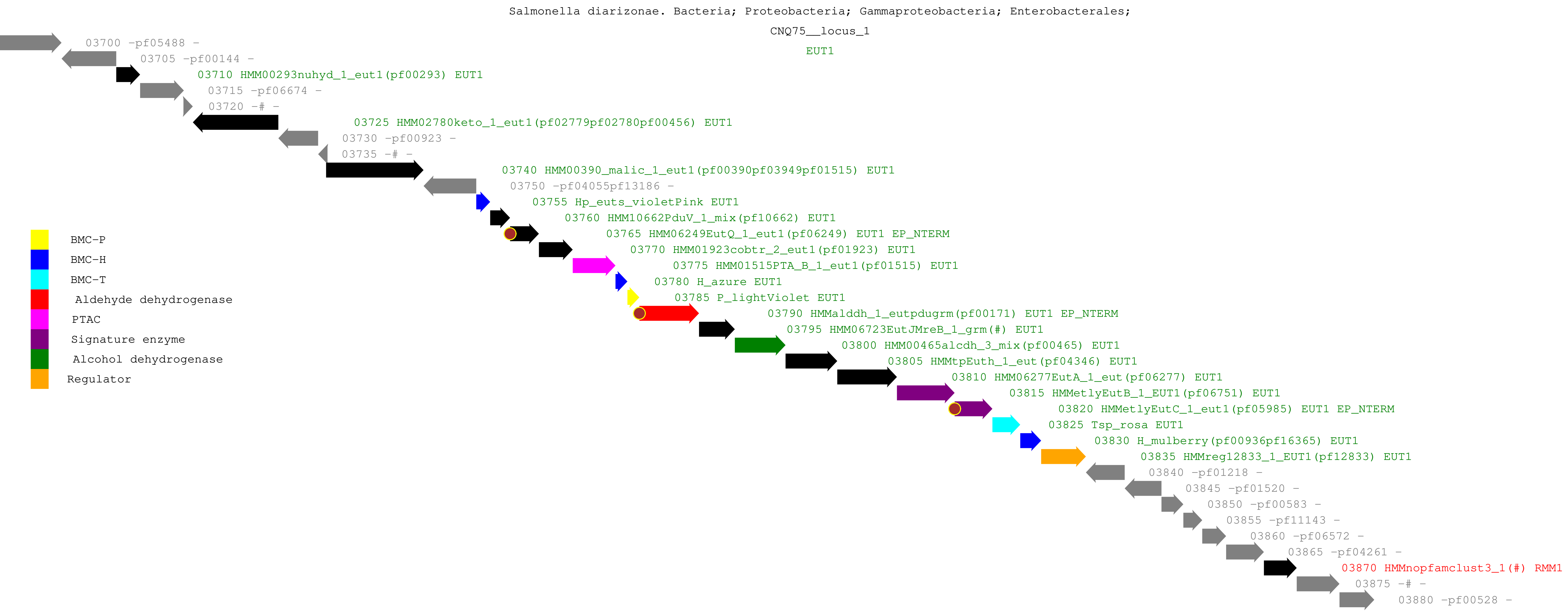

Salmonella diarizonae. Bacteria; Proteobacteria; Gammaproteobacteria; Enterobacterales;
CNQ75__locus_1
EUT1
03700 -pf05488 -
03705 -pf00144 -
03710 HMM00293nuhyd_1_eut1(pf00293) EUT1
03715 -pf06674 -
03720 -# -
03725 HMM02780keto_1_eut1(pf02779pf02780pf00456) EUT1
03730 -pf00923 -
03735 -# -
03740 HMM00390_malic_1_eut1(pf00390pf03949pf01515) EUT1
03750 -pf04055pf13186 -
03755 Hp_euts_violetPink EUT1
03760 HMM10662PduV_1_mix(pf10662) EUT1
03765 HMM06249EutQ_1_eut1(pf06249) EUT1 EP_NTERM
03770 HMM01923cobtr_2_eut1(pf01923) EUT1
03775 HMM01515PTA_B_1_eut1(pf01515) EUT1
03780 H_azure EUT1
03785 P_lightViolet EUT1
03790 HMMalddh_1_eutpdugrm(pf00171) EUT1 EP_NTERM
03795 HMM06723EutJMreB_1_grm(#) EUT1
03800 HMM00465alcdh_3_mix(pf00465) EUT1
03805 HMMtpEuth_1_eut(pf04346) EUT1
03810 HMM06277EutA_1_eut(pf06277) EUT1
03815 HMMetlyEutB_1_EUT1(pf06751) EUT1
03820 HMMetlyEutC_1_eut1(pf05985) EUT1 EP_NTERM
03825 Tsp_rosa EUT1
03830 H_mulberry(pf00936pf16365) EUT1
03835 HMMreg12833_1_EUT1(pf12833) EUT1
03840 -pf01218 -
03845 -pf01520 -
03850 -pf00583 -
03855 -pf11143 -
03860 -pf06572 -
03865 -pf04261 -
03870 HMMnopfamclust3_1(#) RMM1
03875 -# -
03880 -pf00528 -
BMC-P
BMC-H
BMC-T
Aldehyde dehydrogenase
PTAC
Signature enzyme
Alcohol dehydrogenase
Regulator

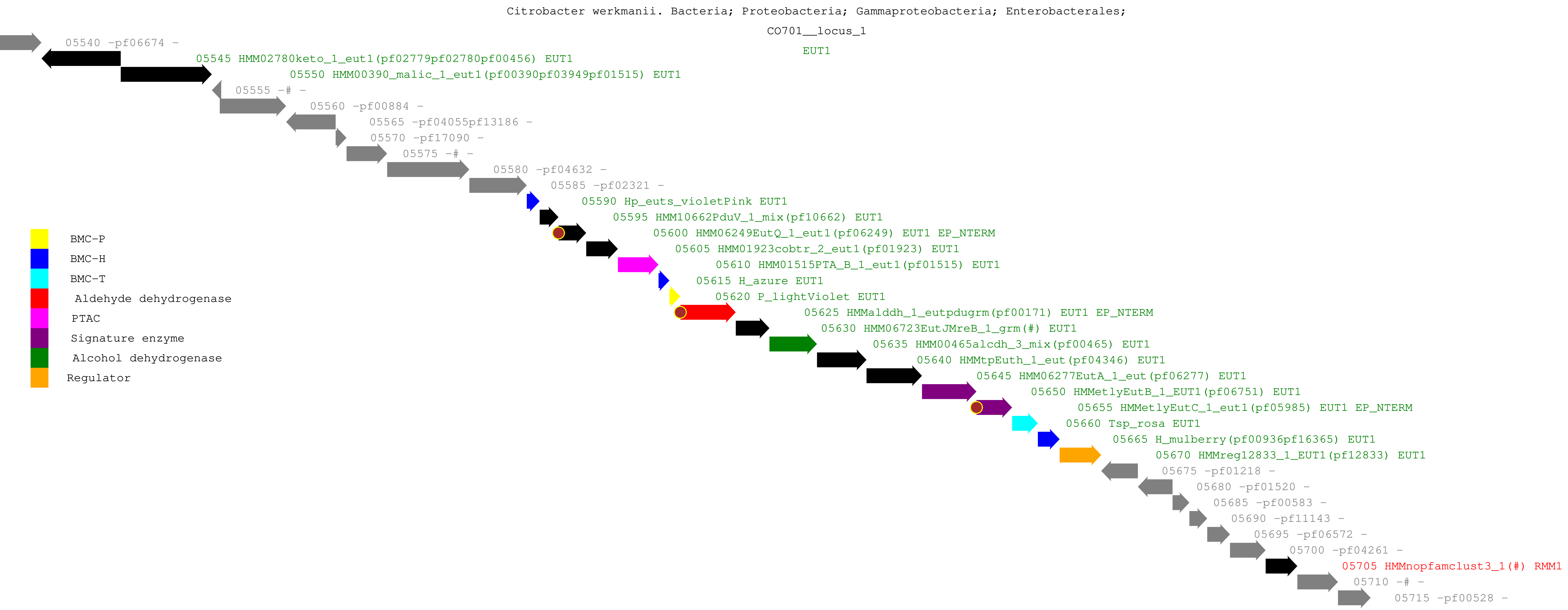

Citrobacter werkmanii. Bacteria; Proteobacteria; Gammaproteobacteria; Enterobacterales;
CO701__locus_1
EUT1
05540 -pf06674 -
05545 HMM02780keto_1_eut1(pf02779pf02780pf00456) EUT1
05550 HMM00390_malic_1_eut1(pf00390pf03949pf01515) EUT1
05555 -# -
05560 -pf00884 -
05565 -pf04055pf13186 -
05570 -pf17090 -
05575 -# -
05580 -pf04632 -
05585 -pf02321 -
05590 Hp_euts_violetPink EUT1
05595 HMM10662PduV_1_mix(pf10662) EUT1
05600 HMM06249EutQ_1_eut1(pf06249) EUT1 EP_NTERM
05605 HMM01923cobtr_2_eut1(pf01923) EUT1
05610 HMM01515PTA_B_1_eut1(pf01515) EUT1
05615 H_azure EUT1
05620 P_lightViolet EUT1
05625 HMMaldh_1_eutpdugrm(pf00171) EUT1 EP_NTERM
05630 HMM06723EutJMreB_1_grm(#) EUT1
05635 HMM00465alcdh_3_mix(pf00465) EUT1
05640 HMMtpEuth_1_eut(pf04346) EUT1
05645 HMM06277EutA_1_eut(pf06277) EUT1
05650 HMMetlyEutB_1_EUT1(pf06751) EUT1
05655 HMMetlyEutC_1_eut1(pf05985) EUT1 EP_NTERM
05660 Tsp_rosa EUT1
05665 H_mulberry(pf00936pf16365) EUT1
05670 HMMreg12833_1_EUT1(pf12833) EUT1
05675 -pf01218 -
05680 -pf01520 -
05685 -pf00583 -
05690 -pf11143 -
05695 -pf06572 -
05700 -pf04261 -
05705 HMMnopfamclust3_1(#) RMM1
05710 -# -
05715 -pf00528 -
BMC-P
BMC-H
BMC-T
Aldehyde dehydrogenase
PTAC
Signature enzyme
Alcohol dehydrogenase
Regulator

Oceanospirillales bacterium. Bacteria; Proteobacteria; Gammaproteobacteria; Oceanospirillales;

COB25__locus_1_main_sat

EUT1

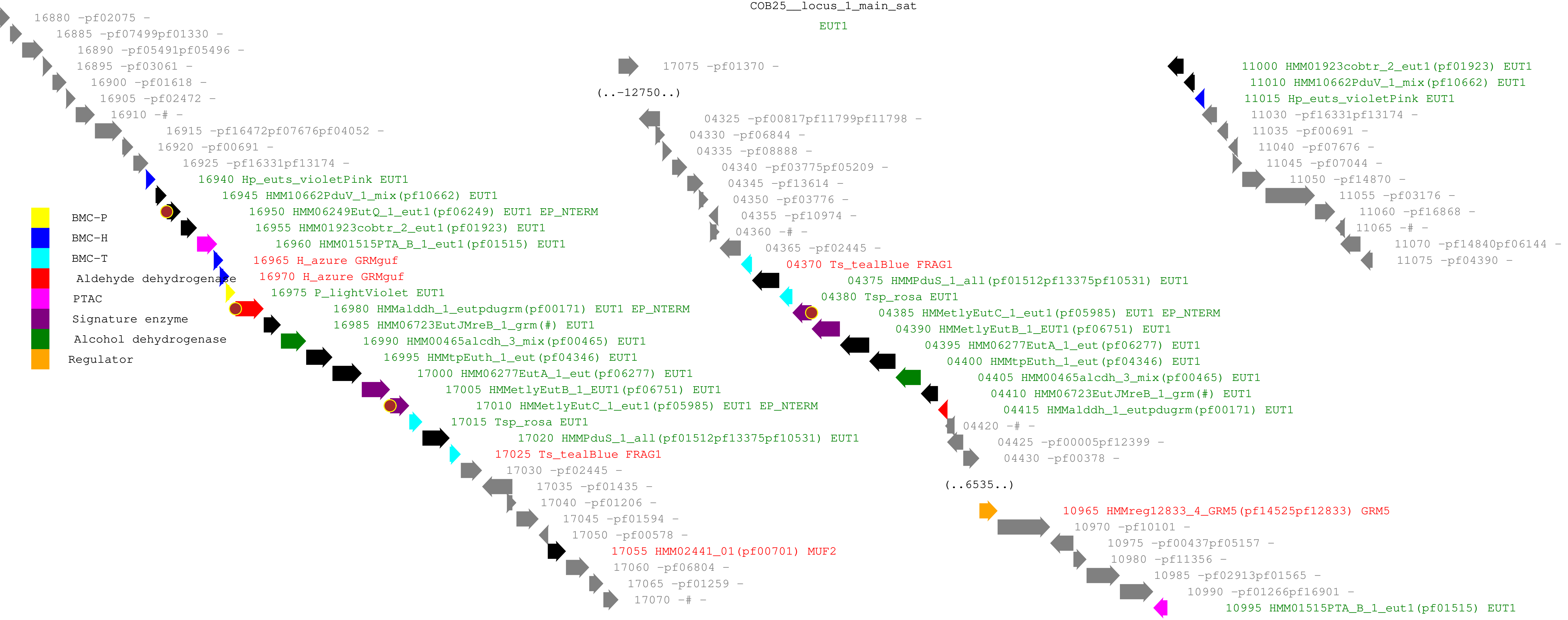

Marinobacter sp. ANT_B65. Bacteria; Proteobacteria; Gammaproteobacteria; Alteromonadales;

CPA50__locus_1

EUT1

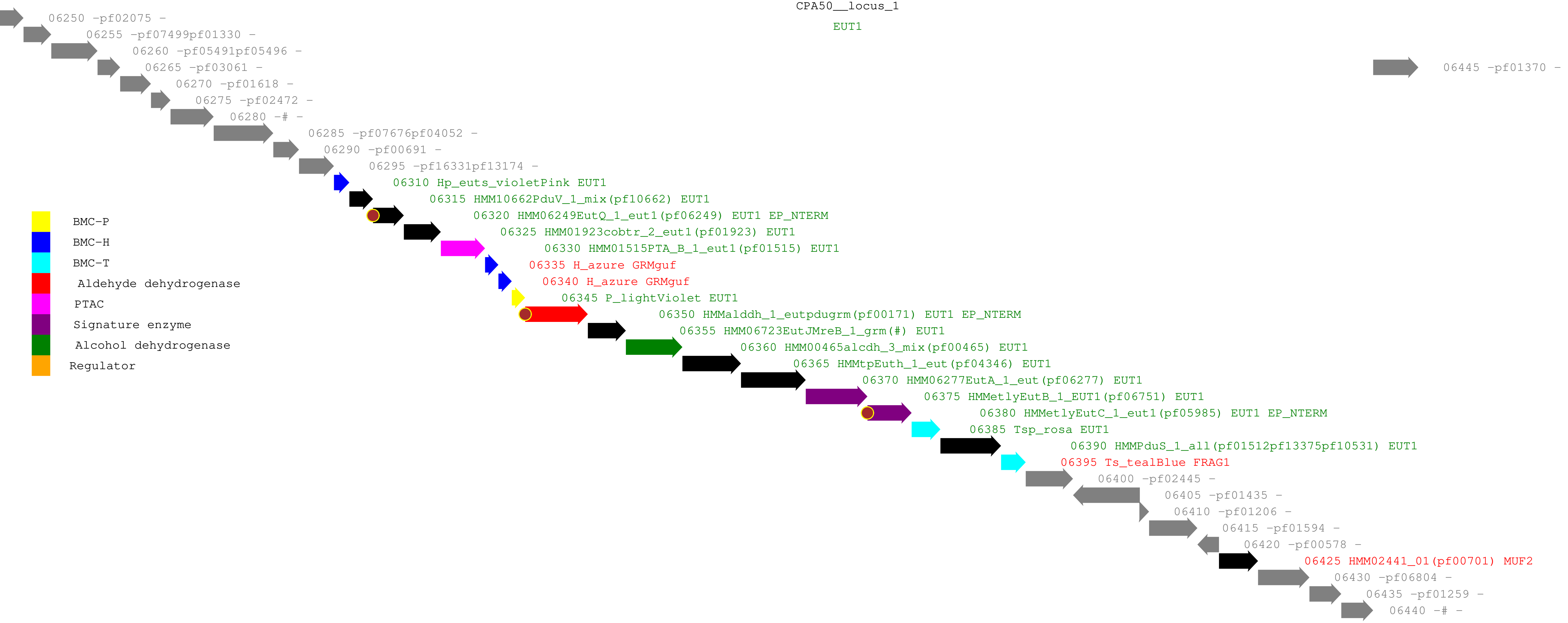

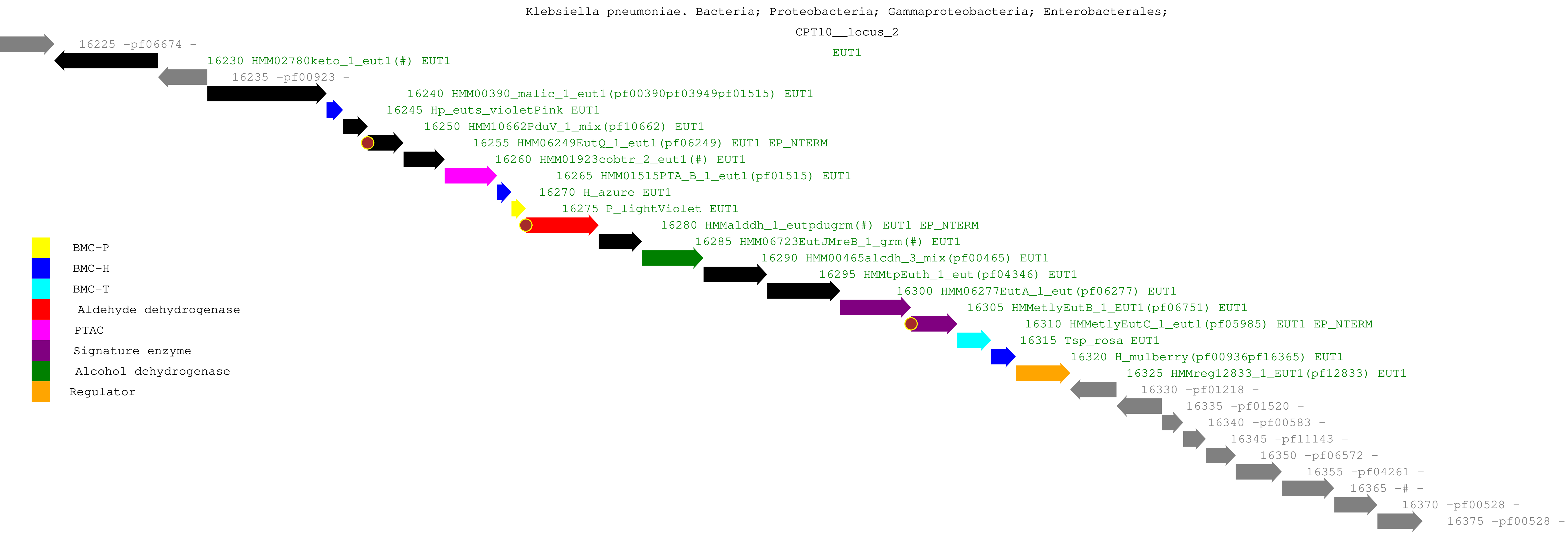

Klebsiella pneumoniae. Bacteria; Proteobacteria; Gammaproteobacteria; Enterobacterales;
CPT10__locus_2
EUT1
16225 -pf06674 -
16230 HMM02780keto_1_eut1(#) EUT1
16235 -pf00923 -
16240 HMM00390_malic_1_eut1(pf00390pf03949pf01515) EUT1
16245 Hp_euts_violetPink EUT1
16250 HMM10662PduV_1_mix(pf10662) EUT1
16255 HMM06249EutQ_1_eut1(pf06249) EUT1 EP_NTERM
16260 HMM01923cobtr_2_eut1(#) EUT1
16265 HMM01515PTA_B_1_eut1(pf01515) EUT1
16270 H_azure EUT1
16275 P_lightViolet EUT1
16280 HMMalddh_1_eutpdugrm(#) EUT1 EP_NTERM
16285 HMM06723EutJMreB_1_grm(#) EUT1
16290 HMM00465alcdh_3_mix(pf00465) EUT1
16295 HMMtpEuth_1_eut(pf04346) EUT1
16300 HMM06277EutA_1_eut(pf06277) EUT1
16305 HMMetlyEutB_1_EUT1(pf06751) EUT1
16310 HMMetlyEutC_1_eut1(pf05985) EUT1 EP_NTERM
16315 Tsp_rosa EUT1
16320 H_mulberry(pf00936pf16365) EUT1
16325 HMMreg12833_1_EUT1(pf12833) EUT1
16330 -pf01218 -
16335 -pf01520 -
16340 -pf00583 -
16345 -pf11143 -
16350 -pf06572 -
16355 -pf04261 -
16365 -# -
16370 -pf00528 -
16375 -pf00528 -
BMC-P
BMC-H
BMC-T
Aldehyde dehydrogenase
PTAC
Signature enzyme
Alcohol dehydrogenase
Regulator

Klebsiella oxytoca. Bacteria; Proteobacteria; Gammaproteobacteria; Enterobacterales;

CPZ29__locus_1

EUT1

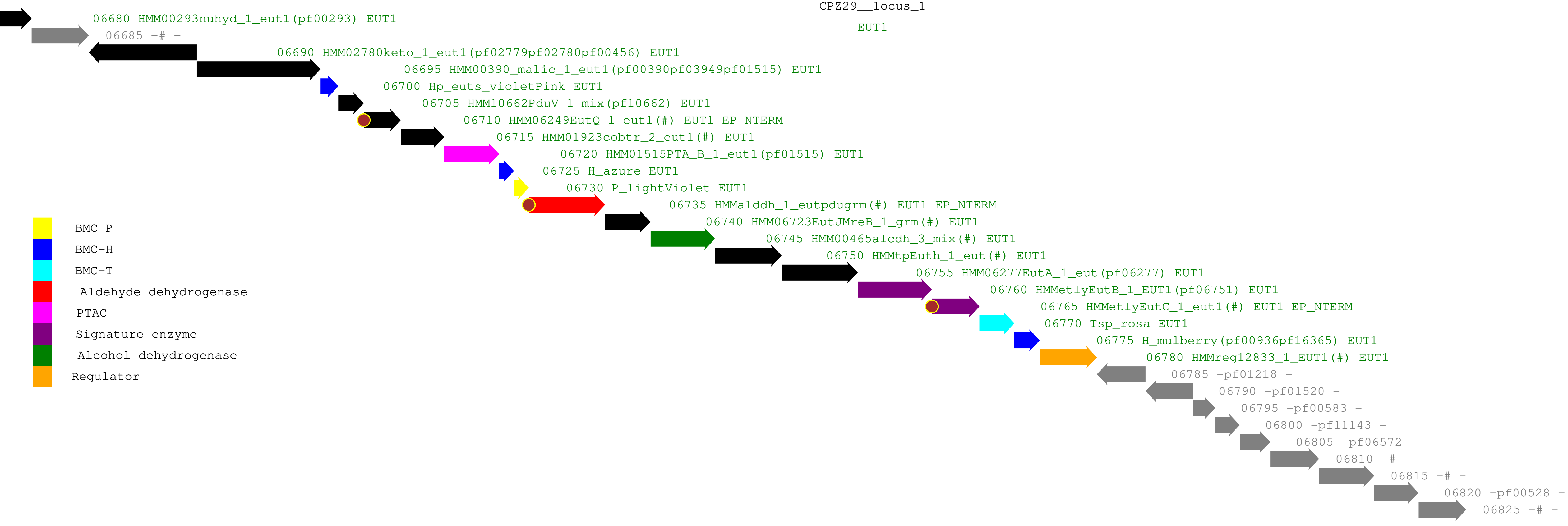

Escherichia coli O26. Bacteria; Proteobacteria; Gammaproteobacteria; Enterobacterales;

CQ146__locus_1

EUT1

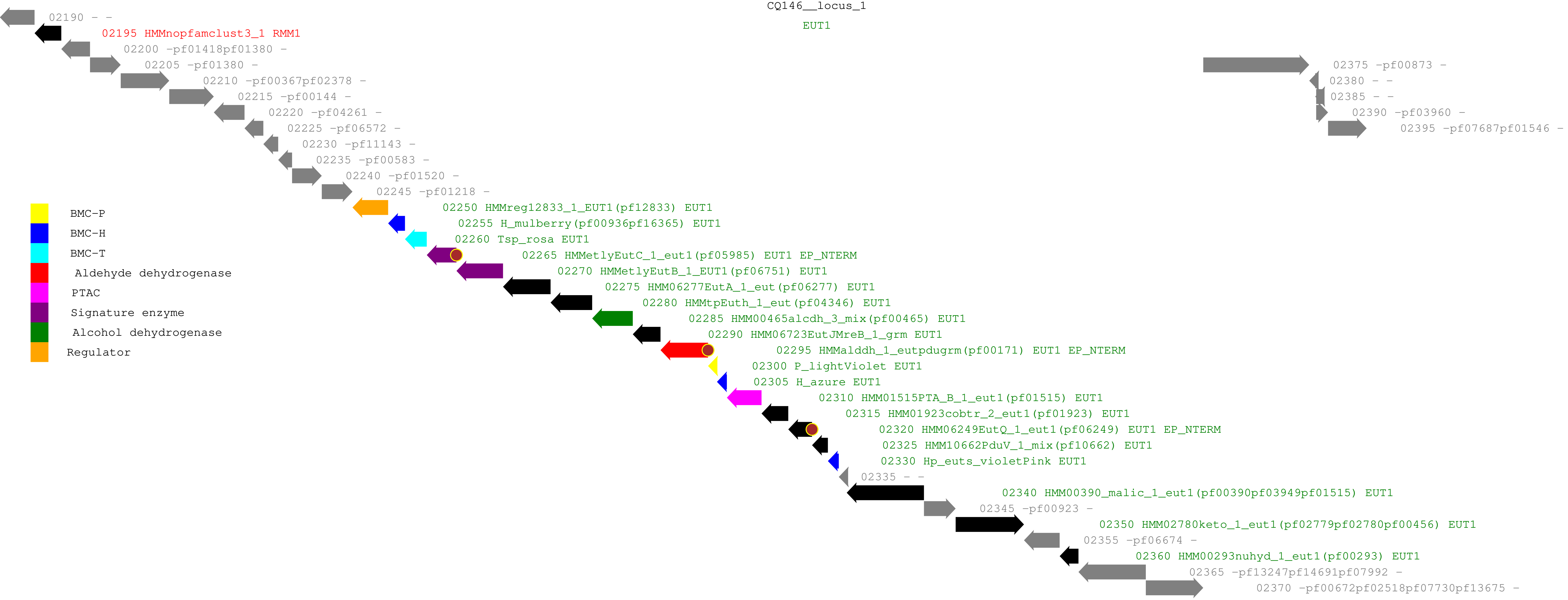

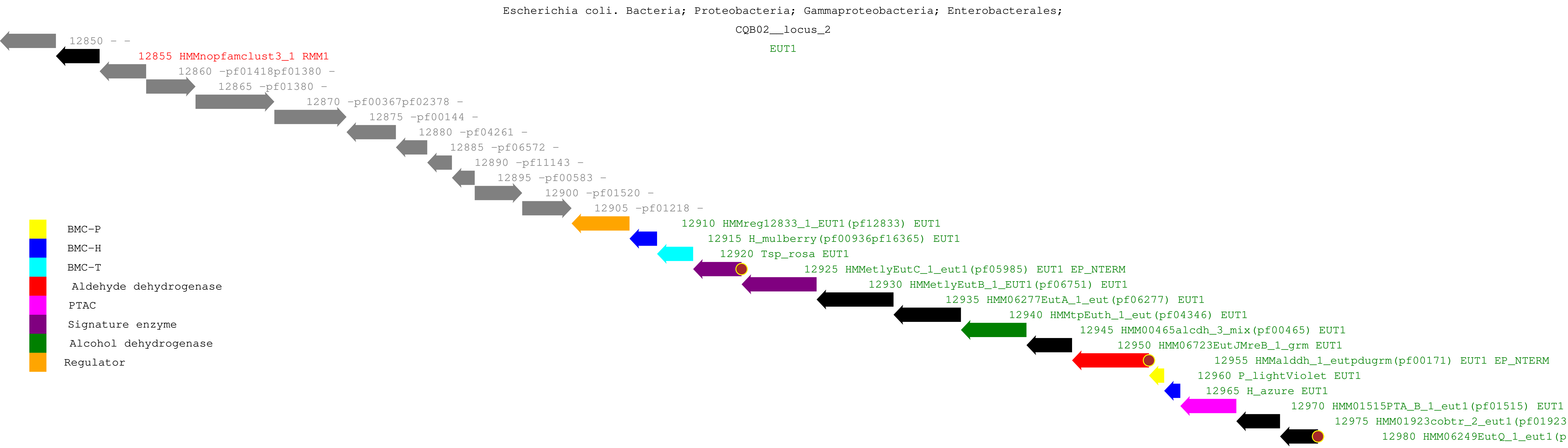
Escherichia coli. Bacteria; Proteobacteria; Gammaproteobacteria; Enterobacterales;
CQB02__locus_2
EUT1
12850 - -
12855 HMMnopfamclust3_1 RMM1
12860 -pf01418pf01380 -
12865 -pf01380 -
12870 -pf00367pf02378 -
12875 -pf00144 -
12880 -pf04261 -
12885 -pf06572 -
12890 -pf11143 -
12895 -pf00583 -
12900 -pf01520 -
12905 -pf01218 -
12910 HMMreg12833_1_EUT1(pf12833) EUT1
12915 H_mulberry(pf00936pf16365) EUT1
12920 Tsp_rosa EUT1
12925 HMMetlyEutC_1_eut1(pf05985) EUT1 EP_NTERM
12930 HMMetlyEutB_1_EUT1(pf06751) EUT1
12935 HMM06277EutA_1_eut(pf06277) EUT1
12940 HMMtpEuth_1_eut(pf04346) EUT1
12945 HMM00465alcdh_3_mix(pf00465) EUT1
12950 HMM06723EutJMreB_1_grm EUT1
12955 HMMalddh_1_eutpdugrm(pf00171) EUT1 EP_NTERM
12960 P_lightViolet EUT1
12965 H_azure EUT1
12970 HMM01515PTA_B_1_eut1(pf01515) EUT1
12975 HMM01923cobtr_2_eut1(pf01923
12980 HMM06249EutQ_1_eut1(p
BMC-P
BMC-H
BMC-T
Aldehyde dehydrogenase
PTAC
Signature enzyme
Alcohol dehydrogenase
Regulator

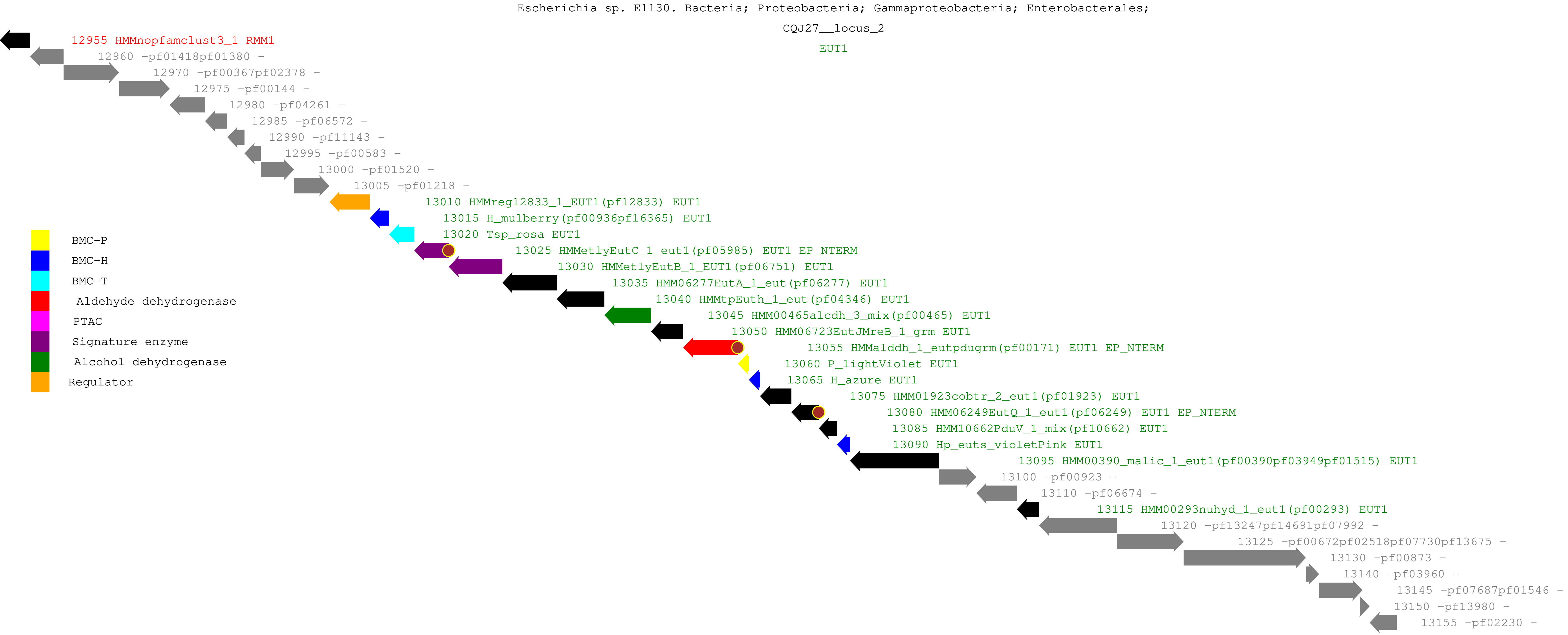
Escherichia sp. E1130. Bacteria; Proteobacteria; Gammaproteobacteria; Enterobacterales;
CQJ27__locus_2
EUT1
12955 HMMnopfamclust3_1 RMM1
12960 -pf01418pf01380 -
12970 -pf00367pf02378 -
12975 -pf00144 -
12980 -pf04261 -
12985 -pf06572 -
12990 -pf11143 -
12995 -pf00583 -
13000 -pf01520 -
13005 -pf01218 -
13010 HMMreg12833_1_EUT1(pf12833) EUT1
13015 H_mulberry(pf00936pf16365) EUT1
13020 Tsp_rosa EUT1
13025 HMMetlyEutC_1_eut1(pf05985) EUT1 EP_NTERM
13030 HMMetlyEutB_1_EUT1(pf06751) EUT1
13035 HMM06277EutA_1_eut(pf06277) EUT1
13040 HMMtpEuth_1_eut(pf04346) EUT1
13045 HMM00465alcdh_3_mix(pf00465) EUT1
13050 HMM06723EutJMreB_1_grm EUT1
13055 HMMalddh_1_eutpdugrm(pf00171) EUT1 EP_NTERM
13060 P_lightViolet EUT1
13065 H_azure EUT1
13075 HMM01923cobtr_2_eut1(pf01923) EUT1
13080 HMM06249EutQ_1_eut1(pf06249) EUT1 EP_NTERM
13085 HMM10662PduV_1_mix(pf10662) EUT1
13090 Hp_euts_violetPink EUT1
13095 HMM00390_malic_1_eut1(pf00390pf03949pf01515) EUT1
13100 -pf00923 -
13110 -pf06674 -
13115 HMM00293nuhyd_1_eut1(pf00293) EUT1
13120 -pf13247pf14691pf07992 -
13125 -pf00672pf02518pf07730pf13675 -
13130 -pf00873 -
13140 -pf03960 -
13145 -pf07687pf01546 -
13150 -pf13980 -
13155 -pf02230 -
BMC-P
BMC-H
BMC-T
Aldehyde dehydrogenase
PTAC
Signature enzyme
Alcohol dehydrogenase
Regulator

Escherichia sp. E2562. Bacteria; Proteobacteria; Gammaproteobacteria; Enterobacterales;

CQJ28__locus_2

EUT1

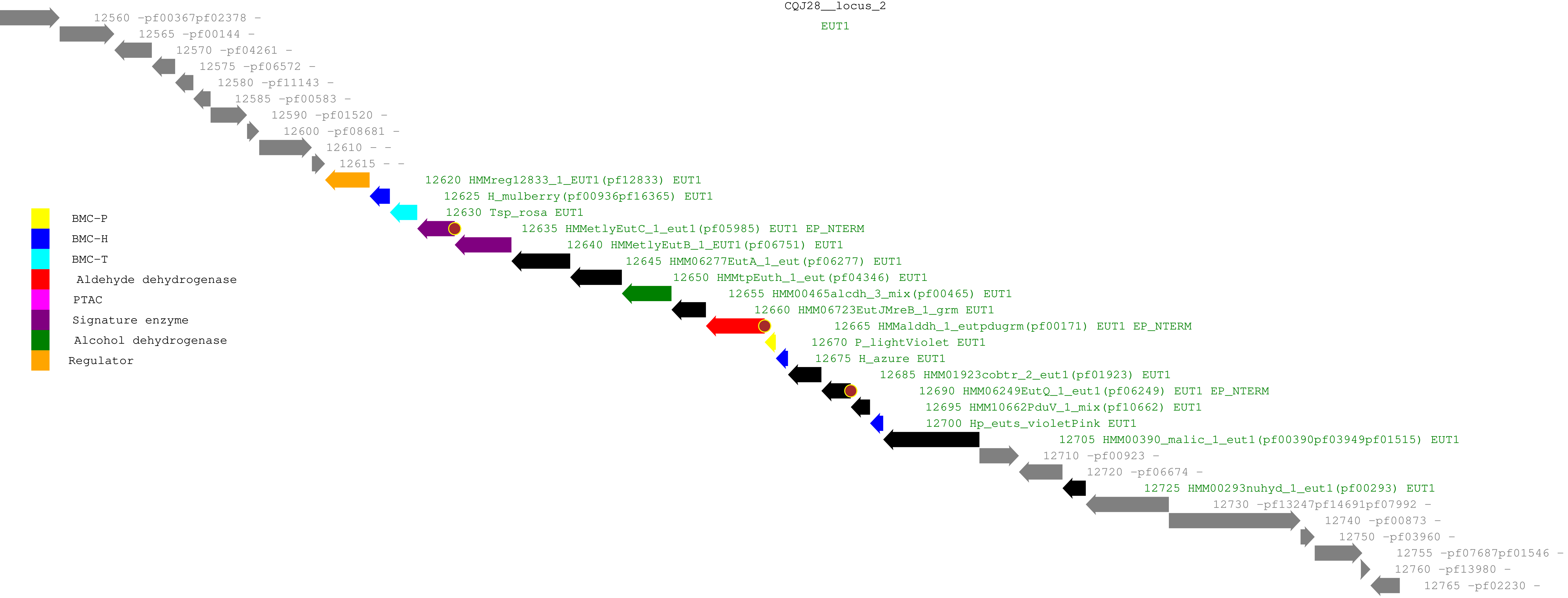

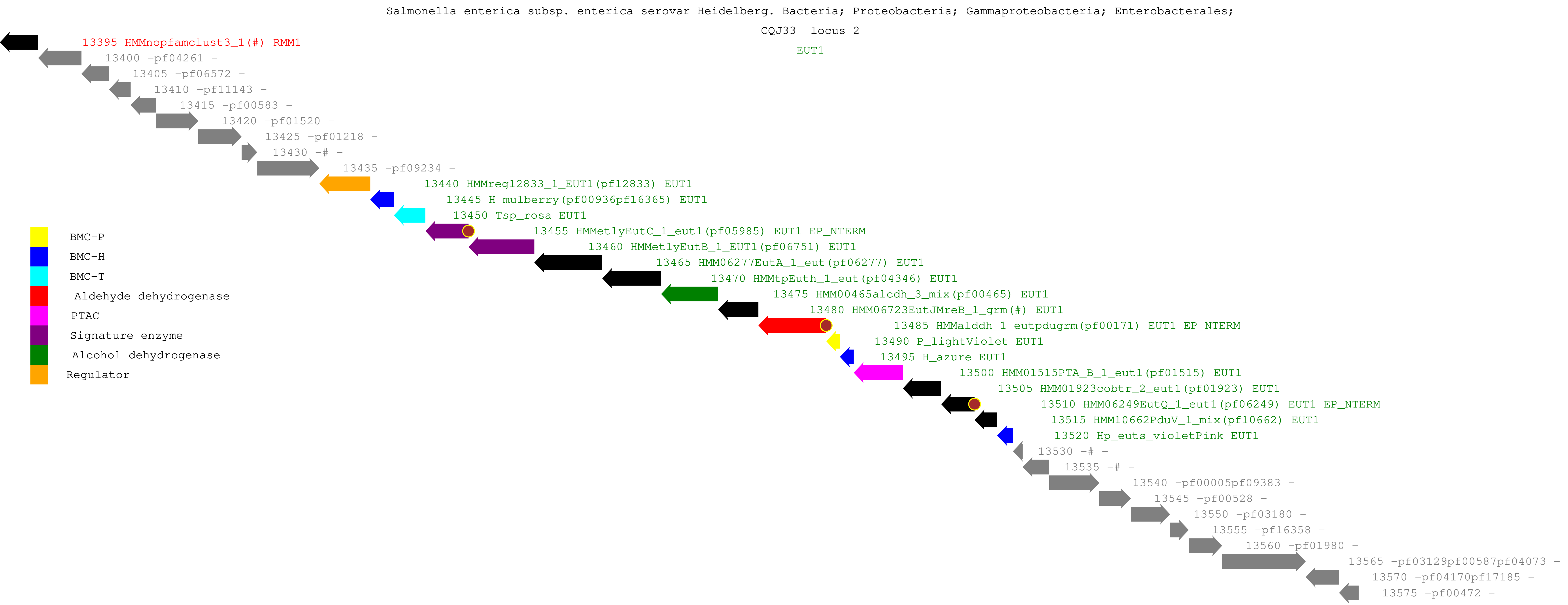

Salmonella enterica subsp. enterica serovar Heidelberg. Bacteria; Proteobacteria; Gammaproteobacteria; Enterobacterales;
CQJ33__locus_2
EUT1
13395 HMMnopfamclust3_1(#) RMM1
13400 -pf04261 -
13405 -pf06572 -
13410 -pf11143 -
13415 -pf00583 -
13420 -pf01520 -
13425 -pf01218 -
13430 -# -
13435 -pf09234 -
13440 HMMreg12833_1_EUT1(pf12833) EUT1
13445 H_mulberry(pf00936pf16365) EUT1
13450 Tsp_rosa EUT1
13455 HMMetlyEutC_1_eut1(pf05985) EUT1 EP_NTERM
13460 HMMetlyEutB_1_EUT1(pf06751) EUT1
13465 HMM06277EutA_1_eut(pf06277) EUT1
13470 HMMtpEuth_1_eut(pf04346) EUT1
13475 HMM00465alcdh_3_mix(pf00465) EUT1
13480 HMM06723EutJMreB_1_grm(#) EUT1
13485 HMMalddh_1_eutpdugrm(pf00171) EUT1 EP_NTERM
13490 P_lightViolet EUT1
13495 H_azure EUT1
13500 HMM01515PTA_B_1_eut1(pf01515) EUT1
13505 HMM01923cobtr_2_eut1(pf01923) EUT1
13510 HMM06249EutQ_1_eut1(pf06249) EUT1 EP_NTERM
13515 HMM10662PduV_1_mix(pf10662) EUT1
13520 Hp_euts_violetPink EUT1
13530 -# -
13535 -# -
13540 -pf00005pf09383 -
13545 -pf00528 -
13550 -pf03180 -
13555 -pf16358 -
13560 -pf01980 -
13565 -pf03129pf00587pf04073 -
13570 -pf04170pf17185 -
13575 -pf00472 -
BMC-P
BMC-H
BMC-T
Aldehyde dehydrogenase
PTAC
Signature enzyme
Alcohol dehydrogenase
Regulator

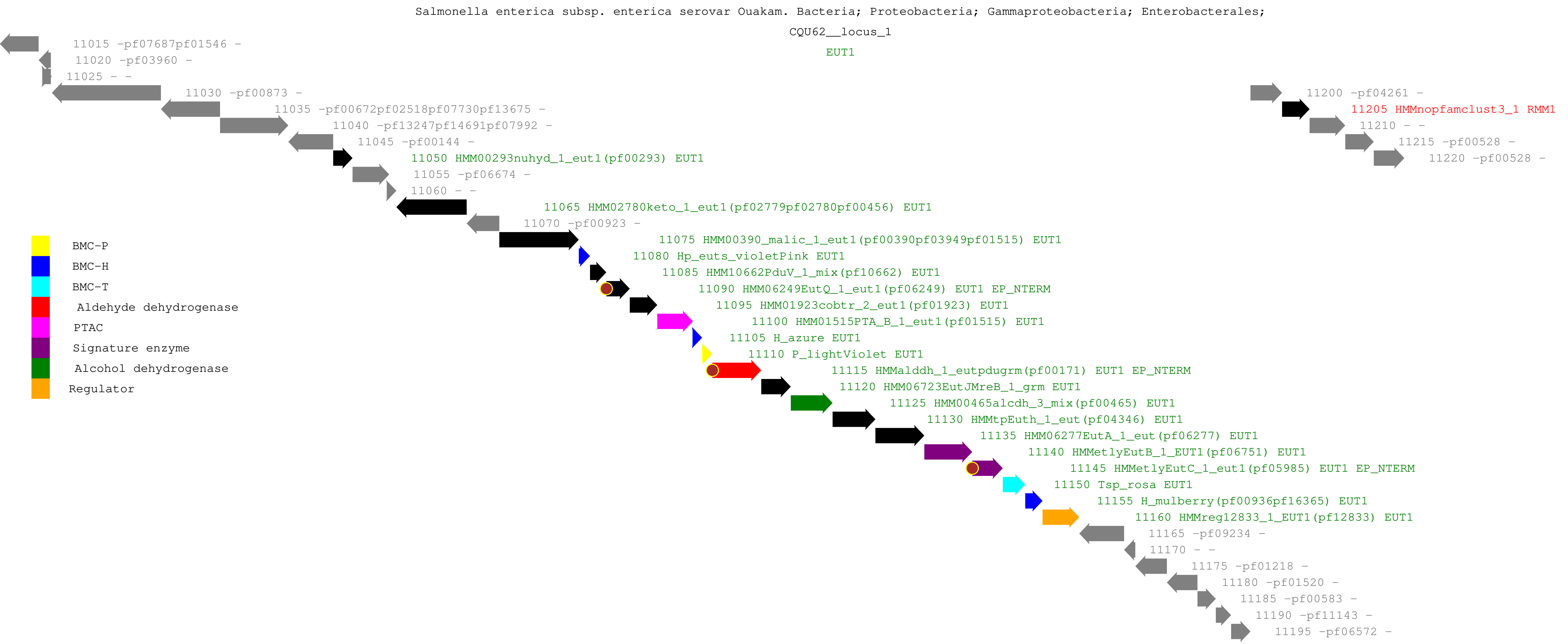
Salmonella enterica subsp. enterica serovar Ouakam. Bacteria; Proteobacteria; Gammaproteobacteria; Enterobacterales;
CQU62__locus_1
EUT1
11015 -pf07687pf01546 -
11020 -pf03960 -
11025 - -
11030 -pf00873 -
11035 -pf00672pf02518pf07730pf13675 -
11040 -pf13247pf14691pf07992 -
11045 -pf00144 -
11050 HMM00293nuhyd_1_eut1(pf00293) EUT1
11055 -pf06674 -
11060 - -
11065 HMM02780keto_1_eut1(pf02779pf02780pf00456) EUT1
11070 -pf00923 -
11075 HMM00390_malic_1_eut1(pf00390pf03949pf01515) EUT1
11080 Hp_euts_violetPink EUT1
11085 HMM10662PduV_1_mix(pf10662) EUT1
11090 HMM06249EutQ_1_eut1(pf06249) EUT1 EP_NTERM
11095 HMM01923cobtr_2_eut1(pf01923) EUT1
11100 HMM01515PTA_B_1_eut1(pf01515) EUT1
11105 H_azure EUT1
11110 P_lightViolet EUT1
11115 HMMalddh_1_eutpdugrm(pf00171) EUT1 EP_NTERM
11120 HMM06723EutJMreB_1_grm EUT1
11125 HMM00465alcdh_3_mix(pf00465) EUT1
11130 HMMtpEuth_1_eut(pf04346) EUT1
11135 HMM06277EutA_1_eut(pf06277) EUT1
11140 HMMetlyEutB_1_EUT1(pf06751) EUT1
11145 HMMetlyEutC_1_eut1(pf05985) EUT1 EP_NTERM
11150 Tsp_rosa EUT1
11155 H_mulberry(pf00936pf16365) EUT1
11160 HMMreg12833_1_EUT1(pf12833) EUT1
11165 -pf09234 -
11170 - -
11175 -pf01218 -
11180 -pf01520 -
11185 -pf00583 -
11190 -pf11143 -
11195 -pf06572 -
11200 -pf04261 -
11205 HMMnopfamclust3_1 RMM1
11210 - -
11215 -pf00528 -
11220 -pf00528 -
BMC-P
BMC-H
BMC-T
Aldehyde dehydrogenase
PTAC
Signature enzyme
Alcohol dehydrogenase
Regulator

Salmonella enterica (Salmonella choleraesuis). Bacteria; Proteobacteria; Gammaproteobacteria; Enterobacterales;

CRE05__locus_1

EUT1

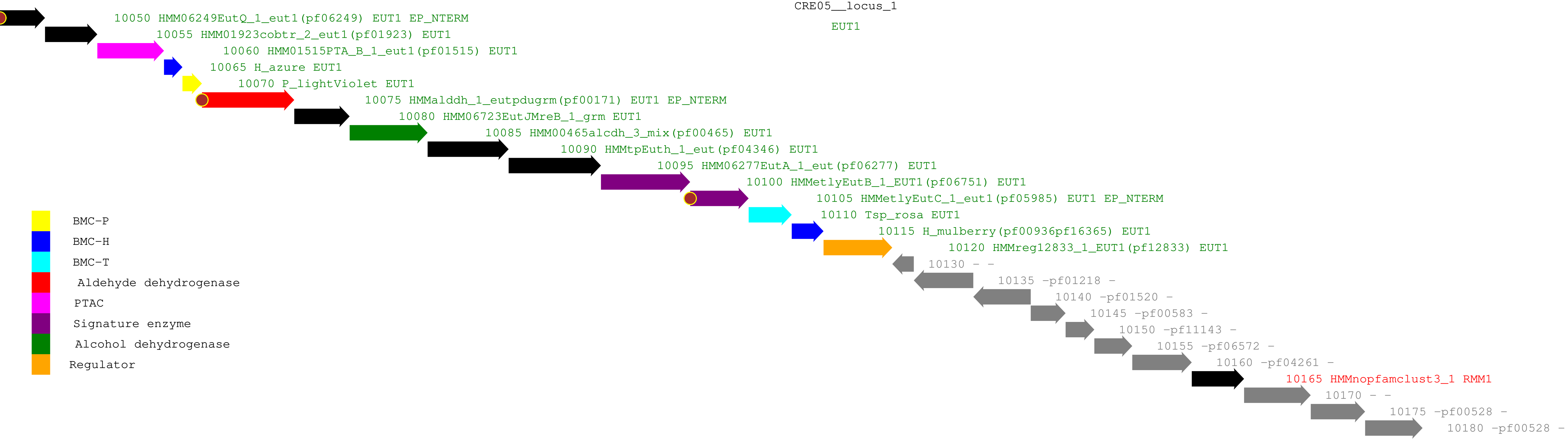

Escherichia sp. E4930. Bacteria; Proteobacteria; Gammaproteobacteria; Enterobacterales;

CRG96__locus_1

EUT1

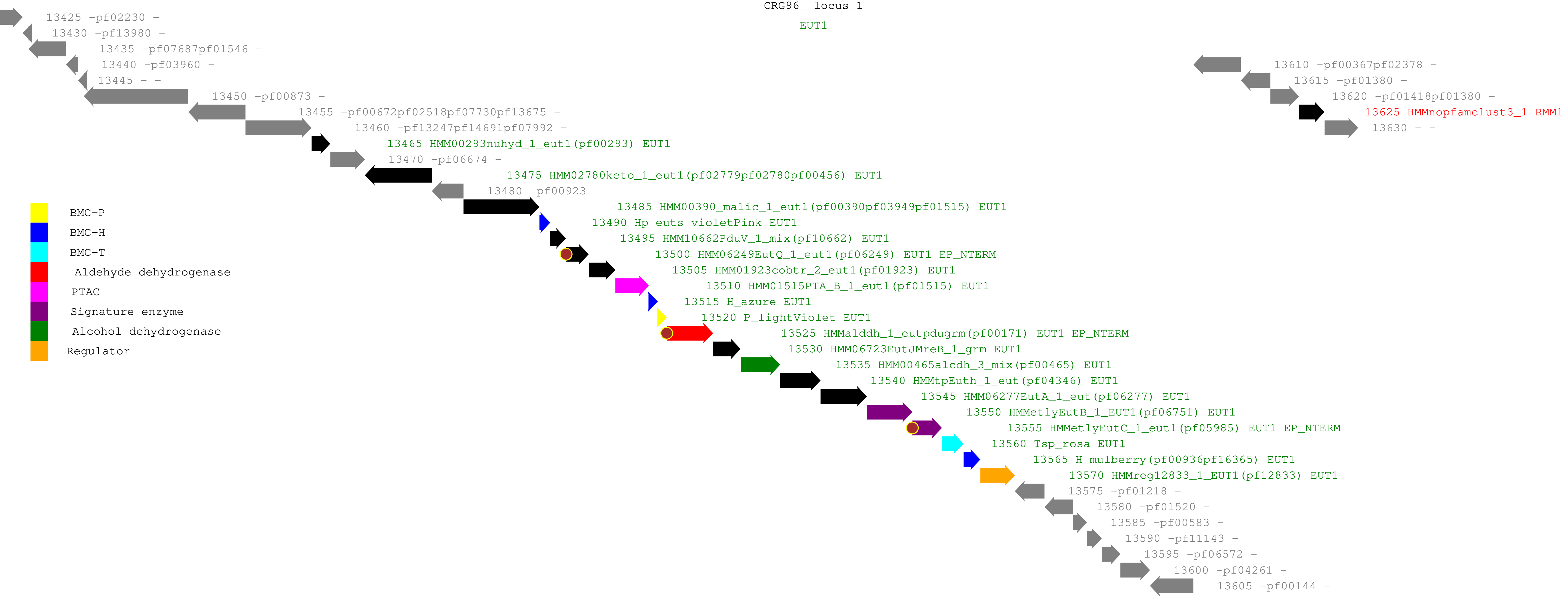

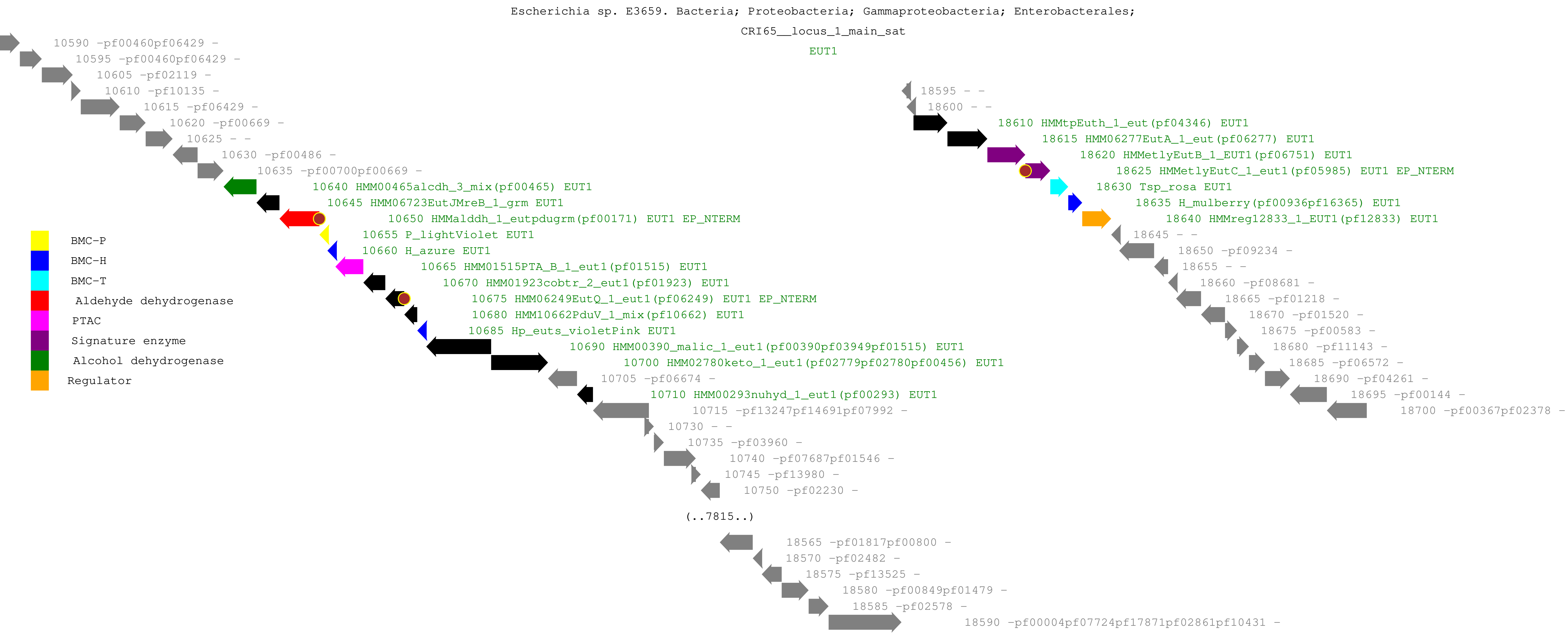
Escherichia sp. E3659. Bacteria; Proteobacteria; Gammaproteobacteria; Enterobacterales;
CRI65__locus_1_main_sat
EUT1
10590 -pf00460pf06429 -
10595 -pf00460pf06429 -
10605 -pf02119 -
10610 -pf10135 -
10615 -pf06429 -
10620 -pf00669 -
10625 - -
10630 -pf00486 -
10635 -pf00700pf00669 -
10640 HMM00465alcdh_3_mix(pf00465) EUT1
10645 HMM06723EutJMreB_1_grm EUT1
10650 HMMalddh_1_eutpdugrm(pf00171) EUT1 EP_NTERM
10655 P_lightViolet EUT1
10660 H_azure EUT1
10665 HMM01515PTA_B_1_eut1(pf01515) EUT1
10670 HMM01923cobtr_2_eut1(pf01923) EUT1
10675 HMM06249EutQ_1_eut1(pf06249) EUT1 EP_NTERM
10680 HMM10662PduV_1_mix(pf10662) EUT1
10685 Hp_euts_violetPink EUT1
10690 HMM00390_malic_1_eut1(pf00390pf03949pf01515) EUT1
10700 HMM02780keto_1_eut1(pf02779pf02780pf00456) EUT1
10705 -pf06674 -
10710 HMM00293nuhyd_1_eut1(pf00293) EUT1
10715 -pf13247pf14691pf07992 -
10730 - -
10735 -pf03960 -
10740 -pf07687pf01546 -
10745 -pf13980 -
10750 -pf02230 -
(..7815..)
18565 -pf01817pf00800 -
18570 -pf02482 -
18575 -pf13525 -
18580 -pf00849pf01479 -
18585 -pf02578 -
18590 -pf00004pf07724pf17871pf02861pf10431 -
18595 - -
18600 - -
18610 HMMtpEuth_1_eut(pf04346) EUT1
18615 HMM06277EutA_1_eut(pf06277) EUT1
18620 HMMetlyEutB_1_EUT1(pf06751) EUT1
18625 HMMetlyEutC_1_eut1(pf05985) EUT1 EP_NTERM
18630 Tsp_rosa EUT1
18635 H_mulberry(pf00936pf16365) EUT1
18640 HMMreg12833_1_EUT1(pf12833) EUT1
18645 - -
18650 -pf09234 -
18655 - -
18660 -pf08681 -
18665 -pf01218 -
18670 -pf01520 -
18675 -pf00583 -
18680 -pf11143 -
18685 -pf06572 -
18690 -pf04261 -
18695 -pf00144 -
18700 -pf00367pf02378 -
BMC-P
BMC-H
BMC-T
Aldehyde dehydrogenase
PTAC
Signature enzyme
Alcohol dehydrogenase
Regulator

Escherichia sp. E4702. Bacteria; Proteobacteria; Gammaproteobacteria; Enterobacterales;

CRI67__locus_1

EUT1

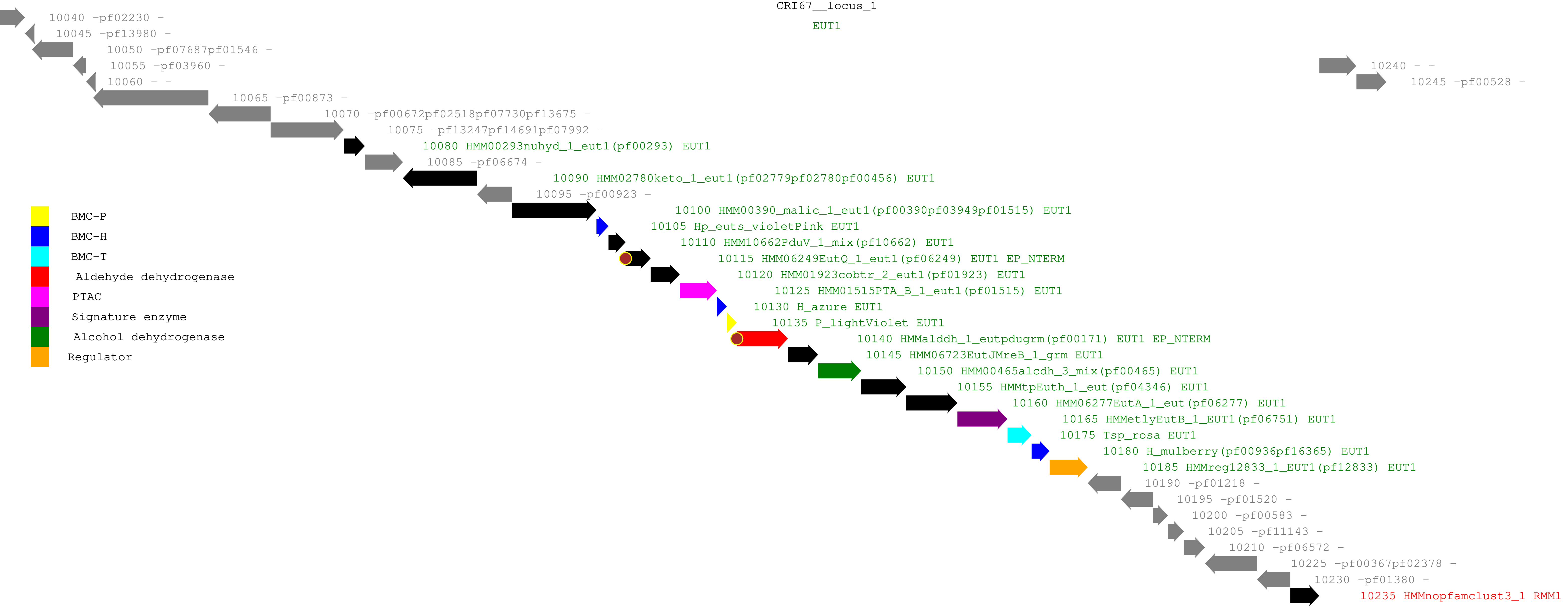

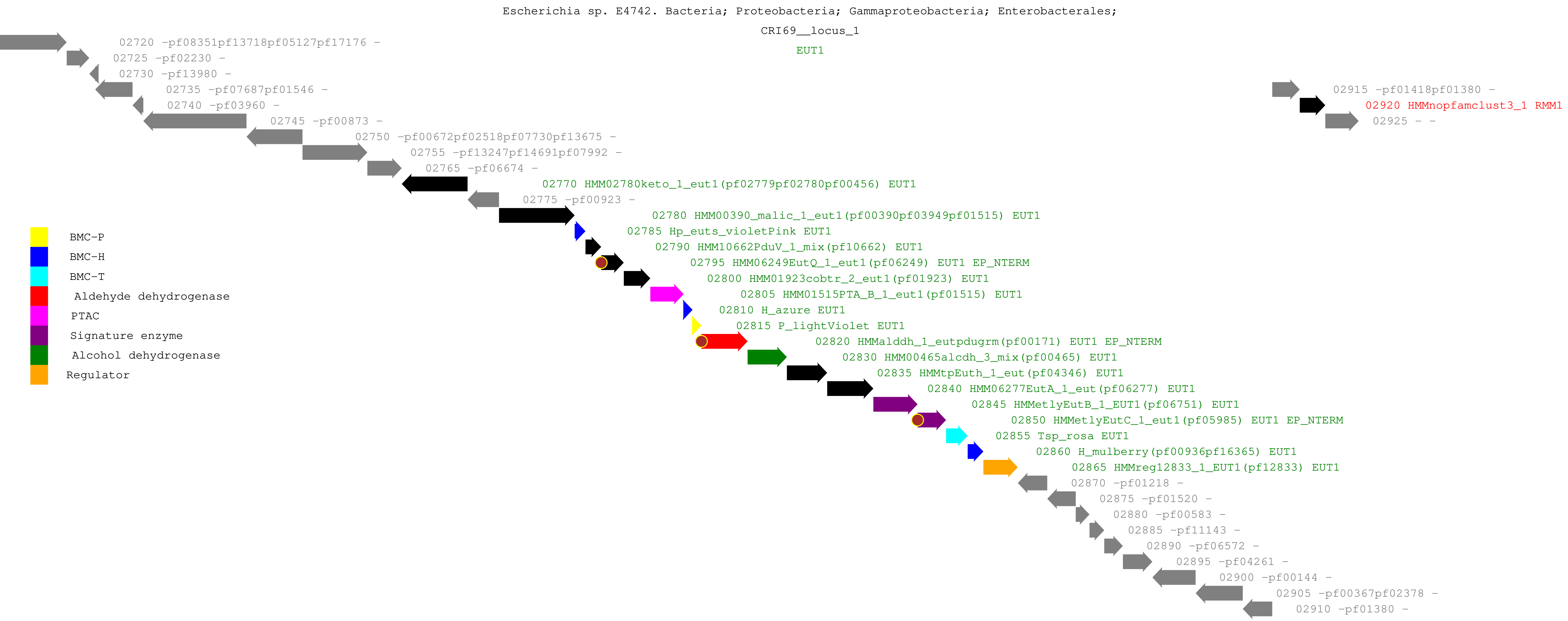
Escherichia sp. E4742. Bacteria; Proteobacteria; Gammaproteobacteria; Enterobacterales;
CRI69__locus_1
EUT1
02720 -pf08351pf13718pf05127pf17176 -
02725 -pf02230 -
02730 -pf13980 -
02735 -pf07687pf01546 -
02740 -pf03960 -
02745 -pf00873 -
02750 -pf00672pf02518pf07730pf13675 -
02755 -pf13247pf14691pf07992 -
02765 -pf06674 -
02770 HMM02780keto_1_eut1(pf02779pf02780pf00456) EUT1
02775 -pf00923 -
02780 HMM00390_malic_1_eut1(pf00390pf03949pf01515) EUT1
02785 Hp_euts_violetPink EUT1
02790 HMM10662PduV_1_mix(pf10662) EUT1
02795 HMM06249EutQ_1_eut1(pf06249) EUT1 EP_NTERM
02800 HMM01923cobtr_2_eut1(pf01923) EUT1
02805 HMM01515PTA_B_1_eut1(pf01515) EUT1
02810 H_azure EUT1
02815 P_lightViolet EUT1
02820 HMMalddh_1_eutpdugrm(pf00171) EUT1 EP_NTERM
02830 HMM00465alcdh_3_mix(pf00465) EUT1
02835 HMMtpEuth_1_eut(pf04346) EUT1
02840 HMM06277EutA_1_eut(pf06277) EUT1
02845 HMMetlyEutB_1_EUT1(pf06751) EUT1
02850 HMMetlyEutC_1_eut1(pf05985) EUT1 EP_NTERM
02855 Tsp_rosa EUT1
02860 H_mulberry(pf00936pf16365) EUT1
02865 HMMreg12833_1_EUT1(pf12833) EUT1
02870 -pf01218 -
02875 -pf01520 -
02880 -pf00583 -
02885 -pf11143 -
02890 -pf06572 -
02895 -pf04261 -
02900 -pf00144 -
02905 -pf00367pf02378 -
02910 -pf01380 -
02915 -pf01418pf01380 -
02920 HMMnopfamclust3_1 RMM1
02925 - -
BMC-P
BMC-H
BMC-T
Aldehyde dehydrogenase
PTAC
Signature enzyme
Alcohol dehydrogenase
Regulator

Escherichia sp. E5028. Bacteria; Proteobacteria; Gammaproteobacteria; Enterobacterales;

CRT22__locus_2

EUT1

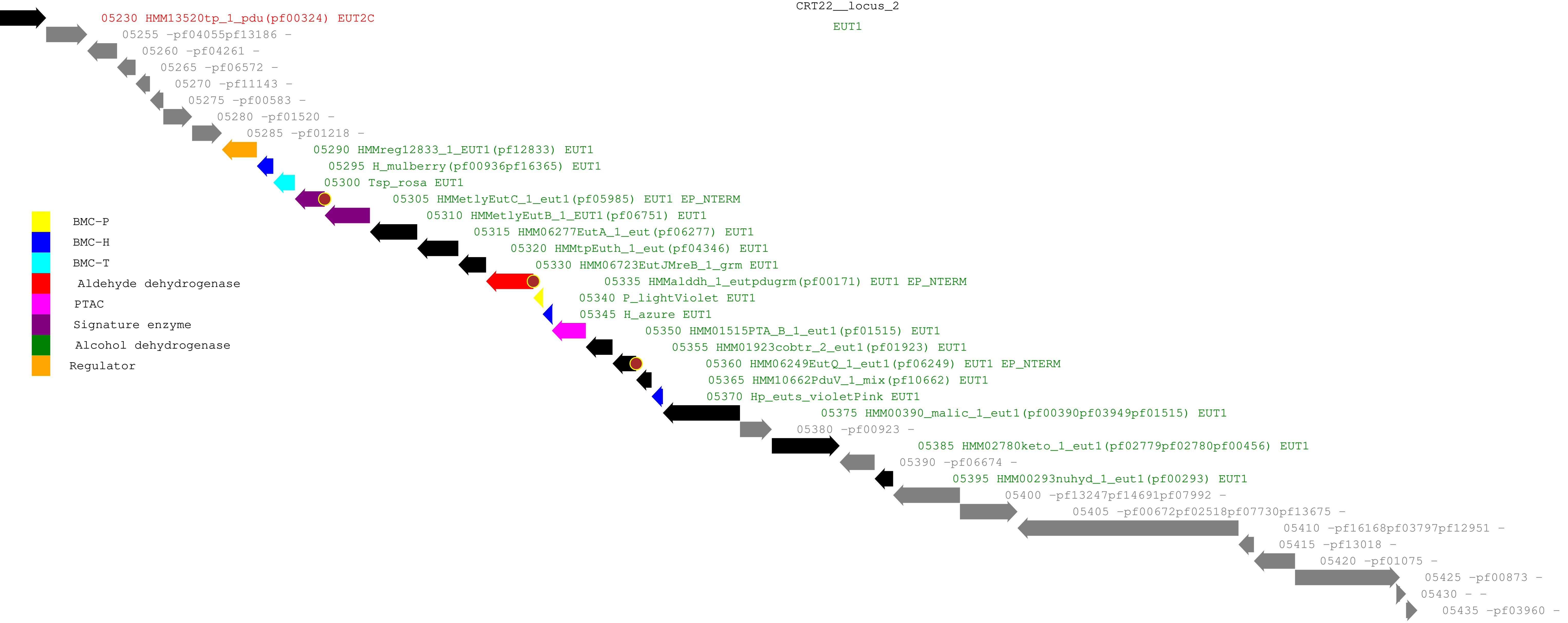

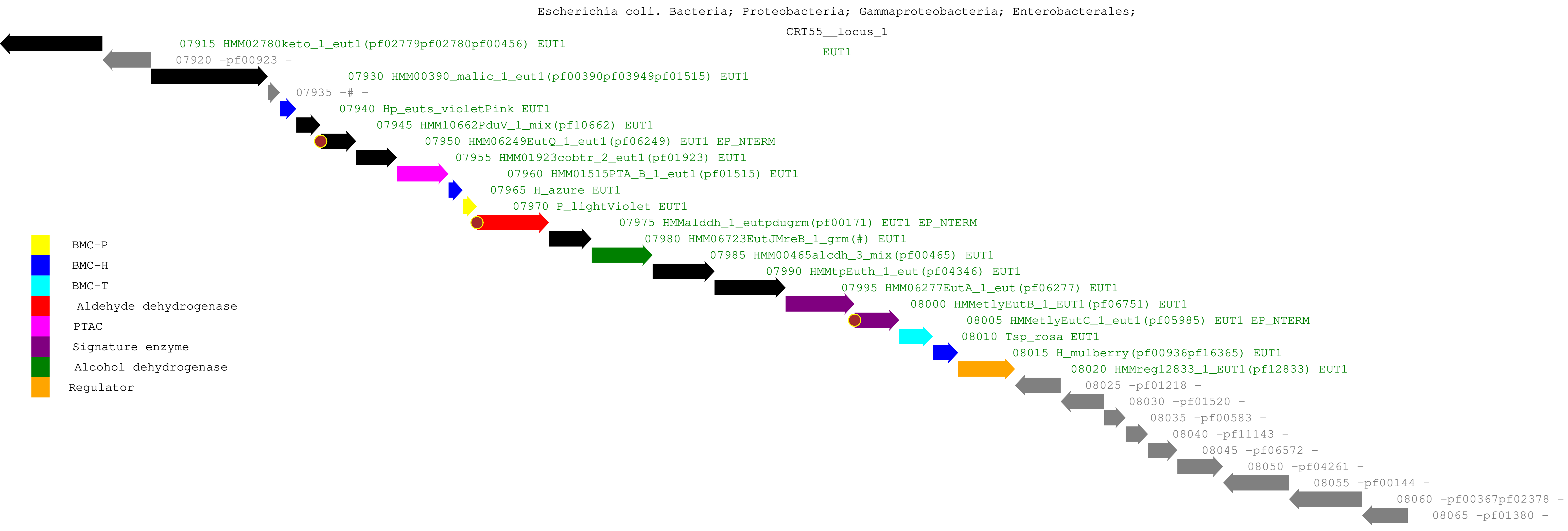
Escherichia coli. Bacteria; Proteobacteria; Gammaproteobacteria; Enterobacterales;
CRT55__locus_1
EUT1
07915 HMM02780keto_1_eut1(pf02779pf02780pf00456) EUT1
07920 -pf00923 -
07930 HMM00390_malic_1_eut1(pf00390pf03949pf01515) EUT1
07935 -# -
07940 Hp_euts_violetPink EUT1
07945 HMM10662PduV_1_mix(pf10662) EUT1
07950 HMM06249EutQ_1_eut1(pf06249) EUT1 EP_NTERM
07955 HMM01923cobtr_2_eut1(pf01923) EUT1
07960 HMM01515PTA_B_1_eut1(pf01515) EUT1
07965 H_azure EUT1
07970 P_lightViolet EUT1
07975 HMMaldh_1_eutpdugrm(pf00171) EUT1 EP_NTERM
07980 HMM06723EutJMreB_1_grm(#) EUT1
07985 HMM00465alcdh_3_mix(pf00465) EUT1
07990 HMMtpEuth_1_eut(pf04346) EUT1
07995 HMM06277EutA_1_eut(pf06277) EUT1
08000 HMMetlyEutB_1_EUT1(pf06751) EUT1
08005 HMMetlyEutC_1_eut1(pf05985) EUT1 EP_NTERM
08010 Tsp_rosa EUT1
08015 H_mulberry(pf00936pf16365) EUT1
08020 HMMreg12833_1_EUT1(pf12833) EUT1
08025 -pf01218 -
08030 -pf01520 -
08035 -pf00583 -
08040 -pf11143 -
08045 -pf06572 -
08050 -pf04261 -
08055 -pf00144 -
08060 -pf00367pf02378 -
08065 -pf01380 -
BMC-P
BMC-H
BMC-T
Aldehyde dehydrogenase
PTAC
Signature enzyme
Alcohol dehydrogenase
Regulator

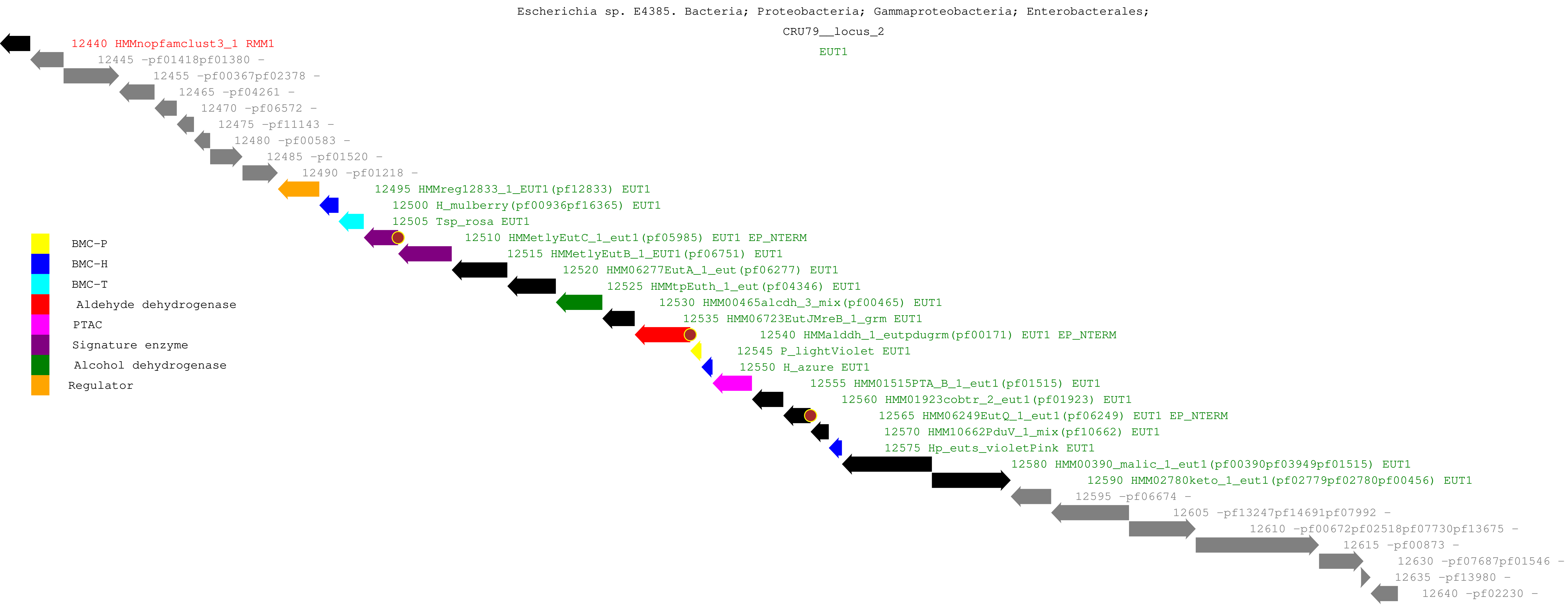
Escherichia sp. E4385. Bacteria; Proteobacteria; Gammaproteobacteria; Enterobacterales;
CRU79__locus_2
EUT1
12440 HMMnopfamclust3_1 RMM1
12445 -pf01418pf01380 -
12455 -pf00367pf02378 -
12465 -pf04261 -
12470 -pf06572 -
12475 -pf11143 -
12480 -pf00583 -
12485 -pf01520 -
12490 -pf01218 -
12495 HMMreg12833_1_EUT1(pf12833) EUT1
12500 H_mulberry(pf00936pf16365) EUT1
12505 Tsp_rosa EUT1
12510 HMMetlyEutC_1_eut1(pf05985) EUT1 EP_NTERM
12515 HMMetlyEutB_1_EUT1(pf06751) EUT1
12520 HMM06277EutA_1_eut(pf06277) EUT1
12525 HMMtpEuth_1_eut(pf04346) EUT1
12530 HMM00465alcdh_3_mix(pf00465) EUT1
12535 HMM06723EutJMreB_1_grm EUT1
12540 HMMaldh_1_eutpdugrm(pf00171) EUT1 EP_NTERM
12545 P_lightViolet EUT1
12550 H_azure EUT1
12555 HMM01515PTA_B_1_eut1(pf01515) EUT1
12560 HMM01923cobtr_2_eut1(pf01923) EUT1
12565 HMM06249EutQ_1_eut1(pf06249) EUT1 EP_NTERM
12570 HMM10662PduV_1_mix(pf10662) EUT1
12575 Hp_euts_violetPink EUT1
12580 HMM00390_malic_1_eut1(pf00390pf03949pf01515) EUT1
12590 HMM02780keto_1_eut1(pf02779pf02780pf00456) EUT1
12595 -pf06674 -
12605 -pf13247pf14691pf07992 -
12610 -pf00672pf02518pf07730pf13675 -
12615 -pf00873 -
12630 -pf07687pf01546 -
12635 -pf13980 -
12640 -pf02230 -
BMC-P
BMC-H
BMC-T
Aldehyde dehydrogenase
PTAC
Signature enzyme
Alcohol dehydrogenase
Regulator

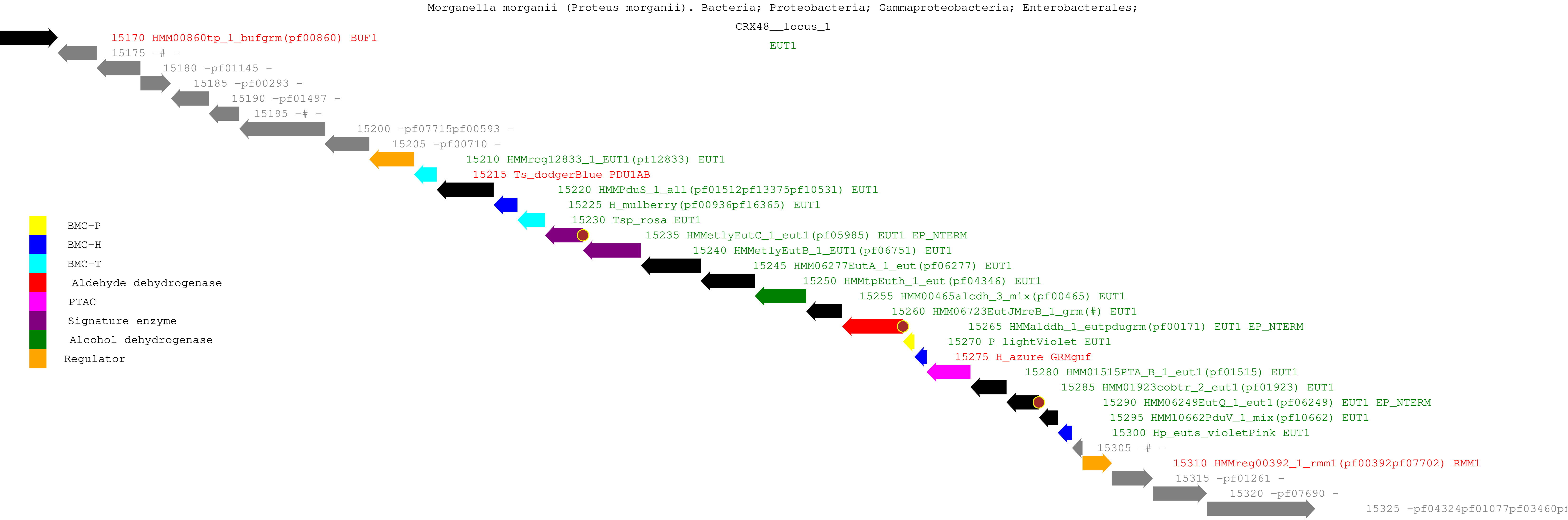
Morganella morganii (Proteus morganii). Bacteria; Proteobacteria; Gammaproteobacteria; Enterobacterales;
CRX48__locus_1
EUT1
15170 HMM00860tp_1_bufgrm(pf00860) BUF1
15175 -# -
15180 -pf01145 -
15185 -pf00293 -
15190 -pf01497 -
15195 -# -
15200 -pf07715pf00593 -
15205 -pf00710 -
15210 HMMreg12833_1_EUT1(pf12833) EUT1
15215 Ts_dodgerBlue PDU1AB
15220 HMMPduS_1_all(pf01512pf13375pf10531) EUT1
15225 H_mulberry(pf00936pf16365) EUT1
15230 Tsp_rosa EUT1
15235 HMMetlyEutC_1_eut1(pf05985) EUT1 EP_NTERM
15240 HMMetlyEutB_1_EUT1(pf06751) EUT1
15245 HMM06277EutA_1_eut(pf06277) EUT1
15250 HMMtpEuth_1_eut(pf04346) EUT1
15255 HMM00465alcdh_3_mix(pf00465) EUT1
15260 HMM06723EutJMreB_1_grm(#) EUT1
15265 HMMalddh_1_eutpdugrm(pf00171) EUT1 EP_NTERM
15270 P_lightViolet EUT1
15275 H_azure GRMguf
15280 HMM01515PTA_B_1_eut1(pf01515) EUT1
15285 HMM01923cobtr_2_eut1(pf01923) EUT1
15290 HMM06249EutQ_1_eut1(pf06249) EUT1 EP_NTERM
15295 HMM10662PduV_1_mix(pf10662) EUT1
15300 Hp_euts_violetPink EUT1
15305 -# -
15310 HMMreg00392_1_rmm1(pf00392pf07702) RMM1
15315 -pf01261 -
15320 -pf07690 -
15325 -pf04324pf01077pf03460p
BMC-P
BMC-H
BMC-T
Aldehyde dehydrogenase
PTAC
Signature enzyme
Alcohol dehydrogenase
Regulator

Pluralibacter gergoviae (Enterobacter gergoviae). Bacteria; Proteobacteria; Gammaproteobacteria; Enterobacterales;

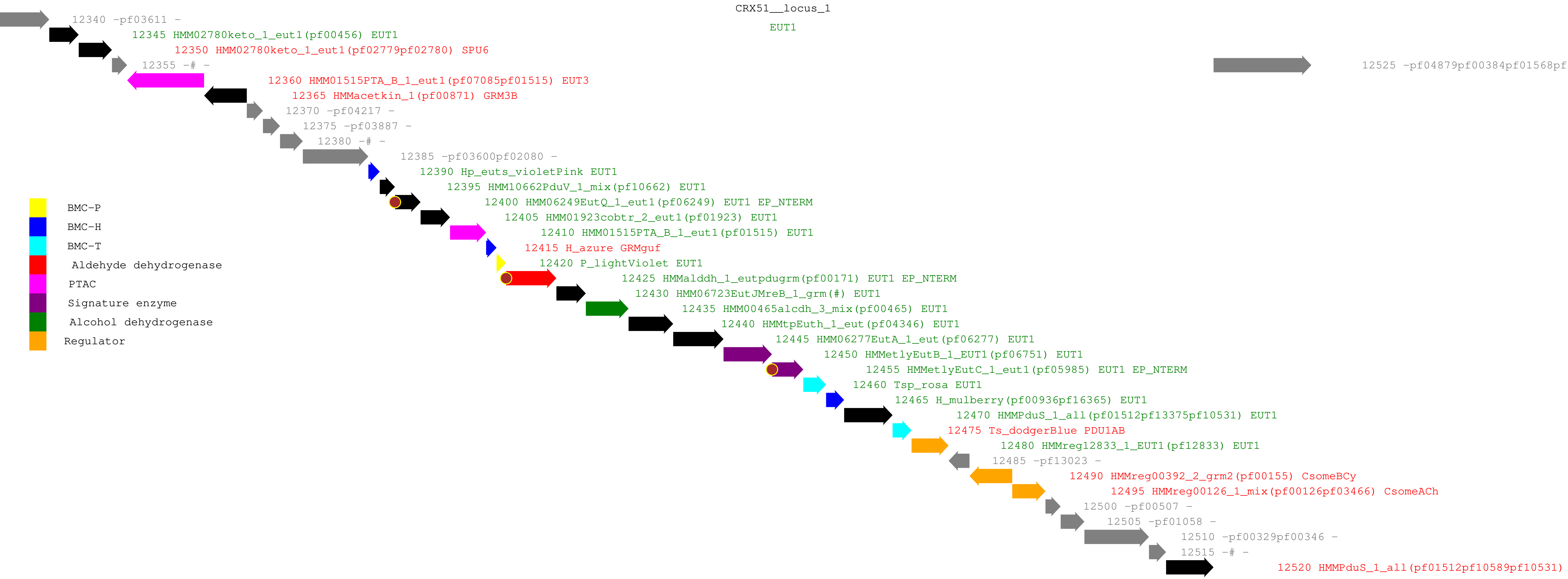

Escherichia coli O103. Bacteria; Proteobacteria; Gammaproteobacteria; Enterobacterales;

CRY61__locus_1

EUT1

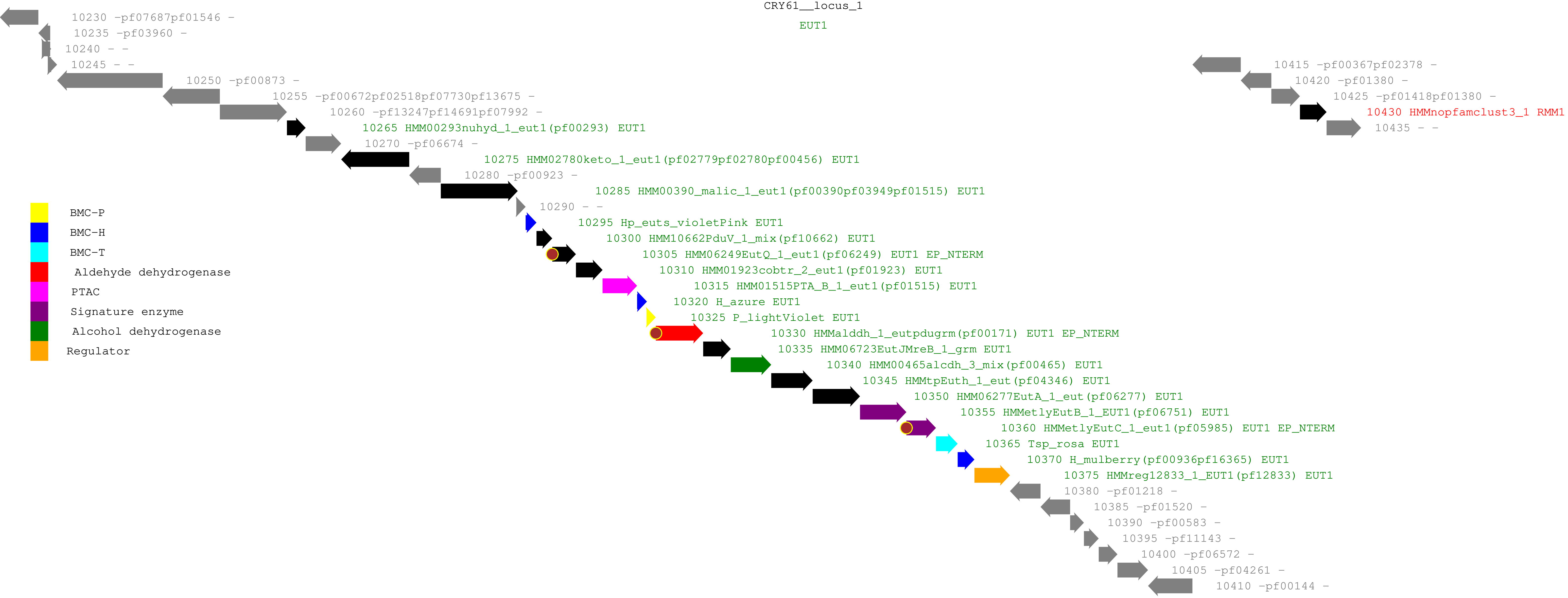

Salmonella enterica subsp. enterica serovar Gaminara. Bacteria; Proteobacteria; Gammaproteobacteria; Enterobacterales;

CS348__locus_1

EUT1

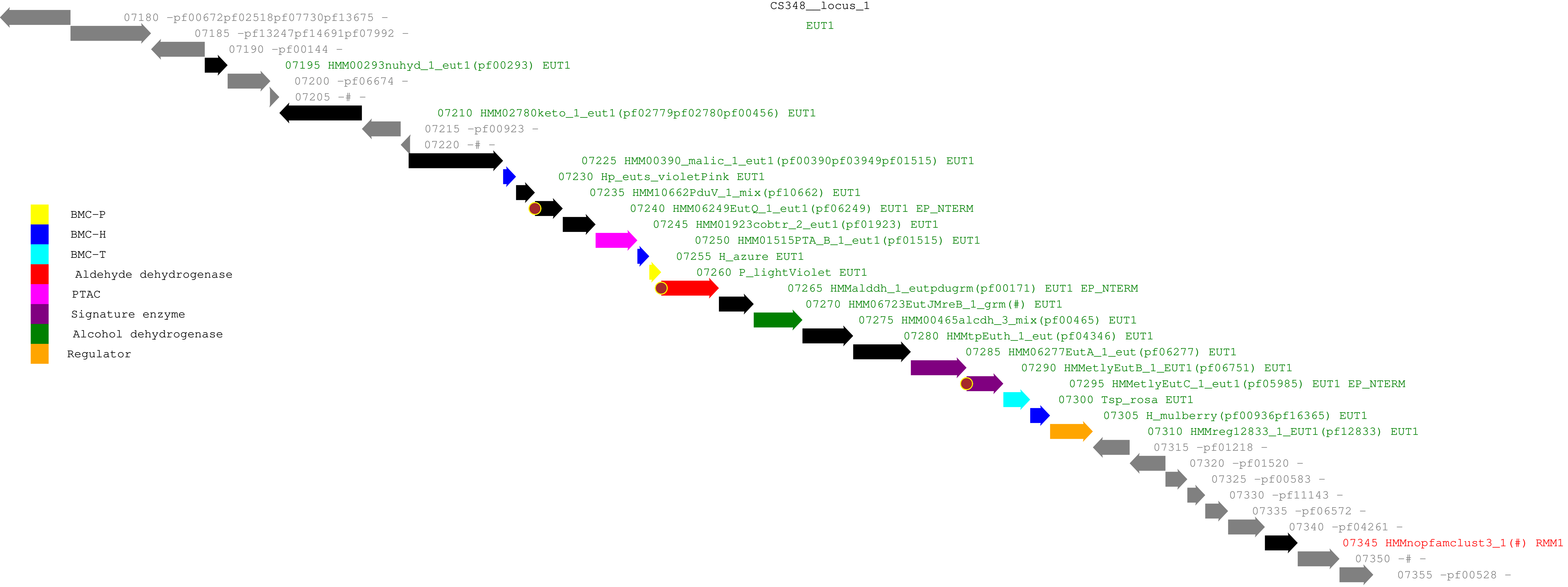

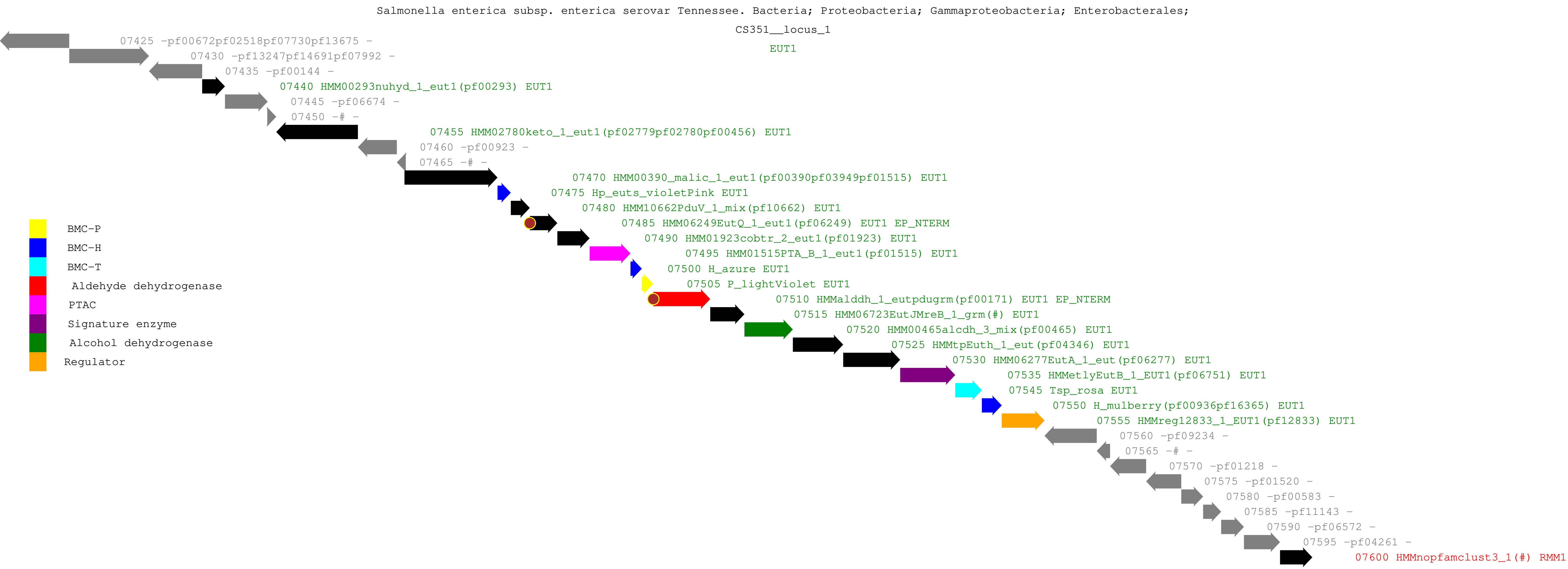

Salmonella enterica subsp. enterica serovar Tennessee. Bacteria; Proteobacteria; Gammaproteobacteria; Enterobacterales;
CS351__locus_1
EUT1
07425 -pf00672pf02518pf07730pf13675 -
07430 -pf13247pf14691pf07992 -
07435 -pf00144 -
07440 HMM00293nuhyd_1_eut1(pf00293) EUT1
07445 -pf06674 -
07450 -# -
07455 HMM02780keto_1_eut1(pf02779pf02780pf00456) EUT1
07460 -pf00923 -
07465 -# -
07470 HMM00390_malic_1_eut1(pf00390pf03949pf01515) EUT1
07475 Hp_euts_violetPink EUT1
07480 HMM10662PduV_1_mix(pf10662) EUT1
07485 HMM06249EutQ_1_eut1(pf06249) EUT1 EP_NTERM
07490 HMM01923cobtr_2_eut1(pf01923) EUT1
07495 HMM01515PTA_B_1_eut1(pf01515) EUT1
07500 H_azure EUT1
07505 P_lightViolet EUT1
07510 HMMalddh_1_eutpdugrm(pf00171) EUT1 EP_NTERM
07515 HMM06723EutJMreB_1_grm(#) EUT1
07520 HMM00465alcdh_3_mix(pf00465) EUT1
07525 HMMtpEuth_1_eut(pf04346) EUT1
07530 HMM06277EutA_1_eut(pf06277) EUT1
07535 HMMetlyEutB_1_EUT1(pf06751) EUT1
07545 Tsp_rosa EUT1
07550 H_mulberry(pf00936pf16365) EUT1
07555 HMMreg12833_1_EUT1(pf12833) EUT1
07560 -pf09234 -
07565 -# -
07570 -pf01218 -
07575 -pf01520 -
07580 -pf00583 -
07585 -pf11143 -
07590 -pf06572 -
07595 -pf04261 -
07600 HMMnopfamclust3_1(#) RMM1
BMC-P
BMC-H
BMC-T
Aldehyde dehydrogenase
PTAC
Signature enzyme
Alcohol dehydrogenase
Regulator

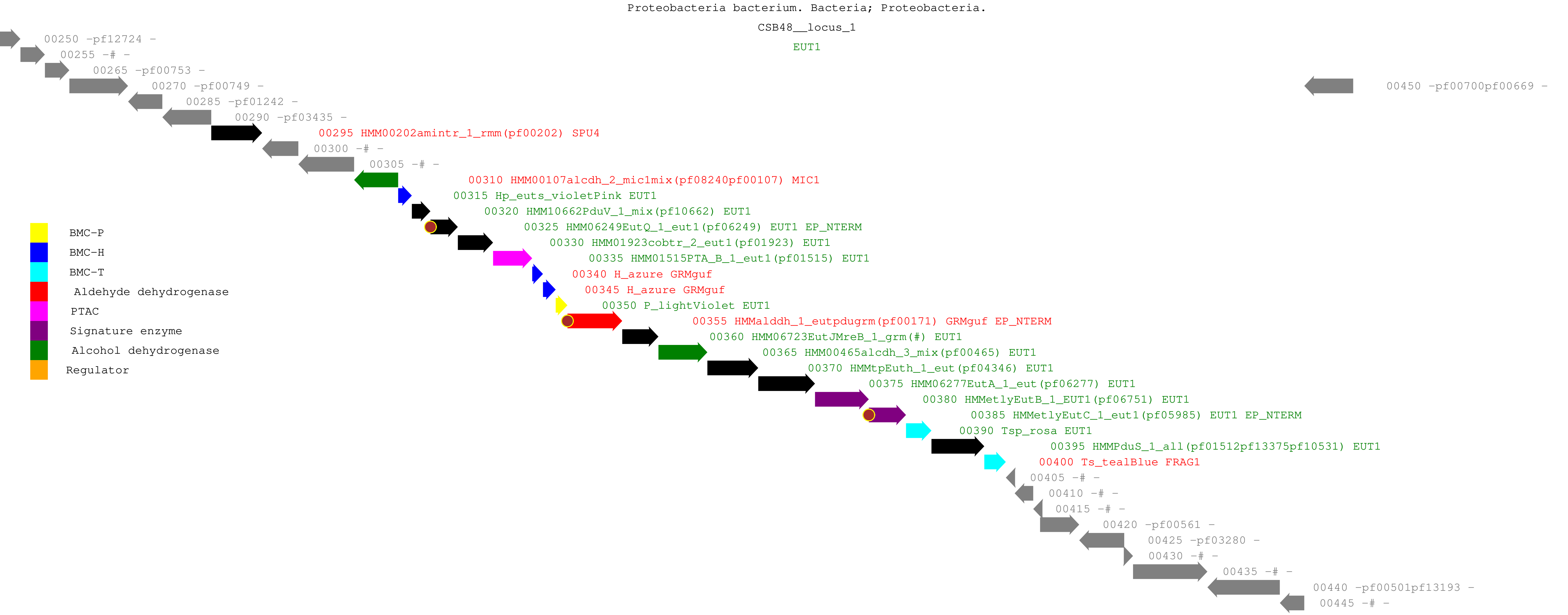

Proteobacteria bacterium. Bacteria; Proteobacteria.
CSB48__locus_1
EUT1
00250 -pf12724 -
00255 -# -
00265 -pf00753 -
00270 -pf00749 -
00285 -pf01242 -
00290 -pf03435 -
00295 HMM00202amintr_1_rmm(pf00202) SPU4
00300 -# -
00305 -# -
00310 HMM00107alcdh_2_mic1mix(pf08240pf00107) MIC1
00315 Hp_euts_violetPink EUT1
00320 HMM10662PduV_1_mix(pf10662) EUT1
00325 HMM06249EutQ_1_eut1(pf06249) EUT1 EP_NTERM
00330 HMM01923cobtr_2_eut1(pf01923) EUT1
00335 HMM01515PTA_B_1_eut1(pf01515) EUT1
00340 H_azure GRMguf
00345 H_azure GRMguf
00350 P_lightViolet EUT1
00355 HMMalddh_1_eutpdugrm(pf00171) GRMguf EP_NTERM
00360 HMM06723EutJMreB_1_grm(#) EUT1
00365 HMM00465alcdh_3_mix(pf00465) EUT1
00370 HMMtpEuth_1_eut(pf04346) EUT1
00375 HMM06277EutA_1_eut(pf06277) EUT1
00380 HMMetlyEutB_1_EUT1(pf06751) EUT1
00385 HMMetlyEutC_1_eut1(pf05985) EUT1 EP_NTERM
00390 Tsp_rosa EUT1
00395 HMMPduS_1_all(pf01512pf13375pf10531) EUT1
00400 Ts_tealBlue FRAG1
00405 -# -
00410 -# -
00415 -# -
00420 -pf00561 -
00425 -pf03280 -
00430 -# -
00435 -# -
00440 -pf00501pf13193 -
00445 -# -
00450 -pf00700pf00669 -
BMC-P
BMC-H
BMC-T
Aldehyde dehydrogenase
PTAC
Signature enzyme
Alcohol dehydrogenase
Regulator

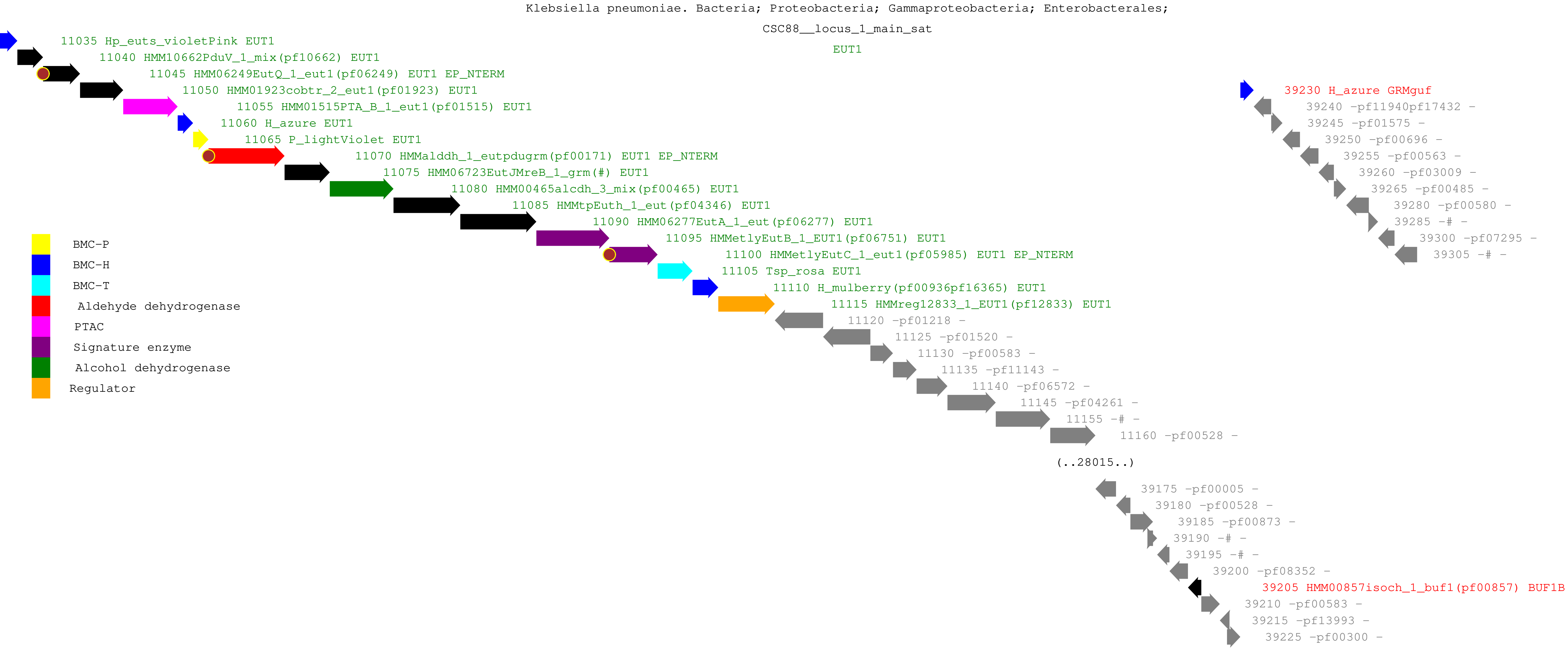
Klebsiella pneumoniae. Bacteria; Proteobacteria; Gammaproteobacteria; Enterobacterales;
CSC88__locus_1_main_sat
EUT1
11035 Hp_euts_violetPink EUT1
11040 HMM10662PduV_1_mix(pf10662) EUT1
11045 HMM06249EutQ_1_eut1(pf06249) EUT1 EP_NTERM
11050 HMM01923cobtr_2_eut1(pf01923) EUT1
11055 HMM01515PTA_B_1_eut1(pf01515) EUT1
11060 H_azure EUT1
11065 P_lightViolet EUT1
11070 HMMalddh_1_eutpdugrm(pf00171) EUT1 EP_NTERM
11075 HMM06723EutJMreB_1_grm(#) EUT1
11080 HMM00465alcdh_3_mix(pf00465) EUT1
11085 HMMtpEuth_1_eut(pf04346) EUT1
11090 HMM06277EutA_1_eut(pf06277) EUT1
11095 HMMetlyEutB_1_EUT1(pf06751) EUT1
11100 HMMetlyEutC_1_eut1(pf05985) EUT1 EP_NTERM
11105 Tsp_rosa EUT1
11110 H_mulberry(pf00936pf16365) EUT1
11115 HMMreg12833_1_EUT1(pf12833) EUT1
11120 -pf01218 -
11125 -pf01520 -
11130 -pf00583 -
11135 -pf11143 -
11140 -pf06572 -
11145 -pf04261 -
11155 -# -
11160 -pf00528 -
(..28015..)
39175 -pf00005 -
39180 -pf00528 -
39185 -pf00873 -
39190 -# -
39195 -# -
39200 -pf08352 -
39205 HMM00857isoch_1_buf1(pf00857) BUF1B
39210 -pf00583 -
39215 -pf13993 -
39225 -pf00300 -
39230 H_azure GRMguf
39240 -pf11940pf17432 -
39245 -pf01575 -
39250 -pf00696 -
39255 -pf00563 -
39260 -pf03009 -
39265 -pf00485 -
39280 -pf00580 -
39285 -# -
39300 -pf07295 -
39305 -# -
BMC-P
BMC-H
BMC-T
Aldehyde dehydrogenase
PTAC
Signature enzyme
Alcohol dehydrogenase
Regulator

Salmonella enterica subsp. arizonae. Bacteria; Proteobacteria; Gammaproteobacteria; Enterobacterales;

CSM93__locus_1

EUT1

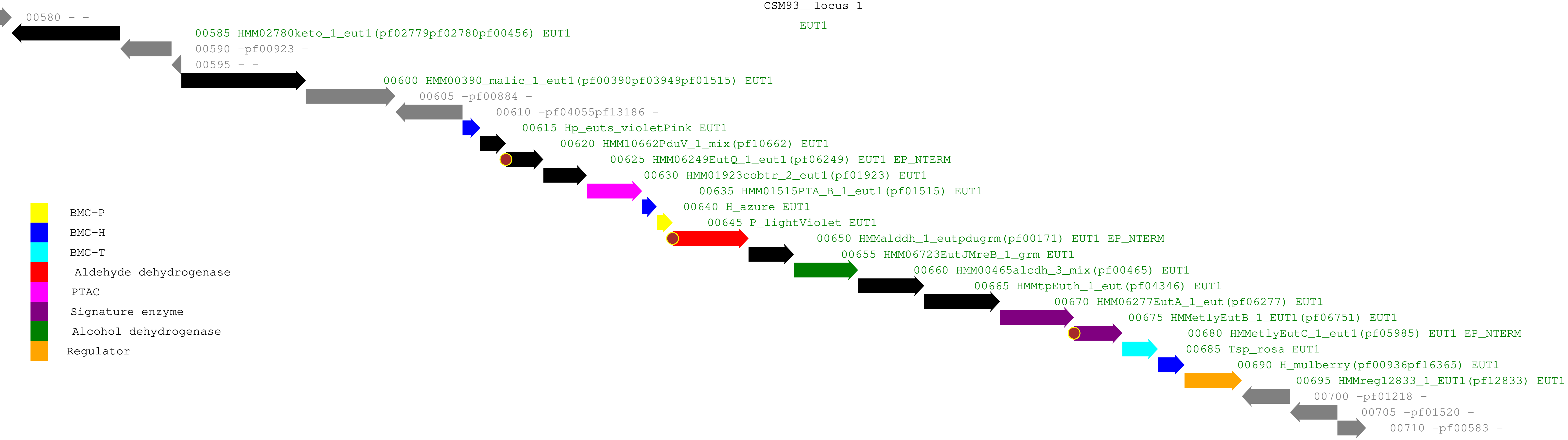

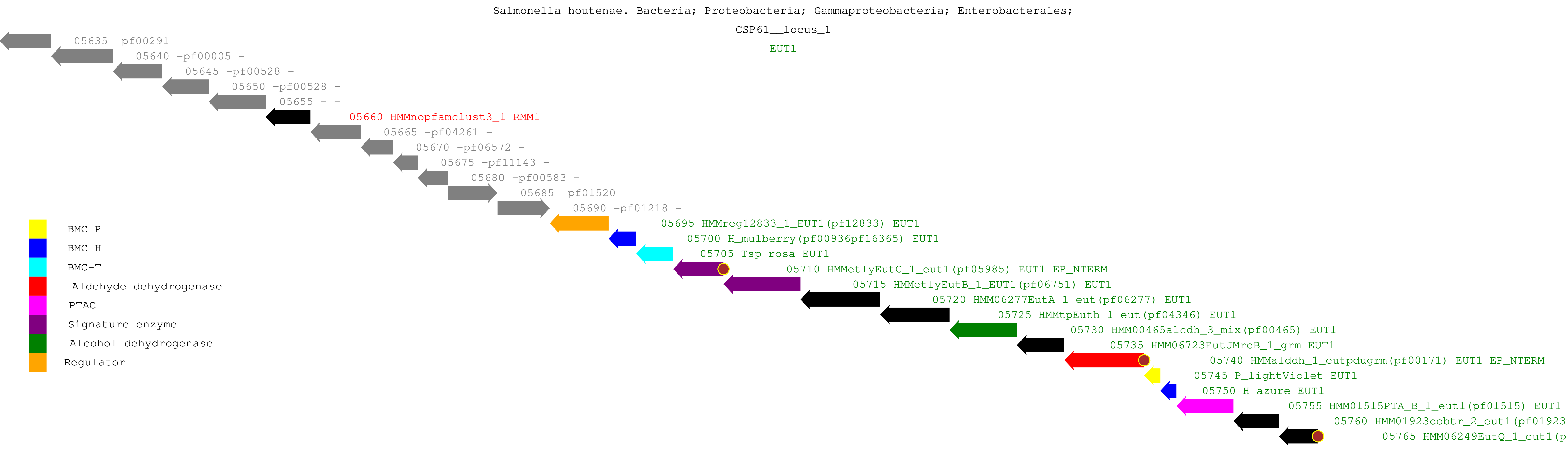
Salmonella houtenae. Bacteria; Proteobacteria; Gammaproteobacteria; Enterobacterales;
CSP61__locus_1
EUT1
05635 -pf00291 -
05640 -pf00005 -
05645 -pf00528 -
05650 -pf00528 -
05655 - -
05660 HMMnopfamclust3_1 RMM1
05665 -pf04261 -
05670 -pf06572 -
05675 -pf11143 -
05680 -pf00583 -
05685 -pf01520 -
05690 -pf01218 -
05695 HMMreg12833_1_EUT1(pf12833) EUT1
05700 H_mulberry(pf00936pf16365) EUT1
05705 Tsp_rosa EUT1
05710 HMMetlyEutC_1_eut1(pf05985) EUT1 EP_NTERM
05715 HMMetlyEutB_1_EUT1(pf06751) EUT1
05720 HMM06277EutA_1_eut(pf06277) EUT1
05725 HMMtpEuth_1_eut(pf04346) EUT1
05730 HMM00465alcdh_3_mix(pf00465) EUT1
05735 HMM06723EutJMreB_1_grm EUT1
05740 HMMaldd h_1_eutpdugrm(pf00171) EUT1 EP_NTERM
05745 P_lightViolet EUT1
05750 H_azure EUT1
05755 HMM01515PTA_B_1_eut1(pf01515) EUT1
05760 HMM01923cobtr_2_eut1(pf01923
05765 HMM06249EutQ_1_eut1(p
BMC-P
BMC-H
BMC-T
Aldehyde dehydrogenase
PTAC
Signature enzyme
Alcohol dehydrogenase
Regulator

Iodobacter sp. BJB302. Bacteria; Proteobacteria; Betaproteobacteria; Neisseriales;

CSQ88__locus_1

EUT1

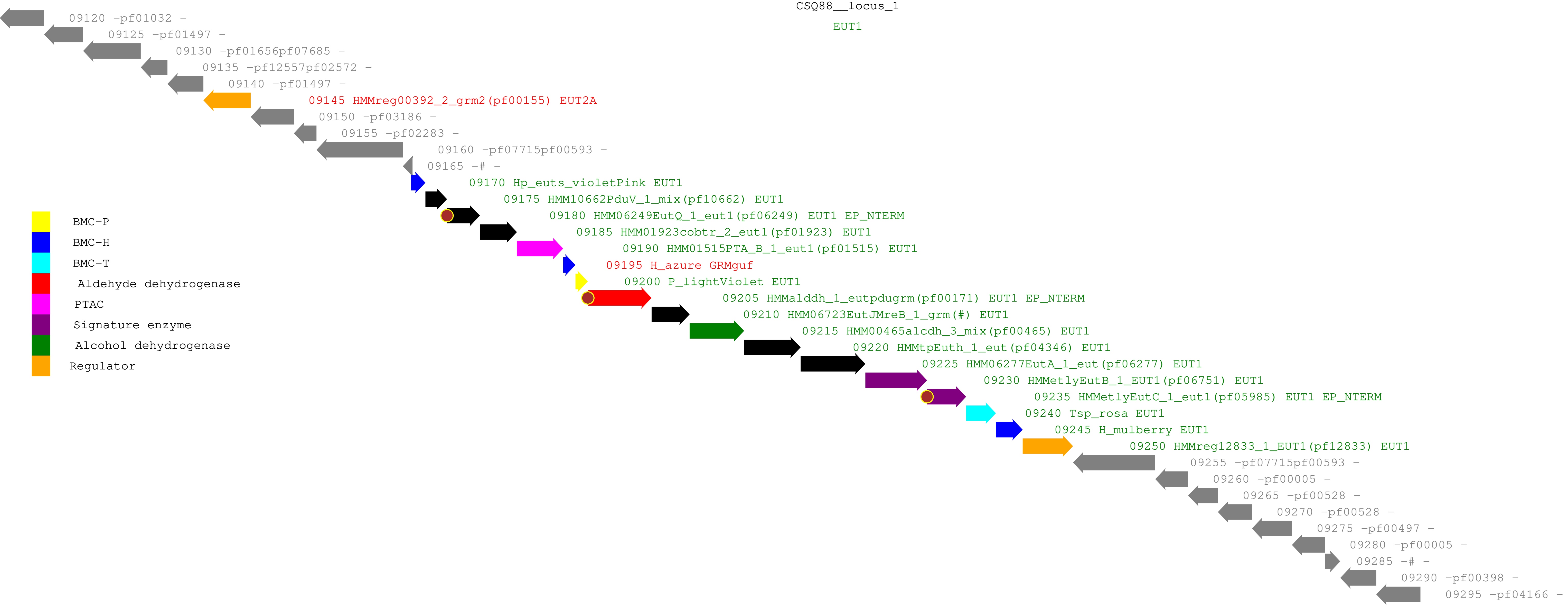

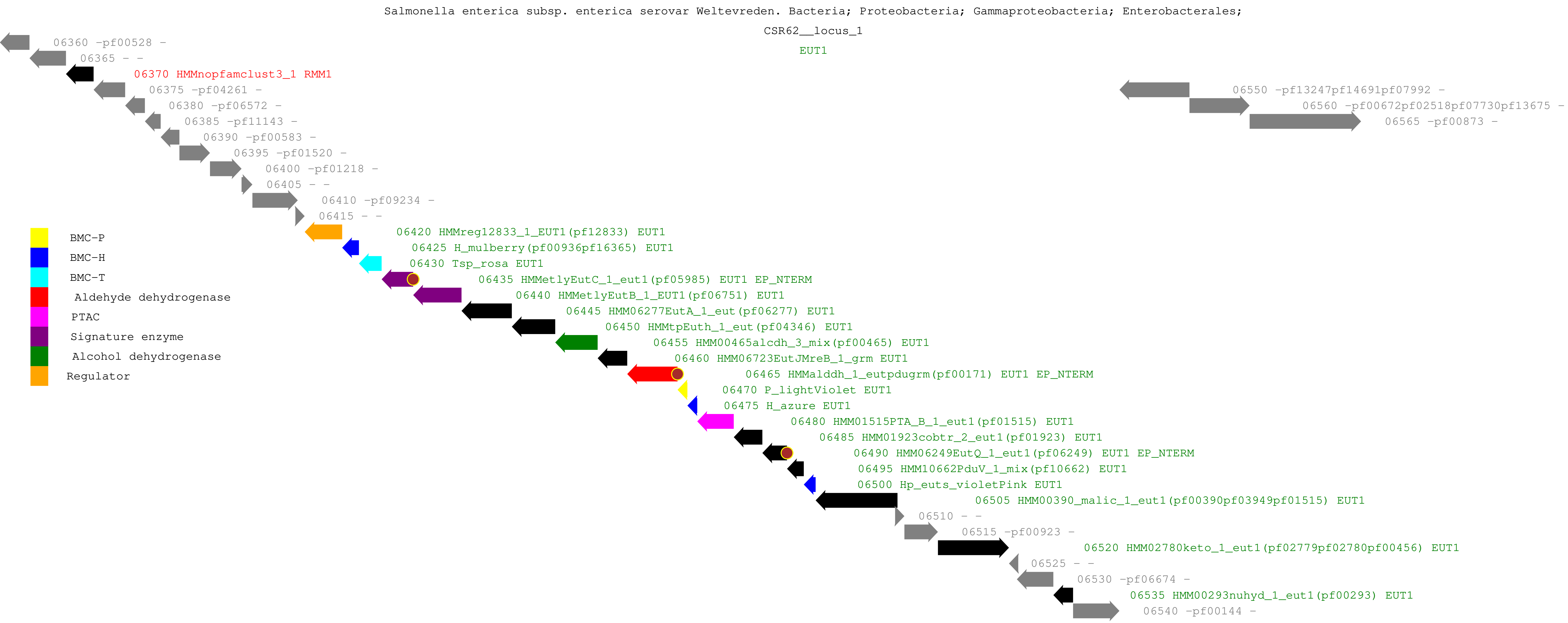
Salmonella enterica subsp. enterica serovar Weltevreden. Bacteria; Proteobacteria; Gammaproteobacteria; Enterobacterales;
CSR62__locus_1
EUT1
06360 -pf00528 -
06365 - -
06370 HMMnopfamclust3_1 RMM1
06375 -pf04261 -
06380 -pf06572 -
06385 -pf11143 -
06390 -pf00583 -
06395 -pf01520 -
06400 -pf01218 -
06405 - -
06410 -pf09234 -
06415 - -
06420 HMMreg12833_1_EUT1(pf12833) EUT1
06425 H_mulberry(pf00936pf16365) EUT1
06430 Tsp_rosa EUT1
06435 HMMetlyEutC_1_eut1(pf05985) EUT1 EP_NTERM
06440 HMMetlyEutB_1_EUT1(pf06751) EUT1
06445 HMM06277EutA_1_eut(pf06277) EUT1
06450 HMMtpEuth_1_eut(pf04346) EUT1
06455 HMM00465alcdh_3_mix(pf00465) EUT1
06460 HMM06723EutJMreB_1_grm EUT1
06465 HMMaldh_1_eutpdugrm(pf00171) EUT1 EP_NTERM
06470 P_lightViolet EUT1
06475 H_azure EUT1
06480 HMM01515PTA_B_1_eut1(pf01515) EUT1
06485 HMM01923cobtr_2_eut1(pf01923) EUT1
06490 HMM06249EutQ_1_eut1(pf06249) EUT1 EP_NTERM
06495 HMM10662PduV_1_mix(pf10662) EUT1
06500 Hp_euts_violetPink EUT1
06505 HMM00390_malic_1_eut1(pf00390pf03949pf01515) EUT1
06510 - -
06515 -pf00923 -
06520 HMM02780keto_1_eut1(pf02779pf02780pf00456) EUT1
06525 - -
06530 -pf06674 -
06535 HMM00293nuhyd_1_eut1(pf00293) EUT1
06540 -pf00144 -
06550 -pf13247pf14691pf07992 -
06560 -pf00672pf02518pf07730pf13675 -
06565 -pf00873 -
BMC-P
BMC-H
BMC-T
Aldehyde dehydrogenase
PTAC
Signature enzyme
Alcohol dehydrogenase
Regulator

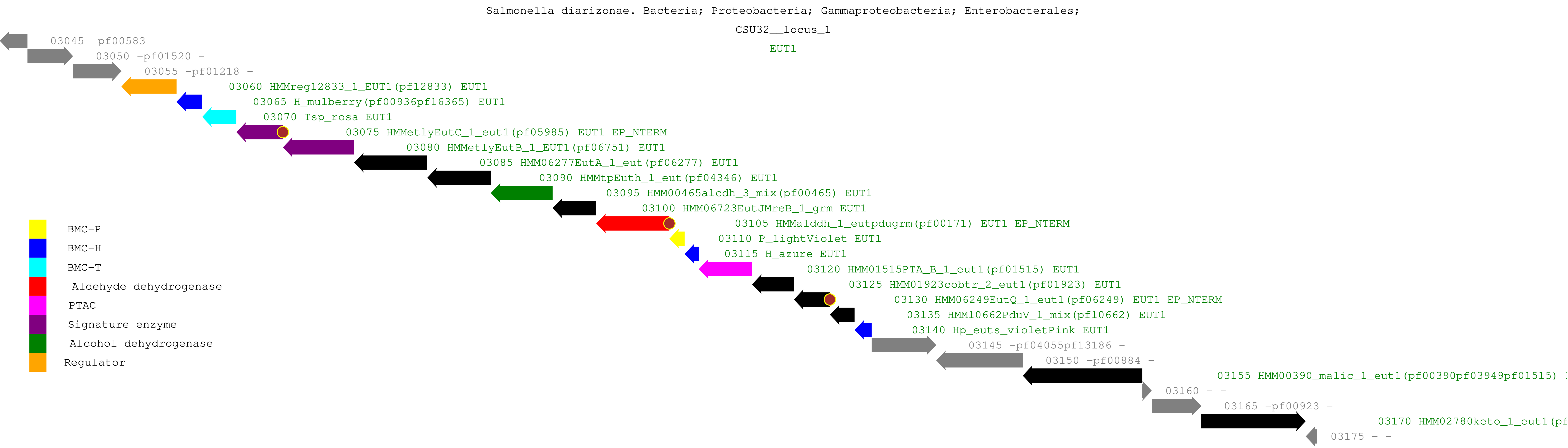
Salmonella diarizonae. Bacteria; Proteobacteria; Gammaproteobacteria; Enterobacterales;
CSU32__locus_1
EUT1
03045 -pf00583 -
03050 -pf01520 -
03055 -pf01218 -
03060 HMMreg12833_1_EUT1(pf12833) EUT1
03065 H_mulberry(pf00936pf16365) EUT1
03070 Tsp_rosa EUT1
03075 HMMetlyEutC_1_eut1(pf05985) EUT1 EP_NTERM
03080 HMMetlyEutB_1_EUT1(pf06751) EUT1
03085 HMM06277EutA_1_eut(pf06277) EUT1
03090 HMMtpEuth_1_eut(pf04346) EUT1
03095 HMM00465alcdh_3_mix(pf00465) EUT1
03100 HMM06723EutJMreB_1_grm EUT1
03105 HMMalddh_1_eutpdugrm(pf00171) EUT1 EP_NTERM
03110 P_lightViolet EUT1
03115 H_azure EUT1
03120 HMM01515PTA_B_1_eut1(pf01515) EUT1
03125 HMM01923cobtr_2_eut1(pf01923) EUT1
03130 HMM06249EutQ_1_eut1(pf06249) EUT1 EP_NTERM
03135 HMM10662PduV_1_mix(pf10662) EUT1
03140 Hp_euts_violetPink EUT1
03145 -pf04055pf13186 -
03150 -pf00884 -
03155 HMM00390_malic_1_eut1(pf00390pf03949pf01515)
03160 - -
03165 -pf00923 -
03170 HMM02780keto_1_eut1(pf
03175 - -
BMC-P
BMC-H
BMC-T
Aldehyde dehydrogenase
PTAC
Signature enzyme
Alcohol dehydrogenase
Regulator

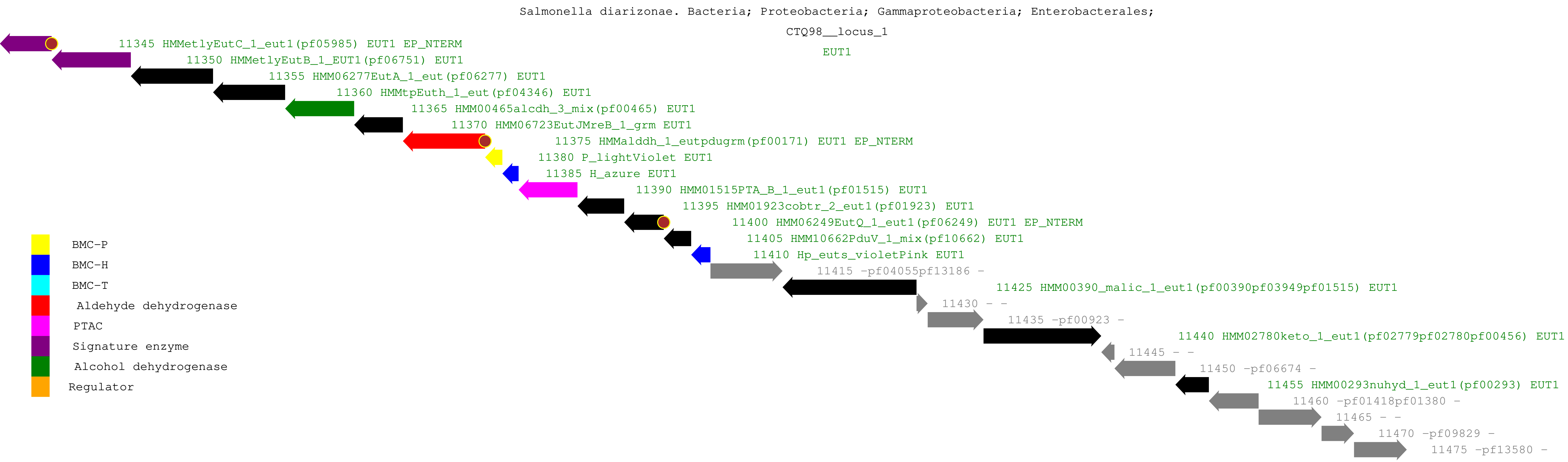
Salmonella diarizonae. Bacteria; Proteobacteria; Gammaproteobacteria; Enterobacterales;
CTQ98__locus_1
EUT1
11345 HMMetlyEutC_1_eut1(pf05985) EUT1 EP_NTERM
11350 HMMetlyEutB_1_EUT1(pf06751) EUT1
11355 HMM06277EutA_1_eut(pf06277) EUT1
11360 HMMtpEuth_1_eut(pf04346) EUT1
11365 HMM00465alcdh_3_mix(pf00465) EUT1
11370 HMM06723EutJMreB_1_grm EUT1
11375 HMMaldh_1_eutpdugrm(pf00171) EUT1 EP_NTERM
11380 P_lightViolet EUT1
11385 H_azure EUT1
11390 HMM01515PTA_B_1_eut1(pf01515) EUT1
11395 HMM01923cobtr_2_eut1(pf01923) EUT1
11400 HMM06249EutQ_1_eut1(pf06249) EUT1 EP_NTERM
11405 HMM10662PduV_1_mix(pf10662) EUT1
11410 Hp_euts_violetPink EUT1
11415 -pf04055pf13186 -
11425 HMM00390_malic_1_eut1(pf00390pf03949pf01515) EUT1
11430 - -
11435 -pf00923 -
11440 HMM02780keto_1_eut1(pf02779pf02780pf00456) EUT1
11445 - -
11450 -pf06674 -
11455 HMM00293nuhyd_1_eut1(pf00293) EUT1
11460 -pf01418pf01380 -
11465 - -
11470 -pf09829 -
11475 -pf13580 -
BMC-P
BMC-H
BMC-T
Aldehyde dehydrogenase
PTAC
Signature enzyme
Alcohol dehydrogenase
Regulator

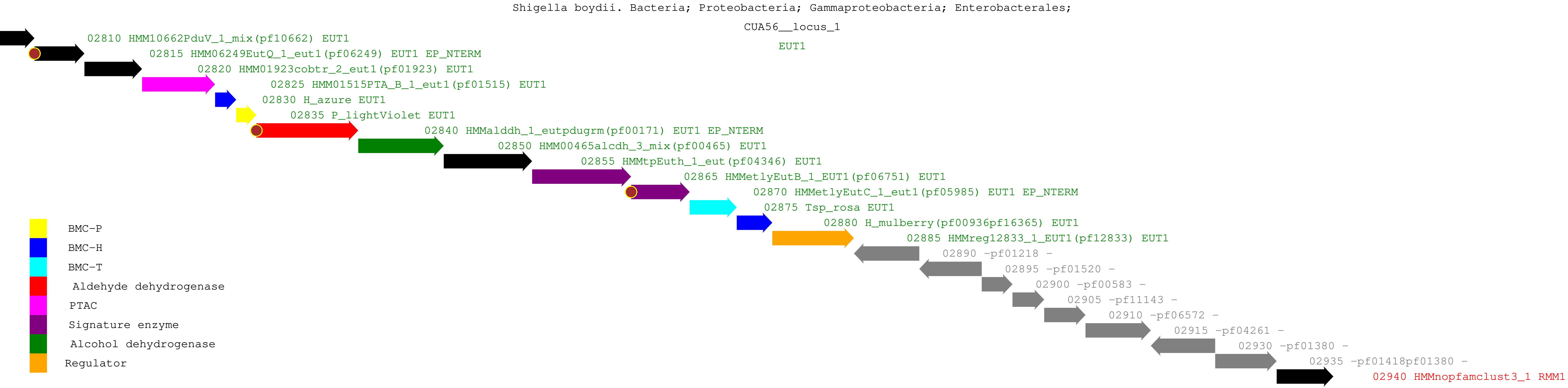
Shigella boydii. Bacteria; Proteobacteria; Gammaproteobacteria; Enterobacterales;
CUA56__locus_1
EUT1
02810 HMM10662PduV_1_mix(pf10662) EUT1
02815 HMM06249EutQ_1_eut1(pf06249) EUT1 EP_NTERM
02820 HMM01923cobtr_2_eut1(pf01923) EUT1
02825 HMM01515PTA_B_1_eut1(pf01515) EUT1
02830 H_azure EUT1
02835 P_lightViolet EUT1
02840 HMMalddh_1_eutpdugrm(pf00171) EUT1 EP_NTERM
02850 HMM00465alcdh_3_mix(pf00465) EUT1
02855 HMMtpEuth_1_eut(pf04346) EUT1
02865 HMMetlyEutB_1_EUT1(pf06751) EUT1
02870 HMMetlyEutC_1_eut1(pf05985) EUT1 EP_NTERM
02875 Tsp_rosa EUT1
02880 H_mulberry(pf00936pf16365) EUT1
02885 HMMreg12833_1_EUT1(pf12833) EUT1
02890 -pf01218 -
02895 -pf01520 -
02900 -pf00583 -
02905 -pf11143 -
02910 -pf06572 -
02915 -pf04261 -
02930 -pf01380 -
02935 -pf01418pf01380 -
02940 HMMnopfamclust3_1 RMM1
BMC-P
BMC-H
BMC-T
Aldehyde dehydrogenase
PTAC
Signature enzyme
Alcohol dehydrogenase
Regulator

Salmonella muenster. Bacteria; Proteobacteria; Gammaproteobacteria; Enterobacterales;

CUR71__locus_2

EUT1

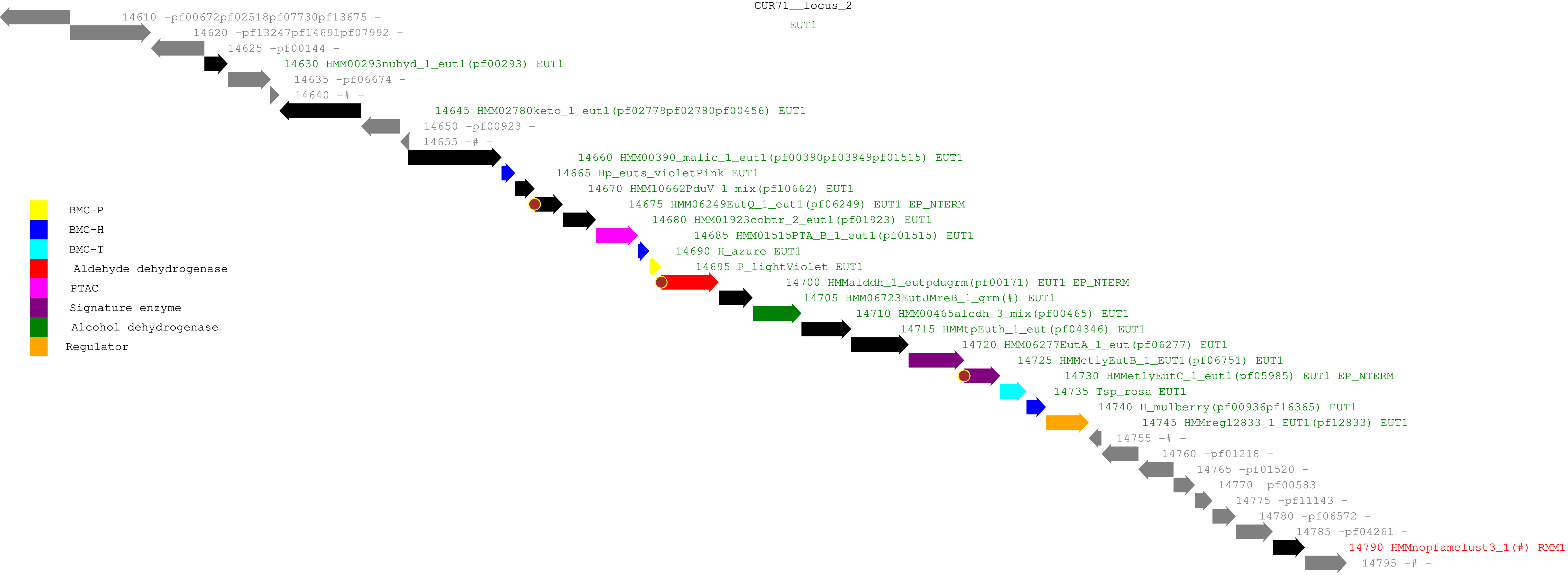

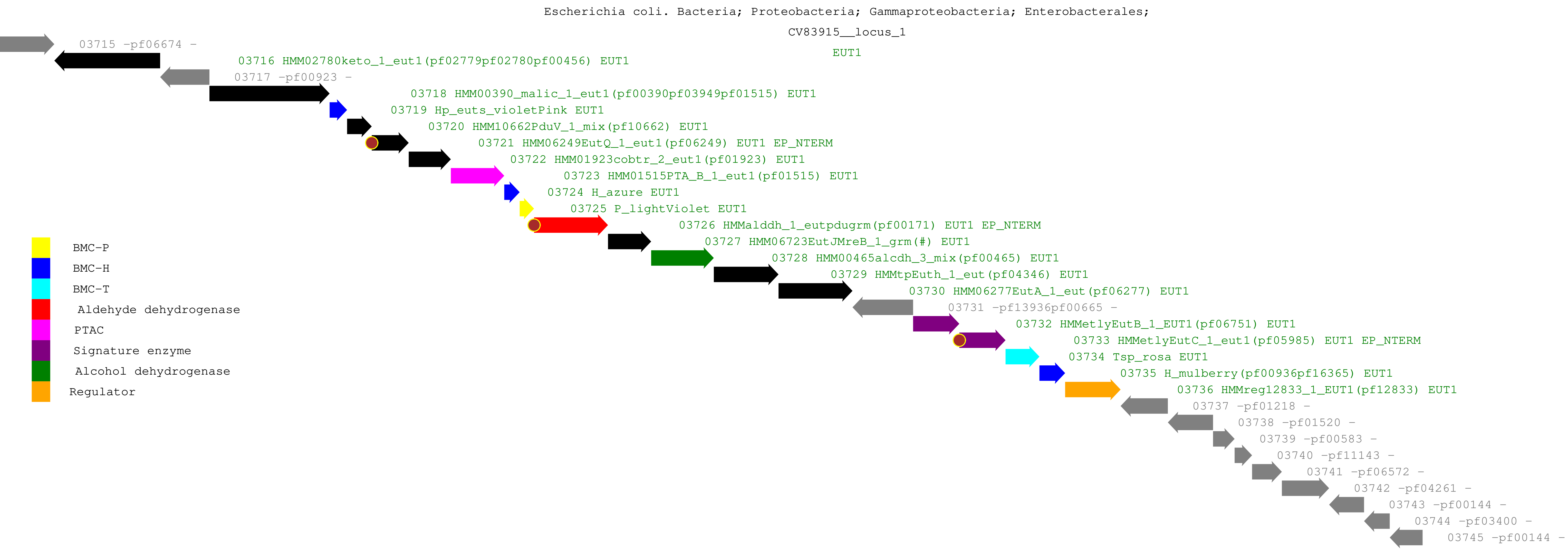
Escherichia coli. Bacteria; Proteobacteria; Gammaproteobacteria; Enterobacterales;
CV83915__locus_1
EUT1
03715 -pf06674 -
03716 HMM02780keto_1_eut1(pf02779pf02780pf00456) EUT1
03717 -pf00923 -
03718 HMM00390_malic_1_eut1(pf00390pf03949pf01515) EUT1
03719 Hp_euts_violetPink EUT1
03720 HMM10662PduV_1_mix(pf10662) EUT1
03721 HMM06249EutQ_1_eut1(pf06249) EUT1 EP_NTERM
03722 HMM01923cobtr_2_eut1(pf01923) EUT1
03723 HMM01515PTA_B_1_eut1(pf01515) EUT1
03724 H_azure EUT1
03725 P_lightViolet EUT1
03726 HMMalddh_1_eutpdugrm(pf00171) EUT1 EP_NTERM
03727 HMM06723EutJMreB_1_grm(#) EUT1
03728 HMM00465alcdh_3_mix(pf00465) EUT1
03729 HMMtpEuth_1_eut(pf04346) EUT1
03730 HMM06277EutA_1_eut(pf06277) EUT1
03731 -pf13936pf00665 -
03732 HMMetlyEutB_1_EUT1(pf06751) EUT1
03733 HMMetlyEutC_1_eut1(pf05985) EUT1 EP_NTERM
03734 Tsp_rosa EUT1
03735 H_mulberry(pf00936pf16365) EUT1
03736 HMMreg12833_1_EUT1(pf12833) EUT1
03737 -pf01218 -
03738 -pf01520 -
03739 -pf00583 -
03740 -pf11143 -
03741 -pf06572 -
03742 -pf04261 -
03743 -pf00144 -
03744 -pf03400 -
03745 -pf00144 -
BMC-P
BMC-H
BMC-T
Aldehyde dehydrogenase
PTAC
Signature enzyme
Alcohol dehydrogenase
Regulator

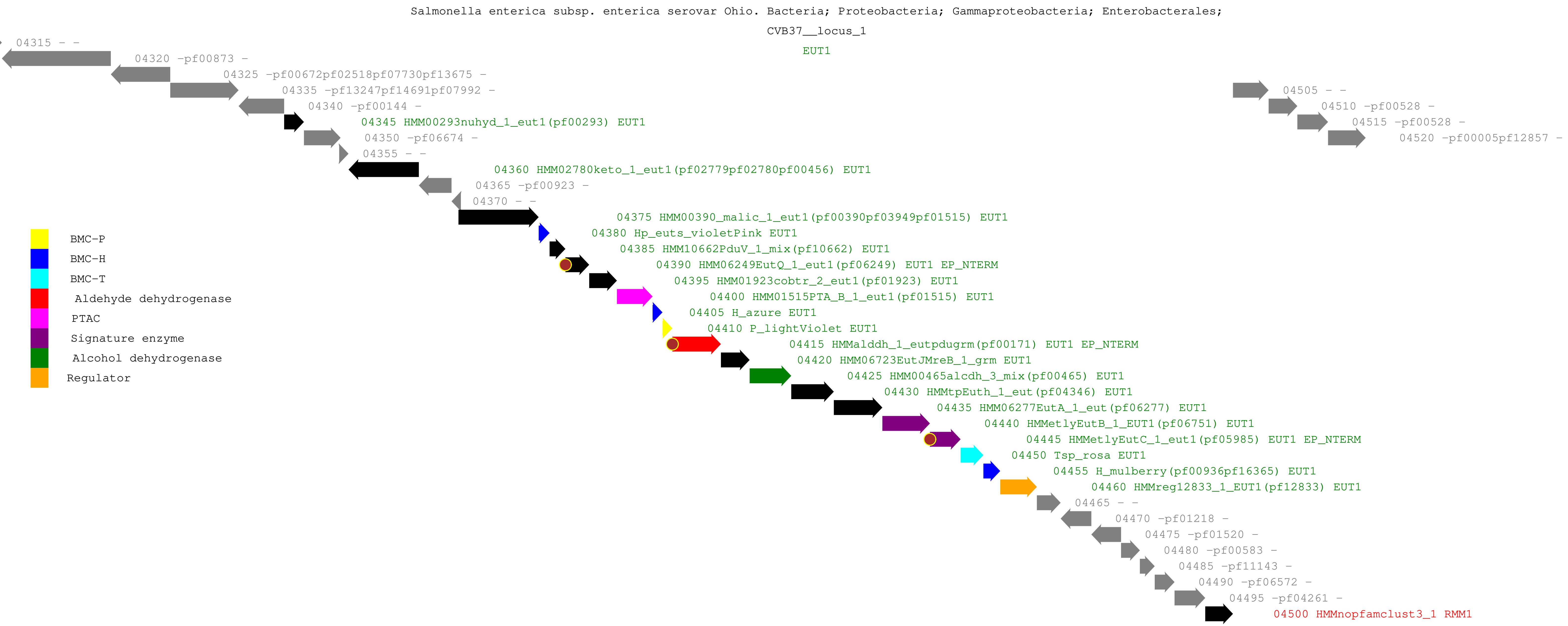

Salmonella enterica subsp. enterica serovar Ohio. Bacteria; Proteobacteria; Gammaproteobacteria; Enterobacterales;
CVB37__locus_1
EUT1
04315 - -
04320 -pf00873 -
04325 -pf00672pf02518pf07730pf13675 -
04335 -pf13247pf14691pf07992 -
04340 -pf00144 -
04345 HMM00293nuhyd_1_eut1(pf00293) EUT1
04350 -pf06674 -
04355 - -
04360 HMM02780keto_1_eut1(pf02779pf02780pf00456) EUT1
04365 -pf00923 -
04370 - -
04375 HMM00390_malic_1_eut1(pf00390pf03949pf01515) EUT1
04380 Hp_euts_violetPink EUT1
04385 HMM10662PduV_1_mix(pf10662) EUT1
04390 HMM06249EutQ_1_eut1(pf06249) EUT1 EP_NTERM
04395 HMM01923cobtr_2_eut1(pf01923) EUT1
04400 HMM01515PTA_B_1_eut1(pf01515) EUT1
04405 H_azure EUT1
04410 P_lightViolet EUT1
04415 HMMaldh_1_eutpdugrm(pf00171) EUT1 EP_NTERM
04420 HMM06723EutJMreB_1_grm EUT1
04425 HMM00465alcdh_3_mix(pf00465) EUT1
04430 HMMtpEuth_1_eut(pf04346) EUT1
04435 HMM06277EutA_1_eut(pf06277) EUT1
04440 HMMetlyEutB_1_EUT1(pf06751) EUT1
04445 HMMetlyEutC_1_eut1(pf05985) EUT1 EP_NTERM
04450 Tsp_rosa EUT1
04455 H_mulberry(pf00936pf16365) EUT1
04460 HMMreg12833_1_EUT1(pf12833) EUT1
04465 - -
04470 -pf01218 -
04475 -pf01520 -
04480 -pf00583 -
04485 -pf11143 -
04490 -pf06572 -
04495 -pf04261 -
04500 HMMnopfamclust3_1 RMM1
04505 - -
04510 -pf00528 -
04515 -pf00528 -
04520 -pf00005pf12857 -
BMC-P
BMC-H
BMC-T
Aldehyde dehydrogenase
PTAC
Signature enzyme
Alcohol dehydrogenase
Regulator

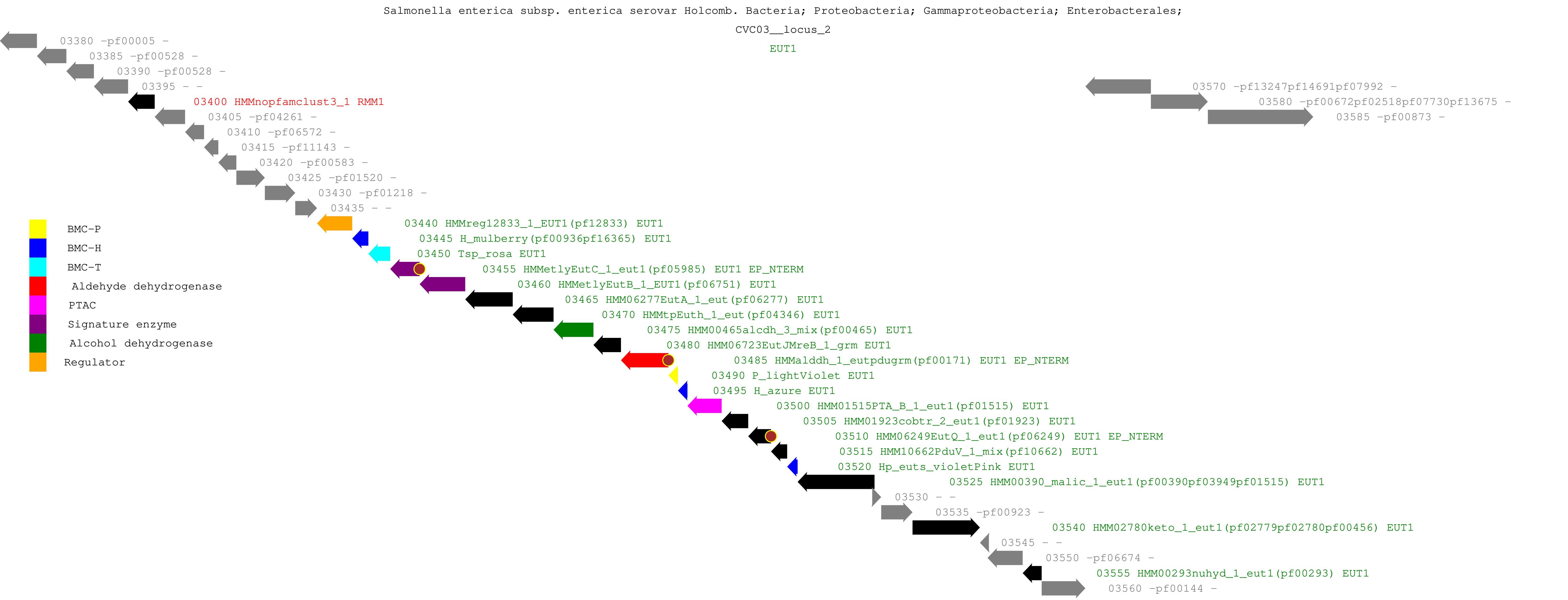

Salmonella enterica subsp. enterica serovar Holcomb. Bacteria; Proteobacteria; Gammaproteobacteria; Enterobacterales;
CVC03__locus_2
EUT1
03380 -pf00005 -
03385 -pf00528 -
03390 -pf00528 -
03395 - -
03400 HMMnopfamclust3_1 RMM1
03405 -pf04261 -
03410 -pf06572 -
03415 -pf11143 -
03420 -pf00583 -
03425 -pf01520 -
03430 -pf01218 -
03435 - -
03440 HMMreg12833_1_EUT1(pf12833) EUT1
03445 H_mulberry(pf00936pf16365) EUT1
03450 Tsp_rosa EUT1
03455 HMMetlyEutC_1_eut1(pf05985) EUT1 EP_NTERM
03460 HMMetlyEutB_1_EUT1(pf06751) EUT1
03465 HMM06277EutA_1_eut(pf06277) EUT1
03470 HMMtpEuth_1_eut(pf04346) EUT1
03475 HMM00465alcdh_3_mix(pf00465) EUT1
03480 HMM06723EutJMreB_1_grm EUT1
03485 HMMaldh_1_eutpdugrm(pf00171) EUT1 EP_NTERM
03490 P_lightViolet EUT1
03495 H_azure EUT1
03500 HMM01515PTA_B_1_eut1(pf01515) EUT1
03505 HMM01923cobtr_2_eut1(pf01923) EUT1
03510 HMM06249EutQ_1_eut1(pf06249) EUT1 EP_NTERM
03515 HMM10662PduV_1_mix(pf10662) EUT1
03520 Hp_euts_violetPink EUT1
03525 HMM00390_malic_1_eut1(pf00390pf03949pf01515) EUT1
03530 - -
03535 -pf00923 -
03540 HMM02780keto_1_eut1(pf02779pf02780pf00456) EUT1
03545 - -
03550 -pf06674 -
03555 HMM00293nuhyd_1_eut1(pf00293) EUT1
03560 -pf00144 -
03570 -pf13247pf14691pf07992 -
03580 -pf00672pf02518pf07730pf13675 -
03585 -pf00873 -
BMC-P
BMC-H
BMC-T
Aldehyde dehydrogenase
PTAC
Signature enzyme
Alcohol dehydrogenase
Regulator

Pseudomonas citronellolis. Bacteria; Proteobacteria; Gammaproteobacteria; Pseudomonadales;

CW310__locus_1

EUT1

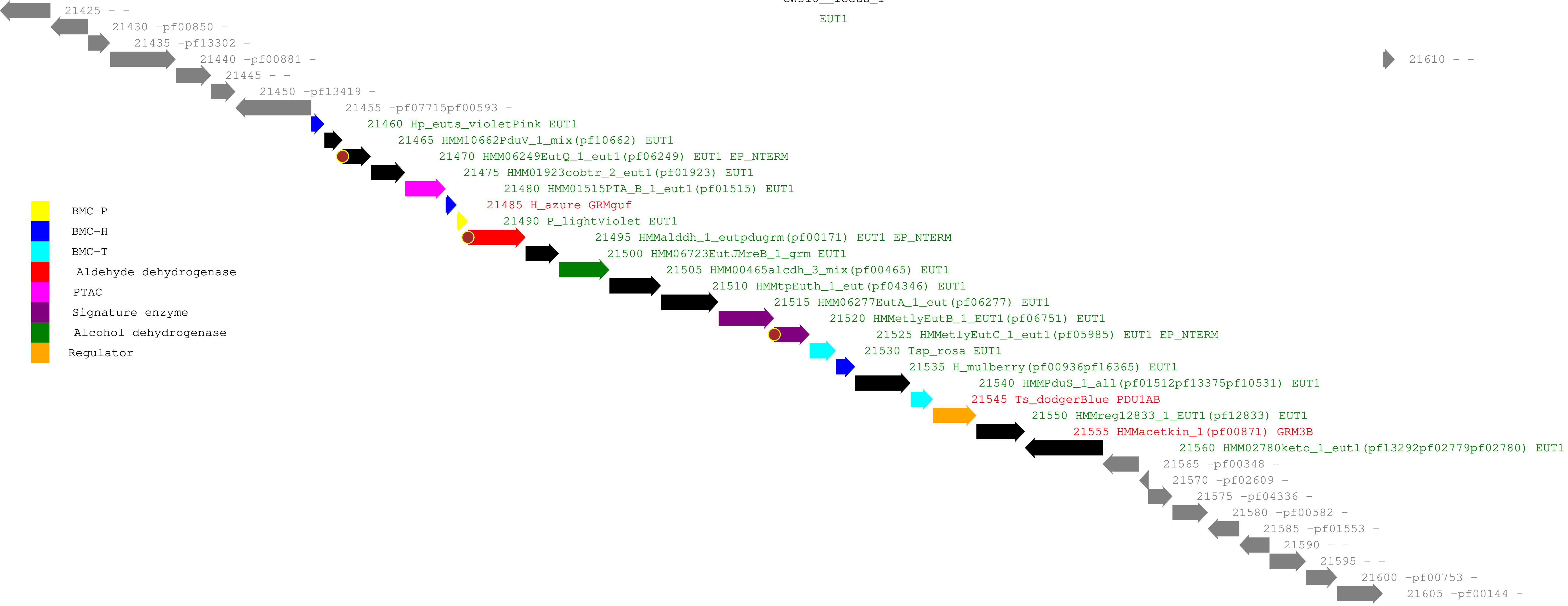

Escherichia coli O11. Bacteria; Proteobacteria; Gammaproteobacteria; Enterobacterales;

CWJ32__locus_1

EUT1

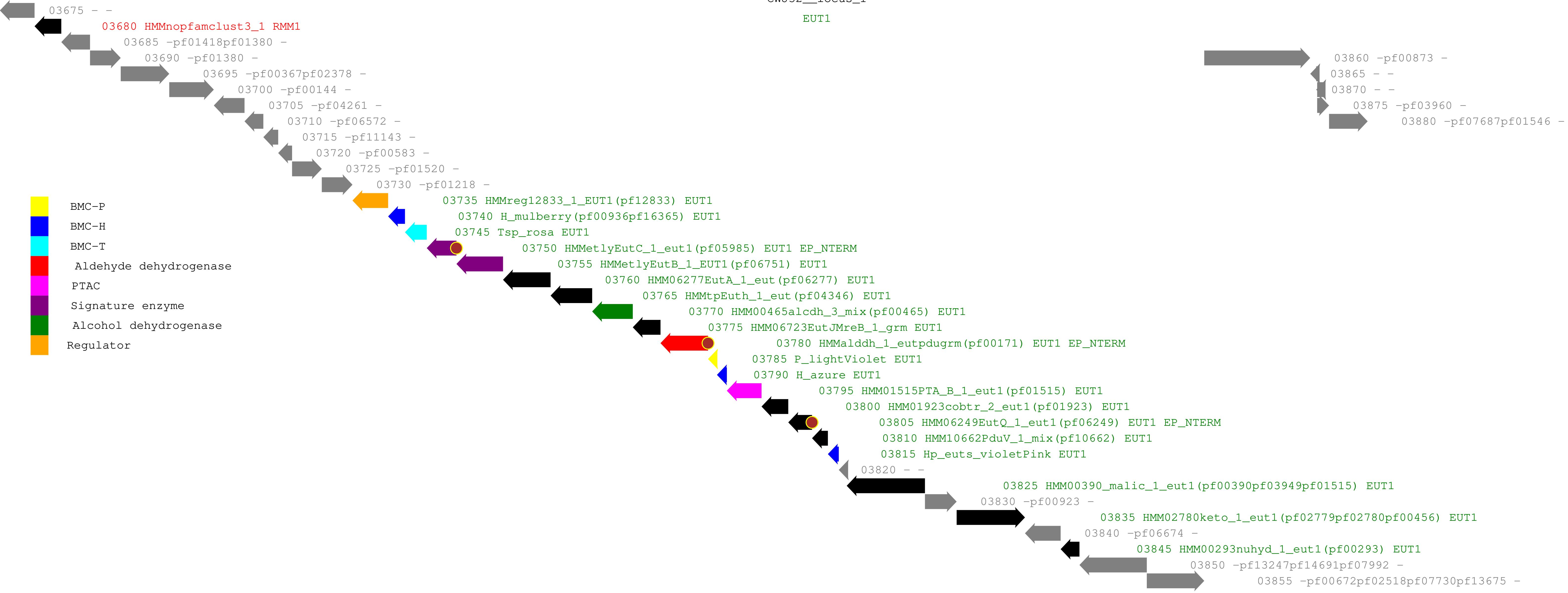

Morganella morganii (Proteus morganii). Bacteria; Proteobacteria; Gammaproteobacteria; Enterobacterales;

CXB74__locus_1

EUT1

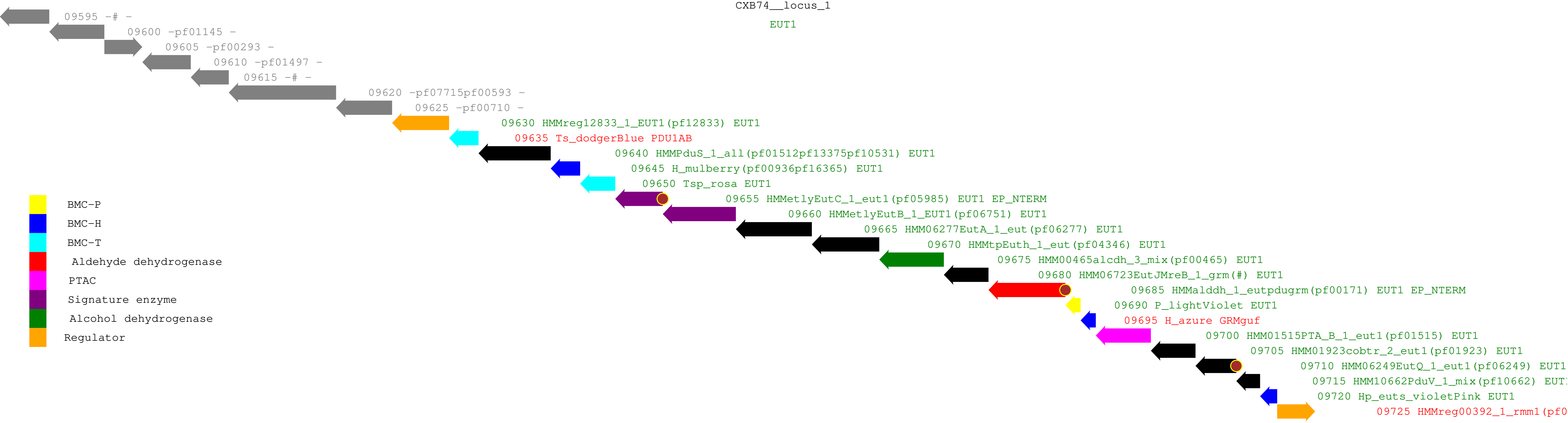

Salmonella enterica subsp. enterica serovar Tennessee. Bacteria; Proteobacteria; Gammaproteobacteria; Enterobacterales;

CXL13__locus_1

EUT1

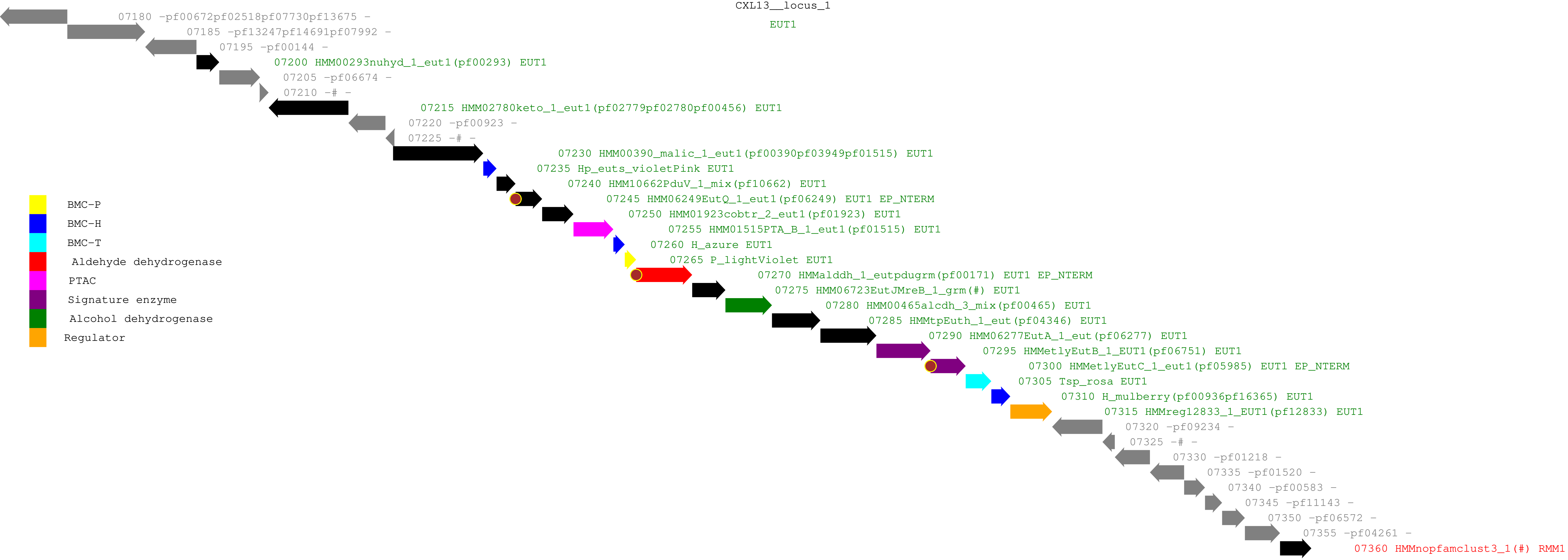

Escherichia albertii. Bacteria; Proteobacteria; Gammaproteobacteria; Enterobacterales;

CXP54__locus_2

EUT1

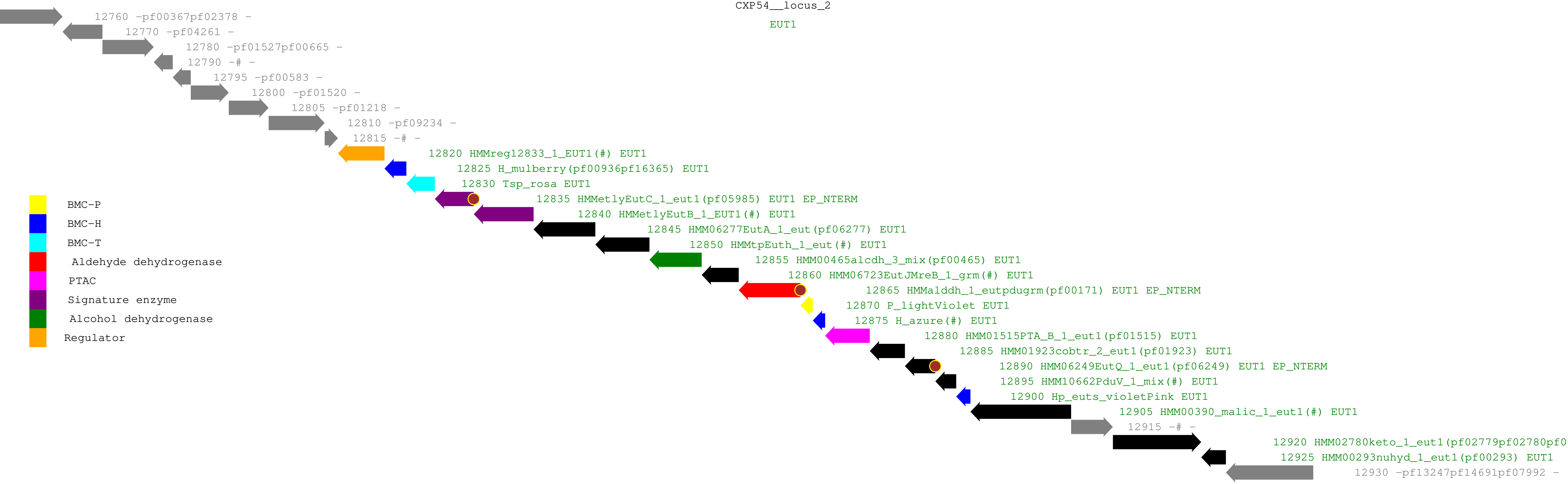

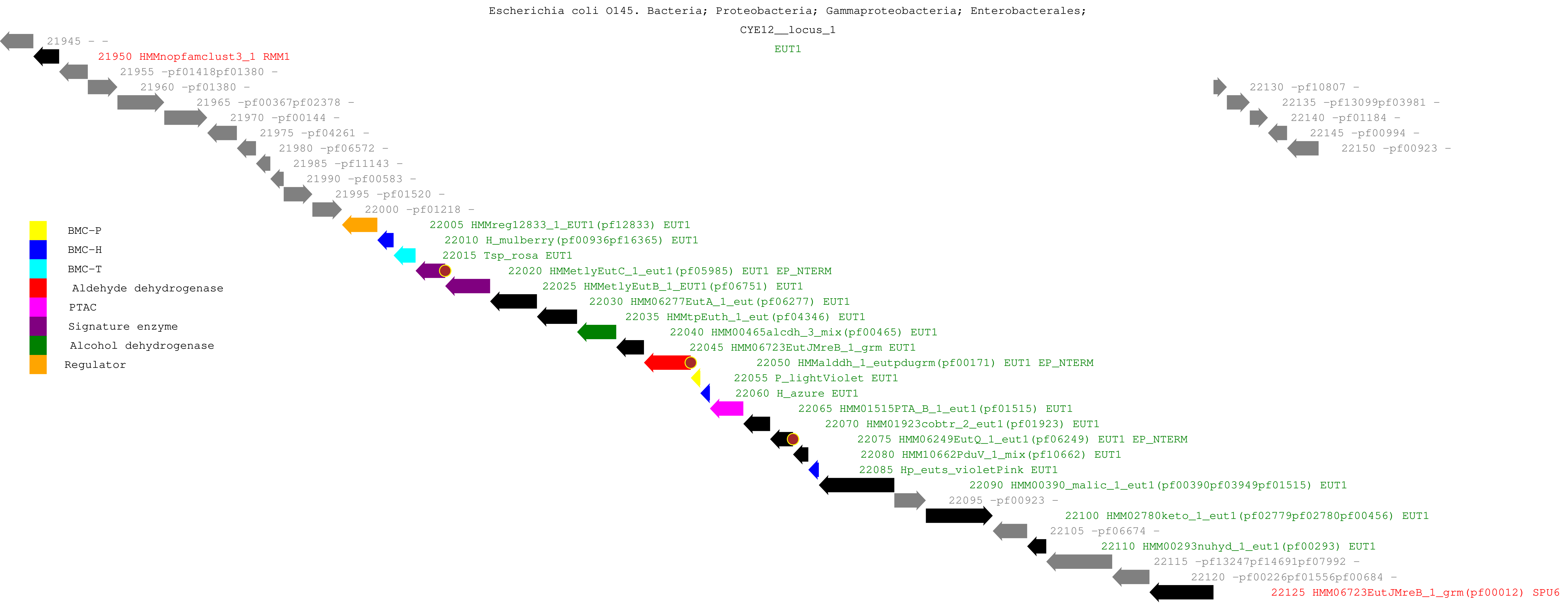

Escherichia coli O145. Bacteria; Proteobacteria; Gammaproteobacteria; Enterobacterales;
CYE12__locus_1
EUT1
21945 - -
21950 HMMnopfamclust3_1 RMM1
21955 -pf01418pf01380 -
21960 -pf01380 -
21965 -pf00367pf02378 -
21970 -pf00144 -
21975 -pf04261 -
21980 -pf06572 -
21985 -pf11143 -
21990 -pf00583 -
21995 -pf01520 -
22000 -pf01218 -
22005 HMMreg12833_1_EUT1(pf12833) EUT1
22010 H_mulberry(pf00936pf16365) EUT1
22015 Tsp_rosa EUT1
22020 HMMetlyEutC_1_eut1(pf05985) EUT1 EP_NTERM
22025 HMMetlyEutB_1_EUT1(pf06751) EUT1
22030 HMM06277EutA_1_eut(pf06277) EUT1
22035 HMMtpEuth_1_eut(pf04346) EUT1
22040 HMM00465alcdh_3_mix(pf00465) EUT1
22045 HMM06723EutJMreB_1_grm EUT1
22050 HMMaldh_1_eutpdugrm(pf00171) EUT1 EP_NTERM
22055 P_lightViolet EUT1
22060 H_azure EUT1
22065 HMM01515PTA_B_1_eut1(pf01515) EUT1
22070 HMM01923cobtr_2_eut1(pf01923) EUT1
22075 HMM06249EutQ_1_eut1(pf06249) EUT1 EP_NTERM
22080 HMM10662PduV_1_mix(pf10662) EUT1
22085 Hp_euts_violetPink EUT1
22090 HMM00390_malic_1_eut1(pf00390pf03949pf01515) EUT1
22095 -pf00923 -
22100 HMM02780keto_1_eut1(pf02779pf02780pf00456) EUT1
22105 -pf06674 -
22110 HMM00293nuhyd_1_eut1(pf00293) EUT1
22115 -pf13247pf14691pf07992 -
22120 -pf00226pf01556pf00684 -
22125 HMM06723EutJMreB_1_grm(pf00012) SPU6
22130 -pf10807 -
22135 -pf13099pf03981 -
22140 -pf01184 -
22145 -pf00994 -
22150 -pf00923 -
BMC-P
BMC-H
BMC-T
Aldehyde dehydrogenase
PTAC
Signature enzyme
Alcohol dehydrogenase
Regulator

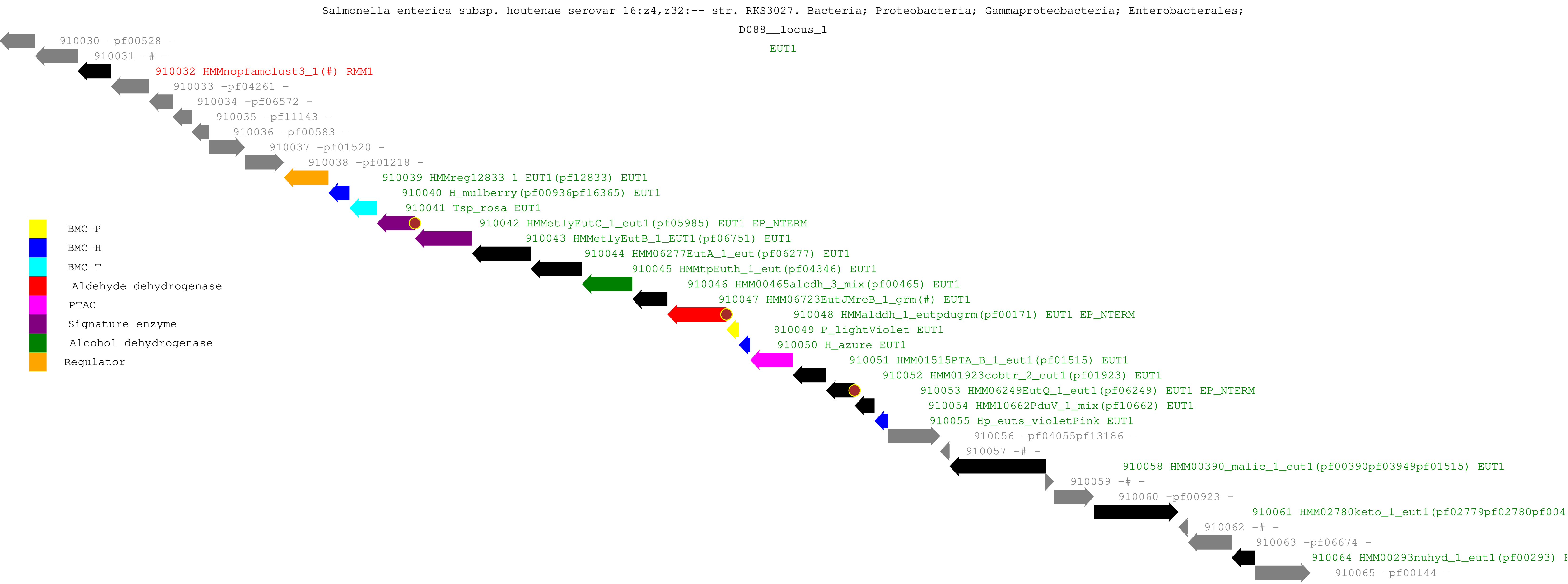

Salmonella enterica subsp. houtenae serovar 16:z4,z32:-- str. RKS3027. Bacteria; Proteobacteria; Gammaproteobacteria; Enterobacterales;
D088__locus_1
EUT1
910030 -pf00528 -
910031 -# -
910032 HMMnopfamclust3_1(#) RMM1
910033 -pf04261 -
910034 -pf06572 -
910035 -pf11143 -
910036 -pf00583 -
910037 -pf01520 -
910038 -pf01218 -
910039 HMMreg12833_1_EUT1(pf12833) EUT1
910040 H_mulberry(pf00936pf16365) EUT1
910041 Tsp_rosa EUT1
910042 HMMetlyEutC_1_eut1(pf05985) EUT1 EP_NTERM
910043 HMMetlyEutB_1_EUT1(pf06751) EUT1
910044 HMM06277EutA_1_eut(pf06277) EUT1
910045 HMMtpEuth_1_eut(pf04346) EUT1
910046 HMM00465alcdh_3_mix(pf00465) EUT1
910047 HMM06723EutJMreB_1_grm(#) EUT1
910048 HMMalddh_1_eutpdugrm(pf00171) EUT1 EP_NTERM
910049 P_lightViolet EUT1
910050 H_azure EUT1
910051 HMM01515PTA_B_1_eut1(pf01515) EUT1
910052 HMM01923cobtr_2_eut1(pf01923) EUT1
910053 HMM06249EutQ_1_eut1(pf06249) EUT1 EP_NTERM
910054 HMM10662PduV_1_mix(pf10662) EUT1
910055 Hp_euts_violetPink EUT1
910056 -pf04055pf13186 -
910057 -# -
910058 HMM00390_malic_1_eut1(pf00390pf03949pf01515) EUT1
910059 -# -
910060 -pf00923 -
910061 HMM02780keto_1_eut1(pf02779pf02780pf004
910062 -# -
910063 -pf06674 -
910064 HMM00293nuhyd_1_eut1(pf00293) E
910065 -pf00144 -
BMC-P
BMC-H
BMC-T
Aldehyde dehydrogenase
PTAC
Signature enzyme
Alcohol dehydrogenase
Regulator

Salmonella enterica subsp. enterica serovar Saintpaul. Bacteria; Proteobacteria; Gammaproteobacteria; Enterobacterales;

D1118__locus_1

EUT1

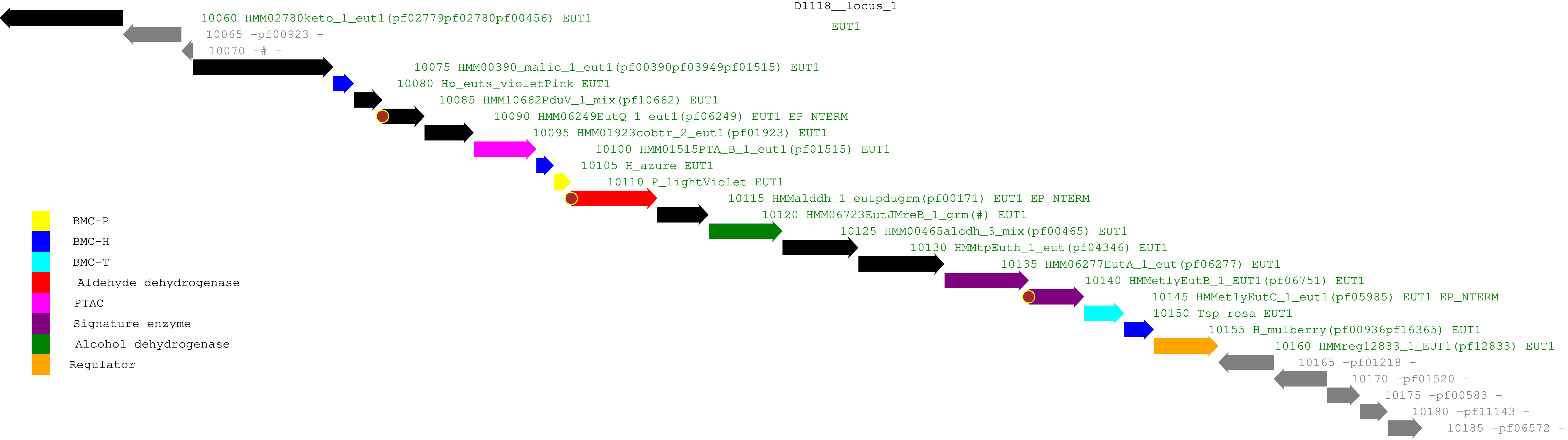

Salmonella enterica subsp. enterica serovar Molade. Bacteria; Proteobacteria; Gammaproteobacteria; Enterobacterales;

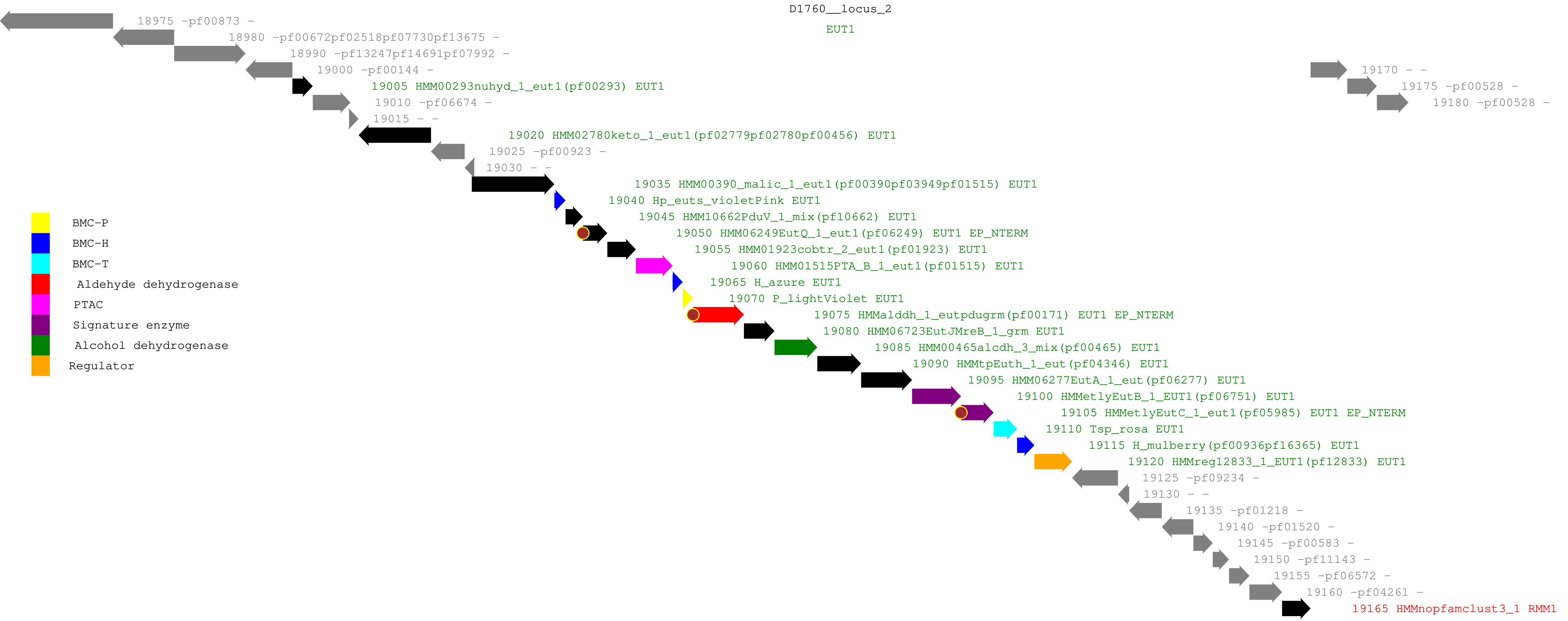

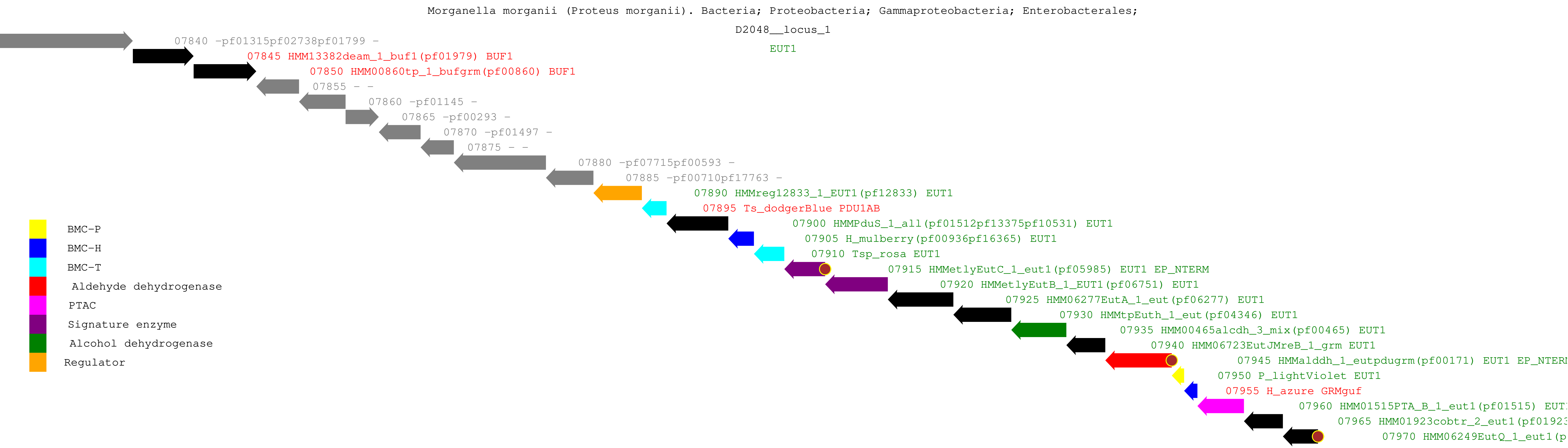

Morganella morganii (Proteus morganii). Bacteria; Proteobacteria; Gammaproteobacteria; Enterobacterales;
D2048__locus_1
EUT1
07840 -pf01315pf02738pf01799 -
07845 HMM13382deam_1_buf1(pf01979) BUF1
07850 HMM00860tp_1_bufgrm(pf00860) BUF1
07855 - -
07860 -pf01145 -
07865 -pf00293 -
07870 -pf01497 -
07875 - -
07880 -pf07715pf00593 -
07885 -pf00710pf17763 -
07890 HMMreg12833_1_EUT1(pf12833) EUT1
07895 Ts_dodgerBlue PDU1AB
07900 HMMPduS_1_all(pf01512pf13375pf10531) EUT1
07905 H_mulberry(pf00936pf16365) EUT1
07910 Tsp_rosa EUT1
07915 HMMetlyEutC_1_eut1(pf05985) EUT1 EP_NTERM
07920 HMMetlyEutB_1_EUT1(pf06751) EUT1
07925 HMM06277EutA_1_eut(pf06277) EUT1
07930 HMMtpEuth_1_eut(pf04346) EUT1
07935 HMM00465alcdh_3_mix(pf00465) EUT1
07940 HMM06723EutJMreB_1_grm EUT1
07945 HMMalddh_1_eutpdugrm(pf00171) EUT1 EP_NTERM
07950 P_lightViolet EUT1
07955 H_azure GRMguf
07960 HMM01515PTA_B_1_eut1(pf01515) EUT
07965 HMM01923cobtr_2_eut1(pf0192
07970 HMM06249EutQ_1_eut1(p
BMC-P
BMC-H
BMC-T
Aldehyde dehydrogenase
PTAC
Signature enzyme
Alcohol dehydrogenase
Regulator

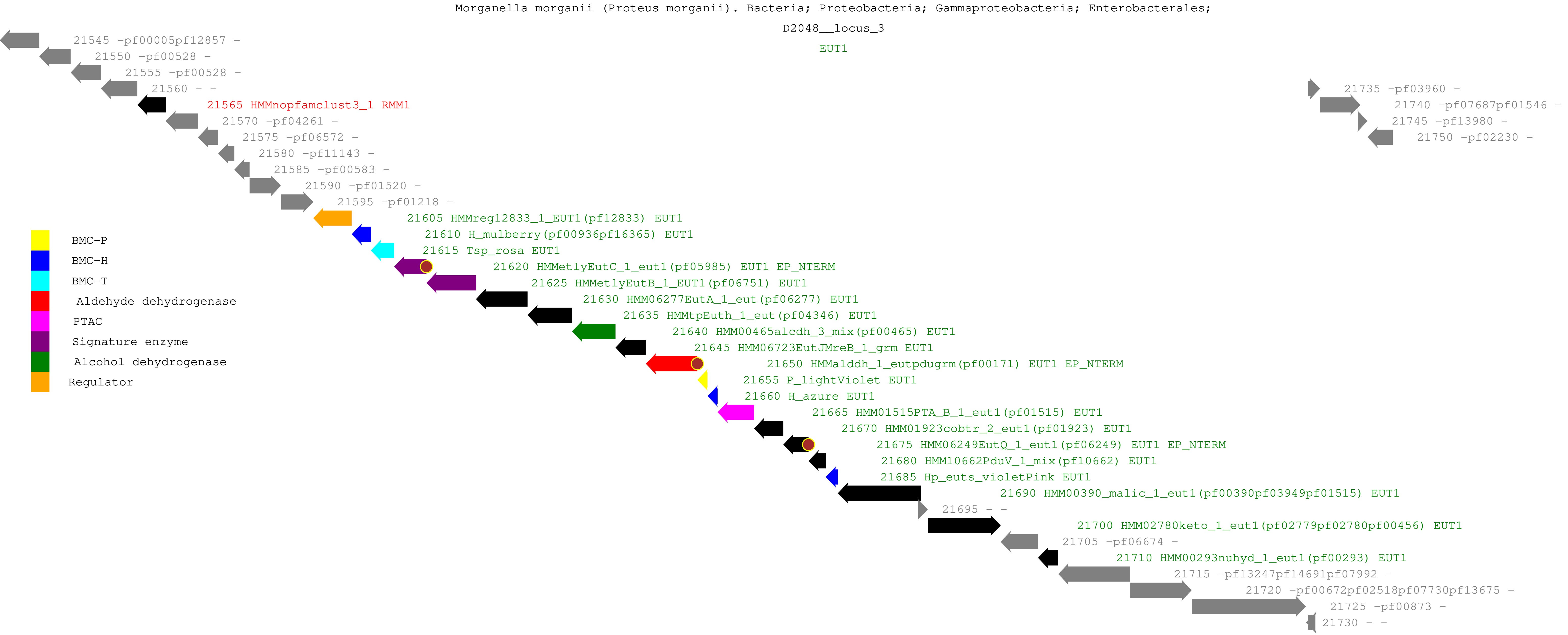

Morganella morganii (Proteus morganii). Bacteria; Proteobacteria; Gammaproteobacteria; Enterobacterales;
D2048__locus_3
EUT1
21545 -pf00005pf12857 -
21550 -pf00528 -
21555 -pf00528 -
21560 - -
21565 HMMnopfamclust3_1 RMM1
21570 -pf04261 -
21575 -pf06572 -
21580 -pf11143 -
21585 -pf00583 -
21590 -pf01520 -
21595 -pf01218 -
21605 HMMreg12833_1_EUT1(pf12833) EUT1
21610 H_mulberry(pf00936pf16365) EUT1
21615 Tsp_rosa EUT1
21620 HMMetlyEutC_1_eut1(pf05985) EUT1 EP_NTERM
21625 HMMetlyEutB_1_EUT1(pf06751) EUT1
21630 HMM06277EutA_1_eut(pf06277) EUT1
21635 HMMtpEuth_1_eut(pf04346) EUT1
21640 HMM00465alcdh_3_mix(pf00465) EUT1
21645 HMM06723EutJMreB_1_grm EUT1
21650 HMMalddh_1_eutpdugrm(pf00171) EUT1 EP_NTERM
21655 P_lightViolet EUT1
21660 H_azure EUT1
21665 HMM01515PTA_B_1_eut1(pf01515) EUT1
21670 HMM01923cobtr_2_eut1(pf01923) EUT1
21675 HMM06249EutQ_1_eut1(pf06249) EUT1 EP_NTERM
21680 HMM10662PduV_1_mix(pf10662) EUT1
21685 Hp_euts_violetPink EUT1
21690 HMM00390_malic_1_eut1(pf00390pf03949pf01515) EUT1
21695 - -
21700 HMM02780keto_1_eut1(pf02779pf02780pf00456) EUT1
21705 -pf06674 -
21710 HMM00293nuhyd_1_eut1(pf00293) EUT1
21715 -pf13247pf14691pf07992 -
21720 -pf00672pf02518pf07730pf13675 -
21725 -pf00873 -
21730 - -
21735 -pf03960 -
21740 -pf07687pf01546 -
21745 -pf13980 -
21750 -pf02230 -
BMC-P
BMC-H
BMC-T
Aldehyde dehydrogenase
PTAC
Signature enzyme
Alcohol dehydrogenase
Regulator

Herbaspirillum sp. K1R23-30. Bacteria; Proteobacteria; Betaproteobacteria; Burkholderiales;

D3871__locus_1

EUT1

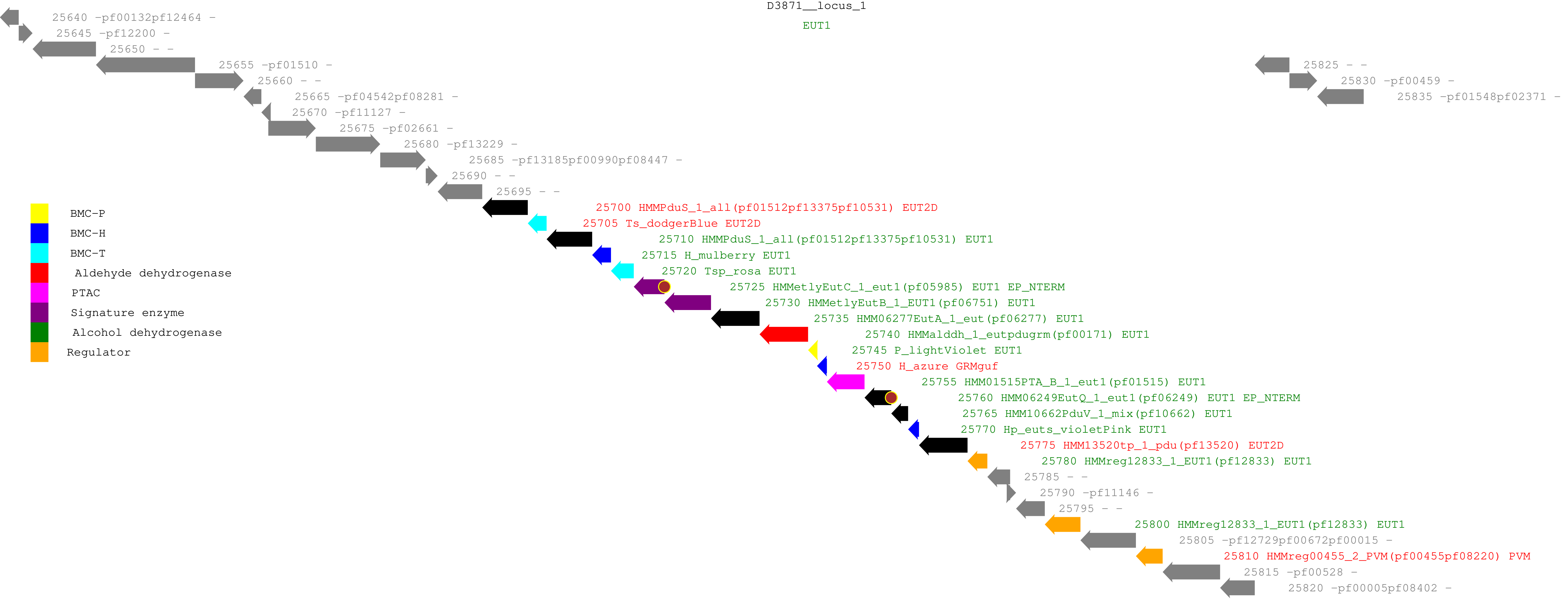

Escherichia coli. Bacteria; Proteobacteria; Gammaproteobacteria; Enterobacterales;

D3O91__locus_1

EUT1

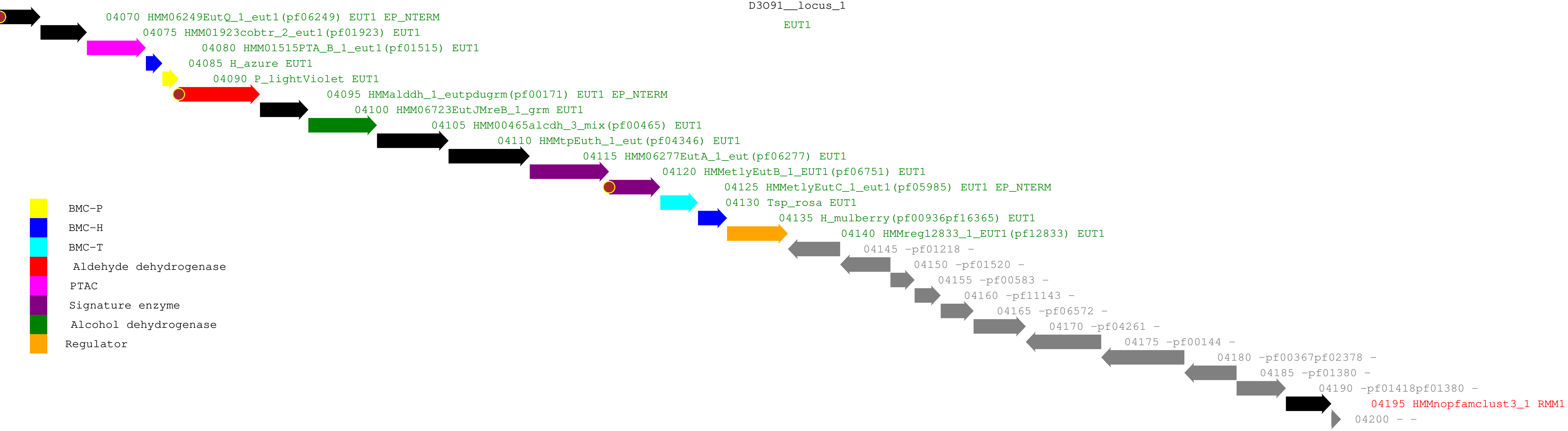

Salmonella enterica subsp. enterica serovar Java. Bacteria; Proteobacteria; Gammaproteobacteria; Enterobacterales;

D4358__locus_1_main_sat

EUT1

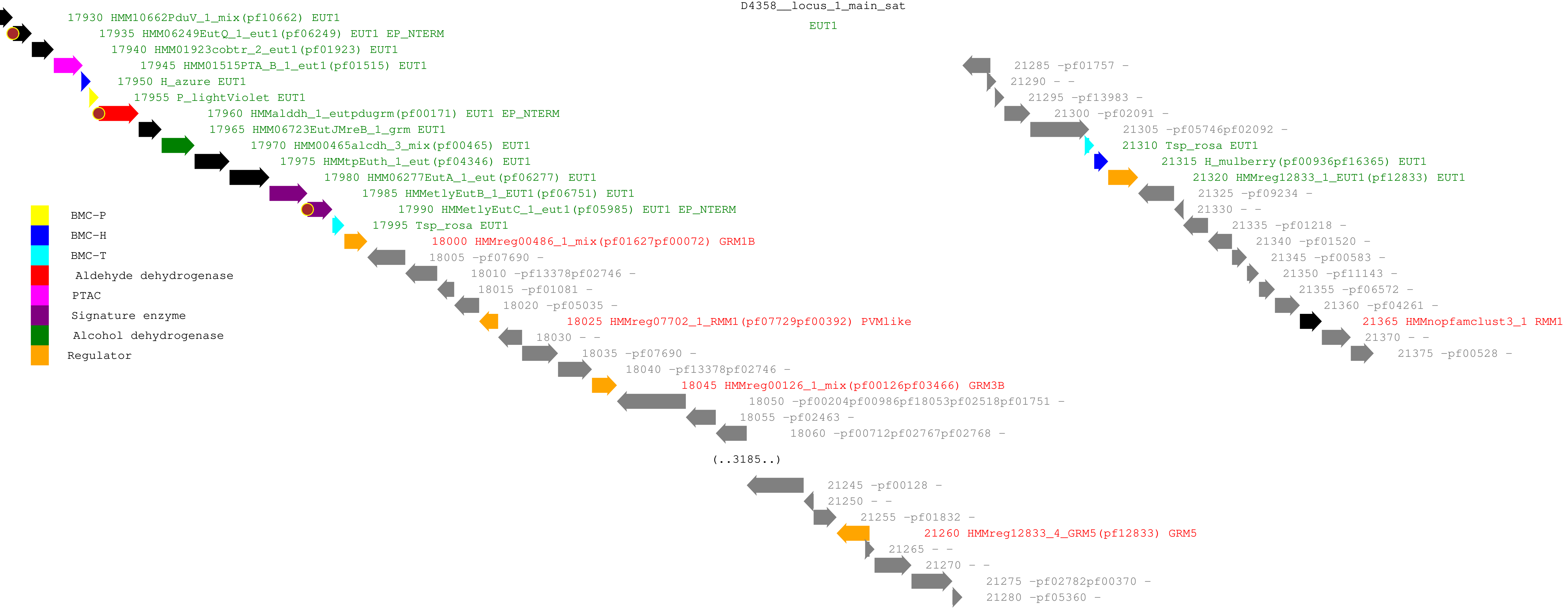

Salmonella typhimurium. Bacteria; Proteobacteria; Gammaproteobacteria; Enterobacterales;

D4380__locus_1

EUT1

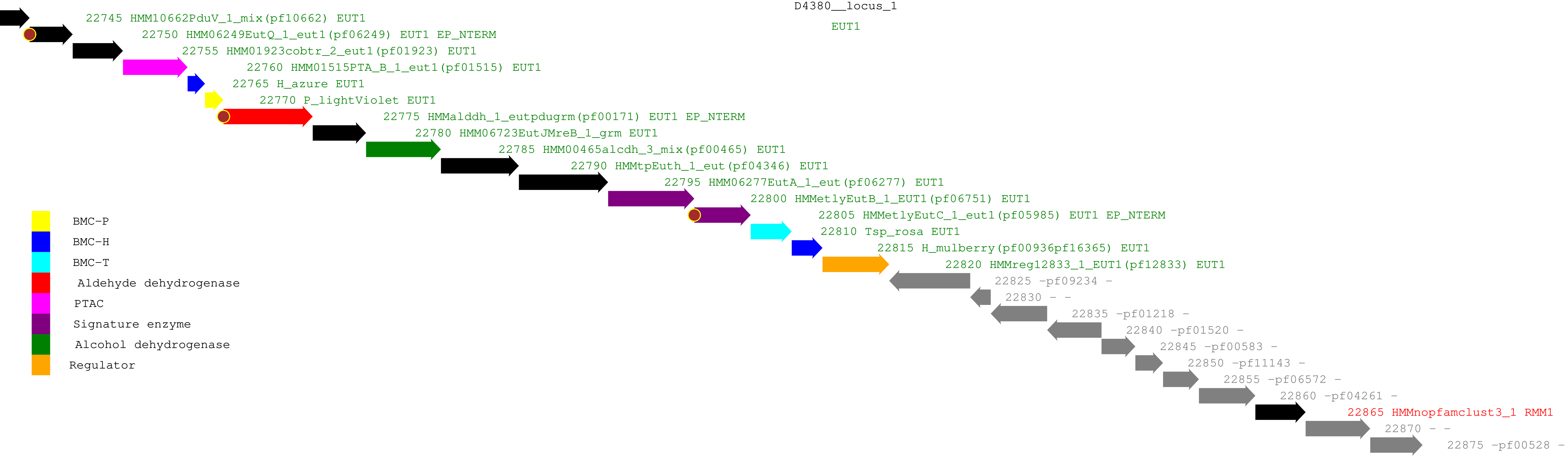

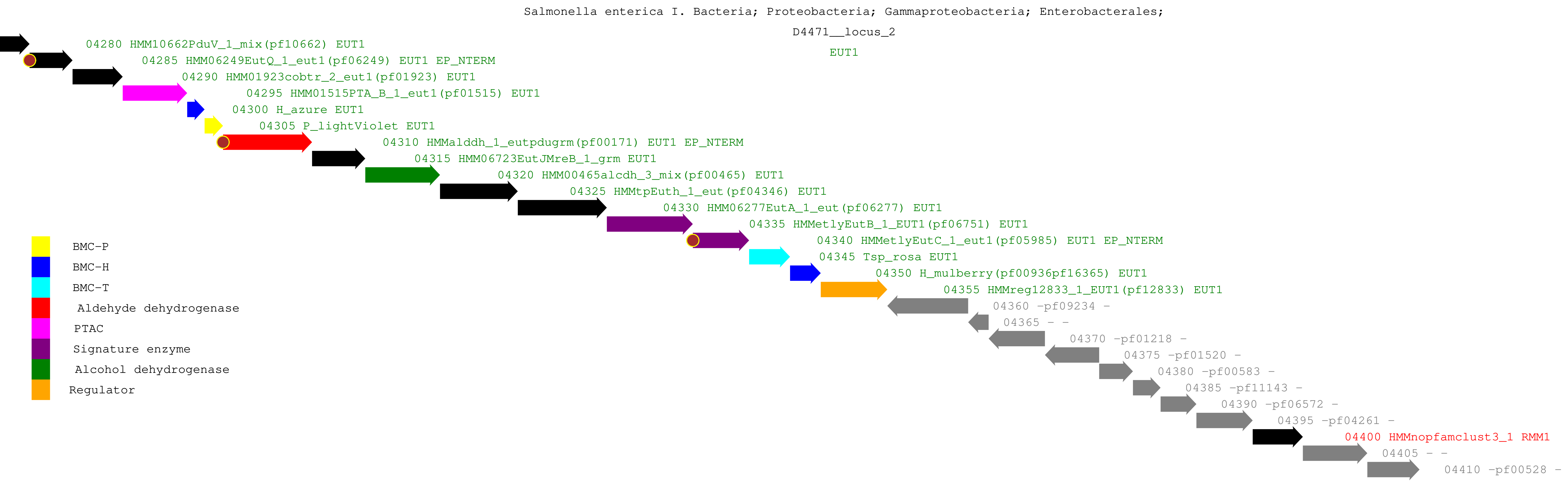
Salmonella enterica I. Bacteria; Proteobacteria; Gammaproteobacteria; Enterobacterales;
D4471__locus_2
EUT1
04280 HMM10662PduV_1_mix(pf10662) EUT1
04285 HMM06249EutQ_1_eut1(pf06249) EUT1 EP_NTERM
04290 HMM01923cobtr_2_eut1(pf01923) EUT1
04295 HMM01515PTA_B_1_eut1(pf01515) EUT1
04300 H_azure EUT1
04305 P_lightViolet EUT1
04310 HMMaldh_1_eutpdugrm(pf00171) EUT1 EP_NTERM
04315 HMM06723EutJMreB_1_grm EUT1
04320 HMM00465alcdh_3_mix(pf00465) EUT1
04325 HMMtpEuth_1_eut(pf04346) EUT1
04330 HMM06277EutA_1_eut(pf06277) EUT1
04335 HMMetlyEutB_1_EUT1(pf06751) EUT1
04340 HMMetlyEutC_1_eut1(pf05985) EUT1 EP_NTERM
04345 Tsp_rosa EUT1
04350 H_mulberry(pf00936pf16365) EUT1
04355 HMMreg12833_1_EUT1(pf12833) EUT1
04360 -pf09234 -
04365 - -
04370 -pf01218 -
04375 -pf01520 -
04380 -pf00583 -
04385 -pf11143 -
04390 -pf06572 -
04395 -pf04261 -
04400 HMMnopfamclust3_1 RMM1
04405 - -
04410 -pf00528 -
BMC-P
BMC-H
BMC-T
Aldehyde dehydrogenase
PTAC
Signature enzyme
Alcohol dehydrogenase
Regulator

Salmonella enterica subsp. enterica serovar Kentucky. Bacteria; Proteobacteria; Gammaproteobacteria; Enterobacterales;

D4523__locus_2

EUT1

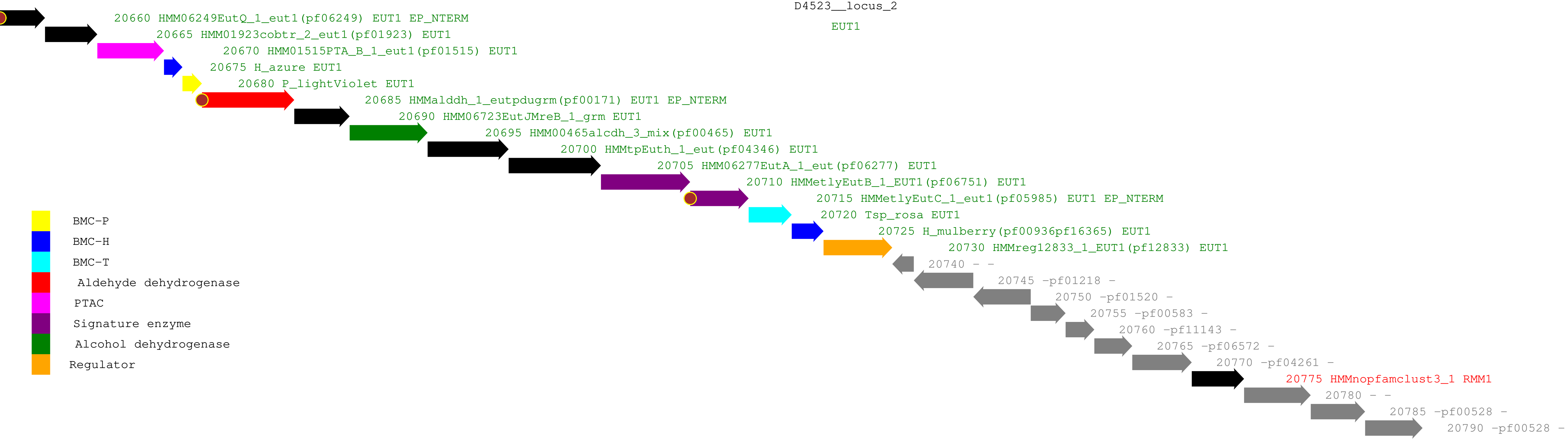

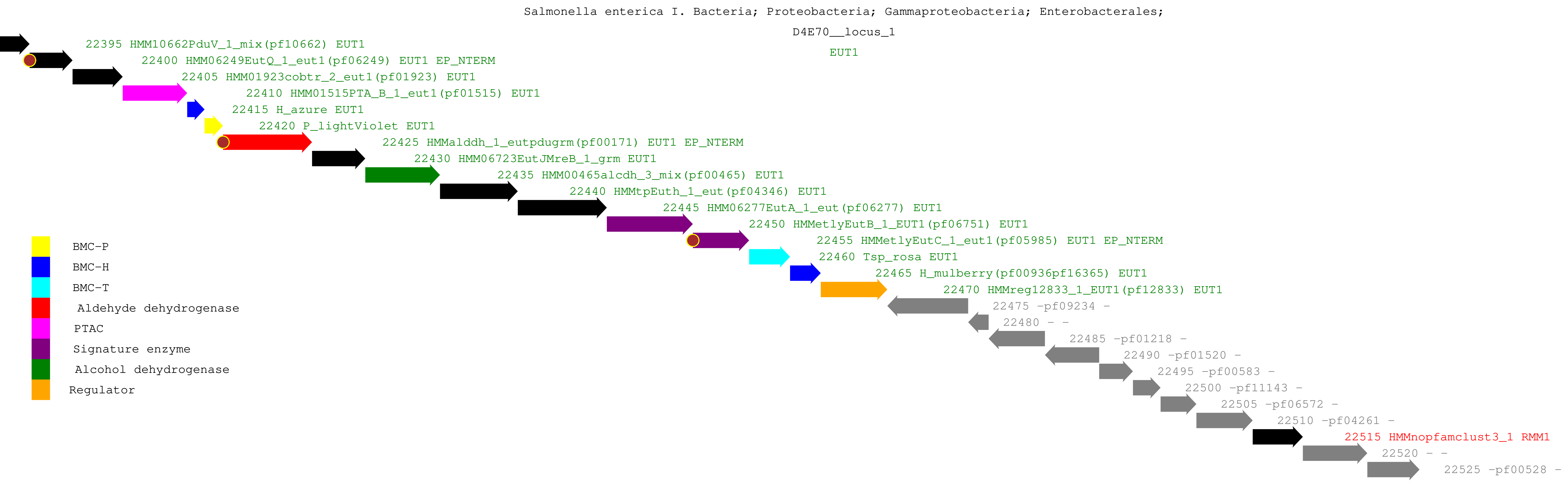
Salmonella enterica I. Bacteria; Proteobacteria; Gammaproteobacteria; Enterobacterales;
D4E70__locus_1
EUT1
22395 HMM10662PduV_1_mix(pf10662) EUT1
22400 HMM06249EutQ_1_eut1(pf06249) EUT1 EP_NTERM
22405 HMM01923cobtr_2_eut1(pf01923) EUT1
22410 HMM01515PTA_B_1_eut1(pf01515) EUT1
22415 H_azure EUT1
22420 P_lightViolet EUT1
22425 HMMalddh_1_eutpdugrm(pf00171) EUT1 EP_NTERM
22430 HMM06723EutJMreB_1_grm EUT1
22435 HMM00465alcdh_3_mix(pf00465) EUT1
22440 HMMtpEuth_1_eut(pf04346) EUT1
22445 HMM06277EutA_1_eut(pf06277) EUT1
22450 HMMetlyEutB_1_EUT1(pf06751) EUT1
22455 HMMetlyEutC_1_eut1(pf05985) EUT1 EP_NTERM
22460 Tsp_rosa EUT1
22465 H_mulberry(pf00936pf16365) EUT1
22470 HMMreg12833_1_EUT1(pf12833) EUT1
22475 -pf09234 -
22480 - -
22485 -pf01218 -
22490 -pf01520 -
22495 -pf00583 -
22500 -pf11143 -
22505 -pf06572 -
22510 -pf04261 -
22515 HMMnopfamclust3_1 RMM1
22520 - -
22525 -pf00528 -
BMC-P
BMC-H
BMC-T
Aldehyde dehydrogenase
PTAC
Signature enzyme
Alcohol dehydrogenase
Regulator

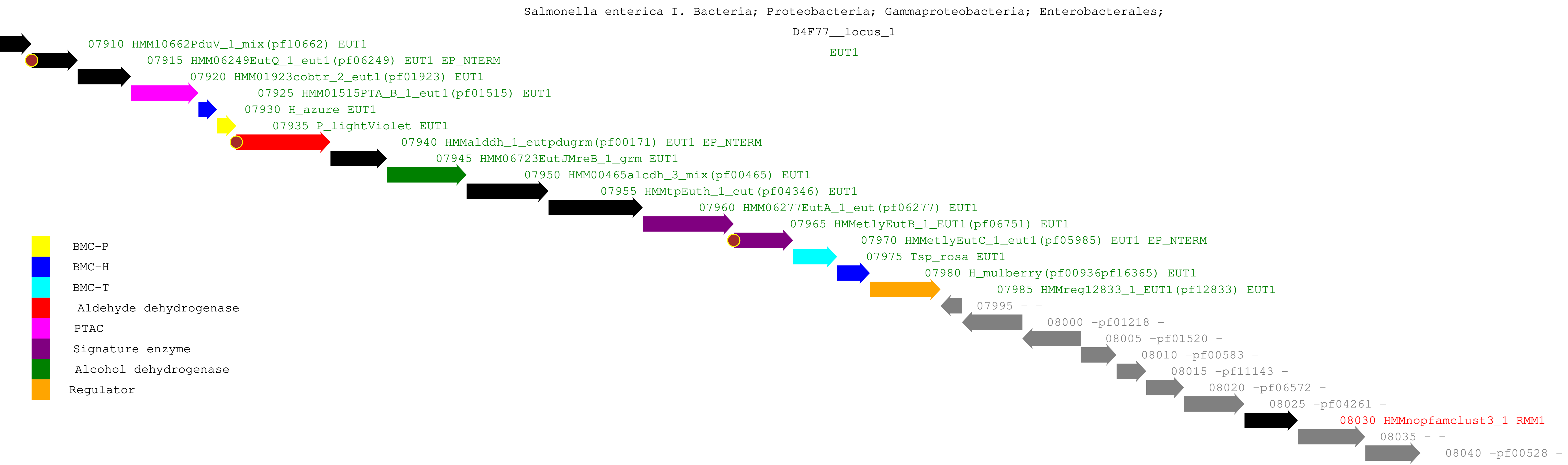

Salmonella enterica I. Bacteria; Proteobacteria; Gammaproteobacteria; Enterobacterales;
D4F77__locus_1
EUT1
07910 HMM10662PduV_1_mix(pf10662) EUT1
07915 HMM06249EutQ_1_eut1(pf06249) EUT1 EP_NTERM
07920 HMM01923cobtr_2_eut1(pf01923) EUT1
07925 HMM01515PTA_B_1_eut1(pf01515) EUT1
07930 H_azure EUT1
07935 P_lightViolet EUT1
07940 HMMalddh_1_eutpdugrm(pf00171) EUT1 EP_NTERM
07945 HMM06723EutJMreB_1_grm EUT1
07950 HMM00465alcdh_3_mix(pf00465) EUT1
07955 HMMtpEuth_1_eut(pf04346) EUT1
07960 HMM06277EutA_1_eut(pf06277) EUT1
07965 HMMetlyEutB_1_EUT1(pf06751) EUT1
07970 HMMetlyEutC_1_eut1(pf05985) EUT1 EP_NTERM
07975 Tsp_rosa EUT1
07980 H_mulberry(pf00936pf16365) EUT1
07985 HMMreg12833_1_EUT1(pf12833) EUT1
07995 - -
08000 -pf01218 -
08005 -pf01520 -
08010 -pf00583 -
08015 -pf11143 -
08020 -pf06572 -
08025 -pf04261 -
08030 HMMnopfamclust3_1 RMM1
08035 - -
08040 -pf00528 -
BMC-P
BMC-H
BMC-T
Aldehyde dehydrogenase
PTAC
Signature enzyme
Alcohol dehydrogenase
Regulator

Salmonella enterica I. Bacteria; Proteobacteria; Gammaproteobacteria; Enterobacterales;

D4G09__locus_1

EUT1

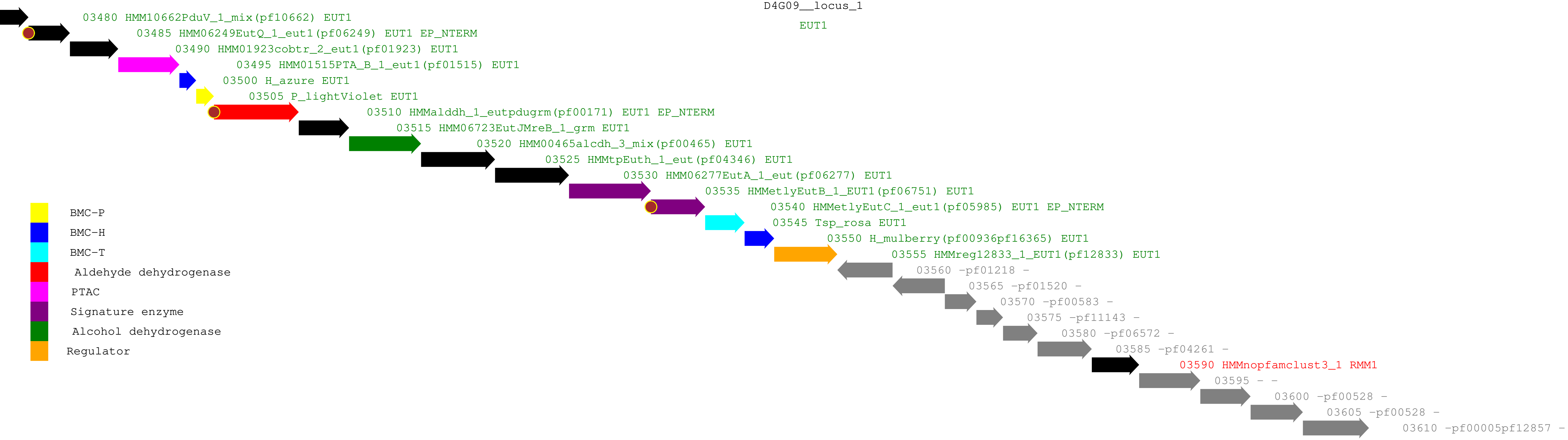

Salmonella enterica I. Bacteria; Proteobacteria; Gammaproteobacteria; Enterobacterales;

D4Y27__locus_1

EUT1

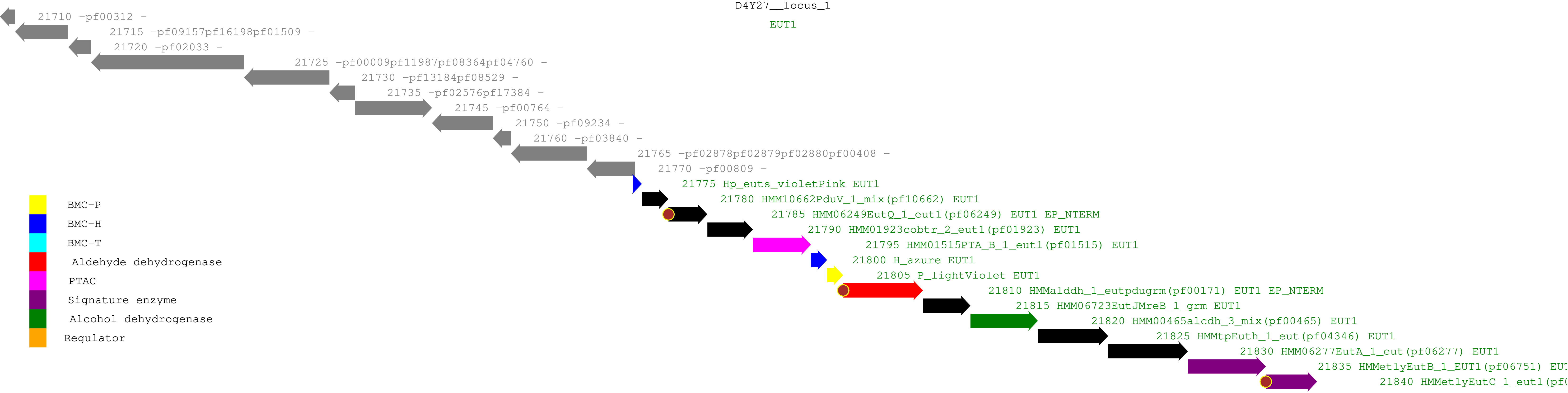

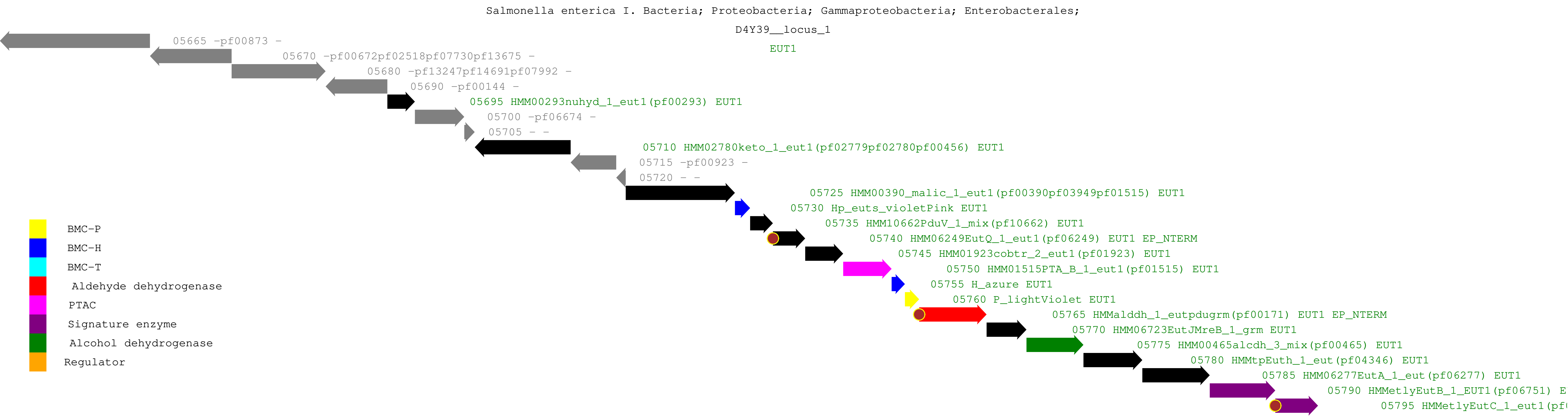

Salmonella enterica I. Bacteria; Proteobacteria; Gammaproteobacteria; Enterobacterales;
D4Y39__locus_1
EUT1
05665 -pf00873 -
05670 -pf00672pf02518pf07730pf13675 -
05680 -pf13247pf14691pf07992 -
05690 -pf00144 -
05695 HMM00293nuhyd_1_eut1(pf00293) EUT1
05700 -pf06674 -
05705 - -
05710 HMM02780keto_1_eut1(pf02779pf02780pf00456) EUT1
05715 -pf00923 -
05720 - -
05725 HMM00390_malic_1_eut1(pf00390pf03949pf01515) EUT1
05730 Hp_euts_violetPink EUT1
05735 HMM10662PduV_1_mix(pf10662) EUT1
05740 HMM06249EutQ_1_eut1(pf06249) EUT1 EP_NTERM
05745 HMM01923cobtr_2_eut1(pf01923) EUT1
05750 HMM01515PTA_B_1_eut1(pf01515) EUT1
05755 H_azure EUT1
05760 P_lightViolet EUT1
05765 HMMalddh_1_eutpdugrm(pf00171) EUT1 EP_NTERM
05770 HMM06723EutJMreB_1_grm EUT1
05775 HMM00465alcdh_3_mix(pf00465) EUT1
05780 HMMtpEuth_1_eut(pf04346) EUT1
05785 HMM06277EutA_1_eut(pf06277) EUT1
05790 HMMetlyEutB_1_EUT1(pf06751) E
05795 HMMetlyEutC_1_eut1(pf
BMC-P
BMC-H
BMC-T
Aldehyde dehydrogenase
PTAC
Signature enzyme
Alcohol dehydrogenase
Regulator

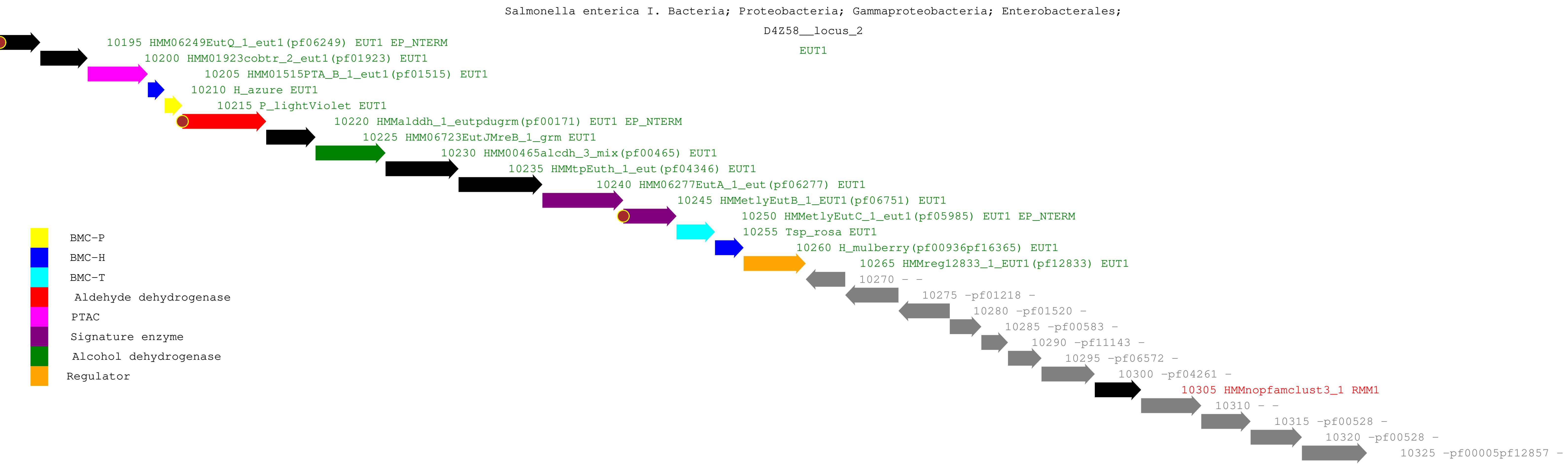
Salmonella enterica I. Bacteria; Proteobacteria; Gammaproteobacteria; Enterobacterales;
D4Z58__locus_2
EUT1
10195 HMM06249EutQ_1_eut1(pf06249) EUT1 EP_NTERM
10200 HMM01923cobtr_2_eut1(pf01923) EUT1
10205 HMM01515PTA_B_1_eut1(pf01515) EUT1
10210 H_azure EUT1
10215 P_lightViolet EUT1
10220 HMMalddh_1_eutpdugrm(pf00171) EUT1 EP_NTERM
10225 HMM06723EutJMreB_1_grm EUT1
10230 HMM00465alcdh_3_mix(pf00465) EUT1
10235 HMMtpEuth_1_eut(pf04346) EUT1
10240 HMM06277EutA_1_eut(pf06277) EUT1
10245 HMMetlyEutB_1_EUT1(pf06751) EUT1
10250 HMMetlyEutC_1_eut1(pf05985) EUT1 EP_NTERM
10255 Tsp_rosa EUT1
10260 H_mulberry(pf00936pf16365) EUT1
10265 HMMreg12833_1_EUT1(pf12833) EUT1
10270 - -
10275 -pf01218 -
10280 -pf01520 -
10285 -pf00583 -
10290 -pf11143 -
10295 -pf06572 -
10300 -pf04261 -
10305 HMMnopfamclust3_1 RMM1
10310 - -
10315 -pf00528 -
10320 -pf00528 -
10325 -pf00005pf12857 -
BMC-P
BMC-H
BMC-T
Aldehyde dehydrogenase
PTAC
Signature enzyme
Alcohol dehydrogenase
Regulator

Salmonella enterica I. Bacteria; Proteobacteria; Gammaproteobacteria; Enterobacterales;

D5810__locus_1

EUT1

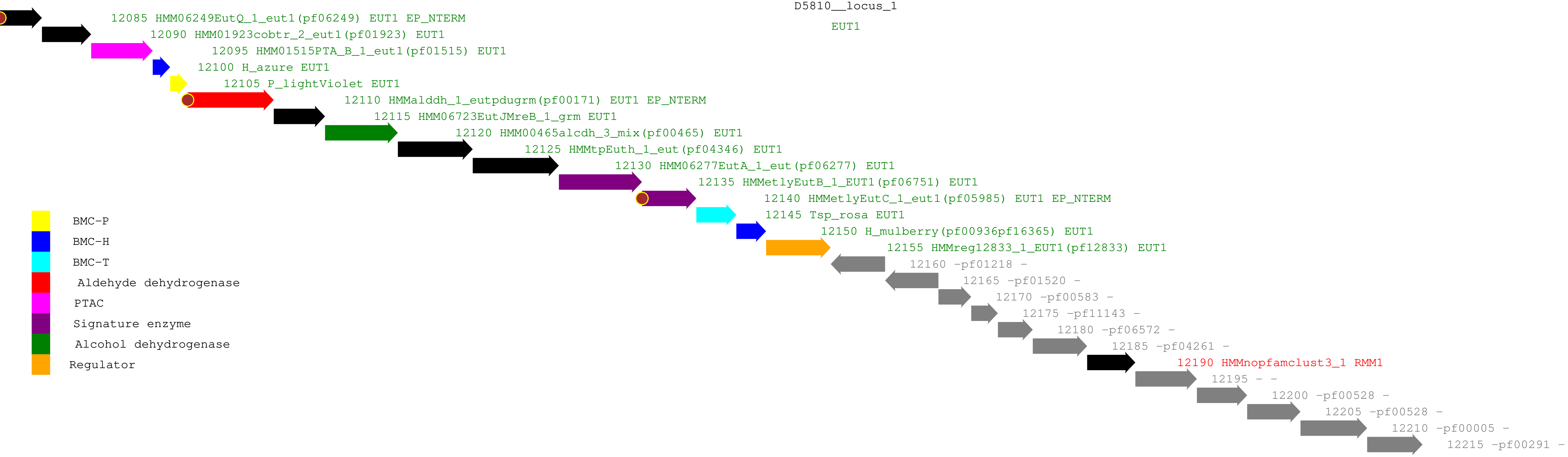

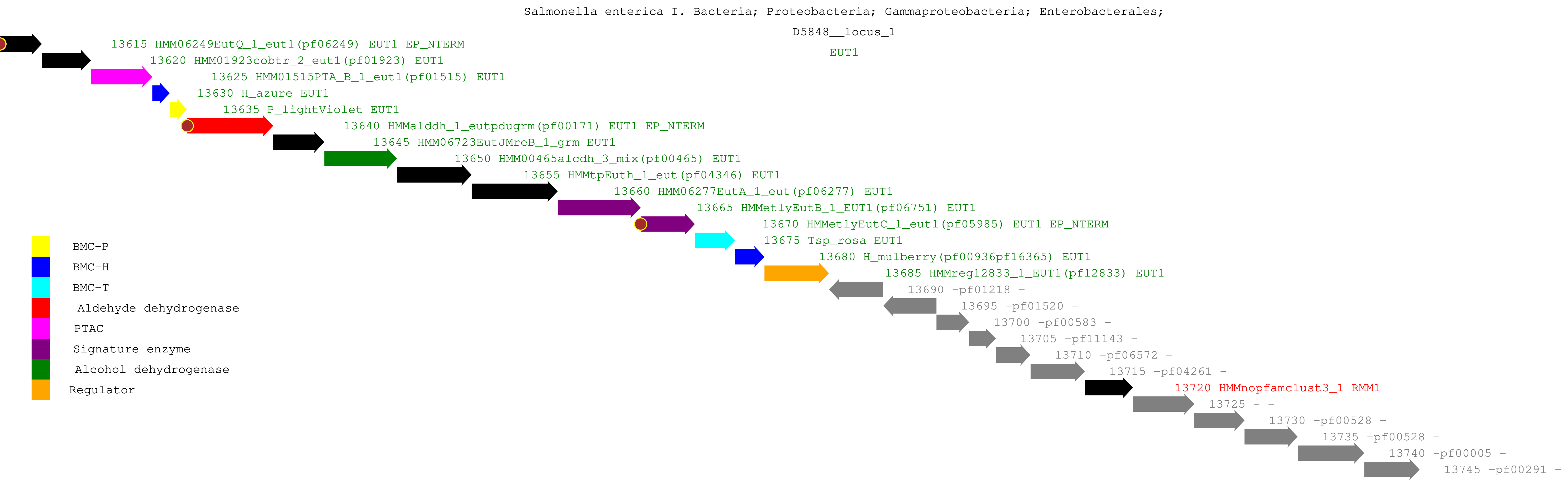
Salmonella enterica I. Bacteria; Proteobacteria; Gammaproteobacteria; Enterobacterales;
D5848__locus_1
EUT1
13615 HMM06249EutQ_1_eut1(pf06249) EUT1 EP_NTERM
13620 HMM01923cobtr_2_eut1(pf01923) EUT1
13625 HMM01515PTA_B_1_eut1(pf01515) EUT1
13630 H_azure EUT1
13635 P_lightViolet EUT1
13640 HMMalddh_1_eutpdugrm(pf00171) EUT1 EP_NTERM
13645 HMM06723EutJMreB_1_grm EUT1
13650 HMM00465alcdh_3_mix(pf00465) EUT1
13655 HMMtpEuth_1_eut(pf04346) EUT1
13660 HMM06277EutA_1_eut(pf06277) EUT1
13665 HMMetlyEutB_1_EUT1(pf06751) EUT1
13670 HMMetlyEutC_1_eut1(pf05985) EUT1 EP_NTERM
13675 Tsp_rosa EUT1
13680 H_mulberry(pf00936pf16365) EUT1
13685 HMMreg12833_1_EUT1(pf12833) EUT1
13690 -pf01218 -
13695 -pf01520 -
13700 -pf00583 -
13705 -pf11143 -
13710 -pf06572 -
13715 -pf04261 -
13720 HMMnopfamclust3_1 RMM1
13725 - -
13730 -pf00528 -
13735 -pf00528 -
13740 -pf00005 -
13745 -pf00291 -
BMC-P
BMC-H
BMC-T
Aldehyde dehydrogenase
PTAC
Signature enzyme
Alcohol dehydrogenase
Regulator

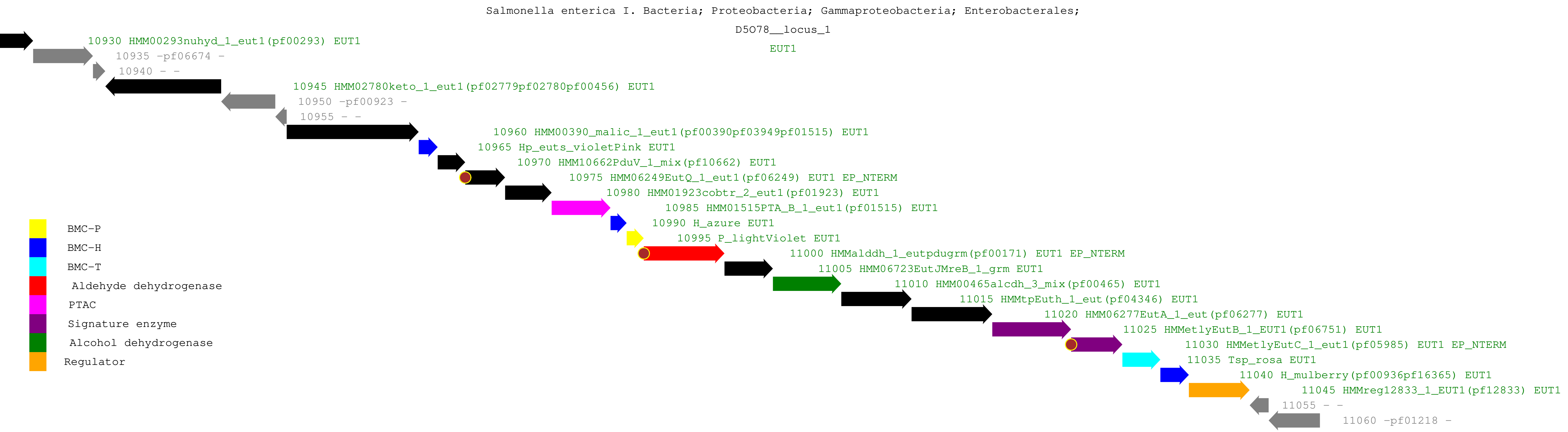
Salmonella enterica I. Bacteria; Proteobacteria; Gammaproteobacteria; Enterobacterales;
D5078__locus_1
EUT1
10930 HMM00293nuhyd_1_eut1(pf00293) EUT1
10935 -pf06674 -
10940 - -
10945 HMM02780keto_1_eut1(pf02779pf02780pf00456) EUT1
10950 -pf00923 -
10955 - -
10960 HMM00390_malic_1_eut1(pf00390pf03949pf01515) EUT1
10965 Hp_euts_violetPink EUT1
10970 HMM10662PduV_1_mix(pf10662) EUT1
10975 HMM06249EutQ_1_eut1(pf06249) EUT1 EP_NTERM
10980 HMM01923cobtr_2_eut1(pf01923) EUT1
10985 HMM01515PTA_B_1_eut1(pf01515) EUT1
10990 H_azure EUT1
10995 P_lightViolet EUT1
11000 HMMalddh_1_eutpdugrm(pf00171) EUT1 EP_NTERM
11005 HMM06723EutJMreB_1_grm EUT1
11010 HMM00465alcdh_3_mix(pf00465) EUT1
11015 HMMtpEuth_1_eut(pf04346) EUT1
11020 HMM06277EutA_1_eut(pf06277) EUT1
11025 HMMetlyEutB_1_EUT1(pf06751) EUT1
11030 HMMetlyEutC_1_eut1(pf05985) EUT1 EP_NTERM
11035 Tsp_rosa EUT1
11040 H_mulberry(pf00936pf16365) EUT1
11045 HMMreg12833_1_EUT1(pf12833) EUT1
11055 - -
11060 -pf01218 -
BMC-P
BMC-H
BMC-T
Aldehyde dehydrogenase
PTAC
Signature enzyme
Alcohol dehydrogenase
Regulator

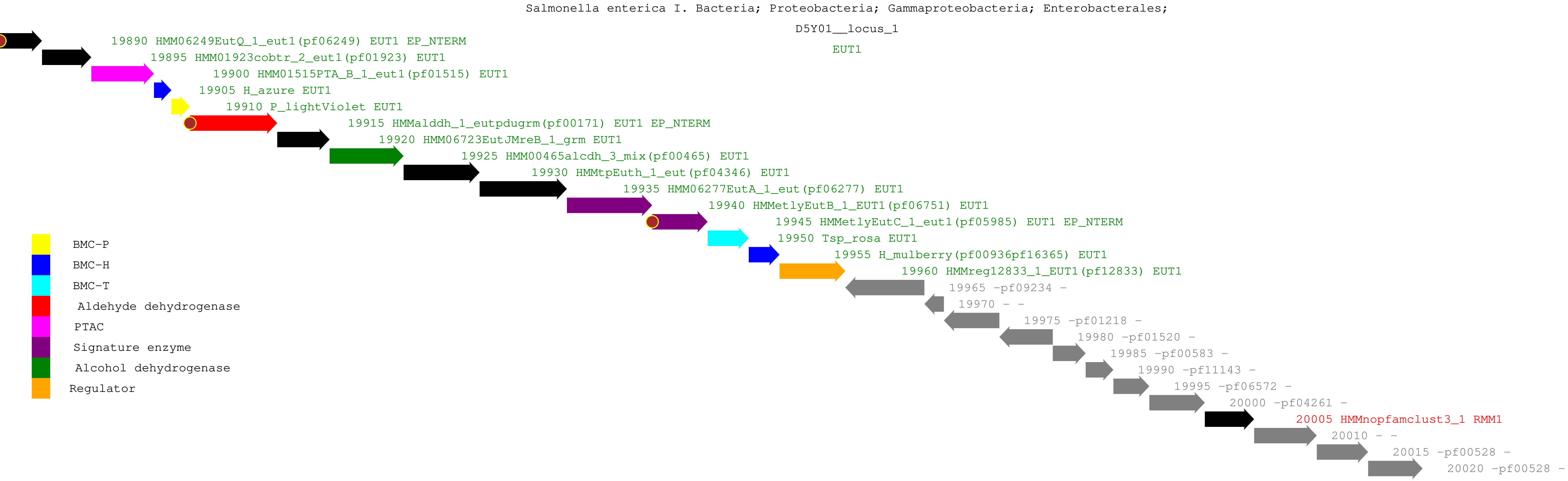
Salmonella enterica I. Bacteria; Proteobacteria; Gammaproteobacteria; Enterobacterales;
D5Y01__locus_1
EUT1
19890 HMM06249EutQ_1_eut1(pf06249) EUT1 EP_NTERM
19895 HMM01923cobtr_2_eut1(pf01923) EUT1
19900 HMM01515PTA_B_1_eut1(pf01515) EUT1
19905 H_azure EUT1
19910 P_lightViolet EUT1
19915 HMMalddh_1_eutpdugrm(pf00171) EUT1 EP_NTERM
19920 HMM06723EutJMreB_1_grm EUT1
19925 HMM00465alcdh_3_mix(pf00465) EUT1
19930 HMMtpEuth_1_eut(pf04346) EUT1
19935 HMM06277EutA_1_eut(pf06277) EUT1
19940 HMMetlyEutB_1_EUT1(pf06751) EUT1
19945 HMMetlyEutC_1_eut1(pf05985) EUT1 EP_NTERM
19950 Tsp_rosa EUT1
19955 H_mulberry(pf00936pf16365) EUT1
19960 HMMreg12833_1_EUT1(pf12833) EUT1
19965 -pf09234 -
19970 - -
19975 -pf01218 -
19980 -pf01520 -
19985 -pf00583 -
19990 -pf11143 -
19995 -pf06572 -
20000 -pf04261 -
20005 HMMnopfamclust3_1 RMM1
20010 - -
20015 -pf00528 -
20020 -pf00528 -
BMC-P
BMC-H
BMC-T
Aldehyde dehydrogenase
PTAC
Signature enzyme
Alcohol dehydrogenase
Regulator

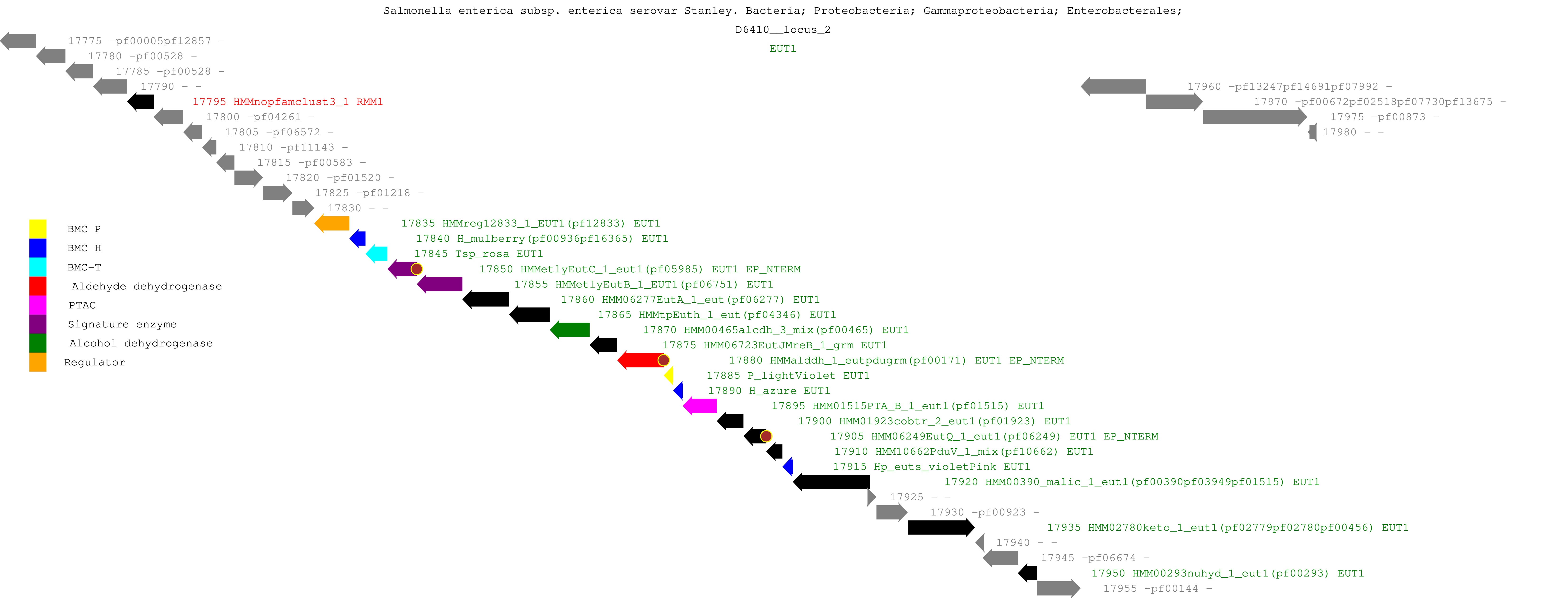
Salmonella enterica subsp. enterica serovar Stanley. Bacteria; Proteobacteria; Gammaproteobacteria; Enterobacterales;
D6410__locus_2
EUT1
17775 -pf00005pf12857 -
17780 -pf00528 -
17785 -pf00528 -
17790 - -
17795 HMMnopfamclust3_1 RMM1
17800 -pf04261 -
17805 -pf06572 -
17810 -pf11143 -
17815 -pf00583 -
17820 -pf01520 -
17825 -pf01218 -
17830 - -
17835 HMMreg12833_1_EUT1(pf12833) EUT1
17840 H_mulberry(pf00936pf16365) EUT1
17845 Tsp_rosa EUT1
17850 HMMetlyEutC_1_eut1(pf05985) EUT1 EP_NTERM
17855 HMMetlyEutB_1_EUT1(pf06751) EUT1
17860 HMM06277EutA_1_eut(pf06277) EUT1
17865 HMMtpEuth_1_eut(pf04346) EUT1
17870 HMM00465alcdh_3_mix(pf00465) EUT1
17875 HMM06723EutJMreB_1_grm EUT1
17880 HMMalddh_1_eutpdugrm(pf00171) EUT1 EP_NTERM
17885 P_lightViolet EUT1
17890 H_azure EUT1
17895 HMM01515PTA_B_1_eut1(pf01515) EUT1
17900 HMM01923cobtr_2_eut1(pf01923) EUT1
17905 HMM06249EutQ_1_eut1(pf06249) EUT1 EP_NTERM
17910 HMM10662PduV_1_mix(pf10662) EUT1
17915 Hp_euts_violetPink EUT1
17920 HMM00390_malic_1_eut1(pf00390pf03949pf01515) EUT1
17925 - -
17930 -pf00923 -
17935 HMM02780keto_1_eut1(pf02779pf02780pf00456) EUT1
17940 - -
17945 -pf06674 -
17950 HMM00293nuhyd_1_eut1(pf00293) EUT1
17955 -pf00144 -
17960 -pf13247pf14691pf07992 -
17970 -pf00672pf02518pf07730pf13675 -
17975 -pf00873 -
17980 - -
BMC-P
BMC-H
BMC-T
Aldehyde dehydrogenase
PTAC
Signature enzyme
Alcohol dehydrogenase
Regulator

Salmonella enterica subsp. enterica serovar Schwarzengrund. Bacteria; Proteobacteria; Gammaproteobacteria; Enterobacterales;

D6A15__locus_1

EUT1

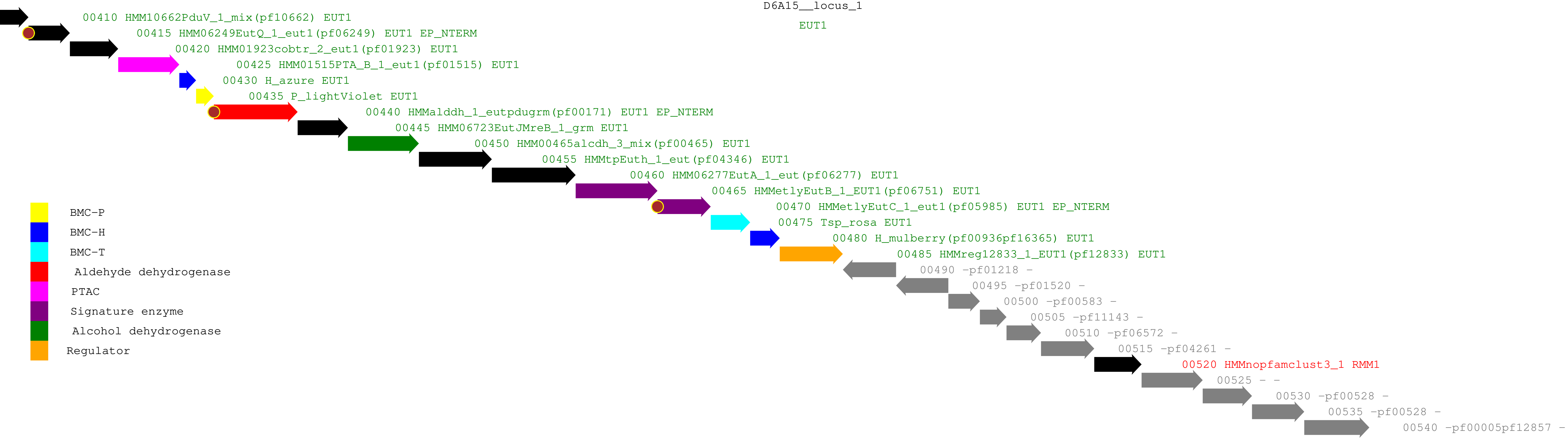

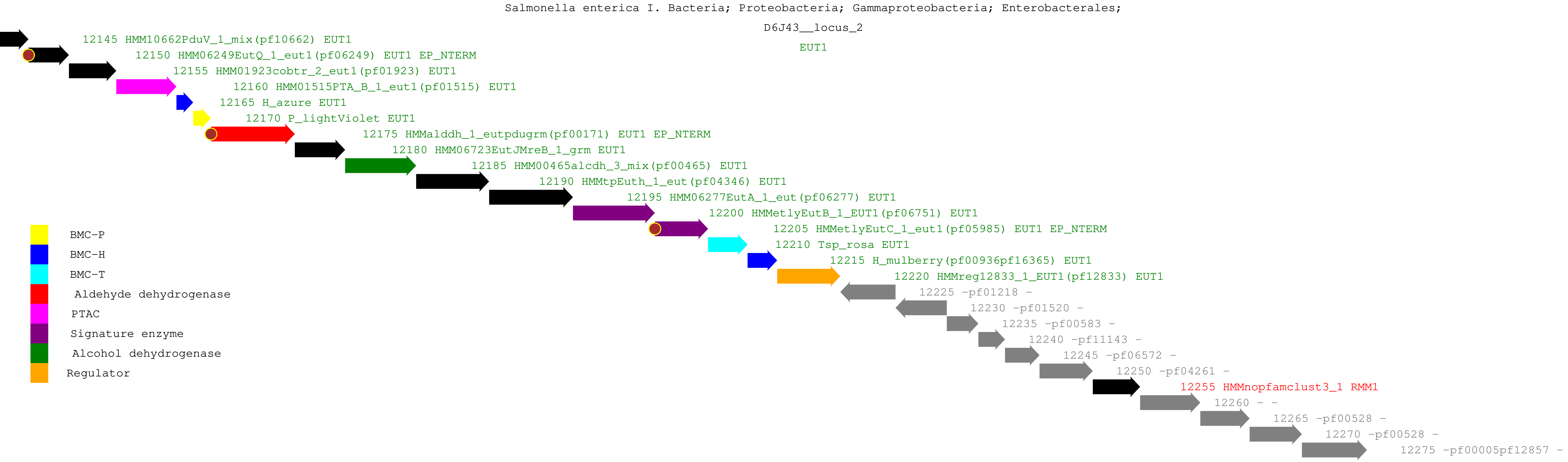

Salmonella enterica I. Bacteria; Proteobacteria; Gammaproteobacteria; Enterobacterales;
D6J43__locus_2
EUT1
12145 HMM10662PduV_1_mix(pf10662) EUT1
12150 HMM06249EutQ_1_eut1(pf06249) EUT1 EP_NTERM
12155 HMM01923cobtr_2_eut1(pf01923) EUT1
12160 HMM01515PTA_B_1_eut1(pf01515) EUT1
12165 H_azure EUT1
12170 P_lightViolet EUT1
12175 HMMalddh_1_eutpdugrm(pf00171) EUT1 EP_NTERM
12180 HMM06723EutJMreB_1_grm EUT1
12185 HMM00465alcdh_3_mix(pf00465) EUT1
12190 HMMtpEuth_1_eut(pf04346) EUT1
12195 HMM06277EutA_1_eut(pf06277) EUT1
12200 HMMetlyEutB_1_EUT1(pf06751) EUT1
12205 HMMetlyEutC_1_eut1(pf05985) EUT1 EP_NTERM
12210 Tsp_rosa EUT1
12215 H_mulberry(pf00936pf16365) EUT1
12220 HMMreg12833_1_EUT1(pf12833) EUT1
12225 -pf01218 -
12230 -pf01520 -
12235 -pf00583 -
12240 -pf11143 -
12245 -pf06572 -
12250 -pf04261 -
12255 HMMnopfamclust3_1 RMM1
12260 - -
12265 -pf00528 -
12270 -pf00528 -
12275 -pf00005pf12857 -
BMC-P
BMC-H
BMC-T
Aldehyde dehydrogenase
PTAC
Signature enzyme
Alcohol dehydrogenase
Regulator

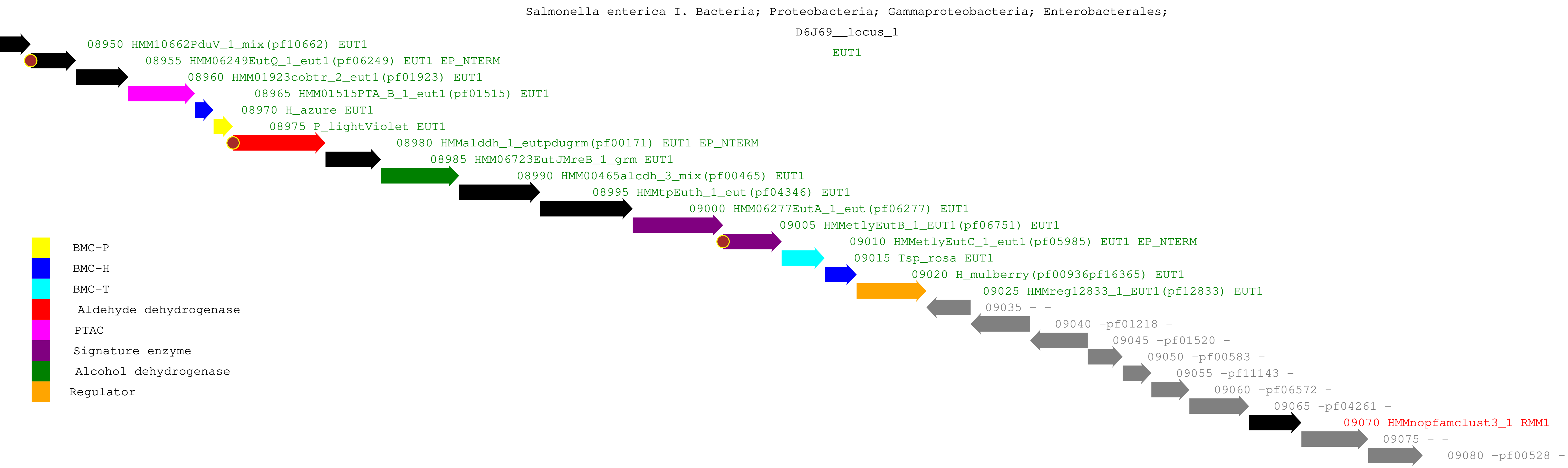

Salmonella enterica I. Bacteria; Proteobacteria; Gammaproteobacteria; Enterobacterales;
D6J69__locus_1
EUT1
08950 HMM10662PduV_1_mix(pf10662) EUT1
08955 HMM06249EutQ_1_eut1(pf06249) EUT1 EP_NTERM
08960 HMM01923cobtr_2_eut1(pf01923) EUT1
08965 HMM01515PTA_B_1_eut1(pf01515) EUT1
08970 H_azure EUT1
08975 P_lightViolet EUT1
08980 HMMalddh_1_eutpdugrm(pf00171) EUT1 EP_NTERM
08985 HMM06723EutJMreB_1_grm EUT1
08990 HMM00465alcdh_3_mix(pf00465) EUT1
08995 HMMtpEuth_1_eut(pf04346) EUT1
09000 HMM06277EutA_1_eut(pf06277) EUT1
09005 HMMetlyEutB_1_EUT1(pf06751) EUT1
09010 HMMetlyEutC_1_eut1(pf05985) EUT1 EP_NTERM
09015 Tsp_rosa EUT1
09020 H_mulberry(pf00936pf16365) EUT1
09025 HMMreg12833_1_EUT1(pf12833) EUT1
09035 - -
09040 -pf01218 -
09045 -pf01520 -
09050 -pf00583 -
09055 -pf11143 -
09060 -pf06572 -
09065 -pf04261 -
09070 HMMnopfamclust3_1 RMM1
09075 - -
09080 -pf00528 -
BMC-P
BMC-H
BMC-T
Aldehyde dehydrogenase
PTAC
Signature enzyme
Alcohol dehydrogenase
Regulator

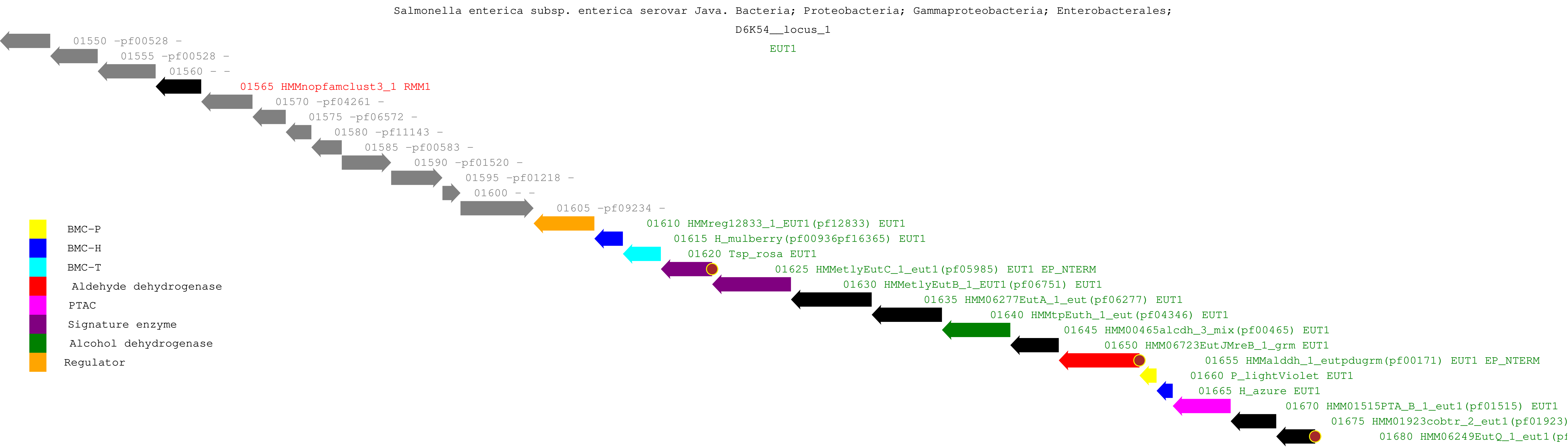

Salmonella enterica subsp. enterica serovar Java. Bacteria; Proteobacteria; Gammaproteobacteria; Enterobacterales;
D6K54__locus_1
EUT1
01550 -pf00528 -
01555 -pf00528 -
01560 - -
01565 HMMnopfamclust3_1 RMM1
01570 -pf04261 -
01575 -pf06572 -
01580 -pf11143 -
01585 -pf00583 -
01590 -pf01520 -
01595 -pf01218 -
01600 - -
01605 -pf09234 -
01610 HMMreg12833_1_EUT1(pf12833) EUT1
01615 H_mulberry(pf00936pf16365) EUT1
01620 Tsp_rosa EUT1
01625 HMMetlyEutC_1_eut1(pf05985) EUT1 EP_NTERM
01630 HMMetlyEutB_1_EUT1(pf06751) EUT1
01635 HMM06277EutA_1_eut(pf06277) EUT1
01640 HMMtpEuth_1_eut(pf04346) EUT1
01645 HMM00465alcdh_3_mix(pf00465) EUT1
01650 HMM06723EutJMreB_1_grm EUT1
01655 HMMaldh_1_eutpdugrm(pf00171) EUT1 EP_NTERM
01660 P_lightViolet EUT1
01665 H_azure EUT1
01670 HMM01515PTA_B_1_eut1(pf01515) EUT1
01675 HMM01923cobtr_2_eut1(pf01923)
01680 HMM06249EutQ_1_eut1(pf
BMC-P
BMC-H
BMC-T
Aldehyde dehydrogenase
PTAC
Signature enzyme
Alcohol dehydrogenase
Regulator

Salmonella enterica I. Bacteria; Proteobacteria; Gammaproteobacteria; Enterobacterales;

D6K86__locus_1

EUT1

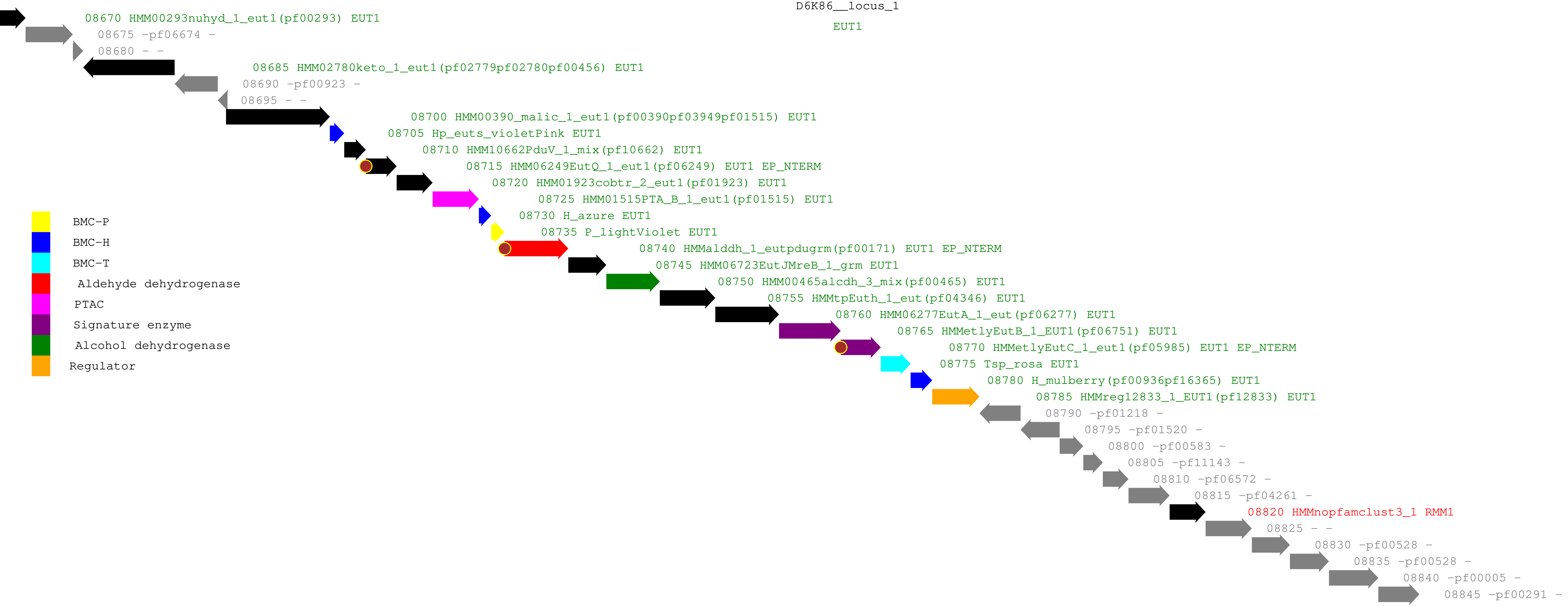

Enterobacteriaceae bacterium (strain FGI 57). Bacteria; Proteobacteria; Gammaproteobacteria; Enterobacterales;

D782__locus_1

EUT1

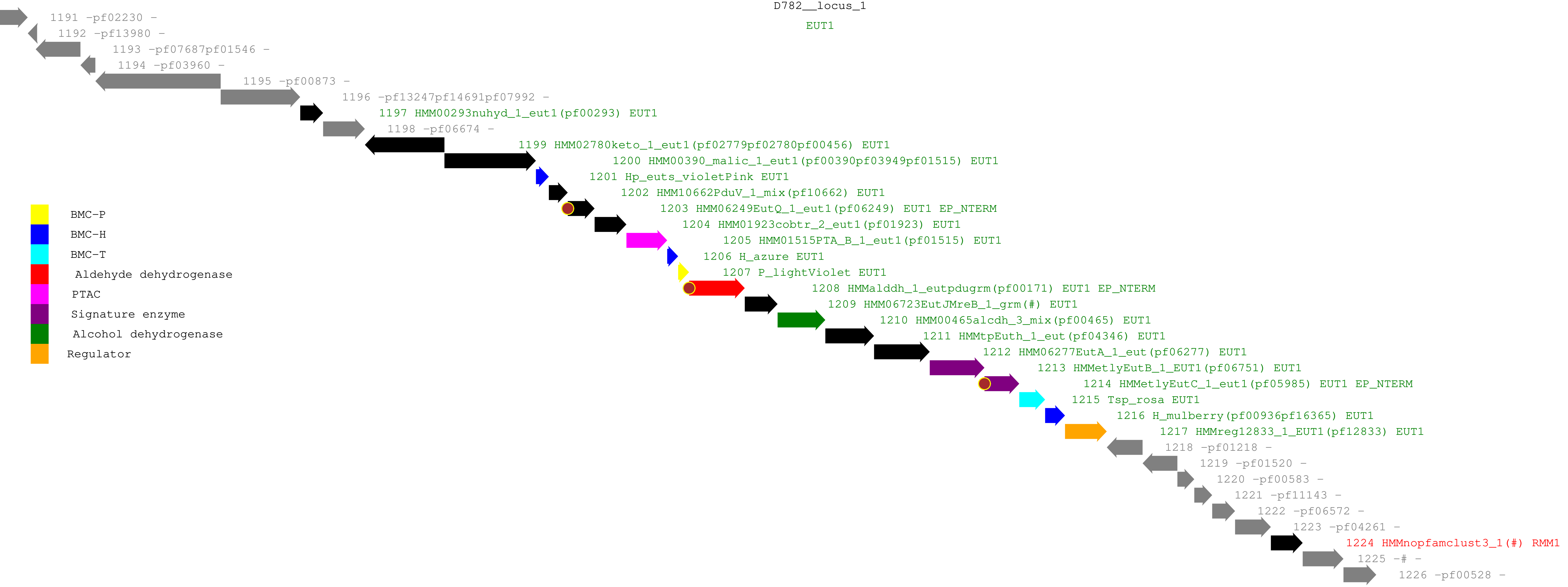

Citrobacter sp. MH181794. Bacteria; Proteobacteria; Gammaproteobacteria; Enterobacterales;

D7I40__locus_2

EUT1

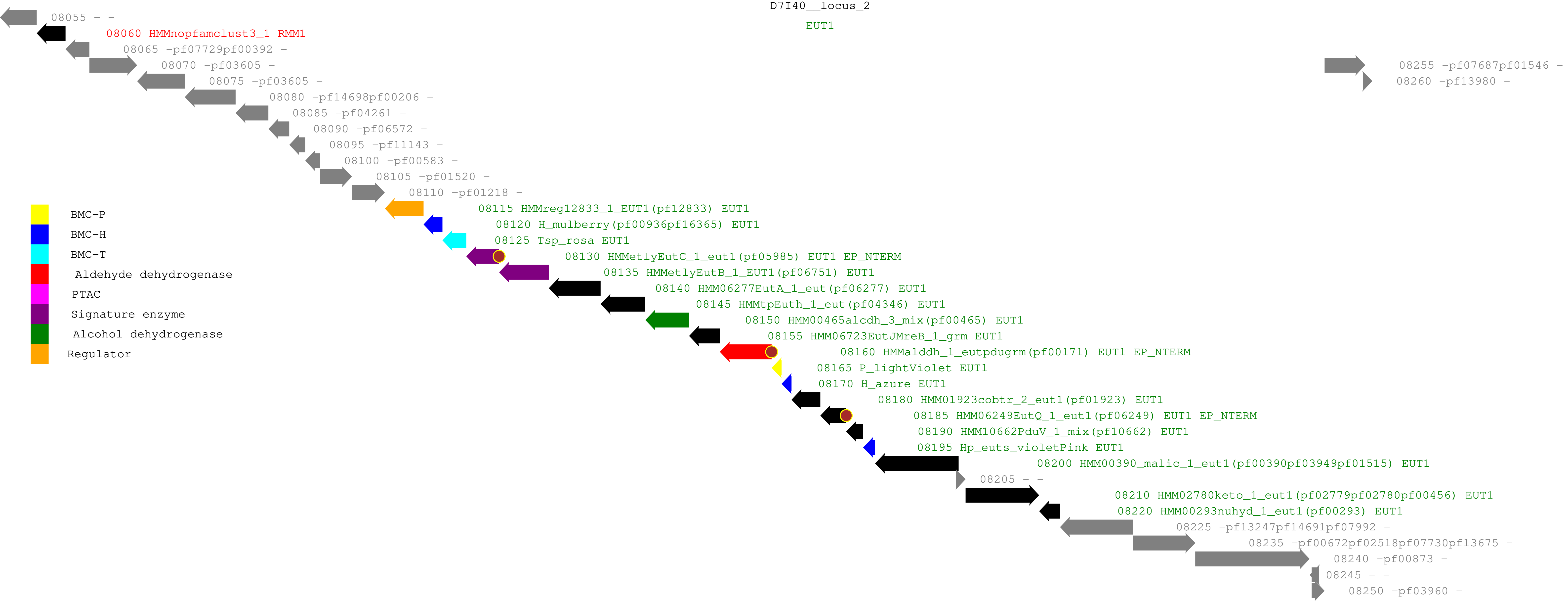

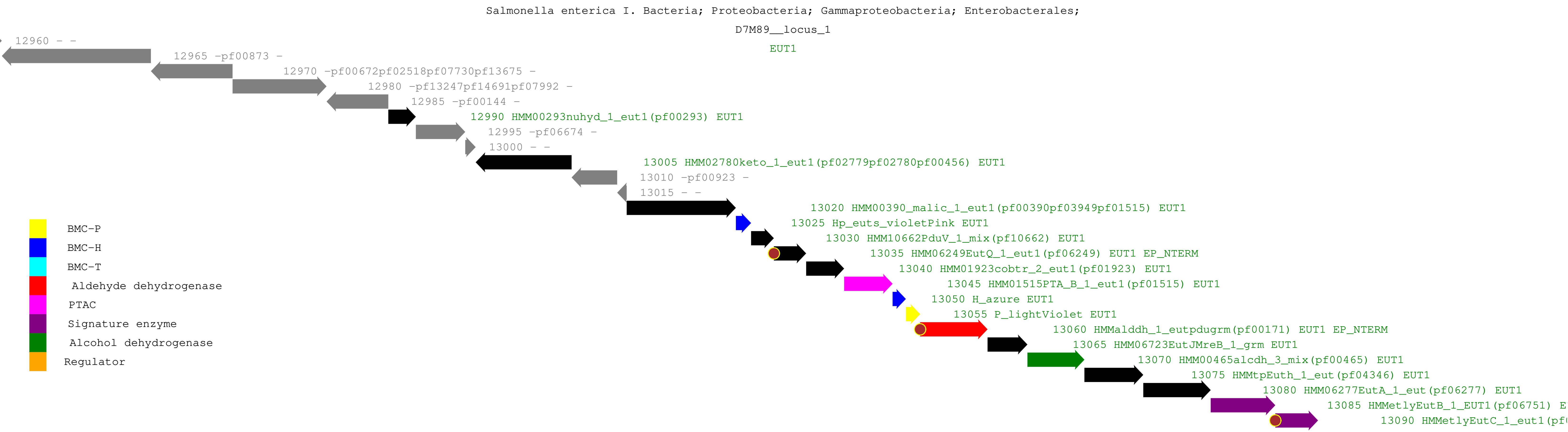

Salmonella enterica I. Bacteria; Proteobacteria; Gammaproteobacteria; Enterobacterales;
D7M89__locus_1
EUT1
12960 - -
12965 -pf00873 -
12970 -pf00672pf02518pf07730pf13675 -
12980 -pf13247pf14691pf07992 -
12985 -pf00144 -
12990 HMM00293nuhyd_1_eut1(pf00293) EUT1
12995 -pf06674 -
13000 - -
13005 HMM02780keto_1_eut1(pf02779pf02780pf00456) EUT1
13010 -pf00923 -
13015 - -
13020 HMM00390_malic_1_eut1(pf00390pf03949pf01515) EUT1
13025 Hp_euts_violetPink EUT1
13030 HMM10662PduV_1_mix(pf10662) EUT1
13035 HMM06249EutQ_1_eut1(pf06249) EUT1 EP_NTERM
13040 HMM01923cobtr_2_eut1(pf01923) EUT1
13045 HMM01515PTA_B_1_eut1(pf01515) EUT1
13050 H_azure EUT1
13055 P_lightViolet EUT1
13060 HMMalddh_1_eutpdugrm(pf00171) EUT1 EP_NTERM
13065 HMM06723EutJMreB_1_grm EUT1
13070 HMM00465alcdh_3_mix(pf00465) EUT1
13075 HMMtpEuth_1_eut(pf04346) EUT1
13080 HMM06277EutA_1_eut(pf06277) EUT1
13085 HMMetlyEutB_1_EUT1(pf06751) E
13090 HMMetlyEutC_1_eut1(pf
BMC-P
BMC-H
BMC-T
Aldehyde dehydrogenase
PTAC
Signature enzyme
Alcohol dehydrogenase
Regulator

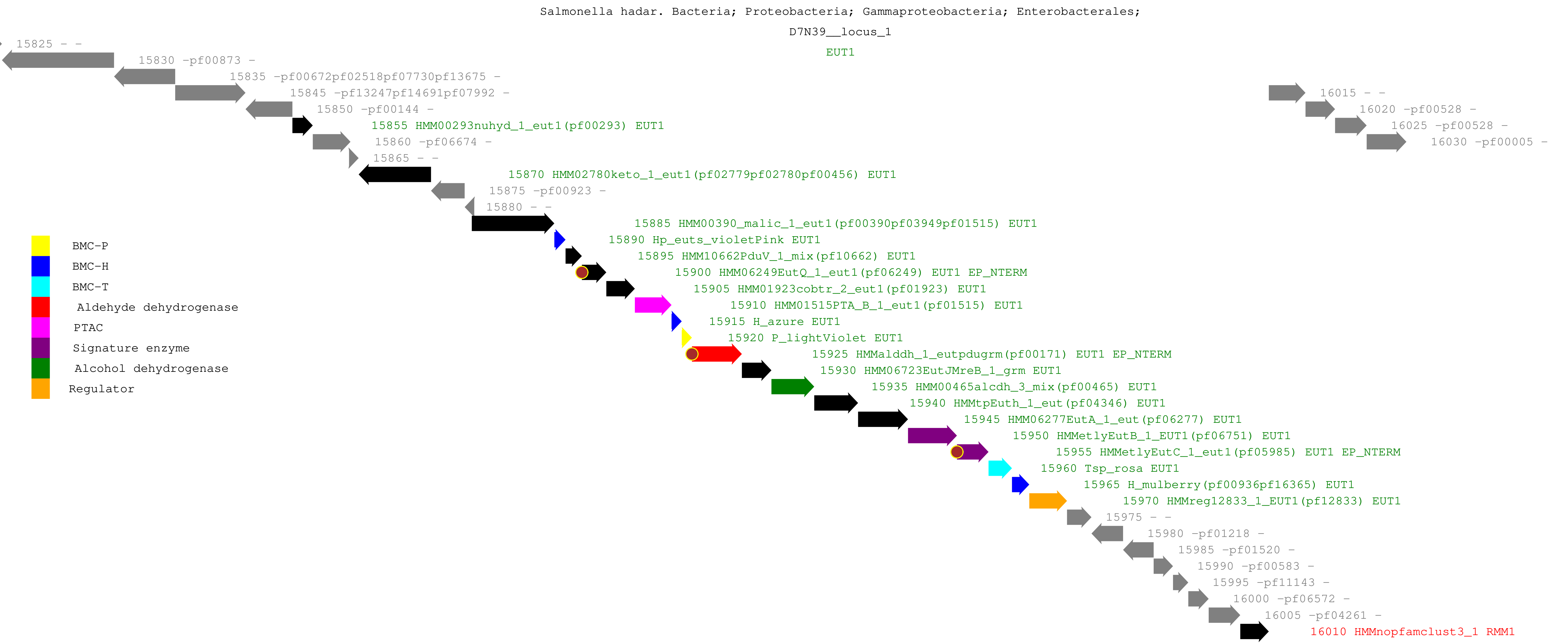

Salmonella hadar. Bacteria; Proteobacteria; Gammaproteobacteria; Enterobacterales;
D7N39__locus_1
EUT1
15825 - -
15830 -pf00873 -
15835 -pf00672pf02518pf07730pf13675 -
15845 -pf13247pf14691pf07992 -
15850 -pf00144 -
15855 HMM00293nuhyd_1_eut1(pf00293) EUT1
15860 -pf06674 -
15865 - -
15870 HMM02780keto_1_eut1(pf02779pf02780pf00456) EUT1
15875 -pf00923 -
15880 - -
15885 HMM00390_malic_1_eut1(pf00390pf03949pf01515) EUT1
15890 Hp_euts_violetPink EUT1
15895 HMM10662PduV_1_mix(pf10662) EUT1
15900 HMM06249EutQ_1_eut1(pf06249) EUT1 EP_NTERM
15905 HMM01923cobtr_2_eut1(pf01923) EUT1
15910 HMM01515PTA_B_1_eut1(pf01515) EUT1
15915 H_azure EUT1
15920 P_lightViolet EUT1
15925 HMMalddh_1_eutpdugrm(pf00171) EUT1 EP_NTERM
15930 HMM06723EutJMreB_1_grm EUT1
15935 HMM00465alcdh_3_mix(pf00465) EUT1
15940 HMMtpEuth_1_eut(pf04346) EUT1
15945 HMM06277EutA_1_eut(pf06277) EUT1
15950 HMMetlyEutB_1_EUT1(pf06751) EUT1
15955 HMMetlyEutC_1_eut1(pf05985) EUT1 EP_NTERM
15960 Tsp_rosa EUT1
15965 H_mulberry(pf00936pf16365) EUT1
15970 HMMreg12833_1_EUT1(pf12833) EUT1
15975 - -
15980 -pf01218 -
15985 -pf01520 -
15990 -pf00583 -
15995 -pf11143 -
16000 -pf06572 -
16005 -pf04261 -
16010 HMMnopfamclust3_1 RMM1
16015 - -
16020 -pf00528 -
16025 -pf00528 -
16030 -pf00005 -
BMC-P
BMC-H
BMC-T
Aldehyde dehydrogenase
PTAC
Signature enzyme
Alcohol dehydrogenase
Regulator

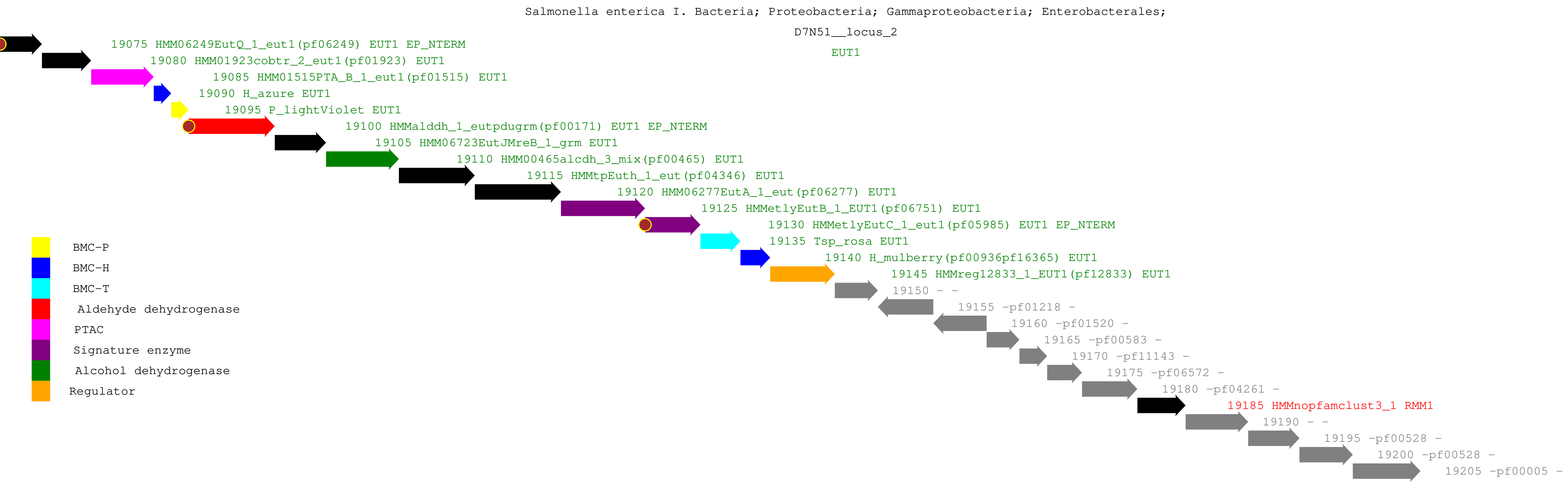

Salmonella enterica I. Bacteria; Proteobacteria; Gammaproteobacteria; Enterobacterales;
D7N51__locus_2
EUT1
19075 HMM06249EutQ_1_eut1(pf06249) EUT1 EP_NTERM
19080 HMM01923cobtr_2_eut1(pf01923) EUT1
19085 HMM01515PTA_B_1_eut1(pf01515) EUT1
19090 H_azure EUT1
19095 P_lightViolet EUT1
19100 HMMalddh_1_eutpdugrm(pf00171) EUT1 EP_NTERM
19105 HMM06723EutJMreB_1_grm EUT1
19110 HMM00465alcdh_3_mix(pf00465) EUT1
19115 HMMtpEuth_1_eut(pf04346) EUT1
19120 HMM06277EutA_1_eut(pf06277) EUT1
19125 HMMetlyEutB_1_EUT1(pf06751) EUT1
19130 HMMetlyEutC_1_eut1(pf05985) EUT1 EP_NTERM
19135 Tsp_rosa EUT1
19140 H_mulberry(pf00936pf16365) EUT1
19145 HMMreg12833_1_EUT1(pf12833) EUT1
19150 - -
19155 -pf01218 -
19160 -pf01520 -
19165 -pf00583 -
19170 -pf11143 -
19175 -pf06572 -
19180 -pf04261 -
19185 HMMnopfamclust3_1 RMM1
19190 - -
19195 -pf00528 -
19200 -pf00528 -
19205 -pf00005 -
BMC-P
BMC-H
BMC-T
Aldehyde dehydrogenase
PTAC
Signature enzyme
Alcohol dehydrogenase
Regulator

Salmonella enterica I. Bacteria; Proteobacteria; Gammaproteobacteria; Enterobacterales;

D7028__locus_1

EUT1

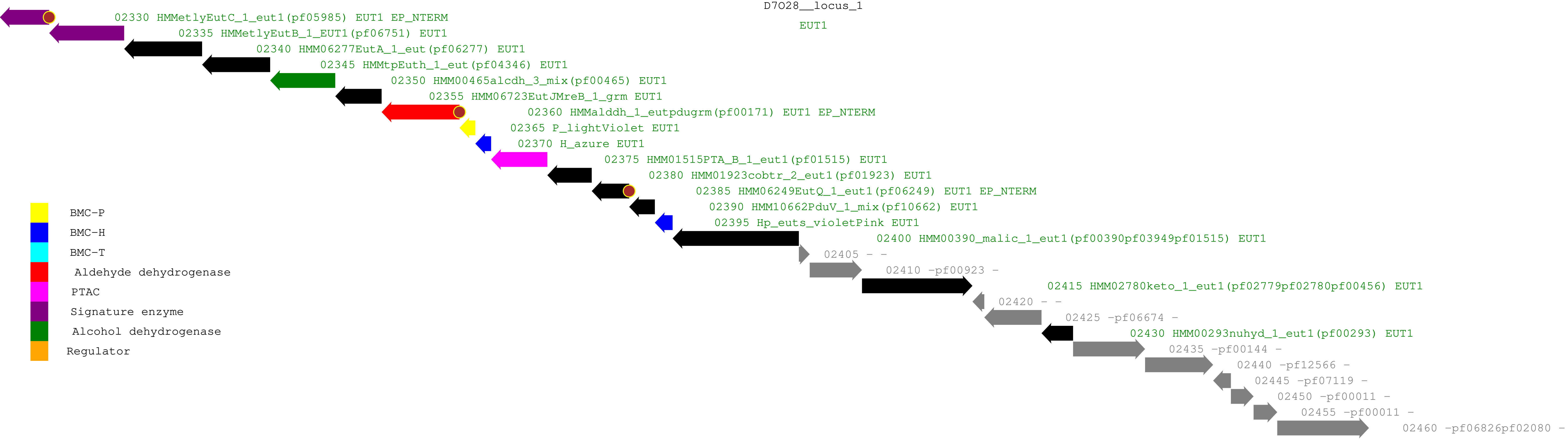

Salmonella enterica subsp. enterica serovar Saintpaul. Bacteria; Proteobacteria; Gammaproteobacteria; Enterobacterales;

D7055__locus_1

EUT1

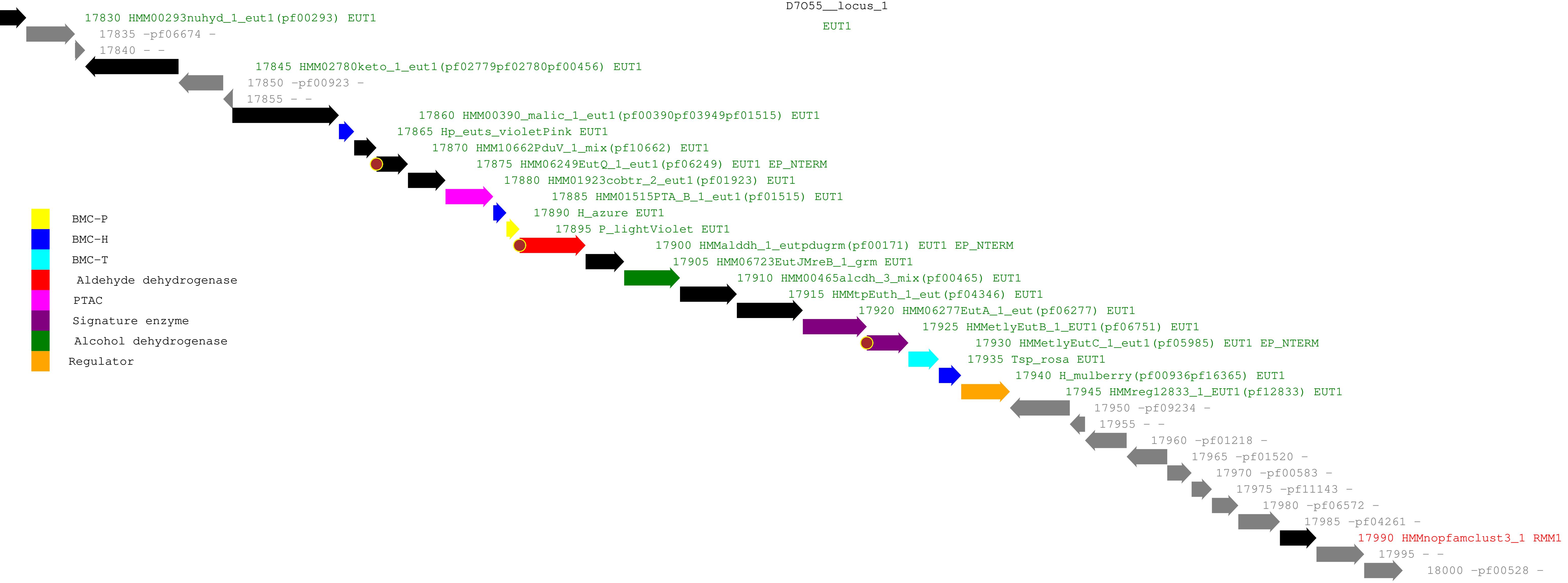

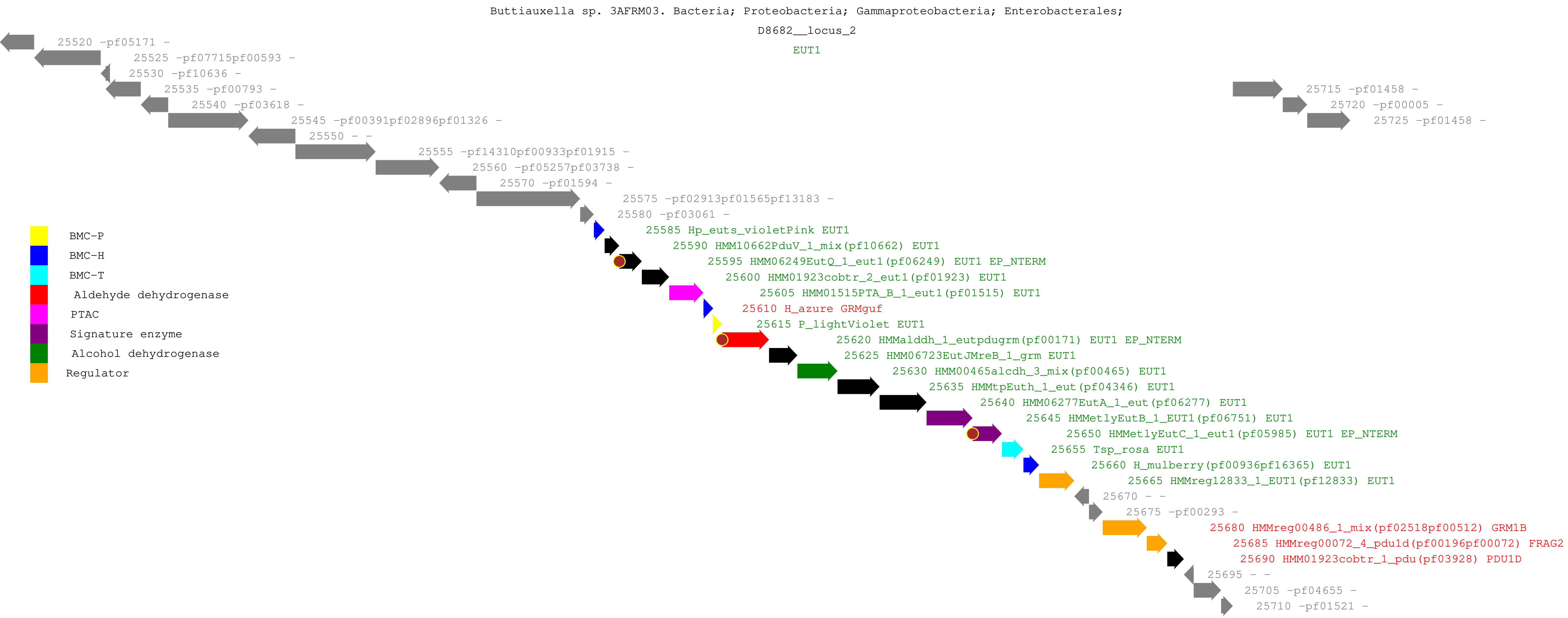
Buttiauxella sp. 3AFRM03. Bacteria; Proteobacteria; Gammaproteobacteria; Enterobacterales;
D8682__locus_2
EUT1
25520 -pf05171 -
25525 -pf07715pf00593 -
25530 -pf10636 -
25535 -pf00793 -
25540 -pf03618 -
25545 -pf00391pf02896pf01326 -
25550 - -
25555 -pf14310pf00933pf01915 -
25560 -pf05257pf03738 -
25570 -pf01594 -
25575 -pf02913pf01565pf13183 -
25580 -pf03061 -
25585 Hp_euts_violetPink EUT1
25590 HMM10662PduV_1_mix(pf10662) EUT1
25595 HMM06249EutQ_1_eut1(pf06249) EUT1 EP_NTERM
25600 HMM01923cobtr_2_eut1(pf01923) EUT1
25605 HMM01515PTA_B_1_eut1(pf01515) EUT1
25610 H_azure GRMguf
25615 P_lightViolet EUT1
25620 HMMaldh_1_eutpdugrm(pf00171) EUT1 EP_NTERM
25625 HMM06723EutJMreB_1_grm EUT1
25630 HMM00465alcdh_3_mix(pf00465) EUT1
25635 HMMtpEuth_1_eut(pf04346) EUT1
25640 HMM06277EutA_1_eut(pf06277) EUT1
25645 HMMetlyEutB_1_EUT1(pf06751) EUT1
25650 HMMetlyEutC_1_eut1(pf05985) EUT1 EP_NTERM
25655 Tsp_rosa EUT1
25660 H_mulberry(pf00936pf16365) EUT1
25665 HMMreg12833_1_EUT1(pf12833) EUT1
25670 - -
25675 -pf00293 -
25680 HMMreg00486_1_mix(pf02518pf00512) GRM1B
25685 HMMreg00072_4_pdu1d(pf00196pf00072) FRAG2
25690 HMM01923cobtr_1_pdu(pf03928) PDU1D
25695 - -
25705 -pf04655 -
25710 -pf01521 -
25715 -pf01458 -
25720 -pf00005 -
25725 -pf01458 -
BMC-P
BMC-H
BMC-T
Aldehyde dehydrogenase
PTAC
Signature enzyme
Alcohol dehydrogenase
Regulator

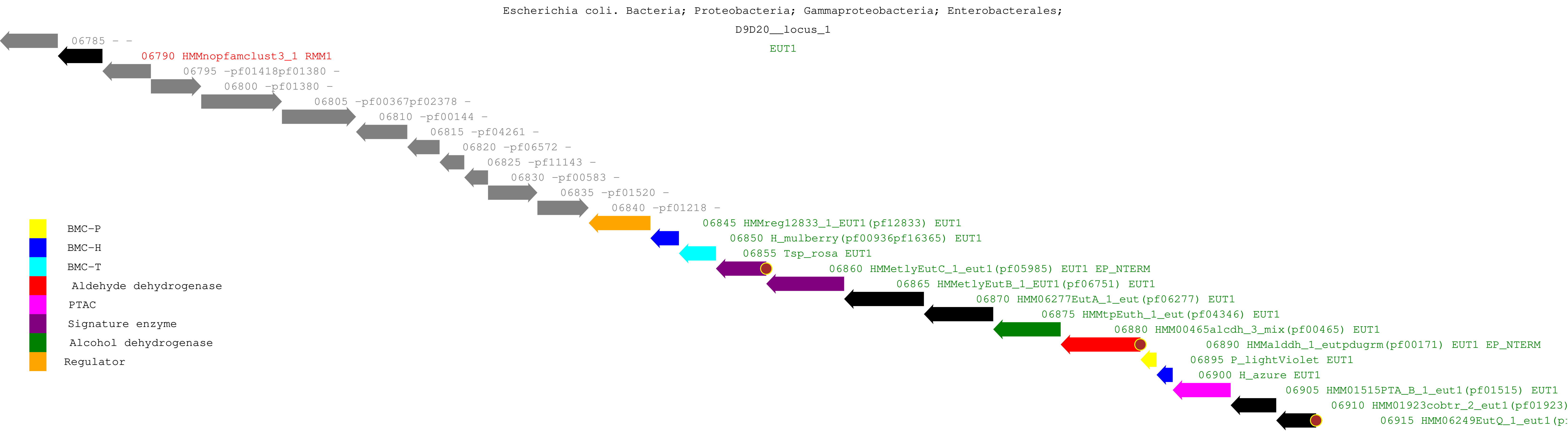

Escherichia coli. Bacteria; Proteobacteria; Gammaproteobacteria; Enterobacterales;
D9D20__locus_1
EUT1
06785 - -
06790 HMMnopfamclust3_1 RMM1
06795 -pf01418pf01380 -
06800 -pf01380 -
06805 -pf00367pf02378 -
06810 -pf00144 -
06815 -pf04261 -
06820 -pf06572 -
06825 -pf11143 -
06830 -pf00583 -
06835 -pf01520 -
06840 -pf01218 -
06845 HMMreg12833_1_EUT1(pf12833) EUT1
06850 H_mulberry(pf00936pf16365) EUT1
06855 Tsp_rosa EUT1
06860 HMMetlyEutC_1_eut1(pf05985) EUT1 EP_NTERM
06865 HMMetlyEutB_1_EUT1(pf06751) EUT1
06870 HMM06277EutA_1_eut(pf06277) EUT1
06875 HMMtpEuth_1_eut(pf04346) EUT1
06880 HMM00465alcdh_3_mix(pf00465) EUT1
06890 HMMaldd h_1_eutpdugrm(pf00171) EUT1 EP_NTERM
06895 P_lightViolet EUT1
06900 H_azure EUT1
06905 HMM01515PTA_B_1_eut1(pf01515) EUT1
06910 HMM01923cobtr_2_eut1(pf01923)
06915 HMM06249EutQ_1_eut1(p
BMC-P
BMC-H
BMC-T
Aldehyde dehydrogenase
PTAC
Signature enzyme
Alcohol dehydrogenase
Regulator

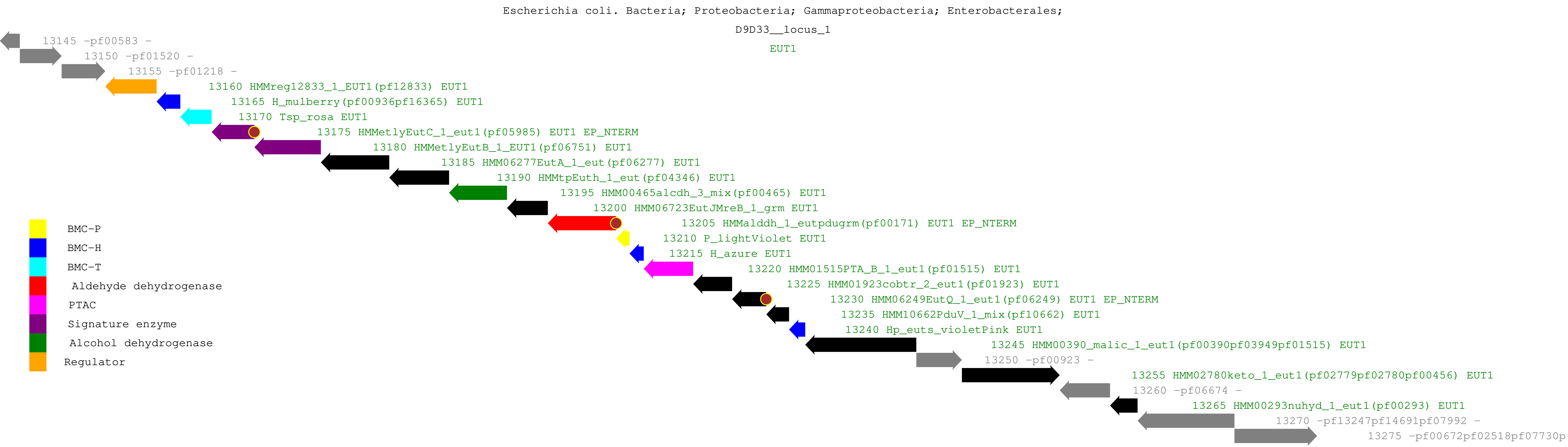
Escherichia coli. Bacteria; Proteobacteria; Gammaproteobacteria; Enterobacterales;
D9D33__locus_1
EUT1
13145 -pf00583 -
13150 -pf01520 -
13155 -pf01218 -
13160 HMMreg12833_1_EUT1(pf12833) EUT1
13165 H_mulberry(pf00936pf16365) EUT1
13170 Tsp_rosa EUT1
13175 HMMetlyEutC_1_eut1(pf05985) EUT1 EP_NTERM
13180 HMMetlyEutB_1_EUT1(pf06751) EUT1
13185 HMM06277EutA_1_eut(pf06277) EUT1
13190 HMMtpEuth_1_eut(pf04346) EUT1
13195 HMM00465alcdh_3_mix(pf00465) EUT1
13200 HMM06723EutJMreB_1_grm EUT1
13205 HMMalddh_1_eutpdugrm(pf00171) EUT1 EP_NTERM
13210 P_lightViolet EUT1
13215 H_azure EUT1
13220 HMM01515PTA_B_1_eut1(pf01515) EUT1
13225 HMM01923cobtr_2_eut1(pf01923) EUT1
13230 HMM06249EutQ_1_eut1(pf06249) EUT1 EP_NTERM
13235 HMM10662PduV_1_mix(pf10662) EUT1
13240 Hp_euts_violetPink EUT1
13245 HMM00390_malic_1_eut1(pf00390pf03949pf01515) EUT1
13250 -pf00923 -
13255 HMM02780keto_1_eut1(pf02779pf02780pf00456) EUT1
13260 -pf06674 -
13265 HMM00293nuhyd_1_eut1(pf00293) EUT1
13270 -pf13247pf14691pf07992 -
13275 -pf00672pf02518pf07730p
BMC-P
BMC-H
BMC-T
Aldehyde dehydrogenase
PTAC
Signature enzyme
Alcohol dehydrogenase
Regulator

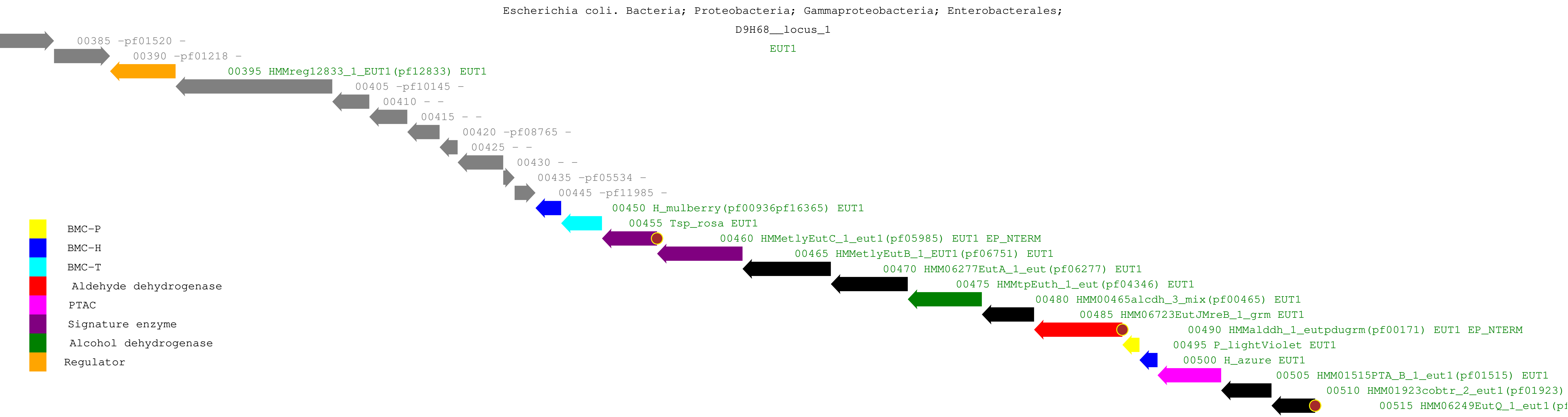
Escherichia coli. Bacteria; Proteobacteria; Gammaproteobacteria; Enterobacterales;
D9H68__locus_1
EUT1
00385 -pf01520 -
00390 -pf01218 -
00395 HMMreg12833_1_EUT1(pf12833) EUT1
00405 -pf10145 -
00410 - -
00415 - -
00420 -pf08765 -
00425 - -
00430 - -
00435 -pf05534 -
00445 -pf11985 -
00450 H_mulberry(pf00936pf16365) EUT1
00455 Tsp_rosa EUT1
00460 HMMetlyEutC_1_eut1(pf05985) EUT1 EP_NTERM
00465 HMMetlyEutB_1_EUT1(pf06751) EUT1
00470 HMM06277EutA_1_eut(pf06277) EUT1
00475 HMMtpEuth_1_eut(pf04346) EUT1
00480 HMM00465alcdh_3_mix(pf00465) EUT1
00485 HMM06723EutJMreB_1_grm EUT1
00490 HMMalddh_1_eutpdugrm(pf00171) EUT1 EP_NTERM
00495 P_lightViolet EUT1
00500 H_azure EUT1
00505 HMM01515PTA_B_1_eut1(pf01515) EUT1
00510 HMM01923cobtr_2_eut1(pf01923)
00515 HMM06249EutQ_1_eut1(p
BMC-P
BMC-H
BMC-T
Aldehyde dehydrogenase
PTAC
Signature enzyme
Alcohol dehydrogenase
Regulator

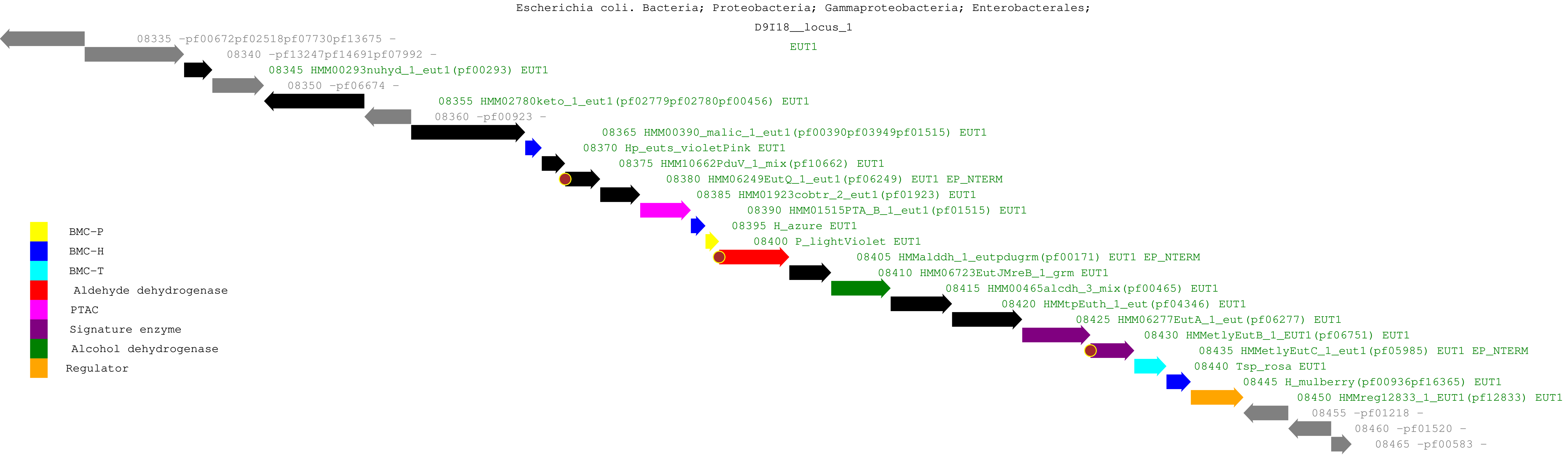
Escherichia coli. Bacteria; Proteobacteria; Gammaproteobacteria; Enterobacterales;
D9I18__locus_1
EUT1
08335 -pf00672pf02518pf07730pf13675 -
08340 -pf13247pf14691pf07992 -
08345 HMM00293nuhyd_1_eut1(pf00293) EUT1
08350 -pf06674 -
08355 HMM02780keto_1_eut1(pf02779pf02780pf00456) EUT1
08360 -pf00923 -
08365 HMM00390_malic_1_eut1(pf00390pf03949pf01515) EUT1
08370 Hp_euts_violetPink EUT1
08375 HMM10662PduV_1_mix(pf10662) EUT1
08380 HMM06249EutQ_1_eut1(pf06249) EUT1 EP_NTERM
08385 HMM01923cobtr_2_eut1(pf01923) EUT1
08390 HMM01515PTA_B_1_eut1(pf01515) EUT1
08395 H_azure EUT1
08400 P_lightViolet EUT1
08405 HMMalddh_1_eutpdugrm(pf00171) EUT1 EP_NTERM
08410 HMM06723EutJMreB_1_grm EUT1
08415 HMM00465alcdh_3_mix(pf00465) EUT1
08420 HMMtpEuth_1_eut(pf04346) EUT1
08425 HMM06277EutA_1_eut(pf06277) EUT1
08430 HMMetlyEutB_1_EUT1(pf06751) EUT1
08435 HMMetlyEutC_1_eut1(pf05985) EUT1 EP_NTERM
08440 Tsp_rosa EUT1
08445 H_mulberry(pf00936pf16365) EUT1
08450 HMMreg12833_1_EUT1(pf12833) EUT1
08455 -pf01218 -
08460 -pf01520 -
08465 -pf00583 -
BMC-P
BMC-H
BMC-T
Aldehyde dehydrogenase
PTAC
Signature enzyme
Alcohol dehydrogenase
Regulator

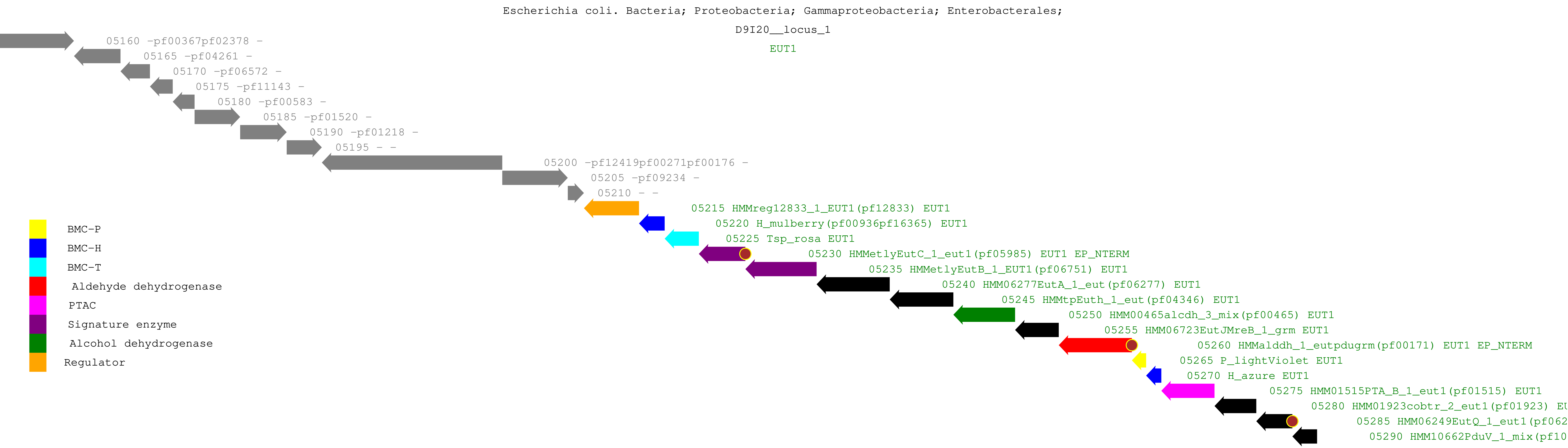

Escherichia coli. Bacteria; Proteobacteria; Gammaproteobacteria; Enterobacterales;
D9I20__locus_1
EUT1
05160 -pf00367pf02378 -
05165 -pf04261 -
05170 -pf06572 -
05175 -pf11143 -
05180 -pf00583 -
05185 -pf01520 -
05190 -pf01218 -
05195 - -
05200 -pf12419pf00271pf00176 -
05205 -pf09234 -
05210 - -
05215 HMMreg12833_1_EUT1(pf12833) EUT1
05220 H_mulberry(pf00936pf16365) EUT1
05225 Tsp_rosa EUT1
05230 HMMetlyEutC_1_eut1(pf05985) EUT1 EP_NTERM
05235 HMMetlyEutB_1_EUT1(pf06751) EUT1
05240 HMM06277EutA_1_eut(pf06277) EUT1
05245 HMMtpEuth_1_eut(pf04346) EUT1
05250 HMM00465alcdh_3_mix(pf00465) EUT1
05255 HMM06723EutJMreB_1_grm EUT1
05260 HMMaldh_1_eutpdugrm(pf00171) EUT1 EP_NTERM
05265 P_lightViolet EUT1
05270 H_azure EUT1
05275 HMM01515PTA_B_1_eut1(pf01515) EUT1
05280 HMM01923cobtr_2_eut1(pf01923) EU
05285 HMM06249EutQ_1_eut1(pf062
05290 HMM10662PduV_1_mix(pf10
BMC-P
BMC-H
BMC-T
Aldehyde dehydrogenase
PTAC
Signature enzyme
Alcohol dehydrogenase
Regulator

Escherichia coli. Bacteria; Proteobacteria; Gammaproteobacteria; Enterobacterales;

D9I88__locus_1

EUT1

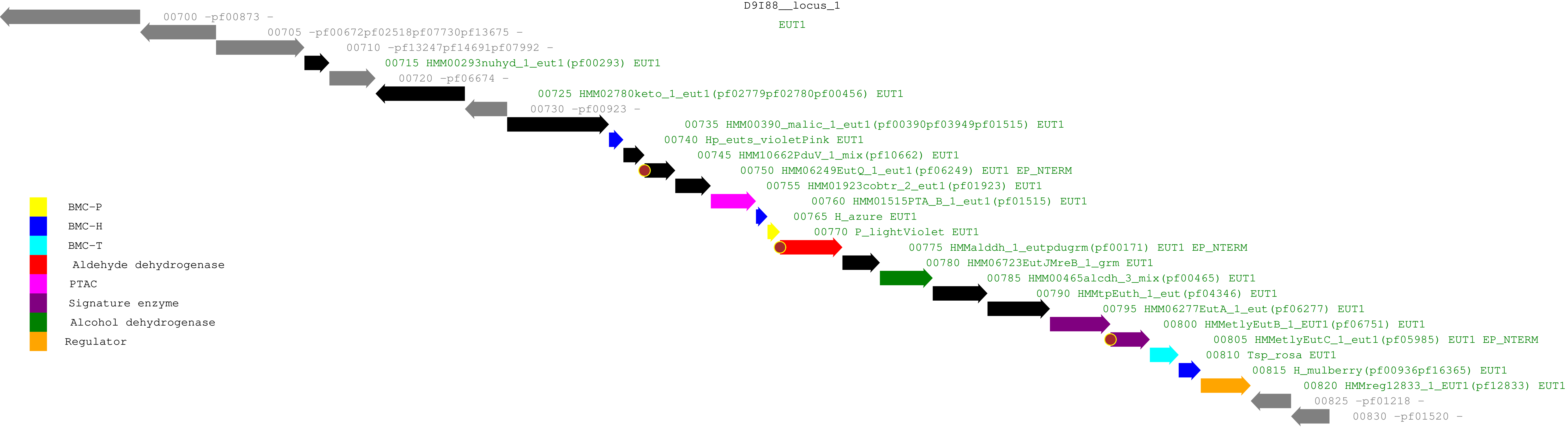

Escherichia coli. Bacteria; Proteobacteria; Gammaproteobacteria; Enterobacterales;

D9J44__locus_1

EUT1

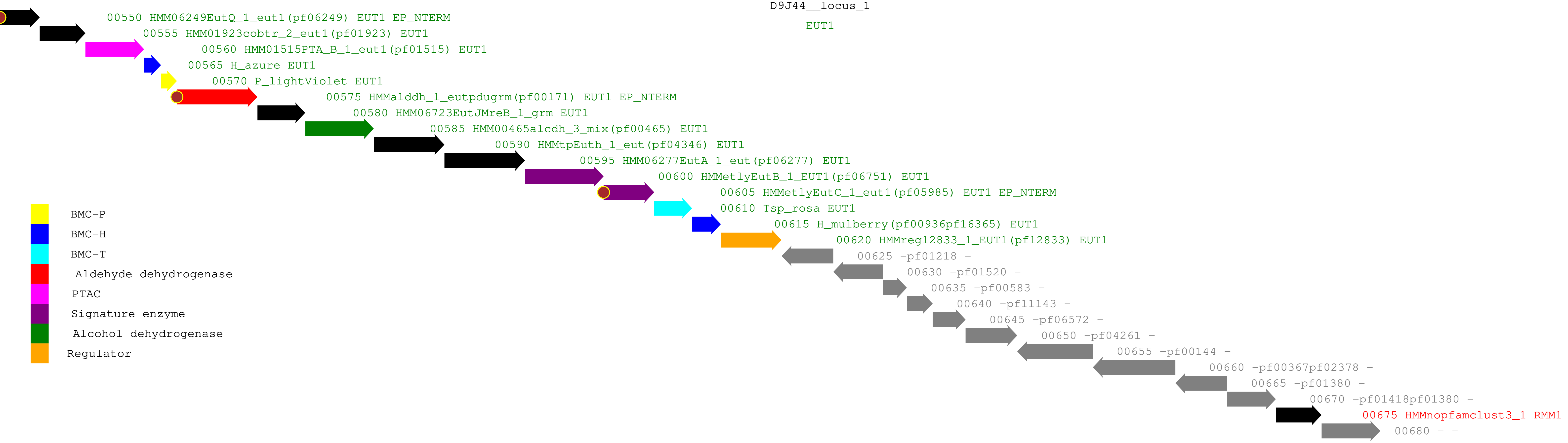

Salmonella enterica (Salmonella choleraesuis). Bacteria; Proteobacteria; Gammaproteobacteria; Enterobacterales;

D9031__locus_2

EUT1

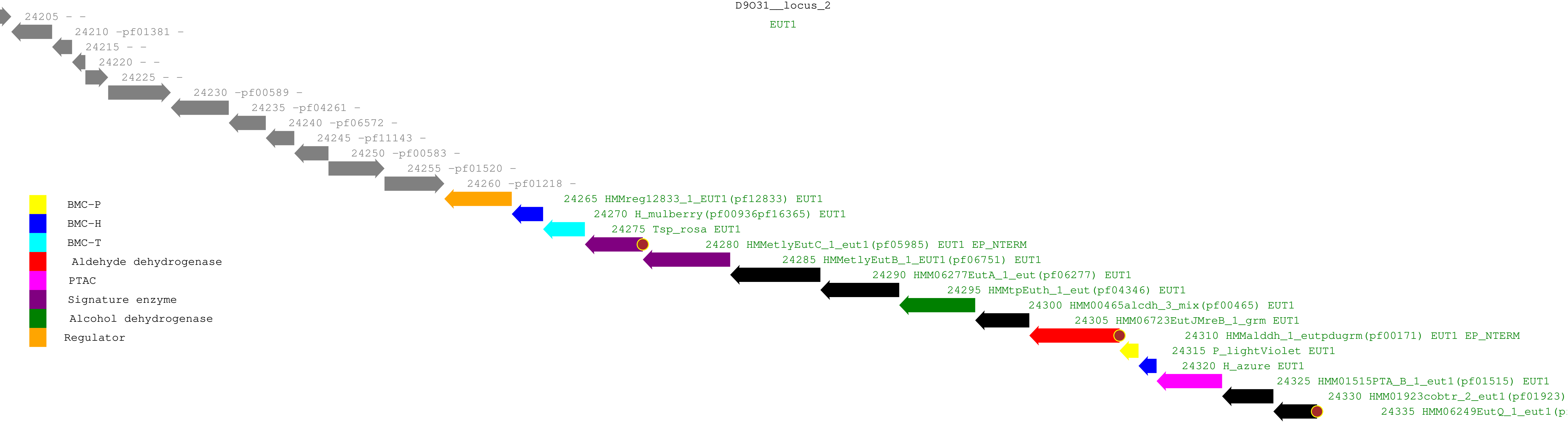

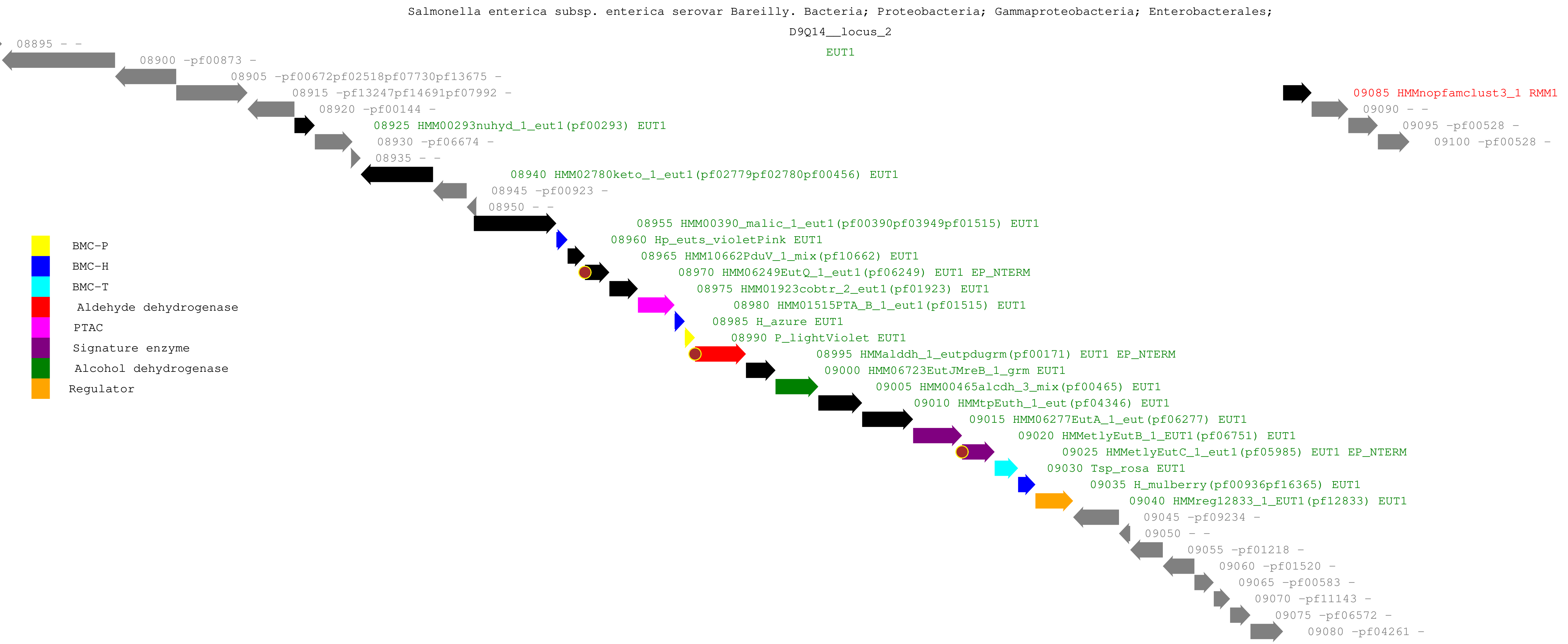
Salmonella enterica subsp. enterica serovar Bareilly. Bacteria; Proteobacteria; Gammaproteobacteria; Enterobacterales;
D9Q14__locus_2
EUT1
08895 - -
08900 -pf00873 -
08905 -pf00672pf02518pf07730pf13675 -
08915 -pf13247pf14691pf07992 -
08920 -pf00144 -
08925 HMM00293nuhyd_1_eut1(pf00293) EUT1
08930 -pf06674 -
08935 - -
08940 HMM02780keto_1_eut1(pf02779pf02780pf00456) EUT1
08945 -pf00923 -
08950 - -
08955 HMM00390_malic_1_eut1(pf00390pf03949pf01515) EUT1
08960 Hp_euts_violetPink EUT1
08965 HMM10662PduV_1_mix(pf10662) EUT1
08970 HMM06249EutQ_1_eut1(pf06249) EUT1 EP_NTERM
08975 HMM01923cobtr_2_eut1(pf01923) EUT1
08980 HMM01515PTA_B_1_eut1(pf01515) EUT1
08985 H_azure EUT1
08990 P_lightViolet EUT1
08995 HMMaldh_1_eutpdugrm(pf00171) EUT1 EP_NTERM
09000 HMM06723EutJMreB_1_grm EUT1
09005 HMM00465alcdh_3_mix(pf00465) EUT1
09010 HMMtpEuth_1_eut(pf04346) EUT1
09015 HMM06277EutA_1_eut(pf06277) EUT1
09020 HMMetlyEutB_1_EUT1(pf06751) EUT1
09025 HMMetlyEutC_1_eut1(pf05985) EUT1 EP_NTERM
09030 Tsp_rosa EUT1
09035 H_mulberry(pf00936pf16365) EUT1
09040 HMMreg12833_1_EUT1(pf12833) EUT1
09045 -pf09234 -
09050 - -
09055 -pf01218 -
09060 -pf01520 -
09065 -pf00583 -
09070 -pf11143 -
09075 -pf06572 -
09080 -pf04261 -
09085 HMMnopfamclust3_1 RMM1
09090 - -
09095 -pf00528 -
09100 -pf00528 -
BMC-P
BMC-H
BMC-T
Aldehyde dehydrogenase
PTAC
Signature enzyme
Alcohol dehydrogenase
Regulator

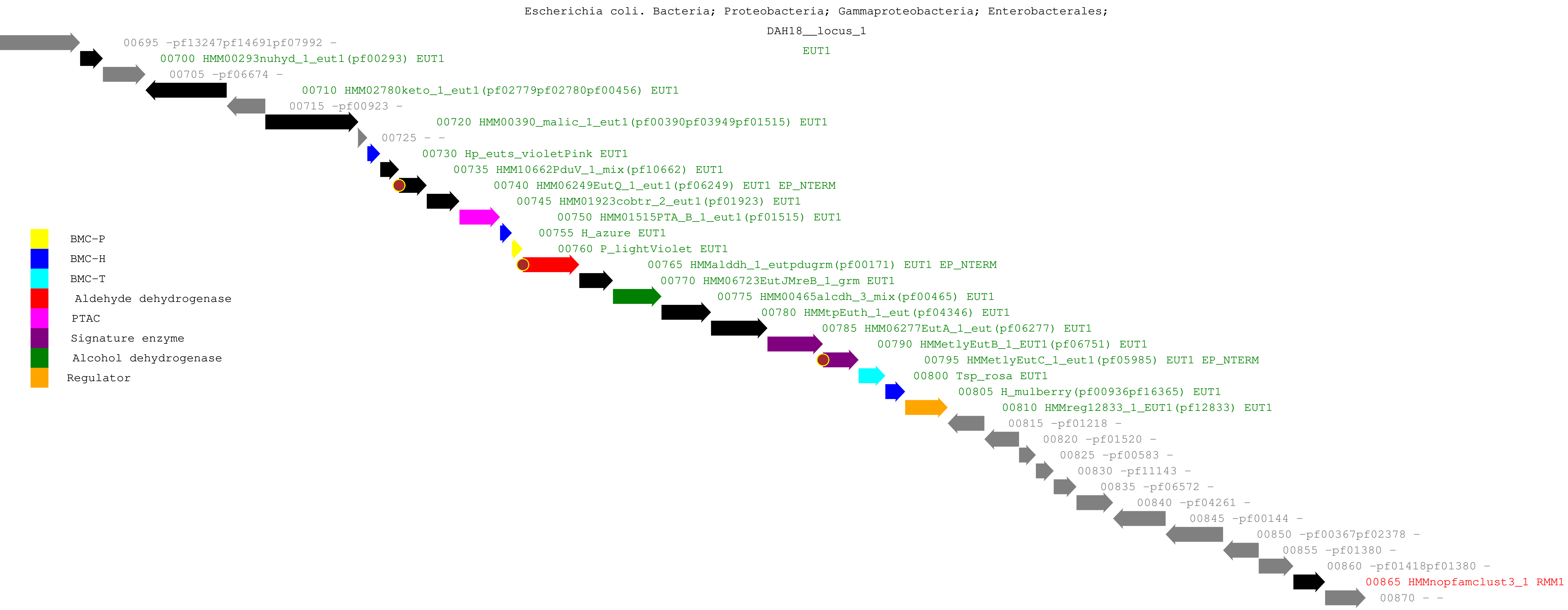

Escherichia coli. Bacteria; Proteobacteria; Gammaproteobacteria; Enterobacterales;
DAH18__locus_1
EUT1
00695 -pf13247pf14691pf07992 -
00700 HMM00293nuhyd_1_eut1(pf00293) EUT1
00705 -pf06674 -
00710 HMM02780keto_1_eut1(pf02779pf02780pf00456) EUT1
00715 -pf00923 -
00720 HMM00390_malic_1_eut1(pf00390pf03949pf01515) EUT1
00725 - -
00730 Hp_euts_violetPink EUT1
00735 HMM10662PduV_1_mix(pf10662) EUT1
00740 HMM06249EutQ_1_eut1(pf06249) EUT1 EP_NTERM
00745 HMM01923cobtr_2_eut1(pf01923) EUT1
00750 HMM01515PTA_B_1_eut1(pf01515) EUT1
00755 H_azure EUT1
00760 P_lightViolet EUT1
00765 HMMalddh_1_eutpdugrm(pf00171) EUT1 EP_NTERM
00770 HMM06723EutJMreB_1_grm EUT1
00775 HMM00465alcdh_3_mix(pf00465) EUT1
00780 HMMtpEuth_1_eut(pf04346) EUT1
00785 HMM06277EutA_1_eut(pf06277) EUT1
00790 HMMetlyEutB_1_EUT1(pf06751) EUT1
00795 HMMetlyEutC_1_eut1(pf05985) EUT1 EP_NTERM
00800 Tsp_rosa EUT1
00805 H_mulberry(pf00936pf16365) EUT1
00810 HMMreg12833_1_EUT1(pf12833) EUT1
00815 -pf01218 -
00820 -pf01520 -
00825 -pf00583 -
00830 -pf11143 -
00835 -pf06572 -
00840 -pf04261 -
00845 -pf00144 -
00850 -pf00367pf02378 -
00855 -pf01380 -
00860 -pf01418pf01380 -
00865 HMMnopfamclust3_1 RMM1
00870 - -
BMC-P
BMC-H
BMC-T
Aldehyde dehydrogenase
PTAC
Signature enzyme
Alcohol dehydrogenase
Regulator

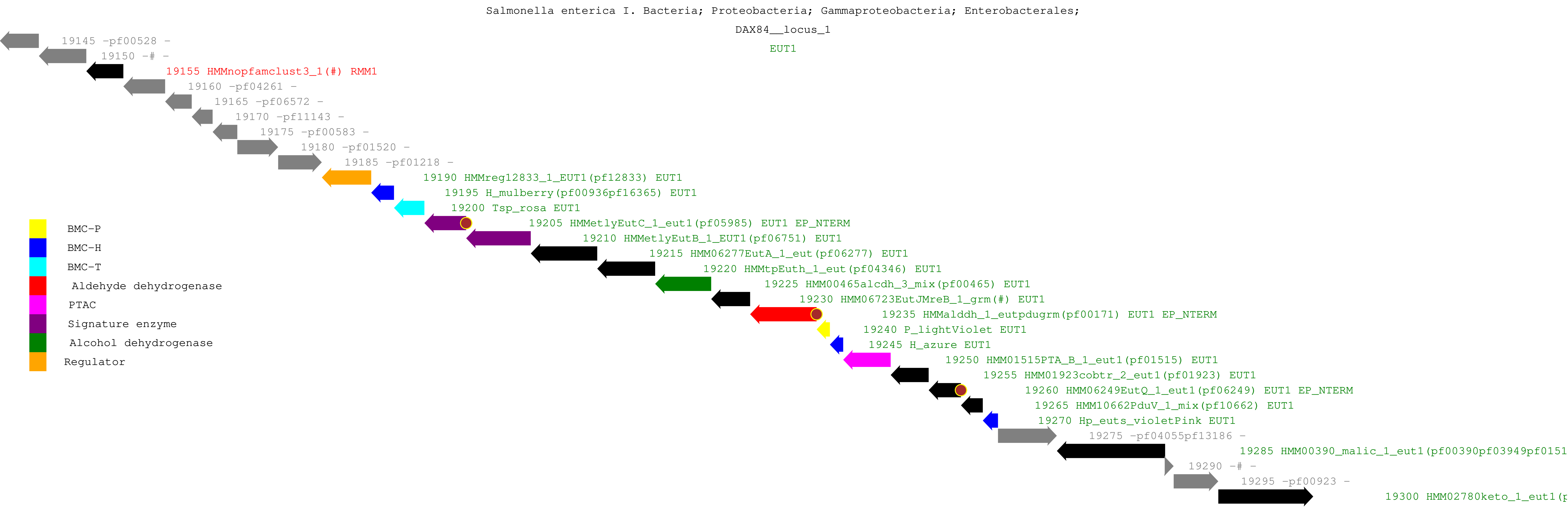

Salmonella enterica I. Bacteria; Proteobacteria; Gammaproteobacteria; Enterobacterales;
DAX84__locus_1
EUT1
19145 -pf00528 -
19150 -# -
19155 HMMnopfamclust3_1(#) RMM1
19160 -pf04261 -
19165 -pf06572 -
19170 -pf11143 -
19175 -pf00583 -
19180 -pf01520 -
19185 -pf01218 -
19190 HMMreg12833_1_EUT1(pf12833) EUT1
19195 H_mulberry(pf00936pf16365) EUT1
19200 Tsp_rosa EUT1
19205 HMMetlyEutC_1_eut1(pf05985) EUT1 EP_NTERM
19210 HMMetlyEutB_1_EUT1(pf06751) EUT1
19215 HMM06277EutA_1_eut(pf06277) EUT1
19220 HMMtpEuth_1_eut(pf04346) EUT1
19225 HMM00465alcdh_3_mix(pf00465) EUT1
19230 HMM06723EutJMreB_1_grm(#) EUT1
19235 HMMalddh_1_eutpdugrm(pf00171) EUT1 EP_NTERM
19240 P_lightViolet EUT1
19245 H_azure EUT1
19250 HMM01515PTA_B_1_eut1(pf01515) EUT1
19255 HMM01923cobtr_2_eut1(pf01923) EUT1
19260 HMM06249EutQ_1_eut1(pf06249) EUT1 EP_NTERM
19265 HMM10662PduV_1_mix(pf10662) EUT1
19270 Hp_euts_violetPink EUT1
19275 -pf04055pf13186 -
19285 HMM00390_malic_1_eut1(pf00390pf03949pf0151
19290 -# -
19295 -pf00923 -
19300 HMM02780keto_1_eut1(p
BMC-P
BMC-H
BMC-T
Aldehyde dehydrogenase
PTAC
Signature enzyme
Alcohol dehydrogenase
Regulator

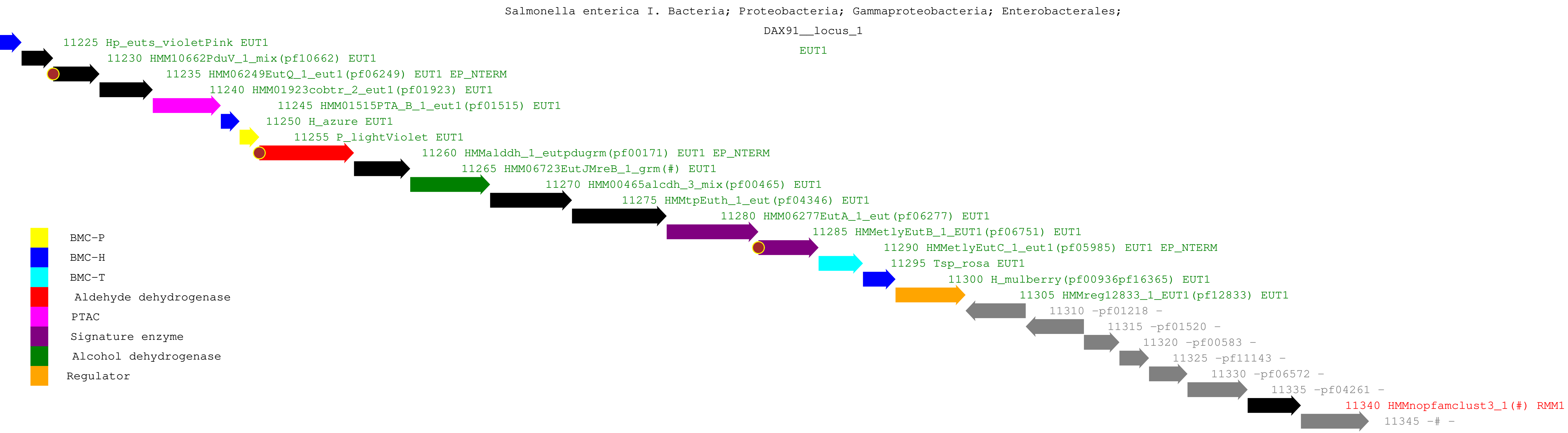

Salmonella enterica I. Bacteria; Proteobacteria; Gammaproteobacteria; Enterobacterales;
DAX91__locus_1
EUT1
11225 Hp_euts_violetPink EUT1
11230 HMM10662PduV_1_mix(pf10662) EUT1
11235 HMM06249EutQ_1_eut1(pf06249) EUT1 EP_NTERM
11240 HMM01923cobtr_2_eut1(pf01923) EUT1
11245 HMM01515PTA_B_1_eut1(pf01515) EUT1
11250 H_azure EUT1
11255 P_lightViolet EUT1
11260 HMMalddh_1_eutpdugrm(pf00171) EUT1 EP_NTERM
11265 HMM06723EutJMreB_1_grm(#) EUT1
11270 HMM00465alcdh_3_mix(pf00465) EUT1
11275 HMMtpEuth_1_eut(pf04346) EUT1
11280 HMM06277EutA_1_eut(pf06277) EUT1
11285 HMMetlyEutB_1_EUT1(pf06751) EUT1
11290 HMMetlyEutC_1_eut1(pf05985) EUT1 EP_NTERM
11295 Tsp_rosa EUT1
11300 H_mulberry(pf00936pf16365) EUT1
11305 HMMreg12833_1_EUT1(pf12833) EUT1
11310 -pf01218 -
11315 -pf01520 -
11320 -pf00583 -
11325 -pf11143 -
11330 -pf06572 -
11335 -pf04261 -
11340 HMMnopfamclust3_1(#) RMM1
11345 -# -
BMC-P
BMC-H
BMC-T
Aldehyde dehydrogenase
PTAC
Signature enzyme
Alcohol dehydrogenase
Regulator

Salmonella enterica subsp. enterica serovar Kingston. Bacteria; Proteobacteria; Gammaproteobacteria; Enterobacterales;

DBB49__locus_2

EUT1

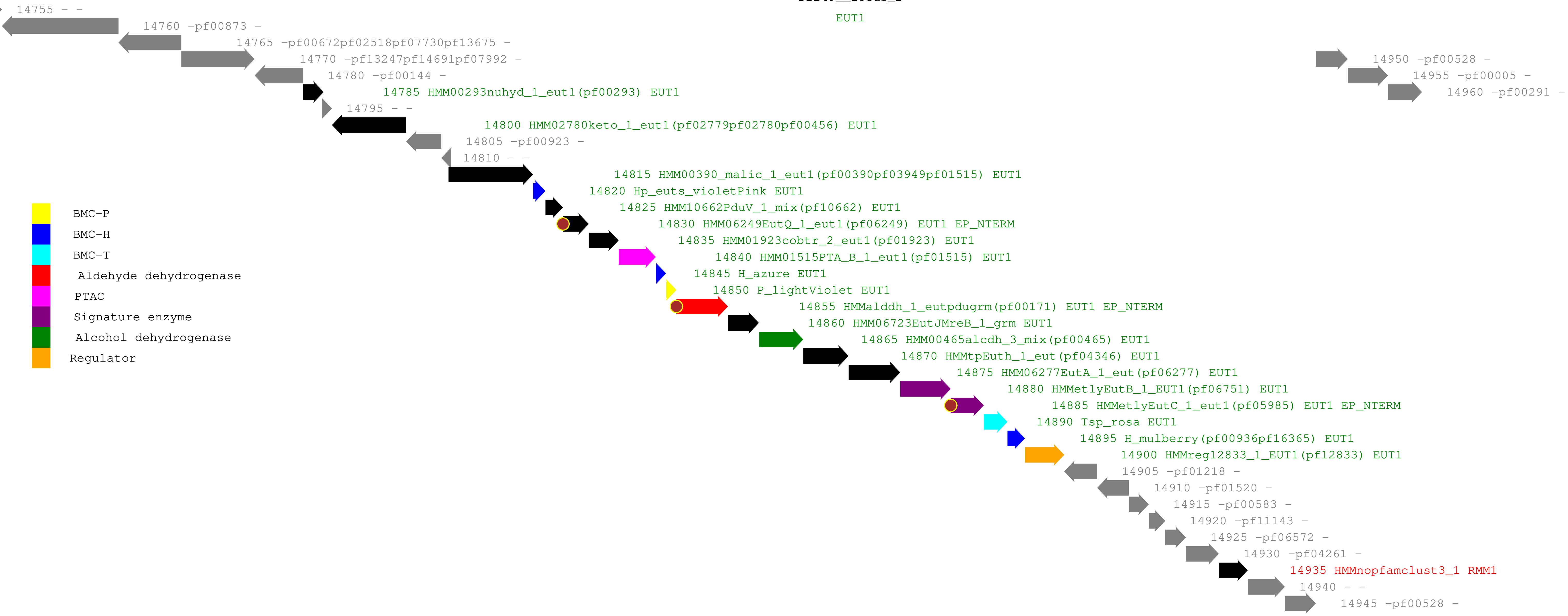

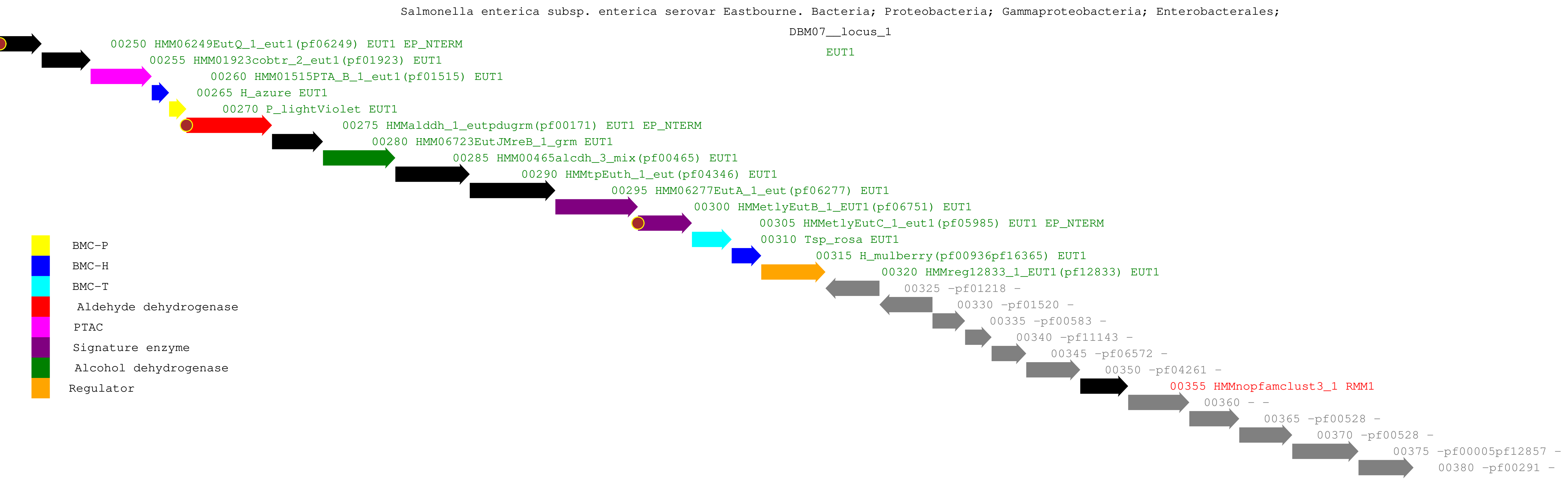

Salmonella enterica subsp. enterica serovar Eastbourne. Bacteria; Proteobacteria; Gammaproteobacteria; Enterobacterales;
DBM07__locus_1
EUT1
00250 HMM06249EutQ_1_eut1(pf06249) EUT1 EP_NTERM
00255 HMM01923cobtr_2_eut1(pf01923) EUT1
00260 HMM01515PTA_B_1_eut1(pf01515) EUT1
00265 H_azure EUT1
00270 P_lightViolet EUT1
00275 HMMalddh_1_eutpdugrm(pf00171) EUT1 EP_NTERM
00280 HMM06723EutJMreB_1_grm EUT1
00285 HMM00465alcdh_3_mix(pf00465) EUT1
00290 HMMtpEuth_1_eut(pf04346) EUT1
00295 HMM06277EutA_1_eut(pf06277) EUT1
00300 HMMetlyEutB_1_EUT1(pf06751) EUT1
00305 HMMetlyEutC_1_eut1(pf05985) EUT1 EP_NTERM
00310 Tsp_rosa EUT1
00315 H_mulberry(pf00936pf16365) EUT1
00320 HMMreg12833_1_EUT1(pf12833) EUT1
00325 -pf01218 -
00330 -pf01520 -
00335 -pf00583 -
00340 -pf11143 -
00345 -pf06572 -
00350 -pf04261 -
00355 HMMnopfamclust3_1 RMM1
00360 - -
00365 -pf00528 -
00370 -pf00528 -
00375 -pf00005pf12857 -
00380 -pf00291 -
BMC-P
BMC-H
BMC-T
Aldehyde dehydrogenase
PTAC
Signature enzyme
Alcohol dehydrogenase
Regulator

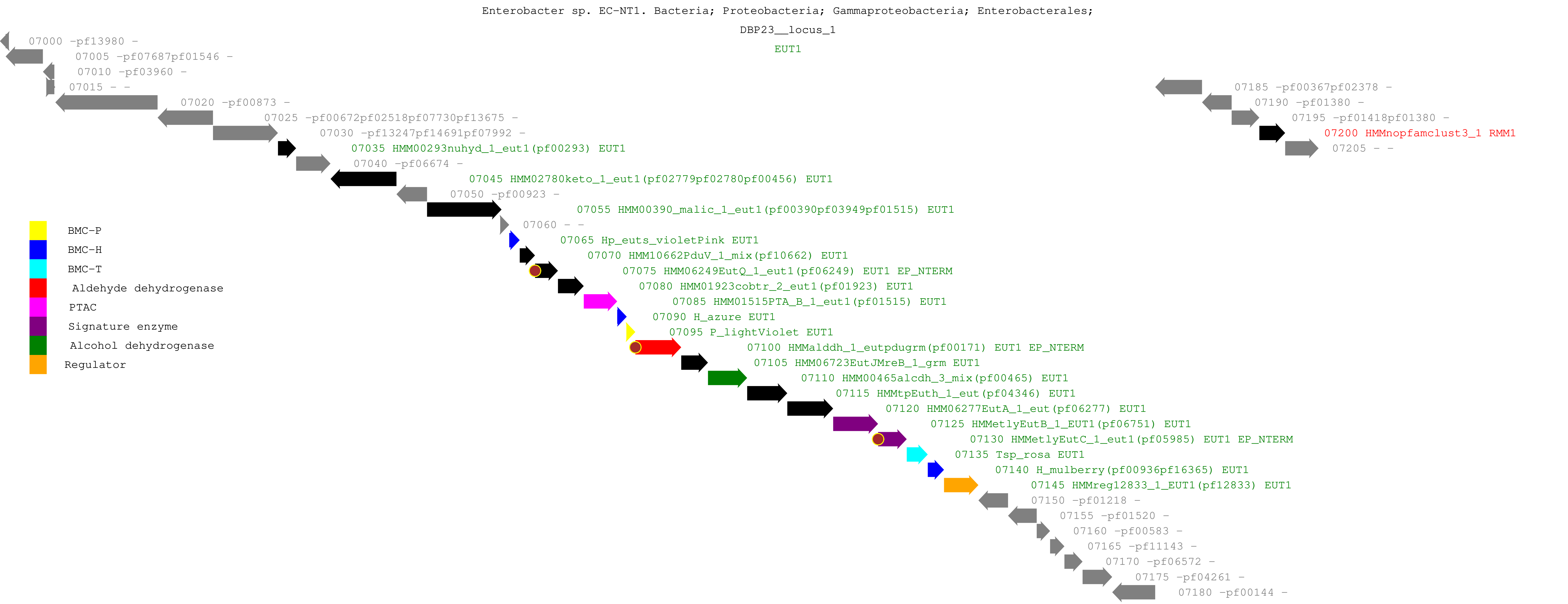
Enterobacter sp. EC-NT1. Bacteria; Proteobacteria; Gammaproteobacteria; Enterobacterales;
DBP23__locus_1
EUT1
07000 -pf13980 -
07005 -pf07687pf01546 -
07010 -pf03960 -
07015 - -
07020 -pf00873 -
07025 -pf00672pf02518pf07730pf13675 -
07030 -pf13247pf14691pf07992 -
07035 HMM00293nuhyd_1_eut1(pf00293) EUT1
07040 -pf06674 -
07045 HMM02780keto_1_eut1(pf02779pf02780pf00456) EUT1
07050 -pf00923 -
07055 HMM00390_malic_1_eut1(pf00390pf03949pf01515) EUT1
07060 - -
07065 Hp_euts_violetPink EUT1
07070 HMM10662PduV_1_mix(pf10662) EUT1
07075 HMM06249EutQ_1_eut1(pf06249) EUT1 EP_NTERM
07080 HMM01923cobtr_2_eut1(pf01923) EUT1
07085 HMM01515PTA_B_1_eut1(pf01515) EUT1
07090 H_azure EUT1
07095 P_lightViolet EUT1
07100 HMMalddh_1_eutpdugrm(pf00171) EUT1 EP_NTERM
07105 HMM06723EutJMreB_1_grm EUT1
07110 HMM00465alcdh_3_mix(pf00465) EUT1
07115 HMMtpEuth_1_eut(pf04346) EUT1
07120 HMM06277EutA_1_eut(pf06277) EUT1
07125 HMMetlyEutB_1_EUT1(pf06751) EUT1
07130 HMMetlyEutC_1_eut1(pf05985) EUT1 EP_NTERM
07135 Tsp_rosa EUT1
07140 H_mulberry(pf00936pf16365) EUT1
07145 HMMreg12833_1_EUT1(pf12833) EUT1
07150 -pf01218 -
07155 -pf01520 -
07160 -pf00583 -
07165 -pf11143 -
07170 -pf06572 -
07175 -pf04261 -
07180 -pf00144 -
07185 -pf00367pf02378 -
07190 -pf01380 -
07195 -pf01418pf01380 -
07200 HMMnopfamclust3_1 RMM1
07205 - -
BMC-P
BMC-H
BMC-T
Aldehyde dehydrogenase
PTAC
Signature enzyme
Alcohol dehydrogenase
Regulator

Salmonella enterica subsp. enterica serovar 4,5,12:i:-. Bacteria; Proteobacteria; Gammaproteobacteria; Enterobacterales;

DBP43__locus_2

EUT1

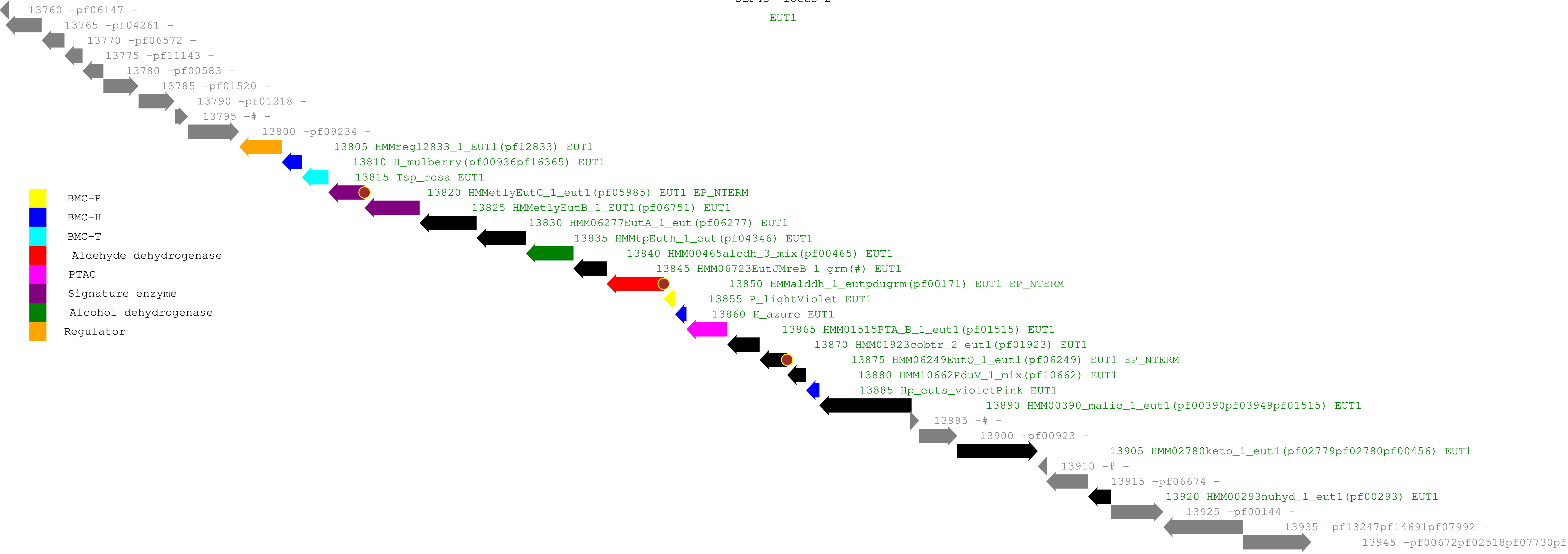

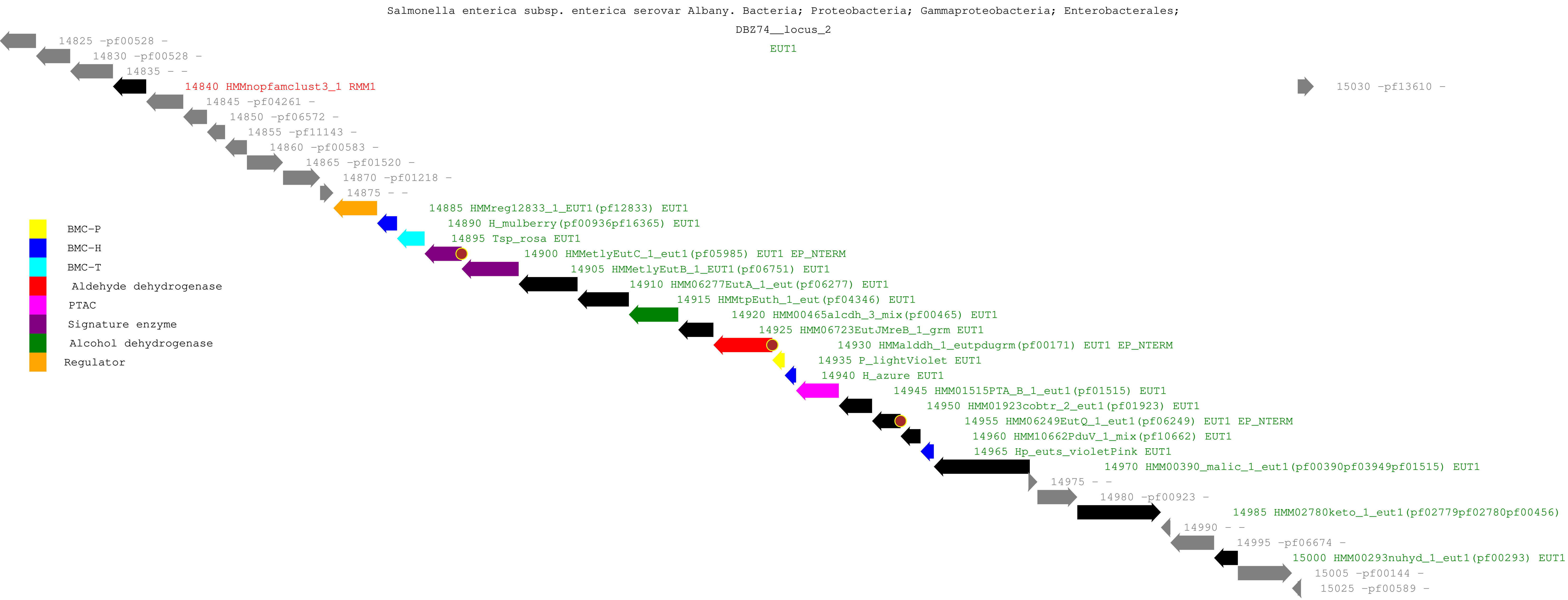
Salmonella enterica subsp. enterica serovar Albany. Bacteria; Proteobacteria; Gammaproteobacteria; Enterobacterales;
DBZ74__locus_2
EUT1
14825 -pf00528 -
14830 -pf00528 -
14835 - -
14840 HMMnopfamclust3_1 RMM1
15030 -pf13610 -
14845 -pf04261 -
14850 -pf06572 -
14855 -pf11143 -
14860 -pf00583 -
14865 -pf01520 -
14870 -pf01218 -
14875 - -
14885 HMMreg12833_1_EUT1(pf12833) EUT1
14890 H_mulberry(pf00936pf16365) EUT1
14895 Tsp_rosa EUT1
14900 HMMetlyEutC_1_eut1(pf05985) EUT1 EP_NTERM
14905 HMMetlyEutB_1_EUT1(pf06751) EUT1
14910 HMM06277EutA_1_eut(pf06277) EUT1
14915 HMMtpEuth_1_eut(pf04346) EUT1
14920 HMM00465alcdh_3_mix(pf00465) EUT1
14925 HMM06723EutJMreB_1_grm EUT1
14930 HMMalddh_1_eutpdugrm(pf00171) EUT1 EP_NTERM
14935 P_lightViolet EUT1
14940 H_azure EUT1
14945 HMM01515PTA_B_1_eut1(pf01515) EUT1
14950 HMM01923cobtr_2_eut1(pf01923) EUT1
14955 HMM06249EutQ_1_eut1(pf06249) EUT1 EP_NTERM
14960 HMM10662PduV_1_mix(pf10662) EUT1
14965 Hp_euts_violetPink EUT1
14970 HMM00390_malic_1_eut1(pf00390pf03949pf01515) EUT1
14975 - -
14980 -pf00923 -
14985 HMM02780keto_1_eut1(pf02779pf02780pf00456)
14990 - -
14995 -pf06674 -
15000 HMM00293nuhyd_1_eut1(pf00293) EUT1
15005 -pf00144 -
15025 -pf00589 -
BMC-P
BMC-H
BMC-T
Aldehyde dehydrogenase
PTAC
Signature enzyme
Alcohol dehydrogenase
Regulator

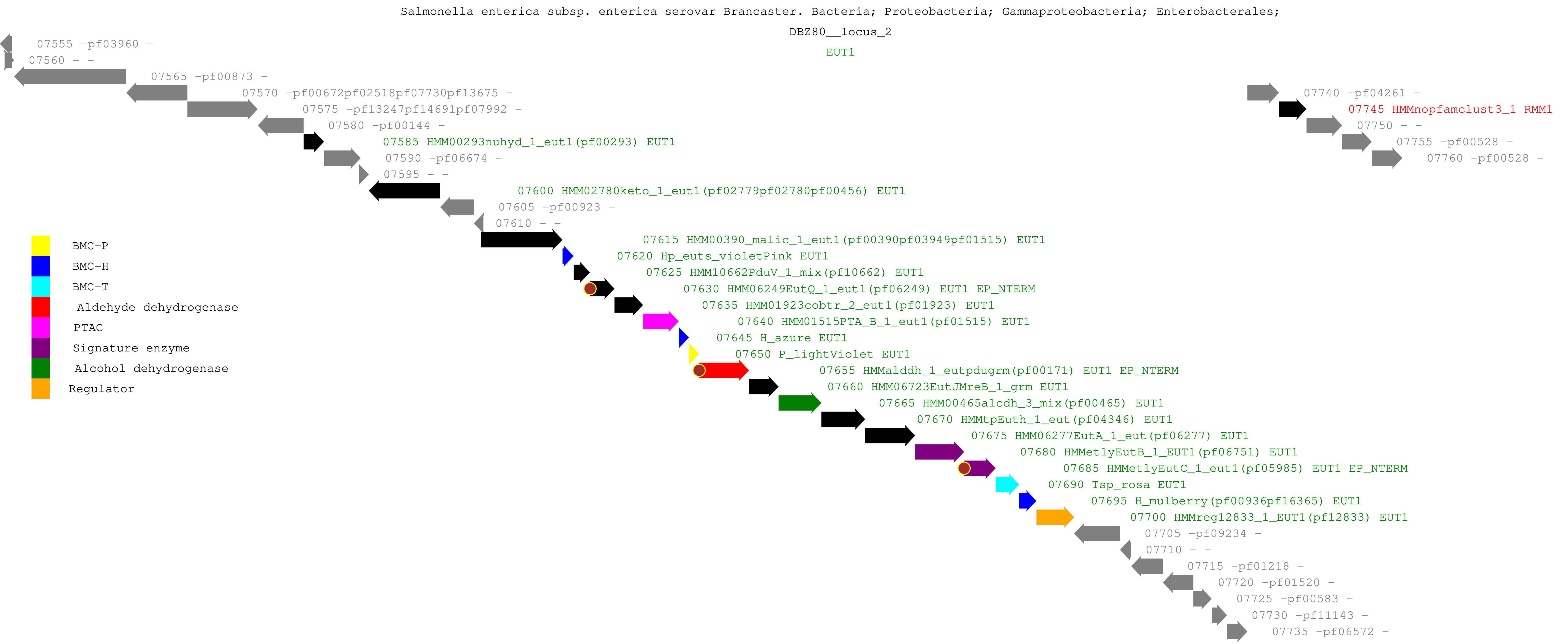

Salmonella enterica subsp. enterica serovar Brancaster. Bacteria; Proteobacteria; Gammaproteobacteria; Enterobacterales;
DBZ80__locus_2
EUT1
07555 -pf03960 -
07560 - -
07565 -pf00873 -
07570 -pf00672pf02518pf07730pf13675 -
07575 -pf13247pf14691pf07992 -
07580 -pf00144 -
07585 HMM00293nuhyd_1_eut1(pf00293) EUT1
07590 -pf06674 -
07595 - -
07600 HMM02780keto_1_eut1(pf02779pf02780pf00456) EUT1
07605 -pf00923 -
07610 - -
07615 HMM00390_malic_1_eut1(pf00390pf03949pf01515) EUT1
07620 Hp_euts_violetPink EUT1
07625 HMM10662PduV_1_mix(pf10662) EUT1
07630 HMM06249EutQ_1_eut1(pf06249) EUT1 EP_NTERM
07635 HMM01923cobtr_2_eut1(pf01923) EUT1
07640 HMM01515PTA_B_1_eut1(pf01515) EUT1
07645 H_azure EUT1
07650 P_lightViolet EUT1
07655 HMMaldh_1_eutpdugrm(pf00171) EUT1 EP_NTERM
07660 HMM06723EutJMreB_1_grm EUT1
07665 HMM00465alcdh_3_mix(pf00465) EUT1
07670 HMMtpEuth_1_eut(pf04346) EUT1
07675 HMM06277EutA_1_eut(pf06277) EUT1
07680 HMMetlyEutB_1_EUT1(pf06751) EUT1
07685 HMMetlyEutC_1_eut1(pf05985) EUT1 EP_NTERM
07690 Tsp_rosa EUT1
07695 H_mulberry(pf00936pf16365) EUT1
07700 HMMreg12833_1_EUT1(pf12833) EUT1
07705 -pf09234 -
07710 - -
07715 -pf01218 -
07720 -pf01520 -
07725 -pf00583 -
07730 -pf11143 -
07735 -pf06572 -
07740 -pf04261 -
07745 HMMnopfamclust3_1 RMM1
07750 - -
07755 -pf00528 -
07760 -pf00528 -
BMC-P
BMC-H
BMC-T
Aldehyde dehydrogenase
PTAC
Signature enzyme
Alcohol dehydrogenase
Regulator

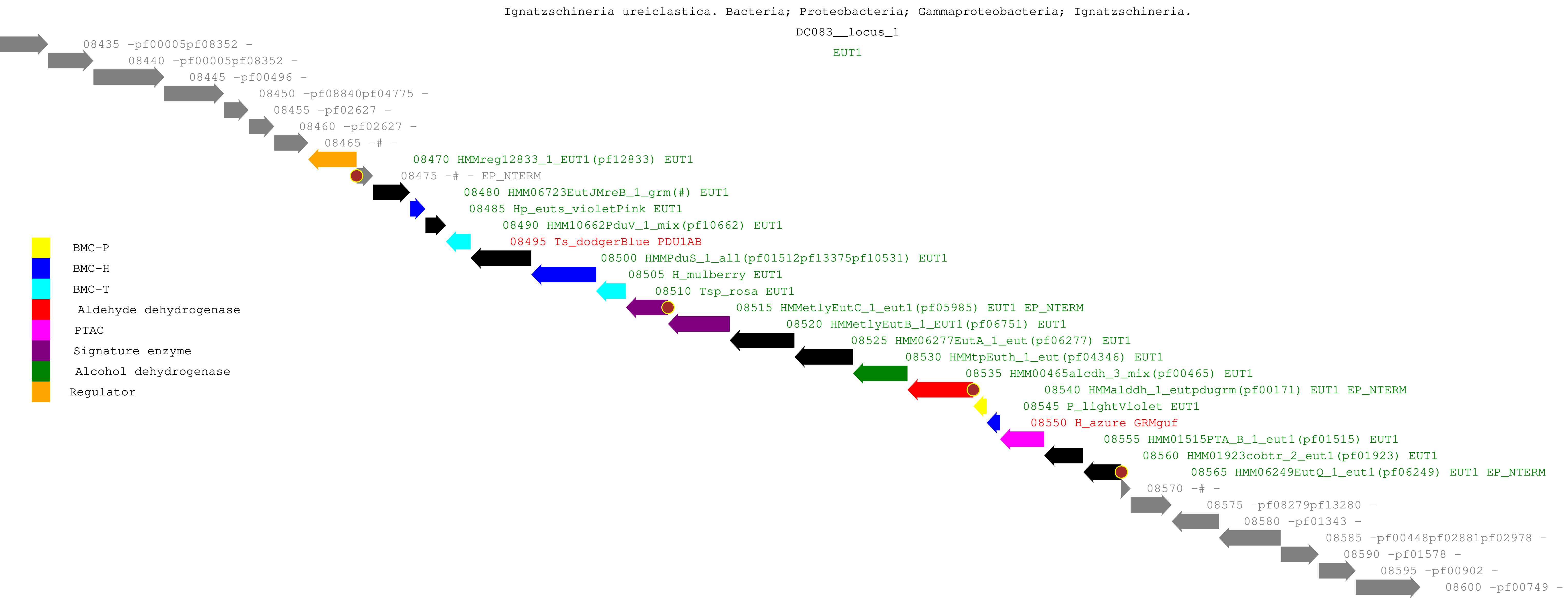
Ignatzschineria ureiclastica. Bacteria; Proteobacteria; Gammaproteobacteria; Ignatzschineria.
DC083__locus_1
EUT1
08435 -pf00005pf08352 -
08440 -pf00005pf08352 -
08445 -pf00496 -
08450 -pf08840pf04775 -
08455 -pf02627 -
08460 -pf02627 -
08465 -# -
08470 HMMreg12833_1_EUT1(pf12833) EUT1
08475 -# - EP_NTERM
08480 HMM06723EutJMreB_1_grm(#) EUT1
08485 Hp_euts_violetPink EUT1
08490 HMM10662PduV_1_mix(pf10662) EUT1
08495 Ts_dodgerBlue PDU1AB
08500 HMMPduS_1_all(pf01512pf13375pf10531) EUT1
08505 H_mulberry EUT1
08510 Tsp_rosa EUT1
08515 HMMetlyEutC_1_eut1(pf05985) EUT1 EP_NTERM
08520 HMMetlyEutB_1_EUT1(pf06751) EUT1
08525 HMM06277EutA_1_eut(pf06277) EUT1
08530 HMMtpEuth_1_eut(pf04346) EUT1
08535 HMM00465alcdh_3_mix(pf00465) EUT1
08540 HMMaldh_1_eutpdugrm(pf00171) EUT1 EP_NTERM
08545 P_lightViolet EUT1
08550 H_azure GRMguf
08555 HMM01515PTA_B_1_eut1(pf01515) EUT1
08560 HMM01923cobtr_2_eut1(pf01923) EUT1
08565 HMM06249EutQ_1_eut1(pf06249) EUT1 EP_NTERM
08570 -# -
08575 -pf08279pf13280 -
08580 -pf01343 -
08585 -pf00448pf02881pf02978 -
08590 -pf01578 -
08595 -pf00902 -
08600 -pf00749 -
BMC-P
BMC-H
BMC-T
Aldehyde dehydrogenase
PTAC
Signature enzyme
Alcohol dehydrogenase
Regulator

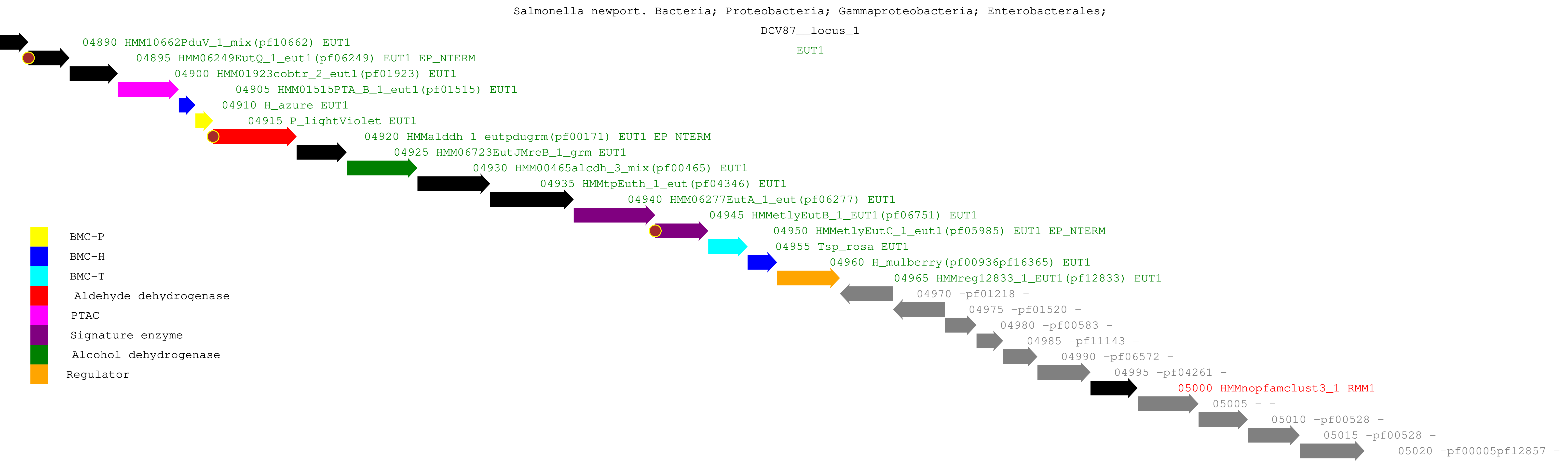
Salmonella newport. Bacteria; Proteobacteria; Gammaproteobacteria; Enterobacterales;
DCV87__locus_1
EUT1
04890 HMM10662PduV_1_mix(pf10662) EUT1
04895 HMM06249EutQ_1_eut1(pf06249) EUT1 EP_NTERM
04900 HMM01923cobtr_2_eut1(pf01923) EUT1
04905 HMM01515PTA_B_1_eut1(pf01515) EUT1
04910 H_azure EUT1
04915 P_lightViolet EUT1
04920 HMMalddh_1_eutpdugrm(pf00171) EUT1 EP_NTERM
04925 HMM06723EutJMreB_1_grm EUT1
04930 HMM00465alcdh_3_mix(pf00465) EUT1
04935 HMMtpEuth_1_eut(pf04346) EUT1
04940 HMM06277EutA_1_eut(pf06277) EUT1
04945 HMMetlyEutB_1_EUT1(pf06751) EUT1
04950 HMMetlyEutC_1_eut1(pf05985) EUT1 EP_NTERM
04955 Tsp_rosa EUT1
04960 H_mulberry(pf00936pf16365) EUT1
04965 HMMreg12833_1_EUT1(pf12833) EUT1
04970 -pf01218 -
04975 -pf01520 -
04980 -pf00583 -
04985 -pf11143 -
04990 -pf06572 -
04995 -pf04261 -
05000 HMMnopfamclust3_1 RMM1
05005 - -
05010 -pf00528 -
05015 -pf00528 -
05020 -pf00005pf12857 -
BMC-P
BMC-H
BMC-T
Aldehyde dehydrogenase
PTAC
Signature enzyme
Alcohol dehydrogenase
Regulator

Salmonella enterica subsp. enterica serovar Orion. Bacteria; Proteobacteria; Gammaproteobacteria; Enterobacterales;

DCV89__locus_1

EUT1

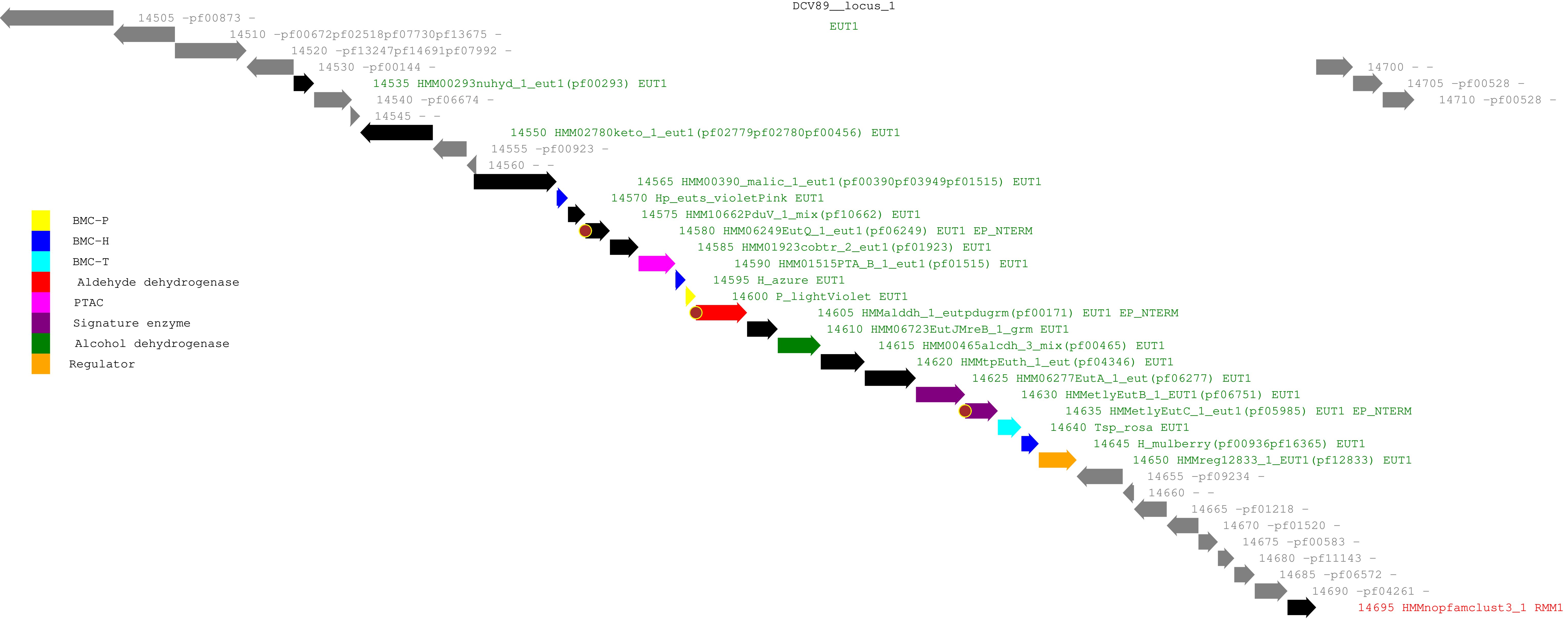

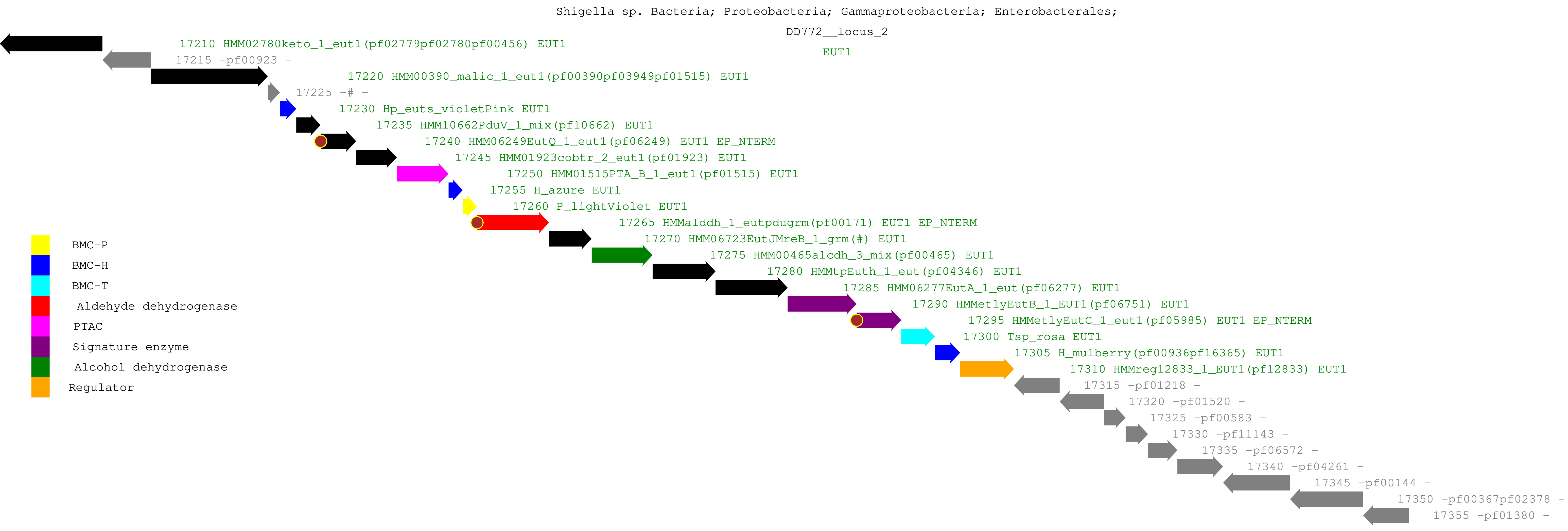
Shigella sp. Bacteria; Proteobacteria; Gammaproteobacteria; Enterobacterales;
DD772__locus_2
EUT1
17210 HMM02780keto_1_eut1(pf02779pf02780pf00456) EUT1
17215 -pf00923 -
17220 HMM00390_malic_1_eut1(pf00390pf03949pf01515) EUT1
17225 -# -
17230 Hp_euts_violetPink EUT1
17235 HMM10662PduV_1_mix(pf10662) EUT1
17240 HMM06249EutQ_1_eut1(pf06249) EUT1 EP_NTERM
17245 HMM01923cobtr_2_eut1(pf01923) EUT1
17250 HMM01515PTA_B_1_eut1(pf01515) EUT1
17255 H_azure EUT1
17260 P_lightViolet EUT1
17265 HMMalddh_1_eutpdugrm(pf00171) EUT1 EP_NTERM
17270 HMM06723EutJMreB_1_grm(#) EUT1
17275 HMM00465alcdh_3_mix(pf00465) EUT1
17280 HMMtpEuth_1_eut(pf04346) EUT1
17285 HMM06277EutA_1_eut(pf06277) EUT1
17290 HMMetlyEutB_1_EUT1(pf06751) EUT1
17295 HMMetlyEutC_1_eut1(pf05985) EUT1 EP_NTERM
17300 Tsp_rosa EUT1
17305 H_mulberry(pf00936pf16365) EUT1
17310 HMMreg12833_1_EUT1(pf12833) EUT1
17315 -pf01218 -
17320 -pf01520 -
17325 -pf00583 -
17330 -pf11143 -
17335 -pf06572 -
17340 -pf04261 -
17345 -pf00144 -
17350 -pf00367pf02378 -
17355 -pf01380 -
BMC-P
BMC-H
BMC-T
Aldehyde dehydrogenase
PTAC
Signature enzyme
Alcohol dehydrogenase
Regulator

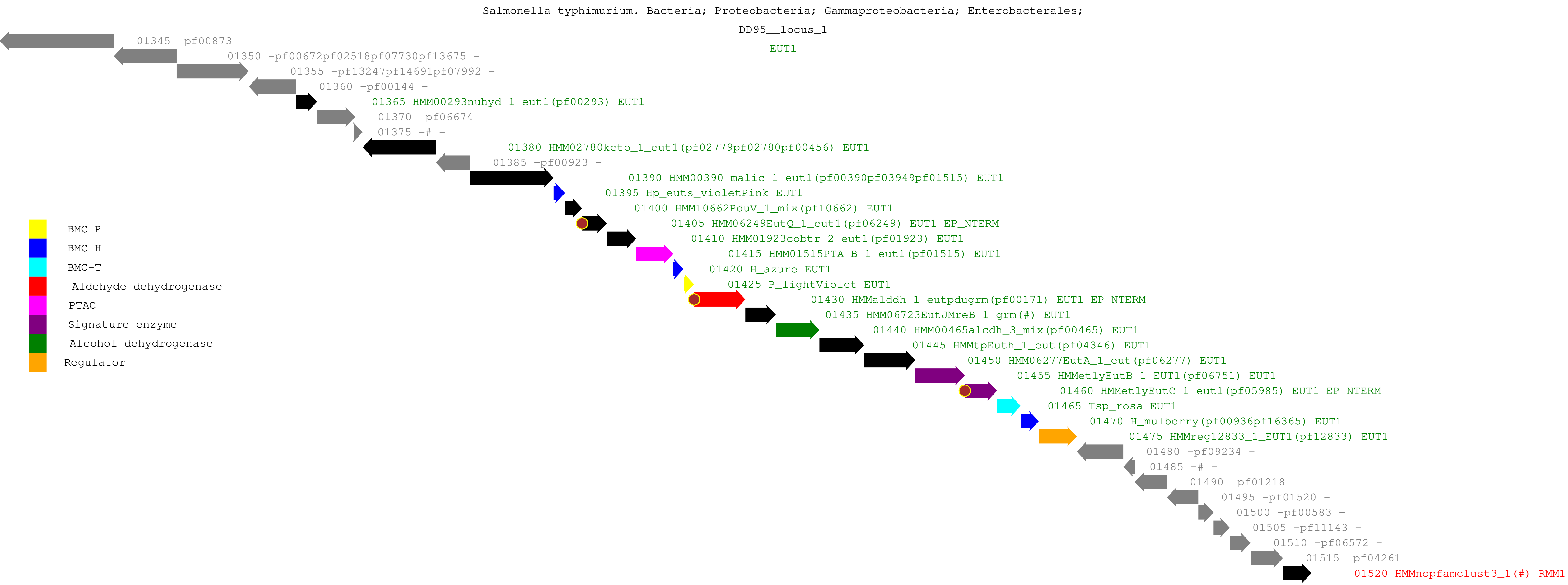
Salmonella typhimurium. Bacteria; Proteobacteria; Gammaproteobacteria; Enterobacterales;
DD95__locus_1
EUT1
01345 -pf00873 -
01350 -pf00672pf02518pf07730pf13675 -
01355 -pf13247pf14691pf07992 -
01360 -pf00144 -
01365 HMM00293nuhyd_1_eut1(pf00293) EUT1
01370 -pf06674 -
01375 -# -
01380 HMM02780keto_1_eut1(pf02779pf02780pf00456) EUT1
01385 -pf00923 -
01390 HMM00390_malic_1_eut1(pf00390pf03949pf01515) EUT1
01395 Hp_euts_violetPink EUT1
01400 HMM10662PduV_1_mix(pf10662) EUT1
01405 HMM06249EutQ_1_eut1(pf06249) EUT1 EP_NTERM
01410 HMM01923cobtr_2_eut1(pf01923) EUT1
01415 HMM01515PTA_B_1_eut1(pf01515) EUT1
01420 H_azure EUT1
01425 P_lightViolet EUT1
01430 HMMaldh_1_eutpdugrm(pf00171) EUT1 EP_NTERM
01435 HMM06723EutJMreB_1_grm(#) EUT1
01440 HMM00465alcdh_3_mix(pf00465) EUT1
01445 HMMtpEuth_1_eut(pf04346) EUT1
01450 HMM06277EutA_1_eut(pf06277) EUT1
01455 HMMetlyEutB_1_EUT1(pf06751) EUT1
01460 HMMetlyEutC_1_eut1(pf05985) EUT1 EP_NTERM
01465 Tsp_rosa EUT1
01470 H_mulberry(pf00936pf16365) EUT1
01475 HMMreg12833_1_EUT1(pf12833) EUT1
01480 -pf09234 -
01485 -# -
01490 -pf01218 -
01495 -pf01520 -
01500 -pf00583 -
01505 -pf11143 -
01510 -pf06572 -
01515 -pf04261 -
01520 HMMnopfamclust3_1(#) RMM1
BMC-P
BMC-H
BMC-T
Aldehyde dehydrogenase
PTAC
Signature enzyme
Alcohol dehydrogenase
Regulator

Klebsiella oxytoca. Bacteria; Proteobacteria; Gammaproteobacteria; Enterobacterales;

DDJ66__locus_1

EUT1

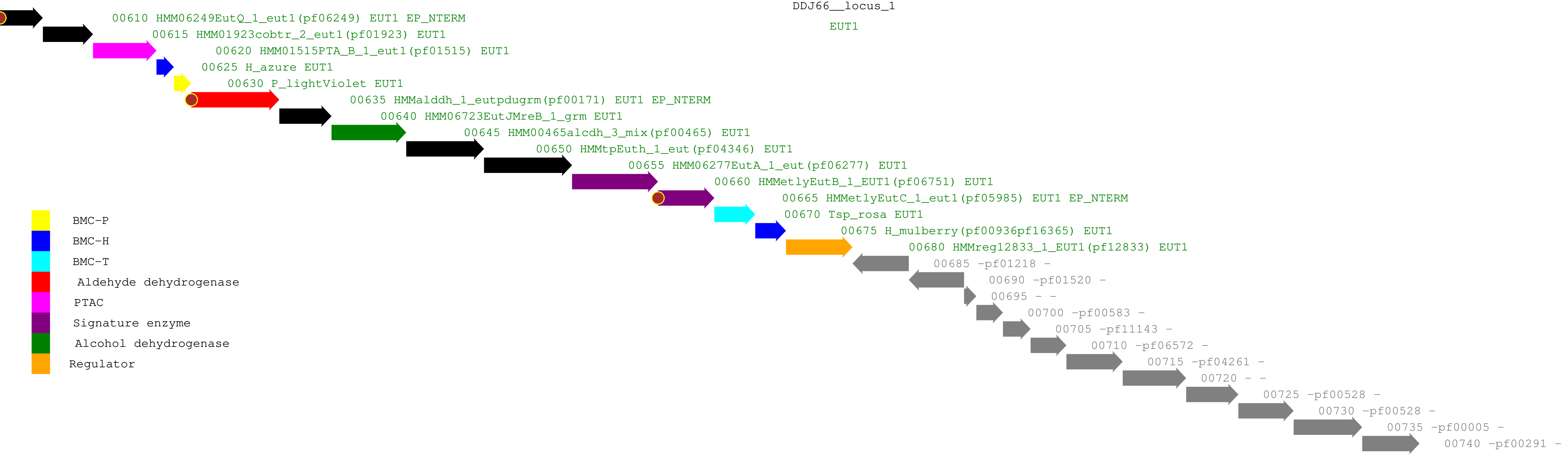

Shigella sp. Bacteria; Proteobacteria; Gammaproteobacteria; Enterobacterales;

DEF11__locus_1

EUT1

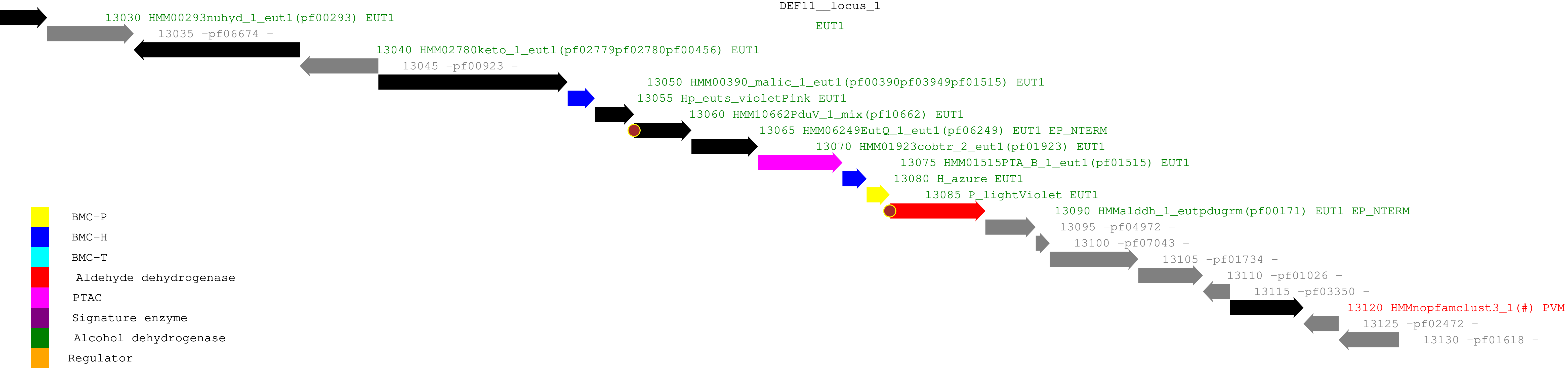

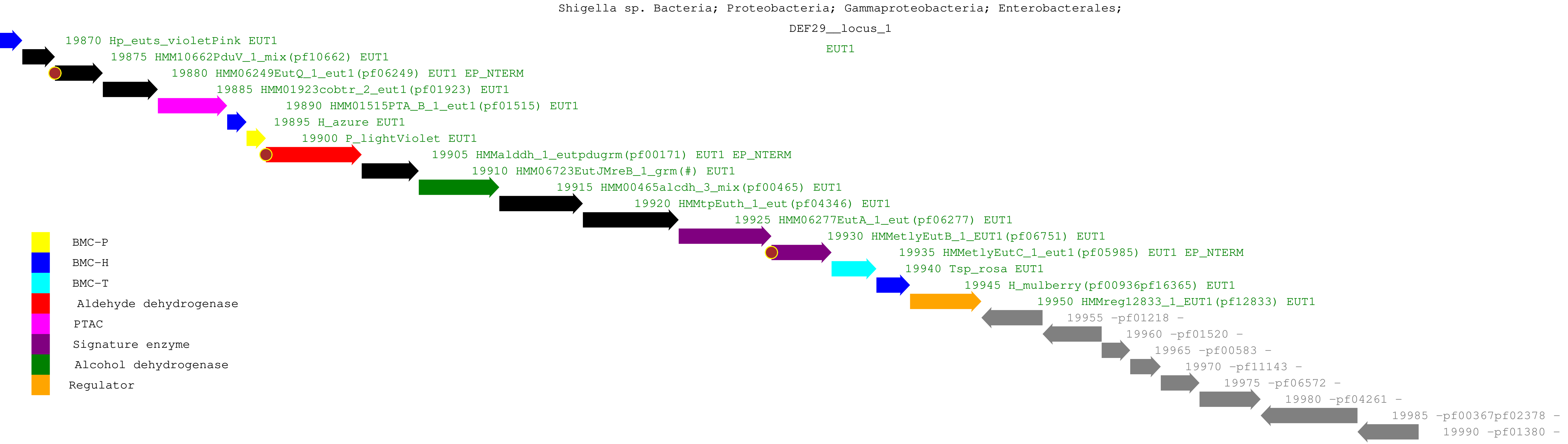
Shigella sp. Bacteria; Proteobacteria; Gammaproteobacteria; Enterobacterales;
DEF29__locus_1
EUT1
19870 Hp_euts_violetPink EUT1
19875 HMM10662PduV_1_mix(pf10662) EUT1
19880 HMM06249EutQ_1_eut1(pf06249) EUT1 EP_NTERM
19885 HMM01923cobtr_2_eut1(pf01923) EUT1
19890 HMM01515PTA_B_1_eut1(pf01515) EUT1
19895 H_azure EUT1
19900 P_lightViolet EUT1
19905 HMMalddh_1_eutpdugrm(pf00171) EUT1 EP_NTERM
19910 HMM06723EutJMreB_1_grm(#) EUT1
19915 HMM00465alcdh_3_mix(pf00465) EUT1
19920 HMMtpEuth_1_eut(pf04346) EUT1
19925 HMM06277EutA_1_eut(pf06277) EUT1
19930 HMMetlyEutB_1_EUT1(pf06751) EUT1
19935 HMMetlyEutC_1_eut1(pf05985) EUT1 EP_NTERM
19940 Tsp_rosa EUT1
19945 H_mulberry(pf00936pf16365) EUT1
19950 HMMreg12833_1_EUT1(pf12833) EUT1
19955 -pf01218 -
19960 -pf01520 -
19965 -pf00583 -
19970 -pf11143 -
19975 -pf06572 -
19980 -pf04261 -
19985 -pf00367pf02378 -
19990 -pf01380 -
BMC-P
BMC-H
BMC-T
Aldehyde dehydrogenase
PTAC
Signature enzyme
Alcohol dehydrogenase
Regulator

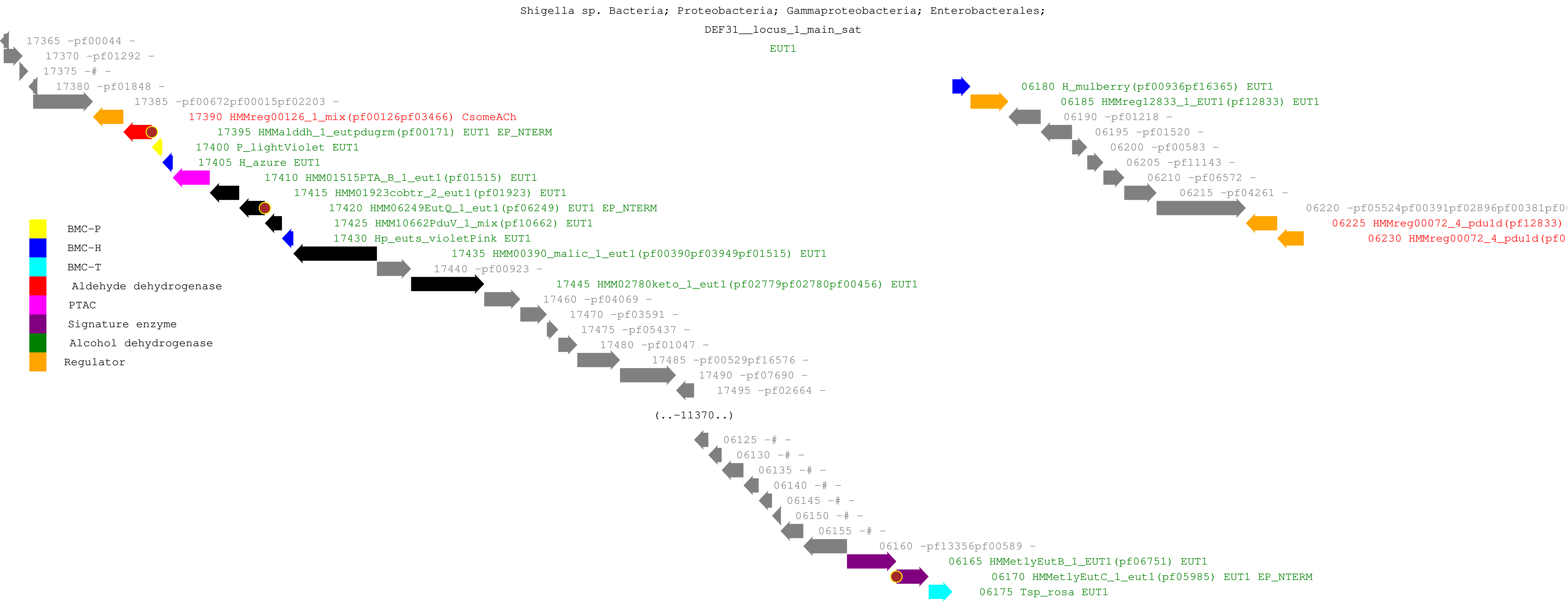

Shigella sp. Bacteria; Proteobacteria; Gammaproteobacteria; Enterobacterales;
DEF31__locus_1_main_sat
EUT1
17365 -pf00044 -
17370 -pf01292 -
17375 -# -
17380 -pf01848 -
17385 -pf00672pf00015pf02203 -
17390 HMMreg00126_1_mix(pf00126pf03466) CsomeACh
17395 HMMaldh_1_eutpdugrm(pf00171) EUT1 EP_NTERM
17400 P_lightViolet EUT1
17405 H_azure EUT1
17410 HMM01515PTA_B_1_eut1(pf01515) EUT1
17415 HMM01923cobtr_2_eut1(pf01923) EUT1
17420 HMM06249EutQ_1_eut1(pf06249) EUT1 EP_NTERM
17425 HMM10662PduV_1_mix(pf10662) EUT1
17430 Hp_euts_violetPink EUT1
17435 HMM00390_malic_1_eut1(pf00390pf03949pf01515) EUT1
17440 -pf00923 -
17445 HMM02780keto_1_eut1(pf02779pf02780pf00456) EUT1
17460 -pf04069 -
17470 -pf03591 -
17475 -pf05437 -
17480 -pf01047 -
17485 -pf00529pf16576 -
17490 -pf07690 -
17495 -pf02664 -
(..-11370..)
06125 -# -
06130 -# -
06135 -# -
06140 -# -
06145 -# -
06150 -# -
06155 -# -
06160 -pf13356pf00589 -
06165 HMMetlyEutB_1_EUT1(pf06751) EUT1
06170 HMMetlyEutC_1_eut1(pf05985) EUT1 EP_NTERM
06175 Tsp_rosa EUT1
06180 H_mulberry(pf00936pf16365) EUT1
06185 HMMreg12833_1_EUT1(pf12833) EUT1
06190 -pf01218 -
06195 -pf01520 -
06200 -pf00583 -
06205 -pf11143 -
06210 -pf06572 -
06215 -pf04261 -
06220 -pf05524pf00391pf02896pf00381pf0
06225 HMMreg00072_4_pdu1d(pf12833)
06230 HMMreg00072_4_pdu1d(pf0
BMC-P
BMC-H
BMC-T
Aldehyde dehydrogenase
PTAC
Signature enzyme
Alcohol dehydrogenase
Regulator

Klebsiella oxytoca. Bacteria; Proteobacteria; Gammaproteobacteria; Enterobacterales;

DET57__locus_3

EUT1

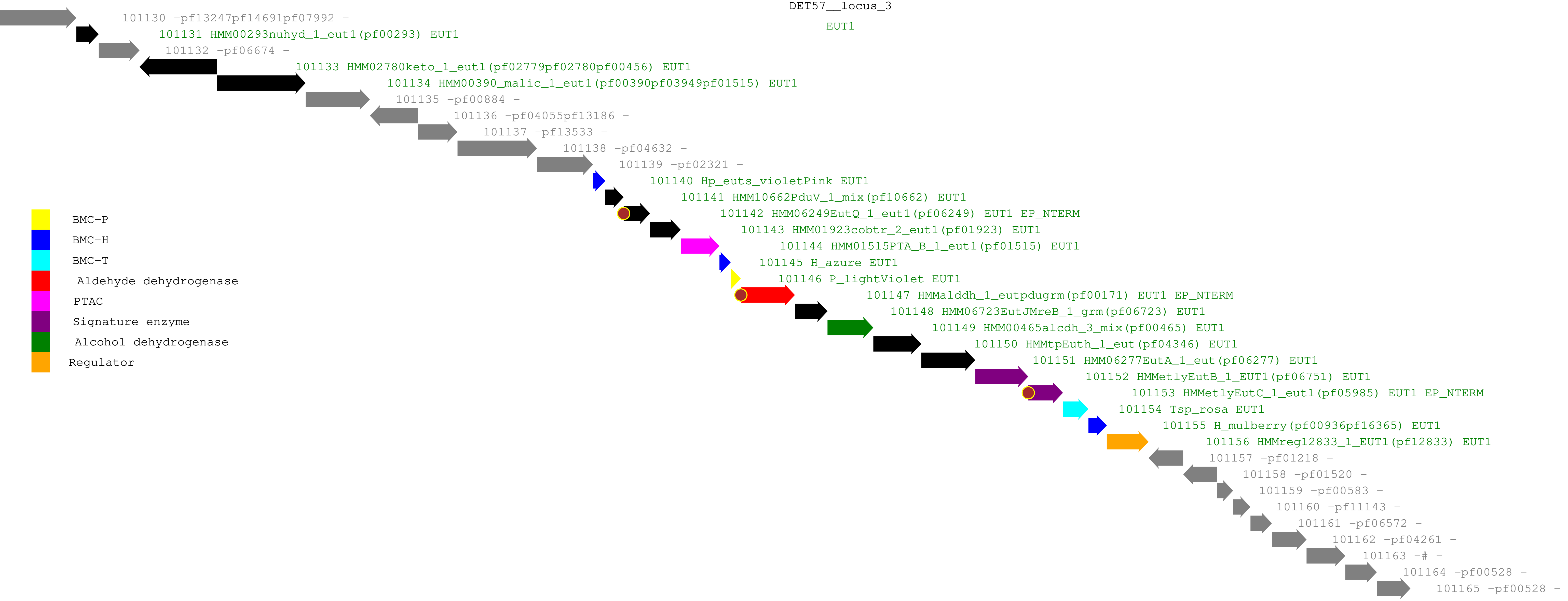

Salmonella enterica I. Bacteria; Proteobacteria; Gammaproteobacteria; Enterobacterales;

DFQ85__locus_2

EUT1

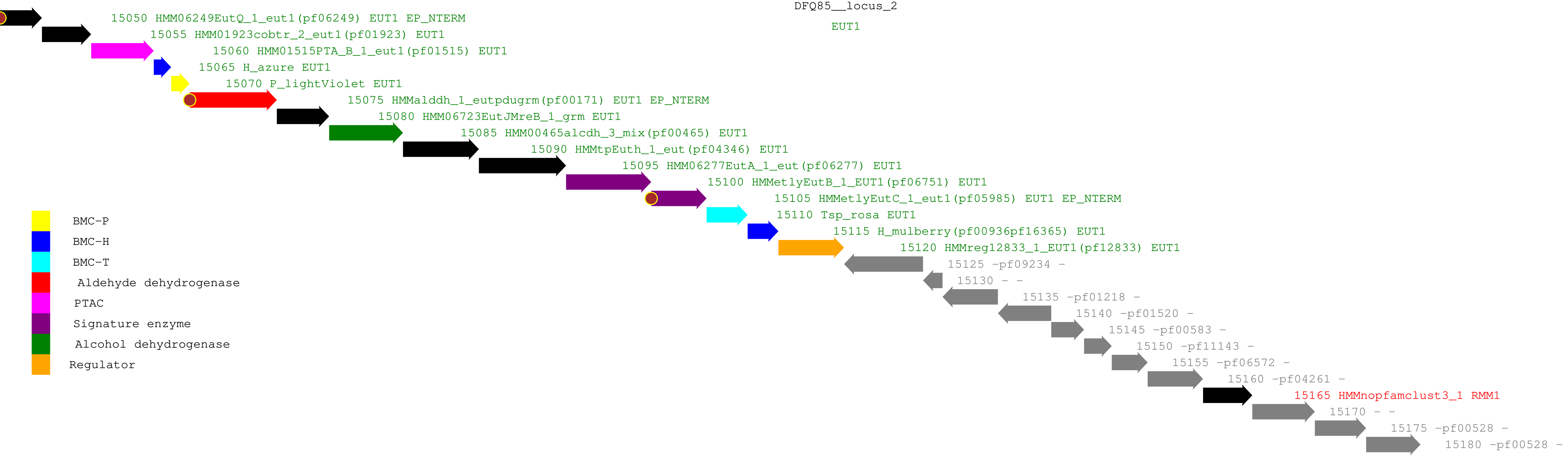

Salmonella thompson. Bacteria; Proteobacteria; Gammaproteobacteria; Enterobacterales;

DFV51__locus_1

EUT1

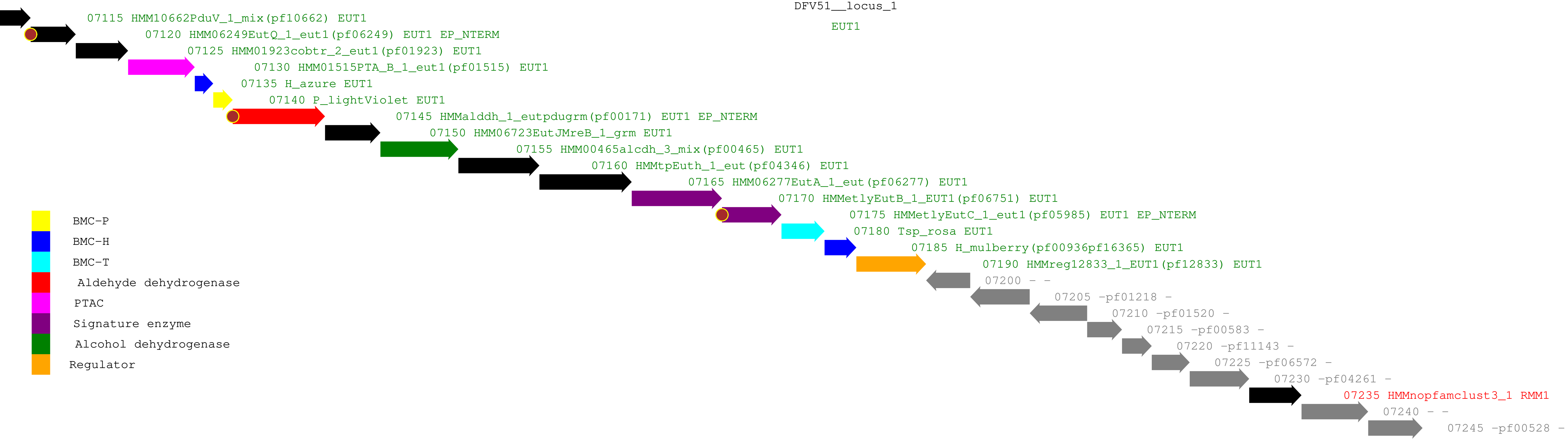

Morganella sp. (in: Bacteria). Bacteria; Proteobacteria; Gammaproteobacteria; Enterobacterales;

DIT05__locus_1

EUT1

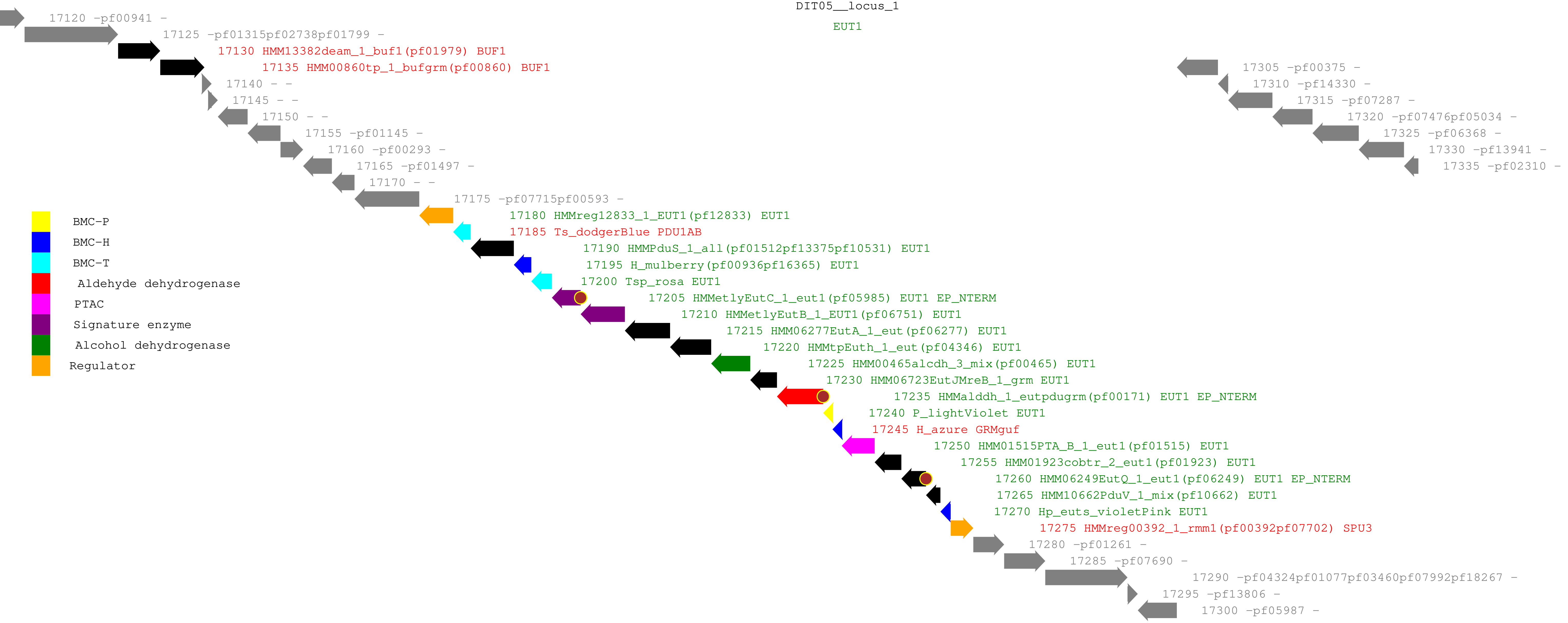

Salmonella enterica I. Bacteria; Proteobacteria; Gammaproteobacteria; Enterobacterales;

DJ273__locus_2

EUT1

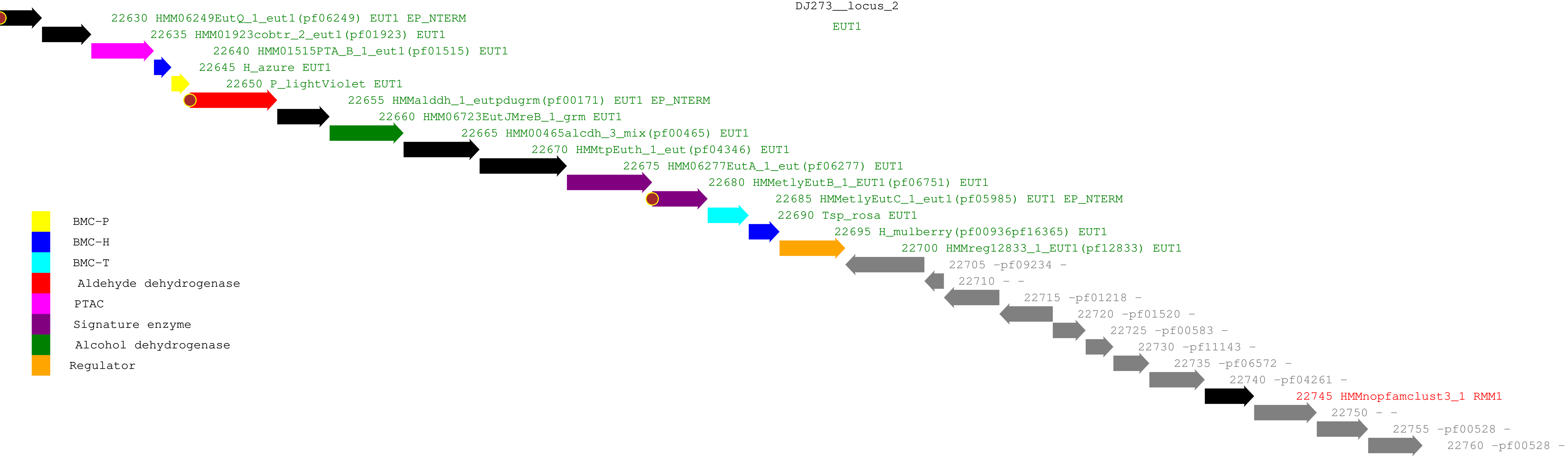

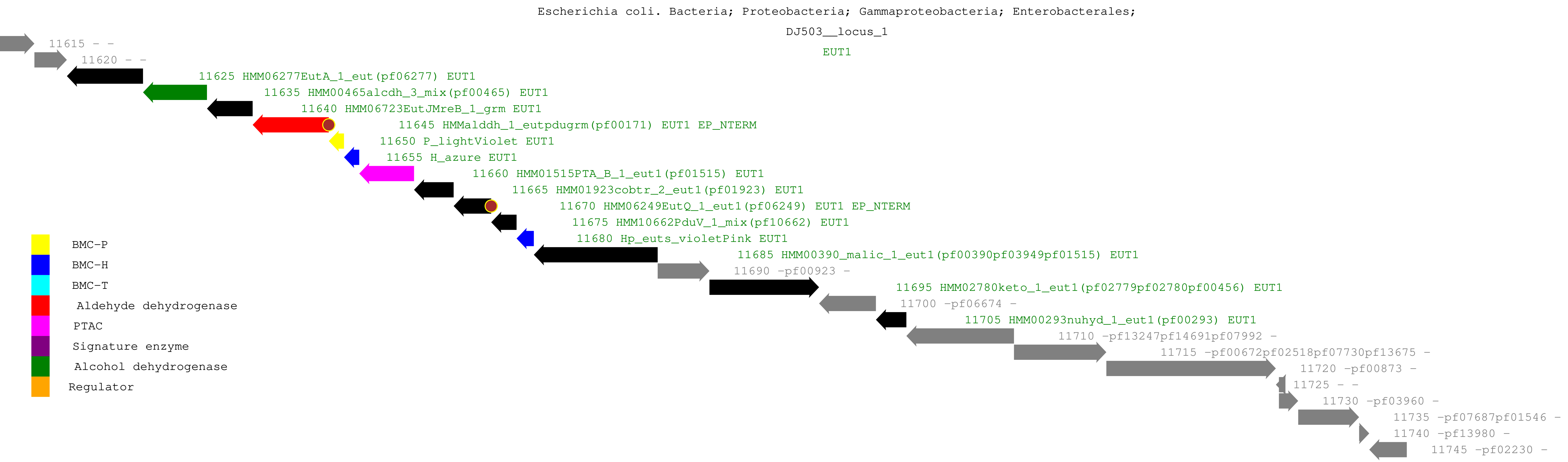
Escherichia coli. Bacteria; Proteobacteria; Gammaproteobacteria; Enterobacterales;
DJ503__locus_1
EUT1
11615 - -
11620 - -
11625 HMM06277EutA_1_eut(pf06277) EUT1
11635 HMM00465alcdh_3_mix(pf00465) EUT1
11640 HMM06723EutJMreB_1_grm EUT1
11645 HMMalddh_1_eutpdugrm(pf00171) EUT1 EP_NTERM
11650 P_lightViolet EUT1
11655 H_azure EUT1
11660 HMM01515PTA_B_1_eut1(pf01515) EUT1
11665 HMM01923cobtr_2_eut1(pf01923) EUT1
11670 HMM06249EutQ_1_eut1(pf06249) EUT1 EP_NTERM
11675 HMM10662PduV_1_mix(pf10662) EUT1
11680 Hp_euts_violetPink EUT1
11685 HMM00390_malic_1_eut1(pf00390pf03949pf01515) EUT1
11690 -pf00923 -
11695 HMM02780keto_1_eut1(pf02779pf02780pf00456) EUT1
11700 -pf06674 -
11705 HMM00293nuhyd_1_eut1(pf00293) EUT1
11710 -pf13247pf14691pf07992 -
11715 -pf00672pf02518pf07730pf13675 -
11720 -pf00873 -
11725 - -
11730 -pf03960 -
11735 -pf07687pf01546 -
11740 -pf13980 -
11745 -pf02230 -
BMC-P
BMC-H
BMC-T
Aldehyde dehydrogenase
PTAC
Signature enzyme
Alcohol dehydrogenase
Regulator

Klebsiella grimontii. Bacteria; Proteobacteria; Gammaproteobacteria; Enterobacterales;

DJ542__locus_2

EUT1

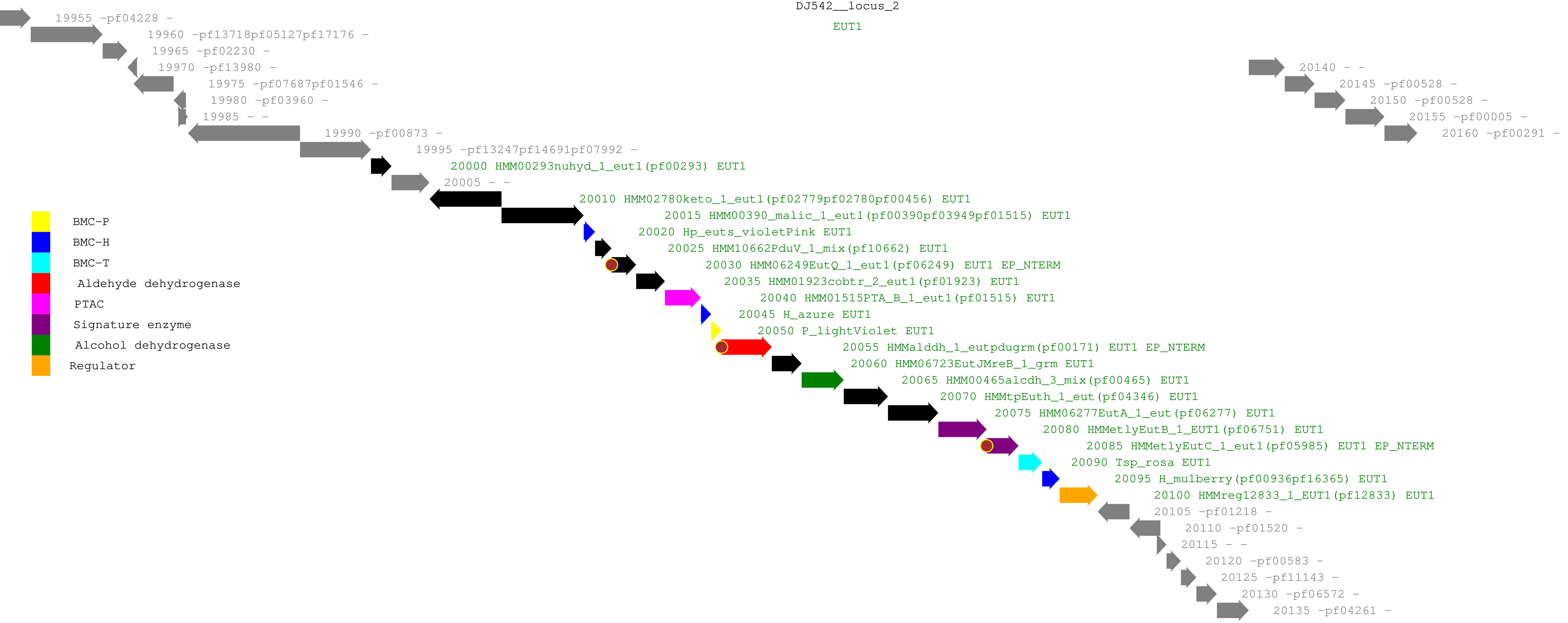

Salmonella enteritidis. Bacteria; Proteobacteria; Gammaproteobacteria; Enterobacterales;

DK092__locus_2

EUT1

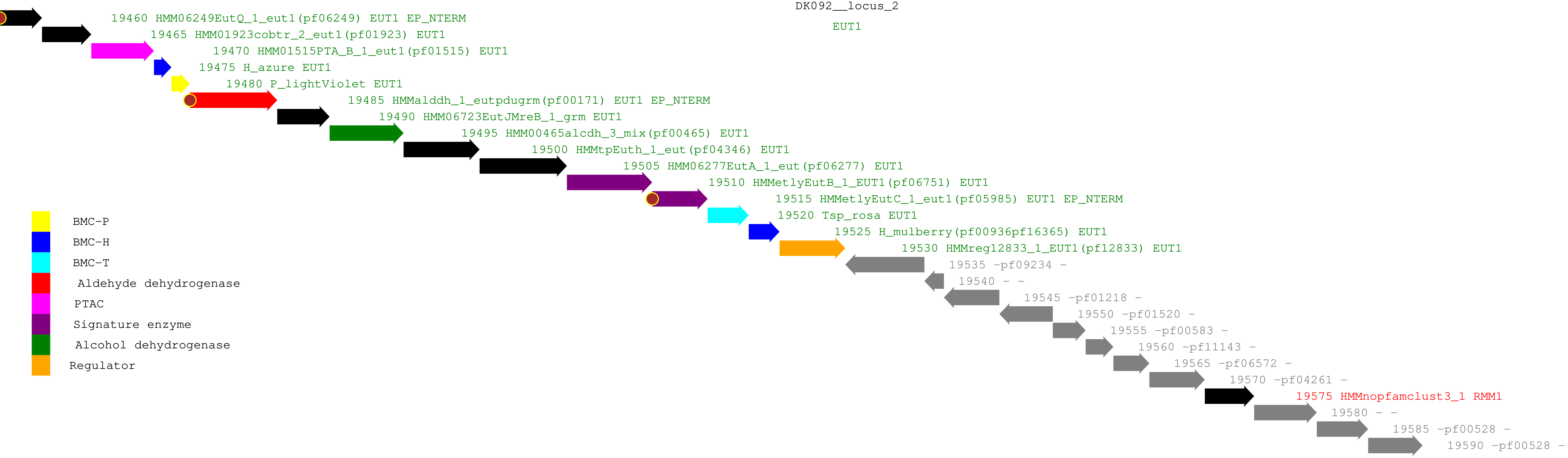

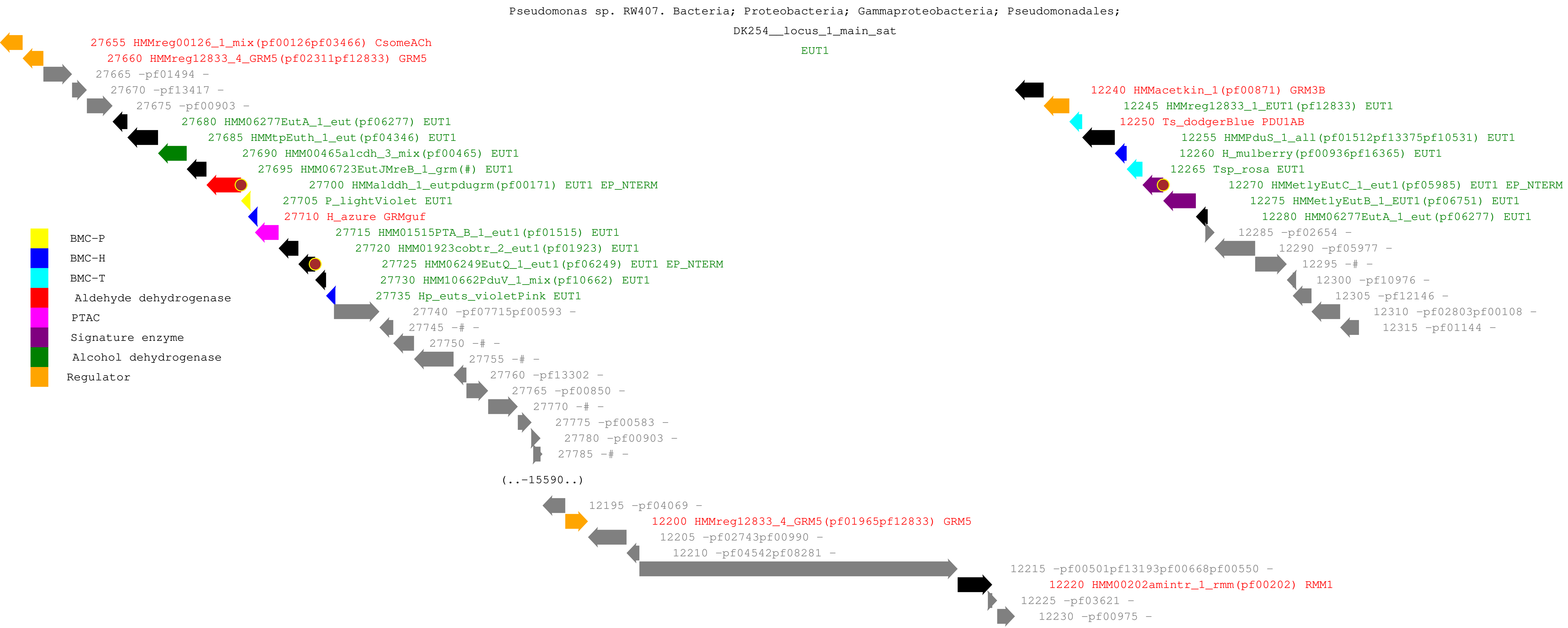

Pseudomonas sp. RW407. Bacteria; Proteobacteria; Gammaproteobacteria; Pseudomonadales;
DK254__locus_1_main_sat
EUT1
27655 HMMreg00126_1_mix(pf00126pf03466) CsomeACh
27660 HMMreg12833_4_GRM5(pf02311pf12833) GRM5
27665 -pf01494 -
27670 -pf13417 -
27675 -pf00903 -
27680 HMM06277EutA_1_eut(pf06277) EUT1
27685 HMMtpEuth_1_eut(pf04346) EUT1
27690 HMM00465alcdh_3_mix(pf00465) EUT1
27695 HMM06723EutJMreB_1_grm(#) EUT1
27700 HMMaldh_1_eutpdugrm(pf00171) EUT1 EP_NTERM
27705 P_lightViolet EUT1
27710 H_azure GRMguf
27715 HMM01515PTA_B_1_eut1(pf01515) EUT1
27720 HMM01923cobtr_2_eut1(pf01923) EUT1
27725 HMM06249EutQ_1_eut1(pf06249) EUT1 EP_NTERM
27730 HMM10662PduV_1_mix(pf10662) EUT1
27735 Hp_euts_violetPink EUT1
27740 -pf07715pf00593 -
27745 -# -
27750 -# -
27755 -# -
27760 -pf13302 -
27765 -pf00850 -
27770 -# -
27775 -pf00583 -
27780 -pf00903 -
27785 -# -
(..-15590..)
12195 -pf04069 -
12200 HMMreg12833_4_GRM5(pf01965pf12833) GRM5
12205 -pf02743pf00990 -
12210 -pf04542pf08281 -
12215 -pf00501pf13193pf00668pf00550 -
12220 HMM00202amintr_1_rmm(pf00202) RMM1
12225 -pf03621 -
12230 -pf00975 -
12240 HMMacetkin_1(pf00871) GRM3B
12245 HMMreg12833_1_EUT1(pf12833) EUT1
12250 Ts_dodgerBlue PDU1AB
12255 HMMPduS_1_all(pf01512pf13375pf10531) EUT1
12260 H_mulberry(pf00936pf16365) EUT1
12265 Tsp_rosa EUT1
12270 HMMetlyEutC_1_eut1(pf05985) EUT1 EP_NTERM
12275 HMMetlyEutB_1_EUT1(pf06751) EUT1
12280 HMM06277EutA_1_eut(pf06277) EUT1
12285 -pf02654 -
12290 -pf05977 -
12295 -# -
12300 -pf10976 -
12305 -pf12146 -
12310 -pf02803pf00108 -
12315 -pf01144 -
BMC-P
BMC-H
BMC-T
Aldehyde dehydrogenase
PTAC
Signature enzyme
Alcohol dehydrogenase
Regulator

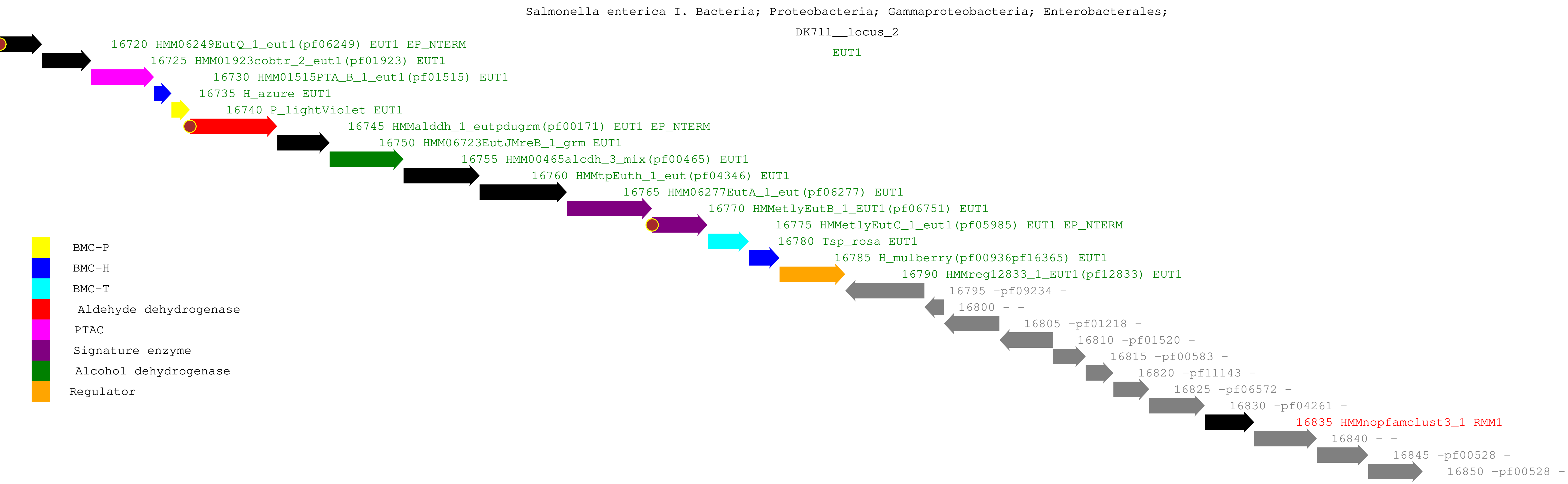

Salmonella enterica I. Bacteria; Proteobacteria; Gammaproteobacteria; Enterobacterales;
DK711__locus_2
EUT1
16720 HMM06249EutQ_1_eut1(pf06249) EUT1 EP_NTERM
16725 HMM01923cobtr_2_eut1(pf01923) EUT1
16730 HMM01515PTA_B_1_eut1(pf01515) EUT1
16735 H_azure EUT1
16740 P_lightViolet EUT1
16745 HMMalddh_1_eutpdugrm(pf00171) EUT1 EP_NTERM
16750 HMM06723EutJMreB_1_grm EUT1
16755 HMM00465alcdh_3_mix(pf00465) EUT1
16760 HMMtpEuth_1_eut(pf04346) EUT1
16765 HMM06277EutA_1_eut(pf06277) EUT1
16770 HMMetlyEutB_1_EUT1(pf06751) EUT1
16775 HMMetlyEutC_1_eut1(pf05985) EUT1 EP_NTERM
16780 Tsp_rosa EUT1
16785 H_mulberry(pf00936pf16365) EUT1
16790 HMMreg12833_1_EUT1(pf12833) EUT1
16795 -pf09234 -
16800 - -
16805 -pf01218 -
16810 -pf01520 -
16815 -pf00583 -
16820 -pf11143 -
16825 -pf06572 -
16830 -pf04261 -
16835 HMMnopfamclust3_1 RMM1
16840 - -
16845 -pf00528 -
16850 -pf00528 -
BMC-P
BMC-H
BMC-T
Aldehyde dehydrogenase
PTAC
Signature enzyme
Alcohol dehydrogenase
Regulator

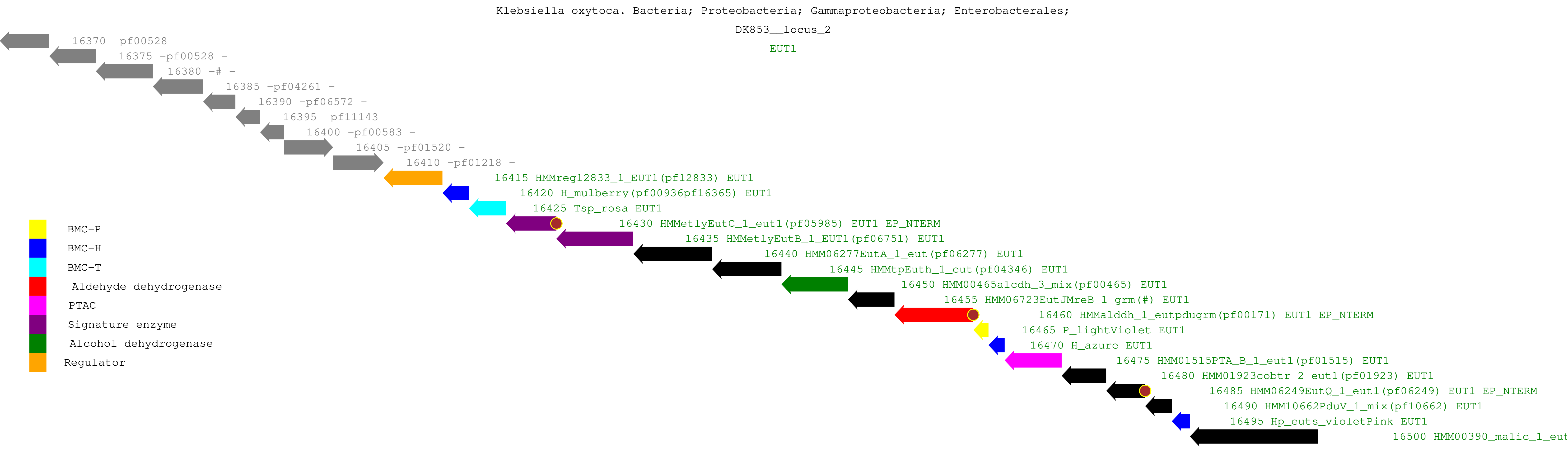
Klebsiella oxytoca. Bacteria; Proteobacteria; Gammaproteobacteria; Enterobacterales;
DK853__locus_2
EUT1
16370 -pf00528 -
16375 -pf00528 -
16380 -# -
16385 -pf04261 -
16390 -pf06572 -
16395 -pf11143 -
16400 -pf00583 -
16405 -pf01520 -
16410 -pf01218 -
16415 HMMreg12833_1_EUT1(pf12833) EUT1
16420 H_mulberry(pf00936pf16365) EUT1
16425 Tsp_rosa EUT1
16430 HMMetlyEutC_1_eut1(pf05985) EUT1 EP_NTERM
16435 HMMetlyEutB_1_EUT1(pf06751) EUT1
16440 HMM06277EutA_1_eut(pf06277) EUT1
16445 HMMtpEuth_1_eut(pf04346) EUT1
16450 HMM00465alcdh_3_mix(pf00465) EUT1
16455 HMM06723EutJMreB_1_grm(#) EUT1
16460 HMMalddh_1_eutpdugrm(pf00171) EUT1 EP_NTERM
16465 P_lightViolet EUT1
16470 H_azure EUT1
16475 HMM01515PTA_B_1_eut1(pf01515) EUT1
16480 HMM01923cobtr_2_eut1(pf01923) EUT1
16485 HMM06249EutQ_1_eut1(pf06249) EUT1 EP_NTERM
16490 HMM10662PduV_1_mix(pf10662) EUT1
16495 Hp_euts_violetPink EUT1
16500 HMM00390_malic_1_eut
BMC-P
BMC-H
BMC-T
Aldehyde dehydrogenase
PTAC
Signature enzyme
Alcohol dehydrogenase
Regulator

Salmonella enterica subsp. enterica serovar Mishmarhaemek. Bacteria; Proteobacteria; Gammaproteobacteria; Enterobacterales;

DKA99__locus_1

EUT1

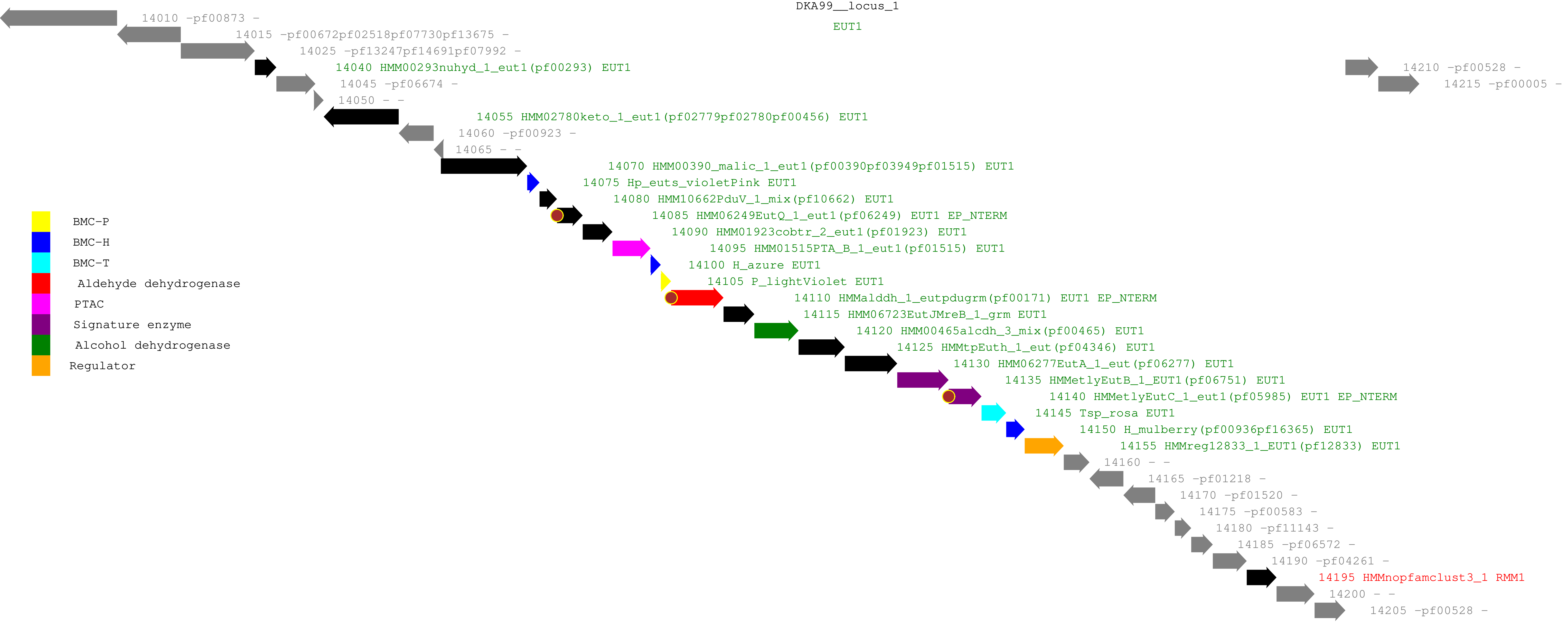

Salmonella enterica subsp. enterica serovar Lexington. Bacteria; Proteobacteria; Gammaproteobacteria; Enterobacterales;

DKG82__locus_2

EUT1

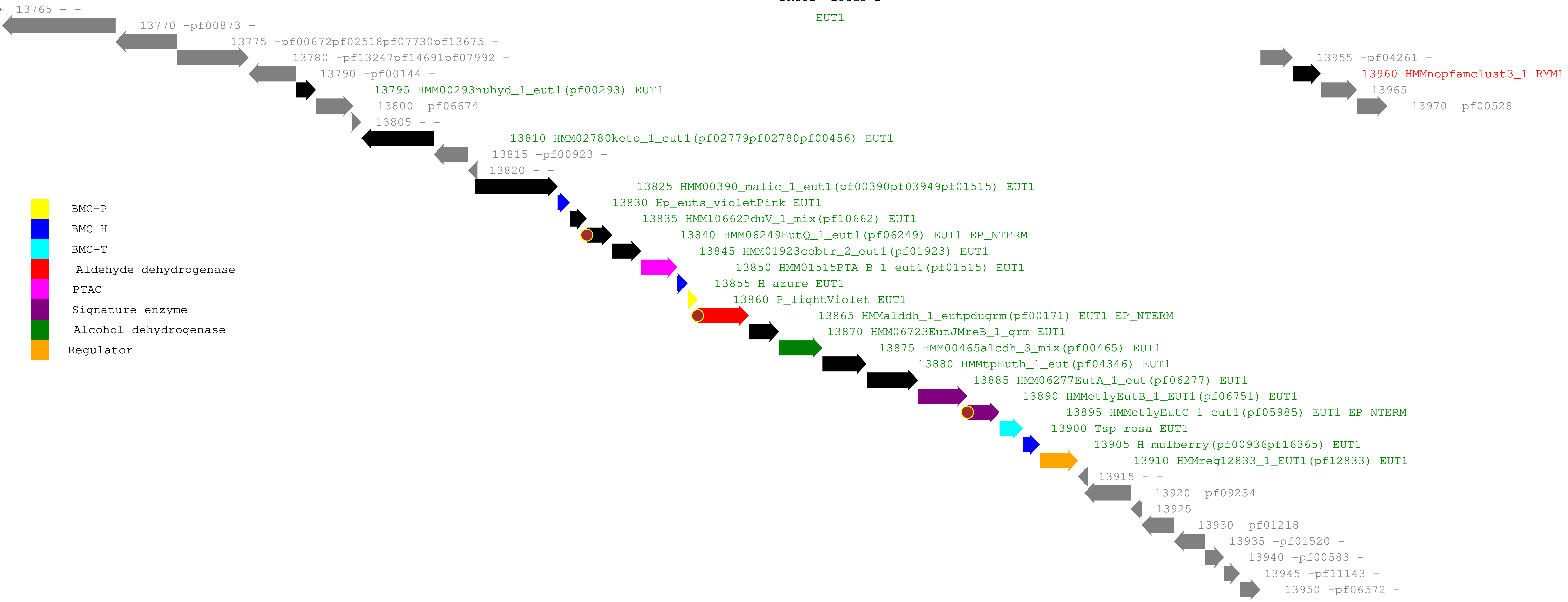

Salmonella typhimurium. Bacteria; Proteobacteria; Gammaproteobacteria; Enterobacterales;

DKJ11__locus_1

EUT1

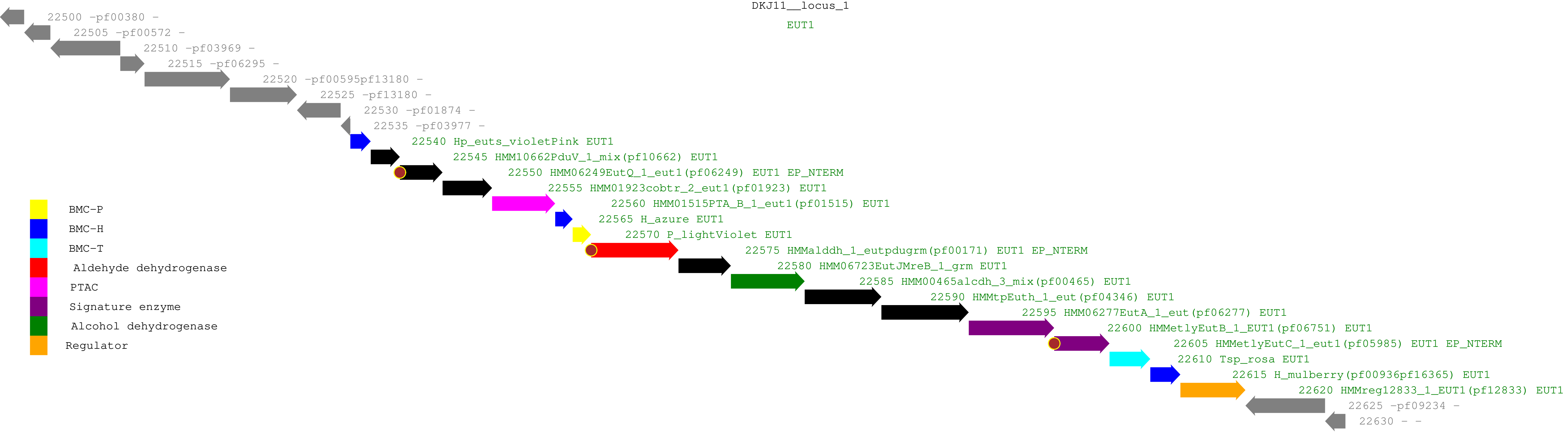

Gammaproteobacteria bacterium ESL0073. Bacteria; Proteobacteria; Gammaproteobacteria.

DKL61__locus_1

EUT1

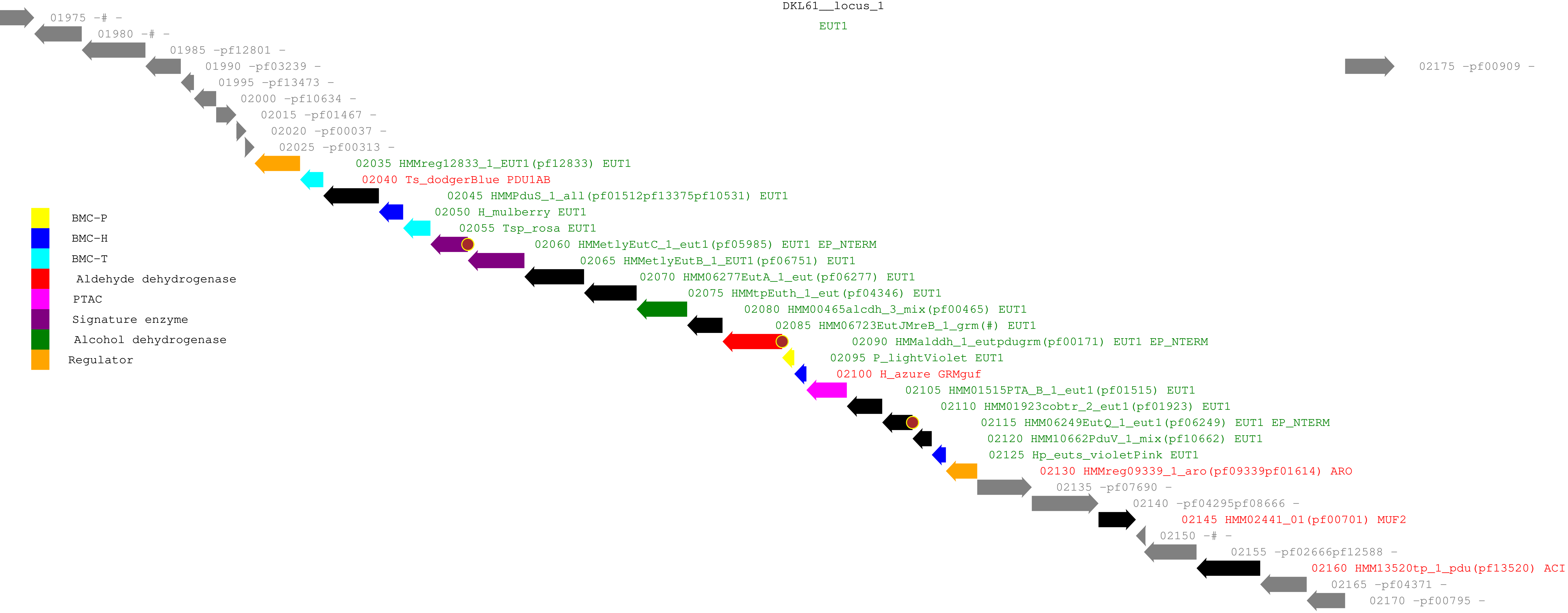

Salmonella diarizonae. Bacteria; Proteobacteria; Gammaproteobacteria; Enterobacterales;

DKP45__locus_1

EUT1

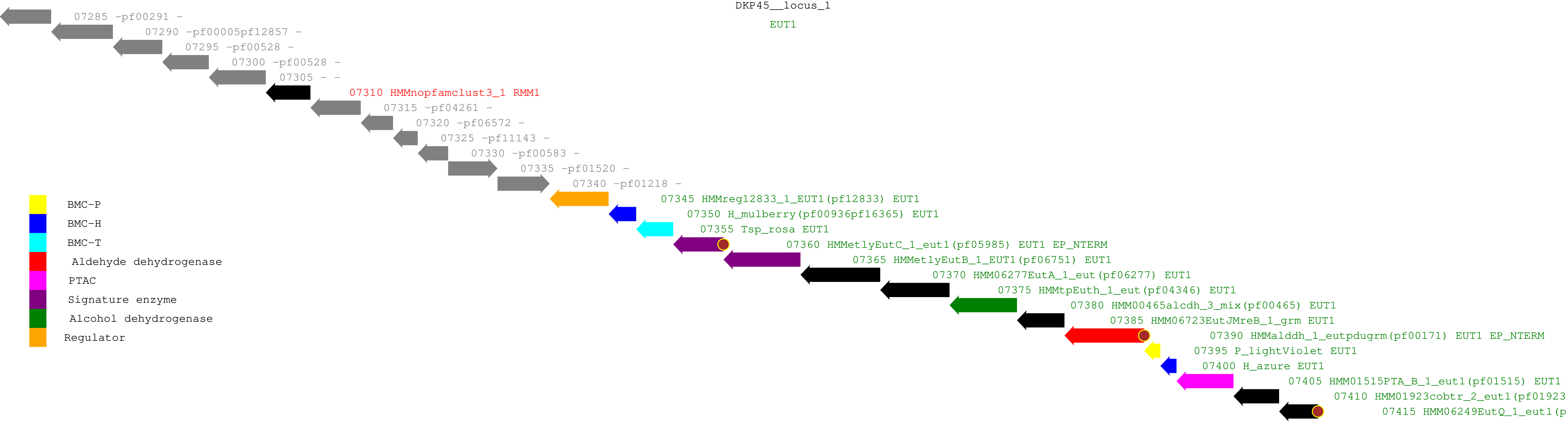

Salmonella enterica subsp. enterica serovar Mountpleasant. Bacteria; Proteobacteria; Gammaproteobacteria; Enterobacterales;

DKT33__locus_2

EUT1

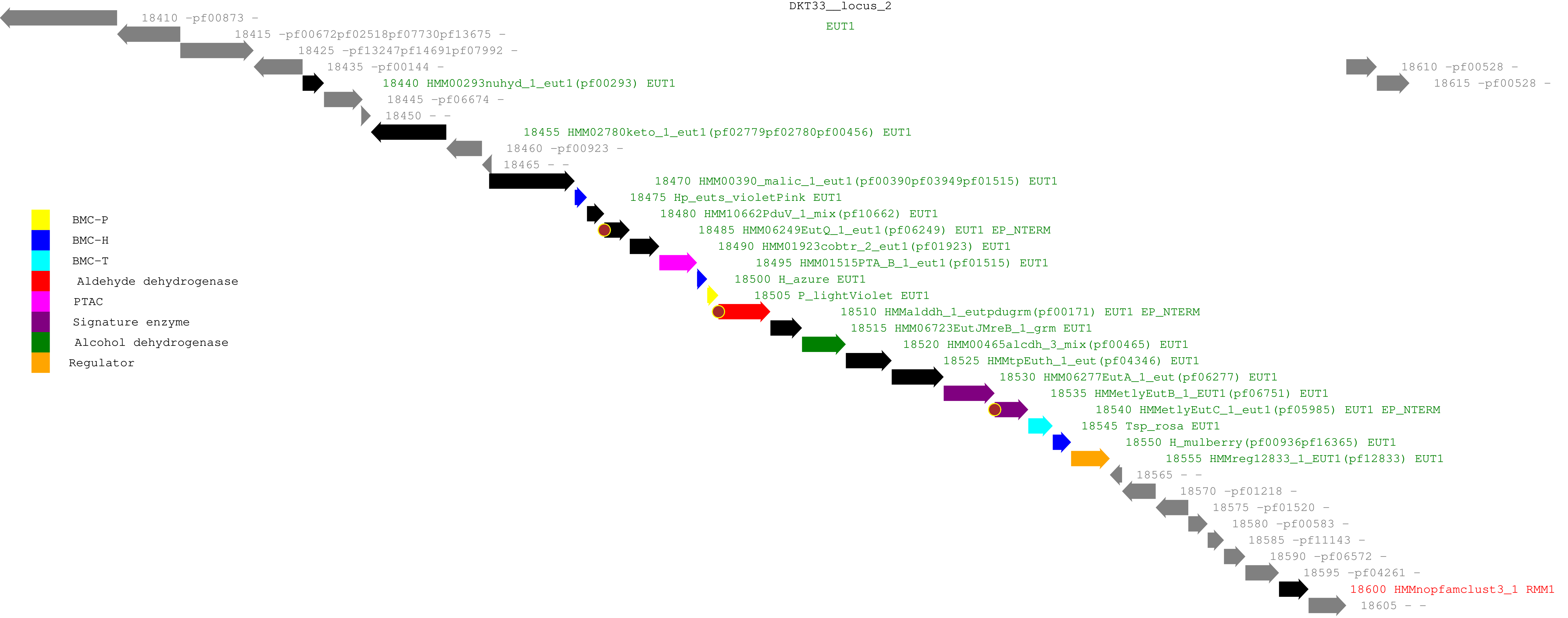

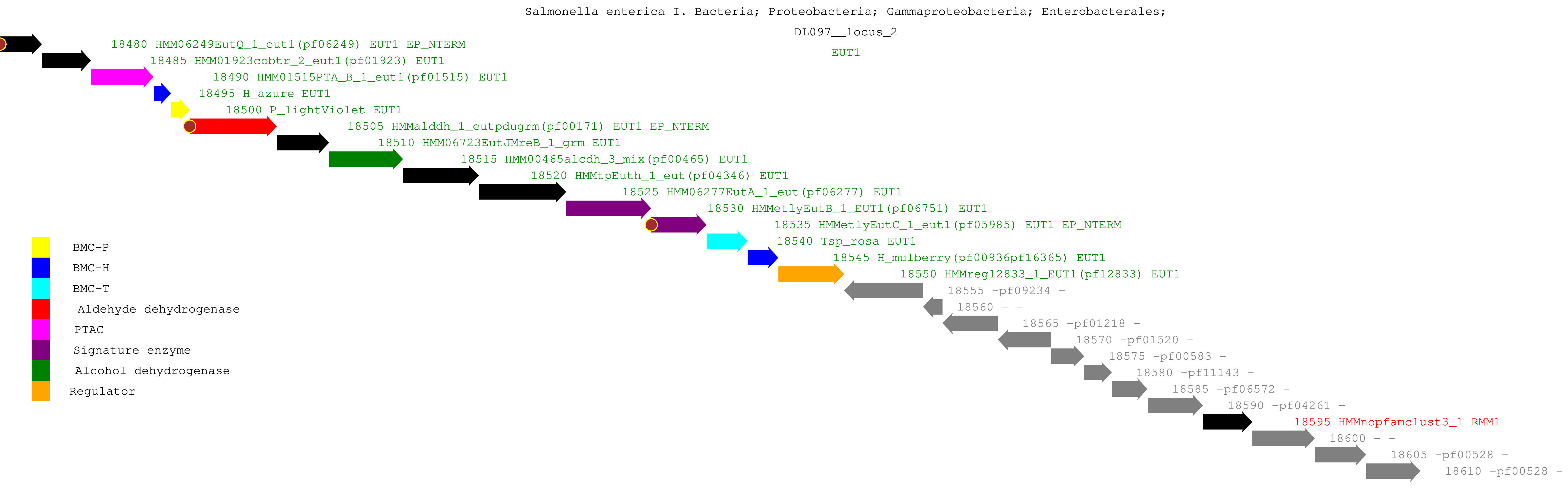

Salmonella enterica I. Bacteria; Proteobacteria; Gammaproteobacteria; Enterobacterales;
DL097__locus_2
EUT1
18480 HMM06249EutQ_1_eut1(pf06249) EUT1 EP_NTERM
18485 HMM01923cobtr_2_eut1(pf01923) EUT1
18490 HMM01515PTA_B_1_eut1(pf01515) EUT1
18495 H_azure EUT1
18500 P_lightViolet EUT1
18505 HMMalddh_1_eutpdugrm(pf00171) EUT1 EP_NTERM
18510 HMM06723EutJMreB_1_grm EUT1
18515 HMM00465alcdh_3_mix(pf00465) EUT1
18520 HMMtpEuth_1_eut(pf04346) EUT1
18525 HMM06277EutA_1_eut(pf06277) EUT1
18530 HMMetlyEutB_1_EUT1(pf06751) EUT1
18535 HMMetlyEutC_1_eut1(pf05985) EUT1 EP_NTERM
18540 Tsp_rosa EUT1
18545 H_mulberry(pf00936pf16365) EUT1
18550 HMMreg12833_1_EUT1(pf12833) EUT1
18555 -pf09234 -
18560 - -
18565 -pf01218 -
18570 -pf01520 -
18575 -pf00583 -
18580 -pf11143 -
18585 -pf06572 -
18590 -pf04261 -
18595 HMMnopfamclust3_1 RMM1
18600 - -
18605 -pf00528 -
18610 -pf00528 -
BMC-P
BMC-H
BMC-T
Aldehyde dehydrogenase
PTAC
Signature enzyme
Alcohol dehydrogenase
Regulator

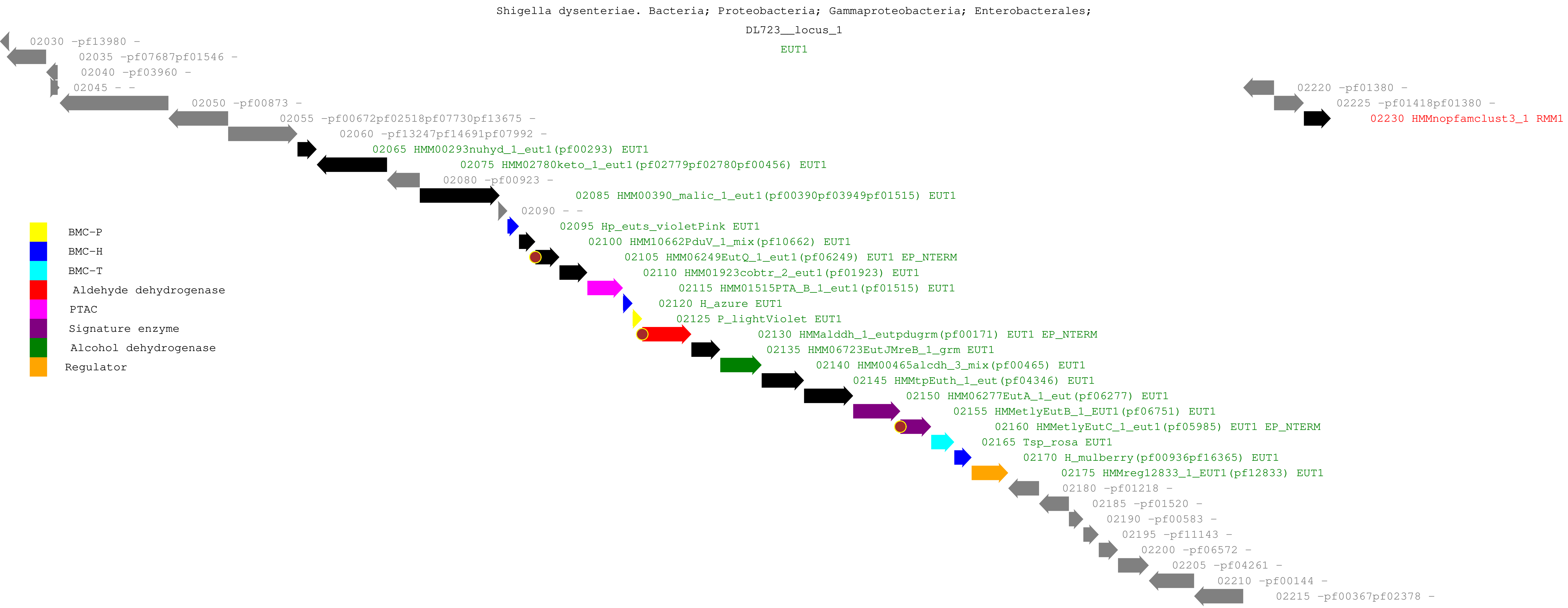
Shigella dysenteriae. Bacteria; Proteobacteria; Gammaproteobacteria; Enterobacterales;
DL723__locus_1
EUT1
02030 -pf13980 -
02035 -pf07687pf01546 -
02040 -pf03960 -
02045 - -
02050 -pf00873 -
02055 -pf00672pf02518pf07730pf13675 -
02060 -pf13247pf14691pf07992 -
02065 HMM00293nuhyd_1_eut1(pf00293) EUT1
02075 HMM02780keto_1_eut1(pf02779pf02780pf00456) EUT1
02080 -pf00923 -
02085 HMM00390_malic_1_eut1(pf00390pf03949pf01515) EUT1
02090 - -
02095 Hp_euts_violetPink EUT1
02100 HMM10662PduV_1_mix(pf10662) EUT1
02105 HMM06249EutQ_1_eut1(pf06249) EUT1 EP_NTERM
02110 HMM01923cobtr_2_eut1(pf01923) EUT1
02115 HMM01515PTA_B_1_eut1(pf01515) EUT1
02120 H_azure EUT1
02125 P_lightViolet EUT1
02130 HMMalddh_1_eutpdugrm(pf00171) EUT1 EP_NTERM
02135 HMM06723EutJMreB_1_grm EUT1
02140 HMM00465alcdh_3_mix(pf00465) EUT1
02145 HMMtpEuth_1_eut(pf04346) EUT1
02150 HMM06277EutA_1_eut(pf06277) EUT1
02155 HMMetlyEutB_1_EUT1(pf06751) EUT1
02160 HMMetlyEutC_1_eut1(pf05985) EUT1 EP_NTERM
02165 Tsp_rosa EUT1
02170 H_mulberry(pf00936pf16365) EUT1
02175 HMMreg12833_1_EUT1(pf12833) EUT1
02180 -pf01218 -
02185 -pf01520 -
02190 -pf00583 -
02195 -pf11143 -
02200 -pf06572 -
02205 -pf04261 -
02210 -pf00144 -
02215 -pf00367pf02378 -
02220 -pf01380 -
02225 -pf01418pf01380 -
02230 HMMnopfamclust3_1 RMM1
BMC-P
BMC-H
BMC-T
Aldehyde dehydrogenase
PTAC
Signature enzyme
Alcohol dehydrogenase
Regulator

Salmonella typhimurium. Bacteria; Proteobacteria; Gammaproteobacteria; Enterobacterales;

DLM31__locus_1

EUT1

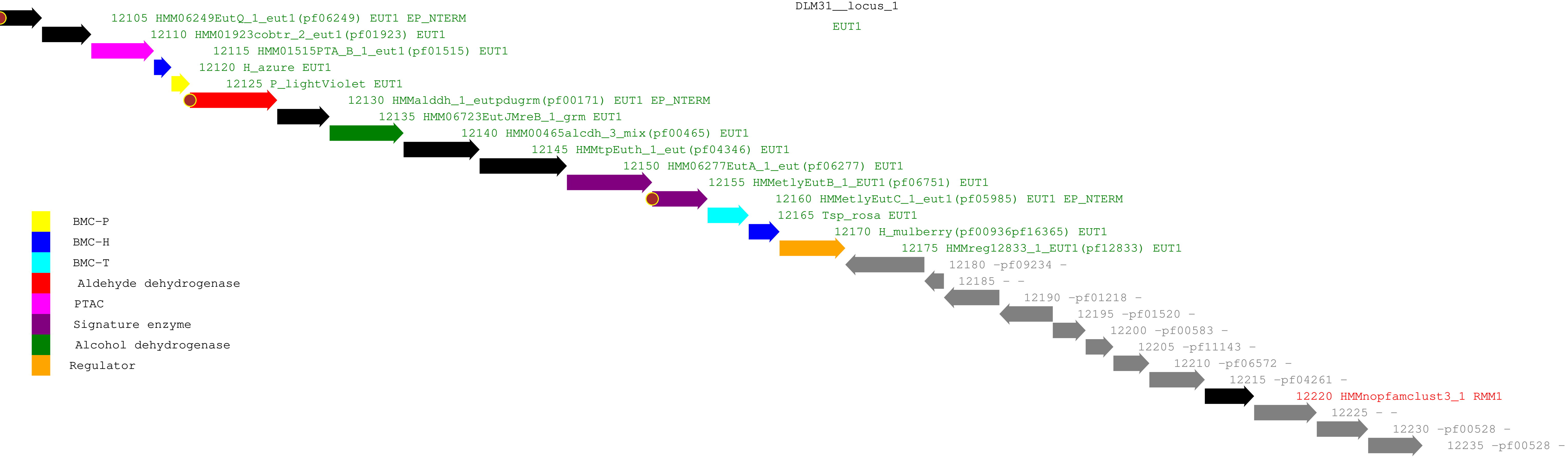

Klebsiella pneumoniae. Bacteria; Proteobacteria; Gammaproteobacteria; Enterobacterales;

DM059__locus_1

EUT1

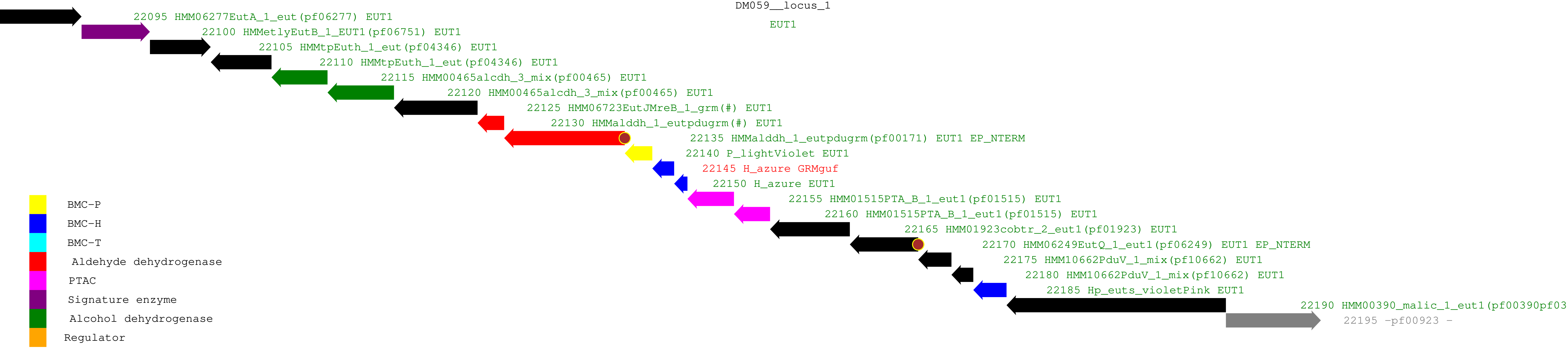

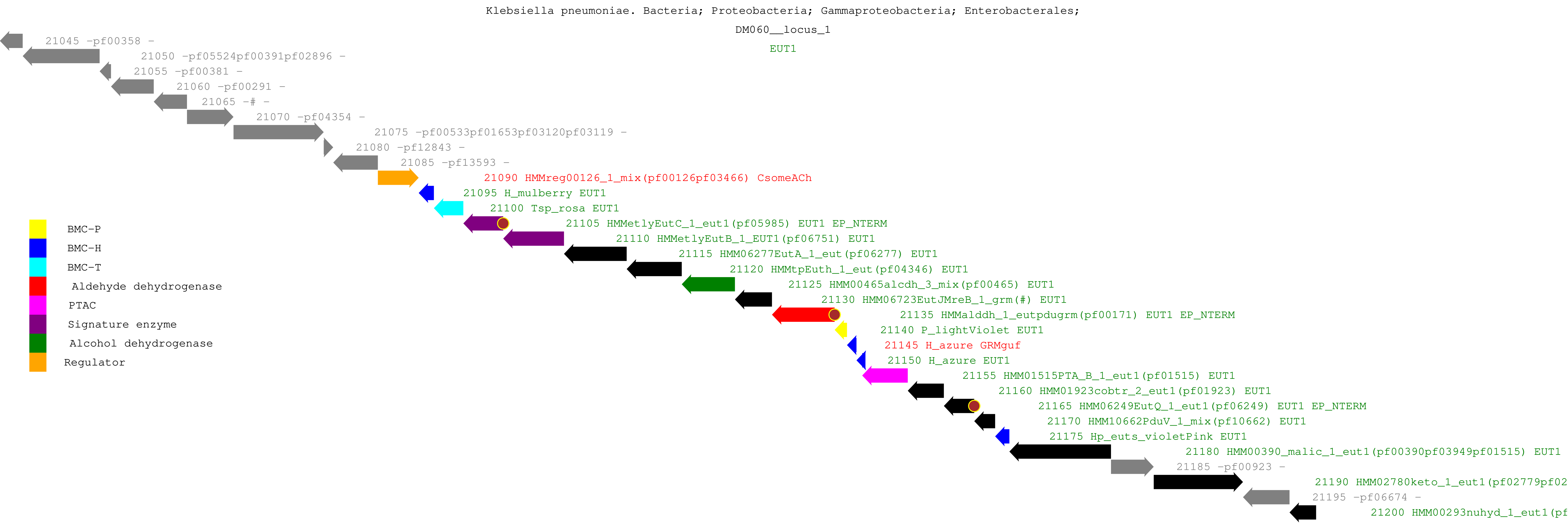
Klebsiella pneumoniae. Bacteria; Proteobacteria; Gammaproteobacteria; Enterobacterales;
DM060__locus_1
EUT1
21045 -pf00358 -
21050 -pf05524pf00391pf02896 -
21055 -pf00381 -
21060 -pf00291 -
21065 -# -
21070 -pf04354 -
21075 -pf00533pf01653pf03120pf03119 -
21080 -pf12843 -
21085 -pf13593 -
21090 HMMreg00126_1_mix(pf00126pf03466) CsomeACh
21095 H_mulberry EUT1
21100 Tsp_rosa EUT1
21105 HMMetlyEutC_1_eut1(pf05985) EUT1 EP_NTERM
21110 HMMetlyEutB_1_EUT1(pf06751) EUT1
21115 HMM06277EutA_1_eut(pf06277) EUT1
21120 HMMtpEuth_1_eut(pf04346) EUT1
21125 HMM00465alcdh_3_mix(pf00465) EUT1
21130 HMM06723EutJMreB_1_grm(#) EUT1
21135 HMMalddh_1_eutpdugrm(pf00171) EUT1 EP_NTERM
21140 P_lightViolet EUT1
21145 H_azure GRMguf
21150 H_azure EUT1
21155 HMM01515PTA_B_1_eut1(pf01515) EUT1
21160 HMM01923cobtr_2_eut1(pf01923) EUT1
21165 HMM06249EutQ_1_eut1(pf06249) EUT1 EP_NTERM
21170 HMM10662PduV_1_mix(pf10662) EUT1
21175 Hp_euts_violetPink EUT1
21180 HMM00390_malic_1_eut1(pf00390pf03949pf01515) EUT1
21185 -pf00923 -
21190 HMM02780keto_1_eut1(pf02779pf02
21195 -pf06674 -
21200 HMM00293nuhyd_1_eut1(pf
BMC-P
BMC-H
BMC-T
Aldehyde dehydrogenase
PTAC
Signature enzyme
Alcohol dehydrogenase
Regulator

Klebsiella pneumoniae. Bacteria; Proteobacteria; Gammaproteobacteria; Enterobacterales;

DM062__locus_1

EUT1

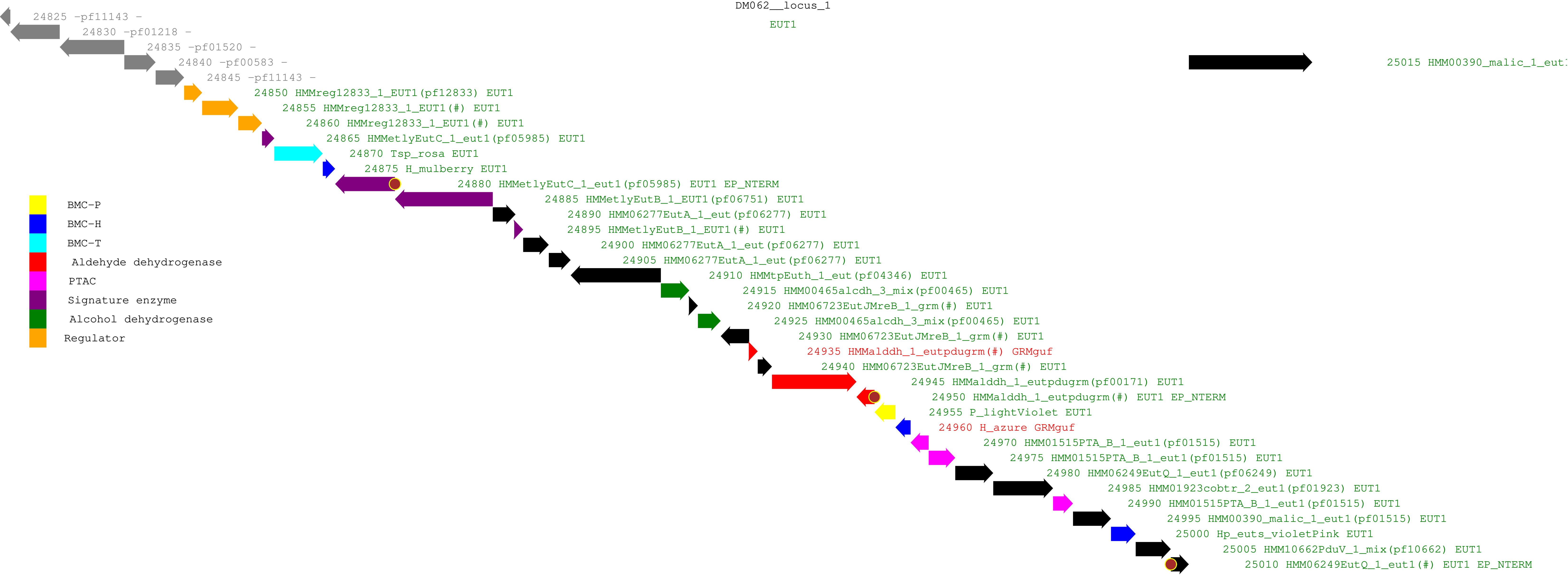

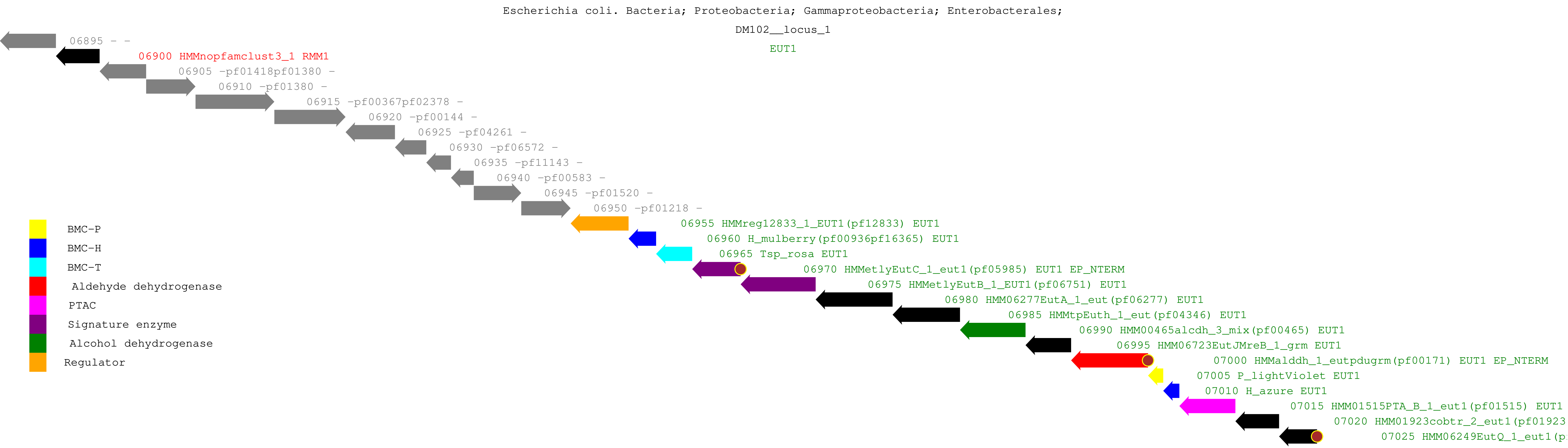
Escherichia coli. Bacteria; Proteobacteria; Gammaproteobacteria; Enterobacterales;
DM102__locus_1
EUT1
06895 - -
06900 HMMnopfamclust3_1 RMM1
06905 -pf01418pf01380 -
06910 -pf01380 -
06915 -pf00367pf02378 -
06920 -pf00144 -
06925 -pf04261 -
06930 -pf06572 -
06935 -pf11143 -
06940 -pf00583 -
06945 -pf01520 -
06950 -pf01218 -
06955 HMMreg12833_1_EUT1(pf12833) EUT1
06960 H_mulberry(pf00936pf16365) EUT1
06965 Tsp_rosa EUT1
06970 HMMetlyEutC_1_eut1(pf05985) EUT1 EP_NTERM
06975 HMMetlyEutB_1_EUT1(pf06751) EUT1
06980 HMM06277EutA_1_eut(pf06277) EUT1
06985 HMMtpEuth_1_eut(pf04346) EUT1
06990 HMM00465alcdh_3_mix(pf00465) EUT1
06995 HMM06723EutJMreB_1_grm EUT1
07000 HMMalddh_1_eutpdugrm(pf00171) EUT1 EP_NTERM
07005 P_lightViolet EUT1
07010 H_azure EUT1
07015 HMM01515PTA_B_1_eut1(pf01515) EUT1
07020 HMM01923cobtr_2_eut1(pf01923
07025 HMM06249EutQ_1_eut1(p
BMC-P
BMC-H
BMC-T
Aldehyde dehydrogenase
PTAC
Signature enzyme
Alcohol dehydrogenase
Regulator

Salmonella enterica I. Bacteria; Proteobacteria; Gammaproteobacteria; Enterobacterales;

DM358__locus_1

EUT1

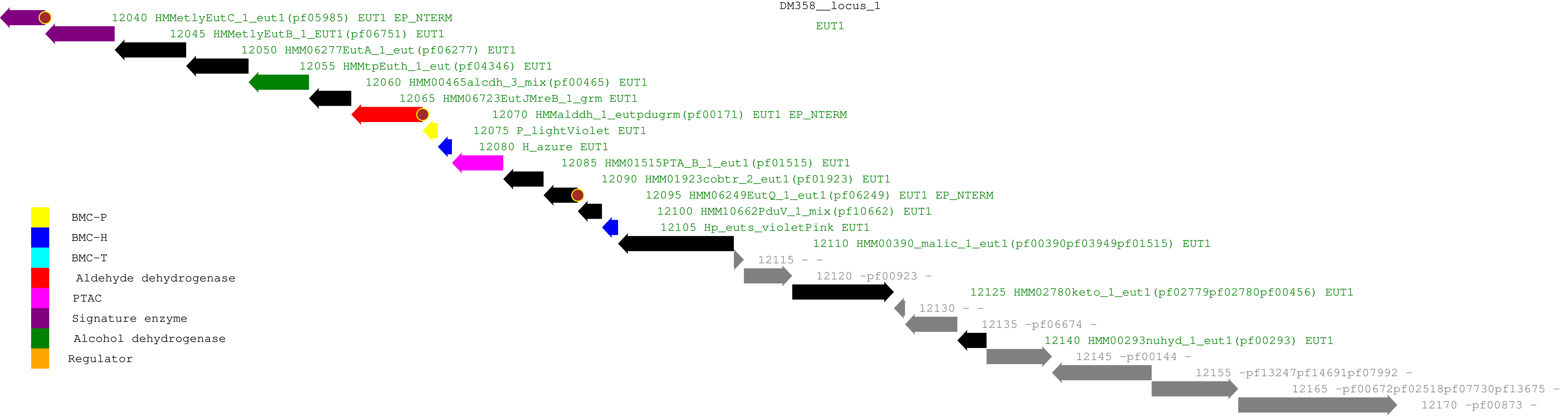

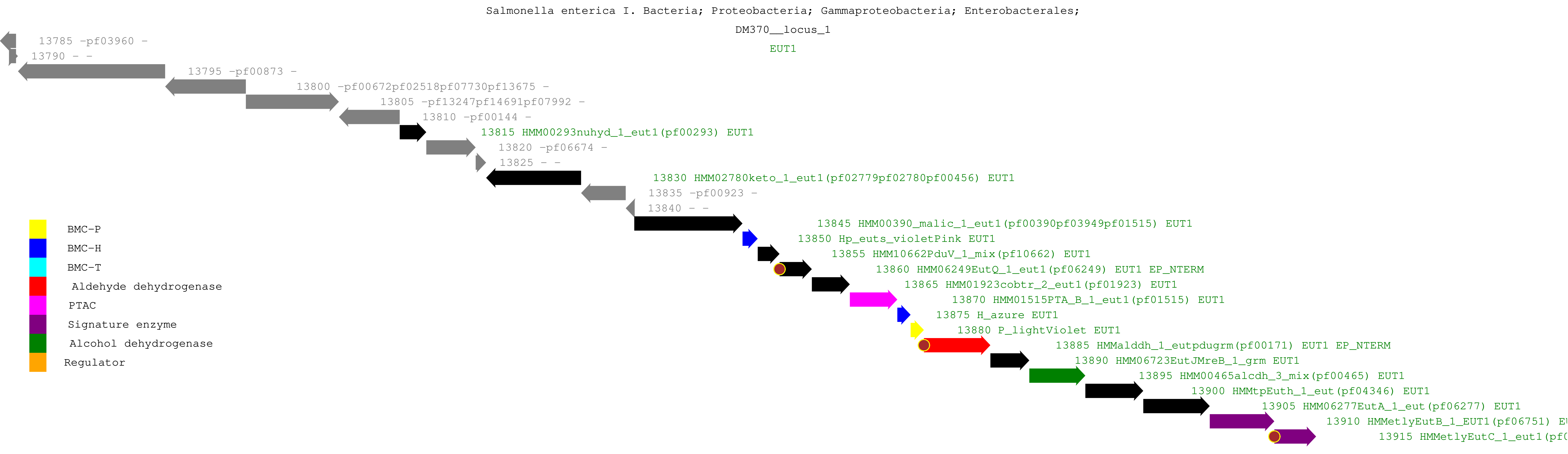

Salmonella enterica I. Bacteria; Proteobacteria; Gammaproteobacteria; Enterobacterales;
DM370__locus_1
EUT1
13785 -pf03960 -
13790 - -
13795 -pf00873 -
13800 -pf00672pf02518pf07730pf13675 -
13805 -pf13247pf14691pf07992 -
13810 -pf00144 -
13815 HMM00293nuhyd_1_eut1(pf00293) EUT1
13820 -pf06674 -
13825 - -
13830 HMM02780keto_1_eut1(pf02779pf02780pf00456) EUT1
13835 -pf00923 -
13840 - -
13845 HMM00390_malic_1_eut1(pf00390pf03949pf01515) EUT1
13850 Hp_euts_violetPink EUT1
13855 HMM10662PduV_1_mix(pf10662) EUT1
13860 HMM06249EutQ_1_eut1(pf06249) EUT1 EP_NTERM
13865 HMM01923cobtr_2_eut1(pf01923) EUT1
13870 HMM01515PTA_B_1_eut1(pf01515) EUT1
13875 H_azure EUT1
13880 P_lightViolet EUT1
13885 HMMalddh_1_eutpdugrm(pf00171) EUT1 EP_NTERM
13890 HMM06723EutJMreB_1_grm EUT1
13895 HMM00465alcdh_3_mix(pf00465) EUT1
13900 HMMtpEuth_1_eut(pf04346) EUT1
13905 HMM06277EutA_1_eut(pf06277) EUT1
13910 HMMetlyEutB_1_EUT1(pf06751) EU
13915 HMMetlyEutC_1_eut1(pf(
BMC-P
BMC-H
BMC-T
Aldehyde dehydrogenase
PTAC
Signature enzyme
Alcohol dehydrogenase
Regulator

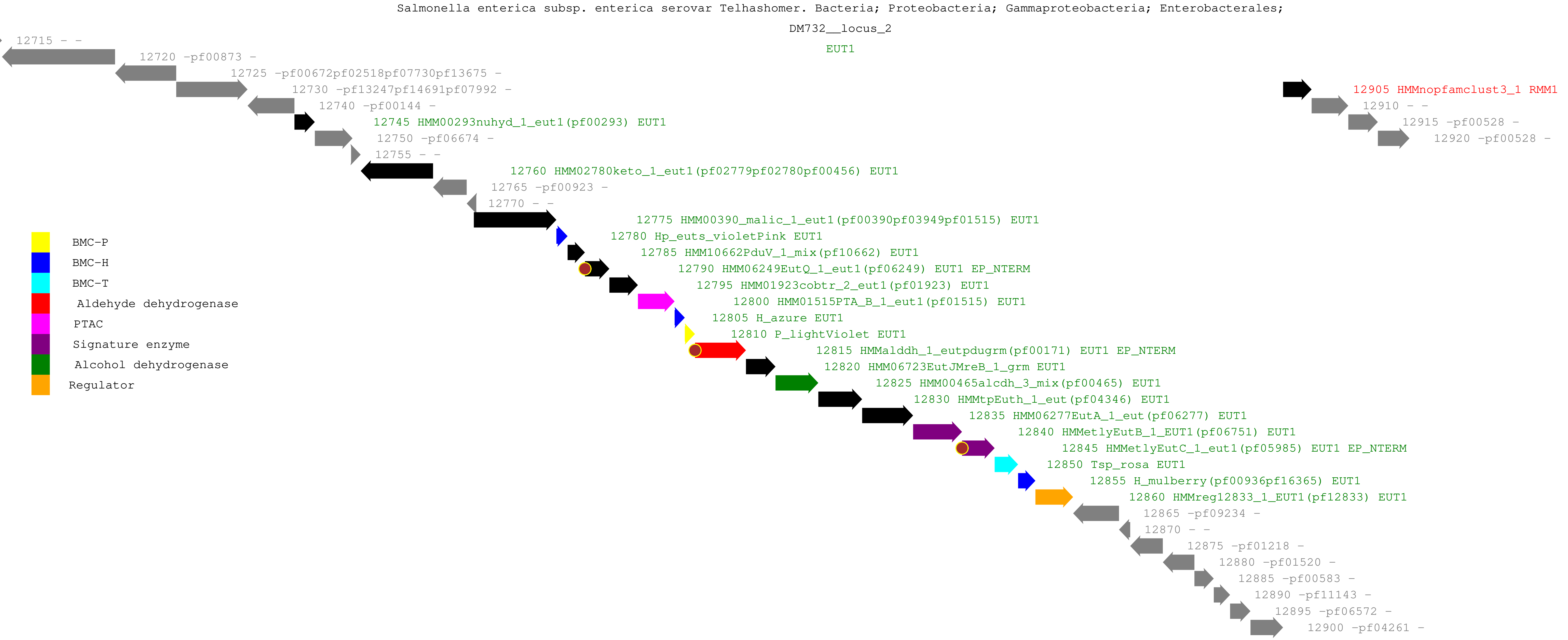

Salmonella enterica subsp. enterica serovar Telhashomer. Bacteria; Proteobacteria; Gammaproteobacteria; Enterobacterales;
DM732__locus_2
EUT1
12715 - -
12720 -pf00873 -
12725 -pf00672pf02518pf07730pf13675 -
12730 -pf13247pf14691pf07992 -
12740 -pf00144 -
12745 HMM00293nuhyd_1_eut1(pf00293) EUT1
12750 -pf06674 -
12755 - -
12760 HMM02780keto_1_eut1(pf02779pf02780pf00456) EUT1
12765 -pf00923 -
12770 - -
12775 HMM00390_malic_1_eut1(pf00390pf03949pf01515) EUT1
12780 Hp_euts_violetPink EUT1
12785 HMM10662PduV_1_mix(pf10662) EUT1
12790 HMM06249EutQ_1_eut1(pf06249) EUT1 EP_NTERM
12795 HMM01923cobtr_2_eut1(pf01923) EUT1
12800 HMM01515PTA_B_1_eut1(pf01515) EUT1
12805 H_azure EUT1
12810 P_lightViolet EUT1
12815 HMMaldh_1_eutpdugrm(pf00171) EUT1 EP_NTERM
12820 HMM06723EutJMreB_1_grm EUT1
12825 HMM00465alcdh_3_mix(pf00465) EUT1
12830 HMMtpEuth_1_eut(pf04346) EUT1
12835 HMM06277EutA_1_eut(pf06277) EUT1
12840 HMMetlyEutB_1_EUT1(pf06751) EUT1
12845 HMMetlyEutC_1_eut1(pf05985) EUT1 EP_NTERM
12850 Tsp_rosa EUT1
12855 H_mulberry(pf00936pf16365) EUT1
12860 HMMreg12833_1_EUT1(pf12833) EUT1
12865 -pf09234 -
12870 - -
12875 -pf01218 -
12880 -pf01520 -
12885 -pf00583 -
12890 -pf11143 -
12895 -pf06572 -
12900 -pf04261 -
12905 HMMnopfamclust3_1 RMM1
12910 - -
12915 -pf00528 -
12920 -pf00528 -
BMC-P
BMC-H
BMC-T
Aldehyde dehydrogenase
PTAC
Signature enzyme
Alcohol dehydrogenase
Regulator

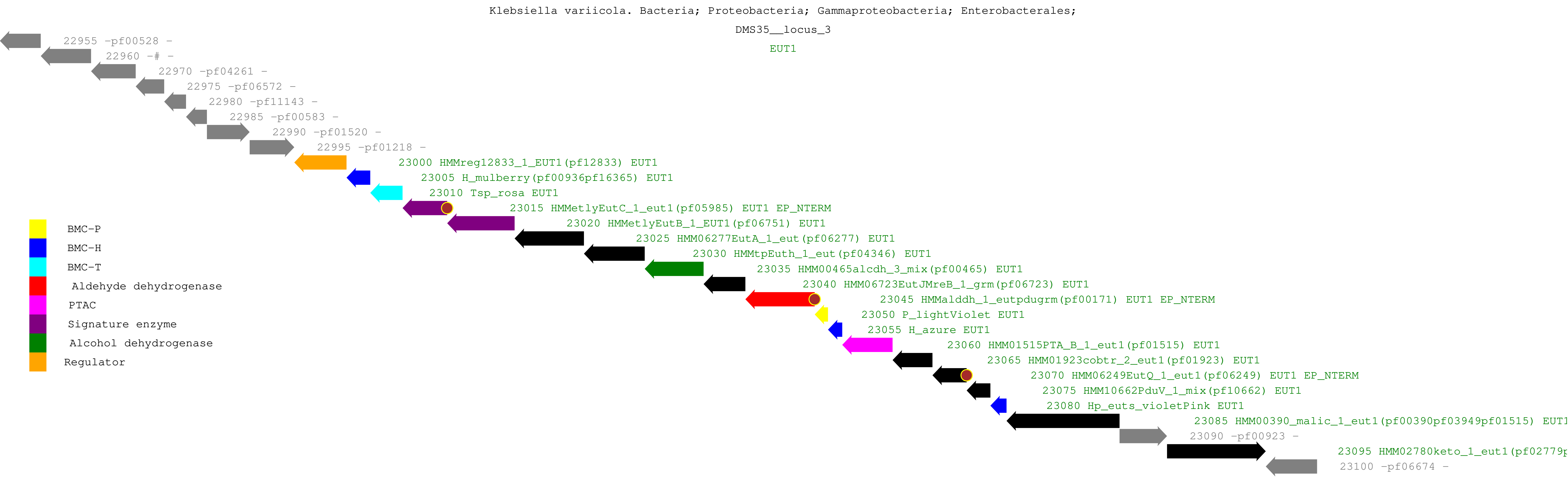

Klebsiella variicola. Bacteria; Proteobacteria; Gammaproteobacteria; Enterobacterales;
DMS35__locus_3
EUT1
22955 -pf00528 -
22960 -# -
22970 -pf04261 -
22975 -pf06572 -
22980 -pf11143 -
22985 -pf00583 -
22990 -pf01520 -
22995 -pf01218 -
23000 HMMreg12833_1_EUT1(pf12833) EUT1
23005 H_mulberry(pf00936pf16365) EUT1
23010 Tsp_rosa EUT1
23015 HMMetlyEutC_1_eut1(pf05985) EUT1 EP_NTERM
23020 HMMetlyEutB_1_EUT1(pf06751) EUT1
23025 HMM06277EutA_1_eut(pf06277) EUT1
23030 HMMtpEuth_1_eut(pf04346) EUT1
23035 HMM00465alcdh_3_mix(pf00465) EUT1
23040 HMM06723EutJMreB_1_grm(pf06723) EUT1
23045 HMMalddh_1_eutpdugrm(pf00171) EUT1 EP_NTERM
23050 P_lightViolet EUT1
23055 H_azure EUT1
23060 HMM01515PTA_B_1_eut1(pf01515) EUT1
23065 HMM01923cobtr_2_eut1(pf01923) EUT1
23070 HMM06249EutQ_1_eut1(pf06249) EUT1 EP_NTERM
23075 HMM10662PduV_1_mix(pf10662) EUT1
23080 Hp_euts_violetPink EUT1
23085 HMM00390_malic_1_eut1(pf00390pf03949pf01515) EUT1
23090 -pf00923 -
23095 HMM02780keto_1_eut1(pf02779p
23100 -pf06674 -
BMC-P
BMC-H
BMC-T
Aldehyde dehydrogenase
PTAC
Signature enzyme
Alcohol dehydrogenase
Regulator

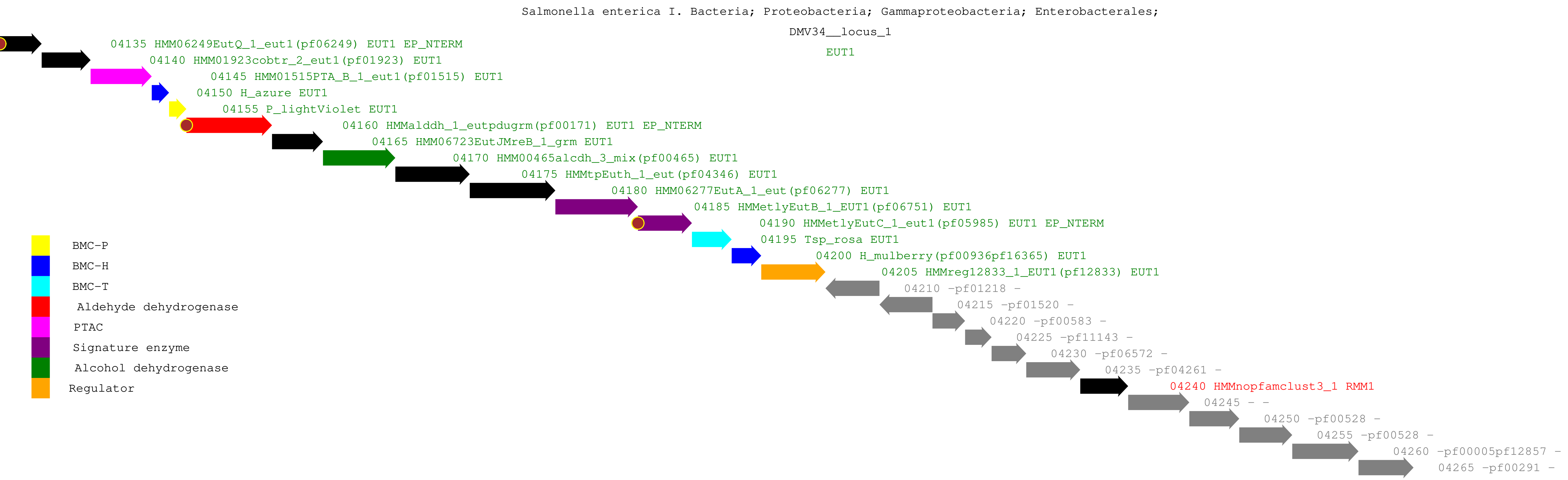
Salmonella enterica I. Bacteria; Proteobacteria; Gammaproteobacteria; Enterobacterales;
DMV34__locus_1
EUT1
04135 HMM06249EutQ_1_eut1(pf06249) EUT1 EP_NTERM
04140 HMM01923cobtr_2_eut1(pf01923) EUT1
04145 HMM01515PTA_B_1_eut1(pf01515) EUT1
04150 H_azure EUT1
04155 P_lightViolet EUT1
04160 HMMalddh_1_eutpdugrm(pf00171) EUT1 EP_NTERM
04165 HMM06723EutJMreB_1_grm EUT1
04170 HMM00465alcdh_3_mix(pf00465) EUT1
04175 HMMtpEuth_1_eut(pf04346) EUT1
04180 HMM06277EutA_1_eut(pf06277) EUT1
04185 HMMetlyEutB_1_EUT1(pf06751) EUT1
04190 HMMetlyEutC_1_eut1(pf05985) EUT1 EP_NTERM
04195 Tsp_rosa EUT1
04200 H_mulberry(pf00936pf16365) EUT1
04205 HMMreg12833_1_EUT1(pf12833) EUT1
04210 -pf01218 -
04215 -pf01520 -
04220 -pf00583 -
04225 -pf11143 -
04230 -pf06572 -
04235 -pf04261 -
04240 HMMnopfamclust3_1 RMM1
04245 - -
04250 -pf00528 -
04255 -pf00528 -
04260 -pf00005pf12857 -
04265 -pf00291 -
BMC-P
BMC-H
BMC-T
Aldehyde dehydrogenase
PTAC
Signature enzyme
Alcohol dehydrogenase
Regulator

Salmonella enterica I. Bacteria; Proteobacteria; Gammaproteobacteria; Enterobacterales;

DN322__locus_1

EUT1

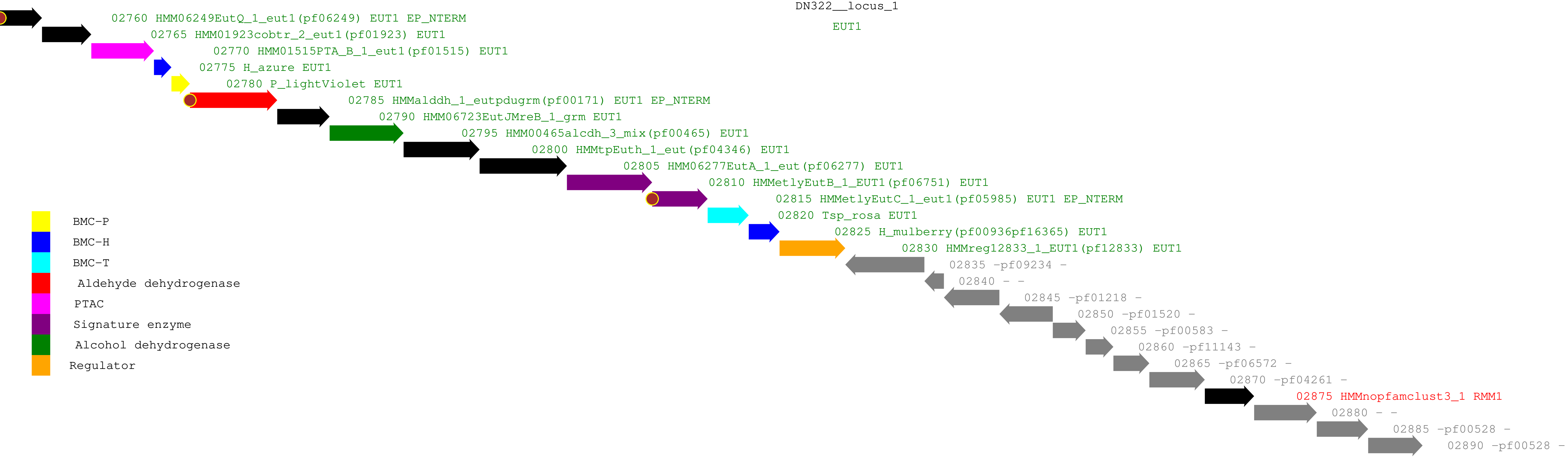

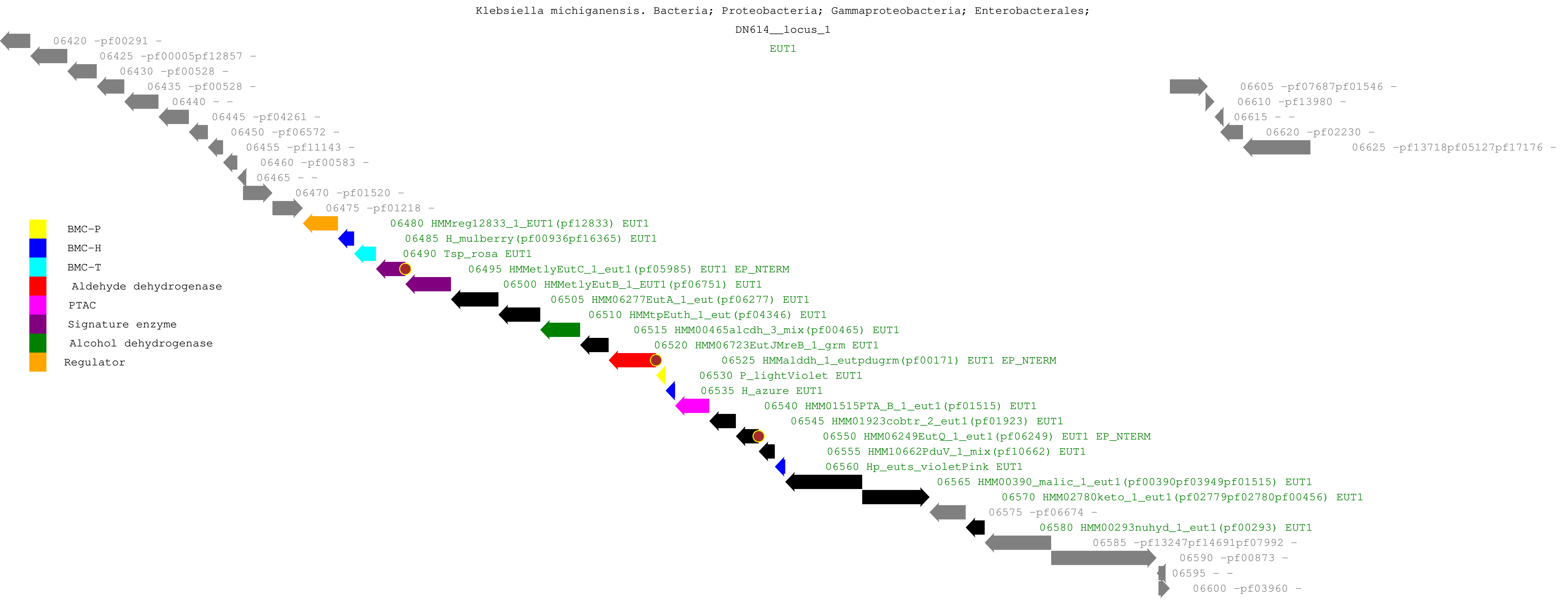
Klebsiella michiganensis. Bacteria; Proteobacteria; Gammaproteobacteria; Enterobacterales;
DN614__locus_1
EUT1
06420 -pf00291 -
06425 -pf00005pf12857 -
06430 -pf00528 -
06435 -pf00528 -
06440 - -
06445 -pf04261 -
06450 -pf06572 -
06455 -pf11143 -
06460 -pf00583 -
06465 - -
06470 -pf01520 -
06475 -pf01218 -
06480 HMMreg12833_1_EUT1(pf12833) EUT1
06485 H_mulberry(pf00936pf16365) EUT1
06490 Tsp_rosa EUT1
06495 HMMetlyEutC_1_eut1(pf05985) EUT1 EP_NTERM
06500 HMMetlyEutB_1_EUT1(pf06751) EUT1
06505 HMM06277EutA_1_eut(pf06277) EUT1
06510 HMMtpEuth_1_eut(pf04346) EUT1
06515 HMM00465alcdh_3_mix(pf00465) EUT1
06520 HMM06723EutJMreB_1_grm EUT1
06525 HMMalddh_1_eutpdugrm(pf00171) EUT1 EP_NTERM
06530 P_lightViolet EUT1
06535 H_azure EUT1
06540 HMM01515PTA_B_1_eut1(pf01515) EUT1
06545 HMM01923cobtr_2_eut1(pf01923) EUT1
06550 HMM06249EutQ_1_eut1(pf06249) EUT1 EP_NTERM
06555 HMM10662PduV_1_mix(pf10662) EUT1
06560 Hp_euts_violetPink EUT1
06565 HMM00390_malic_1_eut1(pf00390pf03949pf01515) EUT1
06570 HMM02780keto_1_eut1(pf02779pf02780pf00456) EUT1
06575 -pf06674 -
06580 HMM00293nuhyd_1_eut1(pf00293) EUT1
06585 -pf13247pf14691pf07992 -
06590 -pf00873 -
06595 - -
06600 -pf03960 -
06605 -pf07687pf01546 -
06610 -pf13980 -
06615 - -
06620 -pf02230 -
06625 -pf13718pf05127pf17176 -
BMC-P
BMC-H
BMC-T
Aldehyde dehydrogenase
PTAC
Signature enzyme
Alcohol dehydrogenase
Regulator

Klebsiella michiganensis. Bacteria; Proteobacteria; Gammaproteobacteria; Enterobacterales;

DN619__locus_1

EUT1

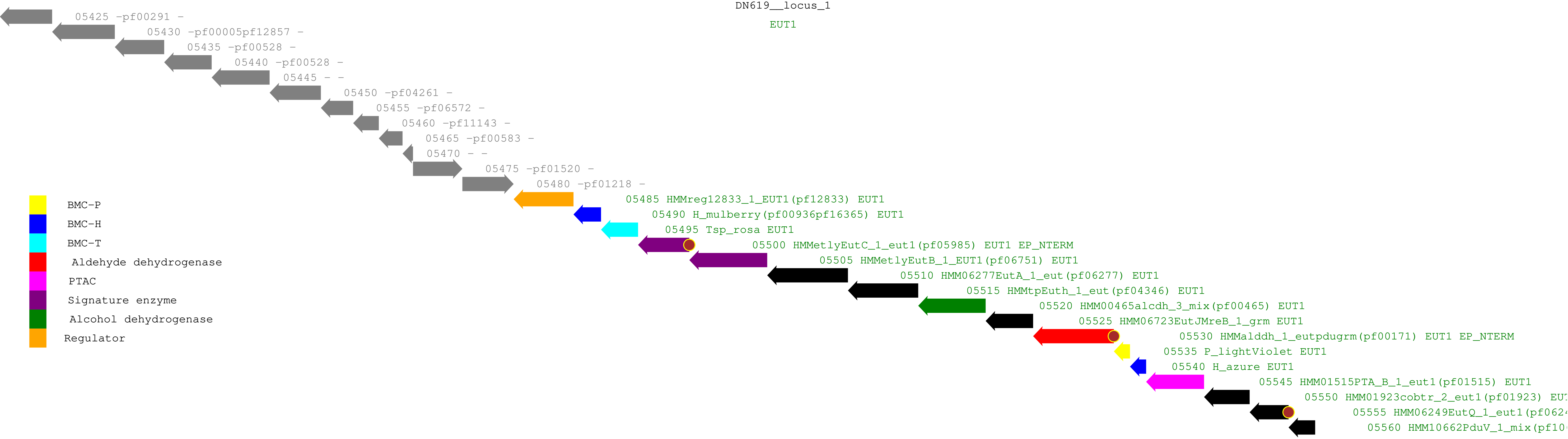

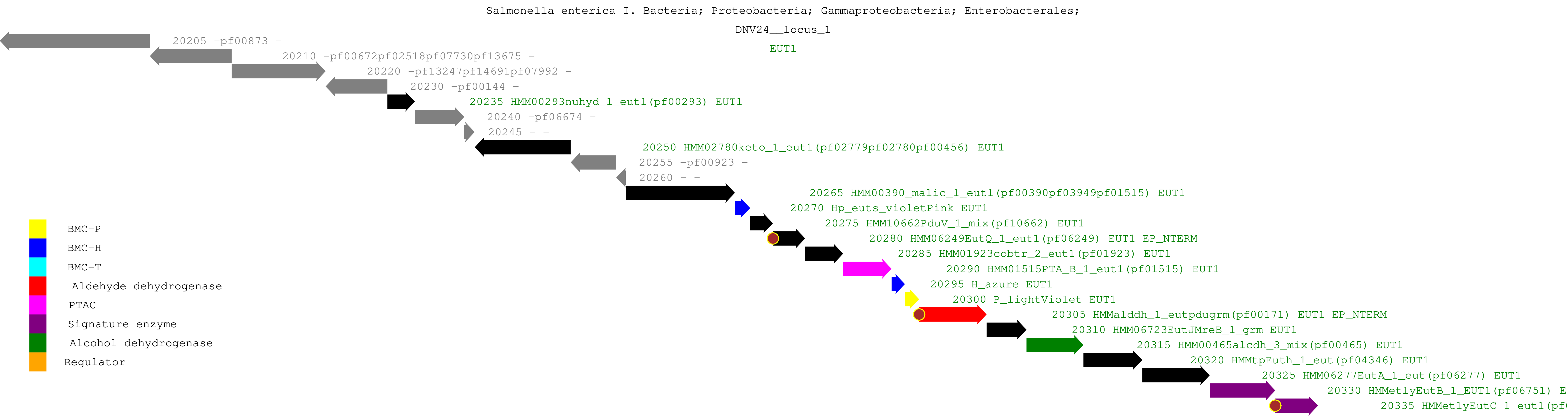

Salmonella enterica I. Bacteria; Proteobacteria; Gammaproteobacteria; Enterobacterales;
DNV24__locus_1
EUT1
20205 -pf00873 -
20210 -pf00672pf02518pf07730pf13675 -
20220 -pf13247pf14691pf07992 -
20230 -pf00144 -
20235 HMM00293nuhyd_1_eut1(pf00293) EUT1
20240 -pf06674 -
20245 - -
20250 HMM02780keto_1_eut1(pf02779pf02780pf00456) EUT1
20255 -pf00923 -
20260 - -
20265 HMM00390_malic_1_eut1(pf00390pf03949pf01515) EUT1
20270 Hp_euts_violetPink EUT1
20275 HMM10662PduV_1_mix(pf10662) EUT1
20280 HMM06249EutQ_1_eut1(pf06249) EUT1 EP_NTERM
20285 HMM01923cobtr_2_eut1(pf01923) EUT1
20290 HMM01515PTA_B_1_eut1(pf01515) EUT1
20295 H_azure EUT1
20300 P_lightViolet EUT1
20305 HMMalddh_1_eutpdugrm(pf00171) EUT1 EP_NTERM
20310 HMM06723EutJMreB_1_grm EUT1
20315 HMM00465alcdh_3_mix(pf00465) EUT1
20320 HMMtpEuth_1_eut(pf04346) EUT1
20325 HMM06277EutA_1_eut(pf06277) EUT1
20330 HMMetlyEutB_1_EUT1(pf06751) E
20335 HMMetlyEutC_1_eut1(pf
BMC-P
BMC-H
BMC-T
Aldehyde dehydrogenase
PTAC
Signature enzyme
Alcohol dehydrogenase
Regulator

Salmonella enterica subsp. salamae. Bacteria; Proteobacteria; Gammaproteobacteria; Enterobacterales;

DO628__locus_1

EUT1

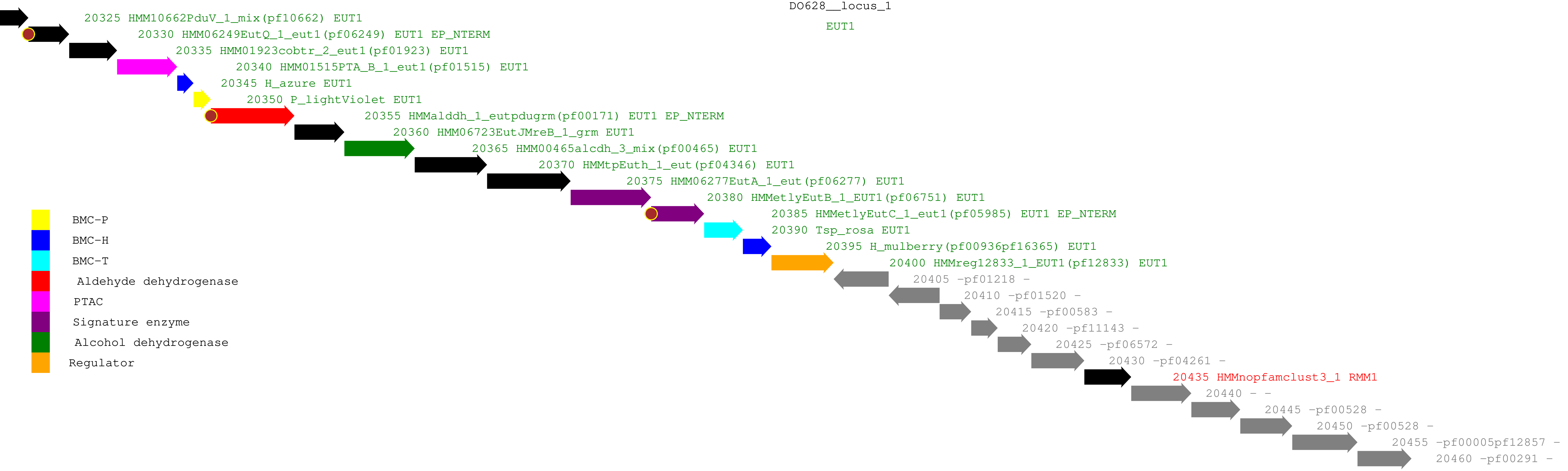

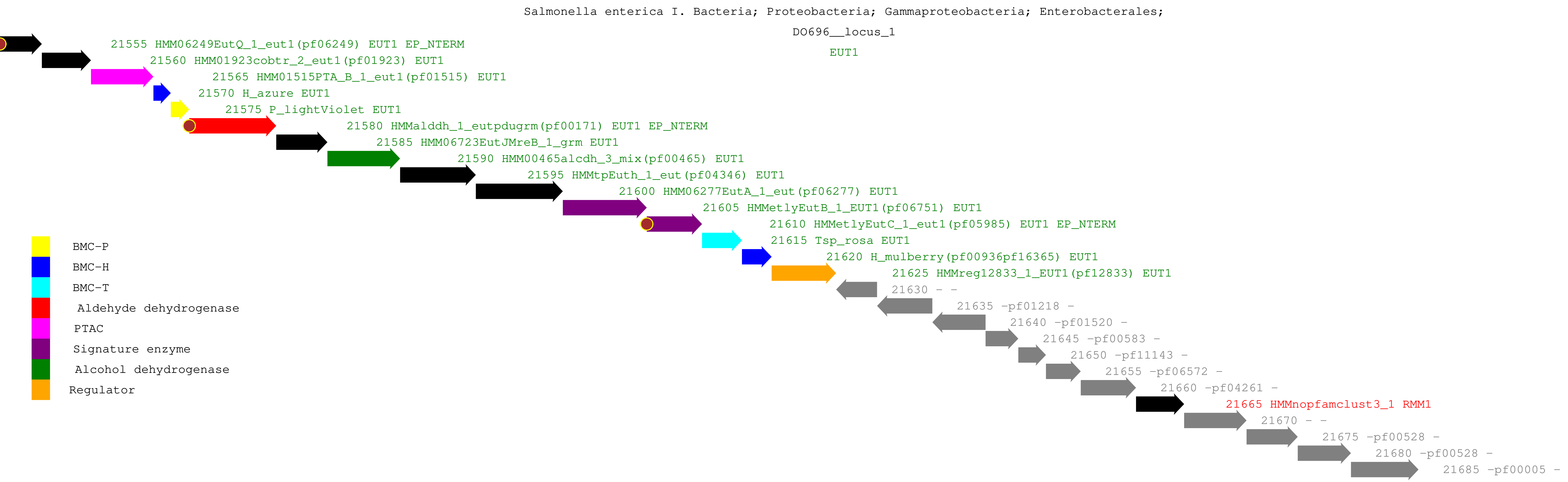
Salmonella enterica I. Bacteria; Proteobacteria; Gammaproteobacteria; Enterobacterales;
DO696__locus_1
EUT1
21555 HMM06249EutQ_1_eut1(pf06249) EUT1 EP_NTERM
21560 HMM01923cobtr_2_eut1(pf01923) EUT1
21565 HMM01515PTA_B_1_eut1(pf01515) EUT1
21570 H_azure EUT1
21575 P_lightViolet EUT1
21580 HMMalddh_1_eutpdugrm(pf00171) EUT1 EP_NTERM
21585 HMM06723EutJMreB_1_grm EUT1
21590 HMM00465alcdh_3_mix(pf00465) EUT1
21595 HMMtpEuth_1_eut(pf04346) EUT1
21600 HMM06277EutA_1_eut(pf06277) EUT1
21605 HMMetlyEutB_1_EUT1(pf06751) EUT1
21610 HMMetlyEutC_1_eut1(pf05985) EUT1 EP_NTERM
21615 Tsp_rosa EUT1
21620 H_mulberry(pf00936pf16365) EUT1
21625 HMMreg12833_1_EUT1(pf12833) EUT1
21630 - -
21635 -pf01218 -
21640 -pf01520 -
21645 -pf00583 -
21650 -pf11143 -
21655 -pf06572 -
21660 -pf04261 -
21665 HMMnopfamclust3_1 RMM1
21670 - -
21675 -pf00528 -
21680 -pf00528 -
21685 -pf00005 -
BMC-P
BMC-H
BMC-T
Aldehyde dehydrogenase
PTAC
Signature enzyme
Alcohol dehydrogenase
Regulator

Salmonella enteritidis. Bacteria; Proteobacteria; Gammaproteobacteria; Enterobacterales;

DO961__locus_1

EUT1

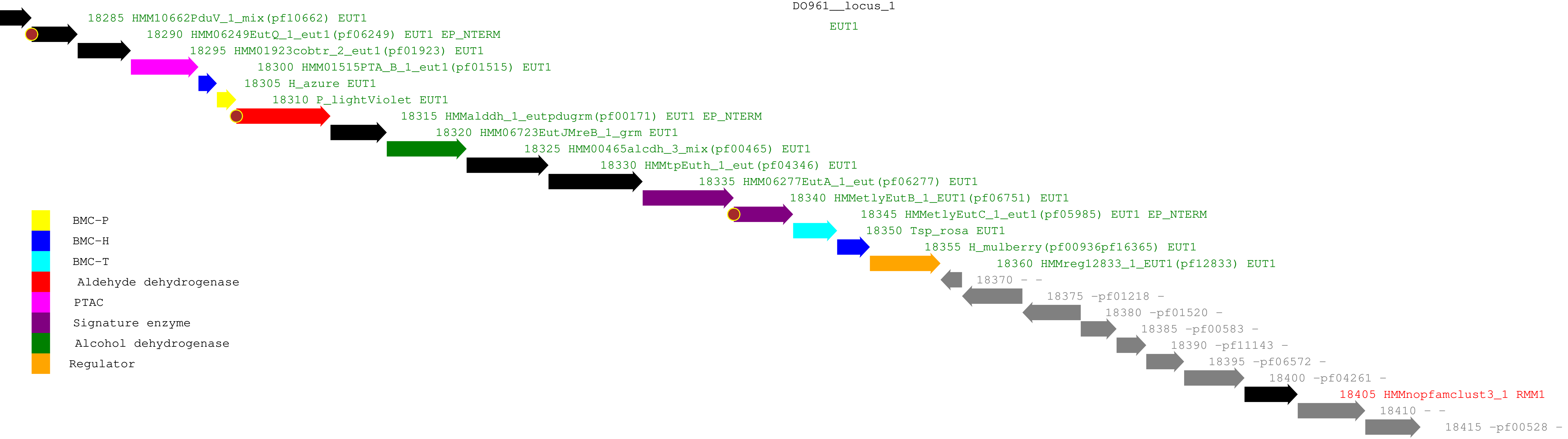

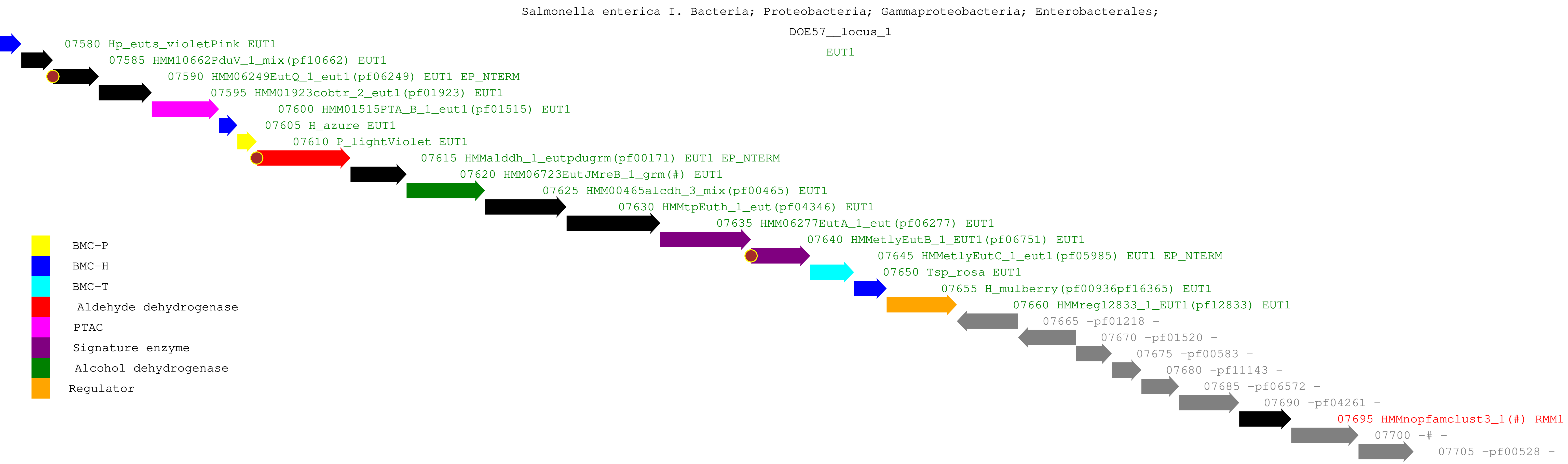

Salmonella enterica I. Bacteria; Proteobacteria; Gammaproteobacteria; Enterobacterales;
DOE57__locus_1
EUT1
07580 Hp_euts_violetPink EUT1
07585 HMM10662PduV_1_mix(pf10662) EUT1
07590 HMM06249EutQ_1_eut1(pf06249) EUT1 EP_NTERM
07595 HMM01923cobtr_2_eut1(pf01923) EUT1
07600 HMM01515PTA_B_1_eut1(pf01515) EUT1
07605 H_azure EUT1
07610 P_lightViolet EUT1
07615 HMMalddh_1_eutpdugrm(pf00171) EUT1 EP_NTERM
07620 HMM06723EutJMreB_1_grm(#) EUT1
07625 HMM00465alcdh_3_mix(pf00465) EUT1
07630 HMMtpEuth_1_eut(pf04346) EUT1
07635 HMM06277EutA_1_eut(pf06277) EUT1
07640 HMMetlyEutB_1_EUT1(pf06751) EUT1
07645 HMMetlyEutC_1_eut1(pf05985) EUT1 EP_NTERM
07650 Tsp_rosa EUT1
07655 H_mulberry(pf00936pf16365) EUT1
07660 HMMreg12833_1_EUT1(pf12833) EUT1
07665 -pf01218 -
07670 -pf01520 -
07675 -pf00583 -
07680 -pf11143 -
07685 -pf06572 -
07690 -pf04261 -
07695 HMMnopfamclust3_1(#) RMM1
07700 -# -
07705 -pf00528 -
BMC-P
BMC-H
BMC-T
Aldehyde dehydrogenase
PTAC
Signature enzyme
Alcohol dehydrogenase
Regulator

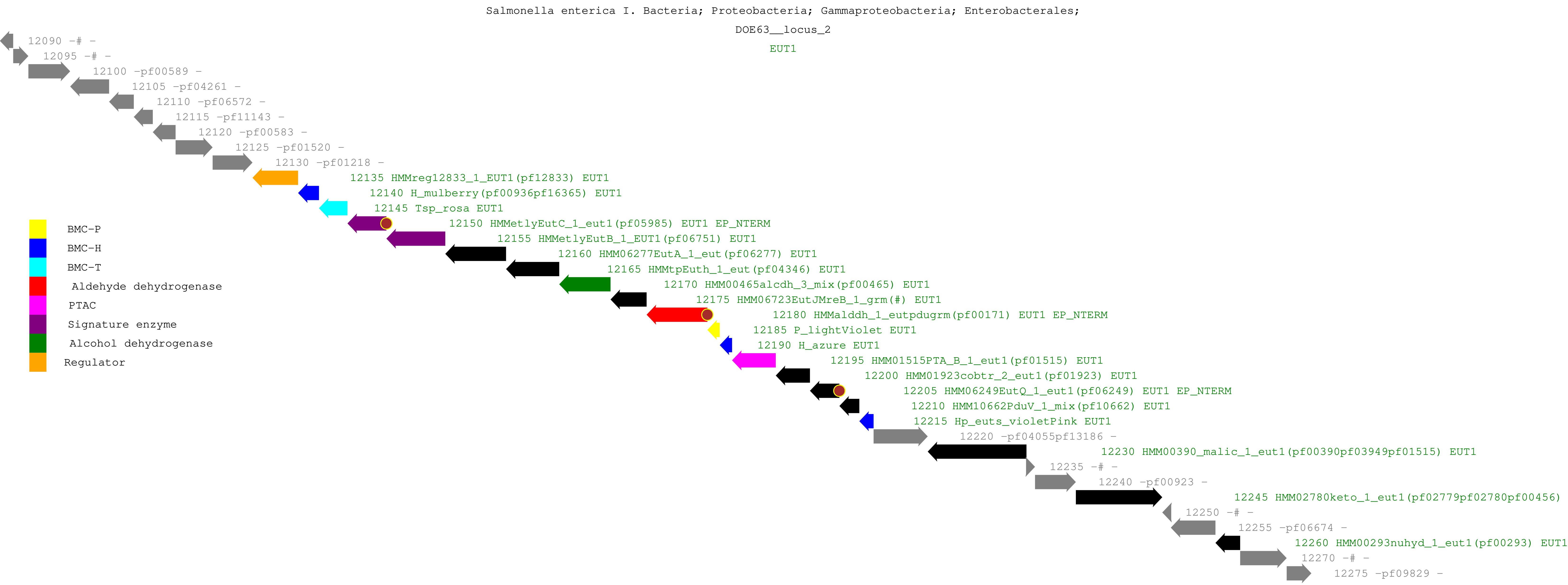
Salmonella enterica I. Bacteria; Proteobacteria; Gammaproteobacteria; Enterobacterales;
DOE63__locus_2
EUT1
12090 -# -
12095 -# -
12100 -pf00589 -
12105 -pf04261 -
12110 -pf06572 -
12115 -pf11143 -
12120 -pf00583 -
12125 -pf01520 -
12130 -pf01218 -
12135 HMMreg12833_1_EUT1(pf12833) EUT1
12140 H_mulberry(pf00936pf16365) EUT1
12145 Tsp_rosa EUT1
12150 HMMetlyEutC_1_eut1(pf05985) EUT1 EP_NTERM
12155 HMMetlyEutB_1_EUT1(pf06751) EUT1
12160 HMM06277EutA_1_eut(pf06277) EUT1
12165 HMMtpEuth_1_eut(pf04346) EUT1
12170 HMM00465alcdh_3_mix(pf00465) EUT1
12175 HMM06723EutJMreB_1_grm(#) EUT1
12180 HMMalddh_1_eutpdugrm(pf00171) EUT1 EP_NTERM
12185 P_lightViolet EUT1
12190 H_azure EUT1
12195 HMM01515PTA_B_1_eut1(pf01515) EUT1
12200 HMM01923cobtr_2_eut1(pf01923) EUT1
12205 HMM06249EutQ_1_eut1(pf06249) EUT1 EP_NTERM
12210 HMM10662PduV_1_mix(pf10662) EUT1
12215 Hp_euts_violetPink EUT1
12220 -pf04055pf13186 -
12230 HMM00390_malic_1_eut1(pf00390pf03949pf01515) EUT1
12235 -# -
12240 -pf00923 -
12245 HMM02780keto_1_eut1(pf02779pf02780pf00456)
12250 -# -
12255 -pf06674 -
12260 HMM00293nuhyd_1_eut1(pf00293) EUT1
12270 -# -
12275 -pf09829 -
BMC-P
BMC-H
BMC-T
Aldehyde dehydrogenase
PTAC
Signature enzyme
Alcohol dehydrogenase
Regulator

Salmonella enterica I. Bacteria; Proteobacteria; Gammaproteobacteria; Enterobacterales;

DOG94__locus_1

EUT1

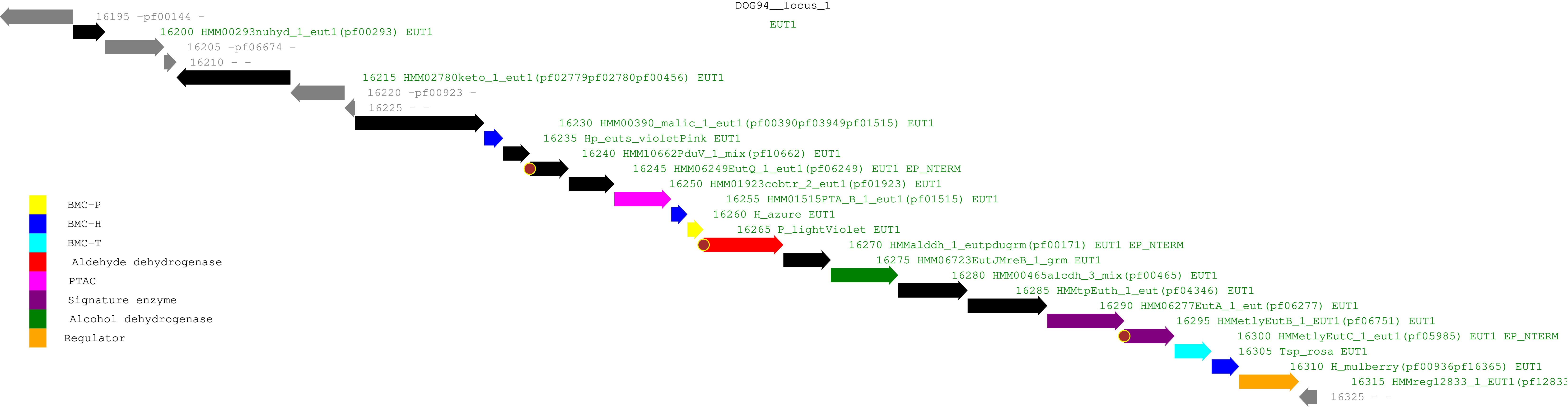

Salmonella enterica I. Bacteria; Proteobacteria; Gammaproteobacteria; Enterobacterales;

DOH53__locus_1

EUT1

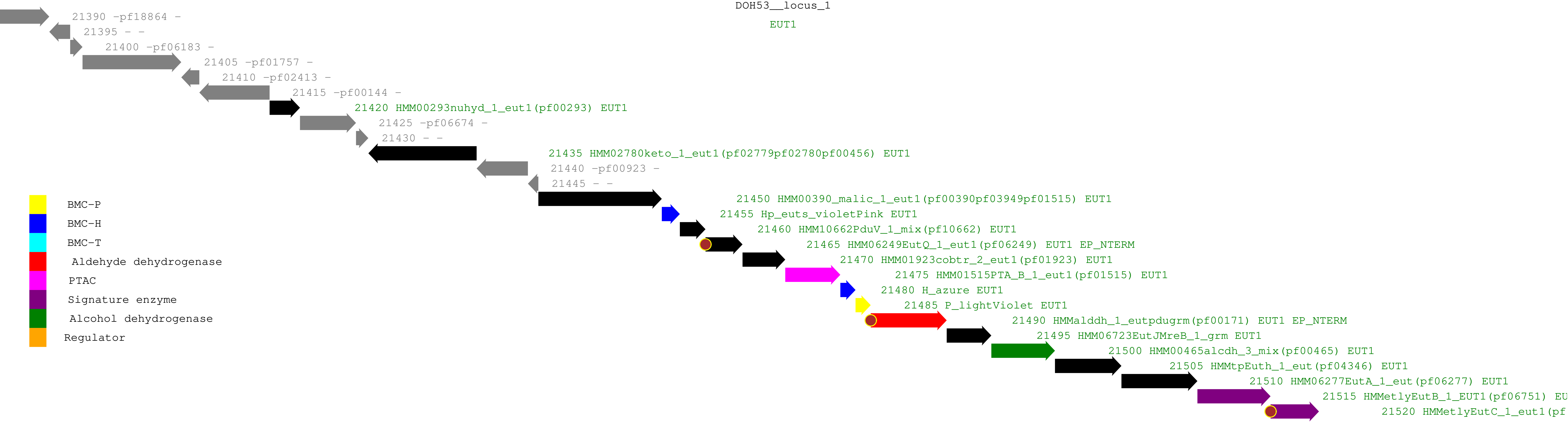

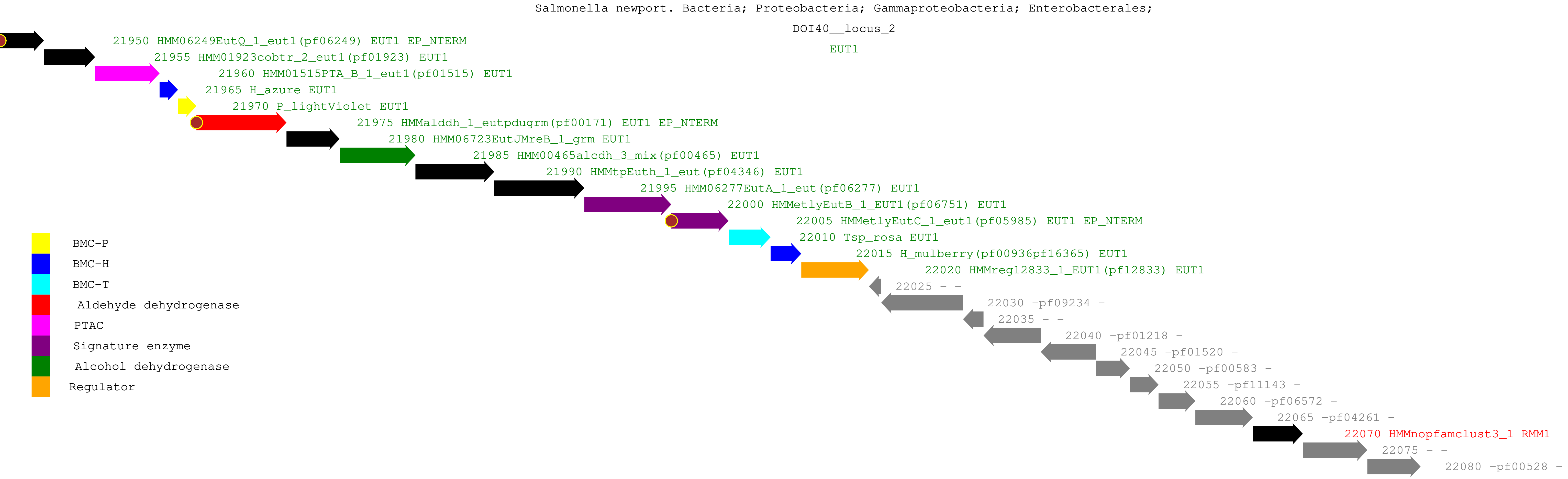

Salmonella newport. Bacteria; Proteobacteria; Gammaproteobacteria; Enterobacterales;
DOI40__locus_2
EUT1
21950 HMM06249EutQ_1_eut1(pf06249) EUT1 EP_NTERM
21955 HMM01923cobtr_2_eut1(pf01923) EUT1
21960 HMM01515PTA_B_1_eut1(pf01515) EUT1
21965 H_azure EUT1
21970 P_lightViolet EUT1
21975 HMMalddh_1_eutpdugrm(pf00171) EUT1 EP_NTERM
21980 HMM06723EutJMreB_1_grm EUT1
21985 HMM00465alcdh_3_mix(pf00465) EUT1
21990 HMMtpEuth_1_eut(pf04346) EUT1
21995 HMM06277EutA_1_eut(pf06277) EUT1
22000 HMMetlyEutB_1_EUT1(pf06751) EUT1
22005 HMMetlyEutC_1_eut1(pf05985) EUT1 EP_NTERM
22010 Tsp_rosa EUT1
22015 H_mulberry(pf00936pf16365) EUT1
22020 HMMreg12833_1_EUT1(pf12833) EUT1
22025 - -
22030 -pf09234 -
22035 - -
22040 -pf01218 -
22045 -pf01520 -
22050 -pf00583 -
22055 -pf11143 -
22060 -pf06572 -
22065 -pf04261 -
22070 HMMnopfamclust3_1 RMM1
22075 - -
22080 -pf00528 -
BMC-P
BMC-H
BMC-T
Aldehyde dehydrogenase
PTAC
Signature enzyme
Alcohol dehydrogenase
Regulator

Shigella flexneri. Bacteria; Proteobacteria; Gammaproteobacteria; Enterobacterales;

DOU91__locus_1_main_sat

EUT1

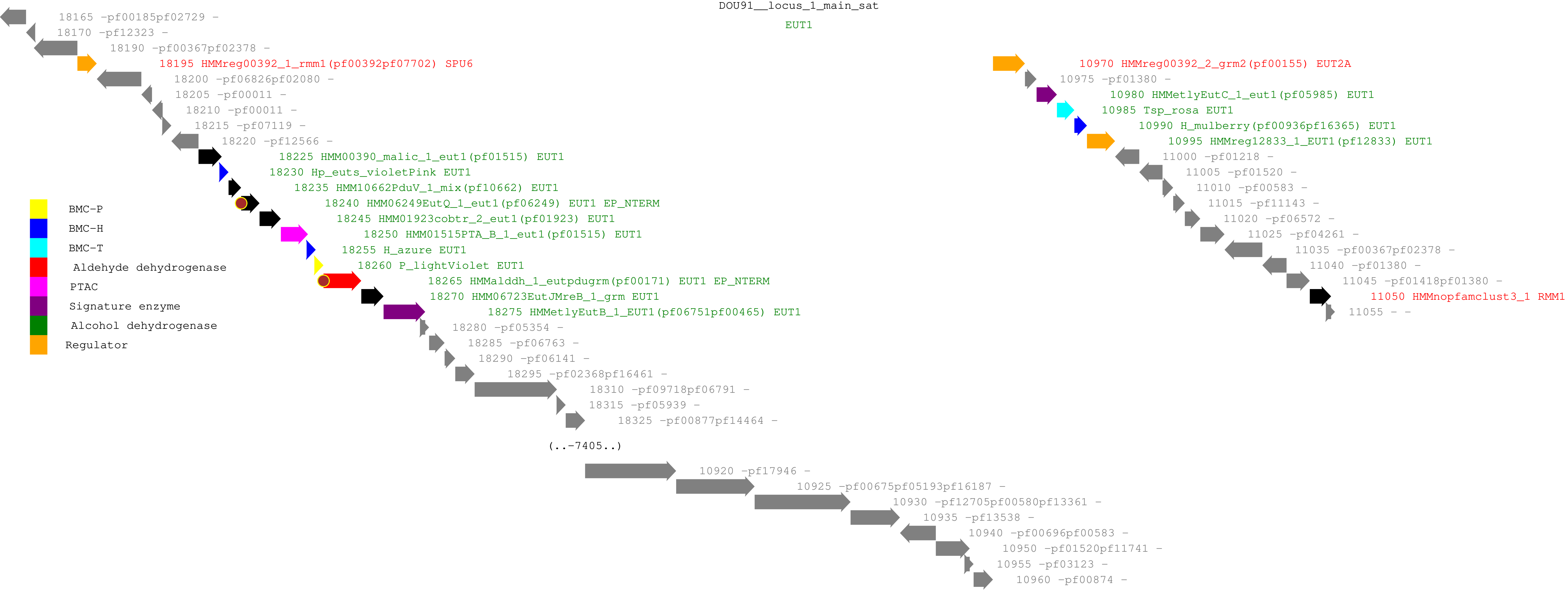

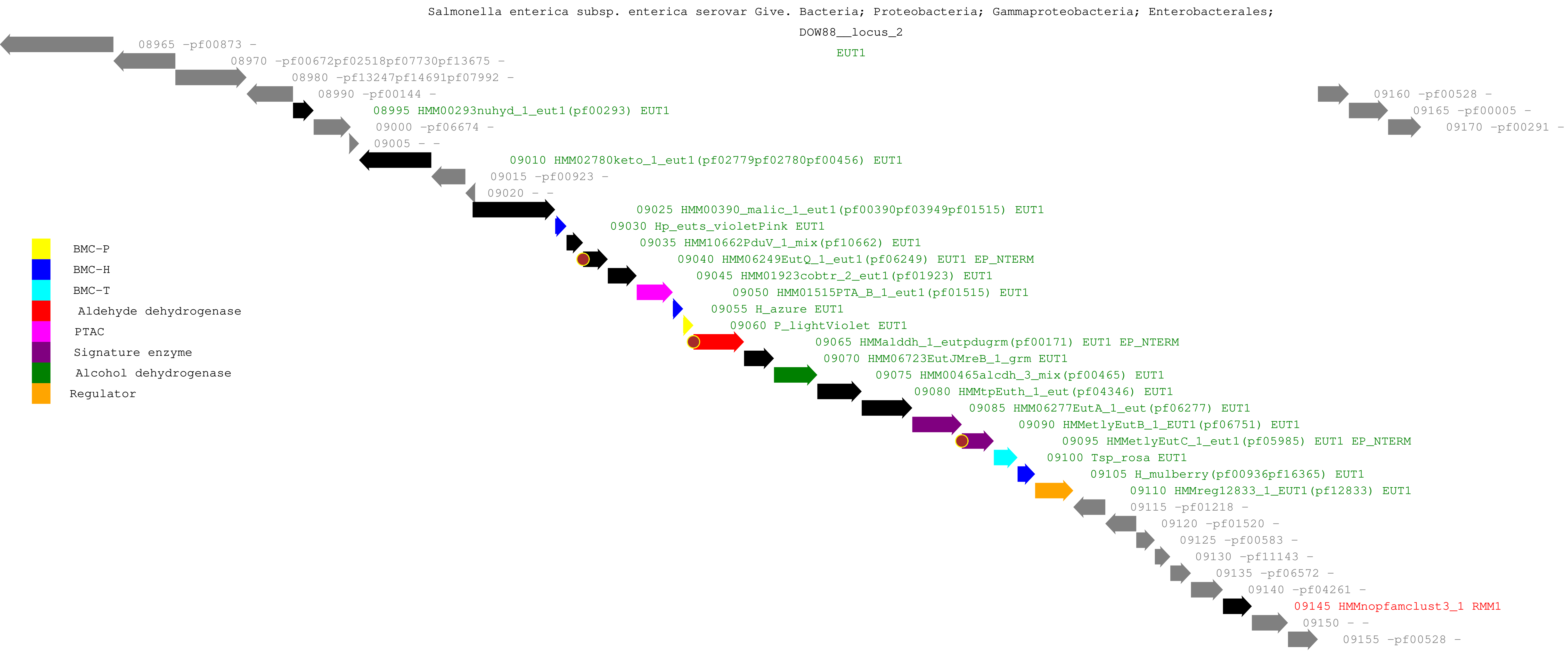
Salmonella enterica subsp. enterica serovar Give. Bacteria; Proteobacteria; Gammaproteobacteria; Enterobacterales;
DOW88__locus_2
EUT1
08965 -pf00873 -
08970 -pf00672pf02518pf07730pf13675 -
08980 -pf13247pf14691pf07992 -
08990 -pf00144 -
08995 HMM00293nuhyd_1_eut1(pf00293) EUT1
09000 -pf06674 -
09005 - -
09010 HMM02780keto_1_eut1(pf02779pf02780pf00456) EUT1
09015 -pf00923 -
09020 - -
09025 HMM00390_malic_1_eut1(pf00390pf03949pf01515) EUT1
09030 Hp_euts_violetPink EUT1
09035 HMM10662PduV_1_mix(pf10662) EUT1
09040 HMM06249EutQ_1_eut1(pf06249) EUT1 EP_NTERM
09045 HMM01923cobtr_2_eut1(pf01923) EUT1
09050 HMM01515PTA_B_1_eut1(pf01515) EUT1
09055 H_azure EUT1
09060 P_lightViolet EUT1
09065 HMMalddh_1_eutpdugrm(pf00171) EUT1 EP_NTERM
09070 HMM06723EutJMreB_1_grm EUT1
09075 HMM00465alcdh_3_mix(pf00465) EUT1
09080 HMMtpEuth_1_eut(pf04346) EUT1
09085 HMM06277EutA_1_eut(pf06277) EUT1
09090 HMMetlyEutB_1_EUT1(pf06751) EUT1
09095 HMMetlyEutC_1_eut1(pf05985) EUT1 EP_NTERM
09100 Tsp_rosa EUT1
09105 H_mulberry(pf00936pf16365) EUT1
09110 HMMreg12833_1_EUT1(pf12833) EUT1
09115 -pf01218 -
09120 -pf01520 -
09125 -pf00583 -
09130 -pf11143 -
09135 -pf06572 -
09140 -pf04261 -
09145 HMMnopfamclust3_1 RMM1
09150 - -
09155 -pf00528 -
09160 -pf00528 -
09165 -pf00005 -
09170 -pf00291 -
BMC-P
BMC-H
BMC-T
Aldehyde dehydrogenase
PTAC
Signature enzyme
Alcohol dehydrogenase
Regulator

Escherichia coli. Bacteria; Proteobacteria; Gammaproteobacteria; Enterobacterales;

DP277__locus_1

EUT1

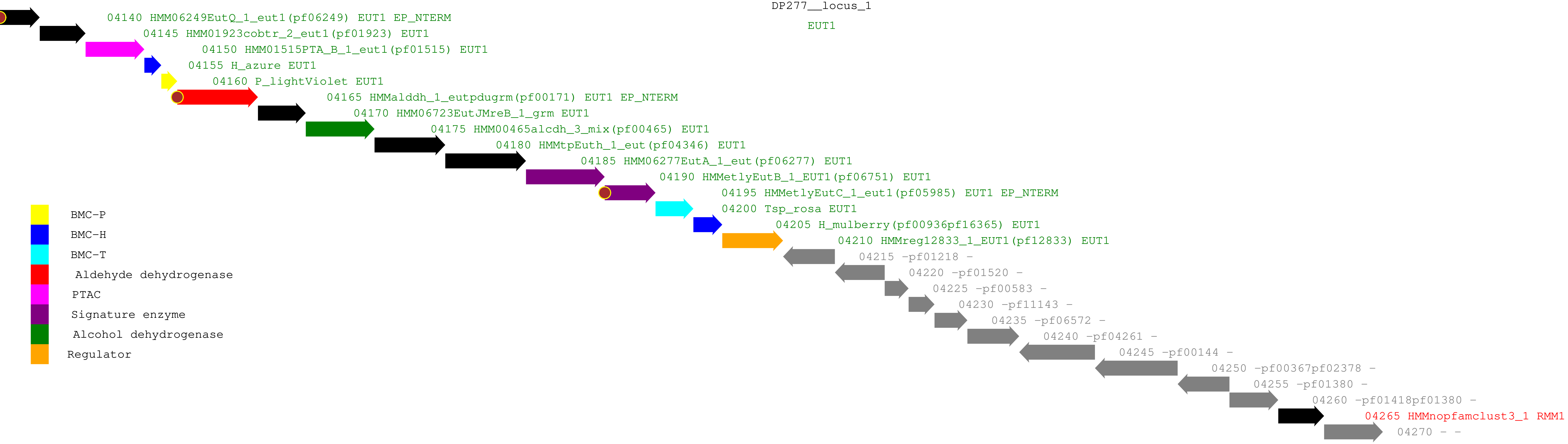

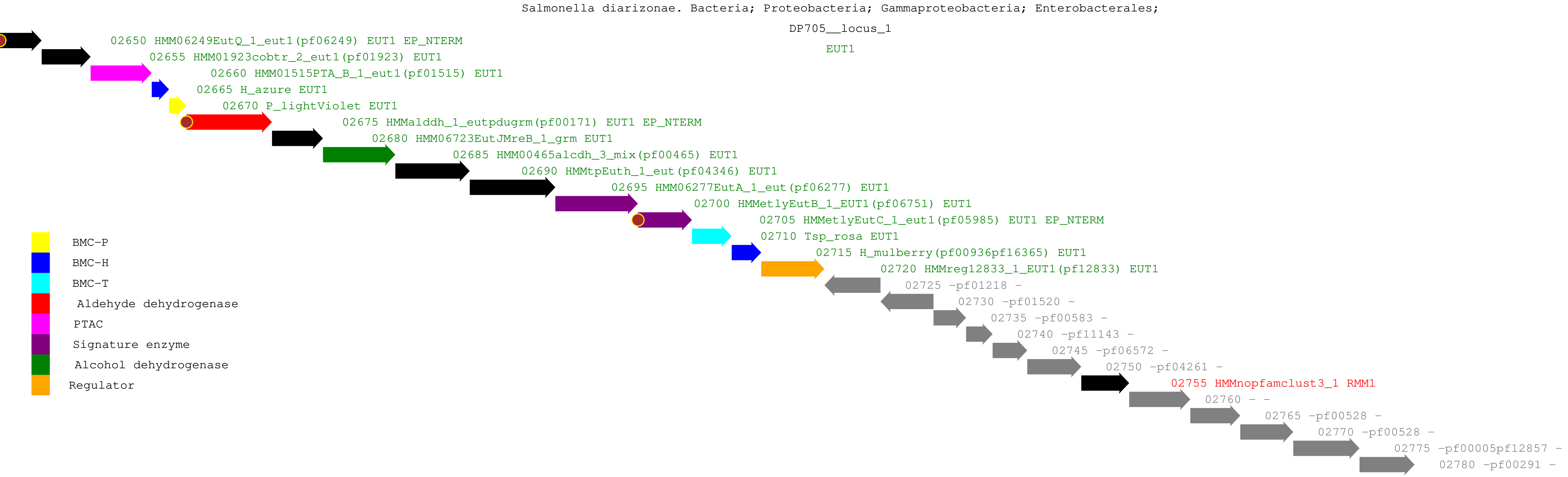
Salmonella diarizonae. Bacteria; Proteobacteria; Gammaproteobacteria; Enterobacterales;
DP705__locus_1
EUT1
02650 HMM06249EutQ_1_eut1(pf06249) EUT1 EP_NTERM
02655 HMM01923cobtr_2_eut1(pf01923) EUT1
02660 HMM01515PTA_B_1_eut1(pf01515) EUT1
02665 H_azure EUT1
02670 P_lightViolet EUT1
02675 HMMalddh_1_eutpdugrm(pf00171) EUT1 EP_NTERM
02680 HMM06723EutJMreB_1_grm EUT1
02685 HMM00465alcdh_3_mix(pf00465) EUT1
02690 HMMtpEuth_1_eut(pf04346) EUT1
02695 HMM06277EutA_1_eut(pf06277) EUT1
02700 HMMetlyEutB_1_EUT1(pf06751) EUT1
02705 HMMetlyEutC_1_eut1(pf05985) EUT1 EP_NTERM
02710 Tsp_rosa EUT1
02715 H_mulberry(pf00936pf16365) EUT1
02720 HMMreg12833_1_EUT1(pf12833) EUT1
02725 -pf01218 -
02730 -pf01520 -
02735 -pf00583 -
02740 -pf11143 -
02745 -pf06572 -
02750 -pf04261 -
02755 HMMnopfamclust3_1 RMM1
02760 - -
02765 -pf00528 -
02770 -pf00528 -
02775 -pf00005pf12857 -
02780 -pf00291 -
BMC-P
BMC-H
BMC-T
Aldehyde dehydrogenase
PTAC
Signature enzyme
Alcohol dehydrogenase
Regulator

Salmonella enterica I. Bacteria; Proteobacteria; Gammaproteobacteria; Enterobacterales;

DP727__locus_1

EUT1

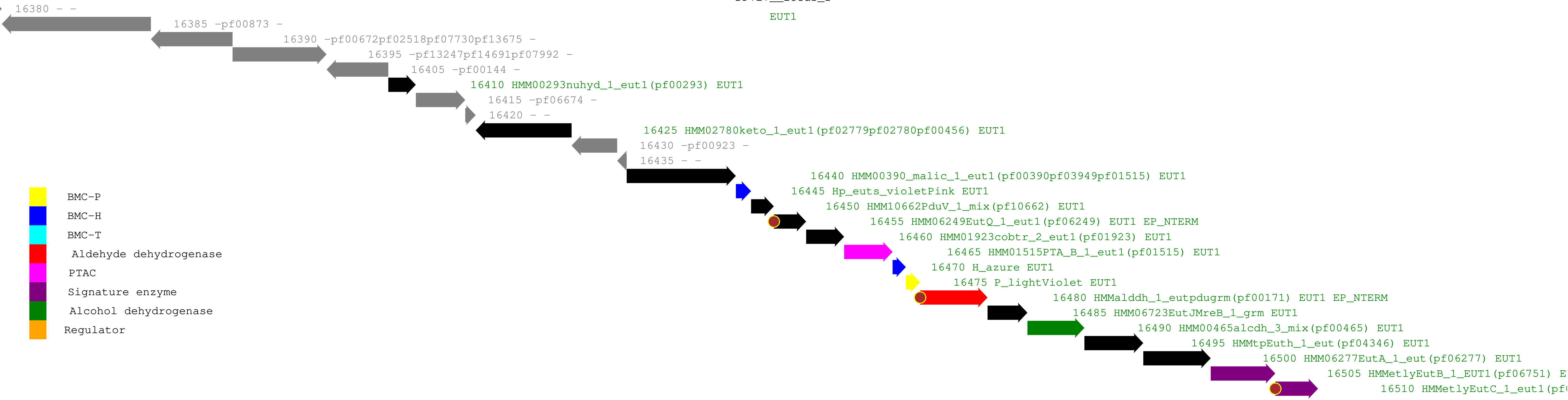

Salmonella enterica subsp. arizonae. Bacteria; Proteobacteria; Gammaproteobacteria; Enterobacterales;

DP773__locus_1

EUT1

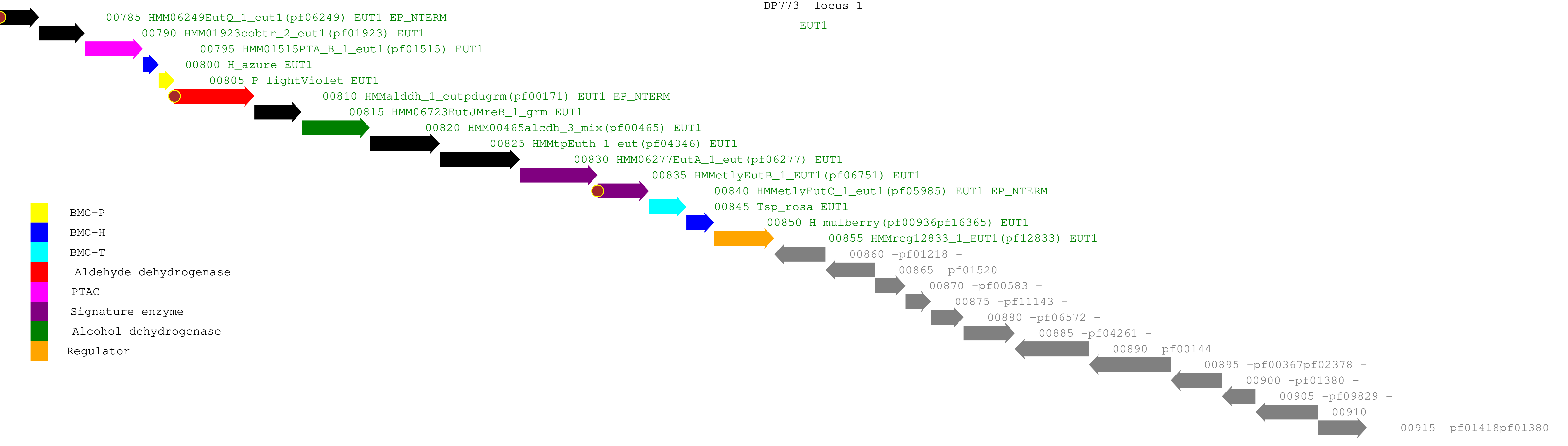

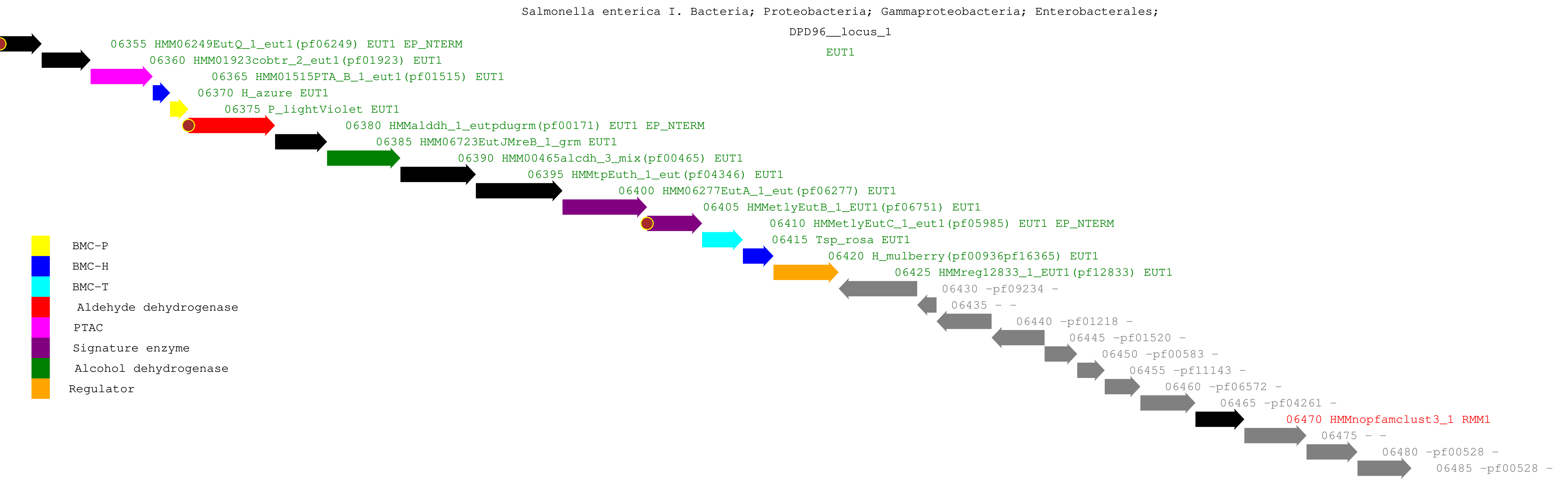

Salmonella enterica I. Bacteria; Proteobacteria; Gammaproteobacteria; Enterobacterales;
DPD96__locus_1
EUT1
06355 HMM06249EutQ_1_eut1(pf06249) EUT1 EP_NTERM
06360 HMM01923cobtr_2_eut1(pf01923) EUT1
06365 HMM01515PTA_B_1_eut1(pf01515) EUT1
06370 H_azure EUT1
06375 P_lightViolet EUT1
06380 HMMalddh_1_eutpdugrm(pf00171) EUT1 EP_NTERM
06385 HMM06723EutJMreB_1_grm EUT1
06390 HMM00465alcdh_3_mix(pf00465) EUT1
06395 HMMtpEuth_1_eut(pf04346) EUT1
06400 HMM06277EutA_1_eut(pf06277) EUT1
06405 HMMetlyEutB_1_EUT1(pf06751) EUT1
06410 HMMetlyEutC_1_eut1(pf05985) EUT1 EP_NTERM
06415 Tsp_rosa EUT1
06420 H_mulberry(pf00936pf16365) EUT1
06425 HMMreg12833_1_EUT1(pf12833) EUT1
06430 -pf09234 -
06435 - -
06440 -pf01218 -
06445 -pf01520 -
06450 -pf00583 -
06455 -pf11143 -
06460 -pf06572 -
06465 -pf04261 -
06470 HMMnopfamclust3_1 RMM1
06475 - -
06480 -pf00528 -
06485 -pf00528 -
BMC-P
BMC-H
BMC-T
Aldehyde dehydrogenase
PTAC
Signature enzyme
Alcohol dehydrogenase
Regulator

Salmonella diarizonae. Bacteria; Proteobacteria; Gammaproteobacteria; Enterobacterales;

DPF13__locus_1

EUT1

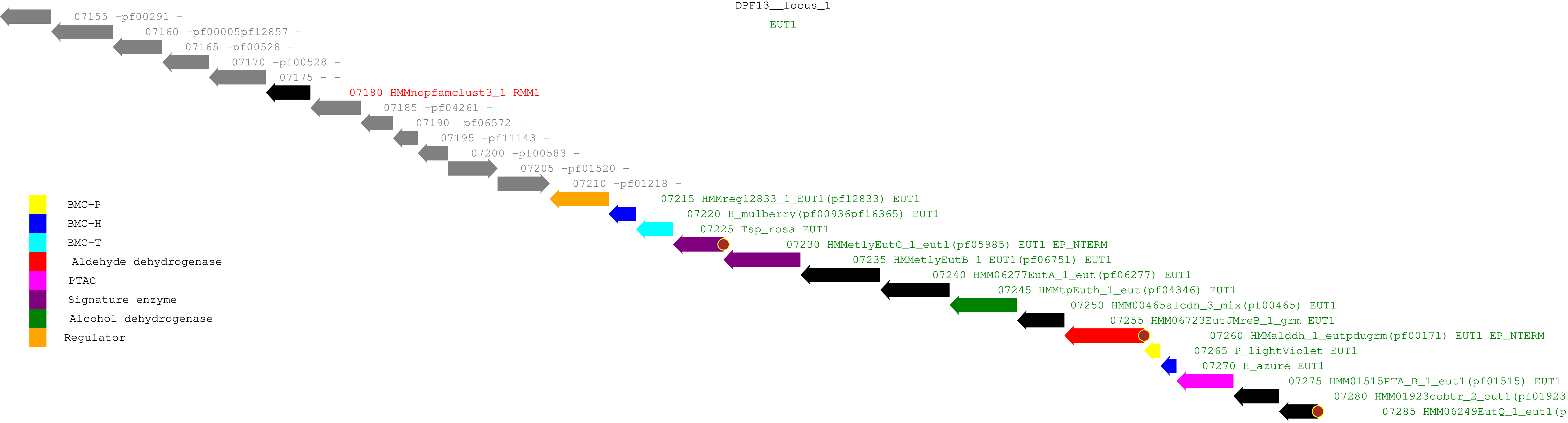

Salmonella enterica I. Bacteria; Proteobacteria; Gammaproteobacteria; Enterobacterales;

DPK49__locus_1

EUT1

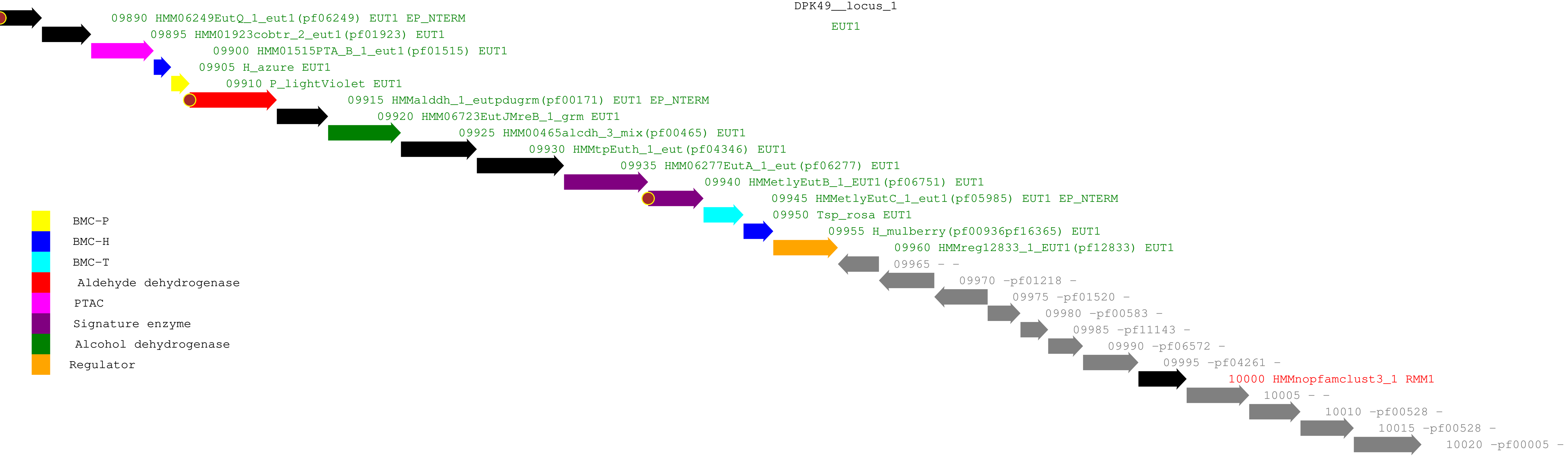

Salmonella enteritidis. Bacteria; Proteobacteria; Gammaproteobacteria; Enterobacterales;

DPK75__locus_1

EUT1

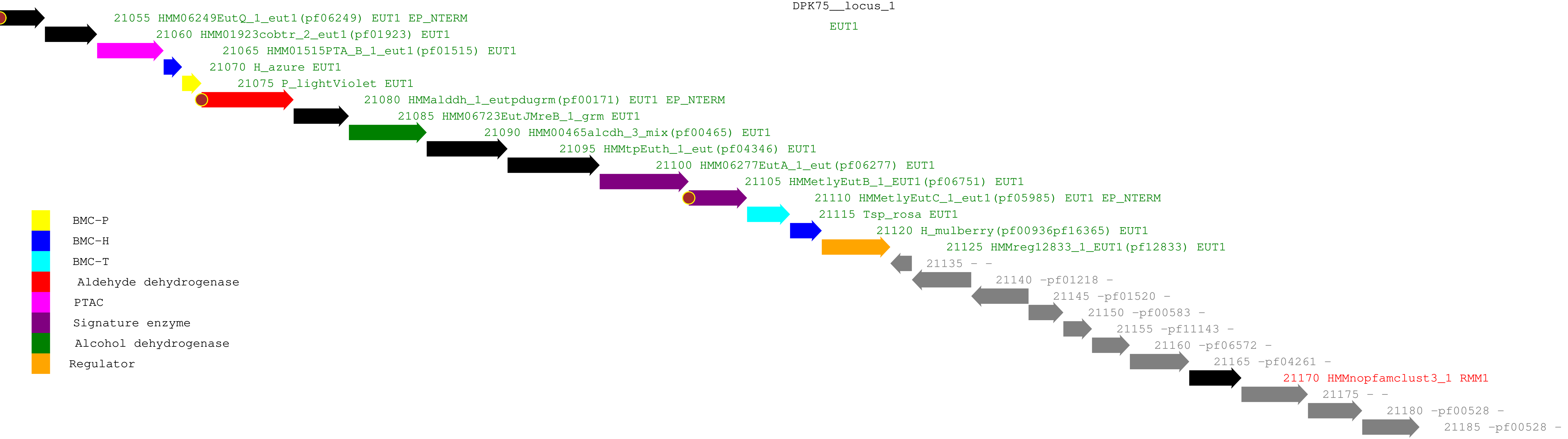

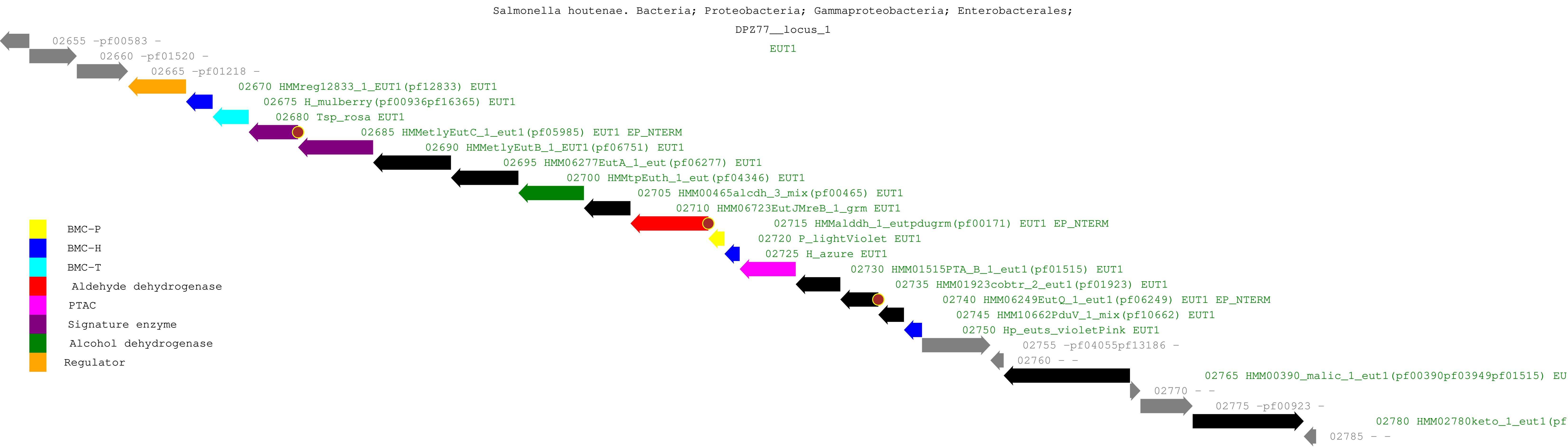

Salmonella houtenae. Bacteria; Proteobacteria; Gammaproteobacteria; Enterobacterales;
DPZ77__locus_1
EUT1
02655 -pf00583 -
02660 -pf01520 -
02665 -pf01218 -
02670 HMMreg12833_1_EUT1(pf12833) EUT1
02675 H_mulberry(pf00936pf16365) EUT1
02680 Tsp_rosa EUT1
02685 HMMetlyEutC_1_eut1(pf05985) EUT1 EP_NTERM
02690 HMMetlyEutB_1_EUT1(pf06751) EUT1
02695 HMM06277EutA_1_eut(pf06277) EUT1
02700 HMMtpEuth_1_eut(pf04346) EUT1
02705 HMM00465alcdh_3_mix(pf00465) EUT1
02710 HMM06723EutJmreB_1_grm EUT1
02715 HMMalddh_1_eutpdugrm(pf00171) EUT1 EP_NTERM
02720 P_lightViolet EUT1
02725 H_azure EUT1
02730 HMM01515PTA_B_1_eut1(pf01515) EUT1
02735 HMM01923cobtr_2_eut1(pf01923) EUT1
02740 HMM06249EutQ_1_eut1(pf06249) EUT1 EP_NTERM
02745 HMM10662PduV_1_mix(pf10662) EUT1
02750 Hp_euts_violetPink EUT1
02755 -pf04055pf13186 -
02760 - -
02765 HMM00390_malic_1_eut1(pf00390pf03949pf01515) EU
02770 - -
02775 -pf00923 -
02780 HMM02780keto_1_eut1(pf
02785 - -
BMC-P
BMC-H
BMC-T
Aldehyde dehydrogenase
PTAC
Signature enzyme
Alcohol dehydrogenase
Regulator

Salmonella enterica subsp. salamae. Bacteria; Proteobacteria; Gammaproteobacteria; Enterobacterales;

DQ004__locus_1

EUT1

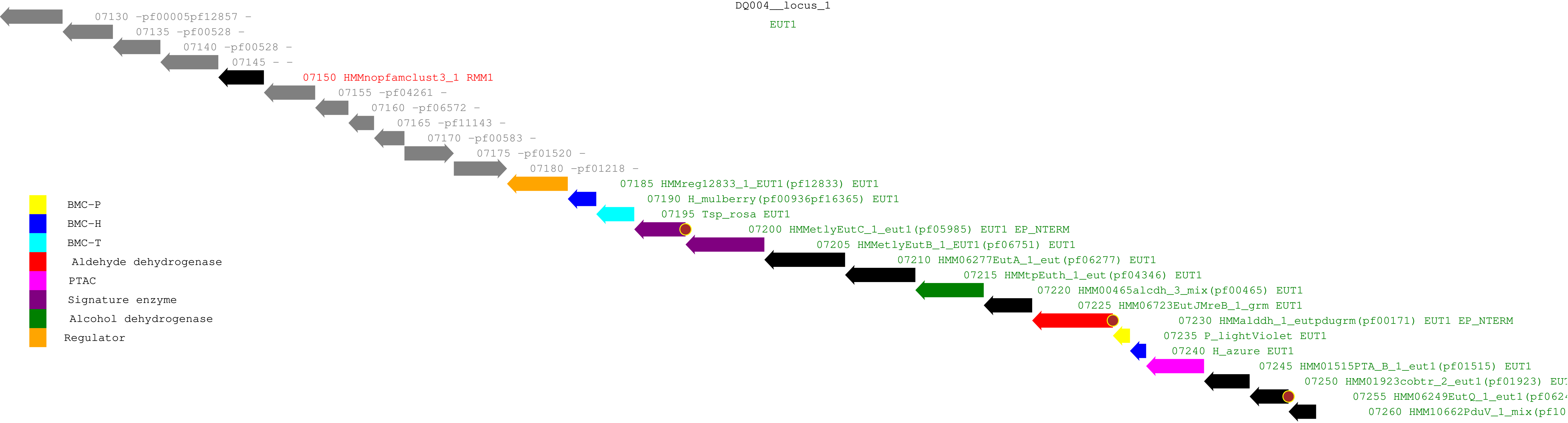

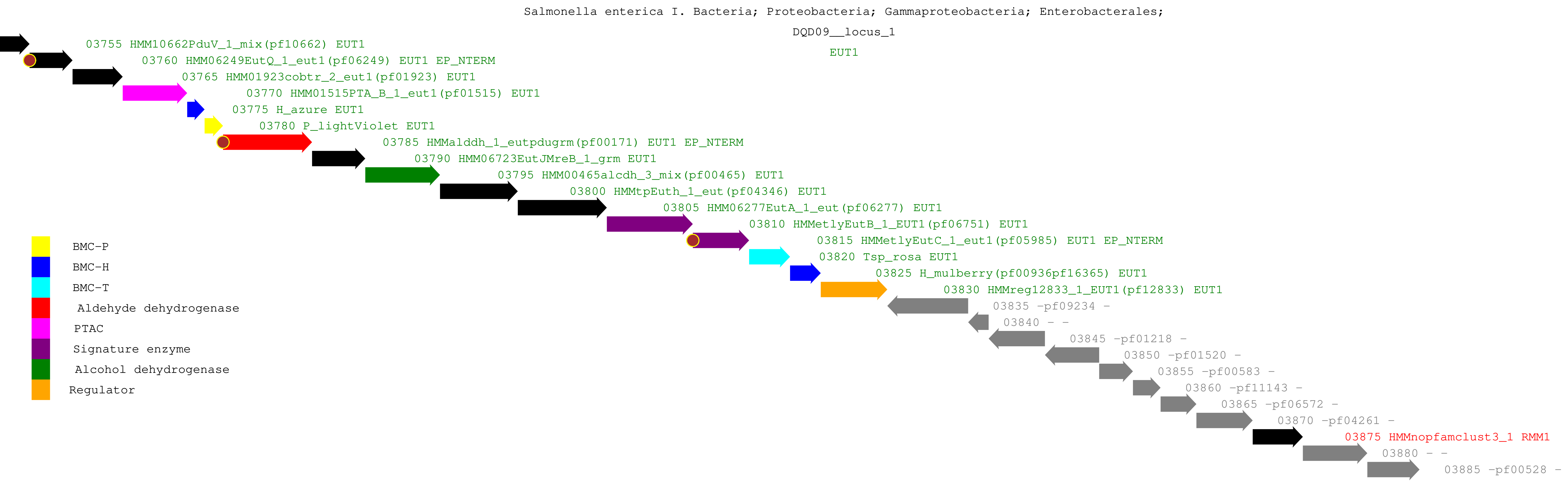
Salmonella enterica I. Bacteria; Proteobacteria; Gammaproteobacteria; Enterobacterales;
DQD09__locus_1
EUT1
03755 HMM10662PduV_1_mix(pf10662) EUT1
03760 HMM06249EutQ_1_eut1(pf06249) EUT1 EP_NTERM
03765 HMM01923cobtr_2_eut1(pf01923) EUT1
03770 HMM01515PTA_B_1_eut1(pf01515) EUT1
03775 H_azure EUT1
03780 P_lightViolet EUT1
03785 HMMalddh_1_eutpdugrm(pf00171) EUT1 EP_NTERM
03790 HMM06723EutJMreB_1_grm EUT1
03795 HMM00465alcdh_3_mix(pf00465) EUT1
03800 HMMtpEuth_1_eut(pf04346) EUT1
03805 HMM06277EutA_1_eut(pf06277) EUT1
03810 HMMetlyEutB_1_EUT1(pf06751) EUT1
03815 HMMetlyEutC_1_eut1(pf05985) EUT1 EP_NTERM
03820 Tsp_rosa EUT1
03825 H_mulberry(pf00936pf16365) EUT1
03830 HMMreg12833_1_EUT1(pf12833) EUT1
03835 -pf09234 -
03840 - -
03845 -pf01218 -
03850 -pf01520 -
03855 -pf00583 -
03860 -pf11143 -
03865 -pf06572 -
03870 -pf04261 -
03875 HMMnopfamclust3_1 RMM1
03880 - -
03885 -pf00528 -
BMC-P
BMC-H
BMC-T
Aldehyde dehydrogenase
PTAC
Signature enzyme
Alcohol dehydrogenase
Regulator

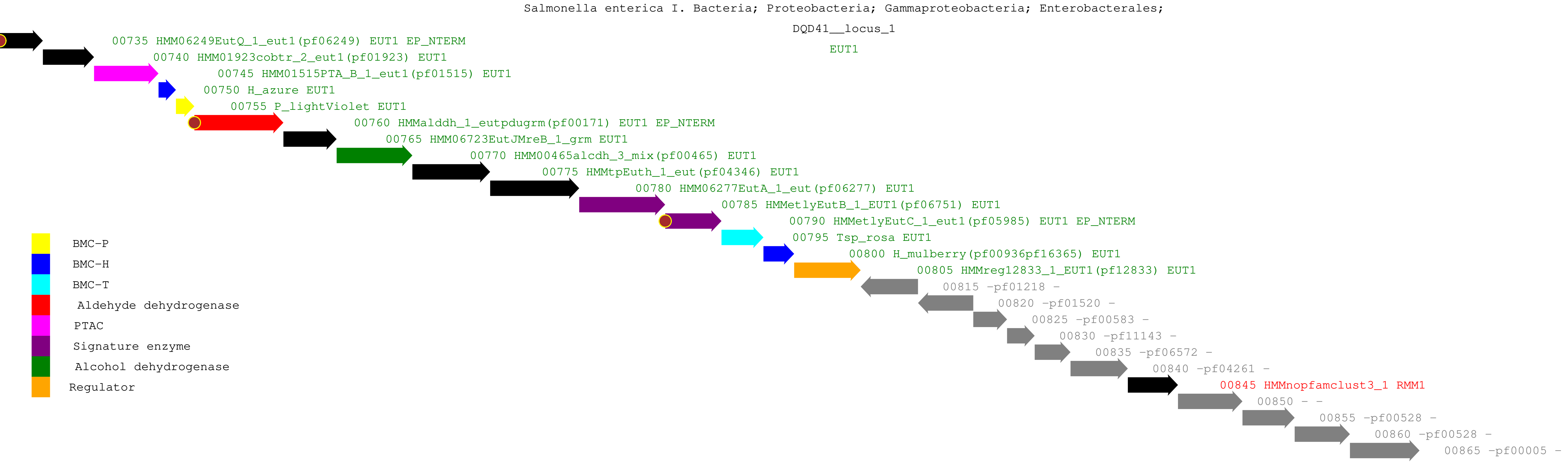
Salmonella enterica I. Bacteria; Proteobacteria; Gammaproteobacteria; Enterobacterales;
DQD41__locus_1
EUT1
00735 HMM06249EutQ_1_eut1(pf06249) EUT1 EP_NTERM
00740 HMM01923cobtr_2_eut1(pf01923) EUT1
00745 HMM01515PTA_B_1_eut1(pf01515) EUT1
00750 H_azure EUT1
00755 P_lightViolet EUT1
00760 HMMaldh_1_eutpdugrm(pf00171) EUT1 EP_NTERM
00765 HMM06723EutJMreB_1_grm EUT1
00770 HMM00465alcdh_3_mix(pf00465) EUT1
00775 HMMtpEuth_1_eut(pf04346) EUT1
00780 HMM06277EutA_1_eut(pf06277) EUT1
00785 HMMetlyEutB_1_EUT1(pf06751) EUT1
00790 HMMetlyEutC_1_eut1(pf05985) EUT1 EP_NTERM
00795 Tsp_rosa EUT1
00800 H_mulberry(pf00936pf16365) EUT1
00805 HMMreg12833_1_EUT1(pf12833) EUT1
00815 -pf01218 -
00820 -pf01520 -
00825 -pf00583 -
00830 -pf11143 -
00835 -pf06572 -
00840 -pf04261 -
00845 HMMnopfamclust3_1 RMM1
00850 - -
00855 -pf00528 -
00860 -pf00528 -
00865 -pf00005 -
BMC-P
BMC-H
BMC-T
Aldehyde dehydrogenase
PTAC
Signature enzyme
Alcohol dehydrogenase
Regulator

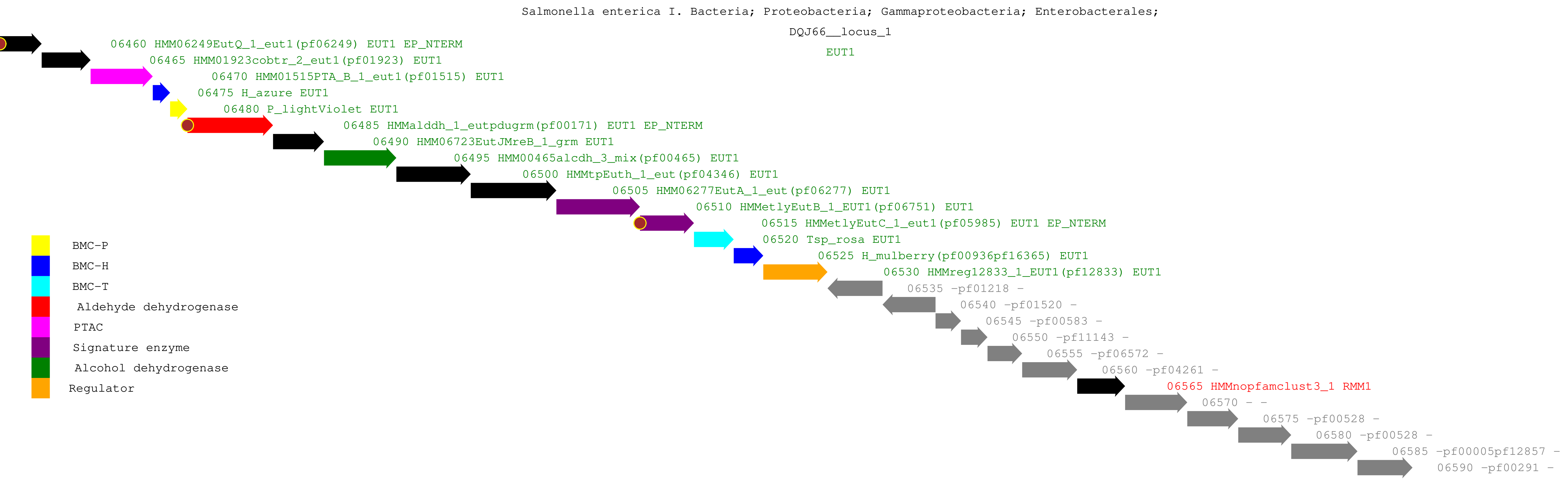

Salmonella enterica I. Bacteria; Proteobacteria; Gammaproteobacteria; Enterobacterales;
DQJ66__locus_1
EUT1
06460 HMM06249EutQ_1_eut1(pf06249) EUT1 EP_NTERM
06465 HMM01923cobtr_2_eut1(pf01923) EUT1
06470 HMM01515PTA_B_1_eut1(pf01515) EUT1
06475 H_azure EUT1
06480 P_lightViolet EUT1
06485 HMMalddh_1_eutpdugrm(pf00171) EUT1 EP_NTERM
06490 HMM06723EutJMreB_1_grm EUT1
06495 HMM00465alcdh_3_mix(pf00465) EUT1
06500 HMMtpEuth_1_eut(pf04346) EUT1
06505 HMM06277EutA_1_eut(pf06277) EUT1
06510 HMMetlyEutB_1_EUT1(pf06751) EUT1
06515 HMMetlyEutC_1_eut1(pf05985) EUT1 EP_NTERM
06520 Tsp_rosa EUT1
06525 H_mulberry(pf00936pf16365) EUT1
06530 HMMreg12833_1_EUT1(pf12833) EUT1
06535 -pf01218 -
06540 -pf01520 -
06545 -pf00583 -
06550 -pf11143 -
06555 -pf06572 -
06560 -pf04261 -
06565 HMMnopfamclust3_1 RMM1
06570 - -
06575 -pf00528 -
06580 -pf00528 -
06585 -pf00005pf12857 -
06590 -pf00291 -
BMC-P
BMC-H
BMC-T
Aldehyde dehydrogenase
PTAC
Signature enzyme
Alcohol dehydrogenase
Regulator

Salmonella enterica I. Bacteria; Proteobacteria; Gammaproteobacteria; Enterobacterales;

DQJ71__locus_1

EUT1

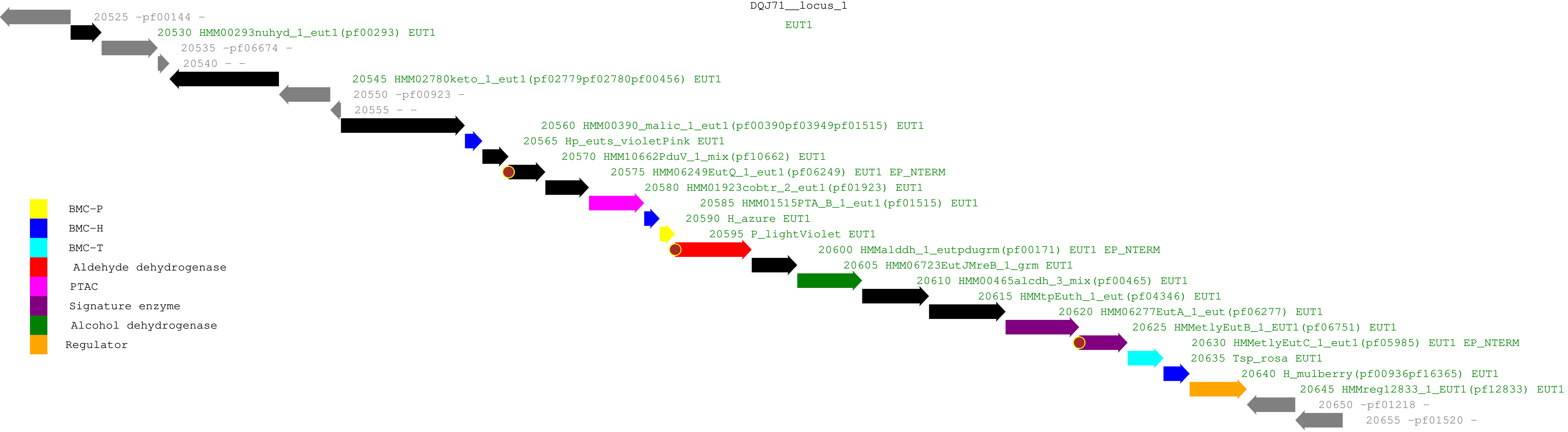

Salmonella enterica subsp. enterica serovar Havana. Bacteria; Proteobacteria; Gammaproteobacteria; Enterobacterales;

DQK02__locus_1

EUT1

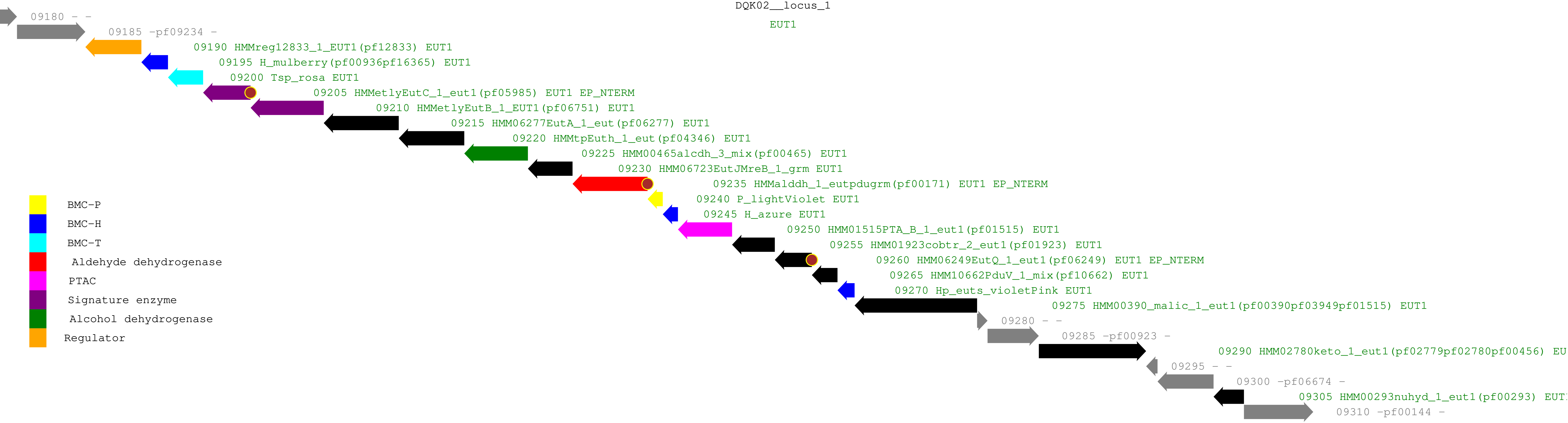

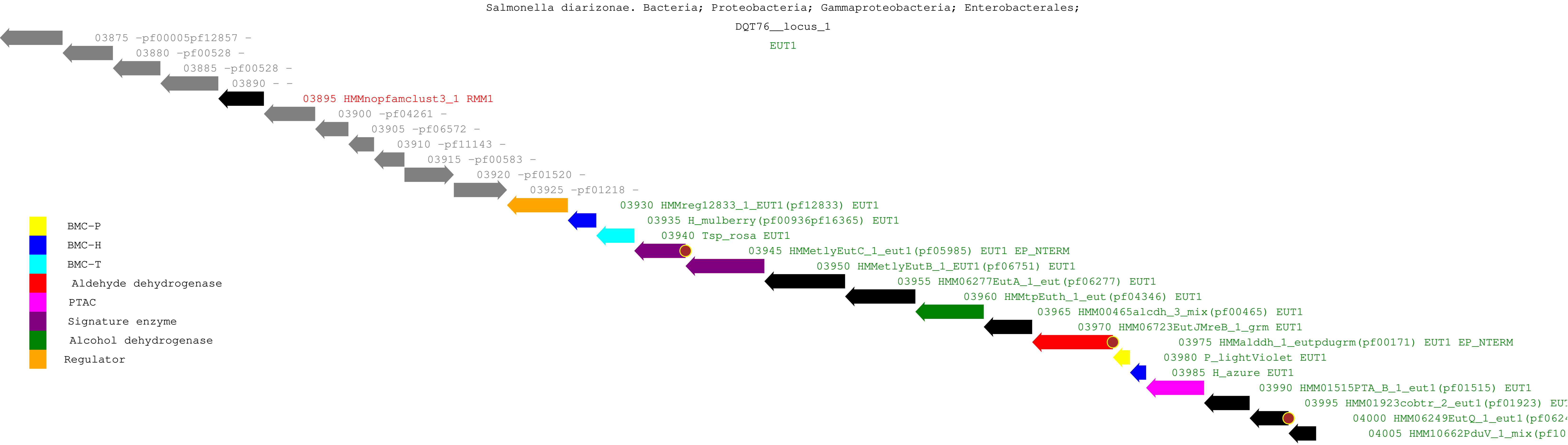

Salmonella diarizonae. Bacteria; Proteobacteria; Gammaproteobacteria; Enterobacterales;
DQT76__locus_1
EUT1
03875 -pf00005pf12857 -
03880 -pf00528 -
03885 -pf00528 -
03890 - -
03895 HMMnopfamclust3_1 RMM1
03900 -pf04261 -
03905 -pf06572 -
03910 -pf11143 -
03915 -pf00583 -
03920 -pf01520 -
03925 -pf01218 -
03930 HMMreg12833_1_EUT1(pf12833) EUT1
03935 H_mulberry(pf00936pf16365) EUT1
03940 Tsp_rosa EUT1
03945 HMMetlyEutC_1_eut1(pf05985) EUT1 EP_NTERM
03950 HMMetlyEutB_1_EUT1(pf06751) EUT1
03955 HMM06277EutA_1_eut(pf06277) EUT1
03960 HMMtpEuth_1_eut(pf04346) EUT1
03965 HMM00465alcdh_3_mix(pf00465) EUT1
03970 HMM06723EutJMreB_1_grm EUT1
03975 HMMaldh_1_eutpdugrm(pf00171) EUT1 EP_NTERM
03980 P_lightViolet EUT1
03985 H_azure EUT1
03990 HMM01515PTA_B_1_eut1(pf01515) EUT1
03995 HMM01923cobtr_2_eut1(pf01923) EU
04000 HMM06249EutQ_1_eut1(pf062
04005 HMM10662PduV_1_mix(pf10
BMC-P
BMC-H
BMC-T
Aldehyde dehydrogenase
PTAC
Signature enzyme
Alcohol dehydrogenase
Regulator

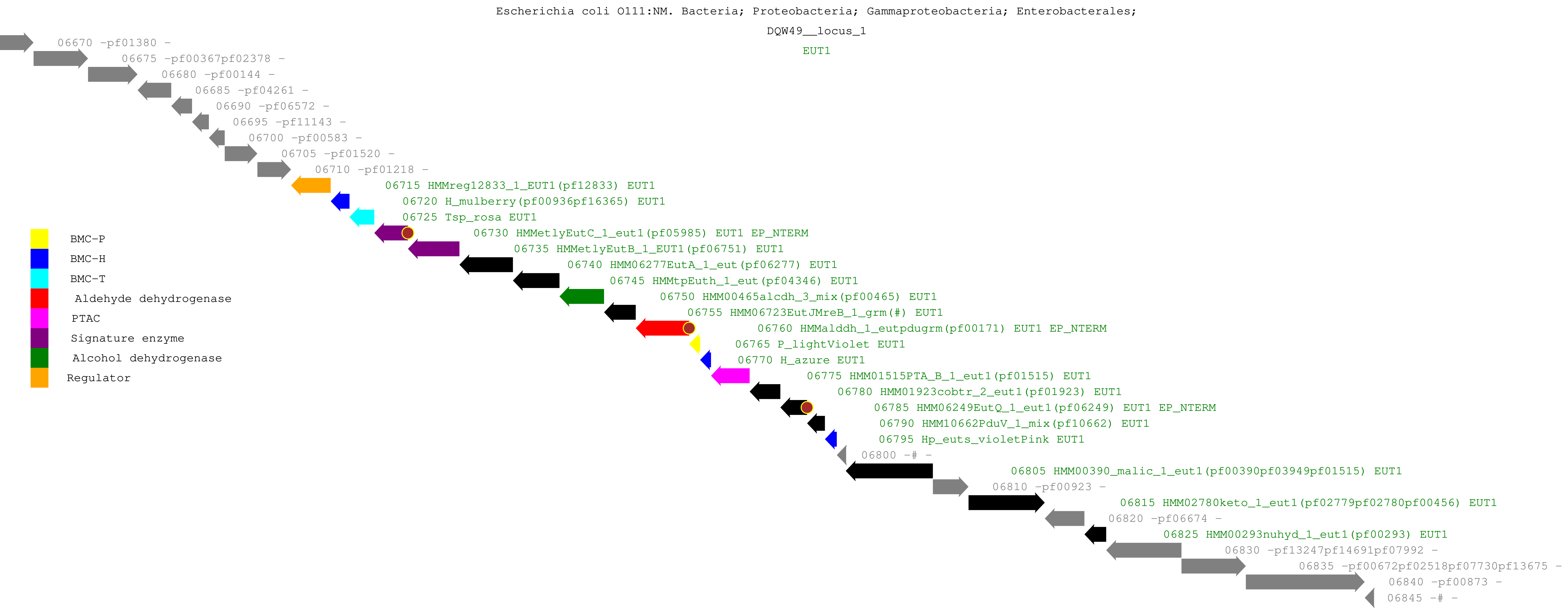

Escherichia coli O111:NM. Bacteria; Proteobacteria; Gammaproteobacteria; Enterobacterales;
DQW49__locus_1
EUT1
06670 -pf01380 -
06675 -pf00367pf02378 -
06680 -pf00144 -
06685 -pf04261 -
06690 -pf06572 -
06695 -pf11143 -
06700 -pf00583 -
06705 -pf01520 -
06710 -pf01218 -
06715 HMMreg12833_1_EUT1(pf12833) EUT1
06720 H_mulberry(pf00936pf16365) EUT1
06725 Tsp_rosa EUT1
06730 HMMetlyEutC_1_eut1(pf05985) EUT1 EP_NTERM
06735 HMMetlyEutB_1_EUT1(pf06751) EUT1
06740 HMM06277EutA_1_eut(pf06277) EUT1
06745 HMMtpEuth_1_eut(pf04346) EUT1
06750 HMM00465alcdh_3_mix(pf00465) EUT1
06755 HMM06723EutJMreB_1_grm(#) EUT1
06760 HMMalddh_1_eutpdugrm(pf00171) EUT1 EP_NTERM
06765 P_lightViolet EUT1
06770 H_azure EUT1
06775 HMM01515PTA_B_1_eut1(pf01515) EUT1
06780 HMM01923cobtr_2_eut1(pf01923) EUT1
06785 HMM06249EutQ_1_eut1(pf06249) EUT1 EP_NTERM
06790 HMM10662PduV_1_mix(pf10662) EUT1
06795 Hp_euts_violetPink EUT1
06800 -# -
06805 HMM00390_malic_1_eut1(pf00390pf03949pf01515) EUT1
06810 -pf00923 -
06815 HMM02780keto_1_eut1(pf02779pf02780pf00456) EUT1
06820 -pf06674 -
06825 HMM00293nuhyd_1_eut1(pf00293) EUT1
06830 -pf13247pf14691pf07992 -
06835 -pf00672pf02518pf07730pf13675 -
06840 -pf00873 -
06845 -# -
BMC-P
BMC-H
BMC-T
Aldehyde dehydrogenase
PTAC
Signature enzyme
Alcohol dehydrogenase
Regulator

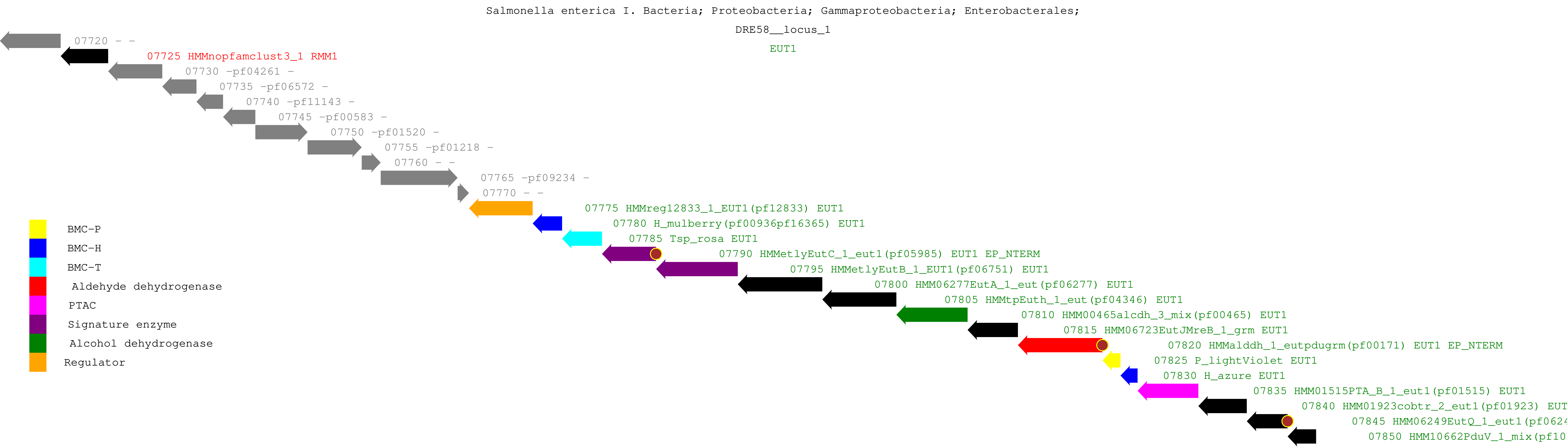
Salmonella enterica I. Bacteria; Proteobacteria; Gammaproteobacteria; Enterobacterales;
DRE58__locus_1
EUT1
07720 - -
07725 HMMnopfamclust3_1 RMM1
07730 -pf04261 -
07735 -pf06572 -
07740 -pf11143 -
07745 -pf00583 -
07750 -pf01520 -
07755 -pf01218 -
07760 - -
07765 -pf09234 -
07770 - -
07775 HMMreg12833_1_EUT1(pf12833) EUT1
07780 H_mulberry(pf00936pf16365) EUT1
07785 Tsp_rosa EUT1
07790 HMMetlyEutC_1_eut1(pf05985) EUT1 EP_NTERM
07795 HMMetlyEutB_1_EUT1(pf06751) EUT1
07800 HMM06277EutA_1_eut(pf06277) EUT1
07805 HMMtpEuth_1_eut(pf04346) EUT1
07810 HMM00465alcdh_3_mix(pf00465) EUT1
07815 HMM06723EutJMreB_1_grm EUT1
07820 HMMaldh_1_eutpdugrm(pf00171) EUT1 EP_NTERM
07825 P_lightViolet EUT1
07830 H_azure EUT1
07835 HMM01515PTA_B_1_eut1(pf01515) EUT1
07840 HMM01923cobtr_2_eut1(pf01923) EUT
07845 HMM06249EutQ_1_eut1(pf062
07850 HMM10662PduV_1_mix(pf10
BMC-P
BMC-H
BMC-T
Aldehyde dehydrogenase
PTAC
Signature enzyme
Alcohol dehydrogenase
Regulator

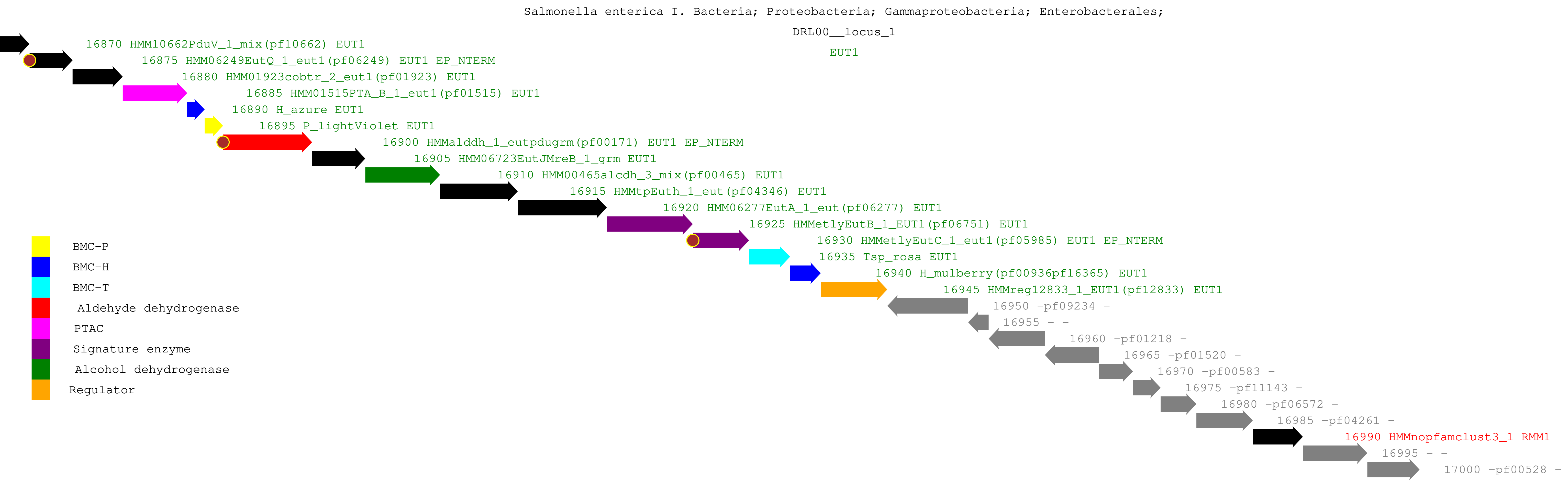

Salmonella enterica I. Bacteria; Proteobacteria; Gammaproteobacteria; Enterobacterales;
DRL00__locus_1
EUT1
16870 HMM10662PduV_1_mix(pf10662) EUT1
16875 HMM06249EutQ_1_eut1(pf06249) EUT1 EP_NTERM
16880 HMM01923cobtr_2_eut1(pf01923) EUT1
16885 HMM01515PTA_B_1_eut1(pf01515) EUT1
16890 H_azure EUT1
16895 P_lightViolet EUT1
16900 HMMalddh_1_eutpdugrm(pf00171) EUT1 EP_NTERM
16905 HMM06723EutJMreB_1_grm EUT1
16910 HMM00465alcdh_3_mix(pf00465) EUT1
16915 HMMtpEuth_1_eut(pf04346) EUT1
16920 HMM06277EutA_1_eut(pf06277) EUT1
16925 HMMetlyEutB_1_EUT1(pf06751) EUT1
16930 HMMetlyEutC_1_eut1(pf05985) EUT1 EP_NTERM
16935 Tsp_rosa EUT1
16940 H_mulberry(pf00936pf16365) EUT1
16945 HMMreg12833_1_EUT1(pf12833) EUT1
16950 -pf09234 -
16955 - -
16960 -pf01218 -
16965 -pf01520 -
16970 -pf00583 -
16975 -pf11143 -
16980 -pf06572 -
16985 -pf04261 -
16990 HMMnopfamclust3_1 RMM1
16995 - -
17000 -pf00528 -
BMC-P
BMC-H
BMC-T
Aldehyde dehydrogenase
PTAC
Signature enzyme
Alcohol dehydrogenase
Regulator

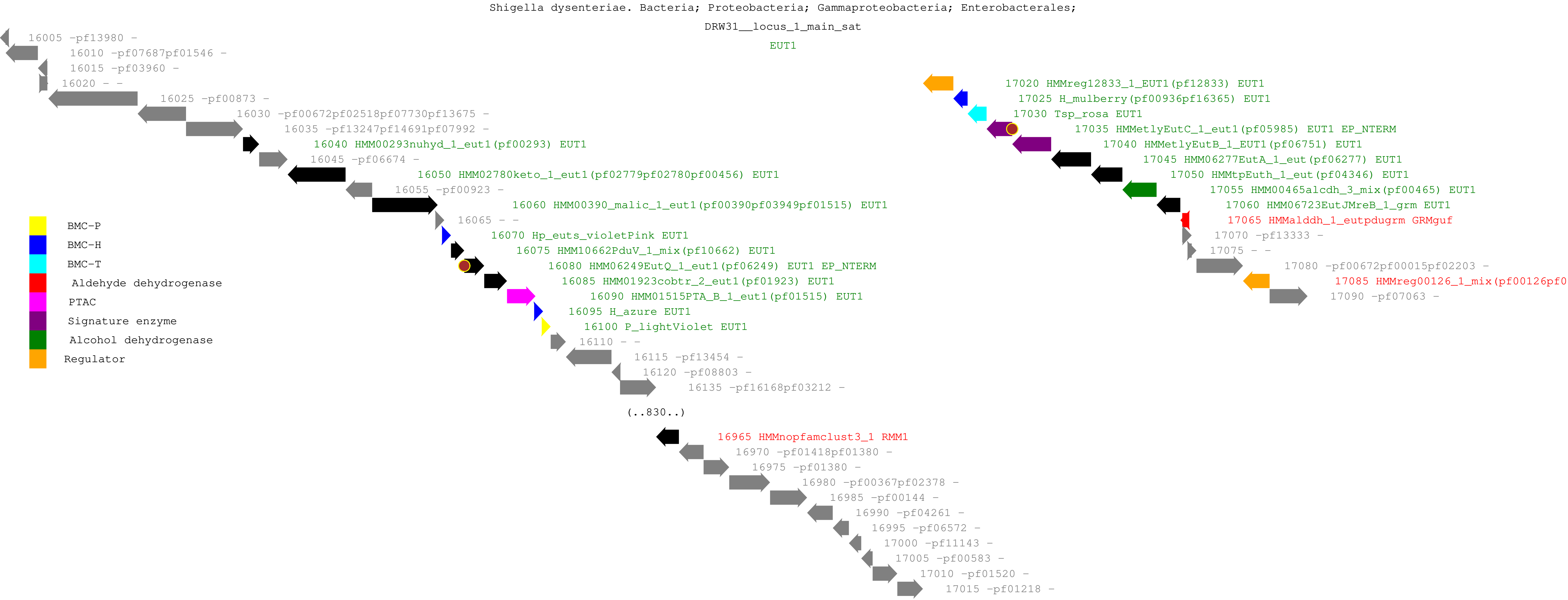

Shigella dysenteriae. Bacteria; Proteobacteria; Gammaproteobacteria; Enterobacterales;
DRW31__locus_1_main_sat
EUT1
16005 -pf13980 -
16010 -pf07687pf01546 -
16015 -pf03960 -
16020 - -
16025 -pf00873 -
16030 -pf00672pf02518pf07730pf13675 -
16035 -pf13247pf14691pf07992 -
16040 HMM00293nuhyd_1_eut1(pf00293) EUT1
16045 -pf06674 -
16050 HMM02780keto_1_eut1(pf02779pf02780pf00456) EUT1
16055 -pf00923 -
16060 HMM00390_malic_1_eut1(pf00390pf03949pf01515) EUT1
16065 - -
16070 Hp_euts_violetPink EUT1
16075 HMM10662PduV_1_mix(pf10662) EUT1
16080 HMM06249EutQ_1_eut1(pf06249) EUT1 EP_NTERM
16085 HMM01923cobtr_2_eut1(pf01923) EUT1
16090 HMM01515PTA_B_1_eut1(pf01515) EUT1
16095 H_azure EUT1
16100 P_lightViolet EUT1
16110 - -
16115 -pf13454 -
16120 -pf08803 -
16135 -pf16168pf03212 -
(..830..)
16965 HMMnopfamclust3_1 RMM1
16970 -pf01418pf01380 -
16975 -pf01380 -
16980 -pf00367pf02378 -
16985 -pf00144 -
16990 -pf04261 -
16995 -pf06572 -
17000 -pf11143 -
17005 -pf00583 -
17010 -pf01520 -
17015 -pf01218 -
17020 HMMreg12833_1_EUT1(pf12833) EUT1
17025 H_mulberry(pf00936pf16365) EUT1
17030 Tsp_rosa EUT1
17035 HMMetlyEutC_1_eut1(pf05985) EUT1 EP_NTERM
17040 HMMetlyEutB_1_EUT1(pf06751) EUT1
17045 HMM06277EutA_1_eut(pf06277) EUT1
17050 HMMtpEuth_1_eut(pf04346) EUT1
17055 HMM00465alcdh_3_mix(pf00465) EUT1
17060 HMM06723EutJMreB_1_grm EUT1
17065 HMMalddh_1_eutpdugrm GRMguf
17070 -pf13333 -
17075 - -
17080 -pf00672pf00015pf02203 -
17085 HMMreg00126_1_mix(pf00126pf0
17090 -pf07063 -
BMC-P
BMC-H
BMC-T
Aldehyde dehydrogenase
PTAC
Signature enzyme
Alcohol dehydrogenase
Regulator

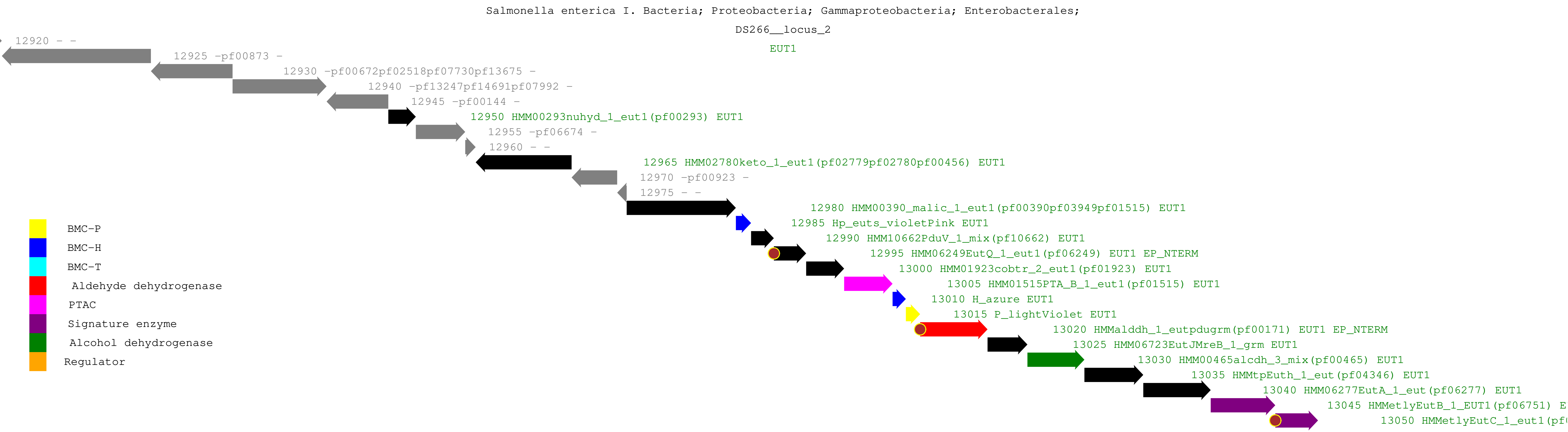

Salmonella enterica I. Bacteria; Proteobacteria; Gammaproteobacteria; Enterobacterales;
DS266__locus_2
EUT1
12920 - -
12925 -pf00873 -
12930 -pf00672pf02518pf07730pf13675 -
12940 -pf13247pf14691pf07992 -
12945 -pf00144 -
12950 HMM00293nuhyd_1_eut1(pf00293) EUT1
12955 -pf06674 -
12960 - -
12965 HMM02780keto_1_eut1(pf02779pf02780pf00456) EUT1
12970 -pf00923 -
12975 - -
12980 HMM00390_malic_1_eut1(pf00390pf03949pf01515) EUT1
12985 Hp_euts_violetPink EUT1
12990 HMM10662PduV_1_mix(pf10662) EUT1
12995 HMM06249EutQ_1_eut1(pf06249) EUT1 EP_NTERM
13000 HMM01923cobtr_2_eut1(pf01923) EUT1
13005 HMM01515PTA_B_1_eut1(pf01515) EUT1
13010 H_azure EUT1
13015 P_lightViolet EUT1
13020 HMMalddh_1_eutpdugrm(pf00171) EUT1 EP_NTERM
13025 HMM06723EutJMreB_1_grm EUT1
13030 HMM00465alcdh_3_mix(pf00465) EUT1
13035 HMMtpEuth_1_eut(pf04346) EUT1
13040 HMM06277EutA_1_eut(pf06277) EUT1
13045 HMMetlyEutB_1_EUT1(pf06751) E
13050 HMMetlyEutC_1_eut1(pf
BMC-P
BMC-H
BMC-T
Aldehyde dehydrogenase
PTAC
Signature enzyme
Alcohol dehydrogenase
Regulator

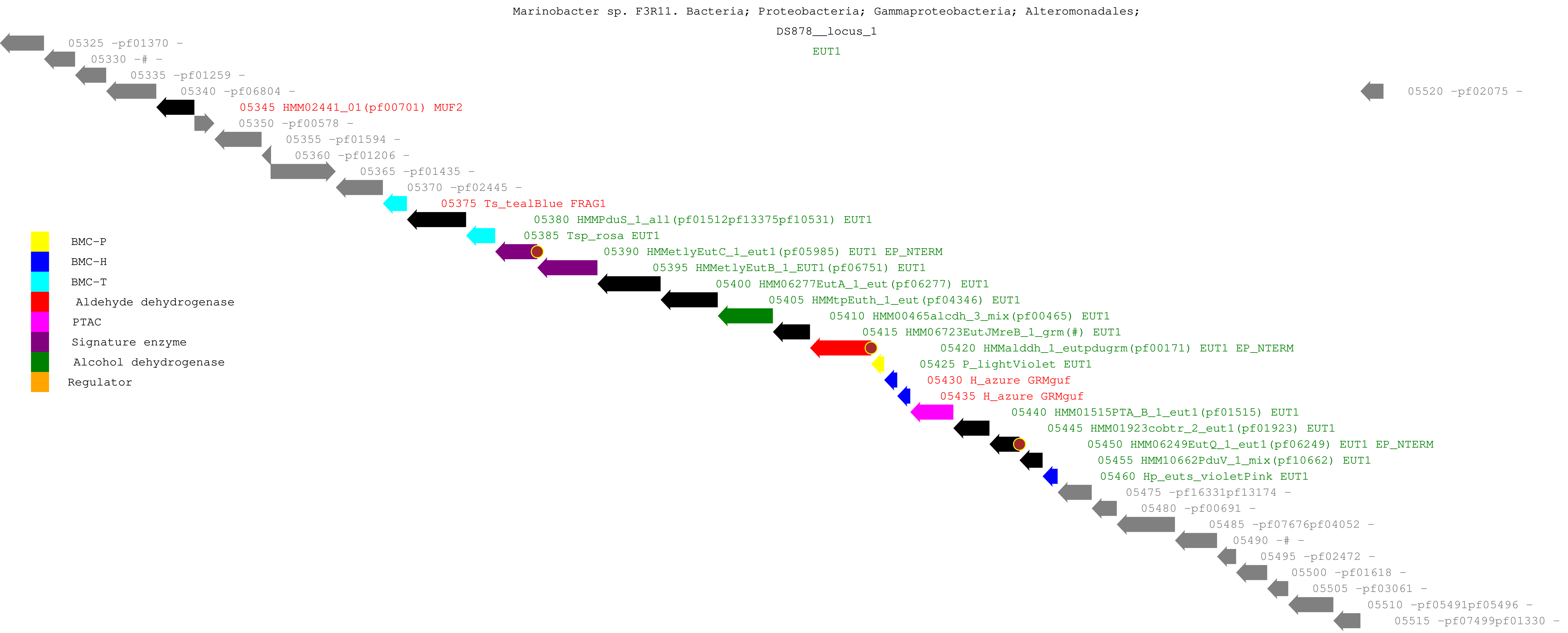
Marinobacter sp. F3R11. Bacteria; Proteobacteria; Gammaproteobacteria; Alteromonadales;
DS878__locus_1
EUT1
05325 -pf01370 -
05330 -# -
05335 -pf01259 -
05340 -pf06804 -
05345 HMM02441_01(pf00701) MUF2
05350 -pf00578 -
05355 -pf01594 -
05360 -pf01206 -
05365 -pf01435 -
05370 -pf02445 -
05375 Ts_tealBlue FRAG1
05380 HMMPduS_1_all(pf01512pf13375pf10531) EUT1
05385 Tsp_rosa EUT1
05390 HMMetlyEutC_1_eut1(pf05985) EUT1 EP_NTERM
05395 HMMetlyEutB_1_EUT1(pf06751) EUT1
05400 HMM06277EutA_1_eut(pf06277) EUT1
05405 HMMtpEuth_1_eut(pf04346) EUT1
05410 HMM00465alcdh_3_mix(pf00465) EUT1
05415 HMM06723EutJMreB_1_grm(#) EUT1
05420 HMMaldh_1_eutpdugrm(pf00171) EUT1 EP_NTERM
05425 P_lightViolet EUT1
05430 H_azure GRMguf
05435 H_azure GRMguf
05440 HMM01515PTA_B_1_eut1(pf01515) EUT1
05445 HMM01923cobtr_2_eut1(pf01923) EUT1
05450 HMM06249EutQ_1_eut1(pf06249) EUT1 EP_NTERM
05455 HMM10662PduV_1_mix(pf10662) EUT1
05460 Hp_euts_violetPink EUT1
05475 -pf16331pf13174 -
05480 -pf00691 -
05485 -pf07676pf04052 -
05490 -# -
05495 -pf02472 -
05500 -pf01618 -
05505 -pf03061 -
05510 -pf05491pf05496 -
05515 -pf07499pf01330 -
05520 -pf02075 -
BMC-P
BMC-H
BMC-T
Aldehyde dehydrogenase
PTAC
Signature enzyme
Alcohol dehydrogenase
Regulator

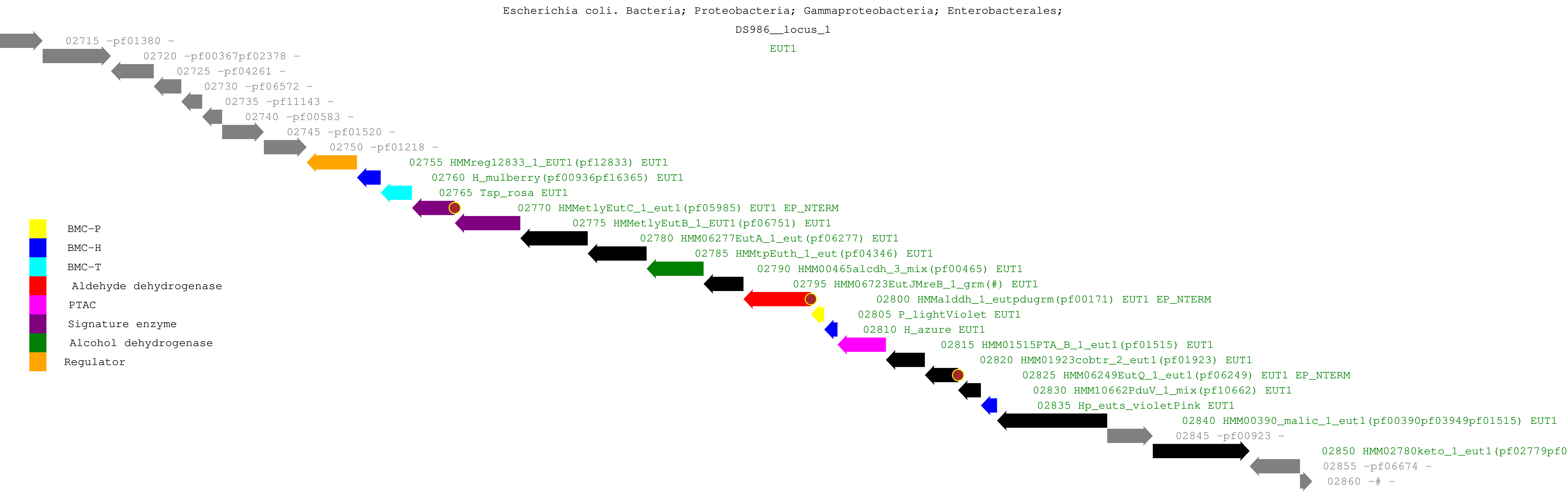
Escherichia coli. Bacteria; Proteobacteria; Gammaproteobacteria; Enterobacterales;
DS986__locus_1
EUT1
02715 -pf01380 -
02720 -pf00367pf02378 -
02725 -pf04261 -
02730 -pf06572 -
02735 -pf11143 -
02740 -pf00583 -
02745 -pf01520 -
02750 -pf01218 -
02755 HMMreg12833_1_EUT1(pf12833) EUT1
02760 H_mulberry(pf00936pf16365) EUT1
02765 Tsp_rosa EUT1
02770 HMMetlyEutC_1_eut1(pf05985) EUT1 EP_NTERM
02775 HMMetlyEutB_1_EUT1(pf06751) EUT1
02780 HMM06277EutA_1_eut(pf06277) EUT1
02785 HMMtpEuth_1_eut(pf04346) EUT1
02790 HMM00465alcdh_3_mix(pf00465) EUT1
02795 HMM06723EutJMreB_1_grm(#) EUT1
02800 HMMaldh_1_eutpdugrm(pf00171) EUT1 EP_NTERM
02805 P_lightViolet EUT1
02810 H_azure EUT1
02815 HMM01515PTA_B_1_eut1(pf01515) EUT1
02820 HMM01923cobtr_2_eut1(pf01923) EUT1
02825 HMM06249EutQ_1_eut1(pf06249) EUT1 EP_NTERM
02830 HMM10662PduV_1_mix(pf10662) EUT1
02835 Hp_euts_violetPink EUT1
02840 HMM00390_malic_1_eut1(pf00390pf03949pf01515) EUT1
02845 -pf00923 -
02850 HMM02780keto_1_eut1(pf02779pf0
02855 -pf06674 -
02860 -# -
BMC-P
BMC-H
BMC-T
Aldehyde dehydrogenase
PTAC
Signature enzyme
Alcohol dehydrogenase
Regulator

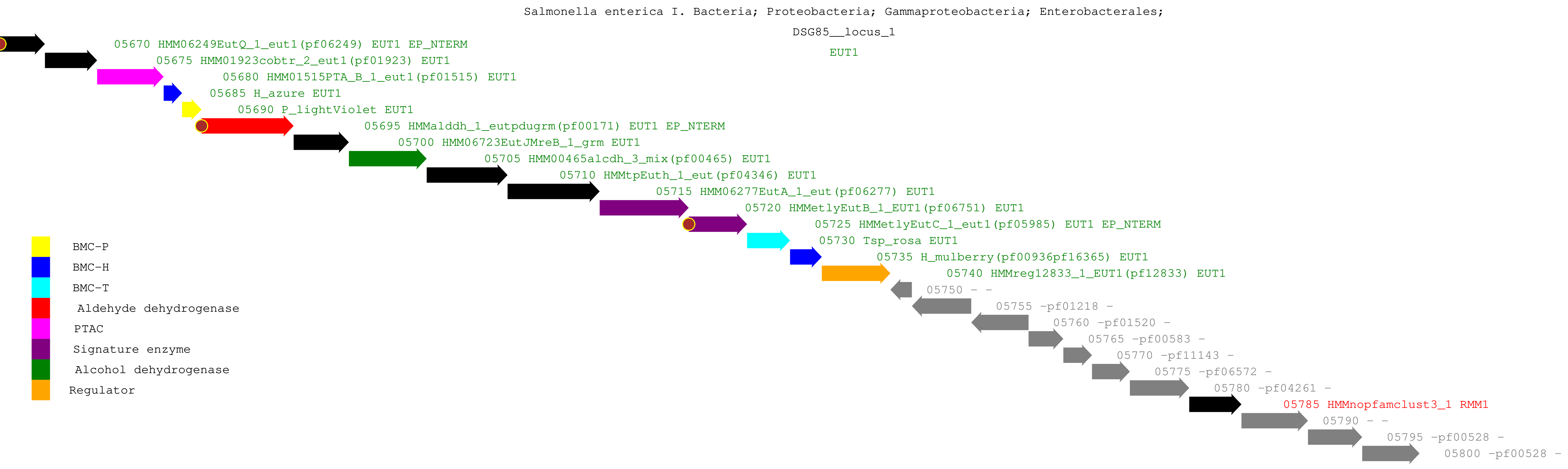
Salmonella enterica I. Bacteria; Proteobacteria; Gammaproteobacteria; Enterobacterales;
DSG85__locus_1
EUT1
05670 HMM06249EutQ_1_eut1(pf06249) EUT1 EP_NTERM
05675 HMM01923cobtr_2_eut1(pf01923) EUT1
05680 HMM01515PTA_B_1_eut1(pf01515) EUT1
05685 H_azure EUT1
05690 P_lightViolet EUT1
05695 HMMalddh_1_eutpdugrm(pf00171) EUT1 EP_NTERM
05700 HMM06723EutJMreB_1_grm EUT1
05705 HMM00465alcdh_3_mix(pf00465) EUT1
05710 HMMtpEuth_1_eut(pf04346) EUT1
05715 HMM06277EutA_1_eut(pf06277) EUT1
05720 HMMetlyEutB_1_EUT1(pf06751) EUT1
05725 HMMetlyEutC_1_eut1(pf05985) EUT1 EP_NTERM
05730 Tsp_rosa EUT1
05735 H_mulberry(pf00936pf16365) EUT1
05740 HMMreg12833_1_EUT1(pf12833) EUT1
05750 - -
05755 -pf01218 -
05760 -pf01520 -
05765 -pf00583 -
05770 -pf11143 -
05775 -pf06572 -
05780 -pf04261 -
05785 HMMnopfamclust3_1 RMM1
05790 - -
05795 -pf00528 -
05800 -pf00528 -
BMC-P
BMC-H
BMC-T
Aldehyde dehydrogenase
PTAC
Signature enzyme
Alcohol dehydrogenase
Regulator

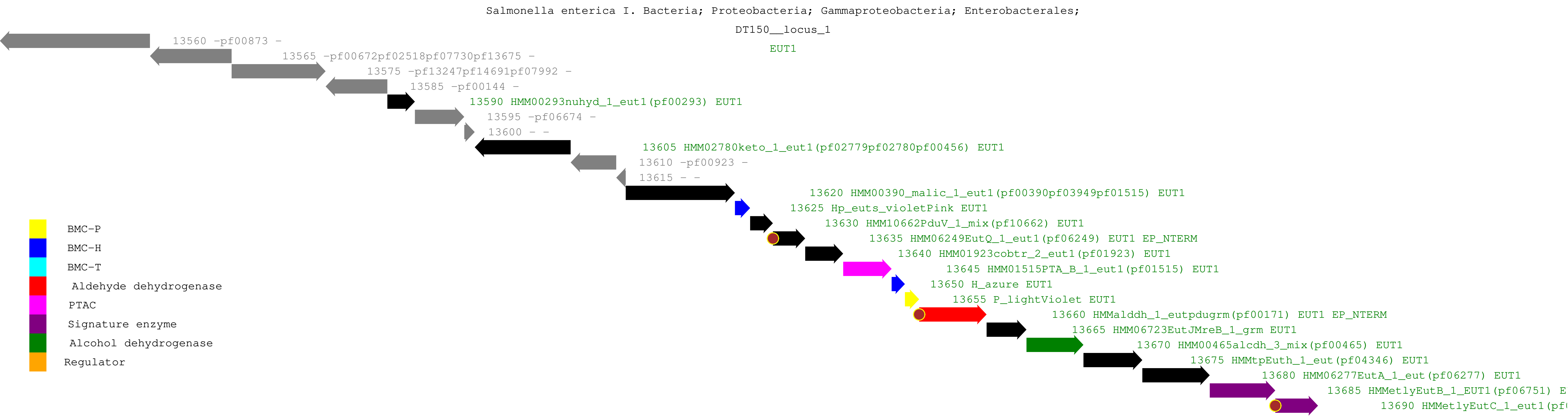
Salmonella enterica I. Bacteria; Proteobacteria; Gammaproteobacteria; Enterobacterales;
DT150__locus_1
EUT1
13560 -pf00873 -
13565 -pf00672pf02518pf07730pf13675 -
13575 -pf13247pf14691pf07992 -
13585 -pf00144 -
13590 HMM00293nuhyd_1_eut1(pf00293) EUT1
13595 -pf06674 -
13600 - -
13605 HMM02780keto_1_eut1(pf02779pf02780pf00456) EUT1
13610 -pf00923 -
13615 - -
13620 HMM00390_malic_1_eut1(pf00390pf03949pf01515) EUT1
13625 Hp_euts_violetPink EUT1
13630 HMM10662PduV_1_mix(pf10662) EUT1
13635 HMM06249EutQ_1_eut1(pf06249) EUT1 EP_NTERM
13640 HMM01923cobtr_2_eut1(pf01923) EUT1
13645 HMM01515PTA_B_1_eut1(pf01515) EUT1
13650 H_azure EUT1
13655 P_lightViolet EUT1
13660 HMMalddh_1_eutpdugrm(pf00171) EUT1 EP_NTERM
13665 HMM06723EutJMreB_1_grm EUT1
13670 HMM00465alcdh_3_mix(pf00465) EUT1
13675 HMMtpEuth_1_eut(pf04346) EUT1
13680 HMM06277EutA_1_eut(pf06277) EUT1
13685 HMMetlyEutB_1_EUT1(pf06751) E
13690 HMMetlyEutC_1_eut1(pf
BMC-P
BMC-H
BMC-T
Aldehyde dehydrogenase
PTAC
Signature enzyme
Alcohol dehydrogenase
Regulator

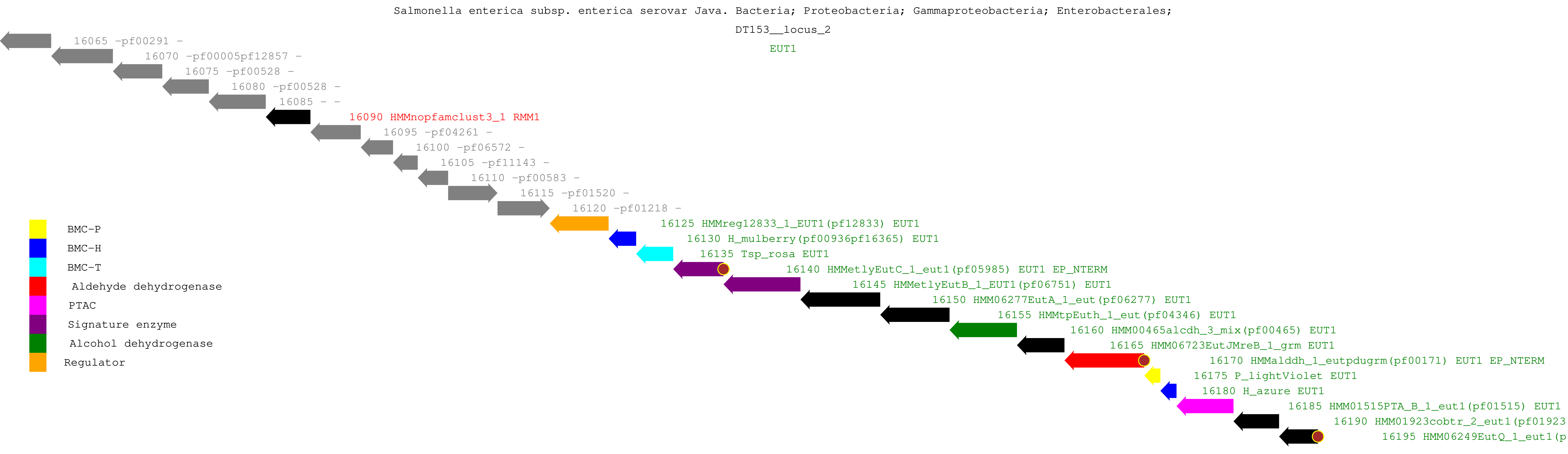

Salmonella enterica subsp. enterica serovar Java. Bacteria; Proteobacteria; Gammaproteobacteria; Enterobacterales;
DT153__locus_2
EUT1
16065 -pf00291 -
16070 -pf00005pf12857 -
16075 -pf00528 -
16080 -pf00528 -
16085 - -
16090 HMMnopfamclust3_1 RMM1
16095 -pf04261 -
16100 -pf06572 -
16105 -pf11143 -
16110 -pf00583 -
16115 -pf01520 -
16120 -pf01218 -
16125 HMMreg12833_1_EUT1(pf12833) EUT1
16130 H_mulberry(pf00936pf16365) EUT1
16135 Tsp_rosa EUT1
16140 HMMetlyEutC_1_eut1(pf05985) EUT1 EP_NTERM
16145 HMMetlyEutB_1_EUT1(pf06751) EUT1
16150 HMM06277EutA_1_eut(pf06277) EUT1
16155 HMMtpEuth_1_eut(pf04346) EUT1
16160 HMM00465alcdh_3_mix(pf00465) EUT1
16165 HMM06723EutJMreB_1_grm EUT1
16170 HMMalddh_1_eutpdugrm(pf00171) EUT1 EP_NTERM
16175 P_lightViolet EUT1
16180 H_azure EUT1
16185 HMM01515PTA_B_1_eut1(pf01515) EUT1
16190 HMM01923cobtr_2_eut1(pf01923
16195 HMM06249EutQ_1_eut1(p
BMC-P
BMC-H
BMC-T
Aldehyde dehydrogenase
PTAC
Signature enzyme
Alcohol dehydrogenase
Regulator

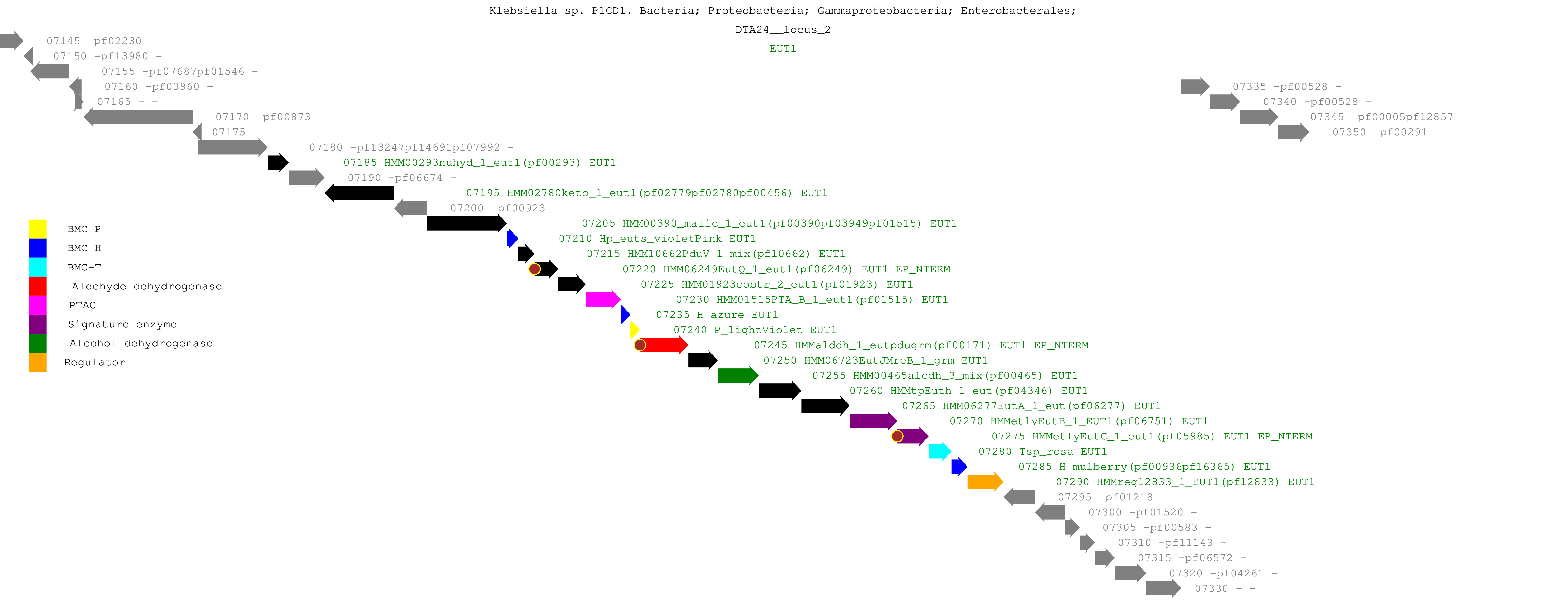

Klebsiella sp. P1CD1. Bacteria; Proteobacteria; Gammaproteobacteria; Enterobacterales;
DTA24__locus_2
EUT1
07145 -pf02230 -
07150 -pf13980 -
07155 -pf07687pf01546 -
07160 -pf03960 -
07165 - -
07170 -pf00873 -
07175 - -
07180 -pf13247pf14691pf07992 -
07185 HMM00293nuhyd_1_eut1(pf00293) EUT1
07190 -pf06674 -
07195 HMM02780keto_1_eut1(pf02779pf02780pf00456) EUT1
07200 -pf00923 -
07205 HMM00390_malic_1_eut1(pf00390pf03949pf01515) EUT1
07210 Hp_euts_violetPink EUT1
07215 HMM10662PduV_1_mix(pf10662) EUT1
07220 HMM06249EutQ_1_eut1(pf06249) EUT1 EP_NTERM
07225 HMM01923cobtr_2_eut1(pf01923) EUT1
07230 HMM01515PTA_B_1_eut1(pf01515) EUT1
07235 H_azure EUT1
07240 P_lightViolet EUT1
07245 HMMalddh_1_eutpdugrm(pf00171) EUT1 EP_NTERM
07250 HMM06723EutJMreB_1_grm EUT1
07255 HMM00465alcdh_3_mix(pf00465) EUT1
07260 HMMtpEuth_1_eut(pf04346) EUT1
07265 HMM06277EutA_1_eut(pf06277) EUT1
07270 HMMetlyEutB_1_EUT1(pf06751) EUT1
07275 HMMetlyEutC_1_eut1(pf05985) EUT1 EP_NTERM
07280 Tsp_rosa EUT1
07285 H_mulberry(pf00936pf16365) EUT1
07290 HMMreg12833_1_EUT1(pf12833) EUT1
07295 -pf01218 -
07300 -pf01520 -
07305 -pf00583 -
07310 -pf11143 -
07315 -pf06572 -
07320 -pf04261 -
07330 - -
07335 -pf00528 -
07340 -pf00528 -
07345 -pf00005pf12857 -
07350 -pf00291 -
BMC-P
BMC-H
BMC-T
Aldehyde dehydrogenase
PTAC
Signature enzyme
Alcohol dehydrogenase
Regulator

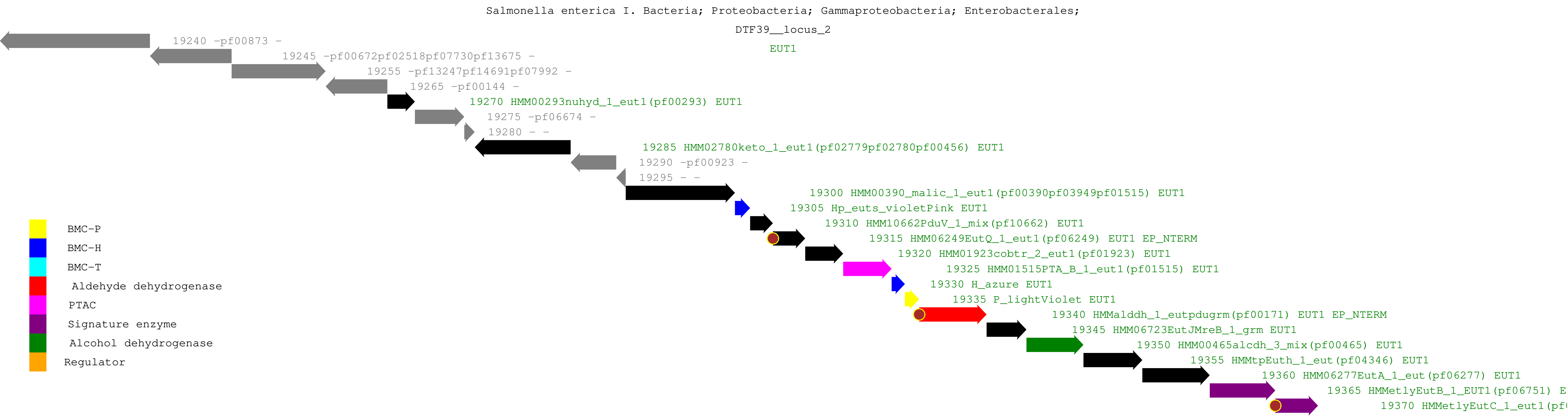
Salmonella enterica I. Bacteria; Proteobacteria; Gammaproteobacteria; Enterobacterales;
DTF39__locus_2
EUT1
19240 -pf00873 -
19245 -pf00672pf02518pf07730pf13675 -
19255 -pf13247pf14691pf07992 -
19265 -pf00144 -
19270 HMM00293nuhyd_1_eut1(pf00293) EUT1
19275 -pf06674 -
19280 - -
19285 HMM02780keto_1_eut1(pf02779pf02780pf00456) EUT1
19290 -pf00923 -
19295 - -
19300 HMM00390_malic_1_eut1(pf00390pf03949pf01515) EUT1
19305 Hp_euts_violetPink EUT1
19310 HMM10662PduV_1_mix(pf10662) EUT1
19315 HMM06249EutQ_1_eut1(pf06249) EUT1 EP_NTERM
19320 HMM01923cobtr_2_eut1(pf01923) EUT1
19325 HMM01515PTA_B_1_eut1(pf01515) EUT1
19330 H_azure EUT1
19335 P_lightViolet EUT1
19340 HMMalddh_1_eutpdugrm(pf00171) EUT1 EP_NTERM
19345 HMM06723EutJMreB_1_grm EUT1
19350 HMM00465alcdh_3_mix(pf00465) EUT1
19355 HMMtpEuth_1_eut(pf04346) EUT1
19360 HMM06277EutA_1_eut(pf06277) EUT1
19365 HMMetlyEutB_1_EUT1(pf06751) E
19370 HMMetlyEutC_1_eut1(pf
BMC-P
BMC-H
BMC-T
Aldehyde dehydrogenase
PTAC
Signature enzyme
Alcohol dehydrogenase
Regulator

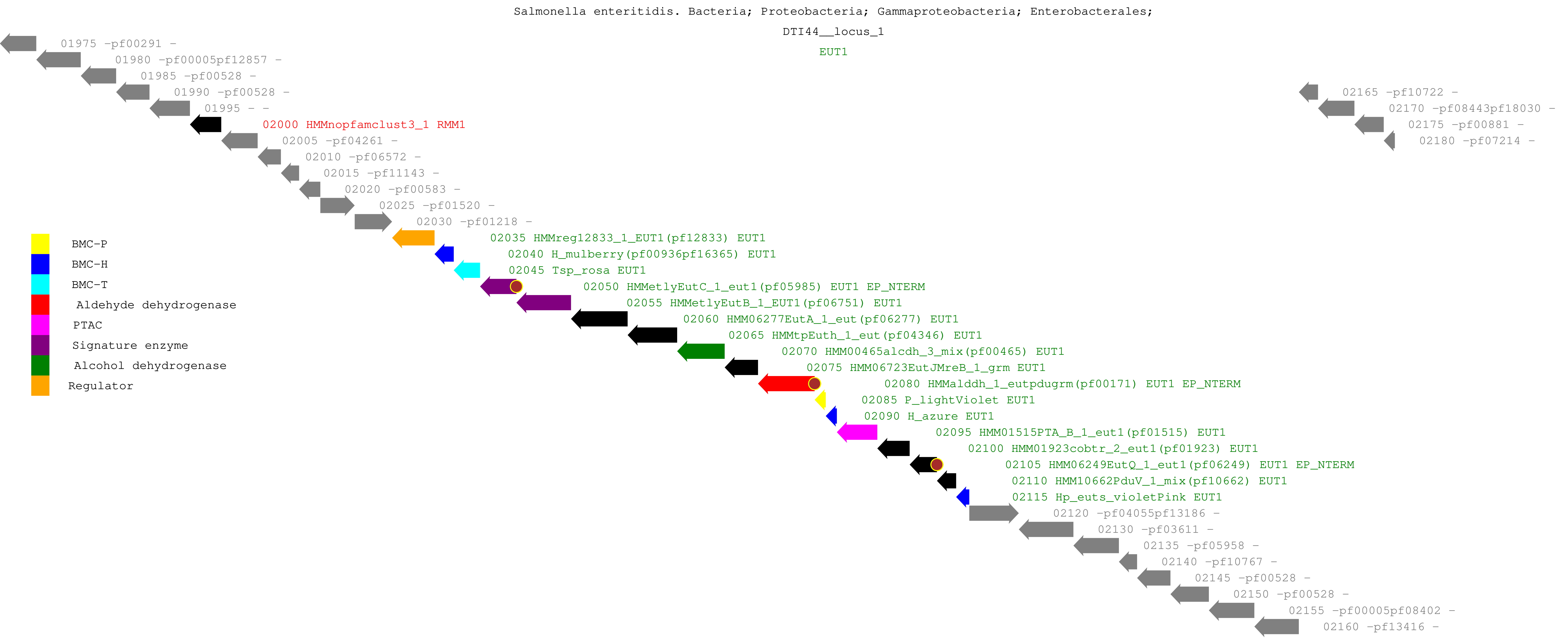

Salmonella enteritidis. Bacteria; Proteobacteria; Gammaproteobacteria; Enterobacterales;
DTI44__locus_1
EUT1
01975 -pf00291 -
01980 -pf00005pf12857 -
01985 -pf00528 -
01990 -pf00528 -
01995 - -
02000 HMMnopfamclust3_1 RMM1
02005 -pf04261 -
02010 -pf06572 -
02015 -pf11143 -
02020 -pf00583 -
02025 -pf01520 -
02030 -pf01218 -
02035 HMMreg12833_1_EUT1(pf12833) EUT1
02040 H_mulberry(pf00936pf16365) EUT1
02045 Tsp_rosa EUT1
02050 HMMetlyEutC_1_eut1(pf05985) EUT1 EP_NTERM
02055 HMMetlyEutB_1_EUT1(pf06751) EUT1
02060 HMM06277EutA_1_eut(pf06277) EUT1
02065 HMMtpEuth_1_eut(pf04346) EUT1
02070 HMM00465alcdh_3_mix(pf00465) EUT1
02075 HMM06723EutJMreB_1_grm EUT1
02080 HMMalddh_1_eutpdugrm(pf00171) EUT1 EP_NTERM
02085 P_lightViolet EUT1
02090 H_azure EUT1
02095 HMM01515PTA_B_1_eut1(pf01515) EUT1
02100 HMM01923cobtr_2_eut1(pf01923) EUT1
02105 HMM06249EutQ_1_eut1(pf06249) EUT1 EP_NTERM
02110 HMM10662PduV_1_mix(pf10662) EUT1
02115 Hp_euts_violetPink EUT1
02120 -pf04055pf13186 -
02130 -pf03611 -
02135 -pf05958 -
02140 -pf10767 -
02145 -pf00528 -
02150 -pf00528 -
02155 -pf00005pf08402 -
02160 -pf13416 -
02165 -pf10722 -
02170 -pf08443pf18030 -
02175 -pf00881 -
02180 -pf07214 -
BMC-P
BMC-H
BMC-T
Aldehyde dehydrogenase
PTAC
Signature enzyme
Alcohol dehydrogenase
Regulator

Escherichia coli. Bacteria; Proteobacteria; Gammaproteobacteria; Enterobacterales;

DTL41__locus_1

EUT1

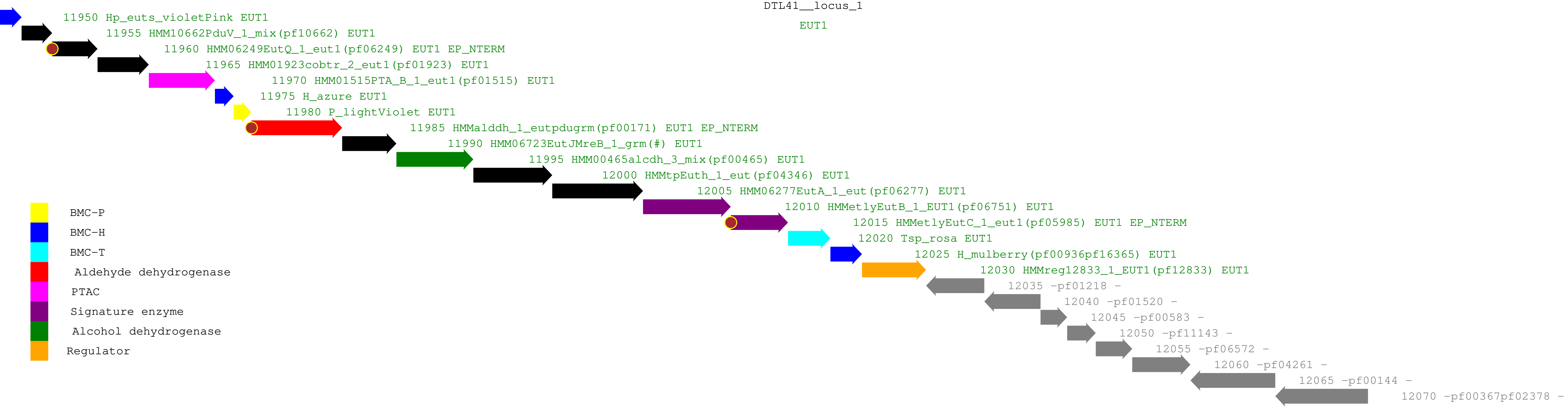

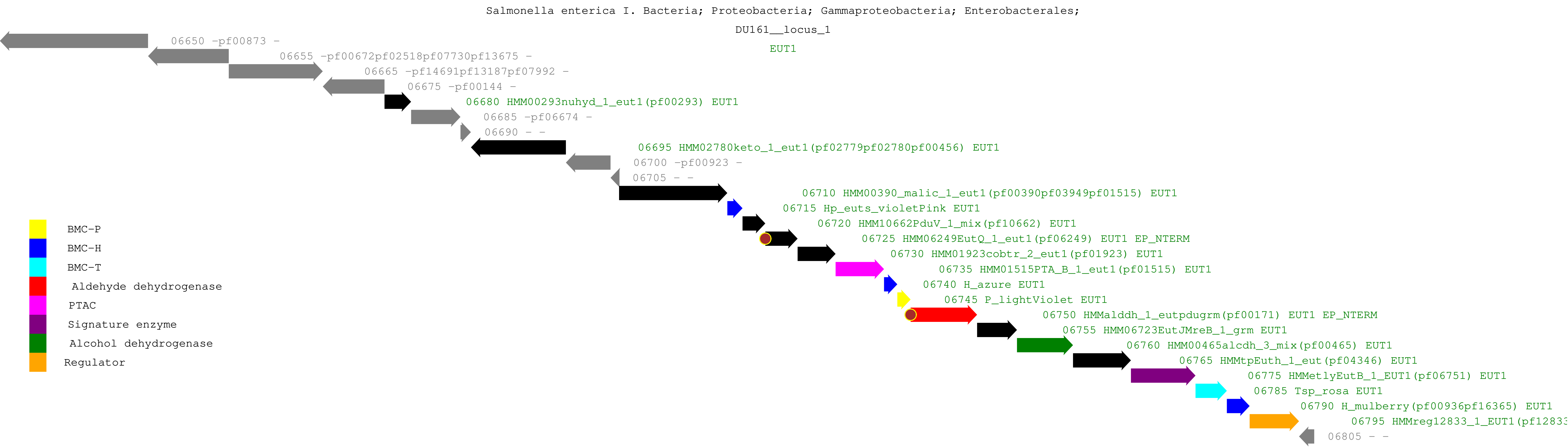
Salmonella enterica I. Bacteria; Proteobacteria; Gammaproteobacteria; Enterobacterales;
DU161__locus_1
EUT1
06650 -pf00873 -
06655 -pf00672pf02518pf07730pf13675 -
06665 -pf14691pf13187pf07992 -
06675 -pf00144 -
06680 HMM00293nuhyd_1_eut1(pf00293) EUT1
06685 -pf06674 -
06690 - -
06695 HMM02780keto_1_eut1(pf02779pf02780pf00456) EUT1
06700 -pf00923 -
06705 - -
06710 HMM00390_malic_1_eut1(pf00390pf03949pf01515) EUT1
06715 Hp_euts_violetPink EUT1
06720 HMM10662PduV_1_mix(pf10662) EUT1
06725 HMM06249EutQ_1_eut1(pf06249) EUT1 EP_NTERM
06730 HMM01923cobtr_2_eut1(pf01923) EUT1
06735 HMM01515PTA_B_1_eut1(pf01515) EUT1
06740 H_azure EUT1
06745 P_lightViolet EUT1
06750 HMMalddh_1_eutpdugrm(pf00171) EUT1 EP_NTERM
06755 HMM06723EutJMreB_1_grm EUT1
06760 HMM00465alcdh_3_mix(pf00465) EUT1
06765 HMMtpEuth_1_eut(pf04346) EUT1
06775 HMMetlyEutB_1_EUT1(pf06751) EUT1
06785 Tsp_rosa EUT1
06790 H_mulberry(pf00936pf16365) EUT1
06795 HMMreg12833_1_EUT1(pf1283
06805 - -
BMC-P
BMC-H
BMC-T
Aldehyde dehydrogenase
PTAC
Signature enzyme
Alcohol dehydrogenase
Regulator

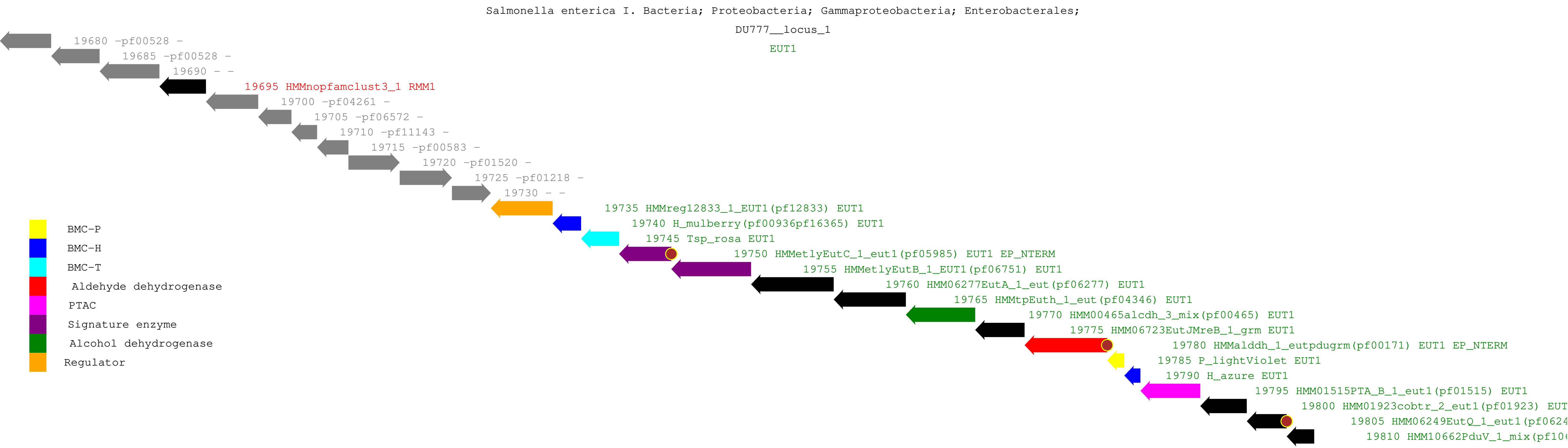

Salmonella enterica I. Bacteria; Proteobacteria; Gammaproteobacteria; Enterobacterales;
DU777__locus_1
EUT1
19680 -pf00528 -
19685 -pf00528 -
19690 - -
19695 HMMnopfamclust3_1 RMM1
19700 -pf04261 -
19705 -pf06572 -
19710 -pf11143 -
19715 -pf00583 -
19720 -pf01520 -
19725 -pf01218 -
19730 - -
19735 HMMreg12833_1_EUT1(pf12833) EUT1
19740 H_mulberry(pf00936pf16365) EUT1
19745 Tsp_rosa EUT1
19750 HMMetlyEutC_1_eut1(pf05985) EUT1 EP_NTERM
19755 HMMetlyEutB_1_EUT1(pf06751) EUT1
19760 HMM06277EutA_1_eut(pf06277) EUT1
19765 HMMtpEuth_1_eut(pf04346) EUT1
19770 HMM00465alcdh_3_mix(pf00465) EUT1
19775 HMM06723EutJMreB_1_grm EUT1
19780 HMMalddh_1_eutpdugrm(pf00171) EUT1 EP_NTERM
19785 P_lightViolet EUT1
19790 H_azure EUT1
19795 HMM01515PTA_B_1_eut1(pf01515) EUT1
19800 HMM01923cobtr_2_eut1(pf01923) EUT
19805 HMM06249EutQ_1_eut1(pf0624
19810 HMM10662PduV_1_mix(pf10
BMC-P
BMC-H
BMC-T
Aldehyde dehydrogenase
PTAC
Signature enzyme
Alcohol dehydrogenase
Regulator

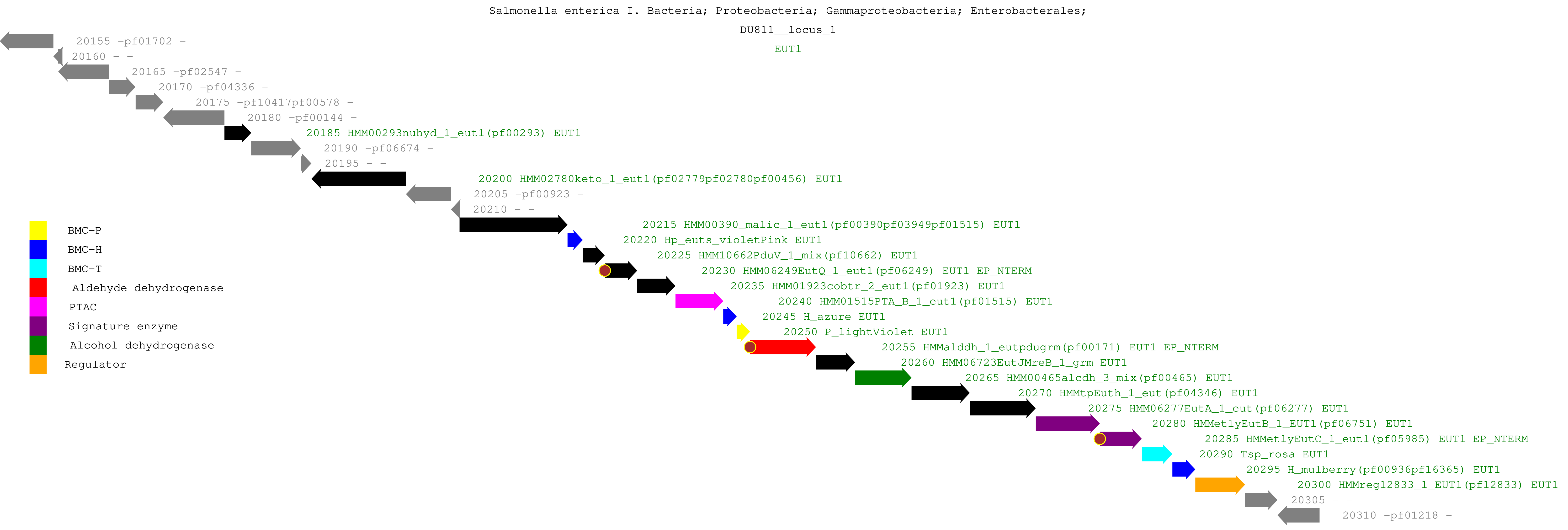
Salmonella enterica I. Bacteria; Proteobacteria; Gammaproteobacteria; Enterobacterales;
DU811__locus_1
EUT1
20155 -pf01702 -
20160 - -
20165 -pf02547 -
20170 -pf04336 -
20175 -pf10417pf00578 -
20180 -pf00144 -
20185 HMM00293nuhyd_1_eut1(pf00293) EUT1
20190 -pf06674 -
20195 - -
20200 HMM02780keto_1_eut1(pf02779pf02780pf00456) EUT1
20205 -pf00923 -
20210 - -
20215 HMM00390_malic_1_eut1(pf00390pf03949pf01515) EUT1
20220 Hp_euts_violetPink EUT1
20225 HMM10662PduV_1_mix(pf10662) EUT1
20230 HMM06249EutQ_1_eut1(pf06249) EUT1 EP_NTERM
20235 HMM01923cobtr_2_eut1(pf01923) EUT1
20240 HMM01515PTA_B_1_eut1(pf01515) EUT1
20245 H_azure EUT1
20250 P_lightViolet EUT1
20255 HMMalddh_1_eutpdugrm(pf00171) EUT1 EP_NTERM
20260 HMM06723EutJMreB_1_grm EUT1
20265 HMM00465alcdh_3_mix(pf00465) EUT1
20270 HMMtpEuth_1_eut(pf04346) EUT1
20275 HMM06277EutA_1_eut(pf06277) EUT1
20280 HMMetlyEutB_1_EUT1(pf06751) EUT1
20285 HMMetlyEutC_1_eut1(pf05985) EUT1 EP_NTERM
20290 Tsp_rosa EUT1
20295 H_mulberry(pf00936pf16365) EUT1
20300 HMMreg12833_1_EUT1(pf12833) EUT1
20305 - -
20310 -pf01218 -
BMC-P
BMC-H
BMC-T
Aldehyde dehydrogenase
PTAC
Signature enzyme
Alcohol dehydrogenase
Regulator

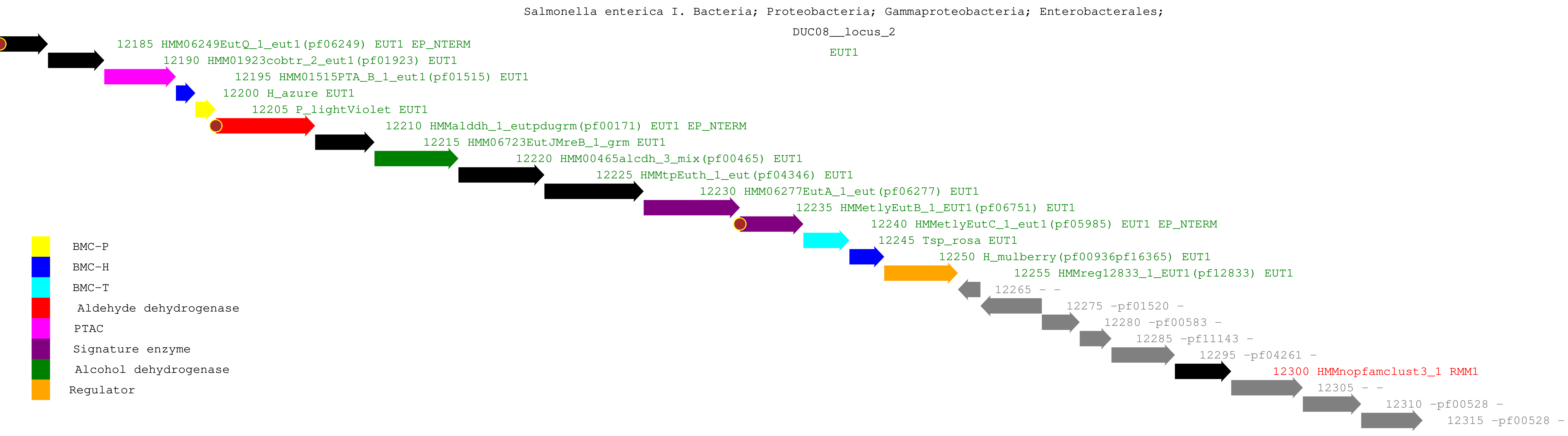
Salmonella enterica I. Bacteria; Proteobacteria; Gammaproteobacteria; Enterobacterales;
DUC08__locus_2
EUT1
12185 HMM06249EutQ_1_eut1(pf06249) EUT1 EP_NTERM
12190 HMM01923cobtr_2_eut1(pf01923) EUT1
12195 HMM01515PTA_B_1_eut1(pf01515) EUT1
12200 H_azure EUT1
12205 P_lightViolet EUT1
12210 HMMalddh_1_eutpdugrm(pf00171) EUT1 EP_NTERM
12215 HMM06723EutJMreB_1_grm EUT1
12220 HMM00465alcdh_3_mix(pf00465) EUT1
12225 HMMtpEuth_1_eut(pf04346) EUT1
12230 HMM06277EutA_1_eut(pf06277) EUT1
12235 HMMetlyEutB_1_EUT1(pf06751) EUT1
12240 HMMetlyEutC_1_eut1(pf05985) EUT1 EP_NTERM
12245 Tsp_rosa EUT1
12250 H_mulberry(pf00936pf16365) EUT1
12255 HMMreg12833_1_EUT1(pf12833) EUT1
12265 - -
12275 -pf01520 -
12280 -pf00583 -
12285 -pf11143 -
12295 -pf04261 -
12300 HMMnopfamclust3_1 RMM1
12305 - -
12310 -pf00528 -
12315 -pf00528 -
BMC-P
BMC-H
BMC-T
Aldehyde dehydrogenase
PTAC
Signature enzyme
Alcohol dehydrogenase
Regulator

Salmonella enterica I. Bacteria; Proteobacteria; Gammaproteobacteria; Enterobacterales;

DUQ60__locus_1

EUT1

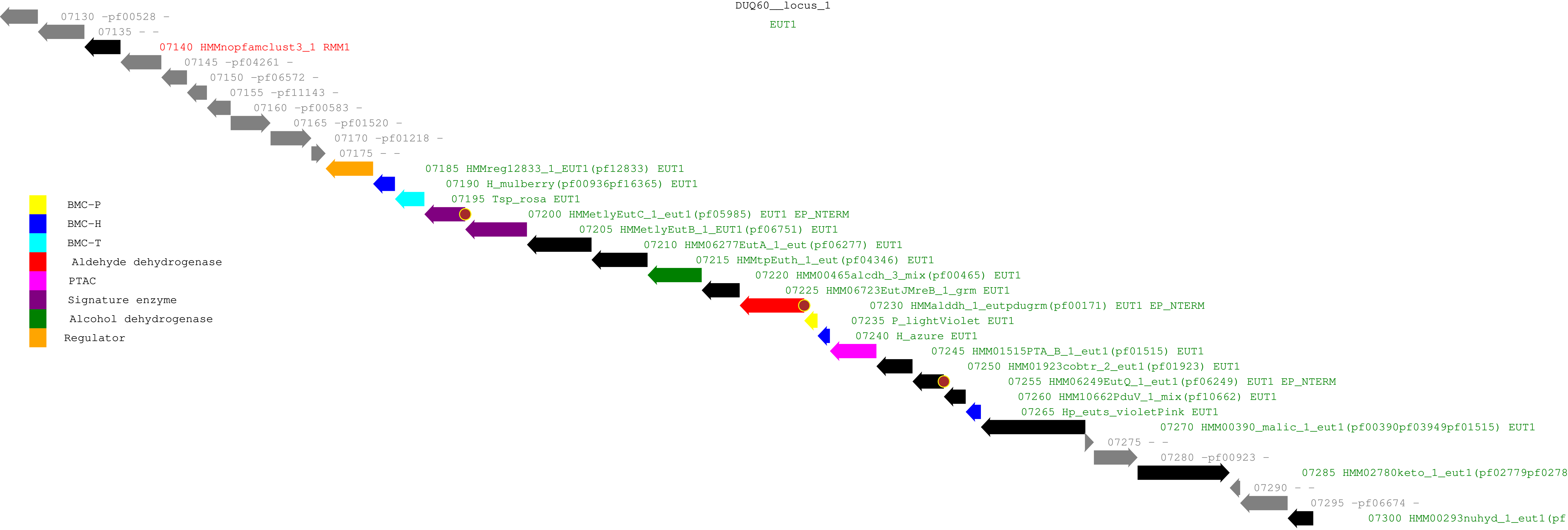

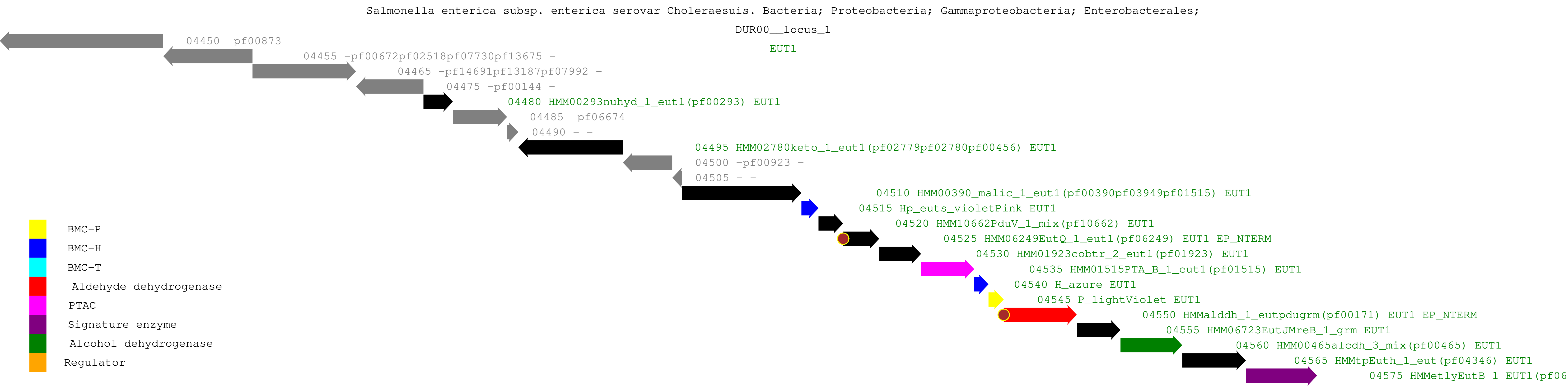
Salmonella enterica subsp. enterica serovar Choleraesuis. Bacteria; Proteobacteria; Gammaproteobacteria; Enterobacterales;
DUR00__locus_1
EUT1
04450 -pf00873 -
04455 -pf00672pf02518pf07730pf13675 -
04465 -pf14691pf13187pf07992 -
04475 -pf00144 -
04480 HMM00293nuhyd_1_eut1(pf00293) EUT1
04485 -pf06674 -
04490 - -
04495 HMM02780keto_1_eut1(pf02779pf02780pf00456) EUT1
04500 -pf00923 -
04505 - -
04510 HMM00390_malic_1_eut1(pf00390pf03949pf01515) EUT1
04515 Hp_euts_violetPink EUT1
04520 HMM10662PduV_1_mix(pf10662) EUT1
04525 HMM06249EutQ_1_eut1(pf06249) EUT1 EP_NTERM
04530 HMM01923cobtr_2_eut1(pf01923) EUT1
04535 HMM01515PTA_B_1_eut1(pf01515) EUT1
04540 H_azure EUT1
04545 P_lightViolet EUT1
04550 HMMalddh_1_eutpdugrm(pf00171) EUT1 EP_NTERM
04555 HMM06723EutJMreB_1_grm EUT1
04560 HMM00465alcdh_3_mix(pf00465) EUT1
04565 HMMtpEuth_1_eut(pf04346) EUT1
04575 HMMetlyEutB_1_EUT1(pf06
BMC-P
BMC-H
BMC-T
Aldehyde dehydrogenase
PTAC
Signature enzyme
Alcohol dehydrogenase
Regulator

Salmonella enterica I. Bacteria; Proteobacteria; Gammaproteobacteria; Enterobacterales;

DUR90__locus_1

EUT1

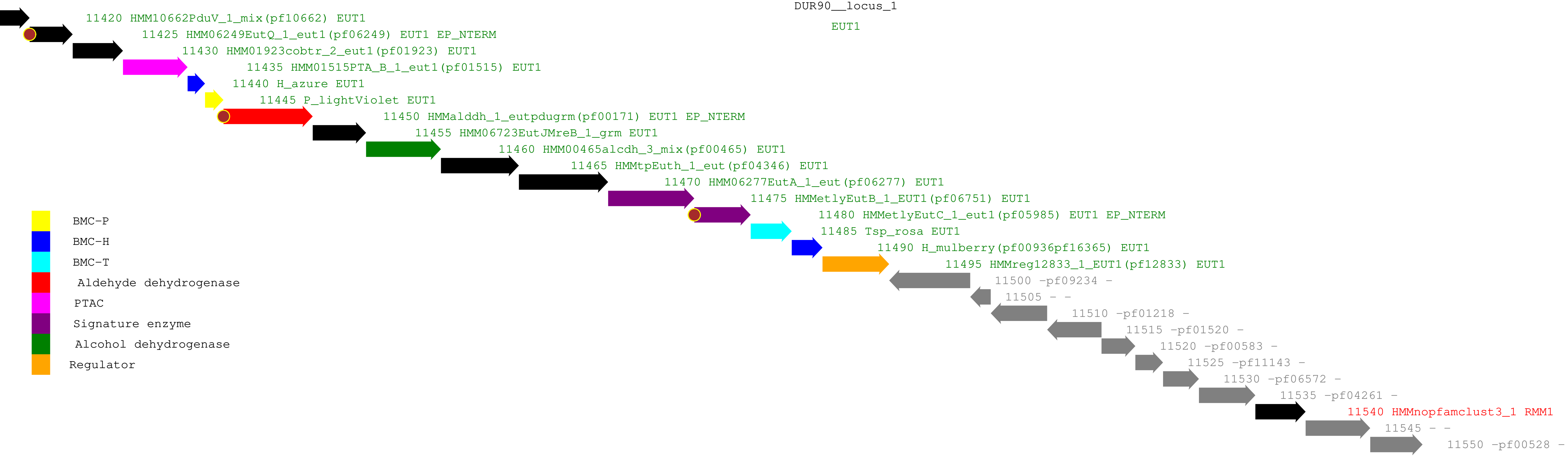

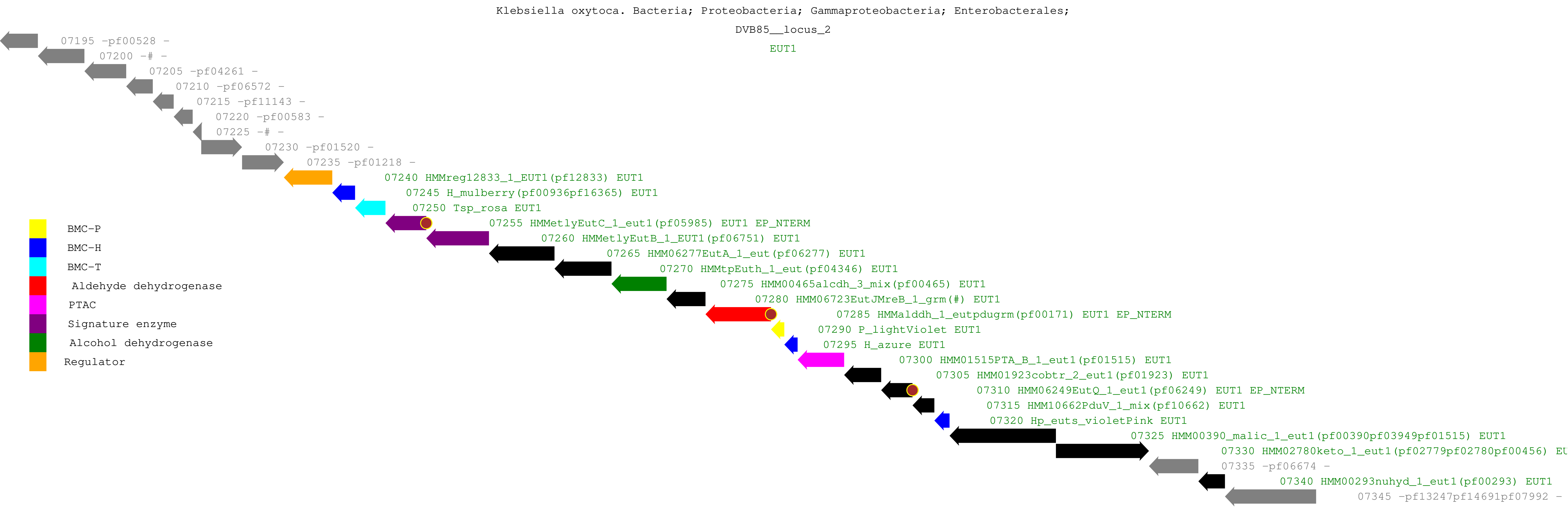
Klebsiella oxytoca. Bacteria; Proteobacteria; Gammaproteobacteria; Enterobacterales;
DVB85__locus_2
EUT1
07195 -pf00528 -
07200 -# -
07205 -pf04261 -
07210 -pf06572 -
07215 -pf11143 -
07220 -pf00583 -
07225 -# -
07230 -pf01520 -
07235 -pf01218 -
07240 HMMreg12833_1_EUT1(pf12833) EUT1
07245 H_mulberry(pf00936pf16365) EUT1
07250 Tsp_rosa EUT1
07255 HMMetlyEutC_1_eut1(pf05985) EUT1 EP_NTERM
07260 HMMetlyEutB_1_EUT1(pf06751) EUT1
07265 HMM06277EutA_1_eut(pf06277) EUT1
07270 HMMtpEuth_1_eut(pf04346) EUT1
07275 HMM00465alcdh_3_mix(pf00465) EUT1
07280 HMM06723EutJMreB_1_grm(#) EUT1
07285 HMMalddh_1_eutpdugrm(pf00171) EUT1 EP_NTERM
07290 P_lightViolet EUT1
07295 H_azure EUT1
07300 HMM01515PTA_B_1_eut1(pf01515) EUT1
07305 HMM01923cobtr_2_eut1(pf01923) EUT1
07310 HMM06249EutQ_1_eut1(pf06249) EUT1 EP_NTERM
07315 HMM10662PduV_1_mix(pf10662) EUT1
07320 Hp_euts_violetPink EUT1
07325 HMM00390_malic_1_eut1(pf00390pf03949pf01515) EUT1
07330 HMM02780keto_1_eut1(pf02779pf02780pf00456) EU
07335 -pf06674 -
07340 HMM00293nuhyd_1_eut1(pf00293) EUT1
07345 -pf13247pf14691pf07992 -
BMC-P
BMC-H
BMC-T
Aldehyde dehydrogenase
PTAC
Signature enzyme
Alcohol dehydrogenase
Regulator

Salmonella enterica I. Bacteria; Proteobacteria; Gammaproteobacteria; Enterobacterales;

DVF43__locus_1

EUT1

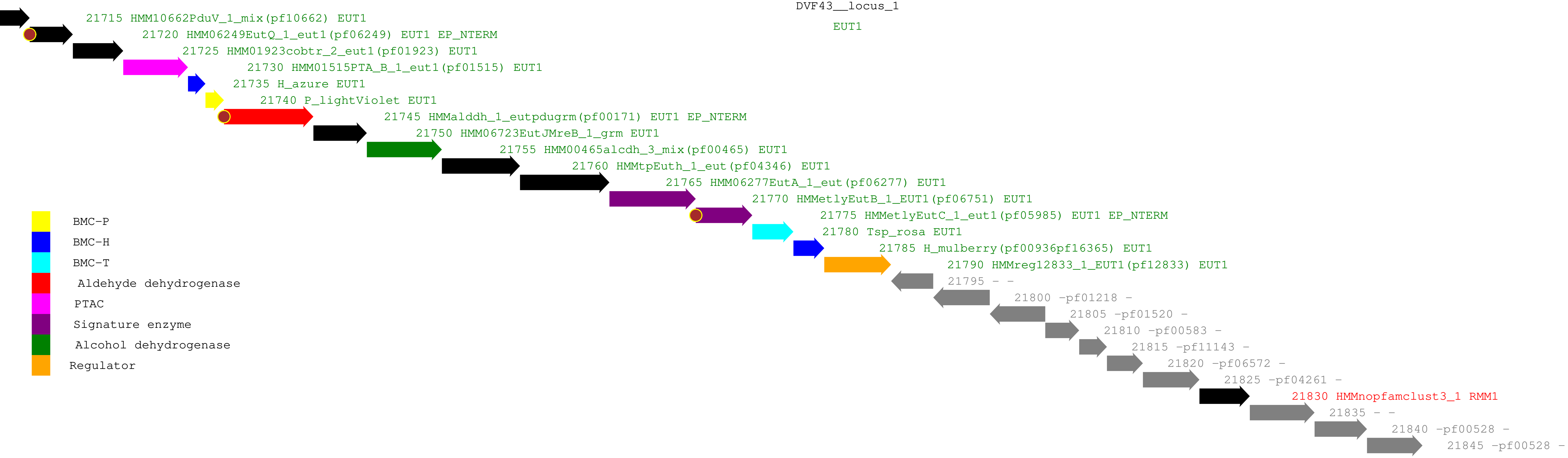

Salmonella enterica I. Bacteria; Proteobacteria; Gammaproteobacteria; Enterobacterales;

DVF58__locus_1

EUT1

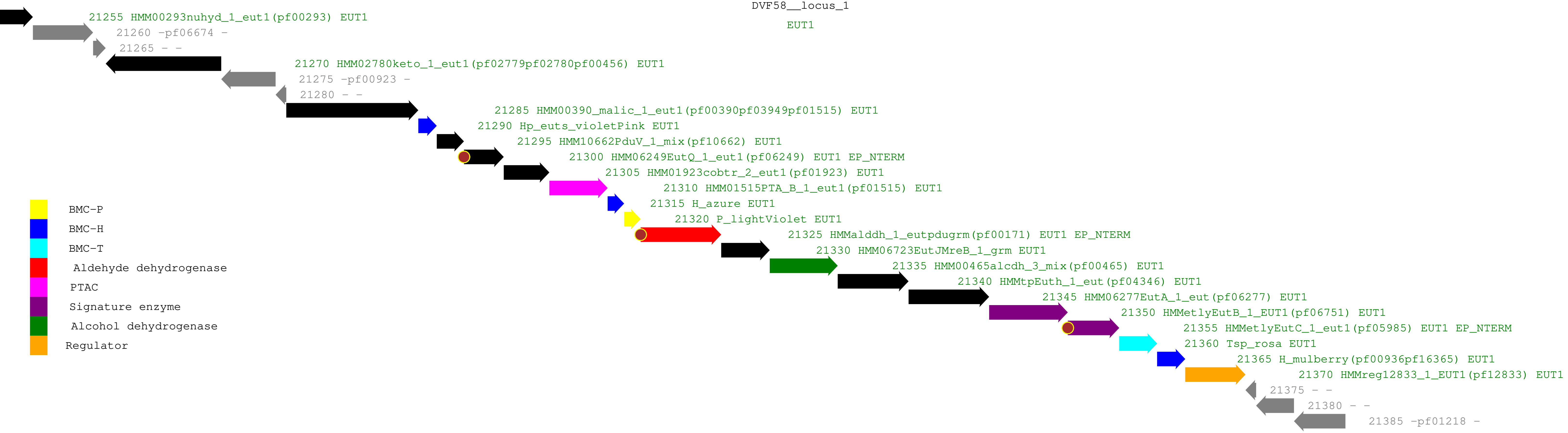

Citrobacter gillenii. Bacteria; Proteobacteria; Gammaproteobacteria; Enterobacterales;

DZA29__locus_2

EUT1

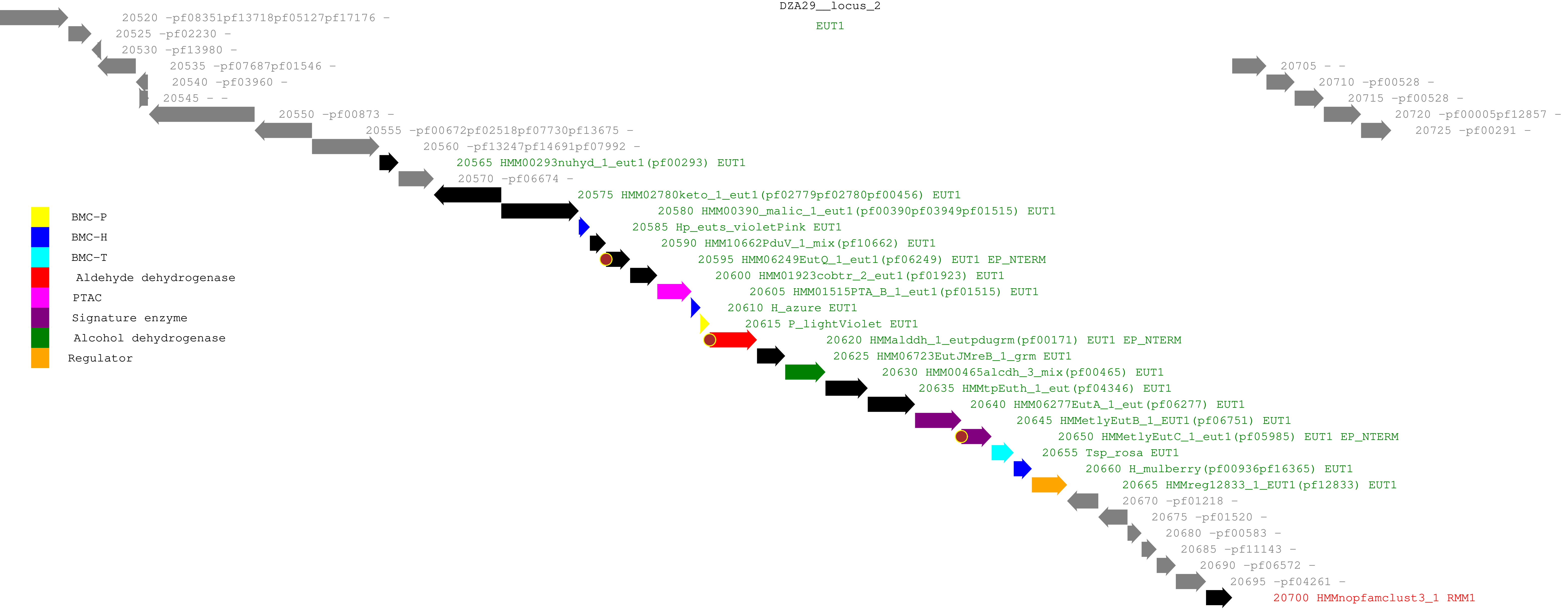

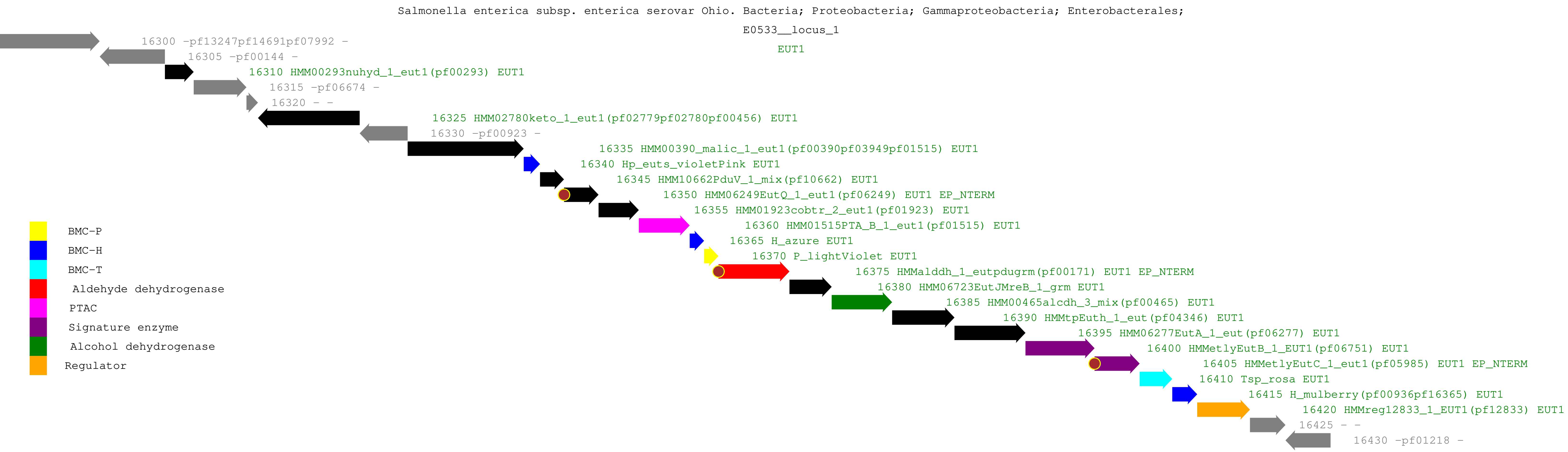

Salmonella enterica subsp. enterica serovar Ohio. Bacteria; Proteobacteria; Gammaproteobacteria; Enterobacterales;
E0533__locus_1
EUT1
16300 -pf13247pf14691pf07992 -
16305 -pf00144 -
16310 HMM00293nuhyd_1_eut1(pf00293) EUT1
16315 -pf06674 -
16320 - -
16325 HMM02780keto_1_eut1(pf02779pf02780pf00456) EUT1
16330 -pf00923 -
16335 HMM00390_malic_1_eut1(pf00390pf03949pf01515) EUT1
16340 Hp_euts_violetPink EUT1
16345 HMM10662PduV_1_mix(pf10662) EUT1
16350 HMM06249EutQ_1_eut1(pf06249) EUT1 EP_NTERM
16355 HMM01923cobtr_2_eut1(pf01923) EUT1
16360 HMM01515PTA_B_1_eut1(pf01515) EUT1
16365 H_azure EUT1
16370 P_lightViolet EUT1
16375 HMMalddh_1_eutpdugrm(pf00171) EUT1 EP_NTERM
16380 HMM06723EutJMreB_1_grm EUT1
16385 HMM00465alcdh_3_mix(pf00465) EUT1
16390 HMMtpEuth_1_eut(pf04346) EUT1
16395 HMM06277EutA_1_eut(pf06277) EUT1
16400 HMMetlyEutB_1_EUT1(pf06751) EUT1
16405 HMMetlyEutC_1_eut1(pf05985) EUT1 EP_NTERM
16410 Tsp_rosa EUT1
16415 H_mulberry(pf00936pf16365) EUT1
16420 HMMreg12833_1_EUT1(pf12833) EUT1
16425 - -
16430 -pf01218 -
BMC-P
BMC-H
BMC-T
Aldehyde dehydrogenase
PTAC
Signature enzyme
Alcohol dehydrogenase
Regulator

Salmonella enterica subsp. enterica serovar Tilene. Bacteria; Proteobacteria; Gammaproteobacteria; Enterobacterales;

E0539__locus_1

EUT1

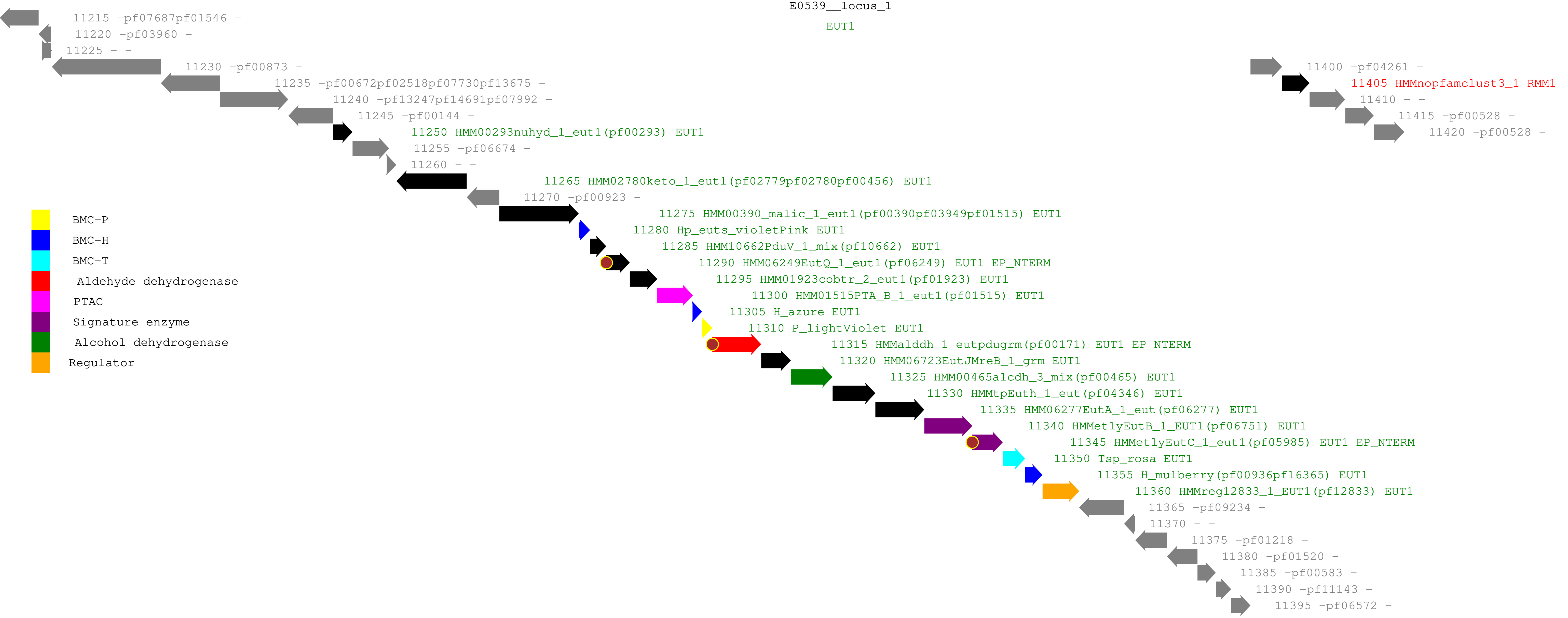

Salmonella enterica subsp. enterica serovar London. Bacteria; Proteobacteria; Gammaproteobacteria; Enterobacterales;

E0541__locus_2

EUT1

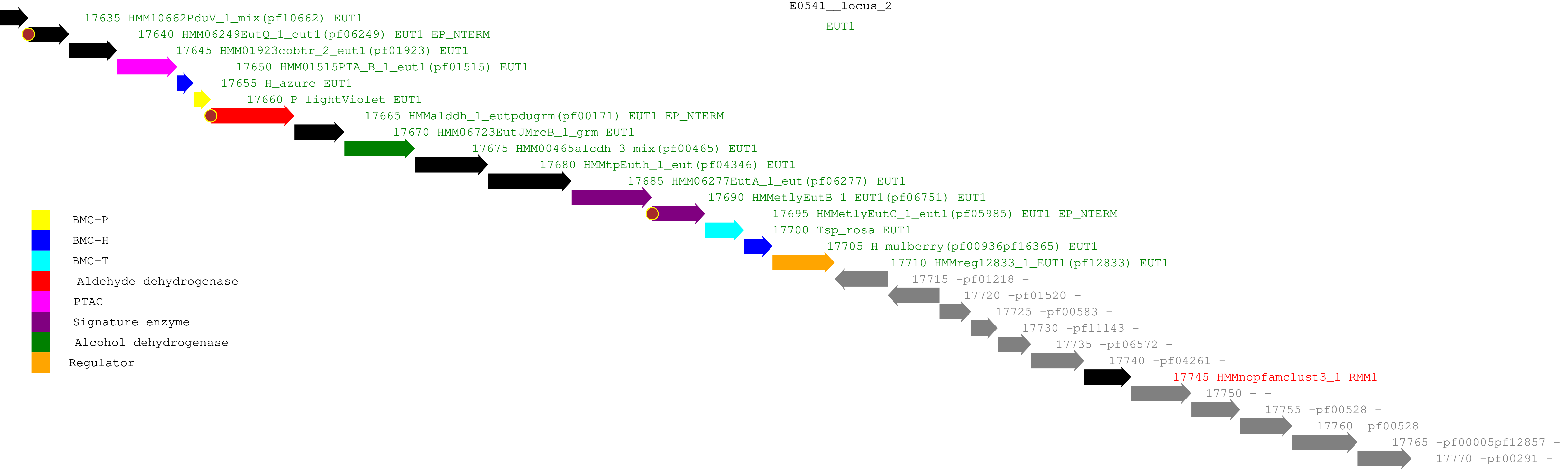

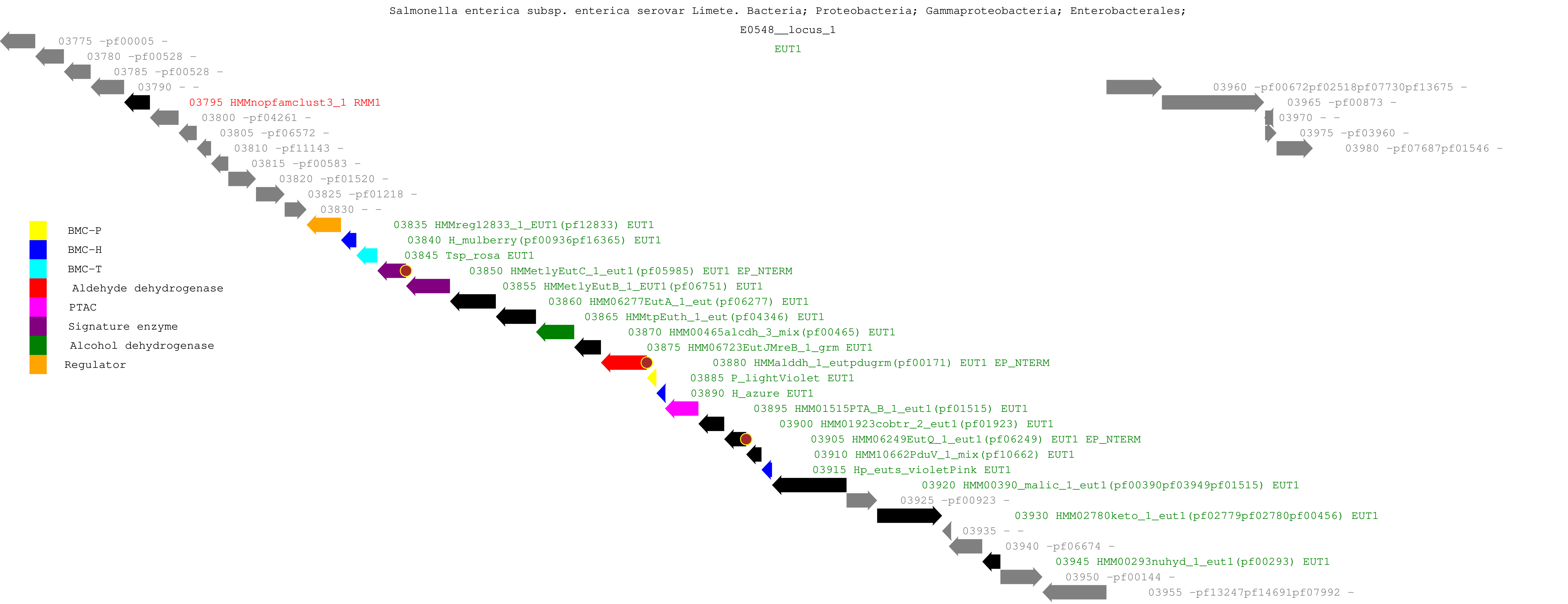
Salmonella enterica subsp. enterica serovar Limete. Bacteria; Proteobacteria; Gammaproteobacteria; Enterobacterales;
E0548__locus_1
EUT1
03775 -pf00005 -
03780 -pf00528 -
03785 -pf00528 -
03790 - -
03795 HMMnopfamclust3_1 RMM1
03800 -pf04261 -
03805 -pf06572 -
03810 -pf11143 -
03815 -pf00583 -
03820 -pf01520 -
03825 -pf01218 -
03830 - -
03835 HMMreg12833_1_EUT1(pf12833) EUT1
03840 H_mulberry(pf00936pf16365) EUT1
03845 Tsp_rosa EUT1
03850 HMMetlyEutC_1_eut1(pf05985) EUT1 EP_NTERM
03855 HMMetlyEutB_1_EUT1(pf06751) EUT1
03860 HMM06277EutA_1_eut(pf06277) EUT1
03865 HMMtpEuth_1_eut(pf04346) EUT1
03870 HMM00465alcdh_3_mix(pf00465) EUT1
03875 HMM06723EutJMreB_1_grm EUT1
03880 HMMalddh_1_eutpdugrm(pf00171) EUT1 EP_NTERM
03885 P_lightViolet EUT1
03890 H_azure EUT1
03895 HMM01515PTA_B_1_eut1(pf01515) EUT1
03900 HMM01923cobtr_2_eut1(pf01923) EUT1
03905 HMM06249EutQ_1_eut1(pf06249) EUT1 EP_NTERM
03910 HMM10662PduV_1_mix(pf10662) EUT1
03915 Hp_euts_violetPink EUT1
03920 HMM00390_malic_1_eut1(pf00390pf03949pf01515) EUT1
03925 -pf00923 -
03930 HMM02780keto_1_eut1(pf02779pf02780pf00456) EUT1
03935 - -
03940 -pf06674 -
03945 HMM00293nuhyd_1_eut1(pf00293) EUT1
03950 -pf00144 -
03955 -pf13247pf14691pf07992 -
03960 -pf00672pf02518pf07730pf13675 -
03965 -pf00873 -
03970 - -
03975 -pf03960 -
03980 -pf07687pf01546 -
BMC-P
BMC-H
BMC-T
Aldehyde dehydrogenase
PTAC
Signature enzyme
Alcohol dehydrogenase
Regulator

Salmonella enterica subsp. enterica serovar Richmond. Bacteria; Proteobacteria; Gammaproteobacteria; Enterobacterales;

E0577__locus_1

EUT1

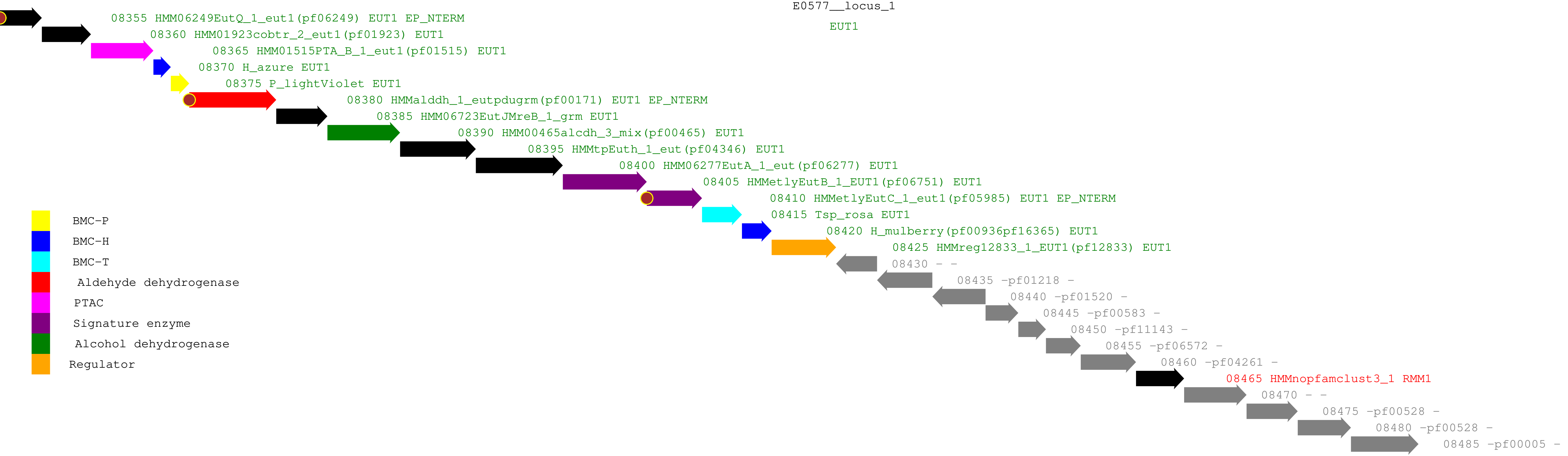

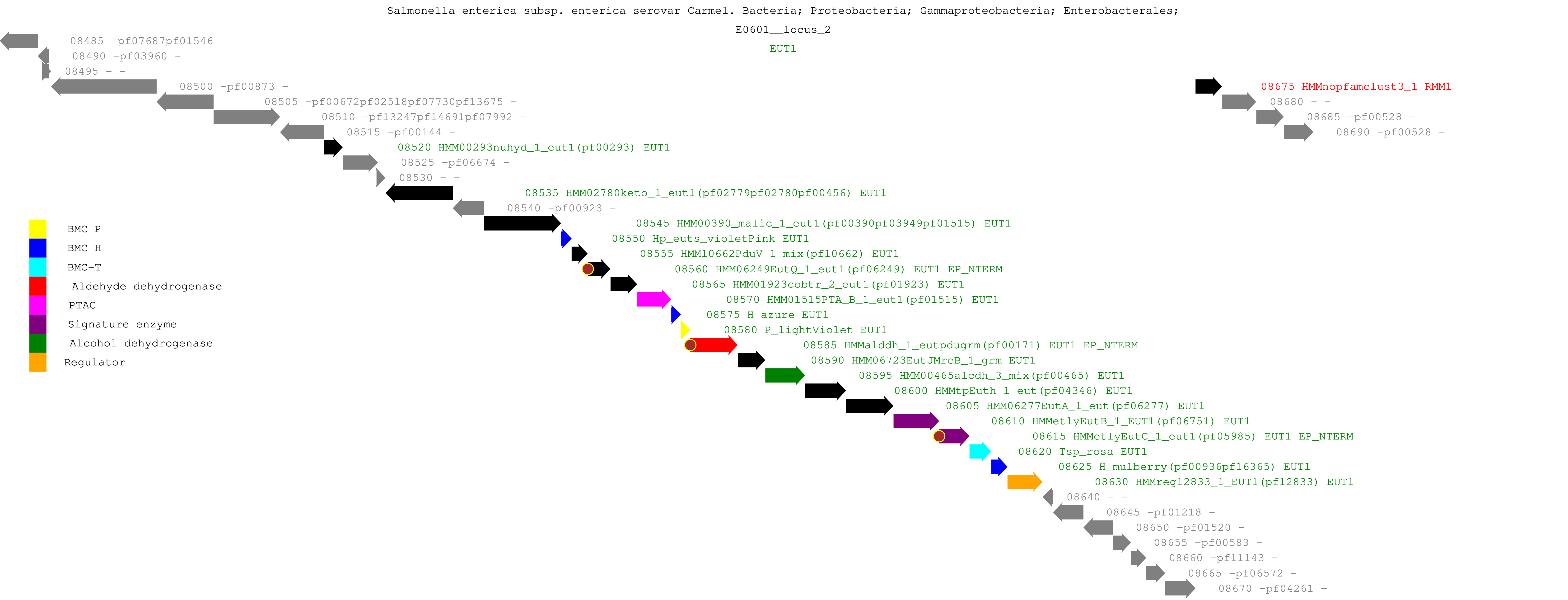
Salmonella enterica subsp. enterica serovar Carmel. Bacteria; Proteobacteria; Gammaproteobacteria; Enterobacterales;
E0601__locus_2
EUT1
08485 -pf07687pf01546 -
08490 -pf03960 -
08495 - -
08500 -pf00873 -
08505 -pf00672pf02518pf07730pf13675 -
08510 -pf13247pf14691pf07992 -
08515 -pf00144 -
08520 HMM00293nuhyd_1_eut1(pf00293) EUT1
08525 -pf06674 -
08530 - -
08535 HMM02780keto_1_eut1(pf02779pf02780pf00456) EUT1
08540 -pf00923 -
08545 HMM00390_malic_1_eut1(pf00390pf03949pf01515) EUT1
08550 Hp_euts_violetPink EUT1
08555 HMM10662PduV_1_mix(pf10662) EUT1
08560 HMM06249EutQ_1_eut1(pf06249) EUT1 EP_NTERM
08565 HMM01923cobtr_2_eut1(pf01923) EUT1
08570 HMM01515PTA_B_1_eut1(pf01515) EUT1
08575 H_azure EUT1
08580 P_lightViolet EUT1
08585 HMMalddh_1_eutpdugrm(pf00171) EUT1 EP_NTERM
08590 HMM06723EutJMreB_1_grm EUT1
08595 HMM00465alcdh_3_mix(pf00465) EUT1
08600 HMMtpEuth_1_eut(pf04346) EUT1
08605 HMM06277EutA_1_eut(pf06277) EUT1
08610 HMMetlyEutB_1_EUT1(pf06751) EUT1
08615 HMMetlyEutC_1_eut1(pf05985) EUT1 EP_NTERM
08620 Tsp_rosa EUT1
08625 H_mulberry(pf00936pf16365) EUT1
08630 HMMreg12833_1_EUT1(pf12833) EUT1
08640 - -
08645 -pf01218 -
08650 -pf01520 -
08655 -pf00583 -
08660 -pf11143 -
08665 -pf06572 -
08670 -pf04261 -
08675 HMMnopfamclust3_1 RMM1
08680 - -
08685 -pf00528 -
08690 -pf00528 -
BMC-P
BMC-H
BMC-T
Aldehyde dehydrogenase
PTAC
Signature enzyme
Alcohol dehydrogenase
Regulator

Salmonella abony. Bacteria; Proteobacteria; Gammaproteobacteria; Enterobacterales;

E0657__locus_2

EUT1

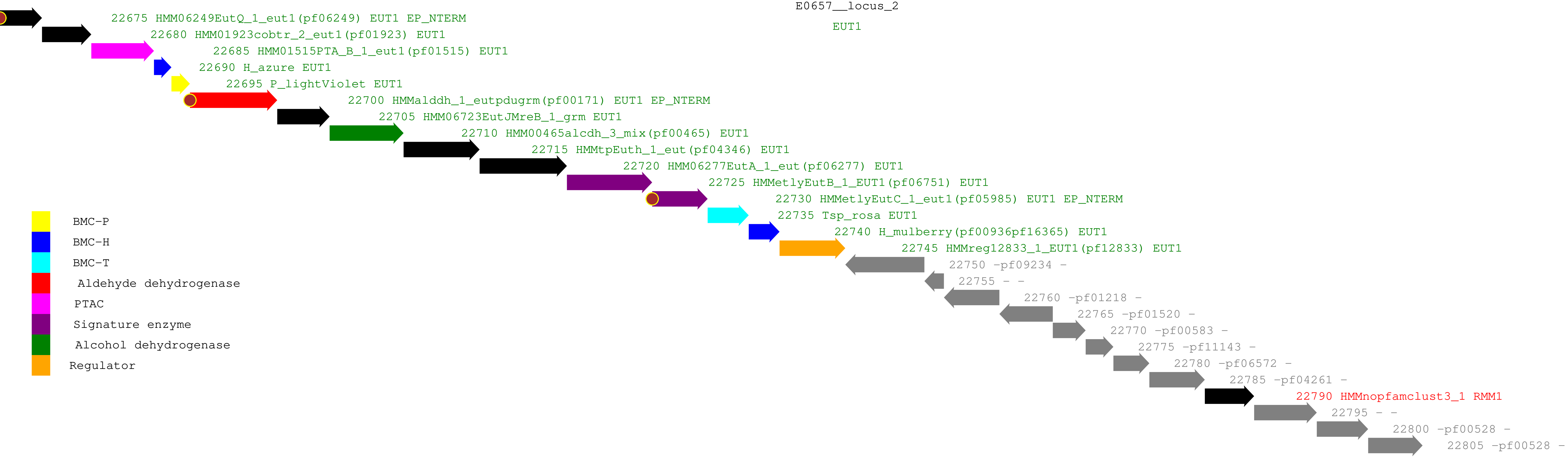

Salmonella enterica subsp. enterica serovar Oslo. Bacteria; Proteobacteria; Gammaproteobacteria; Enterobacterales;

E0660__locus_1

EUT1

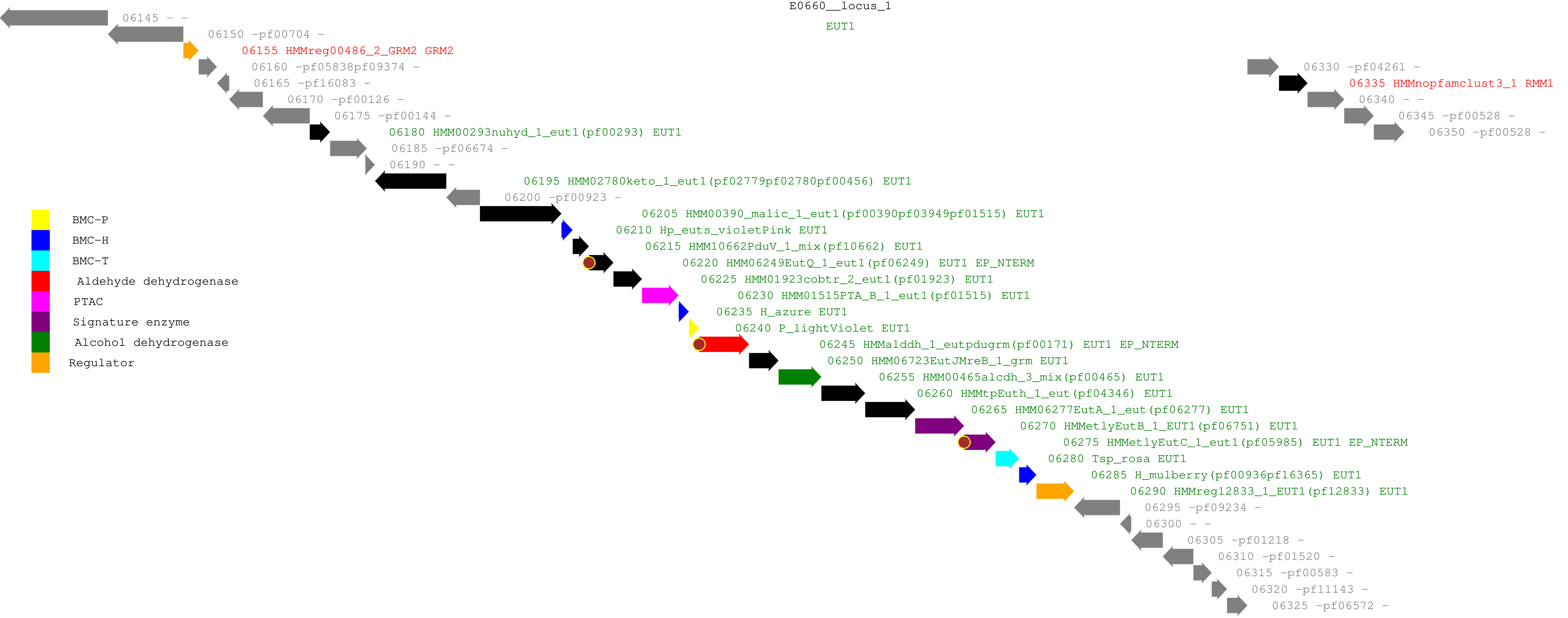

Salmonella berta. Bacteria; Proteobacteria; Gammaproteobacteria; Enterobacterales;

E0917__locus_2

EUT1

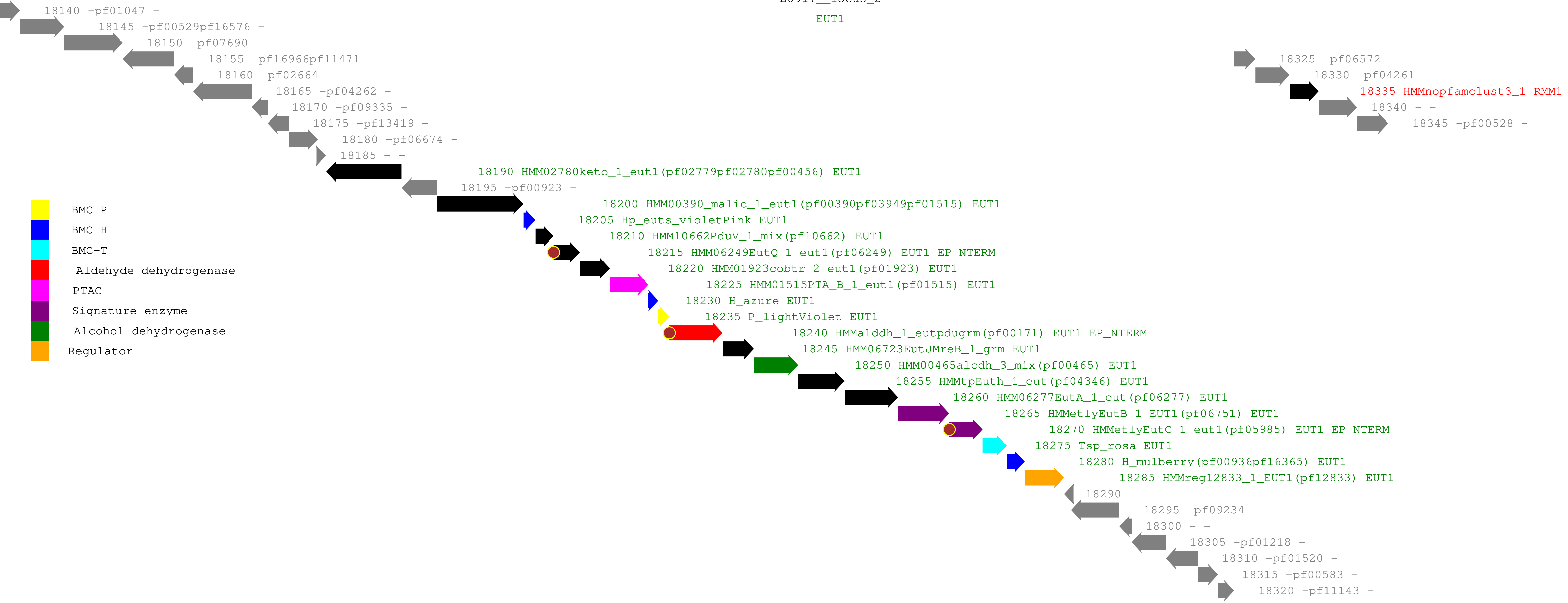

Salmonella enterica subsp. enterica serovar Sundsvall. Bacteria; Proteobacteria; Gammaproteobacteria; Enterobacterales;

E0918__locus_1

EUT1

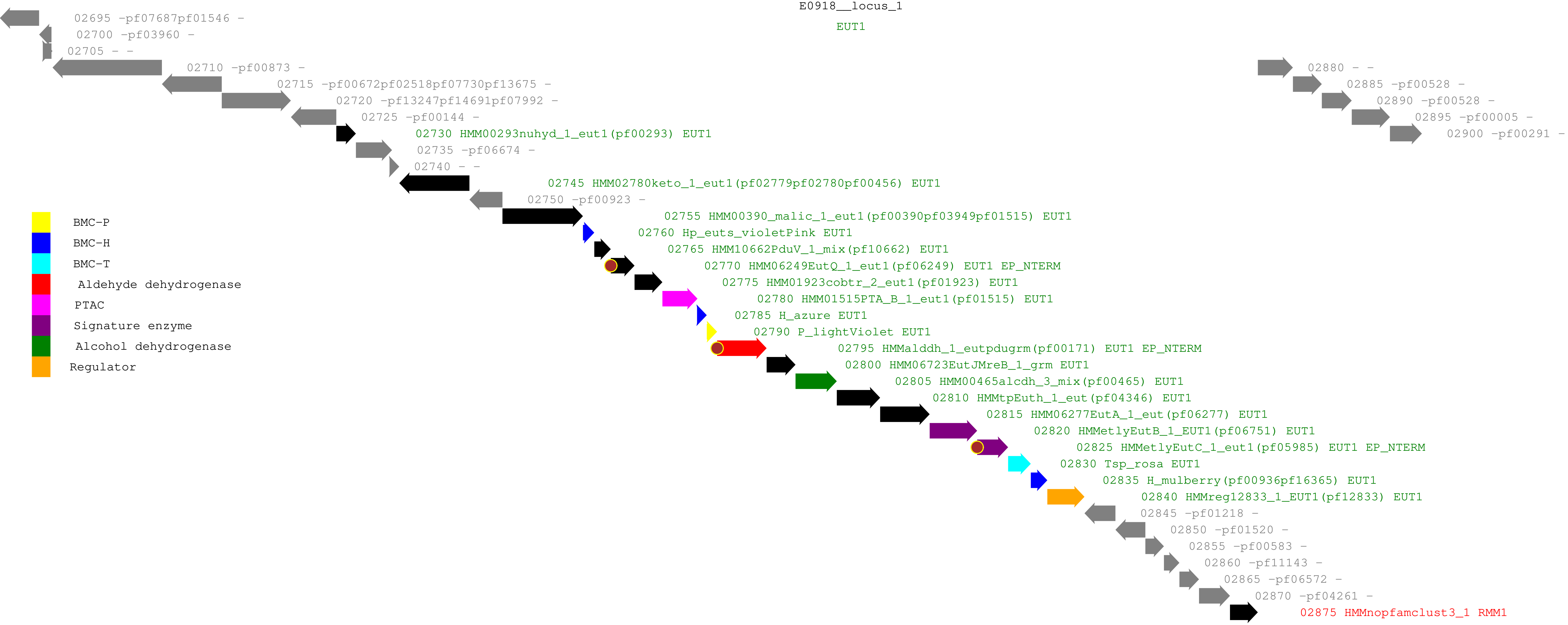

Salmonella enterica subsp. enterica serovar Szentes. Bacteria; Proteobacteria; Gammaproteobacteria; Enterobacterales;

E0931__locus_2

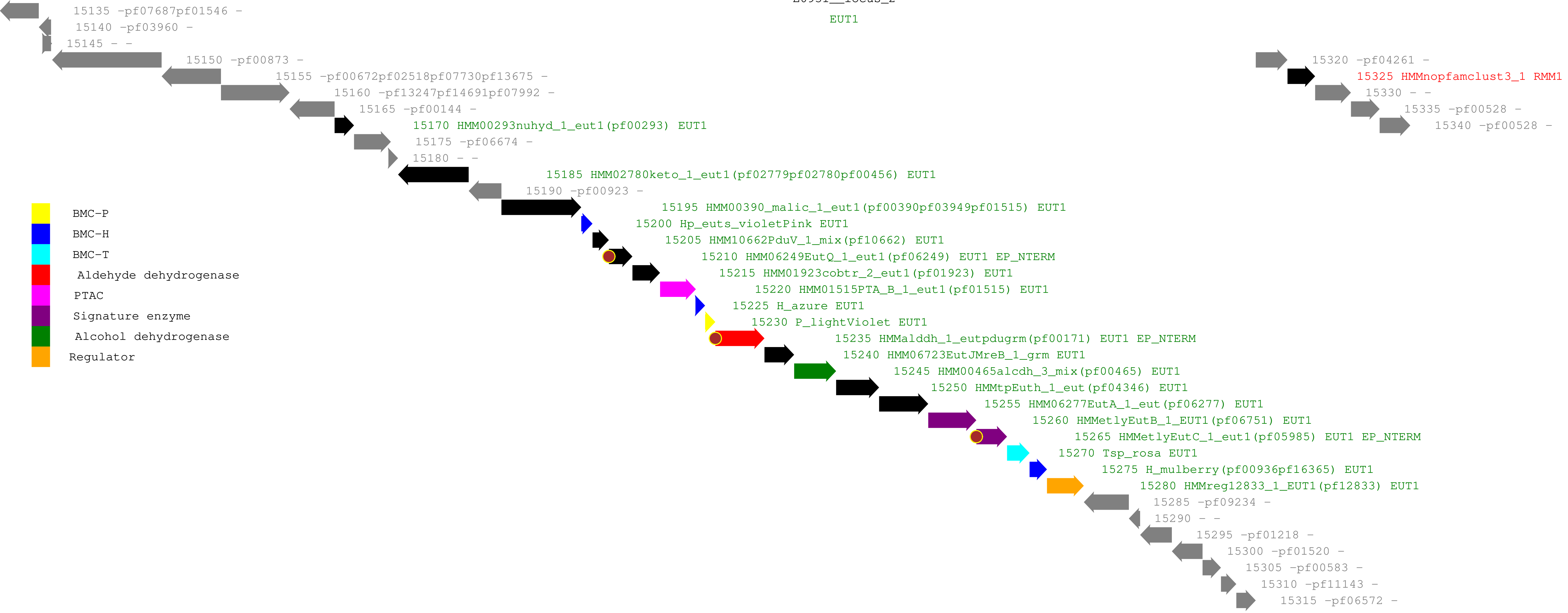

Salmonella enterica subsp. enterica serovar Mapo. Bacteria; Proteobacteria; Gammaproteobacteria; Enterobacterales;

E0940__locus_2

EUT1

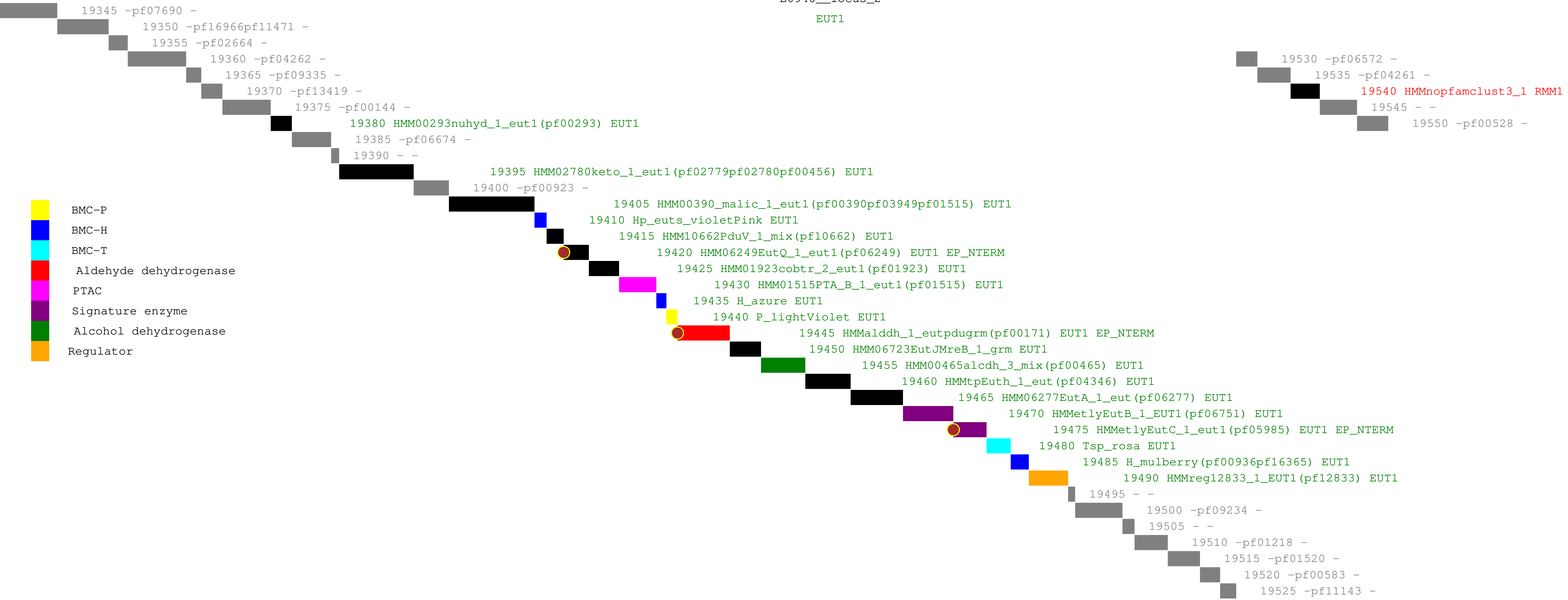

Salmonella enterica subsp. enterica serovar Manhattan. Bacteria; Proteobacteria; Gammaproteobacteria; Enterobacterales;

E0960__locus_2

EUT1

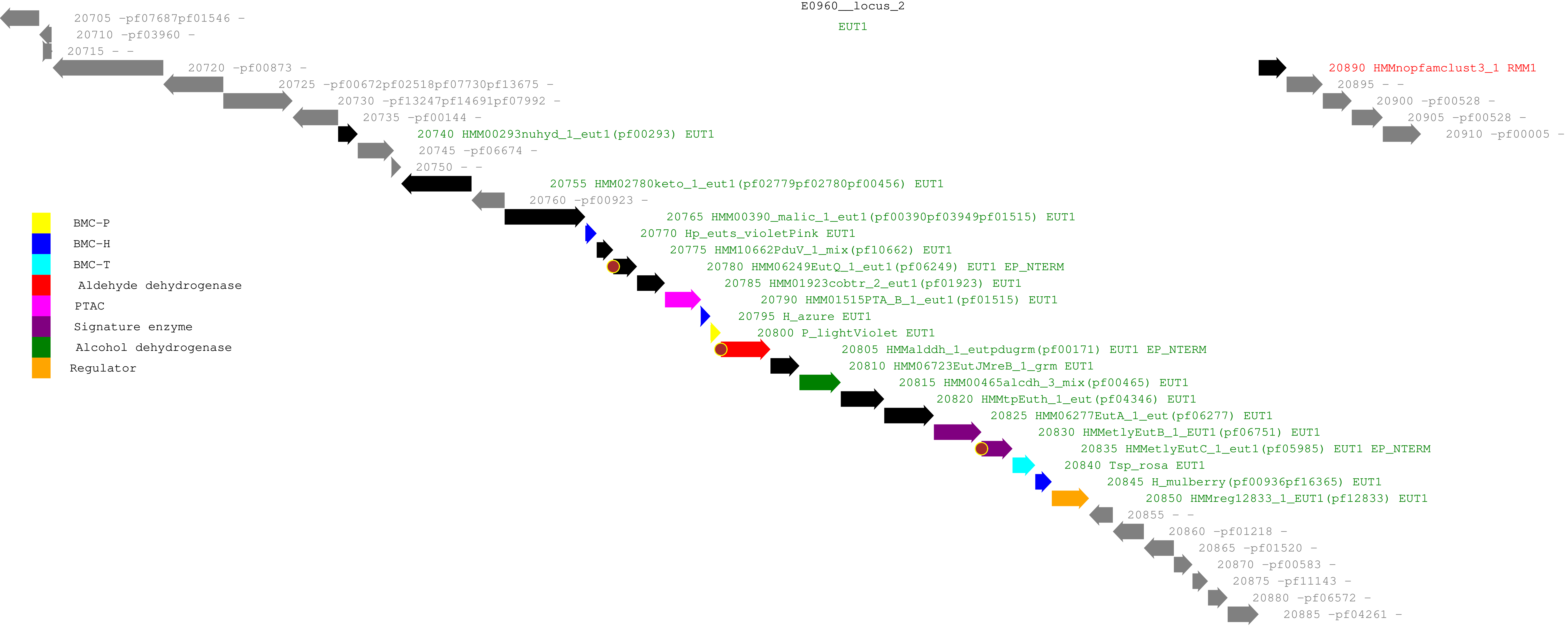

Salmonella enterica subsp. enterica serovar Telaviv. Bacteria; Proteobacteria; Gammaproteobacteria; Enterobacterales;

E0968__locus_2

EUT1

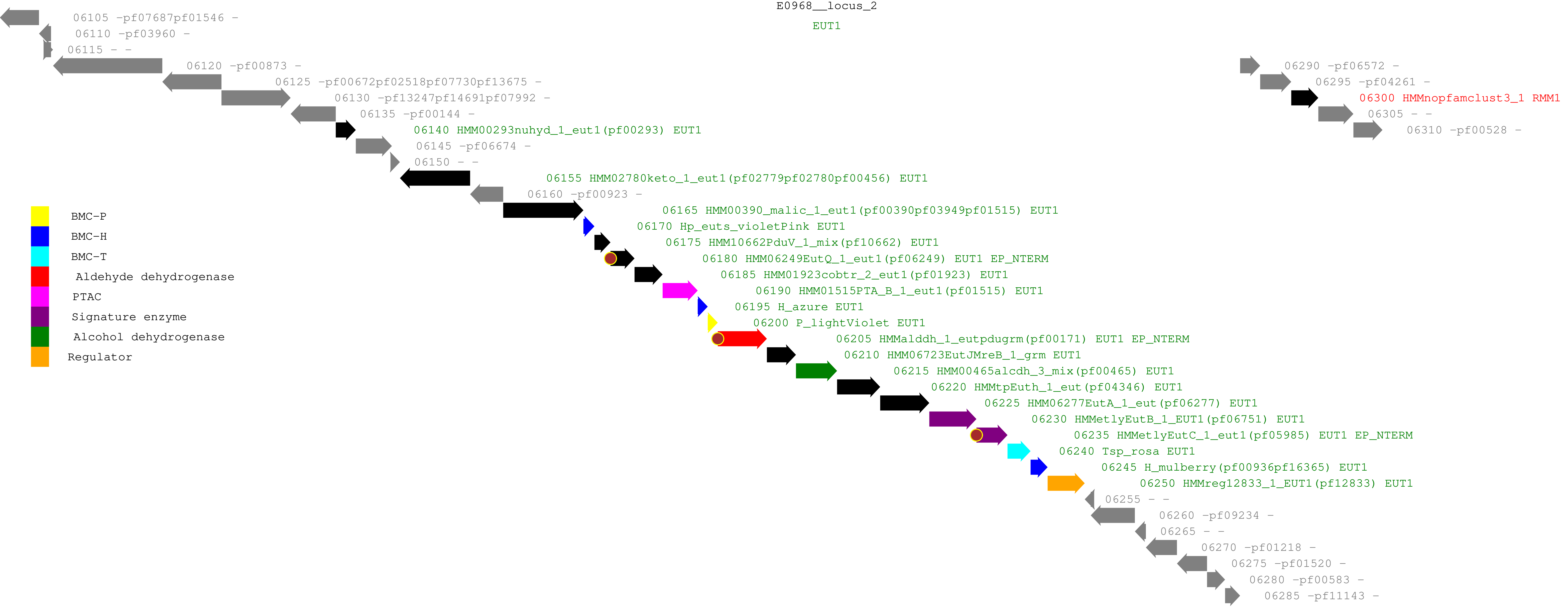

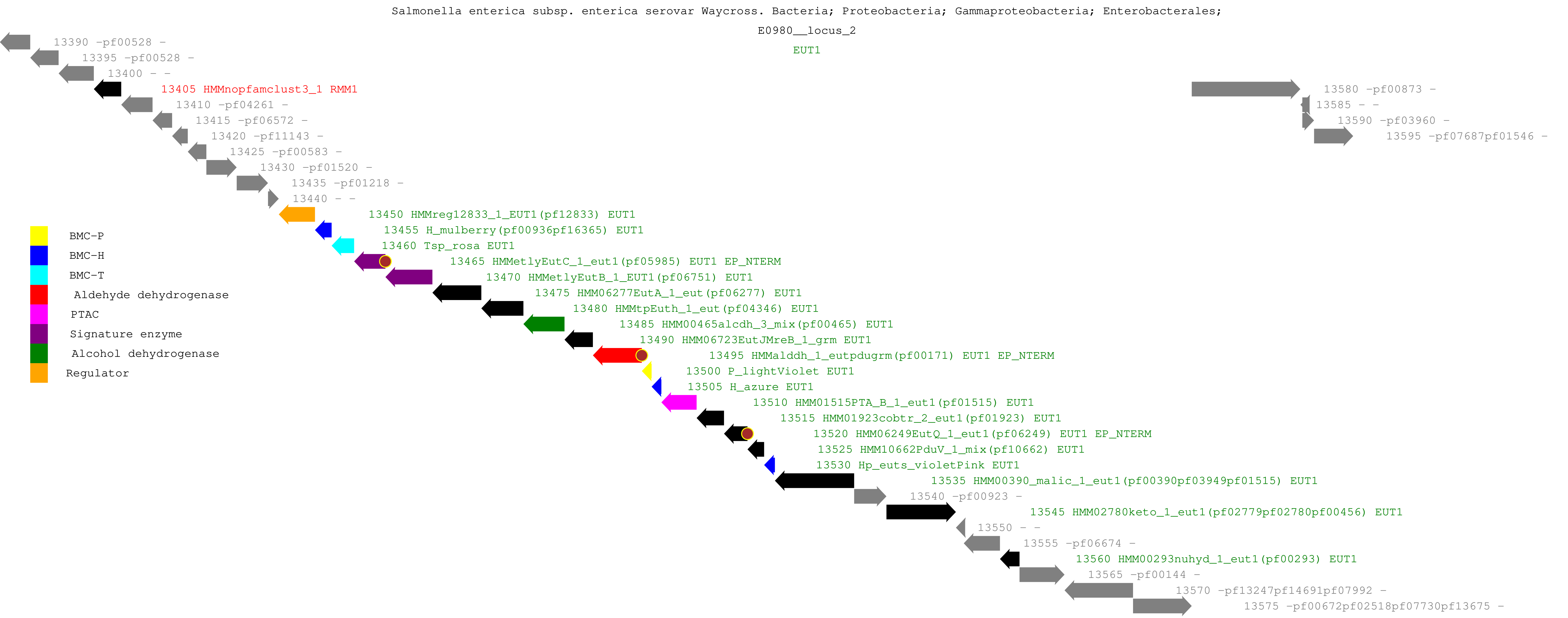
Salmonella enterica subsp. enterica serovar Waycross. Bacteria; Proteobacteria; Gammaproteobacteria; Enterobacterales;
E0980__locus_2
EUT1
13390 -pf00528 -
13395 -pf00528 -
13400 - -
13405 HMMnopfamclust3_1 RMM1
13410 -pf04261 -
13415 -pf06572 -
13420 -pf11143 -
13425 -pf00583 -
13430 -pf01520 -
13435 -pf01218 -
13440 - -
13450 HMMreg12833_1_EUT1(pf12833) EUT1
13455 H_mulberry(pf00936pf16365) EUT1
13460 Tsp_rosa EUT1
13465 HMMetlyEutC_1_eut1(pf05985) EUT1 EP_NTERM
13470 HMMetlyEutB_1_EUT1(pf06751) EUT1
13475 HMM06277EutA_1_eut(pf06277) EUT1
13480 HMMtpEuth_1_eut(pf04346) EUT1
13485 HMM00465alcdh_3_mix(pf00465) EUT1
13490 HMM06723EutJMreB_1_grm EUT1
13495 HMMalddh_1_eutpdugrm(pf00171) EUT1 EP_NTERM
13500 P_lightViolet EUT1
13505 H_azure EUT1
13510 HMM01515PTA_B_1_eut1(pf01515) EUT1
13515 HMM01923cobtr_2_eut1(pf01923) EUT1
13520 HMM06249EutQ_1_eut1(pf06249) EUT1 EP_NTERM
13525 HMM10662PduV_1_mix(pf10662) EUT1
13530 Hp_euts_violetPink EUT1
13535 HMM00390_malic_1_eut1(pf00390pf03949pf01515) EUT1
13540 -pf00923 -
13545 HMM02780keto_1_eut1(pf02779pf02780pf00456) EUT1
13550 - -
13555 -pf06674 -
13560 HMM00293nuhyd_1_eut1(pf00293) EUT1
13565 -pf00144 -
13570 -pf13247pf14691pf07992 -
13575 -pf00672pf02518pf07730pf13675 -
13580 -pf00873 -
13585 - -
13590 -pf03960 -
13595 -pf07687pf01546 -
BMC-P
BMC-H
BMC-T
Aldehyde dehydrogenase
PTAC
Signature enzyme
Alcohol dehydrogenase
Regulator

Salmonella enterica subsp. enterica serovar Newington. Bacteria; Proteobacteria; Gammaproteobacteria; Enterobacterales;

E0991__locus_2

EUT1

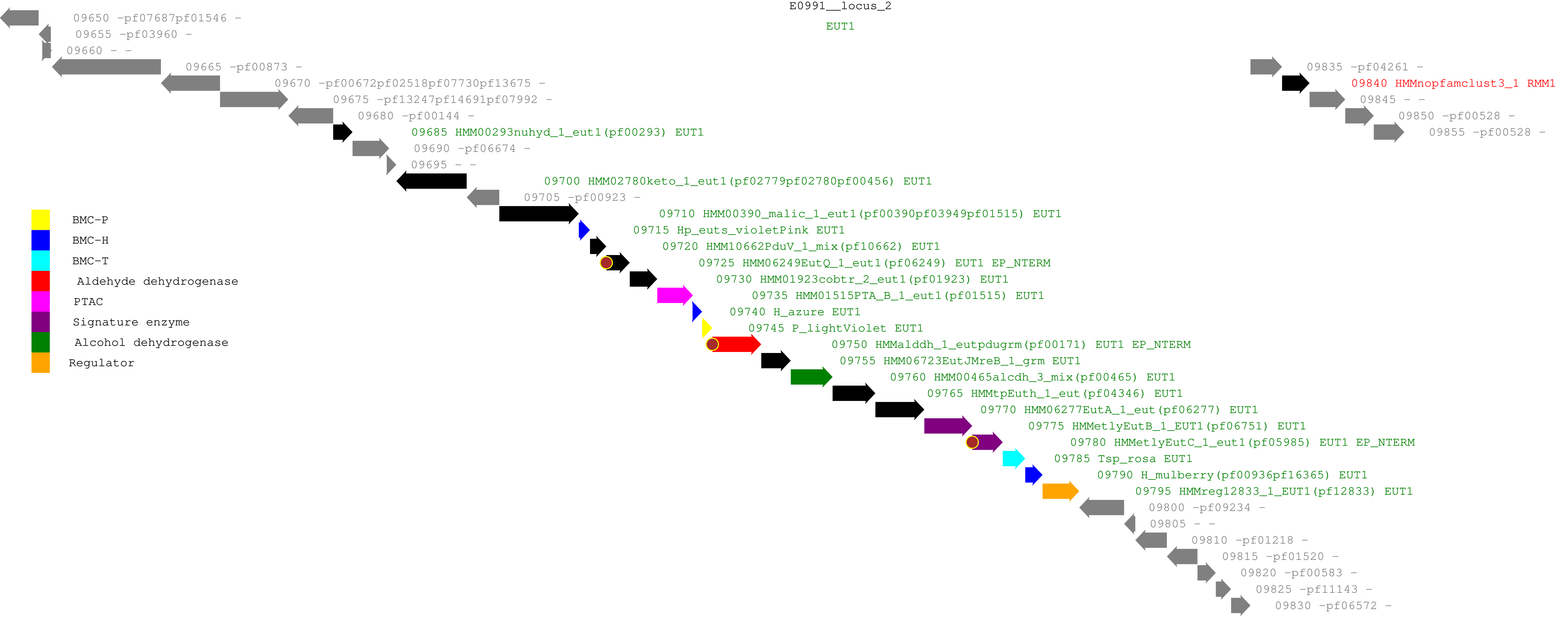

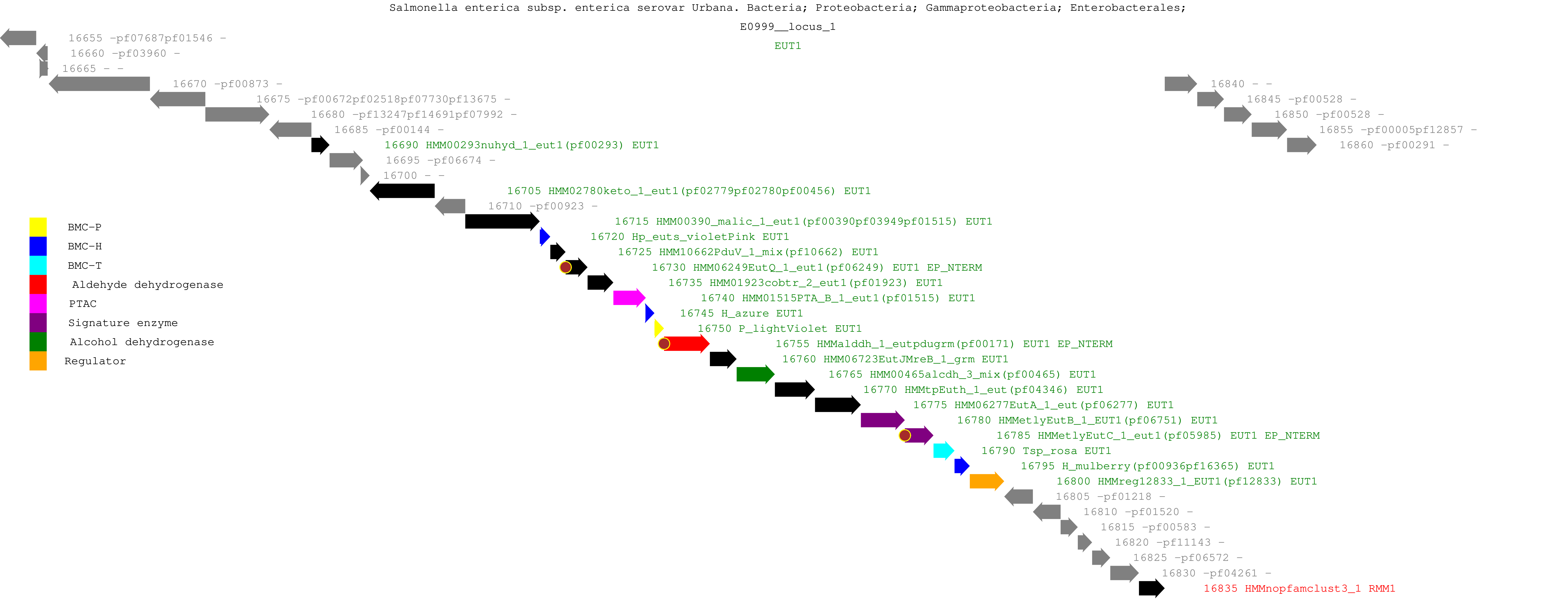
Salmonella enterica subsp. enterica serovar Urbana. Bacteria; Proteobacteria; Gammaproteobacteria; Enterobacterales;
E0999__locus_1
EUT1
16655 -pf07687pf01546 -
16660 -pf03960 -
16665 - -
16670 -pf00873 -
16675 -pf00672pf02518pf07730pf13675 -
16680 -pf13247pf14691pf07992 -
16685 -pf00144 -
16690 HMM00293nuhyd_1_eut1(pf00293) EUT1
16695 -pf06674 -
16700 - -
16705 HMM02780keto_1_eut1(pf02779pf02780pf00456) EUT1
16710 -pf00923 -
16715 HMM00390_malic_1_eut1(pf00390pf03949pf01515) EUT1
16720 Hp_euts_violetPink EUT1
16725 HMM10662PduV_1_mix(pf10662) EUT1
16730 HMM06249EutQ_1_eut1(pf06249) EUT1 EP_NTERM
16735 HMM01923cobtr_2_eut1(pf01923) EUT1
16740 HMM01515PTA_B_1_eut1(pf01515) EUT1
16745 H_azure EUT1
16750 P_lightViolet EUT1
16755 HMMalddh_1_eutpdugrm(pf00171) EUT1 EP_NTERM
16760 HMM06723EutJMreB_1_grm EUT1
16765 HMM00465alcdh_3_mix(pf00465) EUT1
16770 HMMtpEuth_1_eut(pf04346) EUT1
16775 HMM06277EutA_1_eut(pf06277) EUT1
16780 HMMetlyEutB_1_EUT1(pf06751) EUT1
16785 HMMetlyEutC_1_eut1(pf05985) EUT1 EP_NTERM
16790 Tsp_rosa EUT1
16795 H_mulberry(pf00936pf16365) EUT1
16800 HMMreg12833_1_EUT1(pf12833) EUT1
16805 -pf01218 -
16810 -pf01520 -
16815 -pf00583 -
16820 -pf11143 -
16825 -pf06572 -
16830 -pf04261 -
16835 HMMnopfamclust3_1 RMM1
16840 - -
16845 -pf00528 -
16850 -pf00528 -
16855 -pf00005pf12857 -
16860 -pf00291 -
BMC-P
BMC-H
BMC-T
Aldehyde dehydrogenase
PTAC
Signature enzyme
Alcohol dehydrogenase
Regulator

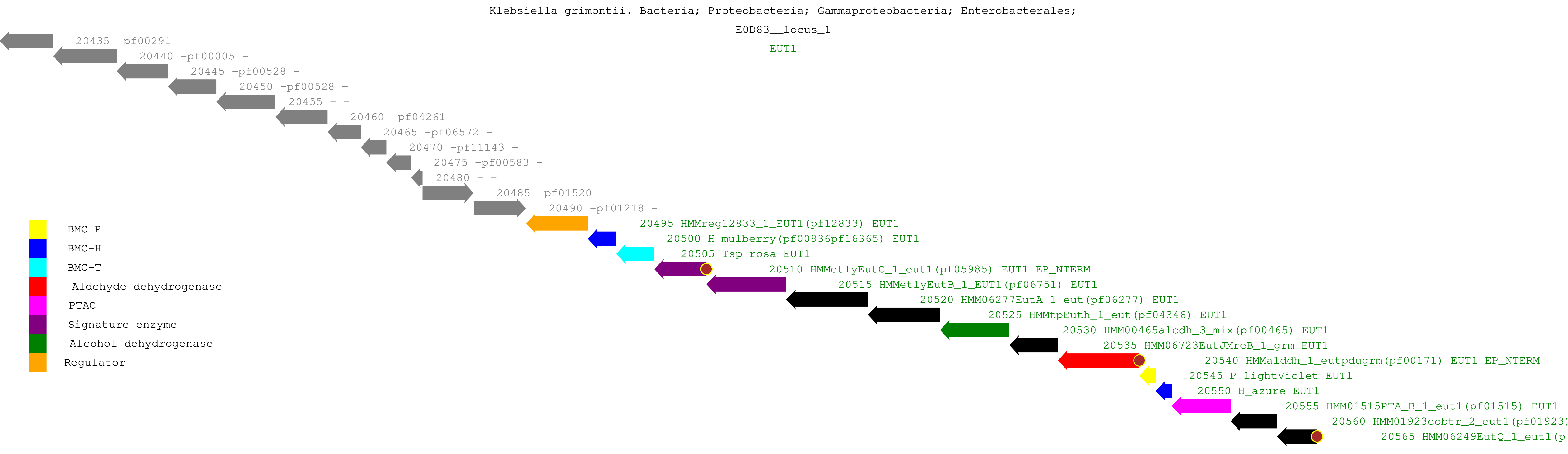
Klebsiella grimontii. Bacteria; Proteobacteria; Gammaproteobacteria; Enterobacterales;
E0D83__locus_1
EUT1
20435 -pf00291 -
20440 -pf00005 -
20445 -pf00528 -
20450 -pf00528 -
20455 - -
20460 -pf04261 -
20465 -pf06572 -
20470 -pf11143 -
20475 -pf00583 -
20480 - -
20485 -pf01520 -
20490 -pf01218 -
20495 HMMreg12833_1_EUT1(pf12833) EUT1
20500 H_mulberry(pf00936pf16365) EUT1
20505 Tsp_rosa EUT1
20510 HMMetlyEutC_1_eut1(pf05985) EUT1 EP_NTERM
20515 HMMetlyEutB_1_EUT1(pf06751) EUT1
20520 HMM06277EutA_1_eut(pf06277) EUT1
20525 HMMtpEuth_1_eut(pf04346) EUT1
20530 HMM00465alcdh_3_mix(pf00465) EUT1
20535 HMM06723EutJMreB_1_grm EUT1
20540 HMMaldh_1_eutpdugrm(pf00171) EUT1 EP_NTERM
20545 P_lightViolet EUT1
20550 H_azure EUT1
20555 HMM01515PTA_B_1_eut1(pf01515) EUT1
20560 HMM01923cobtr_2_eut1(pf01923)
20565 HMM06249EutQ_1_eut1(p
BMC-P
BMC-H
BMC-T
Aldehyde dehydrogenase
PTAC
Signature enzyme
Alcohol dehydrogenase
Regulator

Salmonella enterica subsp. enterica serovar Heidelberg. Bacteria; Proteobacteria; Gammaproteobacteria; Enterobacterales;

E0J14__locus_1

EUT1

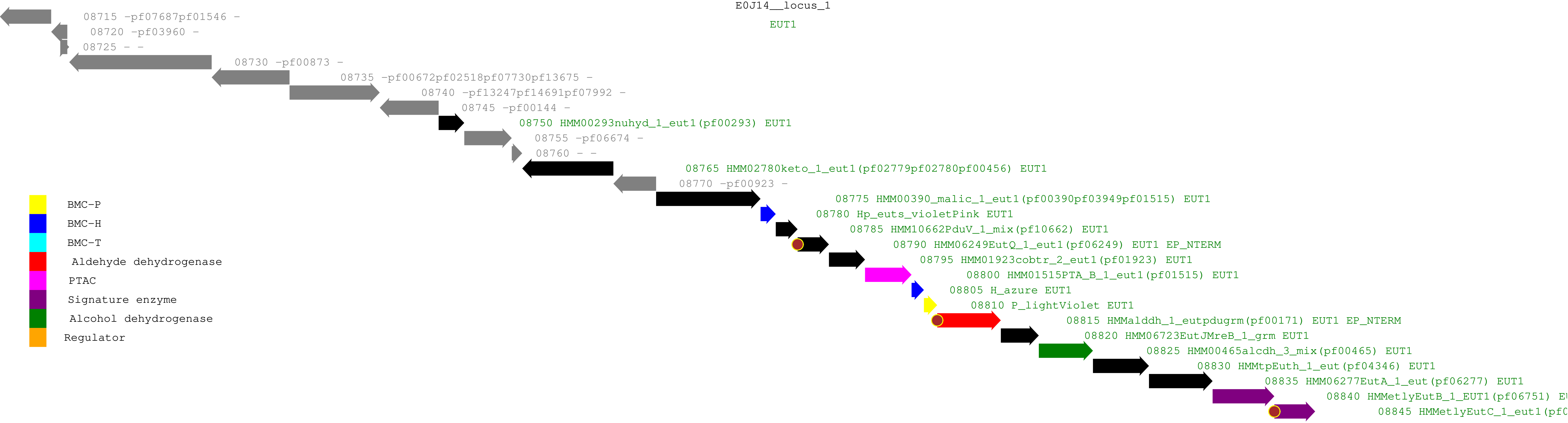

Salmonella enterica subsp. enterica serovar Kisangani. Bacteria; Proteobacteria; Gammaproteobacteria; Enterobacterales;

E0J50__locus_2

EUT1

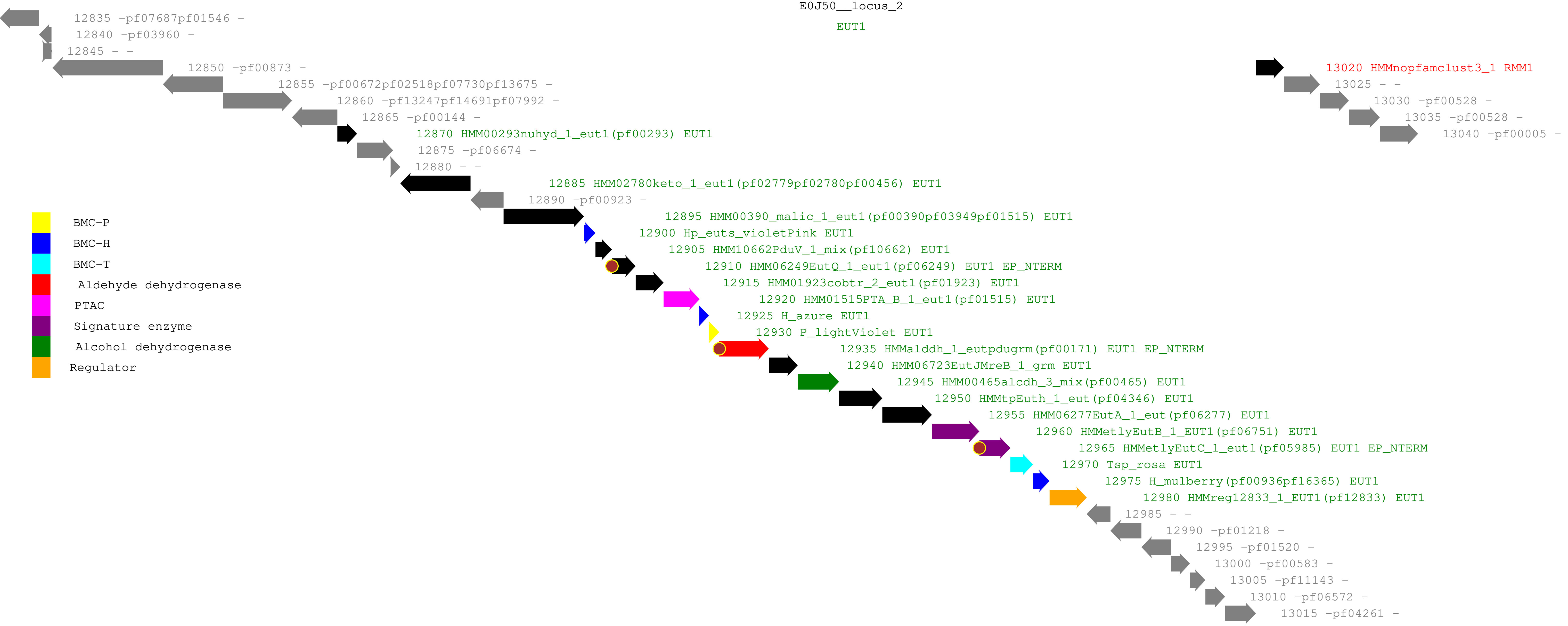

Salmonella enterica subsp. enterica serovar Javiana. Bacteria; Proteobacteria; Gammaproteobacteria; Enterobacterales;

E0S35__locus_1

EUT1

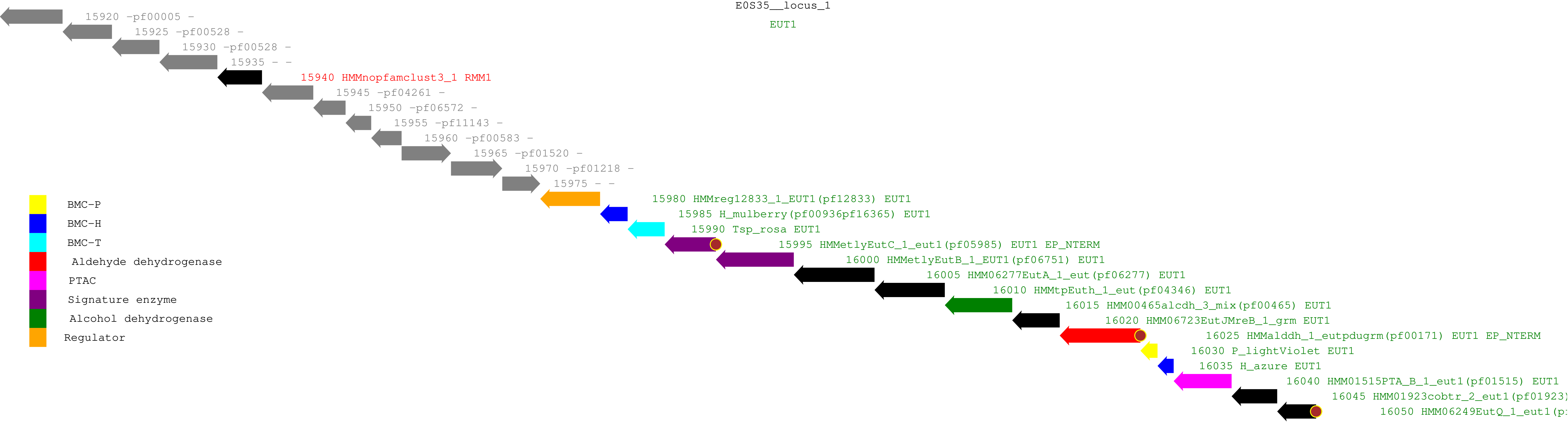

Salmonella enterica subsp. enterica serovar Kandla. Bacteria; Proteobacteria; Gammaproteobacteria; Enterobacterales;

E0S43__locus_2

EUT1

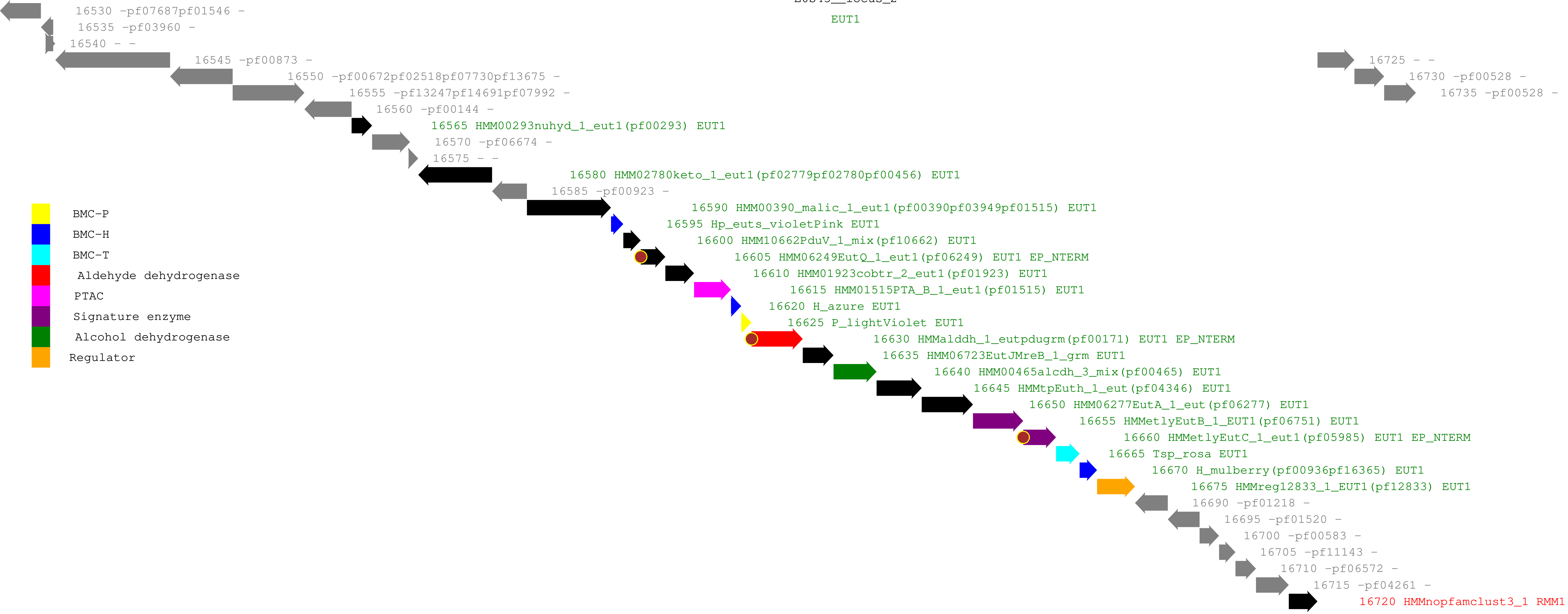

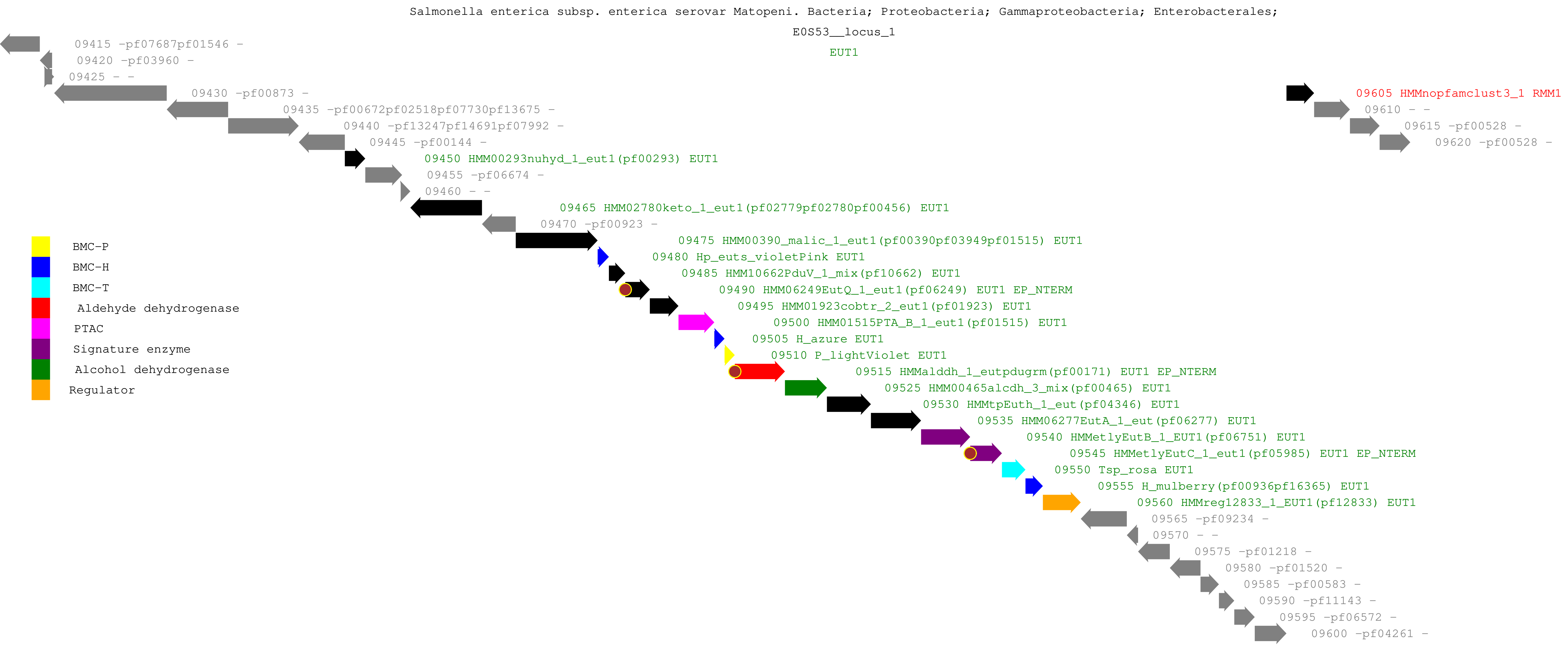

Salmonella enterica subsp. enterica serovar Matopeni. Bacteria; Proteobacteria; Gammaproteobacteria; Enterobacterales;
E0S53__locus_1
EUT1
09415 -pf07687pf01546 -
09420 -pf03960 -
09425 - -
09430 -pf00873 -
09435 -pf00672pf02518pf07730pf13675 -
09440 -pf13247pf14691pf07992 -
09445 -pf00144 -
09450 HMM00293nuhyd_1_eut1(pf00293) EUT1
09455 -pf06674 -
09460 - -
09465 HMM02780keto_1_eut1(pf02779pf02780pf00456) EUT1
09470 -pf00923 -
09475 HMM00390_malic_1_eut1(pf00390pf03949pf01515) EUT1
09480 Hp_euts_violetPink EUT1
09485 HMM10662PduV_1_mix(pf10662) EUT1
09490 HMM06249EutQ_1_eut1(pf06249) EUT1 EP_NTERM
09495 HMM01923cobtr_2_eut1(pf01923) EUT1
09500 HMM01515PTA_B_1_eut1(pf01515) EUT1
09505 H_azure EUT1
09510 P_lightViolet EUT1
09515 HMMalddh_1_eutpdugrm(pf00171) EUT1 EP_NTERM
09525 HMM00465alcdh_3_mix(pf00465) EUT1
09530 HMMtpEuth_1_eut(pf04346) EUT1
09535 HMM06277EutA_1_eut(pf06277) EUT1
09540 HMMetlyEutB_1_EUT1(pf06751) EUT1
09545 HMMetlyEutC_1_eut1(pf05985) EUT1 EP_NTERM
09550 Tsp_rosa EUT1
09555 H_mulberry(pf00936pf16365) EUT1
09560 HMMreg12833_1_EUT1(pf12833) EUT1
09565 -pf09234 -
09570 - -
09575 -pf01218 -
09580 -pf01520 -
09585 -pf00583 -
09590 -pf11143 -
09595 -pf06572 -
09600 -pf04261 -
09605 HMMnopfamclust3_1 RMM1
09610 - -
09615 -pf00528 -
09620 -pf00528 -
BMC-P
BMC-H
BMC-T
Aldehyde dehydrogenase
PTAC
Signature enzyme
Alcohol dehydrogenase
Regulator

Salmonella enterica subsp. enterica serovar Butantan. Bacteria; Proteobacteria; Gammaproteobacteria; Enterobacterales;

E0S81__locus_2

EUT1

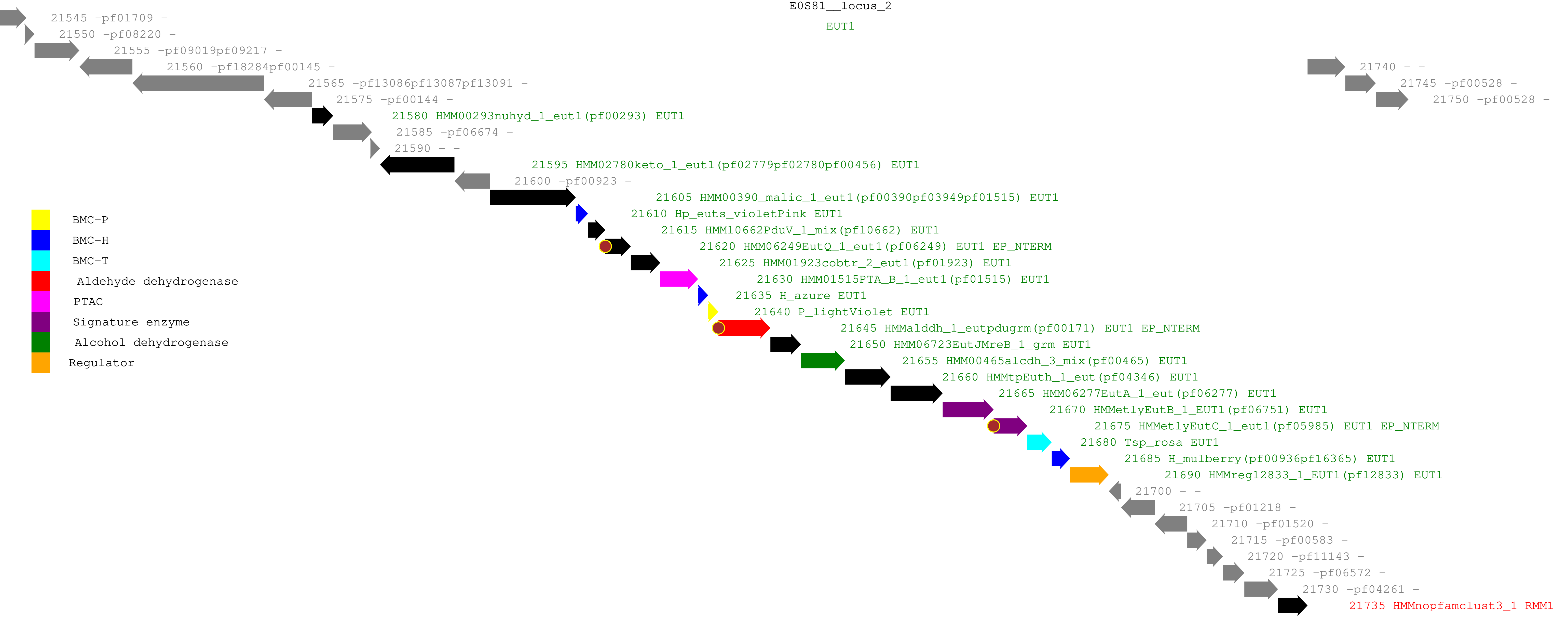

Salmonella enterica subsp. enterica serovar Galiema. Bacteria; Proteobacteria; Gammaproteobacteria; Enterobacterales;

E0U28__locus_2

EUT1

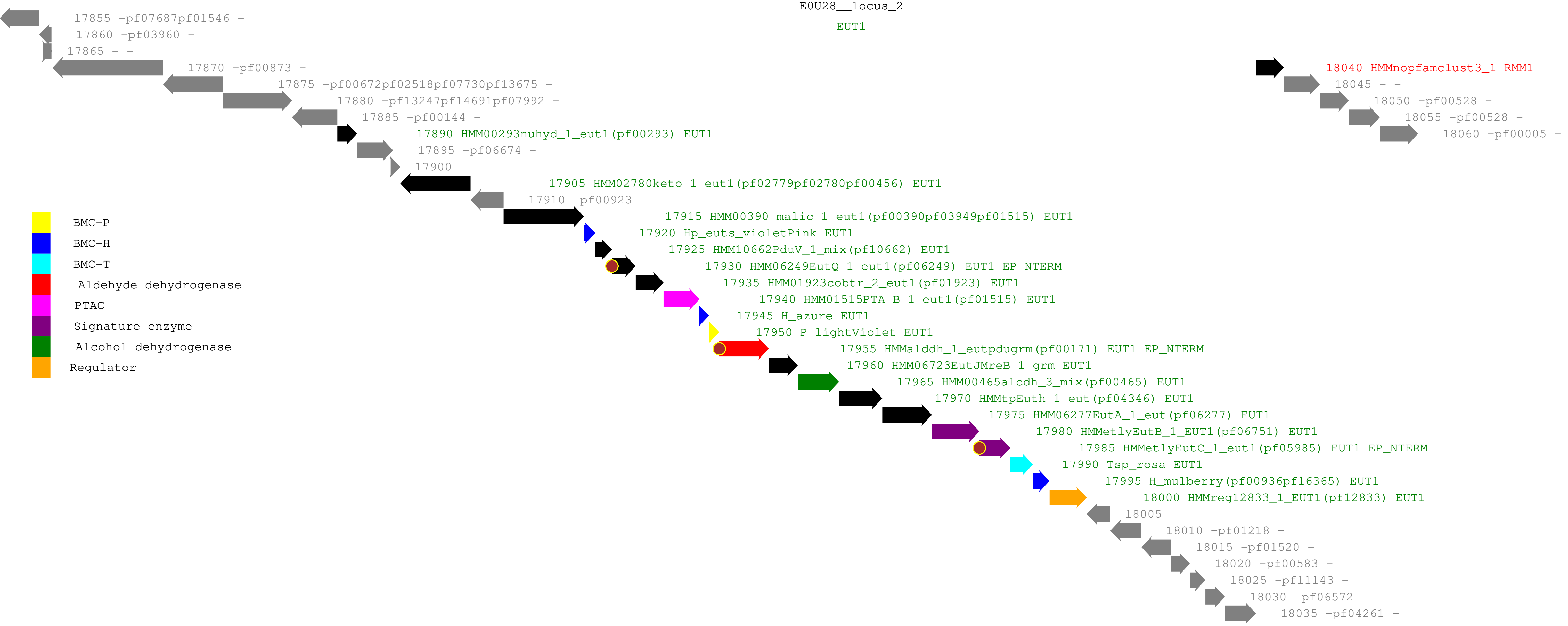

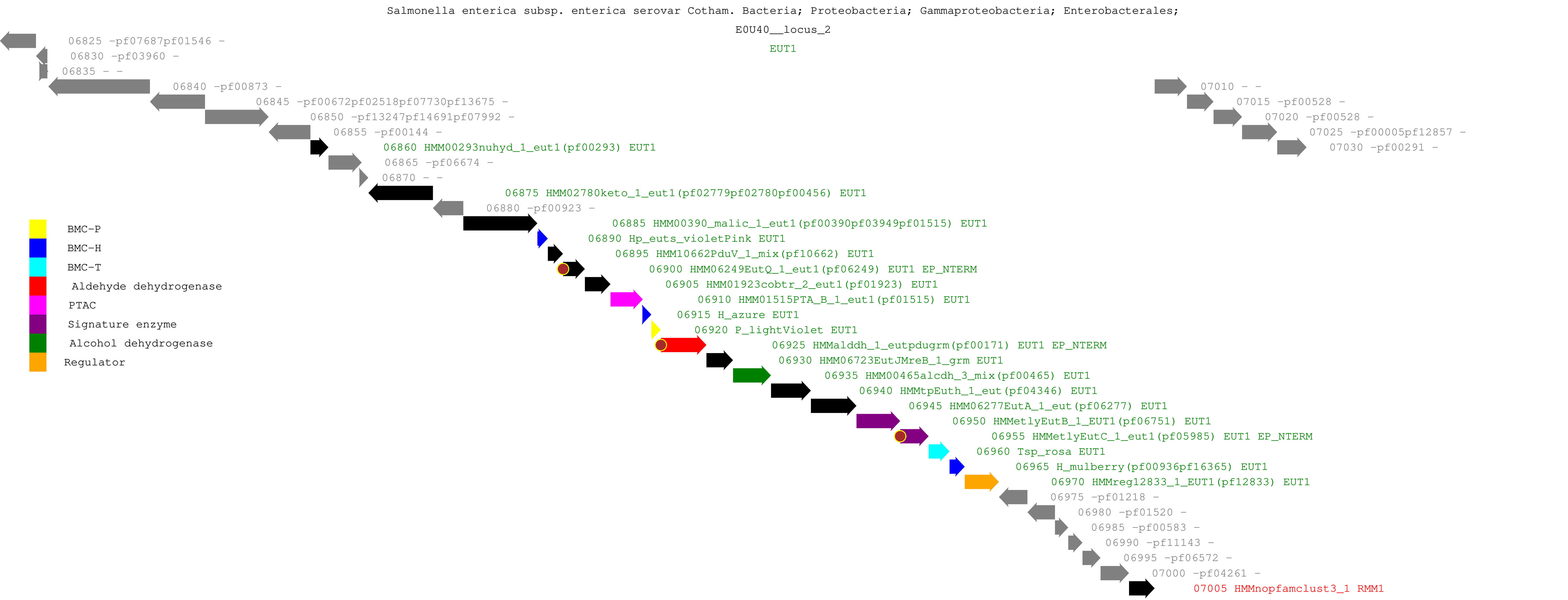

Salmonella enterica subsp. enterica serovar Cotham. Bacteria; Proteobacteria; Gammaproteobacteria; Enterobacterales;
E0U40__locus_2
EUT1
06825 -pf07687pf01546 -
06830 -pf03960 -
06835 - -
06840 -pf00873 -
06845 -pf00672pf02518pf07730pf13675 -
06850 -pf13247pf14691pf07992 -
06855 -pf00144 -
06860 HMM00293nuhyd_1_eut1(pf00293) EUT1
06865 -pf06674 -
06870 - -
06875 HMM02780keto_1_eut1(pf02779pf02780pf00456) EUT1
06880 -pf00923 -
06885 HMM00390_malic_1_eut1(pf00390pf03949pf01515) EUT1
06890 Hp_euts_violetPink EUT1
06895 HMM10662PduV_1_mix(pf10662) EUT1
06900 HMM06249EutQ_1_eut1(pf06249) EUT1 EP_NTERM
06905 HMM01923cobtr_2_eut1(pf01923) EUT1
06910 HMM01515PTA_B_1_eut1(pf01515) EUT1
06915 H_azure EUT1
06920 P_lightViolet EUT1
06925 HMMalddh_1_eutpdugrm(pf00171) EUT1 EP_NTERM
06930 HMM06723EutJMreB_1_grm EUT1
06935 HMM00465alcdh_3_mix(pf00465) EUT1
06940 HMMtpEuth_1_eut(pf04346) EUT1
06945 HMM06277EutA_1_eut(pf06277) EUT1
06950 HMMetlyEutB_1_EUT1(pf06751) EUT1
06955 HMMetlyEutC_1_eut1(pf05985) EUT1 EP_NTERM
06960 Tsp_rosa EUT1
06965 H_mulberry(pf00936pf16365) EUT1
06970 HMMreg12833_1_EUT1(pf12833) EUT1
06975 -pf01218 -
06980 -pf01520 -
06985 -pf00583 -
06990 -pf11143 -
06995 -pf06572 -
07000 -pf04261 -
07005 HMMnopfamclust3_1 RMM1
07010 - -
07015 -pf00528 -
07020 -pf00528 -
07025 -pf00005pf12857 -
07030 -pf00291 -
BMC-P
BMC-H
BMC-T
Aldehyde dehydrogenase
PTAC
Signature enzyme
Alcohol dehydrogenase
Regulator

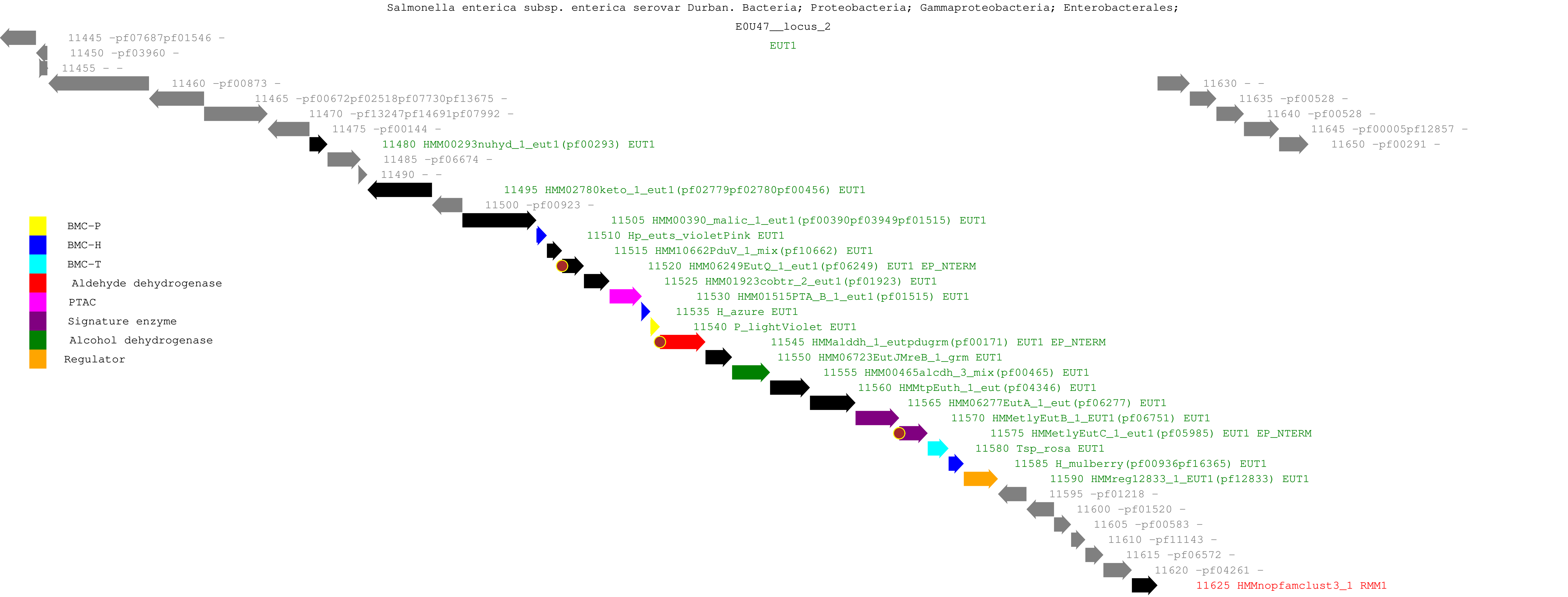

Salmonella enterica subsp. enterica serovar Durban. Bacteria; Proteobacteria; Gammaproteobacteria; Enterobacterales;
E0U47__locus_2
EUT1
11445 -pf07687pf01546 -
11450 -pf03960 -
11455 - -
11460 -pf00873 -
11465 -pf00672pf02518pf07730pf13675 -
11470 -pf13247pf14691pf07992 -
11475 -pf00144 -
11480 HMM00293nuhyd_1_eut1(pf00293) EUT1
11485 -pf06674 -
11490 - -
11495 HMM02780keto_1_eut1(pf02779pf02780pf00456) EUT1
11500 -pf00923 -
11505 HMM00390_malic_1_eut1(pf00390pf03949pf01515) EUT1
11510 Hp_euts_violetPink EUT1
11515 HMM10662PduV_1_mix(pf10662) EUT1
11520 HMM06249EutQ_1_eut1(pf06249) EUT1 EP_NTERM
11525 HMM01923cobtr_2_eut1(pf01923) EUT1
11530 HMM01515PTA_B_1_eut1(pf01515) EUT1
11535 H_azure EUT1
11540 P_lightViolet EUT1
11545 HMMalddh_1_eutpdugrm(pf00171) EUT1 EP_NTERM
11550 HMM06723EutJMreB_1_grm EUT1
11555 HMM00465alcdh_3_mix(pf00465) EUT1
11560 HMMtpEuth_1_eut(pf04346) EUT1
11565 HMM06277EutA_1_eut(pf06277) EUT1
11570 HMMetlyEutB_1_EUT1(pf06751) EUT1
11575 HMMetlyEutC_1_eut1(pf05985) EUT1 EP_NTERM
11580 Tsp_rosa EUT1
11585 H_mulberry(pf00936pf16365) EUT1
11590 HMMreg12833_1_EUT1(pf12833) EUT1
11595 -pf01218 -
11600 -pf01520 -
11605 -pf00583 -
11610 -pf11143 -
11615 -pf06572 -
11620 -pf04261 -
11625 HMMnopfamclust3_1 RMM1
11630 - -
11635 -pf00528 -
11640 -pf00528 -
11645 -pf00005pf12857 -
11650 -pf00291 -
BMC-P
BMC-H
BMC-T
Aldehyde dehydrogenase
PTAC
Signature enzyme
Alcohol dehydrogenase
Regulator

Salmonella enterica subsp. enterica serovar Haga. Bacteria; Proteobacteria; Gammaproteobacteria; Enterobacterales;

E0U59__locus_2

EUT1

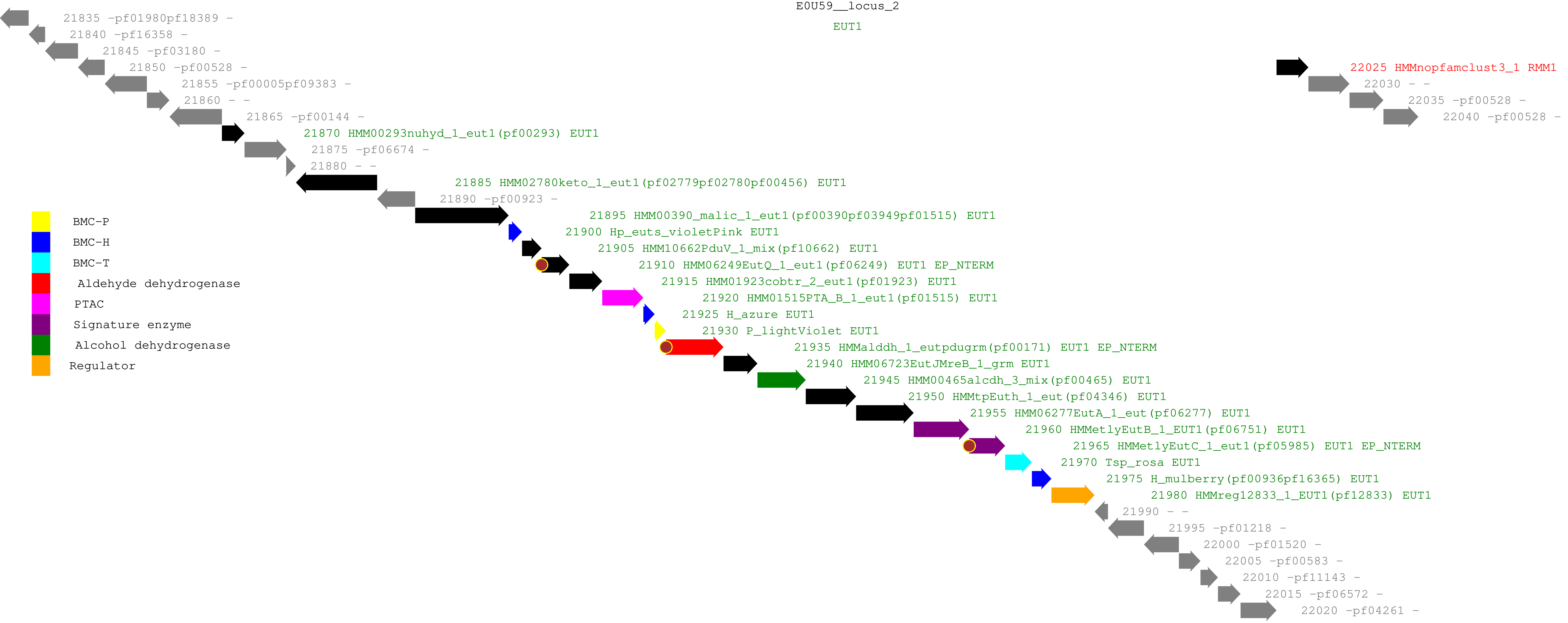

Salmonella enterica subsp. enterica serovar Reading. Bacteria; Proteobacteria; Gammaproteobacteria; Enterobacterales;

E0U64__locus_2

EUT1

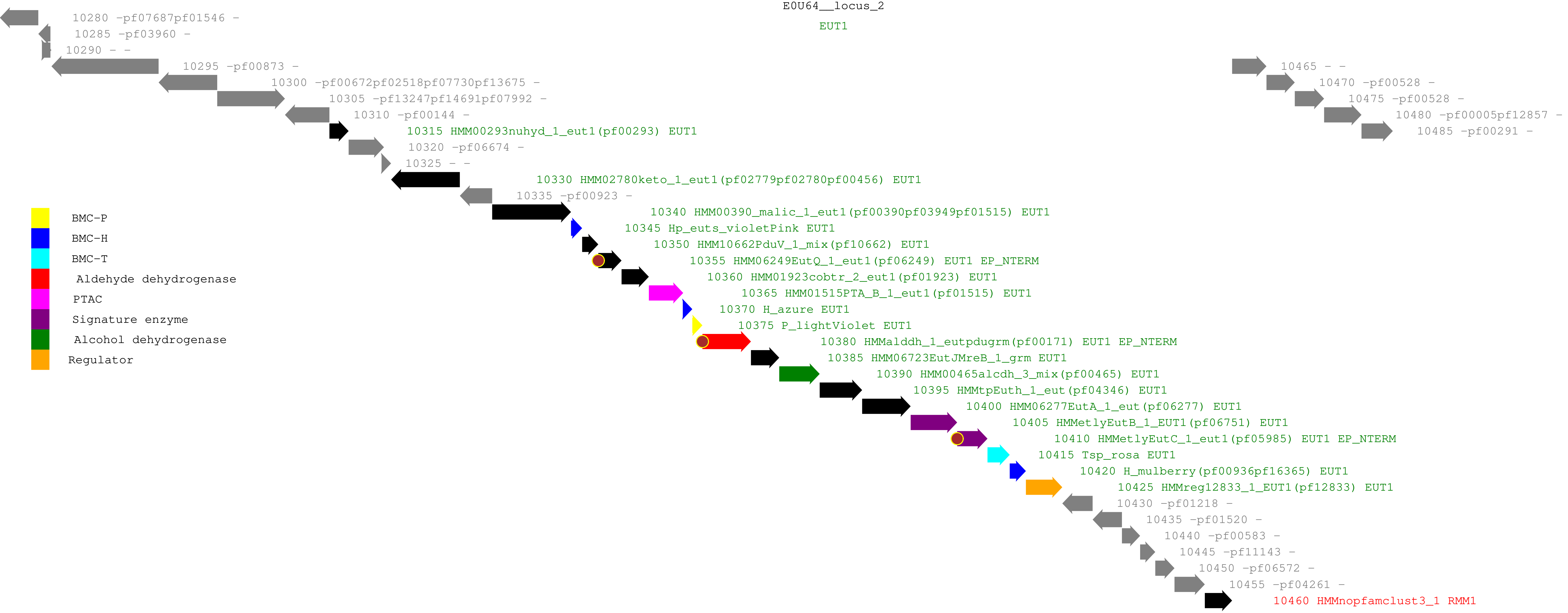

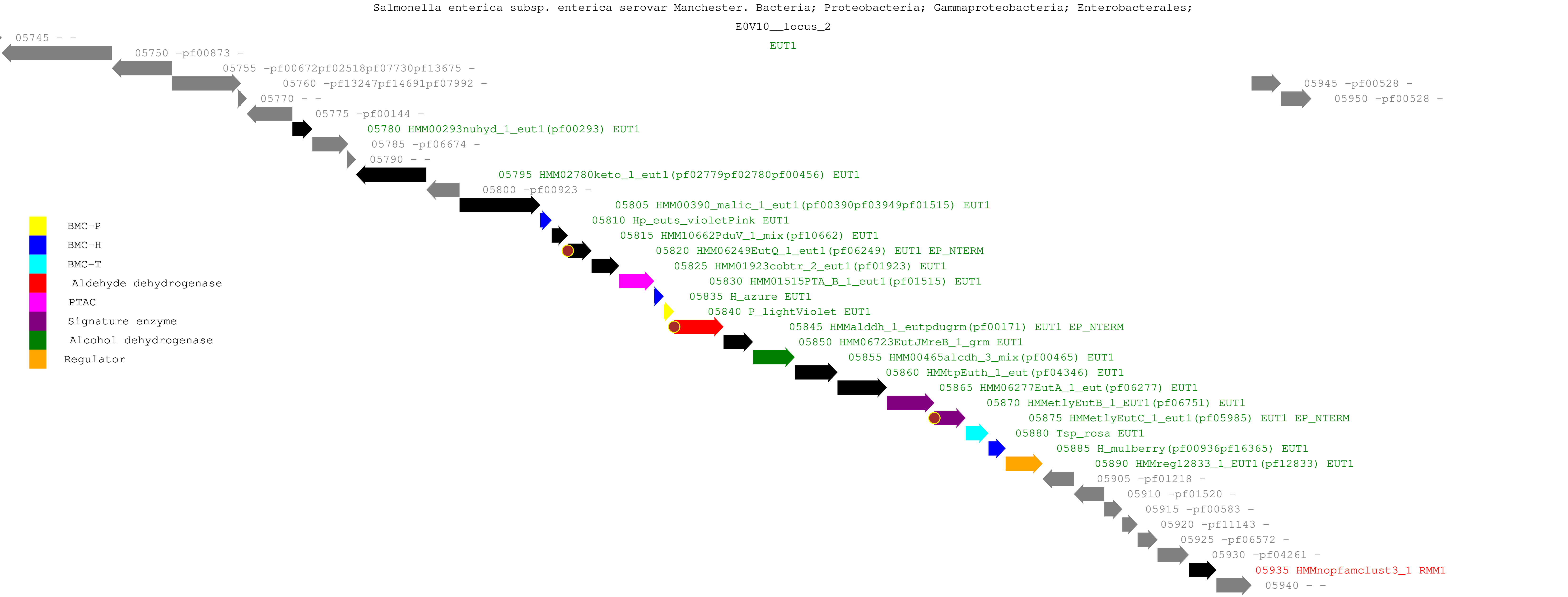
Salmonella enterica subsp. enterica serovar Manchester. Bacteria; Proteobacteria; Gammaproteobacteria; Enterobacterales;
E0V10__locus_2
EUT1
05745 - -
05750 -pf00873 -
05755 -pf00672pf02518pf07730pf13675 -
05760 -pf13247pf14691pf07992 -
05770 - -
05775 -pf00144 -
05780 HMM00293nuhyd_1_eut1(pf00293) EUT1
05785 -pf06674 -
05790 - -
05795 HMM02780keto_1_eut1(pf02779pf02780pf00456) EUT1
05800 -pf00923 -
05805 HMM00390_malic_1_eut1(pf00390pf03949pf01515) EUT1
05810 Hp_euts_violetPink EUT1
05815 HMM10662PduV_1_mix(pf10662) EUT1
05820 HMM06249EutQ_1_eut1(pf06249) EUT1 EP_NTERM
05825 HMM01923cobtr_2_eut1(pf01923) EUT1
05830 HMM01515PTA_B_1_eut1(pf01515) EUT1
05835 H_azure EUT1
05840 P_lightViolet EUT1
05845 HMMaldh_1_eutpdugrm(pf00171) EUT1 EP_NTERM
05850 HMM06723EutJMreB_1_grm EUT1
05855 HMM00465alcdh_3_mix(pf00465) EUT1
05860 HMMtpEuth_1_eut(pf04346) EUT1
05865 HMM06277EutA_1_eut(pf06277) EUT1
05870 HMMetlyEutB_1_EUT1(pf06751) EUT1
05875 HMMetlyEutC_1_eut1(pf05985) EUT1 EP_NTERM
05880 Tsp_rosa EUT1
05885 H_mulberry(pf00936pf16365) EUT1
05890 HMMreg12833_1_EUT1(pf12833) EUT1
05905 -pf01218 -
05910 -pf01520 -
05915 -pf00583 -
05920 -pf11143 -
05925 -pf06572 -
05930 -pf04261 -
05935 HMMnopfamclust3_1 RMM1
05940 - -
05945 -pf00528 -
05950 -pf00528 -
BMC-P
BMC-H
BMC-T
Aldehyde dehydrogenase
PTAC
Signature enzyme
Alcohol dehydrogenase
Regulator

Salmonella enterica subsp. enterica serovar Wangata. Bacteria; Proteobacteria; Gammaproteobacteria; Enterobacterales;

E0V16__locus_1

EUT1

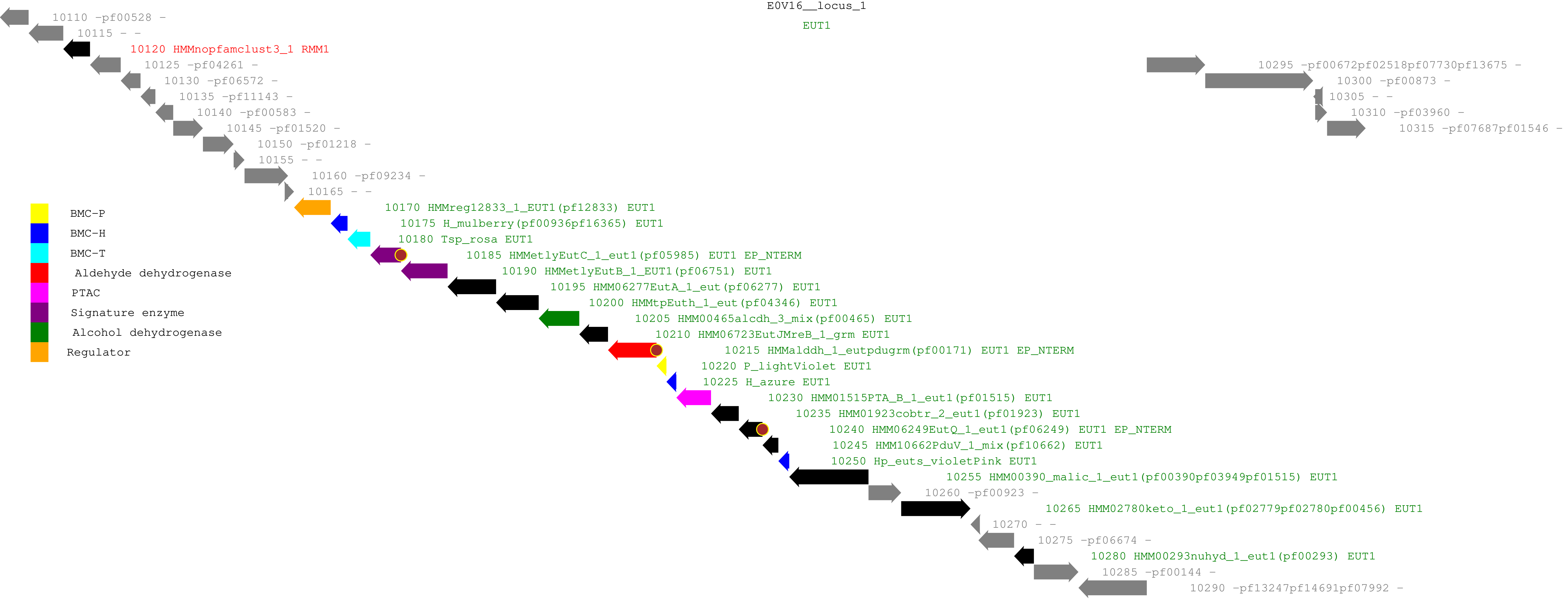

Salmonella enterica subsp. enterica serovar Nottingham. Bacteria; Proteobacteria; Gammaproteobacteria; Enterobacterales;

E0V23__locus_2

EUT1

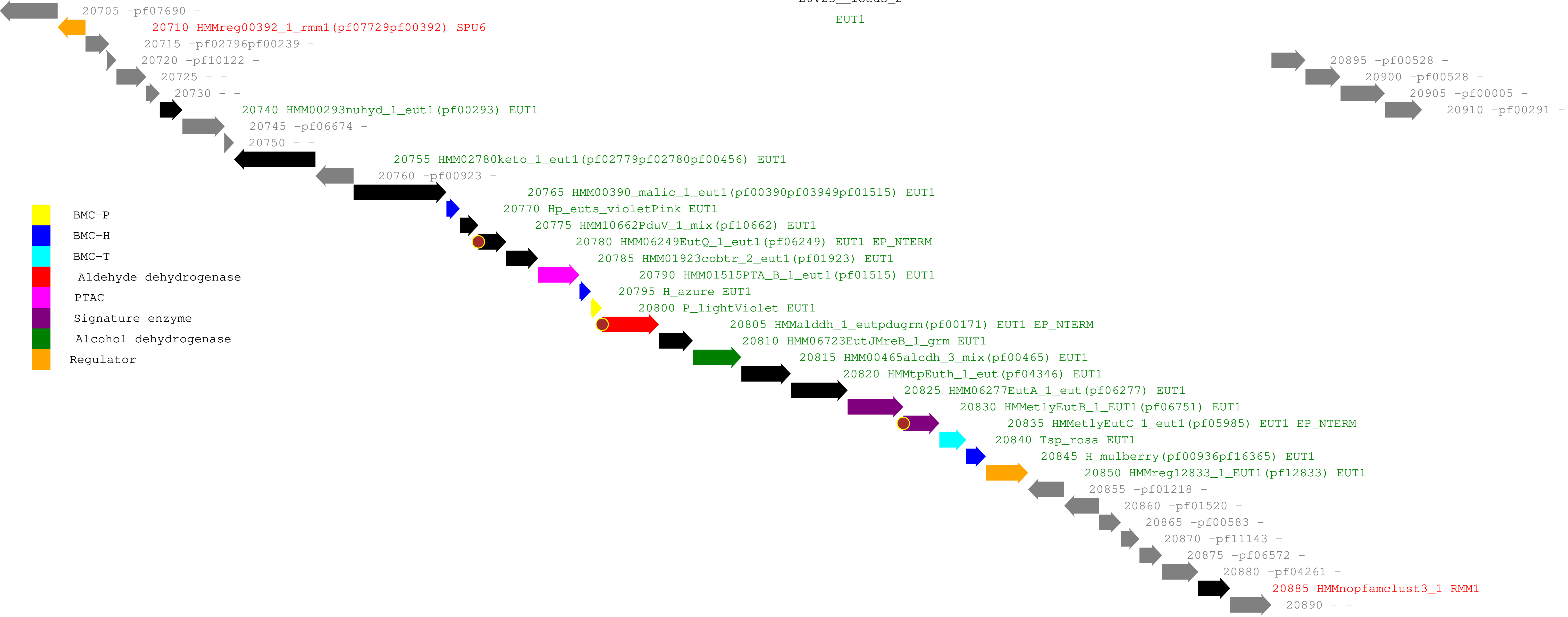

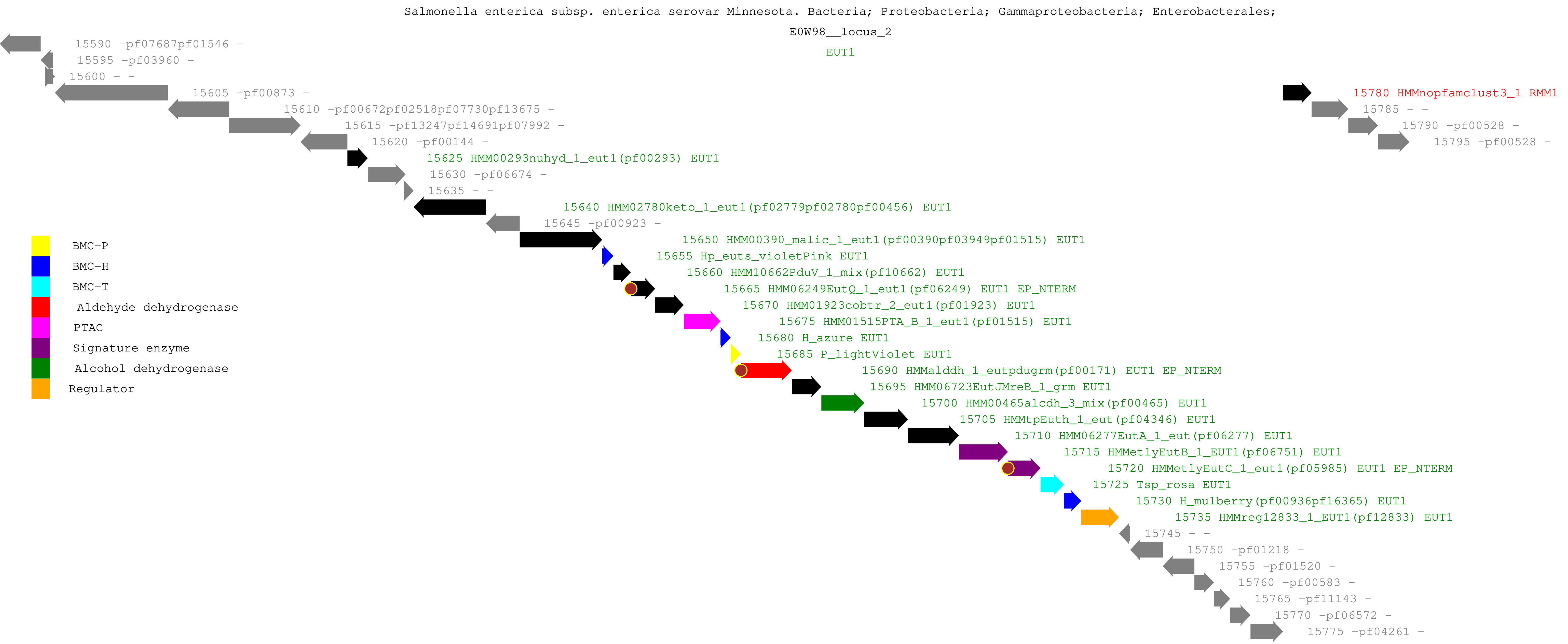
Salmonella enterica subsp. enterica serovar Minnesota. Bacteria; Proteobacteria; Gammaproteobacteria; Enterobacterales;
E0W98__locus_2
EUT1
15590 -pf07687pf01546 -
15595 -pf03960 -
15600 - -
15605 -pf00873 -
15610 -pf00672pf02518pf07730pf13675 -
15615 -pf13247pf14691pf07992 -
15620 -pf00144 -
15625 HMM00293nuhyd_1_eut1(pf00293) EUT1
15630 -pf06674 -
15635 - -
15640 HMM02780keto_1_eut1(pf02779pf02780pf00456) EUT1
15645 -pf00923 -
15650 HMM00390_malic_1_eut1(pf00390pf03949pf01515) EUT1
15655 Hp_euts_violetPink EUT1
15660 HMM10662PduV_1_mix(pf10662) EUT1
15665 HMM06249EutQ_1_eut1(pf06249) EUT1 EP_NTERM
15670 HMM01923cobtr_2_eut1(pf01923) EUT1
15675 HMM01515PTA_B_1_eut1(pf01515) EUT1
15680 H_azure EUT1
15685 P_lightViolet EUT1
15690 HMMalddh_1_eutpdugrm(pf00171) EUT1 EP_NTERM
15695 HMM06723EutJMreB_1_grm EUT1
15700 HMM00465alcdh_3_mix(pf00465) EUT1
15705 HMMtpEuth_1_eut(pf04346) EUT1
15710 HMM06277EutA_1_eut(pf06277) EUT1
15715 HMMetlyEutB_1_EUT1(pf06751) EUT1
15720 HMMetlyEutC_1_eut1(pf05985) EUT1 EP_NTERM
15725 Tsp_rosa EUT1
15730 H_mulberry(pf00936pf16365) EUT1
15735 HMMreg12833_1_EUT1(pf12833) EUT1
15745 - -
15750 -pf01218 -
15755 -pf01520 -
15760 -pf00583 -
15765 -pf11143 -
15770 -pf06572 -
15775 -pf04261 -
15780 HMMnopfamclust3_1 RMM1
15785 - -
15790 -pf00528 -
15795 -pf00528 -
BMC-P
BMC-H
BMC-T
Aldehyde dehydrogenase
PTAC
Signature enzyme
Alcohol dehydrogenase
Regulator

Salmonella enterica subsp. enterica serovar Tudu. Bacteria; Proteobacteria; Gammaproteobacteria; Enterobacterales;

E0X18__locus_2

EUT1

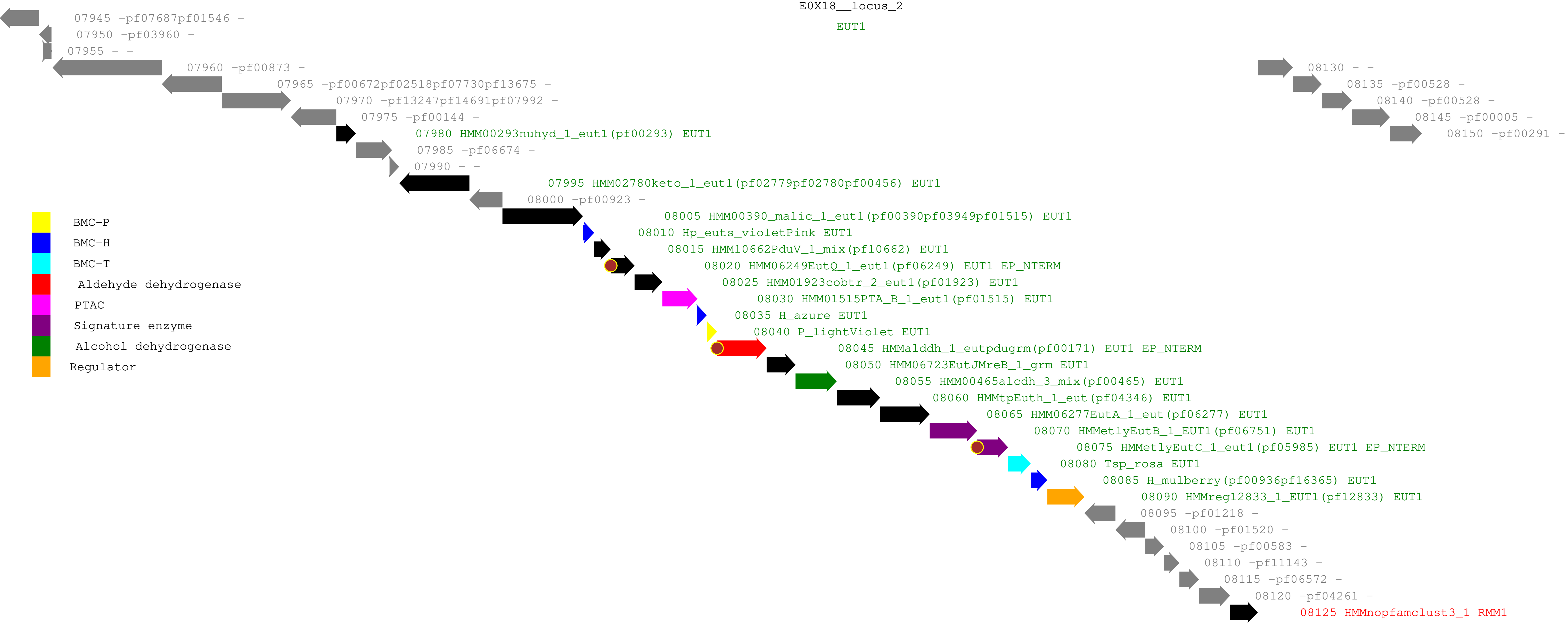

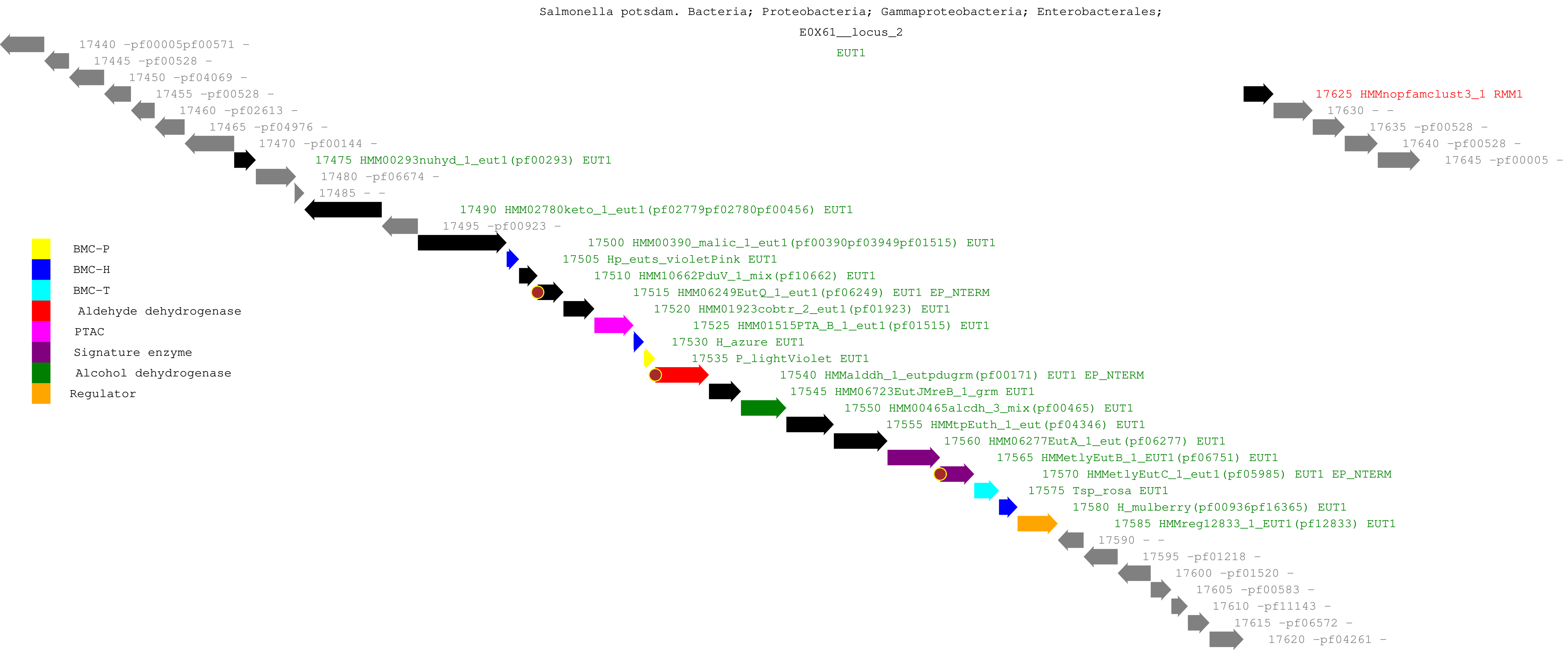
Salmonella potsdam. Bacteria; Proteobacteria; Gammaproteobacteria; Enterobacterales;
E0X61__locus_2
EUT1
17440 -pf00005pf00571 -
17445 -pf00528 -
17450 -pf04069 -
17455 -pf00528 -
17460 -pf02613 -
17465 -pf04976 -
17470 -pf00144 -
17475 HMM00293nuhyd_1_eut1(pf00293) EUT1
17480 -pf06674 -
17485 - -
17490 HMM02780keto_1_eut1(pf02779pf02780pf00456) EUT1
17495 -pf00923 -
17500 HMM00390_malic_1_eut1(pf00390pf03949pf01515) EUT1
17505 Hp_euts_violetPink EUT1
17510 HMM10662PduV_1_mix(pf10662) EUT1
17515 HMM06249EutQ_1_eut1(pf06249) EUT1 EP_NTERM
17520 HMM01923cobtr_2_eut1(pf01923) EUT1
17525 HMM01515PTA_B_1_eut1(pf01515) EUT1
17530 H_azure EUT1
17535 P_lightViolet EUT1
17540 HMMalddh_1_eutpdugrm(pf00171) EUT1 EP_NTERM
17545 HMM06723EutJMreB_1_grm EUT1
17550 HMM00465alcdh_3_mix(pf00465) EUT1
17555 HMMtpEuth_1_eut(pf04346) EUT1
17560 HMM06277EutA_1_eut(pf06277) EUT1
17565 HMMetlyEutB_1_EUT1(pf06751) EUT1
17570 HMMetlyEutC_1_eut1(pf05985) EUT1 EP_NTERM
17575 Tsp_rosa EUT1
17580 H_mulberry(pf00936pf16365) EUT1
17585 HMMreg12833_1_EUT1(pf12833) EUT1
17590 - -
17595 -pf01218 -
17600 -pf01520 -
17605 -pf00583 -
17610 -pf11143 -
17615 -pf06572 -
17620 -pf04261 -
17625 HMMnopfamclust3_1 RMM1
17630 - -
17635 -pf00528 -
17640 -pf00528 -
17645 -pf00005 -
BMC-P
BMC-H
BMC-T
Aldehyde dehydrogenase
PTAC
Signature enzyme
Alcohol dehydrogenase
Regulator

Salmonella enterica subsp. enterica serovar Miami. Bacteria; Proteobacteria; Gammaproteobacteria; Enterobacterales;

E0Y77__locus_2

EUT1

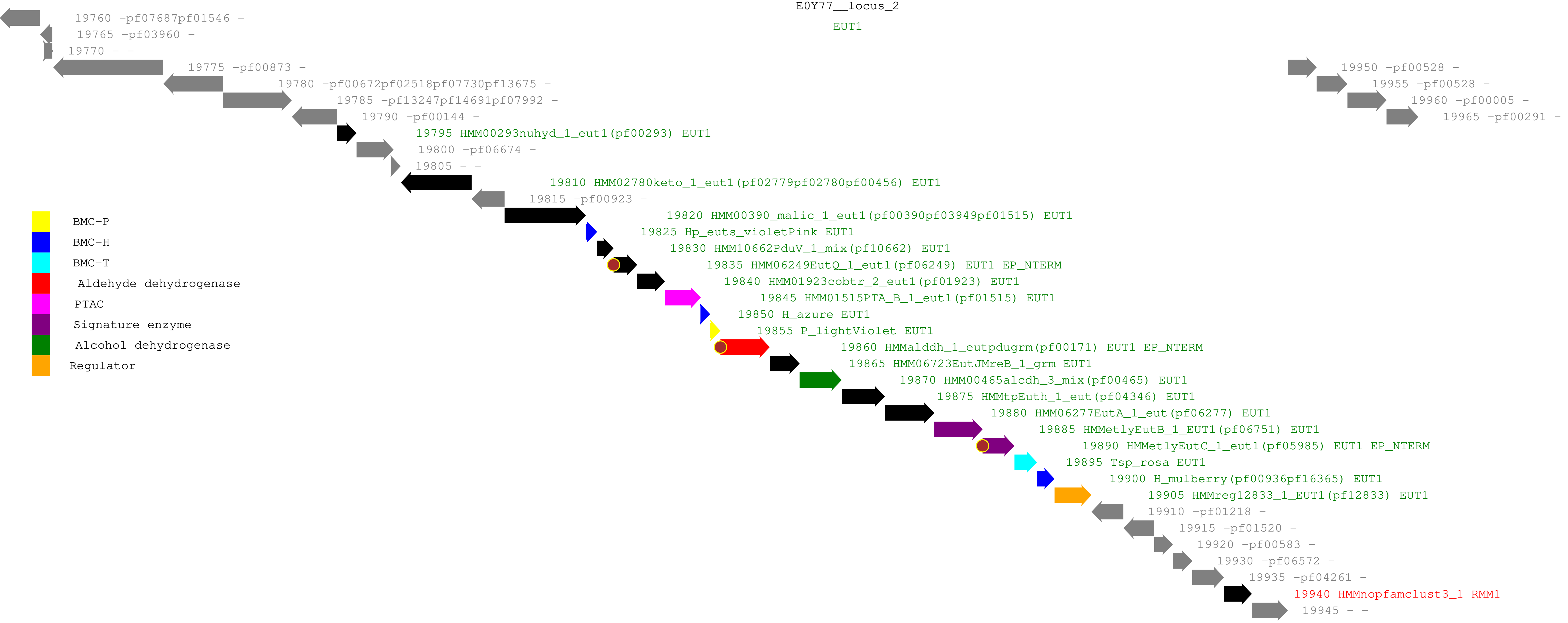

Salmonella enterica subsp. enterica serovar Wentworth. Bacteria; Proteobacteria; Gammaproteobacteria; Enterobacterales;

E1361__locus_1

EUT1

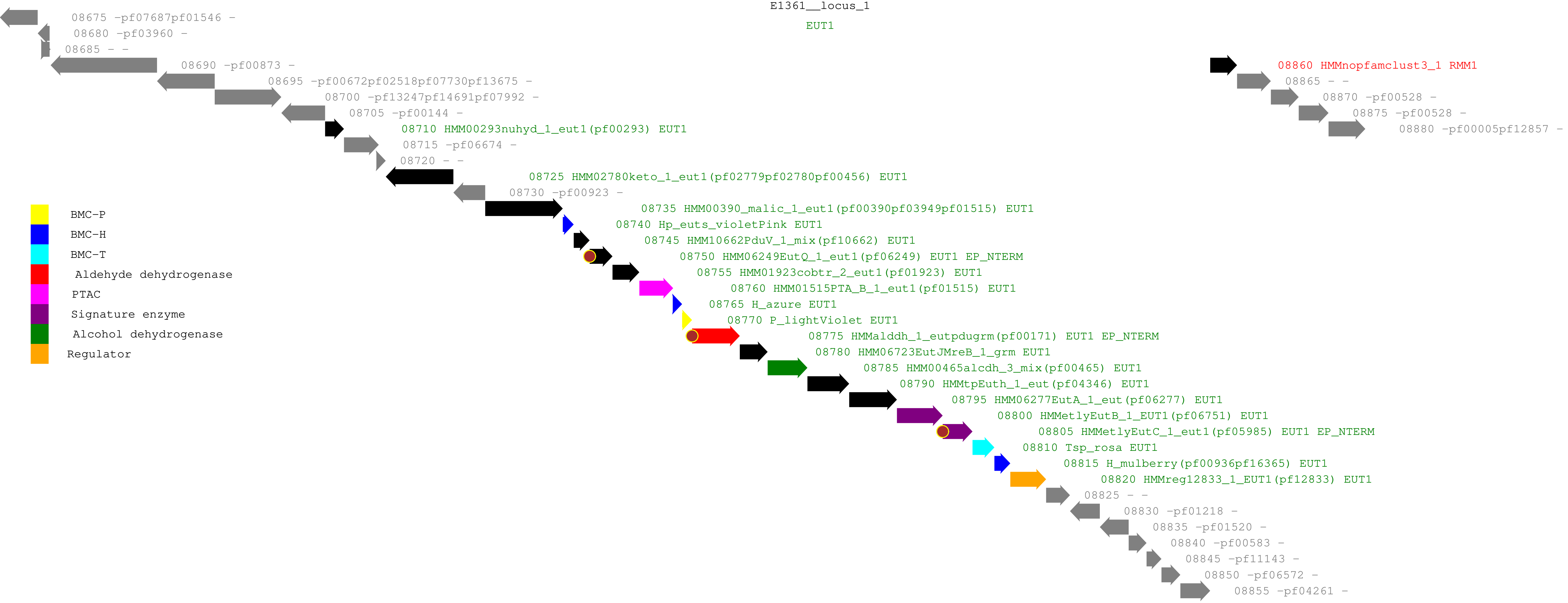

Salmonella enterica subsp. enterica serovar Falkensee. Bacteria; Proteobacteria; Gammaproteobacteria; Enterobacterales;

E1364__locus_1

EUT1

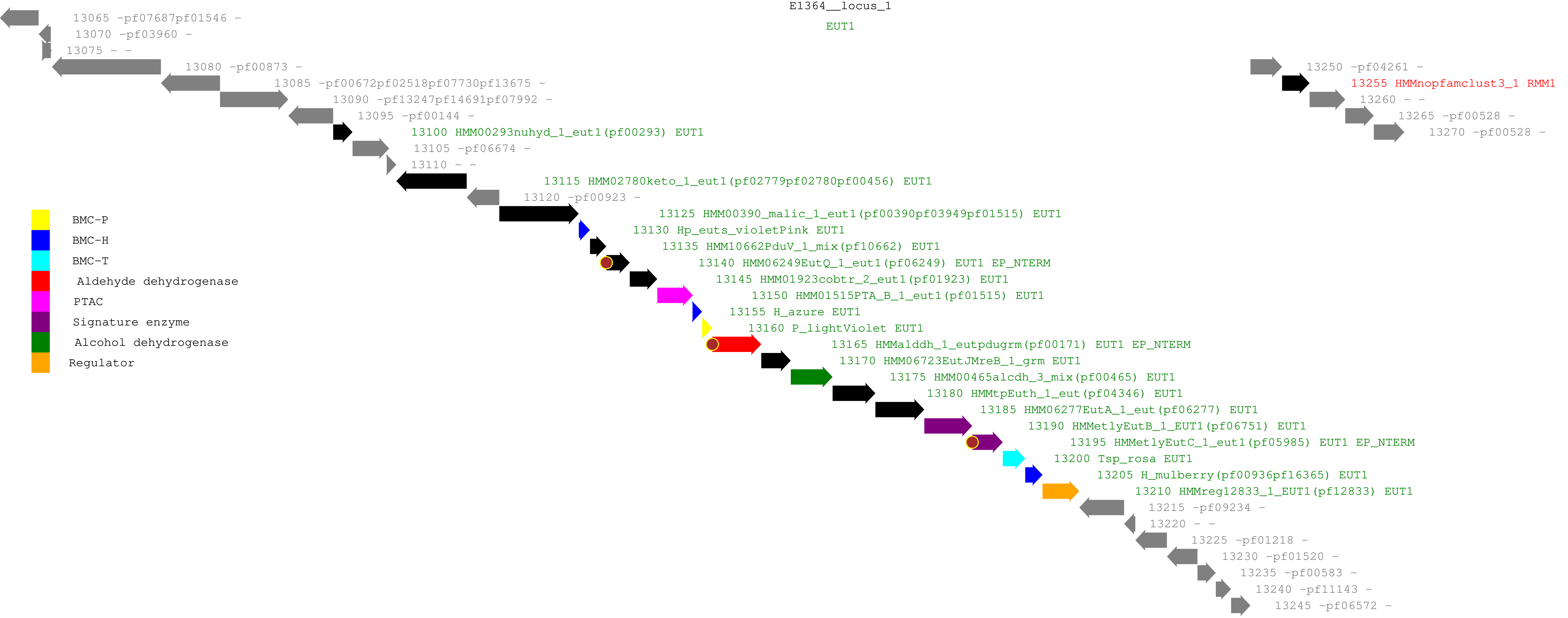

Salmonella enterica subsp. enterica serovar Brunei. Bacteria; Proteobacteria; Gammaproteobacteria; Enterobacterales;

E1379__locus_1

EUT1

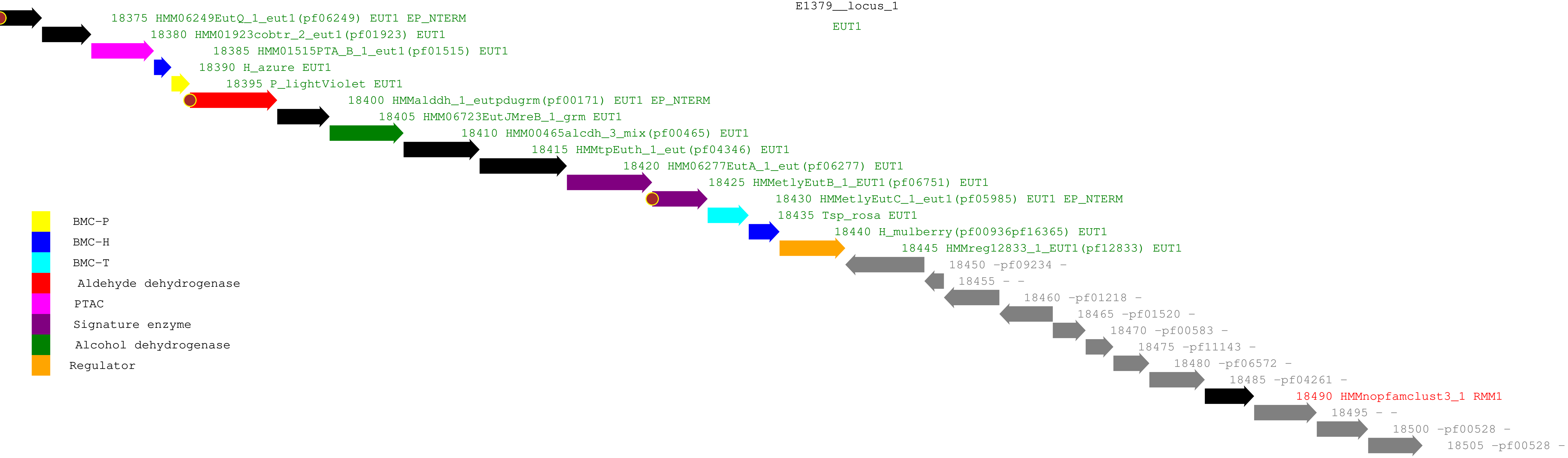

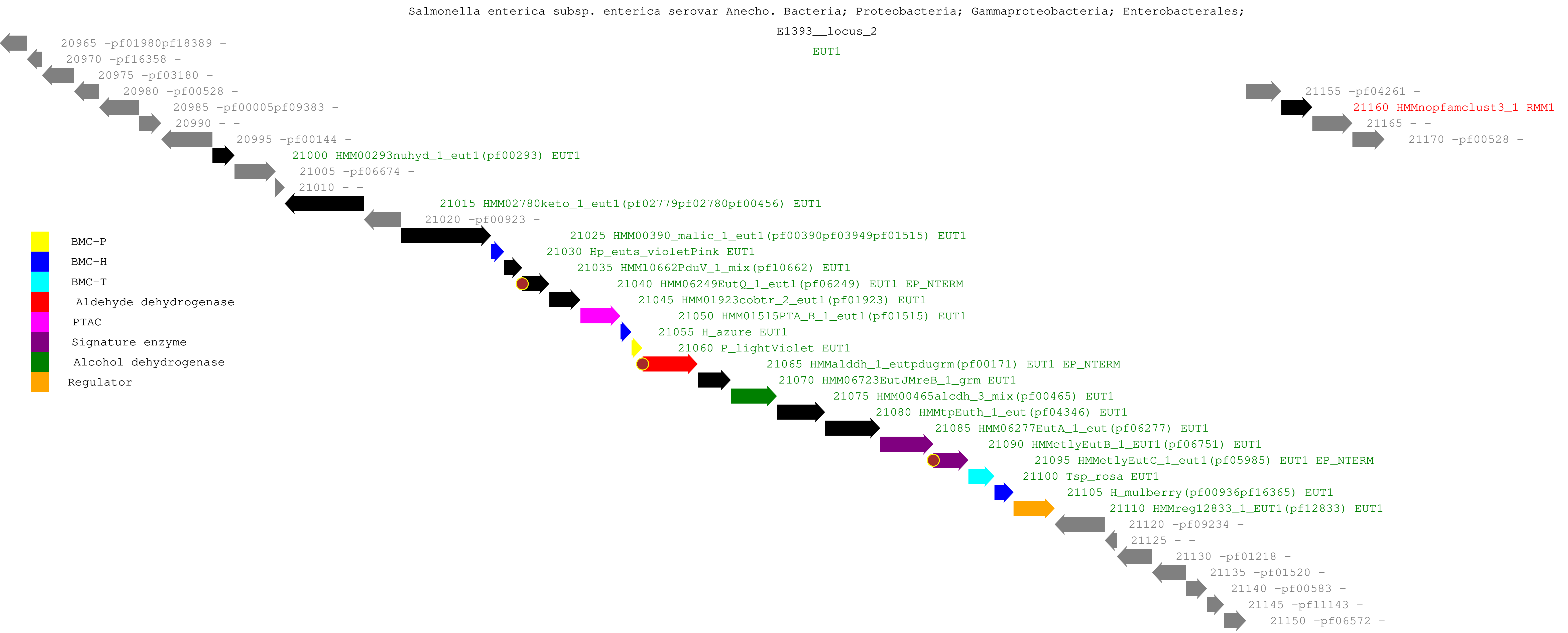

Salmonella enterica subsp. enterica serovar Anecho. Bacteria; Proteobacteria; Gammaproteobacteria; Enterobacterales;
E1393__locus_2
EUT1
20965 -pf01980pf18389 -
20970 -pf16358 -
20975 -pf03180 -
20980 -pf00528 -
20985 -pf00005pf09383 -
20990 - -
20995 -pf00144 -
21000 HMM00293nuhyd_1_eut1(pf00293) EUT1
21005 -pf06674 -
21010 - -
21015 HMM02780keto_1_eut1(pf02779pf02780pf00456) EUT1
21020 -pf00923 -
21025 HMM00390_malic_1_eut1(pf00390pf03949pf01515) EUT1
21030 Hp_euts_violetPink EUT1
21035 HMM10662PduV_1_mix(pf10662) EUT1
21040 HMM06249EutQ_1_eut1(pf06249) EUT1 EP_NTERM
21045 HMM01923cobtr_2_eut1(pf01923) EUT1
21050 HMM01515PTA_B_1_eut1(pf01515) EUT1
21055 H_azure EUT1
21060 P_lightViolet EUT1
21065 HMMalddh_1_eutpdugrm(pf00171) EUT1 EP_NTERM
21070 HMM06723EutJMreB_1_grm EUT1
21075 HMM00465alcdh_3_mix(pf00465) EUT1
21080 HMMtpEuth_1_eut(pf04346) EUT1
21085 HMM06277EutA_1_eut(pf06277) EUT1
21090 HMMetlyEutB_1_EUT1(pf06751) EUT1
21095 HMMetlyEutC_1_eut1(pf05985) EUT1 EP_NTERM
21100 Tsp_rosa EUT1
21105 H_mulberry(pf00936pf16365) EUT1
21110 HMMreg12833_1_EUT1(pf12833) EUT1
21120 -pf09234 -
21125 - -
21130 -pf01218 -
21135 -pf01520 -
21140 -pf00583 -
21145 -pf11143 -
21150 -pf06572 -
21155 -pf04261 -
21160 HMMnopfamclust3_1 RMM1
21165 - -
21170 -pf00528 -
BMC-P
BMC-H
BMC-T
Aldehyde dehydrogenase
PTAC
Signature enzyme
Alcohol dehydrogenase
Regulator

Salmonella enterica subsp. enterica serovar Lille. Bacteria; Proteobacteria; Gammaproteobacteria; Enterobacterales;

E1399__locus_1

EUT1

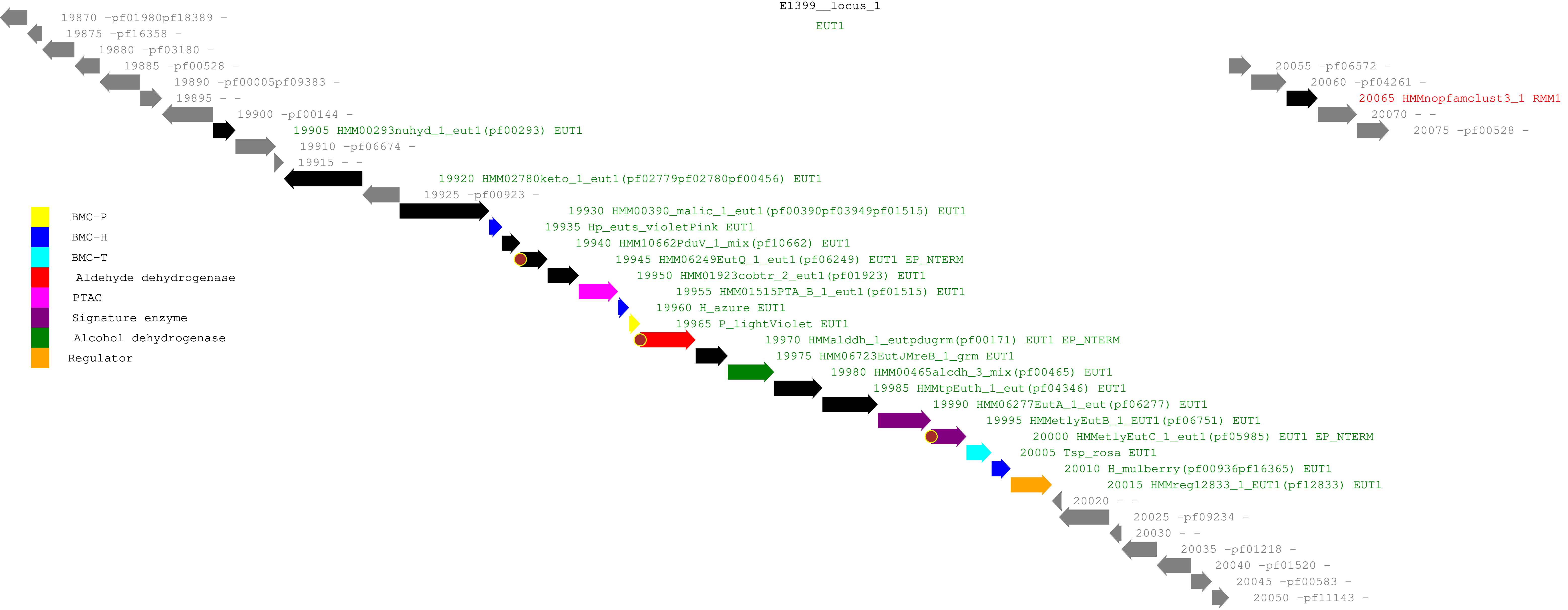

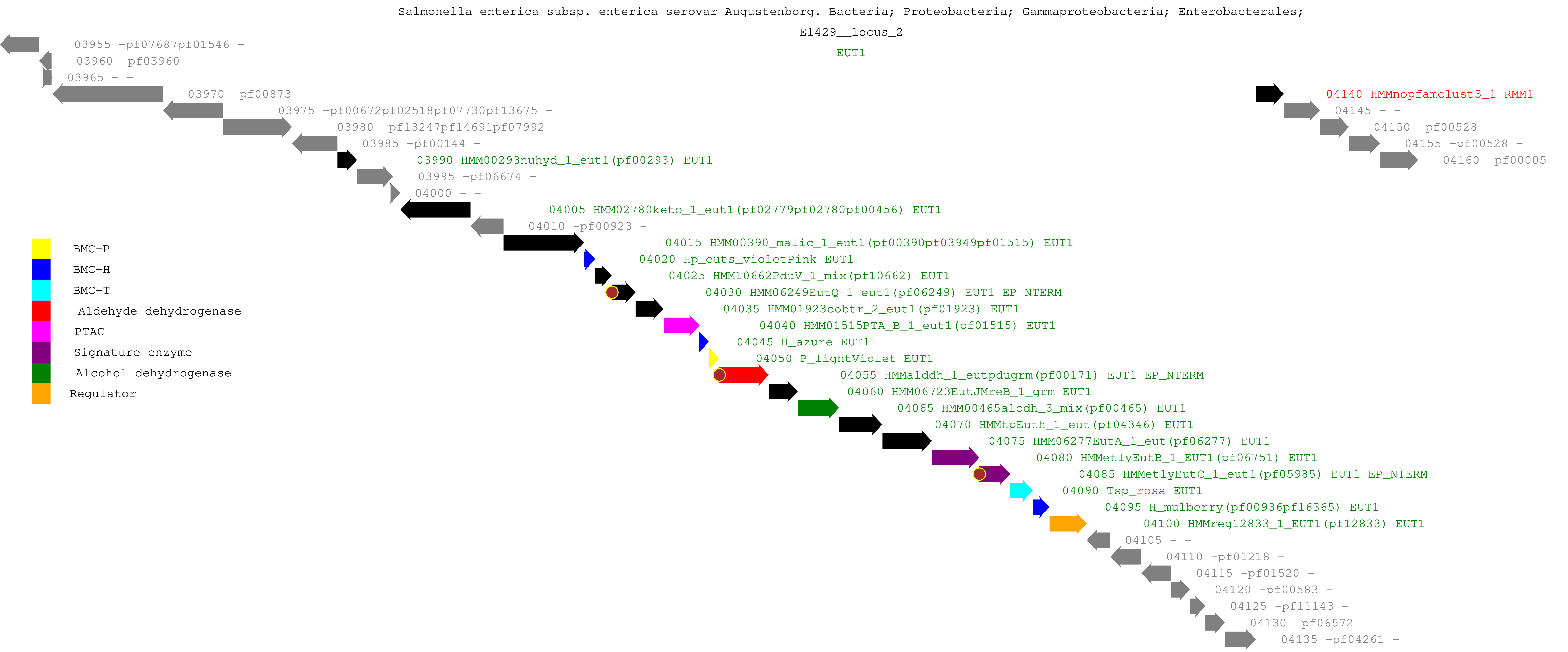

Salmonella enterica subsp. enterica serovar Augustenborg. Bacteria; Proteobacteria; Gammaproteobacteria; Enterobacterales;
E1429__locus_2
EUT1
03955 -pf07687pf01546 -
03960 -pf03960 -
03965 - -
03970 -pf00873 -
03975 -pf00672pf02518pf07730pf13675 -
03980 -pf13247pf14691pf07992 -
03985 -pf00144 -
03990 HMM00293nuhyd_1_eut1(pf00293) EUT1
03995 -pf06674 -
04000 - -
04005 HMM02780keto_1_eut1(pf02779pf02780pf00456) EUT1
04010 -pf00923 -
04015 HMM00390_malic_1_eut1(pf00390pf03949pf01515) EUT1
04020 Hp_euts_violetPink EUT1
04025 HMM10662PduV_1_mix(pf10662) EUT1
04030 HMM06249EutQ_1_eut1(pf06249) EUT1 EP_NTERM
04035 HMM01923cobtr_2_eut1(pf01923) EUT1
04040 HMM01515PTA_B_1_eut1(pf01515) EUT1
04045 H_azure EUT1
04050 P_lightViolet EUT1
04055 HMMalddh_1_eutpdugrm(pf00171) EUT1 EP_NTERM
04060 HMM06723EutJMreB_1_grm EUT1
04065 HMM00465alcdh_3_mix(pf00465) EUT1
04070 HMMtpEuth_1_eut(pf04346) EUT1
04075 HMM06277EutA_1_eut(pf06277) EUT1
04080 HMMetlyEutB_1_EUT1(pf06751) EUT1
04085 HMMetlyEutC_1_eut1(pf05985) EUT1 EP_NTERM
04090 Tsp_rosa EUT1
04095 H_mulberry(pf00936pf16365) EUT1
04100 HMMreg12833_1_EUT1(pf12833) EUT1
04105 - -
04110 -pf01218 -
04115 -pf01520 -
04120 -pf00583 -
04125 -pf11143 -
04130 -pf06572 -
04135 -pf04261 -
04140 HMMnopfamclust3_1 RMM1
04145 - -
04150 -pf00528 -
04155 -pf00528 -
04160 -pf00005 -
BMC-P
BMC-H
BMC-T
Aldehyde dehydrogenase
PTAC
Signature enzyme
Alcohol dehydrogenase
Regulator

Salmonella enterica subsp. enterica serovar Durham. Bacteria; Proteobacteria; Gammaproteobacteria; Enterobacterales;

E1433__locus_1

EUT1

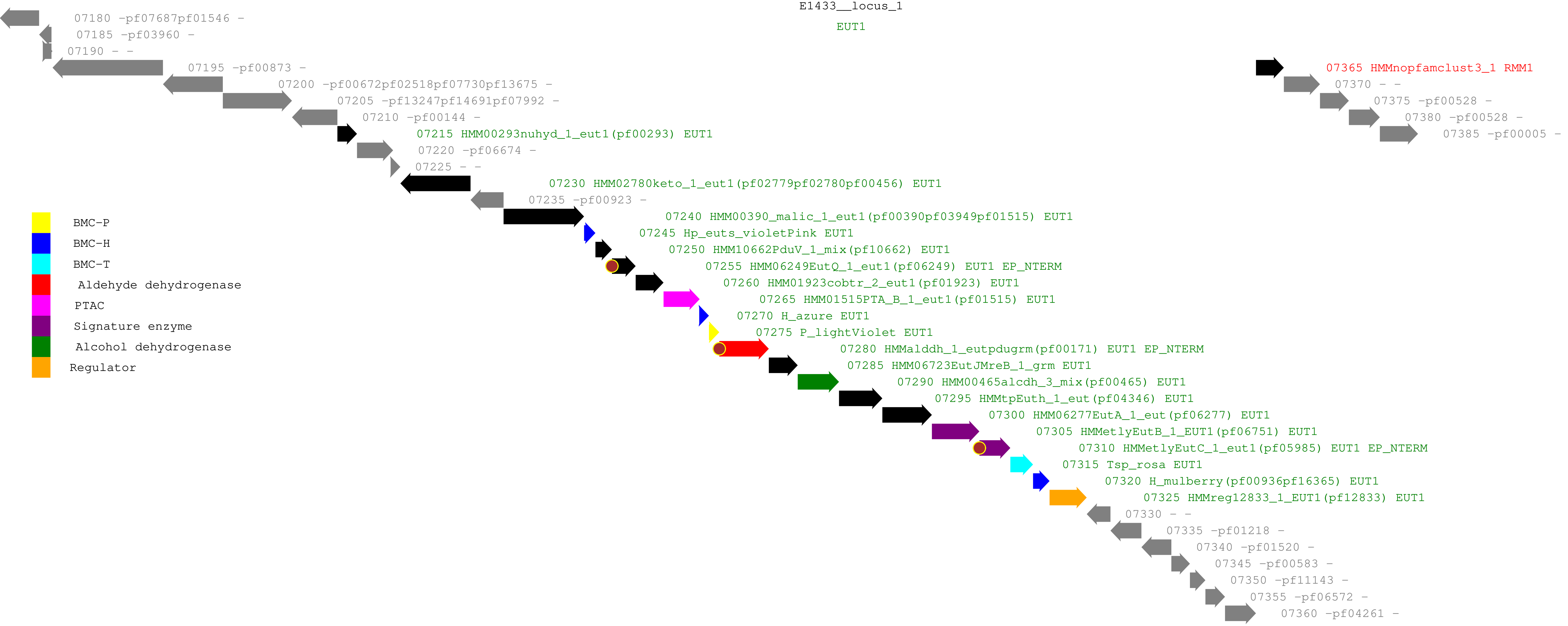

Salmonella enterica subsp. enterica serovar Kimuenza. Bacteria; Proteobacteria; Gammaproteobacteria; Enterobacterales;

E1444__locus_1

EUT1

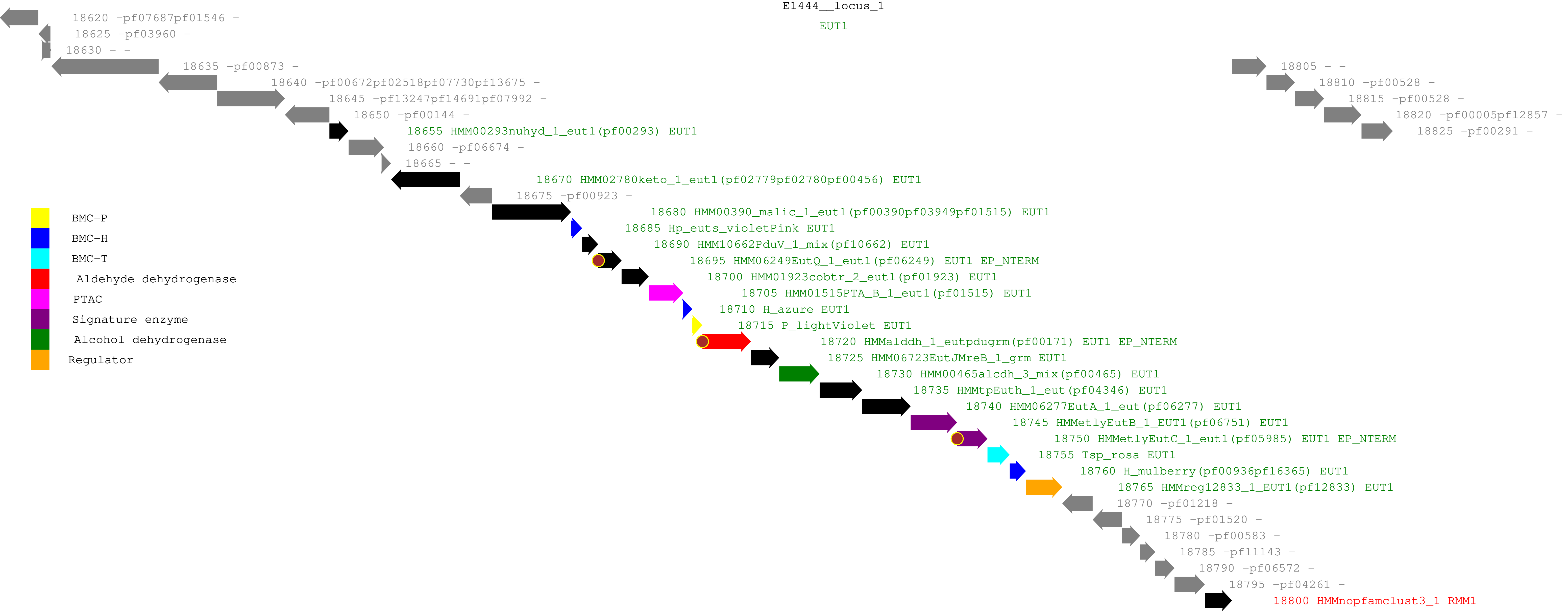

Salmonella enterica subsp. enterica serovar Caracas. Bacteria; Proteobacteria; Gammaproteobacteria; Enterobacterales;

E1460__locus_1

EUT1

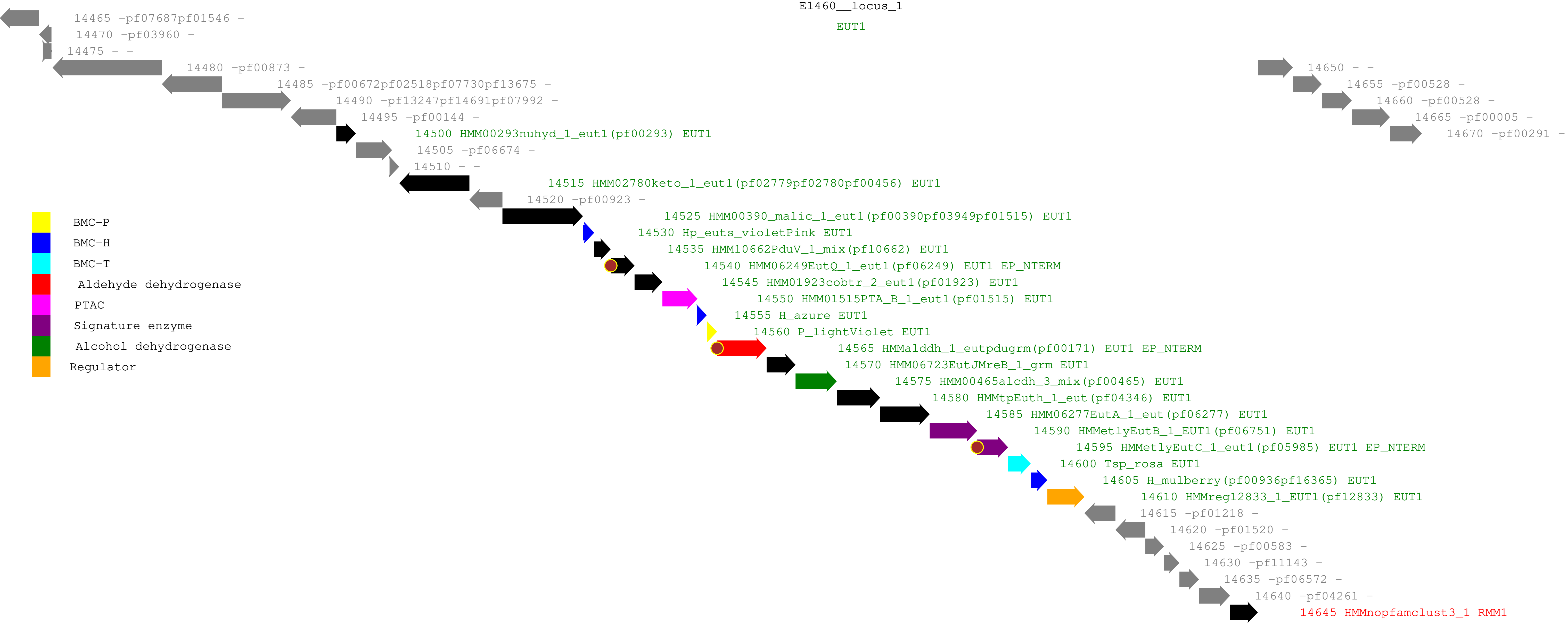

Salmonella enterica subsp. enterica serovar Ilala. Bacteria; Proteobacteria; Gammaproteobacteria; Enterobacterales;

E1617__locus_1

EUT1

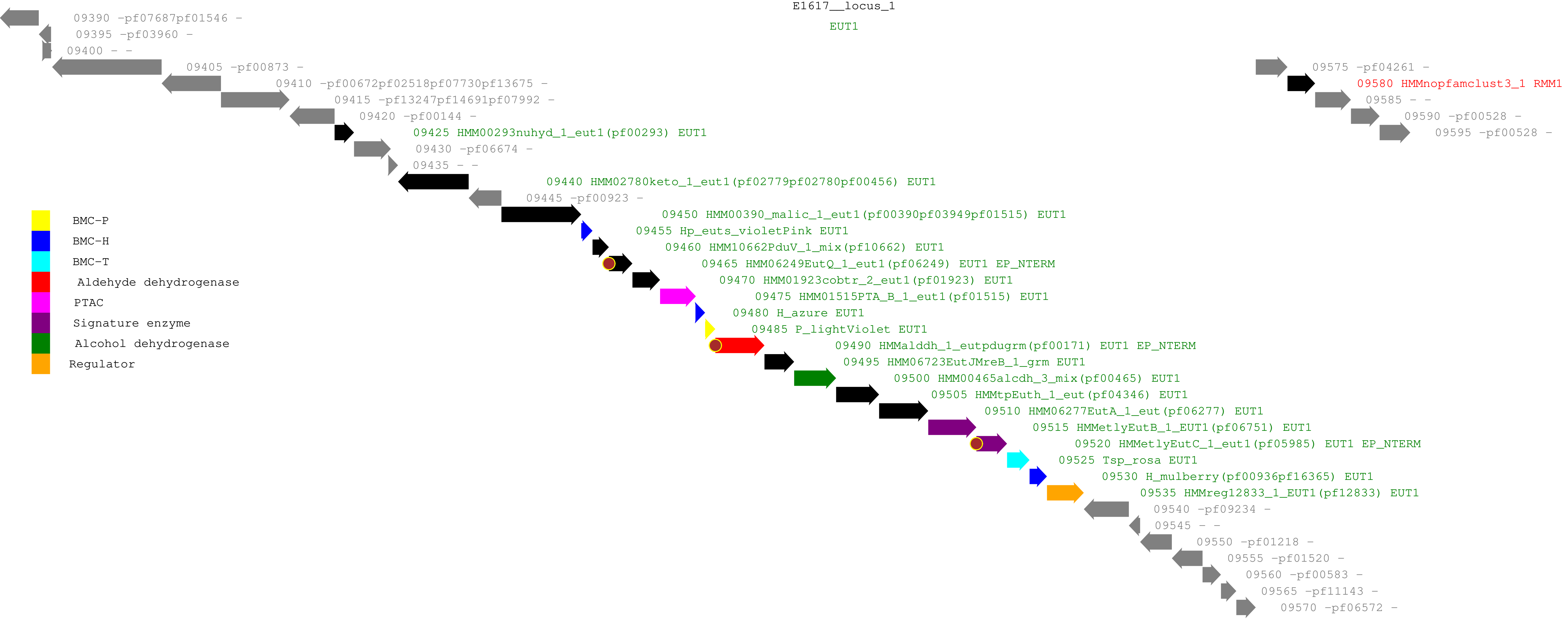

Salmonella enterica subsp. enterica serovar Baguida. Bacteria; Proteobacteria; Gammaproteobacteria; Enterobacterales;

E1630__locus_1

EUT1

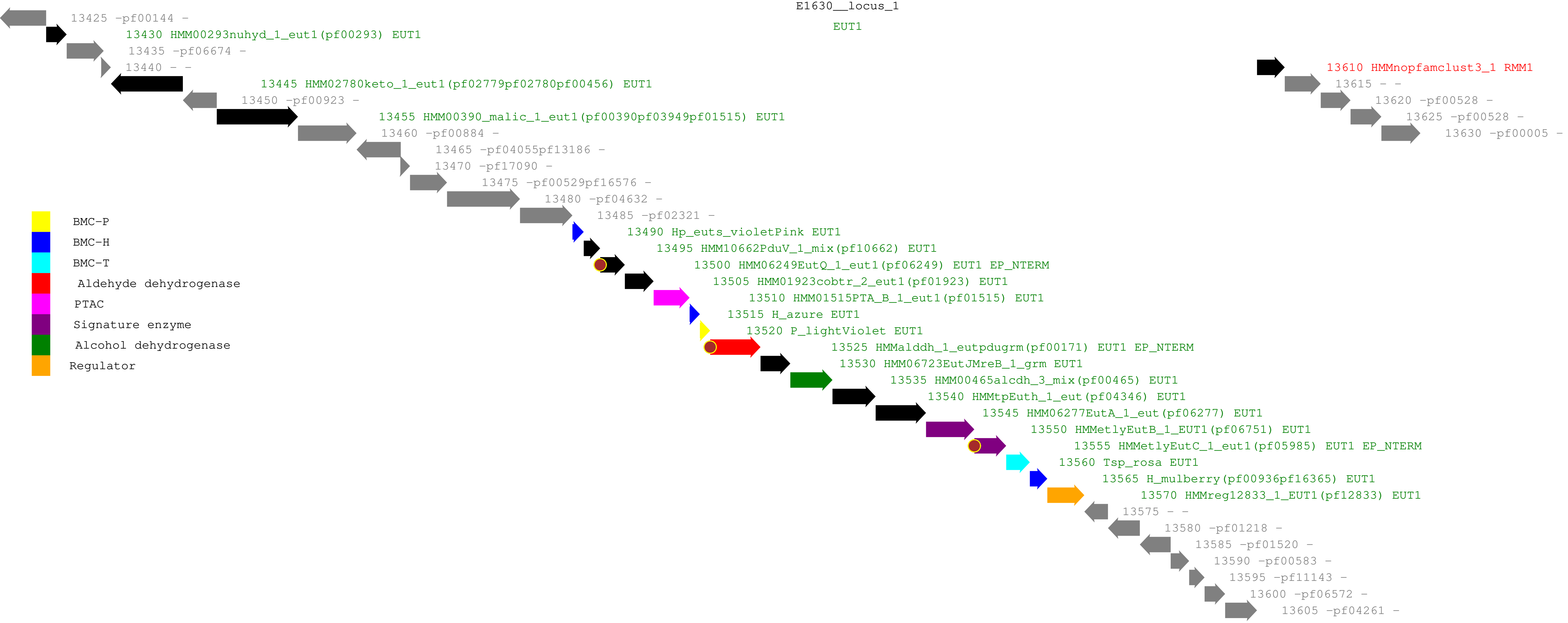

Salmonella enterica subsp. enterica serovar Umbilo. Bacteria; Proteobacteria; Gammaproteobacteria; Enterobacterales;

E1665__locus_1

EUT1

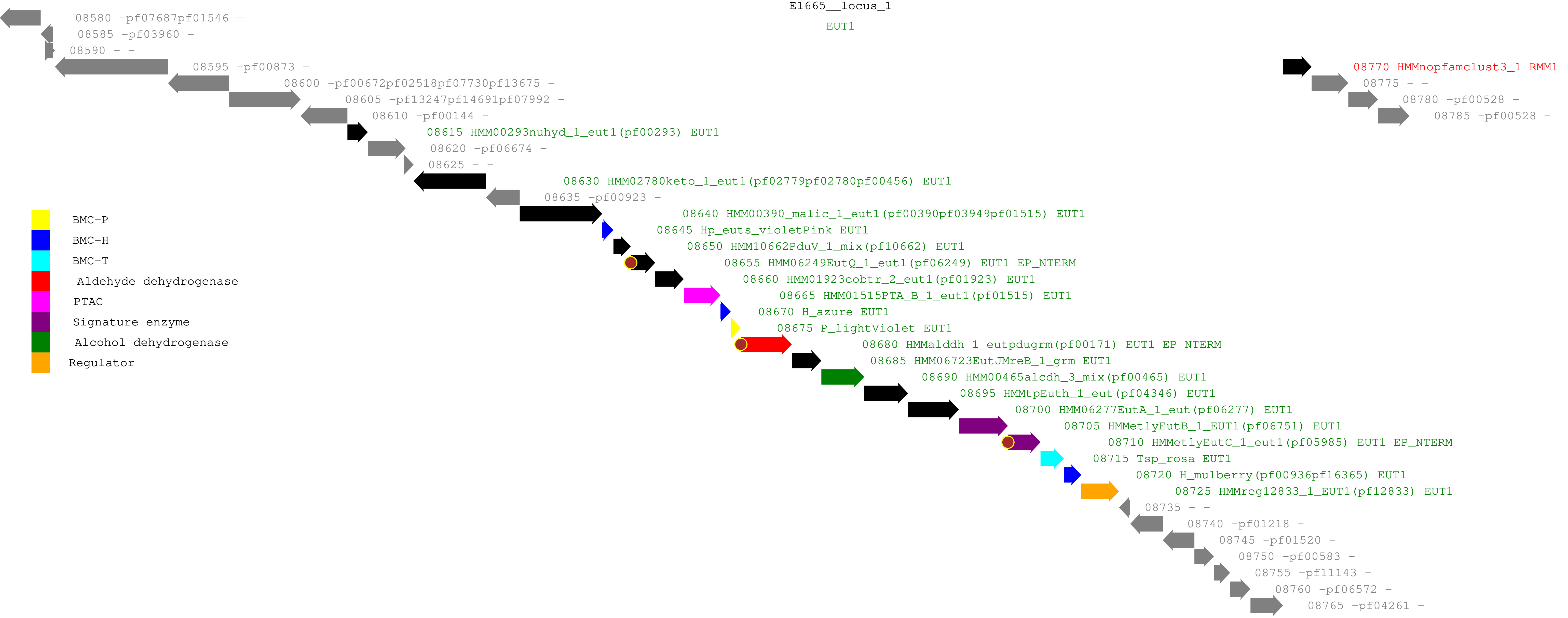

Salmonella blockley. Bacteria; Proteobacteria; Gammaproteobacteria; Enterobacterales;

E1672__locus_2

EUT1

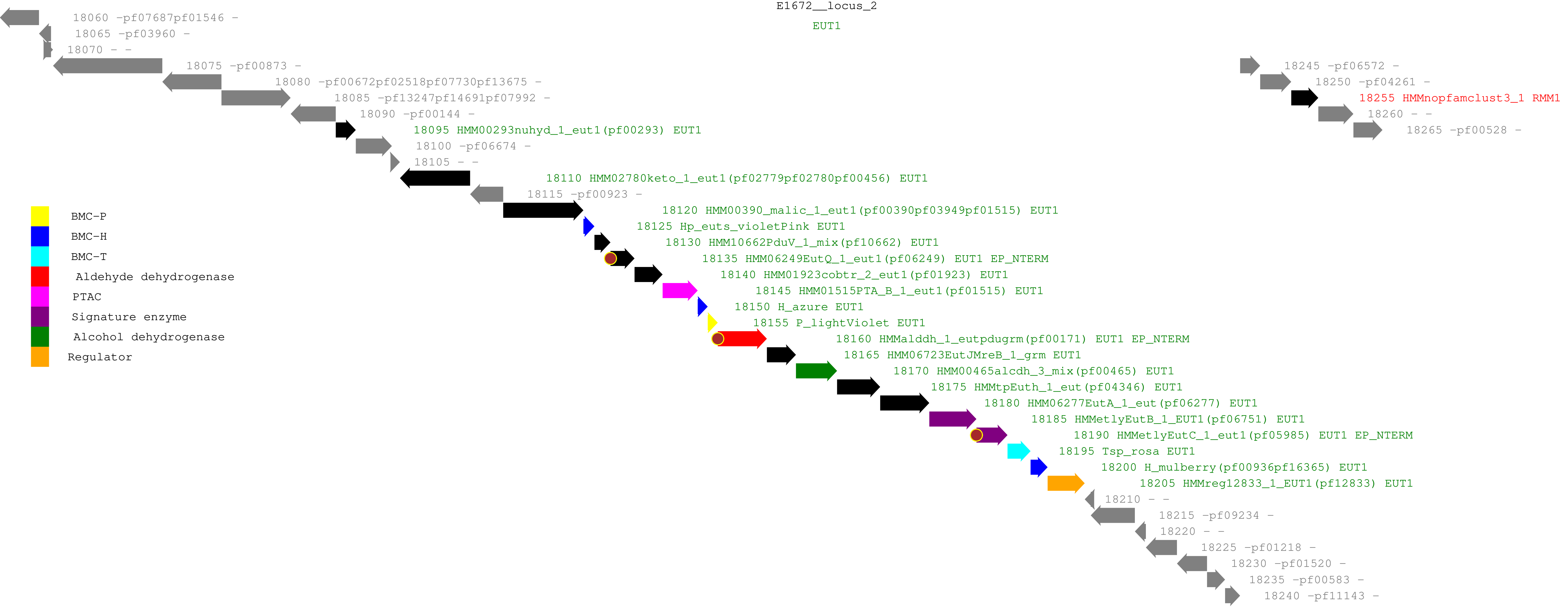

Salmonella enterica subsp. enterica serovar Duesseldorf. Bacteria; Proteobacteria; Gammaproteobacteria; Enterobacterales;

E1759__locus_1

EUT1

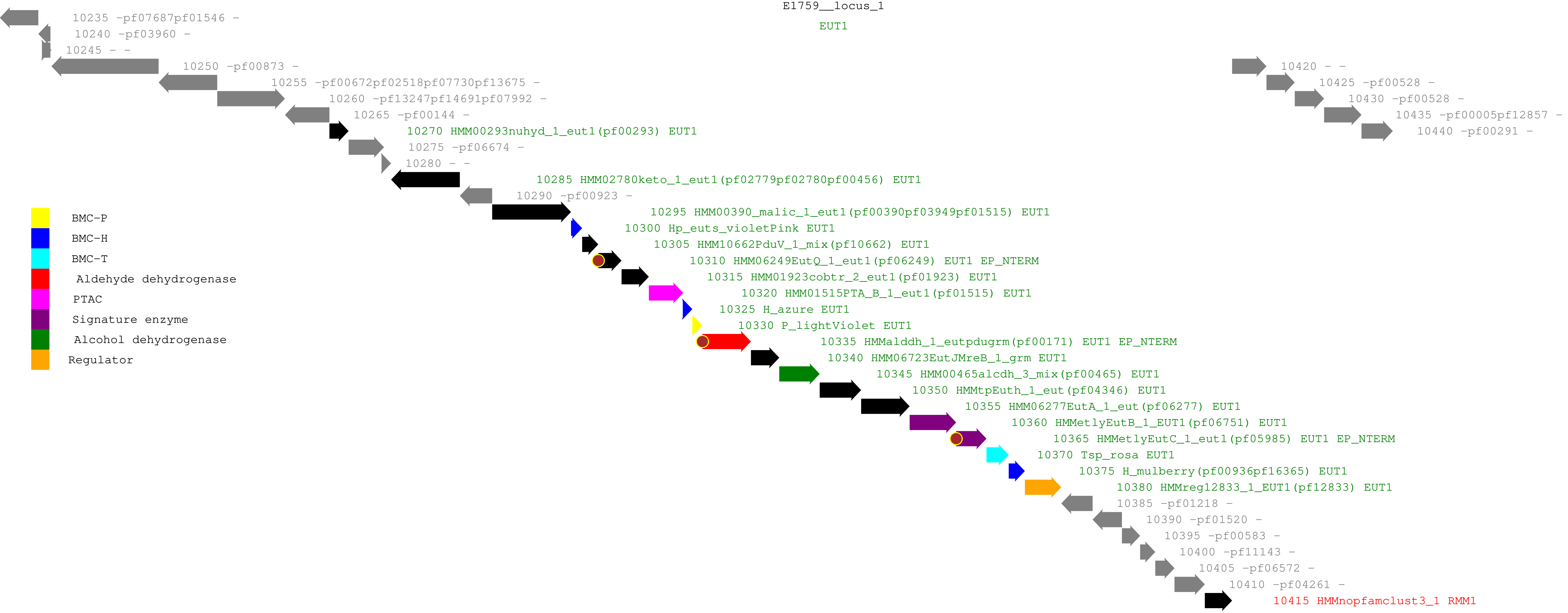

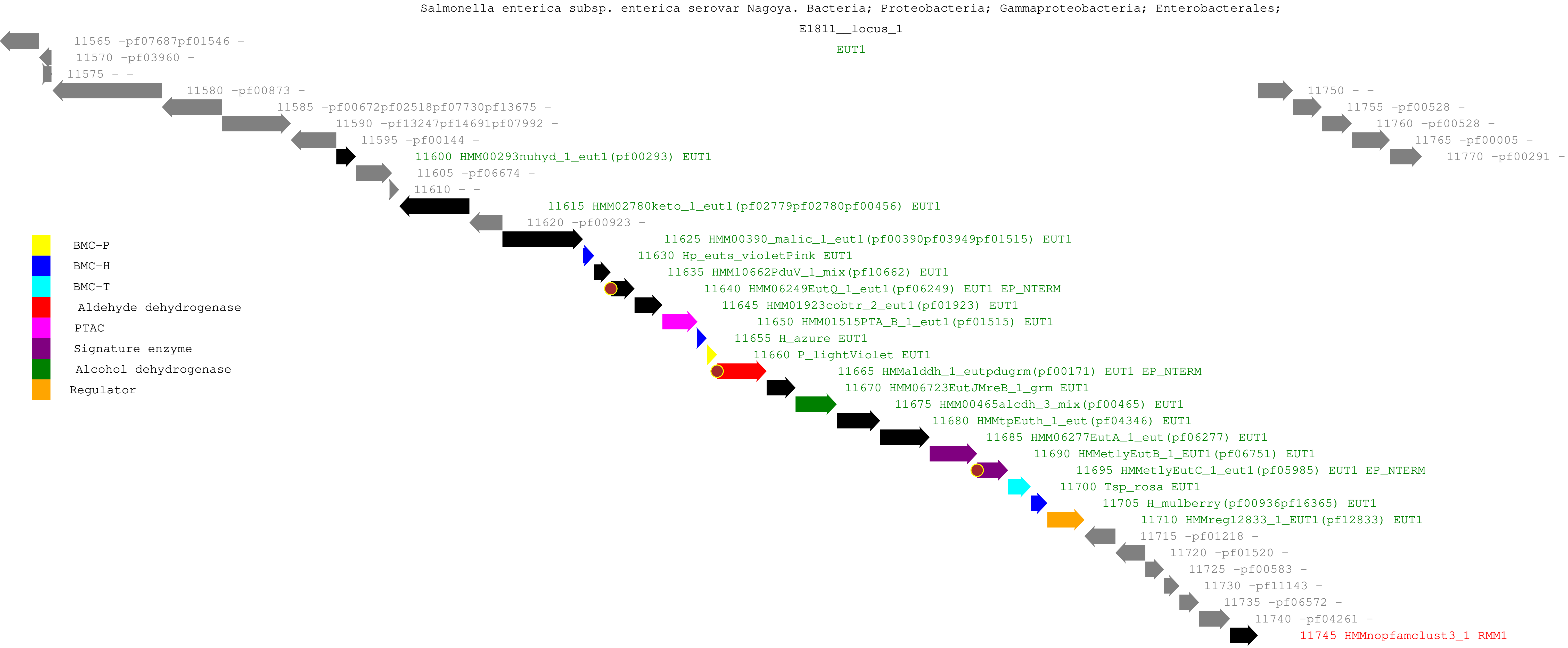
Salmonella enterica subsp. enterica serovar Nagoya. Bacteria; Proteobacteria; Gammaproteobacteria; Enterobacterales;
E1811__locus_1
EUT1
11565 -pf07687pf01546 -
11570 -pf03960 -
11575 - -
11580 -pf00873 -
11585 -pf00672pf02518pf07730pf13675 -
11590 -pf13247pf14691pf07992 -
11595 -pf00144 -
11600 HMM00293nuhyd_1_eut1(pf00293) EUT1
11605 -pf06674 -
11610 - -
11615 HMM02780keto_1_eut1(pf02779pf02780pf00456) EUT1
11620 -pf00923 -
11625 HMM00390_malic_1_eut1(pf00390pf03949pf01515) EUT1
11630 Hp_euts_violetPink EUT1
11635 HMM10662PduV_1_mix(pf10662) EUT1
11640 HMM06249EutQ_1_eut1(pf06249) EUT1 EP_NTERM
11645 HMM01923cobtr_2_eut1(pf01923) EUT1
11650 HMM01515PTA_B_1_eut1(pf01515) EUT1
11655 H_azure EUT1
11660 P_lightViolet EUT1
11665 HMMalddh_1_eutpdugrm(pf00171) EUT1 EP_NTERM
11670 HMM06723EutJMreB_1_grm EUT1
11675 HMM00465alcdh_3_mix(pf00465) EUT1
11680 HMMtpEuth_1_eut(pf04346) EUT1
11685 HMM06277EutA_1_eut(pf06277) EUT1
11690 HMMetlyEutB_1_EUT1(pf06751) EUT1
11695 HMMetlyEutC_1_eut1(pf05985) EUT1 EP_NTERM
11700 Tsp_rosa EUT1
11705 H_mulberry(pf00936pf16365) EUT1
11710 HMMreg12833_1_EUT1(pf12833) EUT1
11715 -pf01218 -
11720 -pf01520 -
11725 -pf00583 -
11730 -pf11143 -
11735 -pf06572 -
11740 -pf04261 -
11745 HMMnopfamclust3_1 RMM1
11750 - -
11755 -pf00528 -
11760 -pf00528 -
11765 -pf00005 -
11770 -pf00291 -
BMC-P
BMC-H
BMC-T
Aldehyde dehydrogenase
PTAC
Signature enzyme
Alcohol dehydrogenase
Regulator

Salmonella enterica subsp. enterica serovar Javiana. Bacteria; Proteobacteria; Gammaproteobacteria; Enterobacterales;

E1843__locus_1

EUT1

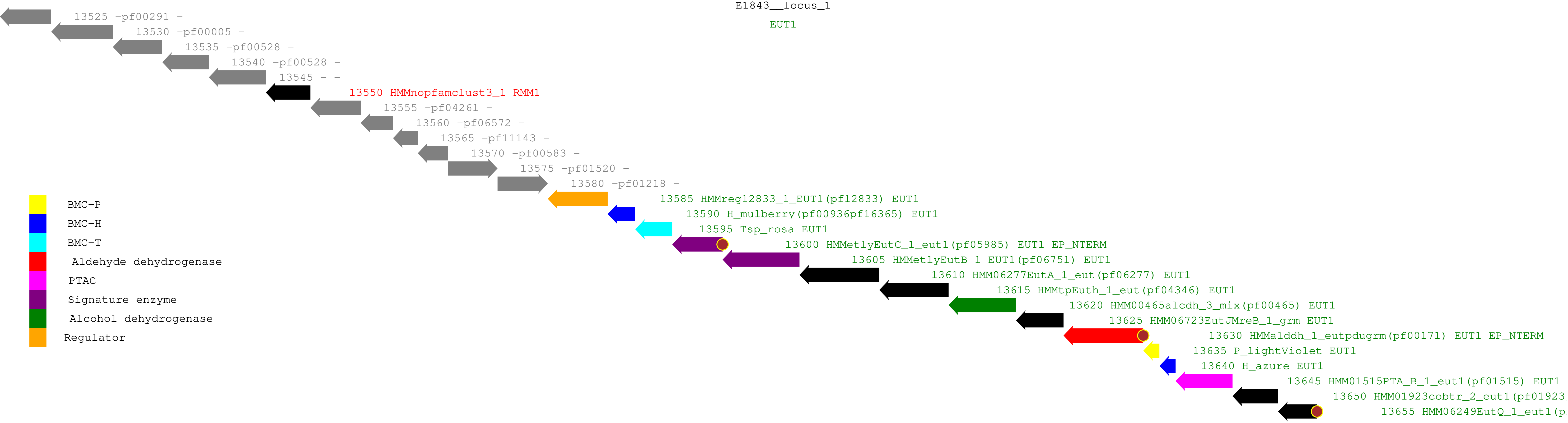

Salmonella enterica subsp. enterica serovar Glostrup. Bacteria; Proteobacteria; Gammaproteobacteria; Enterobacterales;

E1853__locus_2

EUT1

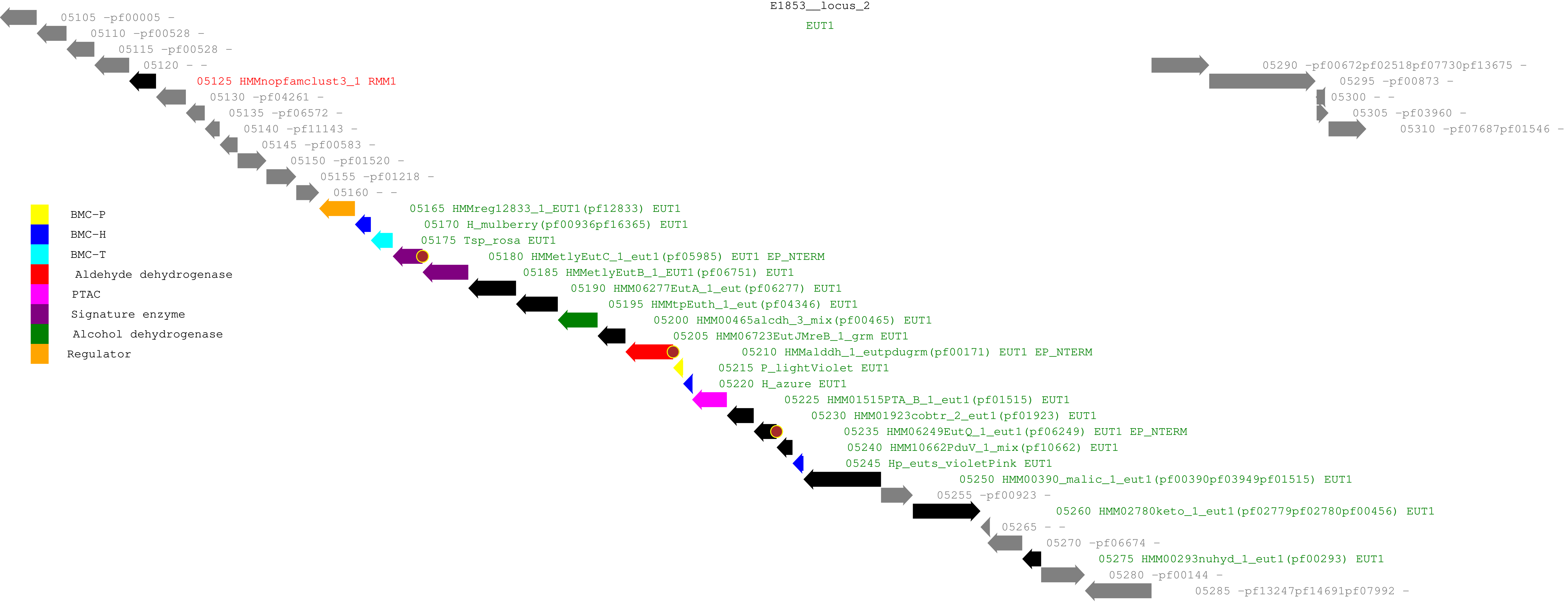

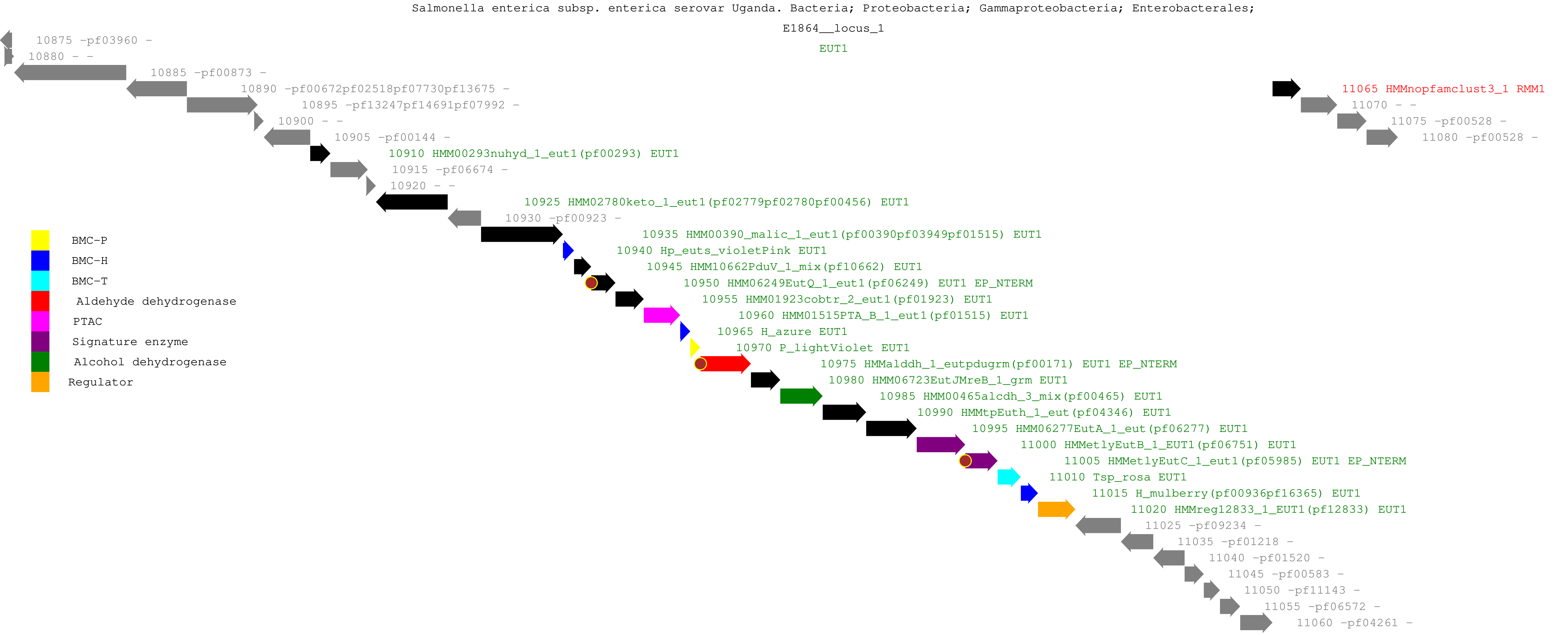

Salmonella enterica subsp. enterica serovar Uganda. Bacteria; Proteobacteria; Gammaproteobacteria; Enterobacterales;
E1864__locus_1
EUT1
10875 -pf03960 -
10880 - -
10885 -pf00873 -
10890 -pf00672pf02518pf07730pf13675 -
10895 -pf13247pf14691pf07992 -
10900 - -
10905 -pf00144 -
10910 HMM00293nuhyd_1_eut1(pf00293) EUT1
10915 -pf06674 -
10920 - -
10925 HMM02780keto_1_eut1(pf02779pf02780pf00456) EUT1
10930 -pf00923 -
10935 HMM00390_malic_1_eut1(pf00390pf03949pf01515) EUT1
10940 Hp_euts_violetPink EUT1
10945 HMM10662PduV_1_mix(pf10662) EUT1
10950 HMM06249EutQ_1_eut1(pf06249) EUT1 EP_NTERM
10955 HMM01923cobtr_2_eut1(pf01923) EUT1
10960 HMM01515PTA_B_1_eut1(pf01515) EUT1
10965 H_azure EUT1
10970 P_lightViolet EUT1
10975 HMMalddh_1_eutpdugrm(pf00171) EUT1 EP_NTERM
10980 HMM06723EutJMreB_1_grm EUT1
10985 HMM00465alcdh_3_mix(pf00465) EUT1
10990 HMMtpEuth_1_eut(pf04346) EUT1
10995 HMM06277EutA_1_eut(pf06277) EUT1
11000 HMMetlyEutB_1_EUT1(pf06751) EUT1
11005 HMMetlyEutC_1_eut1(pf05985) EUT1 EP_NTERM
11010 Tsp_rosa EUT1
11015 H_mulberry(pf00936pf16365) EUT1
11020 HMMreg12833_1_EUT1(pf12833) EUT1
11025 -pf09234 -
11035 -pf01218 -
11040 -pf01520 -
11045 -pf00583 -
11050 -pf11143 -
11055 -pf06572 -
11060 -pf04261 -
11065 HMMnopfamclust3_1 RMM1
11070 - -
11075 -pf00528 -
11080 -pf00528 -
BMC-P
BMC-H
BMC-T
Aldehyde dehydrogenase
PTAC
Signature enzyme
Alcohol dehydrogenase
Regulator

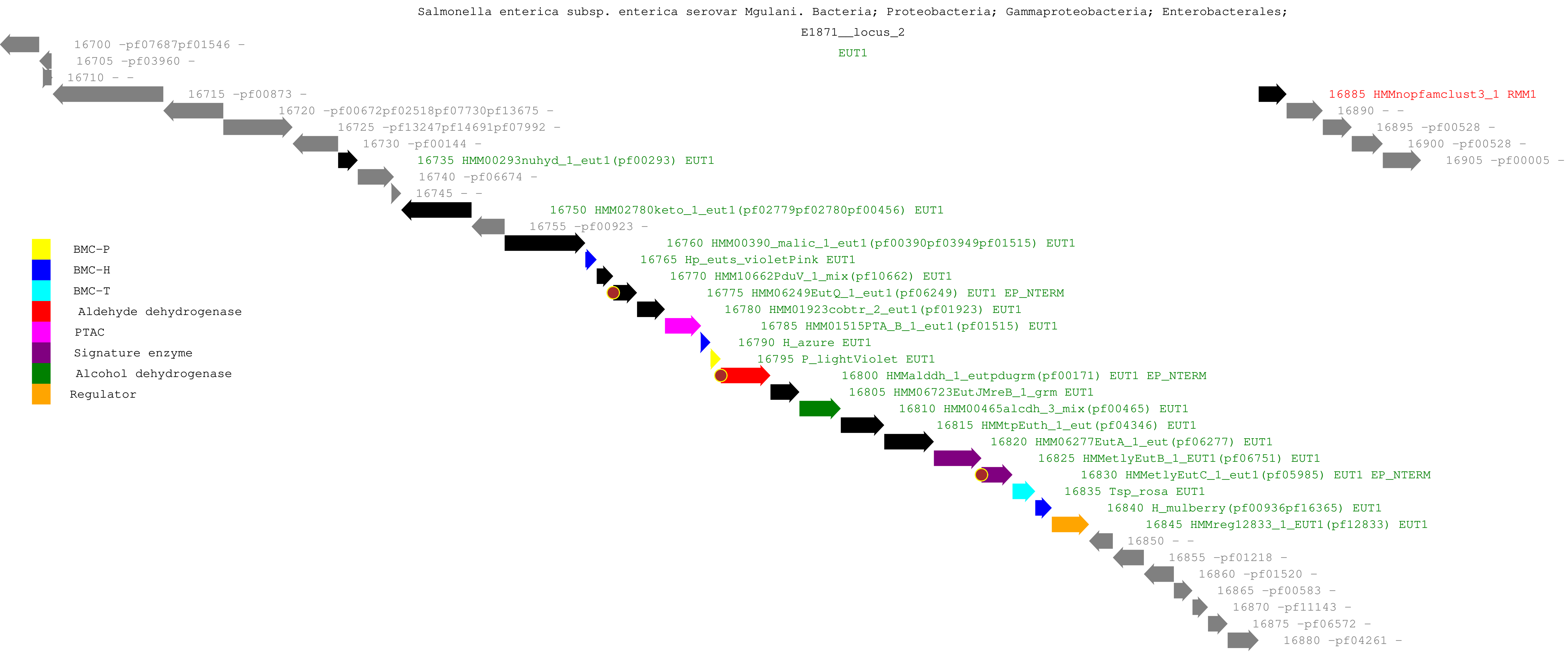
Salmonella enterica subsp. enterica serovar Mgulani. Bacteria; Proteobacteria; Gammaproteobacteria; Enterobacterales;
E1871__locus_2
EUT1
16700 -pf07687pf01546 -
16705 -pf03960 -
16710 - -
16715 -pf00873 -
16720 -pf00672pf02518pf07730pf13675 -
16725 -pf13247pf14691pf07992 -
16730 -pf00144 -
16735 HMM00293nuhyd_1_eut1(pf00293) EUT1
16740 -pf06674 -
16745 - -
16750 HMM02780keto_1_eut1(pf02779pf02780pf00456) EUT1
16755 -pf00923 -
16760 HMM00390_malic_1_eut1(pf00390pf03949pf01515) EUT1
16765 Hp_euts_violetPink EUT1
16770 HMM10662PduV_1_mix(pf10662) EUT1
16775 HMM06249EutQ_1_eut1(pf06249) EUT1 EP_NTERM
16780 HMM01923cobtr_2_eut1(pf01923) EUT1
16785 HMM01515PTA_B_1_eut1(pf01515) EUT1
16790 H_azure EUT1
16795 P_lightViolet EUT1
16800 HMMalddh_1_eutpdugrm(pf00171) EUT1 EP_NTERM
16805 HMM06723EutJMreB_1_grm EUT1
16810 HMM00465alcdh_3_mix(pf00465) EUT1
16815 HMMtpEuth_1_eut(pf04346) EUT1
16820 HMM06277EutA_1_eut(pf06277) EUT1
16825 HMMetlyEutB_1_EUT1(pf06751) EUT1
16830 HMMetlyEutC_1_eut1(pf05985) EUT1 EP_NTERM
16835 Tsp_rosa EUT1
16840 H_mulberry(pf00936pf16365) EUT1
16845 HMMreg12833_1_EUT1(pf12833) EUT1
16850 - -
16855 -pf01218 -
16860 -pf01520 -
16865 -pf00583 -
16870 -pf11143 -
16875 -pf06572 -
16880 -pf04261 -
16885 HMMnopfamclust3_1 RMM1
16890 - -
16895 -pf00528 -
16900 -pf00528 -
16905 -pf00005 -
BMC-P
BMC-H
BMC-T
Aldehyde dehydrogenase
PTAC
Signature enzyme
Alcohol dehydrogenase
Regulator

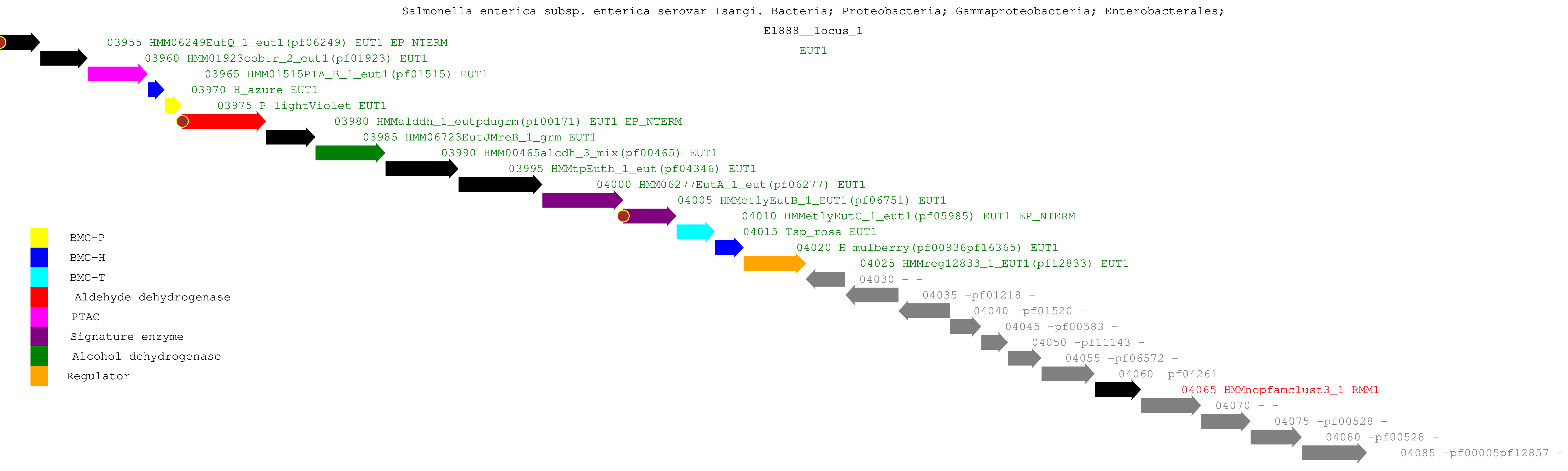

Salmonella enterica subsp. enterica serovar Isangi. Bacteria; Proteobacteria; Gammaproteobacteria; Enterobacterales;
E1888__locus_1
EUT1
03955 HMM06249EutQ_1_eut1(pf06249) EUT1 EP_NTERM
03960 HMM01923cobtr_2_eut1(pf01923) EUT1
03965 HMM01515PTA_B_1_eut1(pf01515) EUT1
03970 H_azure EUT1
03975 P_lightViolet EUT1
03980 HMMalddh_1_eutpdugrm(pf00171) EUT1 EP_NTERM
03985 HMM06723EutJMreB_1_grm EUT1
03990 HMM00465alcdh_3_mix(pf00465) EUT1
03995 HMMtpEuth_1_eut(pf04346) EUT1
04000 HMM06277EutA_1_eut(pf06277) EUT1
04005 HMMetlyEutB_1_EUT1(pf06751) EUT1
04010 HMMetlyEutC_1_eut1(pf05985) EUT1 EP_NTERM
04015 Tsp_rosa EUT1
04020 H_mulberry(pf00936pf16365) EUT1
04025 HMMreg12833_1_EUT1(pf12833) EUT1
04030 - -
04035 -pf01218 -
04040 -pf01520 -
04045 -pf00583 -
04050 -pf11143 -
04055 -pf06572 -
04060 -pf04261 -
04065 HMMnopfamclust3_1 RMM1
04070 - -
04075 -pf00528 -
04080 -pf00528 -
04085 -pf00005pf12857 -
BMC-P
BMC-H
BMC-T
Aldehyde dehydrogenase
PTAC
Signature enzyme
Alcohol dehydrogenase
Regulator

Salmonella enterica subsp. enterica serovar Brijbhumi. Bacteria; Proteobacteria; Gammaproteobacteria; Enterobacterales;

E1916__locus_1

EUT1

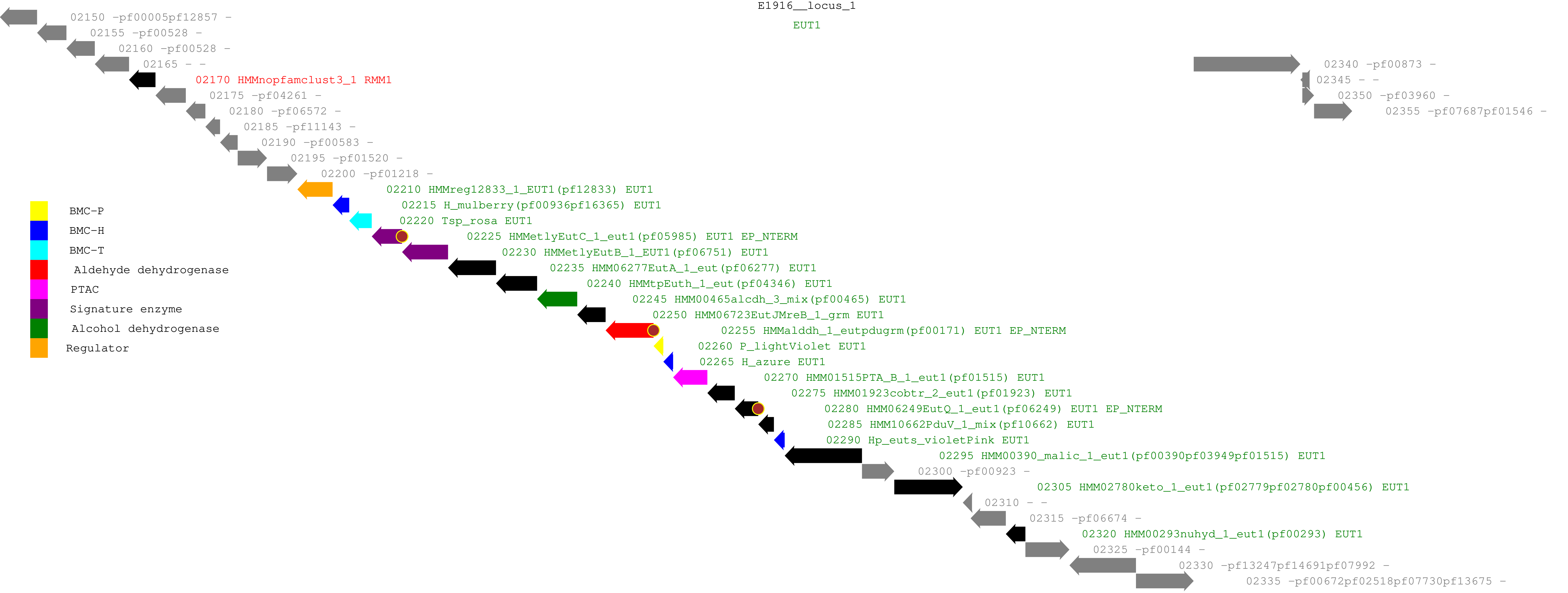

Salmonella enterica subsp. enterica serovar Ahuza. Bacteria; Proteobacteria; Gammaproteobacteria; Enterobacterales;

E1944__locus_2

EUT1

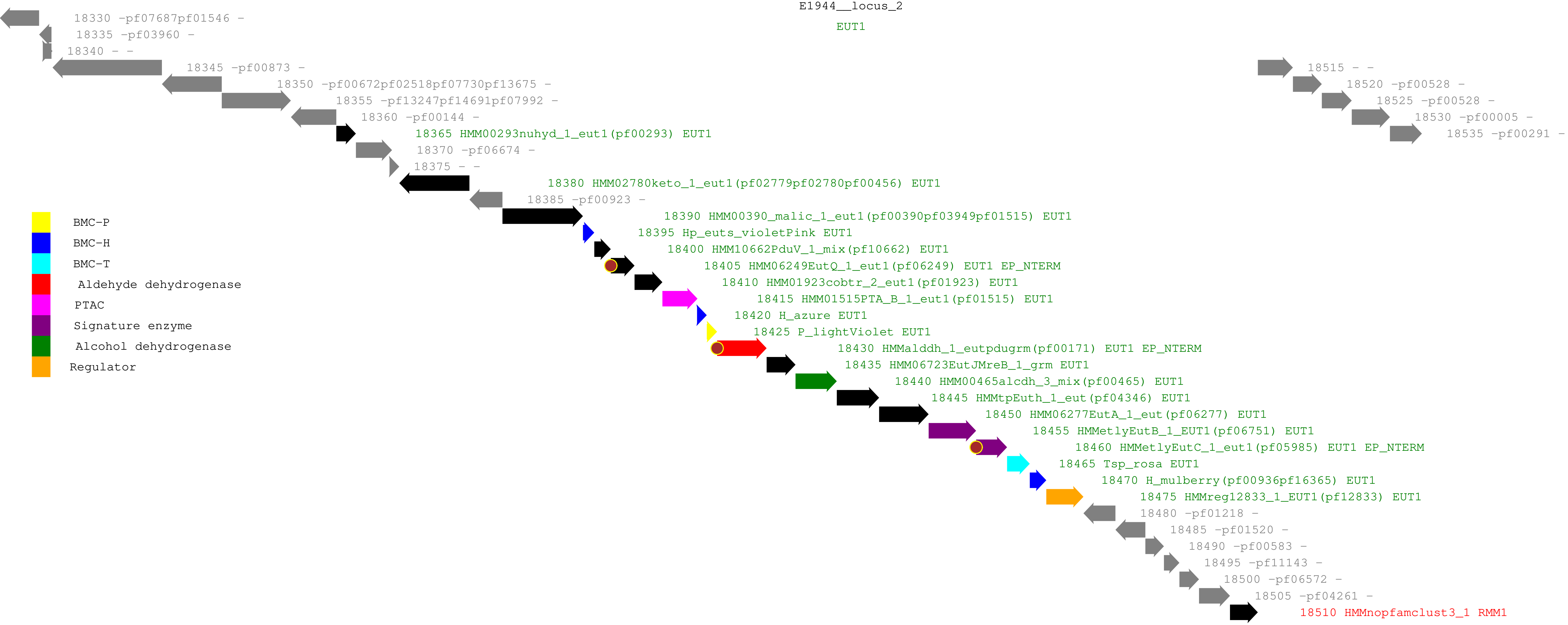

Salmonella enterica subsp. enterica serovar Cubana. Bacteria; Proteobacteria; Gammaproteobacteria; Enterobacterales;

E1A24__locus_2

EUT1

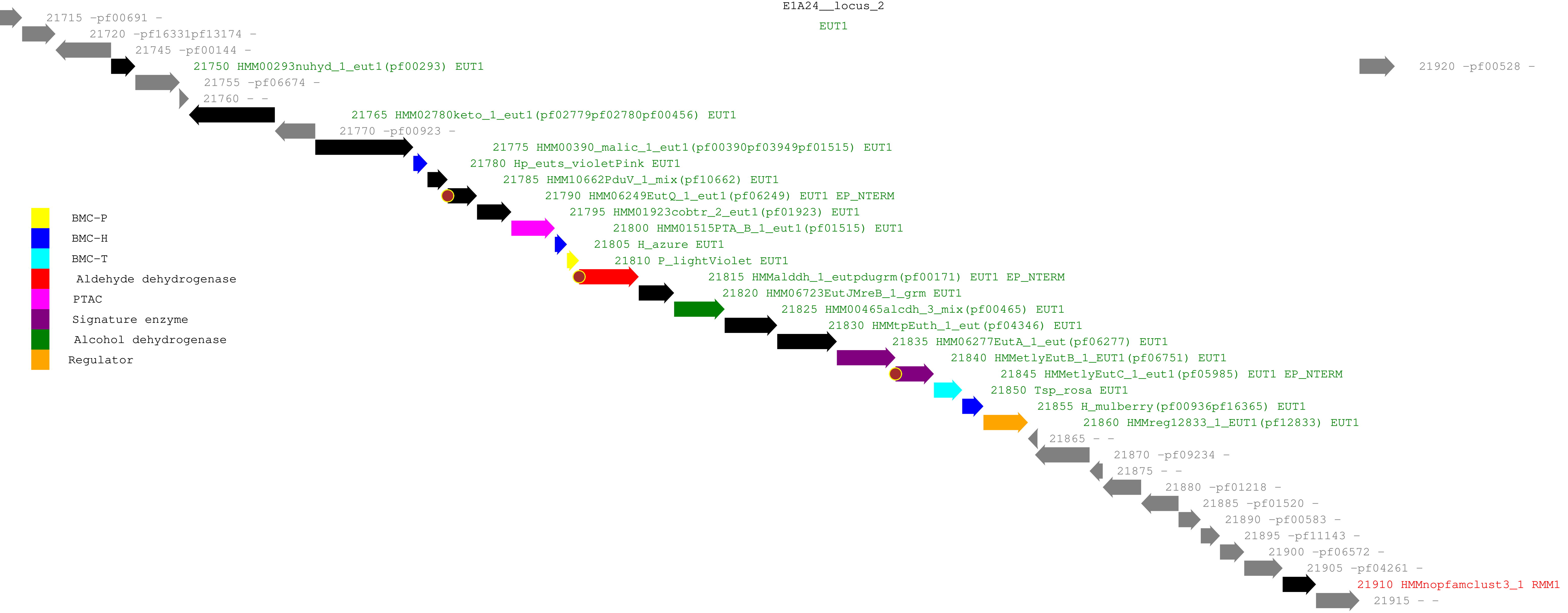

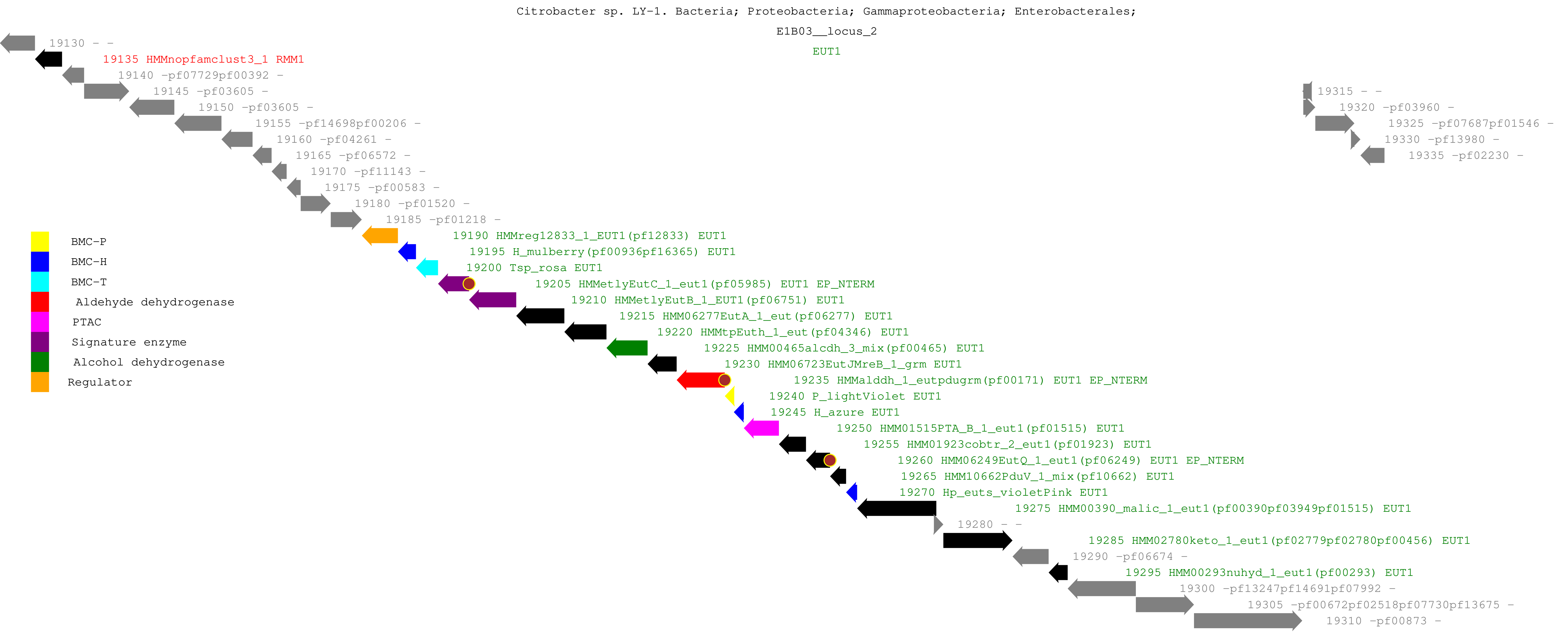

Citrobacter sp. LY-1. Bacteria; Proteobacteria; Gammaproteobacteria; Enterobacterales;
E1B03__locus_2
EUT1
19130 - -
19135 HMMnopfamclust3_1 RMM1
19140 -pf07729pf00392 -
19145 -pf03605 -
19150 -pf03605 -
19155 -pf14698pf00206 -
19160 -pf04261 -
19165 -pf06572 -
19170 -pf11143 -
19175 -pf00583 -
19180 -pf01520 -
19185 -pf01218 -
19190 HMMreg12833_1_EUT1(pf12833) EUT1
19195 H_mulberry(pf00936pf16365) EUT1
19200 Tsp_rosa EUT1
19205 HMMetlyEutC_1_eut1(pf05985) EUT1 EP_NTERM
19210 HMMetlyEutB_1_EUT1(pf06751) EUT1
19215 HMM06277EutA_1_eut(pf06277) EUT1
19220 HMMtpEuth_1_eut(pf04346) EUT1
19225 HMM00465alcdh_3_mix(pf00465) EUT1
19230 HMM06723EutJMreB_1_grm EUT1
19235 HMMalddh_1_eutpdugrm(pf00171) EUT1 EP_NTERM
19240 P_lightViolet EUT1
19245 H_azure EUT1
19250 HMM01515PTA_B_1_eut1(pf01515) EUT1
19255 HMM01923cobtr_2_eut1(pf01923) EUT1
19260 HMM06249EutQ_1_eut1(pf06249) EUT1 EP_NTERM
19265 HMM10662PduV_1_mix(pf10662) EUT1
19270 Hp_euts_violetPink EUT1
19275 HMM00390_malic_1_eut1(pf00390pf03949pf01515) EUT1
19280 - -
19285 HMM02780keto_1_eut1(pf02779pf02780pf00456) EUT1
19290 -pf06674 -
19295 HMM00293nuhyd_1_eut1(pf00293) EUT1
19300 -pf13247pf14691pf07992 -
19305 -pf00672pf02518pf07730pf13675 -
19310 -pf00873 -
19315 - -
19320 -pf03960 -
19325 -pf07687pf01546 -
19330 -pf13980 -
19335 -pf02230 -
BMC-P
BMC-H
BMC-T
Aldehyde dehydrogenase
PTAC
Signature enzyme
Alcohol dehydrogenase
Regulator

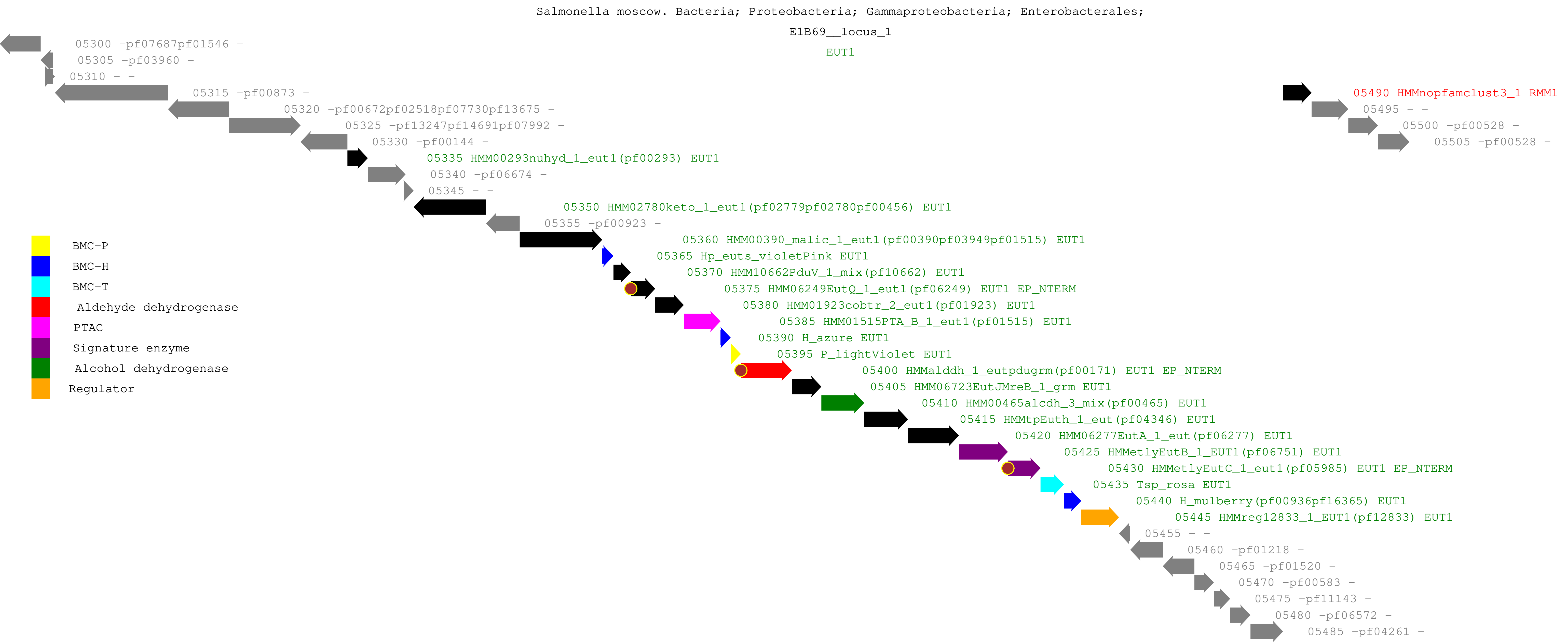

Salmonella moscow. Bacteria; Proteobacteria; Gammaproteobacteria; Enterobacterales;
E1B69__locus_1
EUT1
05300 -pf07687pf01546 -
05305 -pf03960 -
05310 - -
05315 -pf00873 -
05320 -pf00672pf02518pf07730pf13675 -
05325 -pf13247pf14691pf07992 -
05330 -pf00144 -
05335 HMM00293nuhyd_1_eut1(pf00293) EUT1
05340 -pf06674 -
05345 - -
05350 HMM02780keto_1_eut1(pf02779pf02780pf00456) EUT1
05355 -pf00923 -
05360 HMM00390_malic_1_eut1(pf00390pf03949pf01515) EUT1
05365 Hp_euts_violetPink EUT1
05370 HMM10662PduV_1_mix(pf10662) EUT1
05375 HMM06249EutQ_1_eut1(pf06249) EUT1 EP_NTERM
05380 HMM01923cobtr_2_eut1(pf01923) EUT1
05385 HMM01515PTA_B_1_eut1(pf01515) EUT1
05390 H_azure EUT1
05395 P_lightViolet EUT1
05400 HMMalddh_1_eutpdugrm(pf00171) EUT1 EP_NTERM
05405 HMM06723EutJMreB_1_grm EUT1
05410 HMM00465alcdh_3_mix(pf00465) EUT1
05415 HMMtpEuth_1_eut(pf04346) EUT1
05420 HMM06277EutA_1_eut(pf06277) EUT1
05425 HMMetlyEutB_1_EUT1(pf06751) EUT1
05430 HMMetlyEutC_1_eut1(pf05985) EUT1 EP_NTERM
05435 Tsp_rosa EUT1
05440 H_mulberry(pf00936pf16365) EUT1
05445 HMMreg12833_1_EUT1(pf12833) EUT1
05455 - -
05460 -pf01218 -
05465 -pf01520 -
05470 -pf00583 -
05475 -pf11143 -
05480 -pf06572 -
05485 -pf04261 -
05490 HMMnopfamclust3_1 RMM1
05495 - -
05500 -pf00528 -
05505 -pf00528 -
BMC-P
BMC-H
BMC-T
Aldehyde dehydrogenase
PTAC
Signature enzyme
Alcohol dehydrogenase
Regulator

Salmonella enterica subsp. enterica serovar Bonariensis. Bacteria; Proteobacteria; Gammaproteobacteria; Enterobacterales;

E1C95__locus_2

EUT1

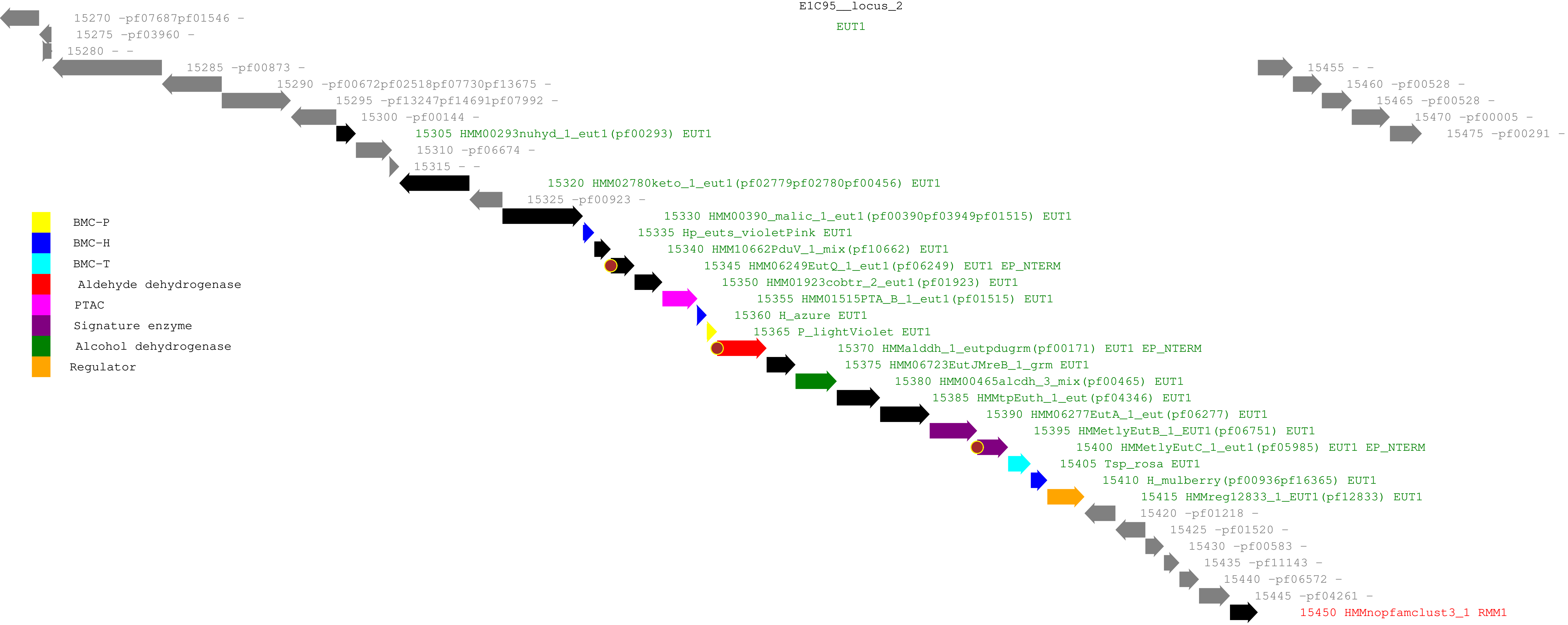

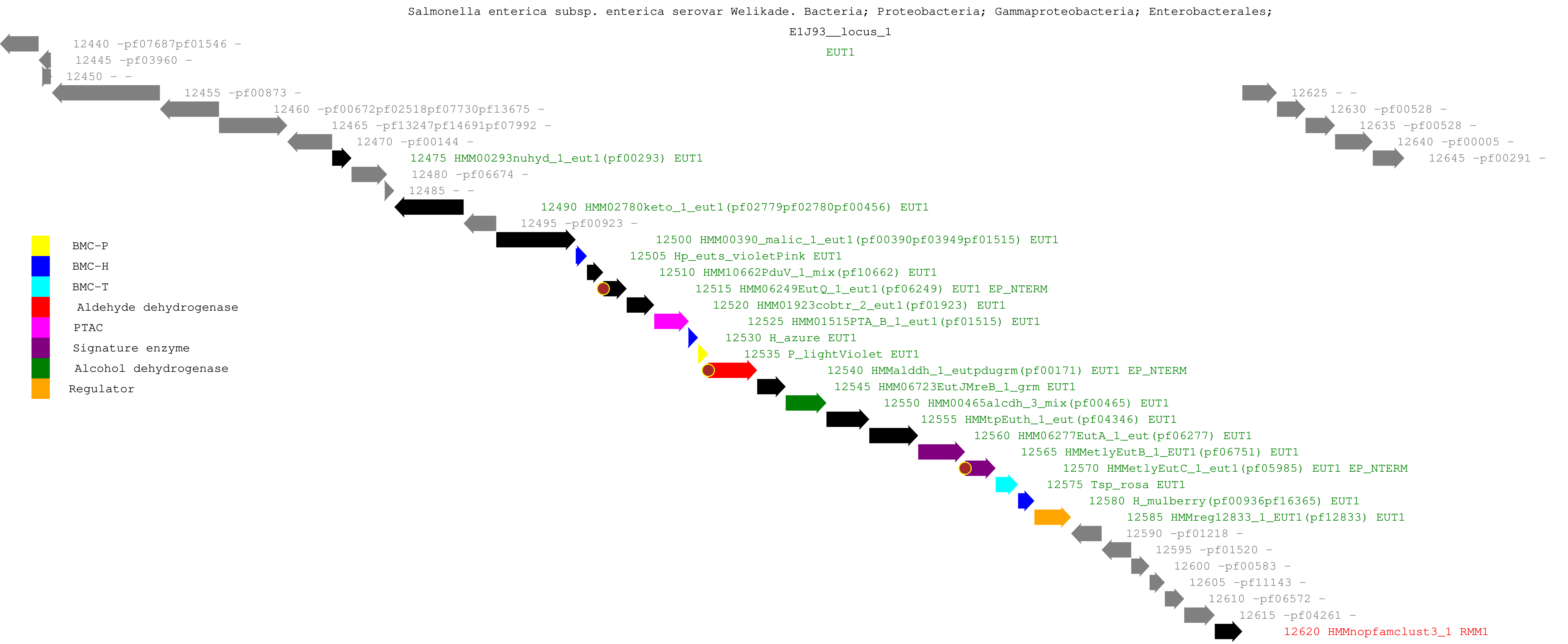

Salmonella enterica subsp. enterica serovar Welikade. Bacteria; Proteobacteria; Gammaproteobacteria; Enterobacterales;
E1J93__locus_1
EUT1
12440 -pf07687pf01546 -
12445 -pf03960 -
12450 - -
12455 -pf00873 -
12460 -pf00672pf02518pf07730pf13675 -
12465 -pf13247pf14691pf07992 -
12470 -pf00144 -
12475 HMM00293nuhyd_1_eut1(pf00293) EUT1
12480 -pf06674 -
12485 - -
12490 HMM02780keto_1_eut1(pf02779pf02780pf00456) EUT1
12495 -pf00923 -
12500 HMM00390_malic_1_eut1(pf00390pf03949pf01515) EUT1
12505 Hp_euts_violetPink EUT1
12510 HMM10662PduV_1_mix(pf10662) EUT1
12515 HMM06249EutQ_1_eut1(pf06249) EUT1 EP_NTERM
12520 HMM01923cobtr_2_eut1(pf01923) EUT1
12525 HMM01515PTA_B_1_eut1(pf01515) EUT1
12530 H_azure EUT1
12535 P_lightViolet EUT1
12540 HMMalddh_1_eutpdugrm(pf00171) EUT1 EP_NTERM
12545 HMM06723EutJMreB_1_grm EUT1
12550 HMM00465alcdh_3_mix(pf00465) EUT1
12555 HMMtpEuth_1_eut(pf04346) EUT1
12560 HMM06277EutA_1_eut(pf06277) EUT1
12565 HMMetlyEutB_1_EUT1(pf06751) EUT1
12570 HMMetlyEutC_1_eut1(pf05985) EUT1 EP_NTERM
12575 Tsp_rosa EUT1
12580 H_mulberry(pf00936pf16365) EUT1
12585 HMMreg12833_1_EUT1(pf12833) EUT1
12590 -pf01218 -
12595 -pf01520 -
12600 -pf00583 -
12605 -pf11143 -
12610 -pf06572 -
12615 -pf04261 -
12620 HMMnopfamclust3_1 RMM1
12625 - -
12630 -pf00528 -
12635 -pf00528 -
12640 -pf00005 -
12645 -pf00291 -
BMC-P
BMC-H
BMC-T
Aldehyde dehydrogenase
PTAC
Signature enzyme
Alcohol dehydrogenase
Regulator

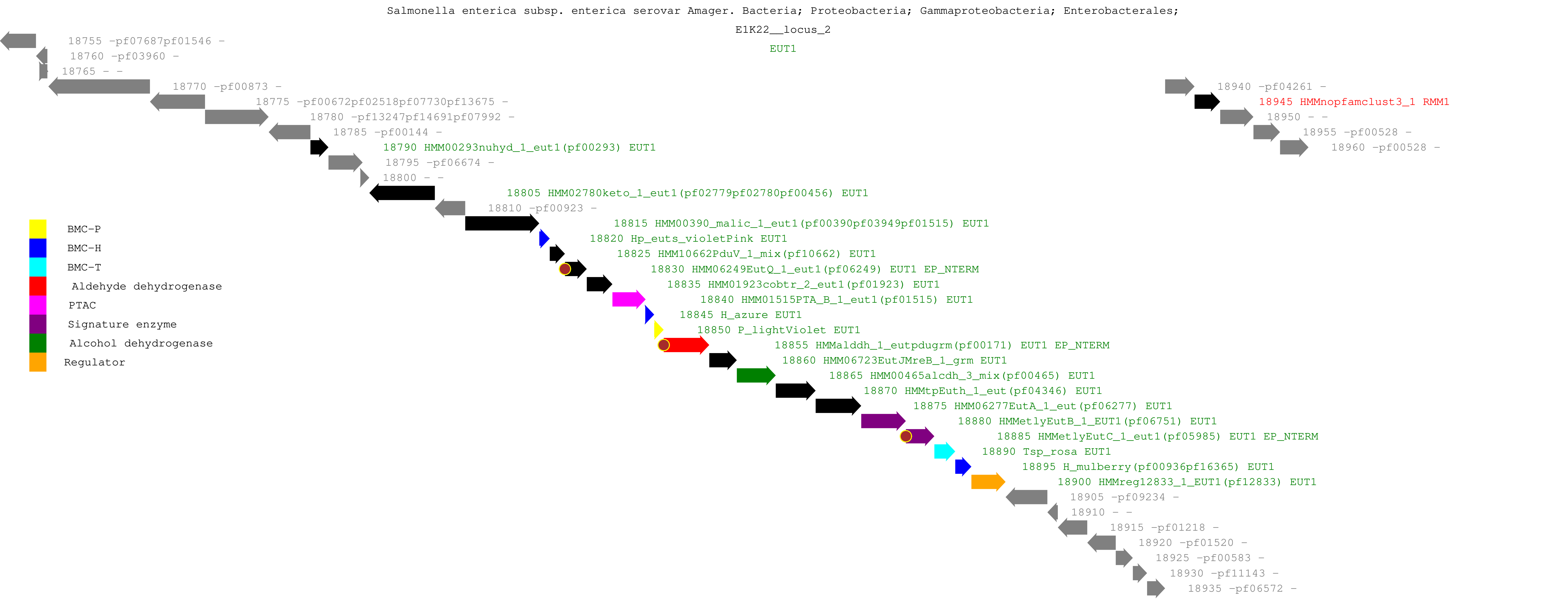

Salmonella enterica subsp. enterica serovar Amager. Bacteria; Proteobacteria; Gammaproteobacteria; Enterobacterales;
E1K22__locus_2
EUT1
18755 -pf07687pf01546 -
18760 -pf03960 -
18765 - -
18770 -pf00873 -
18775 -pf00672pf02518pf07730pf13675 -
18780 -pf13247pf14691pf07992 -
18785 -pf00144 -
18790 HMM00293nuhyd_1_eut1(pf00293) EUT1
18795 -pf06674 -
18800 - -
18805 HMM02780keto_1_eut1(pf02779pf02780pf00456) EUT1
18810 -pf00923 -
18815 HMM00390_malic_1_eut1(pf00390pf03949pf01515) EUT1
18820 Hp_euts_violetPink EUT1
18825 HMM10662PduV_1_mix(pf10662) EUT1
18830 HMM06249EutQ_1_eut1(pf06249) EUT1 EP_NTERM
18835 HMM01923cobtr_2_eut1(pf01923) EUT1
18840 HMM01515PTA_B_1_eut1(pf01515) EUT1
18845 H_azure EUT1
18850 P_lightViolet EUT1
18855 HMMalddh_1_eutpdugrm(pf00171) EUT1 EP_NTERM
18860 HMM06723EutJMreB_1_grm EUT1
18865 HMM00465alcdh_3_mix(pf00465) EUT1
18870 HMMtpEuth_1_eut(pf04346) EUT1
18875 HMM06277EutA_1_eut(pf06277) EUT1
18880 HMMetlyEutB_1_EUT1(pf06751) EUT1
18885 HMMetlyEutC_1_eut1(pf05985) EUT1 EP_NTERM
18890 Tsp_rosa EUT1
18895 H_mulberry(pf00936pf16365) EUT1
18900 HMMreg12833_1_EUT1(pf12833) EUT1
18905 -pf09234 -
18910 - -
18915 -pf01218 -
18920 -pf01520 -
18925 -pf00583 -
18930 -pf11143 -
18935 -pf06572 -
18940 -pf04261 -
18945 HMMnopfamclust3_1 RMM1
18950 - -
18955 -pf00528 -
18960 -pf00528 -
BMC-P
BMC-H
BMC-T
Aldehyde dehydrogenase
PTAC
Signature enzyme
Alcohol dehydrogenase
Regulator

Salmonella enterica subsp. enterica serovar Ruiru. Bacteria; Proteobacteria; Gammaproteobacteria; Enterobacterales;

E1K44__locus_1

EUT1

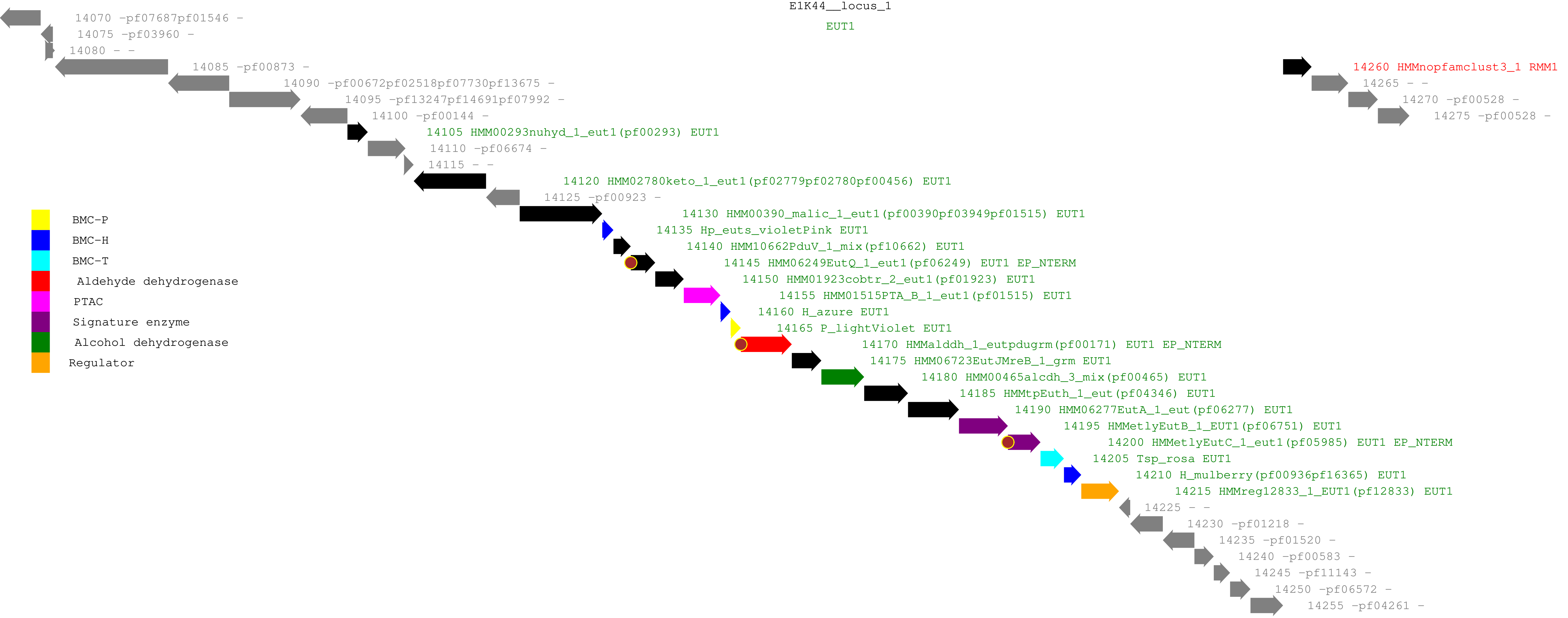

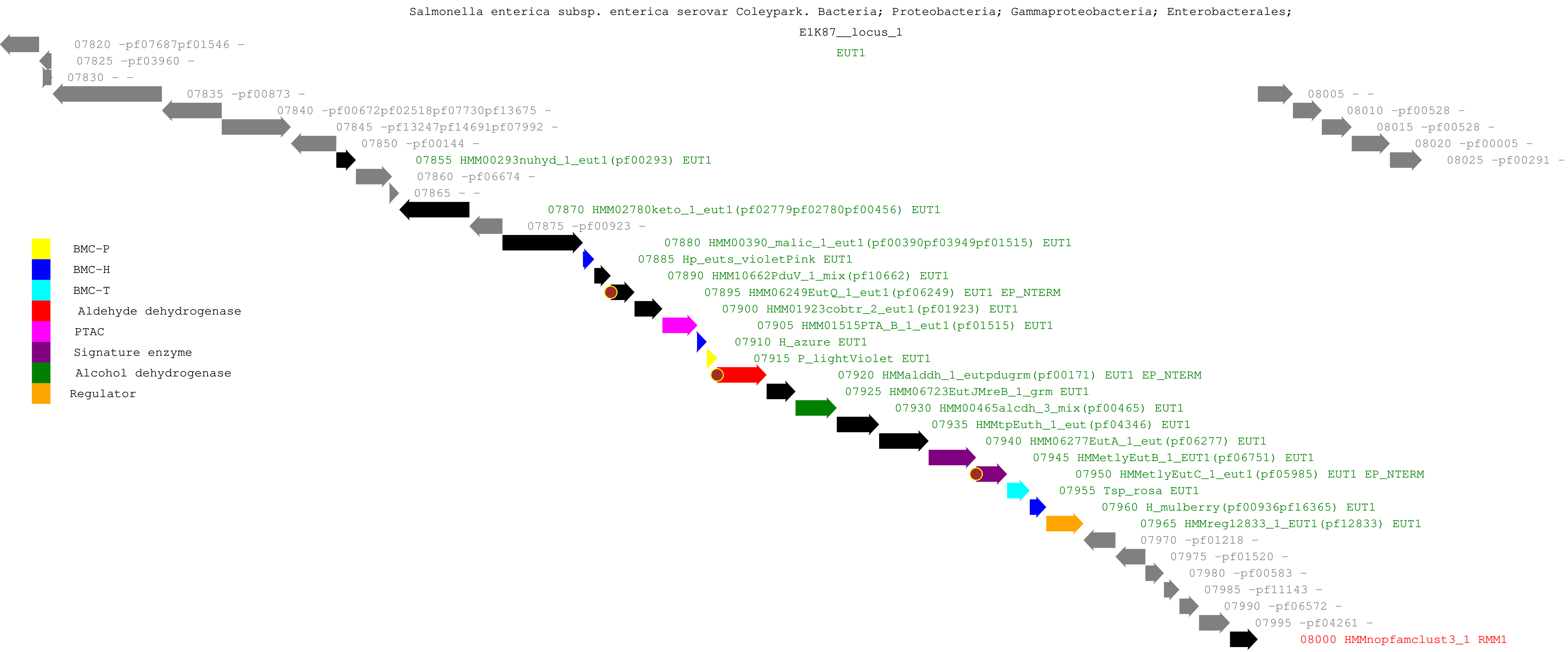
Salmonella enterica subsp. enterica serovar Coleypark. Bacteria; Proteobacteria; Gammaproteobacteria; Enterobacterales;
E1K87__locus_1
EUT1
07820 -pf07687pf01546 -
07825 -pf03960 -
07830 - -
07835 -pf00873 -
07840 -pf00672pf02518pf07730pf13675 -
07845 -pf13247pf14691pf07992 -
07850 -pf00144 -
07855 HMM00293nuhyd_1_eut1(pf00293) EUT1
07860 -pf06674 -
07865 - -
07870 HMM02780keto_1_eut1(pf02779pf02780pf00456) EUT1
07875 -pf00923 -
07880 HMM00390_malic_1_eut1(pf00390pf03949pf01515) EUT1
07885 Hp_euts_violetPink EUT1
07890 HMM10662PduV_1_mix(pf10662) EUT1
07895 HMM06249EutQ_1_eut1(pf06249) EUT1 EP_NTERM
07900 HMM01923cobtr_2_eut1(pf01923) EUT1
07905 HMM01515PTA_B_1_eut1(pf01515) EUT1
07910 H_azure EUT1
07915 P_lightViolet EUT1
07920 HMMalddh_1_eutpdugrm(pf00171) EUT1 EP_NTERM
07925 HMM06723EutJMreB_1_grm EUT1
07930 HMM00465alcdh_3_mix(pf00465) EUT1
07935 HMMtpEuth_1_eut(pf04346) EUT1
07940 HMM06277EutA_1_eut(pf06277) EUT1
07945 HMMetlyEutB_1_EUT1(pf06751) EUT1
07950 HMMetlyEutC_1_eut1(pf05985) EUT1 EP_NTERM
07955 Tsp_rosa EUT1
07960 H_mulberry(pf00936pf16365) EUT1
07965 HMMreg12833_1_EUT1(pf12833) EUT1
07970 -pf01218 -
07975 -pf01520 -
07980 -pf00583 -
07985 -pf11143 -
07990 -pf06572 -
07995 -pf04261 -
08000 HMMnopfamclust3_1 RMM1
08005 - -
08010 -pf00528 -
08015 -pf00528 -
08020 -pf00005 -
08025 -pf00291 -
BMC-P
BMC-H
BMC-T
Aldehyde dehydrogenase
PTAC
Signature enzyme
Alcohol dehydrogenase
Regulator

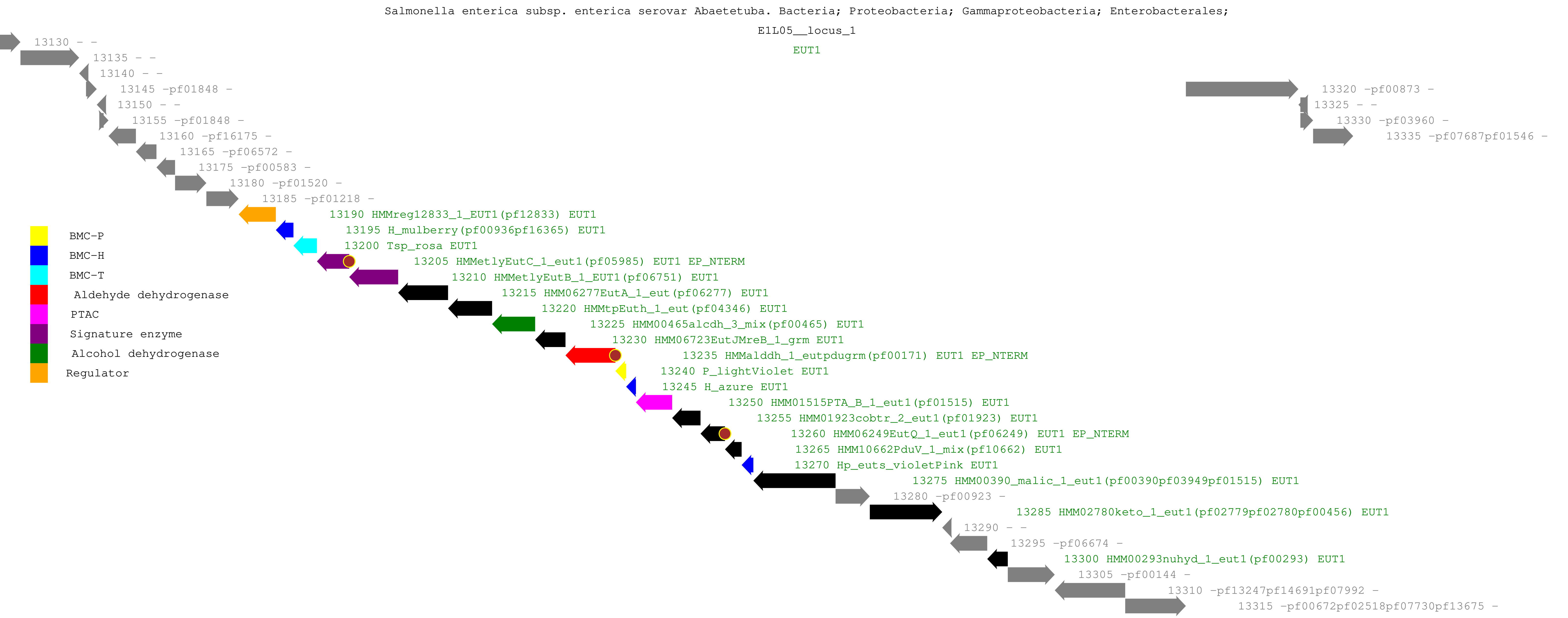
Salmonella enterica subsp. enterica serovar Abaetetuba. Bacteria; Proteobacteria; Gammaproteobacteria; Enterobacterales;
E1L05__locus_1
EUT1
13130 - -
13135 - -
13140 - -
13145 -pf01848 -
13150 - -
13155 -pf01848 -
13160 -pf16175 -
13165 -pf06572 -
13175 -pf00583 -
13180 -pf01520 -
13185 -pf01218 -
13190 HMMreg12833_1_EUT1(pf12833) EUT1
13195 H_mulberry(pf00936pf16365) EUT1
13200 Tsp_rosa EUT1
13205 HMMetlyEutC_1_eut1(pf05985) EUT1 EP_NTERM
13210 HMMetlyEutB_1_EUT1(pf06751) EUT1
13215 HMM06277EutA_1_eut(pf06277) EUT1
13220 HMMtpEuth_1_eut(pf04346) EUT1
13225 HMM00465alcdh_3_mix(pf00465) EUT1
13230 HMM06723EutJMreB_1_grm EUT1
13235 HMMalddh_1_eutpdugrm(pf00171) EUT1 EP_NTERM
13240 P_lightViolet EUT1
13245 H_azure EUT1
13250 HMM01515PTA_B_1_eut1(pf01515) EUT1
13255 HMM01923cobtr_2_eut1(pf01923) EUT1
13260 HMM06249EutQ_1_eut1(pf06249) EUT1 EP_NTERM
13265 HMM10662PduV_1_mix(pf10662) EUT1
13270 Hp_euts_violetPink EUT1
13275 HMM00390_malic_1_eut1(pf00390pf03949pf01515) EUT1
13280 -pf00923 -
13285 HMM02780keto_1_eut1(pf02779pf02780pf00456) EUT1
13290 - -
13295 -pf06674 -
13300 HMM00293nuhyd_1_eut1(pf00293) EUT1
13305 -pf00144 -
13310 -pf13247pf14691pf07992 -
13315 -pf00672pf02518pf07730pf13675 -
13320 -pf00873 -
13325 - -
13330 -pf03960 -
13335 -pf07687pf01546 -
BMC-P
BMC-H
BMC-T
Aldehyde dehydrogenase
PTAC
Signature enzyme
Alcohol dehydrogenase
Regulator

Salmonella enterica subsp. enterica serovar Everleigh. Bacteria; Proteobacteria; Gammaproteobacteria; Enterobacterales;

E1L13__locus_2

EUT1

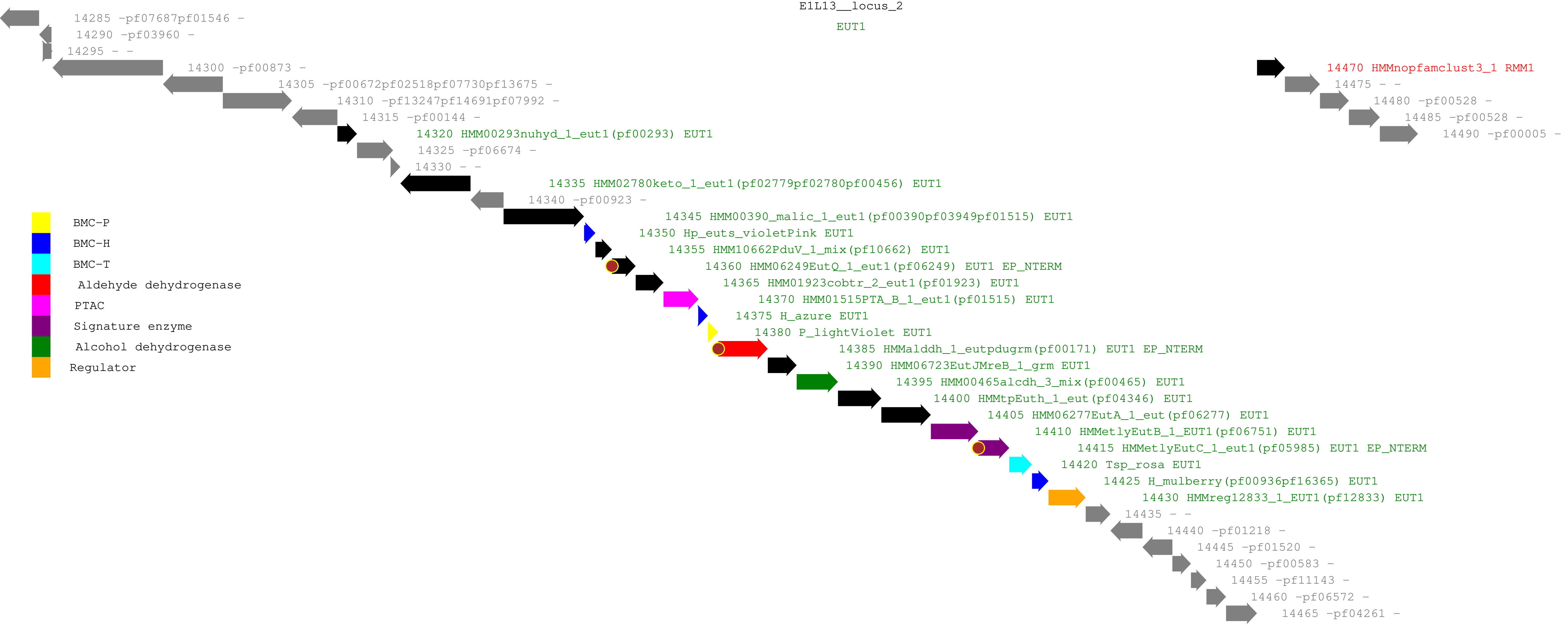

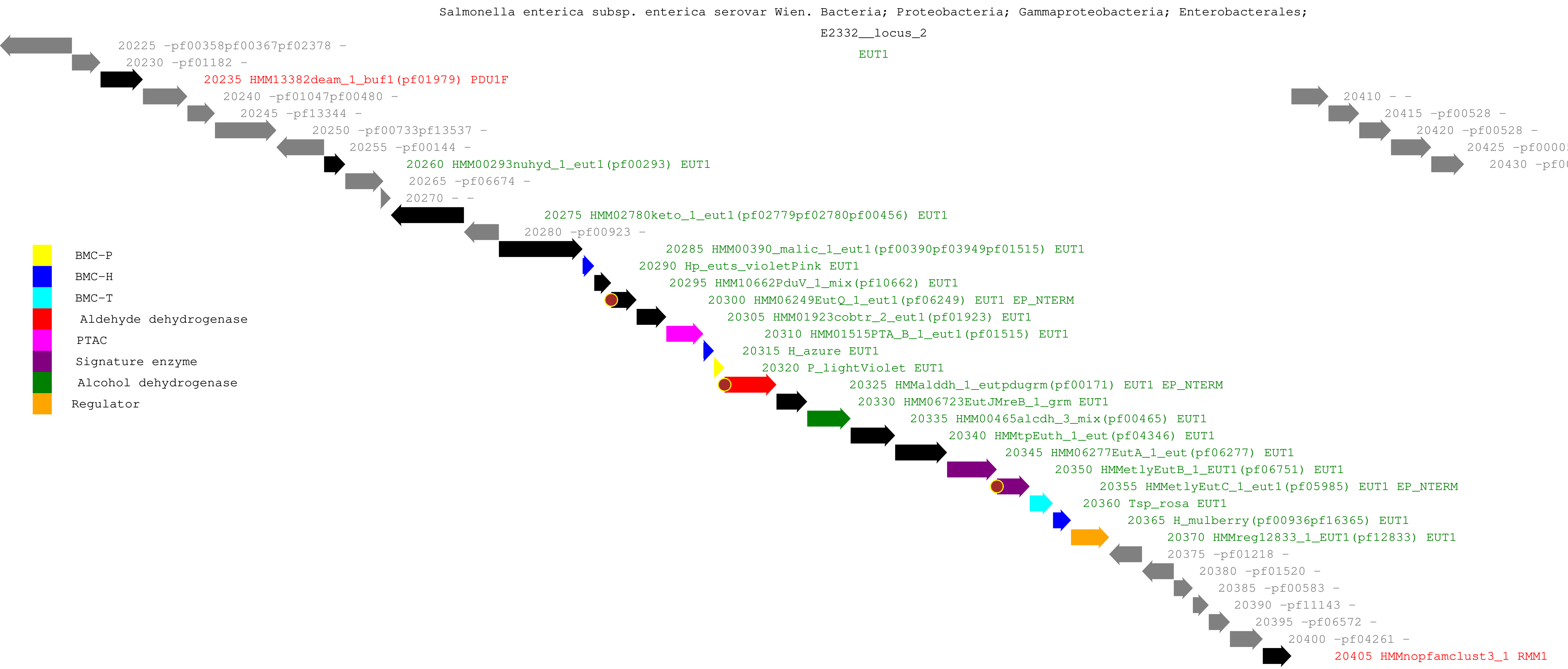

Salmonella enterica subsp. enterica serovar Wien. Bacteria; Proteobacteria; Gammaproteobacteria; Enterobacterales;
E2332__locus_2
EUT1
20225 -pf00358pf00367pf02378 -
20230 -pf01182 -
20235 HMM13382deam_1_buf1(pf01979) PDU1F
20240 -pf01047pf00480 -
20245 -pf13344 -
20250 -pf00733pf13537 -
20255 -pf00144 -
20260 HMM00293nuhyd_1_eut1(pf00293) EUT1
20265 -pf06674 -
20270 - -
20275 HMM02780keto_1_eut1(pf02779pf02780pf00456) EUT1
20280 -pf00923 -
20285 HMM00390_malic_1_eut1(pf00390pf03949pf01515) EUT1
20290 Hp_euts_violetPink EUT1
20295 HMM10662PduV_1_mix(pf10662) EUT1
20300 HMM06249EutQ_1_eut1(pf06249) EUT1 EP_NTERM
20305 HMM01923cobtr_2_eut1(pf01923) EUT1
20310 HMM01515PTA_B_1_eut1(pf01515) EUT1
20315 H_azure EUT1
20320 P_lightViolet EUT1
20325 HMMalddh_1_eutpdugrm(pf00171) EUT1 EP_NTERM
20330 HMM06723EutJMreB_1_grm EUT1
20335 HMM00465alcdh_3_mix(pf00465) EUT1
20340 HMMtpEuth_1_eut(pf04346) EUT1
20345 HMM06277EutA_1_eut(pf06277) EUT1
20350 HMMetlyEutB_1_EUT1(pf06751) EUT1
20355 HMMetlyEutC_1_eut1(pf05985) EUT1 EP_NTERM
20360 Tsp_rosa EUT1
20365 H_mulberry(pf00936pf16365) EUT1
20370 HMMreg12833_1_EUT1(pf12833) EUT1
20375 -pf01218 -
20380 -pf01520 -
20385 -pf00583 -
20390 -pf11143 -
20395 -pf06572 -
20400 -pf04261 -
20405 HMMnopfamclust3_1 RMM1
20410 - -
20415 -pf00528 -
20420 -pf00528 -
20425 -pf00005pf12857 -
20430 -pf00291 -
BMC-P
BMC-H
BMC-T
Aldehyde dehydrogenase
PTAC
Signature enzyme
Alcohol dehydrogenase
Regulator

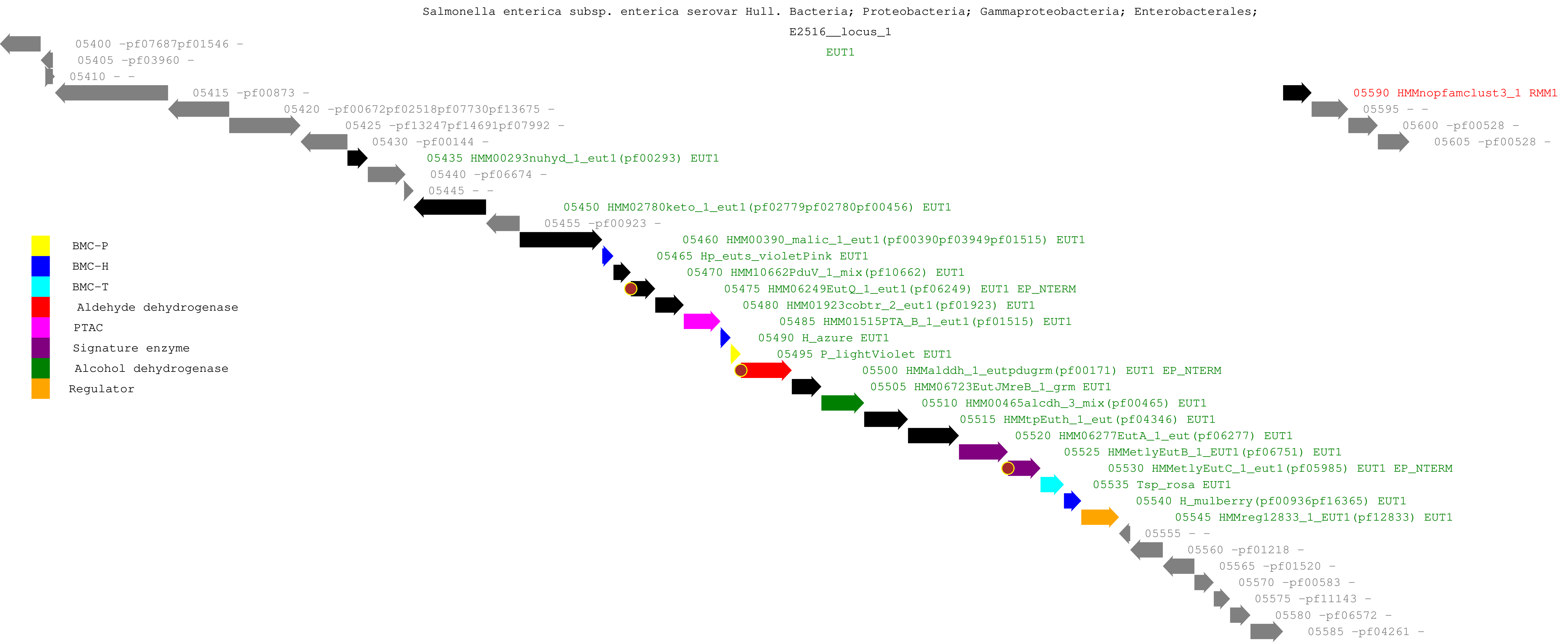
Salmonella enterica subsp. enterica serovar Hull. Bacteria; Proteobacteria; Gammaproteobacteria; Enterobacterales;
E2516__locus_1
EUT1
05400 -pf07687pf01546 -
05405 -pf03960 -
05410 - -
05415 -pf00873 -
05420 -pf00672pf02518pf07730pf13675 -
05425 -pf13247pf14691pf07992 -
05430 -pf00144 -
05435 HMM00293nuhyd_1_eut1(pf00293) EUT1
05440 -pf06674 -
05445 - -
05450 HMM02780keto_1_eut1(pf02779pf02780pf00456) EUT1
05455 -pf00923 -
05460 HMM00390_malic_1_eut1(pf00390pf03949pf01515) EUT1
05465 Hp_euts_violetPink EUT1
05470 HMM10662PduV_1_mix(pf10662) EUT1
05475 HMM06249EutQ_1_eut1(pf06249) EUT1 EP_NTERM
05480 HMM01923cobtr_2_eut1(pf01923) EUT1
05485 HMM01515PTA_B_1_eut1(pf01515) EUT1
05490 H_azure EUT1
05495 P_lightViolet EUT1
05500 HMMalddh_1_eutpdugrm(pf00171) EUT1 EP_NTERM
05505 HMM06723EutJMreB_1_grm EUT1
05510 HMM00465alcdh_3_mix(pf00465) EUT1
05515 HMMtpEuth_1_eut(pf04346) EUT1
05520 HMM06277EutA_1_eut(pf06277) EUT1
05525 HMMetlyEutB_1_EUT1(pf06751) EUT1
05530 HMMetlyEutC_1_eut1(pf05985) EUT1 EP_NTERM
05535 Tsp_rosa EUT1
05540 H_mulberry(pf00936pf16365) EUT1
05545 HMMreg12833_1_EUT1(pf12833) EUT1
05555 - -
05560 -pf01218 -
05565 -pf01520 -
05570 -pf00583 -
05575 -pf11143 -
05580 -pf06572 -
05585 -pf04261 -
05590 HMMnopfamclust3_1 RMM1
05595 - -
05600 -pf00528 -
05605 -pf00528 -
BMC-P
BMC-H
BMC-T
Aldehyde dehydrogenase
PTAC
Signature enzyme
Alcohol dehydrogenase
Regulator

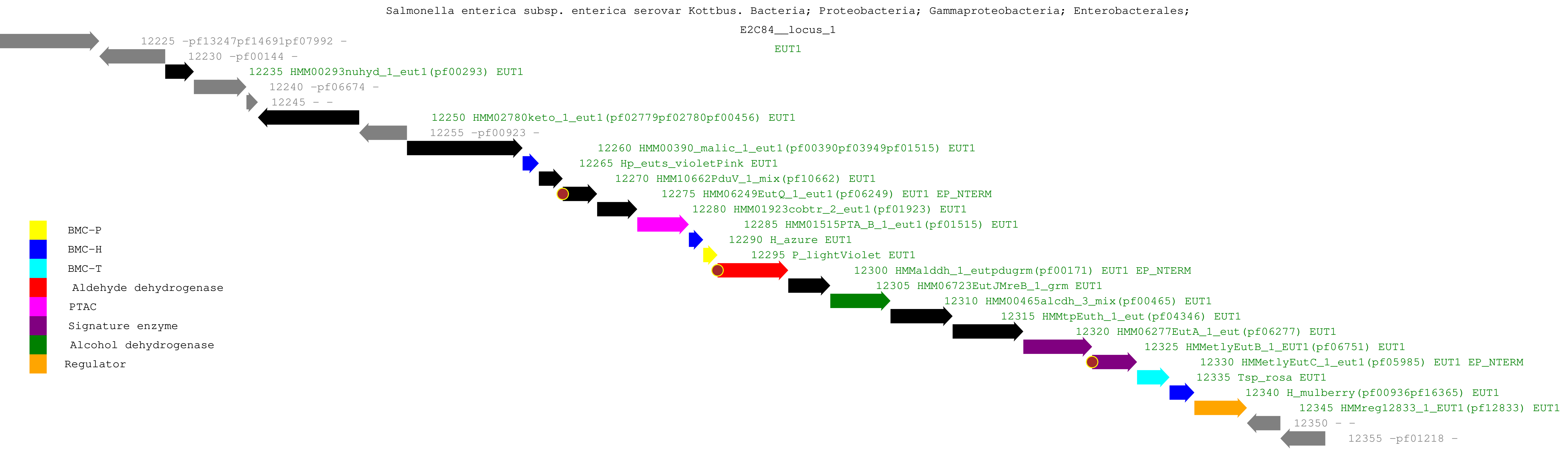

Salmonella enterica subsp. enterica serovar Kottbus. Bacteria; Proteobacteria; Gammaproteobacteria; Enterobacterales;
E2C84__locus_1
EUT1
12225 -pf13247pf14691pf07992 -
12230 -pf00144 -
12235 HMM00293nuhyd_1_eut1(pf00293) EUT1
12240 -pf06674 -
12245 - -
12250 HMM02780keto_1_eut1(pf02779pf02780pf00456) EUT1
12255 -pf00923 -
12260 HMM00390_malic_1_eut1(pf00390pf03949pf01515) EUT1
12265 Hp_euts_violetPink EUT1
12270 HMM10662PduV_1_mix(pf10662) EUT1
12275 HMM06249EutQ_1_eut1(pf06249) EUT1 EP_NTERM
12280 HMM01923cobtr_2_eut1(pf01923) EUT1
12285 HMM01515PTA_B_1_eut1(pf01515) EUT1
12290 H_azure EUT1
12295 P_lightViolet EUT1
12300 HMMalddh_1_eutpdugrm(pf00171) EUT1 EP_NTERM
12305 HMM06723EutJMreB_1_grm EUT1
12310 HMM00465alcdh_3_mix(pf00465) EUT1
12315 HMMtpEuth_1_eut(pf04346) EUT1
12320 HMM06277EutA_1_eut(pf06277) EUT1
12325 HMMetlyEutB_1_EUT1(pf06751) EUT1
12330 HMMetlyEutC_1_eut1(pf05985) EUT1 EP_NTERM
12335 Tsp_rosa EUT1
12340 H_mulberry(pf00936pf16365) EUT1
12345 HMMreg12833_1_EUT1(pf12833) EUT1
12350 - -
12355 -pf01218 -
BMC-P
BMC-H
BMC-T
Aldehyde dehydrogenase
PTAC
Signature enzyme
Alcohol dehydrogenase
Regulator

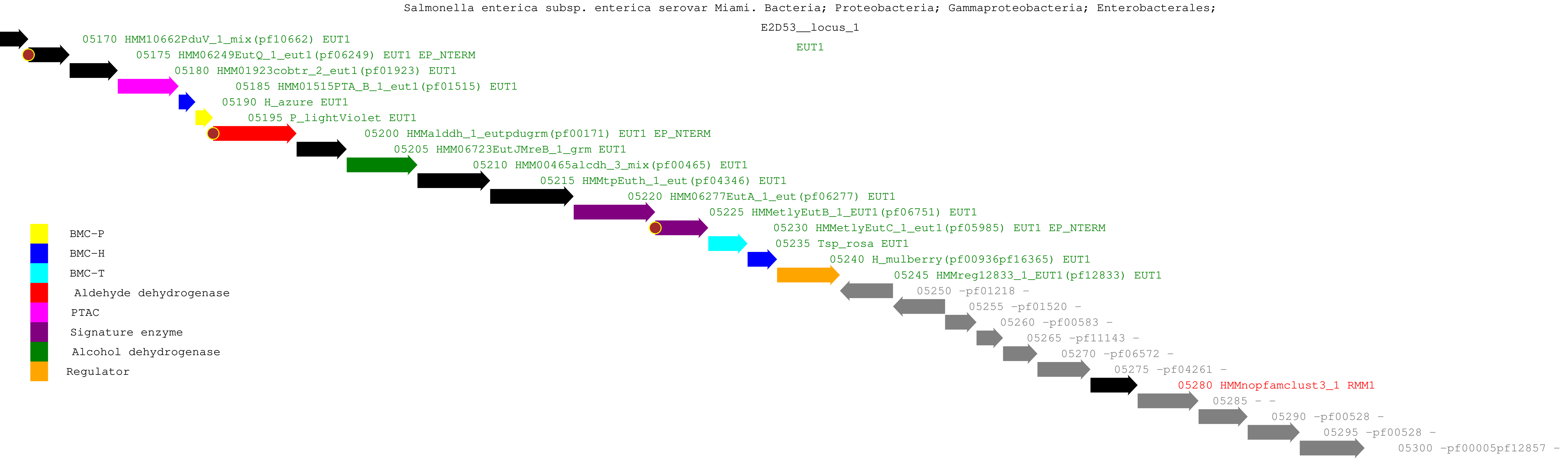

Salmonella enterica subsp. enterica serovar Miami. Bacteria; Proteobacteria; Gammaproteobacteria; Enterobacterales;
E2D53__locus_1
EUT1
05170 HMM10662PduV_1_mix(pf10662) EUT1
05175 HMM06249EutQ_1_eut1(pf06249) EUT1 EP_NTERM
05180 HMM01923cobtr_2_eut1(pf01923) EUT1
05185 HMM01515PTA_B_1_eut1(pf01515) EUT1
05190 H_azure EUT1
05195 P_lightViolet EUT1
05200 HMMaldh_1_eutpdugrm(pf00171) EUT1 EP_NTERM
05205 HMM06723EutJMreB_1_grm EUT1
05210 HMM00465alcdh_3_mix(pf00465) EUT1
05215 HMMtpEuth_1_eut(pf04346) EUT1
05220 HMM06277EutA_1_eut(pf06277) EUT1
05225 HMMetlyEutB_1_EUT1(pf06751) EUT1
05230 HMMetlyEutC_1_eut1(pf05985) EUT1 EP_NTERM
05235 Tsp_rosa EUT1
05240 H_mulberry(pf00936pf16365) EUT1
05245 HMMreg12833_1_EUT1(pf12833) EUT1
05250 -pf01218 -
05255 -pf01520 -
05260 -pf00583 -
05265 -pf11143 -
05270 -pf06572 -
05275 -pf04261 -
05280 HMMnopfamclust3_1 RMM1
05285 - -
05290 -pf00528 -
05295 -pf00528 -
05300 -pf00005pf12857 -
BMC-P
BMC-H
BMC-T
Aldehyde dehydrogenase
PTAC
Signature enzyme
Alcohol dehydrogenase
Regulator

Salmonella enterica subsp. enterica serovar Kokomlemle. Bacteria; Proteobacteria; Gammaproteobacteria; Enterobacterales;

E2D59__locus_1

EUT1

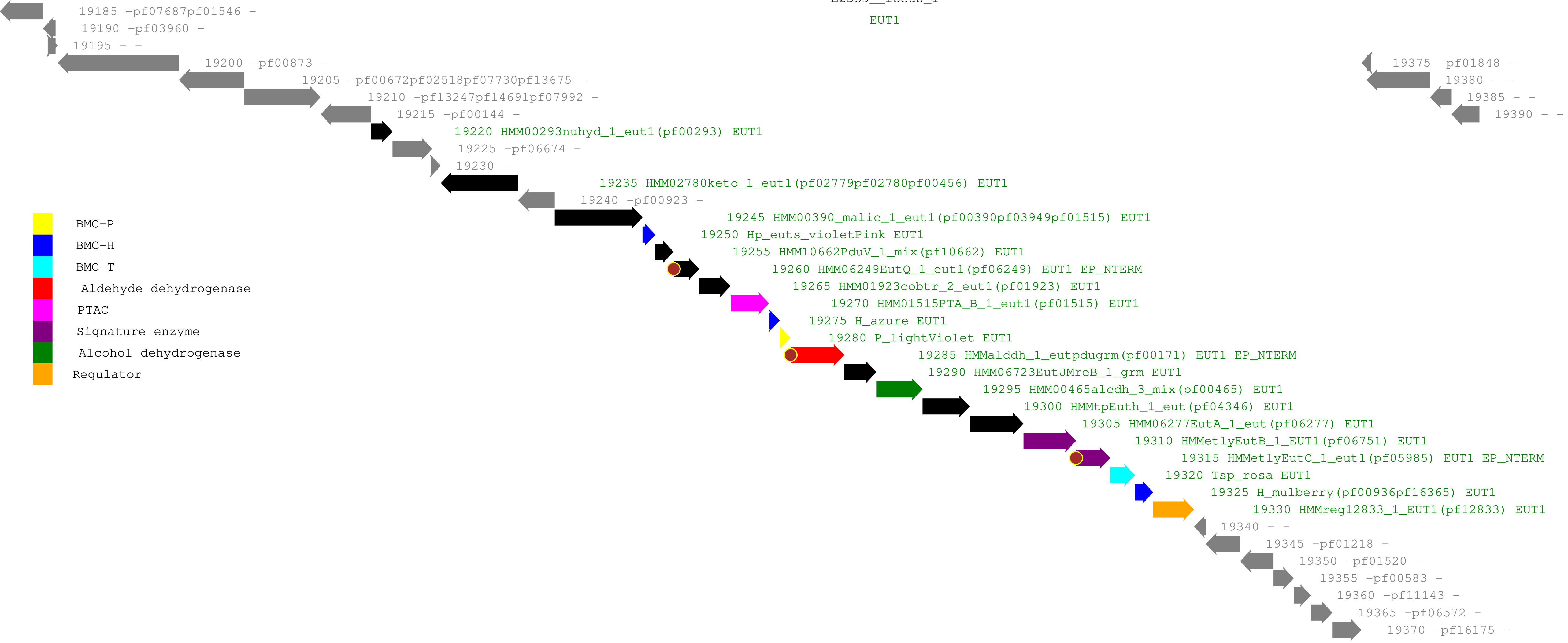

Salmonella enterica subsp. enterica serovar Weltevreden. Bacteria; Proteobacteria; Gammaproteobacteria; Enterobacterales;

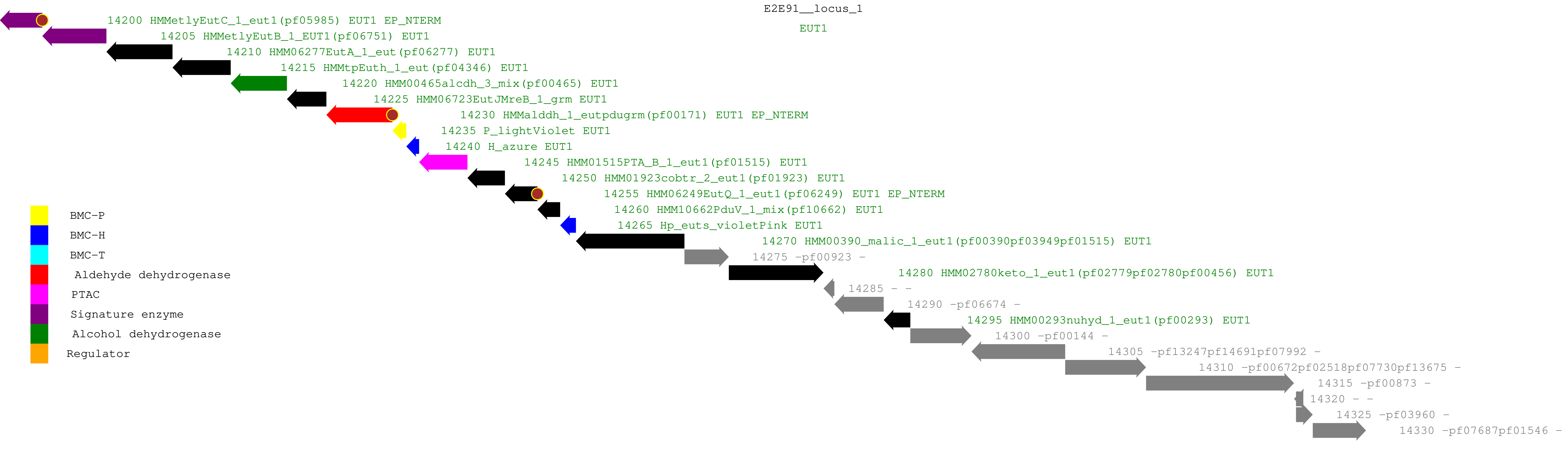

Salmonella enterica subsp. enterica serovar Liverpool. Bacteria; Proteobacteria; Gammaproteobacteria; Enterobacterales;

E2G40__locus_2

EUT1

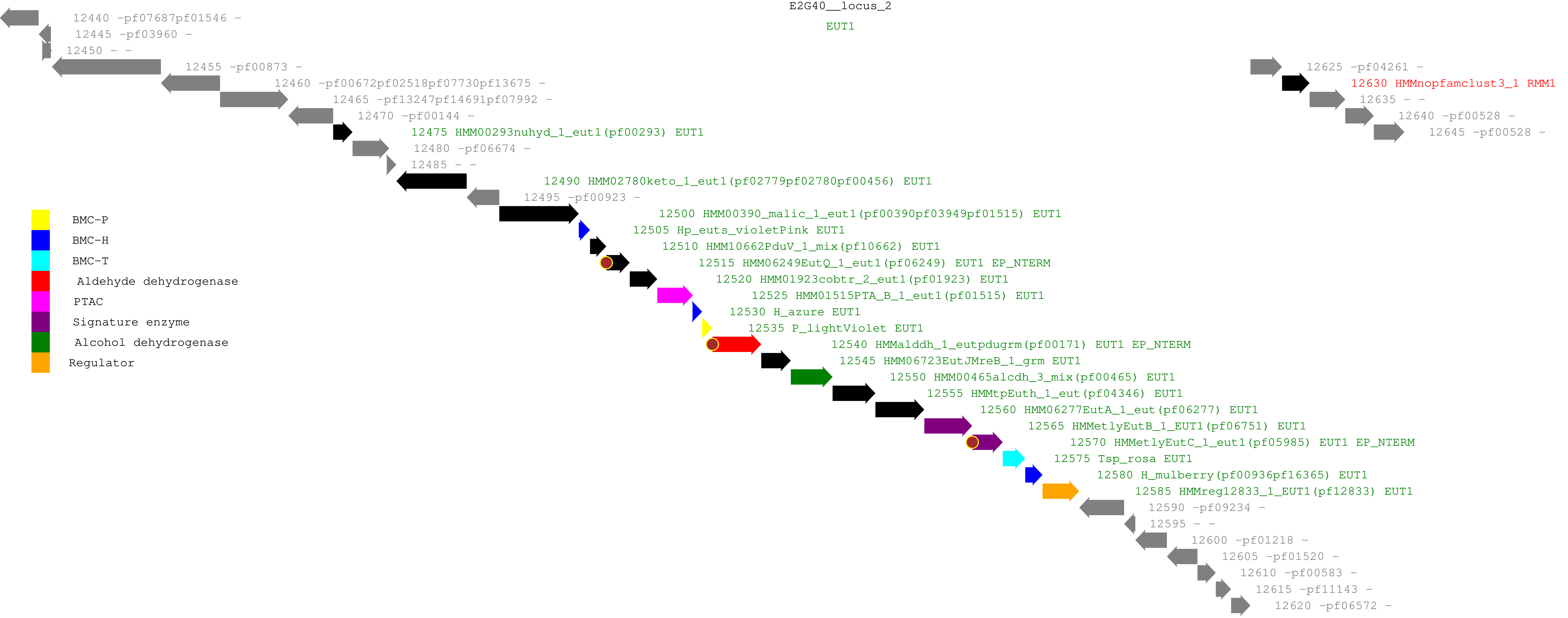

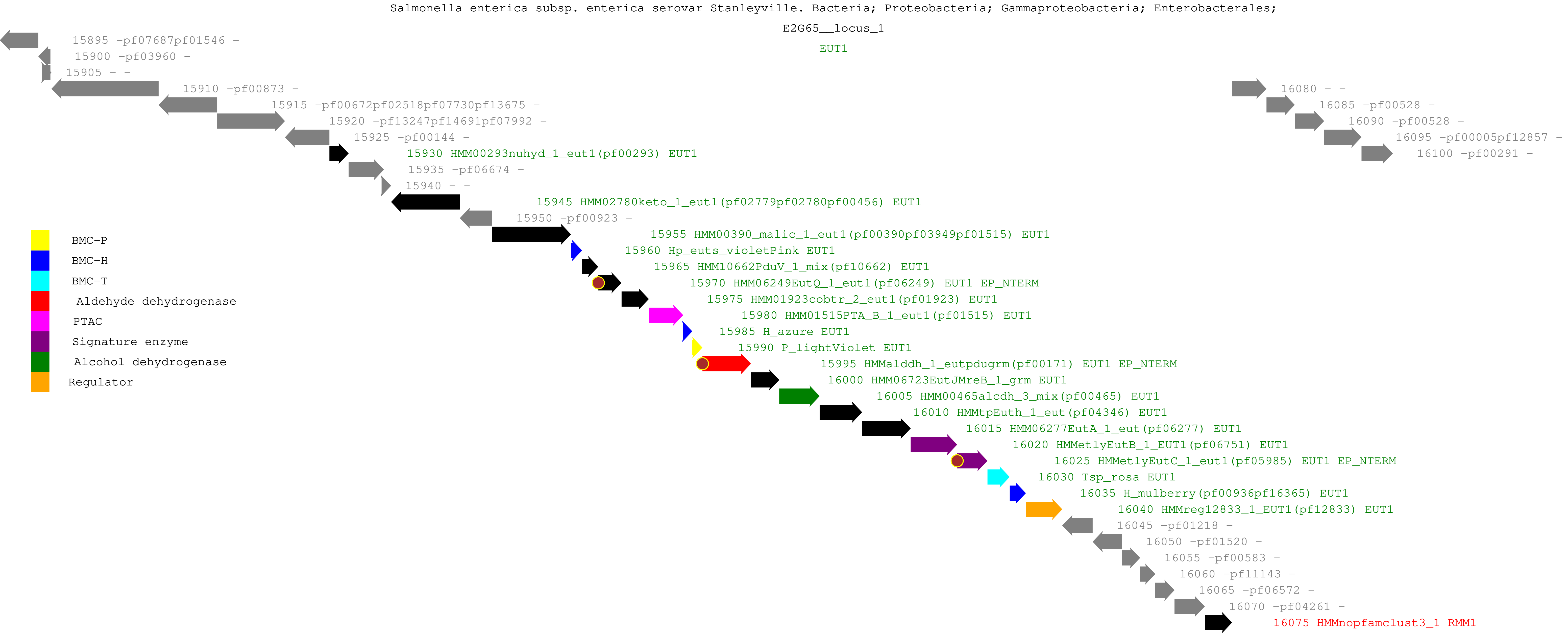
Salmonella enterica subsp. enterica serovar Stanleyville. Bacteria; Proteobacteria; Gammaproteobacteria; Enterobacterales;
E2G65__locus_1
EUT1
15895 -pf07687pf01546 -
15900 -pf03960 -
15905 - -
15910 -pf00873 -
15915 -pf00672pf02518pf07730pf13675 -
15920 -pf13247pf14691pf07992 -
15925 -pf00144 -
15930 HMM00293nuhyd_1_eut1(pf00293) EUT1
15935 -pf06674 -
15940 - -
15945 HMM02780keto_1_eut1(pf02779pf02780pf00456) EUT1
15950 -pf00923 -
15955 HMM00390_malic_1_eut1(pf00390pf03949pf01515) EUT1
15960 Hp_euts_violetPink EUT1
15965 HMM10662PduV_1_mix(pf10662) EUT1
15970 HMM06249EutQ_1_eut1(pf06249) EUT1 EP_NTERM
15975 HMM01923cobtr_2_eut1(pf01923) EUT1
15980 HMM01515PTA_B_1_eut1(pf01515) EUT1
15985 H_azure EUT1
15990 P_lightViolet EUT1
15995 HMMalddh_1_eutpdugrm(pf00171) EUT1 EP_NTERM
16000 HMM06723EutJMreB_1_grm EUT1
16005 HMM00465alcdh_3_mix(pf00465) EUT1
16010 HMMtpEuth_1_eut(pf04346) EUT1
16015 HMM06277EutA_1_eut(pf06277) EUT1
16020 HMMetlyEutB_1_EUT1(pf06751) EUT1
16025 HMMetlyEutC_1_eut1(pf05985) EUT1 EP_NTERM
16030 Tsp_rosa EUT1
16035 H_mulberry(pf00936pf16365) EUT1
16040 HMMreg12833_1_EUT1(pf12833) EUT1
16045 -pf01218 -
16050 -pf01520 -
16055 -pf00583 -
16060 -pf11143 -
16065 -pf06572 -
16070 -pf04261 -
16075 HMMnopfamclust3_1 RMM1
16080 - -
16085 -pf00528 -
16090 -pf00528 -
16095 -pf00005pf12857 -
16100 -pf00291 -
BMC-P
BMC-H
BMC-T
Aldehyde dehydrogenase
PTAC
Signature enzyme
Alcohol dehydrogenase
Regulator

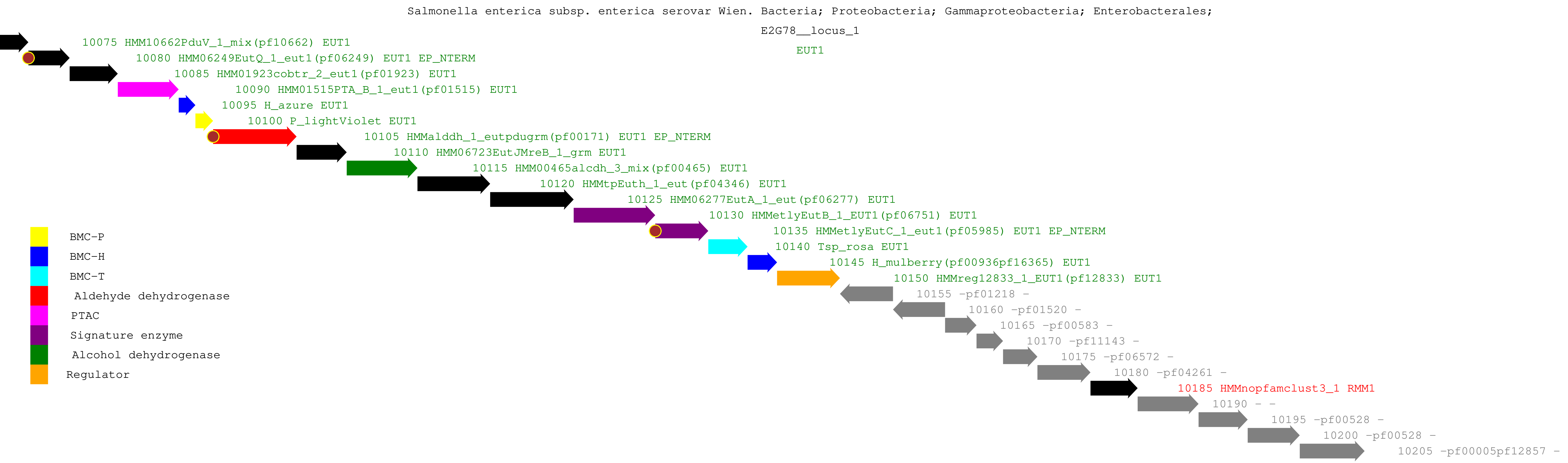
Salmonella enterica subsp. enterica serovar Wien. Bacteria; Proteobacteria; Gammaproteobacteria; Enterobacterales;
E2G78__locus_1
EUT1
10075 HMM10662PduV_1_mix(pf10662) EUT1
10080 HMM06249EutQ_1_eut1(pf06249) EUT1 EP_NTERM
10085 HMM01923cobtr_2_eut1(pf01923) EUT1
10090 HMM01515PTA_B_1_eut1(pf01515) EUT1
10095 H_azure EUT1
10100 P_lightViolet EUT1
10105 HMMalddh_1_eutpdugrm(pf00171) EUT1 EP_NTERM
10110 HMM06723EutJMreB_1_grm EUT1
10115 HMM00465alcdh_3_mix(pf00465) EUT1
10120 HMMtpEuth_1_eut(pf04346) EUT1
10125 HMM06277EutA_1_eut(pf06277) EUT1
10130 HMMetlyEutB_1_EUT1(pf06751) EUT1
10135 HMMetlyEutC_1_eut1(pf05985) EUT1 EP_NTERM
10140 Tsp_rosa EUT1
10145 H_mulberry(pf00936pf16365) EUT1
10150 HMMreg12833_1_EUT1(pf12833) EUT1
10155 -pf01218 -
10160 -pf01520 -
10165 -pf00583 -
10170 -pf11143 -
10175 -pf06572 -
10180 -pf04261 -
10185 HMMnopfamclust3_1 RMM1
10190 - -
10195 -pf00528 -
10200 -pf00528 -
10205 -pf00005pf12857 -
BMC-P
BMC-H
BMC-T
Aldehyde dehydrogenase
PTAC
Signature enzyme
Alcohol dehydrogenase
Regulator

Salmonella enterica subsp. enterica serovar Ramatgan. Bacteria; Proteobacteria; Gammaproteobacteria; Enterobacterales;

E2G82__locus_1

EUT1

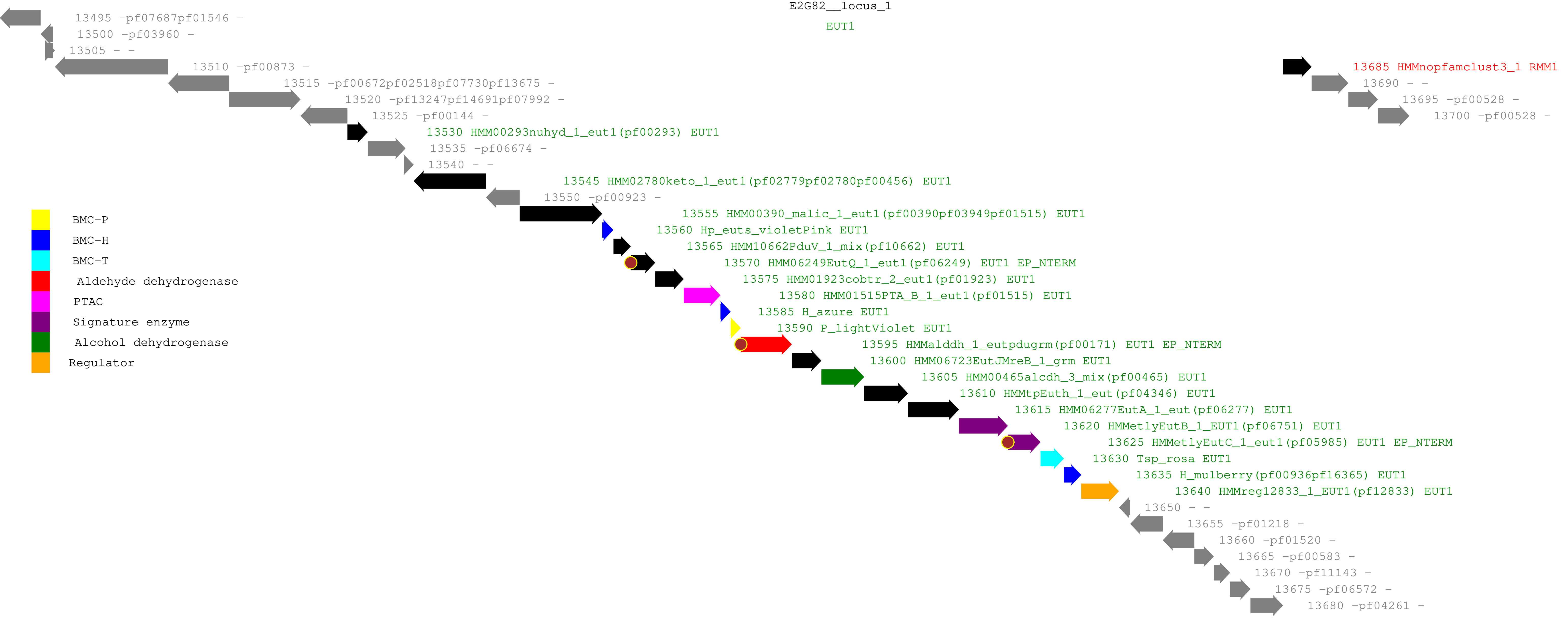

Salmonella enterica subsp. enterica serovar Volkmarsdorf. Bacteria; Proteobacteria; Gammaproteobacteria; Enterobacterales;

E2H20__locus_2

EUT1

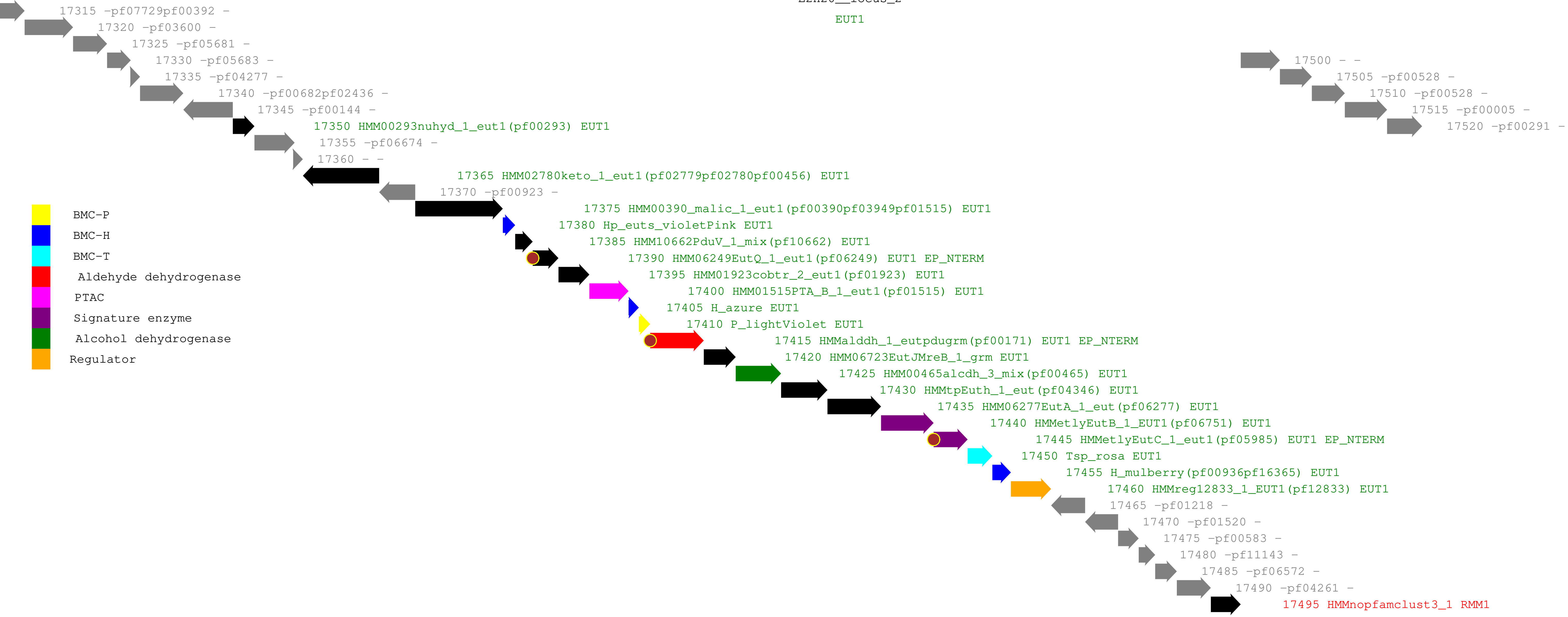

Salmonella enterica subsp. enterica serovar Alachua. Bacteria; Proteobacteria; Gammaproteobacteria; Enterobacterales;

E2H46__locus_2

EUT1

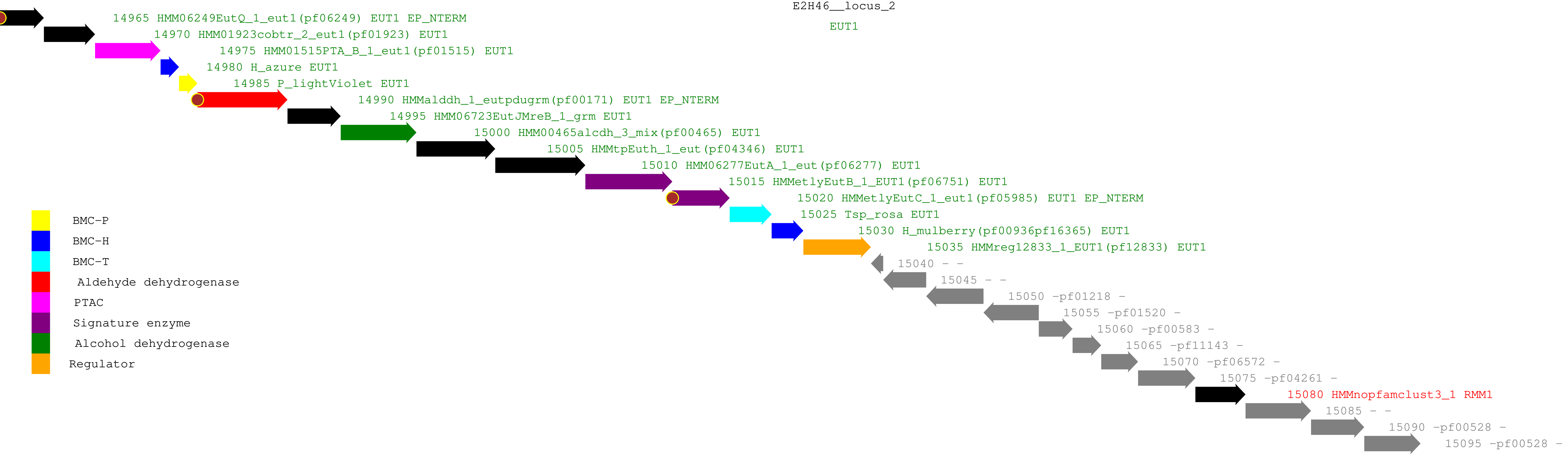

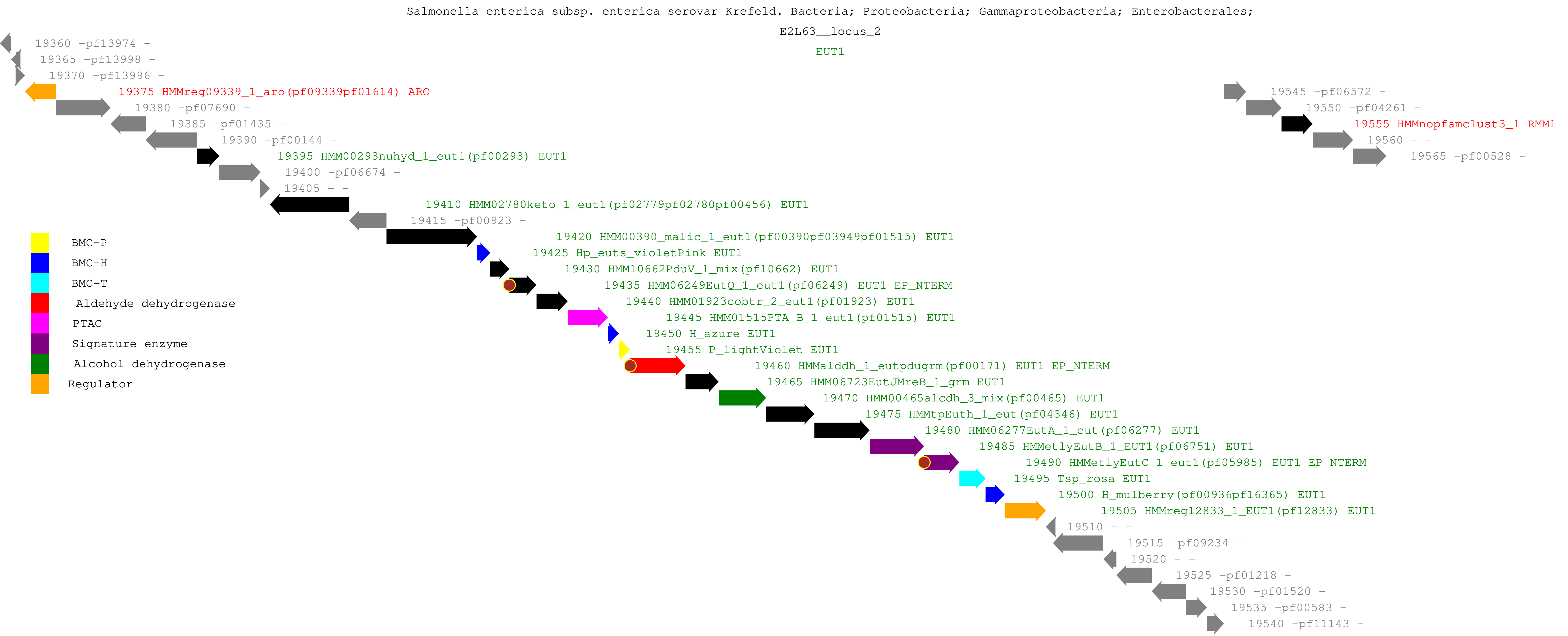

Salmonella enterica subsp. enterica serovar Krefeld. Bacteria; Proteobacteria; Gammaproteobacteria; Enterobacterales;
E2L63__locus_2
EUT1
19360 -pf13974 -
19365 -pf13998 -
19370 -pf13996 -
19375 HMMreg09339_1_aro(pf09339pf01614) ARO
19380 -pf07690 -
19385 -pf01435 -
19390 -pf00144 -
19395 HMM00293nuhyd_1_eut1(pf00293) EUT1
19400 -pf06674 -
19405 - -
19410 HMM02780keto_1_eut1(pf02779pf02780pf00456) EUT1
19415 -pf00923 -
19420 HMM00390_malic_1_eut1(pf00390pf03949pf01515) EUT1
19425 Hp_euts_violetPink EUT1
19430 HMM10662PduV_1_mix(pf10662) EUT1
19435 HMM06249EutQ_1_eut1(pf06249) EUT1 EP_NTERM
19440 HMM01923cobtr_2_eut1(pf01923) EUT1
19445 HMM01515PTA_B_1_eut1(pf01515) EUT1
19450 H_azure EUT1
19455 P_lightViolet EUT1
19460 HMMalddh_1_eutpdugrm(pf00171) EUT1 EP_NTERM
19465 HMM06723EutJMreB_1_grm EUT1
19470 HMM00465alcdh_3_mix(pf00465) EUT1
19475 HMMtpEuth_1_eut(pf04346) EUT1
19480 HMM06277EutA_1_eut(pf06277) EUT1
19485 HMMetlyEutB_1_EUT1(pf06751) EUT1
19490 HMMetlyEutC_1_eut1(pf05985) EUT1 EP_NTERM
19495 Tsp_rosa EUT1
19500 H_mulberry(pf00936pf16365) EUT1
19505 HMMreg12833_1_EUT1(pf12833) EUT1
19510 - -
19515 -pf09234 -
19520 - -
19525 -pf01218 -
19530 -pf01520 -
19535 -pf00583 -
19540 -pf11143 -
19545 -pf06572 -
19550 -pf04261 -
19555 HMMnopfamclust3_1 RMM1
19560 - -
19565 -pf00528 -
BMC-P
BMC-H
BMC-T
Aldehyde dehydrogenase
PTAC
Signature enzyme
Alcohol dehydrogenase
Regulator

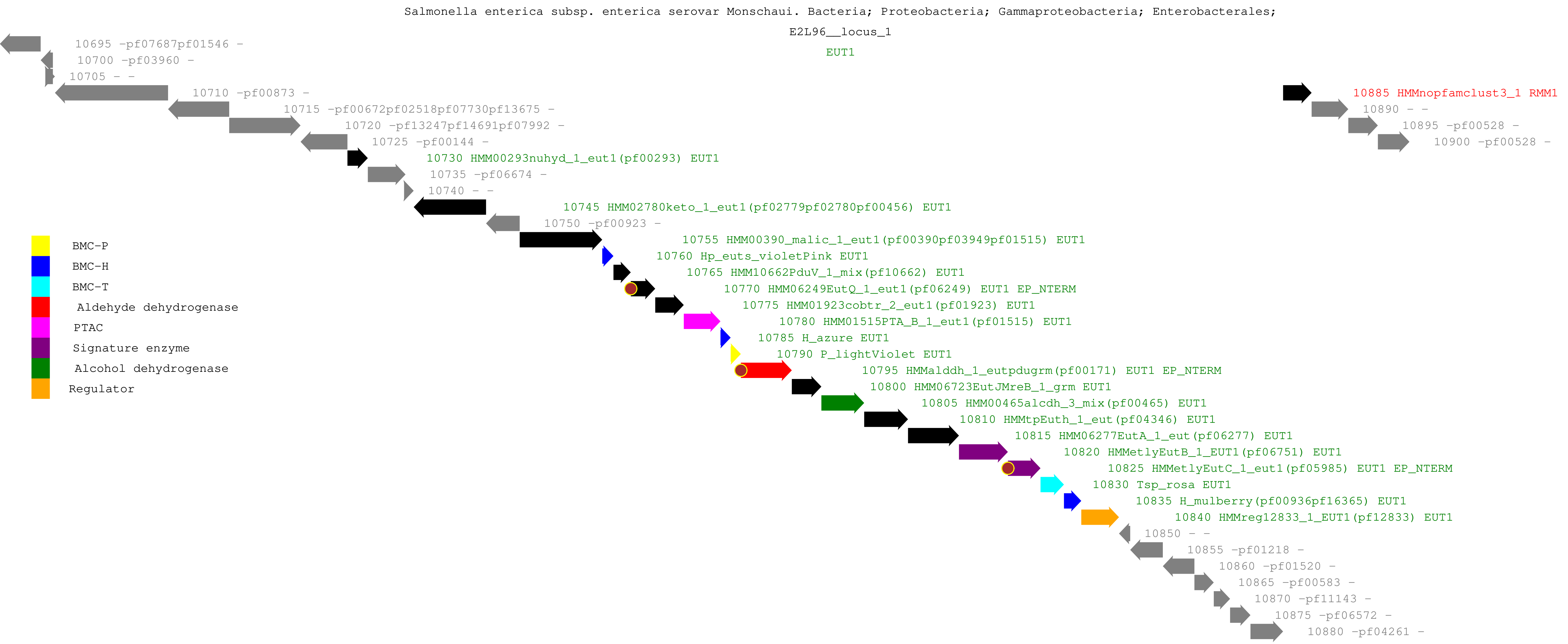
Salmonella enterica subsp. enterica serovar Monschaui. Bacteria; Proteobacteria; Gammaproteobacteria; Enterobacterales;
E2L96__locus_1
EUT1
10695 -pf07687pf01546 -
10700 -pf03960 -
10705 - -
10710 -pf00873 -
10715 -pf00672pf02518pf07730pf13675 -
10720 -pf13247pf14691pf07992 -
10725 -pf00144 -
10730 HMM00293nuhyd_1_eut1(pf00293) EUT1
10735 -pf06674 -
10740 - -
10745 HMM02780keto_1_eut1(pf02779pf02780pf00456) EUT1
10750 -pf00923 -
10755 HMM00390_malic_1_eut1(pf00390pf03949pf01515) EUT1
10760 Hp_euts_violetPink EUT1
10765 HMM10662PduV_1_mix(pf10662) EUT1
10770 HMM06249EutQ_1_eut1(pf06249) EUT1 EP_NTERM
10775 HMM01923cobtr_2_eut1(pf01923) EUT1
10780 HMM01515PTA_B_1_eut1(pf01515) EUT1
10785 H_azure EUT1
10790 P_lightViolet EUT1
10795 HMMalddh_1_eutpdugrm(pf00171) EUT1 EP_NTERM
10800 HMM06723EutJMreB_1_grm EUT1
10805 HMM00465alcdh_3_mix(pf00465) EUT1
10810 HMMtpEuth_1_eut(pf04346) EUT1
10815 HMM06277EutA_1_eut(pf06277) EUT1
10820 HMMetlyEutB_1_EUT1(pf06751) EUT1
10825 HMMetlyEutC_1_eut1(pf05985) EUT1 EP_NTERM
10830 Tsp_rosa EUT1
10835 H_mulberry(pf00936pf16365) EUT1
10840 HMMreg12833_1_EUT1(pf12833) EUT1
10850 - -
10855 -pf01218 -
10860 -pf01520 -
10865 -pf00583 -
10870 -pf11143 -
10875 -pf06572 -
10880 -pf04261 -
10885 HMMnopfamclust3_1 RMM1
10890 - -
10895 -pf00528 -
10900 -pf00528 -
BMC-P
BMC-H
BMC-T
Aldehyde dehydrogenase
PTAC
Signature enzyme
Alcohol dehydrogenase
Regulator

Salmonella enterica subsp. enterica serovar Arechavaleta. Bacteria; Proteobacteria; Gammaproteobacteria; Enterobacterales;

E2M22__locus_1_main_sat

EUT1

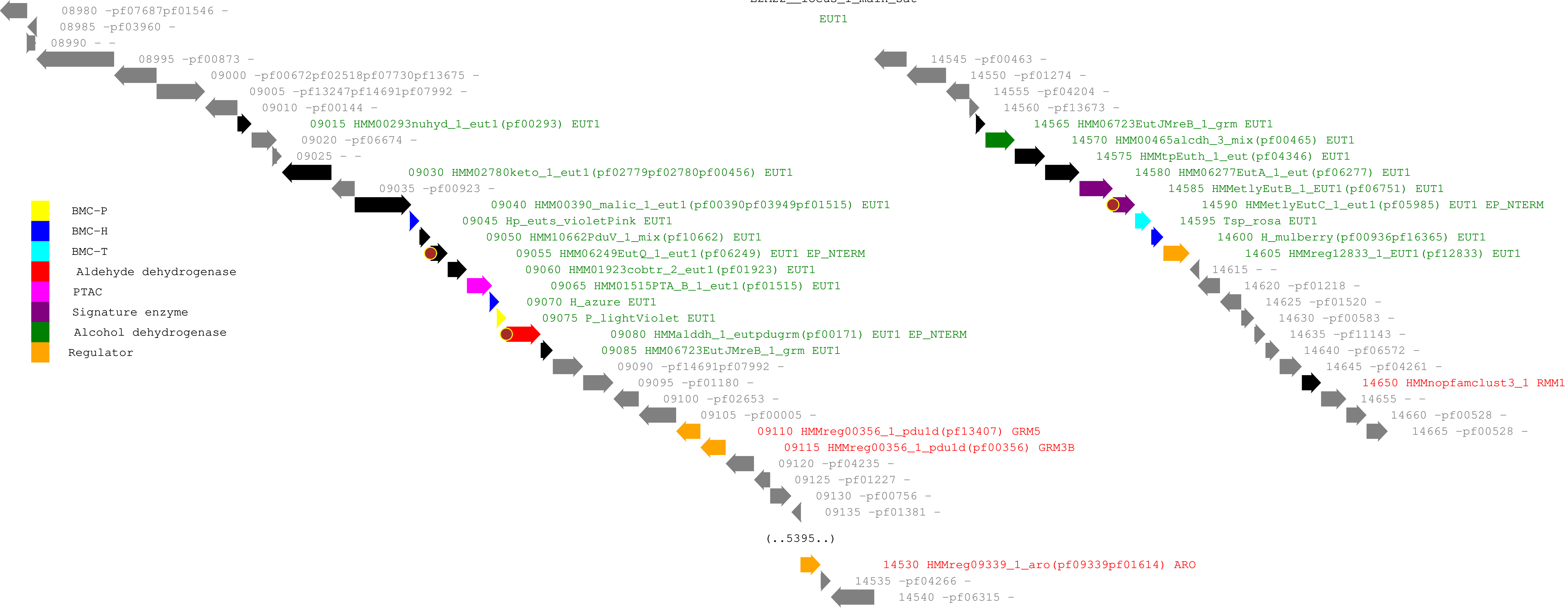

Salmonella enterica subsp. enterica serovar Nima. Bacteria; Proteobacteria; Gammaproteobacteria; Enterobacterales;

E2M36__locus_2

EUT1

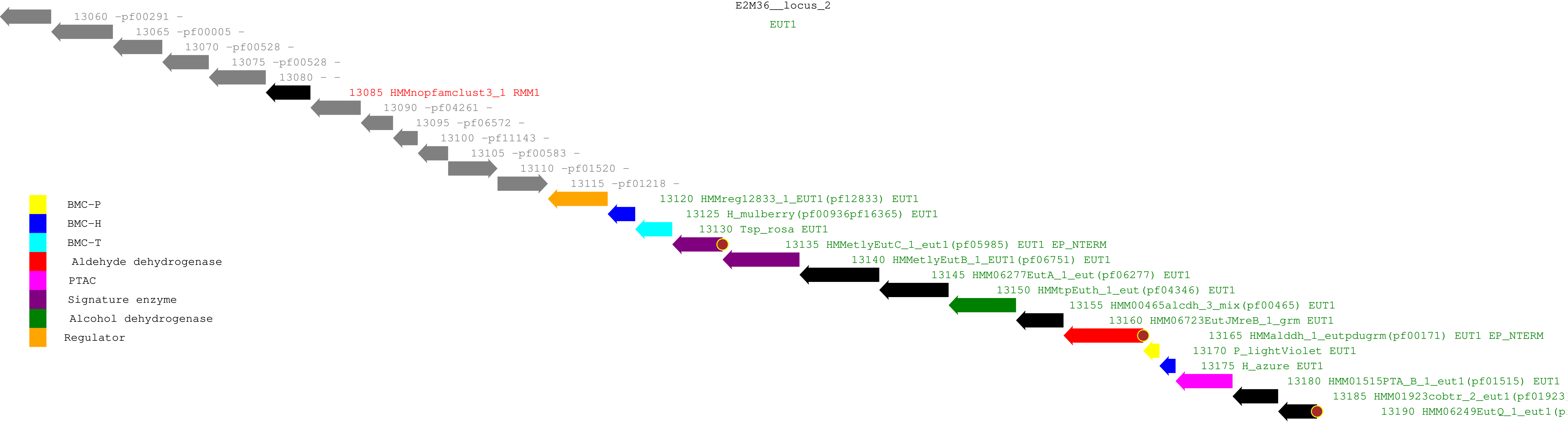

Salmonella enterica subsp. enterica serovar Othmarschen. Bacteria; Proteobacteria; Gammaproteobacteria; Enterobacterales;

E2M46__locus_2

EUT1

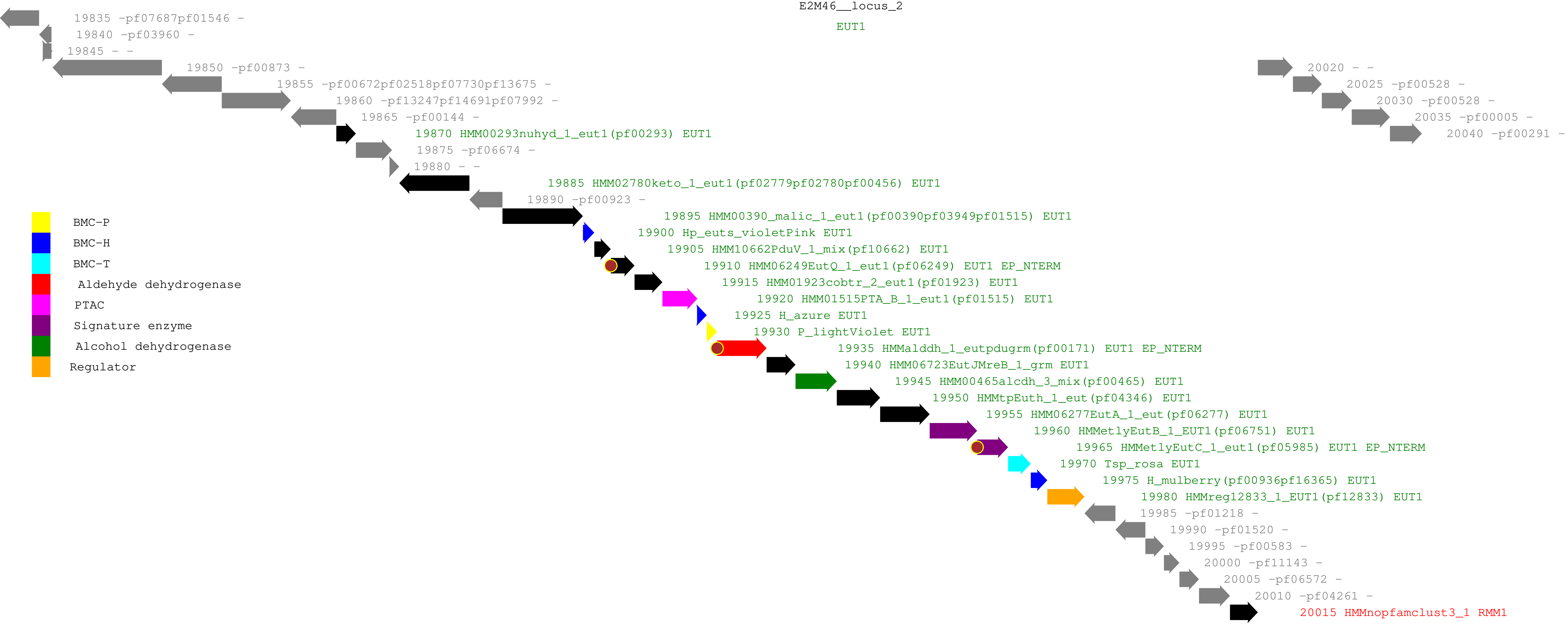

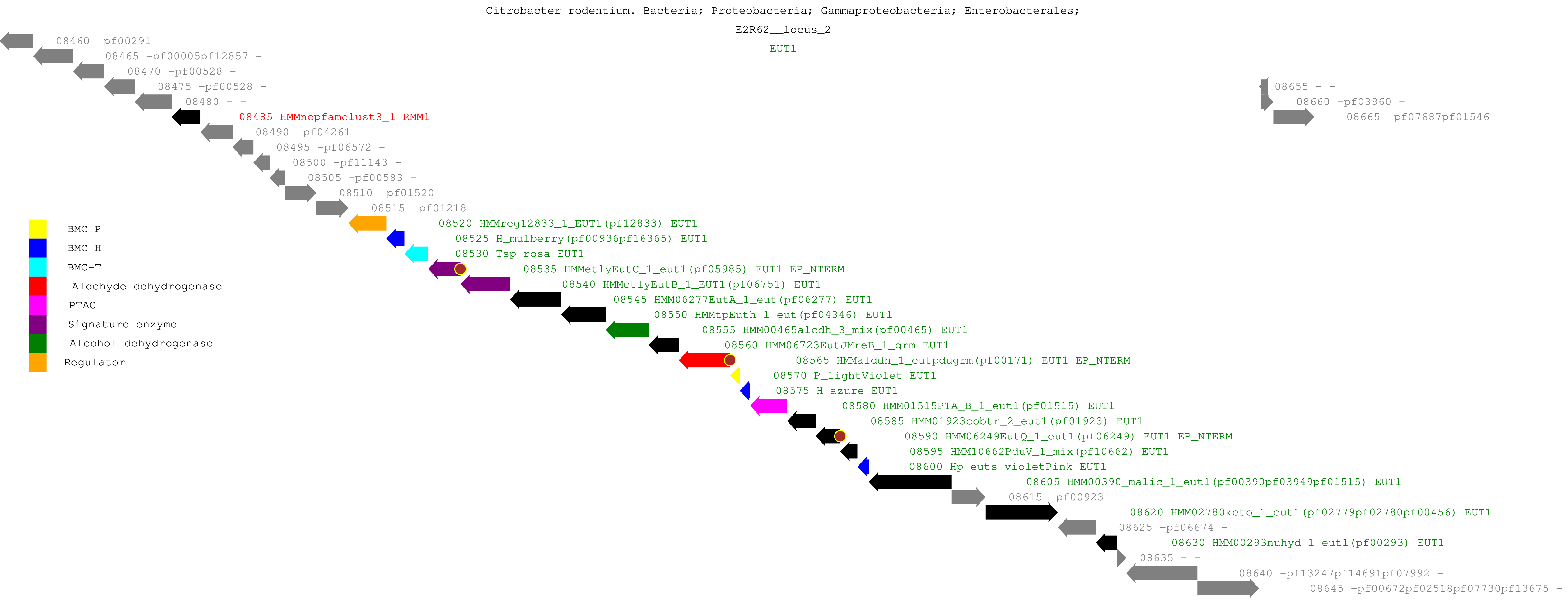

Citrobacter rodentium. Bacteria; Proteobacteria; Gammaproteobacteria; Enterobacterales;
E2R62__locus_2
EUT1
08460 -pf00291 -
08465 -pf00005pf12857 -
08470 -pf00528 -
08475 -pf00528 -
08480 - -
08485 HMMnopfamclust3_1 RMM1
08490 -pf04261 -
08495 -pf06572 -
08500 -pf11143 -
08505 -pf00583 -
08510 -pf01520 -
08515 -pf01218 -
08520 HMMreg12833_1_EUT1(pf12833) EUT1
08525 H_mulberry(pf00936pf16365) EUT1
08530 Tsp_rosa EUT1
08535 HMMetlyEutC_1_eut1(pf05985) EUT1 EP_NTERM
08540 HMMetlyEutB_1_EUT1(pf06751) EUT1
08545 HMM06277EutA_1_eut(pf06277) EUT1
08550 HMMtpEuth_1_eut(pf04346) EUT1
08555 HMM00465alcdh_3_mix(pf00465) EUT1
08560 HMM06723EutJMreB_1_grm EUT1
08565 HMMalddh_1_eutpdugrm(pf00171) EUT1 EP_NTERM
08570 P_lightViolet EUT1
08575 H_azure EUT1
08580 HMM01515PTA_B_1_eut1(pf01515) EUT1
08585 HMM01923cobtr_2_eut1(pf01923) EUT1
08590 HMM06249EutQ_1_eut1(pf06249) EUT1 EP_NTERM
08595 HMM10662PduV_1_mix(pf10662) EUT1
08600 Hp_euts_violetPink EUT1
08605 HMM00390_malic_1_eut1(pf00390pf03949pf01515) EUT1
08615 -pf00923 -
08620 HMM02780keto_1_eut1(pf02779pf02780pf00456) EUT1
08625 -pf06674 -
08630 HMM00293nuhyd_1_eut1(pf00293) EUT1
08635 - -
08640 -pf13247pf14691pf07992 -
08645 -pf00672pf02518pf07730pf13675 -
08655 - -
08660 -pf03960 -
08665 -pf07687pf01546 -
BMC-P
BMC-H
BMC-T
Aldehyde dehydrogenase
PTAC
Signature enzyme
Alcohol dehydrogenase
Regulator

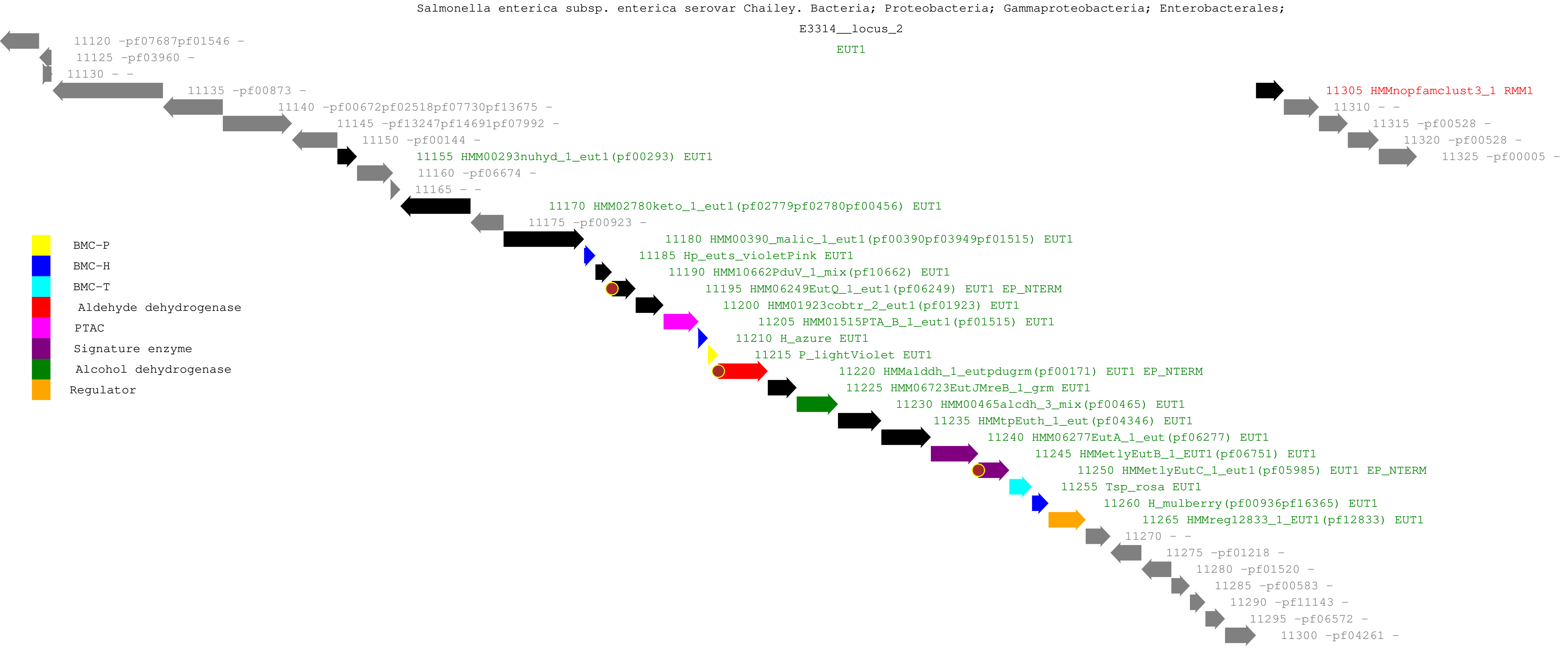
Salmonella enterica subsp. enterica serovar Chailey. Bacteria; Proteobacteria; Gammaproteobacteria; Enterobacterales;
E3314__locus_2
EUT1
11120 -pf07687pf01546 -
11125 -pf03960 -
11130 - -
11135 -pf00873 -
11140 -pf00672pf02518pf07730pf13675 -
11145 -pf13247pf14691pf07992 -
11150 -pf00144 -
11155 HMM00293nuhyd_1_eut1(pf00293) EUT1
11160 -pf06674 -
11165 - -
11170 HMM02780keto_1_eut1(pf02779pf02780pf00456) EUT1
11175 -pf00923 -
11180 HMM00390_malic_1_eut1(pf00390pf03949pf01515) EUT1
11185 Hp_euts_violetPink EUT1
11190 HMM10662PduV_1_mix(pf10662) EUT1
11195 HMM06249EutQ_1_eut1(pf06249) EUT1 EP_NTERM
11200 HMM01923cobtr_2_eut1(pf01923) EUT1
11205 HMM01515PTA_B_1_eut1(pf01515) EUT1
11210 H_azure EUT1
11215 P_lightViolet EUT1
11220 HMMaldh_1_eutpdugrm(pf00171) EUT1 EP_NTERM
11225 HMM06723EutJMreB_1_grm EUT1
11230 HMM00465alcdh_3_mix(pf00465) EUT1
11235 HMMtpEuth_1_eut(pf04346) EUT1
11240 HMM06277EutA_1_eut(pf06277) EUT1
11245 HMMetlyEutB_1_EUT1(pf06751) EUT1
11250 HMMetlyEutC_1_eut1(pf05985) EUT1 EP_NTERM
11255 Tsp_rosa EUT1
11260 H_mulberry(pf00936pf16365) EUT1
11265 HMMreg12833_1_EUT1(pf12833) EUT1
11270 - -
11275 -pf01218 -
11280 -pf01520 -
11285 -pf00583 -
11290 -pf11143 -
11295 -pf06572 -
11300 -pf04261 -
11305 HMMnopfamclust3_1 RMM1
11310 - -
11315 -pf00528 -
11320 -pf00528 -
11325 -pf00005 -
BMC-P
BMC-H
BMC-T
Aldehyde dehydrogenase
PTAC
Signature enzyme
Alcohol dehydrogenase
Regulator

Salmonella enterica subsp. enterica serovar Stourbridge. Bacteria; Proteobacteria; Gammaproteobacteria; Enterobacterales;

E3348__locus_2

EUT1

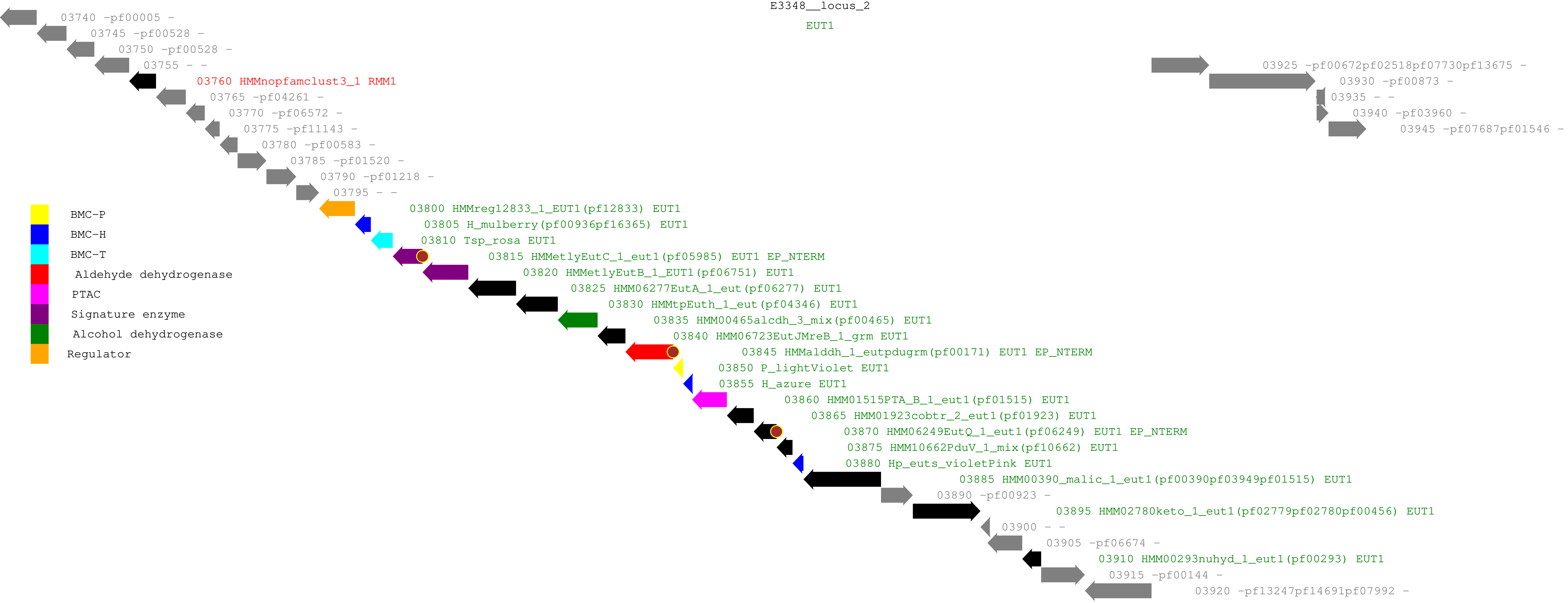

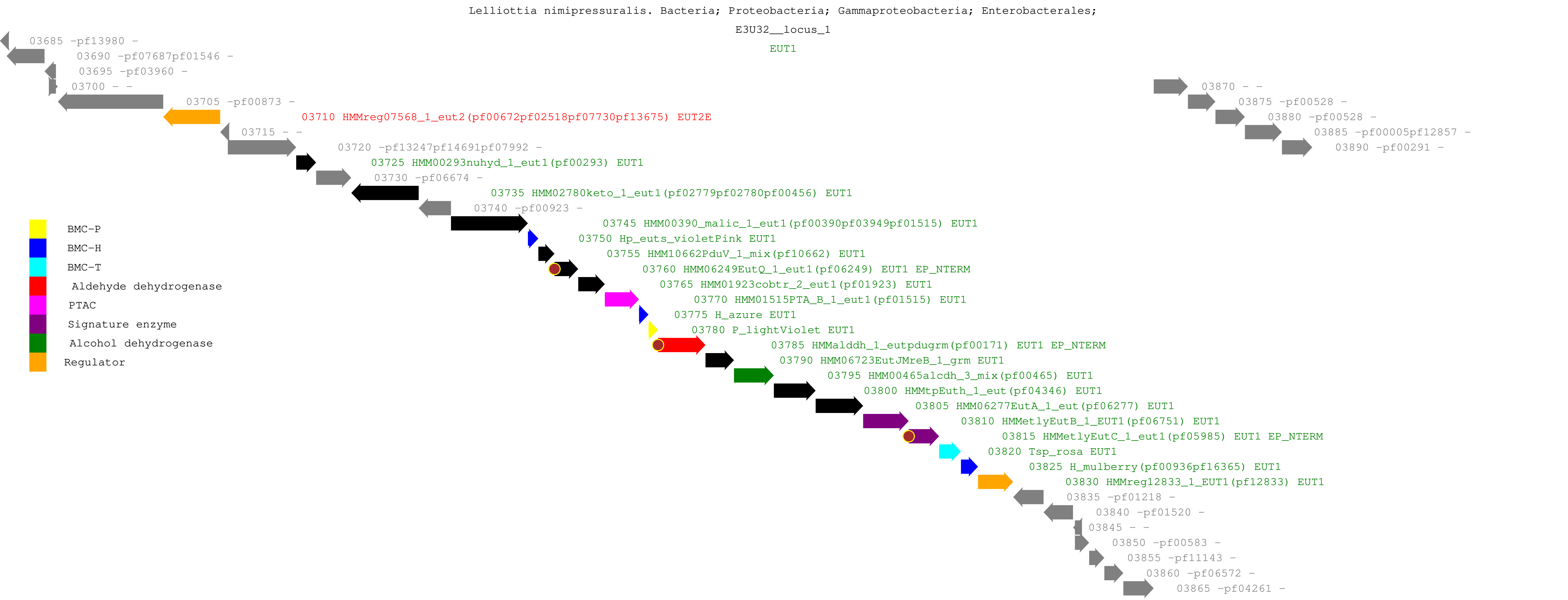
Lelliottia nimipressuralis. Bacteria; Proteobacteria; Gammaproteobacteria; Enterobacterales;
E3U32__locus_1
EUT1
03685 -pf13980 -
03690 -pf07687pf01546 -
03695 -pf03960 -
03700 - -
03705 -pf00873 -
03710 HMMreg07568_1_eut2(pf00672pf02518pf07730pf13675) EUT2E
03715 - -
03720 -pf13247pf14691pf07992 -
03725 HMM00293nuhyd_1_eut1(pf00293) EUT1
03730 -pf06674 -
03735 HMM02780keto_1_eut1(pf02779pf02780pf00456) EUT1
03740 -pf00923 -
03745 HMM00390_malic_1_eut1(pf00390pf03949pf01515) EUT1
03750 Hp_euts_violetPink EUT1
03755 HMM10662PduV_1_mix(pf10662) EUT1
03760 HMM06249EutQ_1_eut1(pf06249) EUT1 EP_NTERM
03765 HMM01923cobtr_2_eut1(pf01923) EUT1
03770 HMM01515PTA_B_1_eut1(pf01515) EUT1
03775 H_azure EUT1
03780 P_lightViolet EUT1
03785 HMMalddh_1_eutpdugrm(pf00171) EUT1 EP_NTERM
03790 HMM06723EutJMreB_1_grm EUT1
03795 HMM00465alcdh_3_mix(pf00465) EUT1
03800 HMMtpEuth_1_eut(pf04346) EUT1
03805 HMM06277EutA_1_eut(pf06277) EUT1
03810 HMMetlyEutB_1_EUT1(pf06751) EUT1
03815 HMMetlyEutC_1_eut1(pf05985) EUT1 EP_NTERM
03820 Tsp_rosa EUT1
03825 H_mulberry(pf00936pf16365) EUT1
03830 HMMreg12833_1_EUT1(pf12833) EUT1
03835 -pf01218 -
03840 -pf01520 -
03845 - -
03850 -pf00583 -
03855 -pf11143 -
03860 -pf06572 -
03865 -pf04261 -
03870 - -
03875 -pf00528 -
03880 -pf00528 -
03885 -pf00005pf12857 -
03890 -pf00291 -
BMC-P
BMC-H
BMC-T
Aldehyde dehydrogenase
PTAC
Signature enzyme
Alcohol dehydrogenase
Regulator

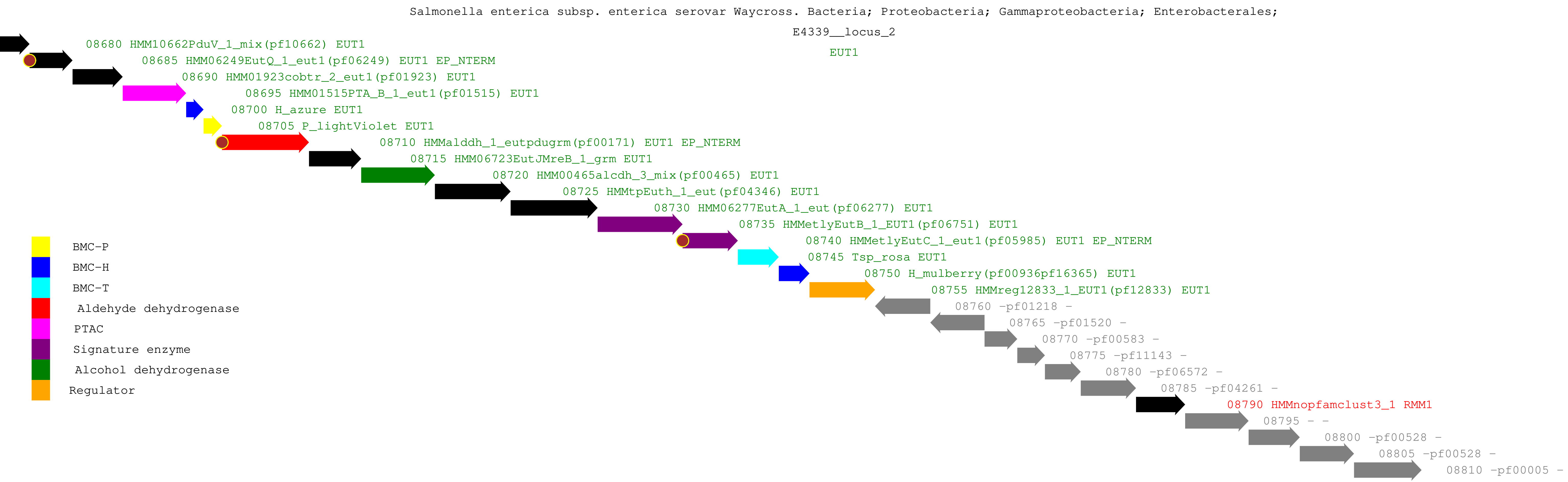
Salmonella enterica subsp. enterica serovar Waycross. Bacteria; Proteobacteria; Gammaproteobacteria; Enterobacterales;
E4339__locus_2
EUT1
08680 HMM10662PduV_1_mix(pf10662) EUT1
08685 HMM06249EutQ_1_eut1(pf06249) EUT1 EP_NTERM
08690 HMM01923cobtr_2_eut1(pf01923) EUT1
08695 HMM01515PTA_B_1_eut1(pf01515) EUT1
08700 H_azure EUT1
08705 P_lightViolet EUT1
08710 HMMaldh_1_eutpdugrm(pf00171) EUT1 EP_NTERM
08715 HMM06723EutJMreB_1_grm EUT1
08720 HMM00465alcdh_3_mix(pf00465) EUT1
08725 HMMtpEuth_1_eut(pf04346) EUT1
08730 HMM06277EutA_1_eut(pf06277) EUT1
08735 HMMetlyEutB_1_EUT1(pf06751) EUT1
08740 HMMetlyEutC_1_eut1(pf05985) EUT1 EP_NTERM
08745 Tsp_rosa EUT1
08750 H_mulberry(pf00936pf16365) EUT1
08755 HMMreg12833_1_EUT1(pf12833) EUT1
08760 -pf01218 -
08765 -pf01520 -
08770 -pf00583 -
08775 -pf11143 -
08780 -pf06572 -
08785 -pf04261 -
08790 HMMnopfamclust3_1 RMM1
08795 - -
08800 -pf00528 -
08805 -pf00528 -
08810 -pf00005 -
BMC-P
BMC-H
BMC-T
Aldehyde dehydrogenase
PTAC
Signature enzyme
Alcohol dehydrogenase
Regulator

Salmonella enterica subsp. enterica serovar Apeyeme. Bacteria; Proteobacteria; Gammaproteobacteria; Enterobacterales;

E4443__locus_2

EUT1

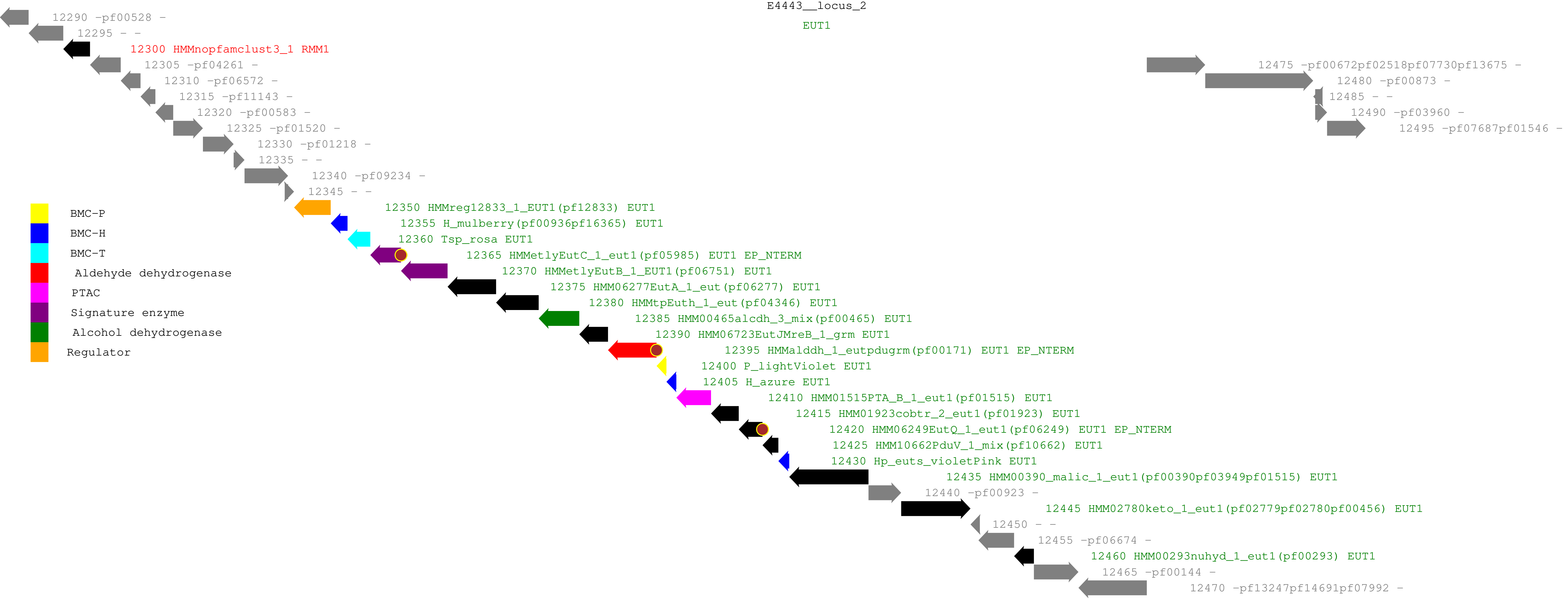

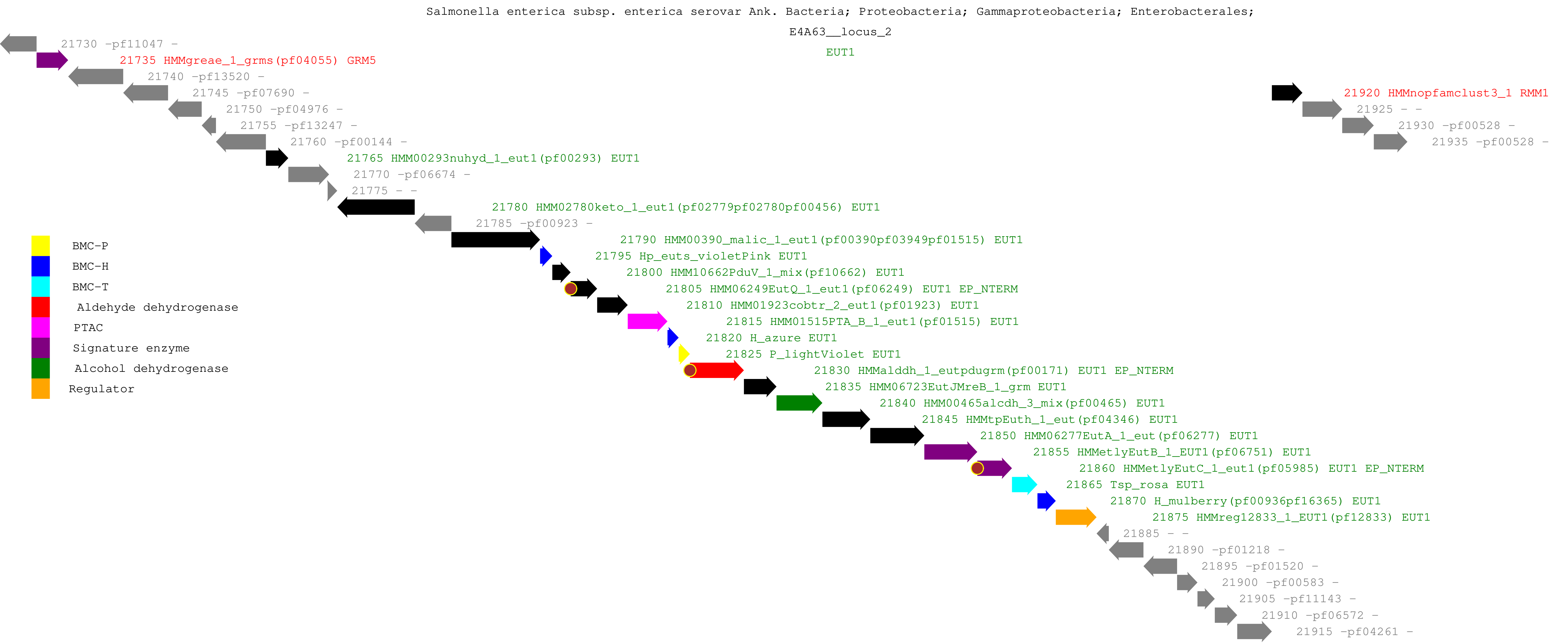
Salmonella enterica subsp. enterica serovar Ank. Bacteria; Proteobacteria; Gammaproteobacteria; Enterobacterales;
E4A63__locus_2
EUT1
21730 -pf11047 -
21735 HMMgreae_1_grms(pf04055) GRM5
21740 -pf13520 -
21745 -pf07690 -
21750 -pf04976 -
21755 -pf13247 -
21760 -pf00144 -
21765 HMM00293nuhyd_1_eut1(pf00293) EUT1
21770 -pf06674 -
21775 - -
21780 HMM02780keto_1_eut1(pf02779pf02780pf00456) EUT1
21785 -pf00923 -
21790 HMM00390_malic_1_eut1(pf00390pf03949pf01515) EUT1
21795 Hp_euts_violetPink EUT1
21800 HMM10662PduV_1_mix(pf10662) EUT1
21805 HMM06249EutQ_1_eut1(pf06249) EUT1 EP_NTERM
21810 HMM01923cobtr_2_eut1(pf01923) EUT1
21815 HMM01515PTA_B_1_eut1(pf01515) EUT1
21820 H_azure EUT1
21825 P_lightViolet EUT1
21830 HMMaldh_1_eutpdugrm(pf00171) EUT1 EP_NTERM
21835 HMM06723EutJMreB_1_grm EUT1
21840 HMM00465alcdh_3_mix(pf00465) EUT1
21845 HMMtpEuth_1_eut(pf04346) EUT1
21850 HMM06277EutA_1_eut(pf06277) EUT1
21855 HMMetlyEutB_1_EUT1(pf06751) EUT1
21860 HMMetlyEutC_1_eut1(pf05985) EUT1 EP_NTERM
21865 Tsp_rosa EUT1
21870 H_mulberry(pf00936pf16365) EUT1
21875 HMMreg12833_1_EUT1(pf12833) EUT1
21885 - -
21890 -pf01218 -
21895 -pf01520 -
21900 -pf00583 -
21905 -pf11143 -
21910 -pf06572 -
21915 -pf04261 -
21920 HMMnopfamclust3_1 RMM1
21925 - -
21930 -pf00528 -
21935 -pf00528 -
BMC-P
BMC-H
BMC-T
Aldehyde dehydrogenase
PTAC
Signature enzyme
Alcohol dehydrogenase
Regulator

Salmonella enterica subsp. enterica serovar Adelaide. Bacteria; Proteobacteria; Gammaproteobacteria; Enterobacterales;

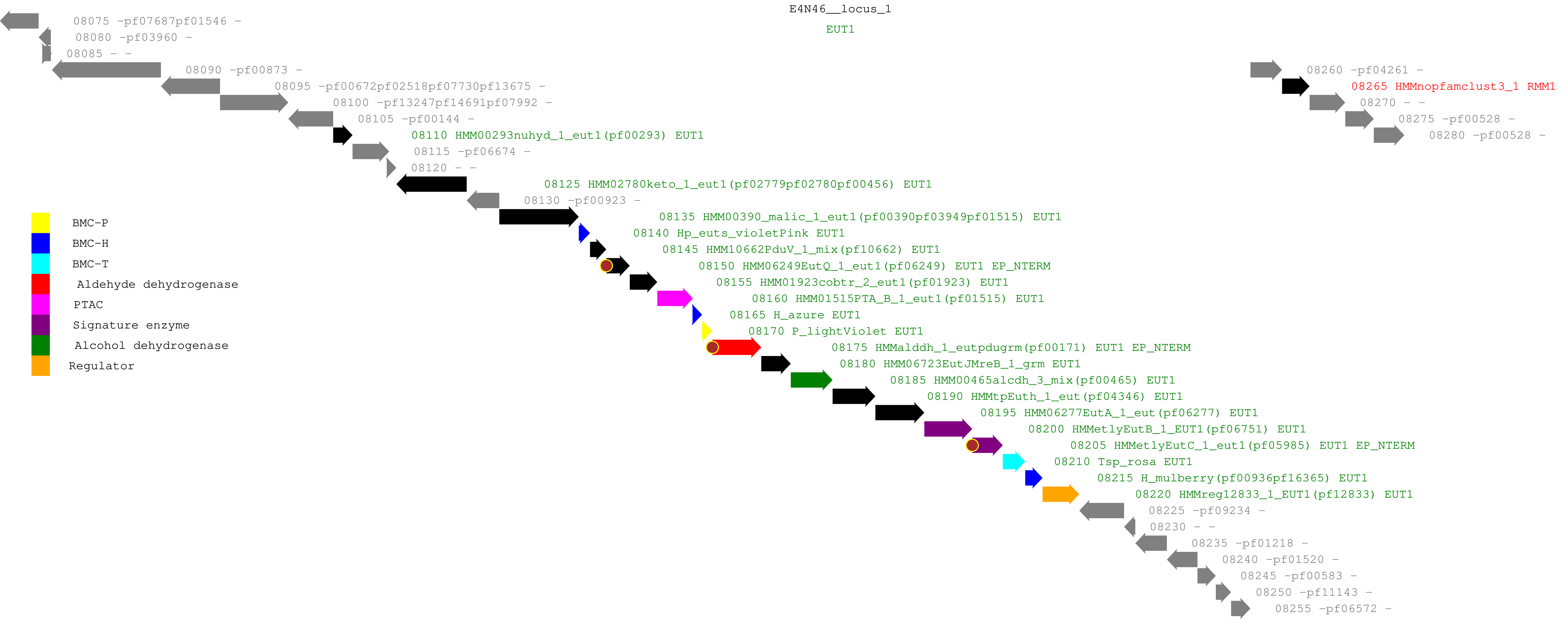

Citrobacter sp. SNU WT2. Bacteria; Proteobacteria; Gammaproteobacteria; Enterobacterales;

E4Z61__locus_2

EUT1

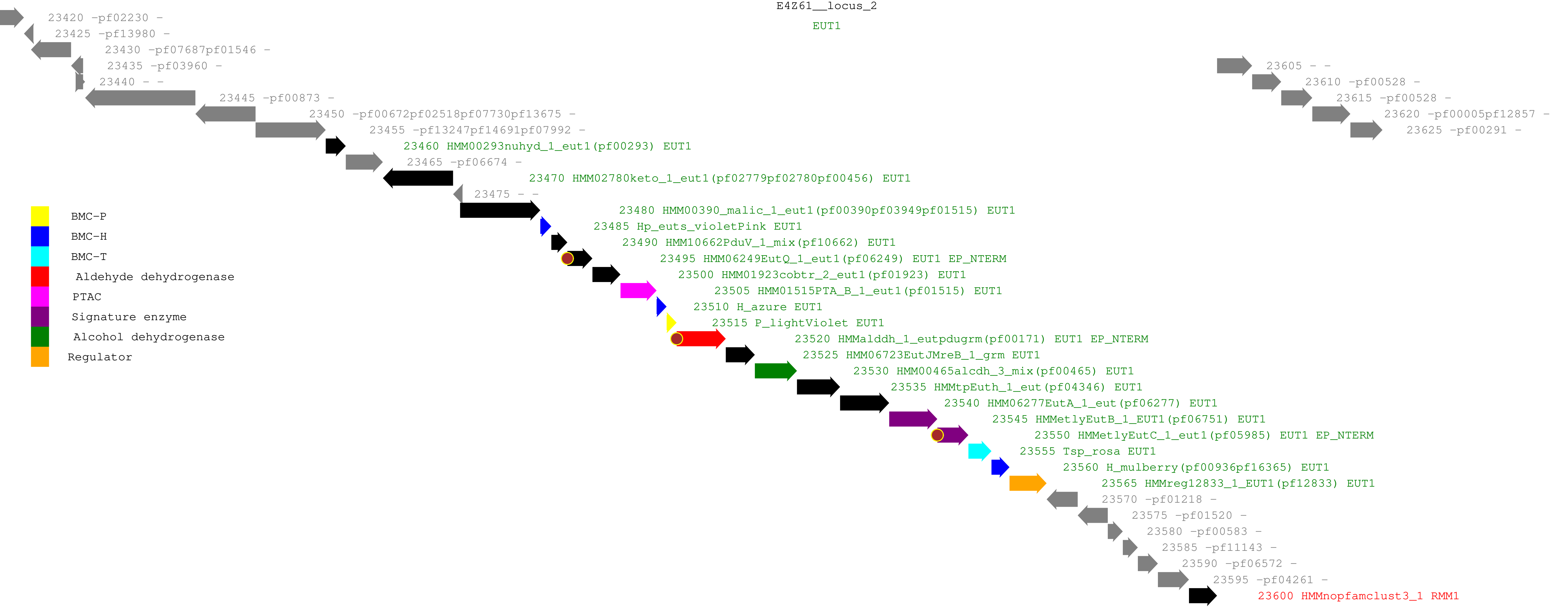

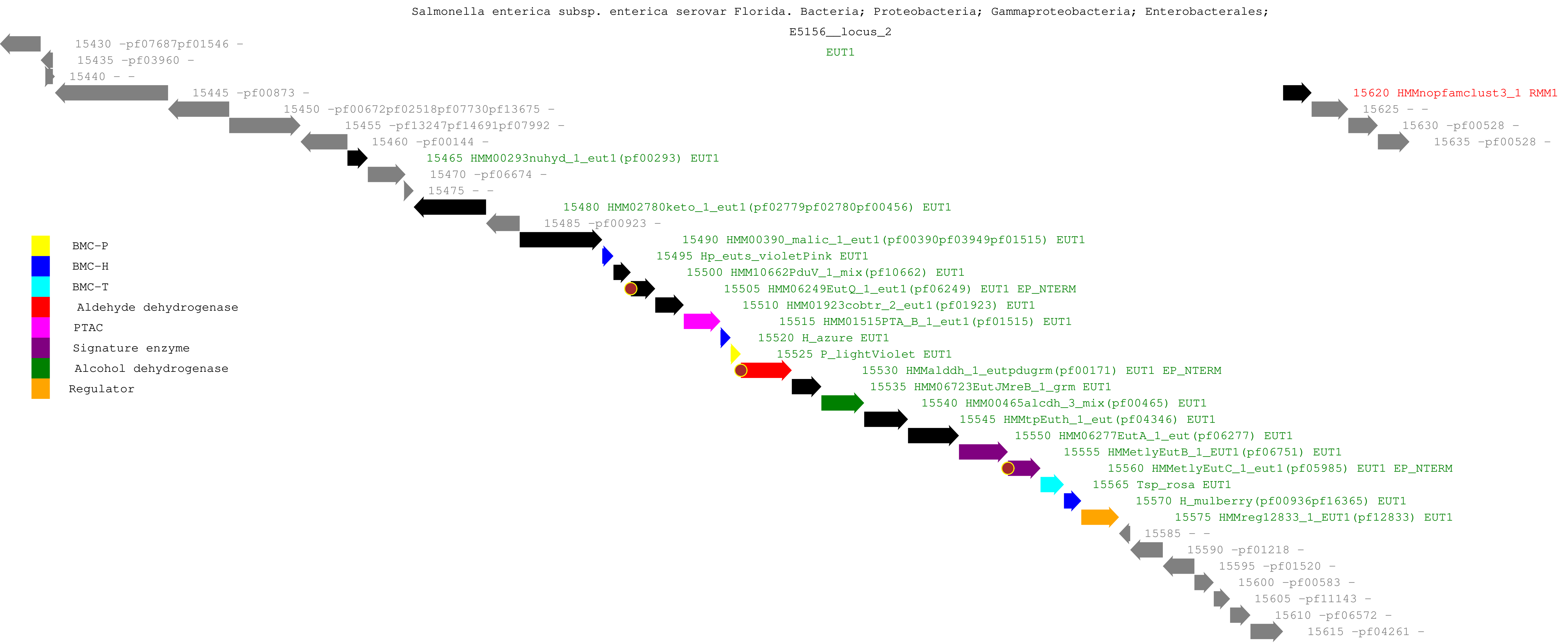

Salmonella enterica subsp. enterica serovar Florida. Bacteria; Proteobacteria; Gammaproteobacteria; Enterobacterales;
E5156__locus_2
EUT1
15430 -pf07687pf01546 -
15435 -pf03960 -
15440 - -
15445 -pf00873 -
15450 -pf00672pf02518pf07730pf13675 -
15455 -pf13247pf14691pf07992 -
15460 -pf00144 -
15465 HMM00293nuhyd_1_eut1(pf00293) EUT1
15470 -pf06674 -
15475 - -
15480 HMM02780keto_1_eut1(pf02779pf02780pf00456) EUT1
15485 -pf00923 -
15490 HMM00390_malic_1_eut1(pf00390pf03949pf01515) EUT1
15495 Hp_euts_violetPink EUT1
15500 HMM10662PduV_1_mix(pf10662) EUT1
15505 HMM06249EutQ_1_eut1(pf06249) EUT1 EP_NTERM
15510 HMM01923cobtr_2_eut1(pf01923) EUT1
15515 HMM01515PTA_B_1_eut1(pf01515) EUT1
15520 H_azure EUT1
15525 P_lightViolet EUT1
15530 HMMalddh_1_eutpdugrm(pf00171) EUT1 EP_NTERM
15535 HMM06723EutJMreB_1_grm EUT1
15540 HMM00465alcdh_3_mix(pf00465) EUT1
15545 HMMtpEuth_1_eut(pf04346) EUT1
15550 HMM06277EutA_1_eut(pf06277) EUT1
15555 HMMetlyEutB_1_EUT1(pf06751) EUT1
15560 HMMetlyEutC_1_eut1(pf05985) EUT1 EP_NTERM
15565 Tsp_rosa EUT1
15570 H_mulberry(pf00936pf16365) EUT1
15575 HMMreg12833_1_EUT1(pf12833) EUT1
15585 - -
15590 -pf01218 -
15595 -pf01520 -
15600 -pf00583 -
15605 -pf11143 -
15610 -pf06572 -
15615 -pf04261 -
15620 HMMnopfamclust3_1 RMM1
15625 - -
15630 -pf00528 -
15635 -pf00528 -
BMC-P
BMC-H
BMC-T
Aldehyde dehydrogenase
PTAC
Signature enzyme
Alcohol dehydrogenase
Regulator

Salmonella enterica subsp. enterica serovar Westhampton. Bacteria; Proteobacteria; Gammaproteobacteria; Enterobacterales;

E5213__locus_1

EUT1

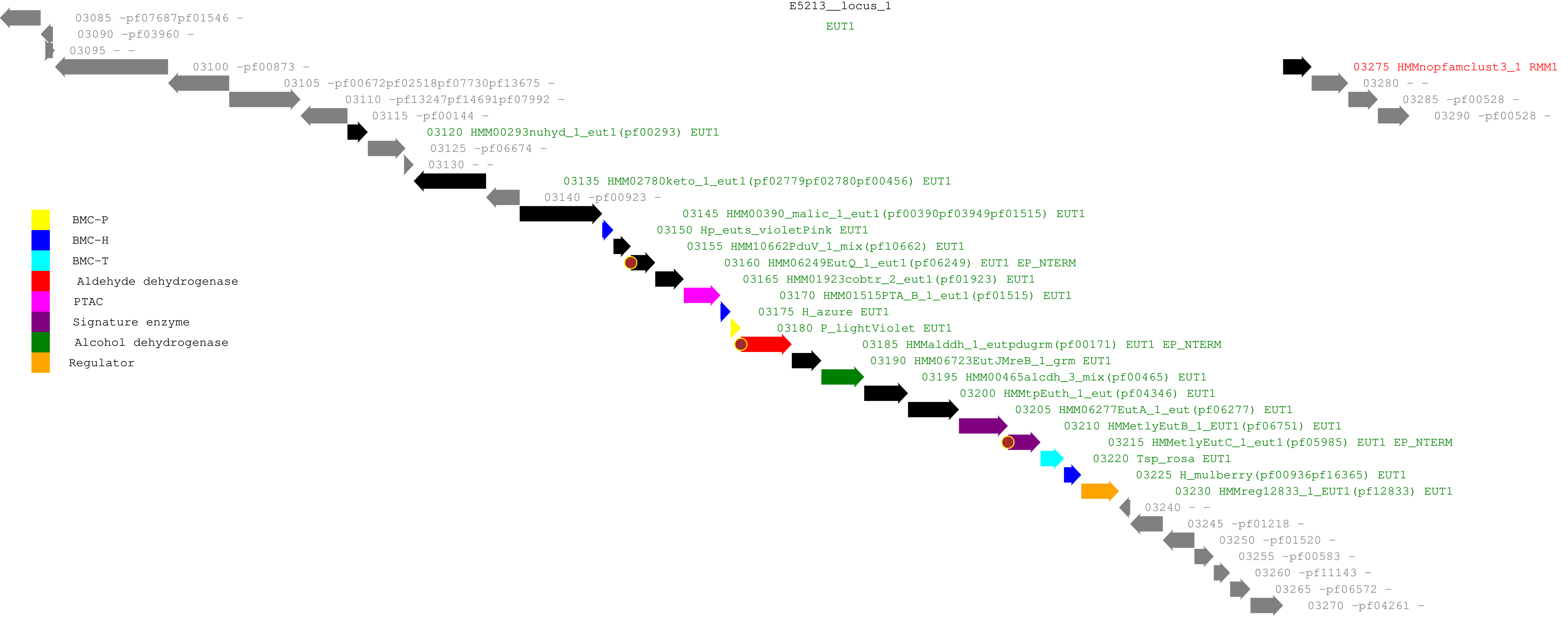

Citrobacter freundii. Bacteria; Proteobacteria; Gammaproteobacteria; Enterobacterales;

E5284__locus_1

EUT1

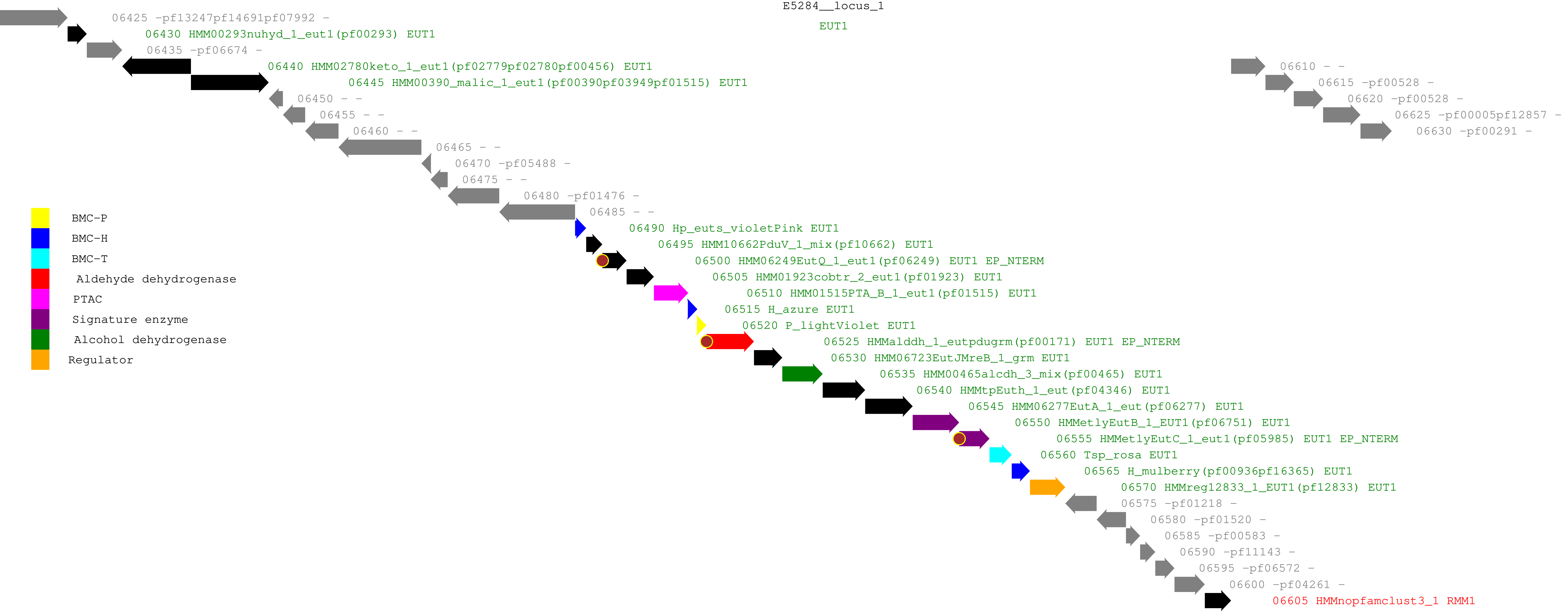

Escherichia coli O157:H7. Bacteria; Proteobacteria; Gammaproteobacteria; Enterobacterales;

E5F07__locus_1

EUT1

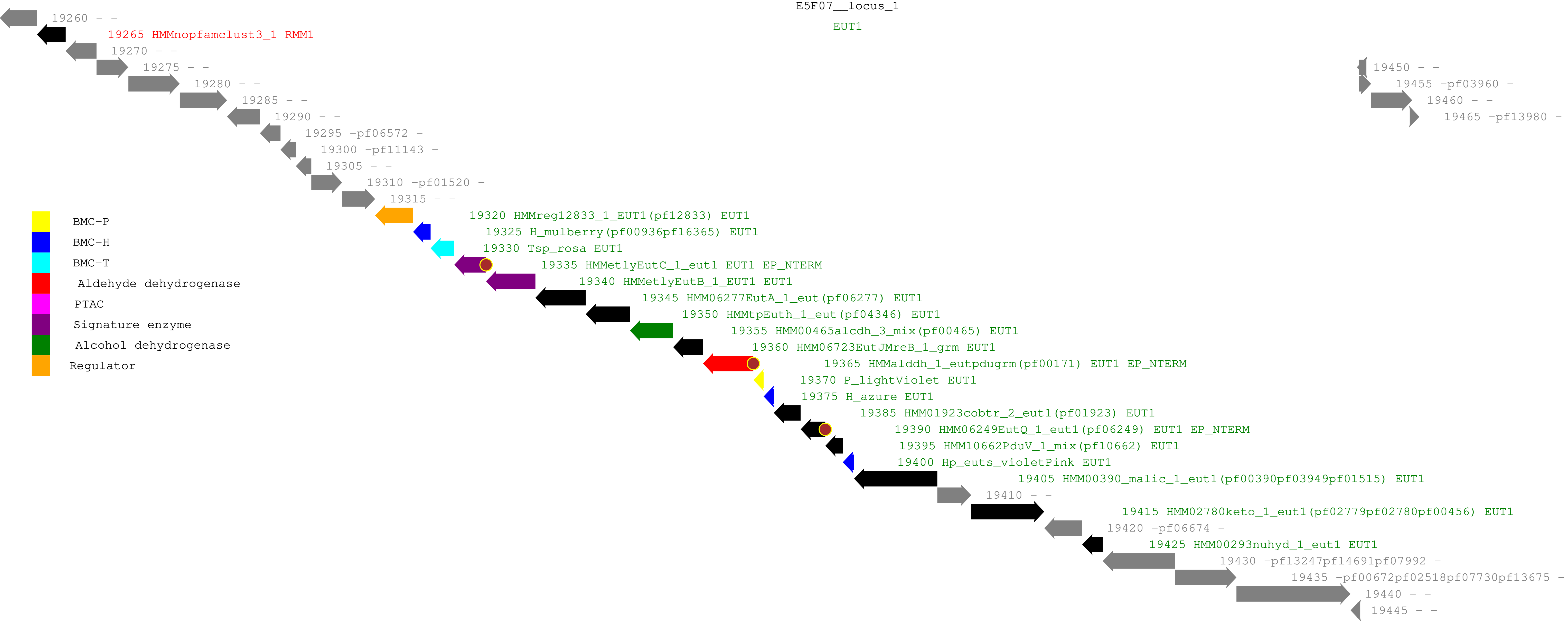

Salmonella enterica subsp. enterica serovar Lagos. Bacteria; Proteobacteria; Gammaproteobacteria; Enterobacterales;

E5J91__locus_2

EUT1

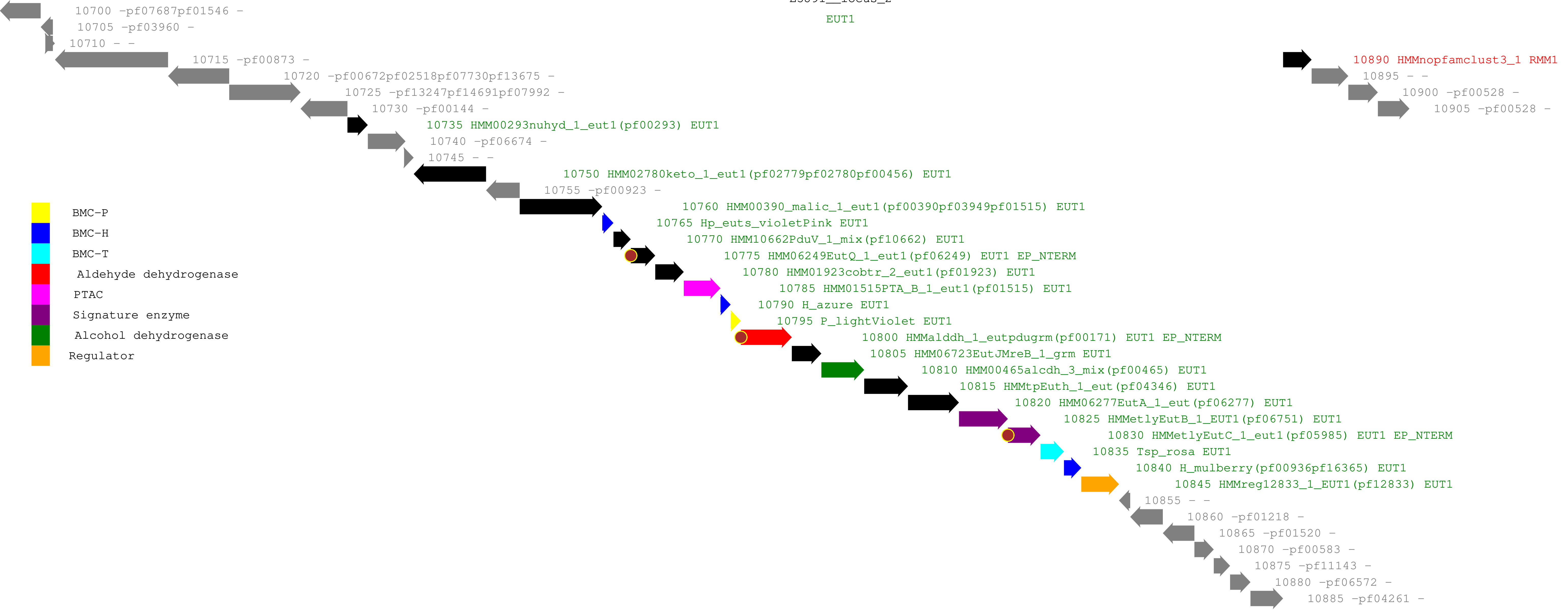

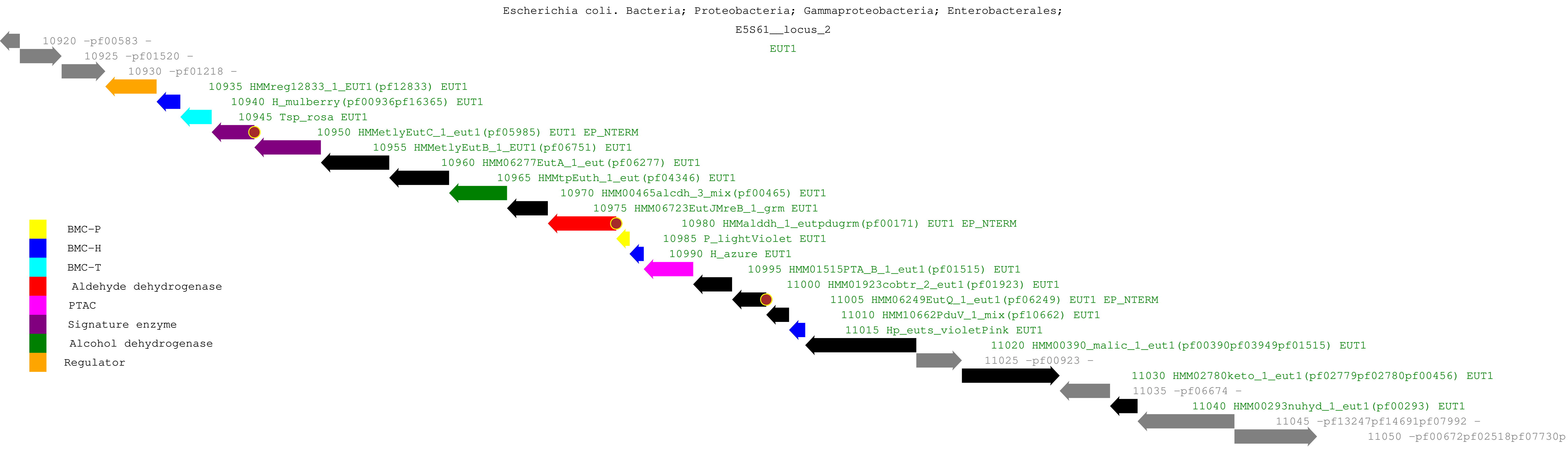
Escherichia coli. Bacteria; Proteobacteria; Gammaproteobacteria; Enterobacterales;
E5S61__locus_2
EUT1
10920 -pf00583 -
10925 -pf01520 -
10930 -pf01218 -
10935 HMMreg12833_1_EUT1(pf12833) EUT1
10940 H_mulberry(pf00936pf16365) EUT1
10945 Tsp_rosa EUT1
10950 HMMetlyEutC_1_eut1(pf05985) EUT1 EP_NTERM
10955 HMMetlyEutB_1_EUT1(pf06751) EUT1
10960 HMM06277EutA_1_eut(pf06277) EUT1
10965 HMMtpEuth_1_eut(pf04346) EUT1
10970 HMM00465alcdh_3_mix(pf00465) EUT1
10975 HMM06723EutJMreB_1_grm EUT1
10980 HMMalddh_1_eutpdugrm(pf00171) EUT1 EP_NTERM
10985 P_lightViolet EUT1
10990 H_azure EUT1
10995 HMM01515PTA_B_1_eut1(pf01515) EUT1
11000 HMM01923cobtr_2_eut1(pf01923) EUT1
11005 HMM06249EutQ_1_eut1(pf06249) EUT1 EP_NTERM
11010 HMM10662PduV_1_mix(pf10662) EUT1
11015 Hp_euts_violetPink EUT1
11020 HMM00390_malic_1_eut1(pf00390pf03949pf01515) EUT1
11025 -pf00923 -
11030 HMM02780keto_1_eut1(pf02779pf02780pf00456) EUT1
11035 -pf06674 -
11040 HMM00293nuhyd_1_eut1(pf00293) EUT1
11045 -pf13247pf14691pf07992 -
11050 -pf00672pf02518pf07730p
BMC-P
BMC-H
BMC-T
Aldehyde dehydrogenase
PTAC
Signature enzyme
Alcohol dehydrogenase
Regulator

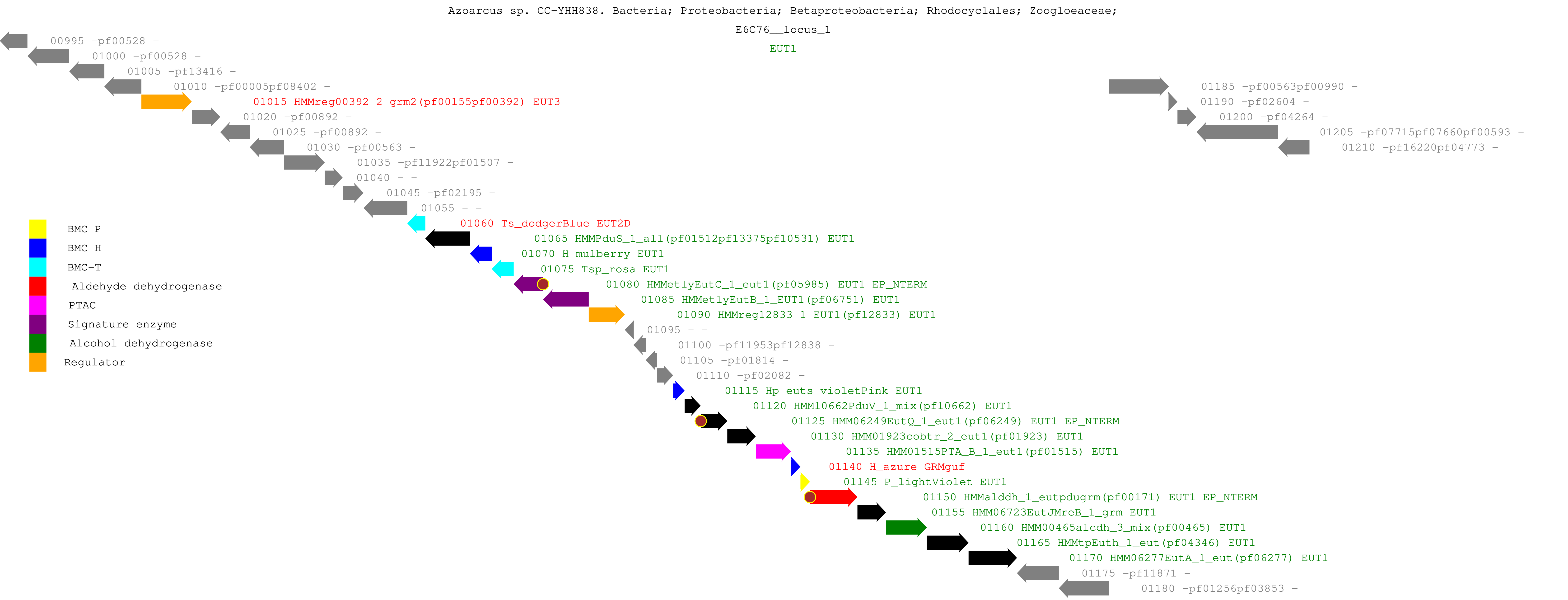
Azoarcus sp. CC-YHH838. Bacteria; Proteobacteria; Betaproteobacteria; Rhodocyclales; Zoogloeaceae;
E6C76__locus_1
EUT1
00995 -pf00528 -
01000 -pf00528 -
01005 -pf13416 -
01010 -pf00005pf08402 -
01015 HMMreg00392_2_grm2(pf00155pf00392) EUT3
01020 -pf00892 -
01025 -pf00892 -
01030 -pf00563 -
01035 -pf11922pf01507 -
01040 - -
01045 -pf02195 -
01055 - -
01060 Ts_dodgerBlue EUT2D
01065 HMMPduS_1_all(pf01512pf13375pf10531) EUT1
01070 H_mulberry EUT1
01075 Tsp_rosa EUT1
01080 HMMetlyEutC_1_eut1(pf05985) EUT1 EP_NTERM
01085 HMMetlyEutB_1_EUT1(pf06751) EUT1
01090 HMMreg12833_1_EUT1(pf12833) EUT1
01095 - -
01100 -pf11953pf12838 -
01105 -pf01814 -
01110 -pf02082 -
01115 Hp_euts_violetPink EUT1
01120 HMM10662PduV_1_mix(pf10662) EUT1
01125 HMM06249EutQ_1_eut1(pf06249) EUT1 EP_NTERM
01130 HMM01923cobtr_2_eut1(pf01923) EUT1
01135 HMM01515PTA_B_1_eut1(pf01515) EUT1
01140 H_azure GRMguf
01145 P_lightViolet EUT1
01150 HMMaldh_1_eutpdugrm(pf00171) EUT1 EP_NTERM
01155 HMM06723EutJMreB_1_grm EUT1
01160 HMM00465alcdh_3_mix(pf00465) EUT1
01165 HMMtpEuth_1_eut(pf04346) EUT1
01170 HMM06277EutA_1_eut(pf06277) EUT1
01175 -pf11871 -
01180 -pf01256pf03853 -
01185 -pf00563pf00990 -
01190 -pf02604 -
01200 -pf04264 -
01205 -pf07715pf07660pf00593 -
01210 -pf16220pf04773 -
BMC-P
BMC-H
BMC-T
Aldehyde dehydrogenase
PTAC
Signature enzyme
Alcohol dehydrogenase
Regulator

Azoarcus sp. CC-YHH848. Bacteria; Proteobacteria; Betaproteobacteria; Rhodocyclales; Zoogloeaceae;

E6051__locus_1

EUT1

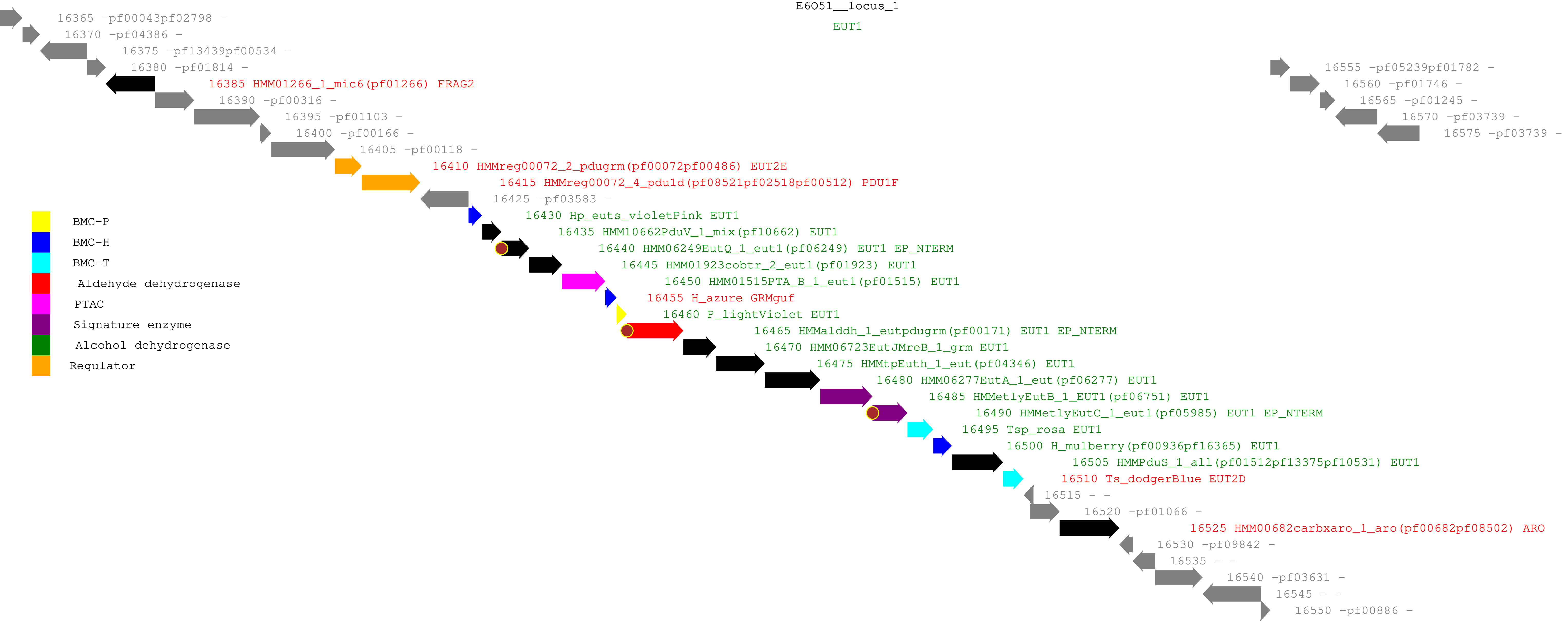

Citrobacter sp. CF971. Bacteria; Proteobacteria; Gammaproteobacteria; Enterobacterales;

E6P06__locus_2

EUT1

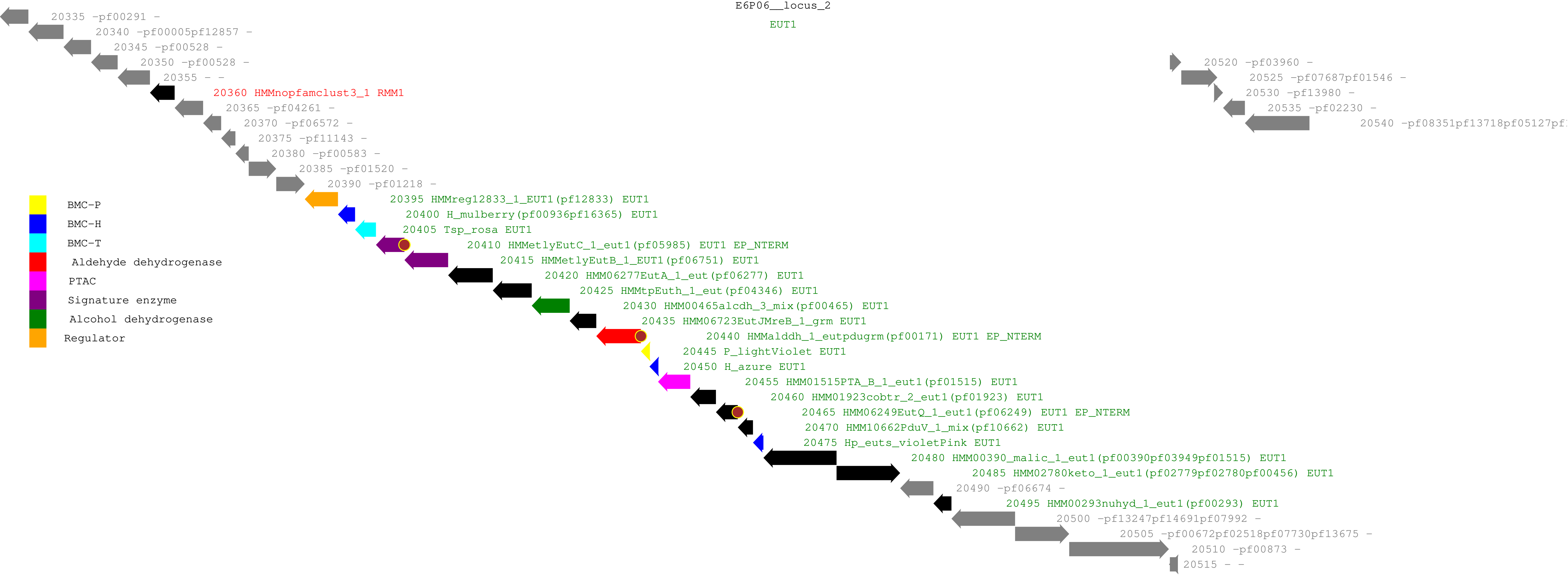

Salmonella enterica subsp. enterica serovar Berkeley. Bacteria; Proteobacteria; Gammaproteobacteria; Enterobacterales;

E6W86__locus_1

EUT1

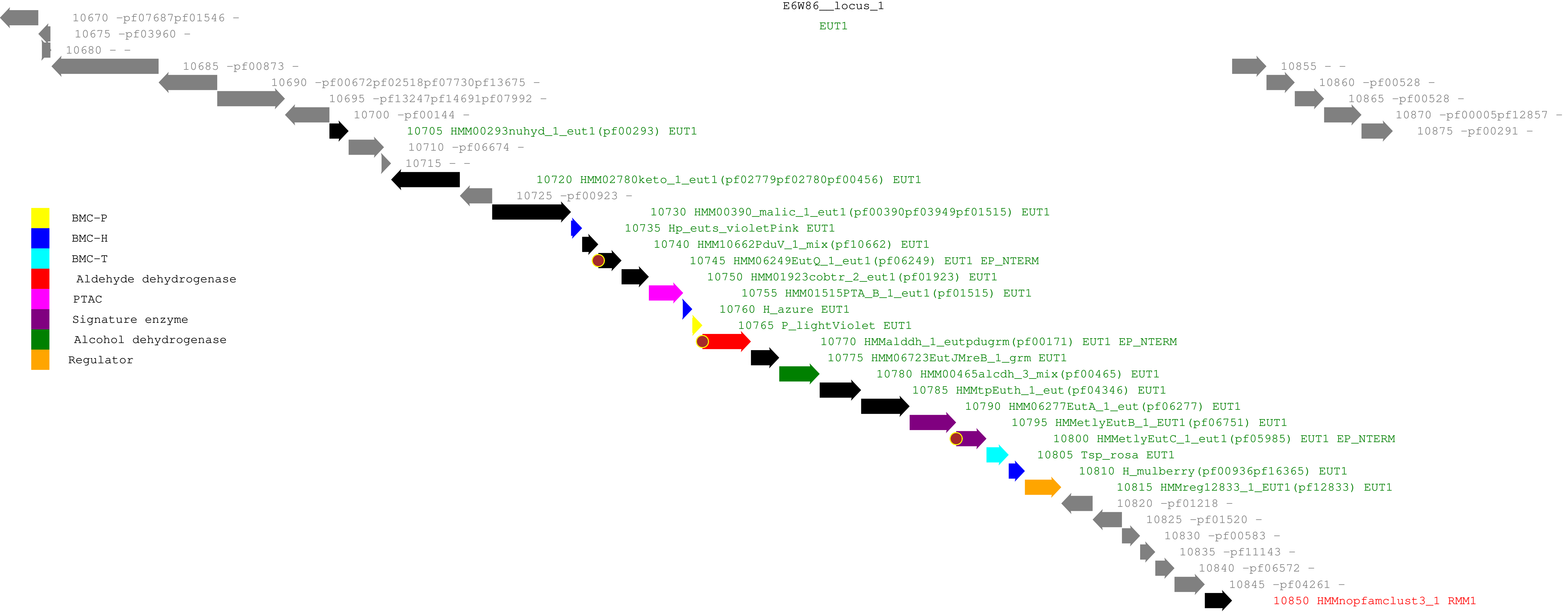

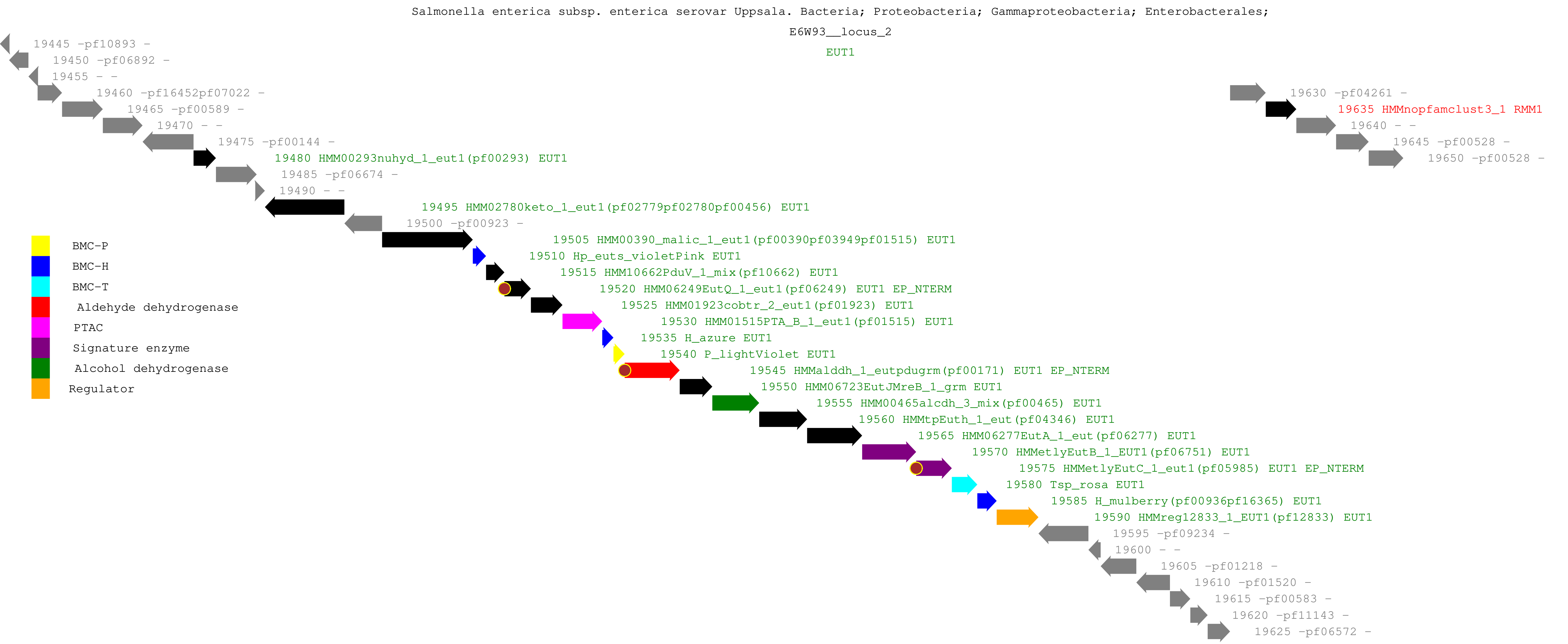
Salmonella enterica subsp. enterica serovar Uppsala. Bacteria; Proteobacteria; Gammaproteobacteria; Enterobacterales;
E6W93__locus_2
EUT1
19445 -pf10893 -
19450 -pf06892 -
19455 - -
19460 -pf16452pf07022 -
19465 -pf00589 -
19470 - -
19475 -pf00144 -
19480 HMM00293nuhyd_1_eut1(pf00293) EUT1
19485 -pf06674 -
19490 - -
19495 HMM02780keto_1_eut1(pf02779pf02780pf00456) EUT1
19500 -pf00923 -
19505 HMM00390_malic_1_eut1(pf00390pf03949pf01515) EUT1
19510 Hp_euts_violetPink EUT1
19515 HMM10662PduV_1_mix(pf10662) EUT1
19520 HMM06249EutQ_1_eut1(pf06249) EUT1 EP_NTERM
19525 HMM01923cobtr_2_eut1(pf01923) EUT1
19530 HMM01515PTA_B_1_eut1(pf01515) EUT1
19535 H_azure EUT1
19540 P_lightViolet EUT1
19545 HMMalddh_1_eutpdugrm(pf00171) EUT1 EP_NTERM
19550 HMM06723EutJMreB_1_grm EUT1
19555 HMM00465alcdh_3_mix(pf00465) EUT1
19560 HMMtpEuth_1_eut(pf04346) EUT1
19565 HMM06277EutA_1_eut(pf06277) EUT1
19570 HMMetlyEutB_1_EUT1(pf06751) EUT1
19575 HMMetlyEutC_1_eut1(pf05985) EUT1 EP_NTERM
19580 Tsp_rosa EUT1
19585 H_mulberry(pf00936pf16365) EUT1
19590 HMMreg12833_1_EUT1(pf12833) EUT1
19595 -pf09234 -
19600 - -
19605 -pf01218 -
19610 -pf01520 -
19615 -pf00583 -
19620 -pf11143 -
19625 -pf06572 -
19630 -pf04261 -
19635 HMMnopfamclust3_1 RMM1
19640 - -
19645 -pf00528 -
19650 -pf00528 -
BMC-P
BMC-H
BMC-T
Aldehyde dehydrogenase
PTAC
Signature enzyme
Alcohol dehydrogenase
Regulator

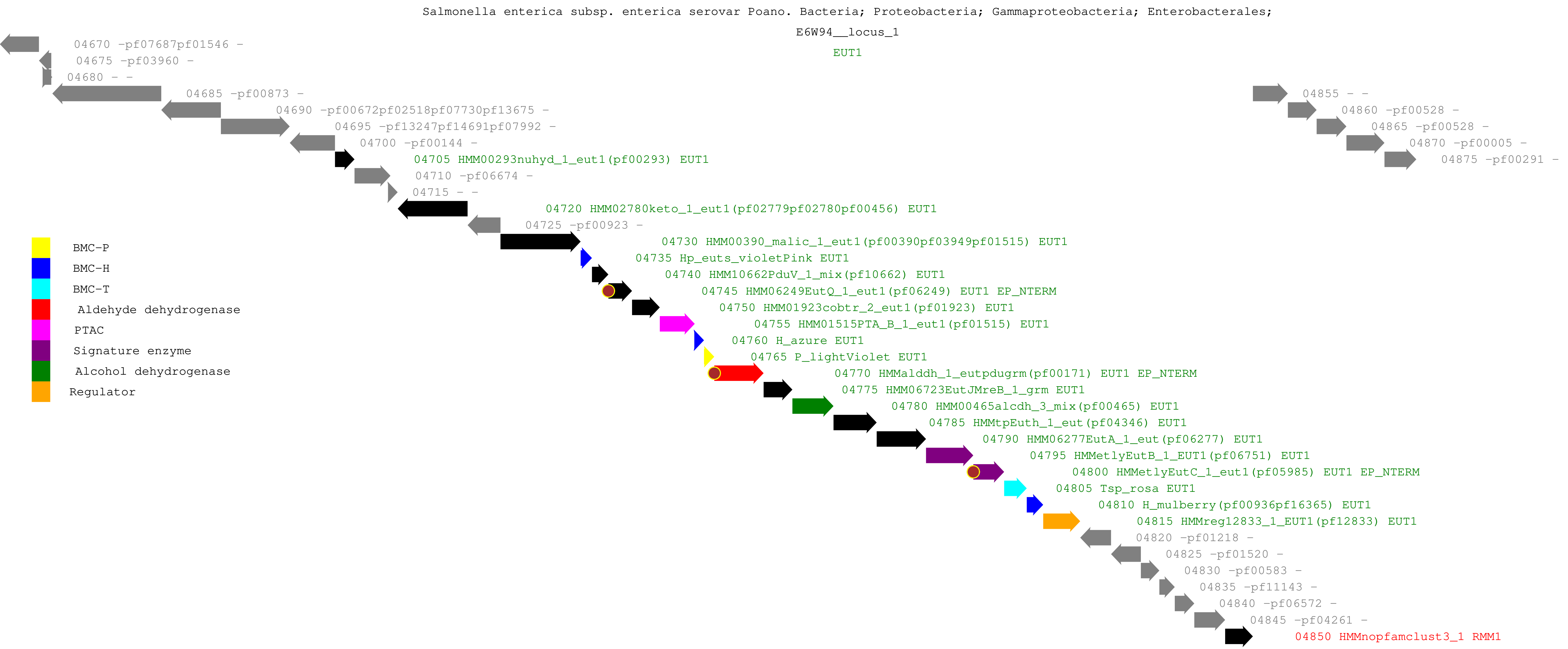

Salmonella enterica subsp. enterica serovar Poano. Bacteria; Proteobacteria; Gammaproteobacteria; Enterobacterales;
E6W94__locus_1
EUT1
04670 -pf07687pf01546 -
04675 -pf03960 -
04680 - -
04685 -pf00873 -
04690 -pf00672pf02518pf07730pf13675 -
04695 -pf13247pf14691pf07992 -
04700 -pf00144 -
04705 HMM00293nuhyd_1_eut1(pf00293) EUT1
04710 -pf06674 -
04715 - -
04720 HMM02780keto_1_eut1(pf02779pf02780pf00456) EUT1
04725 -pf00923 -
04730 HMM00390_malic_1_eut1(pf00390pf03949pf01515) EUT1
04735 Hp_euts_violetPink EUT1
04740 HMM10662PduV_1_mix(pf10662) EUT1
04745 HMM06249EutQ_1_eut1(pf06249) EUT1 EP_NTERM
04750 HMM01923cobtr_2_eut1(pf01923) EUT1
04755 HMM01515PTA_B_1_eut1(pf01515) EUT1
04760 H_azure EUT1
04765 P_lightViolet EUT1
04770 HMMaldh_1_eutpdugrm(pf00171) EUT1 EP_NTERM
04775 HMM06723EutJMreB_1_grm EUT1
04780 HMM00465alcdh_3_mix(pf00465) EUT1
04785 HMMtpEuth_1_eut(pf04346) EUT1
04790 HMM06277EutA_1_eut(pf06277) EUT1
04795 HMMetlyEutB_1_EUT1(pf06751) EUT1
04800 HMMetlyEutC_1_eut1(pf05985) EUT1 EP_NTERM
04805 Tsp_rosa EUT1
04810 H_mulberry(pf00936pf16365) EUT1
04815 HMMreg12833_1_EUT1(pf12833) EUT1
04820 -pf01218 -
04825 -pf01520 -
04830 -pf00583 -
04835 -pf11143 -
04840 -pf06572 -
04845 -pf04261 -
04850 HMMnopfamclust3_1 RMM1
04855 - -
04860 -pf00528 -
04865 -pf00528 -
04870 -pf00005 -
04875 -pf00291 -
BMC-P
BMC-H
BMC-T
Aldehyde dehydrogenase
PTAC
Signature enzyme
Alcohol dehydrogenase
Regulator

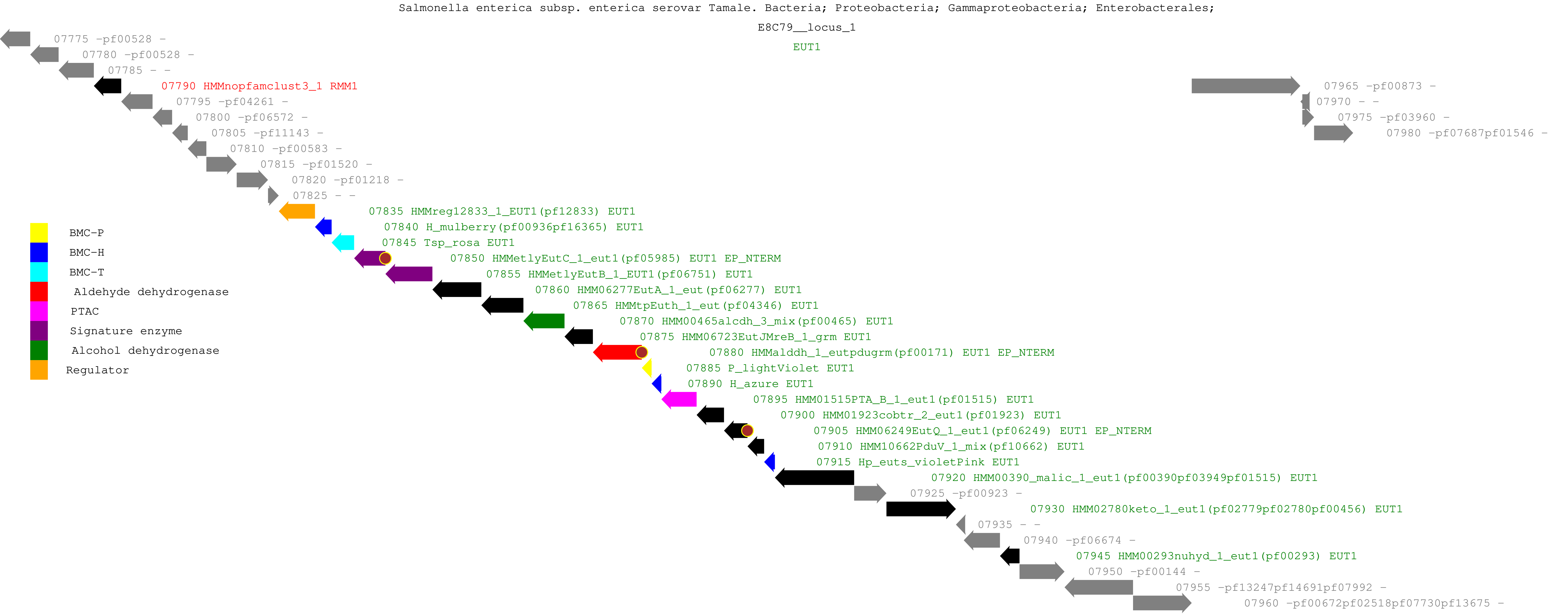

Salmonella enterica subsp. enterica serovar Tamale. Bacteria; Proteobacteria; Gammaproteobacteria; Enterobacterales;
E8C79__locus_1
EUT1
07775 -pf00528 -
07780 -pf00528 -
07785 - -
07790 HMMnopfamclust3_1 RMM1
07795 -pf04261 -
07800 -pf06572 -
07805 -pf11143 -
07810 -pf00583 -
07815 -pf01520 -
07820 -pf01218 -
07825 - -
07835 HMMreg12833_1_EUT1(pf12833) EUT1
07840 H_mulberry(pf00936pf16365) EUT1
07845 Tsp_rosa EUT1
07850 HMMetlyEutC_1_eut1(pf05985) EUT1 EP_NTERM
07855 HMMetlyEutB_1_EUT1(pf06751) EUT1
07860 HMM06277EutA_1_eut(pf06277) EUT1
07865 HMMtpEuth_1_eut(pf04346) EUT1
07870 HMM00465alcdh_3_mix(pf00465) EUT1
07875 HMM06723EutJMreB_1_grm EUT1
07880 HMMalddh_1_eutpdugrm(pf00171) EUT1 EP_NTERM
07885 P_lightViolet EUT1
07890 H_azure EUT1
07895 HMM01515PTA_B_1_eut1(pf01515) EUT1
07900 HMM01923cobtr_2_eut1(pf01923) EUT1
07905 HMM06249EutQ_1_eut1(pf06249) EUT1 EP_NTERM
07910 HMM10662PduV_1_mix(pf10662) EUT1
07915 Hp_euts_violetPink EUT1
07920 HMM00390_malic_1_eut1(pf00390pf03949pf01515) EUT1
07925 -pf00923 -
07930 HMM02780keto_1_eut1(pf02779pf02780pf00456) EUT1
07935 - -
07940 -pf06674 -
07945 HMM00293nuhyd_1_eut1(pf00293) EUT1
07950 -pf00144 -
07955 -pf13247pf14691pf07992 -
07960 -pf00672pf02518pf07730pf13675 -
07965 -pf00873 -
07970 - -
07975 -pf03960 -
07980 -pf07687pf01546 -
BMC-P
BMC-H
BMC-T
Aldehyde dehydrogenase
PTAC
Signature enzyme
Alcohol dehydrogenase
Regulator

Salmonella enterica subsp. enterica serovar Amsterdam. Bacteria; Proteobacteria; Gammaproteobacteria; Enterobacterales;

E8Q13__locus_2

EUT1

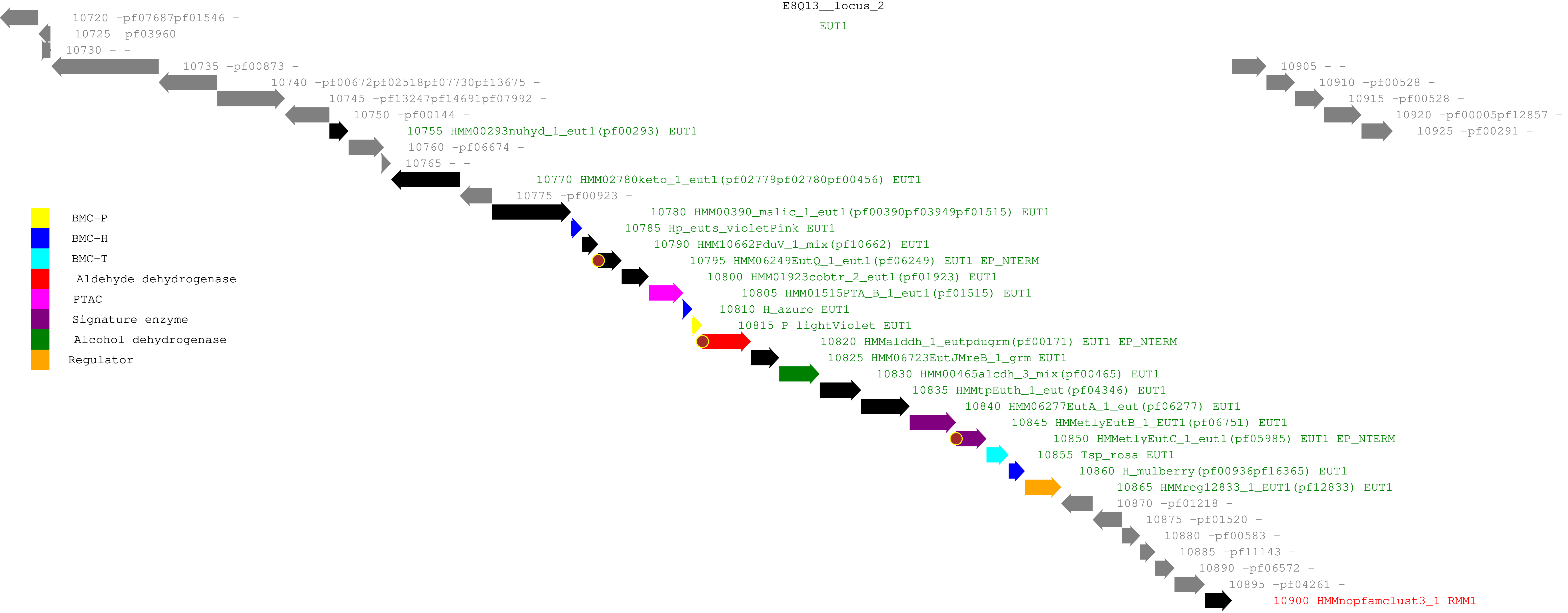

Salmonella enterica subsp. enterica serovar Goldcoast. Bacteria; Proteobacteria; Gammaproteobacteria; Enterobacterales;

E9126__locus_2

EUT1

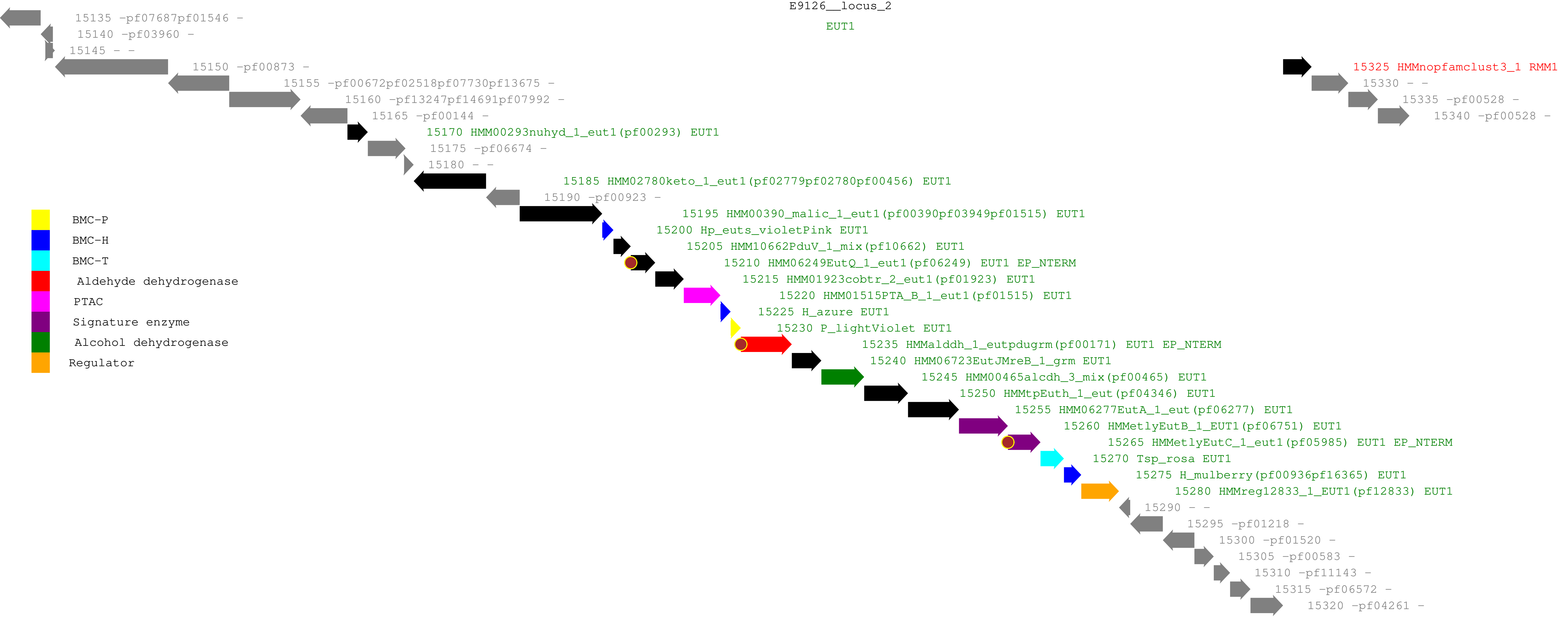

Klebsiella pneumoniae subsp. pneumoniae. Bacteria; Proteobacteria; Gammaproteobacteria; Enterobacterales;

E9161__locus_2

EUT1

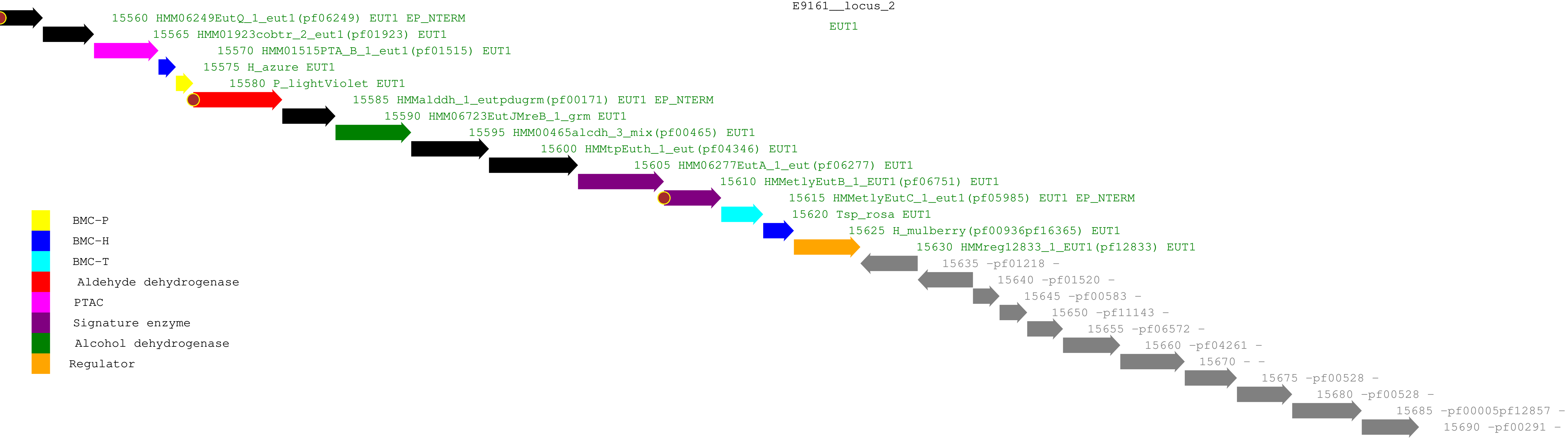

Escherichia albertii. Bacteria; Proteobacteria; Gammaproteobacteria; Enterobacterales;

EAB3F__locus_2

EUT1

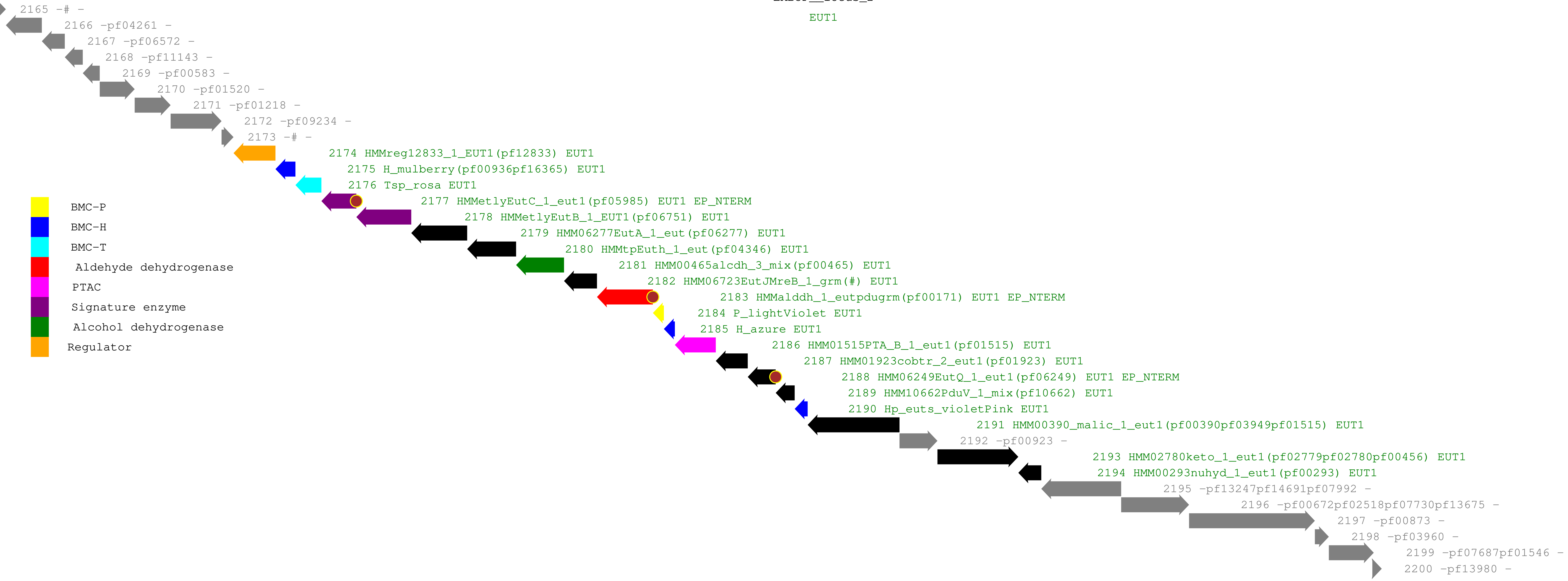

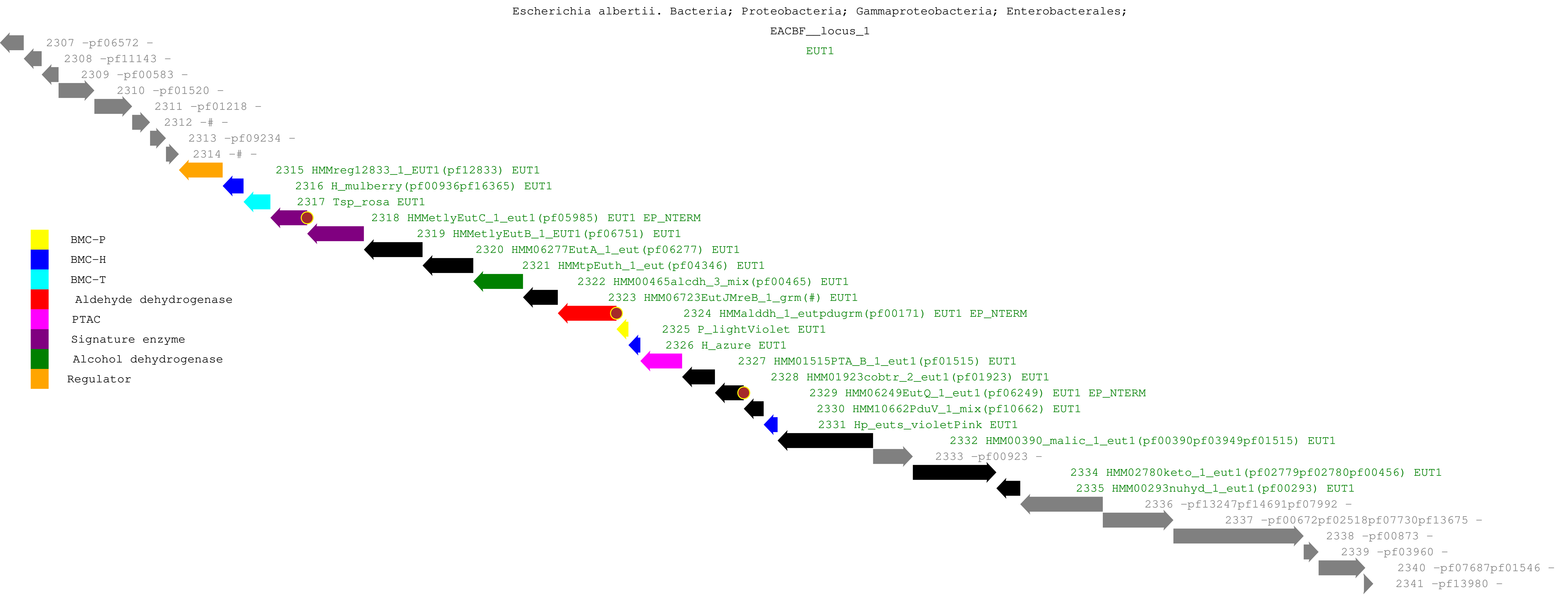

Escherichia albertii. Bacteria; Proteobacteria; Gammaproteobacteria; Enterobacterales;
EACBF__locus_1
EUT1
2307 -pf06572 -
2308 -pf11143 -
2309 -pf00583 -
2310 -pf01520 -
2311 -pf01218 -
2312 -# -
2313 -pf09234 -
2314 -# -
2315 HMMreg12833_1_EUT1(pf12833) EUT1
2316 H_mulberry(pf00936pf16365) EUT1
2317 Tsp_rosa EUT1
2318 HMMetlyEutC_1_eut1(pf05985) EUT1 EP_NTERM
2319 HMMetlyEutB_1_EUT1(pf06751) EUT1
2320 HMM06277EutA_1_eut(pf06277) EUT1
2321 HMMtpEuth_1_eut(pf04346) EUT1
2322 HMM00465alcdh_3_mix(pf00465) EUT1
2323 HMM06723EutJMreB_1_grm(#) EUT1
2324 HMMalddh_1_eutpdugrm(pf00171) EUT1 EP_NTERM
2325 P_lightViolet EUT1
2326 H_azure EUT1
2327 HMM01515PTA_B_1_eut1(pf01515) EUT1
2328 HMM01923cobtr_2_eut1(pf01923) EUT1
2329 HMM06249EutQ_1_eut1(pf06249) EUT1 EP_NTERM
2330 HMM10662PduV_1_mix(pf10662) EUT1
2331 Hp_euts_violetPink EUT1
2332 HMM00390_malic_1_eut1(pf00390pf03949pf01515) EUT1
2333 -pf00923 -
2334 HMM02780keto_1_eut1(pf02779pf02780pf00456) EUT1
2335 HMM00293nuhyd_1_eut1(pf00293) EUT1
2336 -pf13247pf14691pf07992 -
2337 -pf00672pf02518pf07730pf13675 -
2338 -pf00873 -
2339 -pf03960 -
2340 -pf07687pf01546 -
2341 -pf13980 -
BMC-P
BMC-H
BMC-T
Aldehyde dehydrogenase
PTAC
Signature enzyme
Alcohol dehydrogenase
Regulator

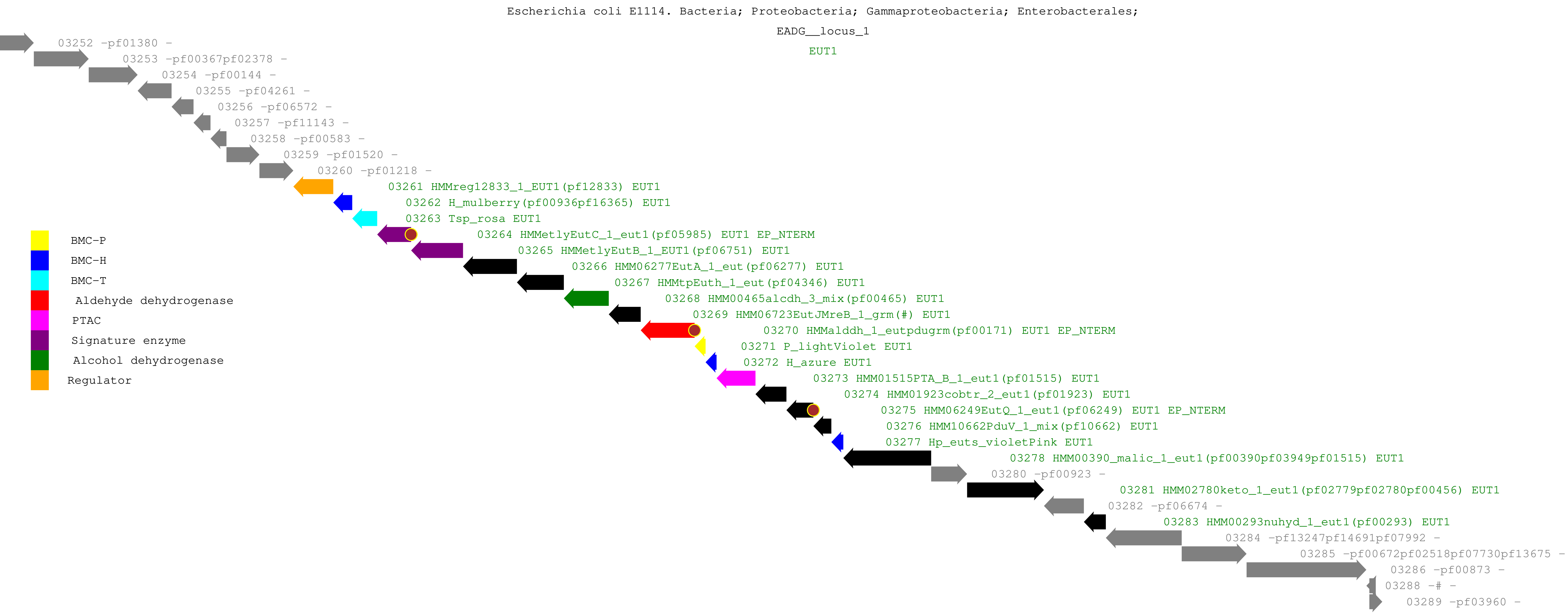

Escherichia coli E1114. Bacteria; Proteobacteria; Gammaproteobacteria; Enterobacterales;
EADG__locus_1
EUT1
03252 -pf01380 -
03253 -pf00367pf02378 -
03254 -pf00144 -
03255 -pf04261 -
03256 -pf06572 -
03257 -pf11143 -
03258 -pf00583 -
03259 -pf01520 -
03260 -pf01218 -
03261 HMMreg12833_1_EUT1(pf12833) EUT1
03262 H_mulberry(pf00936pf16365) EUT1
03263 Tsp_rosa EUT1
03264 HMMetlyEutC_1_eut1(pf05985) EUT1 EP_NTERM
03265 HMMetlyEutB_1_EUT1(pf06751) EUT1
03266 HMM06277EutA_1_eut(pf06277) EUT1
03267 HMMtpEuth_1_eut(pf04346) EUT1
03268 HMM00465alcdh_3_mix(pf00465) EUT1
03269 HMM06723EutJMreB_1_grm(#) EUT1
03270 HMMalddh_1_eutpdugrm(pf00171) EUT1 EP_NTERM
03271 P_lightViolet EUT1
03272 H_azure EUT1
03273 HMM01515PTA_B_1_eut1(pf01515) EUT1
03274 HMM01923cobtr_2_eut1(pf01923) EUT1
03275 HMM06249EutQ_1_eut1(pf06249) EUT1 EP_NTERM
03276 HMM10662PduV_1_mix(pf10662) EUT1
03277 Hp_euts_violetPink EUT1
03278 HMM00390_malic_1_eut1(pf00390pf03949pf01515) EUT1
03280 -pf00923 -
03281 HMM02780keto_1_eut1(pf02779pf02780pf00456) EUT1
03282 -pf06674 -
03283 HMM00293nuhyd_1_eut1(pf00293) EUT1
03284 -pf13247pf14691pf07992 -
03285 -pf00672pf02518pf07730pf13675 -
03286 -pf00873 -
03288 -# -
03289 -pf03960 -
BMC-P
BMC-H
BMC-T
Aldehyde dehydrogenase
PTAC
Signature enzyme
Alcohol dehydrogenase
Regulator

Escherichia coli. Bacteria; Proteobacteria; Gammaproteobacteria; Enterobacterales;

EAI42__locus_1

EUT1

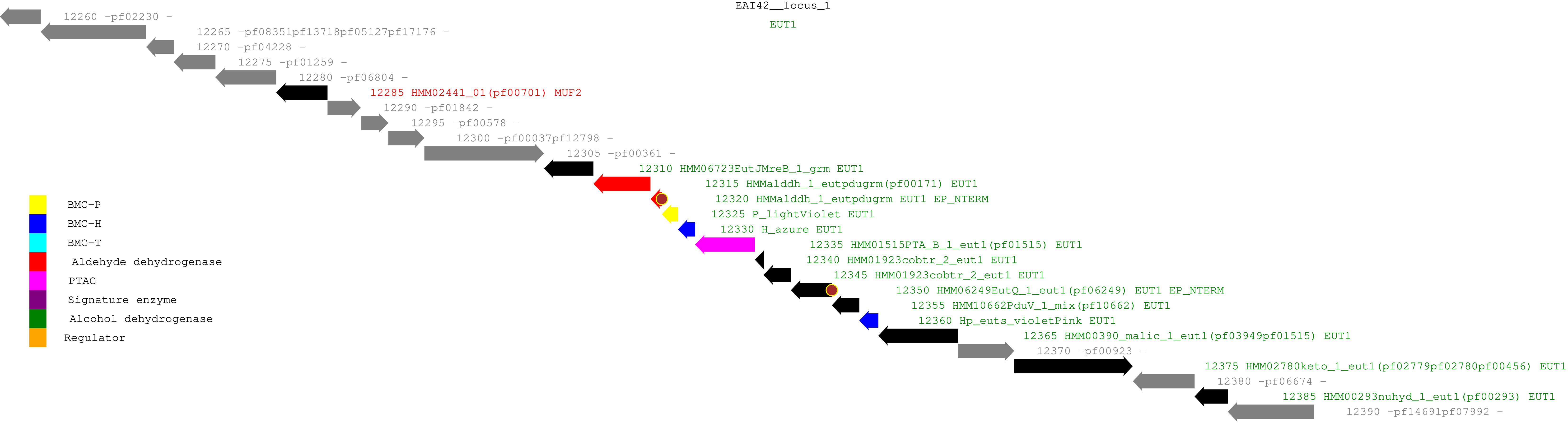

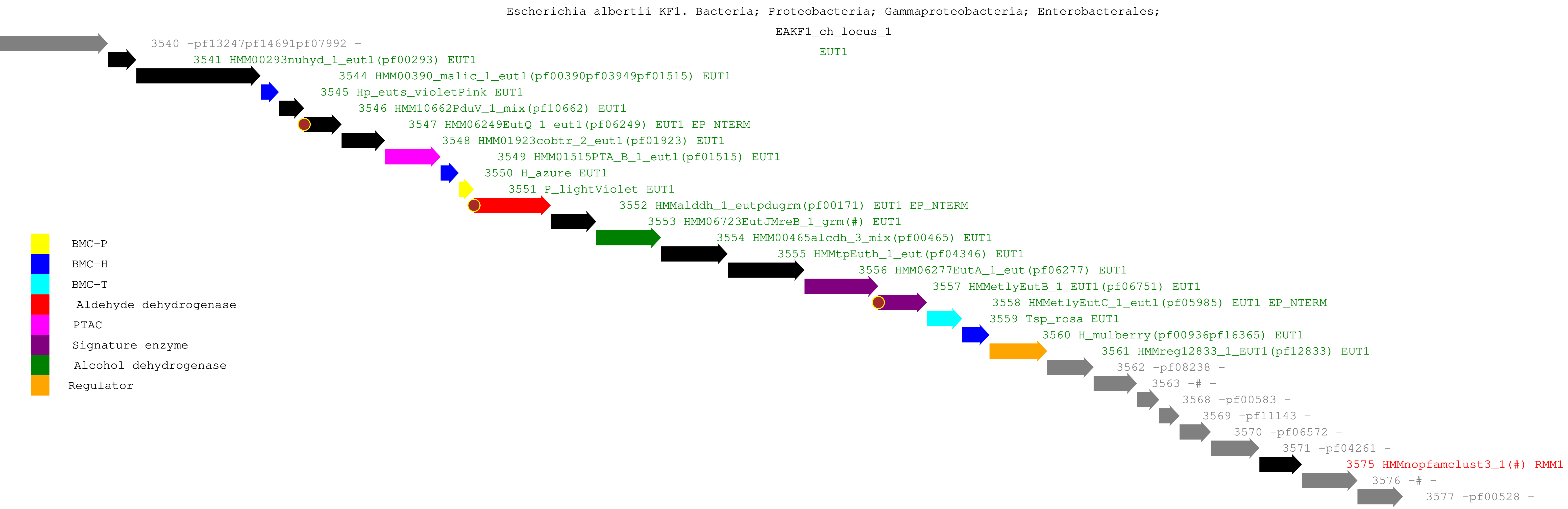
Escherichia albertii KF1. Bacteria; Proteobacteria; Gammaproteobacteria; Enterobacterales;
EAKF1_ch_locus_1
EUT1
3540 -pf13247pf14691pf07992 -
3541 HMM00293nuhyd_1_eut1(pf00293) EUT1
3544 HMM00390_malic_1_eut1(pf00390pf03949pf01515) EUT1
3545 Hp_euts_violetPink EUT1
3546 HMM10662PduV_1_mix(pf10662) EUT1
3547 HMM06249EutQ_1_eut1(pf06249) EUT1 EP_NTERM
3548 HMM01923cobtr_2_eut1(pf01923) EUT1
3549 HMM01515PTA_B_1_eut1(pf01515) EUT1
3550 H_azure EUT1
3551 P_lightViolet EUT1
3552 HMMalddh_1_eutpdugrm(pf00171) EUT1 EP_NTERM
3553 HMM06723EutJMreB_1_grm(#) EUT1
3554 HMM00465alcdh_3_mix(pf00465) EUT1
3555 HMMtpEuth_1_eut(pf04346) EUT1
3556 HMM06277EutA_1_eut(pf06277) EUT1
3557 HMMetlyEutB_1_EUT1(pf06751) EUT1
3558 HMMetlyEutC_1_eut1(pf05985) EUT1 EP_NTERM
3559 Tsp_rosa EUT1
3560 H_mulberry(pf00936pf16365) EUT1
3561 HMMreg12833_1_EUT1(pf12833) EUT1
3562 -pf08238 -
3563 -# -
3568 -pf00583 -
3569 -pf11143 -
3570 -pf06572 -
3571 -pf04261 -
3575 HMMnopfamclust3_1(#) RMM1
3576 -# -
3577 -pf00528 -
BMC-P
BMC-H
BMC-T
Aldehyde dehydrogenase
PTAC
Signature enzyme
Alcohol dehydrogenase
Regulator

Escherichia coli M056. Bacteria; Proteobacteria; Gammaproteobacteria; Enterobacterales;

EAMG__locus_2

EUT1

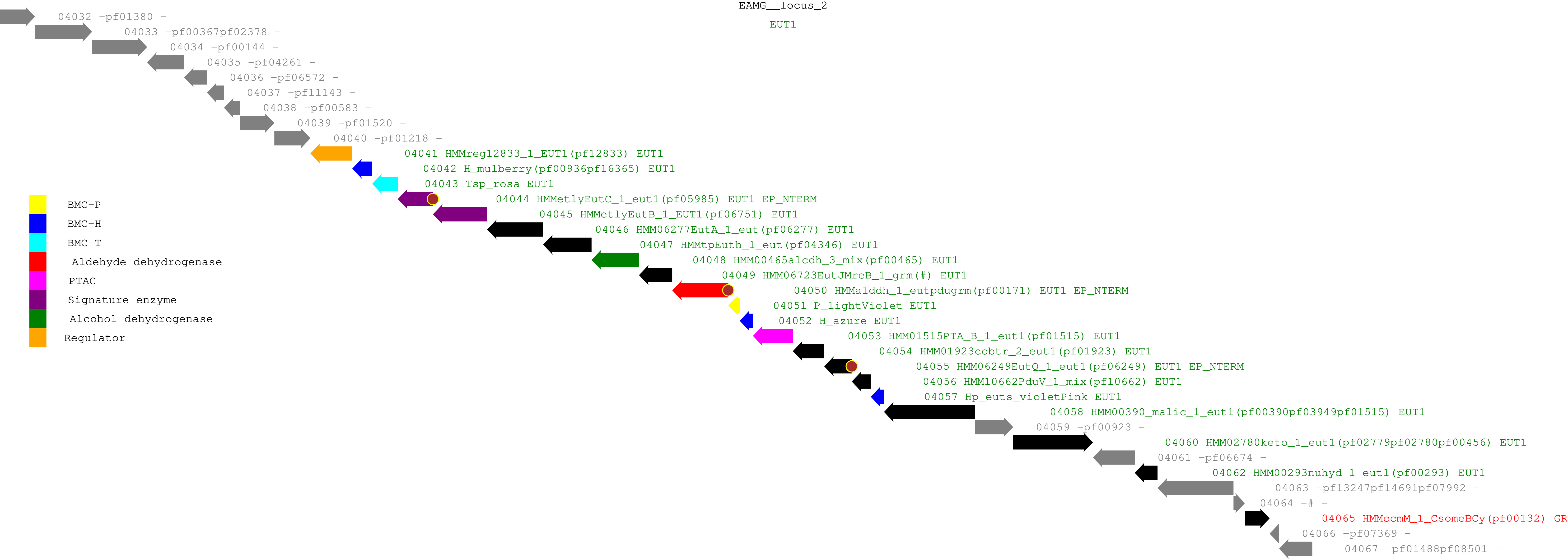

Escherichia albertii B156. Bacteria; Proteobacteria; Gammaproteobacteria; Enterobacterales;

EAPG__locus_2

EUT1

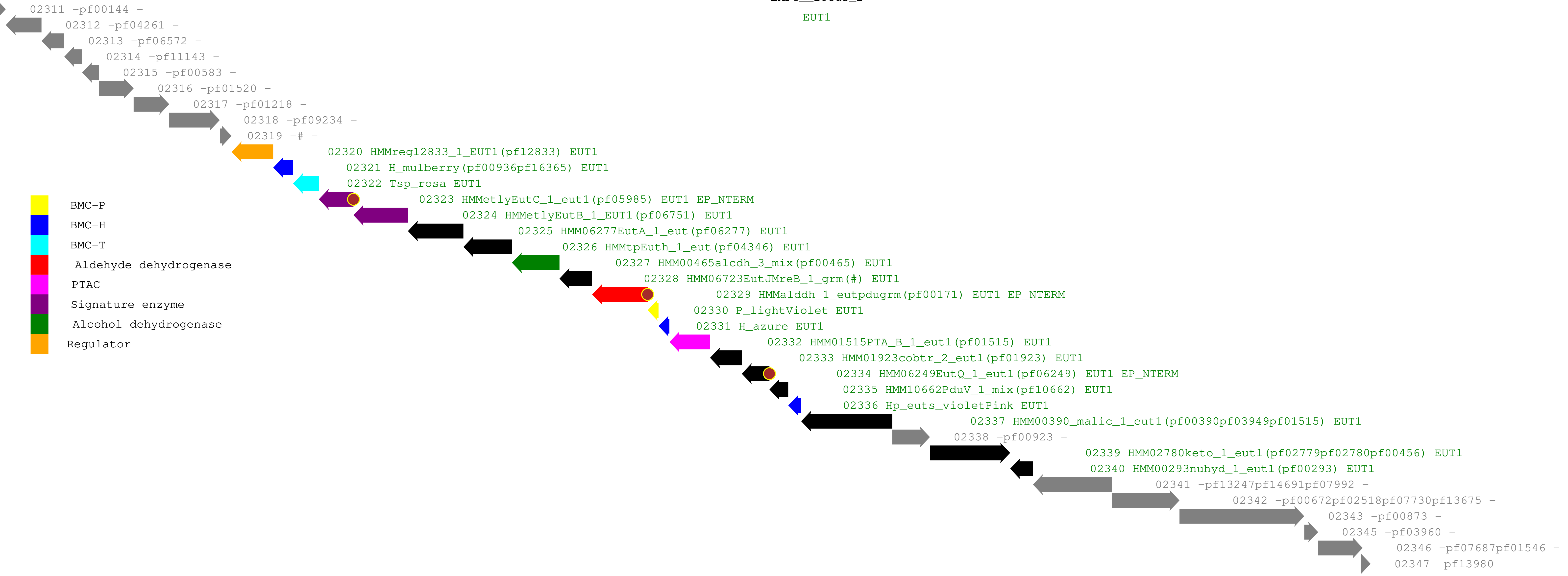

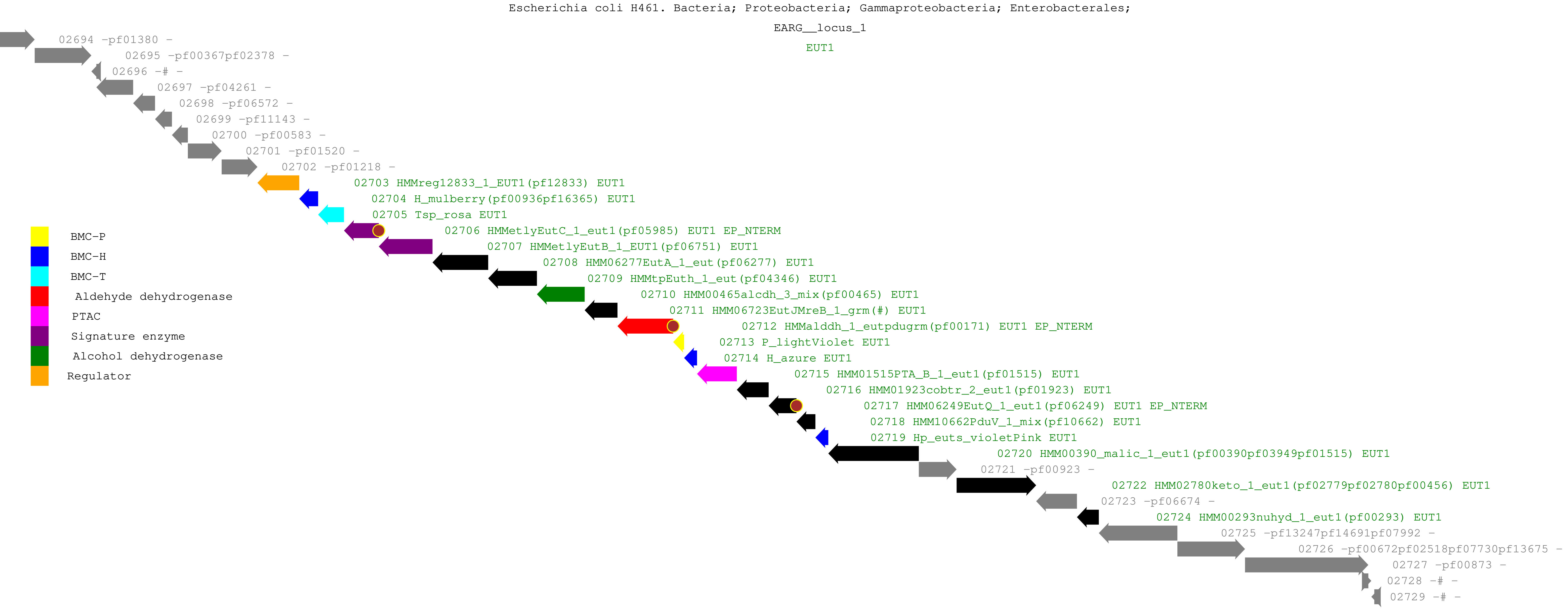

Escherichia coli H461. Bacteria; Proteobacteria; Gammaproteobacteria; Enterobacterales;
EARG__locus_1
EUT1
02694 -pf01380 -
02695 -pf00367pf02378 -
02696 -# -
02697 -pf04261 -
02698 -pf06572 -
02699 -pf11143 -
02700 -pf00583 -
02701 -pf01520 -
02702 -pf01218 -
02703 HMMreg12833_1_EUT1(pf12833) EUT1
02704 H_mulberry(pf00936pf16365) EUT1
02705 Tsp_rosa EUT1
02706 HMMetlyEutC_1_eut1(pf05985) EUT1 EP_NTERM
02707 HMMetlyEutB_1_EUT1(pf06751) EUT1
02708 HMM06277EutA_1_eut(pf06277) EUT1
02709 HMMtpEuth_1_eut(pf04346) EUT1
02710 HMM00465alcdh_3_mix(pf00465) EUT1
02711 HMM06723EutJMreB_1_grm(#) EUT1
02712 HMMalddh_1_eutpdugrm(pf00171) EUT1 EP_NTERM
02713 P_lightViolet EUT1
02714 H_azure EUT1
02715 HMM01515PTA_B_1_eut1(pf01515) EUT1
02716 HMM01923cobtr_2_eut1(pf01923) EUT1
02717 HMM06249EutQ_1_eut1(pf06249) EUT1 EP_NTERM
02718 HMM10662PduV_1_mix(pf10662) EUT1
02719 Hp_euts_violetPink EUT1
02720 HMM00390_malic_1_eut1(pf00390pf03949pf01515) EUT1
02721 -pf00923 -
02722 HMM02780keto_1_eut1(pf02779pf02780pf00456) EUT1
02723 -pf06674 -
02724 HMM00293nuhyd_1_eut1(pf00293) EUT1
02725 -pf13247pf14691pf07992 -
02726 -pf00672pf02518pf07730pf13675 -
02727 -pf00873 -
02728 -# -
02729 -# -
BMC-P
BMC-H
BMC-T
Aldehyde dehydrogenase
PTAC
Signature enzyme
Alcohol dehydrogenase
Regulator

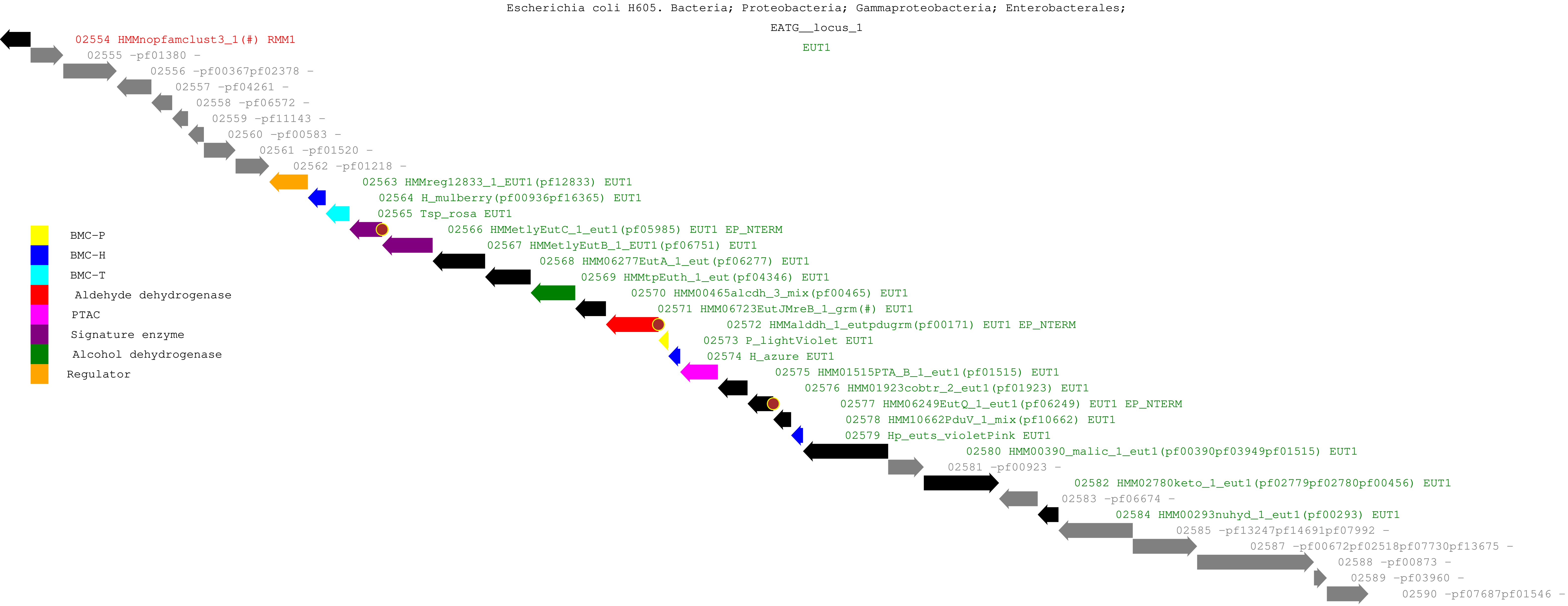

Escherichia coli H605. Bacteria; Proteobacteria; Gammaproteobacteria; Enterobacterales;
EATG__locus_1
EUT1
02554 HMMnopfamclust3_1(#) RMM1
02555 -pf01380 -
02556 -pf00367pf02378 -
02557 -pf04261 -
02558 -pf06572 -
02559 -pf11143 -
02560 -pf00583 -
02561 -pf01520 -
02562 -pf01218 -
02563 HMMreg12833_1_EUT1(pf12833) EUT1
02564 H_mulberry(pf00936pf16365) EUT1
02565 Tsp_rosa EUT1
02566 HMMetlyEutC_1_eut1(pf05985) EUT1 EP_NTERM
02567 HMMetlyEutB_1_EUT1(pf06751) EUT1
02568 HMM06277EutA_1_eut(pf06277) EUT1
02569 HMMtpEuth_1_eut(pf04346) EUT1
02570 HMM00465alcdh_3_mix(pf00465) EUT1
02571 HMM06723EutJMreB_1_grm(#) EUT1
02572 HMMalddh_1_eutpdugrm(pf00171) EUT1 EP_NTERM
02573 P_lightViolet EUT1
02574 H_azure EUT1
02575 HMM01515PTA_B_1_eut1(pf01515) EUT1
02576 HMM01923cobtr_2_eut1(pf01923) EUT1
02577 HMM06249EutQ_1_eut1(pf06249) EUT1 EP_NTERM
02578 HMM10662PduV_1_mix(pf10662) EUT1
02579 Hp_euts_violetPink EUT1
02580 HMM00390_malic_1_eut1(pf00390pf03949pf01515) EUT1
02581 -pf00923 -
02582 HMM02780keto_1_eut1(pf02779pf02780pf00456) EUT1
02583 -pf06674 -
02584 HMM00293nuhyd_1_eut1(pf00293) EUT1
02585 -pf13247pf14691pf07992 -
02587 -pf00672pf02518pf07730pf13675 -
02588 -pf00873 -
02589 -pf03960 -
02590 -pf07687pf01546 -
BMC-P
BMC-H
BMC-T
Aldehyde dehydrogenase
PTAC
Signature enzyme
Alcohol dehydrogenase
Regulator

Escherichia coli H420. Bacteria; Proteobacteria; Gammaproteobacteria; Enterobacterales;

EAVG__locus_1

EUT1

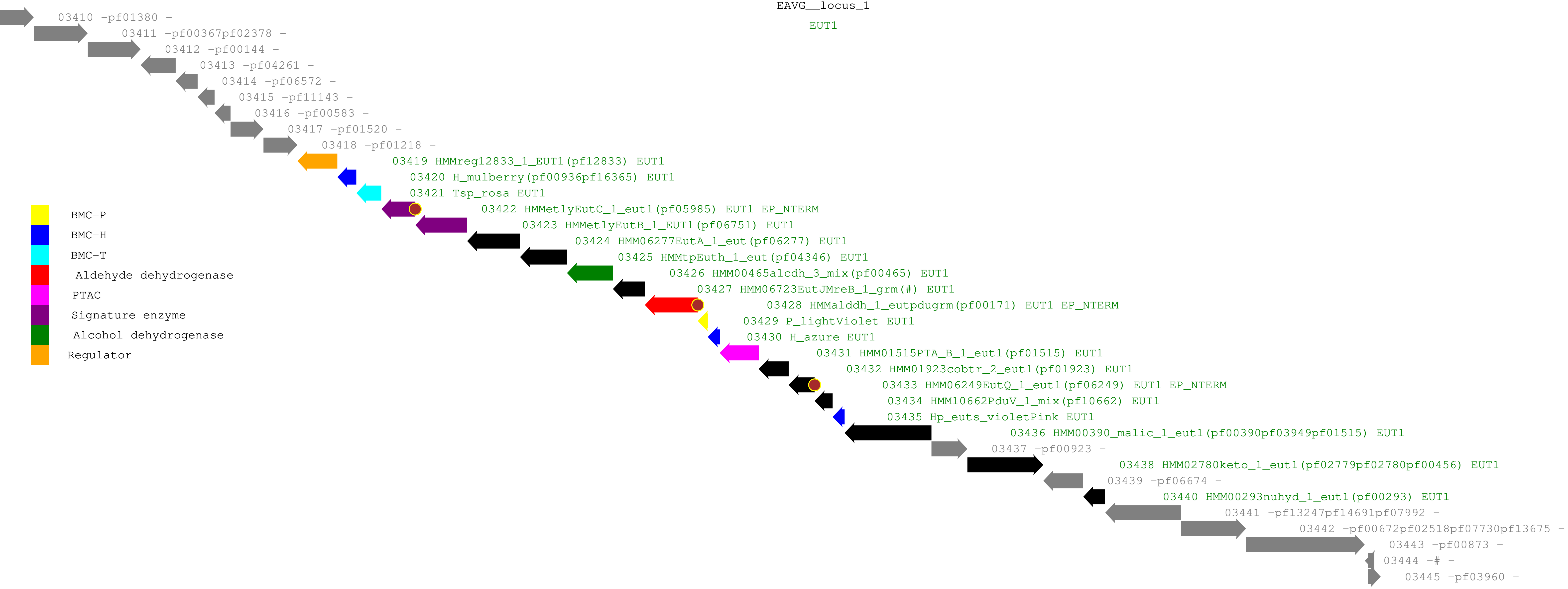

Escherichia coli TA054. Bacteria; Proteobacteria; Gammaproteobacteria; Enterobacterales;

EAXG__locus_1

EUT1

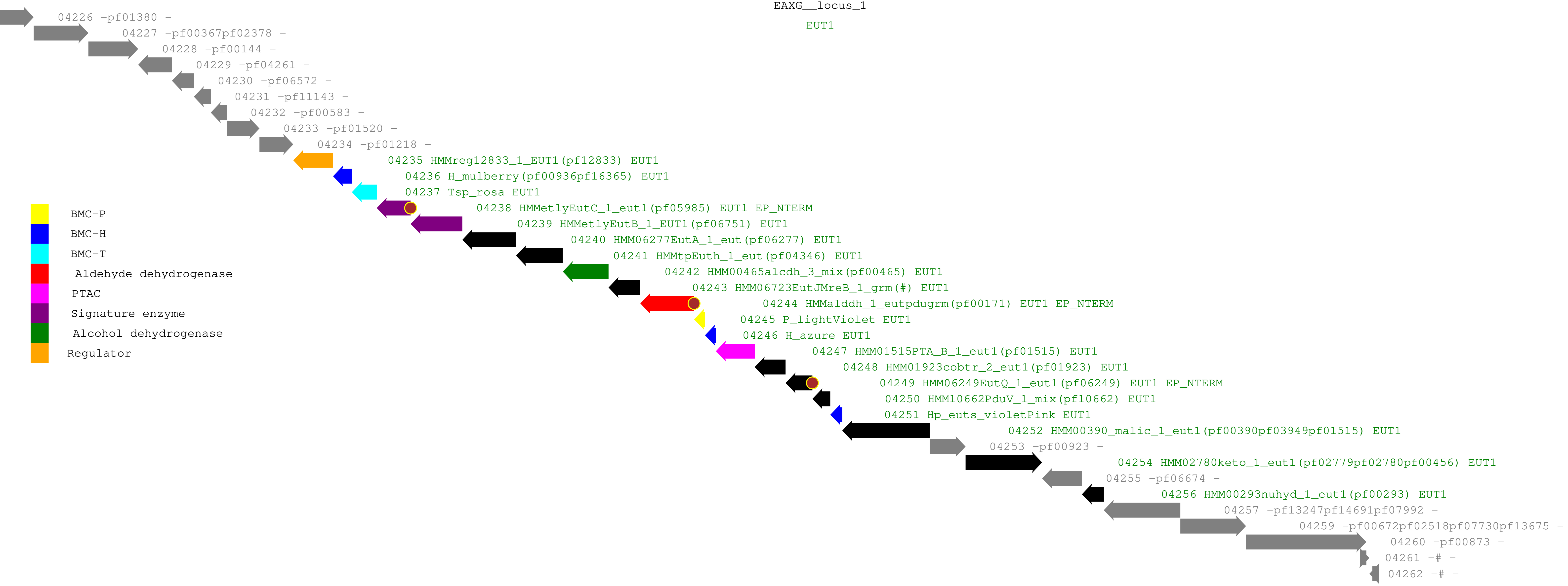

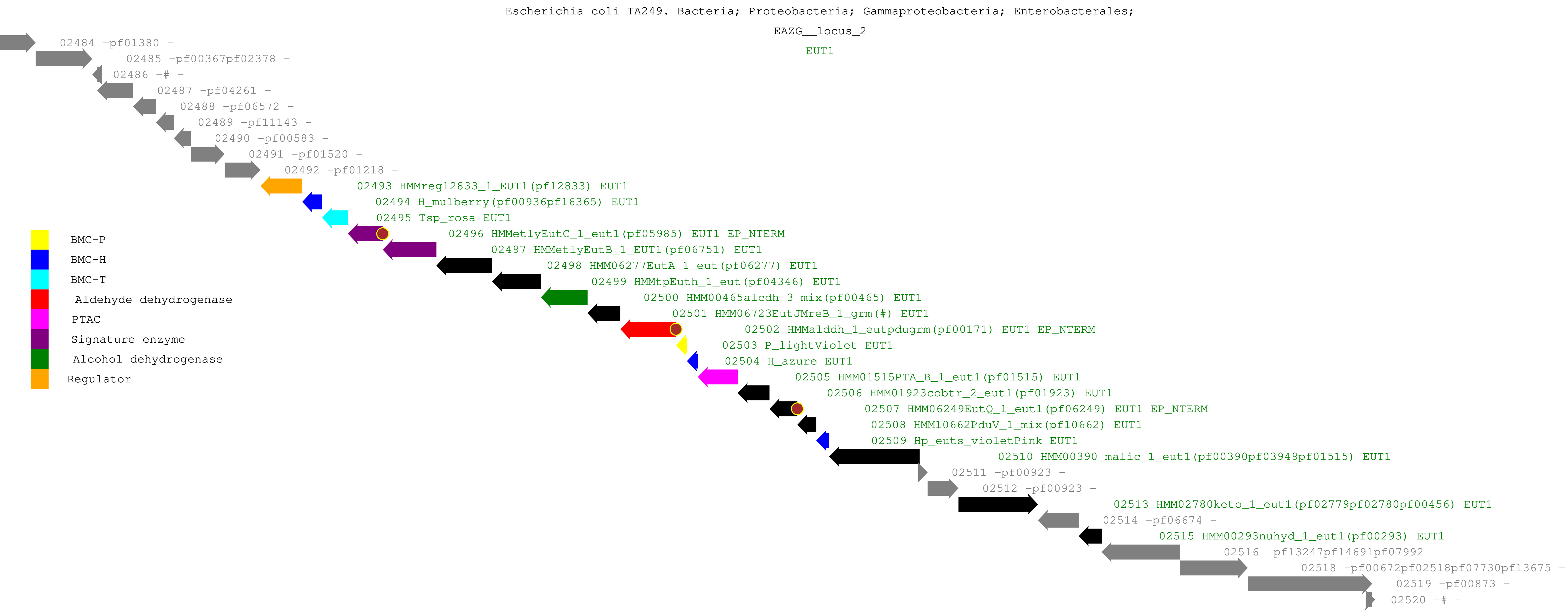
Escherichia coli TA249. Bacteria; Proteobacteria; Gammaproteobacteria; Enterobacterales;
EAZG__locus_2
EUT1
02484 -pf01380 -
02485 -pf00367pf02378 -
02486 -# -
02487 -pf04261 -
02488 -pf06572 -
02489 -pf11143 -
02490 -pf00583 -
02491 -pf01520 -
02492 -pf01218 -
02493 HMMreg12833_1_EUT1(pf12833) EUT1
02494 H_mulberry(pf00936pf16365) EUT1
02495 Tsp_rosa EUT1
02496 HMMetlyEutC_1_eut1(pf05985) EUT1 EP_NTERM
02497 HMMetlyEutB_1_EUT1(pf06751) EUT1
02498 HMM06277EutA_1_eut(pf06277) EUT1
02499 HMMtpEuth_1_eut(pf04346) EUT1
02500 HMM00465alcdh_3_mix(pf00465) EUT1
02501 HMM06723EutJMreB_1_grm(#) EUT1
02502 HMMalddh_1_eutpdugrm(pf00171) EUT1 EP_NTERM
02503 P_lightViolet EUT1
02504 H_azure EUT1
02505 HMM01515PTA_B_1_eut1(pf01515) EUT1
02506 HMM01923cobtr_2_eut1(pf01923) EUT1
02507 HMM06249EutQ_1_eut1(pf06249) EUT1 EP_NTERM
02508 HMM10662PduV_1_mix(pf10662) EUT1
02509 Hp_euts_violetPink EUT1
02510 HMM00390_malic_1_eut1(pf00390pf03949pf01515) EUT1
02511 -pf00923 -
02512 -pf00923 -
02513 HMM02780keto_1_eut1(pf02779pf02780pf00456) EUT1
02514 -pf06674 -
02515 HMM00293nuhyd_1_eut1(pf00293) EUT1
02516 -pf13247pf14691pf07992 -
02518 -pf00672pf02518pf07730pf13675 -
02519 -pf00873 -
02520 -# -
BMC-P
BMC-H
BMC-T
Aldehyde dehydrogenase
PTAC
Signature enzyme
Alcohol dehydrogenase
Regulator

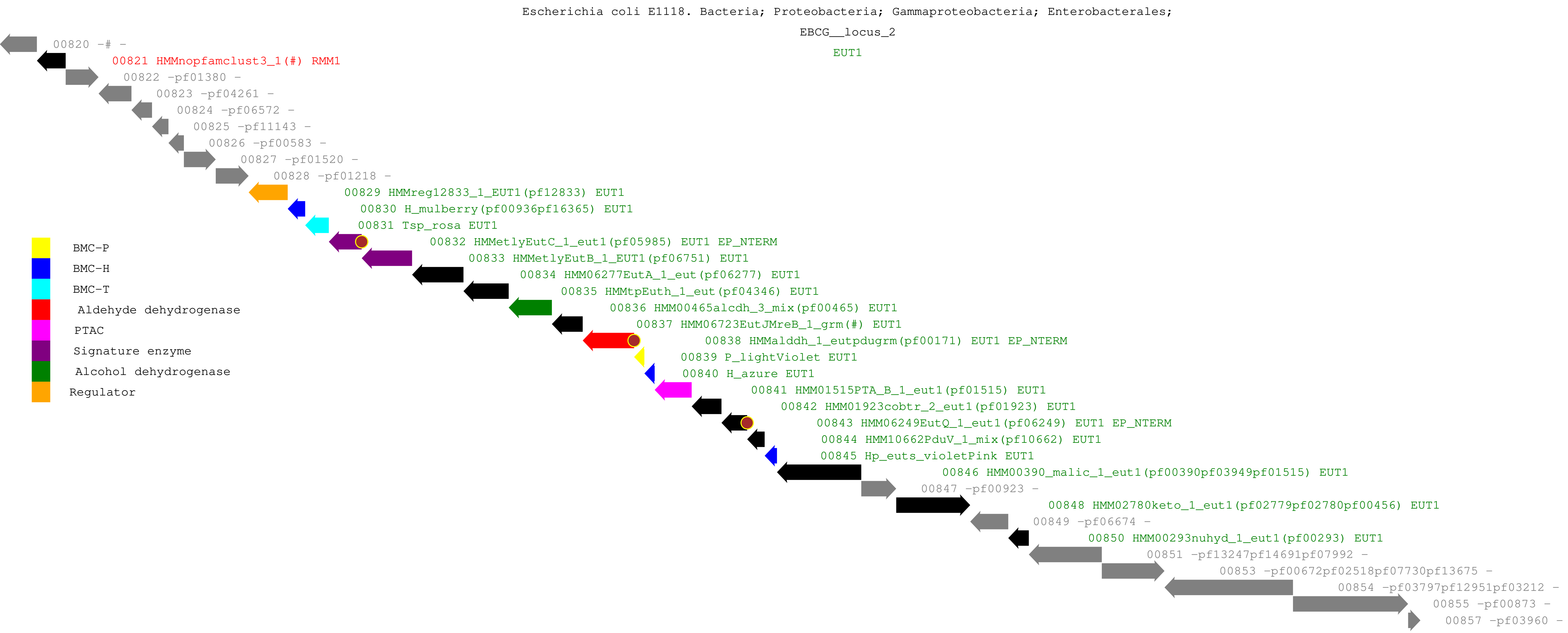

Escherichia coli E1118. Bacteria; Proteobacteria; Gammaproteobacteria; Enterobacterales;
EBCG__locus_2
EUT1
00820 -# -
00821 HMMnopfamclust3_1(#) RMM1
00822 -pf01380 -
00823 -pf04261 -
00824 -pf06572 -
00825 -pf11143 -
00826 -pf00583 -
00827 -pf01520 -
00828 -pf01218 -
00829 HMMreg12833_1_EUT1(pf12833) EUT1
00830 H_mulberry(pf00936pf16365) EUT1
00831 Tsp_rosa EUT1
00832 HMMetlyEutC_1_eut1(pf05985) EUT1 EP_NTERM
00833 HMMetlyEutB_1_EUT1(pf06751) EUT1
00834 HMM06277EutA_1_eut(pf06277) EUT1
00835 HMMtpEuth_1_eut(pf04346) EUT1
00836 HMM00465alcdh_3_mix(pf00465) EUT1
00837 HMM06723EutJMreB_1_grm(#) EUT1
00838 HMMalddh_1_eutpdugrm(pf00171) EUT1 EP_NTERM
00839 P_lightViolet EUT1
00840 H_azure EUT1
00841 HMM01515PTA_B_1_eut1(pf01515) EUT1
00842 HMM01923cobtr_2_eut1(pf01923) EUT1
00843 HMM06249EutQ_1_eut1(pf06249) EUT1 EP_NTERM
00844 HMM10662PduV_1_mix(pf10662) EUT1
00845 Hp_euts_violetPink EUT1
00846 HMM00390_malic_1_eut1(pf00390pf03949pf01515) EUT1
00847 -pf00923 -
00848 HMM02780keto_1_eut1(pf02779pf02780pf00456) EUT1
00849 -pf06674 -
00850 HMM00293nuhyd_1_eut1(pf00293) EUT1
00851 -pf13247pf14691pf07992 -
00853 -pf00672pf02518pf07730pf13675 -
00854 -pf03797pf12951pf03212 -
00855 -pf00873 -
00857 -pf03960 -
BMC-P
BMC-H
BMC-T
Aldehyde dehydrogenase
PTAC
Signature enzyme
Alcohol dehydrogenase
Regulator

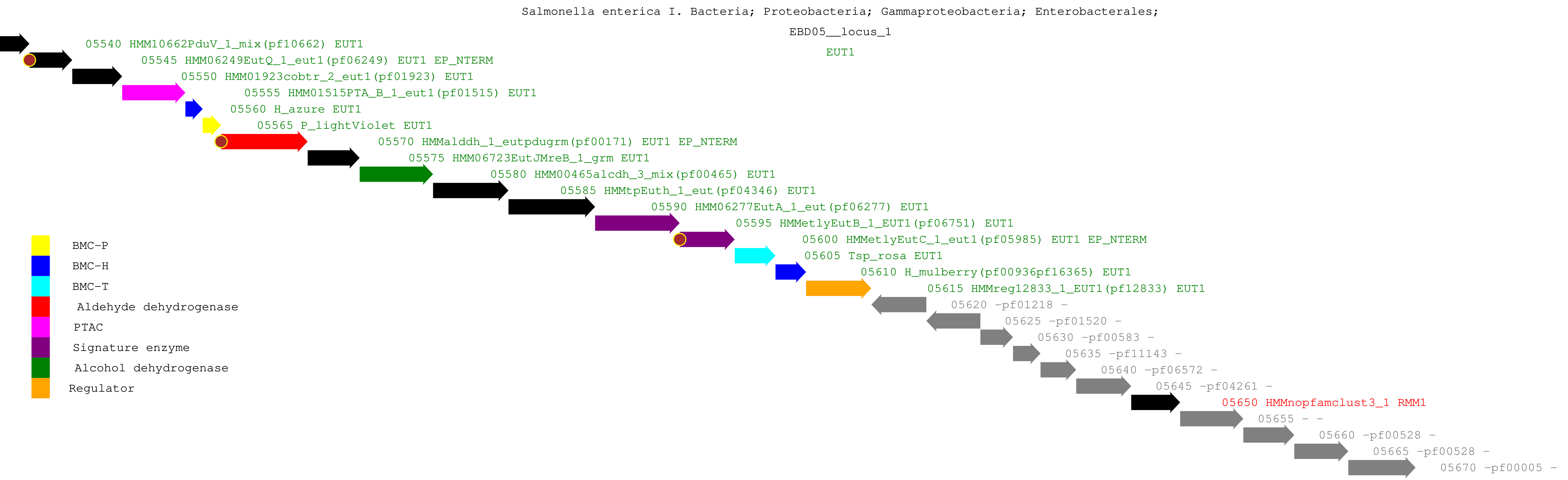

Salmonella enterica I. Bacteria; Proteobacteria; Gammaproteobacteria; Enterobacterales;
EBD05__locus_1
EUT1
05540 HMM10662PduV_1_mix(pf10662) EUT1
05545 HMM06249EutQ_1_eut1(pf06249) EUT1 EP_NTERM
05550 HMM01923cobtr_2_eut1(pf01923) EUT1
05555 HMM01515PTA_B_1_eut1(pf01515) EUT1
05560 H_azure EUT1
05565 P_lightViolet EUT1
05570 HMMalddh_1_eutpdugrm(pf00171) EUT1 EP_NTERM
05575 HMM06723EutJMreB_1_grm EUT1
05580 HMM00465alcdh_3_mix(pf00465) EUT1
05585 HMMtpEuth_1_eut(pf04346) EUT1
05590 HMM06277EutA_1_eut(pf06277) EUT1
05595 HMMetlyEutB_1_EUT1(pf06751) EUT1
05600 HMMetlyEutC_1_eut1(pf05985) EUT1 EP_NTERM
05605 Tsp_rosa EUT1
05610 H_mulberry(pf00936pf16365) EUT1
05615 HMMreg12833_1_EUT1(pf12833) EUT1
05620 -pf01218 -
05625 -pf01520 -
05630 -pf00583 -
05635 -pf11143 -
05640 -pf06572 -
05645 -pf04261 -
05650 HMMnopfamclust3_1 RMM1
05655 - -
05660 -pf00528 -
05665 -pf00528 -
05670 -pf00005 -
BMC-P
BMC-H
BMC-T
Aldehyde dehydrogenase
PTAC
Signature enzyme
Alcohol dehydrogenase
Regulator

Escherichia coli O44:H18 (strain 042 / EAEC). Bacteria; Proteobacteria; Gammaproteobacteria; Enterobacterales;

EC042__locus_1

EUT1

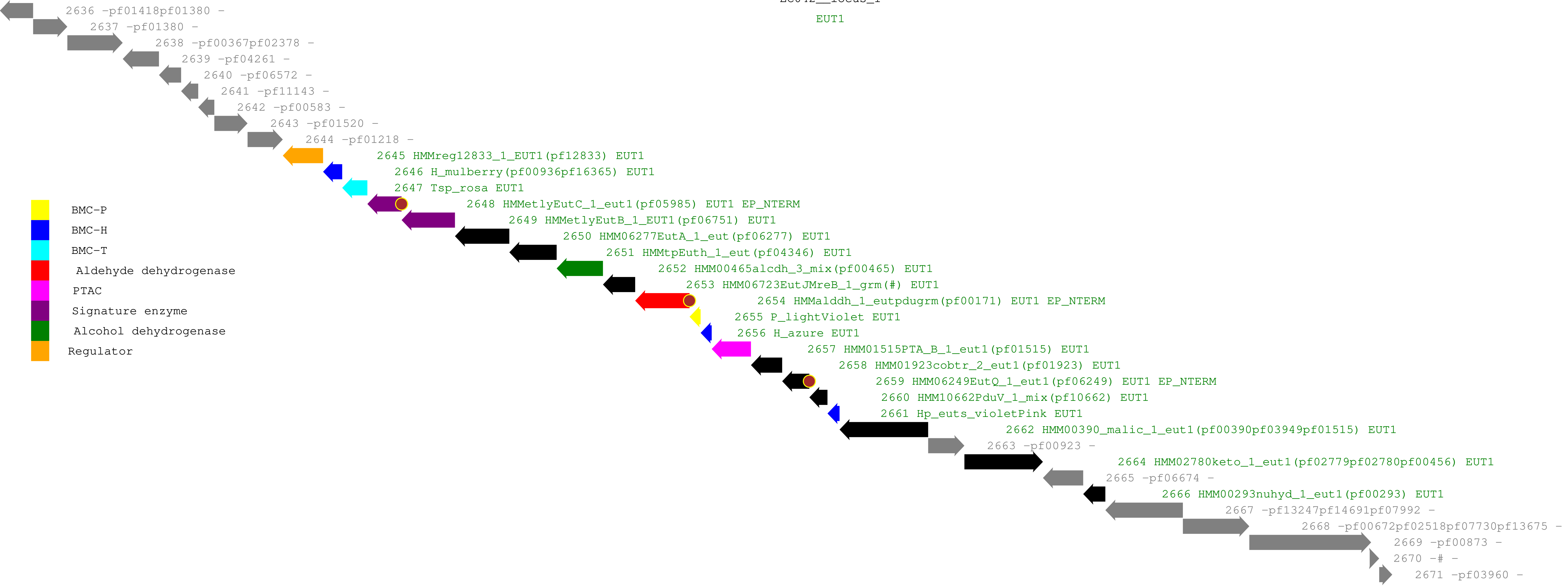

Escherichia coli O145:H28. Bacteria; Proteobacteria; Gammaproteobacteria; Enterobacterales;

EC10942__locus_1

EUT1

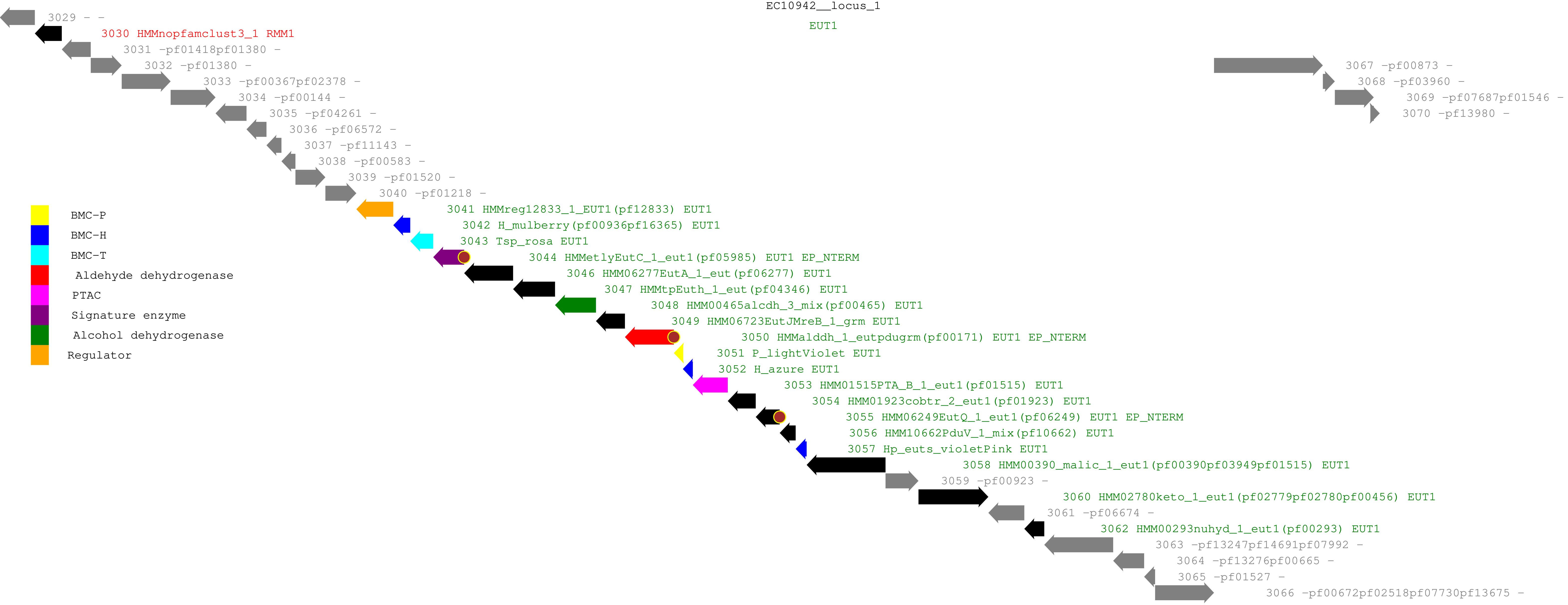

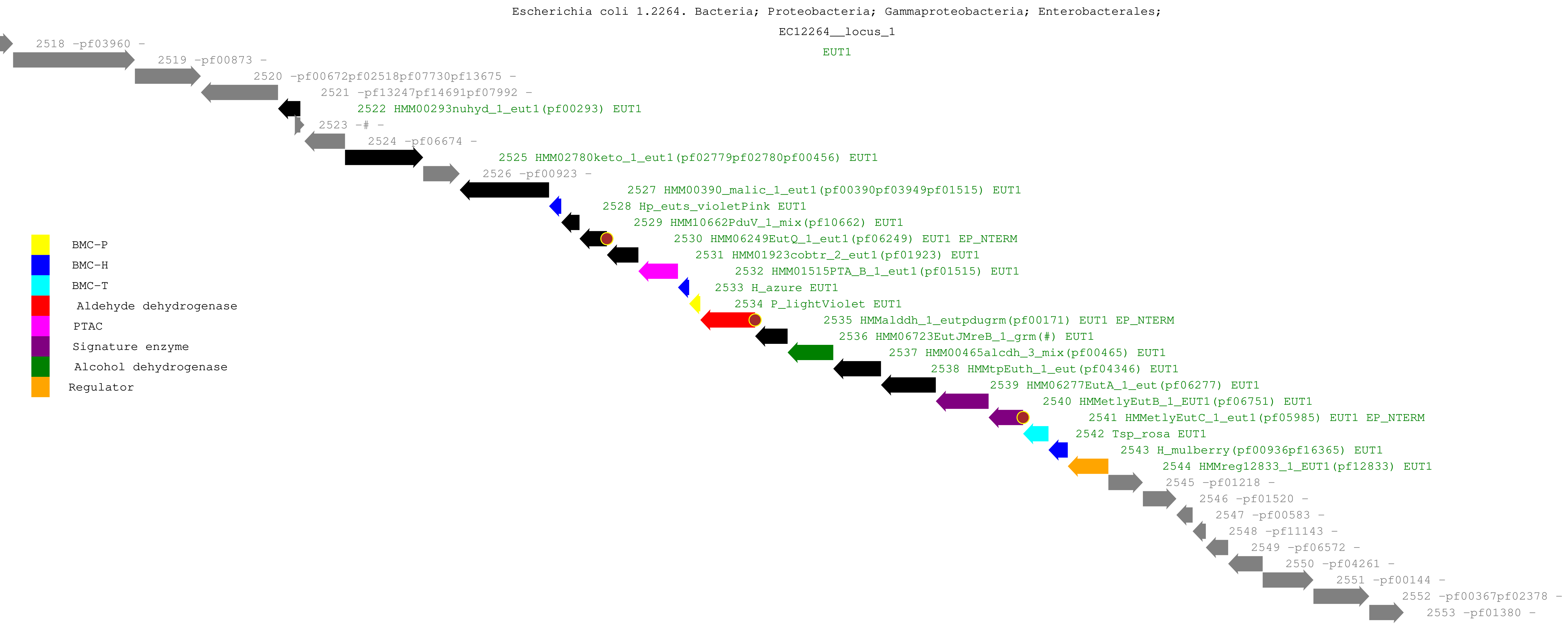
Escherichia coli 1.2264. Bacteria; Proteobacteria; Gammaproteobacteria; Enterobacterales;
EC12264__locus_1
EUT1
2518 -pf03960 -
2519 -pf00873 -
2520 -pf00672pf02518pf07730pf13675 -
2521 -pf13247pf14691pf07992 -
2522 HMM00293nuhyd_1_eut1(pf00293) EUT1
2523 -# -
2524 -pf06674 -
2525 HMM02780keto_1_eut1(pf02779pf02780pf00456) EUT1
2526 -pf00923 -
2527 HMM00390_malic_1_eut1(pf00390pf03949pf01515) EUT1
2528 Hp_euts_violetPink EUT1
2529 HMM10662PduV_1_mix(pf10662) EUT1
2530 HMM06249EutQ_1_eut1(pf06249) EUT1 EP_NTERM
2531 HMM01923cobtr_2_eut1(pf01923) EUT1
2532 HMM01515PTA_B_1_eut1(pf01515) EUT1
2533 H_azure EUT1
2534 P_lightViolet EUT1
2535 HMMalddh_1_eutpdugrm(pf00171) EUT1 EP_NTERM
2536 HMM06723EutJMreB_1_grm(#) EUT1
2537 HMM00465alcdh_3_mix(pf00465) EUT1
2538 HMMtpEuth_1_eut(pf04346) EUT1
2539 HMM06277EutA_1_eut(pf06277) EUT1
2540 HMMetlyEutB_1_EUT1(pf06751) EUT1
2541 HMMetlyEutC_1_eut1(pf05985) EUT1 EP_NTERM
2542 Tsp_rosa EUT1
2543 H_mulberry(pf00936pf16365) EUT1
2544 HMMreg12833_1_EUT1(pf12833) EUT1
2545 -pf01218 -
2546 -pf01520 -
2547 -pf00583 -
2548 -pf11143 -
2549 -pf06572 -
2550 -pf04261 -
2551 -pf00144 -
2552 -pf00367pf02378 -
2553 -pf01380 -
BMC-P
BMC-H
BMC-T
Aldehyde dehydrogenase
PTAC
Signature enzyme
Alcohol dehydrogenase
Regulator

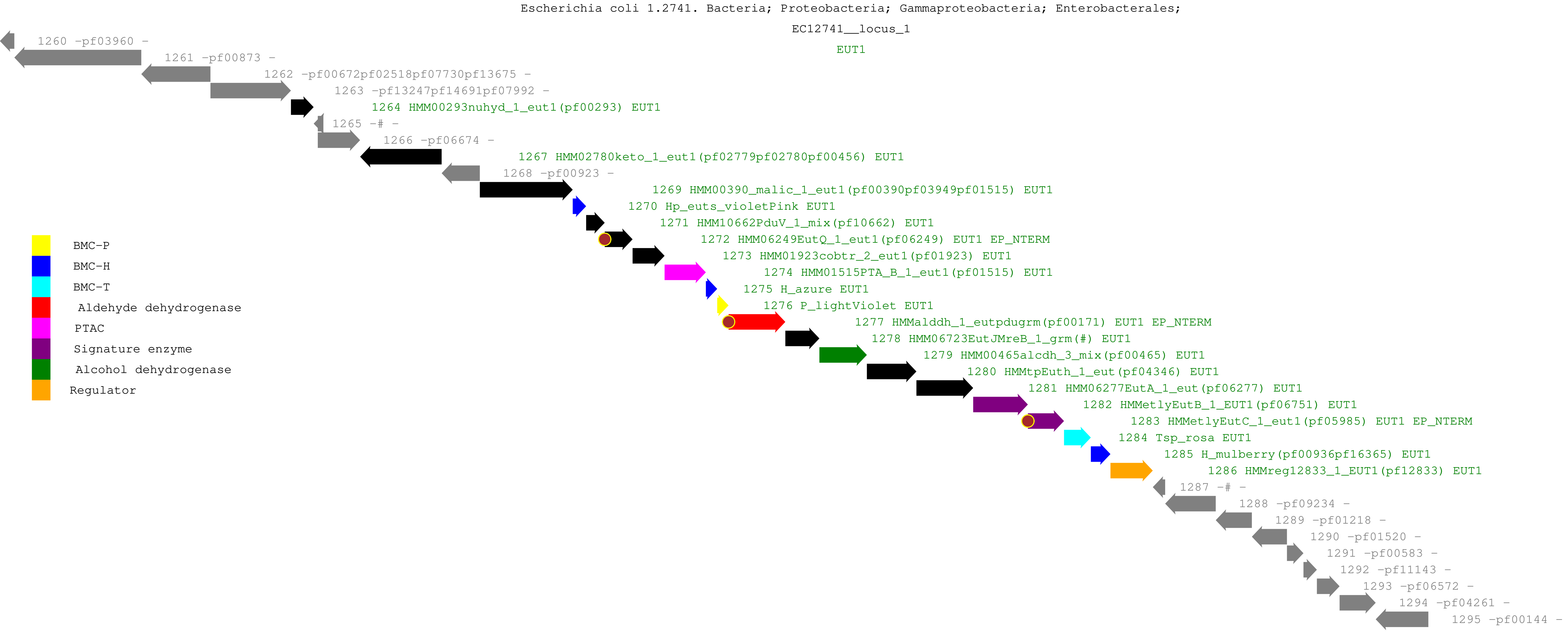
Escherichia coli 1.2741. Bacteria; Proteobacteria; Gammaproteobacteria; Enterobacterales;
EC12741__locus_1
EUT1
1260 -pf03960 -
1261 -pf00873 -
1262 -pf00672pf02518pf07730pf13675 -
1263 -pf13247pf14691pf07992 -
1264 HMM00293nuhyd_1_eut1(pf00293) EUT1
1265 -# -
1266 -pf06674 -
1267 HMM02780keto_1_eut1(pf02779pf02780pf00456) EUT1
1268 -pf00923 -
1269 HMM00390_malic_1_eut1(pf00390pf03949pf01515) EUT1
1270 Hp_euts_violetPink EUT1
1271 HMM10662PduV_1_mix(pf10662) EUT1
1272 HMM06249EutQ_1_eut1(pf06249) EUT1 EP_NTERM
1273 HMM01923cobtr_2_eut1(pf01923) EUT1
1274 HMM01515PTA_B_1_eut1(pf01515) EUT1
1275 H_azure EUT1
1276 P_lightViolet EUT1
1277 HMMalddh_1_eutpdugrm(pf00171) EUT1 EP_NTERM
1278 HMM06723EutJMreB_1_grm(#) EUT1
1279 HMM00465alcdh_3_mix(pf00465) EUT1
1280 HMMtpEuth_1_eut(pf04346) EUT1
1281 HMM06277EutA_1_eut(pf06277) EUT1
1282 HMMetlyEutB_1_EUT1(pf06751) EUT1
1283 HMMetlyEutC_1_eut1(pf05985) EUT1 EP_NTERM
1284 Tsp_rosa EUT1
1285 H_mulberry(pf00936pf16365) EUT1
1286 HMMreg12833_1_EUT1(pf12833) EUT1
1287 -# -
1288 -pf09234 -
1289 -pf01218 -
1290 -pf01520 -
1291 -pf00583 -
1292 -pf11143 -
1293 -pf06572 -
1294 -pf04261 -
1295 -pf00144 -
BMC-P
BMC-H
BMC-T
Aldehyde dehydrogenase
PTAC
Signature enzyme
Alcohol dehydrogenase
Regulator

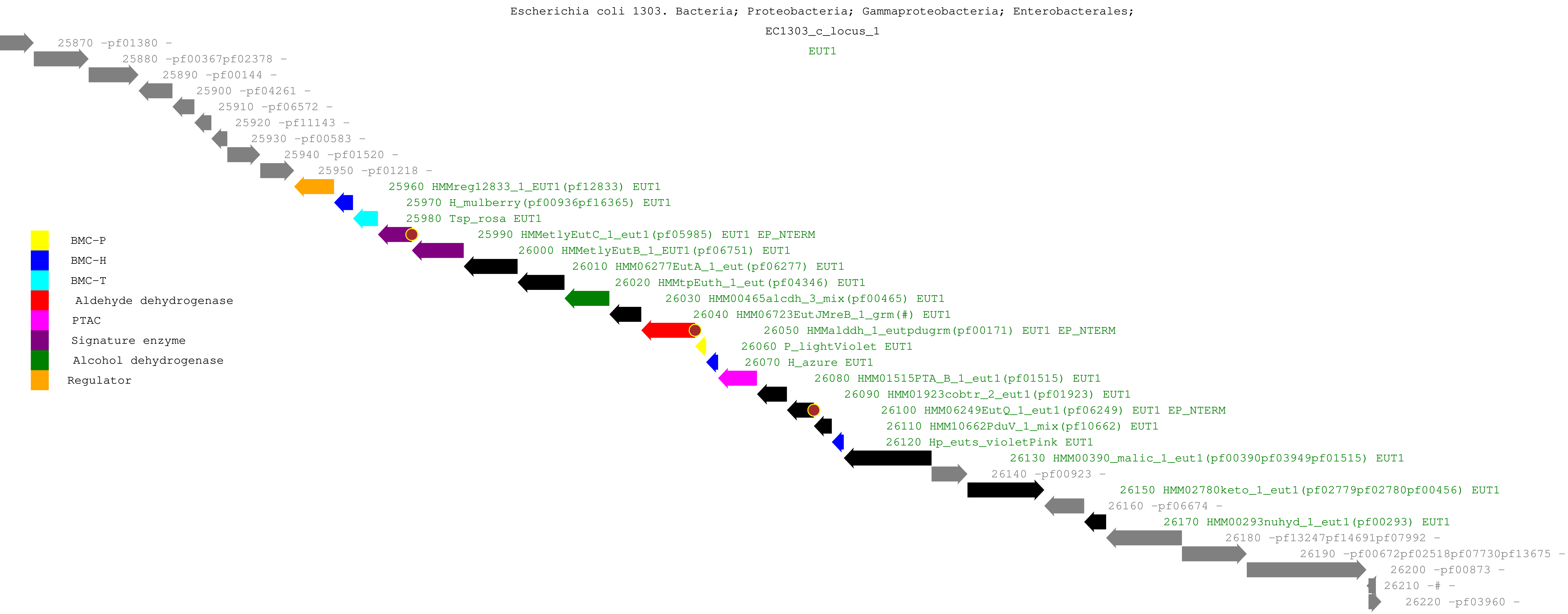
Escherichia coli 1303. Bacteria; Proteobacteria; Gammaproteobacteria; Enterobacterales;
EC1303_c_locus_1
EUT1
25870 -pf01380 -
25880 -pf00367pf02378 -
25890 -pf00144 -
25900 -pf04261 -
25910 -pf06572 -
25920 -pf11143 -
25930 -pf00583 -
25940 -pf01520 -
25950 -pf01218 -
25960 HMMreg12833_1_EUT1(pf12833) EUT1
25970 H_mulberry(pf00936pf16365) EUT1
25980 Tsp_rosa EUT1
25990 HMMetlyEutC_1_eut1(pf05985) EUT1 EP_NTERM
26000 HMMetlyEutB_1_EUT1(pf06751) EUT1
26010 HMM06277EutA_1_eut(pf06277) EUT1
26020 HMMtpEuth_1_eut(pf04346) EUT1
26030 HMM00465alcdh_3_mix(pf00465) EUT1
26040 HMM06723EutJMreB_1_grm(#) EUT1
26050 HMMalddh_1_eutpdugrm(pf00171) EUT1 EP_NTERM
26060 P_lightViolet EUT1
26070 H_azure EUT1
26080 HMM01515PTA_B_1_eut1(pf01515) EUT1
26090 HMM01923cobtr_2_eut1(pf01923) EUT1
26100 HMM06249EutQ_1_eut1(pf06249) EUT1 EP_NTERM
26110 HMM10662PduV_1_mix(pf10662) EUT1
26120 Hp_euts_violetPink EUT1
26130 HMM00390_malic_1_eut1(pf00390pf03949pf01515) EUT1
26140 -pf00923 -
26150 HMM02780keto_1_eut1(pf02779pf02780pf00456) EUT1
26160 -pf06674 -
26170 HMM00293nuhyd_1_eut1(pf00293) EUT1
26180 -pf13247pf14691pf07992 -
26190 -pf00672pf02518pf07730pf13675 -
26200 -pf00873 -
26210 -# -
26220 -pf03960 -
BMC-P
BMC-H
BMC-T
Aldehyde dehydrogenase
PTAC
Signature enzyme
Alcohol dehydrogenase
Regulator

Escherichia coli O145:H28. Bacteria; Proteobacteria; Gammaproteobacteria; Enterobacterales;

EC133313__locus_1

EUT1

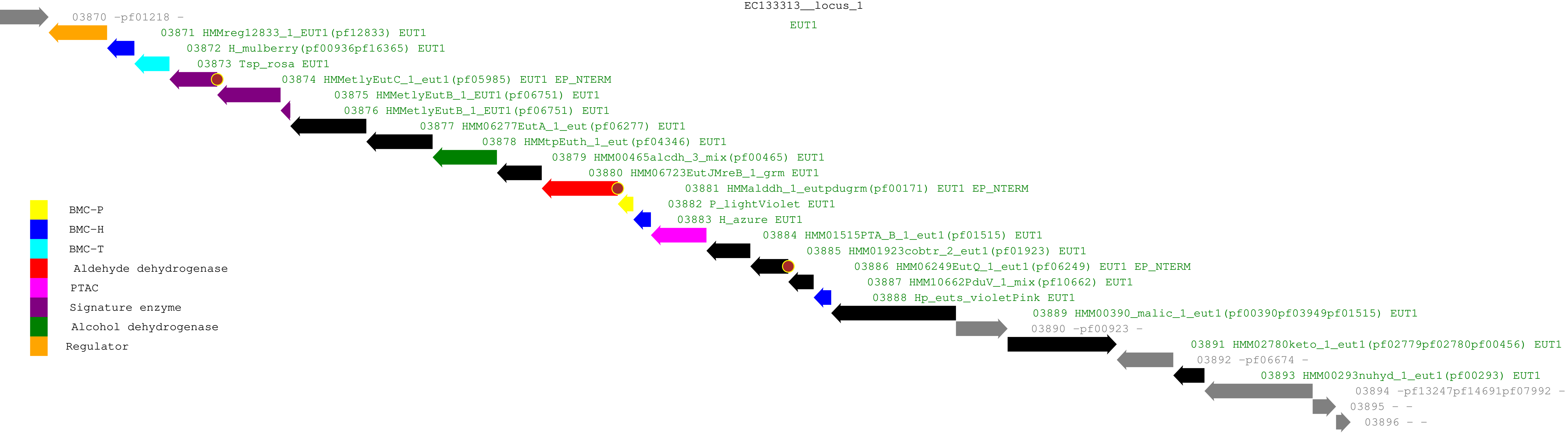

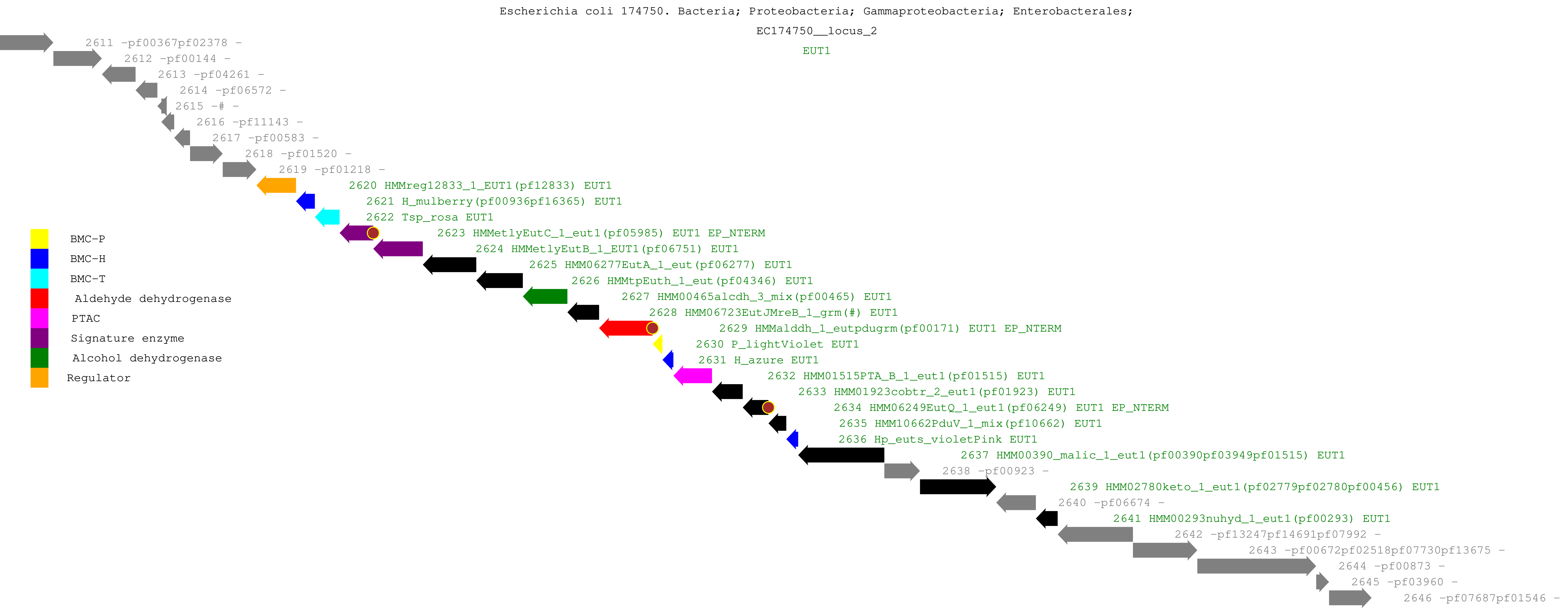
Escherichia coli 174750. Bacteria; Proteobacteria; Gammaproteobacteria; Enterobacterales;
EC174750__locus_2
EUT1
2611 -pf00367pf02378 -
2612 -pf00144 -
2613 -pf04261 -
2614 -pf06572 -
2615 -# -
2616 -pf11143 -
2617 -pf00583 -
2618 -pf01520 -
2619 -pf01218 -
2620 HMMreg12833_1_EUT1(pf12833) EUT1
2621 H_mulberry(pf00936pf16365) EUT1
2622 Tsp_rosa EUT1
2623 HMMetlyEutC_1_eut1(pf05985) EUT1 EP_NTERM
2624 HMMetlyEutB_1_EUT1(pf06751) EUT1
2625 HMM06277EutA_1_eut(pf06277) EUT1
2626 HMMtpEuth_1_eut(pf04346) EUT1
2627 HMM00465alcdh_3_mix(pf00465) EUT1
2628 HMM06723EutJMreB_1_grm(#) EUT1
2629 HMMaldh_1_eutpdugrm(pf00171) EUT1 EP_NTERM
2630 P_lightViolet EUT1
2631 H_azure EUT1
2632 HMM01515PTA_B_1_eut1(pf01515) EUT1
2633 HMM01923cobtr_2_eut1(pf01923) EUT1
2634 HMM06249EutQ_1_eut1(pf06249) EUT1 EP_NTERM
2635 HMM10662PduV_1_mix(pf10662) EUT1
2636 Hp_euts_violetPink EUT1
2637 HMM00390_malic_1_eut1(pf00390pf03949pf01515) EUT1
2638 -pf00923 -
2639 HMM02780keto_1_eut1(pf02779pf02780pf00456) EUT1
2640 -pf06674 -
2641 HMM00293nuhyd_1_eut1(pf00293) EUT1
2642 -pf13247pf14691pf07992 -
2643 -pf00672pf02518pf07730pf13675 -
2644 -pf00873 -
2645 -pf03960 -
2646 -pf07687pf01546 -
BMC-P
BMC-H
BMC-T
Aldehyde dehydrogenase
PTAC
Signature enzyme
Alcohol dehydrogenase
Regulator

Escherichia coli 2362-75. Bacteria; Proteobacteria; Gammaproteobacteria; Enterobacterales;

EC236275__locus_2

EUT1

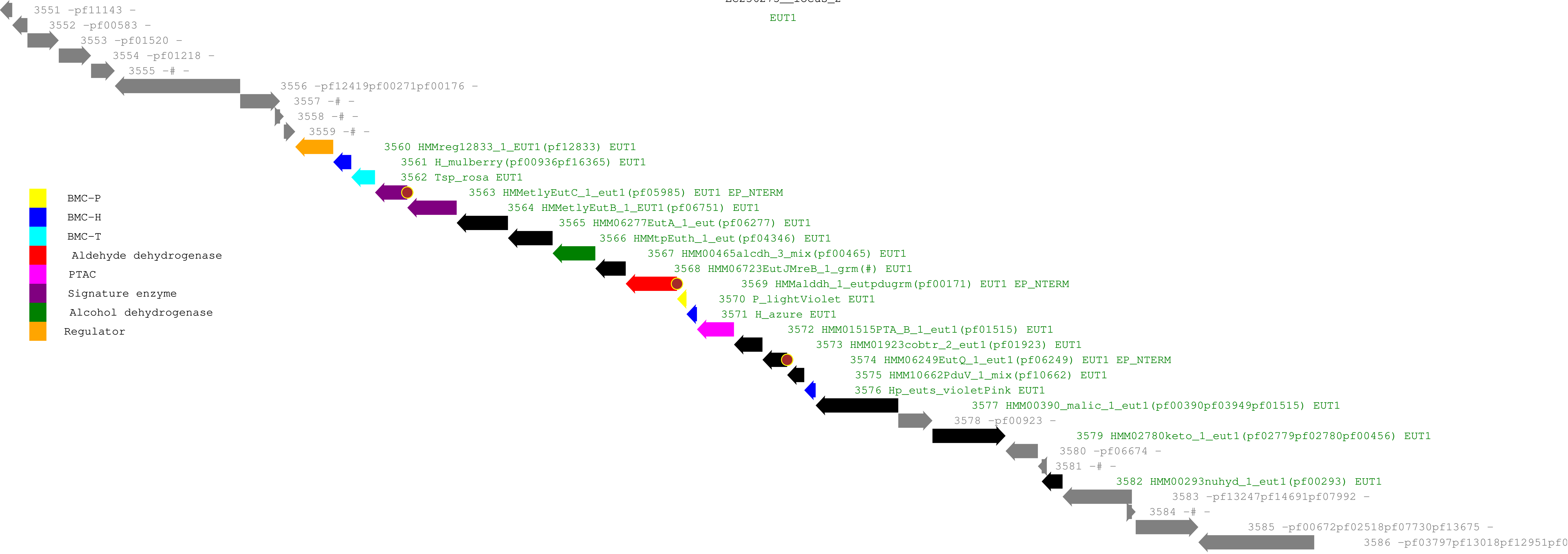

Escherichia coli 2.3916. Bacteria; Proteobacteria; Gammaproteobacteria; Enterobacterales;

EC23916__locus_1

EUT1

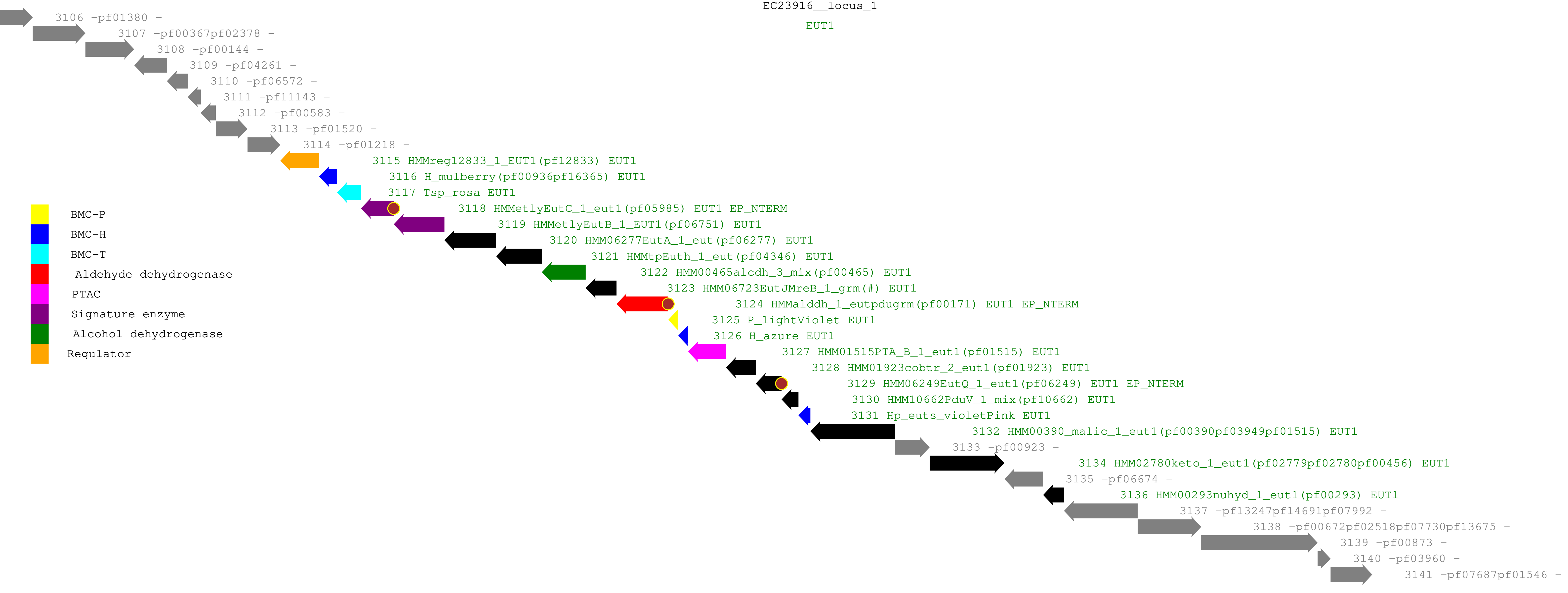

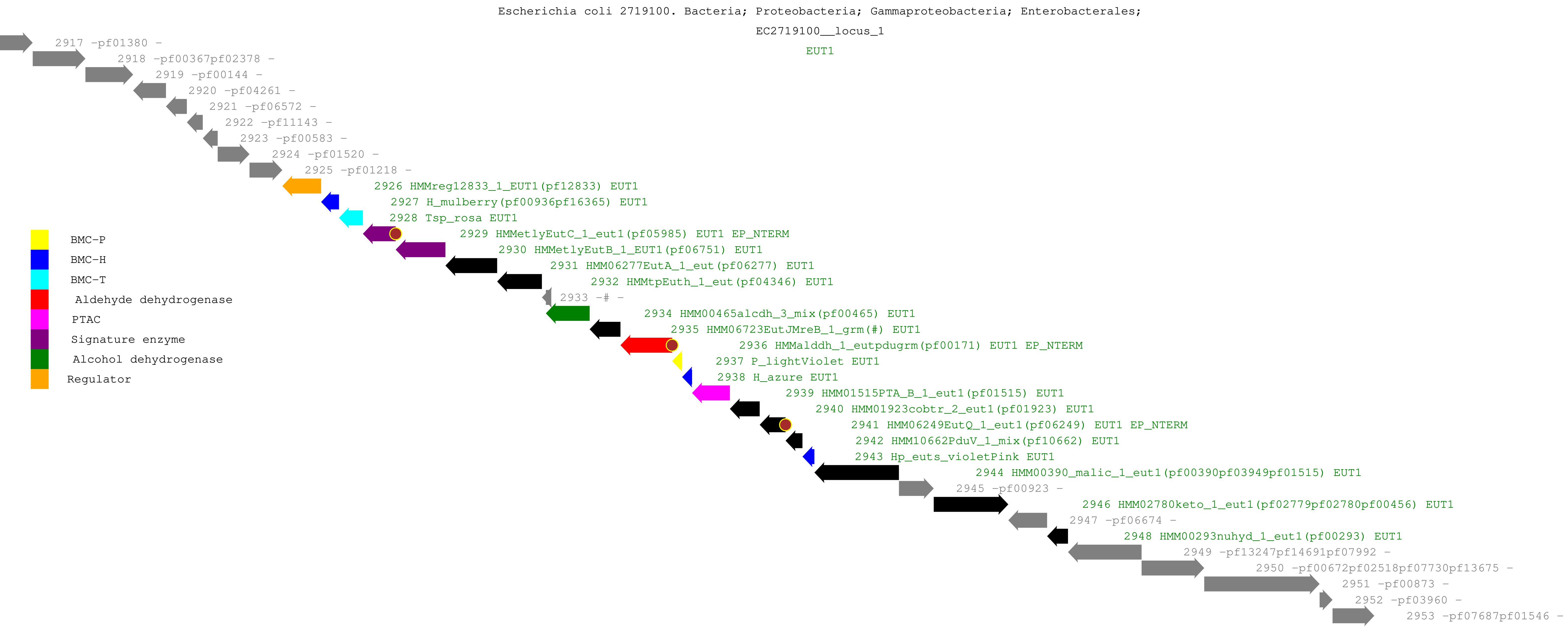

Escherichia coli 2719100. Bacteria; Proteobacteria; Gammaproteobacteria; Enterobacterales;
EC2719100__locus_1
EUT1
2917 -pf01380 -
2918 -pf00367pf02378 -
2919 -pf00144 -
2920 -pf04261 -
2921 -pf06572 -
2922 -pf11143 -
2923 -pf00583 -
2924 -pf01520 -
2925 -pf01218 -
2926 HMMreg12833_1_EUT1(pf12833) EUT1
2927 H_mulberry(pf00936pf16365) EUT1
2928 Tsp_rosa EUT1
2929 HMMetlyEutC_1_eut1(pf05985) EUT1 EP_NTERM
2930 HMMetlyEutB_1_EUT1(pf06751) EUT1
2931 HMM06277EutA_1_eut(pf06277) EUT1
2932 HMMtpEuth_1_eut(pf04346) EUT1
2933 -# -
2934 HMM00465alcdh_3_mix(pf00465) EUT1
2935 HMM06723EutJMreB_1_grm(#) EUT1
2936 HMMaldh_1_eutpdugrm(pf00171) EUT1 EP_NTERM
2937 P_lightViolet EUT1
2938 H_azure EUT1
2939 HMM01515PTA_B_1_eut1(pf01515) EUT1
2940 HMM01923cobtr_2_eut1(pf01923) EUT1
2941 HMM06249EutQ_1_eut1(pf06249) EUT1 EP_NTERM
2942 HMM10662PduV_1_mix(pf10662) EUT1
2943 Hp_euts_violetPink EUT1
2944 HMM00390_malic_1_eut1(pf00390pf03949pf01515) EUT1
2945 -pf00923 -
2946 HMM02780keto_1_eut1(pf02779pf02780pf00456) EUT1
2947 -pf06674 -
2948 HMM00293nuhyd_1_eut1(pf00293) EUT1
2949 -pf13247pf14691pf07992 -
2950 -pf00672pf02518pf07730pf13675 -
2951 -pf00873 -
2952 -pf03960 -
2953 -pf07687pf01546 -
BMC-P
BMC-H
BMC-T
Aldehyde dehydrogenase
PTAC
Signature enzyme
Alcohol dehydrogenase
Regulator

Escherichia coli 3.4880. Bacteria; Proteobacteria; Gammaproteobacteria; Enterobacterales;

EC34880__locus_1

EUT1

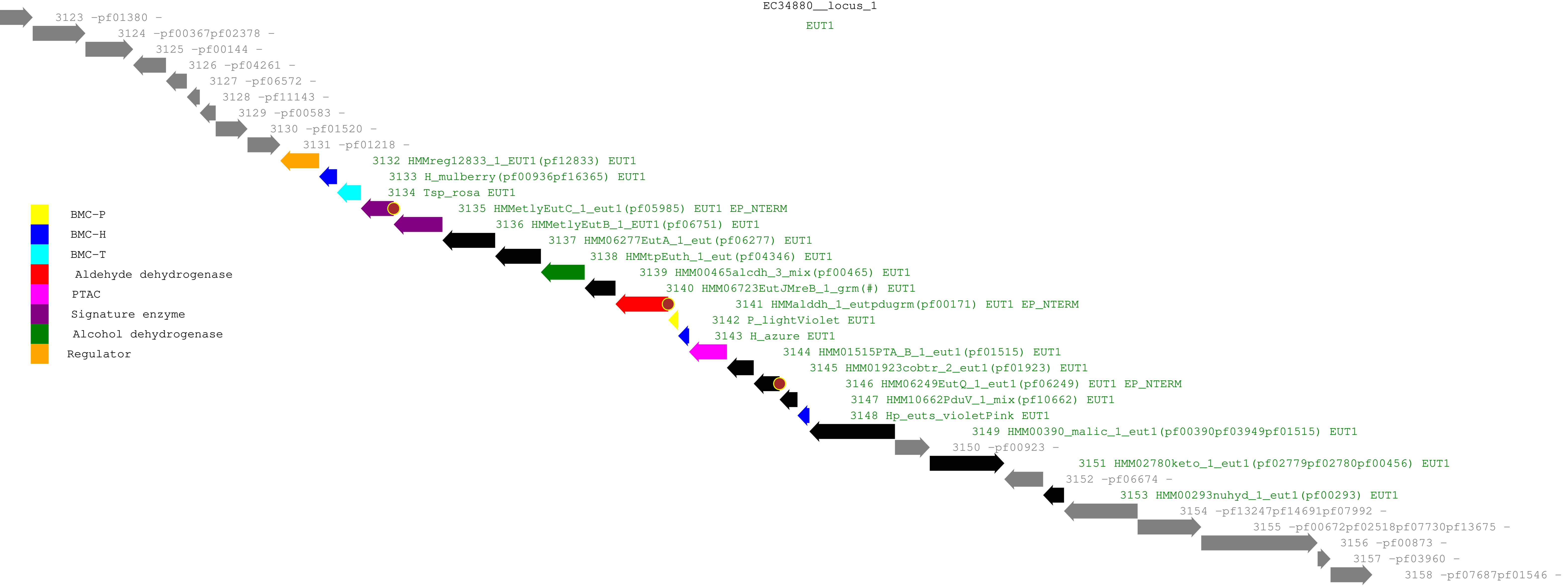

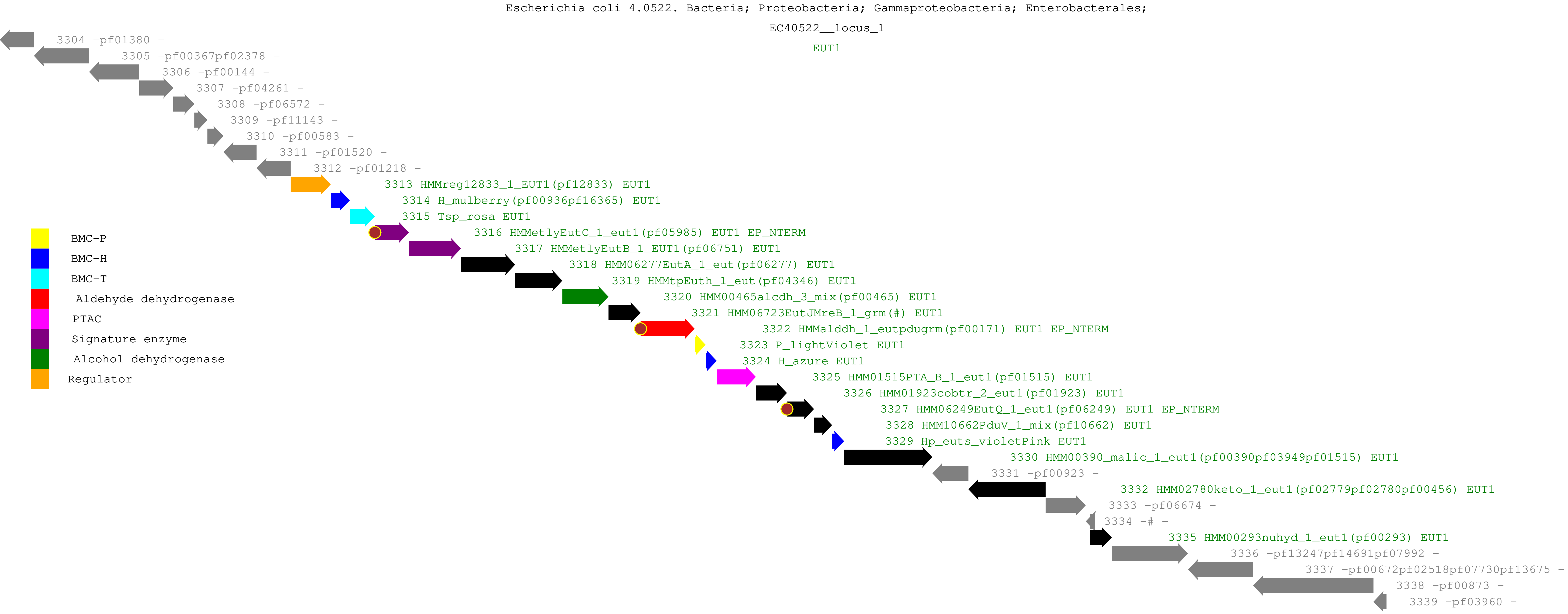
Escherichia coli 4.0522. Bacteria; Proteobacteria; Gammaproteobacteria; Enterobacterales;
EC40522__locus_1
EUT1
3304 -pf01380 -
3305 -pf00367pf02378 -
3306 -pf00144 -
3307 -pf04261 -
3308 -pf06572 -
3309 -pf11143 -
3310 -pf00583 -
3311 -pf01520 -
3312 -pf01218 -
3313 HMMreg12833_1_EUT1(pf12833) EUT1
3314 H_mulberry(pf00936pf16365) EUT1
3315 Tsp_rosa EUT1
3316 HMMetlyEutC_1_eut1(pf05985) EUT1 EP_NTERM
3317 HMMetlyEutB_1_EUT1(pf06751) EUT1
3318 HMM06277EutA_1_eut(pf06277) EUT1
3319 HMMtpEuth_1_eut(pf04346) EUT1
3320 HMM00465alcdh_3_mix(pf00465) EUT1
3321 HMM06723EutJMreB_1_grm(#) EUT1
3322 HMMalddh_1_eutpdugrm(pf00171) EUT1 EP_NTERM
3323 P_lightViolet EUT1
3324 H_azure EUT1
3325 HMM01515PTA_B_1_eut1(pf01515) EUT1
3326 HMM01923cobtr_2_eut1(pf01923) EUT1
3327 HMM06249EutQ_1_eut1(pf06249) EUT1 EP_NTERM
3328 HMM10662PduV_1_mix(pf10662) EUT1
3329 Hp_euts_violetPink EUT1
3330 HMM00390_malic_1_eut1(pf00390pf03949pf01515) EUT1
3331 -pf00923 -
3332 HMM02780keto_1_eut1(pf02779pf02780pf00456) EUT1
3333 -pf06674 -
3334 -# -
3335 HMM00293nuhyd_1_eut1(pf00293) EUT1
3336 -pf13247pf14691pf07992 -
3337 -pf00672pf02518pf07730pf13675 -
3338 -pf00873 -
3339 -pf03960 -
BMC-P
BMC-H
BMC-T
Aldehyde dehydrogenase
PTAC
Signature enzyme
Alcohol dehydrogenase
Regulator

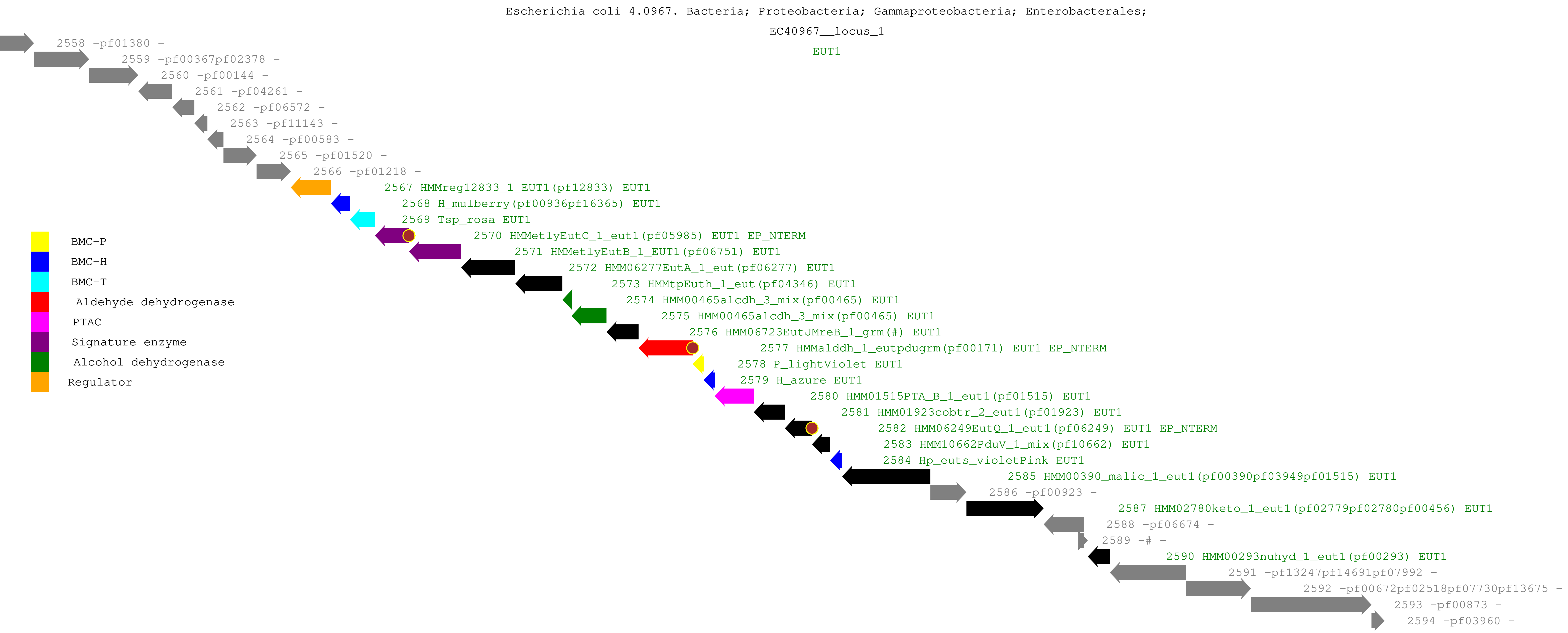

Escherichia coli 4.0967. Bacteria; Proteobacteria; Gammaproteobacteria; Enterobacterales;
EC40967__locus_1
EUT1
2558 -pf01380 -
2559 -pf00367pf02378 -
2560 -pf00144 -
2561 -pf04261 -
2562 -pf06572 -
2563 -pf11143 -
2564 -pf00583 -
2565 -pf01520 -
2566 -pf01218 -
2567 HMMreg12833_1_EUT1(pf12833) EUT1
2568 H_mulberry(pf00936pf16365) EUT1
2569 Tsp_rosa EUT1
2570 HMMetlyEutC_1_eut1(pf05985) EUT1 EP_NTERM
2571 HMMetlyEutB_1_EUT1(pf06751) EUT1
2572 HMM06277EutA_1_eut(pf06277) EUT1
2573 HMMtpEuth_1_eut(pf04346) EUT1
2574 HMM00465alcdh_3_mix(pf00465) EUT1
2575 HMM00465alcdh_3_mix(pf00465) EUT1
2576 HMM06723EutJMreB_1_grm(#) EUT1
2577 HMMalddh_1_eutpdugrm(pf00171) EUT1 EP_NTERM
2578 P_lightViolet EUT1
2579 H_azure EUT1
2580 HMM01515PTA_B_1_eut1(pf01515) EUT1
2581 HMM01923cobtr_2_eut1(pf01923) EUT1
2582 HMM06249EutQ_1_eut1(pf06249) EUT1 EP_NTERM
2583 HMM10662PduV_1_mix(pf10662) EUT1
2584 Hp_euts_violetPink EUT1
2585 HMM00390_malic_1_eut1(pf00390pf03949pf01515) EUT1
2586 -pf00923 -
2587 HMM02780keto_1_eut1(pf02779pf02780pf00456) EUT1
2588 -pf06674 -
2589 -# -
2590 HMM00293nuhyd_1_eut1(pf00293) EUT1
2591 -pf13247pf14691pf07992 -
2592 -pf00672pf02518pf07730pf13675 -
2593 -pf00873 -
2594 -pf03960 -
BMC-P
BMC-H
BMC-T
Aldehyde dehydrogenase
PTAC
Signature enzyme
Alcohol dehydrogenase
Regulator

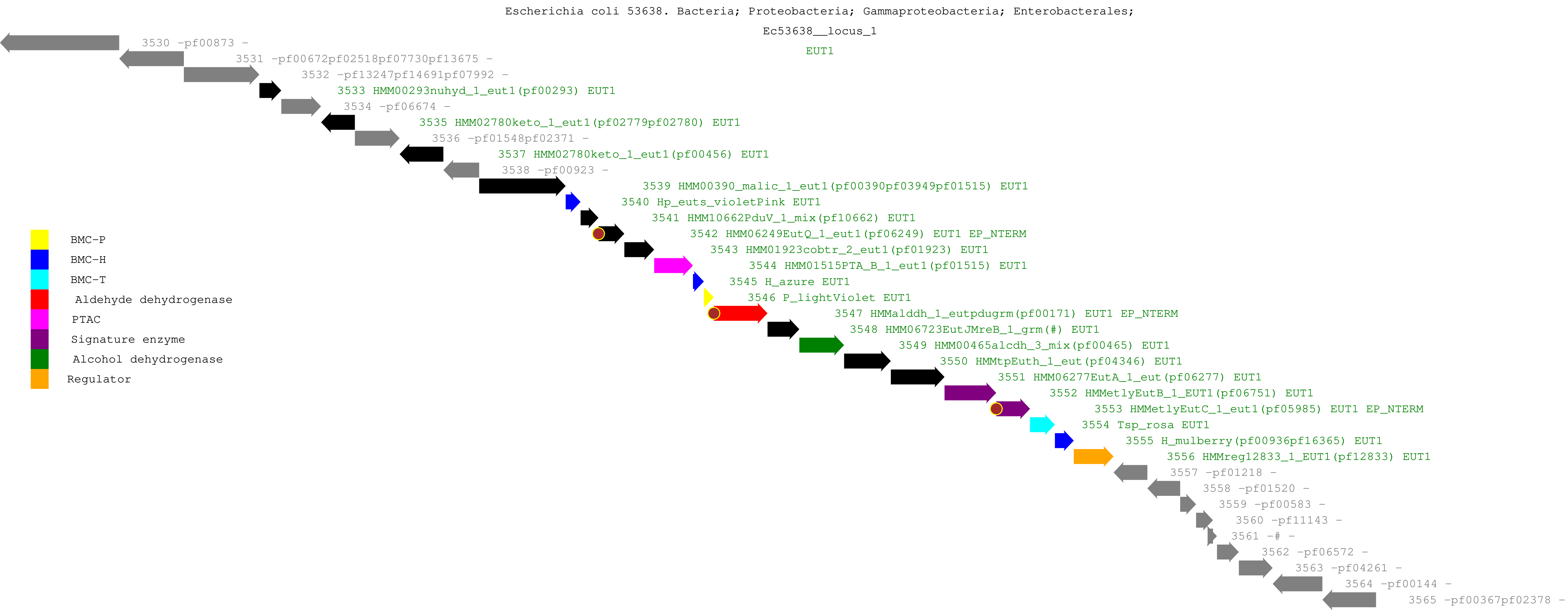
Escherichia coli 53638. Bacteria; Proteobacteria; Gammaproteobacteria; Enterobacterales;
Ec53638__locus_1
EUT1
3530 -pf00873 -
3531 -pf00672pf02518pf07730pf13675 -
3532 -pf13247pf14691pf07992 -
3533 HMM00293nuhyd_1_eut1(pf00293) EUT1
3534 -pf06674 -
3535 HMM02780keto_1_eut1(pf02779pf02780) EUT1
3536 -pf01548pf02371 -
3537 HMM02780keto_1_eut1(pf00456) EUT1
3538 -pf00923 -
3539 HMM00390_malic_1_eut1(pf00390pf03949pf01515) EUT1
3540 Hp_euts_violetPink EUT1
3541 HMM10662PduV_1_mix(pf10662) EUT1
3542 HMM06249EutQ_1_eut1(pf06249) EUT1 EP_NTERM
3543 HMM01923cobtr_2_eut1(pf01923) EUT1
3544 HMM01515PTA_B_1_eut1(pf01515) EUT1
3545 H_azure EUT1
3546 P_lightViolet EUT1
3547 HMMalddh_1_eutpdugrm(pf00171) EUT1 EP_NTERM
3548 HMM06723EutJMreB_1_grm(#) EUT1
3549 HMM00465alcdh_3_mix(pf00465) EUT1
3550 HMMtpEuth_1_eut(pf04346) EUT1
3551 HMM06277EutA_1_eut(pf06277) EUT1
3552 HMMetlyEutB_1_EUT1(pf06751) EUT1
3553 HMMetlyEutC_1_eut1(pf05985) EUT1 EP_NTERM
3554 Tsp_rosa EUT1
3555 H_mulberry(pf00936pf16365) EUT1
3556 HMMreg12833_1_EUT1(pf12833) EUT1
3557 -pf01218 -
3558 -pf01520 -
3559 -pf00583 -
3560 -pf11143 -
3561 -# -
3562 -pf06572 -
3563 -pf04261 -
3564 -pf00144 -
3565 -pf00367pf02378 -
BMC-P
BMC-H
BMC-T
Aldehyde dehydrogenase
PTAC
Signature enzyme
Alcohol dehydrogenase
Regulator

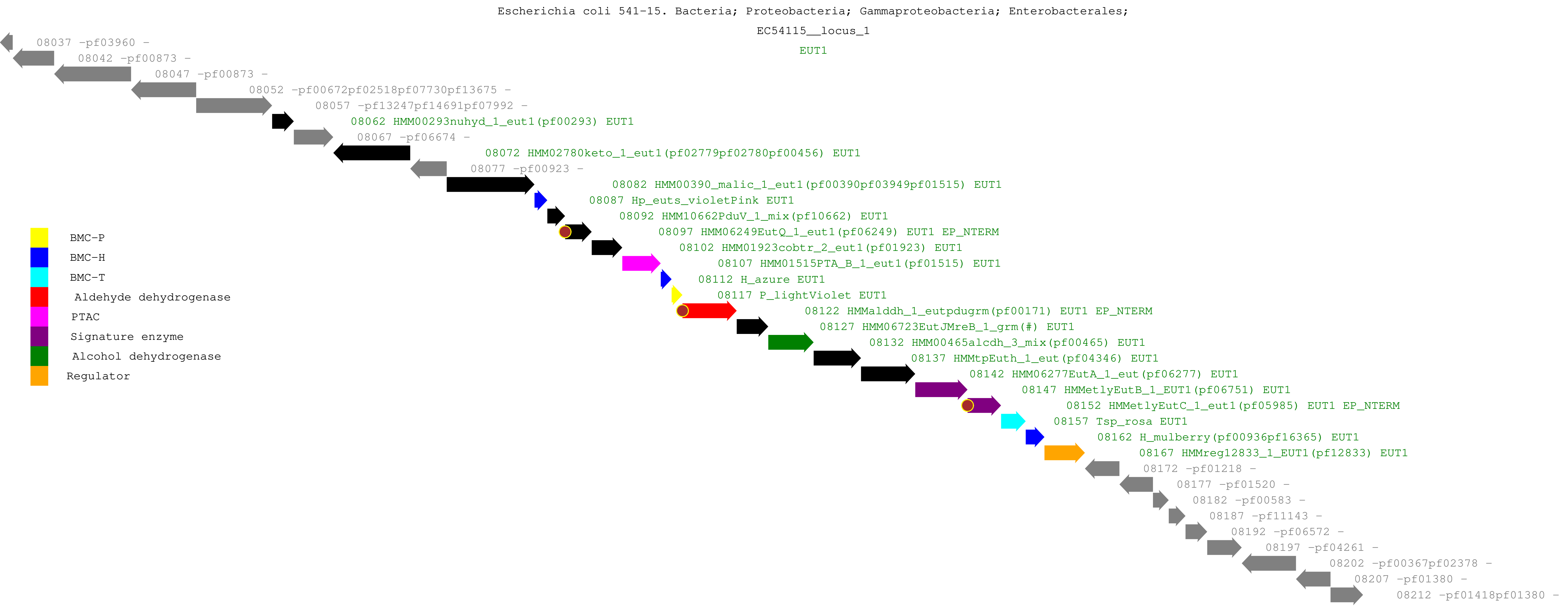

Escherichia coli 541-15. Bacteria; Proteobacteria; Gammaproteobacteria; Enterobacterales;
EC54115__locus_1
EUT1
08037 -pf03960 -
08042 -pf00873 -
08047 -pf00873 -
08052 -pf00672pf02518pf07730pf13675 -
08057 -pf13247pf14691pf07992 -
08062 HMM00293nuhyd_1_eut1(pf00293) EUT1
08067 -pf06674 -
08072 HMM02780keto_1_eut1(pf02779pf02780pf00456) EUT1
08077 -pf00923 -
08082 HMM00390_malic_1_eut1(pf00390pf03949pf01515) EUT1
08087 Hp_euts_violetPink EUT1
08092 HMM10662PduV_1_mix(pf10662) EUT1
08097 HMM06249EutQ_1_eut1(pf06249) EUT1 EP_NTERM
08102 HMM01923cobtr_2_eut1(pf01923) EUT1
08107 HMM01515PTA_B_1_eut1(pf01515) EUT1
08112 H_azure EUT1
08117 P_lightViolet EUT1
08122 HMMalddh_1_eutpdugrm(pf00171) EUT1 EP_NTERM
08127 HMM06723EutJMreB_1_grm(#) EUT1
08132 HMM00465alcdh_3_mix(pf00465) EUT1
08137 HMMtpEuth_1_eut(pf04346) EUT1
08142 HMM06277EutA_1_eut(pf06277) EUT1
08147 HMMetlyEutB_1_EUT1(pf06751) EUT1
08152 HMMetlyEutC_1_eut1(pf05985) EUT1 EP_NTERM
08157 Tsp_rosa EUT1
08162 H_mulberry(pf00936pf16365) EUT1
08167 HMMreg12833_1_EUT1(pf12833) EUT1
08172 -pf01218 -
08177 -pf01520 -
08182 -pf00583 -
08187 -pf11143 -
08192 -pf06572 -
08197 -pf04261 -
08202 -pf00367pf02378 -
08207 -pf01380 -
08212 -pf01418pf01380 -
BMC-P
BMC-H
BMC-T
Aldehyde dehydrogenase
PTAC
Signature enzyme
Alcohol dehydrogenase
Regulator

Escherichia coli (strain 55989 / EAEC). Bacteria; Proteobacteria; Gammaproteobacteria; Enterobacterales;

EC55989__locus_1

EUT1

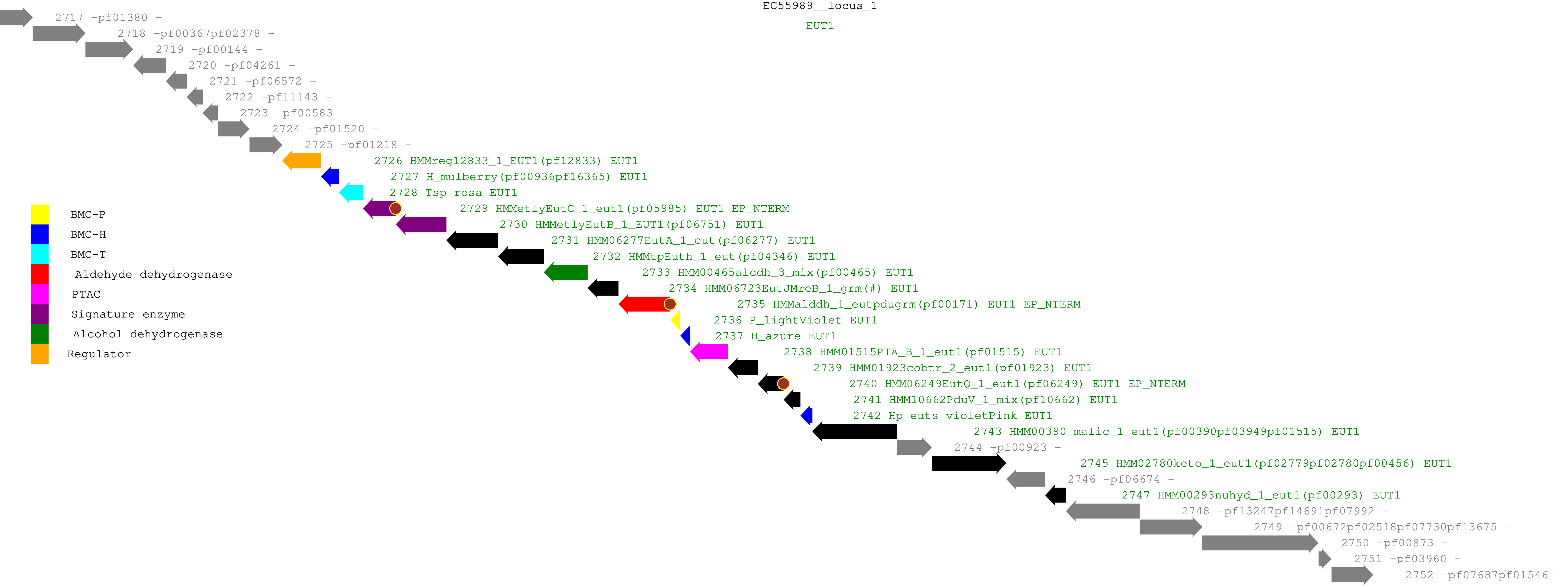

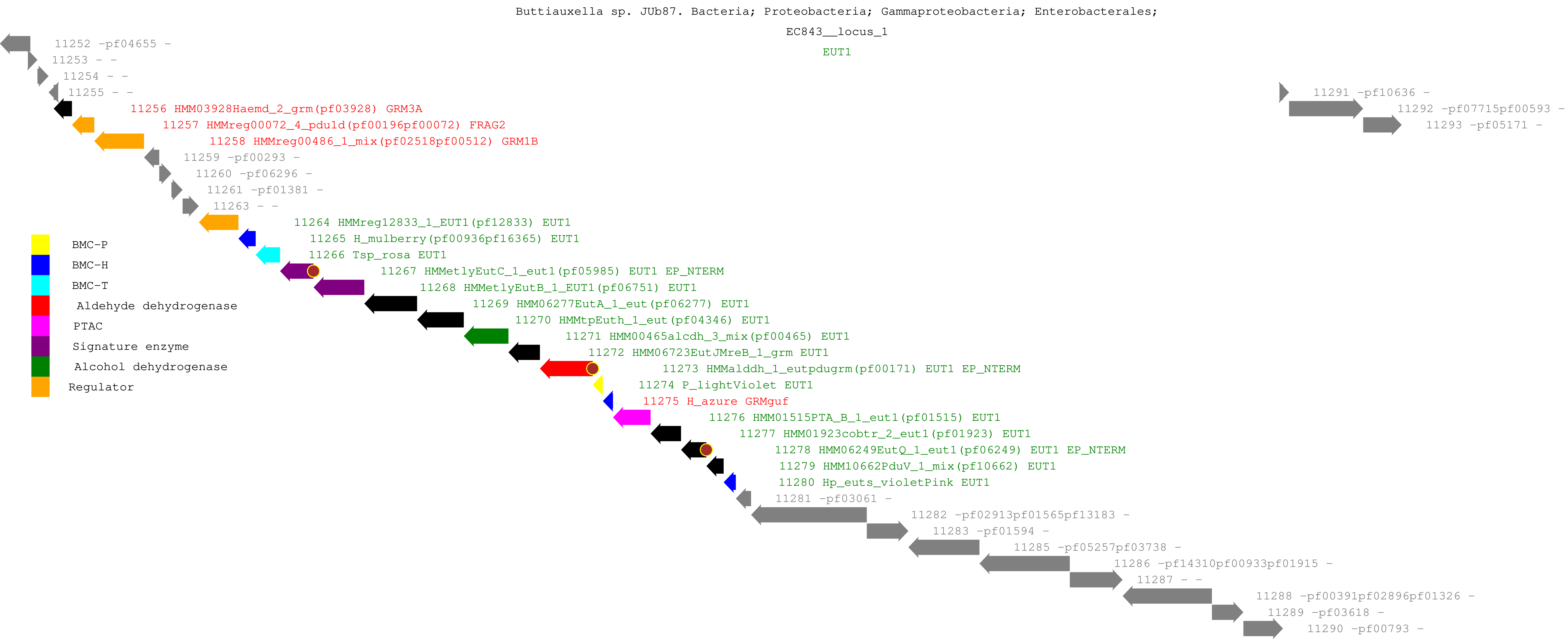
Buttiauxella sp. JUb87. Bacteria; Proteobacteria; Gammaproteobacteria; Enterobacterales;
EC843__locus_1
EUT1
11252 -pf04655 -
11253 - -
11254 - -
11255 - -
11256 HMM03928Haemd_2_grm(pf03928) GRM3A
11257 HMMreg00072_4_pdu1d(pf00196pf00072) FRAG2
11258 HMMreg00486_1_mix(pf02518pf00512) GRM1B
11259 -pf00293 -
11260 -pf06296 -
11261 -pf01381 -
11263 - -
11264 HMMreg12833_1_EUT1(pf12833) EUT1
11265 H_mulberry(pf00936pf16365) EUT1
11266 Tsp_rosa EUT1
11267 HMMetlyEutC_1_eut1(pf05985) EUT1 EP_NTERM
11268 HMMetlyEutB_1_EUT1(pf06751) EUT1
11269 HMM06277EutA_1_eut(pf06277) EUT1
11270 HMMtpEuth_1_eut(pf04346) EUT1
11271 HMM00465alcdh_3_mix(pf00465) EUT1
11272 HMM06723EutJMreB_1_grm EUT1
11273 HMMaldh_1_eutpdugrm(pf00171) EUT1 EP_NTERM
11274 P_lightViolet EUT1
11275 H_azure GRMguf
11276 HMM01515PTA_B_1_eut1(pf01515) EUT1
11277 HMM01923cobtr_2_eut1(pf01923) EUT1
11278 HMM06249EutQ_1_eut1(pf06249) EUT1 EP_NTERM
11279 HMM10662PduV_1_mix(pf10662) EUT1
11280 Hp_euts_violetPink EUT1
11281 -pf03061 -
11282 -pf02913pf01565pf13183 -
11283 -pf01594 -
11285 -pf05257pf03738 -
11286 -pf14310pf00933pf01915 -
11287 - -
11288 -pf00391pf02896pf01326 -
11289 -pf03618 -
11290 -pf00793 -
11291 -pf10636 -
11292 -pf07715pf00593 -
11293 -pf05171 -
BMC-P
BMC-H
BMC-T
Aldehyde dehydrogenase
PTAC
Signature enzyme
Alcohol dehydrogenase
Regulator

Escherichia coli 9.0111. Bacteria; Proteobacteria; Gammaproteobacteria; Enterobacterales;

EC90111__locus_1

EUT1

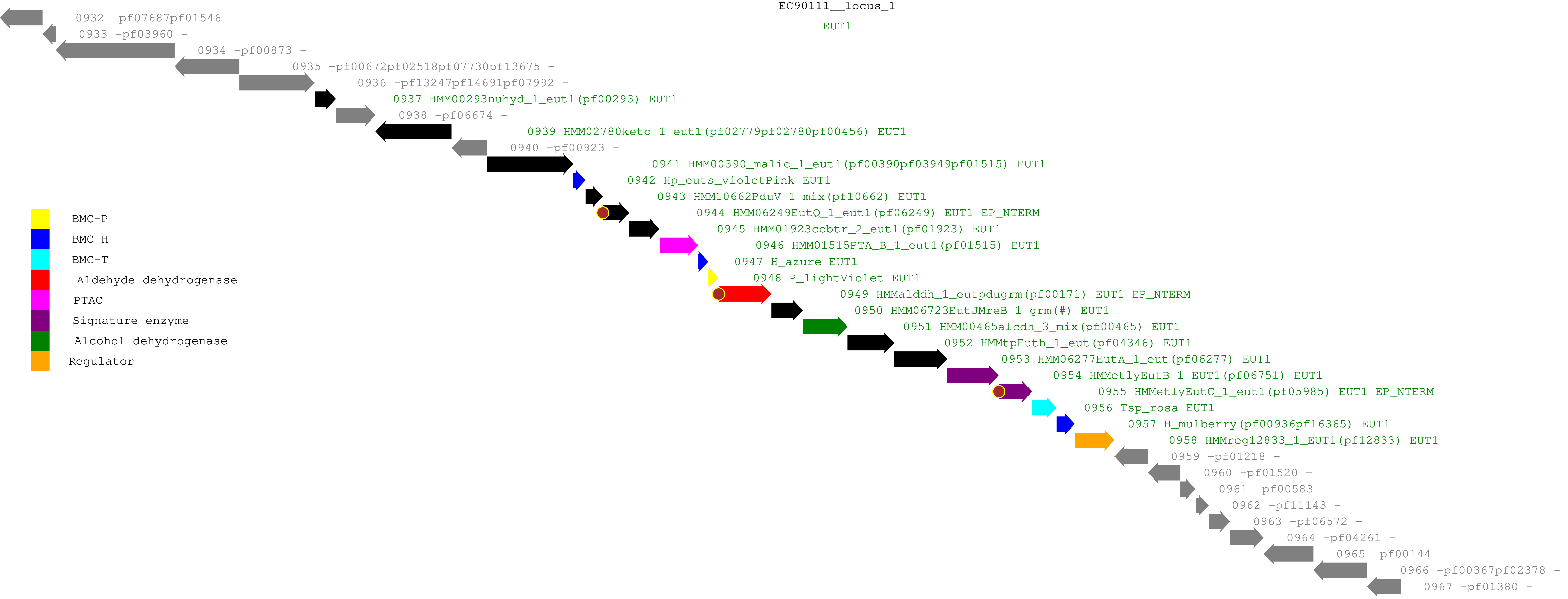

Escherichia coli O25b:H4-ST131. Bacteria; Proteobacteria; Gammaproteobacteria; Enterobacterales;

EC958__locus_1

EUT1

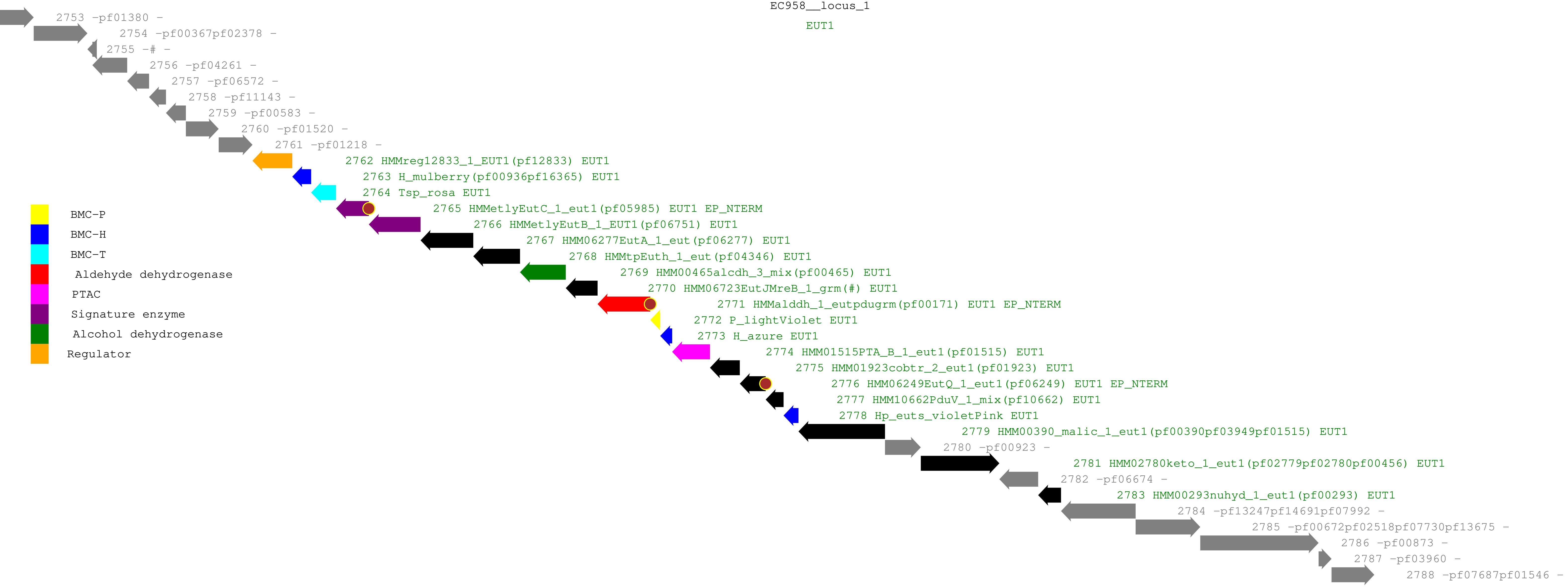

Escherichia coli 97.0246. Bacteria; Proteobacteria; Gammaproteobacteria; Enterobacterales;

EC970246__locus_1

EUT1

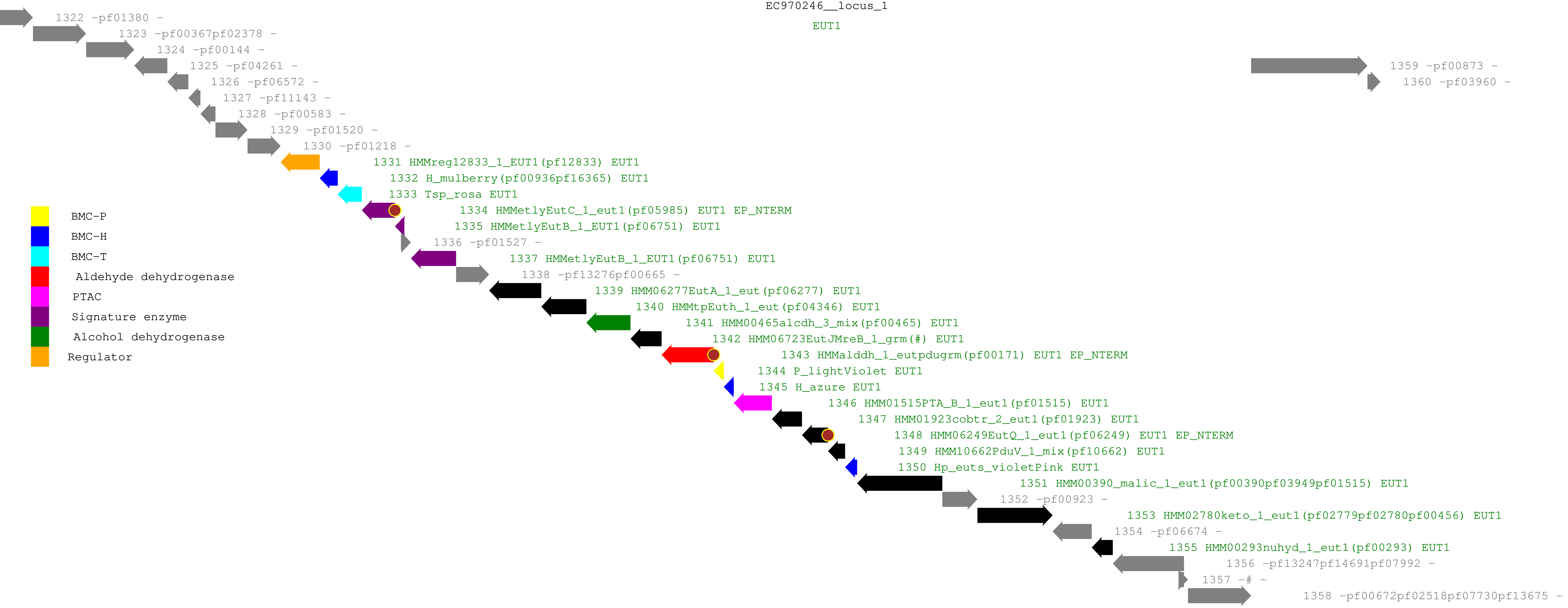

Escherichia coli 99.0741. Bacteria; Proteobacteria; Gammaproteobacteria; Enterobacterales;

EC970259__locus_1

EUT1

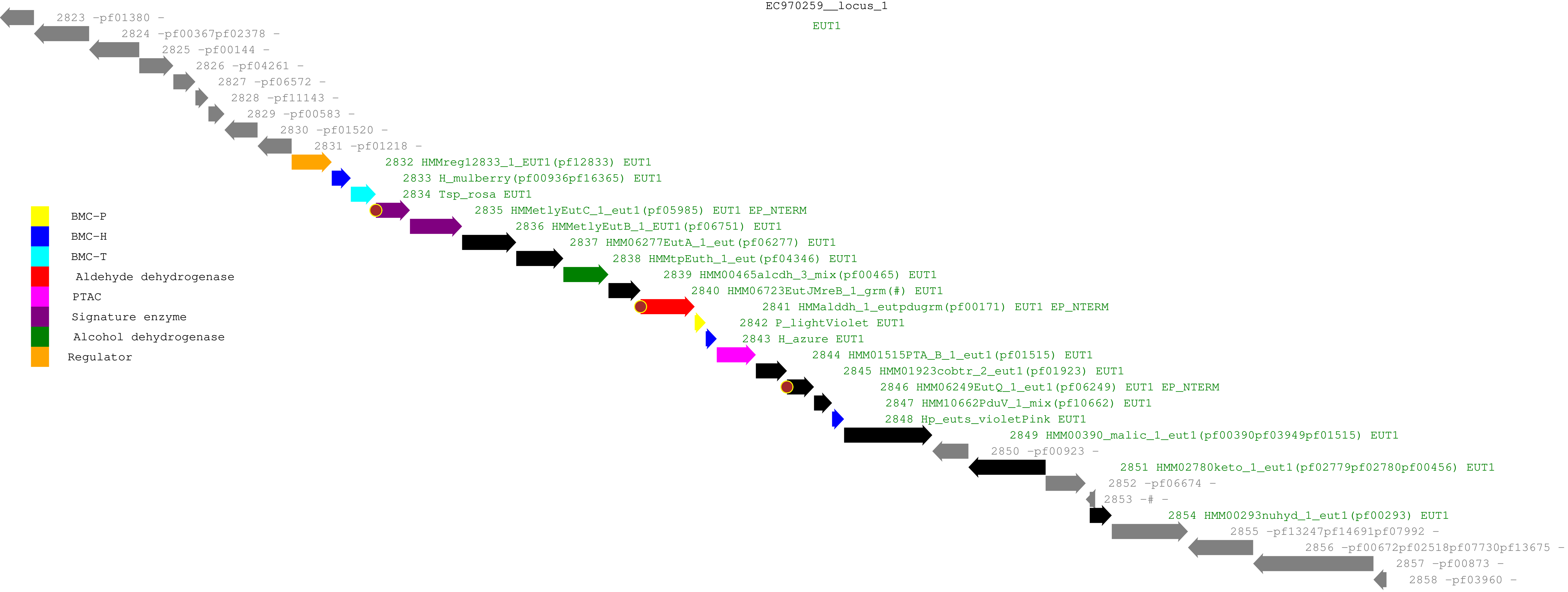

Escherichia coli 97.0259. Bacteria; Proteobacteria; Gammaproteobacteria; Enterobacterales;

EC990741__locus_1

EUT1

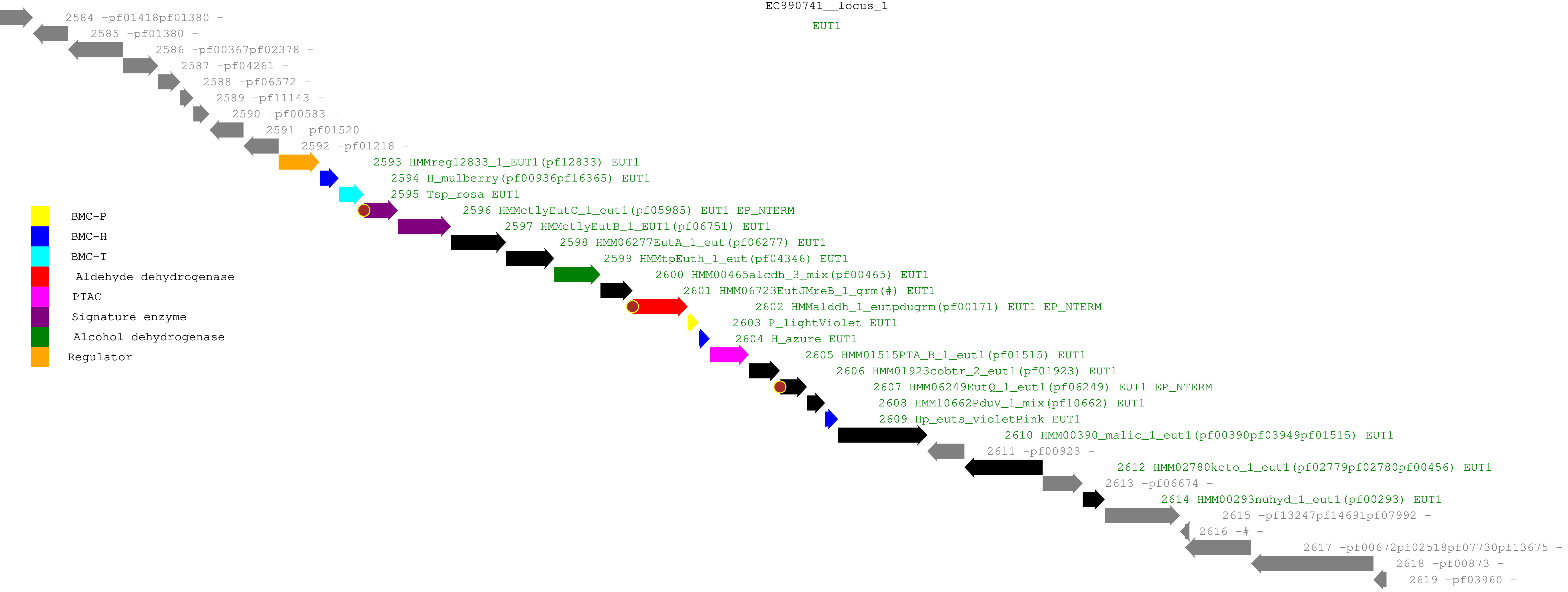

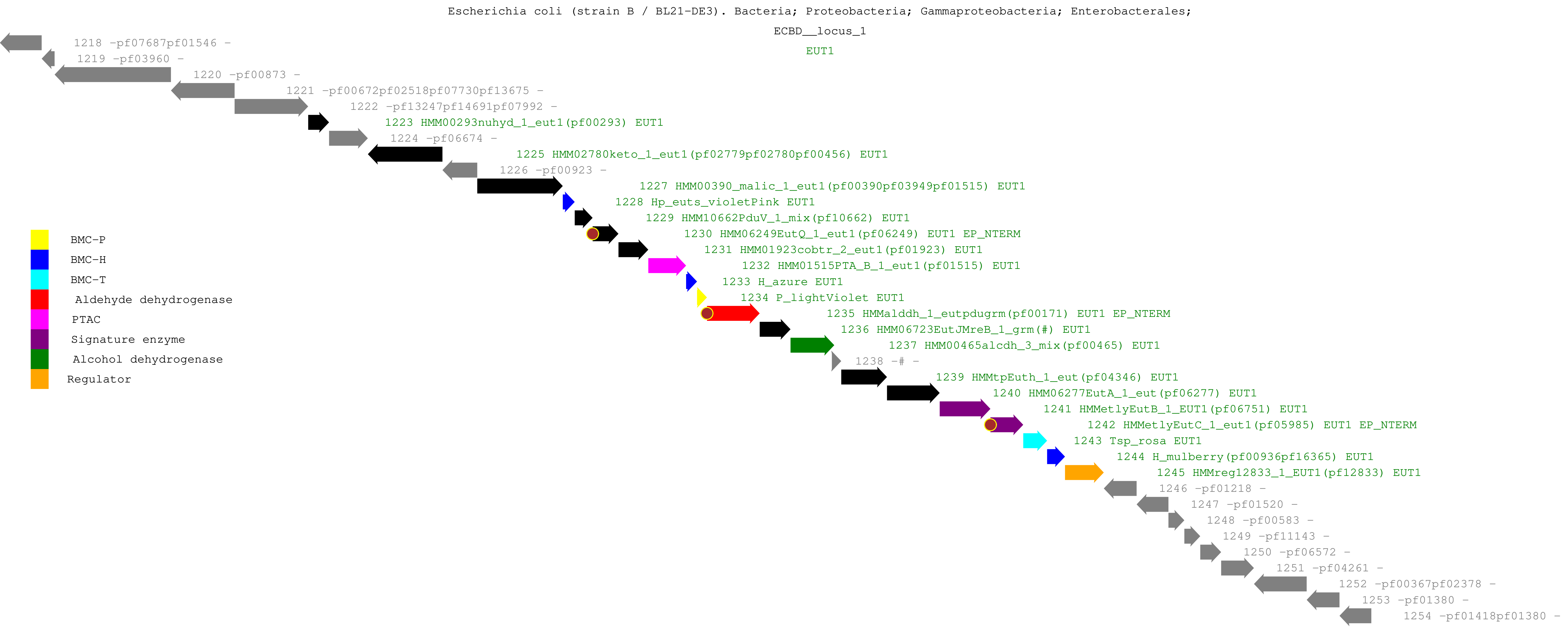

Escherichia coli (strain B / BL21-DE3). Bacteria; Proteobacteria; Gammaproteobacteria; Enterobacterales;
ECBD__locus_1
EUT1
1218 -pf07687pf01546 -
1219 -pf03960 -
1220 -pf00873 -
1221 -pf00672pf02518pf07730pf13675 -
1222 -pf13247pf14691pf07992 -
1223 HMM00293nuhyd_1_eut1(pf00293) EUT1
1224 -pf06674 -
1225 HMM02780keto_1_eut1(pf02779pf02780pf00456) EUT1
1226 -pf00923 -
1227 HMM00390_malic_1_eut1(pf00390pf03949pf01515) EUT1
1228 Hp_euts_violetPink EUT1
1229 HMM10662PduV_1_mix(pf10662) EUT1
1230 HMM06249EutQ_1_eut1(pf06249) EUT1 EP_NTERM
1231 HMM01923cobtr_2_eut1(pf01923) EUT1
1232 HMM01515PTA_B_1_eut1(pf01515) EUT1
1233 H_azure EUT1
1234 P_lightViolet EUT1
1235 HMMalddh_1_eutpdugrm(pf00171) EUT1 EP_NTERM
1236 HMM06723EutJMreB_1_grm(#) EUT1
1237 HMM00465alcdh_3_mix(pf00465) EUT1
1238 -# -
1239 HMMtpEuth_1_eut(pf04346) EUT1
1240 HMM06277EutA_1_eut(pf06277) EUT1
1241 HMMetlyEutB_1_EUT1(pf06751) EUT1
1242 HMMetlyEutC_1_eut1(pf05985) EUT1 EP_NTERM
1243 Tsp_rosa EUT1
1244 H_mulberry(pf00936pf16365) EUT1
1245 HMMreg12833_1_EUT1(pf12833) EUT1
1246 -pf01218 -
1247 -pf01520 -
1248 -pf00583 -
1249 -pf11143 -
1250 -pf06572 -
1251 -pf04261 -
1252 -pf00367pf02378 -
1253 -pf01380 -
1254 -pf01418pf01380 -
BMC-P
BMC-H
BMC-T
Aldehyde dehydrogenase
PTAC
Signature enzyme
Alcohol dehydrogenase
Regulator

Escherichia coli C-34666. Bacteria; Proteobacteria; Gammaproteobacteria; Enterobacterales;

ECC34666__locus_1

EUT1

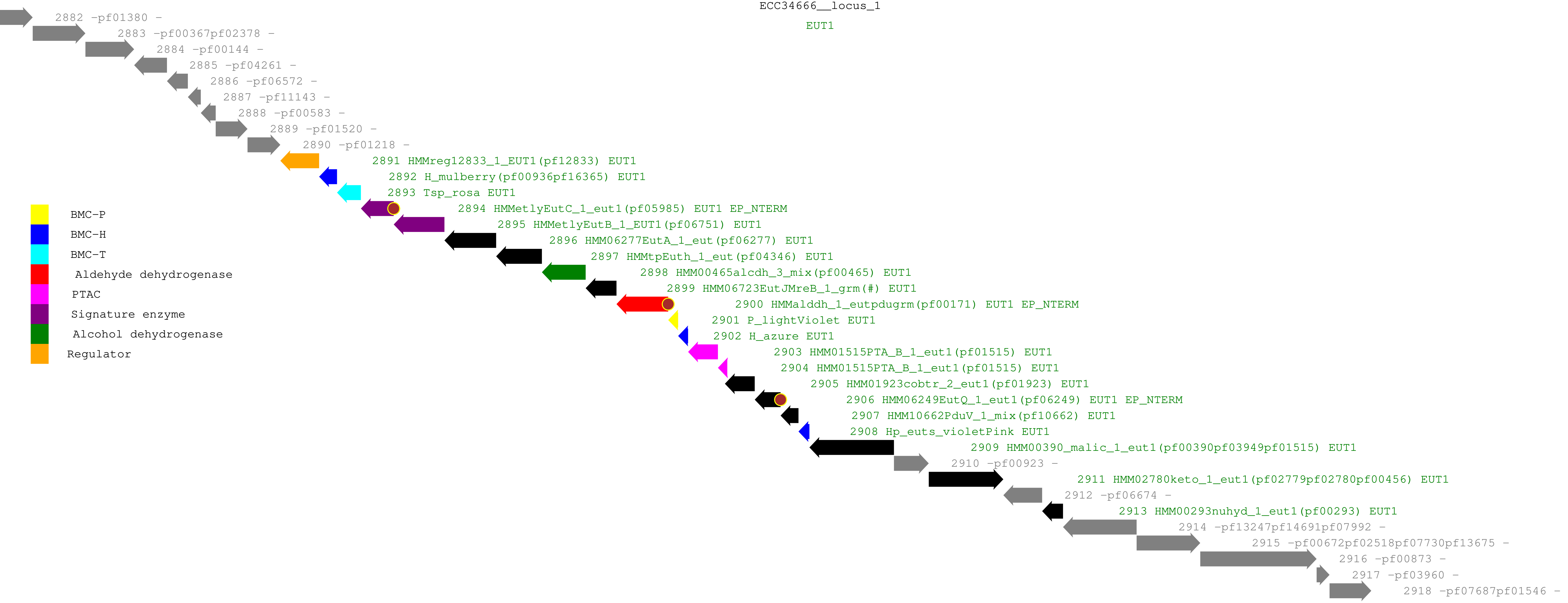

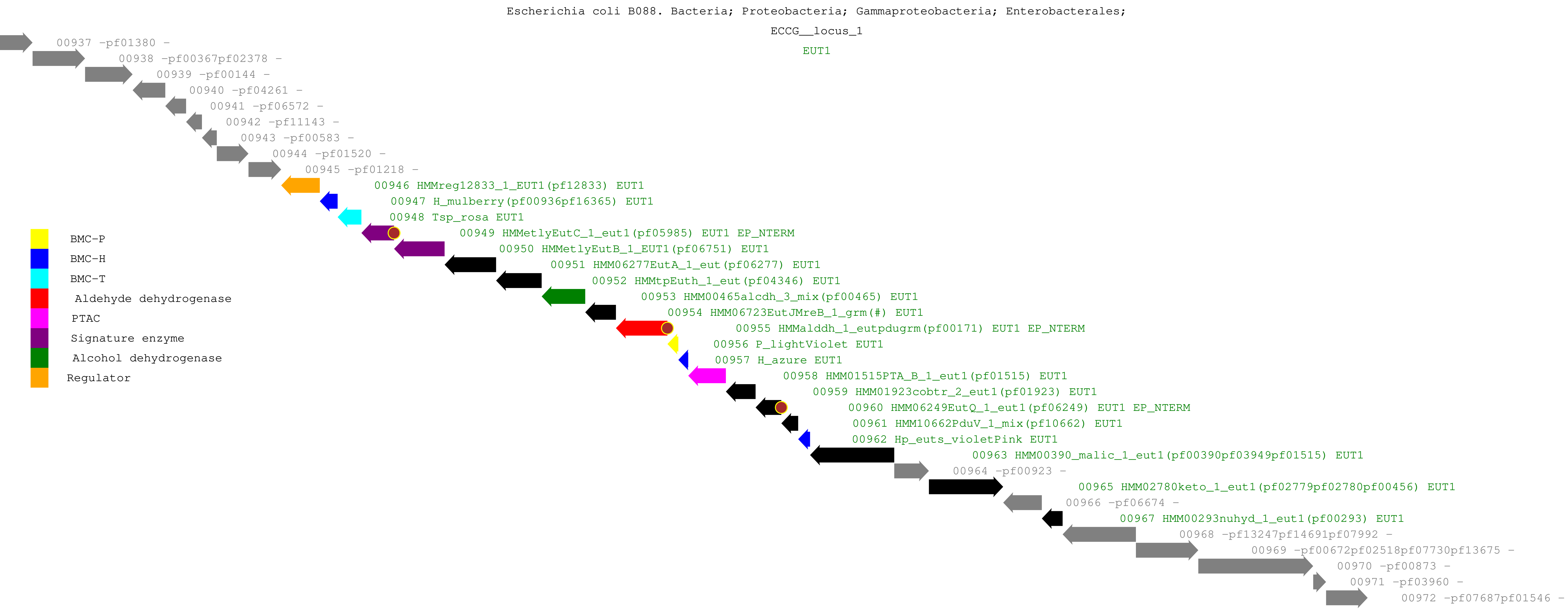

Escherichia coli B088. Bacteria; Proteobacteria; Gammaproteobacteria; Enterobacterales;
ECCG__locus_1
EUT1
00937 -pf01380 -
00938 -pf00367pf02378 -
00939 -pf00144 -
00940 -pf04261 -
00941 -pf06572 -
00942 -pf11143 -
00943 -pf00583 -
00944 -pf01520 -
00945 -pf01218 -
00946 HMMreg12833_1_EUT1(pf12833) EUT1
00947 H_mulberry(pf00936pf16365) EUT1
00948 Tsp_rosa EUT1
00949 HMMetlyEutC_1_eut1(pf05985) EUT1 EP_NTERM
00950 HMMetlyEutB_1_EUT1(pf06751) EUT1
00951 HMM06277EutA_1_eut(pf06277) EUT1
00952 HMMtpEuth_1_eut(pf04346) EUT1
00953 HMM00465alcdh_3_mix(pf00465) EUT1
00954 HMM06723EutJMreB_1_grm(#) EUT1
00955 HMMalddh_1_eutpdugrm(pf00171) EUT1 EP_NTERM
00956 P_lightViolet EUT1
00957 H_azure EUT1
00958 HMM01515PTA_B_1_eut1(pf01515) EUT1
00959 HMM01923cobtr_2_eut1(pf01923) EUT1
00960 HMM06249EutQ_1_eut1(pf06249) EUT1 EP_NTERM
00961 HMM10662PduV_1_mix(pf10662) EUT1
00962 Hp_euts_violetPink EUT1
00963 HMM00390_malic_1_eut1(pf00390pf03949pf01515) EUT1
00964 -pf00923 -
00965 HMM02780keto_1_eut1(pf02779pf02780pf00456) EUT1
00966 -pf06674 -
00967 HMM00293nuhyd_1_eut1(pf00293) EUT1
00968 -pf13247pf14691pf07992 -
00969 -pf00672pf02518pf07730pf13675 -
00970 -pf00873 -
00971 -pf03960 -
00972 -pf07687pf01546 -
BMC-P
BMC-H
BMC-T
Aldehyde dehydrogenase
PTAC
Signature enzyme
Alcohol dehydrogenase
Regulator

Escherichia coli DEC1B. Bacteria; Proteobacteria; Gammaproteobacteria; Enterobacterales;

ECDEC1B__locus_2

EUT1

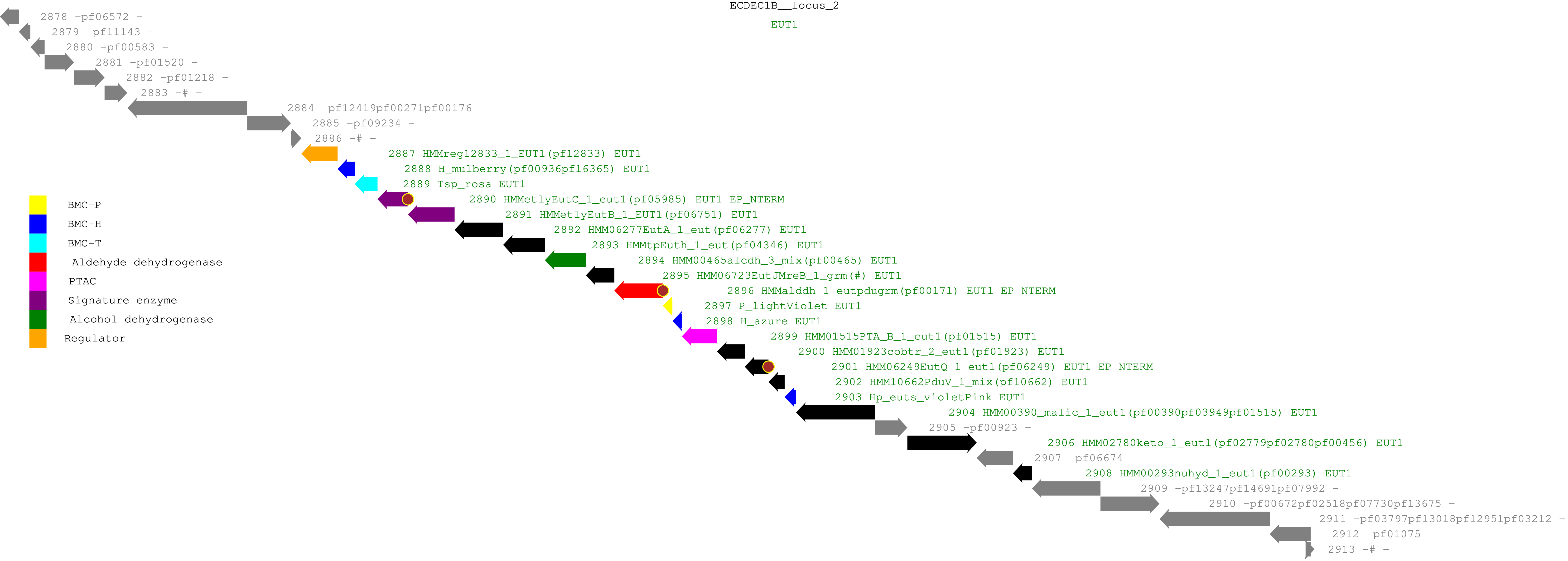

Escherichia coli DEC1C. Bacteria; Proteobacteria; Gammaproteobacteria; Enterobacterales;

ECDEC1C__locus_2

EUT1

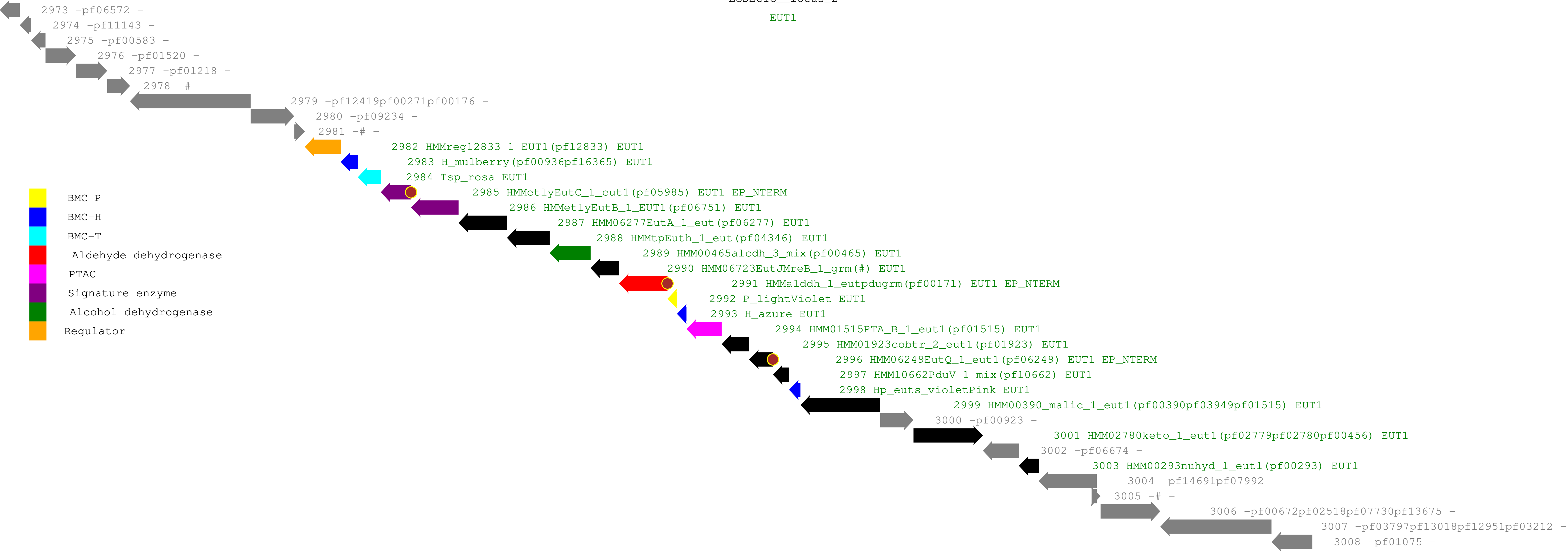

Escherichia coli DEC1D. Bacteria; Proteobacteria; Gammaproteobacteria; Enterobacterales;

ECDEC1D__locus_2

EUT1

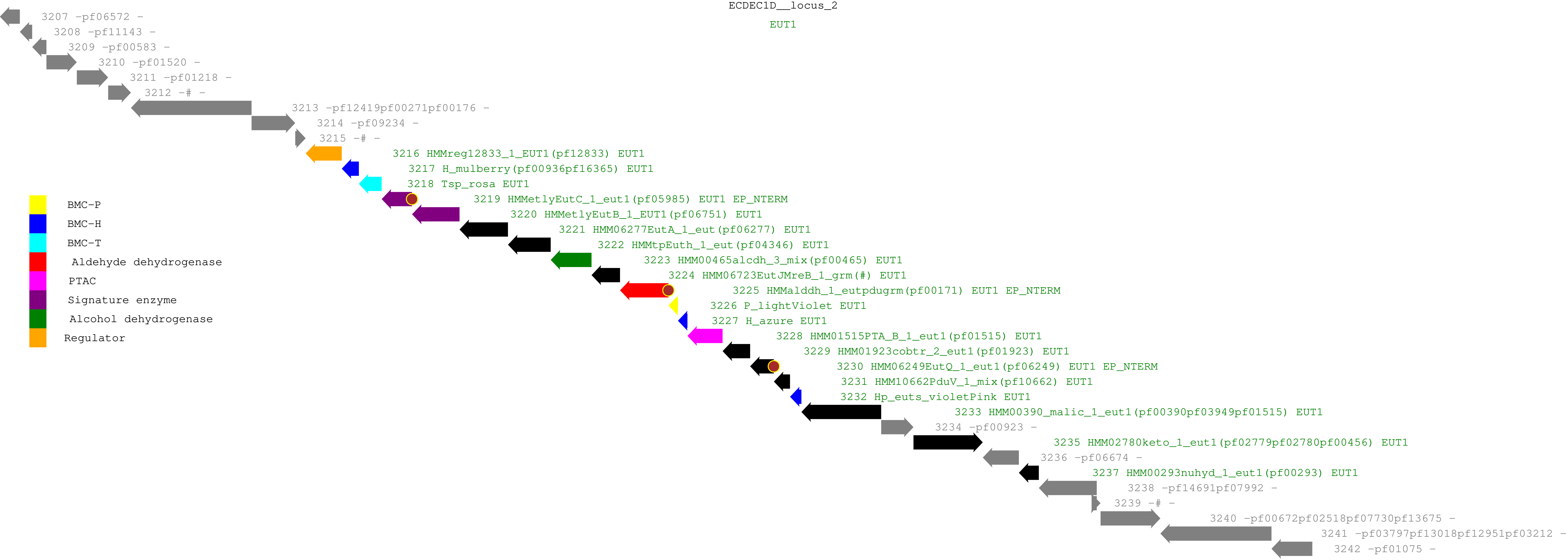

Escherichia coli DEC2C. Bacteria; Proteobacteria; Gammaproteobacteria; Enterobacterales;

ECDEC2C__locus_2

EUT1

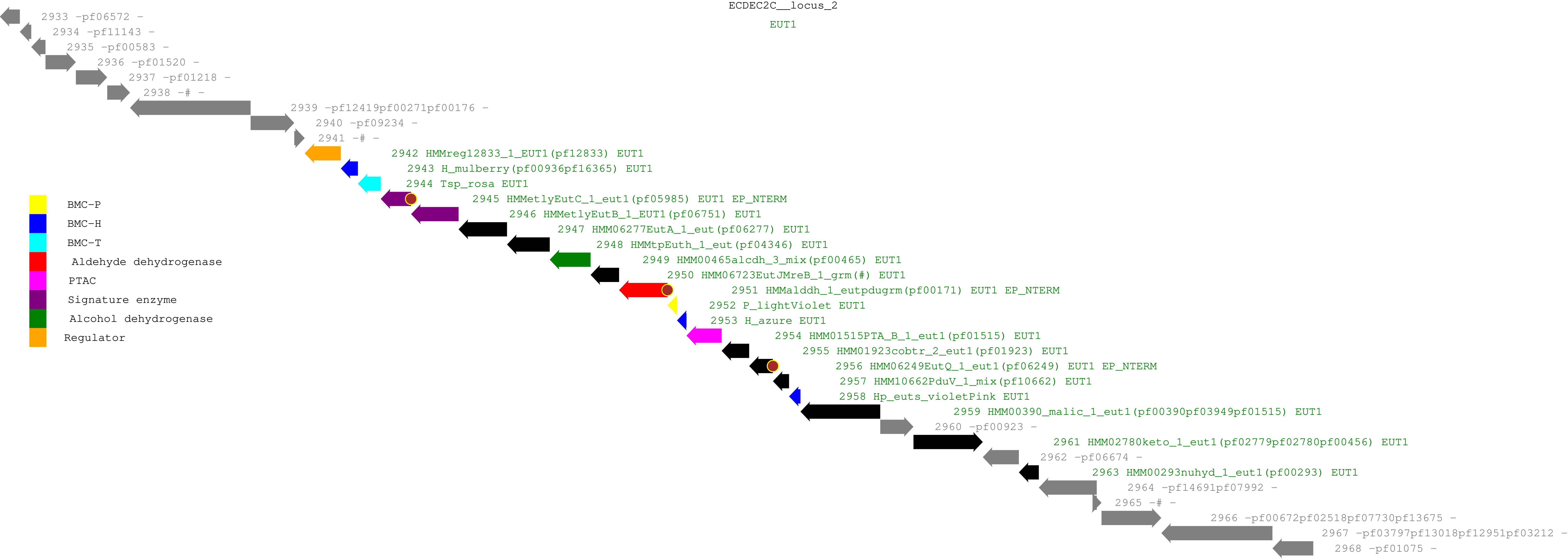

Escherichia coli DEC2E. Bacteria; Proteobacteria; Gammaproteobacteria; Enterobacterales;

ECDEC2E__locus_2

EUT1

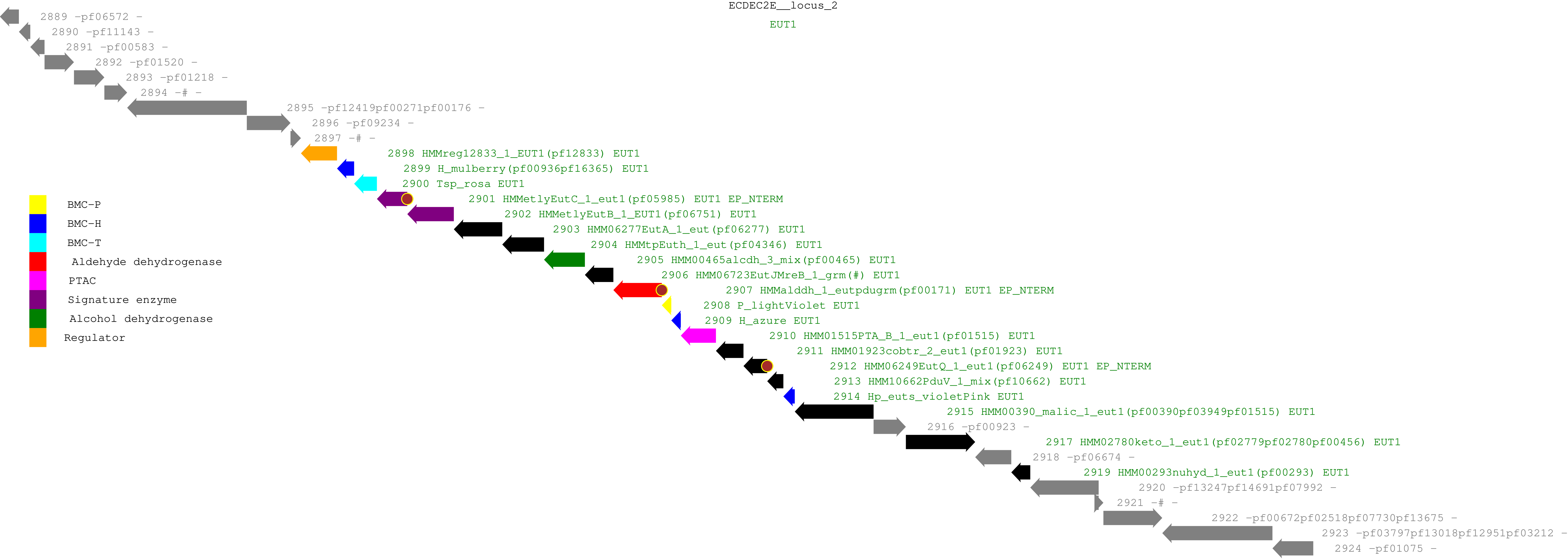

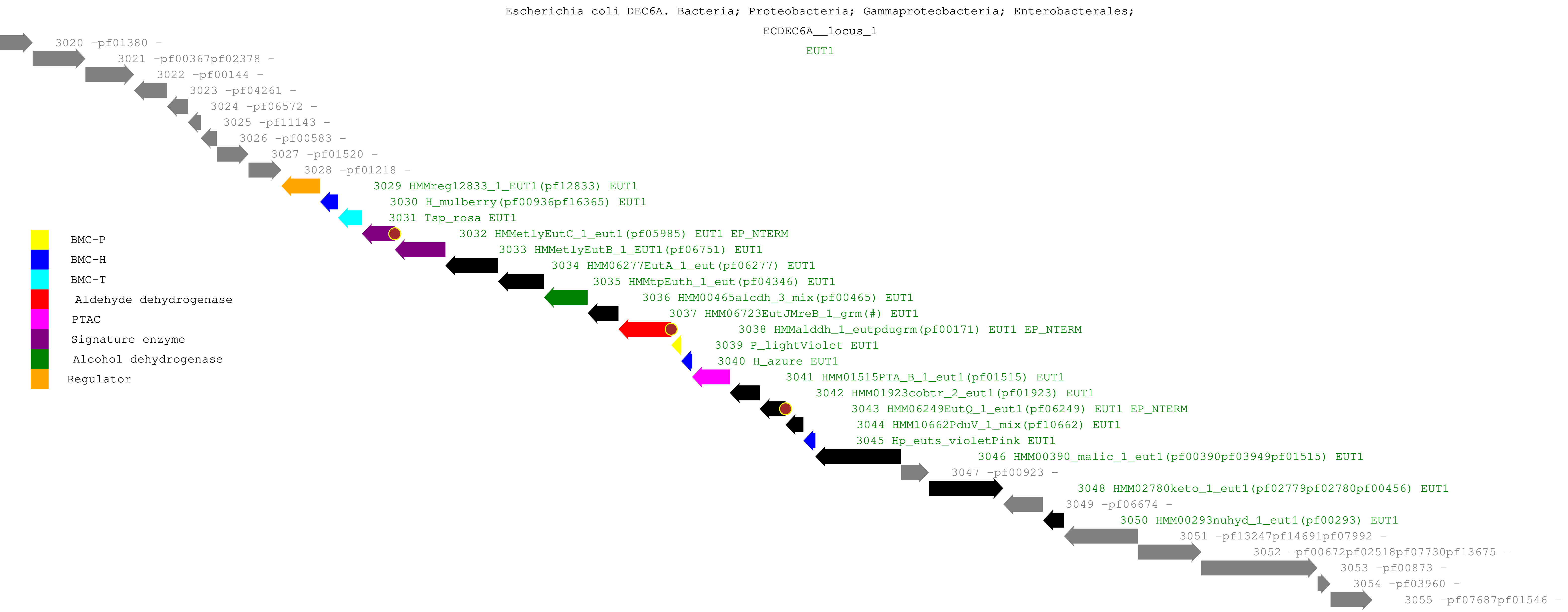

Escherichia coli DEC6A. Bacteria; Proteobacteria; Gammaproteobacteria; Enterobacterales;
ECDEC6A__locus_1
EUT1
3020 -pf01380 -
3021 -pf00367pf02378 -
3022 -pf00144 -
3023 -pf04261 -
3024 -pf06572 -
3025 -pf11143 -
3026 -pf00583 -
3027 -pf01520 -
3028 -pf01218 -
3029 HMMreg12833_1_EUT1(pf12833) EUT1
3030 H_mulberry(pf00936pf16365) EUT1
3031 Tsp_rosa EUT1
3032 HMMetlyEutC_1_eut1(pf05985) EUT1 EP_NTERM
3033 HMMetlyEutB_1_EUT1(pf06751) EUT1
3034 HMM06277EutA_1_eut(pf06277) EUT1
3035 HMMtpEuth_1_eut(pf04346) EUT1
3036 HMM00465alcdh_3_mix(pf00465) EUT1
3037 HMM06723EutJMreB_1_grm(#) EUT1
3038 HMMalddh_1_eutpdugrm(pf00171) EUT1 EP_NTERM
3039 P_lightViolet EUT1
3040 H_azure EUT1
3041 HMM01515PTA_B_1_eut1(pf01515) EUT1
3042 HMM01923cobtr_2_eut1(pf01923) EUT1
3043 HMM06249EutQ_1_eut1(pf06249) EUT1 EP_NTERM
3044 HMM10662PduV_1_mix(pf10662) EUT1
3045 Hp_euts_violetPink EUT1
3046 HMM00390_malic_1_eut1(pf00390pf03949pf01515) EUT1
3047 -pf00923 -
3048 HMM02780keto_1_eut1(pf02779pf02780pf00456) EUT1
3049 -pf06674 -
3050 HMM00293nuhyd_1_eut1(pf00293) EUT1
3051 -pf13247pf14691pf07992 -
3052 -pf00672pf02518pf07730pf13675 -
3053 -pf00873 -
3054 -pf03960 -
3055 -pf07687pf01546 -
BMC-P
BMC-H
BMC-T
Aldehyde dehydrogenase
PTAC
Signature enzyme
Alcohol dehydrogenase
Regulator

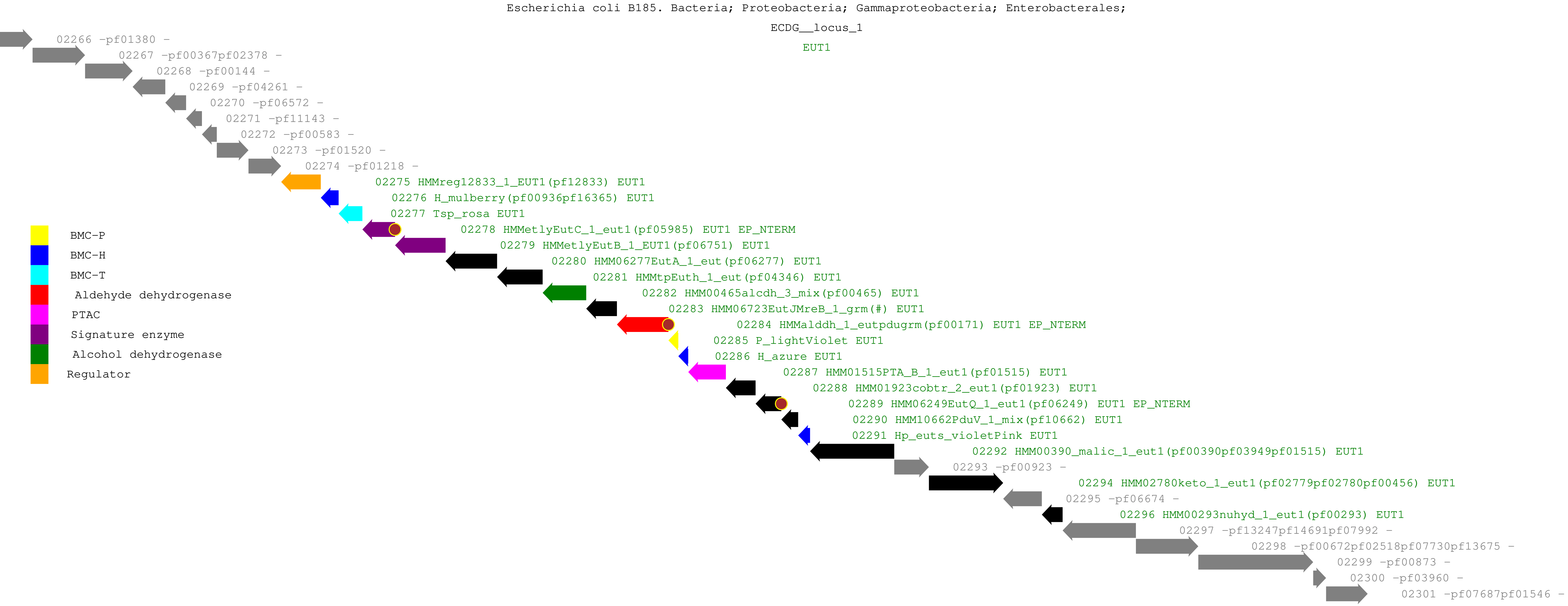
Escherichia coli B185. Bacteria; Proteobacteria; Gammaproteobacteria; Enterobacterales;
ECDG__locus_1
EUT1
02266 -pf01380 -
02267 -pf00367pf02378 -
02268 -pf00144 -
02269 -pf04261 -
02270 -pf06572 -
02271 -pf11143 -
02272 -pf00583 -
02273 -pf01520 -
02274 -pf01218 -
02275 HMMreg12833_1_EUT1(pf12833) EUT1
02276 H_mulberry(pf00936pf16365) EUT1
02277 Tsp_rosa EUT1
02278 HMMetlyEutC_1_eut1(pf05985) EUT1 EP_NTERM
02279 HMMetlyEutB_1_EUT1(pf06751) EUT1
02280 HMM06277EutA_1_eut(pf06277) EUT1
02281 HMMtpEuth_1_eut(pf04346) EUT1
02282 HMM00465alcdh_3_mix(pf00465) EUT1
02283 HMM06723EutJMreB_1_grm(#) EUT1
02284 HMMalddh_1_eutpdugrm(pf00171) EUT1 EP_NTERM
02285 P_lightViolet EUT1
02286 H_azure EUT1
02287 HMM01515PTA_B_1_eut1(pf01515) EUT1
02288 HMM01923cobtr_2_eut1(pf01923) EUT1
02289 HMM06249EutQ_1_eut1(pf06249) EUT1 EP_NTERM
02290 HMM10662PduV_1_mix(pf10662) EUT1
02291 Hp_euts_violetPink EUT1
02292 HMM00390_malic_1_eut1(pf00390pf03949pf01515) EUT1
02293 -pf00923 -
02294 HMM02780keto_1_eut1(pf02779pf02780pf00456) EUT1
02295 -pf06674 -
02296 HMM00293nuhyd_1_eut1(pf00293) EUT1
02297 -pf13247pf14691pf07992 -
02298 -pf00672pf02518pf07730pf13675 -
02299 -pf00873 -
02300 -pf03960 -
02301 -pf07687pf01546 -
BMC-P
BMC-H
BMC-T
Aldehyde dehydrogenase
PTAC
Signature enzyme
Alcohol dehydrogenase
Regulator

Escherichia coli O139:H28 (strain E24377A / ETEC). Bacteria; Proteobacteria; Gammaproteobacteria; Enterobacterales;

EcE24377A__locus_2

EUT1

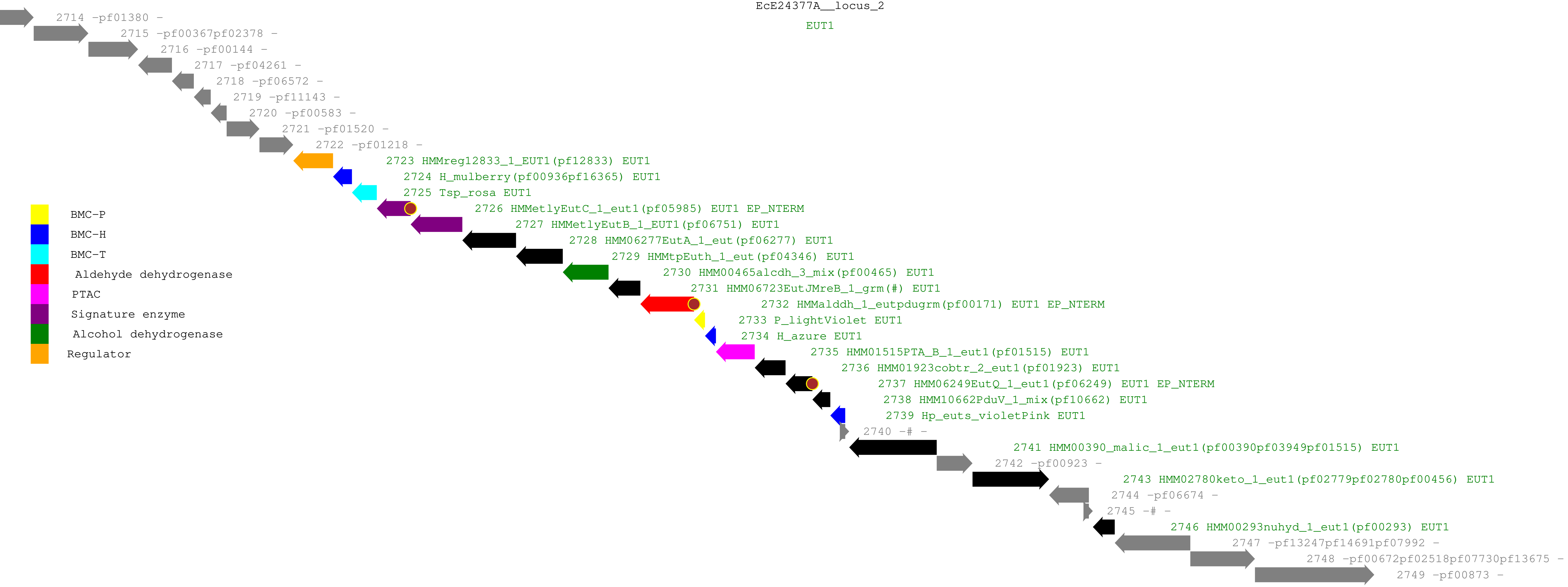

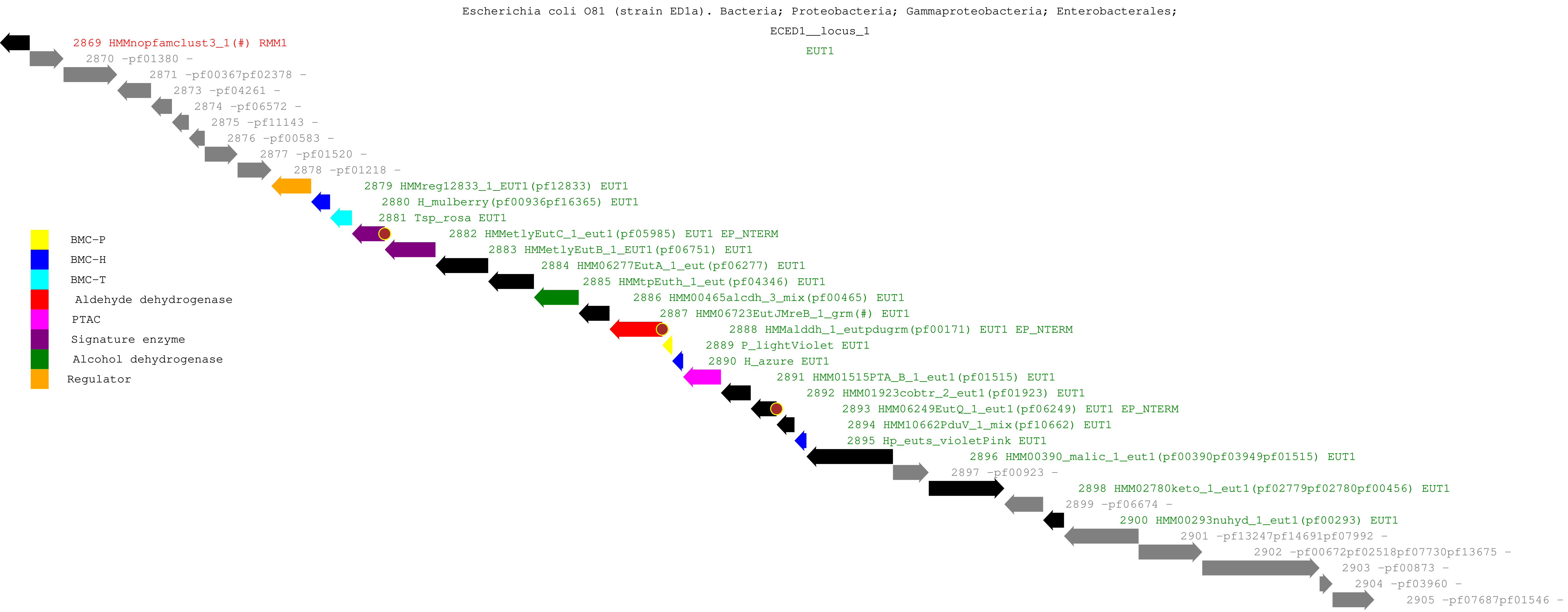

Escherichia coli O81 (strain ED1a). Bacteria; Proteobacteria; Gammaproteobacteria; Enterobacterales;
ECED1__locus_1
EUT1
2869 HMMnopfamclust3_1(#) RMM1
2870 -pf01380 -
2871 -pf00367pf02378 -
2873 -pf04261 -
2874 -pf06572 -
2875 -pf11143 -
2876 -pf00583 -
2877 -pf01520 -
2878 -pf01218 -
2879 HMMreg12833_1_EUT1(pf12833) EUT1
2880 H_mulberry(pf00936pf16365) EUT1
2881 Tsp_rosa EUT1
2882 HMMetlyEutC_1_eut1(pf05985) EUT1 EP_NTERM
2883 HMMetlyEutB_1_EUT1(pf06751) EUT1
2884 HMM06277EutA_1_eut(pf06277) EUT1
2885 HMMtpEuth_1_eut(pf04346) EUT1
2886 HMM00465alcdh_3_mix(pf00465) EUT1
2887 HMM06723EutJMreB_1_grm(#) EUT1
2888 HMMalddh_1_eutpdugrm(pf00171) EUT1 EP_NTERM
2889 P_lightViolet EUT1
2890 H_azure EUT1
2891 HMM01515PTA_B_1_eut1(pf01515) EUT1
2892 HMM01923cobtr_2_eut1(pf01923) EUT1
2893 HMM06249EutQ_1_eut1(pf06249) EUT1 EP_NTERM
2894 HMM10662PduV_1_mix(pf10662) EUT1
2895 Hp_euts_violetPink EUT1
2896 HMM00390_malic_1_eut1(pf00390pf03949pf01515) EUT1
2897 -pf00923 -
2898 HMM02780keto_1_eut1(pf02779pf02780pf00456) EUT1
2899 -pf06674 -
2900 HMM00293nuhyd_1_eut1(pf00293) EUT1
2901 -pf13247pf14691pf07992 -
2902 -pf00672pf02518pf07730pf13675 -
2903 -pf00873 -
2904 -pf03960 -
2905 -pf07687pf01546 -
BMC-P
BMC-H
BMC-T
Aldehyde dehydrogenase
PTAC
Signature enzyme
Alcohol dehydrogenase
Regulator

Escherichia coli B354. Bacteria; Proteobacteria; Gammaproteobacteria; Enterobacterales;

ECEG__locus_2

EUT1

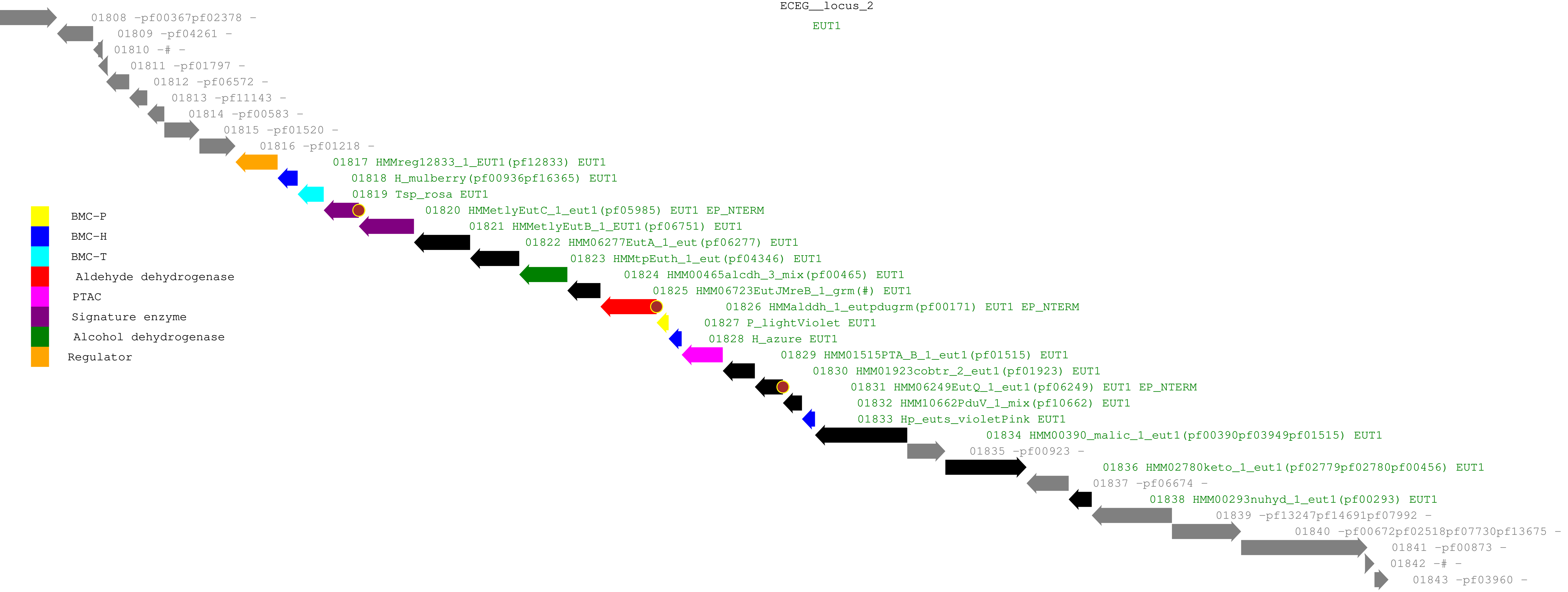

Escherichia coli O157:H7 (strain EC869). Bacteria; Proteobacteria; Gammaproteobacteria; Enterobacterales;

ECH7EC869__locus_1

EUT1

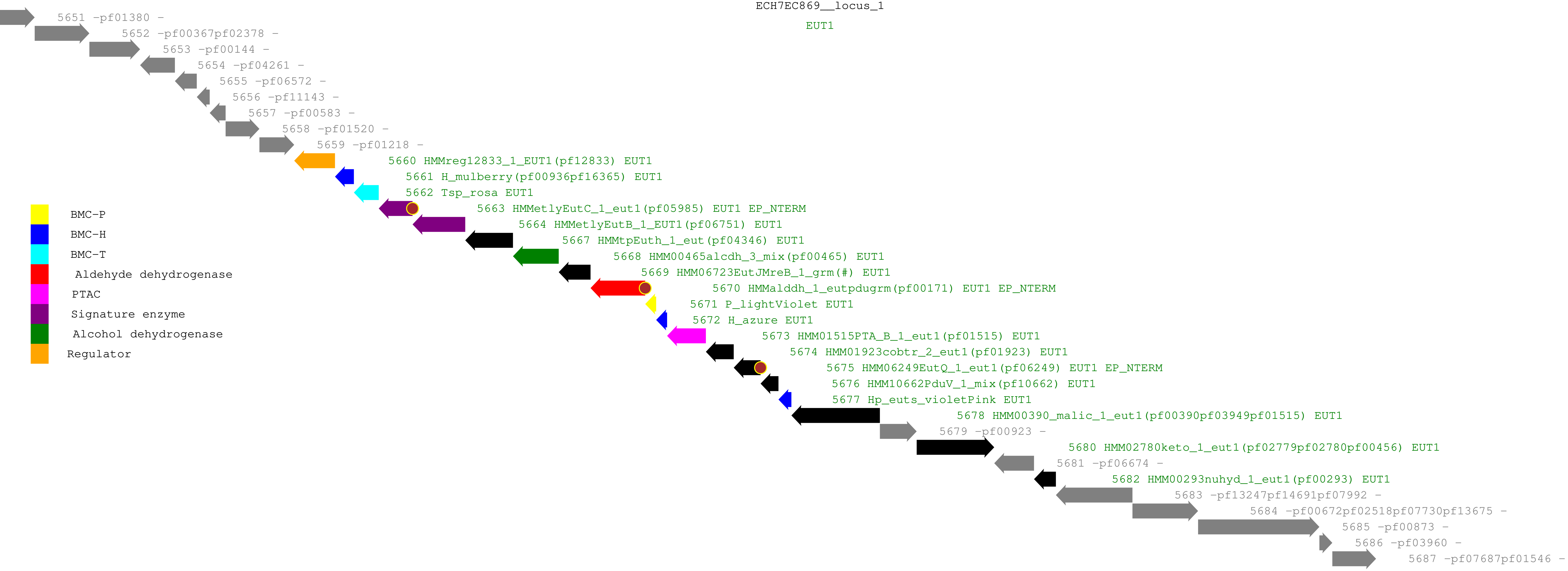

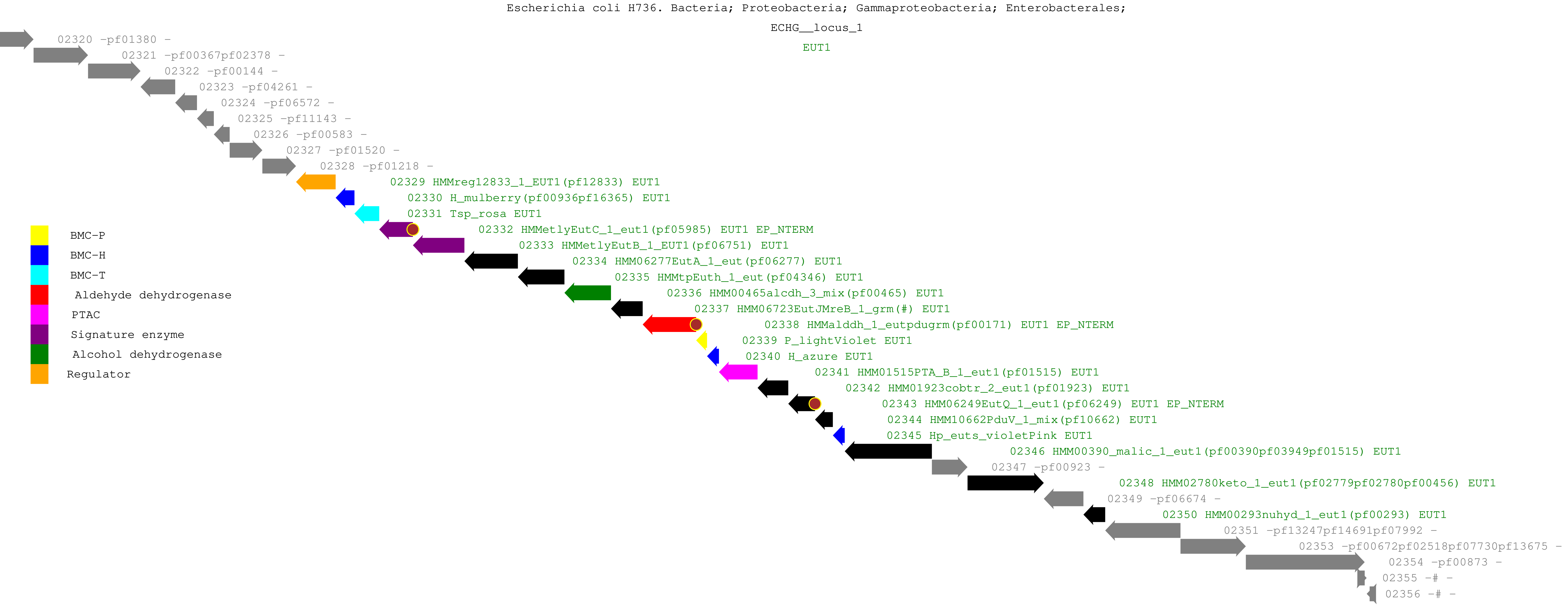

Escherichia coli H736. Bacteria; Proteobacteria; Gammaproteobacteria; Enterobacterales;
ECHG__locus_1
EUT1
02320 -pf01380 -
02321 -pf00367pf02378 -
02322 -pf00144 -
02323 -pf04261 -
02324 -pf06572 -
02325 -pf11143 -
02326 -pf00583 -
02327 -pf01520 -
02328 -pf01218 -
02329 HMMreg12833_1_EUT1(pf12833) EUT1
02330 H_mulberry(pf00936pf16365) EUT1
02331 Tsp_rosa EUT1
02332 HMMetlyEutC_1_eut1(pf05985) EUT1 EP_NTERM
02333 HMMetlyEutB_1_EUT1(pf06751) EUT1
02334 HMM06277EutA_1_eut(pf06277) EUT1
02335 HMMtpEuth_1_eut(pf04346) EUT1
02336 HMM00465alcdh_3_mix(pf00465) EUT1
02337 HMM06723EutJMreB_1_grm(#) EUT1
02338 HMMalddh_1_eutpdugrm(pf00171) EUT1 EP_NTERM
02339 P_lightViolet EUT1
02340 H_azure EUT1
02341 HMM01515PTA_B_1_eut1(pf01515) EUT1
02342 HMM01923cobtr_2_eut1(pf01923) EUT1
02343 HMM06249EutQ_1_eut1(pf06249) EUT1 EP_NTERM
02344 HMM10662PduV_1_mix(pf10662) EUT1
02345 Hp_euts_violetPink EUT1
02346 HMM00390_malic_1_eut1(pf00390pf03949pf01515) EUT1
02347 -pf00923 -
02348 HMM02780keto_1_eut1(pf02779pf02780pf00456) EUT1
02349 -pf06674 -
02350 HMM00293nuhyd_1_eut1(pf00293) EUT1
02351 -pf13247pf14691pf07992 -
02353 -pf00672pf02518pf07730pf13675 -
02354 -pf00873 -
02355 -# -
02356 -# -
BMC-P
BMC-H
BMC-T
Aldehyde dehydrogenase
PTAC
Signature enzyme
Alcohol dehydrogenase
Regulator

Escherichia coli O7:K1 (strain IAI39 / ExPEC). Bacteria; Proteobacteria; Gammaproteobacteria; Enterobacterales;

ECIAI39__locus_1

EUT1

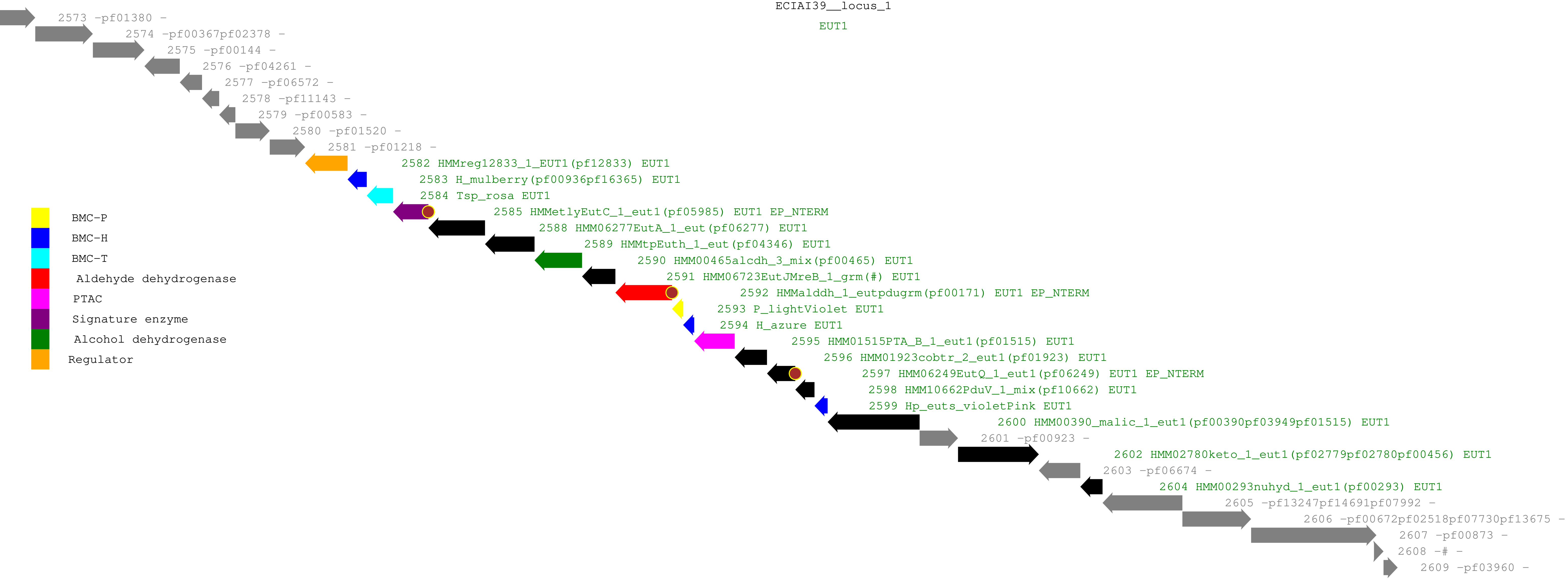

Escherichia coli M605. Bacteria; Proteobacteria; Gammaproteobacteria; Enterobacterales;

ECIG__locus_1

EUT1

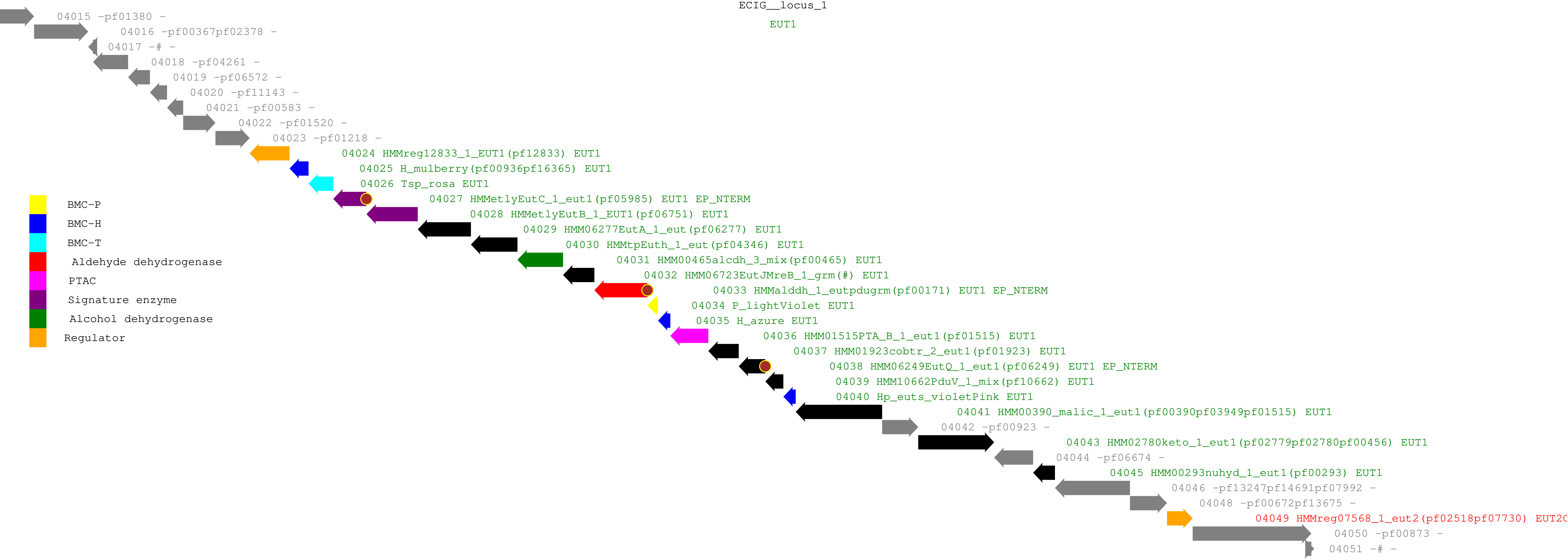

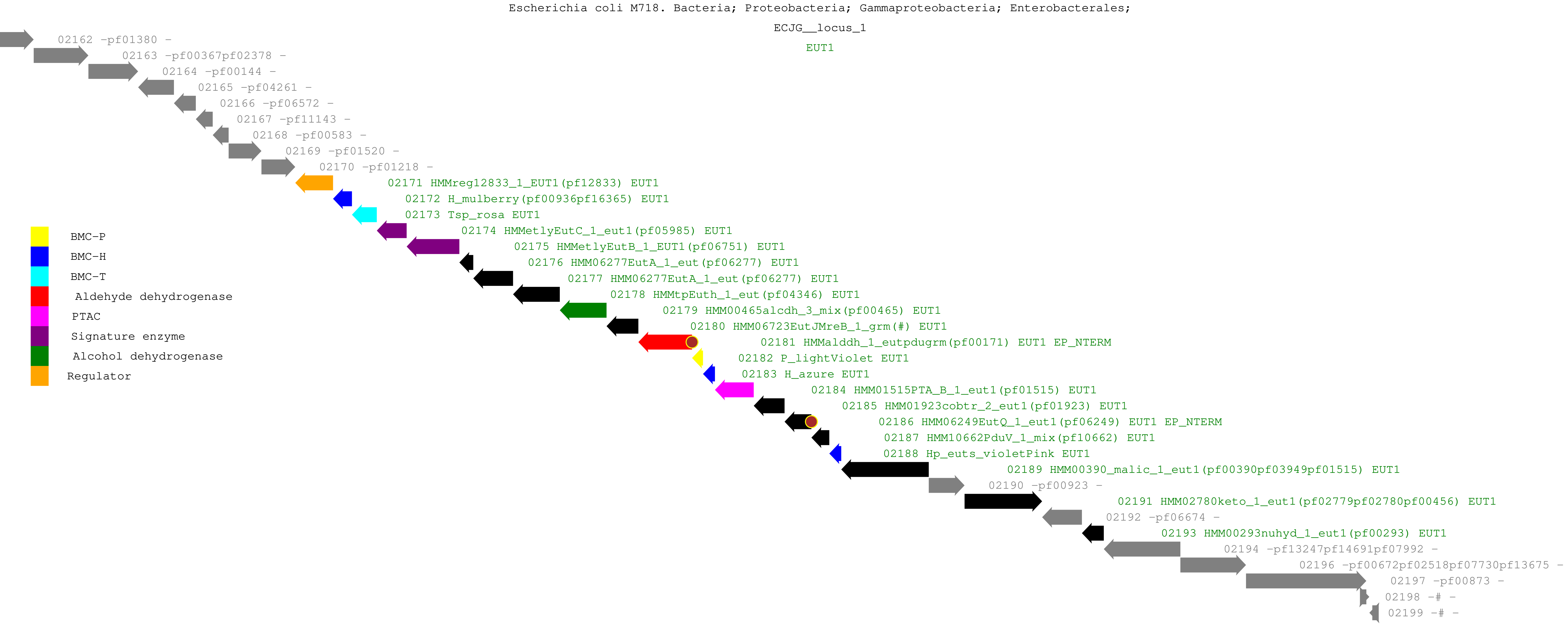
Escherichia coli M718. Bacteria; Proteobacteria; Gammaproteobacteria; Enterobacterales;
ECJG__locus_1
EUT1
02162 -pf01380 -
02163 -pf00367pf02378 -
02164 -pf00144 -
02165 -pf04261 -
02166 -pf06572 -
02167 -pf11143 -
02168 -pf00583 -
02169 -pf01520 -
02170 -pf01218 -
02171 HMMreg12833_1_EUT1(pf12833) EUT1
02172 H_mulberry(pf00936pf16365) EUT1
02173 Tsp_rosa EUT1
02174 HMMetlyEutC_1_eut1(pf05985) EUT1
02175 HMMetlyEutB_1_EUT1(pf06751) EUT1
02176 HMM06277EutA_1_eut(pf06277) EUT1
02177 HMM06277EutA_1_eut(pf06277) EUT1
02178 HMMtpEuth_1_eut(pf04346) EUT1
02179 HMM00465alcdh_3_mix(pf00465) EUT1
02180 HMM06723EutJMreB_1_grm(#) EUT1
02181 HMMalddh_1_eutpdugrm(pf00171) EUT1 EP_NTERM
02182 P_lightViolet EUT1
02183 H_azure EUT1
02184 HMM01515PTA_B_1_eut1(pf01515) EUT1
02185 HMM01923cobtr_2_eut1(pf01923) EUT1
02186 HMM06249EutQ_1_eut1(pf06249) EUT1 EP_NTERM
02187 HMM10662PduV_1_mix(pf10662) EUT1
02188 Hp_euts_violetPink EUT1
02189 HMM00390_malic_1_eut1(pf00390pf03949pf01515) EUT1
02190 -pf00923 -
02191 HMM02780keto_1_eut1(pf02779pf02780pf00456) EUT1
02192 -pf06674 -
02193 HMM00293nuhyd_1_eut1(pf00293) EUT1
02194 -pf13247pf14691pf07992 -
02196 -pf00672pf02518pf07730pf13675 -
02197 -pf00873 -
02198 -# -
02199 -# -
BMC-P
BMC-H
BMC-T
Aldehyde dehydrogenase
PTAC
Signature enzyme
Alcohol dehydrogenase
Regulator

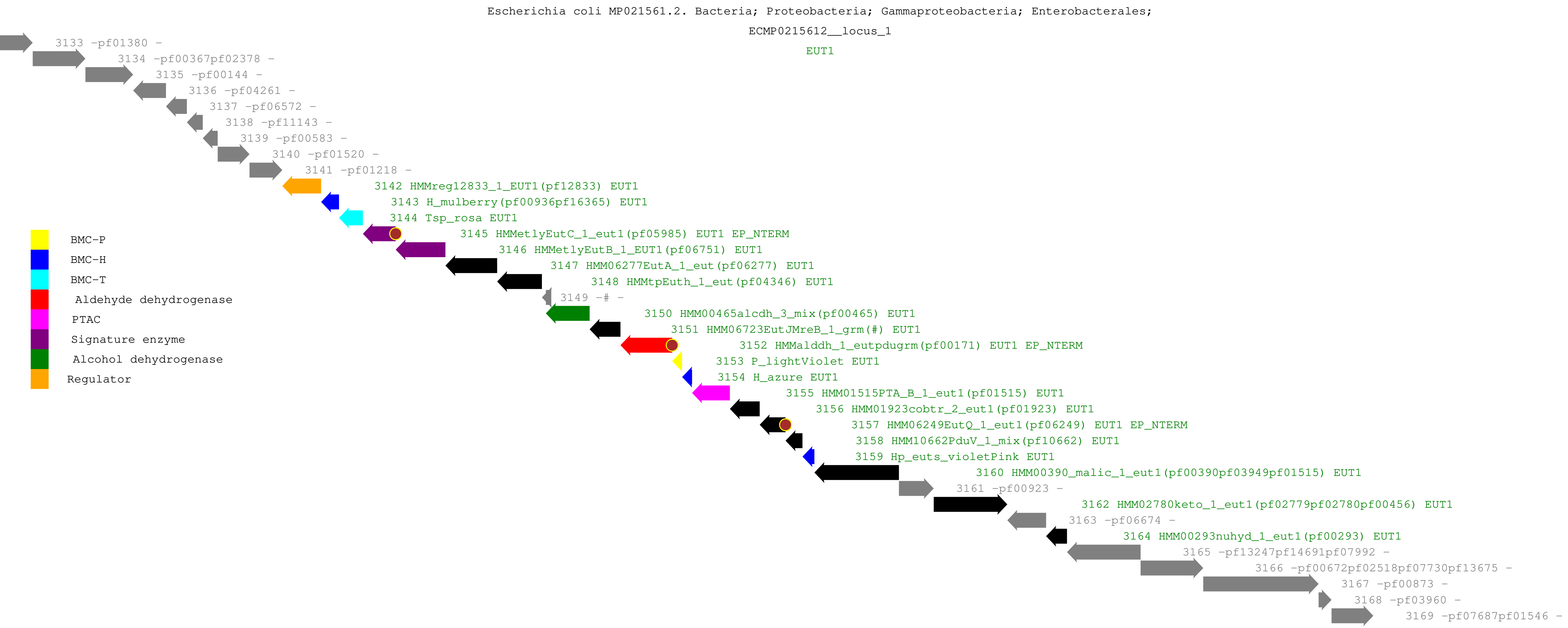
Escherichia coli MP021561.2. Bacteria; Proteobacteria; Gammaproteobacteria; Enterobacterales;
ECMP0215612__locus_1
EUT1
3133 -pf01380 -
3134 -pf00367pf02378 -
3135 -pf00144 -
3136 -pf04261 -
3137 -pf06572 -
3138 -pf11143 -
3139 -pf00583 -
3140 -pf01520 -
3141 -pf01218 -
3142 HMMreg12833_1_EUT1(pf12833) EUT1
3143 H_mulberry(pf00936pf16365) EUT1
3144 Tsp_rosa EUT1
3145 HMMetlyEutC_1_eut1(pf05985) EUT1 EP_NTERM
3146 HMMetlyEutB_1_EUT1(pf06751) EUT1
3147 HMM06277EutA_1_eut(pf06277) EUT1
3148 HMMtpEuth_1_eut(pf04346) EUT1
3149 -# -
3150 HMM00465alcdh_3_mix(pf00465) EUT1
3151 HMM06723EutJMreB_1_grm(#) EUT1
3152 HMMalddh_1_eutpdugrm(pf00171) EUT1 EP_NTERM
3153 P_lightViolet EUT1
3154 H_azure EUT1
3155 HMM01515PTA_B_1_eut1(pf01515) EUT1
3156 HMM01923cobtr_2_eut1(pf01923) EUT1
3157 HMM06249EutQ_1_eut1(pf06249) EUT1 EP_NTERM
3158 HMM10662PduV_1_mix(pf10662) EUT1
3159 Hp_euts_violetPink EUT1
3160 HMM00390_malic_1_eut1(pf00390pf03949pf01515) EUT1
3161 -pf00923 -
3162 HMM02780keto_1_eut1(pf02779pf02780pf00456) EUT1
3163 -pf06674 -
3164 HMM00293nuhyd_1_eut1(pf00293) EUT1
3165 -pf13247pf14691pf07992 -
3166 -pf00672pf02518pf07730pf13675 -
3167 -pf00873 -
3168 -pf03960 -
3169 -pf07687pf01546 -
BMC-P
BMC-H
BMC-T
Aldehyde dehydrogenase
PTAC
Signature enzyme
Alcohol dehydrogenase
Regulator

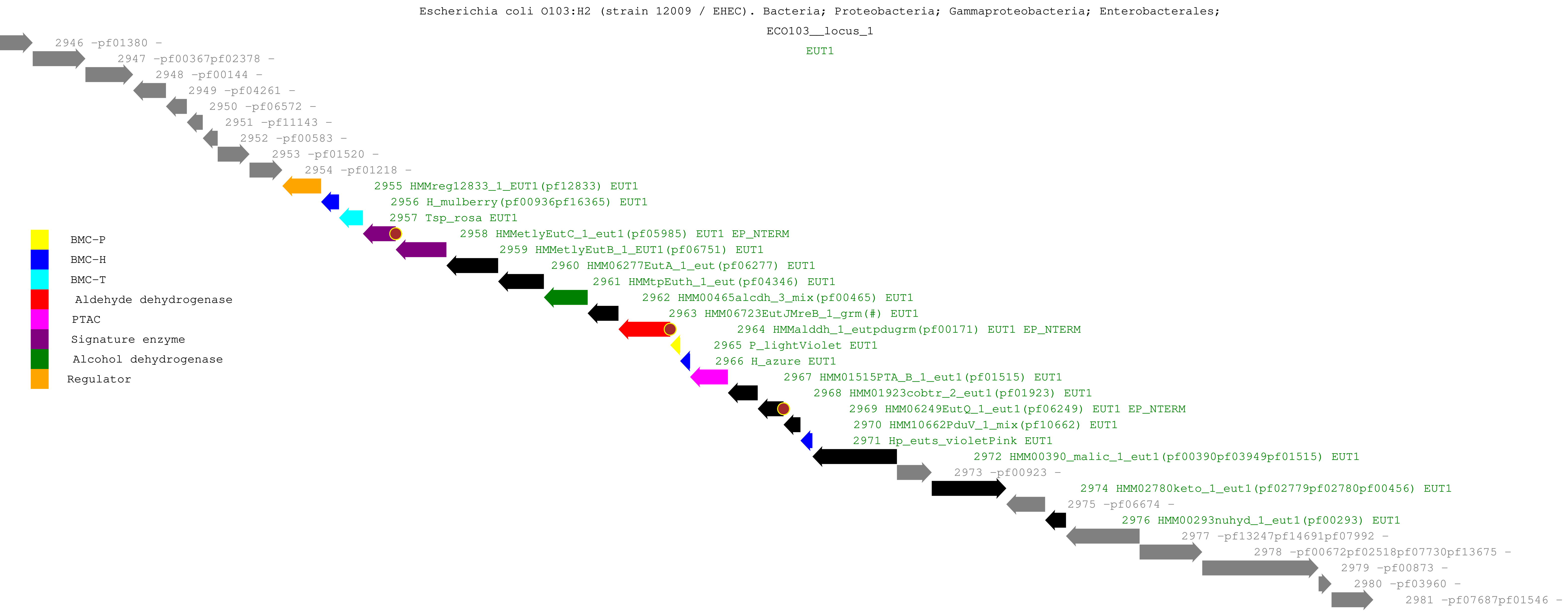

Escherichia coli O103:H2 (strain 12009 / EHEC). Bacteria; Proteobacteria; Gammaproteobacteria; Enterobacterales;
ECO103__locus_1
EUT1
2946 -pf01380 -
2947 -pf00367pf02378 -
2948 -pf00144 -
2949 -pf04261 -
2950 -pf06572 -
2951 -pf11143 -
2952 -pf00583 -
2953 -pf01520 -
2954 -pf01218 -
2955 HMMreg12833_1_EUT1(pf12833) EUT1
2956 H_mulberry(pf00936pf16365) EUT1
2957 Tsp_rosa EUT1
2958 HMMetlyEutC_1_eut1(pf05985) EUT1 EP_NTERM
2959 HMMetlyEutB_1_EUT1(pf06751) EUT1
2960 HMM06277EutA_1_eut(pf06277) EUT1
2961 HMMtpEuth_1_eut(pf04346) EUT1
2962 HMM00465alcdh_3_mix(pf00465) EUT1
2963 HMM06723EutJMreB_1_grm(#) EUT1
2964 HMMalddh_1_eutpdugrm(pf00171) EUT1 EP_NTERM
2965 P_lightViolet EUT1
2966 H_azure EUT1
2967 HMM01515PTA_B_1_eut1(pf01515) EUT1
2968 HMM01923cobtr_2_eut1(pf01923) EUT1
2969 HMM06249EutQ_1_eut1(pf06249) EUT1 EP_NTERM
2970 HMM10662PduV_1_mix(pf10662) EUT1
2971 Hp_euts_violetPink EUT1
2972 HMM00390_malic_1_eut1(pf00390pf03949pf01515) EUT1
2973 -pf00923 -
2974 HMM02780keto_1_eut1(pf02779pf02780pf00456) EUT1
2975 -pf06674 -
2976 HMM00293nuhyd_1_eut1(pf00293) EUT1
2977 -pf13247pf14691pf07992 -
2978 -pf00672pf02518pf07730pf13675 -
2979 -pf00873 -
2980 -pf03960 -
2981 -pf07687pf01546 -
BMC-P
BMC-H
BMC-T
Aldehyde dehydrogenase
PTAC
Signature enzyme
Alcohol dehydrogenase
Regulator

Escherichia coli O111:H- (strain 11128 / EHEC). Bacteria; Proteobacteria; Gammaproteobacteria; Enterobacterales;

ECO111__locus_1

EUT1

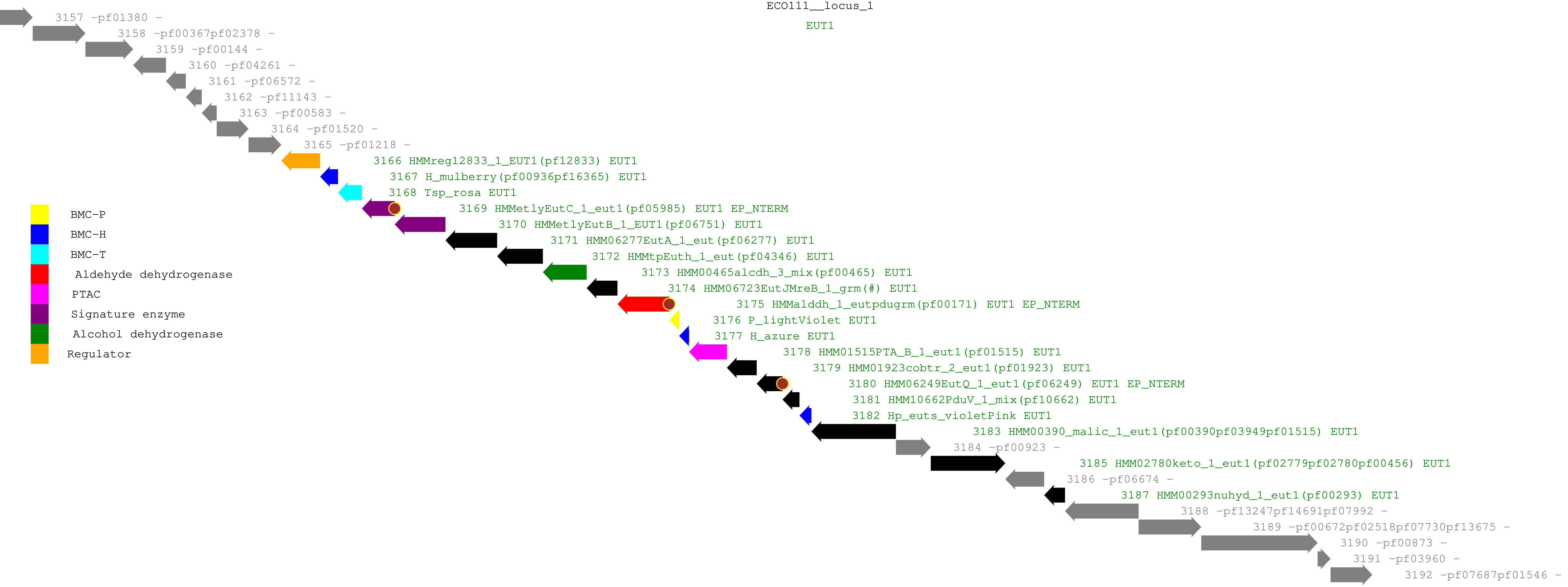

Escherichia coli O26:H11. Bacteria; Proteobacteria; Gammaproteobacteria; Enterobacterales;

ECO26H___locus_1

EUT1

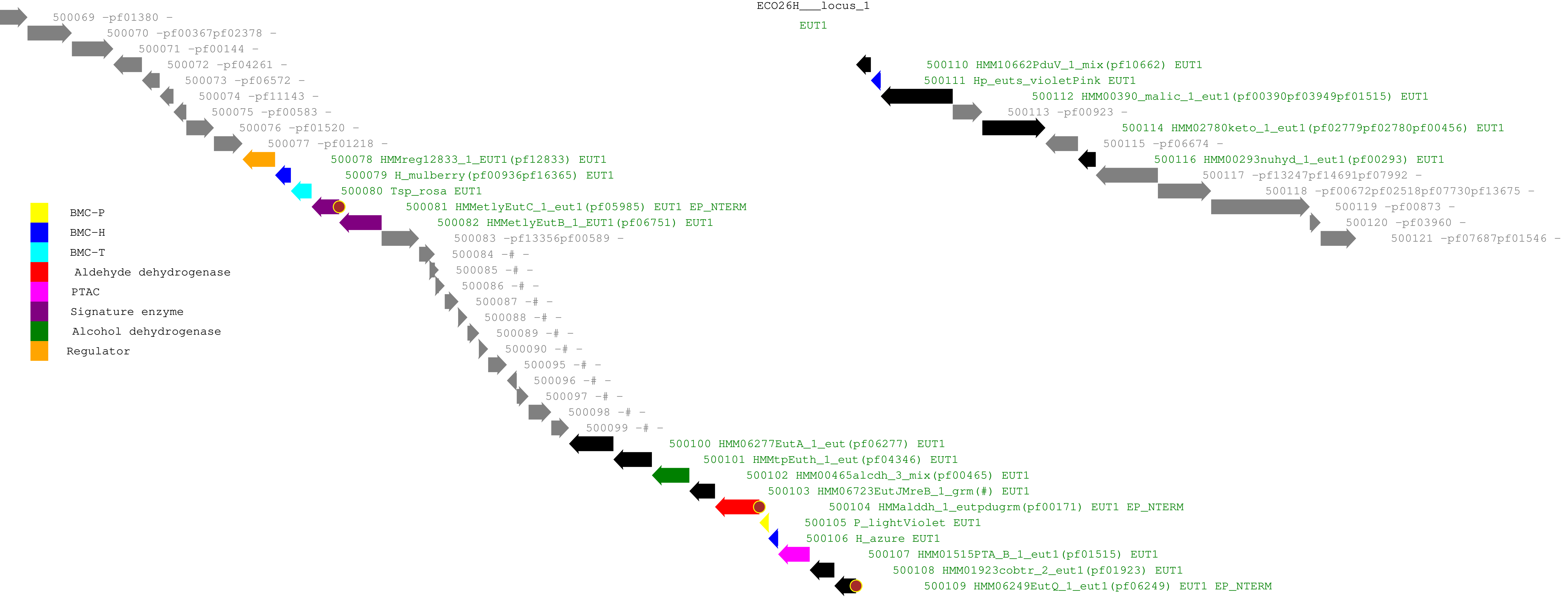

Escherichia coli O111:H11 str. CVM9455. Bacteria; Proteobacteria; Gammaproteobacteria; Enterobacterales;

ECO9455__locus_1

EUT1

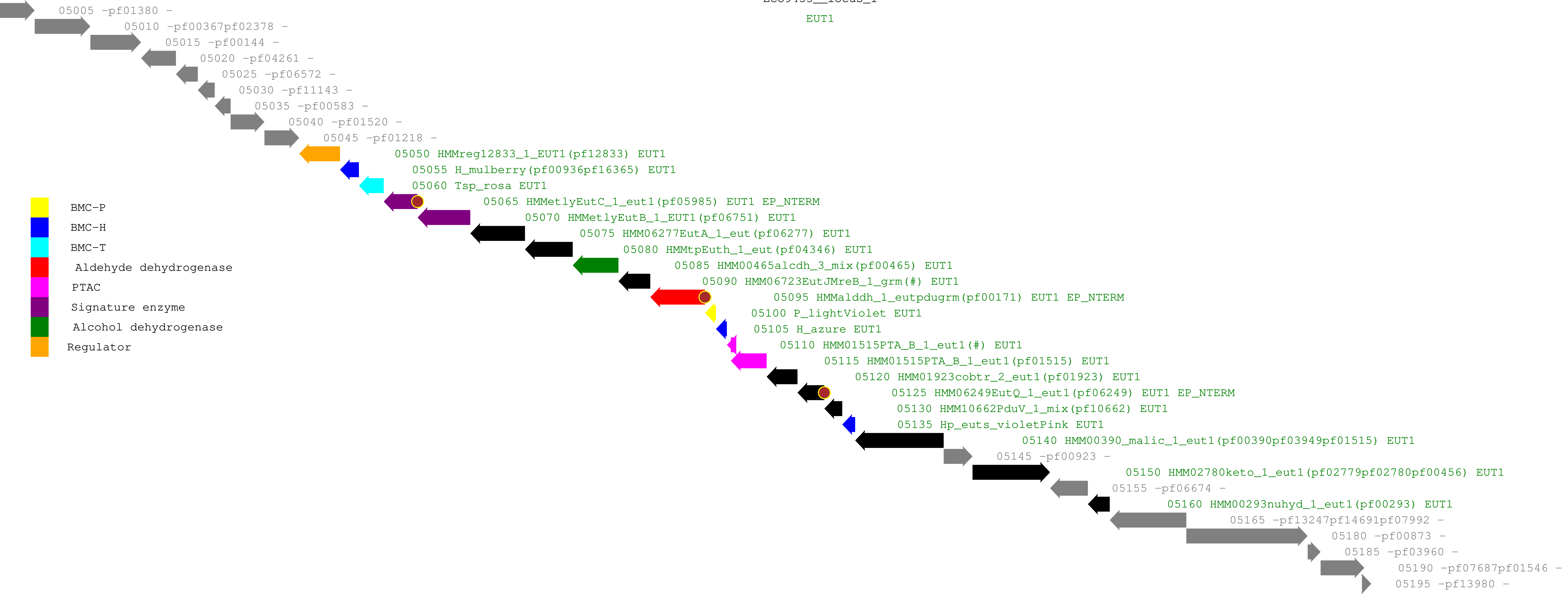

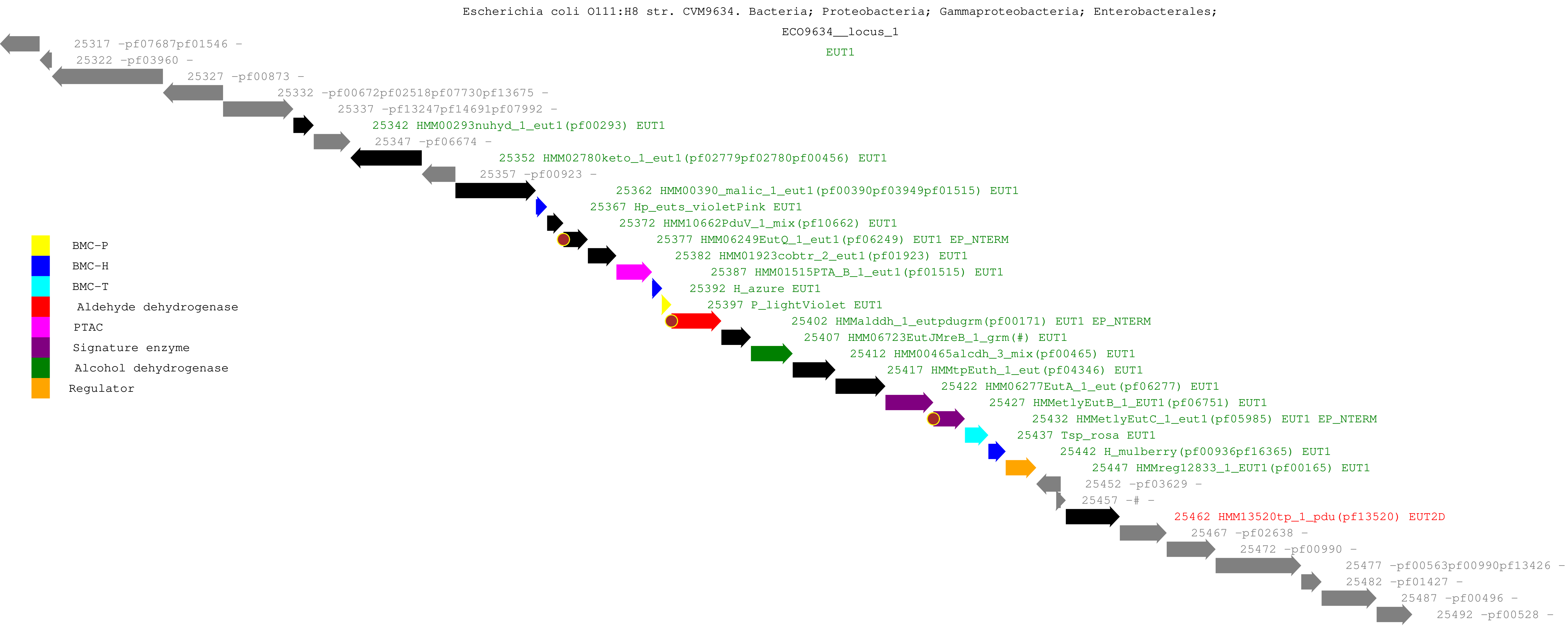
Escherichia coli O111:H8 str. CVM9634. Bacteria; Proteobacteria; Gammaproteobacteria; Enterobacterales;
ECO9634__locus_1
EUT1
25317 -pf07687pf01546 -
25322 -pf03960 -
25327 -pf00873 -
25332 -pf00672pf02518pf07730pf13675 -
25337 -pf13247pf14691pf07992 -
25342 HMM00293nuhyd_1_eut1(pf00293) EUT1
25347 -pf06674 -
25352 HMM02780keto_1_eut1(pf02779pf02780pf00456) EUT1
25357 -pf00923 -
25362 HMM00390_malic_1_eut1(pf00390pf03949pf01515) EUT1
25367 Hp_euts_violetPink EUT1
25372 HMM10662PduV_1_mix(pf10662) EUT1
25377 HMM06249EutQ_1_eut1(pf06249) EUT1 EP_NTERM
25382 HMM01923cobtr_2_eut1(pf01923) EUT1
25387 HMM01515PTA_B_1_eut1(pf01515) EUT1
25392 H_azure EUT1
25397 P_lightViolet EUT1
25402 HMMaldh_1_eutpdugrm(pf00171) EUT1 EP_NTERM
25407 HMM06723EutJMreB_1_grm(#) EUT1
25412 HMM00465alcdh_3_mix(pf00465) EUT1
25417 HMMtpEuth_1_eut(pf04346) EUT1
25422 HMM06277EutA_1_eut(pf06277) EUT1
25427 HMMetlyEutB_1_EUT1(pf06751) EUT1
25432 HMMetlyEutC_1_eut1(pf05985) EUT1 EP_NTERM
25437 Tsp_rosa EUT1
25442 H_mulberry(pf00936pf16365) EUT1
25447 HMMreg12833_1_EUT1(pf00165) EUT1
25452 -pf03629 -
25457 -# -
25462 HMM13520tp_1_pdu(pf13520) EUT2D
25467 -pf02638 -
25472 -pf00990 -
25477 -pf00563pf00990pf13426 -
25482 -pf01427 -
25487 -pf00496 -
25492 -pf00528 -
BMC-P
BMC-H
BMC-T
Aldehyde dehydrogenase
PTAC
Signature enzyme
Alcohol dehydrogenase
Regulator

Escherichia coli P0301867.11. Bacteria; Proteobacteria; Gammaproteobacteria; Enterobacterales;

ECP030186711__locus_1

EUT1

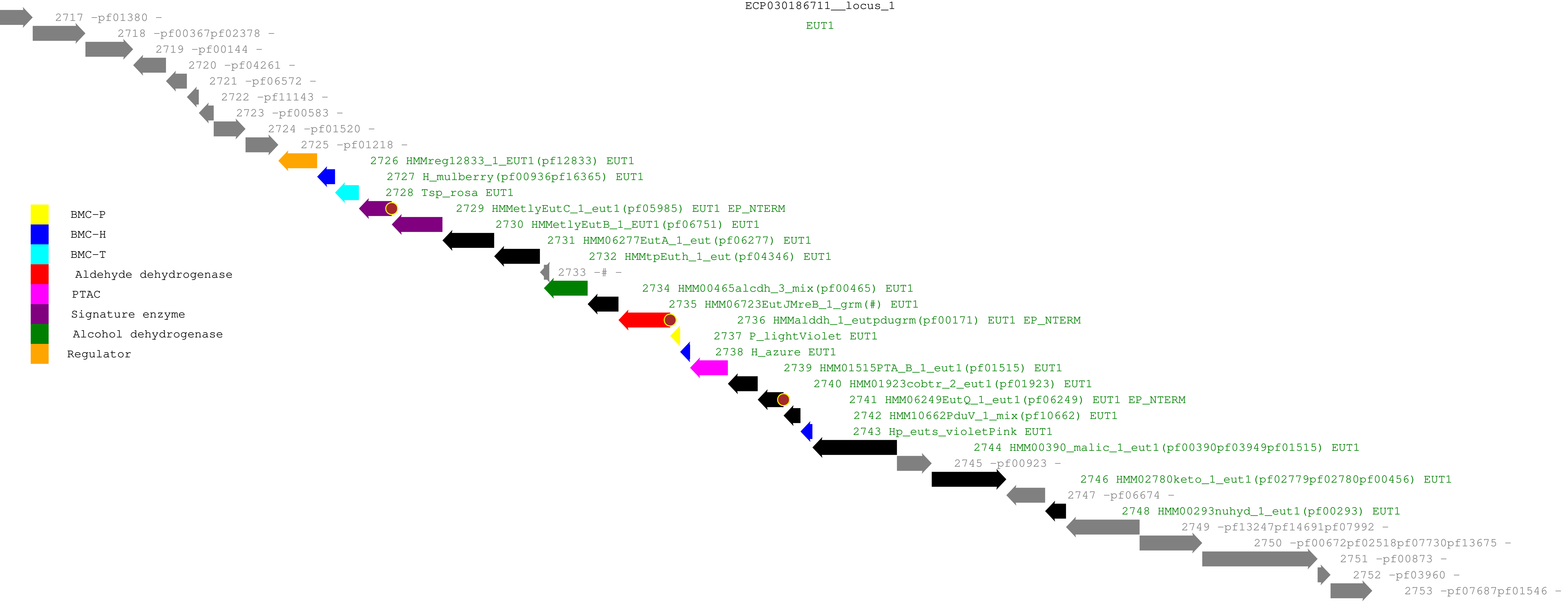

Escherichia coli P0301867.1. Bacteria; Proteobacteria; Gammaproteobacteria; Enterobacterales;

ECP03018671__locus_1

EUT1

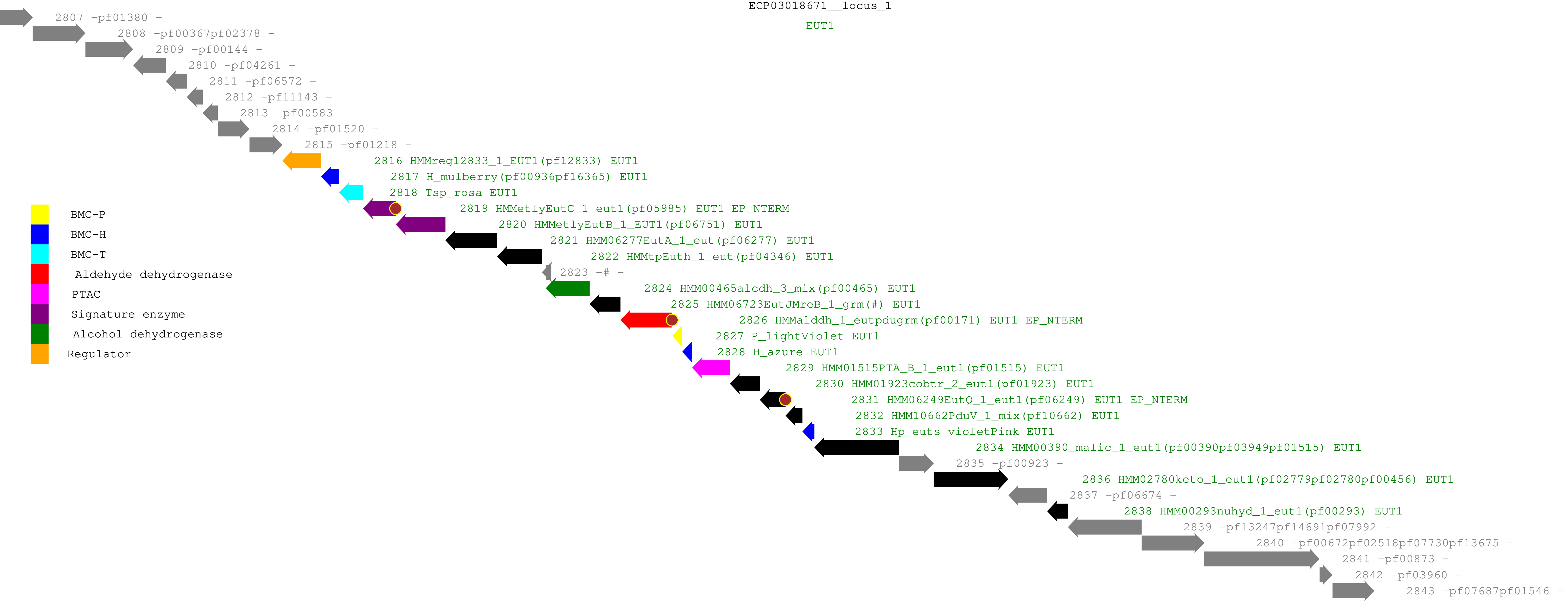

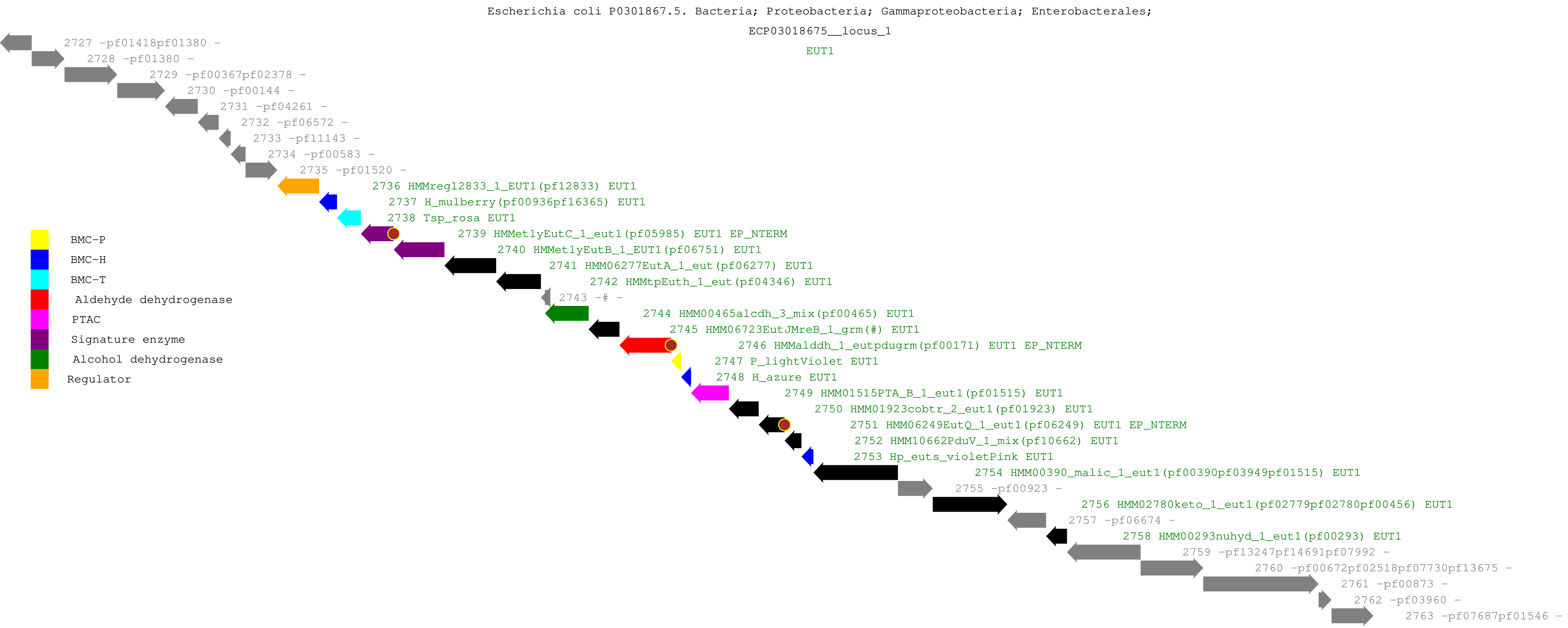

Escherichia coli P0301867.5. Bacteria; Proteobacteria; Gammaproteobacteria; Enterobacterales;
ECP03018675__locus_1
EUT1
2727 -pf01418pf01380 -
2728 -pf01380 -
2729 -pf00367pf02378 -
2730 -pf00144 -
2731 -pf04261 -
2732 -pf06572 -
2733 -pf11143 -
2734 -pf00583 -
2735 -pf01520 -
2736 HMMreg12833_1_EUT1(pf12833) EUT1
2737 H_mulberry(pf00936pf16365) EUT1
2738 Tsp_rosa EUT1
2739 HMMetlyEutC_1_eut1(pf05985) EUT1 EP_NTERM
2740 HMMetlyEutB_1_EUT1(pf06751) EUT1
2741 HMM06277EutA_1_eut(pf06277) EUT1
2742 HMMtpEuth_1_eut(pf04346) EUT1
2743 -# -
2744 HMM00465alcdh_3_mix(pf00465) EUT1
2745 HMM06723EutJMreB_1_grm(#) EUT1
2746 HMMalddh_1_eutpdugrm(pf00171) EUT1 EP_NTERM
2747 P_lightViolet EUT1
2748 H_azure EUT1
2749 HMM01515PTA_B_1_eut1(pf01515) EUT1
2750 HMM01923cobtr_2_eut1(pf01923) EUT1
2751 HMM06249EutQ_1_eut1(pf06249) EUT1 EP_NTERM
2752 HMM10662PduV_1_mix(pf10662) EUT1
2753 Hp_euts_violetPink EUT1
2754 HMM00390_malic_1_eut1(pf00390pf03949pf01515) EUT1
2755 -pf00923 -
2756 HMM02780keto_1_eut1(pf02779pf02780pf00456) EUT1
2757 -pf06674 -
2758 HMM00293nuhyd_1_eut1(pf00293) EUT1
2759 -pf13247pf14691pf07992 -
2760 -pf00672pf02518pf07730pf13675 -
2761 -pf00873 -
2762 -pf03960 -
2763 -pf07687pf01546 -
BMC-P
BMC-H
BMC-T
Aldehyde dehydrogenase
PTAC
Signature enzyme
Alcohol dehydrogenase
Regulator

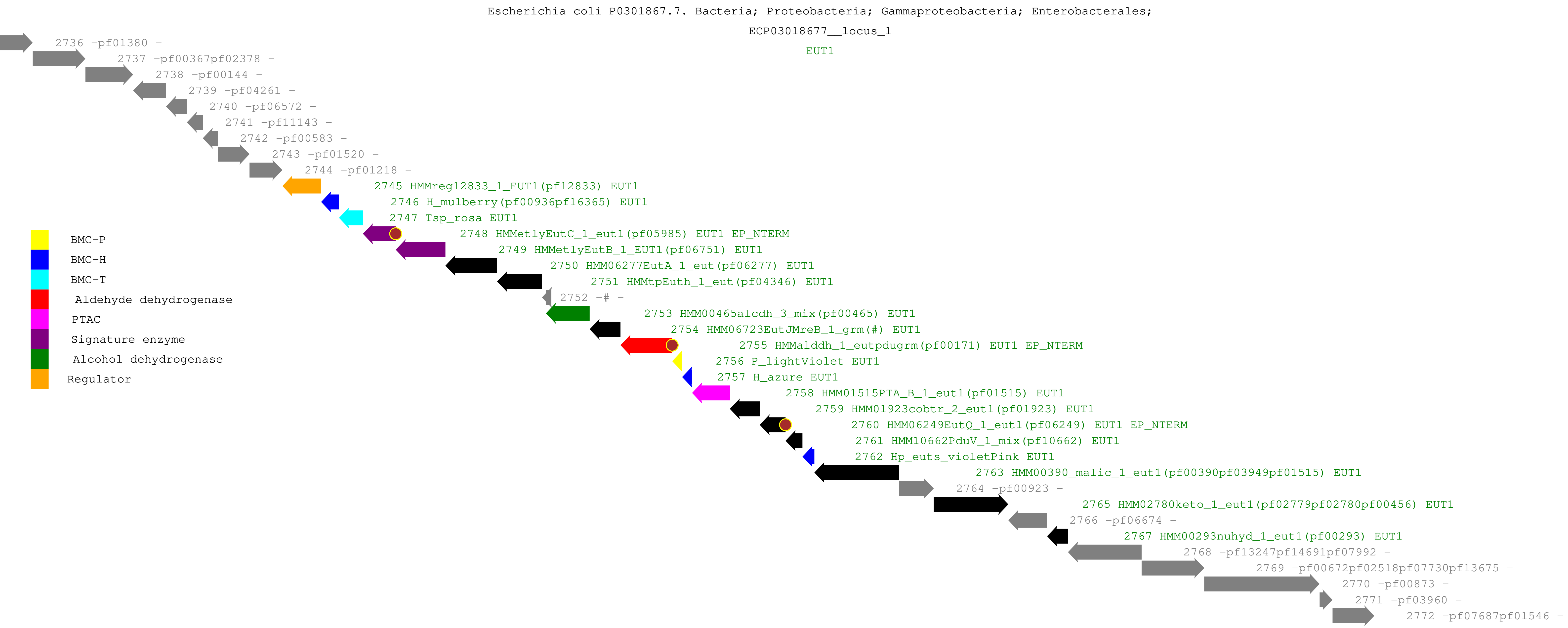

Escherichia coli P0301867.7. Bacteria; Proteobacteria; Gammaproteobacteria; Enterobacterales;
ECP03018677__locus_1
EUT1
2736 -pf01380 -
2737 -pf00367pf02378 -
2738 -pf00144 -
2739 -pf04261 -
2740 -pf06572 -
2741 -pf11143 -
2742 -pf00583 -
2743 -pf01520 -
2744 -pf01218 -
2745 HMMreg12833_1_EUT1(pf12833) EUT1
2746 H_mulberry(pf00936pf16365) EUT1
2747 Tsp_rosa EUT1
2748 HMMetlyEutC_1_eut1(pf05985) EUT1 EP_NTERM
2749 HMMetlyEutB_1_EUT1(pf06751) EUT1
2750 HMM06277EutA_1_eut(pf06277) EUT1
2751 HMMtpEuth_1_eut(pf04346) EUT1
2752 -# -
2753 HMM00465alcdh_3_mix(pf00465) EUT1
2754 HMM06723EutJMreB_1_grm(#) EUT1
2755 HMMaldh_1_eutpdugrm(pf00171) EUT1 EP_NTERM
2756 P_lightViolet EUT1
2757 H_azure EUT1
2758 HMM01515PTA_B_1_eut1(pf01515) EUT1
2759 HMM01923cobtr_2_eut1(pf01923) EUT1
2760 HMM06249EutQ_1_eut1(pf06249) EUT1 EP_NTERM
2761 HMM10662PduV_1_mix(pf10662) EUT1
2762 Hp_euts_violetPink EUT1
2763 HMM00390_malic_1_eut1(pf00390pf03949pf01515) EUT1
2764 -pf00923 -
2765 HMM02780keto_1_eut1(pf02779pf02780pf00456) EUT1
2766 -pf06674 -
2767 HMM00293nuhyd_1_eut1(pf00293) EUT1
2768 -pf13247pf14691pf07992 -
2769 -pf00672pf02518pf07730pf13675 -
2770 -pf00873 -
2771 -pf03960 -
2772 -pf07687pf01546 -
BMC-P
BMC-H
BMC-T
Aldehyde dehydrogenase
PTAC
Signature enzyme
Alcohol dehydrogenase
Regulator

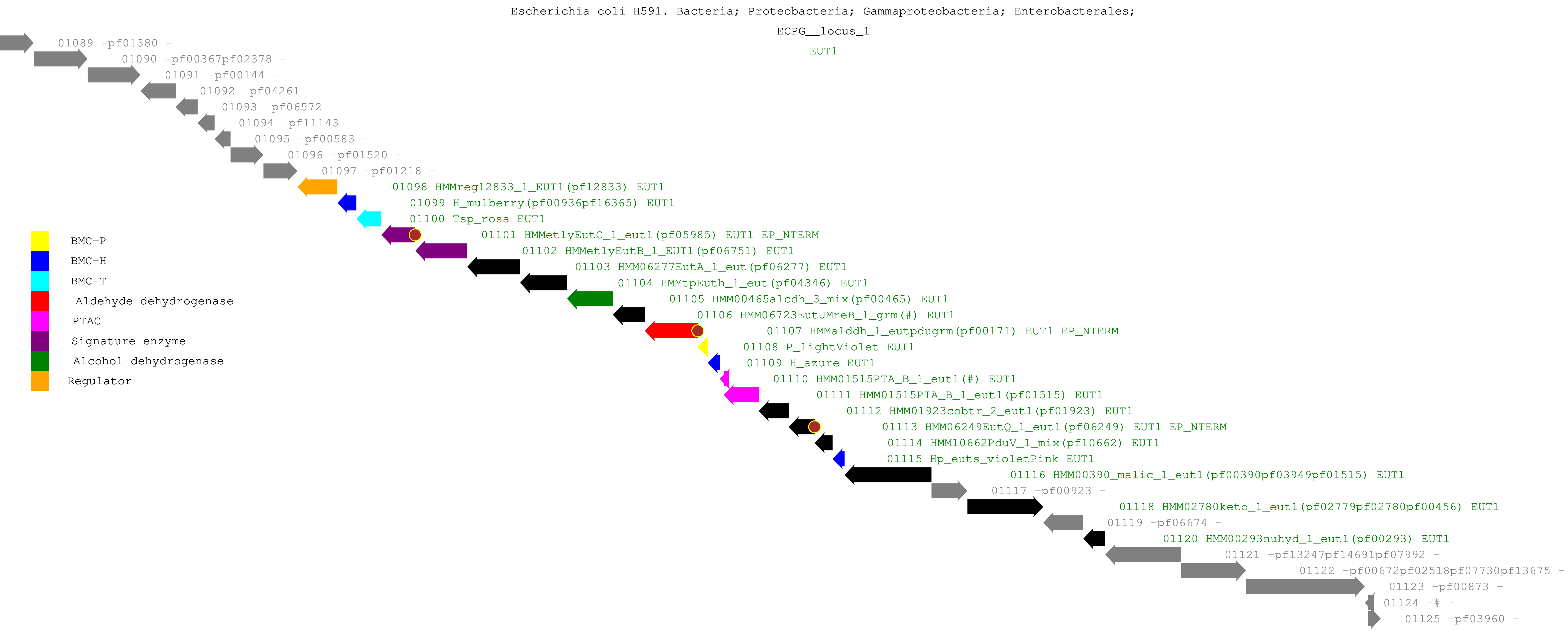
Escherichia coli H591. Bacteria; Proteobacteria; Gammaproteobacteria; Enterobacterales;
ECPG__locus_1
EUT1
01089 -pf01380 -
01090 -pf00367pf02378 -
01091 -pf00144 -
01092 -pf04261 -
01093 -pf06572 -
01094 -pf11143 -
01095 -pf00583 -
01096 -pf01520 -
01097 -pf01218 -
01098 HMMreg12833_1_EUT1(pf12833) EUT1
01099 H_mulberry(pf00936pf16365) EUT1
01100 Tsp_rosa EUT1
01101 HMMetlyEutC_1_eut1(pf05985) EUT1 EP_NTERM
01102 HMMetlyEutB_1_EUT1(pf06751) EUT1
01103 HMM06277EutA_1_eut(pf06277) EUT1
01104 HMMtpEuth_1_eut(pf04346) EUT1
01105 HMM00465alcdh_3_mix(pf00465) EUT1
01106 HMM06723EutJMreB_1_grm(#) EUT1
01107 HMMalddh_1_eutpdugrm(pf00171) EUT1 EP_NTERM
01108 P_lightViolet EUT1
01109 H_azure EUT1
01110 HMM01515PTA_B_1_eut1(#) EUT1
01111 HMM01515PTA_B_1_eut1(pf01515) EUT1
01112 HMM01923cobtr_2_eut1(pf01923) EUT1
01113 HMM06249EutQ_1_eut1(pf06249) EUT1 EP_NTERM
01114 HMM10662PduV_1_mix(pf10662) EUT1
01115 Hp_euts_violetPink EUT1
01116 HMM00390_malic_1_eut1(pf00390pf03949pf01515) EUT1
01117 -pf00923 -
01118 HMM02780keto_1_eut1(pf02779pf02780pf00456) EUT1
01119 -pf06674 -
01120 HMM00293nuhyd_1_eut1(pf00293) EUT1
01121 -pf13247pf14691pf07992 -
01122 -pf00672pf02518pf07730pf13675 -
01123 -pf00873 -
01124 -# -
01125 -pf03960 -
BMC-P
BMC-H
BMC-T
Aldehyde dehydrogenase
PTAC
Signature enzyme
Alcohol dehydrogenase
Regulator

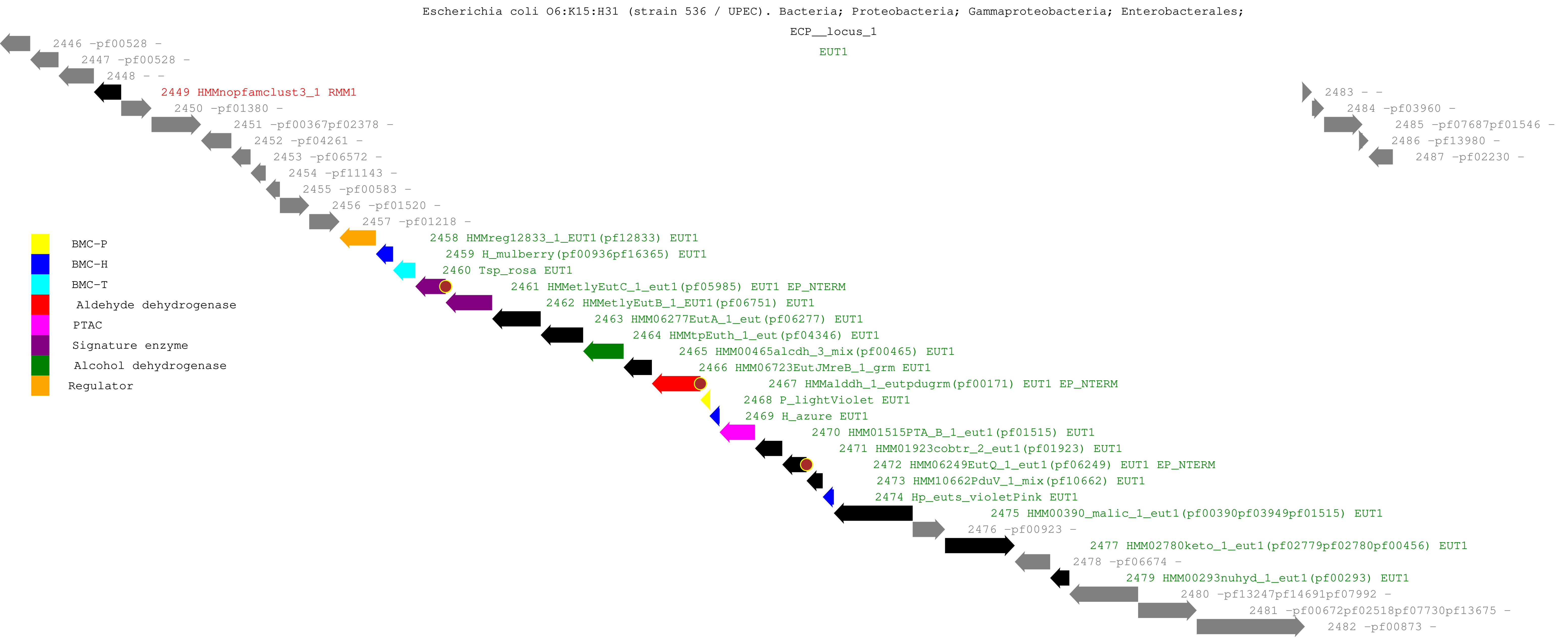
Escherichia coli O6:K15:H31 (strain 536 / UPEC). Bacteria; Proteobacteria; Gammaproteobacteria; Enterobacterales;
ECP__locus_1
EUT1
2446 -pf00528 -
2447 -pf00528 -
2448 - -
2449 HMMnopfamclust3_1 RMM1
2450 -pf01380 -
2451 -pf00367pf02378 -
2452 -pf04261 -
2453 -pf06572 -
2454 -pf11143 -
2455 -pf00583 -
2456 -pf01520 -
2457 -pf01218 -
2458 HMMreg12833_1_EUT1(pf12833) EUT1
2459 H_mulberry(pf00936pf16365) EUT1
2460 Tsp_rosa EUT1
2461 HMMetlyEutC_1_eut1(pf05985) EUT1 EP_NTERM
2462 HMMetlyEutB_1_EUT1(pf06751) EUT1
2463 HMM06277EutA_1_eut(pf06277) EUT1
2464 HMMtpEuth_1_eut(pf04346) EUT1
2465 HMM00465alcdh_3_mix(pf00465) EUT1
2466 HMM06723EutJMreB_1_grm EUT1
2467 HMMalddh_1_eutpdugrm(pf00171) EUT1 EP_NTERM
2468 P_lightViolet EUT1
2469 H_azure EUT1
2470 HMM01515PTA_B_1_eut1(pf01515) EUT1
2471 HMM01923cobtr_2_eut1(pf01923) EUT1
2472 HMM06249EutQ_1_eut1(pf06249) EUT1 EP_NTERM
2473 HMM10662PduV_1_mix(pf10662) EUT1
2474 Hp_euts_violetPink EUT1
2475 HMM00390_malic_1_eut1(pf00390pf03949pf01515) EUT1
2476 -pf00923 -
2477 HMM02780keto_1_eut1(pf02779pf02780pf00456) EUT1
2478 -pf06674 -
2479 HMM00293nuhyd_1_eut1(pf00293) EUT1
2480 -pf13247pf14691pf07992 -
2481 -pf00672pf02518pf07730pf13675 -
2482 -pf00873 -
2483 - -
2484 -pf03960 -
2485 -pf07687pf01546 -
2486 -pf13980 -
2487 -pf02230 -
BMC-P
BMC-H
BMC-T
Aldehyde dehydrogenase
PTAC
Signature enzyme
Alcohol dehydrogenase
Regulator

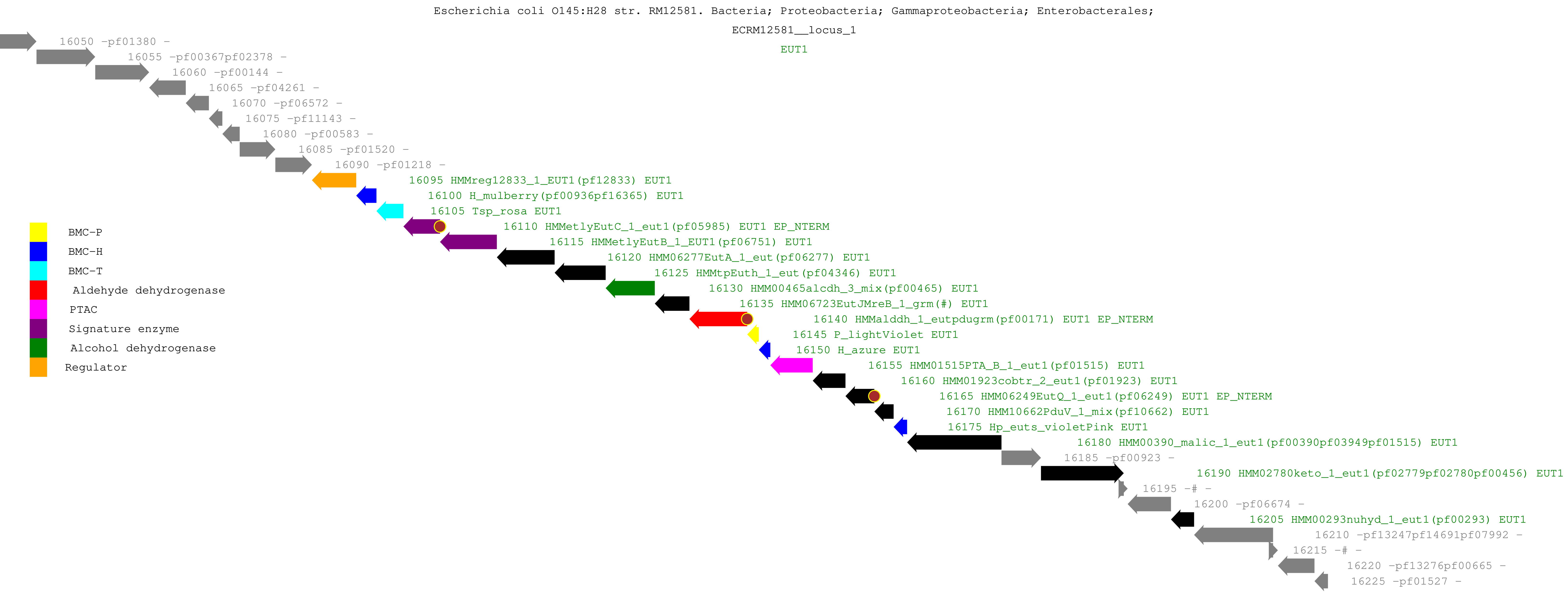
Escherichia coli O145:H28 str. RM12581. Bacteria; Proteobacteria; Gammaproteobacteria; Enterobacterales;
ECRM12581__locus_1
EUT1
16050 -pf01380 -
16055 -pf00367pf02378 -
16060 -pf00144 -
16065 -pf04261 -
16070 -pf06572 -
16075 -pf11143 -
16080 -pf00583 -
16085 -pf01520 -
16090 -pf01218 -
16095 HMMreg12833_1_EUT1(pf12833) EUT1
16100 H_mulberry(pf00936pf16365) EUT1
16105 Tsp_rosa EUT1
16110 HMMetlyEutC_1_eut1(pf05985) EUT1 EP_NTERM
16115 HMMetlyEutB_1_EUT1(pf06751) EUT1
16120 HMM06277EutA_1_eut(pf06277) EUT1
16125 HMMtpEuth_1_eut(pf04346) EUT1
16130 HMM00465alcdh_3_mix(pf00465) EUT1
16135 HMM06723EutJMreB_1_grm(#) EUT1
16140 HMMaldh_1_eutpdugrm(pf00171) EUT1 EP_NTERM
16145 P_lightViolet EUT1
16150 H_azure EUT1
16155 HMM01515PTA_B_1_eut1(pf01515) EUT1
16160 HMM01923cobtr_2_eut1(pf01923) EUT1
16165 HMM06249EutQ_1_eut1(pf06249) EUT1 EP_NTERM
16170 HMM10662PduV_1_mix(pf10662) EUT1
16175 Hp_euts_violetPink EUT1
16180 HMM00390_malic_1_eut1(pf00390pf03949pf01515) EUT1
16185 -pf00923 -
16190 HMM02780keto_1_eut1(pf02779pf02780pf00456) EUT1
16195 -# -
16200 -pf06674 -
16205 HMM00293nuhyd_1_eut1(pf00293) EUT1
16210 -pf13247pf14691pf07992 -
16215 -# -
16220 -pf13276pf00665 -
16225 -pf01527 -
BMC-P
BMC-H
BMC-T
Aldehyde dehydrogenase
PTAC
Signature enzyme
Alcohol dehydrogenase
Regulator

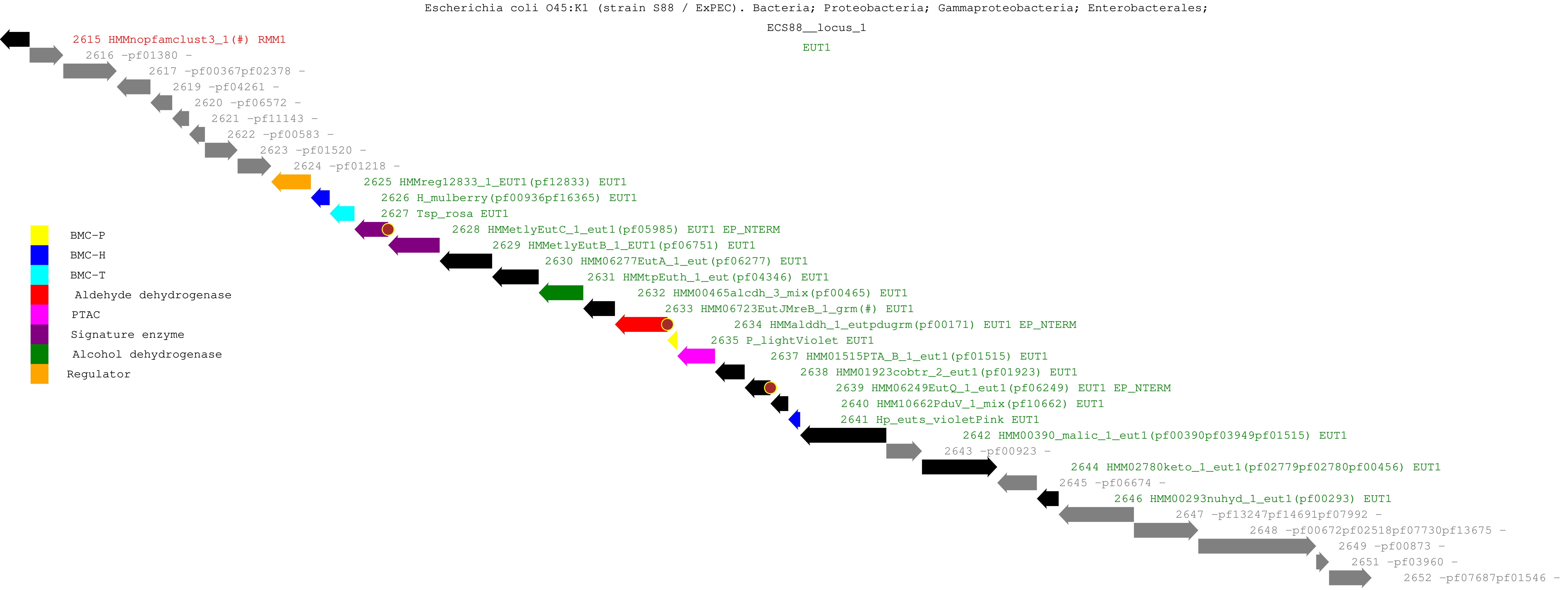

Escherichia coli O45:K1 (strain S88 / ExPEC). Bacteria; Proteobacteria; Gammaproteobacteria; Enterobacterales;
ECS88__locus_1
EUT1
2615 HMMnopfamclust3_1(#) RMM1
2616 -pf01380 -
2617 -pf00367pf02378 -
2619 -pf04261 -
2620 -pf06572 -
2621 -pf11143 -
2622 -pf00583 -
2623 -pf01520 -
2624 -pf01218 -
2625 HMMreg12833_1_EUT1(pf12833) EUT1
2626 H_mulberry(pf00936pf16365) EUT1
2627 Tsp_rosa EUT1
2628 HMMetlyEutC_1_eut1(pf05985) EUT1 EP_NTERM
2629 HMMetlyEutB_1_EUT1(pf06751) EUT1
2630 HMM06277EutA_1_eut(pf06277) EUT1
2631 HMMtpEuth_1_eut(pf04346) EUT1
2632 HMM00465alcdh_3_mix(pf00465) EUT1
2633 HMM06723EutJMreB_1_grm(#) EUT1
2634 HMMaldh_1_eutpdugrm(pf00171) EUT1 EP_NTERM
2635 P_lightViolet EUT1
2637 HMM01515PTA_B_1_eut1(pf01515) EUT1
2638 HMM01923cobtr_2_eut1(pf01923) EUT1
2639 HMM06249EutQ_1_eut1(pf06249) EUT1 EP_NTERM
2640 HMM10662PduV_1_mix(pf10662) EUT1
2641 Hp_euts_violetPink EUT1
2642 HMM00390_malic_1_eut1(pf00390pf03949pf01515) EUT1
2643 -pf00923 -
2644 HMM02780keto_1_eut1(pf02779pf02780pf00456) EUT1
2645 -pf06674 -
2646 HMM00293nuhyd_1_eut1(pf00293) EUT1
2647 -pf13247pf14691pf07992 -
2648 -pf00672pf02518pf07730pf13675 -
2649 -pf00873 -
2651 -pf03960 -
2652 -pf07687pf01546 -
BMC-P
BMC-H
BMC-T
Aldehyde dehydrogenase
PTAC
Signature enzyme
Alcohol dehydrogenase
Regulator

Escherichia coli (strain SE11). Bacteria; Proteobacteria; Gammaproteobacteria; Enterobacterales;

ECSE__locus_1

EUT1

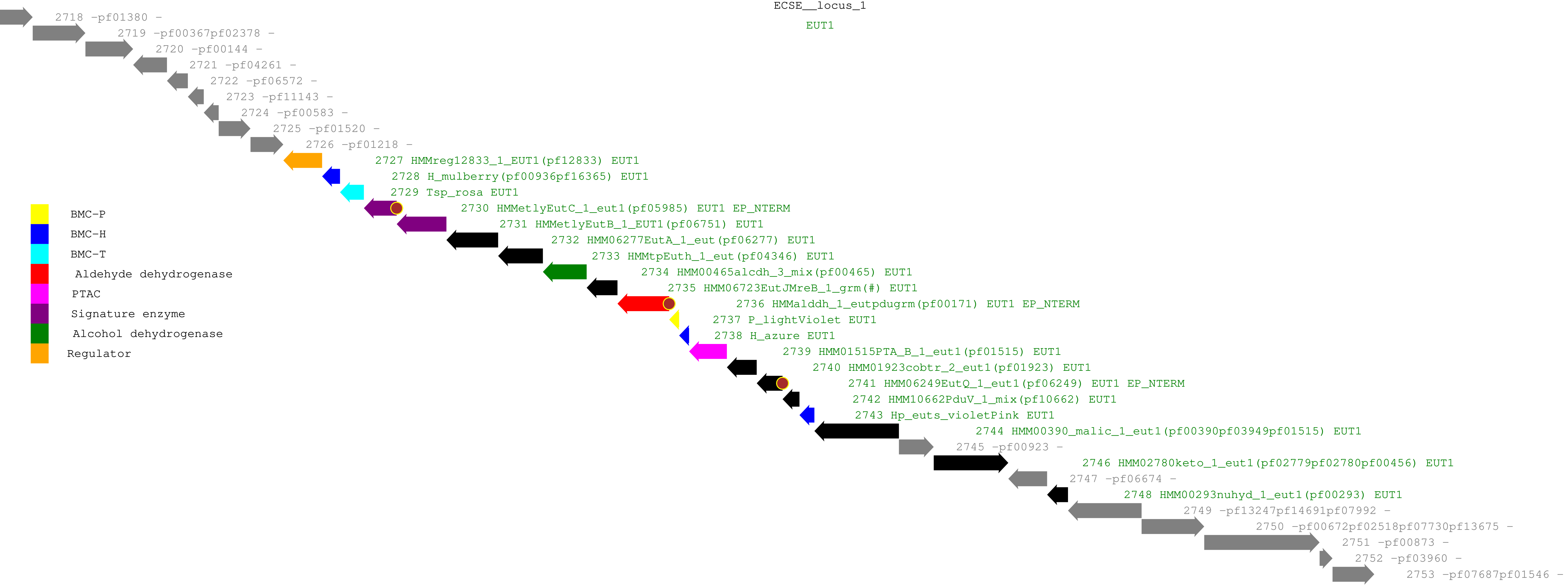

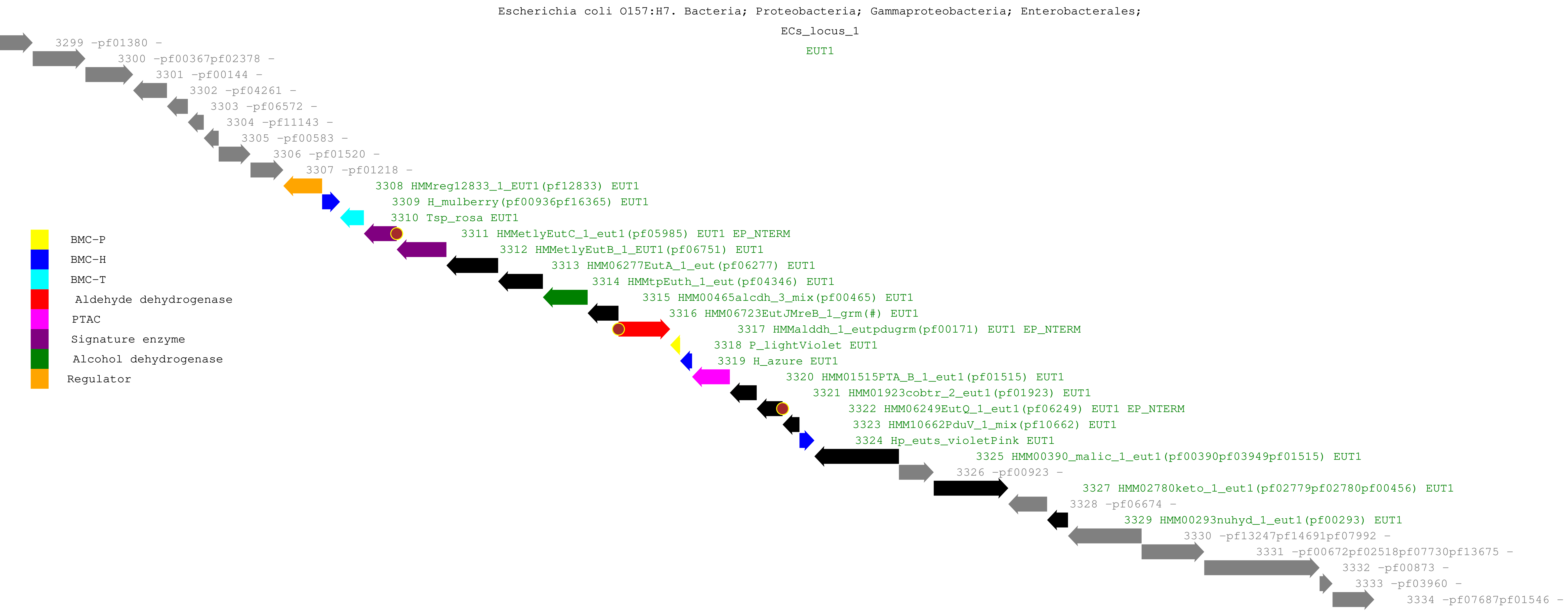
Escherichia coli O157:H7. Bacteria; Proteobacteria; Gammaproteobacteria; Enterobacterales;
ECs_locus_1
EUT1
3299 -pf01380 -
3300 -pf00367pf02378 -
3301 -pf00144 -
3302 -pf04261 -
3303 -pf06572 -
3304 -pf11143 -
3305 -pf00583 -
3306 -pf01520 -
3307 -pf01218 -
3308 HMMreg12833_1_EUT1(pf12833) EUT1
3309 H_mulberry(pf00936pf16365) EUT1
3310 Tsp_rosa EUT1
3311 HMMetlyEutC_1_eut1(pf05985) EUT1 EP_NTERM
3312 HMMetlyEutB_1_EUT1(pf06751) EUT1
3313 HMM06277EutA_1_eut(pf06277) EUT1
3314 HMMtpEuth_1_eut(pf04346) EUT1
3315 HMM00465alcdh_3_mix(pf00465) EUT1
3316 HMM06723EutJMreB_1_grm(#) EUT1
3317 HMMaldh_1_eutpdugrm(pf00171) EUT1 EP_NTERM
3318 P_lightViolet EUT1
3319 H_azure EUT1
3320 HMM01515PTA_B_1_eut1(pf01515) EUT1
3321 HMM01923cobtr_2_eut1(pf01923) EUT1
3322 HMM06249EutQ_1_eut1(pf06249) EUT1 EP_NTERM
3323 HMM10662PduV_1_mix(pf10662) EUT1
3324 Hp_euts_violetPink EUT1
3325 HMM00390_malic_1_eut1(pf00390pf03949pf01515) EUT1
3326 -pf00923 -
3327 HMM02780keto_1_eut1(pf02779pf02780pf00456) EUT1
3328 -pf06674 -
3329 HMM00293nuhyd_1_eut1(pf00293) EUT1
3330 -pf13247pf14691pf07992 -
3331 -pf00672pf02518pf07730pf13675 -
3332 -pf00873 -
3333 -pf03960 -
3334 -pf07687pf01546 -
BMC-P
BMC-H
BMC-T
Aldehyde dehydrogenase
PTAC
Signature enzyme
Alcohol dehydrogenase
Regulator

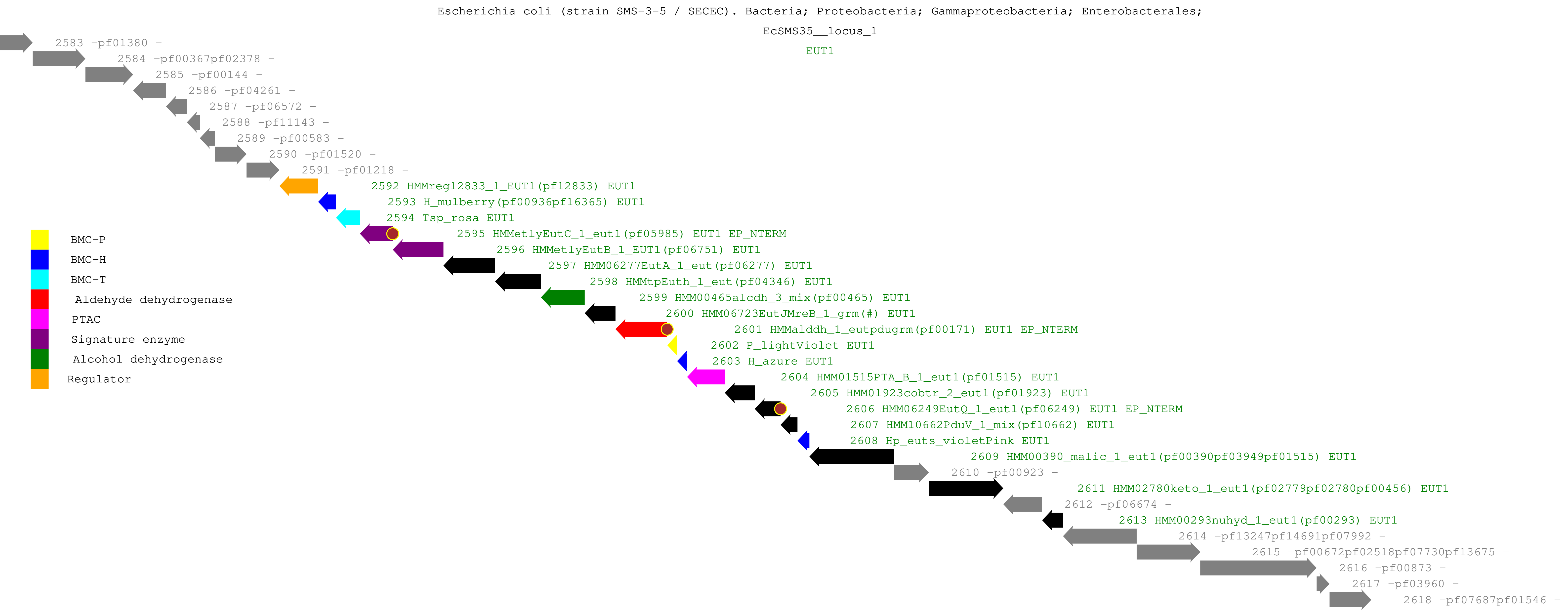
Escherichia coli (strain SMS-3-5 / SECEC). Bacteria; Proteobacteria; Gammaproteobacteria; Enterobacterales;
EcSMS35__locus_1
EUT1
2583 -pf01380 -
2584 -pf00367pf02378 -
2585 -pf00144 -
2586 -pf04261 -
2587 -pf06572 -
2588 -pf11143 -
2589 -pf00583 -
2590 -pf01520 -
2591 -pf01218 -
2592 HMMreg12833_1_EUT1(pf12833) EUT1
2593 H_mulberry(pf00936pf16365) EUT1
2594 Tsp_rosa EUT1
2595 HMMetlyEutC_1_eut1(pf05985) EUT1 EP_NTERM
2596 HMMetlyEutB_1_EUT1(pf06751) EUT1
2597 HMM06277EutA_1_eut(pf06277) EUT1
2598 HMMtpEuth_1_eut(pf04346) EUT1
2599 HMM00465alcdh_3_mix(pf00465) EUT1
2600 HMM06723EutJMreB_1_grm(#) EUT1
2601 HMMaldh_1_eutpdugrm(pf00171) EUT1 EP_NTERM
2602 P_lightViolet EUT1
2603 H_azure EUT1
2604 HMM01515PTA_B_1_eut1(pf01515) EUT1
2605 HMM01923cobtr_2_eut1(pf01923) EUT1
2606 HMM06249EutQ_1_eut1(pf06249) EUT1 EP_NTERM
2607 HMM10662PduV_1_mix(pf10662) EUT1
2608 Hp_euts_violetPink EUT1
2609 HMM00390_malic_1_eut1(pf00390pf03949pf01515) EUT1
2610 -pf00923 -
2611 HMM02780keto_1_eut1(pf02779pf02780pf00456) EUT1
2612 -pf06674 -
2613 HMM00293nuhyd_1_eut1(pf00293) EUT1
2614 -pf13247pf14691pf07992 -
2615 -pf00672pf02518pf07730pf13675 -
2616 -pf00873 -
2617 -pf03960 -
2618 -pf07687pf01546 -
BMC-P
BMC-H
BMC-T
Aldehyde dehydrogenase
PTAC
Signature enzyme
Alcohol dehydrogenase
Regulator

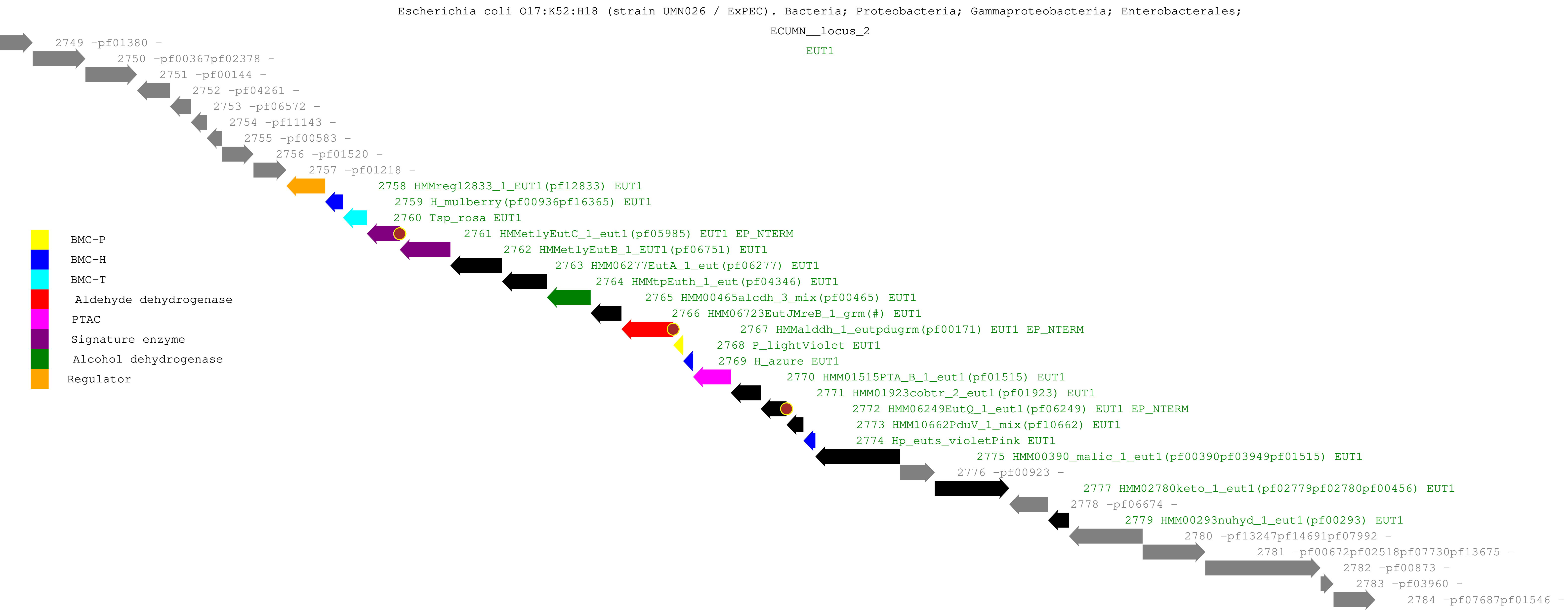
Escherichia coli O17:K52:H18 (strain UMN026 / ExPEC). Bacteria; Proteobacteria; Gammaproteobacteria; Enterobacterales;
ECUMN__locus_2
EUT1
2749 -pf01380 -
2750 -pf00367pf02378 -
2751 -pf00144 -
2752 -pf04261 -
2753 -pf06572 -
2754 -pf11143 -
2755 -pf00583 -
2756 -pf01520 -
2757 -pf01218 -
2758 HMMreg12833_1_EUT1(pf12833) EUT1
2759 H_mulberry(pf00936pf16365) EUT1
2760 Tsp_rosa EUT1
2761 HMMetlyEutC_1_eut1(pf05985) EUT1 EP_NTERM
2762 HMMetlyEutB_1_EUT1(pf06751) EUT1
2763 HMM06277EutA_1_eut(pf06277) EUT1
2764 HMMtpEuth_1_eut(pf04346) EUT1
2765 HMM00465alcdh_3_mix(pf00465) EUT1
2766 HMM06723EutJMreB_1_grm(#) EUT1
2767 HMMalddh_1_eutpdugrm(pf00171) EUT1 EP_NTERM
2768 P_lightViolet EUT1
2769 H_azure EUT1
2770 HMM01515PTA_B_1_eut1(pf01515) EUT1
2771 HMM01923cobtr_2_eut1(pf01923) EUT1
2772 HMM06249EutQ_1_eut1(pf06249) EUT1 EP_NTERM
2773 HMM10662PduV_1_mix(pf10662) EUT1
2774 Hp_euts_violetPink EUT1
2775 HMM00390_malic_1_eut1(pf00390pf03949pf01515) EUT1
2776 -pf00923 -
2777 HMM02780keto_1_eut1(pf02779pf02780pf00456) EUT1
2778 -pf06674 -
2779 HMM00293nuhyd_1_eut1(pf00293) EUT1
2780 -pf13247pf14691pf07992 -
2781 -pf00672pf02518pf07730pf13675 -
2782 -pf00873 -
2783 -pf03960 -
2784 -pf07687pf01546 -
BMC-P
BMC-H
BMC-T
Aldehyde dehydrogenase
PTAC
Signature enzyme
Alcohol dehydrogenase
Regulator

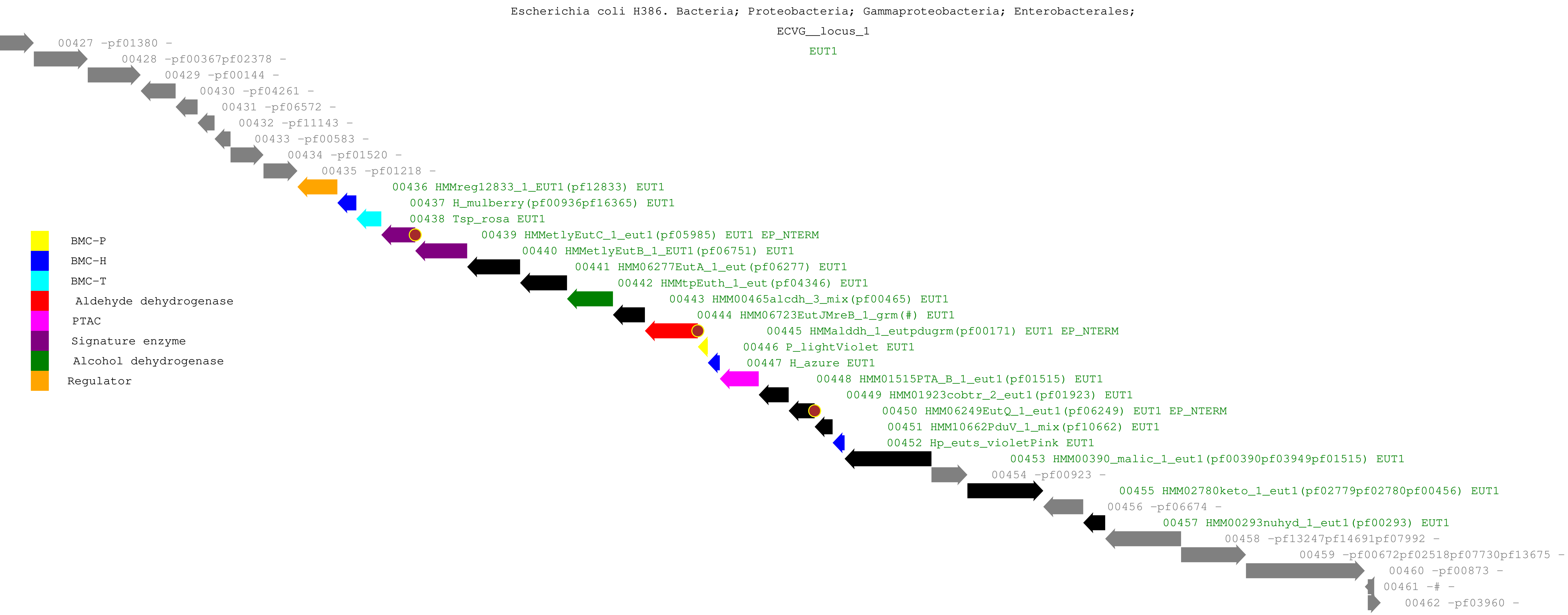
Escherichia coli H386. Bacteria; Proteobacteria; Gammaproteobacteria; Enterobacterales;
ECVG__locus_1
EUT1
00427 -pf01380 -
00428 -pf00367pf02378 -
00429 -pf00144 -
00430 -pf04261 -
00431 -pf06572 -
00432 -pf11143 -
00433 -pf00583 -
00434 -pf01520 -
00435 -pf01218 -
00436 HMMreg12833_1_EUT1(pf12833) EUT1
00437 H_mulberry(pf00936pf16365) EUT1
00438 Tsp_rosa EUT1
00439 HMMetlyEutC_1_eut1(pf05985) EUT1 EP_NTERM
00440 HMMetlyEutB_1_EUT1(pf06751) EUT1
00441 HMM06277EutA_1_eut(pf06277) EUT1
00442 HMMtpEuth_1_eut(pf04346) EUT1
00443 HMM00465alcdh_3_mix(pf00465) EUT1
00444 HMM06723EutJMreB_1_grm(#) EUT1
00445 HMMalddh_1_eutpdugrm(pf00171) EUT1 EP_NTERM
00446 P_lightViolet EUT1
00447 H_azure EUT1
00448 HMM01515PTA_B_1_eut1(pf01515) EUT1
00449 HMM01923cobtr_2_eut1(pf01923) EUT1
00450 HMM06249EutQ_1_eut1(pf06249) EUT1 EP_NTERM
00451 HMM10662PduV_1_mix(pf10662) EUT1
00452 Hp_euts_violetPink EUT1
00453 HMM00390_malic_1_eut1(pf00390pf03949pf01515) EUT1
00454 -pf00923 -
00455 HMM02780keto_1_eut1(pf02779pf02780pf00456) EUT1
00456 -pf06674 -
00457 HMM00293nuhyd_1_eut1(pf00293) EUT1
00458 -pf13247pf14691pf07992 -
00459 -pf00672pf02518pf07730pf13675 -
00460 -pf00873 -
00461 -# -
00462 -pf03960 -
BMC-P
BMC-H
BMC-T
Aldehyde dehydrogenase
PTAC
Signature enzyme
Alcohol dehydrogenase
Regulator

Escherichia coli W26. Bacteria; Proteobacteria; Gammaproteobacteria; Enterobacterales;

ECW26__locus_1

EUT1

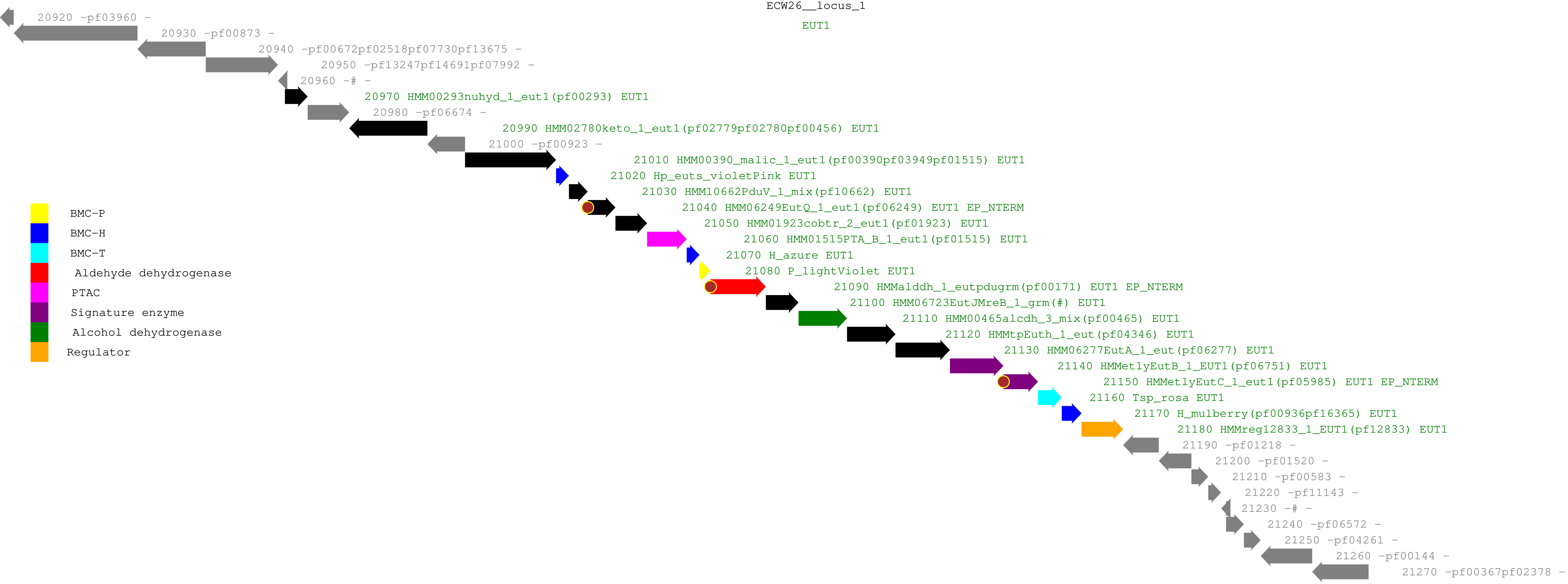

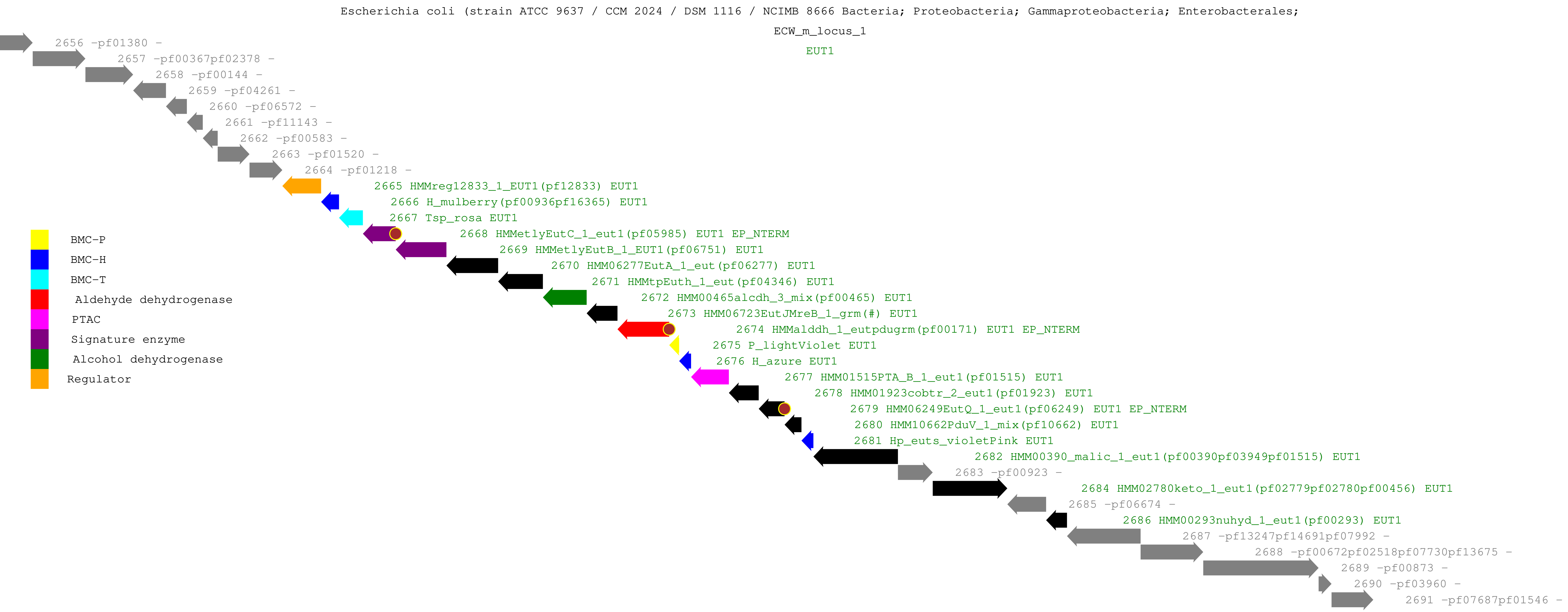
Escherichia coli (strain ATCC 9637 / CCM 2024 / DSM 1116 / NCIMB 8666 Bacteria; Proteobacteria; Gammaproteobacteria; Enterobacterales;
ECW_m_locus_1
EUT1
2656 -pf01380 -
2657 -pf00367pf02378 -
2658 -pf00144 -
2659 -pf04261 -
2660 -pf06572 -
2661 -pf11143 -
2662 -pf00583 -
2663 -pf01520 -
2664 -pf01218 -
2665 HMMreg12833_1_EUT1(pf12833) EUT1
2666 H_mulberry(pf00936pf16365) EUT1
2667 Tsp_rosa EUT1
2668 HMMetlyEutC_1_eut1(pf05985) EUT1 EP_NTERM
2669 HMMetlyEutB_1_EUT1(pf06751) EUT1
2670 HMM06277EutA_1_eut(pf06277) EUT1
2671 HMMtpEuth_1_eut(pf04346) EUT1
2672 HMM00465alcdh_3_mix(pf00465) EUT1
2673 HMM06723EutJMreB_1_grm(#) EUT1
2674 HMMaldh_1_eutpdugrm(pf00171) EUT1 EP_NTERM
2675 P_lightViolet EUT1
2676 H_azure EUT1
2677 HMM01515PTA_B_1_eut1(pf01515) EUT1
2678 HMM01923cobtr_2_eut1(pf01923) EUT1
2679 HMM06249EutQ_1_eut1(pf06249) EUT1 EP_NTERM
2680 HMM10662PduV_1_mix(pf10662) EUT1
2681 Hp_euts_violetPink EUT1
2682 HMM00390_malic_1_eut1(pf00390pf03949pf01515) EUT1
2683 -pf00923 -
2684 HMM02780keto_1_eut1(pf02779pf02780pf00456) EUT1
2685 -pf06674 -
2686 HMM00293nuhyd_1_eut1(pf00293) EUT1
2687 -pf13247pf14691pf07992 -
2688 -pf00672pf02518pf07730pf13675 -
2689 -pf00873 -
2690 -pf03960 -
2691 -pf07687pf01546 -
BMC-P
BMC-H
BMC-T
Aldehyde dehydrogenase
PTAC
Signature enzyme
Alcohol dehydrogenase
Regulator

Escherichia coli TA447. Bacteria; Proteobacteria; Gammaproteobacteria; Enterobacterales;

ECXG__locus_1

EUT1

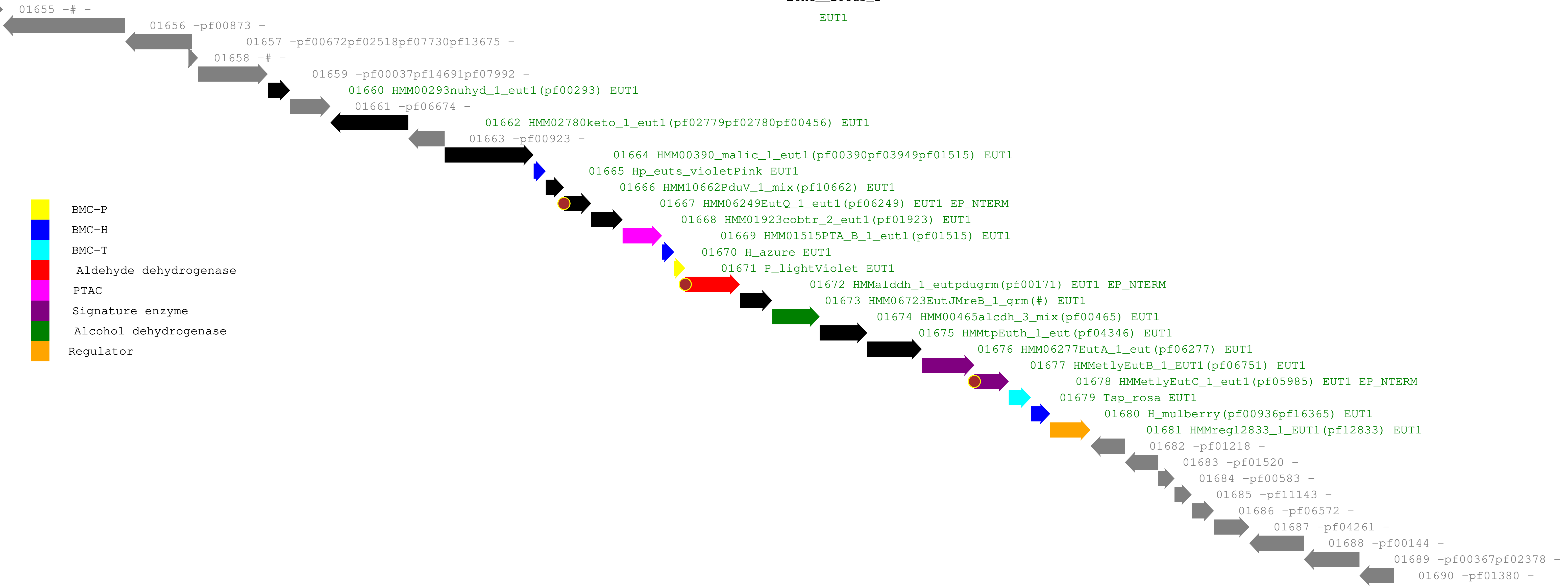

Escherichia coli. Bacteria; Proteobacteria; Gammaproteobacteria; Enterobacterales;

ED607__locus_1

EUT1

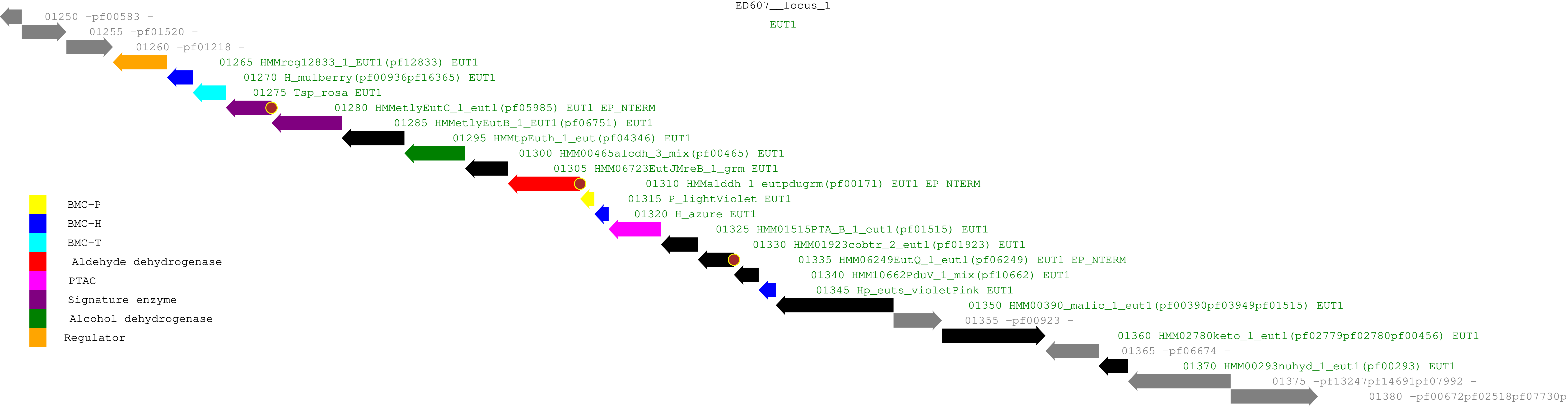

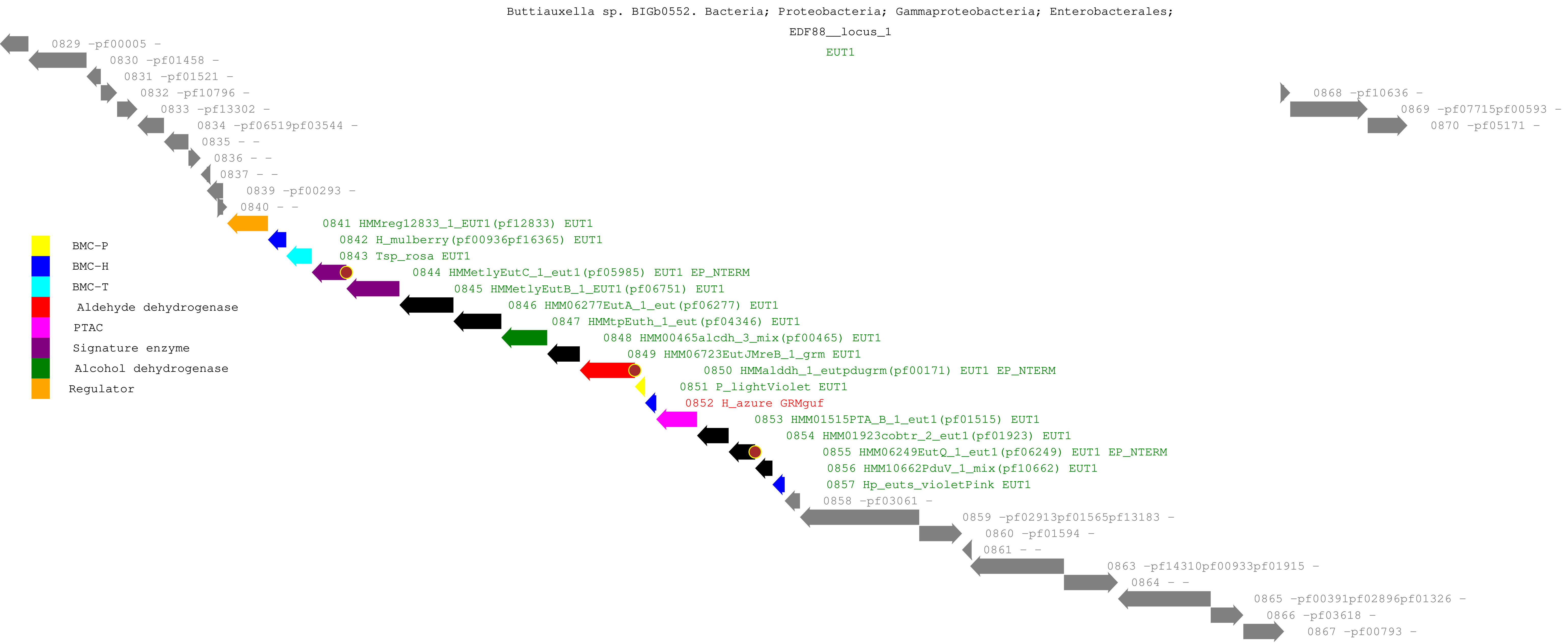
Buttiauxella sp. BIGb0552. Bacteria; Proteobacteria; Gammaproteobacteria; Enterobacterales;
EDF88__locus_1
EUT1
0829 -pf00005 -
0830 -pf01458 -
0831 -pf01521 -
0832 -pf10796 -
0833 -pf13302 -
0834 -pf06519pf03544 -
0835 - -
0836 - -
0837 - -
0839 -pf00293 -
0840 - -
0841 HMMreg12833_1_EUT1(pf12833) EUT1
0842 H_mulberry(pf00936pf16365) EUT1
0843 Tsp_rosa EUT1
0844 HMMetlyEutC_1_eut1(pf05985) EUT1 EP_NTERM
0845 HMMetlyEutB_1_EUT1(pf06751) EUT1
0846 HMM06277EutA_1_eut(pf06277) EUT1
0847 HMMtpEuth_1_eut(pf04346) EUT1
0848 HMM00465alcdh_3_mix(pf00465) EUT1
0849 HMM06723EutJMreB_1_grm EUT1
0850 HMMalddh_1_eutpdugrm(pf00171) EUT1 EP_NTERM
0851 P_lightViolet EUT1
0852 H_azure GRMguf
0853 HMM01515PTA_B_1_eut1(pf01515) EUT1
0854 HMM01923cobtr_2_eut1(pf01923) EUT1
0855 HMM06249EutQ_1_eut1(pf06249) EUT1 EP_NTERM
0856 HMM10662PduV_1_mix(pf10662) EUT1
0857 Hp_euts_violetPink EUT1
0858 -pf03061 -
0859 -pf02913pf01565pf13183 -
0860 -pf01594 -
0861 - -
0863 -pf14310pf00933pf01915 -
0864 - -
0865 -pf00391pf02896pf01326 -
0866 -pf03618 -
0867 -pf00793 -
0868 -pf10636 -
0869 -pf07715pf00593 -
0870 -pf05171 -
BMC-P
BMC-H
BMC-T
Aldehyde dehydrogenase
PTAC
Signature enzyme
Alcohol dehydrogenase
Regulator

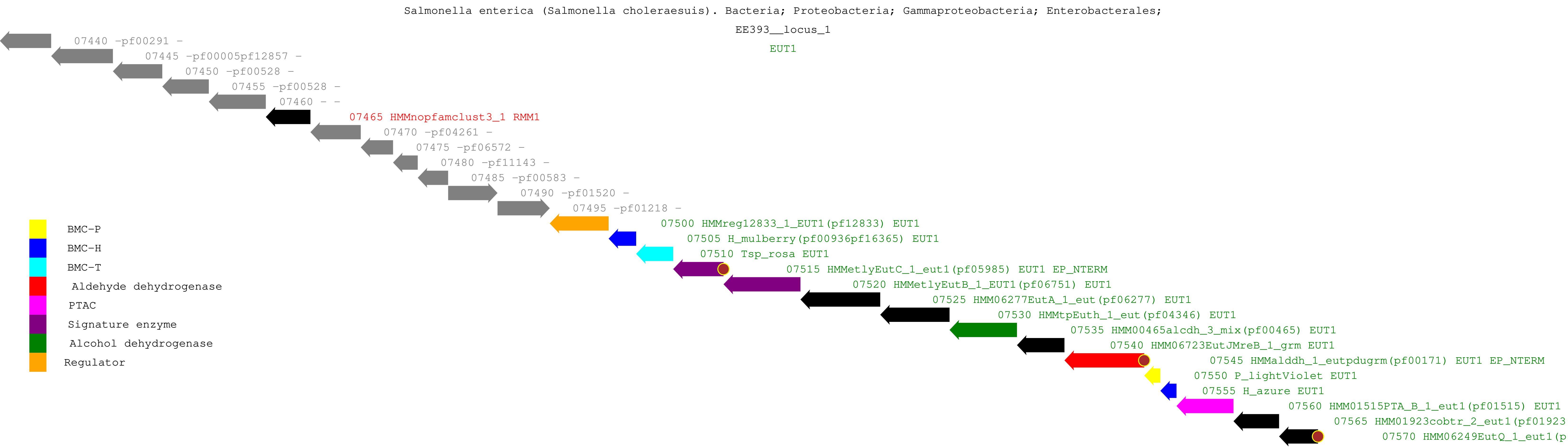
Salmonella enterica (Salmonella choleraesuis). Bacteria; Proteobacteria; Gammaproteobacteria; Enterobacterales;
EE393__locus_1
EUT1
07440 -pf00291 -
07445 -pf00005pf12857 -
07450 -pf00528 -
07455 -pf00528 -
07460 - -
07465 HMMnopfamclust3_1 RMM1
07470 -pf04261 -
07475 -pf06572 -
07480 -pf11143 -
07485 -pf00583 -
07490 -pf01520 -
07495 -pf01218 -
07500 HMMreg12833_1_EUT1(pf12833) EUT1
07505 H_mulberry(pf00936pf16365) EUT1
07510 Tsp_rosa EUT1
07515 HMMetlyEutC_1_eut1(pf05985) EUT1 EP_NTERM
07520 HMMetlyEutB_1_EUT1(pf06751) EUT1
07525 HMM06277EutA_1_eut(pf06277) EUT1
07530 HMMtpEuth_1_eut(pf04346) EUT1
07535 HMM00465alcdh_3_mix(pf00465) EUT1
07540 HMM06723EutJMreB_1_grm EUT1
07545 HMMalddh_1_eutpdugrm(pf00171) EUT1 EP_NTERM
07550 P_lightViolet EUT1
07555 H_azure EUT1
07560 HMM01515PTA_B_1_eut1(pf01515) EUT1
07565 HMM01923cobtr_2_eut1(pf01923
07570 HMM06249EutQ_1_eut1(p
BMC-P
BMC-H
BMC-T
Aldehyde dehydrogenase
PTAC
Signature enzyme
Alcohol dehydrogenase
Regulator

Salmonella enterica subsp. enterica serovar Agona. Bacteria; Proteobacteria; Gammaproteobacteria; Enterobacterales;

EEA82__locus_2

EUT1

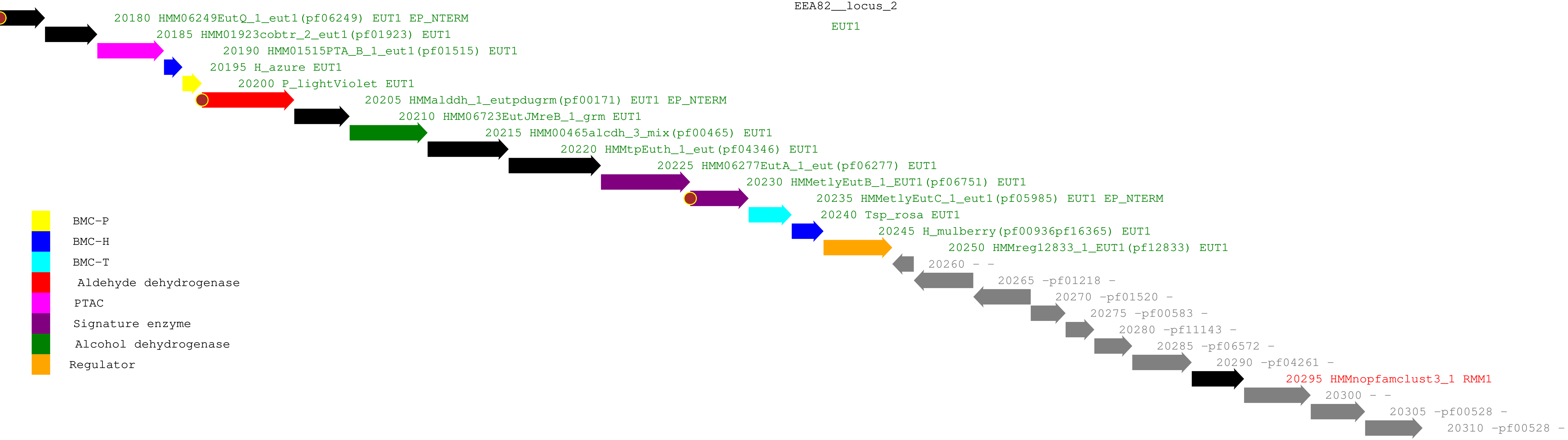

Salmonella enterica (Salmonella choleraesuis). Bacteria; Proteobacteria; Gammaproteobacteria; Enterobacterales;

EEM01__locus_1

EUT1

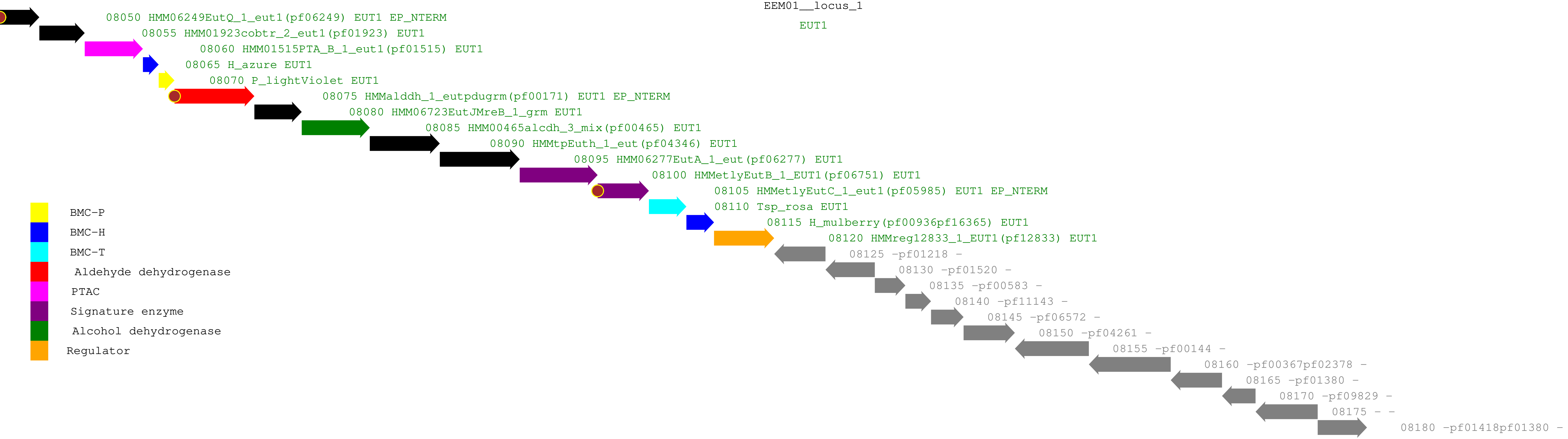

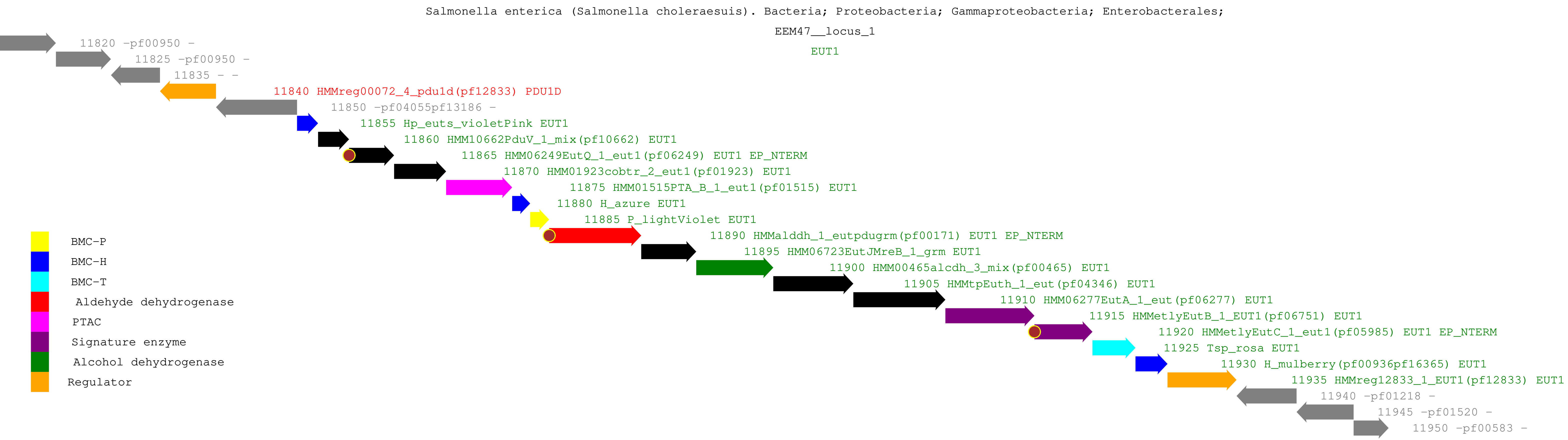

Salmonella enterica (Salmonella choleraesuis). Bacteria; Proteobacteria; Gammaproteobacteria; Enterobacterales;
EEM47__locus_1
EUT1
11820 -pf00950 -
11825 -pf00950 -
11835 - -
11840 HMMreg00072_4_pdu1d(pf12833) PDU1D
11850 -pf04055pf13186 -
11855 Hp_euts_violetPink EUT1
11860 HMM10662PduV_1_mix(pf10662) EUT1
11865 HMM06249EutQ_1_eut1(pf06249) EUT1 EP_NTERM
11870 HMM01923cobtr_2_eut1(pf01923) EUT1
11875 HMM01515PTA_B_1_eut1(pf01515) EUT1
11880 H_azure EUT1
11885 P_lightViolet EUT1
11890 HMMalddh_1_eutpdugrm(pf00171) EUT1 EP_NTERM
11895 HMM06723EutJMreB_1_grm EUT1
11900 HMM00465alcdh_3_mix(pf00465) EUT1
11905 HMMtpEuth_1_eut(pf04346) EUT1
11910 HMM06277EutA_1_eut(pf06277) EUT1
11915 HMMetlyEutB_1_EUT1(pf06751) EUT1
11920 HMMetlyEutC_1_eut1(pf05985) EUT1 EP_NTERM
11925 Tsp_rosa EUT1
11930 H_mulberry(pf00936pf16365) EUT1
11935 HMMreg12833_1_EUT1(pf12833) EUT1
11940 -pf01218 -
11945 -pf01520 -
11950 -pf00583 -
BMC-P
BMC-H
BMC-T
Aldehyde dehydrogenase
PTAC
Signature enzyme
Alcohol dehydrogenase
Regulator

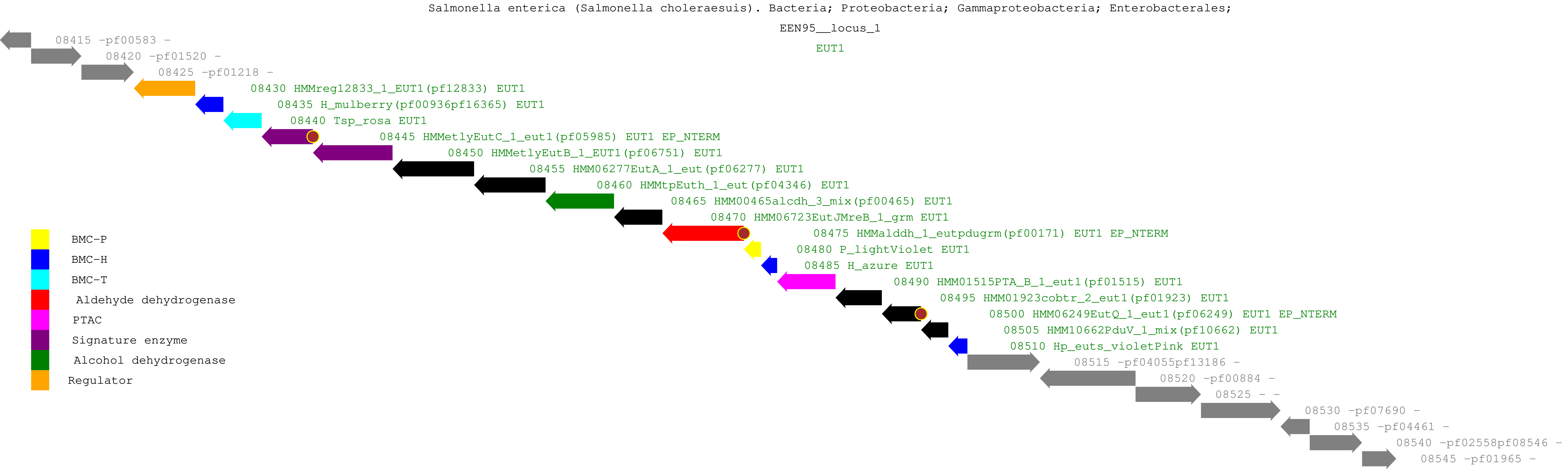
Salmonella enterica (Salmonella choleraesuis). Bacteria; Proteobacteria; Gammaproteobacteria; Enterobacterales;
EEN95__locus_1
EUT1
08415 -pf00583 -
08420 -pf01520 -
08425 -pf01218 -
08430 HMMreg12833_1_EUT1(pf12833) EUT1
08435 H_mulberry(pf00936pf16365) EUT1
08440 Tsp_rosa EUT1
08445 HMMetlyEutC_1_eut1(pf05985) EUT1 EP_NTERM
08450 HMMetlyEutB_1_EUT1(pf06751) EUT1
08455 HMM06277EutA_1_eut(pf06277) EUT1
08460 HMMtpEuth_1_eut(pf04346) EUT1
08465 HMM00465alcdh_3_mix(pf00465) EUT1
08470 HMM06723EutJMreB_1_grm EUT1
08475 HMMalddh_1_eutpdugrm(pf00171) EUT1 EP_NTERM
08480 P_lightViolet EUT1
08485 H_azure EUT1
08490 HMM01515PTA_B_1_eut1(pf01515) EUT1
08495 HMM01923cobtr_2_eut1(pf01923) EUT1
08500 HMM06249EutQ_1_eut1(pf06249) EUT1 EP_NTERM
08505 HMM10662PduV_1_mix(pf10662) EUT1
08510 Hp_euts_violetPink EUT1
08515 -pf04055pf13186 -
08520 -pf00884 -
08525 - -
08530 -pf07690 -
08535 -pf04461 -
08540 -pf02558pf08546 -
08545 -pf01965 -
BMC-P
BMC-H
BMC-T
Aldehyde dehydrogenase
PTAC
Signature enzyme
Alcohol dehydrogenase
Regulator

Salmonella enterica I. Bacteria; Proteobacteria; Gammaproteobacteria; Enterobacterales;

EER80__locus_1

EUT1

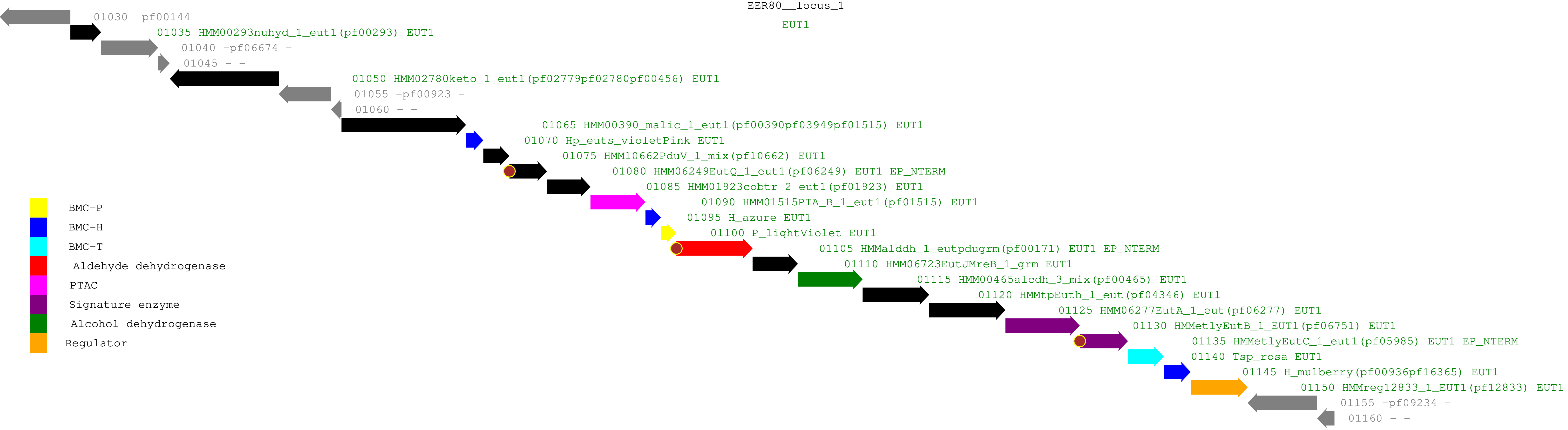

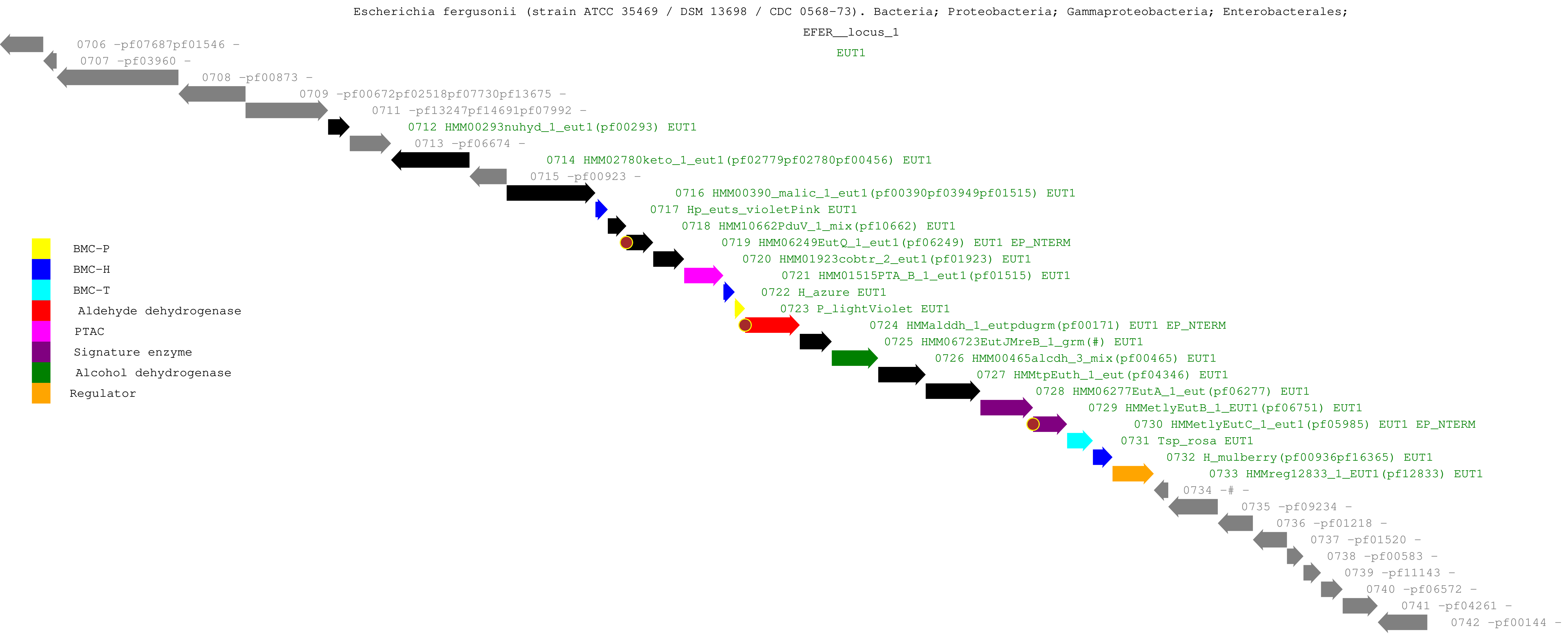
Escherichia fergusonii (strain ATCC 35469 / DSM 13698 / CDC 0568-73). Bacteria; Proteobacteria; Gammaproteobacteria; Enterobacterales;
EFER__locus_1
EUT1
0706 -pf07687pf01546 -
0707 -pf03960 -
0708 -pf00873 -
0709 -pf00672pf02518pf07730pf13675 -
0711 -pf13247pf14691pf07992 -
0712 HMM00293nuhyd_1_eut1(pf00293) EUT1
0713 -pf06674 -
0714 HMM02780keto_1_eut1(pf02779pf02780pf00456) EUT1
0715 -pf00923 -
0716 HMM00390_malic_1_eut1(pf00390pf03949pf01515) EUT1
0717 Hp_euts_violetPink EUT1
0718 HMM10662PduV_1_mix(pf10662) EUT1
0719 HMM06249EutQ_1_eut1(pf06249) EUT1 EP_NTERM
0720 HMM01923cobtr_2_eut1(pf01923) EUT1
0721 HMM01515PTA_B_1_eut1(pf01515) EUT1
0722 H_azure EUT1
0723 P_lightViolet EUT1
0724 HMMalddh_1_eutpdugrm(pf00171) EUT1 EP_NTERM
0725 HMM06723EutJMreB_1_grm(#) EUT1
0726 HMM00465alcdh_3_mix(pf00465) EUT1
0727 HMMtpEuth_1_eut(pf04346) EUT1
0728 HMM06277EutA_1_eut(pf06277) EUT1
0729 HMMetlyEutB_1_EUT1(pf06751) EUT1
0730 HMMetlyEutC_1_eut1(pf05985) EUT1 EP_NTERM
0731 Tsp_rosa EUT1
0732 H_mulberry(pf00936pf16365) EUT1
0733 HMMreg12833_1_EUT1(pf12833) EUT1
0734 -# -
0735 -pf09234 -
0736 -pf01218 -
0737 -pf01520 -
0738 -pf00583 -
0739 -pf11143 -
0740 -pf06572 -
0741 -pf04261 -
0742 -pf00144 -
BMC-P
BMC-H
BMC-T
Aldehyde dehydrogenase
PTAC
Signature enzyme
Alcohol dehydrogenase
Regulator

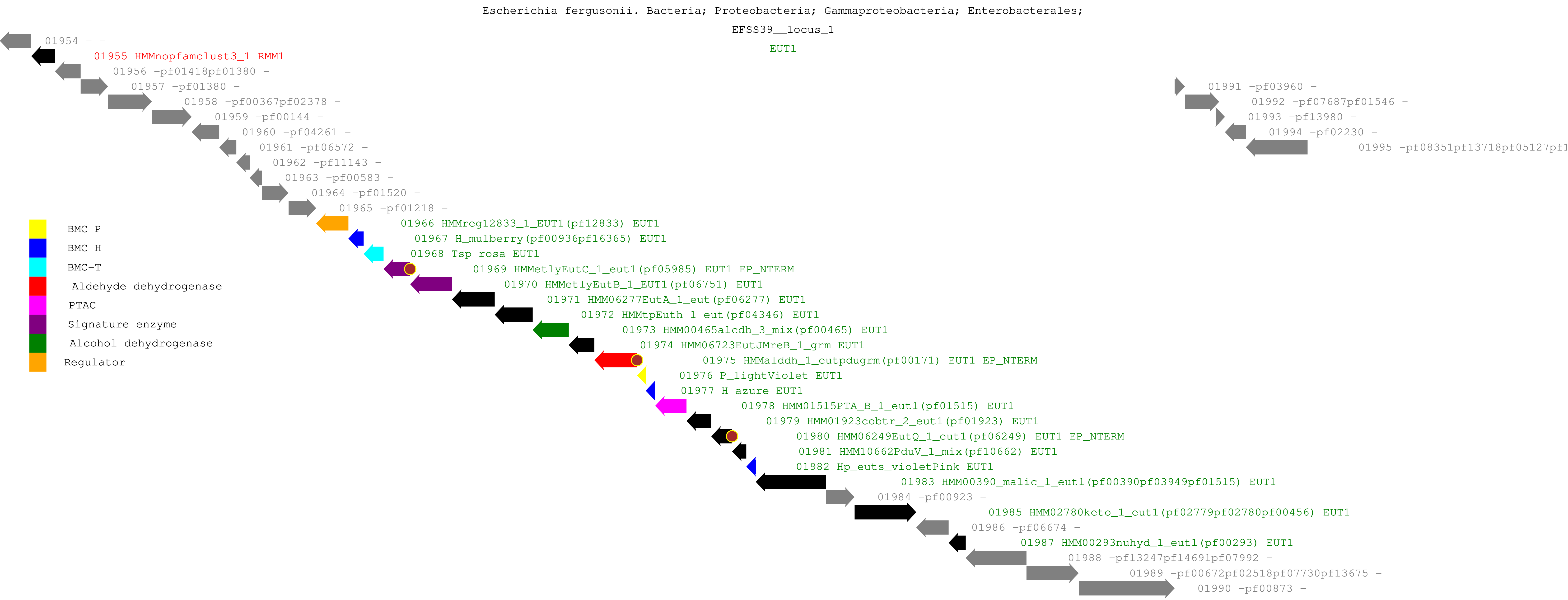

Escherichia fergusonii. Bacteria; Proteobacteria; Gammaproteobacteria; Enterobacterales;
EFSS39__locus_1
EUT1
01954 - -
01955 HMMnopfamclust3_1 RMM1
01956 -pf01418pf01380 -
01957 -pf01380 -
01958 -pf00367pf02378 -
01959 -pf00144 -
01960 -pf04261 -
01961 -pf06572 -
01962 -pf11143 -
01963 -pf00583 -
01964 -pf01520 -
01965 -pf01218 -
01966 HMMreg12833_1_EUT1(pf12833) EUT1
01967 H_mulberry(pf00936pf16365) EUT1
01968 Tsp_rosa EUT1
01969 HMMetlyEutC_1_eut1(pf05985) EUT1 EP_NTERM
01970 HMMetlyEutB_1_EUT1(pf06751) EUT1
01971 HMM06277EutA_1_eut(pf06277) EUT1
01972 HMMtpEuth_1_eut(pf04346) EUT1
01973 HMM00465alcdh_3_mix(pf00465) EUT1
01974 HMM06723EutJMreB_1_grm EUT1
01975 HMMalddh_1_eutpdugrm(pf00171) EUT1 EP_NTERM
01976 P_lightViolet EUT1
01977 H_azure EUT1
01978 HMM01515PTA_B_1_eut1(pf01515) EUT1
01979 HMM01923cobtr_2_eut1(pf01923) EUT1
01980 HMM06249EutQ_1_eut1(pf06249) EUT1 EP_NTERM
01981 HMM10662PduV_1_mix(pf10662) EUT1
01982 Hp_euts_violetPink EUT1
01983 HMM00390_malic_1_eut1(pf00390pf03949pf01515) EUT1
01984 -pf00923 -
01985 HMM02780keto_1_eut1(pf02779pf02780pf00456) EUT1
01986 -pf06674 -
01987 HMM00293nuhyd_1_eut1(pf00293) EUT1
01988 -pf13247pf14691pf07992 -
01989 -pf00672pf02518pf07730pf13675 -
01990 -pf00873 -
01991 -pf03960 -
01992 -pf07687pf01546 -
01993 -pf13980 -
01994 -pf02230 -
01995 -pf08351pf13718pf05127pf1
BMC-P
BMC-H
BMC-T
Aldehyde dehydrogenase
PTAC
Signature enzyme
Alcohol dehydrogenase
Regulator

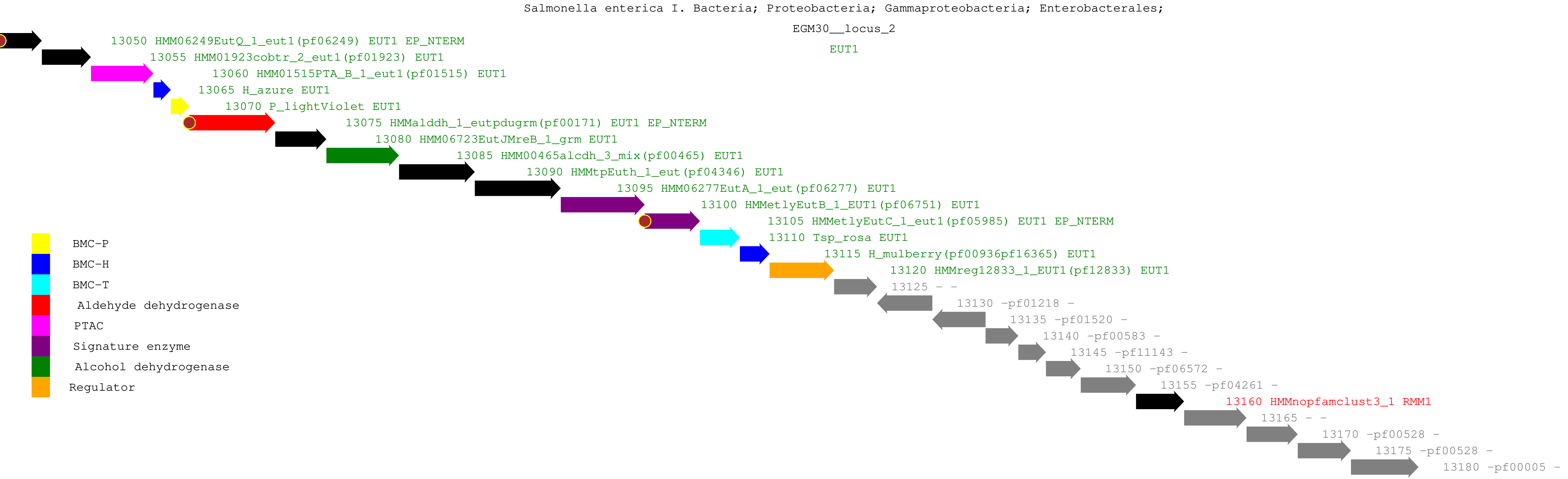
Salmonella enterica I. Bacteria; Proteobacteria; Gammaproteobacteria; Enterobacterales;
EGM30__locus_2
EUT1
13050 HMM06249EutQ_1_eut1(pf06249) EUT1 EP_NTERM
13055 HMM01923cobtr_2_eut1(pf01923) EUT1
13060 HMM01515PTA_B_1_eut1(pf01515) EUT1
13065 H_azure EUT1
13070 P_lightViolet EUT1
13075 HMMalddh_1_eutpdugrm(pf00171) EUT1 EP_NTERM
13080 HMM06723EutJMreB_1_grm EUT1
13085 HMM00465alcdh_3_mix(pf00465) EUT1
13090 HMMtpEuth_1_eut(pf04346) EUT1
13095 HMM06277EutA_1_eut(pf06277) EUT1
13100 HMMetlyEutB_1_EUT1(pf06751) EUT1
13105 HMMetlyEutC_1_eut1(pf05985) EUT1 EP_NTERM
13110 Tsp_rosa EUT1
13115 H_mulberry(pf00936pf16365) EUT1
13120 HMMreg12833_1_EUT1(pf12833) EUT1
13125 - -
13130 -pf01218 -
13135 -pf01520 -
13140 -pf00583 -
13145 -pf11143 -
13150 -pf06572 -
13155 -pf04261 -
13160 HMMnopfamclust3_1 RMM1
13165 - -
13170 -pf00528 -
13175 -pf00528 -
13180 -pf00005 -
BMC-P
BMC-H
BMC-T
Aldehyde dehydrogenase
PTAC
Signature enzyme
Alcohol dehydrogenase
Regulator

Escherichia coli. Bacteria; Proteobacteria; Gammaproteobacteria; Enterobacterales;

EIA21__locus_1

EUT1

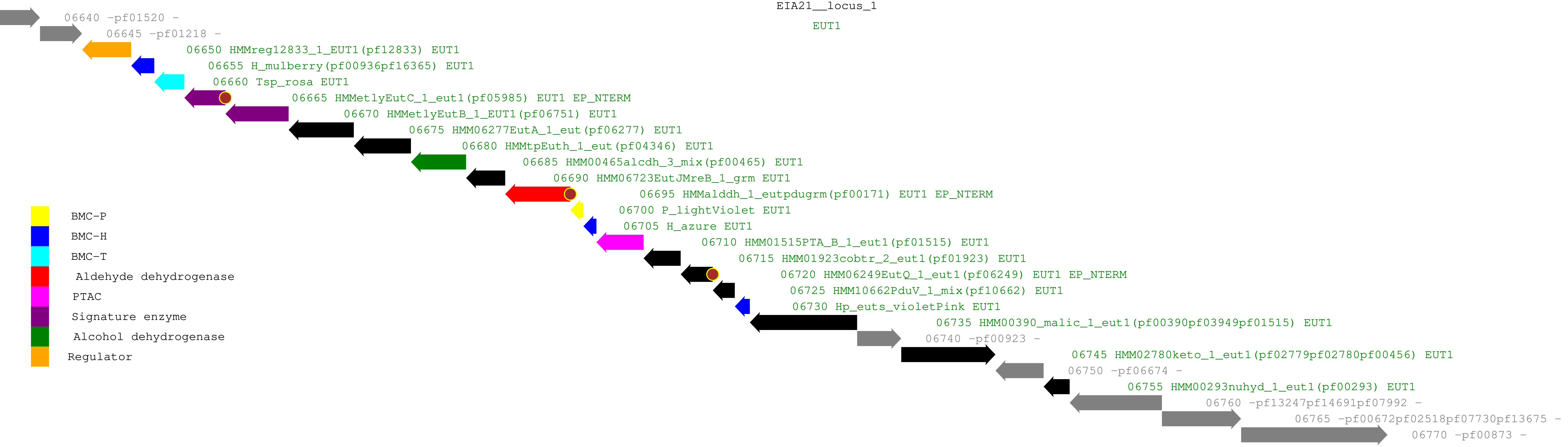

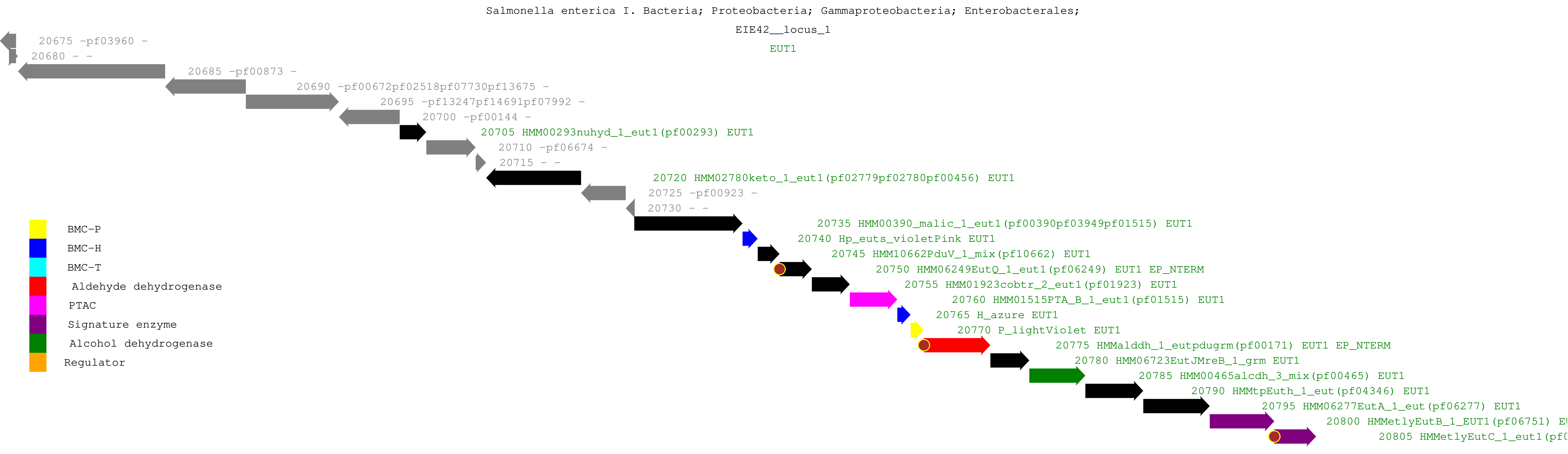
Salmonella enterica I. Bacteria; Proteobacteria; Gammaproteobacteria; Enterobacterales;
EIE42__locus_1
EUT1
20675 -pf03960 -
20680 - -
20685 -pf00873 -
20690 -pf00672pf02518pf07730pf13675 -
20695 -pf13247pf14691pf07992 -
20700 -pf00144 -
20705 HMM00293nuhyd_1_eut1(pf00293) EUT1
20710 -pf06674 -
20715 - -
20720 HMM02780keto_1_eut1(pf02779pf02780pf00456) EUT1
20725 -pf00923 -
20730 - -
20735 HMM00390_malic_1_eut1(pf00390pf03949pf01515) EUT1
20740 Hp_euts_violetPink EUT1
20745 HMM10662PduV_1_mix(pf10662) EUT1
20750 HMM06249EutQ_1_eut1(pf06249) EUT1 EP_NTERM
20755 HMM01923cobtr_2_eut1(pf01923) EUT1
20760 HMM01515PTA_B_1_eut1(pf01515) EUT1
20765 H_azure EUT1
20770 P_lightViolet EUT1
20775 HMMalddh_1_eutpdugrm(pf00171) EUT1 EP_NTERM
20780 HMM06723EutJMreB_1_grm EUT1
20785 HMM00465alcdh_3_mix(pf00465) EUT1
20790 HMMtpEuth_1_eut(pf04346) EUT1
20795 HMM06277EutA_1_eut(pf06277) EUT1
20800 HMMetlyEutB_1_EUT1(pf06751) EU
20805 HMMetlyEutC_1_eut1(pf0
BMC-P
BMC-H
BMC-T
Aldehyde dehydrogenase
PTAC
Signature enzyme
Alcohol dehydrogenase
Regulator

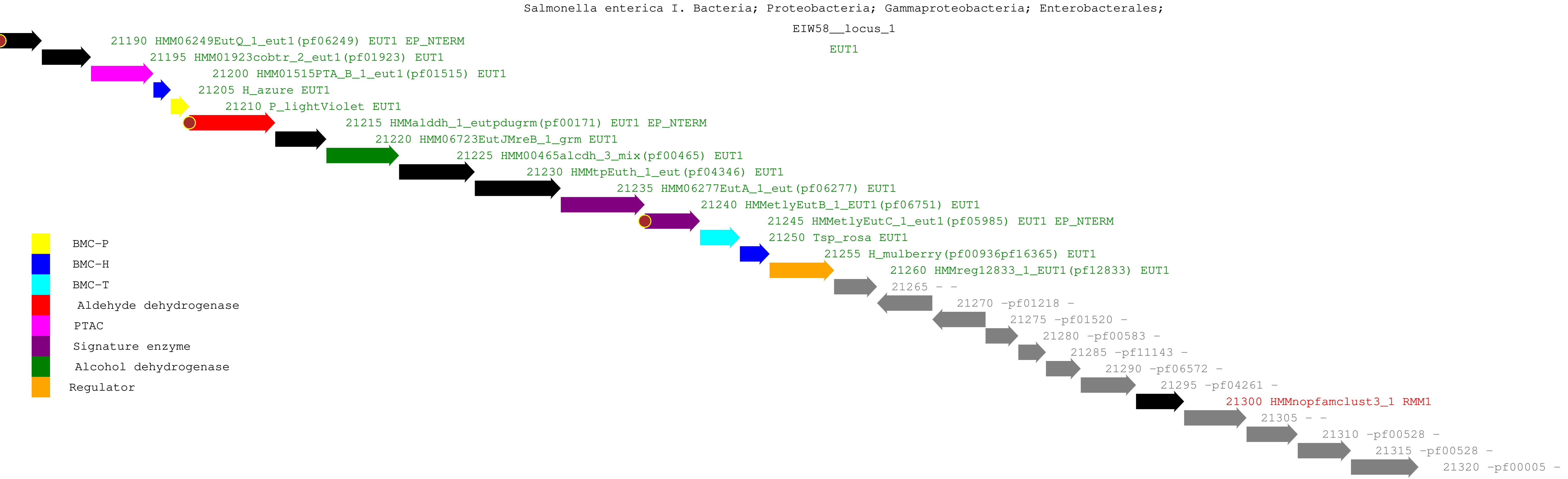

Salmonella enterica I. Bacteria; Proteobacteria; Gammaproteobacteria; Enterobacterales;
EIW58__locus_1
EUT1
21190 HMM06249EutQ_1_eut1(pf06249) EUT1 EP_NTERM
21195 HMM01923cobtr_2_eut1(pf01923) EUT1
21200 HMM01515PTA_B_1_eut1(pf01515) EUT1
21205 H_azure EUT1
21210 P_lightViolet EUT1
21215 HMMalddh_1_eutpdugrm(pf00171) EUT1 EP_NTERM
21220 HMM06723EutJMreB_1_grm EUT1
21225 HMM00465alcdh_3_mix(pf00465) EUT1
21230 HMMtpEuth_1_eut(pf04346) EUT1
21235 HMM06277EutA_1_eut(pf06277) EUT1
21240 HMMetlyEutB_1_EUT1(pf06751) EUT1
21245 HMMetlyEutC_1_eut1(pf05985) EUT1 EP_NTERM
21250 Tsp_rosa EUT1
21255 H_mulberry(pf00936pf16365) EUT1
21260 HMMreg12833_1_EUT1(pf12833) EUT1
21265 - -
21270 -pf01218 -
21275 -pf01520 -
21280 -pf00583 -
21285 -pf11143 -
21290 -pf06572 -
21295 -pf04261 -
21300 HMMnopfamclust3_1 RMM1
21305 - -
21310 -pf00528 -
21315 -pf00528 -
21320 -pf00005 -
BMC-P
BMC-H
BMC-T
Aldehyde dehydrogenase
PTAC
Signature enzyme
Alcohol dehydrogenase
Regulator

Salmonella paratyphi A. Bacteria; Proteobacteria; Gammaproteobacteria; Enterobacterales;

EJI24__locus_1

EUT1

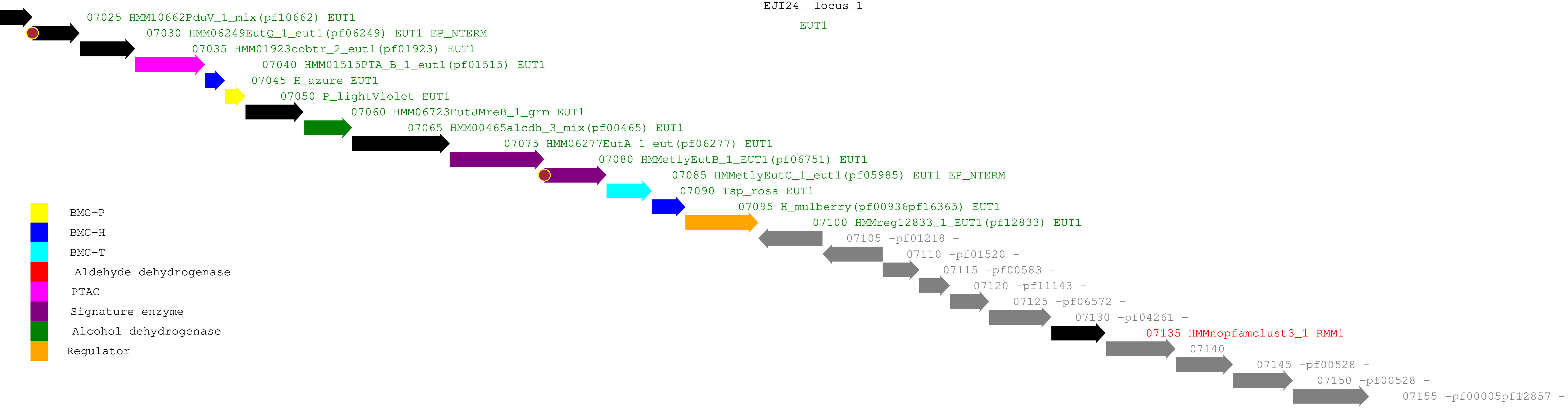

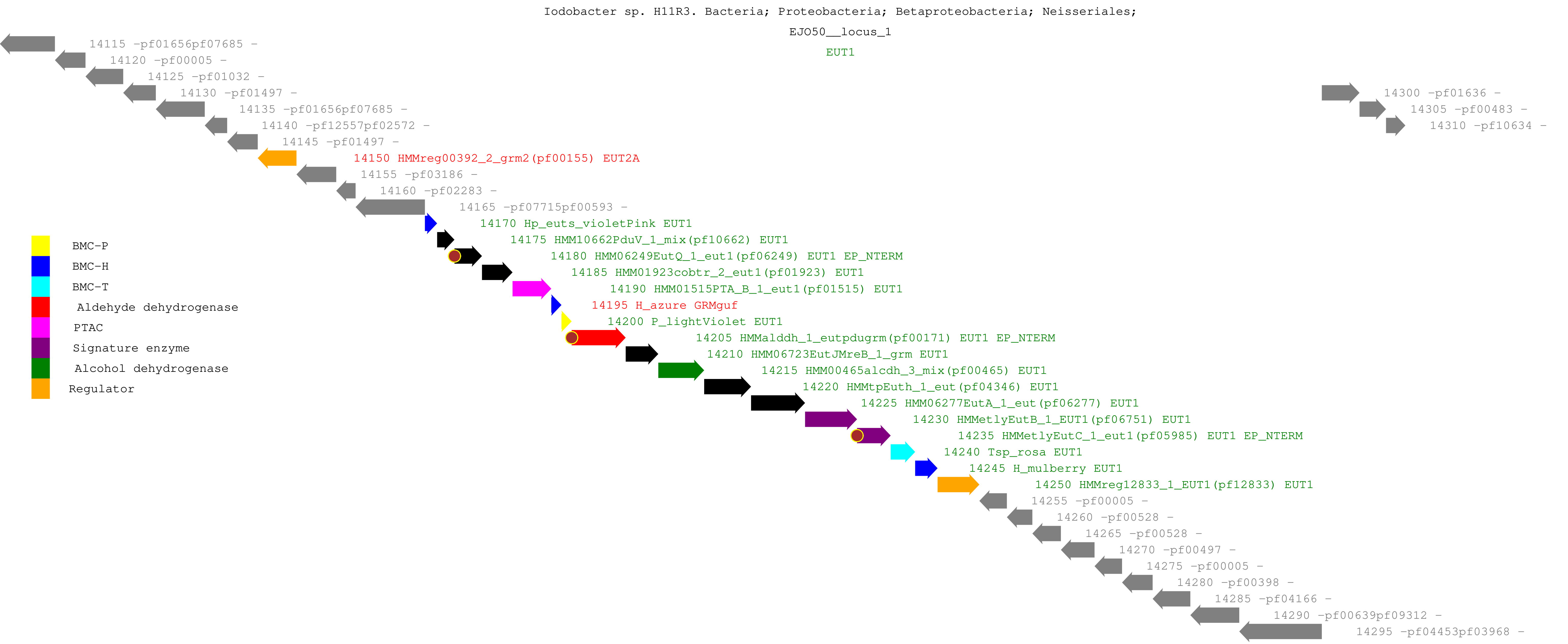
Iodobacter sp. H11R3. Bacteria; Proteobacteria; Betaproteobacteria; Neisseriales;
EJO50__locus_1
EUT1
14115 -pf01656pf07685 -
14120 -pf00005 -
14125 -pf01032 -
14130 -pf01497 -
14135 -pf01656pf07685 -
14140 -pf12557pf02572 -
14145 -pf01497 -
14150 HMMreg00392_2_grm2(pf00155) EUT2A
14155 -pf03186 -
14160 -pf02283 -
14165 -pf07715pf00593 -
14170 Hp_euts_violetPink EUT1
14175 HMM10662PduV_1_mix(pf10662) EUT1
14180 HMM06249EutQ_1_eut1(pf06249) EUT1 EP_NTERM
14185 HMM01923cobtr_2_eut1(pf01923) EUT1
14190 HMM01515PTA_B_1_eut1(pf01515) EUT1
14195 H_azure GRMguf
14200 P_lightViolet EUT1
14205 HMMaldh_1_eutpdugrm(pf00171) EUT1 EP_NTERM
14210 HMM06723EutJMreB_1_grm EUT1
14215 HMM00465alcdh_3_mix(pf00465) EUT1
14220 HMMtpEuth_1_eut(pf04346) EUT1
14225 HMM06277EutA_1_eut(pf06277) EUT1
14230 HMMetlyEutB_1_EUT1(pf06751) EUT1
14235 HMMetlyEutC_1_eut1(pf05985) EUT1 EP_NTERM
14240 Tsp_rosa EUT1
14245 H_mulberry EUT1
14250 HMMreg12833_1_EUT1(pf12833) EUT1
14255 -pf00005 -
14260 -pf00528 -
14265 -pf00528 -
14270 -pf00497 -
14275 -pf00005 -
14280 -pf00398 -
14285 -pf04166 -
14290 -pf00639pf09312 -
14295 -pf04453pf03968 -
14300 -pf01636 -
14305 -pf00483 -
14310 -pf10634 -
BMC-P
BMC-H
BMC-T
Aldehyde dehydrogenase
PTAC
Signature enzyme
Alcohol dehydrogenase
Regulator

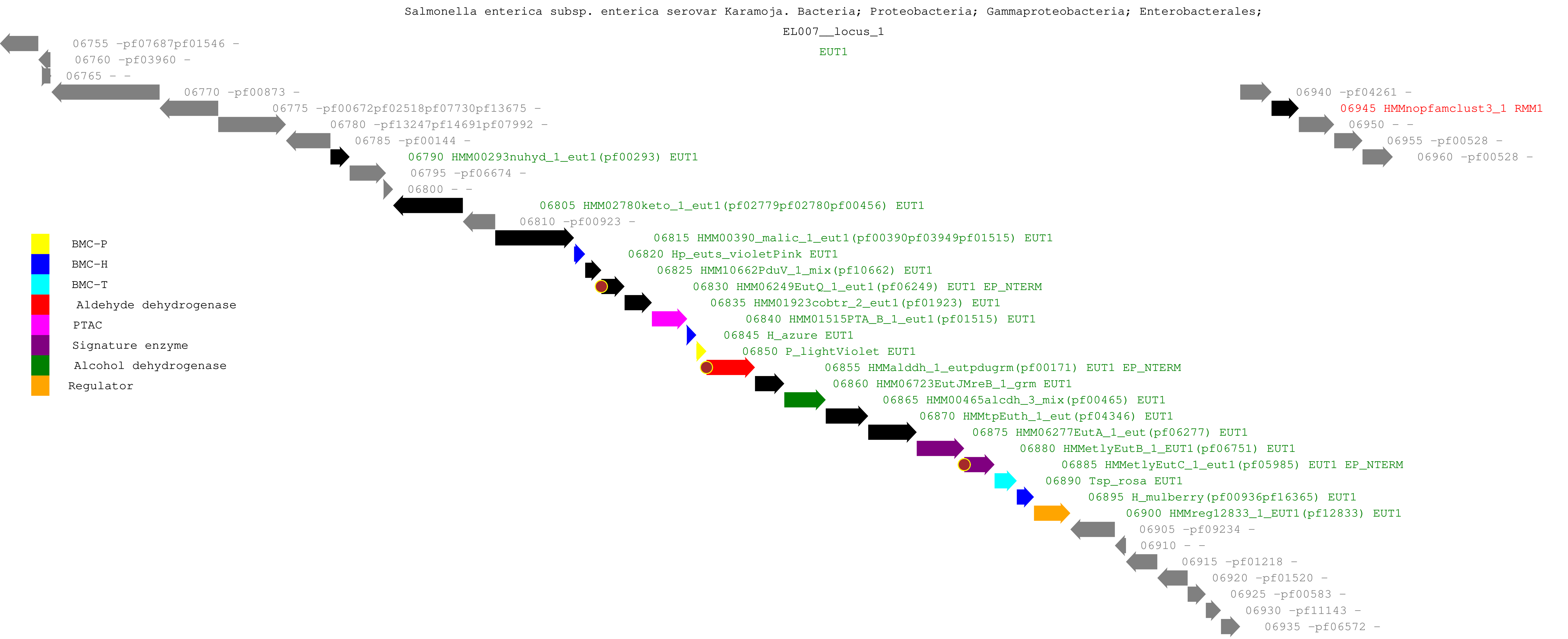

Salmonella enterica subsp. enterica serovar Karamoja. Bacteria; Proteobacteria; Gammaproteobacteria; Enterobacterales;
EL007__locus_1
EUT1
06755 -pf07687pf01546 -
06760 -pf03960 -
06765 - -
06770 -pf00873 -
06775 -pf00672pf02518pf07730pf13675 -
06780 -pf13247pf14691pf07992 -
06785 -pf00144 -
06790 HMM00293nuhyd_1_eut1(pf00293) EUT1
06795 -pf06674 -
06800 - -
06805 HMM02780keto_1_eut1(pf02779pf02780pf00456) EUT1
06810 -pf00923 -
06815 HMM00390_malic_1_eut1(pf00390pf03949pf01515) EUT1
06820 Hp_euts_violetPink EUT1
06825 HMM10662PduV_1_mix(pf10662) EUT1
06830 HMM06249EutQ_1_eut1(pf06249) EUT1 EP_NTERM
06835 HMM01923cobtr_2_eut1(pf01923) EUT1
06840 HMM01515PTA_B_1_eut1(pf01515) EUT1
06845 H_azure EUT1
06850 P_lightViolet EUT1
06855 HMMalddh_1_eutpdugrm(pf00171) EUT1 EP_NTERM
06860 HMM06723EutJMreB_1_grm EUT1
06865 HMM00465alcdh_3_mix(pf00465) EUT1
06870 HMMtpEuth_1_eut(pf04346) EUT1
06875 HMM06277EutA_1_eut(pf06277) EUT1
06880 HMMetlyEutB_1_EUT1(pf06751) EUT1
06885 HMMetlyEutC_1_eut1(pf05985) EUT1 EP_NTERM
06890 Tsp_rosa EUT1
06895 H_mulberry(pf00936pf16365) EUT1
06900 HMMreg12833_1_EUT1(pf12833) EUT1
06905 -pf09234 -
06910 - -
06915 -pf01218 -
06920 -pf01520 -
06925 -pf00583 -
06930 -pf11143 -
06935 -pf06572 -
06940 -pf04261 -
06945 HMMnopfamclust3_1 RMM1
06950 - -
06955 -pf00528 -
06960 -pf00528 -
BMC-P
BMC-H
BMC-T
Aldehyde dehydrogenase
PTAC
Signature enzyme
Alcohol dehydrogenase
Regulator

Salmonella diarizonae. Bacteria; Proteobacteria; Gammaproteobacteria; Enterobacterales;

EL06__locus_1

EUT1

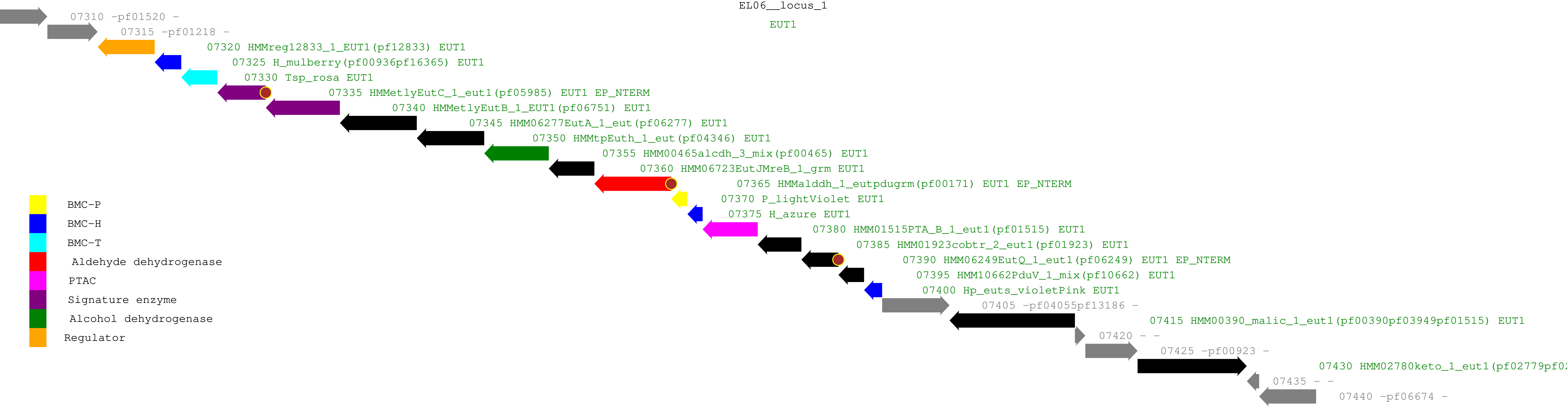

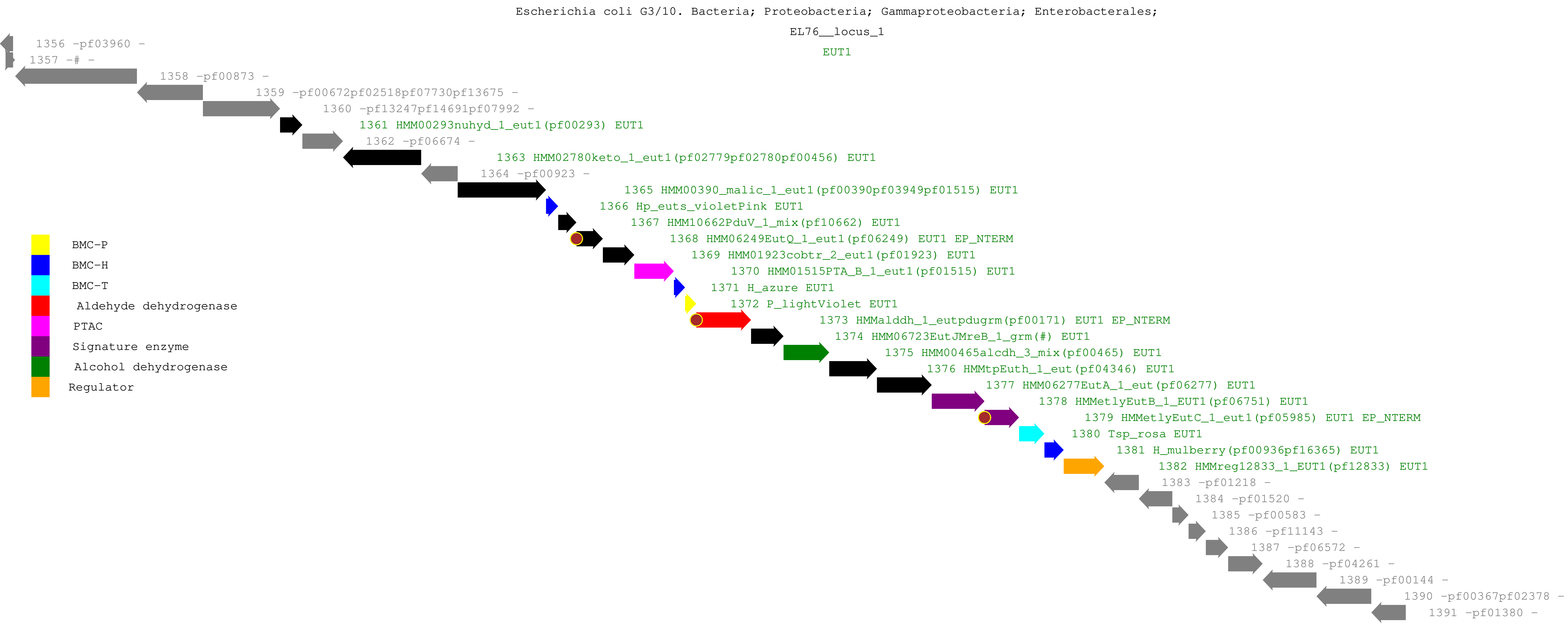
Escherichia coli G3/10. Bacteria; Proteobacteria; Gammaproteobacteria; Enterobacterales;
EL76__locus_1
EUT1
1356 -pf03960 -
1357 -# -
1358 -pf00873 -
1359 -pf00672pf02518pf07730pf13675 -
1360 -pf13247pf14691pf07992 -
1361 HMM00293nuhyd_1_eut1(pf00293) EUT1
1362 -pf06674 -
1363 HMM02780keto_1_eut1(pf02779pf02780pf00456) EUT1
1364 -pf00923 -
1365 HMM00390_malic_1_eut1(pf00390pf03949pf01515) EUT1
1366 Hp_euts_violetPink EUT1
1367 HMM10662PduV_1_mix(pf10662) EUT1
1368 HMM06249EutQ_1_eut1(pf06249) EUT1 EP_NTERM
1369 HMM01923cobtr_2_eut1(pf01923) EUT1
1370 HMM01515PTA_B_1_eut1(pf01515) EUT1
1371 H_azure EUT1
1372 P_lightViolet EUT1
1373 HMMalddh_1_eutpdugrm(pf00171) EUT1 EP_NTERM
1374 HMM06723EutJMreB_1_grm(#) EUT1
1375 HMM00465alcdh_3_mix(pf00465) EUT1
1376 HMMtpEuth_1_eut(pf04346) EUT1
1377 HMM06277EutA_1_eut(pf06277) EUT1
1378 HMMetlyEutB_1_EUT1(pf06751) EUT1
1379 HMMetlyEutC_1_eut1(pf05985) EUT1 EP_NTERM
1380 Tsp_rosa EUT1
1381 H_mulberry(pf00936pf16365) EUT1
1382 HMMreg12833_1_EUT1(pf12833) EUT1
1383 -pf01218 -
1384 -pf01520 -
1385 -pf00583 -
1386 -pf11143 -
1387 -pf06572 -
1388 -pf04261 -
1389 -pf00144 -
1390 -pf00367pf02378 -
1391 -pf01380 -
BMC-P
BMC-H
BMC-T
Aldehyde dehydrogenase
PTAC
Signature enzyme
Alcohol dehydrogenase
Regulator

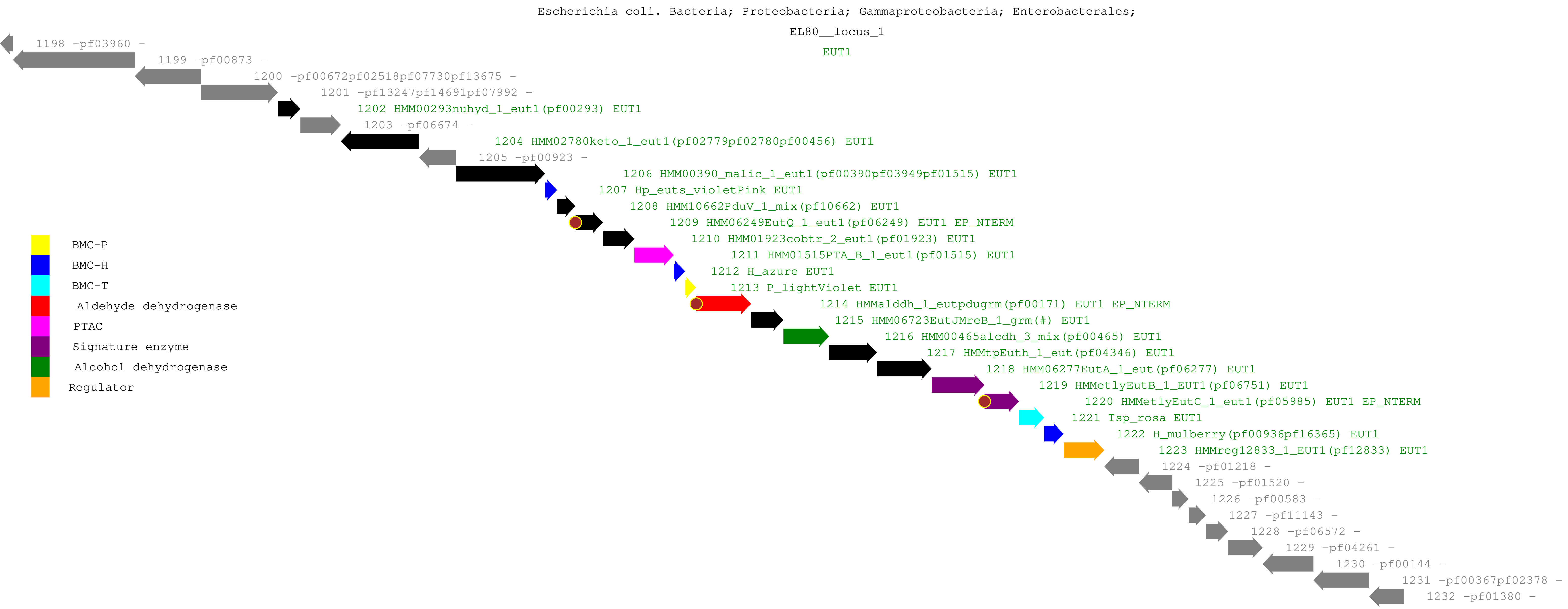

Escherichia coli. Bacteria; Proteobacteria; Gammaproteobacteria; Enterobacterales;
EL80__locus_1
EUT1
1198 -pf03960 -
1199 -pf00873 -
1200 -pf00672pf02518pf07730pf13675 -
1201 -pf13247pf14691pf07992 -
1202 HMM00293nuhyd_1_eut1(pf00293) EUT1
1203 -pf06674 -
1204 HMM02780keto_1_eut1(pf02779pf02780pf00456) EUT1
1205 -pf00923 -
1206 HMM00390_malic_1_eut1(pf00390pf03949pf01515) EUT1
1207 Hp_euts_violetPink EUT1
1208 HMM10662PduV_1_mix(pf10662) EUT1
1209 HMM06249EutQ_1_eut1(pf06249) EUT1 EP_NTERM
1210 HMM01923cobtr_2_eut1(pf01923) EUT1
1211 HMM01515PTA_B_1_eut1(pf01515) EUT1
1212 H_azure EUT1
1213 P_lightViolet EUT1
1214 HMMalddh_1_eutpdugrm(pf00171) EUT1 EP_NTERM
1215 HMM06723EutJMreB_1_grm(#) EUT1
1216 HMM00465alcdh_3_mix(pf00465) EUT1
1217 HMMtpEuth_1_eut(pf04346) EUT1
1218 HMM06277EutA_1_eut(pf06277) EUT1
1219 HMMetlyEutB_1_EUT1(pf06751) EUT1
1220 HMMetlyEutC_1_eut1(pf05985) EUT1 EP_NTERM
1221 Tsp_rosa EUT1
1222 H_mulberry(pf00936pf16365) EUT1
1223 HMMreg12833_1_EUT1(pf12833) EUT1
1224 -pf01218 -
1225 -pf01520 -
1226 -pf00583 -
1227 -pf11143 -
1228 -pf06572 -
1229 -pf04261 -
1230 -pf00144 -
1231 -pf00367pf02378 -
1232 -pf01380 -
BMC-P
BMC-H
BMC-T
Aldehyde dehydrogenase
PTAC
Signature enzyme
Alcohol dehydrogenase
Regulator

Salmonella enterica subsp. enterica serovar Moero. Bacteria; Proteobacteria; Gammaproteobacteria; Enterobacterales;

ELZ69__locus_1

EUT1

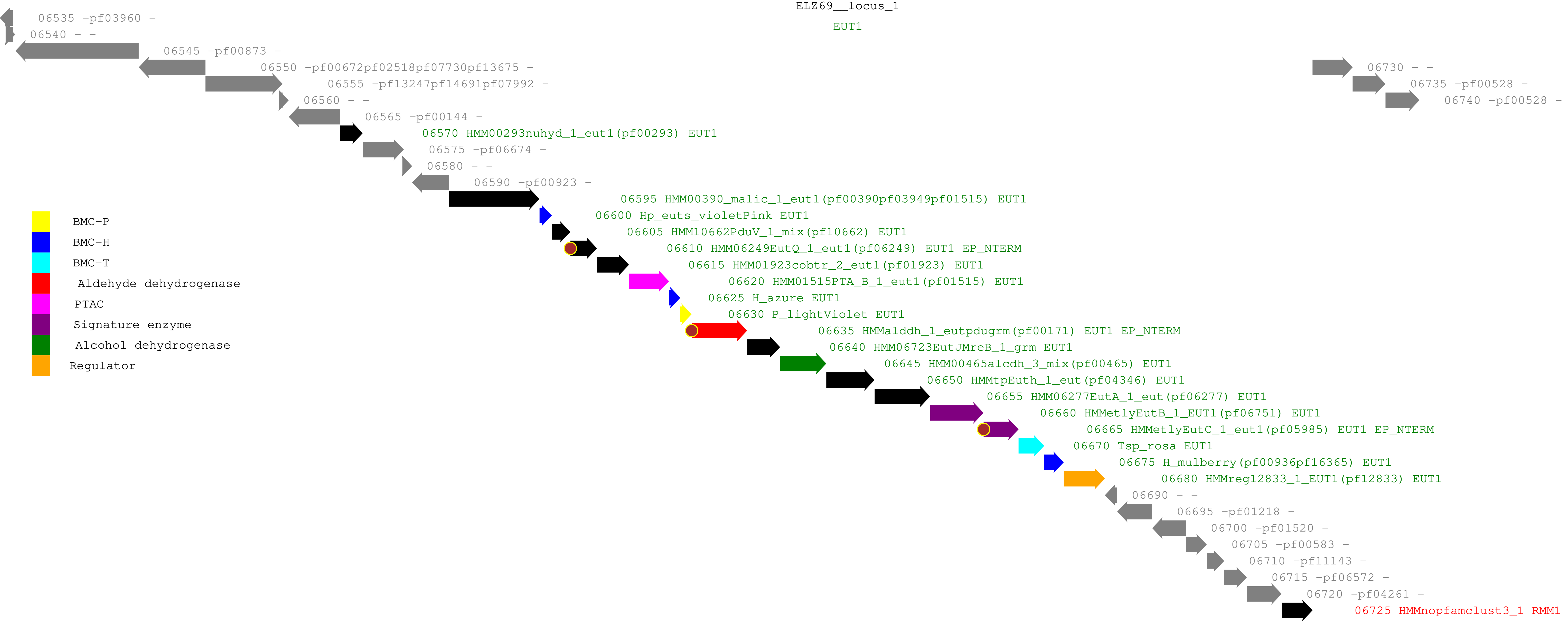

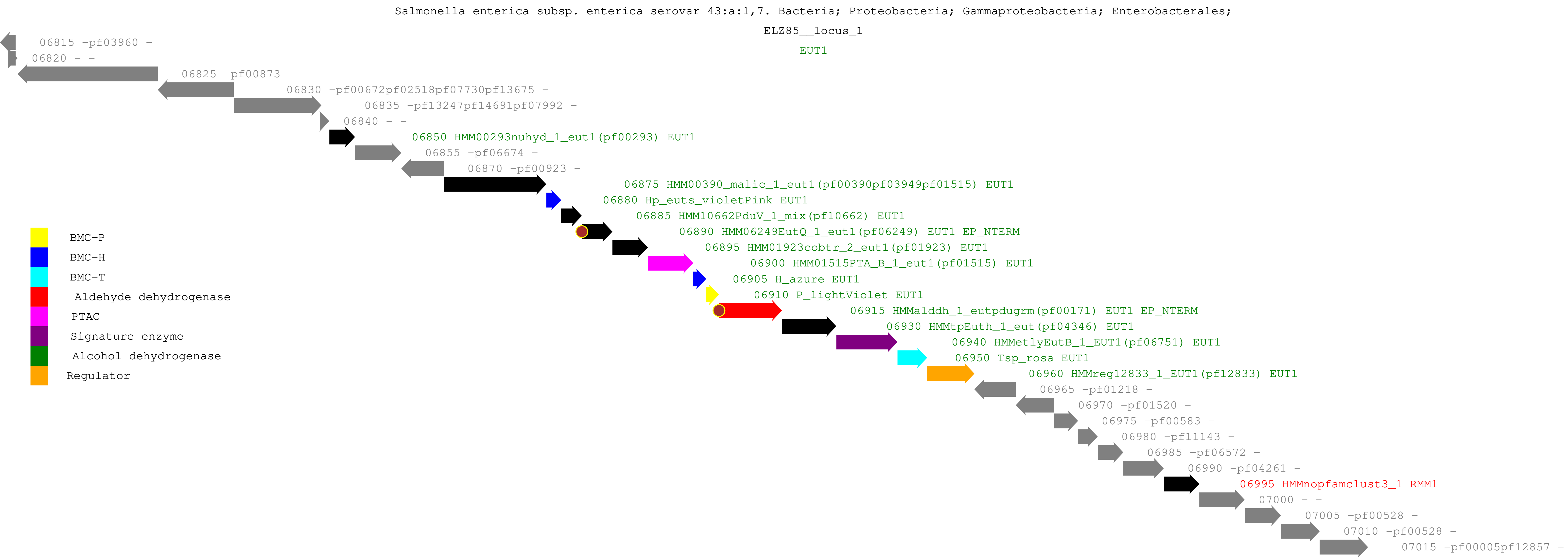
Salmonella enterica subsp. enterica serovar 43:a:1,7. Bacteria; Proteobacteria; Gammaproteobacteria; Enterobacterales;
ELZ85__locus_1
EUT1
06815 -pf03960 -
06820 - -
06825 -pf00873 -
06830 -pf00672pf02518pf07730pf13675 -
06835 -pf13247pf14691pf07992 -
06840 - -
06850 HMM00293nuhyd_1_eut1(pf00293) EUT1
06855 -pf06674 -
06870 -pf00923 -
06875 HMM00390_malic_1_eut1(pf00390pf03949pf01515) EUT1
06880 Hp_euts_violetPink EUT1
06885 HMM10662PduV_1_mix(pf10662) EUT1
06890 HMM06249EutQ_1_eut1(pf06249) EUT1 EP_NTERM
06895 HMM01923cobtr_2_eut1(pf01923) EUT1
06900 HMM01515PTA_B_1_eut1(pf01515) EUT1
06905 H_azure EUT1
06910 P_lightViolet EUT1
06915 HMMalddh_1_eutpdugrm(pf00171) EUT1 EP_NTERM
06930 HMMtpEuth_1_eut(pf04346) EUT1
06940 HMMetlyEutB_1_EUT1(pf06751) EUT1
06950 Tsp_rosa EUT1
06960 HMMreg12833_1_EUT1(pf12833) EUT1
06965 -pf01218 -
06970 -pf01520 -
06975 -pf00583 -
06980 -pf11143 -
06985 -pf06572 -
06990 -pf04261 -
06995 HMMnopfamclust3_1 RMM1
07000 - -
07005 -pf00528 -
07010 -pf00528 -
07015 -pf00005pf12857 -
BMC-P
BMC-H
BMC-T
Aldehyde dehydrogenase
PTAC
Signature enzyme
Alcohol dehydrogenase
Regulator

Salmonella enterica subsp. enterica serovar 43:a:1,7. Bacteria; Proteobacteria; Gammaproteobacteria; Enterobacterales;

ELZ87__locus_1

EUT1

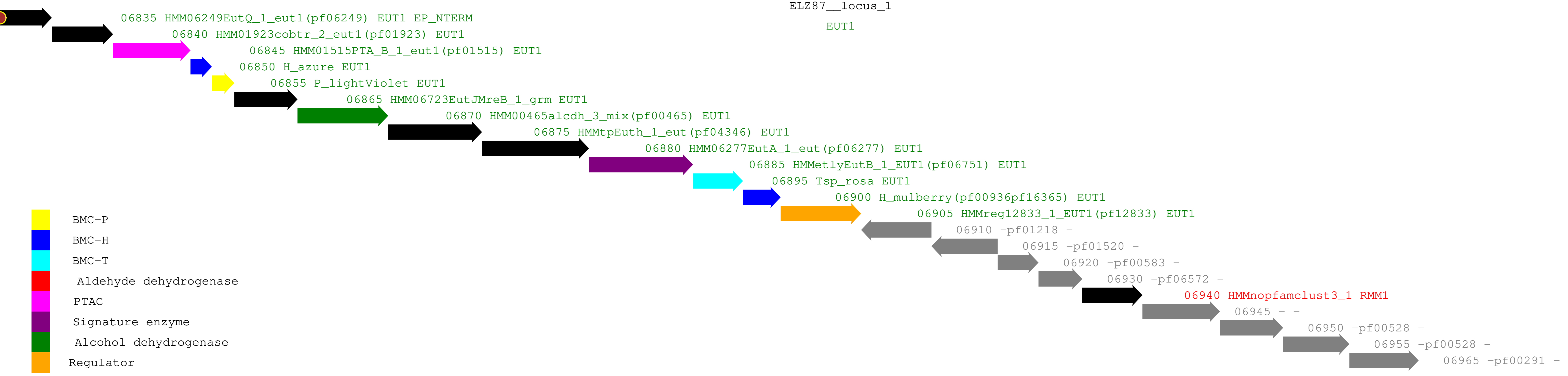

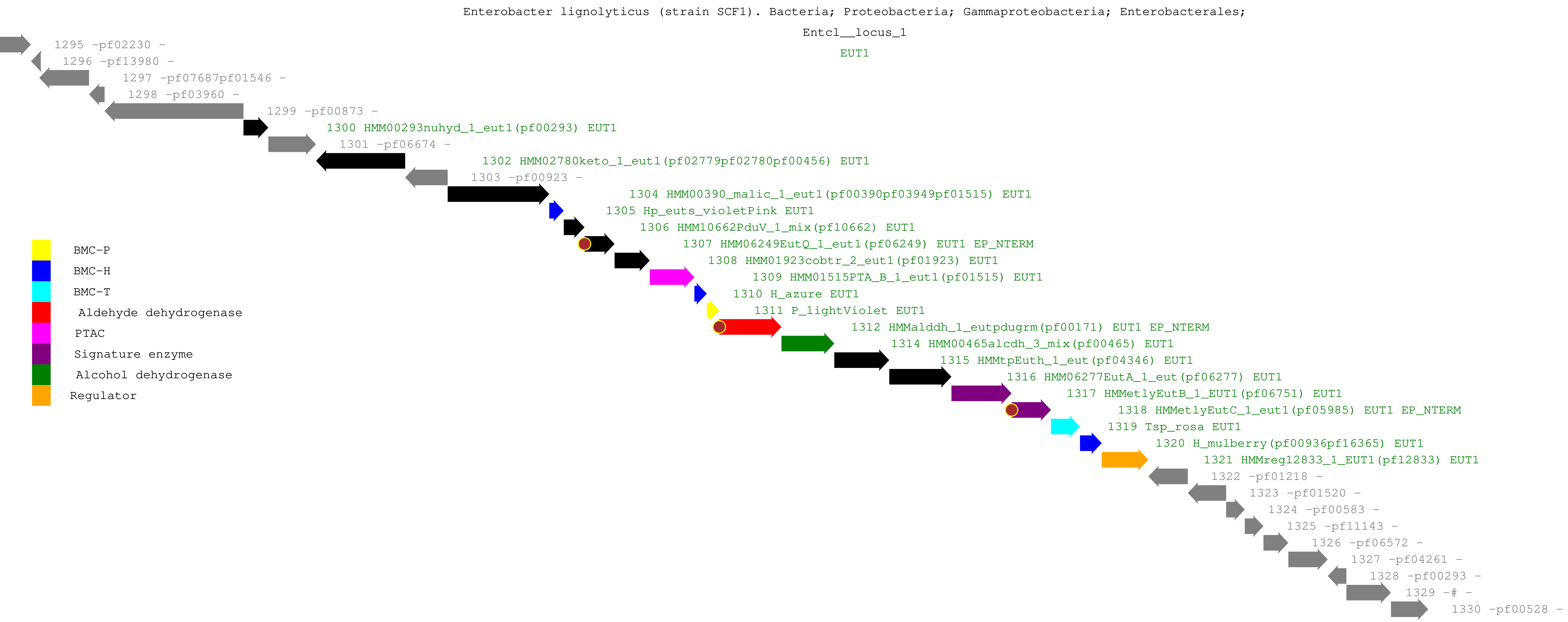

Enterobacter lignolyticus (strain SCF1). Bacteria; Proteobacteria; Gammaproteobacteria; Enterobacterales;
Entcl__locus_1
EUT1
1295 -pf02230 -
1296 -pf13980 -
1297 -pf07687pf01546 -
1298 -pf03960 -
1299 -pf00873 -
1300 HMM00293nuhyd_1_eut1(pf00293) EUT1
1301 -pf06674 -
1302 HMM02780keto_1_eut1(pf02779pf02780pf00456) EUT1
1303 -pf00923 -
1304 HMM00390_malic_1_eut1(pf00390pf03949pf01515) EUT1
1305 Hp_euts_violetPink EUT1
1306 HMM10662PduV_1_mix(pf10662) EUT1
1307 HMM06249EutQ_1_eut1(pf06249) EUT1 EP_NTERM
1308 HMM01923cobtr_2_eut1(pf01923) EUT1
1309 HMM01515PTA_B_1_eut1(pf01515) EUT1
1310 H_azure EUT1
1311 P_lightViolet EUT1
1312 HMMaldh_1_eutpdugrm(pf00171) EUT1 EP_NTERM
1314 HMM00465alcdh_3_mix(pf00465) EUT1
1315 HMMtpEuth_1_eut(pf04346) EUT1
1316 HMM06277EutA_1_eut(pf06277) EUT1
1317 HMMetlyEutB_1_EUT1(pf06751) EUT1
1318 HMMetlyEutC_1_eut1(pf05985) EUT1 EP_NTERM
1319 Tsp_rosa EUT1
1320 H_mulberry(pf00936pf16365) EUT1
1321 HMMreg12833_1_EUT1(pf12833) EUT1
1322 -pf01218 -
1323 -pf01520 -
1324 -pf00583 -
1325 -pf11143 -
1326 -pf06572 -
1327 -pf04261 -
1328 -pf00293 -
1329 -# -
1330 -pf00528 -
BMC-P
BMC-H
BMC-T
Aldehyde dehydrogenase
PTAC
Signature enzyme
Alcohol dehydrogenase
Regulator

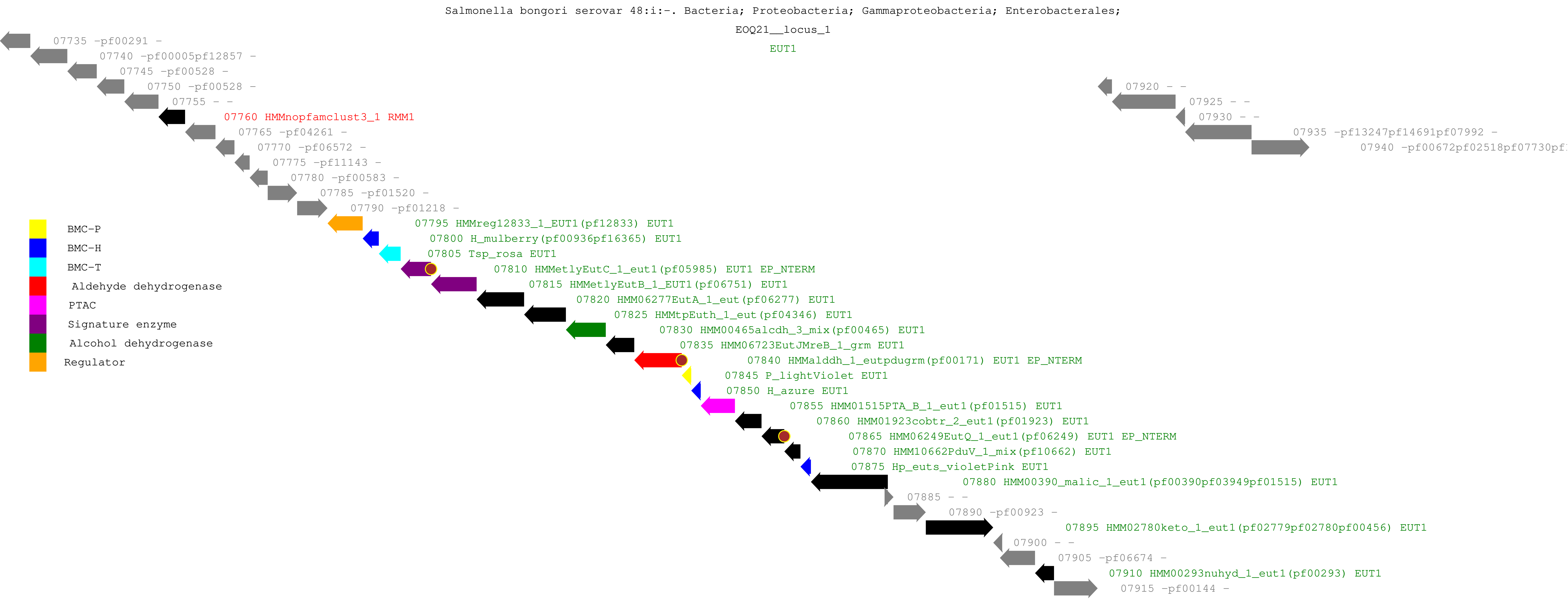

Salmonella bongori serovar 48:i:-. Bacteria; Proteobacteria; Gammaproteobacteria; Enterobacterales;
EOQ21__locus_1
EUT1
07735 -pf00291 -
07740 -pf00005pf12857 -
07745 -pf00528 -
07750 -pf00528 -
07755 - -
07760 HMMnopfamclust3_1 RMM1
07765 -pf04261 -
07770 -pf06572 -
07775 -pf11143 -
07780 -pf00583 -
07785 -pf01520 -
07790 -pf01218 -
07795 HMMreg12833_1_EUT1(pf12833) EUT1
07800 H_mulberry(pf00936pf16365) EUT1
07805 Tsp_rosa EUT1
07810 HMMetlyEutC_1_eut1(pf05985) EUT1 EP_NTERM
07815 HMMetlyEutB_1_EUT1(pf06751) EUT1
07820 HMM06277EutA_1_eut(pf06277) EUT1
07825 HMMtpEuth_1_eut(pf04346) EUT1
07830 HMM00465alcdh_3_mix(pf00465) EUT1
07835 HMM06723EutJMreB_1_grm EUT1
07840 HMMalddh_1_eutpdugrm(pf00171) EUT1 EP_NTERM
07845 P_lightViolet EUT1
07850 H_azure EUT1
07855 HMM01515PTA_B_1_eut1(pf01515) EUT1
07860 HMM01923cobtr_2_eut1(pf01923) EUT1
07865 HMM06249EutQ_1_eut1(pf06249) EUT1 EP_NTERM
07870 HMM10662PduV_1_mix(pf10662) EUT1
07875 Hp_euts_violetPink EUT1
07880 HMM00390_malic_1_eut1(pf00390pf03949pf01515) EUT1
07885 - -
07890 -pf00923 -
07895 HMM02780keto_1_eut1(pf02779pf02780pf00456) EUT1
07900 - -
07905 -pf06674 -
07910 HMM00293nuhyd_1_eut1(pf00293) EUT1
07915 -pf00144 -
07920 - -
07925 - -
07930 - -
07935 -pf13247pf14691pf07992 -
07940 -pf00672pf02518pf07730pf
BMC-P
BMC-H
BMC-T
Aldehyde dehydrogenase
PTAC
Signature enzyme
Alcohol dehydrogenase
Regulator

Escherichia coli N37139PS. Bacteria; Proteobacteria; Gammaproteobacteria; Enterobacterales;

EP55__locus_1

EUT1

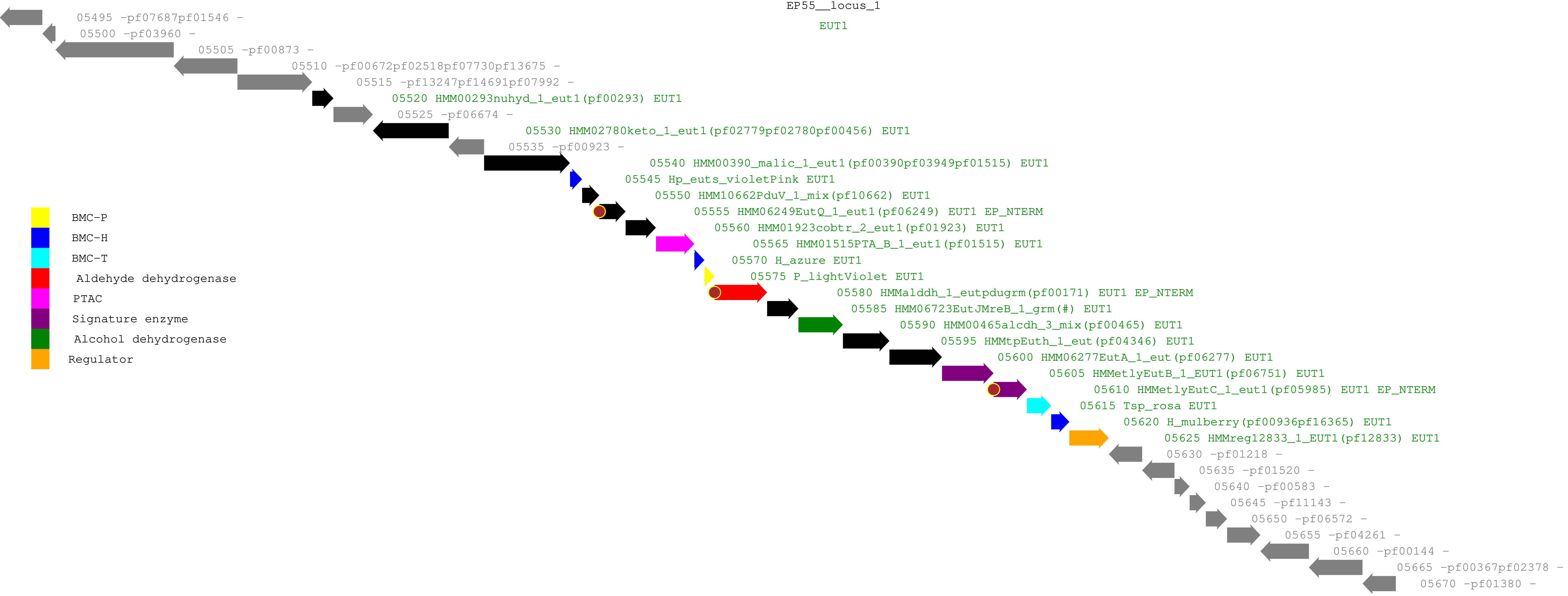

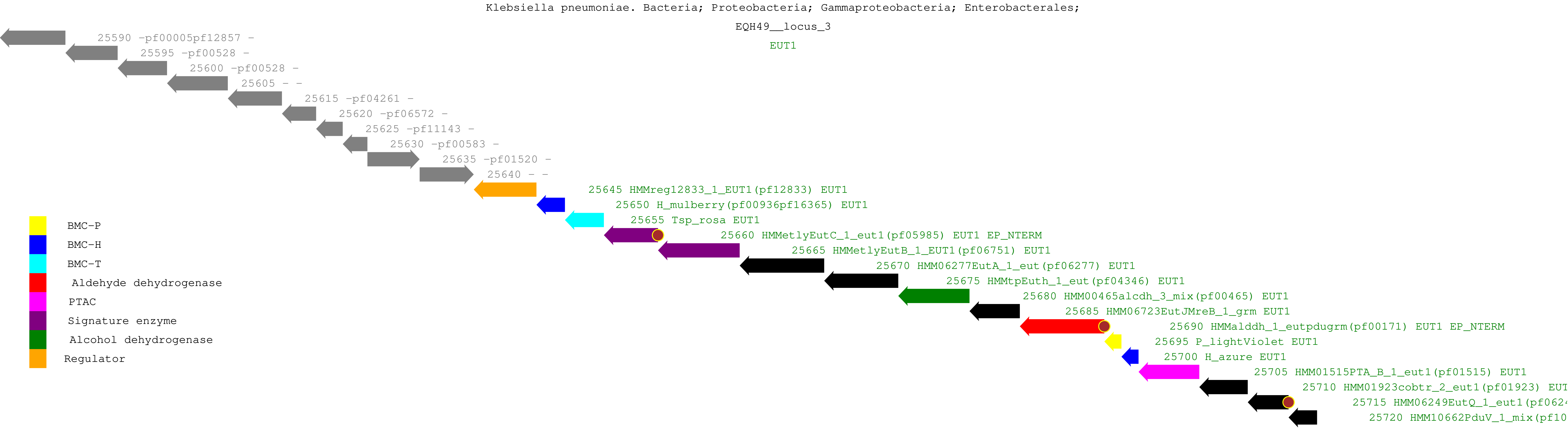
Klebsiella pneumoniae. Bacteria; Proteobacteria; Gammaproteobacteria; Enterobacterales;
EQH49__locus_3
EUT1
25590 -pf00005pf12857 -
25595 -pf00528 -
25600 -pf00528 -
25605 - -
25615 -pf04261 -
25620 -pf06572 -
25625 -pf11143 -
25630 -pf00583 -
25635 -pf01520 -
25640 - -
25645 HMMreg12833_1_EUT1(pf12833) EUT1
25650 H_mulberry(pf00936pf16365) EUT1
25655 Tsp_rosa EUT1
25660 HMMetlyEutC_1_eut1(pf05985) EUT1 EP_NTERM
25665 HMMetlyEutB_1_EUT1(pf06751) EUT1
25670 HMM06277EutA_1_eut(pf06277) EUT1
25675 HMMtpEuth_1_eut(pf04346) EUT1
25680 HMM00465alcdh_3_mix(pf00465) EUT1
25685 HMM06723EutJMreB_1_grm EUT1
25690 HMMalddh_1_eutpdugrm(pf00171) EUT1 EP_NTERM
25695 P_lightViolet EUT1
25700 H_azure EUT1
25705 HMM01515PTA_B_1_eut1(pf01515) EUT1
25710 HMM01923cobtr_2_eut1(pf01923) EUT
25715 HMM06249EutQ_1_eut1(pf062
25720 HMM10662PduV_1_mix(pf10
BMC-P
BMC-H
BMC-T
Aldehyde dehydrogenase
PTAC
Signature enzyme
Alcohol dehydrogenase
Regulator

Escherichia coli TW10509. Bacteria; Proteobacteria; Gammaproteobacteria; Enterobacterales;

ERFG__locus_1

EUT1

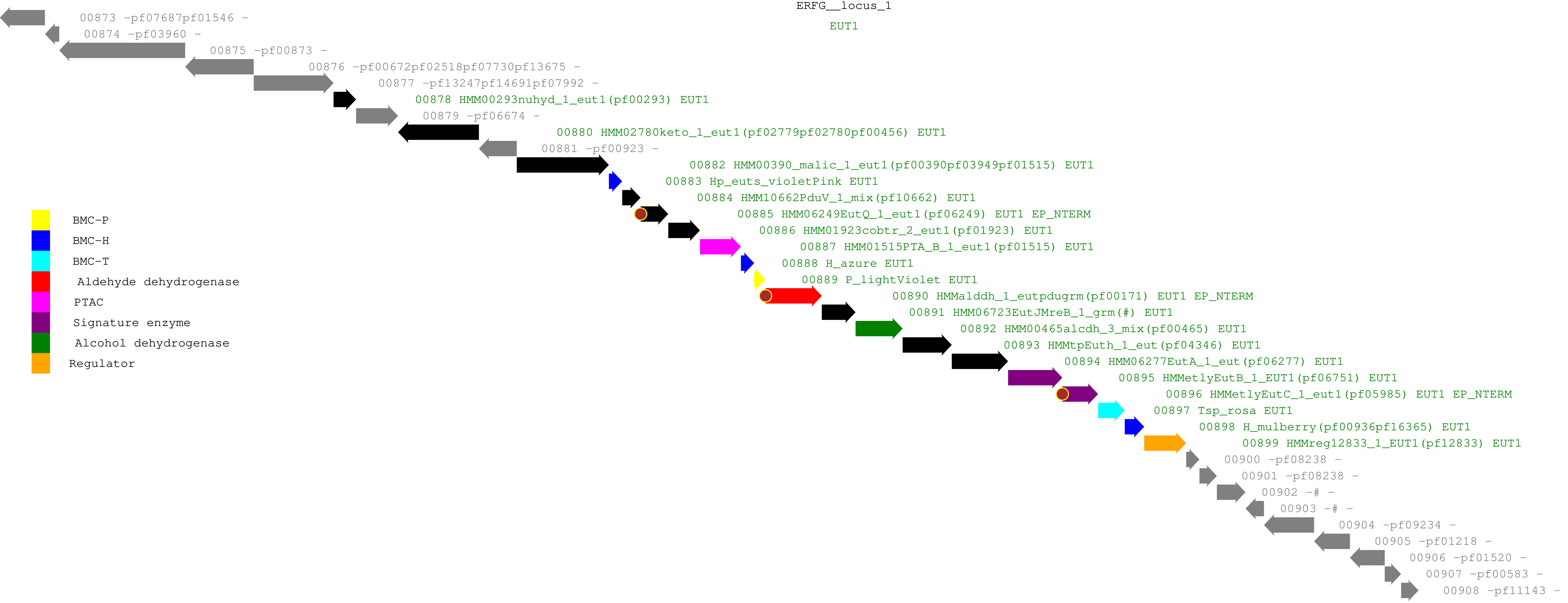

Escherichia coli M863. Bacteria; Proteobacteria; Gammaproteobacteria; Enterobacterales;

ERJG__locus_1

EUT1

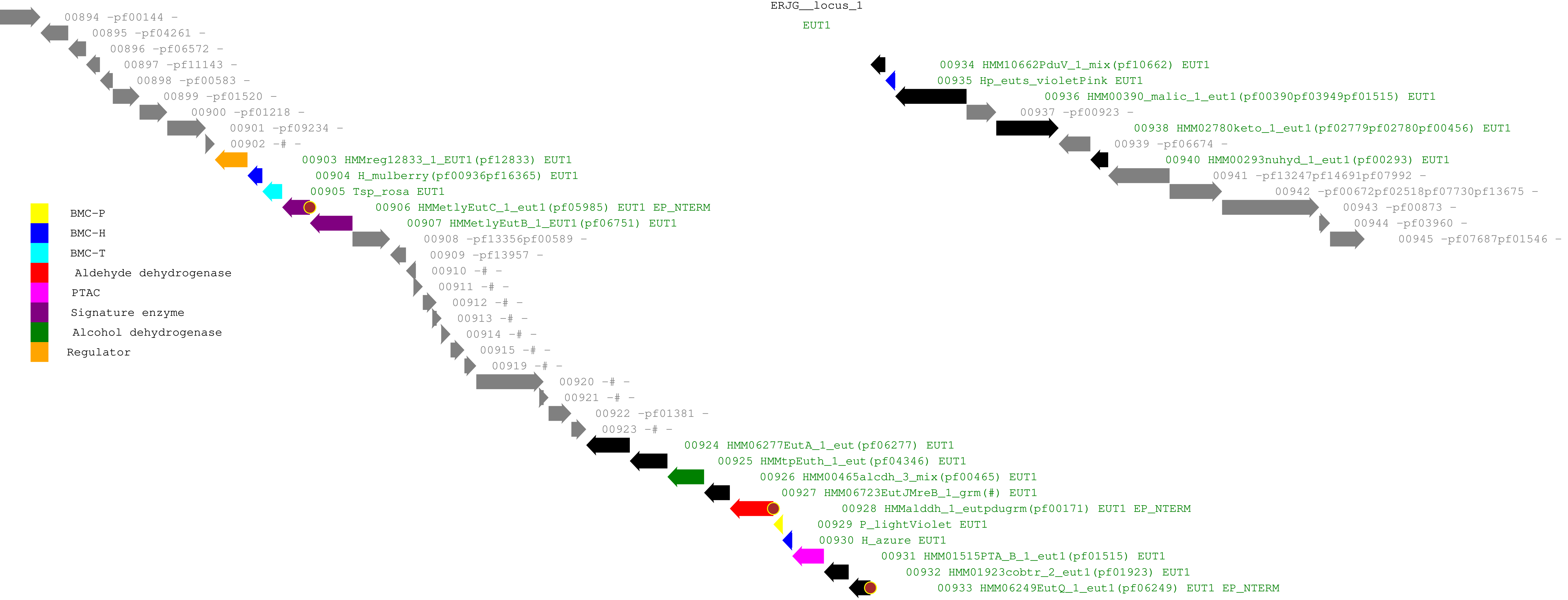

Escherichia coli. Bacteria; Proteobacteria; Gammaproteobacteria; Enterobacterales;

ERL57__locus_1

EUT1

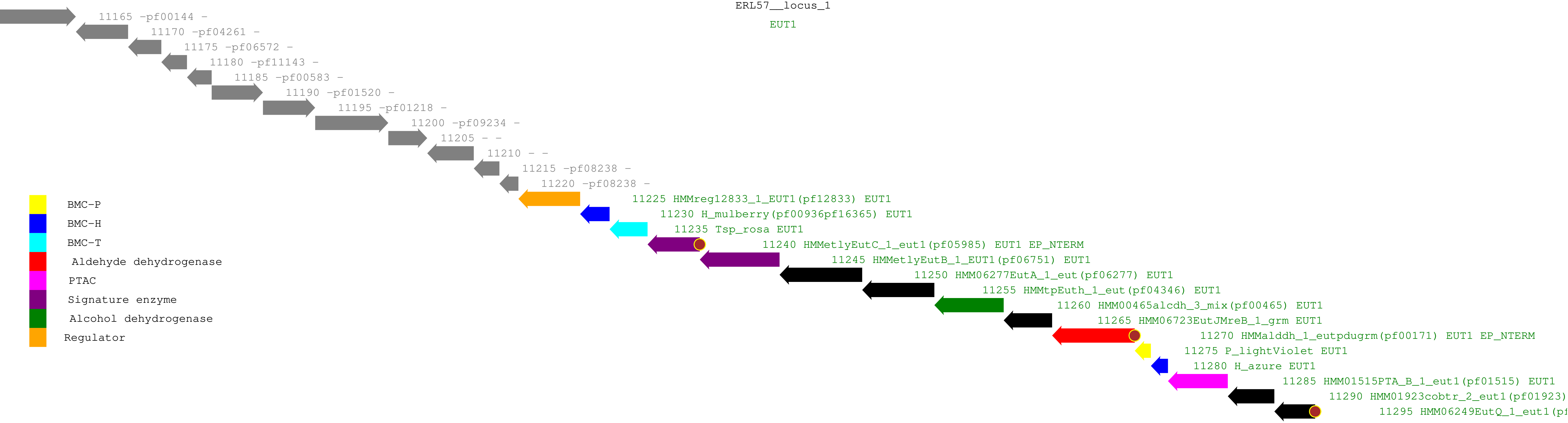

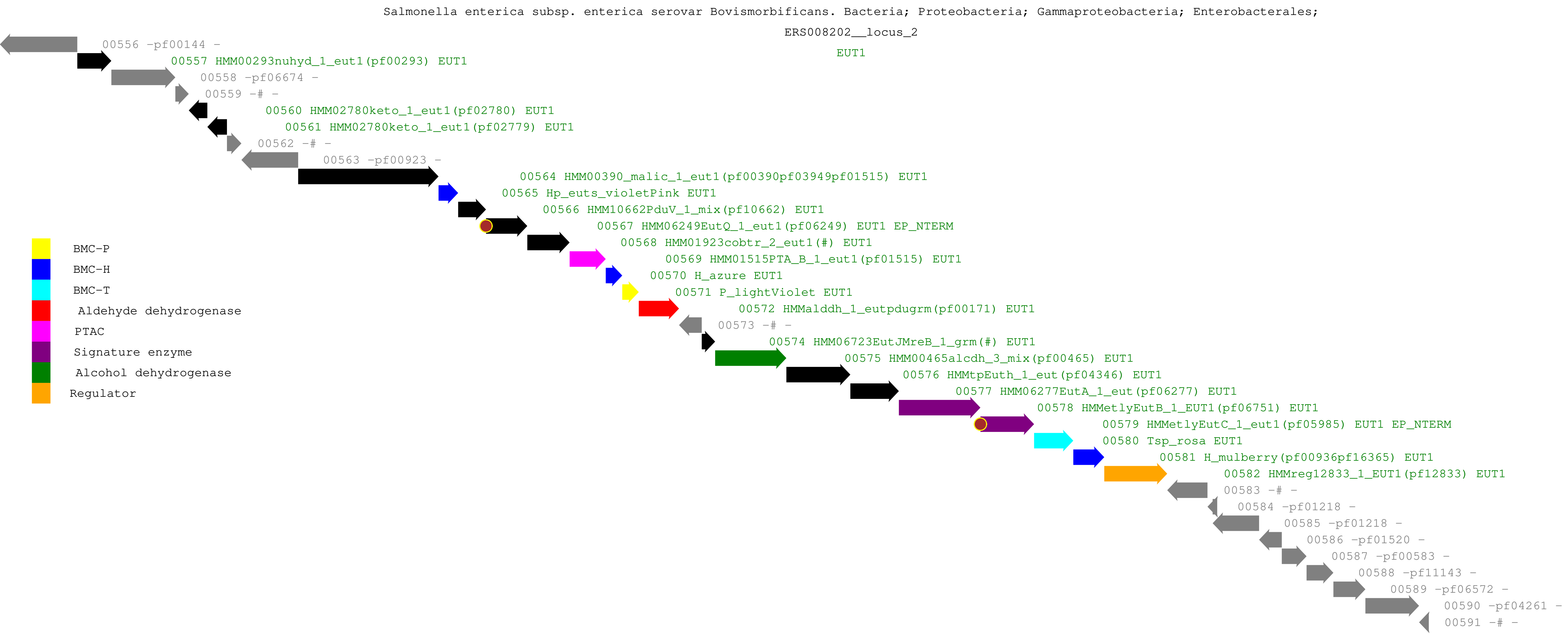
Salmonella enterica subsp. enterica serovar Bovismorbificans. Bacteria; Proteobacteria; Gammaproteobacteria; Enterobacterales;
ERS008202__locus_2
EUT1
00556 -pf00144 -
00557 HMM00293nuhyd_1_eut1(pf00293) EUT1
00558 -pf06674 -
00559 -# -
00560 HMM02780keto_1_eut1(pf02780) EUT1
00561 HMM02780keto_1_eut1(pf02779) EUT1
00562 -# -
00563 -pf00923 -
00564 HMM00390_malic_1_eut1(pf00390pf03949pf01515) EUT1
00565 Hp_euts_violetPink EUT1
00566 HMM10662PduV_1_mix(pf10662) EUT1
00567 HMM06249EutQ_1_eut1(pf06249) EUT1 EP_NTERM
00568 HMM01923cobtr_2_eut1(#) EUT1
00569 HMM01515PTA_B_1_eut1(pf01515) EUT1
00570 H_azure EUT1
00571 P_lightViolet EUT1
00572 HMMalddh_1_eutpdugrm(pf00171) EUT1
00573 -# -
00574 HMM06723EutJMreB_1_grm(#) EUT1
00575 HMM00465alcdh_3_mix(pf00465) EUT1
00576 HMMtpEuth_1_eut(pf04346) EUT1
00577 HMM06277EutA_1_eut(pf06277) EUT1
00578 HMMetlyEutB_1_EUT1(pf06751) EUT1
00579 HMMetlyEutC_1_eut1(pf05985) EUT1 EP_NTERM
00580 Tsp_rosa EUT1
00581 H_mulberry(pf00936pf16365) EUT1
00582 HMMreg12833_1_EUT1(pf12833) EUT1
00583 -# -
00584 -pf01218 -
00585 -pf01218 -
00586 -pf01520 -
00587 -pf00583 -
00588 -pf11143 -
00589 -pf06572 -
00590 -pf04261 -
00591 -# -
BMC-P
BMC-H
BMC-T
Aldehyde dehydrogenase
PTAC
Signature enzyme
Alcohol dehydrogenase
Regulator

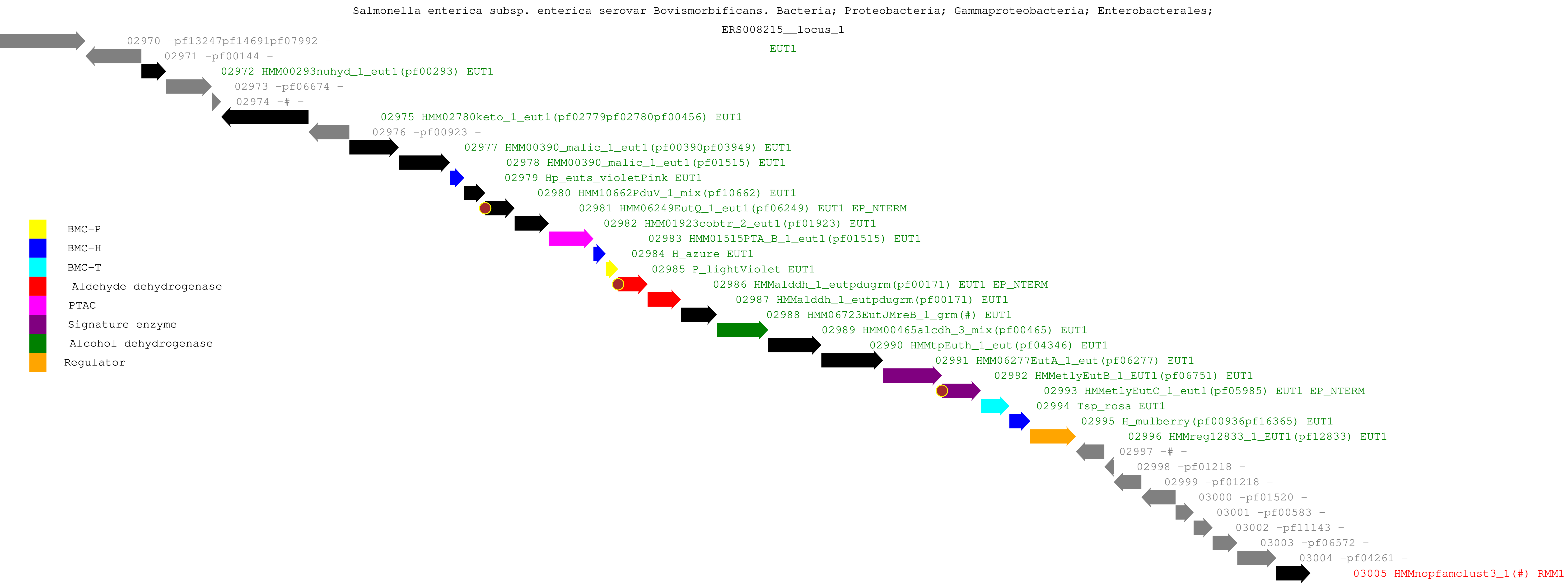

Salmonella enterica subsp. enterica serovar Bovismorbificans. Bacteria; Proteobacteria; Gammaproteobacteria; Enterobacterales;
ERS008215__locus_1
EUT1
02970 -pf13247pf14691pf07992 -
02971 -pf00144 -
02972 HMM00293nuhyd_1_eut1(pf00293) EUT1
02973 -pf06674 -
02974 -# -
02975 HMM02780keto_1_eut1(pf02779pf02780pf00456) EUT1
02976 -pf00923 -
02977 HMM00390_malic_1_eut1(pf00390pf03949) EUT1
02978 HMM00390_malic_1_eut1(pf01515) EUT1
02979 Hp_euts_violetPink EUT1
02980 HMM10662PduV_1_mix(pf10662) EUT1
02981 HMM06249EutQ_1_eut1(pf06249) EUT1 EP_NTERM
02982 HMM01923cobtr_2_eut1(pf01923) EUT1
02983 HMM01515PTA_B_1_eut1(pf01515) EUT1
02984 H_azure EUT1
02985 P_lightViolet EUT1
02986 HMMalddh_1_eutpdugrm(pf00171) EUT1 EP_NTERM
02987 HMMalddh_1_eutpdugrm(pf00171) EUT1
02988 HMM06723EutJMreB_1_grm(#) EUT1
02989 HMM00465alcdh_3_mix(pf00465) EUT1
02990 HMMtpEuth_1_eut(pf04346) EUT1
02991 HMM06277EutA_1_eut(pf06277) EUT1
02992 HMMetlyEutB_1_EUT1(pf06751) EUT1
02993 HMMetlyEutC_1_eut1(pf05985) EUT1 EP_NTERM
02994 Tsp_rosa EUT1
02995 H_mulberry(pf00936pf16365) EUT1
02996 HMMreg12833_1_EUT1(pf12833) EUT1
02997 -# -
02998 -pf01218 -
02999 -pf01218 -
03000 -pf01520 -
03001 -pf00583 -
03002 -pf11143 -
03003 -pf06572 -
03004 -pf04261 -
03005 HMMnopfamclust3_1(#) RMM1
BMC-P
BMC-H
BMC-T
Aldehyde dehydrogenase
PTAC
Signature enzyme
Alcohol dehydrogenase
Regulator

Yersinia intermedia. Bacteria; Proteobacteria; Gammaproteobacteria; Enterobacterales;

ERS008530__locus_2

EUT1

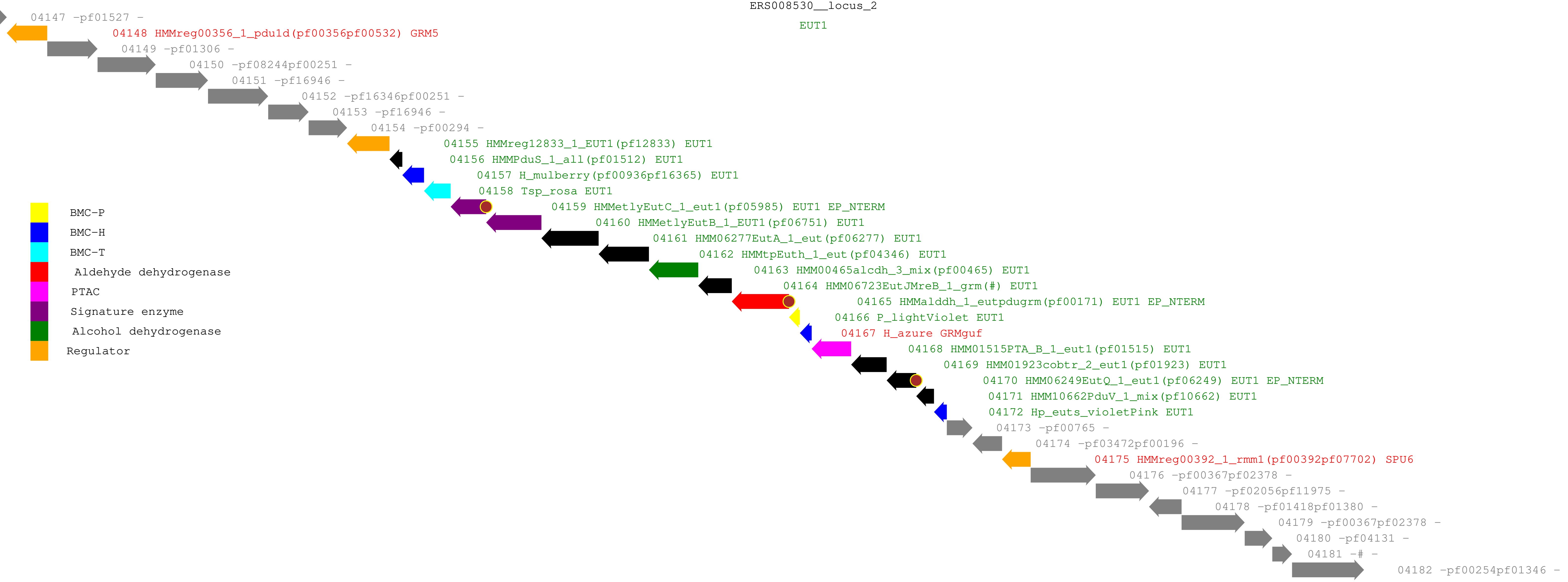

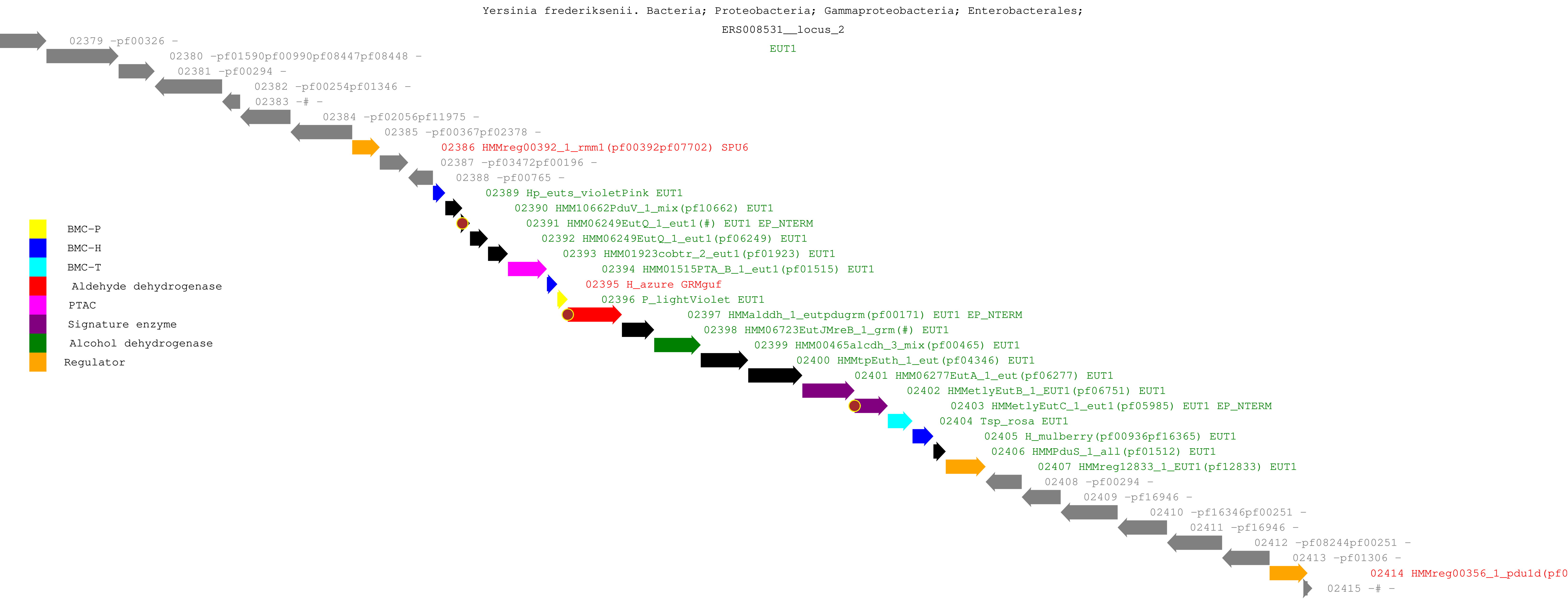

Yersinia frederiksenii. Bacteria; Proteobacteria; Gammaproteobacteria; Enterobacterales;
ERS008531__locus_2
EUT1
02379 -pf00326 -
02380 -pf01590pf00990pf08447pf08448 -
02381 -pf00294 -
02382 -pf00254pf01346 -
02383 -# -
02384 -pf02056pf11975 -
02385 -pf00367pf02378 -
02386 HMMreg00392_1_rmm1(pf00392pf07702) SPU6
02387 -pf03472pf00196 -
02388 -pf00765 -
02389 Hp_euts_violetPink EUT1
02390 HMM10662PduV_1_mix(pf10662) EUT1
02391 HMM06249EutQ_1_eut1(#) EUT1 EP_NTERM
02392 HMM06249EutQ_1_eut1(pf06249) EUT1
02393 HMM01923cobtr_2_eut1(pf01923) EUT1
02394 HMM01515PTA_B_1_eut1(pf01515) EUT1
02395 H_azure GRMguf
02396 P_lightViolet EUT1
02397 HMMaldh_1_eutpdugrm(pf00171) EUT1 EP_NTERM
02398 HMM06723EutJMreB_1_grm(#) EUT1
02399 HMM00465alcdh_3_mix(pf00465) EUT1
02400 HMMtpEuth_1_eut(pf04346) EUT1
02401 HMM06277EutA_1_eut(pf06277) EUT1
02402 HMMetlyEutB_1_EUT1(pf06751) EUT1
02403 HMMetlyEutC_1_eut1(pf05985) EUT1 EP_NTERM
02404 Tsp_rosa EUT1
02405 H_mulberry(pf00936pf16365) EUT1
02406 HMMPduS_1_all(pf01512) EUT1
02407 HMMreg12833_1_EUT1(pf12833) EUT1
02408 -pf00294 -
02409 -pf16946 -
02410 -pf16346pf00251 -
02411 -pf16946 -
02412 -pf08244pf00251 -
02413 -pf01306 -
02414 HMMreg00356_1_pdu1d(pf0
02415 -# -
BMC-P
BMC-H
BMC-T
Aldehyde dehydrogenase
PTAC
Signature enzyme
Alcohol dehydrogenase
Regulator

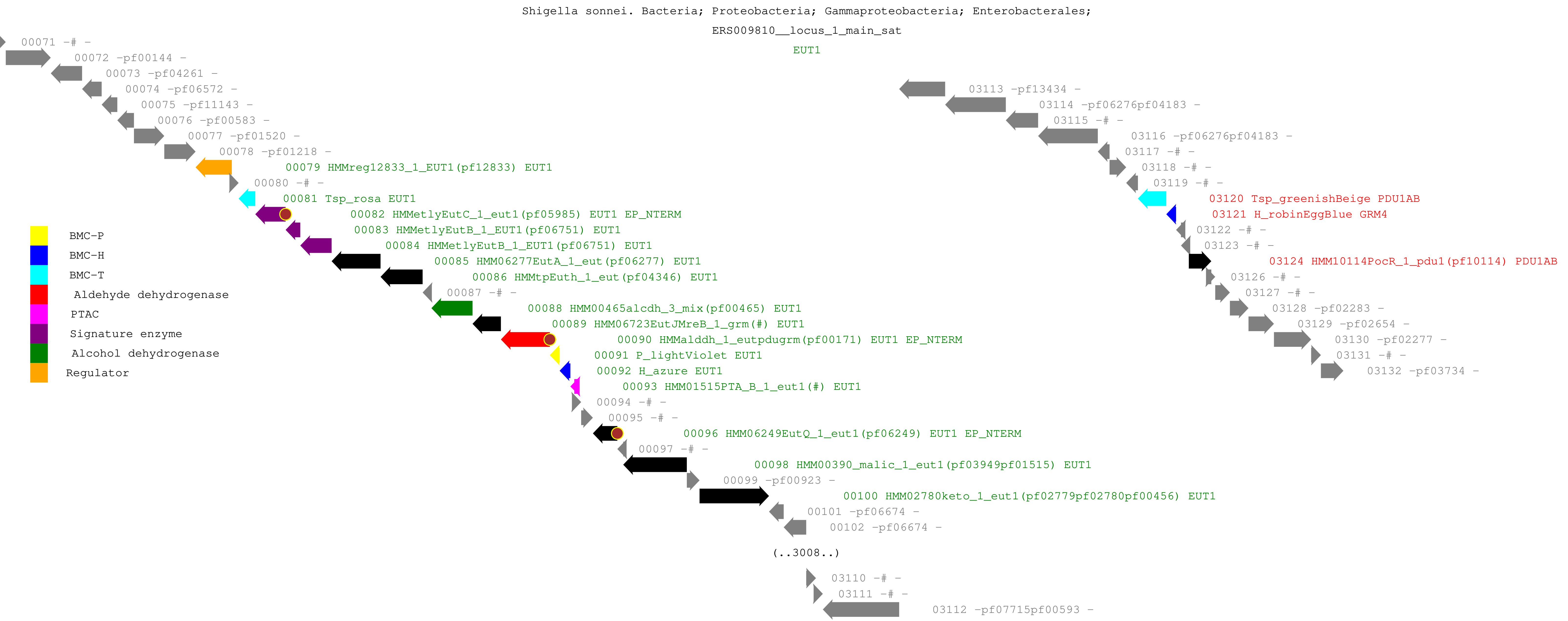
Shigella sonnei. Bacteria; Proteobacteria; Gammaproteobacteria; Enterobacterales;
ERS009810__locus_1_main_sat
EUT1
00071 -# -
00072 -pf00144 -
00073 -pf04261 -
00074 -pf06572 -
00075 -pf11143 -
00076 -pf00583 -
00077 -pf01520 -
00078 -pf01218 -
00079 HMMreg12833_1_EUT1(pf12833) EUT1
00080 -# -
00081 Tsp_rosa EUT1
00082 HMMetlyEutC_1_eut1(pf05985) EUT1 EP_NTERM
00083 HMMetlyEutB_1_EUT1(pf06751) EUT1
00084 HMMetlyEutB_1_EUT1(pf06751) EUT1
00085 HMM06277EutA_1_eut(pf06277) EUT1
00086 HMMtpEuth_1_eut(pf04346) EUT1
00087 -# -
00088 HMM00465alcdh_3_mix(pf00465) EUT1
00089 HMM06723EutJMreB_1_grm(#) EUT1
00090 HMMalddh_1_eutpdugrm(pf00171) EUT1 EP_NTERM
00091 P_lightViolet EUT1
00092 H_azure EUT1
00093 HMM01515PTA_B_1_eut1(#) EUT1
00094 -# -
00095 -# -
00096 HMM06249EutQ_1_eut1(pf06249) EUT1 EP_NTERM
00097 -# -
00098 HMM00390_malic_1_eut1(pf03949pf01515) EUT1
00099 -pf00923 -
00100 HMM02780keto_1_eut1(pf02779pf02780pf00456) EUT1
00101 -pf06674 -
00102 -pf06674 -
(..3008..)
03110 -# -
03111 -# -
03112 -pf07715pf00593 -
03113 -pf13434 -
03114 -pf06276pf04183 -
03115 -# -
03116 -pf06276pf04183 -
03117 -# -
03118 -# -
03119 -# -
03120 Tsp_greenishBeige PDU1AB
03121 H_robinEggBlue GRM4
03122 -# -
03123 -# -
03124 HMM10114PocR_1_pdu1(pf10114) PDU1AB
03126 -# -
03127 -# -
03128 -pf02283 -
03129 -pf02654 -
03130 -pf02277 -
03131 -# -
03132 -pf03734 -
BMC-P
BMC-H
BMC-T
Aldehyde dehydrogenase
PTAC
Signature enzyme
Alcohol dehydrogenase
Regulator

Escherichia coli. Bacteria; Proteobacteria; Gammaproteobacteria; Enterobacterales;

ERS085365__locus_1

EUT1

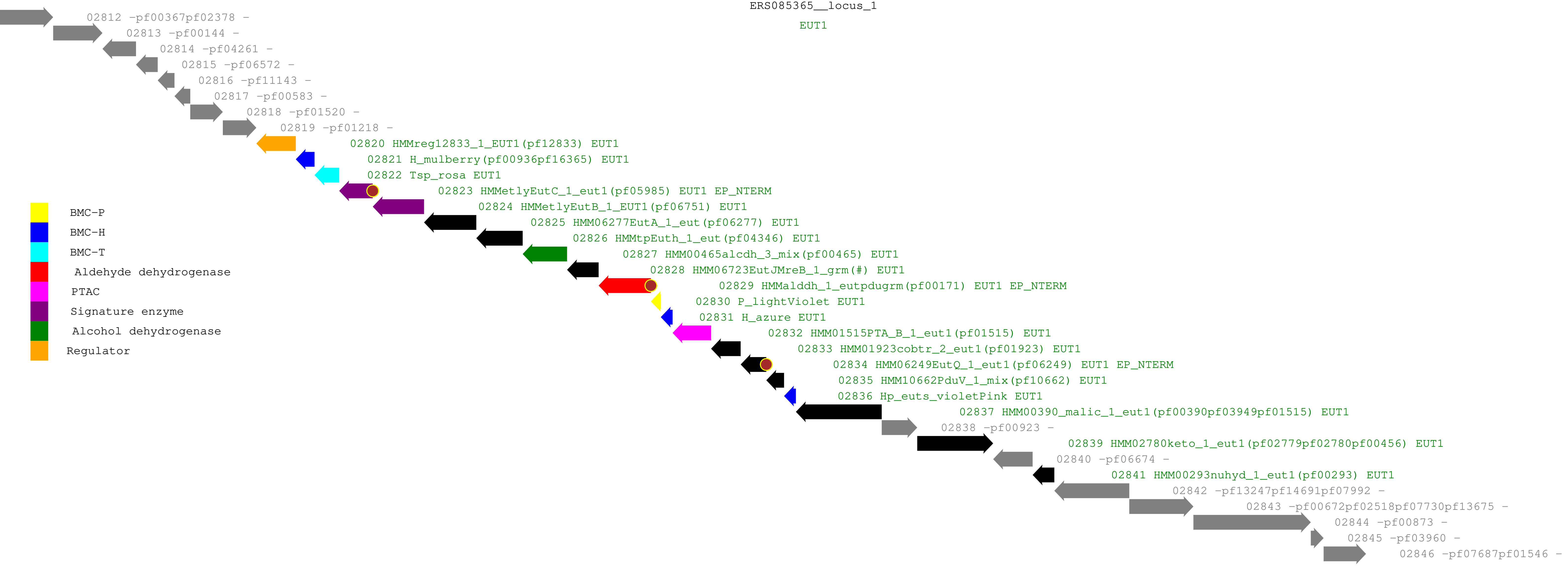

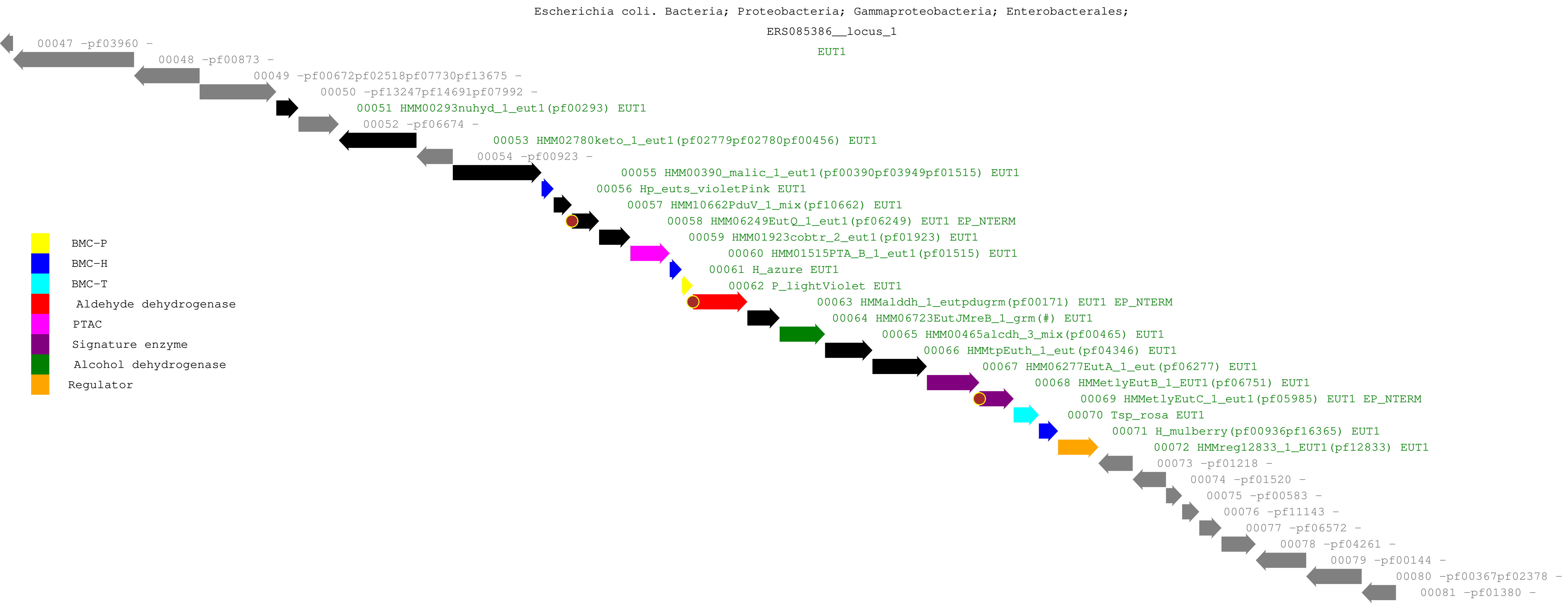
Escherichia coli. Bacteria; Proteobacteria; Gammaproteobacteria; Enterobacterales;
ERS085386__locus_1
EUT1
00047 -pf03960 -
00048 -pf00873 -
00049 -pf00672pf02518pf07730pf13675 -
00050 -pf13247pf14691pf07992 -
00051 HMM00293nuhyd_1_eut1(pf00293) EUT1
00052 -pf06674 -
00053 HMM02780keto_1_eut1(pf02779pf02780pf00456) EUT1
00054 -pf00923 -
00055 HMM00390_malic_1_eut1(pf00390pf03949pf01515) EUT1
00056 Hp_euts_violetPink EUT1
00057 HMM10662PduV_1_mix(pf10662) EUT1
00058 HMM06249EutQ_1_eut1(pf06249) EUT1 EP_NTERM
00059 HMM01923cobtr_2_eut1(pf01923) EUT1
00060 HMM01515PTA_B_1_eut1(pf01515) EUT1
00061 H_azure EUT1
00062 P_lightViolet EUT1
00063 HMMalddh_1_eutpdugrm(pf00171) EUT1 EP_NTERM
00064 HMM06723EutJMreB_1_grm(#) EUT1
00065 HMM00465alcdh_3_mix(pf00465) EUT1
00066 HMMtpEuth_1_eut(pf04346) EUT1
00067 HMM06277EutA_1_eut(pf06277) EUT1
00068 HMMetlyEutB_1_EUT1(pf06751) EUT1
00069 HMMetlyEutC_1_eut1(pf05985) EUT1 EP_NTERM
00070 Tsp_rosa EUT1
00071 H_mulberry(pf00936pf16365) EUT1
00072 HMMreg12833_1_EUT1(pf12833) EUT1
00073 -pf01218 -
00074 -pf01520 -
00075 -pf00583 -
00076 -pf11143 -
00077 -pf06572 -
00078 -pf04261 -
00079 -pf00144 -
00080 -pf00367pf02378 -
00081 -pf01380 -
BMC-P
BMC-H
BMC-T
Aldehyde dehydrogenase
PTAC
Signature enzyme
Alcohol dehydrogenase
Regulator

Escherichia coli. Bacteria; Proteobacteria; Gammaproteobacteria; Enterobacterales;

ERS085406__locus_1

EUT1

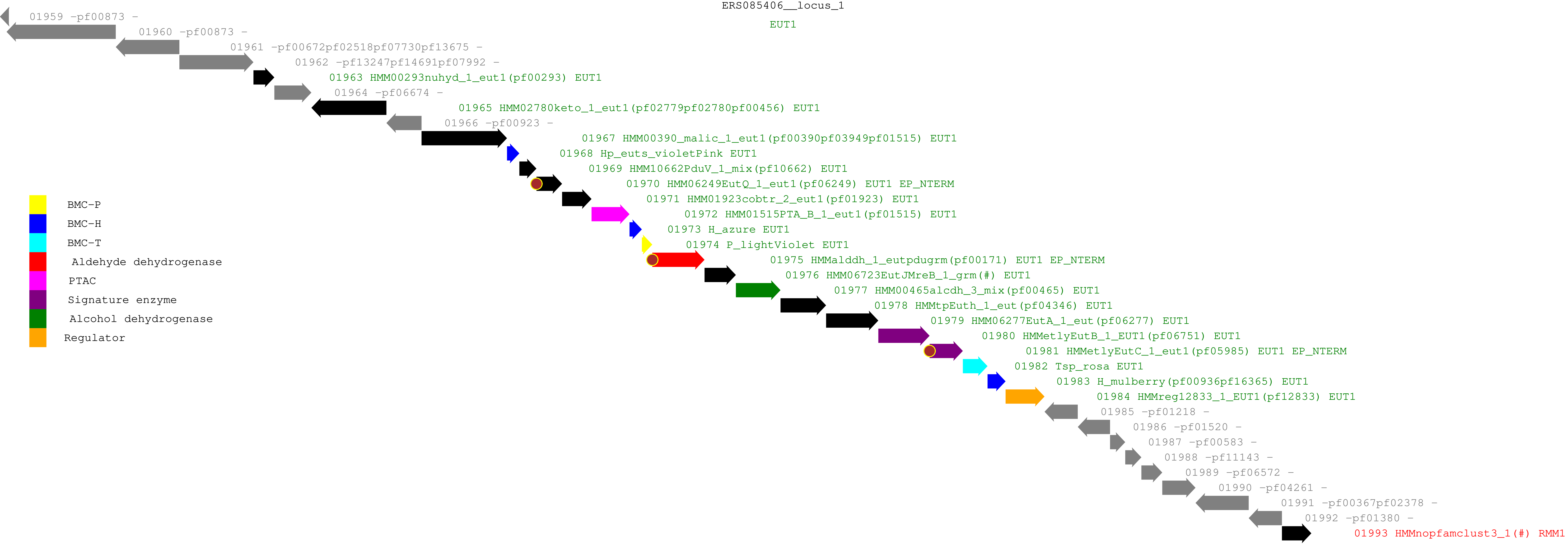

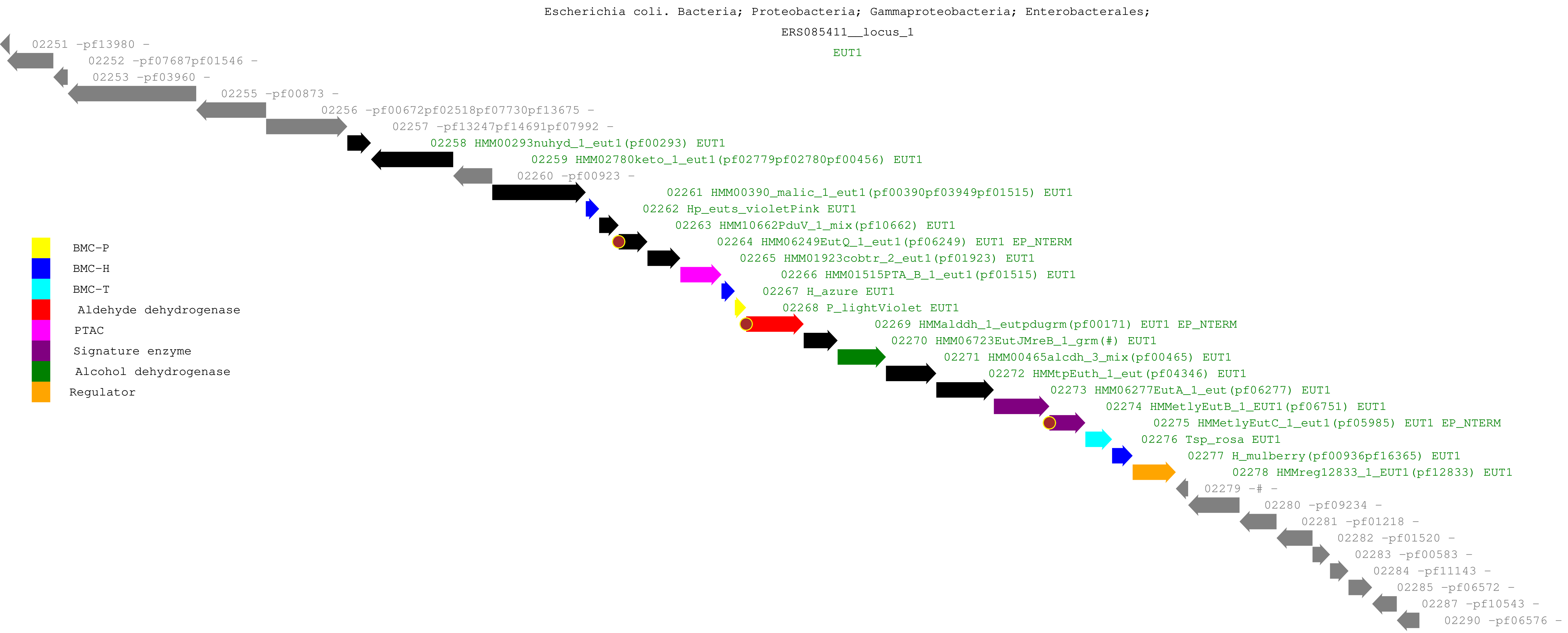

Escherichia coli. Bacteria; Proteobacteria; Gammaproteobacteria; Enterobacterales;
ERS085411__locus_1
EUT1
02251 -pf13980 -
02252 -pf07687pf01546 -
02253 -pf03960 -
02255 -pf00873 -
02256 -pf00672pf02518pf07730pf13675 -
02257 -pf13247pf14691pf07992 -
02258 HMM00293nuhyd_1_eut1(pf00293) EUT1
02259 HMM02780keto_1_eut1(pf02779pf02780pf00456) EUT1
02260 -pf00923 -
02261 HMM00390_malic_1_eut1(pf00390pf03949pf01515) EUT1
02262 Hp_euts_violetPink EUT1
02263 HMM10662PduV_1_mix(pf10662) EUT1
02264 HMM06249EutQ_1_eut1(pf06249) EUT1 EP_NTERM
02265 HMM01923cobtr_2_eut1(pf01923) EUT1
02266 HMM01515PTA_B_1_eut1(pf01515) EUT1
02267 H_azure EUT1
02268 P_lightViolet EUT1
02269 HMMalddh_1_eutpdugrm(pf00171) EUT1 EP_NTERM
02270 HMM06723EutJMreB_1_grm(#) EUT1
02271 HMM00465alcdh_3_mix(pf00465) EUT1
02272 HMMtpEuth_1_eut(pf04346) EUT1
02273 HMM06277EutA_1_eut(pf06277) EUT1
02274 HMMetlyEutB_1_EUT1(pf06751) EUT1
02275 HMMetlyEutC_1_eut1(pf05985) EUT1 EP_NTERM
02276 Tsp_rosa EUT1
02277 H_mulberry(pf00936pf16365) EUT1
02278 HMMreg12833_1_EUT1(pf12833) EUT1
02279 -# -
02280 -pf09234 -
02281 -pf01218 -
02282 -pf01520 -
02283 -pf00583 -
02284 -pf11143 -
02285 -pf06572 -
02287 -pf10543 -
02290 -pf06576 -
BMC-P
BMC-H
BMC-T
Aldehyde dehydrogenase
PTAC
Signature enzyme
Alcohol dehydrogenase
Regulator

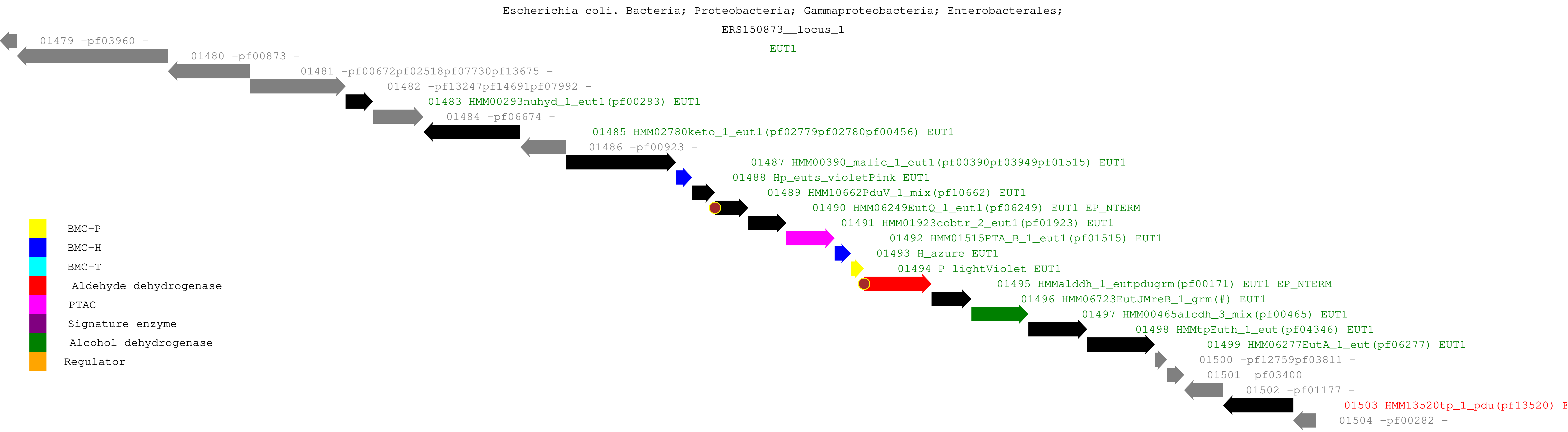

Escherichia coli. Bacteria; Proteobacteria; Gammaproteobacteria; Enterobacterales;
ERS150873__locus_1
EUT1
01479 -pf03960 -
01480 -pf00873 -
01481 -pf00672pf02518pf07730pf13675 -
01482 -pf13247pf14691pf07992 -
01483 HMM00293nuhyd_1_eut1(pf00293) EUT1
01484 -pf06674 -
01485 HMM02780keto_1_eut1(pf02779pf02780pf00456) EUT1
01486 -pf00923 -
01487 HMM00390_malic_1_eut1(pf00390pf03949pf01515) EUT1
01488 Hp_euts_violetPink EUT1
01489 HMM10662PduV_1_mix(pf10662) EUT1
01490 HMM06249EutQ_1_eut1(pf06249) EUT1 EP_NTERM
01491 HMM01923cobtr_2_eut1(pf01923) EUT1
01492 HMM01515PTA_B_1_eut1(pf01515) EUT1
01493 H_azure EUT1
01494 P_lightViolet EUT1
01495 HMMalddh_1_eutpdugrm(pf00171) EUT1 EP_NTERM
01496 HMM06723EutJMreB_1_grm(#) EUT1
01497 HMM00465alcdh_3_mix(pf00465) EUT1
01498 HMMtpEuth_1_eut(pf04346) EUT1
01499 HMM06277EutA_1_eut(pf06277) EUT1
01500 -pf12759pf03811 -
01501 -pf03400 -
01502 -pf01177 -
01503 HMM13520tp_1_pdu(pf13520)
01504 -pf00282 -
BMC-P
BMC-H
BMC-T
Aldehyde dehydrogenase
PTAC
Signature enzyme
Alcohol dehydrogenase
Regulator

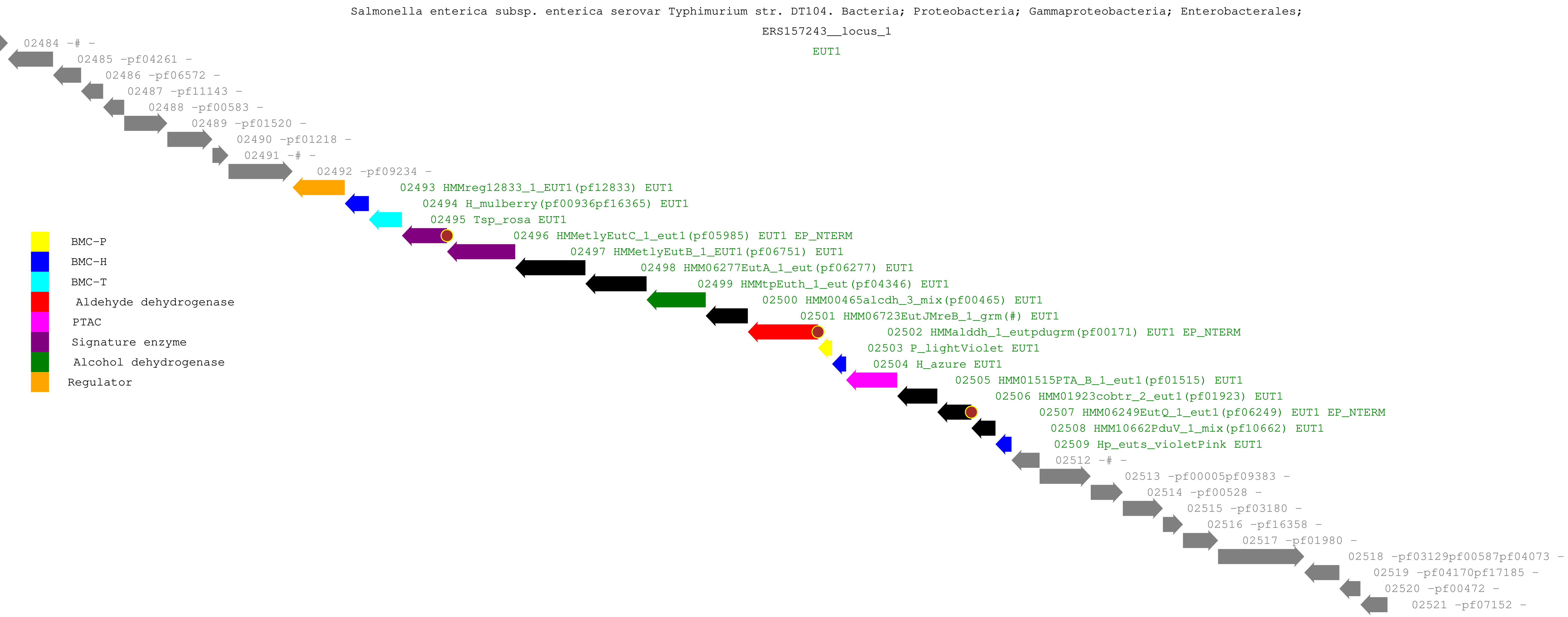
Salmonella enterica subsp. enterica serovar Typhimurium str. DT104. Bacteria; Proteobacteria; Gammaproteobacteria; Enterobacterales;
ERS157243__locus_1
EUT1
02484 -# -
02485 -pf04261 -
02486 -pf06572 -
02487 -pf11143 -
02488 -pf00583 -
02489 -pf01520 -
02490 -pf01218 -
02491 -# -
02492 -pf09234 -
02493 HMMreg12833_1_EUT1(pf12833) EUT1
02494 H_mulberry(pf00936pf16365) EUT1
02495 Tsp_rosa EUT1
02496 HMMetlyEutC_1_eut1(pf05985) EUT1 EP_NTERM
02497 HMMetlyEutB_1_EUT1(pf06751) EUT1
02498 HMM06277EutA_1_eut(pf06277) EUT1
02499 HMMtpEuth_1_eut(pf04346) EUT1
02500 HMM00465alcdh_3_mix(pf00465) EUT1
02501 HMM06723EutJMreB_1_grm(#) EUT1
02502 HMMalddh_1_eutpdugrm(pf00171) EUT1 EP_NTERM
02503 P_lightViolet EUT1
02504 H_azure EUT1
02505 HMM01515PTA_B_1_eut1(pf01515) EUT1
02506 HMM01923cobtr_2_eut1(pf01923) EUT1
02507 HMM06249EutQ_1_eut1(pf06249) EUT1 EP_NTERM
02508 HMM10662PduV_1_mix(pf10662) EUT1
02509 Hp_euts_violetPink EUT1
02512 -# -
02513 -pf00005pf09383 -
02514 -pf00528 -
02515 -pf03180 -
02516 -pf16358 -
02517 -pf01980 -
02518 -pf03129pf00587pf04073 -
02519 -pf04170pf17185 -
02520 -pf00472 -
02521 -pf07152 -
BMC-P
BMC-H
BMC-T
Aldehyde dehydrogenase
PTAC
Signature enzyme
Alcohol dehydrogenase
Regulator

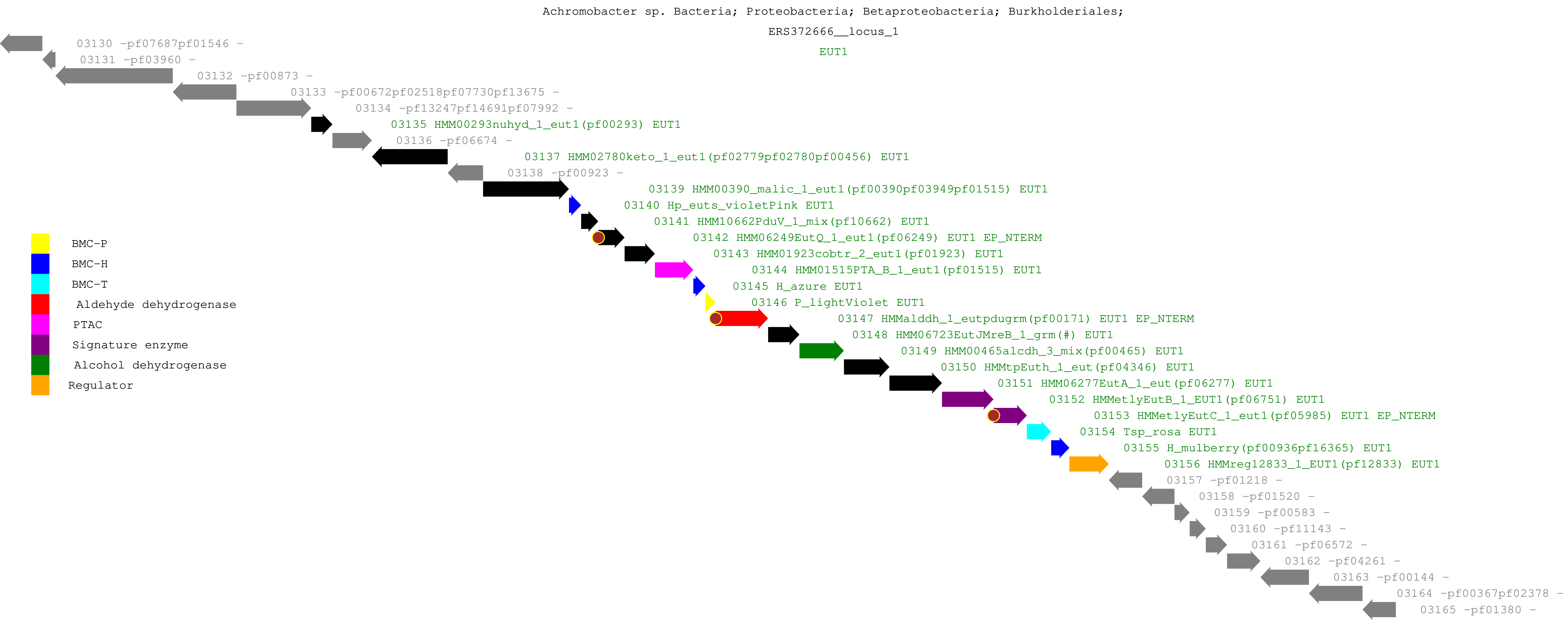
Achromobacter sp. Bacteria; Proteobacteria; Betaproteobacteria; Burkholderiales;
ERS372666__locus_1
EUT1
03130 -pf07687pf01546 -
03131 -pf03960 -
03132 -pf00873 -
03133 -pf00672pf02518pf07730pf13675 -
03134 -pf13247pf14691pf07992 -
03135 HMM00293nuhyd_1_eut1(pf00293) EUT1
03136 -pf06674 -
03137 HMM02780keto_1_eut1(pf02779pf02780pf00456) EUT1
03138 -pf00923 -
03139 HMM00390_malic_1_eut1(pf00390pf03949pf01515) EUT1
03140 Hp_euts_violetPink EUT1
03141 HMM10662PduV_1_mix(pf10662) EUT1
03142 HMM06249EutQ_1_eut1(pf06249) EUT1 EP_NTERM
03143 HMM01923cobtr_2_eut1(pf01923) EUT1
03144 HMM01515PTA_B_1_eut1(pf01515) EUT1
03145 H_azure EUT1
03146 P_lightViolet EUT1
03147 HMMalddh_1_eutpdugrm(pf00171) EUT1 EP_NTERM
03148 HMM06723EutJMreB_1_grm(#) EUT1
03149 HMM00465alcdh_3_mix(pf00465) EUT1
03150 HMMtpEuth_1_eut(pf04346) EUT1
03151 HMM06277EutA_1_eut(pf06277) EUT1
03152 HMMetlyEutB_1_EUT1(pf06751) EUT1
03153 HMMetlyEutC_1_eut1(pf05985) EUT1 EP_NTERM
03154 Tsp_rosa EUT1
03155 H_mulberry(pf00936pf16365) EUT1
03156 HMMreg12833_1_EUT1(pf12833) EUT1
03157 -pf01218 -
03158 -pf01520 -
03159 -pf00583 -
03160 -pf11143 -
03161 -pf06572 -
03162 -pf04261 -
03163 -pf00144 -
03164 -pf00367pf02378 -
03165 -pf01380 -
BMC-P
BMC-H
BMC-T
Aldehyde dehydrogenase
PTAC
Signature enzyme
Alcohol dehydrogenase
Regulator

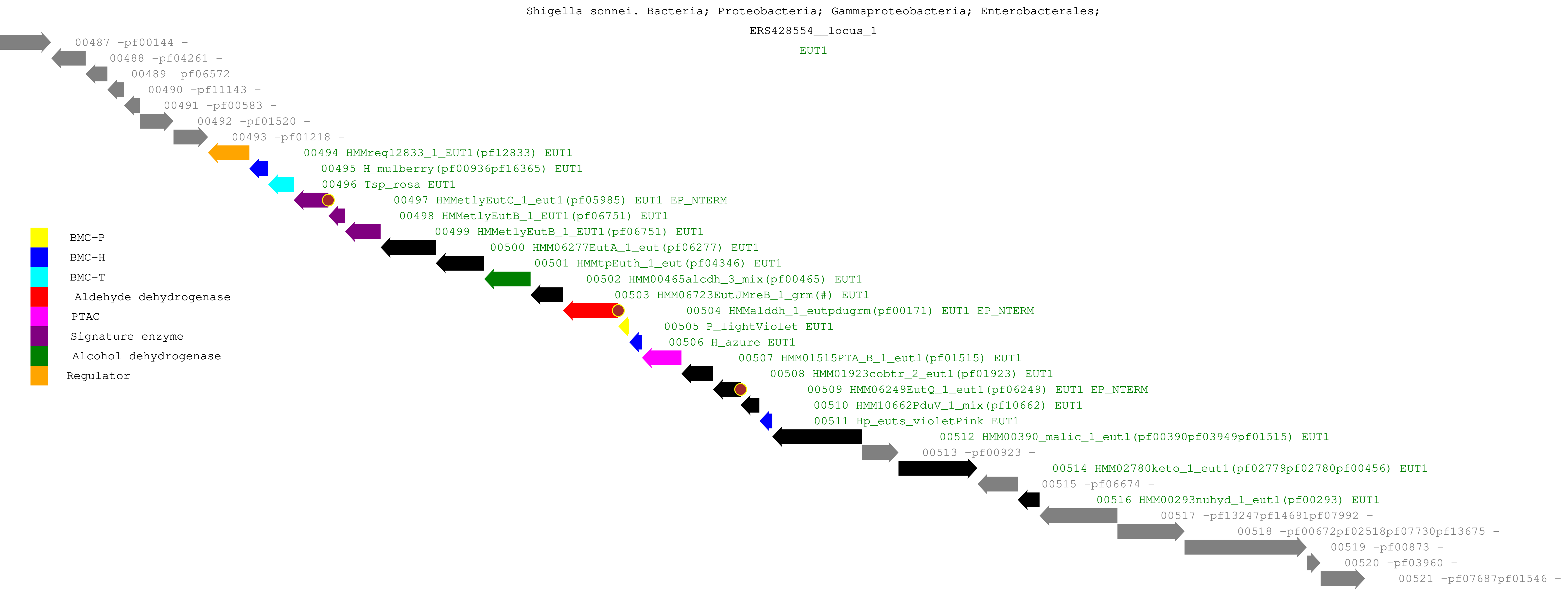

Shigella sonnei. Bacteria; Proteobacteria; Gammaproteobacteria; Enterobacterales;
ERS428554__locus_1
EUT1
00487 -pf00144 -
00488 -pf04261 -
00489 -pf06572 -
00490 -pf11143 -
00491 -pf00583 -
00492 -pf01520 -
00493 -pf01218 -
00494 HMMreg12833_1_EUT1(pf12833) EUT1
00495 H_mulberry(pf00936pf16365) EUT1
00496 Tsp_rosa EUT1
00497 HMMetlyEutC_1_eut1(pf05985) EUT1 EP_NTERM
00498 HMMetlyEutB_1_EUT1(pf06751) EUT1
00499 HMMetlyEutB_1_EUT1(pf06751) EUT1
00500 HMM06277EutA_1_eut(pf06277) EUT1
00501 HMMtpEuth_1_eut(pf04346) EUT1
00502 HMM00465alcdh_3_mix(pf00465) EUT1
00503 HMM06723EutJMreB_1_grm(#) EUT1
00504 HMMalddh_1_eutpdugrm(pf00171) EUT1 EP_NTERM
00505 P_lightViolet EUT1
00506 H_azure EUT1
00507 HMM01515PTA_B_1_eut1(pf01515) EUT1
00508 HMM01923cobtr_2_eut1(pf01923) EUT1
00509 HMM06249EutQ_1_eut1(pf06249) EUT1 EP_NTERM
00510 HMM10662PduV_1_mix(pf10662) EUT1
00511 Hp_euts_violetPink EUT1
00512 HMM00390_malic_1_eut1(pf00390pf03949pf01515) EUT1
00513 -pf00923 -
00514 HMM02780keto_1_eut1(pf02779pf02780pf00456) EUT1
00515 -pf06674 -
00516 HMM00293nuhyd_1_eut1(pf00293) EUT1
00517 -pf13247pf14691pf07992 -
00518 -pf00672pf02518pf07730pf13675 -
00519 -pf00873 -
00520 -pf03960 -
00521 -pf07687pf01546 -
BMC-P
BMC-H
BMC-T
Aldehyde dehydrogenase
PTAC
Signature enzyme
Alcohol dehydrogenase
Regulator

Escherichia coli M114. Bacteria; Proteobacteria; Gammaproteobacteria; Enterobacterales;

ERYG__locus_1

EUT1

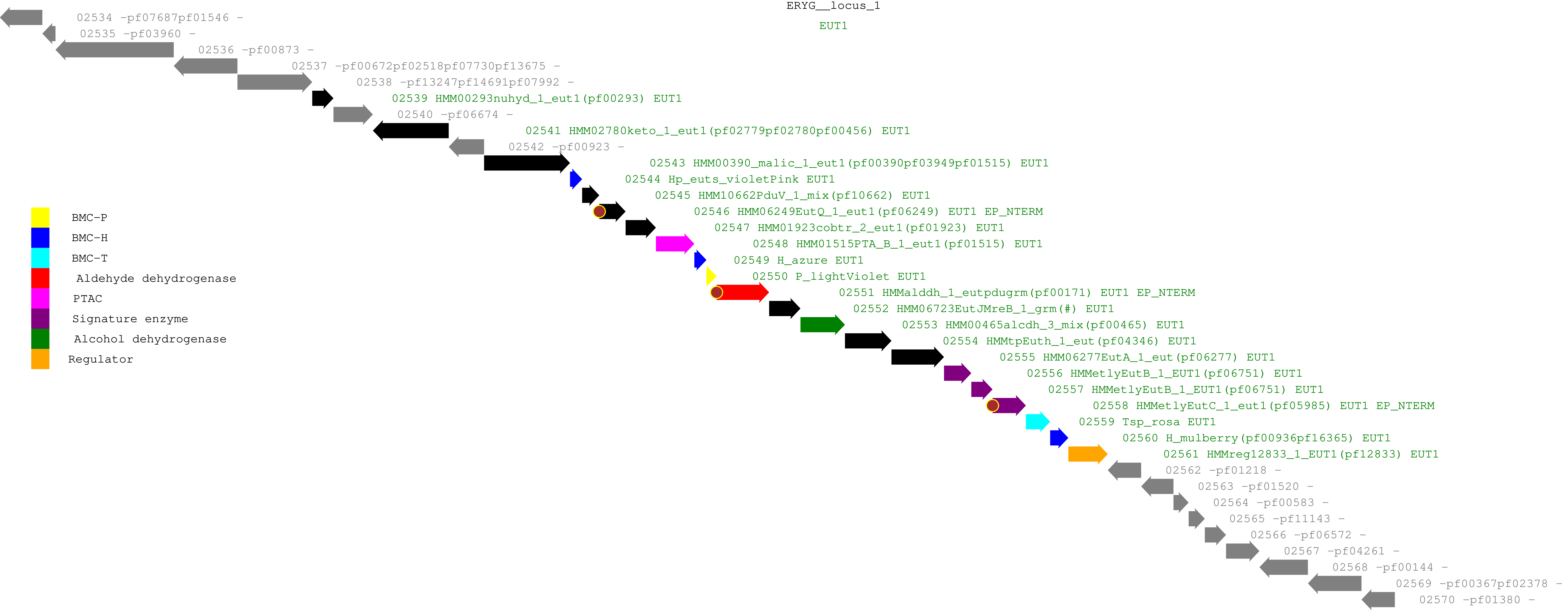

Salmonella enterica subsp. enterica serovar Sandiego. Bacteria; Proteobacteria; Gammaproteobacteria; Enterobacterales;

ES029__locus_1

EUT1

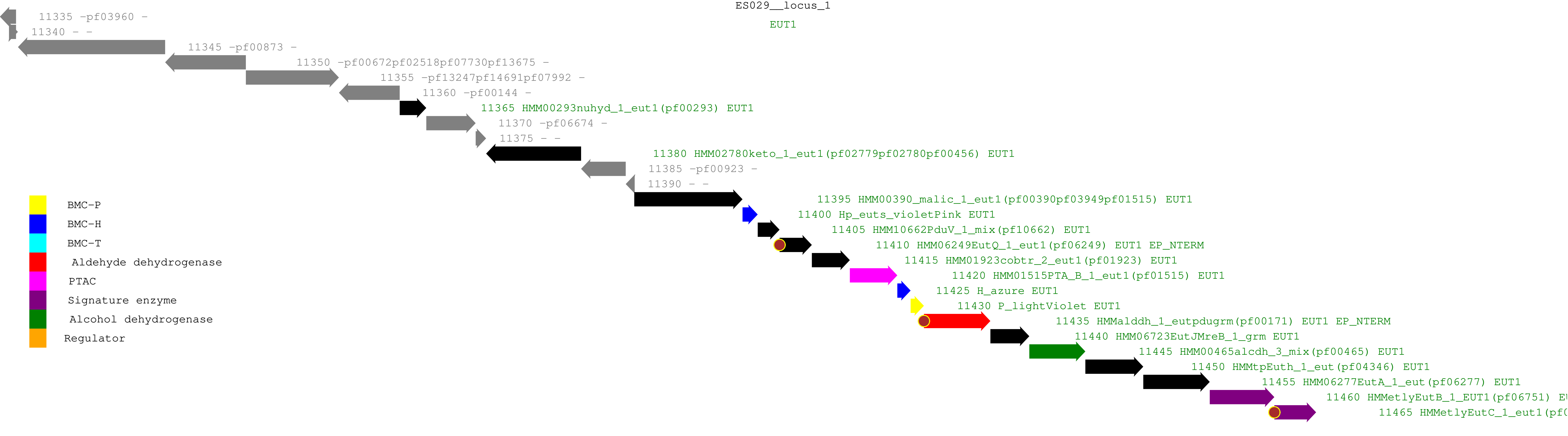

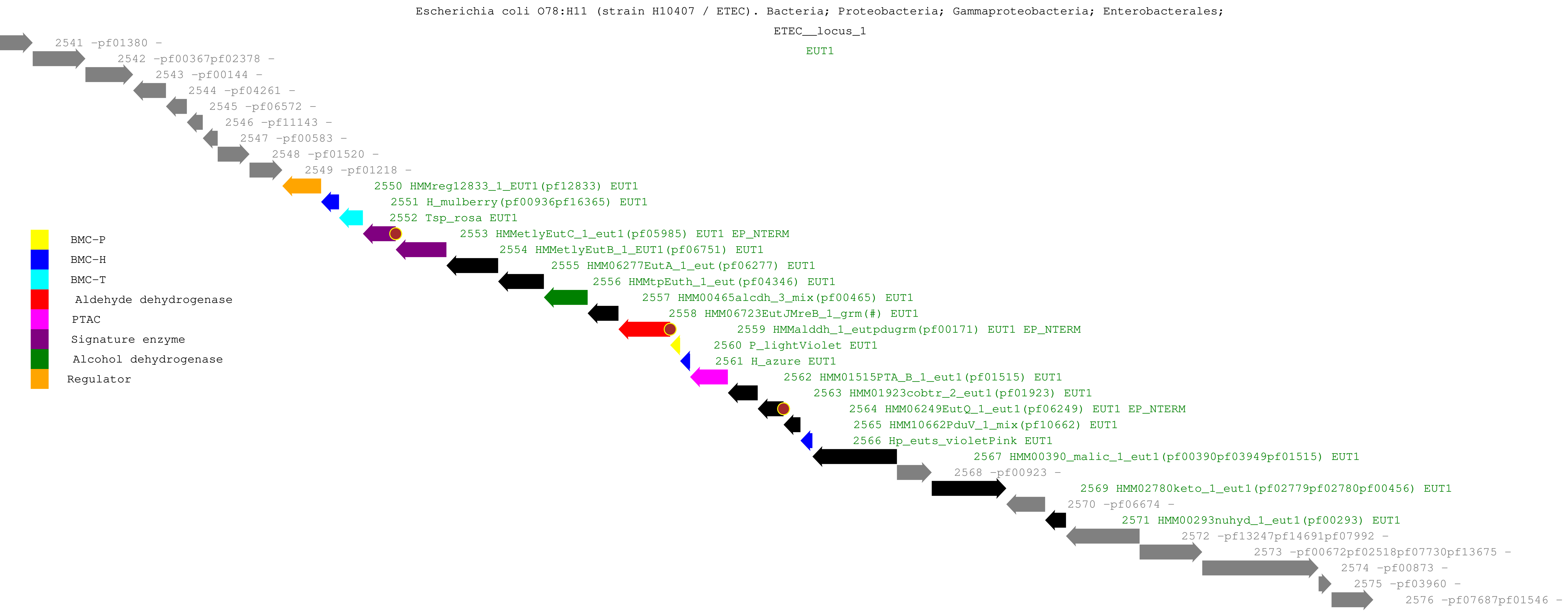
Escherichia coli O78:H11 (strain H10407 / ETEC). Bacteria; Proteobacteria; Gammaproteobacteria; Enterobacterales;
ETEC__locus_1
EUT1
2541 -pf01380 -
2542 -pf00367pf02378 -
2543 -pf00144 -
2544 -pf04261 -
2545 -pf06572 -
2546 -pf11143 -
2547 -pf00583 -
2548 -pf01520 -
2549 -pf01218 -
2550 HMMreg12833_1_EUT1(pf12833) EUT1
2551 H_mulberry(pf00936pf16365) EUT1
2552 Tsp_rosa EUT1
2553 HMMetlyEutC_1_eut1(pf05985) EUT1 EP_NTERM
2554 HMMetlyEutB_1_EUT1(pf06751) EUT1
2555 HMM06277EutA_1_eut(pf06277) EUT1
2556 HMMtpEuth_1_eut(pf04346) EUT1
2557 HMM00465alcdh_3_mix(pf00465) EUT1
2558 HMM06723EutJMreB_1_grm(#) EUT1
2559 HMMalddh_1_eutpdugrm(pf00171) EUT1 EP_NTERM
2560 P_lightViolet EUT1
2561 H_azure EUT1
2562 HMM01515PTA_B_1_eut1(pf01515) EUT1
2563 HMM01923cobtr_2_eut1(pf01923) EUT1
2564 HMM06249EutQ_1_eut1(pf06249) EUT1 EP_NTERM
2565 HMM10662PduV_1_mix(pf10662) EUT1
2566 Hp_euts_violetPink EUT1
2567 HMM00390_malic_1_eut1(pf00390pf03949pf01515) EUT1
2568 -pf00923 -
2569 HMM02780keto_1_eut1(pf02779pf02780pf00456) EUT1
2570 -pf06674 -
2571 HMM00293nuhyd_1_eut1(pf00293) EUT1
2572 -pf13247pf14691pf07992 -
2573 -pf00672pf02518pf07730pf13675 -
2574 -pf00873 -
2575 -pf03960 -
2576 -pf07687pf01546 -
BMC-P
BMC-H
BMC-T
Aldehyde dehydrogenase
PTAC
Signature enzyme
Alcohol dehydrogenase
Regulator

Salmonella enterica subsp. enterica serovar Bonn. Bacteria; Proteobacteria; Gammaproteobacteria; Enterobacterales;

EU363__locus_2

EUT1

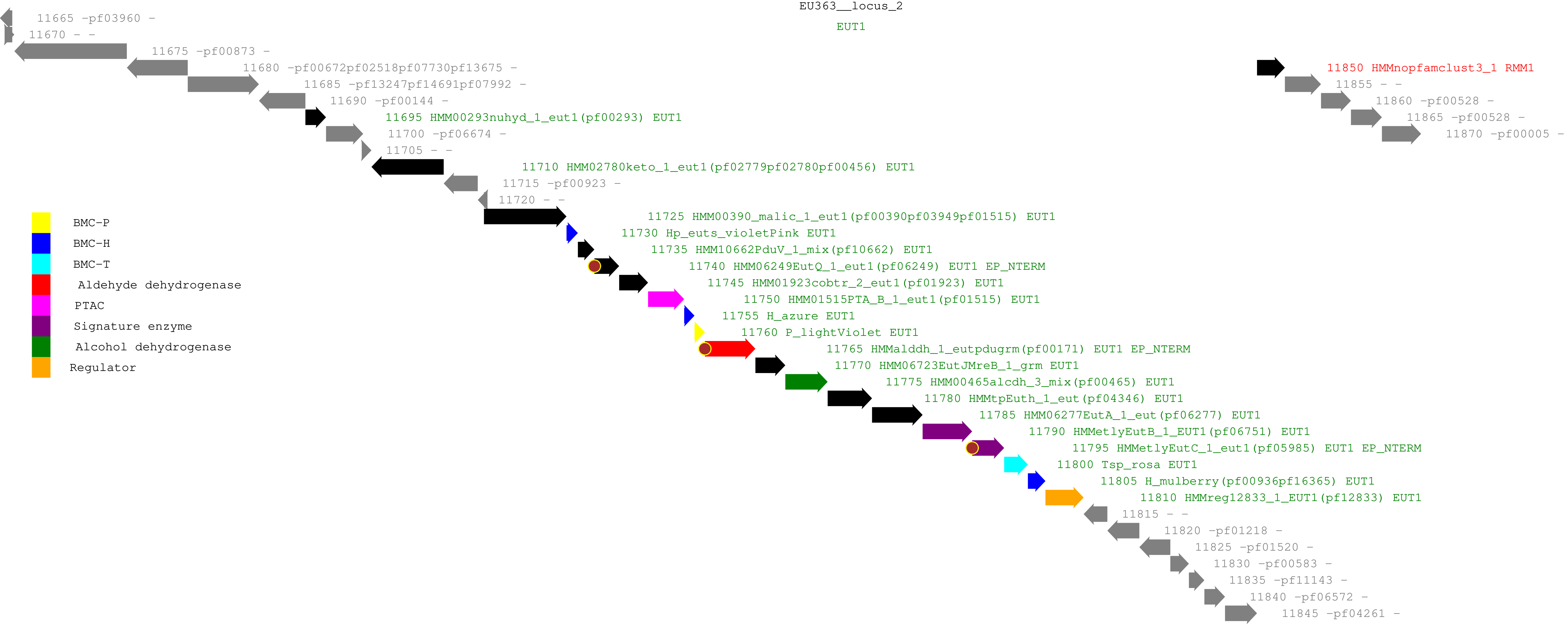

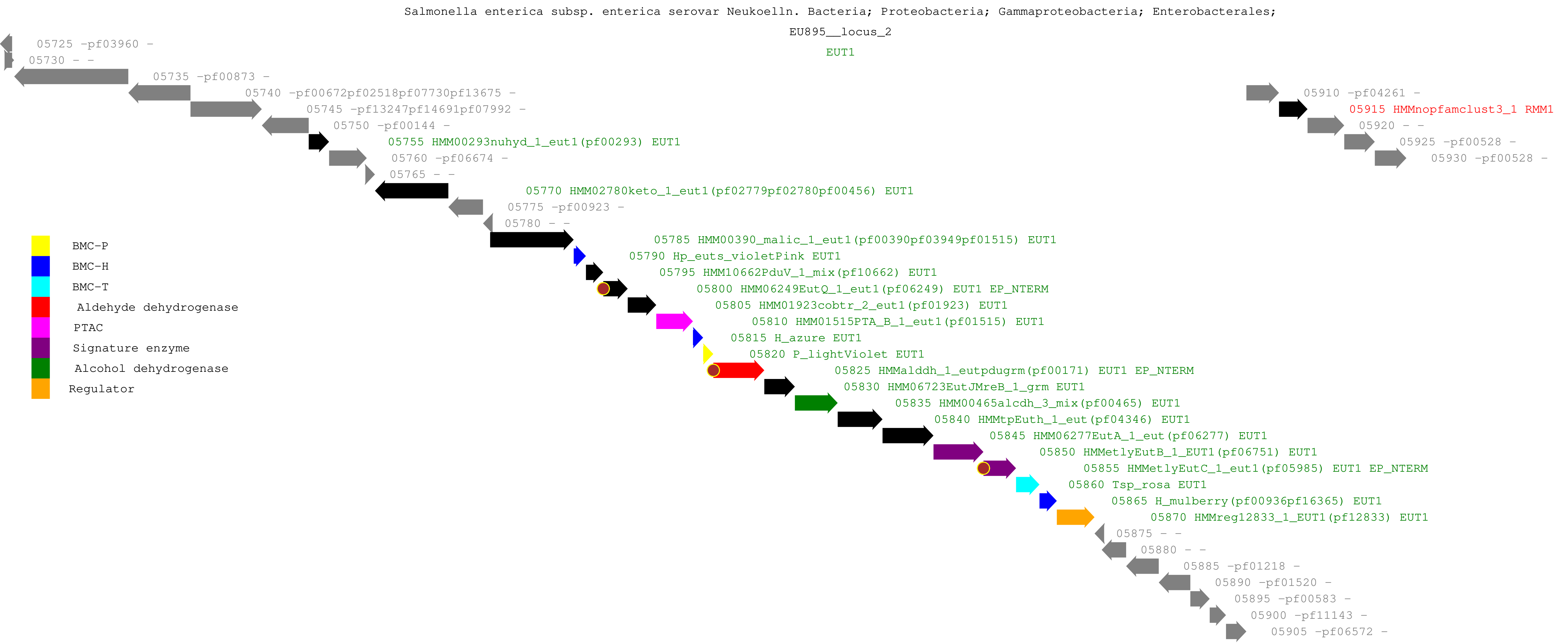

Salmonella enterica subsp. enterica serovar Neukoelln. Bacteria; Proteobacteria; Gammaproteobacteria; Enterobacterales;
EU895__locus_2
EUT1
05725 -pf03960 -
05730 - -
05735 -pf00873 -
05740 -pf00672pf02518pf07730pf13675 -
05745 -pf13247pf14691pf07992 -
05750 -pf00144 -
05755 HMM00293nuhyd_1_eut1(pf00293) EUT1
05760 -pf06674 -
05765 - -
05770 HMM02780keto_1_eut1(pf02779pf02780pf00456) EUT1
05775 -pf00923 -
05780 - -
05785 HMM00390_malic_1_eut1(pf00390pf03949pf01515) EUT1
05790 Hp_euts_violetPink EUT1
05795 HMM10662PduV_1_mix(pf10662) EUT1
05800 HMM06249EutQ_1_eut1(pf06249) EUT1 EP_NTERM
05805 HMM01923cobtr_2_eut1(pf01923) EUT1
05810 HMM01515PTA_B_1_eut1(pf01515) EUT1
05815 H_azure EUT1
05820 P_lightViolet EUT1
05825 HMMalddh_1_eutpdugrm(pf00171) EUT1 EP_NTERM
05830 HMM06723EutJMreB_1_grm EUT1
05835 HMM00465alcdh_3_mix(pf00465) EUT1
05840 HMMtpEuth_1_eut(pf04346) EUT1
05845 HMM06277EutA_1_eut(pf06277) EUT1
05850 HMMetlyEutB_1_EUT1(pf06751) EUT1
05855 HMMetlyEutC_1_eut1(pf05985) EUT1 EP_NTERM
05860 Tsp_rosa EUT1
05865 H_mulberry(pf00936pf16365) EUT1
05870 HMMreg12833_1_EUT1(pf12833) EUT1
05875 - -
05880 - -
05885 -pf01218 -
05890 -pf01520 -
05895 -pf00583 -
05900 -pf11143 -
05905 -pf06572 -
05910 -pf04261 -
05915 HMMnopfamclust3_1 RMM1
05920 - -
05925 -pf00528 -
05930 -pf00528 -
BMC-P
BMC-H
BMC-T
Aldehyde dehydrogenase
PTAC
Signature enzyme
Alcohol dehydrogenase
Regulator

Salmonella enterica subsp. enterica serovar Stanleyville. Bacteria; Proteobacteria; Gammaproteobacteria; Enterobacterales;

EU930__locus_1

EUT1

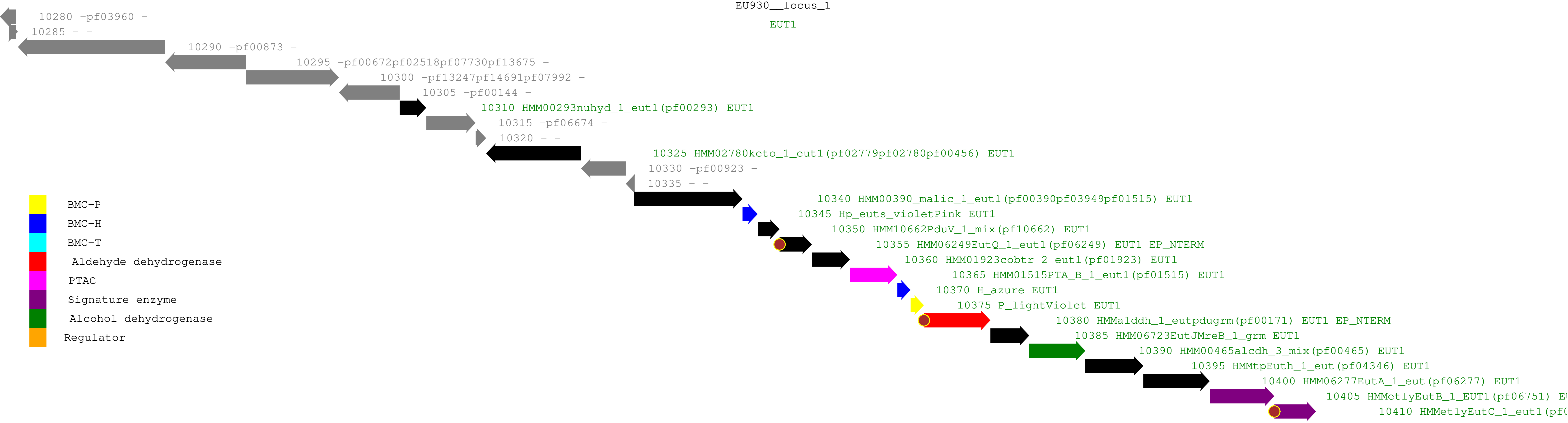

Salmonella enterica subsp. enterica serovar Livingstone. Bacteria; Proteobacteria; Gammaproteobacteria; Enterobacterales;

EU941__locus_1

EUT1

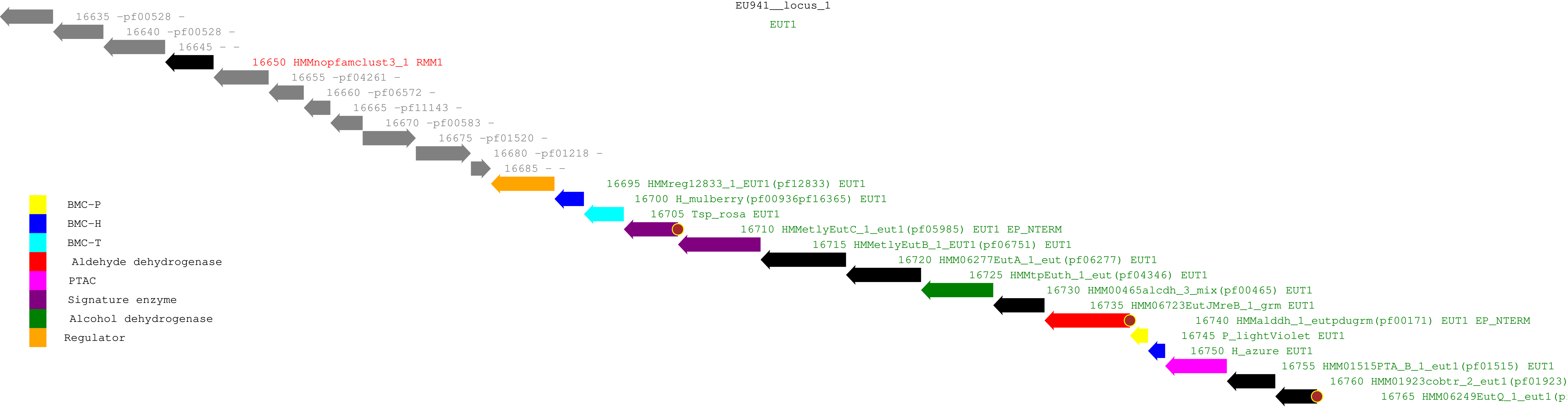

Salmonella enterica subsp. enterica serovar Kinondoni. Bacteria; Proteobacteria; Gammaproteobacteria; Enterobacterales;

EUV21__locus_2

EUT1

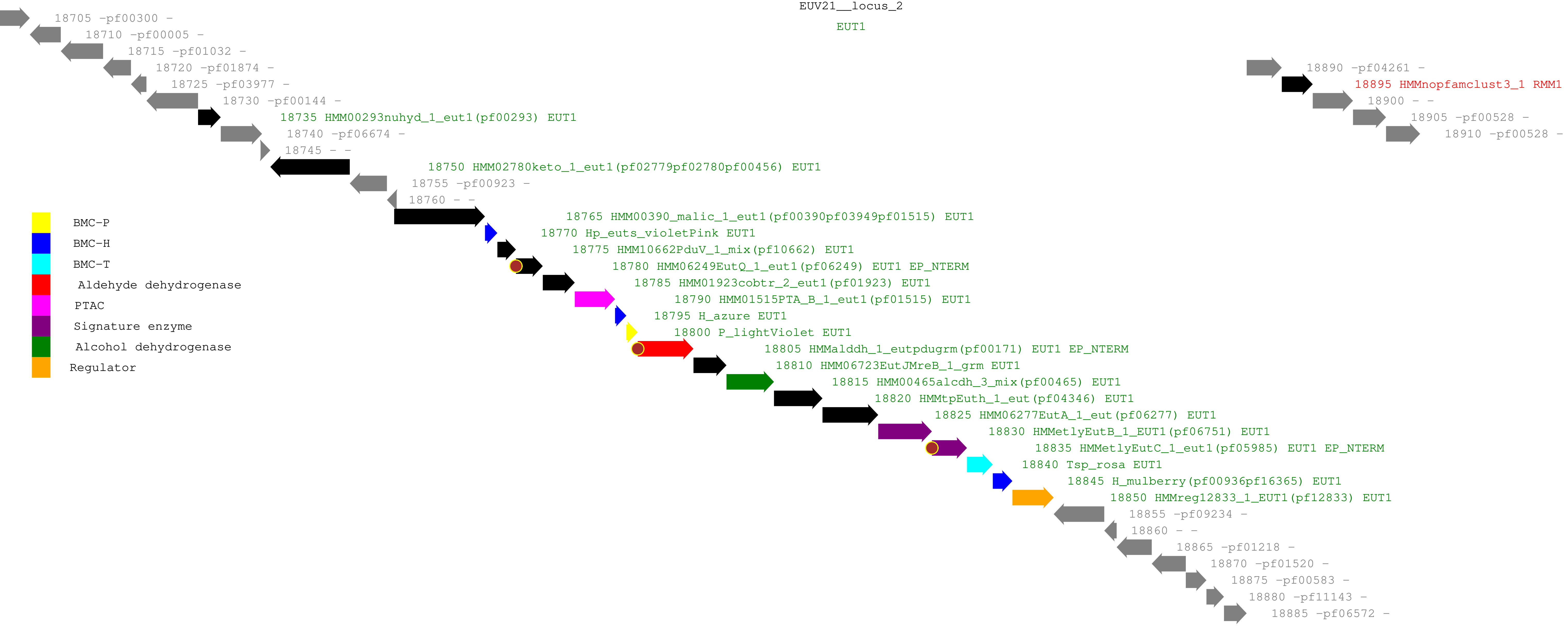

Salmonella enterica subsp. enterica serovar Carmel. Bacteria; Proteobacteria; Gammaproteobacteria; Enterobacterales;

EUW37__locus_2

EUT1

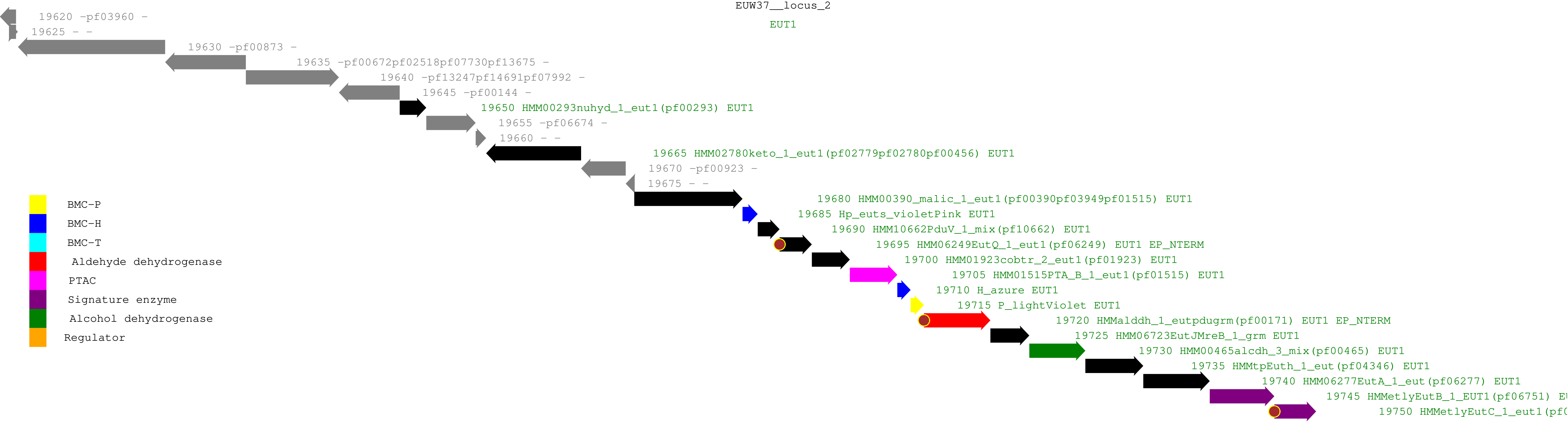

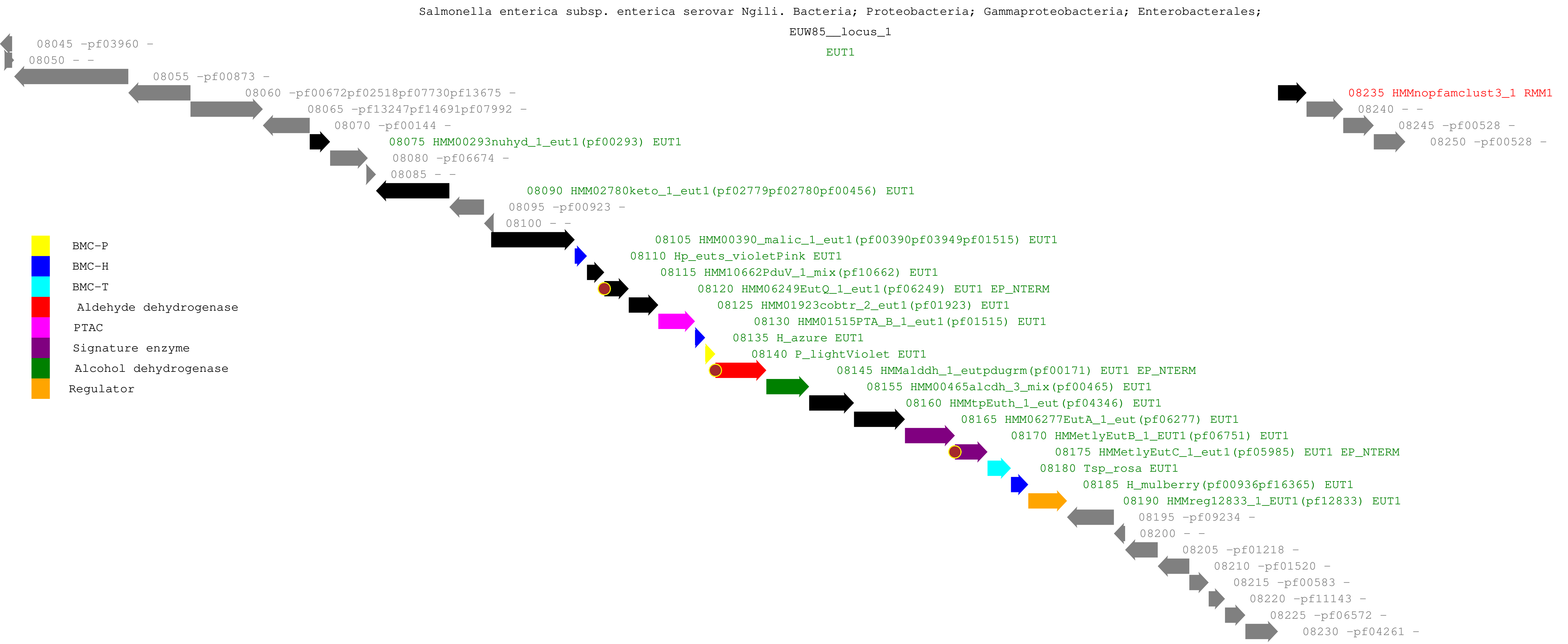

Salmonella enterica subsp. enterica serovar Ngili. Bacteria; Proteobacteria; Gammaproteobacteria; Enterobacterales;
EUW85__locus_1
EUT1
08045 -pf03960 -
08050 - -
08055 -pf00873 -
08060 -pf00672pf02518pf07730pf13675 -
08065 -pf13247pf14691pf07992 -
08070 -pf00144 -
08075 HMM00293nuhyd_1_eut1(pf00293) EUT1
08080 -pf06674 -
08085 - -
08090 HMM02780keto_1_eut1(pf02779pf02780pf00456) EUT1
08095 -pf00923 -
08100 - -
08105 HMM00390_malic_1_eut1(pf00390pf03949pf01515) EUT1
08110 Hp_euts_violetPink EUT1
08115 HMM10662PduV_1_mix(pf10662) EUT1
08120 HMM06249EutQ_1_eut1(pf06249) EUT1 EP_NTERM
08125 HMM01923cobtr_2_eut1(pf01923) EUT1
08130 HMM01515PTA_B_1_eut1(pf01515) EUT1
08135 H_azure EUT1
08140 P_lightViolet EUT1
08145 HMMalddh_1_eutpdugrm(pf00171) EUT1 EP_NTERM
08155 HMM00465alcdh_3_mix(pf00465) EUT1
08160 HMMtpEuth_1_eut(pf04346) EUT1
08165 HMM06277EutA_1_eut(pf06277) EUT1
08170 HMMetlyEutB_1_EUT1(pf06751) EUT1
08175 HMMetlyEutC_1_eut1(pf05985) EUT1 EP_NTERM
08180 Tsp_rosa EUT1
08185 H_mulberry(pf00936pf16365) EUT1
08190 HMMreg12833_1_EUT1(pf12833) EUT1
08195 -pf09234 -
08200 - -
08205 -pf01218 -
08210 -pf01520 -
08215 -pf00583 -
08220 -pf11143 -
08225 -pf06572 -
08230 -pf04261 -
08235 HMMnopfamclust3_1 RMM1
08240 - -
08245 -pf00528 -
08250 -pf00528 -
BMC-P
BMC-H
BMC-T
Aldehyde dehydrogenase
PTAC
Signature enzyme
Alcohol dehydrogenase
Regulator

Salmonella enterica subsp. enterica serovar Reading. Bacteria; Proteobacteria; Gammaproteobacteria; Enterobacterales;

EUX01__locus_2

EUT1

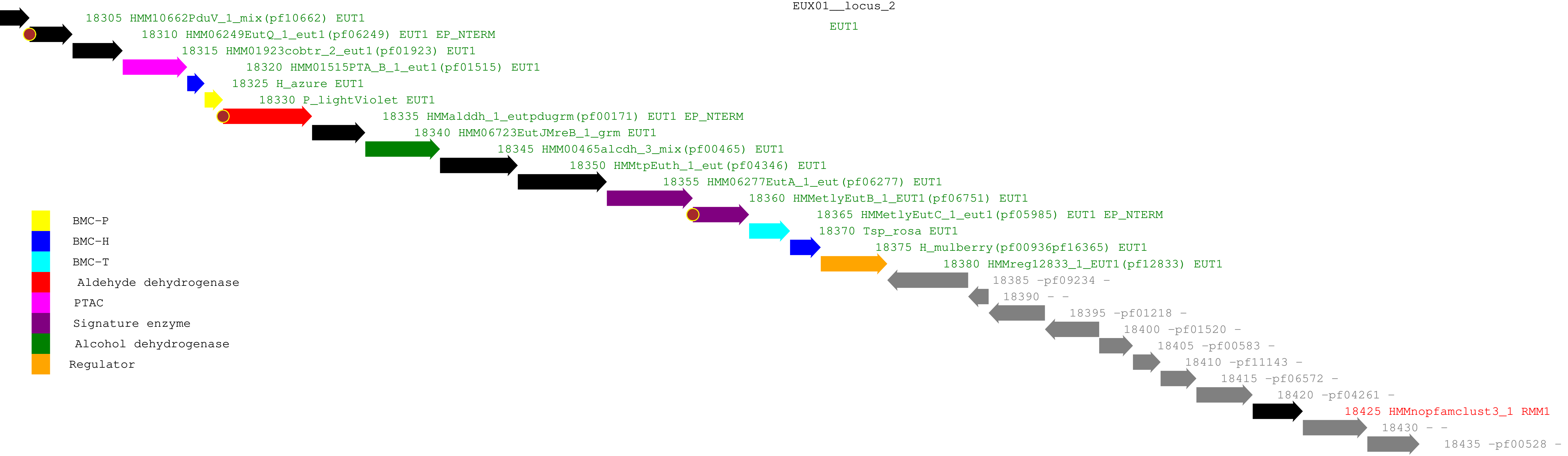

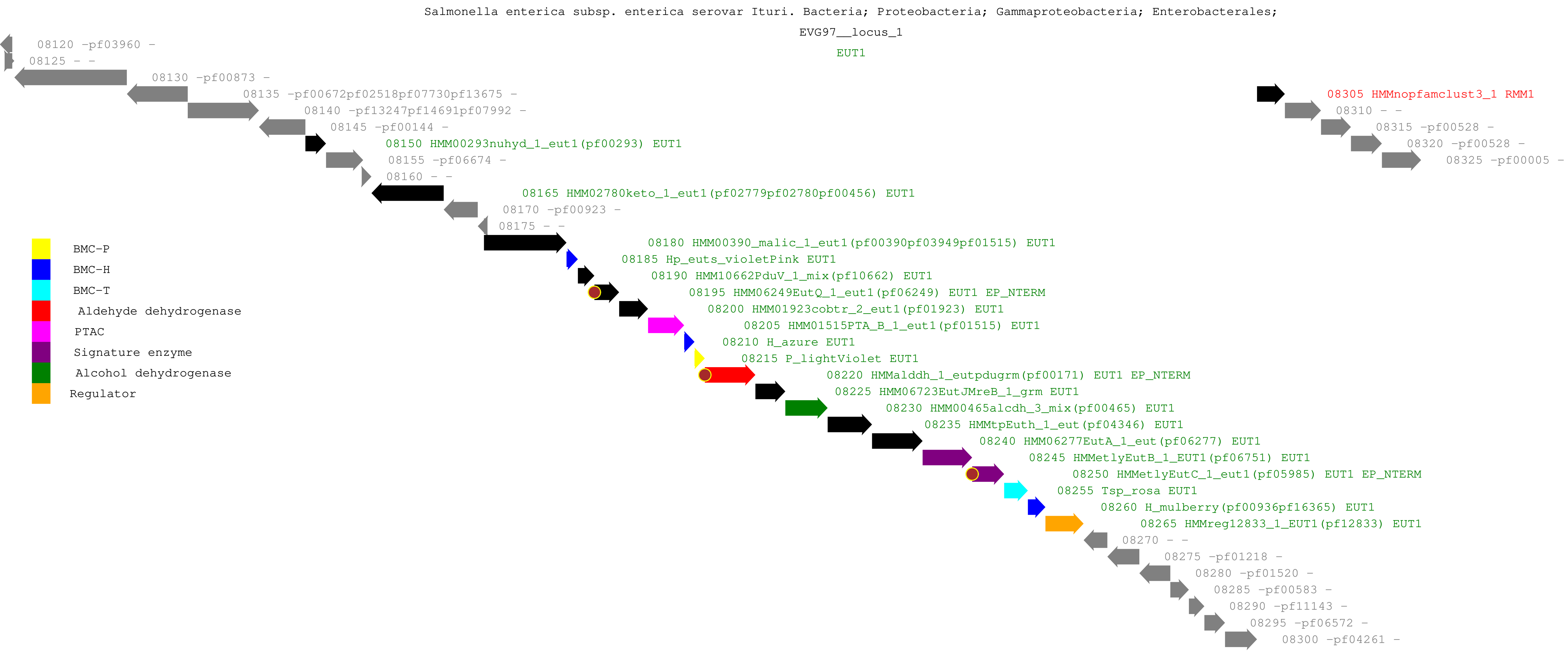

Salmonella enterica subsp. enterica serovar Ituri. Bacteria; Proteobacteria; Gammaproteobacteria; Enterobacterales;
EVG97__locus_1
EUT1
08120 -pf03960 -
08125 - -
08130 -pf00873 -
08135 -pf00672pf02518pf07730pf13675 -
08140 -pf13247pf14691pf07992 -
08145 -pf00144 -
08150 HMM00293nuhyd_1_eut1(pf00293) EUT1
08155 -pf06674 -
08160 - -
08165 HMM02780keto_1_eut1(pf02779pf02780pf00456) EUT1
08170 -pf00923 -
08175 - -
08180 HMM00390_malic_1_eut1(pf00390pf03949pf01515) EUT1
08185 Hp_euts_violetPink EUT1
08190 HMM10662PduV_1_mix(pf10662) EUT1
08195 HMM06249EutQ_1_eut1(pf06249) EUT1 EP_NTERM
08200 HMM01923cobtr_2_eut1(pf01923) EUT1
08205 HMM01515PTA_B_1_eut1(pf01515) EUT1
08210 H_azure EUT1
08215 P_lightViolet EUT1
08220 HMMalddh_1_eutpdugrm(pf00171) EUT1 EP_NTERM
08225 HMM06723EutJMreB_1_grm EUT1
08230 HMM00465alcdh_3_mix(pf00465) EUT1
08235 HMMtpEuth_1_eut(pf04346) EUT1
08240 HMM06277EutA_1_eut(pf06277) EUT1
08245 HMMetlyEutB_1_EUT1(pf06751) EUT1
08250 HMMetlyEutC_1_eut1(pf05985) EUT1 EP_NTERM
08255 Tsp_rosa EUT1
08260 H_mulberry(pf00936pf16365) EUT1
08265 HMMreg12833_1_EUT1(pf12833) EUT1
08270 - -
08275 -pf01218 -
08280 -pf01520 -
08285 -pf00583 -
08290 -pf11143 -
08295 -pf06572 -
08300 -pf04261 -
08305 HMMnopfamclust3_1 RMM1
08310 - -
08315 -pf00528 -
08320 -pf00528 -
08325 -pf00005 -
BMC-P
BMC-H
BMC-T
Aldehyde dehydrogenase
PTAC
Signature enzyme
Alcohol dehydrogenase
Regulator

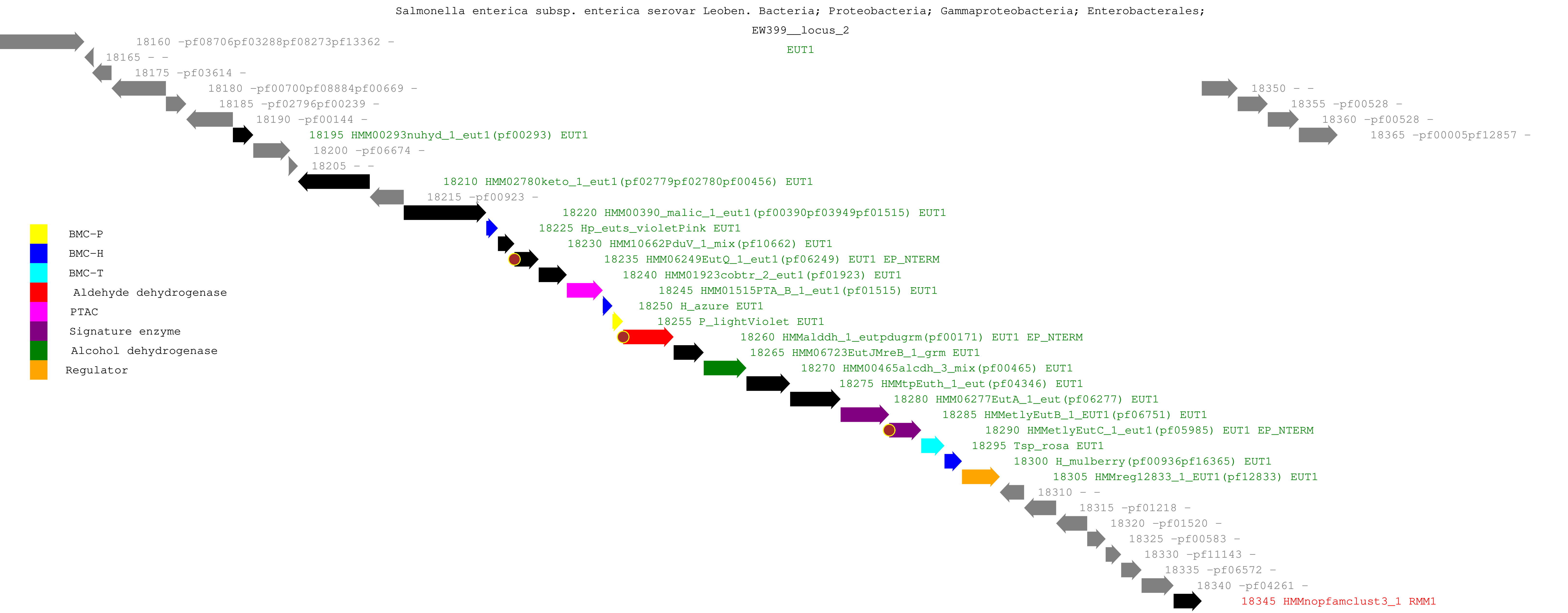

Salmonella enterica subsp. enterica serovar Leoben. Bacteria; Proteobacteria; Gammaproteobacteria; Enterobacterales;
EW399__locus_2
EUT1
18160 -pf08706pf03288pf08273pf13362 -
18165 - -
18175 -pf03614 -
18180 -pf00700pf08884pf00669 -
18185 -pf02796pf00239 -
18190 -pf00144 -
18195 HMM00293nuhyd_1_eut1(pf00293) EUT1
18200 -pf06674 -
18205 - -
18210 HMM02780keto_1_eut1(pf02779pf02780pf00456) EUT1
18215 -pf00923 -
18220 HMM00390_malic_1_eut1(pf00390pf03949pf01515) EUT1
18225 Hp_euts_violetPink EUT1
18230 HMM10662PduV_1_mix(pf10662) EUT1
18235 HMM06249EutQ_1_eut1(pf06249) EUT1 EP_NTERM
18240 HMM01923cobtr_2_eut1(pf01923) EUT1
18245 HMM01515PTA_B_1_eut1(pf01515) EUT1
18250 H_azure EUT1
18255 P_lightViolet EUT1
18260 HMMalddh_1_eutpdugrm(pf00171) EUT1 EP_NTERM
18265 HMM06723EutJMreB_1_grm EUT1
18270 HMM00465alcdh_3_mix(pf00465) EUT1
18275 HMMtpEuth_1_eut(pf04346) EUT1
18280 HMM06277EutA_1_eut(pf06277) EUT1
18285 HMMetlyEutB_1_EUT1(pf06751) EUT1
18290 HMMetlyEutC_1_eut1(pf05985) EUT1 EP_NTERM
18295 Tsp_rosa EUT1
18300 H_mulberry(pf00936pf16365) EUT1
18305 HMMreg12833_1_EUT1(pf12833) EUT1
18310 - -
18315 -pf01218 -
18320 -pf01520 -
18325 -pf00583 -
18330 -pf11143 -
18335 -pf06572 -
18340 -pf04261 -
18345 HMMnopfamclust3_1 RMM1
18350 - -
18355 -pf00528 -
18360 -pf00528 -
18365 -pf00005pf12857 -
BMC-P
BMC-H
BMC-T
Aldehyde dehydrogenase
PTAC
Signature enzyme
Alcohol dehydrogenase
Regulator

Salmonella enterica subsp. enterica serovar Carno. Bacteria; Proteobacteria; Gammaproteobacteria; Enterobacterales;

EW411__locus_2

EUT1

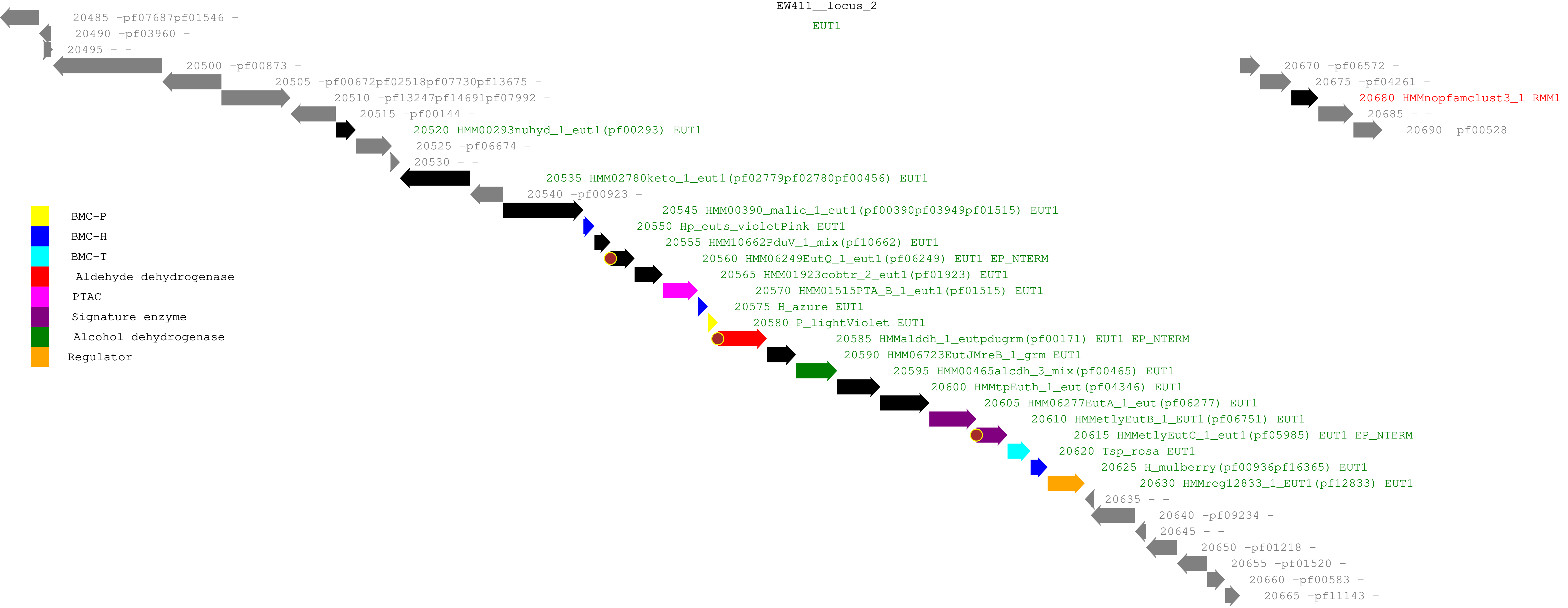

Salmonella enterica subsp. enterica serovar Stockholm. Bacteria; Proteobacteria; Gammaproteobacteria; Enterobacterales;

EW938__locus_2

EUT1

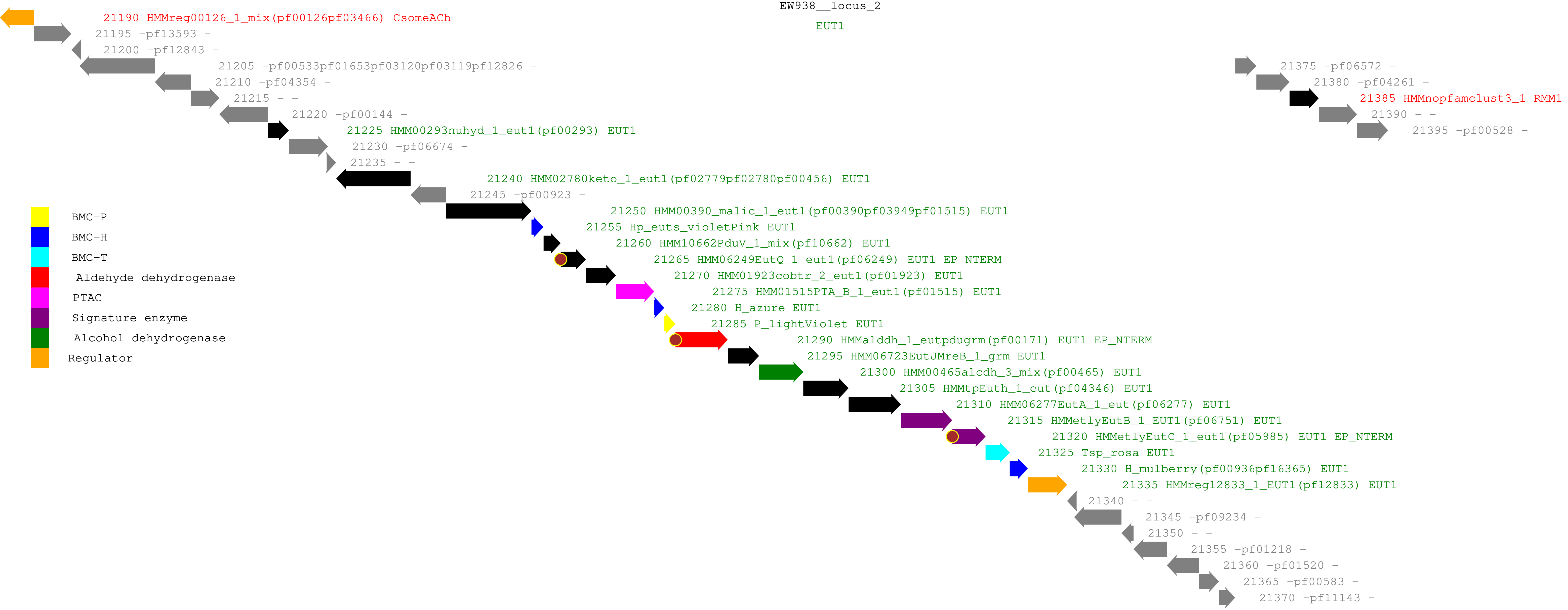

Salmonella enterica subsp. enterica serovar Livingstone. Bacteria; Proteobacteria; Gammaproteobacteria; Enterobacterales;

EW948__locus_1

EUT1

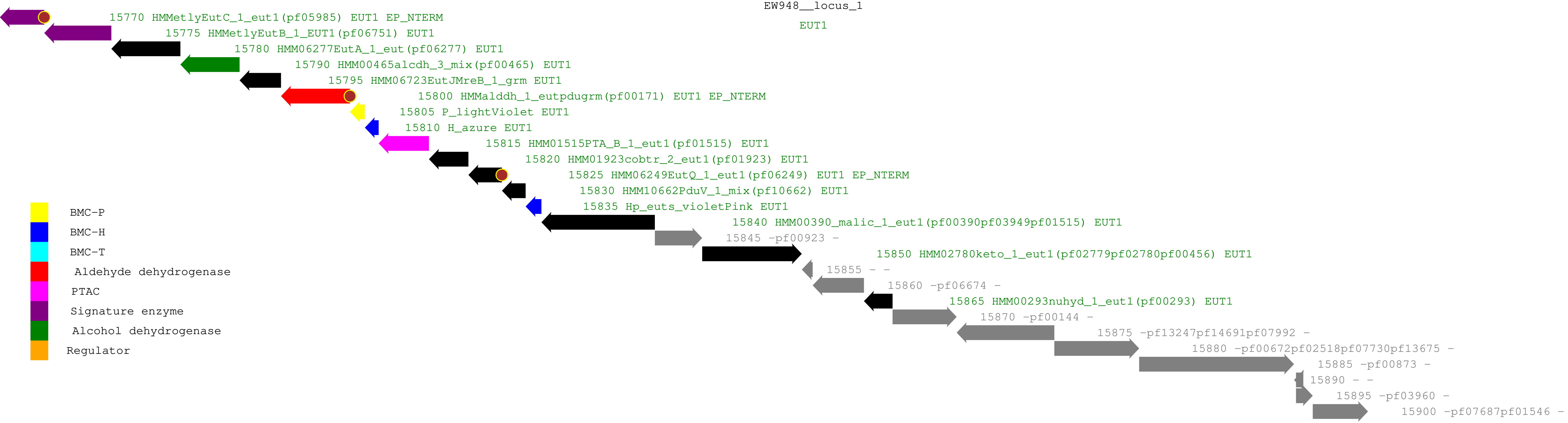

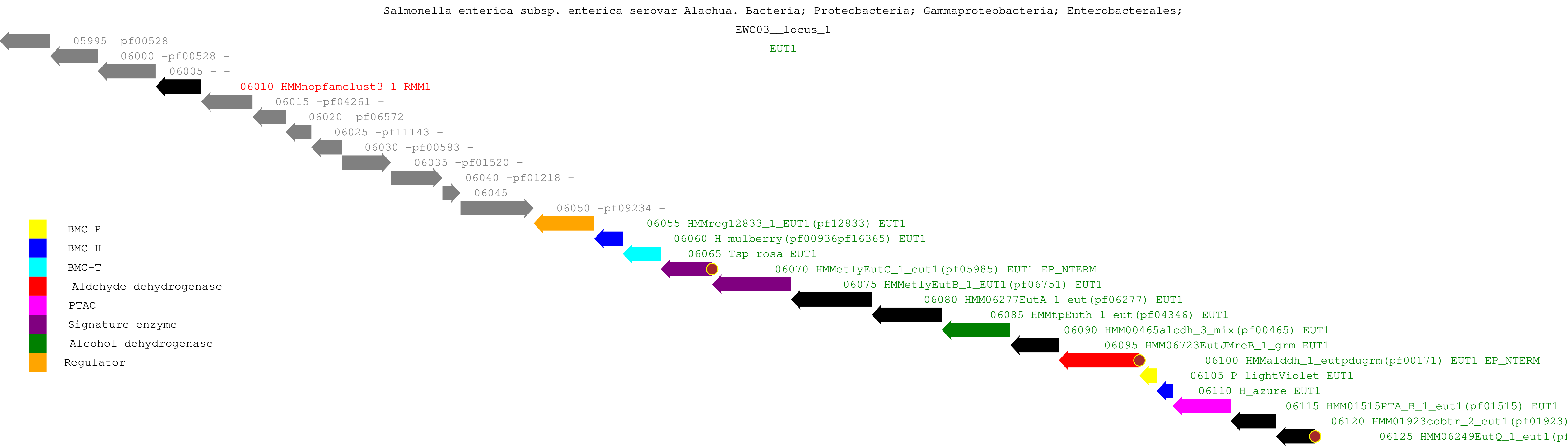
Salmonella enterica subsp. enterica serovar Alachua. Bacteria; Proteobacteria; Gammaproteobacteria; Enterobacterales;
EWC03__locus_1
EUT1
05995 -pf00528 -
06000 -pf00528 -
06005 - -
06010 HMMnopfamclust3_1 RMM1
06015 -pf04261 -
06020 -pf06572 -
06025 -pf11143 -
06030 -pf00583 -
06035 -pf01520 -
06040 -pf01218 -
06045 - -
06050 -pf09234 -
06055 HMMreg12833_1_EUT1(pf12833) EUT1
06060 H_mulberry(pf00936pf16365) EUT1
06065 Tsp_rosa EUT1
06070 HMMetlyEutC_1_eut1(pf05985) EUT1 EP_NTERM
06075 HMMetlyEutB_1_EUT1(pf06751) EUT1
06080 HMM06277EutA_1_eut(pf06277) EUT1
06085 HMMtpEuth_1_eut(pf04346) EUT1
06090 HMM00465alcdh_3_mix(pf00465) EUT1
06095 HMM06723EutJMreB_1_grm EUT1
06100 HMMaldh_1_eutpdugrm(pf00171) EUT1 EP_NTERM
06105 P_lightViolet EUT1
06110 H_azure EUT1
06115 HMM01515PTA_B_1_eut1(pf01515) EUT1
06120 HMM01923cobtr_2_eut1(pf01923)
06125 HMM06249EutQ_1_eut1(pf
BMC-P
BMC-H
BMC-T
Aldehyde dehydrogenase
PTAC
Signature enzyme
Alcohol dehydrogenase
Regulator

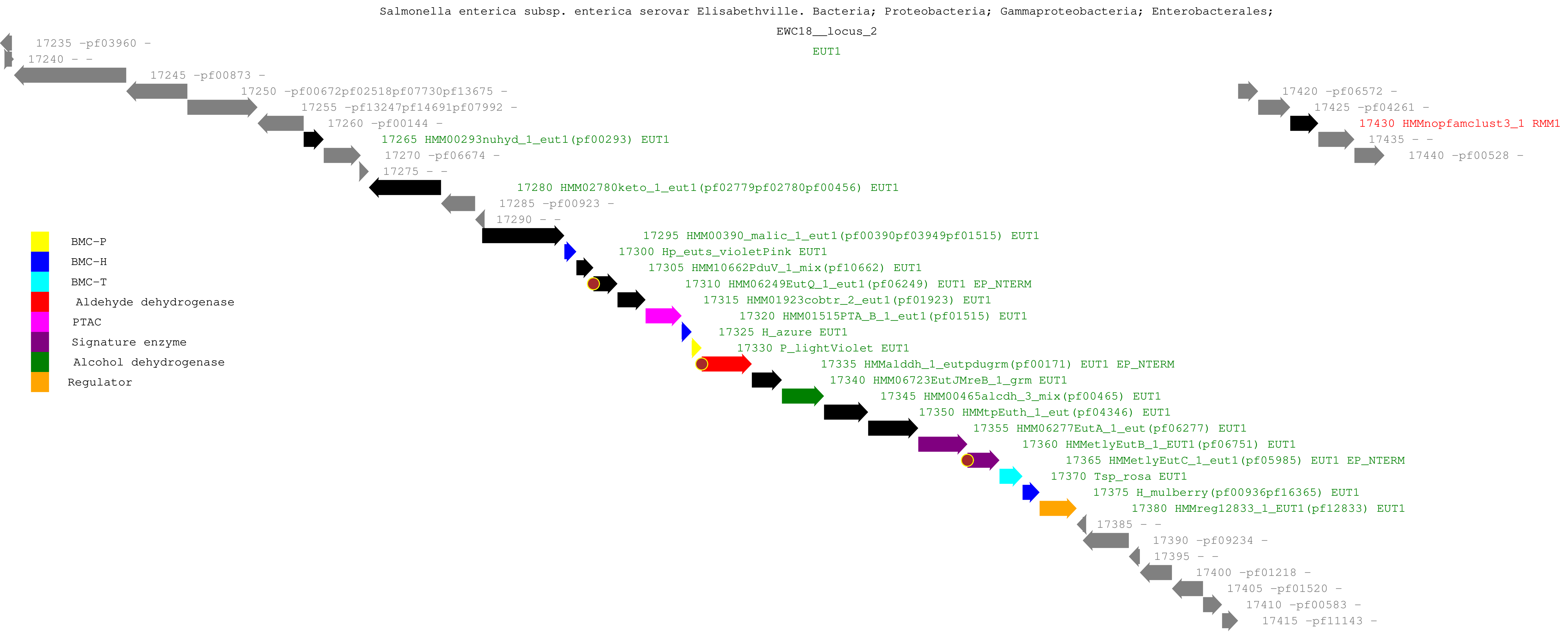

Salmonella enterica subsp. enterica serovar Elisabethville. Bacteria; Proteobacteria; Gammaproteobacteria; Enterobacterales;
EWC18__locus_2
EUT1
17235 -pf03960 -
17240 - -
17245 -pf00873 -
17250 -pf00672pf02518pf07730pf13675 -
17255 -pf13247pf14691pf07992 -
17260 -pf00144 -
17265 HMM00293nuhyd_1_eut1(pf00293) EUT1
17270 -pf06674 -
17275 - -
17280 HMM02780keto_1_eut1(pf02779pf02780pf00456) EUT1
17285 -pf00923 -
17290 - -
17295 HMM00390_malic_1_eut1(pf00390pf03949pf01515) EUT1
17300 Hp_euts_violetPink EUT1
17305 HMM10662PduV_1_mix(pf10662) EUT1
17310 HMM06249EutQ_1_eut1(pf06249) EUT1 EP_NTERM
17315 HMM01923cobtr_2_eut1(pf01923) EUT1
17320 HMM01515PTA_B_1_eut1(pf01515) EUT1
17325 H_azure EUT1
17330 P_lightViolet EUT1
17335 HMMalddh_1_eutpdugrm(pf00171) EUT1 EP_NTERM
17340 HMM06723EutJMreB_1_grm EUT1
17345 HMM00465alcdh_3_mix(pf00465) EUT1
17350 HMMtpEuth_1_eut(pf04346) EUT1
17355 HMM06277EutA_1_eut(pf06277) EUT1
17360 HMMetlyEutB_1_EUT1(pf06751) EUT1
17365 HMMetlyEutC_1_eut1(pf05985) EUT1 EP_NTERM
17370 Tsp_rosa EUT1
17375 H_mulberry(pf00936pf16365) EUT1
17380 HMMreg12833_1_EUT1(pf12833) EUT1
17385 - -
17390 -pf09234 -
17395 - -
17400 -pf01218 -
17405 -pf01520 -
17410 -pf00583 -
17415 -pf11143 -
17420 -pf06572 -
17425 -pf04261 -
17430 HMMnopfamclust3_1 RMM1
17435 - -
17440 -pf00528 -
BMC-P
BMC-H
BMC-T
Aldehyde dehydrogenase
PTAC
Signature enzyme
Alcohol dehydrogenase
Regulator

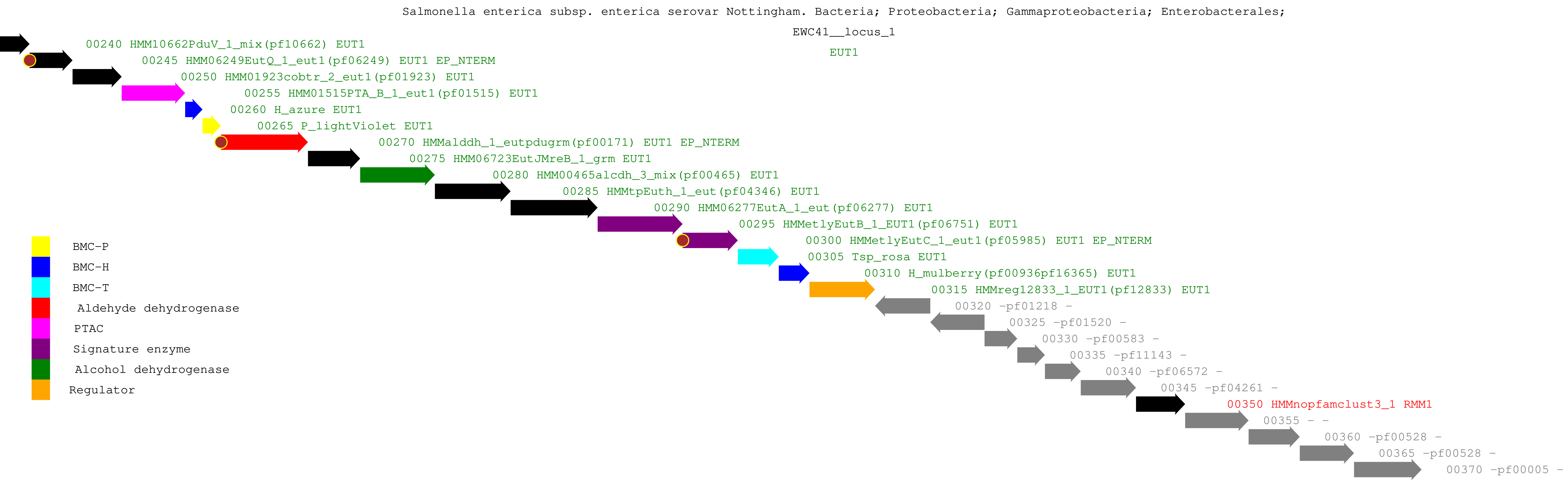
Salmonella enterica subsp. enterica serovar Nottingham. Bacteria; Proteobacteria; Gammaproteobacteria; Enterobacterales;
EWC41__locus_1
EUT1
00240 HMM10662PduV_1_mix(pf10662) EUT1
00245 HMM06249EutQ_1_eut1(pf06249) EUT1 EP_NTERM
00250 HMM01923cobtr_2_eut1(pf01923) EUT1
00255 HMM01515PTA_B_1_eut1(pf01515) EUT1
00260 H_azure EUT1
00265 P_lightViolet EUT1
00270 HMMalddh_1_eutpdugrm(pf00171) EUT1 EP_NTERM
00275 HMM06723EutJMreB_1_grm EUT1
00280 HMM00465alcdh_3_mix(pf00465) EUT1
00285 HMMtpEuth_1_eut(pf04346) EUT1
00290 HMM06277EutA_1_eut(pf06277) EUT1
00295 HMMetlyEutB_1_EUT1(pf06751) EUT1
00300 HMMetlyEutC_1_eut1(pf05985) EUT1 EP_NTERM
00305 Tsp_rosa EUT1
00310 H_mulberry(pf00936pf16365) EUT1
00315 HMMreg12833_1_EUT1(pf12833) EUT1
00320 -pf01218 -
00325 -pf01520 -
00330 -pf00583 -
00335 -pf11143 -
00340 -pf06572 -
00345 -pf04261 -
00350 HMMnopfamclust3_1 RMM1
00355 - -
00360 -pf00528 -
00365 -pf00528 -
00370 -pf00005 -
BMC-P
BMC-H
BMC-T
Aldehyde dehydrogenase
PTAC
Signature enzyme
Alcohol dehydrogenase
Regulator

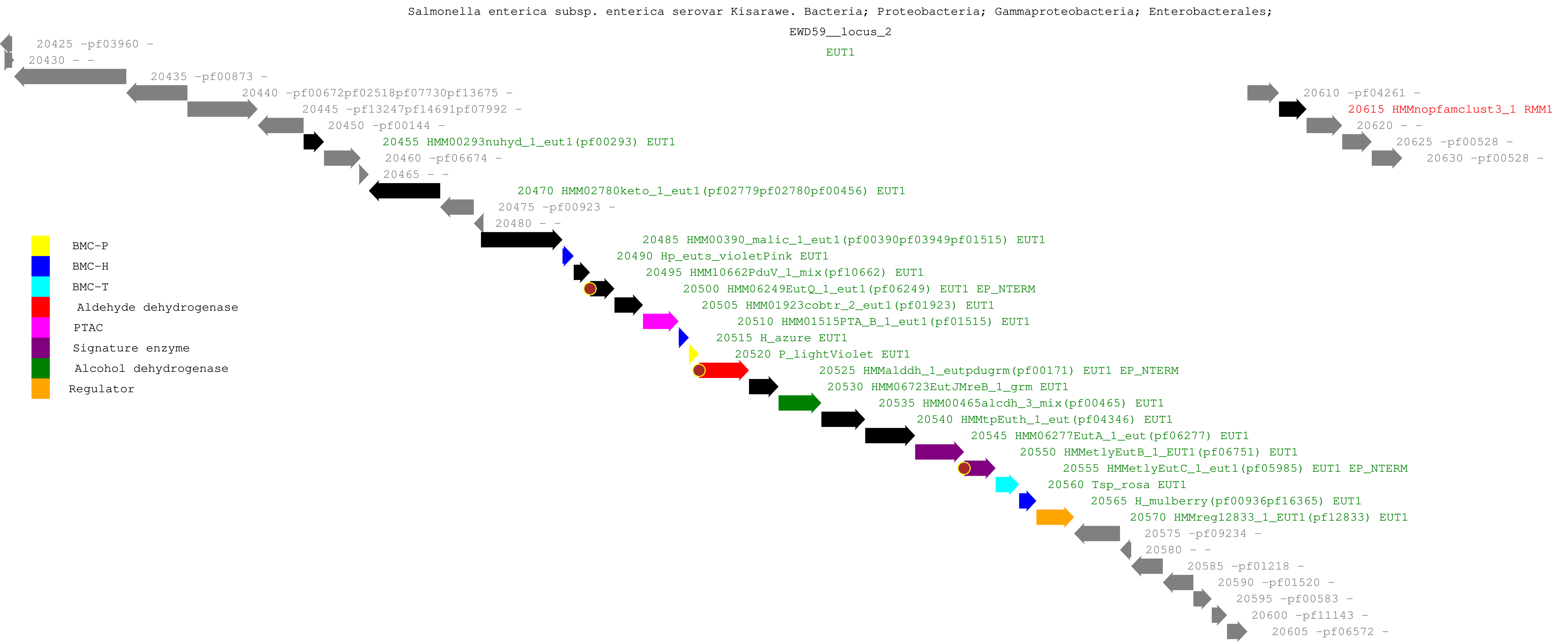
Salmonella enterica subsp. enterica serovar Kisarawe. Bacteria; Proteobacteria; Gammaproteobacteria; Enterobacterales;
EWD59__locus_2
EUT1
20425 -pf03960 -
20430 - -
20435 -pf00873 -
20440 -pf00672pf02518pf07730pf13675 -
20445 -pf13247pf14691pf07992 -
20450 -pf00144 -
20455 HMM00293nuhyd_1_eut1(pf00293) EUT1
20460 -pf06674 -
20465 - -
20470 HMM02780keto_1_eut1(pf02779pf02780pf00456) EUT1
20475 -pf00923 -
20480 - -
20485 HMM00390_malic_1_eut1(pf00390pf03949pf01515) EUT1
20490 Hp_euts_violetPink EUT1
20495 HMM10662PduV_1_mix(pf10662) EUT1
20500 HMM06249EutQ_1_eut1(pf06249) EUT1 EP_NTERM
20505 HMM01923cobtr_2_eut1(pf01923) EUT1
20510 HMM01515PTA_B_1_eut1(pf01515) EUT1
20515 H_azure EUT1
20520 P_lightViolet EUT1
20525 HMMalddh_1_eutpdugrm(pf00171) EUT1 EP_NTERM
20530 HMM06723EutJMreB_1_grm EUT1
20535 HMM00465alcdh_3_mix(pf00465) EUT1
20540 HMMtpEuth_1_eut(pf04346) EUT1
20545 HMM06277EutA_1_eut(pf06277) EUT1
20550 HMMetlyEutB_1_EUT1(pf06751) EUT1
20555 HMMetlyEutC_1_eut1(pf05985) EUT1 EP_NTERM
20560 Tsp_rosa EUT1
20565 H_mulberry(pf00936pf16365) EUT1
20570 HMMreg12833_1_EUT1(pf12833) EUT1
20575 -pf09234 -
20580 - -
20585 -pf01218 -
20590 -pf01520 -
20595 -pf00583 -
20600 -pf11143 -
20605 -pf06572 -
20610 -pf04261 -
20615 HMMnopfamclust3_1 RMM1
20620 - -
20625 -pf00528 -
20630 -pf00528 -
BMC-P
BMC-H
BMC-T
Aldehyde dehydrogenase
PTAC
Signature enzyme
Alcohol dehydrogenase
Regulator

Salmonella enterica subsp. enterica serovar Praha. Bacteria; Proteobacteria; Gammaproteobacteria; Enterobacterales;

EWD62__locus_2

EUT1

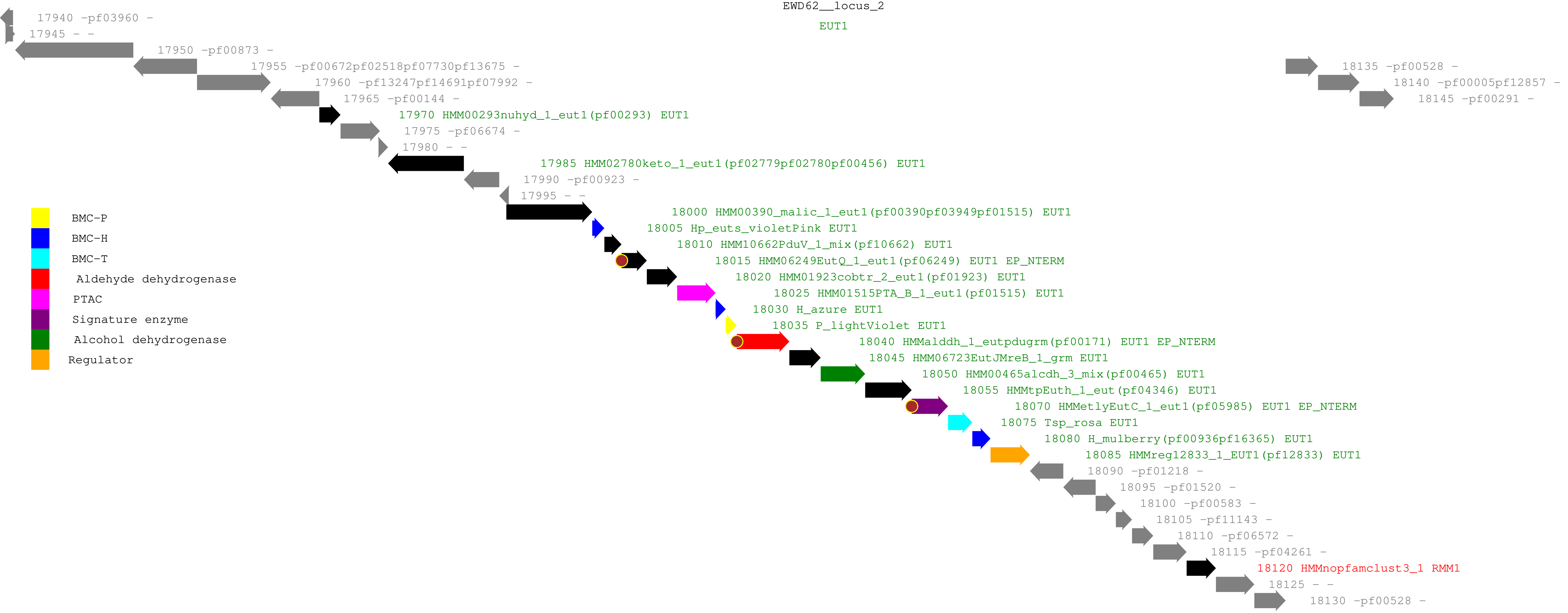

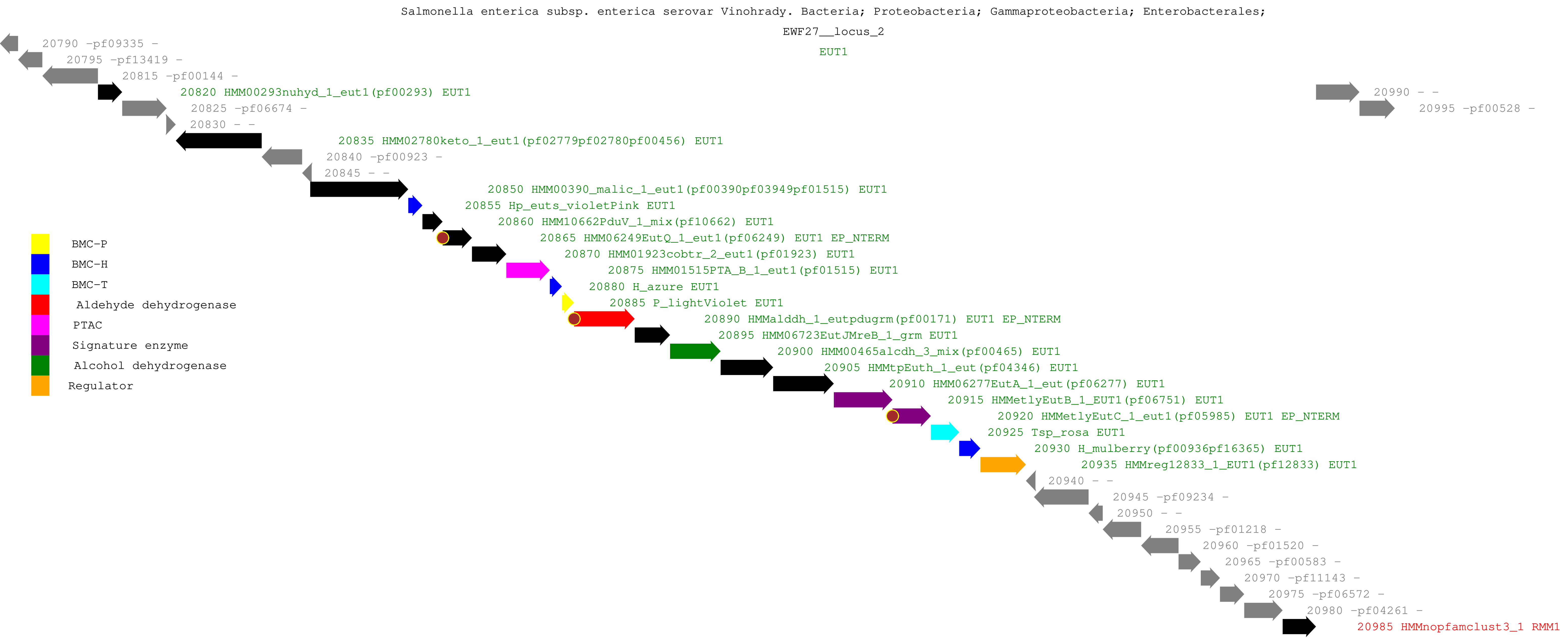

Salmonella enterica subsp. enterica serovar Vinohrady. Bacteria; Proteobacteria; Gammaproteobacteria; Enterobacterales;
EWF27__locus_2
EUT1
20790 -pf09335 -
20795 -pf13419 -
20815 -pf00144 -
20820 HMM00293nuhyd_1_eut1(pf00293) EUT1
20825 -pf06674 -
20830 - -
20835 HMM02780keto_1_eut1(pf02779pf02780pf00456) EUT1
20840 -pf00923 -
20845 - -
20850 HMM00390_malic_1_eut1(pf00390pf03949pf01515) EUT1
20855 Hp_euts_violetPink EUT1
20860 HMM10662PduV_1_mix(pf10662) EUT1
20865 HMM06249EutQ_1_eut1(pf06249) EUT1 EP_NTERM
20870 HMM01923cobtr_2_eut1(pf01923) EUT1
20875 HMM01515PTA_B_1_eut1(pf01515) EUT1
20880 H_azure EUT1
20885 P_lightViolet EUT1
20890 HMMalddh_1_eutpdugrm(pf00171) EUT1 EP_NTERM
20895 HMM06723EutJMreB_1_grm EUT1
20900 HMM00465alcdh_3_mix(pf00465) EUT1
20905 HMMtpEuth_1_eut(pf04346) EUT1
20910 HMM06277EutA_1_eut(pf06277) EUT1
20915 HMMetlyEutB_1_EUT1(pf06751) EUT1
20920 HMMetlyEutC_1_eut1(pf05985) EUT1 EP_NTERM
20925 Tsp_rosa EUT1
20930 H_mulberry(pf00936pf16365) EUT1
20935 HMMreg12833_1_EUT1(pf12833) EUT1
20940 - -
20945 -pf09234 -
20950 - -
20955 -pf01218 -
20960 -pf01520 -
20965 -pf00583 -
20970 -pf11143 -
20975 -pf06572 -
20980 -pf04261 -
20985 HMMnopfamclust3_1 RMM1
20990 - -
20995 -pf00528 -
BMC-P
BMC-H
BMC-T
Aldehyde dehydrogenase
PTAC
Signature enzyme
Alcohol dehydrogenase
Regulator

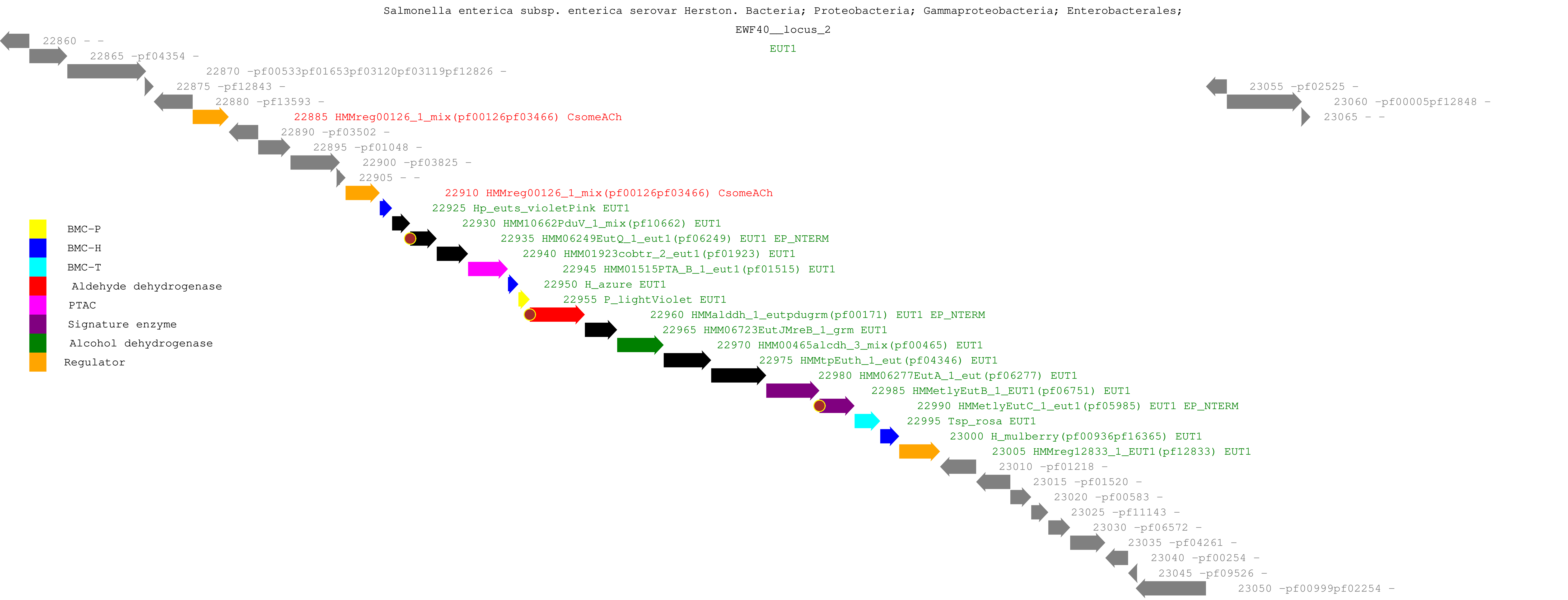
Salmonella enterica subsp. enterica serovar Herston. Bacteria; Proteobacteria; Gammaproteobacteria; Enterobacterales;
EWF40__locus_2
EUT1
22860 - -
22865 -pf04354 -
22870 -pf00533pf01653pf03120pf03119pf12826 -
22875 -pf12843 -
22880 -pf13593 -
22885 HMMreg00126_1_mix(pf00126pf03466) CsomeACh
22890 -pf03502 -
22895 -pf01048 -
22900 -pf03825 -
22905 - -
22910 HMMreg00126_1_mix(pf00126pf03466) CsomeACh
22925 Hp_euts_violetPink EUT1
22930 HMM10662PduV_1_mix(pf10662) EUT1
22935 HMM06249EutQ_1_eut1(pf06249) EUT1 EP_NTERM
22940 HMM01923cobtr_2_eut1(pf01923) EUT1
22945 HMM01515PTA_B_1_eut1(pf01515) EUT1
22950 H_azure EUT1
22955 P_lightViolet EUT1
22960 HMMaldh_1_eutpdugrm(pf00171) EUT1 EP_NTERM
22965 HMM06723EutJMreB_1_grm EUT1
22970 HMM00465alcdh_3_mix(pf00465) EUT1
22975 HMMtpEuth_1_eut(pf04346) EUT1
22980 HMM06277EutA_1_eut(pf06277) EUT1
22985 HMMetlyEutB_1_EUT1(pf06751) EUT1
22990 HMMetlyEutC_1_eut1(pf05985) EUT1 EP_NTERM
22995 Tsp_rosa EUT1
23000 H_mulberry(pf00936pf16365) EUT1
23005 HMMreg12833_1_EUT1(pf12833) EUT1
23010 -pf01218 -
23015 -pf01520 -
23020 -pf00583 -
23025 -pf11143 -
23030 -pf06572 -
23035 -pf04261 -
23040 -pf00254 -
23045 -pf09526 -
23050 -pf00999pf02254 -
23055 -pf02525 -
23060 -pf00005pf12848 -
23065 - -
BMC-P
BMC-H
BMC-T
Aldehyde dehydrogenase
PTAC
Signature enzyme
Alcohol dehydrogenase
Regulator

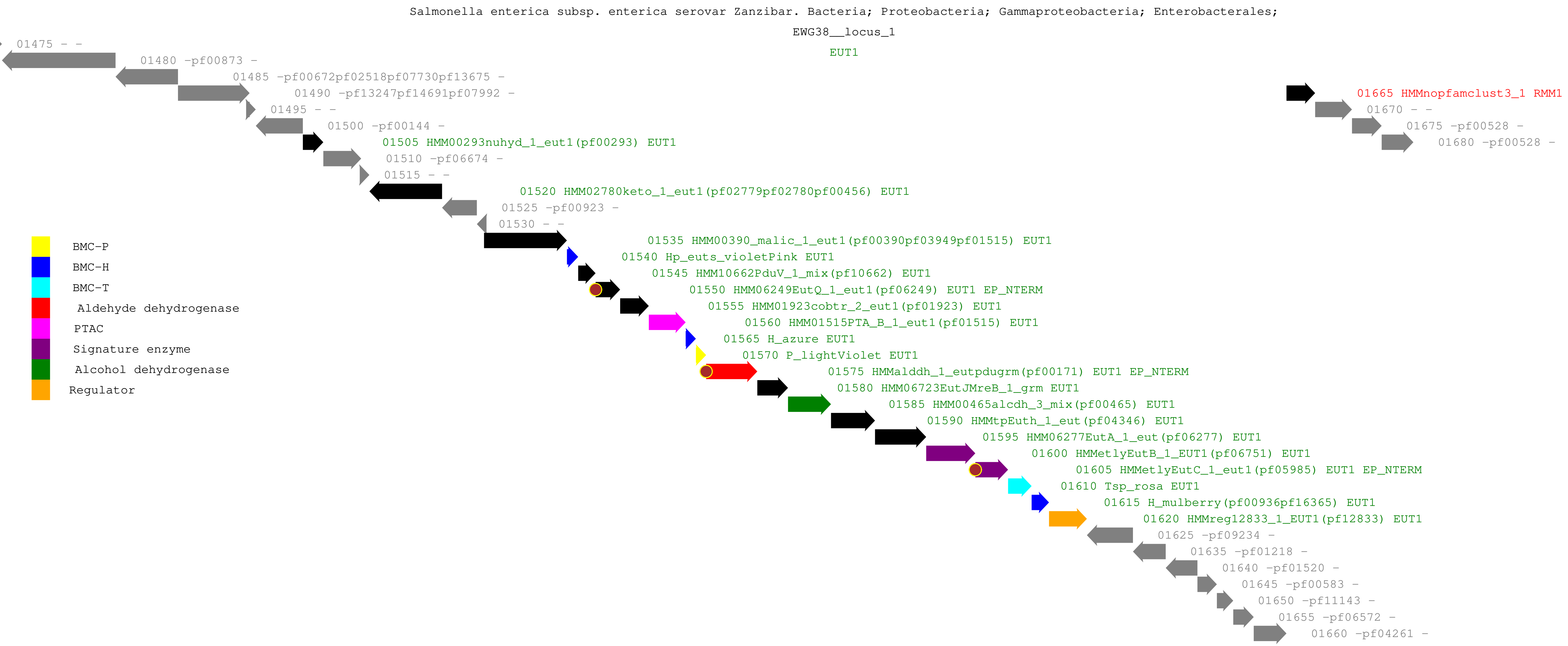

Salmonella enterica subsp. enterica serovar Zanzibar. Bacteria; Proteobacteria; Gammaproteobacteria; Enterobacterales;
EWG38__locus_1
EUT1
01475 - -
01480 -pf00873 -
01485 -pf00672pf02518pf07730pf13675 -
01490 -pf13247pf14691pf07992 -
01495 - -
01500 -pf00144 -
01505 HMM00293nuhyd_1_eut1(pf00293) EUT1
01510 -pf06674 -
01515 - -
01520 HMM02780keto_1_eut1(pf02779pf02780pf00456) EUT1
01525 -pf00923 -
01530 - -
01535 HMM00390_malic_1_eut1(pf00390pf03949pf01515) EUT1
01540 Hp_euts_violetPink EUT1
01545 HMM10662PduV_1_mix(pf10662) EUT1
01550 HMM06249EutQ_1_eut1(pf06249) EUT1 EP_NTERM
01555 HMM01923cobtr_2_eut1(pf01923) EUT1
01560 HMM01515PTA_B_1_eut1(pf01515) EUT1
01565 H_azure EUT1
01570 P_lightViolet EUT1
01575 HMMaldh_1_eutpdugrm(pf00171) EUT1 EP_NTERM
01580 HMM06723EutJMreB_1_grm EUT1
01585 HMM00465alcdh_3_mix(pf00465) EUT1
01590 HMMtpEuth_1_eut(pf04346) EUT1
01595 HMM06277EutA_1_eut(pf06277) EUT1
01600 HMMetlyEutB_1_EUT1(pf06751) EUT1
01605 HMMetlyEutC_1_eut1(pf05985) EUT1 EP_NTERM
01610 Tsp_rosa EUT1
01615 H_mulberry(pf00936pf16365) EUT1
01620 HMMreg12833_1_EUT1(pf12833) EUT1
01625 -pf09234 -
01635 -pf01218 -
01640 -pf01520 -
01645 -pf00583 -
01650 -pf11143 -
01655 -pf06572 -
01660 -pf04261 -
01665 HMMnopfamclust3_1 RMM1
01670 - -
01675 -pf00528 -
01680 -pf00528 -
BMC-P
BMC-H
BMC-T
Aldehyde dehydrogenase
PTAC
Signature enzyme
Alcohol dehydrogenase
Regulator

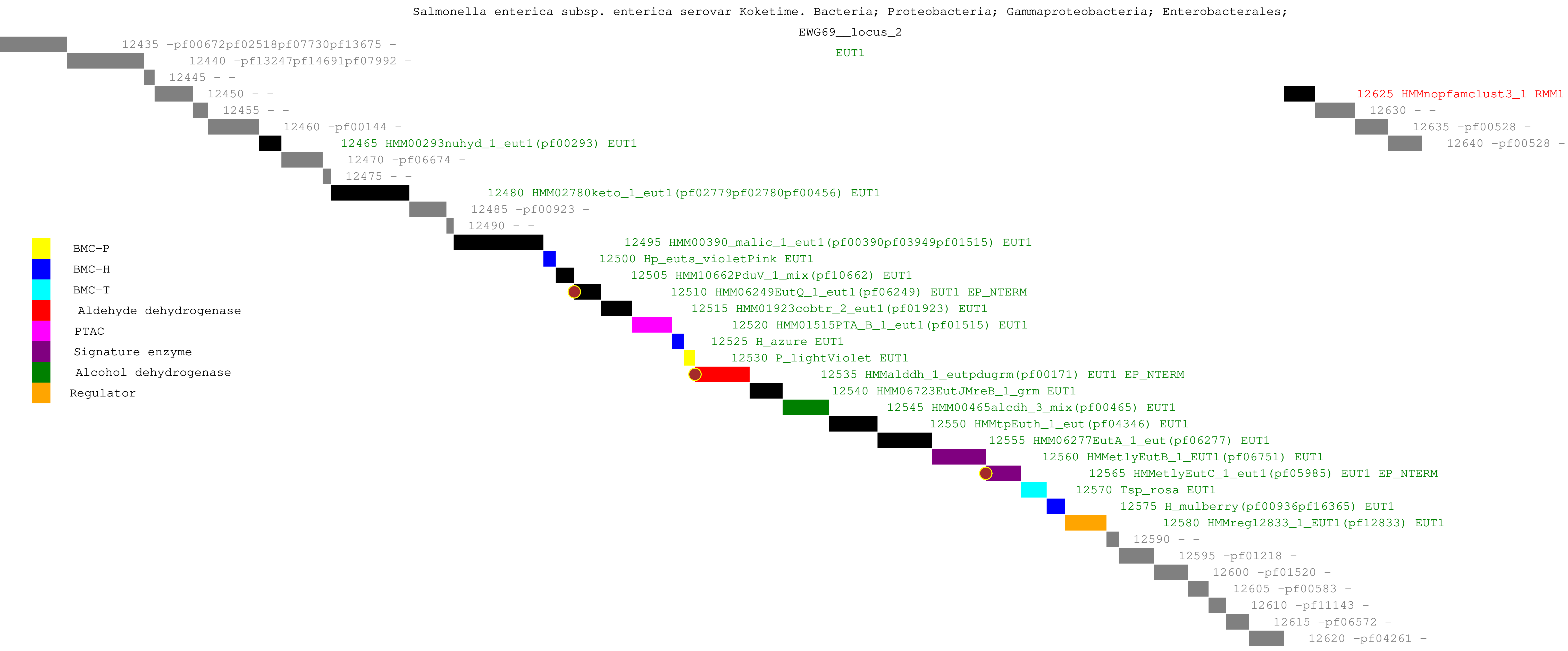
Salmonella enterica subsp. enterica serovar Koketime. Bacteria; Proteobacteria; Gammaproteobacteria; Enterobacterales;
EWG69__locus_2
EUT1
12435 -pf00672pf02518pf07730pf13675 -
12440 -pf13247pf14691pf07992 -
12445 - -
12450 - -
12455 - -
12460 -pf00144 -
12465 HMM00293nuhyd_1_eut1(pf00293) EUT1
12470 -pf06674 -
12475 - -
12480 HMM02780keto_1_eut1(pf02779pf02780pf00456) EUT1
12485 -pf00923 -
12490 - -
12495 HMM00390_malic_1_eut1(pf00390pf03949pf01515) EUT1
12500 Hp_euts_violetPink EUT1
12505 HMM10662PduV_1_mix(pf10662) EUT1
12510 HMM06249EutQ_1_eut1(pf06249) EUT1 EP_NTERM
12515 HMM01923cobtr_2_eut1(pf01923) EUT1
12520 HMM01515PTA_B_1_eut1(pf01515) EUT1
12525 H_azure EUT1
12530 P_lightViolet EUT1
12535 HMMalddh_1_eutpdugrm(pf00171) EUT1 EP_NTERM
12540 HMM06723EutJMreB_1_grm EUT1
12545 HMM00465alcdh_3_mix(pf00465) EUT1
12550 HMMtpEuth_1_eut(pf04346) EUT1
12555 HMM06277EutA_1_eut(pf06277) EUT1
12560 HMMetlyEutB_1_EUT1(pf06751) EUT1
12565 HMMetlyEutC_1_eut1(pf05985) EUT1 EP_NTERM
12570 Tsp_rosa EUT1
12575 H_mulberry(pf00936pf16365) EUT1
12580 HMMreg12833_1_EUT1(pf12833) EUT1
12590 - -
12595 -pf01218 -
12600 -pf01520 -
12605 -pf00583 -
12610 -pf11143 -
12615 -pf06572 -
12620 -pf04261 -
12625 HMMnopfamclust3_1 RMM1
12630 - -
12635 -pf00528 -
12640 -pf00528 -
BMC-P
BMC-H
BMC-T
Aldehyde dehydrogenase
PTAC
Signature enzyme
Alcohol dehydrogenase
Regulator

Enterobacter cloacae. Bacteria; Proteobacteria; Gammaproteobacteria; Enterobacterales;

EWI30__locus_1

EUT1

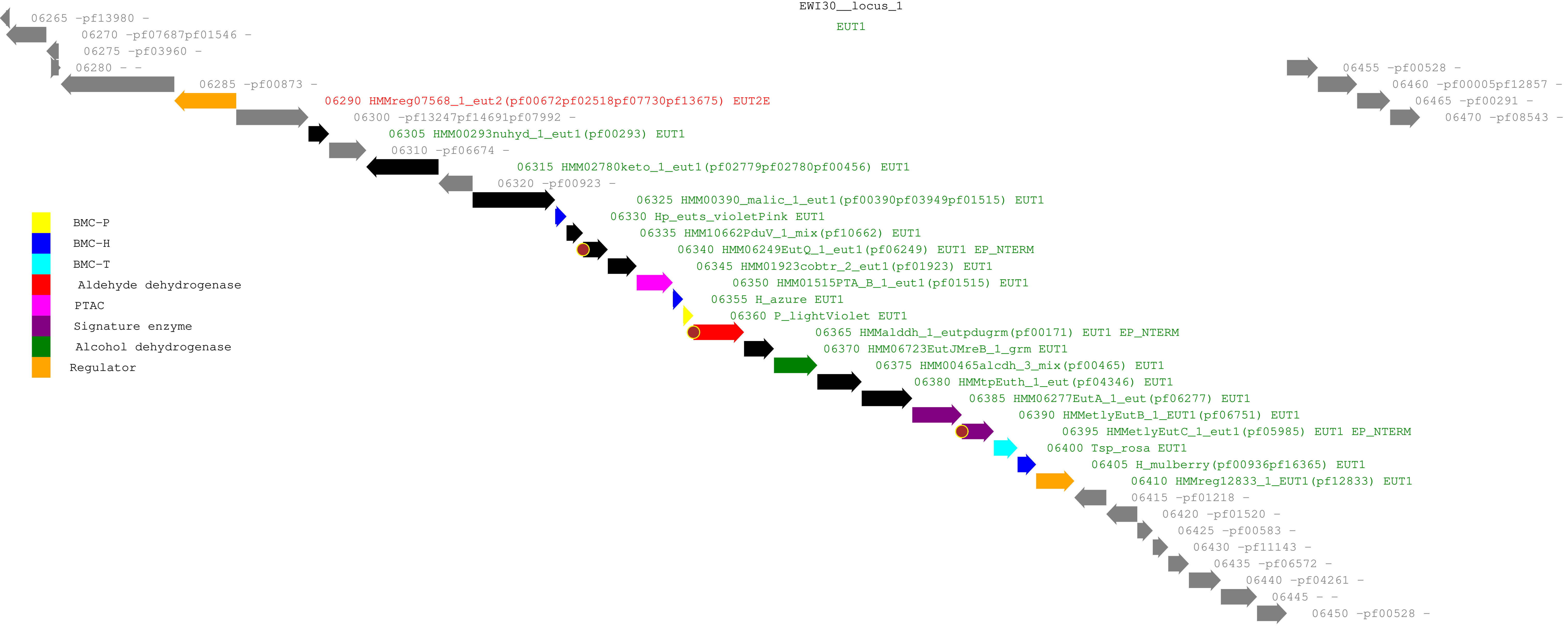

Salmonella enterica subsp. enterica serovar Kenya. Bacteria; Proteobacteria; Gammaproteobacteria; Enterobacterales;

EWJ15__locus_1

EUT1

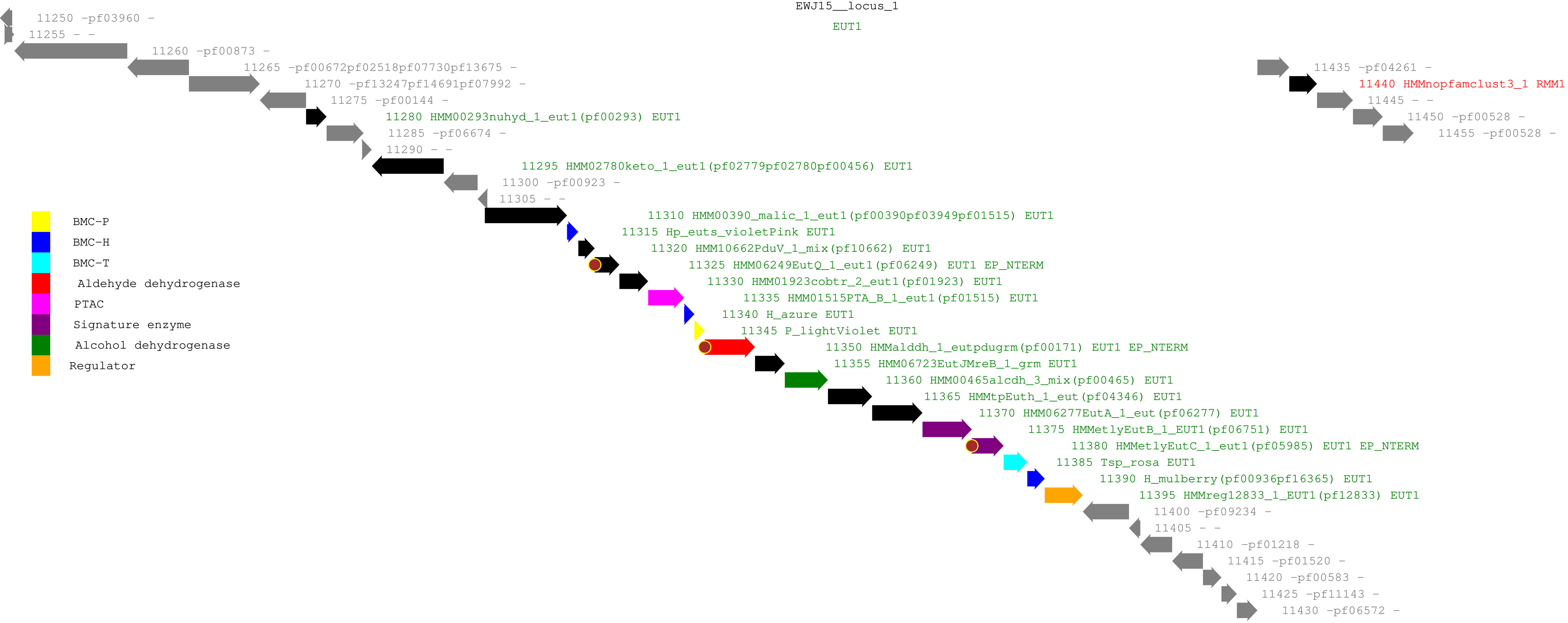

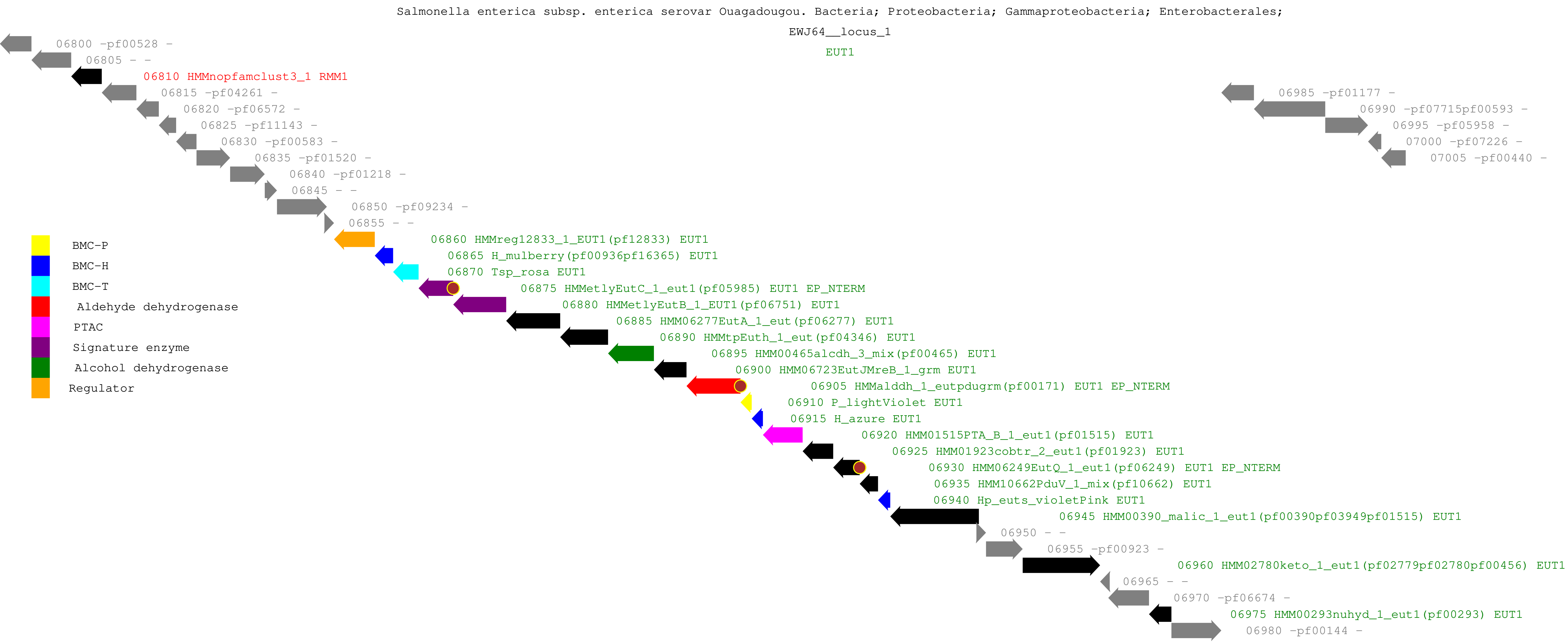

Salmonella enterica subsp. enterica serovar Ouagadougou. Bacteria; Proteobacteria; Gammaproteobacteria; Enterobacterales;
EWJ64__locus_1
EUT1
06800 -pf00528 -
06805 - -
06810 HMMnopfamclust3_1 RMM1
06815 -pf04261 -
06820 -pf06572 -
06825 -pf11143 -
06830 -pf00583 -
06835 -pf01520 -
06840 -pf01218 -
06845 - -
06850 -pf09234 -
06855 - -
06860 HMMreg12833_1_EUT1(pf12833) EUT1
06865 H_mulberry(pf00936pf16365) EUT1
06870 Tsp_rosa EUT1
06875 HMMetlyEutC_1_eut1(pf05985) EUT1 EP_NTERM
06880 HMMetlyEutB_1_EUT1(pf06751) EUT1
06885 HMM06277EutA_1_eut(pf06277) EUT1
06890 HMMtpEuth_1_eut(pf04346) EUT1
06895 HMM00465alcdh_3_mix(pf00465) EUT1
06900 HMM06723EutJMreB_1_grm EUT1
06905 HMMalddh_1_eutpdugrm(pf00171) EUT1 EP_NTERM
06910 P_lightViolet EUT1
06915 H_azure EUT1
06920 HMM01515PTA_B_1_eut1(pf01515) EUT1
06925 HMM01923cobtr_2_eut1(pf01923) EUT1
06930 HMM06249EutQ_1_eut1(pf06249) EUT1 EP_NTERM
06935 HMM10662PduV_1_mix(pf10662) EUT1
06940 Hp_euts_violetPink EUT1
06945 HMM00390_malic_1_eut1(pf00390pf03949pf01515) EUT1
06950 - -
06955 -pf00923 -
06960 HMM02780keto_1_eut1(pf02779pf02780pf00456) EUT1
06965 - -
06970 -pf06674 -
06975 HMM00293nuhyd_1_eut1(pf00293) EUT1
06980 -pf00144 -
06985 -pf01177 -
06990 -pf07715pf00593 -
06995 -pf05958 -
07000 -pf07226 -
07005 -pf00440 -
BMC-P
BMC-H
BMC-T
Aldehyde dehydrogenase
PTAC
Signature enzyme
Alcohol dehydrogenase
Regulator

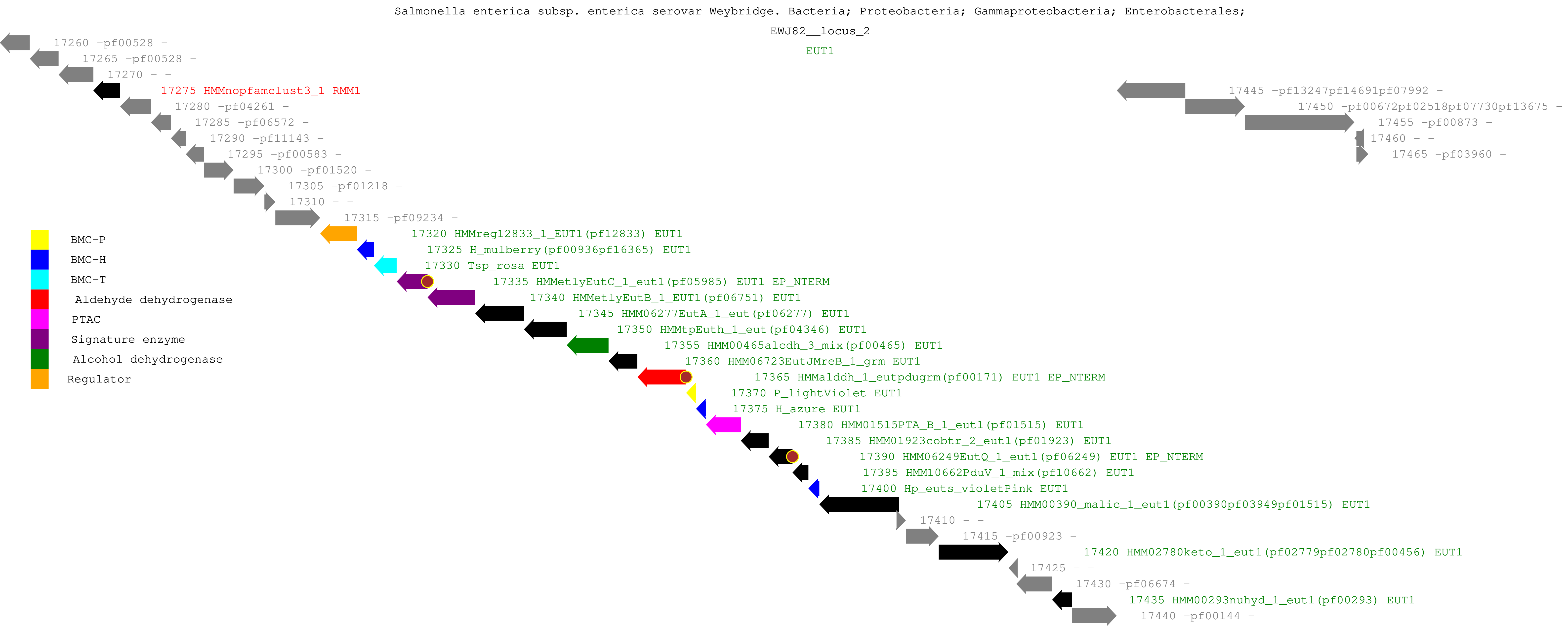
Salmonella enterica subsp. enterica serovar Weybridge. Bacteria; Proteobacteria; Gammaproteobacteria; Enterobacterales;
EWJ82__locus_2
EUT1
17260 -pf00528 -
17265 -pf00528 -
17270 - -
17275 HMMnopfamclust3_1 RMM1
17280 -pf04261 -
17285 -pf06572 -
17290 -pf11143 -
17295 -pf00583 -
17300 -pf01520 -
17305 -pf01218 -
17310 - -
17315 -pf09234 -
17320 HMMreg12833_1_EUT1(pf12833) EUT1
17325 H_mulberry(pf00936pf16365) EUT1
17330 Tsp_rosa EUT1
17335 HMMetlyEutC_1_eut1(pf05985) EUT1 EP_NTERM
17340 HMMetlyEutB_1_EUT1(pf06751) EUT1
17345 HMM06277EutA_1_eut(pf06277) EUT1
17350 HMMtpEuth_1_eut(pf04346) EUT1
17355 HMM00465alcdh_3_mix(pf00465) EUT1
17360 HMM06723EutJMreB_1_grm EUT1
17365 HMMalddh_1_eutpdugrm(pf00171) EUT1 EP_NTERM
17370 P_lightViolet EUT1
17375 H_azure EUT1
17380 HMM01515PTA_B_1_eut1(pf01515) EUT1
17385 HMM01923cobtr_2_eut1(pf01923) EUT1
17390 HMM06249EutQ_1_eut1(pf06249) EUT1 EP_NTERM
17395 HMM10662PduV_1_mix(pf10662) EUT1
17400 Hp_euts_violetPink EUT1
17405 HMM00390_malic_1_eut1(pf00390pf03949pf01515) EUT1
17410 - -
17415 -pf00923 -
17420 HMM02780keto_1_eut1(pf02779pf02780pf00456) EUT1
17425 - -
17430 -pf06674 -
17435 HMM00293nuhyd_1_eut1(pf00293) EUT1
17440 -pf00144 -
17445 -pf13247pf14691pf07992 -
17450 -pf00672pf02518pf07730pf13675 -
17455 -pf00873 -
17460 - -
17465 -pf03960 -
BMC-P
BMC-H
BMC-T
Aldehyde dehydrogenase
PTAC
Signature enzyme
Alcohol dehydrogenase
Regulator

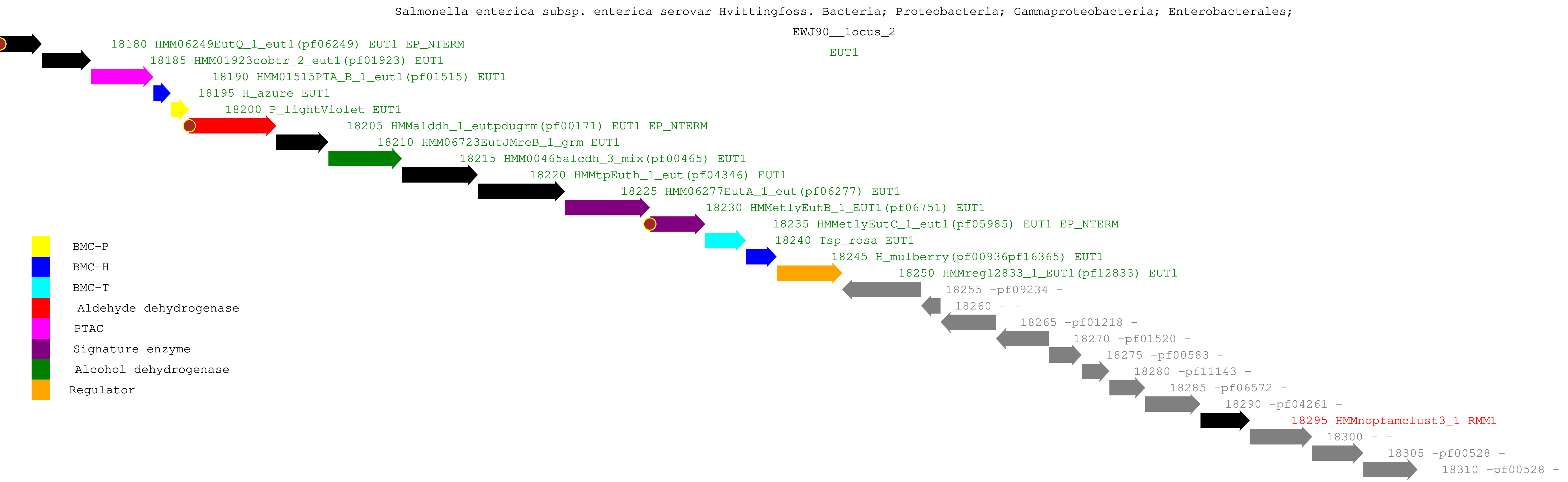
Salmonella enterica subsp. enterica serovar Hvittingfoss. Bacteria; Proteobacteria; Gammaproteobacteria; Enterobacterales;
EWJ90__locus_2
EUT1
18180 HMM06249EutQ_1_eut1(pf06249) EUT1 EP_NTERM
18185 HMM01923cobtr_2_eut1(pf01923) EUT1
18190 HMM01515PTA_B_1_eut1(pf01515) EUT1
18195 H_azure EUT1
18200 P_lightViolet EUT1
18205 HMMalddh_1_eutpdugrm(pf00171) EUT1 EP_NTERM
18210 HMM06723EutJMreB_1_grm EUT1
18215 HMM00465alcdh_3_mix(pf00465) EUT1
18220 HMMtpEuth_1_eut(pf04346) EUT1
18225 HMM06277EutA_1_eut(pf06277) EUT1
18230 HMMetlyEutB_1_EUT1(pf06751) EUT1
18235 HMMetlyEutC_1_eut1(pf05985) EUT1 EP_NTERM
18240 Tsp_rosa EUT1
18245 H_mulberry(pf00936pf16365) EUT1
18250 HMMreg12833_1_EUT1(pf12833) EUT1
18255 -pf09234 -
18260 - -
18265 -pf01218 -
18270 -pf01520 -
18275 -pf00583 -
18280 -pf11143 -
18285 -pf06572 -
18290 -pf04261 -
18295 HMMnopfamclust3_1 RMM1
18300 - -
18305 -pf00528 -
18310 -pf00528 -
BMC-P
BMC-H
BMC-T
Aldehyde dehydrogenase
PTAC
Signature enzyme
Alcohol dehydrogenase
Regulator

Salmonella enterica subsp. enterica serovar Offa. Bacteria; Proteobacteria; Gammaproteobacteria; Enterobacterales;

EWZ14__locus_2

EUT1

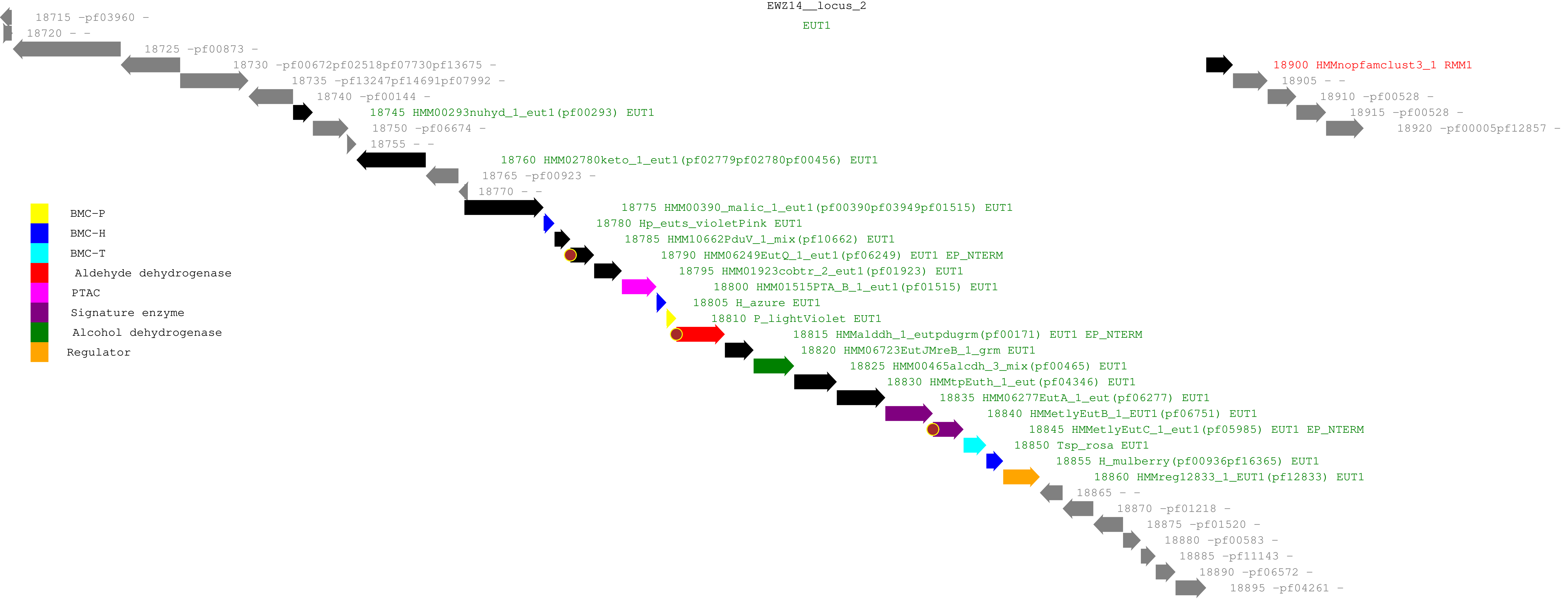

Salmonella enterica subsp. enterica serovar Menston. Bacteria; Proteobacteria; Gammaproteobacteria; Enterobacterales;

EX933__locus_2

EUT1

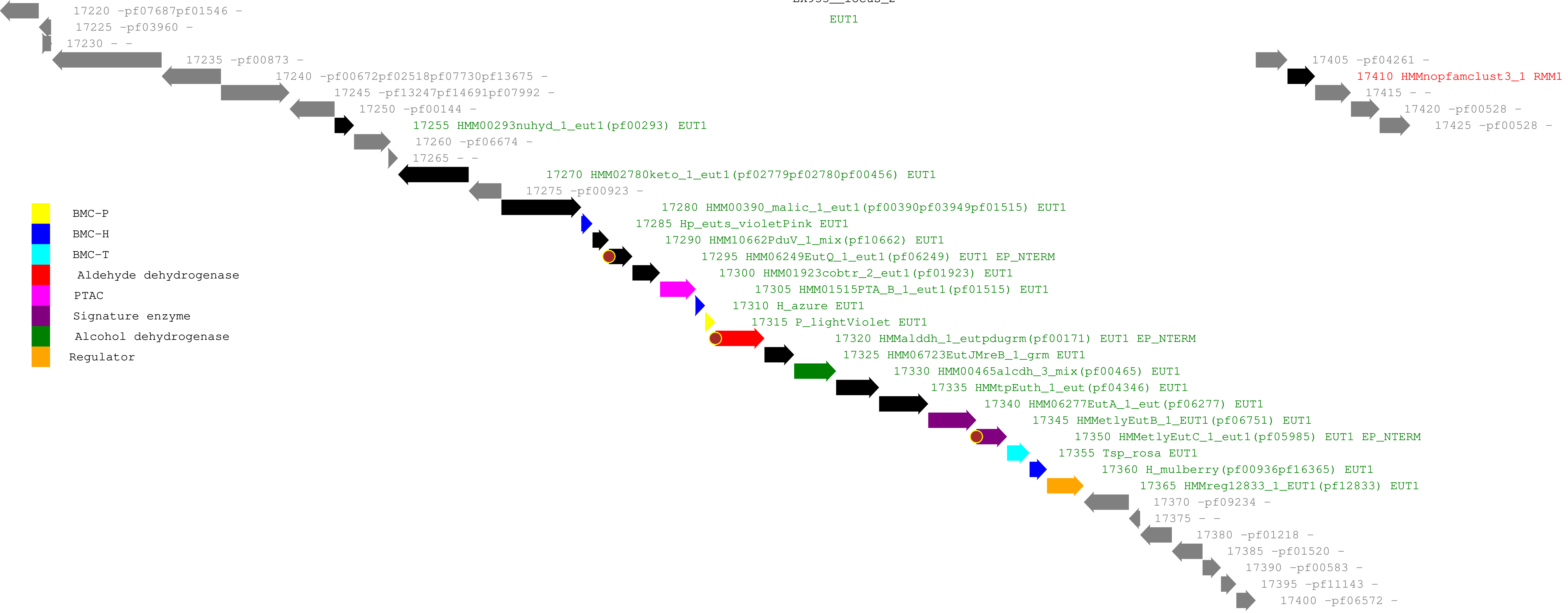

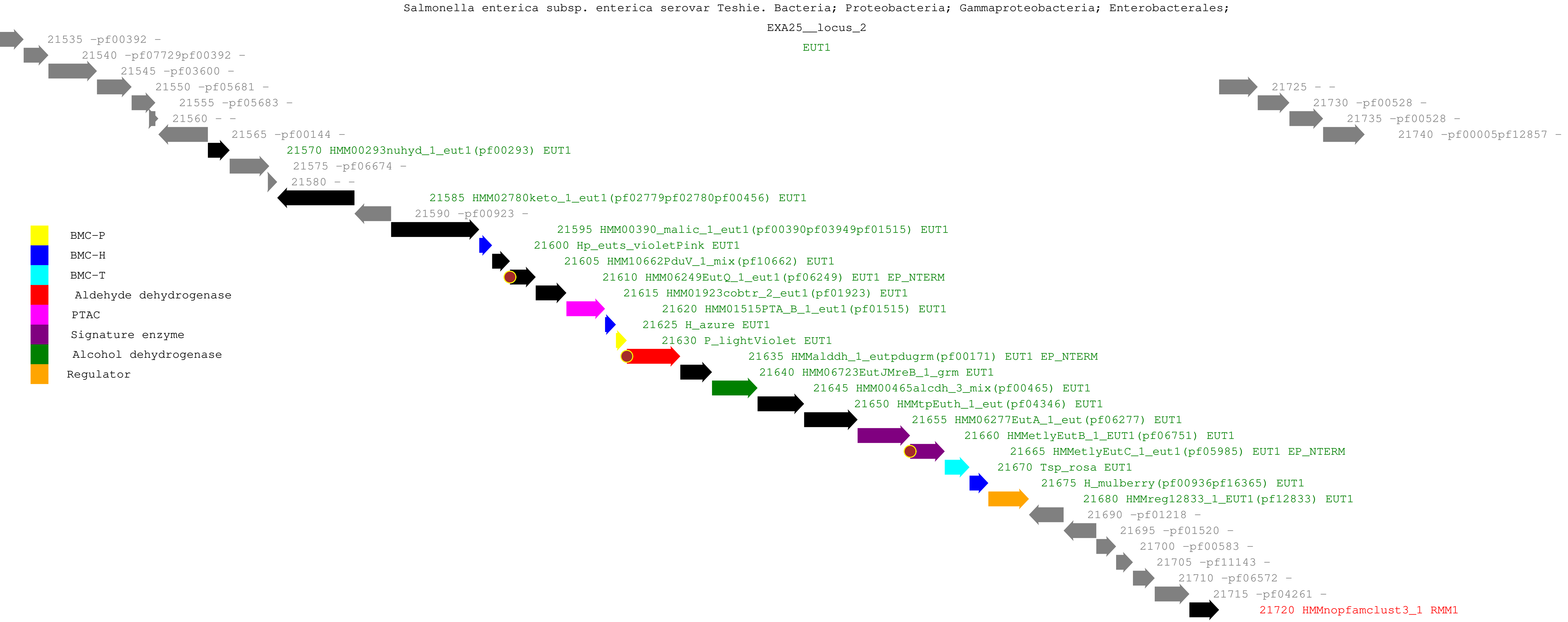

Salmonella enterica subsp. enterica serovar Teshie. Bacteria; Proteobacteria; Gammaproteobacteria; Enterobacterales;
EXA25__locus_2
EUT1
21535 -pf00392 -
21540 -pf07729pf00392 -
21545 -pf03600 -
21550 -pf05681 -
21555 -pf05683 -
21560 - -
21565 -pf00144 -
21570 HMM00293nuhyd_1_eut1(pf00293) EUT1
21575 -pf06674 -
21580 - -
21585 HMM02780keto_1_eut1(pf02779pf02780pf00456) EUT1
21590 -pf00923 -
21595 HMM00390_malic_1_eut1(pf00390pf03949pf01515) EUT1
21600 Hp_euts_violetPink EUT1
21605 HMM10662PduV_1_mix(pf10662) EUT1
21610 HMM06249EutQ_1_eut1(pf06249) EUT1 EP_NTERM
21615 HMM01923cobtr_2_eut1(pf01923) EUT1
21620 HMM01515PTA_B_1_eut1(pf01515) EUT1
21625 H_azure EUT1
21630 P_lightViolet EUT1
21635 HMMalddh_1_eutpdugrm(pf00171) EUT1 EP_NTERM
21640 HMM06723EutJMreB_1_grm EUT1
21645 HMM00465alcdh_3_mix(pf00465) EUT1
21650 HMMtpEuth_1_eut(pf04346) EUT1
21655 HMM06277EutA_1_eut(pf06277) EUT1
21660 HMMetlyEutB_1_EUT1(pf06751) EUT1
21665 HMMetlyEutC_1_eut1(pf05985) EUT1 EP_NTERM
21670 Tsp_rosa EUT1
21675 H_mulberry(pf00936pf16365) EUT1
21680 HMMreg12833_1_EUT1(pf12833) EUT1
21690 -pf01218 -
21695 -pf01520 -
21700 -pf00583 -
21705 -pf11143 -
21710 -pf06572 -
21715 -pf04261 -
21720 HMMnopfamclust3_1 RMM1
21725 - -
21730 -pf00528 -
21735 -pf00528 -
21740 -pf00005pf12857 -
BMC-P
BMC-H
BMC-T
Aldehyde dehydrogenase
PTAC
Signature enzyme
Alcohol dehydrogenase
Regulator

Salmonella enterica subsp. enterica serovar Korlebu. Bacteria; Proteobacteria; Gammaproteobacteria; Enterobacterales;

EXA36__locus_2

EUT1

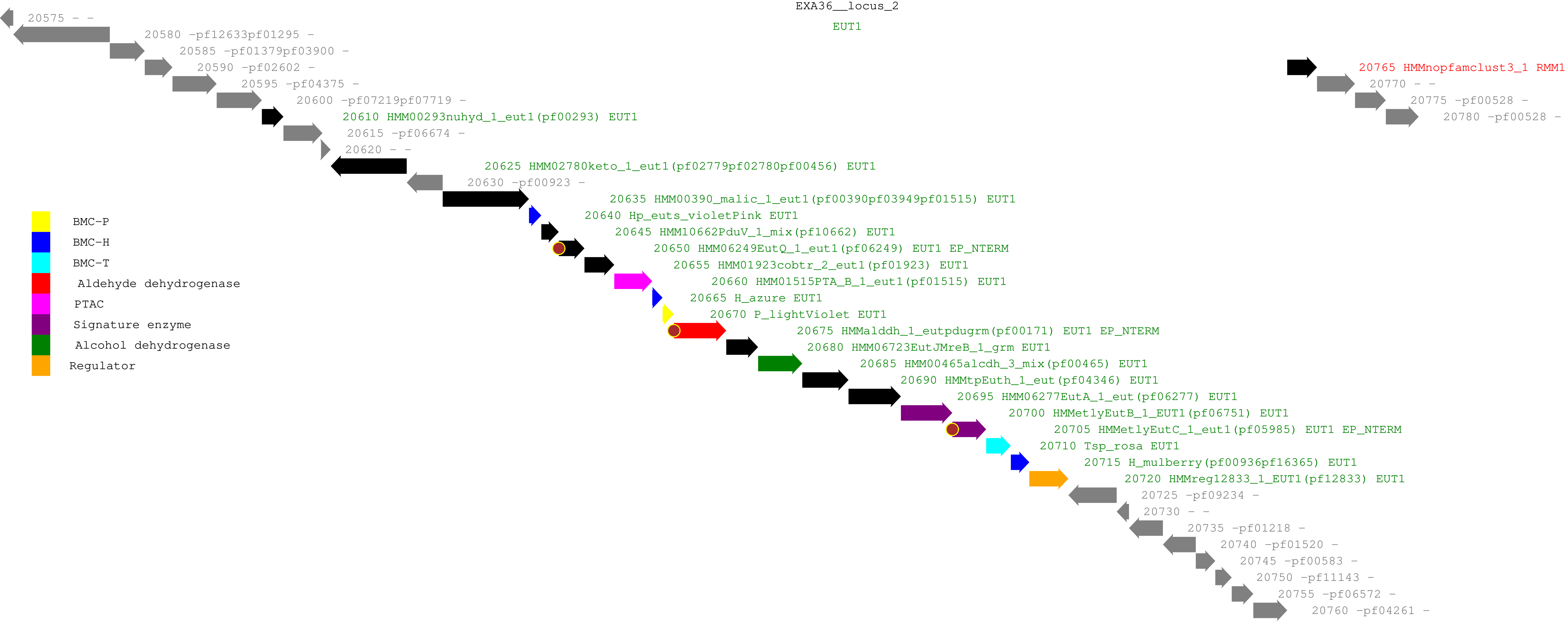

Salmonella virchow. Bacteria; Proteobacteria; Gammaproteobacteria; Enterobacterales;

EXA61__locus_1

EUT1

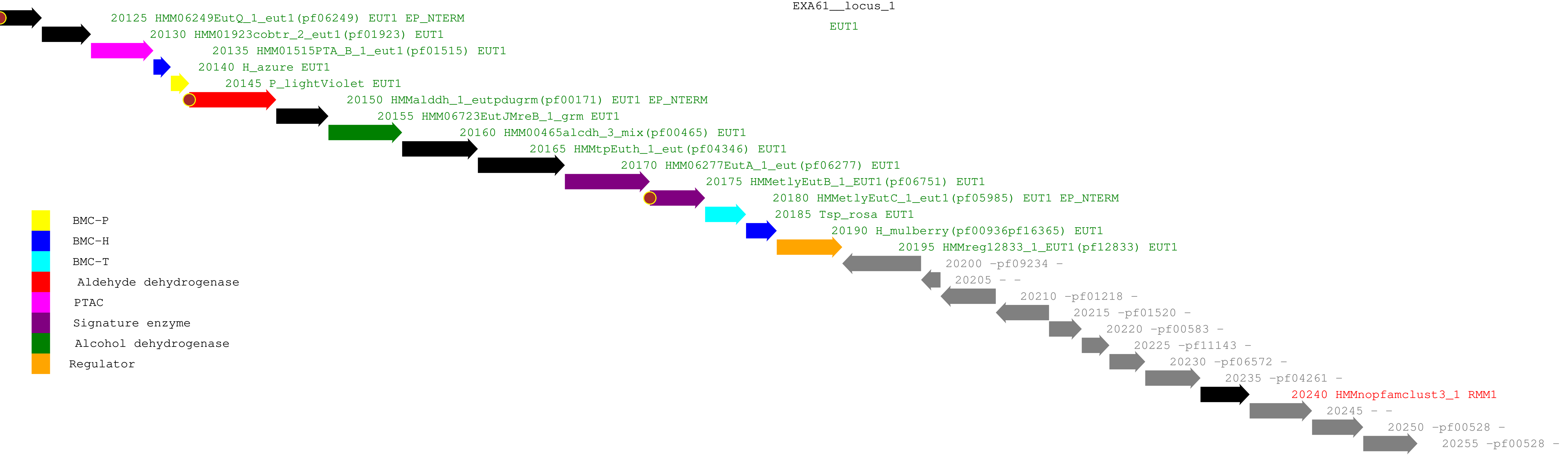

Salmonella enterica subsp. enterica serovar Breukelen. Bacteria; Proteobacteria; Gammaproteobacteria; Enterobacterales;

EXA83__locus_2

EUT1

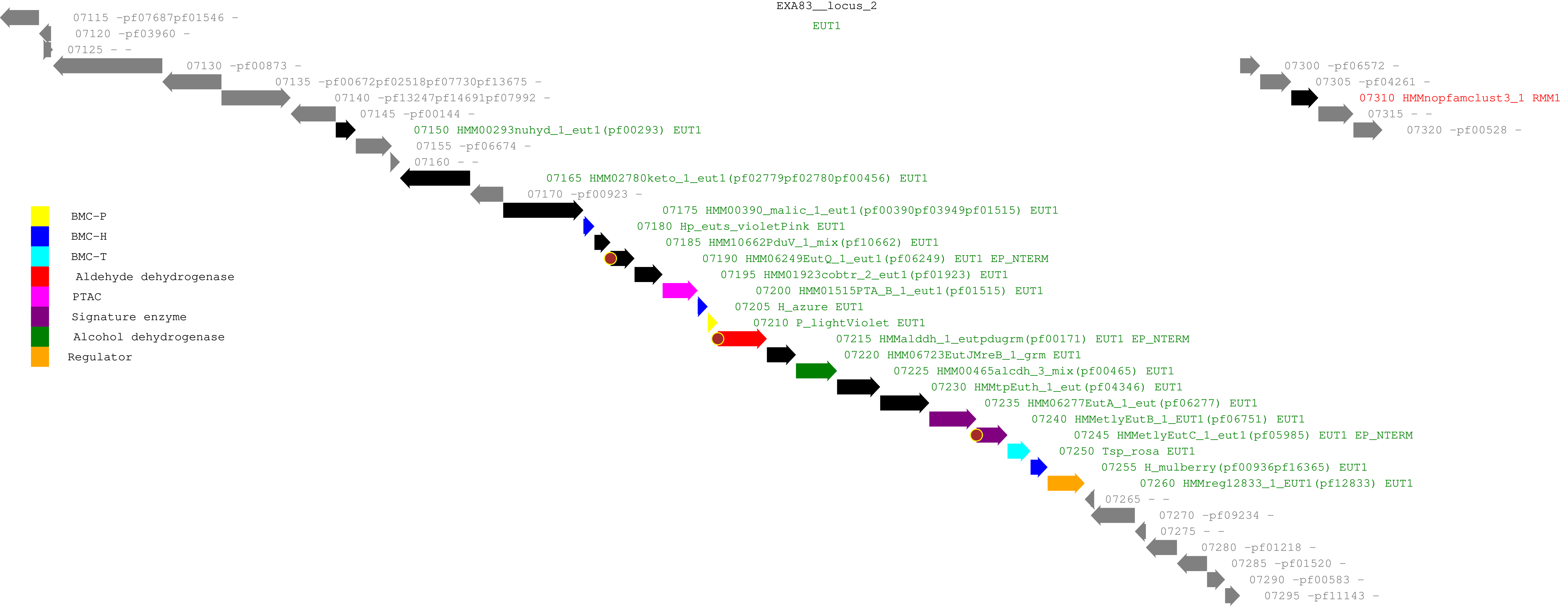

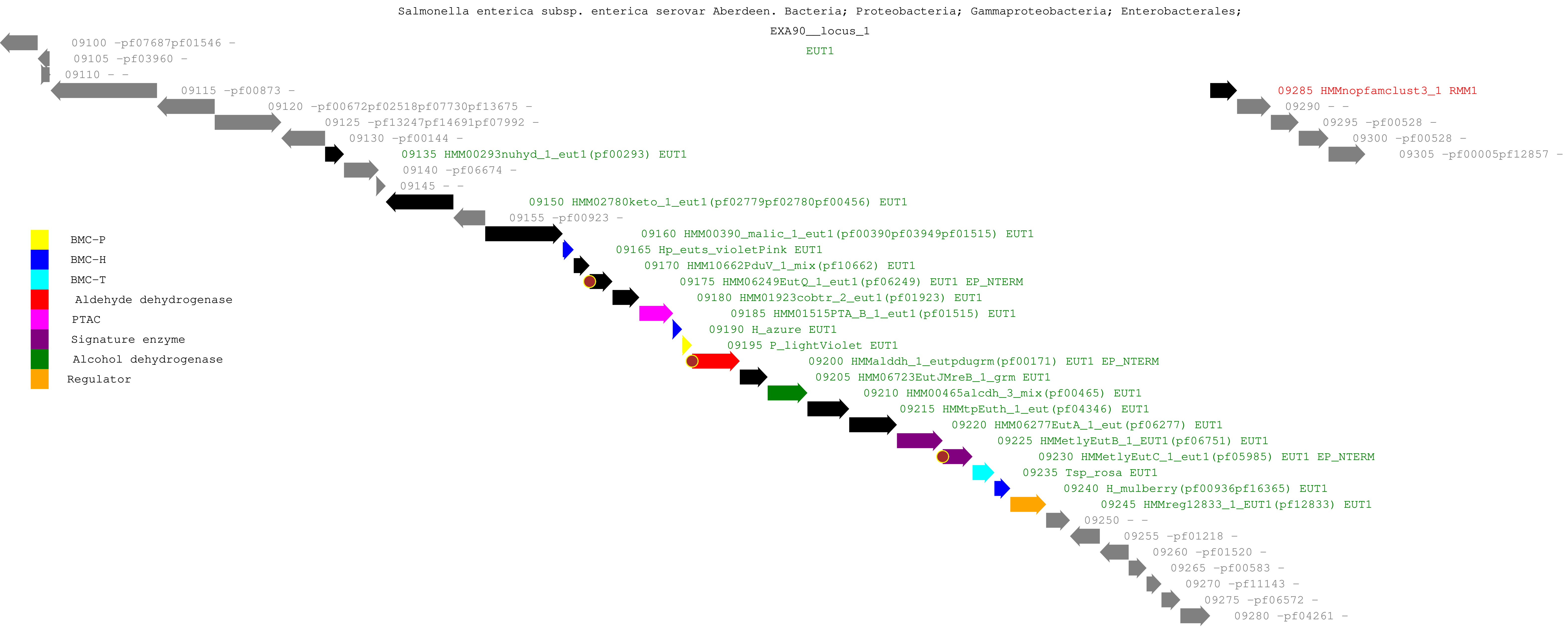

Salmonella enterica subsp. enterica serovar Aberdeen. Bacteria; Proteobacteria; Gammaproteobacteria; Enterobacterales;
EXA90__locus_1
EUT1
09100 -pf07687pf01546 -
09105 -pf03960 -
09110 - -
09115 -pf00873 -
09120 -pf00672pf02518pf07730pf13675 -
09125 -pf13247pf14691pf07992 -
09130 -pf00144 -
09135 HMM00293nuhyd_1_eut1(pf00293) EUT1
09140 -pf06674 -
09145 - -
09150 HMM02780keto_1_eut1(pf02779pf02780pf00456) EUT1
09155 -pf00923 -
09160 HMM00390_malic_1_eut1(pf00390pf03949pf01515) EUT1
09165 Hp_euts_violetPink EUT1
09170 HMM10662PduV_1_mix(pf10662) EUT1
09175 HMM06249EutQ_1_eut1(pf06249) EUT1 EP_NTERM
09180 HMM01923cobtr_2_eut1(pf01923) EUT1
09185 HMM01515PTA_B_1_eut1(pf01515) EUT1
09190 H_azure EUT1
09195 P_lightViolet EUT1
09200 HMMaldh_1_eutpdugrm(pf00171) EUT1 EP_NTERM
09205 HMM06723EutJMreB_1_grm EUT1
09210 HMM00465alcdh_3_mix(pf00465) EUT1
09215 HMMtpEuth_1_eut(pf04346) EUT1
09220 HMM06277EutA_1_eut(pf06277) EUT1
09225 HMMetlyEutB_1_EUT1(pf06751) EUT1
09230 HMMetlyEutC_1_eut1(pf05985) EUT1 EP_NTERM
09235 Tsp_rosa EUT1
09240 H_mulberry(pf00936pf16365) EUT1
09245 HMMreg12833_1_EUT1(pf12833) EUT1
09250 - -
09255 -pf01218 -
09260 -pf01520 -
09265 -pf00583 -
09270 -pf11143 -
09275 -pf06572 -
09280 -pf04261 -
09285 HMMnopfamclust3_1 RMM1
09290 - -
09295 -pf00528 -
09300 -pf00528 -
09305 -pf00005pf12857 -
BMC-P
BMC-H
BMC-T
Aldehyde dehydrogenase
PTAC
Signature enzyme
Alcohol dehydrogenase
Regulator

Salmonella enterica subsp. enterica serovar Fresno. Bacteria; Proteobacteria; Gammaproteobacteria; Enterobacterales;

EXB02__locus_2

EUT1

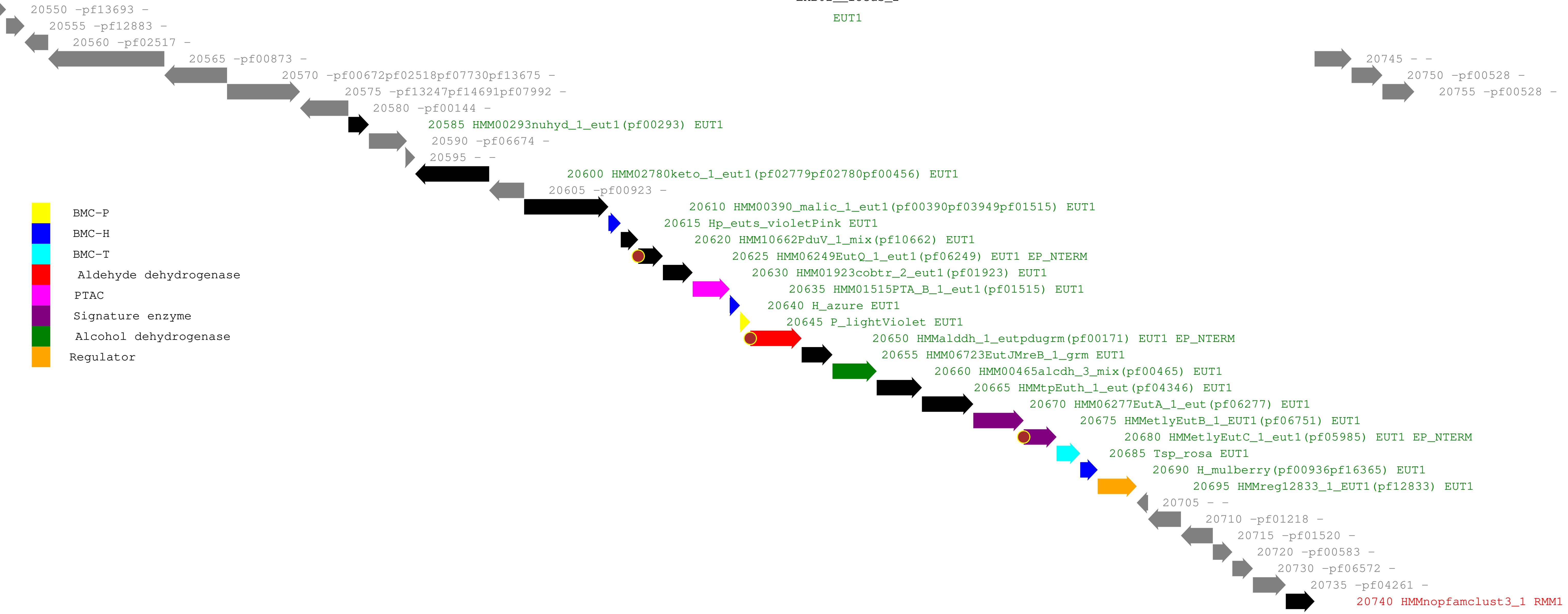

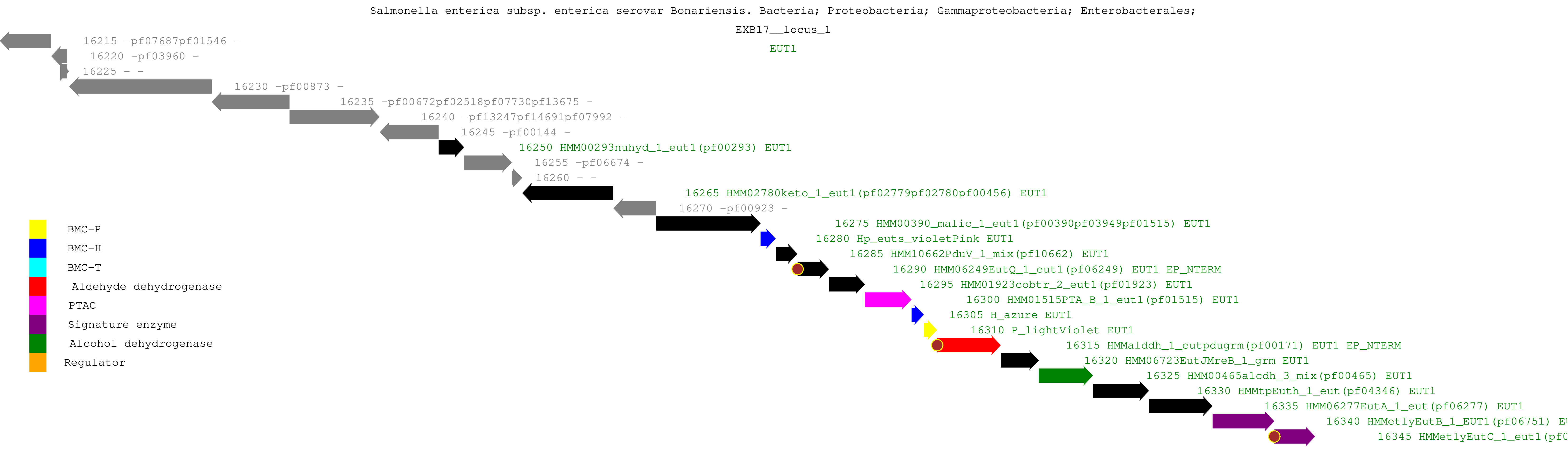

Salmonella enterica subsp. enterica serovar Bonariensis. Bacteria; Proteobacteria; Gammaproteobacteria; Enterobacterales;
EXB17__locus_1
EUT1
16215 -pf07687pf01546 -
16220 -pf03960 -
16225 - -
16230 -pf00873 -
16235 -pf00672pf02518pf07730pf13675 -
16240 -pf13247pf14691pf07992 -
16245 -pf00144 -
16250 HMM00293nuhyd_1_eut1(pf00293) EUT1
16255 -pf06674 -
16260 - -
16265 HMM02780keto_1_eut1(pf02779pf02780pf00456) EUT1
16270 -pf00923 -
16275 HMM00390_malic_1_eut1(pf00390pf03949pf01515) EUT1
16280 Hp_euts_violetPink EUT1
16285 HMM10662PduV_1_mix(pf10662) EUT1
16290 HMM06249EutQ_1_eut1(pf06249) EUT1 EP_NTERM
16295 HMM01923cobtr_2_eut1(pf01923) EUT1
16300 HMM01515PTA_B_1_eut1(pf01515) EUT1
16305 H_azure EUT1
16310 P_lightViolet EUT1
16315 HMMalddh_1_eutpdugrm(pf00171) EUT1 EP_NTERM
16320 HMM06723EutJMreB_1_grm EUT1
16325 HMM00465alcdh_3_mix(pf00465) EUT1
16330 HMMtpEuth_1_eut(pf04346) EUT1
16335 HMM06277EutA_1_eut(pf06277) EUT1
16340 HMMetlyEutB_1_EUT1(pf06751) EU
16345 HMMetlyEutC_1_eut1(pf0
BMC-P
BMC-H
BMC-T
Aldehyde dehydrogenase
PTAC
Signature enzyme
Alcohol dehydrogenase
Regulator

Salmonella enterica subsp. enterica serovar Napoli. Bacteria; Proteobacteria; Gammaproteobacteria; Enterobacterales;

EXB97__locus_1

EUT1

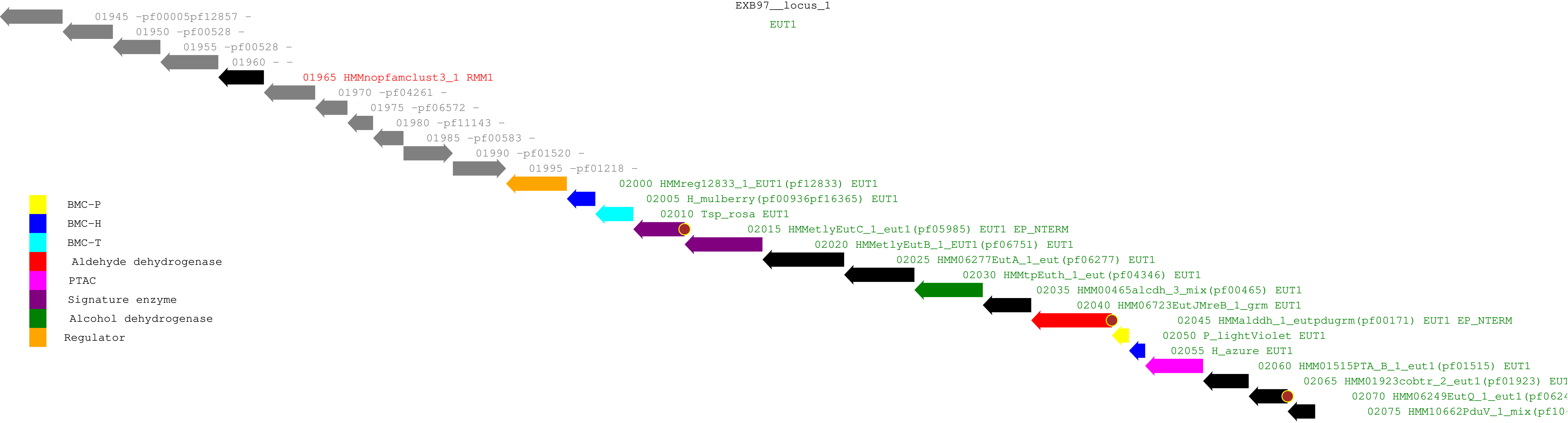

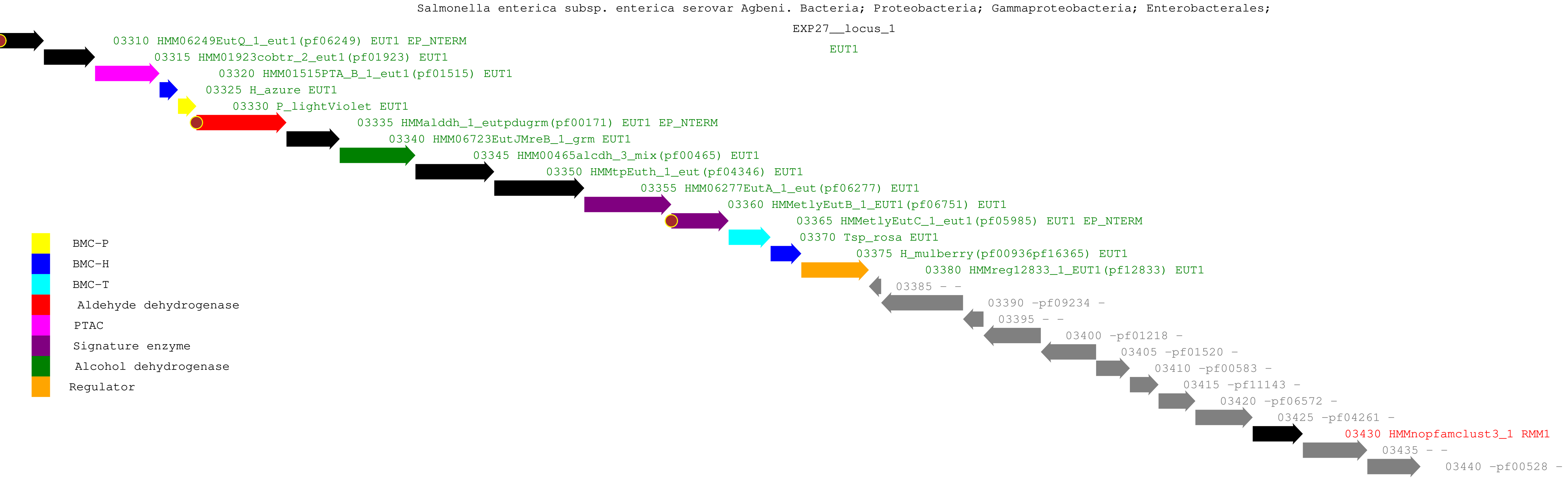
Salmonella enterica subsp. enterica serovar Agbeni. Bacteria; Proteobacteria; Gammaproteobacteria; Enterobacterales;
EXP27__locus_1
EUT1
03310 HMM06249EutQ_1_eut1(pf06249) EUT1 EP_NTERM
03315 HMM01923cobtr_2_eut1(pf01923) EUT1
03320 HMM01515PTA_B_1_eut1(pf01515) EUT1
03325 H_azure EUT1
03330 P_lightViolet EUT1
03335 HMMalddh_1_eutpdugrm(pf00171) EUT1 EP_NTERM
03340 HMM06723EutJMreB_1_grm EUT1
03345 HMM00465alcdh_3_mix(pf00465) EUT1
03350 HMMtpEuth_1_eut(pf04346) EUT1
03355 HMM06277EutA_1_eut(pf06277) EUT1
03360 HMMetlyEutB_1_EUT1(pf06751) EUT1
03365 HMMetlyEutC_1_eut1(pf05985) EUT1 EP_NTERM
03370 Tsp_rosa EUT1
03375 H_mulberry(pf00936pf16365) EUT1
03380 HMMreg12833_1_EUT1(pf12833) EUT1
03385 - -
03390 -pf09234 -
03395 - -
03400 -pf01218 -
03405 -pf01520 -
03410 -pf00583 -
03415 -pf11143 -
03420 -pf06572 -
03425 -pf04261 -
03430 HMMnopfamclust3_1 RMM1
03435 - -
03440 -pf00528 -
BMC-P
BMC-H
BMC-T
Aldehyde dehydrogenase
PTAC
Signature enzyme
Alcohol dehydrogenase
Regulator

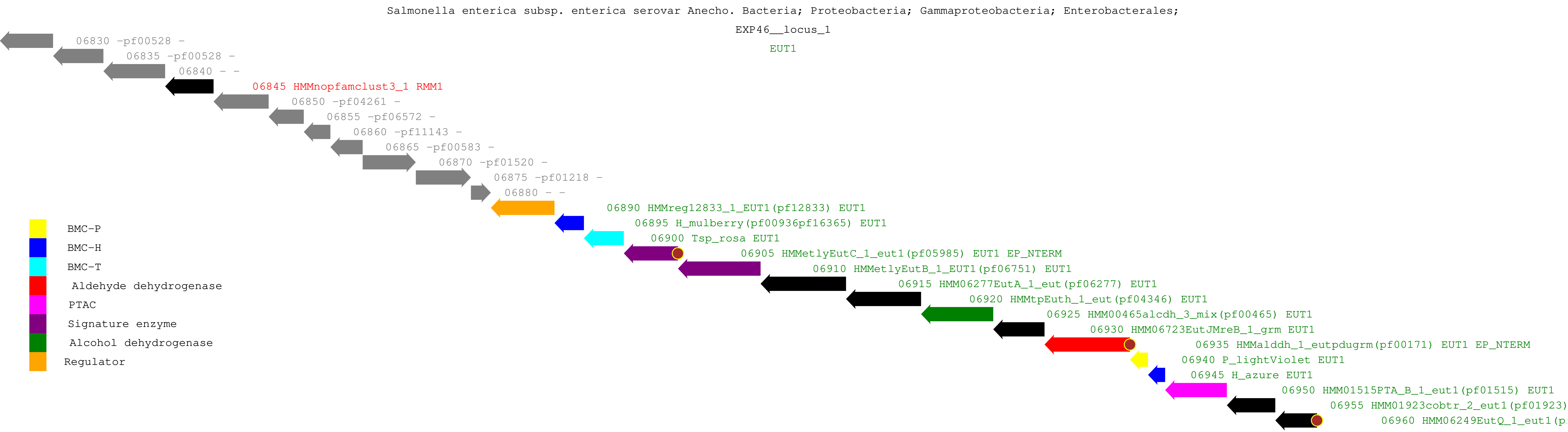

Salmonella enterica subsp. enterica serovar Anecho. Bacteria; Proteobacteria; Gammaproteobacteria; Enterobacterales;
EXP46__locus_1
EUT1
06830 -pf00528 -
06835 -pf00528 -
06840 - -
06845 HMMnopfamclust3_1 RMM1
06850 -pf04261 -
06855 -pf06572 -
06860 -pf11143 -
06865 -pf00583 -
06870 -pf01520 -
06875 -pf01218 -
06880 - -
06890 HMMreg12833_1_EUT1(pf12833) EUT1
06895 H_mulberry(pf00936pf16365) EUT1
06900 Tsp_rosa EUT1
06905 HMMetlyEutC_1_eut1(pf05985) EUT1 EP_NTERM
06910 HMMetlyEutB_1_EUT1(pf06751) EUT1
06915 HMM06277EutA_1_eut(pf06277) EUT1
06920 HMMtpEuth_1_eut(pf04346) EUT1
06925 HMM00465alcdh_3_mix(pf00465) EUT1
06930 HMM06723EutJMreB_1_grm EUT1
06935 HMMalddh_1_eutpdugrm(pf00171) EUT1 EP_NTERM
06940 P_lightViolet EUT1
06945 H_azure EUT1
06950 HMM01515PTA_B_1_eut1(pf01515) EUT1
06955 HMM01923cobtr_2_eut1(pf01923)
06960 HMM06249EutQ_1_eut1(p
BMC-P
BMC-H
BMC-T
Aldehyde dehydrogenase
PTAC
Signature enzyme
Alcohol dehydrogenase
Regulator

Salmonella enterica subsp. enterica serovar Adjame. Bacteria; Proteobacteria; Gammaproteobacteria; Enterobacterales;

EYA23__locus_1

EUT1

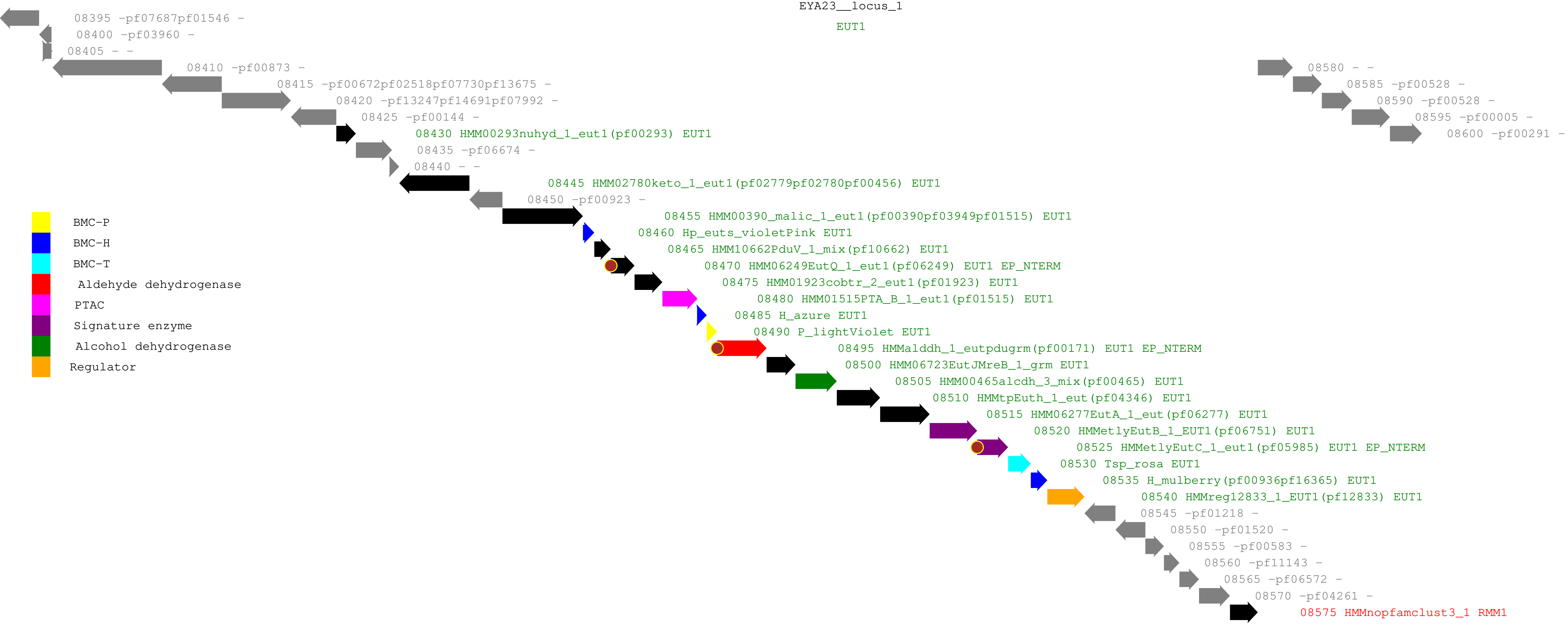

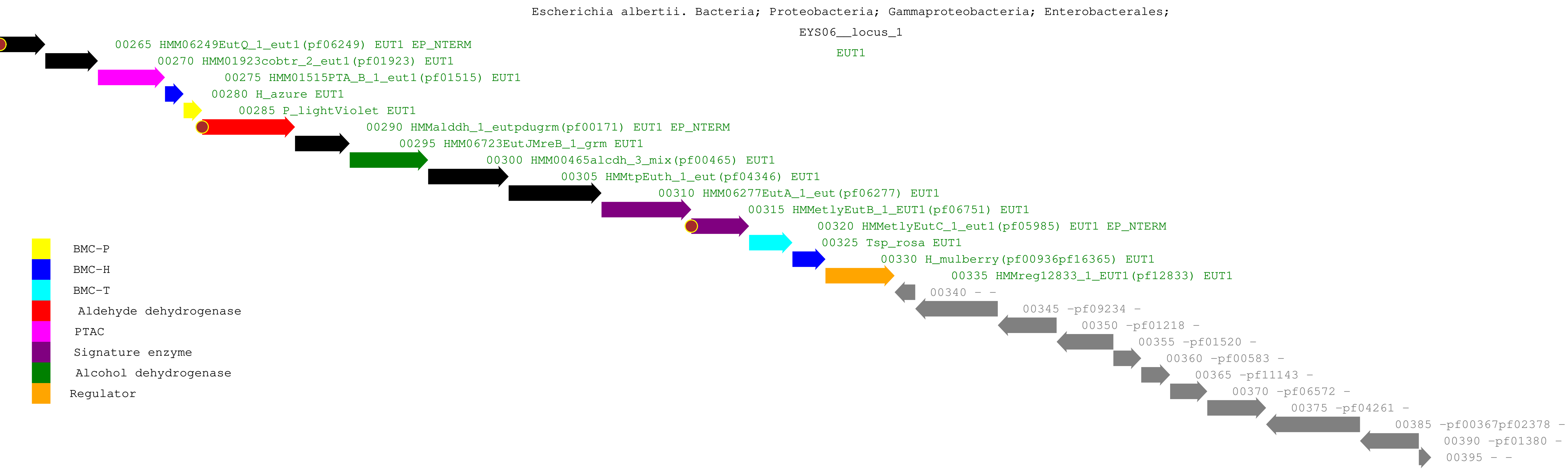
Escherichia albertii. Bacteria; Proteobacteria; Gammaproteobacteria; Enterobacterales;
EYS06__locus_1
EUT1
00265 HMM06249EutQ_1_eut1(pf06249) EUT1 EP_NTERM
00270 HMM01923cobtr_2_eut1(pf01923) EUT1
00275 HMM01515PTA_B_1_eut1(pf01515) EUT1
00280 H_azure EUT1
00285 P_lightViolet EUT1
00290 HMMalddh_1_eutpdugrm(pf00171) EUT1 EP_NTERM
00295 HMM06723EutJMreB_1_grm EUT1
00300 HMM00465alcdh_3_mix(pf00465) EUT1
00305 HMMtpEuth_1_eut(pf04346) EUT1
00310 HMM06277EutA_1_eut(pf06277) EUT1
00315 HMMetlyEutB_1_EUT1(pf06751) EUT1
00320 HMMetlyEutC_1_eut1(pf05985) EUT1 EP_NTERM
00325 Tsp_rosa EUT1
00330 H_mulberry(pf00936pf16365) EUT1
00335 HMMreg12833_1_EUT1(pf12833) EUT1
00340 - -
00345 -pf09234 -
00350 -pf01218 -
00355 -pf01520 -
00360 -pf00583 -
00365 -pf11143 -
00370 -pf06572 -
00375 -pf04261 -
00385 -pf00367pf02378 -
00390 -pf01380 -
00395 - -
BMC-P
BMC-H
BMC-T
Aldehyde dehydrogenase
PTAC
Signature enzyme
Alcohol dehydrogenase
Regulator

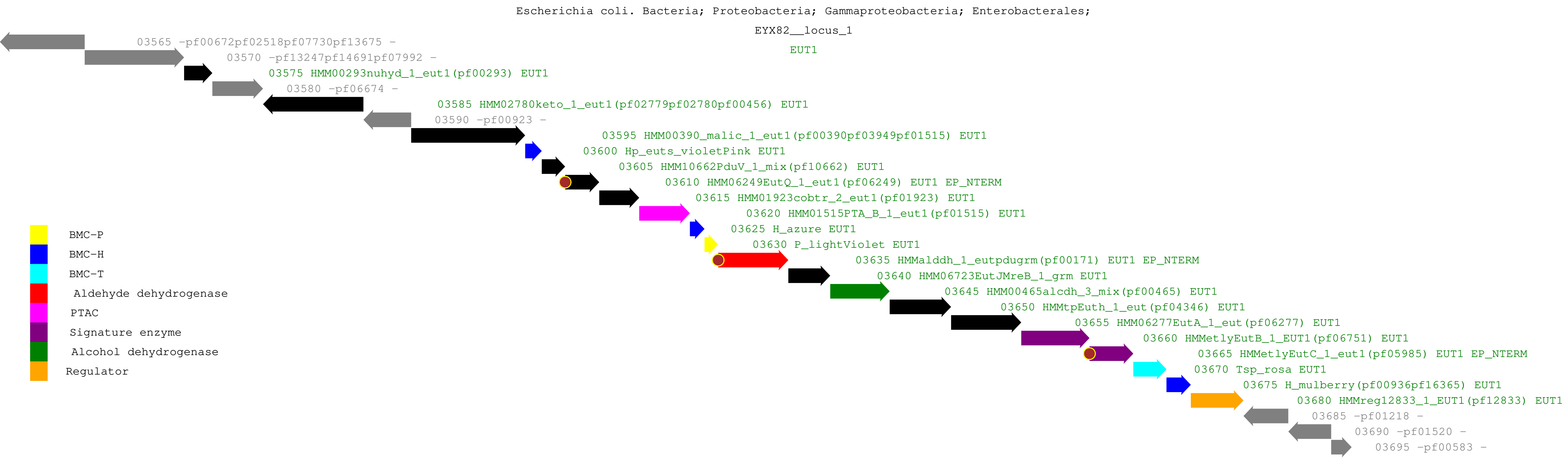
Escherichia coli. Bacteria; Proteobacteria; Gammaproteobacteria; Enterobacterales;
EYX82__locus_1
EUT1
03565 -pf00672pf02518pf07730pf13675 -
03570 -pf13247pf14691pf07992 -
03575 HMM00293nuhyd_1_eut1(pf00293) EUT1
03580 -pf06674 -
03585 HMM02780keto_1_eut1(pf02779pf02780pf00456) EUT1
03590 -pf00923 -
03595 HMM00390_malic_1_eut1(pf00390pf03949pf01515) EUT1
03600 Hp_euts_violetPink EUT1
03605 HMM10662PduV_1_mix(pf10662) EUT1
03610 HMM06249EutQ_1_eut1(pf06249) EUT1 EP_NTERM
03615 HMM01923cobtr_2_eut1(pf01923) EUT1
03620 HMM01515PTA_B_1_eut1(pf01515) EUT1
03625 H_azure EUT1
03630 P_lightViolet EUT1
03635 HMMalddh_1_eutpdugrm(pf00171) EUT1 EP_NTERM
03640 HMM06723EutJMreB_1_grm EUT1
03645 HMM00465alcdh_3_mix(pf00465) EUT1
03650 HMMtpEuth_1_eut(pf04346) EUT1
03655 HMM06277EutA_1_eut(pf06277) EUT1
03660 HMMetlyEutB_1_EUT1(pf06751) EUT1
03665 HMMetlyEutC_1_eut1(pf05985) EUT1 EP_NTERM
03670 Tsp_rosa EUT1
03675 H_mulberry(pf00936pf16365) EUT1
03680 HMMreg12833_1_EUT1(pf12833) EUT1
03685 -pf01218 -
03690 -pf01520 -
03695 -pf00583 -
BMC-P
BMC-H
BMC-T
Aldehyde dehydrogenase
PTAC
Signature enzyme
Alcohol dehydrogenase
Regulator

Obesumbacterium proteus. Bacteria; Proteobacteria; Gammaproteobacteria; Enterobacterales;

EYY98__locus_1

EUT1

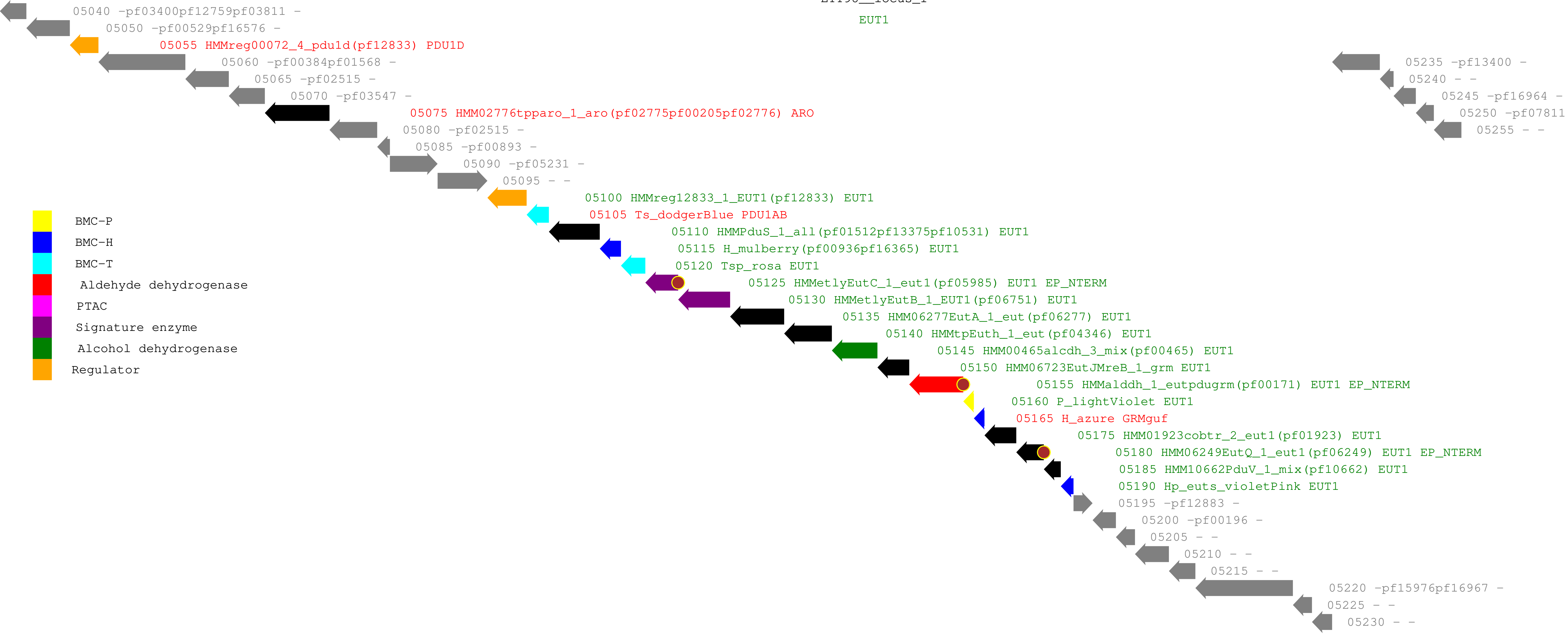

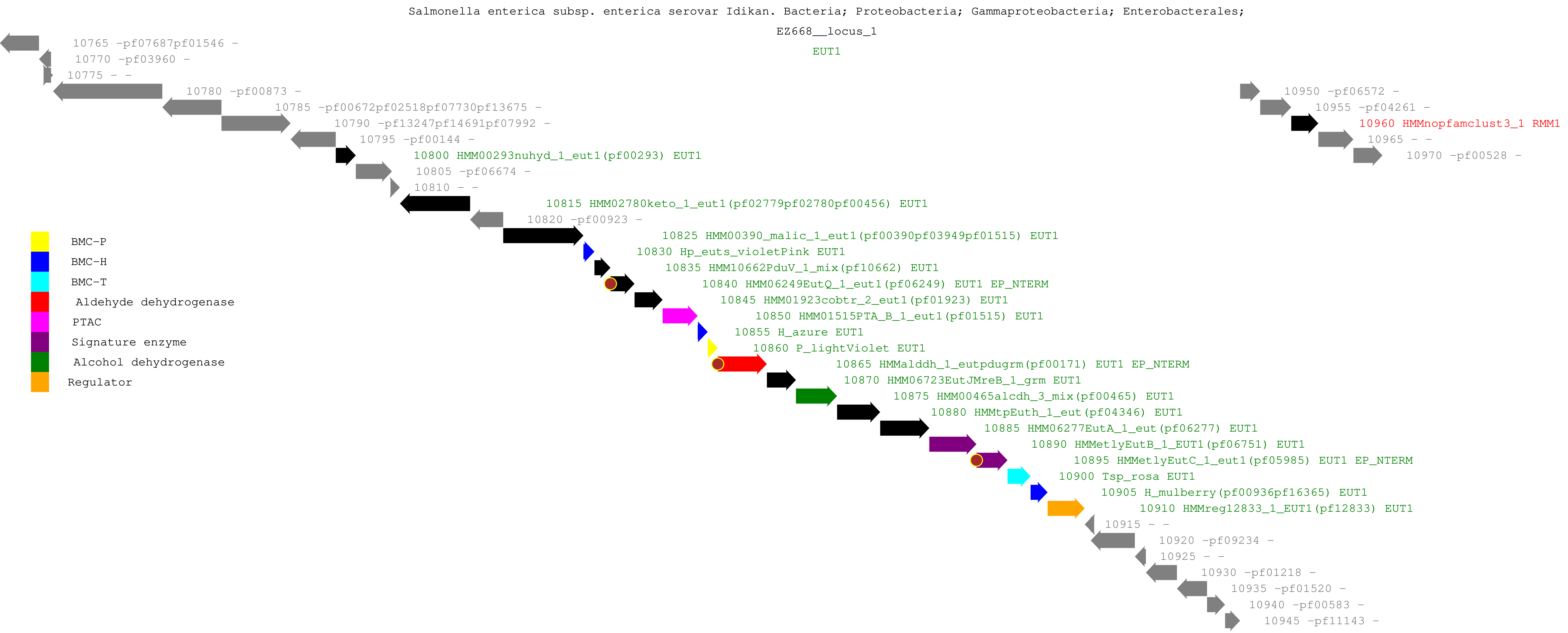

Salmonella enterica subsp. enterica serovar Idikan. Bacteria; Proteobacteria; Gammaproteobacteria; Enterobacterales;
EZ668__locus_1
EUT1
10765 -pf07687pf01546 -
10770 -pf03960 -
10775 - -
10780 -pf00873 -
10785 -pf00672pf02518pf07730pf13675 -
10790 -pf13247pf14691pf07992 -
10795 -pf00144 -
10800 HMM00293nuhyd_1_eut1(pf00293) EUT1
10805 -pf06674 -
10810 - -
10815 HMM02780keto_1_eut1(pf02779pf02780pf00456) EUT1
10820 -pf00923 -
10825 HMM00390_malic_1_eut1(pf00390pf03949pf01515) EUT1
10830 Hp_euts_violetPink EUT1
10835 HMM10662PduV_1_mix(pf10662) EUT1
10840 HMM06249EutQ_1_eut1(pf06249) EUT1 EP_NTERM
10845 HMM01923cobtr_2_eut1(pf01923) EUT1
10850 HMM01515PTA_B_1_eut1(pf01515) EUT1
10855 H_azure EUT1
10860 P_lightViolet EUT1
10865 HMMalddh_1_eutpdugrm(pf00171) EUT1 EP_NTERM
10870 HMM06723EutJMreB_1_grm EUT1
10875 HMM00465alcdh_3_mix(pf00465) EUT1
10880 HMMtpEuth_1_eut(pf04346) EUT1
10885 HMM06277EutA_1_eut(pf06277) EUT1
10890 HMMetlyEutB_1_EUT1(pf06751) EUT1
10895 HMMetlyEutC_1_eut1(pf05985) EUT1 EP_NTERM
10900 Tsp_rosa EUT1
10905 H_mulberry(pf00936pf16365) EUT1
10910 HMMreg12833_1_EUT1(pf12833) EUT1
10915 - -
10920 -pf09234 -
10925 - -
10930 -pf01218 -
10935 -pf01520 -
10940 -pf00583 -
10945 -pf11143 -
10950 -pf06572 -
10955 -pf04261 -
10960 HMMnopfamclust3_1 RMM1
10965 - -
10970 -pf00528 -
BMC-P
BMC-H
BMC-T
Aldehyde dehydrogenase
PTAC
Signature enzyme
Alcohol dehydrogenase
Regulator

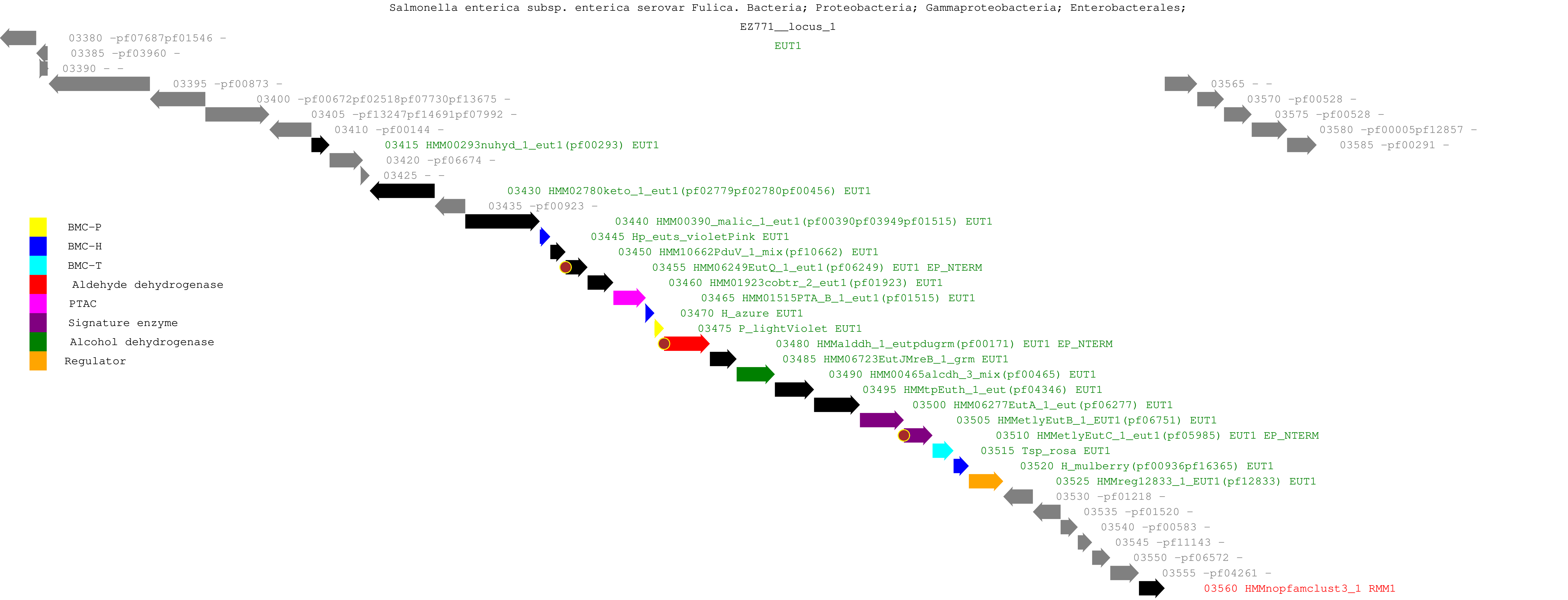
Salmonella enterica subsp. enterica serovar Fulica. Bacteria; Proteobacteria; Gammaproteobacteria; Enterobacterales;
EZ771__locus_1
EUT1
03380 -pf07687pf01546 -
03385 -pf03960 -
03390 - -
03395 -pf00873 -
03400 -pf00672pf02518pf07730pf13675 -
03405 -pf13247pf14691pf07992 -
03410 -pf00144 -
03415 HMM00293nuhyd_1_eut1(pf00293) EUT1
03420 -pf06674 -
03425 - -
03430 HMM02780keto_1_eut1(pf02779pf02780pf00456) EUT1
03435 -pf00923 -
03440 HMM00390_malic_1_eut1(pf00390pf03949pf01515) EUT1
03445 Hp_euts_violetPink EUT1
03450 HMM10662PduV_1_mix(pf10662) EUT1
03455 HMM06249EutQ_1_eut1(pf06249) EUT1 EP_NTERM
03460 HMM01923cobtr_2_eut1(pf01923) EUT1
03465 HMM01515PTA_B_1_eut1(pf01515) EUT1
03470 H_azure EUT1
03475 P_lightViolet EUT1
03480 HMMalddh_1_eutpdugrm(pf00171) EUT1 EP_NTERM
03485 HMM06723EutJMreB_1_grm EUT1
03490 HMM00465alcdh_3_mix(pf00465) EUT1
03495 HMMtpEuth_1_eut(pf04346) EUT1
03500 HMM06277EutA_1_eut(pf06277) EUT1
03505 HMMetlyEutB_1_EUT1(pf06751) EUT1
03510 HMMetlyEutC_1_eut1(pf05985) EUT1 EP_NTERM
03515 Tsp_rosa EUT1
03520 H_mulberry(pf00936pf16365) EUT1
03525 HMMreg12833_1_EUT1(pf12833) EUT1
03530 -pf01218 -
03535 -pf01520 -
03540 -pf00583 -
03545 -pf11143 -
03550 -pf06572 -
03555 -pf04261 -
03560 HMMnopfamclust3_1 RMM1
03565 - -
03570 -pf00528 -
03575 -pf00528 -
03580 -pf00005pf12857 -
03585 -pf00291 -
BMC-P
BMC-H
BMC-T
Aldehyde dehydrogenase
PTAC
Signature enzyme
Alcohol dehydrogenase
Regulator

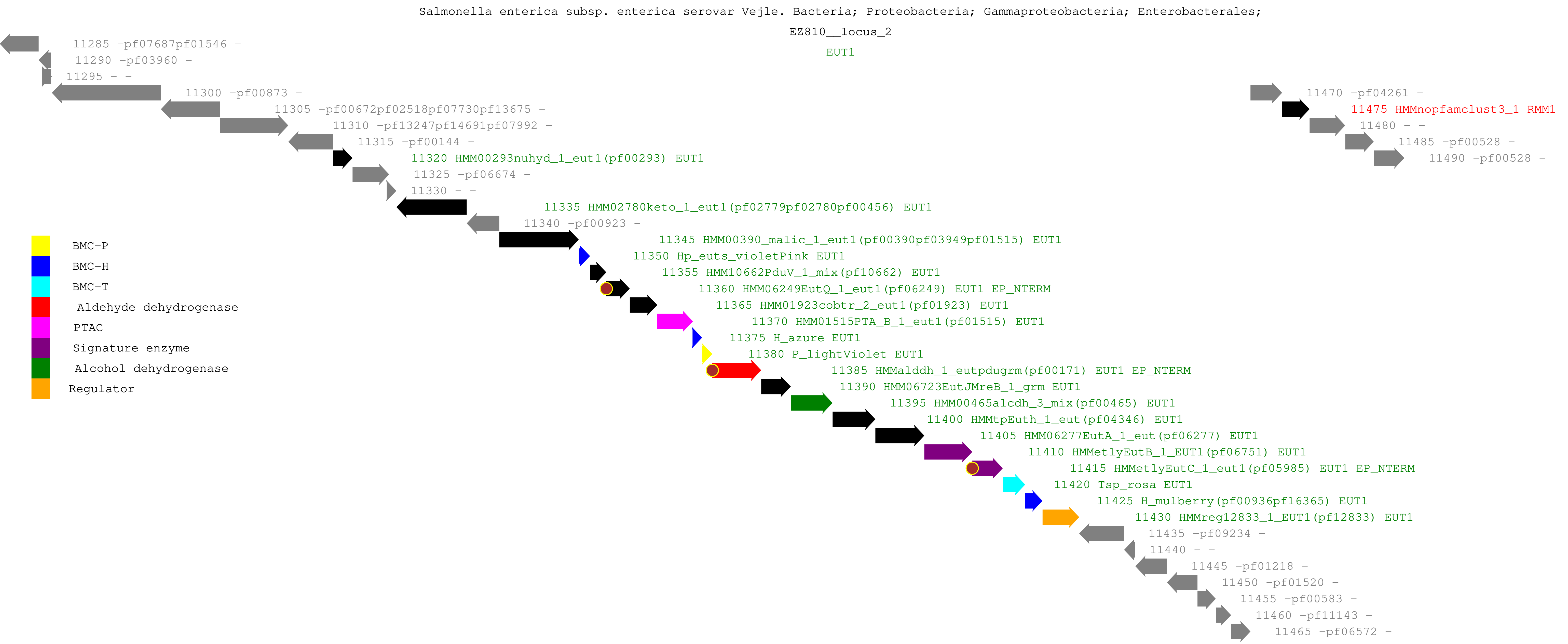
Salmonella enterica subsp. enterica serovar Vejle. Bacteria; Proteobacteria; Gammaproteobacteria; Enterobacterales;
EZ810__locus_2
EUT1
11285 -pf07687pf01546 -
11290 -pf03960 -
11295 - -
11300 -pf00873 -
11305 -pf00672pf02518pf07730pf13675 -
11310 -pf13247pf14691pf07992 -
11315 -pf00144 -
11320 HMM00293nuhyd_1_eut1(pf00293) EUT1
11325 -pf06674 -
11330 - -
11335 HMM02780keto_1_eut1(pf02779pf02780pf00456) EUT1
11340 -pf00923 -
11345 HMM00390_malic_1_eut1(pf00390pf03949pf01515) EUT1
11350 Hp_euts_violetPink EUT1
11355 HMM10662PduV_1_mix(pf10662) EUT1
11360 HMM06249EutQ_1_eut1(pf06249) EUT1 EP_NTERM
11365 HMM01923cobtr_2_eut1(pf01923) EUT1
11370 HMM01515PTA_B_1_eut1(pf01515) EUT1
11375 H_azure EUT1
11380 P_lightViolet EUT1
11385 HMMalddh_1_eutpdugrm(pf00171) EUT1 EP_NTERM
11390 HMM06723EutJMreB_1_grm EUT1
11395 HMM00465alcdh_3_mix(pf00465) EUT1
11400 HMMtpEuth_1_eut(pf04346) EUT1
11405 HMM06277EutA_1_eut(pf06277) EUT1
11410 HMMetlyEutB_1_EUT1(pf06751) EUT1
11415 HMMetlyEutC_1_eut1(pf05985) EUT1 EP_NTERM
11420 Tsp_rosa EUT1
11425 H_mulberry(pf00936pf16365) EUT1
11430 HMMreg12833_1_EUT1(pf12833) EUT1
11435 -pf09234 -
11440 - -
11445 -pf01218 -
11450 -pf01520 -
11455 -pf00583 -
11460 -pf11143 -
11465 -pf06572 -
11470 -pf04261 -
11475 HMMnopfamclust3_1 RMM1
11480 - -
11485 -pf00528 -
11490 -pf00528 -
BMC-P
BMC-H
BMC-T
Aldehyde dehydrogenase
PTAC
Signature enzyme
Alcohol dehydrogenase
Regulator

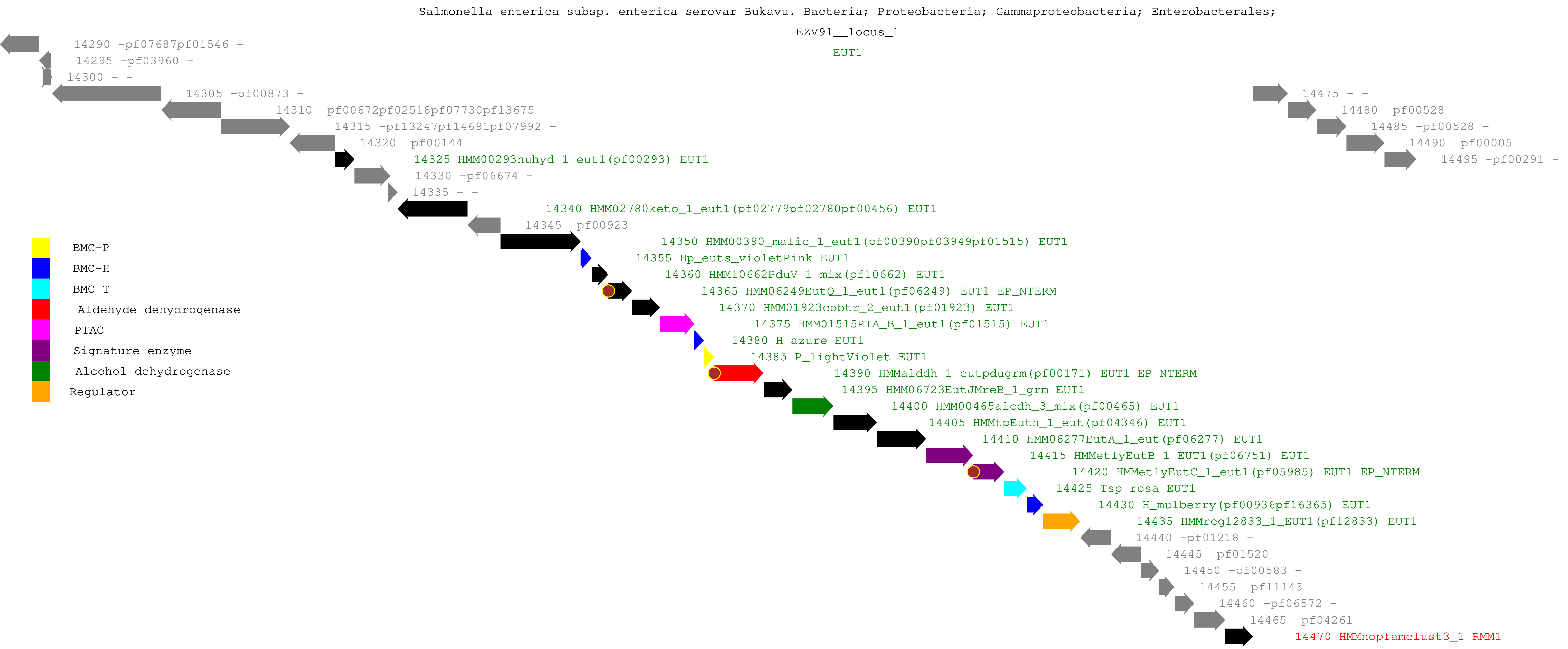
Salmonella enterica subsp. enterica serovar Bukavu. Bacteria; Proteobacteria; Gammaproteobacteria; Enterobacterales;
EZV91__locus_1
EUT1
14290 -pf07687pf01546 -
14295 -pf03960 -
14300 - -
14305 -pf00873 -
14310 -pf00672pf02518pf07730pf13675 -
14315 -pf13247pf14691pf07992 -
14320 -pf00144 -
14325 HMM00293nuhyd_1_eut1(pf00293) EUT1
14330 -pf06674 -
14335 - -
14340 HMM02780keto_1_eut1(pf02779pf02780pf00456) EUT1
14345 -pf00923 -
14350 HMM00390_malic_1_eut1(pf00390pf03949pf01515) EUT1
14355 Hp_euts_violetPink EUT1
14360 HMM10662PduV_1_mix(pf10662) EUT1
14365 HMM06249EutQ_1_eut1(pf06249) EUT1 EP_NTERM
14370 HMM01923cobtr_2_eut1(pf01923) EUT1
14375 HMM01515PTA_B_1_eut1(pf01515) EUT1
14380 H_azure EUT1
14385 P_lightViolet EUT1
14390 HMMalddh_1_eutpdugrm(pf00171) EUT1 EP_NTERM
14395 HMM06723EutJMreB_1_grm EUT1
14400 HMM00465alcdh_3_mix(pf00465) EUT1
14405 HMMtpEuth_1_eut(pf04346) EUT1
14410 HMM06277EutA_1_eut(pf06277) EUT1
14415 HMMetlyEutB_1_EUT1(pf06751) EUT1
14420 HMMetlyEutC_1_eut1(pf05985) EUT1 EP_NTERM
14425 Tsp_rosa EUT1
14430 H_mulberry(pf00936pf16365) EUT1
14435 HMMreg12833_1_EUT1(pf12833) EUT1
14440 -pf01218 -
14445 -pf01520 -
14450 -pf00583 -
14455 -pf11143 -
14460 -pf06572 -
14465 -pf04261 -
14470 HMMnopfamclust3_1 RMM1
14475 - -
14480 -pf00528 -
14485 -pf00528 -
14490 -pf00005 -
14495 -pf00291 -
BMC-P
BMC-H
BMC-T
Aldehyde dehydrogenase
PTAC
Signature enzyme
Alcohol dehydrogenase
Regulator

Salmonella oranienberg. Bacteria; Proteobacteria; Gammaproteobacteria; Enterobacterales;

EZX63__locus_1

EUT1

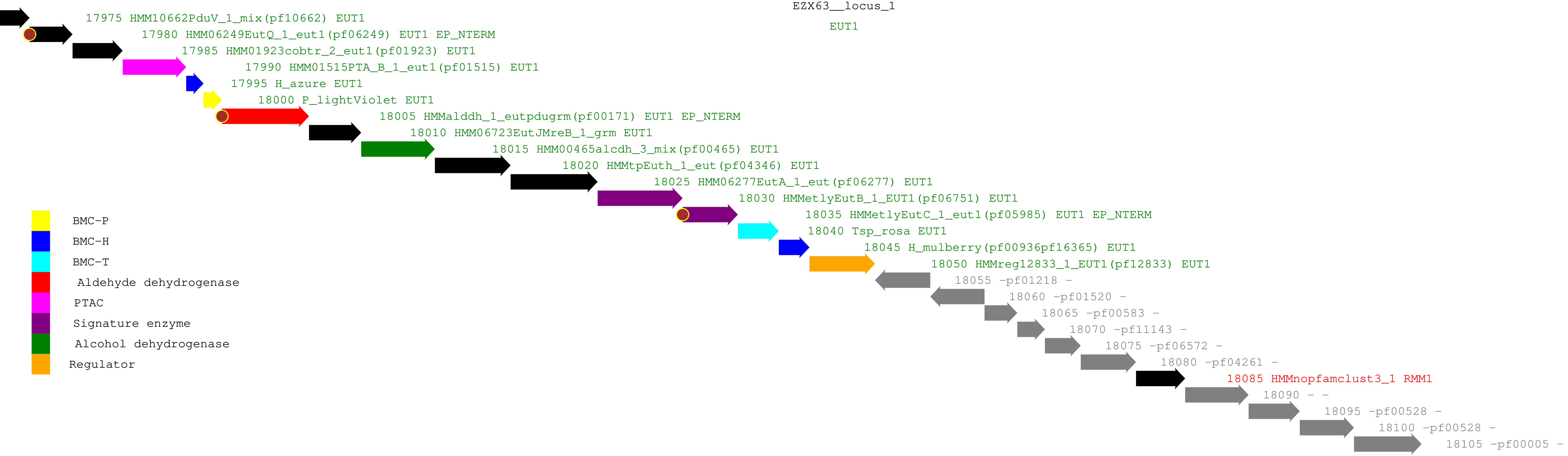

Citrobacter portucalensis. Bacteria; Proteobacteria; Gammaproteobacteria; Enterobacterales;

F0328__locus_2

EUT1

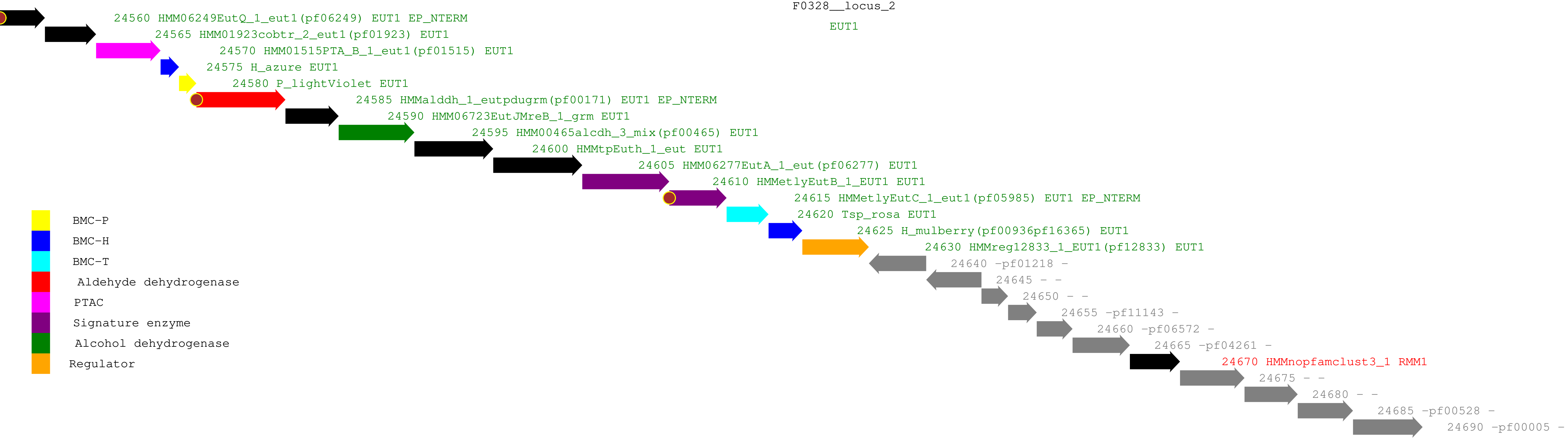

Citrobacter werkmanii. Bacteria; Proteobacteria; Gammaproteobacteria; Enterobacterales;

F0329__locus_2

EUT1

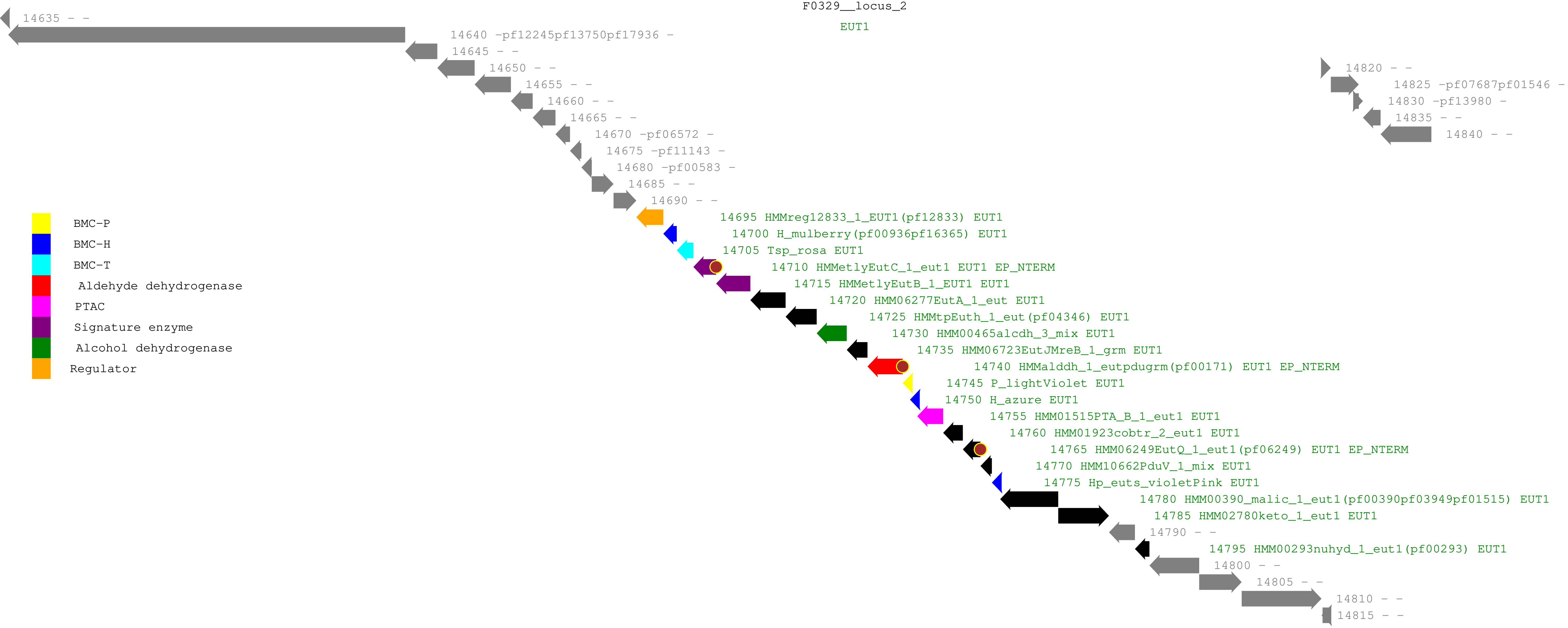

Pseudocitrobacter sp. 73. Bacteria; Proteobacteria; Gammaproteobacteria; Enterobacterales;

F0Q32__locus_1

EUT1

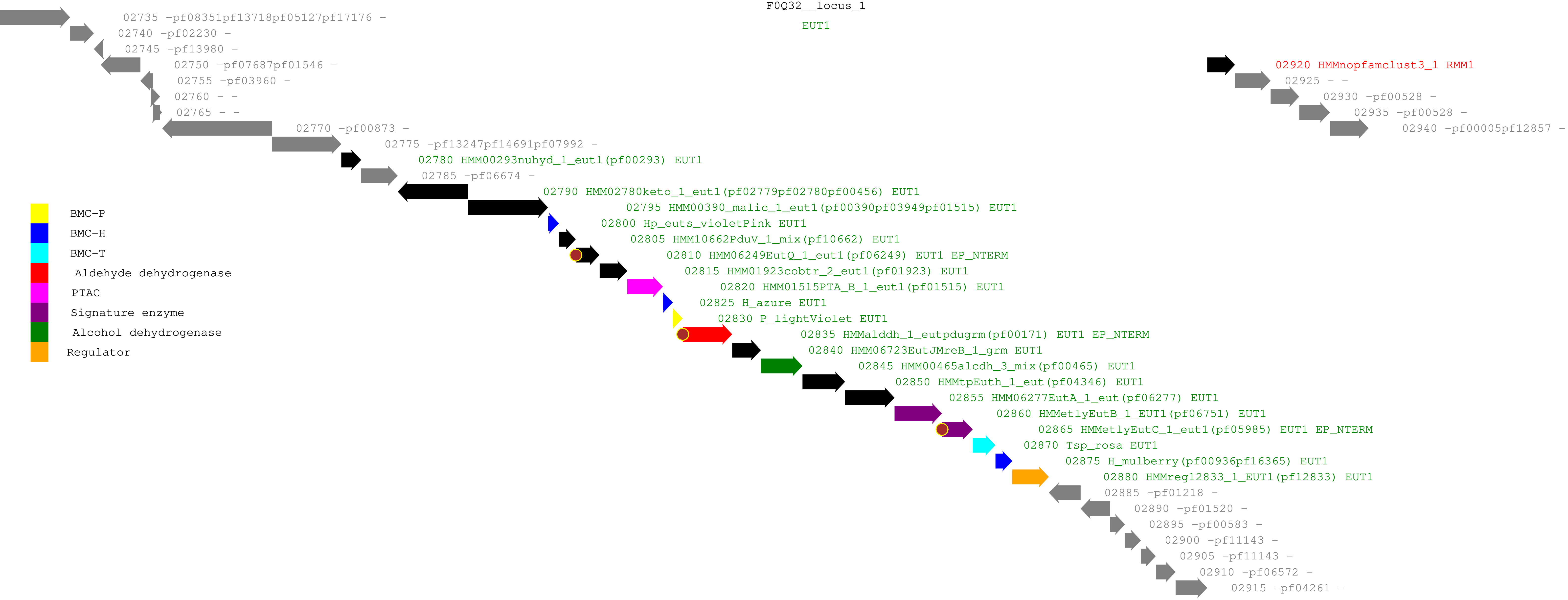

Escherichia coli. Bacteria; Proteobacteria; Gammaproteobacteria; Enterobacterales;

F1E03__locus_1

EUT1

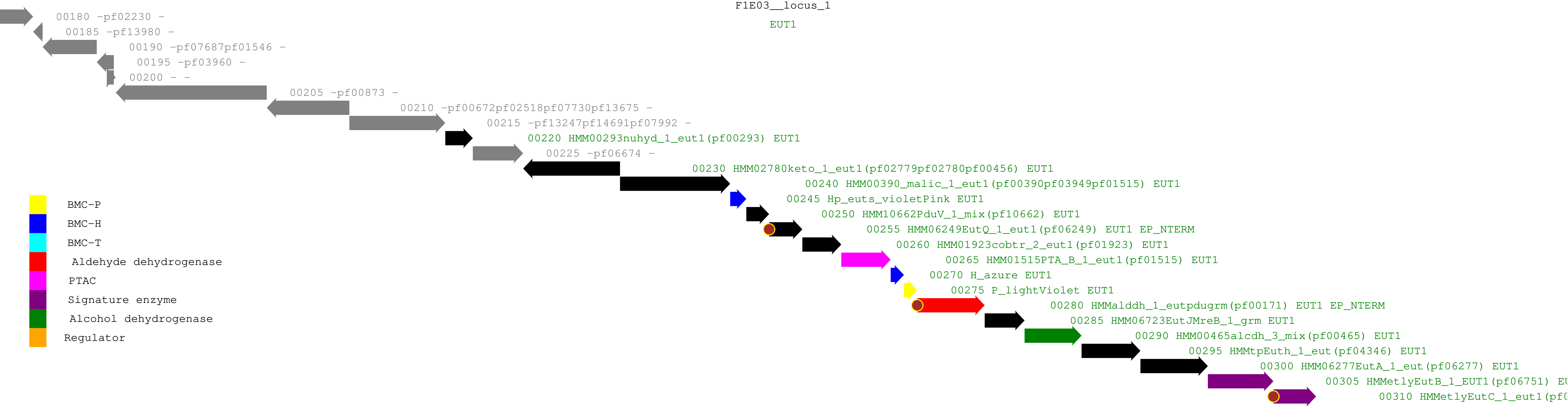

Citrobacter amalonaticus Y19. Bacteria; Proteobacteria; Gammaproteobacteria; Enterobacterales;

F384__locus_2

EUT1

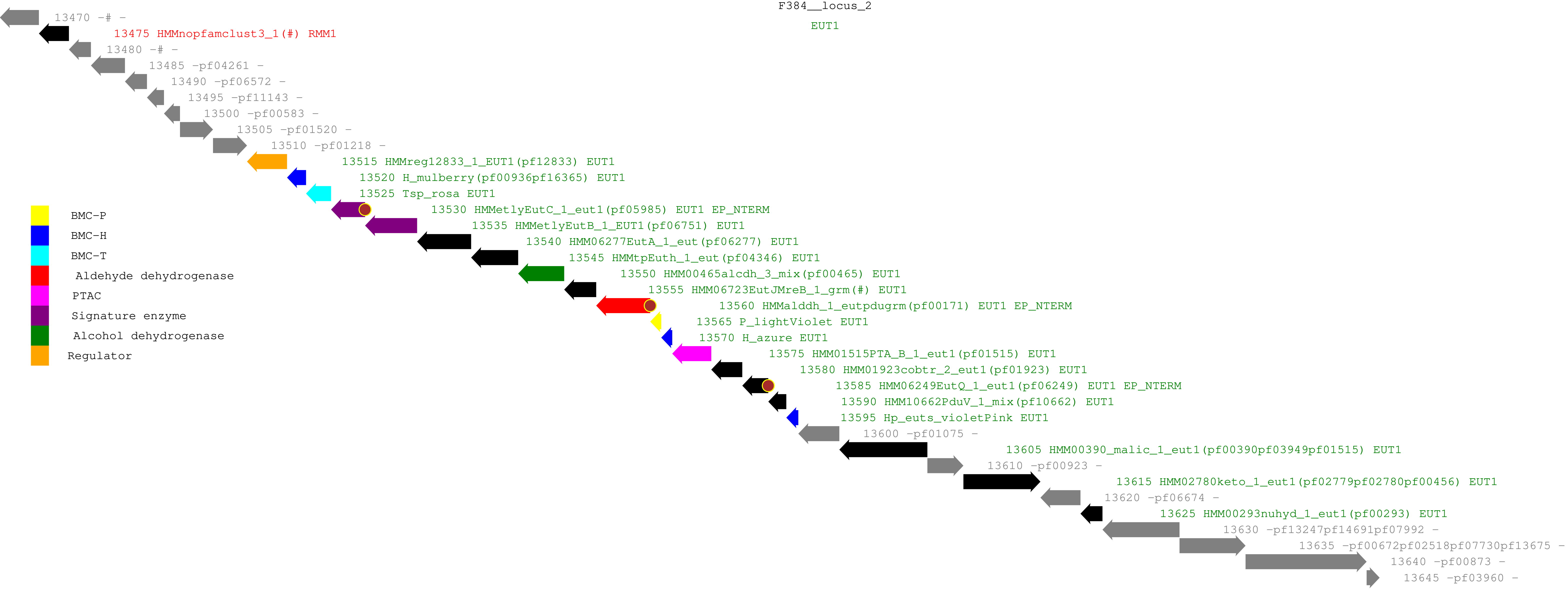

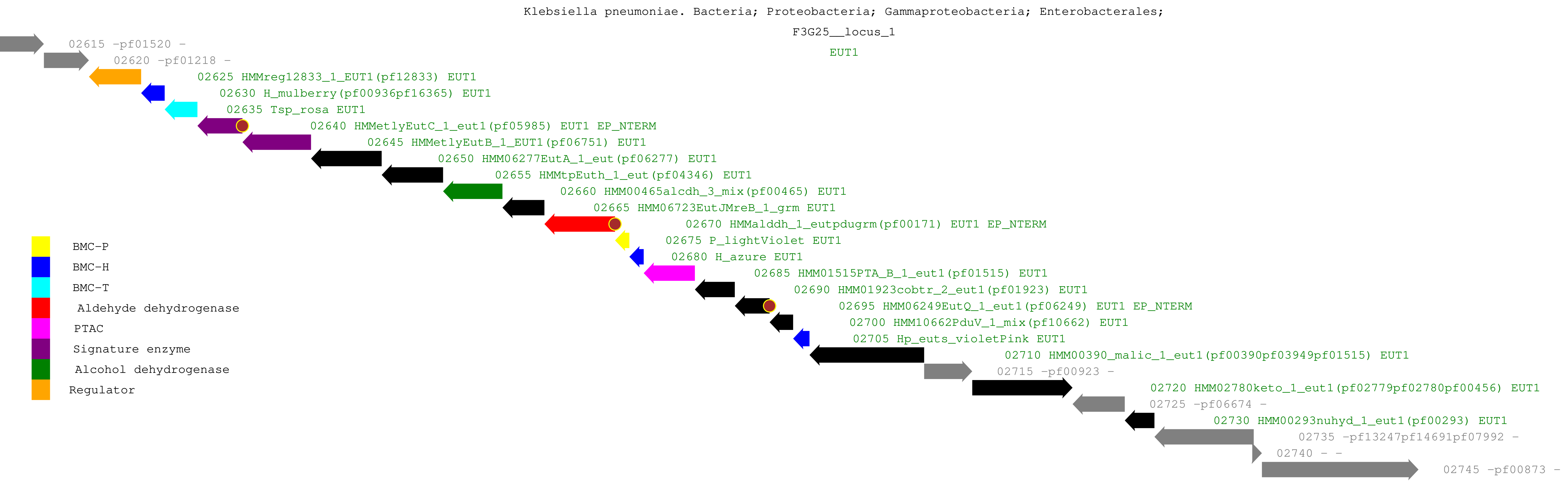
Klebsiella pneumoniae. Bacteria; Proteobacteria; Gammaproteobacteria; Enterobacterales;
F3G25__locus_1
EUT1
02615 -pf01520 -
02620 -pf01218 -
02625 HMMreg12833_1_EUT1(pf12833) EUT1
02630 H_mulberry(pf00936pf16365) EUT1
02635 Tsp_rosa EUT1
02640 HMMetlyEutC_1_eut1(pf05985) EUT1 EP_NTERM
02645 HMMetlyEutB_1_EUT1(pf06751) EUT1
02650 HMM06277EutA_1_eut(pf06277) EUT1
02655 HMMtpEuth_1_eut(pf04346) EUT1
02660 HMM00465alcdh_3_mix(pf00465) EUT1
02665 HMM06723EutJMreB_1_grm EUT1
02670 HMMaldh_1_eutpdugrm(pf00171) EUT1 EP_NTERM
02675 P_lightViolet EUT1
02680 H_azure EUT1
02685 HMM01515PTA_B_1_eut1(pf01515) EUT1
02690 HMM01923cobtr_2_eut1(pf01923) EUT1
02695 HMM06249EutQ_1_eut1(pf06249) EUT1 EP_NTERM
02700 HMM10662PduV_1_mix(pf10662) EUT1
02705 Hp_euts_violetPink EUT1
02710 HMM00390_malic_1_eut1(pf00390pf03949pf01515) EUT1
02715 -pf00923 -
02720 HMM02780keto_1_eut1(pf02779pf02780pf00456) EUT1
02725 -pf06674 -
02730 HMM00293nuhyd_1_eut1(pf00293) EUT1
02735 -pf13247pf14691pf07992 -
02740 - -
02745 -pf00873 -
BMC-P
BMC-H
BMC-T
Aldehyde dehydrogenase
PTAC
Signature enzyme
Alcohol dehydrogenase
Regulator

Salmonella enterica subsp. enterica serovar Eastbourne. Bacteria; Proteobacteria; Gammaproteobacteria; Enterobacterales;

F7754__locus_2

EUT1

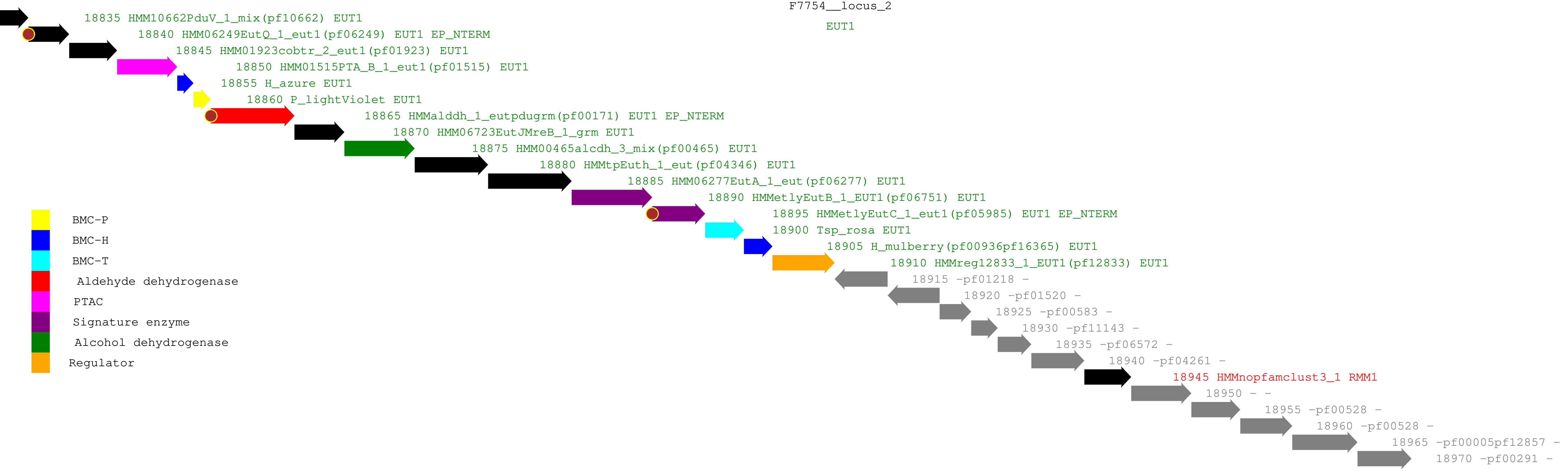

Salmonella enterica subsp. enterica serovar Nchanga. Bacteria; Proteobacteria; Gammaproteobacteria; Enterobacterales;

FA839__locus_2

EUT1

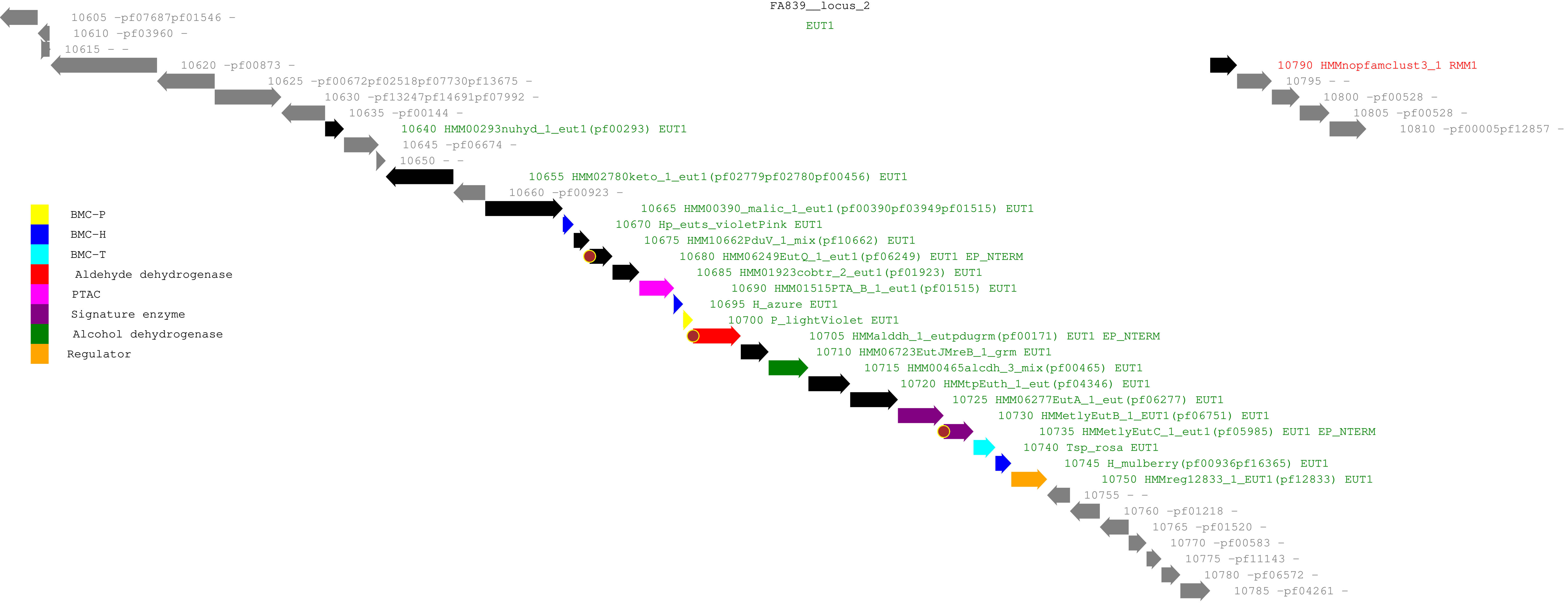

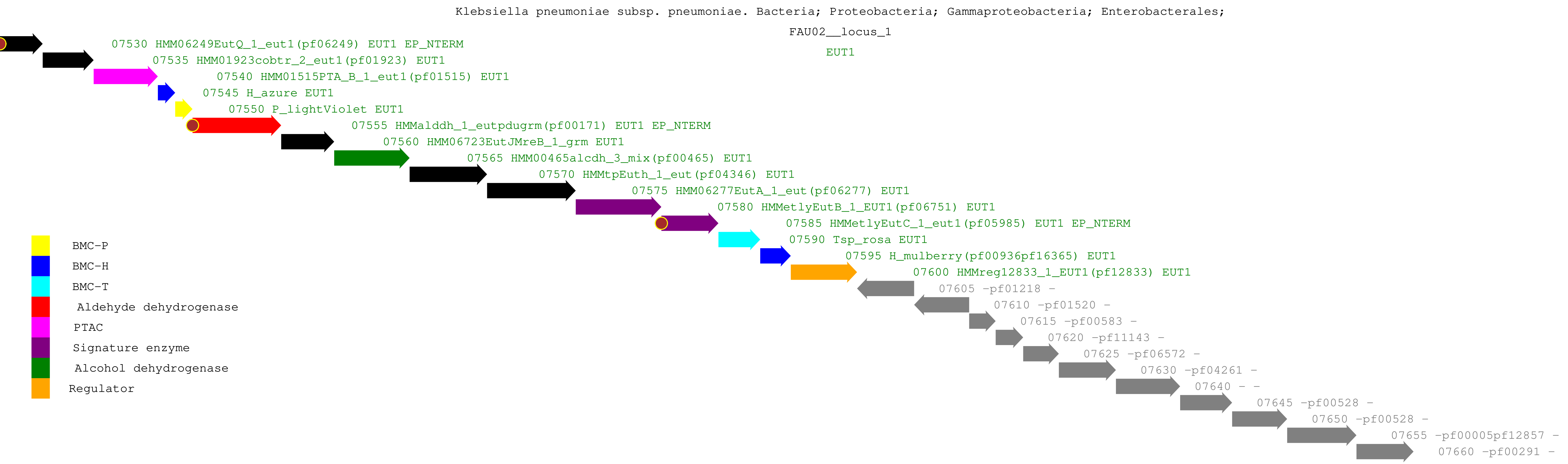
Klebsiella pneumoniae subsp. pneumoniae. Bacteria; Proteobacteria; Gammaproteobacteria; Enterobacterales;
FAU02__locus_1
EUT1
07530 HMM06249EutQ_1_eut1(pf06249) EUT1 EP_NTERM
07535 HMM01923cobtr_2_eut1(pf01923) EUT1
07540 HMM01515PTA_B_1_eut1(pf01515) EUT1
07545 H_azure EUT1
07550 P_lightViolet EUT1
07555 HMMalddh_1_eutpdugrm(pf00171) EUT1 EP_NTERM
07560 HMM06723EutJMreB_1_grm EUT1
07565 HMM00465alcdh_3_mix(pf00465) EUT1
07570 HMMtpEuth_1_eut(pf04346) EUT1
07575 HMM06277EutA_1_eut(pf06277) EUT1
07580 HMMetlyEutB_1_EUT1(pf06751) EUT1
07585 HMMetlyEutC_1_eut1(pf05985) EUT1 EP_NTERM
07590 Tsp_rosa EUT1
07595 H_mulberry(pf00936pf16365) EUT1
07600 HMMreg12833_1_EUT1(pf12833) EUT1
07605 -pf01218 -
07610 -pf01520 -
07615 -pf00583 -
07620 -pf11143 -
07625 -pf06572 -
07630 -pf04261 -
07640 - -
07645 -pf00528 -
07650 -pf00528 -
07655 -pf00005pf12857 -
07660 -pf00291 -
BMC-P
BMC-H
BMC-T
Aldehyde dehydrogenase
PTAC
Signature enzyme
Alcohol dehydrogenase
Regulator

Escherichia coli (strain K12). Bacteria; Proteobacteria; Gammaproteobacteria; Enterobacterales;

FAZ83__locus_1

EUT1

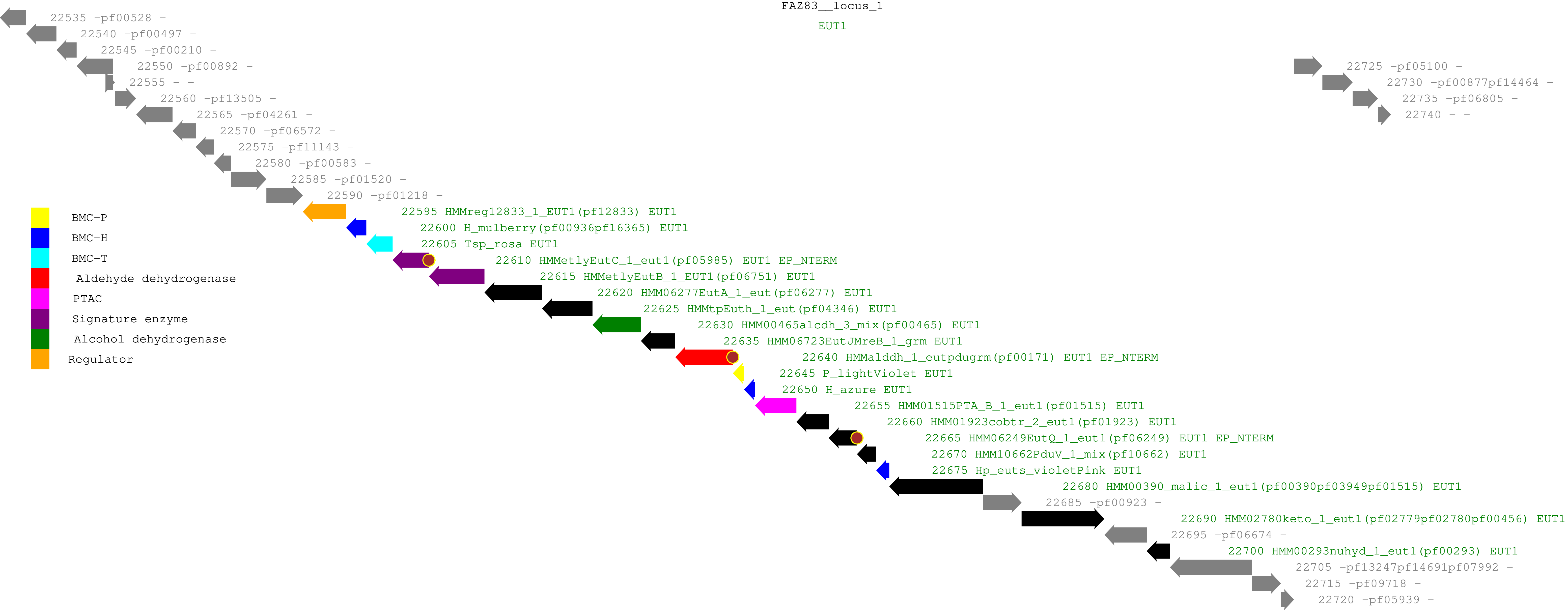

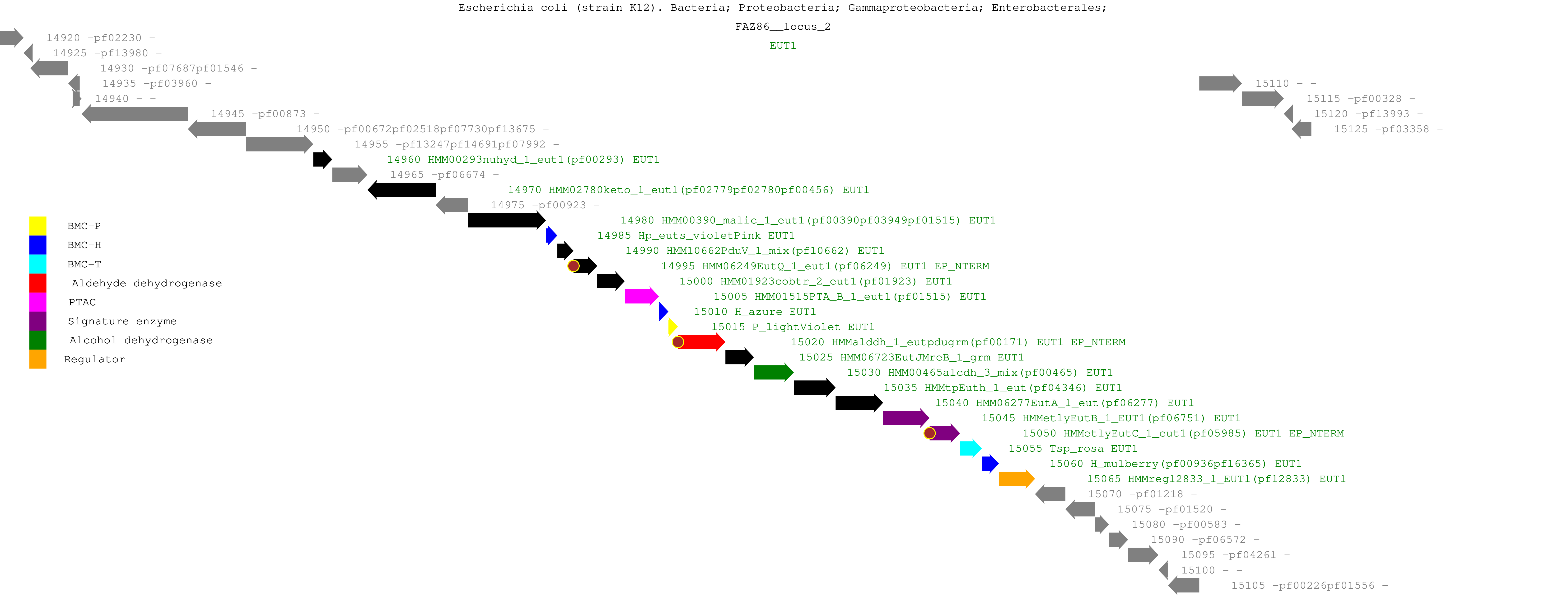

Escherichia coli (strain K12). Bacteria; Proteobacteria; Gammaproteobacteria; Enterobacterales;
FAZ86__locus_2
EUT1
14920 -pf02230 -
14925 -pf13980 -
14930 -pf07687pf01546 -
14935 -pf03960 -
14940 - -
14945 -pf00873 -
14950 -pf00672pf02518pf07730pf13675 -
14955 -pf13247pf14691pf07992 -
14960 HMM00293nuhyd_1_eut1(pf00293) EUT1
14965 -pf06674 -
14970 HMM02780keto_1_eut1(pf02779pf02780pf00456) EUT1
14975 -pf00923 -
14980 HMM00390_malic_1_eut1(pf00390pf03949pf01515) EUT1
14985 Hp_euts_violetPink EUT1
14990 HMM10662PduV_1_mix(pf10662) EUT1
14995 HMM06249EutQ_1_eut1(pf06249) EUT1 EP_NTERM
15000 HMM01923cobtr_2_eut1(pf01923) EUT1
15005 HMM01515PTA_B_1_eut1(pf01515) EUT1
15010 H_azure EUT1
15015 P_lightViolet EUT1
15020 HMMalddh_1_eutpdugrm(pf00171) EUT1 EP_NTERM
15025 HMM06723EutJMreB_1_grm EUT1
15030 HMM00465alcdh_3_mix(pf00465) EUT1
15035 HMMtpEuth_1_eut(pf04346) EUT1
15040 HMM06277EutA_1_eut(pf06277) EUT1
15045 HMMetlyEutB_1_EUT1(pf06751) EUT1
15050 HMMetlyEutC_1_eut1(pf05985) EUT1 EP_NTERM
15055 Tsp_rosa EUT1
15060 H_mulberry(pf00936pf16365) EUT1
15065 HMMreg12833_1_EUT1(pf12833) EUT1
15070 -pf01218 -
15075 -pf01520 -
15080 -pf00583 -
15090 -pf06572 -
15095 -pf04261 -
15100 - -
15105 -pf00226pf01556 -
15110 - -
15115 -pf00328 -
15120 -pf13993 -
15125 -pf03358 -
BMC-P
BMC-H
BMC-T
Aldehyde dehydrogenase
PTAC
Signature enzyme
Alcohol dehydrogenase
Regulator

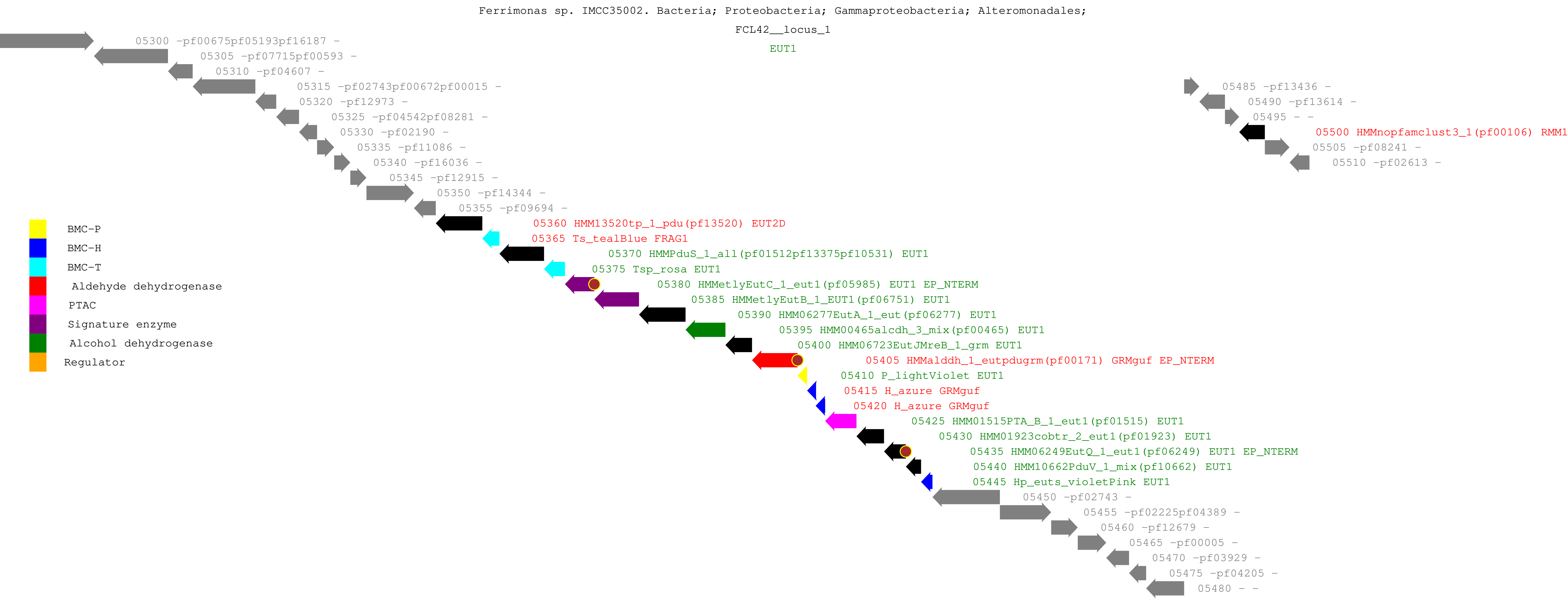

Ferrimonas sp. IMCC35002. Bacteria; Proteobacteria; Gammaproteobacteria; Alteromonadales;
FCL42__locus_1
EUT1
05300 -pf00675pf05193pf16187 -
05305 -pf07715pf00593 -
05310 -pf04607 -
05315 -pf02743pf00672pf00015 -
05320 -pf12973 -
05325 -pf04542pf08281 -
05330 -pf02190 -
05335 -pf11086 -
05340 -pf16036 -
05345 -pf12915 -
05350 -pf14344 -
05355 -pf09694 -
05360 HMM13520tp_1_pdu(pf13520) EUT2D
05365 Ts_tealBlue FRAG1
05370 HMMPduS_1_all(pf01512pf13375pf10531) EUT1
05375 Tsp_rosa EUT1
05380 HMMetlyEutC_1_eut1(pf05985) EUT1 EP_NTERM
05385 HMMetlyEutB_1_EUT1(pf06751) EUT1
05390 HMM06277EutA_1_eut(pf06277) EUT1
05395 HMM00465alcdh_3_mix(pf00465) EUT1
05400 HMM06723EutJMreB_1_grm EUT1
05405 HMMalddh_1_eutpdugrm(pf00171) GRMguf EP_NTERM
05410 P_lightViolet EUT1
05415 H_azure GRMguf
05420 H_azure GRMguf
05425 HMM01515PTA_B_1_eut1(pf01515) EUT1
05430 HMM01923cobtr_2_eut1(pf01923) EUT1
05435 HMM06249EutQ_1_eut1(pf06249) EUT1 EP_NTERM
05440 HMM10662PduV_1_mix(pf10662) EUT1
05445 Hp_euts_violetPink EUT1
05450 -pf02743 -
05455 -pf02225pf04389 -
05460 -pf12679 -
05465 -pf00005 -
05470 -pf03929 -
05475 -pf04205 -
05480 - -
05485 -pf13436 -
05490 -pf13614 -
05495 - -
05500 HMMnopfamclust3_1(pf00106) RMM1
05505 -pf08241 -
05510 -pf02613 -
BMC-P
BMC-H
BMC-T
Aldehyde dehydrogenase
PTAC
Signature enzyme
Alcohol dehydrogenase
Regulator

Comamonadaceae bacterium. Bacteria; Proteobacteria; Betaproteobacteria; Burkholderiales;

FD135__locus_1

EUT1

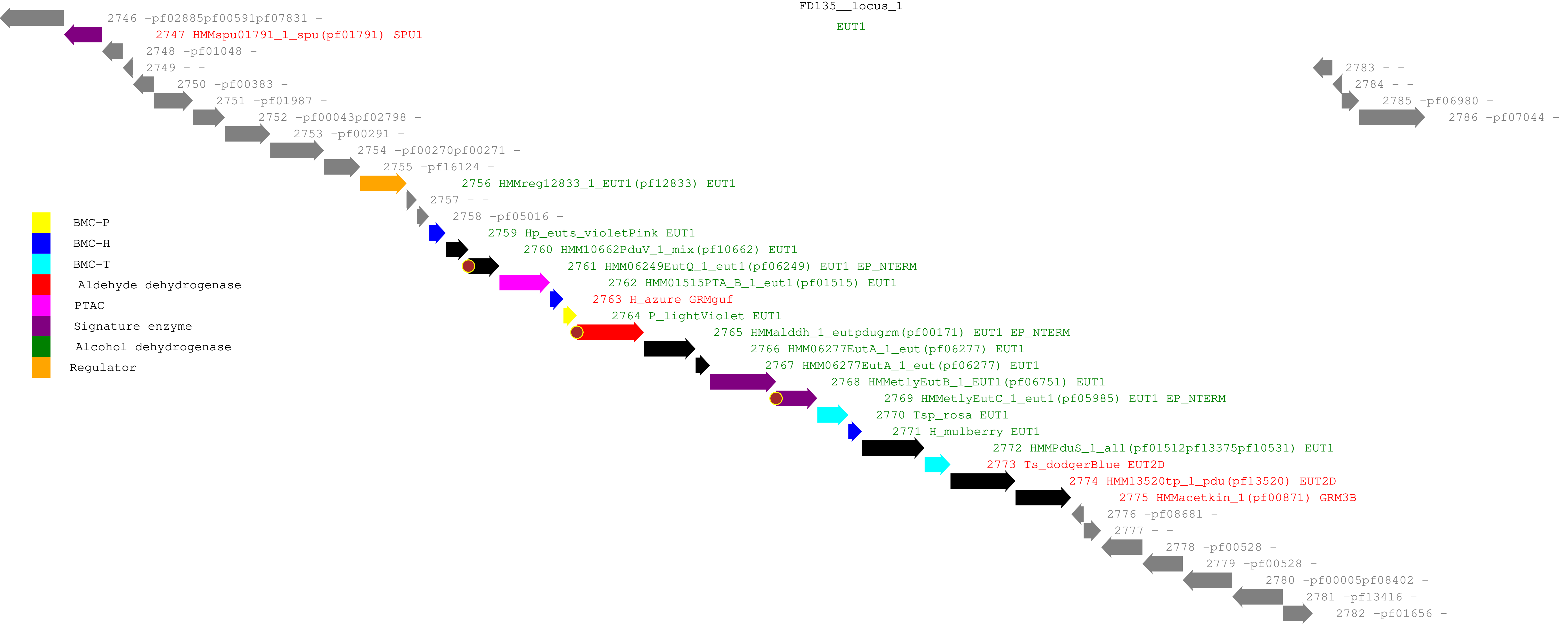

Citrobacter sp. wls715. Bacteria; Proteobacteria; Gammaproteobacteria; Enterobacterales;

FDX05__locus_2

EUT1

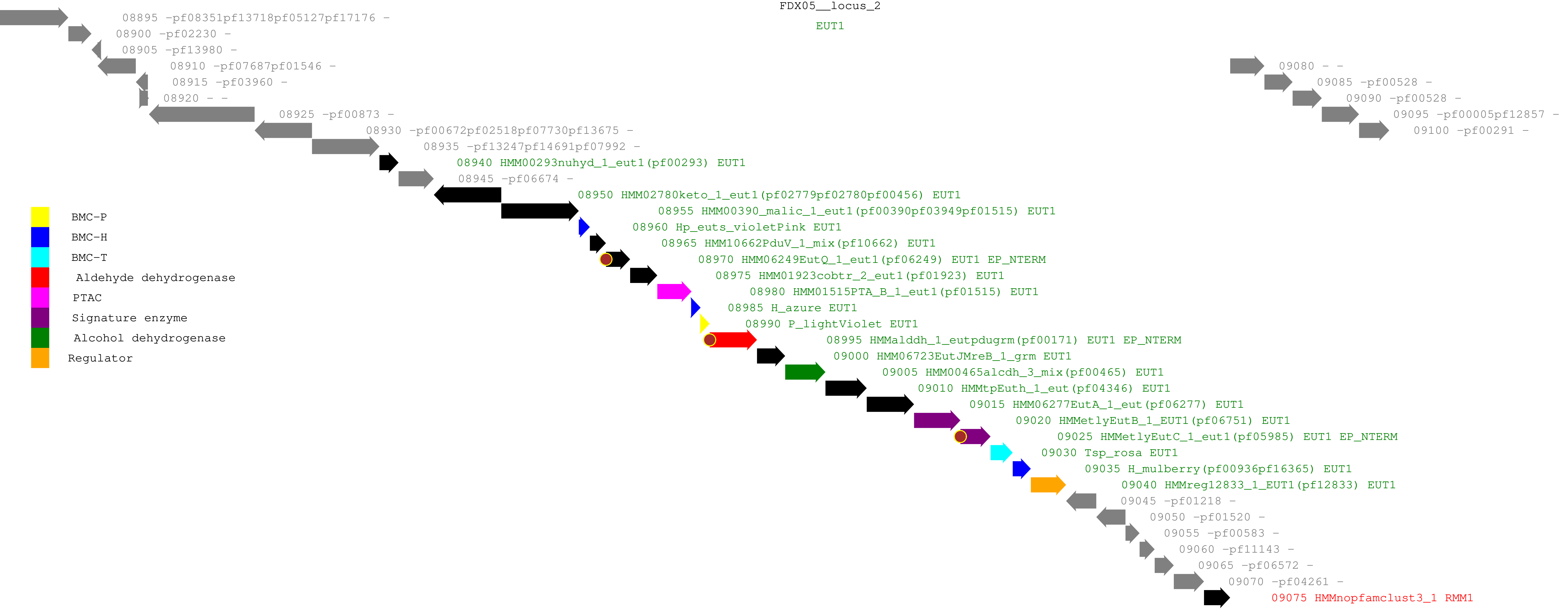

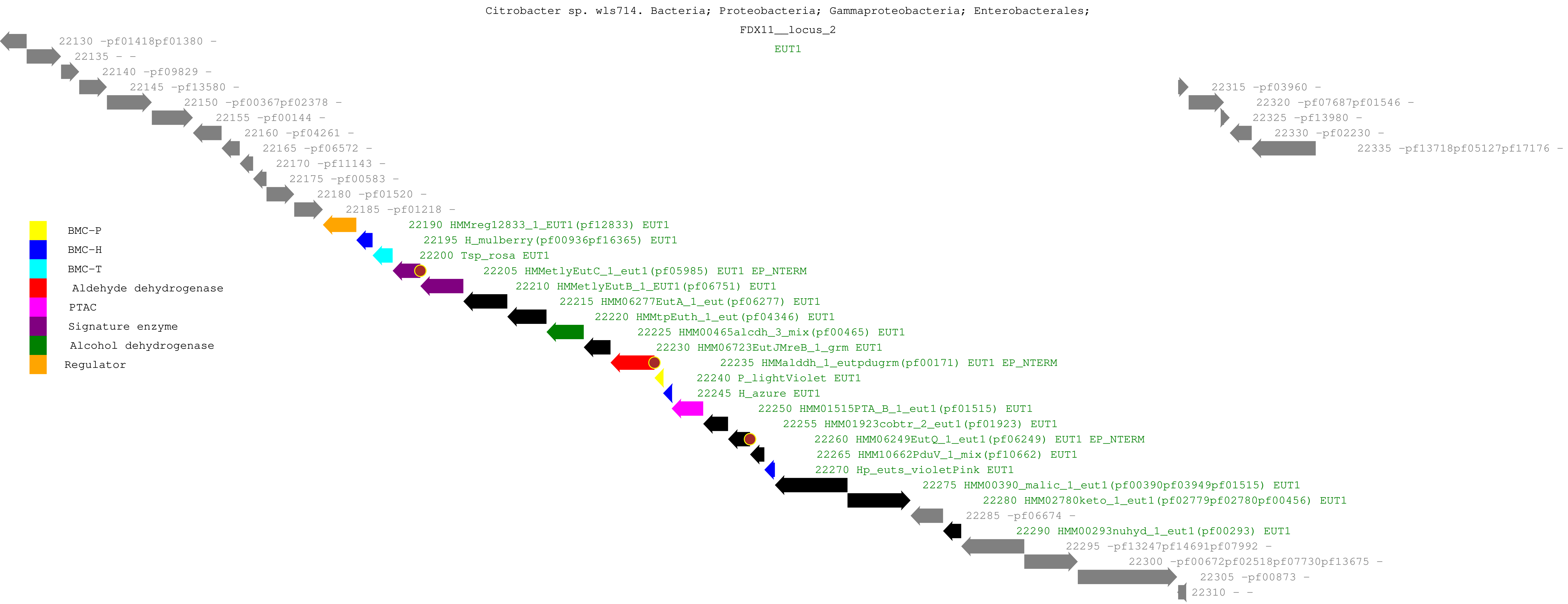

Citrobacter sp. wls714. Bacteria; Proteobacteria; Gammaproteobacteria; Enterobacterales;
FDX11__locus_2
EUT1
22130 -pf01418pf01380 -
22135 - -
22140 -pf09829 -
22145 -pf13580 -
22150 -pf00367pf02378 -
22155 -pf00144 -
22160 -pf04261 -
22165 -pf06572 -
22170 -pf11143 -
22175 -pf00583 -
22180 -pf01520 -
22185 -pf01218 -
22190 HMMreg12833_1_EUT1(pf12833) EUT1
22195 H_mulberry(pf00936pf16365) EUT1
22200 Tsp_rosa EUT1
22205 HMMetlyEutC_1_eut1(pf05985) EUT1 EP_NTERM
22210 HMMetlyEutB_1_EUT1(pf06751) EUT1
22215 HMM06277EutA_1_eut(pf06277) EUT1
22220 HMMtpEuth_1_eut(pf04346) EUT1
22225 HMM00465alcdh_3_mix(pf00465) EUT1
22230 HMM06723EutJMreB_1_grm EUT1
22235 HMMalddh_1_eutpdugrm(pf00171) EUT1 EP_NTERM
22240 P_lightViolet EUT1
22245 H_azure EUT1
22250 HMM01515PTA_B_1_eut1(pf01515) EUT1
22255 HMM01923cobtr_2_eut1(pf01923) EUT1
22260 HMM06249EutQ_1_eut1(pf06249) EUT1 EP_NTERM
22265 HMM10662PduV_1_mix(pf10662) EUT1
22270 Hp_euts_violetPink EUT1
22275 HMM00390_malic_1_eut1(pf00390pf03949pf01515) EUT1
22280 HMM02780keto_1_eut1(pf02779pf02780pf00456) EUT1
22285 -pf06674 -
22290 HMM00293nuhyd_1_eut1(pf00293) EUT1
22295 -pf13247pf14691pf07992 -
22300 -pf00672pf02518pf07730pf13675 -
22305 -pf00873 -
22310 - -
22315 -pf03960 -
22320 -pf07687pf01546 -
22325 -pf13980 -
22330 -pf02230 -
22335 -pf13718pf05127pf17176 -
BMC-P
BMC-H
BMC-T
Aldehyde dehydrogenase
PTAC
Signature enzyme
Alcohol dehydrogenase
Regulator

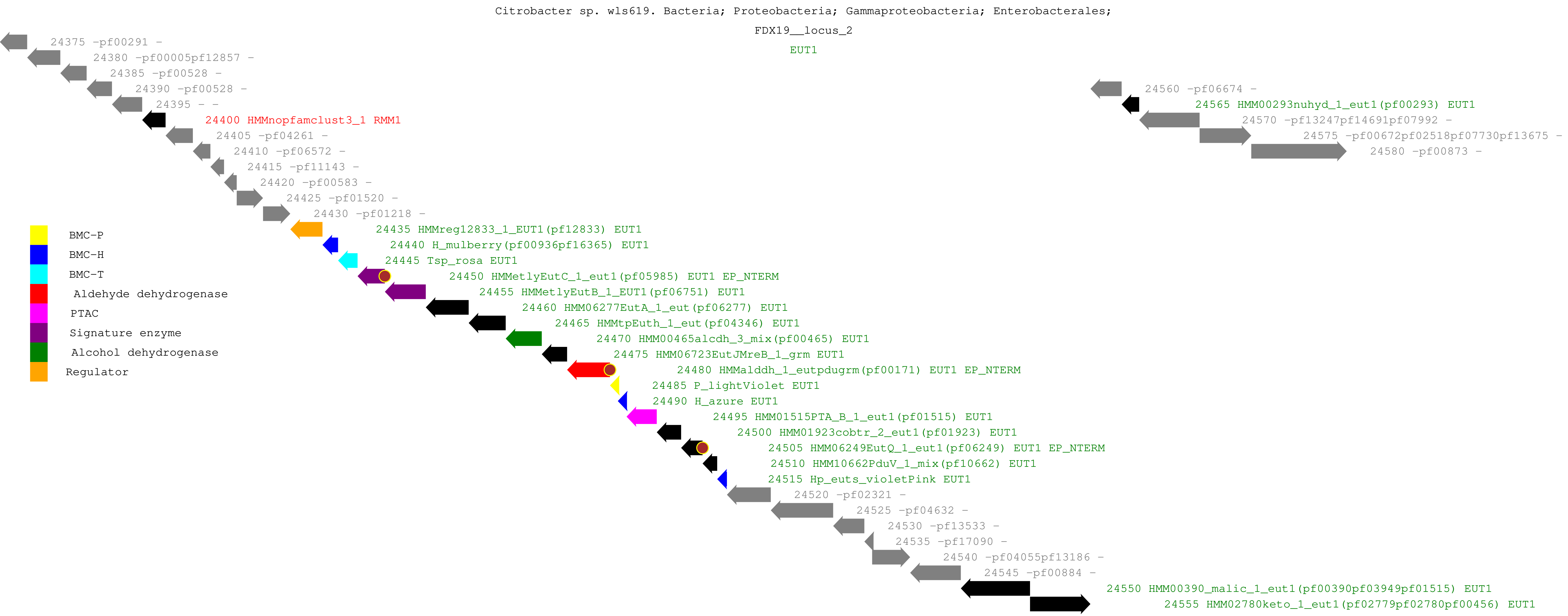
Citrobacter sp. wls619. Bacteria; Proteobacteria; Gammaproteobacteria; Enterobacterales;
FDX19__locus_2
EUT1
24375 -pf00291 -
24380 -pf00005pf12857 -
24385 -pf00528 -
24390 -pf00528 -
24395 - -
24400 HMMnopfamclust3_1 RMM1
24405 -pf04261 -
24410 -pf06572 -
24415 -pf11143 -
24420 -pf00583 -
24425 -pf01520 -
24430 -pf01218 -
24435 HMMreg12833_1_EUT1(pf12833) EUT1
24440 H_mulberry(pf00936pf16365) EUT1
24445 Tsp_rosa EUT1
24450 HMMetlyEutC_1_eut1(pf05985) EUT1 EP_NTERM
24455 HMMetlyEutB_1_EUT1(pf06751) EUT1
24460 HMM06277EutA_1_eut(pf06277) EUT1
24465 HMMtpEuth_1_eut(pf04346) EUT1
24470 HMM00465alcdh_3_mix(pf00465) EUT1
24475 HMM06723EutJMreB_1_grm EUT1
24480 HMMalddh_1_eutpdugrm(pf00171) EUT1 EP_NTERM
24485 P_lightViolet EUT1
24490 H_azure EUT1
24495 HMM01515PTA_B_1_eut1(pf01515) EUT1
24500 HMM01923cobtr_2_eut1(pf01923) EUT1
24505 HMM06249EutQ_1_eut1(pf06249) EUT1 EP_NTERM
24510 HMM10662PduV_1_mix(pf10662) EUT1
24515 Hp_euts_violetPink EUT1
24520 -pf02321 -
24525 -pf04632 -
24530 -pf13533 -
24535 -pf17090 -
24540 -pf04055pf13186 -
24545 -pf00884 -
24550 HMM00390_malic_1_eut1(pf00390pf03949pf01515) EUT1
24555 HMM02780keto_1_eut1(pf02779pf02780pf00456) EUT1
24560 -pf06674 -
24565 HMM00293nuhyd_1_eut1(pf00293) EUT1
24570 -pf13247pf14691pf07992 -
24575 -pf00672pf02518pf07730pf13675 -
24580 -pf00873 -
BMC-P
BMC-H
BMC-T
Aldehyde dehydrogenase
PTAC
Signature enzyme
Alcohol dehydrogenase
Regulator

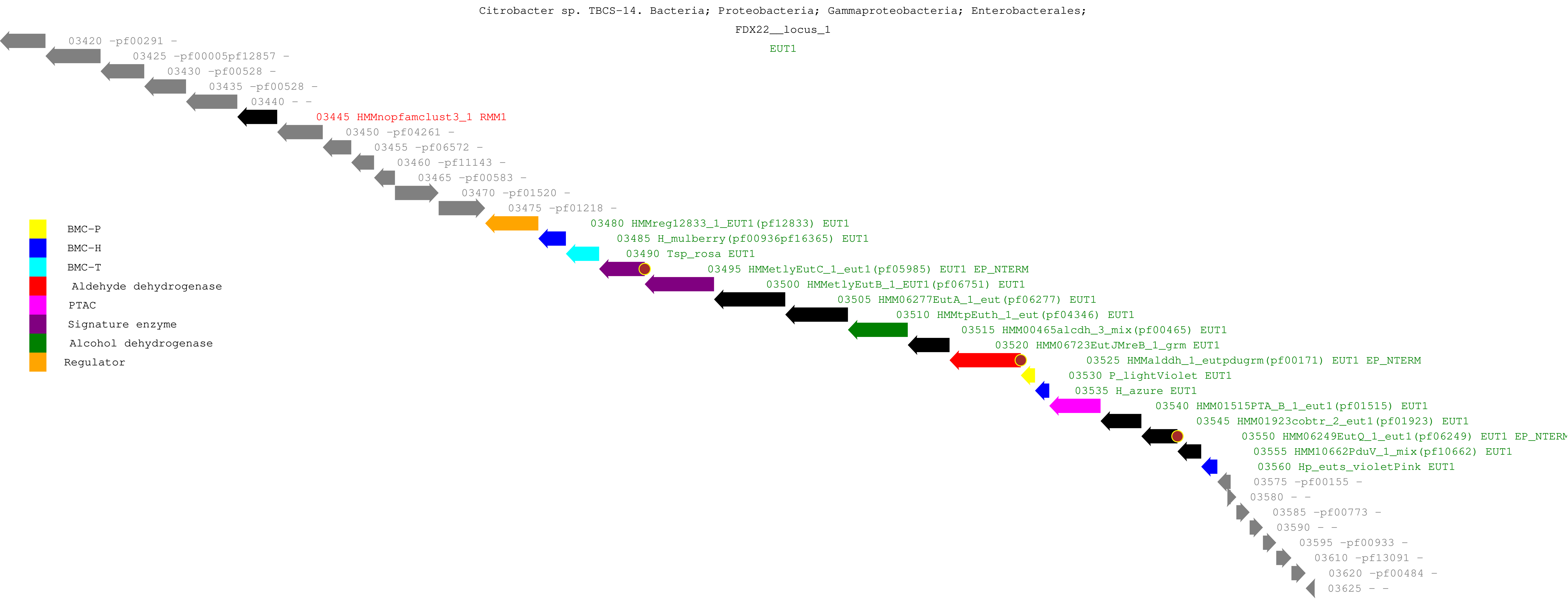

Citrobacter sp. TBCS-14. Bacteria; Proteobacteria; Gammaproteobacteria; Enterobacterales;
FDX22__locus_1
EUT1
03420 -pf00291 -
03425 -pf00005pf12857 -
03430 -pf00528 -
03435 -pf00528 -
03440 - -
03445 HMMnopfamclust3_1 RMM1
03450 -pf04261 -
03455 -pf06572 -
03460 -pf11143 -
03465 -pf00583 -
03470 -pf01520 -
03475 -pf01218 -
03480 HMMreg12833_1_EUT1(pf12833) EUT1
03485 H_mulberry(pf00936pf16365) EUT1
03490 Tsp_rosa EUT1
03495 HMMetlyEutC_1_eut1(pf05985) EUT1 EP_NTERM
03500 HMMetlyEutB_1_EUT1(pf06751) EUT1
03505 HMM06277EutA_1_eut(pf06277) EUT1
03510 HMMtpEuth_1_eut(pf04346) EUT1
03515 HMM00465alcdh_3_mix(pf00465) EUT1
03520 HMM06723EutJMreB_1_grm EUT1
03525 HMMalddh_1_eutpdugrm(pf00171) EUT1 EP_NTERM
03530 P_lightViolet EUT1
03535 H_azure EUT1
03540 HMM01515PTA_B_1_eut1(pf01515) EUT1
03545 HMM01923cobtr_2_eut1(pf01923) EUT1
03550 HMM06249EutQ_1_eut1(pf06249) EUT1 EP_NTERM
03555 HMM10662PduV_1_mix(pf10662) EUT1
03560 Hp_euts_violetPink EUT1
03575 -pf00155 -
03580 - -
03585 -pf00773 -
03590 - -
03595 -pf00933 -
03610 -pf13091 -
03620 -pf00484 -
03625 - -
BMC-P
BMC-H
BMC-T
Aldehyde dehydrogenase
PTAC
Signature enzyme
Alcohol dehydrogenase
Regulator

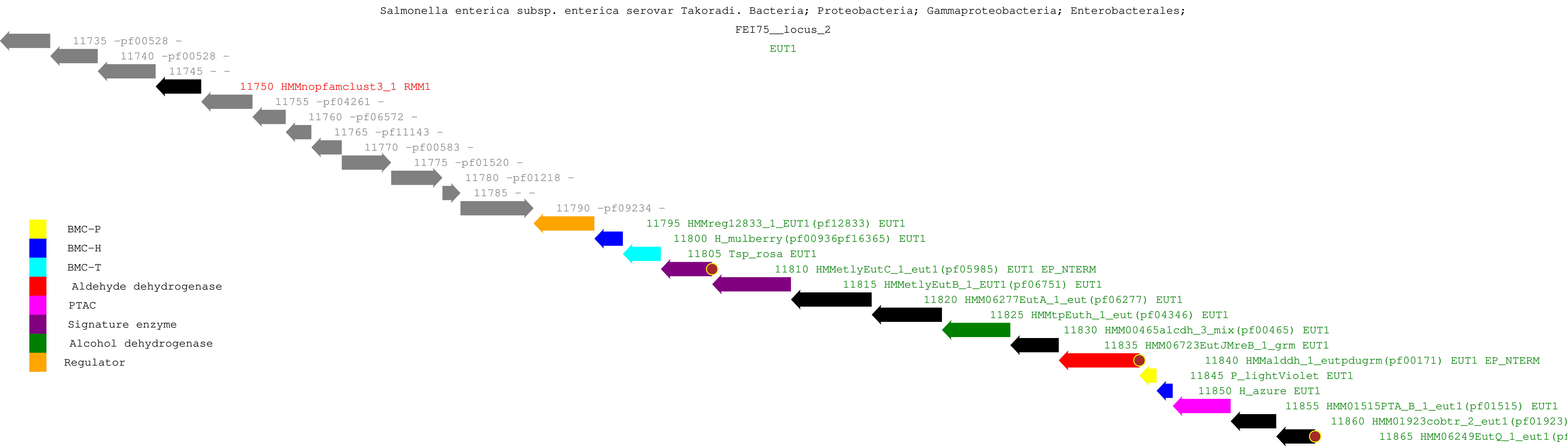

Salmonella enterica subsp. enterica serovar Takoradi. Bacteria; Proteobacteria; Gammaproteobacteria; Enterobacterales;
FEI75__locus_2
EUT1
11735 -pf00528 -
11740 -pf00528 -
11745 - -
11750 HMMnopfamclust3_1 RMM1
11755 -pf04261 -
11760 -pf06572 -
11765 -pf11143 -
11770 -pf00583 -
11775 -pf01520 -
11780 -pf01218 -
11785 - -
11790 -pf09234 -
11795 HMMreg12833_1_EUT1(pf12833) EUT1
11800 H_mulberry(pf00936pf16365) EUT1
11805 Tsp_rosa EUT1
11810 HMMetlyEutC_1_eut1(pf05985) EUT1 EP_NTERM
11815 HMMetlyEutB_1_EUT1(pf06751) EUT1
11820 HMM06277EutA_1_eut(pf06277) EUT1
11825 HMMtpEuth_1_eut(pf04346) EUT1
11830 HMM00465alcdh_3_mix(pf00465) EUT1
11835 HMM06723EutJMreB_1_grm EUT1
11840 HMMaldh_1_eutpdugrm(pf00171) EUT1 EP_NTERM
11845 P_lightViolet EUT1
11850 H_azure EUT1
11855 HMM01515PTA_B_1_eut1(pf01515) EUT1
11860 HMM01923cobtr_2_eut1(pf01923)
11865 HMM06249EutQ_1_eut1(pf
BMC-P
BMC-H
BMC-T
Aldehyde dehydrogenase
PTAC
Signature enzyme
Alcohol dehydrogenase
Regulator

Citrobacter amalonaticus. Bacteria; Proteobacteria; Gammaproteobacteria; Enterobacterales;

FEO47__locus_2

EUT1

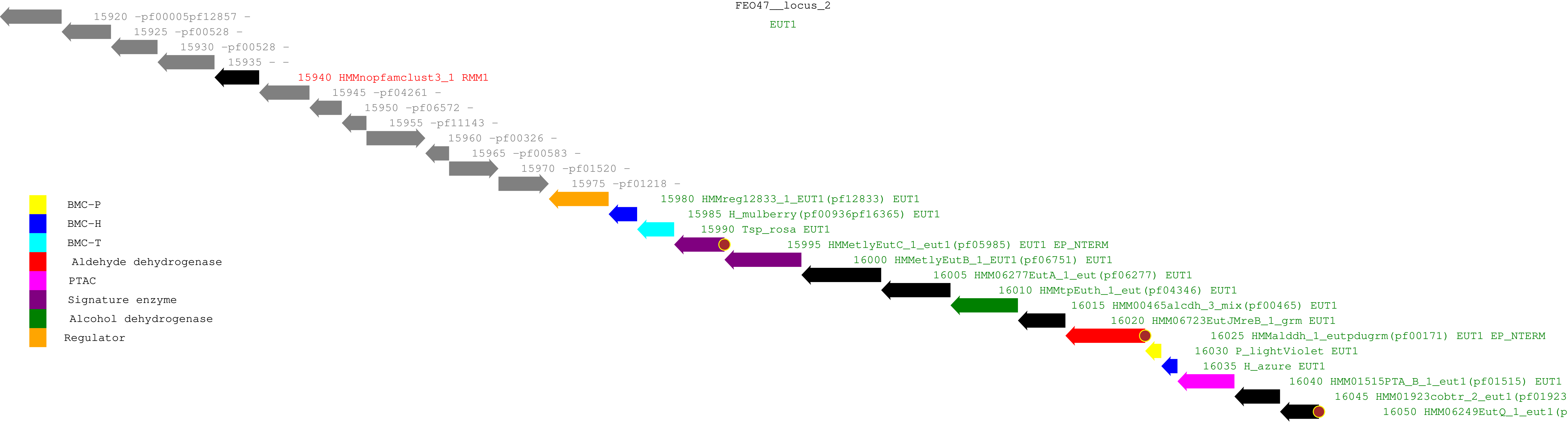

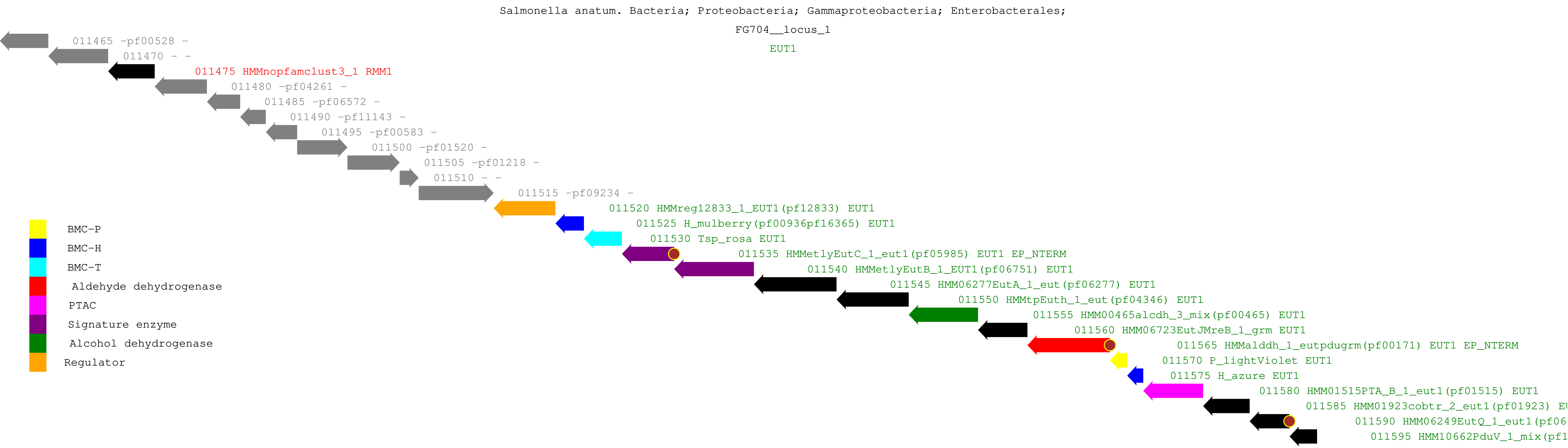

Salmonella anatum. Bacteria; Proteobacteria; Gammaproteobacteria; Enterobacterales;
FG704__locus_1
EUT1
011465 -pf00528 -
011470 - -
011475 HMMnopfamclust3_1 RMM1
011480 -pf04261 -
011485 -pf06572 -
011490 -pf11143 -
011495 -pf00583 -
011500 -pf01520 -
011505 -pf01218 -
011510 - -
011515 -pf09234 -
011520 HMMreg12833_1_EUT1(pf12833) EUT1
011525 H_mulberry(pf00936pf16365) EUT1
011530 Tsp_rosa EUT1
011535 HMMetlyEutC_1_eut1(pf05985) EUT1 EP_NTERM
011540 HMMetlyEutB_1_EUT1(pf06751) EUT1
011545 HMM06277EutA_1_eut(pf06277) EUT1
011550 HMMtpEuth_1_eut(pf04346) EUT1
011555 HMM00465alcdh_3_mix(pf00465) EUT1
011560 HMM06723EutJMreB_1_grm EUT1
011565 HMMalddh_1_eutpdugrm(pf00171) EUT1 EP_NTERM
011570 P_lightViolet EUT1
011575 H_azure EUT1
011580 HMM01515PTA_B_1_eut1(pf01515) EUT1
011585 HMM01923cobtr_2_eut1(pf01923) E
011590 HMM06249EutQ_1_eut1(pf06
011595 HMM10662PduV_1_mix(pf1
BMC-P
BMC-H
BMC-T
Aldehyde dehydrogenase
PTAC
Signature enzyme
Alcohol dehydrogenase
Regulator

Pseudomonas sp. AAC. Bacteria; Proteobacteria; Gammaproteobacteria; Pseudomonadales;

FG99__locus_1

EUT1

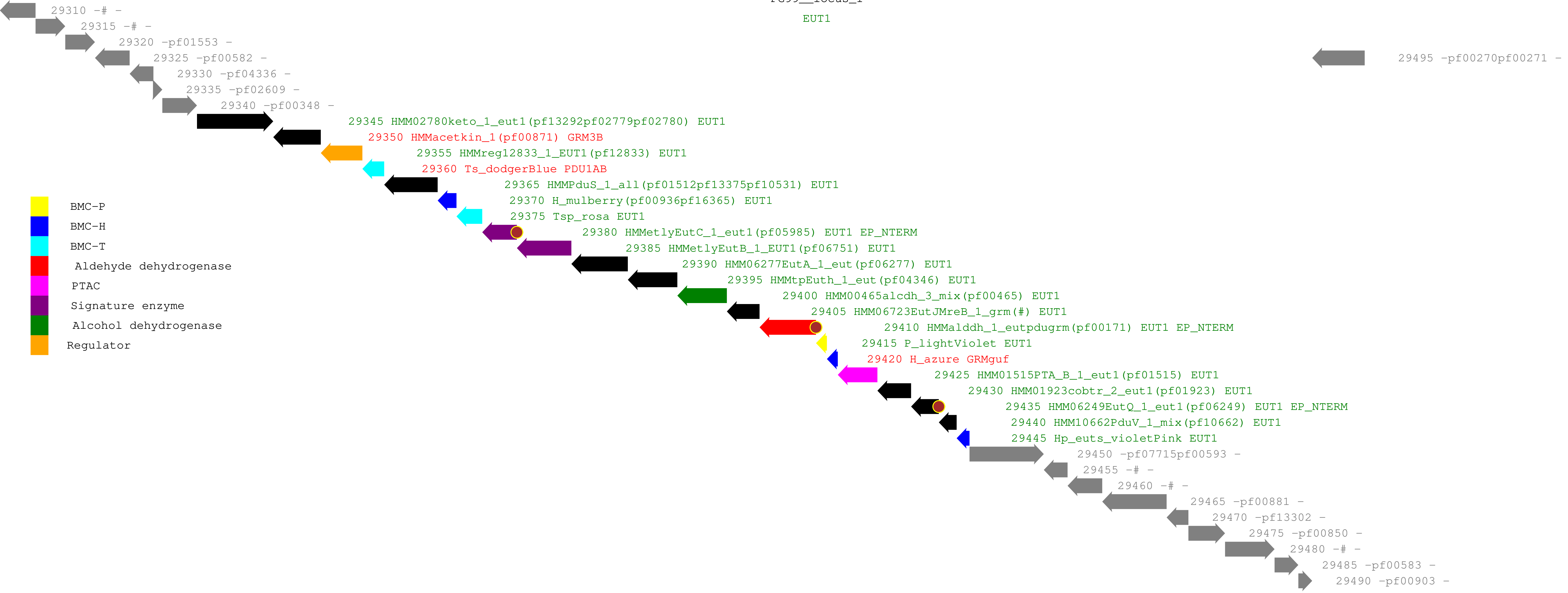

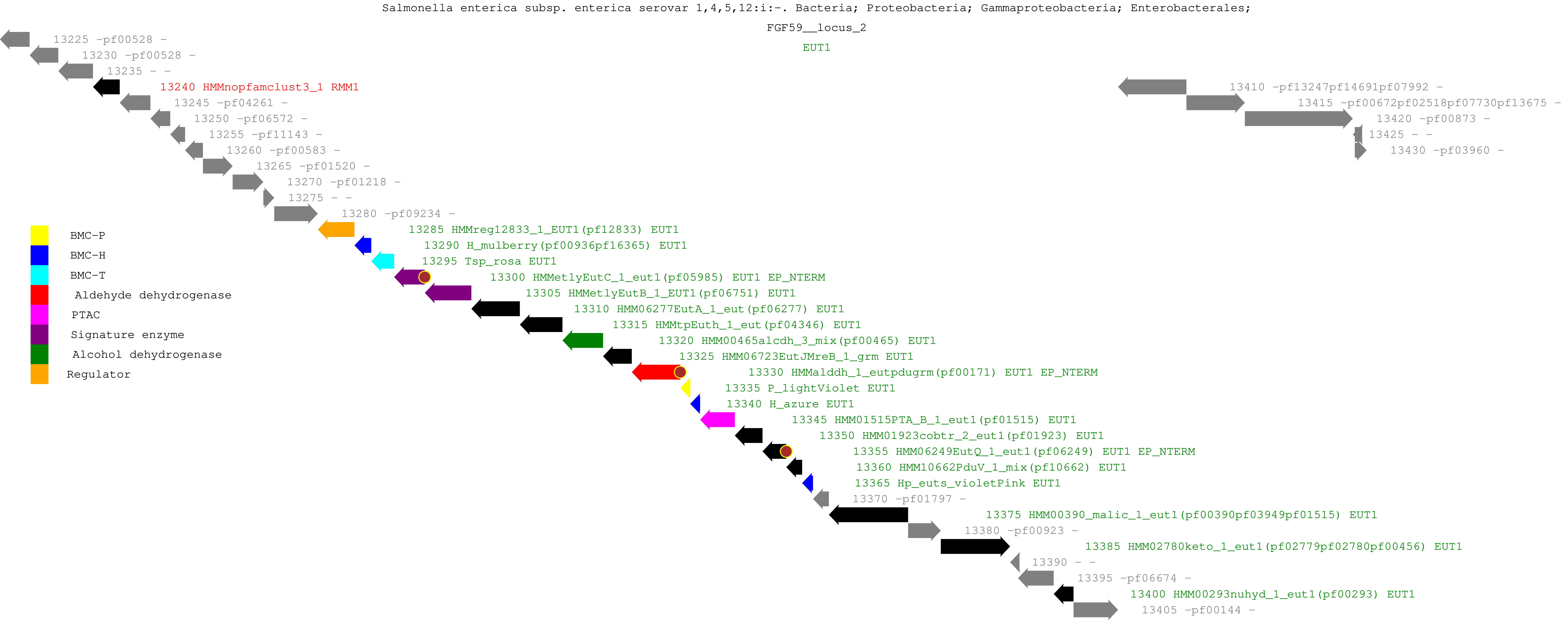
Salmonella enterica subsp. enterica serovar 1,4,5,12:i:-. Bacteria; Proteobacteria; Gammaproteobacteria; Enterobacterales;
FGF59__locus_2
EUT1
13225 -pf00528 -
13230 -pf00528 -
13235 - -
13240 HMMnopfamclust3_1 RMM1
13245 -pf04261 -
13250 -pf06572 -
13255 -pf11143 -
13260 -pf00583 -
13265 -pf01520 -
13270 -pf01218 -
13275 - -
13280 -pf09234 -
13285 HMMreg12833_1_EUT1(pf12833) EUT1
13290 H_mulberry(pf00936pf16365) EUT1
13295 Tsp_rosa EUT1
13300 HMMetlyEutC_1_eut1(pf05985) EUT1 EP_NTERM
13305 HMMetlyEutB_1_EUT1(pf06751) EUT1
13310 HMM06277EutA_1_eut(pf06277) EUT1
13315 HMMtpEuth_1_eut(pf04346) EUT1
13320 HMM00465alcdh_3_mix(pf00465) EUT1
13325 HMM06723EutJMreB_1_grm EUT1
13330 HMMalddh_1_eutpdugrm(pf00171) EUT1 EP_NTERM
13335 P_lightViolet EUT1
13340 H_azure EUT1
13345 HMM01515PTA_B_1_eut1(pf01515) EUT1
13350 HMM01923cobtr_2_eut1(pf01923) EUT1
13355 HMM06249EutQ_1_eut1(pf06249) EUT1 EP_NTERM
13360 HMM10662PduV_1_mix(pf10662) EUT1
13365 Hp_euts_violetPink EUT1
13370 -pf01797 -
13375 HMM00390_malic_1_eut1(pf00390pf03949pf01515) EUT1
13380 -pf00923 -
13385 HMM02780keto_1_eut1(pf02779pf02780pf00456) EUT1
13390 - -
13395 -pf06674 -
13400 HMM00293nuhyd_1_eut1(pf00293) EUT1
13405 -pf00144 -
13410 -pf13247pf14691pf07992 -
13415 -pf00672pf02518pf07730pf13675 -
13420 -pf00873 -
13425 - -
13430 -pf03960 -
BMC-P
BMC-H
BMC-T
Aldehyde dehydrogenase
PTAC
Signature enzyme
Alcohol dehydrogenase
Regulator

Salmonella enterica subsp. enterica serovar Eko. Bacteria; Proteobacteria; Gammaproteobacteria; Enterobacterales;

FGJ70__locus_1

EUT1

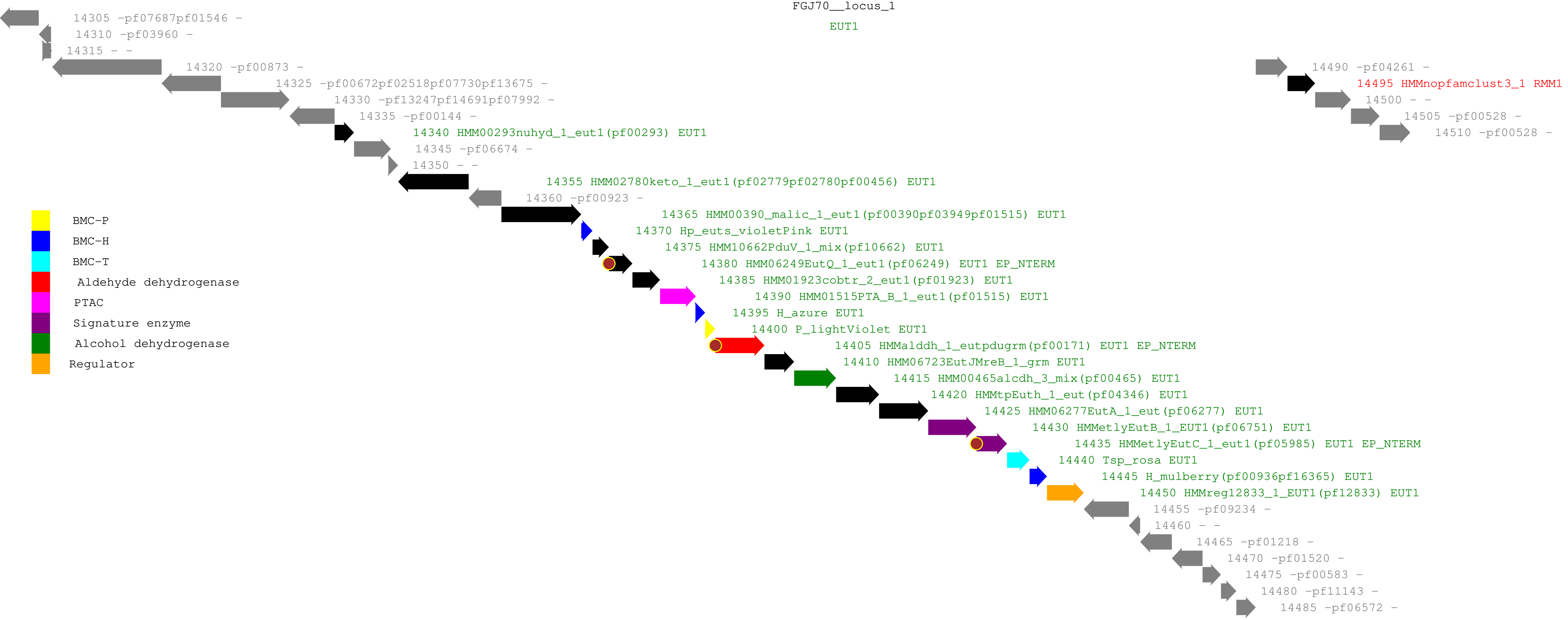

Buttiauxella sp. B2. Bacteria; Proteobacteria; Gammaproteobacteria; Enterobacterales;

FH968__locus_1

EUT1

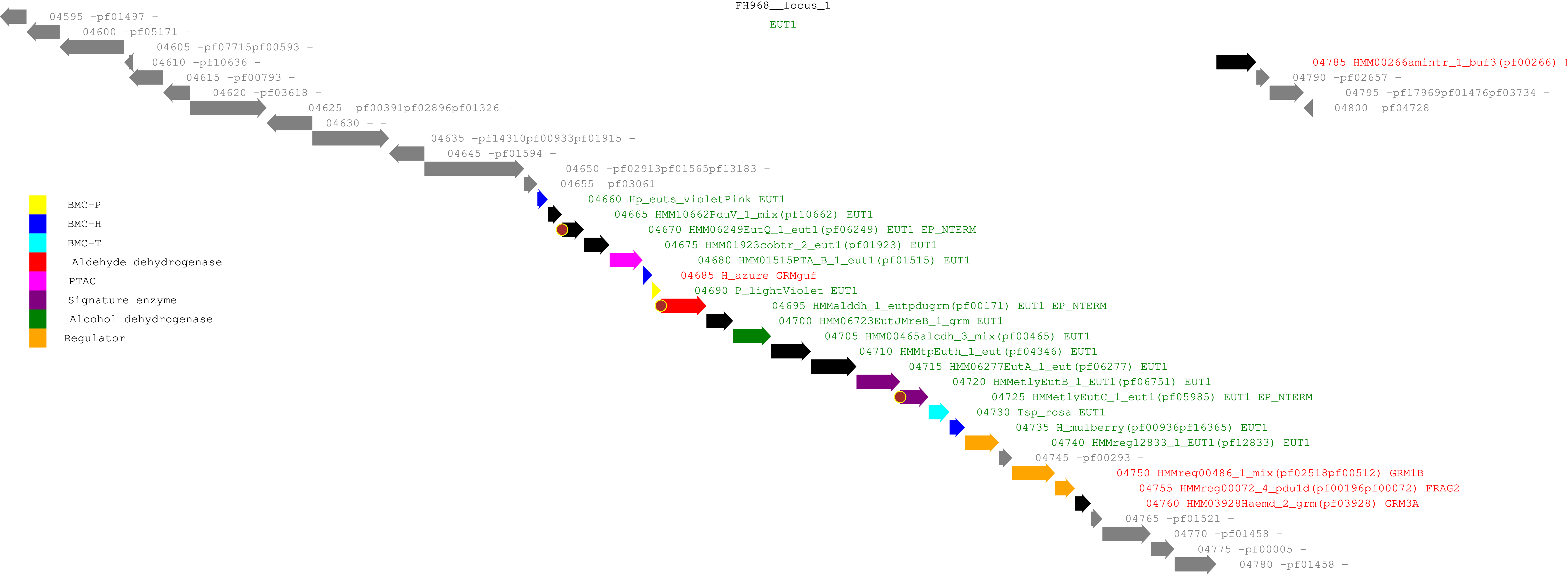

Marinobacter vinifirmus. Bacteria; Proteobacteria; Gammaproteobacteria; Alteromonadales;

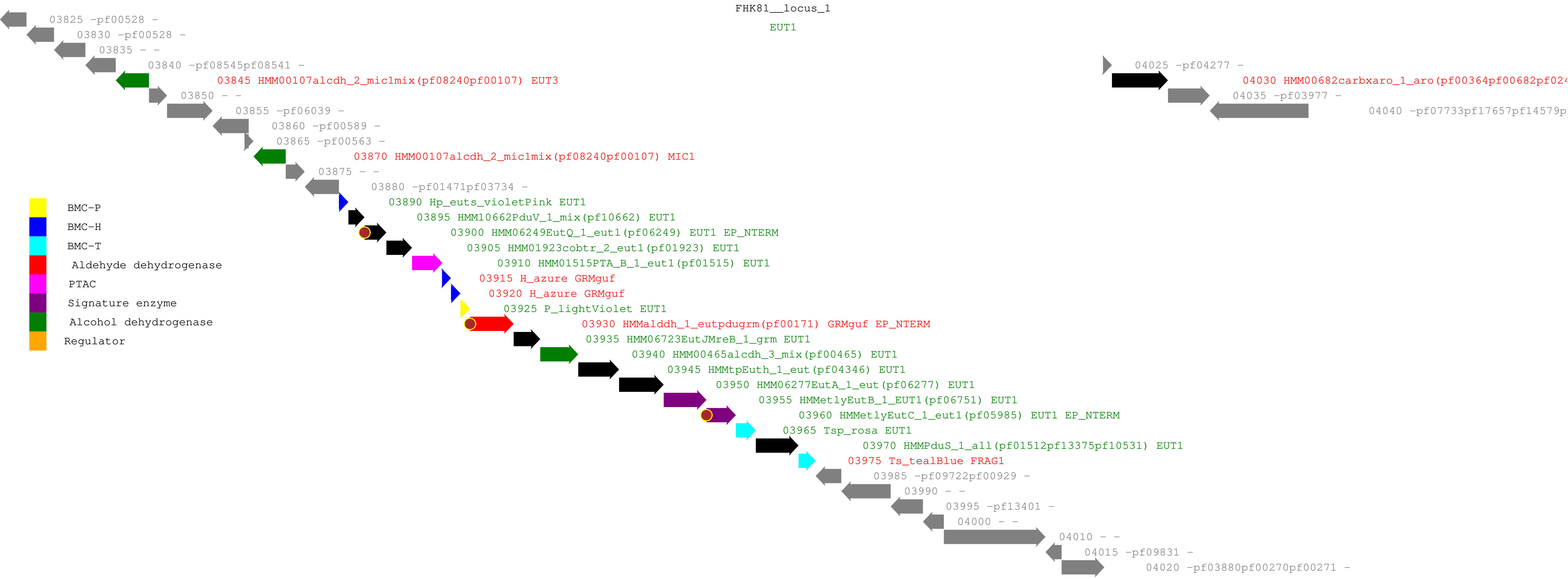

Serratia fonticola. Bacteria; Proteobacteria; Gammaproteobacteria; Enterobacterales;

FHU10__locus_2

EUT1

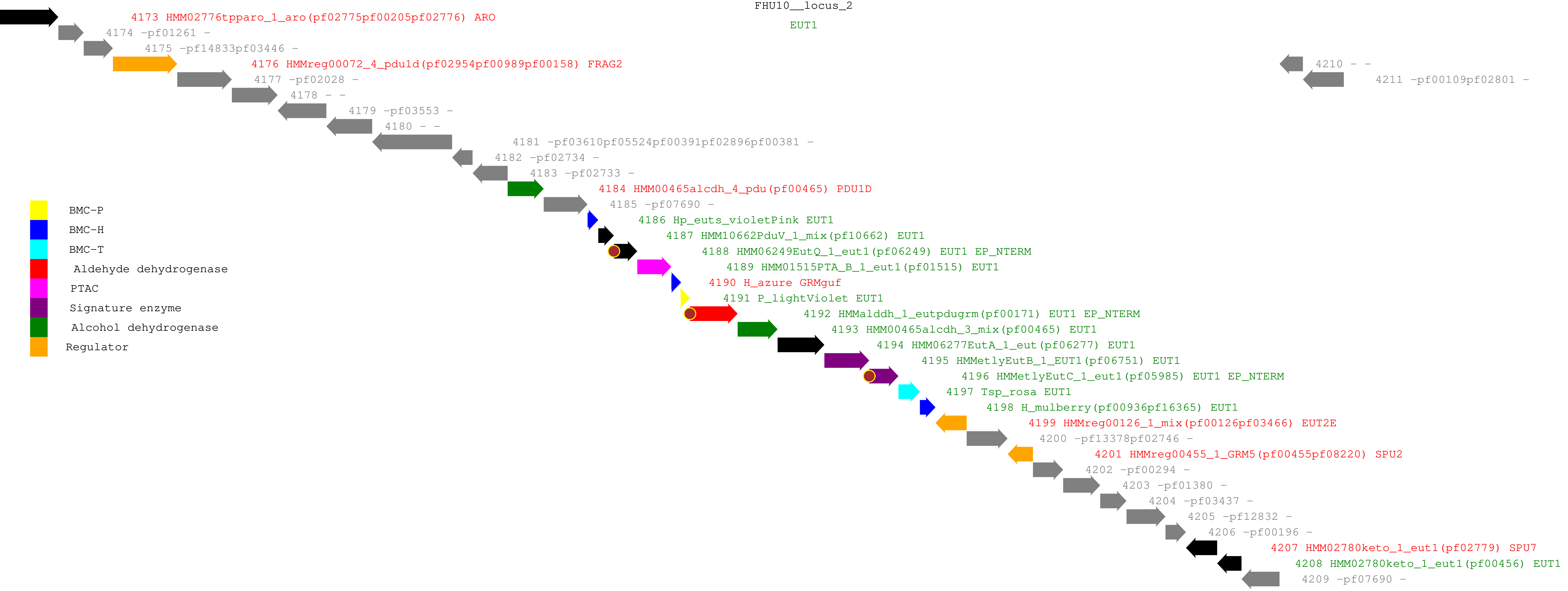

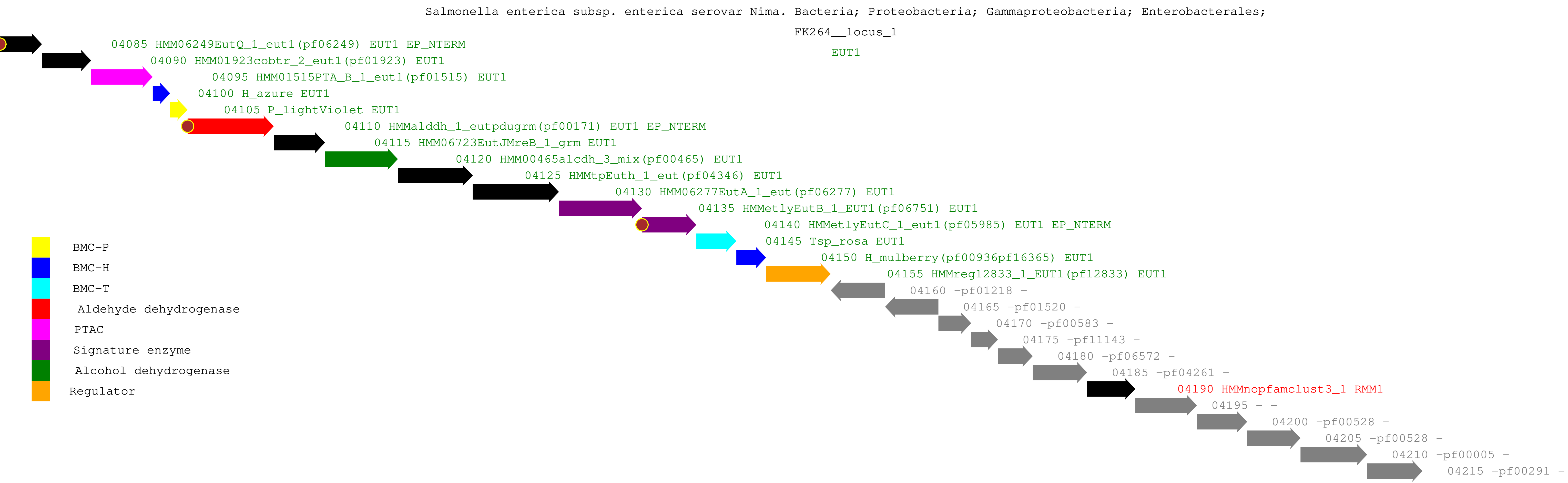
Salmonella enterica subsp. enterica serovar Nima. Bacteria; Proteobacteria; Gammaproteobacteria; Enterobacterales;
FK264__locus_1
EUT1
04085 HMM06249EutQ_1_eut1(pf06249) EUT1 EP_NTERM
04090 HMM01923cobtr_2_eut1(pf01923) EUT1
04095 HMM01515PTA_B_1_eut1(pf01515) EUT1
04100 H_azure EUT1
04105 P_lightViolet EUT1
04110 HMMalddh_1_eutpdugrm(pf00171) EUT1 EP_NTERM
04115 HMM06723EutJMreB_1_grm EUT1
04120 HMM00465alcdh_3_mix(pf00465) EUT1
04125 HMMtpEuth_1_eut(pf04346) EUT1
04130 HMM06277EutA_1_eut(pf06277) EUT1
04135 HMMetlyEutB_1_EUT1(pf06751) EUT1
04140 HMMetlyEutC_1_eut1(pf05985) EUT1 EP_NTERM
04145 Tsp_rosa EUT1
04150 H_mulberry(pf00936pf16365) EUT1
04155 HMMreg12833_1_EUT1(pf12833) EUT1
04160 -pf01218 -
04165 -pf01520 -
04170 -pf00583 -
04175 -pf11143 -
04180 -pf06572 -
04185 -pf04261 -
04190 HMMnopfamclust3_1 RMM1
04195 - -
04200 -pf00528 -
04205 -pf00528 -
04210 -pf00005 -
04215 -pf00291 -
BMC-P
BMC-H
BMC-T
Aldehyde dehydrogenase
PTAC
Signature enzyme
Alcohol dehydrogenase
Regulator

Salmonella enterica subsp. enterica serovar Bahati. Bacteria; Proteobacteria; Gammaproteobacteria; Enterobacterales;

FK693__locus_1

EUT1

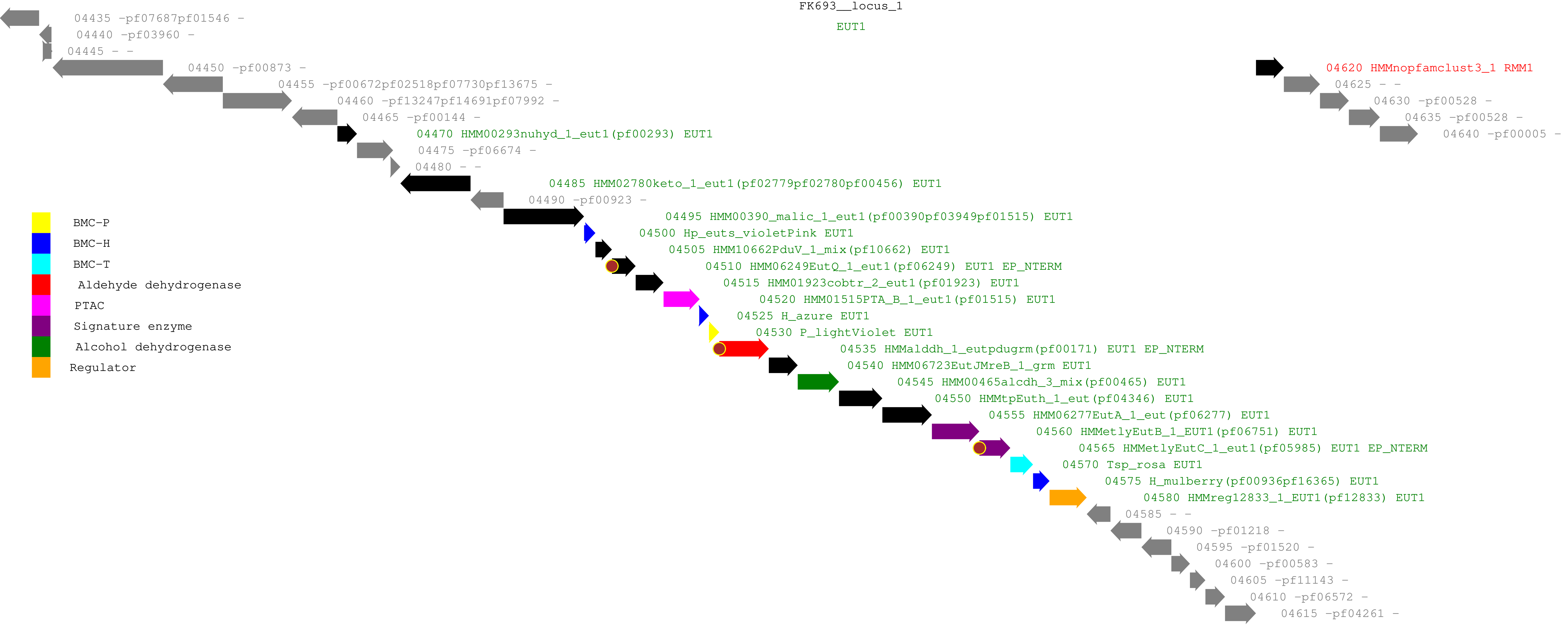

Cellvibrionaceae bacterium R142. Bacteria; Proteobacteria; Gammaproteobacteria; Cellvibrionales;

FKG94__locus_1

EUT1

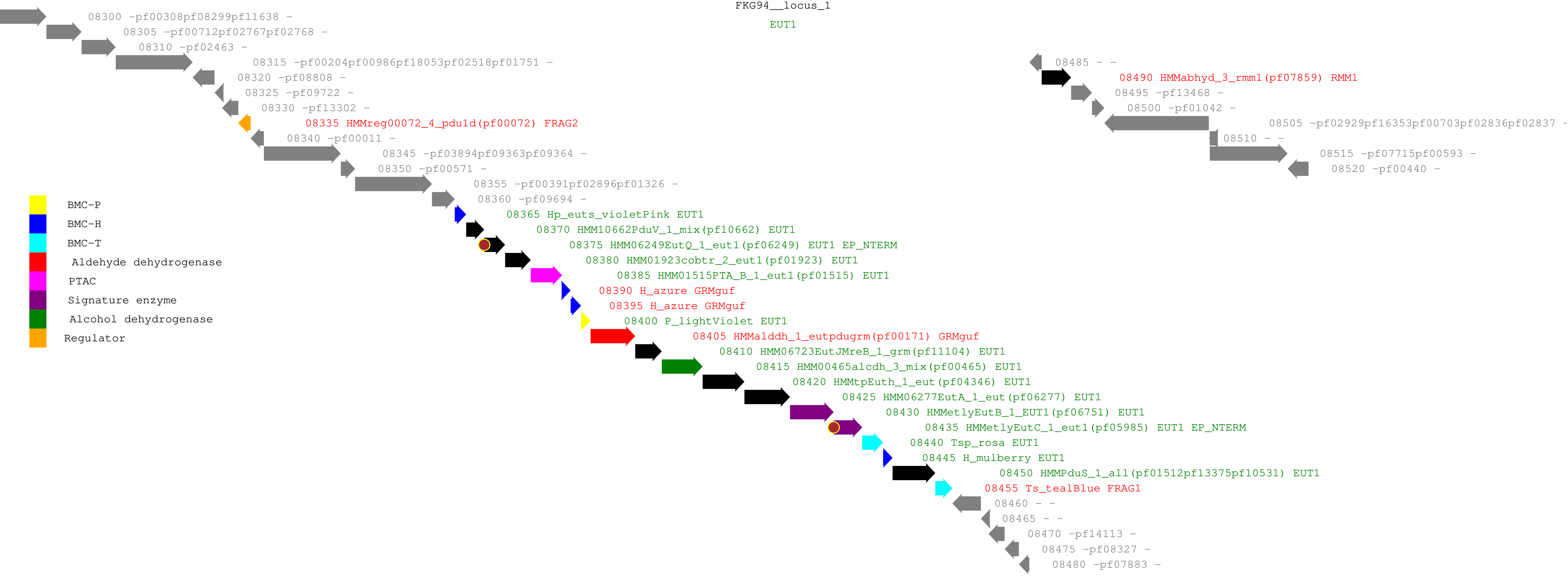

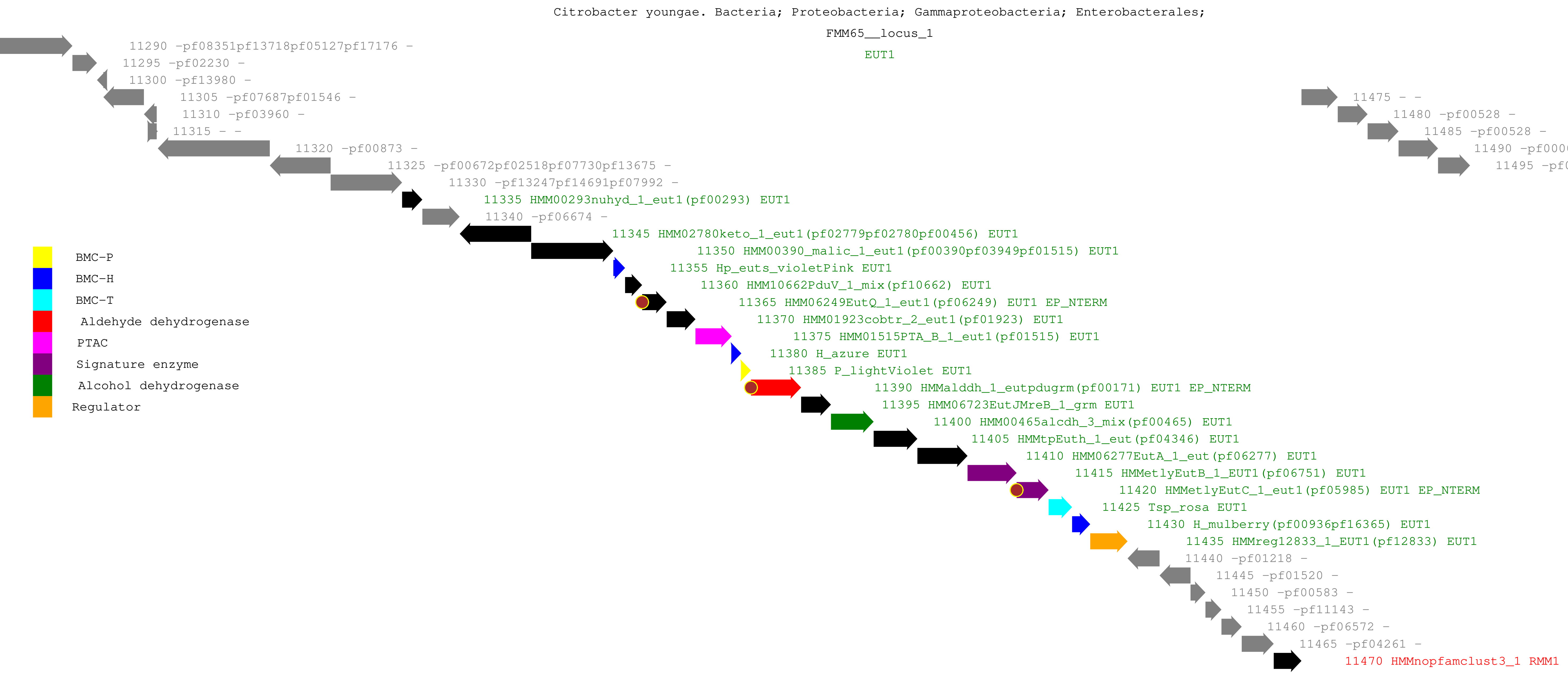

Citrobacter youngae. Bacteria; Proteobacteria; Gammaproteobacteria; Enterobacterales;
FMM65__locus_1
EUT1
11290 -pf08351pf13718pf05127pf17176 -
11295 -pf02230 -
11300 -pf13980 -
11305 -pf07687pf01546 -
11310 -pf03960 -
11315 - -
11320 -pf00873 -
11325 -pf00672pf02518pf07730pf13675 -
11330 -pf13247pf14691pf07992 -
11335 HMM00293nuhyd_1_eut1(pf00293) EUT1
11340 -pf06674 -
11345 HMM02780keto_1_eut1(pf02779pf02780pf00456) EUT1
11350 HMM00390_malic_1_eut1(pf00390pf03949pf01515) EUT1
11355 Hp_euts_violetPink EUT1
11360 HMM10662PduV_1_mix(pf10662) EUT1
11365 HMM06249EutQ_1_eut1(pf06249) EUT1 EP_NTERM
11370 HMM01923cobtr_2_eut1(pf01923) EUT1
11375 HMM01515PTA_B_1_eut1(pf01515) EUT1
11380 H_azure EUT1
11385 P_lightViolet EUT1
11390 HMMaldh_1_eutpdugrm(pf00171) EUT1 EP_NTERM
11395 HMM06723EutJMreB_1_grm EUT1
11400 HMM00465alcdh_3_mix(pf00465) EUT1
11405 HMMtpEuth_1_eut(pf04346) EUT1
11410 HMM06277EutA_1_eut(pf06277) EUT1
11415 HMMetlyEutB_1_EUT1(pf06751) EUT1
11420 HMMetlyEutC_1_eut1(pf05985) EUT1 EP_NTERM
11425 Tsp_rosa EUT1
11430 H_mulberry(pf00936pf16365) EUT1
11435 HMMreg12833_1_EUT1(pf12833) EUT1
11440 -pf01218 -
11445 -pf01520 -
11450 -pf00583 -
11455 -pf11143 -
11460 -pf06572 -
11465 -pf04261 -
11470 HMMnopfamclust3_1 RMM1
11475 - -
11480 -pf00528 -
11485 -pf00528 -
11490 -pf00005pf12857 -
11495 -pf00291 -
BMC-P
BMC-H
BMC-T
Aldehyde dehydrogenase
PTAC
Signature enzyme
Alcohol dehydrogenase
Regulator

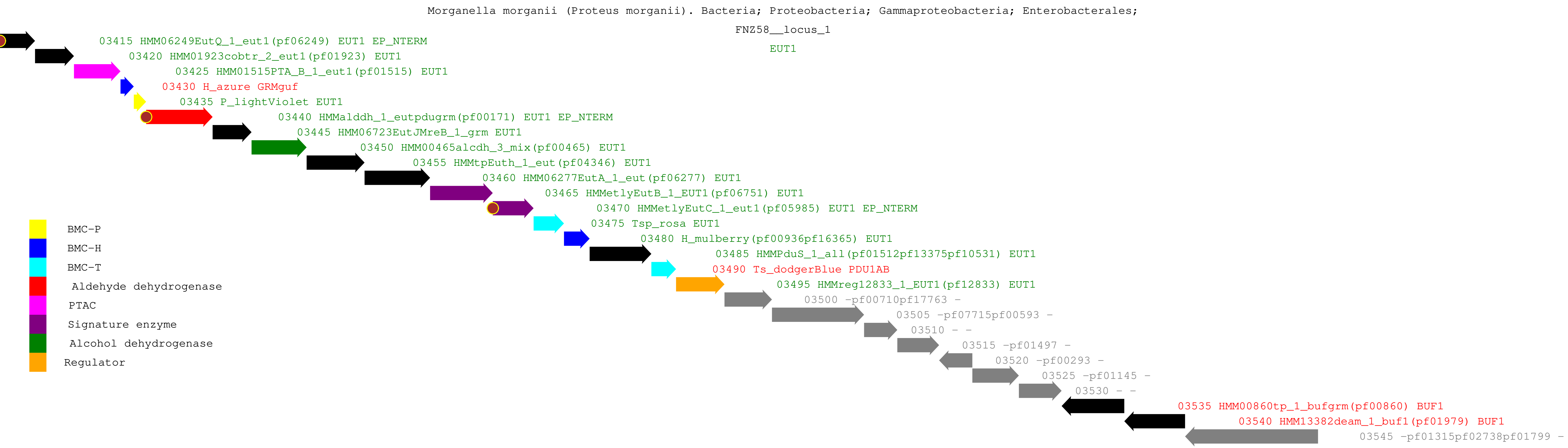
Morganella morganii (Proteus morganii). Bacteria; Proteobacteria; Gammaproteobacteria; Enterobacterales;
FNZ58__locus_1
EUT1
03415 HMM06249EutQ_1_eut1(pf06249) EUT1 EP_NTERM
03420 HMM01923cobtr_2_eut1(pf01923) EUT1
03425 HMM01515PTA_B_1_eut1(pf01515) EUT1
03430 H_azure GRMguf
03435 P_lightViolet EUT1
03440 HMMalddh_1_eutpdugrm(pf00171) EUT1 EP_NTERM
03445 HMM06723EutJMreB_1_grm EUT1
03450 HMM00465alcdh_3_mix(pf00465) EUT1
03455 HMMtpEuth_1_eut(pf04346) EUT1
03460 HMM06277EutA_1_eut(pf06277) EUT1
03465 HMMetlyEutB_1_EUT1(pf06751) EUT1
03470 HMMetlyEutC_1_eut1(pf05985) EUT1 EP_NTERM
03475 Tsp_rosa EUT1
03480 H_mulberry(pf00936pf16365) EUT1
03485 HMMPduS_1_all(pf01512pf13375pf10531) EUT1
03490 Ts_dodgerBlue PDU1AB
03495 HMMreg12833_1_EUT1(pf12833) EUT1
03500 -pf00710pf17763 -
03505 -pf07715pf00593 -
03510 - -
03515 -pf01497 -
03520 -pf00293 -
03525 -pf01145 -
03530 - -
03535 HMM00860tp_1_bufgrm(pf00860) BUF1
03540 HMM13382deam_1_buf1(pf01979) BUF1
03545 -pf01315pf02738pf01799 -
BMC-P
BMC-H
BMC-T
Aldehyde dehydrogenase
PTAC
Signature enzyme
Alcohol dehydrogenase
Regulator

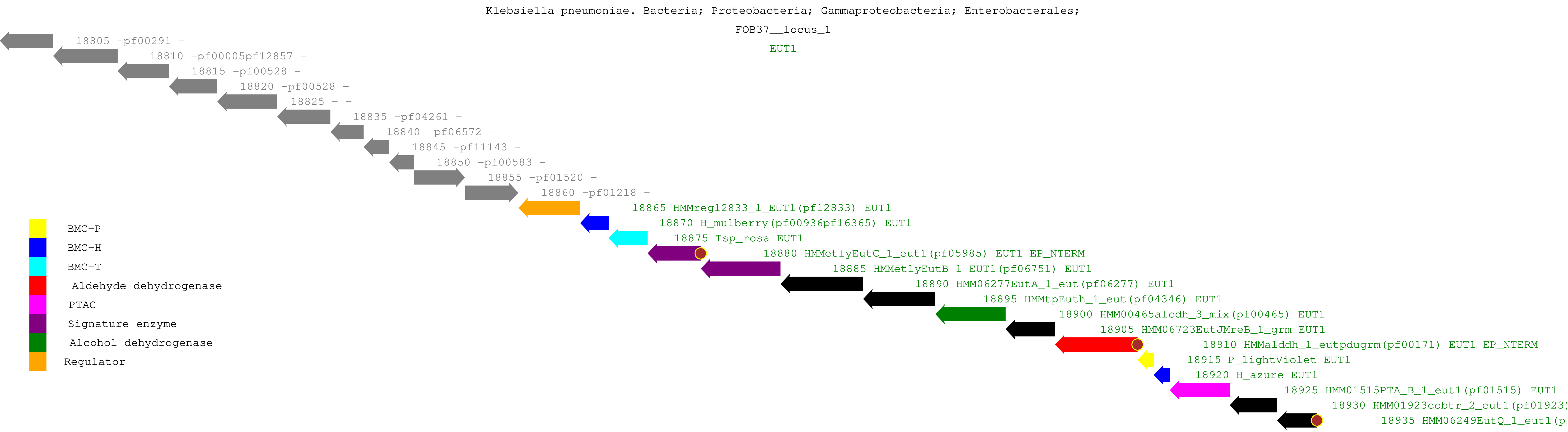
Klebsiella pneumoniae. Bacteria; Proteobacteria; Gammaproteobacteria; Enterobacterales;
FOB37__locus_1
EUT1
18805 -pf00291 -
18810 -pf00005pf12857 -
18815 -pf00528 -
18820 -pf00528 -
18825 - -
18835 -pf04261 -
18840 -pf06572 -
18845 -pf11143 -
18850 -pf00583 -
18855 -pf01520 -
18860 -pf01218 -
18865 HMMreg12833_1_EUT1(pf12833) EUT1
18870 H_mulberry(pf00936pf16365) EUT1
18875 Tsp_rosa EUT1
18880 HMMetlyEutC_1_eut1(pf05985) EUT1 EP_NTERM
18885 HMMetlyEutB_1_EUT1(pf06751) EUT1
18890 HMM06277EutA_1_eut(pf06277) EUT1
18895 HMMtpEuth_1_eut(pf04346) EUT1
18900 HMM00465alcdh_3_mix(pf00465) EUT1
18905 HMM06723EutJMreB_1_grm EUT1
18910 HMMalddh_1_eutpdugrm(pf00171) EUT1 EP_NTERM
18915 P_lightViolet EUT1
18920 H_azure EUT1
18925 HMM01515PTA_B_1_eut1(pf01515) EUT1
18930 HMM01923cobtr_2_eut1(pf01923)
18935 HMM06249EutQ_1_eut1(p
BMC-P
BMC-H
BMC-T
Aldehyde dehydrogenase
PTAC
Signature enzyme
Alcohol dehydrogenase
Regulator

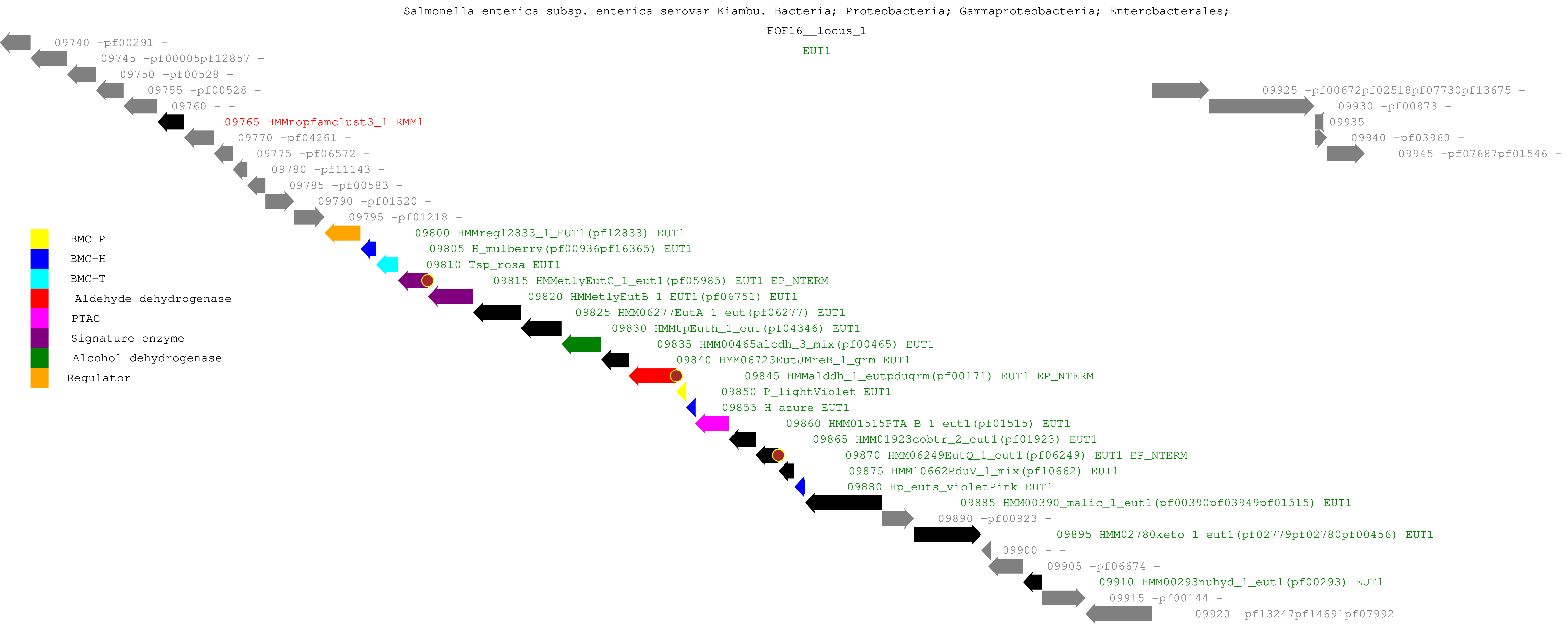
Salmonella enterica subsp. enterica serovar Kiambu. Bacteria; Proteobacteria; Gammaproteobacteria; Enterobacterales;
FOF16__locus_1
EUT1
09740 -pf00291 -
09745 -pf00005pf12857 -
09750 -pf00528 -
09755 -pf00528 -
09760 - -
09765 HMMnopfamclust3_1 RMM1
09770 -pf04261 -
09775 -pf06572 -
09780 -pf11143 -
09785 -pf00583 -
09790 -pf01520 -
09795 -pf01218 -
09800 HMMreg12833_1_EUT1(pf12833) EUT1
09805 H_mulberry(pf00936pf16365) EUT1
09810 Tsp_rosa EUT1
09815 HMMetlyEutC_1_eut1(pf05985) EUT1 EP_NTERM
09820 HMMetlyEutB_1_EUT1(pf06751) EUT1
09825 HMM06277EutA_1_eut(pf06277) EUT1
09830 HMMtpEuth_1_eut(pf04346) EUT1
09835 HMM00465alcdh_3_mix(pf00465) EUT1
09840 HMM06723EutJMreB_1_grm EUT1
09845 HMMalddh_1_eutpdugrm(pf00171) EUT1 EP_NTERM
09850 P_lightViolet EUT1
09855 H_azure EUT1
09860 HMM01515PTA_B_1_eut1(pf01515) EUT1
09865 HMM01923cobtr_2_eut1(pf01923) EUT1
09870 HMM06249EutQ_1_eut1(pf06249) EUT1 EP_NTERM
09875 HMM10662PduV_1_mix(pf10662) EUT1
09880 Hp_euts_violetPink EUT1
09885 HMM00390_malic_1_eut1(pf00390pf03949pf01515) EUT1
09890 -pf00923 -
09895 HMM02780keto_1_eut1(pf02779pf02780pf00456) EUT1
09900 - -
09905 -pf06674 -
09910 HMM00293nuhyd_1_eut1(pf00293) EUT1
09915 -pf00144 -
09920 -pf13247pf14691pf07992 -
09925 -pf00672pf02518pf07730pf13675 -
09930 -pf00873 -
09935 - -
09940 -pf03960 -
09945 -pf07687pf01546 -
BMC-P
BMC-H
BMC-T
Aldehyde dehydrogenase
PTAC
Signature enzyme
Alcohol dehydrogenase
Regulator

Escherichia coli. Bacteria; Proteobacteria; Gammaproteobacteria; Enterobacterales;

FORC31__locus_1

EUT1

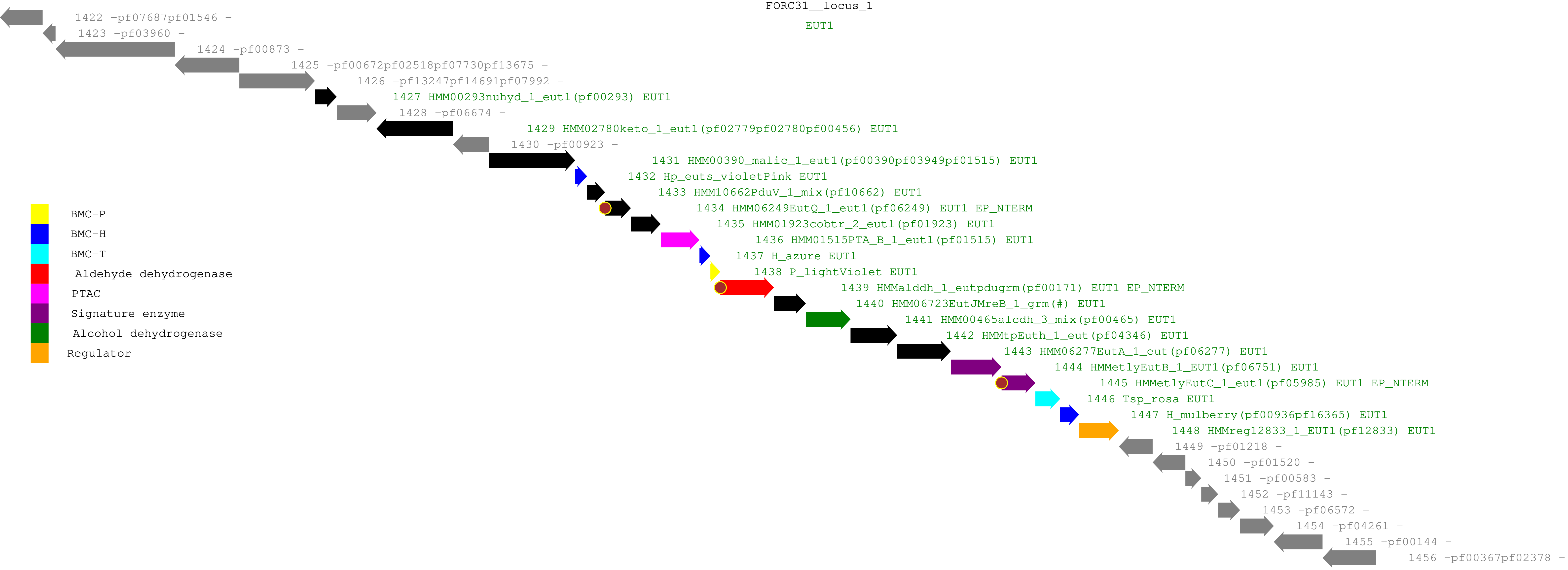

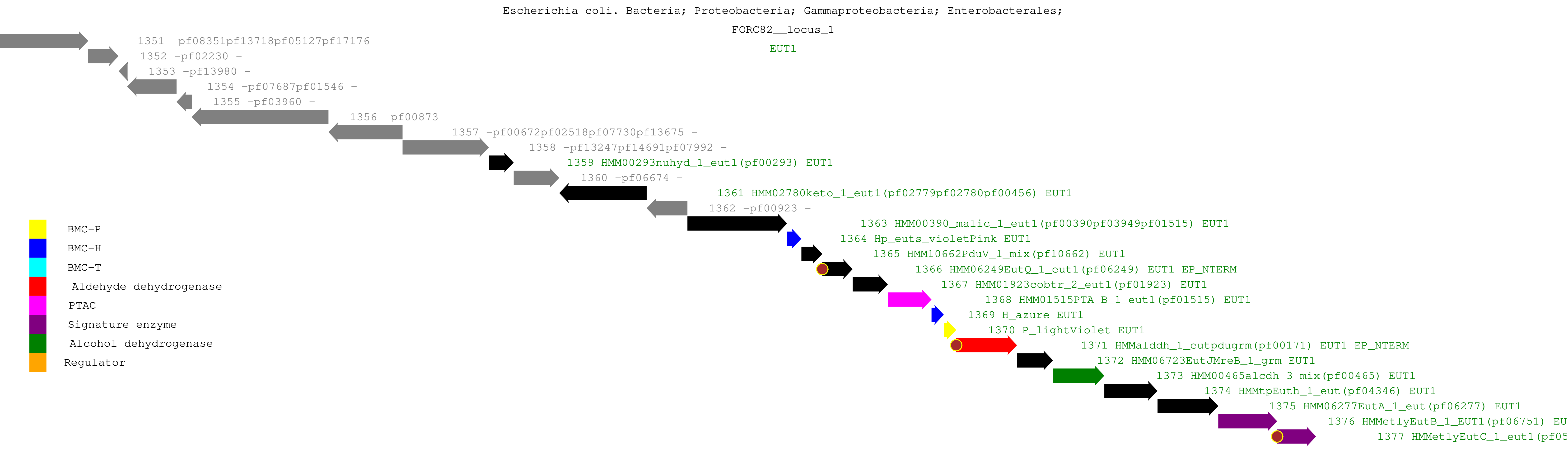
Escherichia coli. Bacteria; Proteobacteria; Gammaproteobacteria; Enterobacterales;
FORC82__locus_1
EUT1
1351 -pf08351pf13718pf05127pf17176 -
1352 -pf02230 -
1353 -pf13980 -
1354 -pf07687pf01546 -
1355 -pf03960 -
1356 -pf00873 -
1357 -pf00672pf02518pf07730pf13675 -
1358 -pf13247pf14691pf07992 -
1359 HMM00293nuhyd_1_eut1(pf00293) EUT1
1360 -pf06674 -
1361 HMM02780keto_1_eut1(pf02779pf02780pf00456) EUT1
1362 -pf00923 -
1363 HMM00390_malic_1_eut1(pf00390pf03949pf01515) EUT1
1364 Hp_euts_violetPink EUT1
1365 HMM10662PduV_1_mix(pf10662) EUT1
1366 HMM06249EutQ_1_eut1(pf06249) EUT1 EP_NTERM
1367 HMM01923cobtr_2_eut1(pf01923) EUT1
1368 HMM01515PTA_B_1_eut1(pf01515) EUT1
1369 H_azure EUT1
1370 P_lightViolet EUT1
1371 HMMaldh_1_eutpdugrm(pf00171) EUT1 EP_NTERM
1372 HMM06723EutJMreB_1_grm EUT1
1373 HMM00465alcdh_3_mix(pf00465) EUT1
1374 HMMtpEuth_1_eut(pf04346) EUT1
1375 HMM06277EutA_1_eut(pf06277) EUT1
1376 HMMetlyEutB_1_EUT1(pf06751) EU
1377 HMMetlyEutC_1_eut1(pf05
BMC-P
BMC-H
BMC-T
Aldehyde dehydrogenase
PTAC
Signature enzyme
Alcohol dehydrogenase
Regulator

Escherichia coli O177. Bacteria; Proteobacteria; Gammaproteobacteria; Enterobacterales;

FPV20__locus_1

EUT1

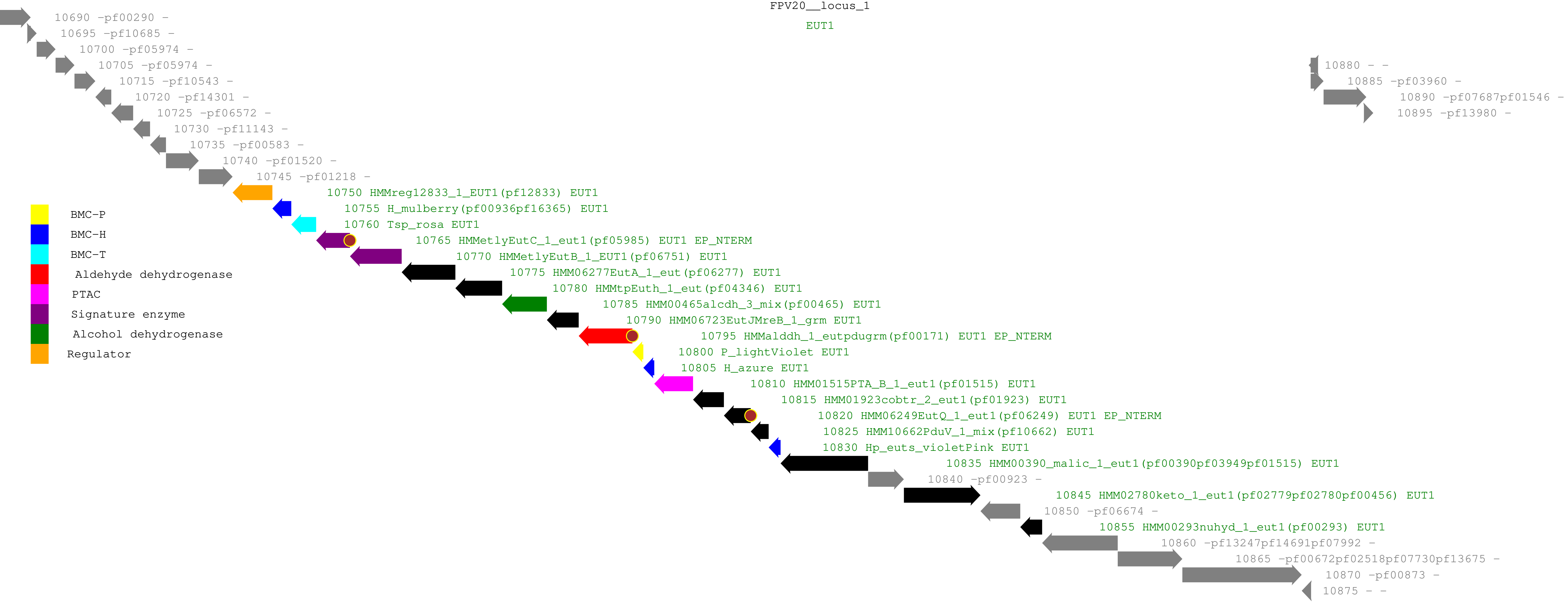

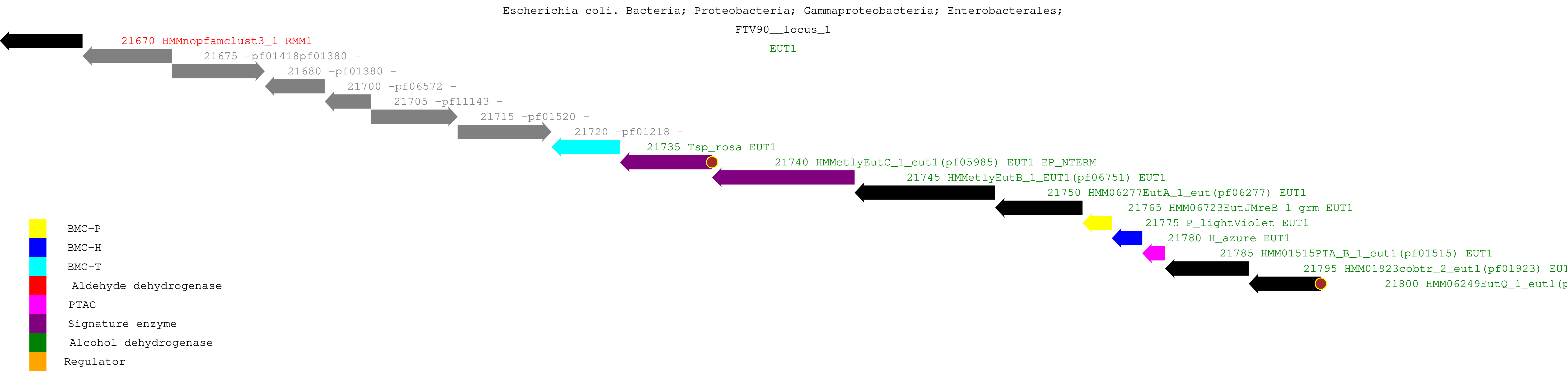

Escherichia coli. Bacteria; Proteobacteria; Gammaproteobacteria; Enterobacterales;
FTV90__locus_1
EUT1
21670 HMMnopfamclust3_1 RMM1
21675 -pf01418pf01380 -
21680 -pf01380 -
21700 -pf06572 -
21705 -pf11143 -
21715 -pf01520 -
21720 -pf01218 -
21735 Tsp_rosa EUT1
21740 HMMetlyEutC_1_eut1(pf05985) EUT1 EP_NTERM
21745 HMMetlyEutB_1_EUT1(pf06751) EUT1
21750 HMM06277EutA_1_eut(pf06277) EUT1
21765 HMM06723EutJMreB_1_grm EUT1
21775 P_lightViolet EUT1
21780 H_azure EUT1
21785 HMM01515PTA_B_1_eut1(pf01515) EUT1
21795 HMM01923cobtr_2_eut1(pf01923) EUT
21800 HMM06249EutQ_1_eut1(p
BMC-P
BMC-H
BMC-T
Aldehyde dehydrogenase
PTAC
Signature enzyme
Alcohol dehydrogenase
Regulator

Escherichia coli. Bacteria; Proteobacteria; Gammaproteobacteria; Enterobacterales;

FTV92__locus_1

EUT1

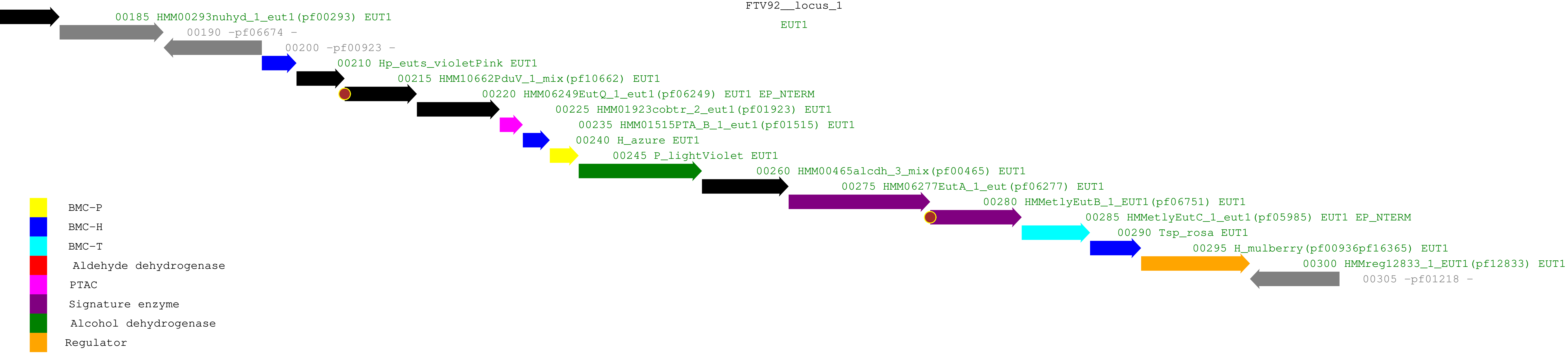

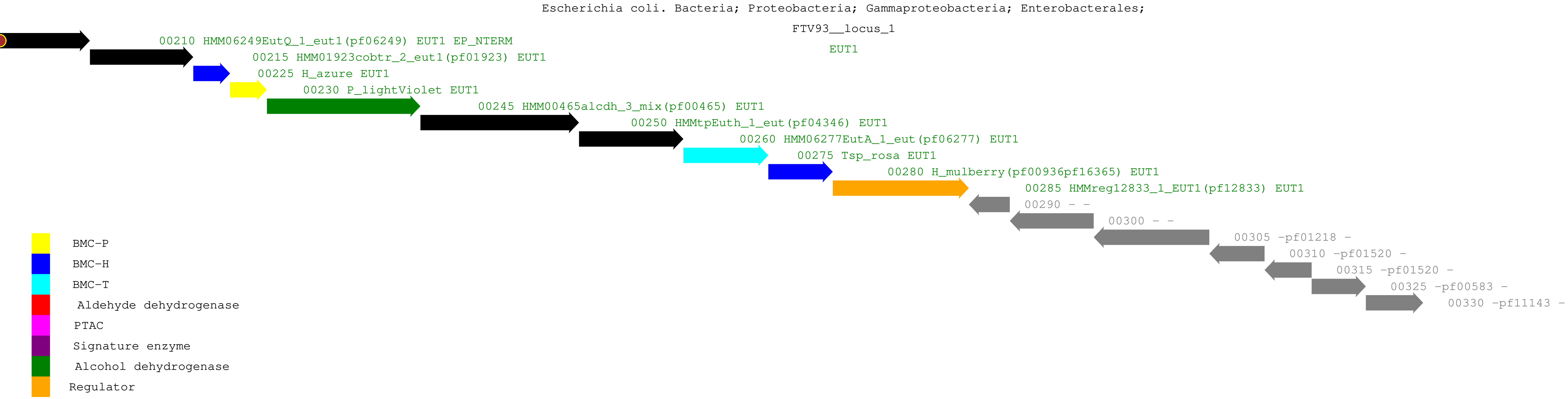

Escherichia coli. Bacteria; Proteobacteria; Gammaproteobacteria; Enterobacterales;
FTV93__locus_1
EUT1
00210 HMM06249EutQ_1_eut1(pf06249) EUT1 EP_NTERM
00215 HMM01923cobtr_2_eut1(pf01923) EUT1
00225 H_azure EUT1
00230 P_lightViolet EUT1
00245 HMM00465alcdh_3_mix(pf00465) EUT1
00250 HMMtpEuth_1_eut(pf04346) EUT1
00260 HMM06277EutA_1_eut(pf06277) EUT1
00275 Tsp_rosa EUT1
00280 H_mulberry(pf00936pf16365) EUT1
00285 HMMreg12833_1_EUT1(pf12833) EUT1
00290 - -
00300 - -
00305 -pf01218 -
00310 -pf01520 -
00315 -pf01520 -
00325 -pf00583 -
00330 -pf11143 -
BMC-P
BMC-H
BMC-T
Aldehyde dehydrogenase
PTAC
Signature enzyme
Alcohol dehydrogenase
Regulator

Klebsiella pneumoniae. Bacteria; Proteobacteria; Gammaproteobacteria; Enterobacterales;

FXN67__locus_1

EUT1

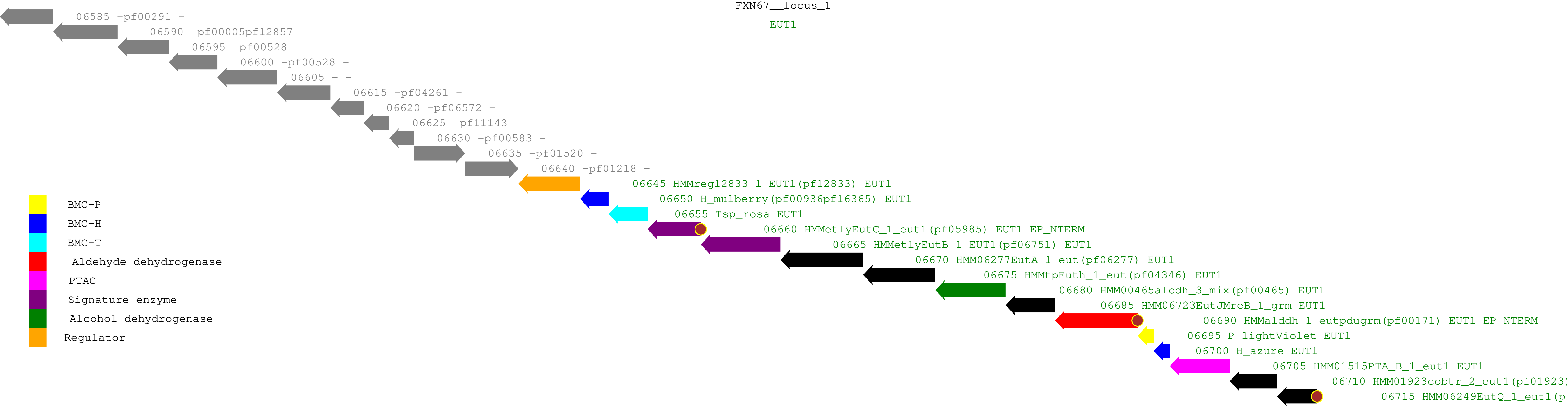

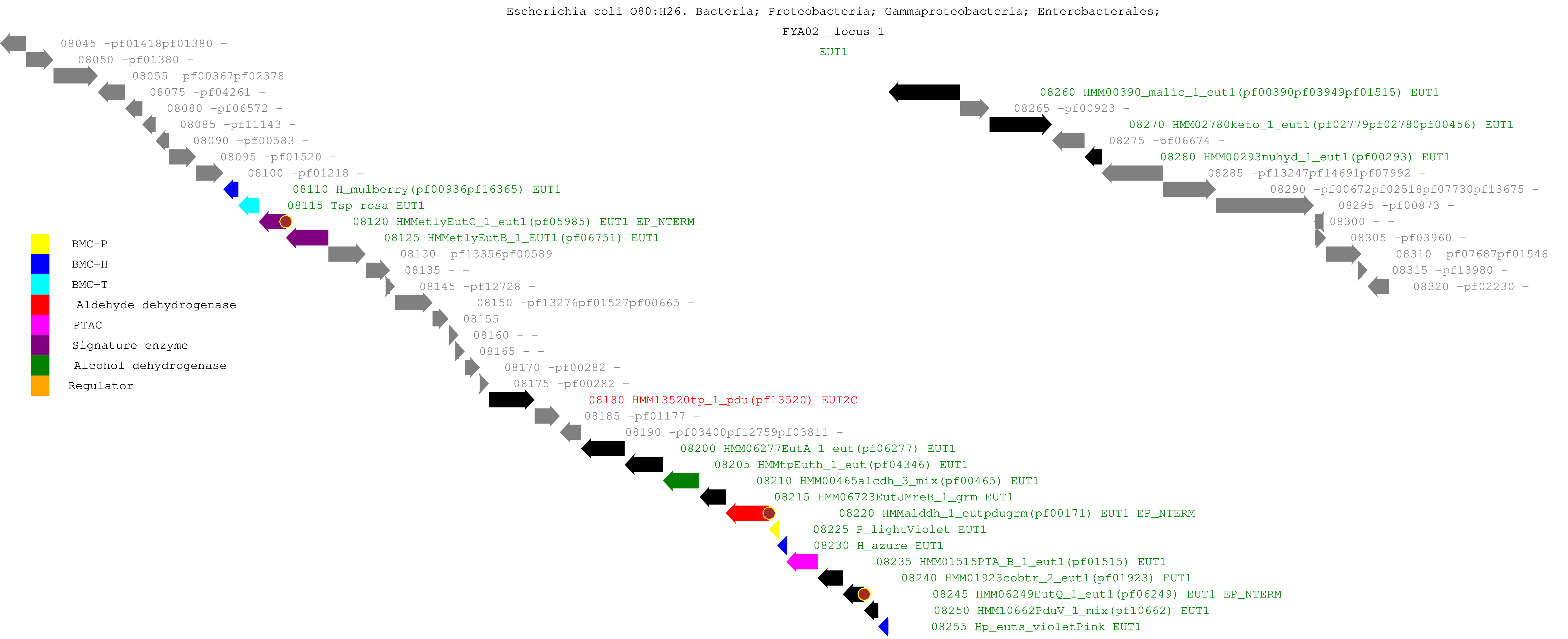
Escherichia coli O80:H26. Bacteria; Proteobacteria; Gammaproteobacteria; Enterobacterales;
FYA02__locus_1
EUT1
08045 -pf01418pf01380 -
08050 -pf01380 -
08055 -pf00367pf02378 -
08075 -pf04261 -
08080 -pf06572 -
08085 -pf11143 -
08090 -pf00583 -
08095 -pf01520 -
08100 -pf01218 -
08110 H_mulberry(pf00936pf16365) EUT1
08115 Tsp_rosa EUT1
08120 HMMetlyEutC_1_eut1(pf05985) EUT1 EP_NTERM
08125 HMMetlyEutB_1_EUT1(pf06751) EUT1
08130 -pf13356pf00589 -
08135 - -
08145 -pf12728 -
08150 -pf13276pf01527pf00665 -
08155 - -
08160 - -
08165 - -
08170 -pf00282 -
08175 -pf00282 -
08180 HMM13520tp_1_pdu(pf13520) EUT2C
08185 -pf01177 -
08190 -pf03400pf12759pf03811 -
08200 HMM06277EutA_1_eut(pf06277) EUT1
08205 HMMtpEuth_1_eut(pf04346) EUT1
08210 HMM00465alcdh_3_mix(pf00465) EUT1
08215 HMM06723EutJMreB_1_grm EUT1
08220 HMMalddh_1_eutpdugrm(pf00171) EUT1 EP_NTERM
08225 P_lightViolet EUT1
08230 H_azure EUT1
08235 HMM01515PTA_B_1_eut1(pf01515) EUT1
08240 HMM01923cobtr_2_eut1(pf01923) EUT1
08245 HMM06249EutQ_1_eut1(pf06249) EUT1 EP_NTERM
08250 HMM10662PduV_1_mix(pf10662) EUT1
08255 Hp_euts_violetPink EUT1
08260 HMM00390_malic_1_eut1(pf00390pf03949pf01515) EUT1
08265 -pf00923 -
08270 HMM02780keto_1_eut1(pf02779pf02780pf00456) EUT1
08275 -pf06674 -
08280 HMM00293nuhyd_1_eut1(pf00293) EUT1
08285 -pf13247pf14691pf07992 -
08290 -pf00672pf02518pf07730pf13675 -
08295 -pf00873 -
08300 - -
08305 -pf03960 -
08310 -pf07687pf01546 -
08315 -pf13980 -
08320 -pf02230 -
BMC-P
BMC-H
BMC-T
Aldehyde dehydrogenase
PTAC
Signature enzyme
Alcohol dehydrogenase
Regulator

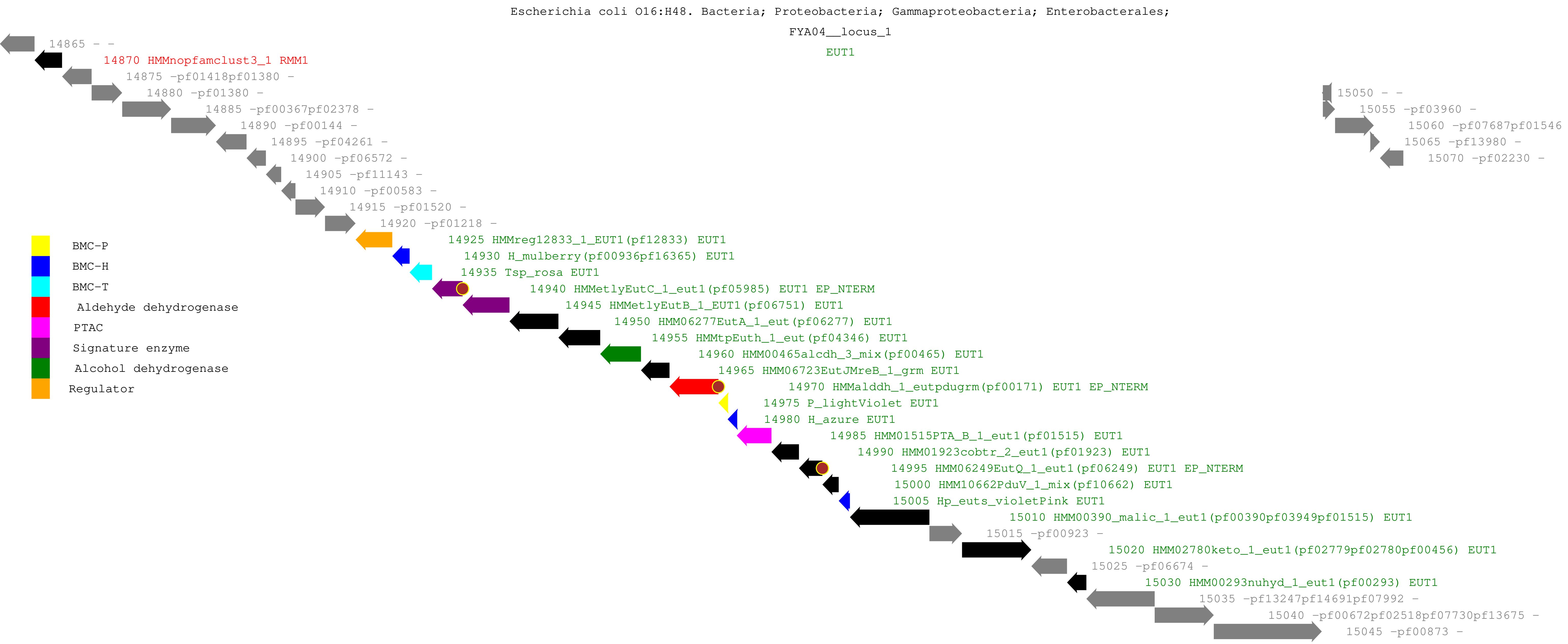
Escherichia coli O16:H48. Bacteria; Proteobacteria; Gammaproteobacteria; Enterobacterales;
FYA04__locus_1
EUT1
14865 - -
14870 HMMnopfamclust3_1 RMM1
14875 -pf01418pf01380 -
14880 -pf01380 -
14885 -pf00367pf02378 -
14890 -pf00144 -
14895 -pf04261 -
14900 -pf06572 -
14905 -pf11143 -
14910 -pf00583 -
14915 -pf01520 -
14920 -pf01218 -
14925 HMMreg12833_1_EUT1(pf12833) EUT1
14930 H_mulberry(pf00936pf16365) EUT1
14935 Tsp_rosa EUT1
14940 HMMetlyEutC_1_eut1(pf05985) EUT1 EP_NTERM
14945 HMMetlyEutB_1_EUT1(pf06751) EUT1
14950 HMM06277EutA_1_eut(pf06277) EUT1
14955 HMMtpEuth_1_eut(pf04346) EUT1
14960 HMM00465alcdh_3_mix(pf00465) EUT1
14965 HMM06723EutJMreB_1_grm EUT1
14970 HMMaldh_1_eutpdugrm(pf00171) EUT1 EP_NTERM
14975 P_lightViolet EUT1
14980 H_azure EUT1
14985 HMM01515PTA_B_1_eut1(pf01515) EUT1
14990 HMM01923cobtr_2_eut1(pf01923) EUT1
14995 HMM06249EutQ_1_eut1(pf06249) EUT1 EP_NTERM
15000 HMM10662PduV_1_mix(pf10662) EUT1
15005 Hp_euts_violetPink EUT1
15010 HMM00390_malic_1_eut1(pf00390pf03949pf01515) EUT1
15015 -pf00923 -
15020 HMM02780keto_1_eut1(pf02779pf02780pf00456) EUT1
15025 -pf06674 -
15030 HMM00293nuhyd_1_eut1(pf00293) EUT1
15035 -pf13247pf14691pf07992 -
15040 -pf00672pf02518pf07730pf13675 -
15045 -pf00873 -
15050 - -
15055 -pf03960 -
15060 -pf07687pf01546 -
15065 -pf13980 -
15070 -pf02230 -
BMC-P
BMC-H
BMC-T
Aldehyde dehydrogenase
PTAC
Signature enzyme
Alcohol dehydrogenase
Regulator

Escherichia coli O2:H6. Bacteria; Proteobacteria; Gammaproteobacteria; Enterobacterales;

FYA20__locus_1

EUT1

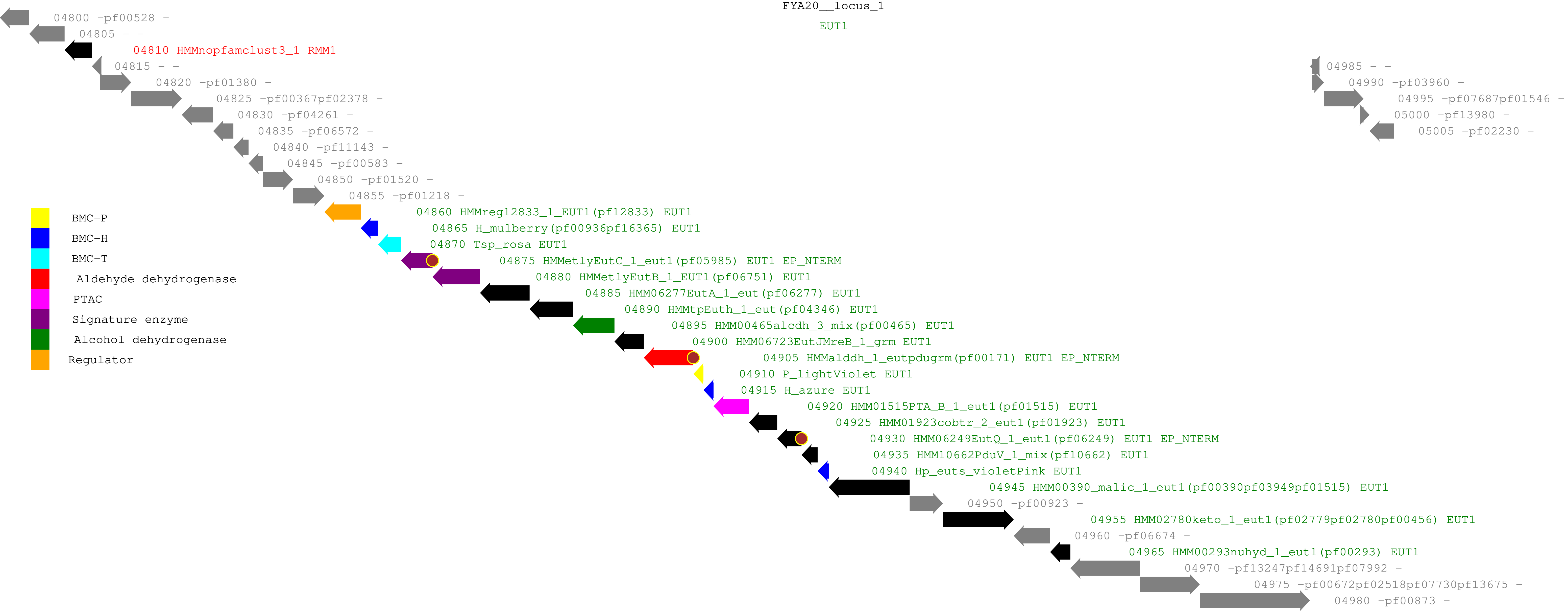

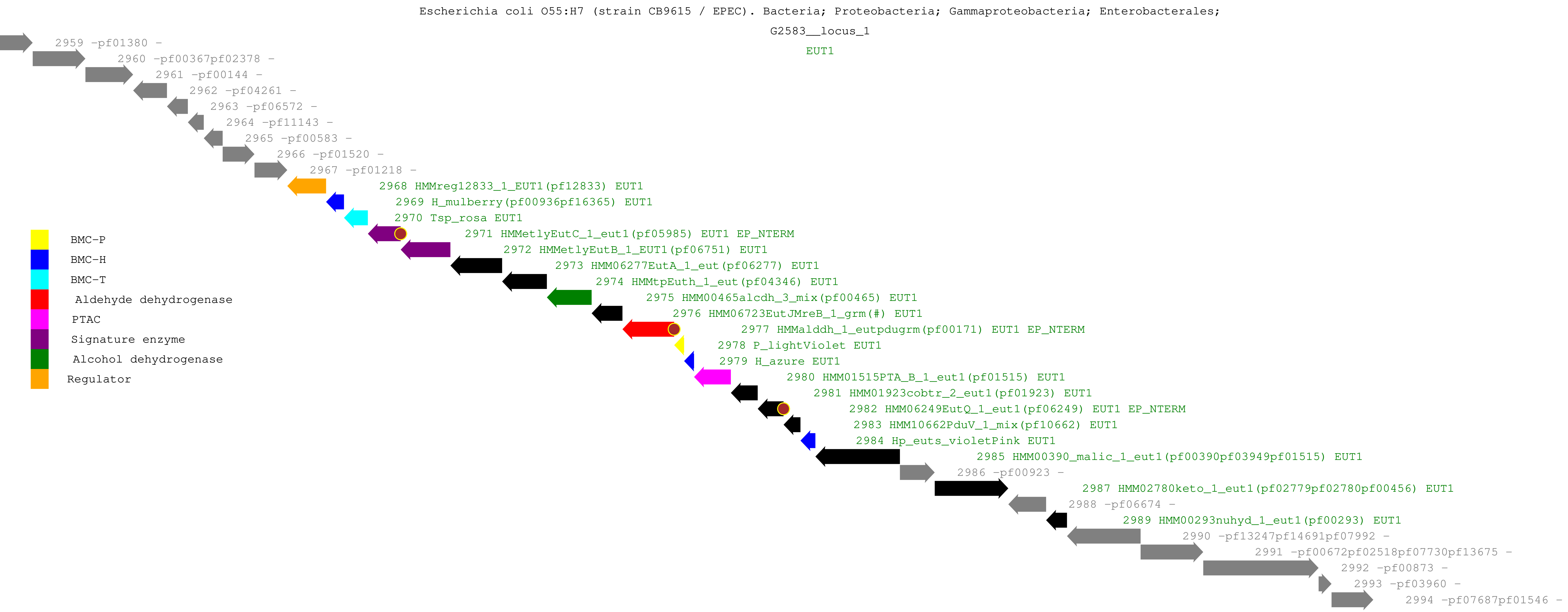

Escherichia coli O55:H7 (strain CB9615 / EPEC). Bacteria; Proteobacteria; Gammaproteobacteria; Enterobacterales;
G2583__locus_1
EUT1
2959 -pf01380 -
2960 -pf00367pf02378 -
2961 -pf00144 -
2962 -pf04261 -
2963 -pf06572 -
2964 -pf11143 -
2965 -pf00583 -
2966 -pf01520 -
2967 -pf01218 -
2968 HMMreg12833_1_EUT1(pf12833) EUT1
2969 H_mulberry(pf00936pf16365) EUT1
2970 Tsp_rosa EUT1
2971 HMMetlyEutC_1_eut1(pf05985) EUT1 EP_NTERM
2972 HMMetlyEutB_1_EUT1(pf06751) EUT1
2973 HMM06277EutA_1_eut(pf06277) EUT1
2974 HMMtpEuth_1_eut(pf04346) EUT1
2975 HMM00465alcdh_3_mix(pf00465) EUT1
2976 HMM06723EutJMreB_1_grm(#) EUT1
2977 HMMaldDH_1_eutpdugrm(pf00171) EUT1 EP_NTERM
2978 P_lightViolet EUT1
2979 H_azure EUT1
2980 HMM01515PTA_B_1_eut1(pf01515) EUT1
2981 HMM01923cobtr_2_eut1(pf01923) EUT1
2982 HMM06249EutQ_1_eut1(pf06249) EUT1 EP_NTERM
2983 HMM10662PduV_1_mix(pf10662) EUT1
2984 Hp_euts_violetPink EUT1
2985 HMM00390_malic_1_eut1(pf00390pf03949pf01515) EUT1
2986 -pf00923 -
2987 HMM02780keto_1_eut1(pf02779pf02780pf00456) EUT1
2988 -pf06674 -
2989 HMM00293nuhyd_1_eut1(pf00293) EUT1
2990 -pf13247pf14691pf07992 -
2991 -pf00672pf02518pf07730pf13675 -
2992 -pf00873 -
2993 -pf03960 -
2994 -pf07687pf01546 -
BMC-P
BMC-H
BMC-T
Aldehyde dehydrogenase
PTAC
Signature enzyme
Alcohol dehydrogenase
Regulator

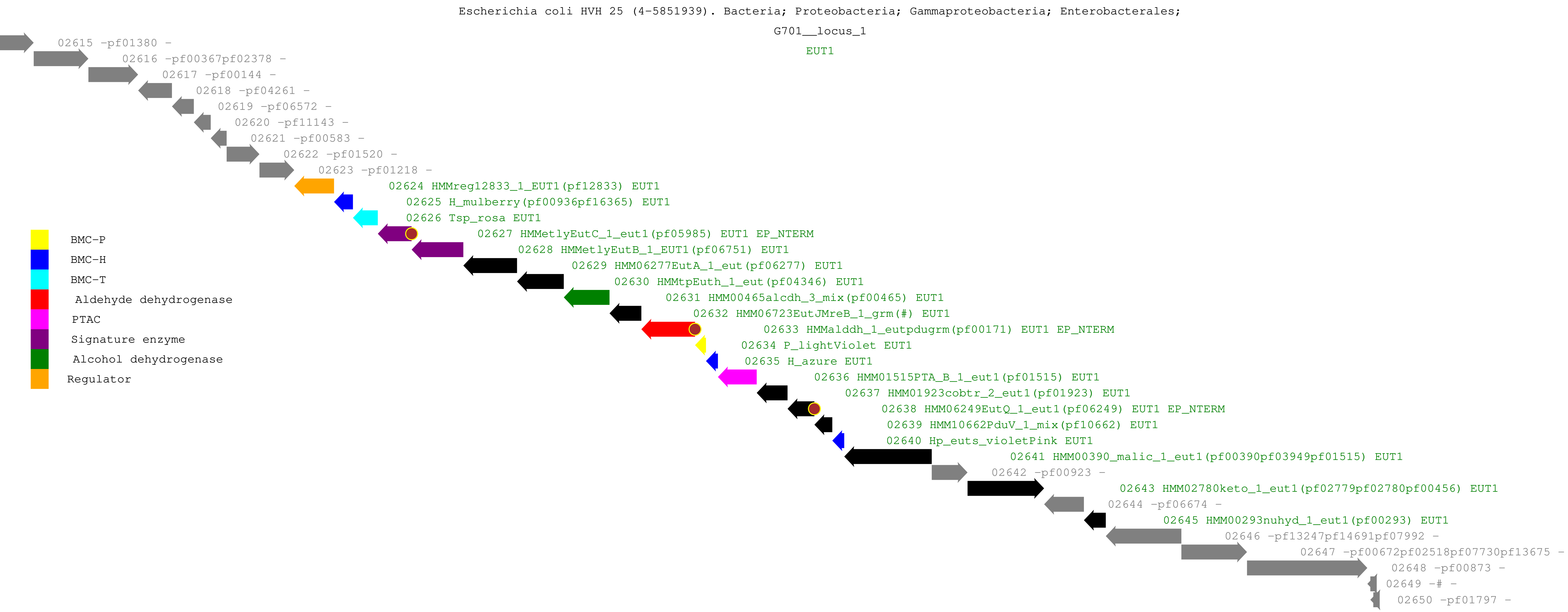
Escherichia coli HVH 25 (4-5851939). Bacteria; Proteobacteria; Gammaproteobacteria; Enterobacterales;
G701__locus_1
EUT1
02615 -pf01380 -
02616 -pf00367pf02378 -
02617 -pf00144 -
02618 -pf04261 -
02619 -pf06572 -
02620 -pf11143 -
02621 -pf00583 -
02622 -pf01520 -
02623 -pf01218 -
02624 HMMreg12833_1_EUT1(pf12833) EUT1
02625 H_mulberry(pf00936pf16365) EUT1
02626 Tsp_rosa EUT1
02627 HMMetlyEutC_1_eut1(pf05985) EUT1 EP_NTERM
02628 HMMetlyEutB_1_EUT1(pf06751) EUT1
02629 HMM06277EutA_1_eut(pf06277) EUT1
02630 HMMtpEuth_1_eut(pf04346) EUT1
02631 HMM00465alcdh_3_mix(pf00465) EUT1
02632 HMM06723EutJMreB_1_grm(#) EUT1
02633 HMMalddh_1_eutpdugrm(pf00171) EUT1 EP_NTERM
02634 P_lightViolet EUT1
02635 H_azure EUT1
02636 HMM01515PTA_B_1_eut1(pf01515) EUT1
02637 HMM01923cobtr_2_eut1(pf01923) EUT1
02638 HMM06249EutQ_1_eut1(pf06249) EUT1 EP_NTERM
02639 HMM10662PduV_1_mix(pf10662) EUT1
02640 Hp_euts_violetPink EUT1
02641 HMM00390_malic_1_eut1(pf00390pf03949pf01515) EUT1
02642 -pf00923 -
02643 HMM02780keto_1_eut1(pf02779pf02780pf00456) EUT1
02644 -pf06674 -
02645 HMM00293nuhyd_1_eut1(pf00293) EUT1
02646 -pf13247pf14691pf07992 -
02647 -pf00672pf02518pf07730pf13675 -
02648 -pf00873 -
02649 -# -
02650 -pf01797 -
BMC-P
BMC-H
BMC-T
Aldehyde dehydrogenase
PTAC
Signature enzyme
Alcohol dehydrogenase
Regulator

Escherichia coli HVH 41 (4-2677849). Bacteria; Proteobacteria; Gammaproteobacteria; Enterobacterales;

G716__locus_1

EUT1

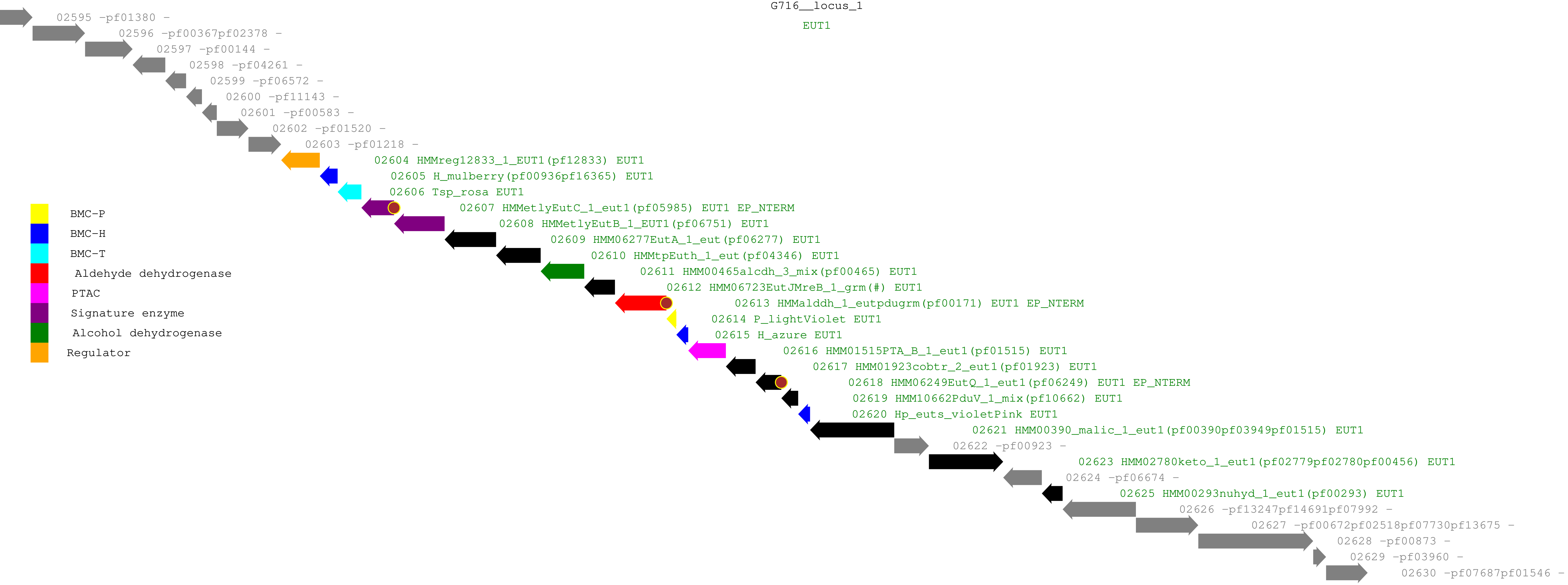

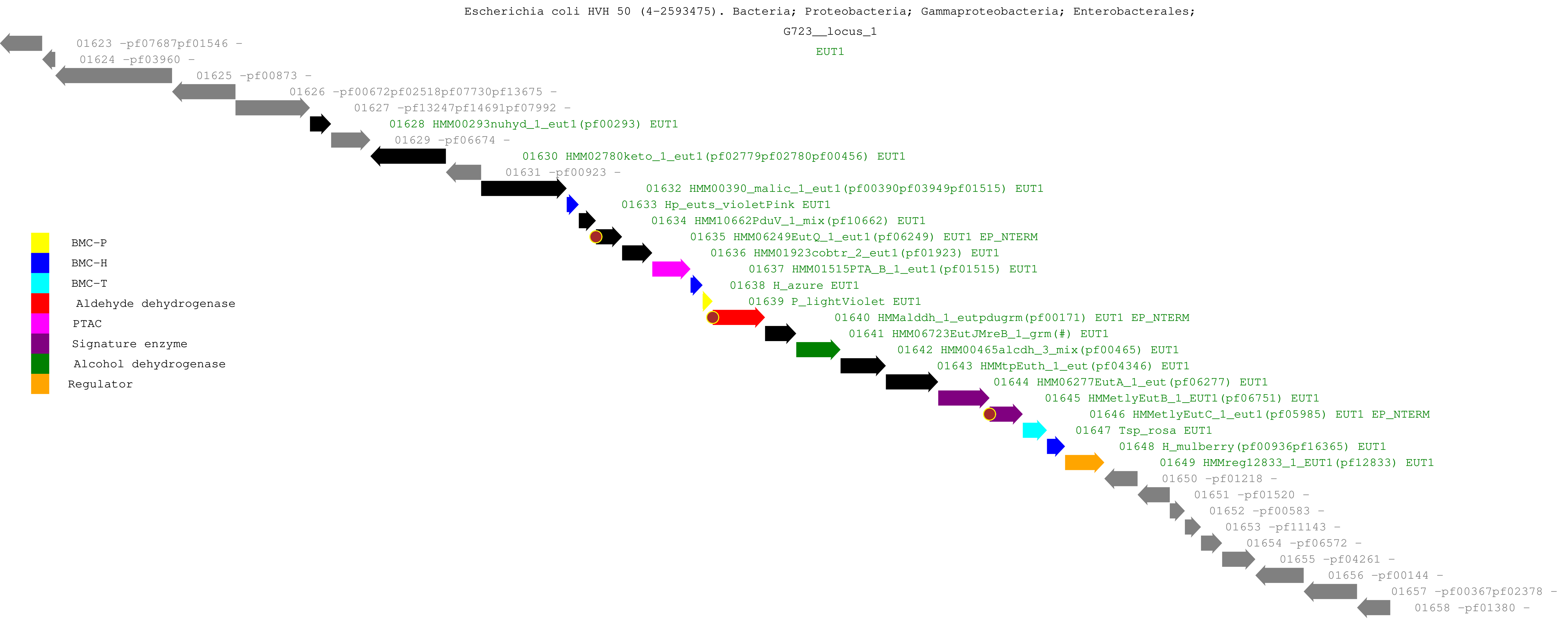
Escherichia coli HVH 50 (4-2593475). Bacteria; Proteobacteria; Gammaproteobacteria; Enterobacterales;
G723__locus_1
EUT1
01623 -pf07687pf01546 -
01624 -pf03960 -
01625 -pf00873 -
01626 -pf00672pf02518pf07730pf13675 -
01627 -pf13247pf14691pf07992 -
01628 HMM00293nuhyd_1_eut1(pf00293) EUT1
01629 -pf06674 -
01630 HMM02780keto_1_eut1(pf02779pf02780pf00456) EUT1
01631 -pf00923 -
01632 HMM00390_malic_1_eut1(pf00390pf03949pf01515) EUT1
01633 Hp_euts_violetPink EUT1
01634 HMM10662PduV_1_mix(pf10662) EUT1
01635 HMM06249EutQ_1_eut1(pf06249) EUT1 EP_NTERM
01636 HMM01923cobtr_2_eut1(pf01923) EUT1
01637 HMM01515PTA_B_1_eut1(pf01515) EUT1
01638 H_azure EUT1
01639 P_lightViolet EUT1
01640 HMMalddh_1_eutpdugrm(pf00171) EUT1 EP_NTERM
01641 HMM06723EutJMreB_1_grm(#) EUT1
01642 HMM00465alcdh_3_mix(pf00465) EUT1
01643 HMMtpEuth_1_eut(pf04346) EUT1
01644 HMM06277EutA_1_eut(pf06277) EUT1
01645 HMMetlyEutB_1_EUT1(pf06751) EUT1
01646 HMMetlyEutC_1_eut1(pf05985) EUT1 EP_NTERM
01647 Tsp_rosa EUT1
01648 H_mulberry(pf00936pf16365) EUT1
01649 HMMreg12833_1_EUT1(pf12833) EUT1
01650 -pf01218 -
01651 -pf01520 -
01652 -pf00583 -
01653 -pf11143 -
01654 -pf06572 -
01655 -pf04261 -
01656 -pf00144 -
01657 -pf00367pf02378 -
01658 -pf01380 -
BMC-P
BMC-H
BMC-T
Aldehyde dehydrogenase
PTAC
Signature enzyme
Alcohol dehydrogenase
Regulator

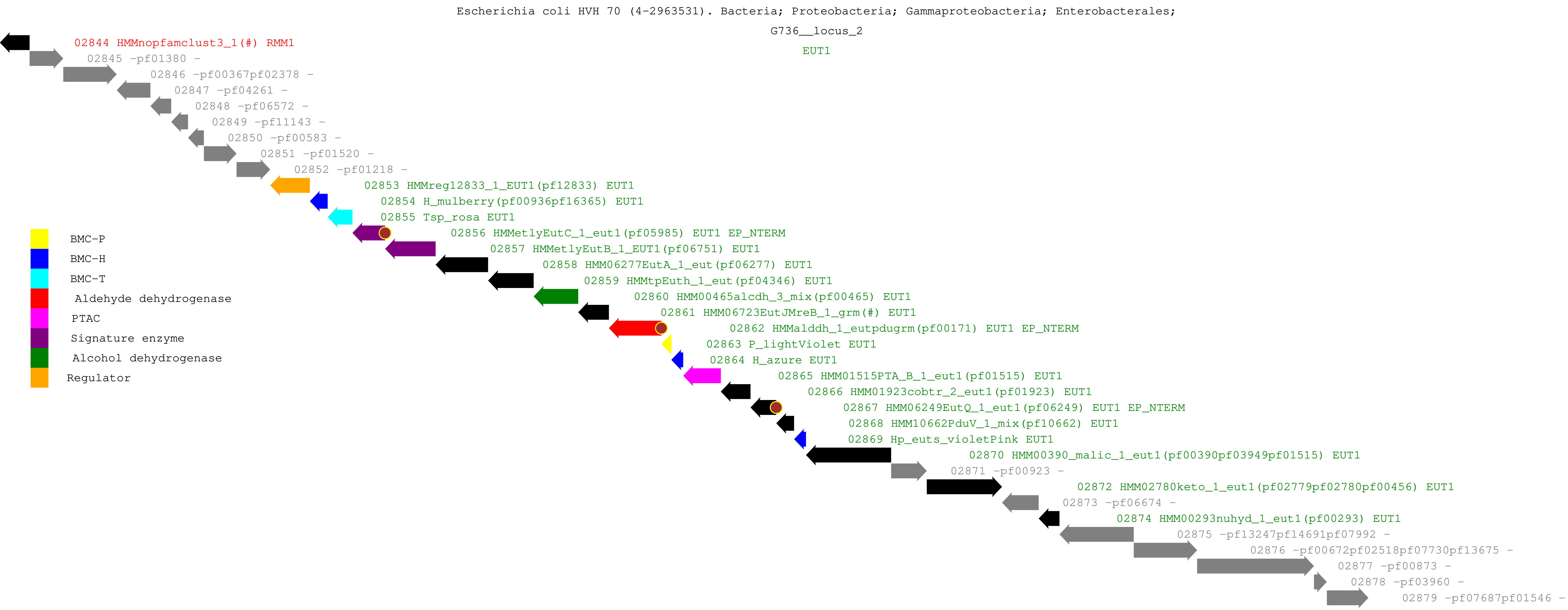
Escherichia coli HVH 70 (4-2963531). Bacteria; Proteobacteria; Gammaproteobacteria; Enterobacterales;
G736__locus_2
EUT1
02844 HMMnopfamclust3_1(#) RMM1
02845 -pf01380 -
02846 -pf00367pf02378 -
02847 -pf04261 -
02848 -pf06572 -
02849 -pf11143 -
02850 -pf00583 -
02851 -pf01520 -
02852 -pf01218 -
02853 HMMreg12833_1_EUT1(pf12833) EUT1
02854 H_mulberry(pf00936pf16365) EUT1
02855 Tsp_rosa EUT1
02856 HMMetlyEutC_1_eut1(pf05985) EUT1 EP_NTERM
02857 HMMetlyEutB_1_EUT1(pf06751) EUT1
02858 HMM06277EutA_1_eut(pf06277) EUT1
02859 HMMtpEuth_1_eut(pf04346) EUT1
02860 HMM00465alcdh_3_mix(pf00465) EUT1
02861 HMM06723EutJMreB_1_grm(#) EUT1
02862 HMMalddh_1_eutpdugrm(pf00171) EUT1 EP_NTERM
02863 P_lightViolet EUT1
02864 H_azure EUT1
02865 HMM01515PTA_B_1_eut1(pf01515) EUT1
02866 HMM01923cobtr_2_eut1(pf01923) EUT1
02867 HMM06249EutQ_1_eut1(pf06249) EUT1 EP_NTERM
02868 HMM10662PduV_1_mix(pf10662) EUT1
02869 Hp_euts_violetPink EUT1
02870 HMM00390_malic_1_eut1(pf00390pf03949pf01515) EUT1
02871 -pf00923 -
02872 HMM02780keto_1_eut1(pf02779pf02780pf00456) EUT1
02873 -pf06674 -
02874 HMM00293nuhyd_1_eut1(pf00293) EUT1
02875 -pf13247pf14691pf07992 -
02876 -pf00672pf02518pf07730pf13675 -
02877 -pf00873 -
02878 -pf03960 -
02879 -pf07687pf01546 -
BMC-P
BMC-H
BMC-T
Aldehyde dehydrogenase
PTAC
Signature enzyme
Alcohol dehydrogenase
Regulator

Escherichia coli KOEGE 71 (186a). Bacteria; Proteobacteria; Gammaproteobacteria; Enterobacterales;

G893__locus_2

EUT1

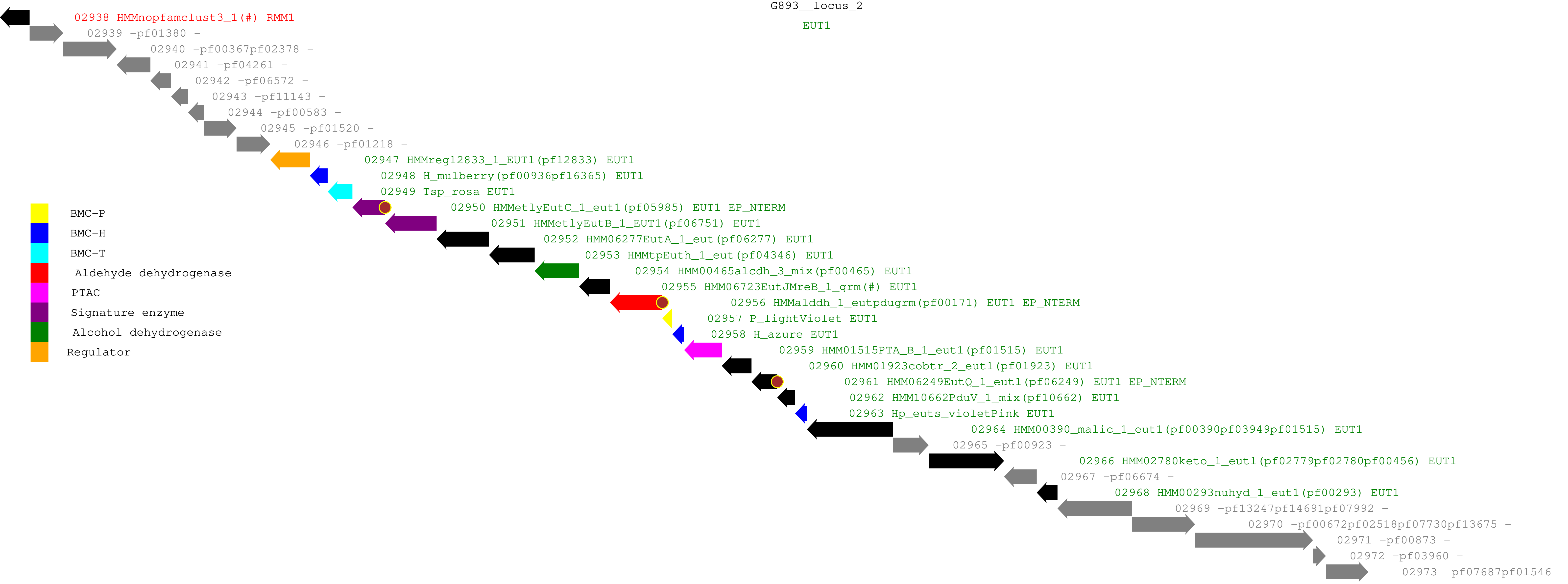

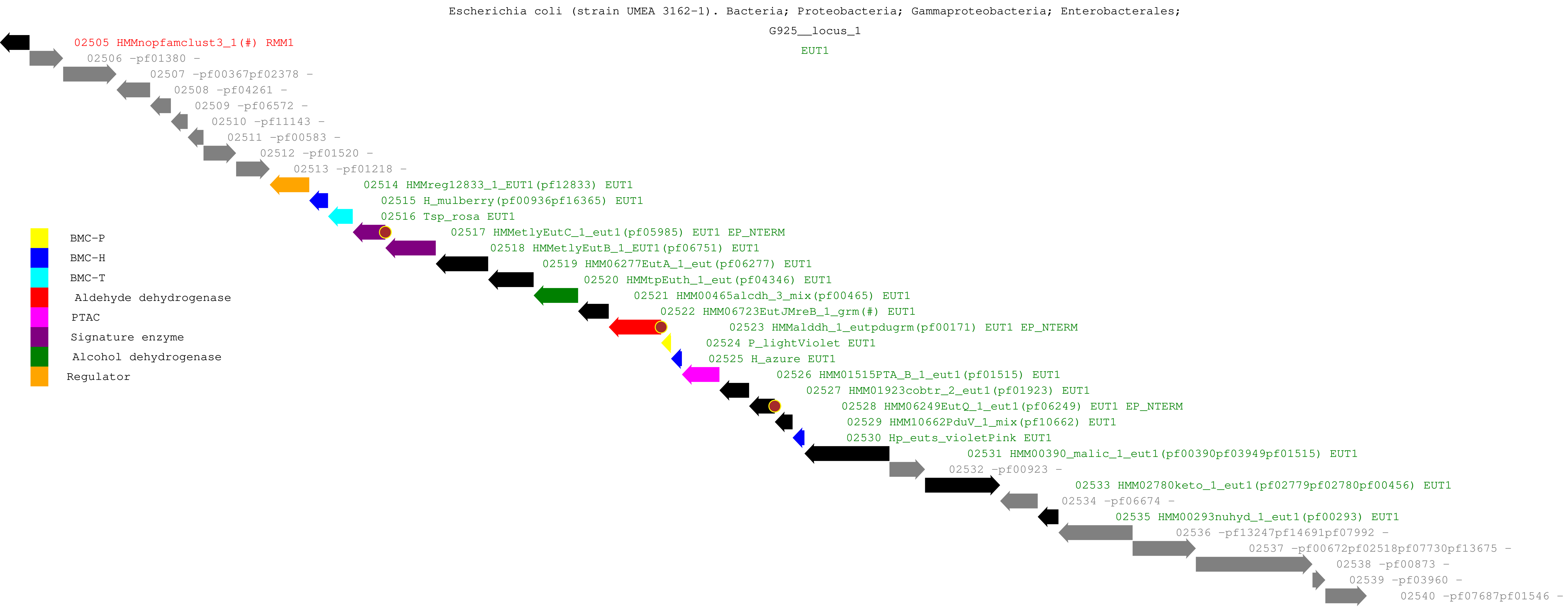

Escherichia coli (strain UMEA 3162-1). Bacteria; Proteobacteria; Gammaproteobacteria; Enterobacterales;
G925__locus_1
EUT1
02505 HMMnopfamclust3_1(#) RMM1
02506 -pf01380 -
02507 -pf00367pf02378 -
02508 -pf04261 -
02509 -pf06572 -
02510 -pf11143 -
02511 -pf00583 -
02512 -pf01520 -
02513 -pf01218 -
02514 HMMreg12833_1_EUT1(pf12833) EUT1
02515 H_mulberry(pf00936pf16365) EUT1
02516 Tsp_rosa EUT1
02517 HMMetlyEutC_1_eut1(pf05985) EUT1 EP_NTERM
02518 HMMetlyEutB_1_EUT1(pf06751) EUT1
02519 HMM06277EutA_1_eut(pf06277) EUT1
02520 HMMtpEuth_1_eut(pf04346) EUT1
02521 HMM00465alcdh_3_mix(pf00465) EUT1
02522 HMM06723EutJMreB_1_grm(#) EUT1
02523 HMMalddh_1_eutpdugrm(pf00171) EUT1 EP_NTERM
02524 P_lightViolet EUT1
02525 H_azure EUT1
02526 HMM01515PTA_B_1_eut1(pf01515) EUT1
02527 HMM01923cobtr_2_eut1(pf01923) EUT1
02528 HMM06249EutQ_1_eut1(pf06249) EUT1 EP_NTERM
02529 HMM10662PduV_1_mix(pf10662) EUT1
02530 Hp_euts_violetPink EUT1
02531 HMM00390_malic_1_eut1(pf00390pf03949pf01515) EUT1
02532 -pf00923 -
02533 HMM02780keto_1_eut1(pf02779pf02780pf00456) EUT1
02534 -pf06674 -
02535 HMM00293nuhyd_1_eut1(pf00293) EUT1
02536 -pf13247pf14691pf07992 -
02537 -pf00672pf02518pf07730pf13675 -
02538 -pf00873 -
02539 -pf03960 -
02540 -pf07687pf01546 -
BMC-P
BMC-H
BMC-T
Aldehyde dehydrogenase
PTAC
Signature enzyme
Alcohol dehydrogenase
Regulator

Escherichia coli UMEA 3200-1. Bacteria; Proteobacteria; Gammaproteobacteria; Enterobacterales;

G938__locus_2

EUT1

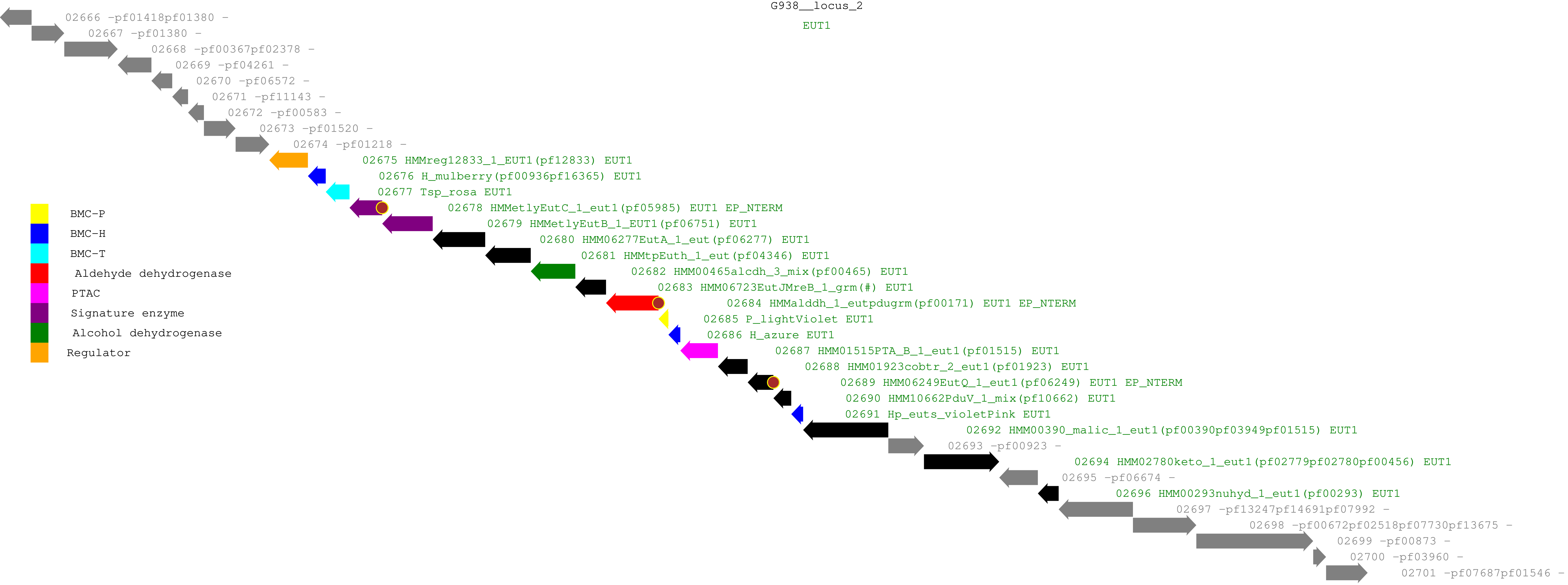

Escherichia coli UMEA 3212-1. Bacteria; Proteobacteria; Gammaproteobacteria; Enterobacterales;

G943__locus_1_main_sat

EUT1

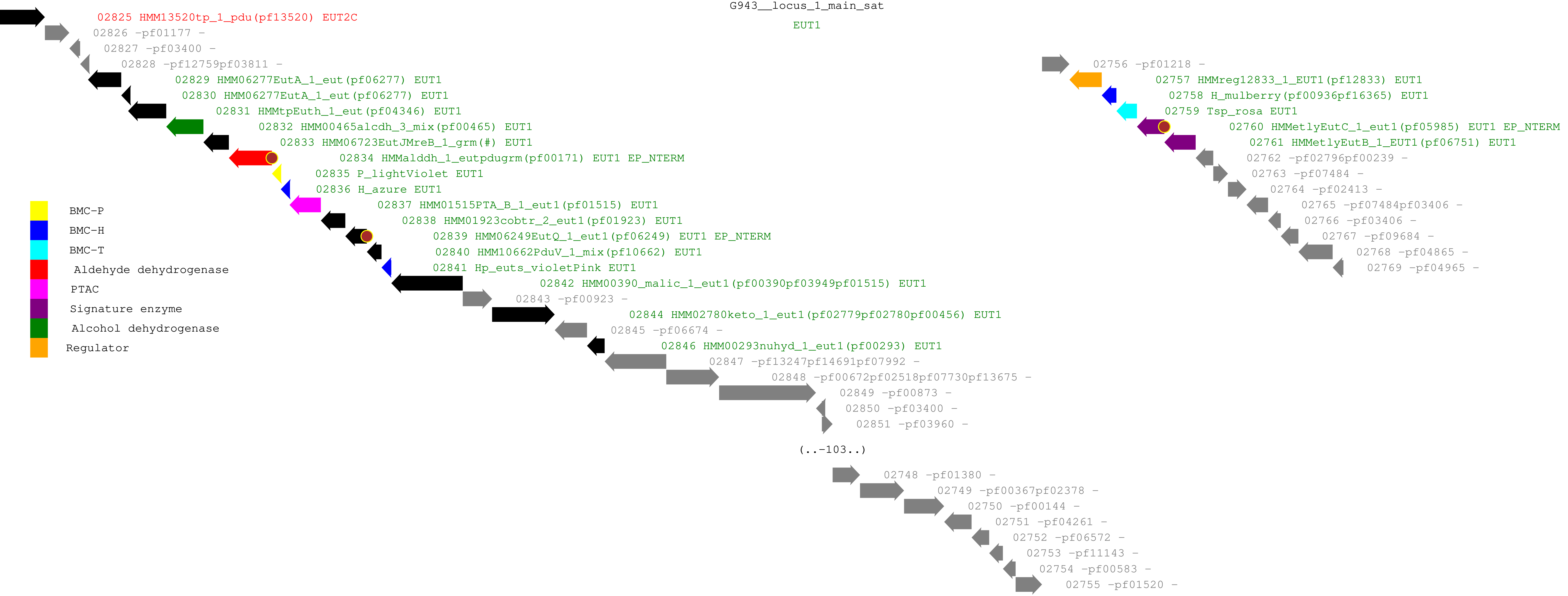

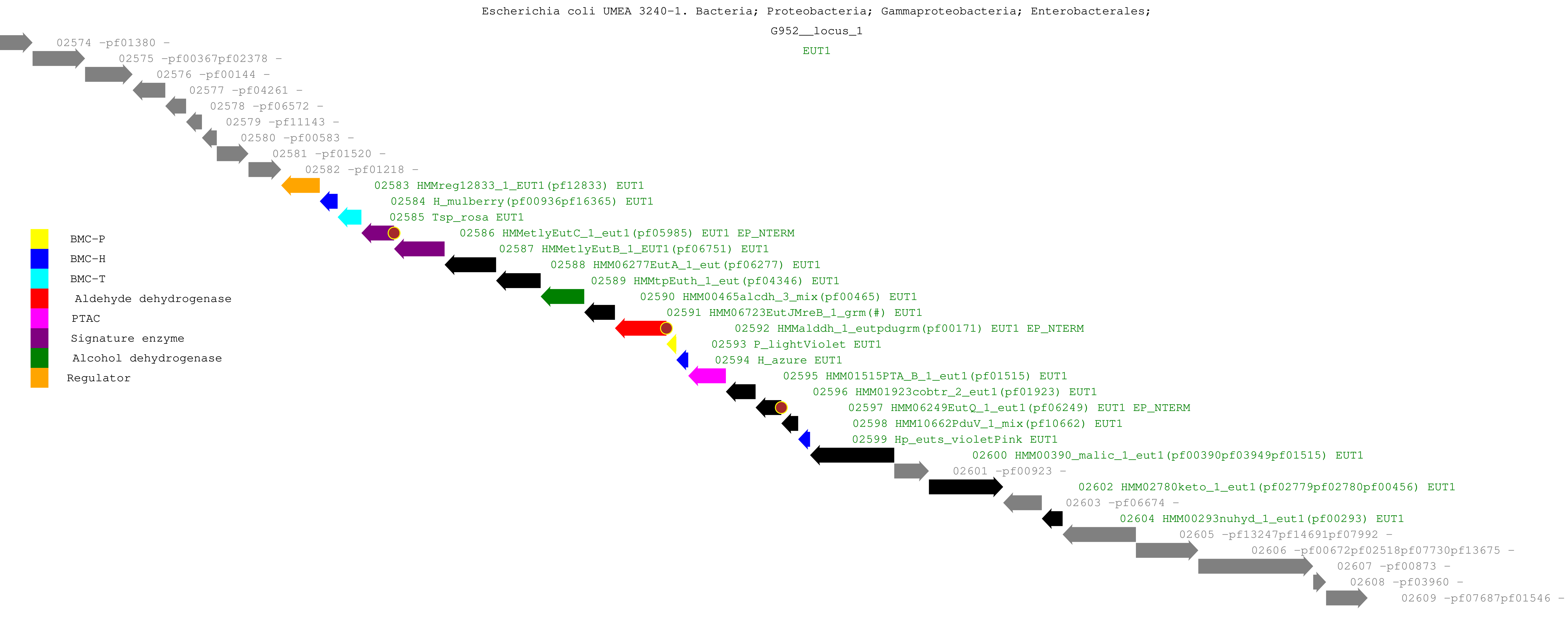
Escherichia coli UMEA 3240-1. Bacteria; Proteobacteria; Gammaproteobacteria; Enterobacterales;
G952__locus_1
EUT1
02574 -pf01380 -
02575 -pf00367pf02378 -
02576 -pf00144 -
02577 -pf04261 -
02578 -pf06572 -
02579 -pf11143 -
02580 -pf00583 -
02581 -pf01520 -
02582 -pf01218 -
02583 HMMreg12833_1_EUT1(pf12833) EUT1
02584 H_mulberry(pf00936pf16365) EUT1
02585 Tsp_rosa EUT1
02586 HMMetlyEutC_1_eut1(pf05985) EUT1 EP_NTERM
02587 HMMetlyEutB_1_EUT1(pf06751) EUT1
02588 HMM06277EutA_1_eut(pf06277) EUT1
02589 HMMtpEuth_1_eut(pf04346) EUT1
02590 HMM00465alcdh_3_mix(pf00465) EUT1
02591 HMM06723EutJMreB_1_grm(#) EUT1
02592 HMMalddh_1_eutpdugrm(pf00171) EUT1 EP_NTERM
02593 P_lightViolet EUT1
02594 H_azure EUT1
02595 HMM01515PTA_B_1_eut1(pf01515) EUT1
02596 HMM01923cobtr_2_eut1(pf01923) EUT1
02597 HMM06249EutQ_1_eut1(pf06249) EUT1 EP_NTERM
02598 HMM10662PduV_1_mix(pf10662) EUT1
02599 Hp_euts_violetPink EUT1
02600 HMM00390_malic_1_eut1(pf00390pf03949pf01515) EUT1
02601 -pf00923 -
02602 HMM02780keto_1_eut1(pf02779pf02780pf00456) EUT1
02603 -pf06674 -
02604 HMM00293nuhyd_1_eut1(pf00293) EUT1
02605 -pf13247pf14691pf07992 -
02606 -pf00672pf02518pf07730pf13675 -
02607 -pf00873 -
02608 -pf03960 -
02609 -pf07687pf01546 -
BMC-P
BMC-H
BMC-T
Aldehyde dehydrogenase
PTAC
Signature enzyme
Alcohol dehydrogenase
Regulator

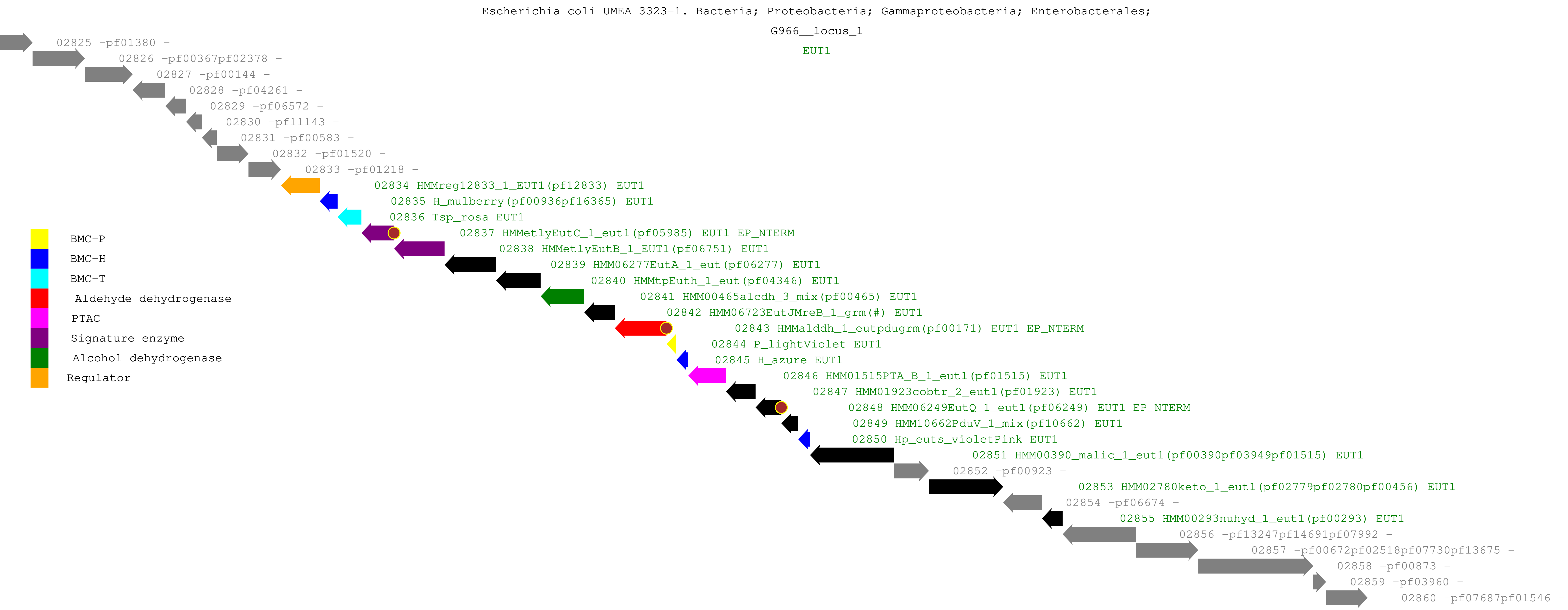

Escherichia coli UMEA 3323-1. Bacteria; Proteobacteria; Gammaproteobacteria; Enterobacterales;
G966__locus_1
EUT1
02825 -pf01380 -
02826 -pf00367pf02378 -
02827 -pf00144 -
02828 -pf04261 -
02829 -pf06572 -
02830 -pf11143 -
02831 -pf00583 -
02832 -pf01520 -
02833 -pf01218 -
02834 HMMreg12833_1_EUT1(pf12833) EUT1
02835 H_mulberry(pf00936pf16365) EUT1
02836 Tsp_rosa EUT1
02837 HMMetlyEutC_1_eut1(pf05985) EUT1 EP_NTERM
02838 HMMetlyEutB_1_EUT1(pf06751) EUT1
02839 HMM06277EutA_1_eut(pf06277) EUT1
02840 HMMtpEuth_1_eut(pf04346) EUT1
02841 HMM00465alcdh_3_mix(pf00465) EUT1
02842 HMM06723EutJMreB_1_grm(#) EUT1
02843 HMMalddh_1_eutpdugrm(pf00171) EUT1 EP_NTERM
02844 P_lightViolet EUT1
02845 H_azure EUT1
02846 HMM01515PTA_B_1_eut1(pf01515) EUT1
02847 HMM01923cobtr_2_eut1(pf01923) EUT1
02848 HMM06249EutQ_1_eut1(pf06249) EUT1 EP_NTERM
02849 HMM10662PduV_1_mix(pf10662) EUT1
02850 Hp_euts_violetPink EUT1
02851 HMM00390_malic_1_eut1(pf00390pf03949pf01515) EUT1
02852 -pf00923 -
02853 HMM02780keto_1_eut1(pf02779pf02780pf00456) EUT1
02854 -pf06674 -
02855 HMM00293nuhyd_1_eut1(pf00293) EUT1
02856 -pf13247pf14691pf07992 -
02857 -pf00672pf02518pf07730pf13675 -
02858 -pf00873 -
02859 -pf03960 -
02860 -pf07687pf01546 -
BMC-P
BMC-H
BMC-T
Aldehyde dehydrogenase
PTAC
Signature enzyme
Alcohol dehydrogenase
Regulator

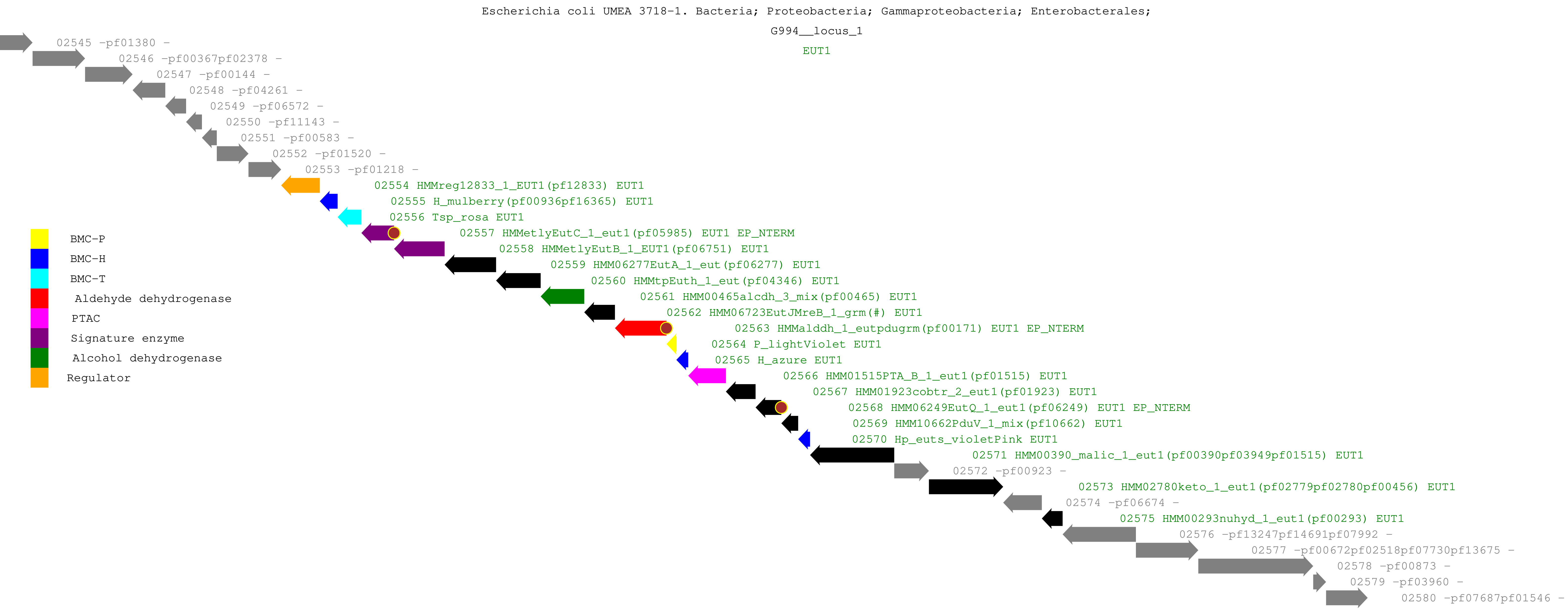

Escherichia coli UMEA 3718-1. Bacteria; Proteobacteria; Gammaproteobacteria; Enterobacterales;
G994__locus_1
EUT1
02545 -pf01380 -
02546 -pf00367pf02378 -
02547 -pf00144 -
02548 -pf04261 -
02549 -pf06572 -
02550 -pf11143 -
02551 -pf00583 -
02552 -pf01520 -
02553 -pf01218 -
02554 HMMreg12833_1_EUT1(pf12833) EUT1
02555 H_mulberry(pf00936pf16365) EUT1
02556 Tsp_rosa EUT1
02557 HMMetlyEutC_1_eut1(pf05985) EUT1 EP_NTERM
02558 HMMetlyEutB_1_EUT1(pf06751) EUT1
02559 HMM06277EutA_1_eut(pf06277) EUT1
02560 HMMtpEuth_1_eut(pf04346) EUT1
02561 HMM00465alcdh_3_mix(pf00465) EUT1
02562 HMM06723EutJMreB_1_grm(#) EUT1
02563 HMMalddh_1_eutpdugrm(pf00171) EUT1 EP_NTERM
02564 P_lightViolet EUT1
02565 H_azure EUT1
02566 HMM01515PTA_B_1_eut1(pf01515) EUT1
02567 HMM01923cobtr_2_eut1(pf01923) EUT1
02568 HMM06249EutQ_1_eut1(pf06249) EUT1 EP_NTERM
02569 HMM10662PduV_1_mix(pf10662) EUT1
02570 Hp_euts_violetPink EUT1
02571 HMM00390_malic_1_eut1(pf00390pf03949pf01515) EUT1
02572 -pf00923 -
02573 HMM02780keto_1_eut1(pf02779pf02780pf00456) EUT1
02574 -pf06674 -
02575 HMM00293nuhyd_1_eut1(pf00293) EUT1
02576 -pf13247pf14691pf07992 -
02577 -pf00672pf02518pf07730pf13675 -
02578 -pf00873 -
02579 -pf03960 -
02580 -pf07687pf01546 -
BMC-P
BMC-H
BMC-T
Aldehyde dehydrogenase
PTAC
Signature enzyme
Alcohol dehydrogenase
Regulator

Paraglaciecola arctica BSs20135. Bacteria; Proteobacteria; Gammaproteobacteria; Alteromonadales;

GARC__locus_1

EUT1

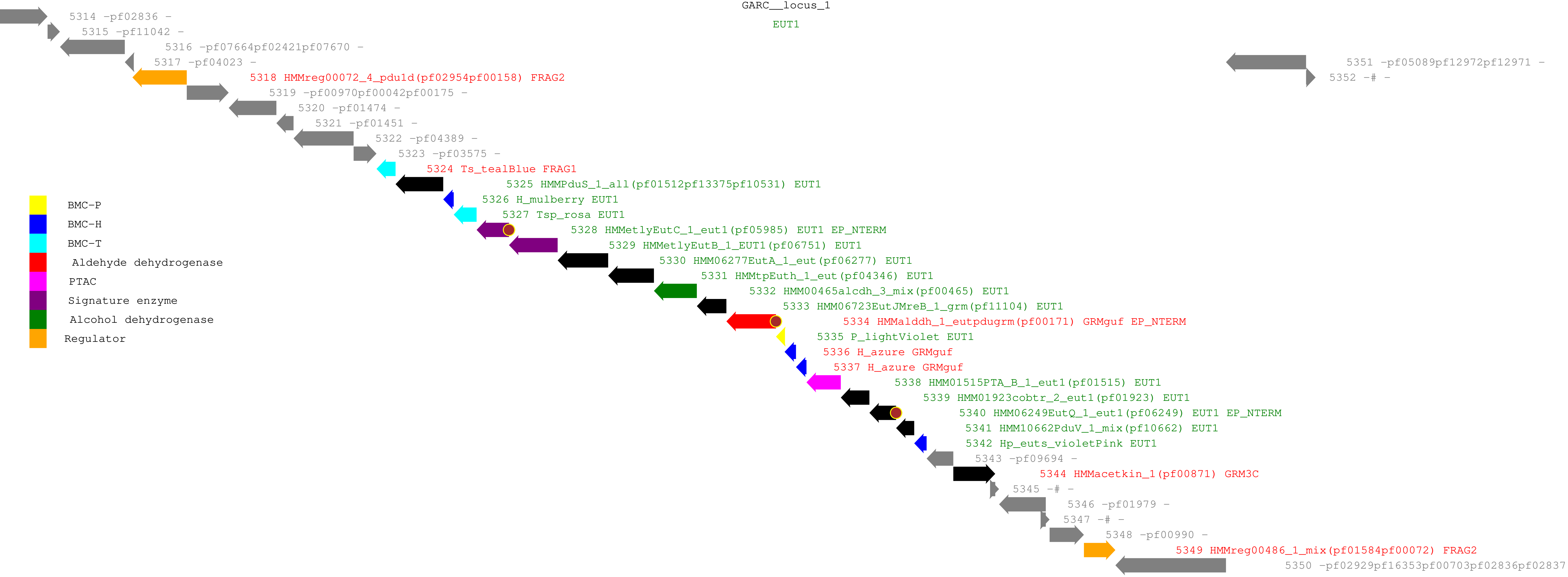

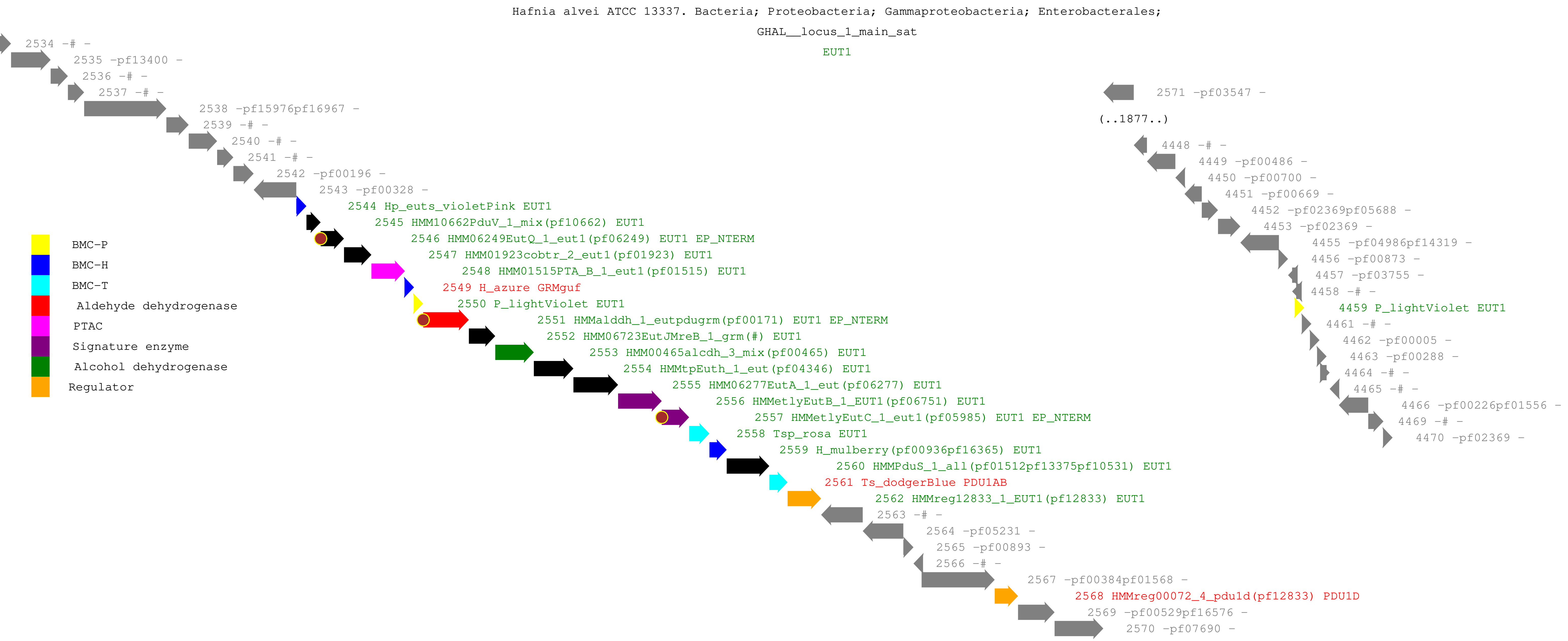
Hafnia alvei ATCC 13337. Bacteria; Proteobacteria; Gammaproteobacteria; Enterobacterales;
GHAL__locus_1_main_sat
EUT1
2534 -# -
2535 -pf13400 -
2536 -# -
2537 -# -
2538 -pf15976pf16967 -
2539 -# -
2540 -# -
2541 -# -
2542 -pf00196 -
2543 -pf00328 -
2544 Hp_euts_violetPink EUT1
2545 HMM10662PduV_1_mix(pf10662) EUT1
2546 HMM06249EutQ_1_eut1(pf06249) EUT1 EP_NTERM
2547 HMM01923cobtr_2_eut1(pf01923) EUT1
2548 HMM01515PTA_B_1_eut1(pf01515) EUT1
2549 H_azure GRMguf
2550 P_lightViolet EUT1
2551 HMMalddh_1_eutpdugrm(pf00171) EUT1 EP_NTERM
2552 HMM06723EutJMreB_1_grm(#) EUT1
2553 HMM00465alcdh_3_mix(pf00465) EUT1
2554 HMMtpEuth_1_eut(pf04346) EUT1
2555 HMM06277EutA_1_eut(pf06277) EUT1
2556 HMMetlyEutB_1_EUT1(pf06751) EUT1
2557 HMMetlyEutC_1_eut1(pf05985) EUT1 EP_NTERM
2558 Tsp_rosa EUT1
2559 H_mulberry(pf00936pf16365) EUT1
2560 HMMPduS_1_all(pf01512pf13375pf10531) EUT1
2561 Ts_dodgerBlue PDU1AB
2562 HMMreg12833_1_EUT1(pf12833) EUT1
2563 -# -
2564 -pf05231 -
2565 -pf00893 -
2566 -# -
2567 -pf00384pf01568 -
2568 HMMreg00072_4_pdu1d(pf12833) PDU1D
2569 -pf00529pf16576 -
2570 -pf07690 -
2571 -pf03547 -
(..1877..)
4448 -# -
4449 -pf00486 -
4450 -pf00700 -
4451 -pf00669 -
4452 -pf02369pf05688 -
4453 -pf02369 -
4455 -pf04986pf14319 -
4456 -pf00873 -
4457 -pf03755 -
4458 -# -
4459 P_lightViolet EUT1
4461 -# -
4462 -pf00005 -
4463 -pf00288 -
4464 -# -
4465 -# -
4466 -pf00226pf01556 -
4469 -# -
4470 -pf02369 -
BMC-P
BMC-H
BMC-T
Aldehyde dehydrogenase
PTAC
Signature enzyme
Alcohol dehydrogenase
Regulator

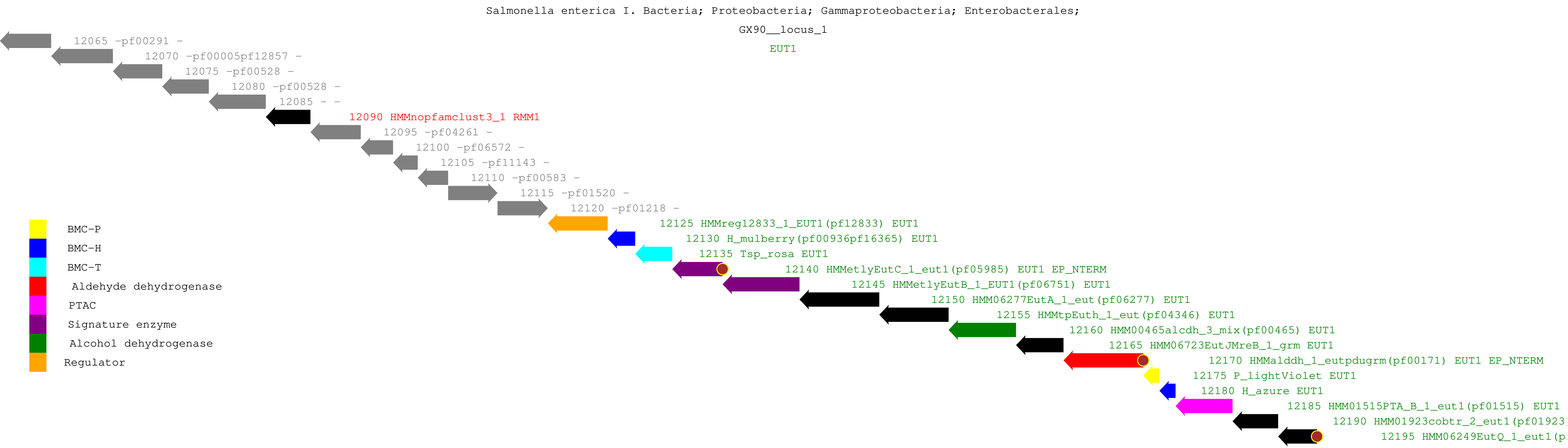

Salmonella enterica I. Bacteria; Proteobacteria; Gammaproteobacteria; Enterobacterales;
GX90__locus_1
EUT1
12065 -pf00291 -
12070 -pf00005pf12857 -
12075 -pf00528 -
12080 -pf00528 -
12085 - -
12090 HMMnopfamclust3_1 RMM1
12095 -pf04261 -
12100 -pf06572 -
12105 -pf11143 -
12110 -pf00583 -
12115 -pf01520 -
12120 -pf01218 -
12125 HMMreg12833_1_EUT1(pf12833) EUT1
12130 H_mulberry(pf00936pf16365) EUT1
12135 Tsp_rosa EUT1
12140 HMMetlyEutC_1_eut1(pf05985) EUT1 EP_NTERM
12145 HMMetlyEutB_1_EUT1(pf06751) EUT1
12150 HMM06277EutA_1_eut(pf06277) EUT1
12155 HMMtpEuth_1_eut(pf04346) EUT1
12160 HMM00465alcdh_3_mix(pf00465) EUT1
12165 HMM06723EutJMreB_1_grm EUT1
12170 HMMaldh_1_eutpdugrm(pf00171) EUT1 EP_NTERM
12175 P_lightViolet EUT1
12180 H_azure EUT1
12185 HMM01515PTA_B_1_eut1(pf01515) EUT1
12190 HMM01923cobtr_2_eut1(pf01923
12195 HMM06249EutQ_1_eut1(p
BMC-P
BMC-H
BMC-T
Aldehyde dehydrogenase
PTAC
Signature enzyme
Alcohol dehydrogenase
Regulator

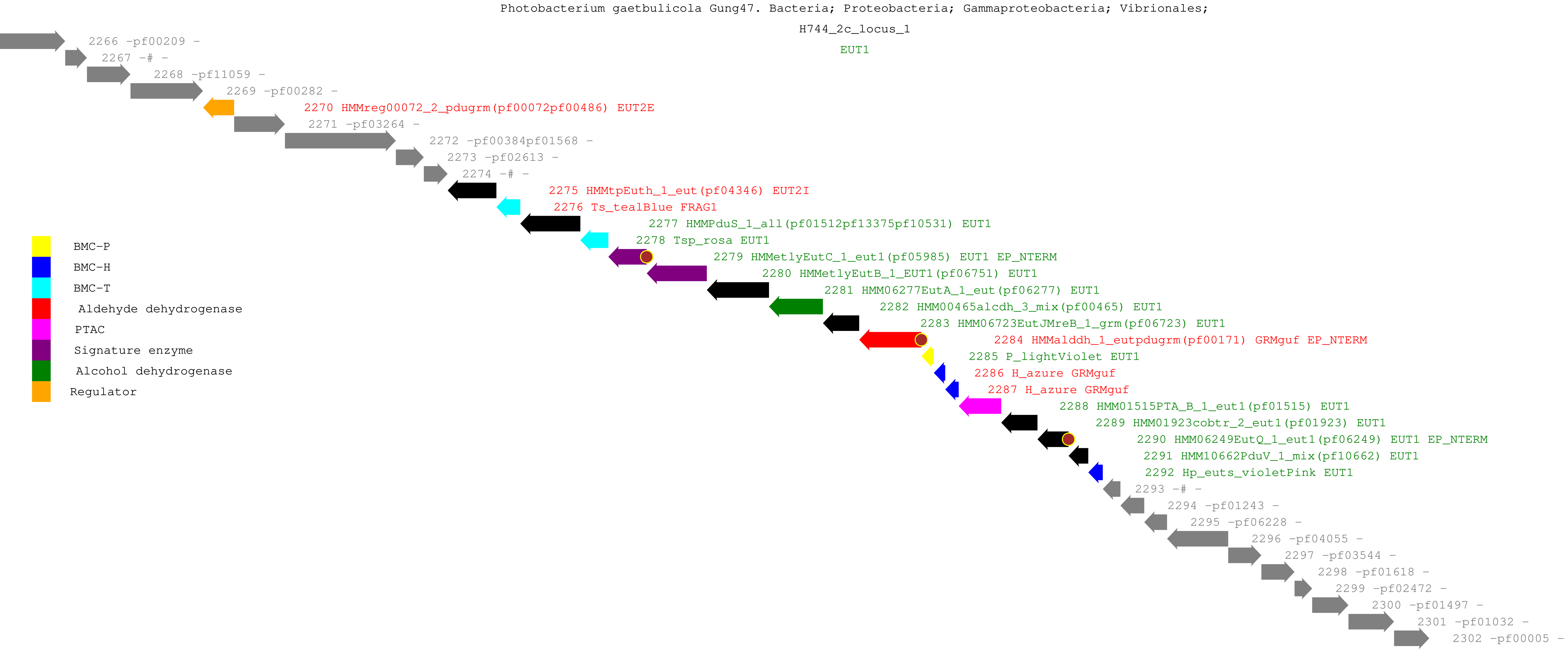

Photobacterium gaetbulicola Gung47. Bacteria; Proteobacteria; Gammaproteobacteria; Vibrionales;
H744_2c_locus_1
EUT1
2266 -pf00209 -
2267 -# -
2268 -pf11059 -
2269 -pf00282 -
2270 HMMreg00072_2_pdugrm(pf00072pf00486) EUT2E
2271 -pf03264 -
2272 -pf00384pf01568 -
2273 -pf02613 -
2274 -# -
2275 HMMtpEuth_1_eut(pf04346) EUT2I
2276 Ts_tealBlue FRAG1
2277 HMMPduS_1_all(pf01512pf13375pf10531) EUT1
2278 Tsp_rosa EUT1
2279 HMMetlyEutC_1_eut1(pf05985) EUT1 EP_NTERM
2280 HMMetlyEutB_1_EUT1(pf06751) EUT1
2281 HMM06277EutA_1_eut(pf06277) EUT1
2282 HMM00465alcdh_3_mix(pf00465) EUT1
2283 HMM06723EutJMreB_1_grm(pf06723) EUT1
2284 HMMaldh_1_eutpdugrm(pf00171) GRMguf EP_NTERM
2285 P_lightViolet EUT1
2286 H_azure GRMguf
2287 H_azure GRMguf
2288 HMM01515PTA_B_1_eut1(pf01515) EUT1
2289 HMM01923cobtr_2_eut1(pf01923) EUT1
2290 HMM06249EutQ_1_eut1(pf06249) EUT1 EP_NTERM
2291 HMM10662PduV_1_mix(pf10662) EUT1
2292 Hp_euts_violetPink EUT1
2293 -# -
2294 -pf01243 -
2295 -pf06228 -
2296 -pf04055 -
2297 -pf03544 -
2298 -pf01618 -
2299 -pf02472 -
2300 -pf01497 -
2301 -pf01032 -
2302 -pf00005 -
BMC-P
BMC-H
BMC-T
Aldehyde dehydrogenase
PTAC
Signature enzyme
Alcohol dehydrogenase
Regulator

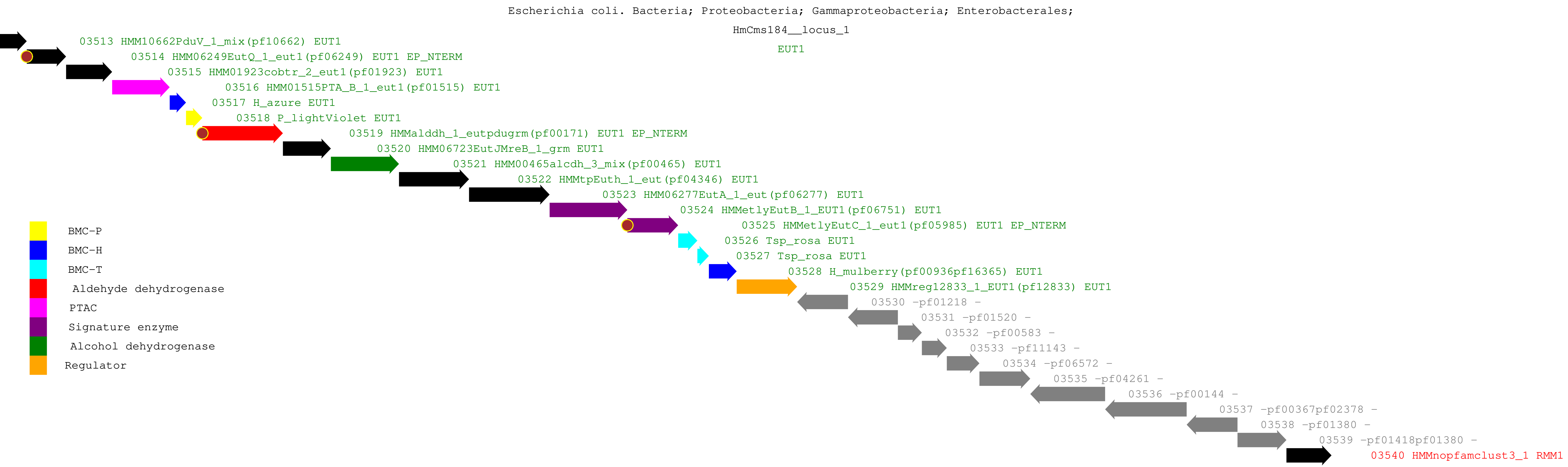

Escherichia coli. Bacteria; Proteobacteria; Gammaproteobacteria; Enterobacterales;
HmCms184__locus_1
EUT1
03513 HMM10662PduV_1_mix(pf10662) EUT1
03514 HMM06249EutQ_1_eut1(pf06249) EUT1 EP_NTERM
03515 HMM01923cobtr_2_eut1(pf01923) EUT1
03516 HMM01515PTA_B_1_eut1(pf01515) EUT1
03517 H_azure EUT1
03518 P_lightViolet EUT1
03519 HMMaldh_1_eutpdugrm(pf00171) EUT1 EP_NTERM
03520 HMM06723EutJMreB_1_grm EUT1
03521 HMM00465alcdh_3_mix(pf00465) EUT1
03522 HMMtpEuth_1_eut(pf04346) EUT1
03523 HMM06277EutA_1_eut(pf06277) EUT1
03524 HMMetlyEutB_1_EUT1(pf06751) EUT1
03525 HMMetlyEutC_1_eut1(pf05985) EUT1 EP_NTERM
03526 Tsp_rosa EUT1
03527 Tsp_rosa EUT1
03528 H_mulberry(pf00936pf16365) EUT1
03529 HMMreg12833_1_EUT1(pf12833) EUT1
03530 -pf01218 -
03531 -pf01520 -
03532 -pf00583 -
03533 -pf11143 -
03534 -pf06572 -
03535 -pf04261 -
03536 -pf00144 -
03537 -pf00367pf02378 -
03538 -pf01380 -
03539 -pf01418pf01380 -
03540 HMMnopfamclust3_1 RMM1
BMC-P
BMC-H
BMC-T
Aldehyde dehydrogenase
PTAC
Signature enzyme
Alcohol dehydrogenase
Regulator

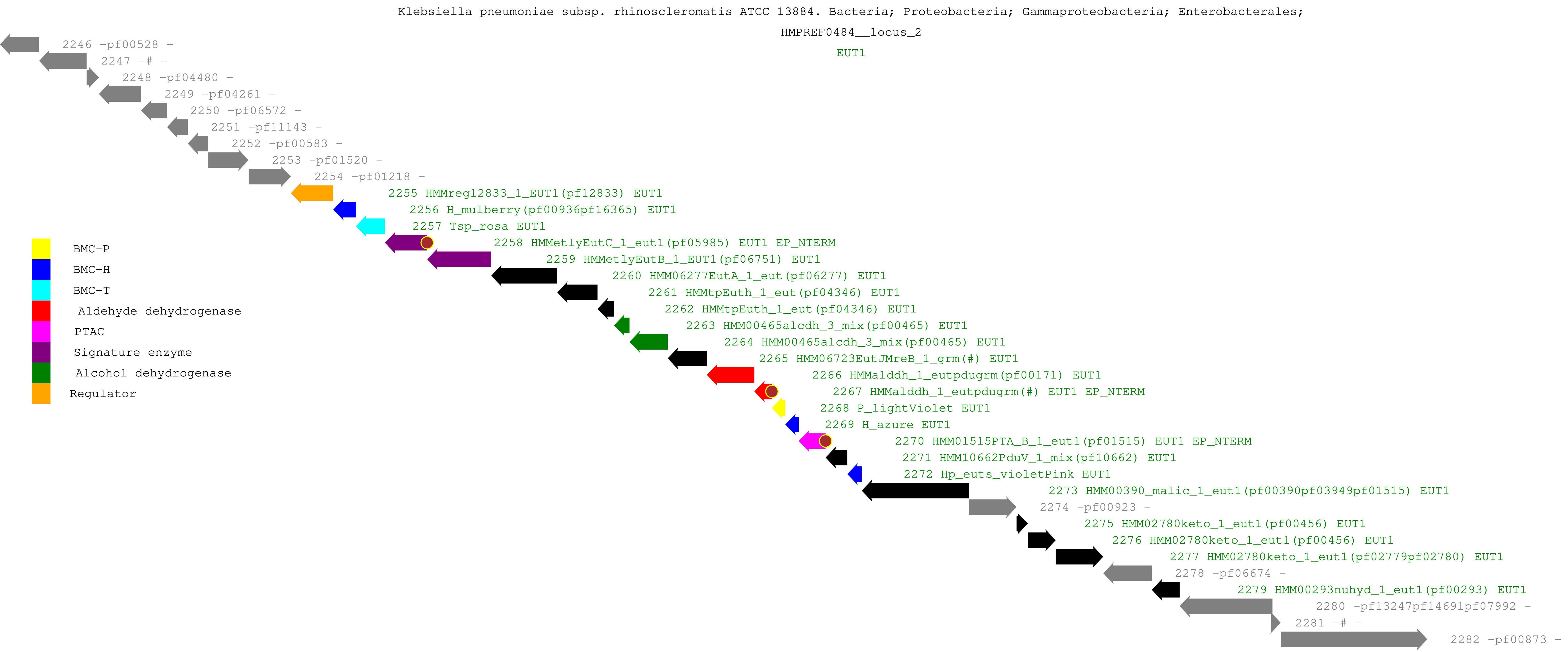

Klebsiella pneumoniae subsp. rhinoscleromatis ATCC 13884. Bacteria; Proteobacteria; Gammaproteobacteria; Enterobacterales;
HMPREF0484__locus_2
EUT1
2246 -pf00528 -
2247 -# -
2248 -pf04480 -
2249 -pf04261 -
2250 -pf06572 -
2251 -pf11143 -
2252 -pf00583 -
2253 -pf01520 -
2254 -pf01218 -
2255 HMMreg12833_1_EUT1(pf12833) EUT1
2256 H_mulberry(pf00936pf16365) EUT1
2257 Tsp_rosa EUT1
2258 HMMetlyEutC_1_eut1(pf05985) EUT1 EP_NTERM
2259 HMMetlyEutB_1_EUT1(pf06751) EUT1
2260 HMM06277EutA_1_eut(pf06277) EUT1
2261 HMMtpEuth_1_eut(pf04346) EUT1
2262 HMMtpEuth_1_eut(pf04346) EUT1
2263 HMM00465alcdh_3_mix(pf00465) EUT1
2264 HMM00465alcdh_3_mix(pf00465) EUT1
2265 HMM06723EutJMreB_1_grm(#) EUT1
2266 HMMalddh_1_eutpdugrm(pf00171) EUT1
2267 HMMalddh_1_eutpdugrm(#) EUT1 EP_NTERM
2268 P_lightViolet EUT1
2269 H_azure EUT1
2270 HMM01515PTA_B_1_eut1(pf01515) EUT1 EP_NTERM
2271 HMM10662PduV_1_mix(pf10662) EUT1
2272 Hp_euts_violetPink EUT1
2273 HMM00390_malic_1_eut1(pf00390pf03949pf01515) EUT1
2274 -pf00923 -
2275 HMM02780keto_1_eut1(pf00456) EUT1
2276 HMM02780keto_1_eut1(pf00456) EUT1
2277 HMM02780keto_1_eut1(pf02779pf02780) EUT1
2278 -pf06674 -
2279 HMM00293nuhyd_1_eut1(pf00293) EUT1
2280 -pf13247pf14691pf07992 -
2281 -# -
2282 -pf00873 -
BMC-P
BMC-H
BMC-T
Aldehyde dehydrogenase
PTAC
Signature enzyme
Alcohol dehydrogenase
Regulator

Escherichia coli 4_1_47FAA. Bacteria; Proteobacteria; Gammaproteobacteria; Enterobacterales;

HMPREF0986__locus_1

EUT1

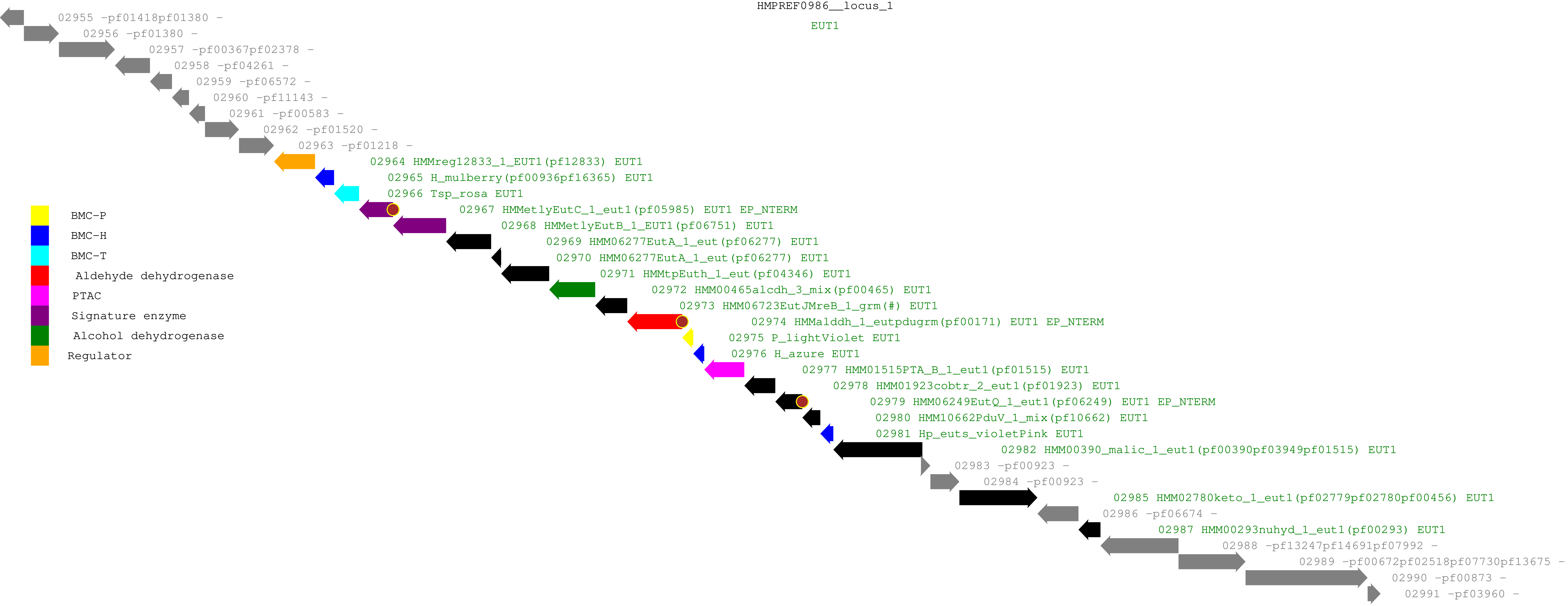

Klebsiella sp. OBRC7. Bacteria; Proteobacteria; Gammaproteobacteria; Enterobacterales;

HMPREF1144__locus_3

EUT1

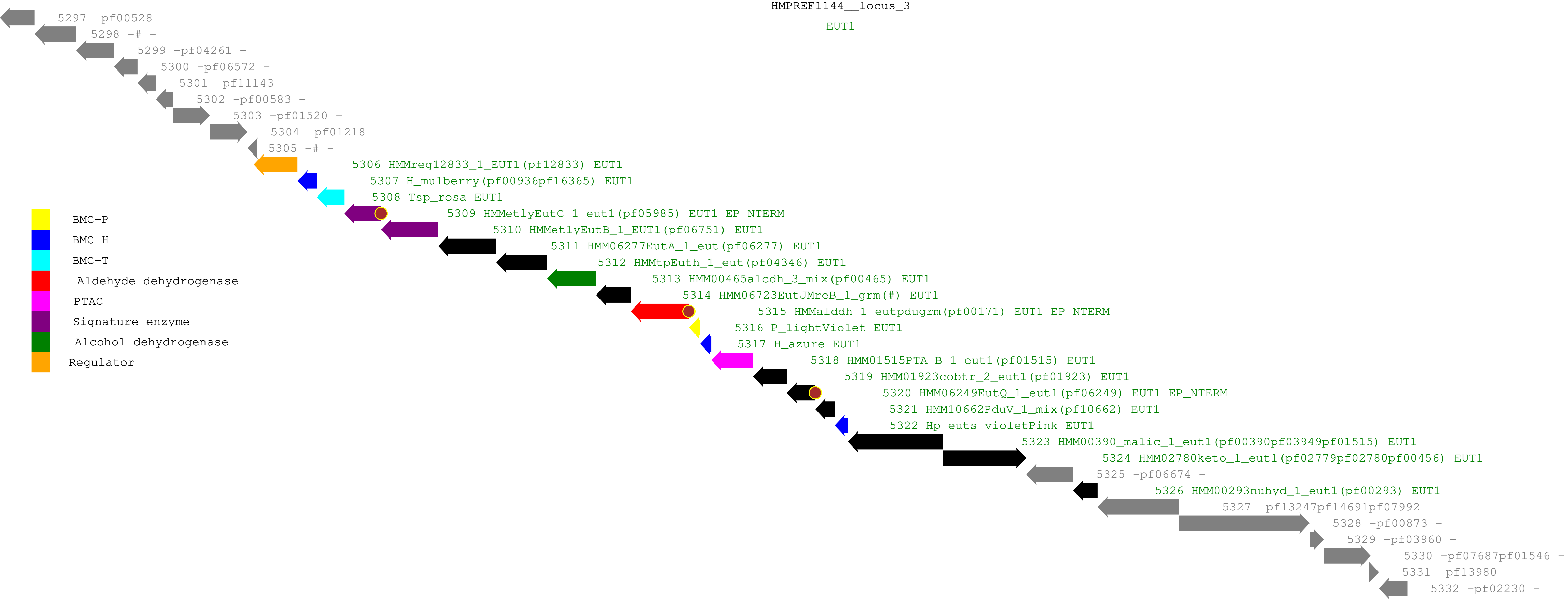

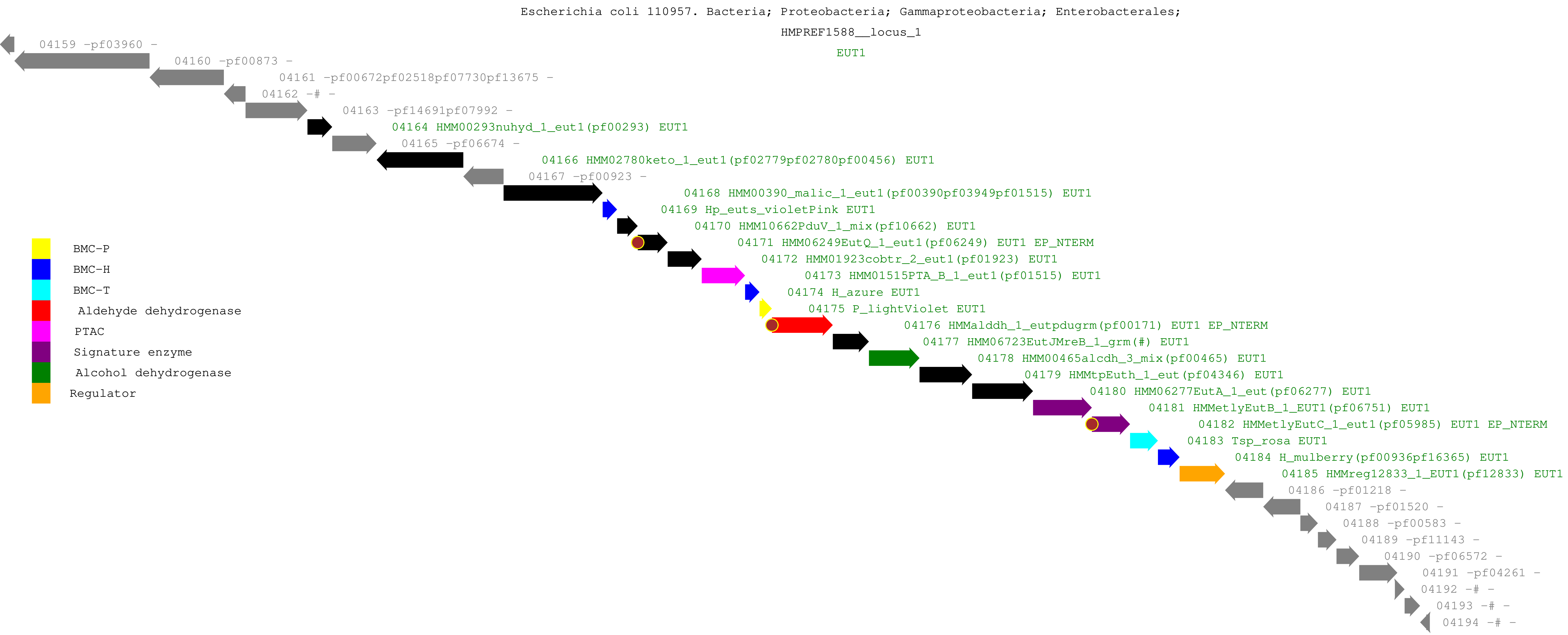

Escherichia coli 110957. Bacteria; Proteobacteria; Gammaproteobacteria; Enterobacterales;
HMPREF1588__locus_1
EUT1
04159 -pf03960 -
04160 -pf00873 -
04161 -pf00672pf02518pf07730pf13675 -
04162 -# -
04163 -pf14691pf07992 -
04164 HMM00293nuhyd_1_eut1(pf00293) EUT1
04165 -pf06674 -
04166 HMM02780keto_1_eut1(pf02779pf02780pf00456) EUT1
04167 -pf00923 -
04168 HMM00390_malic_1_eut1(pf00390pf03949pf01515) EUT1
04169 Hp_euts_violetPink EUT1
04170 HMM10662PduV_1_mix(pf10662) EUT1
04171 HMM06249EutQ_1_eut1(pf06249) EUT1 EP_NTERM
04172 HMM01923cobtr_2_eut1(pf01923) EUT1
04173 HMM01515PTA_B_1_eut1(pf01515) EUT1
04174 H_azure EUT1
04175 P_lightViolet EUT1
04176 HMMalddh_1_eutpdugrm(pf00171) EUT1 EP_NTERM
04177 HMM06723EutJMreB_1_grm(#) EUT1
04178 HMM00465alcdh_3_mix(pf00465) EUT1
04179 HMMtpEuth_1_eut(pf04346) EUT1
04180 HMM06277EutA_1_eut(pf06277) EUT1
04181 HMMetlyEutB_1_EUT1(pf06751) EUT1
04182 HMMetlyEutC_1_eut1(pf05985) EUT1 EP_NTERM
04183 Tsp_rosa EUT1
04184 H_mulberry(pf00936pf16365) EUT1
04185 HMMreg12833_1_EUT1(pf12833) EUT1
04186 -pf01218 -
04187 -pf01520 -
04188 -pf00583 -
04189 -pf11143 -
04190 -pf06572 -
04191 -pf04261 -
04192 -# -
04193 -# -
04194 -# -
BMC-P
BMC-H
BMC-T
Aldehyde dehydrogenase
PTAC
Signature enzyme
Alcohol dehydrogenase
Regulator

Escherichia coli 113290. Bacteria; Proteobacteria; Gammaproteobacteria; Enterobacterales;

HMPREF1589__locus_1

EUT1

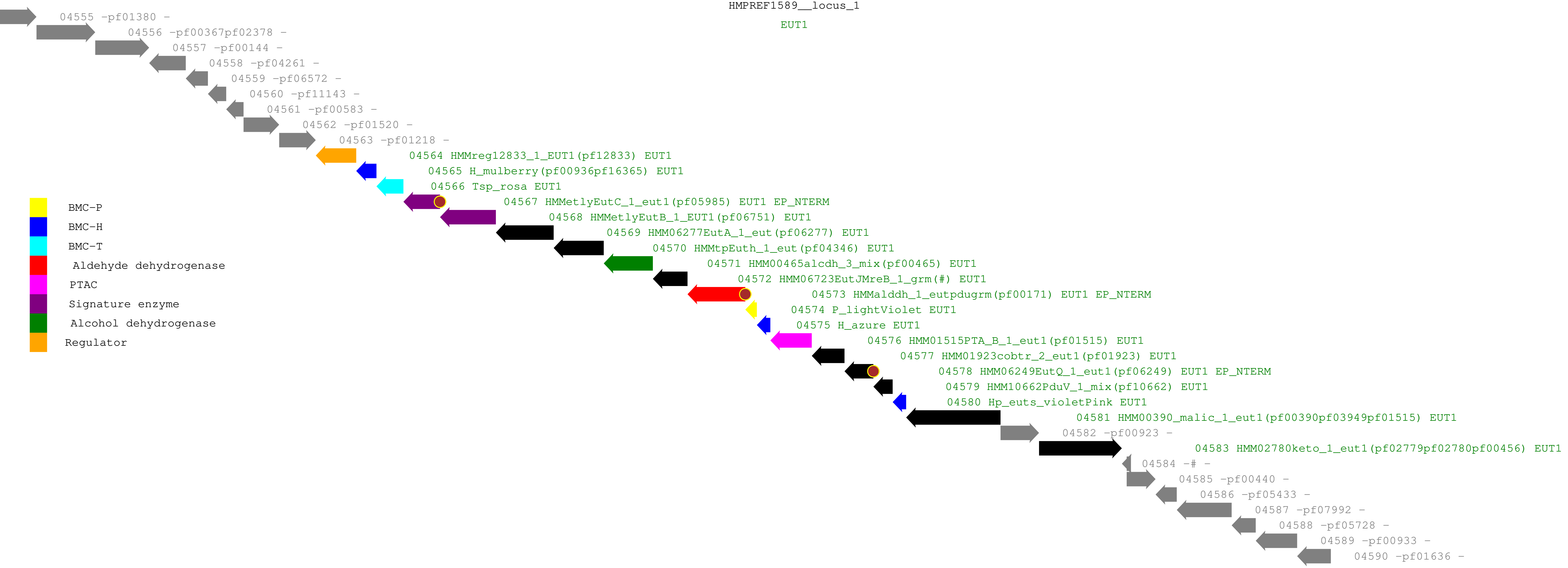

Escherichia coli 113302. Bacteria; Proteobacteria; Gammaproteobacteria; Enterobacterales;

HMPREF1590__locus_1

EUT1

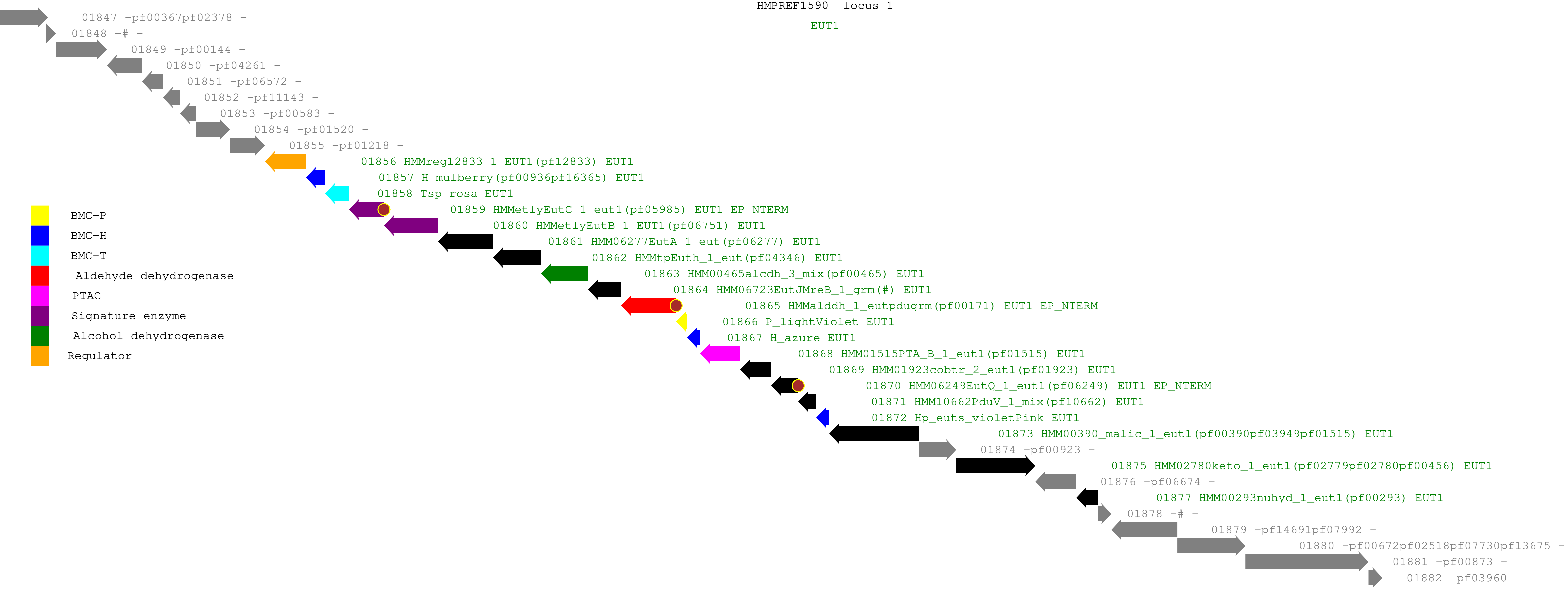

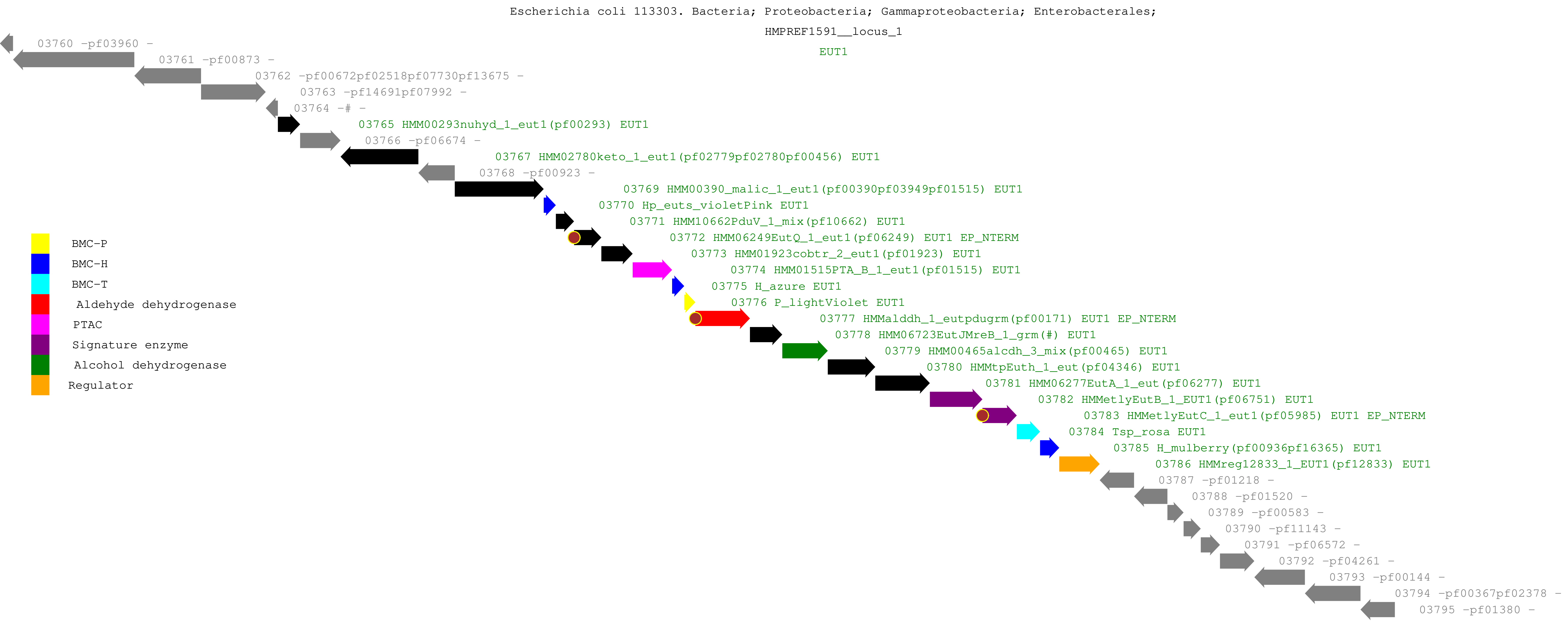
Escherichia coli 113303. Bacteria; Proteobacteria; Gammaproteobacteria; Enterobacterales;
HMPREF1591__locus_1
EUT1
03760 -pf03960 -
03761 -pf00873 -
03762 -pf00672pf02518pf07730pf13675 -
03763 -pf14691pf07992 -
03764 -# -
03765 HMM00293nuhyd_1_eut1(pf00293) EUT1
03766 -pf06674 -
03767 HMM02780keto_1_eut1(pf02779pf02780pf00456) EUT1
03768 -pf00923 -
03769 HMM00390_malic_1_eut1(pf00390pf03949pf01515) EUT1
03770 Hp_euts_violetPink EUT1
03771 HMM10662PduV_1_mix(pf10662) EUT1
03772 HMM06249EutQ_1_eut1(pf06249) EUT1 EP_NTERM
03773 HMM01923cobtr_2_eut1(pf01923) EUT1
03774 HMM01515PTA_B_1_eut1(pf01515) EUT1
03775 H_azure EUT1
03776 P_lightViolet EUT1
03777 HMMalddh_1_eutpdugrm(pf00171) EUT1 EP_NTERM
03778 HMM06723EutJMreB_1_grm(#) EUT1
03779 HMM00465alcdh_3_mix(pf00465) EUT1
03780 HMMtpEuth_1_eut(pf04346) EUT1
03781 HMM06277EutA_1_eut(pf06277) EUT1
03782 HMMetlyEutB_1_EUT1(pf06751) EUT1
03783 HMMetlyEutC_1_eut1(pf05985) EUT1 EP_NTERM
03784 Tsp_rosa EUT1
03785 H_mulberry(pf00936pf16365) EUT1
03786 HMMreg12833_1_EUT1(pf12833) EUT1
03787 -pf01218 -
03788 -pf01520 -
03789 -pf00583 -
03790 -pf11143 -
03791 -pf06572 -
03792 -pf04261 -
03793 -pf00144 -
03794 -pf00367pf02378 -
03795 -pf01380 -
BMC-P
BMC-H
BMC-T
Aldehyde dehydrogenase
PTAC
Signature enzyme
Alcohol dehydrogenase
Regulator

Escherichia coli 907357. Bacteria; Proteobacteria; Gammaproteobacteria; Enterobacterales;

HMPREF1592__locus_2

EUT1

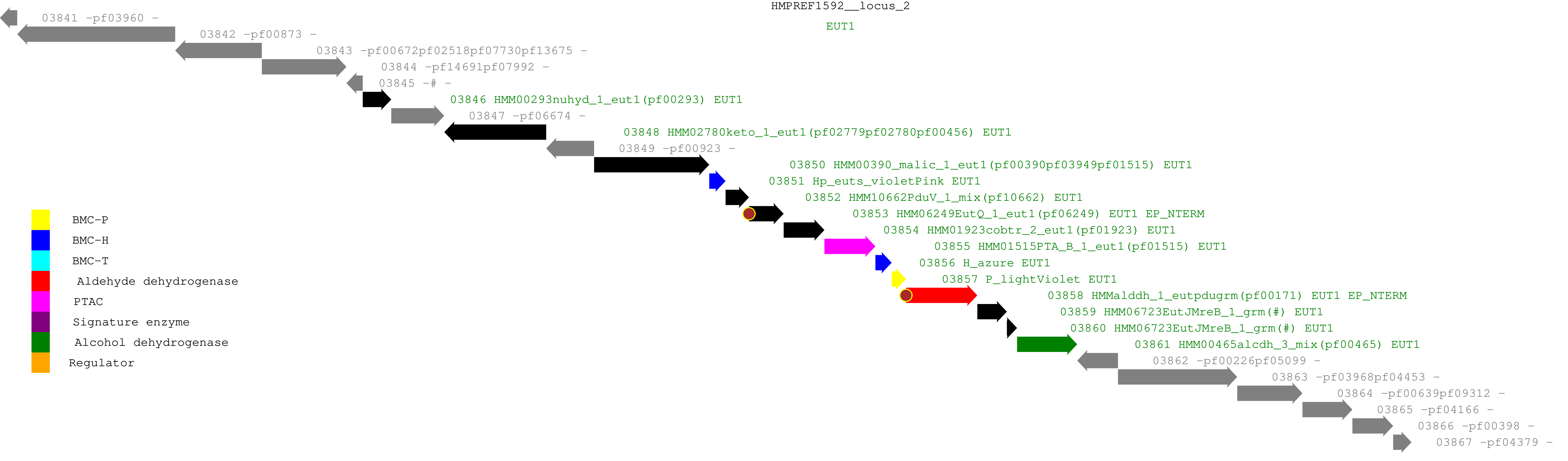

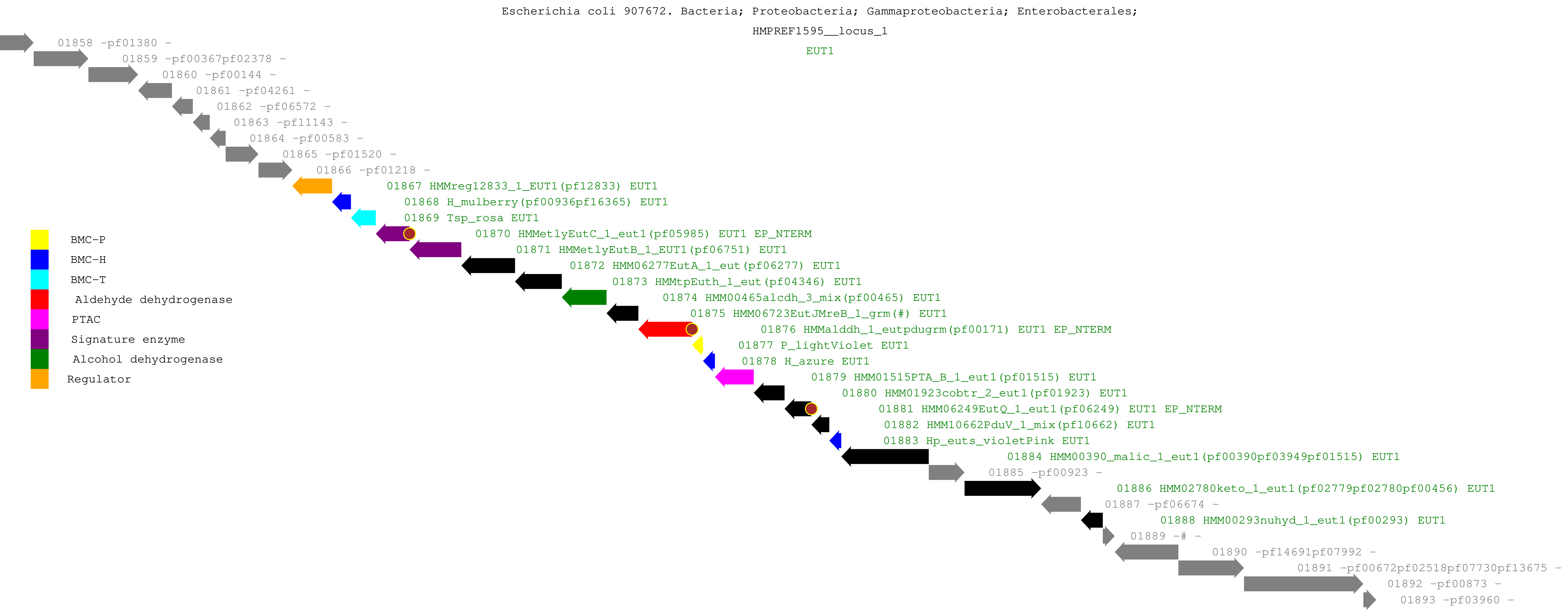
Escherichia coli 907672. Bacteria; Proteobacteria; Gammaproteobacteria; Enterobacterales;
HMPREF1595__locus_1
EUT1
01858 -pf01380 -
01859 -pf00367pf02378 -
01860 -pf00144 -
01861 -pf04261 -
01862 -pf06572 -
01863 -pf11143 -
01864 -pf00583 -
01865 -pf01520 -
01866 -pf01218 -
01867 HMMreg12833_1_EUT1(pf12833) EUT1
01868 H_mulberry(pf00936pf16365) EUT1
01869 Tsp_rosa EUT1
01870 HMMetlyEutC_1_eut1(pf05985) EUT1 EP_NTERM
01871 HMMetlyEutB_1_EUT1(pf06751) EUT1
01872 HMM06277EutA_1_eut(pf06277) EUT1
01873 HMMtpEuth_1_eut(pf04346) EUT1
01874 HMM00465alcdh_3_mix(pf00465) EUT1
01875 HMM06723EutJMreB_1_grm(#) EUT1
01876 HMMalddh_1_eutpdugrm(pf00171) EUT1 EP_NTERM
01877 P_lightViolet EUT1
01878 H_azure EUT1
01879 HMM01515PTA_B_1_eut1(pf01515) EUT1
01880 HMM01923cobtr_2_eut1(pf01923) EUT1
01881 HMM06249EutQ_1_eut1(pf06249) EUT1 EP_NTERM
01882 HMM10662PduV_1_mix(pf10662) EUT1
01883 Hp_euts_violetPink EUT1
01884 HMM00390_malic_1_eut1(pf00390pf03949pf01515) EUT1
01885 -pf00923 -
01886 HMM02780keto_1_eut1(pf02779pf02780pf00456) EUT1
01887 -pf06674 -
01888 HMM00293nuhyd_1_eut1(pf00293) EUT1
01889 -# -
01890 -pf14691pf07992 -
01891 -pf00672pf02518pf07730pf13675 -
01892 -pf00873 -
01893 -pf03960 -
BMC-P
BMC-H
BMC-T
Aldehyde dehydrogenase
PTAC
Signature enzyme
Alcohol dehydrogenase
Regulator

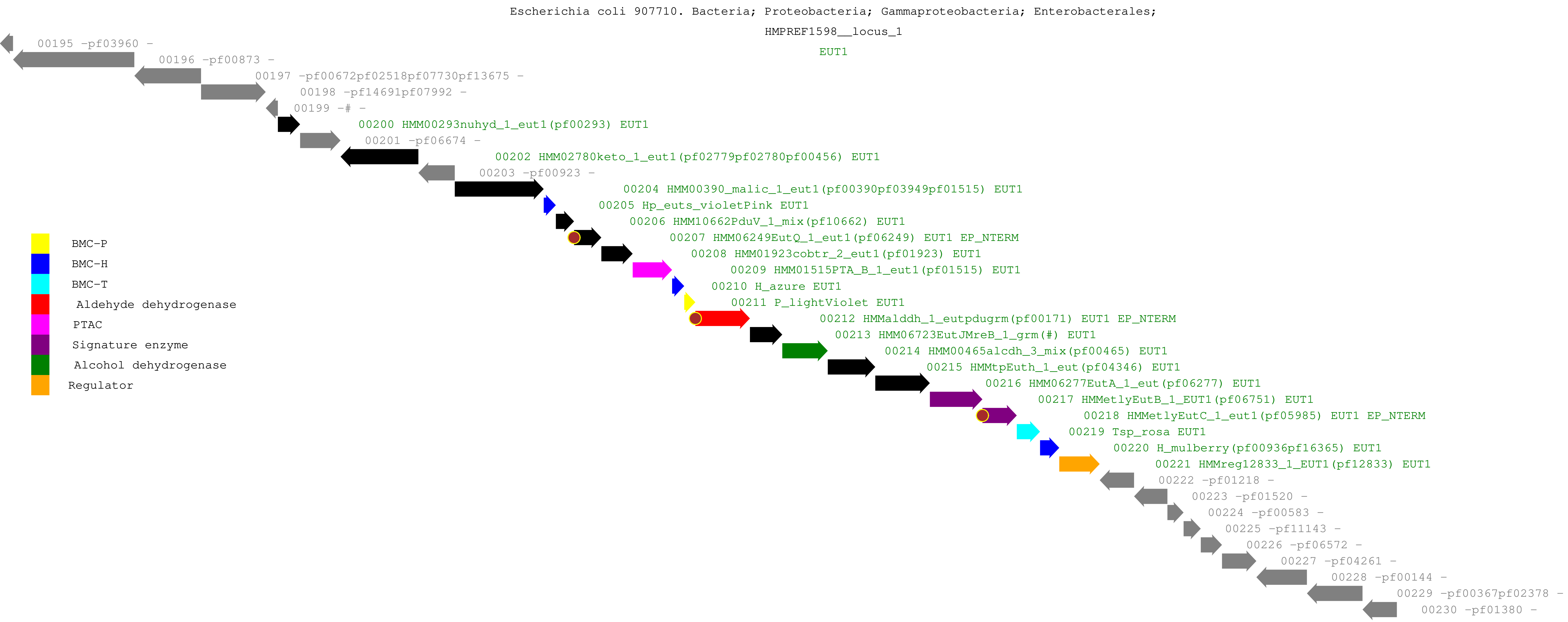

Escherichia coli 907710. Bacteria; Proteobacteria; Gammaproteobacteria; Enterobacterales;
HMPREF1598__locus_1
EUT1
00195 -pf03960 -
00196 -pf00873 -
00197 -pf00672pf02518pf07730pf13675 -
00198 -pf14691pf07992 -
00199 -# -
00200 HMM00293nuhyd_1_eut1(pf00293) EUT1
00201 -pf06674 -
00202 HMM02780keto_1_eut1(pf02779pf02780pf00456) EUT1
00203 -pf00923 -
00204 HMM00390_malic_1_eut1(pf00390pf03949pf01515) EUT1
00205 Hp_euts_violetPink EUT1
00206 HMM10662PduV_1_mix(pf10662) EUT1
00207 HMM06249EutQ_1_eut1(pf06249) EUT1 EP_NTERM
00208 HMM01923cobtr_2_eut1(pf01923) EUT1
00209 HMM01515PTA_B_1_eut1(pf01515) EUT1
00210 H_azure EUT1
00211 P_lightViolet EUT1
00212 HMMaldh_1_eutpdugrm(pf00171) EUT1 EP_NTERM
00213 HMM06723EutJMreB_1_grm(#) EUT1
00214 HMM00465alcdh_3_mix(pf00465) EUT1
00215 HMMtpEuth_1_eut(pf04346) EUT1
00216 HMM06277EutA_1_eut(pf06277) EUT1
00217 HMMetlyEutB_1_EUT1(pf06751) EUT1
00218 HMMetlyEutC_1_eut1(pf05985) EUT1 EP_NTERM
00219 Tsp_rosa EUT1
00220 H_mulberry(pf00936pf16365) EUT1
00221 HMMreg12833_1_EUT1(pf12833) EUT1
00222 -pf01218 -
00223 -pf01520 -
00224 -pf00583 -
00225 -pf11143 -
00226 -pf06572 -
00227 -pf04261 -
00228 -pf00144 -
00229 -pf00367pf02378 -
00230 -pf01380 -
BMC-P
BMC-H
BMC-T
Aldehyde dehydrogenase
PTAC
Signature enzyme
Alcohol dehydrogenase
Regulator

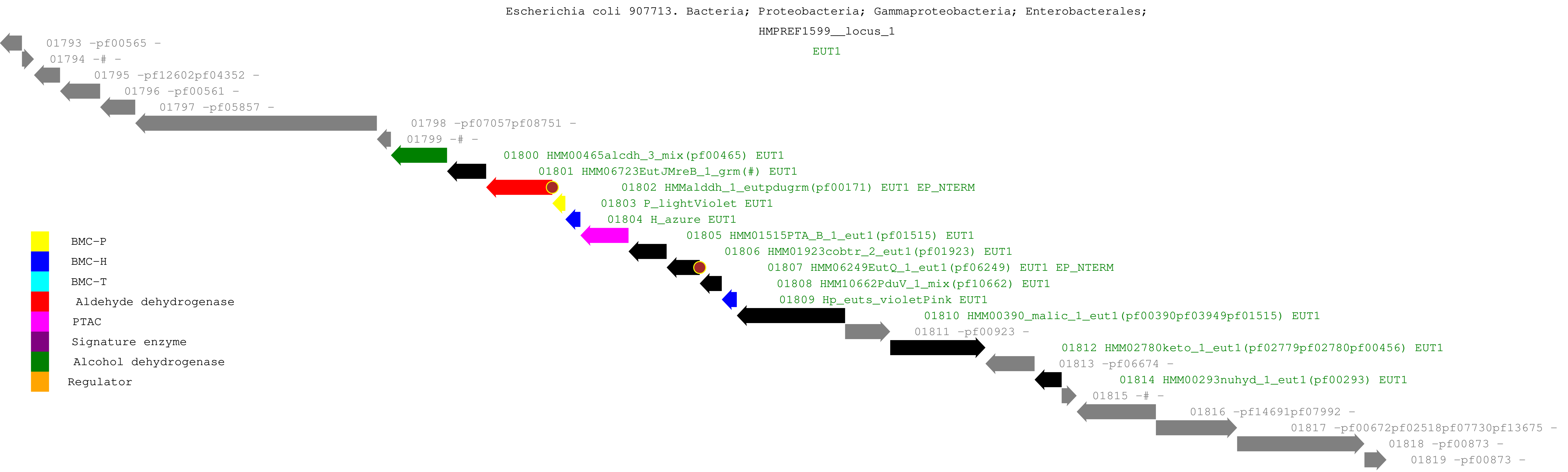

Escherichia coli 907713. Bacteria; Proteobacteria; Gammaproteobacteria; Enterobacterales;
HMPREF1599__locus_1
EUT1
01793 -pf00565 -
01794 -# -
01795 -pf12602pf04352 -
01796 -pf00561 -
01797 -pf05857 -
01798 -pf07057pf08751 -
01799 -# -
01800 HMM00465alcdh_3_mix(pf00465) EUT1
01801 HMM06723EutJMreB_1_grm(#) EUT1
01802 HMMalddh_1_eutpdugrm(pf00171) EUT1 EP_NTERM
01803 P_lightViolet EUT1
01804 H_azure EUT1
01805 HMM01515PTA_B_1_eut1(pf01515) EUT1
01806 HMM01923cobtr_2_eut1(pf01923) EUT1
01807 HMM06249EutQ_1_eut1(pf06249) EUT1 EP_NTERM
01808 HMM10662PduV_1_mix(pf10662) EUT1
01809 Hp_euts_violetPink EUT1
01810 HMM00390_malic_1_eut1(pf00390pf03949pf01515) EUT1
01811 -pf00923 -
01812 HMM02780keto_1_eut1(pf02779pf02780pf00456) EUT1
01813 -pf06674 -
01814 HMM00293nuhyd_1_eut1(pf00293) EUT1
01815 -# -
01816 -pf14691pf07992 -
01817 -pf00672pf02518pf07730pf13675 -
01818 -pf00873 -
01819 -pf00873 -
BMC-P
BMC-H
BMC-T
Aldehyde dehydrogenase
PTAC
Signature enzyme
Alcohol dehydrogenase
Regulator

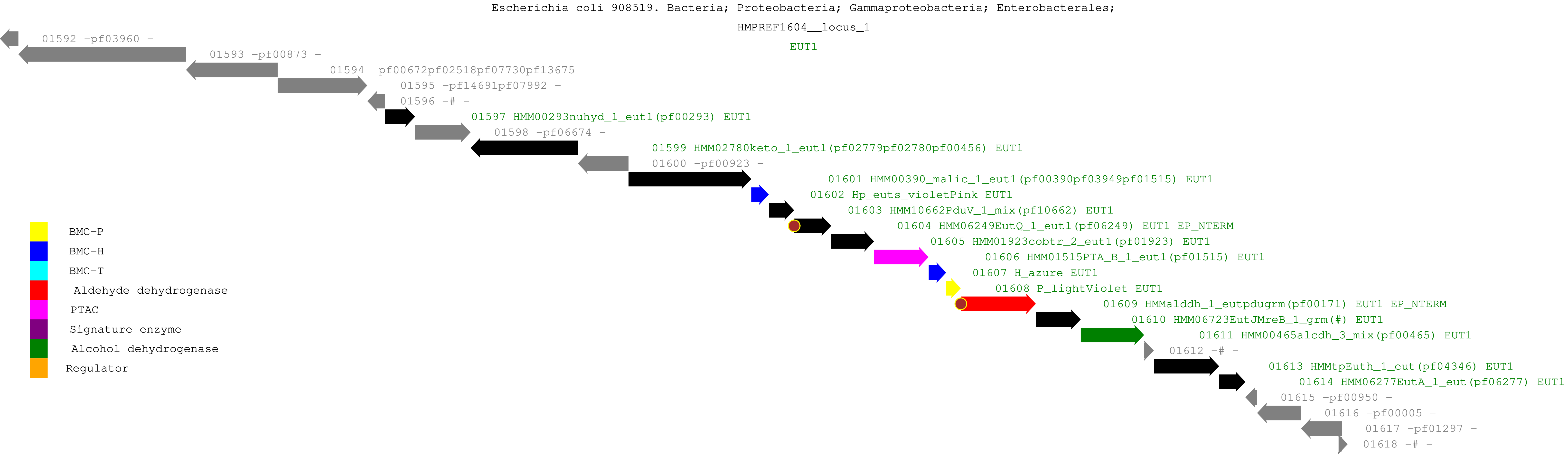
Escherichia coli 908519. Bacteria; Proteobacteria; Gammaproteobacteria; Enterobacterales;
HMPREF1604__locus_1
EUT1
01592 -pf03960 -
01593 -pf00873 -
01594 -pf00672pf02518pf07730pf13675 -
01595 -pf14691pf07992 -
01596 -# -
01597 HMM00293nuhyd_1_eut1(pf00293) EUT1
01598 -pf06674 -
01599 HMM02780keto_1_eut1(pf02779pf02780pf00456) EUT1
01600 -pf00923 -
01601 HMM00390_malic_1_eut1(pf00390pf03949pf01515) EUT1
01602 Hp_euts_violetPink EUT1
01603 HMM10662PduV_1_mix(pf10662) EUT1
01604 HMM06249EutQ_1_eut1(pf06249) EUT1 EP_NTERM
01605 HMM01923cobtr_2_eut1(pf01923) EUT1
01606 HMM01515PTA_B_1_eut1(pf01515) EUT1
01607 H_azure EUT1
01608 P_lightViolet EUT1
01609 HMMalddh_1_eutpdugrm(pf00171) EUT1 EP_NTERM
01610 HMM06723EutJMreB_1_grm(#) EUT1
01611 HMM00465alcdh_3_mix(pf00465) EUT1
01612 -# -
01613 HMMtpEuth_1_eut(pf04346) EUT1
01614 HMM06277EutA_1_eut(pf06277) EUT1
01615 -pf00950 -
01616 -pf00005 -
01617 -pf01297 -
01618 -# -
BMC-P
BMC-H
BMC-T
Aldehyde dehydrogenase
PTAC
Signature enzyme
Alcohol dehydrogenase
Regulator

Escherichia coli 908525. Bacteria; Proteobacteria; Gammaproteobacteria; Enterobacterales;

HMPREF1608__locus_1

EUT1

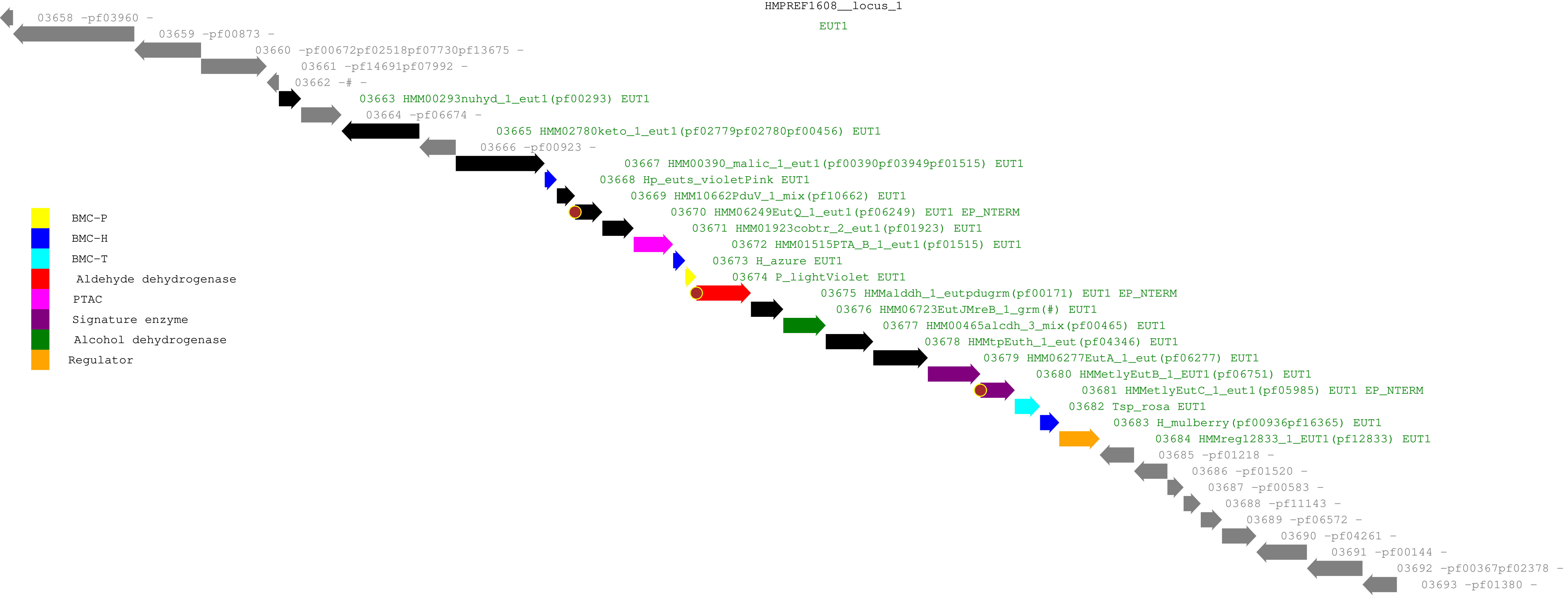

Escherichia coli 908541. Bacteria; Proteobacteria; Gammaproteobacteria; Enterobacterales;

HMPREF1609__locus_1

EUT1

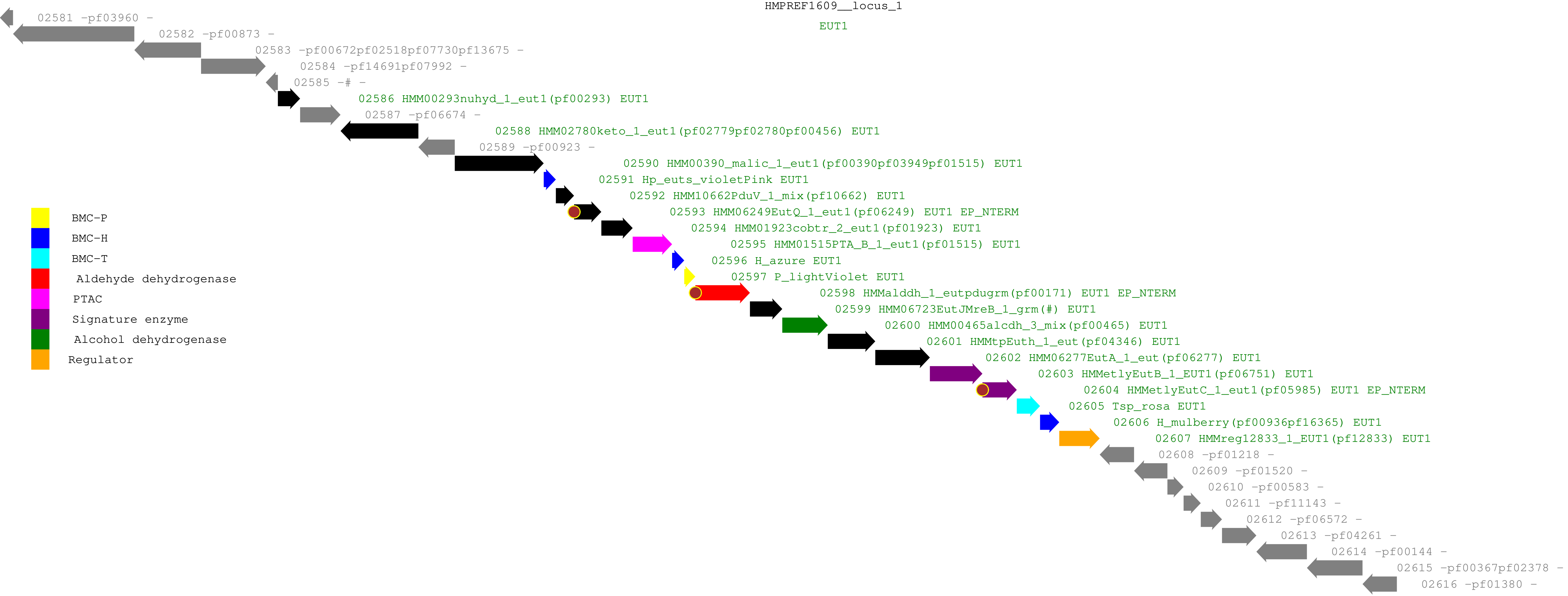

Escherichia coli 908573. Bacteria; Proteobacteria; Gammaproteobacteria; Enterobacterales;

HMPREF1611__locus_1

EUT1

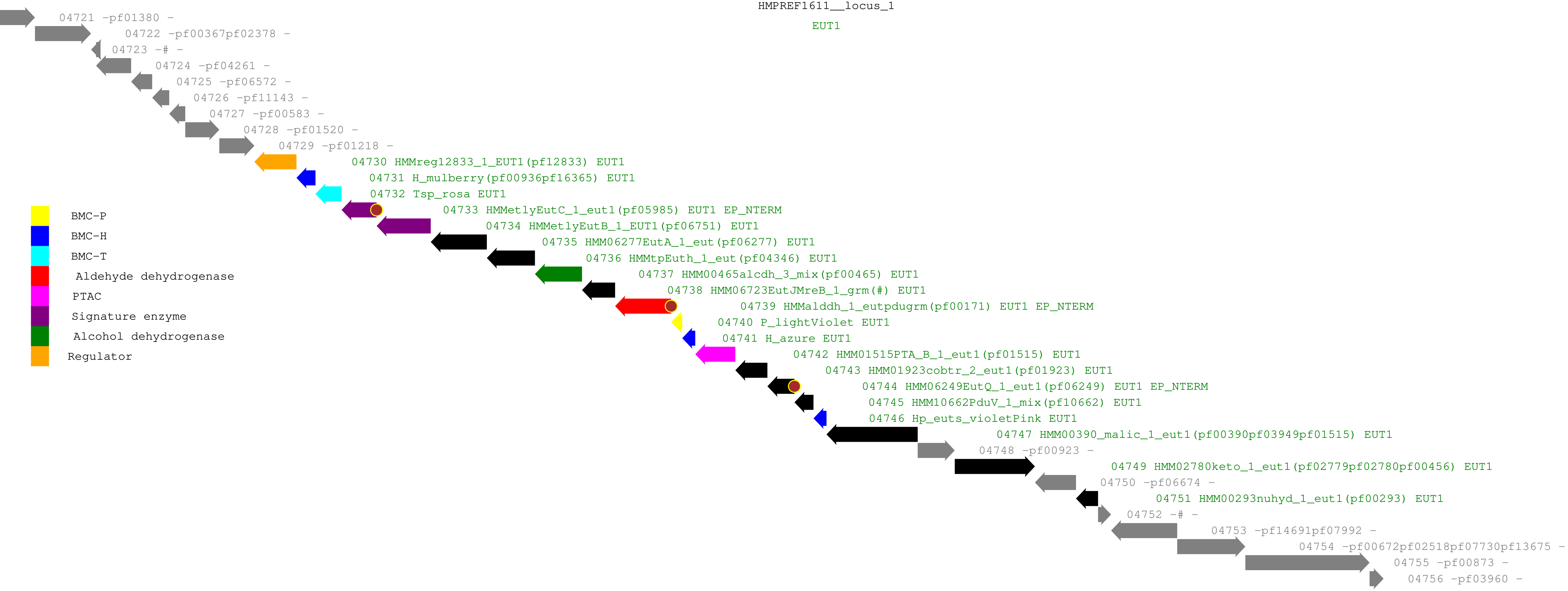

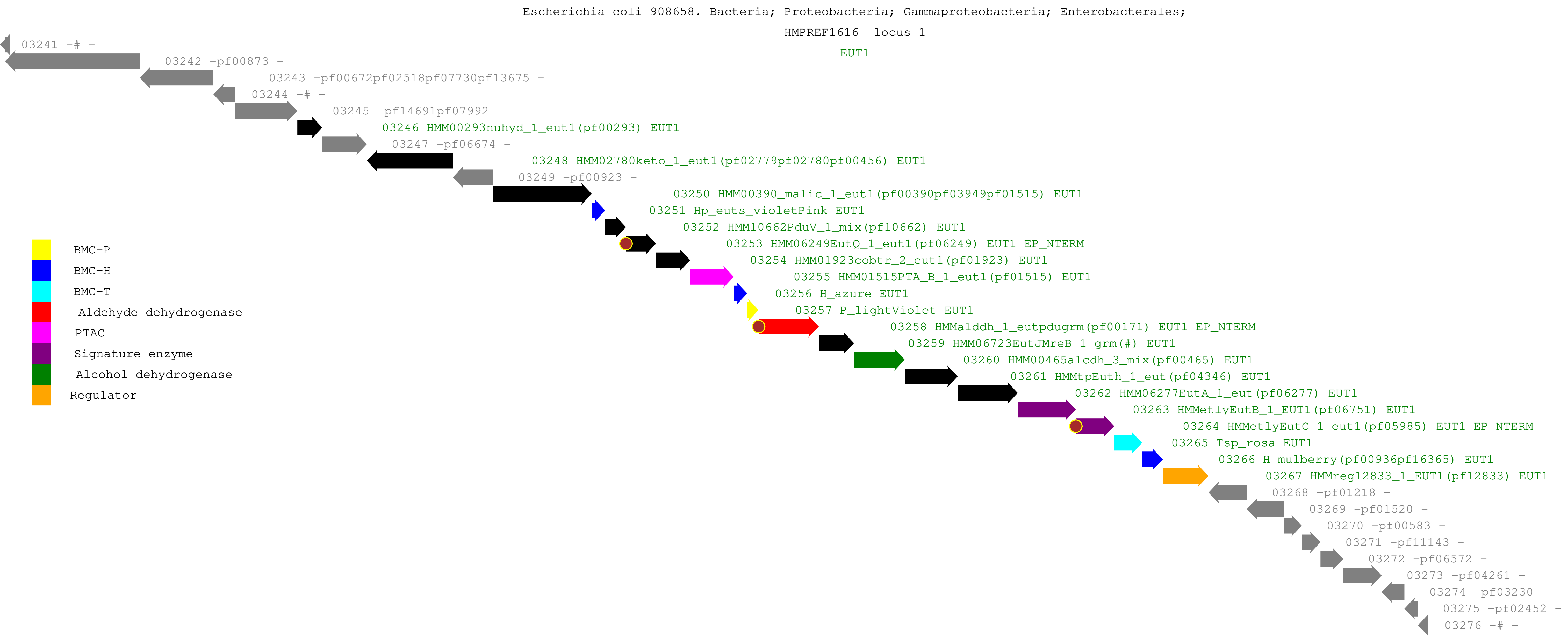

Escherichia coli 908658. Bacteria; Proteobacteria; Gammaproteobacteria; Enterobacterales;
HMPREF1616__locus_1
EUT1
03241 -# -
03242 -pf00873 -
03243 -pf00672pf02518pf07730pf13675 -
03244 -# -
03245 -pf14691pf07992 -
03246 HMM00293nuhyd_1_eut1(pf00293) EUT1
03247 -pf06674 -
03248 HMM02780keto_1_eut1(pf02779pf02780pf00456) EUT1
03249 -pf00923 -
03250 HMM00390_malic_1_eut1(pf00390pf03949pf01515) EUT1
03251 Hp_euts_violetPink EUT1
03252 HMM10662PduV_1_mix(pf10662) EUT1
03253 HMM06249EutQ_1_eut1(pf06249) EUT1 EP_NTERM
03254 HMM01923cobtr_2_eut1(pf01923) EUT1
03255 HMM01515PTA_B_1_eut1(pf01515) EUT1
03256 H_azure EUT1
03257 P_lightViolet EUT1
03258 HMMaldh_1_eutpdugrm(pf00171) EUT1 EP_NTERM
03259 HMM06723EutJMreB_1_grm(#) EUT1
03260 HMM00465alcdh_3_mix(pf00465) EUT1
03261 HMMtpEuth_1_eut(pf04346) EUT1
03262 HMM06277EutA_1_eut(pf06277) EUT1
03263 HMMetlyEutB_1_EUT1(pf06751) EUT1
03264 HMMetlyEutC_1_eut1(pf05985) EUT1 EP_NTERM
03265 Tsp_rosa EUT1
03266 H_mulberry(pf00936pf16365) EUT1
03267 HMMreg12833_1_EUT1(pf12833) EUT1
03268 -pf01218 -
03269 -pf01520 -
03270 -pf00583 -
03271 -pf11143 -
03272 -pf06572 -
03273 -pf04261 -
03274 -pf03230 -
03275 -pf02452 -
03276 -# -
BMC-P
BMC-H
BMC-T
Aldehyde dehydrogenase
PTAC
Signature enzyme
Alcohol dehydrogenase
Regulator

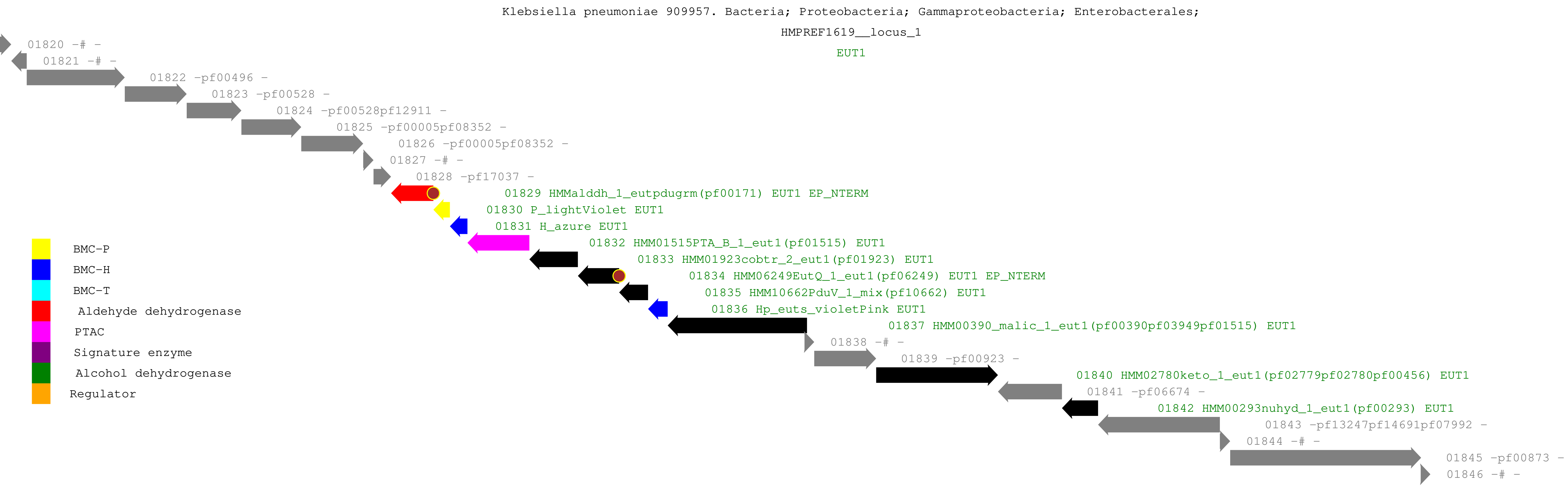

Klebsiella pneumoniae 909957. Bacteria; Proteobacteria; Gammaproteobacteria; Enterobacterales;
HMPREF1619__locus_1
EUT1
01820 -# -
01821 -# -
01822 -pf00496 -
01823 -pf00528 -
01824 -pf00528pf12911 -
01825 -pf00005pf08352 -
01826 -pf00005pf08352 -
01827 -# -
01828 -pf17037 -
01829 HMMalddh_1_eutpdugrm(pf00171) EUT1 EP_NTERM
01830 P_lightViolet EUT1
01831 H_azure EUT1
01832 HMM01515PTA_B_1_eut1(pf01515) EUT1
01833 HMM01923cobtr_2_eut1(pf01923) EUT1
01834 HMM06249EutQ_1_eut1(pf06249) EUT1 EP_NTERM
01835 HMM10662PduV_1_mix(pf10662) EUT1
01836 Hp_euts_violetPink EUT1
01837 HMM00390_malic_1_eut1(pf00390pf03949pf01515) EUT1
01838 -# -
01839 -pf00923 -
01840 HMM02780keto_1_eut1(pf02779pf02780pf00456) EUT1
01841 -pf06674 -
01842 HMM00293nuhyd_1_eut1(pf00293) EUT1
01843 -pf13247pf14691pf07992 -
01844 -# -
01845 -pf00873 -
01846 -# -
BMC-P
BMC-H
BMC-T
Aldehyde dehydrogenase
PTAC
Signature enzyme
Alcohol dehydrogenase
Regulator

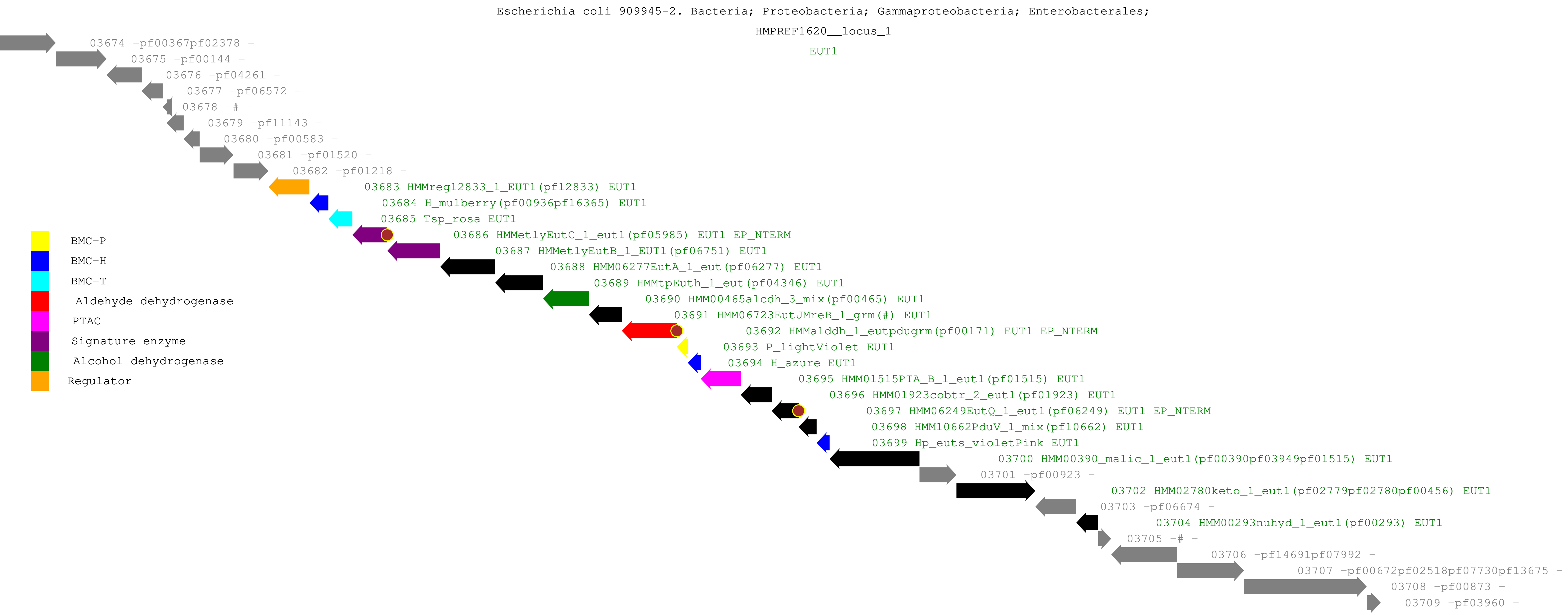

Escherichia coli 909945-2. Bacteria; Proteobacteria; Gammaproteobacteria; Enterobacterales;
HMPREF1620__locus_1
EUT1
03674 -pf00367pf02378 -
03675 -pf00144 -
03676 -pf04261 -
03677 -pf06572 -
03678 -# -
03679 -pf11143 -
03680 -pf00583 -
03681 -pf01520 -
03682 -pf01218 -
03683 HMMreg12833_1_EUT1(pf12833) EUT1
03684 H_mulberry(pf00936pf16365) EUT1
03685 Tsp_rosa EUT1
03686 HMMetlyEutC_1_eut1(pf05985) EUT1 EP_NTERM
03687 HMMetlyEutB_1_EUT1(pf06751) EUT1
03688 HMM06277EutA_1_eut(pf06277) EUT1
03689 HMMtpEuth_1_eut(pf04346) EUT1
03690 HMM00465alcdh_3_mix(pf00465) EUT1
03691 HMM06723EutJMreB_1_grm(#) EUT1
03692 HMMalddh_1_eutpdugrm(pf00171) EUT1 EP_NTERM
03693 P_lightViolet EUT1
03694 H_azure EUT1
03695 HMM01515PTA_B_1_eut1(pf01515) EUT1
03696 HMM01923cobtr_2_eut1(pf01923) EUT1
03697 HMM06249EutQ_1_eut1(pf06249) EUT1 EP_NTERM
03698 HMM10662PduV_1_mix(pf10662) EUT1
03699 Hp_euts_violetPink EUT1
03700 HMM00390_malic_1_eut1(pf00390pf03949pf01515) EUT1
03701 -pf00923 -
03702 HMM02780keto_1_eut1(pf02779pf02780pf00456) EUT1
03703 -pf06674 -
03704 HMM00293nuhyd_1_eut1(pf00293) EUT1
03705 -# -
03706 -pf14691pf07992 -
03707 -pf00672pf02518pf07730pf13675 -
03708 -pf00873 -
03709 -pf03960 -
BMC-P
BMC-H
BMC-T
Aldehyde dehydrogenase
PTAC
Signature enzyme
Alcohol dehydrogenase
Regulator

Enterobacter sp. HMSC055A11. Bacteria; Proteobacteria; Gammaproteobacteria; Enterobacterales;

HMPREF2540__locus_2

EUT1

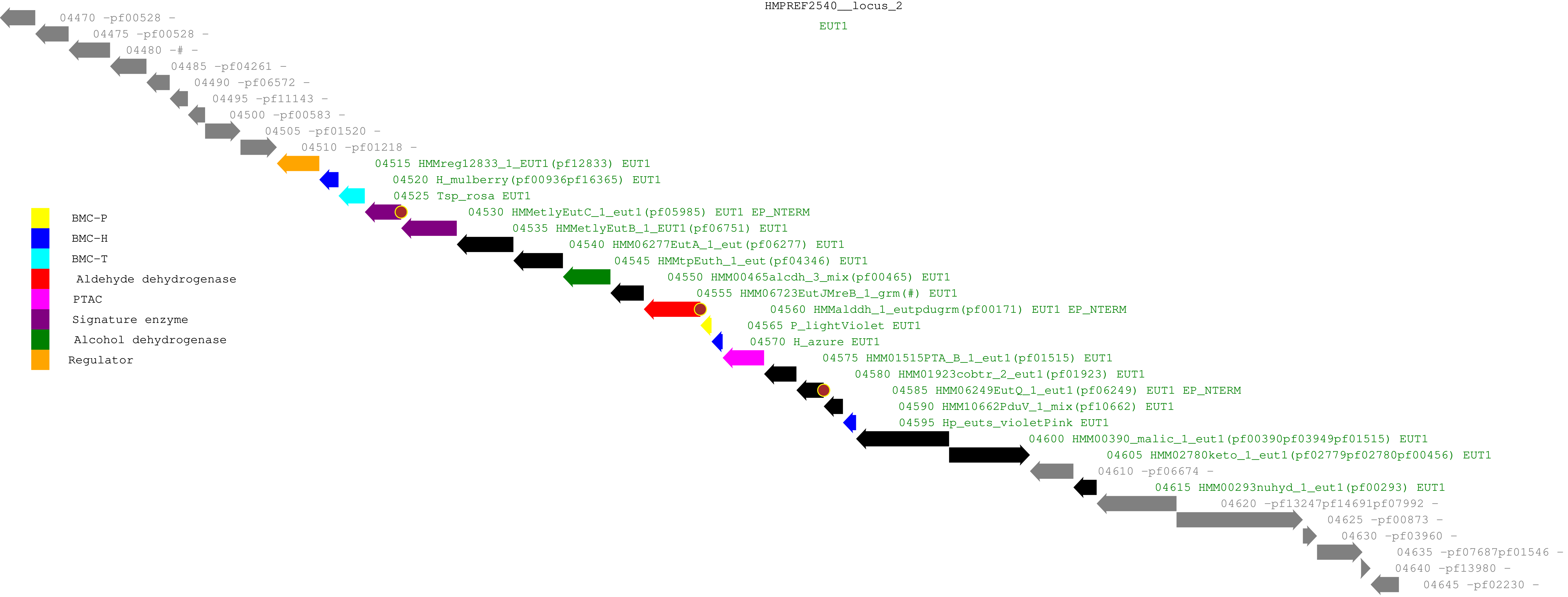

Escherichia coli. Bacteria; Proteobacteria; Gammaproteobacteria; Enterobacterales;

HMPREF3040__locus_2

EUT1

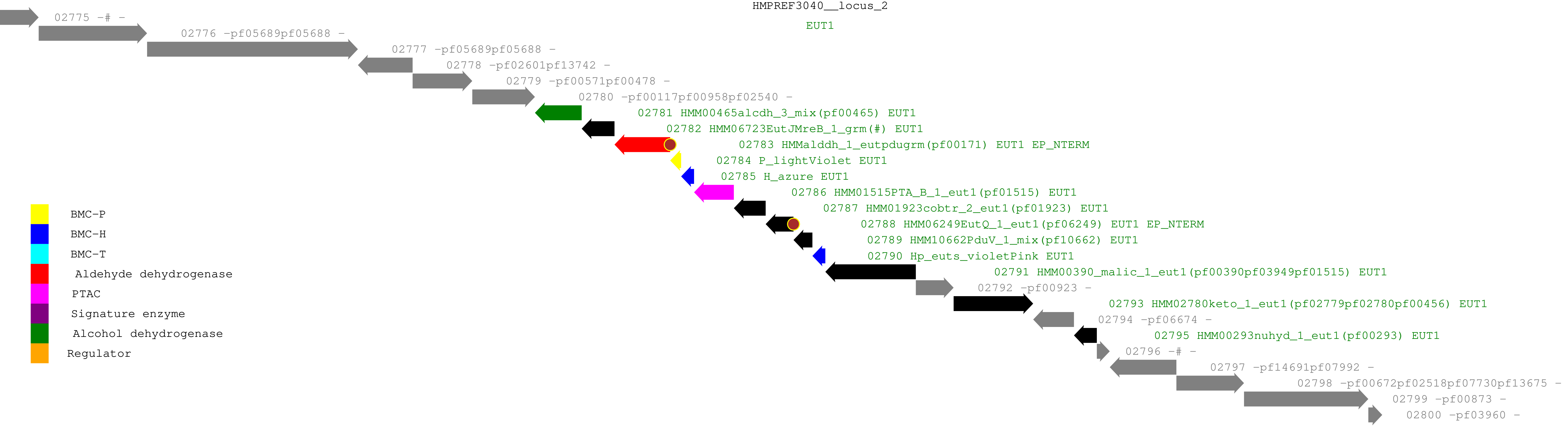

Proteus sp. HMSC10D02. Bacteria; Proteobacteria; Gammaproteobacteria; Enterobacterales;

HMPREF3111__locus_2

EUT1

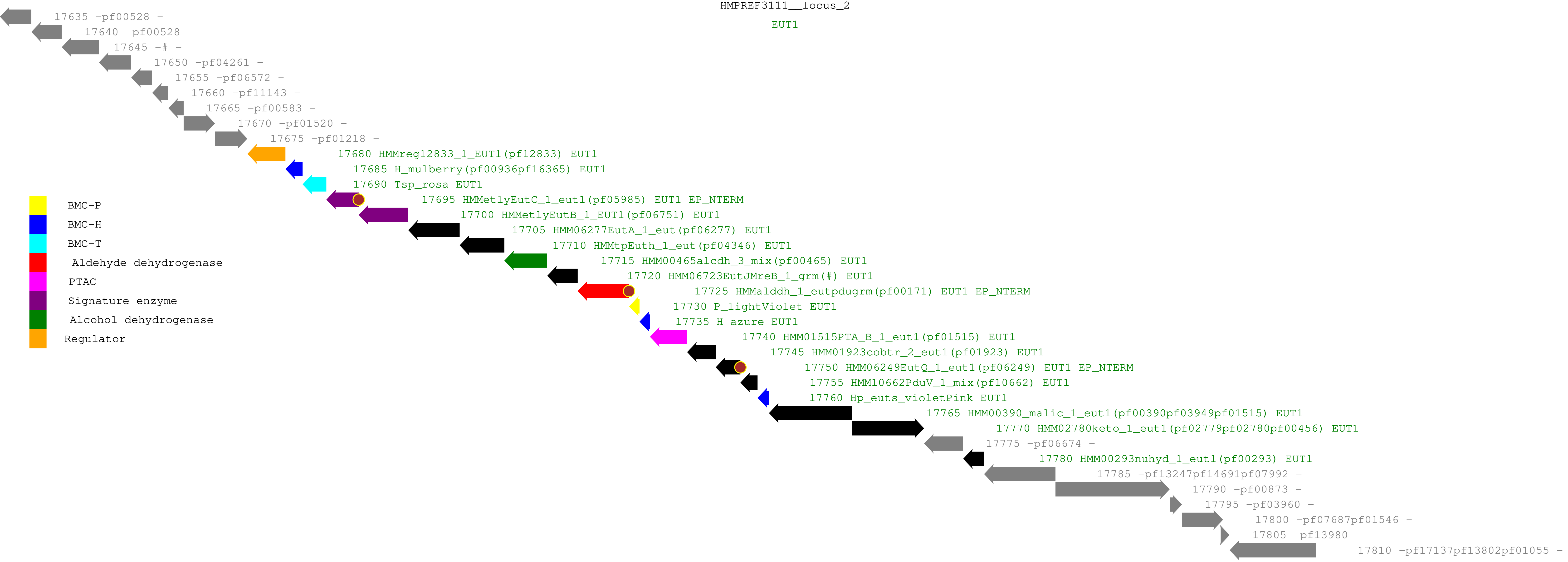

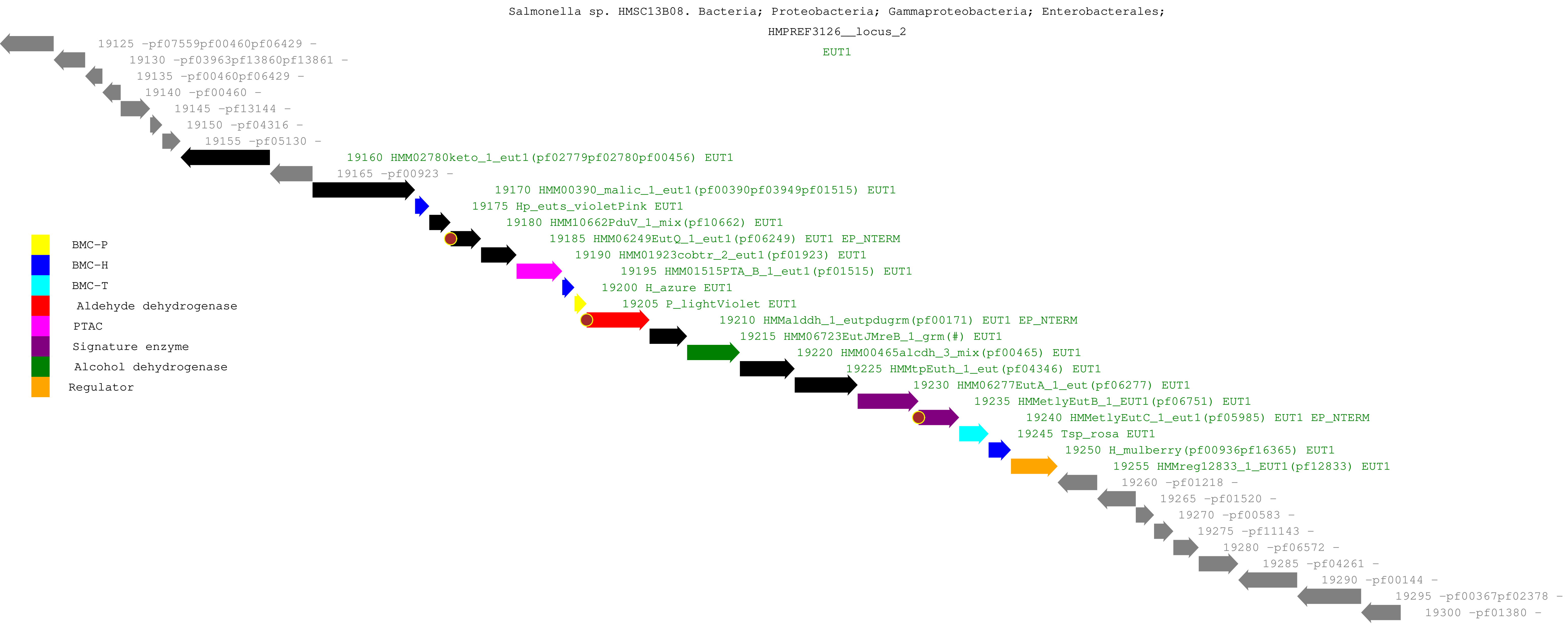

Salmonella sp. HMSC13B08. Bacteria; Proteobacteria; Gammaproteobacteria; Enterobacterales;
HMPREF3126__locus_2
EUT1
19125 -pf07559pf00460pf06429 -
19130 -pf03963pf13860pf13861 -
19135 -pf00460pf06429 -
19140 -pf00460 -
19145 -pf13144 -
19150 -pf04316 -
19155 -pf05130 -
19160 HMM02780keto_1_eut1(pf02779pf02780pf00456) EUT1
19165 -pf00923 -
19170 HMM00390_malic_1_eut1(pf00390pf03949pf01515) EUT1
19175 Hp_euts_violetPink EUT1
19180 HMM10662PduV_1_mix(pf10662) EUT1
19185 HMM06249EutQ_1_eut1(pf06249) EUT1 EP_NTERM
19190 HMM01923cobtr_2_eut1(pf01923) EUT1
19195 HMM01515PTA_B_1_eut1(pf01515) EUT1
19200 H_azure EUT1
19205 P_lightViolet EUT1
19210 HMMalddh_1_eutpdugrm(pf00171) EUT1 EP_NTERM
19215 HMM06723EutJMreB_1_grm(#) EUT1
19220 HMM00465alcdh_3_mix(pf00465) EUT1
19225 HMMtpEuth_1_eut(pf04346) EUT1
19230 HMM06277EutA_1_eut(pf06277) EUT1
19235 HMMetlyEutB_1_EUT1(pf06751) EUT1
19240 HMMetlyEutC_1_eut1(pf05985) EUT1 EP_NTERM
19245 Tsp_rosa EUT1
19250 H_mulberry(pf00936pf16365) EUT1
19255 HMMreg12833_1_EUT1(pf12833) EUT1
19260 -pf01218 -
19265 -pf01520 -
19270 -pf00583 -
19275 -pf11143 -
19280 -pf06572 -
19285 -pf04261 -
19290 -pf00144 -
19295 -pf00367pf02378 -
19300 -pf01380 -
BMC-P
BMC-H
BMC-T
Aldehyde dehydrogenase
PTAC
Signature enzyme
Alcohol dehydrogenase
Regulator

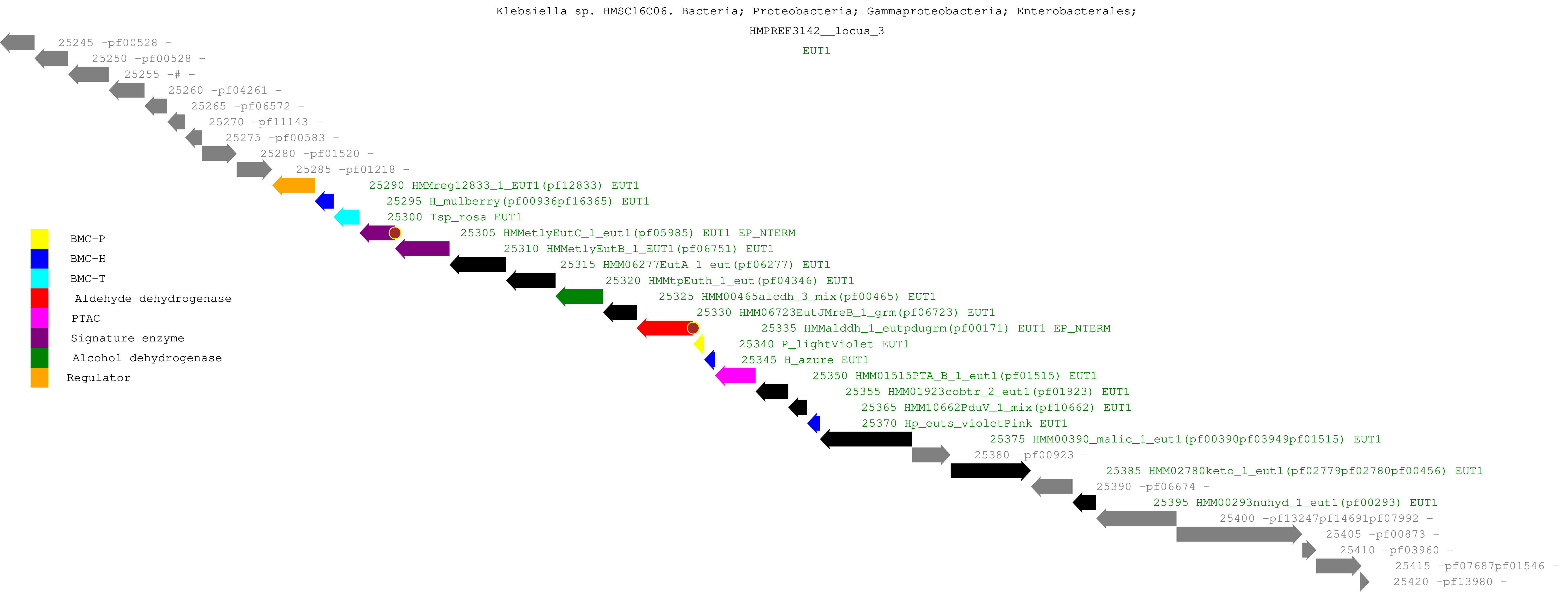
Klebsiella sp. HMSC16C06. Bacteria; Proteobacteria; Gammaproteobacteria; Enterobacterales;
HMPREF3142__locus_3
EUT1
25245 -pf00528 -
25250 -pf00528 -
25255 -# -
25260 -pf04261 -
25265 -pf06572 -
25270 -pf11143 -
25275 -pf00583 -
25280 -pf01520 -
25285 -pf01218 -
25290 HMMreg12833_1_EUT1(pf12833) EUT1
25295 H_mulberry(pf00936pf16365) EUT1
25300 Tsp_rosa EUT1
25305 HMMetlyEutC_1_eut1(pf05985) EUT1 EP_NTERM
25310 HMMetlyEutB_1_EUT1(pf06751) EUT1
25315 HMM06277EutA_1_eut(pf06277) EUT1
25320 HMMtpEuth_1_eut(pf04346) EUT1
25325 HMM00465alcdh_3_mix(pf00465) EUT1
25330 HMM06723EutJMreB_1_grm(pf06723) EUT1
25335 HMMalddh_1_eutpdugrm(pf00171) EUT1 EP_NTERM
25340 P_lightViolet EUT1
25345 H_azure EUT1
25350 HMM01515PTA_B_1_eut1(pf01515) EUT1
25355 HMM01923cobtr_2_eut1(pf01923) EUT1
25365 HMM10662PduV_1_mix(pf10662) EUT1
25370 Hp_euts_violetPink EUT1
25375 HMM00390_malic_1_eut1(pf00390pf03949pf01515) EUT1
25380 -pf00923 -
25385 HMM02780keto_1_eut1(pf02779pf02780pf00456) EUT1
25390 -pf06674 -
25395 HMM00293nuhyd_1_eut1(pf00293) EUT1
25400 -pf13247pf14691pf07992 -
25405 -pf00873 -
25410 -pf03960 -
25415 -pf07687pf01546 -
25420 -pf13980 -
BMC-P
BMC-H
BMC-T
Aldehyde dehydrogenase
PTAC
Signature enzyme
Alcohol dehydrogenase
Regulator

Citrobacter freundii. Bacteria; Proteobacteria; Gammaproteobacteria; Enterobacterales;

HMPREF3212__locus_2

EUT1

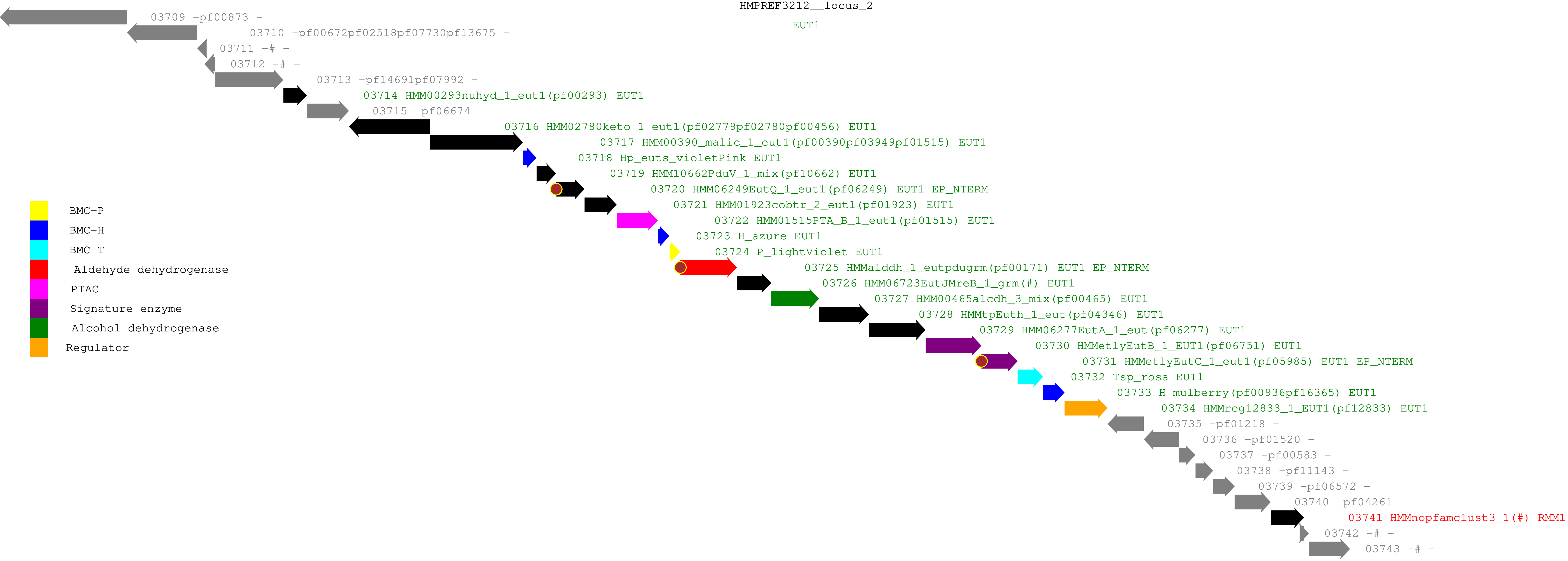

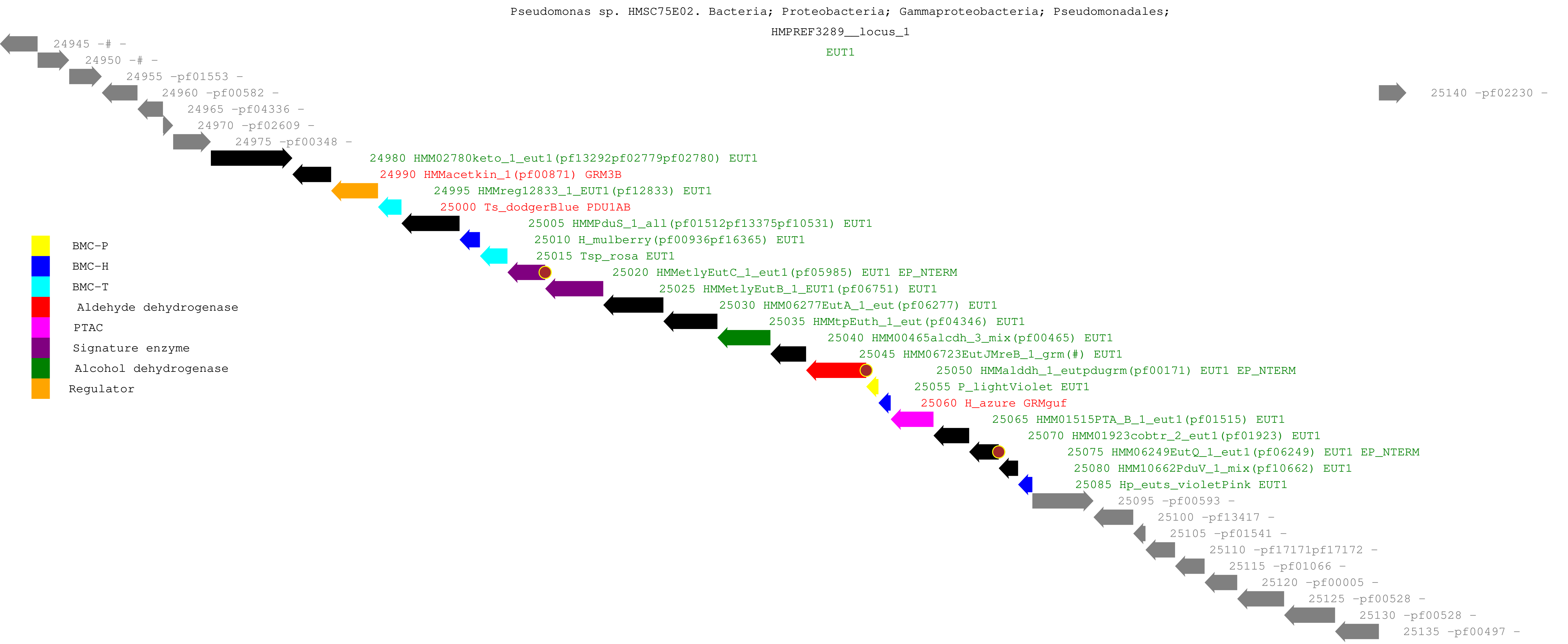
Pseudomonas sp. HMSC75E02. Bacteria; Proteobacteria; Gammaproteobacteria; Pseudomonadales;
HMPREF3289__locus_1
EUT1
24945 -# -
24950 -# -
24955 -pf01553 -
24960 -pf00582 -
24965 -pf04336 -
24970 -pf02609 -
24975 -pf00348 -
24980 HMM02780keto_1_eut1(pf13292pf02779pf02780) EUT1
24990 HMMacetkin_1(pf00871) GRM3B
24995 HMMreg12833_1_EUT1(pf12833) EUT1
25000 Ts_dodgerBlue PDU1AB
25005 HMMPduS_1_all(pf01512pf13375pf10531) EUT1
25010 H_mulberry(pf00936pf16365) EUT1
25015 Tsp_rosa EUT1
25020 HMMetlyEutC_1_eut1(pf05985) EUT1 EP_NTERM
25025 HMMetlyEutB_1_EUT1(pf06751) EUT1
25030 HMM06277EutA_1_eut(pf06277) EUT1
25035 HMMtpEuth_1_eut(pf04346) EUT1
25040 HMM00465alcdh_3_mix(pf00465) EUT1
25045 HMM06723EutJMreB_1_grm(#) EUT1
25050 HMMaldh_1_eutpdugrm(pf00171) EUT1 EP_NTERM
25055 P_lightViolet EUT1
25060 H_azure GRMguf
25065 HMM01515PTA_B_1_eut1(pf01515) EUT1
25070 HMM01923cobtr_2_eut1(pf01923) EUT1
25075 HMM06249EutQ_1_eut1(pf06249) EUT1 EP_NTERM
25080 HMM10662PduV_1_mix(pf10662) EUT1
25085 Hp_euts_violetPink EUT1
25095 -pf00593 -
25100 -pf13417 -
25105 -pf01541 -
25110 -pf17171pf17172 -
25115 -pf01066 -
25120 -pf00005 -
25125 -pf00528 -
25130 -pf00528 -
25135 -pf00497 -
25140 -pf02230 -
BMC-P
BMC-H
BMC-T
Aldehyde dehydrogenase
PTAC
Signature enzyme
Alcohol dehydrogenase
Regulator

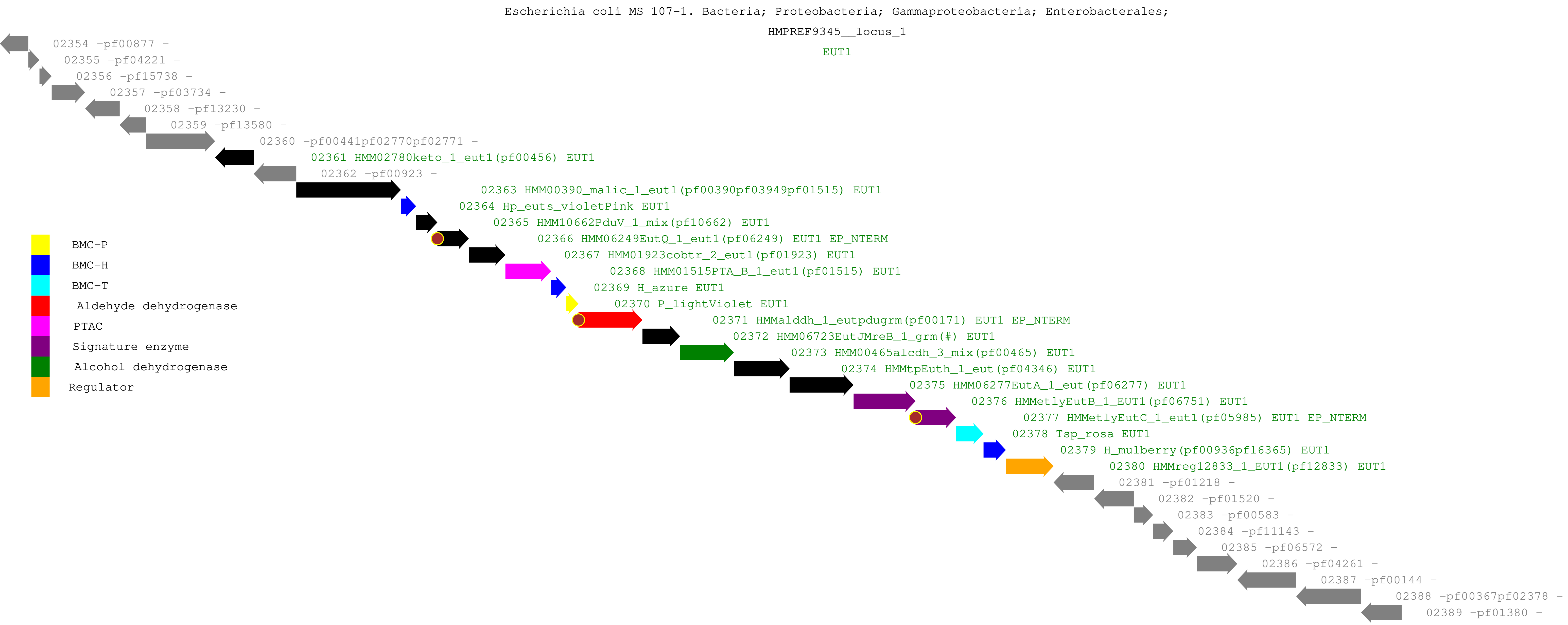

Escherichia coli MS 107-1. Bacteria; Proteobacteria; Gammaproteobacteria; Enterobacterales;
HMPREF9345__locus_1
EUT1
02354 -pf00877 -
02355 -pf04221 -
02356 -pf15738 -
02357 -pf03734 -
02358 -pf13230 -
02359 -pf13580 -
02360 -pf00441pf02770pf02771 -
02361 HMM02780keto_1_eut1(pf00456) EUT1
02362 -pf00923 -
02363 HMM00390_malic_1_eut1(pf00390pf03949pf01515) EUT1
02364 Hp_euts_violetPink EUT1
02365 HMM10662PduV_1_mix(pf10662) EUT1
02366 HMM06249EutQ_1_eut1(pf06249) EUT1 EP_NTERM
02367 HMM01923cobtr_2_eut1(pf01923) EUT1
02368 HMM01515PTA_B_1_eut1(pf01515) EUT1
02369 H_azure EUT1
02370 P_lightViolet EUT1
02371 HMMaldh_1_eutpdugrm(pf00171) EUT1 EP_NTERM
02372 HMM06723EutJMreB_1_grm(#) EUT1
02373 HMM00465alcdh_3_mix(pf00465) EUT1
02374 HMMtpEuth_1_eut(pf04346) EUT1
02375 HMM06277EutA_1_eut(pf06277) EUT1
02376 HMMetlyEutB_1_EUT1(pf06751) EUT1
02377 HMMetlyEutC_1_eut1(pf05985) EUT1 EP_NTERM
02378 Tsp_rosa EUT1
02379 H_mulberry(pf00936pf16365) EUT1
02380 HMMreg12833_1_EUT1(pf12833) EUT1
02381 -pf01218 -
02382 -pf01520 -
02383 -pf00583 -
02384 -pf11143 -
02385 -pf06572 -
02386 -pf04261 -
02387 -pf00144 -
02388 -pf00367pf02378 -
02389 -pf01380 -
BMC-P
BMC-H
BMC-T
Aldehyde dehydrogenase
PTAC
Signature enzyme
Alcohol dehydrogenase
Regulator

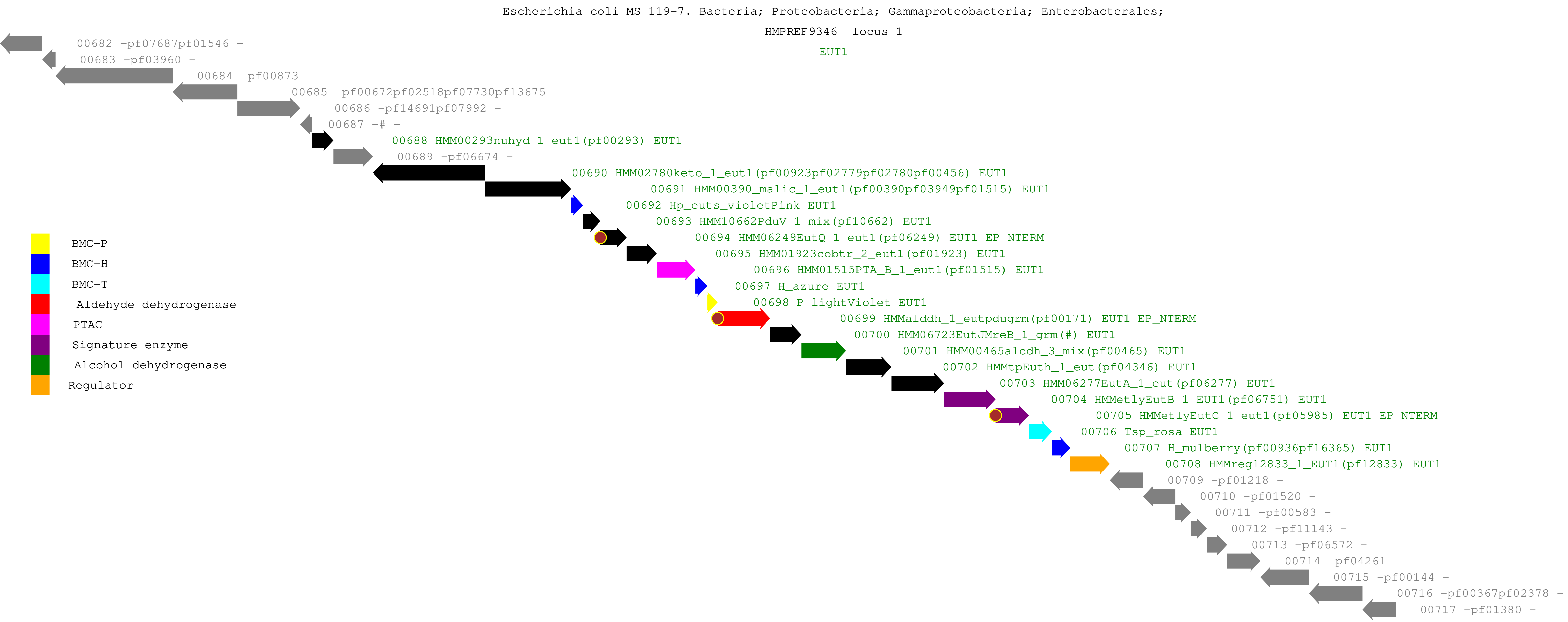
Escherichia coli MS 119-7. Bacteria; Proteobacteria; Gammaproteobacteria; Enterobacterales;
HMPREF9346__locus_1
EUT1
00682 -pf07687pf01546 -
00683 -pf03960 -
00684 -pf00873 -
00685 -pf00672pf02518pf07730pf13675 -
00686 -pf14691pf07992 -
00687 -# -
00688 HMM00293nuhyd_1_eut1(pf00293) EUT1
00689 -pf06674 -
00690 HMM02780keto_1_eut1(pf00923pf02779pf02780pf00456) EUT1
00691 HMM00390_malic_1_eut1(pf00390pf03949pf01515) EUT1
00692 Hp_euts_violetPink EUT1
00693 HMM10662PduV_1_mix(pf10662) EUT1
00694 HMM06249EutQ_1_eut1(pf06249) EUT1 EP_NTERM
00695 HMM01923cobtr_2_eut1(pf01923) EUT1
00696 HMM01515PTA_B_1_eut1(pf01515) EUT1
00697 H_azure EUT1
00698 P_lightViolet EUT1
00699 HMMalddh_1_eutpdugrm(pf00171) EUT1 EP_NTERM
00700 HMM06723EutJMreB_1_grm(#) EUT1
00701 HMM00465alcdh_3_mix(pf00465) EUT1
00702 HMMtpEuth_1_eut(pf04346) EUT1
00703 HMM06277EutA_1_eut(pf06277) EUT1
00704 HMMetlyEutB_1_EUT1(pf06751) EUT1
00705 HMMetlyEutC_1_eut1(pf05985) EUT1 EP_NTERM
00706 Tsp_rosa EUT1
00707 H_mulberry(pf00936pf16365) EUT1
00708 HMMreg12833_1_EUT1(pf12833) EUT1
00709 -pf01218 -
00710 -pf01520 -
00711 -pf00583 -
00712 -pf11143 -
00713 -pf06572 -
00714 -pf04261 -
00715 -pf00144 -
00716 -pf00367pf02378 -
00717 -pf01380 -
BMC-P
BMC-H
BMC-T
Aldehyde dehydrogenase
PTAC
Signature enzyme
Alcohol dehydrogenase
Regulator

Escherichia coli MS 124-1. Bacteria; Proteobacteria; Gammaproteobacteria; Enterobacterales;

HMPREF9347__locus_1

EUT1

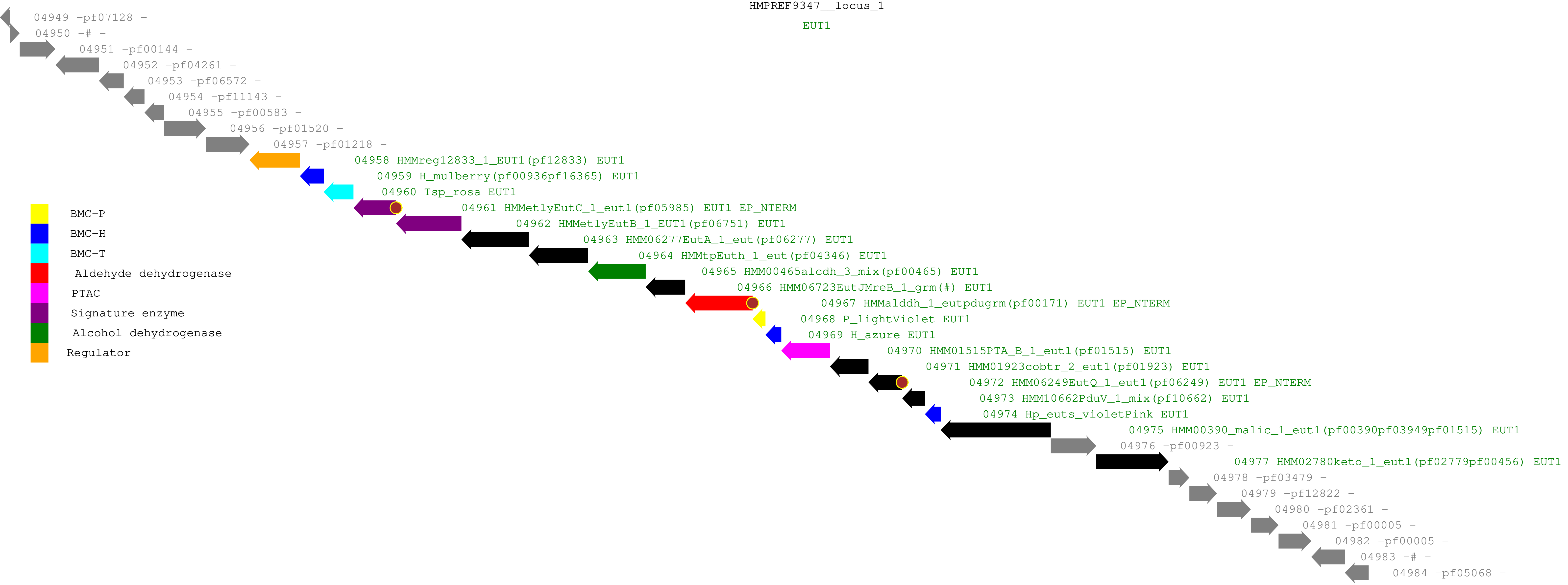

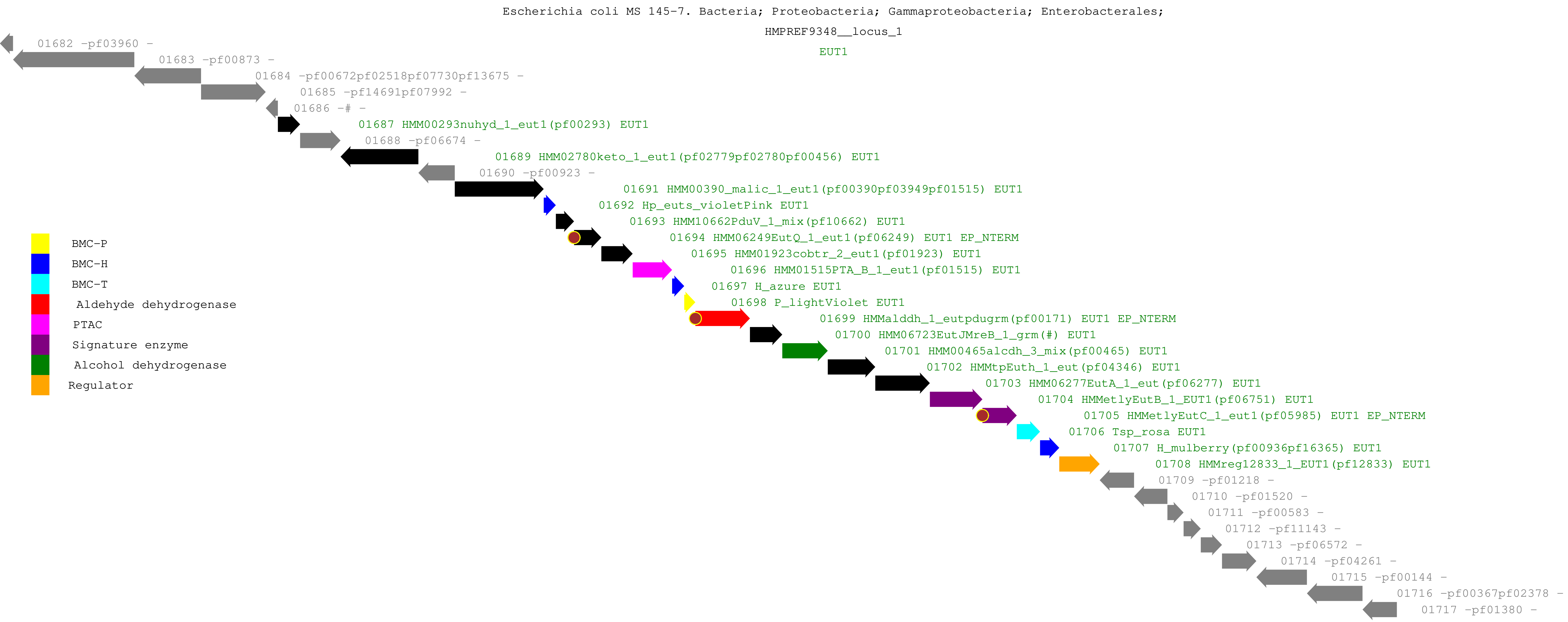
Escherichia coli MS 145-7. Bacteria; Proteobacteria; Gammaproteobacteria; Enterobacterales;
HMPREF9348__locus_1
EUT1
01682 -pf03960 -
01683 -pf00873 -
01684 -pf00672pf02518pf07730pf13675 -
01685 -pf14691pf07992 -
01686 -# -
01687 HMM00293nuhyd_1_eut1(pf00293) EUT1
01688 -pf06674 -
01689 HMM02780keto_1_eut1(pf02779pf02780pf00456) EUT1
01690 -pf00923 -
01691 HMM00390_malic_1_eut1(pf00390pf03949pf01515) EUT1
01692 Hp_euts_violetPink EUT1
01693 HMM10662PduV_1_mix(pf10662) EUT1
01694 HMM06249EutQ_1_eut1(pf06249) EUT1 EP_NTERM
01695 HMM01923cobtr_2_eut1(pf01923) EUT1
01696 HMM01515PTA_B_1_eut1(pf01515) EUT1
01697 H_azure EUT1
01698 P_lightViolet EUT1
01699 HMMaldh_1_eutpdugrm(pf00171) EUT1 EP_NTERM
01700 HMM06723EutJMreB_1_grm(#) EUT1
01701 HMM00465alcdh_3_mix(pf00465) EUT1
01702 HMMtpEuth_1_eut(pf04346) EUT1
01703 HMM06277EutA_1_eut(pf06277) EUT1
01704 HMMetlyEutB_1_EUT1(pf06751) EUT1
01705 HMMetlyEutC_1_eut1(pf05985) EUT1 EP_NTERM
01706 Tsp_rosa EUT1
01707 H_mulberry(pf00936pf16365) EUT1
01708 HMMreg12833_1_EUT1(pf12833) EUT1
01709 -pf01218 -
01710 -pf01520 -
01711 -pf00583 -
01712 -pf11143 -
01713 -pf06572 -
01714 -pf04261 -
01715 -pf00144 -
01716 -pf00367pf02378 -
01717 -pf01380 -
BMC-P
BMC-H
BMC-T
Aldehyde dehydrogenase
PTAC
Signature enzyme
Alcohol dehydrogenase
Regulator

Escherichia coli MS 79-10. Bacteria; Proteobacteria; Gammaproteobacteria; Enterobacterales;

HMPREF9349__locus_1

EUT1

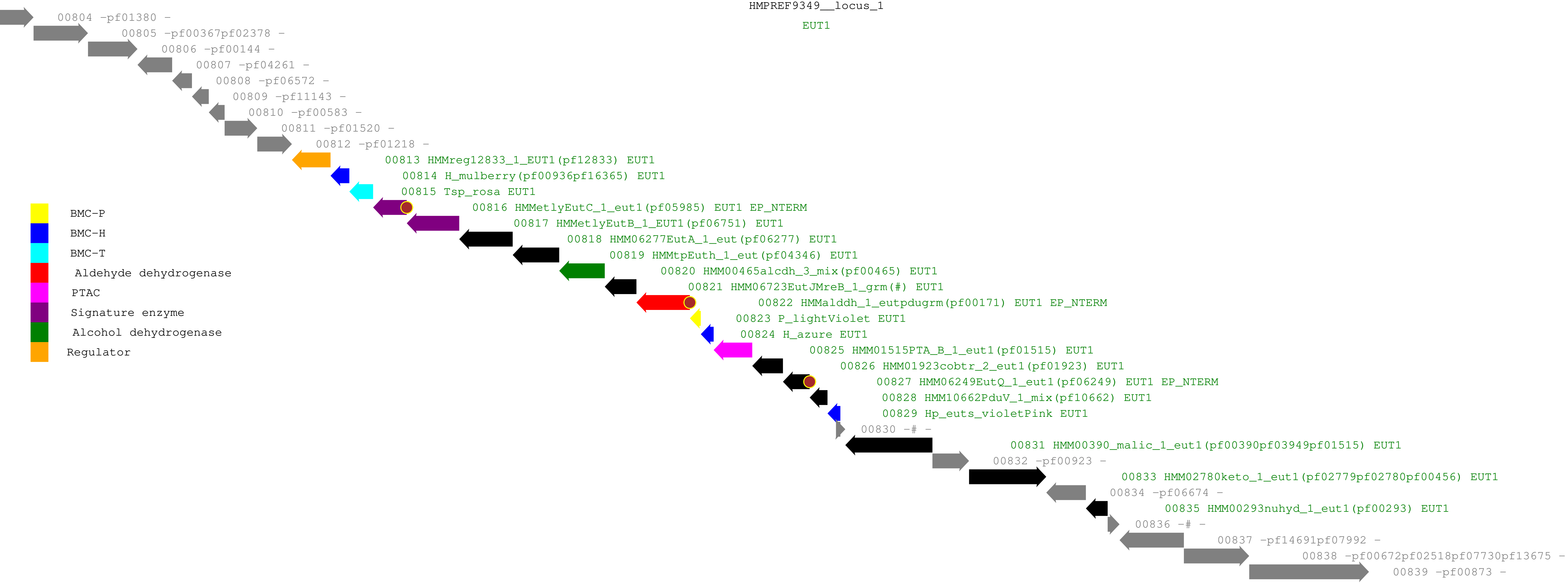

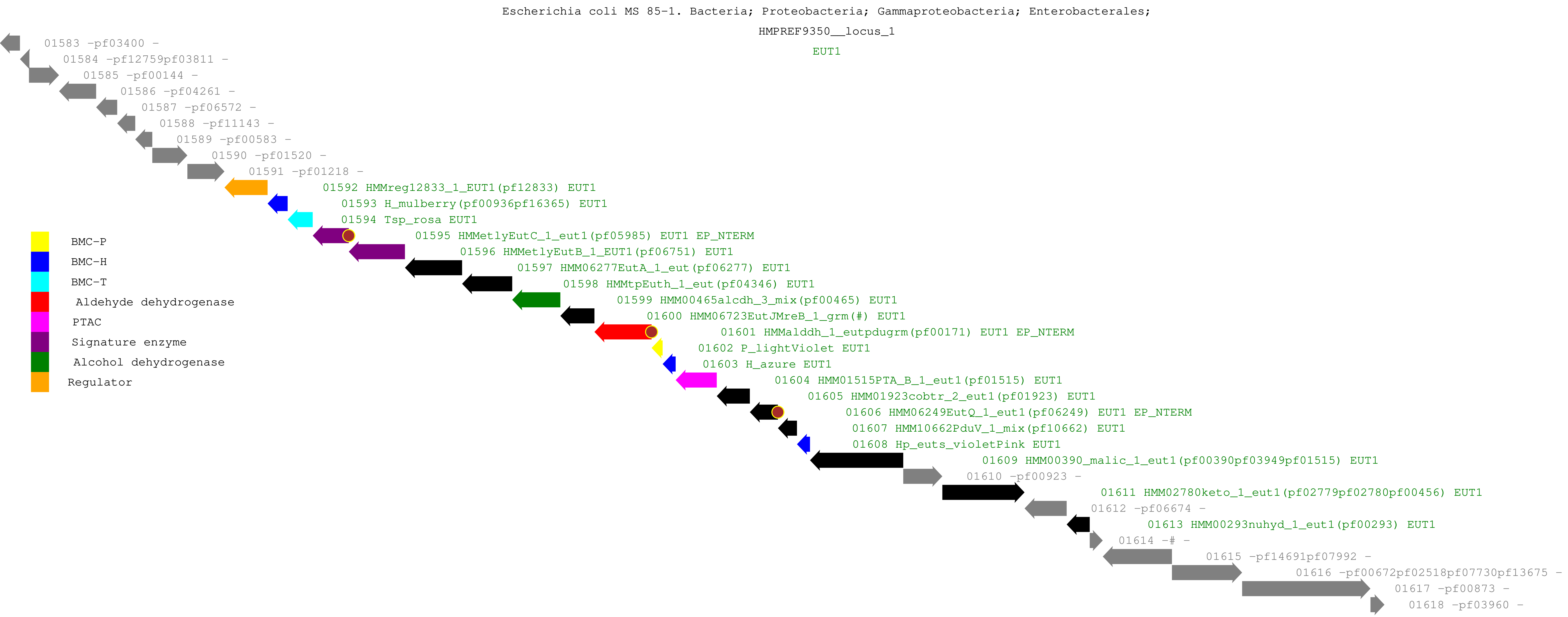

Escherichia coli MS 85-1. Bacteria; Proteobacteria; Gammaproteobacteria; Enterobacterales;
HMPREF9350__locus_1
EUT1
01583 -pf03400 -
01584 -pf12759pf03811 -
01585 -pf00144 -
01586 -pf04261 -
01587 -pf06572 -
01588 -pf11143 -
01589 -pf00583 -
01590 -pf01520 -
01591 -pf01218 -
01592 HMMreg12833_1_EUT1(pf12833) EUT1
01593 H_mulberry(pf00936pf16365) EUT1
01594 Tsp_rosa EUT1
01595 HMMetlyEutC_1_eut1(pf05985) EUT1 EP_NTERM
01596 HMMetlyEutB_1_EUT1(pf06751) EUT1
01597 HMM06277EutA_1_eut(pf06277) EUT1
01598 HMMtpEuth_1_eut(pf04346) EUT1
01599 HMM00465alcdh_3_mix(pf00465) EUT1
01600 HMM06723EutJMreB_1_grm(#) EUT1
01601 HMMalddh_1_eutpdugrm(pf00171) EUT1 EP_NTERM
01602 P_lightViolet EUT1
01603 H_azure EUT1
01604 HMM01515PTA_B_1_eut1(pf01515) EUT1
01605 HMM01923cobtr_2_eut1(pf01923) EUT1
01606 HMM06249EutQ_1_eut1(pf06249) EUT1 EP_NTERM
01607 HMM10662PduV_1_mix(pf10662) EUT1
01608 Hp_euts_violetPink EUT1
01609 HMM00390_malic_1_eut1(pf00390pf03949pf01515) EUT1
01610 -pf00923 -
01611 HMM02780keto_1_eut1(pf02779pf02780pf00456) EUT1
01612 -pf06674 -
01613 HMM00293nuhyd_1_eut1(pf00293) EUT1
01614 -# -
01615 -pf14691pf07992 -
01616 -pf00672pf02518pf07730pf13675 -
01617 -pf00873 -
01618 -pf03960 -
BMC-P
BMC-H
BMC-T
Aldehyde dehydrogenase
PTAC
Signature enzyme
Alcohol dehydrogenase
Regulator

Escherichia coli (strain MS 21-1). Bacteria; Proteobacteria; Gammaproteobacteria; Enterobacterales;

HMPREF9530__locus_2

EUT1

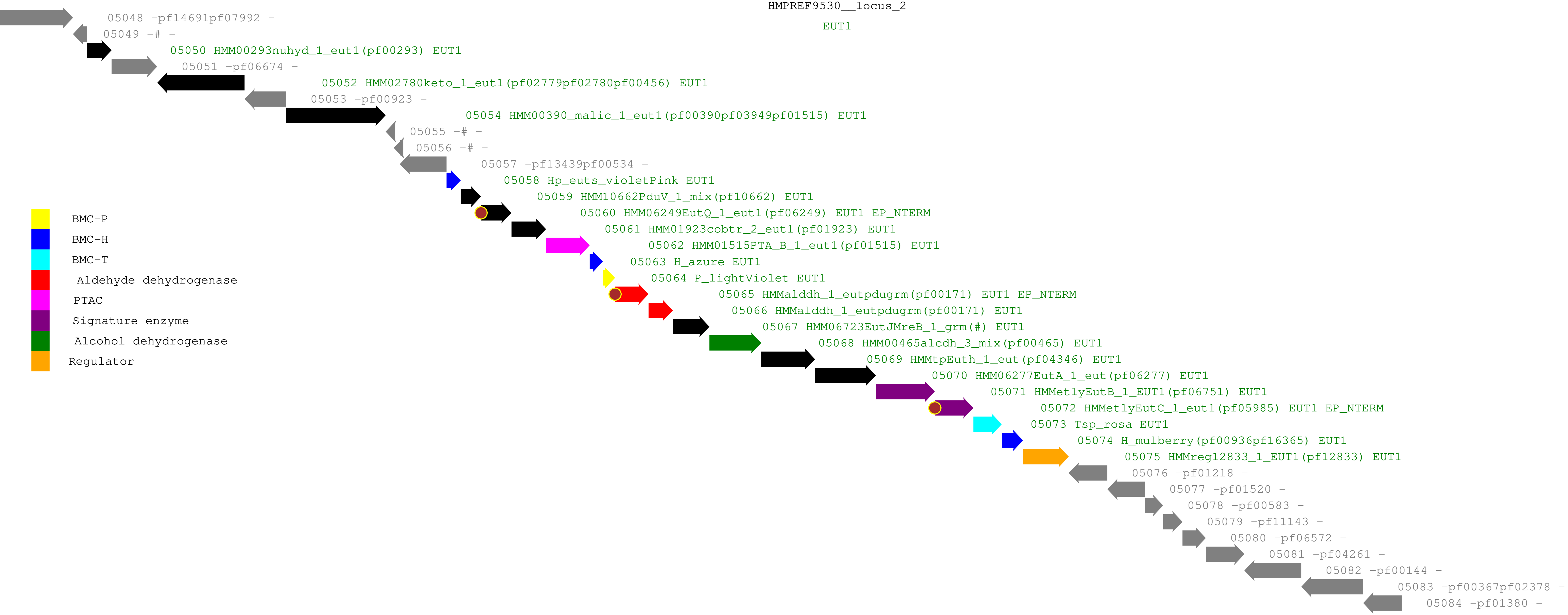

Escherichia coli MS 69-1. Bacteria; Proteobacteria; Gammaproteobacteria; Enterobacterales;

HMPREF9534__locus_2

EUT1

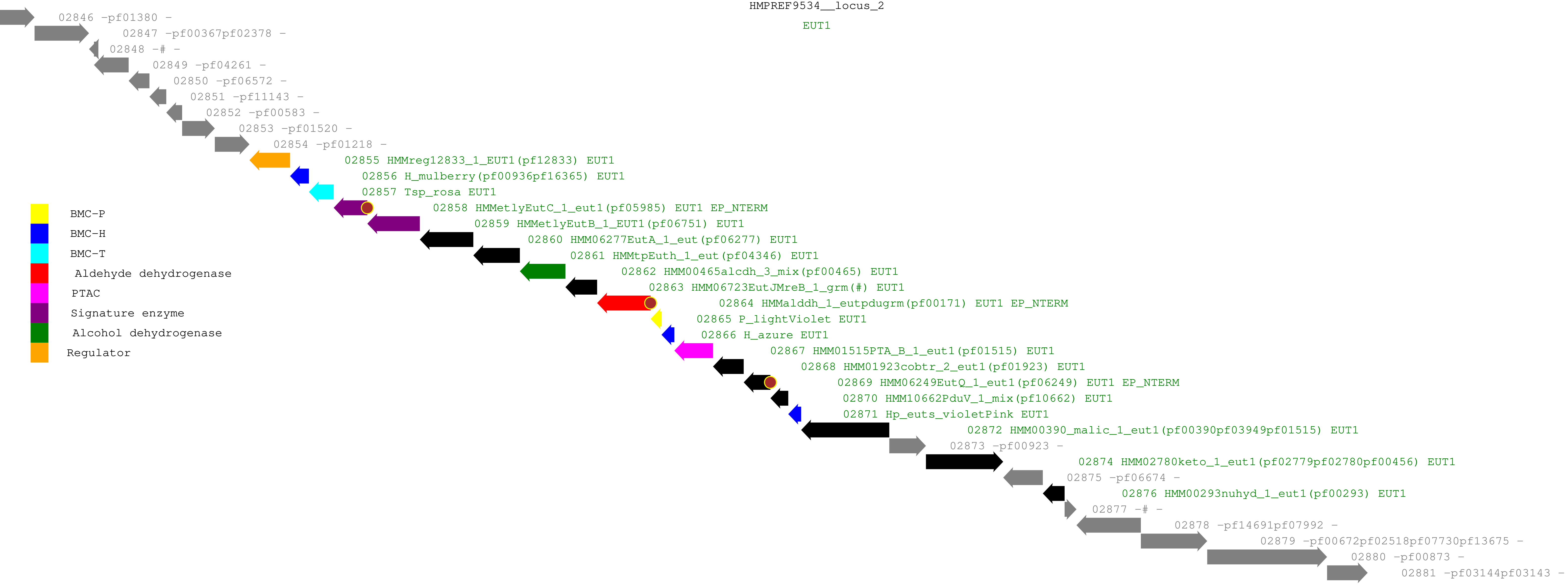

Escherichia coli MS 78-1. Bacteria; Proteobacteria; Gammaproteobacteria; Enterobacterales;

HMPREF9535__locus_1

EUT1

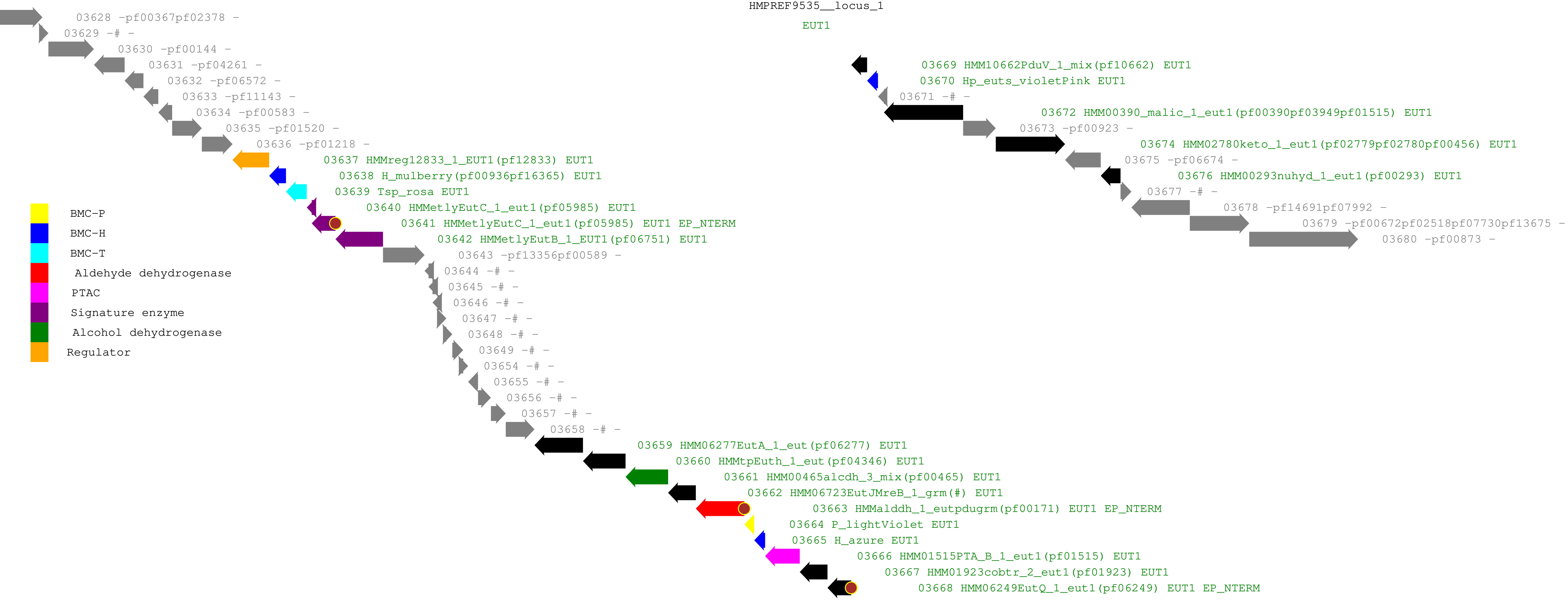

Escherichia coli MS 84-1. Bacteria; Proteobacteria; Gammaproteobacteria; Enterobacterales;

HMPREF9536__locus_1

EUT1

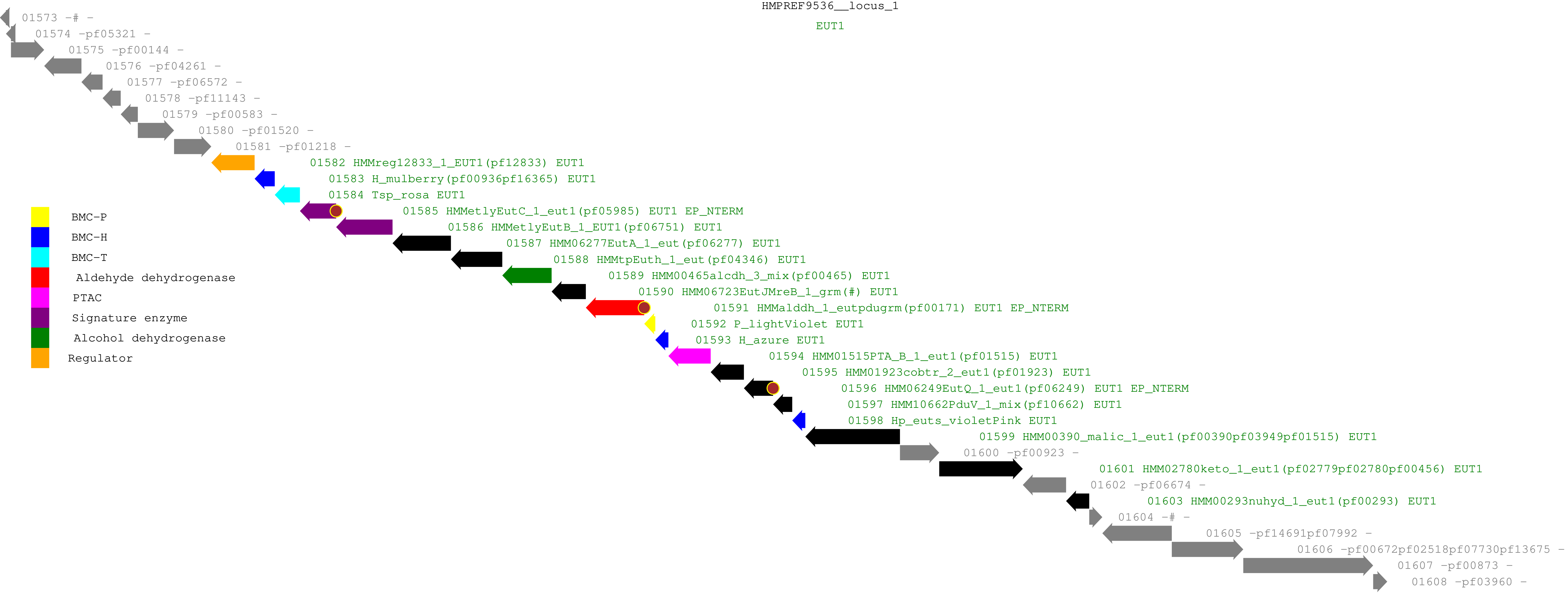

Escherichia coli MS 115-1. Bacteria; Proteobacteria; Gammaproteobacteria; Enterobacterales;

HMPREF9540__locus_2

EUT1

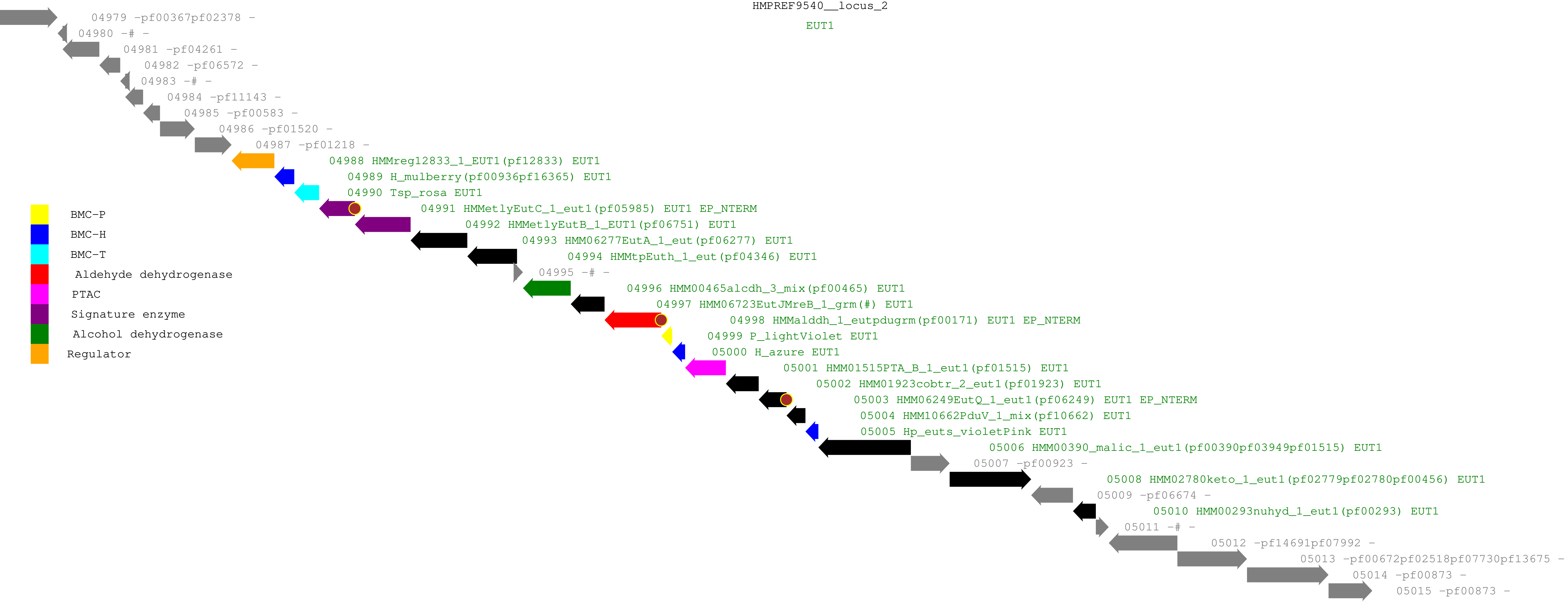

Escherichia coli MS 116-1. Bacteria; Proteobacteria; Gammaproteobacteria; Enterobacterales;

HMPREF9541__locus_2

EUT1

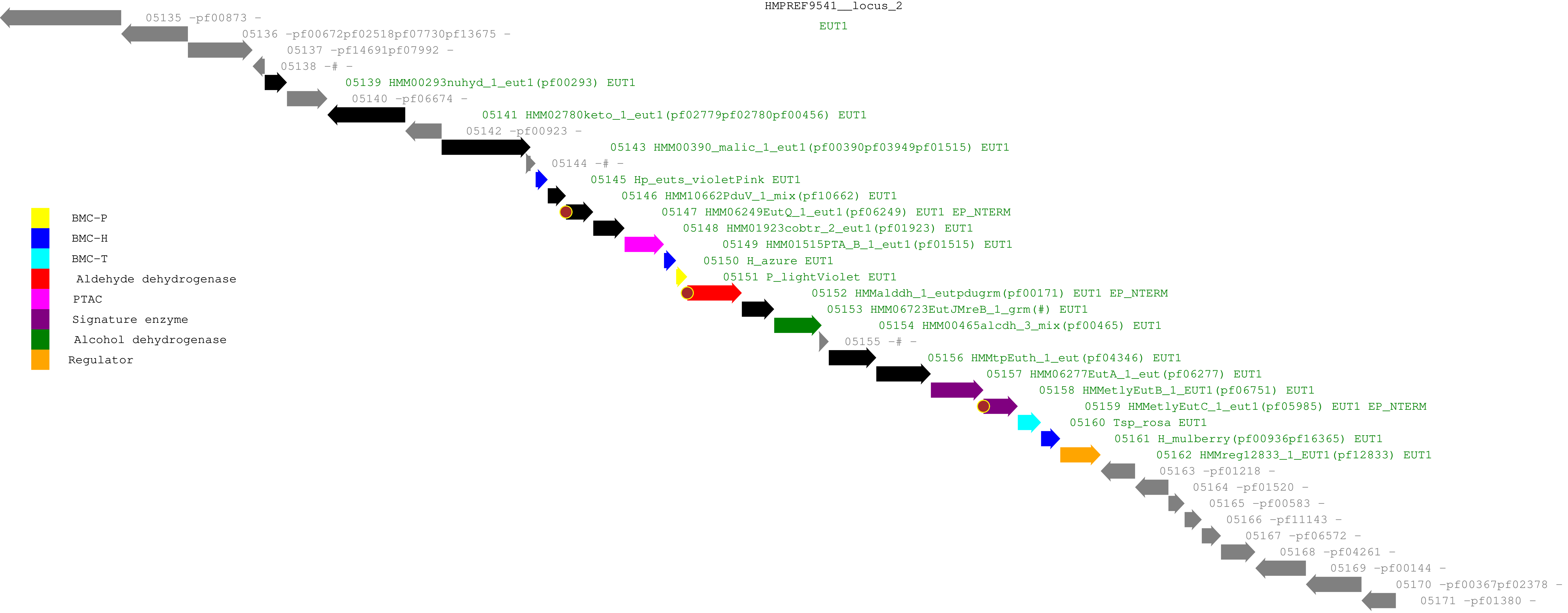

Escherichia coli MS 117-3. Bacteria; Proteobacteria; Gammaproteobacteria; Enterobacterales;

HMPREF9542__locus_1

EUT1

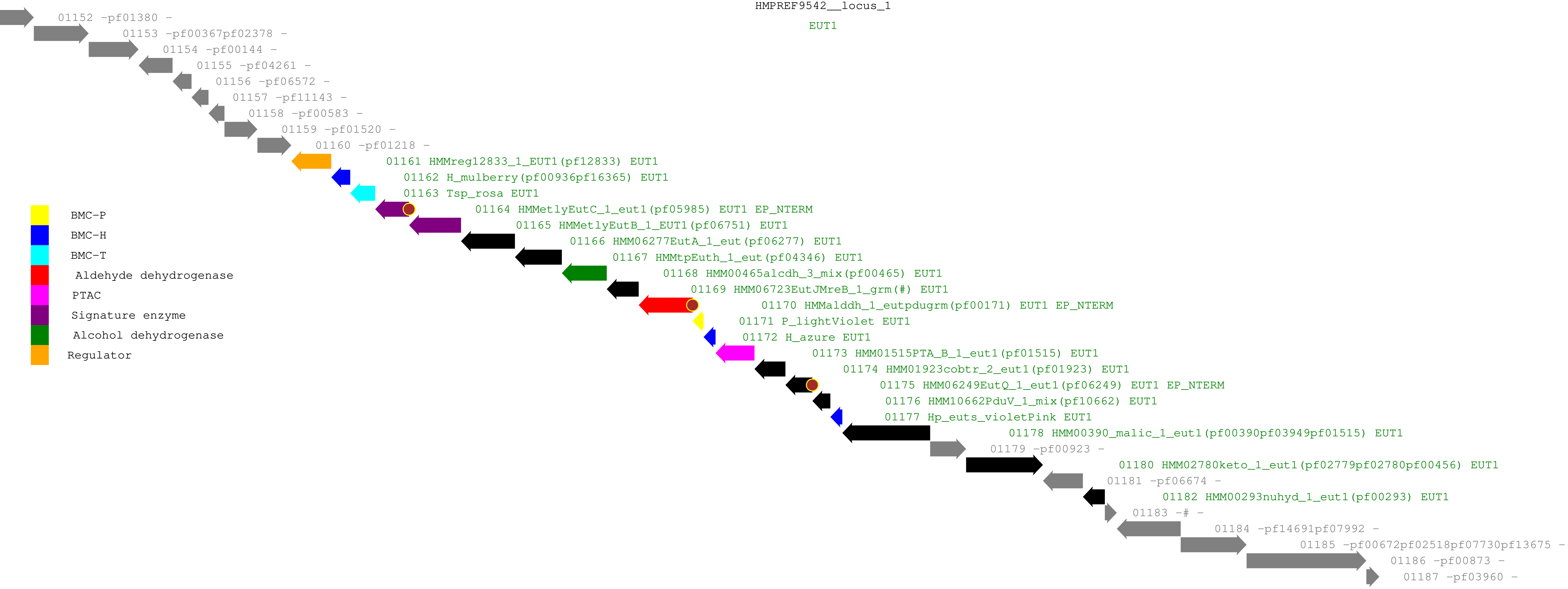

Escherichia coli MS 146-1. Bacteria; Proteobacteria; Gammaproteobacteria; Enterobacterales;

HMPREF9543__locus_1_main_sat

EUT1

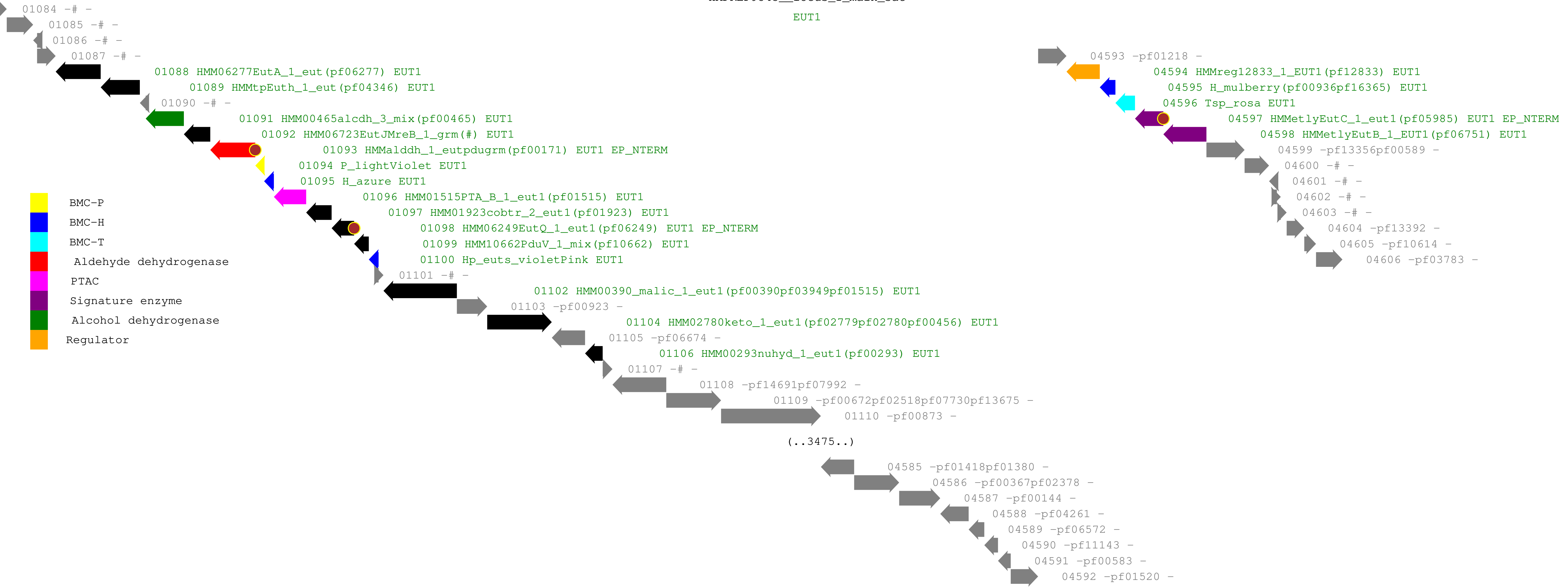

Escherichia coli MS 175-1. Bacteria; Proteobacteria; Gammaproteobacteria; Enterobacterales;

HMPREF9547__locus_1

EUT1

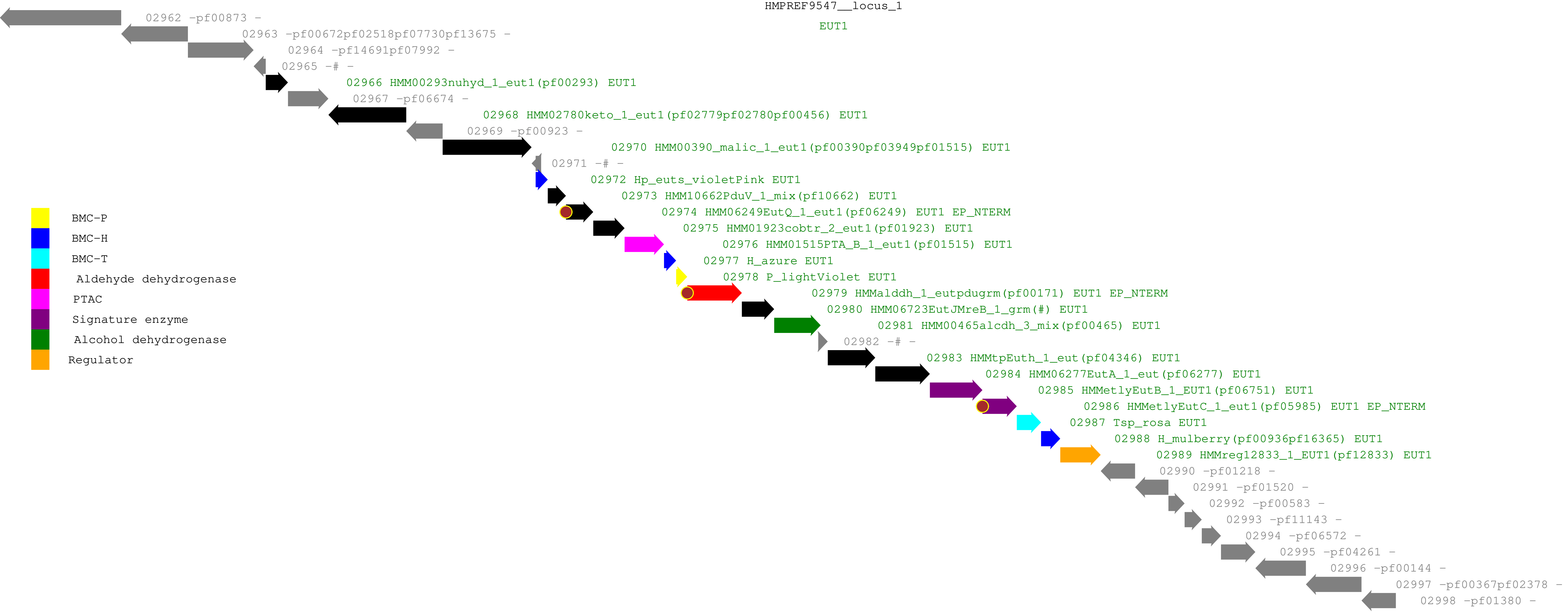

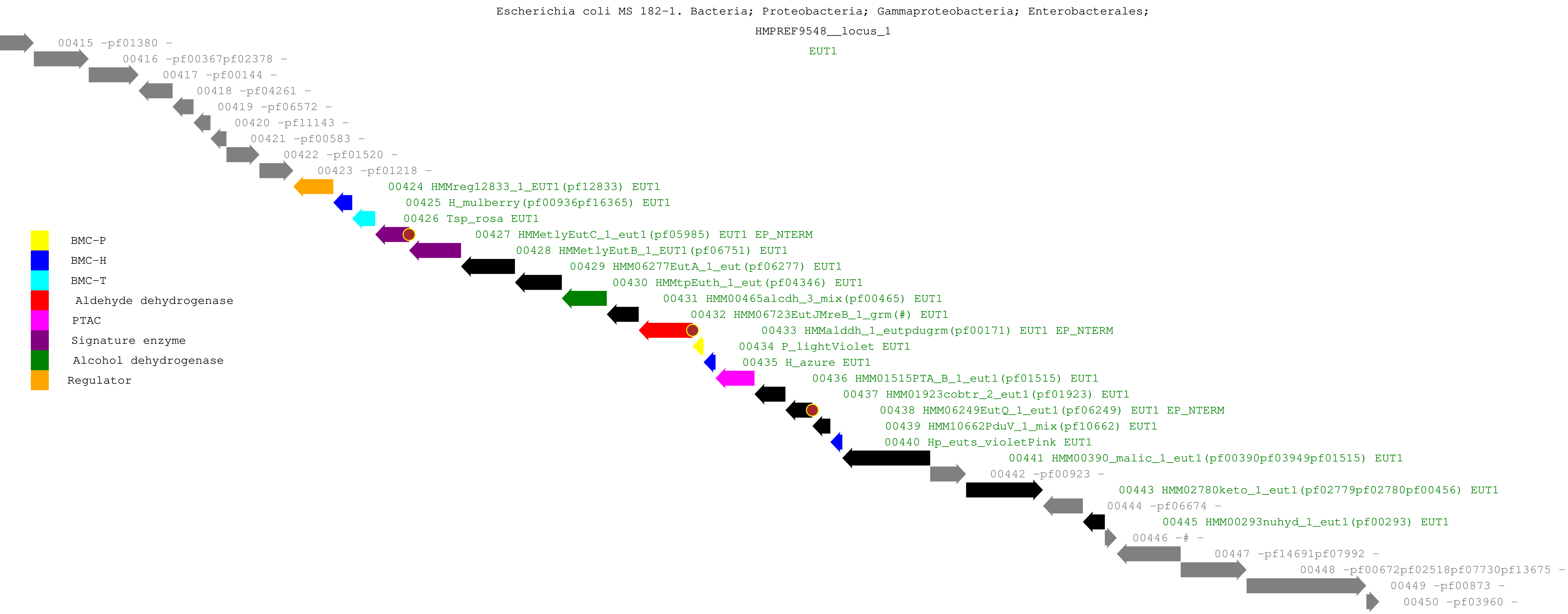
Escherichia coli MS 182-1. Bacteria; Proteobacteria; Gammaproteobacteria; Enterobacterales;
HMPREF9548__locus_1
EUT1
00415 -pf01380 -
00416 -pf00367pf02378 -
00417 -pf00144 -
00418 -pf04261 -
00419 -pf06572 -
00420 -pf11143 -
00421 -pf00583 -
00422 -pf01520 -
00423 -pf01218 -
00424 HMMreg12833_1_EUT1(pf12833) EUT1
00425 H_mulberry(pf00936pf16365) EUT1
00426 Tsp_rosa EUT1
00427 HMMetlyEutC_1_eut1(pf05985) EUT1 EP_NTERM
00428 HMMetlyEutB_1_EUT1(pf06751) EUT1
00429 HMM06277EutA_1_eut(pf06277) EUT1
00430 HMMtpEuth_1_eut(pf04346) EUT1
00431 HMM00465alcdh_3_mix(pf00465) EUT1
00432 HMM06723EutJMreB_1_grm(#) EUT1
00433 HMMalddh_1_eutpdugrm(pf00171) EUT1 EP_NTERM
00434 P_lightViolet EUT1
00435 H_azure EUT1
00436 HMM01515PTA_B_1_eut1(pf01515) EUT1
00437 HMM01923cobtr_2_eut1(pf01923) EUT1
00438 HMM06249EutQ_1_eut1(pf06249) EUT1 EP_NTERM
00439 HMM10662PduV_1_mix(pf10662) EUT1
00440 Hp_euts_violetPink EUT1
00441 HMM00390_malic_1_eut1(pf00390pf03949pf01515) EUT1
00442 -pf00923 -
00443 HMM02780keto_1_eut1(pf02779pf02780pf00456) EUT1
00444 -pf06674 -
00445 HMM00293nuhyd_1_eut1(pf00293) EUT1
00446 -# -
00447 -pf14691pf07992 -
00448 -pf00672pf02518pf07730pf13675 -
00449 -pf00873 -
00450 -pf03960 -
BMC-P
BMC-H
BMC-T
Aldehyde dehydrogenase
PTAC
Signature enzyme
Alcohol dehydrogenase
Regulator

Escherichia coli MS 196-1. Bacteria; Proteobacteria; Gammaproteobacteria; Enterobacterales;

HMPREF9551__locus_1

EUT1

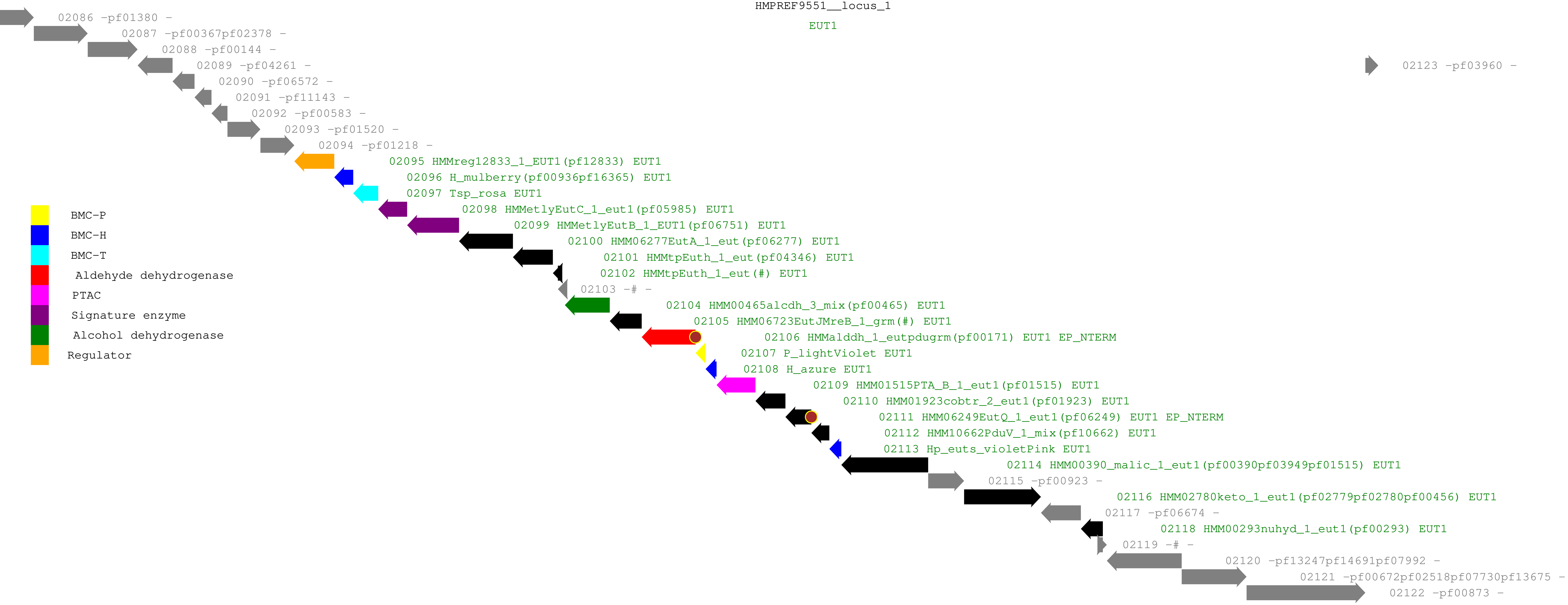

Escherichia coli MS 198-1. Bacteria; Proteobacteria; Gammaproteobacteria; Enterobacterales;

HMPREF9552__locus_1_main_sat

EUT1

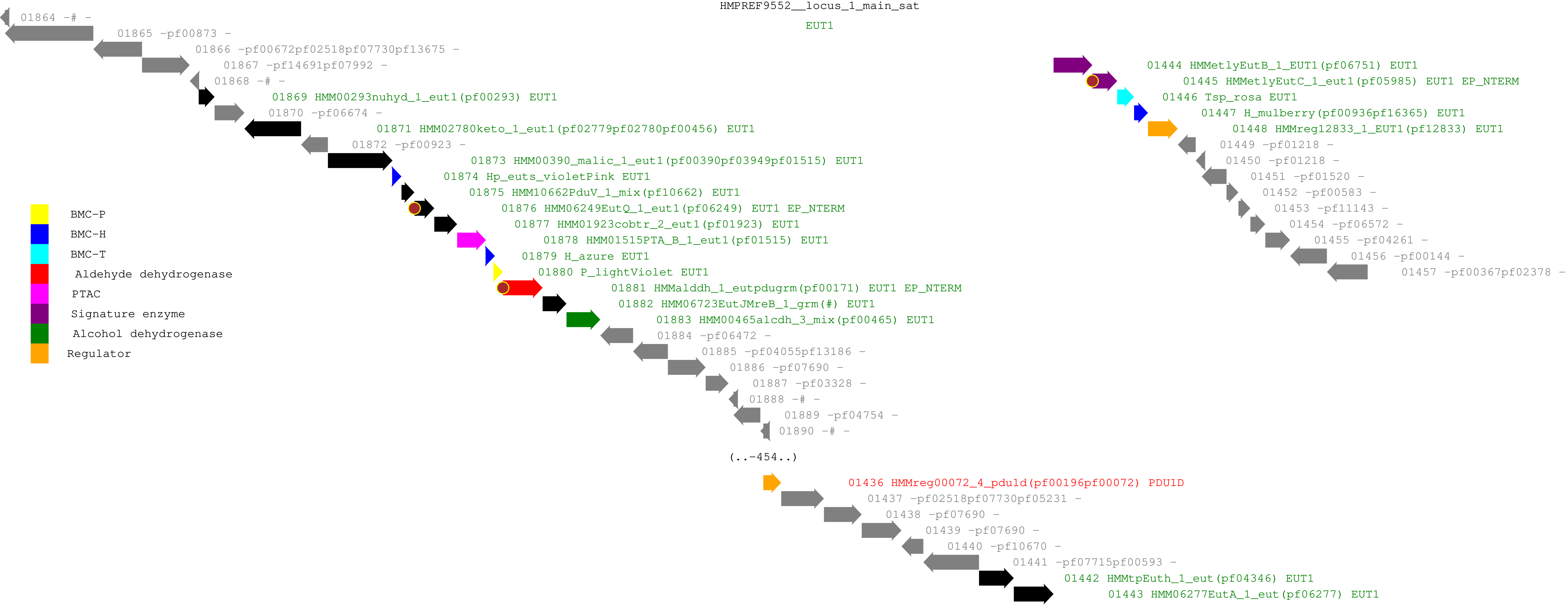

Klebsiella michiganensis. Bacteria; Proteobacteria; Gammaproteobacteria; Enterobacterales;

HMPREF9694__locus_1

EUT1

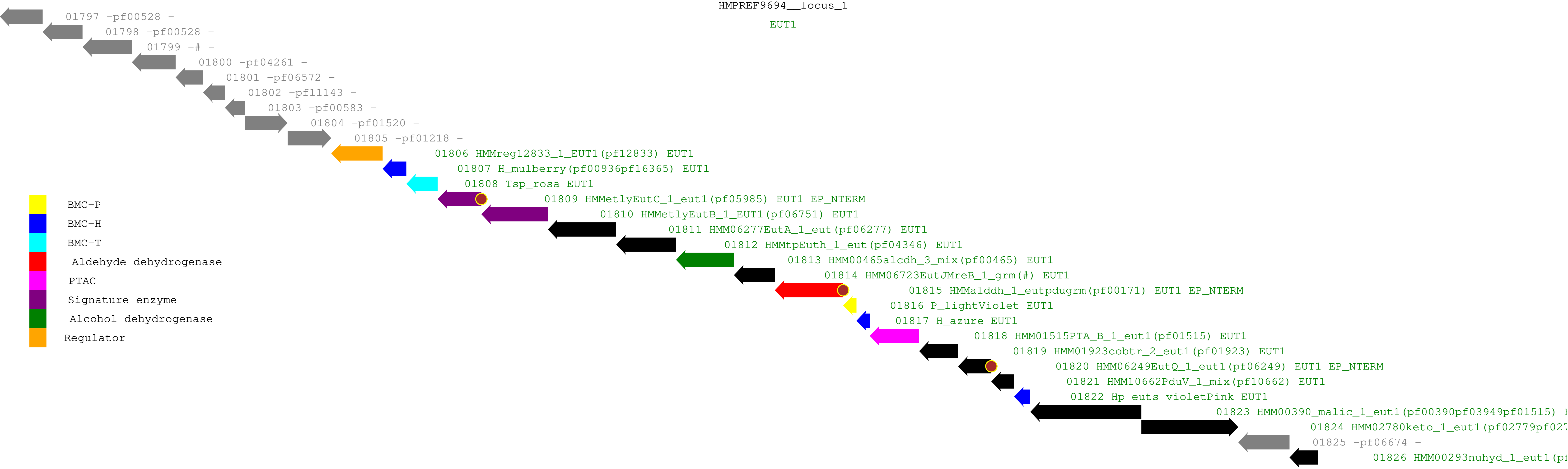

Escherichia coli. Bacteria; Proteobacteria; Gammaproteobacteria; Enterobacterales;

HW43__locus_2

EUT1

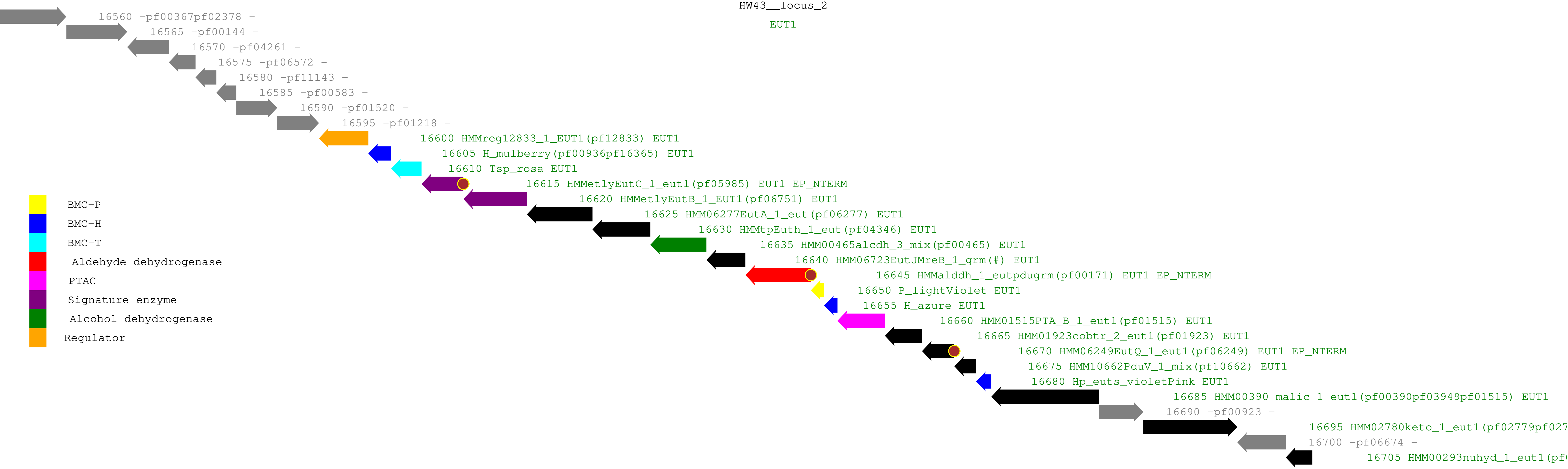

Escherichia coli O26 str. RM10386. Bacteria; Proteobacteria; Gammaproteobacteria; Enterobacterales;

I3U__locus_1

EUT1

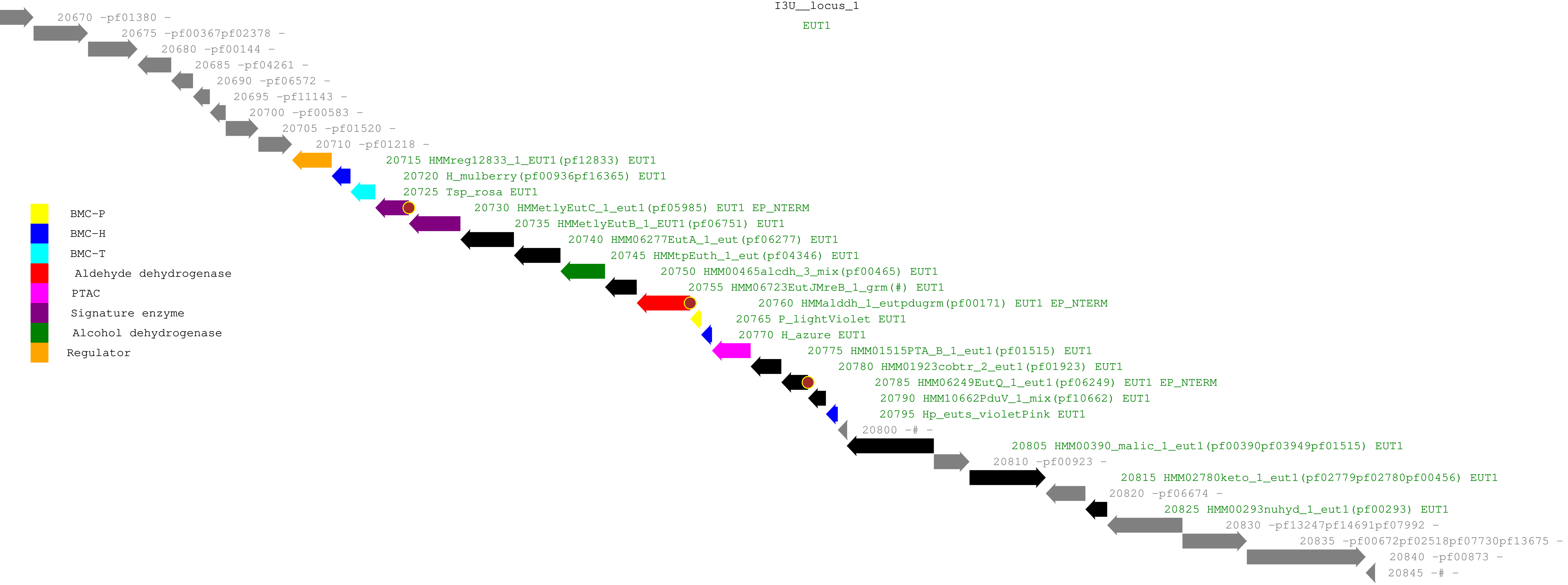

Escherichia coli O145 str. RM9872. Bacteria; Proteobacteria; Gammaproteobacteria; Enterobacterales;

I3Y__locus_1

EUT1

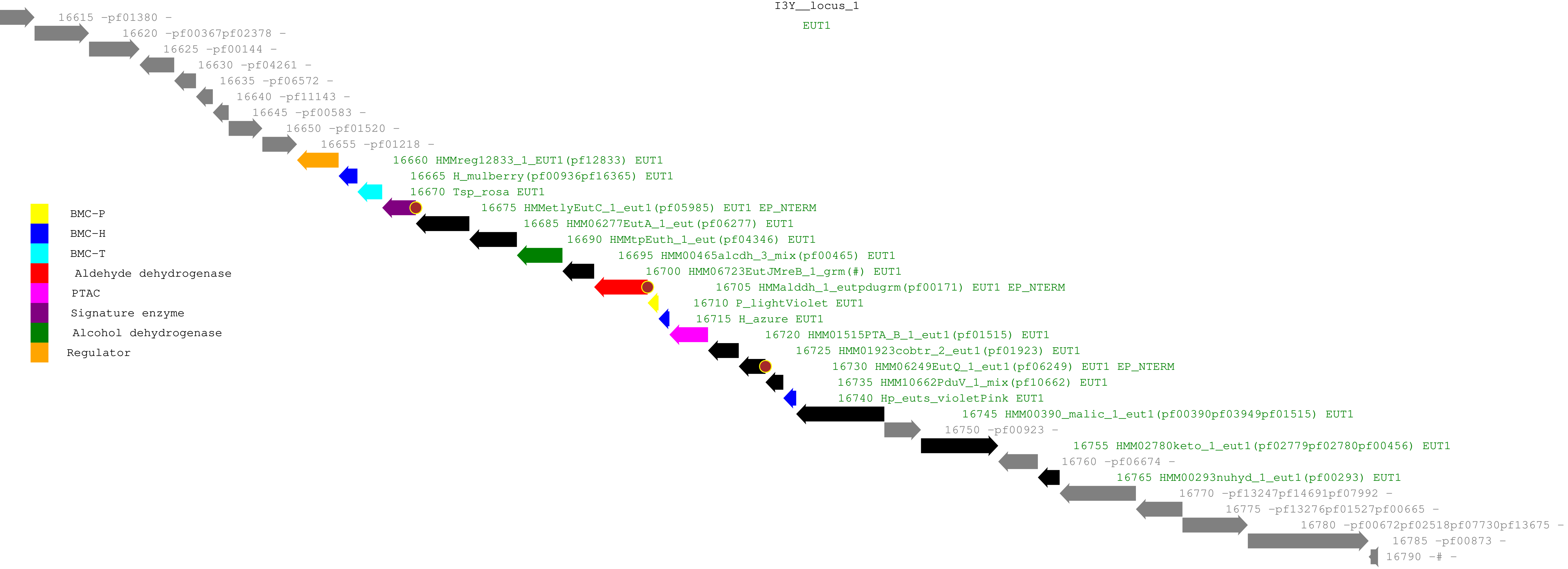

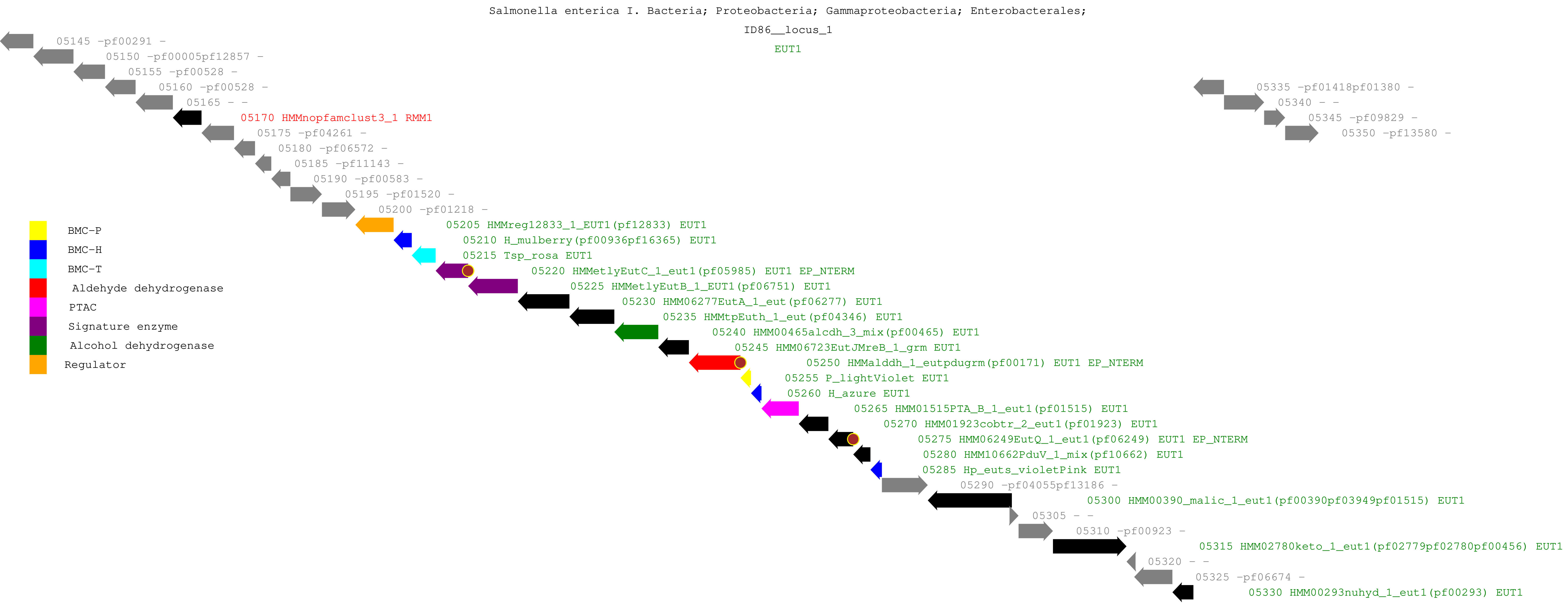
Salmonella enterica I. Bacteria; Proteobacteria; Gammaproteobacteria; Enterobacterales;
ID86__locus_1
EUT1
05145 -pf00291 -
05150 -pf00005pf12857 -
05155 -pf00528 -
05160 -pf00528 -
05165 - -
05170 HMMnopfamclust3_1 RMM1
05175 -pf04261 -
05180 -pf06572 -
05185 -pf11143 -
05190 -pf00583 -
05195 -pf01520 -
05200 -pf01218 -
05205 HMMreg12833_1_EUT1(pf12833) EUT1
05210 H_mulberry(pf00936pf16365) EUT1
05215 Tsp_rosa EUT1
05220 HMMetlyEutC_1_eut1(pf05985) EUT1 EP_NTERM
05225 HMMetlyEutB_1_EUT1(pf06751) EUT1
05230 HMM06277EutA_1_eut(pf06277) EUT1
05235 HMMtpEuth_1_eut(pf04346) EUT1
05240 HMM00465alcdh_3_mix(pf00465) EUT1
05245 HMM06723EutJMreB_1_grm EUT1
05250 HMMalddh_1_eutpdugrm(pf00171) EUT1 EP_NTERM
05255 P_lightViolet EUT1
05260 H_azure EUT1
05265 HMM01515PTA_B_1_eut1(pf01515) EUT1
05270 HMM01923cobtr_2_eut1(pf01923) EUT1
05275 HMM06249EutQ_1_eut1(pf06249) EUT1 EP_NTERM
05280 HMM10662PduV_1_mix(pf10662) EUT1
05285 Hp_euts_violetPink EUT1
05290 -pf04055pf13186 -
05300 HMM00390_malic_1_eut1(pf00390pf03949pf01515) EUT1
05305 - -
05310 -pf00923 -
05315 HMM02780keto_1_eut1(pf02779pf02780pf00456) EUT1
05320 - -
05325 -pf06674 -
05330 HMM00293nuhyd_1_eut1(pf00293) EUT1
05335 -pf01418pf01380 -
05340 - -
05345 -pf09829 -
05350 -pf13580 -
BMC-P
BMC-H
BMC-T
Aldehyde dehydrogenase
PTAC
Signature enzyme
Alcohol dehydrogenase
Regulator

Escherichia coli (strain K12). Bacteria; Proteobacteria; Gammaproteobacteria; Enterobacterales;

JW_locus_1

EUT1

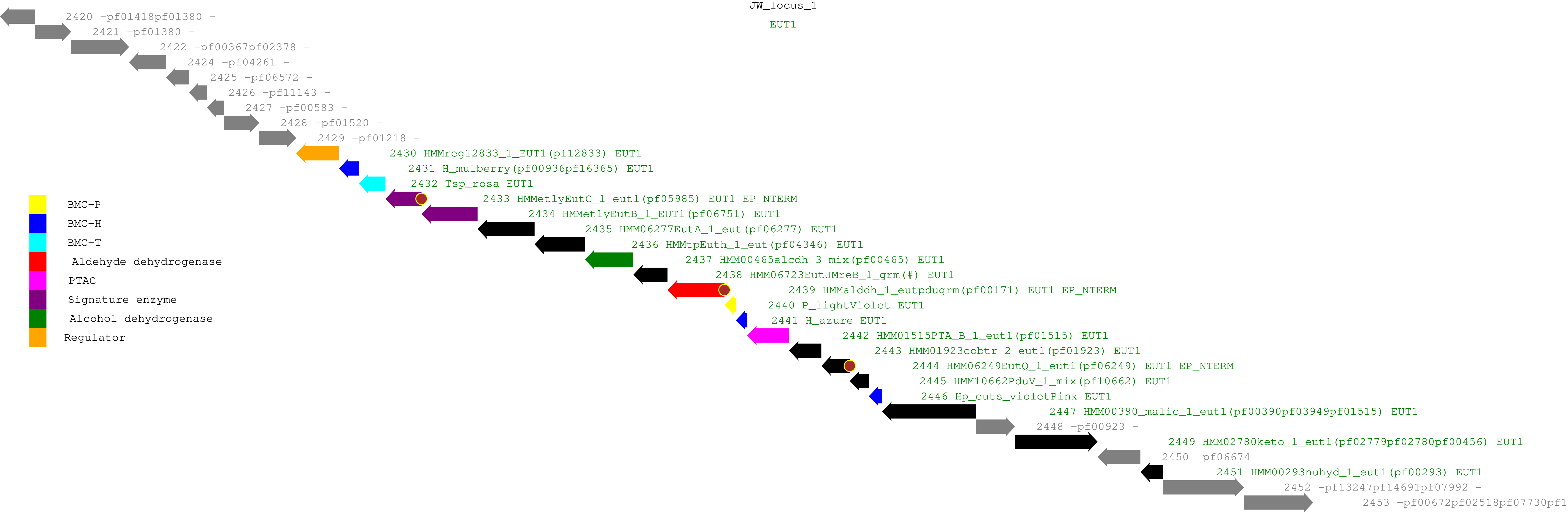

uncultured Citrobacter sp. Bacteria; Proteobacteria; Gammaproteobacteria; Enterobacterales;

KL86CIT2__locus_2

EUT1

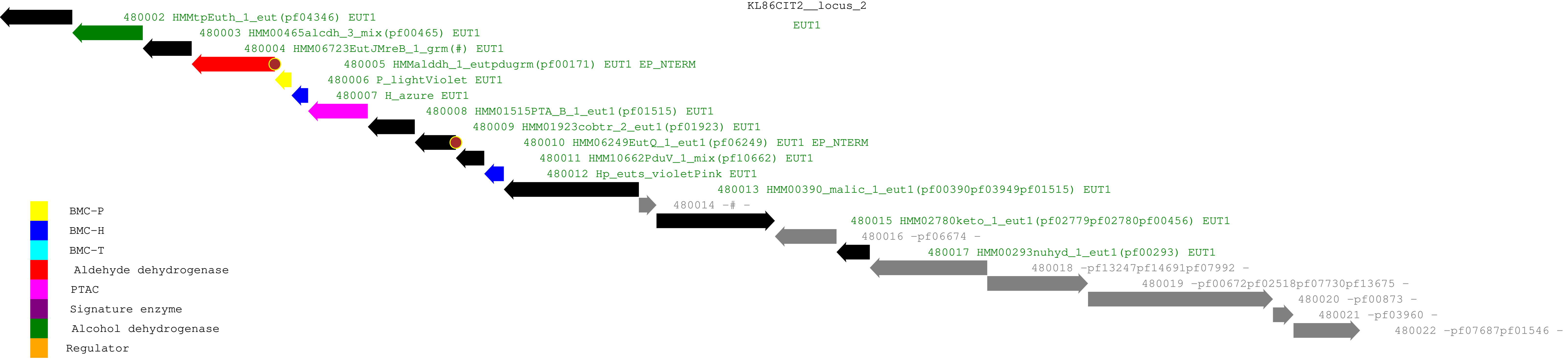

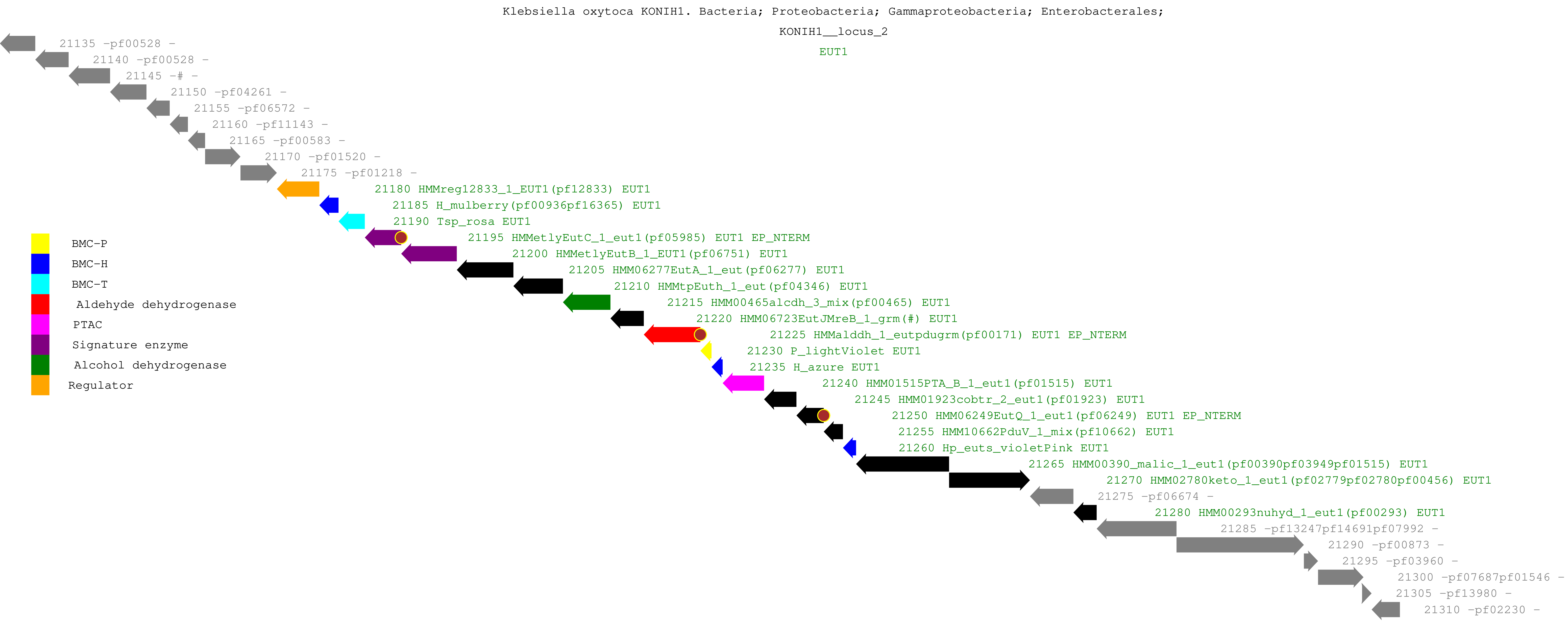
Klebsiella oxytoca KONIH1. Bacteria; Proteobacteria; Gammaproteobacteria; Enterobacterales;
KONIH1__locus_2
EUT1
21135 -pf00528 -
21140 -pf00528 -
21145 -# -
21150 -pf04261 -
21155 -pf06572 -
21160 -pf11143 -
21165 -pf00583 -
21170 -pf01520 -
21175 -pf01218 -
21180 HMMreg12833_1_EUT1(pf12833) EUT1
21185 H_mulberry(pf00936pf16365) EUT1
21190 Tsp_rosa EUT1
21195 HMMetlyEutC_1_eut1(pf05985) EUT1 EP_NTERM
21200 HMMetlyEutB_1_EUT1(pf06751) EUT1
21205 HMM06277EutA_1_eut(pf06277) EUT1
21210 HMMtpEuth_1_eut(pf04346) EUT1
21215 HMM00465alcdh_3_mix(pf00465) EUT1
21220 HMM06723EutJMreB_1_grm(#) EUT1
21225 HMMalddh_1_eutpdugrm(pf00171) EUT1 EP_NTERM
21230 P_lightViolet EUT1
21235 H_azure EUT1
21240 HMM01515PTA_B_1_eut1(pf01515) EUT1
21245 HMM01923cobtr_2_eut1(pf01923) EUT1
21250 HMM06249EutQ_1_eut1(pf06249) EUT1 EP_NTERM
21255 HMM10662PduV_1_mix(pf10662) EUT1
21260 Hp_euts_violetPink EUT1
21265 HMM00390_malic_1_eut1(pf00390pf03949pf01515) EUT1
21270 HMM02780keto_1_eut1(pf02779pf02780pf00456) EUT1
21275 -pf06674 -
21280 HMM00293nuhyd_1_eut1(pf00293) EUT1
21285 -pf13247pf14691pf07992 -
21290 -pf00873 -
21295 -pf03960 -
21300 -pf07687pf01546 -
21305 -pf13980 -
21310 -pf02230 -
BMC-P
BMC-H
BMC-T
Aldehyde dehydrogenase
PTAC
Signature enzyme
Alcohol dehydrogenase
Regulator

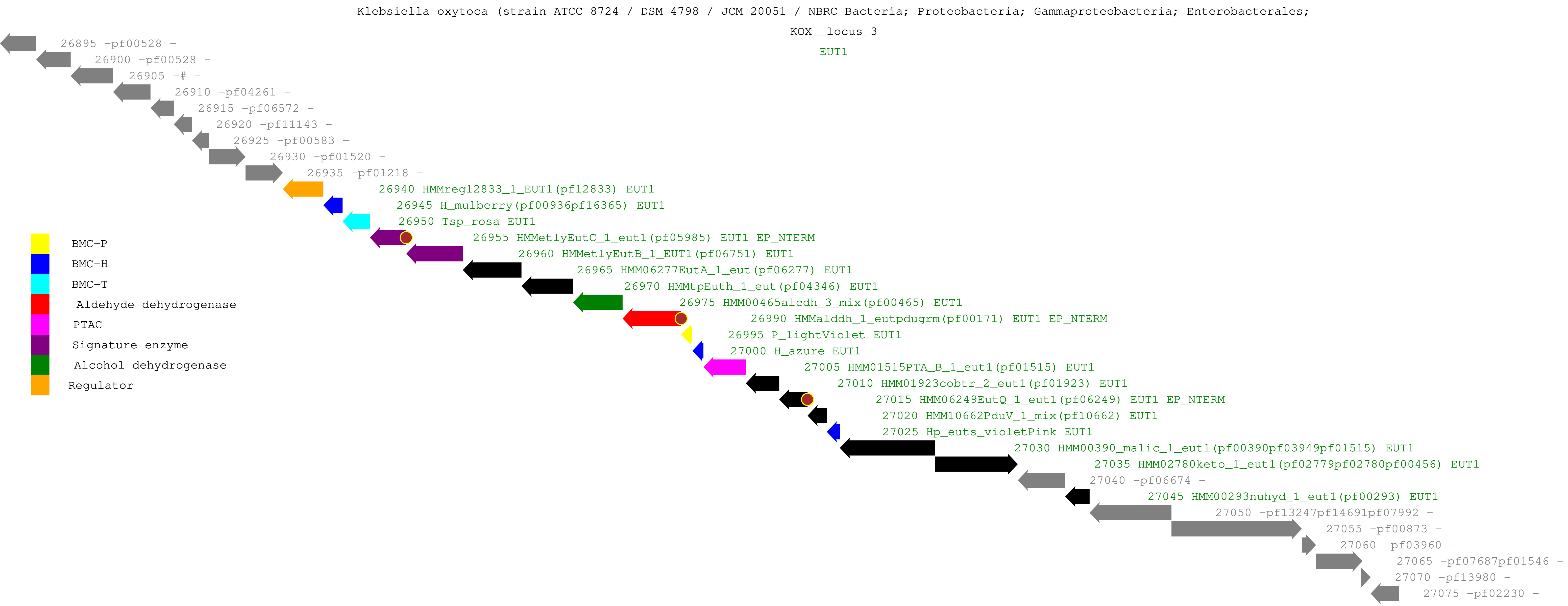
Klebsiella oxytoca (strain ATCC 8724 / DSM 4798 / JCM 20051 / NBRC Bacteria; Proteobacteria; Gammaproteobacteria; Enterobacterales;
KOX__locus_3
EUT1
26895 -pf00528 -
26900 -pf00528 -
26905 -# -
26910 -pf04261 -
26915 -pf06572 -
26920 -pf11143 -
26925 -pf00583 -
26930 -pf01520 -
26935 -pf01218 -
26940 HMMreg12833_1_EUT1(pf12833) EUT1
26945 H_mulberry(pf00936pf16365) EUT1
26950 Tsp_rosa EUT1
26955 HMMetlyEutC_1_eut1(pf05985) EUT1 EP_NTERM
26960 HMMetlyEutB_1_EUT1(pf06751) EUT1
26965 HMM06277EutA_1_eut(pf06277) EUT1
26970 HMMtpEuth_1_eut(pf04346) EUT1
26975 HMM00465alcdh_3_mix(pf00465) EUT1
26990 HMMalddh_1_eutpdugrm(pf00171) EUT1 EP_NTERM
26995 P_lightViolet EUT1
27000 H_azure EUT1
27005 HMM01515PTA_B_1_eut1(pf01515) EUT1
27010 HMM01923cobtr_2_eut1(pf01923) EUT1
27015 HMM06249EutQ_1_eut1(pf06249) EUT1 EP_NTERM
27020 HMM10662PduV_1_mix(pf10662) EUT1
27025 Hp_euts_violetPink EUT1
27030 HMM00390_malic_1_eut1(pf00390pf03949pf01515) EUT1
27035 HMM02780keto_1_eut1(pf02779pf02780pf00456) EUT1
27040 -pf06674 -
27045 HMM00293nuhyd_1_eut1(pf00293) EUT1
27050 -pf13247pf14691pf07992 -
27055 -pf00873 -
27060 -pf03960 -
27065 -pf07687pf01546 -
27070 -pf13980 -
27075 -pf02230 -
BMC-P
BMC-H
BMC-T
Aldehyde dehydrogenase
PTAC
Signature enzyme
Alcohol dehydrogenase
Regulator

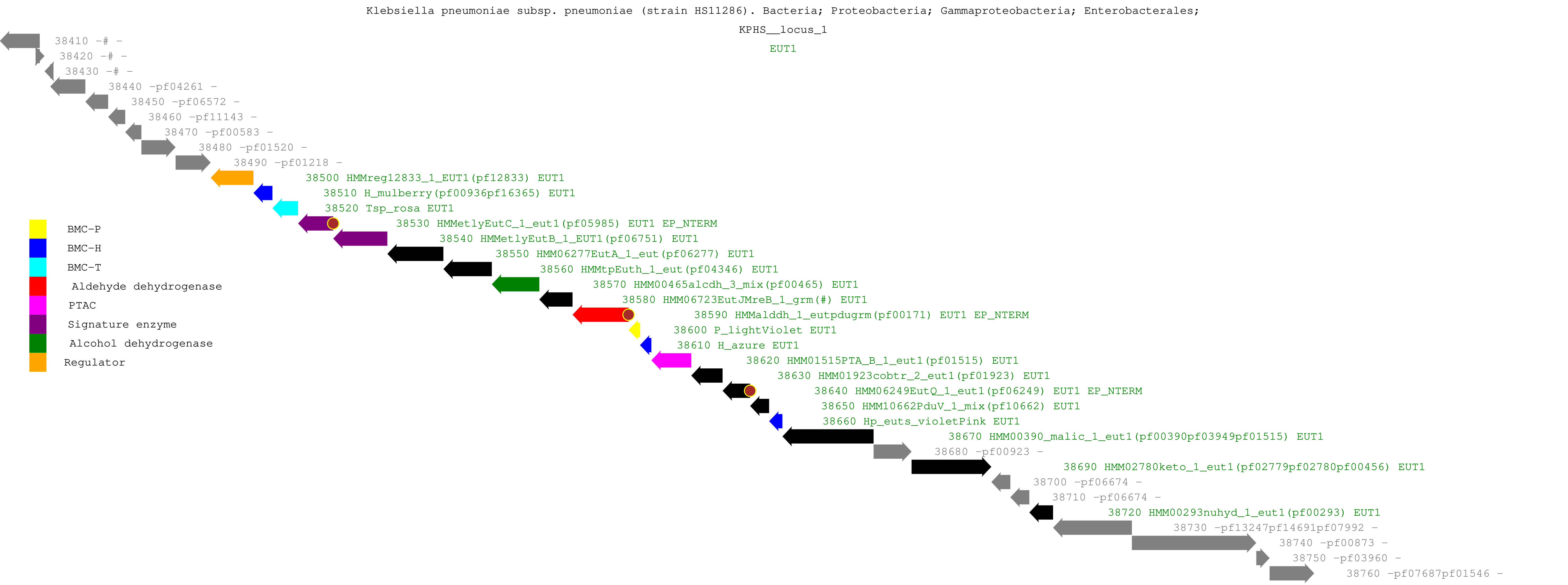
Klebsiella pneumoniae subsp. pneumoniae (strain HS11286). Bacteria; Proteobacteria; Gammaproteobacteria; Enterobacterales;
KPHS__locus_1
EUT1
38410 -# -
38420 -# -
38430 -# -
38440 -pf04261 -
38450 -pf06572 -
38460 -pf11143 -
38470 -pf00583 -
38480 -pf01520 -
38490 -pf01218 -
38500 HMMreg12833_1_EUT1(pf12833) EUT1
38510 H_mulberry(pf00936pf16365) EUT1
38520 Tsp_rosa EUT1
38530 HMMetlyEutC_1_eut1(pf05985) EUT1 EP_NTERM
38540 HMMetlyEutB_1_EUT1(pf06751) EUT1
38550 HMM06277EutA_1_eut(pf06277) EUT1
38560 HMMtpEuth_1_eut(pf04346) EUT1
38570 HMM00465alcdh_3_mix(pf00465) EUT1
38580 HMM06723EutJMreB_1_grm(#) EUT1
38590 HMMalddh_1_eutpdugrm(pf00171) EUT1 EP_NTERM
38600 P_lightViolet EUT1
38610 H_azure EUT1
38620 HMM01515PTA_B_1_eut1(pf01515) EUT1
38630 HMM01923cobtr_2_eut1(pf01923) EUT1
38640 HMM06249EutQ_1_eut1(pf06249) EUT1 EP_NTERM
38650 HMM10662PduV_1_mix(pf10662) EUT1
38660 Hp_euts_violetPink EUT1
38670 HMM00390_malic_1_eut1(pf00390pf03949pf01515) EUT1
38680 -pf00923 -
38690 HMM02780keto_1_eut1(pf02779pf02780pf00456) EUT1
38700 -pf06674 -
38710 -pf06674 -
38720 HMM00293nuhyd_1_eut1(pf00293) EUT1
38730 -pf13247pf14691pf07992 -
38740 -pf00873 -
38750 -pf03960 -
38760 -pf07687pf01546 -
BMC-P
BMC-H
BMC-T
Aldehyde dehydrogenase
PTAC
Signature enzyme
Alcohol dehydrogenase
Regulator

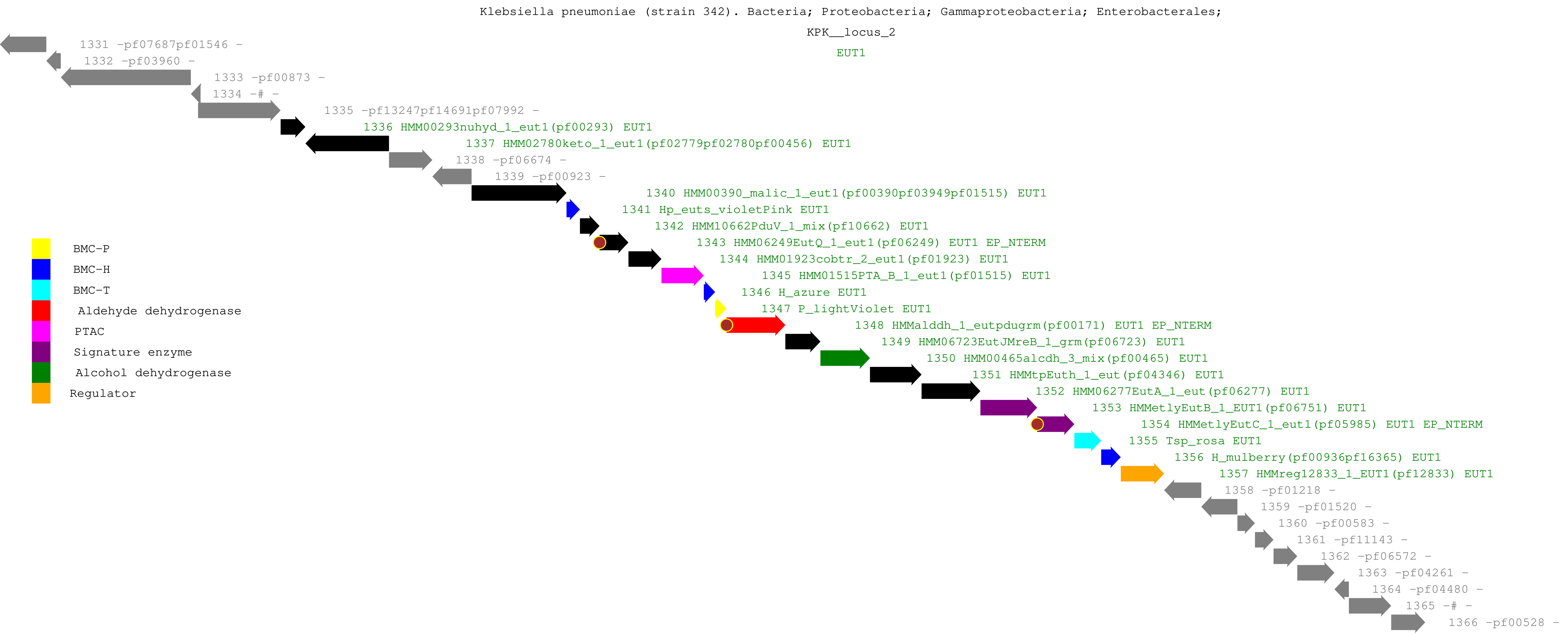
Klebsiella pneumoniae (strain 342). Bacteria; Proteobacteria; Gammaproteobacteria; Enterobacterales;
KPK__locus_2
EUT1
1331 -pf07687pf01546 -
1332 -pf03960 -
1333 -pf00873 -
1334 -# -
1335 -pf13247pf14691pf07992 -
1336 HMM00293nuhyd_1_eut1(pf00293) EUT1
1337 HMM02780keto_1_eut1(pf02779pf02780pf00456) EUT1
1338 -pf06674 -
1339 -pf00923 -
1340 HMM00390_malic_1_eut1(pf00390pf03949pf01515) EUT1
1341 Hp_euts_violetPink EUT1
1342 HMM10662PduV_1_mix(pf10662) EUT1
1343 HMM06249EutQ_1_eut1(pf06249) EUT1 EP_NTERM
1344 HMM01923cobtr_2_eut1(pf01923) EUT1
1345 HMM01515PTA_B_1_eut1(pf01515) EUT1
1346 H_azure EUT1
1347 P_lightViolet EUT1
1348 HMMalddh_1_eutpdugrm(pf00171) EUT1 EP_NTERM
1349 HMM06723EutJMreB_1_grm(pf06723) EUT1
1350 HMM00465alcdh_3_mix(pf00465) EUT1
1351 HMMtpEuth_1_eut(pf04346) EUT1
1352 HMM06277EutA_1_eut(pf06277) EUT1
1353 HMMetlyEutB_1_EUT1(pf06751) EUT1
1354 HMMetlyEutC_1_eut1(pf05985) EUT1 EP_NTERM
1355 Tsp_rosa EUT1
1356 H_mulberry(pf00936pf16365) EUT1
1357 HMMreg12833_1_EUT1(pf12833) EUT1
1358 -pf01218 -
1359 -pf01520 -
1360 -pf00583 -
1361 -pf11143 -
1362 -pf06572 -
1363 -pf04261 -
1364 -pf04480 -
1365 -# -
1366 -pf00528 -
BMC-P
BMC-H
BMC-T
Aldehyde dehydrogenase
PTAC
Signature enzyme
Alcohol dehydrogenase
Regulator

Klebsiella pneumoniae subsp. pneumoniae. Bacteria; Proteobacteria; Gammaproteobacteria; Enterobacterales;

KPNIH32__locus_1

EUT1

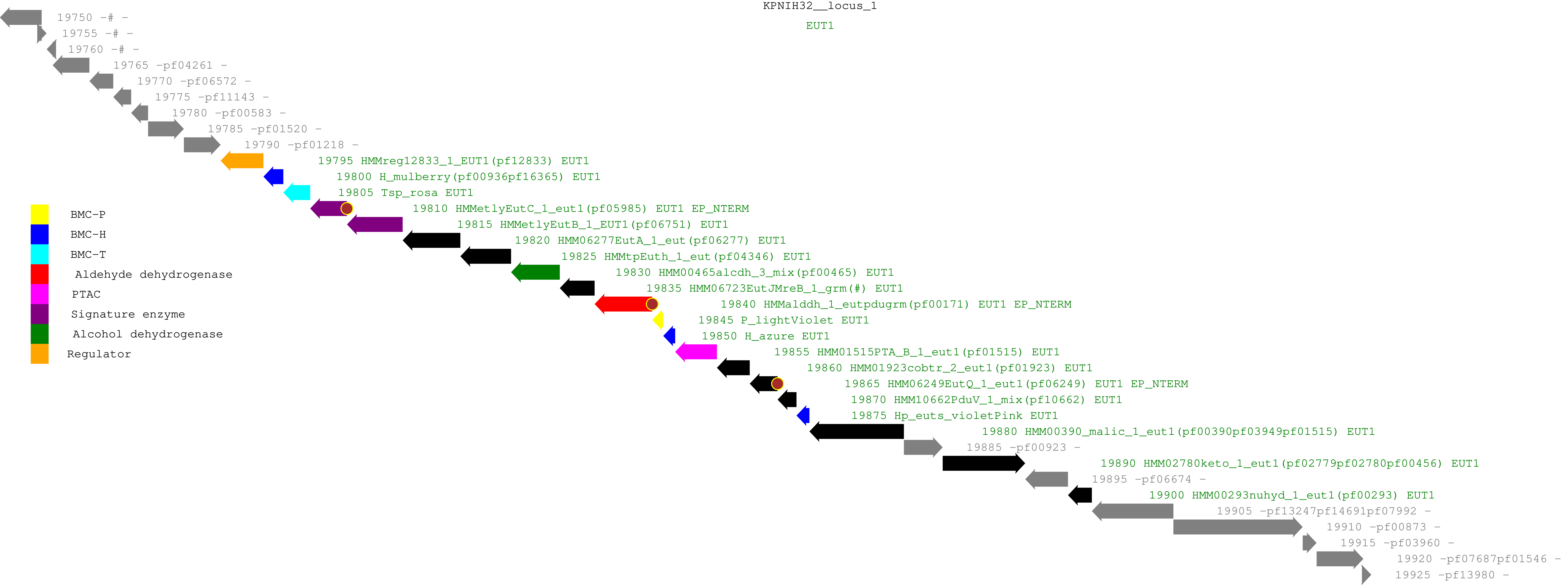

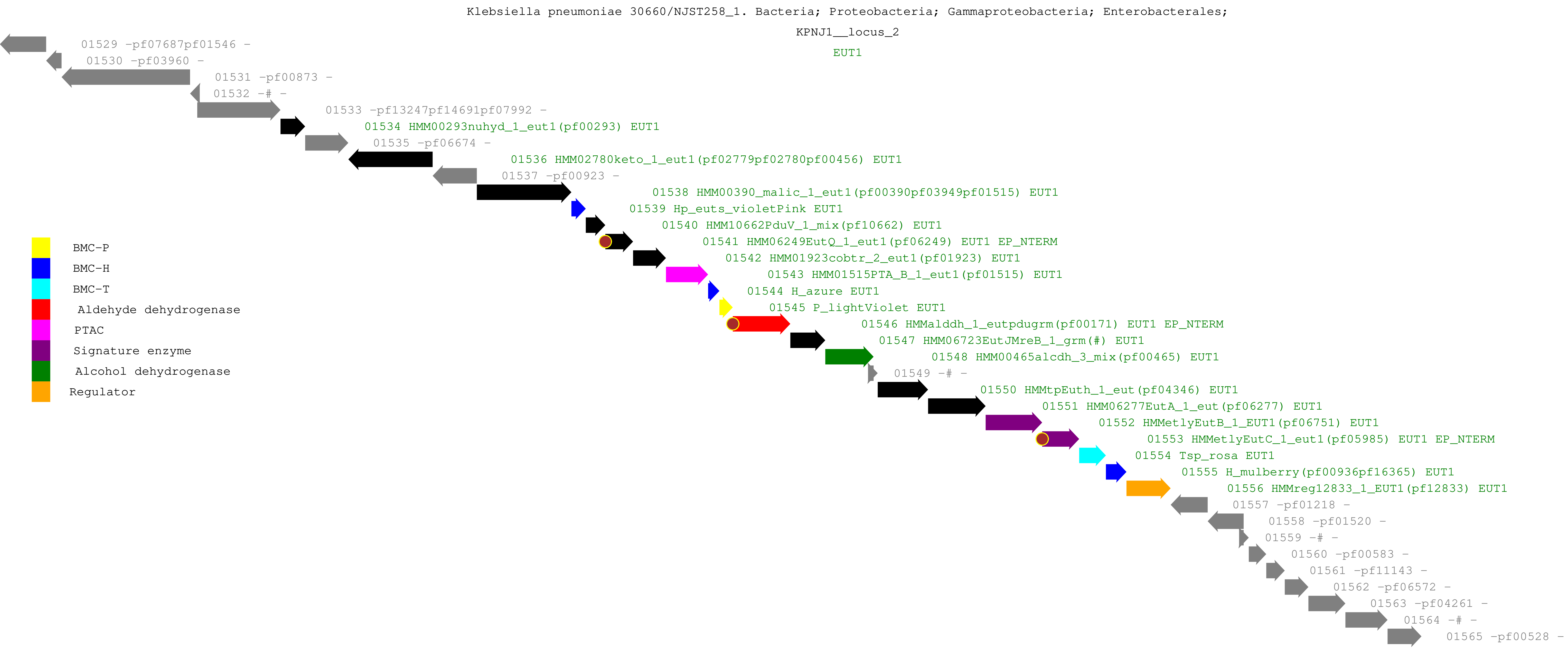
Klebsiella pneumoniae 30660/NJST258_1. Bacteria; Proteobacteria; Gammaproteobacteria; Enterobacterales;
KPNJ1__locus_2
EUT1
01529 -pf07687pf01546 -
01530 -pf03960 -
01531 -pf00873 -
01532 -# -
01533 -pf13247pf14691pf07992 -
01534 HMM00293nuhyd_1_eut1(pf00293) EUT1
01535 -pf06674 -
01536 HMM02780keto_1_eut1(pf02779pf02780pf00456) EUT1
01537 -pf00923 -
01538 HMM00390_malic_1_eut1(pf00390pf03949pf01515) EUT1
01539 Hp_euts_violetPink EUT1
01540 HMM10662PduV_1_mix(pf10662) EUT1
01541 HMM06249EutQ_1_eut1(pf06249) EUT1 EP_NTERM
01542 HMM01923cobtr_2_eut1(pf01923) EUT1
01543 HMM01515PTA_B_1_eut1(pf01515) EUT1
01544 H_azure EUT1
01545 P_lightViolet EUT1
01546 HMMalddh_1_eutpdugrm(pf00171) EUT1 EP_NTERM
01547 HMM06723EutJMreB_1_grm(#) EUT1
01548 HMM00465alcdh_3_mix(pf00465) EUT1
01549 -# -
01550 HMMtpEuth_1_eut(pf04346) EUT1
01551 HMM06277EutA_1_eut(pf06277) EUT1
01552 HMMetlyEutB_1_EUT1(pf06751) EUT1
01553 HMMetlyEutC_1_eut1(pf05985) EUT1 EP_NTERM
01554 Tsp_rosa EUT1
01555 H_mulberry(pf00936pf16365) EUT1
01556 HMMreg12833_1_EUT1(pf12833) EUT1
01557 -pf01218 -
01558 -pf01520 -
01559 -# -
01560 -pf00583 -
01561 -pf11143 -
01562 -pf06572 -
01563 -pf04261 -
01564 -# -
01565 -pf00528 -
BMC-P
BMC-H
BMC-T
Aldehyde dehydrogenase
PTAC
Signature enzyme
Alcohol dehydrogenase
Regulator

Klebsiella pneumoniae 30684/NJST258_2. Bacteria; Proteobacteria; Gammaproteobacteria; Enterobacterales;

KPNJ2__locus_2

EUT1

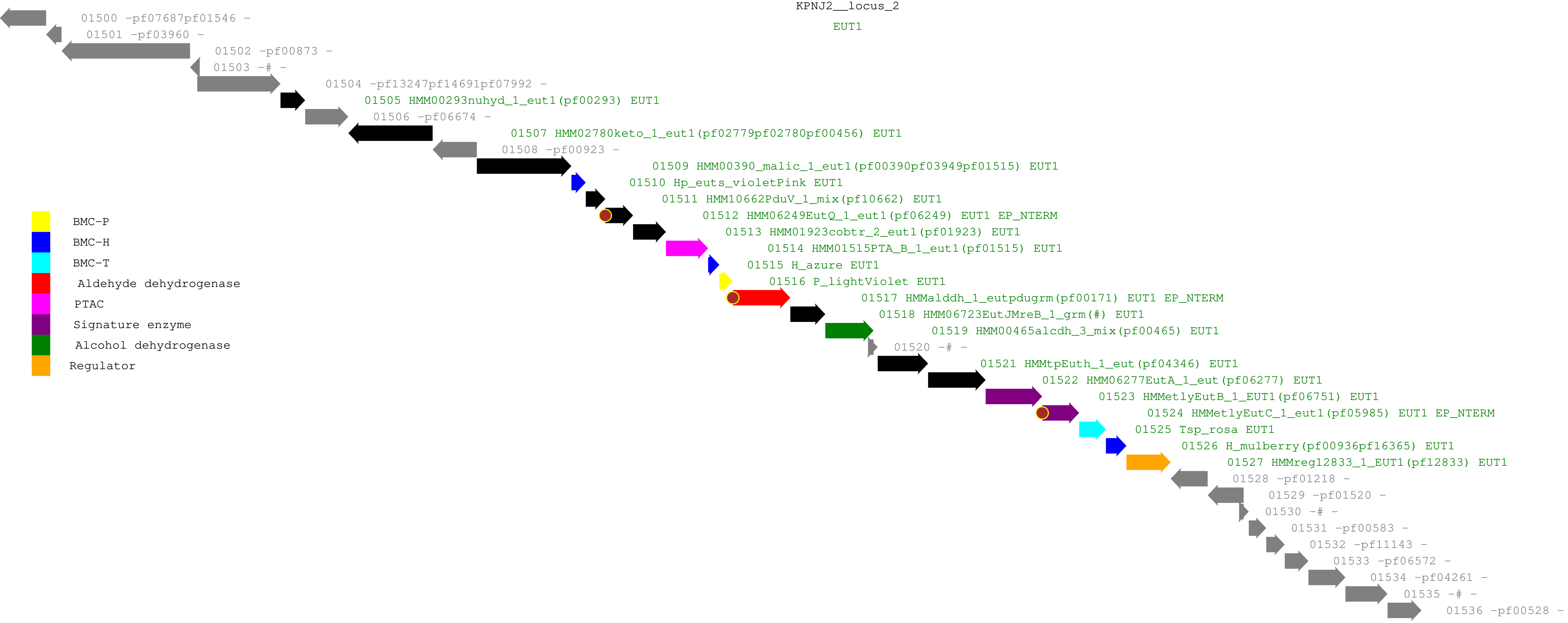

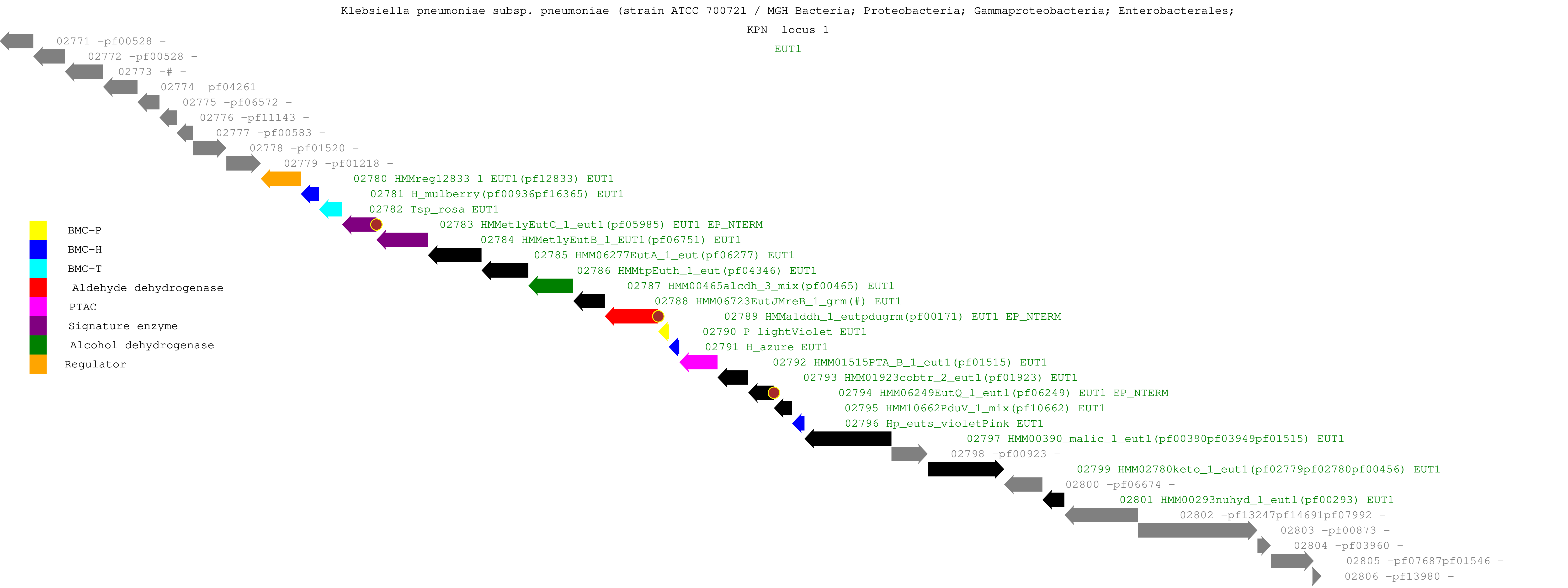

Klebsiella pneumoniae subsp. pneumoniae (strain ATCC 700721 / MGH Bacteria; Proteobacteria; Gammaproteobacteria; Enterobacterales;
KPN__locus_1
EUT1
02771 -pf00528 -
02772 -pf00528 -
02773 -# -
02774 -pf04261 -
02775 -pf06572 -
02776 -pf11143 -
02777 -pf00583 -
02778 -pf01520 -
02779 -pf01218 -
02780 HMMreg12833_1_EUT1(pf12833) EUT1
02781 H_mulberry(pf00936pf16365) EUT1
02782 Tsp_rosa EUT1
02783 HMMetlyEutC_1_eut1(pf05985) EUT1 EP_NTERM
02784 HMMetlyEutB_1_EUT1(pf06751) EUT1
02785 HMM06277EutA_1_eut(pf06277) EUT1
02786 HMMtpEuth_1_eut(pf04346) EUT1
02787 HMM00465alcdh_3_mix(pf00465) EUT1
02788 HMM06723EutJMreB_1_grm(#) EUT1
02789 HMMalddh_1_eutpdugrm(pf00171) EUT1 EP_NTERM
02790 P_lightViolet EUT1
02791 H_azure EUT1
02792 HMM01515PTA_B_1_eut1(pf01515) EUT1
02793 HMM01923cobtr_2_eut1(pf01923) EUT1
02794 HMM06249EutQ_1_eut1(pf06249) EUT1 EP_NTERM
02795 HMM10662PduV_1_mix(pf10662) EUT1
02796 Hp_euts_violetPink EUT1
02797 HMM00390_malic_1_eut1(pf00390pf03949pf01515) EUT1
02798 -pf00923 -
02799 HMM02780keto_1_eut1(pf02779pf02780pf00456) EUT1
02800 -pf06674 -
02801 HMM00293nuhyd_1_eut1(pf00293) EUT1
02802 -pf13247pf14691pf07992 -
02803 -pf00873 -
02804 -pf03960 -
02805 -pf07687pf01546 -
02806 -pf13980 -
BMC-P
BMC-H
BMC-T
Aldehyde dehydrogenase
PTAC
Signature enzyme
Alcohol dehydrogenase
Regulator

Klebsiella pneumoniae subsp. rhinoscleromatis SB3432. Bacteria; Proteobacteria; Gammaproteobacteria; Enterobacterales;

KPR__locus_3

EUT1

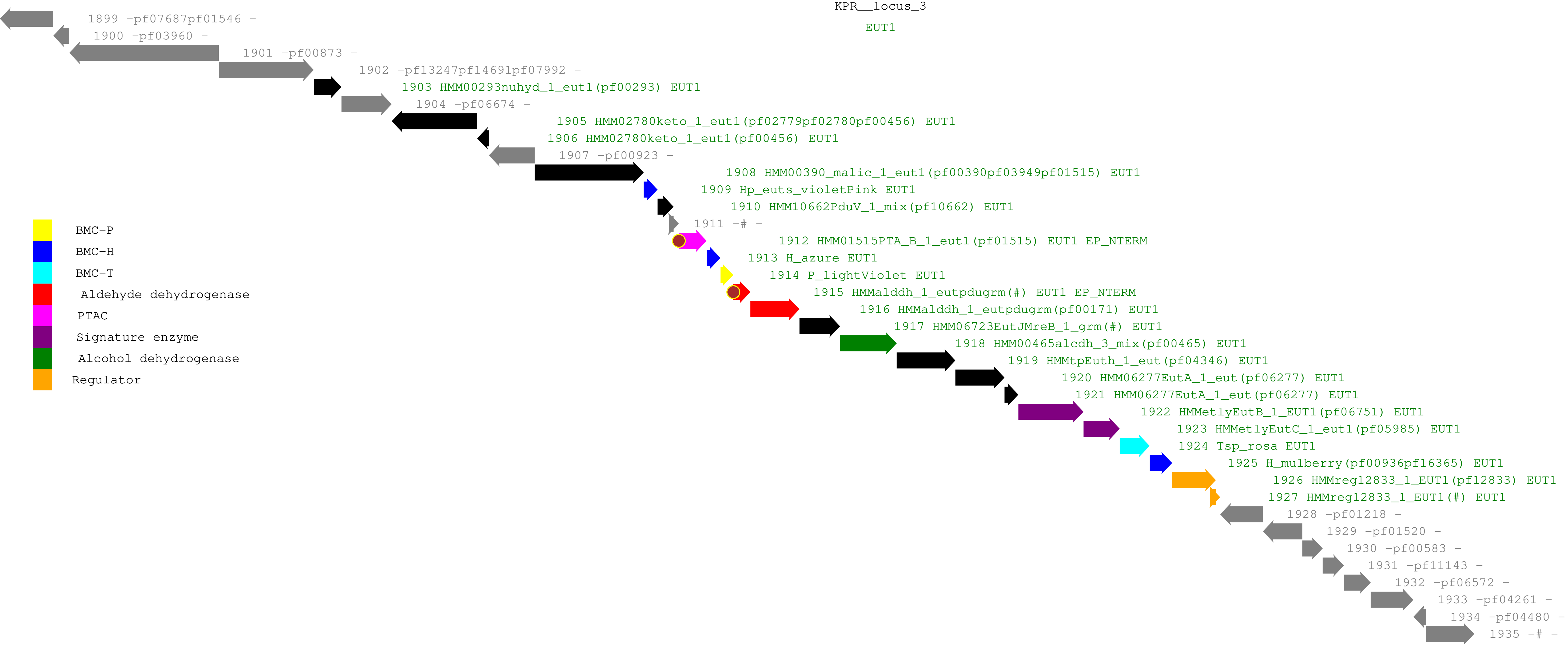

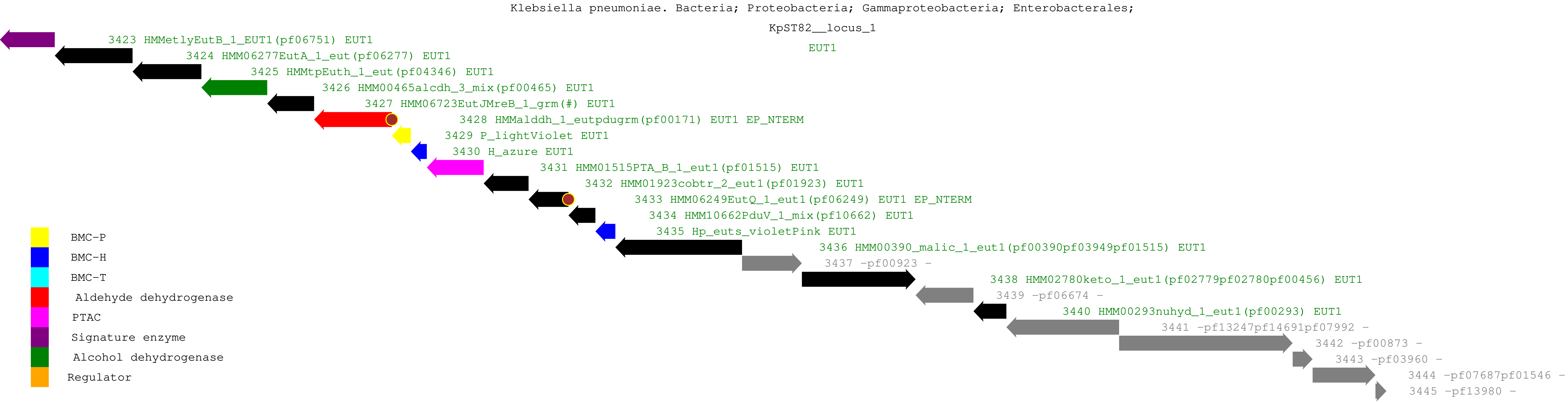

Klebsiella pneumoniae. Bacteria; Proteobacteria; Gammaproteobacteria; Enterobacterales;
KpST82__locus_1
EUT1
3423 HMMetlyEutB_1_EUT1(pf06751) EUT1
3424 HMM06277EutA_1_eut(pf06277) EUT1
3425 HMMtpEuth_1_eut(pf04346) EUT1
3426 HMM00465alcdh_3_mix(pf00465) EUT1
3427 HMM06723EutJMreB_1_grm(#) EUT1
3428 HMMalddh_1_eutpdugrm(pf00171) EUT1 EP_NTERM
3429 P_lightViolet EUT1
3430 H_azure EUT1
3431 HMM01515PTA_B_1_eut1(pf01515) EUT1
3432 HMM01923cobtr_2_eut1(pf01923) EUT1
3433 HMM06249EutQ_1_eut1(pf06249) EUT1 EP_NTERM
3434 HMM10662PduV_1_mix(pf10662) EUT1
3435 Hp_euts_violetPink EUT1
3436 HMM00390_malic_1_eut1(pf00390pf03949pf01515) EUT1
3437 -pf00923 -
3438 HMM02780keto_1_eut1(pf02779pf02780pf00456) EUT1
3439 -pf06674 -
3440 HMM00293nuhyd_1_eut1(pf00293) EUT1
3441 -pf13247pf14691pf07992 -
3442 -pf00873 -
3443 -pf03960 -
3444 -pf07687pf01546 -
3445 -pf13980 -
BMC-P
BMC-H
BMC-T
Aldehyde dehydrogenase
PTAC
Signature enzyme
Alcohol dehydrogenase
Regulator

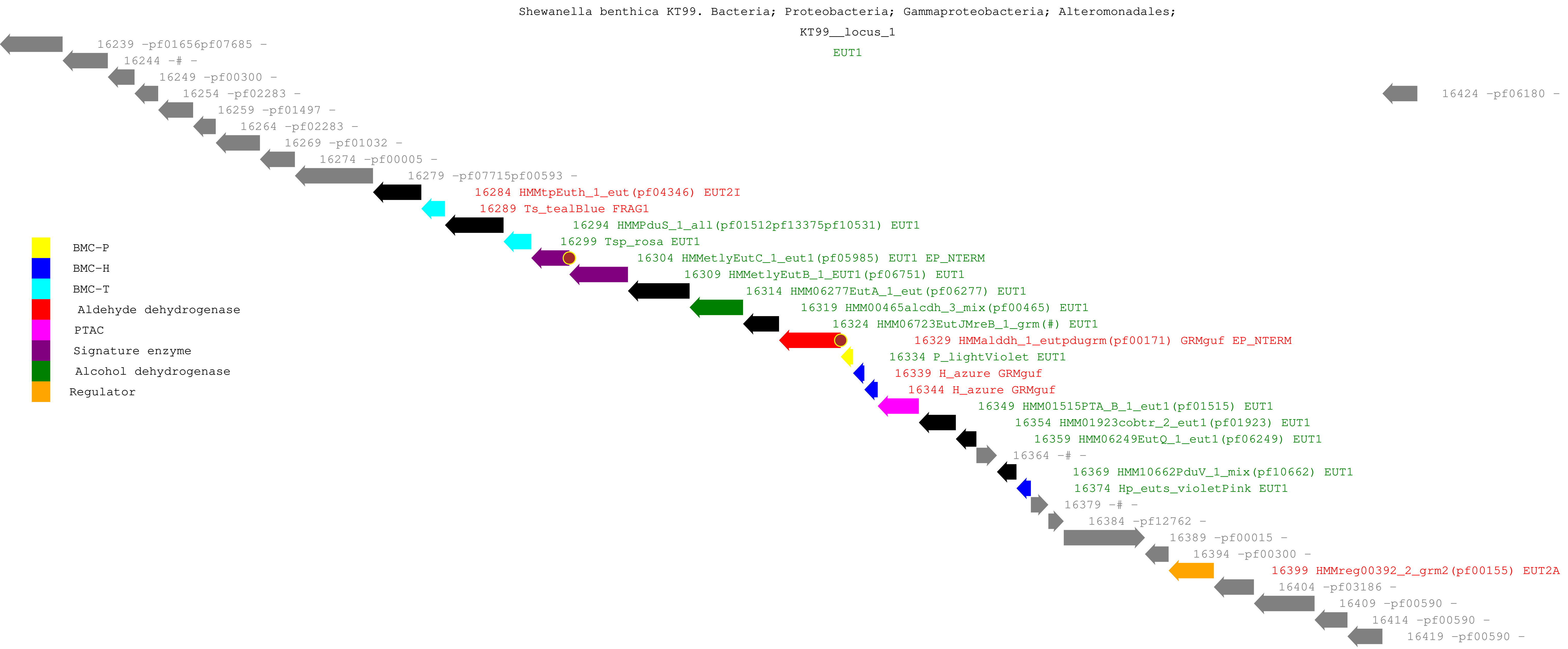
Shewanella benthica KT99. Bacteria; Proteobacteria; Gammaproteobacteria; Alteromonadales;
KT99__locus_1
EUT1
16239 -pf01656pf07685 -
16244 -# -
16249 -pf00300 -
16254 -pf02283 -
16259 -pf01497 -
16264 -pf02283 -
16269 -pf01032 -
16274 -pf00005 -
16279 -pf07715pf00593 -
16284 HMMtpEuth_1_eut(pf04346) EUT2I
16289 Ts_tealBlue FRAG1
16294 HMMPduS_1_all(pf01512pf13375pf10531) EUT1
16299 Tsp_rosa EUT1
16304 HMMetlyEutC_1_eut1(pf05985) EUT1 EP_NTERM
16309 HMMetlyEutB_1_EUT1(pf06751) EUT1
16314 HMM06277EutA_1_eut(pf06277) EUT1
16319 HMM00465alcdh_3_mix(pf00465) EUT1
16324 HMM06723EutJMreB_1_grm(#) EUT1
16329 HMMalddh_1_eutpdugrm(pf00171) GRMguf EP_NTERM
16334 P_lightViolet EUT1
16339 H_azure GRMguf
16344 H_azure GRMguf
16349 HMM01515PTA_B_1_eut1(pf01515) EUT1
16354 HMM01923cobtr_2_eut1(pf01923) EUT1
16359 HMM06249EutQ_1_eut1(pf06249) EUT1
16364 -# -
16369 HMM10662PduV_1_mix(pf10662) EUT1
16374 Hp_euts_violetPink EUT1
16379 -# -
16384 -pf12762 -
16389 -pf00015 -
16394 -pf00300 -
16399 HMMreg00392_2_grm2(pf00155) EUT2A
16404 -pf03186 -
16409 -pf00590 -
16414 -pf00590 -
16419 -pf00590 -
16424 -pf06180 -
BMC-P
BMC-H
BMC-T
Aldehyde dehydrogenase
PTAC
Signature enzyme
Alcohol dehydrogenase
Regulator

Klebsiella variicola. Bacteria; Proteobacteria; Gammaproteobacteria; Enterobacterales;

KVR801__locus_2

EUT1

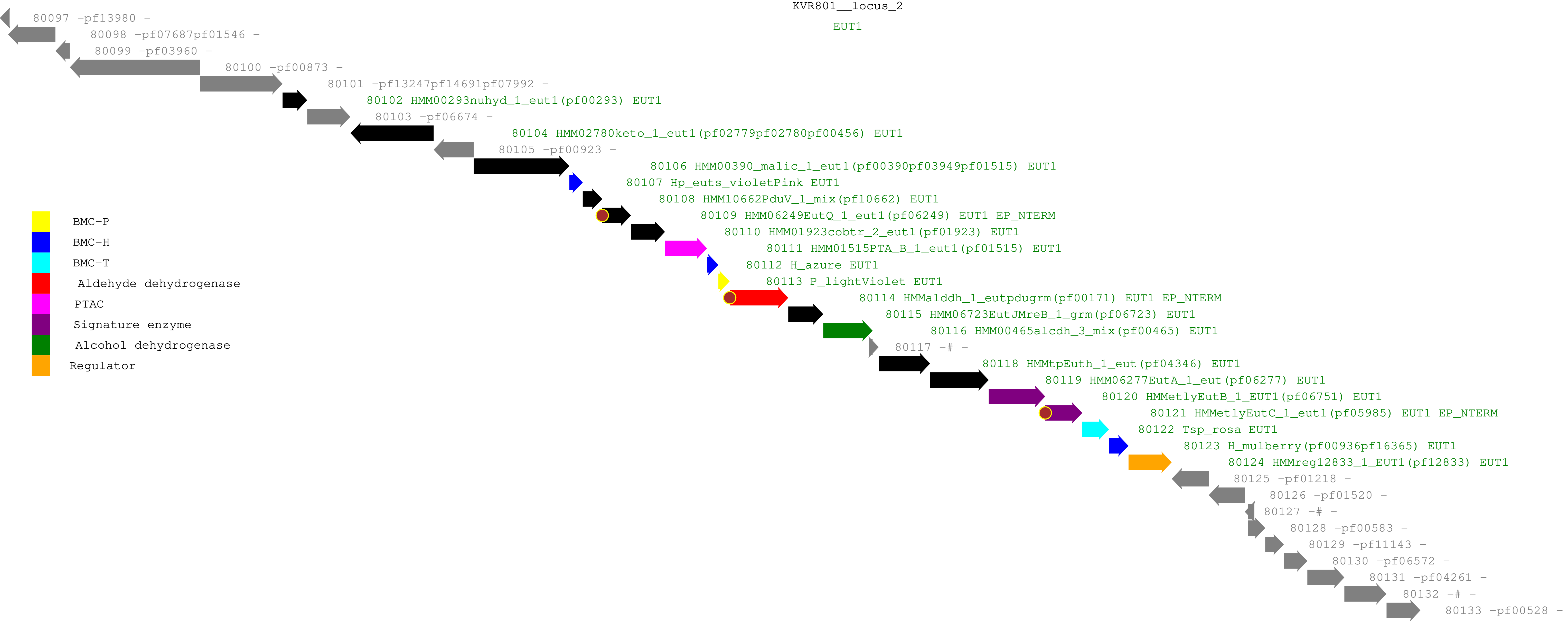

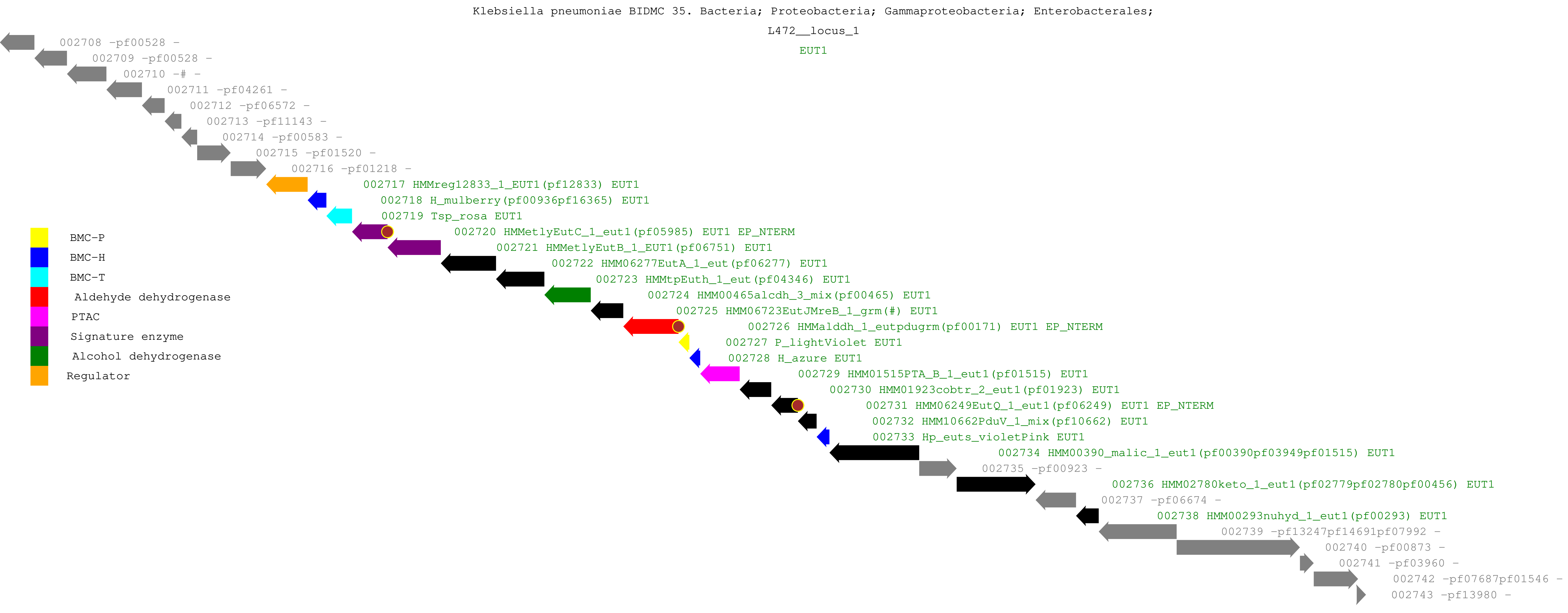

Klebsiella pneumoniae BIDMC 35. Bacteria; Proteobacteria; Gammaproteobacteria; Enterobacterales;
L472__locus_1
EUT1
002708 -pf00528 -
002709 -pf00528 -
002710 -# -
002711 -pf04261 -
002712 -pf06572 -
002713 -pf11143 -
002714 -pf00583 -
002715 -pf01520 -
002716 -pf01218 -
002717 HMMreg12833_1_EUT1(pf12833) EUT1
002718 H_mulberry(pf00936pf16365) EUT1
002719 Tsp_rosa EUT1
002720 HMMetlyEutC_1_eut1(pf05985) EUT1 EP_NTERM
002721 HMMetlyEutB_1_EUT1(pf06751) EUT1
002722 HMM06277EutA_1_eut(pf06277) EUT1
002723 HMMtpEuth_1_eut(pf04346) EUT1
002724 HMM00465alcdh_3_mix(pf00465) EUT1
002725 HMM06723EutJMreB_1_grm(#) EUT1
002726 HMMalddh_1_eutpdugrm(pf00171) EUT1 EP_NTERM
002727 P_lightViolet EUT1
002728 H_azure EUT1
002729 HMM01515PTA_B_1_eut1(pf01515) EUT1
002730 HMM01923cobtr_2_eut1(pf01923) EUT1
002731 HMM06249EutQ_1_eut1(pf06249) EUT1 EP_NTERM
002732 HMM10662PduV_1_mix(pf10662) EUT1
002733 Hp_euts_violetPink EUT1
002734 HMM00390_malic_1_eut1(pf00390pf03949pf01515) EUT1
002735 -pf00923 -
002736 HMM02780keto_1_eut1(pf02779pf02780pf00456) EUT1
002737 -pf06674 -
002738 HMM00293nuhyd_1_eut1(pf00293) EUT1
002739 -pf13247pf14691pf07992 -
002740 -pf00873 -
002741 -pf03960 -
002742 -pf07687pf01546 -
002743 -pf13980 -
BMC-P
BMC-H
BMC-T
Aldehyde dehydrogenase
PTAC
Signature enzyme
Alcohol dehydrogenase
Regulator

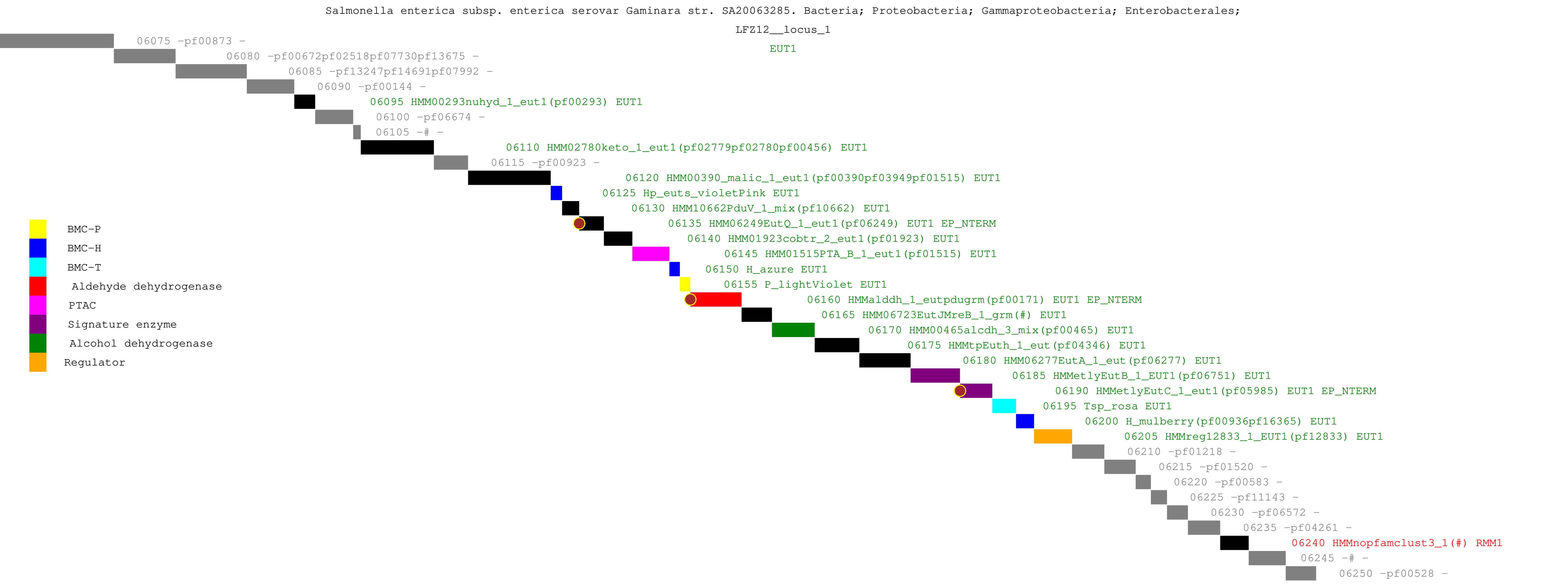
Salmonella enterica subsp. enterica serovar Gaminara str. SA20063285. Bacteria; Proteobacteria; Gammaproteobacteria; Enterobacterales;
LFZ12__locus_1
EUT1
06075 -pf00873 -
06080 -pf00672pf02518pf07730pf13675 -
06085 -pf13247pf14691pf07992 -
06090 -pf00144 -
06095 HMM00293nuhyd_1_eut1(pf00293) EUT1
06100 -pf06674 -
06105 -# -
06110 HMM02780keto_1_eut1(pf02779pf02780pf00456) EUT1
06115 -pf00923 -
06120 HMM00390_malic_1_eut1(pf00390pf03949pf01515) EUT1
06125 Hp_euts_violetPink EUT1
06130 HMM10662PduV_1_mix(pf10662) EUT1
06135 HMM06249EutQ_1_eut1(pf06249) EUT1 EP_NTERM
06140 HMM01923cobtr_2_eut1(pf01923) EUT1
06145 HMM01515PTA_B_1_eut1(pf01515) EUT1
06150 H_azure EUT1
06155 P_lightViolet EUT1
06160 HMMalddh_1_eutpdugrm(pf00171) EUT1 EP_NTERM
06165 HMM06723EutJMreB_1_grm(#) EUT1
06170 HMM00465alcdh_3_mix(pf00465) EUT1
06175 HMMtpEuth_1_eut(pf04346) EUT1
06180 HMM06277EutA_1_eut(pf06277) EUT1
06185 HMMetlyEutB_1_EUT1(pf06751) EUT1
06190 HMMetlyEutC_1_eut1(pf05985) EUT1 EP_NTERM
06195 Tsp_rosa EUT1
06200 H_mulberry(pf00936pf16365) EUT1
06205 HMMreg12833_1_EUT1(pf12833) EUT1
06210 -pf01218 -
06215 -pf01520 -
06220 -pf00583 -
06225 -pf11143 -
06230 -pf06572 -
06235 -pf04261 -
06240 HMMnopfamclust3_1(#) RMM1
06245 -# -
06250 -pf00528 -
BMC-P
BMC-H
BMC-T
Aldehyde dehydrogenase
PTAC
Signature enzyme
Alcohol dehydrogenase
Regulator

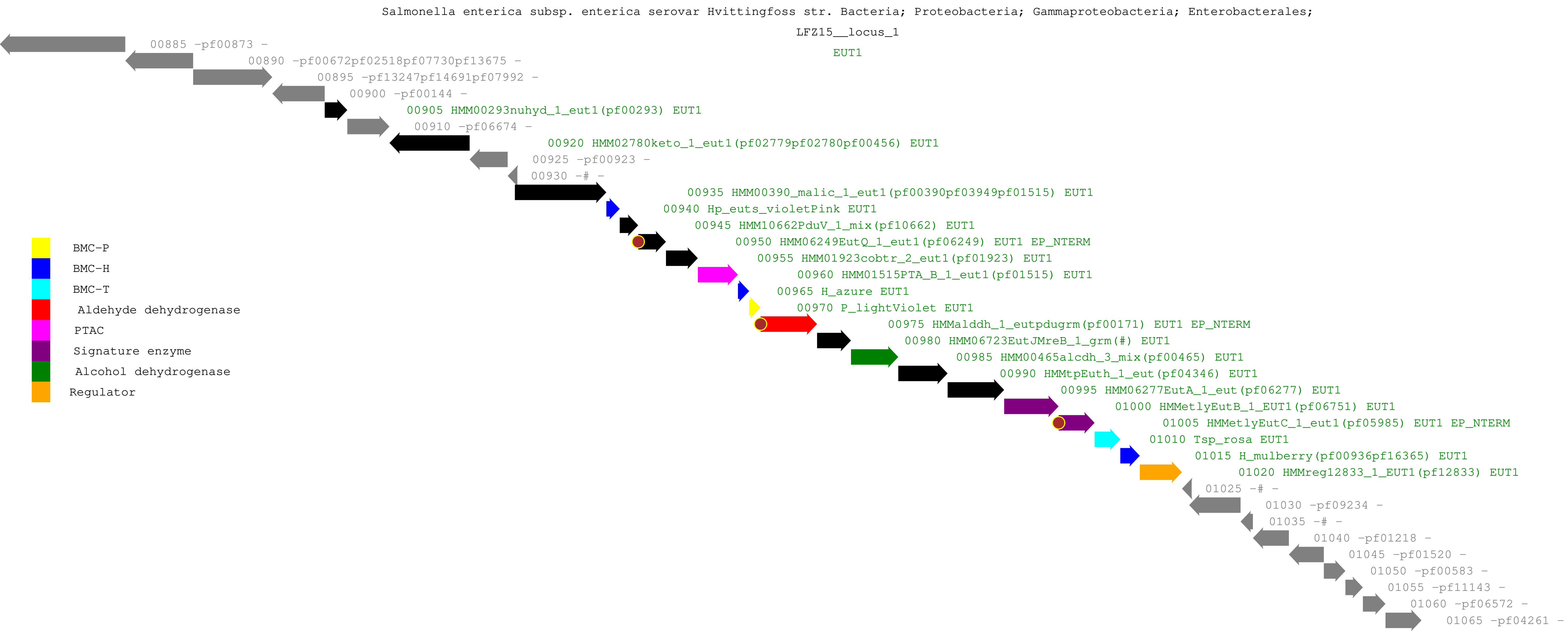
Salmonella enterica subsp. enterica serovar Hvittingfoss str. Bacteria; Proteobacteria; Gammaproteobacteria; Enterobacterales;
LFZ15__locus_1
EUT1
00885 -pf00873 -
00890 -pf00672pf02518pf07730pf13675 -
00895 -pf13247pf14691pf07992 -
00900 -pf00144 -
00905 HMM00293nuhyd_1_eut1(pf00293) EUT1
00910 -pf06674 -
00920 HMM02780keto_1_eut1(pf02779pf02780pf00456) EUT1
00925 -pf00923 -
00930 -# -
00935 HMM00390_malic_1_eut1(pf00390pf03949pf01515) EUT1
00940 Hp_euts_violetPink EUT1
00945 HMM10662PduV_1_mix(pf10662) EUT1
00950 HMM06249EutQ_1_eut1(pf06249) EUT1 EP_NTERM
00955 HMM01923cobtr_2_eut1(pf01923) EUT1
00960 HMM01515PTA_B_1_eut1(pf01515) EUT1
00965 H_azure EUT1
00970 P_lightViolet EUT1
00975 HMMaldh_1_eutpdugrm(pf00171) EUT1 EP_NTERM
00980 HMM06723EutJMreB_1_grm(#) EUT1
00985 HMM00465alcdh_3_mix(pf00465) EUT1
00990 HMMtpEuth_1_eut(pf04346) EUT1
00995 HMM06277EutA_1_eut(pf06277) EUT1
01000 HMMetlyEutB_1_EUT1(pf06751) EUT1
01005 HMMetlyEutC_1_eut1(pf05985) EUT1 EP_NTERM
01010 Tsp_rosa EUT1
01015 H_mulberry(pf00936pf16365) EUT1
01020 HMMreg12833_1_EUT1(pf12833) EUT1
01025 -# -
01030 -pf09234 -
01035 -# -
01040 -pf01218 -
01045 -pf01520 -
01050 -pf00583 -
01055 -pf11143 -
01060 -pf06572 -
01065 -pf04261 -
BMC-P
BMC-H
BMC-T
Aldehyde dehydrogenase
PTAC
Signature enzyme
Alcohol dehydrogenase
Regulator

Salmonella enterica subsp. enterica serovar India str. SA20085604. Bacteria; Proteobacteria; Gammaproteobacteria; Enterobacterales;

LFZ16__locus_1

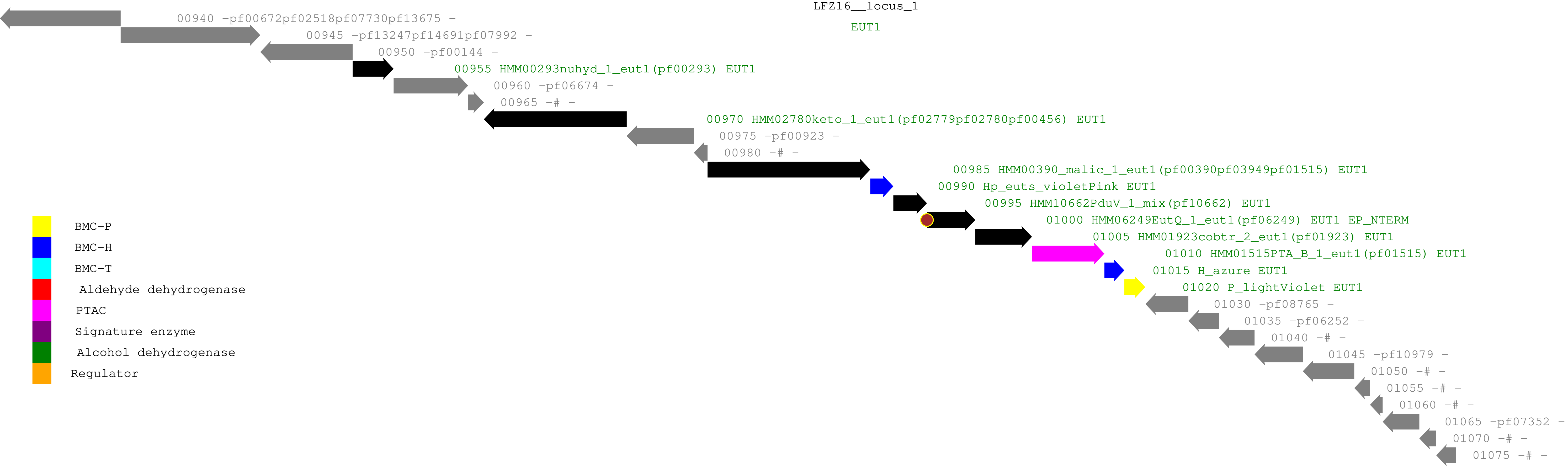

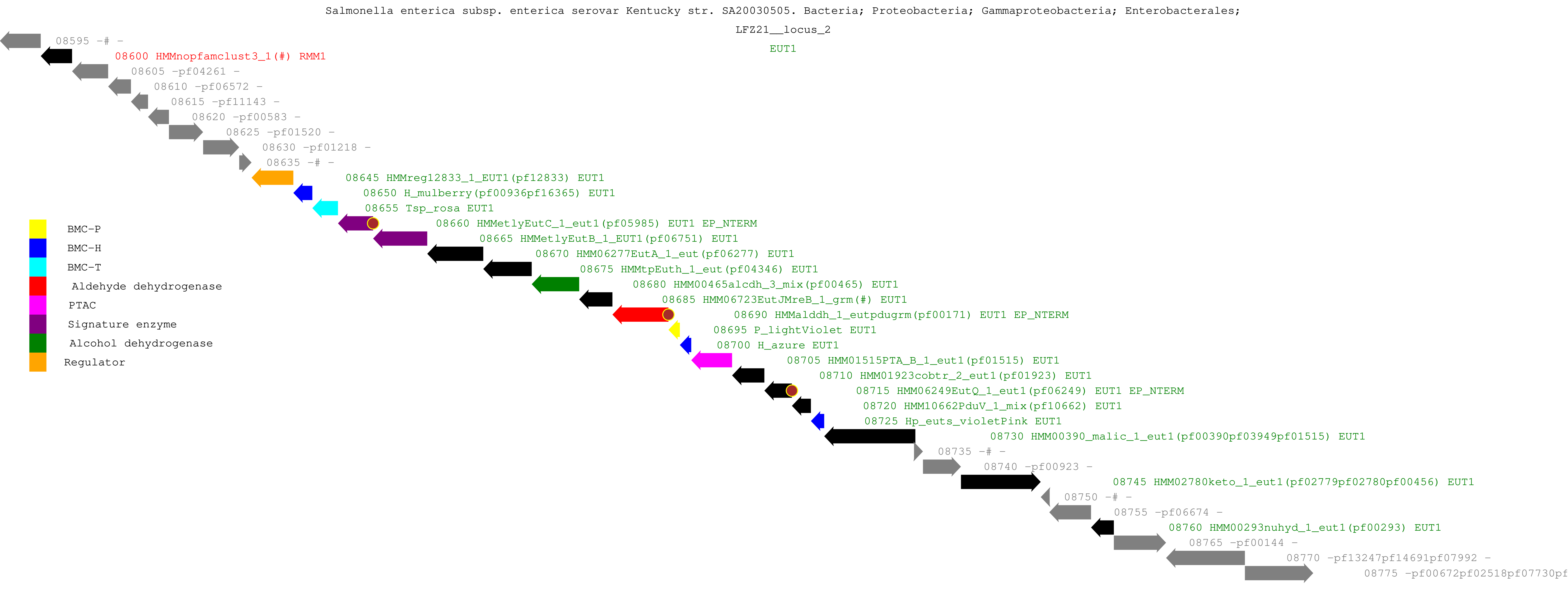

Salmonella enterica subsp. enterica serovar Kentucky str. SA20030505. Bacteria; Proteobacteria; Gammaproteobacteria; Enterobacterales;
LFZ21__locus_2
EUT1
08595 -# -
08600 HMMnopfamclust3_1(#) RMM1
08605 -pf04261 -
08610 -pf06572 -
08615 -pf11143 -
08620 -pf00583 -
08625 -pf01520 -
08630 -pf01218 -
08635 -# -
08645 HMMreg12833_1_EUT1(pf12833) EUT1
08650 H_mulberry(pf00936pf16365) EUT1
08655 Tsp_rosa EUT1
08660 HMMetlyEutC_1_eut1(pf05985) EUT1 EP_NTERM
08665 HMMetlyEutB_1_EUT1(pf06751) EUT1
08670 HMM06277EutA_1_eut(pf06277) EUT1
08675 HMMtpEuth_1_eut(pf04346) EUT1
08680 HMM00465alcdh_3_mix(pf00465) EUT1
08685 HMM06723EutJMreB_1_grm(#) EUT1
08690 HMMalddh_1_eutpdugrm(pf00171) EUT1 EP_NTERM
08695 P_lightViolet EUT1
08700 H_azure EUT1
08705 HMM01515PTA_B_1_eut1(pf01515) EUT1
08710 HMM01923cobtr_2_eut1(pf01923) EUT1
08715 HMM06249EutQ_1_eut1(pf06249) EUT1 EP_NTERM
08720 HMM10662PduV_1_mix(pf10662) EUT1
08725 Hp_euts_violetPink EUT1
08730 HMM00390_malic_1_eut1(pf00390pf03949pf01515) EUT1
08735 -# -
08740 -pf00923 -
08745 HMM02780keto_1_eut1(pf02779pf02780pf00456) EUT1
08750 -# -
08755 -pf06674 -
08760 HMM00293nuhyd_1_eut1(pf00293) EUT1
08765 -pf00144 -
08770 -pf13247pf14691pf07992 -
08775 -pf00672pf02518pf07730pf
BMC-P
BMC-H
BMC-T
Aldehyde dehydrogenase
PTAC
Signature enzyme
Alcohol dehydrogenase
Regulator

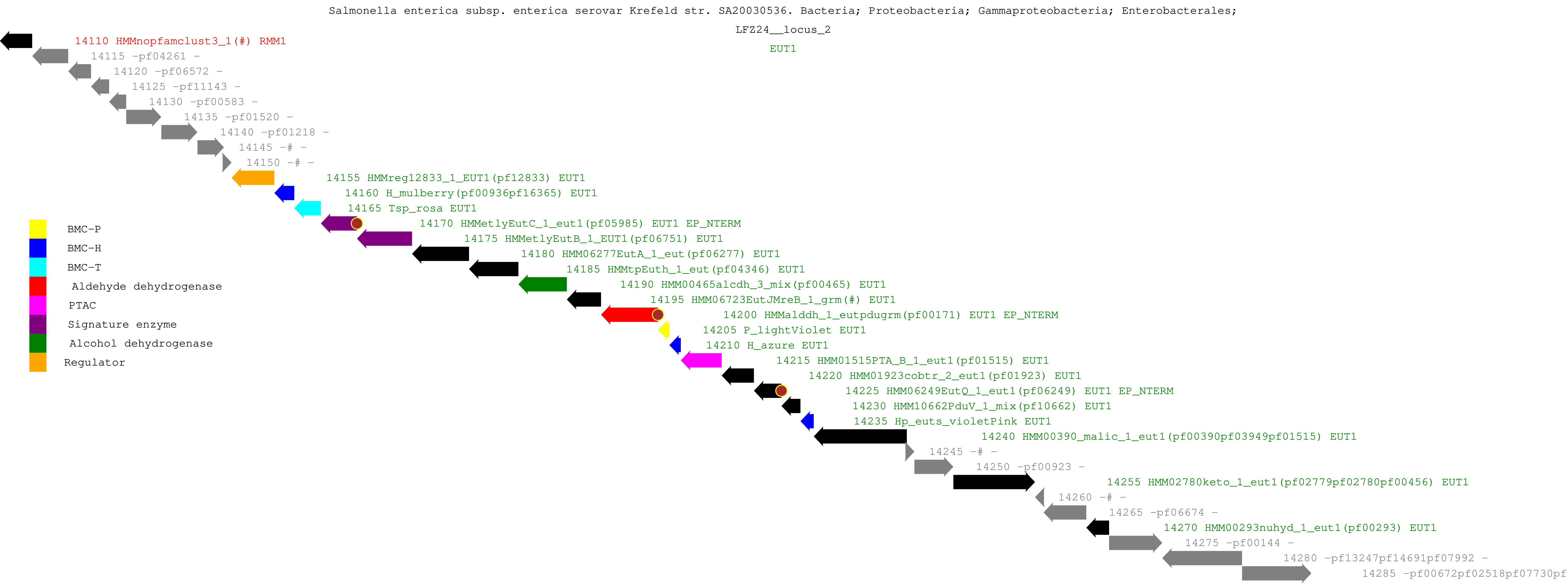
Salmonella enterica subsp. enterica serovar Krefeld str. SA20030536. Bacteria; Proteobacteria; Gammaproteobacteria; Enterobacterales;
LFZ24__locus_2
EUT1
14110 HMMnopfamclust3_1(#) RMM1
14115 -pf04261 -
14120 -pf06572 -
14125 -pf11143 -
14130 -pf00583 -
14135 -pf01520 -
14140 -pf01218 -
14145 -# -
14150 -# -
14155 HMMreg12833_1_EUT1(pf12833) EUT1
14160 H_mulberry(pf00936pf16365) EUT1
14165 Tsp_rosa EUT1
14170 HMMetlyEutC_1_eut1(pf05985) EUT1 EP_NTERM
14175 HMMetlyEutB_1_EUT1(pf06751) EUT1
14180 HMM06277EutA_1_eut(pf06277) EUT1
14185 HMMtpEuth_1_eut(pf04346) EUT1
14190 HMM00465alcdh_3_mix(pf00465) EUT1
14195 HMM06723EutJMreB_1_grm(#) EUT1
14200 HMMalddh_1_eutpdugrm(pf00171) EUT1 EP_NTERM
14205 P_lightViolet EUT1
14210 H_azure EUT1
14215 HMM01515PTA_B_1_eut1(pf01515) EUT1
14220 HMM01923cobtr_2_eut1(pf01923) EUT1
14225 HMM06249EutQ_1_eut1(pf06249) EUT1 EP_NTERM
14230 HMM10662PduV_1_mix(pf10662) EUT1
14235 Hp_euts_violetPink EUT1
14240 HMM00390_malic_1_eut1(pf00390pf03949pf01515) EUT1
14245 -# -
14250 -pf00923 -
14255 HMM02780keto_1_eut1(pf02779pf02780pf00456) EUT1
14260 -# -
14265 -pf06674 -
14270 HMM00293nuhyd_1_eut1(pf00293) EUT1
14275 -pf00144 -
14280 -pf13247pf14691pf07992 -
14285 -pf00672pf02518pf07730pf
BMC-P
BMC-H
BMC-T
Aldehyde dehydrogenase
PTAC
Signature enzyme
Alcohol dehydrogenase
Regulator

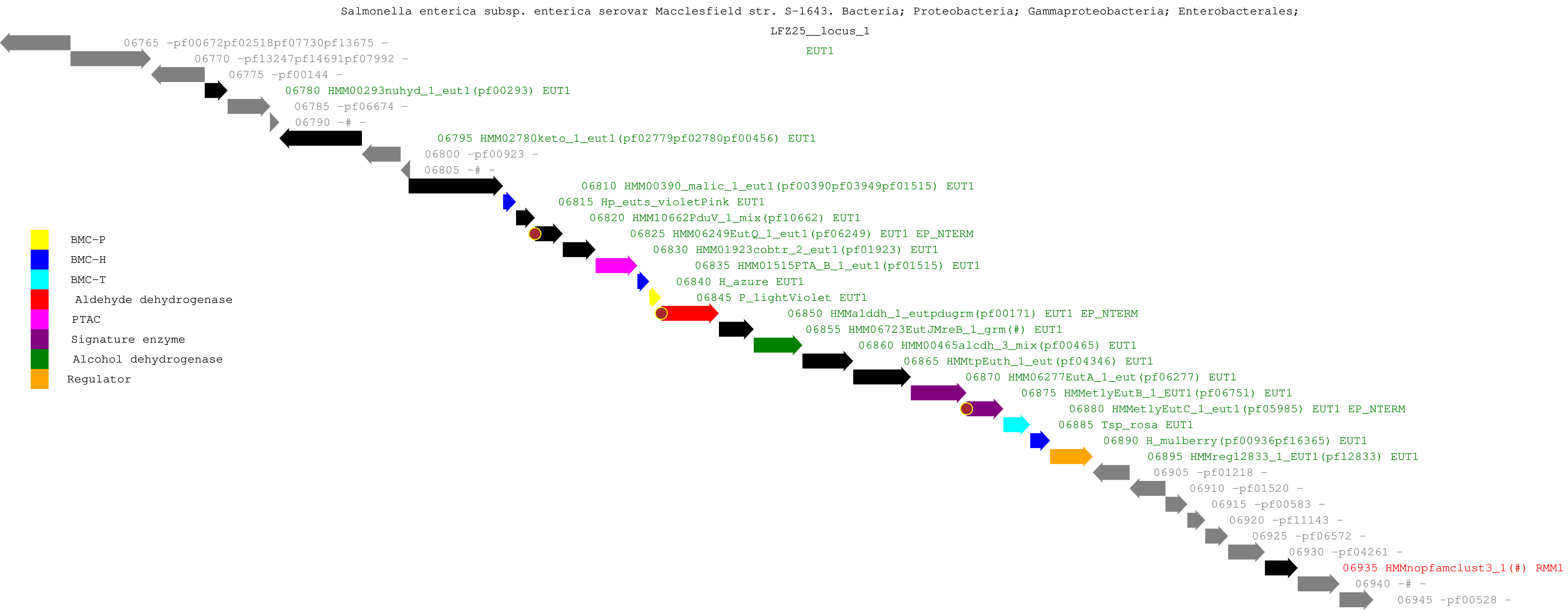
Salmonella enterica subsp. enterica serovar Macclesfield str. S-1643. Bacteria; Proteobacteria; Gammaproteobacteria; Enterobacterales;
LFZ25__locus_1
EUT1
06765 -pf00672pf02518pf07730pf13675 -
06770 -pf13247pf14691pf07992 -
06775 -pf00144 -
06780 HMM00293nuhyd_1_eut1(pf00293) EUT1
06785 -pf06674 -
06790 -# -
06795 HMM02780keto_1_eut1(pf02779pf02780pf00456) EUT1
06800 -pf00923 -
06805 -# -
06810 HMM00390_malic_1_eut1(pf00390pf03949pf01515) EUT1
06815 Hp_euts_violetPink EUT1
06820 HMM10662PduV_1_mix(pf10662) EUT1
06825 HMM06249EutQ_1_eut1(pf06249) EUT1 EP_NTERM
06830 HMM01923cobtr_2_eut1(pf01923) EUT1
06835 HMM01515PTA_B_1_eut1(pf01515) EUT1
06840 H_azure EUT1
06845 P_lightViolet EUT1
06850 HMMalddh_1_eutpdugrm(pf00171) EUT1 EP_NTERM
06855 HMM06723EutJMreB_1_grm(#) EUT1
06860 HMM00465alcdh_3_mix(pf00465) EUT1
06865 HMMtpEuth_1_eut(pf04346) EUT1
06870 HMM06277EutA_1_eut(pf06277) EUT1
06875 HMMetlyEutB_1_EUT1(pf06751) EUT1
06880 HMMetlyEutC_1_eut1(pf05985) EUT1 EP_NTERM
06885 Tsp_rosa EUT1
06890 H_mulberry(pf00936pf16365) EUT1
06895 HMMreg12833_1_EUT1(pf12833) EUT1
06905 -pf01218 -
06910 -pf01520 -
06915 -pf00583 -
06920 -pf11143 -
06925 -pf06572 -
06930 -pf04261 -
06935 HMMnopfamclust3_1(#) RMM1
06940 -# -
06945 -pf00528 -
BMC-P
BMC-H
BMC-T
Aldehyde dehydrogenase
PTAC
Signature enzyme
Alcohol dehydrogenase
Regulator

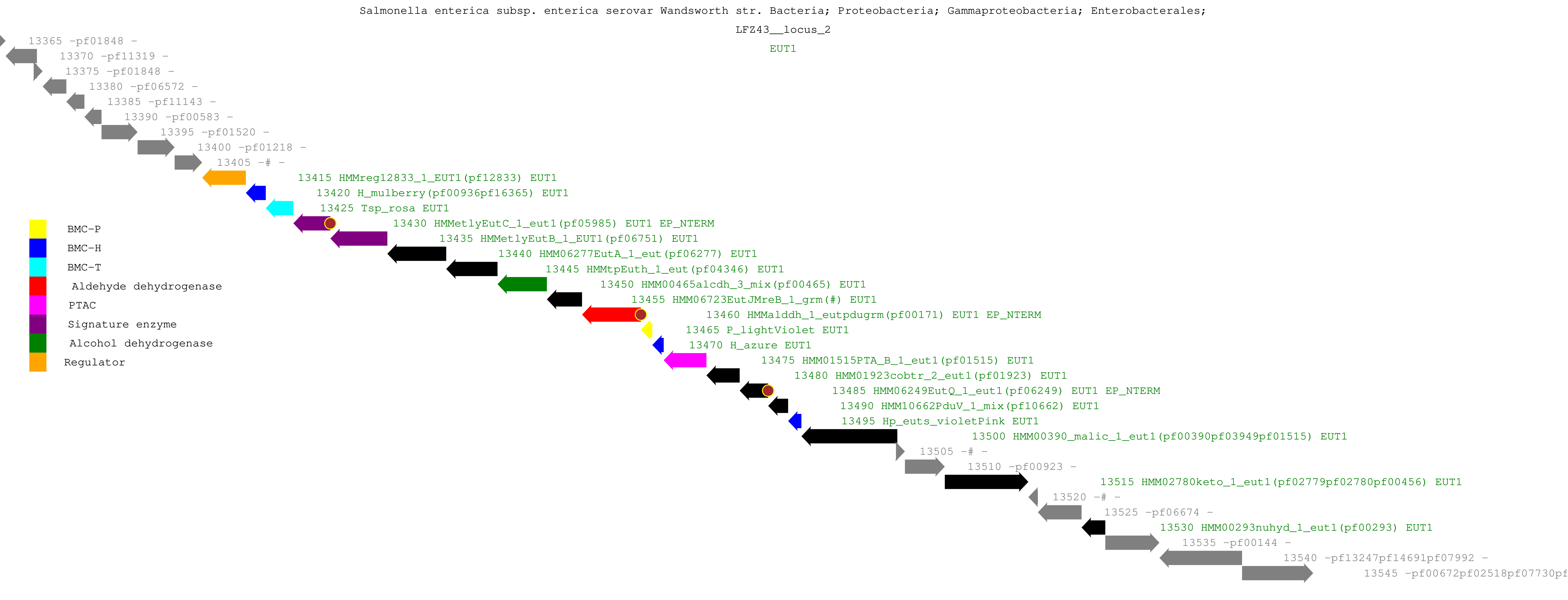

Salmonella enterica subsp. enterica serovar Wandsworth str. Bacteria; Proteobacteria; Gammaproteobacteria; Enterobacterales;
LFZ43__locus_2
EUT1
13365 -pf01848 -
13370 -pf11319 -
13375 -pf01848 -
13380 -pf06572 -
13385 -pf11143 -
13390 -pf00583 -
13395 -pf01520 -
13400 -pf01218 -
13405 -# -
13415 HMMreg12833_1_EUT1(pf12833) EUT1
13420 H_mulberry(pf00936pf16365) EUT1
13425 Tsp_rosa EUT1
13430 HMMetlyEutC_1_eut1(pf05985) EUT1 EP_NTERM
13435 HMMetlyEutB_1_EUT1(pf06751) EUT1
13440 HMM06277EutA_1_eut(pf06277) EUT1
13445 HMMtpEuth_1_eut(pf04346) EUT1
13450 HMM00465alcdh_3_mix(pf00465) EUT1
13455 HMM06723EutJMreB_1_grm(#) EUT1
13460 HMMalddh_1_eutpdugrm(pf00171) EUT1 EP_NTERM
13465 P_lightViolet EUT1
13470 H_azure EUT1
13475 HMM01515PTA_B_1_eut1(pf01515) EUT1
13480 HMM01923cobtr_2_eut1(pf01923) EUT1
13485 HMM06249EutQ_1_eut1(pf06249) EUT1 EP_NTERM
13490 HMM10662PduV_1_mix(pf10662) EUT1
13495 Hp_euts_violetPink EUT1
13500 HMM00390_malic_1_eut1(pf00390pf03949pf01515) EUT1
13505 -# -
13510 -pf00923 -
13515 HMM02780keto_1_eut1(pf02779pf02780pf00456) EUT1
13520 -# -
13525 -pf06674 -
13530 HMM00293nuhyd_1_eut1(pf00293) EUT1
13535 -pf00144 -
13540 -pf13247pf14691pf07992 -
13545 -pf00672pf02518pf07730pf
BMC-P
BMC-H
BMC-T
Aldehyde dehydrogenase
PTAC
Signature enzyme
Alcohol dehydrogenase
Regulator

Salmonella enterica subsp. salamae serovar 55:k:z39 str. 1315K. Bacteria; Proteobacteria; Gammaproteobacteria; Enterobacterales;

LFZ47__locus_1

EUT1

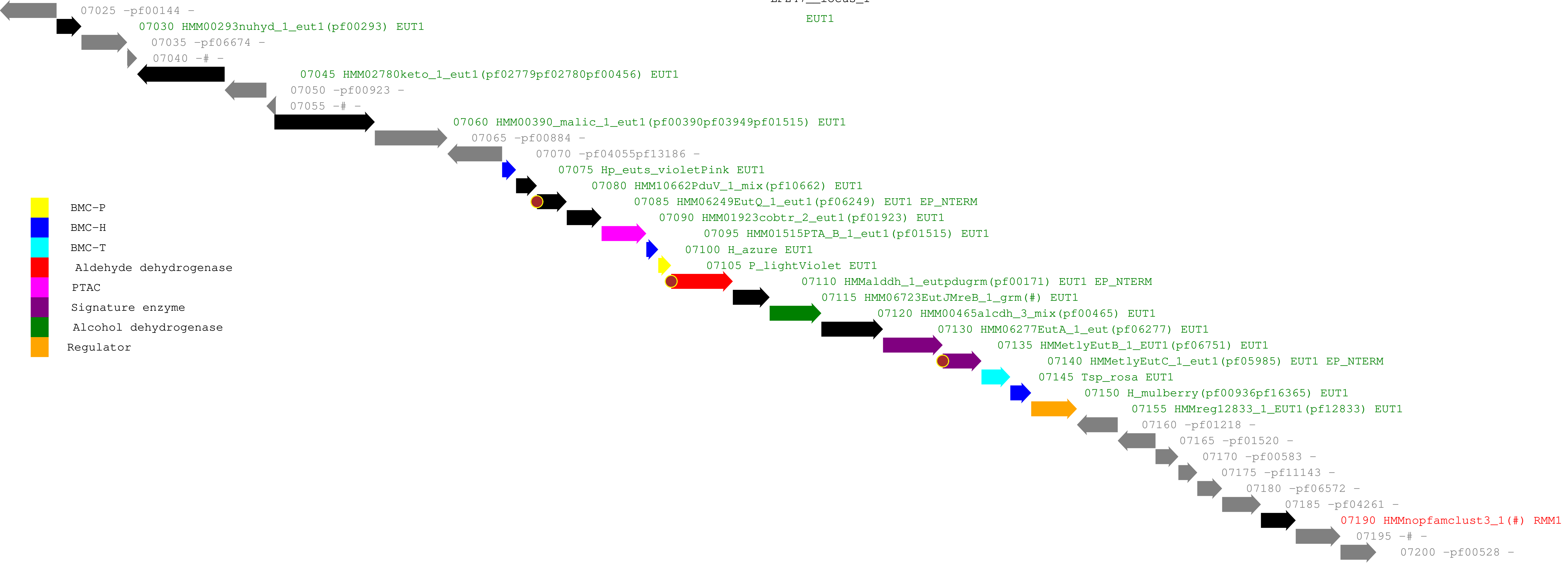

Salmonella enterica subsp. salamae serovar 56:z10:e,n,x str. 1369-73. Bacteria; Proteobacteria; Gammaproteobacteria; Enterobacterales;

LFZ48__locus_2

EUT1

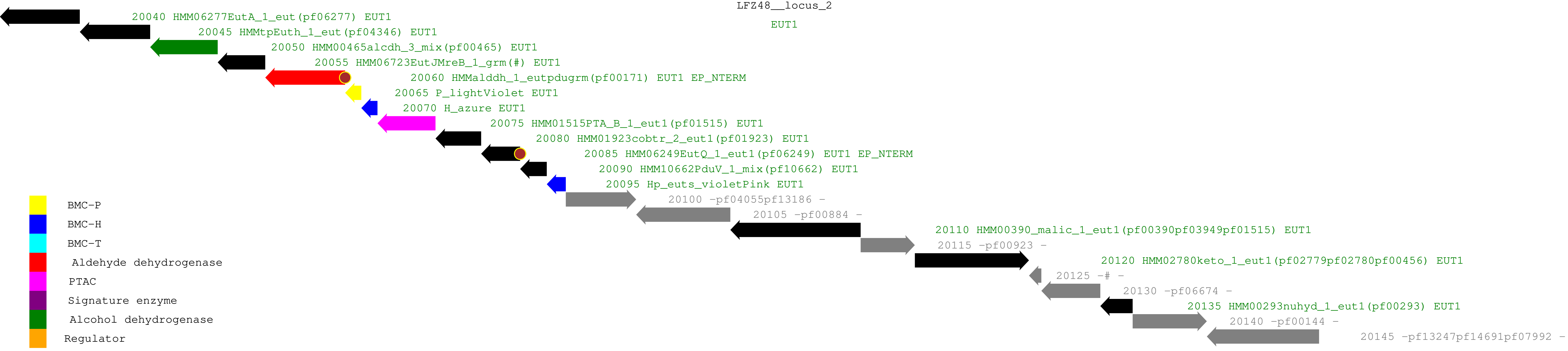

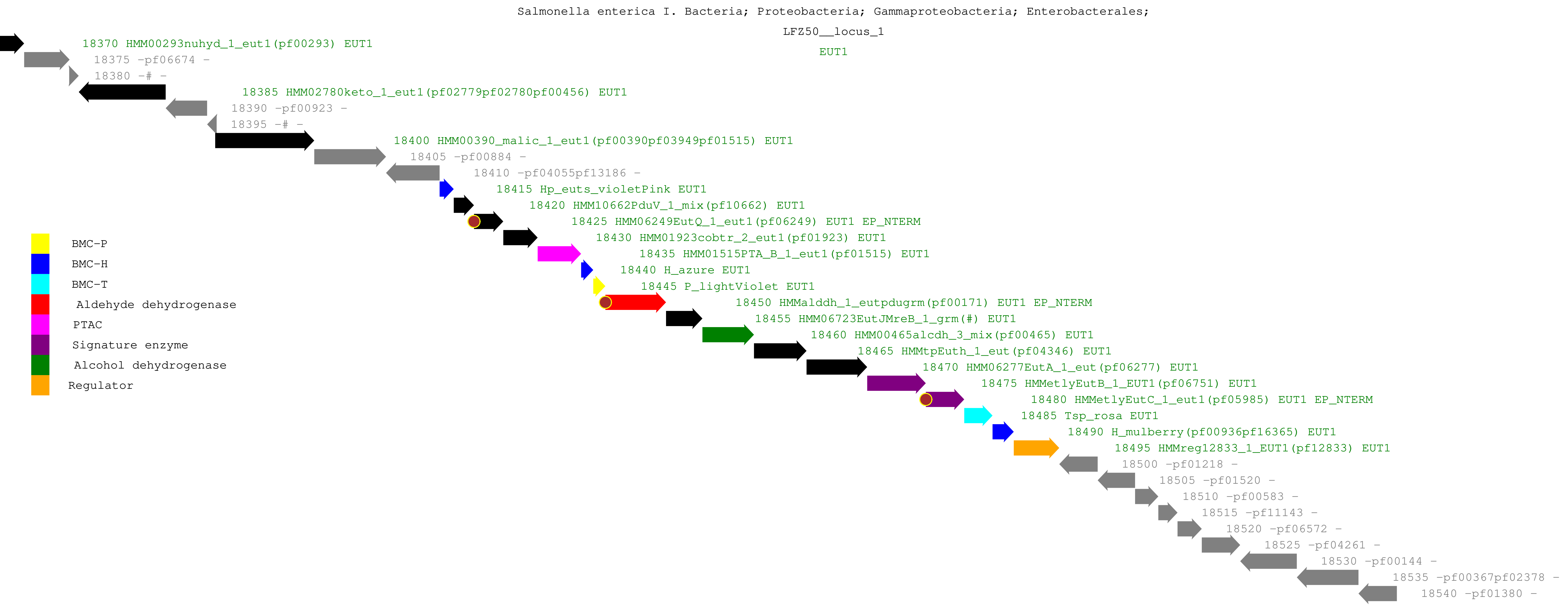
Salmonella enterica I. Bacteria; Proteobacteria; Gammaproteobacteria; Enterobacterales;
LFZ50__locus_1
EUT1
18370 HMM00293nuhyd_1_eut1(pf00293) EUT1
18375 -pf06674 -
18380 -# -
18385 HMM02780keto_1_eut1(pf02779pf02780pf00456) EUT1
18390 -pf00923 -
18395 -# -
18400 HMM00390_malic_1_eut1(pf00390pf03949pf01515) EUT1
18405 -pf00884 -
18410 -pf04055pf13186 -
18415 Hp_euts_violetPink EUT1
18420 HMM10662PduV_1_mix(pf10662) EUT1
18425 HMM06249EutQ_1_eut1(pf06249) EUT1 EP_NTERM
18430 HMM01923cobtr_2_eut1(pf01923) EUT1
18435 HMM01515PTA_B_1_eut1(pf01515) EUT1
18440 H_azure EUT1
18445 P_lightViolet EUT1
18450 HMMalddh_1_eutpdugrm(pf00171) EUT1 EP_NTERM
18455 HMM06723EutJMreB_1_grm(#) EUT1
18460 HMM00465alcdh_3_mix(pf00465) EUT1
18465 HMMtpEuth_1_eut(pf04346) EUT1
18470 HMM06277EutA_1_eut(pf06277) EUT1
18475 HMMetlyEutB_1_EUT1(pf06751) EUT1
18480 HMMetlyEutC_1_eut1(pf05985) EUT1 EP_NTERM
18485 Tsp_rosa EUT1
18490 H_mulberry(pf00936pf16365) EUT1
18495 HMMreg12833_1_EUT1(pf12833) EUT1
18500 -pf01218 -
18505 -pf01520 -
18510 -pf00583 -
18515 -pf11143 -
18520 -pf06572 -
18525 -pf04261 -
18530 -pf00144 -
18535 -pf00367pf02378 -
18540 -pf01380 -
BMC-P
BMC-H
BMC-T
Aldehyde dehydrogenase
PTAC
Signature enzyme
Alcohol dehydrogenase
Regulator

Salmonella enterica subsp. arizonae serovar 63:g,z51:- str. So 20/20. Bacteria; Proteobacteria; Gammaproteobacteria; Enterobacterales;

LFZ51__locus_1

EUT1

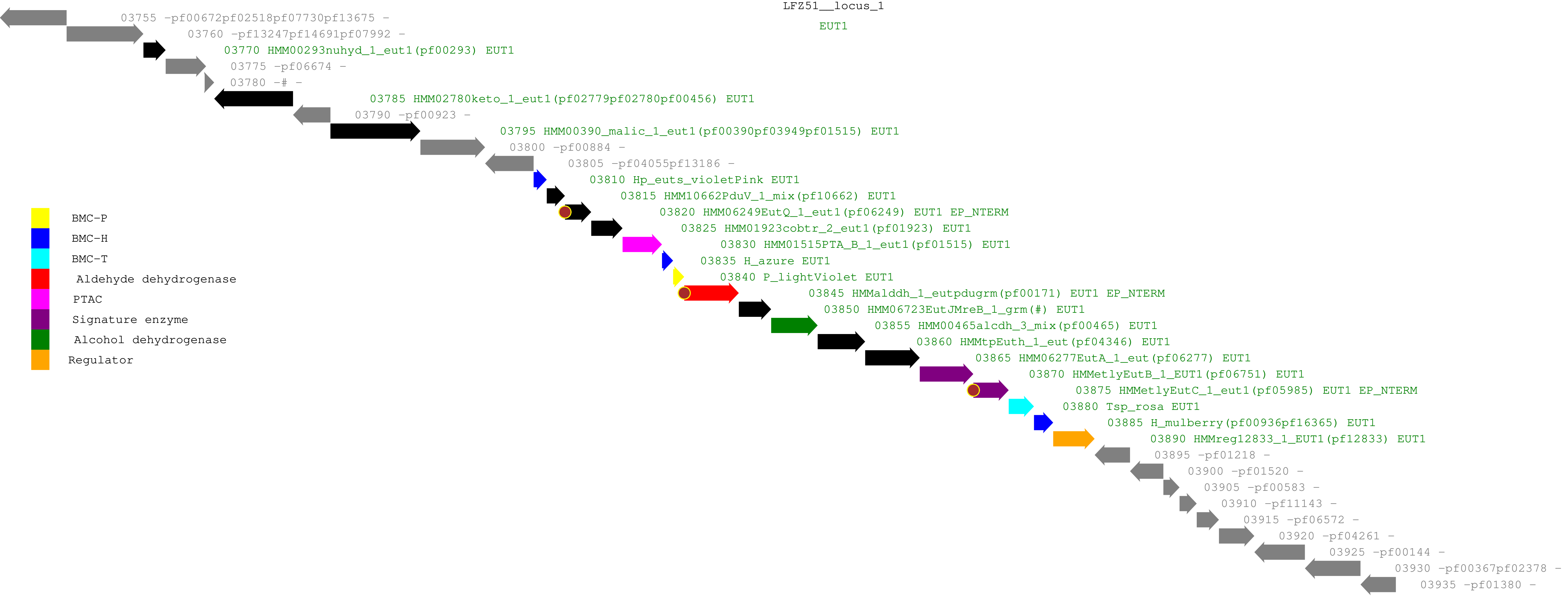

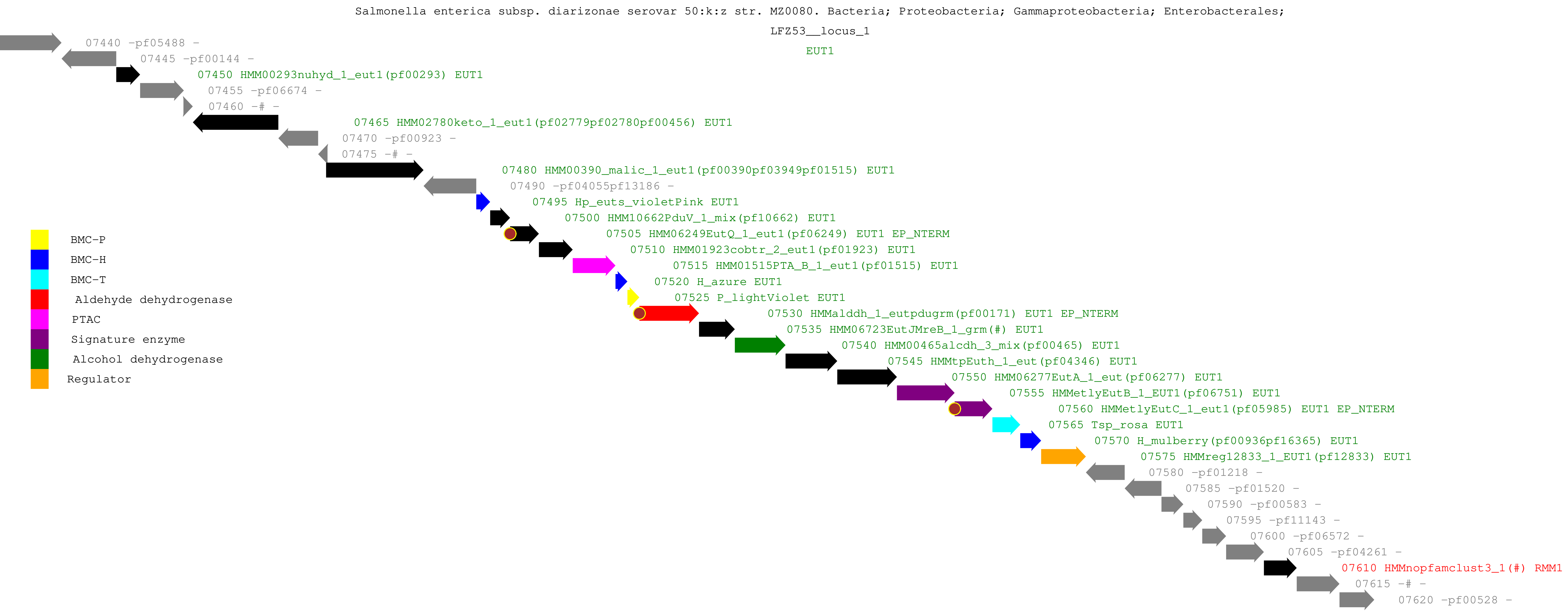
Salmonella enterica subsp. diarizonae serovar 50:k:z str. MZ0080. Bacteria; Proteobacteria; Gammaproteobacteria; Enterobacterales;
LFZ53__locus_1
EUT1
07440 -pf05488 -
07445 -pf00144 -
07450 HMM00293nuhyd_1_eut1(pf00293) EUT1
07455 -pf06674 -
07460 -# -
07465 HMM02780keto_1_eut1(pf02779pf02780pf00456) EUT1
07470 -pf00923 -
07475 -# -
07480 HMM00390_malic_1_eut1(pf00390pf03949pf01515) EUT1
07490 -pf04055pf13186 -
07495 Hp_euts_violetPink EUT1
07500 HMM10662PduV_1_mix(pf10662) EUT1
07505 HMM06249EutQ_1_eut1(pf06249) EUT1 EP_NTERM
07510 HMM01923cobtr_2_eut1(pf01923) EUT1
07515 HMM01515PTA_B_1_eut1(pf01515) EUT1
07520 H_azure EUT1
07525 P_lightViolet EUT1
07530 HMMalddh_1_eutpdugrm(pf00171) EUT1 EP_NTERM
07535 HMM06723EutJMreB_1_grm(#) EUT1
07540 HMM00465alcdh_3_mix(pf00465) EUT1
07545 HMMtpEuth_1_eut(pf04346) EUT1
07550 HMM06277EutA_1_eut(pf06277) EUT1
07555 HMMetlyEutB_1_EUT1(pf06751) EUT1
07560 HMMetlyEutC_1_eut1(pf05985) EUT1 EP_NTERM
07565 Tsp_rosa EUT1
07570 H_mulberry(pf00936pf16365) EUT1
07575 HMMreg12833_1_EUT1(pf12833) EUT1
07580 -pf01218 -
07585 -pf01520 -
07590 -pf00583 -
07595 -pf11143 -
07600 -pf06572 -
07605 -pf04261 -
07610 HMMnopfamclust3_1(#) RMM1
07615 -# -
07620 -pf00528 -
BMC-P
BMC-H
BMC-T
Aldehyde dehydrogenase
PTAC
Signature enzyme
Alcohol dehydrogenase
Regulator

Salmonella enterica subsp. diarizonae serovar 65:c:z str. SA20044251. Bacteria; Proteobacteria; Gammaproteobacteria; Enterobacterales;

LFZ55__locus_1

EUT1

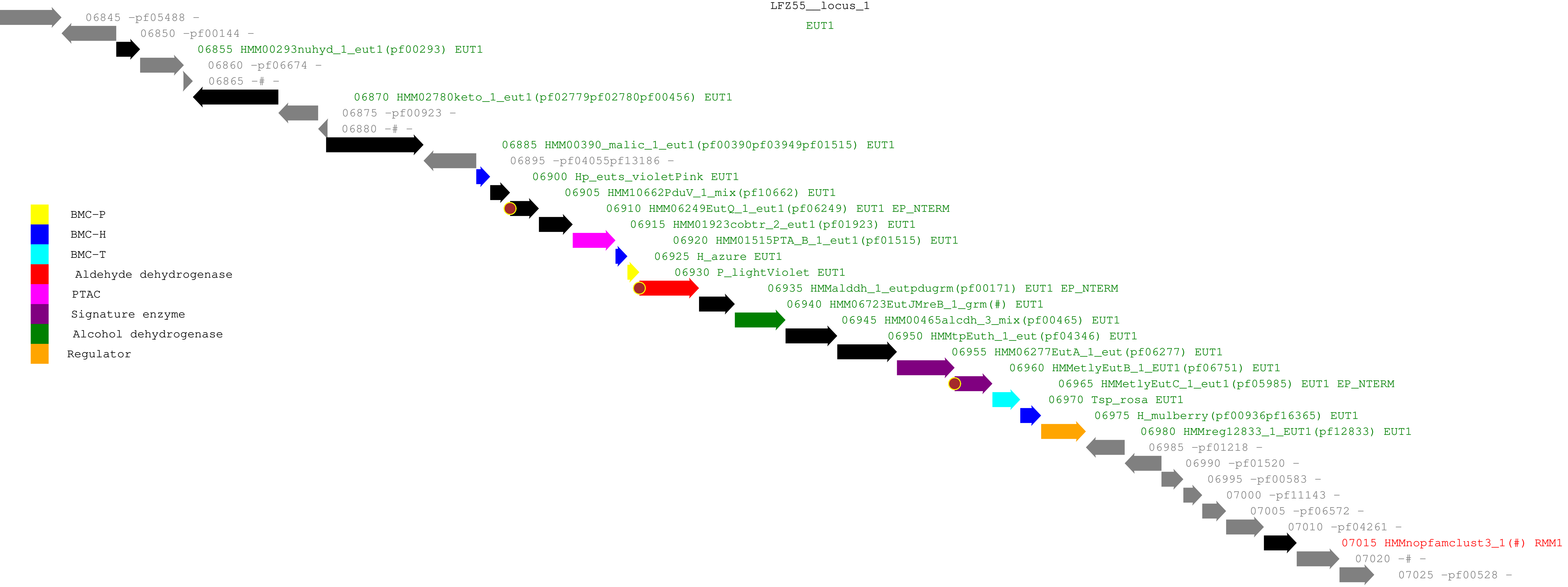

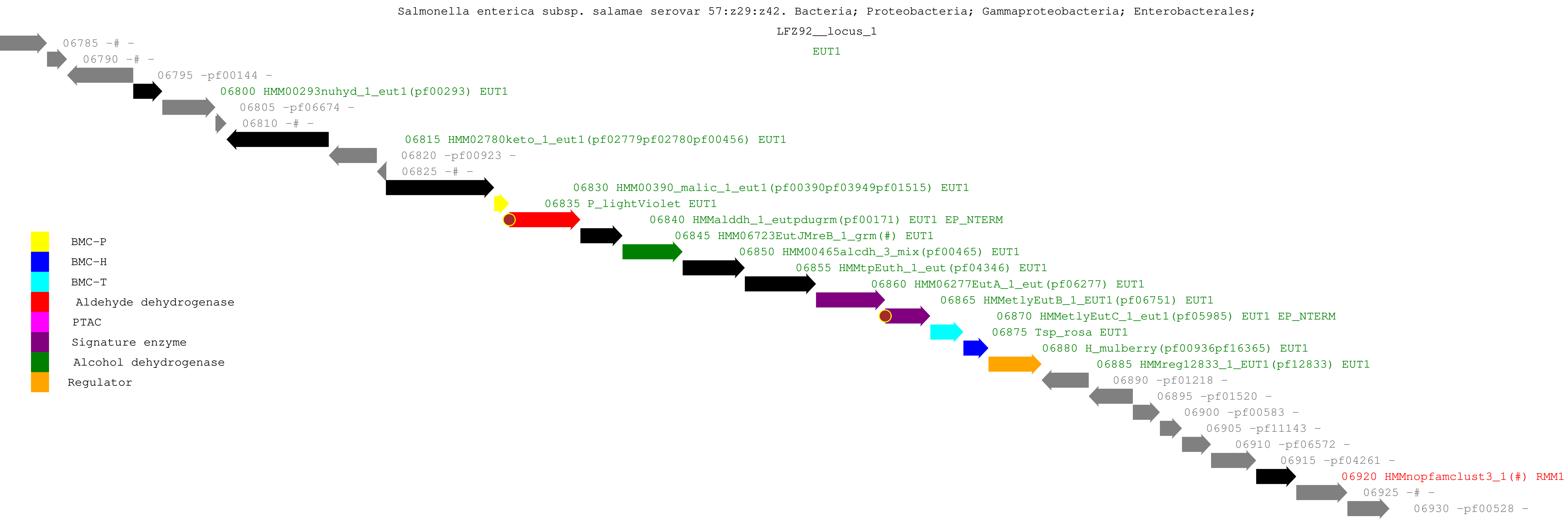
Salmonella enterica subsp. salamae serovar 57:z29:z42. Bacteria; Proteobacteria; Gammaproteobacteria; Enterobacterales;
LFZ92__locus_1
EUT1
06785 -# -
06790 -# -
06795 -pf00144 -
06800 HMM00293nuhyd_1_eut1(pf00293) EUT1
06805 -pf06674 -
06810 -# -
06815 HMM02780keto_1_eut1(pf02779pf02780pf00456) EUT1
06820 -pf00923 -
06825 -# -
06830 HMM00390_malic_1_eut1(pf00390pf03949pf01515) EUT1
06835 P_lightViolet EUT1
06840 HMMaldh_1_eutpdugrm(pf00171) EUT1 EP_NTERM
06845 HMM06723EutJMreB_1_grm(#) EUT1
06850 HMM00465alcdh_3_mix(pf00465) EUT1
06855 HMMtpEuth_1_eut(pf04346) EUT1
06860 HMM06277EutA_1_eut(pf06277) EUT1
06865 HMMetlyEutB_1_EUT1(pf06751) EUT1
06870 HMMetlyEutC_1_eut1(pf05985) EUT1 EP_NTERM
06875 Tsp_rosa EUT1
06880 H_mulberry(pf00936pf16365) EUT1
06885 HMMreg12833_1_EUT1(pf12833) EUT1
06890 -pf01218 -
06895 -pf01520 -
06900 -pf00583 -
06905 -pf11143 -
06910 -pf06572 -
06915 -pf04261 -
06920 HMMnopfamclust3_1(#) RMM1
06925 -# -
06930 -pf00528 -
BMC-P
BMC-H
BMC-T
Aldehyde dehydrogenase
PTAC
Signature enzyme
Alcohol dehydrogenase
Regulator

Morganella morganii (Proteus morganii). Bacteria; Proteobacteria; Gammaproteobacteria; Enterobacterales;

LR61__locus_1

EUT1

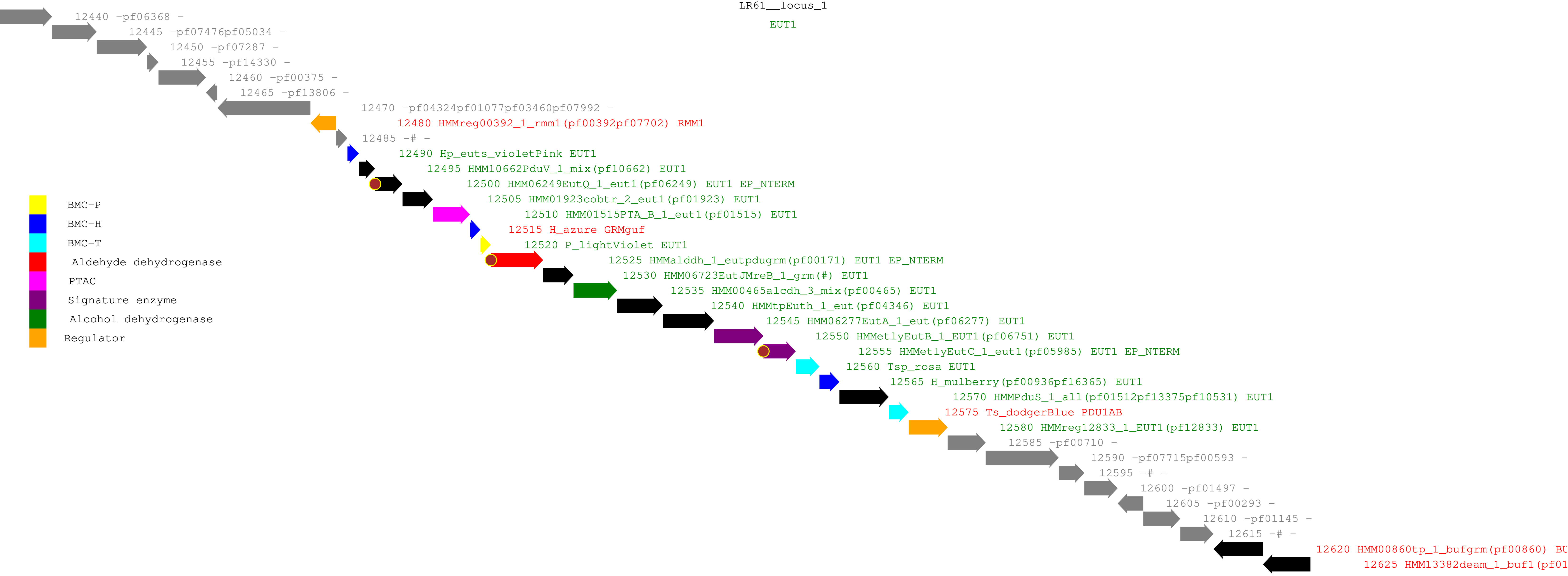

Salmonella enterica subsp. enterica serovar Adelaide str. A4-669. Bacteria; Proteobacteria; Gammaproteobacteria; Enterobacterales;

LTSEADE__locus_2

EUT1

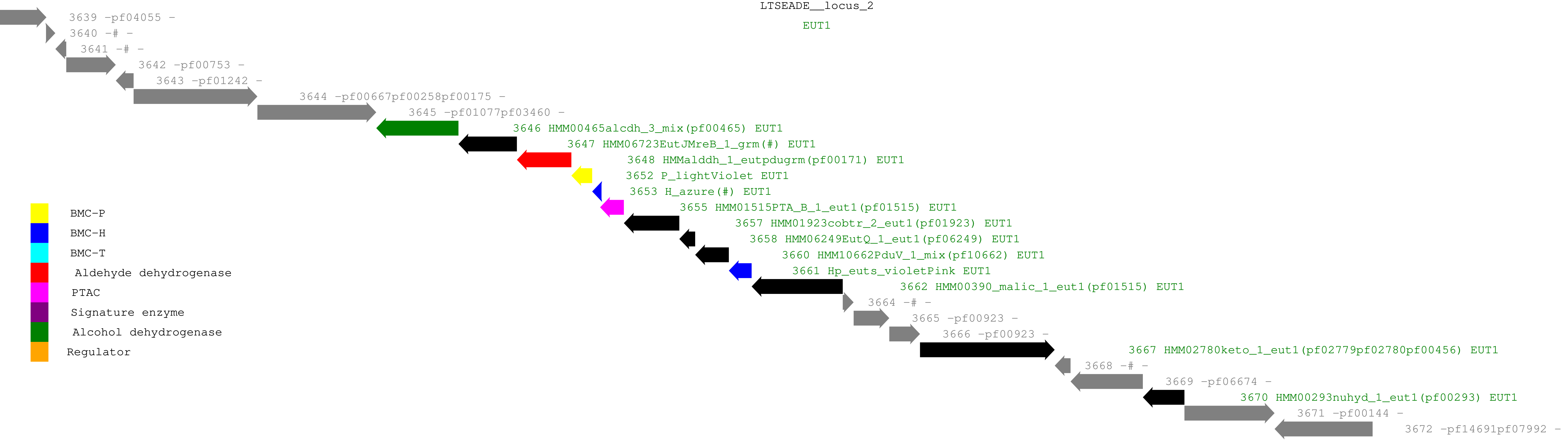

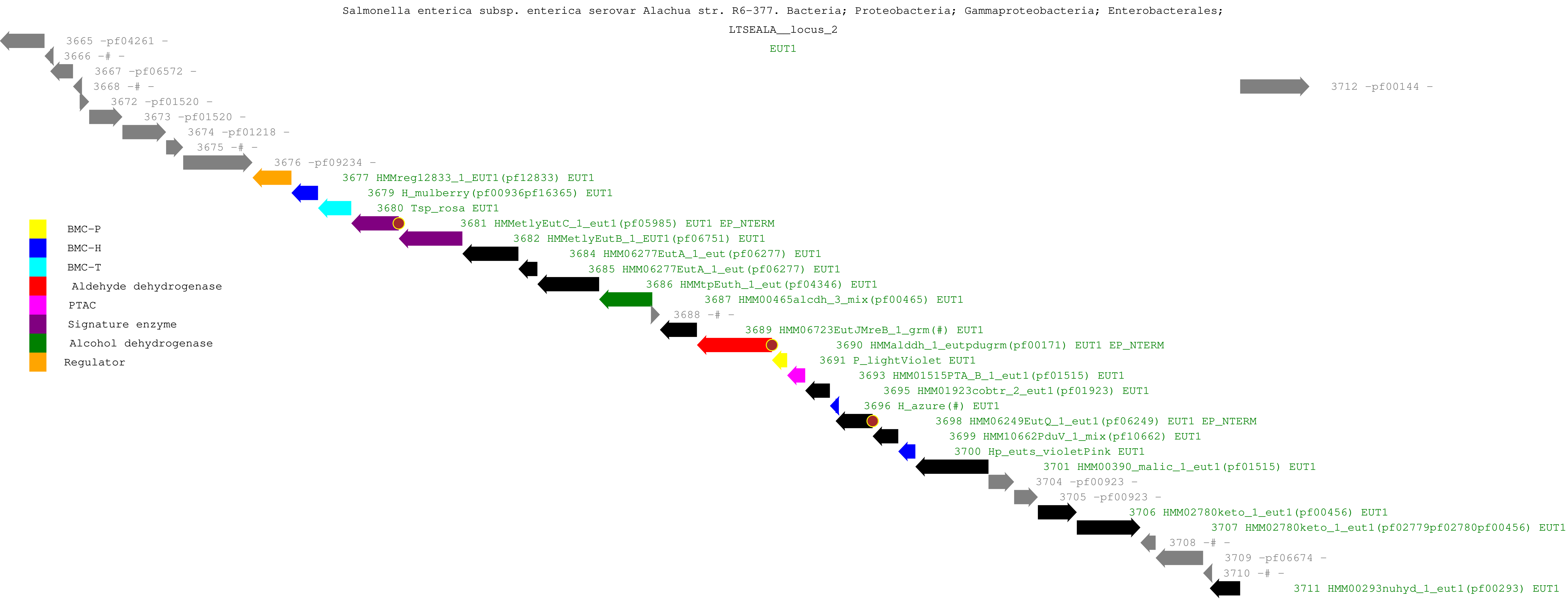
Salmonella enterica subsp. enterica serovar Alachua str. R6-377. Bacteria; Proteobacteria; Gammaproteobacteria; Enterobacterales;
LTSEALA__locus_2
EUT1
3665 -pf04261 -
3666 -# -
3667 -pf06572 -
3668 -# -
3672 -pf01520 -
3673 -pf01520 -
3674 -pf01218 -
3675 -# -
3676 -pf09234 -
3677 HMMreg12833_1_EUT1(pf12833) EUT1
3679 H_mulberry(pf00936pf16365) EUT1
3680 Tsp_rosa EUT1
3681 HMMetlyEutC_1_eut1(pf05985) EUT1 EP_NTERM
3682 HMMetlyEutB_1_EUT1(pf06751) EUT1
3684 HMM06277EutA_1_eut(pf06277) EUT1
3685 HMM06277EutA_1_eut(pf06277) EUT1
3686 HMMtpEuth_1_eut(pf04346) EUT1
3687 HMM00465alcdh_3_mix(pf00465) EUT1
3688 -# -
3689 HMM06723EutJMreB_1_grm(#) EUT1
3690 HMMalddh_1_eutpdugrm(pf00171) EUT1 EP_NTERM
3691 P_lightViolet EUT1
3693 HMM01515PTA_B_1_eut1(pf01515) EUT1
3695 HMM01923cobtr_2_eut1(pf01923) EUT1
3696 H_azure(#) EUT1
3698 HMM06249EutQ_1_eut1(pf06249) EUT1 EP_NTERM
3699 HMM10662PduV_1_mix(pf10662) EUT1
3700 Hp_euts_violetPink EUT1
3701 HMM00390_malic_1_eut1(pf01515) EUT1
3704 -pf00923 -
3705 -pf00923 -
3706 HMM02780keto_1_eut1(pf00456) EUT1
3707 HMM02780keto_1_eut1(pf02779pf02780pf00456) EUT1
3708 -# -
3709 -pf06674 -
3710 -# -
3711 HMM00293nuhyd_1_eut1(pf00293) EUT1
3712 -pf00144 -
BMC-P
BMC-H
BMC-T
Aldehyde dehydrogenase
PTAC
Signature enzyme
Alcohol dehydrogenase
Regulator

Salmonella enterica subsp. enterica serovar Baildon str. R6-199. Bacteria; Proteobacteria; Gammaproteobacteria; Enterobacterales;

LTSEBAI__locus_2

EUT1

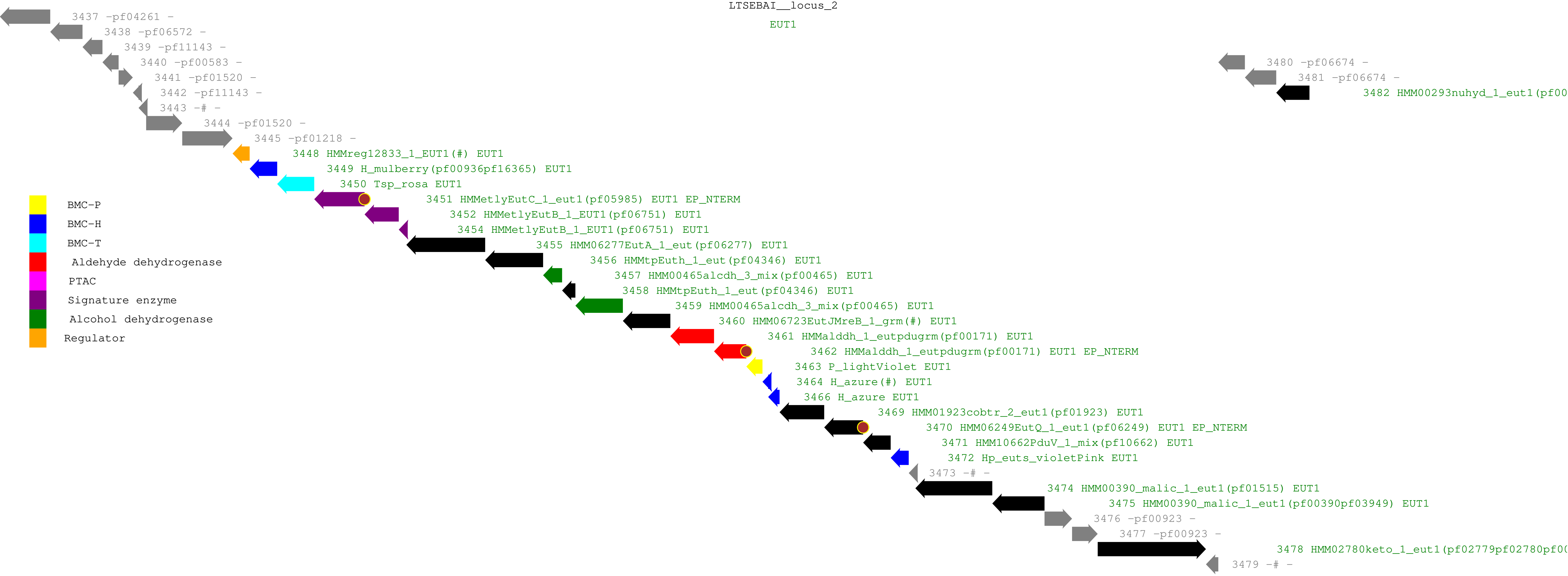

Salmonella enterica subsp. enterica serovar Give str. S5-487. Bacteria; Proteobacteria; Gammaproteobacteria; Enterobacterales;

LTSEGIV__locus_1_main_sat

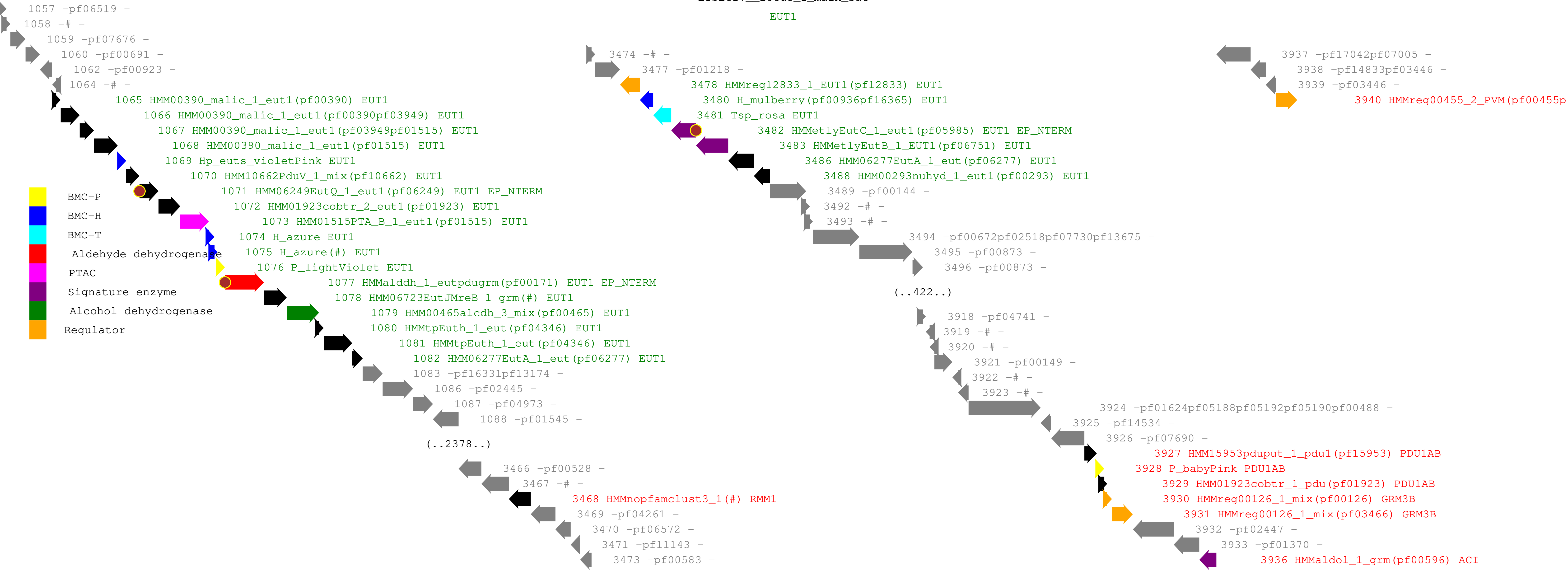

Salmonella enterica subsp. enterica serovar Inverness str. R8-3668. Bacteria; Proteobacteria; Gammaproteobacteria; Enterobacterales;

LTSEINV__locus_2

EUT1

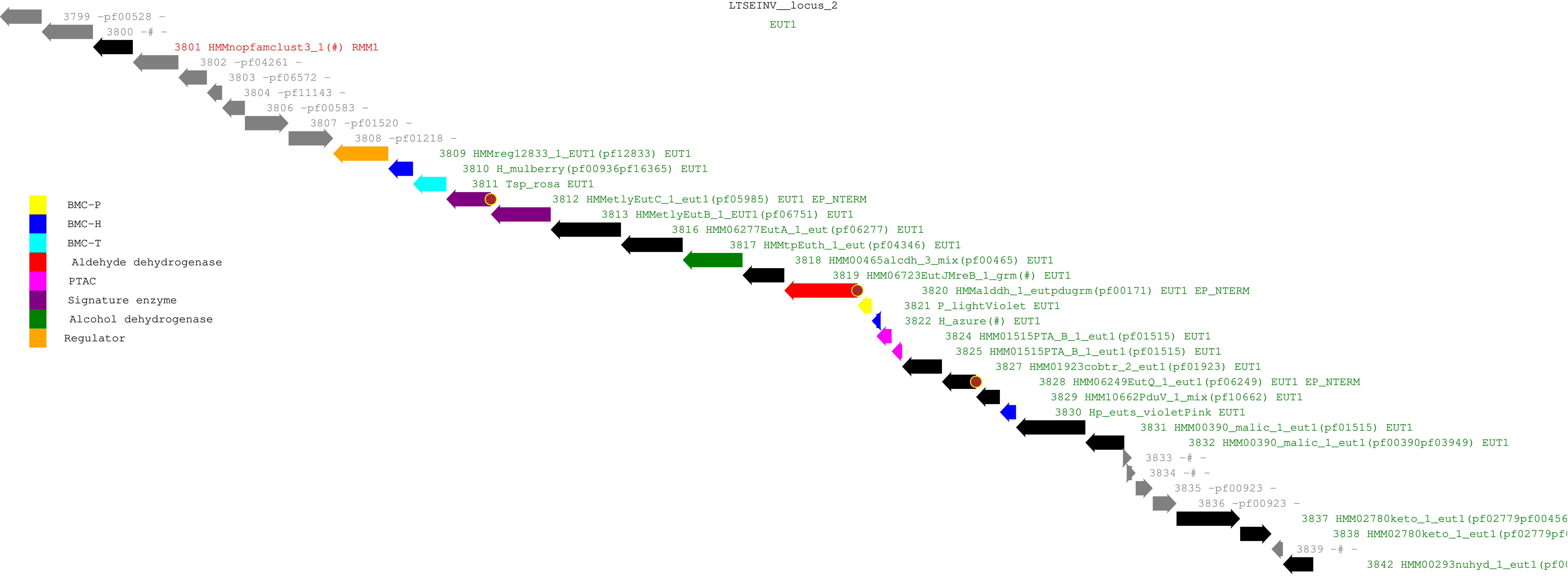

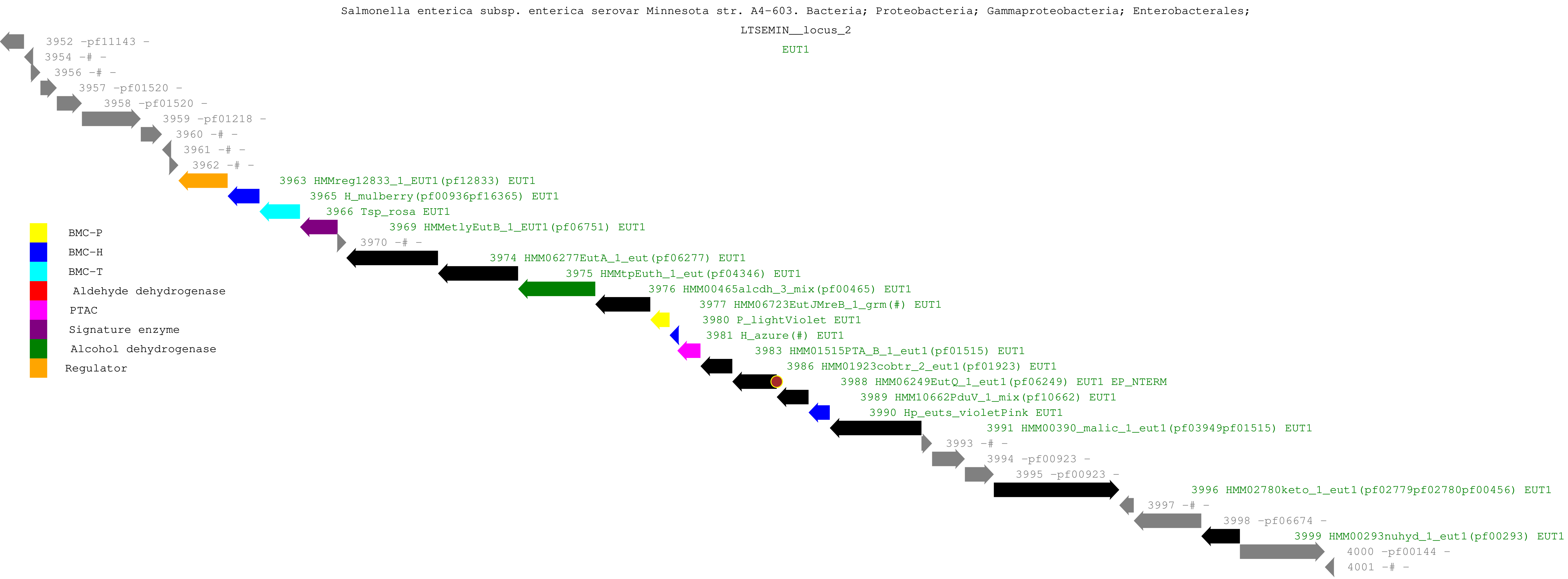
Salmonella enterica subsp. enterica serovar Minnesota str. A4-603. Bacteria; Proteobacteria; Gammaproteobacteria; Enterobacterales;
LTSEMIN__locus_2
EUT1
3952 -pf11143 -
3954 -# -
3956 -# -
3957 -pf01520 -
3958 -pf01520 -
3959 -pf01218 -
3960 -# -
3961 -# -
3962 -# -
3963 HMMreg12833_1_EUT1(pf12833) EUT1
3965 H_mulberry(pf00936pf16365) EUT1
3966 Tsp_rosa EUT1
3969 HMMetlyEutB_1_EUT1(pf06751) EUT1
3970 -# -
3974 HMM06277EutA_1_eut(pf06277) EUT1
3975 HMMtpEuth_1_eut(pf04346) EUT1
3976 HMM00465alcdh_3_mix(pf00465) EUT1
3977 HMM06723EutJMreB_1_grm(#) EUT1
3980 P_lightViolet EUT1
3981 H_azure(#) EUT1
3983 HMM01515PTA_B_1_eut1(pf01515) EUT1
3986 HMM01923cobtr_2_eut1(pf01923) EUT1
3988 HMM06249EutQ_1_eut1(pf06249) EUT1 EP_NTERM
3989 HMM10662PduV_1_mix(pf10662) EUT1
3990 Hp_euts_violetPink EUT1
3991 HMM00390_malic_1_eut1(pf03949pf01515) EUT1
3993 -# -
3994 -pf00923 -
3995 -pf00923 -
3996 HMM02780keto_1_eut1(pf02779pf02780pf00456) EUT1
3997 -# -
3998 -pf06674 -
3999 HMM00293nuhyd_1_eut1(pf00293) EUT1
4000 -pf00144 -
4001 -# -
BMC-P
BMC-H
BMC-T
Aldehyde dehydrogenase
PTAC
Signature enzyme
Alcohol dehydrogenase
Regulator

Salmonella enterica subsp. enterica serovar Montevideo str. S5-403. Bacteria; Proteobacteria; Gammaproteobacteria; Enterobacterales;

LTSEMON__locus_2

EUT1

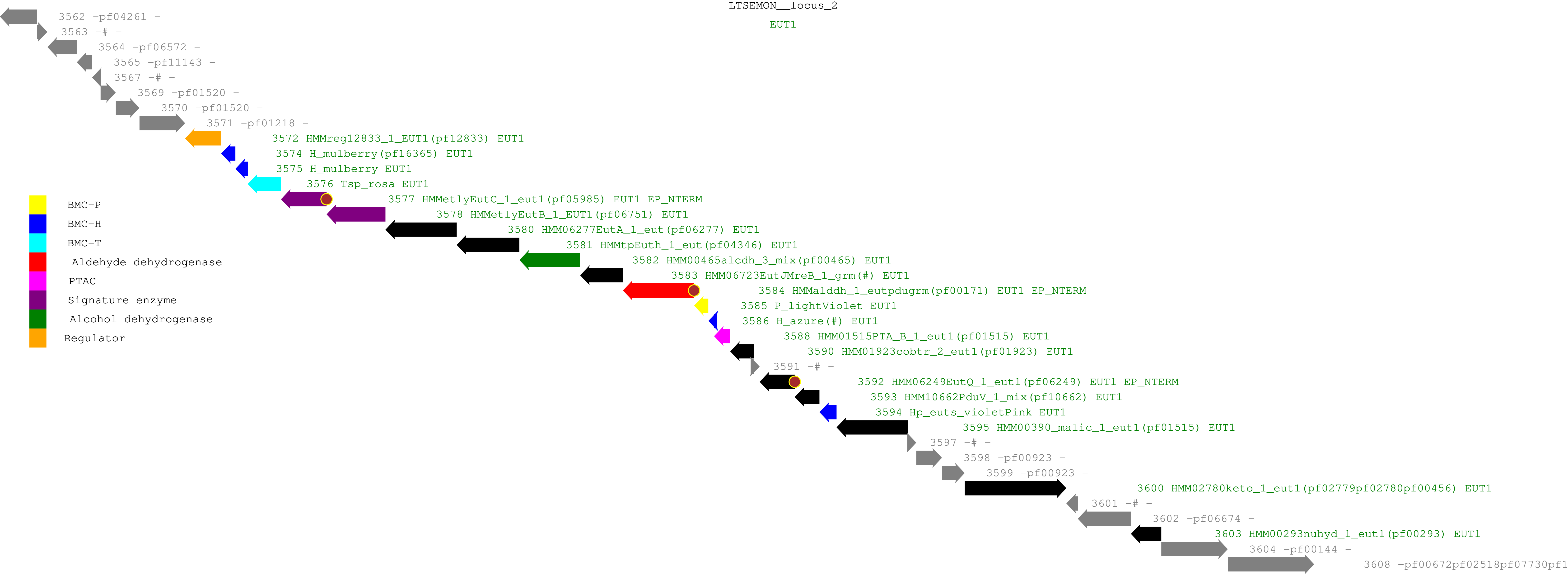

Salmonella enterica subsp. enterica serovar Rubislaw str. A4-653. Bacteria; Proteobacteria; Gammaproteobacteria; Enterobacterales;

LTSERUB__locus_2

EUT1

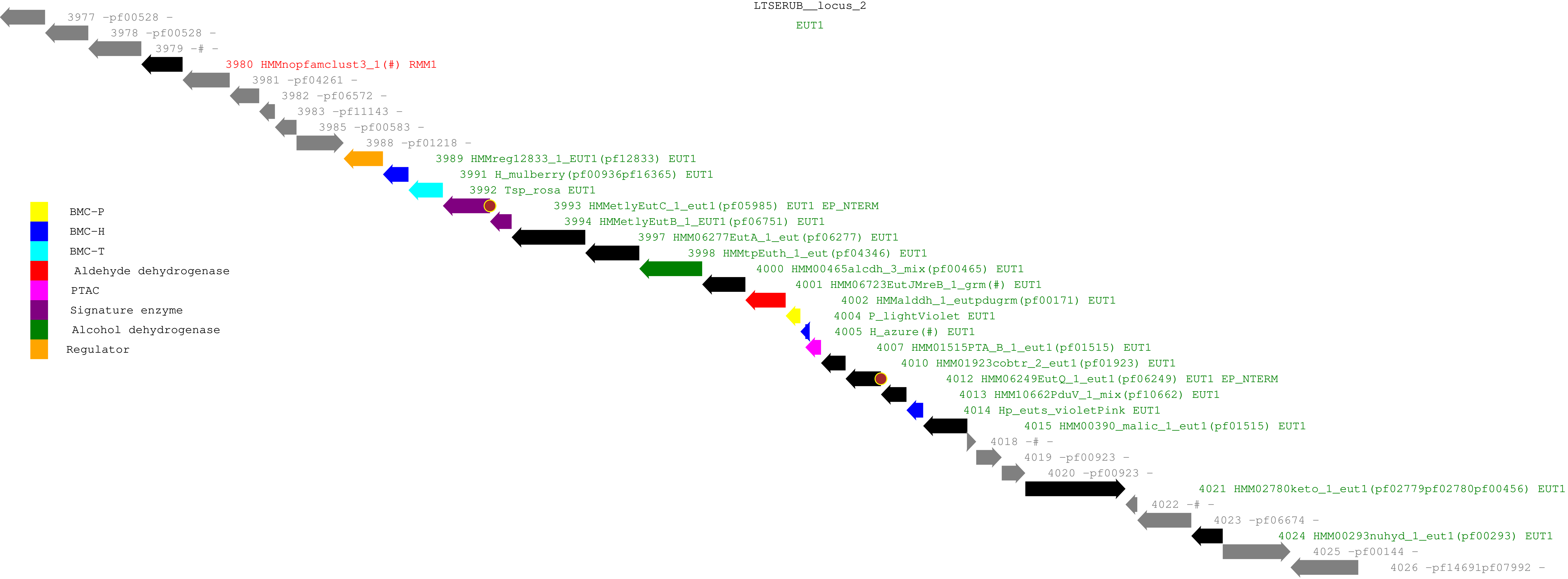

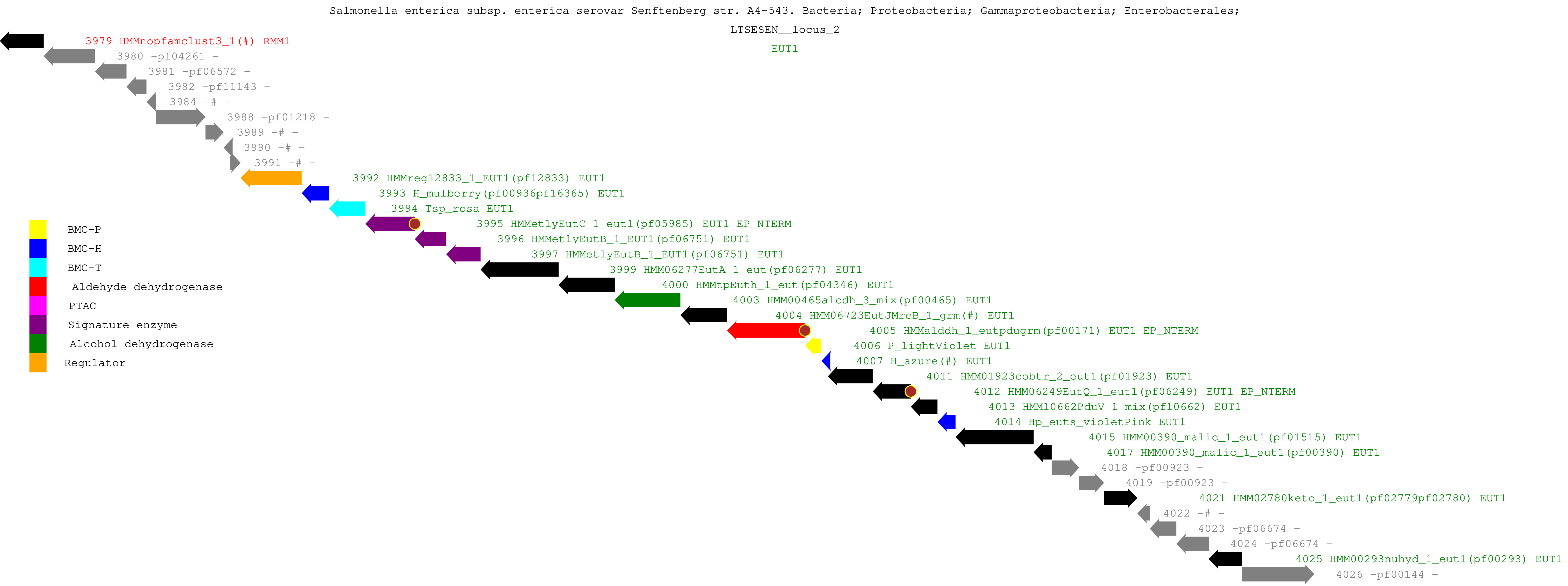
Salmonella enterica subsp. enterica serovar Senftenberg str. A4-543. Bacteria; Proteobacteria; Gammaproteobacteria; Enterobacterales;
LTSESEN__locus_2
EUT1
3979 HMMnopfamclust3_1(#) RMM1
3980 -pf04261 -
3981 -pf06572 -
3982 -pf11143 -
3984 -# -
3988 -pf01218 -
3989 -# -
3990 -# -
3991 -# -
3992 HMMreg12833_1_EUT1(pf12833) EUT1
3993 H_mulberry(pf00936pf16365) EUT1
3994 Tsp_rosa EUT1
3995 HMMetlyEutC_1_eut1(pf05985) EUT1 EP_NTERM
3996 HMMetlyEutB_1_EUT1(pf06751) EUT1
3997 HMMetlyEutB_1_EUT1(pf06751) EUT1
3999 HMM06277EutA_1_eut(pf06277) EUT1
4000 HMMtpEuth_1_eut(pf04346) EUT1
4003 HMM00465alcdh_3_mix(pf00465) EUT1
4004 HMM06723EutJMreB_1_grm(#) EUT1
4005 HMMalddh_1_eutpdugrm(pf00171) EUT1 EP_NTERM
4006 P_lightViolet EUT1
4007 H_azure(#) EUT1
4011 HMM01923cobtr_2_eut1(pf01923) EUT1
4012 HMM06249EutQ_1_eut1(pf06249) EUT1 EP_NTERM
4013 HMM10662PduV_1_mix(pf10662) EUT1
4014 Hp_euts_violetPink EUT1
4015 HMM00390_malic_1_eut1(pf01515) EUT1
4017 HMM00390_malic_1_eut1(pf00390) EUT1
4018 -pf00923 -
4019 -pf00923 -
4021 HMM02780keto_1_eut1(pf02779pf02780) EUT1
4022 -# -
4023 -pf06674 -
4024 -pf06674 -
4025 HMM00293nuhyd_1_eut1(pf00293) EUT1
4026 -pf00144 -
BMC-P
BMC-H
BMC-T
Aldehyde dehydrogenase
PTAC
Signature enzyme
Alcohol dehydrogenase
Regulator

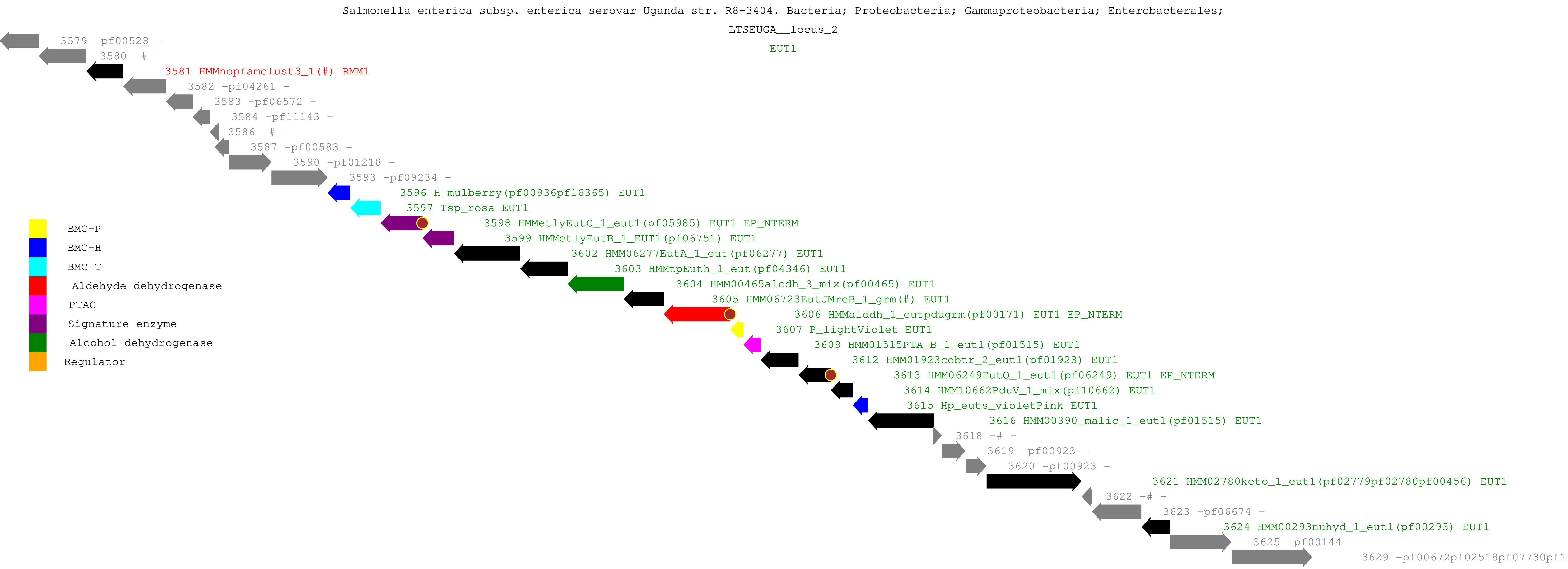
Salmonella enterica subsp. enterica serovar Uganda str. R8-3404. Bacteria; Proteobacteria; Gammaproteobacteria; Enterobacterales;
LTSEUGA__locus_2
EUT1
3579 -pf00528 -
3580 -# -
3581 HMMnopfamclust3_1(#) RMM1
3582 -pf04261 -
3583 -pf06572 -
3584 -pf11143 -
3586 -# -
3587 -pf00583 -
3590 -pf01218 -
3593 -pf09234 -
3596 H_mulberry(pf00936pf16365) EUT1
3597 Tsp_rosa EUT1
3598 HMMetlyEutC_1_eut1(pf05985) EUT1 EP_NTERM
3599 HMMetlyEutB_1_EUT1(pf06751) EUT1
3602 HMM06277EutA_1_eut(pf06277) EUT1
3603 HMMtpEuth_1_eut(pf04346) EUT1
3604 HMM00465alcdh_3_mix(pf00465) EUT1
3605 HMM06723EutJMreB_1_grm(#) EUT1
3606 HMMalddh_1_eutpdugrm(pf00171) EUT1 EP_NTERM
3607 P_lightViolet EUT1
3609 HMM01515PTA_B_1_eut1(pf01515) EUT1
3612 HMM01923cobtr_2_eut1(pf01923) EUT1
3613 HMM06249EutQ_1_eut1(pf06249) EUT1 EP_NTERM
3614 HMM10662PduV_1_mix(pf10662) EUT1
3615 Hp_euts_violetPink EUT1
3616 HMM00390_malic_1_eut1(pf01515) EUT1
3618 -# -
3619 -pf00923 -
3620 -pf00923 -
3621 HMM02780keto_1_eut1(pf02779pf02780pf00456) EUT1
3622 -# -
3623 -pf06674 -
3624 HMM00293nuhyd_1_eut1(pf00293) EUT1
3625 -pf00144 -
3629 -pf00672pf02518pf07730pf1
BMC-P
BMC-H
BMC-T
Aldehyde dehydrogenase
PTAC
Signature enzyme
Alcohol dehydrogenase
Regulator

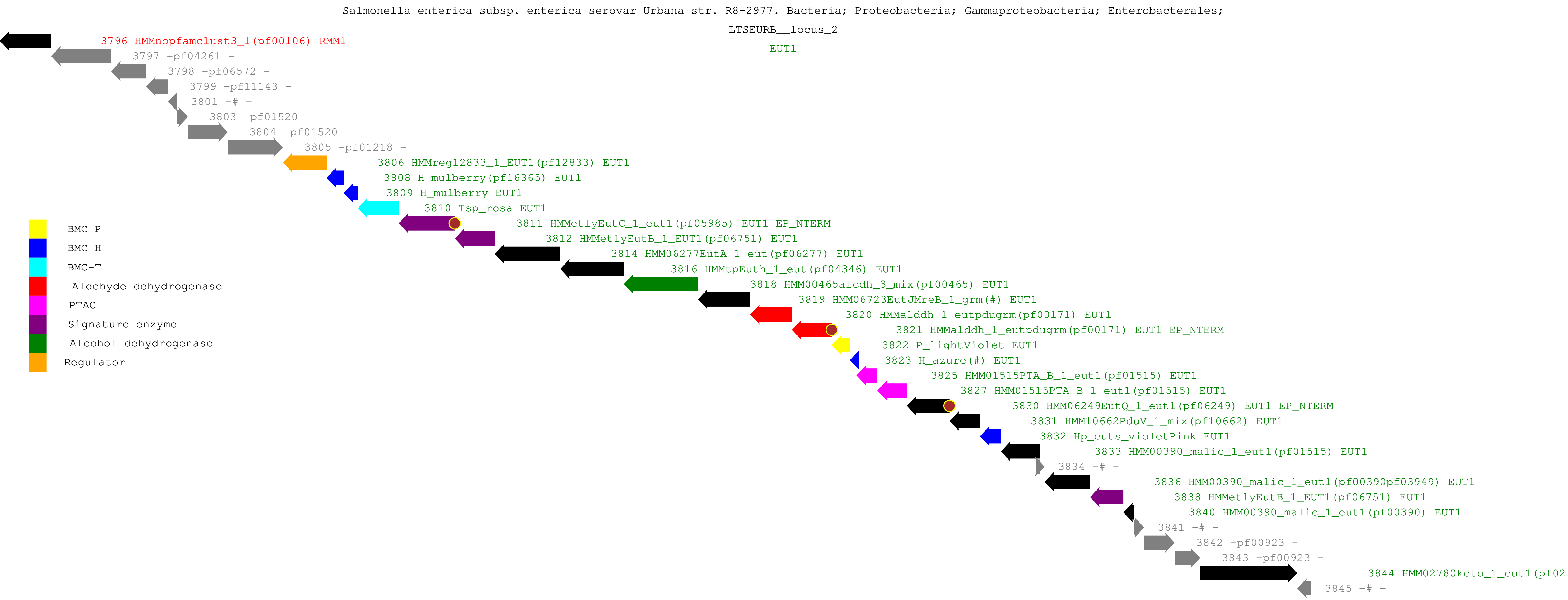
Salmonella enterica subsp. enterica serovar Urbana str. R8-2977. Bacteria; Proteobacteria; Gammaproteobacteria; Enterobacterales;
LTSEURB__locus_2
EUT1
3796 HMMnopfamclust3_1(pf00106) RMM1
3797 -pf04261 -
3798 -pf06572 -
3799 -pf11143 -
3801 -# -
3803 -pf01520 -
3804 -pf01520 -
3805 -pf01218 -
3806 HMMreg12833_1_EUT1(pf12833) EUT1
3808 H_mulberry(pf16365) EUT1
3809 H_mulberry EUT1
3810 Tsp_rosa EUT1
3811 HMMetlyEutC_1_eut1(pf05985) EUT1 EP_NTERM
3812 HMMetlyEutB_1_EUT1(pf06751) EUT1
3814 HMM06277EutA_1_eut(pf06277) EUT1
3816 HMMtpEuth_1_eut(pf04346) EUT1
3818 HMM00465alcdh_3_mix(pf00465) EUT1
3819 HMM06723EutJMreB_1_grm(#) EUT1
3820 HMMalddh_1_eutpdugrm(pf00171) EUT1
3821 HMMalddh_1_eutpdugrm(pf00171) EUT1 EP_NTERM
3822 P_lightViolet EUT1
3823 H_azure(#) EUT1
3825 HMM01515PTA_B_1_eut1(pf01515) EUT1
3827 HMM01515PTA_B_1_eut1(pf01515) EUT1
3830 HMM06249EutQ_1_eut1(pf06249) EUT1 EP_NTERM
3831 HMM10662PduV_1_mix(pf10662) EUT1
3832 Hp_euts_violetPink EUT1
3833 HMM00390_malic_1_eut1(pf01515) EUT1
3834 -# -
3836 HMM00390_malic_1_eut1(pf00390pf03949) EUT1
3838 HMMetlyEutB_1_EUT1(pf06751) EUT1
3840 HMM00390_malic_1_eut1(pf00390) EUT1
3841 -# -
3842 -pf00923 -
3843 -pf00923 -
3844 HMM02780keto_1_eut1(pf02
3845 -# -
BMC-P
BMC-H
BMC-T
Aldehyde dehydrogenase
PTAC
Signature enzyme
Alcohol dehydrogenase
Regulator

Salmonella enterica subsp. enterica serovar Wandsworth str. A4-580. Bacteria; Proteobacteria; Gammaproteobacteria; Enterobacterales;

LTSEWAN__locus_2

EUT1

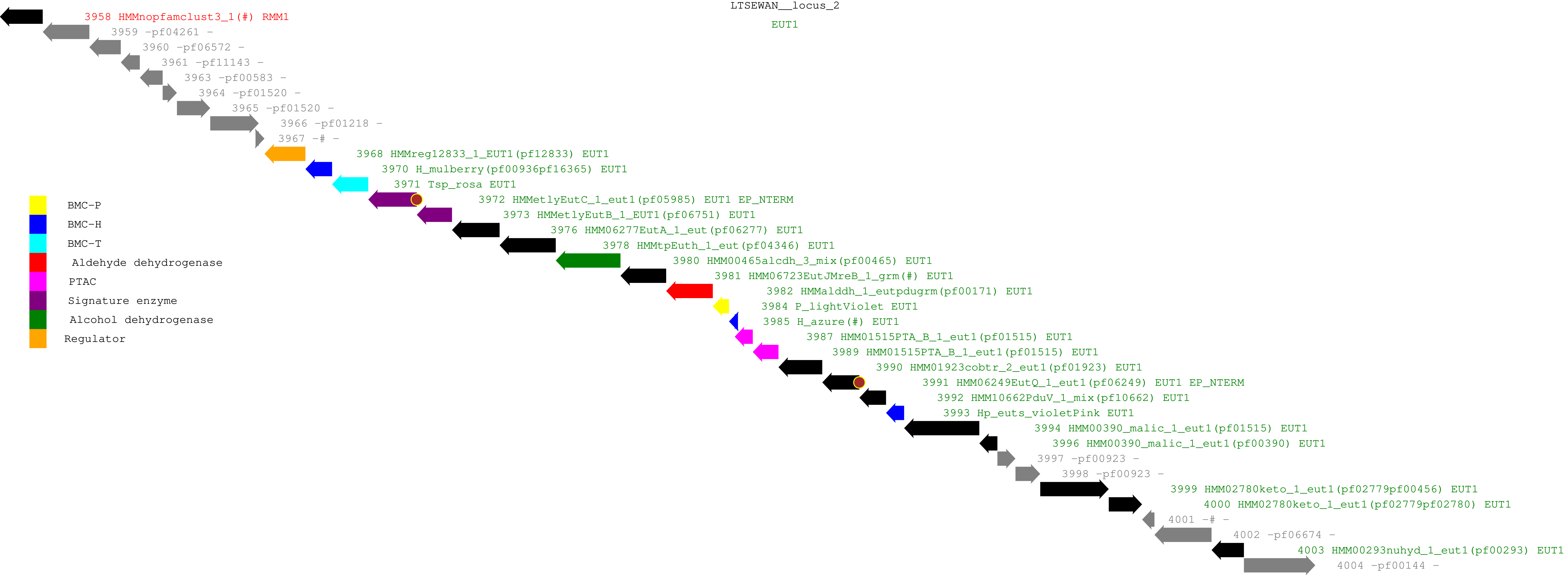

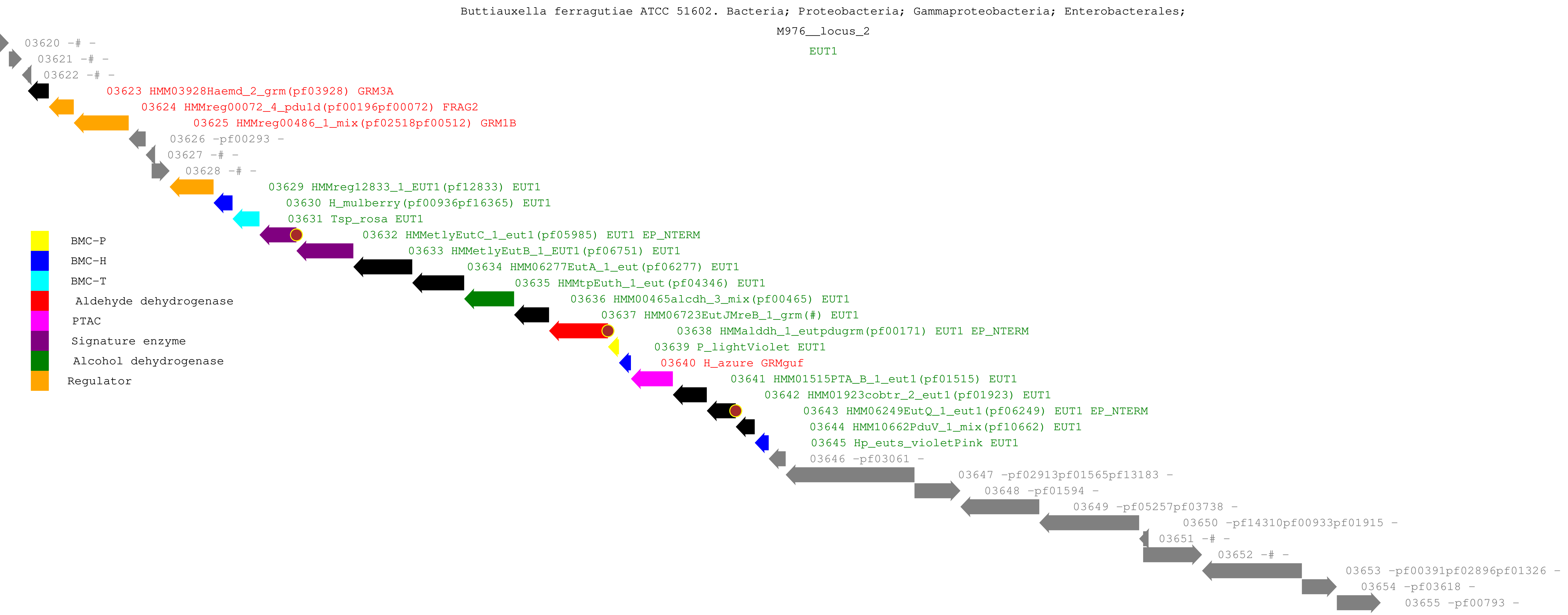

Buttiauxella ferragutiae ATCC 51602. Bacteria; Proteobacteria; Gammaproteobacteria; Enterobacterales;
M976__locus_2
EUT1
03620 -# -
03621 -# -
03622 -# -
03623 HMM03928Haemd_2_grm(pf03928) GRM3A
03624 HMMreg00072_4_pduld(pf00196pf00072) FRAG2
03625 HMMreg00486_1_mix(pf02518pf00512) GRM1B
03626 -pf00293 -
03627 -# -
03628 -# -
03629 HMMreg12833_1_EUT1(pf12833) EUT1
03630 H_mulberry(pf00936pf16365) EUT1
03631 Tsp_rosa EUT1
03632 HMMetlyEutC_1_eut1(pf05985) EUT1 EP_NTERM
03633 HMMetlyEutB_1_EUT1(pf06751) EUT1
03634 HMM06277EutA_1_eut(pf06277) EUT1
03635 HMMtpEuth_1_eut(pf04346) EUT1
03636 HMM00465alcdh_3_mix(pf00465) EUT1
03637 HMM06723EutJMreB_1_grm(#) EUT1
03638 HMMalddh_1_eutpdugrm(pf00171) EUT1 EP_NTERM
03639 P_lightViolet EUT1
03640 H_azure GRMguf
03641 HMM01515PTA_B_1_eut1(pf01515) EUT1
03642 HMM01923cobtr_2_eut1(pf01923) EUT1
03643 HMM06249EutQ_1_eut1(pf06249) EUT1 EP_NTERM
03644 HMM10662PduV_1_mix(pf10662) EUT1
03645 Hp_euts_violetPink EUT1
03646 -pf03061 -
03647 -pf02913pf01565pf13183 -
03648 -pf01594 -
03649 -pf05257pf03738 -
03650 -pf14310pf00933pf01915 -
03651 -# -
03652 -# -
03653 -pf00391pf02896pf01326 -
03654 -pf03618 -
03655 -pf00793 -
BMC-P
BMC-H
BMC-T
Aldehyde dehydrogenase
PTAC
Signature enzyme
Alcohol dehydrogenase
Regulator

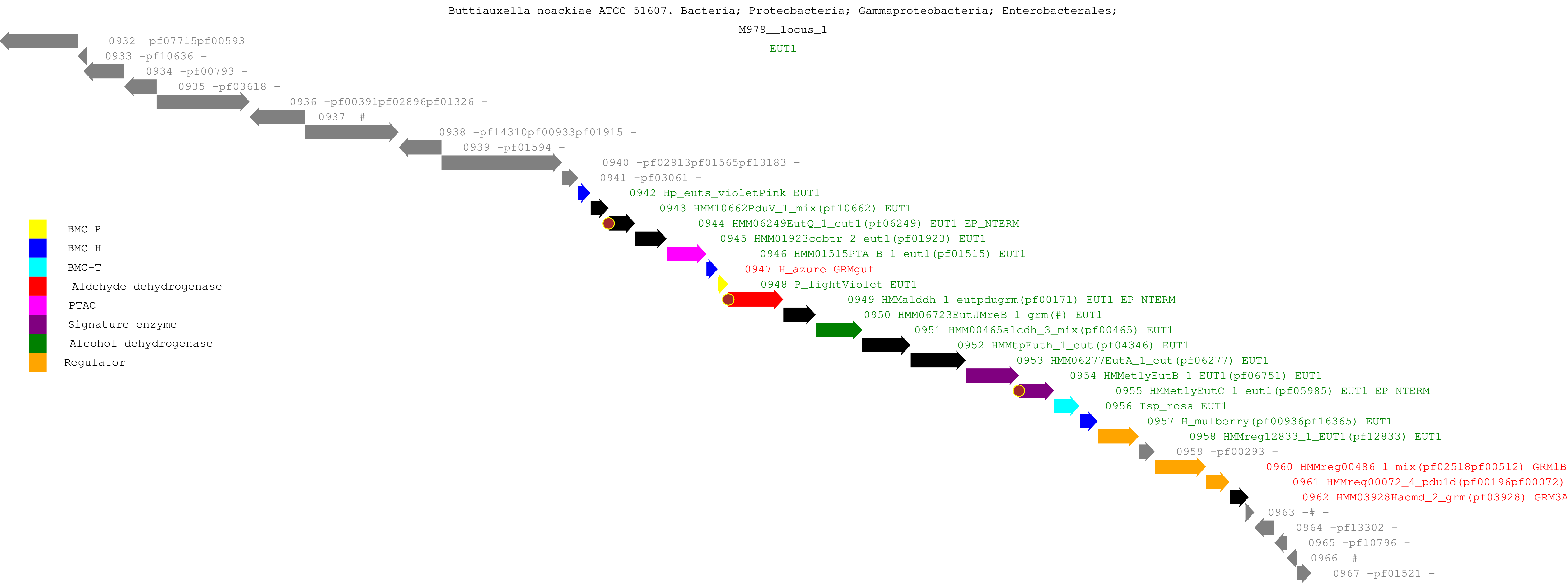

Buttiauxella noackiae ATCC 51607. Bacteria; Proteobacteria; Gammaproteobacteria; Enterobacterales;
M979__locus_1
EUT1
0932 -pf07715pf00593 -
0933 -pf10636 -
0934 -pf00793 -
0935 -pf03618 -
0936 -pf00391pf02896pf01326 -
0937 -# -
0938 -pf14310pf00933pf01915 -
0939 -pf01594 -
0940 -pf02913pf01565pf13183 -
0941 -pf03061 -
0942 Hp_euts_violetPink EUT1
0943 HMM10662PduV_1_mix(pf10662) EUT1
0944 HMM06249EutQ_1_eut1(pf06249) EUT1 EP_NTERM
0945 HMM01923cobtr_2_eut1(pf01923) EUT1
0946 HMM01515PTA_B_1_eut1(pf01515) EUT1
0947 H_azure GRMguf
0948 P_lightViolet EUT1
0949 HMMalddh_1_eutpdugrm(pf00171) EUT1 EP_NTERM
0950 HMM06723EutJMreB_1_grm(#) EUT1
0951 HMM00465alcdh_3_mix(pf00465) EUT1
0952 HMMtpEuth_1_eut(pf04346) EUT1
0953 HMM06277EutA_1_eut(pf06277) EUT1
0954 HMMetlyEutB_1_EUT1(pf06751) EUT1
0955 HMMetlyEutC_1_eut1(pf05985) EUT1 EP_NTERM
0956 Tsp_rosa EUT1
0957 H_mulberry(pf00936pf16365) EUT1
0958 HMMreg12833_1_EUT1(pf12833) EUT1
0959 -pf00293 -
0960 HMMreg00486_1_mix(pf02518pf00512) GRM1B
0961 HMMreg00072_4_pdu1d(pf00196pf00072)
0962 HMM03928Haemd_2_grm(pf03928) GRM3A
0963 -# -
0964 -pf13302 -
0965 -pf10796 -
0966 -# -
0967 -pf01521 -
BMC-P
BMC-H
BMC-T
Aldehyde dehydrogenase
PTAC
Signature enzyme
Alcohol dehydrogenase
Regulator

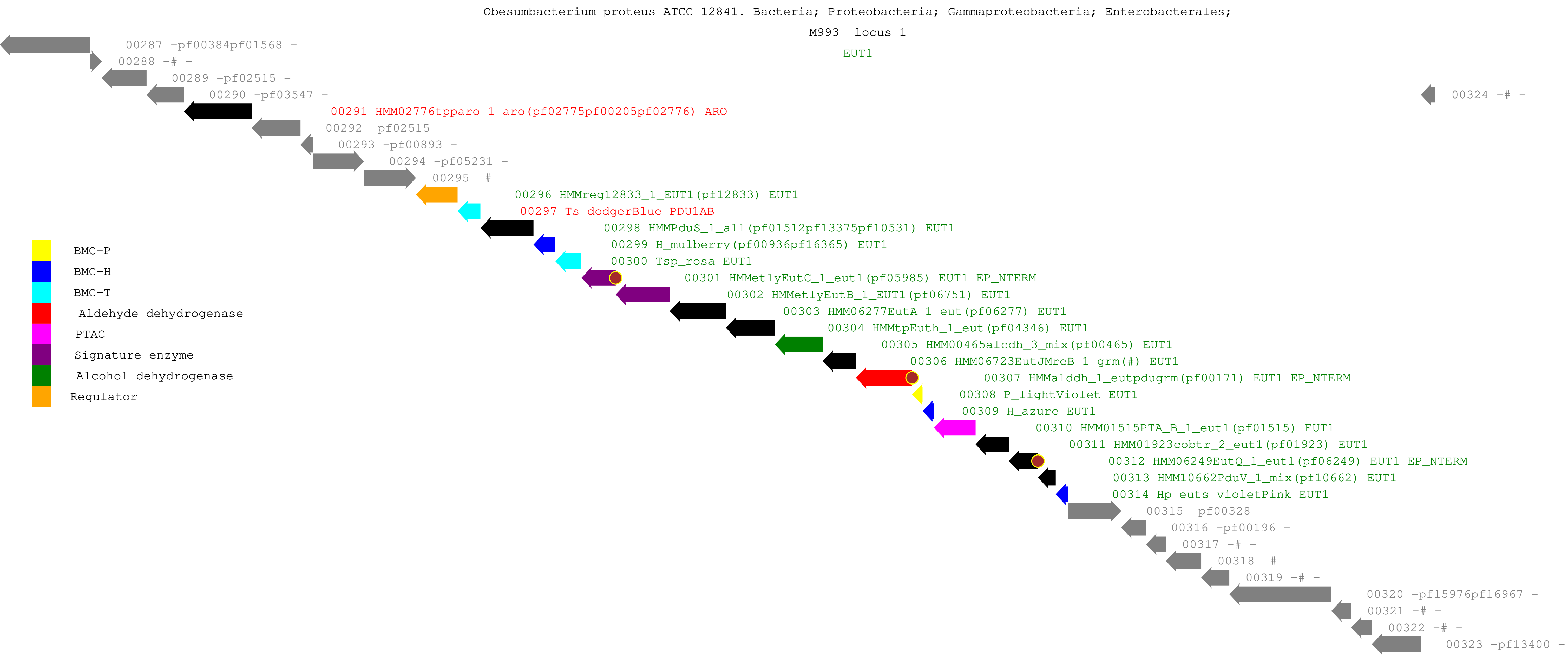
Obesumbacterium proteus ATCC 12841. Bacteria; Proteobacteria; Gammaproteobacteria; Enterobacterales;
M993__locus_1
EUT1
00287 -pf00384pf01568 -
00288 -# -
00289 -pf02515 -
00290 -pf03547 -
00291 HMM02776tpparo_1_aro(pf02775pf00205pf02776) ARO
00292 -pf02515 -
00293 -pf00893 -
00294 -pf05231 -
00295 -# -
00296 HMMreg12833_1_EUT1(pf12833) EUT1
00297 Ts_dodgerBlue PDU1AB
00298 HMMPduS_1_all(pf01512pf13375pf10531) EUT1
00299 H_mulberry(pf00936pf16365) EUT1
00300 Tsp_rosa EUT1
00301 HMMetlyEutC_1_eut1(pf05985) EUT1 EP_NTERM
00302 HMMetlyEutB_1_EUT1(pf06751) EUT1
00303 HMM06277EutA_1_eut(pf06277) EUT1
00304 HMMtpEuth_1_eut(pf04346) EUT1
00305 HMM00465alcdh_3_mix(pf00465) EUT1
00306 HMM06723EutJMreB_1_grm(#) EUT1
00307 HMMalddh_1_eutpdugrm(pf00171) EUT1 EP_NTERM
00308 P_lightViolet EUT1
00309 H_azure EUT1
00310 HMM01515PTA_B_1_eut1(pf01515) EUT1
00311 HMM01923cobtr_2_eut1(pf01923) EUT1
00312 HMM06249EutQ_1_eut1(pf06249) EUT1 EP_NTERM
00313 HMM10662PduV_1_mix(pf10662) EUT1
00314 Hp_euts_violetPink EUT1
00315 -pf00328 -
00316 -pf00196 -
00317 -# -
00318 -# -
00319 -# -
00320 -pf15976pf16967 -
00321 -# -
00322 -# -
00323 -pf13400 -
00324 -# -
BMC-P
BMC-H
BMC-T
Aldehyde dehydrogenase
PTAC
Signature enzyme
Alcohol dehydrogenase
Regulator

Marinobacter hydrocarbonoclasticus (strain ATCC 700491 / DSM 11845 / Bacteria; Proteobacteria; Gammaproteobacteria; Alteromonadales;

Maqu__locus_1

EUT1

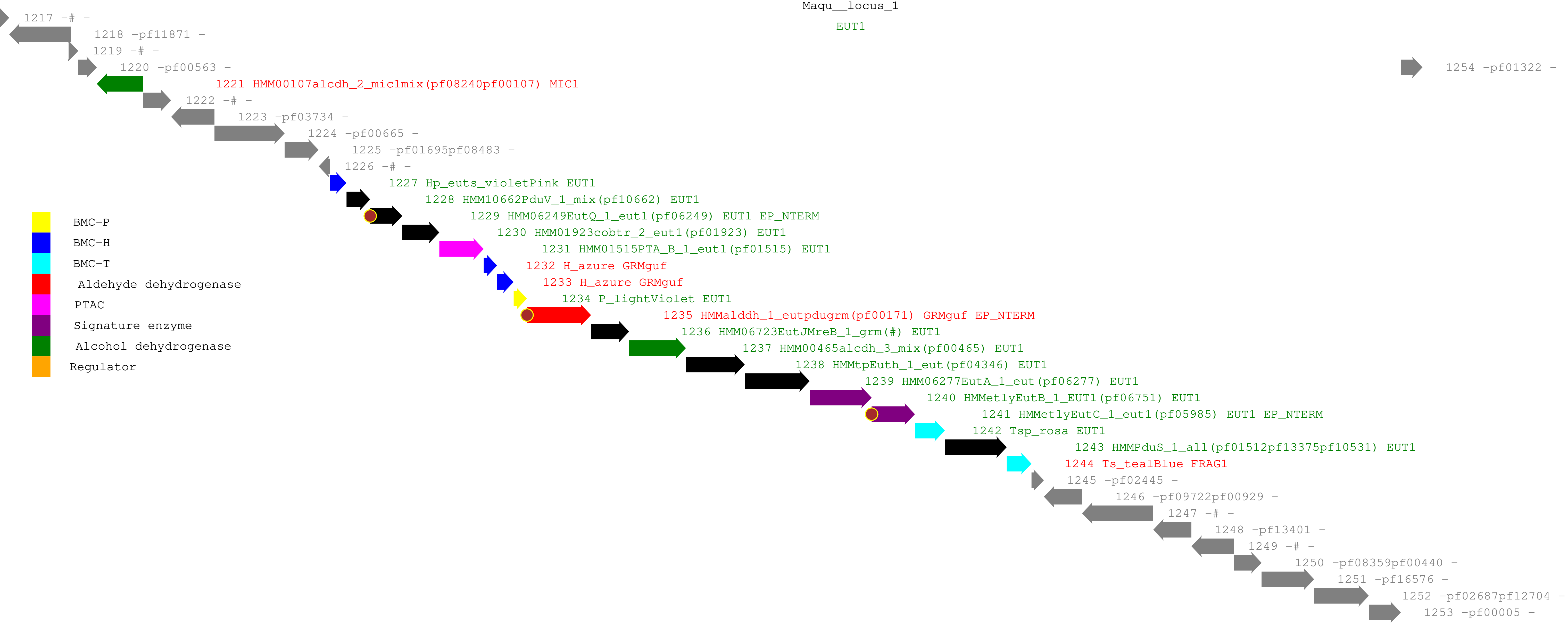

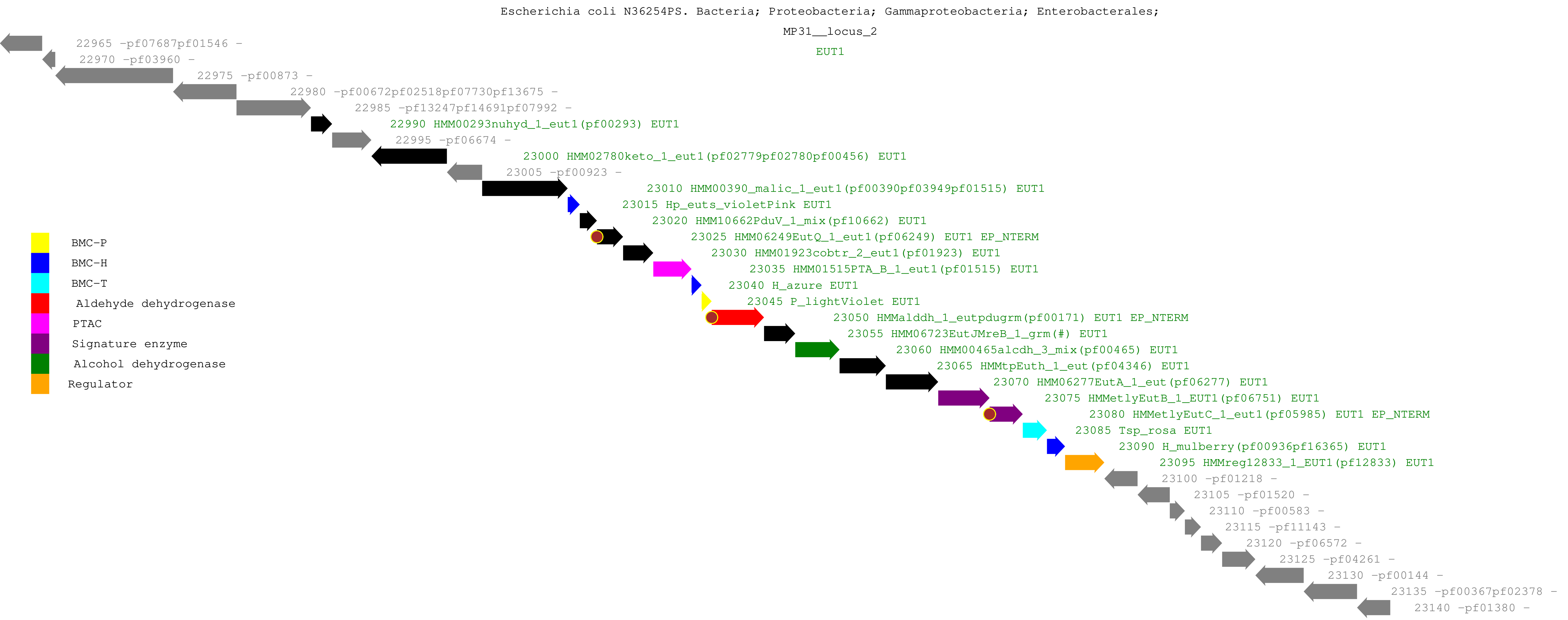
Escherichia coli N36254PS. Bacteria; Proteobacteria; Gammaproteobacteria; Enterobacterales;
MP31__locus_2
EUT1
22965 -pf07687pf01546 -
22970 -pf03960 -
22975 -pf00873 -
22980 -pf00672pf02518pf07730pf13675 -
22985 -pf13247pf14691pf07992 -
22990 HMM00293nuhyd_1_eut1(pf00293) EUT1
22995 -pf06674 -
23000 HMM02780keto_1_eut1(pf02779pf02780pf00456) EUT1
23005 -pf00923 -
23010 HMM00390_malic_1_eut1(pf00390pf03949pf01515) EUT1
23015 Hp_euts_violetPink EUT1
23020 HMM10662PduV_1_mix(pf10662) EUT1
23025 HMM06249EutQ_1_eut1(pf06249) EUT1 EP_NTERM
23030 HMM01923cobtr_2_eut1(pf01923) EUT1
23035 HMM01515PTA_B_1_eut1(pf01515) EUT1
23040 H_azure EUT1
23045 P_lightViolet EUT1
23050 HMMalddh_1_eutpdugrm(pf00171) EUT1 EP_NTERM
23055 HMM06723EutJMreB_1_grm(#) EUT1
23060 HMM00465alcdh_3_mix(pf00465) EUT1
23065 HMMtpEuth_1_eut(pf04346) EUT1
23070 HMM06277EutA_1_eut(pf06277) EUT1
23075 HMMetlyEutB_1_EUT1(pf06751) EUT1
23080 HMMetlyEutC_1_eut1(pf05985) EUT1 EP_NTERM
23085 Tsp_rosa EUT1
23090 H_mulberry(pf00936pf16365) EUT1
23095 HMMreg12833_1_EUT1(pf12833) EUT1
23100 -pf01218 -
23105 -pf01520 -
23110 -pf00583 -
23115 -pf11143 -
23120 -pf06572 -
23125 -pf04261 -
23130 -pf00144 -
23135 -pf00367pf02378 -
23140 -pf01380 -
BMC-P
BMC-H
BMC-T
Aldehyde dehydrogenase
PTAC
Signature enzyme
Alcohol dehydrogenase
Regulator

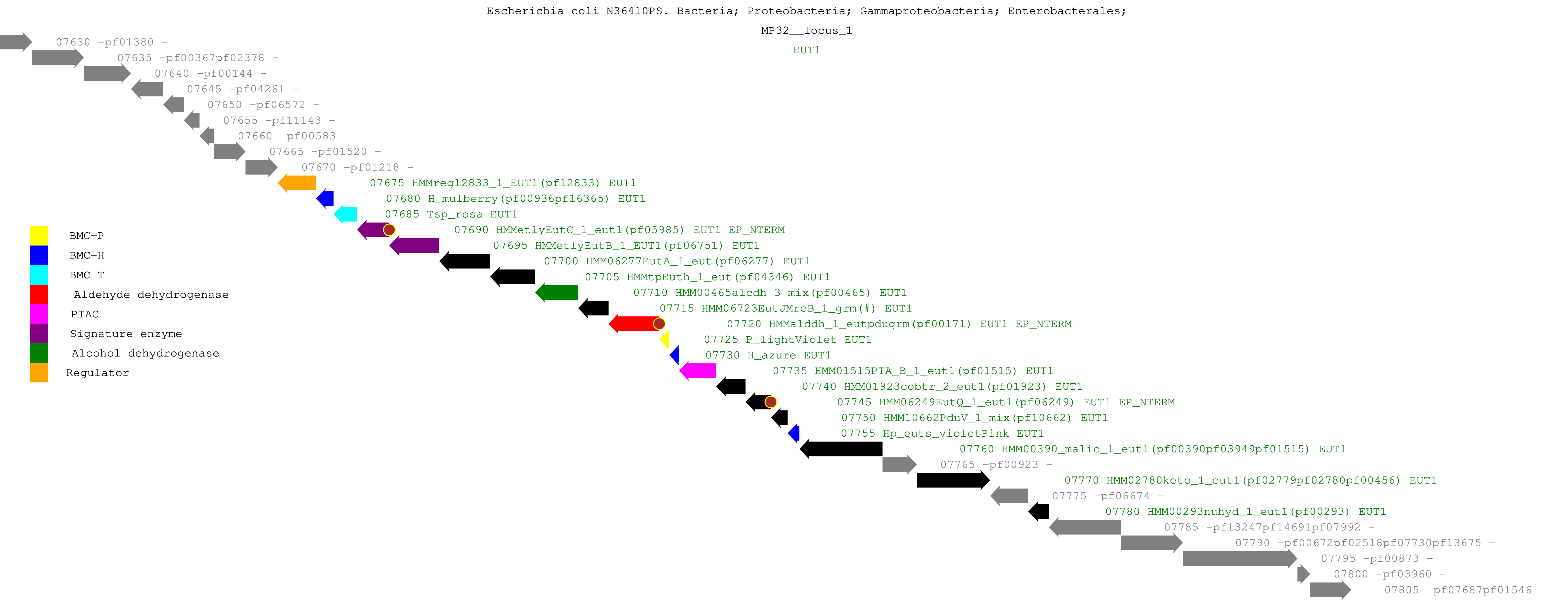

Escherichia coli N36410PS. Bacteria; Proteobacteria; Gammaproteobacteria; Enterobacterales;
MP32__locus_1
EUT1
07630 -pf01380 -
07635 -pf00367pf02378 -
07640 -pf00144 -
07645 -pf04261 -
07650 -pf06572 -
07655 -pf11143 -
07660 -pf00583 -
07665 -pf01520 -
07670 -pf01218 -
07675 HMMreg12833_1_EUT1(pf12833) EUT1
07680 H_mulberry(pf00936pf16365) EUT1
07685 Tsp_rosa EUT1
07690 HMMetlyEutC_1_eut1(pf05985) EUT1 EP_NTERM
07695 HMMetlyEutB_1_EUT1(pf06751) EUT1
07700 HMM06277EutA_1_eut(pf06277) EUT1
07705 HMMtpEuth_1_eut(pf04346) EUT1
07710 HMM00465alcdh_3_mix(pf00465) EUT1
07715 HMM06723EutJMreB_1_grm(#) EUT1
07720 HMMalddh_1_eutpdugrm(pf00171) EUT1 EP_NTERM
07725 P_lightViolet EUT1
07730 H_azure EUT1
07735 HMM01515PTA_B_1_eut1(pf01515) EUT1
07740 HMM01923cobtr_2_eut1(pf01923) EUT1
07745 HMM06249EutQ_1_eut1(pf06249) EUT1 EP_NTERM
07750 HMM10662PduV_1_mix(pf10662) EUT1
07755 Hp_euts_violetPink EUT1
07760 HMM00390_malic_1_eut1(pf00390pf03949pf01515) EUT1
07765 -pf00923 -
07770 HMM02780keto_1_eut1(pf02779pf02780pf00456) EUT1
07775 -pf06674 -
07780 HMM00293nuhyd_1_eut1(pf00293) EUT1
07785 -pf13247pf14691pf07992 -
07790 -pf00672pf02518pf07730pf13675 -
07795 -pf00873 -
07800 -pf03960 -
07805 -pf07687pf01546 -
BMC-P
BMC-H
BMC-T
Aldehyde dehydrogenase
PTAC
Signature enzyme
Alcohol dehydrogenase
Regulator

Escherichia coli N37058PS. Bacteria; Proteobacteria; Gammaproteobacteria; Enterobacterales;

MP33__locus_1

EUT1

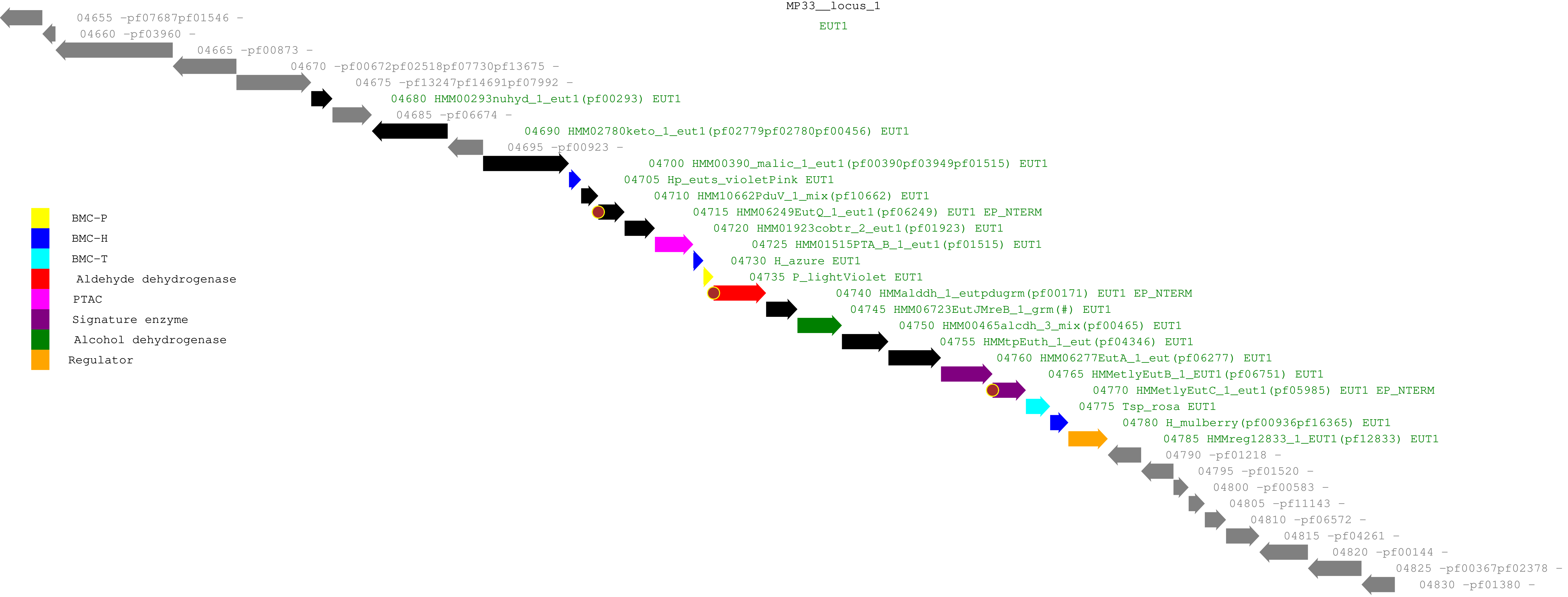

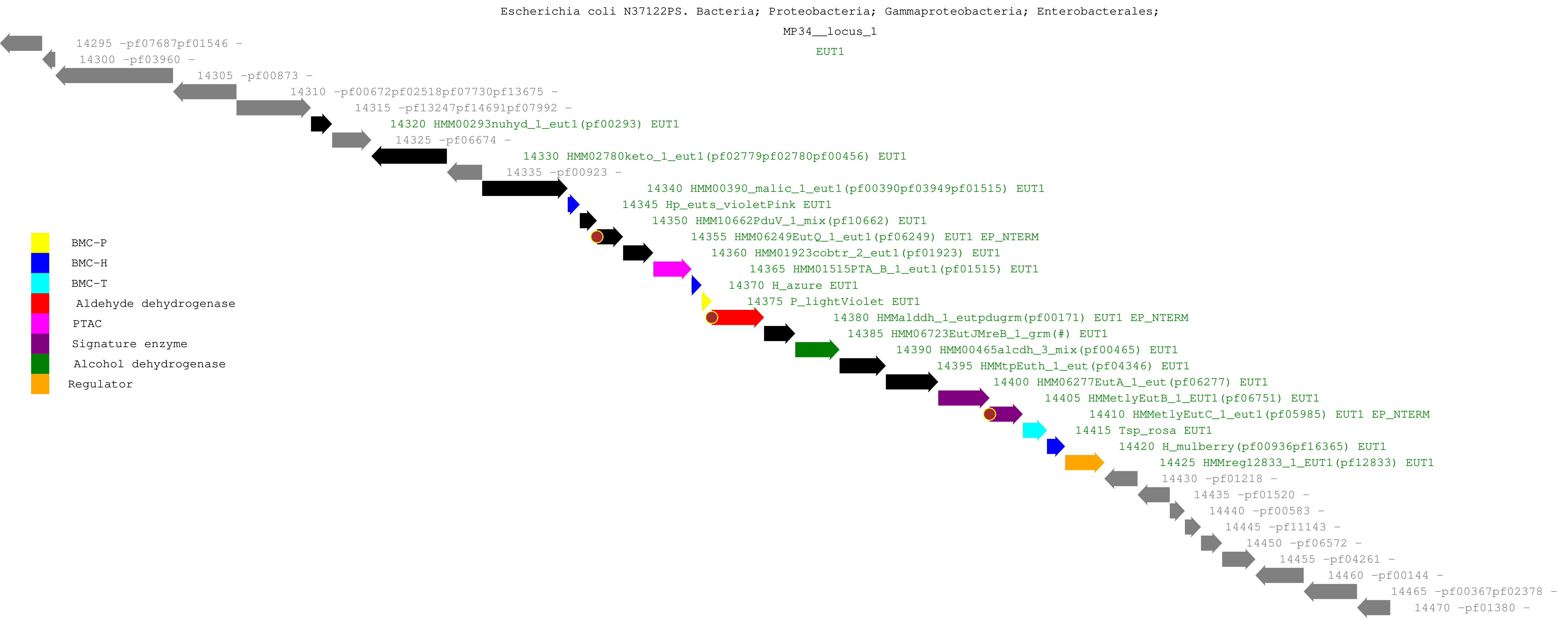
Escherichia coli N37122PS. Bacteria; Proteobacteria; Gammaproteobacteria; Enterobacterales;
MP34__locus_1
EUT1
14295 -pf07687pf01546 -
14300 -pf03960 -
14305 -pf00873 -
14310 -pf00672pf02518pf07730pf13675 -
14315 -pf13247pf14691pf07992 -
14320 HMM00293nuhyd_1_eut1(pf00293) EUT1
14325 -pf06674 -
14330 HMM02780keto_1_eut1(pf02779pf02780pf00456) EUT1
14335 -pf00923 -
14340 HMM00390_malic_1_eut1(pf00390pf03949pf01515) EUT1
14345 Hp_euts_violetPink EUT1
14350 HMM10662PduV_1_mix(pf10662) EUT1
14355 HMM06249EutQ_1_eut1(pf06249) EUT1 EP_NTERM
14360 HMM01923cobtr_2_eut1(pf01923) EUT1
14365 HMM01515PTA_B_1_eut1(pf01515) EUT1
14370 H_azure EUT1
14375 P_lightViolet EUT1
14380 HMMalddh_1_eutpdugrm(pf00171) EUT1 EP_NTERM
14385 HMM06723EutJMreB_1_grm(#) EUT1
14390 HMM00465alcdh_3_mix(pf00465) EUT1
14395 HMMtpEuth_1_eut(pf04346) EUT1
14400 HMM06277EutA_1_eut(pf06277) EUT1
14405 HMMetlyEutB_1_EUT1(pf06751) EUT1
14410 HMMetlyEutC_1_eut1(pf05985) EUT1 EP_NTERM
14415 Tsp_rosa EUT1
14420 H_mulberry(pf00936pf16365) EUT1
14425 HMMreg12833_1_EUT1(pf12833) EUT1
14430 -pf01218 -
14435 -pf01520 -
14440 -pf00583 -
14445 -pf11143 -
14450 -pf06572 -
14455 -pf04261 -
14460 -pf00144 -
14465 -pf00367pf02378 -
14470 -pf01380 -
BMC-P
BMC-H
BMC-T
Aldehyde dehydrogenase
PTAC
Signature enzyme
Alcohol dehydrogenase
Regulator

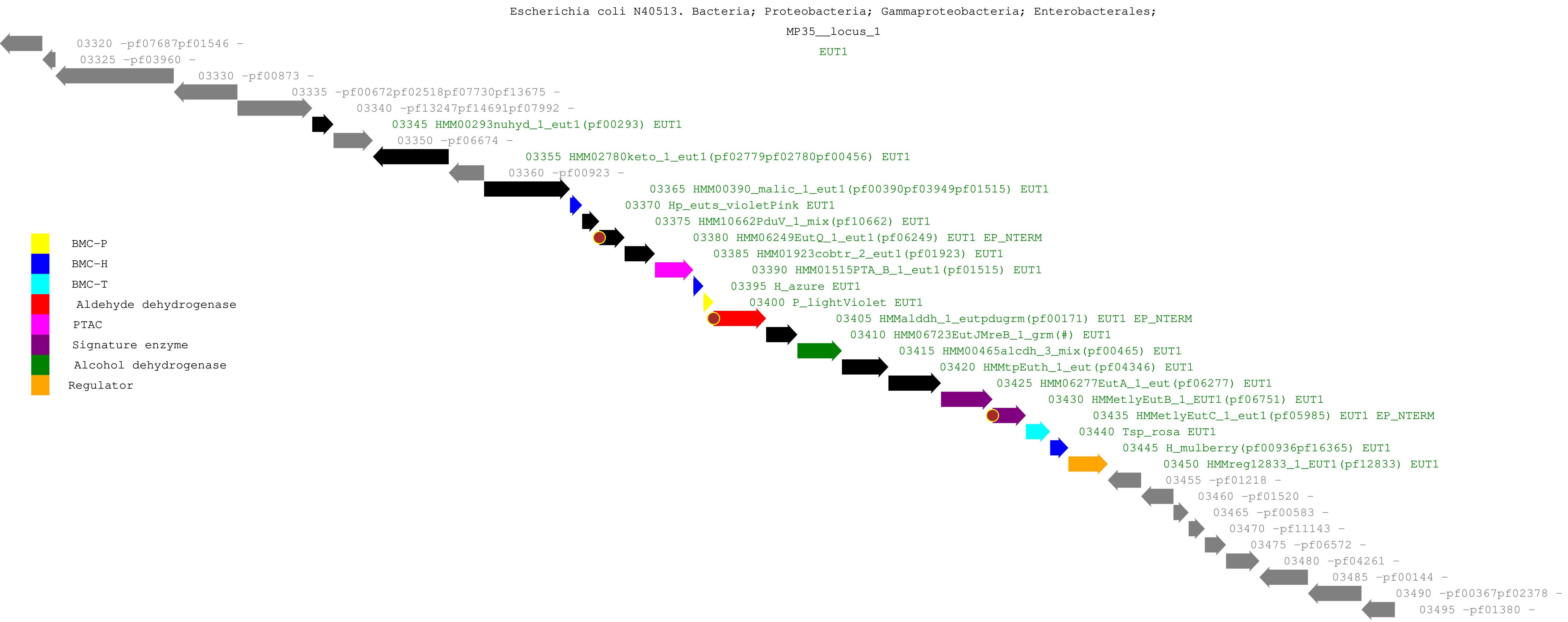
Escherichia coli N40513. Bacteria; Proteobacteria; Gammaproteobacteria; Enterobacterales;
MP35__locus_1
EUT1
03320 -pf07687pf01546 -
03325 -pf03960 -
03330 -pf00873 -
03335 -pf00672pf02518pf07730pf13675 -
03340 -pf13247pf14691pf07992 -
03345 HMM00293nuhyd_1_eut1(pf00293) EUT1
03350 -pf06674 -
03355 HMM02780keto_1_eut1(pf02779pf02780pf00456) EUT1
03360 -pf00923 -
03365 HMM00390_malic_1_eut1(pf00390pf03949pf01515) EUT1
03370 Hp_euts_violetPink EUT1
03375 HMM10662PduV_1_mix(pf10662) EUT1
03380 HMM06249EutQ_1_eut1(pf06249) EUT1 EP_NTERM
03385 HMM01923cobtr_2_eut1(pf01923) EUT1
03390 HMM01515PTA_B_1_eut1(pf01515) EUT1
03395 H_azure EUT1
03400 P_lightViolet EUT1
03405 HMMalddh_1_eutpdugrm(pf00171) EUT1 EP_NTERM
03410 HMM06723EutJMreB_1_grm(#) EUT1
03415 HMM00465alcdh_3_mix(pf00465) EUT1
03420 HMMtpEuth_1_eut(pf04346) EUT1
03425 HMM06277EutA_1_eut(pf06277) EUT1
03430 HMMetlyEutB_1_EUT1(pf06751) EUT1
03435 HMMetlyEutC_1_eut1(pf05985) EUT1 EP_NTERM
03440 Tsp_rosa EUT1
03445 H_mulberry(pf00936pf16365) EUT1
03450 HMMreg12833_1_EUT1(pf12833) EUT1
03455 -pf01218 -
03460 -pf01520 -
03465 -pf00583 -
03470 -pf11143 -
03475 -pf06572 -
03480 -pf04261 -
03485 -pf00144 -
03490 -pf00367pf02378 -
03495 -pf01380 -
BMC-P
BMC-H
BMC-T
Aldehyde dehydrogenase
PTAC
Signature enzyme
Alcohol dehydrogenase
Regulator

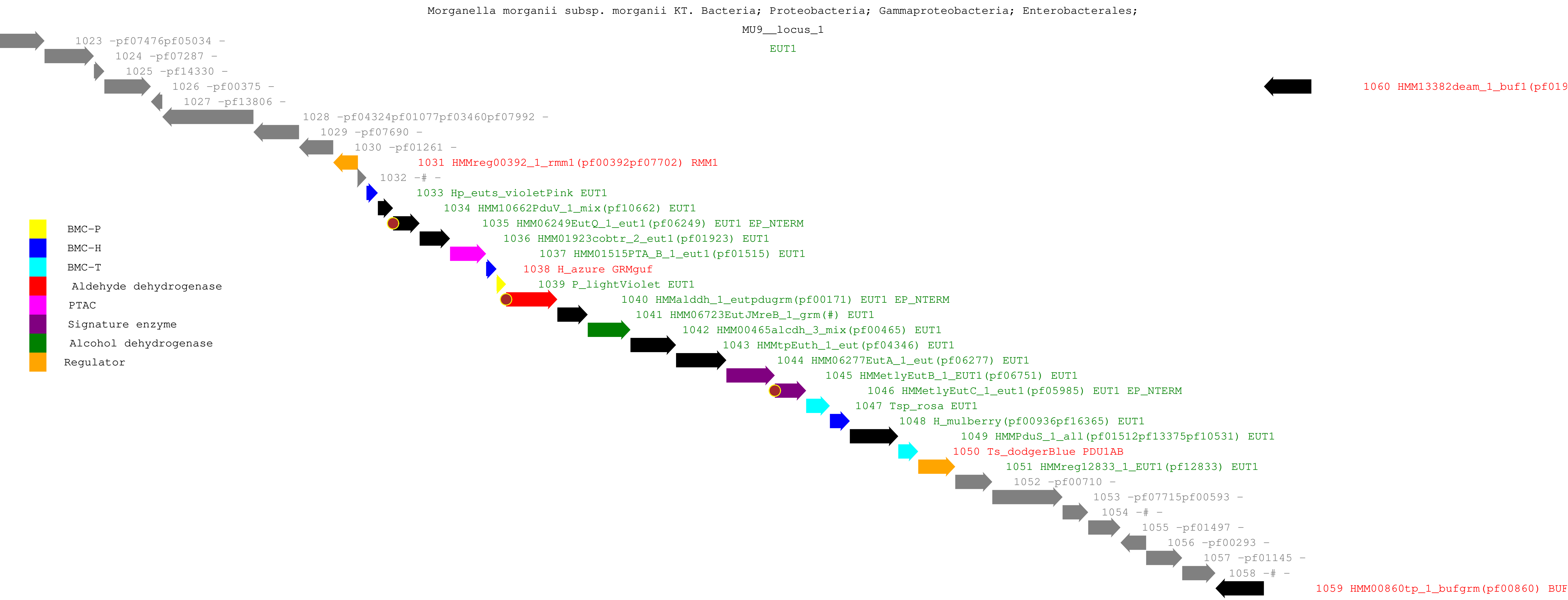
Morganella morganii subsp. morganii KT. Bacteria; Proteobacteria; Gammaproteobacteria; Enterobacterales;
MU9__locus_1
EUT1
1023 -pf07476pf05034 -
1024 -pf07287 -
1025 -pf14330 -
1026 -pf00375 -
1027 -pf13806 -
1028 -pf04324pf01077pf03460pf07992 -
1029 -pf07690 -
1030 -pf01261 -
1031 HMMreg00392_1_rmm1(pf00392pf07702) RMM1
1032 -# -
1033 Hp_euts_violetPink EUT1
1034 HMM10662PduV_1_mix(pf10662) EUT1
1035 HMM06249EutQ_1_eut1(pf06249) EUT1 EP_NTERM
1036 HMM01923cobtr_2_eut1(pf01923) EUT1
1037 HMM01515PTA_B_1_eut1(pf01515) EUT1
1038 H_azure GRMguf
1039 P_lightViolet EUT1
1040 HMMaldh_1_eutpdugrm(pf00171) EUT1 EP_NTERM
1041 HMM06723EutJMreB_1_grm(#) EUT1
1042 HMM00465alcdh_3_mix(pf00465) EUT1
1043 HMMtpEuth_1_eut(pf04346) EUT1
1044 HMM06277EutA_1_eut(pf06277) EUT1
1045 HMMetlyEutB_1_EUT1(pf06751) EUT1
1046 HMMetlyEutC_1_eut1(pf05985) EUT1 EP_NTERM
1047 Tsp_rosa EUT1
1048 H_mulberry(pf00936pf16365) EUT1
1049 HMMPduS_1_all(pf01512pf13375pf10531) EUT1
1050 Ts_dodgerBlue PDU1AB
1051 HMMreg12833_1_EUT1(pf12833) EUT1
1052 -pf00710 -
1053 -pf07715pf00593 -
1054 -# -
1055 -pf01497 -
1056 -pf00293 -
1057 -pf01145 -
1058 -# -
1059 HMM00860tp_1_bufgrm(pf00860) BUF
1060 HMM13382deam_1_buf1(pf019
BMC-P
BMC-H
BMC-T
Aldehyde dehydrogenase
PTAC
Signature enzyme
Alcohol dehydrogenase
Regulator

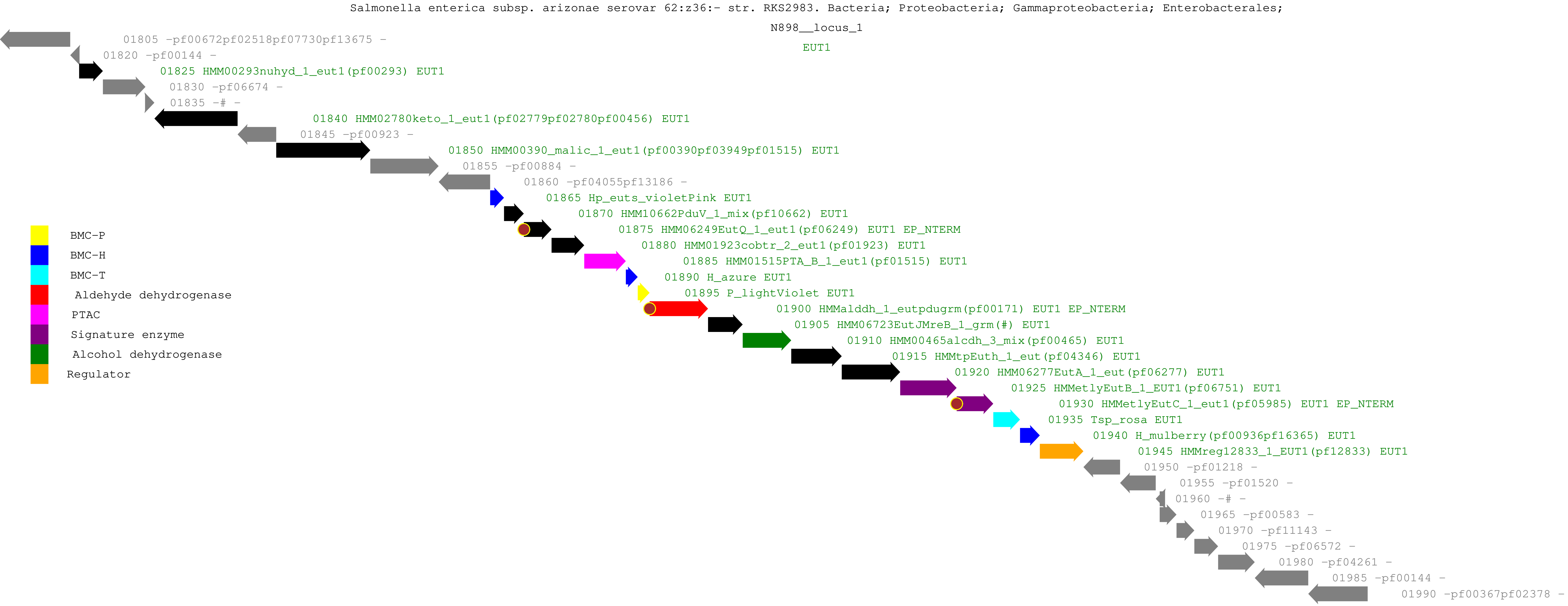
Salmonella enterica subsp. arizonae serovar 62:z36:- str. RKS2983. Bacteria; Proteobacteria; Gammaproteobacteria; Enterobacterales;
N898__locus_1
EUT1
01805 -pf00672pf02518pf07730pf13675 -
01820 -pf00144 -
01825 HMM00293nuhyd_1_eut1(pf00293) EUT1
01830 -pf06674 -
01835 -# -
01840 HMM02780keto_1_eut1(pf02779pf02780pf00456) EUT1
01845 -pf00923 -
01850 HMM00390_malic_1_eut1(pf00390pf03949pf01515) EUT1
01855 -pf00884 -
01860 -pf04055pf13186 -
01865 Hp_euts_violetPink EUT1
01870 HMM10662PduV_1_mix(pf10662) EUT1
01875 HMM06249EutQ_1_eut1(pf06249) EUT1 EP_NTERM
01880 HMM01923cobtr_2_eut1(pf01923) EUT1
01885 HMM01515PTA_B_1_eut1(pf01515) EUT1
01890 H_azure EUT1
01895 P_lightViolet EUT1
01900 HMMalddh_1_eutpdugrm(pf00171) EUT1 EP_NTERM
01905 HMM06723EutJMreB_1_grm(#) EUT1
01910 HMM00465alcdh_3_mix(pf00465) EUT1
01915 HMMtpEuth_1_eut(pf04346) EUT1
01920 HMM06277EutA_1_eut(pf06277) EUT1
01925 HMMetlyEutB_1_EUT1(pf06751) EUT1
01930 HMMetlyEutC_1_eut1(pf05985) EUT1 EP_NTERM
01935 Tsp_rosa EUT1
01940 H_mulberry(pf00936pf16365) EUT1
01945 HMMreg12833_1_EUT1(pf12833) EUT1
01950 -pf01218 -
01955 -pf01520 -
01960 -# -
01965 -pf00583 -
01970 -pf11143 -
01975 -pf06572 -
01980 -pf04261 -
01985 -pf00144 -
01990 -pf00367pf02378 -
BMC-P
BMC-H
BMC-T
Aldehyde dehydrogenase
PTAC
Signature enzyme
Alcohol dehydrogenase
Regulator

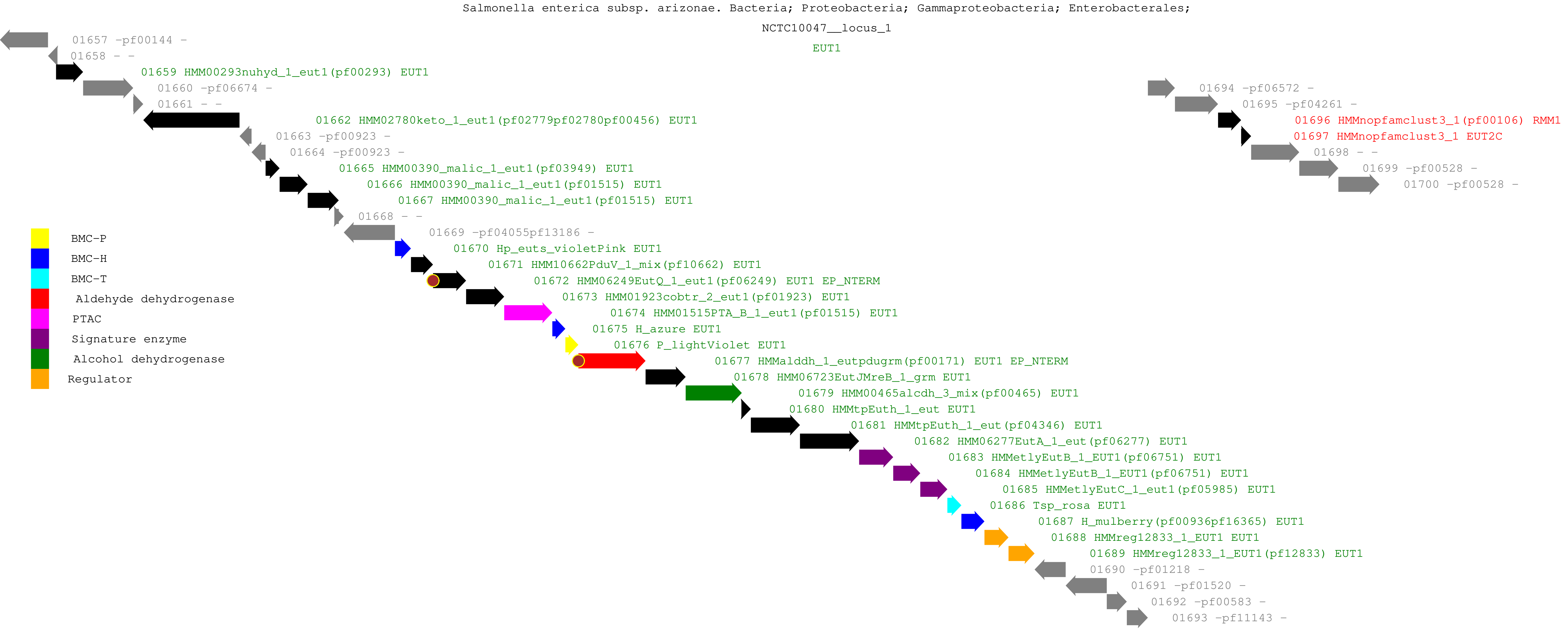
Salmonella enterica subsp. arizonae. Bacteria; Proteobacteria; Gammaproteobacteria; Enterobacterales;
NCTC10047__locus_1
EUT1
01657 -pf00144 -
01658 - -
01659 HMM00293nuhyd_1_eut1(pf00293) EUT1
01660 -pf06674 -
01661 - -
01662 HMM02780keto_1_eut1(pf02779pf02780pf00456) EUT1
01663 -pf00923 -
01664 -pf00923 -
01665 HMM00390_malic_1_eut1(pf03949) EUT1
01666 HMM00390_malic_1_eut1(pf01515) EUT1
01667 HMM00390_malic_1_eut1(pf01515) EUT1
01668 - -
01669 -pf04055pf13186 -
01670 Hp_euts_violetPink EUT1
01671 HMM10662PduV_1_mix(pf10662) EUT1
01672 HMM06249EutQ_1_eut1(pf06249) EUT1 EP_NTERM
01673 HMM01923cobtr_2_eut1(pf01923) EUT1
01674 HMM01515PTA_B_1_eut1(pf01515) EUT1
01675 H_azure EUT1
01676 P_lightViolet EUT1
01677 HMMalddh_1_eutpdugrm(pf00171) EUT1 EP_NTERM
01678 HMM06723EutJMreB_1_grm EUT1
01679 HMM00465alcdh_3_mix(pf00465) EUT1
01680 HMMtpEuth_1_eut EUT1
01681 HMMtpEuth_1_eut(pf04346) EUT1
01682 HMM06277EutA_1_eut(pf06277) EUT1
01683 HMMetlyEutB_1_EUT1(pf06751) EUT1
01684 HMMetlyEutB_1_EUT1(pf06751) EUT1
01685 HMMetlyEutC_1_eut1(pf05985) EUT1
01686 Tsp_rosa EUT1
01687 H_mulberry(pf00936pf16365) EUT1
01688 HMMreg12833_1_EUT1 EUT1
01689 HMMreg12833_1_EUT1(pf12833) EUT1
01690 -pf01218 -
01691 -pf01520 -
01692 -pf00583 -
01693 -pf11143 -
01694 -pf06572 -
01695 -pf04261 -
01696 HMMnopfamclust3_1(pf00106) RMM1
01697 HMMnopfamclust3_1 EUT2C
01698 - -
01699 -pf00528 -
01700 -pf00528 -
BMC-P
BMC-H
BMC-T
Aldehyde dehydrogenase
PTAC
Signature enzyme
Alcohol dehydrogenase
Regulator

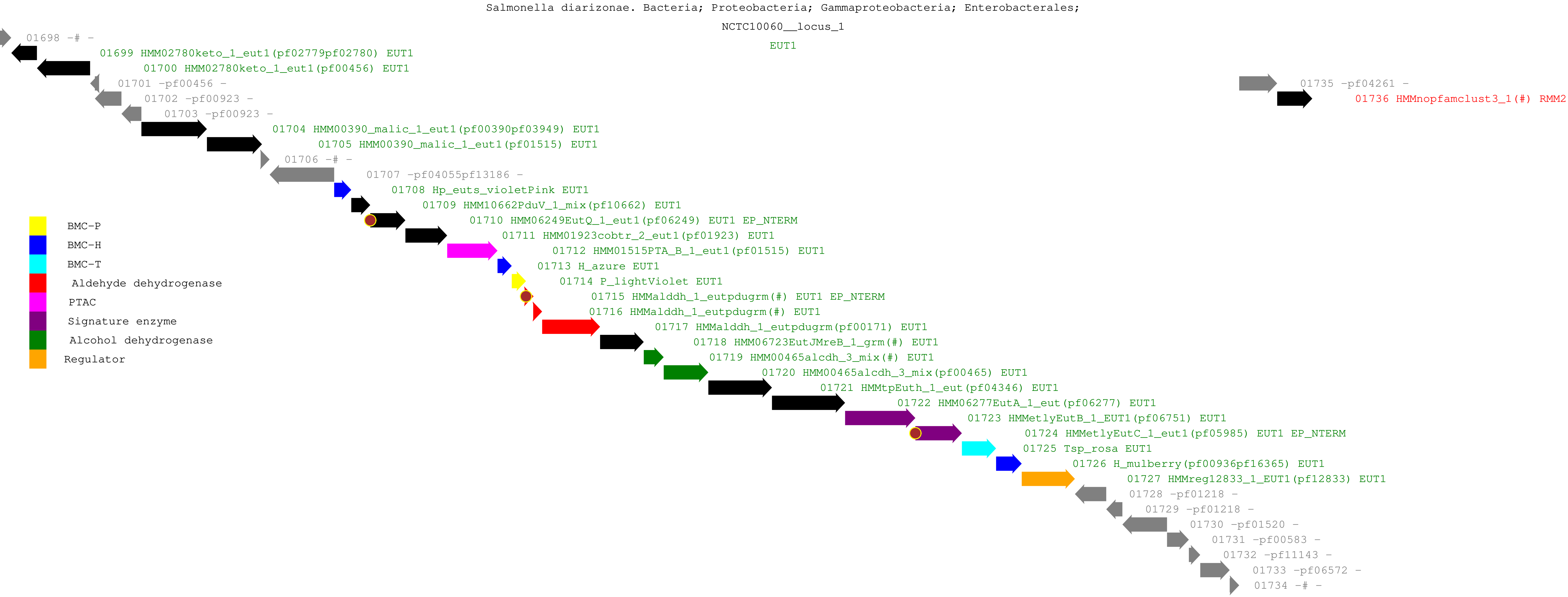

Salmonella diarizonae. Bacteria; Proteobacteria; Gammaproteobacteria; Enterobacterales;
NCTC10060__locus_1
EUT1
01698 -# -
01699 HMM02780keto_1_eut1(pf02779pf02780) EUT1
01700 HMM02780keto_1_eut1(pf00456) EUT1
01701 -pf00456 -
01702 -pf00923 -
01703 -pf00923 -
01704 HMM00390_malic_1_eut1(pf00390pf03949) EUT1
01705 HMM00390_malic_1_eut1(pf01515) EUT1
01706 -# -
01707 -pf04055pf13186 -
01708 Hp_euts_violetPink EUT1
01709 HMM10662PduV_1_mix(pf10662) EUT1
01710 HMM06249EutQ_1_eut1(pf06249) EUT1 EP_NTERM
01711 HMM01923cobtr_2_eut1(pf01923) EUT1
01712 HMM01515PTA_B_1_eut1(pf01515) EUT1
01713 H_azure EUT1
01714 P_lightViolet EUT1
01715 HMMalddh_1_eutpdugrm(#) EUT1 EP_NTERM
01716 HMMalddh_1_eutpdugrm(#) EUT1
01717 HMMalddh_1_eutpdugrm(pf00171) EUT1
01718 HMM06723EutJMreB_1_grm(#) EUT1
01719 HMM00465alcdh_3_mix(#) EUT1
01720 HMM00465alcdh_3_mix(pf00465) EUT1
01721 HMMtpEuth_1_eut(pf04346) EUT1
01722 HMM06277EutA_1_eut(pf06277) EUT1
01723 HMMetlyEutB_1_EUT1(pf06751) EUT1
01724 HMMetlyEutC_1_eut1(pf05985) EUT1 EP_NTERM
01725 Tsp_rosa EUT1
01726 H_mulberry(pf00936pf16365) EUT1
01727 HMMreg12833_1_EUT1(pf12833) EUT1
01728 -pf01218 -
01729 -pf01218 -
01730 -pf01520 -
01731 -pf00583 -
01732 -pf11143 -
01733 -pf06572 -
01734 -# -
01735 -pf04261 -
01736 HMMnopfamclust3_1(#) RMM2
BMC-P
BMC-H
BMC-T
Aldehyde dehydrogenase
PTAC
Signature enzyme
Alcohol dehydrogenase
Regulator

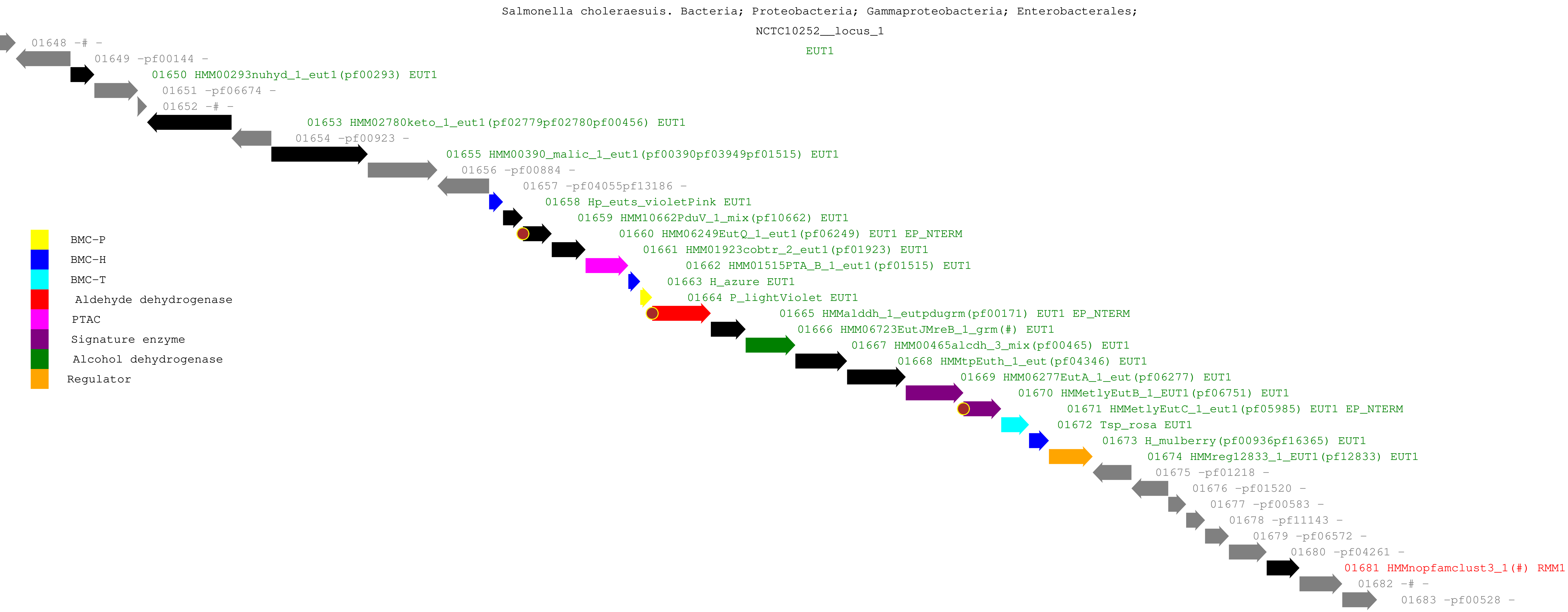
Salmonella choleraesuis. Bacteria; Proteobacteria; Gammaproteobacteria; Enterobacterales;
NCTC10252__locus_1
EUT1
01648 -# -
01649 -pf00144 -
01650 HMM00293nuhyd_1_eut1(pf00293) EUT1
01651 -pf06674 -
01652 -# -
01653 HMM02780keto_1_eut1(pf02779pf02780pf00456) EUT1
01654 -pf00923 -
01655 HMM00390_malic_1_eut1(pf00390pf03949pf01515) EUT1
01656 -pf00884 -
01657 -pf04055pf13186 -
01658 Hp_euts_violetPink EUT1
01659 HMM10662PduV_1_mix(pf10662) EUT1
01660 HMM06249EutQ_1_eut1(pf06249) EUT1 EP_NTERM
01661 HMM01923cobtr_2_eut1(pf01923) EUT1
01662 HMM01515PTA_B_1_eut1(pf01515) EUT1
01663 H_azure EUT1
01664 P_lightViolet EUT1
01665 HMMalddh_1_eutpdugrm(pf00171) EUT1 EP_NTERM
01666 HMM06723EutJMreB_1_grm(#) EUT1
01667 HMM00465alcdh_3_mix(pf00465) EUT1
01668 HMMtpEuth_1_eut(pf04346) EUT1
01669 HMM06277EutA_1_eut(pf06277) EUT1
01670 HMMetlyEutB_1_EUT1(pf06751) EUT1
01671 HMMetlyEutC_1_eut1(pf05985) EUT1 EP_NTERM
01672 Tsp_rosa EUT1
01673 H_mulberry(pf00936pf16365) EUT1
01674 HMMreg12833_1_EUT1(pf12833) EUT1
01675 -pf01218 -
01676 -pf01520 -
01677 -pf00583 -
01678 -pf11143 -
01679 -pf06572 -
01680 -pf04261 -
01681 HMMnopfamclust3_1(#) RMM1
01682 -# -
01683 -pf00528 -
BMC-P
BMC-H
BMC-T
Aldehyde dehydrogenase
PTAC
Signature enzyme
Alcohol dehydrogenase
Regulator

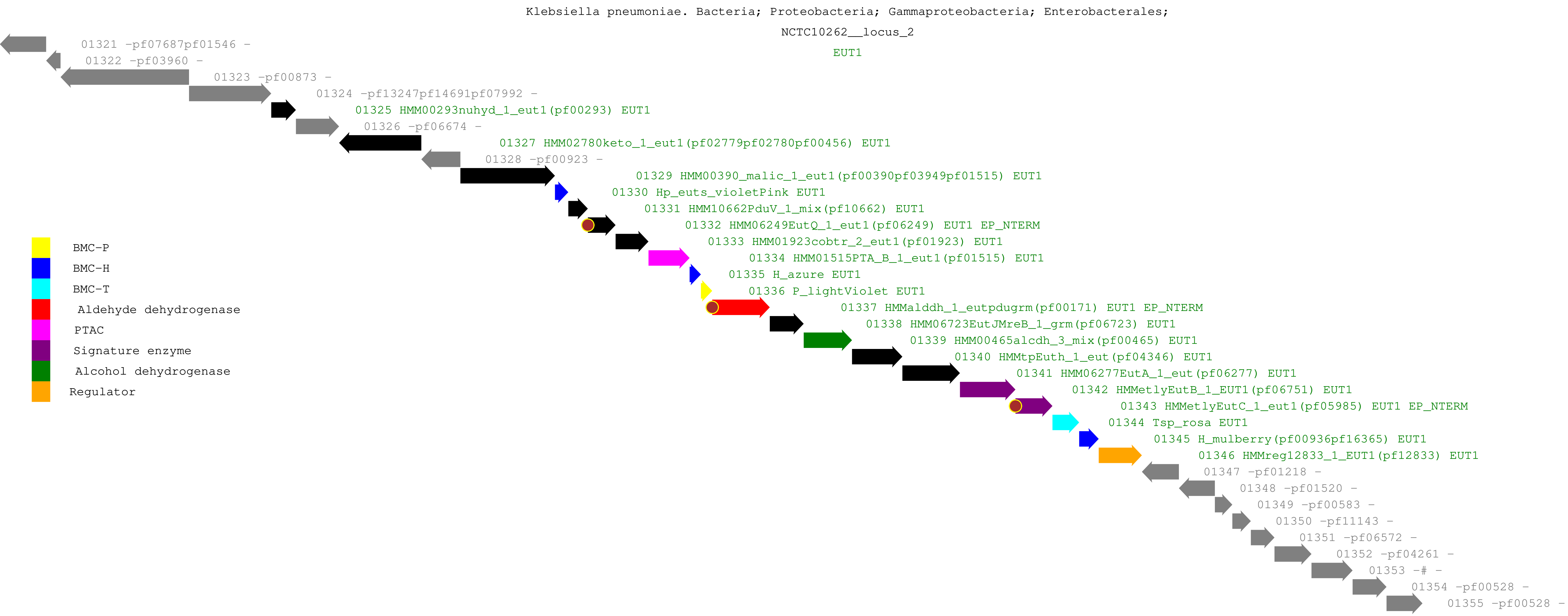
Klebsiella pneumoniae. Bacteria; Proteobacteria; Gammaproteobacteria; Enterobacterales;
NCTC10262__locus_2
EUT1
01321 -pf07687pf01546 -
01322 -pf03960 -
01323 -pf00873 -
01324 -pf13247pf14691pf07992 -
01325 HMM00293nuhyd_1_eut1(pf00293) EUT1
01326 -pf06674 -
01327 HMM02780keto_1_eut1(pf02779pf02780pf00456) EUT1
01328 -pf00923 -
01329 HMM00390_malic_1_eut1(pf00390pf03949pf01515) EUT1
01330 Hp_euts_violetPink EUT1
01331 HMM10662PduV_1_mix(pf10662) EUT1
01332 HMM06249EutQ_1_eut1(pf06249) EUT1 EP_NTERM
01333 HMM01923cobtr_2_eut1(pf01923) EUT1
01334 HMM01515PTA_B_1_eut1(pf01515) EUT1
01335 H_azure EUT1
01336 P_lightViolet EUT1
01337 HMMalddh_1_eutpdugrm(pf00171) EUT1 EP_NTERM
01338 HMM06723EutJMreB_1_grm(pf06723) EUT1
01339 HMM00465alcdh_3_mix(pf00465) EUT1
01340 HMMtpEuth_1_eut(pf04346) EUT1
01341 HMM06277EutA_1_eut(pf06277) EUT1
01342 HMMetlyEutB_1_EUT1(pf06751) EUT1
01343 HMMetlyEutC_1_eut1(pf05985) EUT1 EP_NTERM
01344 Tsp_rosa EUT1
01345 H_mulberry(pf00936pf16365) EUT1
01346 HMMreg12833_1_EUT1(pf12833) EUT1
01347 -pf01218 -
01348 -pf01520 -
01349 -pf00583 -
01350 -pf11143 -
01351 -pf06572 -
01352 -pf04261 -
01353 -# -
01354 -pf00528 -
01355 -pf00528 -
BMC-P
BMC-H
BMC-T
Aldehyde dehydrogenase
PTAC
Signature enzyme
Alcohol dehydrogenase
Regulator

Escherichia coli. Bacteria; Proteobacteria; Gammaproteobacteria; Enterobacterales;

NCTC10418__locus_1

EUT1

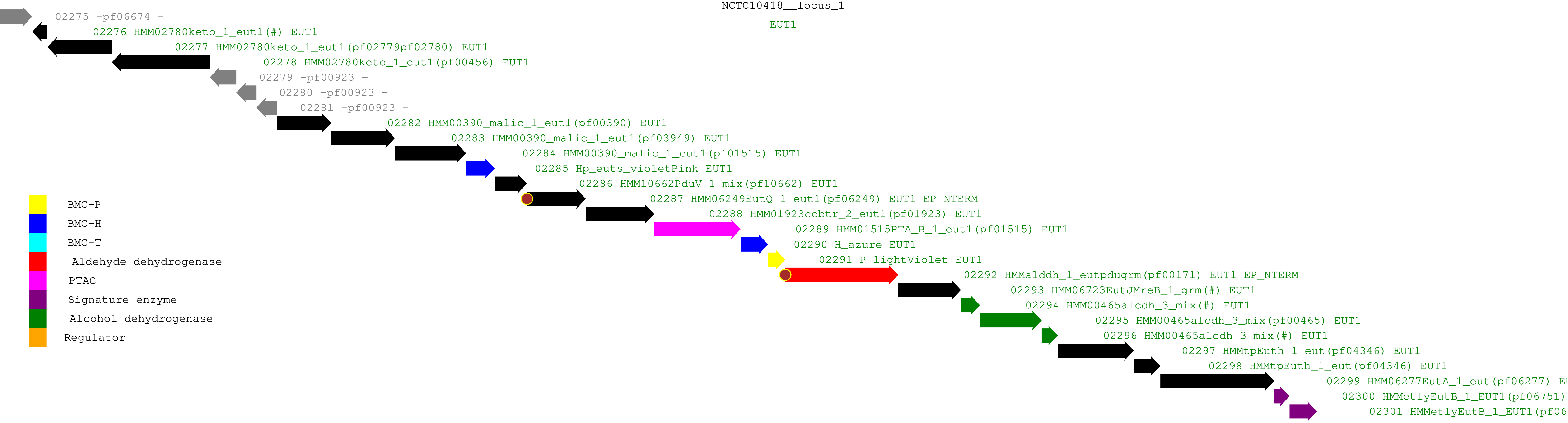

Salmonella choleraesuis. Bacteria; Proteobacteria; Gammaproteobacteria; Enterobacterales;

NCTC10718__locus_1

EUT1

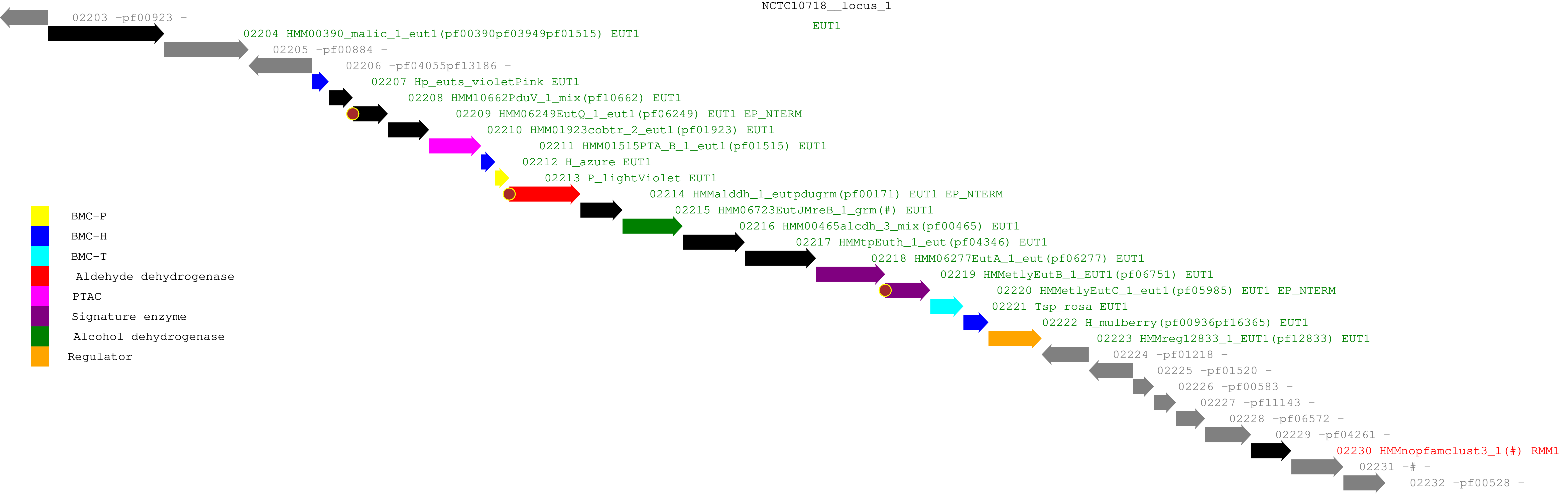

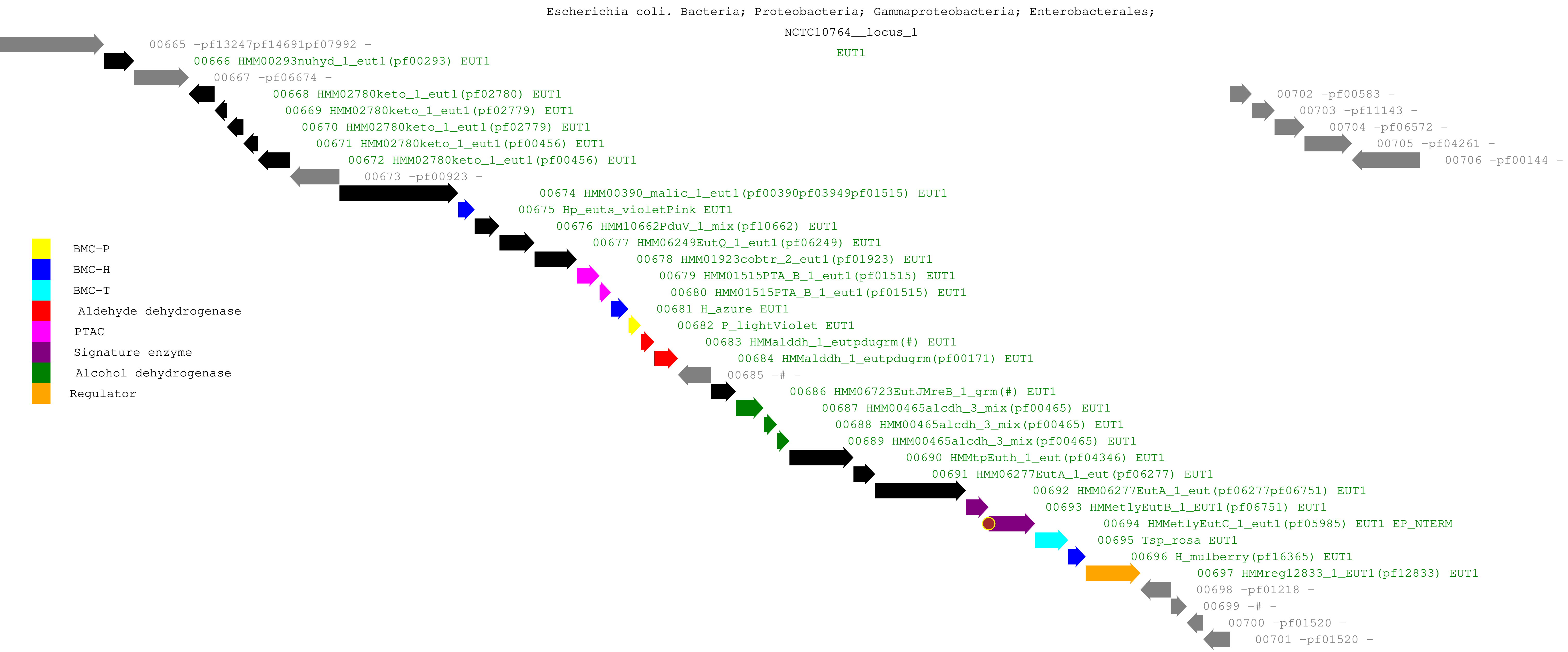

Escherichia coli. Bacteria; Proteobacteria; Gammaproteobacteria; Enterobacterales;
NCTC10764__locus_1
EUT1
00665 -pf13247pf14691pf07992 -
00666 HMM00293nuhyd_1_eut1(pf00293) EUT1
00667 -pf06674 -
00668 HMM02780keto_1_eut1(pf02780) EUT1
00669 HMM02780keto_1_eut1(pf02779) EUT1
00670 HMM02780keto_1_eut1(pf02779) EUT1
00671 HMM02780keto_1_eut1(pf00456) EUT1
00672 HMM02780keto_1_eut1(pf00456) EUT1
00673 -pf00923 -
00674 HMM00390_malic_1_eut1(pf00390pf03949pf01515) EUT1
00675 Hp_euts_violetPink EUT1
00676 HMM10662PduV_1_mix(pf10662) EUT1
00677 HMM06249EutQ_1_eut1(pf06249) EUT1
00678 HMM01923cobtr_2_eut1(pf01923) EUT1
00679 HMM01515PTA_B_1_eut1(pf01515) EUT1
00680 HMM01515PTA_B_1_eut1(pf01515) EUT1
00681 H_azure EUT1
00682 P_lightViolet EUT1
00683 HMMalddh_1_eutpdugrm(#) EUT1
00684 HMMalddh_1_eutpdugrm(pf00171) EUT1
00685 -# -
00686 HMM06723EutJMreB_1_grm(#) EUT1
00687 HMM00465alcdh_3_mix(pf00465) EUT1
00688 HMM00465alcdh_3_mix(pf00465) EUT1
00689 HMM00465alcdh_3_mix(pf00465) EUT1
00690 HMMtpEuth_1_eut(pf04346) EUT1
00691 HMM06277EutA_1_eut(pf06277) EUT1
00692 HMM06277EutA_1_eut(pf06277pf06751) EUT1
00693 HMMetlyEutB_1_EUT1(pf06751) EUT1
00694 HMMetlyEutC_1_eut1(pf05985) EUT1 EP_NTERM
00695 Tsp_rosa EUT1
00696 H_mulberry(pf16365) EUT1
00697 HMMreg12833_1_EUT1(pf12833) EUT1
00698 -pf01218 -
00699 -# -
00700 -pf01520 -
00701 -pf01520 -
00702 -pf00583 -
00703 -pf11143 -
00704 -pf06572 -
00705 -pf04261 -
00706 -pf00144 -
BMC-P
BMC-H
BMC-T
Aldehyde dehydrogenase
PTAC
Signature enzyme
Alcohol dehydrogenase
Regulator

Escherichia coli. Bacteria; Proteobacteria; Gammaproteobacteria; Enterobacterales;

NCTC10766__locus_1

EUT1

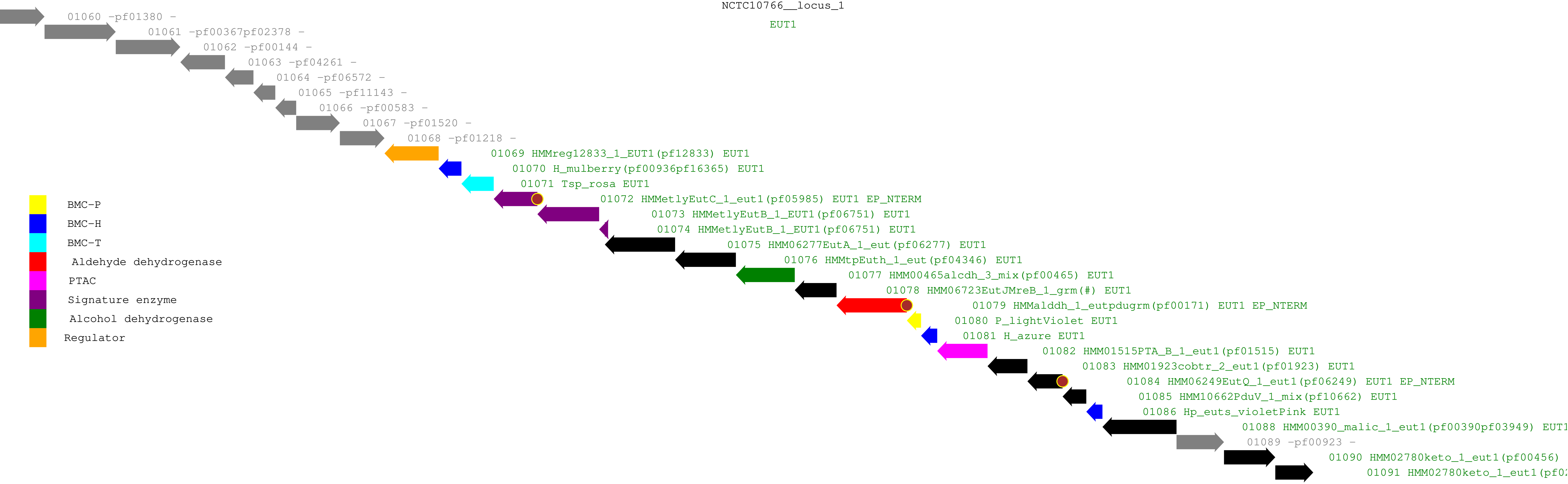

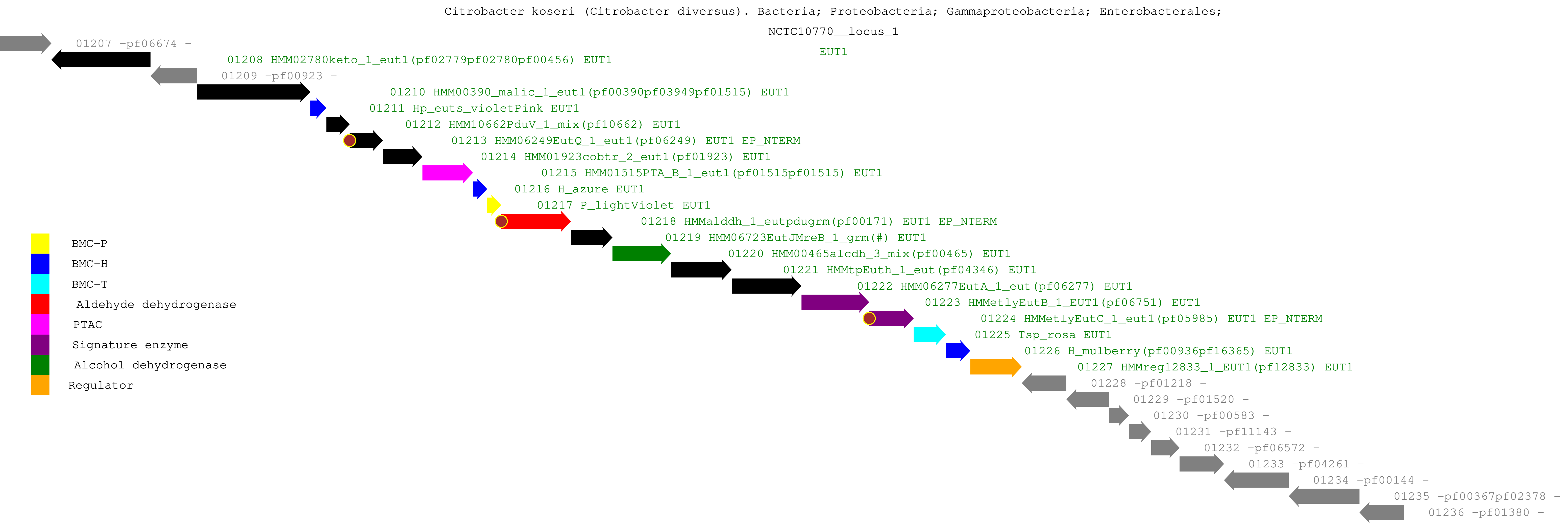
Citrobacter koseri (Citrobacter diversus). Bacteria; Proteobacteria; Gammaproteobacteria; Enterobacterales;
NCTC10770__locus_1
EUT1
01207 -pf06674 -
01208 HMM02780keto_1_eut1(pf02779pf02780pf00456) EUT1
01209 -pf00923 -
01210 HMM00390_malic_1_eut1(pf00390pf03949pf01515) EUT1
01211 Hp_euts_violetPink EUT1
01212 HMM10662PduV_1_mix(pf10662) EUT1
01213 HMM06249EutQ_1_eut1(pf06249) EUT1 EP_NTERM
01214 HMM01923cobtr_2_eut1(pf01923) EUT1
01215 HMM01515PTA_B_1_eut1(pf01515pf01515) EUT1
01216 H_azure EUT1
01217 P_lightViolet EUT1
01218 HMMalddh_1_eutpdugrm(pf00171) EUT1 EP_NTERM
01219 HMM06723EutJMreB_1_grm(#) EUT1
01220 HMM00465alcdh_3_mix(pf00465) EUT1
01221 HMMtpEuth_1_eut(pf04346) EUT1
01222 HMM06277EutA_1_eut(pf06277) EUT1
01223 HMMetlyEutB_1_EUT1(pf06751) EUT1
01224 HMMetlyEutC_1_eut1(pf05985) EUT1 EP_NTERM
01225 Tsp_rosa EUT1
01226 H_mulberry(pf00936pf16365) EUT1
01227 HMMreg12833_1_EUT1(pf12833) EUT1
01228 -pf01218 -
01229 -pf01520 -
01230 -pf00583 -
01231 -pf11143 -
01232 -pf06572 -
01233 -pf04261 -
01234 -pf00144 -
01235 -pf00367pf02378 -
01236 -pf01380 -
BMC-P
BMC-H
BMC-T
Aldehyde dehydrogenase
PTAC
Signature enzyme
Alcohol dehydrogenase
Regulator

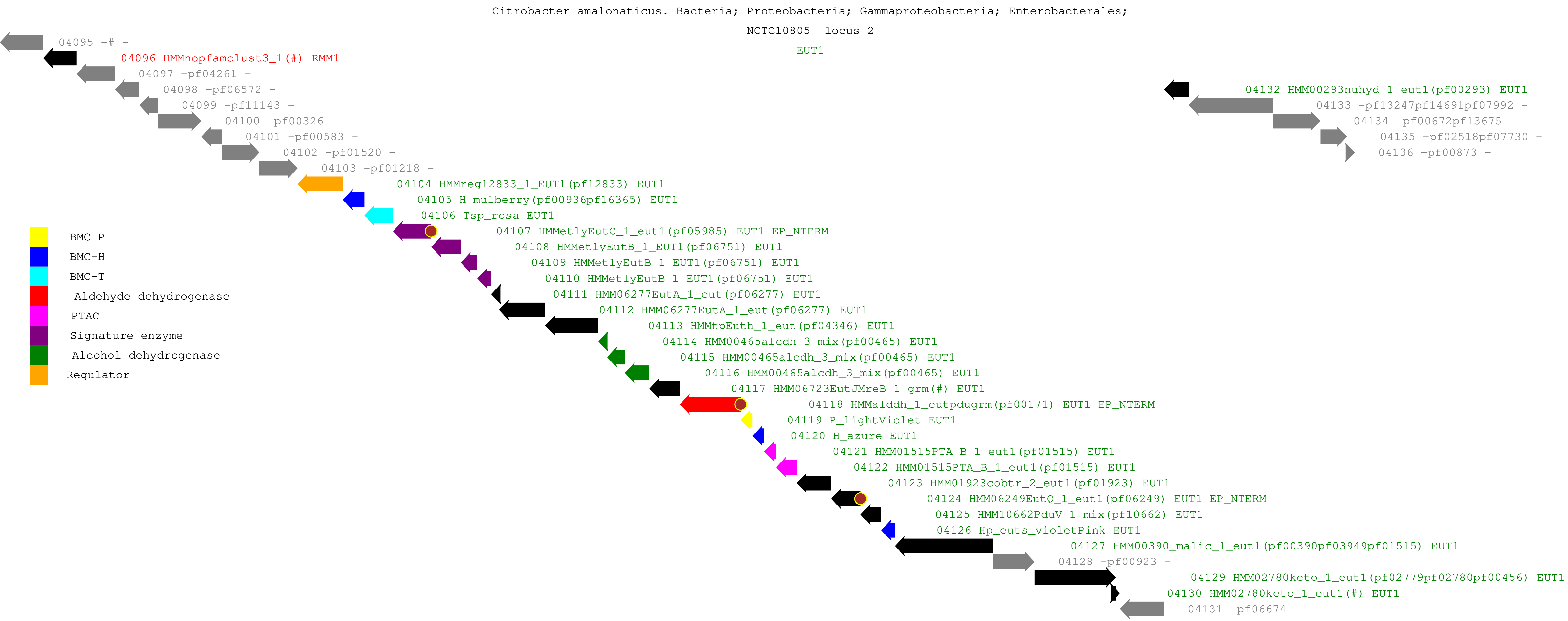
Citrobacter amalonaticus. Bacteria; Proteobacteria; Gammaproteobacteria; Enterobacterales;
NCTC10805__locus_2
EUT1
04095 -# -
04096 HMMnopfamclust3_1(#) RMM1
04097 -pf04261 -
04098 -pf06572 -
04099 -pf11143 -
04100 -pf00326 -
04101 -pf00583 -
04102 -pf01520 -
04103 -pf01218 -
04104 HMMreg12833_1_EUT1(pf12833) EUT1
04105 H_mulberry(pf00936pf16365) EUT1
04106 Tsp_rosa EUT1
04107 HMMetlyEutC_1_eut1(pf05985) EUT1 EP_NTERM
04108 HMMetlyEutB_1_EUT1(pf06751) EUT1
04109 HMMetlyEutB_1_EUT1(pf06751) EUT1
04110 HMMetlyEutB_1_EUT1(pf06751) EUT1
04111 HMM06277EutA_1_eut(pf06277) EUT1
04112 HMM06277EutA_1_eut(pf06277) EUT1
04113 HMMtpEuth_1_eut(pf04346) EUT1
04114 HMM00465alcdh_3_mix(pf00465) EUT1
04115 HMM00465alcdh_3_mix(pf00465) EUT1
04116 HMM00465alcdh_3_mix(pf00465) EUT1
04117 HMM06723EutJMreB_1_grm(#) EUT1
04118 HMMalddh_1_eutpdugrm(pf00171) EUT1 EP_NTERM
04119 P_lightViolet EUT1
04120 H_azure EUT1
04121 HMM01515PTA_B_1_eut1(pf01515) EUT1
04122 HMM01515PTA_B_1_eut1(pf01515) EUT1
04123 HMM01923cobtr_2_eut1(pf01923) EUT1
04124 HMM06249EutQ_1_eut1(pf06249) EUT1 EP_NTERM
04125 HMM10662PduV_1_mix(pf10662) EUT1
04126 Hp_euts_violetPink EUT1
04127 HMM00390_malic_1_eut1(pf00390pf03949pf01515) EUT1
04128 -pf00923 -
04129 HMM02780keto_1_eut1(pf02779pf02780pf00456) EUT1
04130 HMM02780keto_1_eut1(#) EUT1
04131 -pf06674 -
04132 HMM00293nuhyd_1_eut1(pf00293) EUT1
04133 -pf13247pf14691pf07992 -
04134 -pf00672pf13675 -
04135 -pf02518pf07730 -
04136 -pf00873 -
BMC-P
BMC-H
BMC-T
Aldehyde dehydrogenase
PTAC
Signature enzyme
Alcohol dehydrogenase
Regulator

Citrobacter koseri (Citrobacter diversus). Bacteria; Proteobacteria; Gammaproteobacteria; Enterobacterales;

NCTC10810__locus_1

EUT1

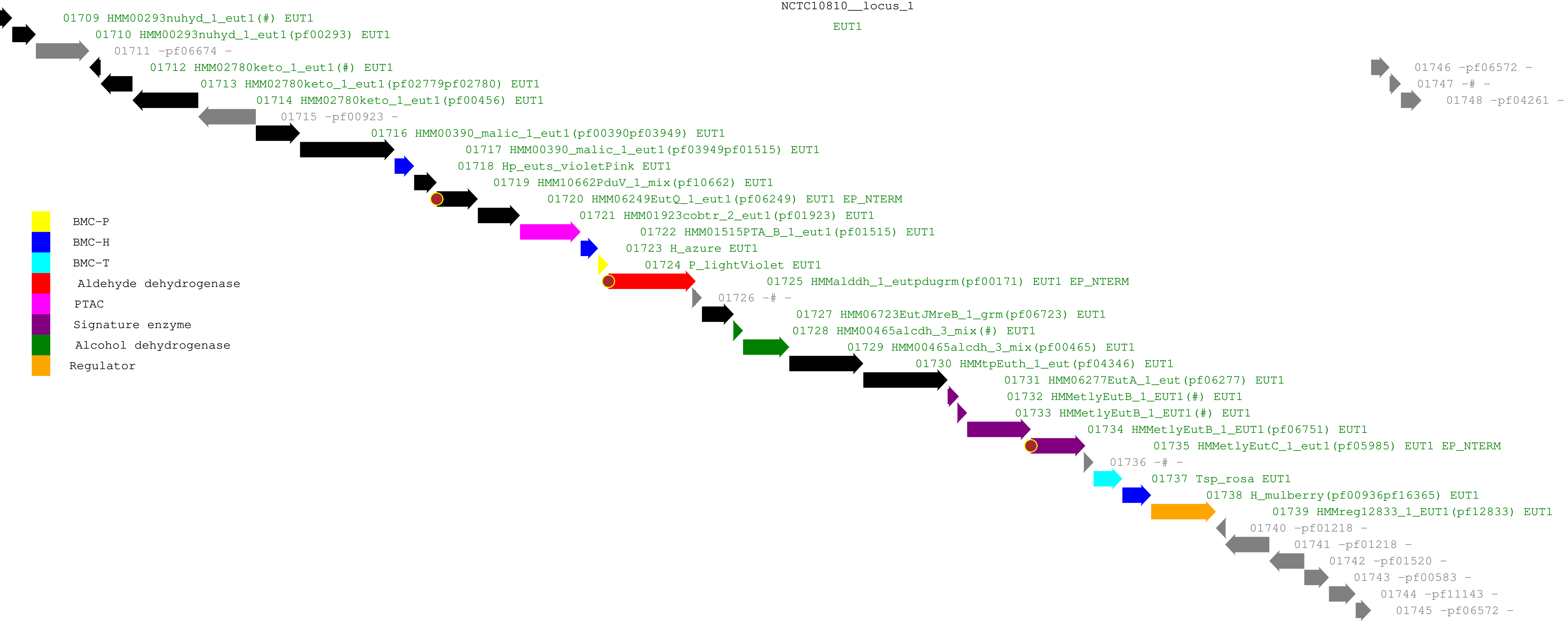

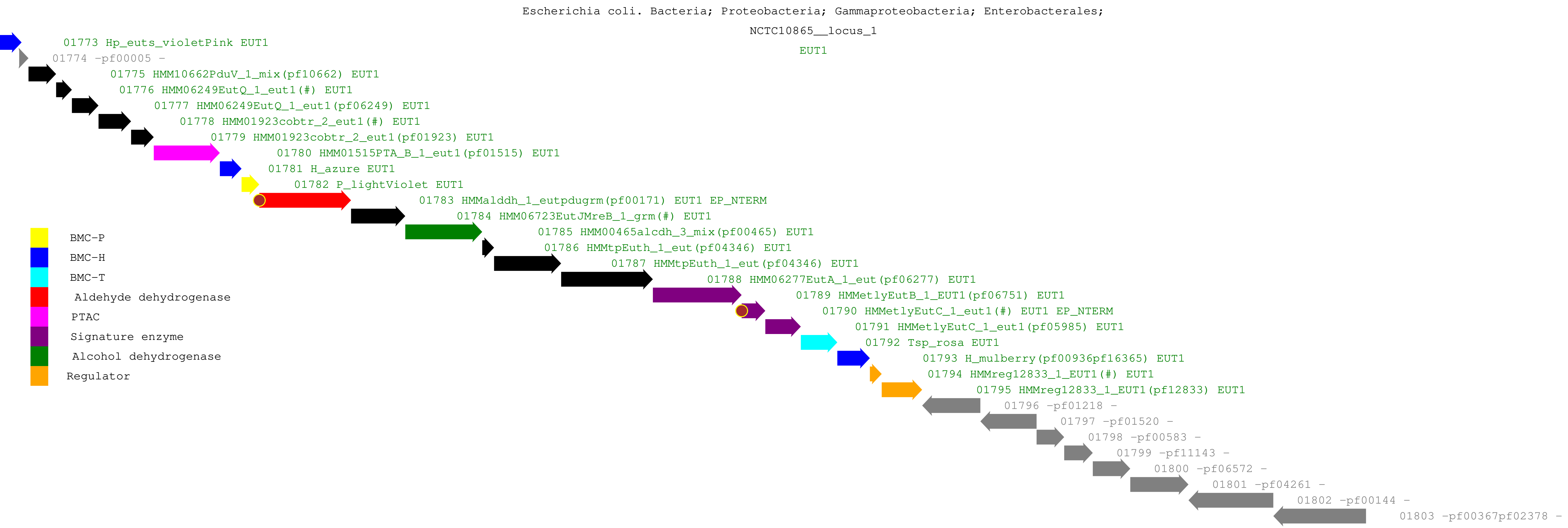

Escherichia coli. Bacteria; Proteobacteria; Gammaproteobacteria; Enterobacterales;
NCTC10865__locus_1
EUT1
01773 Hp_euts_violetPink EUT1
01774 -pf00005 -
01775 HMM10662PduV_1_mix(pf10662) EUT1
01776 HMM06249EutQ_1_eut1(#) EUT1
01777 HMM06249EutQ_1_eut1(pf06249) EUT1
01778 HMM01923cobtr_2_eut1(#) EUT1
01779 HMM01923cobtr_2_eut1(pf01923) EUT1
01780 HMM01515PTA_B_1_eut1(pf01515) EUT1
01781 H_azure EUT1
01782 P_lightViolet EUT1
01783 HMMalddh_1_eutpdugrm(pf00171) EUT1 EP_NTERM
01784 HMM06723EutJMreB_1_grm(#) EUT1
01785 HMM00465alcdh_3_mix(pf00465) EUT1
01786 HMMtpEuth_1_eut(pf04346) EUT1
01787 HMMtpEuth_1_eut(pf04346) EUT1
01788 HMM06277EutA_1_eut(pf06277) EUT1
01789 HMMetlyEutB_1_EUT1(pf06751) EUT1
01790 HMMetlyEutC_1_eut1(#) EUT1 EP_NTERM
01791 HMMetlyEutC_1_eut1(pf05985) EUT1
01792 Tsp_rosa EUT1
01793 H_mulberry(pf00936pf16365) EUT1
01794 HMMreg12833_1_EUT1(#) EUT1
01795 HMMreg12833_1_EUT1(pf12833) EUT1
01796 -pf01218 -
01797 -pf01520 -
01798 -pf00583 -
01799 -pf11143 -
01800 -pf06572 -
01801 -pf04261 -
01802 -pf00144 -
01803 -pf00367pf02378 -
BMC-P
BMC-H
BMC-T
Aldehyde dehydrogenase
PTAC
Signature enzyme
Alcohol dehydrogenase
Regulator

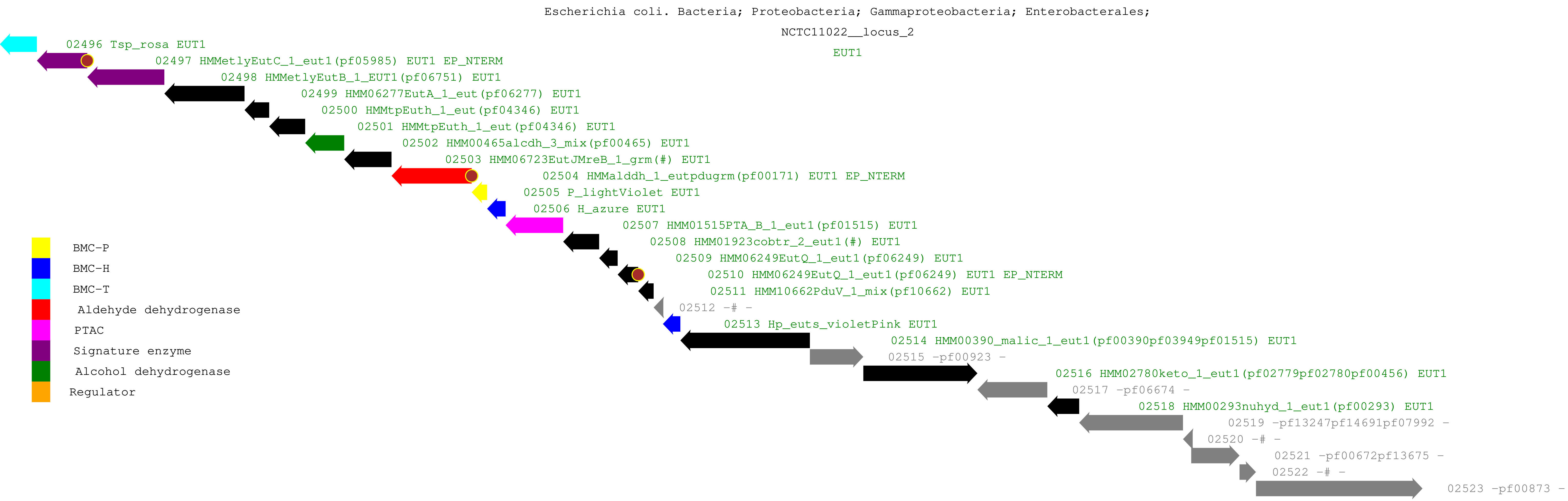

Escherichia coli. Bacteria; Proteobacteria; Gammaproteobacteria; Enterobacterales;
NCTC11022__locus_2
EUT1
02496 Tsp_rosa EUT1
02497 HMMetlyEutC_1_eut1(pf05985) EUT1 EP_NTERM
02498 HMMetlyEutB_1_EUT1(pf06751) EUT1
02499 HMM06277EutA_1_eut(pf06277) EUT1
02500 HMMtpEuth_1_eut(pf04346) EUT1
02501 HMMtpEuth_1_eut(pf04346) EUT1
02502 HMM00465alcdh_3_mix(pf00465) EUT1
02503 HMM06723EutJMreB_1_grm(#) EUT1
02504 HMMalddh_1_eutpdugrm(pf00171) EUT1 EP_NTERM
02505 P_lightViolet EUT1
02506 H_azure EUT1
02507 HMM01515PTA_B_1_eut1(pf01515) EUT1
02508 HMM01923cobtr_2_eut1(#) EUT1
02509 HMM06249EutQ_1_eut1(pf06249) EUT1
02510 HMM06249EutQ_1_eut1(pf06249) EUT1 EP_NTERM
02511 HMM10662PduV_1_mix(pf10662) EUT1
02512 -# -
02513 Hp_euts_violetPink EUT1
02514 HMM00390_malic_1_eut1(pf00390pf03949pf01515) EUT1
02515 -pf00923 -
02516 HMM02780keto_1_eut1(pf02779pf02780pf00456) EUT1
02517 -pf06674 -
02518 HMM00293nuhyd_1_eut1(pf00293) EUT1
02519 -pf13247pf14691pf07992 -
02520 -# -
02521 -pf00672pf13675 -
02522 -# -
02523 -pf00873 -
BMC-P
BMC-H
BMC-T
Aldehyde dehydrogenase
PTAC
Signature enzyme
Alcohol dehydrogenase
Regulator

Citrobacter koseri (Citrobacter diversus). Bacteria; Proteobacteria; Gammaproteobacteria; Enterobacterales;

NCTC11075__locus_2

EUT1

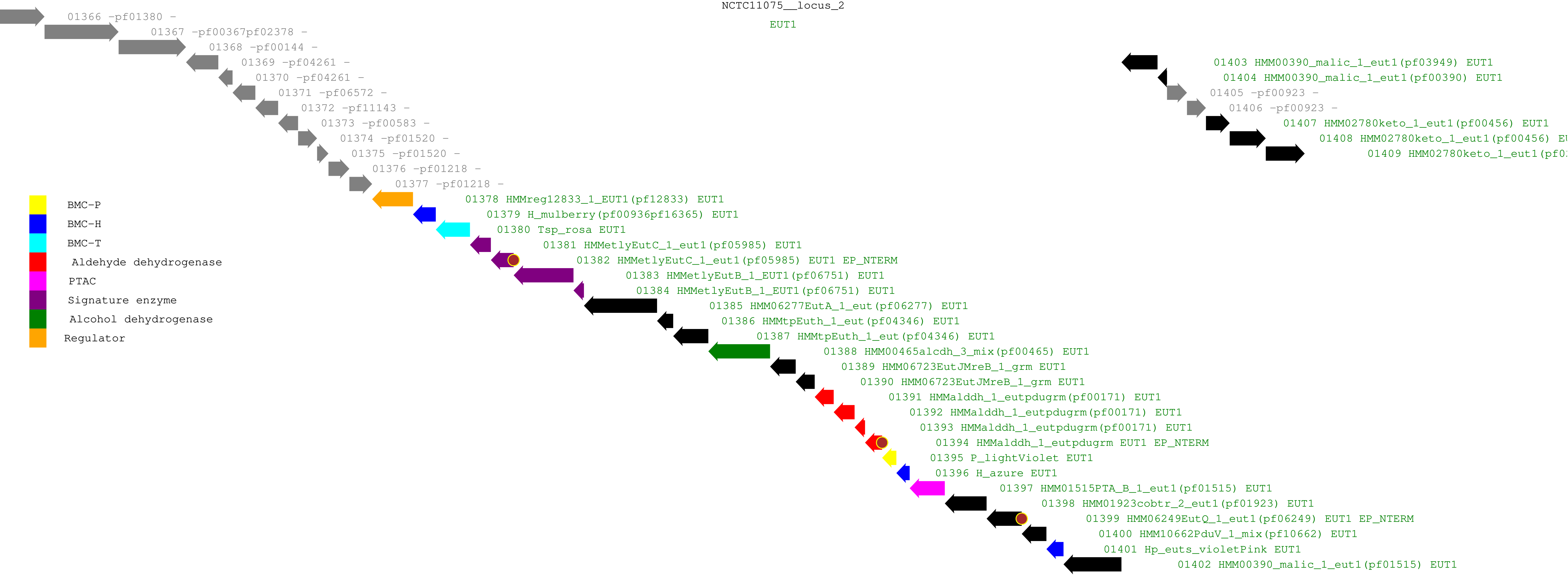

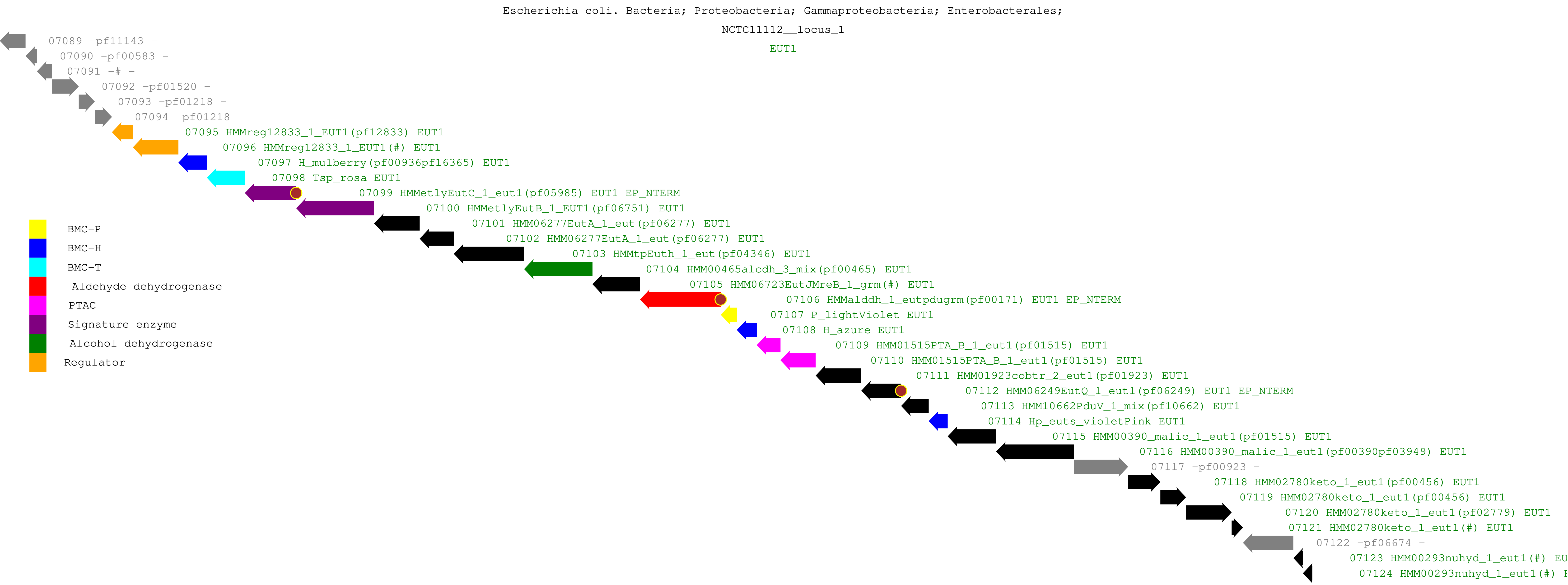

Escherichia coli. Bacteria; Proteobacteria; Gammaproteobacteria; Enterobacterales;
NCTC11112__locus_1
EUT1
07089 -pf11143 -
07090 -pf00583 -
07091 -# -
07092 -pf01520 -
07093 -pf01218 -
07094 -pf01218 -
07095 HMMreg12833_1_EUT1(pf12833) EUT1
07096 HMMreg12833_1_EUT1(#) EUT1
07097 H_mulberry(pf00936pf16365) EUT1
07098 Tsp_rosa EUT1
07099 HMMetlyEutC_1_eut1(pf05985) EUT1 EP_NTERM
07100 HMMetlyEutB_1_EUT1(pf06751) EUT1
07101 HMM06277EutA_1_eut(pf06277) EUT1
07102 HMM06277EutA_1_eut(pf06277) EUT1
07103 HMMtpEuth_1_eut(pf04346) EUT1
07104 HMM00465alcdh_3_mix(pf00465) EUT1
07105 HMM06723EutJMreB_1_grm(#) EUT1
07106 HMMalddh_1_eutpdugrm(pf00171) EUT1 EP_NTERM
07107 P_lightViolet EUT1
07108 H_azure EUT1
07109 HMM01515PTA_B_1_eut1(pf01515) EUT1
07110 HMM01515PTA_B_1_eut1(pf01515) EUT1
07111 HMM01923cobtr_2_eut1(pf01923) EUT1
07112 HMM06249EutQ_1_eut1(pf06249) EUT1 EP_NTERM
07113 HMM10662PduV_1_mix(pf10662) EUT1
07114 Hp_euts_violetPink EUT1
07115 HMM00390_malic_1_eut1(pf01515) EUT1
07116 HMM00390_malic_1_eut1(pf00390pf03949) EUT1
07117 -pf00923 -
07118 HMM02780keto_1_eut1(pf00456) EUT1
07119 HMM02780keto_1_eut1(pf00456) EUT1
07120 HMM02780keto_1_eut1(pf02779) EUT1
07121 HMM02780keto_1_eut1(#) EUT1
07122 -pf06674 -
07123 HMM00293nuhyd_1_eut1(#) EU
07124 HMM00293nuhyd_1_eut1(#) E
BMC-P
BMC-H
BMC-T
Aldehyde dehydrogenase
PTAC
Signature enzyme
Alcohol dehydrogenase
Regulator

Escherichia coli. Bacteria; Proteobacteria; Gammaproteobacteria; Enterobacterales;

NCTC11126__locus_1

EUT1

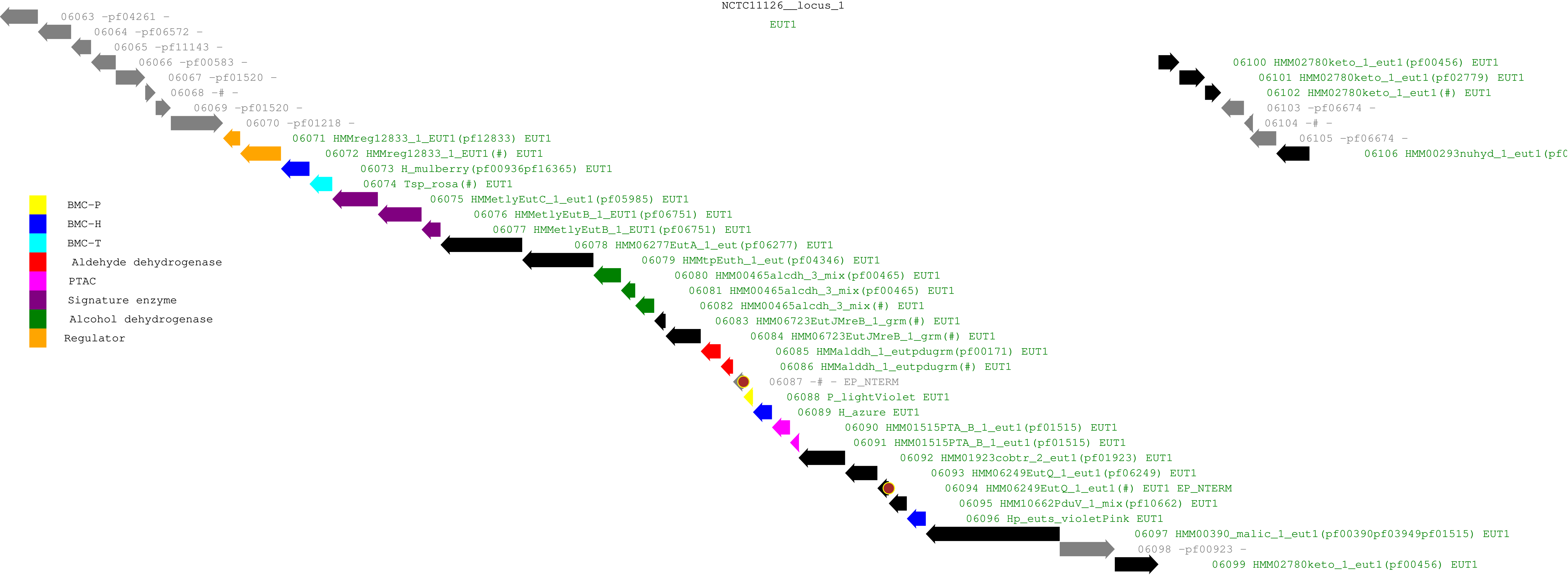

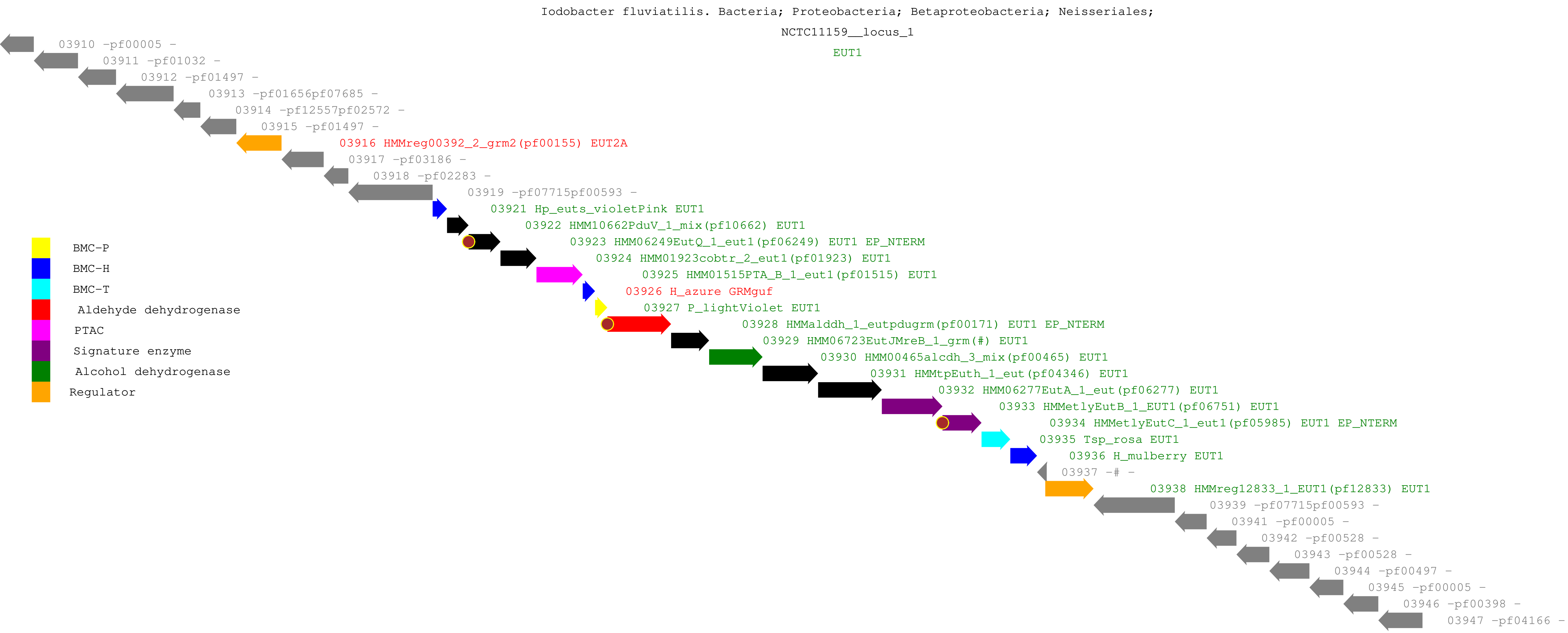
Iodobacter fluviatilis. Bacteria; Proteobacteria; Betaproteobacteria; Neisseriales;
NCTC11159__locus_1
EUT1
03910 -pf00005 -
03911 -pf01032 -
03912 -pf01497 -
03913 -pf01656pf07685 -
03914 -pf12557pf02572 -
03915 -pf01497 -
03916 HMMreg00392_2_grm2(pf00155) EUT2A
03917 -pf03186 -
03918 -pf02283 -
03919 -pf07715pf00593 -
03921 Hp_euts_violetPink EUT1
03922 HMM10662PduV_1_mix(pf10662) EUT1
03923 HMM06249EutQ_1_eut1(pf06249) EUT1 EP_NTERM
03924 HMM01923cobtr_2_eut1(pf01923) EUT1
03925 HMM01515PTA_B_1_eut1(pf01515) EUT1
03926 H_azure GRMguf
03927 P_lightViolet EUT1
03928 HMMaldh_1_eutpdugrm(pf00171) EUT1 EP_NTERM
03929 HMM06723EutJMreB_1_grm(#) EUT1
03930 HMM00465alcdh_3_mix(pf00465) EUT1
03931 HMMtpEuth_1_eut(pf04346) EUT1
03932 HMM06277EutA_1_eut(pf06277) EUT1
03933 HMMetlyEutB_1_EUT1(pf06751) EUT1
03934 HMMetlyEutC_1_eut1(pf05985) EUT1 EP_NTERM
03935 Tsp_rosa EUT1
03936 H_mulberry EUT1
03937 -# -
03938 HMMreg12833_1_EUT1(pf12833) EUT1
03939 -pf07715pf00593 -
03941 -pf00005 -
03942 -pf00528 -
03943 -pf00528 -
03944 -pf00497 -
03945 -pf00005 -
03946 -pf00398 -
03947 -pf04166 -
BMC-P
BMC-H
BMC-T
Aldehyde dehydrogenase
PTAC
Signature enzyme
Alcohol dehydrogenase
Regulator

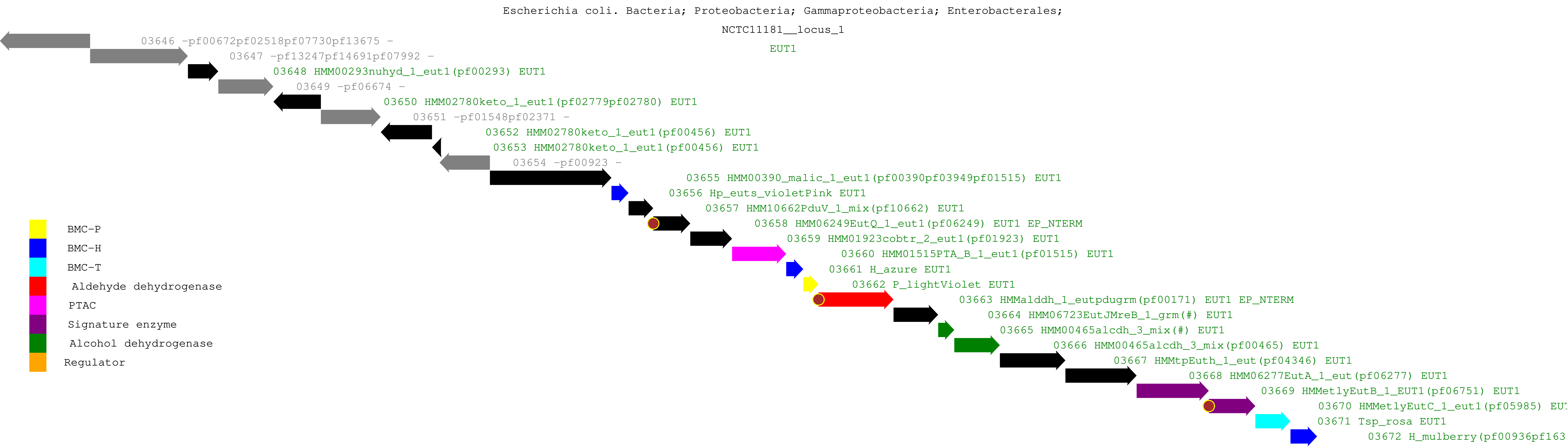

Escherichia coli. Bacteria; Proteobacteria; Gammaproteobacteria; Enterobacterales;
NCTC11181__locus_1
EUT1
03646 -pf00672pf02518pf07730pf13675 -
03647 -pf13247pf14691pf07992 -
03648 HMM00293nuhyd_1_eut1(pf00293) EUT1
03649 -pf06674 -
03650 HMM02780keto_1_eut1(pf02779pf02780) EUT1
03651 -pf01548pf02371 -
03652 HMM02780keto_1_eut1(pf00456) EUT1
03653 HMM02780keto_1_eut1(pf00456) EUT1
03654 -pf00923 -
03655 HMM00390_malic_1_eut1(pf00390pf03949pf01515) EUT1
03656 Hp_euts_violetPink EUT1
03657 HMM10662PduV_1_mix(pf10662) EUT1
03658 HMM06249EutQ_1_eut1(pf06249) EUT1 EP_NTERM
03659 HMM01923cobtr_2_eut1(pf01923) EUT1
03660 HMM01515PTA_B_1_eut1(pf01515) EUT1
03661 H_azure EUT1
03662 P_lightViolet EUT1
03663 HMMaldh_1_eutpdugrm(pf00171) EUT1 EP_NTERM
03664 HMM06723EutJMreB_1_grm(#) EUT1
03665 HMM00465alcdh_3_mix(#) EUT1
03666 HMM00465alcdh_3_mix(pf00465) EUT1
03667 HMMtpEuth_1_eut(pf04346) EUT1
03668 HMM06277EutA_1_eut(pf06277) EUT1
03669 HMMetlyEutB_1_EUT1(pf06751) EUT1
03670 HMMetlyEutC_1_eut1(pf05985) EU
03671 Tsp_rosa EUT1
03672 H_mulberry(pf00936pf163
BMC-P
BMC-H
BMC-T
Aldehyde dehydrogenase
PTAC
Signature enzyme
Alcohol dehydrogenase
Regulator

Klebsiella oxytoca. Bacteria; Proteobacteria; Gammaproteobacteria; Enterobacterales;

NCTC11355__locus_1

EUT1

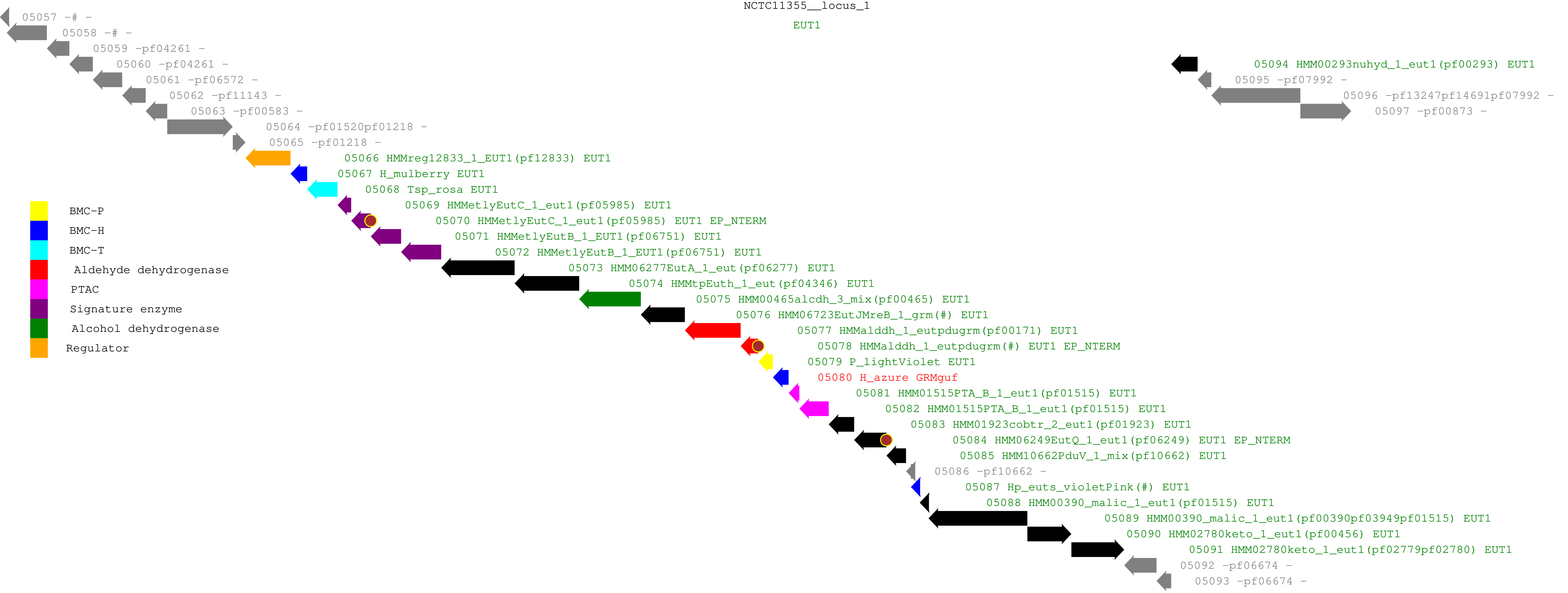

Pluralibacter gergoviae (Enterobacter gergoviae). Bacteria; Proteobacteria; Gammaproteobacteria; Enterobacterales;

NCTC11434__locus_1

EUT1

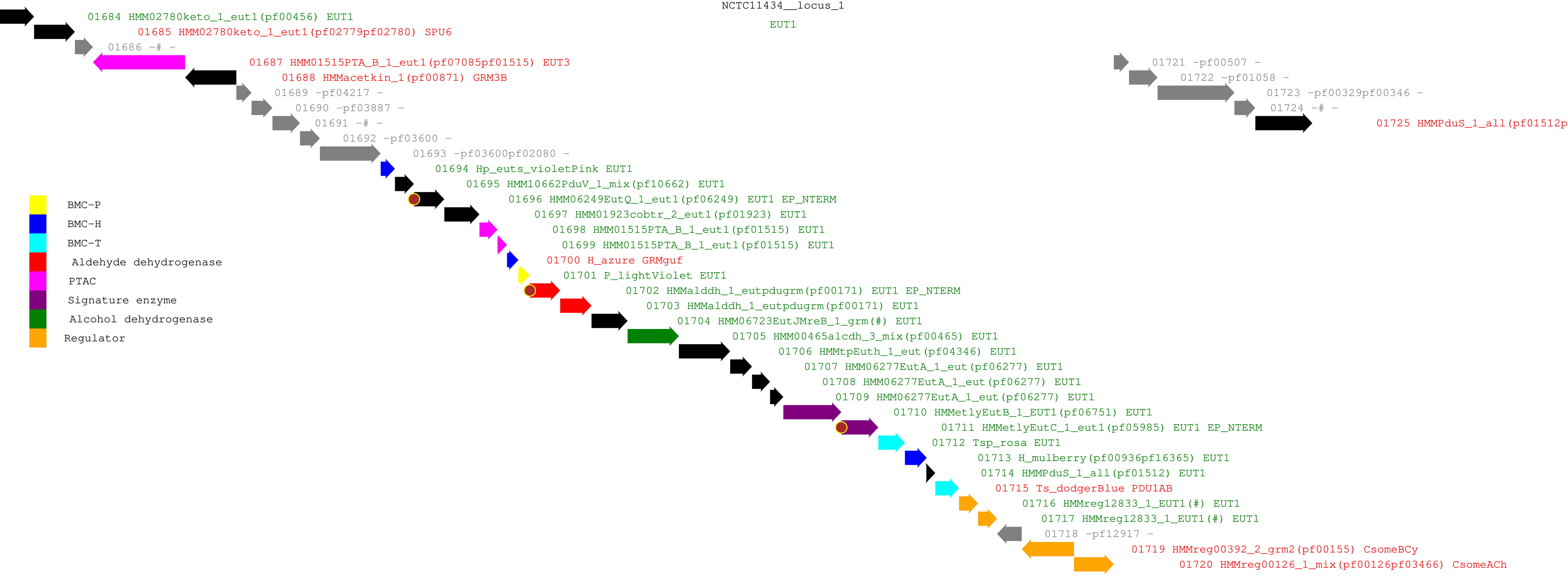

Escherichia coli. Bacteria; Proteobacteria; Gammaproteobacteria; Enterobacterales;

NCTC11560__locus_1

EUT1

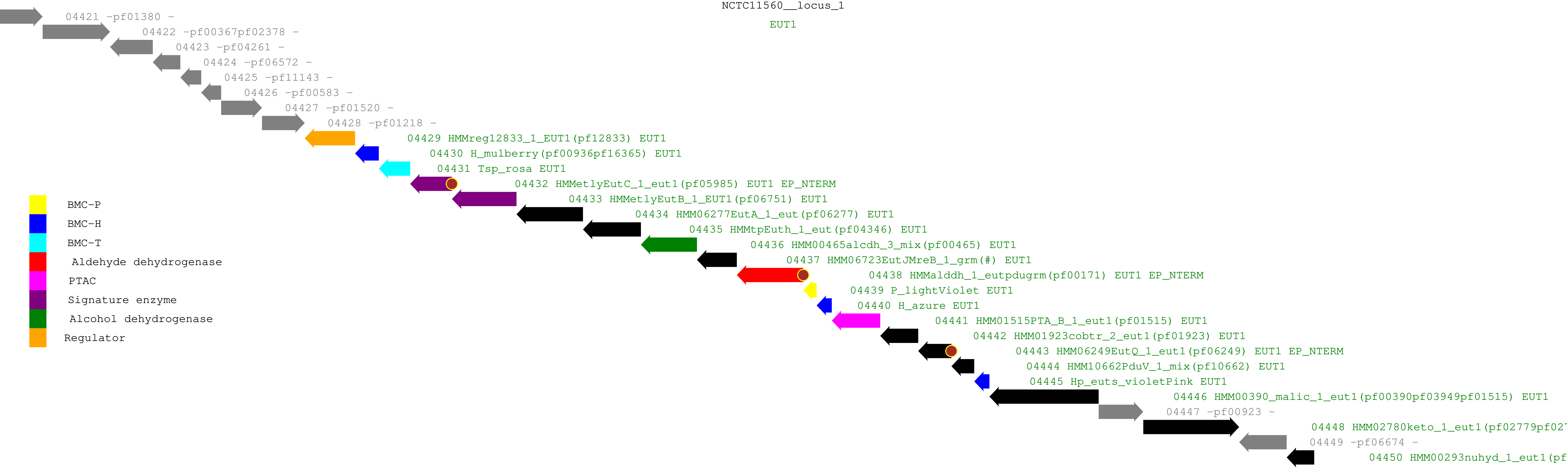

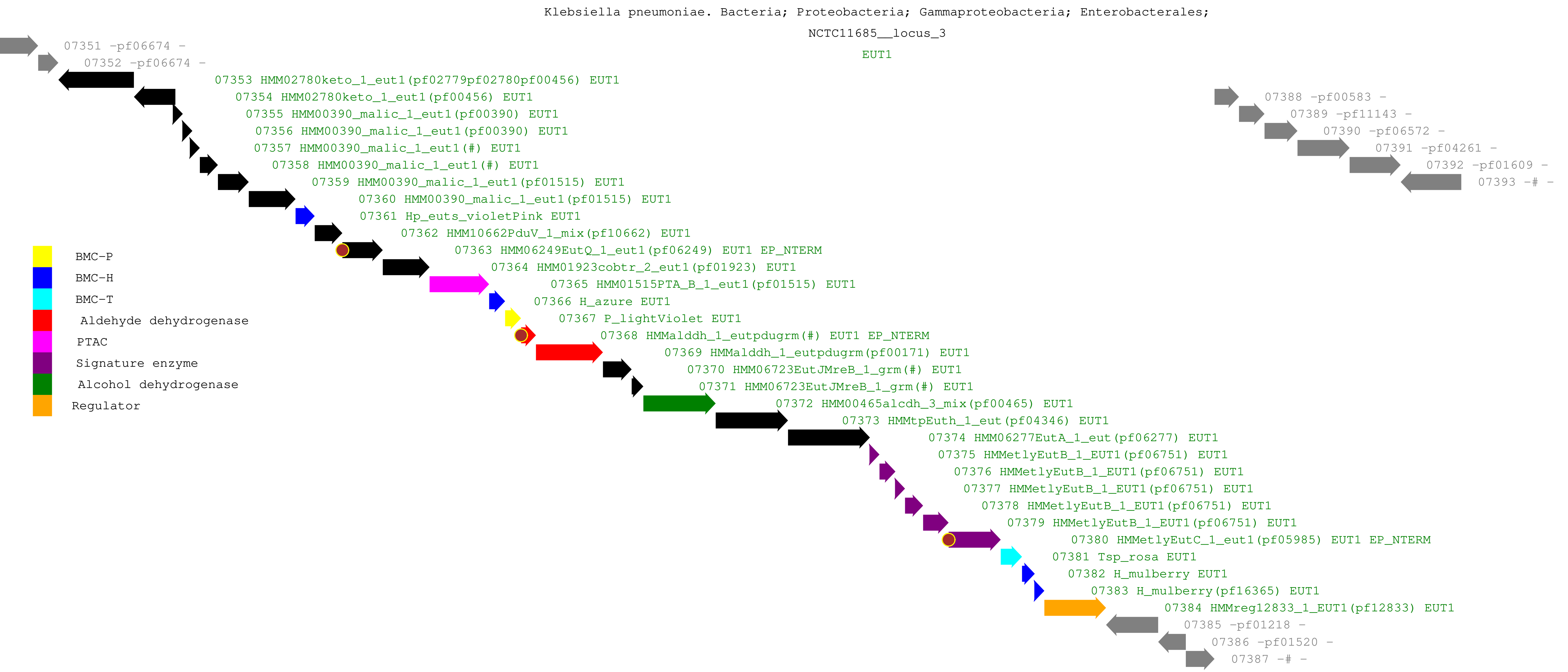
Klebsiella pneumoniae. Bacteria; Proteobacteria; Gammaproteobacteria; Enterobacterales;
NCTC11685__locus_3
EUT1
07351 -pf06674 -
07352 -pf06674 -
07353 HMM02780keto_1_eut1(pf02779pf02780pf00456) EUT1
07354 HMM02780keto_1_eut1(pf00456) EUT1
07355 HMM00390_malic_1_eut1(pf00390) EUT1
07356 HMM00390_malic_1_eut1(pf00390) EUT1
07357 HMM00390_malic_1_eut1(#) EUT1
07358 HMM00390_malic_1_eut1(#) EUT1
07359 HMM00390_malic_1_eut1(pf01515) EUT1
07360 HMM00390_malic_1_eut1(pf01515) EUT1
07361 Hp_euts_violetPink EUT1
07362 HMM10662PduV_1_mix(pf10662) EUT1
07363 HMM06249EutQ_1_eut1(pf06249) EUT1 EP_NTERM
07364 HMM01923cobtr_2_eut1(pf01923) EUT1
07365 HMM01515PTA_B_1_eut1(pf01515) EUT1
07366 H_azure EUT1
07367 P_lightViolet EUT1
07368 HMMalddh_1_eutpdugrm(#) EUT1 EP_NTERM
07369 HMMalddh_1_eutpdugrm(pf00171) EUT1
07370 HMM06723EutJMreB_1_grm(#) EUT1
07371 HMM06723EutJMreB_1_grm(#) EUT1
07372 HMM00465alcdh_3_mix(pf00465) EUT1
07373 HMMtpEuth_1_eut(pf04346) EUT1
07374 HMM06277EutA_1_eut(pf06277) EUT1
07375 HMMetlyEutB_1_EUT1(pf06751) EUT1
07376 HMMetlyEutB_1_EUT1(pf06751) EUT1
07377 HMMetlyEutB_1_EUT1(pf06751) EUT1
07378 HMMetlyEutB_1_EUT1(pf06751) EUT1
07379 HMMetlyEutB_1_EUT1(pf06751) EUT1
07380 HMMetlyEutC_1_eut1(pf05985) EUT1 EP_NTERM
07381 Tsp_rosa EUT1
07382 H_mulberry EUT1
07383 H_mulberry(pf16365) EUT1
07384 HMMreg12833_1_EUT1(pf12833) EUT1
07385 -pf01218 -
07386 -pf01520 -
07387 -# -
07388 -pf00583 -
07389 -pf11143 -
07390 -pf06572 -
07391 -pf04261 -
07392 -pf01609 -
07393 -# -
BMC-P
BMC-H
BMC-T
Aldehyde dehydrogenase
PTAC
Signature enzyme
Alcohol dehydrogenase
Regulator

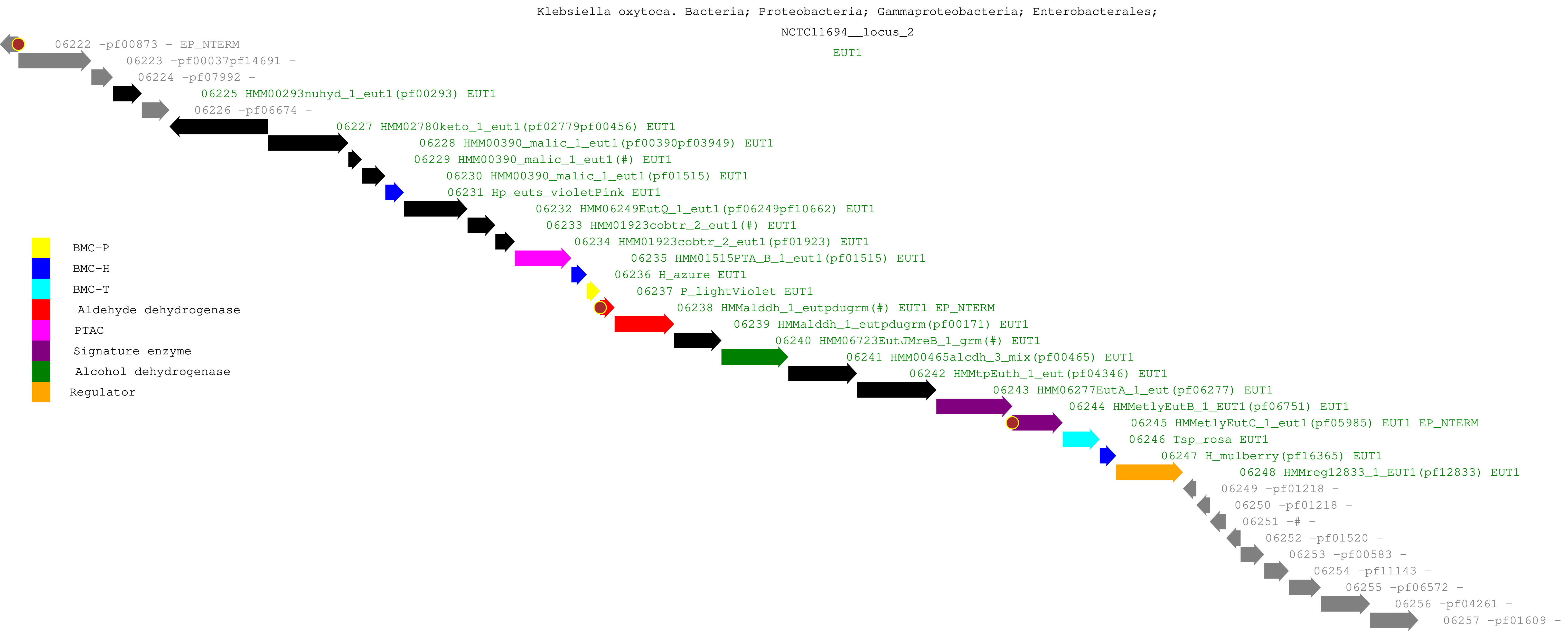
Klebsiella oxytoca. Bacteria; Proteobacteria; Gammaproteobacteria; Enterobacterales;
NCTC11694__locus_2
EUT1
06222 -pf00873 - EP_NTERM
06223 -pf00037pf14691 -
06224 -pf07992 -
06225 HMM00293nuhyd_1_eut1(pf00293) EUT1
06226 -pf06674 -
06227 HMM02780keto_1_eut1(pf02779pf00456) EUT1
06228 HMM00390_malic_1_eut1(pf00390pf03949) EUT1
06229 HMM00390_malic_1_eut1(#) EUT1
06230 HMM00390_malic_1_eut1(pf01515) EUT1
06231 Hp_euts_violetPink EUT1
06232 HMM06249EutQ_1_eut1(pf06249pf10662) EUT1
06233 HMM01923cobtr_2_eut1(#) EUT1
06234 HMM01923cobtr_2_eut1(pf01923) EUT1
06235 HMM01515PTA_B_1_eut1(pf01515) EUT1
06236 H_azure EUT1
06237 P_lightViolet EUT1
06238 HMMalddh_1_eutpdugrm(#) EUT1 EP_NTERM
06239 HMMalddh_1_eutpdugrm(pf00171) EUT1
06240 HMM06723EutJMreB_1_grm(#) EUT1
06241 HMM00465alcdh_3_mix(pf00465) EUT1
06242 HMMtpEuth_1_eut(pf04346) EUT1
06243 HMM06277EutA_1_eut(pf06277) EUT1
06244 HMMetlyEutB_1_EUT1(pf06751) EUT1
06245 HMMetlyEutC_1_eut1(pf05985) EUT1 EP_NTERM
06246 Tsp_rosa EUT1
06247 H_mulberry(pf16365) EUT1
06248 HMMreg12833_1_EUT1(pf12833) EUT1
06249 -pf01218 -
06250 -pf01218 -
06251 -# -
06252 -pf01520 -
06253 -pf00583 -
06254 -pf11143 -
06255 -pf06572 -
06256 -pf04261 -
06257 -pf01609 -
BMC-P
BMC-H
BMC-T
Aldehyde dehydrogenase
PTAC
Signature enzyme
Alcohol dehydrogenase
Regulator

Shigella dysenteriae. Bacteria; Proteobacteria; Gammaproteobacteria; Enterobacterales;

NCTC11868__locus_1

EUT1

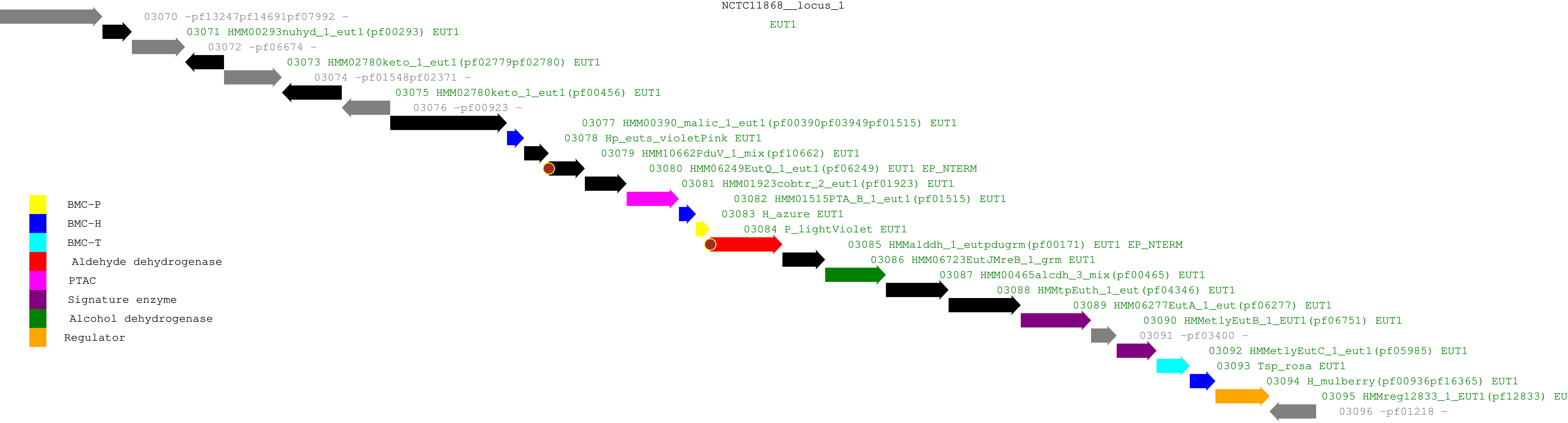

Salmonella sp. NCTC 11881. Bacteria; Proteobacteria; Gammaproteobacteria; Enterobacterales;

NCTC11881__locus_1

EUT1

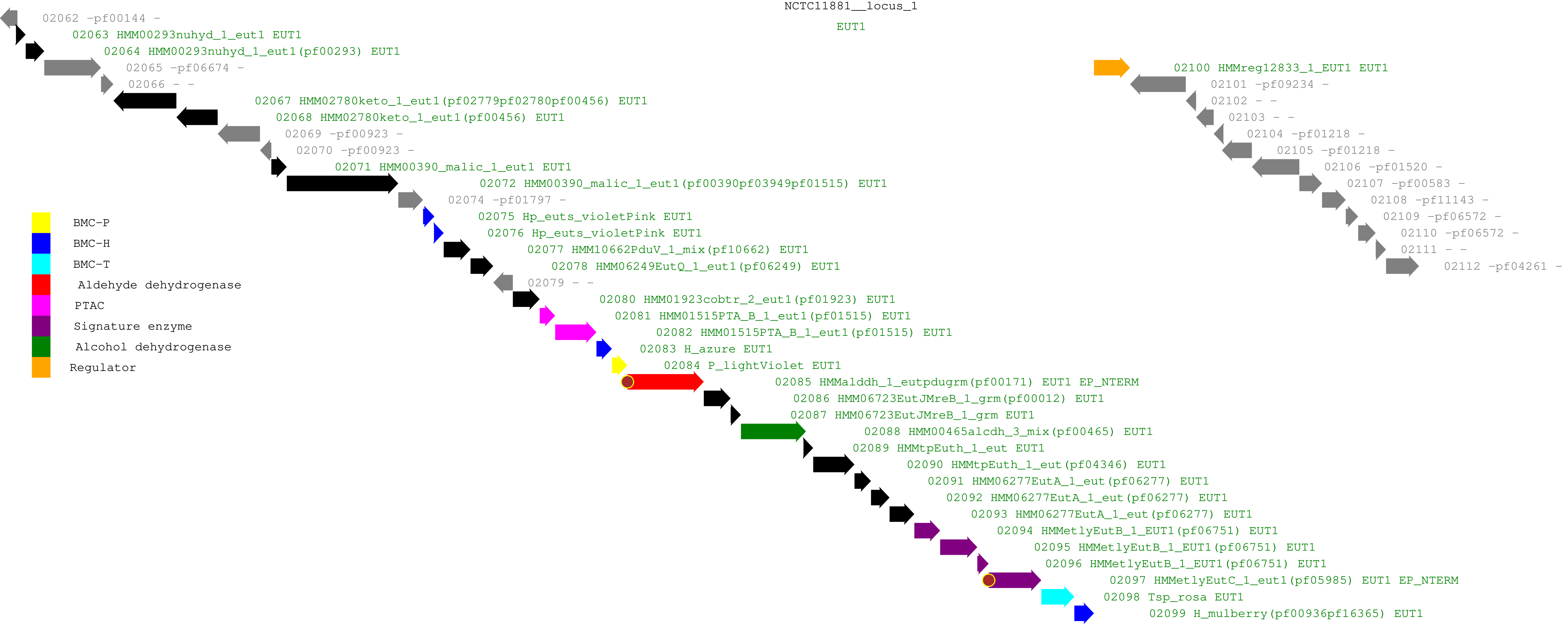

Morganella morganii (Proteus morganii). Bacteria; Proteobacteria; Gammaproteobacteria; Enterobacterales;

NCTC12358__locus_1

EUT1

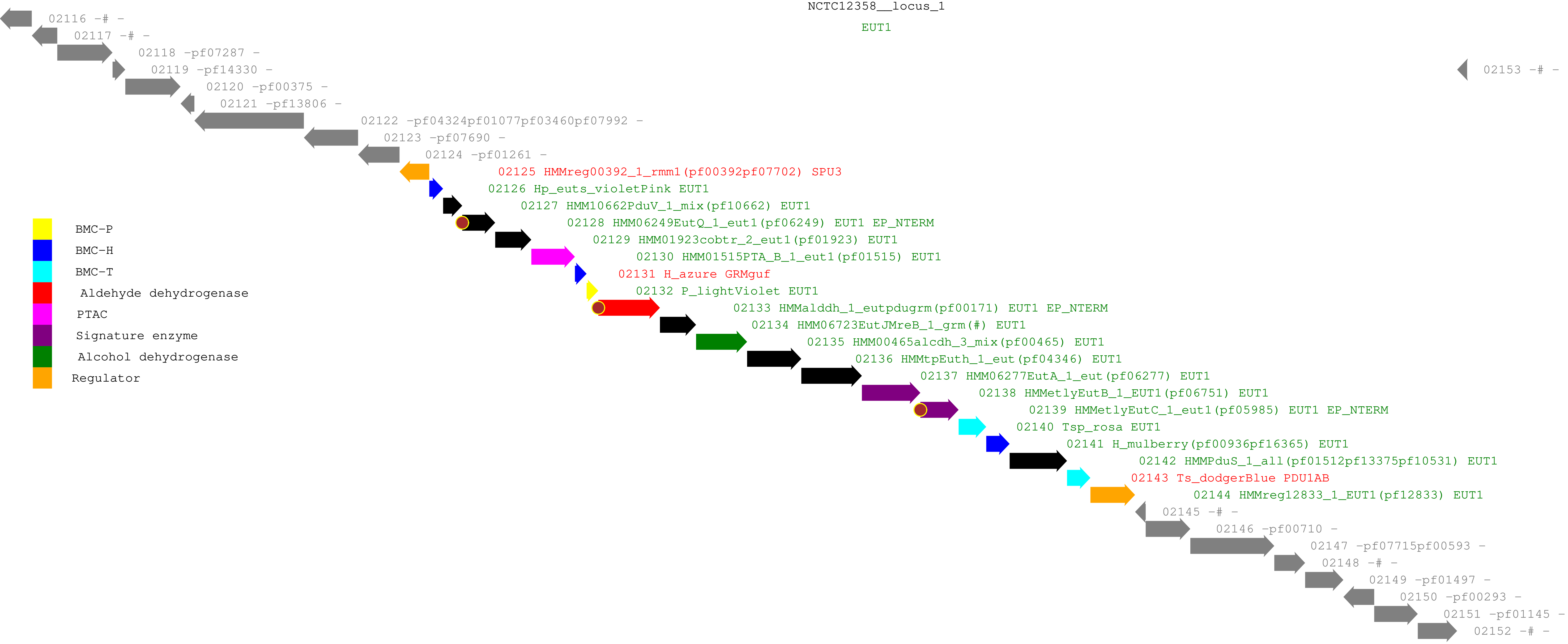

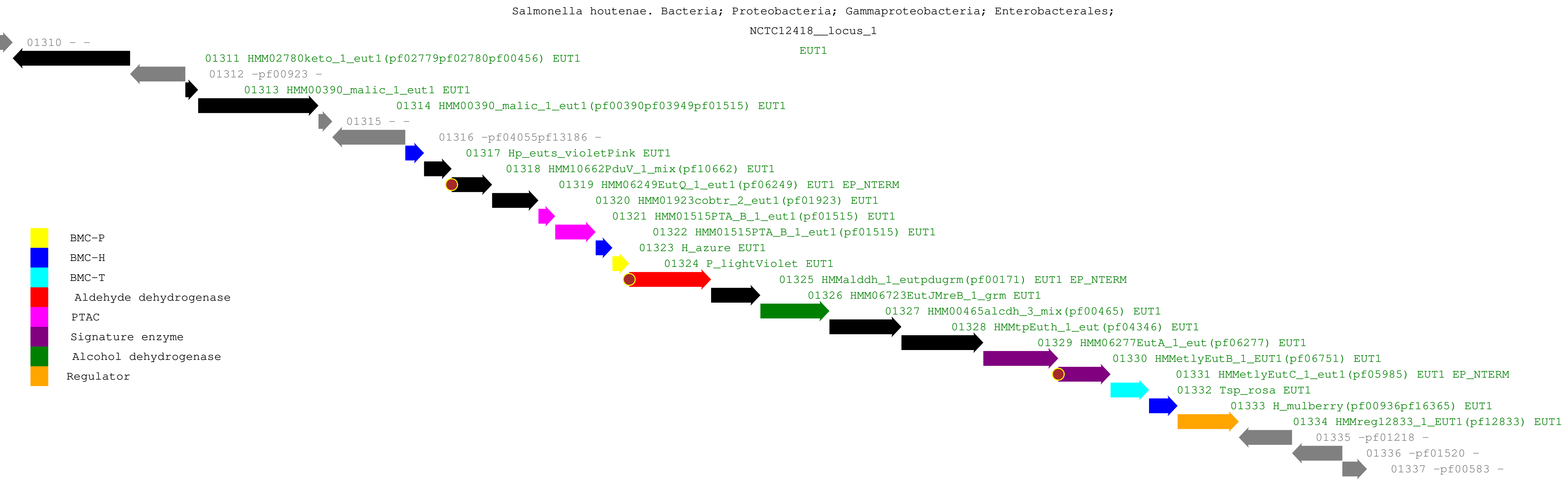
Salmonella houtenae. Bacteria; Proteobacteria; Gammaproteobacteria; Enterobacterales;
NCTC12418__locus_1
EUT1
01310 - -
01311 HMM02780keto_1_eut1(pf02779pf02780pf00456) EUT1
01312 -pf00923 -
01313 HMM00390_malic_1_eut1 EUT1
01314 HMM00390_malic_1_eut1(pf00390pf03949pf01515) EUT1
01315 - -
01316 -pf04055pf13186 -
01317 Hp_euts_violetPink EUT1
01318 HMM10662PduV_1_mix(pf10662) EUT1
01319 HMM06249EutQ_1_eut1(pf06249) EUT1 EP_NTERM
01320 HMM01923cobtr_2_eut1(pf01923) EUT1
01321 HMM01515PTA_B_1_eut1(pf01515) EUT1
01322 HMM01515PTA_B_1_eut1(pf01515) EUT1
01323 H_azure EUT1
01324 P_lightViolet EUT1
01325 HMMalddh_1_eutpdugrm(pf00171) EUT1 EP_NTERM
01326 HMM06723EutJMreB_1_grm EUT1
01327 HMM00465alcdh_3_mix(pf00465) EUT1
01328 HMMtpEuth_1_eut(pf04346) EUT1
01329 HMM06277EutA_1_eut(pf06277) EUT1
01330 HMMetlyEutB_1_EUT1(pf06751) EUT1
01331 HMMetlyEutC_1_eut1(pf05985) EUT1 EP_NTERM
01332 Tsp_rosa EUT1
01333 H_mulberry(pf00936pf16365) EUT1
01334 HMMreg12833_1_EUT1(pf12833) EUT1
01335 -pf01218 -
01336 -pf01520 -
01337 -pf00583 -
BMC-P
BMC-H
BMC-T
Aldehyde dehydrogenase
PTAC
Signature enzyme
Alcohol dehydrogenase
Regulator

Salmonella enterica subsp. indica. Bacteria; Proteobacteria; Gammaproteobacteria; Enterobacterales;
NCTC12420__locus_1
EUT1

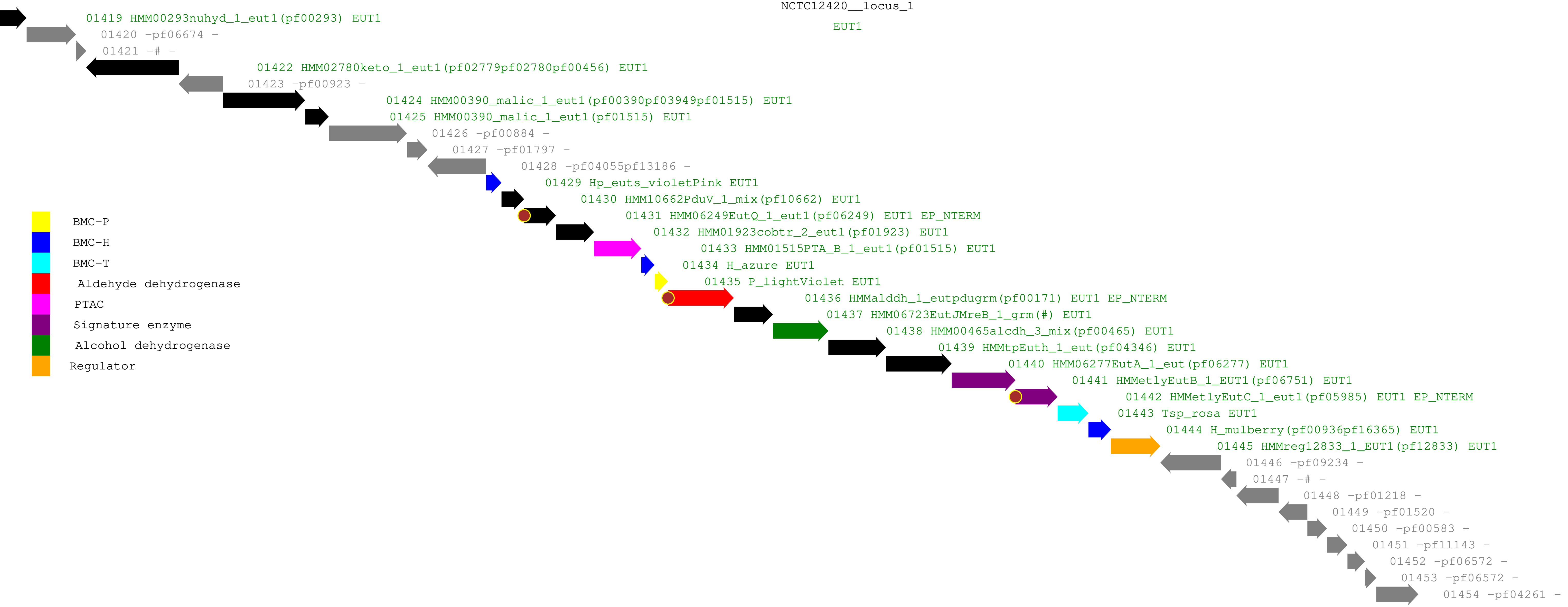

Salmonella dublin. Bacteria; Proteobacteria; Gammaproteobacteria; Enterobacterales;

NCTC12709__locus_1

EUT1

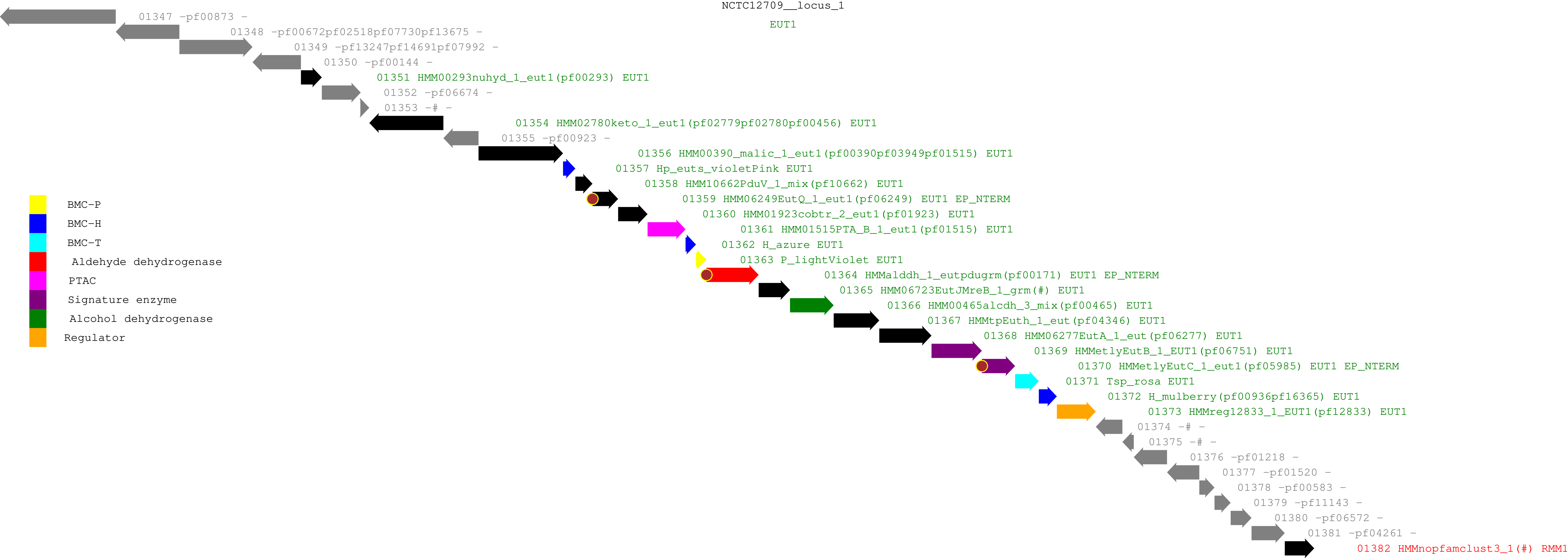

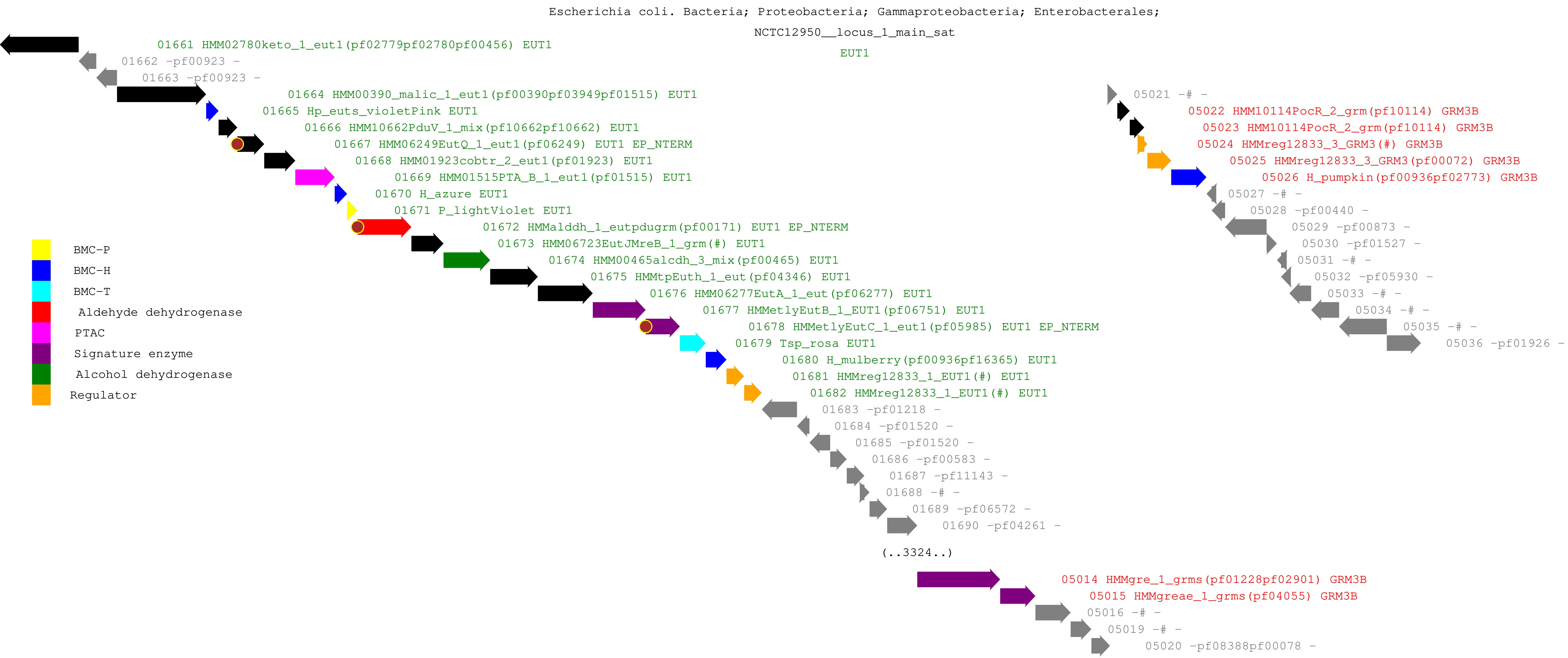
Escherichia coli. Bacteria; Proteobacteria; Gammaproteobacteria; Enterobacterales;
NCTC12950__locus_1_main_sat
EUT1
01661 HMM02780keto_1_eut1(pf02779pf02780pf00456) EUT1
01662 -pf00923 -
01663 -pf00923 -
01664 HMM00390_malic_1_eut1(pf00390pf03949pf01515) EUT1
01665 Hp_euts_violetPink EUT1
01666 HMM10662PduV_1_mix(pf10662pf10662) EUT1
01667 HMM06249EutQ_1_eut1(pf06249) EUT1 EP_NTERM
01668 HMM01923cobtr_2_eut1(pf01923) EUT1
01669 HMM01515PTA_B_1_eut1(pf01515) EUT1
01670 H_azure EUT1
01671 P_lightViolet EUT1
01672 HMMalddh_1_eutpdugrm(pf00171) EUT1 EP_NTERM
01673 HMM06723EutJMreB_1_grm(#) EUT1
01674 HMM00465alcdh_3_mix(pf00465) EUT1
01675 HMMtpEuth_1_eut(pf04346) EUT1
01676 HMM06277EutA_1_eut(pf06277) EUT1
01677 HMMetlyEutB_1_EUT1(pf06751) EUT1
01678 HMMetlyEutC_1_eut1(pf05985) EUT1 EP_NTERM
01679 Tsp_rosa EUT1
01680 H_mulberry(pf00936pf16365) EUT1
01681 HMMreg12833_1_EUT1(#) EUT1
01682 HMMreg12833_1_EUT1(#) EUT1
01683 -pf01218 -
01684 -pf01520 -
01685 -pf01520 -
01686 -pf00583 -
01687 -pf11143 -
01688 -# -
01689 -pf06572 -
01690 -pf04261 -
(..3324..)
05014 HMMgre_1_grms(pf01228pf02901) GRM3B
05015 HMMgreae_1_grms(pf04055) GRM3B
05016 -# -
05019 -# -
05020 -pf08388pf00078 -
05021 -# -
05022 HMM10114PocR_2_grm(pf10114) GRM3B
05023 HMM10114PocR_2_grm(pf10114) GRM3B
05024 HMMreg12833_3_GRM3(#) GRM3B
05025 HMMreg12833_3_GRM3(pf00072) GRM3B
05026 H_pumpkin(pf00936pf02773) GRM3B
05027 -# -
05028 -pf00440 -
05029 -pf00873 -
05030 -pf01527 -
05031 -# -
05032 -pf05930 -
05033 -# -
05034 -# -
05035 -# -
05036 -pf01926 -
BMC-P
BMC-H
BMC-T
Aldehyde dehydrogenase
PTAC
Signature enzyme
Alcohol dehydrogenase
Regulator

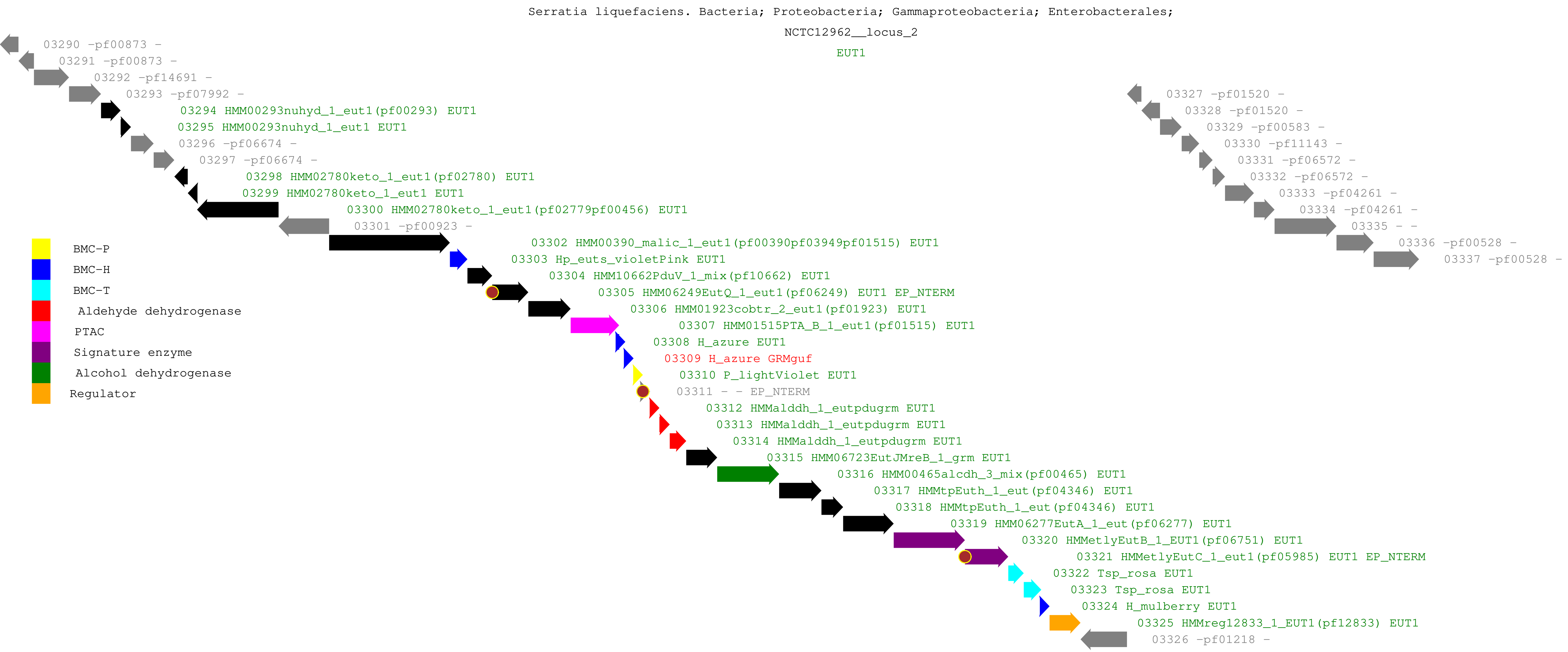

Serratia liquefaciens. Bacteria; Proteobacteria; Gammaproteobacteria; Enterobacterales;
NCTC12962__locus_2
EUT1
03290 -pf00873 -
03291 -pf00873 -
03292 -pf14691 -
03293 -pf07992 -
03294 HMM00293nuhyd_1_eut1(pf00293) EUT1
03295 HMM00293nuhyd_1_eut1 EUT1
03296 -pf06674 -
03297 -pf06674 -
03298 HMM02780keto_1_eut1(pf02780) EUT1
03299 HMM02780keto_1_eut1 EUT1
03300 HMM02780keto_1_eut1(pf02779pf00456) EUT1
03301 -pf00923 -
03302 HMM00390_malic_1_eut1(pf00390pf03949pf01515) EUT1
03303 Hp_euts_violetPink EUT1
03304 HMM10662PduV_1_mix(pf10662) EUT1
03305 HMM06249EutQ_1_eut1(pf06249) EUT1 EP_NTERM
03306 HMM01923cobtr_2_eut1(pf01923) EUT1
03307 HMM01515PTA_B_1_eut1(pf01515) EUT1
03308 H_azure EUT1
03309 H_azure GRMguf
03310 P_lightViolet EUT1
03311 - - EP_NTERM
03312 HMMalddh_1_eutpdugrm EUT1
03313 HMMalddh_1_eutpdugrm EUT1
03314 HMMalddh_1_eutpdugrm EUT1
03315 HMM06723EutJMreB_1_grm EUT1
03316 HMM00465alcdh_3_mix(pf00465) EUT1
03317 HMMtpEuth_1_eut(pf04346) EUT1
03318 HMMtpEuth_1_eut(pf04346) EUT1
03319 HMM06277EutA_1_eut(pf06277) EUT1
03320 HMMetlyEutB_1_EUT1(pf06751) EUT1
03321 HMMetlyEutC_1_eut1(pf05985) EUT1 EP_NTERM
03322 Tsp_rosa EUT1
03323 Tsp_rosa EUT1
03324 H_mulberry EUT1
03325 HMMreg12833_1_EUT1(pf12833) EUT1
03326 -pf01218 -
03327 -pf01520 -
03328 -pf01520 -
03329 -pf00583 -
03330 -pf11143 -
03331 -pf06572 -
03332 -pf06572 -
03333 -pf04261 -
03334 -pf04261 -
03335 - -
03336 -pf00528 -
03337 -pf00528 -
BMC-P
BMC-H
BMC-T
Aldehyde dehydrogenase
PTAC
Signature enzyme
Alcohol dehydrogenase
Regulator

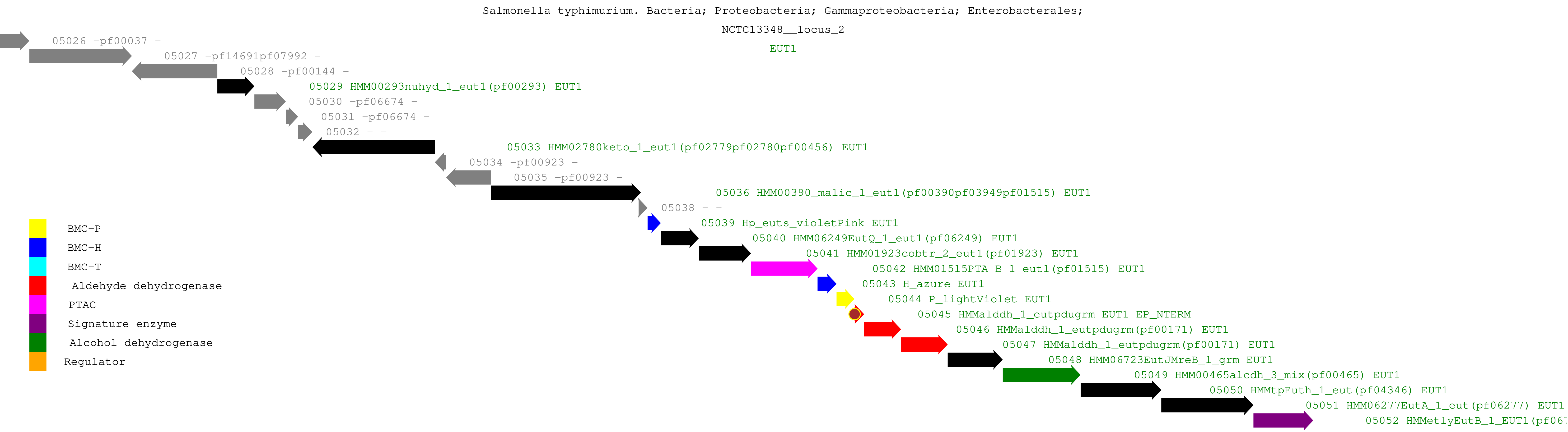
Salmonella typhimurium. Bacteria; Proteobacteria; Gammaproteobacteria; Enterobacterales;
NCTC13348__locus_2
EUT1
05026 -pf00037 -
05027 -pf14691pf07992 -
05028 -pf00144 -
05029 HMM00293nuhyd_1_eut1(pf00293) EUT1
05030 -pf06674 -
05031 -pf06674 -
05032 - -
05033 HMM02780keto_1_eut1(pf02779pf02780pf00456) EUT1
05034 -pf00923 -
05035 -pf00923 -
05036 HMM00390_malic_1_eut1(pf00390pf03949pf01515) EUT1
05038 - -
05039 Hp_euts_violetPink EUT1
05040 HMM06249EutQ_1_eut1(pf06249) EUT1
05041 HMM01923cobtr_2_eut1(pf01923) EUT1
05042 HMM01515PTA_B_1_eut1(pf01515) EUT1
05043 H_azure EUT1
05044 P_lightViolet EUT1
05045 HMMalddh_1_eutpdugrm EUT1 EP_NTERM
05046 HMMalddh_1_eutpdugrm(pf00171) EUT1
05047 HMMalddh_1_eutpdugrm(pf00171) EUT1
05048 HMM06723EutJMreB_1_grm EUT1
05049 HMM00465alcdh_3_mix(pf00465) EUT1
05050 HMMtpEuth_1_eut(pf04346) EUT1
05051 HMM06277EutA_1_eut(pf06277) EUT1
05052 HMMetlyEutB_1_EUT1(pf067
BMC-P
BMC-H
BMC-T
Aldehyde dehydrogenase
PTAC
Signature enzyme
Alcohol dehydrogenase
Regulator

Escherichia coli. Bacteria; Proteobacteria; Gammaproteobacteria; Enterobacterales;

NCTC13353__locus_1

EUT1

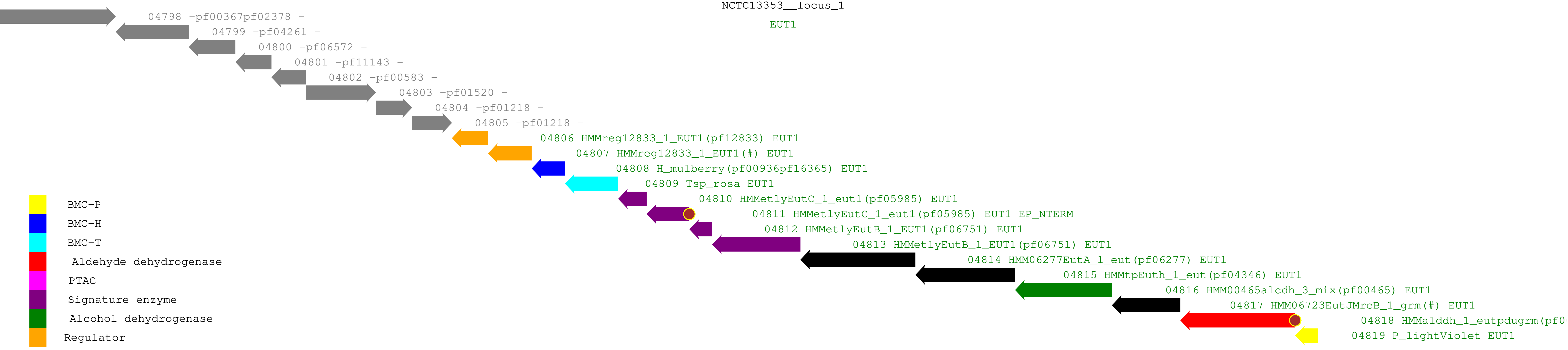

Klebsiella pneumoniae. Bacteria; Proteobacteria; Gammaproteobacteria; Enterobacterales;

NCTC13443__locus_1

EUT1

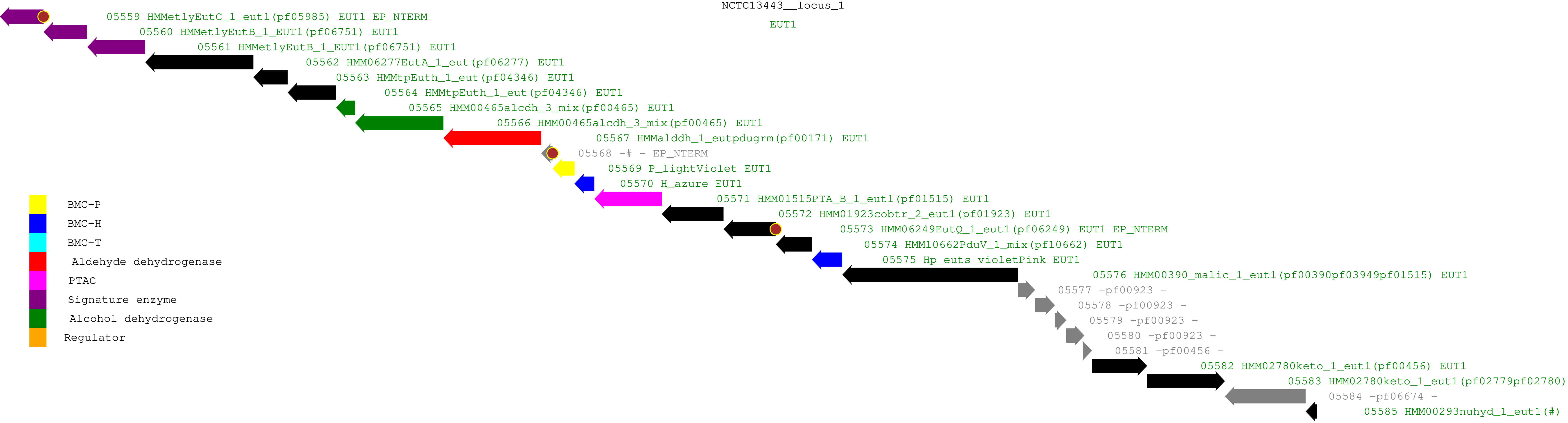

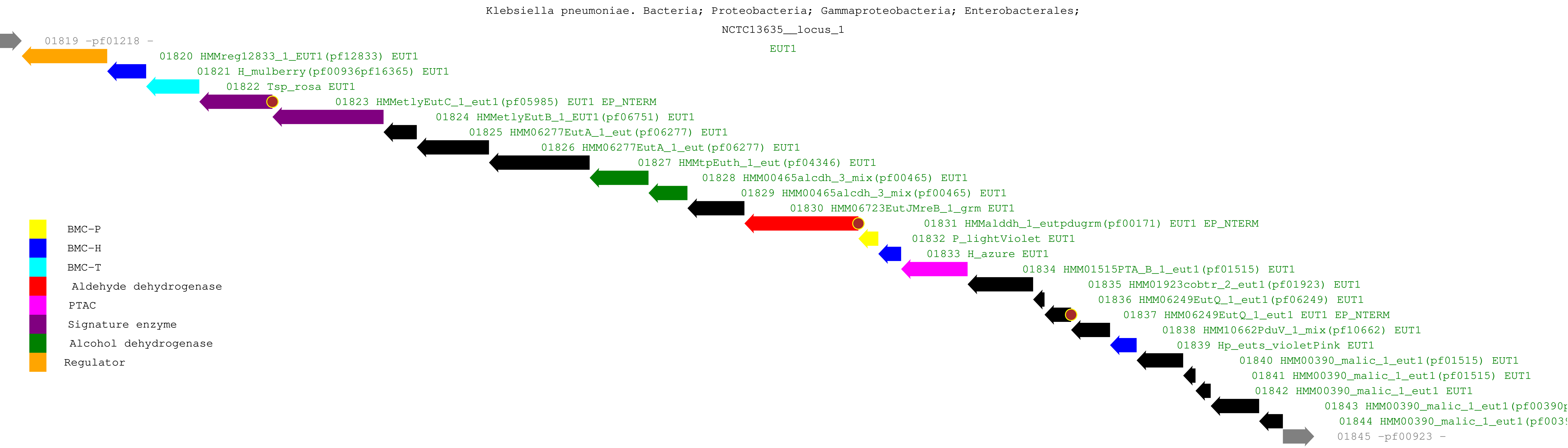
Klebsiella pneumoniae. Bacteria; Proteobacteria; Gammaproteobacteria; Enterobacterales;
NCTC13635__locus_1
EUT1
01819 -pf01218 -
01820 HMMreg12833_1_EUT1(pf12833) EUT1
01821 H_mulberry(pf00936pf16365) EUT1
01822 Tsp_rosa EUT1
01823 HMMetlyEutC_1_eut1(pf05985) EUT1 EP_NTERM
01824 HMMetlyEutB_1_EUT1(pf06751) EUT1
01825 HMM06277EutA_1_eut(pf06277) EUT1
01826 HMM06277EutA_1_eut(pf06277) EUT1
01827 HMMtpEuth_1_eut(pf04346) EUT1
01828 HMM00465alcdh_3_mix(pf00465) EUT1
01829 HMM00465alcdh_3_mix(pf00465) EUT1
01830 HMM06723EutJMreB_1_grm EUT1
01831 HMMalddh_1_eutpdugrm(pf00171) EUT1 EP_NTERM
01832 P_lightViolet EUT1
01833 H_azure EUT1
01834 HMM01515PTA_B_1_eut1(pf01515) EUT1
01835 HMM01923cobtr_2_eut1(pf01923) EUT1
01836 HMM06249EutQ_1_eut1(pf06249) EUT1
01837 HMM06249EutQ_1_eut1 EUT1 EP_NTERM
01838 HMM10662PduV_1_mix(pf10662) EUT1
01839 Hp_euts_violetPink EUT1
01840 HMM00390_malic_1_eut1(pf01515) EUT1
01841 HMM00390_malic_1_eut1(pf01515) EUT1
01842 HMM00390_malic_1_eut1 EUT1
01843 HMM00390_malic_1_eut1(pf00390p
01844 HMM00390_malic_1_eut1(pf003
01845 -pf00923 -
BMC-P
BMC-H
BMC-T
Aldehyde dehydrogenase
PTAC
Signature enzyme
Alcohol dehydrogenase
Regulator

Citrobacter youngae. Bacteria; Proteobacteria; Gammaproteobacteria; Enterobacterales;

NCTC13708__locus_1

EUT1

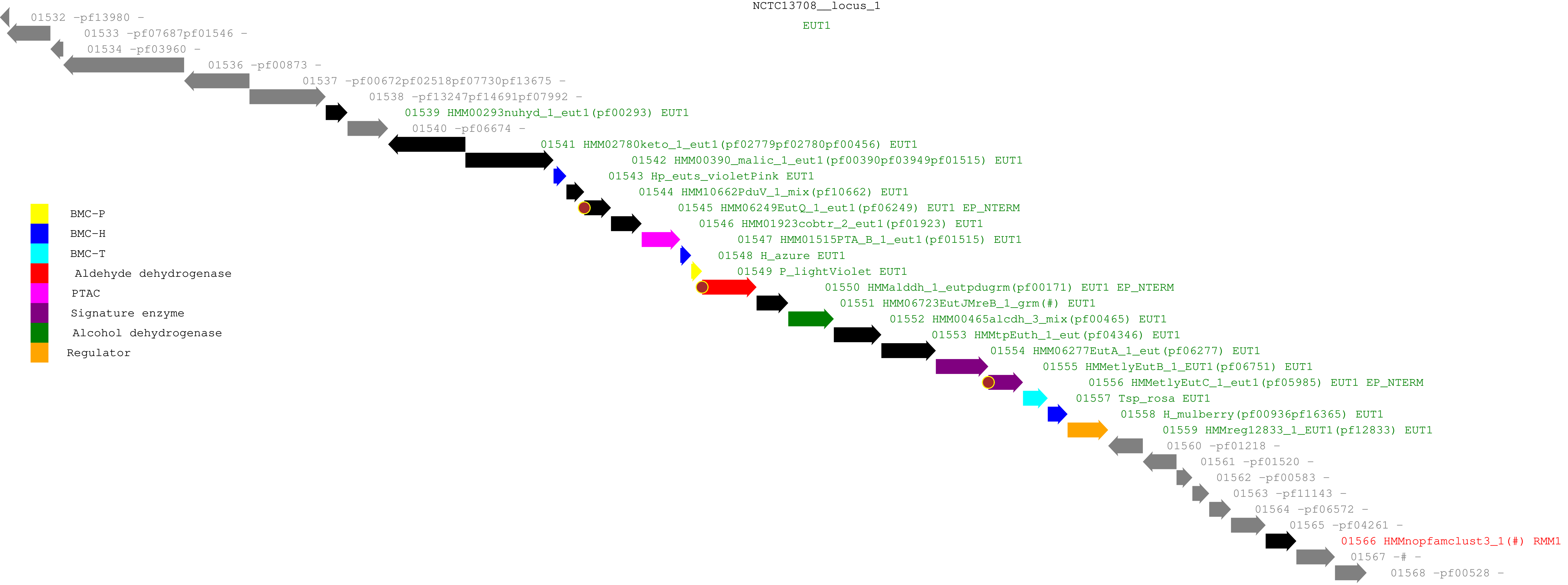

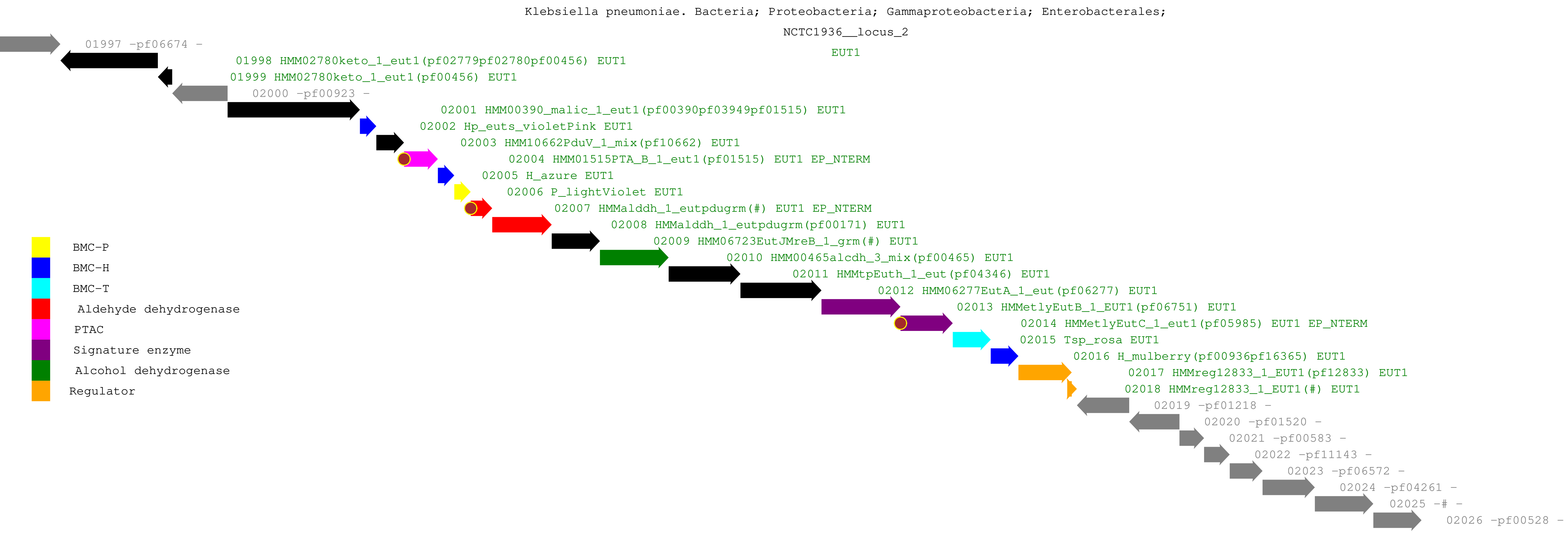
Klebsiella pneumoniae. Bacteria; Proteobacteria; Gammaproteobacteria; Enterobacterales;
NCTC1936__locus_2
EUT1
01997 -pf06674 -
01998 HMM02780keto_1_eut1(pf02779pf02780pf00456) EUT1
01999 HMM02780keto_1_eut1(pf00456) EUT1
02000 -pf00923 -
02001 HMM00390_malic_1_eut1(pf00390pf03949pf01515) EUT1
02002 Hp_euts_violetPink EUT1
02003 HMM10662PduV_1_mix(pf10662) EUT1
02004 HMM01515PTA_B_1_eut1(pf01515) EUT1 EP_NTERM
02005 H_azure EUT1
02006 P_lightViolet EUT1
02007 HMMaldh_1_eutpdugrm(#) EUT1 EP_NTERM
02008 HMMaldh_1_eutpdugrm(pf00171) EUT1
02009 HMM06723EutJMreB_1_grm(#) EUT1
02010 HMM00465alcdh_3_mix(pf00465) EUT1
02011 HMMtpEuth_1_eut(pf04346) EUT1
02012 HMM06277EutA_1_eut(pf06277) EUT1
02013 HMMetlyEutB_1_EUT1(pf06751) EUT1
02014 HMMetlyEutC_1_eut1(pf05985) EUT1 EP_NTERM
02015 Tsp_rosa EUT1
02016 H_mulberry(pf00936pf16365) EUT1
02017 HMMreg12833_1_EUT1(pf12833) EUT1
02018 HMMreg12833_1_EUT1(#) EUT1
02019 -pf01218 -
02020 -pf01520 -
02021 -pf00583 -
02022 -pf11143 -
02023 -pf06572 -
02024 -pf04261 -
02025 -# -
02026 -pf00528 -
BMC-P
BMC-H
BMC-T
Aldehyde dehydrogenase
PTAC
Signature enzyme
Alcohol dehydrogenase
Regulator

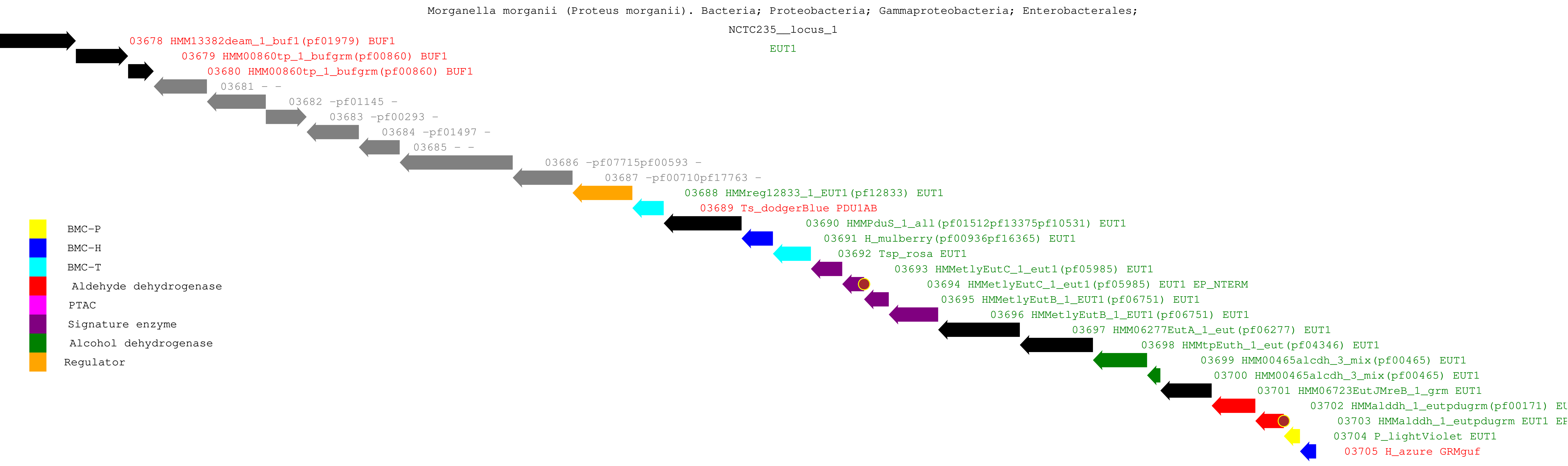

Morganella morganii (Proteus morganii). Bacteria; Proteobacteria; Gammaproteobacteria; Enterobacterales;
NCTC235__locus_1
EUT1
03678 HMM13382deam_1_buf1(pf01979) BUF1
03679 HMM00860tp_1_bufgrm(pf00860) BUF1
03680 HMM00860tp_1_bufgrm(pf00860) BUF1
03681 - -
03682 -pf01145 -
03683 -pf00293 -
03684 -pf01497 -
03685 - -
03686 -pf07715pf00593 -
03687 -pf00710pf17763 -
03688 HMMreg12833_1_EUT1(pf12833) EUT1
03689 Ts_dodgerBlue PDU1AB
03690 HMMPduS_1_all(pf01512pf13375pf10531) EUT1
03691 H_mulberry(pf00936pf16365) EUT1
03692 Tsp_rosa EUT1
03693 HMMetlyEutC_1_eut1(pf05985) EUT1
03694 HMMetlyEutC_1_eut1(pf05985) EUT1 EP_NTERM
03695 HMMetlyEutB_1_EUT1(pf06751) EUT1
03696 HMMetlyEutB_1_EUT1(pf06751) EUT1
03697 HMM06277EutA_1_eut(pf06277) EUT1
03698 HMMtpEuth_1_eut(pf04346) EUT1
03699 HMM00465alcdh_3_mix(pf00465) EUT1
03700 HMM00465alcdh_3_mix(pf00465) EUT1
03701 HMM06723EutJMreB_1_grm EUT1
03702 HMMalddh_1_eutpdugrm(pf00171) EU
03703 HMMalddh_1_eutpdugrm EUT1 EP
03704 P_lightViolet EUT1
03705 H_azure GRMguf
BMC-P
BMC-H
BMC-T
Aldehyde dehydrogenase
PTAC
Signature enzyme
Alcohol dehydrogenase
Regulator

Klebsiella aerogenes (Enterobacter aerogenes). Bacteria; Proteobacteria; Gammaproteobacteria; Enterobacterales;

NCTC243__locus_1

EUT1

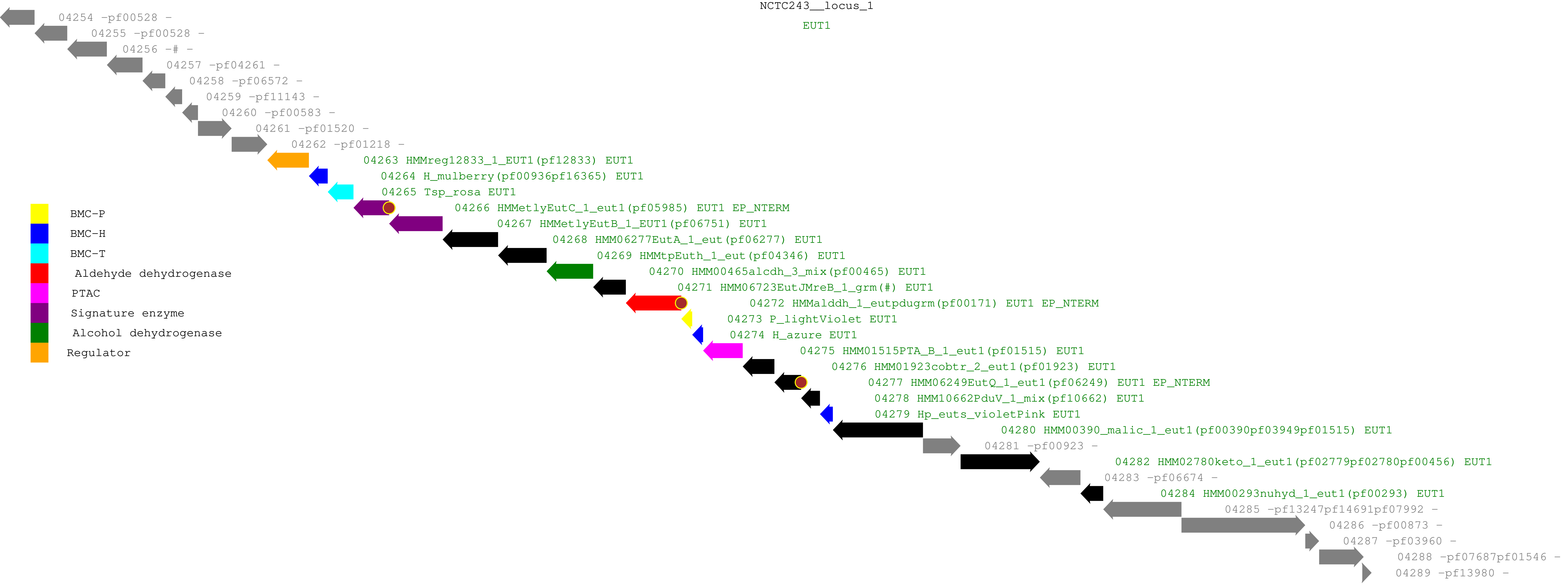

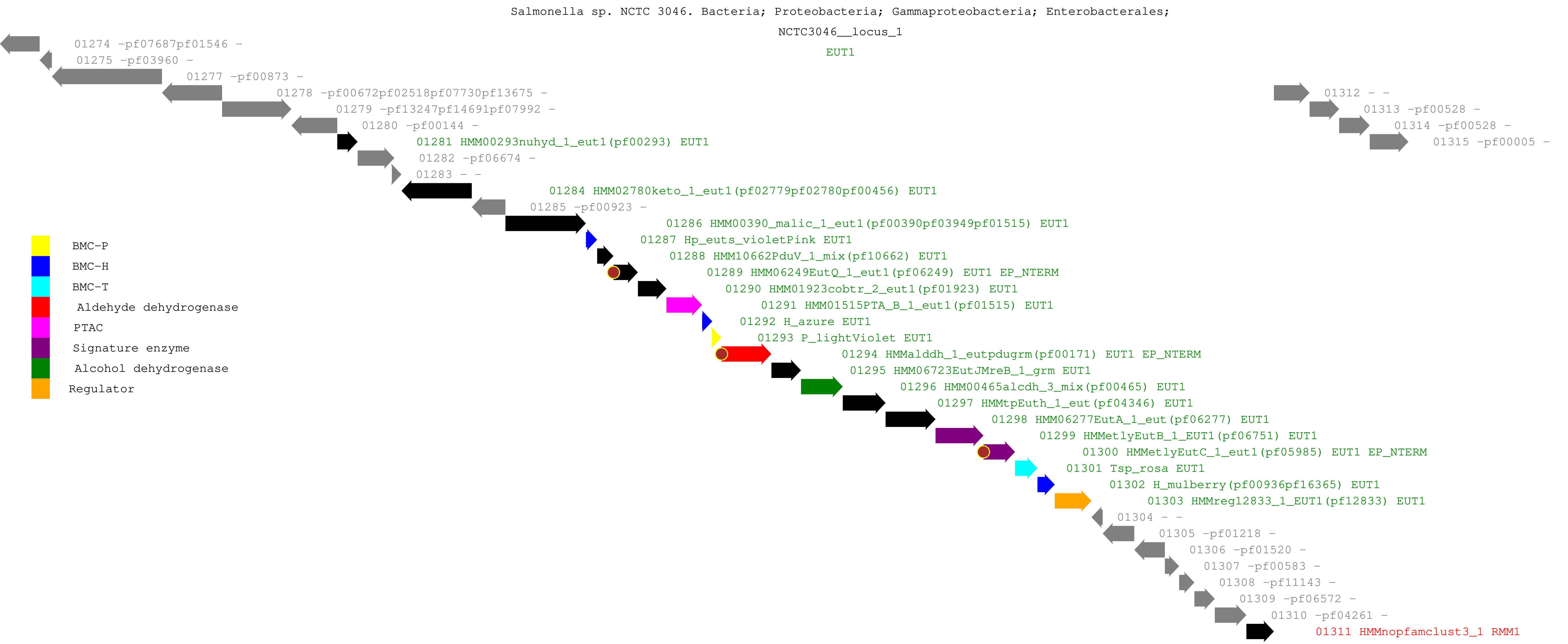
Salmonella sp. NCTC 3046. Bacteria; Proteobacteria; Gammaproteobacteria; Enterobacterales;
NCTC3046__locus_1
EUT1
01274 -pf07687pf01546 -
01275 -pf03960 -
01277 -pf00873 -
01278 -pf00672pf02518pf07730pf13675 -
01279 -pf13247pf14691pf07992 -
01280 -pf00144 -
01281 HMM00293nuhyd_1_eut1(pf00293) EUT1
01282 -pf06674 -
01283 - -
01284 HMM02780keto_1_eut1(pf02779pf02780pf00456) EUT1
01285 -pf00923 -
01286 HMM00390_malic_1_eut1(pf00390pf03949pf01515) EUT1
01287 Hp_euts_violetPink EUT1
01288 HMM10662PduV_1_mix(pf10662) EUT1
01289 HMM06249EutQ_1_eut1(pf06249) EUT1 EP_NTERM
01290 HMM01923cobtr_2_eut1(pf01923) EUT1
01291 HMM01515PTA_B_1_eut1(pf01515) EUT1
01292 H_azure EUT1
01293 P_lightViolet EUT1
01294 HMMaldh_1_eutpdugrm(pf00171) EUT1 EP_NTERM
01295 HMM06723EutJMreB_1_grm EUT1
01296 HMM00465alcdh_3_mix(pf00465) EUT1
01297 HMMtpEuth_1_eut(pf04346) EUT1
01298 HMM06277EutA_1_eut(pf06277) EUT1
01299 HMMetlyEutB_1_EUT1(pf06751) EUT1
01300 HMMetlyEutC_1_eut1(pf05985) EUT1 EP_NTERM
01301 Tsp_rosa EUT1
01302 H_mulberry(pf00936pf16365) EUT1
01303 HMMreg12833_1_EUT1(pf12833) EUT1
01304 - -
01305 -pf01218 -
01306 -pf01520 -
01307 -pf00583 -
01308 -pf11143 -
01309 -pf06572 -
01310 -pf04261 -
01311 HMMnopfamclust3_1 RMM1
01312 - -
01313 -pf00528 -
01314 -pf00528 -
01315 -pf00005 -
BMC-P
BMC-H
BMC-T
Aldehyde dehydrogenase
PTAC
Signature enzyme
Alcohol dehydrogenase
Regulator

Klebsiella pneumoniae subsp. rhinoscleromatis. Bacteria; Proteobacteria; Gammaproteobacteria; Enterobacterales;

NCTC5046__locus_3

EUT1

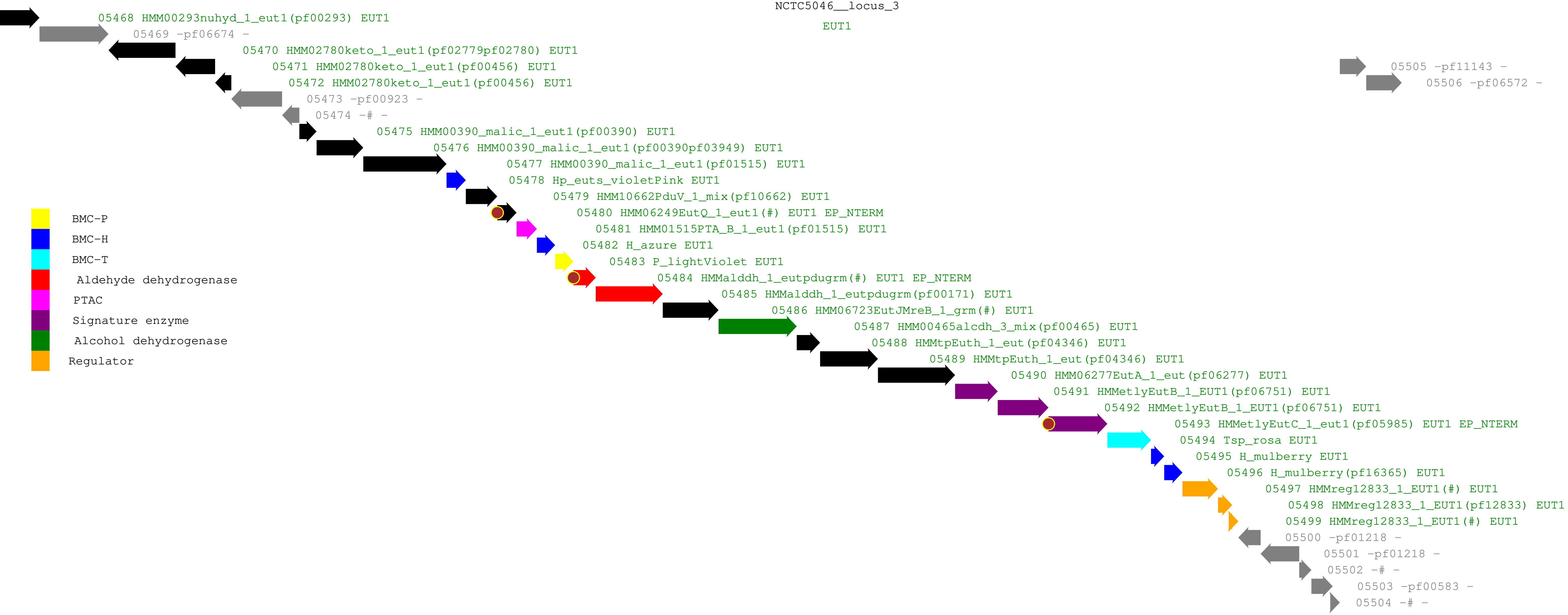

Klebsiella pneumoniae. Bacteria; Proteobacteria; Gammaproteobacteria; Enterobacterales;

NCTC5047__locus_2

EUT1

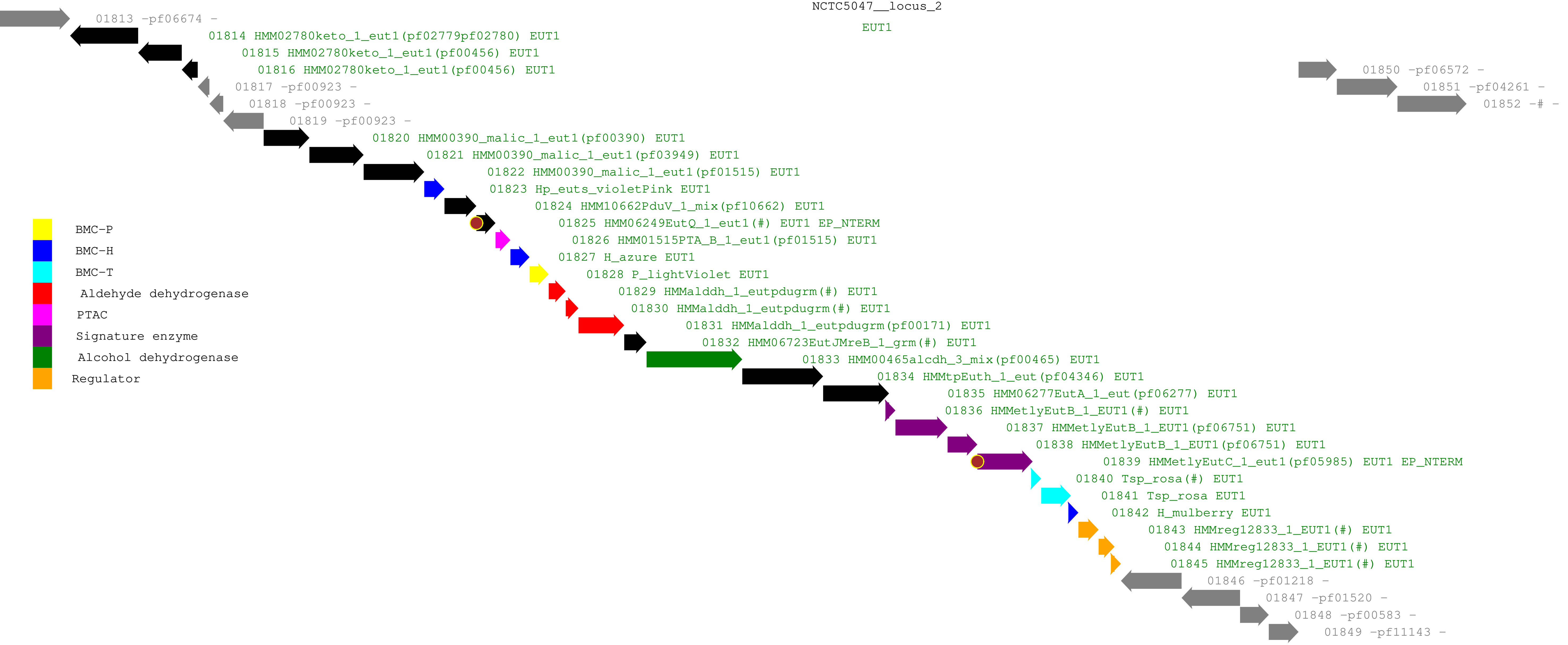

Klebsiella pneumoniae subsp. ozaenae. Bacteria; Proteobacteria; Gammaproteobacteria; Enterobacterales;

NCTC5050__locus_2

EUT1

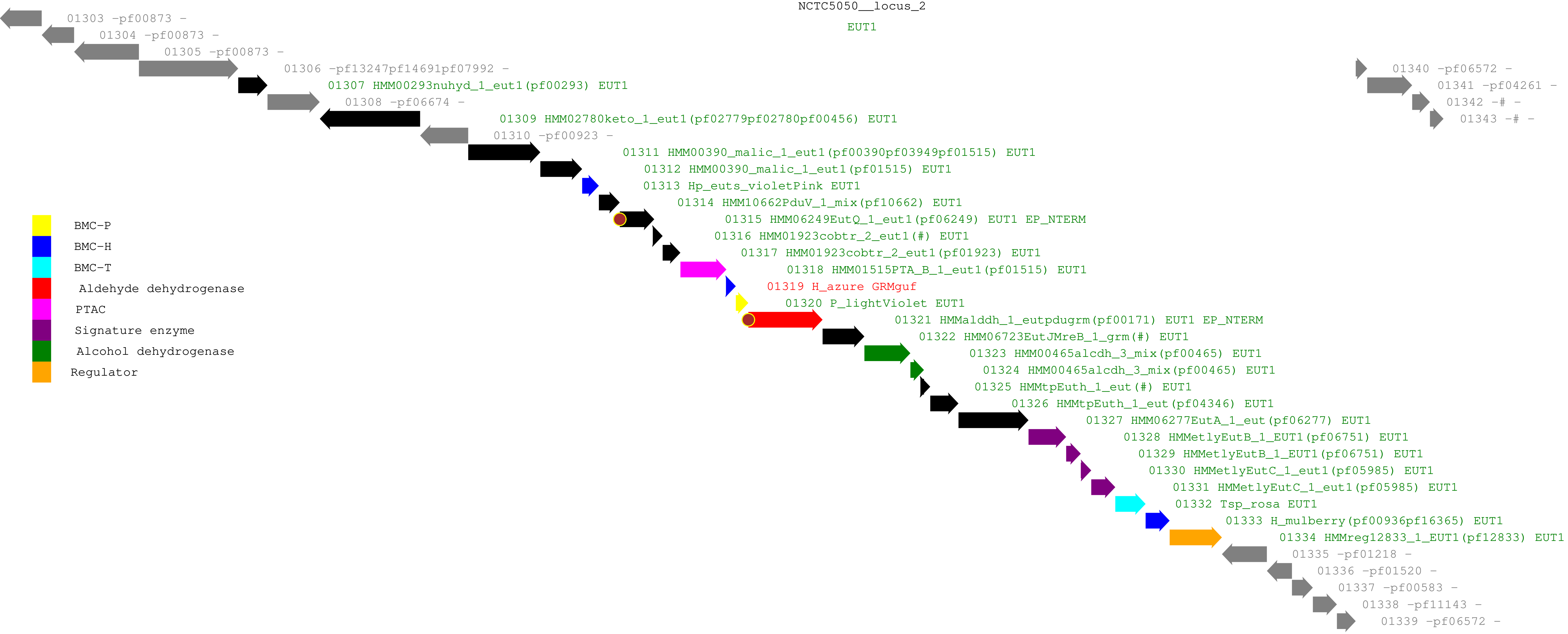

Klebsiella pneumoniae. Bacteria; Proteobacteria; Gammaproteobacteria; Enterobacterales;

NCTC5052__locus_2

EUT1

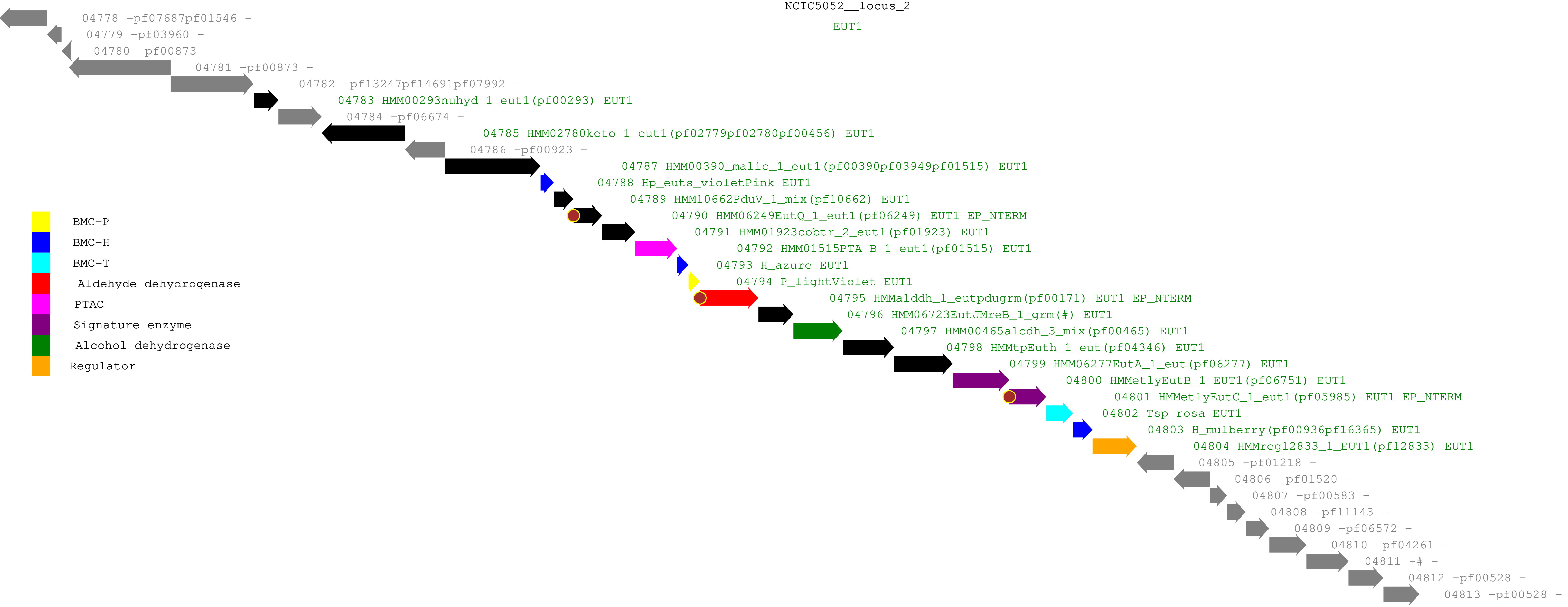

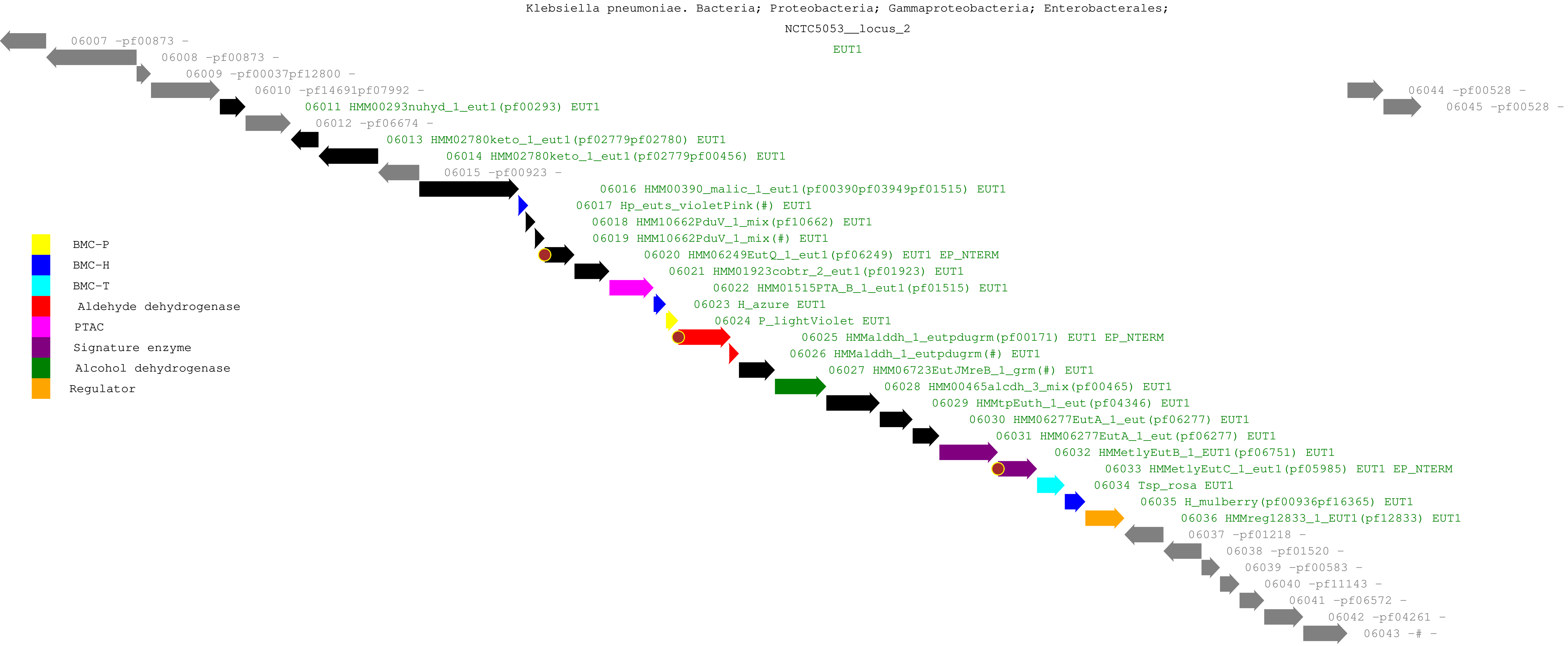

Klebsiella pneumoniae. Bacteria; Proteobacteria; Gammaproteobacteria; Enterobacterales;
NCTC5053__locus_2
EUT1
06007 -pf00873 -
06008 -pf00873 -
06009 -pf00037pf12800 -
06010 -pf14691pf07992 -
06011 HMM00293nuhyd_1_eut1(pf00293) EUT1
06012 -pf06674 -
06013 HMM02780keto_1_eut1(pf02779pf02780) EUT1
06014 HMM02780keto_1_eut1(pf02779pf00456) EUT1
06015 -pf00923 -
06016 HMM00390_malic_1_eut1(pf00390pf03949pf01515) EUT1
06017 Hp_euts_violetPink(#) EUT1
06018 HMM10662PduV_1_mix(pf10662) EUT1
06019 HMM10662PduV_1_mix(#) EUT1
06020 HMM06249EutQ_1_eut1(pf06249) EUT1 EP_NTERM
06021 HMM01923cobtr_2_eut1(pf01923) EUT1
06022 HMM01515PTA_B_1_eut1(pf01515) EUT1
06023 H_azure EUT1
06024 P_lightViolet EUT1
06025 HMMalddh_1_eutpdugrm(pf00171) EUT1 EP_NTERM
06026 HMMalddh_1_eutpdugrm(#) EUT1
06027 HMM06723EutJMreB_1_grm(#) EUT1
06028 HMM00465alcdh_3_mix(pf00465) EUT1
06029 HMMtpEuth_1_eut(pf04346) EUT1
06030 HMM06277EutA_1_eut(pf06277) EUT1
06031 HMM06277EutA_1_eut(pf06277) EUT1
06032 HMMetlyEutB_1_EUT1(pf06751) EUT1
06033 HMMetlyEutC_1_eut1(pf05985) EUT1 EP_NTERM
06034 Tsp_rosa EUT1
06035 H_mulberry(pf00936pf16365) EUT1
06036 HMMreg12833_1_EUT1(pf12833) EUT1
06037 -pf01218 -
06038 -pf01520 -
06039 -pf00583 -
06040 -pf11143 -
06041 -pf06572 -
06042 -pf04261 -
06043 -# -
06044 -pf00528 -
06045 -pf00528 -
BMC-P
BMC-H
BMC-T
Aldehyde dehydrogenase
PTAC
Signature enzyme
Alcohol dehydrogenase
Regulator

Klebsiella pneumoniae. Bacteria; Proteobacteria; Gammaproteobacteria; Enterobacterales;

NCTC5055__locus_1

EUT1

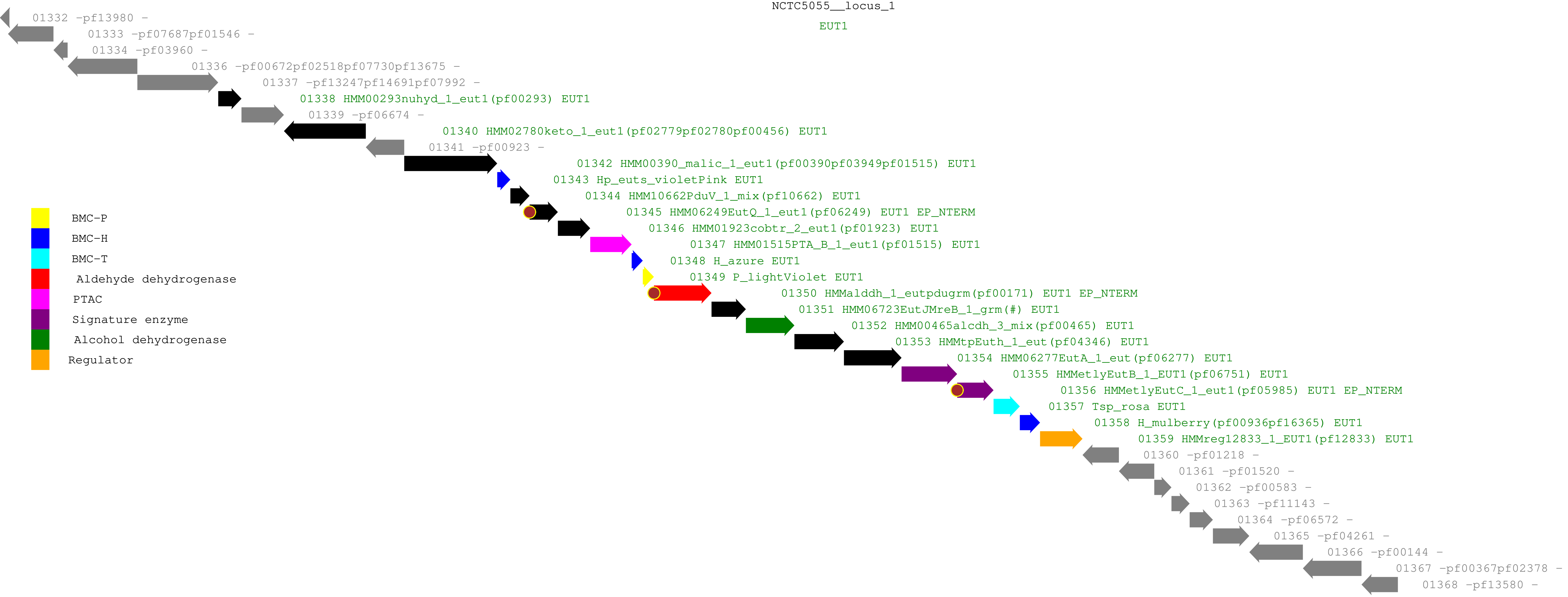

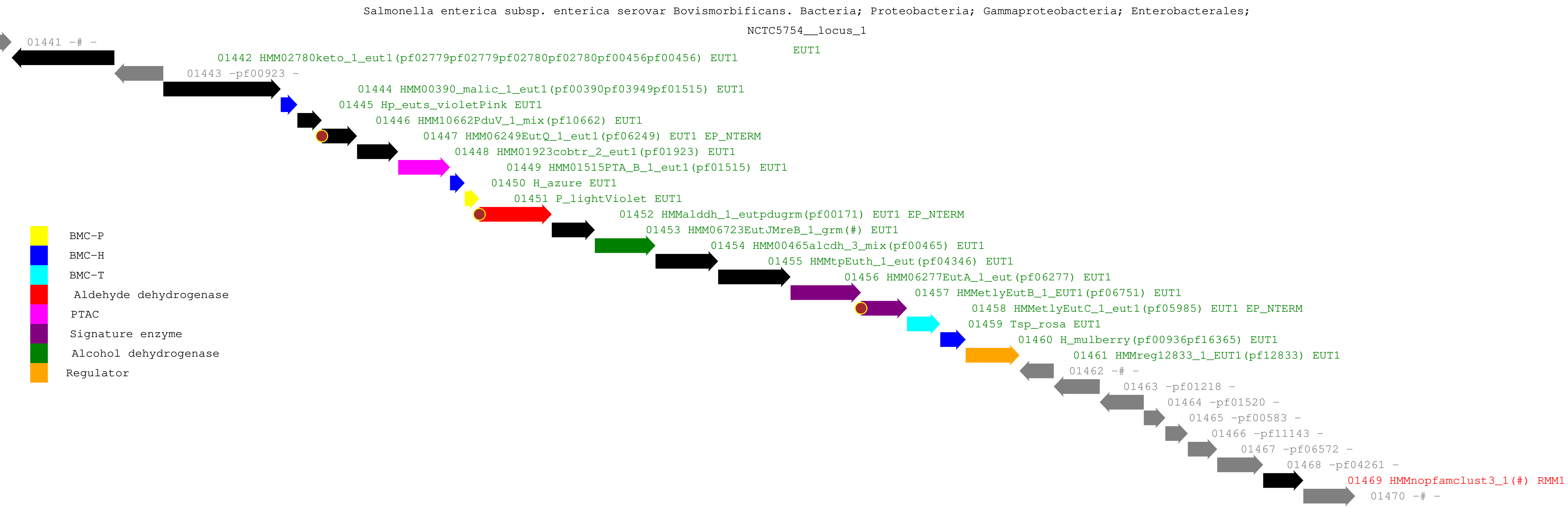

Salmonella enterica subsp. enterica serovar Bovismorbificans. Bacteria; Proteobacteria; Gammaproteobacteria; Enterobacterales;
NCTC5754__locus_1
EUT1
01441 -# -
01442 HMM02780keto_1_eut1(pf02779pf02779pf02780pf02780pf00456pf00456) EUT1
01443 -pf00923 -
01444 HMM00390_malic_1_eut1(pf00390pf03949pf01515) EUT1
01445 Hp_euts_violetPink EUT1
01446 HMM10662PduV_1_mix(pf10662) EUT1
01447 HMM06249EutQ_1_eut1(pf06249) EUT1 EP_NTERM
01448 HMM01923cobtr_2_eut1(pf01923) EUT1
01449 HMM01515PTA_B_1_eut1(pf01515) EUT1
01450 H_azure EUT1
01451 P_lightViolet EUT1
01452 HMMalddh_1_eutpdugrm(pf00171) EUT1 EP_NTERM
01453 HMM06723EutJMreB_1_grm(#) EUT1
01454 HMM00465alcdh_3_mix(pf00465) EUT1
01455 HMMtpEuth_1_eut(pf04346) EUT1
01456 HMM06277EutA_1_eut(pf06277) EUT1
01457 HMMetlyEutB_1_EUT1(pf06751) EUT1
01458 HMMetlyEutC_1_eut1(pf05985) EUT1 EP_NTERM
01459 Tsp_rosa EUT1
01460 H_mulberry(pf00936pf16365) EUT1
01461 HMMreg12833_1_EUT1(pf12833) EUT1
01462 -# -
01463 -pf01218 -
01464 -pf01520 -
01465 -pf00583 -
01466 -pf11143 -
01467 -pf06572 -
01468 -pf04261 -
01469 HMMnopfamclust3_1(#) RMM1
01470 -# -
BMC-P
BMC-H
BMC-T
Aldehyde dehydrogenase
PTAC
Signature enzyme
Alcohol dehydrogenase
Regulator

Salmonella enterica subsp. salamae. Bacteria; Proteobacteria; Gammaproteobacteria; Enterobacterales;

NCTC5773__locus_1

EUT1

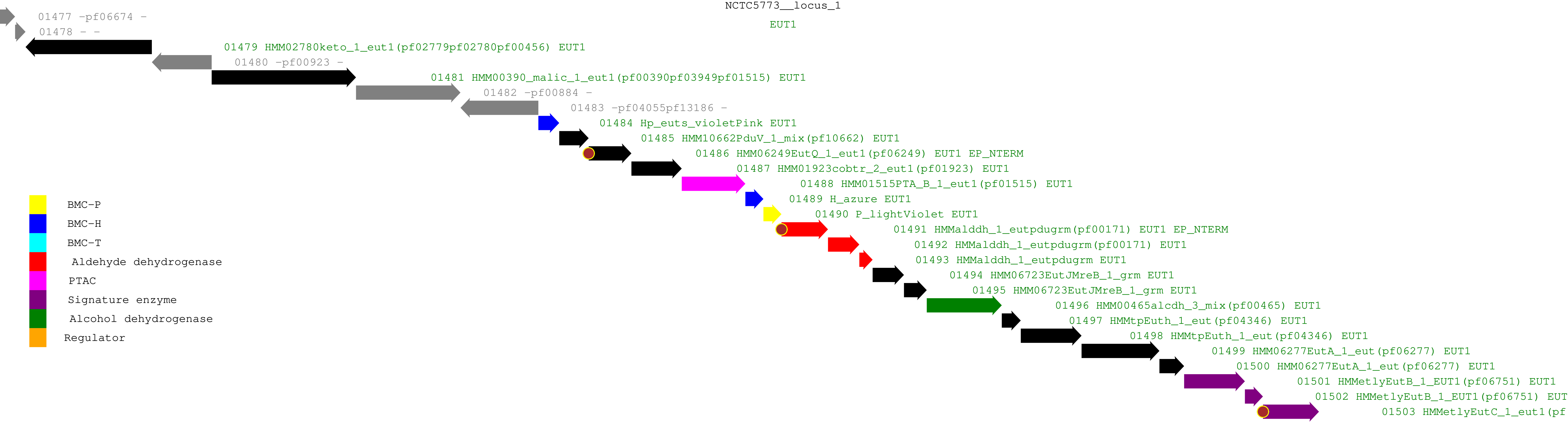

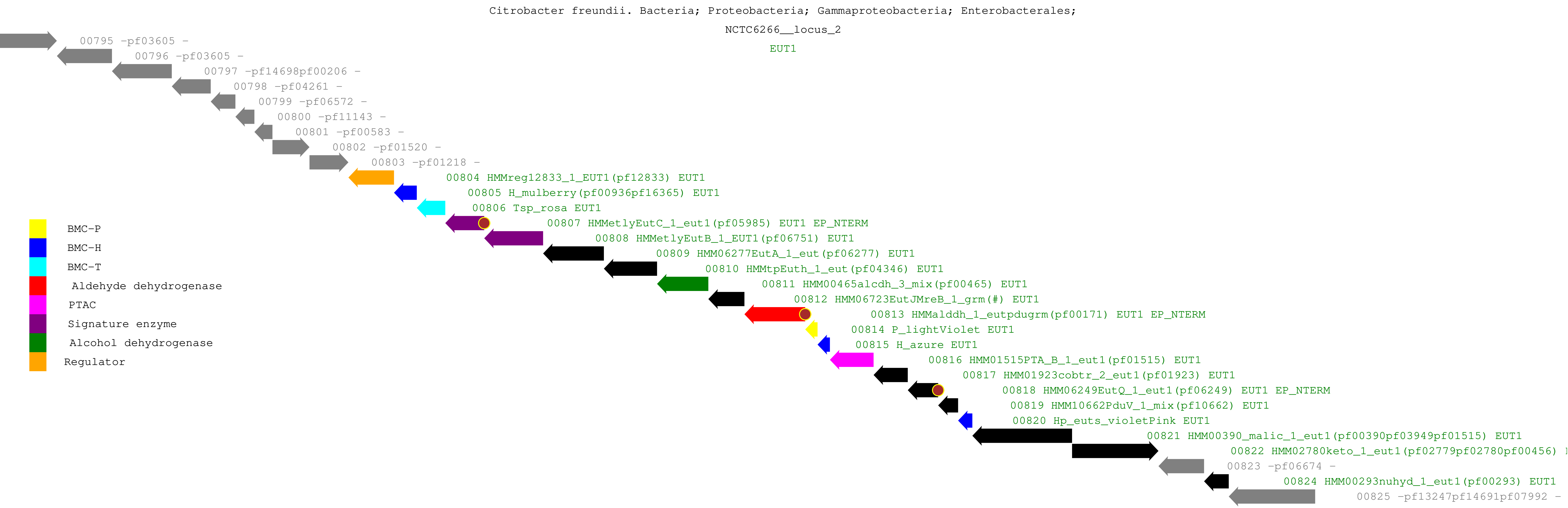
Citrobacter freundii. Bacteria; Proteobacteria; Gammaproteobacteria; Enterobacterales;
NCTC6266__locus_2
EUT1
00795 -pf03605 -
00796 -pf03605 -
00797 -pf14698pf00206 -
00798 -pf04261 -
00799 -pf06572 -
00800 -pf11143 -
00801 -pf00583 -
00802 -pf01520 -
00803 -pf01218 -
00804 HMMreg12833_1_EUT1(pf12833) EUT1
00805 H_mulberry(pf00936pf16365) EUT1
00806 Tsp_rosa EUT1
00807 HMMetlyEutC_1_eut1(pf05985) EUT1 EP_NTERM
00808 HMMetlyEutB_1_EUT1(pf06751) EUT1
00809 HMM06277EutA_1_eut(pf06277) EUT1
00810 HMMtpEuth_1_eut(pf04346) EUT1
00811 HMM00465alcdh_3_mix(pf00465) EUT1
00812 HMM06723EutJMreB_1_grm(#) EUT1
00813 HMMalddh_1_eutpdugrm(pf00171) EUT1 EP_NTERM
00814 P_lightViolet EUT1
00815 H_azure EUT1
00816 HMM01515PTA_B_1_eut1(pf01515) EUT1
00817 HMM01923cobtr_2_eut1(pf01923) EUT1
00818 HMM06249EutQ_1_eut1(pf06249) EUT1 EP_NTERM
00819 HMM10662PduV_1_mix(pf10662) EUT1
00820 Hp_euts_violetPink EUT1
00821 HMM00390_malic_1_eut1(pf00390pf03949pf01515) EUT1
00822 HMM02780keto_1_eut1(pf02779pf02780pf00456)
00823 -pf06674 -
00824 HMM00293nuhyd_1_eut1(pf00293) EUT1
00825 -pf13247pf14691pf07992 -
BMC-P
BMC-H
BMC-T
Aldehyde dehydrogenase
PTAC
Signature enzyme
Alcohol dehydrogenase
Regulator

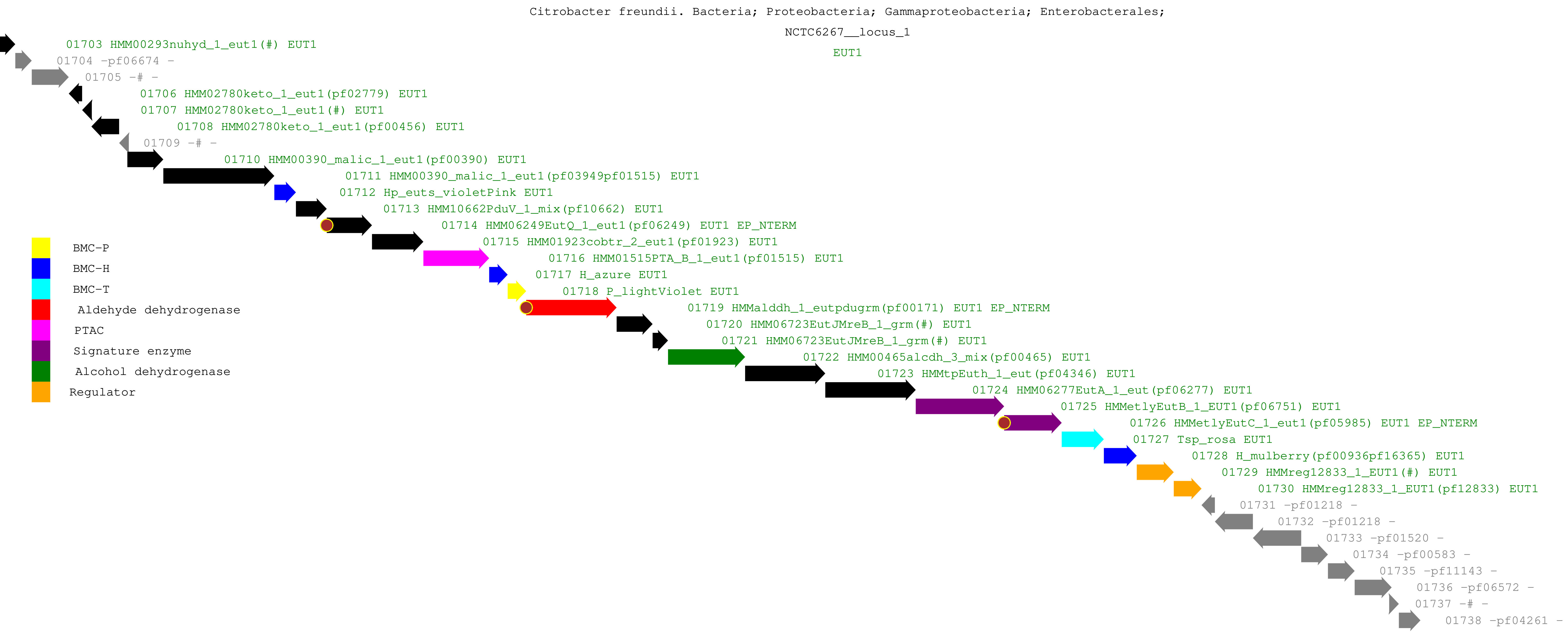
Citrobacter freundii. Bacteria; Proteobacteria; Gammaproteobacteria; Enterobacterales;
NCTC6267__locus_1
EUT1
01703 HMM00293nuhyd_1_eut1(#) EUT1
01704 -pf06674 -
01705 -# -
01706 HMM02780keto_1_eut1(pf02779) EUT1
01707 HMM02780keto_1_eut1(#) EUT1
01708 HMM02780keto_1_eut1(pf00456) EUT1
01709 -# -
01710 HMM00390_malic_1_eut1(pf00390) EUT1
01711 HMM00390_malic_1_eut1(pf03949pf01515) EUT1
01712 Hp_euts_violetPink EUT1
01713 HMM10662PduV_1_mix(pf10662) EUT1
01714 HMM06249EutQ_1_eut1(pf06249) EUT1 EP_NTERM
01715 HMM01923cobtr_2_eut1(pf01923) EUT1
01716 HMM01515PTA_B_1_eut1(pf01515) EUT1
01717 H_azure EUT1
01718 P_lightViolet EUT1
01719 HMMalddh_1_eutpdugrm(pf00171) EUT1 EP_NTERM
01720 HMM06723EutJMreB_1_grm(#) EUT1
01721 HMM06723EutJMreB_1_grm(#) EUT1
01722 HMM00465alcdh_3_mix(pf00465) EUT1
01723 HMMtpEuth_1_eut(pf04346) EUT1
01724 HMM06277EutA_1_eut(pf06277) EUT1
01725 HMMetlyEutB_1_EUT1(pf06751) EUT1
01726 HMMetlyEutC_1_eut1(pf05985) EUT1 EP_NTERM
01727 Tsp_rosa EUT1
01728 H_mulberry(pf00936pf16365) EUT1
01729 HMMreg12833_1_EUT1(#) EUT1
01730 HMMreg12833_1_EUT1(pf12833) EUT1
01731 -pf01218 -
01732 -pf01218 -
01733 -pf01520 -
01734 -pf00583 -
01735 -pf11143 -
01736 -pf06572 -
01737 -# -
01738 -pf04261 -
BMC-P
BMC-H
BMC-T
Aldehyde dehydrogenase
PTAC
Signature enzyme
Alcohol dehydrogenase
Regulator

Salmonella enterica I. Bacteria; Proteobacteria; Gammaproteobacteria; Enterobacterales;

NCTC6754__locus_1

EUT1

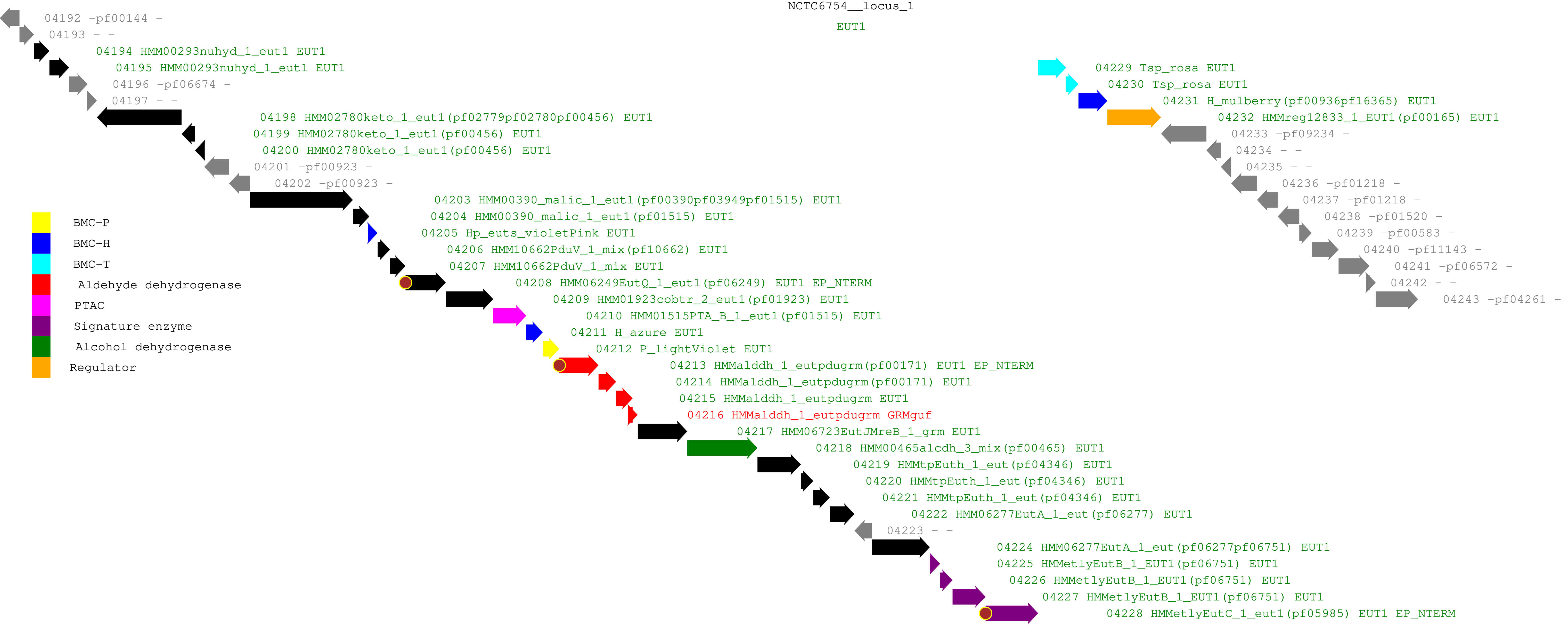

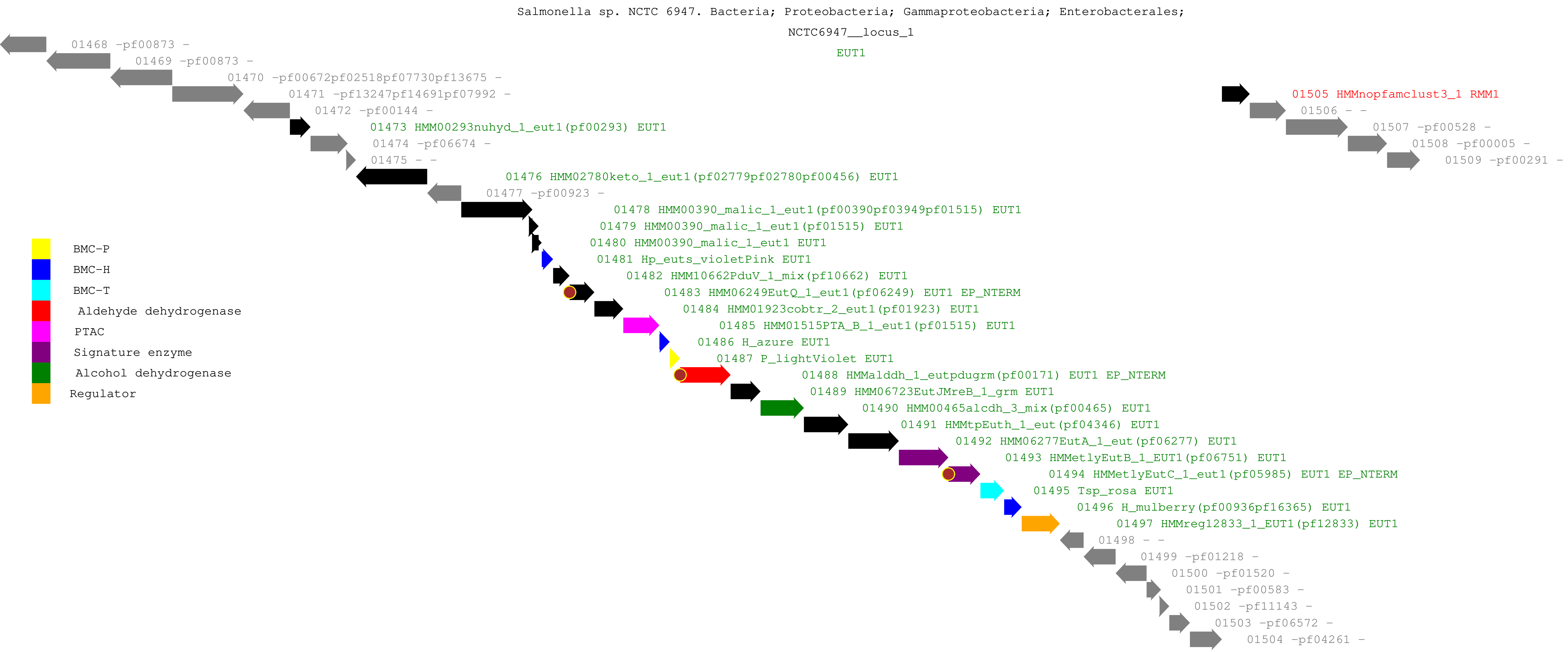
Salmonella sp. NCTC 6947. Bacteria; Proteobacteria; Gammaproteobacteria; Enterobacterales;
NCTC6947__locus_1
EUT1
01468 -pf00873 -
01469 -pf00873 -
01470 -pf00672pf02518pf07730pf13675 -
01471 -pf13247pf14691pf07992 -
01472 -pf00144 -
01473 HMM00293nuhyd_1_eut1(pf00293) EUT1
01474 -pf06674 -
01475 - -
01476 HMM02780keto_1_eut1(pf02779pf02780pf00456) EUT1
01477 -pf00923 -
01478 HMM00390_malic_1_eut1(pf00390pf03949pf01515) EUT1
01479 HMM00390_malic_1_eut1(pf01515) EUT1
01480 HMM00390_malic_1_eut1 EUT1
01481 Hp_euts_violetPink EUT1
01482 HMM10662PduV_1_mix(pf10662) EUT1
01483 HMM06249EutQ_1_eut1(pf06249) EUT1 EP_NTERM
01484 HMM01923cobtr_2_eut1(pf01923) EUT1
01485 HMM01515PTA_B_1_eut1(pf01515) EUT1
01486 H_azure EUT1
01487 P_lightViolet EUT1
01488 HMMalddh_1_eutpdugrm(pf00171) EUT1 EP_NTERM
01489 HMM06723EutJMreB_1_grm EUT1
01490 HMM00465alcdh_3_mix(pf00465) EUT1
01491 HMMtpEuth_1_eut(pf04346) EUT1
01492 HMM06277EutA_1_eut(pf06277) EUT1
01493 HMMetlyEutB_1_EUT1(pf06751) EUT1
01494 HMMetlyEutC_1_eut1(pf05985) EUT1 EP_NTERM
01495 Tsp_rosa EUT1
01496 H_mulberry(pf00936pf16365) EUT1
01497 HMMreg12833_1_EUT1(pf12833) EUT1
01498 - -
01499 -pf01218 -
01500 -pf01520 -
01501 -pf00583 -
01502 -pf11143 -
01503 -pf06572 -
01504 -pf04261 -
01505 HMMnopfamclust3_1 RMM1
01506 - -
01507 -pf00528 -
01508 -pf00005 -
01509 -pf00291 -
BMC-P
BMC-H
BMC-T
Aldehyde dehydrogenase
PTAC
Signature enzyme
Alcohol dehydrogenase
Regulator

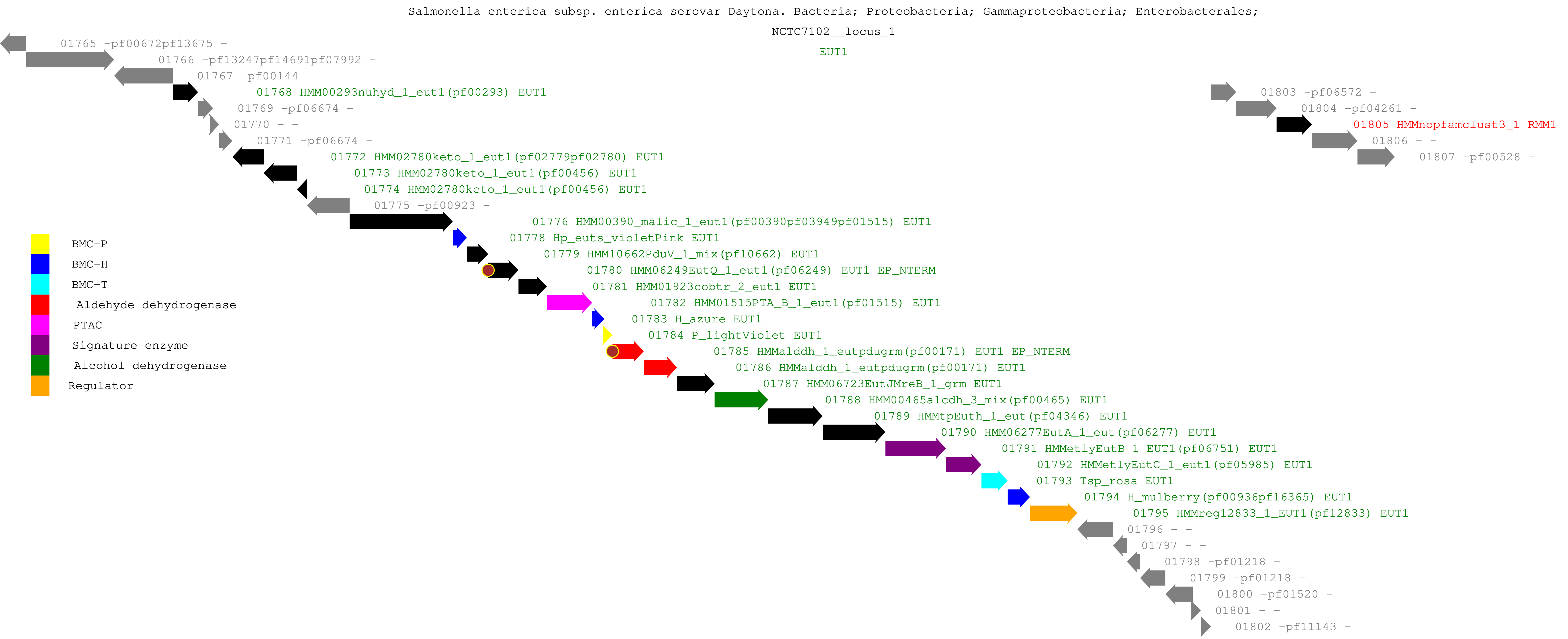
Salmonella enterica subsp. enterica serovar Daytona. Bacteria; Proteobacteria; Gammaproteobacteria; Enterobacterales;
NCTC7102__locus_1
EUT1
01765 -pf00672pf13675 -
01766 -pf13247pf14691pf07992 -
01767 -pf00144 -
01768 HMM00293nuhyd_1_eut1(pf00293) EUT1
01769 -pf06674 -
01770 - -
01771 -pf06674 -
01772 HMM02780keto_1_eut1(pf02779pf02780) EUT1
01773 HMM02780keto_1_eut1(pf00456) EUT1
01774 HMM02780keto_1_eut1(pf00456) EUT1
01775 -pf00923 -
01776 HMM00390_malic_1_eut1(pf00390pf03949pf01515) EUT1
01778 Hp_euts_violetPink EUT1
01779 HMM10662PduV_1_mix(pf10662) EUT1
01780 HMM06249EutQ_1_eut1(pf06249) EUT1 EP_NTERM
01781 HMM01923cobtr_2_eut1 EUT1
01782 HMM01515PTA_B_1_eut1(pf01515) EUT1
01783 H_azure EUT1
01784 P_lightViolet EUT1
01785 HMMalddh_1_eutpdugrm(pf00171) EUT1 EP_NTERM
01786 HMMalddh_1_eutpdugrm(pf00171) EUT1
01787 HMM06723EutJMreB_1_grm EUT1
01788 HMM00465alcdh_3_mix(pf00465) EUT1
01789 HMMtpEuth_1_eut(pf04346) EUT1
01790 HMM06277EutA_1_eut(pf06277) EUT1
01791 HMMetlyEutB_1_EUT1(pf06751) EUT1
01792 HMMetlyEutC_1_eut1(pf05985) EUT1
01793 Tsp_rosa EUT1
01794 H_mulberry(pf00936pf16365) EUT1
01795 HMMreg12833_1_EUT1(pf12833) EUT1
01796 - -
01797 - -
01798 -pf01218 -
01799 -pf01218 -
01800 -pf01520 -
01801 - -
01802 -pf11143 -
01803 -pf06572 -
01804 -pf04261 -
01805 HMMnopfamclust3_1 RMM1
01806 - -
01807 -pf00528 -
BMC-P
BMC-H
BMC-T
Aldehyde dehydrogenase
PTAC
Signature enzyme
Alcohol dehydrogenase
Regulator

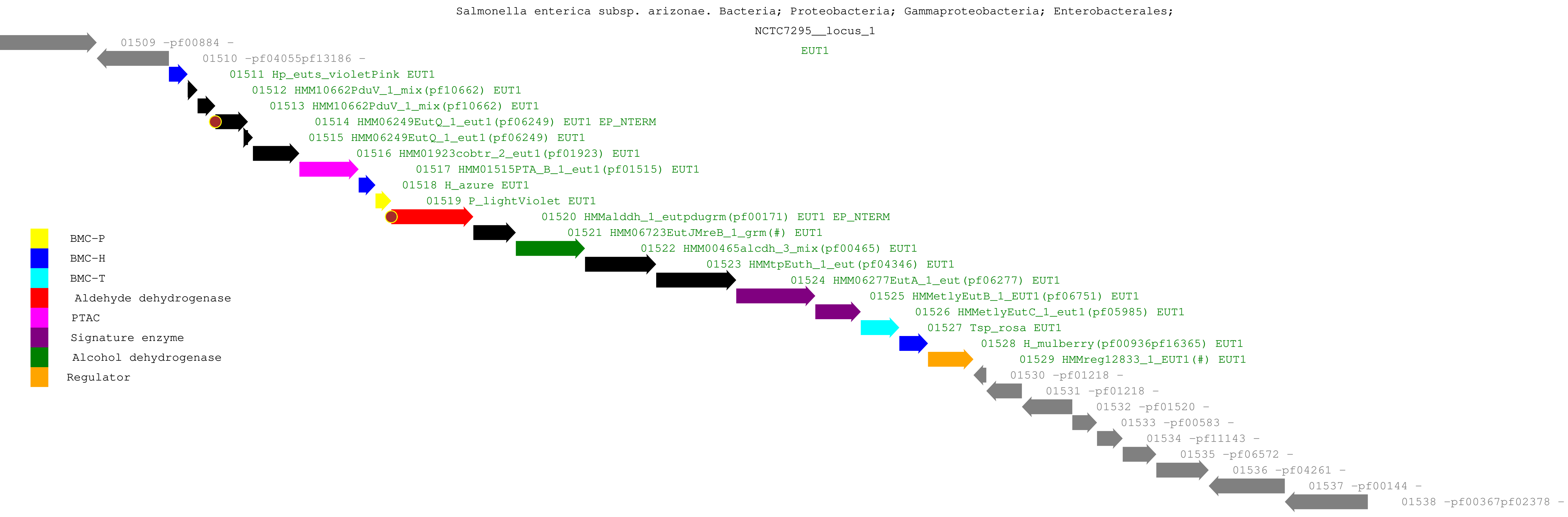
Salmonella enterica subsp. arizonae. Bacteria; Proteobacteria; Gammaproteobacteria; Enterobacterales;
NCTC7295__locus_1
EUT1
01509 -pf00884 -
01510 -pf04055pf13186 -
01511 Hp_euts_violetPink EUT1
01512 HMM10662PduV_1_mix(pf10662) EUT1
01513 HMM10662PduV_1_mix(pf10662) EUT1
01514 HMM06249EutQ_1_eut1(pf06249) EUT1 EP_NTERM
01515 HMM06249EutQ_1_eut1(pf06249) EUT1
01516 HMM01923cobtr_2_eut1(pf01923) EUT1
01517 HMM01515PTA_B_1_eut1(pf01515) EUT1
01518 H_azure EUT1
01519 P_lightViolet EUT1
01520 HMMaldh_1_eutpdugrm(pf00171) EUT1 EP_NTERM
01521 HMM06723EutJMreB_1_grm(#) EUT1
01522 HMM00465alcdh_3_mix(pf00465) EUT1
01523 HMMtpEuth_1_eut(pf04346) EUT1
01524 HMM06277EutA_1_eut(pf06277) EUT1
01525 HMMetlyEutB_1_EUT1(pf06751) EUT1
01526 HMMetlyEutC_1_eut1(pf05985) EUT1
01527 Tsp_rosa EUT1
01528 H_mulberry(pf00936pf16365) EUT1
01529 HMMreg12833_1_EUT1(#) EUT1
01530 -pf01218 -
01531 -pf01218 -
01532 -pf01520 -
01533 -pf00583 -
01534 -pf11143 -
01535 -pf06572 -
01536 -pf04261 -
01537 -pf00144 -
01538 -pf00367pf02378 -
BMC-P
BMC-H
BMC-T
Aldehyde dehydrogenase
PTAC
Signature enzyme
Alcohol dehydrogenase
Regulator

Salmonella sp. NCTC 7297. Bacteria; Proteobacteria; Gammaproteobacteria; Enterobacterales;

NCTC7297__locus_fragmented

EUT1

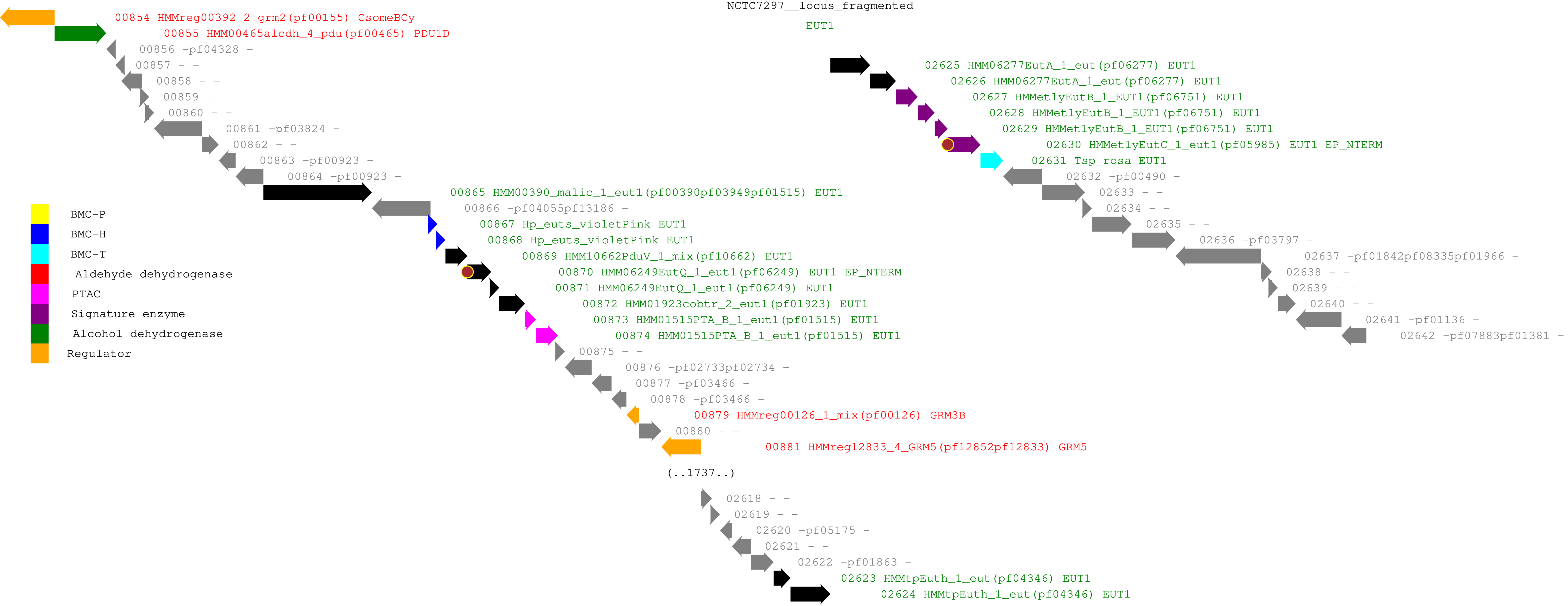

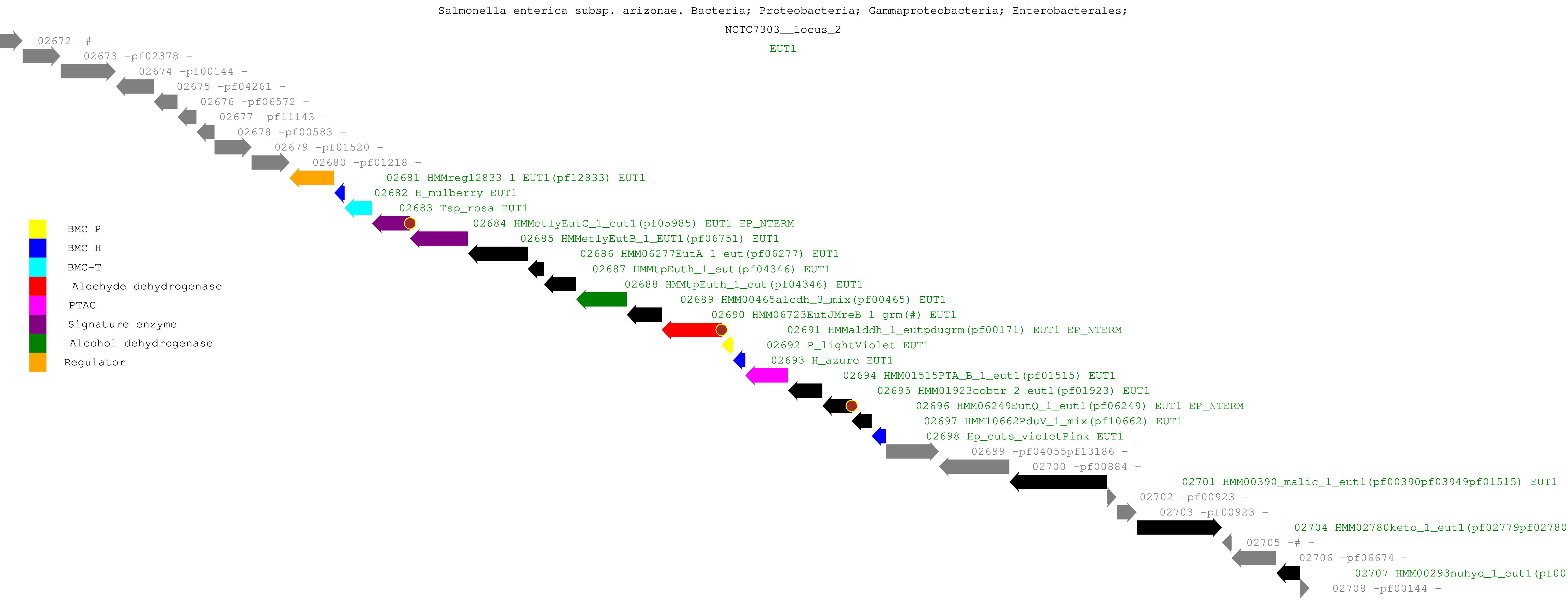
Salmonella enterica subsp. arizonae. Bacteria; Proteobacteria; Gammaproteobacteria; Enterobacterales;
NCTC7303__locus_2
EUT1
02672 -# -
02673 -pf02378 -
02674 -pf00144 -
02675 -pf04261 -
02676 -pf06572 -
02677 -pf11143 -
02678 -pf00583 -
02679 -pf01520 -
02680 -pf01218 -
02681 HMMreg12833_1_EUT1(pf12833) EUT1
02682 H_mulberry EUT1
02683 Tsp_rosa EUT1
02684 HMMetlyEutC_1_eut1(pf05985) EUT1 EP_NTERM
02685 HMMetlyEutB_1_EUT1(pf06751) EUT1
02686 HMM06277EutA_1_eut(pf06277) EUT1
02687 HMMtpEuth_1_eut(pf04346) EUT1
02688 HMMtpEuth_1_eut(pf04346) EUT1
02689 HMM00465alcdh_3_mix(pf00465) EUT1
02690 HMM06723EutJMreB_1_grm(#) EUT1
02691 HMMaldh_1_eutpdugrm(pf00171) EUT1 EP_NTERM
02692 P_lightViolet EUT1
02693 H_azure EUT1
02694 HMM01515PTA_B_1_eut1(pf01515) EUT1
02695 HMM01923cobtr_2_eut1(pf01923) EUT1
02696 HMM06249EutQ_1_eut1(pf06249) EUT1 EP_NTERM
02697 HMM10662PduV_1_mix(pf10662) EUT1
02698 Hp_euts_violetPink EUT1
02699 -pf04055pf13186 -
02700 -pf00884 -
02701 HMM00390_malic_1_eut1(pf00390pf03949pf01515) EUT1
02702 -pf00923 -
02703 -pf00923 -
02704 HMM02780keto_1_eut1(pf02779pf02780
02705 -# -
02706 -pf06674 -
02707 HMM00293nuhyd_1_eut1(pf00
02708 -pf00144 -
BMC-P
BMC-H
BMC-T
Aldehyde dehydrogenase
PTAC
Signature enzyme
Alcohol dehydrogenase
Regulator

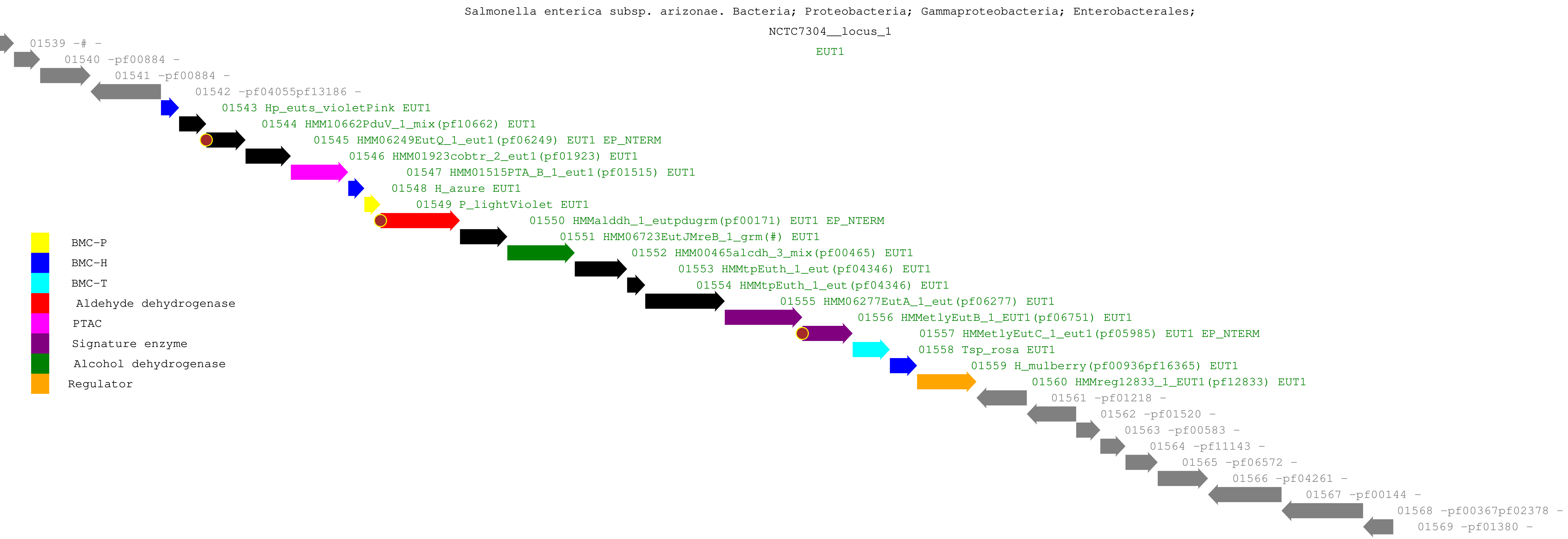
Salmonella enterica subsp. arizonae. Bacteria; Proteobacteria; Gammaproteobacteria; Enterobacterales;
NCTC7304__locus_1
EUT1
01539 -# -
01540 -pf00884 -
01541 -pf00884 -
01542 -pf04055pf13186 -
01543 Hp_euts_violetPink EUT1
01544 HMM10662PduV_1_mix(pf10662) EUT1
01545 HMM06249EutQ_1_eut1(pf06249) EUT1 EP_NTERM
01546 HMM01923cobtr_2_eut1(pf01923) EUT1
01547 HMM01515PTA_B_1_eut1(pf01515) EUT1
01548 H_azure EUT1
01549 P_lightViolet EUT1
01550 HMMaldh_1_eutpdugrm(pf00171) EUT1 EP_NTERM
01551 HMM06723EutJMreB_1_grm(#) EUT1
01552 HMM00465alcdh_3_mix(pf00465) EUT1
01553 HMMtpEuth_1_eut(pf04346) EUT1
01554 HMMtpEuth_1_eut(pf04346) EUT1
01555 HMM06277EutA_1_eut(pf06277) EUT1
01556 HMMetlyEutB_1_EUT1(pf06751) EUT1
01557 HMMetlyEutC_1_eut1(pf05985) EUT1 EP_NTERM
01558 Tsp_rosa EUT1
01559 H_mulberry(pf00936pf16365) EUT1
01560 HMMreg12833_1_EUT1(pf12833) EUT1
01561 -pf01218 -
01562 -pf01520 -
01563 -pf00583 -
01564 -pf11143 -
01565 -pf06572 -
01566 -pf04261 -
01567 -pf00144 -
01568 -pf00367pf02378 -
01569 -pf01380 -
BMC-P
BMC-H
BMC-T
Aldehyde dehydrogenase
PTAC
Signature enzyme
Alcohol dehydrogenase
Regulator

Salmonella enterica subsp. arizonae. Bacteria; Proteobacteria; Gammaproteobacteria; Enterobacterales;

NCTC7307__locus_1

EUT1

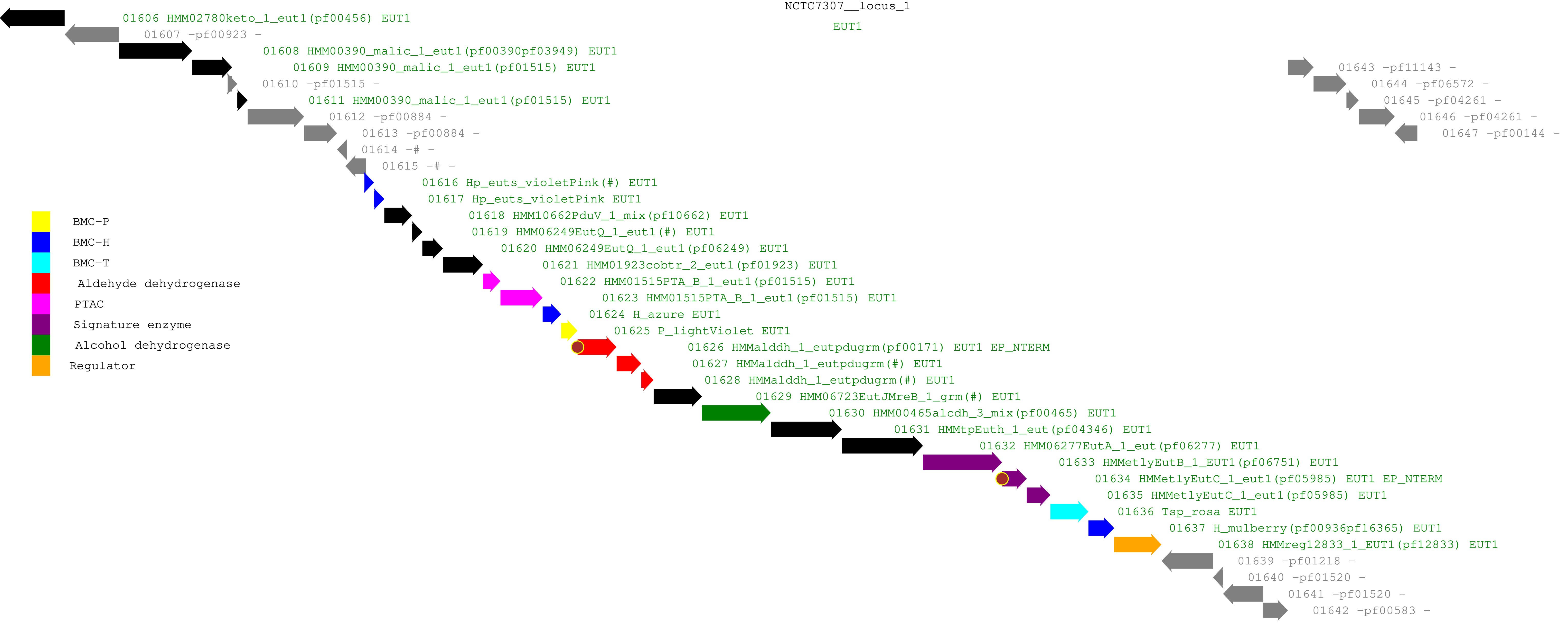

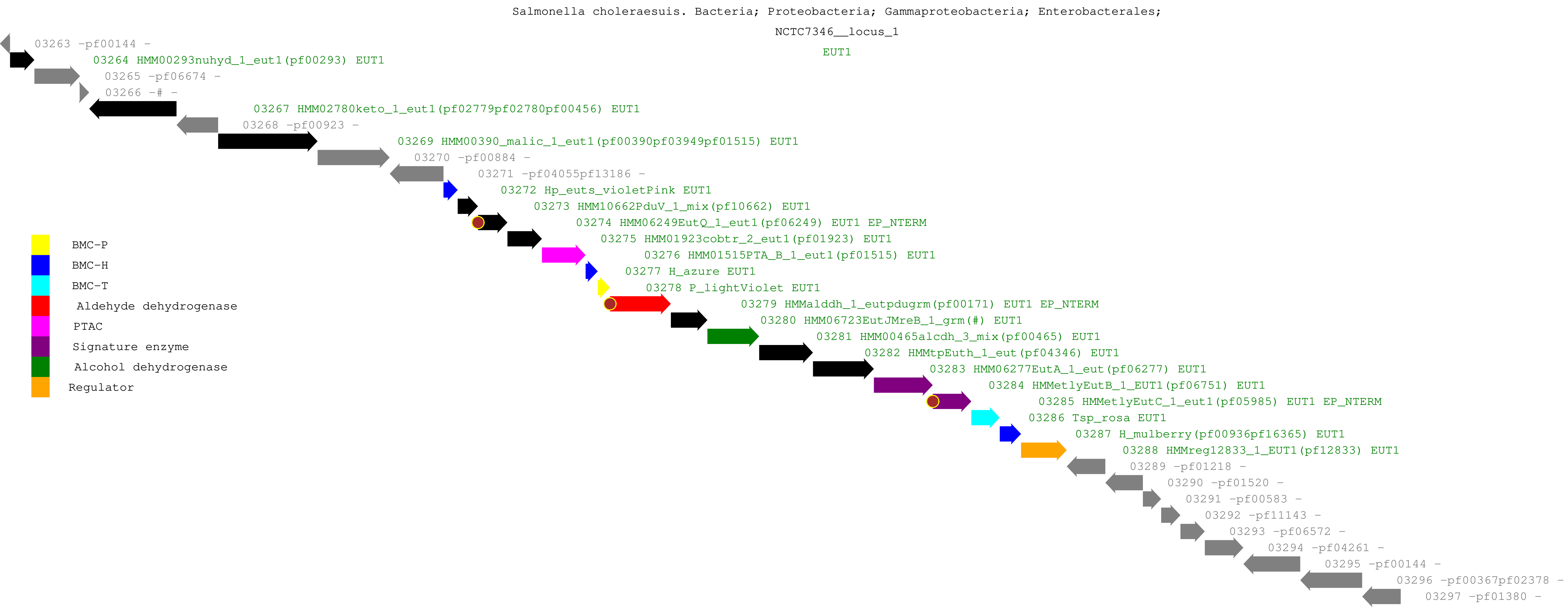

Salmonella choleraesuis. Bacteria; Proteobacteria; Gammaproteobacteria; Enterobacterales;
NCTC7346__locus_1
EUT1
03263 -pf00144 -
03264 HMM00293nuhyd_1_eut1(pf00293) EUT1
03265 -pf06674 -
03266 -# -
03267 HMM02780keto_1_eut1(pf02779pf02780pf00456) EUT1
03268 -pf00923 -
03269 HMM00390_malic_1_eut1(pf00390pf03949pf01515) EUT1
03270 -pf00884 -
03271 -pf04055pf13186 -
03272 Hp_euts_violetPink EUT1
03273 HMM10662PduV_1_mix(pf10662) EUT1
03274 HMM06249EutQ_1_eut1(pf06249) EUT1 EP_NTERM
03275 HMM01923cobtr_2_eut1(pf01923) EUT1
03276 HMM01515PTA_B_1_eut1(pf01515) EUT1
03277 H_azure EUT1
03278 P_lightViolet EUT1
03279 HMMalddh_1_eutpdugrm(pf00171) EUT1 EP_NTERM
03280 HMM06723EutJMreB_1_grm(#) EUT1
03281 HMM00465alcdh_3_mix(pf00465) EUT1
03282 HMMtpEuth_1_eut(pf04346) EUT1
03283 HMM06277EutA_1_eut(pf06277) EUT1
03284 HMMetlyEutB_1_EUT1(pf06751) EUT1
03285 HMMetlyEutC_1_eut1(pf05985) EUT1 EP_NTERM
03286 Tsp_rosa EUT1
03287 H_mulberry(pf00936pf16365) EUT1
03288 HMMreg12833_1_EUT1(pf12833) EUT1
03289 -pf01218 -
03290 -pf01520 -
03291 -pf00583 -
03292 -pf11143 -
03293 -pf06572 -
03294 -pf04261 -
03295 -pf00144 -
03296 -pf00367pf02378 -
03297 -pf01380 -
BMC-P
BMC-H
BMC-T
Aldehyde dehydrogenase
PTAC
Signature enzyme
Alcohol dehydrogenase
Regulator

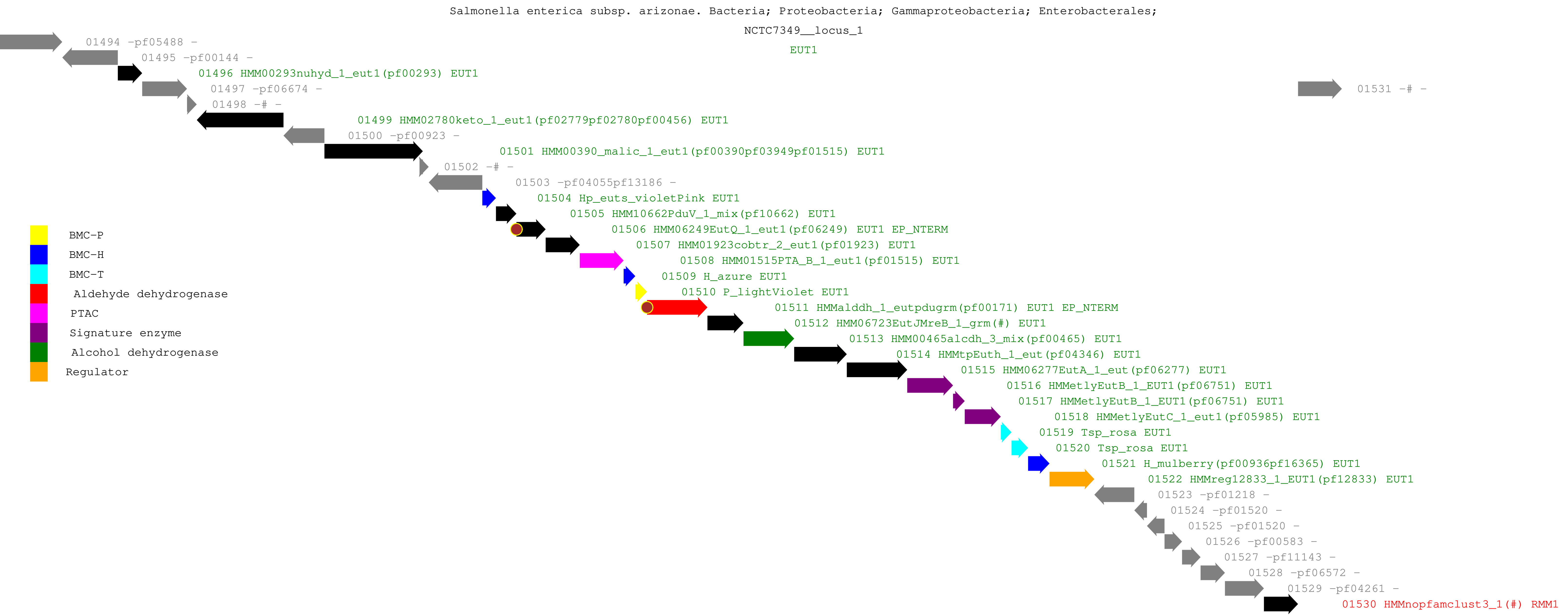
Salmonella enterica subsp. arizonae. Bacteria; Proteobacteria; Gammaproteobacteria; Enterobacterales;
NCTC7349__locus_1
EUT1
01494 -pf05488 -
01495 -pf00144 -
01496 HMM00293nuhyd_1_eut1(pf00293) EUT1
01497 -pf06674 -
01498 -# -
01499 HMM02780keto_1_eut1(pf02779pf02780pf00456) EUT1
01500 -pf00923 -
01501 HMM00390_malic_1_eut1(pf00390pf03949pf01515) EUT1
01502 -# -
01503 -pf04055pf13186 -
01504 Hp_euts_violetPink EUT1
01505 HMM10662PduV_1_mix(pf10662) EUT1
01506 HMM06249EutQ_1_eut1(pf06249) EUT1 EP_NTERM
01507 HMM01923cobtr_2_eut1(pf01923) EUT1
01508 HMM01515PTA_B_1_eut1(pf01515) EUT1
01509 H_azure EUT1
01510 P_lightViolet EUT1
01511 HMMalddh_1_eutpdugrm(pf00171) EUT1 EP_NTERM
01512 HMM06723EutJMreB_1_grm(#) EUT1
01513 HMM00465alcdh_3_mix(pf00465) EUT1
01514 HMMtpEuth_1_eut(pf04346) EUT1
01515 HMM06277EutA_1_eut(pf06277) EUT1
01516 HMMetlyEutB_1_EUT1(pf06751) EUT1
01517 HMMetlyEutB_1_EUT1(pf06751) EUT1
01518 HMMetlyEutC_1_eut1(pf05985) EUT1
01519 Tsp_rosa EUT1
01520 Tsp_rosa EUT1
01521 H_mulberry(pf00936pf16365) EUT1
01522 HMMreg12833_1_EUT1(pf12833) EUT1
01523 -pf01218 -
01524 -pf01520 -
01525 -pf01520 -
01526 -pf00583 -
01527 -pf11143 -
01528 -pf06572 -
01529 -pf04261 -
01530 HMMnopfamclust3_1(#) RMM1
01531 -# -
BMC-P
BMC-H
BMC-T
Aldehyde dehydrogenase
PTAC
Signature enzyme
Alcohol dehydrogenase
Regulator

Salmonella enterica subsp. enterica serovar Sanjuan. Bacteria; Proteobacteria; Gammaproteobacteria; Enterobacterales;

NCTC7406__locus_1

EUT1

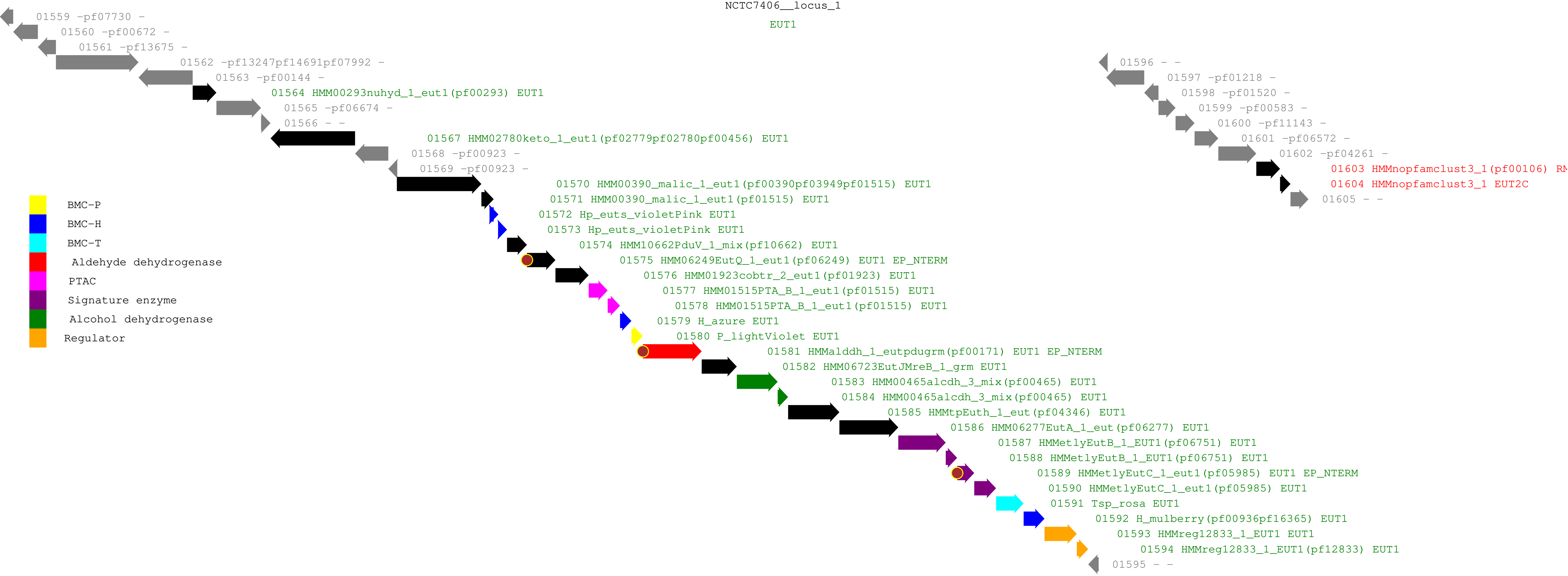

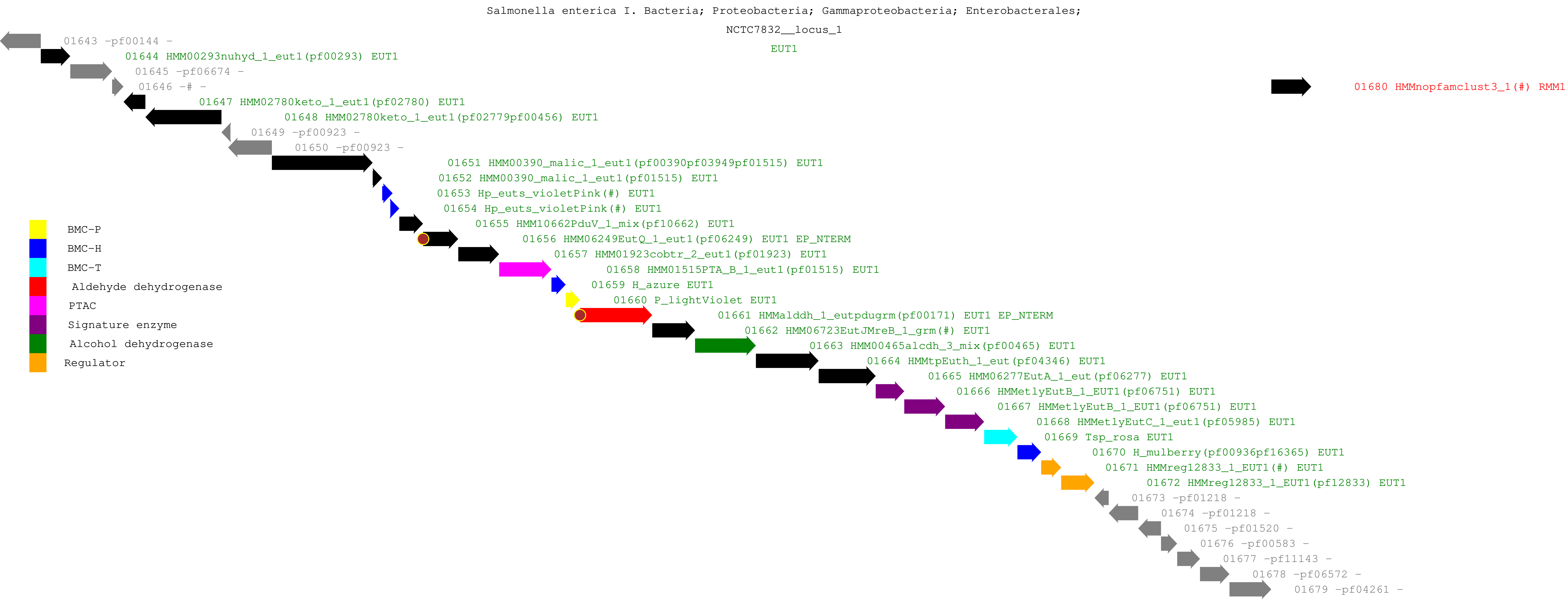
Salmonella enterica I. Bacteria; Proteobacteria; Gammaproteobacteria; Enterobacterales;
NCTC7832__locus_1
EUT1
01643 -pf00144 -
01644 HMM00293nuhyd_1_eut1(pf00293) EUT1
01645 -pf06674 -
01646 -# -
01647 HMM02780keto_1_eut1(pf02780) EUT1
01648 HMM02780keto_1_eut1(pf02779pf00456) EUT1
01649 -pf00923 -
01650 -pf00923 -
01651 HMM00390_malic_1_eut1(pf00390pf03949pf01515) EUT1
01652 HMM00390_malic_1_eut1(pf01515) EUT1
01653 Hp_euts_violetPink(#) EUT1
01654 Hp_euts_violetPink(#) EUT1
01655 HMM10662PduV_1_mix(pf10662) EUT1
01656 HMM06249EutQ_1_eut1(pf06249) EUT1 EP_NTERM
01657 HMM01923cobtr_2_eut1(pf01923) EUT1
01658 HMM01515PTA_B_1_eut1(pf01515) EUT1
01659 H_azure EUT1
01660 P_lightViolet EUT1
01661 HMMalddh_1_eutpdugrm(pf00171) EUT1 EP_NTERM
01662 HMM06723EutJMreB_1_grm(#) EUT1
01663 HMM00465alcdh_3_mix(pf00465) EUT1
01664 HMMtpEuth_1_eut(pf04346) EUT1
01665 HMM06277EutA_1_eut(pf06277) EUT1
01666 HMMetlyEutB_1_EUT1(pf06751) EUT1
01667 HMMetlyEutB_1_EUT1(pf06751) EUT1
01668 HMMetlyEutC_1_eut1(pf05985) EUT1
01669 Tsp_rosa EUT1
01670 H_mulberry(pf00936pf16365) EUT1
01671 HMMreg12833_1_EUT1(#) EUT1
01672 HMMreg12833_1_EUT1(pf12833) EUT1
01673 -pf01218 -
01674 -pf01218 -
01675 -pf01520 -
01676 -pf00583 -
01677 -pf11143 -
01678 -pf06572 -
01679 -pf04261 -
01680 HMMnopfamclust3_1(#) RMM1
BMC-P
BMC-H
BMC-T
Aldehyde dehydrogenase
PTAC
Signature enzyme
Alcohol dehydrogenase
Regulator

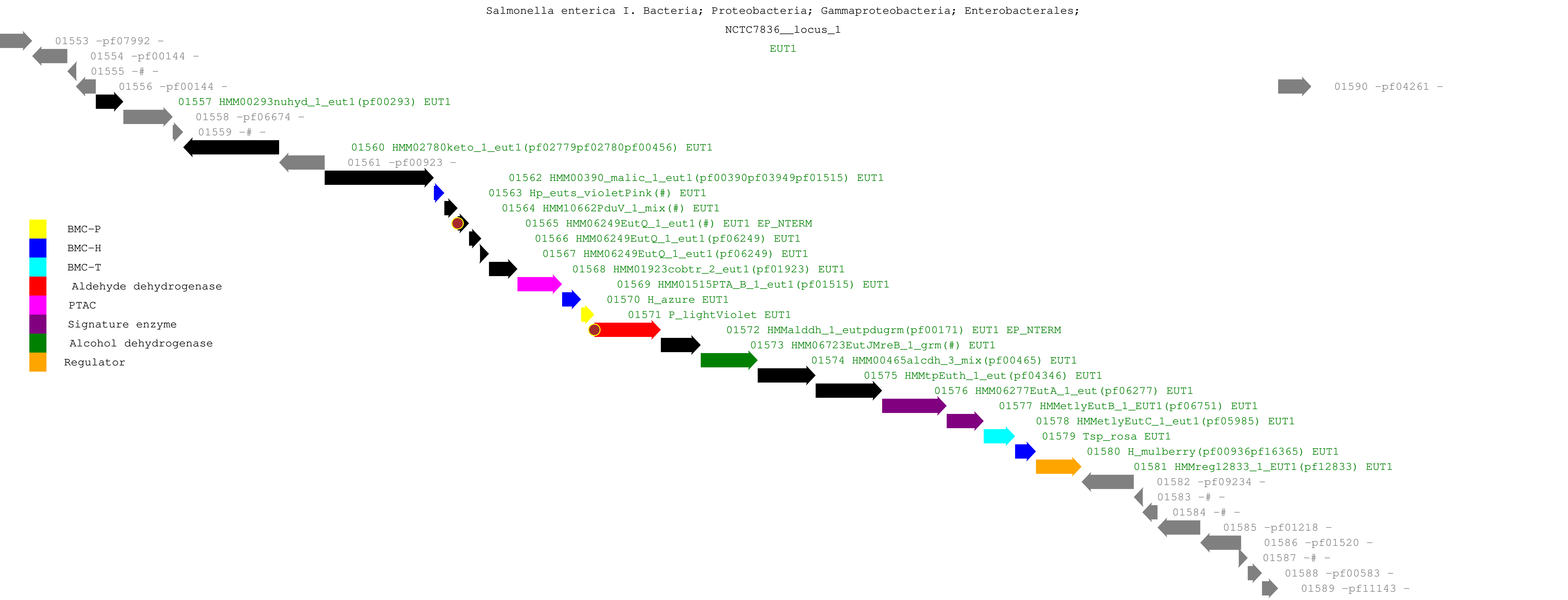

Salmonella enterica I. Bacteria; Proteobacteria; Gammaproteobacteria; Enterobacterales;
NCTC7836__locus_1
EUT1
01553 -pf07992 -
01554 -pf00144 -
01555 -# -
01556 -pf00144 -
01557 HMM00293nuhyd_1_eut1(pf00293) EUT1
01558 -pf06674 -
01559 -# -
01560 HMM02780keto_1_eut1(pf02779pf02780pf00456) EUT1
01561 -pf00923 -
01562 HMM00390_malic_1_eut1(pf00390pf03949pf01515) EUT1
01563 Hp_euts_violetPink(#) EUT1
01564 HMM10662PduV_1_mix(#) EUT1
01565 HMM06249EutQ_1_eut1(#) EUT1 EP_NTERM
01566 HMM06249EutQ_1_eut1(pf06249) EUT1
01567 HMM06249EutQ_1_eut1(pf06249) EUT1
01568 HMM01923cobtr_2_eut1(pf01923) EUT1
01569 HMM01515PTA_B_1_eut1(pf01515) EUT1
01570 H_azure EUT1
01571 P_lightViolet EUT1
01572 HMMalddh_1_eutpdugrm(pf00171) EUT1 EP_NTERM
01573 HMM06723EutJMreB_1_grm(#) EUT1
01574 HMM00465alcdh_3_mix(pf00465) EUT1
01575 HMMtpEuth_1_eut(pf04346) EUT1
01576 HMM06277EutA_1_eut(pf06277) EUT1
01577 HMMetlyEutB_1_EUT1(pf06751) EUT1
01578 HMMetlyEutC_1_eut1(pf05985) EUT1
01579 Tsp_rosa EUT1
01580 H_mulberry(pf00936pf16365) EUT1
01581 HMMreg12833_1_EUT1(pf12833) EUT1
01582 -pf09234 -
01583 -# -
01584 -# -
01585 -pf01218 -
01586 -pf01520 -
01587 -# -
01588 -pf00583 -
01589 -pf11143 -
01590 -pf04261 -
BMC-P
BMC-H
BMC-T
Aldehyde dehydrogenase
PTAC
Signature enzyme
Alcohol dehydrogenase
Regulator

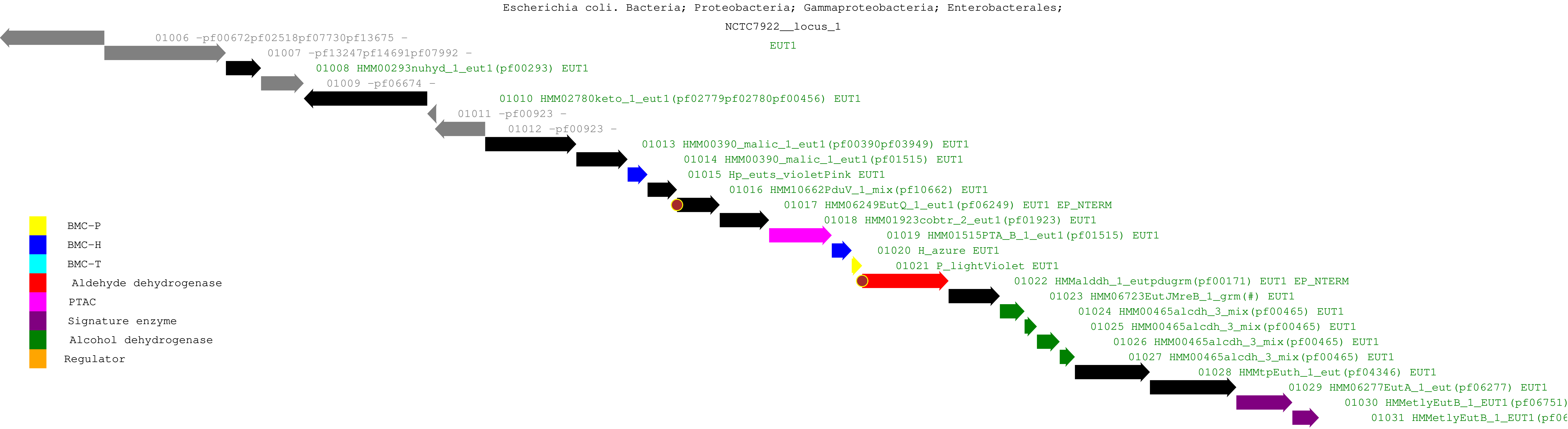
Escherichia coli. Bacteria; Proteobacteria; Gammaproteobacteria; Enterobacterales;
NCTC7922__locus_1
EUT1
01006 -pf00672pf02518pf07730pf13675 -
01007 -pf13247pf14691pf07992 -
01008 HMM00293nuhyd_1_eut1(pf00293) EUT1
01009 -pf06674 -
01010 HMM02780keto_1_eut1(pf02779pf02780pf00456) EUT1
01011 -pf00923 -
01012 -pf00923 -
01013 HMM00390_malic_1_eut1(pf00390pf03949) EUT1
01014 HMM00390_malic_1_eut1(pf01515) EUT1
01015 Hp_euts_violetPink EUT1
01016 HMM10662PduV_1_mix(pf10662) EUT1
01017 HMM06249EutQ_1_eut1(pf06249) EUT1 EP_NTERM
01018 HMM01923cobtr_2_eut1(pf01923) EUT1
01019 HMM01515PTA_B_1_eut1(pf01515) EUT1
01020 H_azure EUT1
01021 P_lightViolet EUT1
01022 HMMalddh_1_eutpdugrm(pf00171) EUT1 EP_NTERM
01023 HMM06723EutJMreB_1_grm(#) EUT1
01024 HMM00465alcdh_3_mix(pf00465) EUT1
01025 HMM00465alcdh_3_mix(pf00465) EUT1
01026 HMM00465alcdh_3_mix(pf00465) EUT1
01027 HMM00465alcdh_3_mix(pf00465) EUT1
01028 HMMtpEuth_1_eut(pf04346) EUT1
01029 HMM06277EutA_1_eut(pf06277) EUT1
01030 HMMetlyEutB_1_EUT1(pf06751)
01031 HMMetlyEutB_1_EUT1(pf06
BMC-P
BMC-H
BMC-T
Aldehyde dehydrogenase
PTAC
Signature enzyme
Alcohol dehydrogenase
Regulator

Escherichia coli. Bacteria; Proteobacteria; Gammaproteobacteria; Enterobacterales;

NCTC7927__locus_1

EUT1

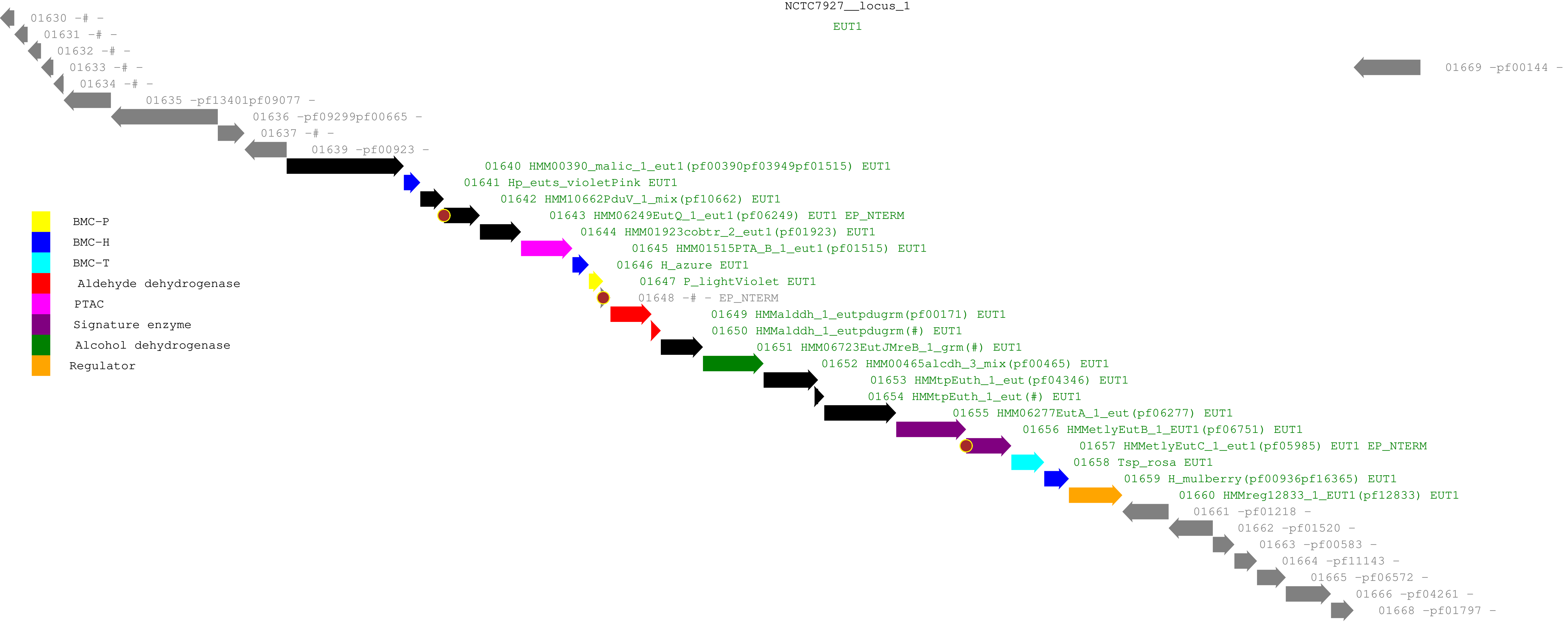

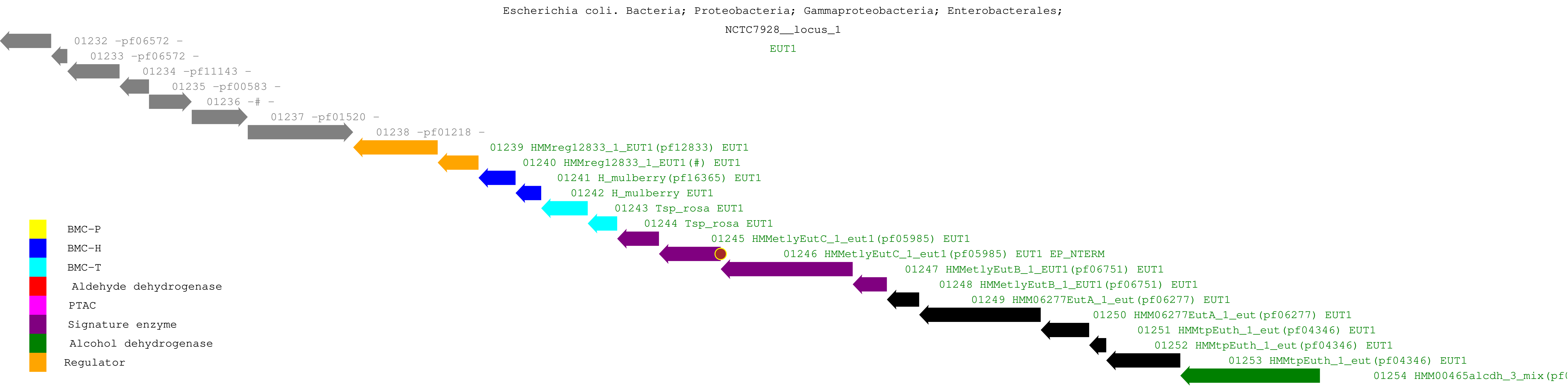
Escherichia coli. Bacteria; Proteobacteria; Gammaproteobacteria; Enterobacterales;
NCTC7928__locus_1
EUT1
01232 -pf06572 -
01233 -pf06572 -
01234 -pf11143 -
01235 -pf00583 -
01236 -# -
01237 -pf01520 -
01238 -pf01218 -
01239 HMMreg12833_1_EUT1(pf12833) EUT1
01240 HMMreg12833_1_EUT1(#) EUT1
01241 H_mulberry(pf16365) EUT1
01242 H_mulberry EUT1
01243 Tsp_rosa EUT1
01244 Tsp_rosa EUT1
01245 HMMetlyEutC_1_eut1(pf05985) EUT1
01246 HMMetlyEutC_1_eut1(pf05985) EUT1 EP_NTERM
01247 HMMetlyEutB_1_EUT1(pf06751) EUT1
01248 HMMetlyEutB_1_EUT1(pf06751) EUT1
01249 HMM06277EutA_1_eut(pf06277) EUT1
01250 HMM06277EutA_1_eut(pf06277) EUT1
01251 HMMtpEuth_1_eut(pf04346) EUT1
01252 HMMtpEuth_1_eut(pf04346) EUT1
01253 HMMtpEuth_1_eut(pf04346) EUT1
01254 HMM00465alcdh_3_mix(pf
BMC-P
BMC-H
BMC-T
Aldehyde dehydrogenase
PTAC
Signature enzyme
Alcohol dehydrogenase
Regulator

Escherichia coli. Bacteria; Proteobacteria; Gammaproteobacteria; Enterobacterales;

NCTC8009__locus_1

EUT1

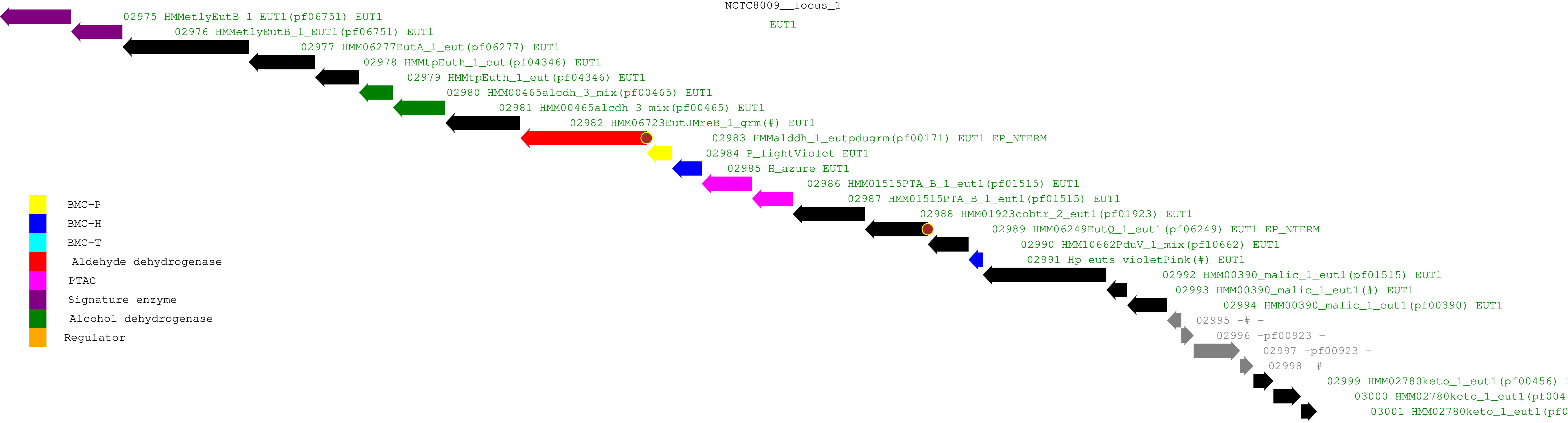

Hafnia alvei. Bacteria; Proteobacteria; Gammaproteobacteria; Enterobacterales;

NCTC8105__locus_1

EUT1

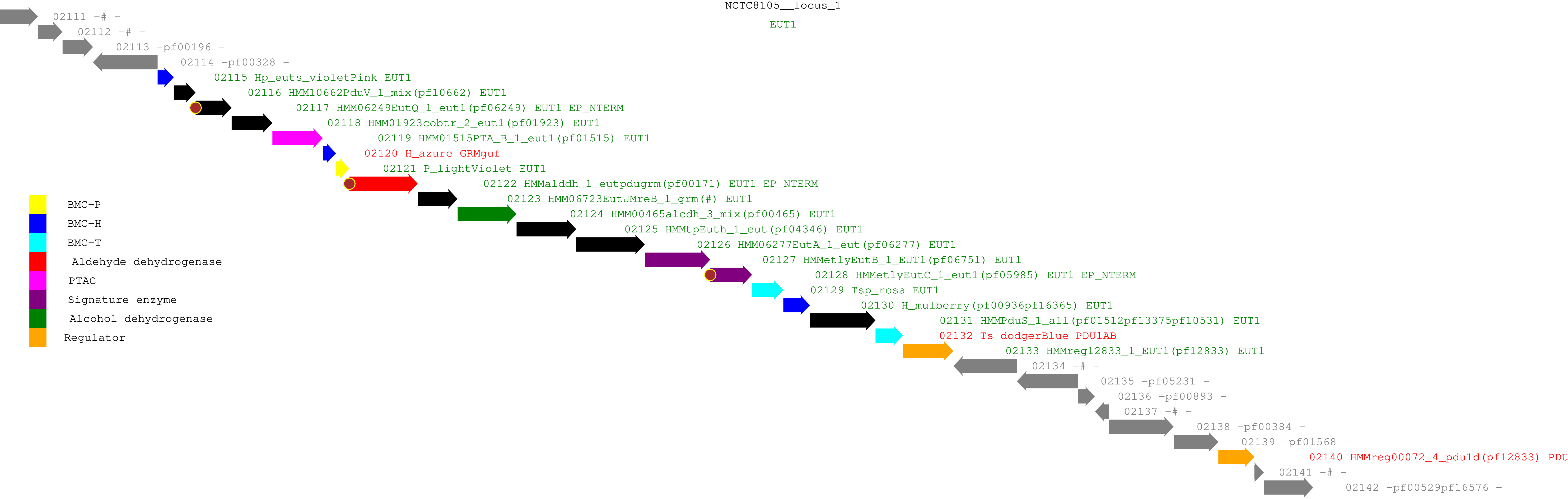

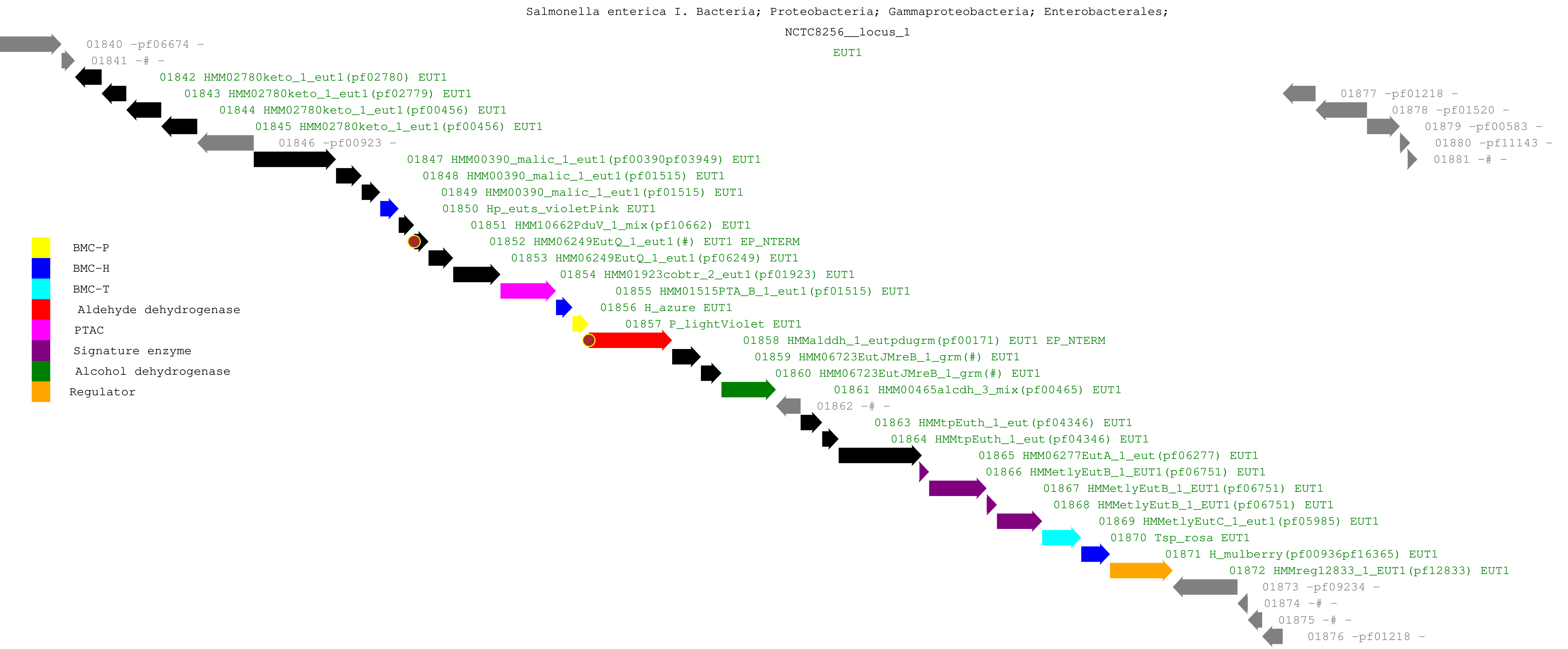
Salmonella enterica I. Bacteria; Proteobacteria; Gammaproteobacteria; Enterobacterales;
NCTC8256__locus_1
EUT1
01840 -pf06674 -
01841 -# -
01842 HMM02780keto_1_eut1(pf02780) EUT1
01843 HMM02780keto_1_eut1(pf02779) EUT1
01844 HMM02780keto_1_eut1(pf00456) EUT1
01845 HMM02780keto_1_eut1(pf00456) EUT1
01846 -pf00923 -
01847 HMM00390_malic_1_eut1(pf00390pf03949) EUT1
01848 HMM00390_malic_1_eut1(pf01515) EUT1
01849 HMM00390_malic_1_eut1(pf01515) EUT1
01850 Hp_euts_violetPink EUT1
01851 HMM10662PduV_1_mix(pf10662) EUT1
01852 HMM06249EutQ_1_eut1(#) EUT1 EP_NTERM
01853 HMM06249EutQ_1_eut1(pf06249) EUT1
01854 HMM01923cobtr_2_eut1(pf01923) EUT1
01855 HMM01515PTA_B_1_eut1(pf01515) EUT1
01856 H_azure EUT1
01857 P_lightViolet EUT1
01858 HMMalddh_1_eutpdugrm(pf00171) EUT1 EP_NTERM
01859 HMM06723EutJMreB_1_grm(#) EUT1
01860 HMM06723EutJMreB_1_grm(#) EUT1
01861 HMM00465alcdh_3_mix(pf00465) EUT1
01862 -# -
01863 HMMtpEuth_1_eut(pf04346) EUT1
01864 HMMtpEuth_1_eut(pf04346) EUT1
01865 HMM06277EutA_1_eut(pf06277) EUT1
01866 HMMetlyEutB_1_EUT1(pf06751) EUT1
01867 HMMetlyEutB_1_EUT1(pf06751) EUT1
01868 HMMetlyEutB_1_EUT1(pf06751) EUT1
01869 HMMetlyEutC_1_eut1(pf05985) EUT1
01870 Tsp_rosa EUT1
01871 H_mulberry(pf00936pf16365) EUT1
01872 HMMreg12833_1_EUT1(pf12833) EUT1
01873 -pf09234 -
01874 -# -
01875 -# -
01876 -pf01218 -
01877 -pf01218 -
01878 -pf01520 -
01879 -pf00583 -
01880 -pf11143 -
01881 -# -
BMC-P
BMC-H
BMC-T
Aldehyde dehydrogenase
PTAC
Signature enzyme
Alcohol dehydrogenase
Regulator

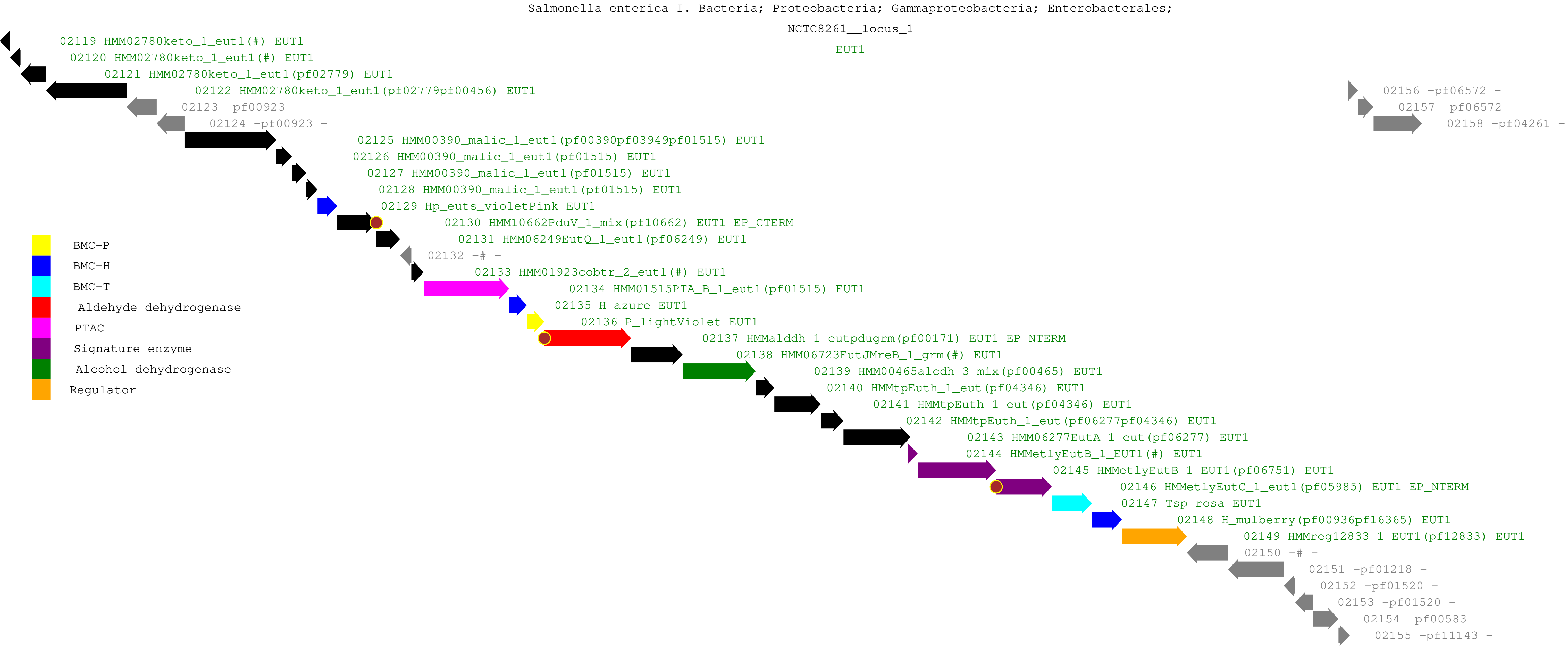

Salmonella enterica I. Bacteria; Proteobacteria; Gammaproteobacteria; Enterobacterales;
NCTC8261__locus_1
EUT1
02119 HMM02780keto_1_eut1(#) EUT1
02120 HMM02780keto_1_eut1(#) EUT1
02121 HMM02780keto_1_eut1(pf02779) EUT1
02122 HMM02780keto_1_eut1(pf02779pf00456) EUT1
02123 -pf00923 -
02124 -pf00923 -
02125 HMM00390_malic_1_eut1(pf00390pf03949pf01515) EUT1
02126 HMM00390_malic_1_eut1(pf01515) EUT1
02127 HMM00390_malic_1_eut1(pf01515) EUT1
02128 HMM00390_malic_1_eut1(pf01515) EUT1
02129 Hp_euts_violetPink EUT1
02130 HMM10662PduV_1_mix(pf10662) EUT1 EP_CTERM
02131 HMM06249EutQ_1_eut1(pf06249) EUT1
02132 -# -
02133 HMM01923cobtr_2_eut1(#) EUT1
02134 HMM01515PTA_B_1_eut1(pf01515) EUT1
02135 H_azure EUT1
02136 P_lightViolet EUT1
02137 HMMalddh_1_eutpdugrm(pf00171) EUT1 EP_NTERM
02138 HMM06723EutJMreB_1_grm(#) EUT1
02139 HMM00465alcdh_3_mix(pf00465) EUT1
02140 HMMtpEuth_1_eut(pf04346) EUT1
02141 HMMtpEuth_1_eut(pf04346) EUT1
02142 HMMtpEuth_1_eut(pf06277pf04346) EUT1
02143 HMM06277EutA_1_eut(pf06277) EUT1
02144 HMMetlyEutB_1_EUT1(#) EUT1
02145 HMMetlyEutB_1_EUT1(pf06751) EUT1
02146 HMMetlyEutC_1_eut1(pf05985) EUT1 EP_NTERM
02147 Tsp_rosa EUT1
02148 H_mulberry(pf00936pf16365) EUT1
02149 HMMreg12833_1_EUT1(pf12833) EUT1
02150 -# -
02151 -pf01218 -
02152 -pf01520 -
02153 -pf01520 -
02154 -pf00583 -
02155 -pf11143 -
02156 -pf06572 -
02157 -pf06572 -
02158 -pf04261 -
BMC-P
BMC-H
BMC-T
Aldehyde dehydrogenase
PTAC
Signature enzyme
Alcohol dehydrogenase
Regulator

Salmonella enterica I. Bacteria; Proteobacteria; Gammaproteobacteria; Enterobacterales;

NCTC8271__locus_1

EUT1

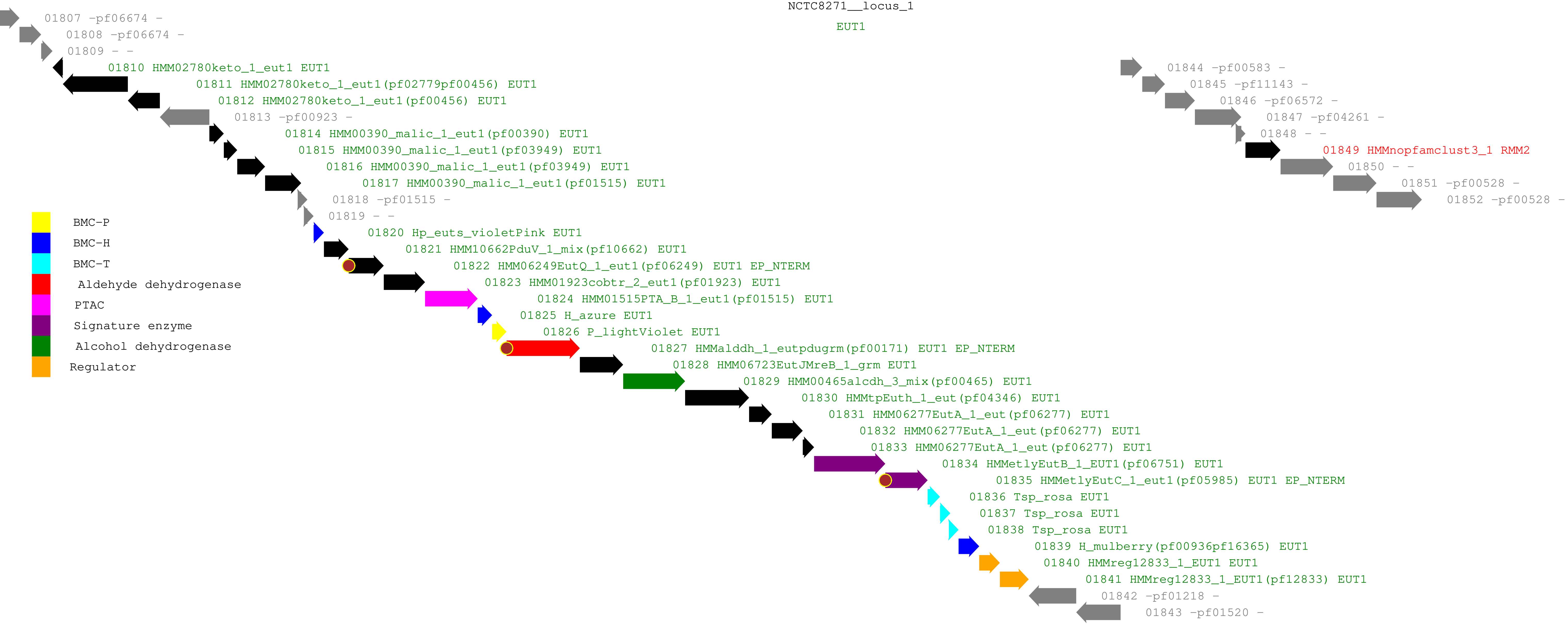

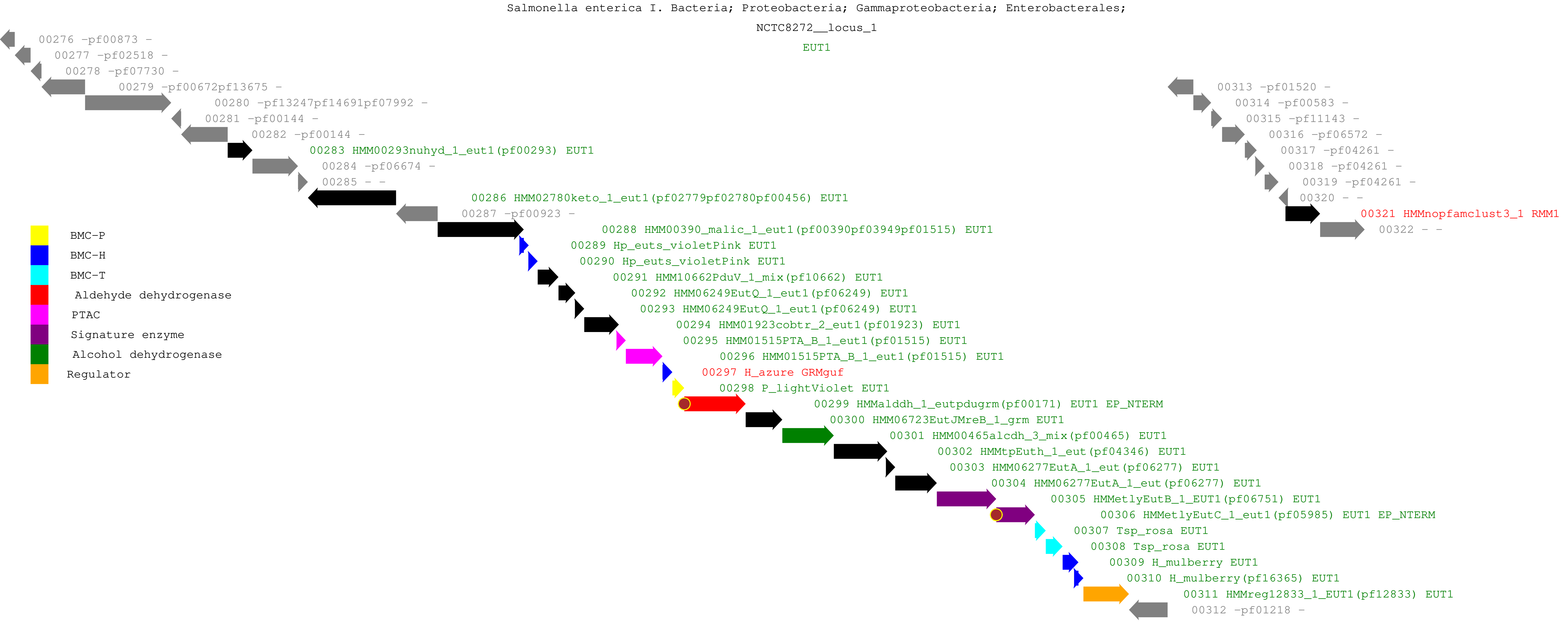
Salmonella enterica I. Bacteria; Proteobacteria; Gammaproteobacteria; Enterobacterales;
NCTC8272__locus_1
EUT1
00276 -pf00873 -
00277 -pf02518 -
00278 -pf07730 -
00279 -pf00672pf13675 -
00280 -pf13247pf14691pf07992 -
00281 -pf00144 -
00282 -pf00144 -
00283 HMM00293nuhyd_1_eut1(pf00293) EUT1
00284 -pf06674 -
00285 - -
00286 HMM02780keto_1_eut1(pf02779pf02780pf00456) EUT1
00287 -pf00923 -
00288 HMM00390_malic_1_eut1(pf00390pf03949pf01515) EUT1
00289 Hp_euts_violetPink EUT1
00290 Hp_euts_violetPink EUT1
00291 HMM10662PduV_1_mix(pf10662) EUT1
00292 HMM06249EutQ_1_eut1(pf06249) EUT1
00293 HMM06249EutQ_1_eut1(pf06249) EUT1
00294 HMM01923cobtr_2_eut1(pf01923) EUT1
00295 HMM01515PTA_B_1_eut1(pf01515) EUT1
00296 HMM01515PTA_B_1_eut1(pf01515) EUT1
00297 H_azure GRMguf
00298 P_lightViolet EUT1
00299 HMMalddh_1_eutpdugrm(pf00171) EUT1 EP_NTERM
00300 HMM06723EutJMreB_1_grm EUT1
00301 HMM00465alcdh_3_mix(pf00465) EUT1
00302 HMMtpEuth_1_eut(pf04346) EUT1
00303 HMM06277EutA_1_eut(pf06277) EUT1
00304 HMM06277EutA_1_eut(pf06277) EUT1
00305 HMMetlyEutB_1_EUT1(pf06751) EUT1
00306 HMMetlyEutC_1_eut1(pf05985) EUT1 EP_NTERM
00307 Tsp_rosa EUT1
00308 Tsp_rosa EUT1
00309 H_mulberry EUT1
00310 H_mulberry(pf16365) EUT1
00311 HMMreg12833_1_EUT1(pf12833) EUT1
00312 -pf01218 -
00313 -pf01520 -
00314 -pf00583 -
00315 -pf11143 -
00316 -pf06572 -
00317 -pf04261 -
00318 -pf04261 -
00319 -pf04261 -
00320 - -
00321 HMMnopfamclust3_1 RMM1
00322 - -
BMC-P
BMC-H
BMC-T
Aldehyde dehydrogenase
PTAC
Signature enzyme
Alcohol dehydrogenase
Regulator

Salmonella enterica subsp. arizonae. Bacteria; Proteobacteria; Gammaproteobacteria; Enterobacterales;

NCTC8297__locus_1

EUT1

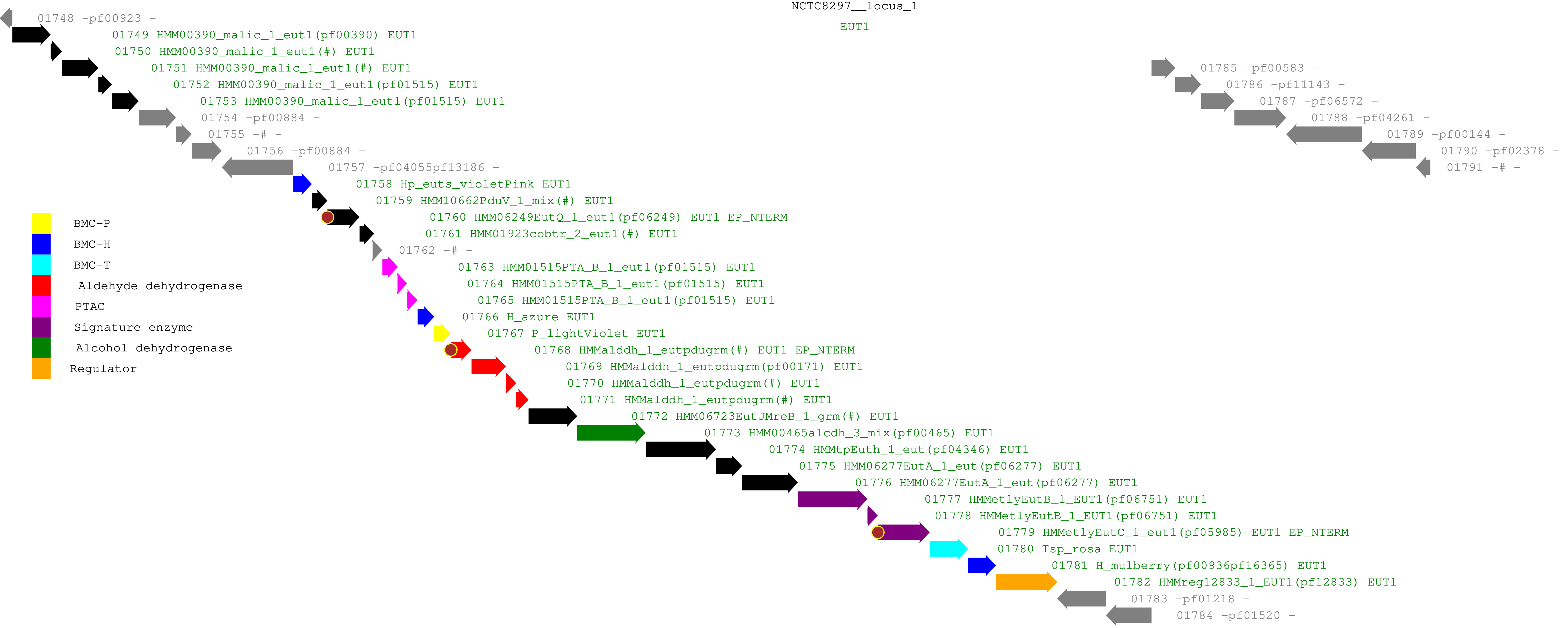

Hafnia alvei. Bacteria; Proteobacteria; Gammaproteobacteria; Enterobacterales;

NCTC8535__locus_1

EUT1

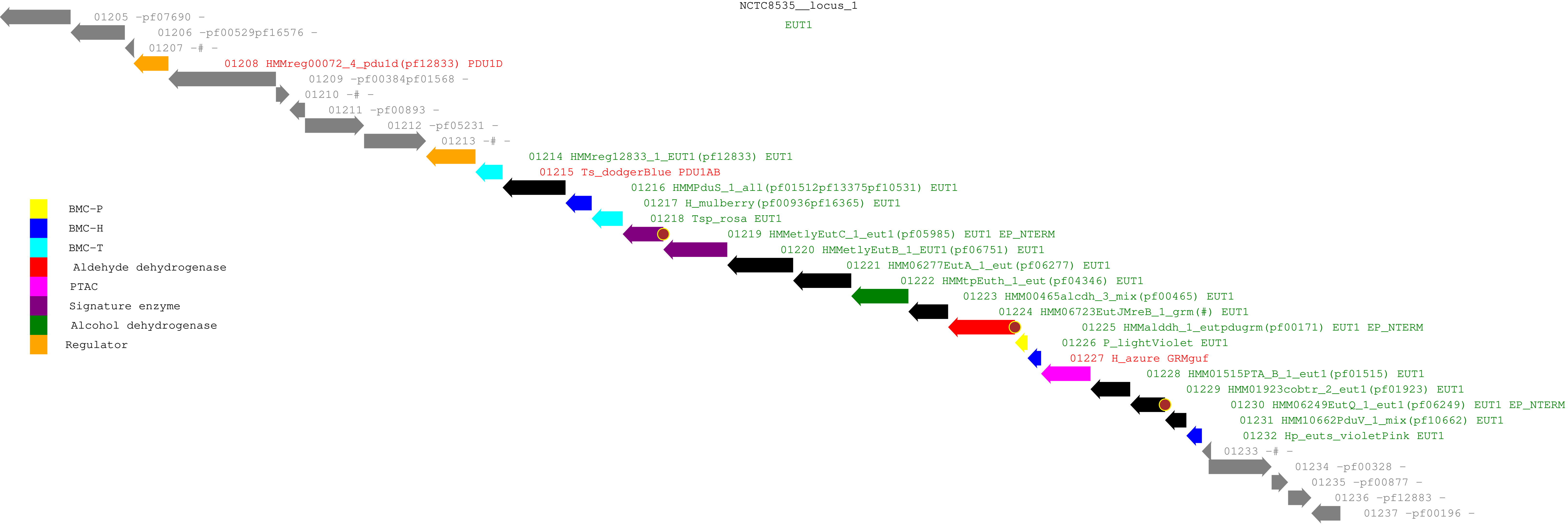

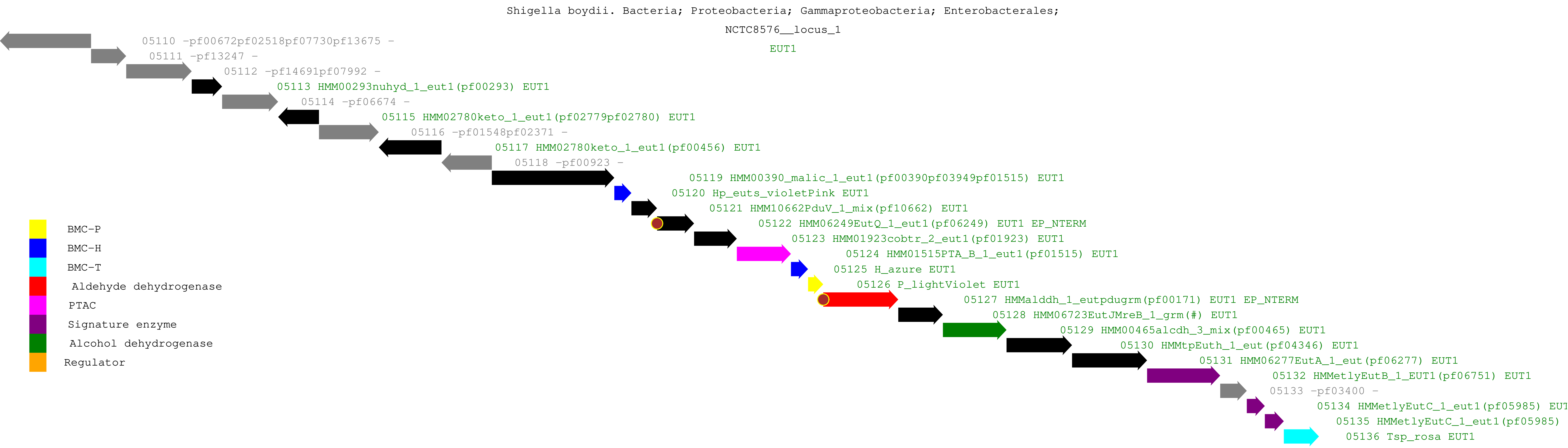

Shigella boydii. Bacteria; Proteobacteria; Gammaproteobacteria; Enterobacterales;
NCTC8576__locus_1
EUT1
05110 -pf00672pf02518pf07730pf13675 -
05111 -pf13247 -
05112 -pf14691pf07992 -
05113 HMM00293nuhyd_1_eut1(pf00293) EUT1
05114 -pf06674 -
05115 HMM02780keto_1_eut1(pf02779pf02780) EUT1
05116 -pf01548pf02371 -
05117 HMM02780keto_1_eut1(pf00456) EUT1
05118 -pf00923 -
05119 HMM00390_malic_1_eut1(pf00390pf03949pf01515) EUT1
05120 Hp_euts_violetPink EUT1
05121 HMM10662PduV_1_mix(pf10662) EUT1
05122 HMM06249EutQ_1_eut1(pf06249) EUT1 EP_NTERM
05123 HMM01923cobtr_2_eut1(pf01923) EUT1
05124 HMM01515PTA_B_1_eut1(pf01515) EUT1
05125 H_azure EUT1
05126 P_lightViolet EUT1
05127 HMMalddh_1_eutpdugrm(pf00171) EUT1 EP_NTERM
05128 HMM06723EutJMreB_1_grm(#) EUT1
05129 HMM00465alcdh_3_mix(pf00465) EUT1
05130 HMMtpEuth_1_eut(pf04346) EUT1
05131 HMM06277EutA_1_eut(pf06277) EUT1
05132 HMMetlyEutB_1_EUT1(pf06751) EUT1
05133 -pf03400 -
05134 HMMetlyEutC_1_eut1(pf05985) EUT
05135 HMMetlyEutC_1_eut1(pf05985)
05136 Tsp_rosa EUT1
BMC-P
BMC-H
BMC-T
Aldehyde dehydrogenase
PTAC
Signature enzyme
Alcohol dehydrogenase
Regulator

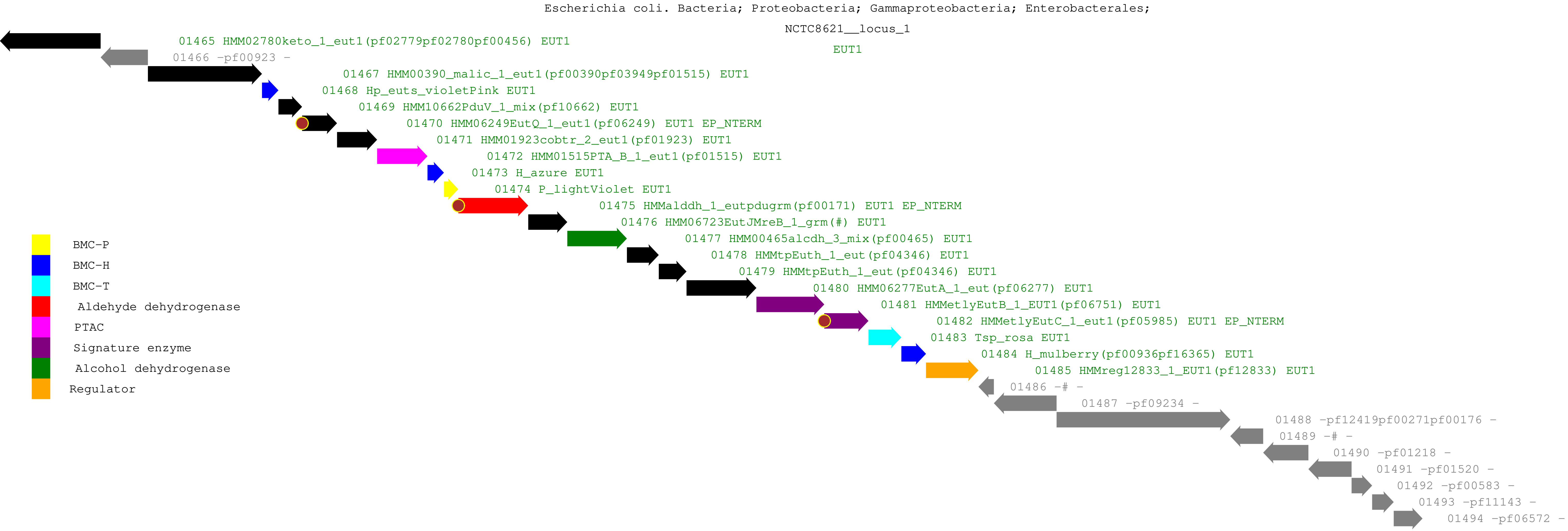
Escherichia coli. Bacteria; Proteobacteria; Gammaproteobacteria; Enterobacterales;
NCTC8621__locus_1
EUT1
01465 HMM02780keto_1_eut1(pf02779pf02780pf00456) EUT1
01466 -pf00923 -
01467 HMM00390_malic_1_eut1(pf00390pf03949pf01515) EUT1
01468 Hp_euts_violetPink EUT1
01469 HMM10662PduV_1_mix(pf10662) EUT1
01470 HMM06249EutQ_1_eut1(pf06249) EUT1 EP_NTERM
01471 HMM01923cobtr_2_eut1(pf01923) EUT1
01472 HMM01515PTA_B_1_eut1(pf01515) EUT1
01473 H_azure EUT1
01474 P_lightViolet EUT1
01475 HMMaldh_1_eutpdugrm(pf00171) EUT1 EP_NTERM
01476 HMM06723EutJMreB_1_grm(#) EUT1
01477 HMM00465alcdh_3_mix(pf00465) EUT1
01478 HMMtpEuth_1_eut(pf04346) EUT1
01479 HMMtpEuth_1_eut(pf04346) EUT1
01480 HMM06277EutA_1_eut(pf06277) EUT1
01481 HMMetlyEutB_1_EUT1(pf06751) EUT1
01482 HMMetlyEutC_1_eut1(pf05985) EUT1 EP_NTERM
01483 Tsp_rosa EUT1
01484 H_mulberry(pf00936pf16365) EUT1
01485 HMMreg12833_1_EUT1(pf12833) EUT1
01486 -# -
01487 -pf09234 -
01488 -pf12419pf00271pf00176 -
01489 -# -
01490 -pf01218 -
01491 -pf01520 -
01492 -pf00583 -
01493 -pf11143 -
01494 -pf06572 -
BMC-P
BMC-H
BMC-T
Aldehyde dehydrogenase
PTAC
Signature enzyme
Alcohol dehydrogenase
Regulator

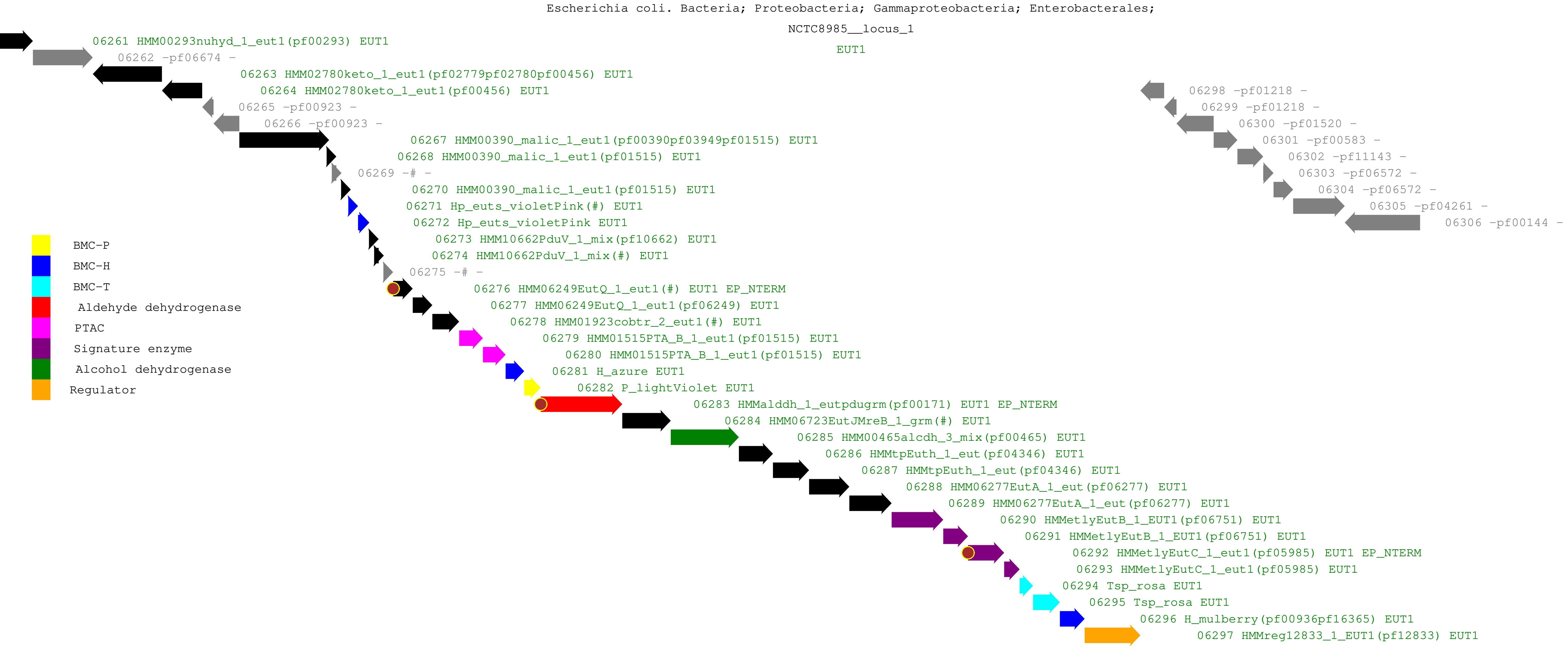
Escherichia coli. Bacteria; Proteobacteria; Gammaproteobacteria; Enterobacterales;
NCTC8985__locus_1
EUT1
06261 HMM00293nuhyd_1_eut1(pf00293) EUT1
06262 -pf06674 -
06263 HMM02780keto_1_eut1(pf02779pf02780pf00456) EUT1
06264 HMM02780keto_1_eut1(pf00456) EUT1
06265 -pf00923 -
06266 -pf00923 -
06267 HMM00390_malic_1_eut1(pf00390pf03949pf01515) EUT1
06268 HMM00390_malic_1_eut1(pf01515) EUT1
06269 -# -
06270 HMM00390_malic_1_eut1(pf01515) EUT1
06271 Hp_euts_violetPink(#) EUT1
06272 Hp_euts_violetPink EUT1
06273 HMM10662PduV_1_mix(pf10662) EUT1
06274 HMM10662PduV_1_mix(#) EUT1
06275 -# -
06276 HMM06249EutQ_1_eut1(#) EUT1 EP_NTERM
06277 HMM06249EutQ_1_eut1(pf06249) EUT1
06278 HMM01923cobtr_2_eut1(#) EUT1
06279 HMM01515PTA_B_1_eut1(pf01515) EUT1
06280 HMM01515PTA_B_1_eut1(pf01515) EUT1
06281 H_azure EUT1
06282 P_lightViolet EUT1
06283 HMMalddh_1_eutpdugrm(pf00171) EUT1 EP_NTERM
06284 HMM06723EutJMreB_1_grm(#) EUT1
06285 HMM00465alcdh_3_mix(pf00465) EUT1
06286 HMMtpEuth_1_eut(pf04346) EUT1
06287 HMMtpEuth_1_eut(pf04346) EUT1
06288 HMM06277EutA_1_eut(pf06277) EUT1
06289 HMM06277EutA_1_eut(pf06277) EUT1
06290 HMMetlyEutB_1_EUT1(pf06751) EUT1
06291 HMMetlyEutB_1_EUT1(pf06751) EUT1
06292 HMMetlyEutC_1_eut1(pf05985) EUT1 EP_NTERM
06293 HMMetlyEutC_1_eut1(pf05985) EUT1
06294 Tsp_rosa EUT1
06295 Tsp_rosa EUT1
06296 H_mulberry(pf00936pf16365) EUT1
06297 HMMreg12833_1_EUT1(pf12833) EUT1
06298 -pf01218 -
06299 -pf01218 -
06300 -pf01520 -
06301 -pf00583 -
06302 -pf11143 -
06303 -pf06572 -
06304 -pf06572 -
06305 -pf04261 -
06306 -pf00144 -
BMC-P
BMC-H
BMC-T
Aldehyde dehydrogenase
PTAC
Signature enzyme
Alcohol dehydrogenase
Regulator

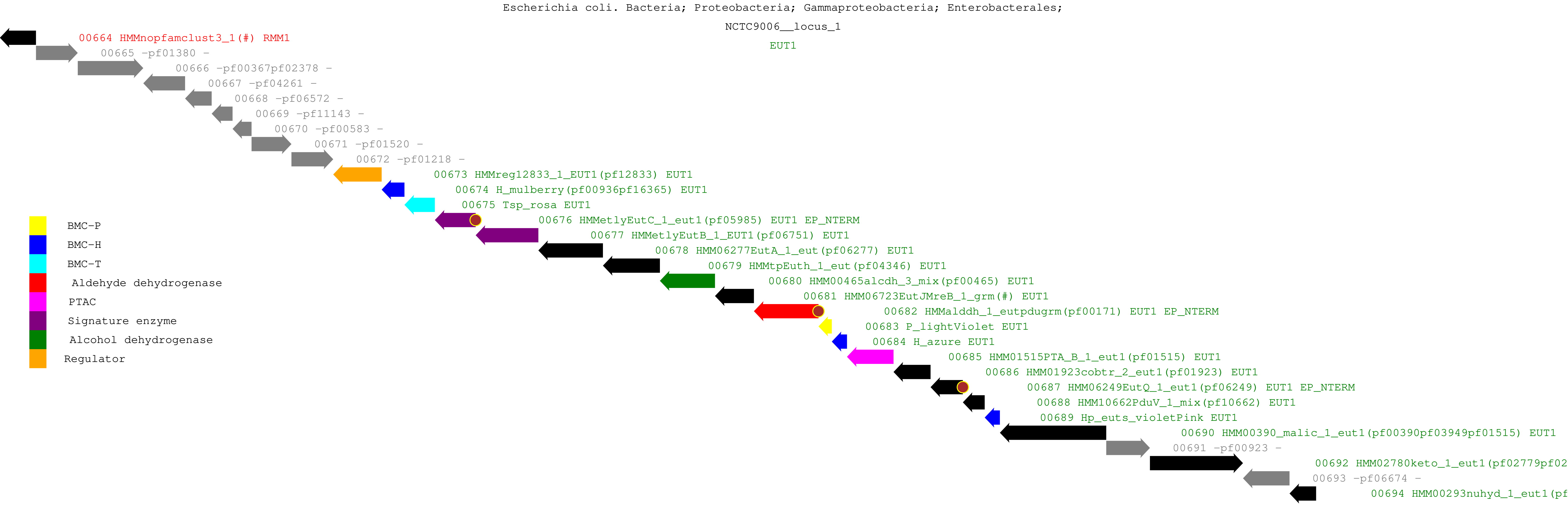
Escherichia coli. Bacteria; Proteobacteria; Gammaproteobacteria; Enterobacterales;
NCTC9006__locus_1
EUT1
00664 HMMnopfamclust3_1(#) RMM1
00665 -pf01380 -
00666 -pf00367pf02378 -
00667 -pf04261 -
00668 -pf06572 -
00669 -pf11143 -
00670 -pf00583 -
00671 -pf01520 -
00672 -pf01218 -
00673 HMMreg12833_1_EUT1(pf12833) EUT1
00674 H_mulberry(pf00936pf16365) EUT1
00675 Tsp_rosa EUT1
00676 HMMetlyEutC_1_eut1(pf05985) EUT1 EP_NTERM
00677 HMMetlyEutB_1_EUT1(pf06751) EUT1
00678 HMM06277EutA_1_eut(pf06277) EUT1
00679 HMMtpEuth_1_eut(pf04346) EUT1
00680 HMM00465alcdh_3_mix(pf00465) EUT1
00681 HMM06723EutJMreB_1_grm(#) EUT1
00682 HMMalddh_1_eutpdugrm(pf00171) EUT1 EP_NTERM
00683 P_lightViolet EUT1
00684 H_azure EUT1
00685 HMM01515PTA_B_1_eut1(pf01515) EUT1
00686 HMM01923cobtr_2_eut1(pf01923) EUT1
00687 HMM06249EutQ_1_eut1(pf06249) EUT1 EP_NTERM
00688 HMM10662PduV_1_mix(pf10662) EUT1
00689 Hp_euts_violetPink EUT1
00690 HMM00390_malic_1_eut1(pf00390pf03949pf01515) EUT1
00691 -pf00923 -
00692 HMM02780keto_1_eut1(pf02779pf02
00693 -pf06674 -
00694 HMM00293nuhyd_1_eut1(pf
BMC-P
BMC-H
BMC-T
Aldehyde dehydrogenase
PTAC
Signature enzyme
Alcohol dehydrogenase
Regulator

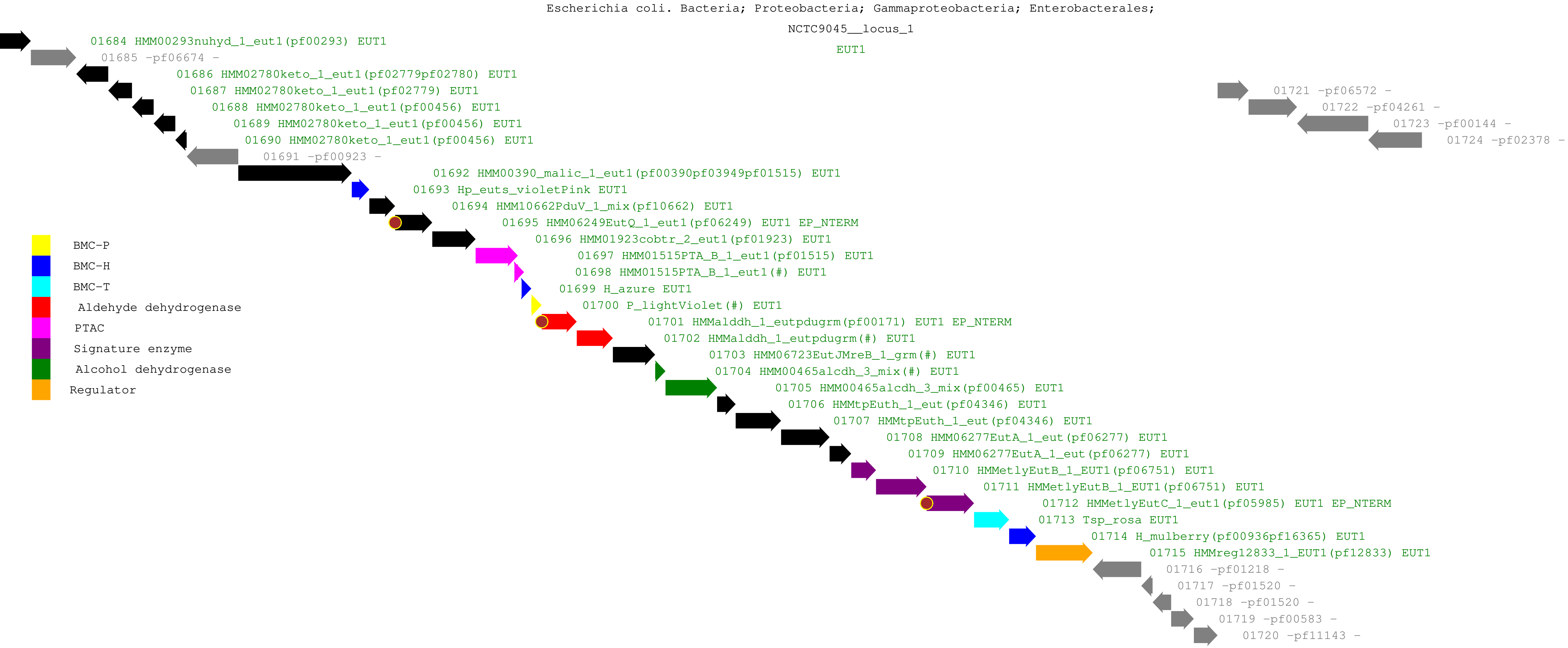

Escherichia coli. Bacteria; Proteobacteria; Gammaproteobacteria; Enterobacterales;
NCTC9045__locus_1
EUT1
01684 HMM00293nuhyd_1_eut1(pf00293) EUT1
01685 -pf06674 -
01686 HMM02780keto_1_eut1(pf02779pf02780) EUT1
01687 HMM02780keto_1_eut1(pf02779) EUT1
01688 HMM02780keto_1_eut1(pf00456) EUT1
01689 HMM02780keto_1_eut1(pf00456) EUT1
01690 HMM02780keto_1_eut1(pf00456) EUT1
01691 -pf00923 -
01692 HMM00390_malic_1_eut1(pf00390pf03949pf01515) EUT1
01693 Hp_euts_violetPink EUT1
01694 HMM10662PduV_1_mix(pf10662) EUT1
01695 HMM06249EutQ_1_eut1(pf06249) EUT1 EP_NTERM
01696 HMM01923cobtr_2_eut1(pf01923) EUT1
01697 HMM01515PTA_B_1_eut1(pf01515) EUT1
01698 HMM01515PTA_B_1_eut1(#) EUT1
01699 H_azure EUT1
01700 P_lightViolet(#) EUT1
01701 HMMalddh_1_eutpdugrm(pf00171) EUT1 EP_NTERM
01702 HMMalddh_1_eutpdugrm(#) EUT1
01703 HMM06723EutJMreB_1_grm(#) EUT1
01704 HMM00465alcdh_3_mix(#) EUT1
01705 HMM00465alcdh_3_mix(pf00465) EUT1
01706 HMMtpEuth_1_eut(pf04346) EUT1
01707 HMMtpEuth_1_eut(pf04346) EUT1
01708 HMM06277EutA_1_eut(pf06277) EUT1
01709 HMM06277EutA_1_eut(pf06277) EUT1
01710 HMMetlyEutB_1_EUT1(pf06751) EUT1
01711 HMMetlyEutB_1_EUT1(pf06751) EUT1
01712 HMMetlyEutC_1_eut1(pf05985) EUT1 EP_NTERM
01713 Tsp_rosa EUT1
01714 H_mulberry(pf00936pf16365) EUT1
01715 HMMreg12833_1_EUT1(pf12833) EUT1
01716 -pf01218 -
01717 -pf01520 -
01718 -pf01520 -
01719 -pf00583 -
01720 -pf11143 -
01721 -pf06572 -
01722 -pf04261 -
01723 -pf00144 -
01724 -pf02378 -
BMC-P
BMC-H
BMC-T
Aldehyde dehydrogenase
PTAC
Signature enzyme
Alcohol dehydrogenase
Regulator

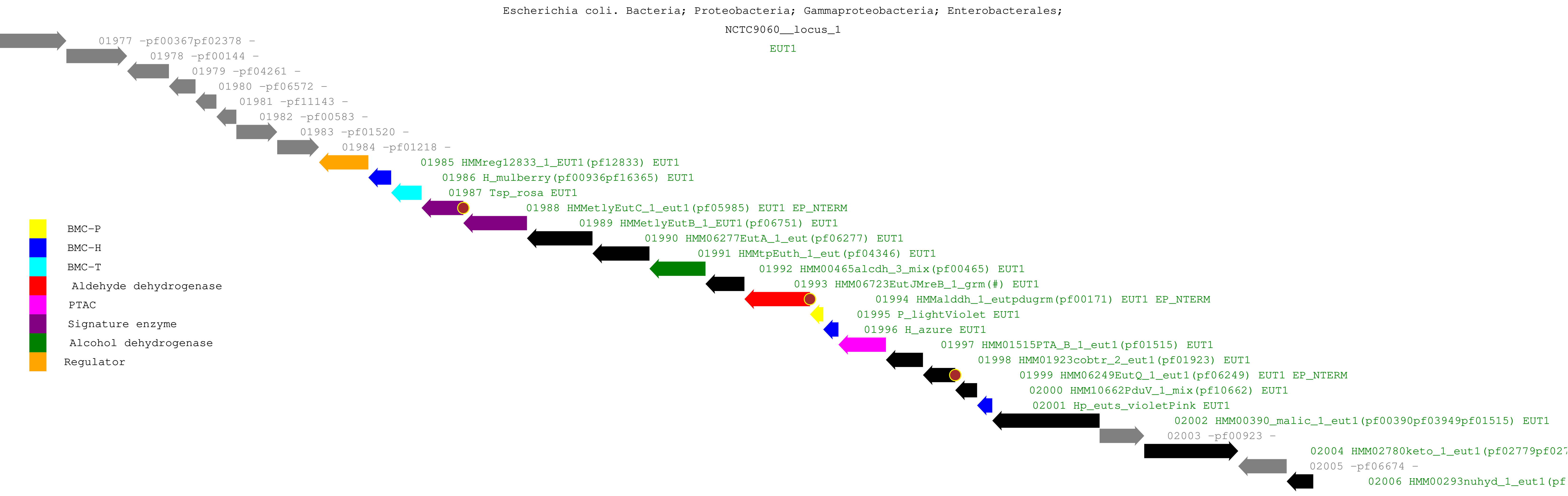

Escherichia coli. Bacteria; Proteobacteria; Gammaproteobacteria; Enterobacterales;
NCTC9060__locus_1
EUT1
01977 -pf00367pf02378 -
01978 -pf00144 -
01979 -pf04261 -
01980 -pf06572 -
01981 -pf11143 -
01982 -pf00583 -
01983 -pf01520 -
01984 -pf01218 -
01985 HMMreg12833_1_EUT1(pf12833) EUT1
01986 H_mulberry(pf00936pf16365) EUT1
01987 Tsp_rosa EUT1
01988 HMMetlyEutC_1_eut1(pf05985) EUT1 EP_NTERM
01989 HMMetlyEutB_1_EUT1(pf06751) EUT1
01990 HMM06277EutA_1_eut(pf06277) EUT1
01991 HMMtpEuth_1_eut(pf04346) EUT1
01992 HMM00465alcdh_3_mix(pf00465) EUT1
01993 HMM06723EutJMreB_1_grm(#) EUT1
01994 HMMalddh_1_eutpdugrm(pf00171) EUT1 EP_NTERM
01995 P_lightViolet EUT1
01996 H_azure EUT1
01997 HMM01515PTA_B_1_eut1(pf01515) EUT1
01998 HMM01923cobtr_2_eut1(pf01923) EUT1
01999 HMM06249EutQ_1_eut1(pf06249) EUT1 EP_NTERM
02000 HMM10662PduV_1_mix(pf10662) EUT1
02001 Hp_euts_violetPink EUT1
02002 HMM00390_malic_1_eut1(pf00390pf03949pf01515) EUT1
02003 -pf00923 -
02004 HMM02780keto_1_eut1(pf0277
02005 -pf06674 -
02006 HMM00293nuhyd_1_eut1(pf
BMC-P
BMC-H
BMC-T
Aldehyde dehydrogenase
PTAC
Signature enzyme
Alcohol dehydrogenase
Regulator

Escherichia coli. Bacteria; Proteobacteria; Gammaproteobacteria; Enterobacterales;

NCTC9067__locus_1

EUT1

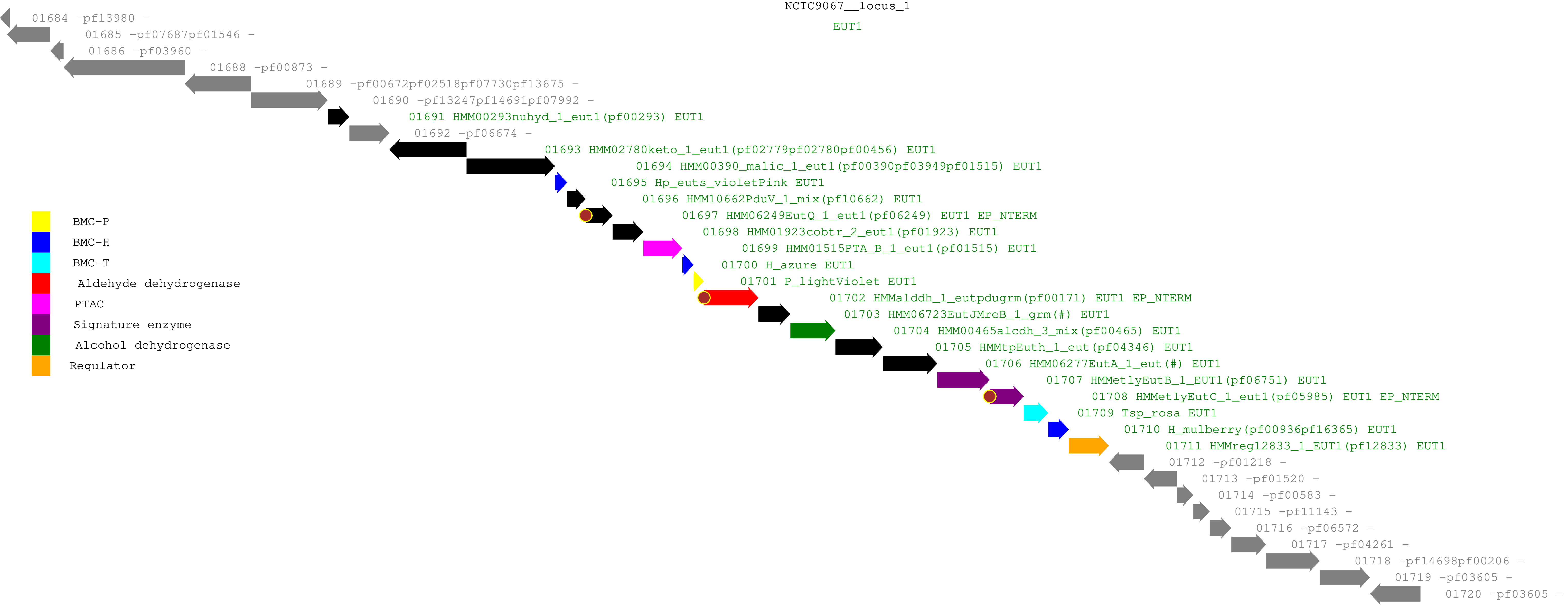

Escherichia coli. Bacteria; Proteobacteria; Gammaproteobacteria; Enterobacterales;

NCTC9094__locus_1

EUT1

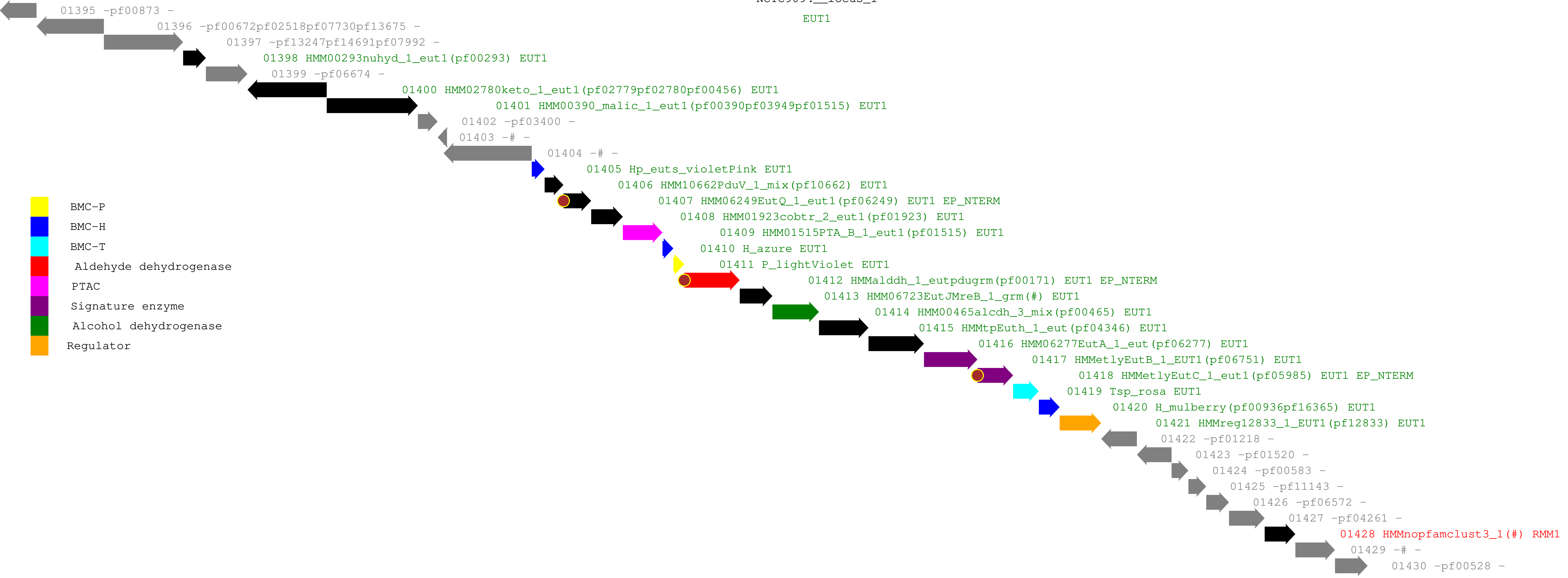

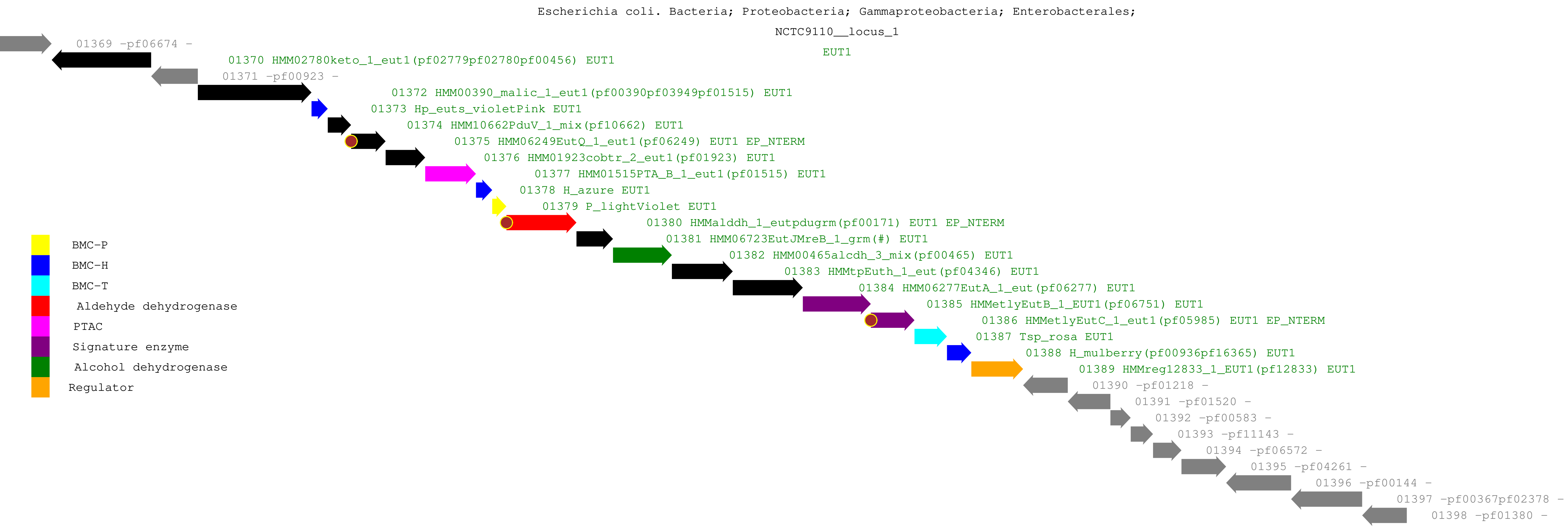

Escherichia coli. Bacteria; Proteobacteria; Gammaproteobacteria; Enterobacterales;
NCTC9110__locus_1
EUT1
01369 -pf06674 -
01370 HMM02780keto_1_eut1(pf02779pf02780pf00456) EUT1
01371 -pf00923 -
01372 HMM00390_malic_1_eut1(pf00390pf03949pf01515) EUT1
01373 Hp_euts_violetPink EUT1
01374 HMM10662PduV_1_mix(pf10662) EUT1
01375 HMM06249EutQ_1_eut1(pf06249) EUT1 EP_NTERM
01376 HMM01923cobtr_2_eut1(pf01923) EUT1
01377 HMM01515PTA_B_1_eut1(pf01515) EUT1
01378 H_azure EUT1
01379 P_lightViolet EUT1
01380 HMMalddh_1_eutpdugrm(pf00171) EUT1 EP_NTERM
01381 HMM06723EutJMreB_1_grm(#) EUT1
01382 HMM00465alcdh_3_mix(pf00465) EUT1
01383 HMMtpEuth_1_eut(pf04346) EUT1
01384 HMM06277EutA_1_eut(pf06277) EUT1
01385 HMMetlyEutB_1_EUT1(pf06751) EUT1
01386 HMMetlyEutC_1_eut1(pf05985) EUT1 EP_NTERM
01387 Tsp_rosa EUT1
01388 H_mulberry(pf00936pf16365) EUT1
01389 HMMreg12833_1_EUT1(pf12833) EUT1
01390 -pf01218 -
01391 -pf01520 -
01392 -pf00583 -
01393 -pf11143 -
01394 -pf06572 -
01395 -pf04261 -
01396 -pf00144 -
01397 -pf00367pf02378 -
01398 -pf01380 -
BMC-P
BMC-H
BMC-T
Aldehyde dehydrogenase
PTAC
Signature enzyme
Alcohol dehydrogenase
Regulator

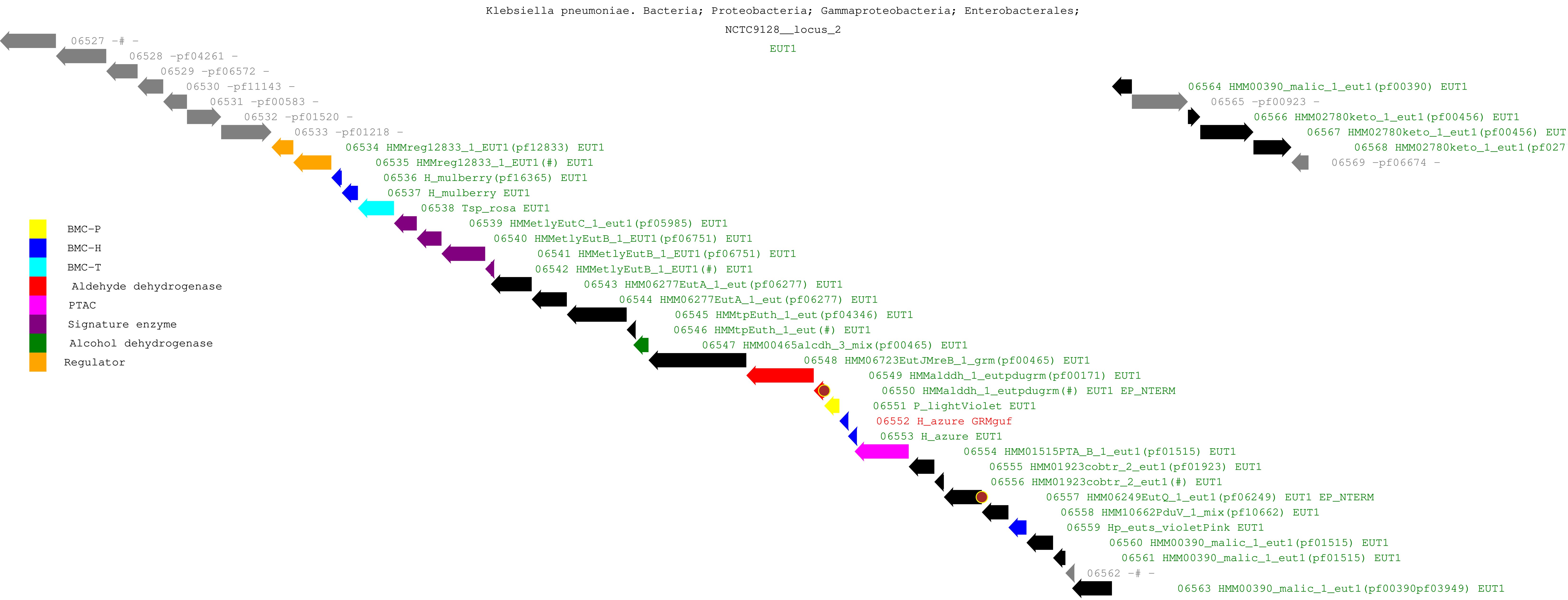

Klebsiella pneumoniae. Bacteria; Proteobacteria; Gammaproteobacteria; Enterobacterales;
NCTC9128__locus_2
EUT1
06527 -# -
06528 -pf04261 -
06529 -pf06572 -
06530 -pf11143 -
06531 -pf00583 -
06532 -pf01520 -
06533 -pf01218 -
06534 HMMreg12833_1_EUT1(pf12833) EUT1
06535 HMMreg12833_1_EUT1(#) EUT1
06536 H_mulberry(pf16365) EUT1
06537 H_mulberry EUT1
06538 Tsp_rosa EUT1
06539 HMMetlyEutC_1_eut1(pf05985) EUT1
06540 HMMetlyEutB_1_EUT1(pf06751) EUT1
06541 HMMetlyEutB_1_EUT1(pf06751) EUT1
06542 HMMetlyEutB_1_EUT1(#) EUT1
06543 HMM06277EutA_1_eut(pf06277) EUT1
06544 HMM06277EutA_1_eut(pf06277) EUT1
06545 HMMtpEuth_1_eut(pf04346) EUT1
06546 HMMtpEuth_1_eut(#) EUT1
06547 HMM00465alcdh_3_mix(pf00465) EUT1
06548 HMM06723EutJMreB_1_grm(pf00465) EUT1
06549 HMMalddh_1_eutpdugrm(pf00171) EUT1
06550 HMMalddh_1_eutpdugrm(#) EUT1 EP_NTERM
06551 P_lightViolet EUT1
06552 H_azure GRMguf
06553 H_azure EUT1
06554 HMM01515PTA_B_1_eut1(pf01515) EUT1
06555 HMM01923cobtr_2_eut1(pf01923) EUT1
06556 HMM01923cobtr_2_eut1(#) EUT1
06557 HMM06249EutQ_1_eut1(pf06249) EUT1 EP_NTERM
06558 HMM10662PduV_1_mix(pf10662) EUT1
06559 Hp_euts_violetPink EUT1
06560 HMM00390_malic_1_eut1(pf01515) EUT1
06561 HMM00390_malic_1_eut1(pf01515) EUT1
06562 -# -
06563 HMM00390_malic_1_eut1(pf00390pf03949) EUT1
06564 HMM00390_malic_1_eut1(pf00390) EUT1
06565 -pf00923 -
06566 HMM02780keto_1_eut1(pf00456) EUT1
06567 HMM02780keto_1_eut1(pf00456) EUT
06568 HMM02780keto_1_eut1(pf027
06569 -pf06674 -
BMC-P
BMC-H
BMC-T
Aldehyde dehydrogenase
PTAC
Signature enzyme
Alcohol dehydrogenase
Regulator

Klebsiella pneumoniae. Bacteria; Proteobacteria; Gammaproteobacteria; Enterobacterales;

NCTC9140__locus_1

EUT1

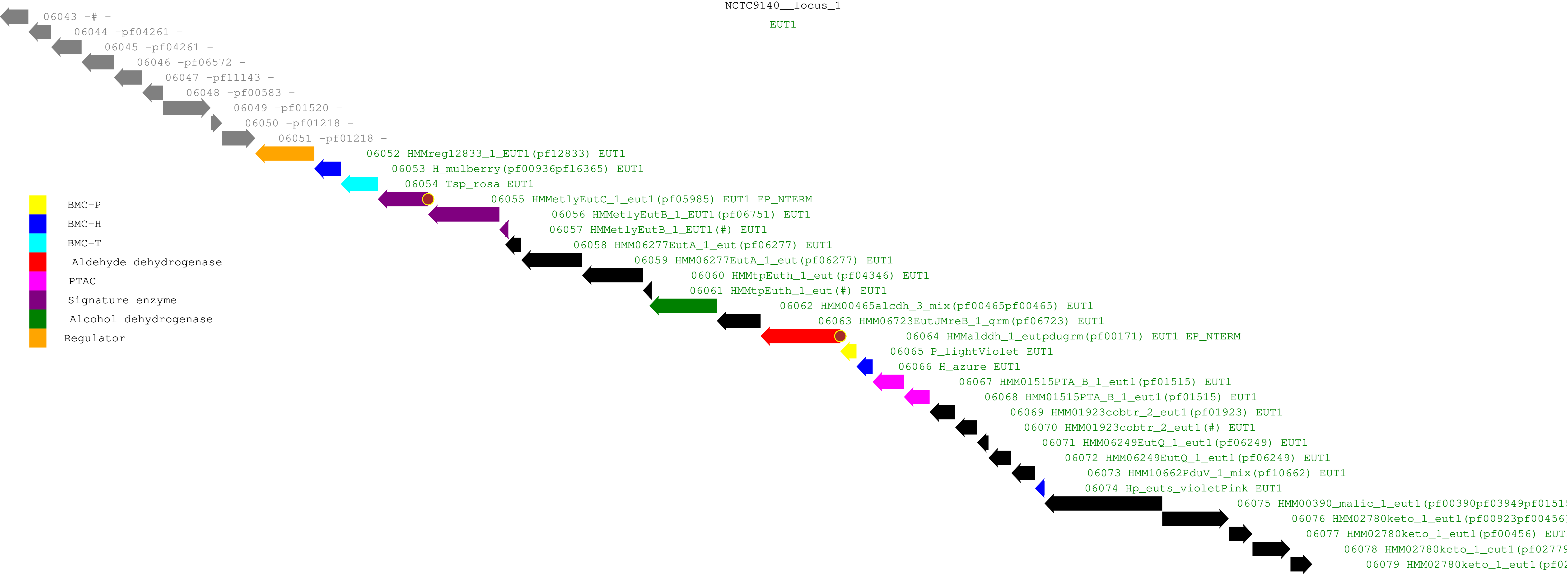

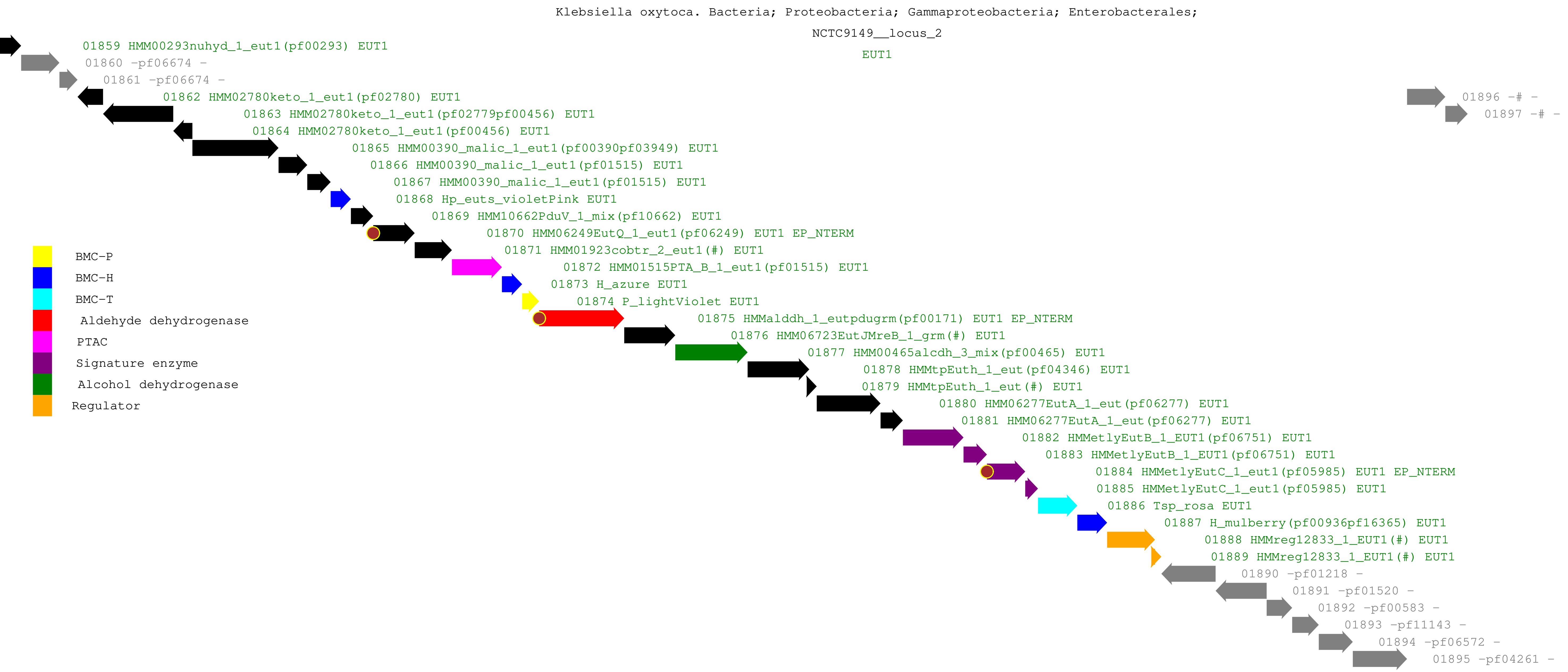
Klebsiella oxytoca. Bacteria; Proteobacteria; Gammaproteobacteria; Enterobacterales;
NCTC9149__locus_2
EUT1
01859 HMM00293nuhyd_1_eut1(pf00293) EUT1
01860 -pf06674 -
01861 -pf06674 -
01862 HMM02780keto_1_eut1(pf02780) EUT1
01863 HMM02780keto_1_eut1(pf02779pf00456) EUT1
01864 HMM02780keto_1_eut1(pf00456) EUT1
01865 HMM00390_malic_1_eut1(pf00390pf03949) EUT1
01866 HMM00390_malic_1_eut1(pf01515) EUT1
01867 HMM00390_malic_1_eut1(pf01515) EUT1
01868 Hp_euts_violetPink EUT1
01869 HMM10662PduV_1_mix(pf10662) EUT1
01870 HMM06249EutQ_1_eut1(pf06249) EUT1 EP_NTERM
01871 HMM01923cobtr_2_eut1(#) EUT1
01872 HMM01515PTA_B_1_eut1(pf01515) EUT1
01873 H_azure EUT1
01874 P_lightViolet EUT1
01875 HMMalddh_1_eutpdugrm(pf00171) EUT1 EP_NTERM
01876 HMM06723EutJMreB_1_grm(#) EUT1
01877 HMM00465alcdh_3_mix(pf00465) EUT1
01878 HMMtpEuth_1_eut(pf04346) EUT1
01879 HMMtpEuth_1_eut(#) EUT1
01880 HMM06277EutA_1_eut(pf06277) EUT1
01881 HMM06277EutA_1_eut(pf06277) EUT1
01882 HMMetlyEutB_1_EUT1(pf06751) EUT1
01883 HMMetlyEutB_1_EUT1(pf06751) EUT1
01884 HMMetlyEutC_1_eut1(pf05985) EUT1 EP_NTERM
01885 HMMetlyEutC_1_eut1(pf05985) EUT1
01886 Tsp_rosa EUT1
01887 H_mulberry(pf00936pf16365) EUT1
01888 HMMreg12833_1_EUT1(#) EUT1
01889 HMMreg12833_1_EUT1(#) EUT1
01890 -pf01218 -
01891 -pf01520 -
01892 -pf00583 -
01893 -pf11143 -
01894 -pf06572 -
01895 -pf04261 -
01896 -# -
01897 -# -
BMC-P
BMC-H
BMC-T
Aldehyde dehydrogenase
PTAC
Signature enzyme
Alcohol dehydrogenase
Regulator

Klebsiella oxytoca. Bacteria; Proteobacteria; Gammaproteobacteria; Enterobacterales;

NCTC9161__locus_2

EUT1

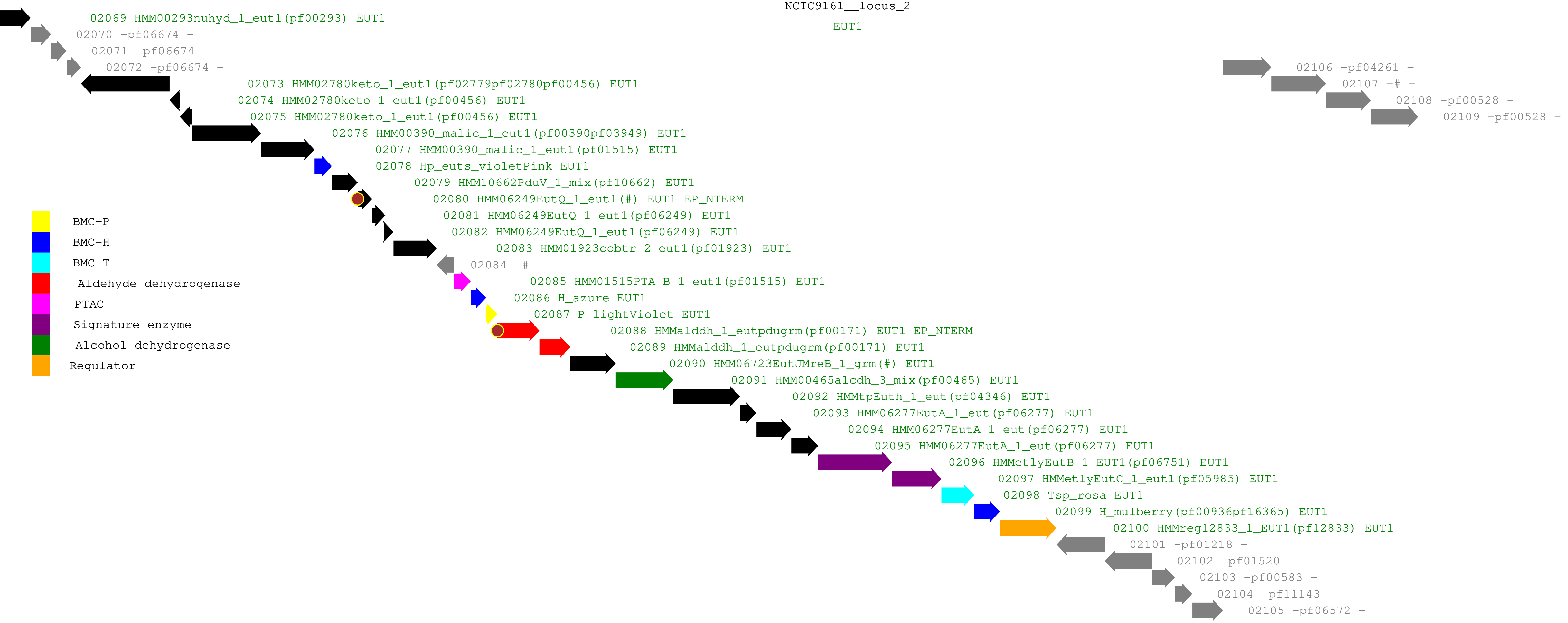

Klebsiella pneumoniae. Bacteria; Proteobacteria; Gammaproteobacteria; Enterobacterales;

NCTC9177__locus_2

EUT1

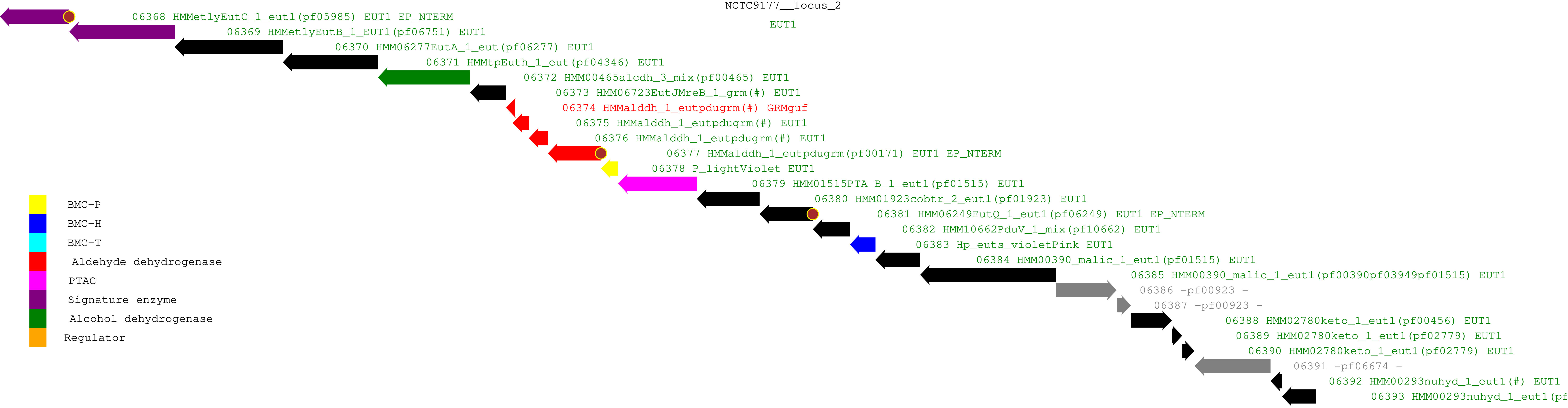

Klebsiella oxytoca. Bacteria; Proteobacteria; Gammaproteobacteria; Enterobacterales;

NCTC9186__locus_2

EUT1

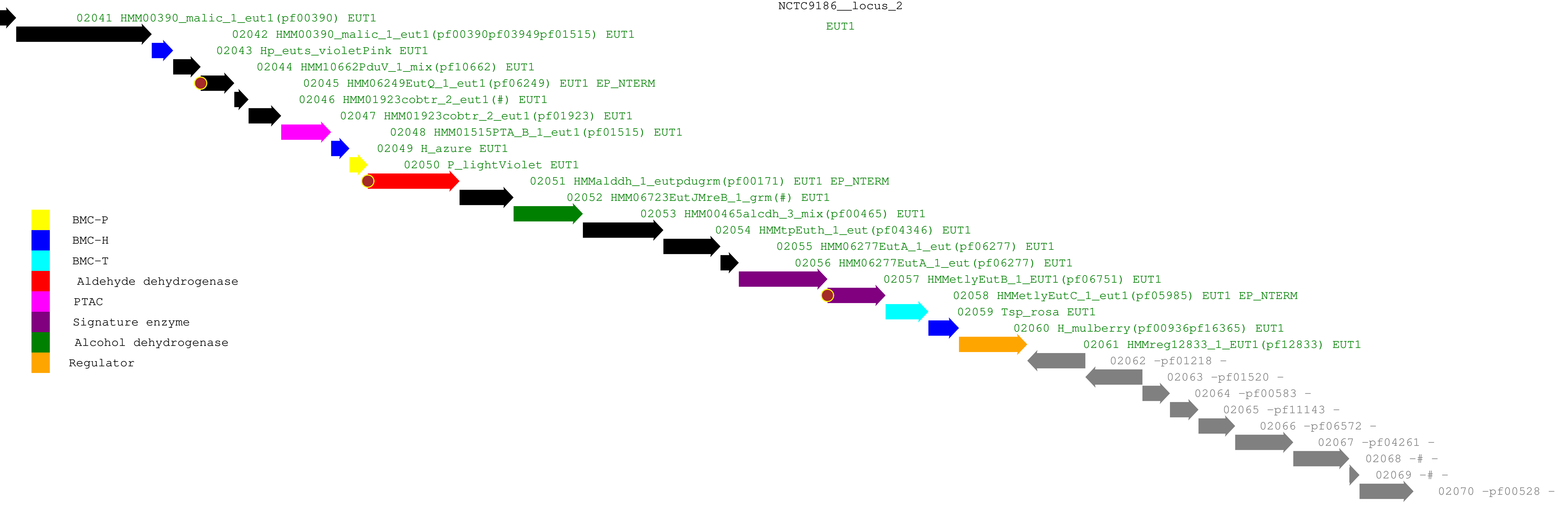

Klebsiella pneumoniae subsp. pneumoniae. Bacteria; Proteobacteria; Gammaproteobacteria; Enterobacterales;

NCTC9494__locus_2

EUT1

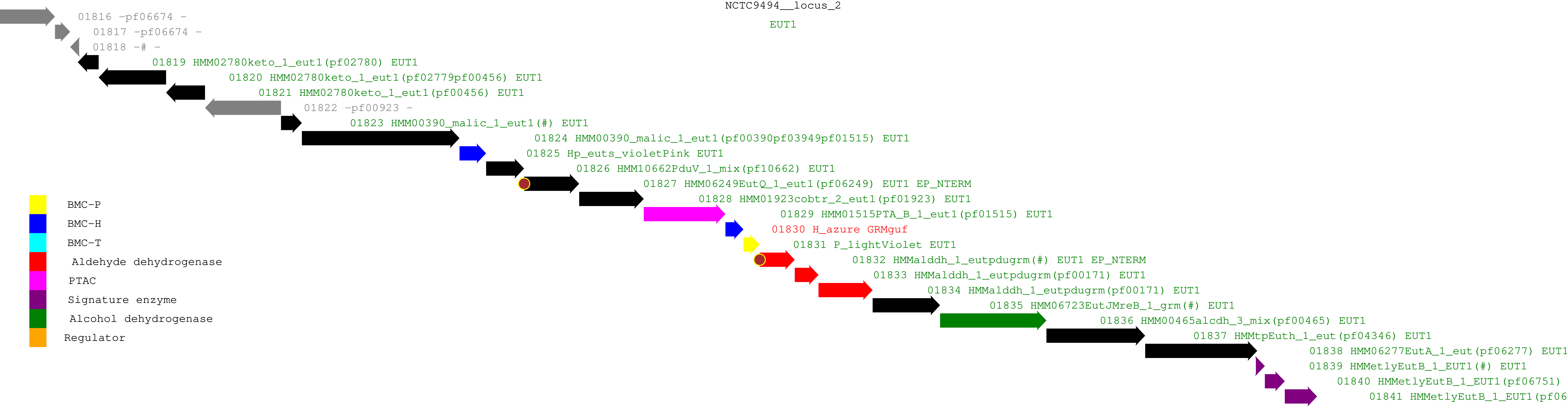

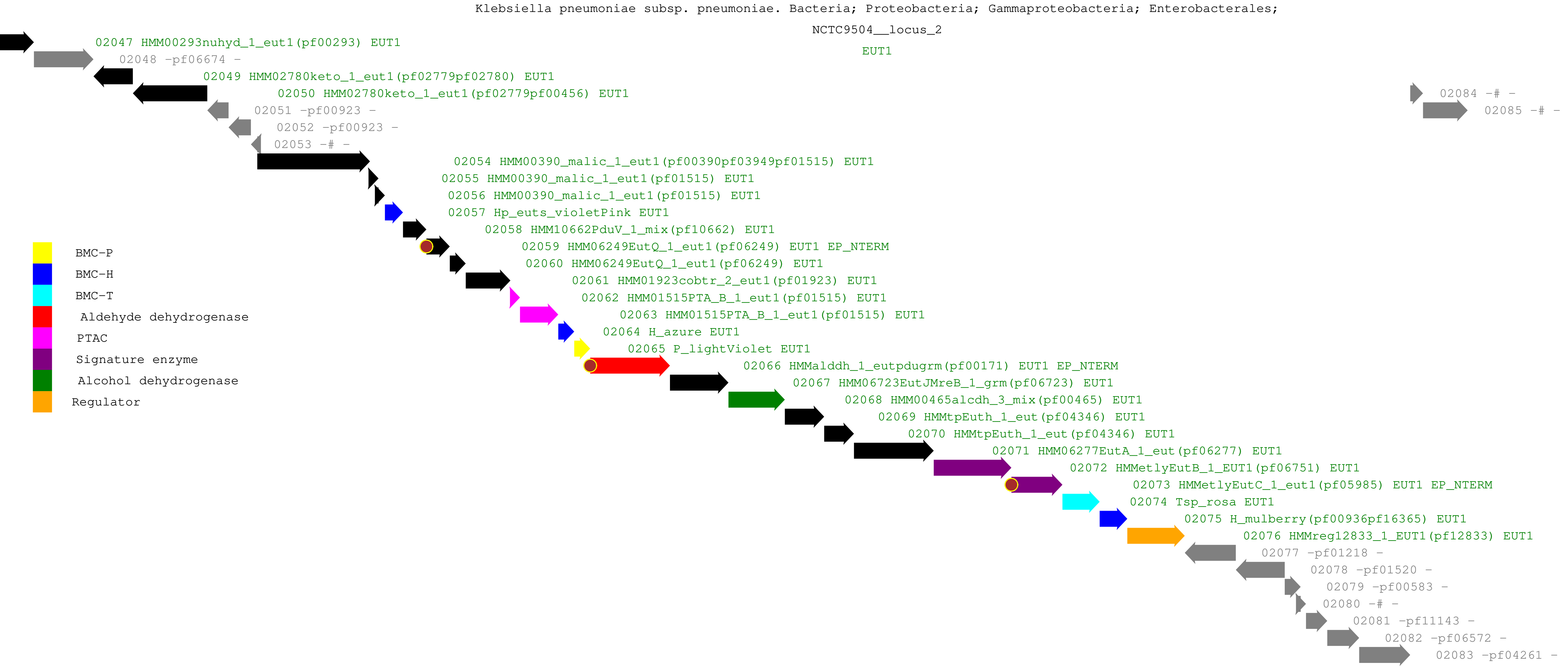

Klebsiella pneumoniae subsp. pneumoniae. Bacteria; Proteobacteria; Gammaproteobacteria; Enterobacterales;
NCTC9504__locus_2
EUT1
02047 HMM00293nuhyd_1_eut1(pf00293) EUT1
02048 -pf06674 -
02049 HMM02780keto_1_eut1(pf02779pf02780) EUT1
02050 HMM02780keto_1_eut1(pf02779pf00456) EUT1
02051 -pf00923 -
02052 -pf00923 -
02053 -# -
02054 HMM00390_malic_1_eut1(pf00390pf03949pf01515) EUT1
02055 HMM00390_malic_1_eut1(pf01515) EUT1
02056 HMM00390_malic_1_eut1(pf01515) EUT1
02057 Hp_euts_violetPink EUT1
02058 HMM10662PduV_1_mix(pf10662) EUT1
02059 HMM06249EutQ_1_eut1(pf06249) EUT1 EP_NTERM
02060 HMM06249EutQ_1_eut1(pf06249) EUT1
02061 HMM01923cobtr_2_eut1(pf01923) EUT1
02062 HMM01515PTA_B_1_eut1(pf01515) EUT1
02063 HMM01515PTA_B_1_eut1(pf01515) EUT1
02064 H_azure EUT1
02065 P_lightViolet EUT1
02066 HMMalddh_1_eutpdugrm(pf00171) EUT1 EP_NTERM
02067 HMM06723EutJMreB_1_grm(pf06723) EUT1
02068 HMM00465alcdh_3_mix(pf00465) EUT1
02069 HMMtpEuth_1_eut(pf04346) EUT1
02070 HMMtpEuth_1_eut(pf04346) EUT1
02071 HMM06277EutA_1_eut(pf06277) EUT1
02072 HMMetlyEutB_1_EUT1(pf06751) EUT1
02073 HMMetlyEutC_1_eut1(pf05985) EUT1 EP_NTERM
02074 Tsp_rosa EUT1
02075 H_mulberry(pf00936pf16365) EUT1
02076 HMMreg12833_1_EUT1(pf12833) EUT1
02077 -pf01218 -
02078 -pf01520 -
02079 -pf00583 -
02080 -# -
02081 -pf11143 -
02082 -pf06572 -
02083 -pf04261 -
02084 -# -
02085 -# -
BMC-P
BMC-H
BMC-T
Aldehyde dehydrogenase
PTAC
Signature enzyme
Alcohol dehydrogenase
Regulator

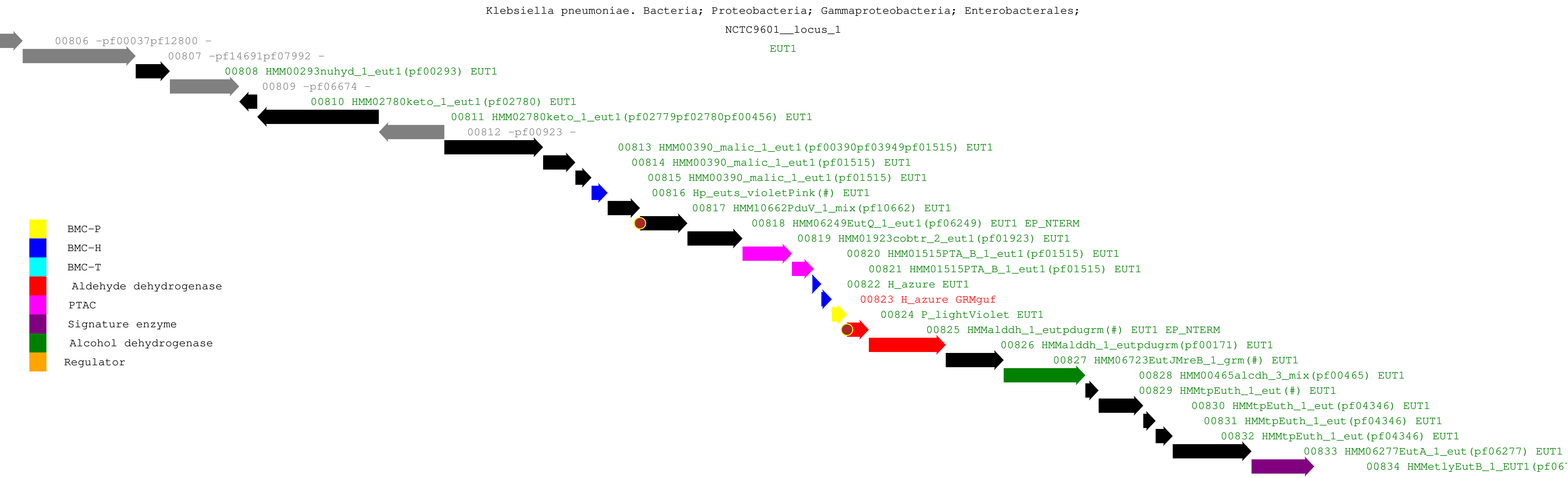

Klebsiella pneumoniae. Bacteria; Proteobacteria; Gammaproteobacteria; Enterobacterales;
NCTC9601__locus_1
EUT1
00806 -pf00037pf12800 -
00807 -pf14691pf07992 -
00808 HMM00293nuhyd_1_eut1(pf00293) EUT1
00809 -pf06674 -
00810 HMM02780keto_1_eut1(pf02780) EUT1
00811 HMM02780keto_1_eut1(pf02779pf02780pf00456) EUT1
00812 -pf00923 -
00813 HMM00390_malic_1_eut1(pf00390pf03949pf01515) EUT1
00814 HMM00390_malic_1_eut1(pf01515) EUT1
00815 HMM00390_malic_1_eut1(pf01515) EUT1
00816 Hp_euts_violetPink(#) EUT1
00817 HMM10662PduV_1_mix(pf10662) EUT1
00818 HMM06249EutQ_1_eut1(pf06249) EUT1 EP_NTERM
00819 HMM01923cobtr_2_eut1(pf01923) EUT1
00820 HMM01515PTA_B_1_eut1(pf01515) EUT1
00821 HMM01515PTA_B_1_eut1(pf01515) EUT1
00822 H_azure EUT1
00823 H_azure GRMguf
00824 P_lightViolet EUT1
00825 HMMalddh_1_eutpdugrm(#) EUT1 EP_NTERM
00826 HMMalddh_1_eutpdugrm(pf00171) EUT1
00827 HMM06723EutJMreB_1_grm(#) EUT1
00828 HMM00465alcdh_3_mix(pf00465) EUT1
00829 HMMtpEuth_1_eut(#) EUT1
00830 HMMtpEuth_1_eut(pf04346) EUT1
00831 HMMtpEuth_1_eut(pf04346) EUT1
00832 HMMtpEuth_1_eut(pf04346) EUT1
00833 HMM06277EutA_1_eut(pf06277) EUT1
00834 HMMetlyEutB_1_EUT1(pf06
BMC-P
BMC-H
BMC-T
Aldehyde dehydrogenase
PTAC
Signature enzyme
Alcohol dehydrogenase
Regulator

Klebsiella pneumoniae. Bacteria; Proteobacteria; Gammaproteobacteria; Enterobacterales;

NCTC9645__locus_1

EUT1

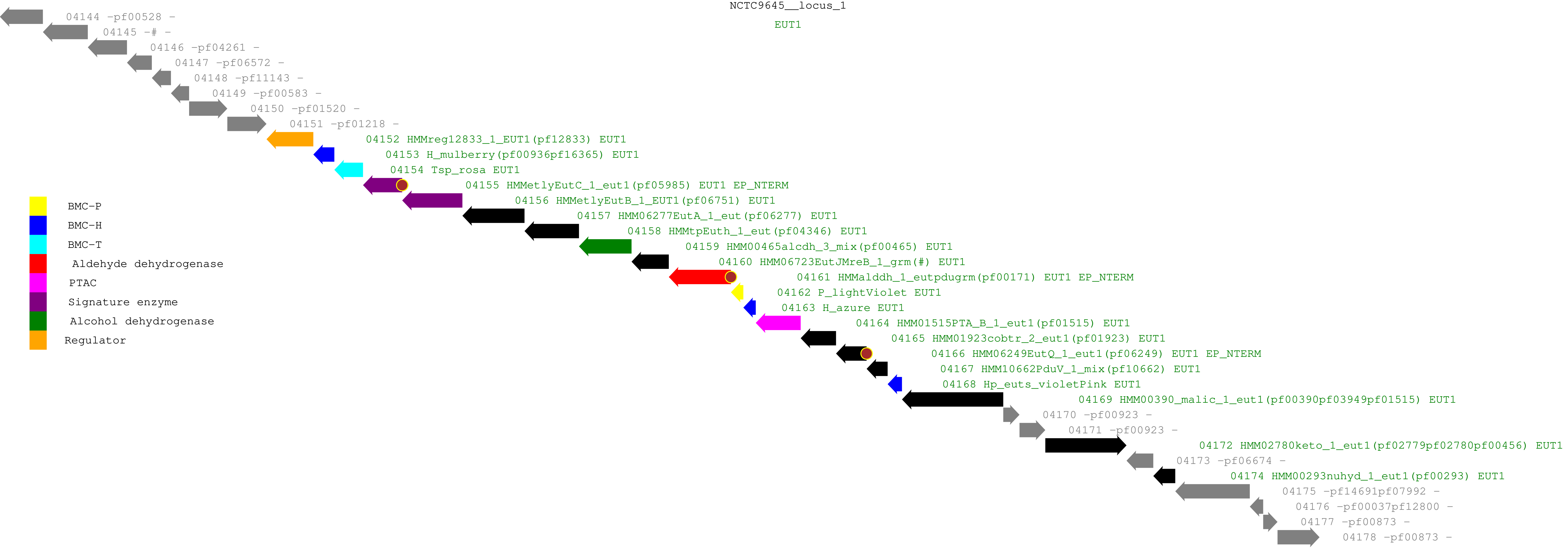

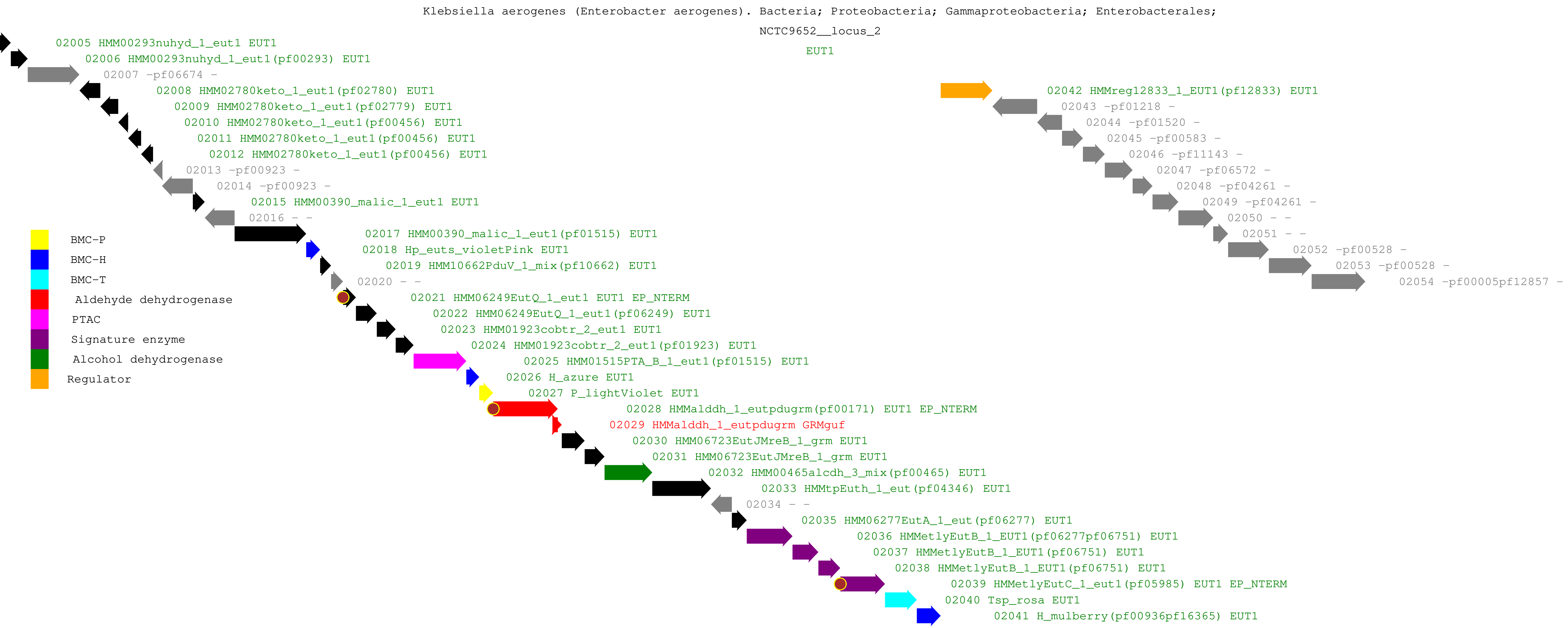

Klebsiella aerogenes (Enterobacter aerogenes). Bacteria; Proteobacteria; Gammaproteobacteria; Enterobacterales;
NCTC9652__locus_2
EUT1
02005 HMM00293nuhyd_1_eut1 EUT1
02006 HMM00293nuhyd_1_eut1(pf00293) EUT1
02007 -pf06674 -
02008 HMM02780keto_1_eut1(pf02780) EUT1
02009 HMM02780keto_1_eut1(pf02779) EUT1
02010 HMM02780keto_1_eut1(pf00456) EUT1
02011 HMM02780keto_1_eut1(pf00456) EUT1
02012 HMM02780keto_1_eut1(pf00456) EUT1
02013 -pf00923 -
02014 -pf00923 -
02015 HMM00390_malic_1_eut1 EUT1
02016 - -
02017 HMM00390_malic_1_eut1(pf01515) EUT1
02018 Hp_euts_violetPink EUT1
02019 HMM10662PduV_1_mix(pf10662) EUT1
02020 - -
02021 HMM06249EutQ_1_eut1 EUT1 EP_NTERM
02022 HMM06249EutQ_1_eut1(pf06249) EUT1
02023 HMM01923cobtr_2_eut1 EUT1
02024 HMM01923cobtr_2_eut1(pf01923) EUT1
02025 HMM01515PTA_B_1_eut1(pf01515) EUT1
02026 H_azure EUT1
02027 P_lightViolet EUT1
02028 HMMalddh_1_eutpdugrm(pf00171) EUT1 EP_NTERM
02029 HMMalddh_1_eutpdugrm GRMguf
02030 HMM06723EutJMreB_1_grm EUT1
02031 HMM06723EutJMreB_1_grm EUT1
02032 HMM00465alcdh_3_mix(pf00465) EUT1
02033 HMMtpEuth_1_eut(pf04346) EUT1
02034 - -
02035 HMM06277EutA_1_eut(pf06277) EUT1
02036 HMMetlyEutB_1_EUT1(pf06277pf06751) EUT1
02037 HMMetlyEutB_1_EUT1(pf06751) EUT1
02038 HMMetlyEutB_1_EUT1(pf06751) EUT1
02039 HMMetlyEutC_1_eut1(pf05985) EUT1 EP_NTERM
02040 Tsp_rosa EUT1
02041 H_mulberry(pf00936pf16365) EUT1
02042 HMMreg12833_1_EUT1(pf12833) EUT1
02043 -pf01218 -
02044 -pf01520 -
02045 -pf00583 -
02046 -pf11143 -
02047 -pf06572 -
02048 -pf04261 -
02049 -pf04261 -
02050 - -
02051 - -
02052 -pf00528 -
02053 -pf00528 -
02054 -pf00005pf12857 -
BMC-P
BMC-H
BMC-T
Aldehyde dehydrogenase
PTAC
Signature enzyme
Alcohol dehydrogenase
Regulator

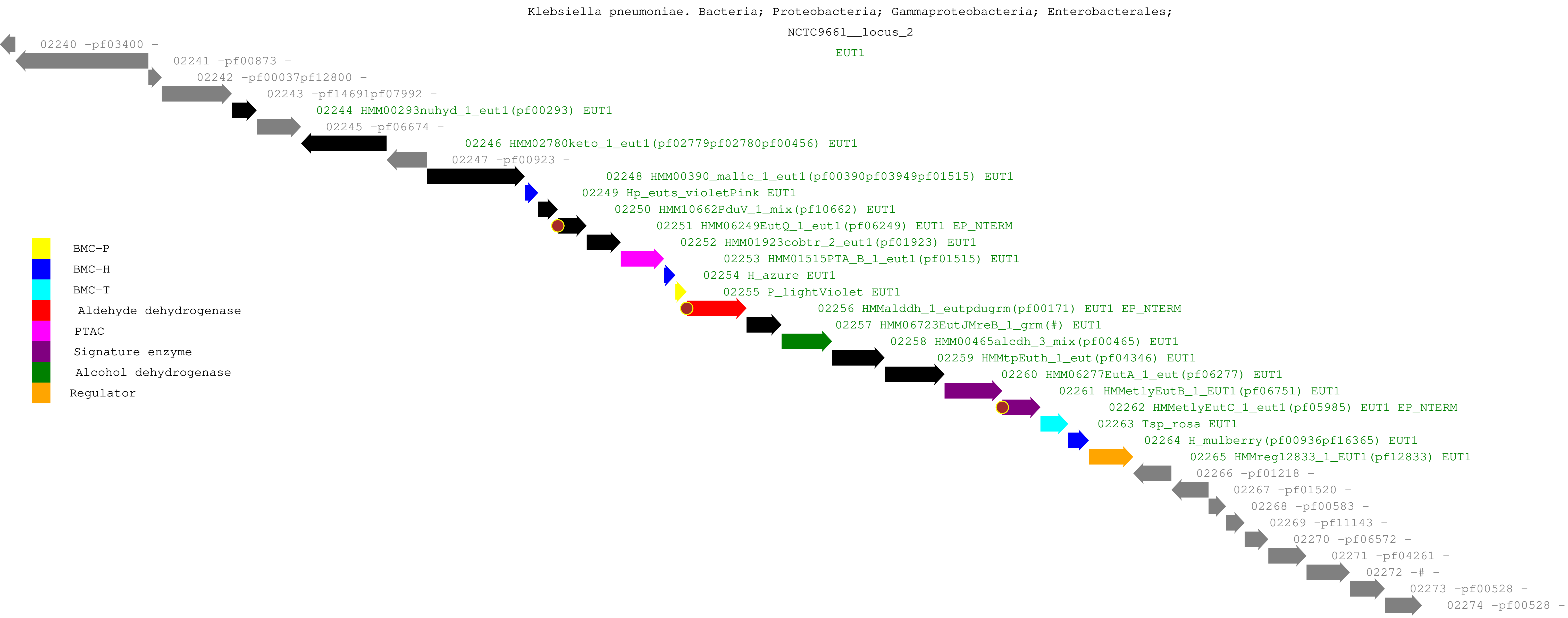

Klebsiella pneumoniae. Bacteria; Proteobacteria; Gammaproteobacteria; Enterobacterales;
NCTC9661__locus_2
EUT1
02240 -pf03400 -
02241 -pf00873 -
02242 -pf00037pf12800 -
02243 -pf14691pf07992 -
02244 HMM00293nuhyd_1_eut1(pf00293) EUT1
02245 -pf06674 -
02246 HMM02780keto_1_eut1(pf02779pf02780pf00456) EUT1
02247 -pf00923 -
02248 HMM00390_malic_1_eut1(pf00390pf03949pf01515) EUT1
02249 Hp_euts_violetPink EUT1
02250 HMM10662PduV_1_mix(pf10662) EUT1
02251 HMM06249EutQ_1_eut1(pf06249) EUT1 EP_NTERM
02252 HMM01923cobtr_2_eut1(pf01923) EUT1
02253 HMM01515PTA_B_1_eut1(pf01515) EUT1
02254 H_azure EUT1
02255 P_lightViolet EUT1
02256 HMMaldh_1_eutpdugrm(pf00171) EUT1 EP_NTERM
02257 HMM06723EutJMreB_1_grm(#) EUT1
02258 HMM00465alcdh_3_mix(pf00465) EUT1
02259 HMMtpEuth_1_eut(pf04346) EUT1
02260 HMM06277EutA_1_eut(pf06277) EUT1
02261 HMMetlyEutB_1_EUT1(pf06751) EUT1
02262 HMMetlyEutC_1_eut1(pf05985) EUT1 EP_NTERM
02263 Tsp_rosa EUT1
02264 H_mulberry(pf00936pf16365) EUT1
02265 HMMreg12833_1_EUT1(pf12833) EUT1
02266 -pf01218 -
02267 -pf01520 -
02268 -pf00583 -
02269 -pf11143 -
02270 -pf06572 -
02271 -pf04261 -
02272 -# -
02273 -pf00528 -
02274 -pf00528 -
BMC-P
BMC-H
BMC-T
Aldehyde dehydrogenase
PTAC
Signature enzyme
Alcohol dehydrogenase
Regulator

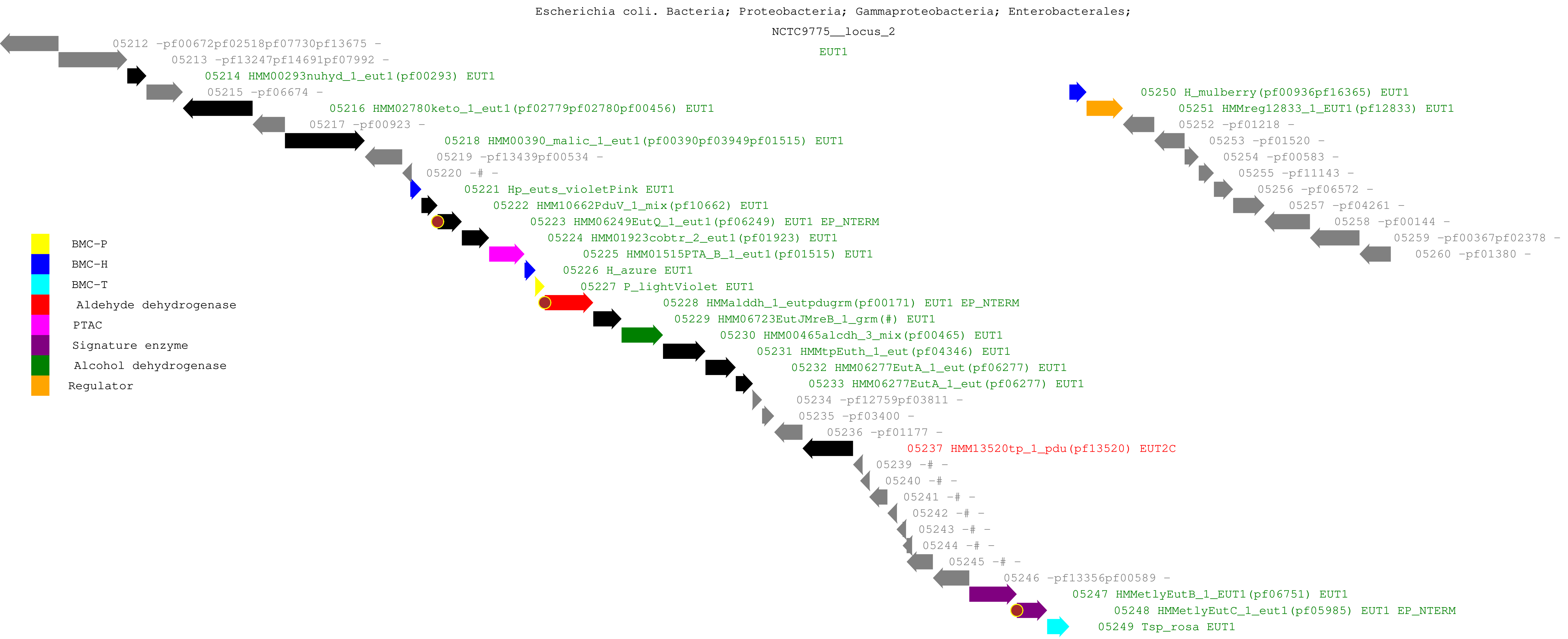
Escherichia coli. Bacteria; Proteobacteria; Gammaproteobacteria; Enterobacterales;
NCTC9775__locus_2
EUT1
05212 -pf00672pf02518pf07730pf13675 -
05213 -pf13247pf14691pf07992 -
05214 HMM00293nuhyd_1_eut1(pf00293) EUT1
05215 -pf06674 -
05216 HMM02780keto_1_eut1(pf02779pf02780pf00456) EUT1
05217 -pf00923 -
05218 HMM00390_malic_1_eut1(pf00390pf03949pf01515) EUT1
05219 -pf13439pf00534 -
05220 -# -
05221 Hp_euts_violetPink EUT1
05222 HMM10662PduV_1_mix(pf10662) EUT1
05223 HMM06249EutQ_1_eut1(pf06249) EUT1 EP_NTERM
05224 HMM01923cobtr_2_eut1(pf01923) EUT1
05225 HMM01515PTA_B_1_eut1(pf01515) EUT1
05226 H_azure EUT1
05227 P_lightViolet EUT1
05228 HMMaldh_1_eutpdugrm(pf00171) EUT1 EP_NTERM
05229 HMM06723EutJMreB_1_grm(#) EUT1
05230 HMM00465alcdh_3_mix(pf00465) EUT1
05231 HMMtpEuth_1_eut(pf04346) EUT1
05232 HMM06277EutA_1_eut(pf06277) EUT1
05233 HMM06277EutA_1_eut(pf06277) EUT1
05234 -pf12759pf03811 -
05235 -pf03400 -
05236 -pf01177 -
05237 HMM13520tp_1_pdu(pf13520) EUT2C
05239 -# -
05240 -# -
05241 -# -
05242 -# -
05243 -# -
05244 -# -
05245 -# -
05246 -pf13356pf00589 -
05247 HMMetlyEutB_1_EUT1(pf06751) EUT1
05248 HMMetlyEutC_1_eut1(pf05985) EUT1 EP_NTERM
05249 Tsp_rosa EUT1
05250 H_mulberry(pf00936pf16365) EUT1
05251 HMMreg12833_1_EUT1(pf12833) EUT1
05252 -pf01218 -
05253 -pf01520 -
05254 -pf00583 -
05255 -pf11143 -
05256 -pf06572 -
05257 -pf04261 -
05258 -pf00144 -
05259 -pf00367pf02378 -
05260 -pf01380 -
BMC-P
BMC-H
BMC-T
Aldehyde dehydrogenase
PTAC
Signature enzyme
Alcohol dehydrogenase
Regulator

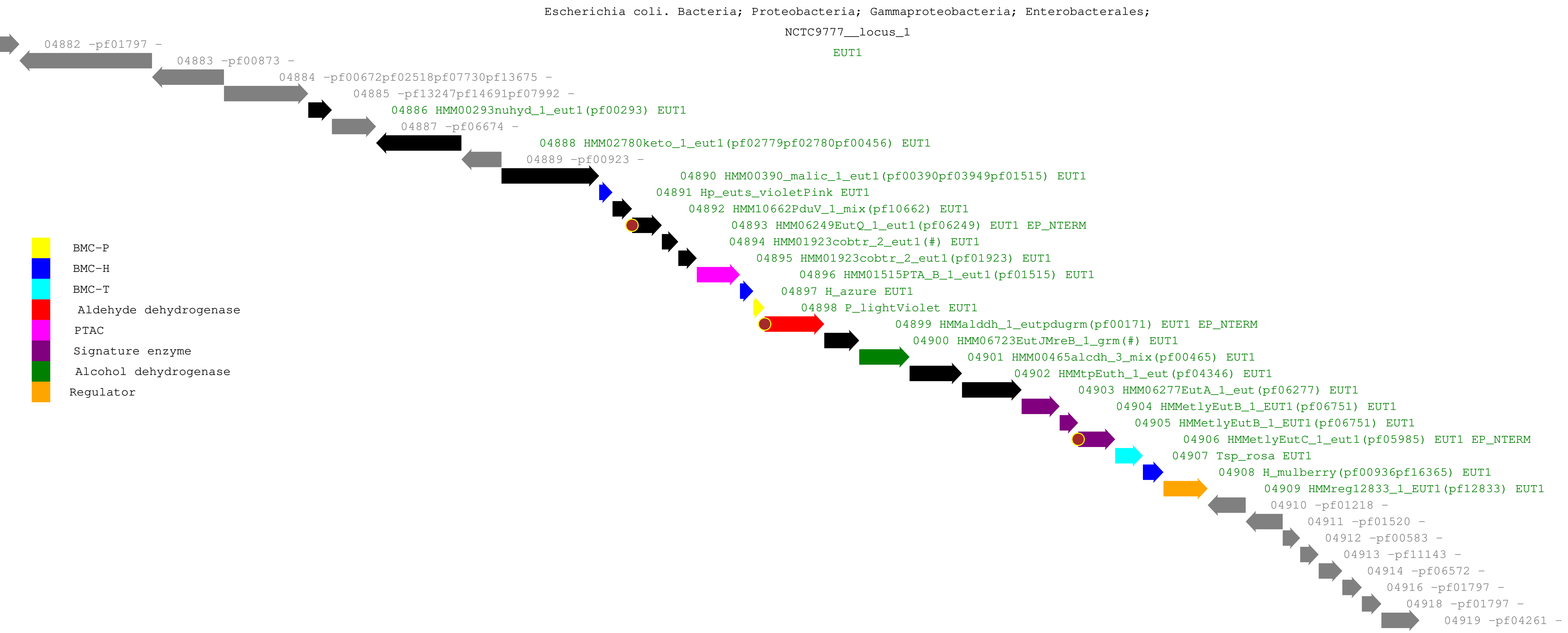
Escherichia coli. Bacteria; Proteobacteria; Gammaproteobacteria; Enterobacterales;
NCTC9777__locus_1
EUT1
04882 -pf01797 -
04883 -pf00873 -
04884 -pf00672pf02518pf07730pf13675 -
04885 -pf13247pf14691pf07992 -
04886 HMM00293nuhyd_1_eut1(pf00293) EUT1
04887 -pf06674 -
04888 HMM02780keto_1_eut1(pf02779pf02780pf00456) EUT1
04889 -pf00923 -
04890 HMM00390_malic_1_eut1(pf00390pf03949pf01515) EUT1
04891 Hp_euts_violetPink EUT1
04892 HMM10662PduV_1_mix(pf10662) EUT1
04893 HMM06249EutQ_1_eut1(pf06249) EUT1 EP_NTERM
04894 HMM01923cobtr_2_eut1(#) EUT1
04895 HMM01923cobtr_2_eut1(pf01923) EUT1
04896 HMM01515PTA_B_1_eut1(pf01515) EUT1
04897 H_azure EUT1
04898 P_lightViolet EUT1
04899 HMMalddh_1_eutpdugrm(pf00171) EUT1 EP_NTERM
04900 HMM06723EutJMreB_1_grm(#) EUT1
04901 HMM00465alcdh_3_mix(pf00465) EUT1
04902 HMMtpEuth_1_eut(pf04346) EUT1
04903 HMM06277EutA_1_eut(pf06277) EUT1
04904 HMMetlyEutB_1_EUT1(pf06751) EUT1
04905 HMMetlyEutB_1_EUT1(pf06751) EUT1
04906 HMMetlyEutC_1_eut1(pf05985) EUT1 EP_NTERM
04907 Tsp_rosa EUT1
04908 H_mulberry(pf00936pf16365) EUT1
04909 HMMreg12833_1_EUT1(pf12833) EUT1
04910 -pf01218 -
04911 -pf01520 -
04912 -pf00583 -
04913 -pf11143 -
04914 -pf06572 -
04916 -pf01797 -
04918 -pf01797 -
04919 -pf04261 -
BMC-P
BMC-H
BMC-T
Aldehyde dehydrogenase
PTAC
Signature enzyme
Alcohol dehydrogenase
Regulator

Salmonella choleraesuis. Bacteria; Proteobacteria; Gammaproteobacteria; Enterobacterales;

NCTC9854__locus_1

EUT1

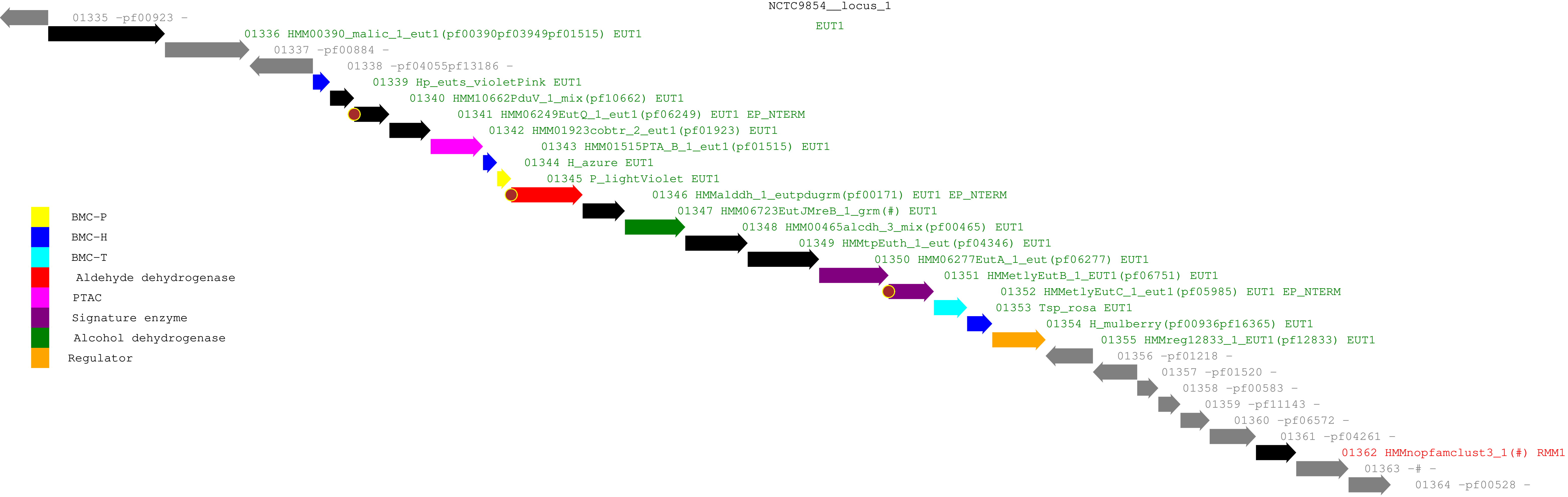

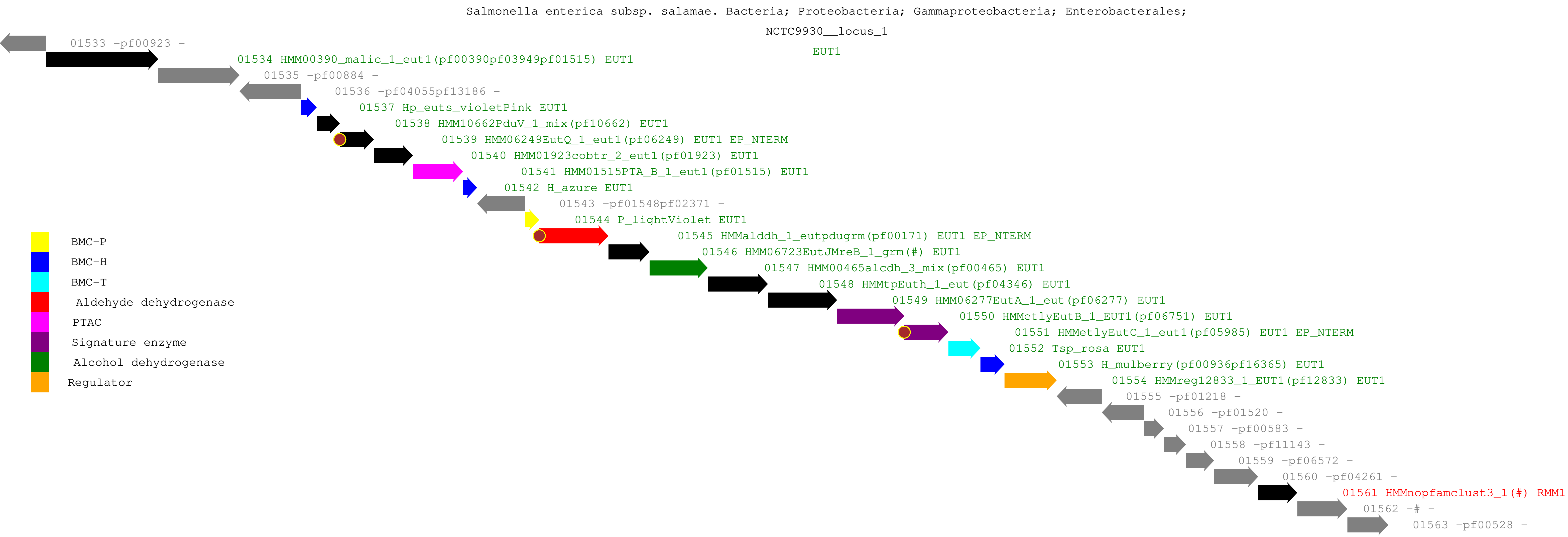
Salmonella enterica subsp. salamae. Bacteria; Proteobacteria; Gammaproteobacteria; Enterobacterales;
NCTC9930__locus_1
EUT1
01533 -pf00923 -
01534 HMM00390_malic_1_eut1(pf00390pf03949pf01515) EUT1
01535 -pf00884 -
01536 -pf04055pf13186 -
01537 Hp_euts_violetPink EUT1
01538 HMM10662PduV_1_mix(pf10662) EUT1
01539 HMM06249EutQ_1_eut1(pf06249) EUT1 EP_NTERM
01540 HMM01923cobtr_2_eut1(pf01923) EUT1
01541 HMM01515PTA_B_1_eut1(pf01515) EUT1
01542 H_azure EUT1
01543 -pf01548pf02371 -
01544 P_lightViolet EUT1
01545 HMMaldh_1_eutpdugrm(pf00171) EUT1 EP_NTERM
01546 HMM06723EutJMreB_1_grm(#) EUT1
01547 HMM00465alcdh_3_mix(pf00465) EUT1
01548 HMMtpEuth_1_eut(pf04346) EUT1
01549 HMM06277EutA_1_eut(pf06277) EUT1
01550 HMMetlyEutB_1_EUT1(pf06751) EUT1
01551 HMMetlyEutC_1_eut1(pf05985) EUT1 EP_NTERM
01552 Tsp_rosa EUT1
01553 H_mulberry(pf00936pf16365) EUT1
01554 HMMreg12833_1_EUT1(pf12833) EUT1
01555 -pf01218 -
01556 -pf01520 -
01557 -pf00583 -
01558 -pf11143 -
01559 -pf06572 -
01560 -pf04261 -
01561 HMMnopfamclust3_1(#) RMM1
01562 -# -
01563 -pf00528 -
BMC-P
BMC-H
BMC-T
Aldehyde dehydrogenase
PTAC
Signature enzyme
Alcohol dehydrogenase
Regulator

Escherichia coli. Bacteria; Proteobacteria; Gammaproteobacteria; Enterobacterales;

NCTC9962__locus_1

EUT1

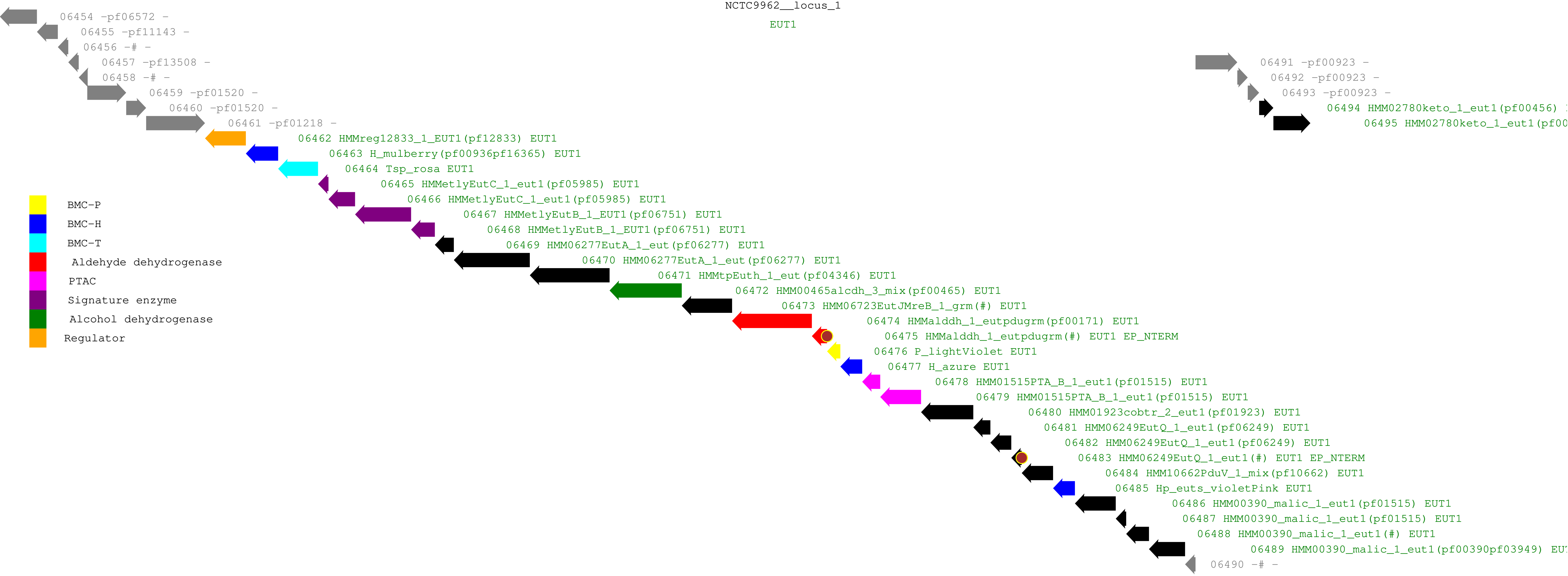

Escherichia coli. Bacteria; Proteobacteria; Gammaproteobacteria; Enterobacterales;

NCTC9965__locus_1

EUT1

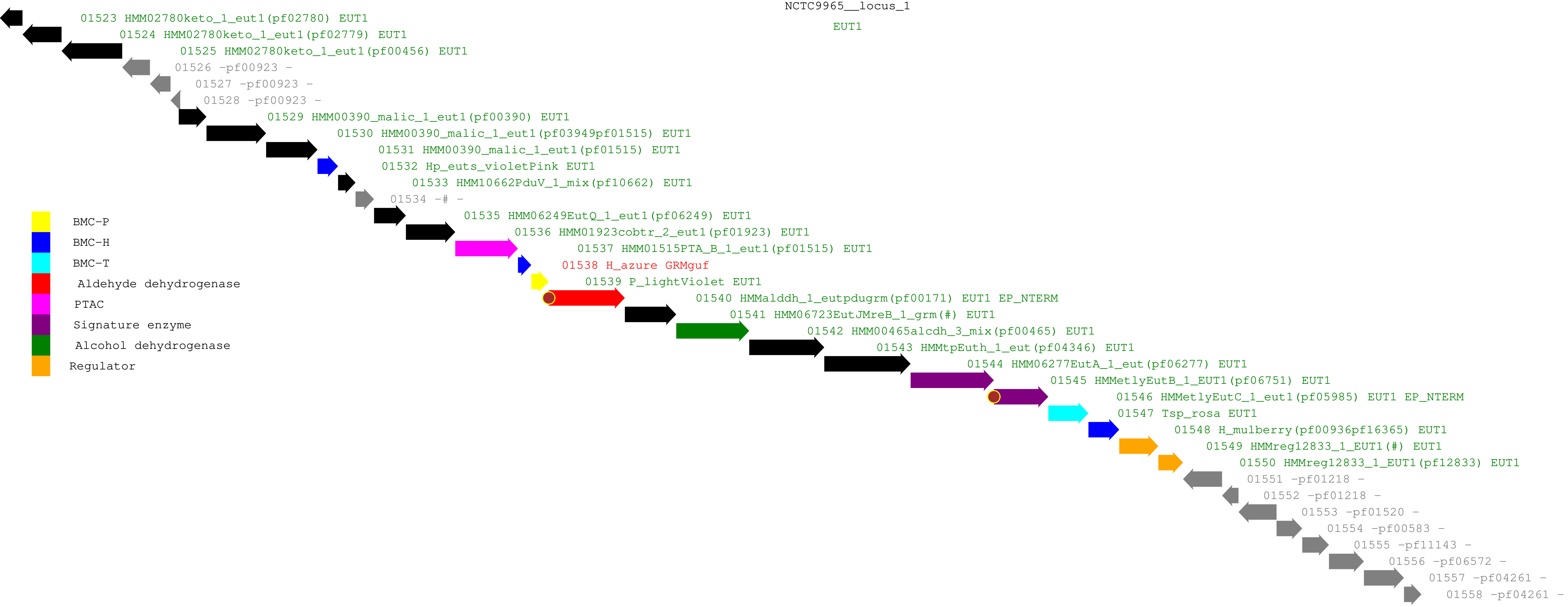

Salmonella choleraesuis. Bacteria; Proteobacteria; Gammaproteobacteria; Enterobacterales;

NGUA18__locus_1

EUT1

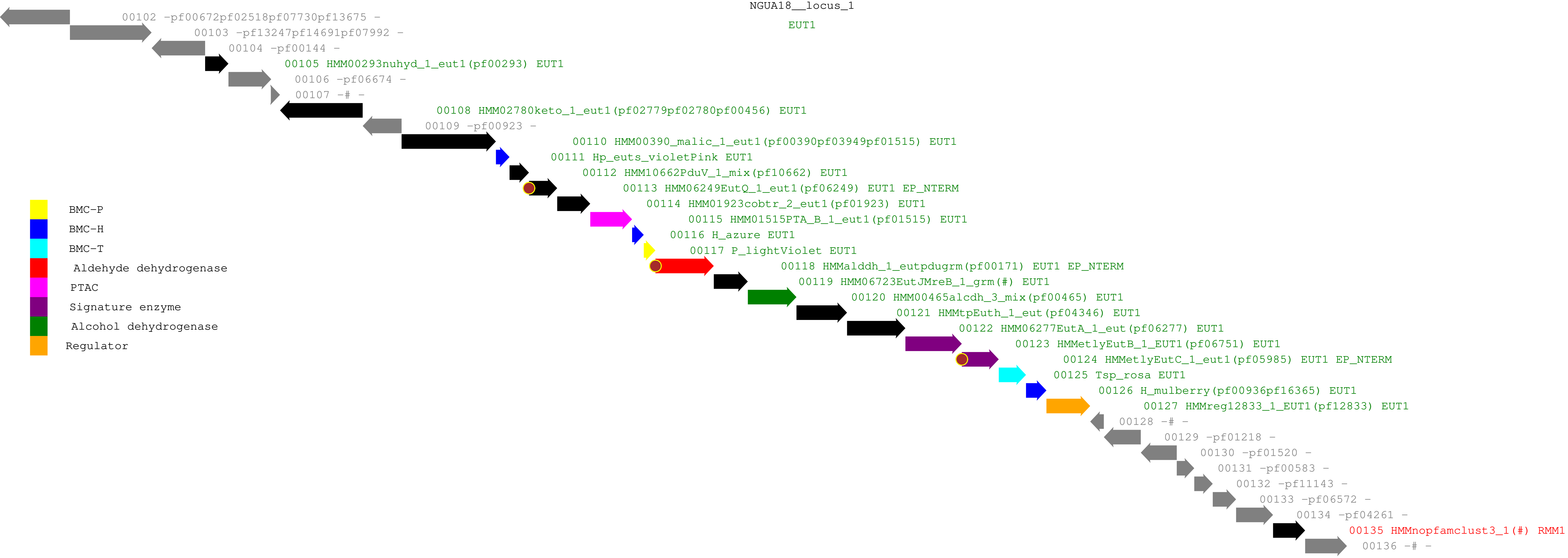

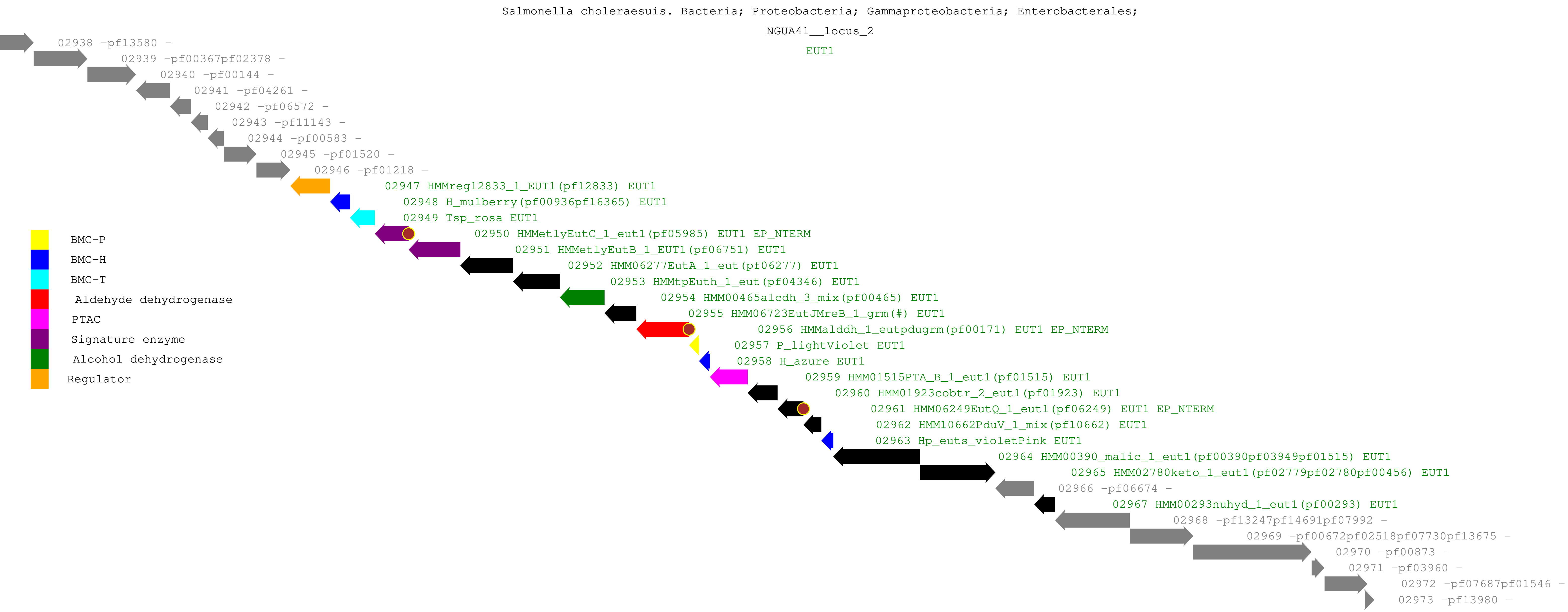

Salmonella choleraesuis. Bacteria; Proteobacteria; Gammaproteobacteria; Enterobacterales;
NGUA41__locus_2
EUT1
02938 -pf13580 -
02939 -pf00367pf02378 -
02940 -pf00144 -
02941 -pf04261 -
02942 -pf06572 -
02943 -pf11143 -
02944 -pf00583 -
02945 -pf01520 -
02946 -pf01218 -
02947 HMMreg12833_1_EUT1(pf12833) EUT1
02948 H_mulberry(pf00936pf16365) EUT1
02949 Tsp_rosa EUT1
02950 HMMetlyEutC_1_eut1(pf05985) EUT1 EP_NTERM
02951 HMMetlyEutB_1_EUT1(pf06751) EUT1
02952 HMM06277EutA_1_eut(pf06277) EUT1
02953 HMMtpEuth_1_eut(pf04346) EUT1
02954 HMM00465alcdh_3_mix(pf00465) EUT1
02955 HMM06723EutJMreB_1_grm(#) EUT1
02956 HMMalddh_1_eutpdugrm(pf00171) EUT1 EP_NTERM
02957 P_lightViolet EUT1
02958 H_azure EUT1
02959 HMM01515PTA_B_1_eut1(pf01515) EUT1
02960 HMM01923cobtr_2_eut1(pf01923) EUT1
02961 HMM06249EutQ_1_eut1(pf06249) EUT1 EP_NTERM
02962 HMM10662PduV_1_mix(pf10662) EUT1
02963 Hp_euts_violetPink EUT1
02964 HMM00390_malic_1_eut1(pf00390pf03949pf01515) EUT1
02965 HMM02780keto_1_eut1(pf02779pf02780pf00456) EUT1
02966 -pf06674 -
02967 HMM00293nuhyd_1_eut1(pf00293) EUT1
02968 -pf13247pf14691pf07992 -
02969 -pf00672pf02518pf07730pf13675 -
02970 -pf00873 -
02971 -pf03960 -
02972 -pf07687pf01546 -
02973 -pf13980 -
BMC-P
BMC-H
BMC-T
Aldehyde dehydrogenase
PTAC
Signature enzyme
Alcohol dehydrogenase
Regulator

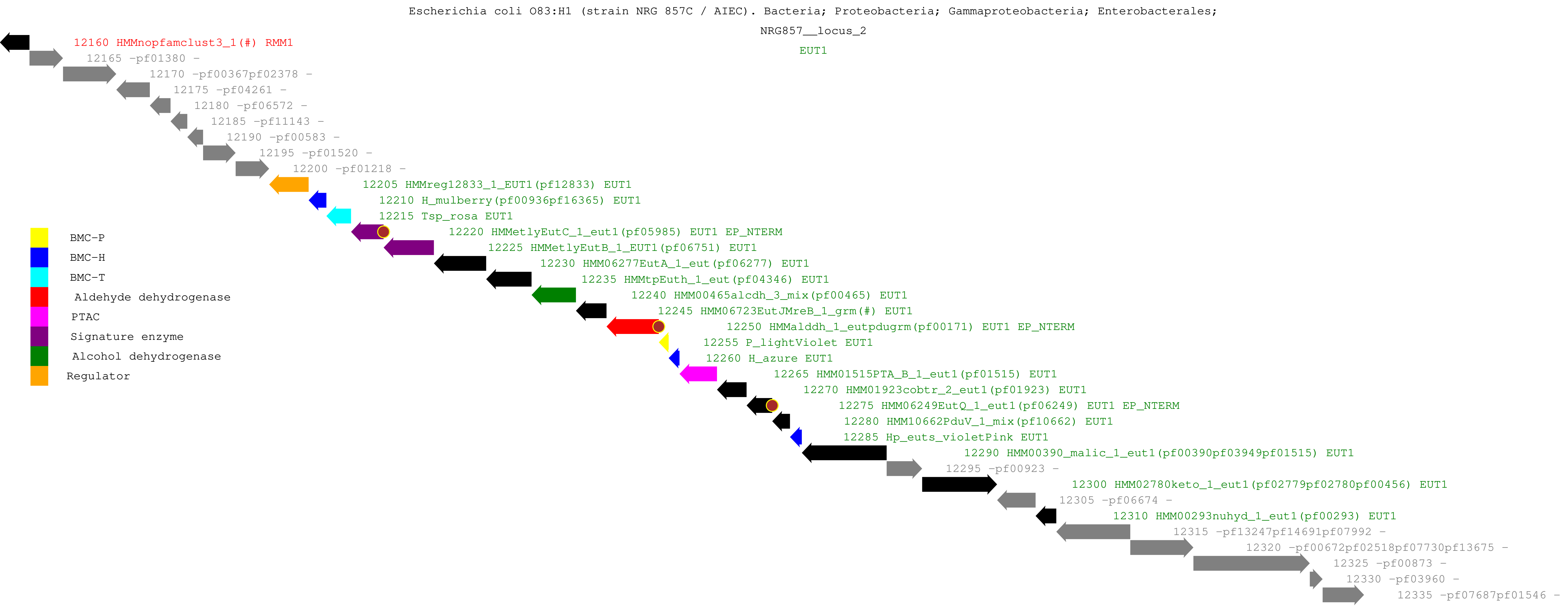
Escherichia coli O83:H1 (strain NRG 857C / AIEC). Bacteria; Proteobacteria; Gammaproteobacteria; Enterobacterales;
NRG857__locus_2
EUT1
12160 HMMnopfamclust3_1(#) RMM1
12165 -pf01380 -
12170 -pf00367pf02378 -
12175 -pf04261 -
12180 -pf06572 -
12185 -pf11143 -
12190 -pf00583 -
12195 -pf01520 -
12200 -pf01218 -
12205 HMMreg12833_1_EUT1(pf12833) EUT1
12210 H_mulberry(pf00936pf16365) EUT1
12215 Tsp_rosa EUT1
12220 HMMetlyEutC_1_eut1(pf05985) EUT1 EP_NTERM
12225 HMMetlyEutB_1_EUT1(pf06751) EUT1
12230 HMM06277EutA_1_eut(pf06277) EUT1
12235 HMMtpEuth_1_eut(pf04346) EUT1
12240 HMM00465alcdh_3_mix(pf00465) EUT1
12245 HMM06723EutJMreB_1_grm(#) EUT1
12250 HMMalddh_1_eutpdugrm(pf00171) EUT1 EP_NTERM
12255 P_lightViolet EUT1
12260 H_azure EUT1
12265 HMM01515PTA_B_1_eut1(pf01515) EUT1
12270 HMM01923cobtr_2_eut1(pf01923) EUT1
12275 HMM06249EutQ_1_eut1(pf06249) EUT1 EP_NTERM
12280 HMM10662PduV_1_mix(pf10662) EUT1
12285 Hp_euts_violetPink EUT1
12290 HMM00390_malic_1_eut1(pf00390pf03949pf01515) EUT1
12295 -pf00923 -
12300 HMM02780keto_1_eut1(pf02779pf02780pf00456) EUT1
12305 -pf06674 -
12310 HMM00293nuhyd_1_eut1(pf00293) EUT1
12315 -pf13247pf14691pf07992 -
12320 -pf00672pf02518pf07730pf13675 -
12325 -pf00873 -
12330 -pf03960 -
12335 -pf07687pf01546 -
BMC-P
BMC-H
BMC-T
Aldehyde dehydrogenase
PTAC
Signature enzyme
Alcohol dehydrogenase
Regulator

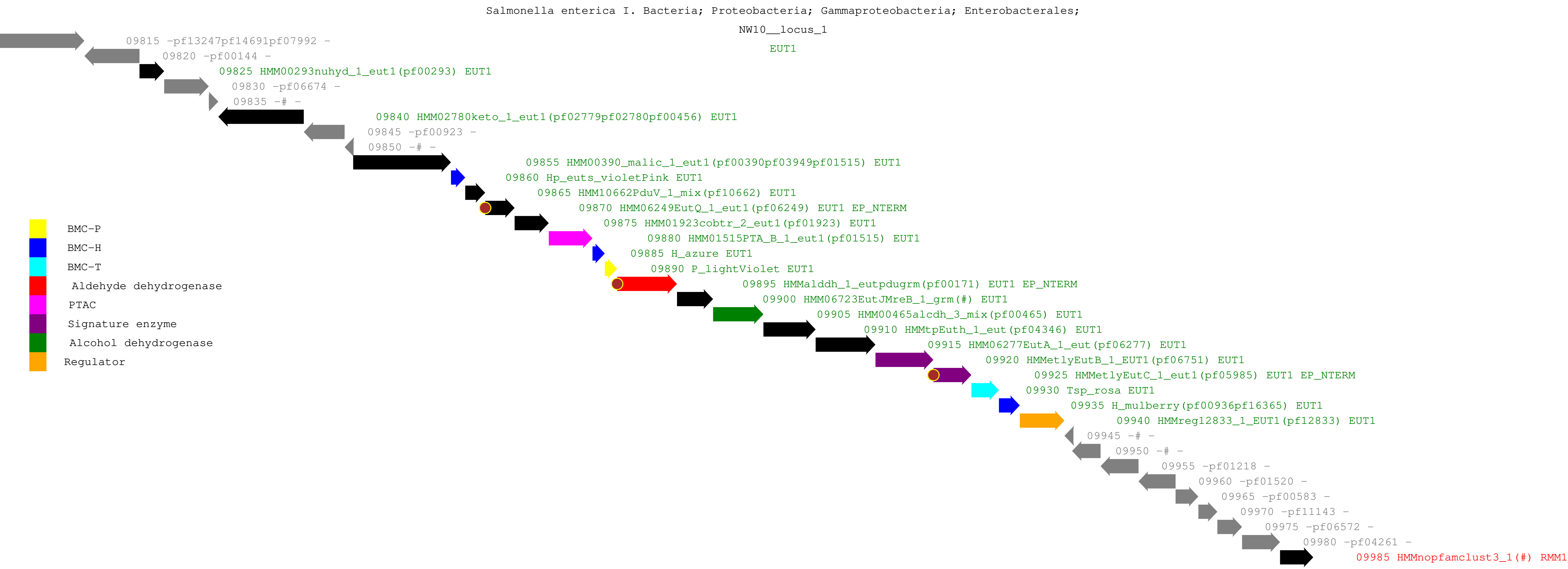
Salmonella enterica I. Bacteria; Proteobacteria; Gammaproteobacteria; Enterobacterales;
NW10__locus_1
EUT1
09815 -pf13247pf14691pf07992 -
09820 -pf00144 -
09825 HMM00293nuhyd_1_eut1(pf00293) EUT1
09830 -pf06674 -
09835 -# -
09840 HMM02780keto_1_eut1(pf02779pf02780pf00456) EUT1
09845 -pf00923 -
09850 -# -
09855 HMM00390_malic_1_eut1(pf00390pf03949pf01515) EUT1
09860 Hp_euts_violetPink EUT1
09865 HMM10662PduV_1_mix(pf10662) EUT1
09870 HMM06249EutQ_1_eut1(pf06249) EUT1 EP_NTERM
09875 HMM01923cobtr_2_eut1(pf01923) EUT1
09880 HMM01515PTA_B_1_eut1(pf01515) EUT1
09885 H_azure EUT1
09890 P_lightViolet EUT1
09895 HMMalddh_1_eutpdugrm(pf00171) EUT1 EP_NTERM
09900 HMM06723EutJmreB_1_grm(#) EUT1
09905 HMM00465alcdh_3_mix(pf00465) EUT1
09910 HMMtpEuth_1_eut(pf04346) EUT1
09915 HMM06277EutA_1_eut(pf06277) EUT1
09920 HMMetlyEutB_1_EUT1(pf06751) EUT1
09925 HMMetlyEutC_1_eut1(pf05985) EUT1 EP_NTERM
09930 Tsp_rosa EUT1
09935 H_mulberry(pf00936pf16365) EUT1
09940 HMMreg12833_1_EUT1(pf12833) EUT1
09945 -# -
09950 -# -
09955 -pf01218 -
09960 -pf01520 -
09965 -pf00583 -
09970 -pf11143 -
09975 -pf06572 -
09980 -pf04261 -
09985 HMMnopfamclust3_1(#) RMM1
BMC-P
BMC-H
BMC-T
Aldehyde dehydrogenase
PTAC
Signature enzyme
Alcohol dehydrogenase
Regulator

Escherichia coli O104:H4 (strain 2011C-3493). Bacteria; Proteobacteria; Gammaproteobacteria; Enterobacterales;

O3K__locus_1

EUT1

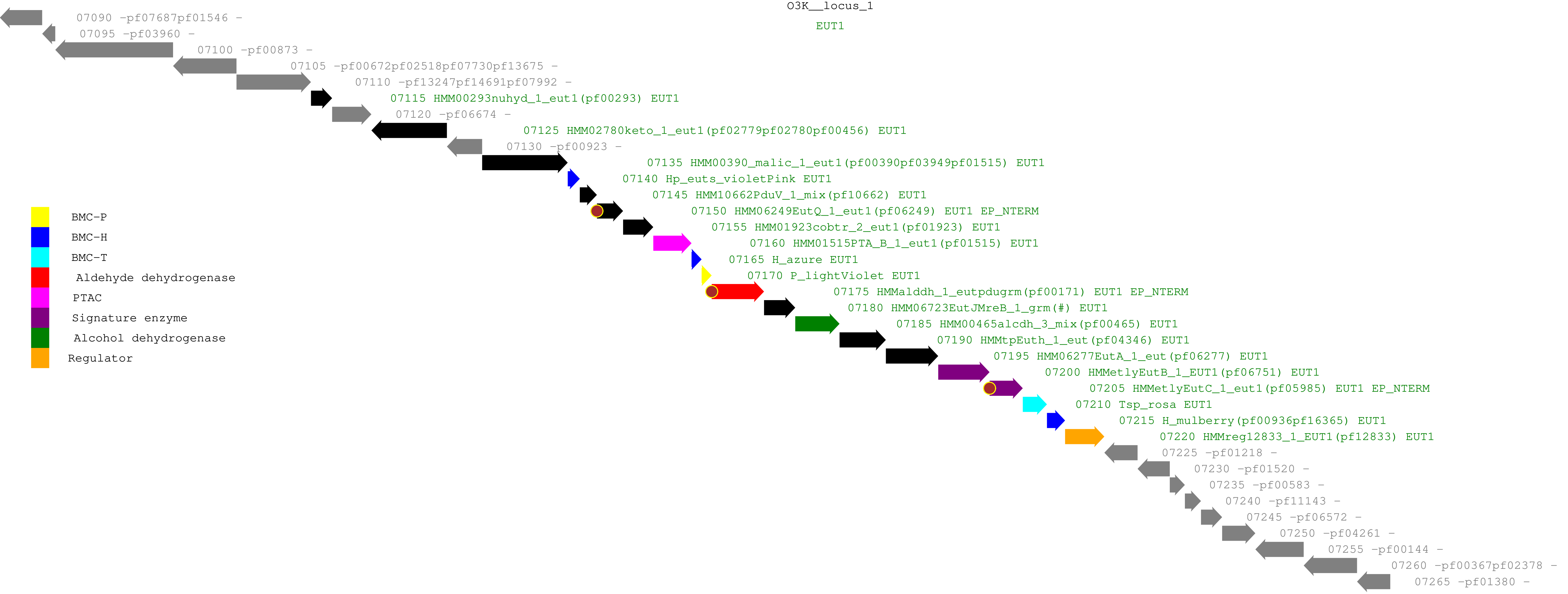

Morganella morganii (Proteus morganii). Bacteria; Proteobacteria; Gammaproteobacteria; Enterobacterales;

OA40__locus_1

EUT1

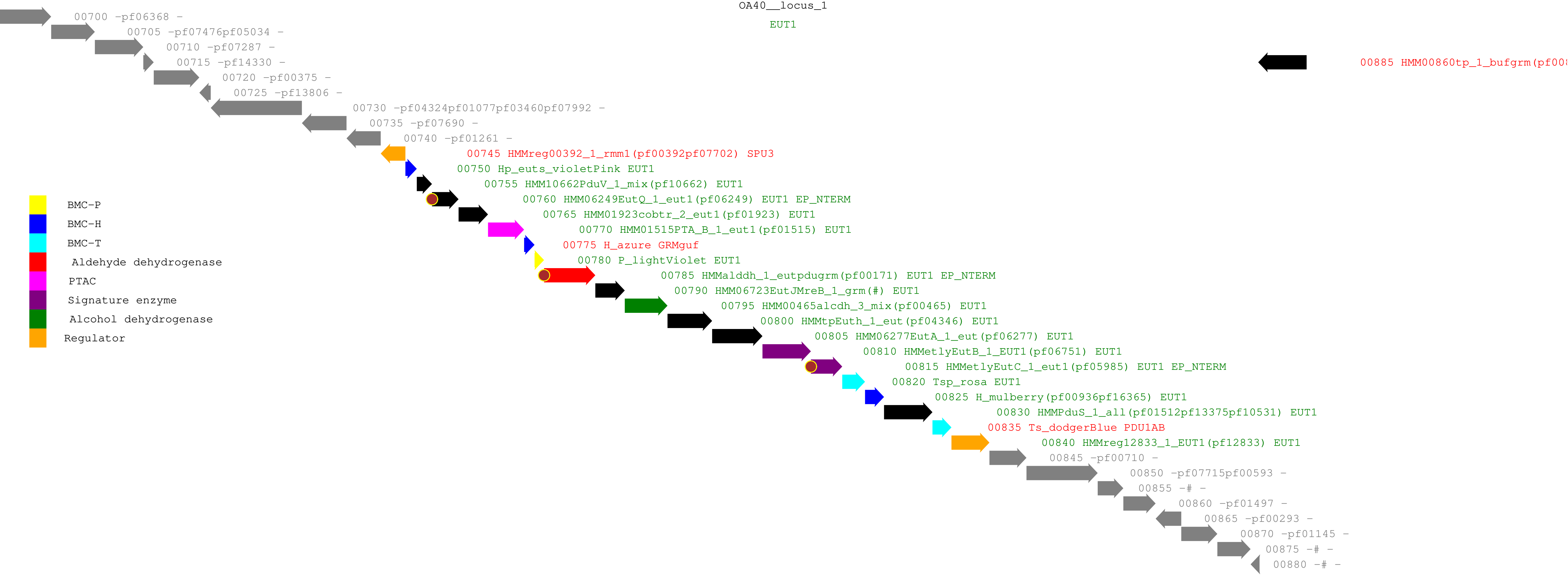

Salmonella enterica (Salmonella choleraesuis). Bacteria; Proteobacteria; Gammaproteobacteria; Enterobacterales;

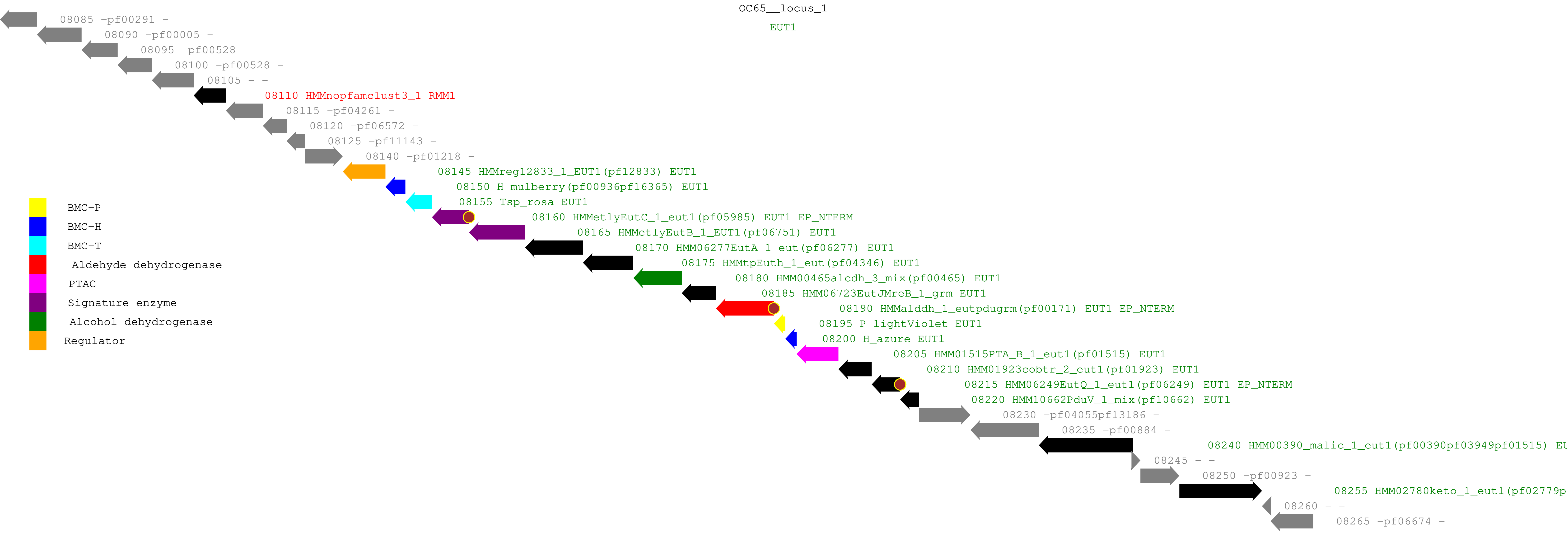

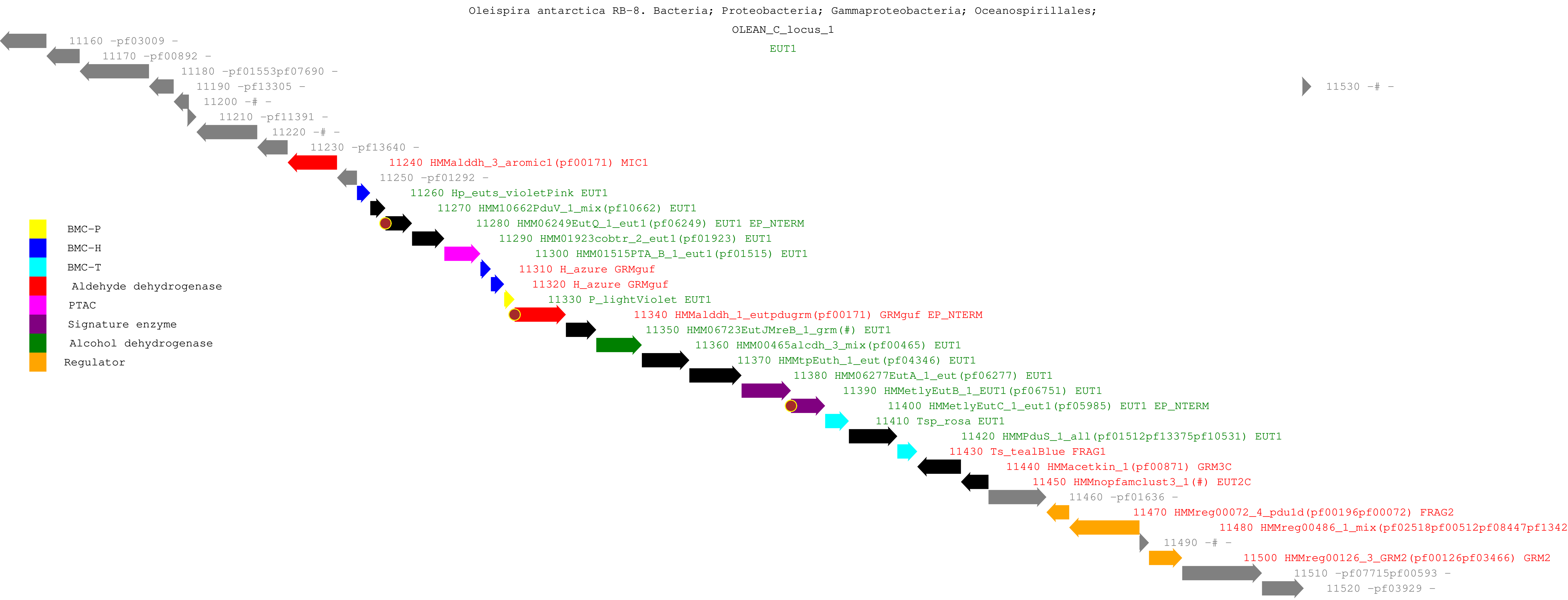
Oleispira antarctica RB-8. Bacteria; Proteobacteria; Gammaproteobacteria; Oceanospirillales;
OLEAN_C_locus_1
EUT1
11160 -pf03009 -
11170 -pf00892 -
11180 -pf01553pf07690 -
11190 -pf13305 -
11200 -# -
11210 -pf11391 -
11220 -# -
11230 -pf13640 -
11240 HMMaldh_3_aromic1(pf00171) MIC1
11250 -pf01292 -
11260 Hp_euts_violetPink EUT1
11270 HMM10662PduV_1_mix(pf10662) EUT1
11280 HMM06249EutQ_1_eut1(pf06249) EUT1 EP_NTERM
11290 HMM01923cobtr_2_eut1(pf01923) EUT1
11300 HMM01515PTA_B_1_eut1(pf01515) EUT1
11310 H_azure GRMguf
11320 H_azure GRMguf
11330 P_lightViolet EUT1
11340 HMMaldh_1_eutpdugrm(pf00171) GRMguf EP_NTERM
11350 HMM06723EutJMreB_1_grm(#) EUT1
11360 HMM00465alcdh_3_mix(pf00465) EUT1
11370 HMMtpEuth_1_eut(pf04346) EUT1
11380 HMM06277EutA_1_eut(pf06277) EUT1
11390 HMMetlyEutB_1_EUT1(pf06751) EUT1
11400 HMMetlyEutC_1_eut1(pf05985) EUT1 EP_NTERM
11410 Tsp_rosa EUT1
11420 HMMPduS_1_all(pf01512pf13375pf10531) EUT1
11430 Ts_tealBlue FRAG1
11440 HMMacetkin_1(pf00871) GRM3C
11450 HMMnopfamclust3_1(#) EUT2C
11460 -pf01636 -
11470 HMMreg00072_4_pdu1d(pf00196pf00072) FRAG2
11480 HMMreg00486_1_mix(pf02518pf00512pf08447pf1342
11490 -# -
11500 HMMreg00126_3_GRM2(pf00126pf03466) GRM2
11510 -pf07715pf00593 -
11520 -pf03929 -
11530 -# -
BMC-P
BMC-H
BMC-T
Aldehyde dehydrogenase
PTAC
Signature enzyme
Alcohol dehydrogenase
Regulator

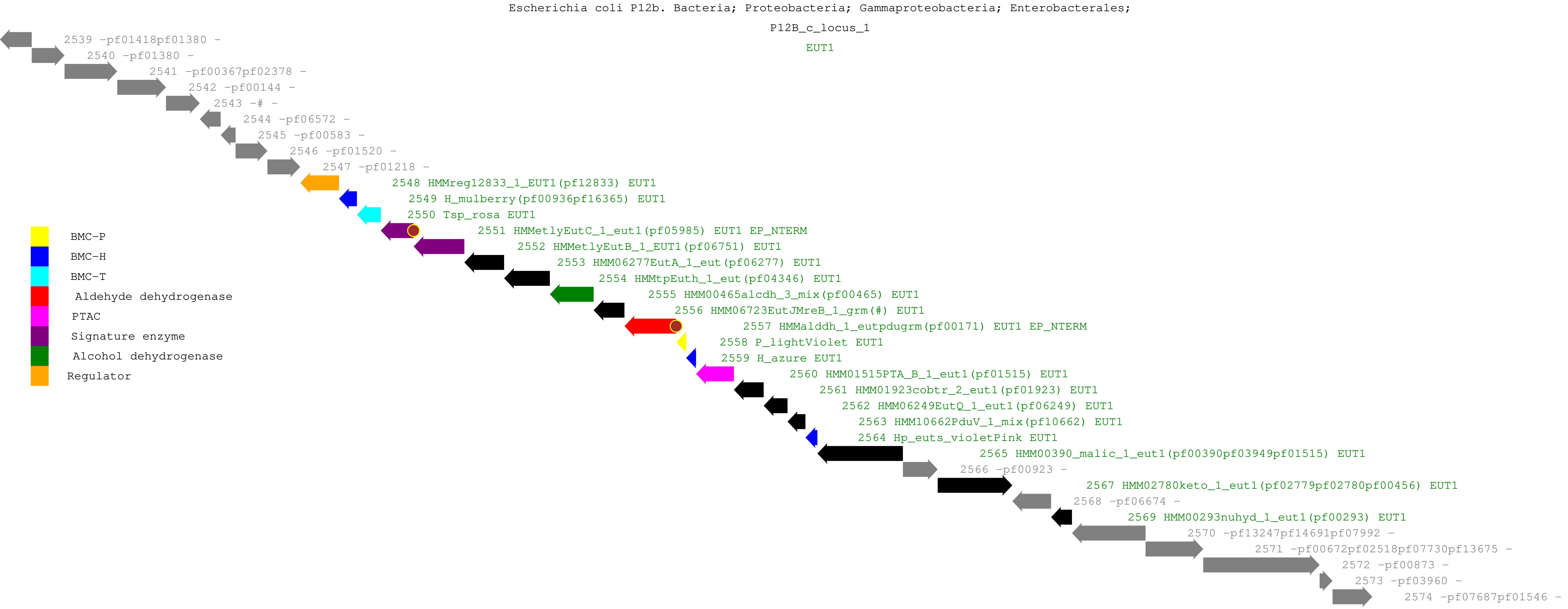
Escherichia coli P12b. Bacteria; Proteobacteria; Gammaproteobacteria; Enterobacterales;
P12B_c_locus_1
EUT1
2539 -pf01418pf01380 -
2540 -pf01380 -
2541 -pf00367pf02378 -
2542 -pf00144 -
2543 -# -
2544 -pf06572 -
2545 -pf00583 -
2546 -pf01520 -
2547 -pf01218 -
2548 HMMreg12833_1_EUT1(pf12833) EUT1
2549 H_mulberry(pf00936pf16365) EUT1
2550 Tsp_rosa EUT1
2551 HMMetlyEutC_1_eut1(pf05985) EUT1 EP_NTERM
2552 HMMetlyEutB_1_EUT1(pf06751) EUT1
2553 HMM06277EutA_1_eut(pf06277) EUT1
2554 HMMtpEuth_1_eut(pf04346) EUT1
2555 HMM00465alcdh_3_mix(pf00465) EUT1
2556 HMM06723EutJMreB_1_grm(#) EUT1
2557 HMMalddh_1_eutpdugrm(pf00171) EUT1 EP_NTERM
2558 P_lightViolet EUT1
2559 H_azure EUT1
2560 HMM01515PTA_B_1_eut1(pf01515) EUT1
2561 HMM01923cobtr_2_eut1(pf01923) EUT1
2562 HMM06249EutQ_1_eut1(pf06249) EUT1
2563 HMM10662PduV_1_mix(pf10662) EUT1
2564 Hp_euts_violetPink EUT1
2565 HMM00390_malic_1_eut1(pf00390pf03949pf01515) EUT1
2566 -pf00923 -
2567 HMM02780keto_1_eut1(pf02779pf02780pf00456) EUT1
2568 -pf06674 -
2569 HMM00293nuhyd_1_eut1(pf00293) EUT1
2570 -pf13247pf14691pf07992 -
2571 -pf00672pf02518pf07730pf13675 -
2572 -pf00873 -
2573 -pf03960 -
2574 -pf07687pf01546 -
BMC-P
BMC-H
BMC-T
Aldehyde dehydrogenase
PTAC
Signature enzyme
Alcohol dehydrogenase
Regulator

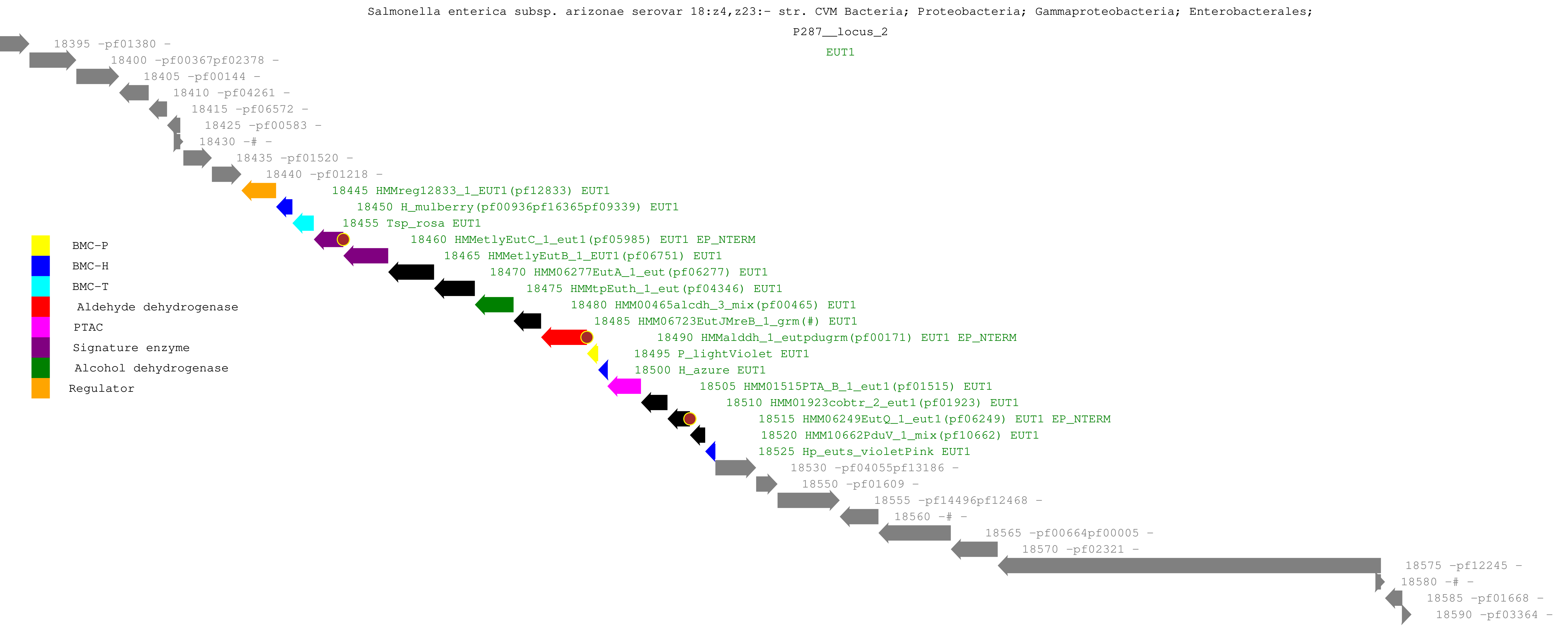

Salmonella enterica subsp. arizonae serovar 18:z4,z23:- str. CVM Bacteria; Proteobacteria; Gammaproteobacteria; Enterobacterales;
P287__locus_2
EUT1
18395 -pf01380 -
18400 -pf00367pf02378 -
18405 -pf00144 -
18410 -pf04261 -
18415 -pf06572 -
18425 -pf00583 -
18430 -# -
18435 -pf01520 -
18440 -pf01218 -
18445 HMMreg12833_1_EUT1(pf12833) EUT1
18450 H_mulberry(pf00936pf16365pf09339) EUT1
18455 Tsp_rosa EUT1
18460 HMMetlyEutC_1_eut1(pf05985) EUT1 EP_NTERM
18465 HMMetlyEutB_1_EUT1(pf06751) EUT1
18470 HMM06277EutA_1_eut(pf06277) EUT1
18475 HMMtpEuth_1_eut(pf04346) EUT1
18480 HMM00465alcdh_3_mix(pf00465) EUT1
18485 HMM06723EutJMreB_1_grm(#) EUT1
18490 HMMalddh_1_eutpdugrm(pf00171) EUT1 EP_NTERM
18495 P_lightViolet EUT1
18500 H_azure EUT1
18505 HMM01515PTA_B_1_eut1(pf01515) EUT1
18510 HMM01923cobtr_2_eut1(pf01923) EUT1
18515 HMM06249EutQ_1_eut1(pf06249) EUT1 EP_NTERM
18520 HMM10662PduV_1_mix(pf10662) EUT1
18525 Hp_euts_violetPink EUT1
18530 -pf04055pf13186 -
18550 -pf01609 -
18555 -pf14496pf12468 -
18560 -# -
18565 -pf00664pf00005 -
18570 -pf02321 -
18575 -pf12245 -
18580 -# -
18585 -pf01668 -
18590 -pf03364 -
BMC-P
BMC-H
BMC-T
Aldehyde dehydrogenase
PTAC
Signature enzyme
Alcohol dehydrogenase
Regulator

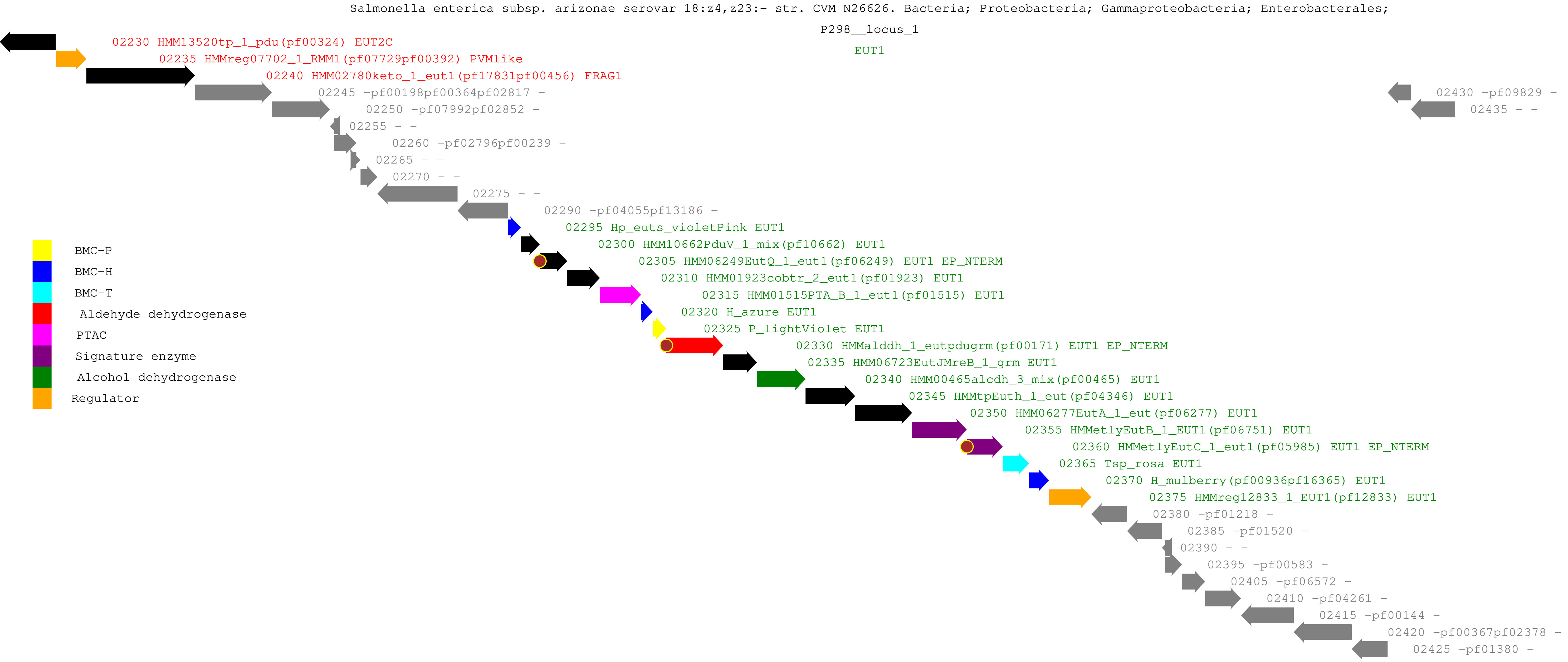

Salmonella enterica subsp. arizonae serovar 18:z4,z23:- str. CVM N26626. Bacteria; Proteobacteria; Gammaproteobacteria; Enterobacterales;
P298__locus_1
EUT1
02230 HMM13520tp_1_pdu(pf00324) EUT2C
02235 HMMreg07702_1_RMM1(pf07729pf00392) PVMlike
02240 HMM02780keto_1_eut1(pf17831pf00456) FRAG1
02245 -pf00198pf00364pf02817 -
02250 -pf07992pf02852 -
02255 - -
02260 -pf02796pf00239 -
02265 - -
02270 - -
02275 - -
02290 -pf04055pf13186 -
02295 Hp_euts_violetPink EUT1
02300 HMM10662PduV_1_mix(pf10662) EUT1
02305 HMM06249EutQ_1_eut1(pf06249) EUT1 EP_NTERM
02310 HMM01923cobtr_2_eut1(pf01923) EUT1
02315 HMM01515PTA_B_1_eut1(pf01515) EUT1
02320 H_azure EUT1
02325 P_lightViolet EUT1
02330 HMMaldh_1_eutpdugrm(pf00171) EUT1 EP_NTERM
02335 HMM06723EutJMreB_1_grm EUT1
02340 HMM00465alcdh_3_mix(pf00465) EUT1
02345 HMMtpEuth_1_eut(pf04346) EUT1
02350 HMM06277EutA_1_eut(pf06277) EUT1
02355 HMMetlyEutB_1_EUT1(pf06751) EUT1
02360 HMMetlyEutC_1_eut1(pf05985) EUT1 EP_NTERM
02365 Tsp_rosa EUT1
02370 H_mulberry(pf00936pf16365) EUT1
02375 HMMreg12833_1_EUT1(pf12833) EUT1
02380 -pf01218 -
02385 -pf01520 -
02390 - -
02395 -pf00583 -
02405 -pf06572 -
02410 -pf04261 -
02415 -pf00144 -
02420 -pf00367pf02378 -
02425 -pf01380 -
02430 -pf09829 -
02435 - -
BMC-P
BMC-H
BMC-T
Aldehyde dehydrogenase
PTAC
Signature enzyme
Alcohol dehydrogenase
Regulator

Enterobacter sp. DC4. Bacteria; Proteobacteria; Gammaproteobacteria; Enterobacterales;

P349__locus_1

EUT1

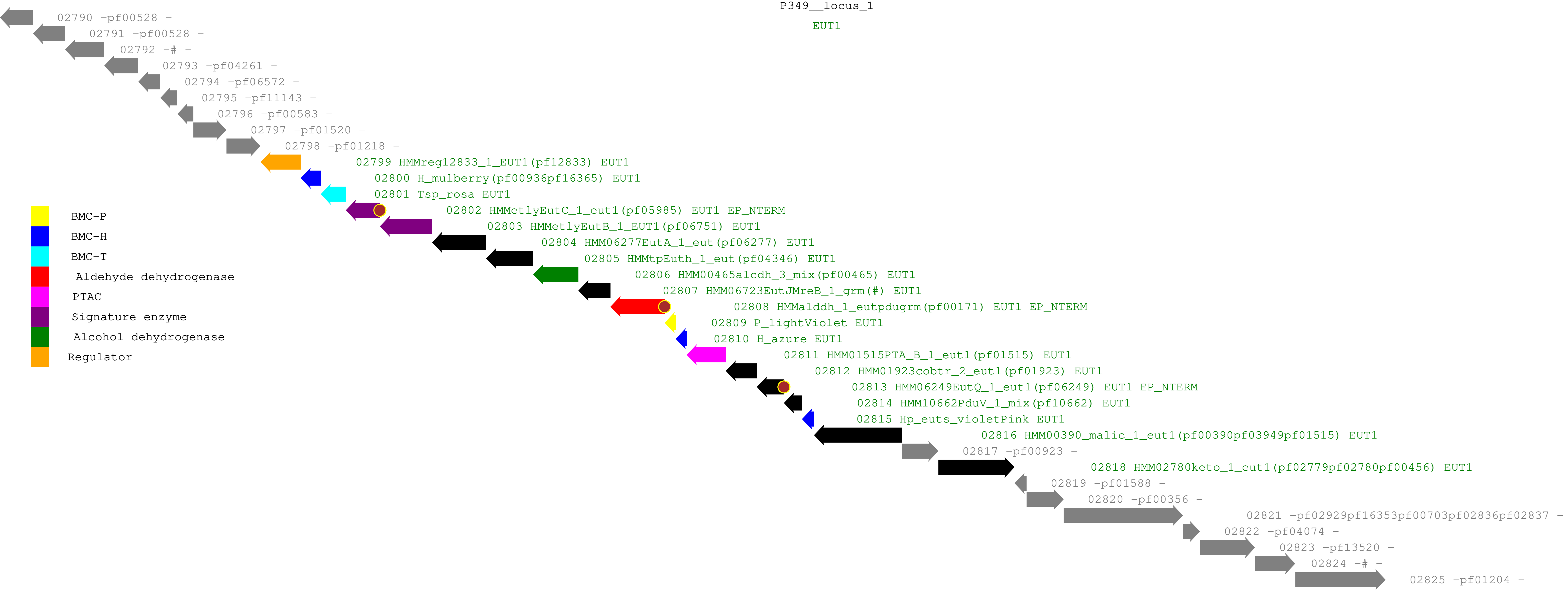

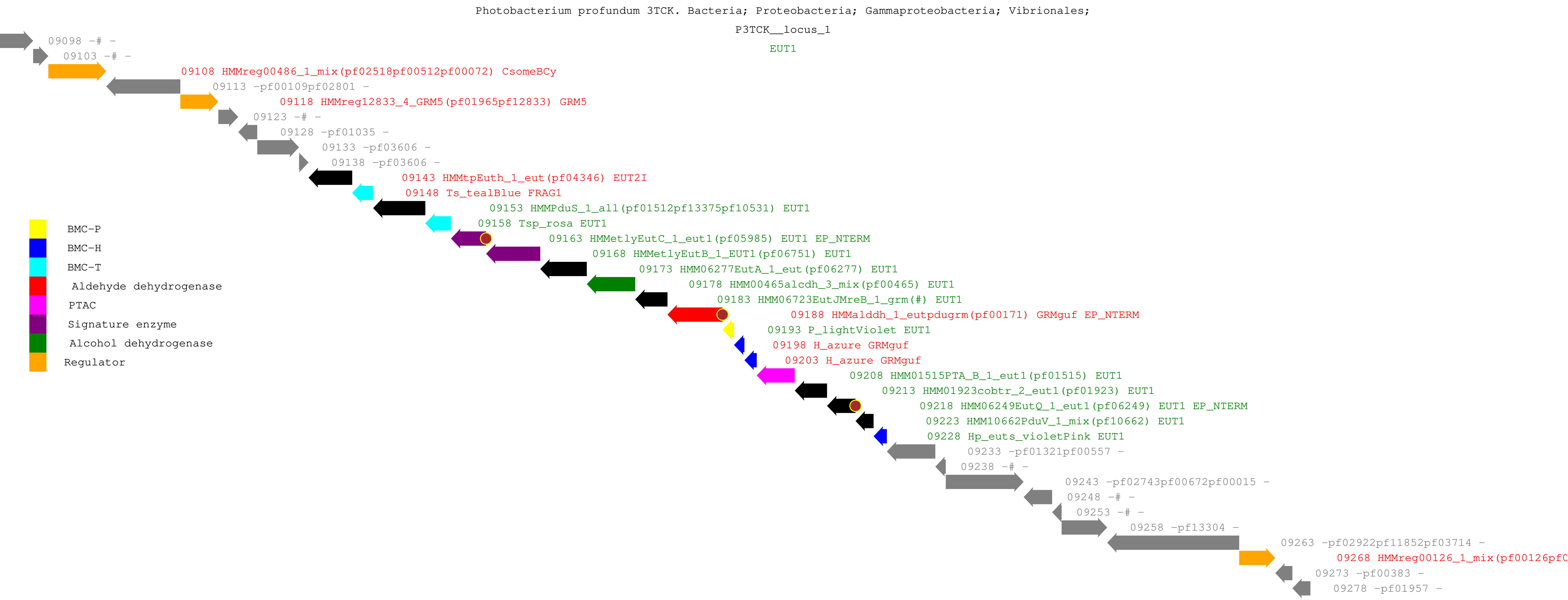

Photobacterium profundum 3TCK. Bacteria; Proteobacteria; Gammaproteobacteria; Vibrionales;
P3TCK__locus_1
EUT1
09098 -# -
09103 -# -
09108 HMMreg00486_1_mix(pf02518pf00512pf00072) CsomeBCy
09113 -pf00109pf02801 -
09118 HMMreg12833_4_GRM5(pf01965pf12833) GRM5
09123 -# -
09128 -pf01035 -
09133 -pf03606 -
09138 -pf03606 -
09143 HMMtpEuth_1_eut(pf04346) EUT2I
09148 Ts_tealBlue FRAG1
09153 HMMPduS_1_all(pf01512pf13375pf10531) EUT1
09158 Tsp_rosa EUT1
09163 HMMetlyEutC_1_eut1(pf05985) EUT1 EP_NTERM
09168 HMMetlyEutB_1_EUT1(pf06751) EUT1
09173 HMM06277EutA_1_eut(pf06277) EUT1
09178 HMM00465alcdh_3_mix(pf00465) EUT1
09183 HMM06723EutJMreB_1_grm(#) EUT1
09188 HMMalddh_1_eutpdugrm(pf00171) GRMguf EP_NTERM
09193 P_lightViolet EUT1
09198 H_azure GRMguf
09203 H_azure GRMguf
09208 HMM01515PTA_B_1_eut1(pf01515) EUT1
09213 HMM01923cobtr_2_eut1(pf01923) EUT1
09218 HMM06249EutQ_1_eut1(pf06249) EUT1 EP_NTERM
09223 HMM10662PduV_1_mix(pf10662) EUT1
09228 Hp_euts_violetPink EUT1
09233 -pf01321pf00557 -
09238 -# -
09243 -pf02743pf00672pf00015 -
09248 -# -
09253 -# -
09258 -pf13304 -
09263 -pf02922pf11852pf03714 -
09268 HMMreg00126_1_mix(pf00126pf0
09273 -pf00383 -
09278 -pf01957 -
BMC-P
BMC-H
BMC-T
Aldehyde dehydrogenase
PTAC
Signature enzyme
Alcohol dehydrogenase
Regulator

Salmonella enterica subsp. enterica serovar Newport str. CVM80_2288. Bacteria; Proteobacteria; Gammaproteobacteria; Enterobacterales;

P701__locus_2

EUT1

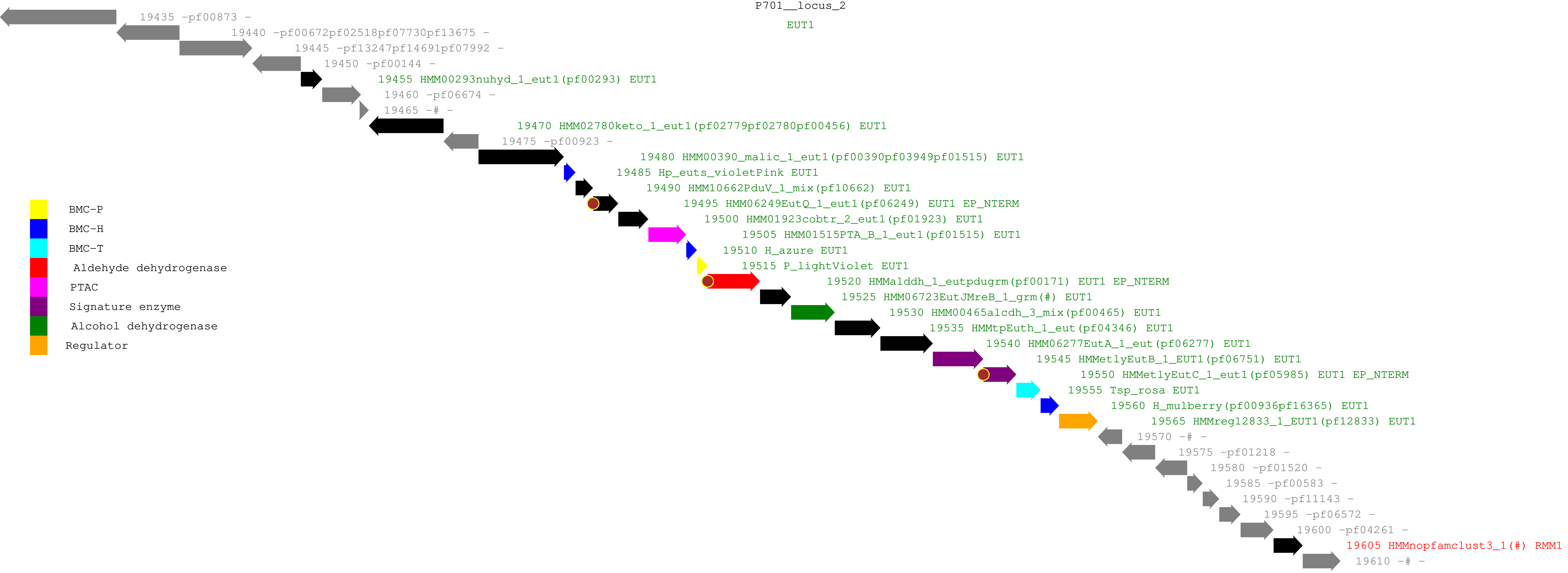

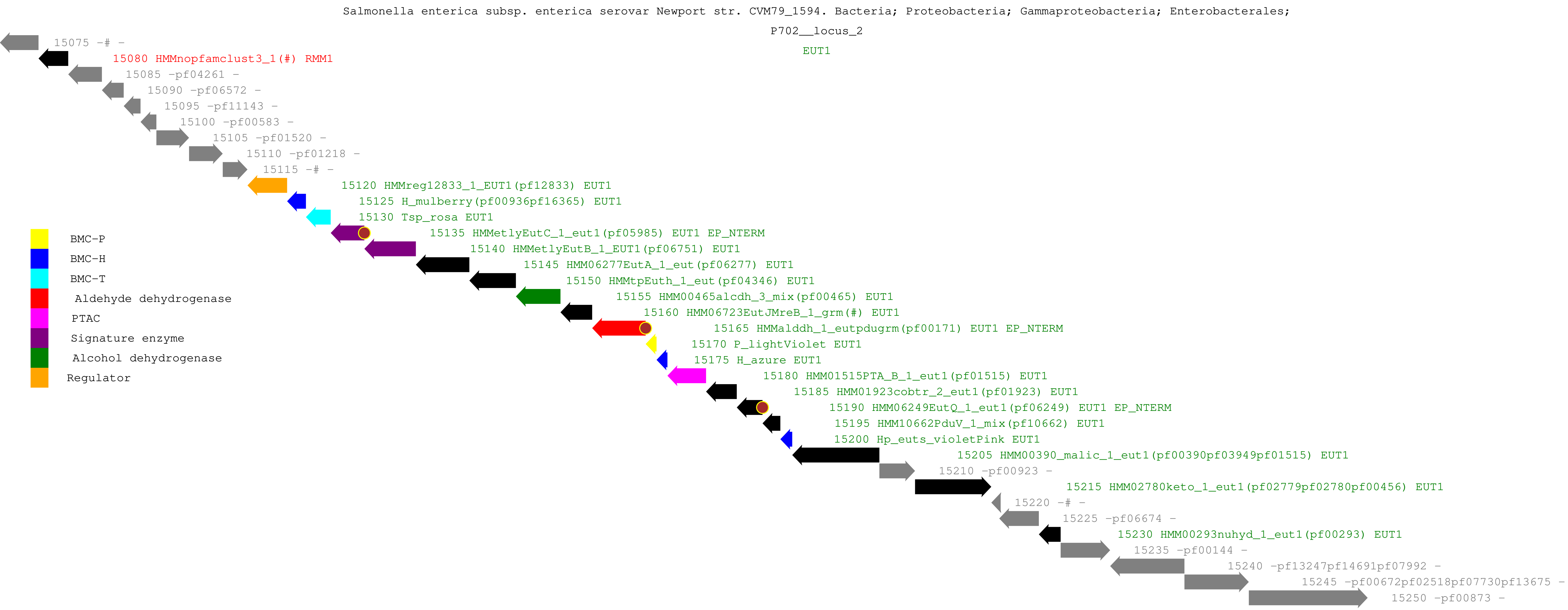

Salmonella enterica subsp. enterica serovar Newport str. CVM79_1594. Bacteria; Proteobacteria; Gammaproteobacteria; Enterobacterales;
P702__locus_2
EUT1
15075 -# -
15080 HMMnopfamclust3_1(#) RMM1
15085 -pf04261 -
15090 -pf06572 -
15095 -pf11143 -
15100 -pf00583 -
15105 -pf01520 -
15110 -pf01218 -
15115 -# -
15120 HMMreg12833_1_EUT1(pf12833) EUT1
15125 H_mulberry(pf00936pf16365) EUT1
15130 Tsp_rosa EUT1
15135 HMMetlyEutC_1_eut1(pf05985) EUT1 EP_NTERM
15140 HMMetlyEutB_1_EUT1(pf06751) EUT1
15145 HMM06277EutA_1_eut(pf06277) EUT1
15150 HMMtpEuth_1_eut(pf04346) EUT1
15155 HMM00465alcdh_3_mix(pf00465) EUT1
15160 HMM06723EutJMreB_1_grm(#) EUT1
15165 HMMalddh_1_eutpdugrm(pf00171) EUT1 EP_NTERM
15170 P_lightViolet EUT1
15175 H_azure EUT1
15180 HMM01515PTA_B_1_eut1(pf01515) EUT1
15185 HMM01923cobtr_2_eut1(pf01923) EUT1
15190 HMM06249EutQ_1_eut1(pf06249) EUT1 EP_NTERM
15195 HMM10662PduV_1_mix(pf10662) EUT1
15200 Hp_euts_violetPink EUT1
15205 HMM00390_malic_1_eut1(pf00390pf03949pf01515) EUT1
15210 -pf00923 -
15215 HMM02780keto_1_eut1(pf02779pf02780pf00456) EUT1
15220 -# -
15225 -pf06674 -
15230 HMM00293nuhyd_1_eut1(pf00293) EUT1
15235 -pf00144 -
15240 -pf13247pf14691pf07992 -
15245 -pf00672pf02518pf07730pf13675 -
15250 -pf00873 -
BMC-P
BMC-H
BMC-T
Aldehyde dehydrogenase
PTAC
Signature enzyme
Alcohol dehydrogenase
Regulator

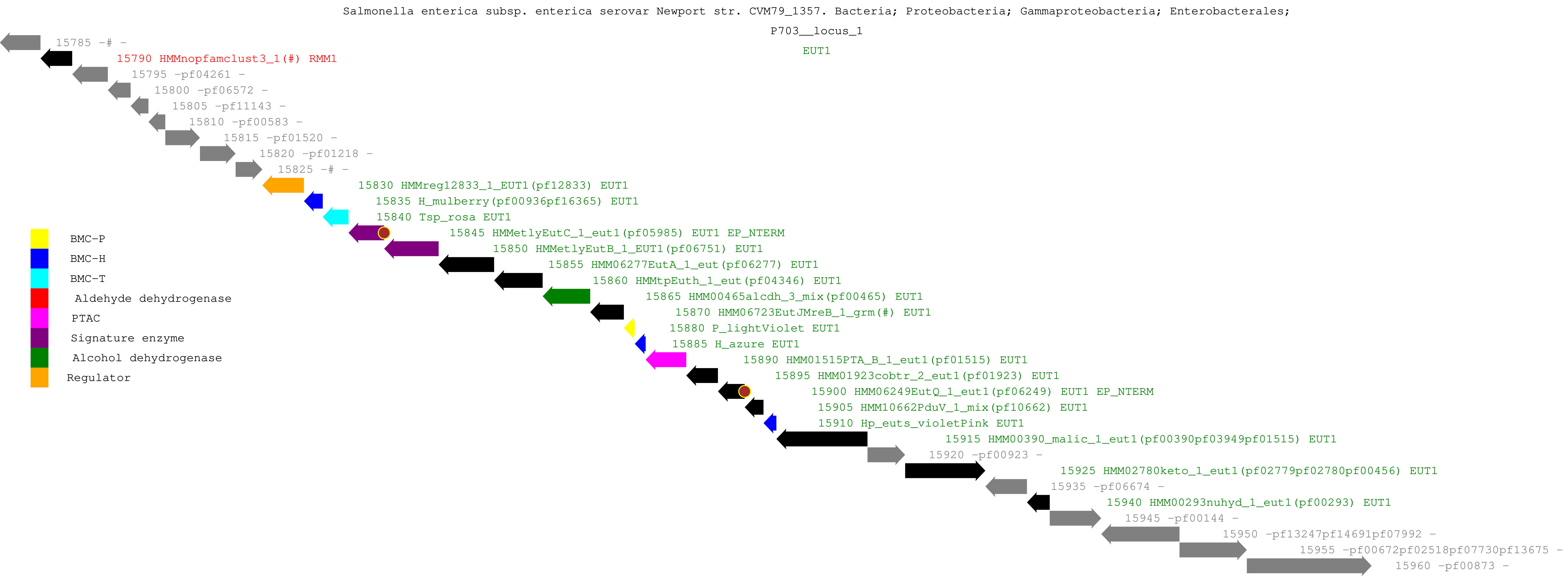
Salmonella enterica subsp. enterica serovar Newport str. CVM79_1357. Bacteria; Proteobacteria; Gammaproteobacteria; Enterobacterales;
P703__locus_1
EUT1
15785 -# -
15790 HMMnopfamclust3_1(#) RMM1
15795 -pf04261 -
15800 -pf06572 -
15805 -pf11143 -
15810 -pf00583 -
15815 -pf01520 -
15820 -pf01218 -
15825 -# -
15830 HMMreg12833_1_EUT1(pf12833) EUT1
15835 H_mulberry(pf00936pf16365) EUT1
15840 Tsp_rosa EUT1
15845 HMMetlyEutC_1_eut1(pf05985) EUT1 EP_NTERM
15850 HMMetlyEutB_1_EUT1(pf06751) EUT1
15855 HMM06277EutA_1_eut(pf06277) EUT1
15860 HMMtpEuth_1_eut(pf04346) EUT1
15865 HMM00465alcdh_3_mix(pf00465) EUT1
15870 HMM06723EutJMreB_1_grm(#) EUT1
15880 P_lightViolet EUT1
15885 H_azure EUT1
15890 HMM01515PTA_B_1_eut1(pf01515) EUT1
15895 HMM01923cobtr_2_eut1(pf01923) EUT1
15900 HMM06249EutQ_1_eut1(pf06249) EUT1 EP_NTERM
15905 HMM10662PduV_1_mix(pf10662) EUT1
15910 Hp_euts_violetPink EUT1
15915 HMM00390_malic_1_eut1(pf00390pf03949pf01515) EUT1
15920 -pf00923 -
15925 HMM02780keto_1_eut1(pf02779pf02780pf00456) EUT1
15935 -pf06674 -
15940 HMM00293nuhyd_1_eut1(pf00293) EUT1
15945 -pf00144 -
15950 -pf13247pf14691pf07992 -
15955 -pf00672pf02518pf07730pf13675 -
15960 -pf00873 -
BMC-P
BMC-H
BMC-T
Aldehyde dehydrogenase
PTAC
Signature enzyme
Alcohol dehydrogenase
Regulator

Salmonella enterica subsp. enterica serovar Newport str. CVM21537. Bacteria; Proteobacteria; Gammaproteobacteria; Enterobacterales;

P704__locus_2

EUT1

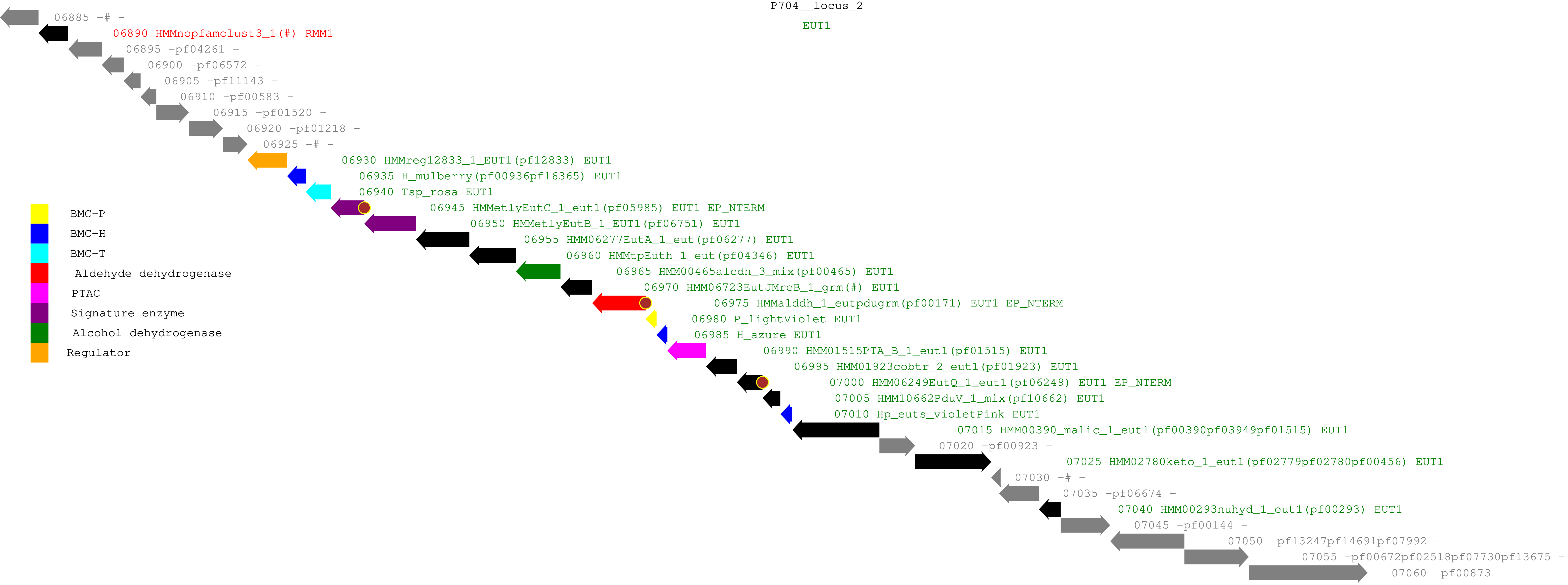

Salmonella enterica subsp. enterica serovar Newport str. CVM18000. Bacteria; Proteobacteria; Gammaproteobacteria; Enterobacterales;

P705__locus_1

EUT1

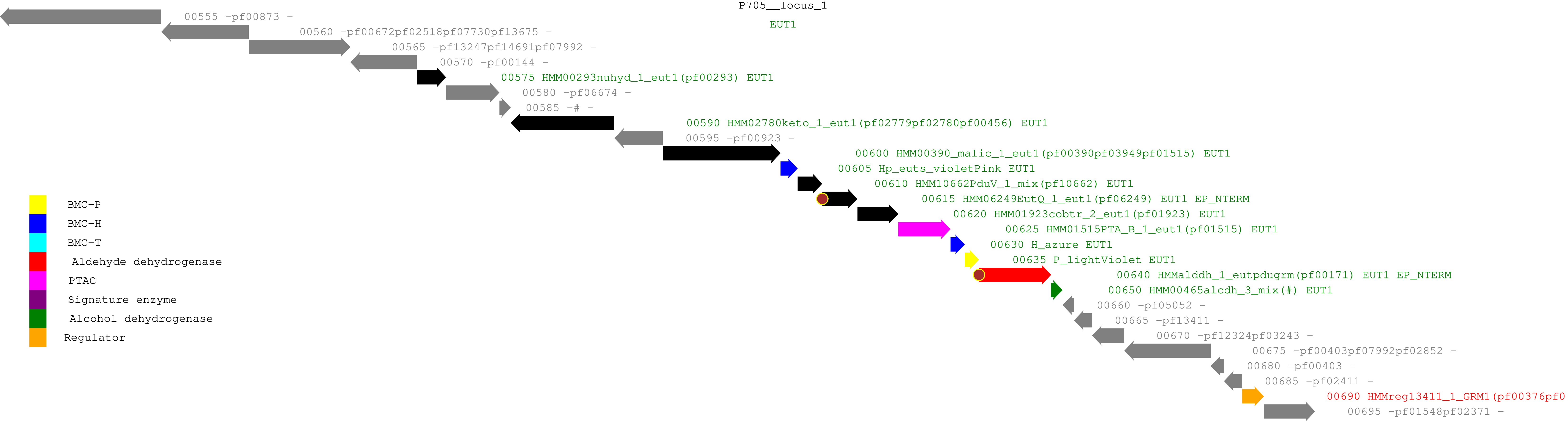

Salmonella enterica subsp. enterica serovar Newport str. CVM20776. Bacteria; Proteobacteria; Gammaproteobacteria; Enterobacterales;

P706__locus_1

EUT1

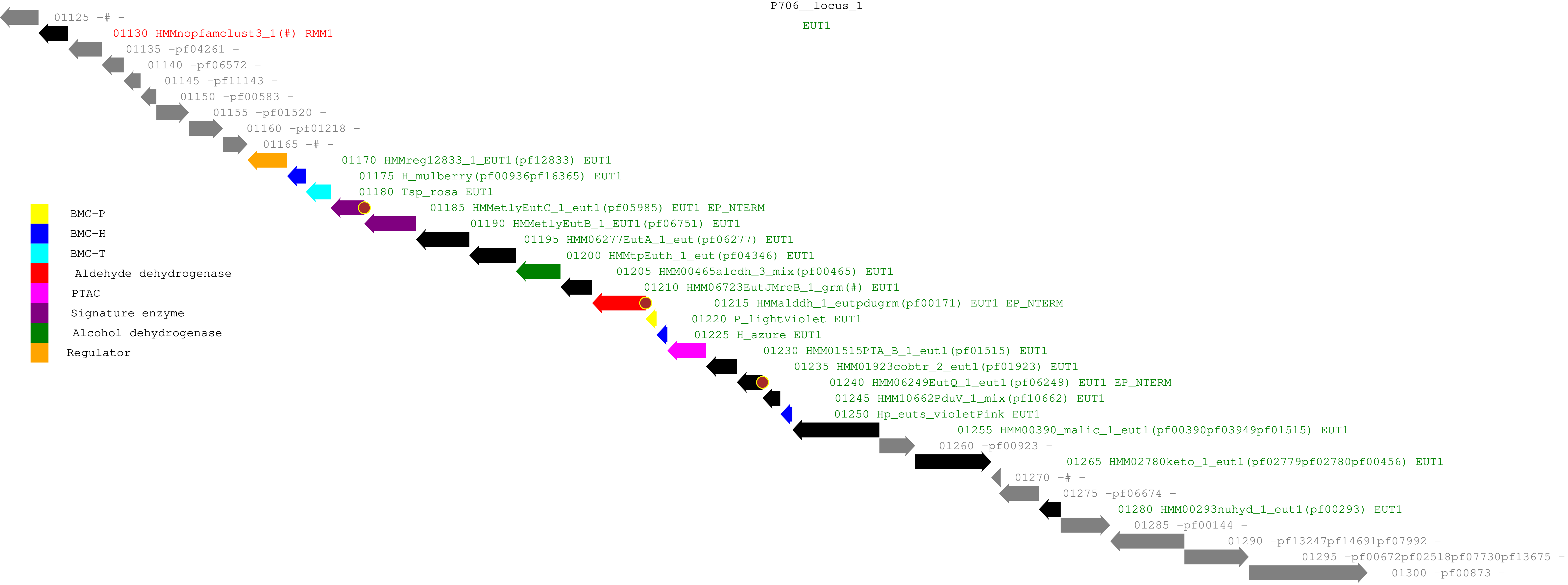

Salmonella enterica subsp. enterica serovar Newport str. CVM22707. Bacteria; Proteobacteria; Gammaproteobacteria; Enterobacterales;

P707__locus_1

EUT1

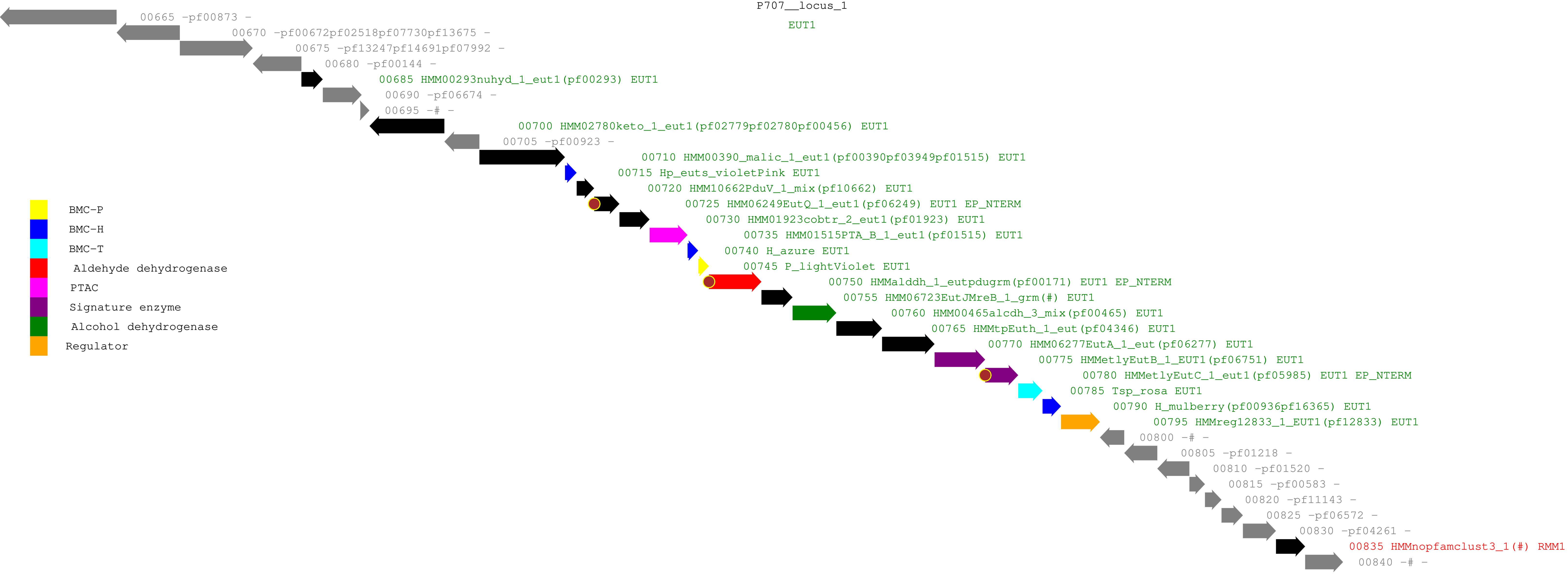

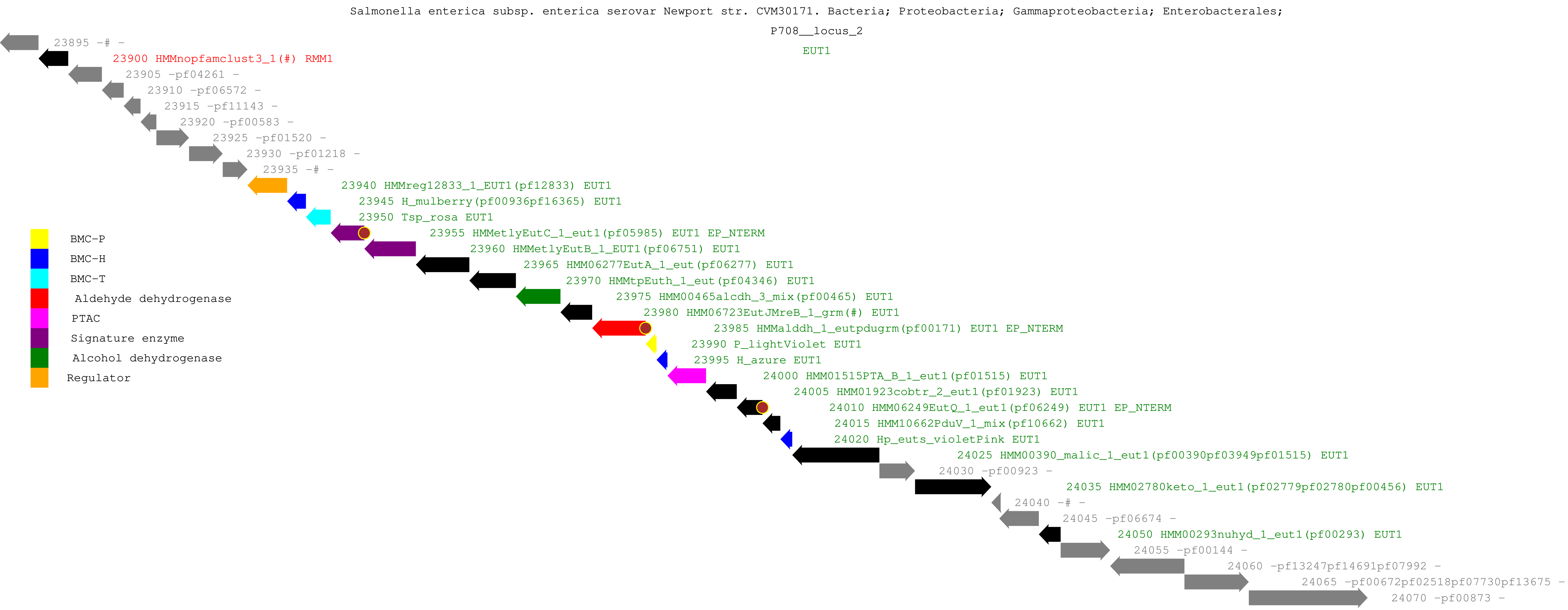
Salmonella enterica subsp. enterica serovar Newport str. CVM30171. Bacteria; Proteobacteria; Gammaproteobacteria; Enterobacterales;
P708__locus_2
EUT1
23895 -# -
23900 HMMnopfamclust3_1(#) RMM1
23905 -pf04261 -
23910 -pf06572 -
23915 -pf11143 -
23920 -pf00583 -
23925 -pf01520 -
23930 -pf01218 -
23935 -# -
23940 HMMreg12833_1_EUT1(pf12833) EUT1
23945 H_mulberry(pf00936pf16365) EUT1
23950 Tsp_rosa EUT1
23955 HMMetlyEutC_1_eut1(pf05985) EUT1 EP_NTERM
23960 HMMetlyEutB_1_EUT1(pf06751) EUT1
23965 HMM06277EutA_1_eut(pf06277) EUT1
23970 HMMtpEuth_1_eut(pf04346) EUT1
23975 HMM00465alcdh_3_mix(pf00465) EUT1
23980 HMM06723EutJMreB_1_grm(#) EUT1
23985 HMMaldh_1_eutpdugrm(pf00171) EUT1 EP_NTERM
23990 P_lightViolet EUT1
23995 H_azure EUT1
24000 HMM01515PTA_B_1_eut1(pf01515) EUT1
24005 HMM01923cobtr_2_eut1(pf01923) EUT1
24010 HMM06249EutQ_1_eut1(pf06249) EUT1 EP_NTERM
24015 HMM10662PduV_1_mix(pf10662) EUT1
24020 Hp_euts_violetPink EUT1
24025 HMM00390_malic_1_eut1(pf00390pf03949pf01515) EUT1
24030 -pf00923 -
24035 HMM02780keto_1_eut1(pf02779pf02780pf00456) EUT1
24040 -# -
24045 -pf06674 -
24050 HMM00293nuhyd_1_eut1(pf00293) EUT1
24055 -pf00144 -
24060 -pf13247pf14691pf07992 -
24065 -pf00672pf02518pf07730pf13675 -
24070 -pf00873 -
BMC-P
BMC-H
BMC-T
Aldehyde dehydrogenase
PTAC
Signature enzyme
Alcohol dehydrogenase
Regulator

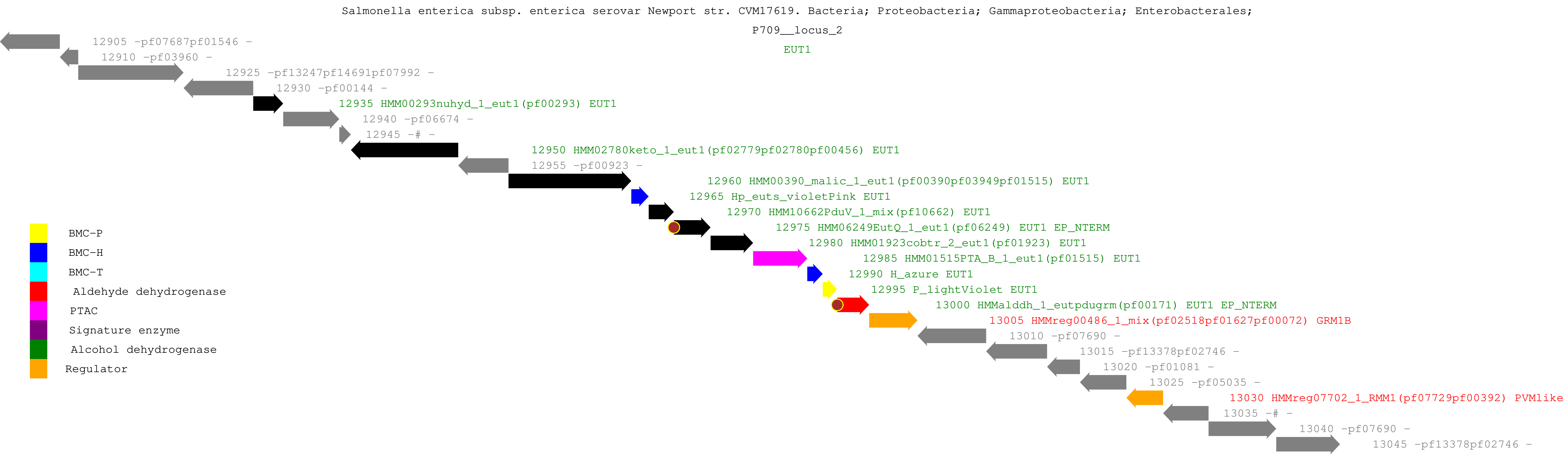

Salmonella enterica subsp. enterica serovar Newport str. CVM17619. Bacteria; Proteobacteria; Gammaproteobacteria; Enterobacterales;
P709__locus_2
EUT1
12905 -pf07687pf01546 -
12910 -pf03960 -
12925 -pf13247pf14691pf07992 -
12930 -pf00144 -
12935 HMM00293nuhyd_1_eut1(pf00293) EUT1
12940 -pf06674 -
12945 -# -
12950 HMM02780keto_1_eut1(pf02779pf02780pf00456) EUT1
12955 -pf00923 -
12960 HMM00390_malic_1_eut1(pf00390pf03949pf01515) EUT1
12965 Hp_euts_violetPink EUT1
12970 HMM10662PduV_1_mix(pf10662) EUT1
12975 HMM06249EutQ_1_eut1(pf06249) EUT1 EP_NTERM
12980 HMM01923cobtr_2_eut1(pf01923) EUT1
12985 HMM01515PTA_B_1_eut1(pf01515) EUT1
12990 H_azure EUT1
12995 P_lightViolet EUT1
13000 HMMalddh_1_eutpdugrm(pf00171) EUT1 EP_NTERM
13005 HMMreg00486_1_mix(pf02518pf01627pf00072) GRM1B
13010 -pf07690 -
13015 -pf13378pf02746 -
13020 -pf01081 -
13025 -pf05035 -
13030 HMMreg07702_1_RMM1(pf07729pf00392) PVMlike
13035 -# -
13040 -pf07690 -
13045 -pf13378pf02746 -
BMC-P
BMC-H
BMC-T
Aldehyde dehydrogenase
PTAC
Signature enzyme
Alcohol dehydrogenase
Regulator

Salmonella enterica subsp. enterica serovar Newport str. CVM20775. Bacteria; Proteobacteria; Gammaproteobacteria; Enterobacterales;

P710__locus_2

EUT1

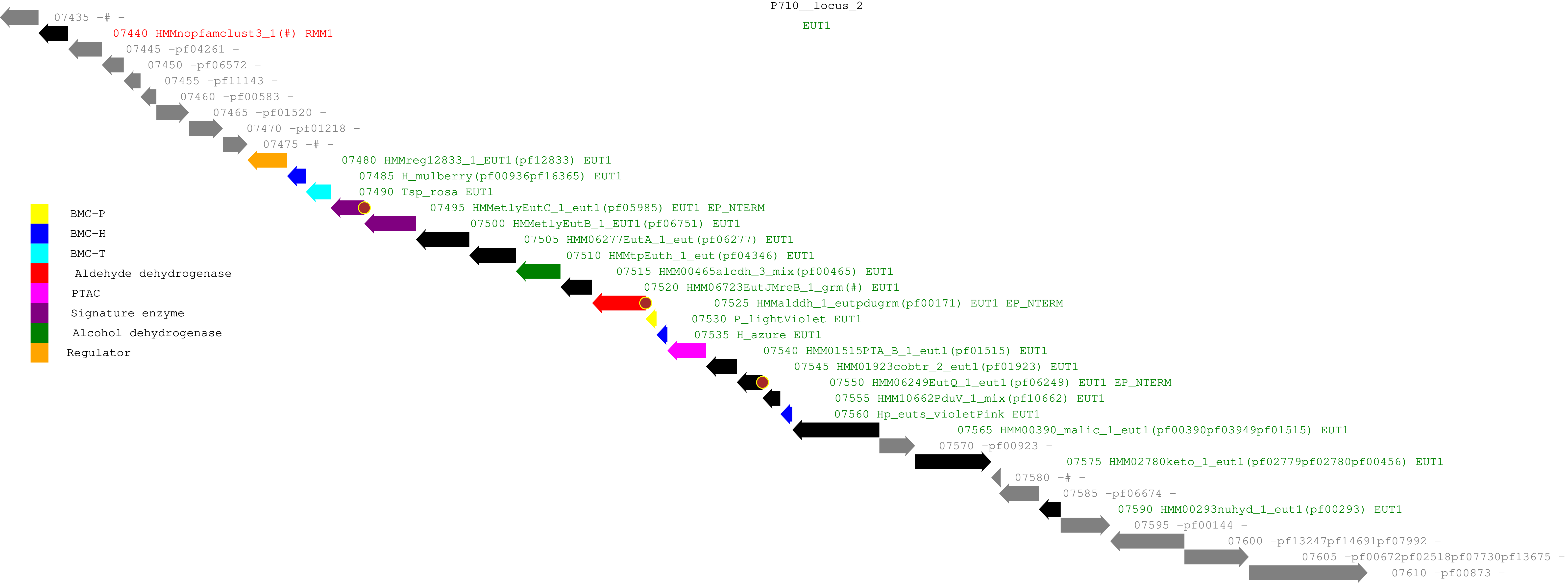

Salmonella enterica subsp. enterica serovar Newport str. CVM22697. Bacteria; Proteobacteria; Gammaproteobacteria; Enterobacterales;

P711__locus_2

EUT1

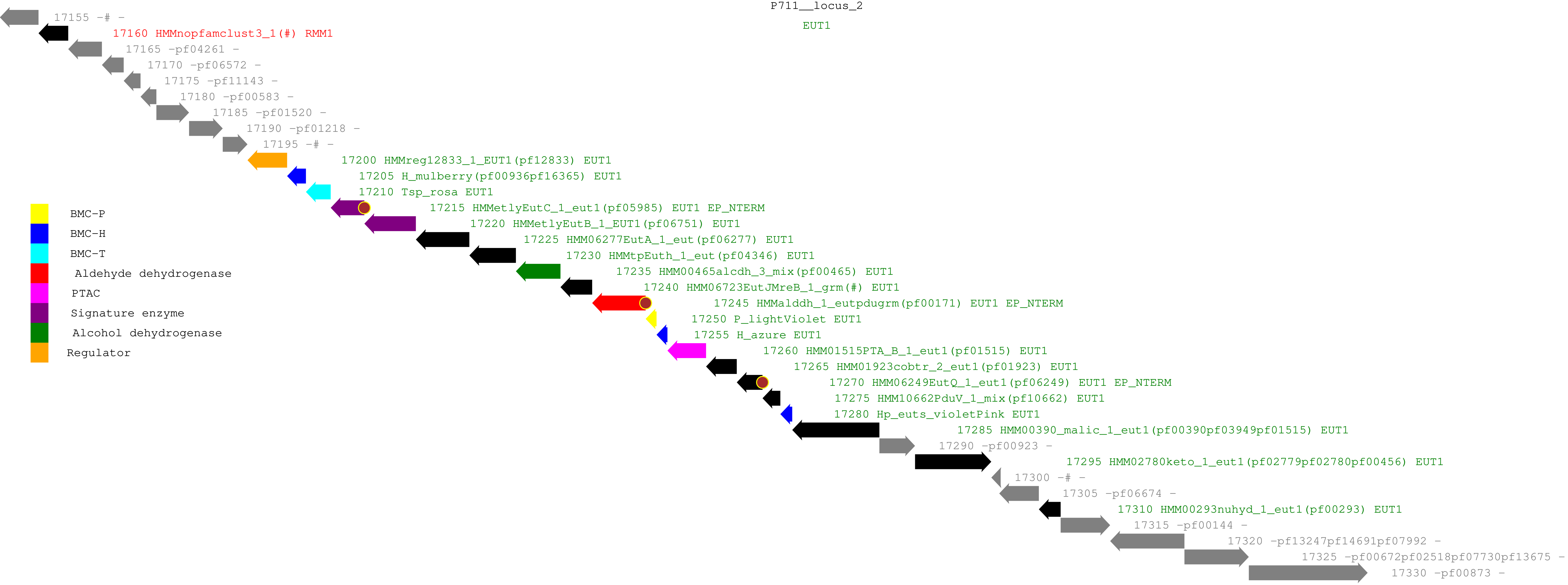

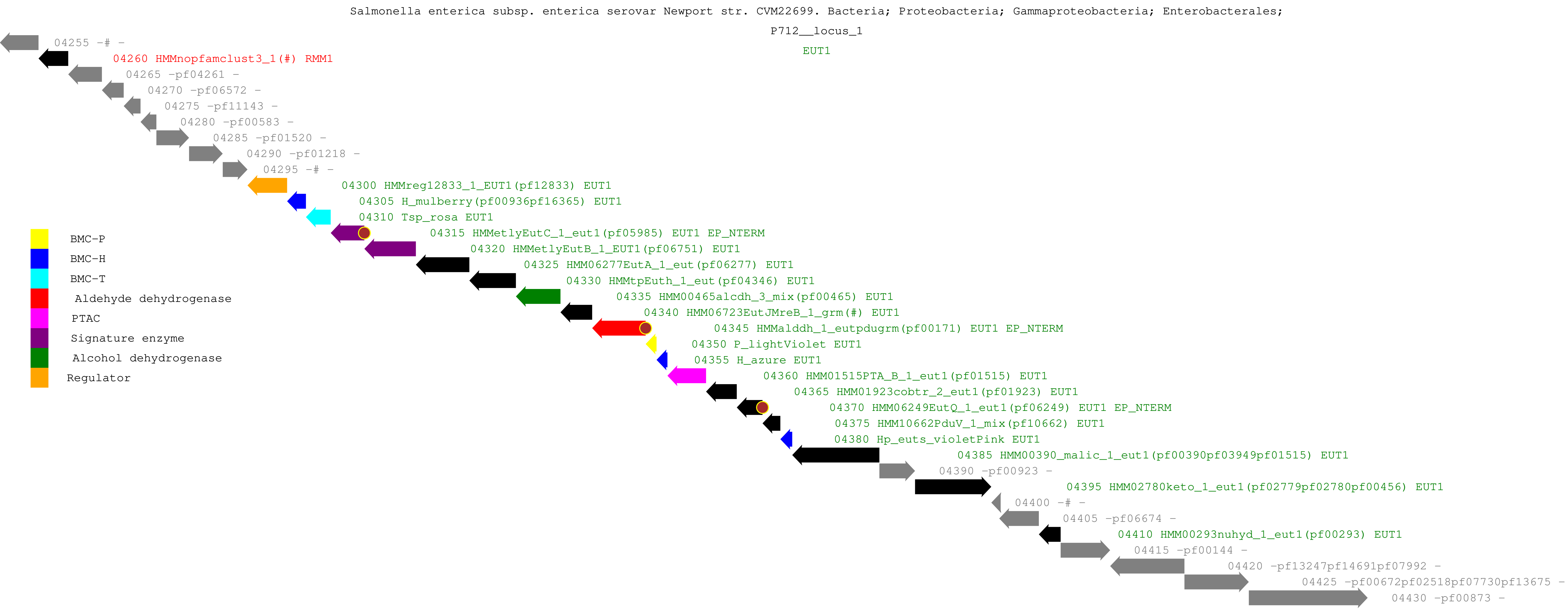
Salmonella enterica subsp. enterica serovar Newport str. CVM22699. Bacteria; Proteobacteria; Gammaproteobacteria; Enterobacterales;
P712__locus_1
EUT1
04255 -# -
04260 HMMnopfamclust3_1(#) RMM1
04265 -pf04261 -
04270 -pf06572 -
04275 -pf11143 -
04280 -pf00583 -
04285 -pf01520 -
04290 -pf01218 -
04295 -# -
04300 HMMreg12833_1_EUT1(pf12833) EUT1
04305 H_mulberry(pf00936pf16365) EUT1
04310 Tsp_rosa EUT1
04315 HMMetlyEutC_1_eut1(pf05985) EUT1 EP_NTERM
04320 HMMetlyEutB_1_EUT1(pf06751) EUT1
04325 HMM06277EutA_1_eut(pf06277) EUT1
04330 HMMtpEuth_1_eut(pf04346) EUT1
04335 HMM00465alcdh_3_mix(pf00465) EUT1
04340 HMM06723EutJMreB_1_grm(#) EUT1
04345 HMMalddh_1_eutpdugrm(pf00171) EUT1 EP_NTERM
04350 P_lightViolet EUT1
04355 H_azure EUT1
04360 HMM01515PTA_B_1_eut1(pf01515) EUT1
04365 HMM01923cobtr_2_eut1(pf01923) EUT1
04370 HMM06249EutQ_1_eut1(pf06249) EUT1 EP_NTERM
04375 HMM10662PduV_1_mix(pf10662) EUT1
04380 Hp_euts_violetPink EUT1
04385 HMM00390_malic_1_eut1(pf00390pf03949pf01515) EUT1
04390 -pf00923 -
04395 HMM02780keto_1_eut1(pf02779pf02780pf00456) EUT1
04400 -# -
04405 -pf06674 -
04410 HMM00293nuhyd_1_eut1(pf00293) EUT1
04415 -pf00144 -
04420 -pf13247pf14691pf07992 -
04425 -pf00672pf02518pf07730pf13675 -
04430 -pf00873 -
BMC-P
BMC-H
BMC-T
Aldehyde dehydrogenase
PTAC
Signature enzyme
Alcohol dehydrogenase
Regulator

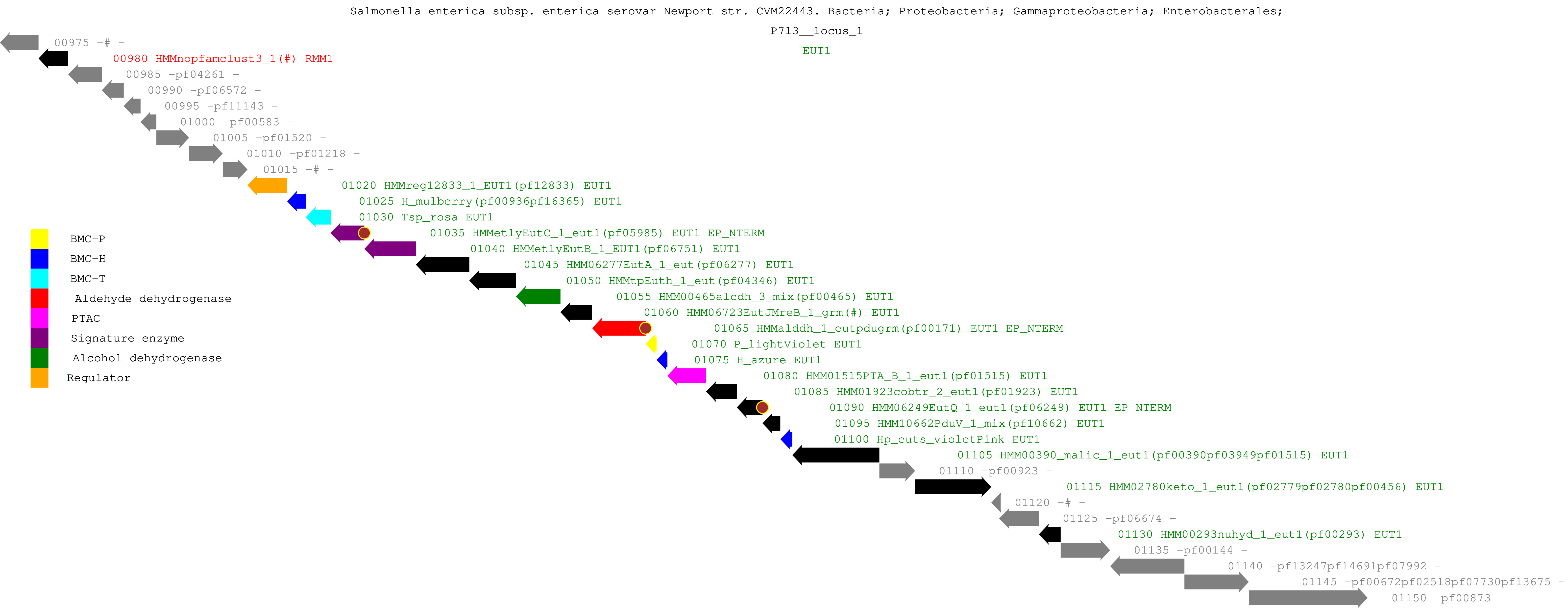
Salmonella enterica subsp. enterica serovar Newport str. CVM22443. Bacteria; Proteobacteria; Gammaproteobacteria; Enterobacterales;
P713__locus_1
EUT1
00975 -# -
00980 HMMnopfamclust3_1(#) RMM1
00985 -pf04261 -
00990 -pf06572 -
00995 -pf11143 -
01000 -pf00583 -
01005 -pf01520 -
01010 -pf01218 -
01015 -# -
01020 HMMreg12833_1_EUT1(pf12833) EUT1
01025 H_mulberry(pf00936pf16365) EUT1
01030 Tsp_rosa EUT1
01035 HMMetlyEutC_1_eut1(pf05985) EUT1 EP_NTERM
01040 HMMetlyEutB_1_EUT1(pf06751) EUT1
01045 HMM06277EutA_1_eut(pf06277) EUT1
01050 HMMtpEuth_1_eut(pf04346) EUT1
01055 HMM00465alcdh_3_mix(pf00465) EUT1
01060 HMM06723EutJMreB_1_grm(#) EUT1
01065 HMMalddh_1_eutpdugrm(pf00171) EUT1 EP_NTERM
01070 P_lightViolet EUT1
01075 H_azure EUT1
01080 HMM01515PTA_B_1_eut1(pf01515) EUT1
01085 HMM01923cobtr_2_eut1(pf01923) EUT1
01090 HMM06249EutQ_1_eut1(pf06249) EUT1 EP_NTERM
01095 HMM10662PduV_1_mix(pf10662) EUT1
01100 Hp_euts_violetPink EUT1
01105 HMM00390_malic_1_eut1(pf00390pf03949pf01515) EUT1
01110 -pf00923 -
01115 HMM02780keto_1_eut1(pf02779pf02780pf00456) EUT1
01120 -# -
01125 -pf06674 -
01130 HMM00293nuhyd_1_eut1(pf00293) EUT1
01135 -pf00144 -
01140 -pf13247pf14691pf07992 -
01145 -pf00672pf02518pf07730pf13675 -
01150 -pf00873 -
BMC-P
BMC-H
BMC-T
Aldehyde dehydrogenase
PTAC
Signature enzyme
Alcohol dehydrogenase
Regulator

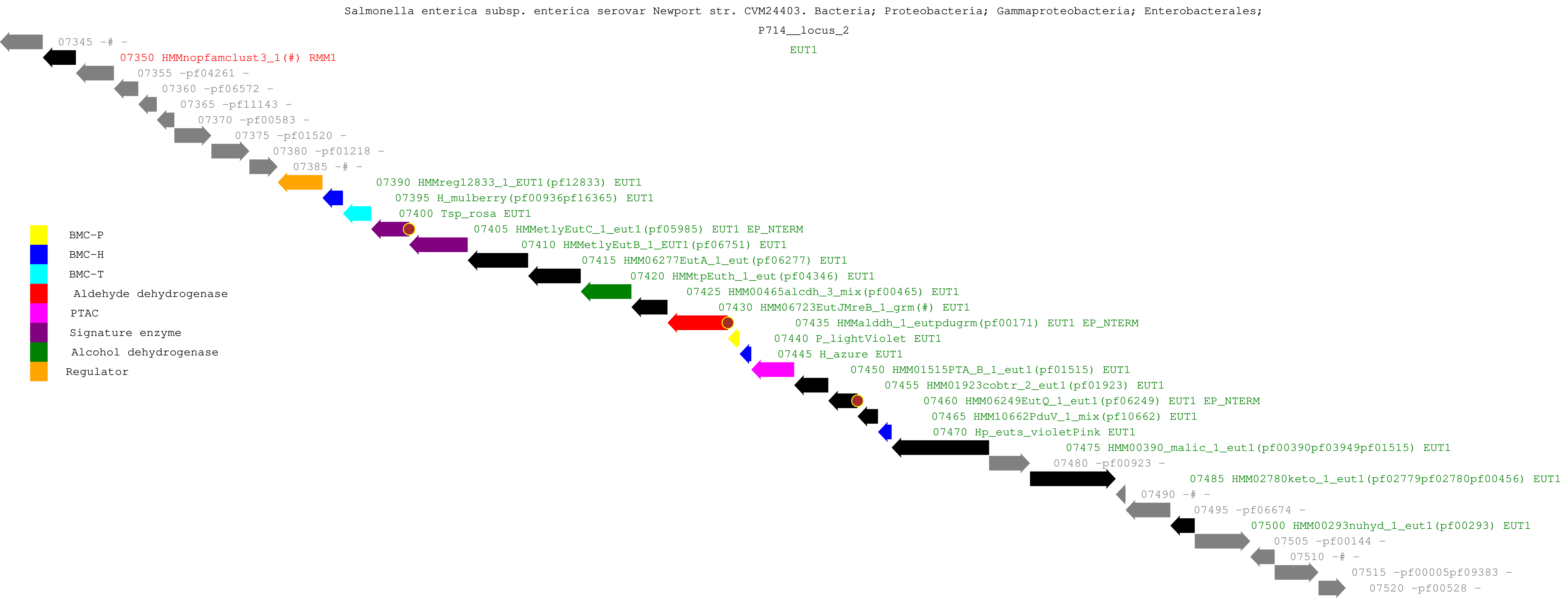

Salmonella enterica subsp. enterica serovar Newport str. CVM24403. Bacteria; Proteobacteria; Gammaproteobacteria; Enterobacterales;
P714__locus_2
EUT1
07345 -# -
07350 HMMnopfamclust3_1(#) RMM1
07355 -pf04261 -
07360 -pf06572 -
07365 -pf11143 -
07370 -pf00583 -
07375 -pf01520 -
07380 -pf01218 -
07385 -# -
07390 HMMreg12833_1_EUT1(pf12833) EUT1
07395 H_mulberry(pf00936pf16365) EUT1
07400 Tsp_rosa EUT1
07405 HMMetlyEutC_1_eut1(pf05985) EUT1 EP_NTERM
07410 HMMetlyEutB_1_EUT1(pf06751) EUT1
07415 HMM06277EutA_1_eut(pf06277) EUT1
07420 HMMtpEuth_1_eut(pf04346) EUT1
07425 HMM00465alcdh_3_mix(pf00465) EUT1
07430 HMM06723EutJMreB_1_grm(#) EUT1
07435 HMMalddh_1_eutpdugrm(pf00171) EUT1 EP_NTERM
07440 P_lightViolet EUT1
07445 H_azure EUT1
07450 HMM01515PTA_B_1_eut1(pf01515) EUT1
07455 HMM01923cobtr_2_eut1(pf01923) EUT1
07460 HMM06249EutQ_1_eut1(pf06249) EUT1 EP_NTERM
07465 HMM10662PduV_1_mix(pf10662) EUT1
07470 Hp_euts_violetPink EUT1
07475 HMM00390_malic_1_eut1(pf00390pf03949pf01515) EUT1
07480 -pf00923 -
07485 HMM02780keto_1_eut1(pf02779pf02780pf00456) EUT1
07490 -# -
07495 -pf06674 -
07500 HMM00293nuhyd_1_eut1(pf00293) EUT1
07505 -pf00144 -
07510 -# -
07515 -pf00005pf09383 -
07520 -pf00528 -
BMC-P
BMC-H
BMC-T
Aldehyde dehydrogenase
PTAC
Signature enzyme
Alcohol dehydrogenase
Regulator

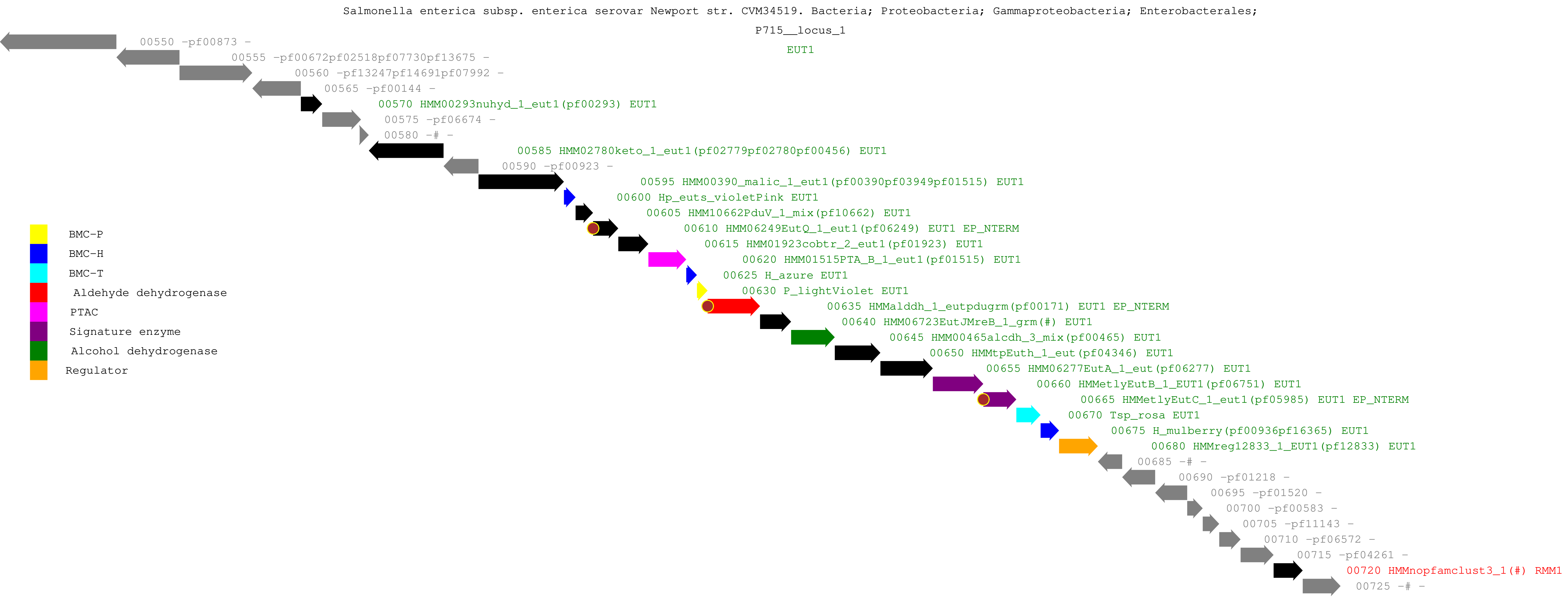

Salmonella enterica subsp. enterica serovar Newport str. CVM34519. Bacteria; Proteobacteria; Gammaproteobacteria; Enterobacterales;
P715__locus_1
EUT1
00550 -pf00873 -
00555 -pf00672pf02518pf07730pf13675 -
00560 -pf13247pf14691pf07992 -
00565 -pf00144 -
00570 HMM00293nuhyd_1_eut1(pf00293) EUT1
00575 -pf06674 -
00580 -# -
00585 HMM02780keto_1_eut1(pf02779pf02780pf00456) EUT1
00590 -pf00923 -
00595 HMM00390_malic_1_eut1(pf00390pf03949pf01515) EUT1
00600 Hp_euts_violetPink EUT1
00605 HMM10662PduV_1_mix(pf10662) EUT1
00610 HMM06249EutQ_1_eut1(pf06249) EUT1 EP_NTERM
00615 HMM01923cobtr_2_eut1(pf01923) EUT1
00620 HMM01515PTA_B_1_eut1(pf01515) EUT1
00625 H_azure EUT1
00630 P_lightViolet EUT1
00635 HMMalddh_1_eutpdugrm(pf00171) EUT1 EP_NTERM
00640 HMM06723EutJMreB_1_grm(#) EUT1
00645 HMM00465alcdh_3_mix(pf00465) EUT1
00650 HMMtpEuth_1_eut(pf04346) EUT1
00655 HMM06277EutA_1_eut(pf06277) EUT1
00660 HMMetlyEutB_1_EUT1(pf06751) EUT1
00665 HMMetlyEutC_1_eut1(pf05985) EUT1 EP_NTERM
00670 Tsp_rosa EUT1
00675 H_mulberry(pf00936pf16365) EUT1
00680 HMMreg12833_1_EUT1(pf12833) EUT1
00685 -# -
00690 -pf01218 -
00695 -pf01520 -
00700 -pf00583 -
00705 -pf11143 -
00710 -pf06572 -
00715 -pf04261 -
00720 HMMnopfamclust3_1(#) RMM1
00725 -# -
BMC-P
BMC-H
BMC-T
Aldehyde dehydrogenase
PTAC
Signature enzyme
Alcohol dehydrogenase
Regulator

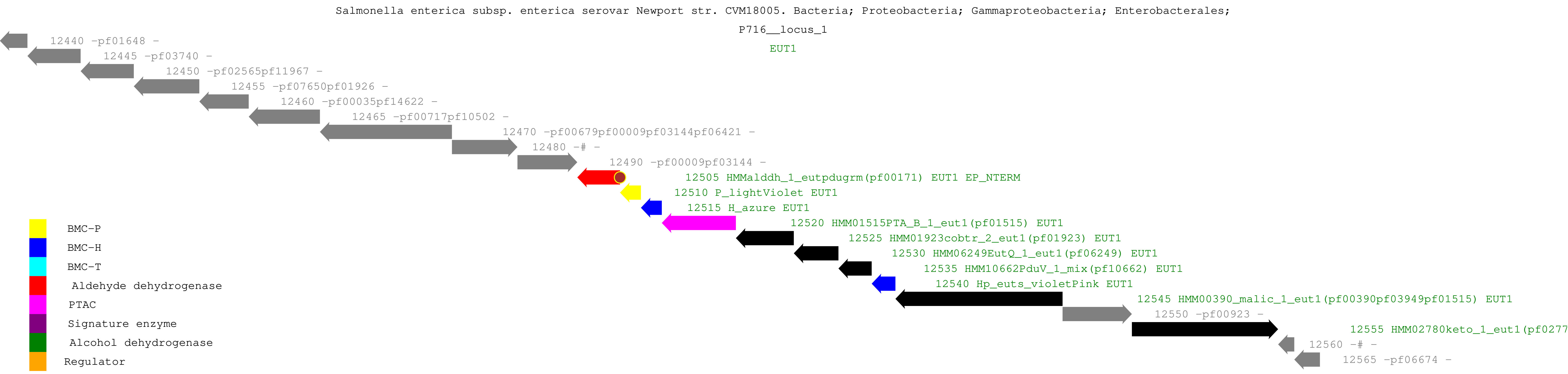

Salmonella enterica subsp. enterica serovar Newport str. CVM18005. Bacteria; Proteobacteria; Gammaproteobacteria; Enterobacterales;
P716__locus_1
EUT1
12440 -pf01648 -
12445 -pf03740 -
12450 -pf02565pf11967 -
12455 -pf07650pf01926 -
12460 -pf00035pf14622 -
12465 -pf00717pf10502 -
12470 -pf00679pf00009pf03144pf06421 -
12480 -# -
12490 -pf00009pf03144 -
12505 HMMalddh_1_eutpdugrm(pf00171) EUT1 EP_NTERM
12510 P_lightViolet EUT1
12515 H_azure EUT1
12520 HMM01515PTA_B_1_eut1(pf01515) EUT1
12525 HMM01923cobtr_2_eut1(pf01923) EUT1
12530 HMM06249EutQ_1_eut1(pf06249) EUT1
12535 HMM10662PduV_1_mix(pf10662) EUT1
12540 Hp_euts_violetPink EUT1
12545 HMM00390_malic_1_eut1(pf00390pf03949pf01515) EUT1
12550 -pf00923 -
12555 HMM02780keto_1_eut1(pf0277
12560 -# -
12565 -pf06674 -
BMC-P
BMC-H
BMC-T
Aldehyde dehydrogenase
PTAC
Signature enzyme
Alcohol dehydrogenase
Regulator

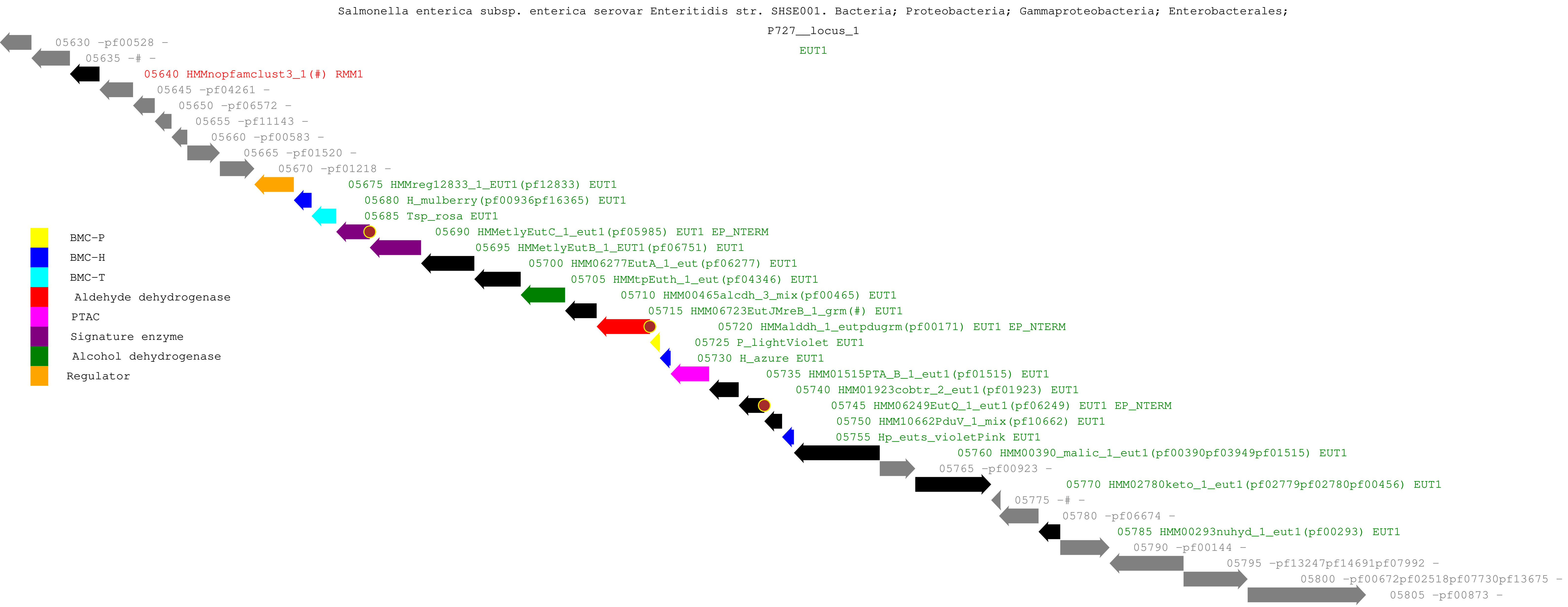

Salmonella enterica subsp. enterica serovar Enteritidis str. SHSE001. Bacteria; Proteobacteria; Gammaproteobacteria; Enterobacterales;
P727__locus_1
EUT1
05630 -pf00528 -
05635 -# -
05640 HMMnopfamclust3_1(#) RMM1
05645 -pf04261 -
05650 -pf06572 -
05655 -pf11143 -
05660 -pf00583 -
05665 -pf01520 -
05670 -pf01218 -
05675 HMMreg12833_1_EUT1(pf12833) EUT1
05680 H_mulberry(pf00936pf16365) EUT1
05685 Tsp_rosa EUT1
05690 HMMetlyEutC_1_eut1(pf05985) EUT1 EP_NTERM
05695 HMMetlyEutB_1_EUT1(pf06751) EUT1
05700 HMM06277EutA_1_eut(pf06277) EUT1
05705 HMMtpEuth_1_eut(pf04346) EUT1
05710 HMM00465alcdh_3_mix(pf00465) EUT1
05715 HMM06723EutJMreB_1_grm(#) EUT1
05720 HMMalddh_1_eutpdugrm(pf00171) EUT1 EP_NTERM
05725 P_lightViolet EUT1
05730 H_azure EUT1
05735 HMM01515PTA_B_1_eut1(pf01515) EUT1
05740 HMM01923cobtr_2_eut1(pf01923) EUT1
05745 HMM06249EutQ_1_eut1(pf06249) EUT1 EP_NTERM
05750 HMM10662PduV_1_mix(pf10662) EUT1
05755 Hp_euts_violetPink EUT1
05760 HMM00390_malic_1_eut1(pf00390pf03949pf01515) EUT1
05765 -pf00923 -
05770 HMM02780keto_1_eut1(pf02779pf02780pf00456) EUT1
05775 -# -
05780 -pf06674 -
05785 HMM00293nuhyd_1_eut1(pf00293) EUT1
05790 -pf00144 -
05795 -pf13247pf14691pf07992 -
05800 -pf00672pf02518pf07730pf13675 -
05805 -pf00873 -
BMC-P
BMC-H
BMC-T
Aldehyde dehydrogenase
PTAC
Signature enzyme
Alcohol dehydrogenase
Regulator

Salmonella enterica subsp. enterica serovar Enteritidis str. SHSE002. Bacteria; Proteobacteria; Gammaproteobacteria; Enterobacterales;

P728__locus_2

EUT1

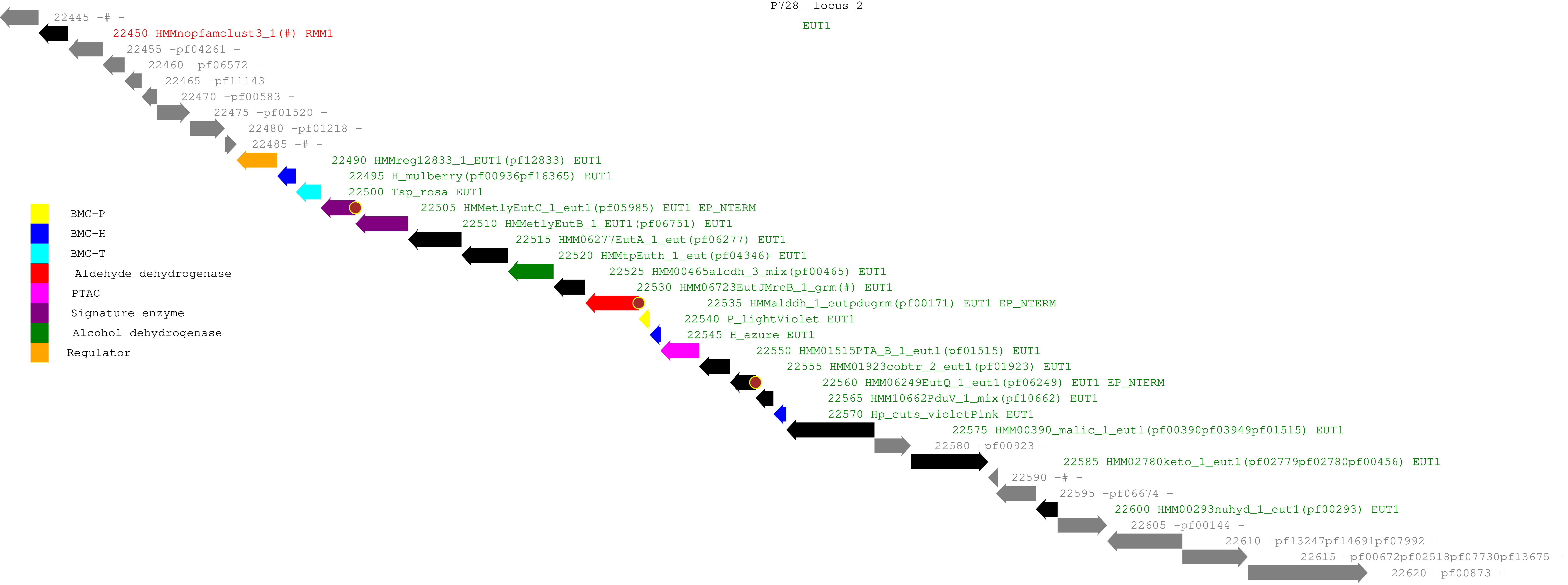

Salmonella enterica subsp. enterica serovar Enteritidis str. SHSE003. Bacteria; Proteobacteria; Gammaproteobacteria; Enterobacterales;

P729__locus_2

EUT1

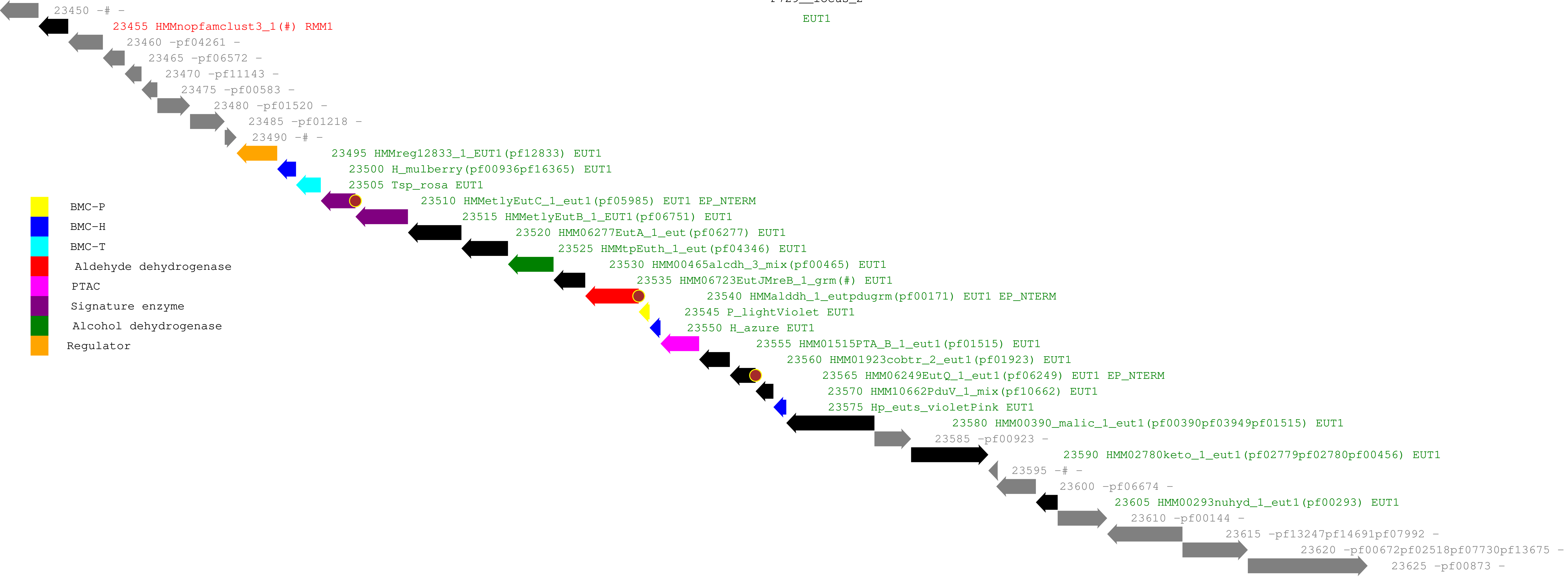

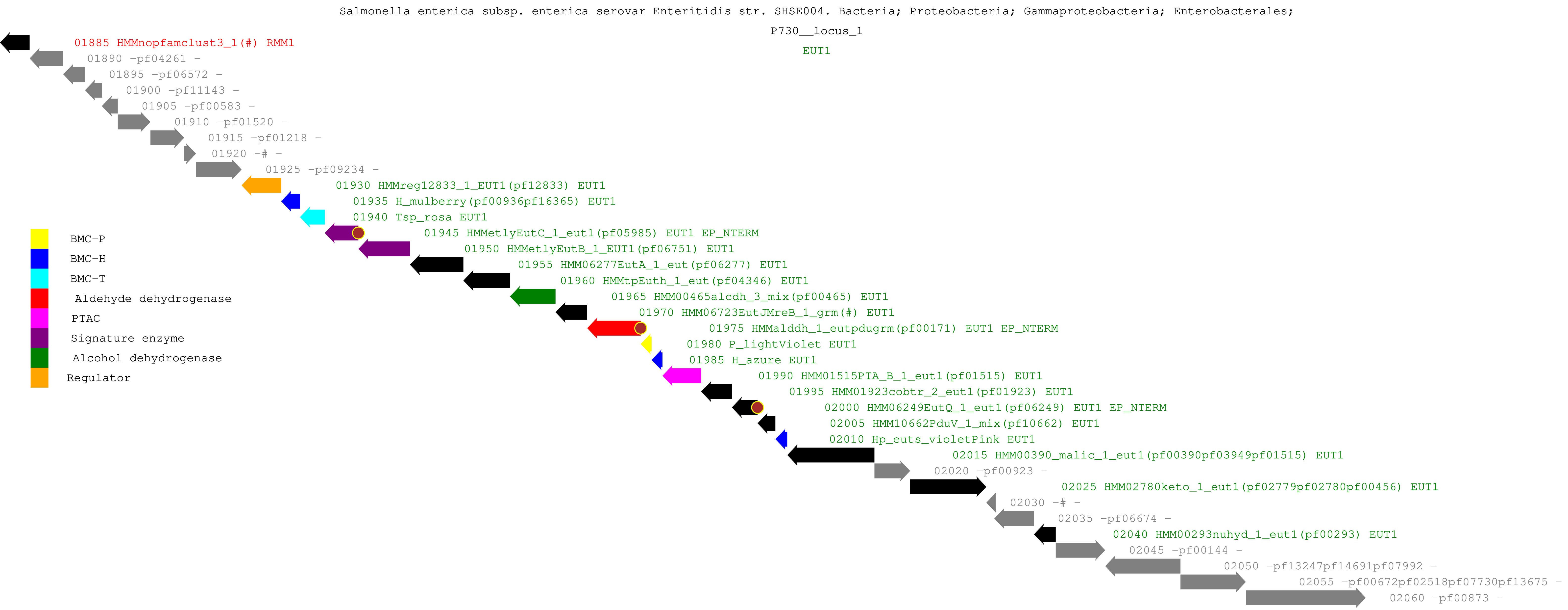
Salmonella enterica subsp. enterica serovar Enteritidis str. SHSE004. Bacteria; Proteobacteria; Gammaproteobacteria; Enterobacterales;
P730__locus_1
EUT1
01885 HMMnopfamclust3_1(#) RMM1
01890 -pf04261 -
01895 -pf06572 -
01900 -pf11143 -
01905 -pf00583 -
01910 -pf01520 -
01915 -pf01218 -
01920 -# -
01925 -pf09234 -
01930 HMMreg12833_1_EUT1(pf12833) EUT1
01935 H_mulberry(pf00936pf16365) EUT1
01940 Tsp_rosa EUT1
01945 HMMetlyEutC_1_eut1(pf05985) EUT1 EP_NTERM
01950 HMMetlyEutB_1_EUT1(pf06751) EUT1
01955 HMM06277EutA_1_eut(pf06277) EUT1
01960 HMMtpEuth_1_eut(pf04346) EUT1
01965 HMM00465alcdh_3_mix(pf00465) EUT1
01970 HMM06723EutJMreB_1_grm(#) EUT1
01975 HMMalddh_1_eutpdugrm(pf00171) EUT1 EP_NTERM
01980 P_lightViolet EUT1
01985 H_azure EUT1
01990 HMM01515PTA_B_1_eut1(pf01515) EUT1
01995 HMM01923cobtr_2_eut1(pf01923) EUT1
02000 HMM06249EutQ_1_eut1(pf06249) EUT1 EP_NTERM
02005 HMM10662PduV_1_mix(pf10662) EUT1
02010 Hp_euts_violetPink EUT1
02015 HMM00390_malic_1_eut1(pf00390pf03949pf01515) EUT1
02020 -pf00923 -
02025 HMM02780keto_1_eut1(pf02779pf02780pf00456) EUT1
02030 -# -
02035 -pf06674 -
02040 HMM00293nuhyd_1_eut1(pf00293) EUT1
02045 -pf00144 -
02050 -pf13247pf14691pf07992 -
02055 -pf00672pf02518pf07730pf13675 -
02060 -pf00873 -
BMC-P
BMC-H
BMC-T
Aldehyde dehydrogenase
PTAC
Signature enzyme
Alcohol dehydrogenase
Regulator

Pseudomonas citronellolis. Bacteria; Proteobacteria; Gammaproteobacteria; Pseudomonadales;

PcP3B5__locus_1

EUT1

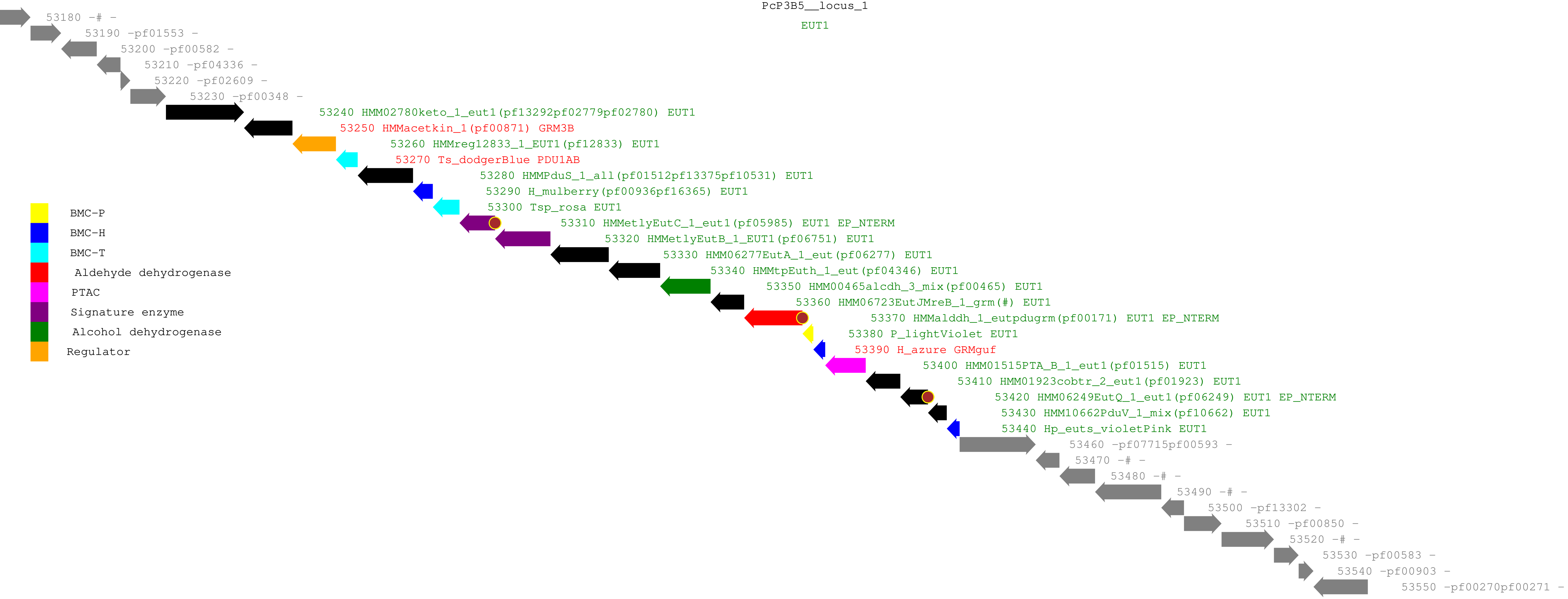

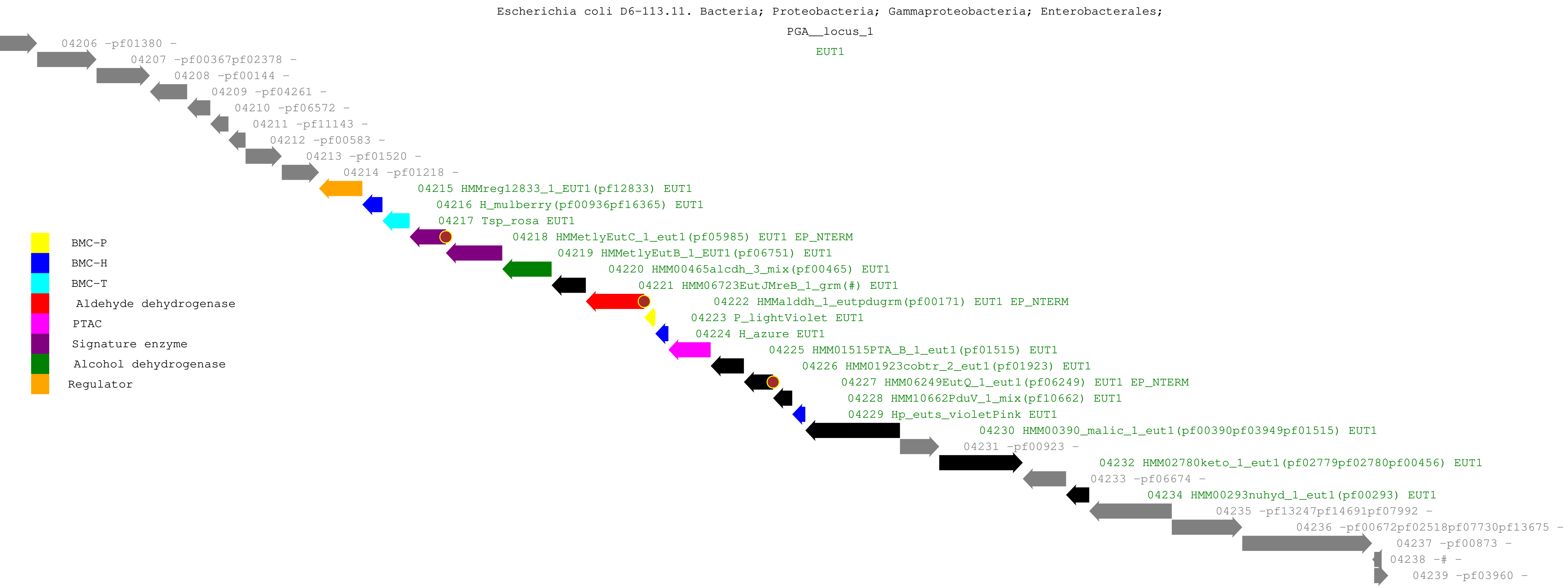
Escherichia coli D6-113.11. Bacteria; Proteobacteria; Gammaproteobacteria; Enterobacterales;
PGA__locus_1
EUT1
04206 -pf01380 -
04207 -pf00367pf02378 -
04208 -pf00144 -
04209 -pf04261 -
04210 -pf06572 -
04211 -pf11143 -
04212 -pf00583 -
04213 -pf01520 -
04214 -pf01218 -
04215 HMMreg12833_1_EUT1(pf12833) EUT1
04216 H_mulberry(pf00936pf16365) EUT1
04217 Tsp_rosa EUT1
04218 HMMetlyEutC_1_eut1(pf05985) EUT1 EP_NTERM
04219 HMMetlyEutB_1_EUT1(pf06751) EUT1
04220 HMM00465alcdh_3_mix(pf00465) EUT1
04221 HMM06723EutJMreB_1_grm(#) EUT1
04222 HMMalddh_1_eutpdugrm(pf00171) EUT1 EP_NTERM
04223 P_lightViolet EUT1
04224 H_azure EUT1
04225 HMM01515PTA_B_1_eut1(pf01515) EUT1
04226 HMM01923cobtr_2_eut1(pf01923) EUT1
04227 HMM06249EutQ_1_eut1(pf06249) EUT1 EP_NTERM
04228 HMM10662PduV_1_mix(pf10662) EUT1
04229 Hp_euts_violetPink EUT1
04230 HMM00390_malic_1_eut1(pf00390pf03949pf01515) EUT1
04231 -pf00923 -
04232 HMM02780keto_1_eut1(pf02779pf02780pf00456) EUT1
04233 -pf06674 -
04234 HMM00293nuhyd_1_eut1(pf00293) EUT1
04235 -pf13247pf14691pf07992 -
04236 -pf00672pf02518pf07730pf13675 -
04237 -pf00873 -
04238 -# -
04239 -pf03960 -
BMC-P
BMC-H
BMC-T
Aldehyde dehydrogenase
PTAC
Signature enzyme
Alcohol dehydrogenase
Regulator

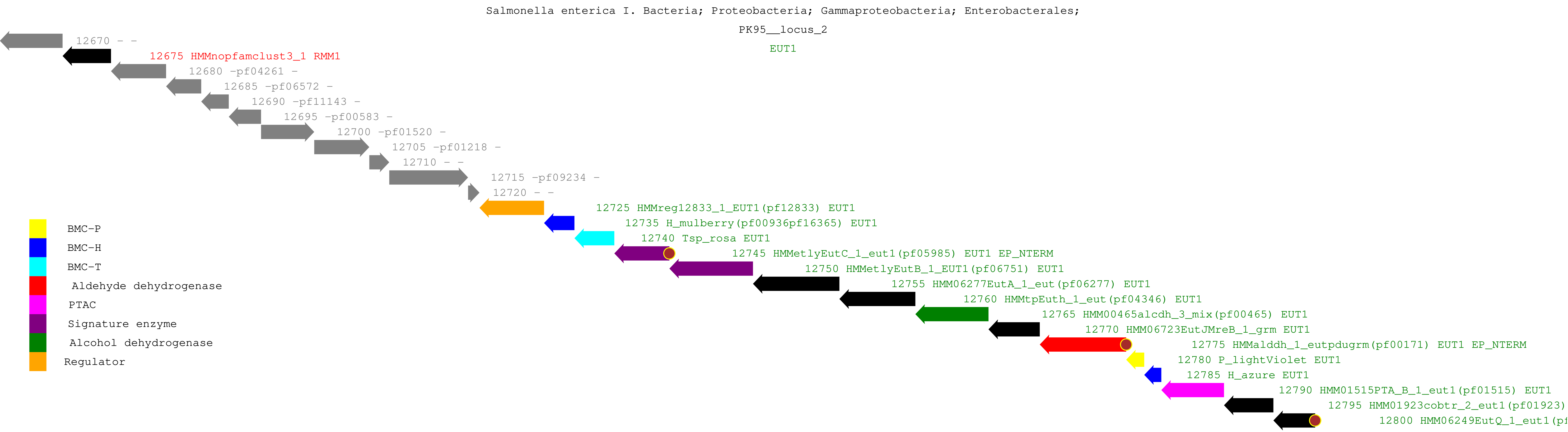
Salmonella enterica I. Bacteria; Proteobacteria; Gammaproteobacteria; Enterobacterales;
PK95__locus_2
EUT1
12670 - -
12675 HMMnopfamclust3_1 RMM1
12680 -pf04261 -
12685 -pf06572 -
12690 -pf11143 -
12695 -pf00583 -
12700 -pf01520 -
12705 -pf01218 -
12710 - -
12715 -pf09234 -
12720 - -
12725 HMMreg12833_1_EUT1(pf12833) EUT1
12735 H_mulberry(pf00936pf16365) EUT1
12740 Tsp_rosa EUT1
12745 HMMetlyEutC_1_eut1(pf05985) EUT1 EP_NTERM
12750 HMMetlyEutB_1_EUT1(pf06751) EUT1
12755 HMM06277EutA_1_eut(pf06277) EUT1
12760 HMMtpEuth_1_eut(pf04346) EUT1
12765 HMM00465alcdh_3_mix(pf00465) EUT1
12770 HMM06723EutJMreB_1_grm EUT1
12775 HMMaldh_1_eutpdugrm(pf00171) EUT1 EP_NTERM
12780 P_lightViolet EUT1
12785 H_azure EUT1
12790 HMM01515PTA_B_1_eut1(pf01515) EUT1
12795 HMM01923cobtr_2_eut1(pf01923)
12800 HMM06249EutQ_1_eut1(pf
BMC-P
BMC-H
BMC-T
Aldehyde dehydrogenase
PTAC
Signature enzyme
Alcohol dehydrogenase
Regulator

Pseudomonas sp. (strain M1). Bacteria; Proteobacteria; Gammaproteobacteria; Pseudomonadales;

PM1__locus_1

EUT1

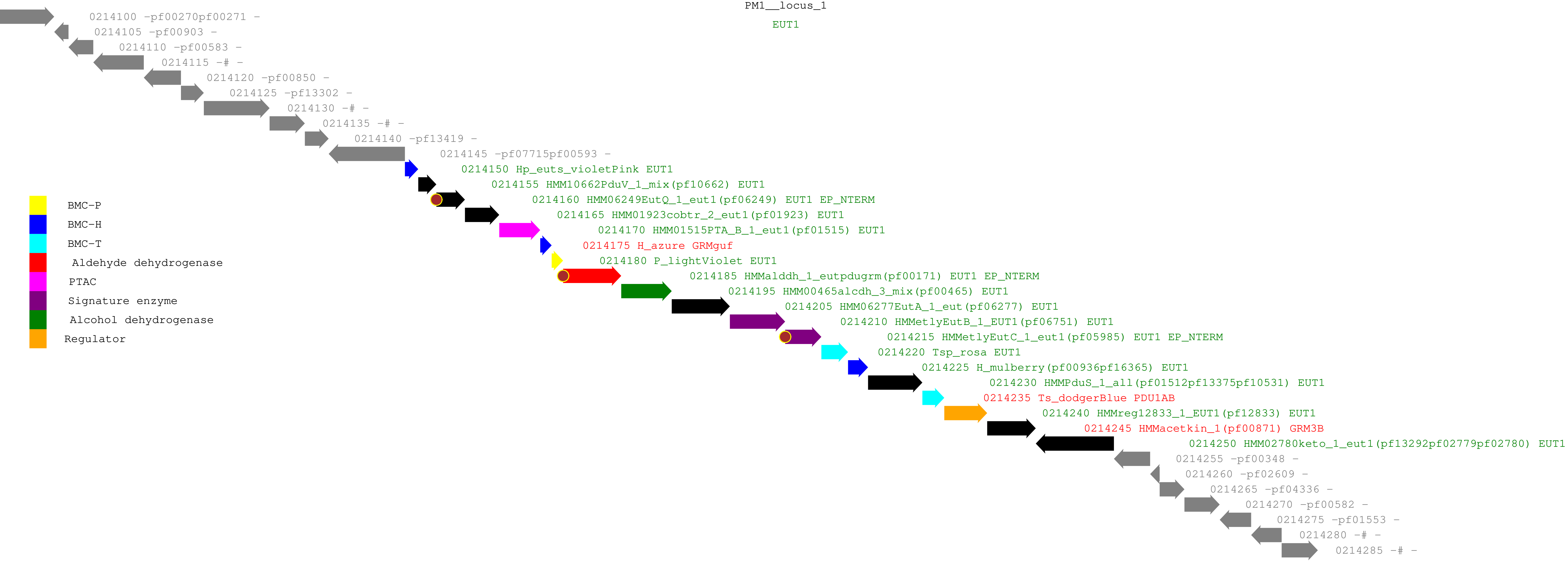

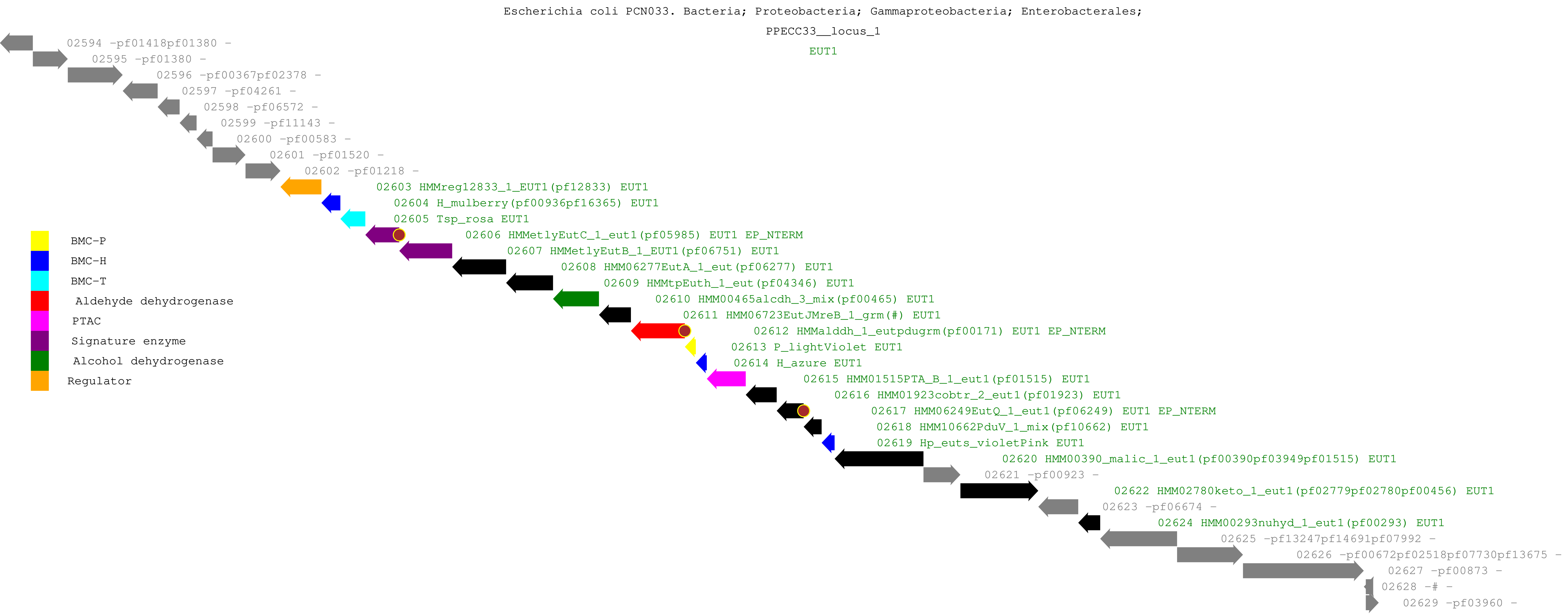

Escherichia coli PCN033. Bacteria; Proteobacteria; Gammaproteobacteria; Enterobacterales;
PPECC33__locus_1
EUT1
02594 -pf01418pf01380 -
02595 -pf01380 -
02596 -pf00367pf02378 -
02597 -pf04261 -
02598 -pf06572 -
02599 -pf11143 -
02600 -pf00583 -
02601 -pf01520 -
02602 -pf01218 -
02603 HMMreg12833_1_EUT1(pf12833) EUT1
02604 H_mulberry(pf00936pf16365) EUT1
02605 Tsp_rosa EUT1
02606 HMMetlyEutC_1_eut1(pf05985) EUT1 EP_NTERM
02607 HMMetlyEutB_1_EUT1(pf06751) EUT1
02608 HMM06277EutA_1_eut(pf06277) EUT1
02609 HMMtpEuth_1_eut(pf04346) EUT1
02610 HMM00465alcdh_3_mix(pf00465) EUT1
02611 HMM06723EutJMreB_1_grm(#) EUT1
02612 HMMaldh_1_eutpdugrm(pf00171) EUT1 EP_NTERM
02613 P_lightViolet EUT1
02614 H_azure EUT1
02615 HMM01515PTA_B_1_eut1(pf01515) EUT1
02616 HMM01923cobtr_2_eut1(pf01923) EUT1
02617 HMM06249EutQ_1_eut1(pf06249) EUT1 EP_NTERM
02618 HMM10662PduV_1_mix(pf10662) EUT1
02619 Hp_euts_violetPink EUT1
02620 HMM00390_malic_1_eut1(pf00390pf03949pf01515) EUT1
02621 -pf00923 -
02622 HMM02780keto_1_eut1(pf02779pf02780pf00456) EUT1
02623 -pf06674 -
02624 HMM00293nuhyd_1_eut1(pf00293) EUT1
02625 -pf13247pf14691pf07992 -
02626 -pf00672pf02518pf07730pf13675 -
02627 -pf00873 -
02628 -# -
02629 -pf03960 -
BMC-P
BMC-H
BMC-T
Aldehyde dehydrogenase
PTAC
Signature enzyme
Alcohol dehydrogenase
Regulator

Hafnia alvei. Bacteria; Proteobacteria; Gammaproteobacteria; Enterobacterales;

PU01__locus_1

EUT1

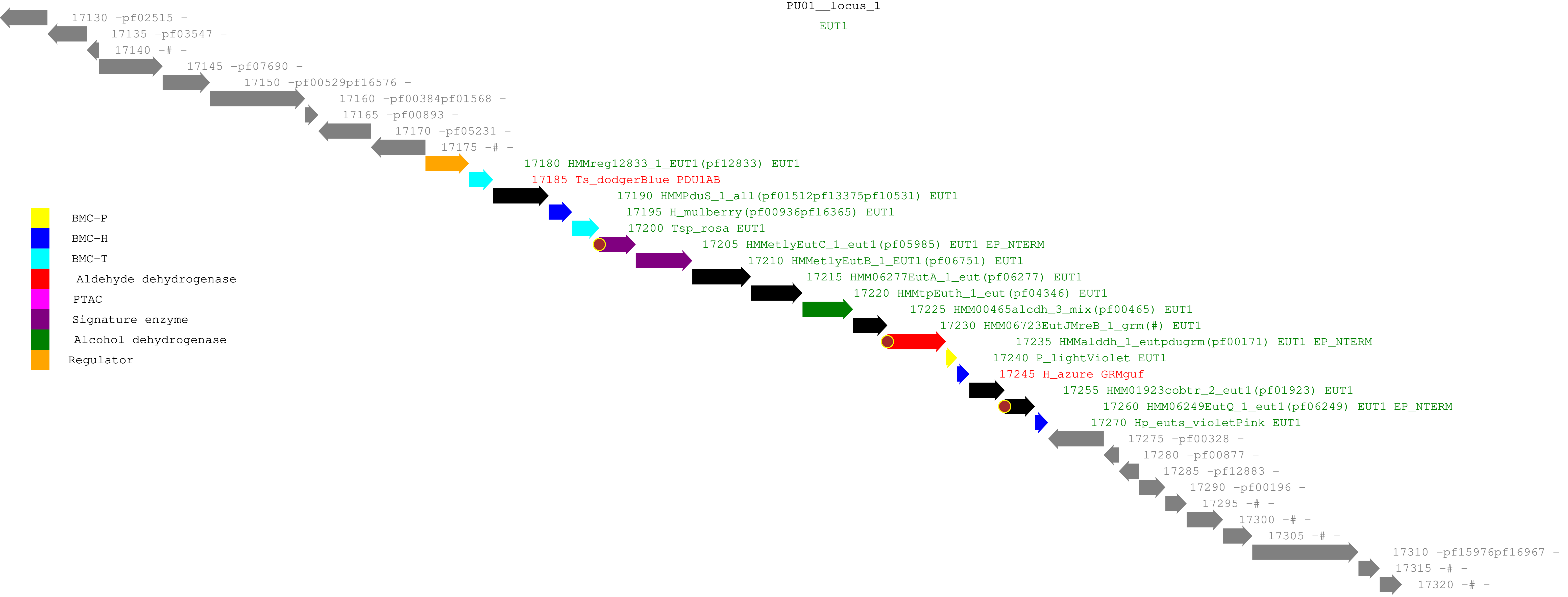

Escherichia coli ATCC BAA-2209. Bacteria; Proteobacteria; Gammaproteobacteria; Enterobacterales;

Q458__locus_1_main_sat

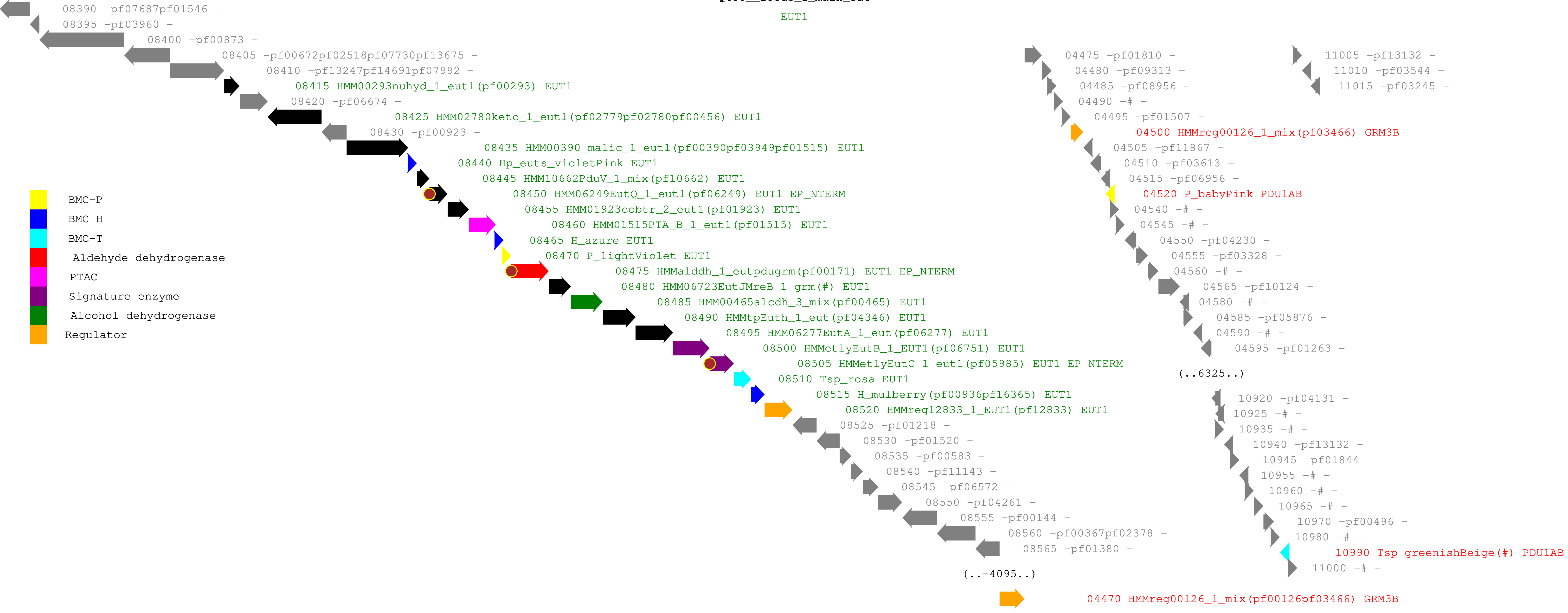

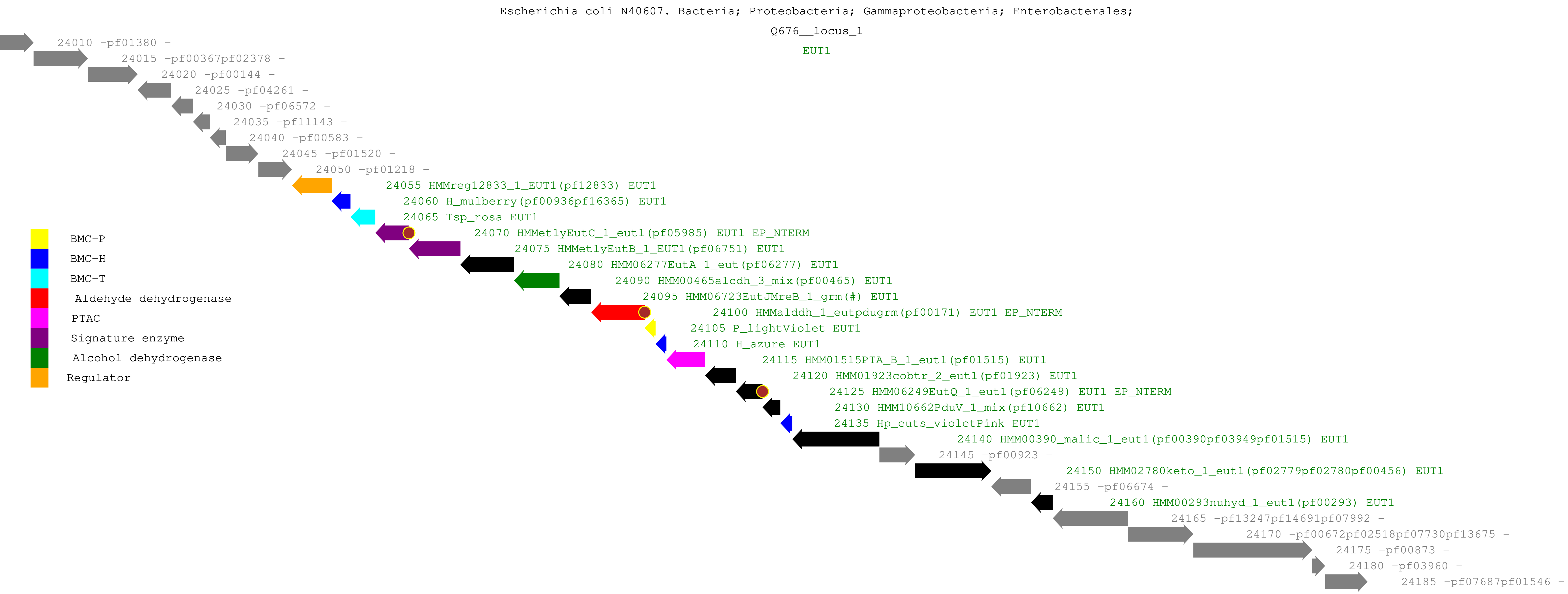

Escherichia coli N40607. Bacteria; Proteobacteria; Gammaproteobacteria; Enterobacterales;
Q676__locus_1
EUT1
24010 -pf01380 -
24015 -pf00367pf02378 -
24020 -pf00144 -
24025 -pf04261 -
24030 -pf06572 -
24035 -pf11143 -
24040 -pf00583 -
24045 -pf01520 -
24050 -pf01218 -
24055 HMMreg12833_1_EUT1(pf12833) EUT1
24060 H_mulberry(pf00936pf16365) EUT1
24065 Tsp_rosa EUT1
24070 HMMetlyEutC_1_eut1(pf05985) EUT1 EP_NTERM
24075 HMMetlyEutB_1_EUT1(pf06751) EUT1
24080 HMM06277EutA_1_eut(pf06277) EUT1
24090 HMM00465alcdh_3_mix(pf00465) EUT1
24095 HMM06723EutJMreB_1_grm(#) EUT1
24100 HMMalddh_1_eutpdugrm(pf00171) EUT1 EP_NTERM
24105 P_lightViolet EUT1
24110 H_azure EUT1
24115 HMM01515PTA_B_1_eut1(pf01515) EUT1
24120 HMM01923cobtr_2_eut1(pf01923) EUT1
24125 HMM06249EutQ_1_eut1(pf06249) EUT1 EP_NTERM
24130 HMM10662PduV_1_mix(pf10662) EUT1
24135 Hp_euts_violetPink EUT1
24140 HMM00390_malic_1_eut1(pf00390pf03949pf01515) EUT1
24145 -pf00923 -
24150 HMM02780keto_1_eut1(pf02779pf02780pf00456) EUT1
24155 -pf06674 -
24160 HMM00293nuhyd_1_eut1(pf00293) EUT1
24165 -pf13247pf14691pf07992 -
24170 -pf00672pf02518pf07730pf13675 -
24175 -pf00873 -
24180 -pf03960 -
24185 -pf07687pf01546 -
BMC-P
BMC-H
BMC-T
Aldehyde dehydrogenase
PTAC
Signature enzyme
Alcohol dehydrogenase
Regulator

Salmonella enterica I. Bacteria; Proteobacteria; Gammaproteobacteria; Enterobacterales;

R118__locus_2

EUT1

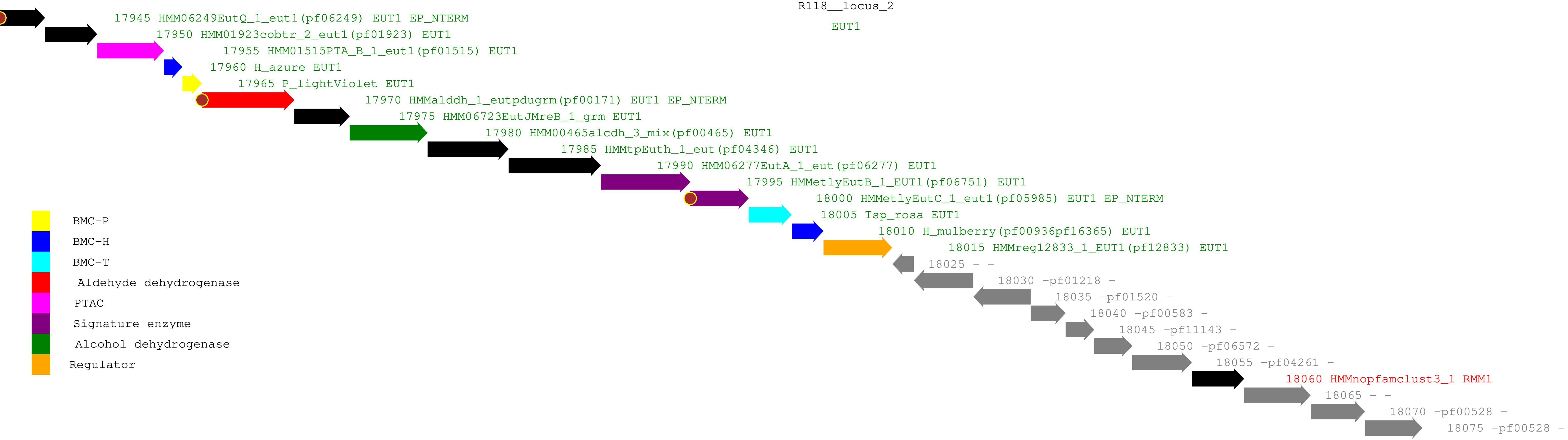

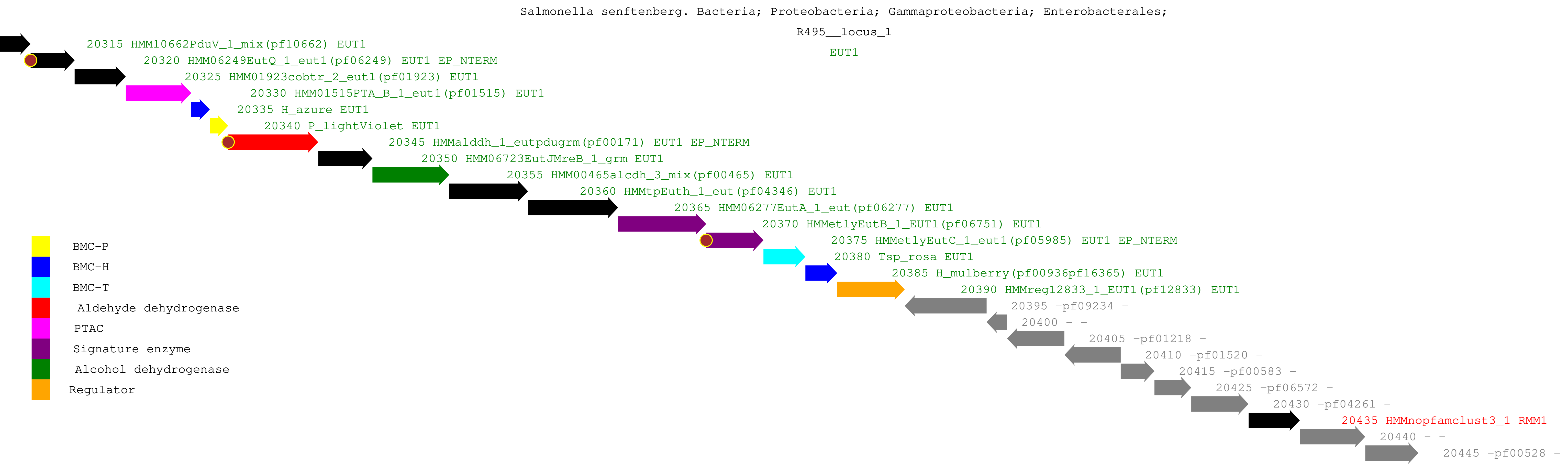
Salmonella senftenberg. Bacteria; Proteobacteria; Gammaproteobacteria; Enterobacterales;
R495__locus_1
EUT1
20315 HMM10662PduV_1_mix(pf10662) EUT1
20320 HMM06249EutQ_1_eut1(pf06249) EUT1 EP_NTERM
20325 HMM01923cobtr_2_eut1(pf01923) EUT1
20330 HMM01515PTA_B_1_eut1(pf01515) EUT1
20335 H_azure EUT1
20340 P_lightViolet EUT1
20345 HMMalddh_1_eutpdugrm(pf00171) EUT1 EP_NTERM
20350 HMM06723EutJMreB_1_grm EUT1
20355 HMM00465alcdh_3_mix(pf00465) EUT1
20360 HMMtpEuth_1_eut(pf04346) EUT1
20365 HMM06277EutA_1_eut(pf06277) EUT1
20370 HMMetlyEutB_1_EUT1(pf06751) EUT1
20375 HMMetlyEutC_1_eut1(pf05985) EUT1 EP_NTERM
20380 Tsp_rosa EUT1
20385 H_mulberry(pf00936pf16365) EUT1
20390 HMMreg12833_1_EUT1(pf12833) EUT1
20395 -pf09234 -
20400 - -
20405 -pf01218 -
20410 -pf01520 -
20415 -pf00583 -
20425 -pf06572 -
20430 -pf04261 -
20435 HMMnopfamclust3_1 RMM1
20440 - -
20445 -pf00528 -
BMC-P
BMC-H
BMC-T
Aldehyde dehydrogenase
PTAC
Signature enzyme
Alcohol dehydrogenase
Regulator

Salmonella enterica subsp. arizonae serovar 41:z4,z23:-. Bacteria; Proteobacteria; Gammaproteobacteria; Enterobacterales;

R515__locus_1

EUT1

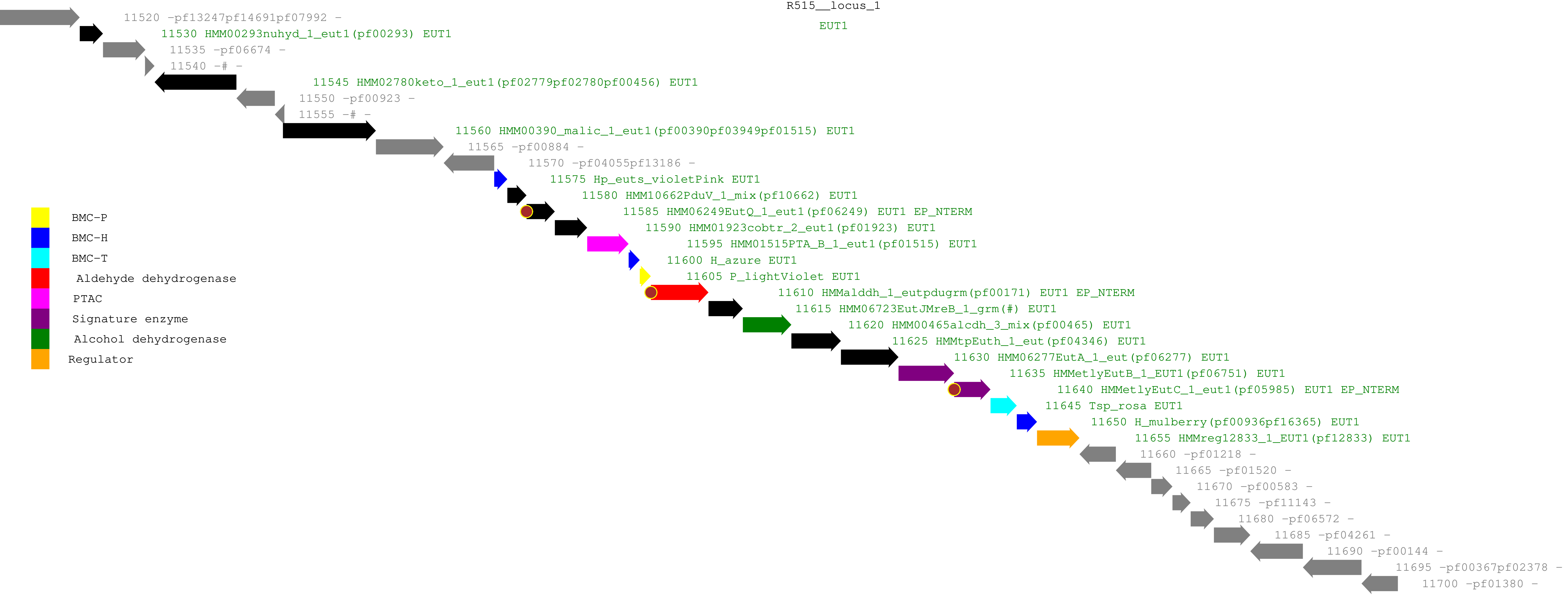

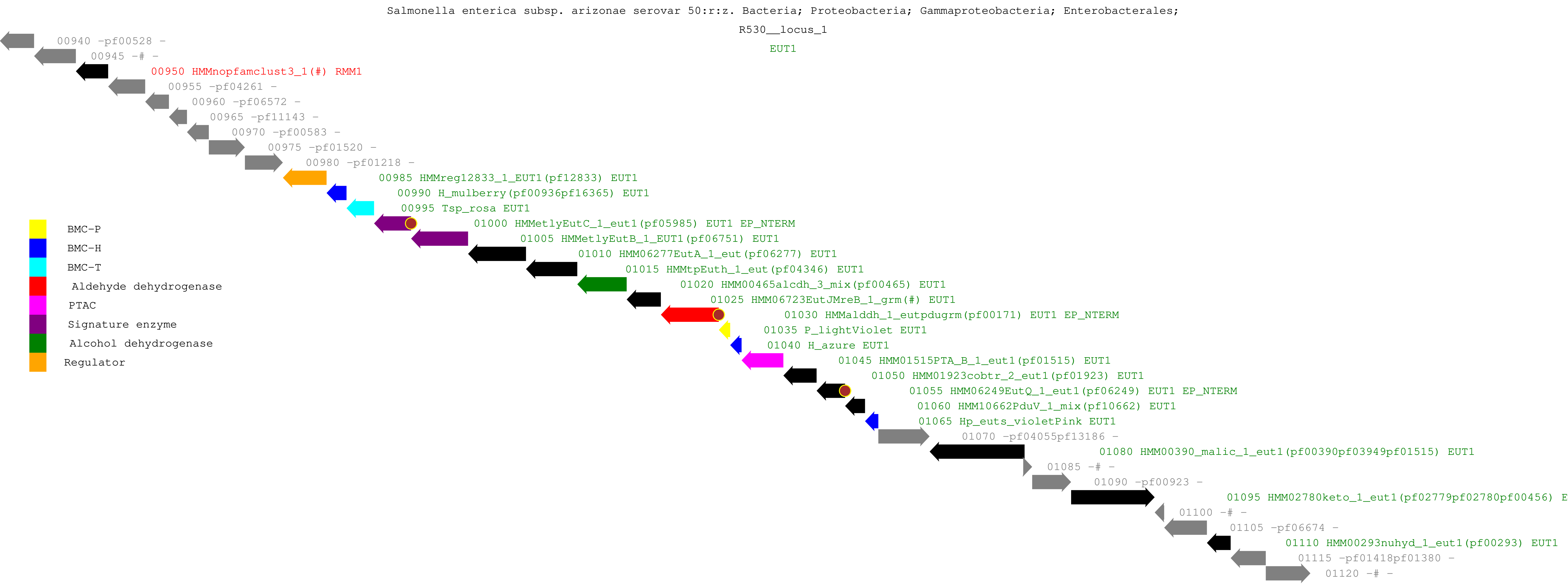
Salmonella enterica subsp. arizonae serovar 50:r:z. Bacteria; Proteobacteria; Gammaproteobacteria; Enterobacterales;
R530__locus_1
EUT1
00940 -pf00528 -
00945 -# -
00950 HMMnopfamclust3_1(#) RMM1
00955 -pf04261 -
00960 -pf06572 -
00965 -pf11143 -
00970 -pf00583 -
00975 -pf01520 -
00980 -pf01218 -
00985 HMMreg12833_1_EUT1(pf12833) EUT1
00990 H_mulberry(pf00936pf16365) EUT1
00995 Tsp_rosa EUT1
01000 HMMetlyEutC_1_eut1(pf05985) EUT1 EP_NTERM
01005 HMMetlyEutB_1_EUT1(pf06751) EUT1
01010 HMM06277EutA_1_eut(pf06277) EUT1
01015 HMMtpEuth_1_eut(pf04346) EUT1
01020 HMM00465alcdh_3_mix(pf00465) EUT1
01025 HMM06723EutJMreB_1_grm(#) EUT1
01030 HMMalddh_1_eutpdugrm(pf00171) EUT1 EP_NTERM
01035 P_lightViolet EUT1
01040 H_azure EUT1
01045 HMM01515PTA_B_1_eut1(pf01515) EUT1
01050 HMM01923cobtr_2_eut1(pf01923) EUT1
01055 HMM06249EutQ_1_eut1(pf06249) EUT1 EP_NTERM
01060 HMM10662PduV_1_mix(pf10662) EUT1
01065 Hp_euts_violetPink EUT1
01070 -pf04055pf13186 -
01080 HMM00390_malic_1_eut1(pf00390pf03949pf01515) EUT1
01085 -# -
01090 -pf00923 -
01095 HMM02780keto_1_eut1(pf02779pf02780pf00456) E
01100 -# -
01105 -pf06674 -
01110 HMM00293nuhyd_1_eut1(pf00293) EUT1
01115 -pf01418pf01380 -
01120 -# -
BMC-P
BMC-H
BMC-T
Aldehyde dehydrogenase
PTAC
Signature enzyme
Alcohol dehydrogenase
Regulator

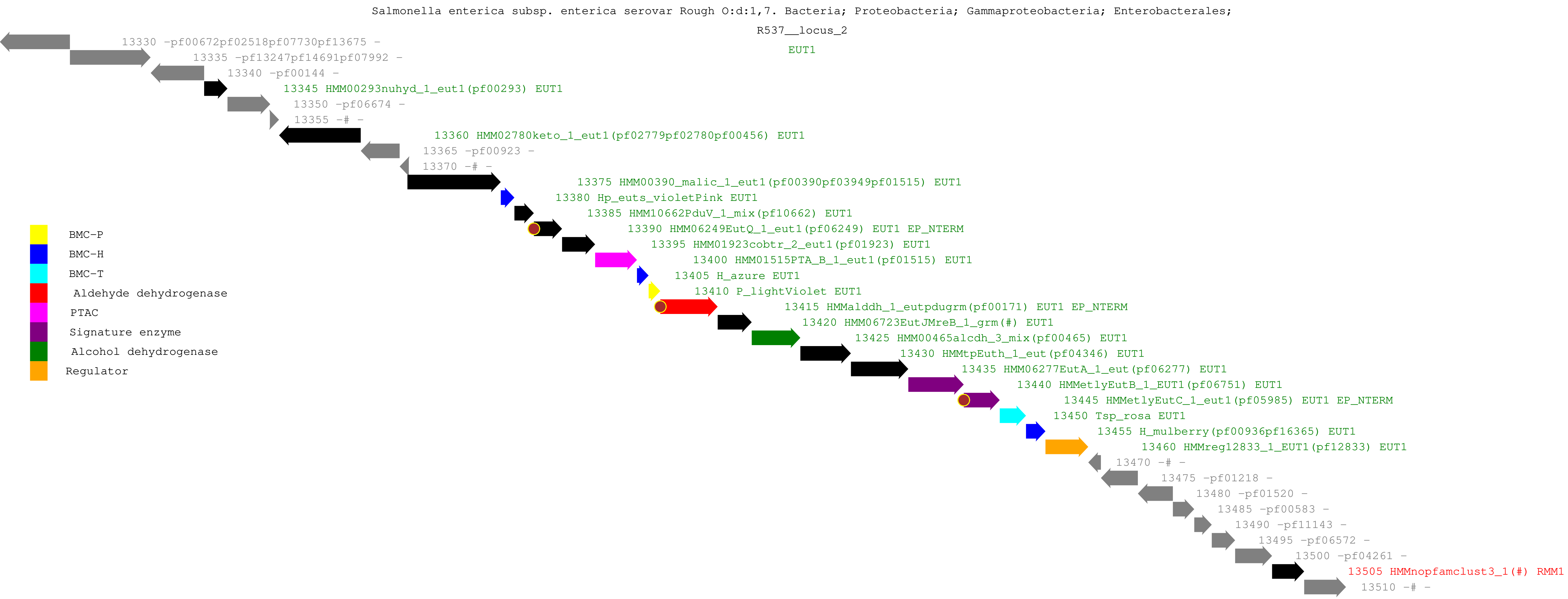
Salmonella enterica subsp. enterica serovar Rough O:d:1,7. Bacteria; Proteobacteria; Gammaproteobacteria; Enterobacterales;
R537__locus_2
EUT1
13330 -pf00672pf02518pf07730pf13675 -
13335 -pf13247pf14691pf07992 -
13340 -pf00144 -
13345 HMM00293nuhyd_1_eut1(pf00293) EUT1
13350 -pf06674 -
13355 -# -
13360 HMM02780keto_1_eut1(pf02779pf02780pf00456) EUT1
13365 -pf00923 -
13370 -# -
13375 HMM00390_malic_1_eut1(pf00390pf03949pf01515) EUT1
13380 Hp_euts_violetPink EUT1
13385 HMM10662PduV_1_mix(pf10662) EUT1
13390 HMM06249EutQ_1_eut1(pf06249) EUT1 EP_NTERM
13395 HMM01923cobtr_2_eut1(pf01923) EUT1
13400 HMM01515PTA_B_1_eut1(pf01515) EUT1
13405 H_azure EUT1
13410 P_lightViolet EUT1
13415 HMMalddh_1_eutpdugrm(pf00171) EUT1 EP_NTERM
13420 HMM06723EutJMreB_1_grm(#) EUT1
13425 HMM00465alcdh_3_mix(pf00465) EUT1
13430 HMMtpEuth_1_eut(pf04346) EUT1
13435 HMM06277EutA_1_eut(pf06277) EUT1
13440 HMMetlyEutB_1_EUT1(pf06751) EUT1
13445 HMMetlyEutC_1_eut1(pf05985) EUT1 EP_NTERM
13450 Tsp_rosa EUT1
13455 H_mulberry(pf00936pf16365) EUT1
13460 HMMreg12833_1_EUT1(pf12833) EUT1
13470 -# -
13475 -pf01218 -
13480 -pf01520 -
13485 -pf00583 -
13490 -pf11143 -
13495 -pf06572 -
13500 -pf04261 -
13505 HMMnopfamclust3_1(#) RMM1
13510 -# -
BMC-P
BMC-H
BMC-T
Aldehyde dehydrogenase
PTAC
Signature enzyme
Alcohol dehydrogenase
Regulator

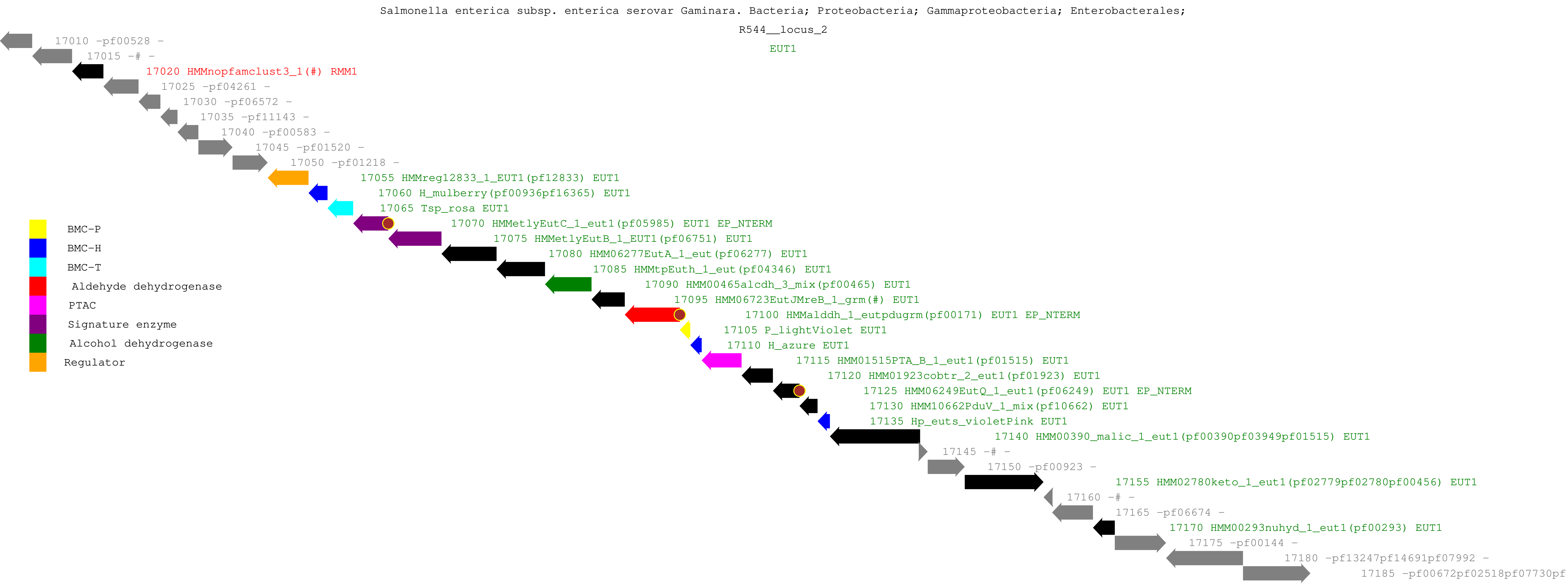
Salmonella enterica subsp. enterica serovar Gaminara. Bacteria; Proteobacteria; Gammaproteobacteria; Enterobacterales;
R544__locus_2
EUT1
17010 -pf00528 -
17015 -# -
17020 HMMnopfamclust3_1(#) RMM1
17025 -pf04261 -
17030 -pf06572 -
17035 -pf11143 -
17040 -pf00583 -
17045 -pf01520 -
17050 -pf01218 -
17055 HMMreg12833_1_EUT1(pf12833) EUT1
17060 H_mulberry(pf00936pf16365) EUT1
17065 Tsp_rosa EUT1
17070 HMMetlyEutC_1_eut1(pf05985) EUT1 EP_NTERM
17075 HMMetlyEutB_1_EUT1(pf06751) EUT1
17080 HMM06277EutA_1_eut(pf06277) EUT1
17085 HMMtpEuth_1_eut(pf04346) EUT1
17090 HMM00465alcdh_3_mix(pf00465) EUT1
17095 HMM06723EutJMreB_1_grm(#) EUT1
17100 HMMalddh_1_eutpdugrm(pf00171) EUT1 EP_NTERM
17105 P_lightViolet EUT1
17110 H_azure EUT1
17115 HMM01515PTA_B_1_eut1(pf01515) EUT1
17120 HMM01923cobtr_2_eut1(pf01923) EUT1
17125 HMM06249EutQ_1_eut1(pf06249) EUT1 EP_NTERM
17130 HMM10662PduV_1_mix(pf10662) EUT1
17135 Hp_euts_violetPink EUT1
17140 HMM00390_malic_1_eut1(pf00390pf03949pf01515) EUT1
17145 -# -
17150 -pf00923 -
17155 HMM02780keto_1_eut1(pf02779pf02780pf00456) EUT1
17160 -# -
17165 -pf06674 -
17170 HMM00293nuhyd_1_eut1(pf00293) EUT1
17175 -pf00144 -
17180 -pf13247pf14691pf07992 -
17185 -pf00672pf02518pf07730pf
BMC-P
BMC-H
BMC-T
Aldehyde dehydrogenase
PTAC
Signature enzyme
Alcohol dehydrogenase
Regulator

Salmonella enterica subsp. enterica serovar Saphra. Bacteria; Proteobacteria; Gammaproteobacteria; Enterobacterales;

R549__locus_2

EUT1

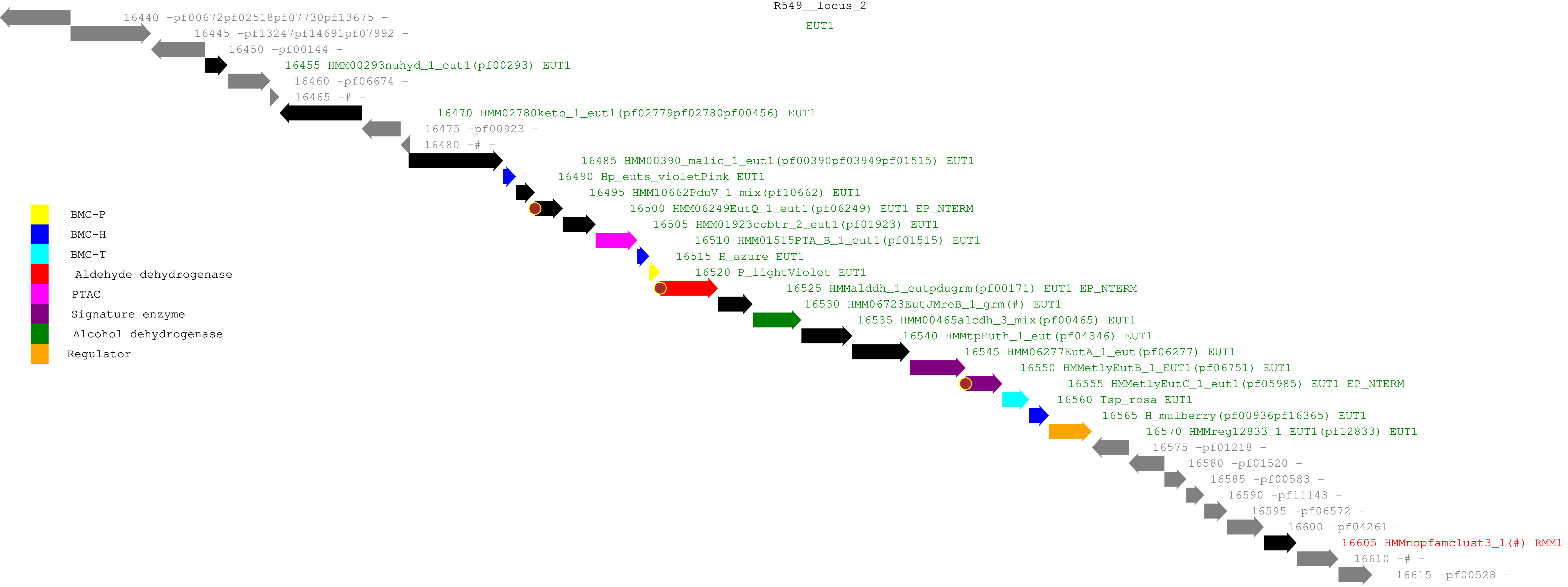

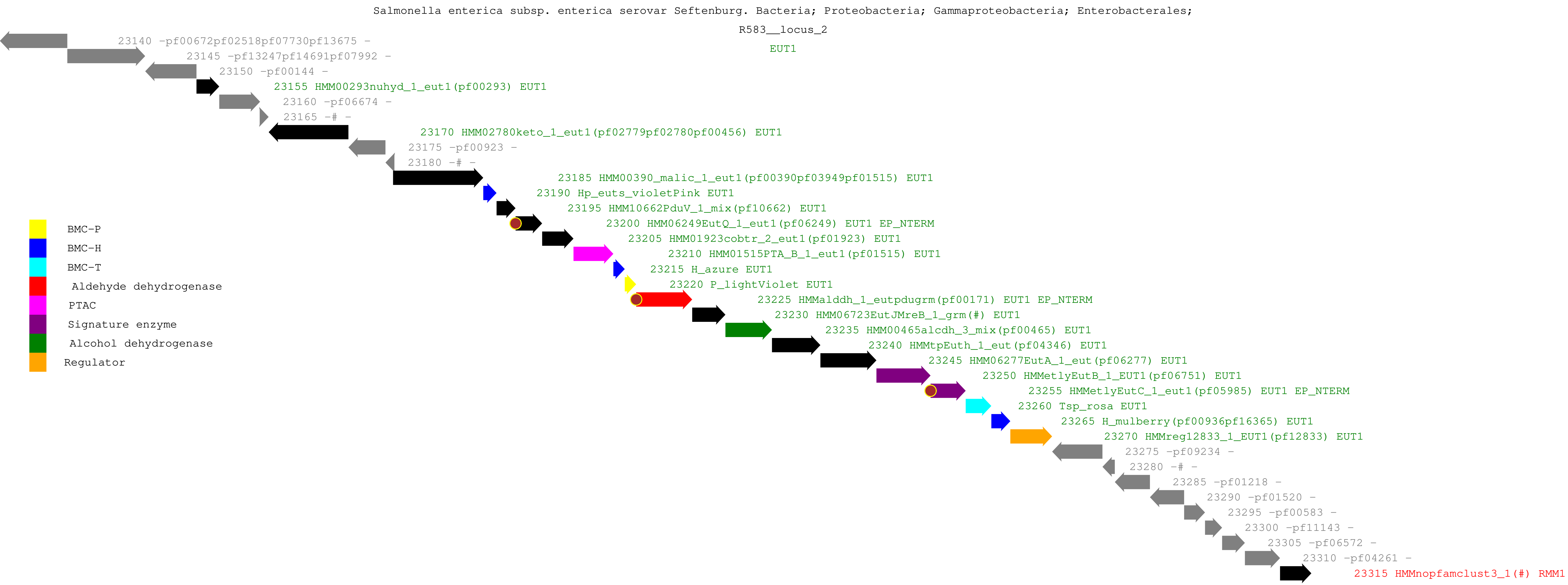

Salmonella enterica subsp. enterica serovar Seftenburg. Bacteria; Proteobacteria; Gammaproteobacteria; Enterobacterales;
R583__locus_2
EUT1
23140 -pf00672pf02518pf07730pf13675 -
23145 -pf13247pf14691pf07992 -
23150 -pf00144 -
23155 HMM00293nuhyd_1_eut1(pf00293) EUT1
23160 -pf06674 -
23165 -# -
23170 HMM02780keto_1_eut1(pf02779pf02780pf00456) EUT1
23175 -pf00923 -
23180 -# -
23185 HMM00390_malic_1_eut1(pf00390pf03949pf01515) EUT1
23190 Hp_euts_violetPink EUT1
23195 HMM10662PduV_1_mix(pf10662) EUT1
23200 HMM06249EutQ_1_eut1(pf06249) EUT1 EP_NTERM
23205 HMM01923cobtr_2_eut1(pf01923) EUT1
23210 HMM01515PTA_B_1_eut1(pf01515) EUT1
23215 H_azure EUT1
23220 P_lightViolet EUT1
23225 HMMalddh_1_eutpdugrm(pf00171) EUT1 EP_NTERM
23230 HMM06723EutJMreB_1_grm(#) EUT1
23235 HMM00465alcdh_3_mix(pf00465) EUT1
23240 HMMtpEuth_1_eut(pf04346) EUT1
23245 HMM06277EutA_1_eut(pf06277) EUT1
23250 HMMetlyEutB_1_EUT1(pf06751) EUT1
23255 HMMetlyEutC_1_eut1(pf05985) EUT1 EP_NTERM
23260 Tsp_rosa EUT1
23265 H_mulberry(pf00936pf16365) EUT1
23270 HMMreg12833_1_EUT1(pf12833) EUT1
23275 -pf09234 -
23280 -# -
23285 -pf01218 -
23290 -pf01520 -
23295 -pf00583 -
23300 -pf11143 -
23305 -pf06572 -
23310 -pf04261 -
23315 HMMnopfamclust3_1(#) RMM1
BMC-P
BMC-H
BMC-T
Aldehyde dehydrogenase
PTAC
Signature enzyme
Alcohol dehydrogenase
Regulator

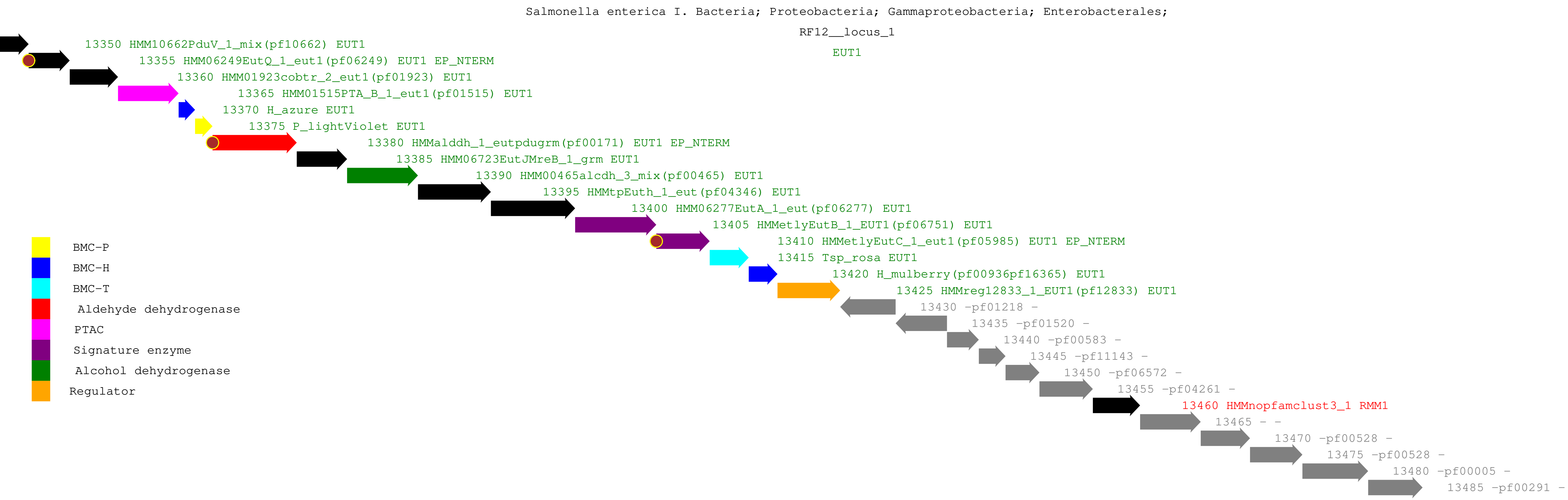

Salmonella enterica I. Bacteria; Proteobacteria; Gammaproteobacteria; Enterobacterales;
RF12__locus_1
EUT1
13350 HMM10662PduV_1_mix(pf10662) EUT1
13355 HMM06249EutQ_1_eut1(pf06249) EUT1 EP_NTERM
13360 HMM01923cobtr_2_eut1(pf01923) EUT1
13365 HMM01515PTA_B_1_eut1(pf01515) EUT1
13370 H_azure EUT1
13375 P_lightViolet EUT1
13380 HMMalddh_1_eutpdugrm(pf00171) EUT1 EP_NTERM
13385 HMM06723EutJMreB_1_grm EUT1
13390 HMM00465alcdh_3_mix(pf00465) EUT1
13395 HMMtpEuth_1_eut(pf04346) EUT1
13400 HMM06277EutA_1_eut(pf06277) EUT1
13405 HMMetlyEutB_1_EUT1(pf06751) EUT1
13410 HMMetlyEutC_1_eut1(pf05985) EUT1 EP_NTERM
13415 Tsp_rosa EUT1
13420 H_mulberry(pf00936pf16365) EUT1
13425 HMMreg12833_1_EUT1(pf12833) EUT1
13430 -pf01218 -
13435 -pf01520 -
13440 -pf00583 -
13445 -pf11143 -
13450 -pf06572 -
13455 -pf04261 -
13460 HMMnopfamclust3_1 RMM1
13465 - -
13470 -pf00528 -
13475 -pf00528 -
13480 -pf00005 -
13485 -pf00291 -
BMC-P
BMC-H
BMC-T
Aldehyde dehydrogenase
PTAC
Signature enzyme
Alcohol dehydrogenase
Regulator

Salmonella enterica I. Bacteria; Proteobacteria; Gammaproteobacteria; Enterobacterales;

RF16__locus_1

EUT1

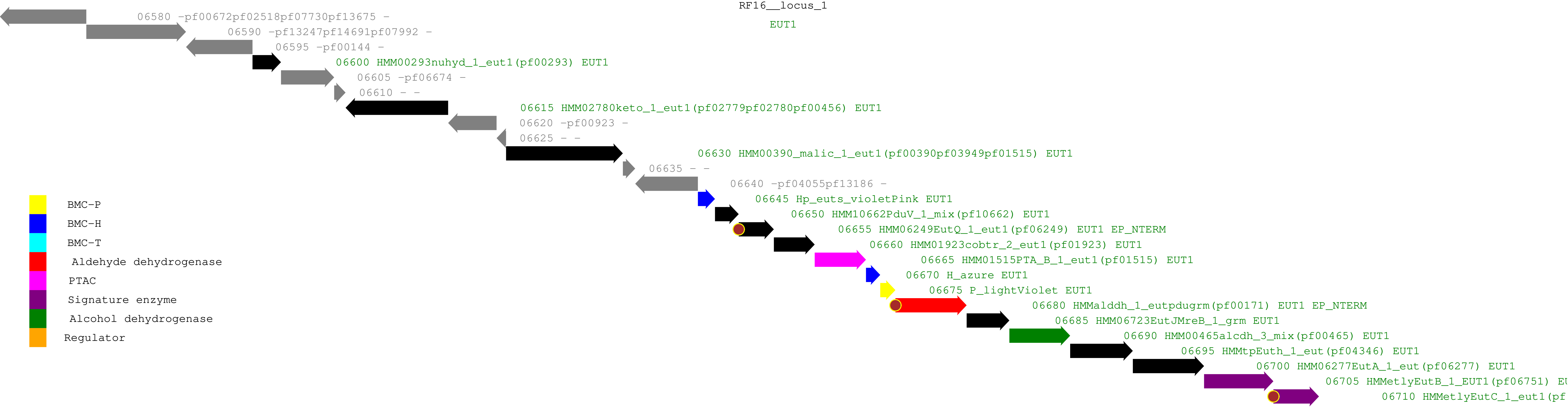

Photobacterium gaetbulicola. Bacteria; Proteobacteria; Gammaproteobacteria; Vibrionales;

RJ45__locus_2

EUT1

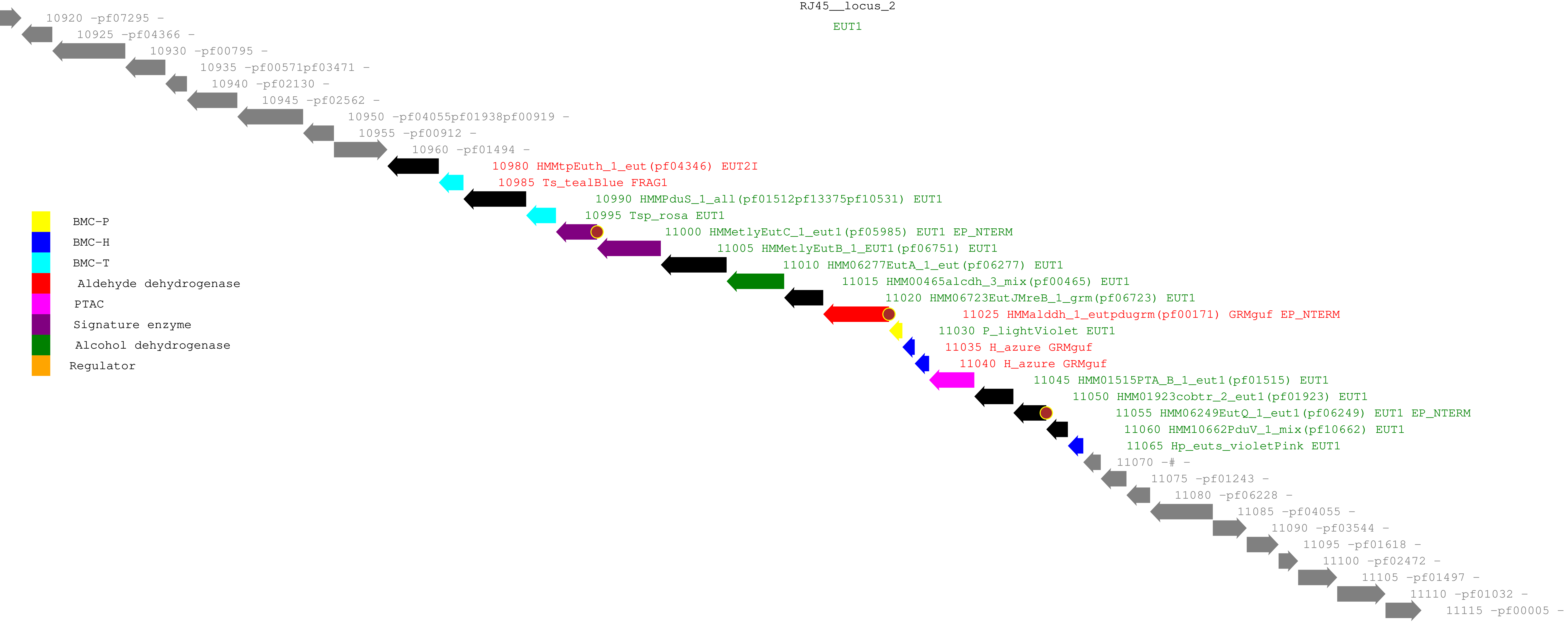

Salmonella enterica I. Bacteria; Proteobacteria; Gammaproteobacteria; Enterobacterales;

RK54__locus_1

EUT1

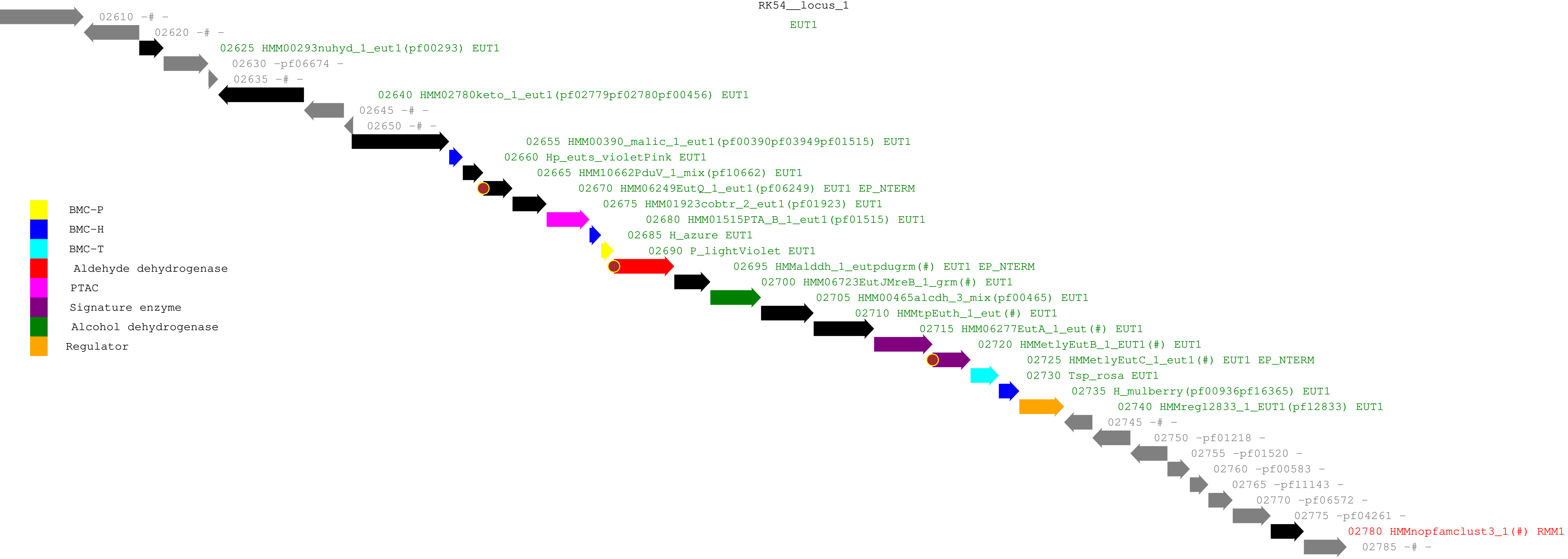

Salmonella houtenae. Bacteria; Proteobacteria; Gammaproteobacteria; Enterobacterales;

RK55__locus_1_main_sat

EUT1

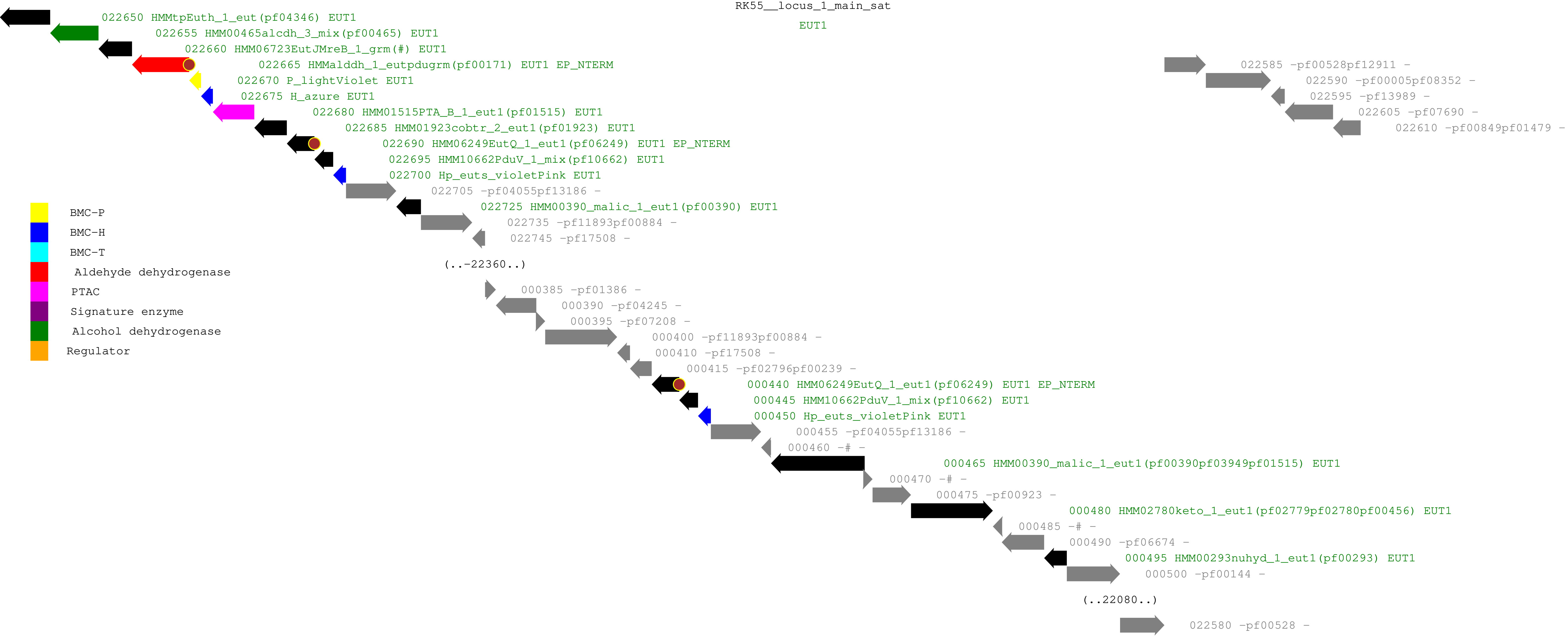

Shigella flexneri. Bacteria; Proteobacteria; Gammaproteobacteria; Enterobacterales;

RK59__locus_1

EUT1

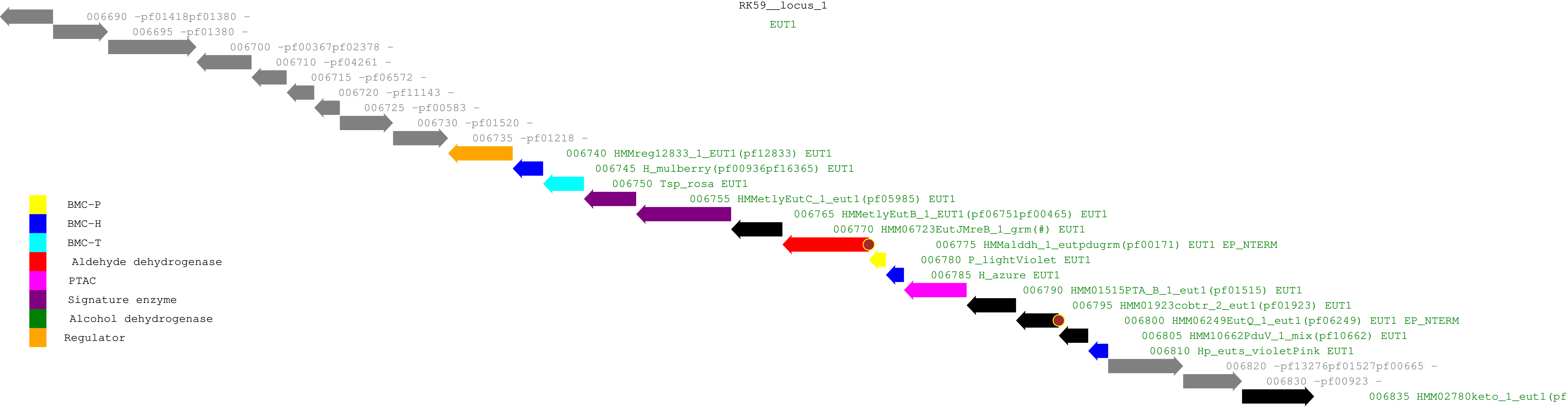

Salmonella enterica subsp. enterica serovar Paratyphi B. Bacteria; Proteobacteria; Gammaproteobacteria; Enterobacterales;

RL96__locus_1

EUT1

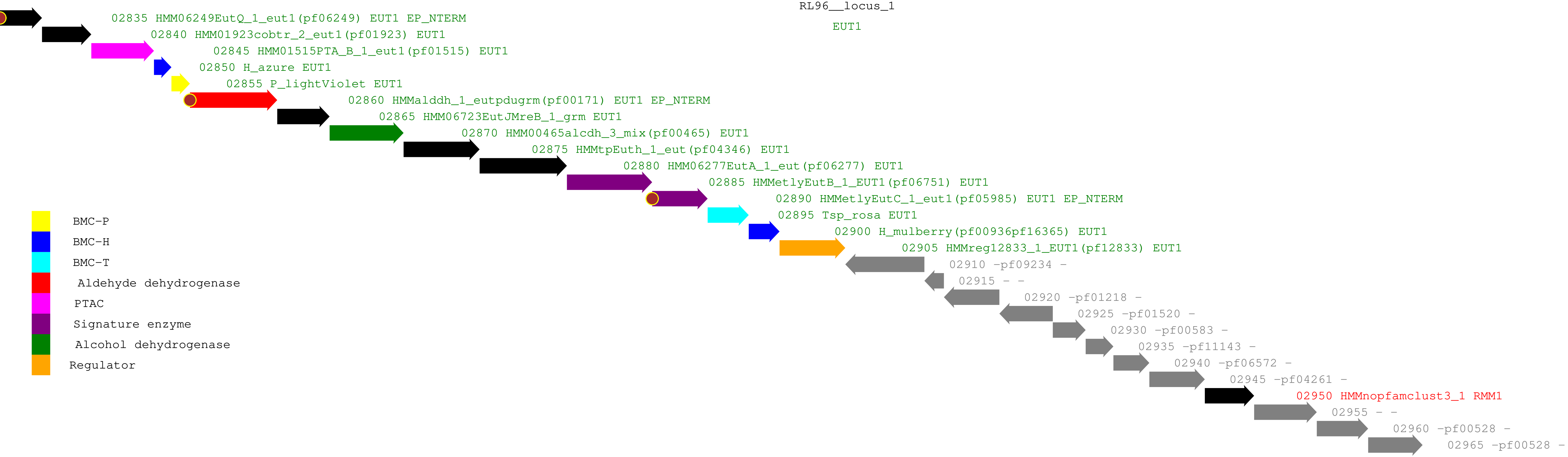

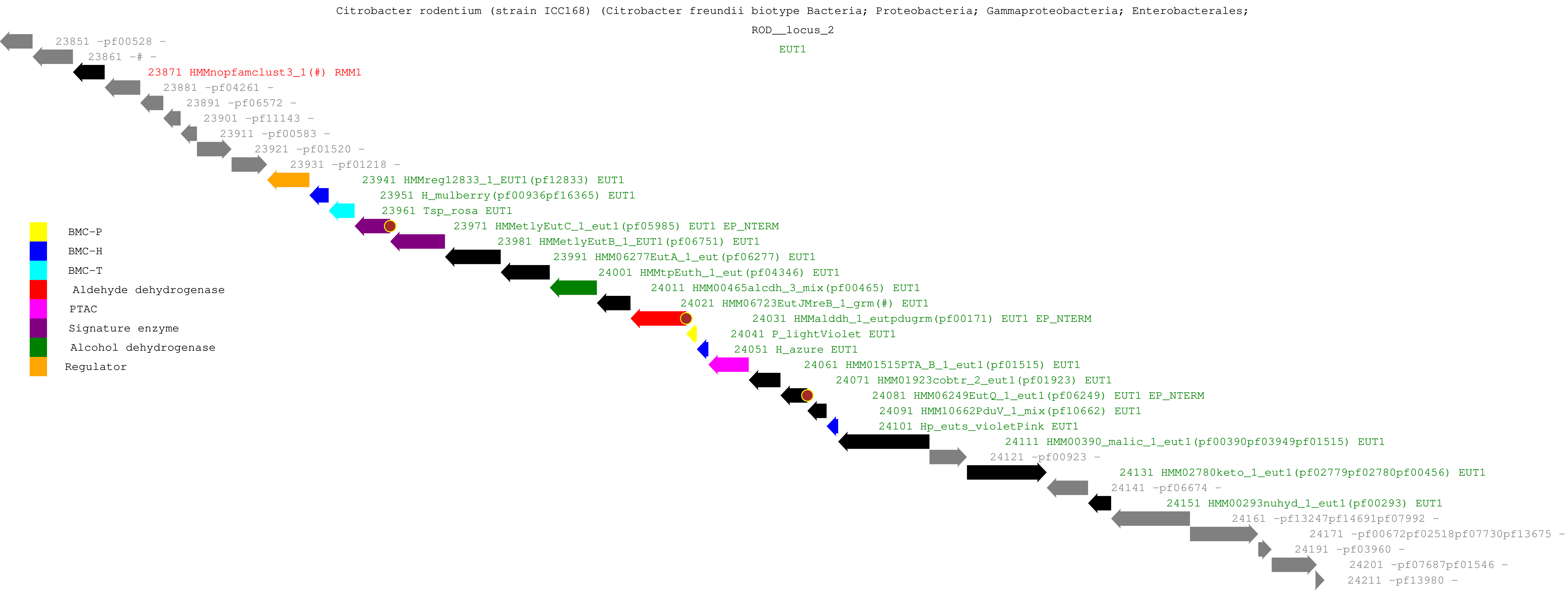
Citrobacter rodentium (strain ICC168) (Citrobacter freundii biotype Bacteria; Proteobacteria; Gammaproteobacteria; Enterobacterales;
ROD__locus_2
EUT1
23851 -pf00528 -
23861 -# -
23871 HMMnopfamclust3_1(#) RMM1
23881 -pf04261 -
23891 -pf06572 -
23901 -pf11143 -
23911 -pf00583 -
23921 -pf01520 -
23931 -pf01218 -
23941 HMMreg12833_1_EUT1(pf12833) EUT1
23951 H_mulberry(pf00936pf16365) EUT1
23961 Tsp_rosa EUT1
23971 HMMetlyEutC_1_eut1(pf05985) EUT1 EP_NTERM
23981 HMMetlyEutB_1_EUT1(pf06751) EUT1
23991 HMM06277EutA_1_eut(pf06277) EUT1
24001 HMMtpEuth_1_eut(pf04346) EUT1
24011 HMM00465alcdh_3_mix(pf00465) EUT1
24021 HMM06723EutJMreB_1_grm(#) EUT1
24031 HMMalddh_1_eutpdugrm(pf00171) EUT1 EP_NTERM
24041 P_lightViolet EUT1
24051 H_azure EUT1
24061 HMM01515PTA_B_1_eut1(pf01515) EUT1
24071 HMM01923cobtr_2_eut1(pf01923) EUT1
24081 HMM06249EutQ_1_eut1(pf06249) EUT1 EP_NTERM
24091 HMM10662PduV_1_mix(pf10662) EUT1
24101 Hp_euts_violetPink EUT1
24111 HMM00390_malic_1_eut1(pf00390pf03949pf01515) EUT1
24121 -pf00923 -
24131 HMM02780keto_1_eut1(pf02779pf02780pf00456) EUT1
24141 -pf06674 -
24151 HMM00293nuhyd_1_eut1(pf00293) EUT1
24161 -pf13247pf14691pf07992 -
24171 -pf00672pf02518pf07730pf13675 -
24191 -pf03960 -
24201 -pf07687pf01546 -
24211 -pf13980 -
BMC-P
BMC-H
BMC-T
Aldehyde dehydrogenase
PTAC
Signature enzyme
Alcohol dehydrogenase
Regulator

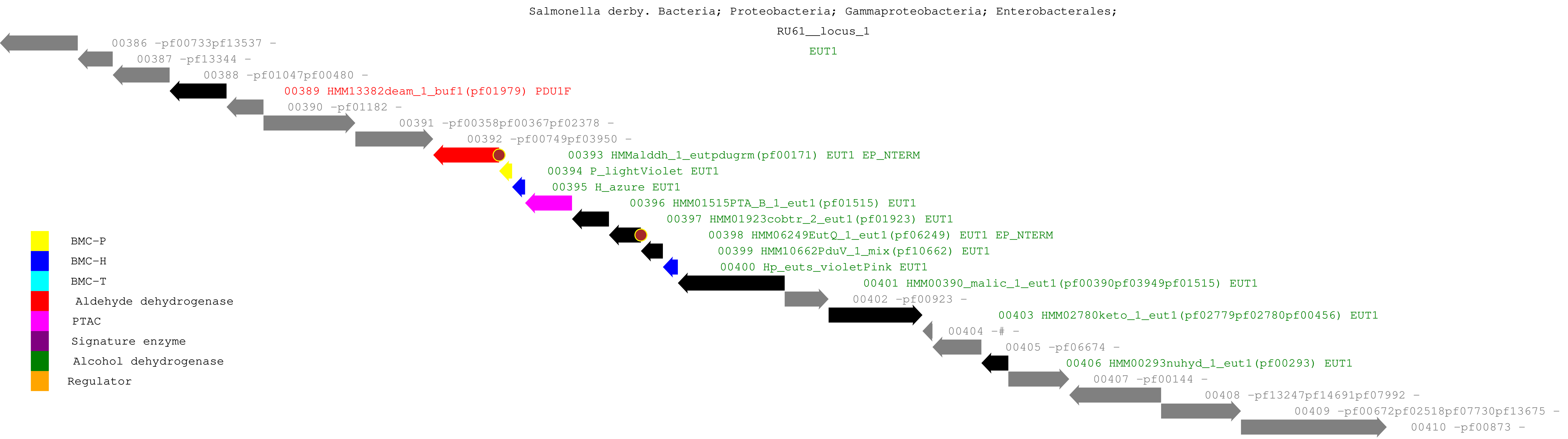

Salmonella derby. Bacteria; Proteobacteria; Gammaproteobacteria; Enterobacterales;
RU61__locus_1
EUT1
00386 -pf00733pf13537 -
00387 -pf13344 -
00388 -pf01047pf00480 -
00389 HMM13382deam_1_buf1(pf01979) PDU1F
00390 -pf01182 -
00391 -pf00358pf00367pf02378 -
00392 -pf00749pf03950 -
00393 HMMalddh_1_eutpdugrm(pf00171) EUT1 EP_NTERM
00394 P_lightViolet EUT1
00395 H_azure EUT1
00396 HMM01515PTA_B_1_eut1(pf01515) EUT1
00397 HMM01923cobtr_2_eut1(pf01923) EUT1
00398 HMM06249EutQ_1_eut1(pf06249) EUT1 EP_NTERM
00399 HMM10662PduV_1_mix(pf10662) EUT1
00400 Hp_euts_violetPink EUT1
00401 HMM00390_malic_1_eut1(pf00390pf03949pf01515) EUT1
00402 -pf00923 -
00403 HMM02780keto_1_eut1(pf02779pf02780pf00456) EUT1
00404 -# -
00405 -pf06674 -
00406 HMM00293nuhyd_1_eut1(pf00293) EUT1
00407 -pf00144 -
00408 -pf13247pf14691pf07992 -
00409 -pf00672pf02518pf07730pf13675 -
00410 -pf00873 -
BMC-P
BMC-H
BMC-T
Aldehyde dehydrogenase
PTAC
Signature enzyme
Alcohol dehydrogenase
Regulator

Salmonella enterica I. Bacteria; Proteobacteria; Gammaproteobacteria; Enterobacterales;

S639__locus_1

EUT1

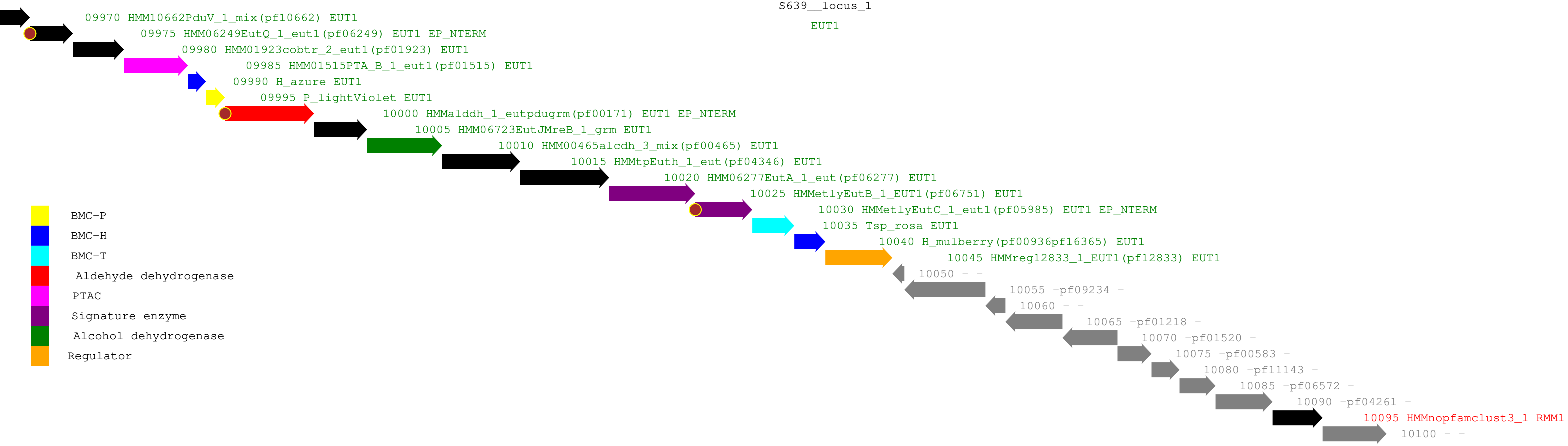

Klebsiella pneumoniae. Bacteria; Proteobacteria; Gammaproteobacteria; Enterobacterales;

SAMEA104567903__locus_1

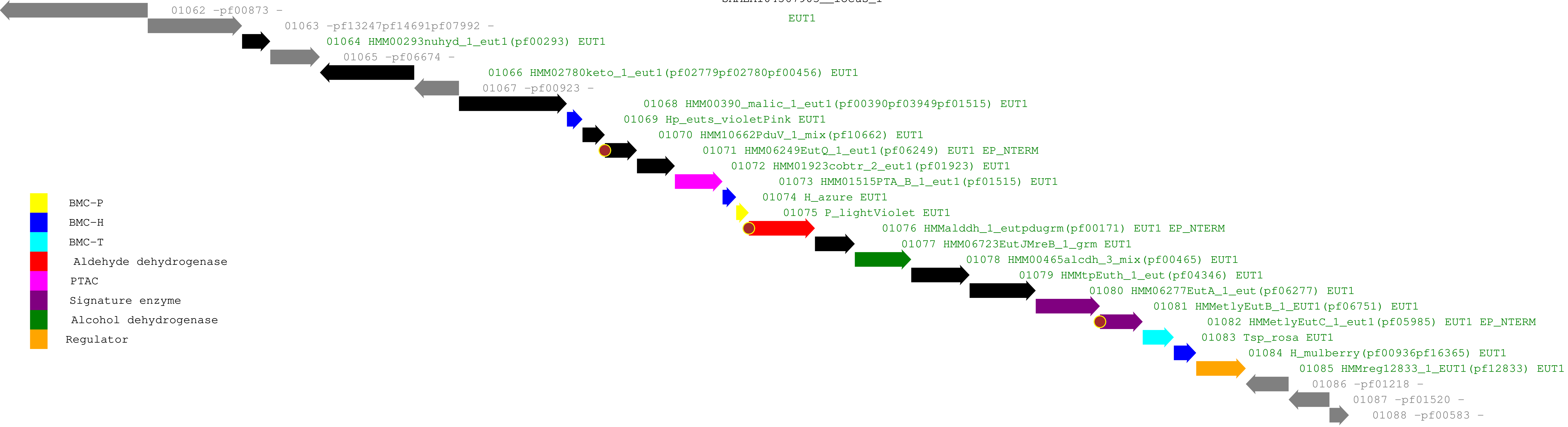

Klebsiella pneumoniae. Bacteria; Proteobacteria; Gammaproteobacteria; Enterobacterales;

SAMEA1713115__locus_1

EUT1

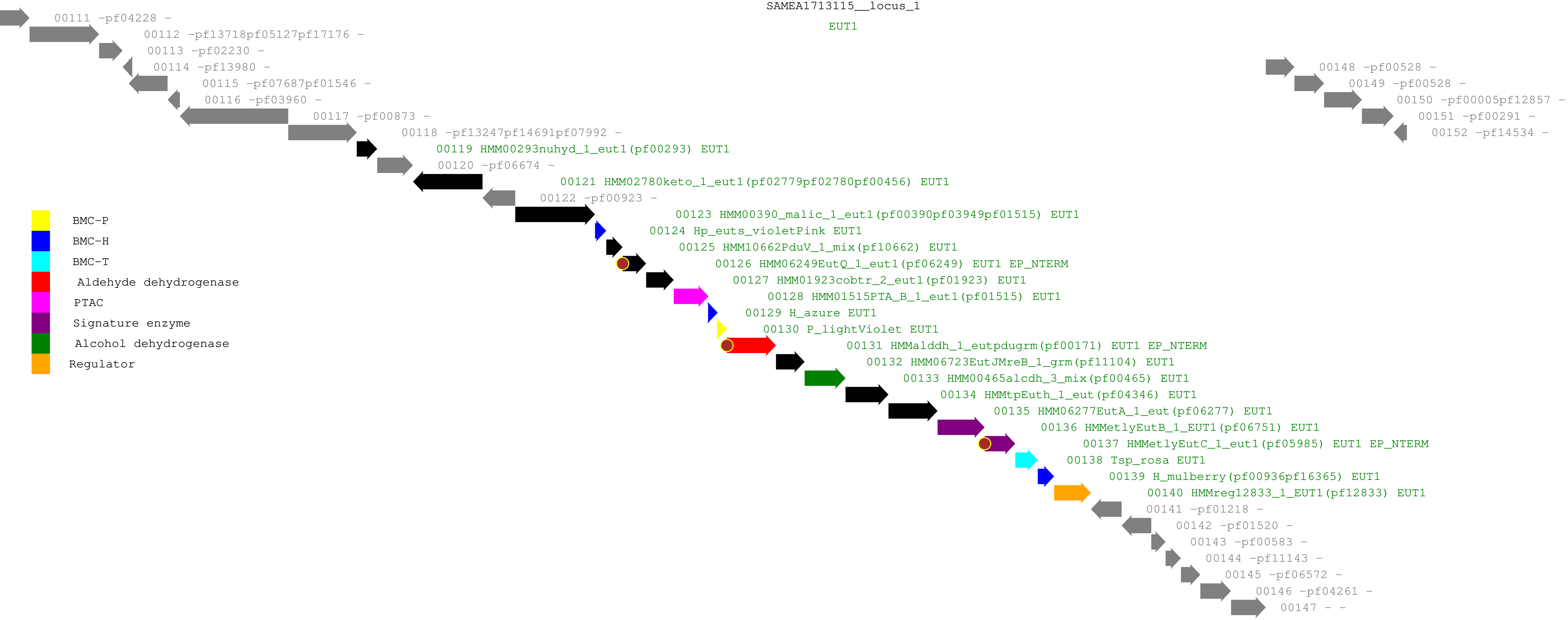

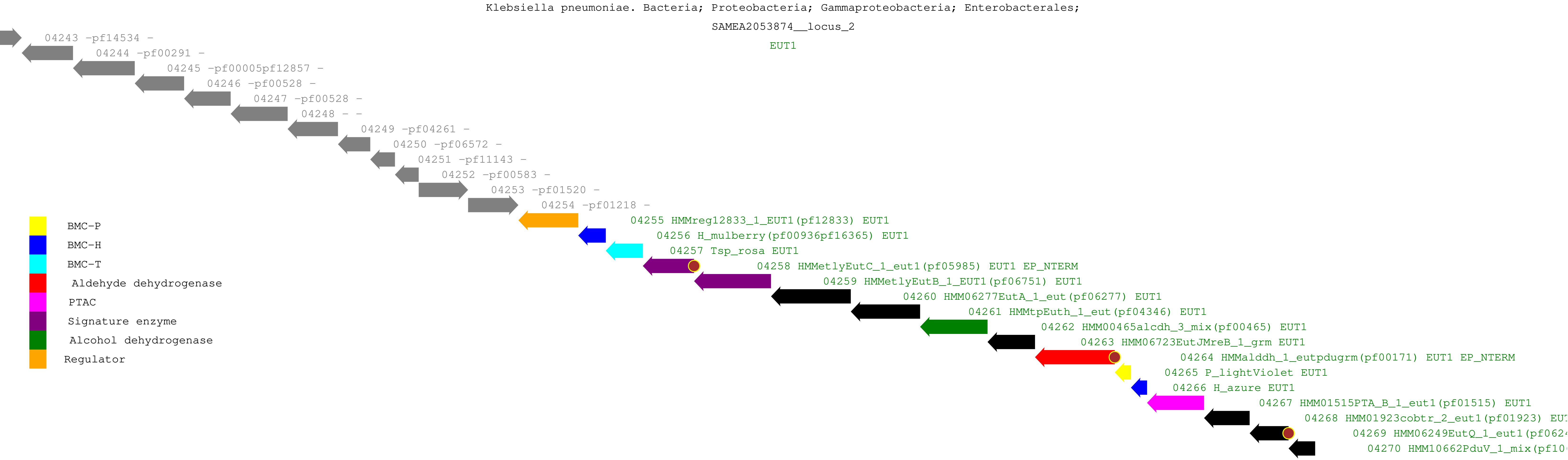
Klebsiella pneumoniae. Bacteria; Proteobacteria; Gammaproteobacteria; Enterobacterales;
SAMEA2053874__locus_2
EUT1
04243 -pf14534 -
04244 -pf00291 -
04245 -pf00005pf12857 -
04246 -pf00528 -
04247 -pf00528 -
04248 - -
04249 -pf04261 -
04250 -pf06572 -
04251 -pf11143 -
04252 -pf00583 -
04253 -pf01520 -
04254 -pf01218 -
04255 HMMreg12833_1_EUT1(pf12833) EUT1
04256 H_mulberry(pf00936pf16365) EUT1
04257 Tsp_rosa EUT1
04258 HMMetlyEutC_1_eut1(pf05985) EUT1 EP_NTERM
04259 HMMetlyEutB_1_EUT1(pf06751) EUT1
04260 HMM06277EutA_1_eut(pf06277) EUT1
04261 HMMtpEuth_1_eut(pf04346) EUT1
04262 HMM00465alcdh_3_mix(pf00465) EUT1
04263 HMM06723EutJMreB_1_grm EUT1
04264 HMMalddh_1_eutpdugrm(pf00171) EUT1 EP_NTERM
04265 P_lightViolet EUT1
04266 H_azure EUT1
04267 HMM01515PTA_B_1_eut1(pf01515) EUT1
04268 HMM01923cobtr_2_eut1(pf01923) EU
04269 HMM06249EutQ_1_eut1(pf062
04270 HMM10662PduV_1_mix(pf10
BMC-P
BMC-H
BMC-T
Aldehyde dehydrogenase
PTAC
Signature enzyme
Alcohol dehydrogenase
Regulator

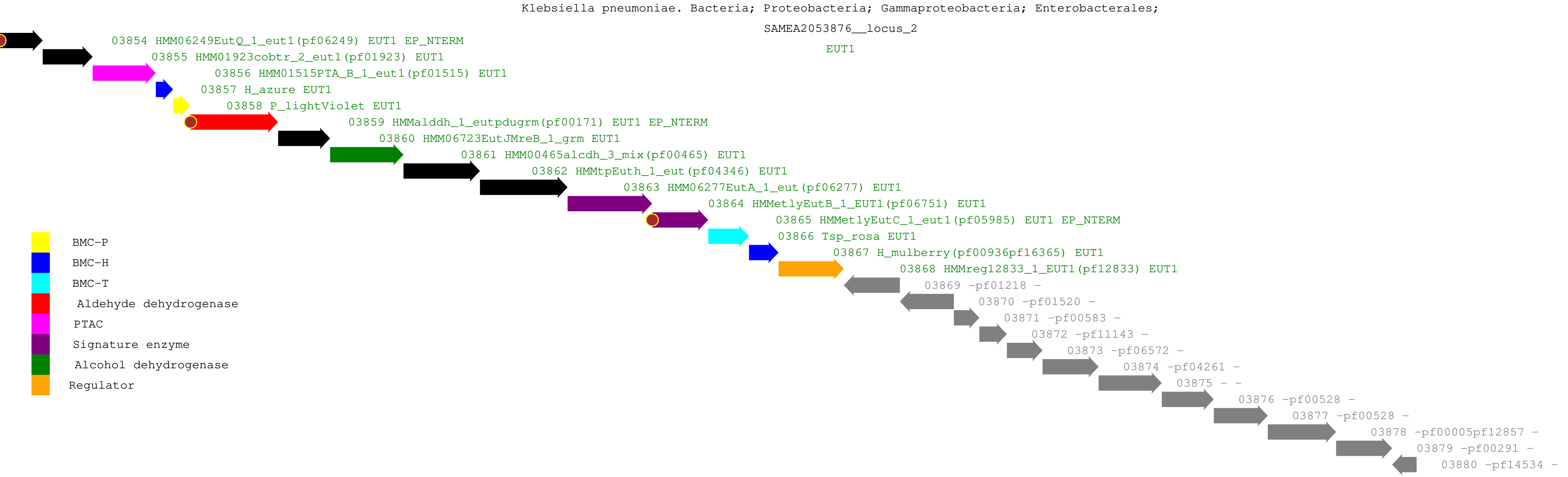
Klebsiella pneumoniae. Bacteria; Proteobacteria; Gammaproteobacteria; Enterobacterales;
SAMEA2053876__locus_2
EUT1
03854 HMM06249EutQ_1_eut1(pf06249) EUT1 EP_NTERM
03855 HMM01923cobtr_2_eut1(pf01923) EUT1
03856 HMM01515PTA_B_1_eut1(pf01515) EUT1
03857 H_azure EUT1
03858 P_lightViolet EUT1
03859 HMMalddh_1_eutpdugrm(pf00171) EUT1 EP_NTERM
03860 HMM06723EutJMreB_1_grm EUT1
03861 HMM00465alcdh_3_mix(pf00465) EUT1
03862 HMMtpEuth_1_eut(pf04346) EUT1
03863 HMM06277EutA_1_eut(pf06277) EUT1
03864 HMMetlyEutB_1_EUT1(pf06751) EUT1
03865 HMMetlyEutC_1_eut1(pf05985) EUT1 EP_NTERM
03866 Tsp_rosa EUT1
03867 H_mulberry(pf00936pf16365) EUT1
03868 HMMreg12833_1_EUT1(pf12833) EUT1
03869 -pf01218 -
03870 -pf01520 -
03871 -pf00583 -
03872 -pf11143 -
03873 -pf06572 -
03874 -pf04261 -
03875 - -
03876 -pf00528 -
03877 -pf00528 -
03878 -pf00005pf12857 -
03879 -pf00291 -
03880 -pf14534 -
BMC-P
BMC-H
BMC-T
Aldehyde dehydrogenase
PTAC
Signature enzyme
Alcohol dehydrogenase
Regulator

Klebsiella pneumoniae. Bacteria; Proteobacteria; Gammaproteobacteria; Enterobacterales;

SAMEA2053943__locus_2

EUT1

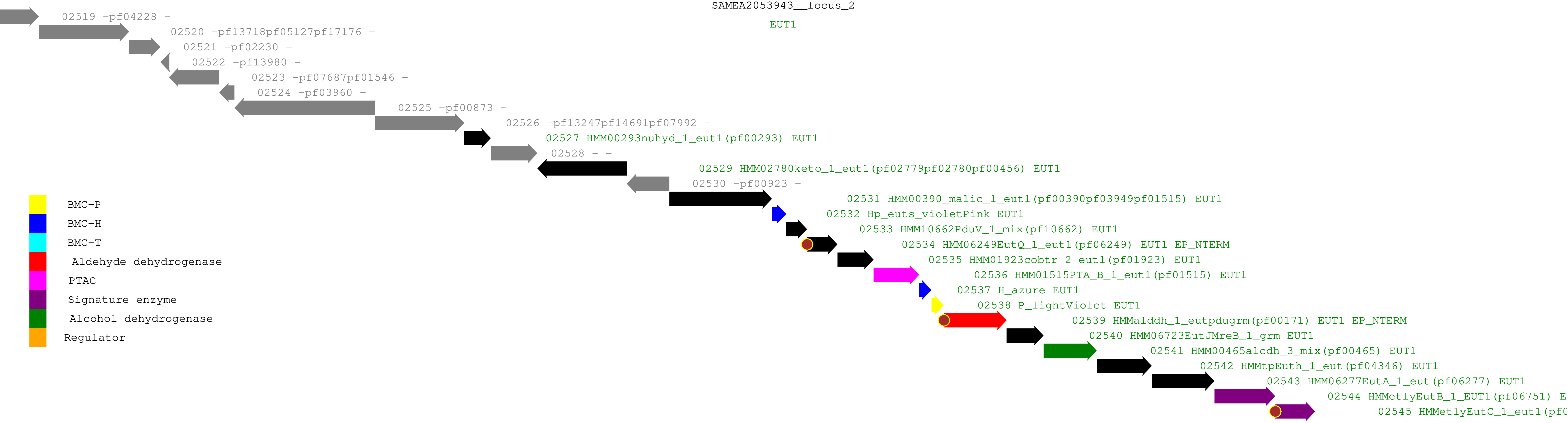

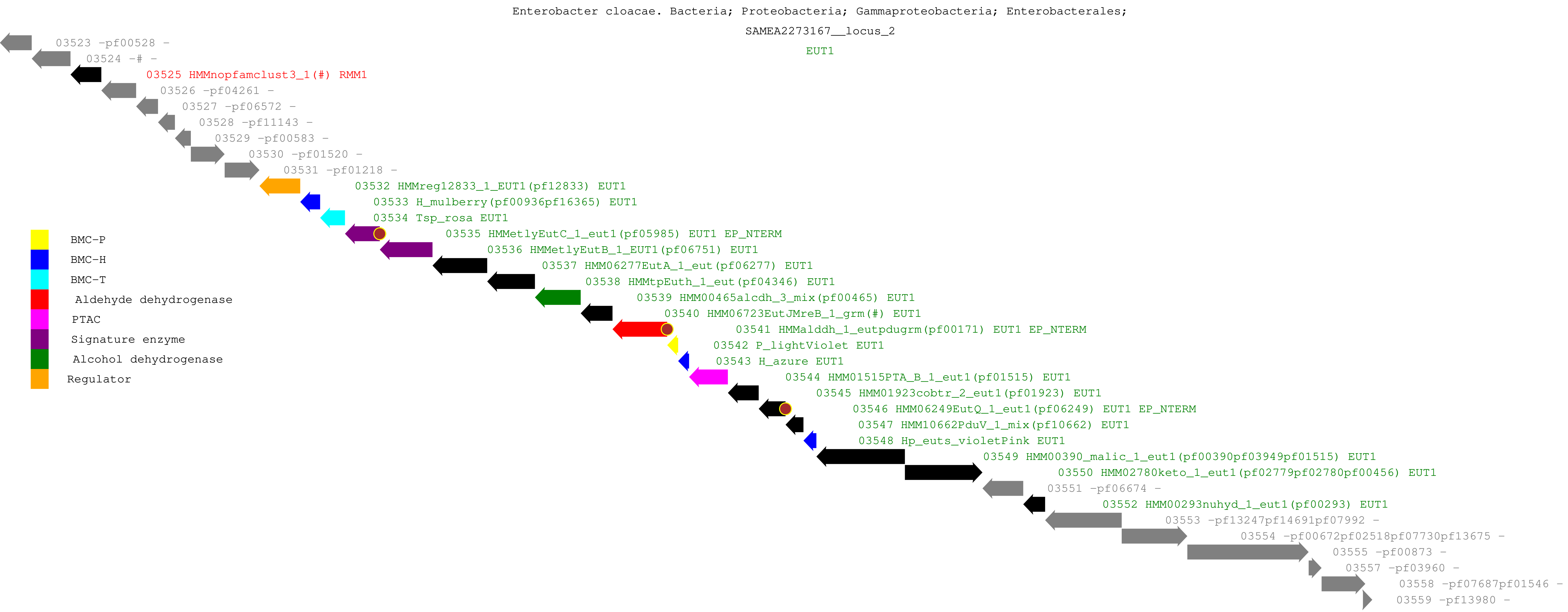

Enterobacter cloacae. Bacteria; Proteobacteria; Gammaproteobacteria; Enterobacterales;
SAMEA2273167__locus_2
EUT1
03523 -pf00528 -
03524 -# -
03525 HMMnopfamclust3_1(#) RMM1
03526 -pf04261 -
03527 -pf06572 -
03528 -pf11143 -
03529 -pf00583 -
03530 -pf01520 -
03531 -pf01218 -
03532 HMMreg12833_1_EUT1(pf12833) EUT1
03533 H_mulberry(pf00936pf16365) EUT1
03534 Tsp_rosa EUT1
03535 HMMetlyEutC_1_eut1(pf05985) EUT1 EP_NTERM
03536 HMMetlyEutB_1_EUT1(pf06751) EUT1
03537 HMM06277EutA_1_eut(pf06277) EUT1
03538 HMMtpEuth_1_eut(pf04346) EUT1
03539 HMM00465alcdh_3_mix(pf00465) EUT1
03540 HMM06723EutJMreB_1_grm(#) EUT1
03541 HMMalddh_1_eutpdugrm(pf00171) EUT1 EP_NTERM
03542 P_lightViolet EUT1
03543 H_azure EUT1
03544 HMM01515PTA_B_1_eut1(pf01515) EUT1
03545 HMM01923cobtr_2_eut1(pf01923) EUT1
03546 HMM06249EutQ_1_eut1(pf06249) EUT1 EP_NTERM
03547 HMM10662PduV_1_mix(pf10662) EUT1
03548 Hp_euts_violetPink EUT1
03549 HMM00390_malic_1_eut1(pf00390pf03949pf01515) EUT1
03550 HMM02780keto_1_eut1(pf02779pf02780pf00456) EUT1
03551 -pf06674 -
03552 HMM00293nuhyd_1_eut1(pf00293) EUT1
03553 -pf13247pf14691pf07992 -
03554 -pf00672pf02518pf07730pf13675 -
03555 -pf00873 -
03557 -pf03960 -
03558 -pf07687pf01546 -
03559 -pf13980 -
BMC-P
BMC-H
BMC-T
Aldehyde dehydrogenase
PTAC
Signature enzyme
Alcohol dehydrogenase
Regulator

Klebsiella oxytoca. Bacteria; Proteobacteria; Gammaproteobacteria; Enterobacterales;

SAMEA2273575__locus_1

EUT1

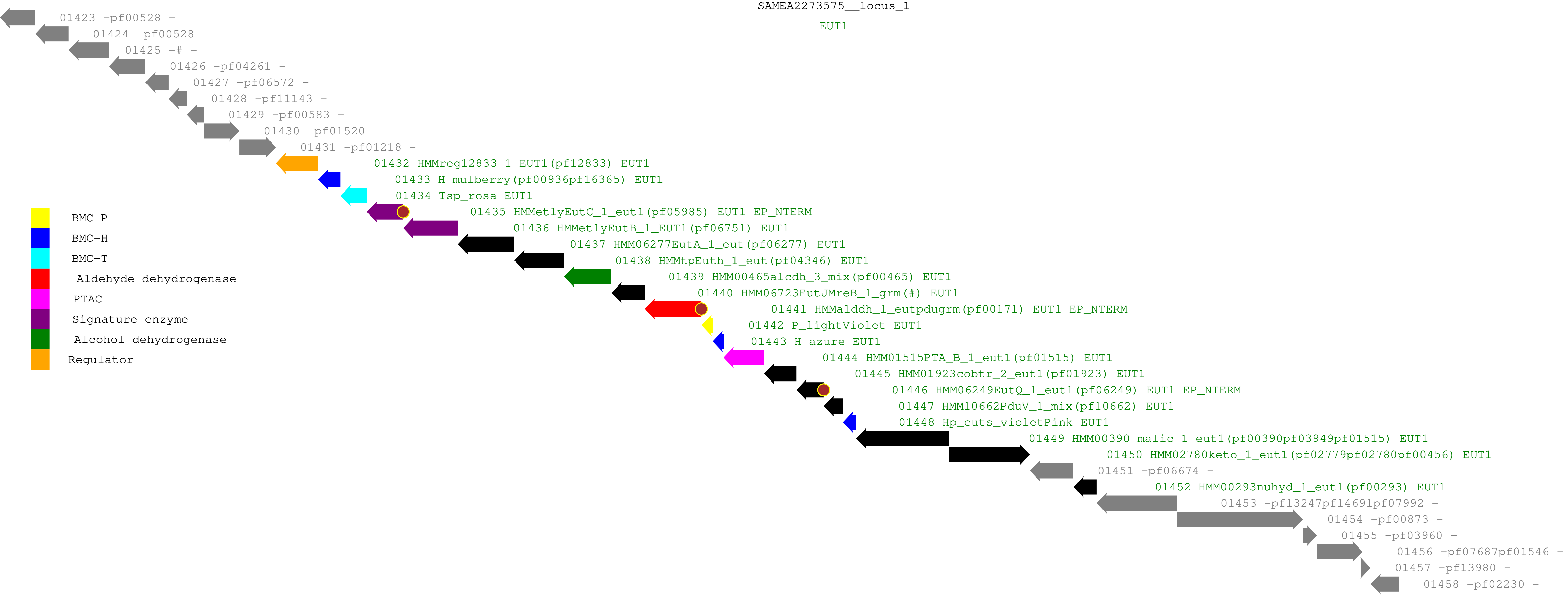

Klebsiella oxytoca. Bacteria; Proteobacteria; Gammaproteobacteria; Enterobacterales;

SAMEA2273639__locus_1

EUT1

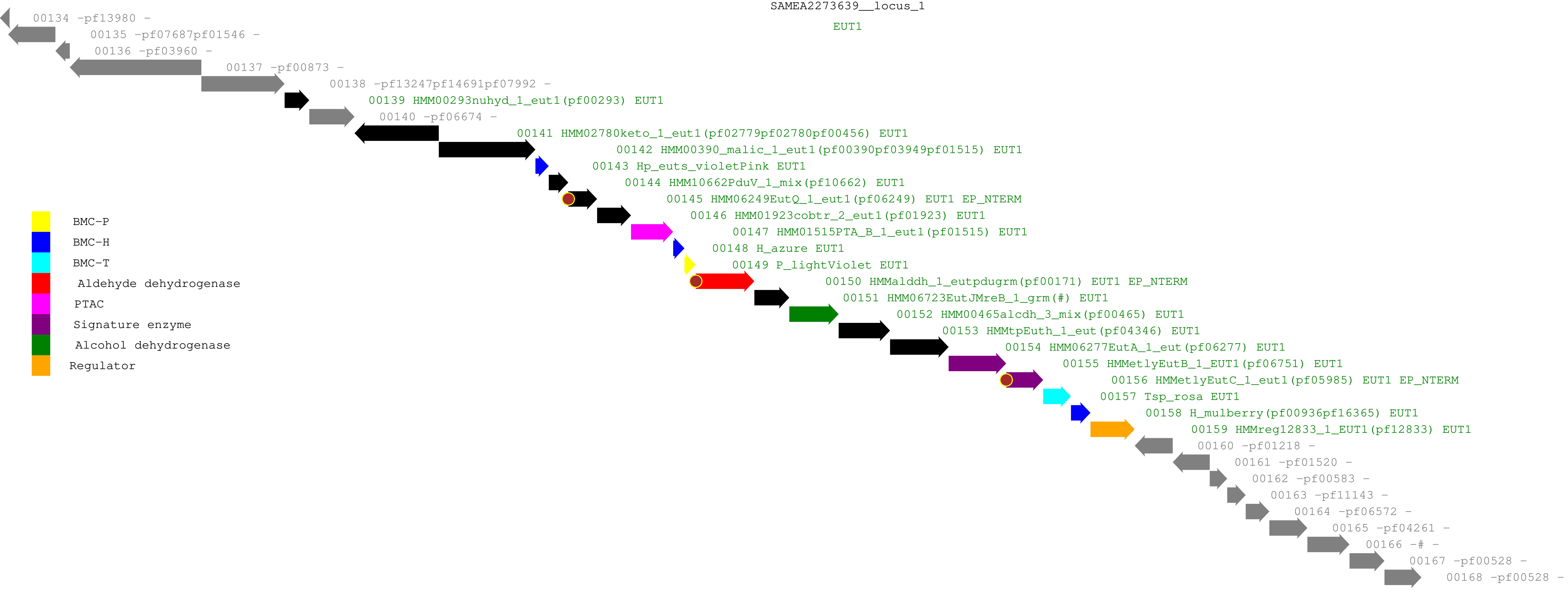

Klebsiella oxytoca. Bacteria; Proteobacteria; Gammaproteobacteria; Enterobacterales;

SAMEA2273697__locus_1

EUT1

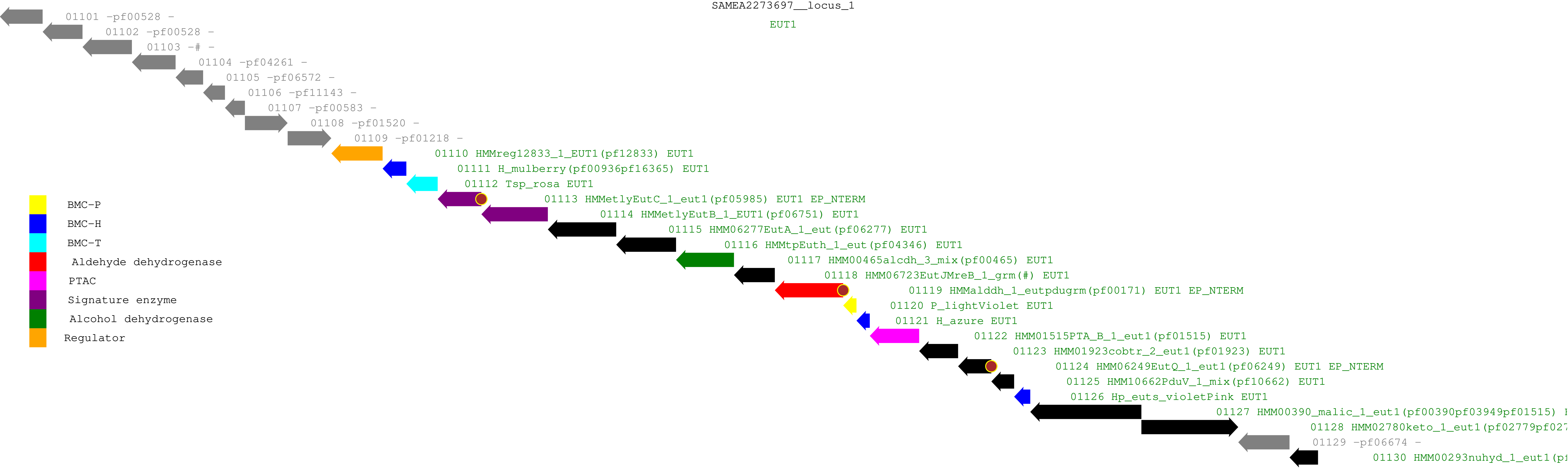

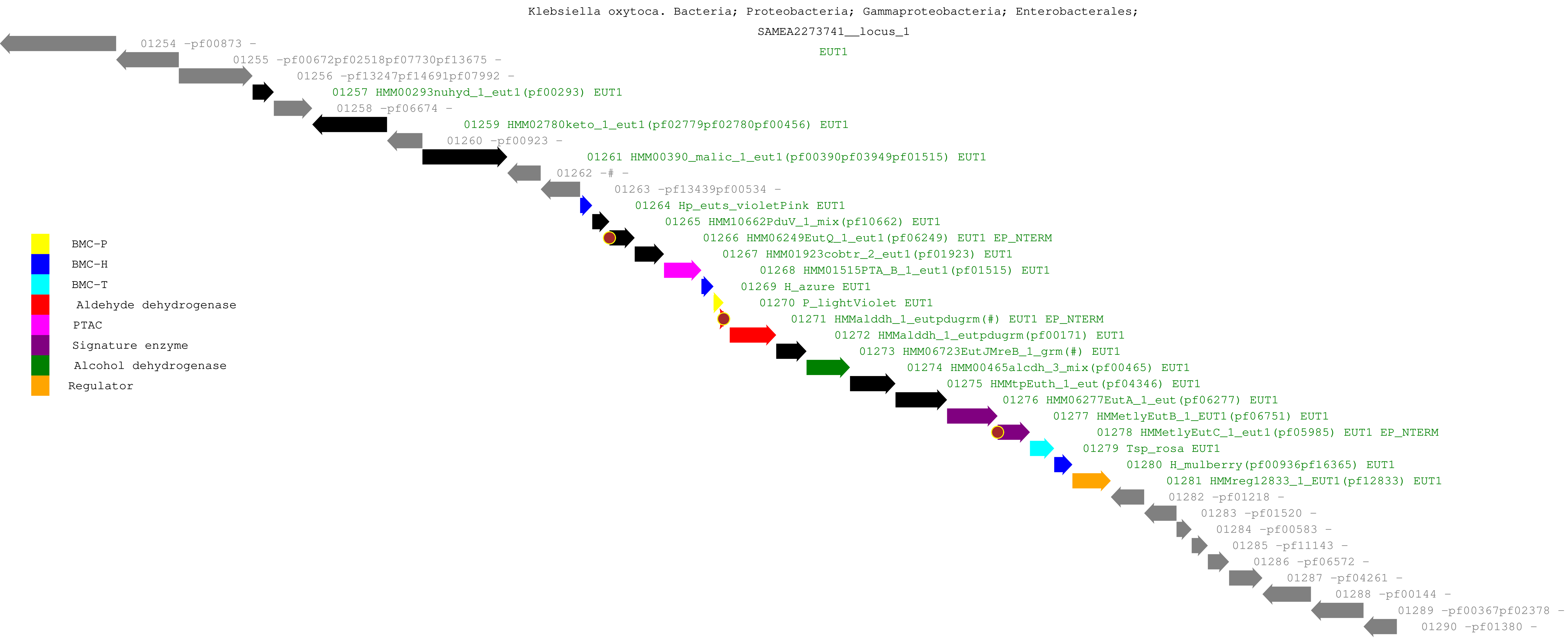
Klebsiella oxytoca. Bacteria; Proteobacteria; Gammaproteobacteria; Enterobacterales;
SAMEA2273741__locus_1
EUT1
01254 -pf00873 -
01255 -pf00672pf02518pf07730pf13675 -
01256 -pf13247pf14691pf07992 -
01257 HMM00293nuhyd_1_eut1(pf00293) EUT1
01258 -pf06674 -
01259 HMM02780keto_1_eut1(pf02779pf02780pf00456) EUT1
01260 -pf00923 -
01261 HMM00390_malic_1_eut1(pf00390pf03949pf01515) EUT1
01262 -# -
01263 -pf13439pf00534 -
01264 Hp_euts_violetPink EUT1
01265 HMM10662PduV_1_mix(pf10662) EUT1
01266 HMM06249EutQ_1_eut1(pf06249) EUT1 EP_NTERM
01267 HMM01923cobtr_2_eut1(pf01923) EUT1
01268 HMM01515PTA_B_1_eut1(pf01515) EUT1
01269 H_azure EUT1
01270 P_lightViolet EUT1
01271 HMMalddh_1_eutpdugrm(#) EUT1 EP_NTERM
01272 HMMalddh_1_eutpdugrm(pf00171) EUT1
01273 HMM06723EutJMreB_1_grm(#) EUT1
01274 HMM00465alcdh_3_mix(pf00465) EUT1
01275 HMMtpEuth_1_eut(pf04346) EUT1
01276 HMM06277EutA_1_eut(pf06277) EUT1
01277 HMMetlyEutB_1_EUT1(pf06751) EUT1
01278 HMMetlyEutC_1_eut1(pf05985) EUT1 EP_NTERM
01279 Tsp_rosa EUT1
01280 H_mulberry(pf00936pf16365) EUT1
01281 HMMreg12833_1_EUT1(pf12833) EUT1
01282 -pf01218 -
01283 -pf01520 -
01284 -pf00583 -
01285 -pf11143 -
01286 -pf06572 -
01287 -pf04261 -
01288 -pf00144 -
01289 -pf00367pf02378 -
01290 -pf01380 -
BMC-P
BMC-H
BMC-T
Aldehyde dehydrogenase
PTAC
Signature enzyme
Alcohol dehydrogenase
Regulator

Klebsiella oxytoca. Bacteria; Proteobacteria; Gammaproteobacteria; Enterobacterales;

SAMEA2273883__locus_1

EUT1

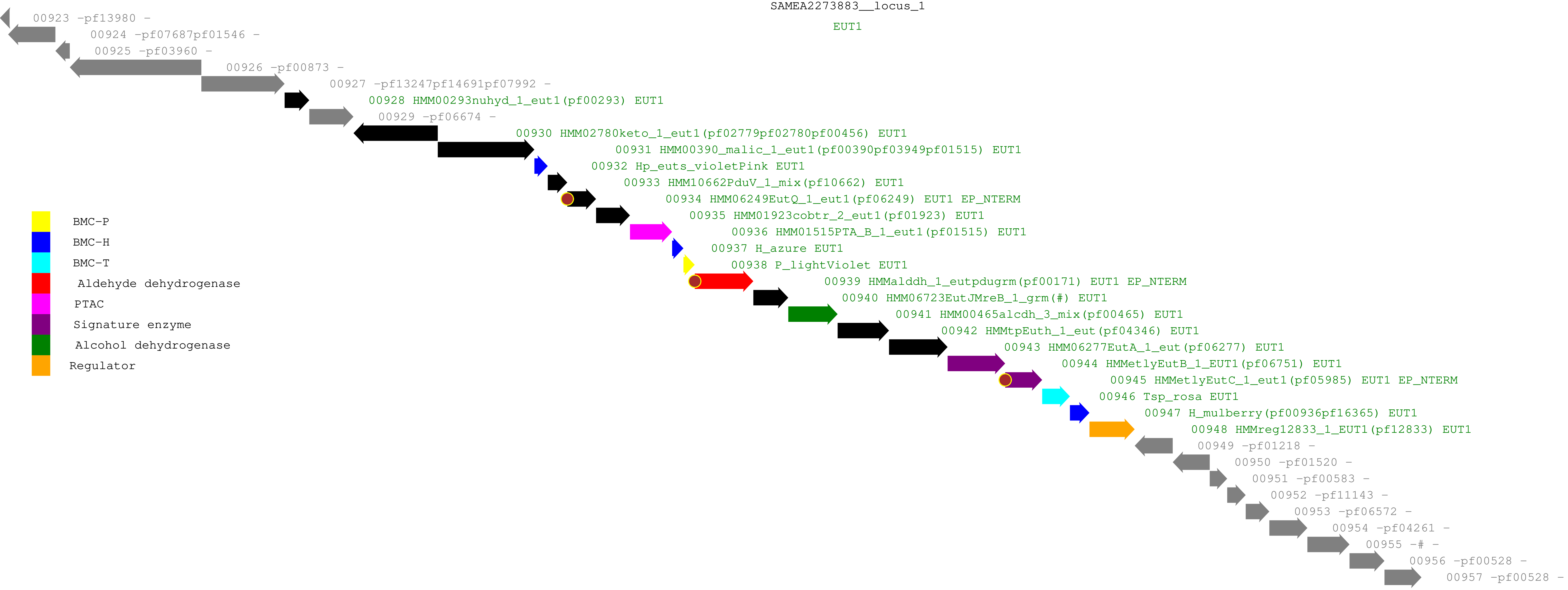

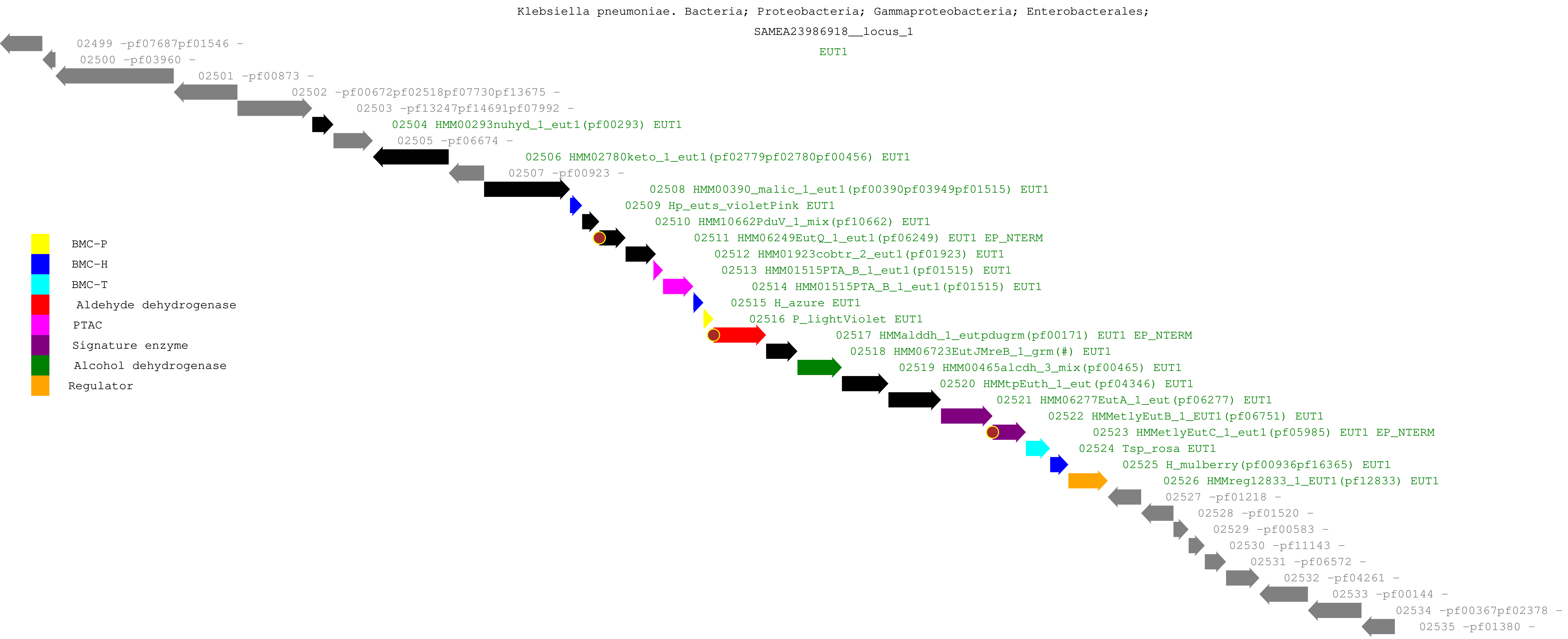
Klebsiella pneumoniae. Bacteria; Proteobacteria; Gammaproteobacteria; Enterobacterales;
SAMEA23986918__locus_1
EUT1
02499 -pf07687pf01546 -
02500 -pf03960 -
02501 -pf00873 -
02502 -pf00672pf02518pf07730pf13675 -
02503 -pf13247pf14691pf07992 -
02504 HMM00293nuhyd_1_eut1(pf00293) EUT1
02505 -pf06674 -
02506 HMM02780keto_1_eut1(pf02779pf02780pf00456) EUT1
02507 -pf00923 -
02508 HMM00390_malic_1_eut1(pf00390pf03949pf01515) EUT1
02509 Hp_euts_violetPink EUT1
02510 HMM10662PduV_1_mix(pf10662) EUT1
02511 HMM06249EutQ_1_eut1(pf06249) EUT1 EP_NTERM
02512 HMM01923cobtr_2_eut1(pf01923) EUT1
02513 HMM01515PTA_B_1_eut1(pf01515) EUT1
02514 HMM01515PTA_B_1_eut1(pf01515) EUT1
02515 H_azure EUT1
02516 P_lightViolet EUT1
02517 HMMaldдh_1_eutpdugrm(pf00171) EUT1 EP_NTERM
02518 HMM06723EutJMreB_1_grm(#) EUT1
02519 HMM00465alcdh_3_mix(pf00465) EUT1
02520 HMMtpEuth_1_eut(pf04346) EUT1
02521 HMM06277EutA_1_eut(pf06277) EUT1
02522 HMMetlyEutB_1_EUT1(pf06751) EUT1
02523 HMMetlyEutC_1_eut1(pf05985) EUT1 EP_NTERM
02524 Tsp_rosa EUT1
02525 H_mulberry(pf00936pf16365) EUT1
02526 HMMreg12833_1_EUT1(pf12833) EUT1
02527 -pf01218 -
02528 -pf01520 -
02529 -pf00583 -
02530 -pf11143 -
02531 -pf06572 -
02532 -pf04261 -
02533 -pf00144 -
02534 -pf00367pf02378 -
02535 -pf01380 -
BMC-P
BMC-H
BMC-T
Aldehyde dehydrogenase
PTAC
Signature enzyme
Alcohol dehydrogenase
Regulator

Escherichia coli. Bacteria; Proteobacteria; Gammaproteobacteria; Enterobacterales;

SAMEA3472047__locus_1

EUT1

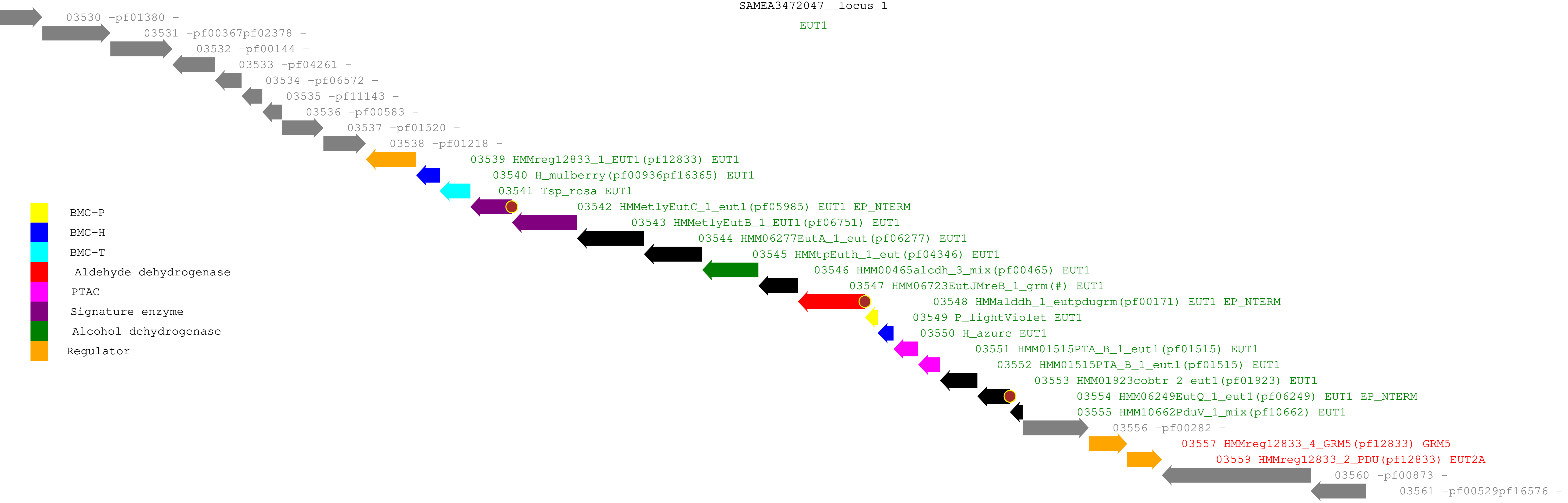

Escherichia coli. Bacteria; Proteobacteria; Gammaproteobacteria; Enterobacterales;

SAMEA3472115__locus_1

EUT1

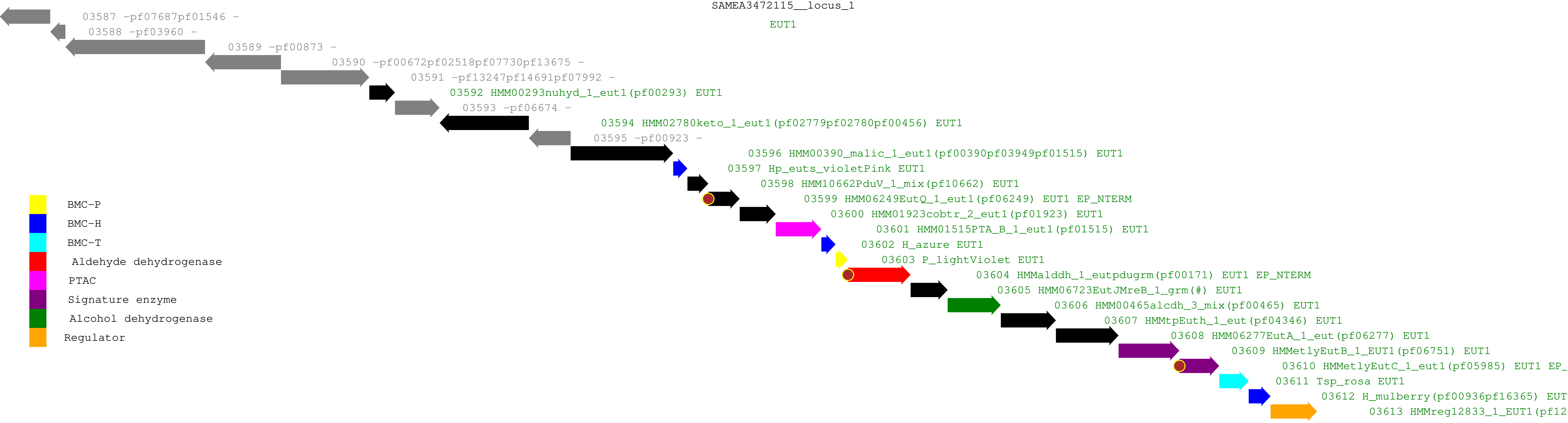

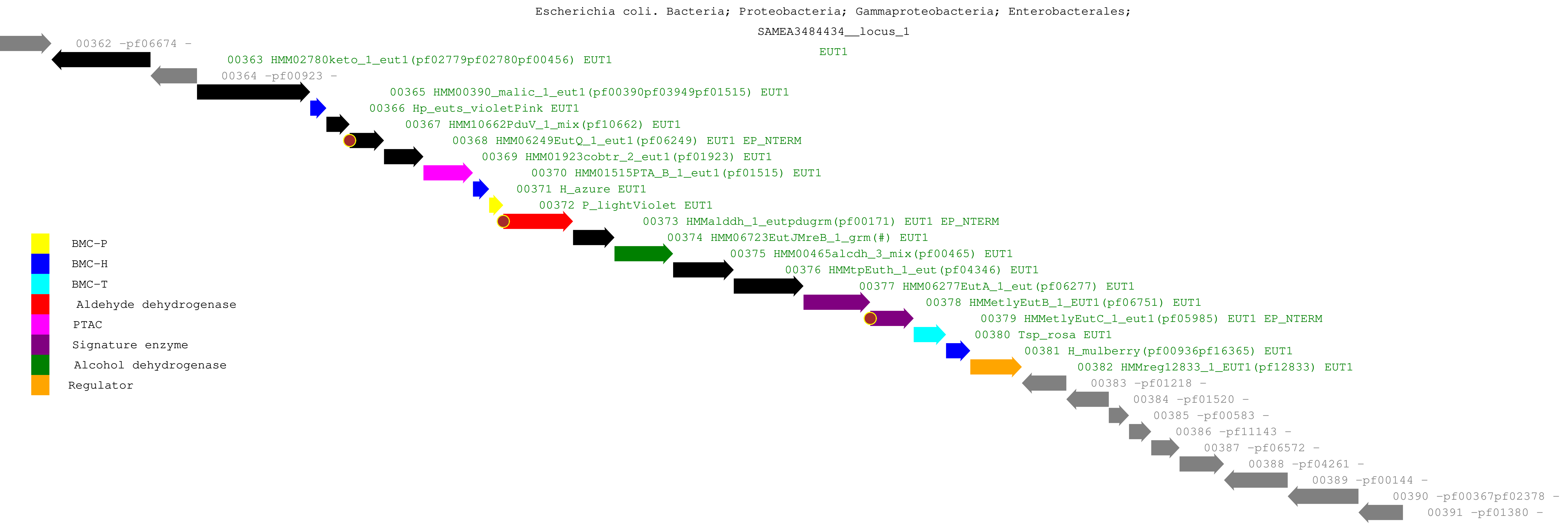
Escherichia coli. Bacteria; Proteobacteria; Gammaproteobacteria; Enterobacterales;
SAMEA3484434__locus_1
EUT1
00362 -pf06674 -
00363 HMM02780keto_1_eut1(pf02779pf02780pf00456) EUT1
00364 -pf00923 -
00365 HMM00390_malic_1_eut1(pf00390pf03949pf01515) EUT1
00366 Hp_euts_violetPink EUT1
00367 HMM10662PduV_1_mix(pf10662) EUT1
00368 HMM06249EutQ_1_eut1(pf06249) EUT1 EP_NTERM
00369 HMM01923cobtr_2_eut1(pf01923) EUT1
00370 HMM01515PTA_B_1_eut1(pf01515) EUT1
00371 H_azure EUT1
00372 P_lightViolet EUT1
00373 HMMalddh_1_eutpdugrm(pf00171) EUT1 EP_NTERM
00374 HMM06723EutJMreB_1_grm(#) EUT1
00375 HMM00465alcdh_3_mix(pf00465) EUT1
00376 HMMtpEuth_1_eut(pf04346) EUT1
00377 HMM06277EutA_1_eut(pf06277) EUT1
00378 HMMetlyEutB_1_EUT1(pf06751) EUT1
00379 HMMetlyEutC_1_eut1(pf05985) EUT1 EP_NTERM
00380 Tsp_rosa EUT1
00381 H_mulberry(pf00936pf16365) EUT1
00382 HMMreg12833_1_EUT1(pf12833) EUT1
00383 -pf01218 -
00384 -pf01520 -
00385 -pf00583 -
00386 -pf11143 -
00387 -pf06572 -
00388 -pf04261 -
00389 -pf00144 -
00390 -pf00367pf02378 -
00391 -pf01380 -
BMC-P
BMC-H
BMC-T
Aldehyde dehydrogenase
PTAC
Signature enzyme
Alcohol dehydrogenase
Regulator

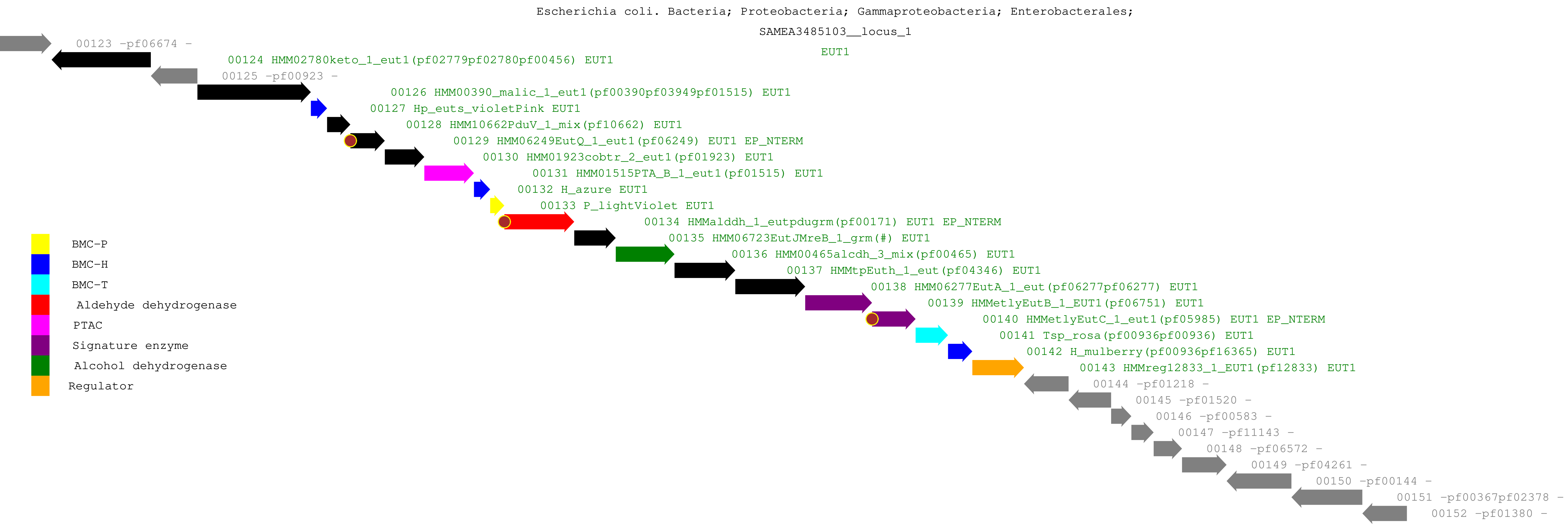
Escherichia coli. Bacteria; Proteobacteria; Gammaproteobacteria; Enterobacterales;
SAMEA3485103__locus_1
EUT1
00123 -pf06674 -
00124 HMM02780keto_1_eut1(pf02779pf02780pf00456) EUT1
00125 -pf00923 -
00126 HMM00390_malic_1_eut1(pf00390pf03949pf01515) EUT1
00127 Hp_euts_violetPink EUT1
00128 HMM10662PduV_1_mix(pf10662) EUT1
00129 HMM06249EutQ_1_eut1(pf06249) EUT1 EP_NTERM
00130 HMM01923cobtr_2_eut1(pf01923) EUT1
00131 HMM01515PTA_B_1_eut1(pf01515) EUT1
00132 H_azure EUT1
00133 P_lightViolet EUT1
00134 HMMalddh_1_eutpdugrm(pf00171) EUT1 EP_NTERM
00135 HMM06723EutJMreB_1_grm(#) EUT1
00136 HMM00465alcdh_3_mix(pf00465) EUT1
00137 HMMtpEuth_1_eut(pf04346) EUT1
00138 HMM06277EutA_1_eut(pf06277pf06277) EUT1
00139 HMMetlyEutB_1_EUT1(pf06751) EUT1
00140 HMMetlyEutC_1_eut1(pf05985) EUT1 EP_NTERM
00141 Tsp_rosa(pf00936pf00936) EUT1
00142 H_mulberry(pf00936pf16365) EUT1
00143 HMMreg12833_1_EUT1(pf12833) EUT1
00144 -pf01218 -
00145 -pf01520 -
00146 -pf00583 -
00147 -pf11143 -
00148 -pf06572 -
00149 -pf04261 -
00150 -pf00144 -
00151 -pf00367pf02378 -
00152 -pf01380 -
BMC-P
BMC-H
BMC-T
Aldehyde dehydrogenase
PTAC
Signature enzyme
Alcohol dehydrogenase
Regulator

Klebsiella variicola. Bacteria; Proteobacteria; Gammaproteobacteria; Enterobacterales;

SAMEA3515120__locus_2

EUT1

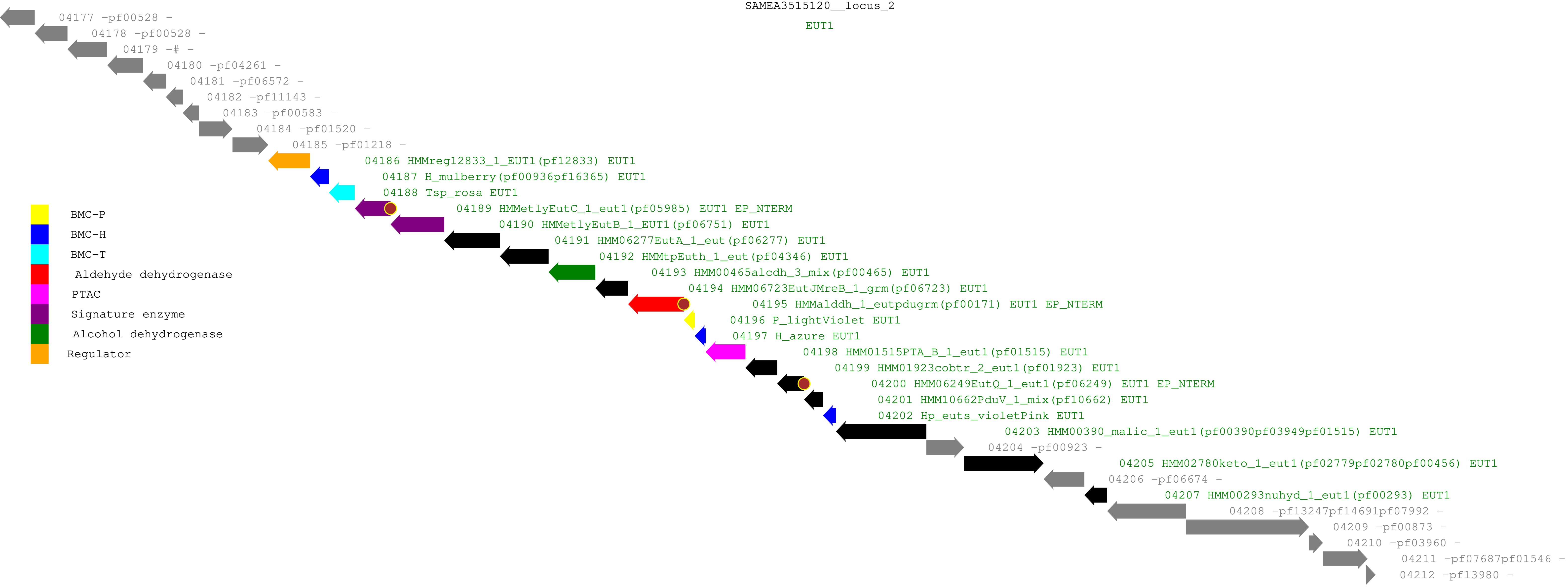

Escherichia coli. Bacteria; Proteobacteria; Gammaproteobacteria; Enterobacterales;

SAMEA3531867__locus_1

EUT1

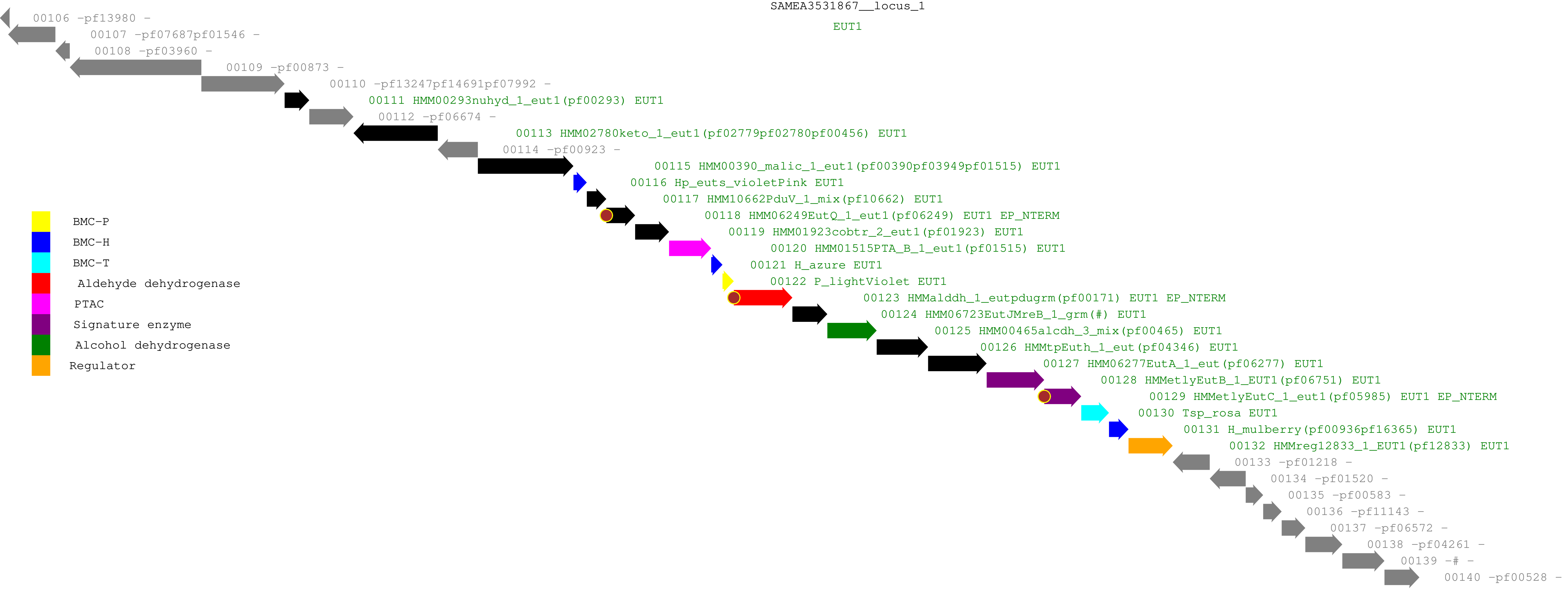

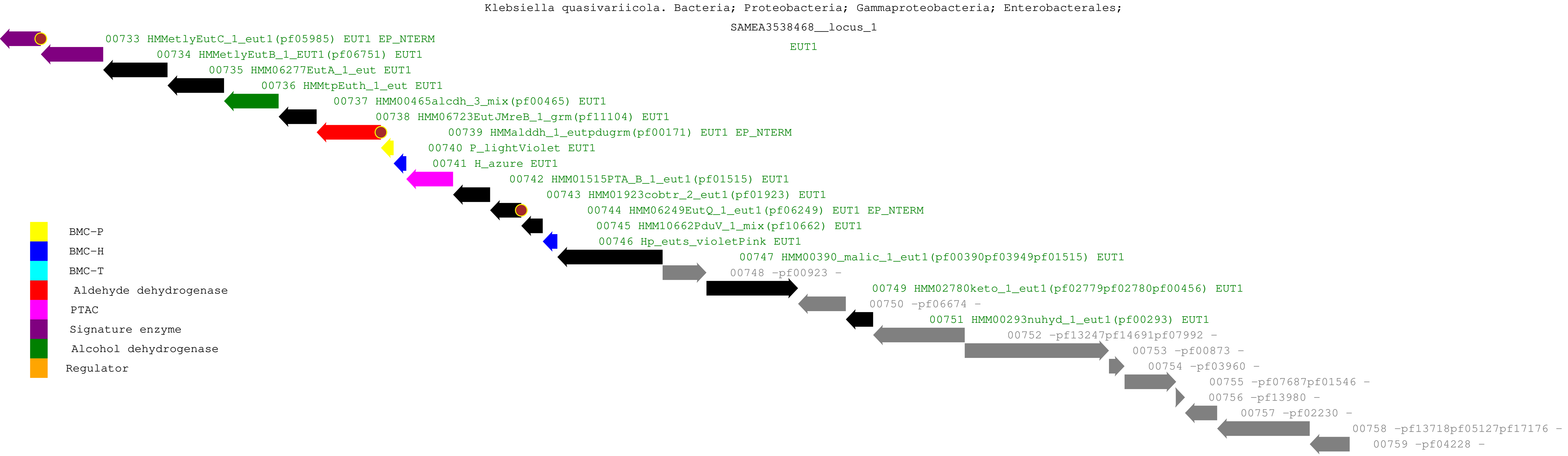
Klebsiella quasivariicola. Bacteria; Proteobacteria; Gammaproteobacteria; Enterobacterales;
SAMEA3538468__locus_1
EUT1
00733 HMMetlyEutC_1_eut1(pf05985) EUT1 EP_NTERM
00734 HMMetlyEutB_1_EUT1(pf06751) EUT1
00735 HMM06277EutA_1_eut EUT1
00736 HMMtpEuth_1_eut EUT1
00737 HMM00465alcdh_3_mix(pf00465) EUT1
00738 HMM06723EutJMreB_1_grm(pf11104) EUT1
00739 HMMalddh_1_eutpdugrm(pf00171) EUT1 EP_NTERM
00740 P_lightViolet EUT1
00741 H_azure EUT1
00742 HMM01515PTA_B_1_eut1(pf01515) EUT1
00743 HMM01923cobtr_2_eut1(pf01923) EUT1
00744 HMM06249EutQ_1_eut1(pf06249) EUT1 EP_NTERM
00745 HMM10662PduV_1_mix(pf10662) EUT1
00746 Hp_euts_violetPink EUT1
00747 HMM00390_malic_1_eut1(pf00390pf03949pf01515) EUT1
00748 -pf00923 -
00749 HMM02780keto_1_eut1(pf02779pf02780pf00456) EUT1
00750 -pf06674 -
00751 HMM00293nuhyd_1_eut1(pf00293) EUT1
00752 -pf13247pf14691pf07992 -
00753 -pf00873 -
00754 -pf03960 -
00755 -pf07687pf01546 -
00756 -pf13980 -
00757 -pf02230 -
00758 -pf13718pf05127pf17176 -
00759 -pf04228 -
BMC-P
BMC-H
BMC-T
Aldehyde dehydrogenase
PTAC
Signature enzyme
Alcohol dehydrogenase
Regulator

Klebsiella pneumoniae. Bacteria; Proteobacteria; Gammaproteobacteria; Enterobacterales;
SAMEA3649709__locus_1
EUT1

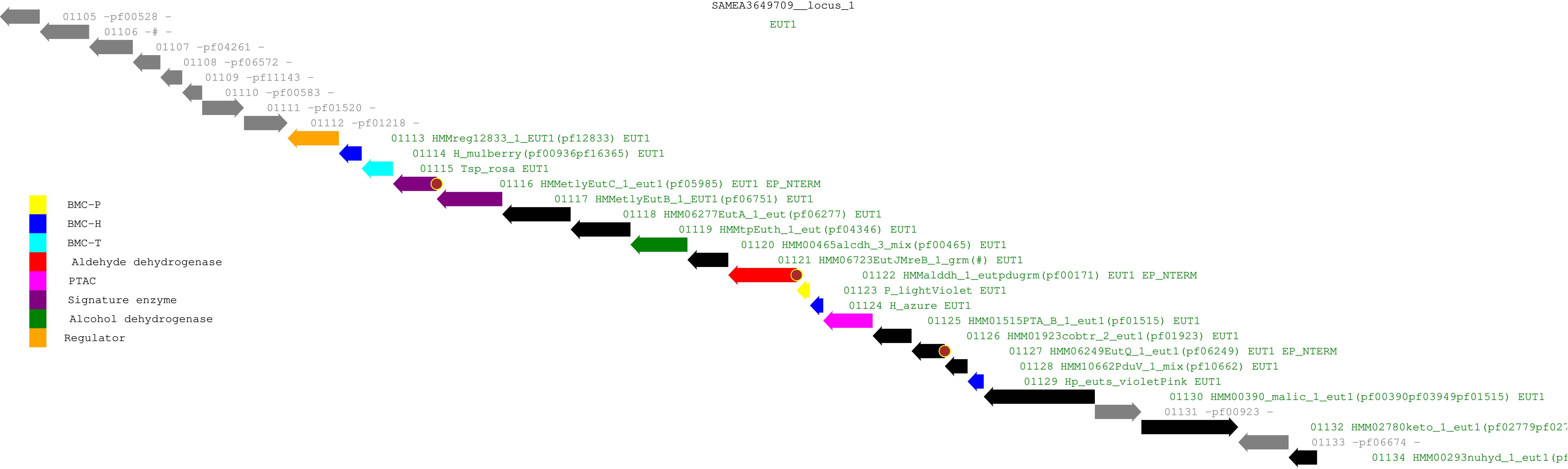

Klebsiella pneumoniae. Bacteria; Proteobacteria; Gammaproteobacteria; Enterobacterales;

SAMEA3729690__locus_1

EUT1

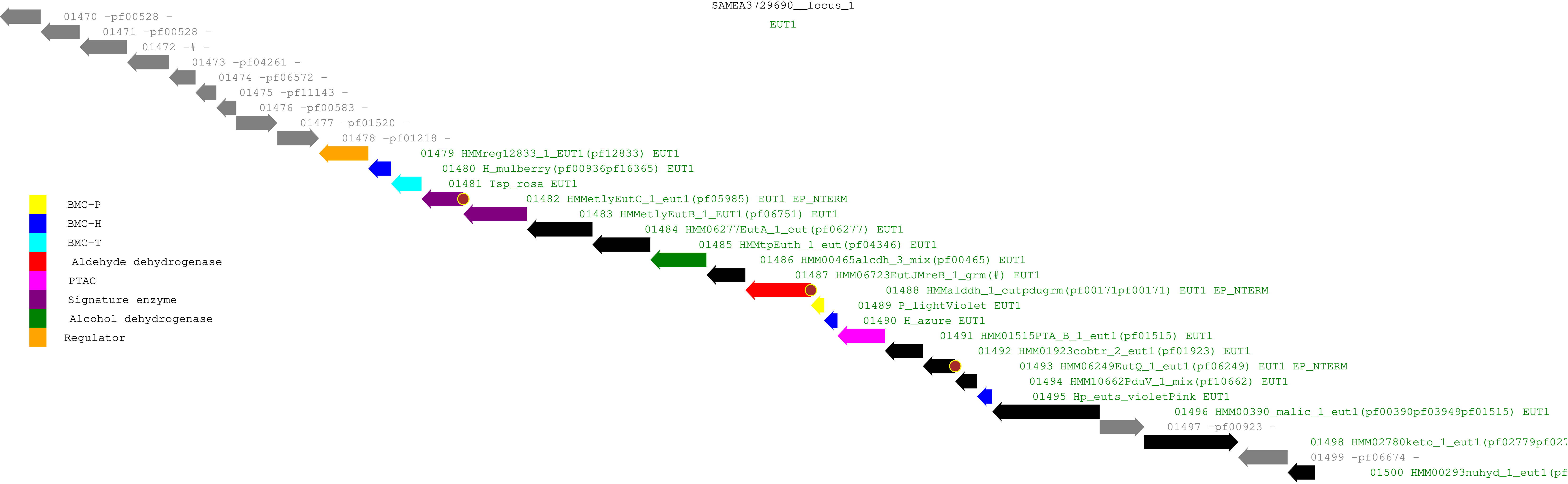

Escherichia coli. Bacteria; Proteobacteria; Gammaproteobacteria; Enterobacterales;

SAMEA3752372__locus_1

EUT1

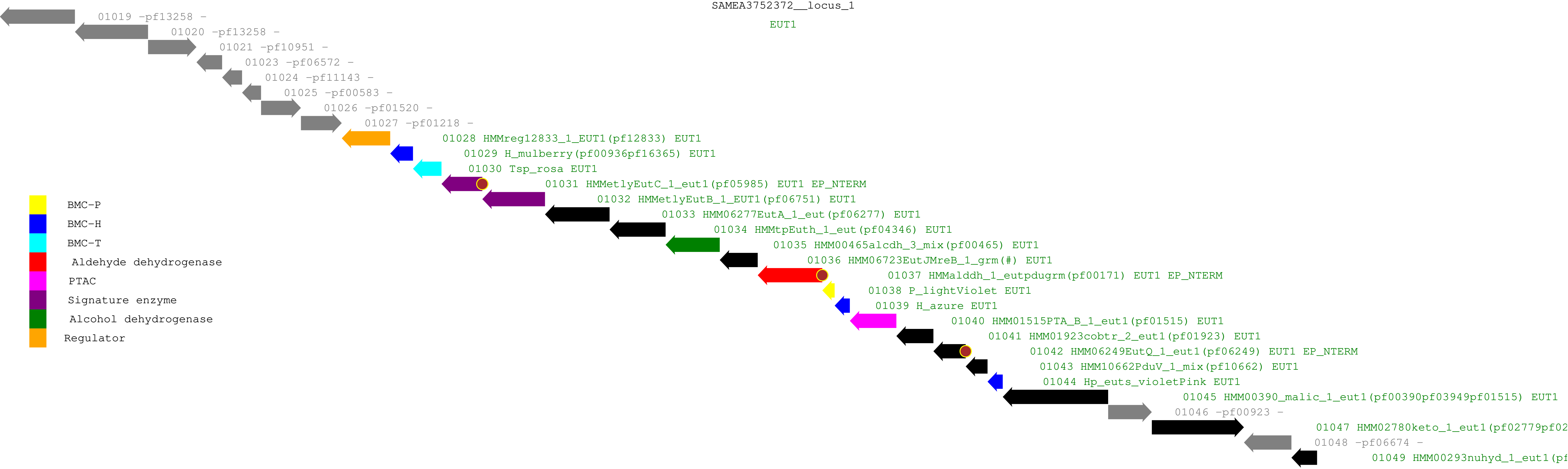

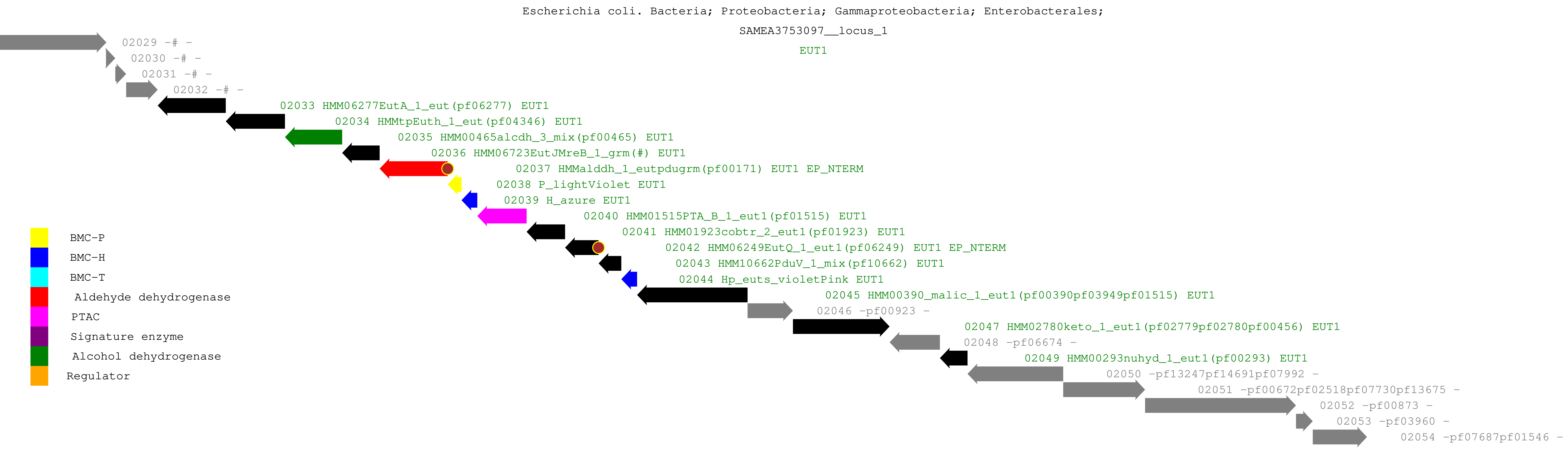
Escherichia coli. Bacteria; Proteobacteria; Gammaproteobacteria; Enterobacterales;
SAMEA3753097__locus_1
EUT1
02029 -# -
02030 -# -
02031 -# -
02032 -# -
02033 HMM06277EutA_1_eut(pf06277) EUT1
02034 HMMtpEuth_1_eut(pf04346) EUT1
02035 HMM00465alcdh_3_mix(pf00465) EUT1
02036 HMM06723EutJMreB_1_grm(#) EUT1
02037 HMMalddh_1_eutpdugrm(pf00171) EUT1 EP_NTERM
02038 P_lightViolet EUT1
02039 H_azure EUT1
02040 HMM01515PTA_B_1_eut1(pf01515) EUT1
02041 HMM01923cobtr_2_eut1(pf01923) EUT1
02042 HMM06249EutQ_1_eut1(pf06249) EUT1 EP_NTERM
02043 HMM10662PduV_1_mix(pf10662) EUT1
02044 Hp_euts_violetPink EUT1
02045 HMM00390_malic_1_eut1(pf00390pf03949pf01515) EUT1
02046 -pf00923 -
02047 HMM02780keto_1_eut1(pf02779pf02780pf00456) EUT1
02048 -pf06674 -
02049 HMM00293nuhyd_1_eut1(pf00293) EUT1
02050 -pf13247pf14691pf07992 -
02051 -pf00672pf02518pf07730pf13675 -
02052 -pf00873 -
02053 -pf03960 -
02054 -pf07687pf01546 -
BMC-P
BMC-H
BMC-T
Aldehyde dehydrogenase
PTAC
Signature enzyme
Alcohol dehydrogenase
Regulator

Escherichia coli. Bacteria; Proteobacteria; Gammaproteobacteria; Enterobacterales;
SAMEA3753304__locus_1
EUT1

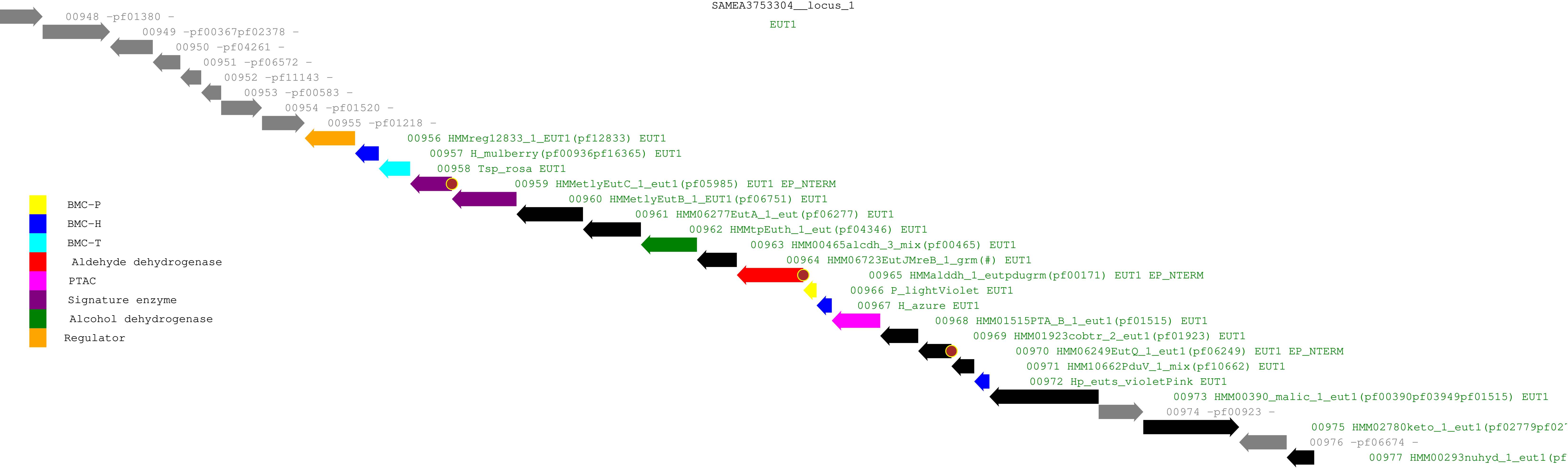

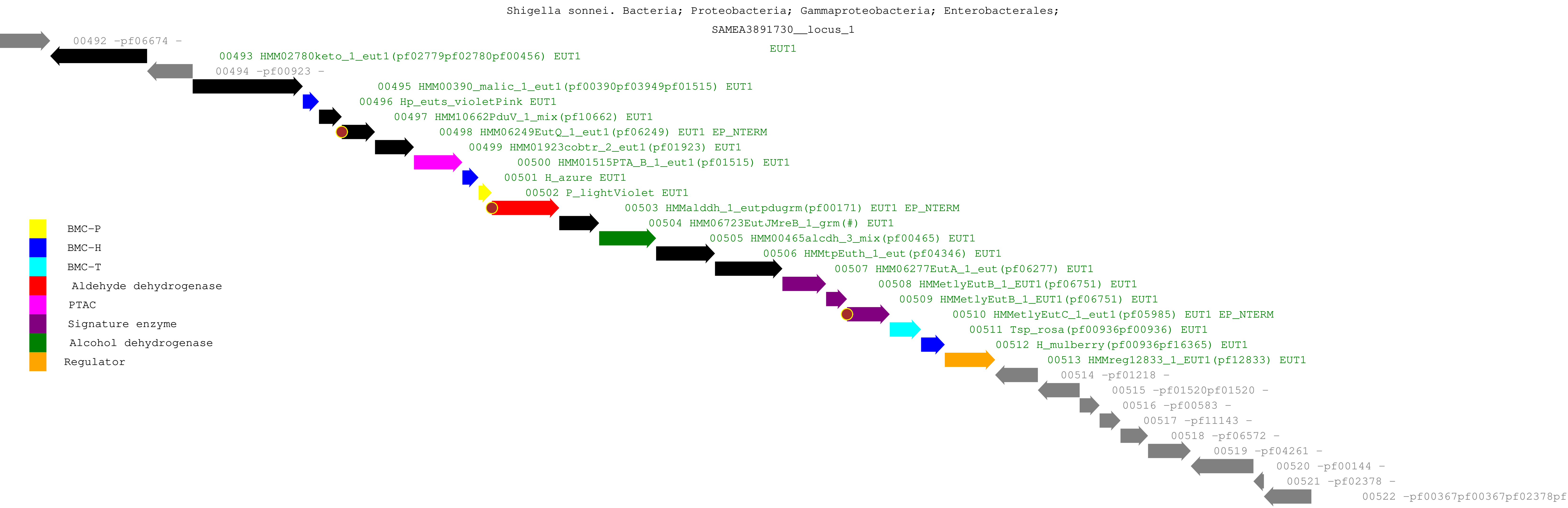

Shigella sonnei. Bacteria; Proteobacteria; Gammaproteobacteria; Enterobacterales;
SAMEA3891730__locus_1
EUT1
00492 -pf06674 -
00493 HMM02780keto_1_eut1(pf02779pf02780pf00456) EUT1
00494 -pf00923 -
00495 HMM00390_malic_1_eut1(pf00390pf03949pf01515) EUT1
00496 Hp_euts_violetPink EUT1
00497 HMM10662PduV_1_mix(pf10662) EUT1
00498 HMM06249EutQ_1_eut1(pf06249) EUT1 EP_NTERM
00499 HMM01923cobtr_2_eut1(pf01923) EUT1
00500 HMM01515PTA_B_1_eut1(pf01515) EUT1
00501 H_azure EUT1
00502 P_lightViolet EUT1
00503 HMMalddh_1_eutpdugrm(pf00171) EUT1 EP_NTERM
00504 HMM06723EutJMreB_1_grm(#) EUT1
00505 HMM00465alcdh_3_mix(pf00465) EUT1
00506 HMMtpEuth_1_eut(pf04346) EUT1
00507 HMM06277EutA_1_eut(pf06277) EUT1
00508 HMMetlyEutB_1_EUT1(pf06751) EUT1
00509 HMMetlyEutB_1_EUT1(pf06751) EUT1
00510 HMMetlyEutC_1_eut1(pf05985) EUT1 EP_NTERM
00511 Tsp_rosa(pf00936pf00936) EUT1
00512 H_mulberry(pf00936pf16365) EUT1
00513 HMMreg12833_1_EUT1(pf12833) EUT1
00514 -pf01218 -
00515 -pf01520pf01520 -
00516 -pf00583 -
00517 -pf11143 -
00518 -pf06572 -
00519 -pf04261 -
00520 -pf00144 -
00521 -pf02378 -
00522 -pf00367pf00367pf02378pf
BMC-P
BMC-H
BMC-T
Aldehyde dehydrogenase
PTAC
Signature enzyme
Alcohol dehydrogenase
Regulator

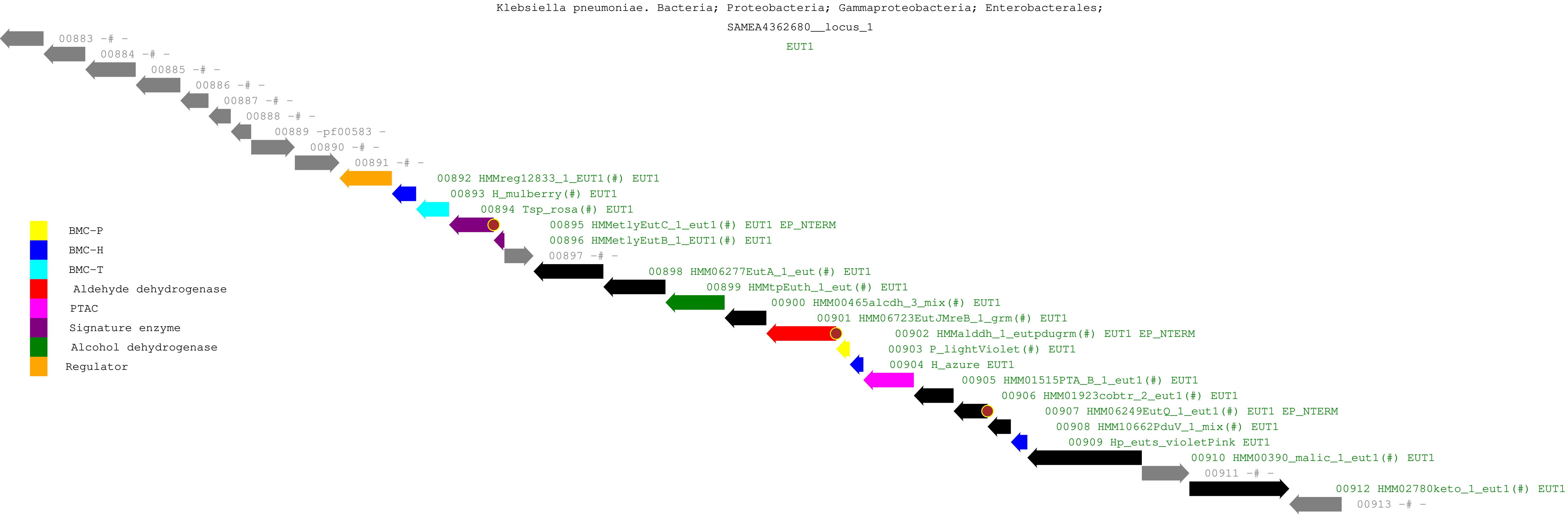
Klebsiella pneumoniae. Bacteria; Proteobacteria; Gammaproteobacteria; Enterobacterales;
SAMEA4362680__locus_1
EUT1
00883 -# -
00884 -# -
00885 -# -
00886 -# -
00887 -# -
00888 -# -
00889 -pf00583 -
00890 -# -
00891 -# -
00892 HMMreg12833_1_EUT1(#) EUT1
00893 H_mulberry(#) EUT1
00894 Tsp_rosa(#) EUT1
00895 HMMetlyEutC_1_eut1(#) EUT1 EP_NTERM
00896 HMMetlyEutB_1_EUT1(#) EUT1
00897 -# -
00898 HMM06277EutA_1_eut(#) EUT1
00899 HMMtpEuth_1_eut(#) EUT1
00900 HMM00465alcdh_3_mix(#) EUT1
00901 HMM06723EutJMreB_1_grm(#) EUT1
00902 HMMalddh_1_eutpdugrm(#) EUT1 EP_NTERM
00903 P_lightViolet(#) EUT1
00904 H_azure EUT1
00905 HMM01515PTA_B_1_eut1(#) EUT1
00906 HMM01923cobtr_2_eut1(#) EUT1
00907 HMM06249EutQ_1_eut1(#) EUT1 EP_NTERM
00908 HMM10662PduV_1_mix(#) EUT1
00909 Hp_euts_violetPink EUT1
00910 HMM00390_malic_1_eut1(#) EUT1
00911 -# -
00912 HMM02780keto_1_eut1(#) EUT1
00913 -# -
BMC-P
BMC-H
BMC-T
Aldehyde dehydrogenase
PTAC
Signature enzyme
Alcohol dehydrogenase
Regulator

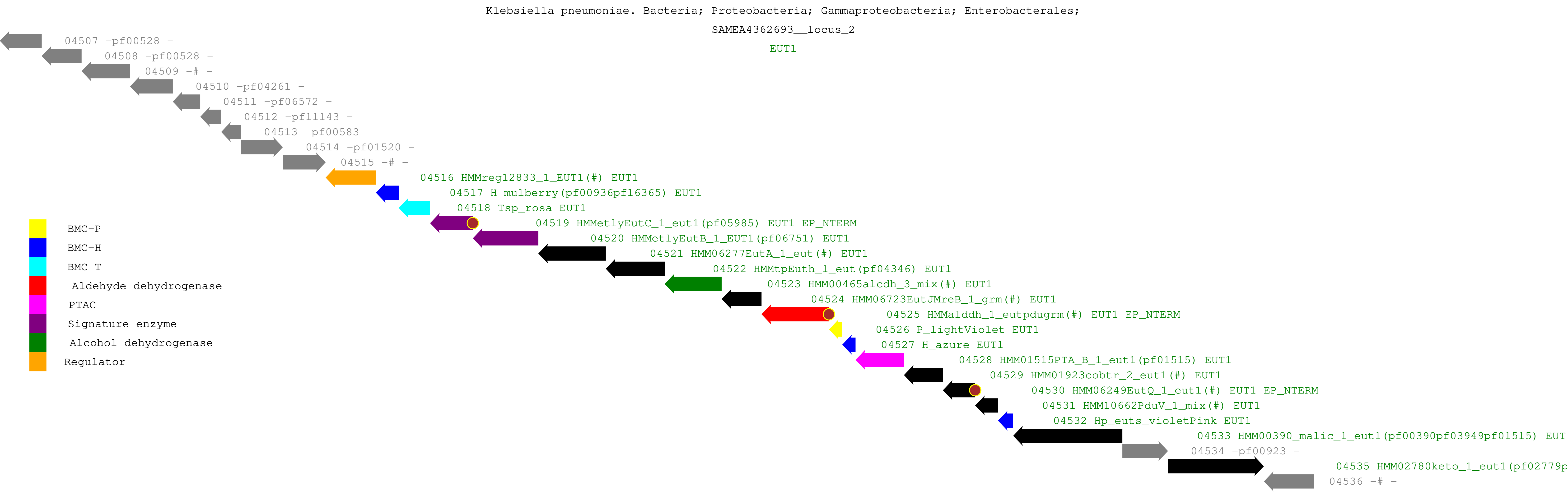
Klebsiella pneumoniae. Bacteria; Proteobacteria; Gammaproteobacteria; Enterobacterales;
SAMEA4362693__locus_2
EUT1
04507 -pf00528 -
04508 -pf00528 -
04509 -# -
04510 -pf04261 -
04511 -pf06572 -
04512 -pf11143 -
04513 -pf00583 -
04514 -pf01520 -
04515 -# -
04516 HMMreg12833_1_EUT1(#) EUT1
04517 H_mulberry(pf00936pf16365) EUT1
04518 Tsp_rosa EUT1
04519 HMMetlyEutC_1_eut1(pf05985) EUT1 EP_NTERM
04520 HMMetlyEutB_1_EUT1(pf06751) EUT1
04521 HMM06277EutA_1_eut(#) EUT1
04522 HMMtpEuth_1_eut(pf04346) EUT1
04523 HMM00465alcdh_3_mix(#) EUT1
04524 HMM06723EutJMreB_1_grm(#) EUT1
04525 HMMalddh_1_eutpdugrm(#) EUT1 EP_NTERM
04526 P_lightViolet EUT1
04527 H_azure EUT1
04528 HMM01515PTA_B_1_eut1(pf01515) EUT1
04529 HMM01923cobtr_2_eut1(#) EUT1
04530 HMM06249EutQ_1_eut1(#) EUT1 EP_NTERM
04531 HMM10662PduV_1_mix(#) EUT1
04532 Hp_euts_violetPink EUT1
04533 HMM00390_malic_1_eut1(pf00390pf03949pf01515) EUT
04534 -pf00923 -
04535 HMM02780keto_1_eut1(pf02779p
04536 -# -
BMC-P
BMC-H
BMC-T
Aldehyde dehydrogenase
PTAC
Signature enzyme
Alcohol dehydrogenase
Regulator

Klebsiella pneumoniae. Bacteria; Proteobacteria; Gammaproteobacteria; Enterobacterales;

SAMEA4364652__locus_1

EUT1

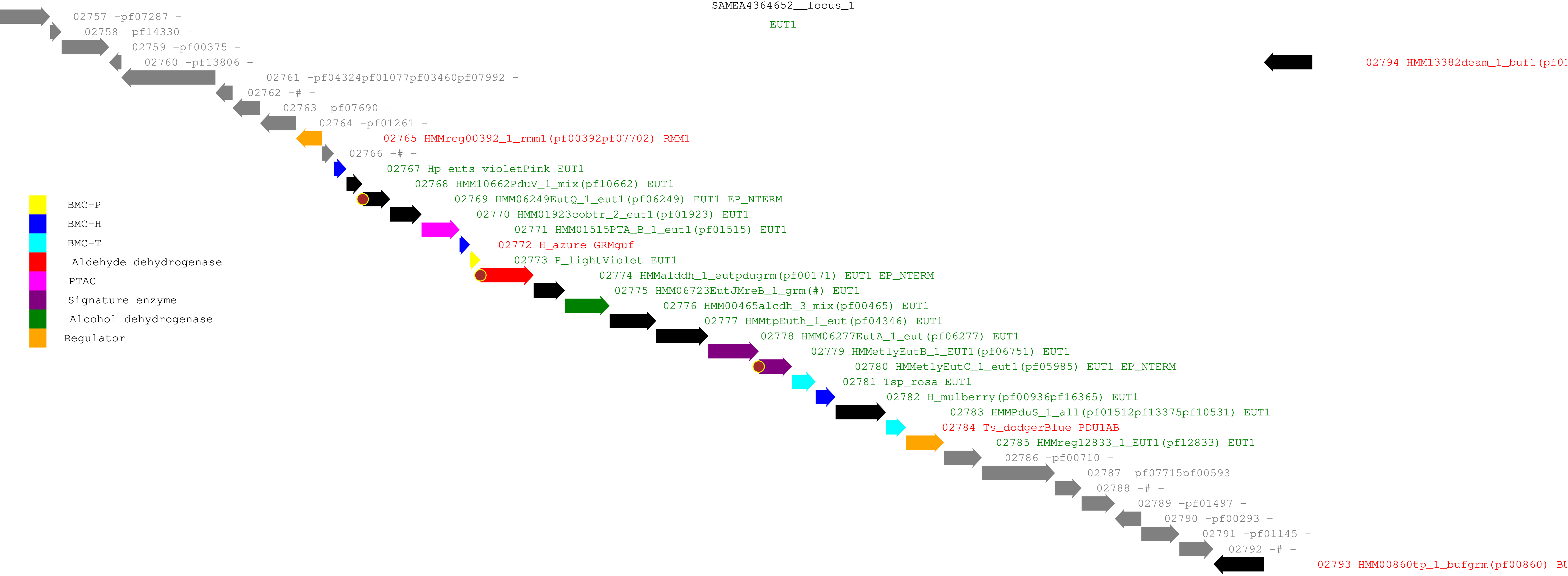

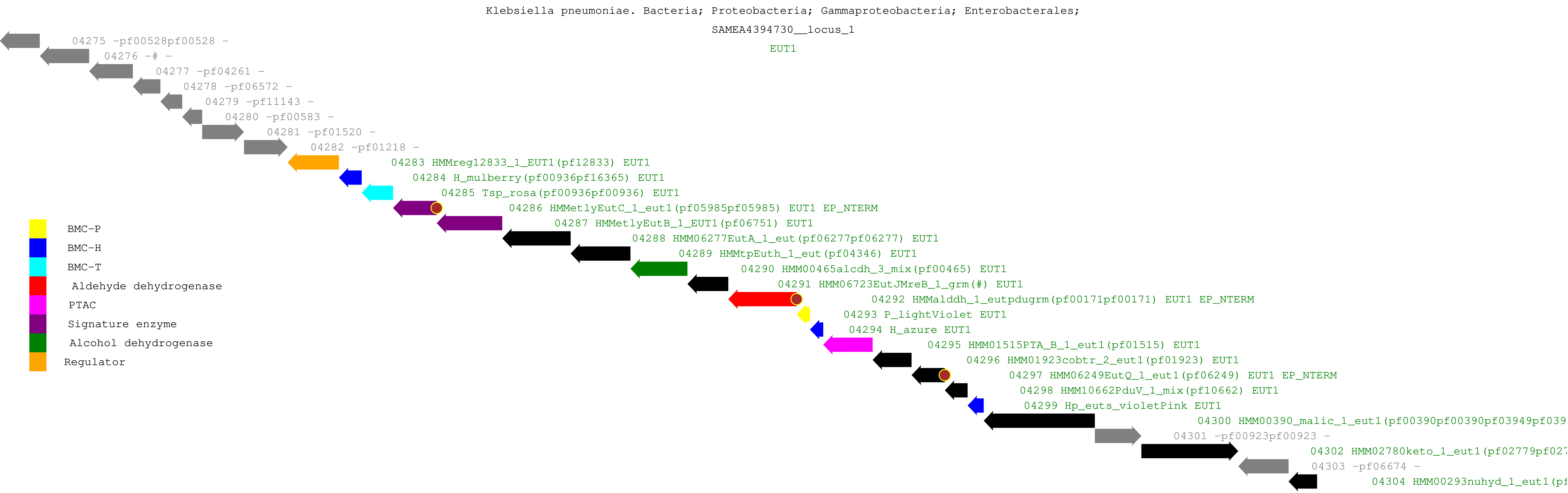
Klebsiella pneumoniae. Bacteria; Proteobacteria; Gammaproteobacteria; Enterobacterales;
SAMEA4394730__locus_1
EUT1
04275 -pf00528pf00528 -
04276 -# -
04277 -pf04261 -
04278 -pf06572 -
04279 -pf11143 -
04280 -pf00583 -
04281 -pf01520 -
04282 -pf01218 -
04283 HMMreg12833_1_EUT1(pf12833) EUT1
04284 H_mulberry(pf00936pf16365) EUT1
04285 Tsp_rosa(pf00936pf00936) EUT1
04286 HMMetlyEutC_1_eut1(pf05985pf05985) EUT1 EP_NTERM
04287 HMMetlyEutB_1_EUT1(pf06751) EUT1
04288 HMM06277EutA_1_eut(pf06277pf06277) EUT1
04289 HMMtpEuth_1_eut(pf04346) EUT1
04290 HMM00465alcdh_3_mix(pf00465) EUT1
04291 HMM06723EutJMreB_1_grm(#) EUT1
04292 HMMalddh_1_eutpdugrm(pf00171pf00171) EUT1 EP_NTERM
04293 P_lightViolet EUT1
04294 H_azure EUT1
04295 HMM01515PTA_B_1_eut1(pf01515) EUT1
04296 HMM01923cobtr_2_eut1(pf01923) EUT1
04297 HMM06249EutQ_1_eut1(pf06249) EUT1 EP_NTERM
04298 HMM10662PduV_1_mix(pf10662) EUT1
04299 Hp_euts_violetPink EUT1
04300 HMM00390_malic_1_eut1(pf00390pf00390pf03949pf039
04301 -pf00923pf00923 -
04302 HMM02780keto_1_eut1(pf02779pf027
04303 -pf06674 -
04304 HMM00293nuhyd_1_eut1(pf
BMC-P
BMC-H
BMC-T
Aldehyde dehydrogenase
PTAC
Signature enzyme
Alcohol dehydrogenase
Regulator

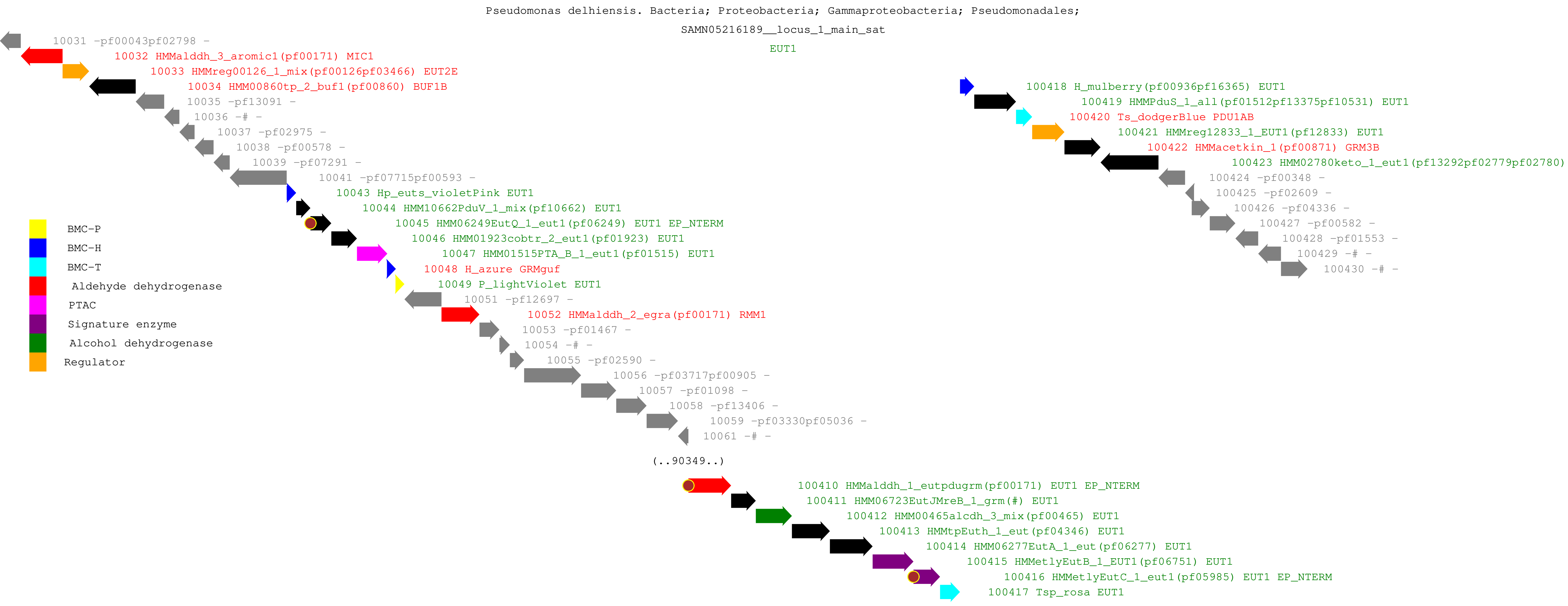

Pseudomonas delhiensis. Bacteria; Proteobacteria; Gammaproteobacteria; Pseudomonadales;
SAMN05216189__locus_1_main_sat
EUT1
10031 -pf00043pf02798 -
10032 HMMalddh_3_aromic1(pf00171) MIC1
10033 HMMreg00126_1_mix(pf00126pf03466) EUT2E
10034 HMM00860tp_2_buf1(pf00860) BUF1B
10035 -pf13091 -
10036 -# -
10037 -pf02975 -
10038 -pf00578 -
10039 -pf07291 -
10041 -pf07715pf00593 -
10043 Hp_euts_violetPink EUT1
10044 HMM10662PduV_1_mix(pf10662) EUT1
10045 HMM06249EutQ_1_eut1(pf06249) EUT1 EP_NTERM
10046 HMM01923cobtr_2_eut1(pf01923) EUT1
10047 HMM01515PTA_B_1_eut1(pf01515) EUT1
10048 H_azure GRMguf
10049 P_lightViolet EUT1
10051 -pf12697 -
10052 HMMalddh_2_egra(pf00171) RMM1
10053 -pf01467 -
10054 -# -
10055 -pf02590 -
10056 -pf03717pf00905 -
10057 -pf01098 -
10058 -pf13406 -
10059 -pf03330pf05036 -
10061 -# -
(..90349..)
100410 HMMalddh_1_eutpdugrm(pf00171) EUT1 EP_NTERM
100411 HMM06723EutJMreB_1_grm(#) EUT1
100412 HMM00465alcdh_3_mix(pf00465) EUT1
100413 HMMtpEuth_1_eut(pf04346) EUT1
100414 HMM06277EutA_1_eut(pf06277) EUT1
100415 HMMetlyEutB_1_EUT1(pf06751) EUT1
100416 HMMetlyEutC_1_eut1(pf05985) EUT1 EP_NTERM
100417 Tsp_rosa EUT1
100418 H_mulberry(pf00936pf16365) EUT1
100419 HMMPduS_1_all(pf01512pf13375pf10531) EUT1
100420 Ts_dodgerBlue PDU1AB
100421 HMMreg12833_1_EUT1(pf12833) EUT1
100422 HMMacetkin_1(pf00871) GRM3B
100423 HMM02780keto_1_eut1(pf13292pf02779pf02780)
100424 -pf00348 -
100425 -pf02609 -
100426 -pf04336 -
100427 -pf00582 -
100428 -pf01553 -
100429 -# -
100430 -# -
BMC-P
BMC-H
BMC-T
Aldehyde dehydrogenase
PTAC
Signature enzyme
Alcohol dehydrogenase
Regulator

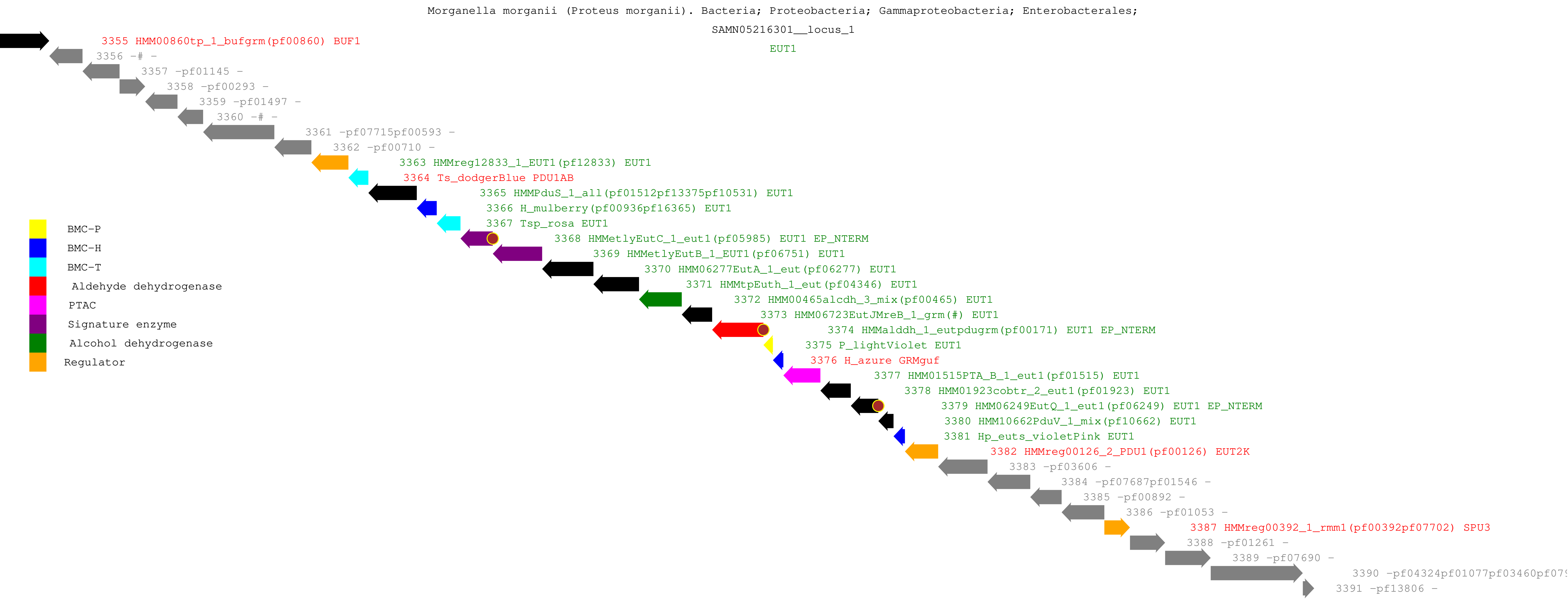
Morganella morganii (Proteus morganii). Bacteria; Proteobacteria; Gammaproteobacteria; Enterobacterales;
SAMN05216301__locus_1
EUT1
3355 HMM00860tp_1_bufgrm(pf00860) BUF1
3356 -# -
3357 -pf01145 -
3358 -pf00293 -
3359 -pf01497 -
3360 -# -
3361 -pf07715pf00593 -
3362 -pf00710 -
3363 HMMreg12833_1_EUT1(pf12833) EUT1
3364 Ts_dodgerBlue PDU1AB
3365 HMMPduS_1_all(pf01512pf13375pf10531) EUT1
3366 H_mulberry(pf00936pf16365) EUT1
3367 Tsp_rosa EUT1
3368 HMMetlyEutC_1_eut1(pf05985) EUT1 EP_NTERM
3369 HMMetlyEutB_1_EUT1(pf06751) EUT1
3370 HMM06277EutA_1_eut(pf06277) EUT1
3371 HMMtpEuth_1_eut(pf04346) EUT1
3372 HMM00465alcdh_3_mix(pf00465) EUT1
3373 HMM06723EutJMreB_1_grm(#) EUT1
3374 HMMalddh_1_eutpdugrm(pf00171) EUT1 EP_NTERM
3375 P_lightViolet EUT1
3376 H_azure GRMguf
3377 HMM01515PTA_B_1_eut1(pf01515) EUT1
3378 HMM01923cobtr_2_eut1(pf01923) EUT1
3379 HMM06249EutQ_1_eut1(pf06249) EUT1 EP_NTERM
3380 HMM10662PduV_1_mix(pf10662) EUT1
3381 Hp_euts_violetPink EUT1
3382 HMMreg00126_2_PDU1(pf00126) EUT2K
3383 -pf03606 -
3384 -pf07687pf01546 -
3385 -pf00892 -
3386 -pf01053 -
3387 HMMreg00392_1_rmm1(pf00392pf07702) SPU3
3388 -pf01261 -
3389 -pf07690 -
3390 -pf04324pf01077pf03460pf07
3391 -pf13806 -
BMC-P
BMC-H
BMC-T
Aldehyde dehydrogenase
PTAC
Signature enzyme
Alcohol dehydrogenase
Regulator

Shigella sonnei. Bacteria; Proteobacteria; Gammaproteobacteria; Enterobacterales;

SAMN05216485__locus_1

EUT1

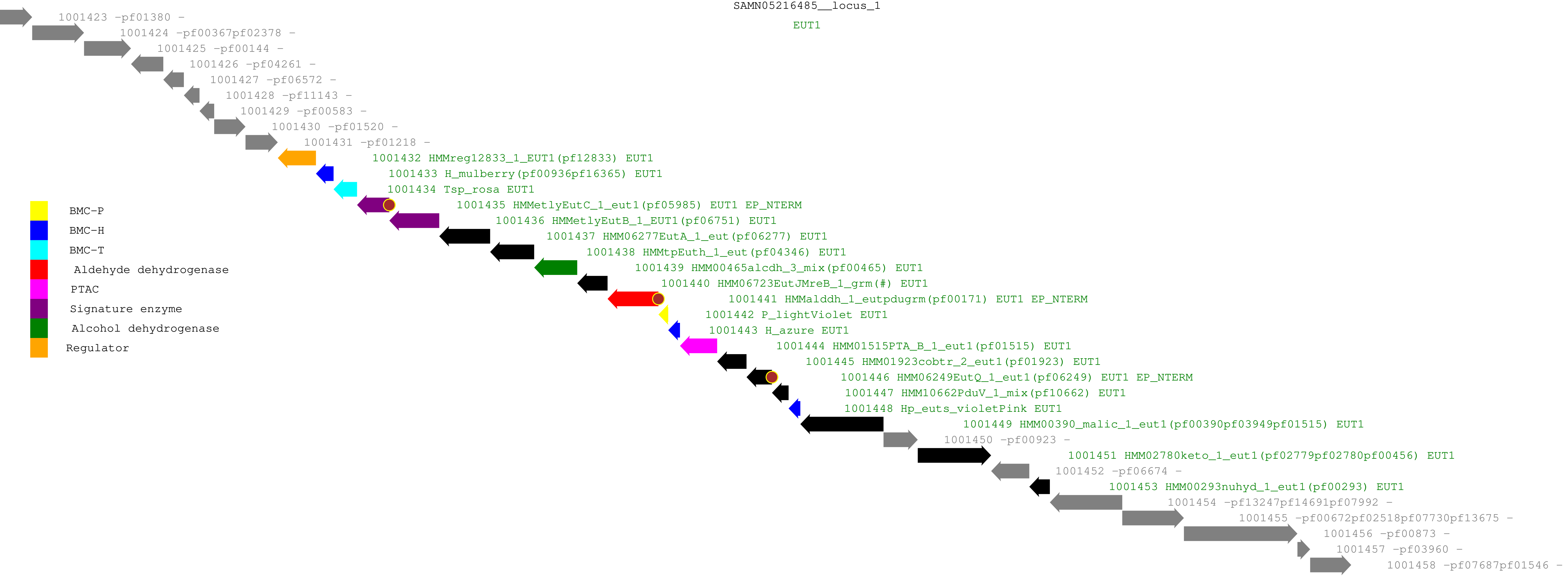

Citrobacter sp. NLAE-zl-C269. Bacteria; Proteobacteria; Gammaproteobacteria; Enterobacterales;

SAMN05216502__locus_2

EUT1

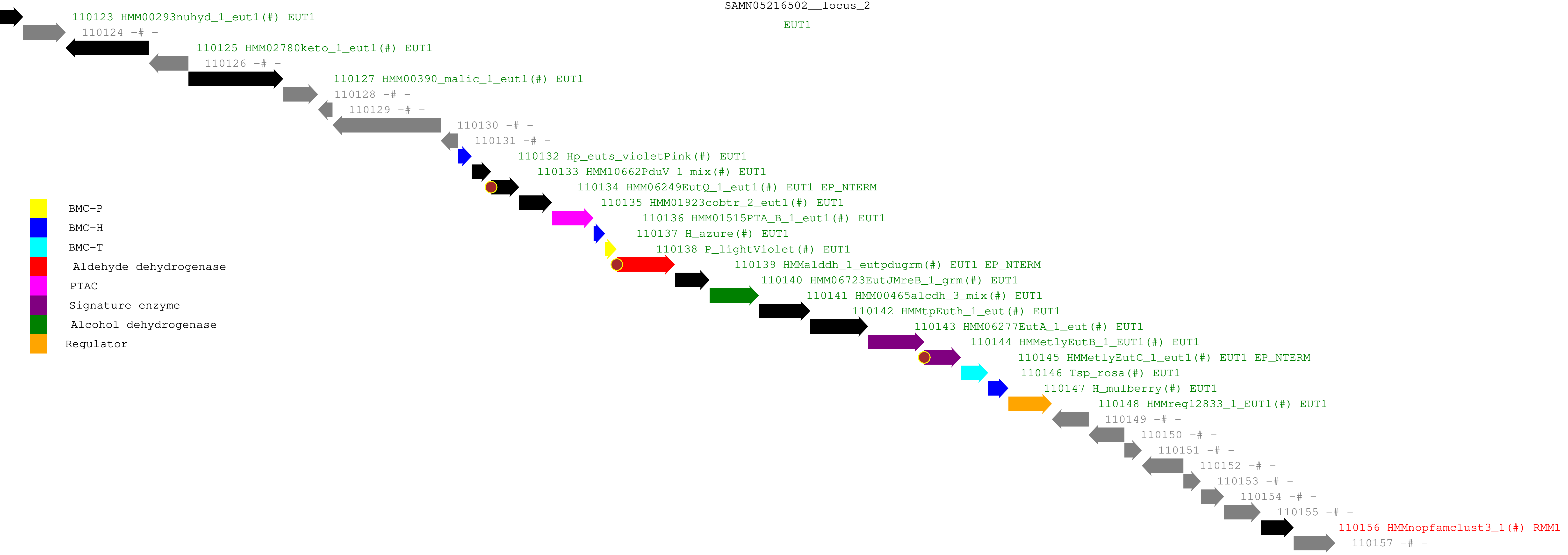

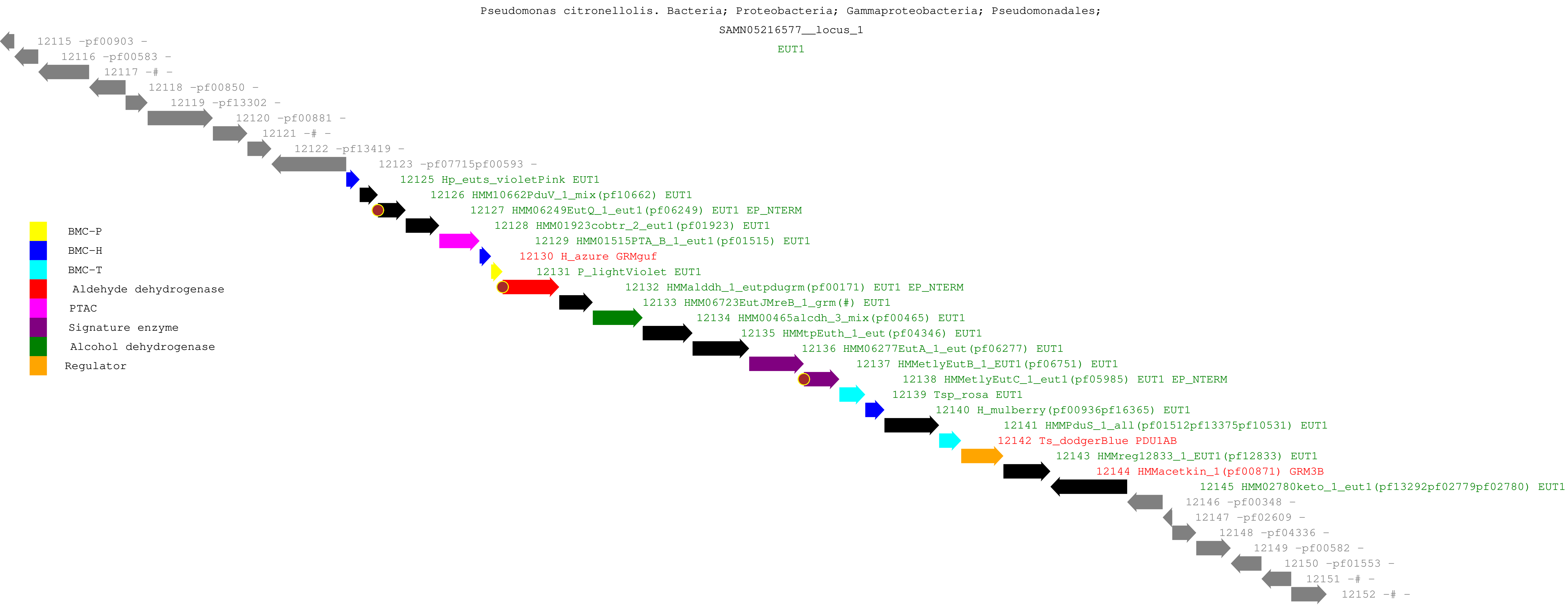
Pseudomonas citronellolis. Bacteria; Proteobacteria; Gammaproteobacteria; Pseudomonadales;
SAMN05216577__locus_1
EUT1
12115 -pf00903 -
12116 -pf00583 -
12117 -# -
12118 -pf00850 -
12119 -pf13302 -
12120 -pf00881 -
12121 -# -
12122 -pf13419 -
12123 -pf07715pf00593 -
12125 Hp_euts_violetPink EUT1
12126 HMM10662PduV_1_mix(pf10662) EUT1
12127 HMM06249EutQ_1_eut1(pf06249) EUT1 EP_NTERM
12128 HMM01923cobtr_2_eut1(pf01923) EUT1
12129 HMM01515PTA_B_1_eut1(pf01515) EUT1
12130 H_azure GRMguf
12131 P_lightViolet EUT1
12132 HMMalddh_1_eutpdugrm(pf00171) EUT1 EP_NTERM
12133 HMM06723EutJMreB_1_grm(#) EUT1
12134 HMM00465alcdh_3_mix(pf00465) EUT1
12135 HMMtpEuth_1_eut(pf04346) EUT1
12136 HMM06277EutA_1_eut(pf06277) EUT1
12137 HMMetlyEutB_1_EUT1(pf06751) EUT1
12138 HMMetlyEutC_1_eut1(pf05985) EUT1 EP_NTERM
12139 Tsp_rosa EUT1
12140 H_mulberry(pf00936pf16365) EUT1
12141 HMMPduS_1_all(pf01512pf13375pf10531) EUT1
12142 Ts_dodgerBlue PDU1AB
12143 HMMreg12833_1_EUT1(pf12833) EUT1
12144 HMMacetkin_1(pf00871) GRM3B
12145 HMM02780keto_1_eut1(pf13292pf02779pf02780) EUT1
12146 -pf00348 -
12147 -pf02609 -
12148 -pf04336 -
12149 -pf00582 -
12150 -pf01553 -
12151 -# -
12152 -# -
BMC-P
BMC-H
BMC-T
Aldehyde dehydrogenase
PTAC
Signature enzyme
Alcohol dehydrogenase
Regulator

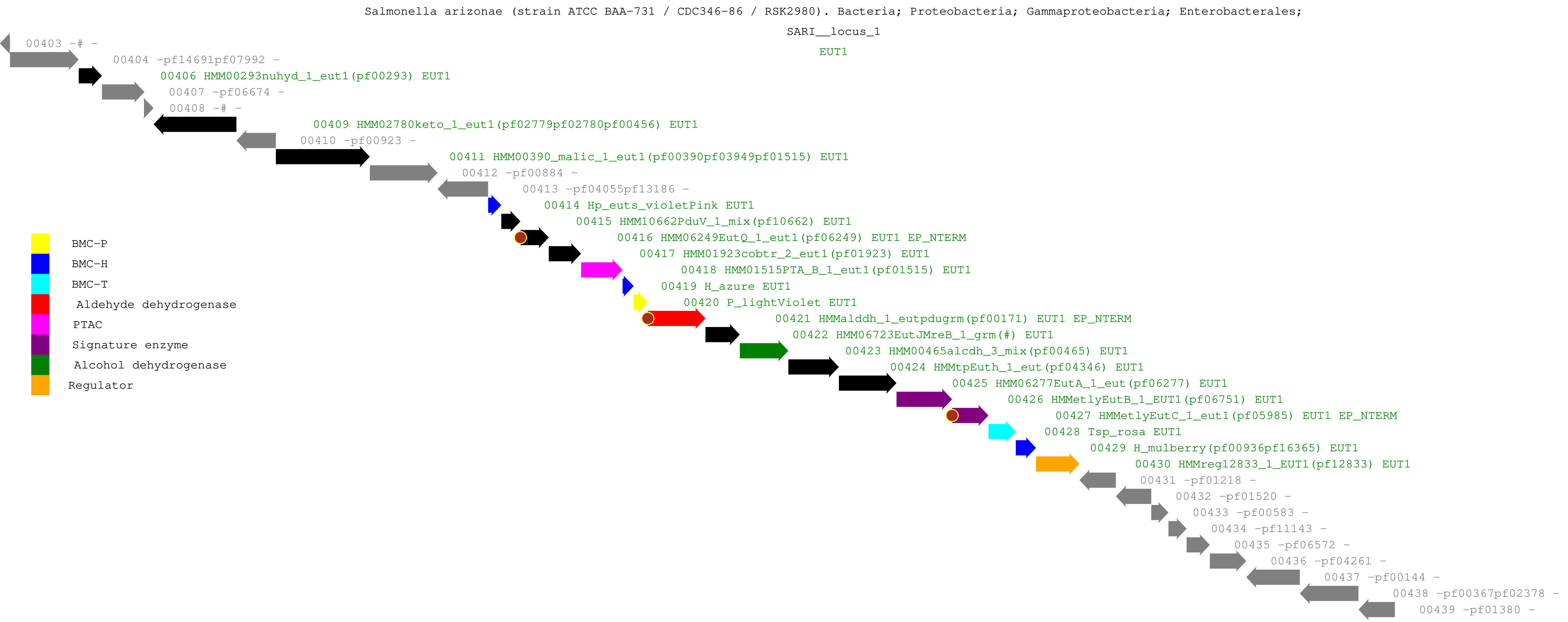

Salmonella arizonae (strain ATCC BAA-731 / CDC346-86 / RSK2980). Bacteria; Proteobacteria; Gammaproteobacteria; Enterobacterales;
SARI__locus_1
EUT1
00403 -# -
00404 -pf14691pf07992 -
00406 HMM00293nuhyd_1_eut1(pf00293) EUT1
00407 -pf06674 -
00408 -# -
00409 HMM02780keto_1_eut1(pf02779pf02780pf00456) EUT1
00410 -pf00923 -
00411 HMM00390_malic_1_eut1(pf00390pf03949pf01515) EUT1
00412 -pf00884 -
00413 -pf04055pf13186 -
00414 Hp_euts_violetPink EUT1
00415 HMM10662PduV_1_mix(pf10662) EUT1
00416 HMM06249EutQ_1_eut1(pf06249) EUT1 EP_NTERM
00417 HMM01923cobtr_2_eut1(pf01923) EUT1
00418 HMM01515PTA_B_1_eut1(pf01515) EUT1
00419 H_azure EUT1
00420 P_lightViolet EUT1
00421 HMMalddh_1_eutpdugrm(pf00171) EUT1 EP_NTERM
00422 HMM06723EutJMreB_1_grm(#) EUT1
00423 HMM00465alcdh_3_mix(pf00465) EUT1
00424 HMMtpEuth_1_eut(pf04346) EUT1
00425 HMM06277EutA_1_eut(pf06277) EUT1
00426 HMMetlyEutB_1_EUT1(pf06751) EUT1
00427 HMMetlyEutC_1_eut1(pf05985) EUT1 EP_NTERM
00428 Tsp_rosa EUT1
00429 H_mulberry(pf00936pf16365) EUT1
00430 HMMreg12833_1_EUT1(pf12833) EUT1
00431 -pf01218 -
00432 -pf01520 -
00433 -pf00583 -
00434 -pf11143 -
00435 -pf06572 -
00436 -pf04261 -
00437 -pf00144 -
00438 -pf00367pf02378 -
00439 -pf01380 -
BMC-P
BMC-H
BMC-T
Aldehyde dehydrogenase
PTAC
Signature enzyme
Alcohol dehydrogenase
Regulator

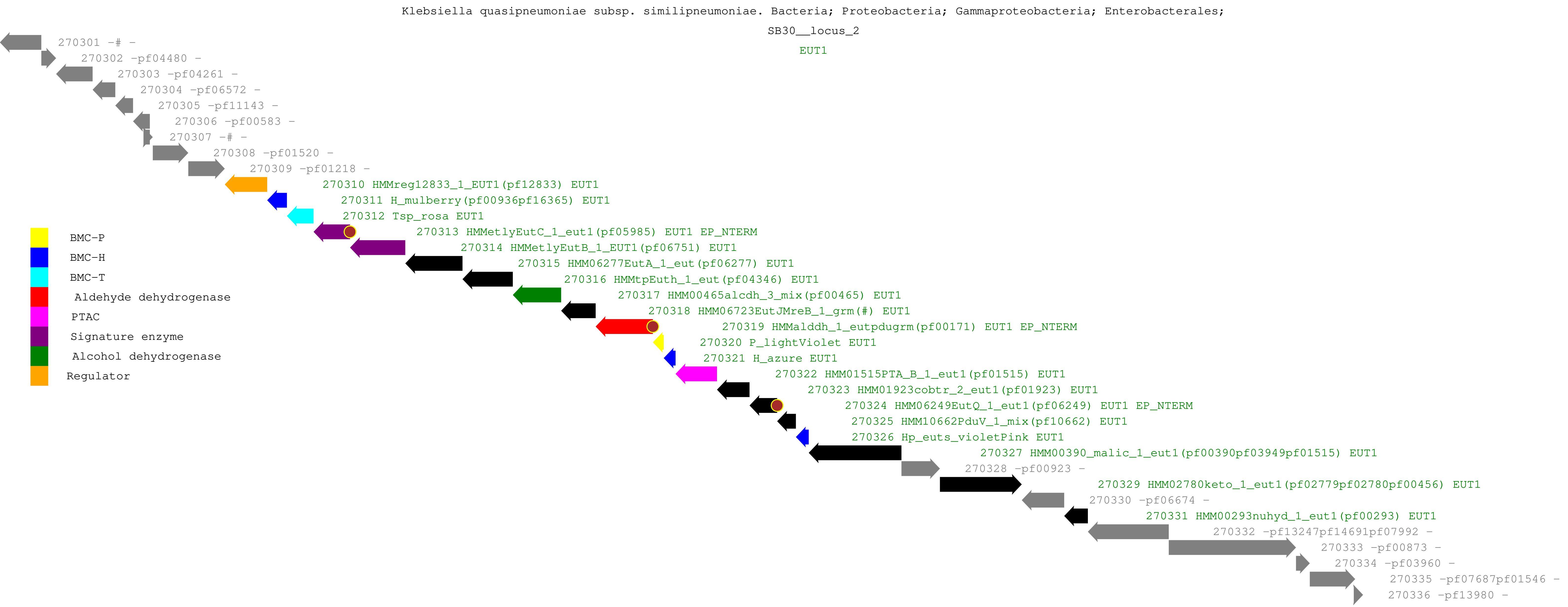
Klebsiella quasipneumoniae subsp. similipneumoniae. Bacteria; Proteobacteria; Gammaproteobacteria; Enterobacterales;
SB30__locus_2
EUT1
270301 -# -
270302 -pf04480 -
270303 -pf04261 -
270304 -pf06572 -
270305 -pf11143 -
270306 -pf00583 -
270307 -# -
270308 -pf01520 -
270309 -pf01218 -
270310 HMMreg12833_1_EUT1(pf12833) EUT1
270311 H_mulberry(pf00936pf16365) EUT1
270312 Tsp_rosa EUT1
270313 HMMetlyEutC_1_eut1(pf05985) EUT1 EP_NTERM
270314 HMMetlyEutB_1_EUT1(pf06751) EUT1
270315 HMM06277EutA_1_eut(pf06277) EUT1
270316 HMMtpEuth_1_eut(pf04346) EUT1
270317 HMM00465alcdh_3_mix(pf00465) EUT1
270318 HMM06723EutJMreB_1_grm(#) EUT1
270319 HMMaldh_1_eutpdugrm(pf00171) EUT1 EP_NTERM
270320 P_lightViolet EUT1
270321 H_azure EUT1
270322 HMM01515PTA_B_1_eut1(pf01515) EUT1
270323 HMM01923cobtr_2_eut1(pf01923) EUT1
270324 HMM06249EutQ_1_eut1(pf06249) EUT1 EP_NTERM
270325 HMM10662PduV_1_mix(pf10662) EUT1
270326 Hp_euts_violetPink EUT1
270327 HMM00390_malic_1_eut1(pf00390pf03949pf01515) EUT1
270328 -pf00923 -
270329 HMM02780keto_1_eut1(pf02779pf02780pf00456) EUT1
270330 -pf06674 -
270331 HMM00293nuhyd_1_eut1(pf00293) EUT1
270332 -pf13247pf14691pf07992 -
270333 -pf00873 -
270334 -pf03960 -
270335 -pf07687pf01546 -
270336 -pf13980 -
BMC-P
BMC-H
BMC-T
Aldehyde dehydrogenase
PTAC
Signature enzyme
Alcohol dehydrogenase
Regulator

Shigella boydii 4444-74. Bacteria; Proteobacteria; Gammaproteobacteria; Enterobacterales;

SB444474__locus_1

EUT1

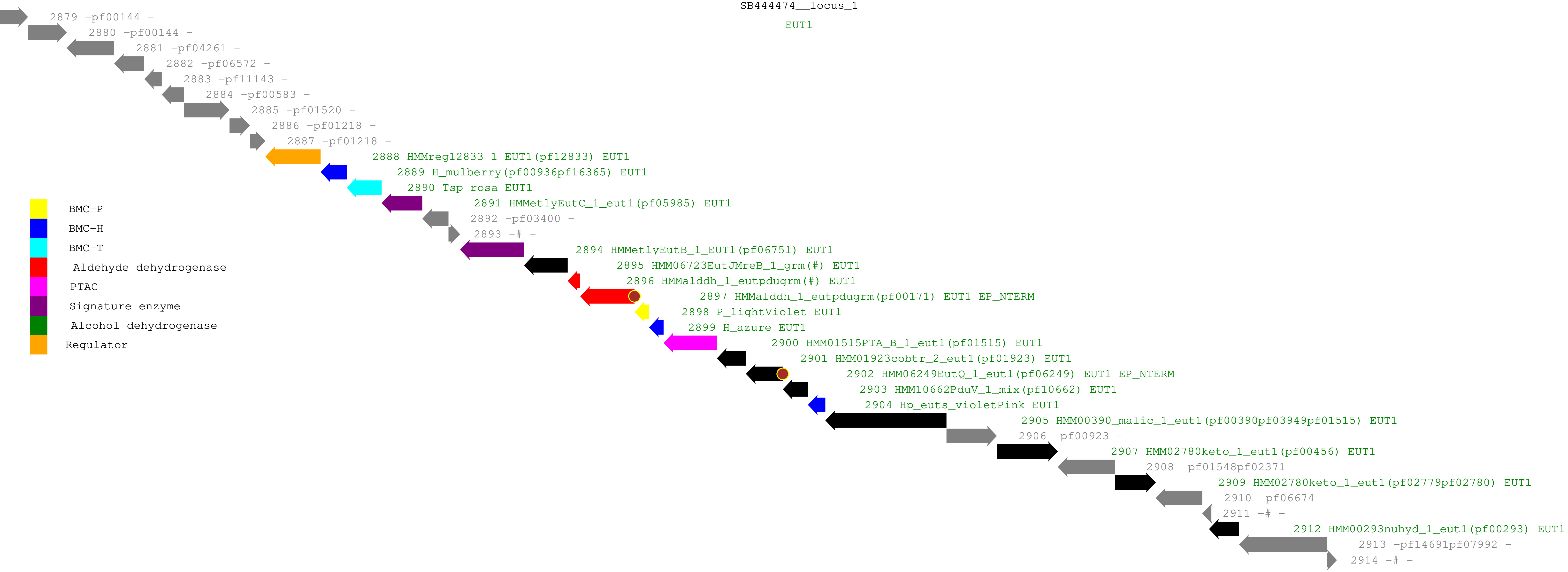

Shigella boydii 5216-82. Bacteria; Proteobacteria; Gammaproteobacteria; Enterobacterales;

SB521682__locus_1

EUT1

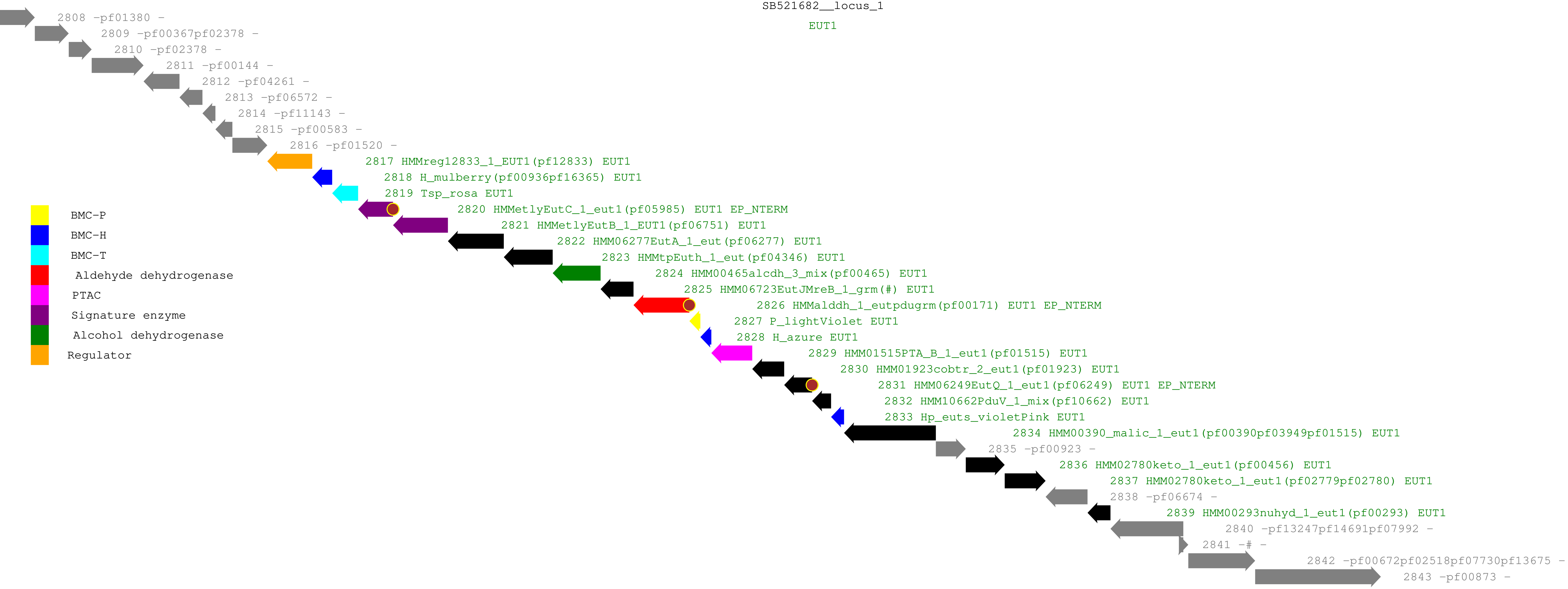

Klebsiella michiganensis. Bacteria; Proteobacteria; Gammaproteobacteria; Enterobacterales;

SB6408__locus_3

EUT1

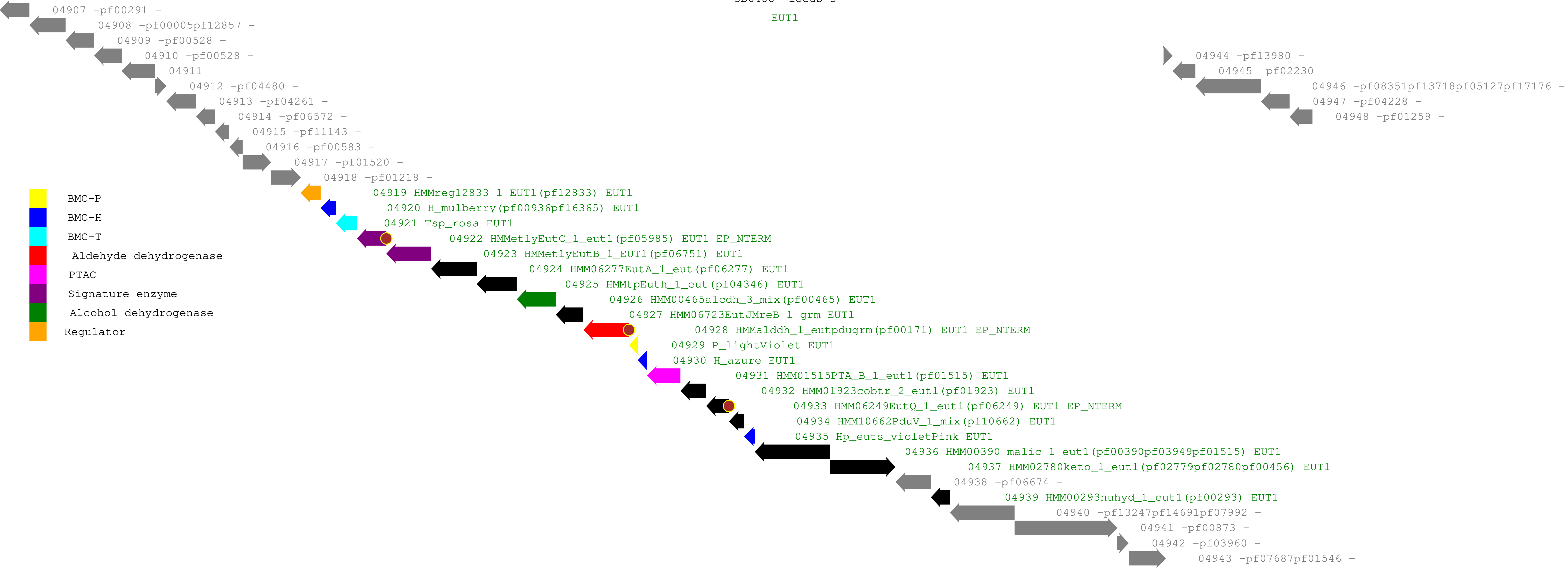

Klebsiella huaxiensis. Bacteria; Proteobacteria; Gammaproteobacteria; Enterobacterales;

SB6422__locus_1

EUT1

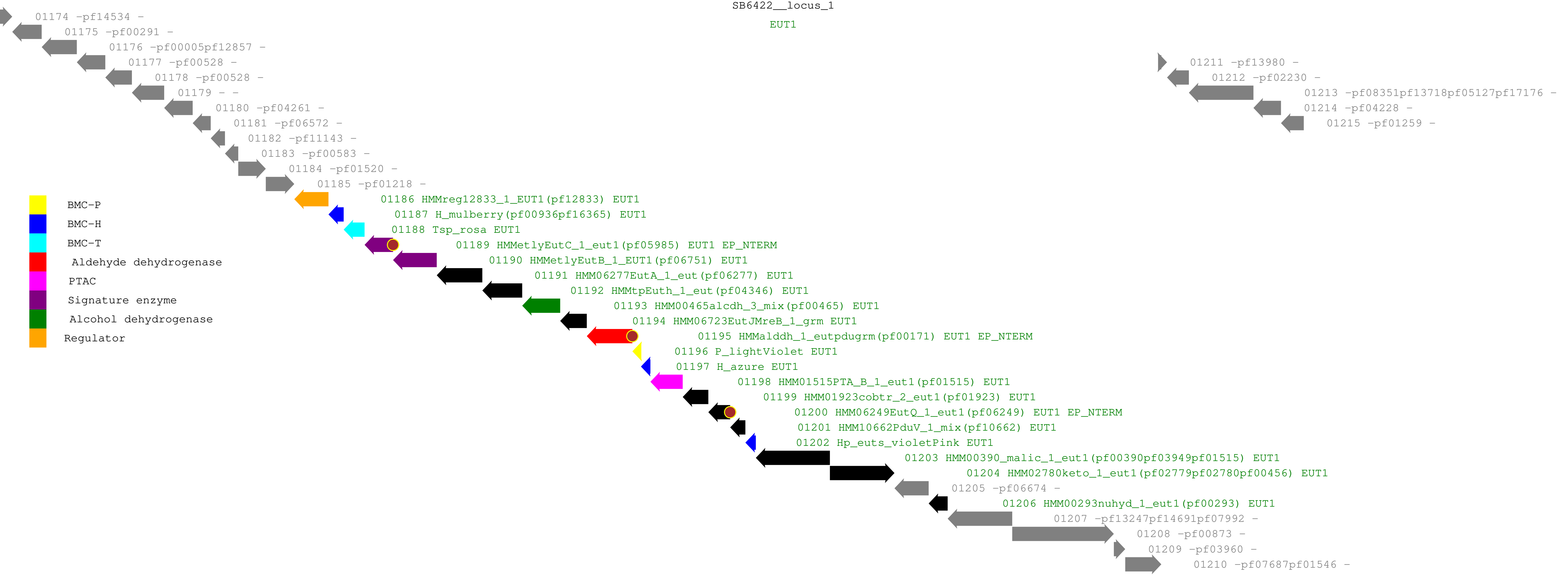

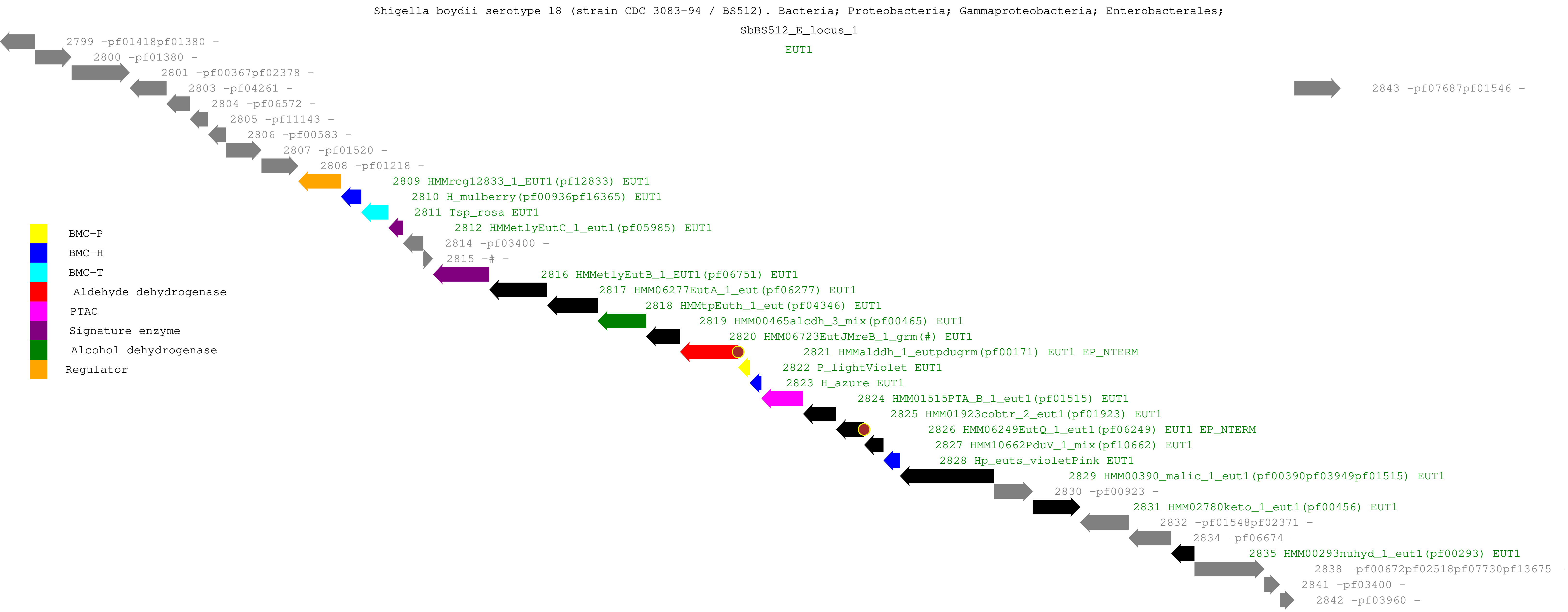
Shigella boydii serotype 18 (strain CDC 3083-94 / BS512). Bacteria; Proteobacteria; Gammaproteobacteria; Enterobacterales;
SbBS512_E_locus_1
EUT1
2799 -pf01418pf01380 -
2800 -pf01380 -
2801 -pf00367pf02378 -
2803 -pf04261 -
2804 -pf06572 -
2805 -pf11143 -
2806 -pf00583 -
2807 -pf01520 -
2808 -pf01218 -
2809 HMMreg12833_1_EUT1(pf12833) EUT1
2810 H_mulberry(pf00936pf16365) EUT1
2811 Tsp_rosa EUT1
2812 HMMetlyEutC_1_eut1(pf05985) EUT1
2814 -pf03400 -
2815 -# -
2816 HMMetlyEutB_1_EUT1(pf06751) EUT1
2817 HMM06277EutA_1_eut(pf06277) EUT1
2818 HMMtpEuth_1_eut(pf04346) EUT1
2819 HMM00465alcdh_3_mix(pf00465) EUT1
2820 HMM06723EutJMreB_1_grm(#) EUT1
2821 HMMalddh_1_eutpdugrm(pf00171) EUT1 EP_NTERM
2822 P_lightViolet EUT1
2823 H_azure EUT1
2824 HMM01515PTA_B_1_eut1(pf01515) EUT1
2825 HMM01923cobtr_2_eut1(pf01923) EUT1
2826 HMM06249EutQ_1_eut1(pf06249) EUT1 EP_NTERM
2827 HMM10662PduV_1_mix(pf10662) EUT1
2828 Hp_euts_violetPink EUT1
2829 HMM00390_malic_1_eut1(pf00390pf03949pf01515) EUT1
2830 -pf00923 -
2831 HMM02780keto_1_eut1(pf00456) EUT1
2832 -pf01548pf02371 -
2834 -pf06674 -
2835 HMM00293nuhyd_1_eut1(pf00293) EUT1
2838 -pf00672pf02518pf07730pf13675 -
2841 -pf03400 -
2842 -pf03960 -
2843 -pf07687pf01546 -
BMC-P
BMC-H
BMC-T
Aldehyde dehydrogenase
PTAC
Signature enzyme
Alcohol dehydrogenase
Regulator

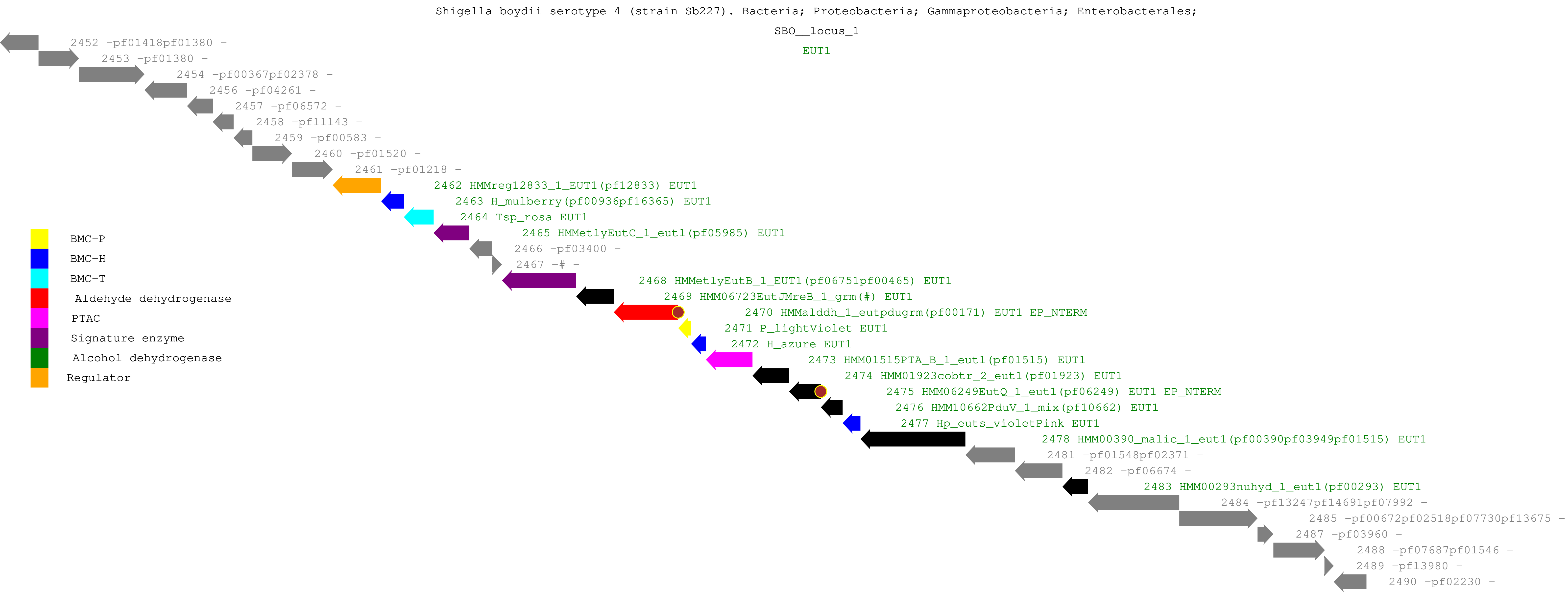
Shigella boydii serotype 4 (strain Sb227). Bacteria; Proteobacteria; Gammaproteobacteria; Enterobacterales;
SBO__locus_1
EUT1
2452 -pf01418pf01380 -
2453 -pf01380 -
2454 -pf00367pf02378 -
2456 -pf04261 -
2457 -pf06572 -
2458 -pf11143 -
2459 -pf00583 -
2460 -pf01520 -
2461 -pf01218 -
2462 HMMreg12833_1_EUT1(pf12833) EUT1
2463 H_mulberry(pf00936pf16365) EUT1
2464 Tsp_rosa EUT1
2465 HMMetlyEutC_1_eut1(pf05985) EUT1
2466 -pf03400 -
2467 -# -
2468 HMMetlyEutB_1_EUT1(pf06751pf00465) EUT1
2469 HMM06723EutJMreB_1_grm(#) EUT1
2470 HMMaldh_1_eutpdugrm(pf00171) EUT1 EP_NTERM
2471 P_lightViolet EUT1
2472 H_azure EUT1
2473 HMM01515PTA_B_1_eut1(pf01515) EUT1
2474 HMM01923cobtr_2_eut1(pf01923) EUT1
2475 HMM06249EutQ_1_eut1(pf06249) EUT1 EP_NTERM
2476 HMM10662PduV_1_mix(pf10662) EUT1
2477 Hp_euts_violetPink EUT1
2478 HMM00390_malic_1_eut1(pf00390pf03949pf01515) EUT1
2481 -pf01548pf02371 -
2482 -pf06674 -
2483 HMM00293nuhyd_1_eut1(pf00293) EUT1
2484 -pf13247pf14691pf07992 -
2485 -pf00672pf02518pf07730pf13675 -
2487 -pf03960 -
2488 -pf07687pf01546 -
2489 -pf13980 -
2490 -pf02230 -
BMC-P
BMC-H
BMC-T
Aldehyde dehydrogenase
PTAC
Signature enzyme
Alcohol dehydrogenase
Regulator

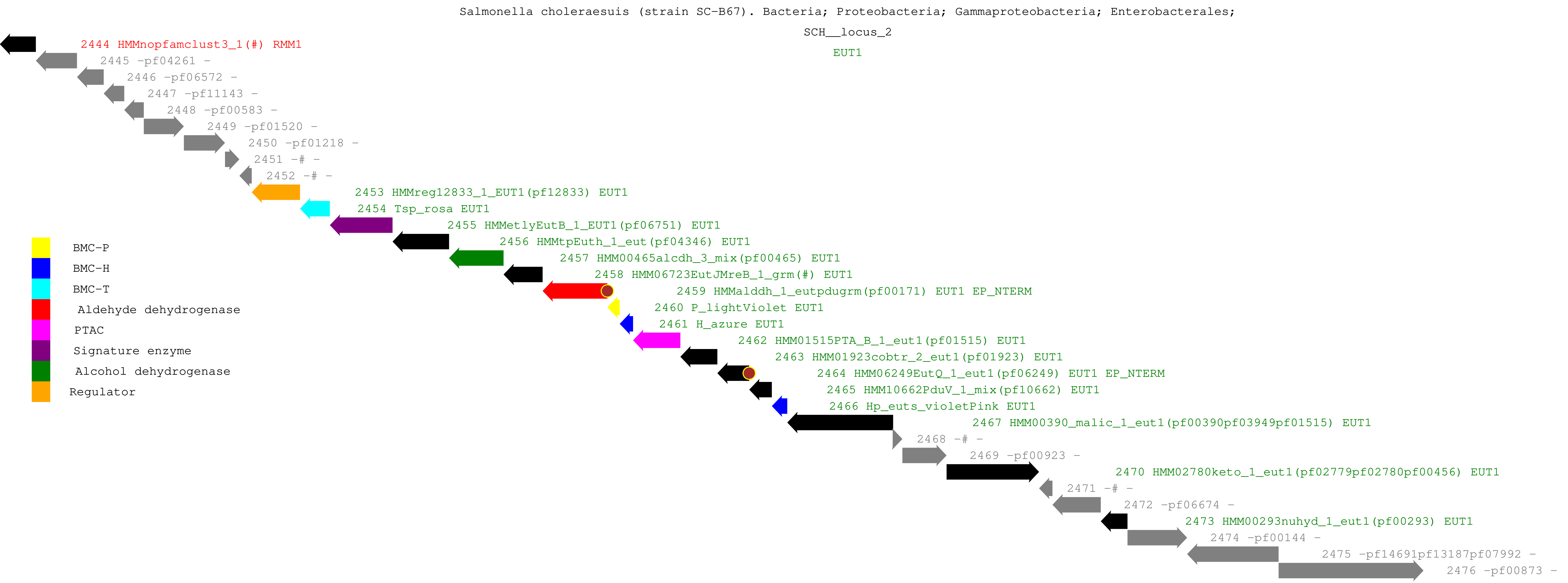
Salmonella choleraesuis (strain SC-B67). Bacteria; Proteobacteria; Gammaproteobacteria; Enterobacterales;
SCH__locus_2
EUT1
2444 HMMnopfamclust3_1(#) RMM1
2445 -pf04261 -
2446 -pf06572 -
2447 -pf11143 -
2448 -pf00583 -
2449 -pf01520 -
2450 -pf01218 -
2451 -# -
2452 -# -
2453 HMMreg12833_1_EUT1(pf12833) EUT1
2454 Tsp_rosa EUT1
2455 HMMetlyEutB_1_EUT1(pf06751) EUT1
2456 HMMtpEuth_1_eut(pf04346) EUT1
2457 HMM00465alcdh_3_mix(pf00465) EUT1
2458 HMM06723EutJMreB_1_grm(#) EUT1
2459 HMMalddh_1_eutpdugrm(pf00171) EUT1 EP_NTERM
2460 P_lightViolet EUT1
2461 H_azure EUT1
2462 HMM01515PTA_B_1_eut1(pf01515) EUT1
2463 HMM01923cobtr_2_eut1(pf01923) EUT1
2464 HMM06249EutQ_1_eut1(pf06249) EUT1 EP_NTERM
2465 HMM10662PduV_1_mix(pf10662) EUT1
2466 Hp_euts_violetPink EUT1
2467 HMM00390_malic_1_eut1(pf00390pf03949pf01515) EUT1
2468 -# -
2469 -pf00923 -
2470 HMM02780keto_1_eut1(pf02779pf02780pf00456) EUT1
2471 -# -
2472 -pf06674 -
2473 HMM00293nuhyd_1_eut1(pf00293) EUT1
2474 -pf00144 -
2475 -pf14691pf13187pf07992 -
2476 -pf00873 -
BMC-P
BMC-H
BMC-T
Aldehyde dehydrogenase
PTAC
Signature enzyme
Alcohol dehydrogenase
Regulator

Shigella dysenteriae 1012. Bacteria; Proteobacteria; Gammaproteobacteria; Enterobacterales;

Sd1012__locus_1

EUT1

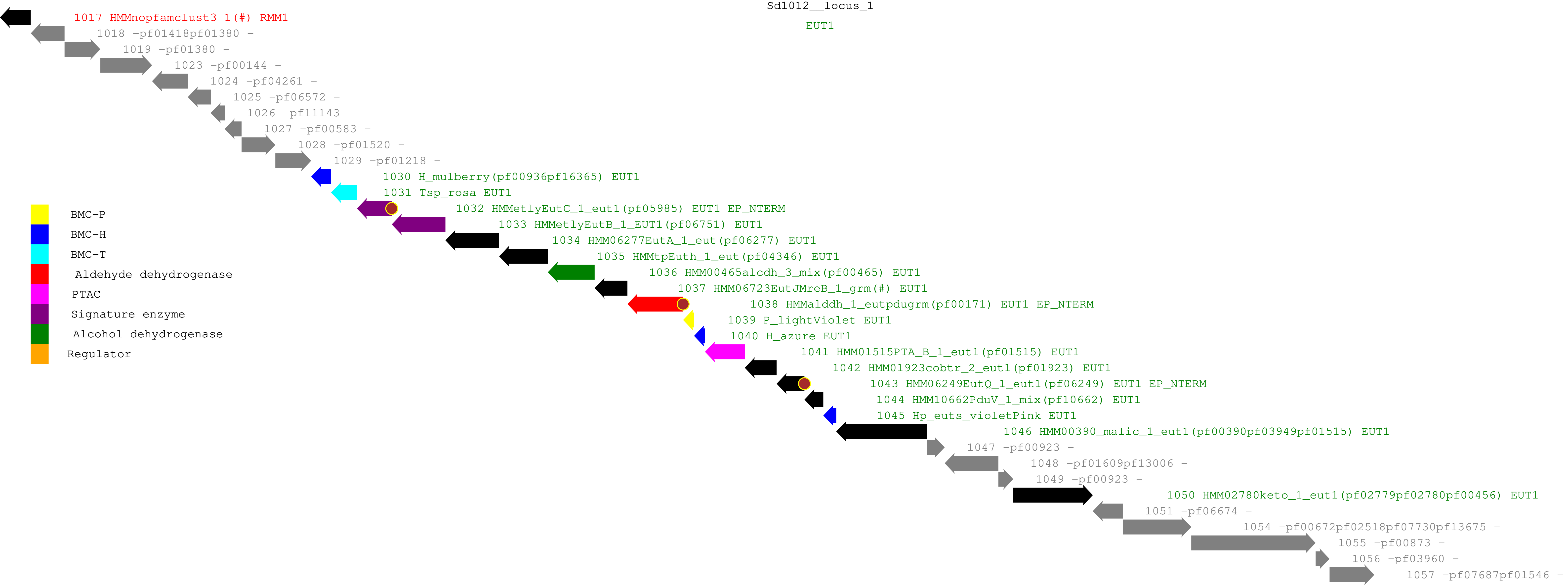

Shigella dysenteriae 155-74. Bacteria; Proteobacteria; Gammaproteobacteria; Enterobacterales;

SD15574__locus_1

EUT1

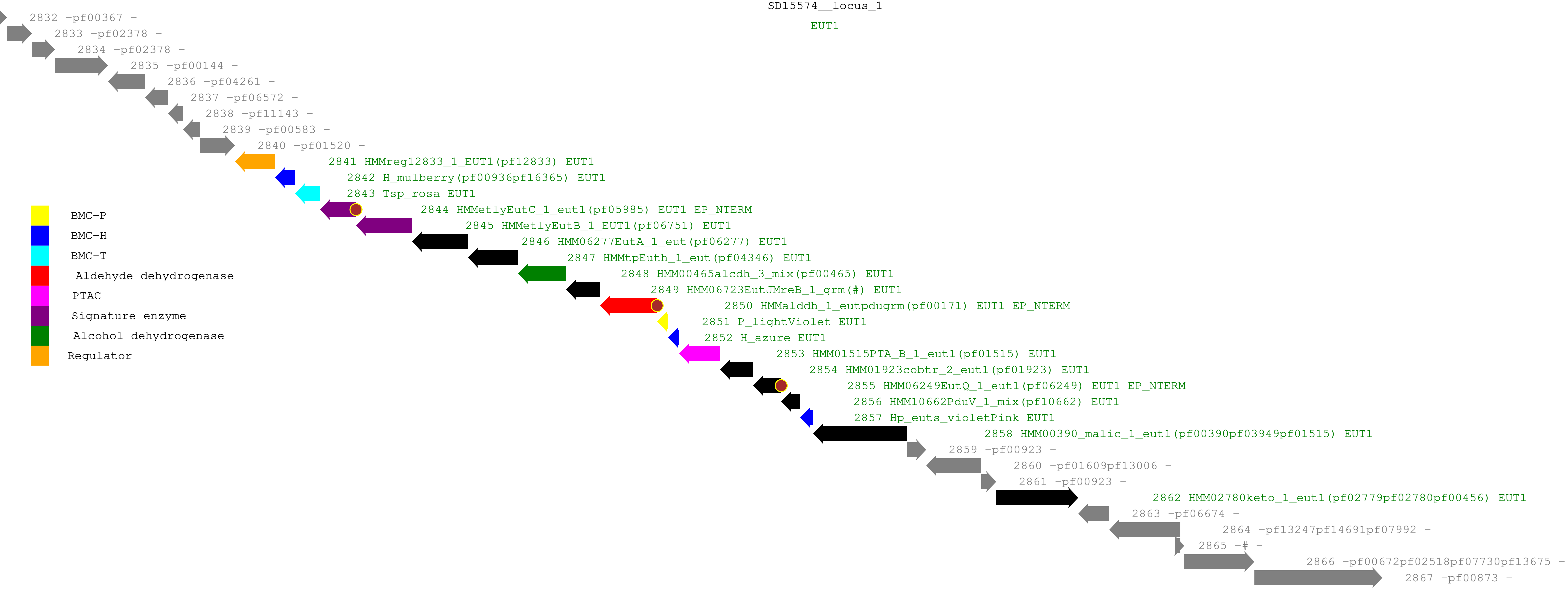

Shigella dysenteriae 225-75. Bacteria; Proteobacteria; Gammaproteobacteria; Enterobacterales;

SD22575__locus_1

EUT1

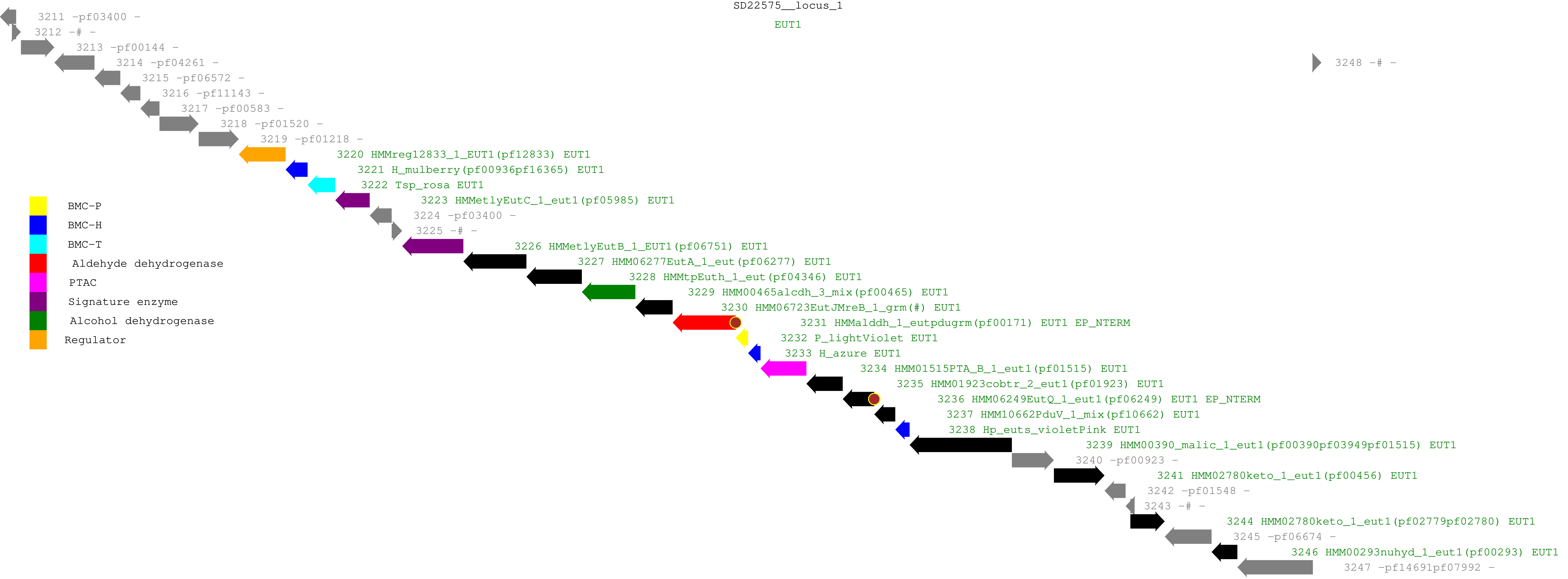

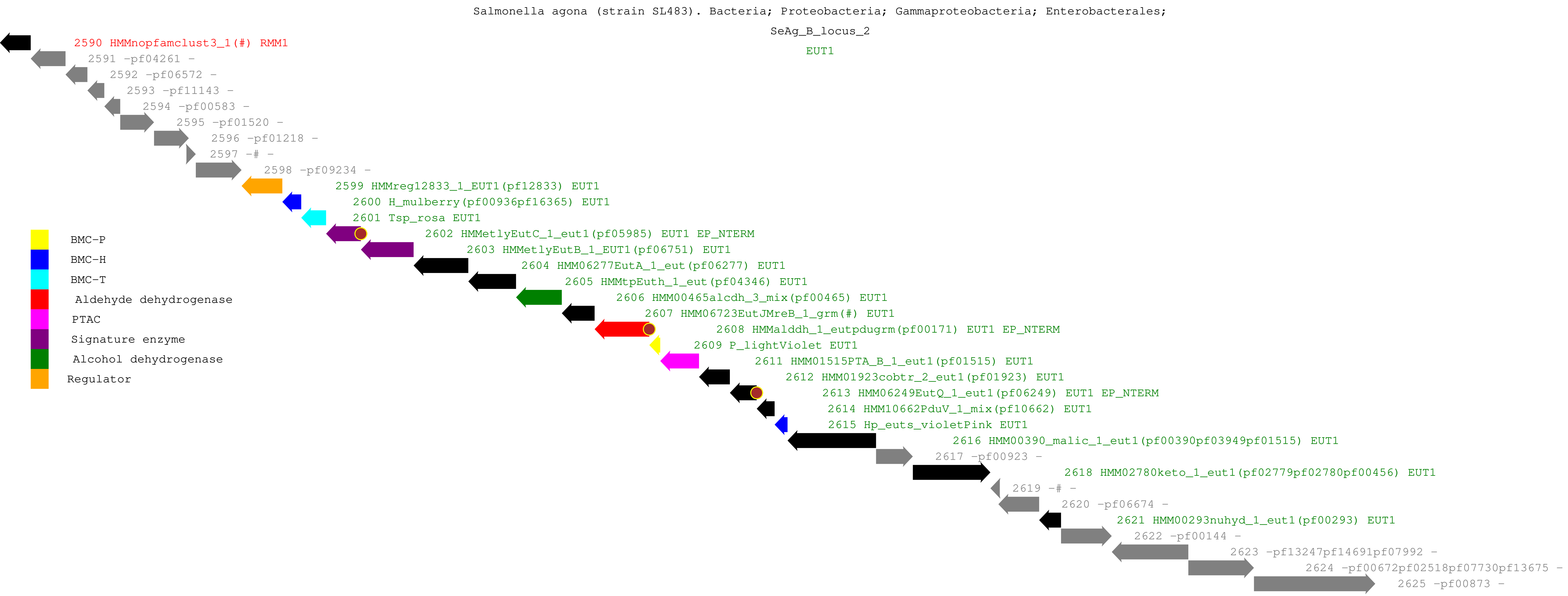

Salmonella agona (strain SL483). Bacteria; Proteobacteria; Gammaproteobacteria; Enterobacterales;
SeAg_B_locus_2
EUT1
2590 HMMnopfamclust3_1(#) RMM1
2591 -pf04261 -
2592 -pf06572 -
2593 -pf11143 -
2594 -pf00583 -
2595 -pf01520 -
2596 -pf01218 -
2597 -# -
2598 -pf09234 -
2599 HMMreg12833_1_EUT1(pf12833) EUT1
2600 H_mulberry(pf00936pf16365) EUT1
2601 Tsp_rosa EUT1
2602 HMMetlyEutC_1_eut1(pf05985) EUT1 EP_NTERM
2603 HMMetlyEutB_1_EUT1(pf06751) EUT1
2604 HMM06277EutA_1_eut(pf06277) EUT1
2605 HMMtpEuth_1_eut(pf04346) EUT1
2606 HMM00465alcdh_3_mix(pf00465) EUT1
2607 HMM06723EutJMreB_1_grm(#) EUT1
2608 HMMalddh_1_eutpdugrm(pf00171) EUT1 EP_NTERM
2609 P_lightViolet EUT1
2611 HMM01515PTA_B_1_eut1(pf01515) EUT1
2612 HMM01923cobtr_2_eut1(pf01923) EUT1
2613 HMM06249EutQ_1_eut1(pf06249) EUT1 EP_NTERM
2614 HMM10662PduV_1_mix(pf10662) EUT1
2615 Hp_euts_violetPink EUT1
2616 HMM00390_malic_1_eut1(pf00390pf03949pf01515) EUT1
2617 -pf00923 -
2618 HMM02780keto_1_eut1(pf02779pf02780pf00456) EUT1
2619 -# -
2620 -pf06674 -
2621 HMM00293nuhyd_1_eut1(pf00293) EUT1
2622 -pf00144 -
2623 -pf13247pf14691pf07992 -
2624 -pf00672pf02518pf07730pf13675 -
2625 -pf00873 -
BMC-P
BMC-H
BMC-T
Aldehyde dehydrogenase
PTAC
Signature enzyme
Alcohol dehydrogenase
Regulator

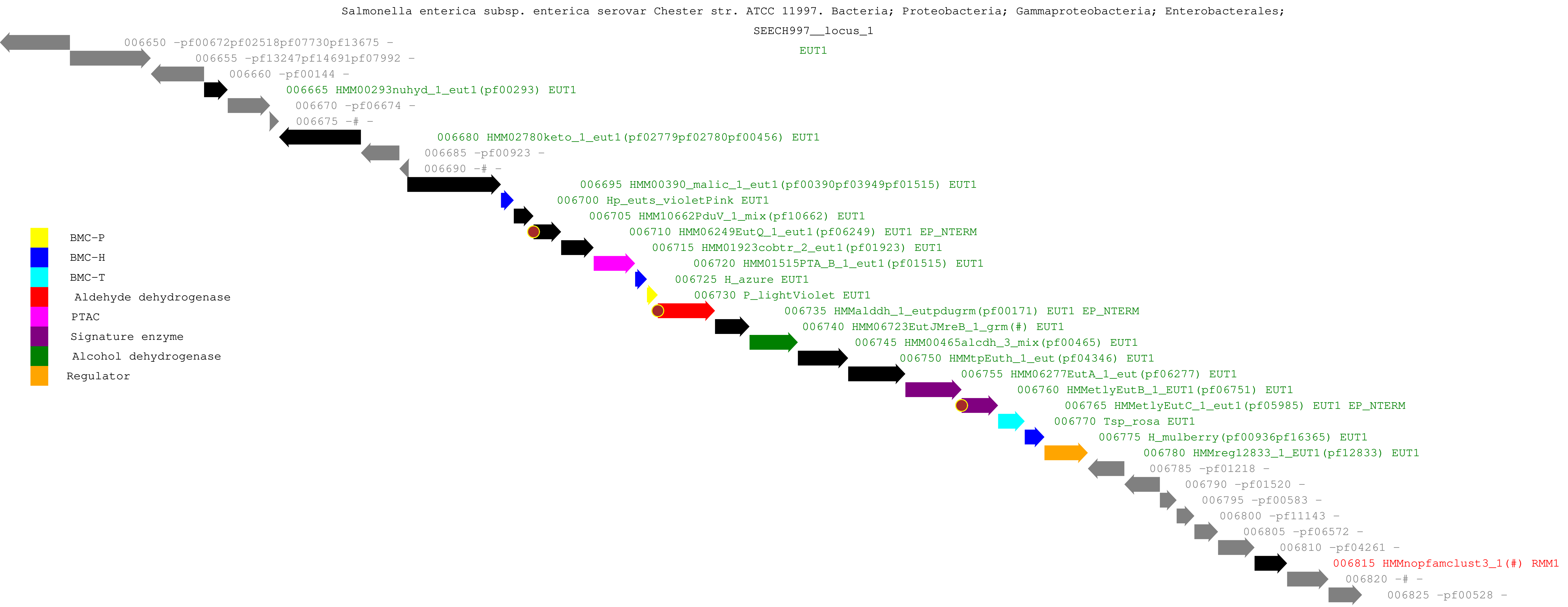
Salmonella enterica subsp. enterica serovar Chester str. ATCC 11997. Bacteria; Proteobacteria; Gammaproteobacteria; Enterobacterales;
SEECH997__locus_1
EUT1
006650 -pf00672pf02518pf07730pf13675 -
006655 -pf13247pf14691pf07992 -
006660 -pf00144 -
006665 HMM00293nuhyd_1_eut1(pf00293) EUT1
006670 -pf06674 -
006675 -# -
006680 HMM02780keto_1_eut1(pf02779pf02780pf00456) EUT1
006685 -pf00923 -
006690 -# -
006695 HMM00390_malic_1_eut1(pf00390pf03949pf01515) EUT1
006700 Hp_euts_violetPink EUT1
006705 HMM10662PduV_1_mix(pf10662) EUT1
006710 HMM06249EutQ_1_eut1(pf06249) EUT1 EP_NTERM
006715 HMM01923cobtr_2_eut1(pf01923) EUT1
006720 HMM01515PTA_B_1_eut1(pf01515) EUT1
006725 H_azure EUT1
006730 P_lightViolet EUT1
006735 HMMalddh_1_eutpdugrm(pf00171) EUT1 EP_NTERM
006740 HMM06723EutJMreB_1_grm(#) EUT1
006745 HMM00465alcdh_3_mix(pf00465) EUT1
006750 HMMtpEuth_1_eut(pf04346) EUT1
006755 HMM06277EutA_1_eut(pf06277) EUT1
006760 HMMetlyEutB_1_EUT1(pf06751) EUT1
006765 HMMetlyEutC_1_eut1(pf05985) EUT1 EP_NTERM
006770 Tsp_rosa EUT1
006775 H_mulberry(pf00936pf16365) EUT1
006780 HMMreg12833_1_EUT1(pf12833) EUT1
006785 -pf01218 -
006790 -pf01520 -
006795 -pf00583 -
006800 -pf11143 -
006805 -pf06572 -
006810 -pf04261 -
006815 HMMnopfamclust3_1(#) RMM1
006820 -# -
006825 -pf00528 -
BMC-P
BMC-H
BMC-T
Aldehyde dehydrogenase
PTAC
Signature enzyme
Alcohol dehydrogenase
Regulator

Salmonella enterica subsp. enterica serovar Muenster str. 0315. Bacteria; Proteobacteria; Gammaproteobacteria; Enterobacterales;

SEEM0315__locus_1

EUT1

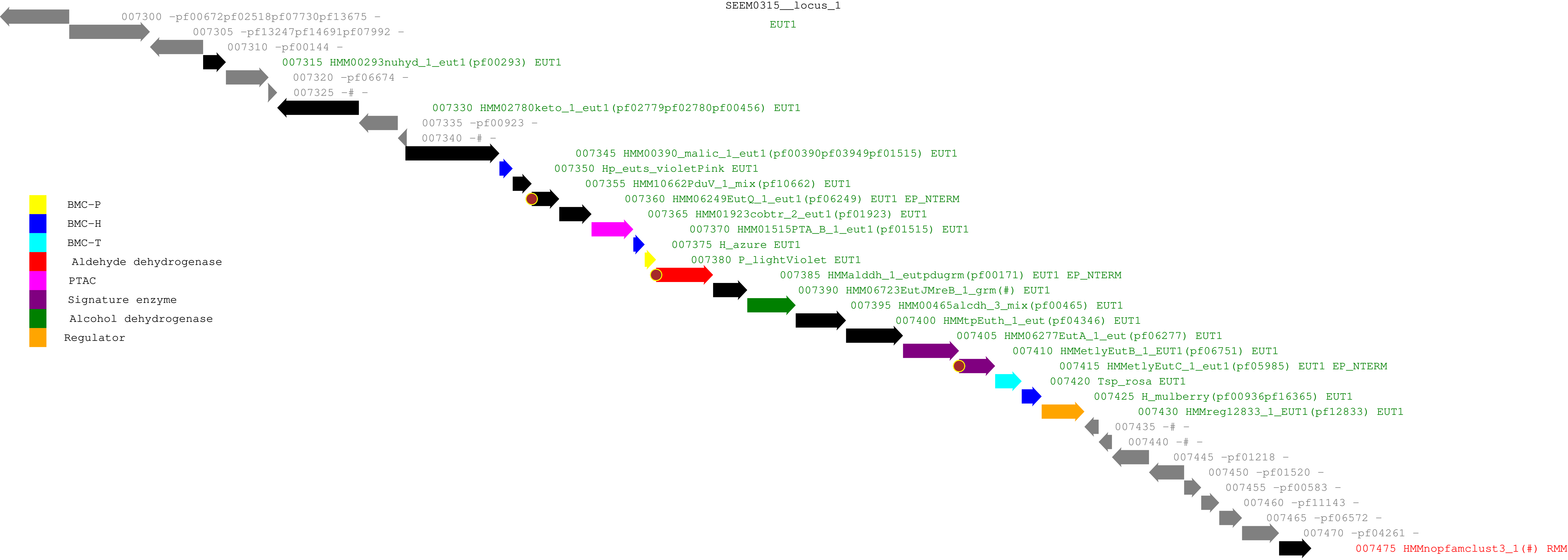

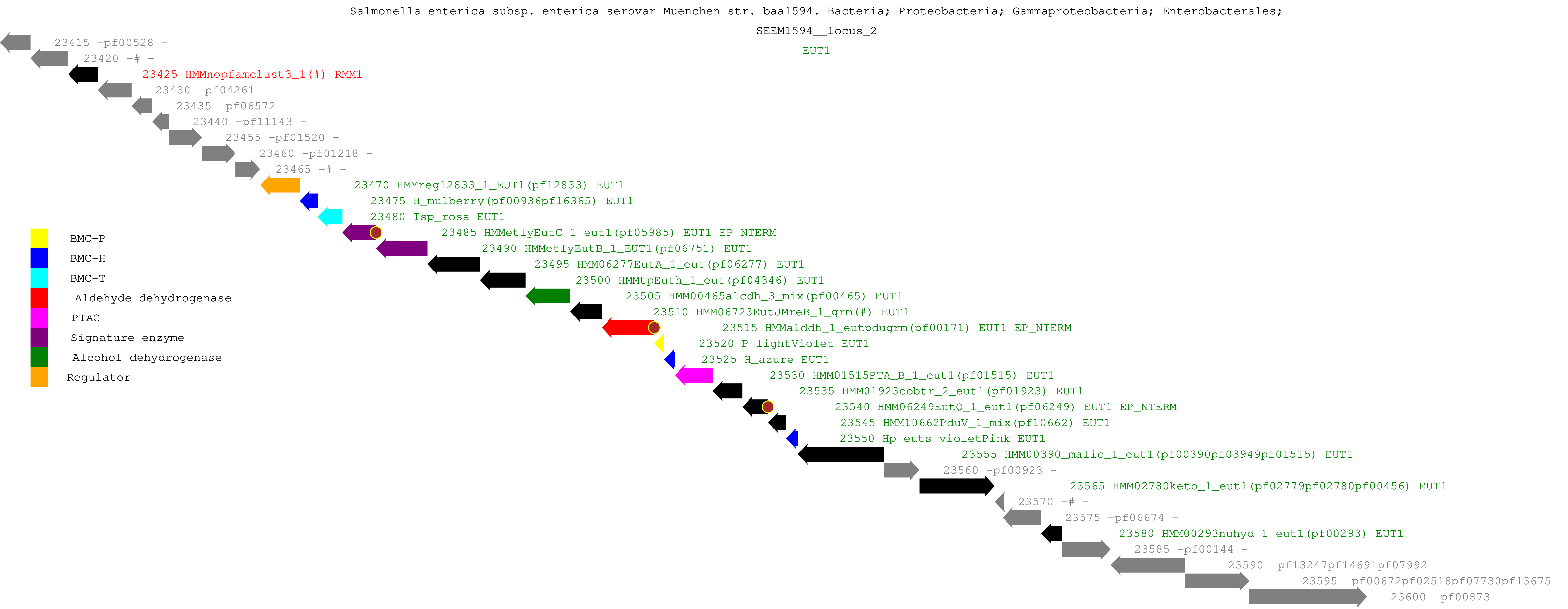

Salmonella enterica subsp. enterica serovar Muenchen str. baa1594. Bacteria; Proteobacteria; Gammaproteobacteria; Enterobacterales;
SEEM1594__locus_2
EUT1
23415 -pf00528 -
23420 -# -
23425 HMMnopfamclust3_1(#) RMM1
23430 -pf04261 -
23435 -pf06572 -
23440 -pf11143 -
23455 -pf01520 -
23460 -pf01218 -
23465 -# -
23470 HMMreg12833_1_EUT1(pf12833) EUT1
23475 H_mulberry(pf00936pf16365) EUT1
23480 Tsp_rosa EUT1
23485 HMMetlyEutC_1_eut1(pf05985) EUT1 EP_NTERM
23490 HMMetlyEutB_1_EUT1(pf06751) EUT1
23495 HMM06277EutA_1_eut(pf06277) EUT1
23500 HMMtpEuth_1_eut(pf04346) EUT1
23505 HMM00465alcdh_3_mix(pf00465) EUT1
23510 HMM06723EutJMreB_1_grm(#) EUT1
23515 HMMalddh_1_eutpdugrm(pf00171) EUT1 EP_NTERM
23520 P_lightViolet EUT1
23525 H_azure EUT1
23530 HMM01515PTA_B_1_eut1(pf01515) EUT1
23535 HMM01923cobtr_2_eut1(pf01923) EUT1
23540 HMM06249EutQ_1_eut1(pf06249) EUT1 EP_NTERM
23545 HMM10662PduV_1_mix(pf10662) EUT1
23550 Hp_euts_violetPink EUT1
23555 HMM00390_malic_1_eut1(pf00390pf03949pf01515) EUT1
23560 -pf00923 -
23565 HMM02780keto_1_eut1(pf02779pf02780pf00456) EUT1
23570 -# -
23575 -pf06674 -
23580 HMM00293nuhyd_1_eut1(pf00293) EUT1
23585 -pf00144 -
23590 -pf13247pf14691pf07992 -
23595 -pf00672pf02518pf07730pf13675 -
23600 -pf00873 -
BMC-P
BMC-H
BMC-T
Aldehyde dehydrogenase
PTAC
Signature enzyme
Alcohol dehydrogenase
Regulator

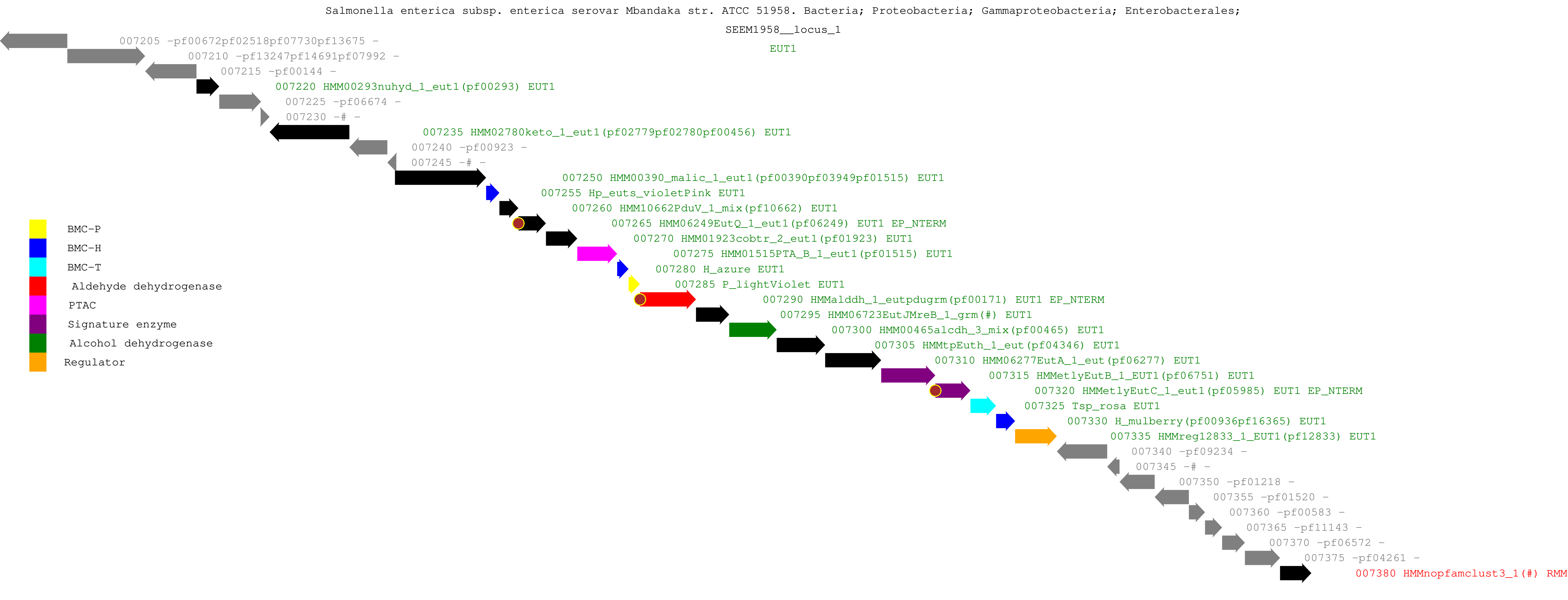
Salmonella enterica subsp. enterica serovar Mbandaka str. ATCC 51958. Bacteria; Proteobacteria; Gammaproteobacteria; Enterobacterales;
SEEM1958__locus_1
EUT1
007205 -pf00672pf02518pf07730pf13675 -
007210 -pf13247pf14691pf07992 -
007215 -pf00144 -
007220 HMM00293nuhyd_1_eut1(pf00293) EUT1
007225 -pf06674 -
007230 -# -
007235 HMM02780keto_1_eut1(pf02779pf02780pf00456) EUT1
007240 -pf00923 -
007245 -# -
007250 HMM00390_malic_1_eut1(pf00390pf03949pf01515) EUT1
007255 Hp_euts_violetPink EUT1
007260 HMM10662PduV_1_mix(pf10662) EUT1
007265 HMM06249EutQ_1_eut1(pf06249) EUT1 EP_NTERM
007270 HMM01923cobtr_2_eut1(pf01923) EUT1
007275 HMM01515PTA_B_1_eut1(pf01515) EUT1
007280 H_azure EUT1
007285 P_lightViolet EUT1
007290 HMMaldh_1_eutpdugrm(pf00171) EUT1 EP_NTERM
007295 HMM06723EutJMreB_1_grm(#) EUT1
007300 HMM00465alcdh_3_mix(pf00465) EUT1
007305 HMMtpEuth_1_eut(pf04346) EUT1
007310 HMM06277EutA_1_eut(pf06277) EUT1
007315 HMMetlyEutB_1_EUT1(pf06751) EUT1
007320 HMMetlyEutC_1_eut1(pf05985) EUT1 EP_NTERM
007325 Tsp_rosa EUT1
007330 H_mulberry(pf00936pf16365) EUT1
007335 HMMreg12833_1_EUT1(pf12833) EUT1
007340 -pf09234 -
007345 -# -
007350 -pf01218 -
007355 -pf01520 -
007360 -pf00583 -
007365 -pf11143 -
007370 -pf06572 -
007375 -pf04261 -
007380 HMMnopfamclust3_1(#) RMM
BMC-P
BMC-H
BMC-T
Aldehyde dehydrogenase
PTAC
Signature enzyme
Alcohol dehydrogenase
Regulator

Salmonella enterica subsp. enterica serovar Montevideo str. 42N. Bacteria; Proteobacteria; Gammaproteobacteria; Enterobacterales;

SEEM42N__locus_1

EUT1

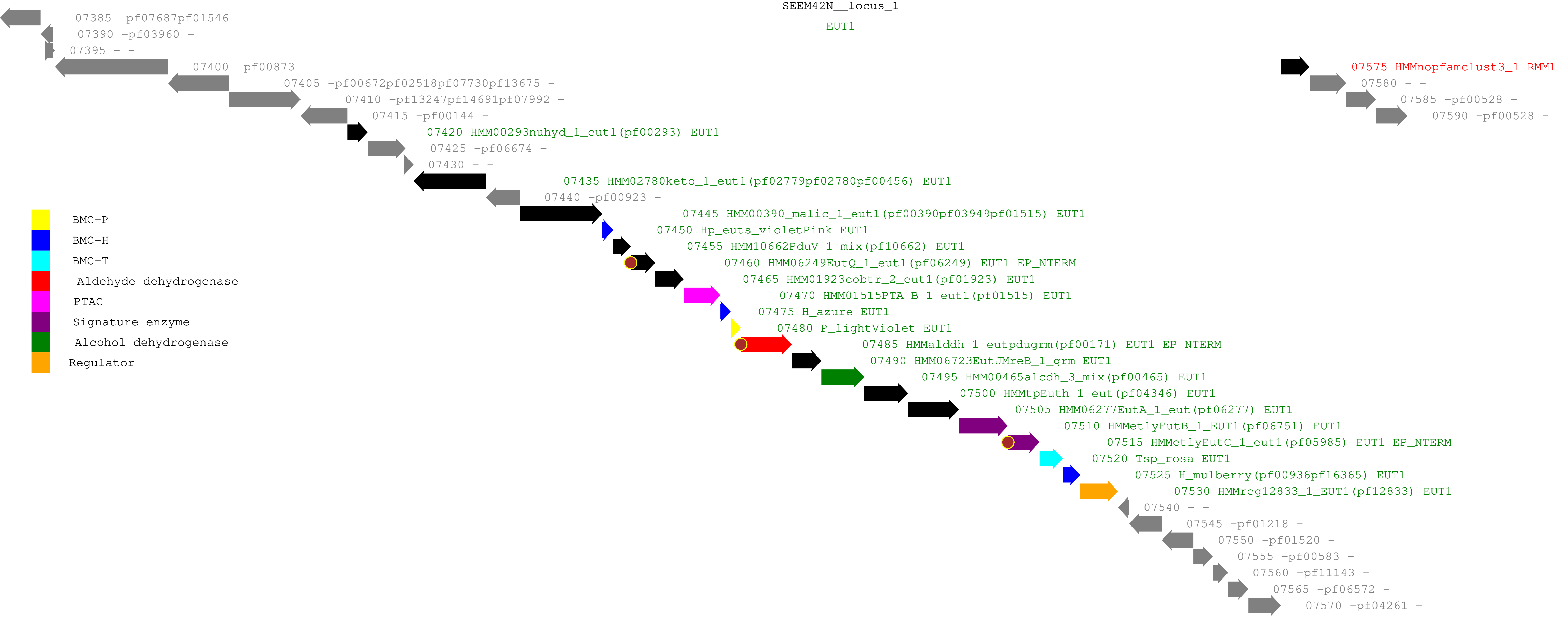

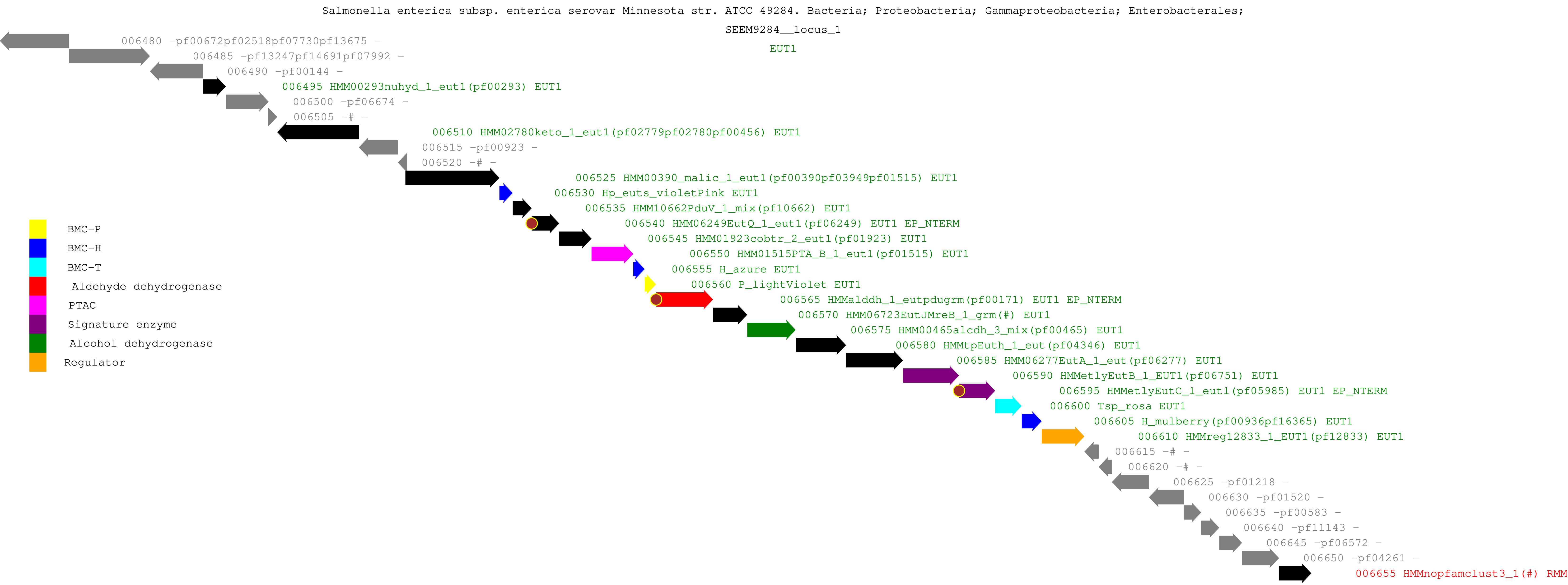
Salmonella enterica subsp. enterica serovar Minnesota str. ATCC 49284. Bacteria; Proteobacteria; Gammaproteobacteria; Enterobacterales;
SEEM9284__locus_1
EUT1
006480 -pf00672pf02518pf07730pf13675 -
006485 -pf13247pf14691pf07992 -
006490 -pf00144 -
006495 HMM00293nuhyd_1_eut1(pf00293) EUT1
006500 -pf06674 -
006505 -# -
006510 HMM02780keto_1_eut1(pf02779pf02780pf00456) EUT1
006515 -pf00923 -
006520 -# -
006525 HMM00390_malic_1_eut1(pf00390pf03949pf01515) EUT1
006530 Hp_euts_violetPink EUT1
006535 HMM10662PduV_1_mix(pf10662) EUT1
006540 HMM06249EutQ_1_eut1(pf06249) EUT1 EP_NTERM
006545 HMM01923cobtr_2_eut1(pf01923) EUT1
006550 HMM01515PTA_B_1_eut1(pf01515) EUT1
006555 H_azure EUT1
006560 P_lightViolet EUT1
006565 HMMalddh_1_eutpdugrm(pf00171) EUT1 EP_NTERM
006570 HMM06723EutJMreB_1_grm(#) EUT1
006575 HMM00465alcdh_3_mix(pf00465) EUT1
006580 HMMtpEuth_1_eut(pf04346) EUT1
006585 HMM06277EutA_1_eut(pf06277) EUT1
006590 HMMetlyEutB_1_EUT1(pf06751) EUT1
006595 HMMetlyEutC_1_eut1(pf05985) EUT1 EP_NTERM
006600 Tsp_rosa EUT1
006605 H_mulberry(pf00936pf16365) EUT1
006610 HMMreg12833_1_EUT1(pf12833) EUT1
006615 -# -
006620 -# -
006625 -pf01218 -
006630 -pf01520 -
006635 -pf00583 -
006640 -pf11143 -
006645 -pf06572 -
006650 -pf04261 -
006655 HMMnopfamclust3_1(#) RMM
BMC-P
BMC-H
BMC-T
Aldehyde dehydrogenase
PTAC
Signature enzyme
Alcohol dehydrogenase
Regulator

Salmonella enterica subsp. enterica serovar Infantis str. SARB27. Bacteria; Proteobacteria; Gammaproteobacteria; Enterobacterales;

SEENIN0B__locus_2

EUT1

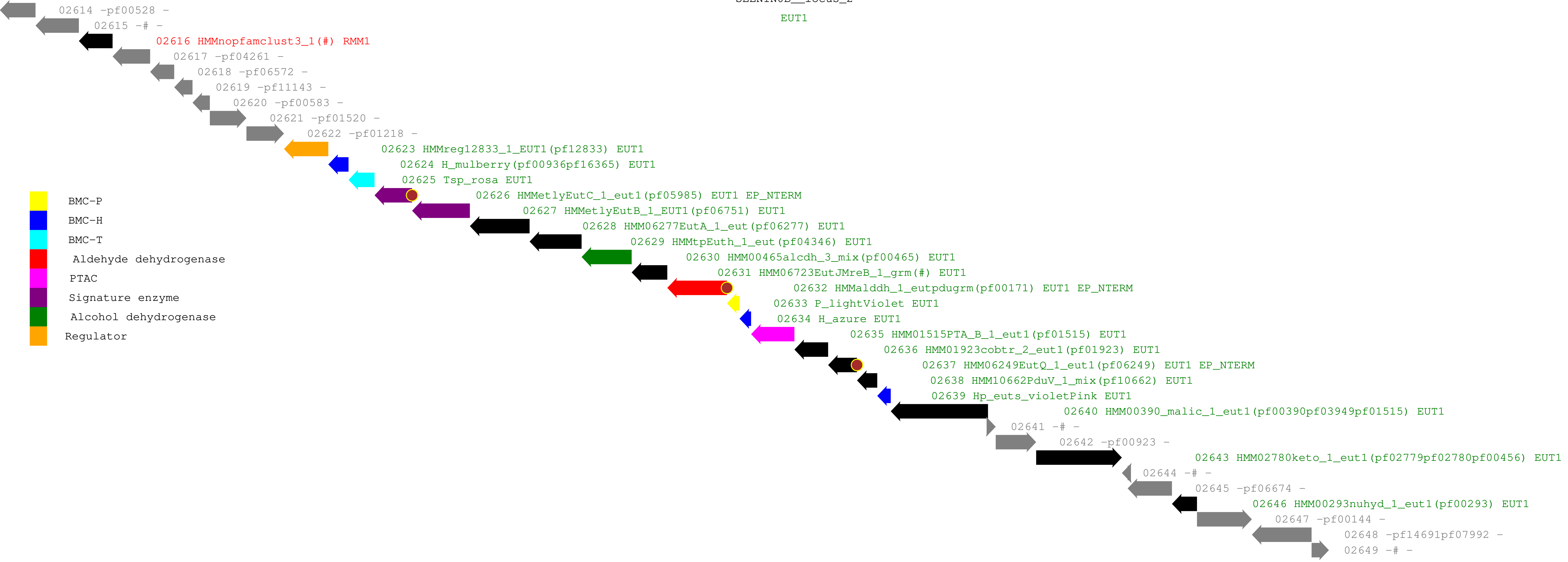

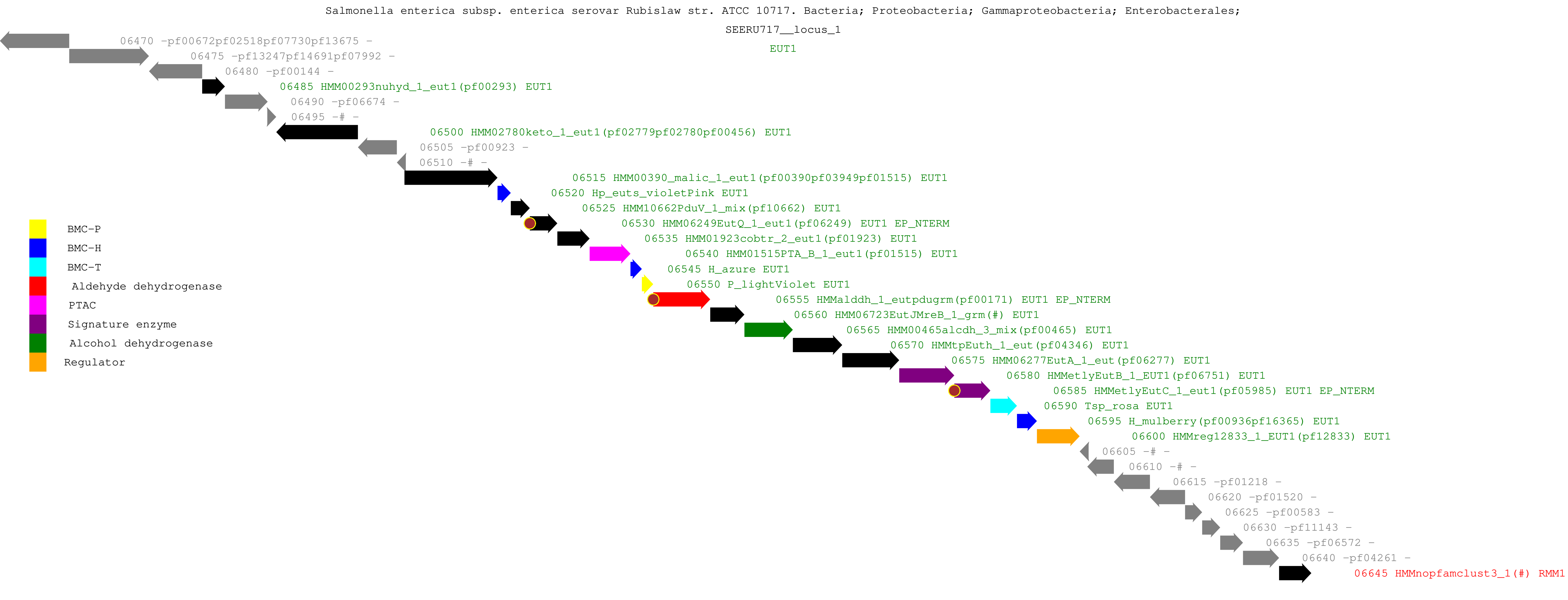
Salmonella enterica subsp. enterica serovar Rubislaw str. ATCC 10717. Bacteria; Proteobacteria; Gammaproteobacteria; Enterobacterales;
SEERU717__locus_1
EUT1
06470 -pf00672pf02518pf07730pf13675 -
06475 -pf13247pf14691pf07992 -
06480 -pf00144 -
06485 HMM00293nuhyd_1_eut1(pf00293) EUT1
06490 -pf06674 -
06495 -# -
06500 HMM02780keto_1_eut1(pf02779pf02780pf00456) EUT1
06505 -pf00923 -
06510 -# -
06515 HMM00390_malic_1_eut1(pf00390pf03949pf01515) EUT1
06520 Hp_euts_violetPink EUT1
06525 HMM10662PduV_1_mix(pf10662) EUT1
06530 HMM06249EutQ_1_eut1(pf06249) EUT1 EP_NTERM
06535 HMM01923cobtr_2_eut1(pf01923) EUT1
06540 HMM01515PTA_B_1_eut1(pf01515) EUT1
06545 H_azure EUT1
06550 P_lightViolet EUT1
06555 HMMalddh_1_eutpdugrm(pf00171) EUT1 EP_NTERM
06560 HMM06723EutJMreB_1_grm(#) EUT1
06565 HMM00465alcdh_3_mix(pf00465) EUT1
06570 HMMtpEuth_1_eut(pf04346) EUT1
06575 HMM06277EutA_1_eut(pf06277) EUT1
06580 HMMetlyEutB_1_EUT1(pf06751) EUT1
06585 HMMetlyEutC_1_eut1(pf05985) EUT1 EP_NTERM
06590 Tsp_rosa EUT1
06595 H_mulberry(pf00936pf16365) EUT1
06600 HMMreg12833_1_EUT1(pf12833) EUT1
06605 -# -
06610 -# -
06615 -pf01218 -
06620 -pf01520 -
06625 -pf00583 -
06630 -pf11143 -
06635 -pf06572 -
06640 -pf04261 -
06645 HMMnopfamclust3_1(#) RMM1
BMC-P
BMC-H
BMC-T
Aldehyde dehydrogenase
PTAC
Signature enzyme
Alcohol dehydrogenase
Regulator

Salmonella enterica subsp. enterica serovar Senftenberg str. ATCC Bacteria; Proteobacteria; Gammaproteobacteria; Enterobacterales;

SEES3845__locus_1

EUT1

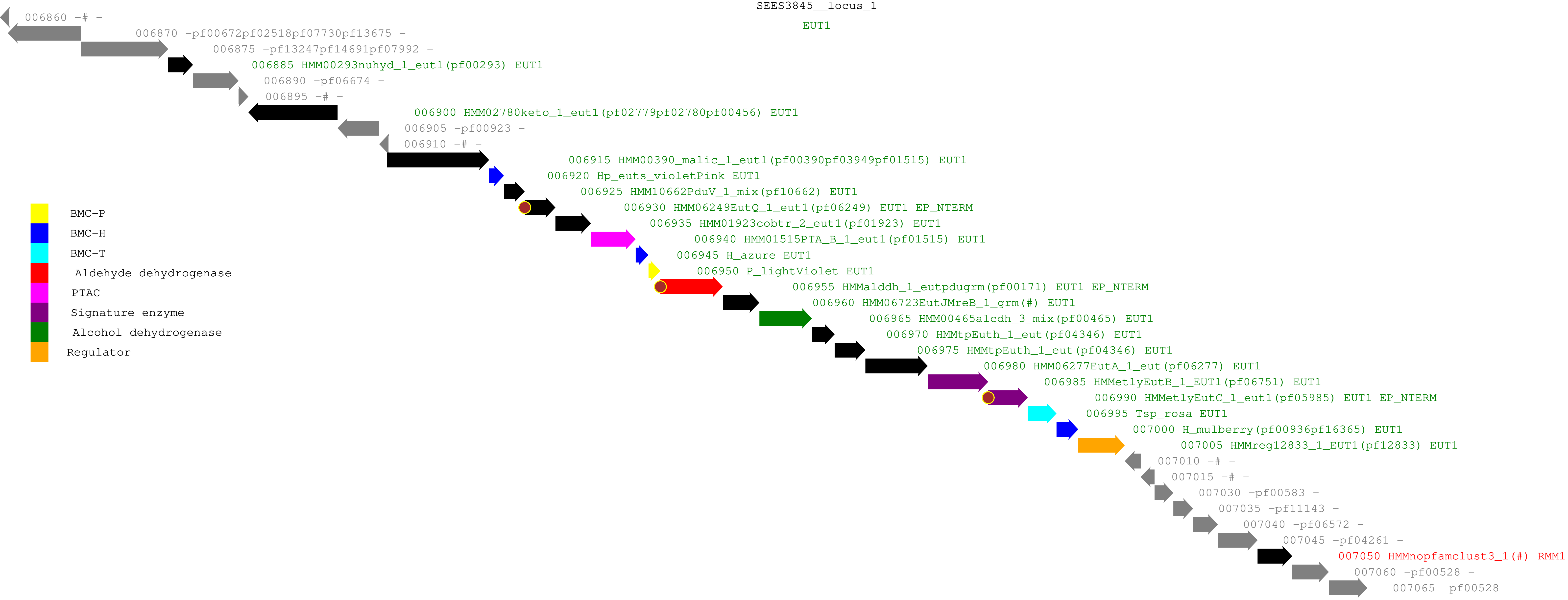

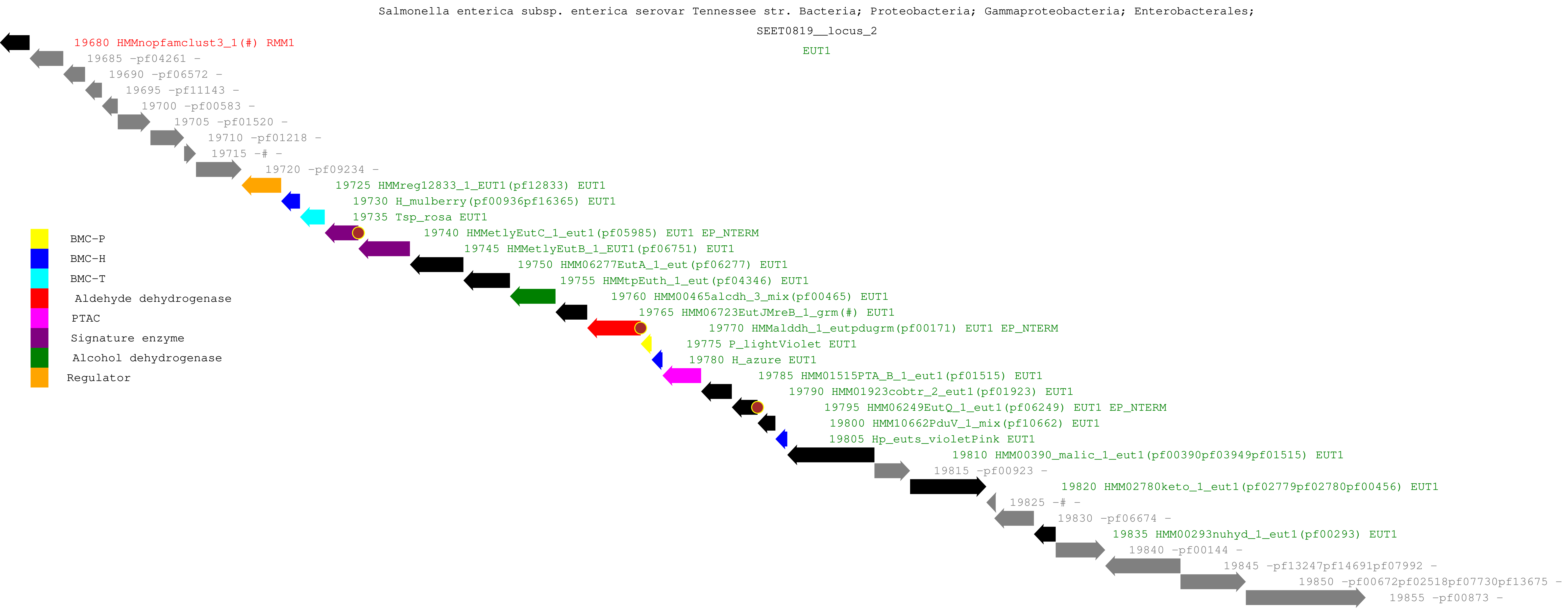
Salmonella enterica subsp. enterica serovar Tennessee str. Bacteria; Proteobacteria; Gammaproteobacteria; Enterobacterales;
SEET0819__locus_2
EUT1
19680 HMMnopfamclust3_1(#) RMM1
19685 -pf04261 -
19690 -pf06572 -
19695 -pf11143 -
19700 -pf00583 -
19705 -pf01520 -
19710 -pf01218 -
19715 -# -
19720 -pf09234 -
19725 HMMreg12833_1_EUT1(pf12833) EUT1
19730 H_mulberry(pf00936pf16365) EUT1
19735 Tsp_rosa EUT1
19740 HMMetlyEutC_1_eut1(pf05985) EUT1 EP_NTERM
19745 HMMetlyEutB_1_EUT1(pf06751) EUT1
19750 HMM06277EutA_1_eut(pf06277) EUT1
19755 HMMtpEuth_1_eut(pf04346) EUT1
19760 HMM00465alcdh_3_mix(pf00465) EUT1
19765 HMM06723EutJMreB_1_grm(#) EUT1
19770 HMMalddh_1_eutpdugrm(pf00171) EUT1 EP_NTERM
19775 P_lightViolet EUT1
19780 H_azure EUT1
19785 HMM01515PTA_B_1_eut1(pf01515) EUT1
19790 HMM01923cobtr_2_eut1(pf01923) EUT1
19795 HMM06249EutQ_1_eut1(pf06249) EUT1 EP_NTERM
19800 HMM10662PduV_1_mix(pf10662) EUT1
19805 Hp_euts_violetPink EUT1
19810 HMM00390_malic_1_eut1(pf00390pf03949pf01515) EUT1
19815 -pf00923 -
19820 HMM02780keto_1_eut1(pf02779pf02780pf00456) EUT1
19825 -# -
19830 -pf06674 -
19835 HMM00293nuhyd_1_eut1(pf00293) EUT1
19840 -pf00144 -
19845 -pf13247pf14691pf07992 -
19850 -pf00672pf02518pf07730pf13675 -
19855 -pf00873 -
BMC-P
BMC-H
BMC-T
Aldehyde dehydrogenase
PTAC
Signature enzyme
Alcohol dehydrogenase
Regulator

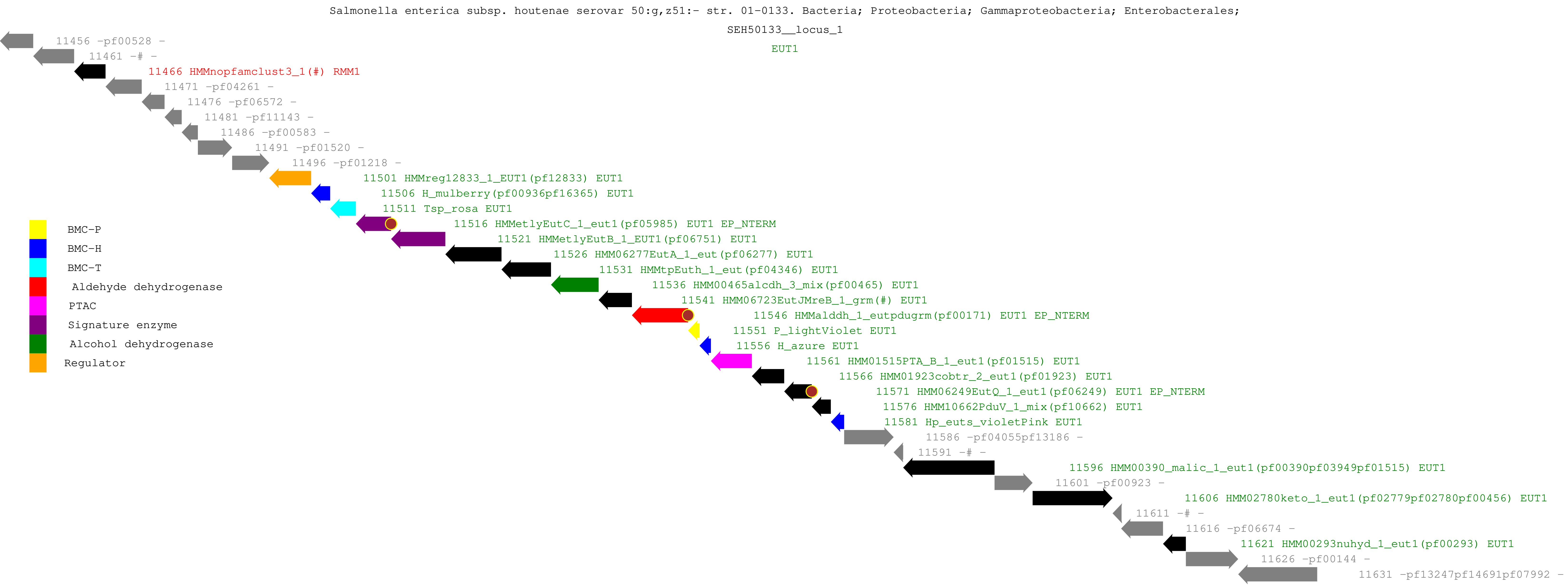
Salmonella enterica subsp. houtenae serovar 50:g,z51:- str. 01-0133. Bacteria; Proteobacteria; Gammaproteobacteria; Enterobacterales;
SEH50133__locus_1
EUT1
11456 -pf00528 -
11461 -# -
11466 HMMnopfamclust3_1(#) RMM1
11471 -pf04261 -
11476 -pf06572 -
11481 -pf11143 -
11486 -pf00583 -
11491 -pf01520 -
11496 -pf01218 -
11501 HMMreg12833_1_EUT1(pf12833) EUT1
11506 H_mulberry(pf00936pf16365) EUT1
11511 Tsp_rosa EUT1
11516 HMMetlyEutC_1_eut1(pf05985) EUT1 EP_NTERM
11521 HMMetlyEutB_1_EUT1(pf06751) EUT1
11526 HMM06277EutA_1_eut(pf06277) EUT1
11531 HMMtpEuth_1_eut(pf04346) EUT1
11536 HMM00465alcdh_3_mix(pf00465) EUT1
11541 HMM06723EutJMreB_1_grm(#) EUT1
11546 HMMalddh_1_eutpdugrm(pf00171) EUT1 EP_NTERM
11551 P_lightViolet EUT1
11556 H_azure EUT1
11561 HMM01515PTA_B_1_eut1(pf01515) EUT1
11566 HMM01923cobtr_2_eut1(pf01923) EUT1
11571 HMM06249EutQ_1_eut1(pf06249) EUT1 EP_NTERM
11576 HMM10662PduV_1_mix(pf10662) EUT1
11581 Hp_euts_violetPink EUT1
11586 -pf04055pf13186 -
11591 -# -
11596 HMM00390_malic_1_eut1(pf00390pf03949pf01515) EUT1
11601 -pf00923 -
11606 HMM02780keto_1_eut1(pf02779pf02780pf00456) EUT1
11611 -# -
11616 -pf06674 -
11621 HMM00293nuhyd_1_eut1(pf00293) EUT1
11626 -pf00144 -
11631 -pf13247pf14691pf07992 -
BMC-P
BMC-H
BMC-T
Aldehyde dehydrogenase
PTAC
Signature enzyme
Alcohol dehydrogenase
Regulator

Salmonella enterica subsp. houtenae str. ATCC BAA-1581. Bacteria; Proteobacteria; Gammaproteobacteria; Enterobacterales;

SEHO0A__locus_1

EUT1

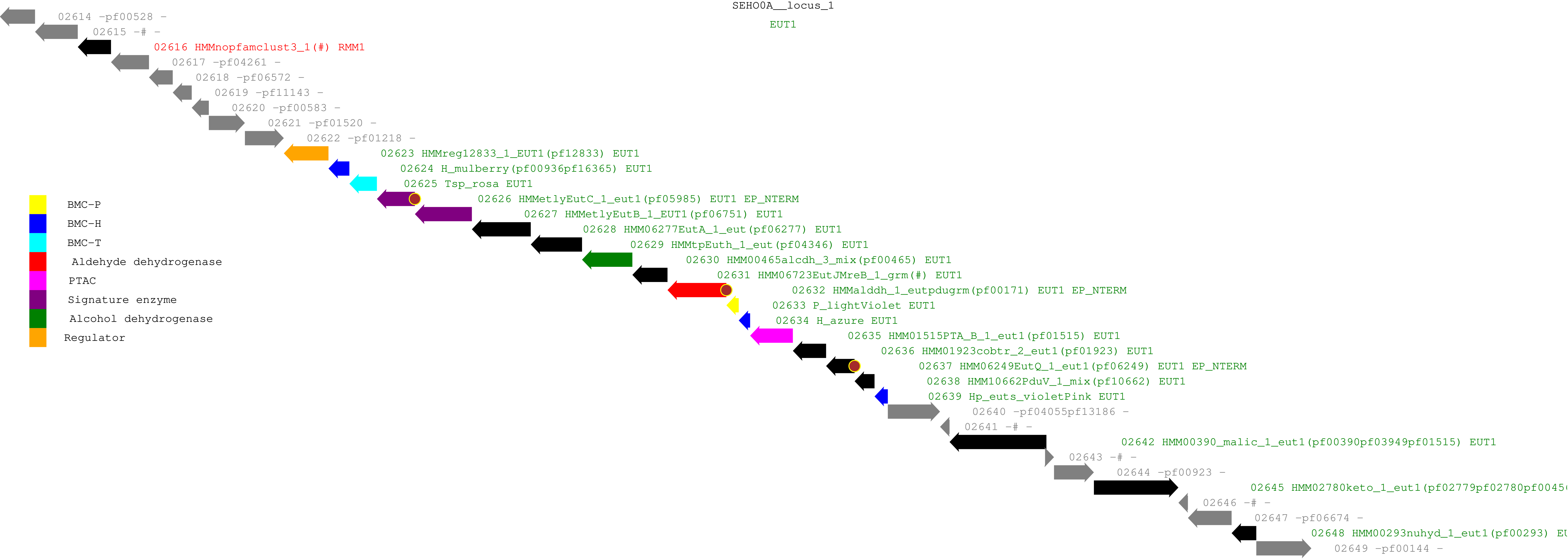

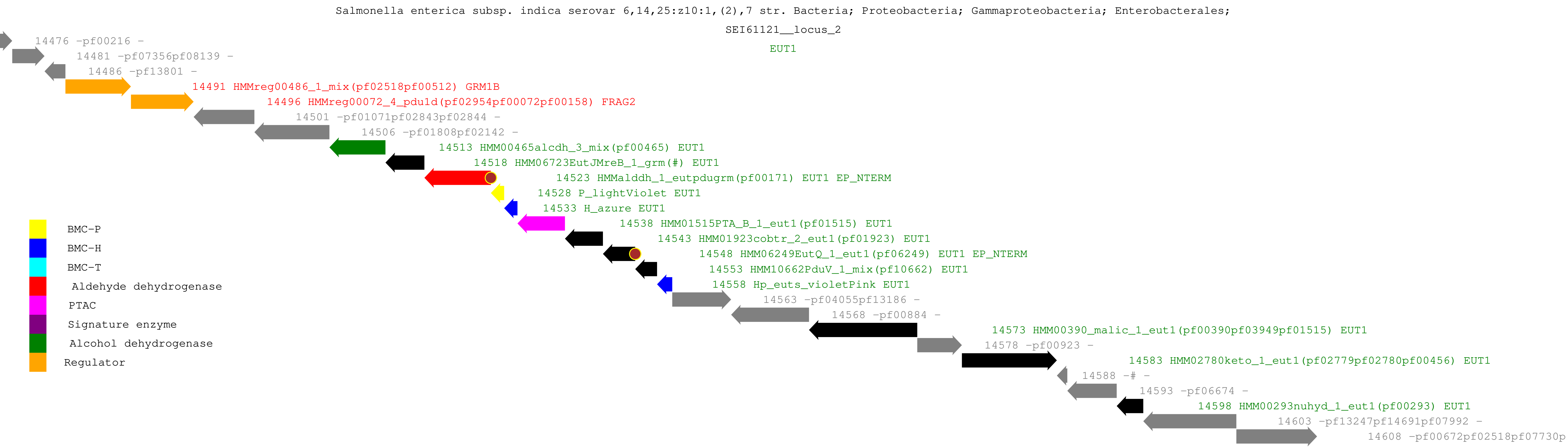
Salmonella enterica subsp. indica serovar 6,14,25:z10:1,(2),7 str. Bacteria; Proteobacteria; Gammaproteobacteria; Enterobacterales;
SEI61121__locus_2
EUT1
14476 -pf00216 -
14481 -pf07356pf08139 -
14486 -pf13801 -
14491 HMMreg00486_1_mix(pf02518pf00512) GRM1B
14496 HMMreg00072_4_pdu1d(pf02954pf00072pf00158) FRAG2
14501 -pf01071pf02843pf02844 -
14506 -pf01808pf02142 -
14513 HMM00465alcdh_3_mix(pf00465) EUT1
14518 HMM06723EutJMreB_1_grm(#) EUT1
14523 HMMalddh_1_eutpdugrm(pf00171) EUT1 EP_NTERM
14528 P_lightViolet EUT1
14533 H_azure EUT1
14538 HMM01515PTA_B_1_eut1(pf01515) EUT1
14543 HMM01923cobtr_2_eut1(pf01923) EUT1
14548 HMM06249EutQ_1_eut1(pf06249) EUT1 EP_NTERM
14553 HMM10662PduV_1_mix(pf10662) EUT1
14558 Hp_euts_violetPink EUT1
14563 -pf04055pf13186 -
14568 -pf00884 -
14573 HMM00390_malic_1_eut1(pf00390pf03949pf01515) EUT1
14578 -pf00923 -
14583 HMM02780keto_1_eut1(pf02779pf02780pf00456) EUT1
14588 -# -
14593 -pf06674 -
14598 HMM00293nuhyd_1_eut1(pf00293) EUT1
14603 -pf13247pf14691pf07992 -
14608 -pf00672pf02518pf07730p
BMC-P
BMC-H
BMC-T
Aldehyde dehydrogenase
PTAC
Signature enzyme
Alcohol dehydrogenase
Regulator

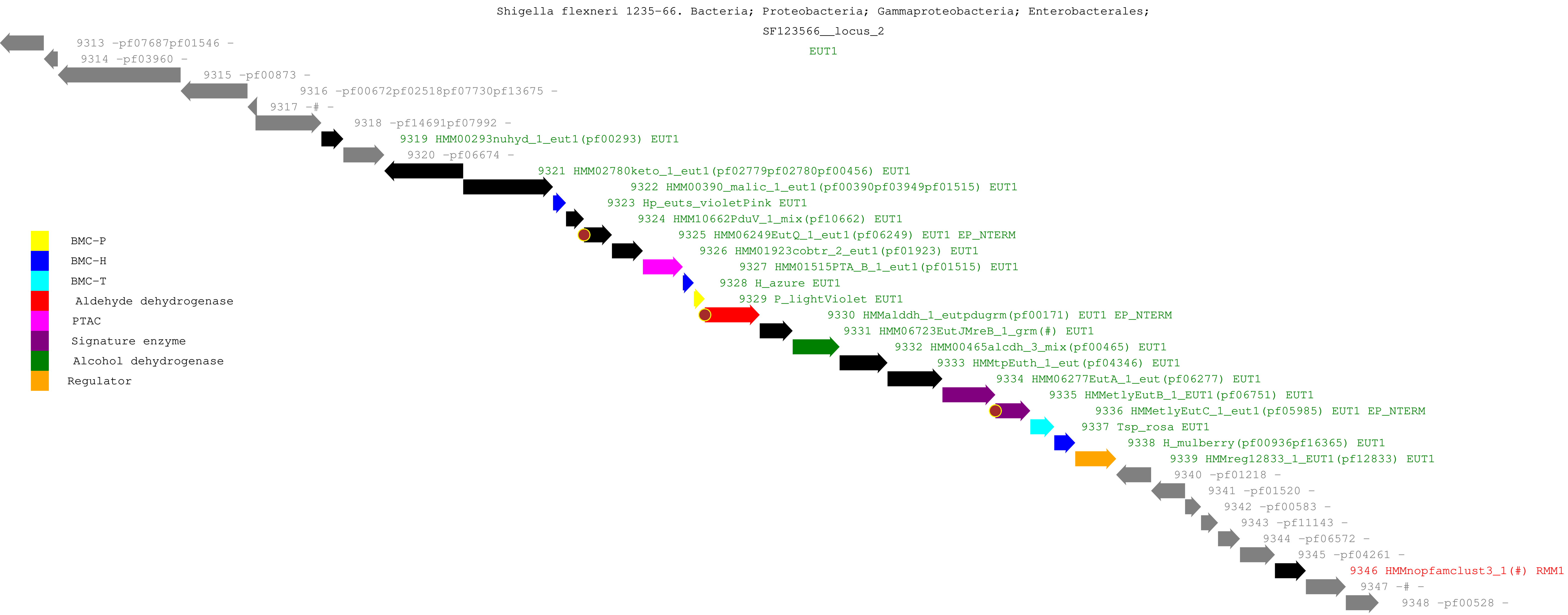
Shigella flexneri 1235-66. Bacteria; Proteobacteria; Gammaproteobacteria; Enterobacterales;
SF123566__locus_2
EUT1
9313 -pf07687pf01546 -
9314 -pf03960 -
9315 -pf00873 -
9316 -pf00672pf02518pf07730pf13675 -
9317 -# -
9318 -pf14691pf07992 -
9319 HMM00293nuhyd_1_eut1(pf00293) EUT1
9320 -pf06674 -
9321 HMM02780keto_1_eut1(pf02779pf02780pf00456) EUT1
9322 HMM00390_malic_1_eut1(pf00390pf03949pf01515) EUT1
9323 Hp_euts_violetPink EUT1
9324 HMM10662PduV_1_mix(pf10662) EUT1
9325 HMM06249EutQ_1_eut1(pf06249) EUT1 EP_NTERM
9326 HMM01923cobtr_2_eut1(pf01923) EUT1
9327 HMM01515PTA_B_1_eut1(pf01515) EUT1
9328 H_azure EUT1
9329 P_lightViolet EUT1
9330 HMMalddh_1_eutpdugrm(pf00171) EUT1 EP_NTERM
9331 HMM06723EutJMreB_1_grm(#) EUT1
9332 HMM00465alcdh_3_mix(pf00465) EUT1
9333 HMMtpEuth_1_eut(pf04346) EUT1
9334 HMM06277EutA_1_eut(pf06277) EUT1
9335 HMMetlyEutB_1_EUT1(pf06751) EUT1
9336 HMMetlyEutC_1_eut1(pf05985) EUT1 EP_NTERM
9337 Tsp_rosa EUT1
9338 H_mulberry(pf00936pf16365) EUT1
9339 HMMreg12833_1_EUT1(pf12833) EUT1
9340 -pf01218 -
9341 -pf01520 -
9342 -pf00583 -
9343 -pf11143 -
9344 -pf06572 -
9345 -pf04261 -
9346 HMMnopfamclust3_1(#) RMM1
9347 -# -
9348 -pf00528 -
BMC-P
BMC-H
BMC-T
Aldehyde dehydrogenase
PTAC
Signature enzyme
Alcohol dehydrogenase
Regulator

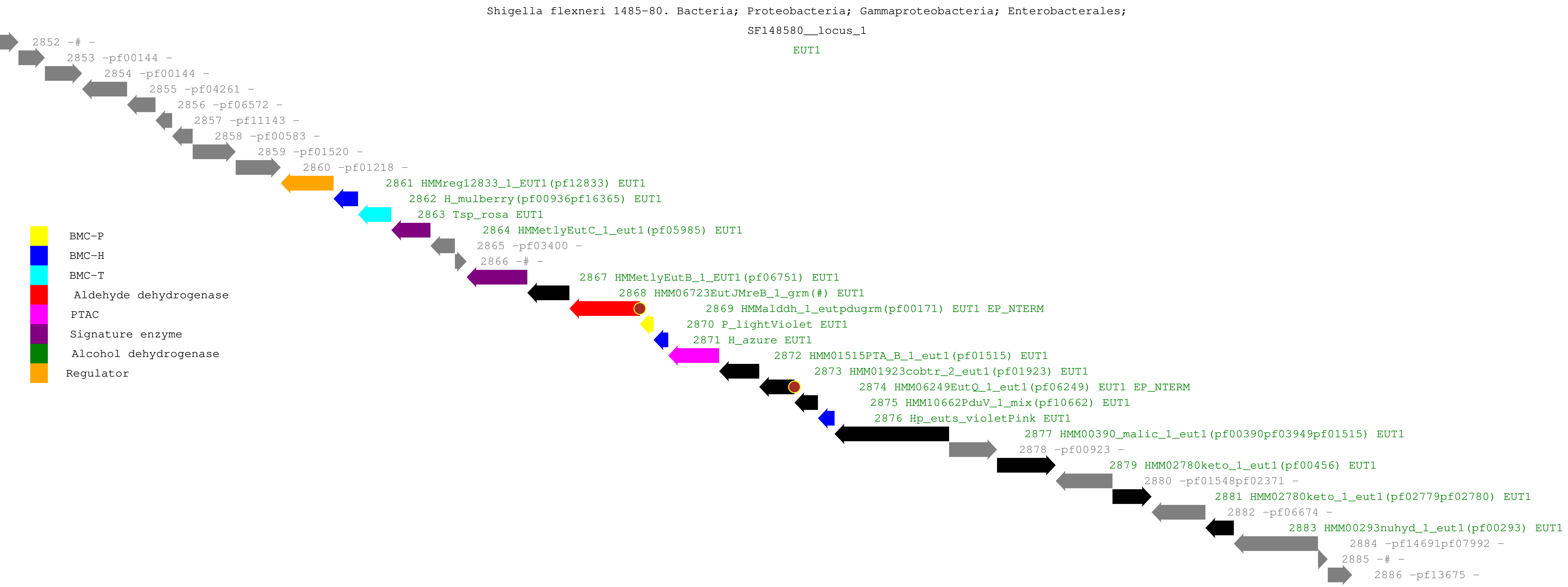

Shigella flexneri 1485-80. Bacteria; Proteobacteria; Gammaproteobacteria; Enterobacterales;
SF148580__locus_1
EUT1
2852 -# -
2853 -pf00144 -
2854 -pf00144 -
2855 -pf04261 -
2856 -pf06572 -
2857 -pf11143 -
2858 -pf00583 -
2859 -pf01520 -
2860 -pf01218 -
2861 HMMreg12833_1_EUT1(pf12833) EUT1
2862 H_mulberry(pf00936pf16365) EUT1
2863 Tsp_rosa EUT1
2864 HMMetlyEutC_1_eut1(pf05985) EUT1
2865 -pf03400 -
2866 -# -
2867 HMMetlyEutB_1_EUT1(pf06751) EUT1
2868 HMM06723EutJMreB_1_grm(#) EUT1
2869 HMMalddh_1_eutpdugrm(pf00171) EUT1 EP_NTERM
2870 P_lightViolet EUT1
2871 H_azure EUT1
2872 HMM01515PTA_B_1_eut1(pf01515) EUT1
2873 HMM01923cobtr_2_eut1(pf01923) EUT1
2874 HMM06249EutQ_1_eut1(pf06249) EUT1 EP_NTERM
2875 HMM10662PduV_1_mix(pf10662) EUT1
2876 Hp_euts_violetPink EUT1
2877 HMM00390_malic_1_eut1(pf00390pf03949pf01515) EUT1
2878 -pf00923 -
2879 HMM02780keto_1_eut1(pf00456) EUT1
2880 -pf01548pf02371 -
2881 HMM02780keto_1_eut1(pf02779pf02780) EUT1
2882 -pf06674 -
2883 HMM00293nuhyd_1_eut1(pf00293) EUT1
2884 -pf14691pf07992 -
2885 -# -
2886 -pf13675 -
BMC-P
BMC-H
BMC-T
Aldehyde dehydrogenase
PTAC
Signature enzyme
Alcohol dehydrogenase
Regulator

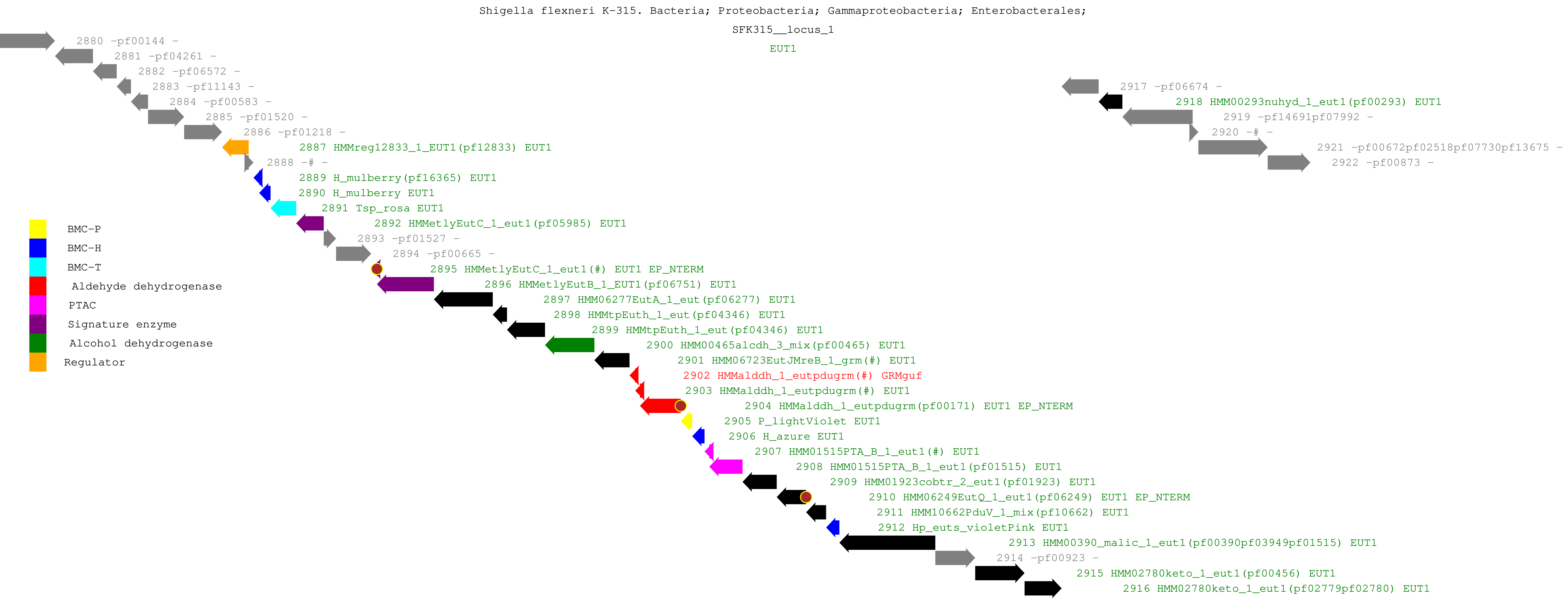
Shigella flexneri K-315. Bacteria; Proteobacteria; Gammaproteobacteria; Enterobacterales;
SFK315__locus_1
EUT1
2880 -pf00144 -
2881 -pf04261 -
2882 -pf06572 -
2883 -pf11143 -
2884 -pf00583 -
2885 -pf01520 -
2886 -pf01218 -
2887 HMMreg12833_1_EUT1(pf12833) EUT1
2888 -# -
2889 H_mulberry(pf16365) EUT1
2890 H_mulberry EUT1
2891 Tsp_rosa EUT1
2892 HMMetlyEutC_1_eut1(pf05985) EUT1
2893 -pf01527 -
2894 -pf00665 -
2895 HMMetlyEutC_1_eut1(#) EUT1 EP_NTERM
2896 HMMetlyEutB_1_EUT1(pf06751) EUT1
2897 HMM06277EutA_1_eut(pf06277) EUT1
2898 HMMtpEuth_1_eut(pf04346) EUT1
2899 HMMtpEuth_1_eut(pf04346) EUT1
2900 HMM00465alcdh_3_mix(pf00465) EUT1
2901 HMM06723EutJMreB_1_grm(#) EUT1
2902 HMMalddh_1_eutpdugrm(#) GRMguf
2903 HMMalddh_1_eutpdugrm(#) EUT1
2904 HMMalddh_1_eutpdugrm(pf00171) EUT1 EP_NTERM
2905 P_lightViolet EUT1
2906 H_azure EUT1
2907 HMM01515PTA_B_1_eut1(#) EUT1
2908 HMM01515PTA_B_1_eut1(pf01515) EUT1
2909 HMM01923cobtr_2_eut1(pf01923) EUT1
2910 HMM06249EutQ_1_eut1(pf06249) EUT1 EP_NTERM
2911 HMM10662PduV_1_mix(pf10662) EUT1
2912 Hp_euts_violetPink EUT1
2913 HMM00390_malic_1_eut1(pf00390pf03949pf01515) EUT1
2914 -pf00923 -
2915 HMM02780keto_1_eut1(pf00456) EUT1
2916 HMM02780keto_1_eut1(pf02779pf02780) EUT1
2917 -pf06674 -
2918 HMM00293nuhyd_1_eut1(pf00293) EUT1
2919 -pf14691pf07992 -
2920 -# -
2921 -pf00672pf02518pf07730pf13675 -
2922 -pf00873 -
BMC-P
BMC-H
BMC-T
Aldehyde dehydrogenase
PTAC
Signature enzyme
Alcohol dehydrogenase
Regulator

Salmonella enterica subsp. enterica serovar Gallinarum str. SG9. Bacteria; Proteobacteria; Gammaproteobacteria; Enterobacterales;

SG9__locus_2

EUT1

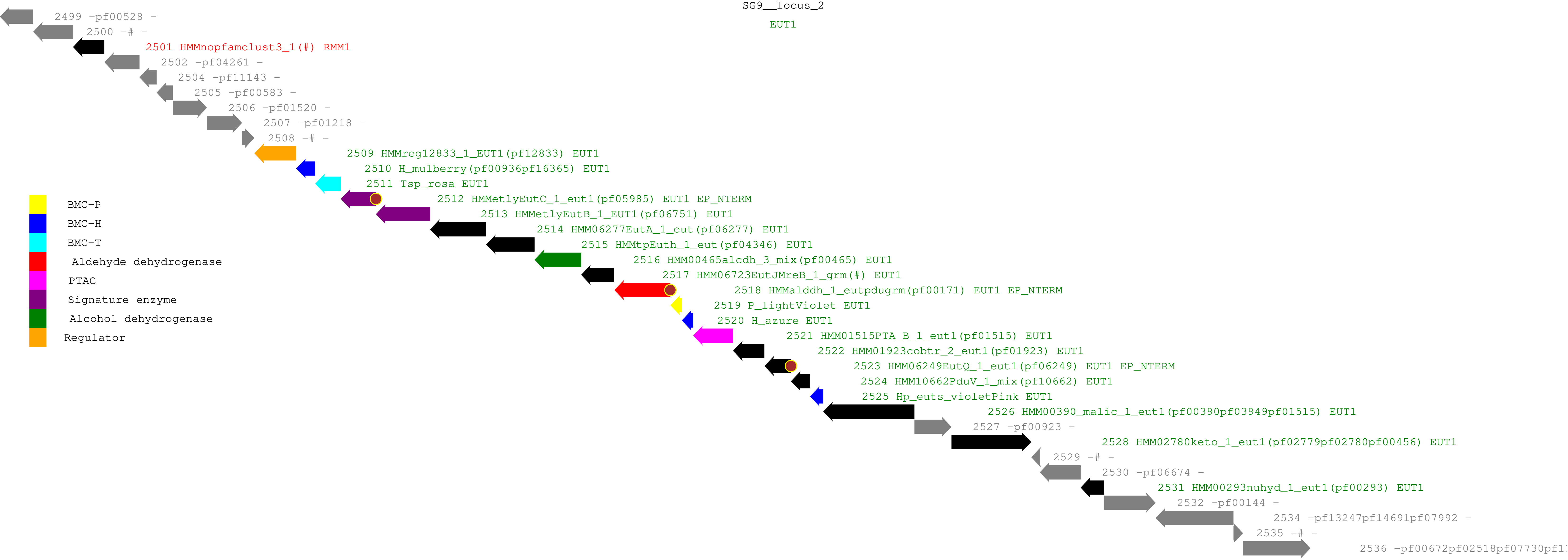

Shigella boydii ATCC 9905. Bacteria; Proteobacteria; Gammaproteobacteria; Enterobacterales;

SGB__locus_1

EUT1

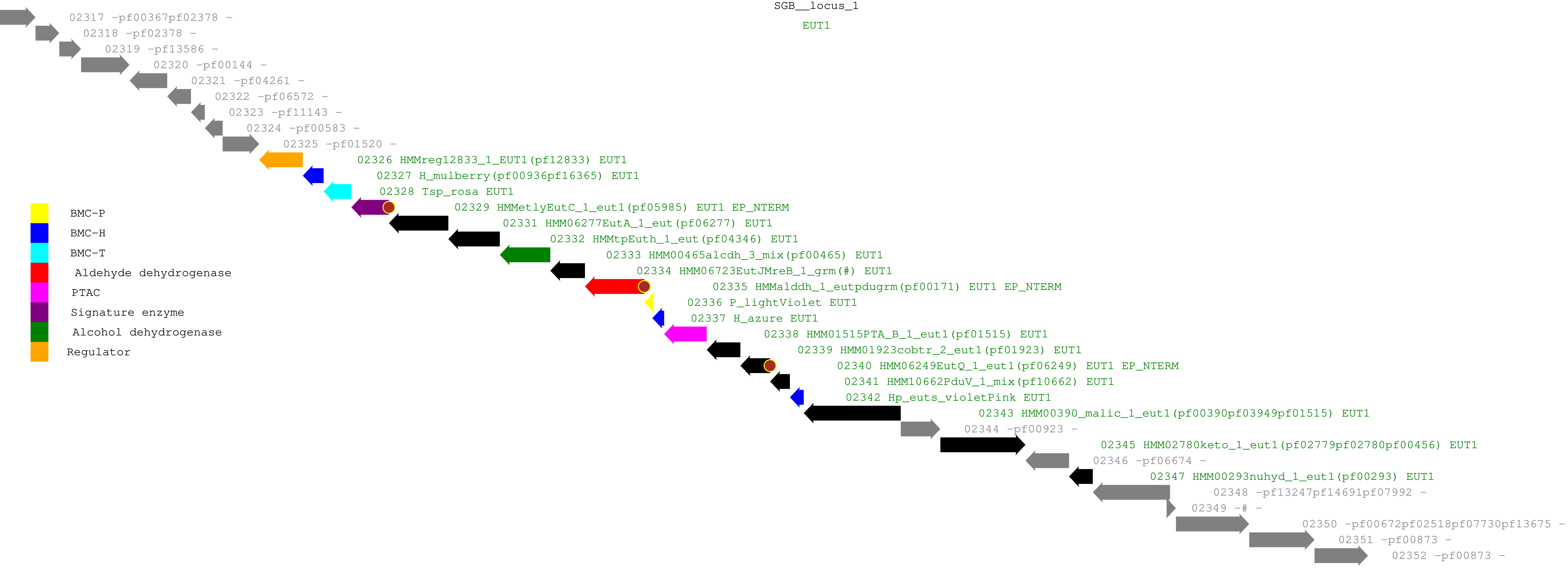

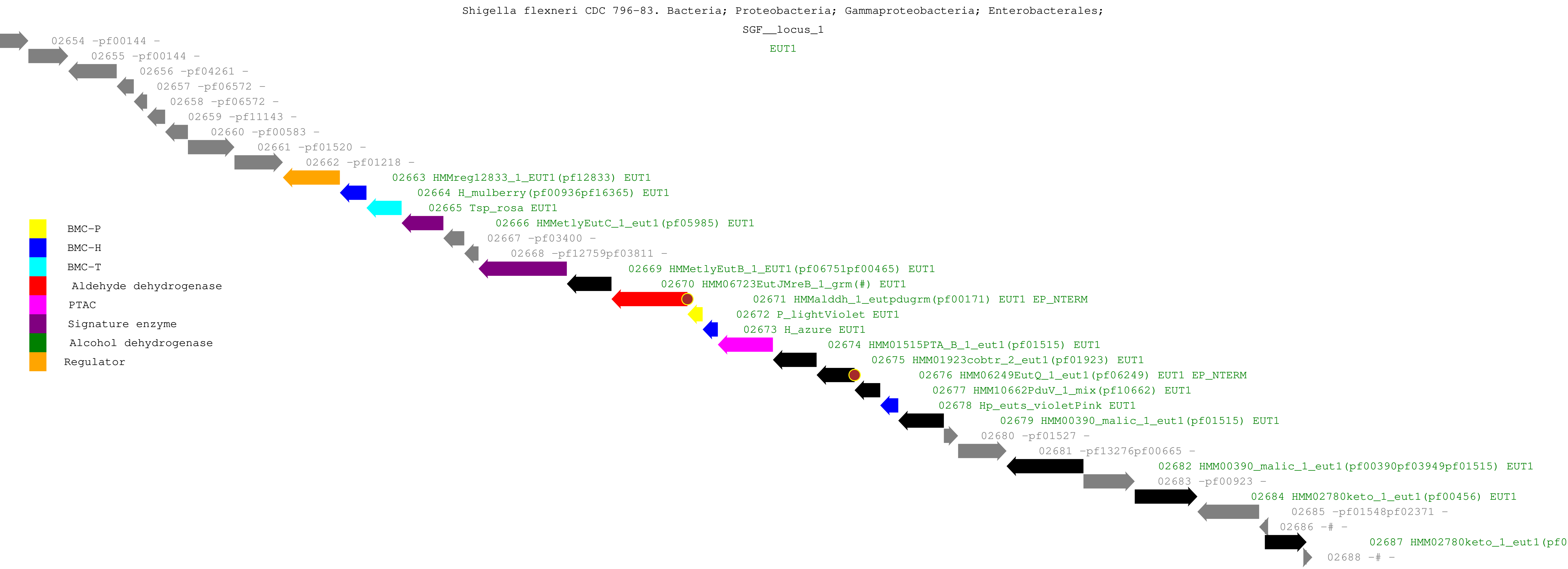

Shigella flexneri CDC 796-83. Bacteria; Proteobacteria; Gammaproteobacteria; Enterobacterales;
SGF__locus_1
EUT1
02654 -pf00144 -
02655 -pf00144 -
02656 -pf04261 -
02657 -pf06572 -
02658 -pf06572 -
02659 -pf11143 -
02660 -pf00583 -
02661 -pf01520 -
02662 -pf01218 -
02663 HMMreg12833_1_EUT1(pf12833) EUT1
02664 H_mulberry(pf00936pf16365) EUT1
02665 Tsp_rosa EUT1
02666 HMMetlyEutC_1_eut1(pf05985) EUT1
02667 -pf03400 -
02668 -pf12759pf03811 -
02669 HMMetlyEutB_1_EUT1(pf06751pf00465) EUT1
02670 HMM06723EutJMreB_1_grm(#) EUT1
02671 HMMalddh_1_eutpdugrm(pf00171) EUT1 EP_NTERM
02672 P_lightViolet EUT1
02673 H_azure EUT1
02674 HMM01515PTA_B_1_eut1(pf01515) EUT1
02675 HMM01923cobtr_2_eut1(pf01923) EUT1
02676 HMM06249EutQ_1_eut1(pf06249) EUT1 EP_NTERM
02677 HMM10662PduV_1_mix(pf10662) EUT1
02678 Hp_euts_violetPink EUT1
02679 HMM00390_malic_1_eut1(pf01515) EUT1
02680 -pf01527 -
02681 -pf13276pf00665 -
02682 HMM00390_malic_1_eut1(pf00390pf03949pf01515) EUT1
02683 -pf00923 -
02684 HMM02780keto_1_eut1(pf00456) EUT1
02685 -pf01548pf02371 -
02686 -# -
02687 HMM02780keto_1_eut1(pf0
02688 -# -
BMC-P
BMC-H
BMC-T
Aldehyde dehydrogenase
PTAC
Signature enzyme
Alcohol dehydrogenase
Regulator

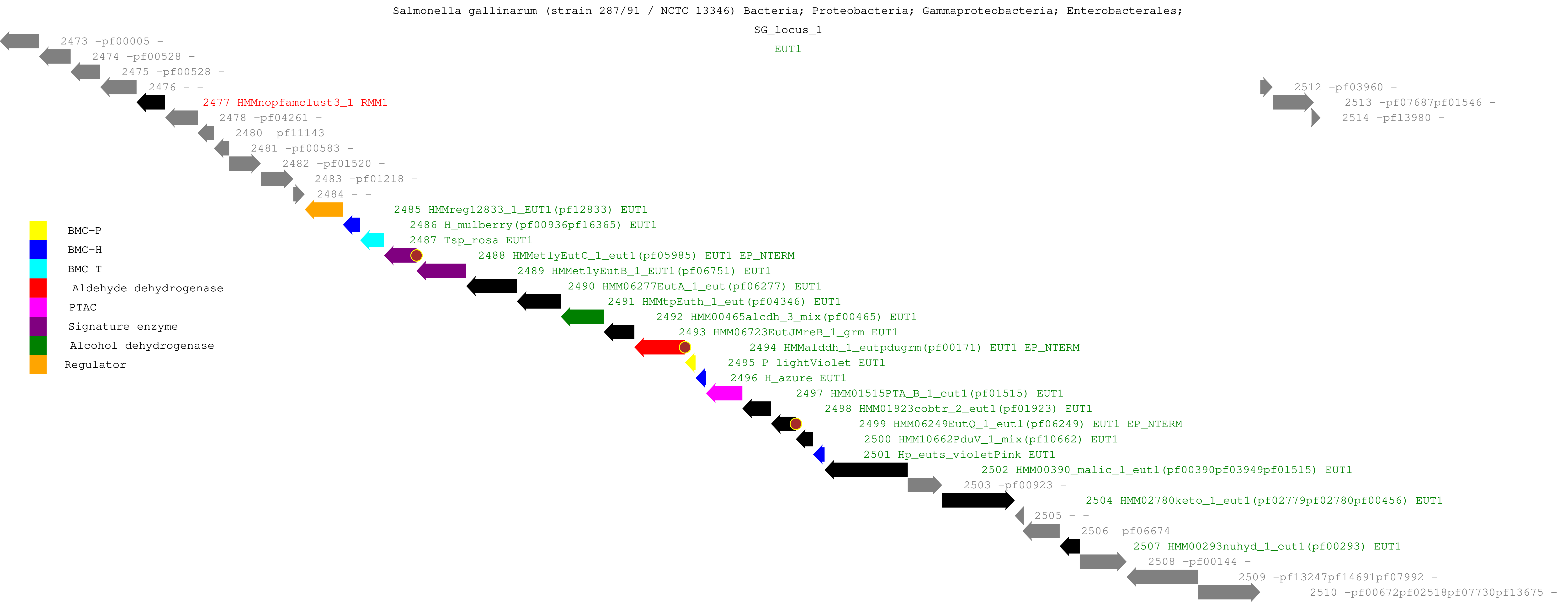
Salmonella gallinarum (strain 287/91 / NCTC 13346) Bacteria; Proteobacteria; Gammaproteobacteria; Enterobacterales;
SG_locus_1
EUT1
2473 -pf00005 -
2474 -pf00528 -
2475 -pf00528 -
2476 - -
2477 HMMnopfamclust3_1 RMM1
2478 -pf04261 -
2480 -pf11143 -
2481 -pf00583 -
2482 -pf01520 -
2483 -pf01218 -
2484 - -
2485 HMMreg12833_1_EUT1(pf12833) EUT1
2486 H_mulberry(pf00936pf16365) EUT1
2487 Tsp_rosa EUT1
2488 HMMetlyEutC_1_eut1(pf05985) EUT1 EP_NTERM
2489 HMMetlyEutB_1_EUT1(pf06751) EUT1
2490 HMM06277EutA_1_eut(pf06277) EUT1
2491 HMMtpEuth_1_eut(pf04346) EUT1
2492 HMM00465alcdh_3_mix(pf00465) EUT1
2493 HMM06723EutJMreB_1_grm EUT1
2494 HMMalddh_1_eutpdugrm(pf00171) EUT1 EP_NTERM
2495 P_lightViolet EUT1
2496 H_azure EUT1
2497 HMM01515PTA_B_1_eut1(pf01515) EUT1
2498 HMM01923cobtr_2_eut1(pf01923) EUT1
2499 HMM06249EutQ_1_eut1(pf06249) EUT1 EP_NTERM
2500 HMM10662PduV_1_mix(pf10662) EUT1
2501 Hp_euts_violetPink EUT1
2502 HMM00390_malic_1_eut1(pf00390pf03949pf01515) EUT1
2503 -pf00923 -
2504 HMM02780keto_1_eut1(pf02779pf02780pf00456) EUT1
2505 - -
2506 -pf06674 -
2507 HMM00293nuhyd_1_eut1(pf00293) EUT1
2508 -pf00144 -
2509 -pf13247pf14691pf07992 -
2510 -pf00672pf02518pf07730pf13675 -
2512 -pf03960 -
2513 -pf07687pf01546 -
2514 -pf13980 -
BMC-P
BMC-H
BMC-T
Aldehyde dehydrogenase
PTAC
Signature enzyme
Alcohol dehydrogenase
Regulator

Citrobacter sp. MGH100. Bacteria; Proteobacteria; Gammaproteobacteria; Enterobacterales;

SK32__locus_1

EUT1

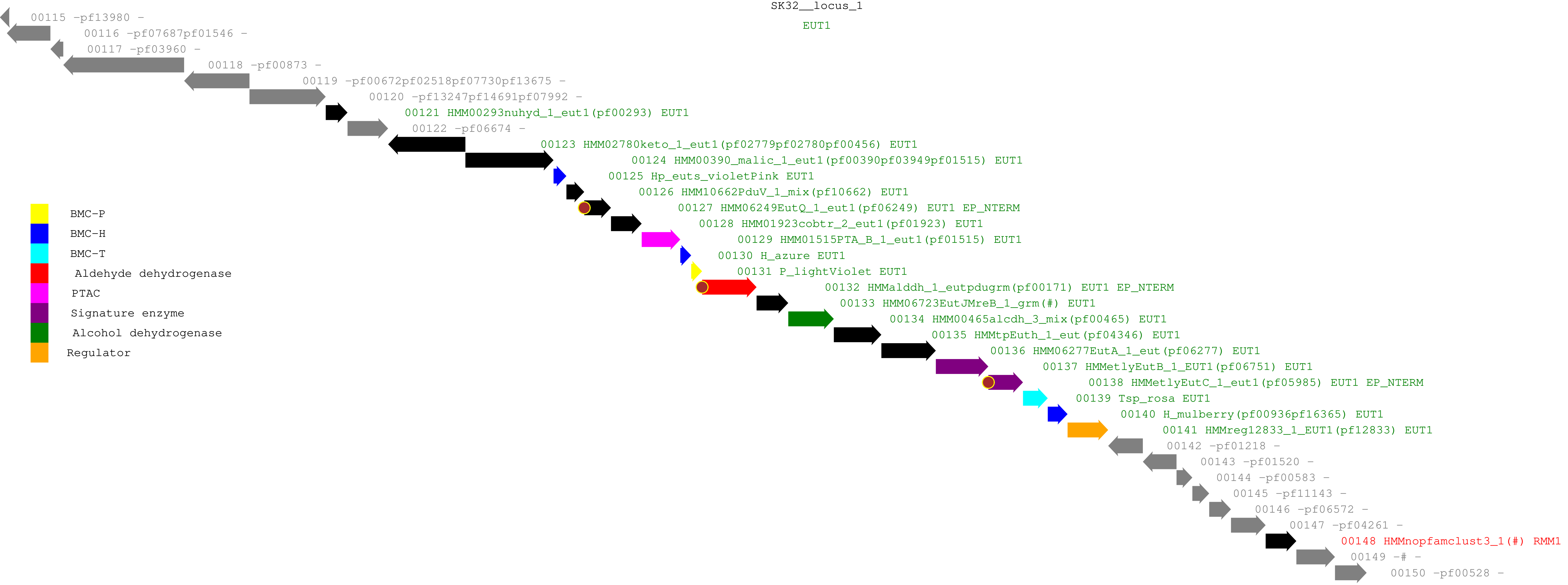

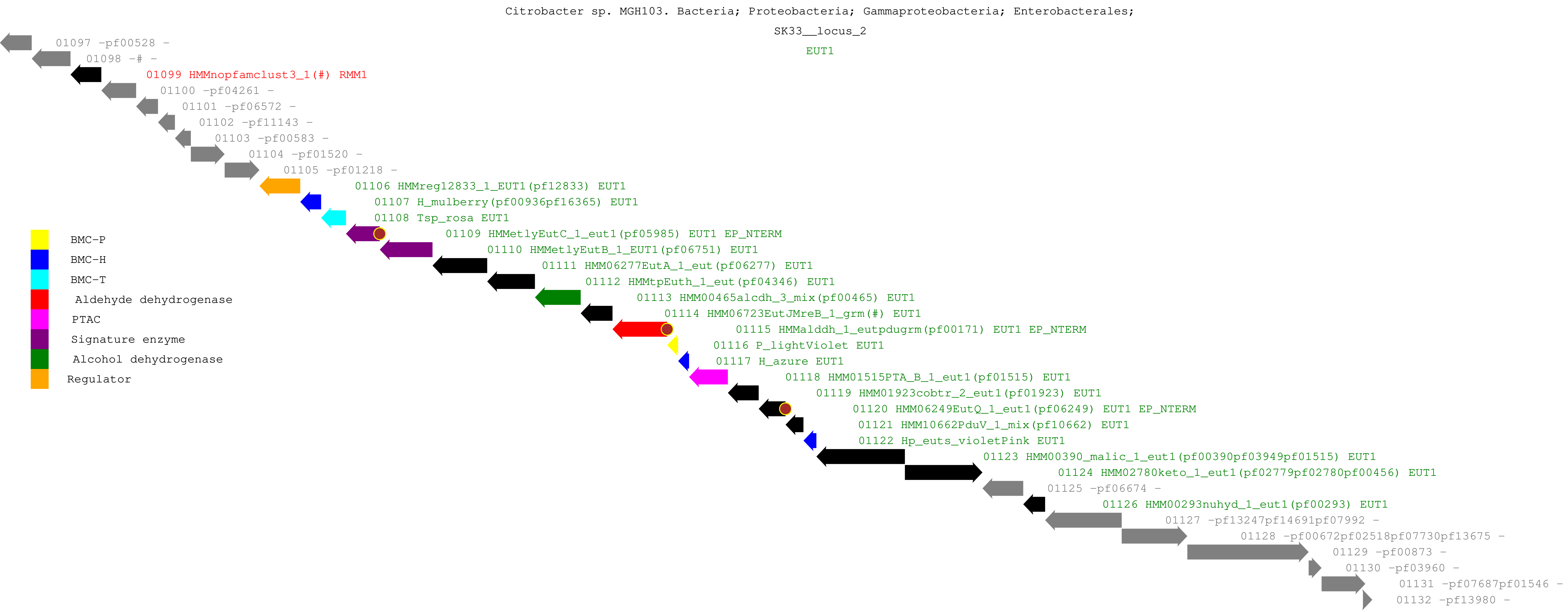
Citrobacter sp. MGH103. Bacteria; Proteobacteria; Gammaproteobacteria; Enterobacterales;
SK33__locus_2
EUT1
01097 -pf00528 -
01098 -# -
01099 HMMnopfamclust3_1(#) RMM1
01100 -pf04261 -
01101 -pf06572 -
01102 -pf11143 -
01103 -pf00583 -
01104 -pf01520 -
01105 -pf01218 -
01106 HMMreg12833_1_EUT1(pf12833) EUT1
01107 H_mulberry(pf00936pf16365) EUT1
01108 Tsp_rosa EUT1
01109 HMMetlyEutC_1_eut1(pf05985) EUT1 EP_NTERM
01110 HMMetlyEutB_1_EUT1(pf06751) EUT1
01111 HMM06277EutA_1_eut(pf06277) EUT1
01112 HMMtpEuth_1_eut(pf04346) EUT1
01113 HMM00465alcdh_3_mix(pf00465) EUT1
01114 HMM06723EutJMreB_1_grm(#) EUT1
01115 HMMaldddh_1_eutpdugrm(pf00171) EUT1 EP_NTERM
01116 P_lightViolet EUT1
01117 H_azure EUT1
01118 HMM01515PTA_B_1_eut1(pf01515) EUT1
01119 HMM01923cobtr_2_eut1(pf01923) EUT1
01120 HMM06249EutQ_1_eut1(pf06249) EUT1 EP_NTERM
01121 HMM10662PduV_1_mix(pf10662) EUT1
01122 Hp_euts_violetPink EUT1
01123 HMM00390_malic_1_eut1(pf00390pf03949pf01515) EUT1
01124 HMM02780keto_1_eut1(pf02779pf02780pf00456) EUT1
01125 -pf06674 -
01126 HMM00293nuhyd_1_eut1(pf00293) EUT1
01127 -pf13247pf14691pf07992 -
01128 -pf00672pf02518pf07730pf13675 -
01129 -pf00873 -
01130 -pf03960 -
01131 -pf07687pf01546 -
01132 -pf13980 -
BMC-P
BMC-H
BMC-T
Aldehyde dehydrogenase
PTAC
Signature enzyme
Alcohol dehydrogenase
Regulator

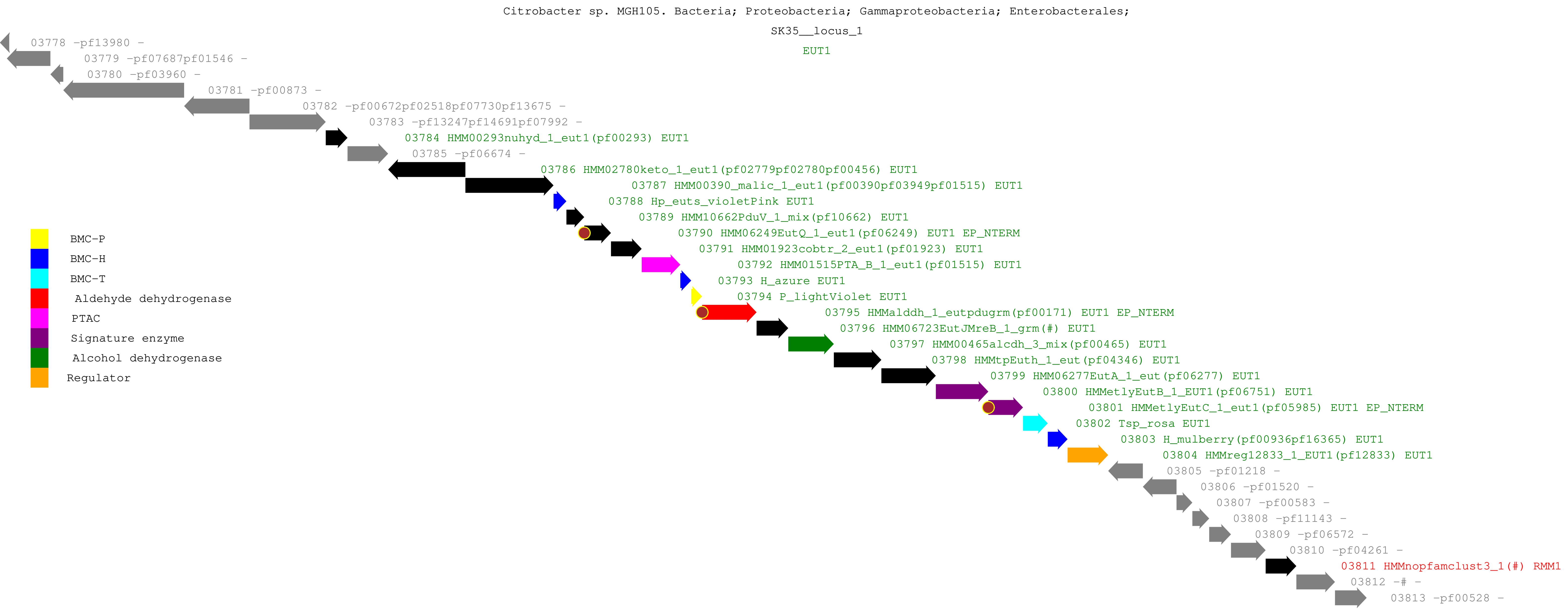
Citrobacter sp. MGH105. Bacteria; Proteobacteria; Gammaproteobacteria; Enterobacterales;
SK35__locus_1
EUT1
03778 -pf13980 -
03779 -pf07687pf01546 -
03780 -pf03960 -
03781 -pf00873 -
03782 -pf00672pf02518pf07730pf13675 -
03783 -pf13247pf14691pf07992 -
03784 HMM00293nuhyd_1_eut1(pf00293) EUT1
03785 -pf06674 -
03786 HMM02780keto_1_eut1(pf02779pf02780pf00456) EUT1
03787 HMM00390_malic_1_eut1(pf00390pf03949pf01515) EUT1
03788 Hp_euts_violetPink EUT1
03789 HMM10662PduV_1_mix(pf10662) EUT1
03790 HMM06249EutQ_1_eut1(pf06249) EUT1 EP_NTERM
03791 HMM01923cobtr_2_eut1(pf01923) EUT1
03792 HMM01515PTA_B_1_eut1(pf01515) EUT1
03793 H_azure EUT1
03794 P_lightViolet EUT1
03795 HMMalddh_1_eutpdugrm(pf00171) EUT1 EP_NTERM
03796 HMM06723EutJMreB_1_grm(#) EUT1
03797 HMM00465alcdh_3_mix(pf00465) EUT1
03798 HMMtpEuth_1_eut(pf04346) EUT1
03799 HMM06277EutA_1_eut(pf06277) EUT1
03800 HMMetlyEutB_1_EUT1(pf06751) EUT1
03801 HMMetlyEutC_1_eut1(pf05985) EUT1 EP_NTERM
03802 Tsp_rosa EUT1
03803 H_mulberry(pf00936pf16365) EUT1
03804 HMMreg12833_1_EUT1(pf12833) EUT1
03805 -pf01218 -
03806 -pf01520 -
03807 -pf00583 -
03808 -pf11143 -
03809 -pf06572 -
03810 -pf04261 -
03811 HMMnopfamclust3_1(#) RMM1
03812 -# -
03813 -pf00528 -
BMC-P
BMC-H
BMC-T
Aldehyde dehydrogenase
PTAC
Signature enzyme
Alcohol dehydrogenase
Regulator

Citrobacter sp. MGH106. Bacteria; Proteobacteria; Gammaproteobacteria; Enterobacterales;

SK36__locus_1

EUT1

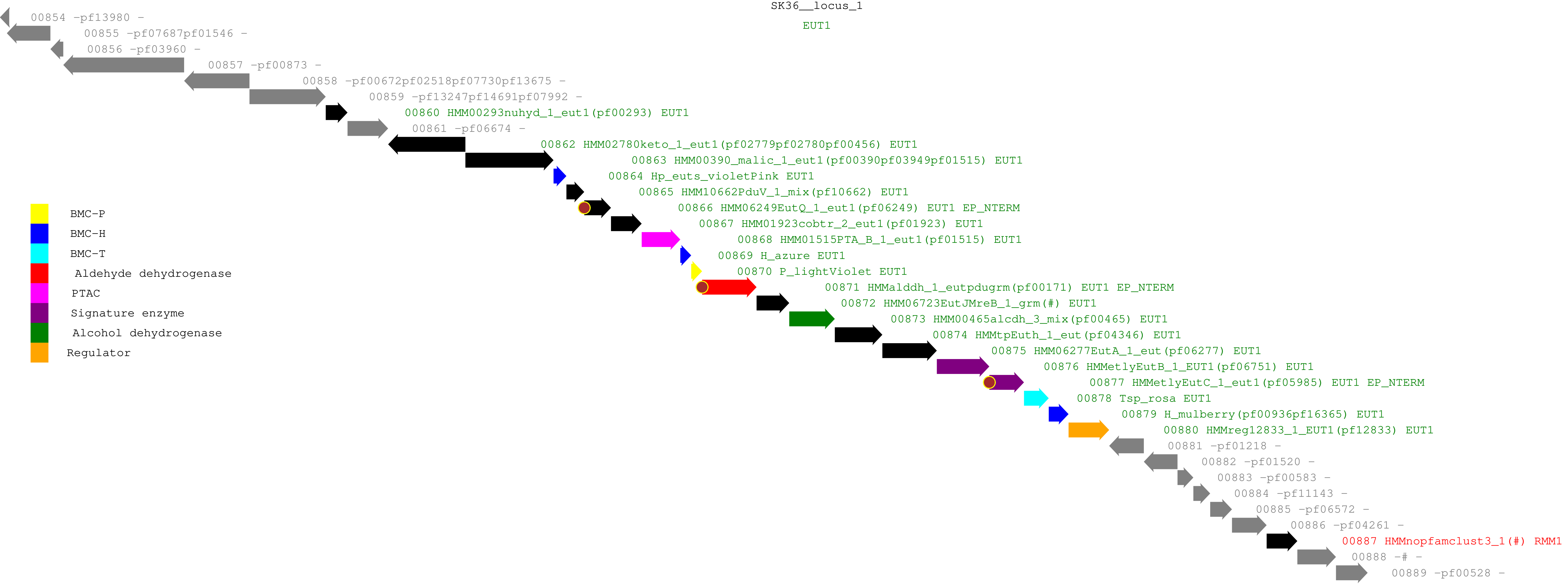

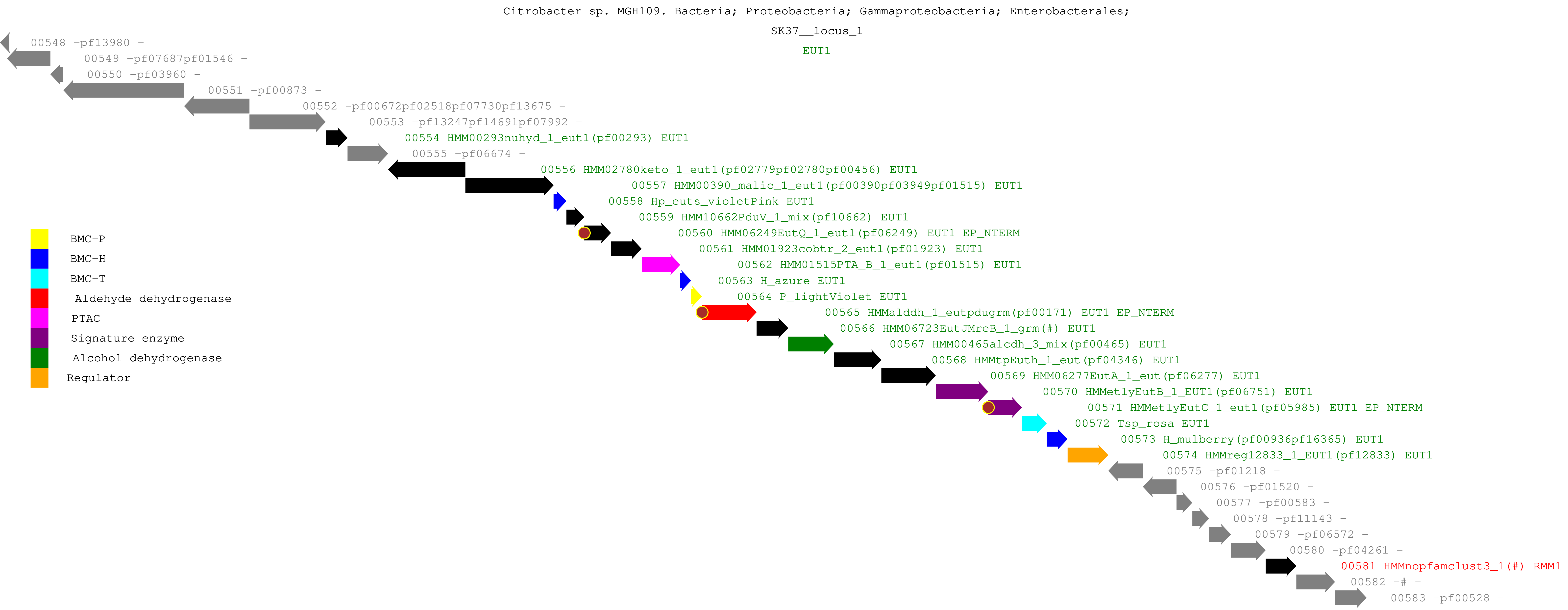
Citrobacter sp. MGH109. Bacteria; Proteobacteria; Gammaproteobacteria; Enterobacterales;
SK37__locus_1
EUT1
00548 -pf13980 -
00549 -pf07687pf01546 -
00550 -pf03960 -
00551 -pf00873 -
00552 -pf00672pf02518pf07730pf13675 -
00553 -pf13247pf14691pf07992 -
00554 HMM00293nuhyd_1_eut1(pf00293) EUT1
00555 -pf06674 -
00556 HMM02780keto_1_eut1(pf02779pf02780pf00456) EUT1
00557 HMM00390_malic_1_eut1(pf00390pf03949pf01515) EUT1
00558 Hp_euts_violetPink EUT1
00559 HMM10662PduV_1_mix(pf10662) EUT1
00560 HMM06249EutQ_1_eut1(pf06249) EUT1 EP_NTERM
00561 HMM01923cobtr_2_eut1(pf01923) EUT1
00562 HMM01515PTA_B_1_eut1(pf01515) EUT1
00563 H_azure EUT1
00564 P_lightViolet EUT1
00565 HMMalddh_1_eutpdugrm(pf00171) EUT1 EP_NTERM
00566 HMM06723EutJMreB_1_grm(#) EUT1
00567 HMM00465alcdh_3_mix(pf00465) EUT1
00568 HMMtpEuth_1_eut(pf04346) EUT1
00569 HMM06277EutA_1_eut(pf06277) EUT1
00570 HMMetlyEutB_1_EUT1(pf06751) EUT1
00571 HMMetlyEutC_1_eut1(pf05985) EUT1 EP_NTERM
00572 Tsp_rosa EUT1
00573 H_mulberry(pf00936pf16365) EUT1
00574 HMMreg12833_1_EUT1(pf12833) EUT1
00575 -pf01218 -
00576 -pf01520 -
00577 -pf00583 -
00578 -pf11143 -
00579 -pf06572 -
00580 -pf04261 -
00581 HMMnopfamclust3_1(#) RMM1
00582 -# -
00583 -pf00528 -
BMC-P
BMC-H
BMC-T
Aldehyde dehydrogenase
PTAC
Signature enzyme
Alcohol dehydrogenase
Regulator

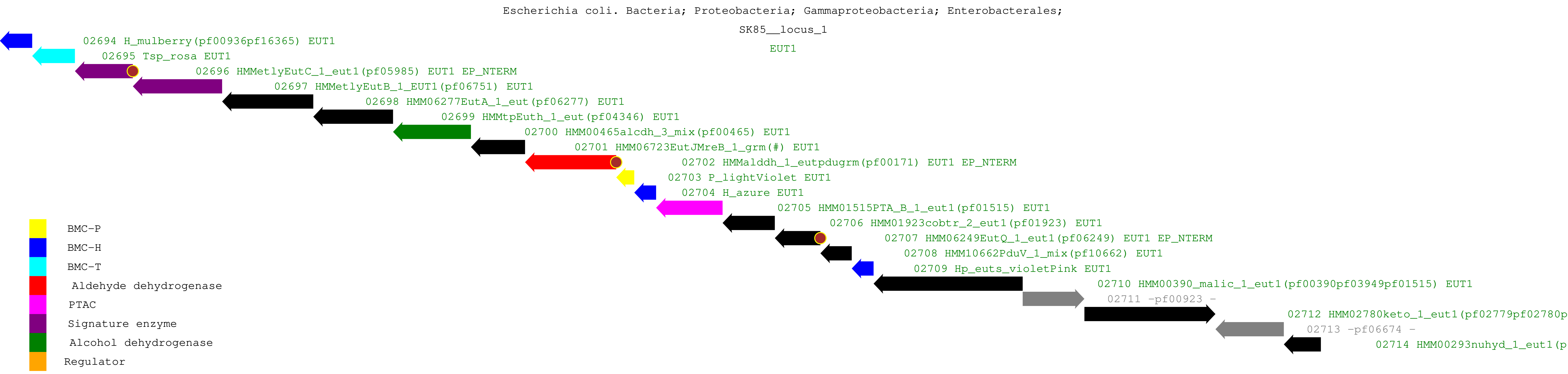

Escherichia coli. Bacteria; Proteobacteria; Gammaproteobacteria; Enterobacterales;
SK85__locus_1
EUT1
02694 H_mulberry(pf00936pf16365) EUT1
02695 Tsp_rosa EUT1
02696 HMMetlyEutC_1_eut1(pf05985) EUT1 EP_NTERM
02697 HMMetlyEutB_1_EUT1(pf06751) EUT1
02698 HMM06277EutA_1_eut(pf06277) EUT1
02699 HMMtpEuth_1_eut(pf04346) EUT1
02700 HMM00465alcdh_3_mix(pf00465) EUT1
02701 HMM06723EutJMreB_1_grm(#) EUT1
02702 HMMalddh_1_eutpdugrm(pf00171) EUT1 EP_NTERM
02703 P_lightViolet EUT1
02704 H_azure EUT1
02705 HMM01515PTA_B_1_eut1(pf01515) EUT1
02706 HMM01923cobtr_2_eut1(pf01923) EUT1
02707 HMM06249EutQ_1_eut1(pf06249) EUT1 EP_NTERM
02708 HMM10662PduV_1_mix(pf10662) EUT1
02709 Hp_euts_violetPink EUT1
02710 HMM00390_malic_1_eut1(pf00390pf03949pf01515) EUT1
02711 -pf00923 -
02712 HMM02780keto_1_eut1(pf02779pf02780p
02713 -pf06674 -
02714 HMM00293nuhyd_1_eut1(p
BMC-P
BMC-H
BMC-T
Aldehyde dehydrogenase
PTAC
Signature enzyme
Alcohol dehydrogenase
Regulator

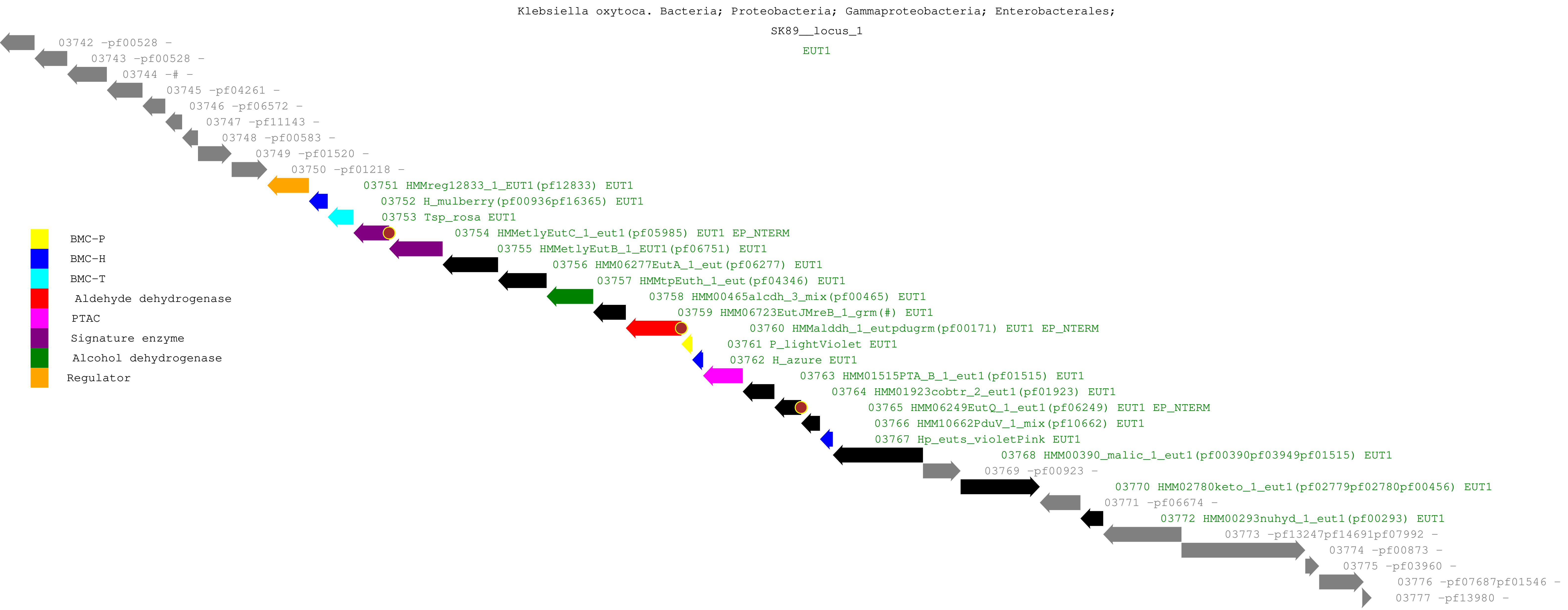
Klebsiella oxytoca. Bacteria; Proteobacteria; Gammaproteobacteria; Enterobacterales;
SK89__locus_1
EUT1
03742 -pf00528 -
03743 -pf00528 -
03744 -# -
03745 -pf04261 -
03746 -pf06572 -
03747 -pf11143 -
03748 -pf00583 -
03749 -pf01520 -
03750 -pf01218 -
03751 HMMreg12833_1_EUT1(pf12833) EUT1
03752 H_mulberry(pf00936pf16365) EUT1
03753 Tsp_rosa EUT1
03754 HMMetlyEutC_1_eut1(pf05985) EUT1 EP_NTERM
03755 HMMetlyEutB_1_EUT1(pf06751) EUT1
03756 HMM06277EutA_1_eut(pf06277) EUT1
03757 HMMtpEuth_1_eut(pf04346) EUT1
03758 HMM00465alcdh_3_mix(pf00465) EUT1
03759 HMM06723EutJMreB_1_grm(#) EUT1
03760 HMMaldh_1_eutpdugrm(pf00171) EUT1 EP_NTERM
03761 P_lightViolet EUT1
03762 H_azure EUT1
03763 HMM01515PTA_B_1_eut1(pf01515) EUT1
03764 HMM01923cobtr_2_eut1(pf01923) EUT1
03765 HMM06249EutQ_1_eut1(pf06249) EUT1 EP_NTERM
03766 HMM10662PduV_1_mix(pf10662) EUT1
03767 Hp_euts_violetPink EUT1
03768 HMM00390_malic_1_eut1(pf00390pf03949pf01515) EUT1
03769 -pf00923 -
03770 HMM02780keto_1_eut1(pf02779pf02780pf00456) EUT1
03771 -pf06674 -
03772 HMM00293nuhyd_1_eut1(pf00293) EUT1
03773 -pf13247pf14691pf07992 -
03774 -pf00873 -
03775 -pf03960 -
03776 -pf07687pf01546 -
03777 -pf13980 -
BMC-P
BMC-H
BMC-T
Aldehyde dehydrogenase
PTAC
Signature enzyme
Alcohol dehydrogenase
Regulator

Klebsiella michiganensis. Bacteria; Proteobacteria; Gammaproteobacteria; Enterobacterales;

SK91__locus_3

EUT1

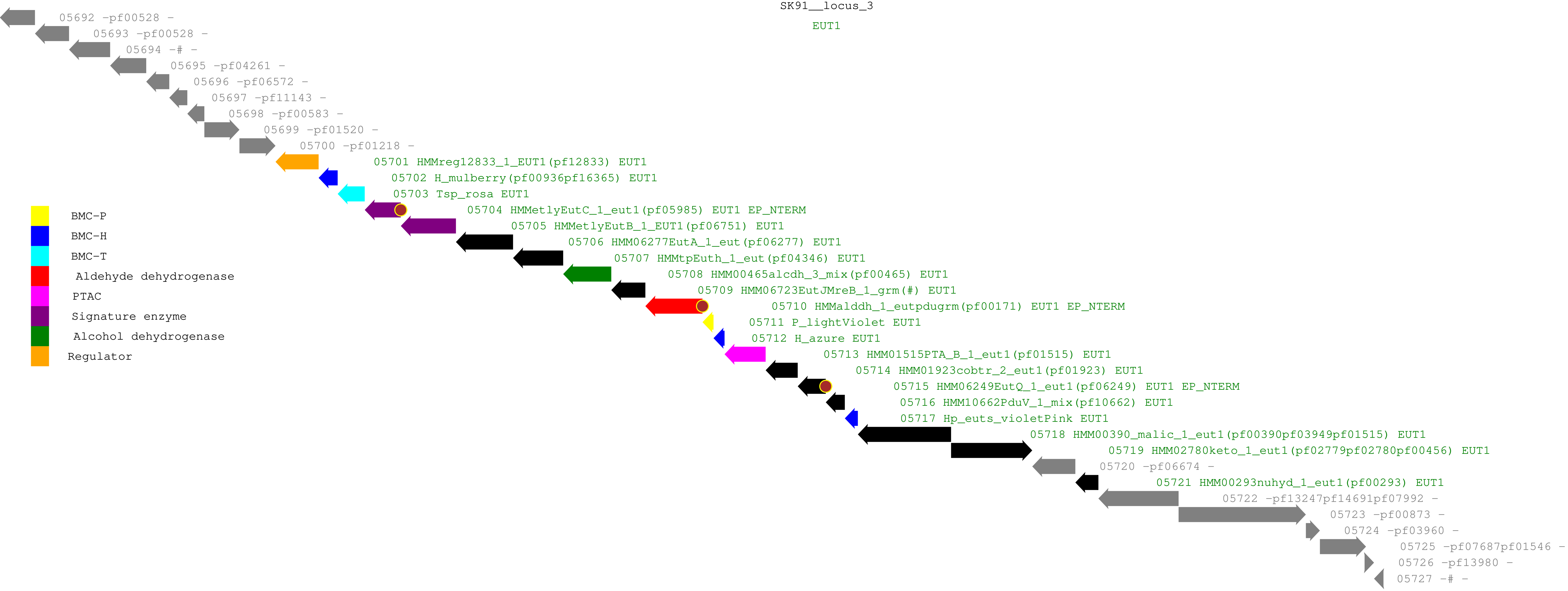

Salmonella typhimurium (strain SL1344). Bacteria; Proteobacteria; Gammaproteobacteria; Enterobacterales;

SL1344__locus_2

EUT1

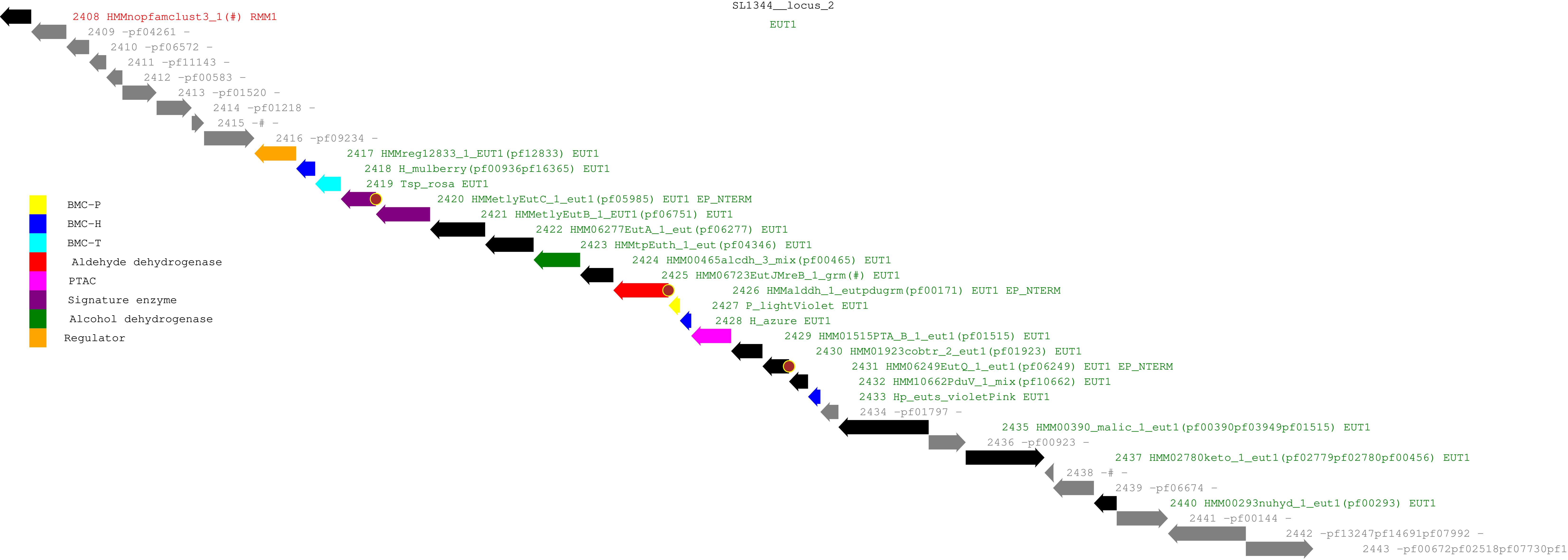

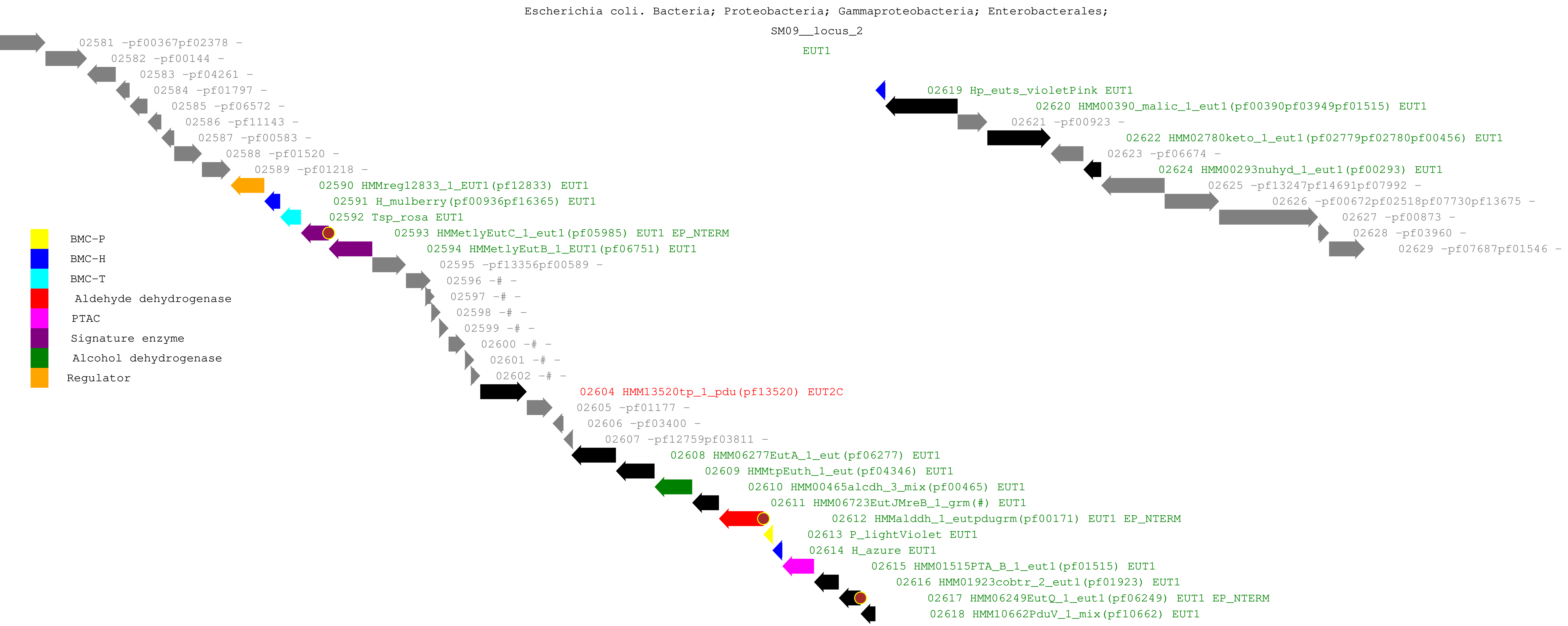
Escherichia coli. Bacteria; Proteobacteria; Gammaproteobacteria; Enterobacterales;
SM09__locus_2
EUT1
02581 -pf00367pf02378 -
02582 -pf00144 -
02583 -pf04261 -
02584 -pf01797 -
02585 -pf06572 -
02586 -pf11143 -
02587 -pf00583 -
02588 -pf01520 -
02589 -pf01218 -
02590 HMMreg12833_1_EUT1(pf12833) EUT1
02591 H_mulberry(pf00936pf16365) EUT1
02592 Tsp_rosa EUT1
02593 HMMetlyEutC_1_eut1(pf05985) EUT1 EP_NTERM
02594 HMMetlyEutB_1_EUT1(pf06751) EUT1
02595 -pf13356pf00589 -
02596 -# -
02597 -# -
02598 -# -
02599 -# -
02600 -# -
02601 -# -
02602 -# -
02604 HMM13520tp_1_pdu(pf13520) EUT2C
02605 -pf01177 -
02606 -pf03400 -
02607 -pf12759pf03811 -
02608 HMM06277EutA_1_eut(pf06277) EUT1
02609 HMMtpEuth_1_eut(pf04346) EUT1
02610 HMM00465alcdh_3_mix(pf00465) EUT1
02611 HMM06723EutJMreB_1_grm(#) EUT1
02612 HMMalddh_1_eutpdugrm(pf00171) EUT1 EP_NTERM
02613 P_lightViolet EUT1
02614 H_azure EUT1
02615 HMM01515PTA_B_1_eut1(pf01515) EUT1
02616 HMM01923cobtr_2_eut1(pf01923) EUT1
02617 HMM06249EutQ_1_eut1(pf06249) EUT1 EP_NTERM
02618 HMM10662PduV_1_mix(pf10662) EUT1
02619 Hp_euts_violetPink EUT1
02620 HMM00390_malic_1_eut1(pf00390pf03949pf01515) EUT1
02621 -pf00923 -
02622 HMM02780keto_1_eut1(pf02779pf02780pf00456) EUT1
02623 -pf06674 -
02624 HMM00293nuhyd_1_eut1(pf00293) EUT1
02625 -pf13247pf14691pf07992 -
02626 -pf00672pf02518pf07730pf13675 -
02627 -pf00873 -
02628 -pf03960 -
02629 -pf07687pf01546 -
BMC-P
BMC-H
BMC-T
Aldehyde dehydrogenase
PTAC
Signature enzyme
Alcohol dehydrogenase
Regulator

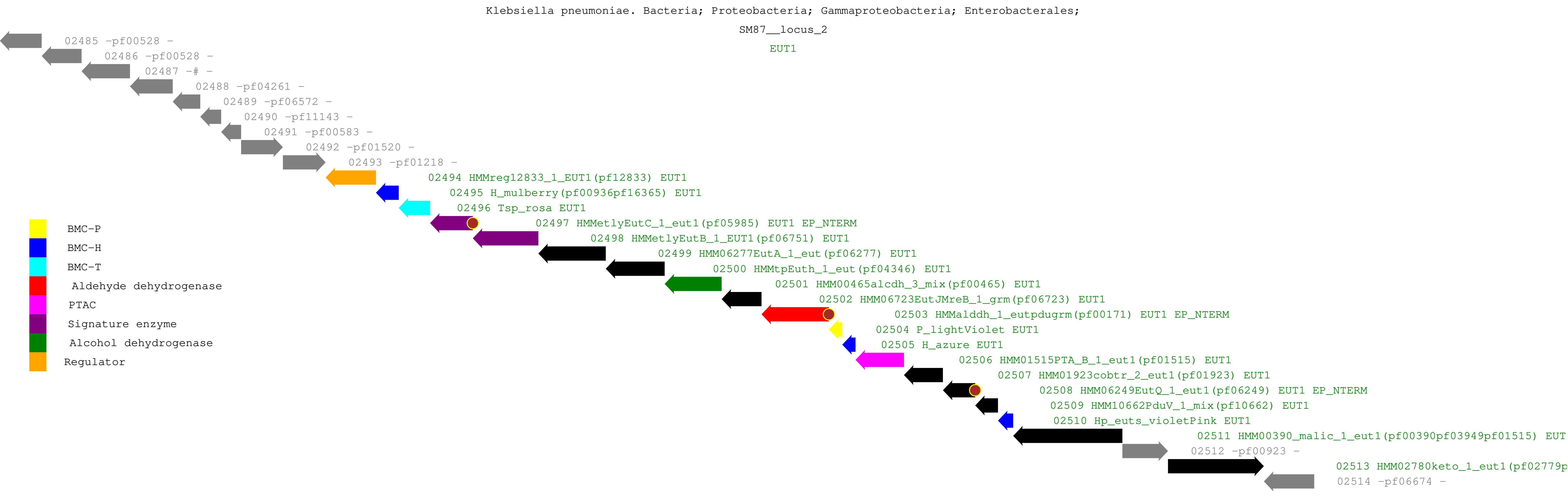

Klebsiella pneumoniae. Bacteria; Proteobacteria; Gammaproteobacteria; Enterobacterales;
SM87__locus_2
EUT1
02485 -pf00528 -
02486 -pf00528 -
02487 -# -
02488 -pf04261 -
02489 -pf06572 -
02490 -pf11143 -
02491 -pf00583 -
02492 -pf01520 -
02493 -pf01218 -
02494 HMMreg12833_1_EUT1(pf12833) EUT1
02495 H_mulberry(pf00936pf16365) EUT1
02496 Tsp_rosa EUT1
02497 HMMetlyEutC_1_eut1(pf05985) EUT1 EP_NTERM
02498 HMMetlyEutB_1_EUT1(pf06751) EUT1
02499 HMM06277EutA_1_eut(pf06277) EUT1
02500 HMMtpEuth_1_eut(pf04346) EUT1
02501 HMM00465alcdh_3_mix(pf00465) EUT1
02502 HMM06723EutJMreB_1_grm(pf06723) EUT1
02503 HMMalddh_1_eutpdugrm(pf00171) EUT1 EP_NTERM
02504 P_lightViolet EUT1
02505 H_azure EUT1
02506 HMM01515PTA_B_1_eut1(pf01515) EUT1
02507 HMM01923cobtr_2_eut1(pf01923) EUT1
02508 HMM06249EutQ_1_eut1(pf06249) EUT1 EP_NTERM
02509 HMM10662PduV_1_mix(pf10662) EUT1
02510 Hp_euts_violetPink EUT1
02511 HMM00390_malic_1_eut1(pf00390pf03949pf01515) EUT
02512 -pf00923 -
02513 HMM02780keto_1_eut1(pf02779p
02514 -pf06674 -
BMC-P
BMC-H
BMC-T
Aldehyde dehydrogenase
PTAC
Signature enzyme
Alcohol dehydrogenase
Regulator

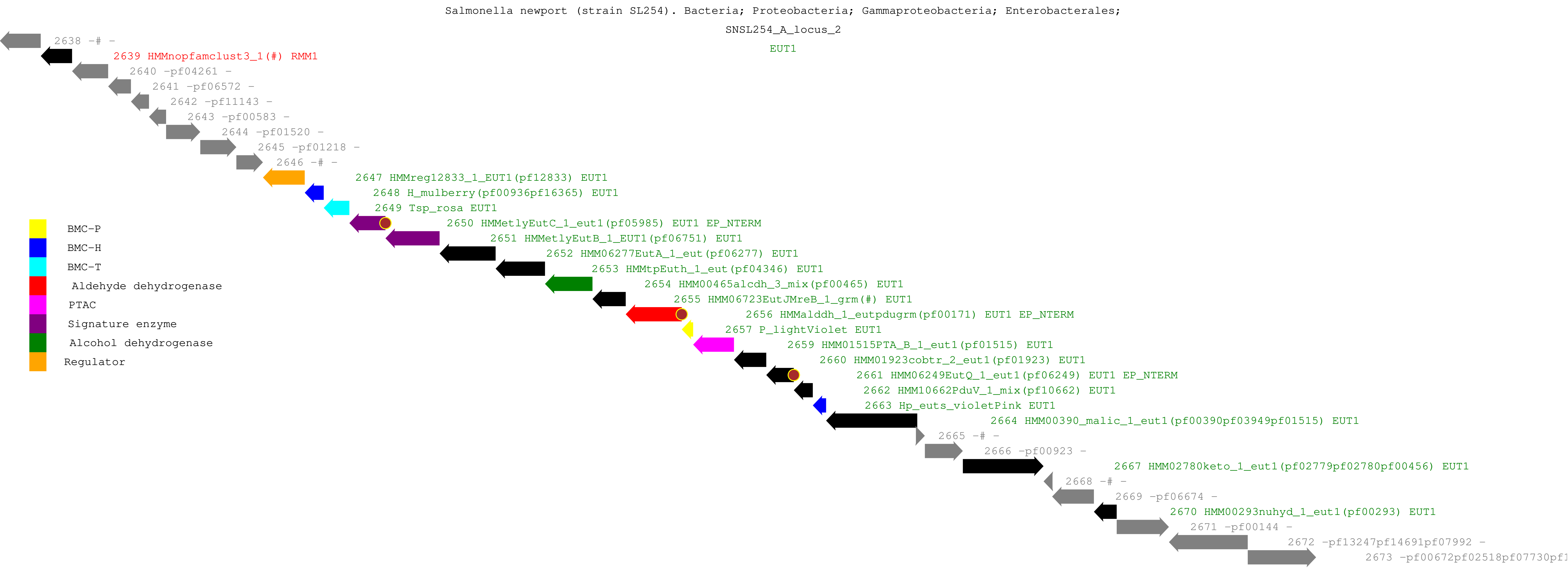
Salmonella newport (strain SL254). Bacteria; Proteobacteria; Gammaproteobacteria; Enterobacterales;
SNSL254_A_locus_2
EUT1
2638 -# -
2639 HMMnopfamclust3_1(#) RMM1
2640 -pf04261 -
2641 -pf06572 -
2642 -pf11143 -
2643 -pf00583 -
2644 -pf01520 -
2645 -pf01218 -
2646 -# -
2647 HMMreg12833_1_EUT1(pf12833) EUT1
2648 H_mulberry(pf00936pf16365) EUT1
2649 Tsp_rosa EUT1
2650 HMMetlyEutC_1_eut1(pf05985) EUT1 EP_NTERM
2651 HMMetlyEutB_1_EUT1(pf06751) EUT1
2652 HMM06277EutA_1_eut(pf06277) EUT1
2653 HMMtpEuth_1_eut(pf04346) EUT1
2654 HMM00465alcdh_3_mix(pf00465) EUT1
2655 HMM06723EutJMreB_1_grm(#) EUT1
2656 HMMalddh_1_eutpdugrm(pf00171) EUT1 EP_NTERM
2657 P_lightViolet EUT1
2659 HMM01515PTA_B_1_eut1(pf01515) EUT1
2660 HMM01923cobtr_2_eut1(pf01923) EUT1
2661 HMM06249EutQ_1_eut1(pf06249) EUT1 EP_NTERM
2662 HMM10662PduV_1_mix(pf10662) EUT1
2663 Hp_euts_violetPink EUT1
2664 HMM00390_malic_1_eut1(pf00390pf03949pf01515) EUT1
2665 -# -
2666 -pf00923 -
2667 HMM02780keto_1_eut1(pf02779pf02780pf00456) EUT1
2668 -# -
2669 -pf06674 -
2670 HMM00293nuhyd_1_eut1(pf00293) EUT1
2671 -pf00144 -
2672 -pf13247pf14691pf07992 -
2673 -pf00672pf02518pf07730pf1
BMC-P
BMC-H
BMC-T
Aldehyde dehydrogenase
PTAC
Signature enzyme
Alcohol dehydrogenase
Regulator

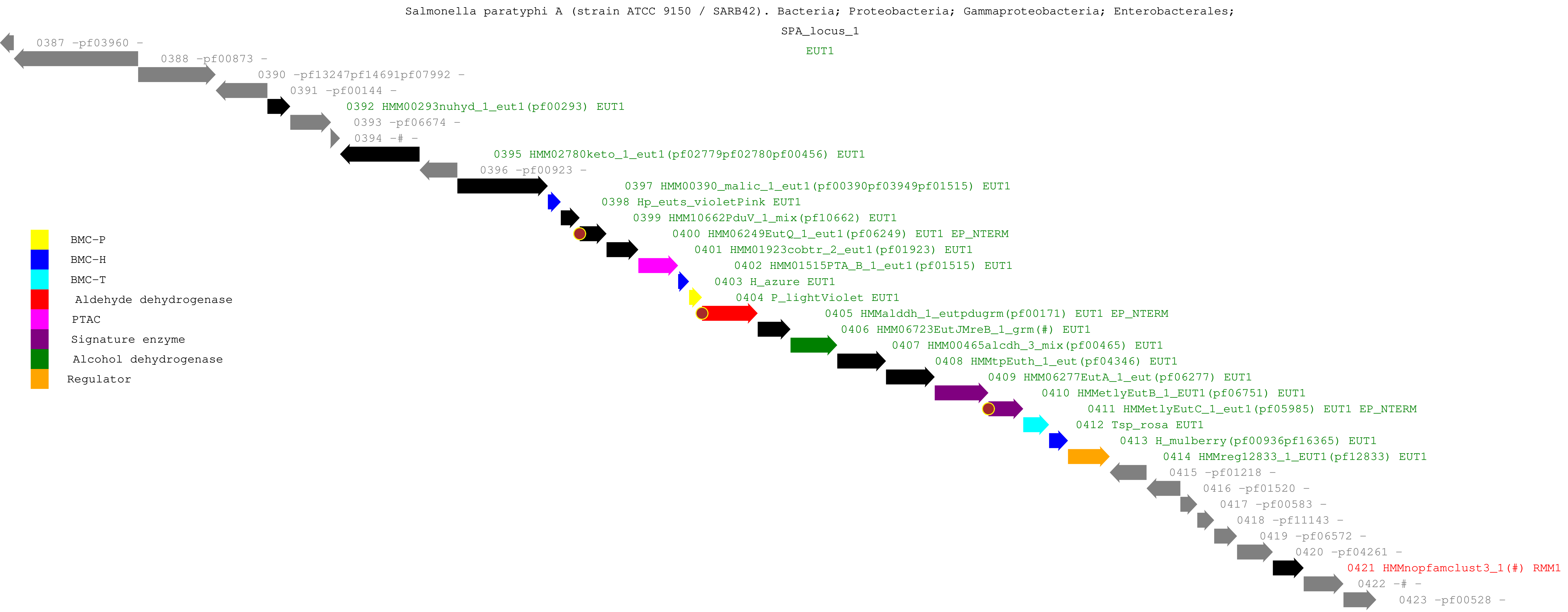
Salmonella paratyphi A (strain ATCC 9150 / SARB42). Bacteria; Proteobacteria; Gammaproteobacteria; Enterobacterales;
SPA_locus_1
EUT1
0387 -pf03960 -
0388 -pf00873 -
0390 -pf13247pf14691pf07992 -
0391 -pf00144 -
0392 HMM00293nuhyd_1_eut1(pf00293) EUT1
0393 -pf06674 -
0394 -# -
0395 HMM02780keto_1_eut1(pf02779pf02780pf00456) EUT1
0396 -pf00923 -
0397 HMM00390_malic_1_eut1(pf00390pf03949pf01515) EUT1
0398 Hp_euts_violetPink EUT1
0399 HMM10662PduV_1_mix(pf10662) EUT1
0400 HMM06249EutQ_1_eut1(pf06249) EUT1 EP_NTERM
0401 HMM01923cobtr_2_eut1(pf01923) EUT1
0402 HMM01515PTA_B_1_eut1(pf01515) EUT1
0403 H_azure EUT1
0404 P_lightViolet EUT1
0405 HMMalddh_1_eutpdugrm(pf00171) EUT1 EP_NTERM
0406 HMM06723EutJMreB_1_grm(#) EUT1
0407 HMM00465alcdh_3_mix(pf00465) EUT1
0408 HMMtpEuth_1_eut(pf04346) EUT1
0409 HMM06277EutA_1_eut(pf06277) EUT1
0410 HMMetlyEutB_1_EUT1(pf06751) EUT1
0411 HMMetlyEutC_1_eut1(pf05985) EUT1 EP_NTERM
0412 Tsp_rosa EUT1
0413 H_mulberry(pf00936pf16365) EUT1
0414 HMMreg12833_1_EUT1(pf12833) EUT1
0415 -pf01218 -
0416 -pf01520 -
0417 -pf00583 -
0418 -pf11143 -
0419 -pf06572 -
0420 -pf04261 -
0421 HMMnopfamclust3_1(#) RMM1
0422 -# -
0423 -pf00528 -
BMC-P
BMC-H
BMC-T
Aldehyde dehydrogenase
PTAC
Signature enzyme
Alcohol dehydrogenase
Regulator

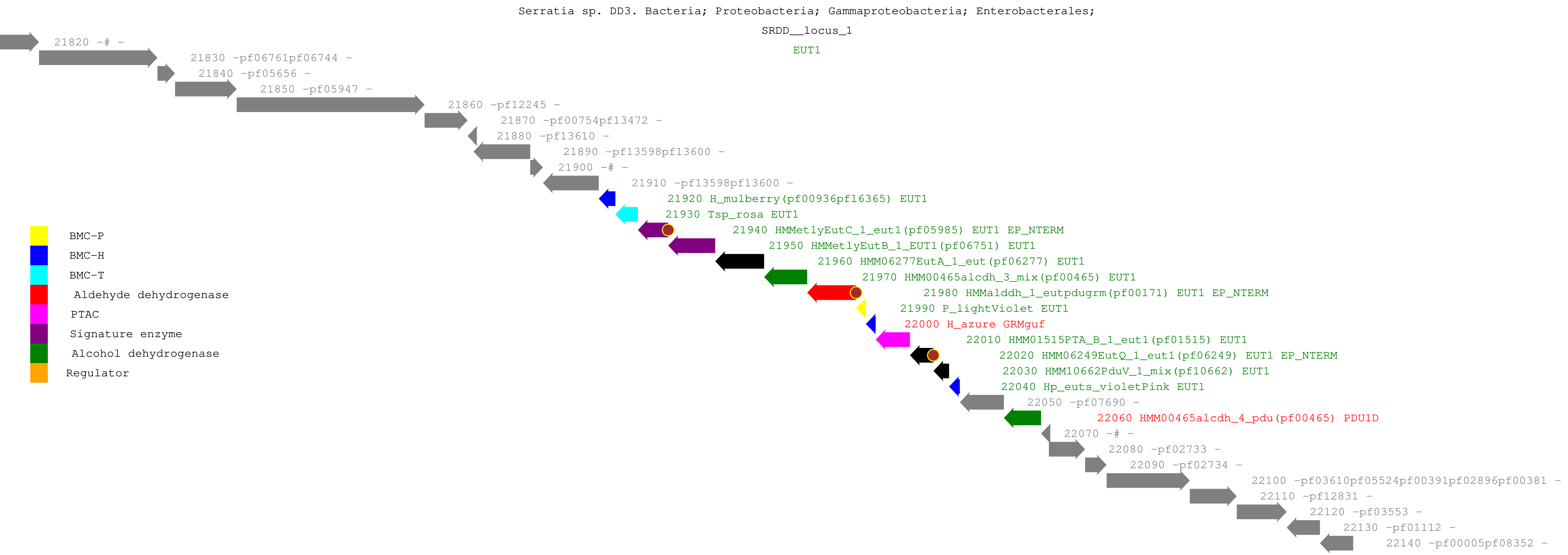

Serratia sp. DD3. Bacteria; Proteobacteria; Gammaproteobacteria; Enterobacterales;
SRDD__locus_1
EUT1
21820 -# -
21830 -pf06761pf06744 -
21840 -pf05656 -
21850 -pf05947 -
21860 -pf12245 -
21870 -pf00754pf13472 -
21880 -pf13610 -
21890 -pf13598pf13600 -
21900 -# -
21910 -pf13598pf13600 -
21920 H_mulberry(pf00936pf16365) EUT1
21930 Tsp_rosa EUT1
21940 HMMetlyEutC_1_eut1(pf05985) EUT1 EP_NTERM
21950 HMMetlyEutB_1_EUT1(pf06751) EUT1
21960 HMM06277EutA_1_eut(pf06277) EUT1
21970 HMM00465alcdh_3_mix(pf00465) EUT1
21980 HMMaldh_1_eutpdugrm(pf00171) EUT1 EP_NTERM
21990 P_lightViolet EUT1
22000 H_azure GRMguf
22010 HMM01515PTA_B_1_eut1(pf01515) EUT1
22020 HMM06249EutQ_1_eut1(pf06249) EUT1 EP_NTERM
22030 HMM10662PduV_1_mix(pf10662) EUT1
22040 Hp_euts_violetPink EUT1
22050 -pf07690 -
22060 HMM00465alcdh_4_pdu(pf00465) PDU1D
22070 -# -
22080 -pf02733 -
22090 -pf02734 -
22100 -pf03610pf05524pf00391pf02896pf00381 -
22110 -pf12831 -
22120 -pf03553 -
22130 -pf01112 -
22140 -pf00005pf08352 -
BMC-P
BMC-H
BMC-T
Aldehyde dehydrogenase
PTAC
Signature enzyme
Alcohol dehydrogenase
Regulator

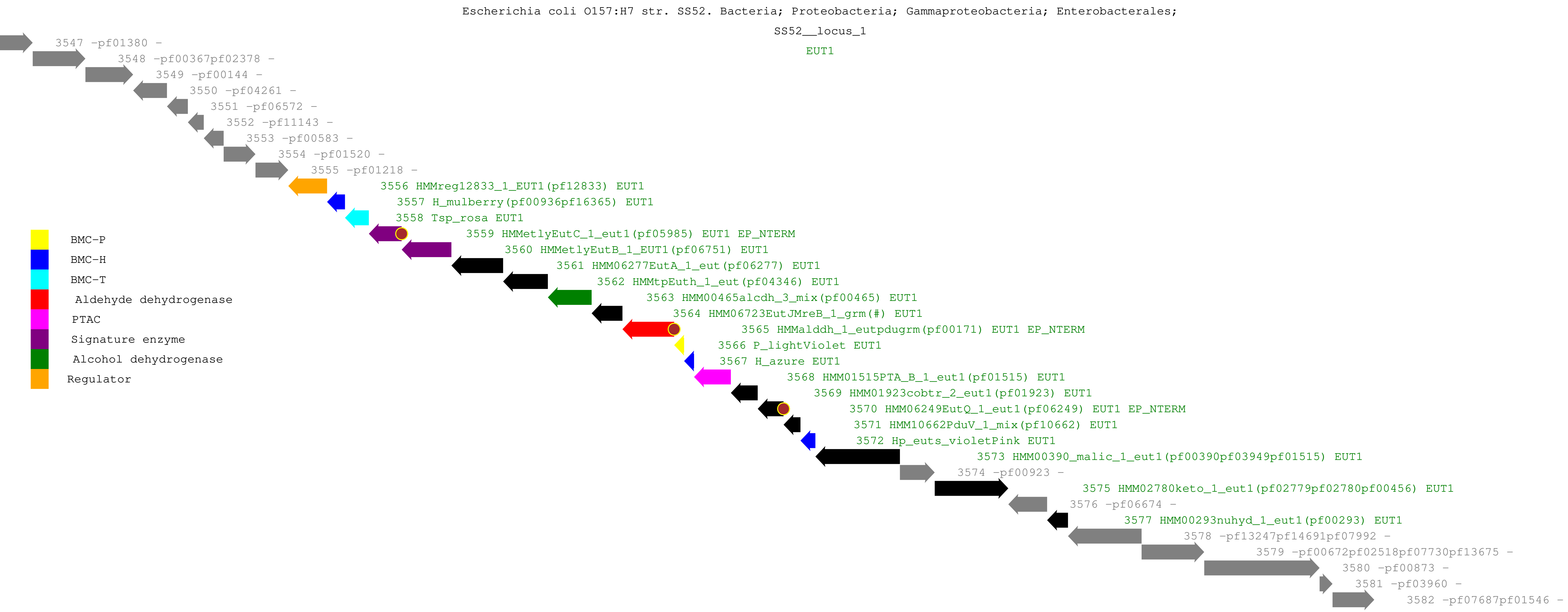

Escherichia coli O157:H7 str. SS52. Bacteria; Proteobacteria; Gammaproteobacteria; Enterobacterales;
SS52__locus_1
EUT1
3547 -pf01380 -
3548 -pf00367pf02378 -
3549 -pf00144 -
3550 -pf04261 -
3551 -pf06572 -
3552 -pf11143 -
3553 -pf00583 -
3554 -pf01520 -
3555 -pf01218 -
3556 HMMreg12833_1_EUT1(pf12833) EUT1
3557 H_mulberry(pf00936pf16365) EUT1
3558 Tsp_rosa EUT1
3559 HMMetlyEutC_1_eut1(pf05985) EUT1 EP_NTERM
3560 HMMetlyEutB_1_EUT1(pf06751) EUT1
3561 HMM06277EutA_1_eut(pf06277) EUT1
3562 HMMtpEuth_1_eut(pf04346) EUT1
3563 HMM00465alcdh_3_mix(pf00465) EUT1
3564 HMM06723EutJMreB_1_grm(#) EUT1
3565 HMMalddh_1_eutpdugrm(pf00171) EUT1 EP_NTERM
3566 P_lightViolet EUT1
3567 H_azure EUT1
3568 HMM01515PTA_B_1_eut1(pf01515) EUT1
3569 HMM01923cobtr_2_eut1(pf01923) EUT1
3570 HMM06249EutQ_1_eut1(pf06249) EUT1 EP_NTERM
3571 HMM10662PduV_1_mix(pf10662) EUT1
3572 Hp_euts_violetPink EUT1
3573 HMM00390_malic_1_eut1(pf00390pf03949pf01515) EUT1
3574 -pf00923 -
3575 HMM02780keto_1_eut1(pf02779pf02780pf00456) EUT1
3576 -pf06674 -
3577 HMM00293nuhyd_1_eut1(pf00293) EUT1
3578 -pf13247pf14691pf07992 -
3579 -pf00672pf02518pf07730pf13675 -
3580 -pf00873 -
3581 -pf03960 -
3582 -pf07687pf01546 -
BMC-P
BMC-H
BMC-T
Aldehyde dehydrogenase
PTAC
Signature enzyme
Alcohol dehydrogenase
Regulator

Escherichia coli D9. Bacteria; Proteobacteria; Gammaproteobacteria; Enterobacterales;

SSJG__locus_1

EUT1

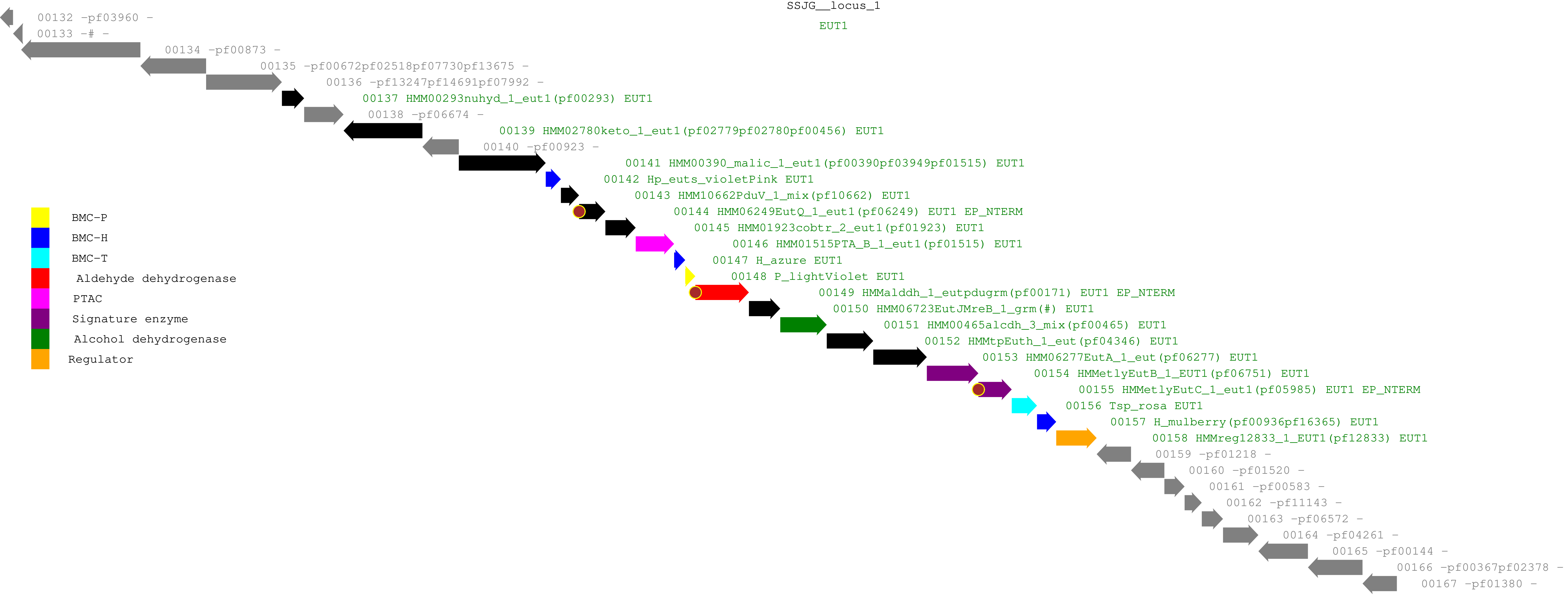

Shigella sonnei str. Moseley. Bacteria; Proteobacteria; Gammaproteobacteria; Enterobacterales;

SSMOSELEY__locus_2

EUT1

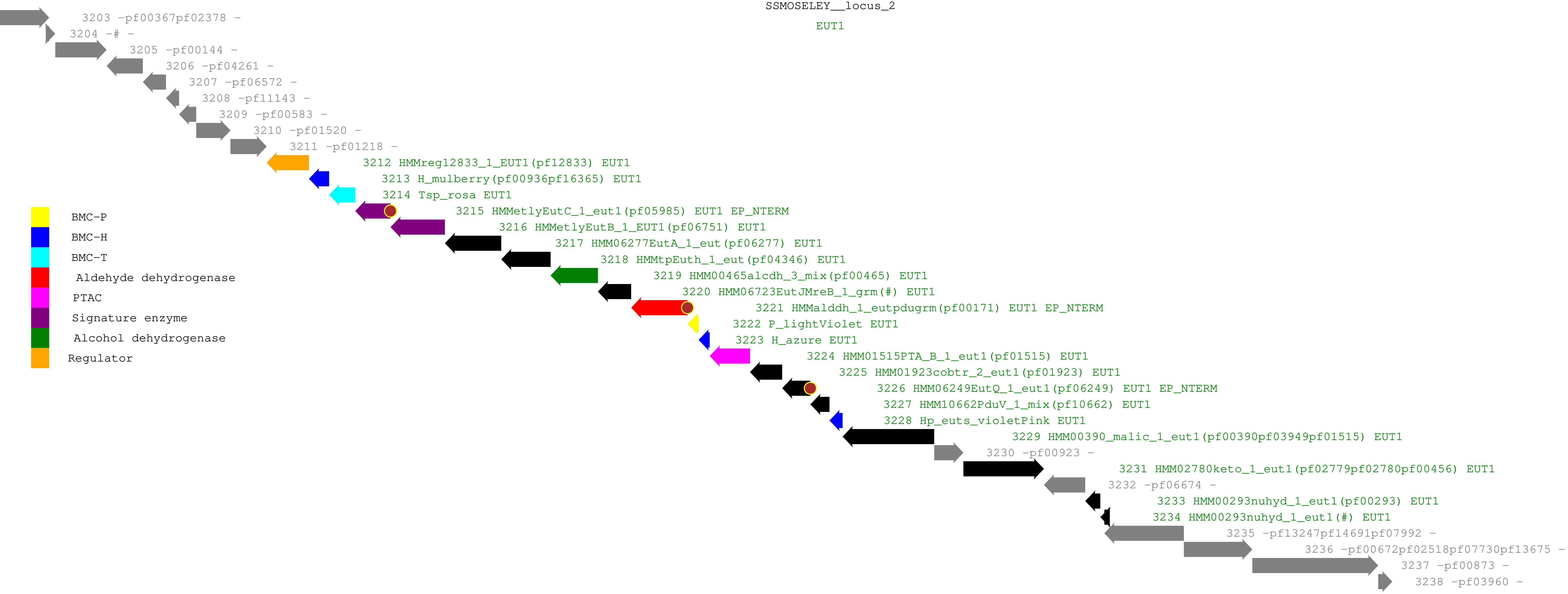

Shigella sonnei (strain Ss046). Bacteria; Proteobacteria; Gammaproteobacteria; Enterobacterales;

SSON__locus_2

EUT1

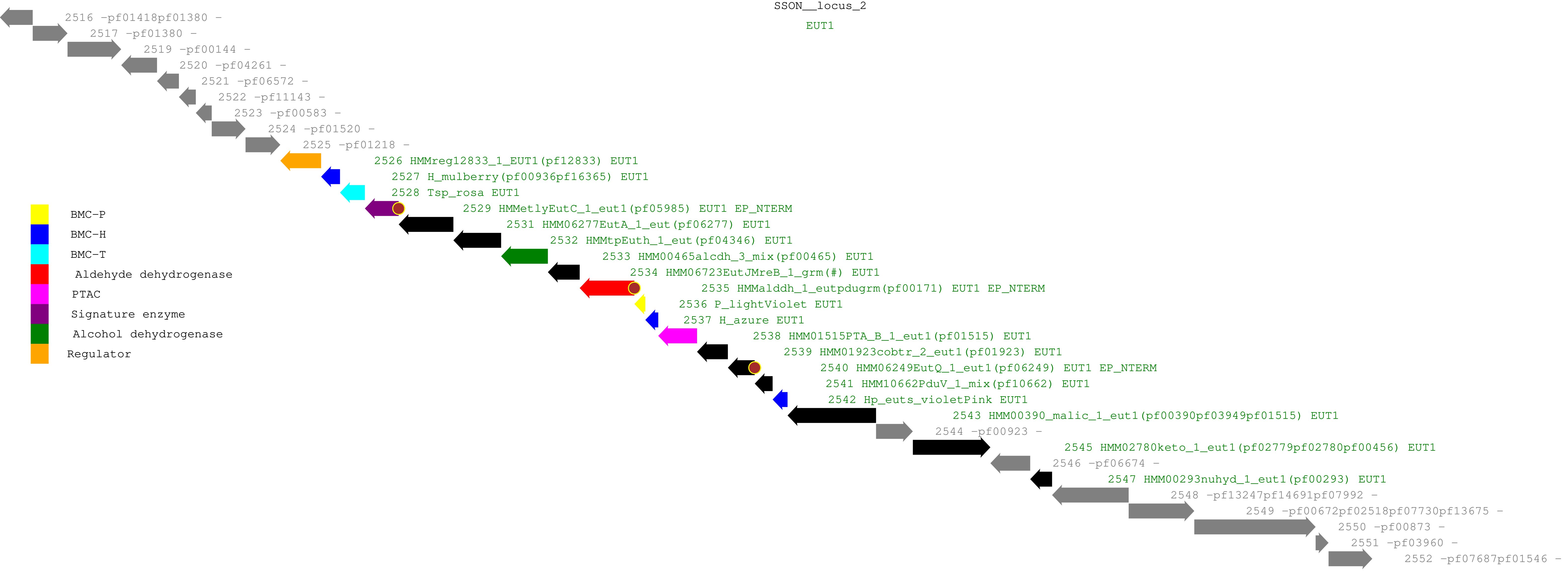

Salmonella enterica subsp. enterica serovar 4,5,12:i:-. Bacteria; Proteobacteria; Gammaproteobacteria; Enterobacterales;

STL3838__locus_1

EUT1

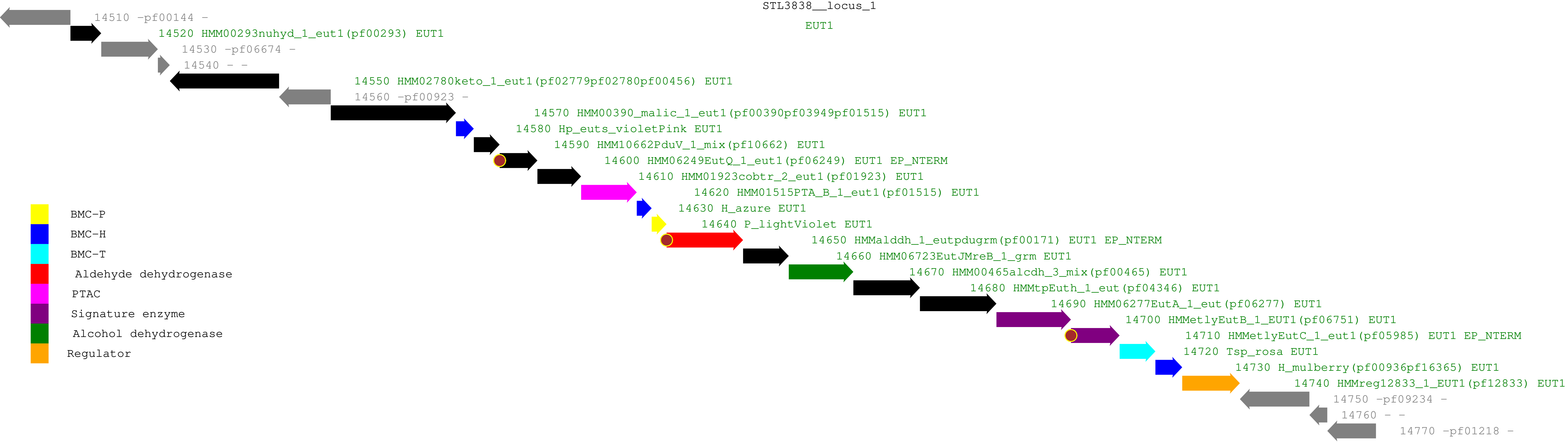

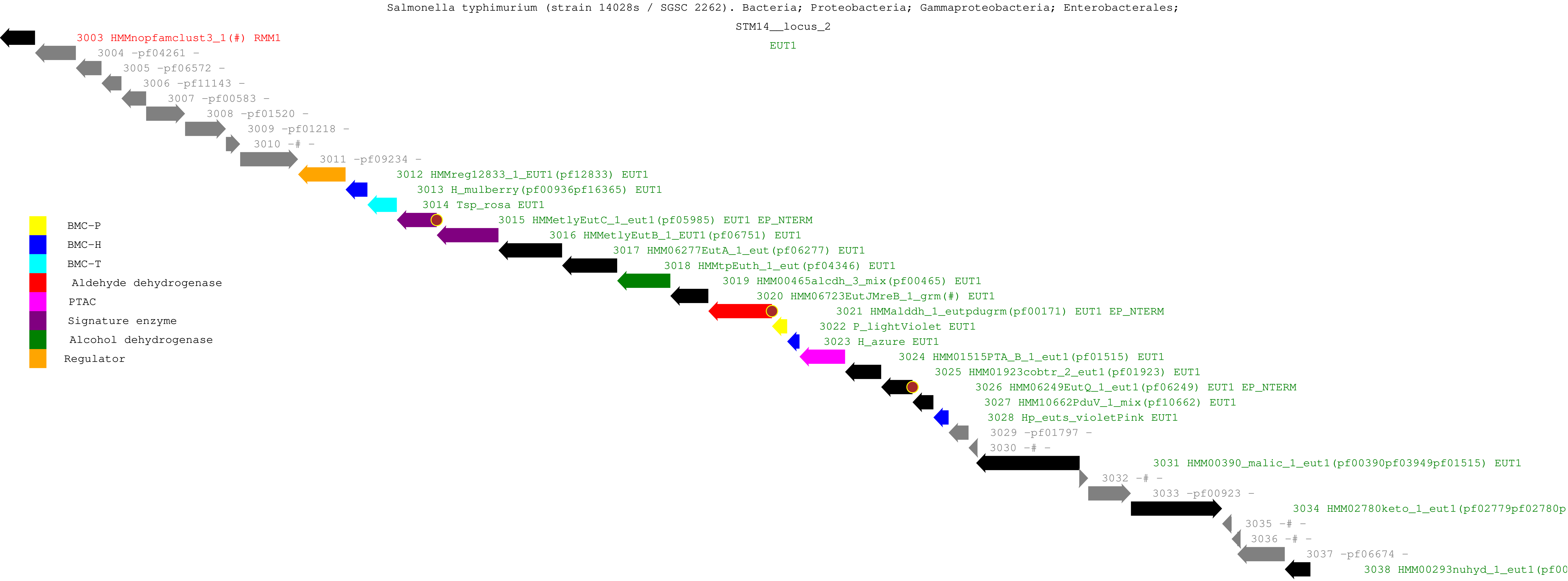
Salmonella typhimurium (strain 14028s / SGSC 2262). Bacteria; Proteobacteria; Gammaproteobacteria; Enterobacterales;
STM14__locus_2
EUT1
3003 HMMnopfamclust3_1(#) RMM1
3004 -pf04261 -
3005 -pf06572 -
3006 -pf11143 -
3007 -pf00583 -
3008 -pf01520 -
3009 -pf01218 -
3010 -# -
3011 -pf09234 -
3012 HMMreg12833_1_EUT1(pf12833) EUT1
3013 H_mulberry(pf00936pf16365) EUT1
3014 Tsp_rosa EUT1
3015 HMMetlyEutC_1_eut1(pf05985) EUT1 EP_NTERM
3016 HMMetlyEutB_1_EUT1(pf06751) EUT1
3017 HMM06277EutA_1_eut(pf06277) EUT1
3018 HMMtpEuth_1_eut(pf04346) EUT1
3019 HMM00465alcdh_3_mix(pf00465) EUT1
3020 HMM06723EutJMreB_1_grm(#) EUT1
3021 HMMalddh_1_eutpdugrm(pf00171) EUT1 EP_NTERM
3022 P_lightViolet EUT1
3023 H_azure EUT1
3024 HMM01515PTA_B_1_eut1(pf01515) EUT1
3025 HMM01923cobtr_2_eut1(pf01923) EUT1
3026 HMM06249EutQ_1_eut1(pf06249) EUT1 EP_NTERM
3027 HMM10662PduV_1_mix(pf10662) EUT1
3028 Hp_euts_violetPink EUT1
3029 -pf01797 -
3030 -# -
3031 HMM00390_malic_1_eut1(pf00390pf03949pf01515) EUT1
3032 -# -
3033 -pf00923 -
3034 HMM02780keto_1_eut1(pf02779pf02780p
3035 -# -
3036 -# -
3037 -pf06674 -
3038 HMM00293nuhyd_1_eut1(pf00
BMC-P
BMC-H
BMC-T
Aldehyde dehydrogenase
PTAC
Signature enzyme
Alcohol dehydrogenase
Regulator

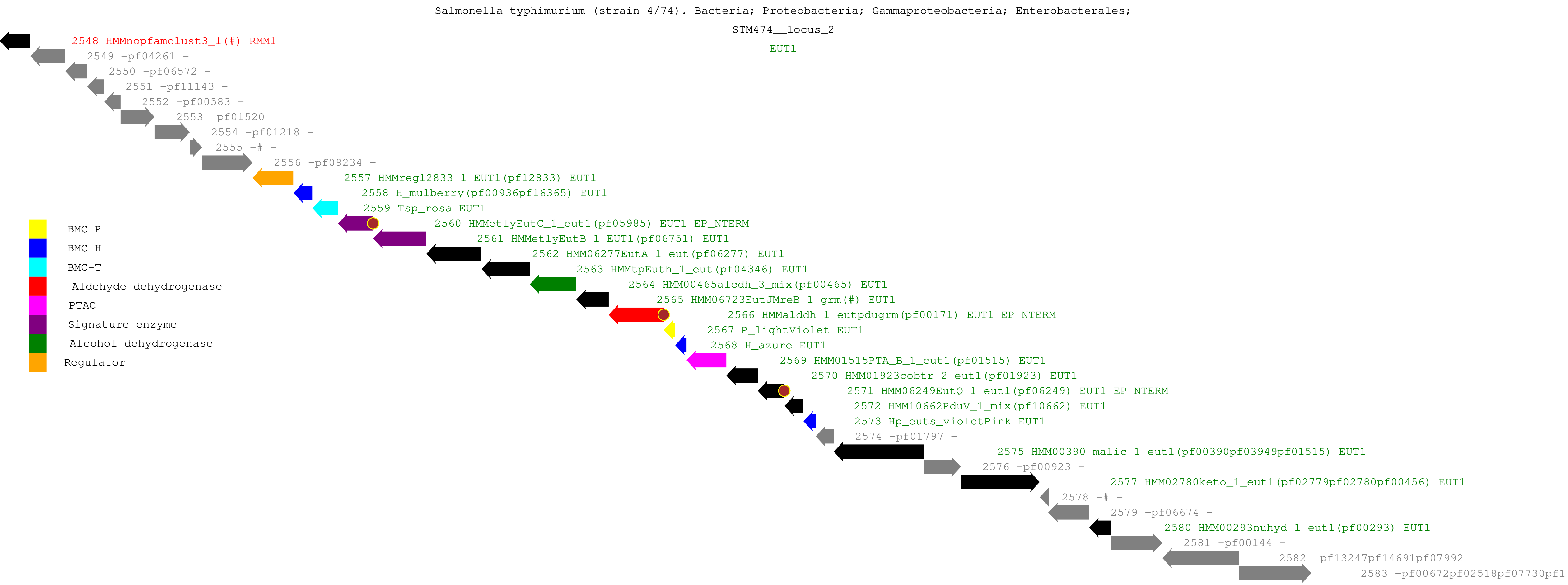
Salmonella typhimurium (strain 4/74). Bacteria; Proteobacteria; Gammaproteobacteria; Enterobacterales;
STM474__locus_2
EUT1
2548 HMMnopfamclust3_1(#) RMM1
2549 -pf04261 -
2550 -pf06572 -
2551 -pf11143 -
2552 -pf00583 -
2553 -pf01520 -
2554 -pf01218 -
2555 -# -
2556 -pf09234 -
2557 HMMreg12833_1_EUT1(pf12833) EUT1
2558 H_mulberry(pf00936pf16365) EUT1
2559 Tsp_rosa EUT1
2560 HMMetlyEutC_1_eut1(pf05985) EUT1 EP_NTERM
2561 HMMetlyEutB_1_EUT1(pf06751) EUT1
2562 HMM06277EutA_1_eut(pf06277) EUT1
2563 HMMtpEuth_1_eut(pf04346) EUT1
2564 HMM00465alcdh_3_mix(pf00465) EUT1
2565 HMM06723EutJMreB_1_grm(#) EUT1
2566 HMMalddh_1_eutpdugrm(pf00171) EUT1 EP_NTERM
2567 P_lightViolet EUT1
2568 H_azure EUT1
2569 HMM01515PTA_B_1_eut1(pf01515) EUT1
2570 HMM01923cobtr_2_eut1(pf01923) EUT1
2571 HMM06249EutQ_1_eut1(pf06249) EUT1 EP_NTERM
2572 HMM10662PduV_1_mix(pf10662) EUT1
2573 Hp_euts_violetPink EUT1
2574 -pf01797 -
2575 HMM00390_malic_1_eut1(pf00390pf03949pf01515) EUT1
2576 -pf00923 -
2577 HMM02780keto_1_eut1(pf02779pf02780pf00456) EUT1
2578 -# -
2579 -pf06674 -
2580 HMM00293nuhyd_1_eut1(pf00293) EUT1
2581 -pf00144 -
2582 -pf13247pf14691pf07992 -
2583 -pf00672pf02518pf07730pf1
BMC-P
BMC-H
BMC-T
Aldehyde dehydrogenase
PTAC
Signature enzyme
Alcohol dehydrogenase
Regulator

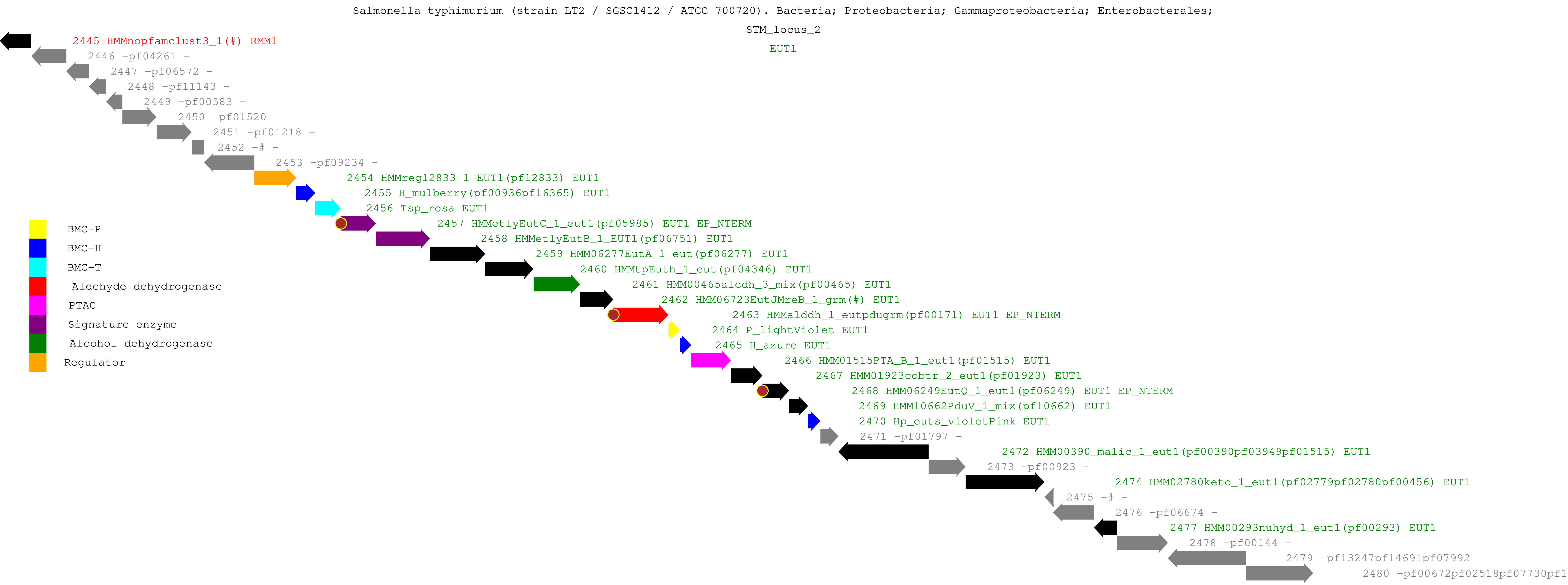
Salmonella typhimurium (strain LT2 / SGSC1412 / ATCC 700720). Bacteria; Proteobacteria; Gammaproteobacteria; Enterobacterales;
STM_locus_2
EUT1
2445 HMMnopfamclust3_1(#) RMM1
2446 -pf04261 -
2447 -pf06572 -
2448 -pf11143 -
2449 -pf00583 -
2450 -pf01520 -
2451 -pf01218 -
2452 -# -
2453 -pf09234 -
2454 HMMreg12833_1_EUT1(pf12833) EUT1
2455 H_mulberry(pf00936pf16365) EUT1
2456 Tsp_rosa EUT1
2457 HMMetlyEutC_1_eut1(pf05985) EUT1 EP_NTERM
2458 HMMetlyEutB_1_EUT1(pf06751) EUT1
2459 HMM06277EutA_1_eut(pf06277) EUT1
2460 HMMtpEuth_1_eut(pf04346) EUT1
2461 HMM00465alcdh_3_mix(pf00465) EUT1
2462 HMM06723EutJMreB_1_grm(#) EUT1
2463 HMMaldh_1_eutpdugrm(pf00171) EUT1 EP_NTERM
2464 P_lightViolet EUT1
2465 H_azure EUT1
2466 HMM01515PTA_B_1_eut1(pf01515) EUT1
2467 HMM01923cobtr_2_eut1(pf01923) EUT1
2468 HMM06249EutQ_1_eut1(pf06249) EUT1 EP_NTERM
2469 HMM10662PduV_1_mix(pf10662) EUT1
2470 Hp_euts_violetPink EUT1
2471 -pf01797 -
2472 HMM00390_malic_1_eut1(pf00390pf03949pf01515) EUT1
2473 -pf00923 -
2474 HMM02780keto_1_eut1(pf02779pf02780pf00456) EUT1
2475 -# -
2476 -pf06674 -
2477 HMM00293nuhyd_1_eut1(pf00293) EUT1
2478 -pf00144 -
2479 -pf13247pf14691pf07992 -
2480 -pf00672pf02518pf07730pf1
BMC-P
BMC-H
BMC-T
Aldehyde dehydrogenase
PTAC
Signature enzyme
Alcohol dehydrogenase
Regulator

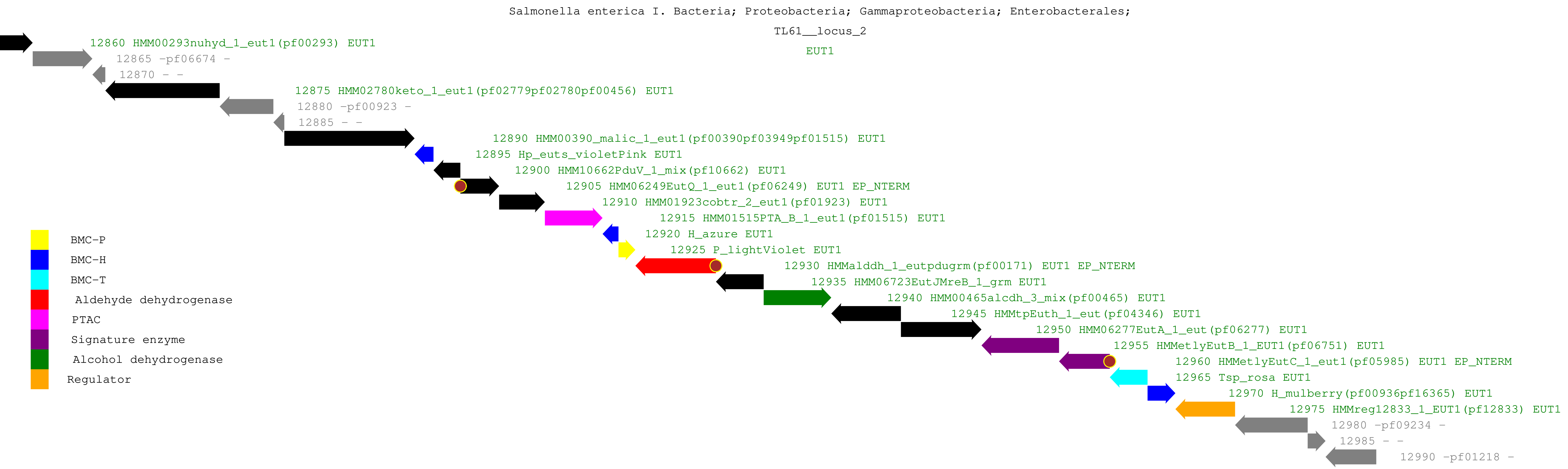

Salmonella enterica I. Bacteria; Proteobacteria; Gammaproteobacteria; Enterobacterales;
TL61__locus_2
EUT1
12860 HMM00293nuhyd_1_eut1(pf00293) EUT1
12865 -pf06674 -
12870 - -
12875 HMM02780keto_1_eut1(pf02779pf02780pf00456) EUT1
12880 -pf00923 -
12885 - -
12890 HMM00390_malic_1_eut1(pf00390pf03949pf01515) EUT1
12895 Hp_euts_violetPink EUT1
12900 HMM10662PduV_1_mix(pf10662) EUT1
12905 HMM06249EutQ_1_eut1(pf06249) EUT1 EP_NTERM
12910 HMM01923cobtr_2_eut1(pf01923) EUT1
12915 HMM01515PTA_B_1_eut1(pf01515) EUT1
12920 H_azure EUT1
12925 P_lightViolet EUT1
12930 HMMalddh_1_eutpdugrm(pf00171) EUT1 EP_NTERM
12935 HMM06723EutJMreB_1_grm EUT1
12940 HMM00465alcdh_3_mix(pf00465) EUT1
12945 HMMtpEuth_1_eut(pf04346) EUT1
12950 HMM06277EutA_1_eut(pf06277) EUT1
12955 HMMetlyEutB_1_EUT1(pf06751) EUT1
12960 HMMetlyEutC_1_eut1(pf05985) EUT1 EP_NTERM
12965 Tsp_rosa EUT1
12970 H_mulberry(pf00936pf16365) EUT1
12975 HMMreg12833_1_EUT1(pf12833) EUT1
12980 -pf09234 -
12985 - -
12990 -pf01218 -
BMC-P
BMC-H
BMC-T
Aldehyde dehydrogenase
PTAC
Signature enzyme
Alcohol dehydrogenase
Regulator

Salmonella enterica subsp. salamae. Bacteria; Proteobacteria; Gammaproteobacteria; Enterobacterales;

TM63__locus_1

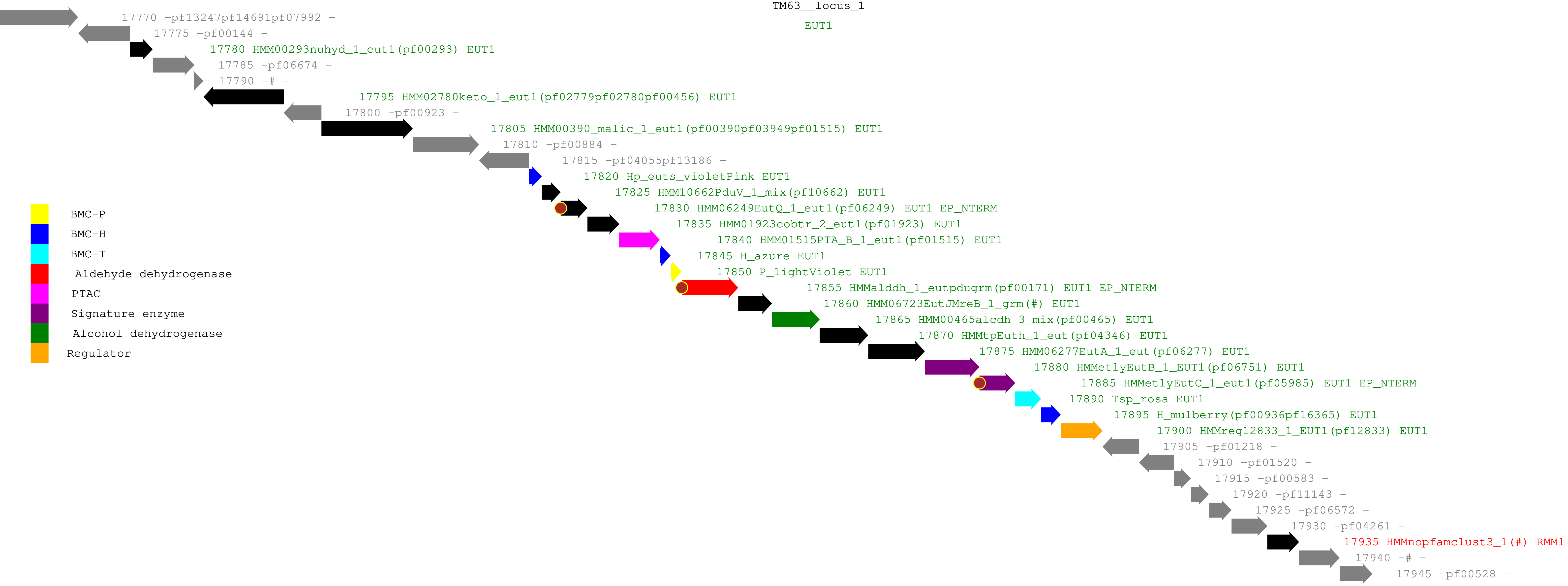

Escherichia coli TW10598. Bacteria; Proteobacteria; Gammaproteobacteria; Enterobacterales;

TW10598__locus_1

EUT1

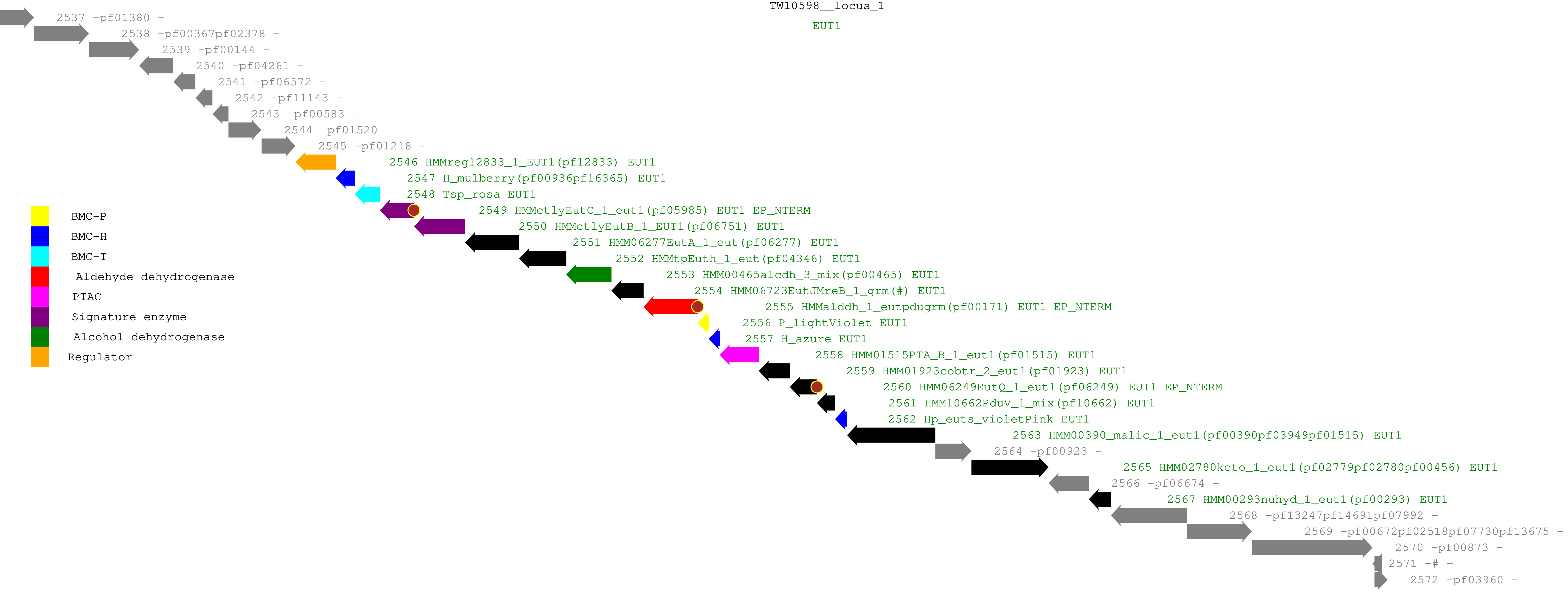

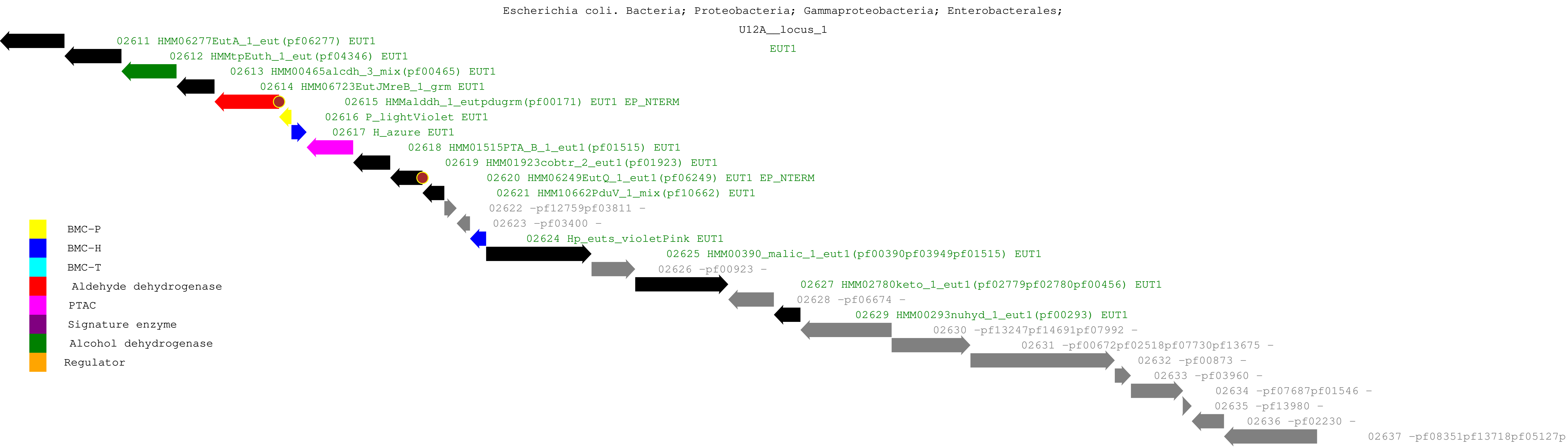

Escherichia coli. Bacteria; Proteobacteria; Gammaproteobacteria; Enterobacterales;
U12A__locus_1
EUT1
02611 HMM06277EutA_1_eut(pf06277) EUT1
02612 HMMtpEuth_1_eut(pf04346) EUT1
02613 HMM00465alcdh_3_mix(pf00465) EUT1
02614 HMM06723EutJMreB_1_grm EUT1
02615 HMMalddh_1_eutpdugrm(pf00171) EUT1 EP_NTERM
02616 P_lightViolet EUT1
02617 H_azure EUT1
02618 HMM01515PTA_B_1_eut1(pf01515) EUT1
02619 HMM01923cobtr_2_eut1(pf01923) EUT1
02620 HMM06249EutQ_1_eut1(pf06249) EUT1 EP_NTERM
02621 HMM10662PduV_1_mix(pf10662) EUT1
02622 -pf12759pf03811 -
02623 -pf03400 -
02624 Hp_euts_violetPink EUT1
02625 HMM00390_malic_1_eut1(pf00390pf03949pf01515) EUT1
02626 -pf00923 -
02627 HMM02780keto_1_eut1(pf02779pf02780pf00456) EUT1
02628 -pf06674 -
02629 HMM00293nuhyd_1_eut1(pf00293) EUT1
02630 -pf13247pf14691pf07992 -
02631 -pf00672pf02518pf07730pf13675 -
02632 -pf00873 -
02633 -pf03960 -
02634 -pf07687pf01546 -
02635 -pf13980 -
02636 -pf02230 -
02637 -pf08351pf13718pf05127p
BMC-P
BMC-H
BMC-T
Aldehyde dehydrogenase
PTAC
Signature enzyme
Alcohol dehydrogenase
Regulator

Escherichia coli O25b:H4-ST131. Bacteria; Proteobacteria; Gammaproteobacteria; Enterobacterales;

UG58__locus_1

EUT1

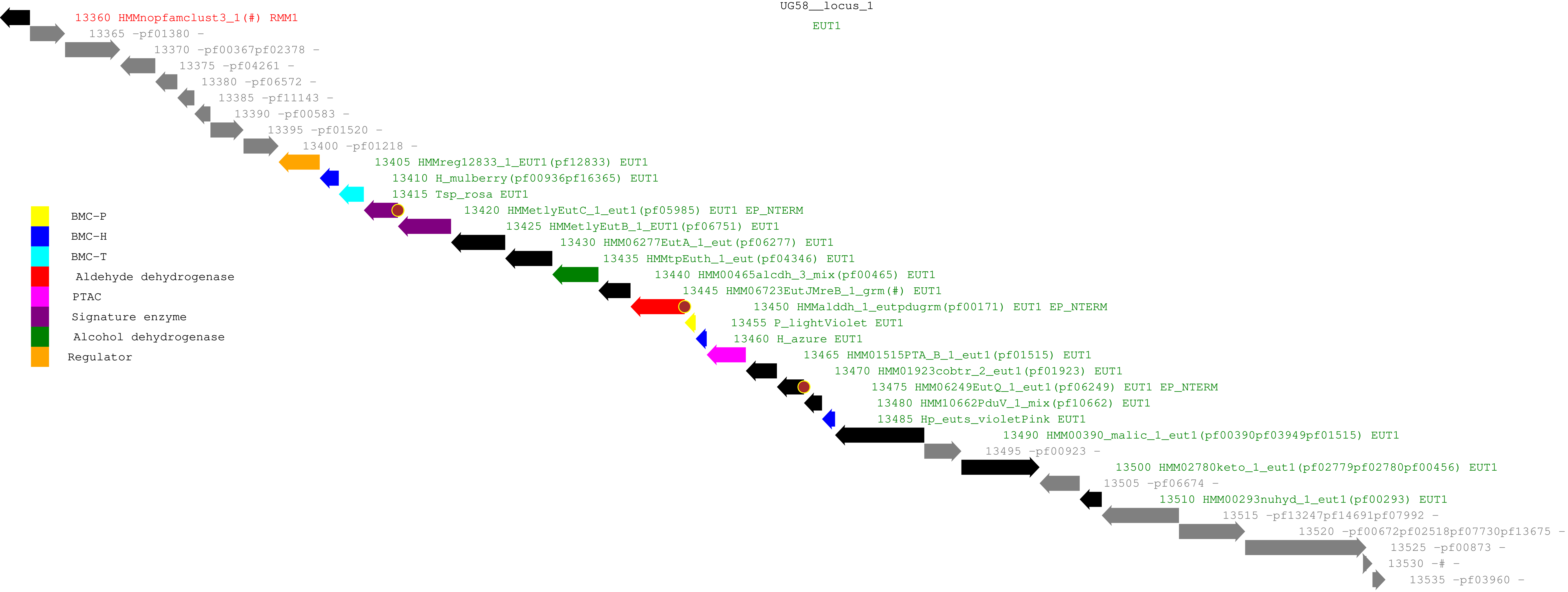

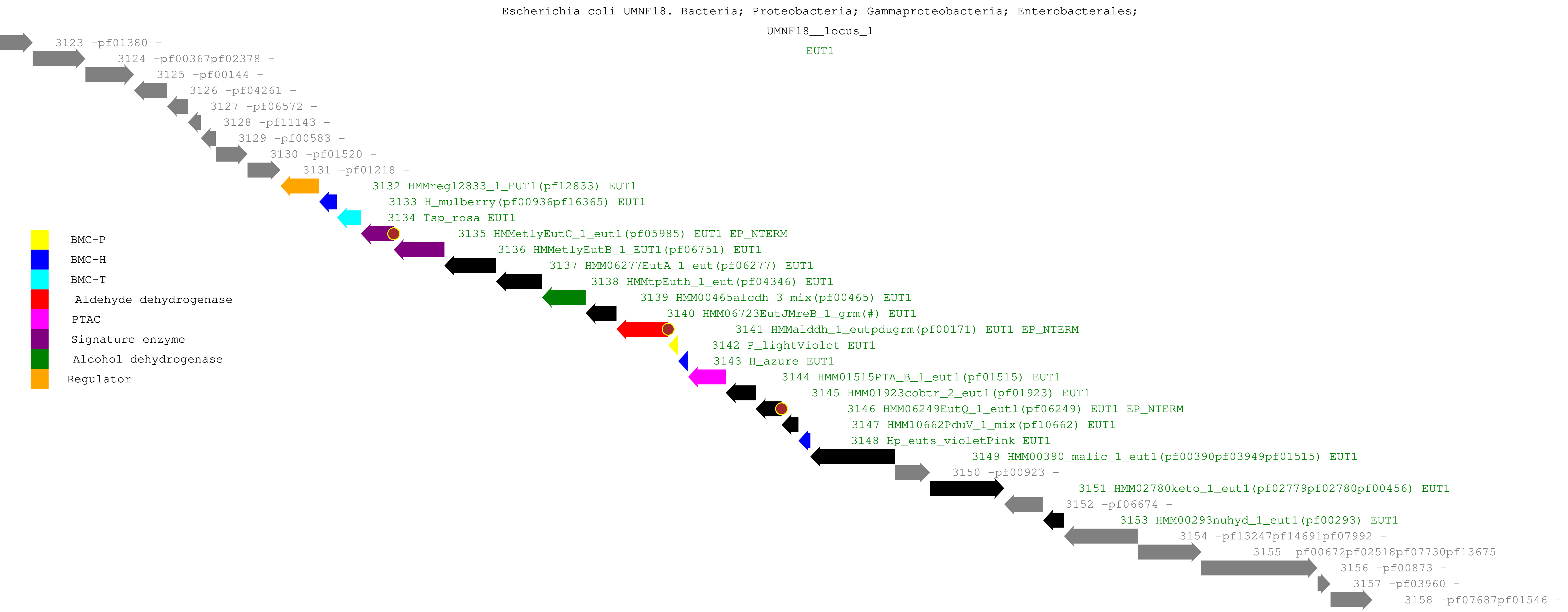

Escherichia coli UMNF18. Bacteria; Proteobacteria; Gammaproteobacteria; Enterobacterales;
UMNF18__locus_1
EUT1
3123 -pf01380 -
3124 -pf00367pf02378 -
3125 -pf00144 -
3126 -pf04261 -
3127 -pf06572 -
3128 -pf11143 -
3129 -pf00583 -
3130 -pf01520 -
3131 -pf01218 -
3132 HMMreg12833_1_EUT1(pf12833) EUT1
3133 H_mulberry(pf00936pf16365) EUT1
3134 Tsp_rosa EUT1
3135 HMMetlyEutC_1_eut1(pf05985) EUT1 EP_NTERM
3136 HMMetlyEutB_1_EUT1(pf06751) EUT1
3137 HMM06277EutA_1_eut(pf06277) EUT1
3138 HMMtpEuth_1_eut(pf04346) EUT1
3139 HMM00465alcdh_3_mix(pf00465) EUT1
3140 HMM06723EutJMreB_1_grm(#) EUT1
3141 HMMaldh_1_eutpdugrm(pf00171) EUT1 EP_NTERM
3142 P_lightViolet EUT1
3143 H_azure EUT1
3144 HMM01515PTA_B_1_eut1(pf01515) EUT1
3145 HMM01923cobtr_2_eut1(pf01923) EUT1
3146 HMM06249EutQ_1_eut1(pf06249) EUT1 EP_NTERM
3147 HMM10662PduV_1_mix(pf10662) EUT1
3148 Hp_euts_violetPink EUT1
3149 HMM00390_malic_1_eut1(pf00390pf03949pf01515) EUT1
3150 -pf00923 -
3151 HMM02780keto_1_eut1(pf02779pf02780pf00456) EUT1
3152 -pf06674 -
3153 HMM00293nuhyd_1_eut1(pf00293) EUT1
3154 -pf13247pf14691pf07992 -
3155 -pf00672pf02518pf07730pf13675 -
3156 -pf00873 -
3157 -pf03960 -
3158 -pf07687pf01546 -
BMC-P
BMC-H
BMC-T
Aldehyde dehydrogenase
PTAC
Signature enzyme
Alcohol dehydrogenase
Regulator

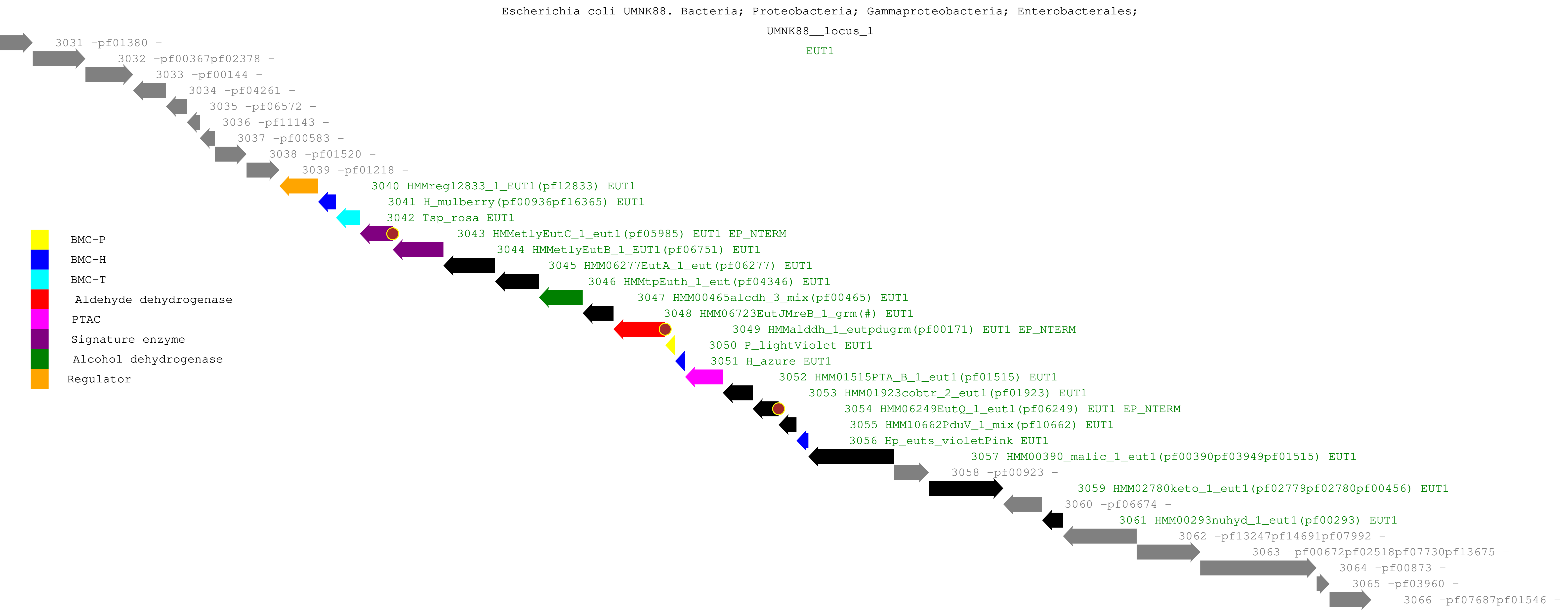

Escherichia coli UMNK88. Bacteria; Proteobacteria; Gammaproteobacteria; Enterobacterales;
UMNK88__locus_1
EUT1
3031 -pf01380 -
3032 -pf00367pf02378 -
3033 -pf00144 -
3034 -pf04261 -
3035 -pf06572 -
3036 -pf11143 -
3037 -pf00583 -
3038 -pf01520 -
3039 -pf01218 -
3040 HMMreg12833_1_EUT1(pf12833) EUT1
3041 H_mulberry(pf00936pf16365) EUT1
3042 Tsp_rosa EUT1
3043 HMMetlyEutC_1_eut1(pf05985) EUT1 EP_NTERM
3044 HMMetlyEutB_1_EUT1(pf06751) EUT1
3045 HMM06277EutA_1_eut(pf06277) EUT1
3046 HMMtpEuth_1_eut(pf04346) EUT1
3047 HMM00465alcdh_3_mix(pf00465) EUT1
3048 HMM06723EutJMreB_1_grm(#) EUT1
3049 HMMalddh_1_eutpdugrm(pf00171) EUT1 EP_NTERM
3050 P_lightViolet EUT1
3051 H_azure EUT1
3052 HMM01515PTA_B_1_eut1(pf01515) EUT1
3053 HMM01923cobtr_2_eut1(pf01923) EUT1
3054 HMM06249EutQ_1_eut1(pf06249) EUT1 EP_NTERM
3055 HMM10662PduV_1_mix(pf10662) EUT1
3056 Hp_euts_violetPink EUT1
3057 HMM00390_malic_1_eut1(pf00390pf03949pf01515) EUT1
3058 -pf00923 -
3059 HMM02780keto_1_eut1(pf02779pf02780pf00456) EUT1
3060 -pf06674 -
3061 HMM00293nuhyd_1_eut1(pf00293) EUT1
3062 -pf13247pf14691pf07992 -
3063 -pf00672pf02518pf07730pf13675 -
3064 -pf00873 -
3065 -pf03960 -
3066 -pf07687pf01546 -
BMC-P
BMC-H
BMC-T
Aldehyde dehydrogenase
PTAC
Signature enzyme
Alcohol dehydrogenase
Regulator

Escherichia coli. Bacteria; Proteobacteria; Gammaproteobacteria; Enterobacterales;

UN86__locus_1

EUT1

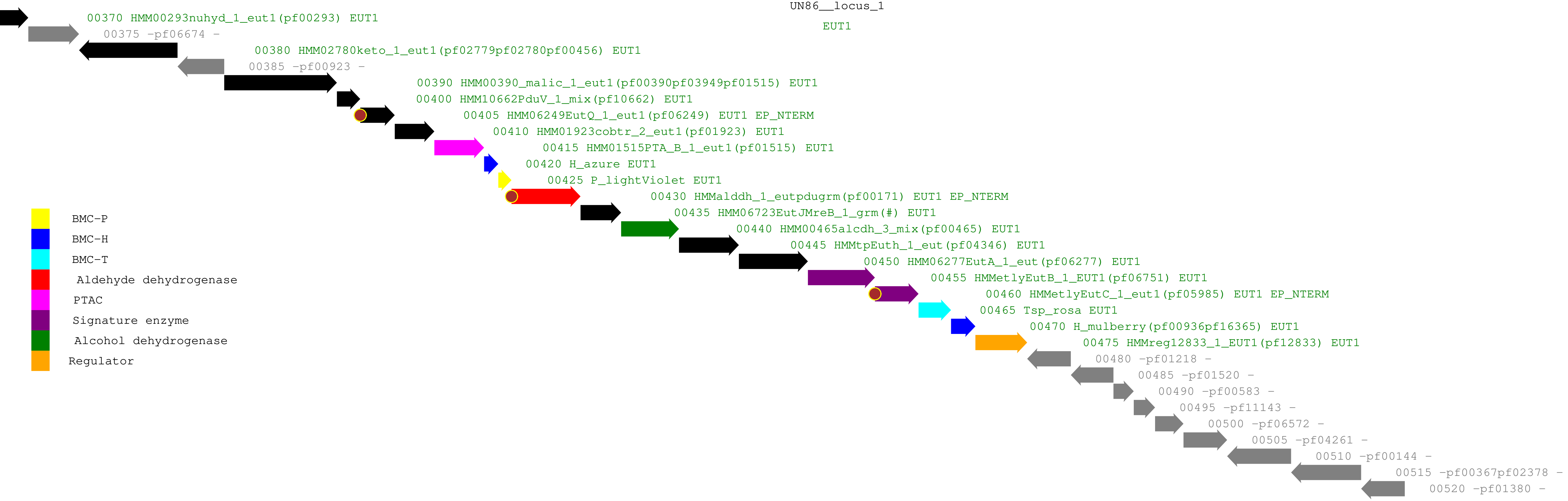

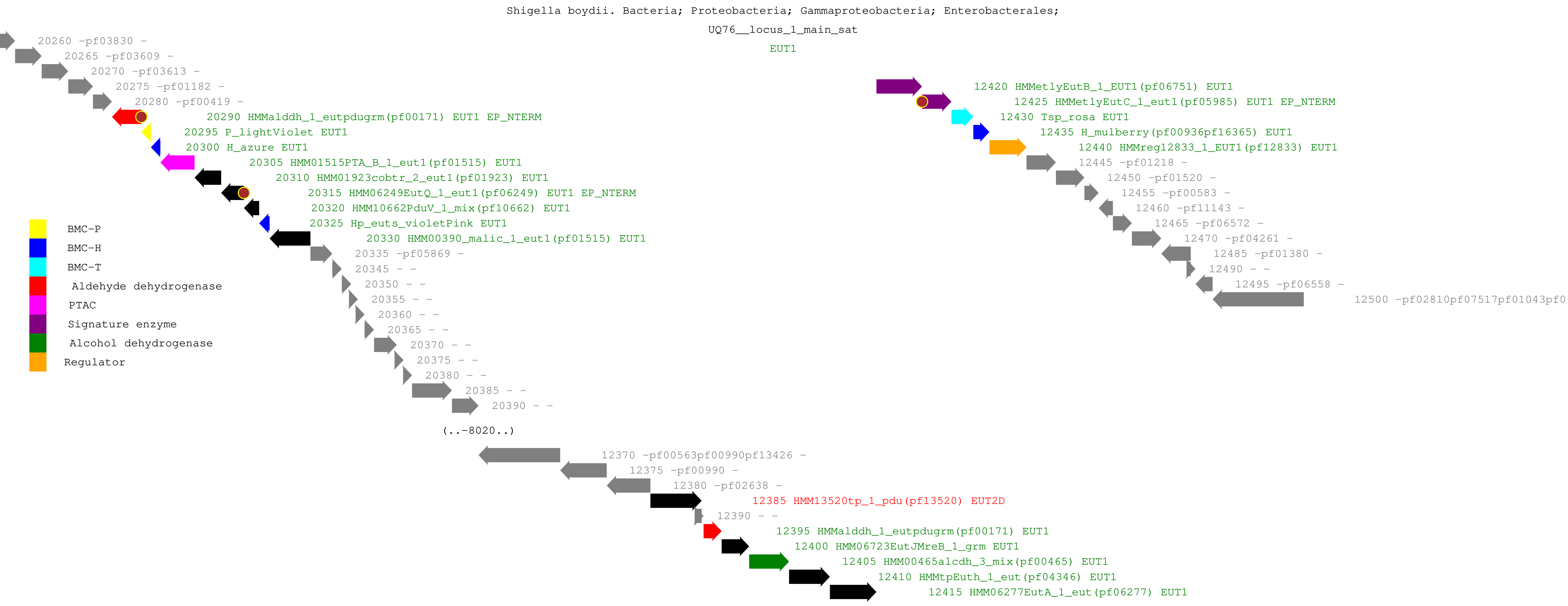

Shigella boydii. Bacteria; Proteobacteria; Gammaproteobacteria; Enterobacterales;
UQ76__locus_1_main_sat
EUT1
20260 -pf03830 -
20265 -pf03609 -
20270 -pf03613 -
20275 -pf01182 -
20280 -pf00419 -
20290 HMMaldh_1_eutpdugrm(pf00171) EUT1 EP_NTERM
20295 P_lightViolet EUT1
20300 H_azure EUT1
20305 HMM01515PTA_B_1_eut1(pf01515) EUT1
20310 HMM01923cobtr_2_eut1(pf01923) EUT1
20315 HMM06249EutQ_1_eut1(pf06249) EUT1 EP_NTERM
20320 HMM10662PduV_1_mix(pf10662) EUT1
20325 Hp_euts_violetPink EUT1
20330 HMM00390_malic_1_eut1(pf01515) EUT1
20335 -pf05869 -
20345 - -
20350 - -
20355 - -
20360 - -
20365 - -
20370 - -
20375 - -
20380 - -
20385 - -
20390 - -
(..-8020..)
12370 -pf00563pf00990pf13426 -
12375 -pf00990 -
12380 -pf02638 -
12385 HMM13520tp_1_pdu(pf13520) EUT2D
12390 - -
12395 HMMaldh_1_eutpdugrm(pf00171) EUT1
12400 HMM06723EutJMreB_1_grm EUT1
12405 HMM00465alcdh_3_mix(pf00465) EUT1
12410 HMMtpEuth_1_eut(pf04346) EUT1
12415 HMM06277EutA_1_eut(pf06277) EUT1
12420 HMMetlyEutB_1_EUT1(pf06751) EUT1
12425 HMMetlyEutC_1_eut1(pf05985) EUT1 EP_NTERM
12430 Tsp_rosa EUT1
12435 H_mulberry(pf00936pf16365) EUT1
12440 HMMreg12833_1_EUT1(pf12833) EUT1
12445 -pf01218 -
12450 -pf01520 -
12455 -pf00583 -
12460 -pf11143 -
12465 -pf06572 -
12470 -pf04261 -
12485 -pf01380 -
12490 - -
12495 -pf06558 -
12500 -pf02810pf07517pf01043pf0
BMC-P
BMC-H
BMC-T
Aldehyde dehydrogenase
PTAC
Signature enzyme
Alcohol dehydrogenase
Regulator

Escherichia coli (strain UTI89 / UPEC). Bacteria; Proteobacteria; Gammaproteobacteria; Enterobacterales;

UTI89_C_locus_1

EUT1

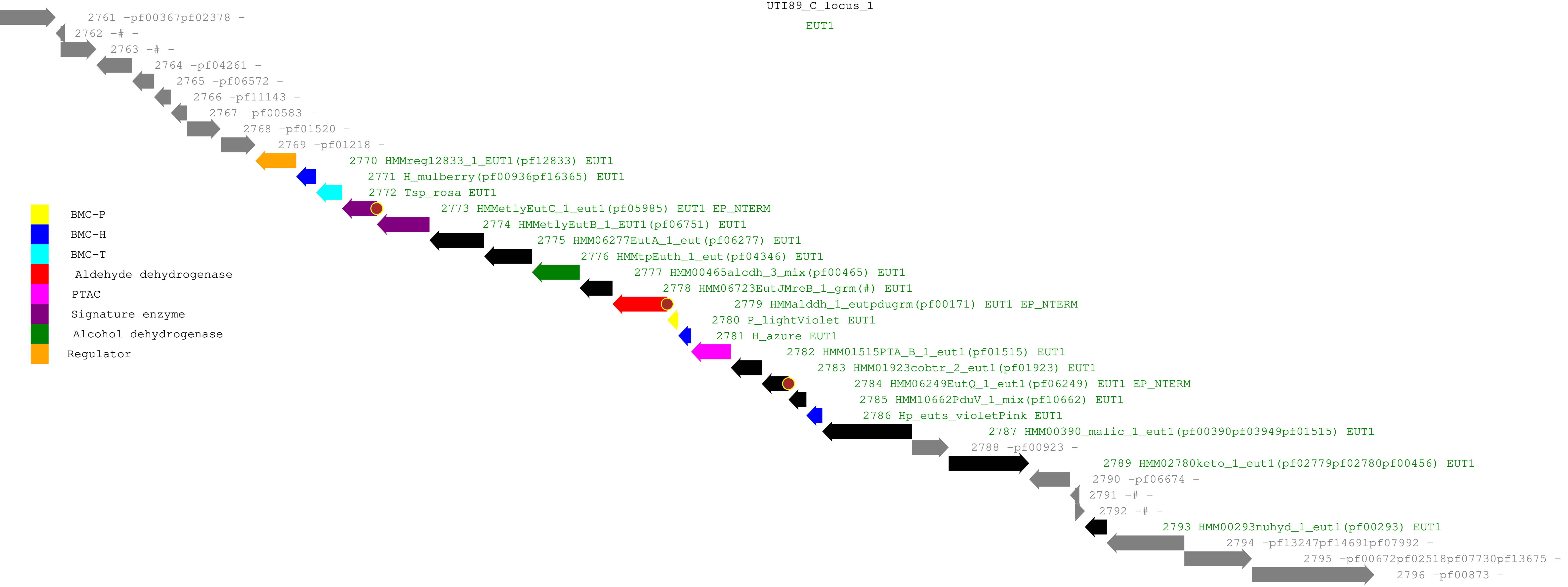

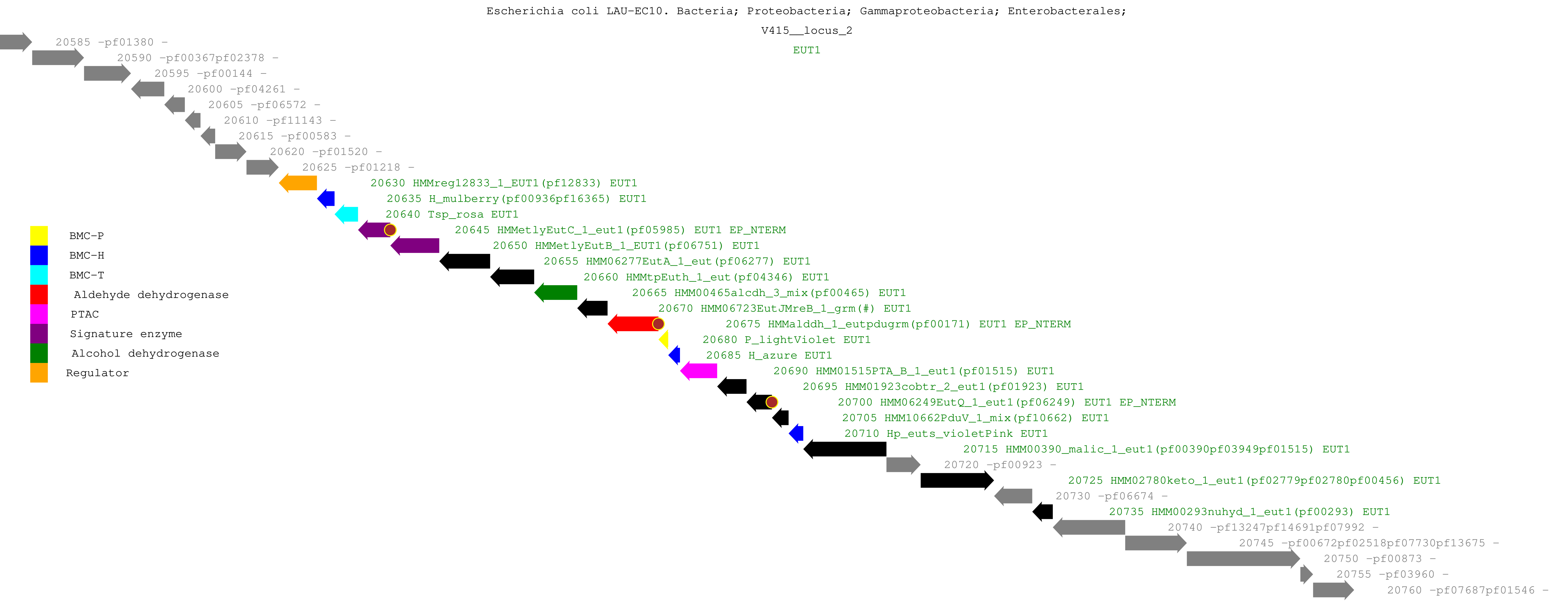

Escherichia coli LAU-EC10. Bacteria; Proteobacteria; Gammaproteobacteria; Enterobacterales;
V415__locus_2
EUT1
20585 -pf01380 -
20590 -pf00367pf02378 -
20595 -pf00144 -
20600 -pf04261 -
20605 -pf06572 -
20610 -pf11143 -
20615 -pf00583 -
20620 -pf01520 -
20625 -pf01218 -
20630 HMMreg12833_1_EUT1(pf12833) EUT1
20635 H_mulberry(pf00936pf16365) EUT1
20640 Tsp_rosa EUT1
20645 HMMetlyEutC_1_eut1(pf05985) EUT1 EP_NTERM
20650 HMMetlyEutB_1_EUT1(pf06751) EUT1
20655 HMM06277EutA_1_eut(pf06277) EUT1
20660 HMMtpEuth_1_eut(pf04346) EUT1
20665 HMM00465alcdh_3_mix(pf00465) EUT1
20670 HMM06723EutJMreB_1_grm(#) EUT1
20675 HMMaldh_1_eutpdugrm(pf00171) EUT1 EP_NTERM
20680 P_lightViolet EUT1
20685 H_azure EUT1
20690 HMM01515PTA_B_1_eut1(pf01515) EUT1
20695 HMM01923cobtr_2_eut1(pf01923) EUT1
20700 HMM06249EutQ_1_eut1(pf06249) EUT1 EP_NTERM
20705 HMM10662PduV_1_mix(pf10662) EUT1
20710 Hp_euts_violetPink EUT1
20715 HMM00390_malic_1_eut1(pf00390pf03949pf01515) EUT1
20720 -pf00923 -
20725 HMM02780keto_1_eut1(pf02779pf02780pf00456) EUT1
20730 -pf06674 -
20735 HMM00293nuhyd_1_eut1(pf00293) EUT1
20740 -pf13247pf14691pf07992 -
20745 -pf00672pf02518pf07730pf13675 -
20750 -pf00873 -
20755 -pf03960 -
20760 -pf07687pf01546 -
BMC-P
BMC-H
BMC-T
Aldehyde dehydrogenase
PTAC
Signature enzyme
Alcohol dehydrogenase
Regulator

Chromobacterium sp. LK1. Bacteria; Proteobacteria; Betaproteobacteria; Neisseriales;

VI26__locus_1

EUT1

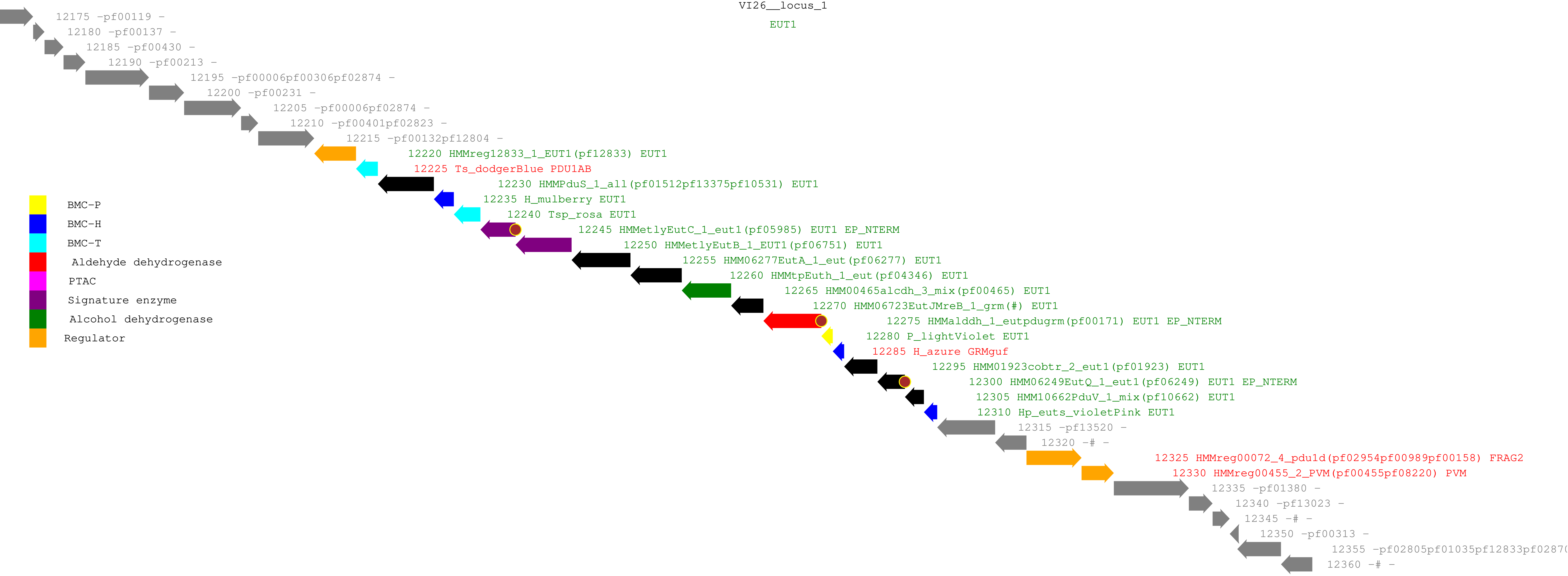

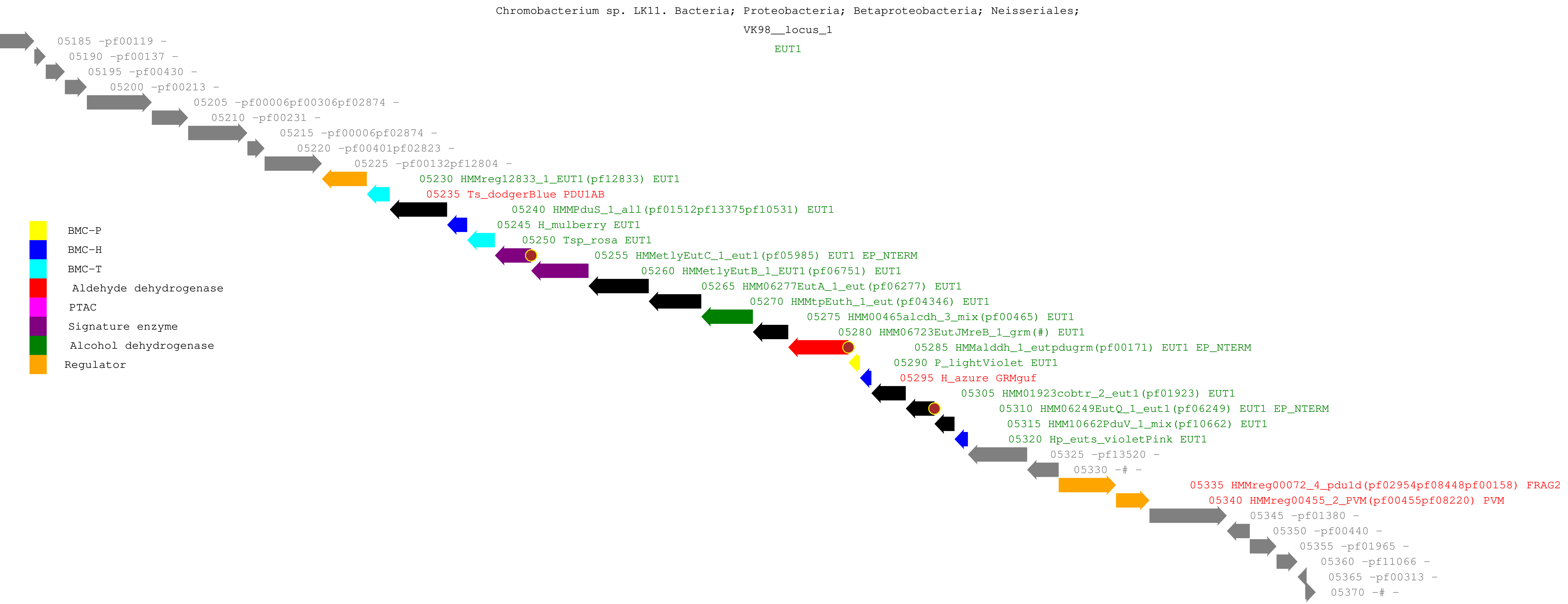
Chromobacterium sp. LK11. Bacteria; Proteobacteria; Betaproteobacteria; Neisseriales;
VK98__locus_1
EUT1
05185 -pf00119 -
05190 -pf00137 -
05195 -pf00430 -
05200 -pf00213 -
05205 -pf00006pf00306pf02874 -
05210 -pf00231 -
05215 -pf00006pf02874 -
05220 -pf00401pf02823 -
05225 -pf00132pf12804 -
05230 HMMreg12833_1_EUT1(pf12833) EUT1
05235 Ts_dodgerBlue PDU1AB
05240 HMMPduS_1_all(pf01512pf13375pf10531) EUT1
05245 H_mulberry EUT1
05250 Tsp_rosa EUT1
05255 HMMetlyEutC_1_eut1(pf05985) EUT1 EP_NTERM
05260 HMMetlyEutB_1_EUT1(pf06751) EUT1
05265 HMM06277EutA_1_eut(pf06277) EUT1
05270 HMMtpEuth_1_eut(pf04346) EUT1
05275 HMM00465alcdh_3_mix(pf00465) EUT1
05280 HMM06723EutJMreB_1_grm(#) EUT1
05285 HMMalddh_1_eutpdugrm(pf00171) EUT1 EP_NTERM
05290 P_lightViolet EUT1
05295 H_azure GRMguf
05305 HMM01923cobtr_2_eut1(pf01923) EUT1
05310 HMM06249EutQ_1_eut1(pf06249) EUT1 EP_NTERM
05315 HMM10662PduV_1_mix(pf10662) EUT1
05320 Hp_euts_violetPink EUT1
05325 -pf13520 -
05330 -# -
05335 HMMreg00072_4_pdu1d(pf02954pf08448pf00158) FRAG2
05340 HMMreg00455_2_PVM(pf00455pf08220) PVM
05345 -pf01380 -
05350 -pf00440 -
05355 -pf01965 -
05360 -pf11066 -
05365 -pf00313 -
05370 -# -
BMC-P
BMC-H
BMC-T
Aldehyde dehydrogenase
PTAC
Signature enzyme
Alcohol dehydrogenase
Regulator

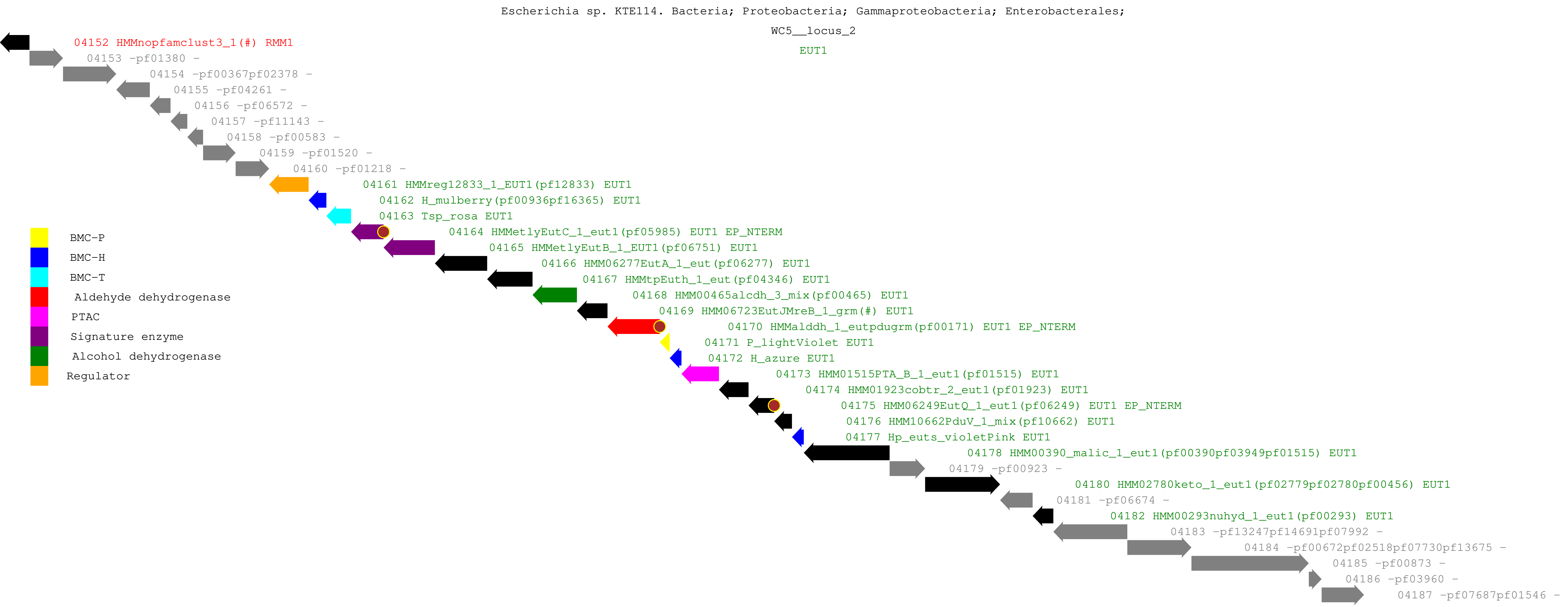

Escherichia sp. KTE114. Bacteria; Proteobacteria; Gammaproteobacteria; Enterobacterales;
WC5__locus_2
EUT1
04152 HMMnopfamclust3_1(#) RMM1
04153 -pf01380 -
04154 -pf00367pf02378 -
04155 -pf04261 -
04156 -pf06572 -
04157 -pf11143 -
04158 -pf00583 -
04159 -pf01520 -
04160 -pf01218 -
04161 HMMreg12833_1_EUT1(pf12833) EUT1
04162 H_mulberry(pf00936pf16365) EUT1
04163 Tsp_rosa EUT1
04164 HMMetlyEutC_1_eut1(pf05985) EUT1 EP_NTERM
04165 HMMetlyEutB_1_EUT1(pf06751) EUT1
04166 HMM06277EutA_1_eut(pf06277) EUT1
04167 HMMtpEuth_1_eut(pf04346) EUT1
04168 HMM00465alcdh_3_mix(pf00465) EUT1
04169 HMM06723EutJMreB_1_grm(#) EUT1
04170 HMMalddh_1_eutpdugrm(pf00171) EUT1 EP_NTERM
04171 P_lightViolet EUT1
04172 H_azure EUT1
04173 HMM01515PTA_B_1_eut1(pf01515) EUT1
04174 HMM01923cobtr_2_eut1(pf01923) EUT1
04175 HMM06249EutQ_1_eut1(pf06249) EUT1 EP_NTERM
04176 HMM10662PduV_1_mix(pf10662) EUT1
04177 Hp_euts_violetPink EUT1
04178 HMM00390_malic_1_eut1(pf00390pf03949pf01515) EUT1
04179 -pf00923 -
04180 HMM02780keto_1_eut1(pf02779pf02780pf00456) EUT1
04181 -pf06674 -
04182 HMM00293nuhyd_1_eut1(pf00293) EUT1
04183 -pf13247pf14691pf07992 -
04184 -pf00672pf02518pf07730pf13675 -
04185 -pf00873 -
04186 -pf03960 -
04187 -pf07687pf01546 -
BMC-P
BMC-H
BMC-T
Aldehyde dehydrogenase
PTAC
Signature enzyme
Alcohol dehydrogenase
Regulator

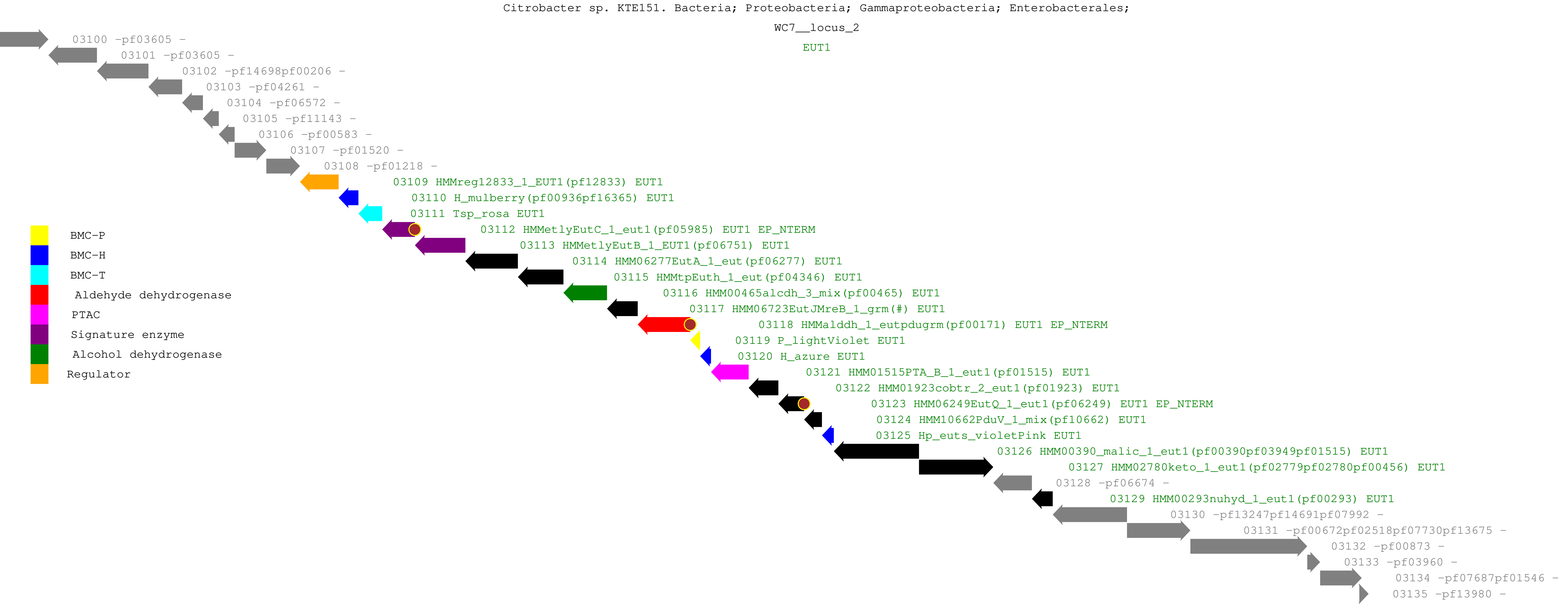
Citrobacter sp. KTE151. Bacteria; Proteobacteria; Gammaproteobacteria; Enterobacterales;
WC7__locus_2
EUT1
03100 -pf03605 -
03101 -pf03605 -
03102 -pf14698pf00206 -
03103 -pf04261 -
03104 -pf06572 -
03105 -pf11143 -
03106 -pf00583 -
03107 -pf01520 -
03108 -pf01218 -
03109 HMMreg12833_1_EUT1(pf12833) EUT1
03110 H_mulberry(pf00936pf16365) EUT1
03111 Tsp_rosa EUT1
03112 HMMetlyEutC_1_eut1(pf05985) EUT1 EP_NTERM
03113 HMMetlyEutB_1_EUT1(pf06751) EUT1
03114 HMM06277EutA_1_eut(pf06277) EUT1
03115 HMMtpEuth_1_eut(pf04346) EUT1
03116 HMM00465alcdh_3_mix(pf00465) EUT1
03117 HMM06723EutJMreB_1_grm(#) EUT1
03118 HMMalddh_1_eutpdugrm(pf00171) EUT1 EP_NTERM
03119 P_lightViolet EUT1
03120 H_azure EUT1
03121 HMM01515PTA_B_1_eut1(pf01515) EUT1
03122 HMM01923cobtr_2_eut1(pf01923) EUT1
03123 HMM06249EutQ_1_eut1(pf06249) EUT1 EP_NTERM
03124 HMM10662PduV_1_mix(pf10662) EUT1
03125 Hp_euts_violetPink EUT1
03126 HMM00390_malic_1_eut1(pf00390pf03949pf01515) EUT1
03127 HMM02780keto_1_eut1(pf02779pf02780pf00456) EUT1
03128 -pf06674 -
03129 HMM00293nuhyd_1_eut1(pf00293) EUT1
03130 -pf13247pf14691pf07992 -
03131 -pf00672pf02518pf07730pf13675 -
03132 -pf00873 -
03133 -pf03960 -
03134 -pf07687pf01546 -
03135 -pf13980 -
BMC-P
BMC-H
BMC-T
Aldehyde dehydrogenase
PTAC
Signature enzyme
Alcohol dehydrogenase
Regulator

Escherichia coli KTE10. Bacteria; Proteobacteria; Gammaproteobacteria; Enterobacterales;

WCM__locus_1

EUT1

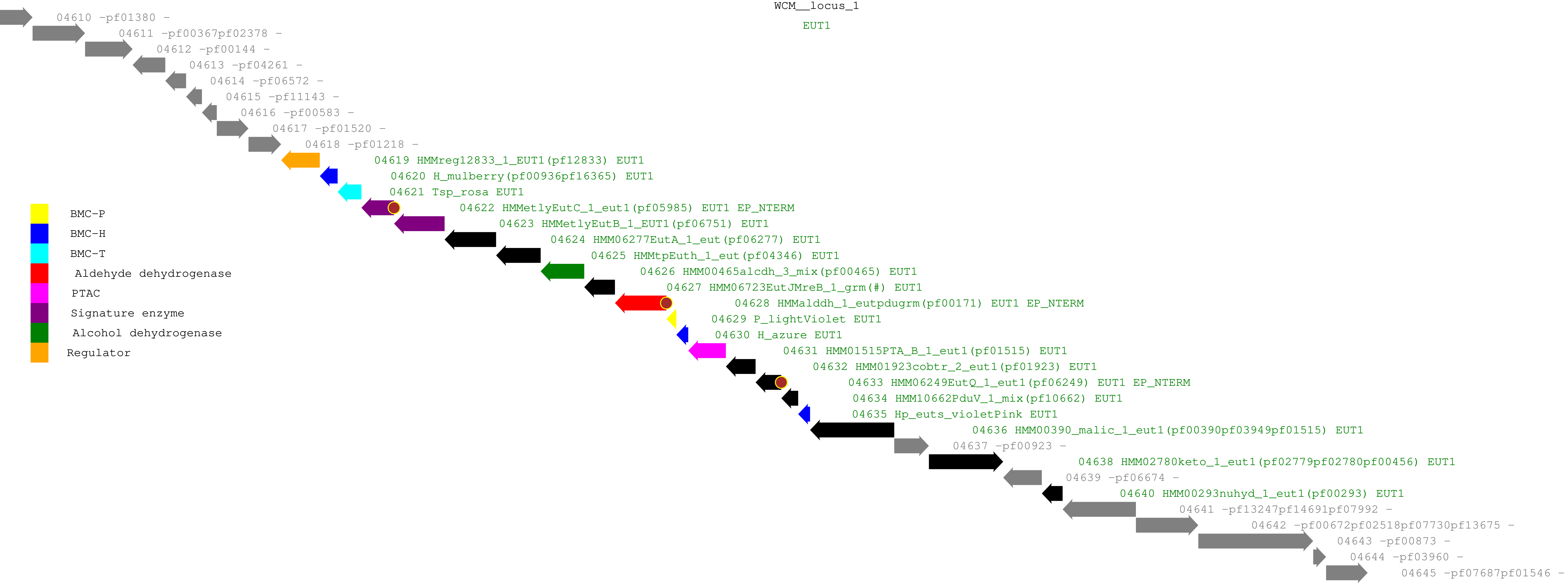

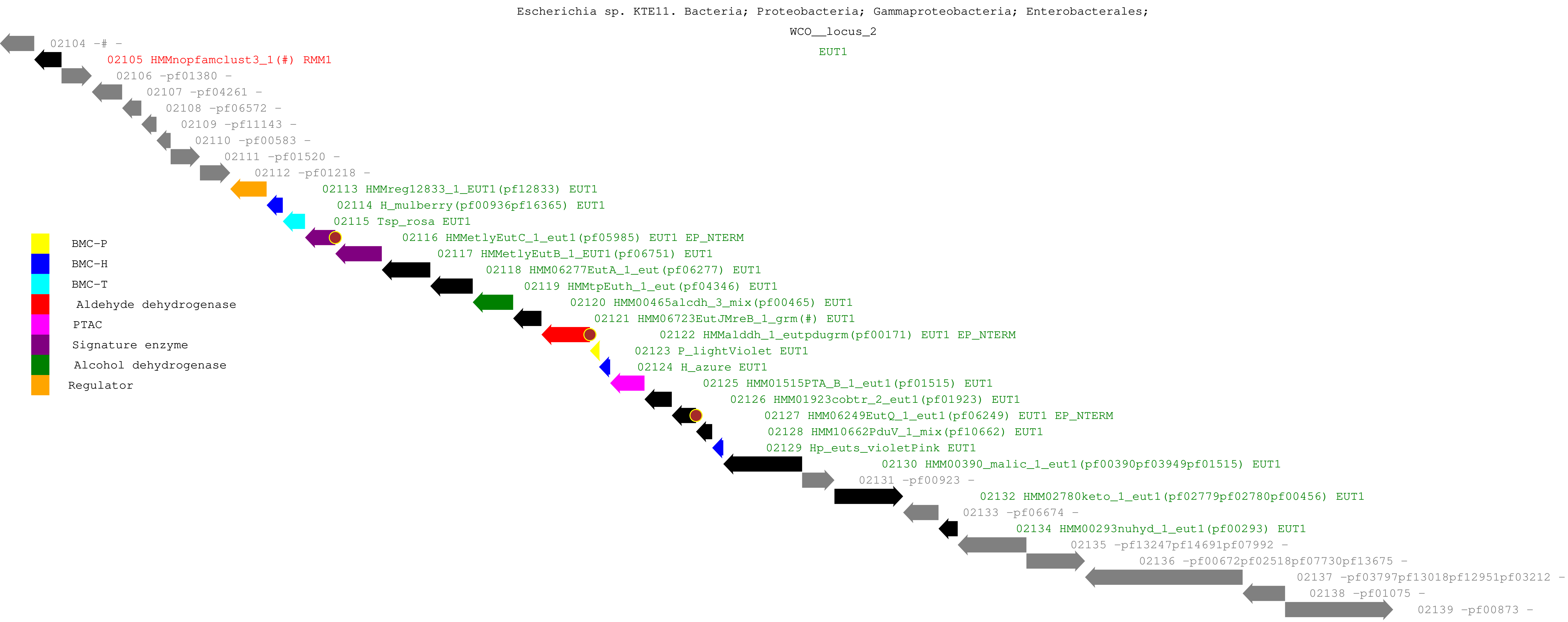
Escherichia sp. KTE11. Bacteria; Proteobacteria; Gammaproteobacteria; Enterobacterales;
WCO__locus_2
EUT1
02104 -# -
02105 HMMnopfamclust3_1(#) RMM1
02106 -pf01380 -
02107 -pf04261 -
02108 -pf06572 -
02109 -pf11143 -
02110 -pf00583 -
02111 -pf01520 -
02112 -pf01218 -
02113 HMMreg12833_1_EUT1(pf12833) EUT1
02114 H_mulberry(pf00936pf16365) EUT1
02115 Tsp_rosa EUT1
02116 HMMetlyEutC_1_eut1(pf05985) EUT1 EP_NTERM
02117 HMMetlyEutB_1_EUT1(pf06751) EUT1
02118 HMM06277EutA_1_eut(pf06277) EUT1
02119 HMMtpEuth_1_eut(pf04346) EUT1
02120 HMM00465alcdh_3_mix(pf00465) EUT1
02121 HMM06723EutJMreB_1_grm(#) EUT1
02122 HMMalddh_1_eutpdugrm(pf00171) EUT1 EP_NTERM
02123 P_lightViolet EUT1
02124 H_azure EUT1
02125 HMM01515PTA_B_1_eut1(pf01515) EUT1
02126 HMM01923cobtr_2_eut1(pf01923) EUT1
02127 HMM06249EutQ_1_eut1(pf06249) EUT1 EP_NTERM
02128 HMM10662PduV_1_mix(pf10662) EUT1
02129 Hp_euts_violetPink EUT1
02130 HMM00390_malic_1_eut1(pf00390pf03949pf01515) EUT1
02131 -pf00923 -
02132 HMM02780keto_1_eut1(pf02779pf02780pf00456) EUT1
02133 -pf06674 -
02134 HMM00293nuhyd_1_eut1(pf00293) EUT1
02135 -pf13247pf14691pf07992 -
02136 -pf00672pf02518pf07730pf13675 -
02137 -pf03797pf13018pf12951pf03212 -
02138 -pf01075 -
02139 -pf00873 -
BMC-P
BMC-H
BMC-T
Aldehyde dehydrogenase
PTAC
Signature enzyme
Alcohol dehydrogenase
Regulator

Escherichia coli KTE20. Bacteria; Proteobacteria; Gammaproteobacteria; Enterobacterales;

WE7__locus_1_main_sat

EUT1

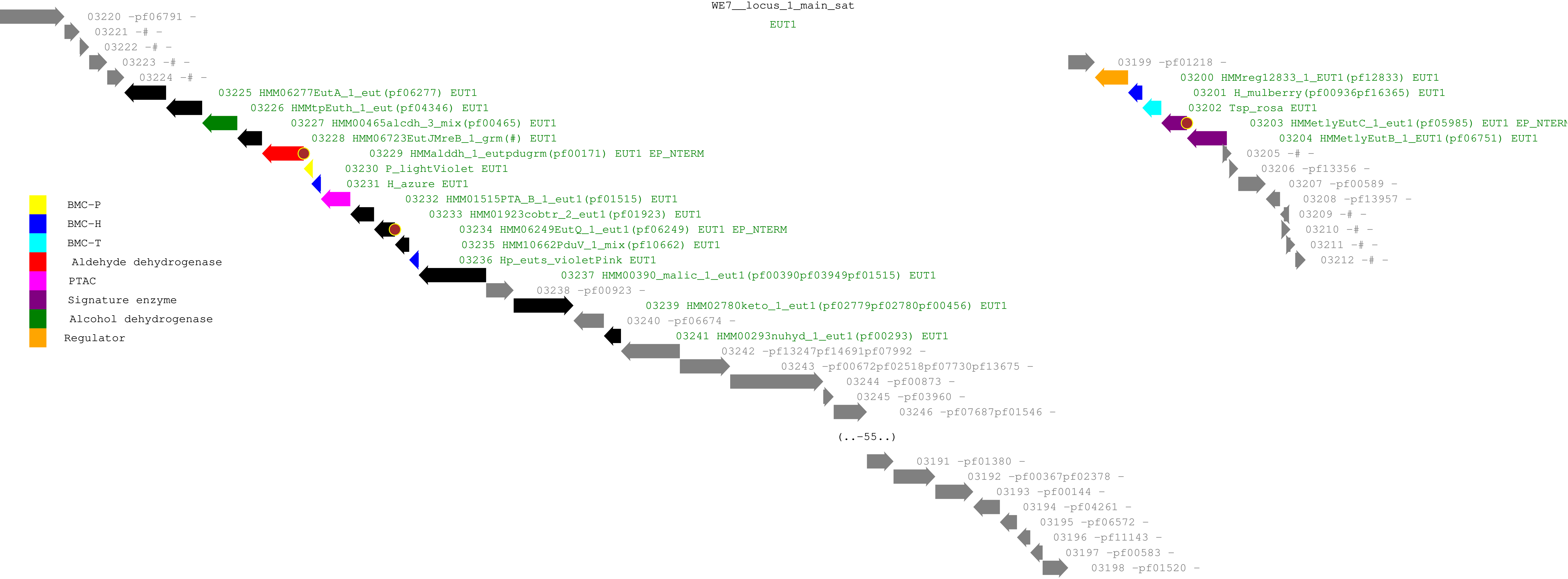

Escherichia coli KTE21. Bacteria; Proteobacteria; Gammaproteobacteria; Enterobacterales;

WE9__locus_1_main_sat

EUT1

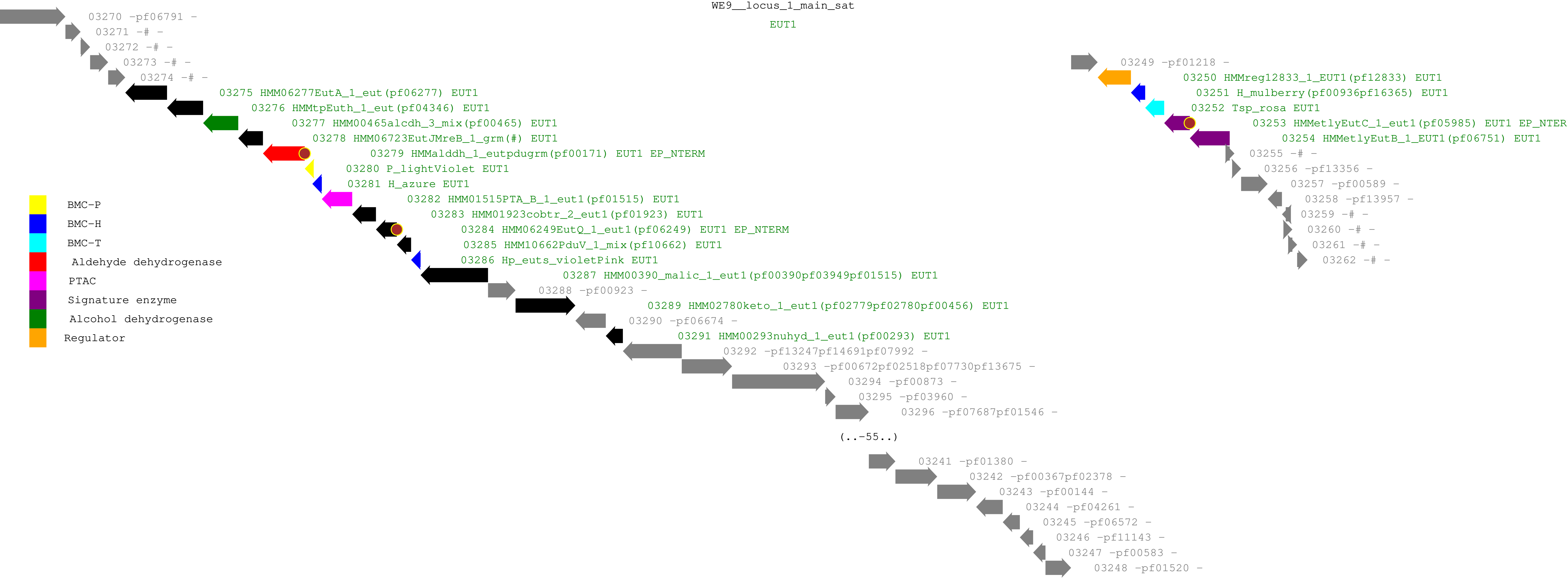

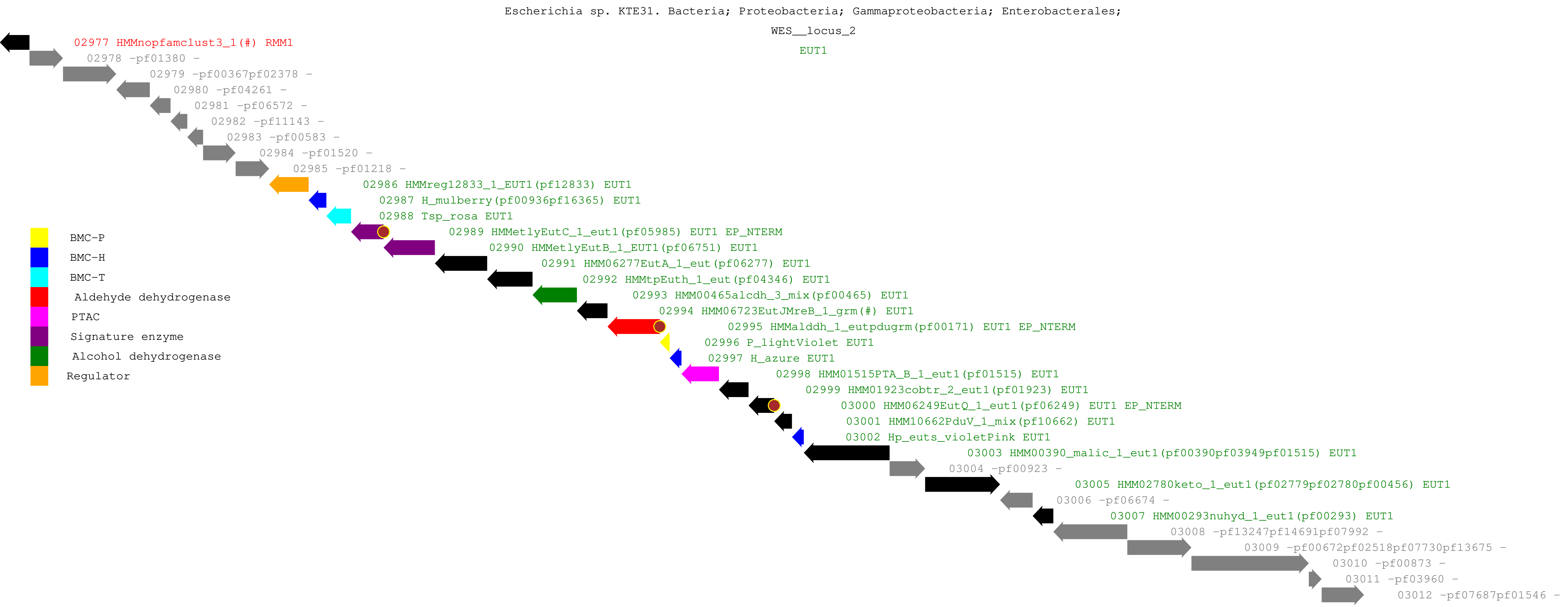
Escherichia sp. KTE31. Bacteria; Proteobacteria; Gammaproteobacteria; Enterobacterales;
WES__locus_2
EUT1
02977 HMMnopfamclust3_1(#) RMM1
02978 -pf01380 -
02979 -pf00367pf02378 -
02980 -pf04261 -
02981 -pf06572 -
02982 -pf11143 -
02983 -pf00583 -
02984 -pf01520 -
02985 -pf01218 -
02986 HMMreg12833_1_EUT1(pf12833) EUT1
02987 H_mulberry(pf00936pf16365) EUT1
02988 Tsp_rosa EUT1
02989 HMMetlyEutC_1_eut1(pf05985) EUT1 EP_NTERM
02990 HMMetlyEutB_1_EUT1(pf06751) EUT1
02991 HMM06277EutA_1_eut(pf06277) EUT1
02992 HMMtpEuth_1_eut(pf04346) EUT1
02993 HMM00465alcdh_3_mix(pf00465) EUT1
02994 HMM06723EutJMreB_1_grm(#) EUT1
02995 HMMalddh_1_eutpdugrm(pf00171) EUT1 EP_NTERM
02996 P_lightViolet EUT1
02997 H_azure EUT1
02998 HMM01515PTA_B_1_eut1(pf01515) EUT1
02999 HMM01923cobtr_2_eut1(pf01923) EUT1
03000 HMM06249EutQ_1_eut1(pf06249) EUT1 EP_NTERM
03001 HMM10662PduV_1_mix(pf10662) EUT1
03002 Hp_euts_violetPink EUT1
03003 HMM00390_malic_1_eut1(pf00390pf03949pf01515) EUT1
03004 -pf00923 -
03005 HMM02780keto_1_eut1(pf02779pf02780pf00456) EUT1
03006 -pf06674 -
03007 HMM00293nuhyd_1_eut1(pf00293) EUT1
03008 -pf13247pf14691pf07992 -
03009 -pf00672pf02518pf07730pf13675 -
03010 -pf00873 -
03011 -pf03960 -
03012 -pf07687pf01546 -
BMC-P
BMC-H
BMC-T
Aldehyde dehydrogenase
PTAC
Signature enzyme
Alcohol dehydrogenase
Regulator

Escherichia coli KTE33. Bacteria; Proteobacteria; Gammaproteobacteria; Enterobacterales;

WEW__locus_2

EUT1

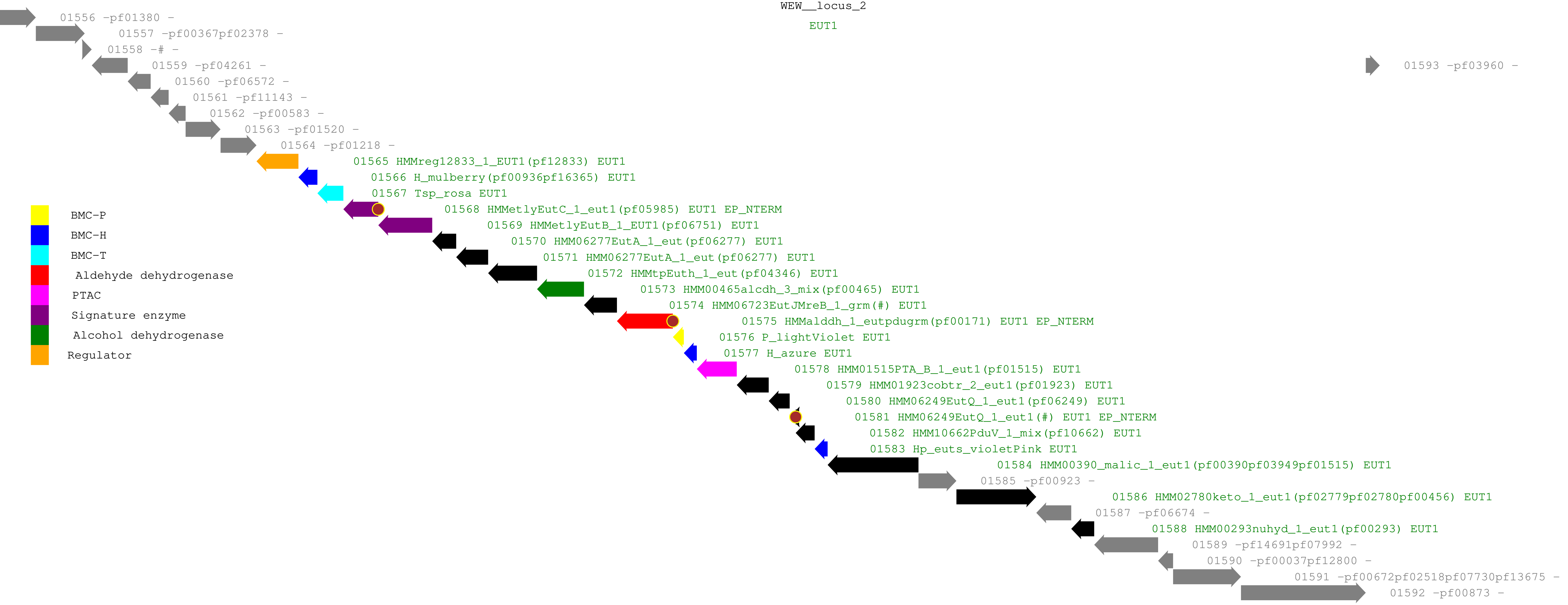

Escherichia coli KTE37. Bacteria; Proteobacteria; Gammaproteobacteria; Enterobacterales;

WG5__locus_1_main_sat

EUT1

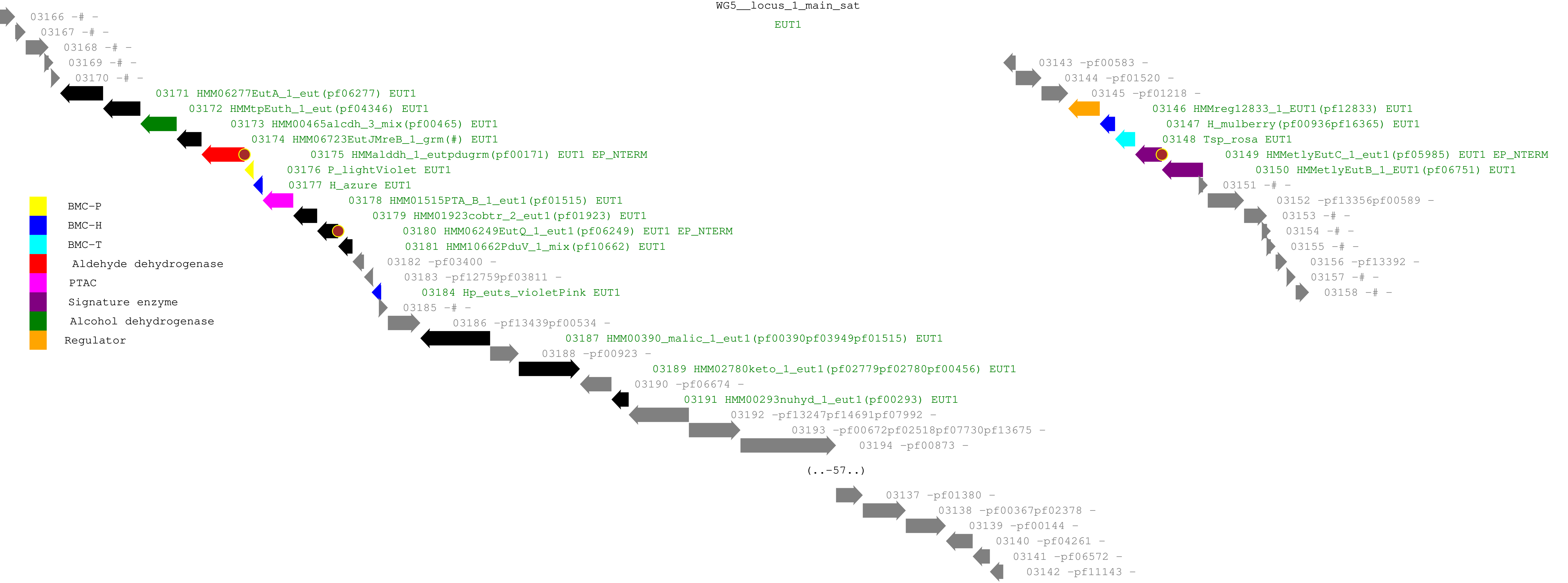

Escherichia coli KTE38. Bacteria; Proteobacteria; Gammaproteobacteria; Enterobacterales;

WG7__locus_1_main_sat

EUT1

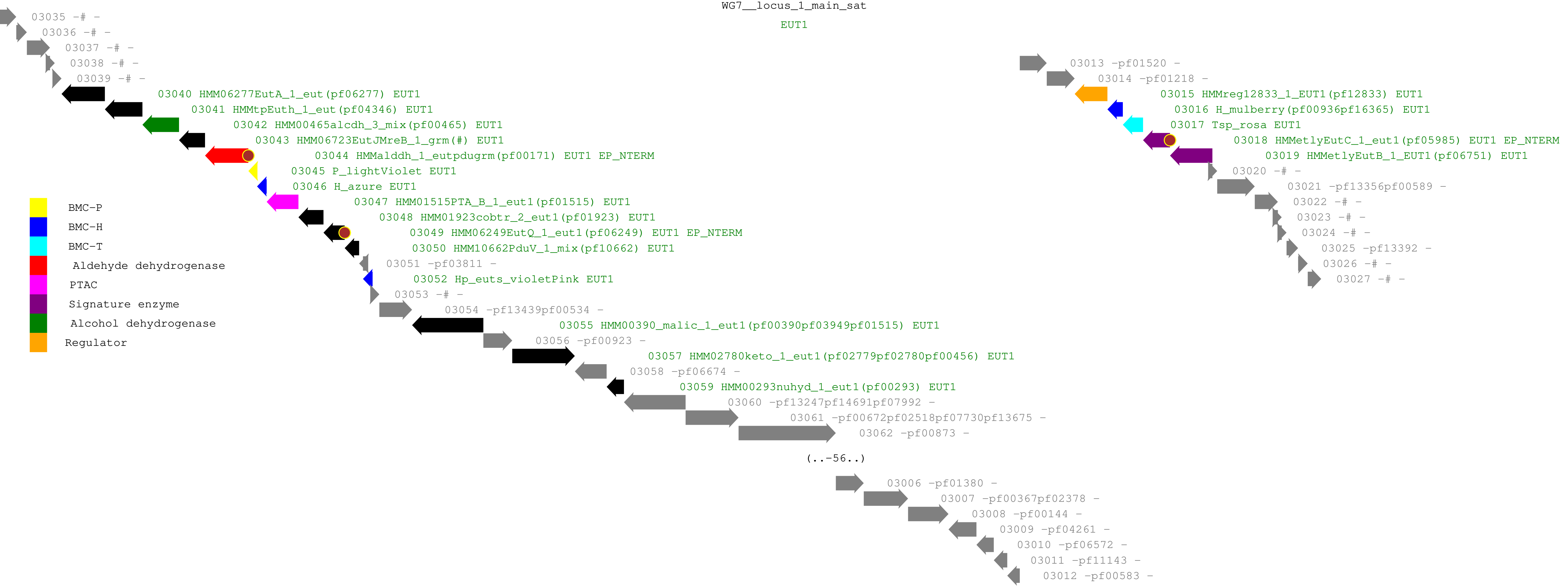

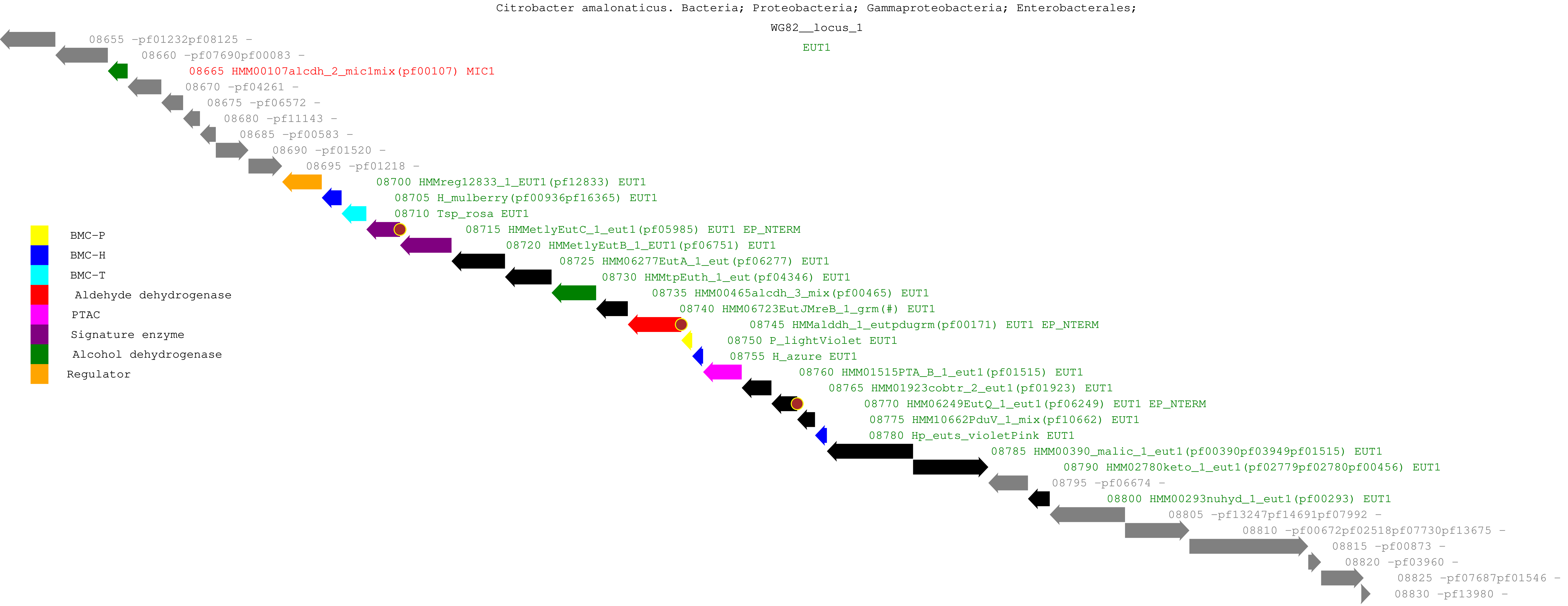
Citrobacter amalonaticus. Bacteria; Proteobacteria; Gammaproteobacteria; Enterobacterales;
WG82__locus_1
EUT1
08655 -pf01232pf08125 -
08660 -pf07690pf00083 -
08665 HMM00107alcdh_2_mic1mix(pf00107) MIC1
08670 -pf04261 -
08675 -pf06572 -
08680 -pf11143 -
08685 -pf00583 -
08690 -pf01520 -
08695 -pf01218 -
08700 HMMreg12833_1_EUT1(pf12833) EUT1
08705 H_mulberry(pf00936pf16365) EUT1
08710 Tsp_rosa EUT1
08715 HMMetlyEutC_1_eut1(pf05985) EUT1 EP_NTERM
08720 HMMetlyEutB_1_EUT1(pf06751) EUT1
08725 HMM06277EutA_1_eut(pf06277) EUT1
08730 HMMtpEuth_1_eut(pf04346) EUT1
08735 HMM00465alcdh_3_mix(pf00465) EUT1
08740 HMM06723EutJMreB_1_grm(#) EUT1
08745 HMMalddh_1_eutpdugrm(pf00171) EUT1 EP_NTERM
08750 P_lightViolet EUT1
08755 H_azure EUT1
08760 HMM01515PTA_B_1_eut1(pf01515) EUT1
08765 HMM01923cobtr_2_eut1(pf01923) EUT1
08770 HMM06249EutQ_1_eut1(pf06249) EUT1 EP_NTERM
08775 HMM10662PduV_1_mix(pf10662) EUT1
08780 Hp_euts_violetPink EUT1
08785 HMM00390_malic_1_eut1(pf00390pf03949pf01515) EUT1
08790 HMM02780keto_1_eut1(pf02779pf02780pf00456) EUT1
08795 -pf06674 -
08800 HMM00293nuhyd_1_eut1(pf00293) EUT1
08805 -pf13247pf14691pf07992 -
08810 -pf00672pf02518pf07730pf13675 -
08815 -pf00873 -
08820 -pf03960 -
08825 -pf07687pf01546 -
08830 -pf13980 -
BMC-P
BMC-H
BMC-T
Aldehyde dehydrogenase
PTAC
Signature enzyme
Alcohol dehydrogenase
Regulator

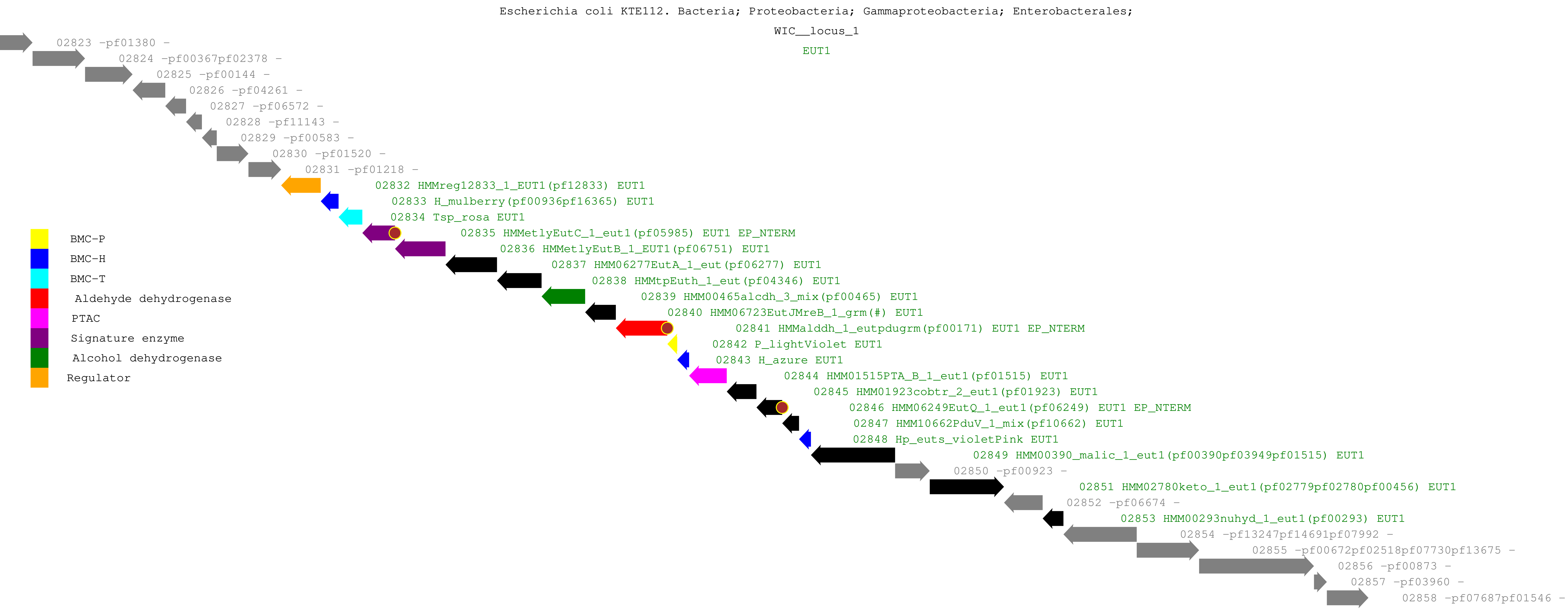

Escherichia coli KTE112. Bacteria; Proteobacteria; Gammaproteobacteria; Enterobacterales;
WIC__locus_1
EUT1
02823 -pf01380 -
02824 -pf00367pf02378 -
02825 -pf00144 -
02826 -pf04261 -
02827 -pf06572 -
02828 -pf11143 -
02829 -pf00583 -
02830 -pf01520 -
02831 -pf01218 -
02832 HMMreg12833_1_EUT1(pf12833) EUT1
02833 H_mulberry(pf00936pf16365) EUT1
02834 Tsp_rosa EUT1
02835 HMMetlyEutC_1_eut1(pf05985) EUT1 EP_NTERM
02836 HMMetlyEutB_1_EUT1(pf06751) EUT1
02837 HMM06277EutA_1_eut(pf06277) EUT1
02838 HMMtpEuth_1_eut(pf04346) EUT1
02839 HMM00465alcdh_3_mix(pf00465) EUT1
02840 HMM06723EutJMreB_1_grm(#) EUT1
02841 HMMalddh_1_eutpdugrm(pf00171) EUT1 EP_NTERM
02842 P_lightViolet EUT1
02843 H_azure EUT1
02844 HMM01515PTA_B_1_eut1(pf01515) EUT1
02845 HMM01923cobtr_2_eut1(pf01923) EUT1
02846 HMM06249EutQ_1_eut1(pf06249) EUT1 EP_NTERM
02847 HMM10662PduV_1_mix(pf10662) EUT1
02848 Hp_euts_violetPink EUT1
02849 HMM00390_malic_1_eut1(pf00390pf03949pf01515) EUT1
02850 -pf00923 -
02851 HMM02780keto_1_eut1(pf02779pf02780pf00456) EUT1
02852 -pf06674 -
02853 HMM00293nuhyd_1_eut1(pf00293) EUT1
02854 -pf13247pf14691pf07992 -
02855 -pf00672pf02518pf07730pf13675 -
02856 -pf00873 -
02857 -pf03960 -
02858 -pf07687pf01546 -
BMC-P
BMC-H
BMC-T
Aldehyde dehydrogenase
PTAC
Signature enzyme
Alcohol dehydrogenase
Regulator

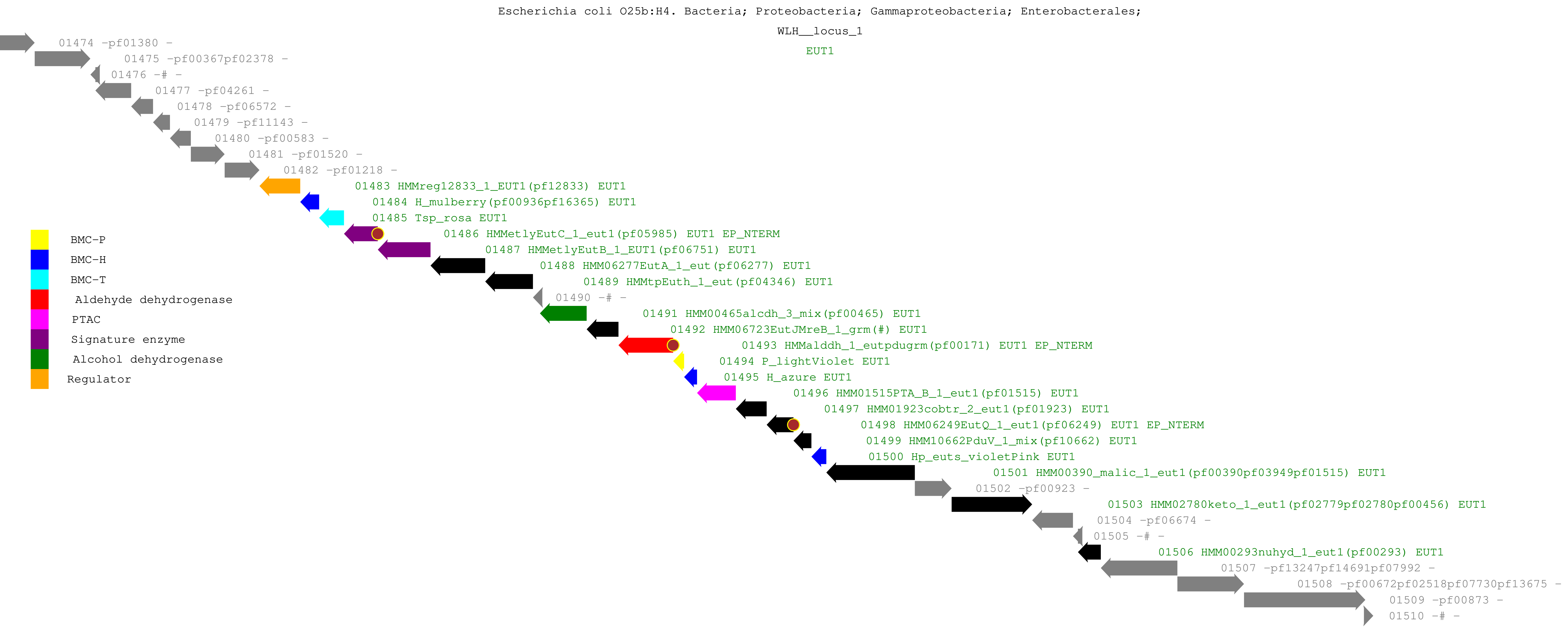
Escherichia coli O25b:H4. Bacteria; Proteobacteria; Gammaproteobacteria; Enterobacterales;
WLH__locus_1
EUT1
01474 -pf01380 -
01475 -pf00367pf02378 -
01476 -# -
01477 -pf04261 -
01478 -pf06572 -
01479 -pf11143 -
01480 -pf00583 -
01481 -pf01520 -
01482 -pf01218 -
01483 HMMreg12833_1_EUT1(pf12833) EUT1
01484 H_mulberry(pf00936pf16365) EUT1
01485 Tsp_rosa EUT1
01486 HMMetlyEutC_1_eut1(pf05985) EUT1 EP_NTERM
01487 HMMetlyEutB_1_EUT1(pf06751) EUT1
01488 HMM06277EutA_1_eut(pf06277) EUT1
01489 HMMtpEuth_1_eut(pf04346) EUT1
01490 -# -
01491 HMM00465alcdh_3_mix(pf00465) EUT1
01492 HMM06723EutJMreB_1_grm(#) EUT1
01493 HMMalddh_1_eutpdugrm(pf00171) EUT1 EP_NTERM
01494 P_lightViolet EUT1
01495 H_azure EUT1
01496 HMM01515PTA_B_1_eut1(pf01515) EUT1
01497 HMM01923cobtr_2_eut1(pf01923) EUT1
01498 HMM06249EutQ_1_eut1(pf06249) EUT1 EP_NTERM
01499 HMM10662PduV_1_mix(pf10662) EUT1
01500 Hp_euts_violetPink EUT1
01501 HMM00390_malic_1_eut1(pf00390pf03949pf01515) EUT1
01502 -pf00923 -
01503 HMM02780keto_1_eut1(pf02779pf02780pf00456) EUT1
01504 -pf06674 -
01505 -# -
01506 HMM00293nuhyd_1_eut1(pf00293) EUT1
01507 -pf13247pf14691pf07992 -
01508 -pf00672pf02518pf07730pf13675 -
01509 -pf00873 -
01510 -# -
BMC-P
BMC-H
BMC-T
Aldehyde dehydrogenase
PTAC
Signature enzyme
Alcohol dehydrogenase
Regulator

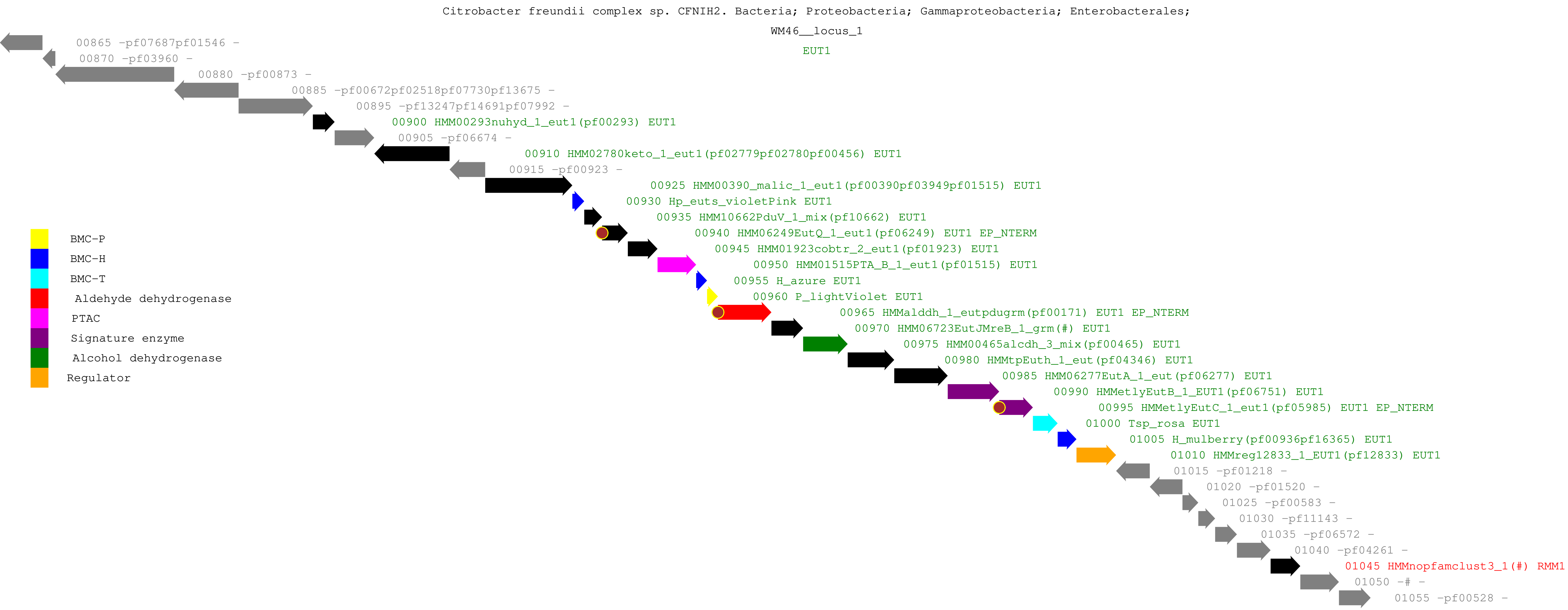
Citrobacter freundii complex sp. CFNIH2. Bacteria; Proteobacteria; Gammaproteobacteria; Enterobacterales;
WM46__locus_1
EUT1
00865 -pf07687pf01546 -
00870 -pf03960 -
00880 -pf00873 -
00885 -pf00672pf02518pf07730pf13675 -
00895 -pf13247pf14691pf07992 -
00900 HMM00293nuhyd_1_eut1(pf00293) EUT1
00905 -pf06674 -
00910 HMM02780keto_1_eut1(pf02779pf02780pf00456) EUT1
00915 -pf00923 -
00925 HMM00390_malic_1_eut1(pf00390pf03949pf01515) EUT1
00930 Hp_euts_violetPink EUT1
00935 HMM10662PduV_1_mix(pf10662) EUT1
00940 HMM06249EutQ_1_eut1(pf06249) EUT1 EP_NTERM
00945 HMM01923cobtr_2_eut1(pf01923) EUT1
00950 HMM01515PTA_B_1_eut1(pf01515) EUT1
00955 H_azure EUT1
00960 P_lightViolet EUT1
00965 HMMalddh_1_eutpdugrm(pf00171) EUT1 EP_NTERM
00970 HMM06723EutJMreB_1_grm(#) EUT1
00975 HMM00465alcdh_3_mix(pf00465) EUT1
00980 HMMtpEuth_1_eut(pf04346) EUT1
00985 HMM06277EutA_1_eut(pf06277) EUT1
00990 HMMetlyEutB_1_EUT1(pf06751) EUT1
00995 HMMetlyEutC_1_eut1(pf05985) EUT1 EP_NTERM
01000 Tsp_rosa EUT1
01005 H_mulberry(pf00936pf16365) EUT1
01010 HMMreg12833_1_EUT1(pf12833) EUT1
01015 -pf01218 -
01020 -pf01520 -
01025 -pf00583 -
01030 -pf11143 -
01035 -pf06572 -
01040 -pf04261 -
01045 HMMnopfamclust3_1(#) RMM1
01050 -# -
01055 -pf00528 -
BMC-P
BMC-H
BMC-T
Aldehyde dehydrogenase
PTAC
Signature enzyme
Alcohol dehydrogenase
Regulator

Escherichia coli. Bacteria; Proteobacteria; Gammaproteobacteria; Enterobacterales;

WQ89__locus_1

EUT1

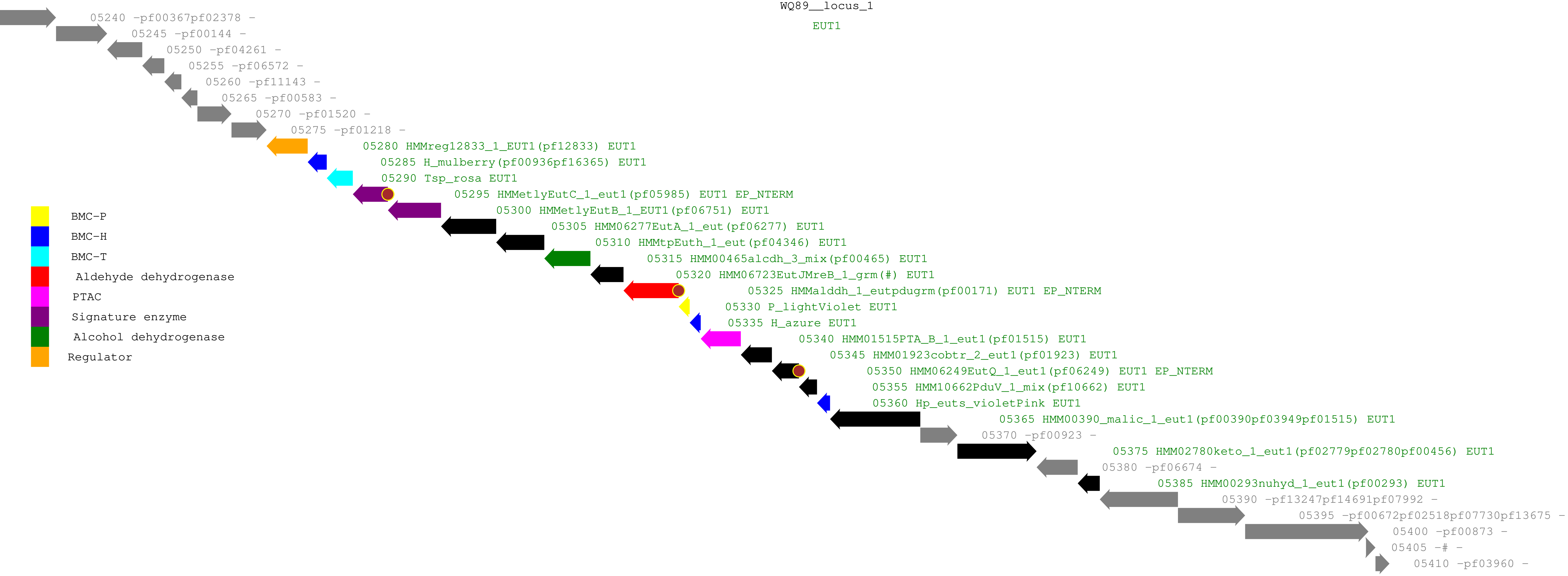

Escherichia coli. Bacteria; Proteobacteria; Gammaproteobacteria; Enterobacterales;

WR15__locus_1

EUT1

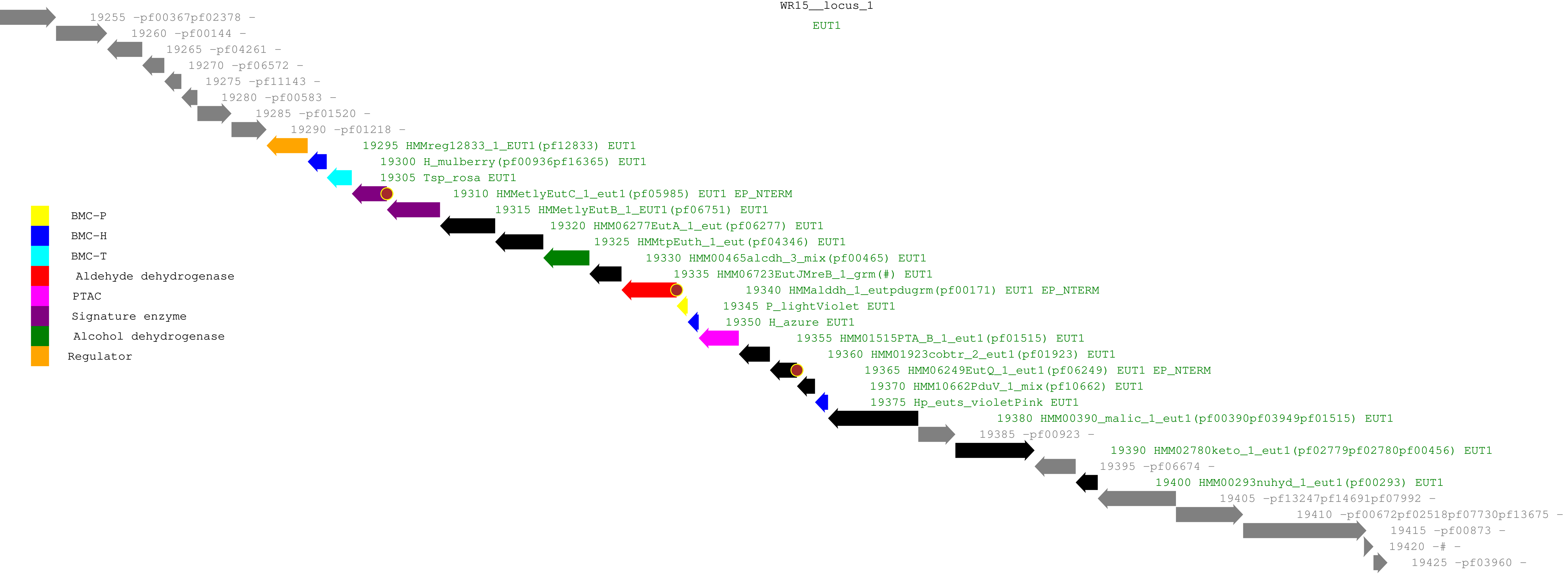

Shigella dysenteriae WRSd3. Bacteria; Proteobacteria; Gammaproteobacteria; Enterobacterales;

WRSd3__locus_fragmented

EUT1

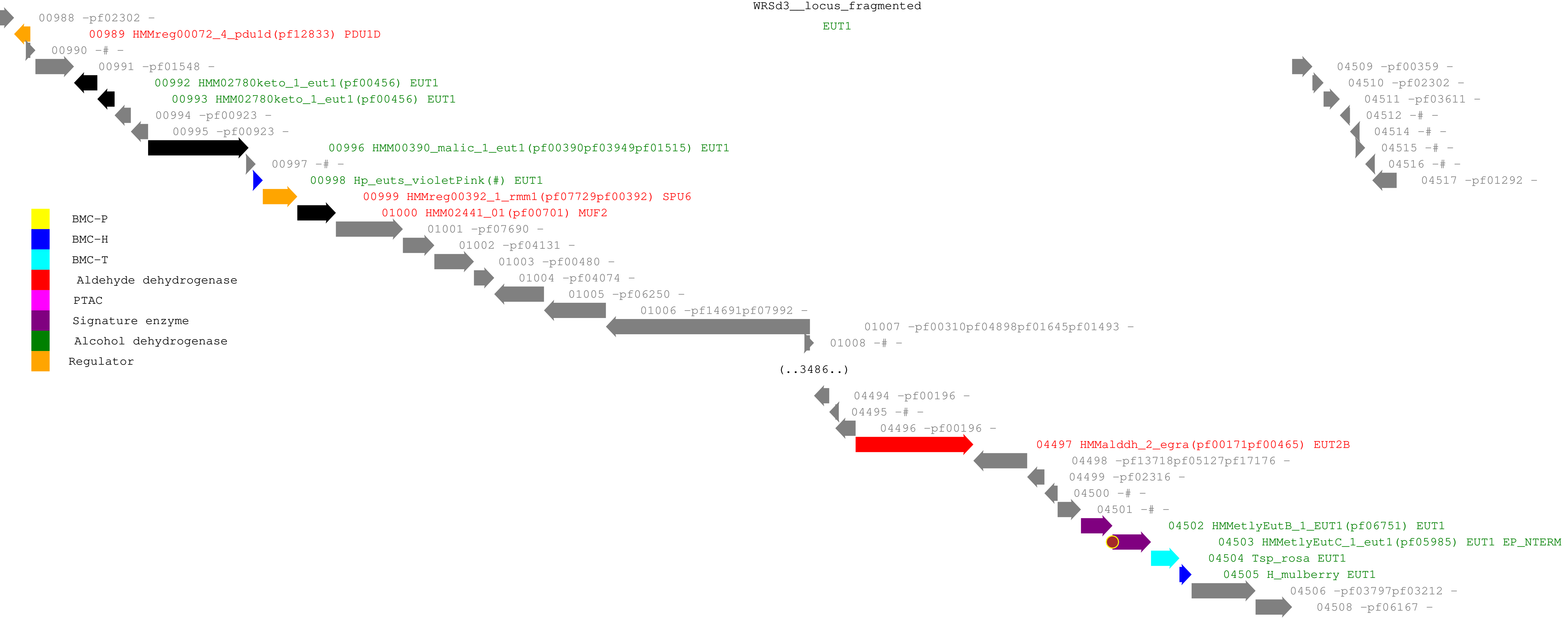

Hafnia alvei. Bacteria; Proteobacteria; Gammaproteobacteria; Enterobacterales;

XK97__locus_1

EUT1

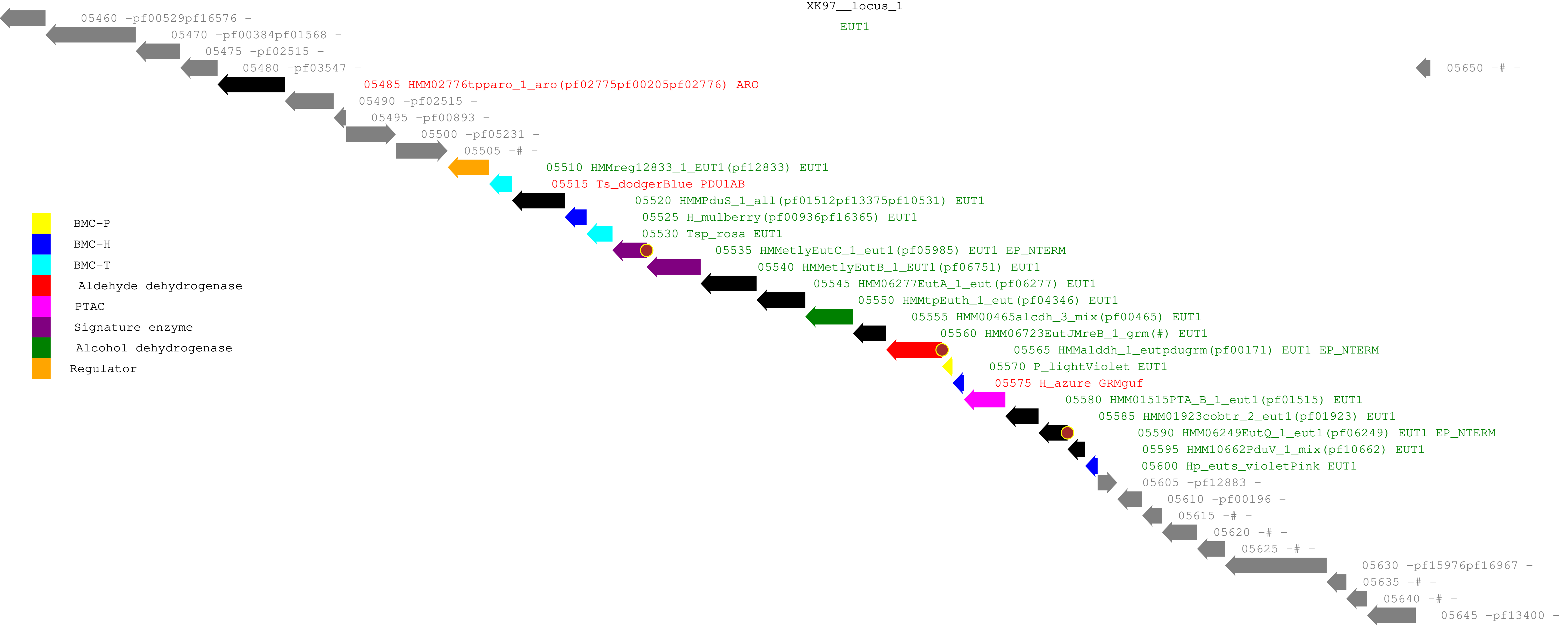

Salmonella berta. Bacteria; Proteobacteria; Gammaproteobacteria; Enterobacterales;

XR79__locus_2

EUT1

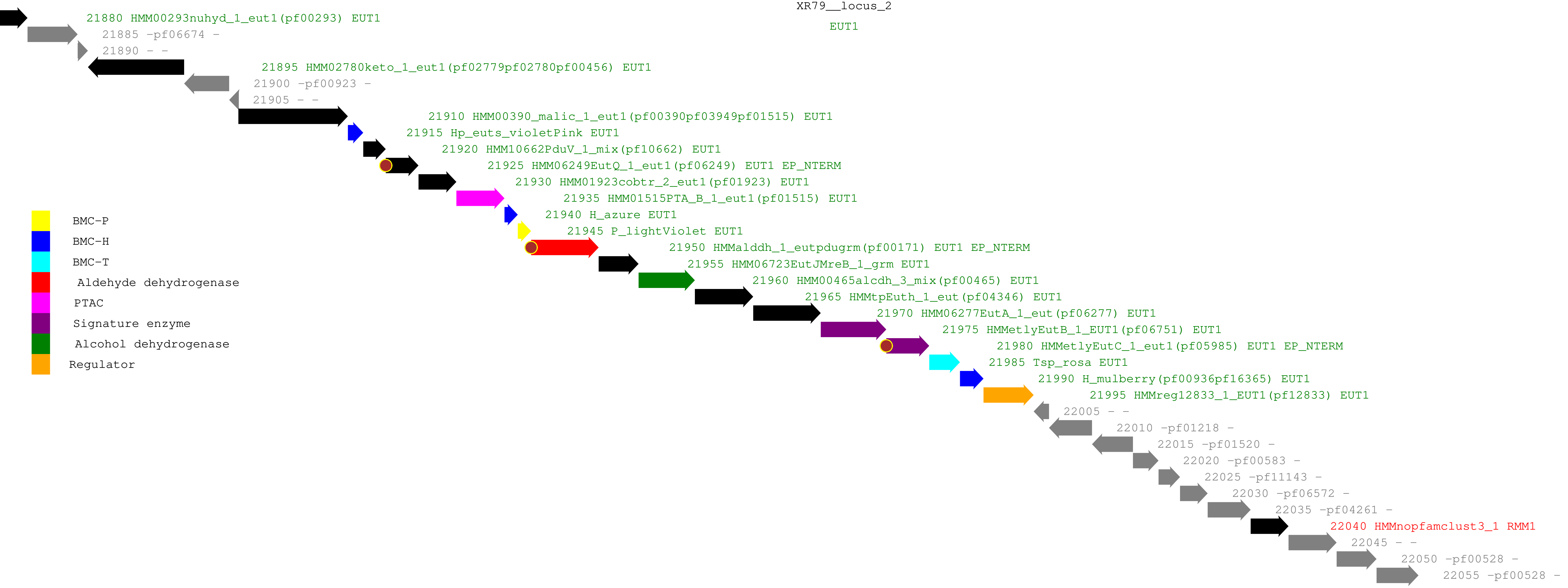

Salmonella enterica subsp. enterica serovar Infantis str. CVM Bacteria; Proteobacteria; Gammaproteobacteria; Enterobacterales;

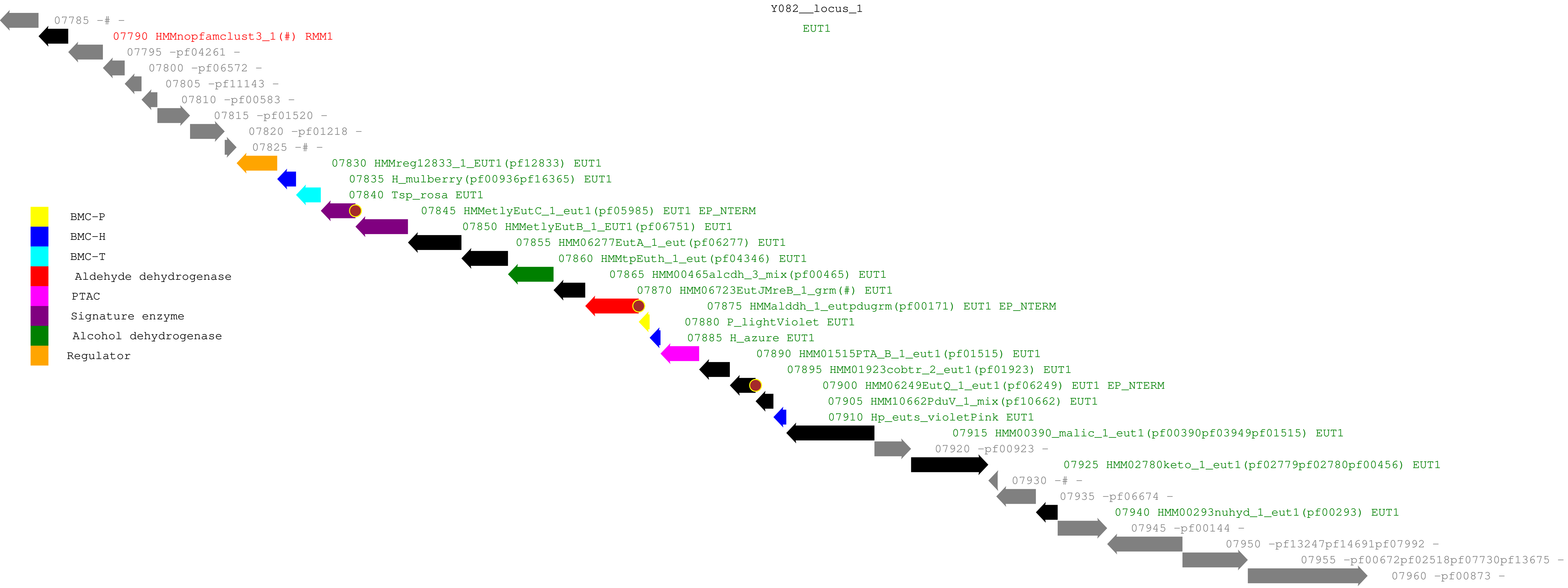

Marinobacter hydrocarbonoclasticus (Pseudomonas nautica). Bacteria; Proteobacteria; Gammaproteobacteria; Alteromonadales;

YBY__locus_1

EUT1

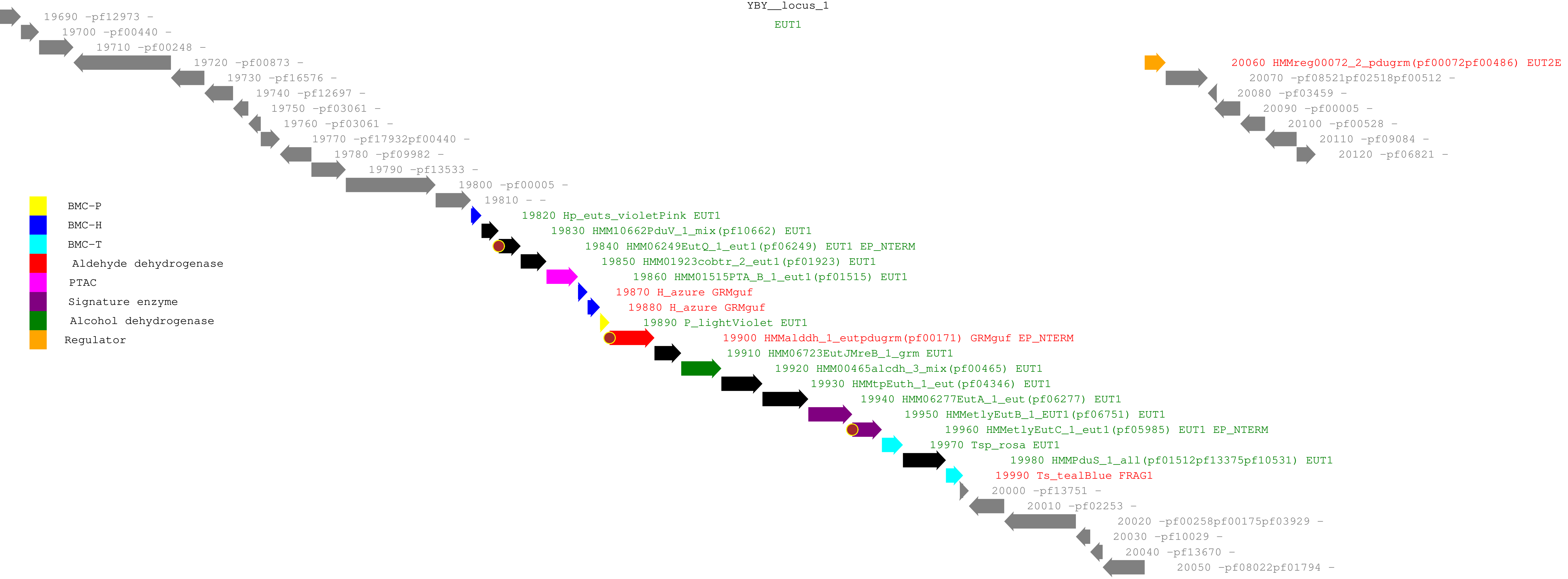

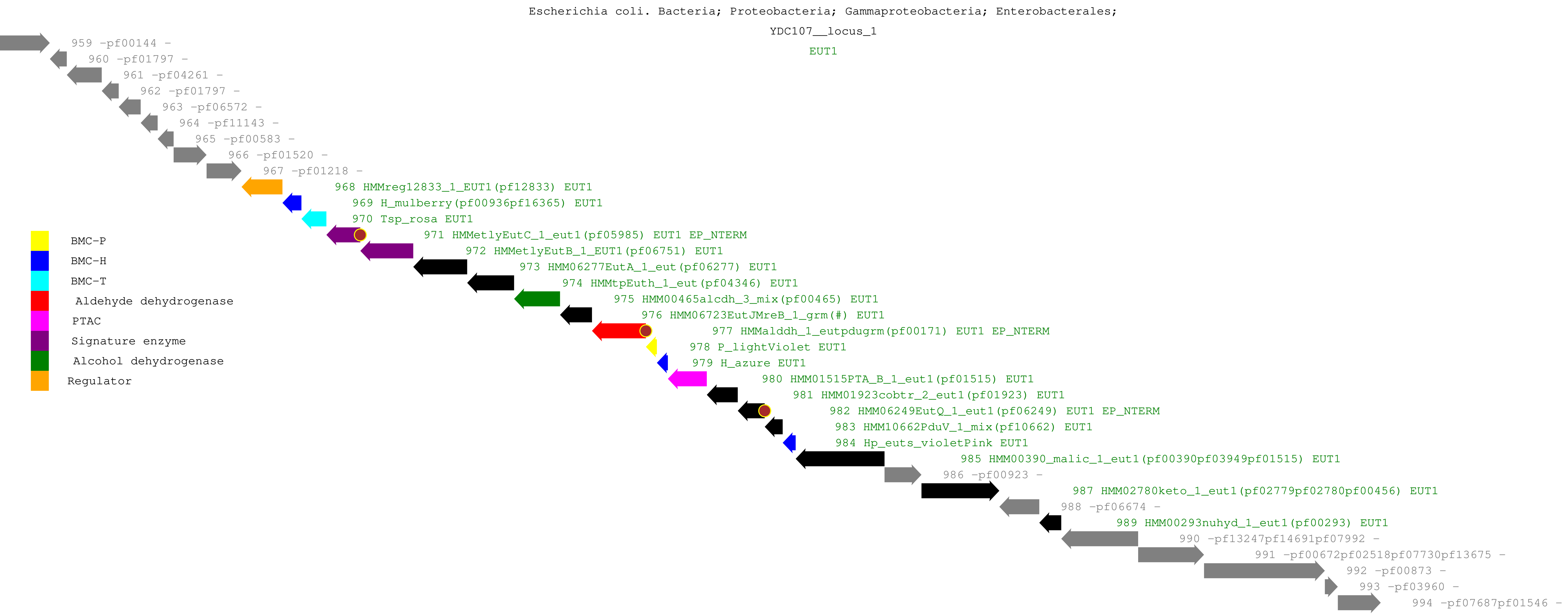
Escherichia coli. Bacteria; Proteobacteria; Gammaproteobacteria; Enterobacterales;
YDC107__locus_1
EUT1
959 -pf00144 -
960 -pf01797 -
961 -pf04261 -
962 -pf01797 -
963 -pf06572 -
964 -pf11143 -
965 -pf00583 -
966 -pf01520 -
967 -pf01218 -
968 HMMreg12833_1_EUT1(pf12833) EUT1
969 H_mulberry(pf00936pf16365) EUT1
970 Tsp_rosa EUT1
971 HMMetlyEutC_1_eut1(pf05985) EUT1 EP_NTERM
972 HMMetlyEutB_1_EUT1(pf06751) EUT1
973 HMM06277EutA_1_eut(pf06277) EUT1
974 HMMtpEuth_1_eut(pf04346) EUT1
975 HMM00465alcdh_3_mix(pf00465) EUT1
976 HMM06723EutJMreB_1_grm(#) EUT1
977 HMMalddh_1_eutpdugrm(pf00171) EUT1 EP_NTERM
978 P_lightViolet EUT1
979 H_azure EUT1
980 HMM01515PTA_B_1_eut1(pf01515) EUT1
981 HMM01923cobtr_2_eut1(pf01923) EUT1
982 HMM06249EutQ_1_eut1(pf06249) EUT1 EP_NTERM
983 HMM10662PduV_1_mix(pf10662) EUT1
984 Hp_euts_violetPink EUT1
985 HMM00390_malic_1_eut1(pf00390pf03949pf01515) EUT1
986 -pf00923 -
987 HMM02780keto_1_eut1(pf02779pf02780pf00456) EUT1
988 -pf06674 -
989 HMM00293nuhyd_1_eut1(pf00293) EUT1
990 -pf13247pf14691pf07992 -
991 -pf00672pf02518pf07730pf13675 -
992 -pf00873 -
993 -pf03960 -
994 -pf07687pf01546 -
BMC-P
BMC-H
BMC-T
Aldehyde dehydrogenase
PTAC
Signature enzyme
Alcohol dehydrogenase
Regulator

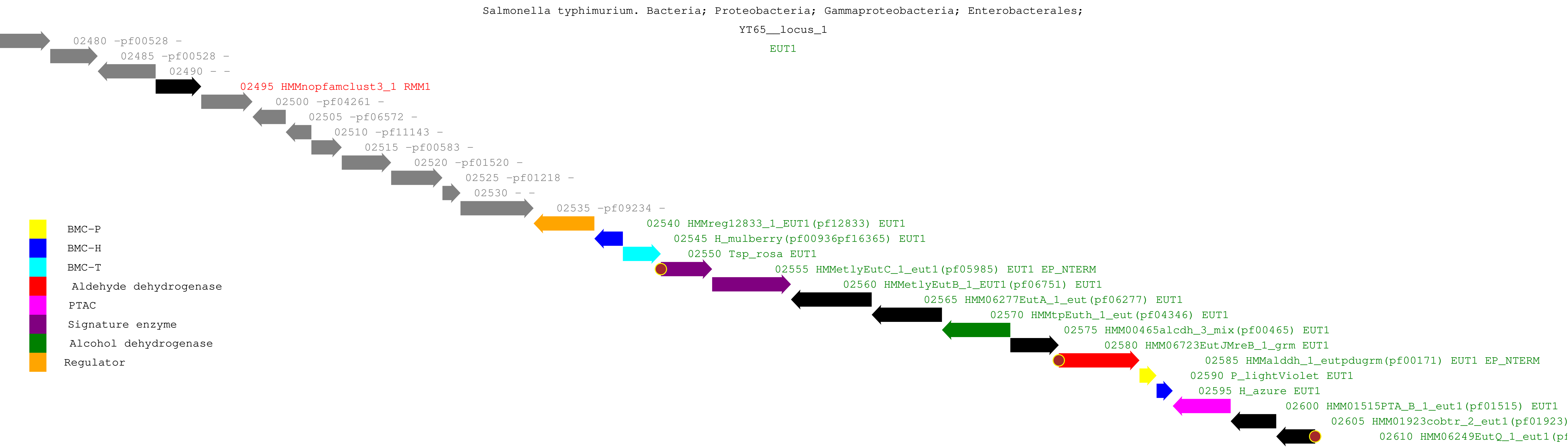
Salmonella typhimurium. Bacteria; Proteobacteria; Gammaproteobacteria; Enterobacterales;
YT65__locus_1
EUT1
02480 -pf00528 -
02485 -pf00528 -
02490 - -
02495 HMMnopfamclust3_1 RMM1
02500 -pf04261 -
02505 -pf06572 -
02510 -pf11143 -
02515 -pf00583 -
02520 -pf01520 -
02525 -pf01218 -
02530 - -
02535 -pf09234 -
02540 HMMreg12833_1_EUT1(pf12833) EUT1
02545 H_mulberry(pf00936pf16365) EUT1
02550 Tsp_rosa EUT1
02555 HMMetlyEutC_1_eut1(pf05985) EUT1 EP_NTERM
02560 HMMetlyEutB_1_EUT1(pf06751) EUT1
02565 HMM06277EutA_1_eut(pf06277) EUT1
02570 HMMtpEuth_1_eut(pf04346) EUT1
02575 HMM00465alcdh_3_mix(pf00465) EUT1
02580 HMM06723EutJMreB_1_grm EUT1
02585 HMMalddh_1_eutpdugrm(pf00171) EUT1 EP_NTERM
02590 P_lightViolet EUT1
02595 H_azure EUT1
02600 HMM01515PTA_B_1_eut1(pf01515) EUT1
02605 HMM01923cobtr_2_eut1(pf01923)
02610 HMM06249EutQ_1_eut1(pf
BMC-P
BMC-H
BMC-T
Aldehyde dehydrogenase
PTAC
Signature enzyme
Alcohol dehydrogenase
Regulator

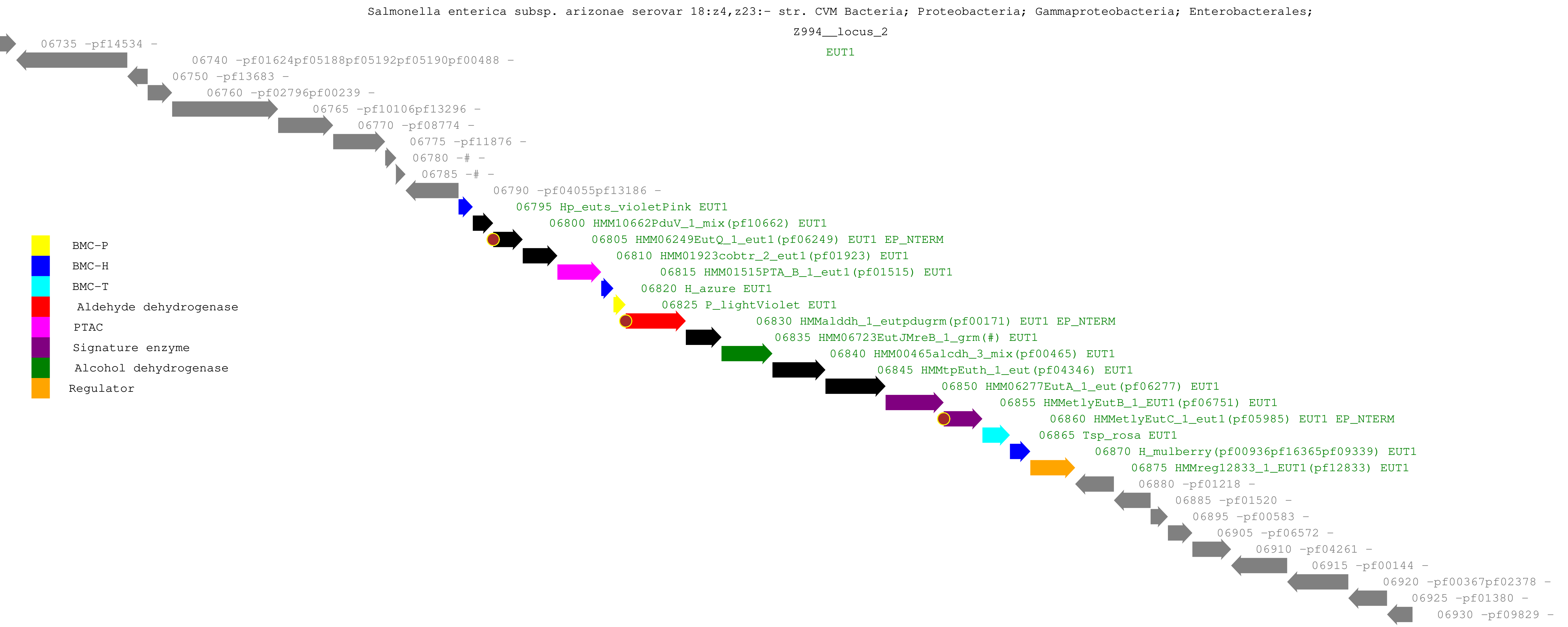
Salmonella enterica subsp. arizonae serovar 18:z4,z23:- str. CVM Bacteria; Proteobacteria; Gammaproteobacteria; Enterobacterales;
Z994__locus_2
EUT1
06735 -pf14534 -
06740 -pf01624pf05188pf05192pf05190pf00488 -
06750 -pf13683 -
06760 -pf02796pf00239 -
06765 -pf10106pf13296 -
06770 -pf08774 -
06775 -pf11876 -
06780 -# -
06785 -# -
06790 -pf04055pf13186 -
06795 Hp_euts_violetPink EUT1
06800 HMM10662PduV_1_mix(pf10662) EUT1
06805 HMM06249EutQ_1_eut1(pf06249) EUT1 EP_NTERM
06810 HMM01923cobtr_2_eut1(pf01923) EUT1
06815 HMM01515PTA_B_1_eut1(pf01515) EUT1
06820 H_azure EUT1
06825 P_lightViolet EUT1
06830 HMMalddh_1_eutpdugrm(pf00171) EUT1 EP_NTERM
06835 HMM06723EutJMreB_1_grm(#) EUT1
06840 HMM00465alcdh_3_mix(pf00465) EUT1
06845 HMMtpEuth_1_eut(pf04346) EUT1
06850 HMM06277EutA_1_eut(pf06277) EUT1
06855 HMMetlyEutB_1_EUT1(pf06751) EUT1
06860 HMMetlyEutC_1_eut1(pf05985) EUT1 EP_NTERM
06865 Tsp_rosa EUT1
06870 H_mulberry(pf00936pf16365pf09339) EUT1
06875 HMMreg12833_1_EUT1(pf12833) EUT1
06880 -pf01218 -
06885 -pf01520 -
06895 -pf00583 -
06905 -pf06572 -
06910 -pf04261 -
06915 -pf00144 -
06920 -pf00367pf02378 -
06925 -pf01380 -
06930 -pf09829 -
BMC-P
BMC-H
BMC-T
Aldehyde dehydrogenase
PTAC
Signature enzyme
Alcohol dehydrogenase
Regulator

Salmonella enterica subsp. enterica serovar Corvallis. Bacteria; Proteobacteria; Gammaproteobacteria; Enterobacterales;

ZU68__locus_2_main_sat

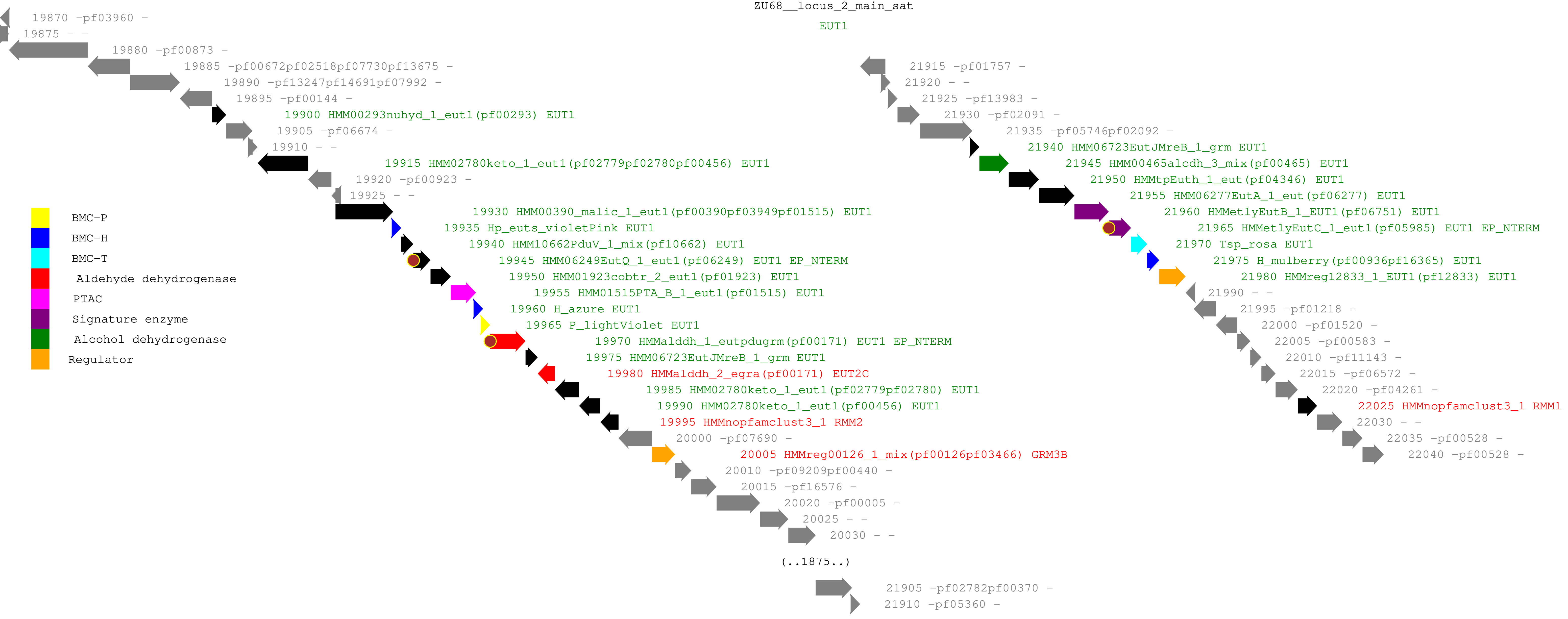

Enterococcus mundtii. Bacteria; Firmicutes; Bacilli; Lactobacillales; Enterococcaceae;

A5802__locus_2

EUT2A

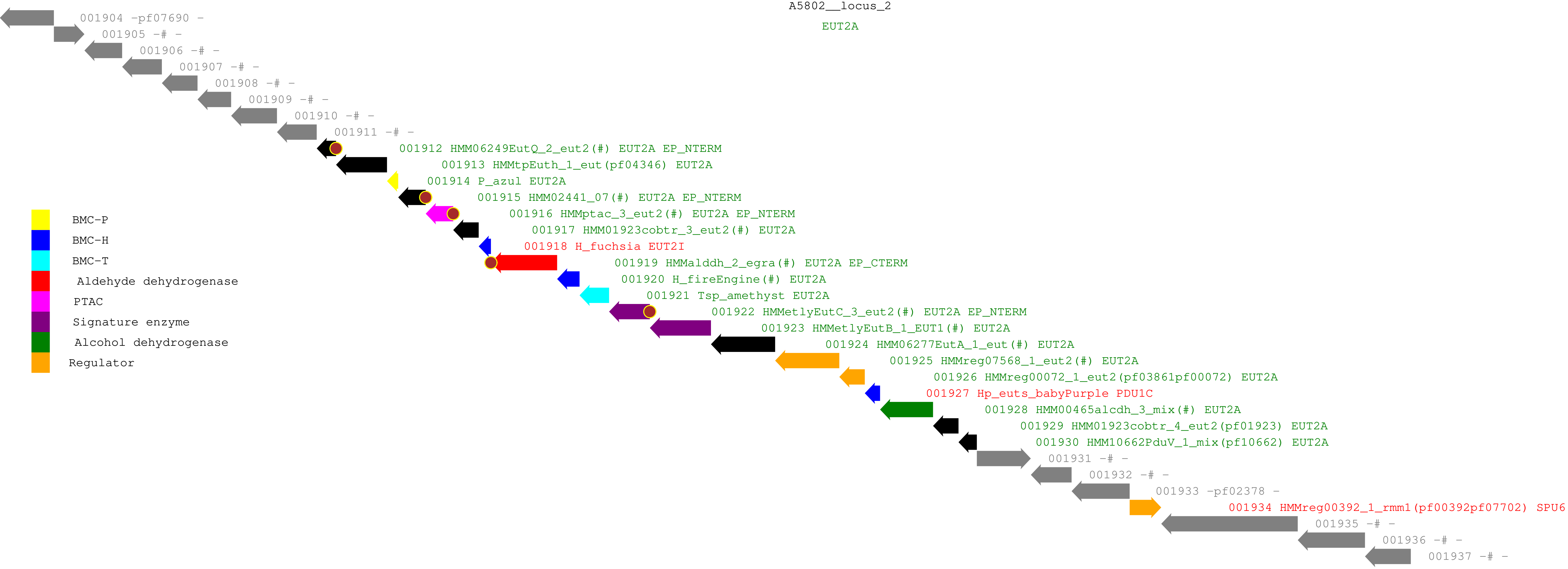

Enterococcus sp. 7F3_DIV0205. Bacteria; Firmicutes; Bacilli; Lactobacillales; Enterococcaceae;

A5821__locus_1

EUT2A

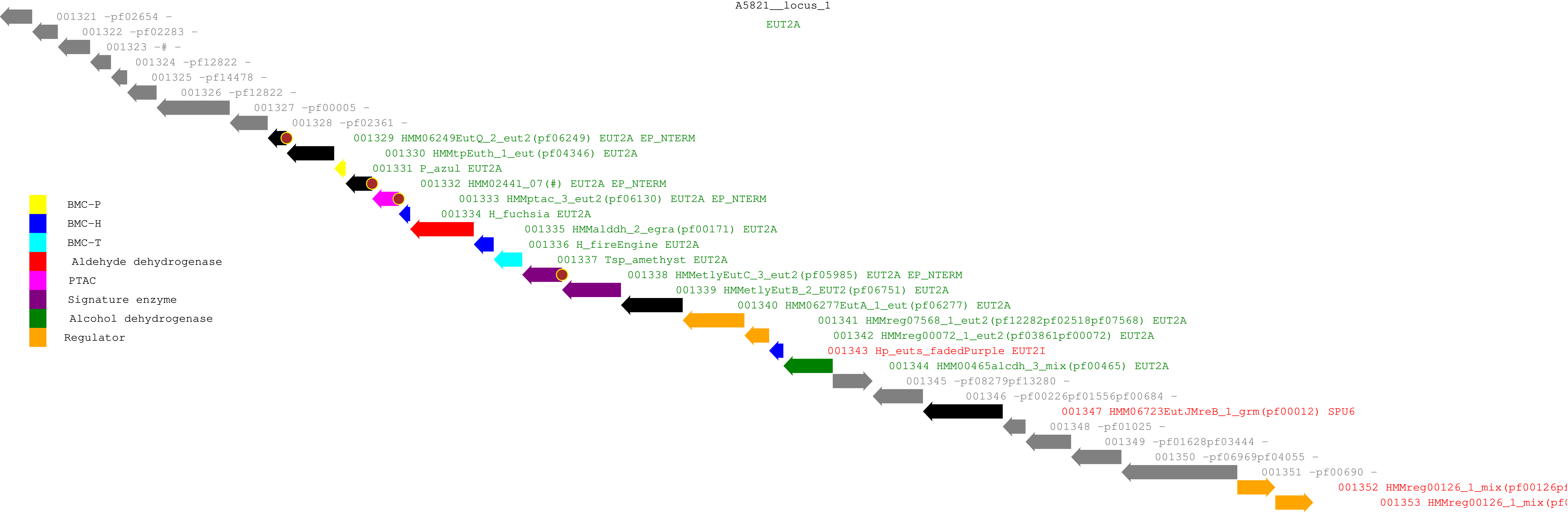

Enterococcus sp. 9D6_DIV0238. Bacteria; Firmicutes; Bacilli; Lactobacillales; Enterococcaceae;

A5889__locus_2

EUT2A

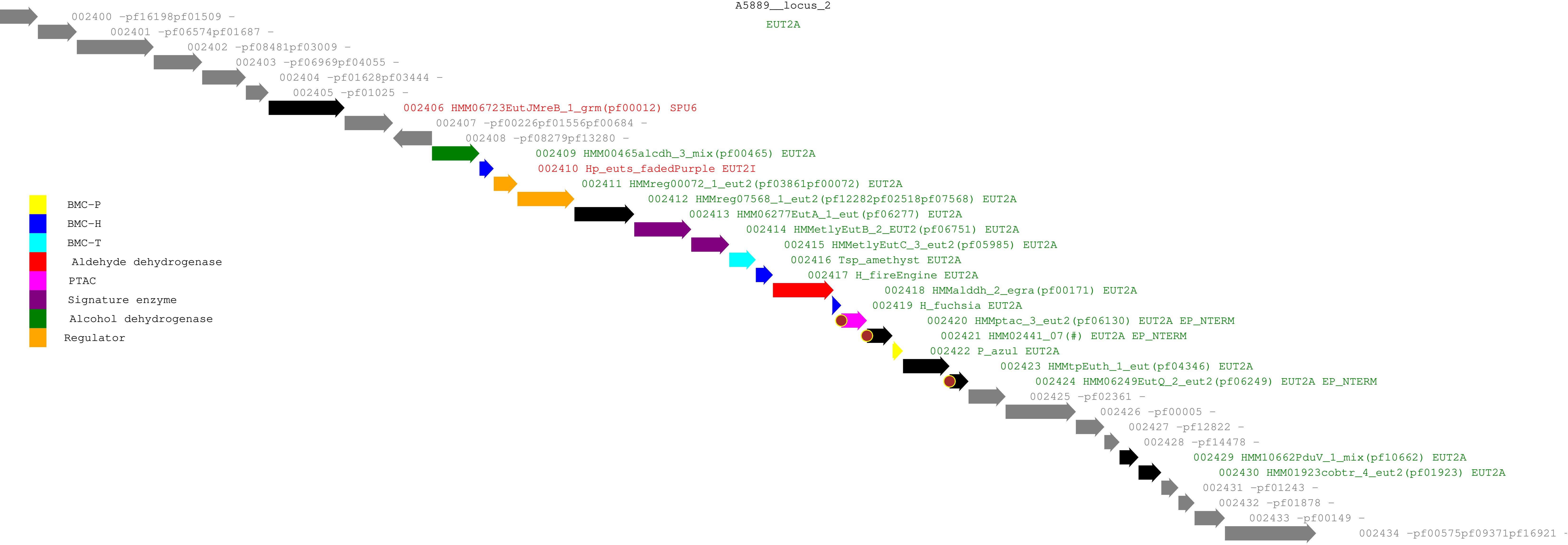

Enterococcus faecalis (Streptococcus faecalis). Bacteria; Firmicutes; Bacilli; Lactobacillales; Enterococcaceae;

A6B47__locus_1

EUT2A

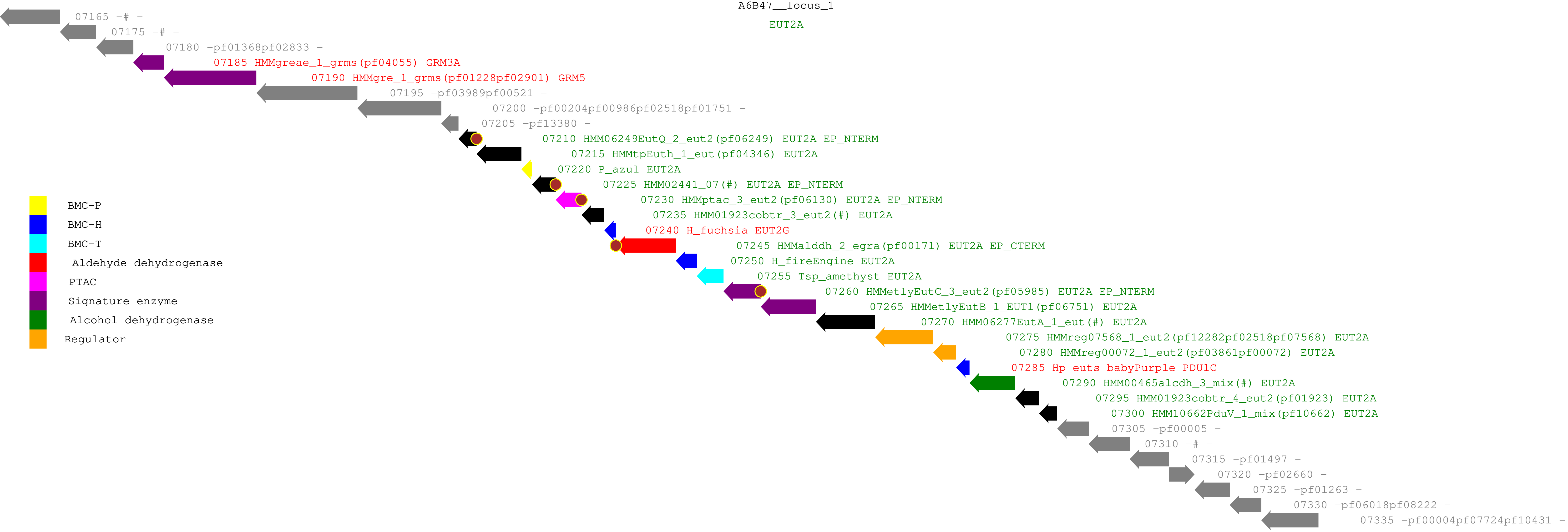

Enterococcus avium (Streptococcus avium). Bacteria; Firmicutes; Bacilli; Lactobacillales; Enterococcaceae;

AUF12__locus_1

EUT2A

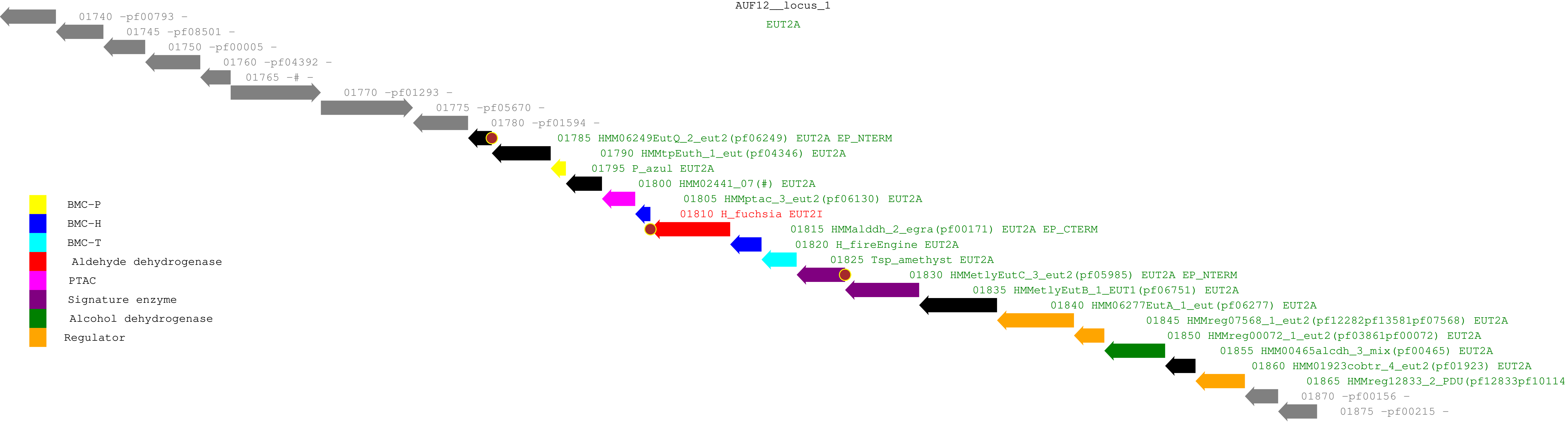

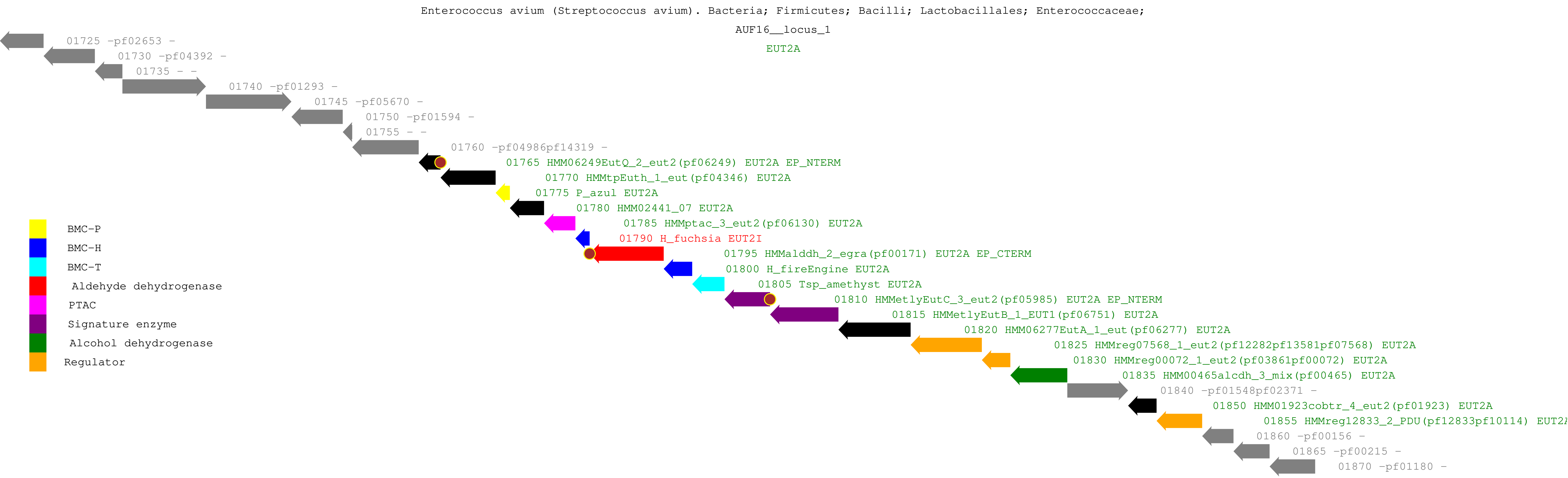
Enterococcus avium (Streptococcus avium). Bacteria; Firmicutes; Bacilli; Lactobacillales; Enterococcaceae;
AUF16__locus_1
EUT2A
01725 -pf02653 -
01730 -pf04392 -
01735 - -
01740 -pf01293 -
01745 -pf05670 -
01750 -pf01594 -
01755 - -
01760 -pf04986pf14319 -
01765 HMM06249EutQ_2_eut2(pf06249) EUT2A EP_NTERM
01770 HMMtpEuth_1_eut(pf04346) EUT2A
01775 P_azul EUT2A
01780 HMM02441_07 EUT2A
01785 HMMptac_3_eut2(pf06130) EUT2A
01790 H_fuchsia EUT2I
01795 HMMalddh_2_egra(pf00171) EUT2A EP_CTERM
01800 H_fireEngine EUT2A
01805 Tsp_amethyst EUT2A
01810 HMMetlyEutC_3_eut2(pf05985) EUT2A EP_NTERM
01815 HMMetlyEutB_1_EUT1(pf06751) EUT2A
01820 HMM06277EutA_1_eut(pf06277) EUT2A
01825 HMMreg07568_1_eut2(pf12282pf13581pf07568) EUT2A
01830 HMMreg00072_1_eut2(pf03861pf00072) EUT2A
01835 HMM00465alcdh_3_mix(pf00465) EUT2A
01840 -pf01548pf02371 -
01850 HMM01923cobtr_4_eut2(pf01923) EUT2A
01855 HMMreg12833_2_PDU(pf12833pf10114) EUT2A
01860 -pf00156 -
01865 -pf00215 -
01870 -pf01180 -
BMC-P
BMC-H
BMC-T
Aldehyde dehydrogenase
PTAC
Signature enzyme
Alcohol dehydrogenase
Regulator

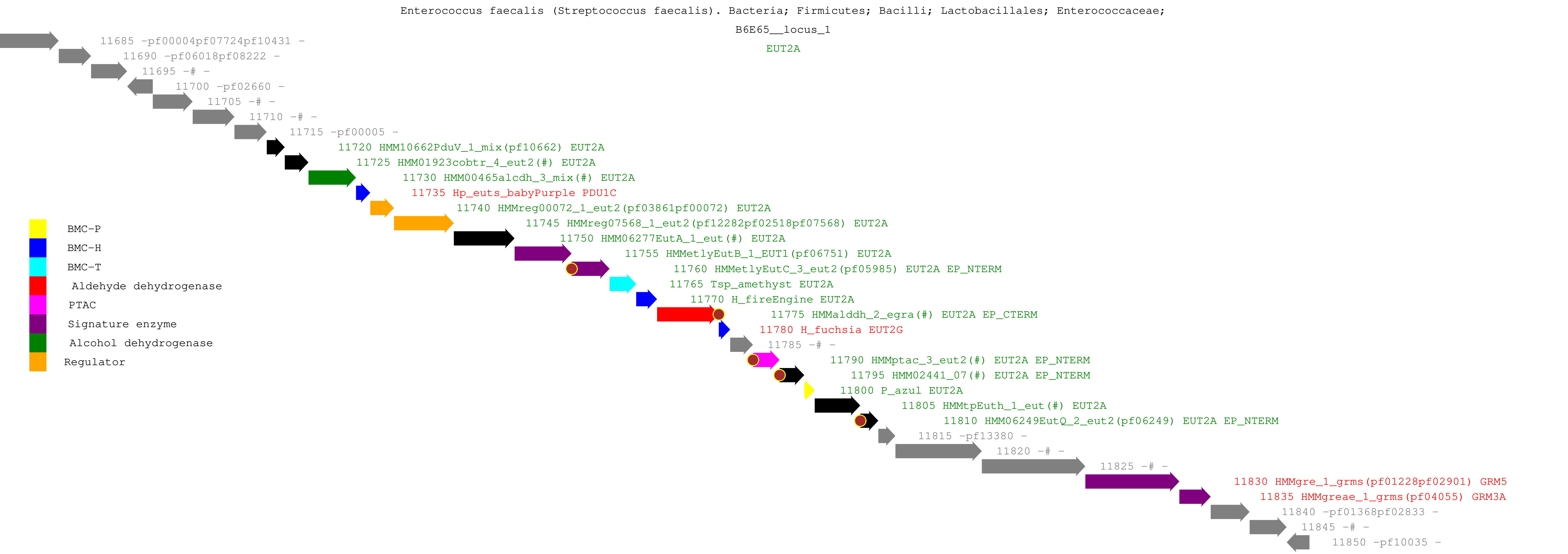

Enterococcus faecalis (Streptococcus faecalis). Bacteria; Firmicutes; Bacilli; Lactobacillales; Enterococcaceae;
B6E65__locus_1
EUT2A
11685 -pf00004pf07724pf10431 -
11690 -pf06018pf08222 -
11695 -# -
11700 -pf02660 -
11705 -# -
11710 -# -
11715 -pf00005 -
11720 HMM10662PduV_1_mix(pf10662) EUT2A
11725 HMM01923cobtr_4_eut2(#) EUT2A
11730 HMM00465alcdh_3_mix(#) EUT2A
11735 Hp_euts_babyPurple PDU1C
11740 HMMreg00072_1_eut2(pf03861pf00072) EUT2A
11745 HMMreg07568_1_eut2(pf12282pf02518pf07568) EUT2A
11750 HMM06277EutA_1_eut(#) EUT2A
11755 HMMetlyEutB_1_EUT1(pf06751) EUT2A
11760 HMMetlyEutC_3_eut2(pf05985) EUT2A EP_NTERM
11765 Tsp_amethyst EUT2A
11770 H_fireEngine EUT2A
11775 HMMalddh_2_egra(#) EUT2A EP_CTERM
11780 H_fuchsia EUT2G
11785 -# -
11790 HMMptac_3_eut2(#) EUT2A EP_NTERM
11795 HMM02441_07(#) EUT2A EP_NTERM
11800 P_azul EUT2A
11805 HMMtpEuth_1_eut(#) EUT2A
11810 HMM06249EutQ_2_eut2(pf06249) EUT2A EP_NTERM
11815 -pf13380 -
11820 -# -
11825 -# -
11830 HMMgre_1_grms(pf01228pf02901) GRM5
11835 HMMgreae_1_grms(pf04055) GRM3A
11840 -pf01368pf02833 -
11845 -# -
11850 -pf10035 -
BMC-P
BMC-H
BMC-T
Aldehyde dehydrogenase
PTAC
Signature enzyme
Alcohol dehydrogenase
Regulator

Enterococcus faecalis (Streptococcus faecalis). Bacteria; Firmicutes; Bacilli; Lactobacillales; Enterococcaceae;

B6S42__locus_1

EUT2A

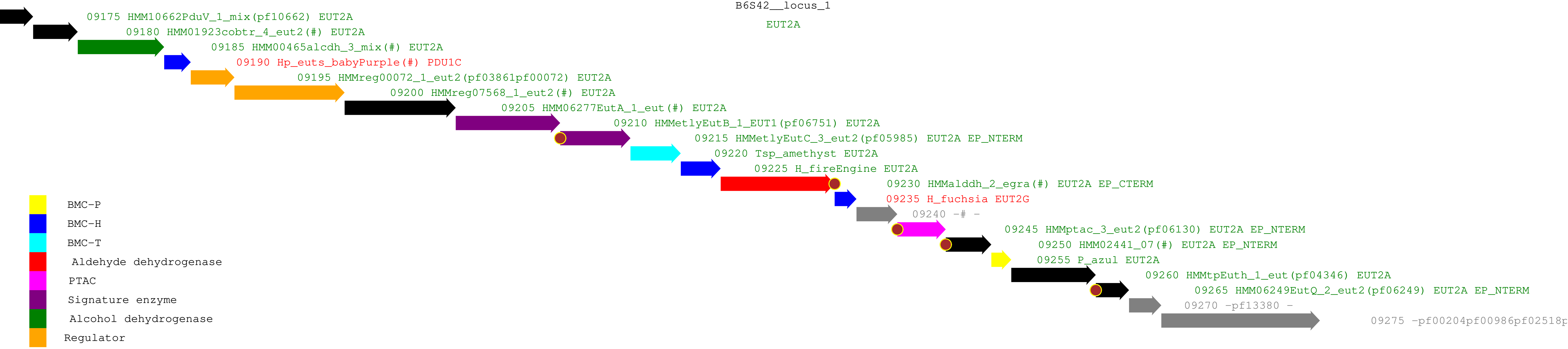

Brochothrix campestris FSL F6-1037. Bacteria; Firmicutes; Bacilli; Bacillales; Listeriaceae; Brochothrix.

BCAMP__locus_1

EUT2A

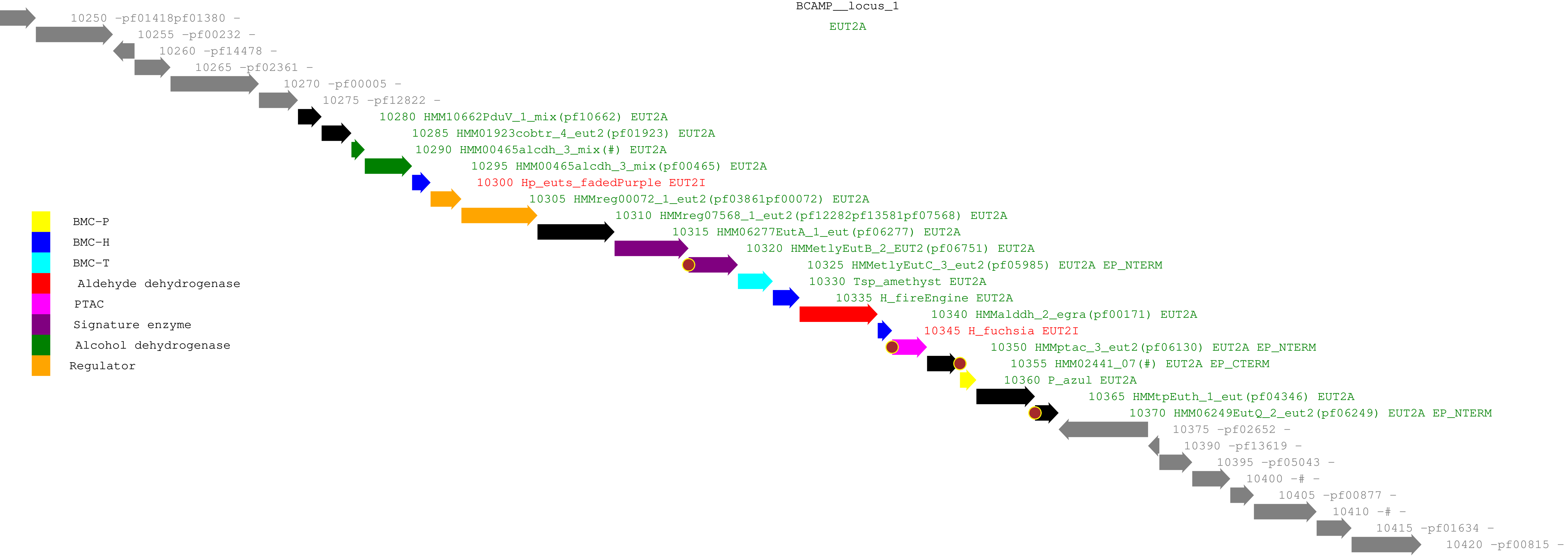

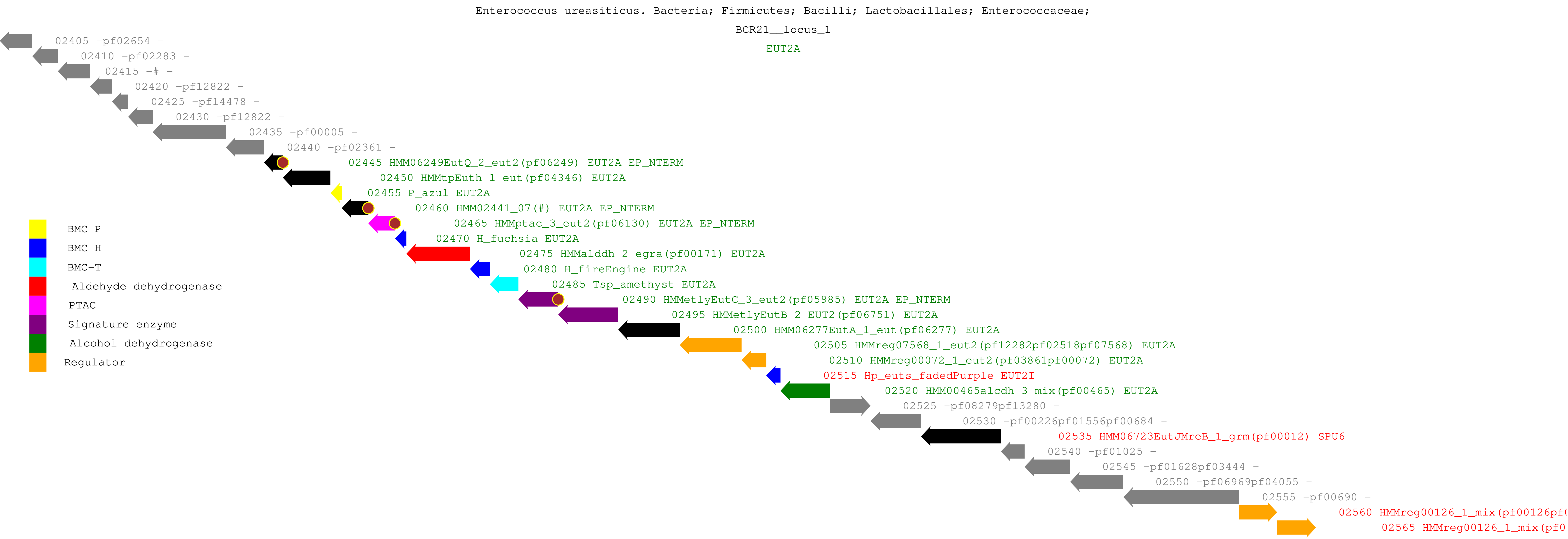

Enterococcus ureasiticus. Bacteria; Firmicutes; Bacilli; Lactobacillales; Enterococcaceae;
BCR21__locus_1
EUT2A
02405 -pf02654 -
02410 -pf02283 -
02415 -# -
02420 -pf12822 -
02425 -pf14478 -
02430 -pf12822 -
02435 -pf00005 -
02440 -pf02361 -
02445 HMM06249EutQ_2_eut2(pf06249) EUT2A EP_NTERM
02450 HMMtpEuth_1_eut(pf04346) EUT2A
02455 P_azul EUT2A
02460 HMM02441_07(#) EUT2A EP_NTERM
02465 HMMptac_3_eut2(pf06130) EUT2A EP_NTERM
02470 H_fuchsia EUT2A
02475 HMMalddh_2_egra(pf00171) EUT2A
02480 H_fireEngine EUT2A
02485 Tsp_amethyst EUT2A
02490 HMMetlyEutC_3_eut2(pf05985) EUT2A EP_NTERM
02495 HMMetlyEutB_2_EUT2(pf06751) EUT2A
02500 HMM06277EutA_1_eut(pf06277) EUT2A
02505 HMMreg07568_1_eut2(pf12282pf02518pf07568) EUT2A
02510 HMMreg00072_1_eut2(pf03861pf00072) EUT2A
02515 Hp_euts_fadedPurple EUT2I
02520 HMM00465alcdh_3_mix(pf00465) EUT2A
02525 -pf08279pf13280 -
02530 -pf00226pf01556pf00684 -
02535 HMM06723EutJMreB_1_grm(pf00012) SPU6
02540 -pf01025 -
02545 -pf01628pf03444 -
02550 -pf06969pf04055 -
02555 -pf00690 -
02560 HMMreg00126_1_mix(pf00126pf0
02565 HMMreg00126_1_mix(pf0
BMC-P
BMC-H
BMC-T
Aldehyde dehydrogenase
PTAC
Signature enzyme
Alcohol dehydrogenase
Regulator

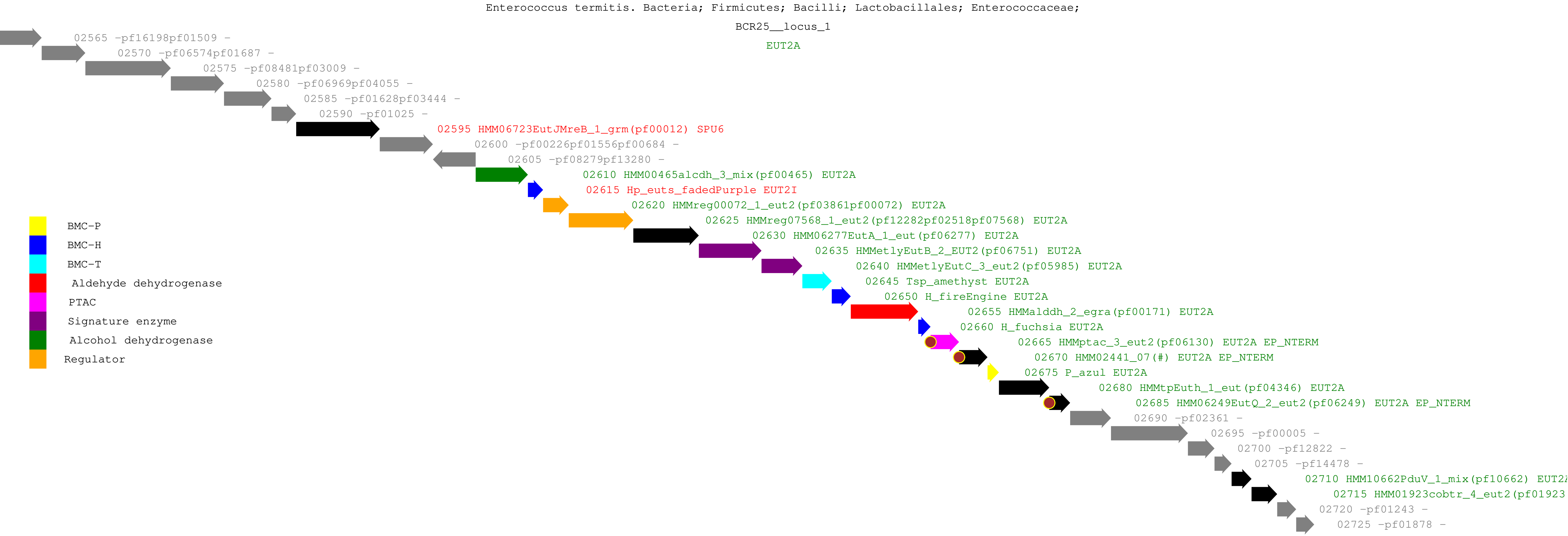
Enterococcus termitis. Bacteria; Firmicutes; Bacilli; Lactobacillales; Enterococcaceae;
BCR25__locus_1
EUT2A
02565 -pf16198pf01509 -
02570 -pf06574pf01687 -
02575 -pf08481pf03009 -
02580 -pf06969pf04055 -
02585 -pf01628pf03444 -
02590 -pf01025 -
02595 HMM06723EutJMreB_1_grm(pf00012) SPU6
02600 -pf00226pf01556pf00684 -
02605 -pf08279pf13280 -
02610 HMM00465alcdh_3_mix(pf00465) EUT2A
02615 Hp_euts_fadedPurple EUT2I
02620 HMMreg00072_1_eut2(pf03861pf00072) EUT2A
02625 HMMreg07568_1_eut2(pf12282pf02518pf07568) EUT2A
02630 HMM06277EutA_1_eut(pf06277) EUT2A
02635 HMMetlyEutB_2_EUT2(pf06751) EUT2A
02640 HMMetlyEutC_3_eut2(pf05985) EUT2A
02645 Tsp_amethyst EUT2A
02650 H_fireEngine EUT2A
02655 HMMalddh_2_egra(pf00171) EUT2A
02660 H_fuchsia EUT2A
02665 HMMptac_3_eut2(pf06130) EUT2A EP_NTERM
02670 HMM02441_07(#) EUT2A EP_NTERM
02675 P_azul EUT2A
02680 HMMtpEuth_1_eut(pf04346) EUT2A
02685 HMM06249EutQ_2_eut2(pf06249) EUT2A EP_NTERM
02690 -pf02361 -
02695 -pf00005 -
02700 -pf12822 -
02705 -pf14478 -
02710 HMM10662PduV_1_mix(pf10662) EUT2
02715 HMM01923cobtr_4_eut2(pf01923
02720 -pf01243 -
02725 -pf01878 -
BMC-P
BMC-H
BMC-T
Aldehyde dehydrogenase
PTAC
Signature enzyme
Alcohol dehydrogenase
Regulator

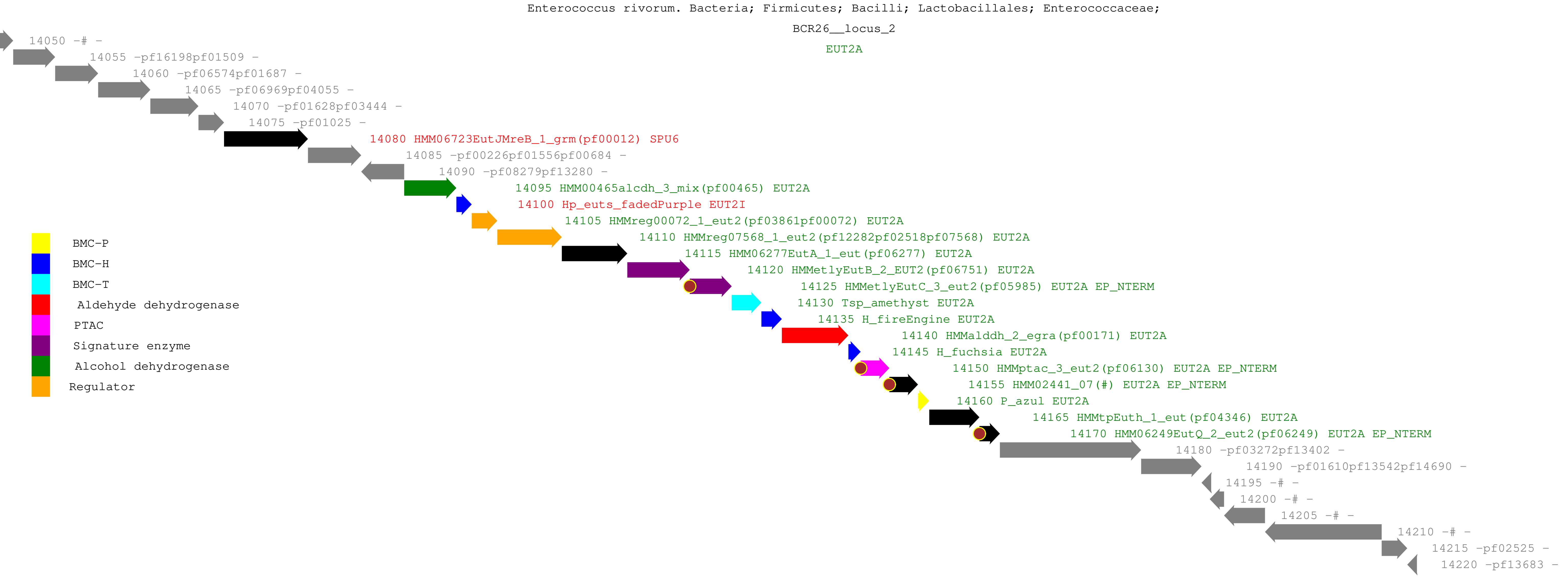
Enterococcus rivorum. Bacteria; Firmicutes; Bacilli; Lactobacillales; Enterococcaceae;
BCR26__locus_2
EUT2A
14050 -# -
14055 -pf16198pf01509 -
14060 -pf06574pf01687 -
14065 -pf06969pf04055 -
14070 -pf01628pf03444 -
14075 -pf01025 -
14080 HMM06723EutJMreB_1_grm(pf00012) SPU6
14085 -pf00226pf01556pf00684 -
14090 -pf08279pf13280 -
14095 HMM00465alcdh_3_mix(pf00465) EUT2A
14100 Hp_euts_fadedPurple EUT2I
14105 HMMreg00072_1_eut2(pf03861pf00072) EUT2A
14110 HMMreg07568_1_eut2(pf12282pf02518pf07568) EUT2A
14115 HMM06277EutA_1_eut(pf06277) EUT2A
14120 HMMetlyEutB_2_EUT2(pf06751) EUT2A
14125 HMMetlyEutC_3_eut2(pf05985) EUT2A EP_NTERM
14130 Tsp_amethyst EUT2A
14135 H_fireEngine EUT2A
14140 HMMalddh_2_egra(pf00171) EUT2A
14145 H_fuchsia EUT2A
14150 HMMptac_3_eut2(pf06130) EUT2A EP_NTERM
14155 HMM02441_07(#) EUT2A EP_NTERM
14160 P_azul EUT2A
14165 HMMtpEuth_1_eut(pf04346) EUT2A
14170 HMM06249EutQ_2_eut2(pf06249) EUT2A EP_NTERM
14180 -pf03272pf13402 -
14190 -pf01610pf13542pf14690 -
14195 -# -
14200 -# -
14205 -# -
14210 -# -
14215 -pf02525 -
14220 -pf13683 -
BMC-P
BMC-H
BMC-T
Aldehyde dehydrogenase
PTAC
Signature enzyme
Alcohol dehydrogenase
Regulator

Carnobacterium maltaromaticum (Carnobacterium piscicola). Bacteria; Firmicutes; Bacilli; Lactobacillales; Carnobacteriaceae;

BFC23__locus_1

EUT2A

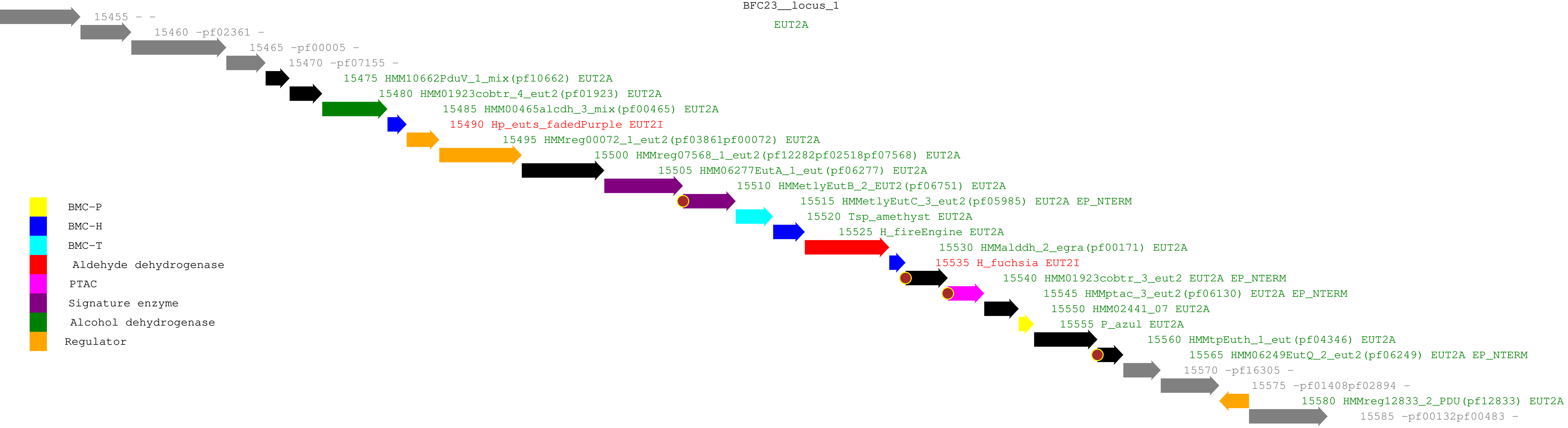

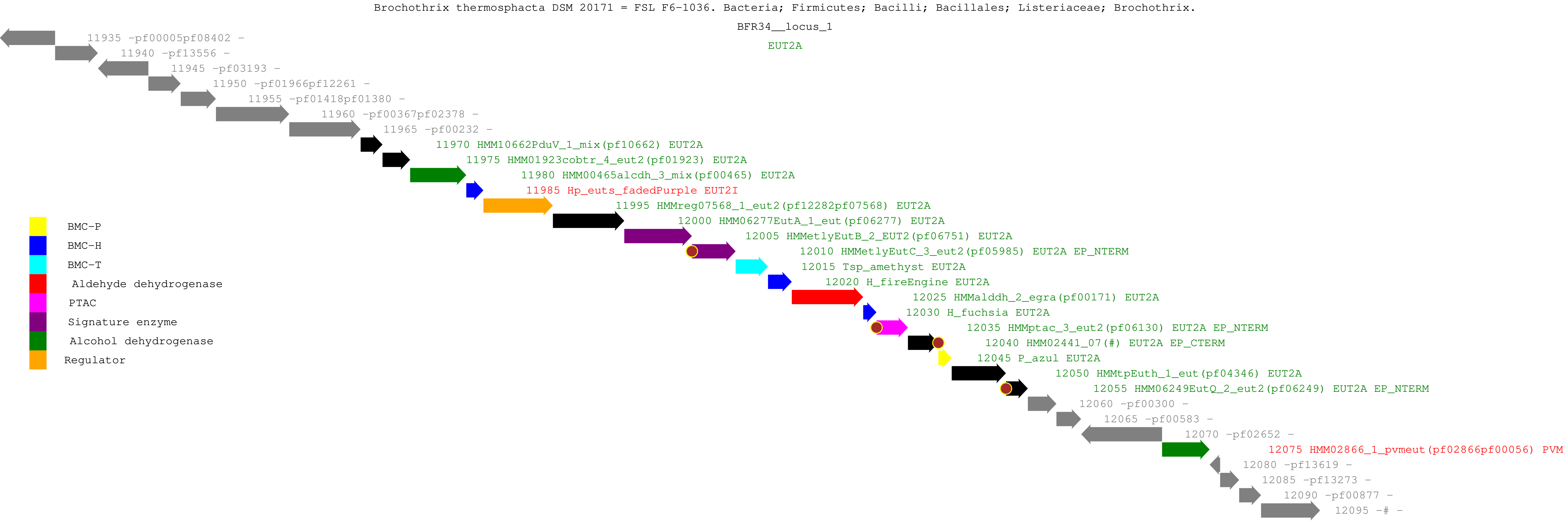
Brochothrix thermosphacta DSM 20171 = FSL F6-1036. Bacteria; Firmicutes; Bacilli; Bacillales; Listeriaceae; Brochothrix.
BFR34__locus_1
EUT2A
11935 -pf00005pf08402 -
11940 -pf13556 -
11945 -pf03193 -
11950 -pf01966pf12261 -
11955 -pf01418pf01380 -
11960 -pf00367pf02378 -
11965 -pf00232 -
11970 HMM10662PduV_1_mix(pf10662) EUT2A
11975 HMM01923cobtr_4_eut2(pf01923) EUT2A
11980 HMM00465alcdh_3_mix(pf00465) EUT2A
11985 Hp_euts_fadedPurple EUT2I
11995 HMMreg07568_1_eut2(pf12282pf07568) EUT2A
12000 HMM06277EutA_1_eut(pf06277) EUT2A
12005 HMMetlyEutB_2_EUT2(pf06751) EUT2A
12010 HMMetlyEutC_3_eut2(pf05985) EUT2A EP_NTERM
12015 Tsp_amethyst EUT2A
12020 H_fireEngine EUT2A
12025 HMMalddh_2_egra(pf00171) EUT2A
12030 H_fuchsia EUT2A
12035 HMMptac_3_eut2(pf06130) EUT2A EP_NTERM
12040 HMM02441_07(#) EUT2A EP_CTERM
12045 P_azul EUT2A
12050 HMMtpEuth_1_eut(pf04346) EUT2A
12055 HMM06249EutQ_2_eut2(pf06249) EUT2A EP_NTERM
12060 -pf00300 -
12065 -pf00583 -
12070 -pf02652 -
12075 HMM02866_1_pvmeut(pf02866pf00056) PVM
12080 -pf13619 -
12085 -pf13273 -
12090 -pf00877 -
12095 -# -
BMC-P
BMC-H
BMC-T
Aldehyde dehydrogenase
PTAC
Signature enzyme
Alcohol dehydrogenase
Regulator

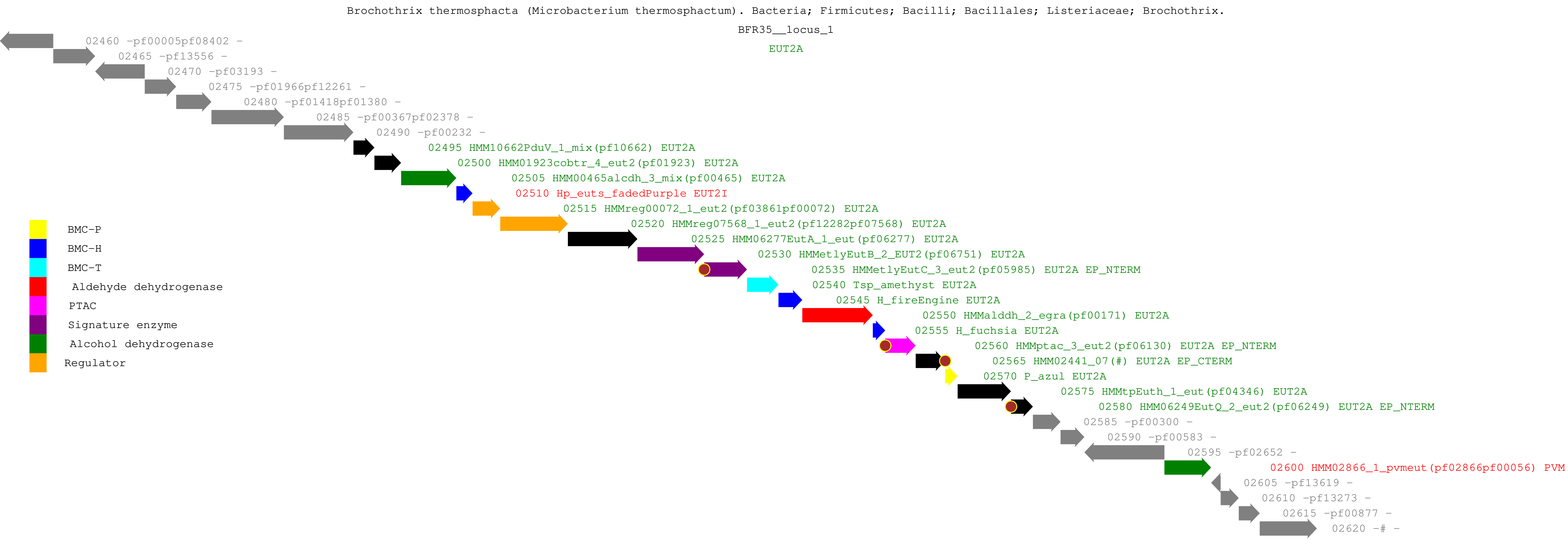
Brochothrix thermosphacta (Microbacterium thermosphactum). Bacteria; Firmicutes; Bacilli; Bacillales; Listeriaceae; Brochothrix.
BFR35__locus_1
EUT2A
02460 -pf00005pf08402 -
02465 -pf13556 -
02470 -pf03193 -
02475 -pf01966pf12261 -
02480 -pf01418pf01380 -
02485 -pf00367pf02378 -
02490 -pf00232 -
02495 HMM10662PduV_1_mix(pf10662) EUT2A
02500 HMM01923cobtr_4_eut2(pf01923) EUT2A
02505 HMM00465alcdh_3_mix(pf00465) EUT2A
02510 Hp_euts_fadedPurple EUT2I
02515 HMMreg00072_1_eut2(pf03861pf00072) EUT2A
02520 HMMreg07568_1_eut2(pf12282pf07568) EUT2A
02525 HMM06277EutA_1_eut(pf06277) EUT2A
02530 HMMetlyEutB_2_EUT2(pf06751) EUT2A
02535 HMMetlyEutC_3_eut2(pf05985) EUT2A EP_NTERM
02540 Tsp_amethyst EUT2A
02545 H_fireEngine EUT2A
02550 HMMalddh_2_egra(pf00171) EUT2A
02555 H_fuchsia EUT2A
02560 HMMptac_3_eut2(pf06130) EUT2A EP_NTERM
02565 HMM02441_07(#) EUT2A EP_CTERM
02570 P_azul EUT2A
02575 HMMtpEuth_1_eut(pf04346) EUT2A
02580 HMM06249EutQ_2_eut2(pf06249) EUT2A EP_NTERM
02585 -pf00300 -
02590 -pf00583 -
02595 -pf02652 -
02600 HMM02866_1_pvmeut(pf02866pf00056) PVM
02605 -pf13619 -
02610 -pf13273 -
02615 -pf00877 -
02620 -# -
BMC-P
BMC-H
BMC-T
Aldehyde dehydrogenase
PTAC
Signature enzyme
Alcohol dehydrogenase
Regulator

Brochothrix thermosphacta (Microbacterium thermosphactum). Bacteria; Firmicutes; Bacilli; Bacillales; Listeriaceae; Brochothrix.

BFR40__locus_1

EUT2A

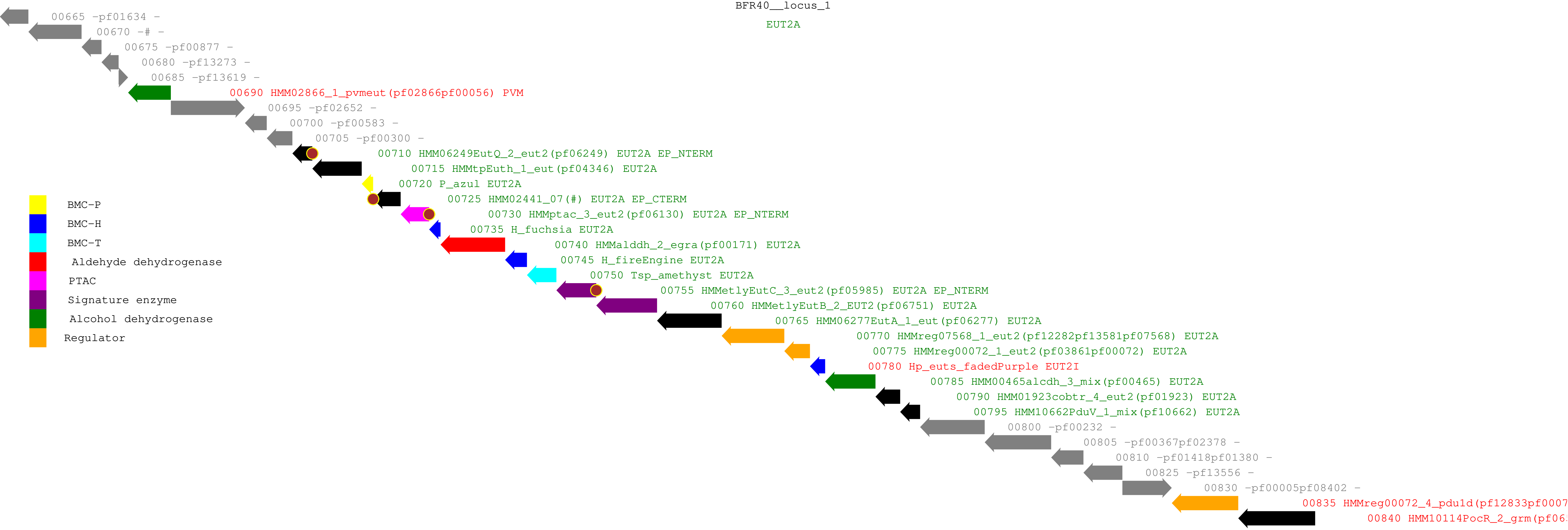

Brochothrix thermosphacta (Microbacterium thermosphactum). Bacteria; Firmicutes; Bacilli; Bacillales; Listeriaceae; Brochothrix.

BFR41__locus_1

EUT2A

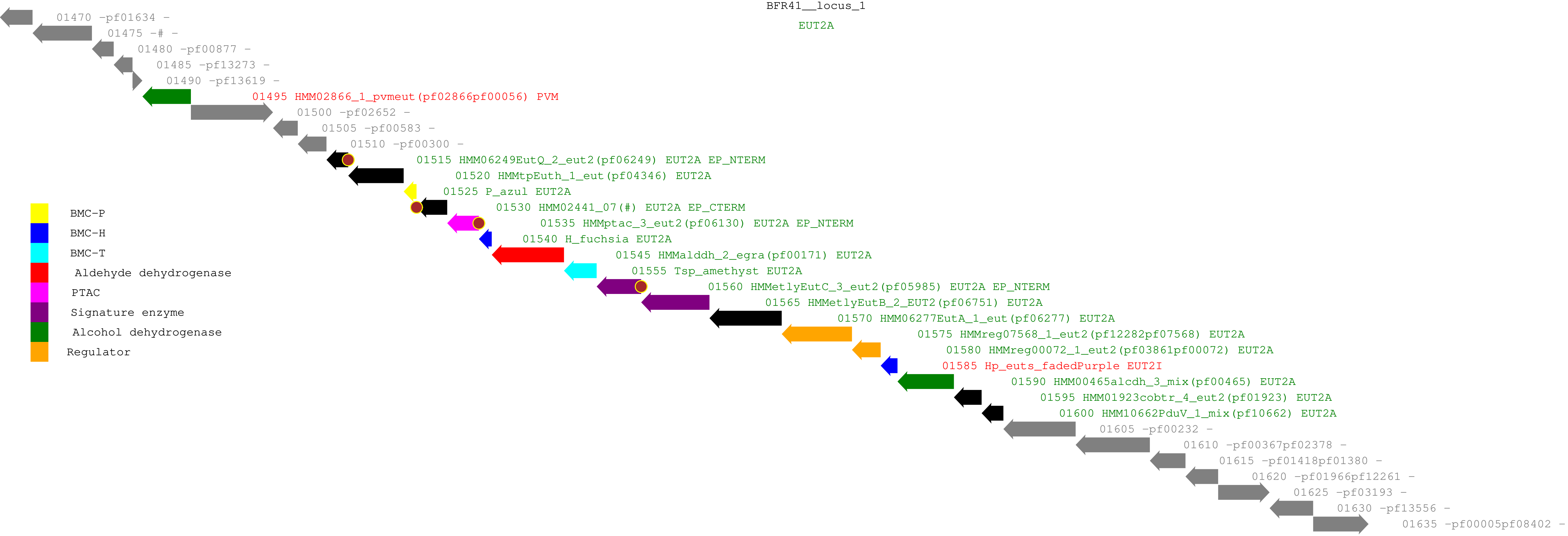

Brochothrix thermosphacta (Microbacterium thermosphactum). Bacteria; Firmicutes; Bacilli; Bacillales; Listeriaceae; Brochothrix.

BFR44__locus_1

EUT2A

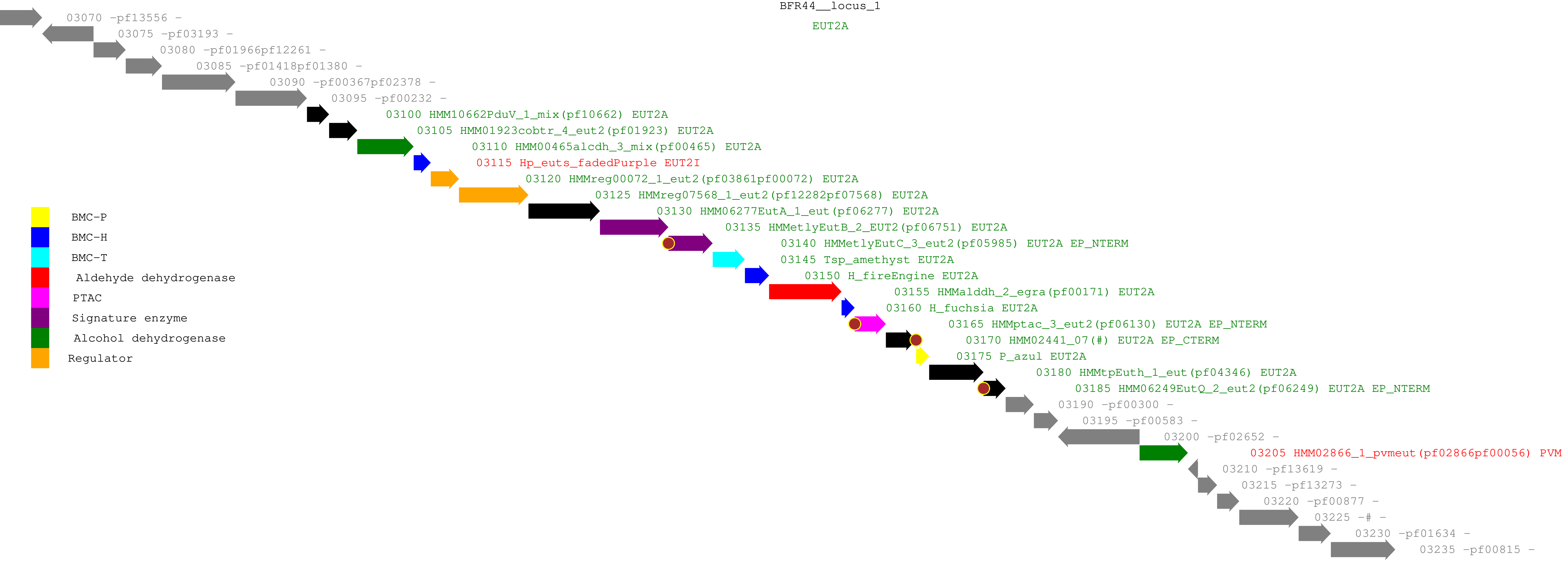

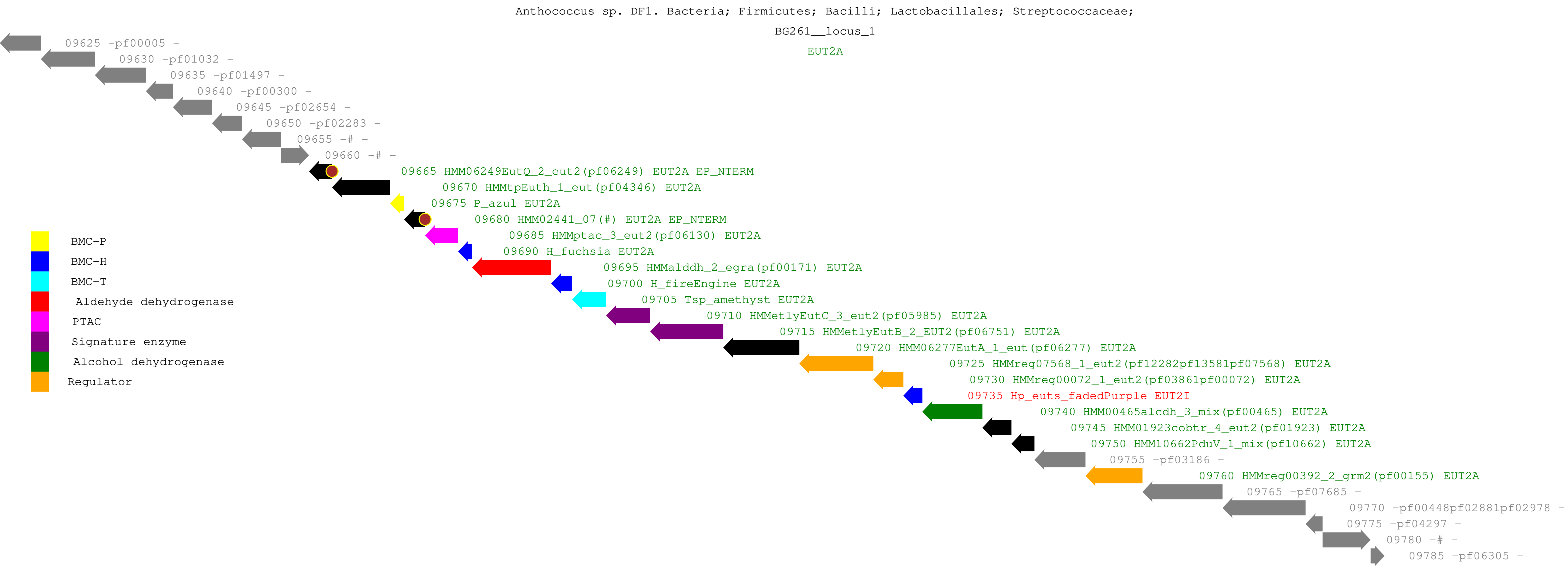

Anthococcus sp. DF1. Bacteria; Firmicutes; Bacilli; Lactobacillales; Streptococcaceae;
BG261__locus_1
EUT2A
09625 -pf00005 -
09630 -pf01032 -
09635 -pf01497 -
09640 -pf00300 -
09645 -pf02654 -
09650 -pf02283 -
09655 -# -
09660 -# -
09665 HMM06249EutQ_2_eut2(pf06249) EUT2A EP_NTERM
09670 HMMtpEuth_1_eut(pf04346) EUT2A
09675 P_azul EUT2A
09680 HMM02441_07(#) EUT2A EP_NTERM
09685 HMMptac_3_eut2(pf06130) EUT2A
09690 H_fuchsia EUT2A
09695 HMMalddh_2_egra(pf00171) EUT2A
09700 H_fireEngine EUT2A
09705 Tsp_amethyst EUT2A
09710 HMMetlyEutC_3_eut2(pf05985) EUT2A
09715 HMMetlyEutB_2_EUT2(pf06751) EUT2A
09720 HMM06277EutA_1_eut(pf06277) EUT2A
09725 HMMreg07568_1_eut2(pf12282pf13581pf07568) EUT2A
09730 HMMreg00072_1_eut2(pf03861pf00072) EUT2A
09735 Hp_euts_fadedPurple EUT2I
09740 HMM00465alcdh_3_mix(pf00465) EUT2A
09745 HMM01923cobtr_4_eut2(pf01923) EUT2A
09750 HMM10662PduV_1_mix(pf10662) EUT2A
09755 -pf03186 -
09760 HMMreg00392_2_grm2(pf00155) EUT2A
09765 -pf07685 -
09770 -pf00448pf02881pf02978 -
09775 -pf04297 -
09780 -# -
09785 -pf06305 -
BMC-P
BMC-H
BMC-T
Aldehyde dehydrogenase
PTAC
Signature enzyme
Alcohol dehydrogenase
Regulator

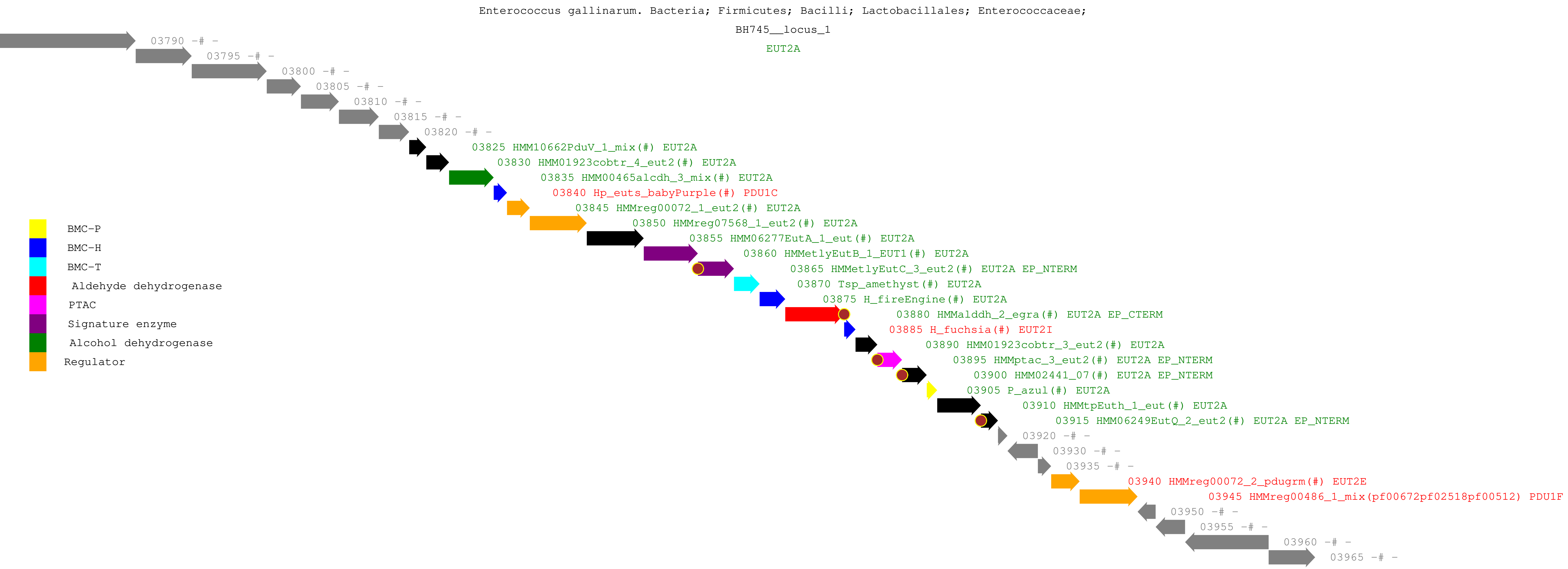

Enterococcus gallinarum. Bacteria; Firmicutes; Bacilli; Lactobacillales; Enterococcaceae;
BH745__locus_1
EUT2A
03790 -# -
03795 -# -
03800 -# -
03805 -# -
03810 -# -
03815 -# -
03820 -# -
03825 HMM10662PduV_1_mix(#) EUT2A
03830 HMM01923cobtr_4_eut2(#) EUT2A
03835 HMM00465alcdh_3_mix(#) EUT2A
03840 Hp_euts_babyPurple(#) PDU1C
03845 HMMreg00072_1_eut2(#) EUT2A
03850 HMMreg07568_1_eut2(#) EUT2A
03855 HMM06277EutA_1_eut(#) EUT2A
03860 HMMetlyEutB_1_EUT1(#) EUT2A
03865 HMMetlyEutC_3_eut2(#) EUT2A EP_NTERM
03870 Tsp_amethyst(#) EUT2A
03875 H_fireEngine(#) EUT2A
03880 HMMalddh_2_egra(#) EUT2A EP_CTERM
03885 H_fuchsia(#) EUT2I
03890 HMM01923cobtr_3_eut2(#) EUT2A
03895 HMMptac_3_eut2(#) EUT2A EP_NTERM
03900 HMM02441_07(#) EUT2A EP_NTERM
03905 P_azul(#) EUT2A
03910 HMMtpEuth_1_eut(#) EUT2A
03915 HMM06249EutQ_2_eut2(#) EUT2A EP_NTERM
03920 -# -
03930 -# -
03935 -# -
03940 HMMreg00072_2_pdugrm(#) EUT2E
03945 HMMreg00486_1_mix(pf00672pf02518pf00512) PDU1F
03950 -# -
03955 -# -
03960 -# -
03965 -# -
BMC-P
BMC-H
BMC-T
Aldehyde dehydrogenase
PTAC
Signature enzyme
Alcohol dehydrogenase
Regulator

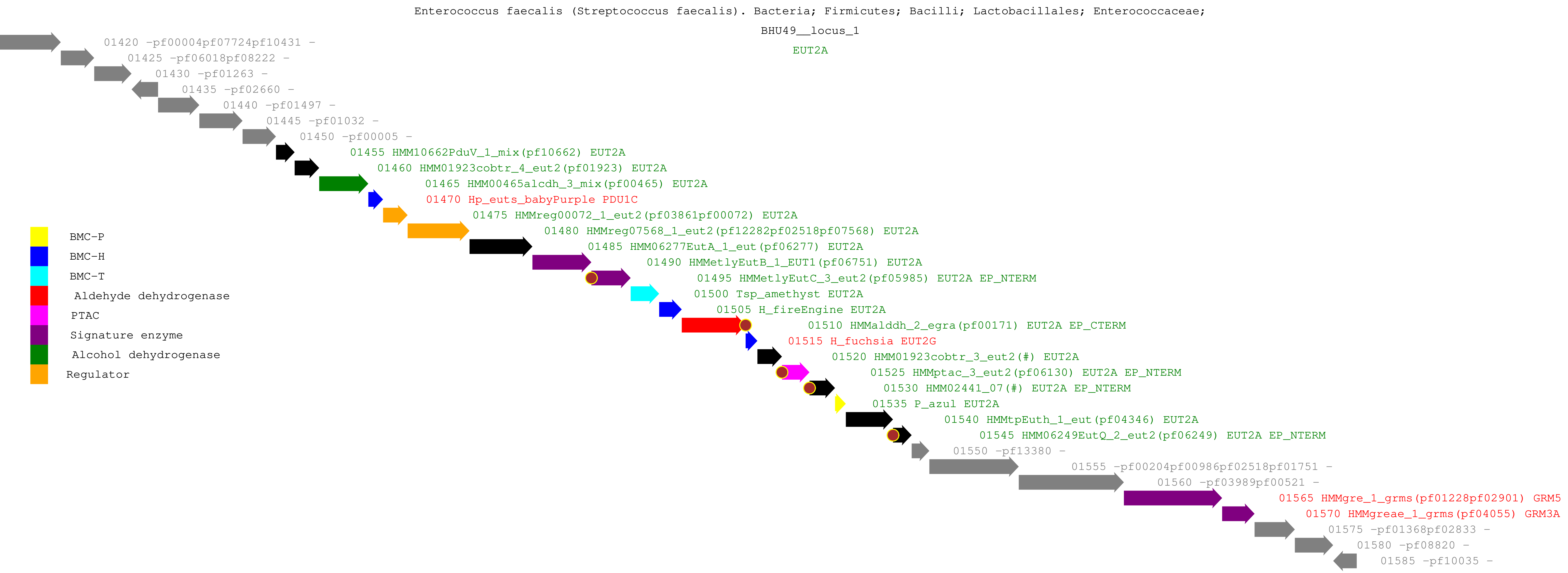
Enterococcus faecalis (Streptococcus faecalis). Bacteria; Firmicutes; Bacilli; Lactobacillales; Enterococcaceae;
BHU49__locus_1
EUT2A
01420 -pf00004pf07724pf10431 -
01425 -pf06018pf08222 -
01430 -pf01263 -
01435 -pf02660 -
01440 -pf01497 -
01445 -pf01032 -
01450 -pf00005 -
01455 HMM10662PduV_1_mix(pf10662) EUT2A
01460 HMM01923cobtr_4_eut2(pf01923) EUT2A
01465 HMM00465alcdh_3_mix(pf00465) EUT2A
01470 Hp_euts_babyPurple PDU1C
01475 HMMreg00072_1_eut2(pf03861pf00072) EUT2A
01480 HMMreg07568_1_eut2(pf12282pf02518pf07568) EUT2A
01485 HMM06277EutA_1_eut(pf06277) EUT2A
01490 HMMetlyEutB_1_EUT1(pf06751) EUT2A
01495 HMMetlyEutC_3_eut2(pf05985) EUT2A EP_NTERM
01500 Tsp_amethyst EUT2A
01505 H_fireEngine EUT2A
01510 HMMaldh_2_egra(pf00171) EUT2A EP_CTERM
01515 H_fuchsia EUT2G
01520 HMM01923cobtr_3_eut2(#) EUT2A
01525 HMMptac_3_eut2(pf06130) EUT2A EP_NTERM
01530 HMM02441_07(#) EUT2A EP_NTERM
01535 P_azul EUT2A
01540 HMMtpEuth_1_eut(pf04346) EUT2A
01545 HMM06249EutQ_2_eut2(pf06249) EUT2A EP_NTERM
01550 -pf13380 -
01555 -pf00204pf00986pf02518pf01751 -
01560 -pf03989pf00521 -
01565 HMMgre_1_grms(pf01228pf02901) GRM5
01570 HMMgreae_1_grms(pf04055) GRM3A
01575 -pf01368pf02833 -
01580 -pf08820 -
01585 -pf10035 -
BMC-P
BMC-H
BMC-T
Aldehyde dehydrogenase
PTAC
Signature enzyme
Alcohol dehydrogenase
Regulator

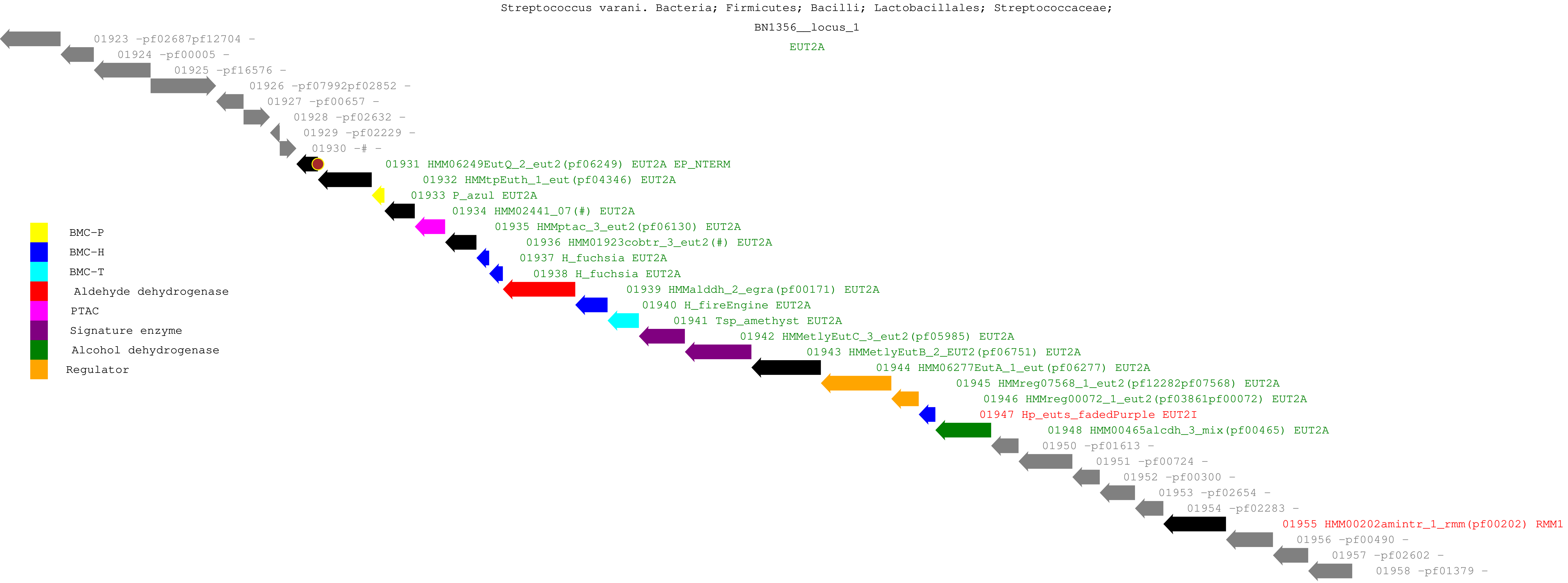

Streptococcus varani. Bacteria; Firmicutes; Bacilli; Lactobacillales; Streptococcaceae;
BN1356__locus_1
EUT2A
01923 -pf02687pf12704 -
01924 -pf00005 -
01925 -pf16576 -
01926 -pf07992pf02852 -
01927 -pf00657 -
01928 -pf02632 -
01929 -pf02229 -
01930 -# -
01931 HMM06249EutQ_2_eut2(pf06249) EUT2A EP_NTERM
01932 HMMtpEuth_1_eut(pf04346) EUT2A
01933 P_azul EUT2A
01934 HMM02441_07(#) EUT2A
01935 HMMptac_3_eut2(pf06130) EUT2A
01936 HMM01923cobtr_3_eut2(#) EUT2A
01937 H_fuchsia EUT2A
01938 H_fuchsia EUT2A
01939 HMMalddh_2_egra(pf00171) EUT2A
01940 H_fireEngine EUT2A
01941 Tsp_amethyst EUT2A
01942 HMMetlyEutC_3_eut2(pf05985) EUT2A
01943 HMMetlyEutB_2_EUT2(pf06751) EUT2A
01944 HMM06277EutA_1_eut(pf06277) EUT2A
01945 HMMreg07568_1_eut2(pf12282pf07568) EUT2A
01946 HMMreg00072_1_eut2(pf03861pf00072) EUT2A
01947 Hp_euts_fadedPurple EUT2I
01948 HMM00465alcdh_3_mix(pf00465) EUT2A
01950 -pf01613 -
01951 -pf00724 -
01952 -pf00300 -
01953 -pf02654 -
01954 -pf02283 -
01955 HMM00202amintr_1_rmm(pf00202) RMM1
01956 -pf00490 -
01957 -pf02602 -
01958 -pf01379 -
BMC-P
BMC-H
BMC-T
Aldehyde dehydrogenase
PTAC
Signature enzyme
Alcohol dehydrogenase
Regulator

Clostridium sp. C105KSO15. Bacteria; Firmicutes; Clostridia; Clostridiales; Clostridiaceae;

BN3590__locus_1

EUT2A

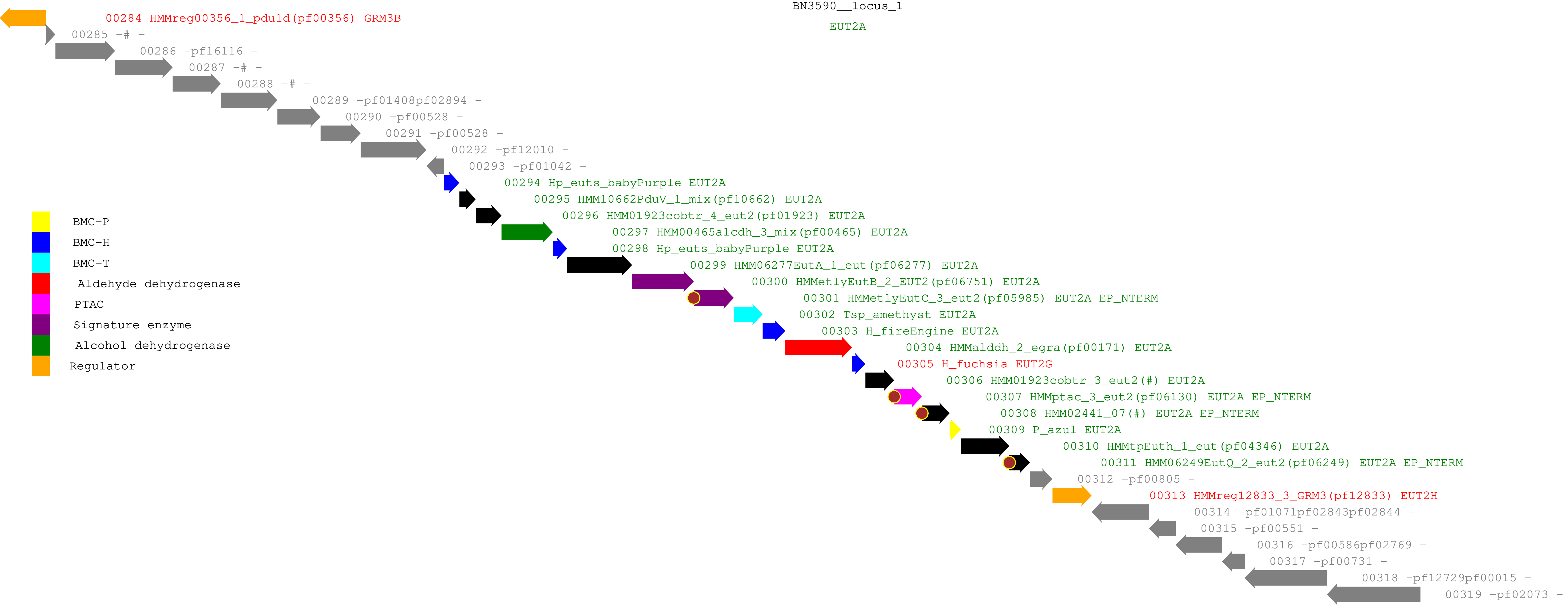

Carnobacterium maltaromaticum LMA28. Bacteria; Firmicutes; Bacilli; Lactobacillales; Carnobacteriaceae;

BN424__locus_1

EUT2A

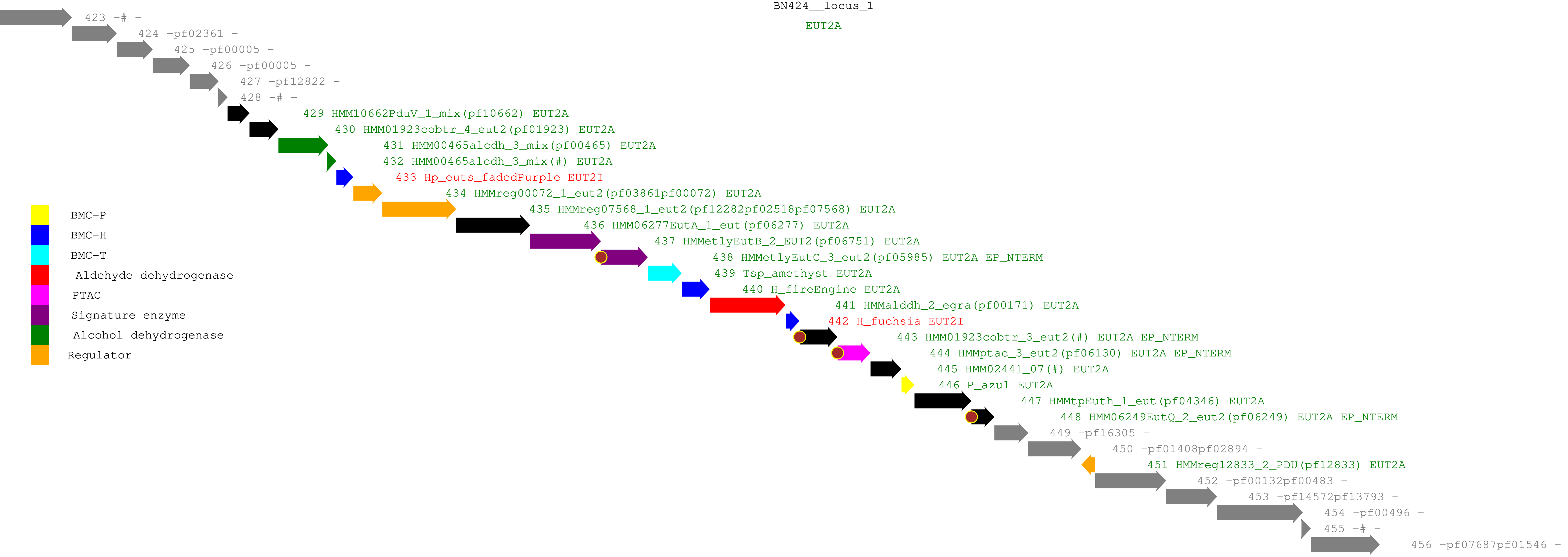

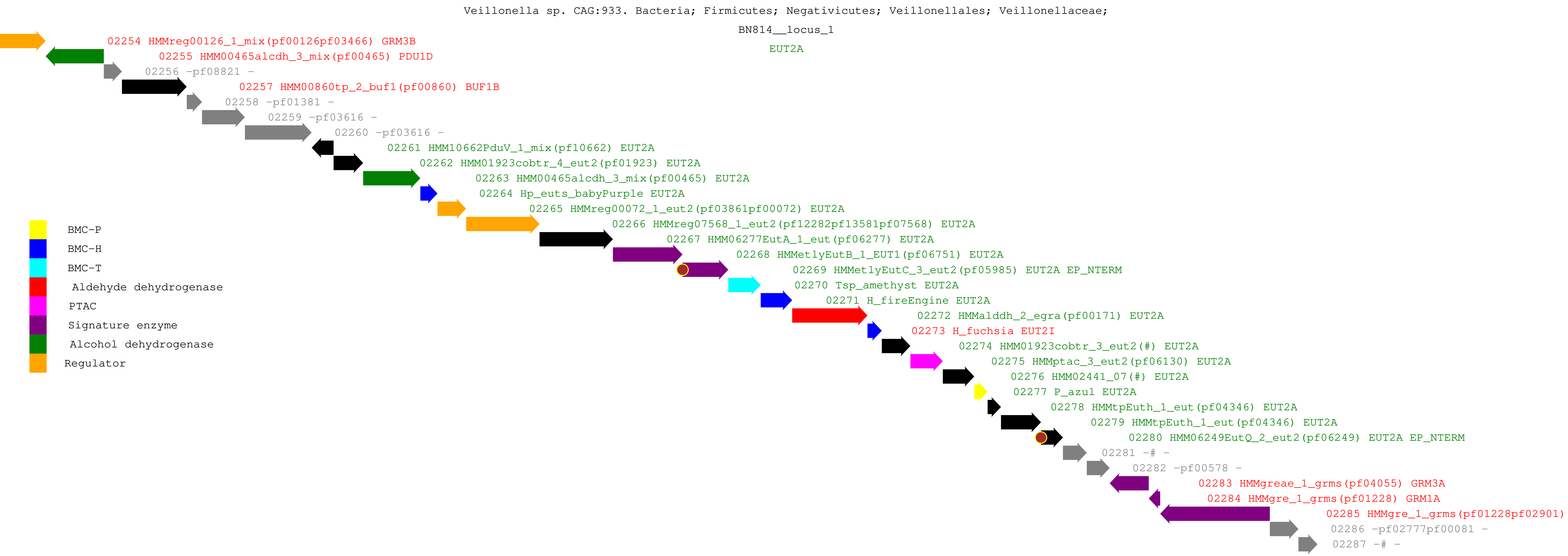
Veillonella sp. CAG:933. Bacteria; Firmicutes; Negativicutes; Veillonellales; Veillonellaceae;
BN814__locus_1
EUT2A
02254 HMMreg00126_1_mix(pf00126pf03466) GRM3B
02255 HMM00465alcdh_3_mix(pf00465) PDU1D
02256 -pf08821 -
02257 HMM00860tp_2_buf1(pf00860) BUF1B
02258 -pf01381 -
02259 -pf03616 -
02260 -pf03616 -
02261 HMM10662PduV_1_mix(pf10662) EUT2A
02262 HMM01923cobtr_4_eut2(pf01923) EUT2A
02263 HMM00465alcdh_3_mix(pf00465) EUT2A
02264 Hp_euts_babyPurple EUT2A
02265 HMMreg00072_1_eut2(pf03861pf00072) EUT2A
02266 HMMreg07568_1_eut2(pf12282pf13581pf07568) EUT2A
02267 HMM06277EutA_1_eut(pf06277) EUT2A
02268 HMMetlyEutB_1_EUT1(pf06751) EUT2A
02269 HMMetlyEutC_3_eut2(pf05985) EUT2A EP_NTERM
02270 Tsp_amethyst EUT2A
02271 H_fireEngine EUT2A
02272 HMMalddh_2_egra(pf00171) EUT2A
02273 H_fuchsia EUT2I
02274 HMM01923cobtr_3_eut2(#) EUT2A
02275 HMMptac_3_eut2(pf06130) EUT2A
02276 HMM02441_07(#) EUT2A
02277 P_azul EUT2A
02278 HMMtpEuth_1_eut(pf04346) EUT2A
02279 HMMtpEuth_1_eut(pf04346) EUT2A
02280 HMM06249EutQ_2_eut2(pf06249) EUT2A EP_NTERM
02281 -# -
02282 -pf00578 -
02283 HMMgreae_1_grms(pf04055) GRM3A
02284 HMMgre_1_grms(pf01228) GRM1A
02285 HMMgre_1_grms(pf01228pf02901) GRM1A
02286 -pf02777pf00081 -
02287 -# -
BMC-P
BMC-H
BMC-T
Aldehyde dehydrogenase
PTAC
Signature enzyme
Alcohol dehydrogenase
Regulator

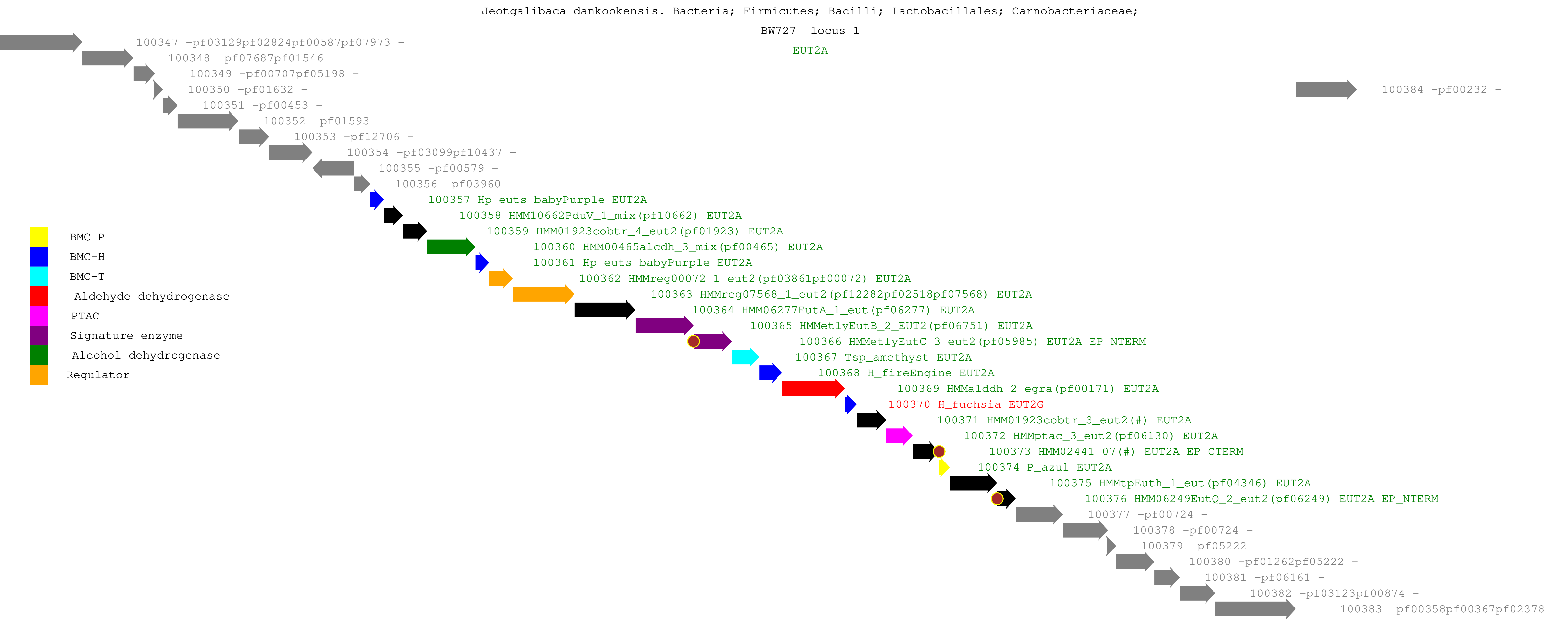

Jeotgalibaca dankookensis. Bacteria; Firmicutes; Bacilli; Lactobacillales; Carnobacteriaceae;
BW727__locus_1
EUT2A
100347 -pf03129pf02824pf00587pf07973 -
100348 -pf07687pf01546 -
100349 -pf00707pf05198 -
100350 -pf01632 -
100351 -pf00453 -
100352 -pf01593 -
100353 -pf12706 -
100354 -pf03099pf10437 -
100355 -pf00579 -
100356 -pf03960 -
100357 Hp_euts_babyPurple EUT2A
100358 HMM10662PduV_1_mix(pf10662) EUT2A
100359 HMM01923cobtr_4_eut2(pf01923) EUT2A
100360 HMM00465alcdh_3_mix(pf00465) EUT2A
100361 Hp_euts_babyPurple EUT2A
100362 HMMreg00072_1_eut2(pf03861pf00072) EUT2A
100363 HMMreg07568_1_eut2(pf12282pf02518pf07568) EUT2A
100364 HMM06277EutA_1_eut(pf06277) EUT2A
100365 HMMetlyEutB_2_EUT2(pf06751) EUT2A
100366 HMMetlyEutC_3_eut2(pf05985) EUT2A EP_NTERM
100367 Tsp_amethyst EUT2A
100368 H_fireEngine EUT2A
100369 HMMalddh_2_egra(pf00171) EUT2A
100370 H_fuchsia EUT2G
100371 HMM01923cobtr_3_eut2(#) EUT2A
100372 HMMptac_3_eut2(pf06130) EUT2A
100373 HMM02441_07(#) EUT2A EP_CTERM
100374 P_azul EUT2A
100375 HMMtpEuth_1_eut(pf04346) EUT2A
100376 HMM06249EutQ_2_eut2(pf06249) EUT2A EP_NTERM
100377 -pf00724 -
100378 -pf00724 -
100379 -pf05222 -
100380 -pf01262pf05222 -
100381 -pf06161 -
100382 -pf03123pf00874 -
100383 -pf00358pf00367pf02378 -
100384 -pf00232 -
BMC-P
BMC-H
BMC-T
Aldehyde dehydrogenase
PTAC
Signature enzyme
Alcohol dehydrogenase
Regulator

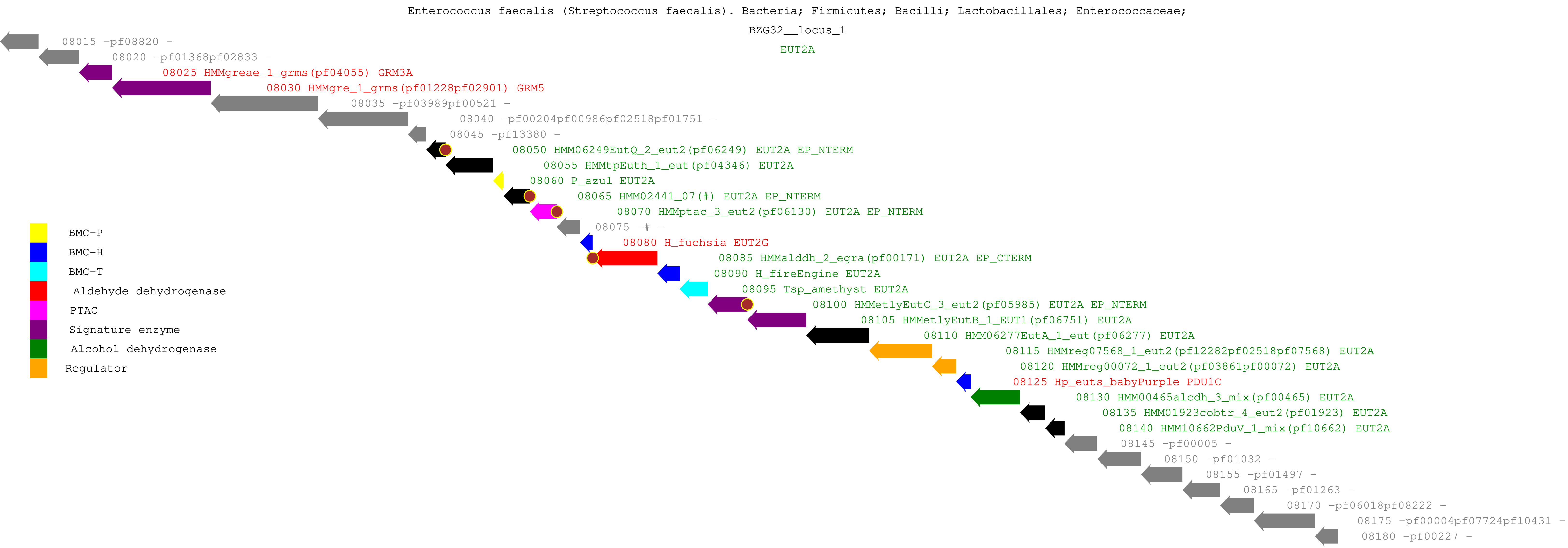

Enterococcus faecalis (Streptococcus faecalis). Bacteria; Firmicutes; Bacilli; Lactobacillales; Enterococcaceae;
BZG32__locus_1
EUT2A
08015 -pf08820 -
08020 -pf01368pf02833 -
08025 HMMgreae_1_grms(pf04055) GRM3A
08030 HMMgre_1_grms(pf01228pf02901) GRM5
08035 -pf03989pf00521 -
08040 -pf00204pf00986pf02518pf01751 -
08045 -pf13380 -
08050 HMM06249EutQ_2_eut2(pf06249) EUT2A EP_NTERM
08055 HMMtpEuth_1_eut(pf04346) EUT2A
08060 P_azul EUT2A
08065 HMM02441_07(#) EUT2A EP_NTERM
08070 HMMptac_3_eut2(pf06130) EUT2A EP_NTERM
08075 -# -
08080 H_fuchsia EUT2G
08085 HMMalddh_2_egra(pf00171) EUT2A EP_CTERM
08090 H_fireEngine EUT2A
08095 Tsp_amethyst EUT2A
08100 HMMetlyEutC_3_eut2(pf05985) EUT2A EP_NTERM
08105 HMMetlyEutB_1_EUT1(pf06751) EUT2A
08110 HMM06277EutA_1_eut(pf06277) EUT2A
08115 HMMreg07568_1_eut2(pf12282pf02518pf07568) EUT2A
08120 HMMreg00072_1_eut2(pf03861pf00072) EUT2A
08125 Hp_euts_babyPurple PDU1C
08130 HMM00465alcdh_3_mix(pf00465) EUT2A
08135 HMM01923cobtr_4_eut2(pf01923) EUT2A
08140 HMM10662PduV_1_mix(pf10662) EUT2A
08145 -pf00005 -
08150 -pf01032 -
08155 -pf01497 -
08165 -pf01263 -
08170 -pf06018pf08222 -
08175 -pf00004pf07724pf10431 -
08180 -pf00227 -
BMC-P
BMC-H
BMC-T
Aldehyde dehydrogenase
PTAC
Signature enzyme
Alcohol dehydrogenase
Regulator

Enterococcus casseliflavus (Enterococcus flavescens). Bacteria; Firmicutes; Bacilli; Lactobacillales; Enterococcaceae;

BZK37__locus_1

EUT2A

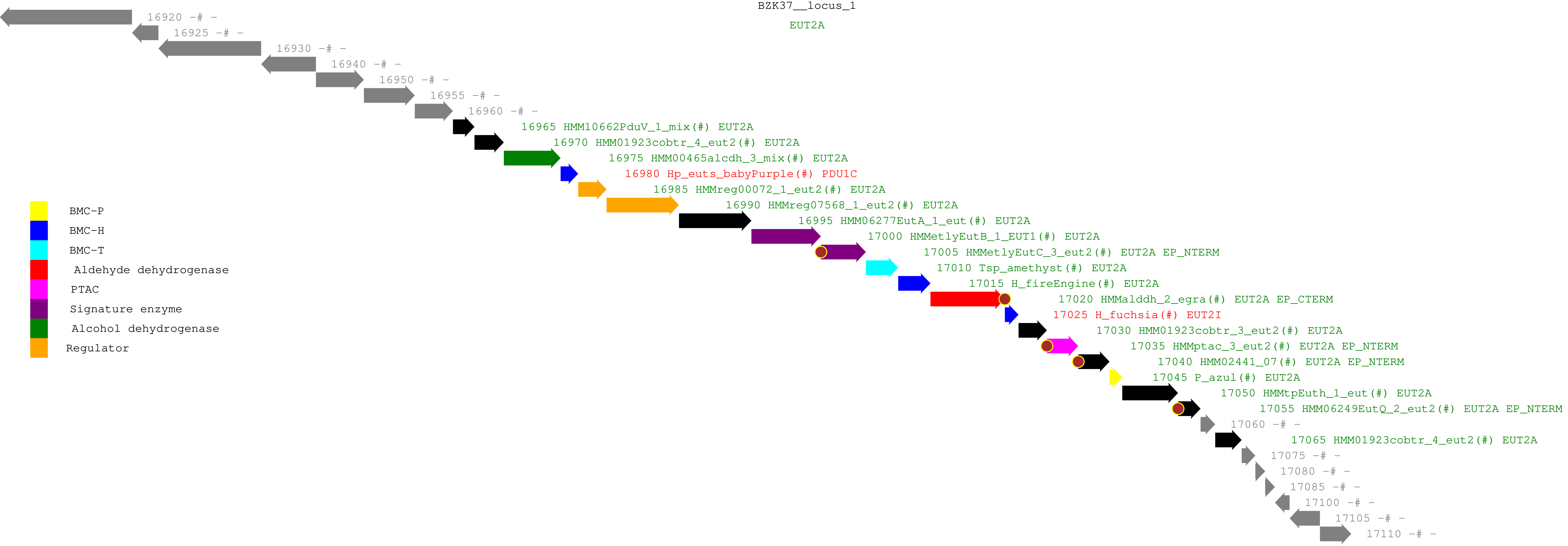

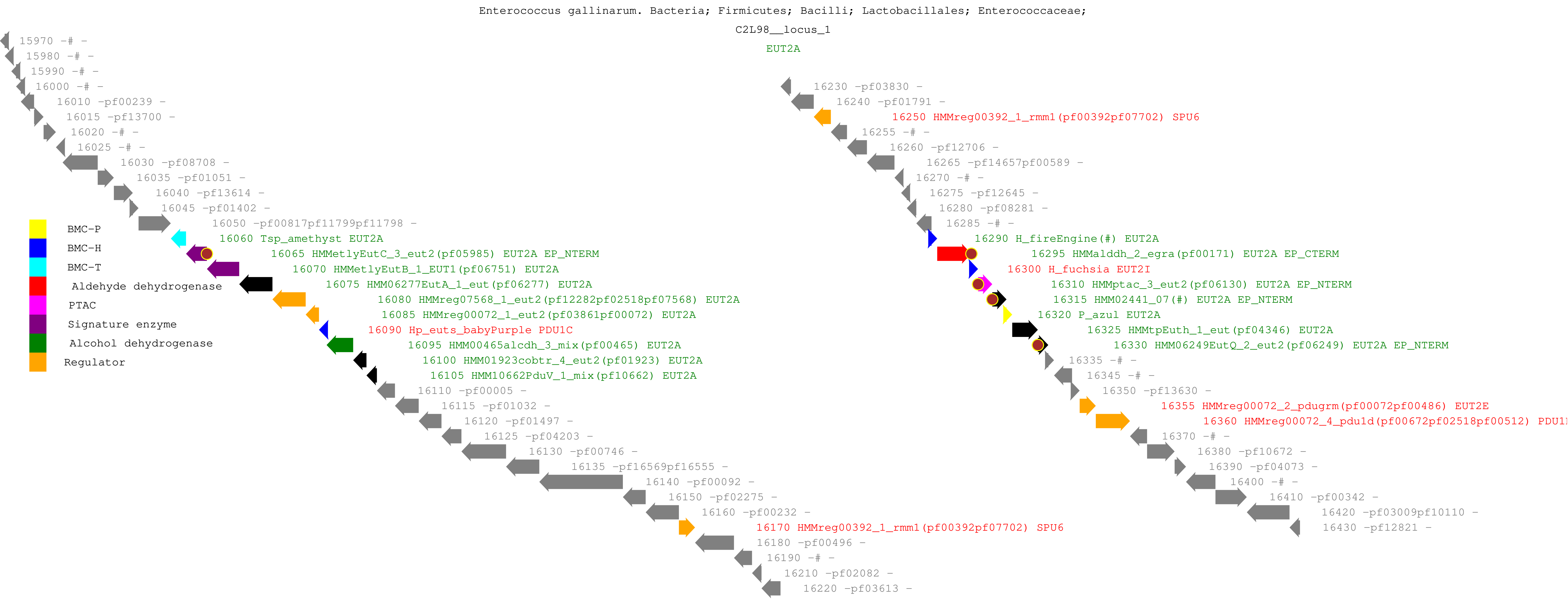

Enterococcus gallinarum. Bacteria; Firmicutes; Bacilli; Lactobacillales; Enterococcaceae;
C2L98__locus_1
EUT2A
15970 -# -
15980 -# -
15990 -# -
16000 -# -
16010 -pf00239 -
16015 -pf13700 -
16020 -# -
16025 -# -
16030 -pf08708 -
16035 -pf01051 -
16040 -pf13614 -
16045 -pf01402 -
16050 -pf00817pf11799pf11798 -
16060 Tsp_amethyst EUT2A
16065 HMMetlyEutC_3_eut2(pf05985) EUT2A EP_NTERM
16070 HMMetlyEutB_1_EUT1(pf06751) EUT2A
16075 HMM06277EutA_1_eut(pf06277) EUT2A
16080 HMMreg07568_1_eut2(pf12282pf02518pf07568) EUT2A
16085 HMMreg00072_1_eut2(pf03861pf00072) EUT2A
16090 Hp_euts_babyPurple PDU1C
16095 HMM00465alcdh_3_mix(pf00465) EUT2A
16100 HMM01923cobtr_4_eut2(pf01923) EUT2A
16105 HMM10662PduV_1_mix(pf10662) EUT2A
16110 -pf00005 -
16115 -pf01032 -
16120 -pf01497 -
16125 -pf04203 -
16130 -pf00746 -
16135 -pf16569pf16555 -
16140 -pf00092 -
16150 -pf02275 -
16160 -pf00232 -
16170 HMMreg00392_1_rmm1(pf00392pf07702) SPU6
16180 -pf00496 -
16190 -# -
16210 -pf02082 -
16220 -pf03613 -
16230 -pf03830 -
16240 -pf01791 -
16250 HMMreg00392_1_rmm1(pf00392pf07702) SPU6
16255 -# -
16260 -pf12706 -
16265 -pf14657pf00589 -
16270 -# -
16275 -pf12645 -
16280 -pf08281 -
16285 -# -
16290 H_fireEngine(#) EUT2A
16295 HMMalddh_2_egra(pf00171) EUT2A EP_CTERM
16300 H_fuchsia EUT2I
16310 HMMptac_3_eut2(pf06130) EUT2A EP_NTERM
16315 HMM02441_07(#) EUT2A EP_NTERM
16320 P_azul EUT2A
16325 HMMtpEuth_1_eut(pf04346) EUT2A
16330 HMM06249EutQ_2_eut2(pf06249) EUT2A EP_NTERM
16335 -# -
16345 -# -
16350 -pf13630 -
16355 HMMreg00072_2_pdugrm(pf00072pf00486) EUT2E
16360 HMMreg00072_4_pdu1d(pf00672pf02518pf00512) PDU1
16370 -# -
16380 -pf10672 -
16390 -pf04073 -
16400 -# -
16410 -pf00342 -
16420 -pf03009pf10110 -
16430 -pf12821 -
BMC-P
BMC-H
BMC-T
Aldehyde dehydrogenase
PTAC
Signature enzyme
Alcohol dehydrogenase
Regulator

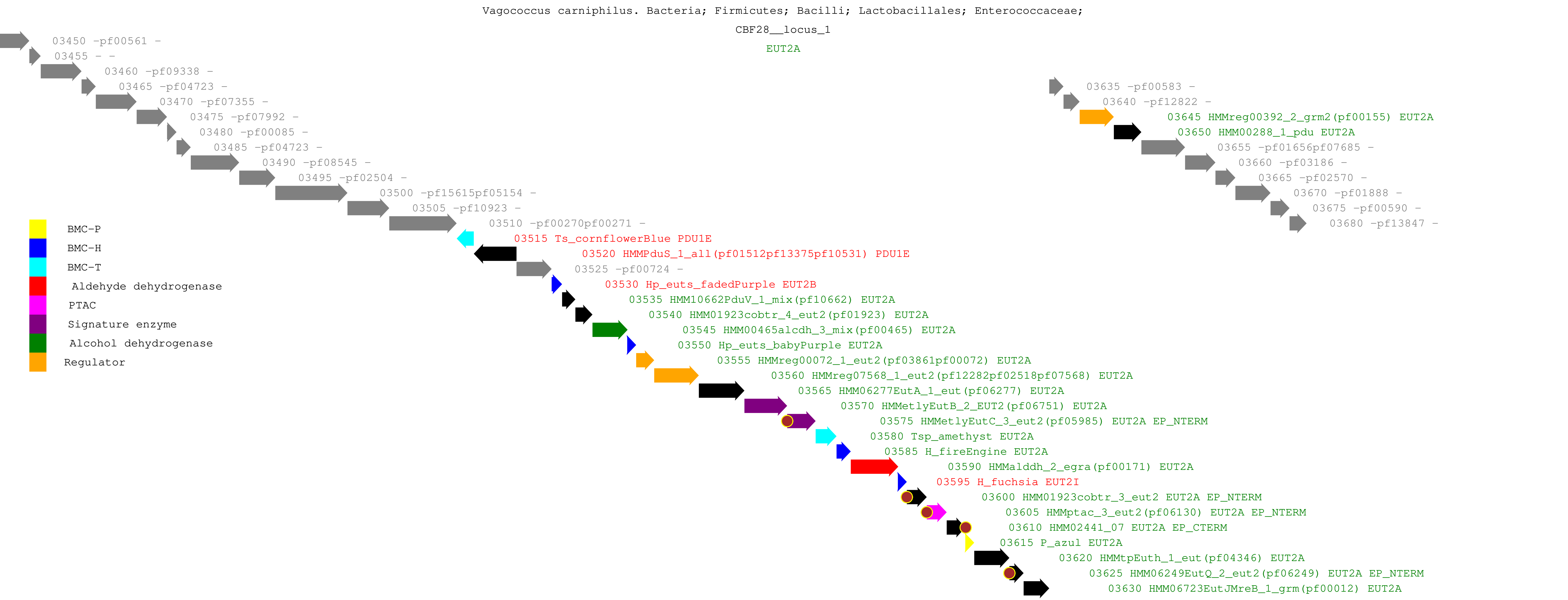
Vagococcus carniphilus. Bacteria; Firmicutes; Bacilli; Lactobacillales; Enterococcaceae;
CBF28__locus_1
EUT2A
03450 -pf00561 -
03455 - -
03460 -pf09338 -
03465 -pf04723 -
03470 -pf07355 -
03475 -pf07992 -
03480 -pf00085 -
03485 -pf04723 -
03490 -pf08545 -
03495 -pf02504 -
03500 -pf15615pf05154 -
03505 -pf10923 -
03510 -pf00270pf00271 -
03515 Ts_cornflowerBlue PDU1E
03520 HMMPduS_1_all(pf01512pf13375pf10531) PDU1E
03525 -pf00724 -
03530 Hp_euts_fadedPurple EUT2B
03535 HMM10662PduV_1_mix(pf10662) EUT2A
03540 HMM01923cobtr_4_eut2(pf01923) EUT2A
03545 HMM00465alcdh_3_mix(pf00465) EUT2A
03550 Hp_euts_babyPurple EUT2A
03555 HMMreg00072_1_eut2(pf03861pf00072) EUT2A
03560 HMMreg07568_1_eut2(pf12282pf02518pf07568) EUT2A
03565 HMM06277EutA_1_eut(pf06277) EUT2A
03570 HMMetlyEutB_2_EUT2(pf06751) EUT2A
03575 HMMetlyEutC_3_eut2(pf05985) EUT2A EP_NTERM
03580 Tsp_amethyst EUT2A
03585 H_fireEngine EUT2A
03590 HMMalddh_2_egra(pf00171) EUT2A
03595 H_fuchsia EUT2I
03600 HMM01923cobtr_3_eut2 EUT2A EP_NTERM
03605 HMMptac_3_eut2(pf06130) EUT2A EP_NTERM
03610 HMM02441_07 EUT2A EP_CTERM
03615 P_azul EUT2A
03620 HMMtpEuth_1_eut(pf04346) EUT2A
03625 HMM06249EutQ_2_eut2(pf06249) EUT2A EP_NTERM
03630 HMM06723EutJMreB_1_grm(pf00012) EUT2A
03635 -pf00583 -
03640 -pf12822 -
03645 HMMreg00392_2_grm2(pf00155) EUT2A
03650 HMM00288_1_pdu EUT2A
03655 -pf01656pf07685 -
03660 -pf03186 -
03665 -pf02570 -
03670 -pf01888 -
03675 -pf00590 -
03680 -pf13847 -
BMC-P
BMC-H
BMC-T
Aldehyde dehydrogenase
PTAC
Signature enzyme
Alcohol dehydrogenase
Regulator

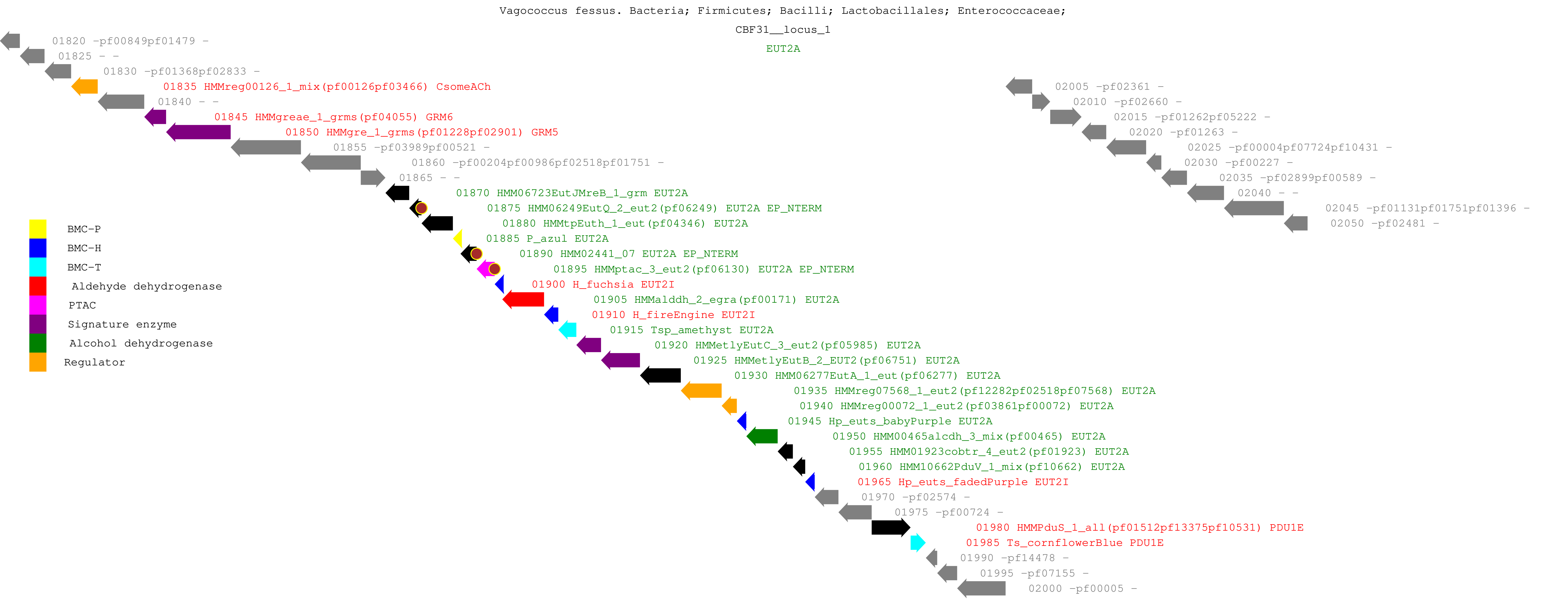

Vagococcus fessus. Bacteria; Firmicutes; Bacilli; Lactobacillales; Enterococcaceae;
CBF31__locus_1
EUT2A
01820 -pf00849pf01479 -
01825 - -
01830 -pf01368pf02833 -
01835 HMMreg00126_1_mix(pf00126pf03466) CsomeACh
01840 - -
01845 HMMgreae_1_grms(pf04055) GRM6
01850 HMMgre_1_grms(pf01228pf02901) GRM5
01855 -pf03989pf00521 -
01860 -pf00204pf00986pf02518pf01751 -
01865 - -
01870 HMM06723EutJMreB_1_grm EUT2A
01875 HMM06249EutQ_2_eut2(pf06249) EUT2A EP_NTERM
01880 HMMtpEuth_1_eut(pf04346) EUT2A
01885 P_azul EUT2A
01890 HMM02441_07 EUT2A EP_NTERM
01895 HMMptac_3_eut2(pf06130) EUT2A EP_NTERM
01900 H_fuchsia EUT2I
01905 HMMalddh_2_egra(pf00171) EUT2A
01910 H_fireEngine EUT2I
01915 Tsp_amethyst EUT2A
01920 HMMetlyEutC_3_eut2(pf05985) EUT2A
01925 HMMetlyEutB_2_EUT2(pf06751) EUT2A
01930 HMM06277EutA_1_eut(pf06277) EUT2A
01935 HMMreg07568_1_eut2(pf12282pf02518pf07568) EUT2A
01940 HMMreg00072_1_eut2(pf03861pf00072) EUT2A
01945 Hp_euts_babyPurple EUT2A
01950 HMM00465alcdh_3_mix(pf00465) EUT2A
01955 HMM01923cobtr_4_eut2(pf01923) EUT2A
01960 HMM10662PduV_1_mix(pf10662) EUT2A
01965 Hp_euts_fadedPurple EUT2I
01970 -pf02574 -
01975 -pf00724 -
01980 HMMPduS_1_all(pf01512pf13375pf10531) PDU1E
01985 Ts_cornflowerBlue PDU1E
01990 -pf14478 -
01995 -pf07155 -
02000 -pf00005 -
02005 -pf02361 -
02010 -pf02660 -
02015 -pf01262pf05222 -
02020 -pf01263 -
02025 -pf00004pf07724pf10431 -
02030 -pf00227 -
02035 -pf02899pf00589 -
02040 - -
02045 -pf01131pf01751pf01396 -
02050 -pf02481 -
BMC-P
BMC-H
BMC-T
Aldehyde dehydrogenase
PTAC
Signature enzyme
Alcohol dehydrogenase
Regulator

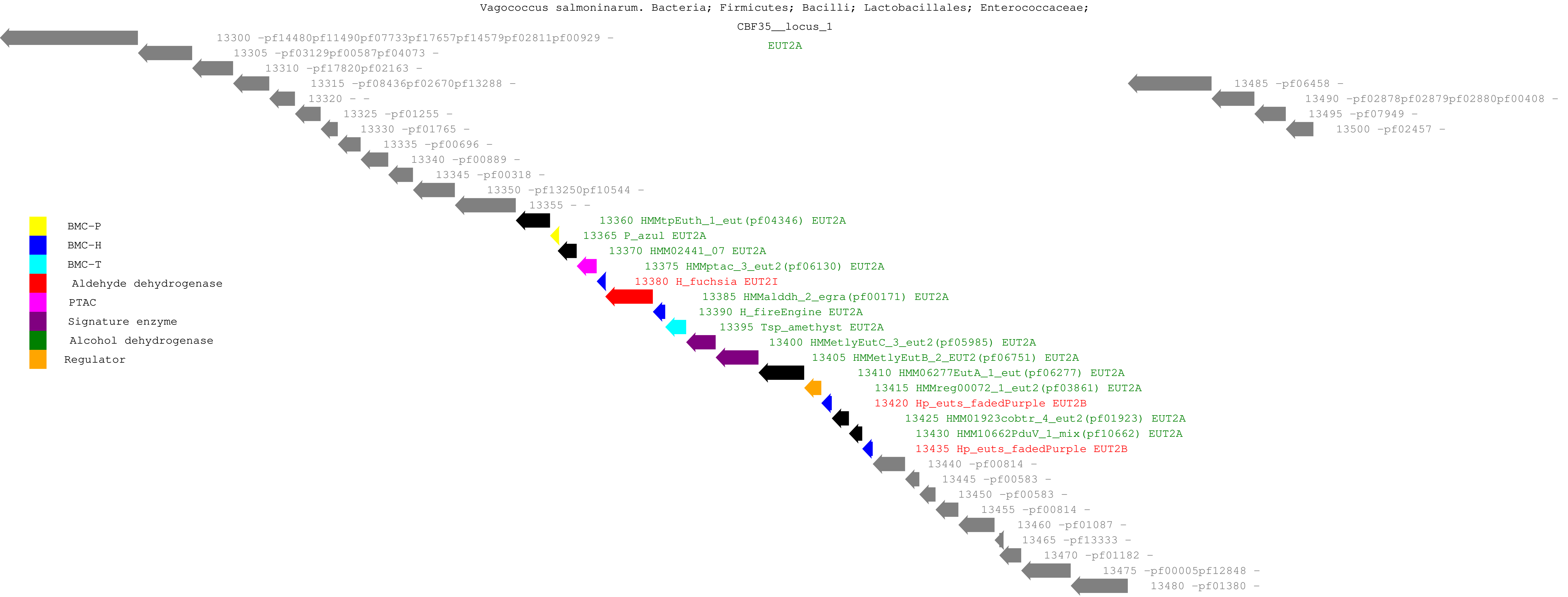
Vagococcus salmoninarum. Bacteria; Firmicutes; Bacilli; Lactobacillales; Enterococcaceae;
CBF35__locus_1
EUT2A
13300 -pf14480pf11490pf07733pf17657pf14579pf02811pf00929 -
13305 -pf03129pf00587pf04073 -
13310 -pf17820pf02163 -
13315 -pf08436pf02670pf13288 -
13320 - -
13325 -pf01255 -
13330 -pf01765 -
13335 -pf00696 -
13340 -pf00889 -
13345 -pf00318 -
13350 -pf13250pf10544 -
13355 - -
13360 HMMtpEuth_1_eut(pf04346) EUT2A
13365 P_azul EUT2A
13370 HMM02441_07 EUT2A
13375 HMMptac_3_eut2(pf06130) EUT2A
13380 H_fuchsia EUT2I
13385 HMMaldh_2_egra(pf00171) EUT2A
13390 H_fireEngine EUT2A
13395 Tsp_amethyst EUT2A
13400 HMMetlyEutC_3_eut2(pf05985) EUT2A
13405 HMMetlyEutB_2_EUT2(pf06751) EUT2A
13410 HMM06277EutA_1_eut(pf06277) EUT2A
13415 HMMreg00072_1_eut2(pf03861) EUT2A
13420 Hp_euts_fadedPurple EUT2B
13425 HMM01923cobtr_4_eut2(pf01923) EUT2A
13430 HMM10662PduV_1_mix(pf10662) EUT2A
13435 Hp_euts_fadedPurple EUT2B
13440 -pf00814 -
13445 -pf00583 -
13450 -pf00583 -
13455 -pf00814 -
13460 -pf01087 -
13465 -pf13333 -
13470 -pf01182 -
13475 -pf00005pf12848 -
13480 -pf01380 -
13485 -pf06458 -
13490 -pf02878pf02879pf02880pf00408 -
13495 -pf07949 -
13500 -pf02457 -
BMC-P
BMC-H
BMC-T
Aldehyde dehydrogenase
PTAC
Signature enzyme
Alcohol dehydrogenase
Regulator

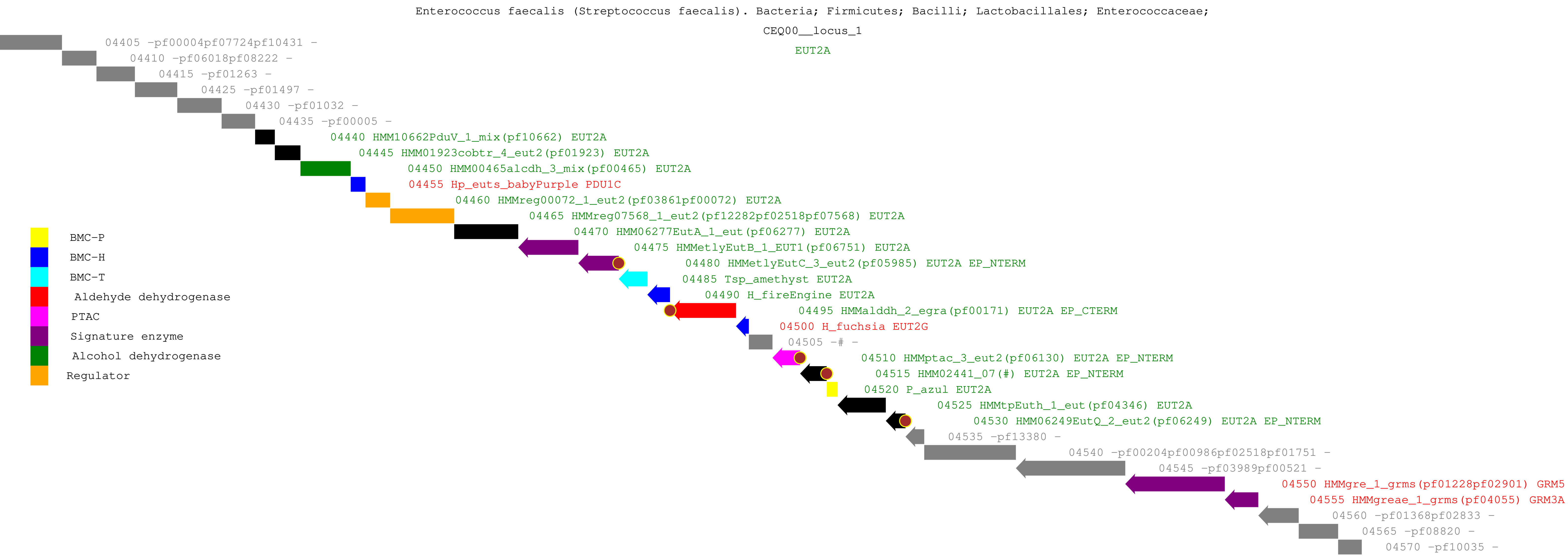
Enterococcus faecalis (Streptococcus faecalis). Bacteria; Firmicutes; Bacilli; Lactobacillales; Enterococcaceae;
CEQ00__locus_1
EUT2A
04405 -pf00004pf07724pf10431 -
04410 -pf06018pf08222 -
04415 -pf01263 -
04425 -pf01497 -
04430 -pf01032 -
04435 -pf00005 -
04440 HMM10662PduV_1_mix(pf10662) EUT2A
04445 HMM01923cobtr_4_eut2(pf01923) EUT2A
04450 HMM00465alcdh_3_mix(pf00465) EUT2A
04455 Hp_euts_babyPurple PDU1C
04460 HMMreg00072_1_eut2(pf03861pf00072) EUT2A
04465 HMMreg07568_1_eut2(pf12282pf02518pf07568) EUT2A
04470 HMM06277EutA_1_eut(pf06277) EUT2A
04475 HMMetlyEutB_1_EUT1(pf06751) EUT2A
04480 HMMetlyEutC_3_eut2(pf05985) EUT2A EP_NTERM
04485 Tsp_amethyst EUT2A
04490 H_fireEngine EUT2A
04495 HMMaldh_2_egra(pf00171) EUT2A EP_CTERM
04500 H_fuchsia EUT2G
04505 -# -
04510 HMMptac_3_eut2(pf06130) EUT2A EP_NTERM
04515 HMM02441_07(#) EUT2A EP_NTERM
04520 P_azul EUT2A
04525 HMMtpEuth_1_eut(pf04346) EUT2A
04530 HMM06249EutQ_2_eut2(pf06249) EUT2A EP_NTERM
04535 -pf13380 -
04540 -pf00204pf00986pf02518pf01751 -
04545 -pf03989pf00521 -
04550 HMMgre_1_grms(pf01228pf02901) GRM5
04555 HMMgreae_1_grms(pf04055) GRM3A
04560 -pf01368pf02833 -
04565 -pf08820 -
04570 -pf10035 -
BMC-P
BMC-H
BMC-T
Aldehyde dehydrogenase
PTAC
Signature enzyme
Alcohol dehydrogenase
Regulator

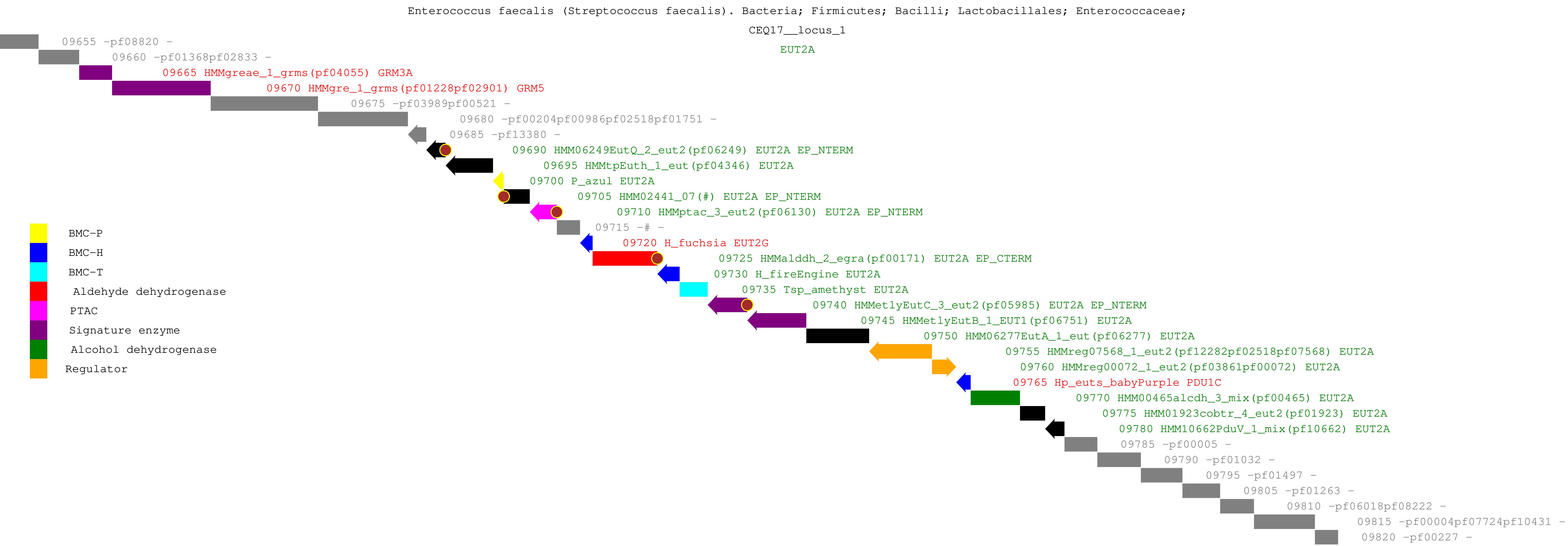

Enterococcus faecalis (Streptococcus faecalis). Bacteria; Firmicutes; Bacilli; Lactobacillales; Enterococcaceae;
CEQ17__locus_1
EUT2A
09655 -pf08820 -
09660 -pf01368pf02833 -
09665 HMMgreae_1_grms(pf04055) GRM3A
09670 HMMgre_1_grms(pf01228pf02901) GRM5
09675 -pf03989pf00521 -
09680 -pf00204pf00986pf02518pf01751 -
09685 -pf13380 -
09690 HMM06249EutQ_2_eut2(pf06249) EUT2A EP_NTERM
09695 HMMtpEuth_1_eut(pf04346) EUT2A
09700 P_azul EUT2A
09705 HMM02441_07(#) EUT2A EP_NTERM
09710 HMMptac_3_eut2(pf06130) EUT2A EP_NTERM
09715 -# -
09720 H_fuchsia EUT2G
09725 HMMalddh_2_egra(pf00171) EUT2A EP_CTERM
09730 H_fireEngine EUT2A
09735 Tsp_amethyst EUT2A
09740 HMMetlyEutC_3_eut2(pf05985) EUT2A EP_NTERM
09745 HMMetlyEutB_1_EUT1(pf06751) EUT2A
09750 HMM06277EutA_1_eut(pf06277) EUT2A
09755 HMMreg07568_1_eut2(pf12282pf02518pf07568) EUT2A
09760 HMMreg00072_1_eut2(pf03861pf00072) EUT2A
09765 Hp_euts_babyPurple PDU1C
09770 HMM00465alcdh_3_mix(pf00465) EUT2A
09775 HMM01923cobtr_4_eut2(pf01923) EUT2A
09780 HMM10662PduV_1_mix(pf10662) EUT2A
09785 -pf00005 -
09790 -pf01032 -
09795 -pf01497 -
09805 -pf01263 -
09810 -pf06018pf08222 -
09815 -pf00004pf07724pf10431 -
09820 -pf00227 -
BMC-P
BMC-H
BMC-T
Aldehyde dehydrogenase
PTAC
Signature enzyme
Alcohol dehydrogenase
Regulator

Enterococcus faecalis (Streptococcus faecalis). Bacteria; Firmicutes; Bacilli; Lactobacillales; Enterococcaceae;

CEQ25__locus_1

EUT2A

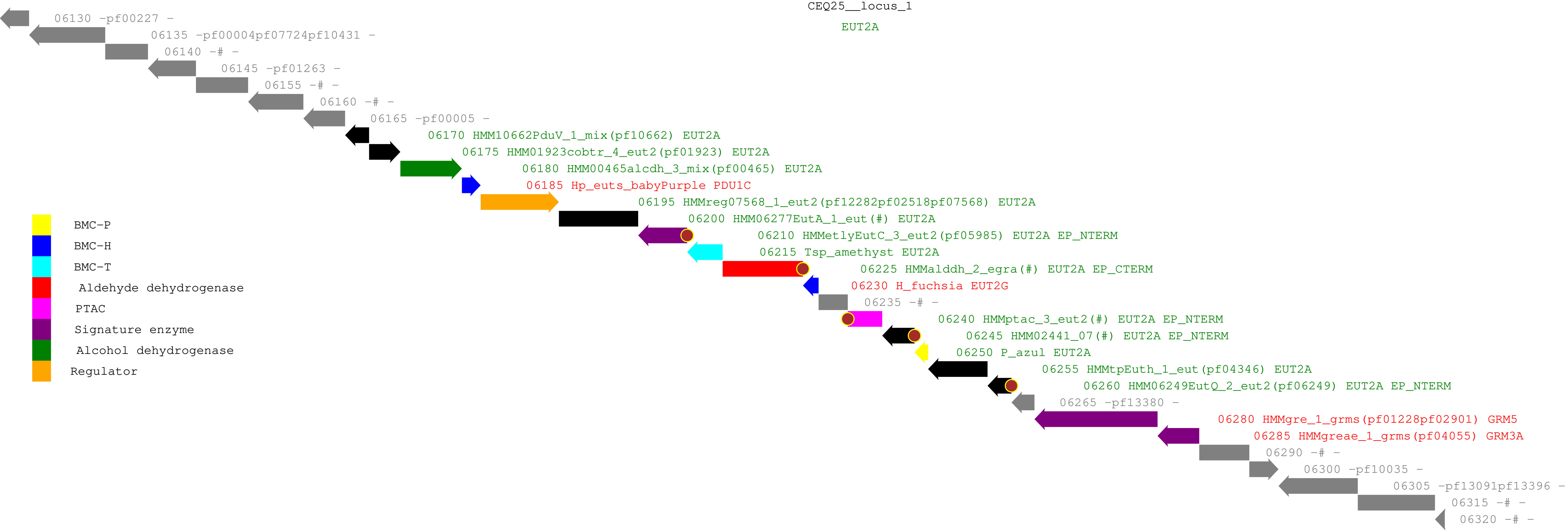

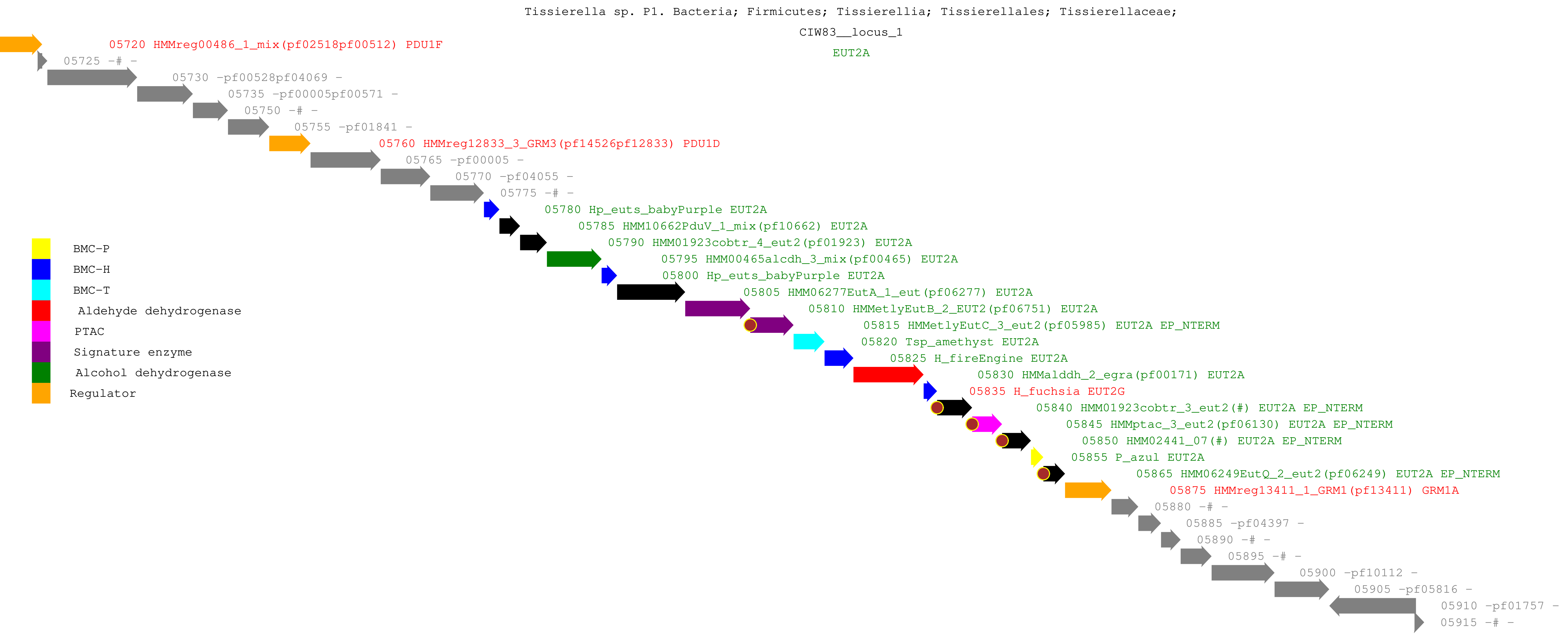
Tissierella sp. P1. Bacteria; Firmicutes; Tissierellia; Tissierellales; Tissierellaceae;
CIW83__locus_1
EUT2A
05720 HMMreg00486_1_mix(pf02518pf00512) PDU1F
05725 -# -
05730 -pf00528pf04069 -
05735 -pf00005pf00571 -
05750 -# -
05755 -pf01841 -
05760 HMMreg12833_3_GRM3(pf14526pf12833) PDU1D
05765 -pf00005 -
05770 -pf04055 -
05775 -# -
05780 Hp_euts_babyPurple EUT2A
05785 HMM10662PduV_1_mix(pf10662) EUT2A
05790 HMM01923cobtr_4_eut2(pf01923) EUT2A
05795 HMM00465alcdh_3_mix(pf00465) EUT2A
05800 Hp_euts_babyPurple EUT2A
05805 HMM06277EutA_1_eut(pf06277) EUT2A
05810 HMMetlyEutB_2_EUT2(pf06751) EUT2A
05815 HMMetlyEutC_3_eut2(pf05985) EUT2A EP_NTERM
05820 Tsp_amethyst EUT2A
05825 H_fireEngine EUT2A
05830 HMMalddh_2_egra(pf00171) EUT2A
05835 H_fuchsia EUT2G
05840 HMM01923cobtr_3_eut2(#) EUT2A EP_NTERM
05845 HMMptac_3_eut2(pf06130) EUT2A EP_NTERM
05850 HMM02441_07(#) EUT2A EP_NTERM
05855 P_azul EUT2A
05865 HMM06249EutQ_2_eut2(pf06249) EUT2A EP_NTERM
05875 HMMreg13411_1_GRM1(pf13411) GRM1A
05880 -# -
05885 -pf04397 -
05890 -# -
05895 -# -
05900 -pf10112 -
05905 -pf05816 -
05910 -pf01757 -
05915 -# -
BMC-P
BMC-H
BMC-T
Aldehyde dehydrogenase
PTAC
Signature enzyme
Alcohol dehydrogenase
Regulator

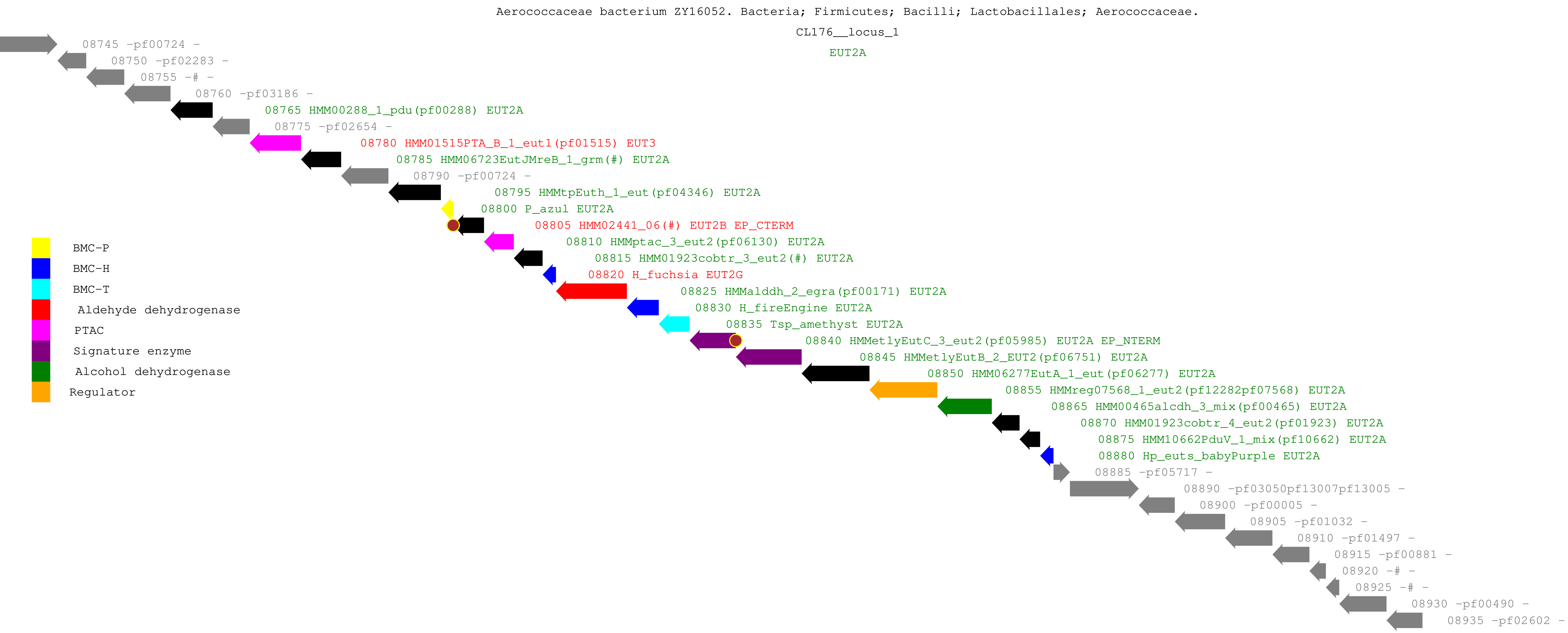

Aerococcaceae bacterium ZY16052. Bacteria; Firmicutes; Bacilli; Lactobacillales; Aerococcaceae.
CL176__locus_1
EUT2A
08745 -pf00724 -
08750 -pf02283 -
08755 -# -
08760 -pf03186 -
08765 HMM00288_1_pdu(pf00288) EUT2A
08775 -pf02654 -
08780 HMM01515PTA_B_1_eut1(pf01515) EUT3
08785 HMM06723EutJMreB_1_grm(#) EUT2A
08790 -pf00724 -
08795 HMMtpEuth_1_eut(pf04346) EUT2A
08800 P_azul EUT2A
08805 HMM02441_06(#) EUT2B EP_CTERM
08810 HMMptac_3_eut2(pf06130) EUT2A
08815 HMM01923cobtr_3_eut2(#) EUT2A
08820 H_fuchsia EUT2G
08825 HMMalddh_2_egra(pf00171) EUT2A
08830 H_fireEngine EUT2A
08835 Tsp_amethyst EUT2A
08840 HMMetlyEutC_3_eut2(pf05985) EUT2A EP_NTERM
08845 HMMetlyEutB_2_EUT2(pf06751) EUT2A
08850 HMM06277EutA_1_eut(pf06277) EUT2A
08855 HMMreg07568_1_eut2(pf12282pf07568) EUT2A
08865 HMM00465alcdh_3_mix(pf00465) EUT2A
08870 HMM01923cobtr_4_eut2(pf01923) EUT2A
08875 HMM10662PduV_1_mix(pf10662) EUT2A
08880 Hp_euts_babyPurple EUT2A
08885 -pf05717 -
08890 -pf03050pf13007pf13005 -
08900 -pf00005 -
08905 -pf01032 -
08910 -pf01497 -
08915 -pf00881 -
08920 -# -
08925 -# -
08930 -pf00490 -
08935 -pf02602 -
BMC-P
BMC-H
BMC-T
Aldehyde dehydrogenase
PTAC
Signature enzyme
Alcohol dehydrogenase
Regulator

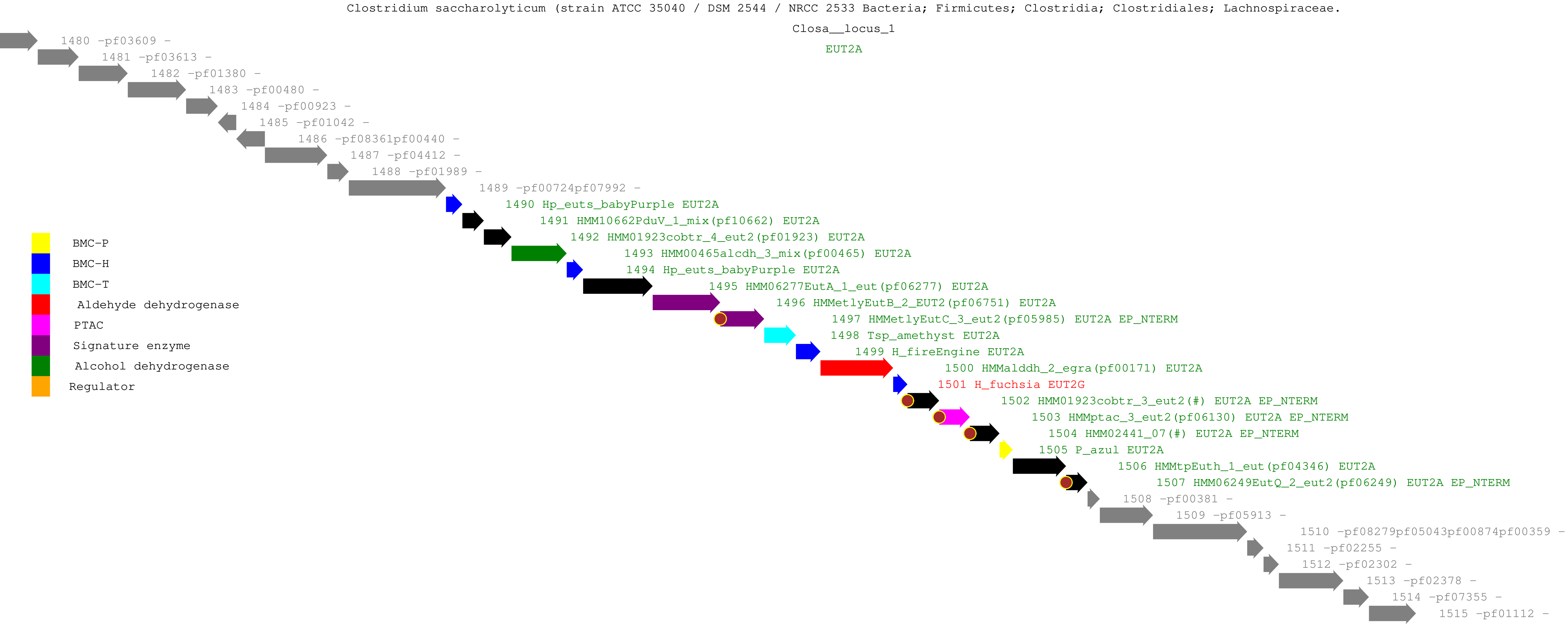
Clostridium saccharolyticum (strain ATCC 35040 / DSM 2544 / NRCC 2533 Bacteria; Firmicutes; Clostridia; Clostridiales; Lachnospiraceae.
Closa__locus_1
EUT2A
1480 -pf03609 -
1481 -pf03613 -
1482 -pf01380 -
1483 -pf00480 -
1484 -pf00923 -
1485 -pf01042 -
1486 -pf08361pf00440 -
1487 -pf04412 -
1488 -pf01989 -
1489 -pf00724pf07992 -
1490 Hp_euts_babyPurple EUT2A
1491 HMM10662PduV_1_mix(pf10662) EUT2A
1492 HMM01923cobtr_4_eut2(pf01923) EUT2A
1493 HMM00465alcdh_3_mix(pf00465) EUT2A
1494 Hp_euts_babyPurple EUT2A
1495 HMM06277EutA_1_eut(pf06277) EUT2A
1496 HMMetlyEutB_2_EUT2(pf06751) EUT2A
1497 HMMetlyEutC_3_eut2(pf05985) EUT2A EP_NTERM
1498 Tsp_amethyst EUT2A
1499 H_fireEngine EUT2A
1500 HMMalddh_2_egra(pf00171) EUT2A
1501 H_fuchsia EUT2G
1502 HMM01923cobtr_3_eut2(#) EUT2A EP_NTERM
1503 HMMptac_3_eut2(pf06130) EUT2A EP_NTERM
1504 HMM02441_07(#) EUT2A EP_NTERM
1505 P_azul EUT2A
1506 HMMtpEuth_1_eut(pf04346) EUT2A
1507 HMM06249EutQ_2_eut2(pf06249) EUT2A EP_NTERM
1508 -pf00381 -
1509 -pf05913 -
1510 -pf08279pf05043pf00874pf00359 -
1511 -pf02255 -
1512 -pf02302 -
1513 -pf02378 -
1514 -pf07355 -
1515 -pf01112 -
BMC-P
BMC-H
BMC-T
Aldehyde dehydrogenase
PTAC
Signature enzyme
Alcohol dehydrogenase
Regulator

Alkaliphilus oremlandii (strain OhILAs) (Clostridium oremlandii Bacteria; Firmicutes; Clostridia; Clostridiales; Clostridiaceae;

Clos__locus_2

EUT2A

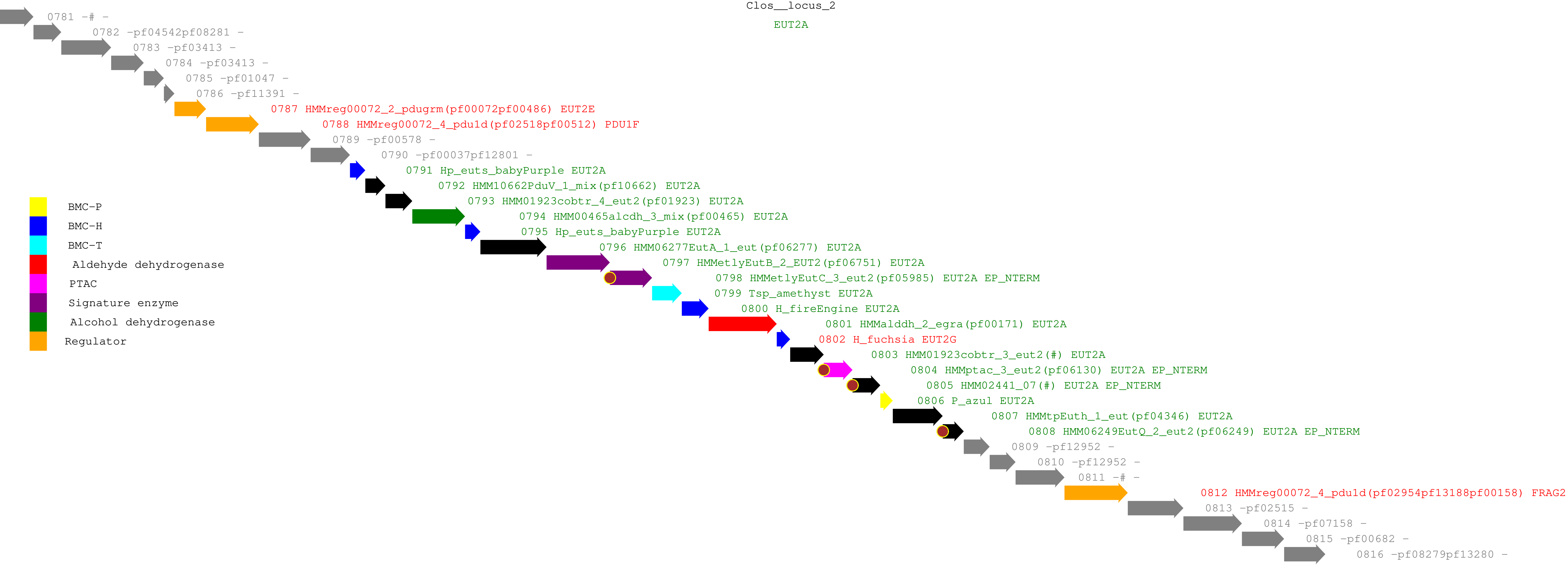

Lactobacillus brevis. Bacteria; Firmicutes; Bacilli; Lactobacillales; Lactobacillaceae;

CNR29__locus_2

EUT2A

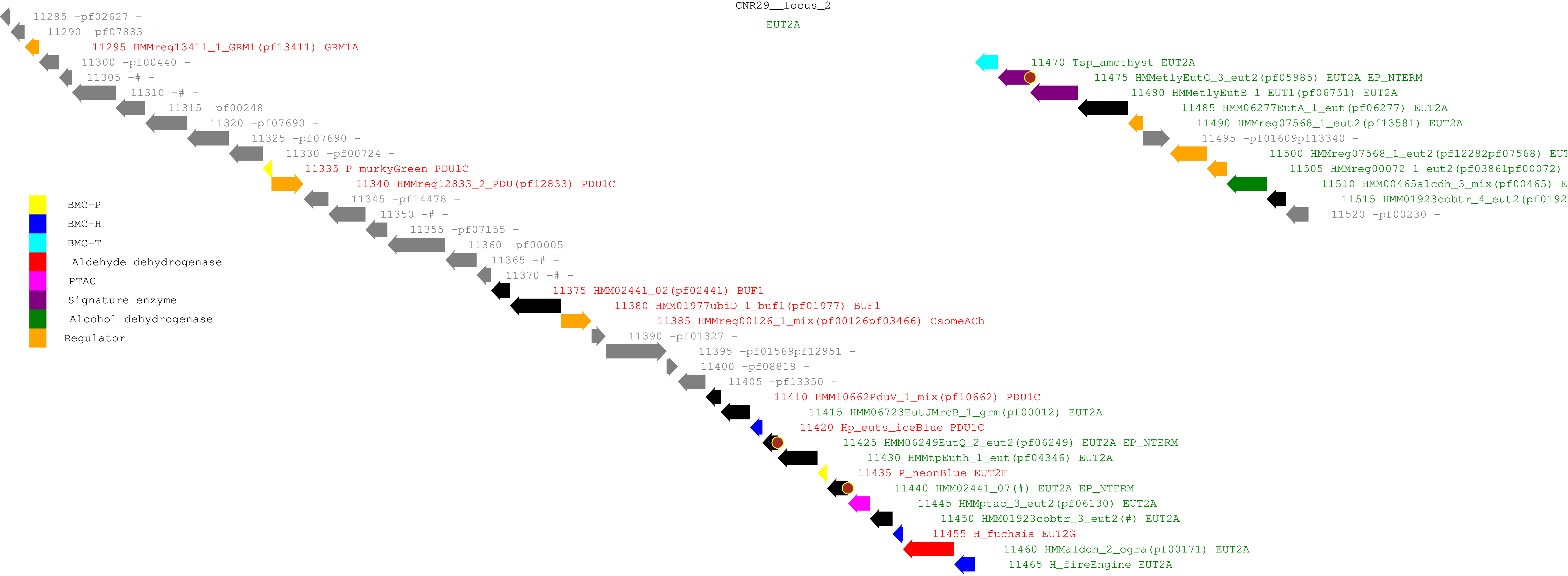

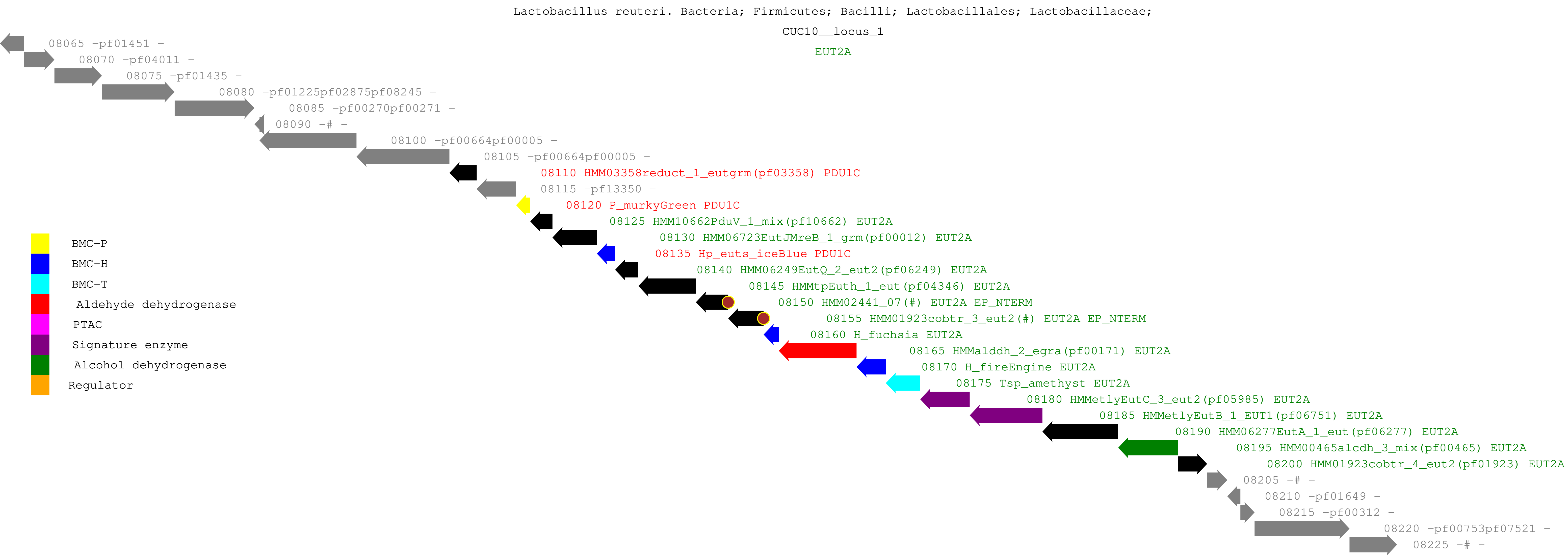
Lactobacillus reuteri. Bacteria; Firmicutes; Bacilli; Lactobacillales; Lactobacillaceae;
CUC10__locus_1
EUT2A
08065 -pf01451 -
08070 -pf04011 -
08075 -pf01435 -
08080 -pf01225pf02875pf08245 -
08085 -pf00270pf00271 -
08090 -# -
08100 -pf00664pf00005 -
08105 -pf00664pf00005 -
08110 HMM03358reduct_1_eutgrm(pf03358) PDU1C
08115 -pf13350 -
08120 P_murkyGreen PDU1C
08125 HMM10662PduV_1_mix(pf10662) EUT2A
08130 HMM06723EutJMreB_1_grm(pf00012) EUT2A
08135 Hp_euts_iceBlue PDU1C
08140 HMM06249EutQ_2_eut2(pf06249) EUT2A
08145 HMMtpEuth_1_eut(pf04346) EUT2A
08150 HMM02441_07(#) EUT2A EP_NTERM
08155 HMM01923cobtr_3_eut2(#) EUT2A EP_NTERM
08160 H_fuchsia EUT2A
08165 HMMalddh_2_egra(pf00171) EUT2A
08170 H_fireEngine EUT2A
08175 Tsp_amethyst EUT2A
08180 HMMetlyEutC_3_eut2(pf05985) EUT2A
08185 HMMetlyEutB_1_EUT1(pf06751) EUT2A
08190 HMM06277EutA_1_eut(pf06277) EUT2A
08195 HMM00465alcdh_3_mix(pf00465) EUT2A
08200 HMM01923cobtr_4_eut2(pf01923) EUT2A
08205 -# -
08210 -pf01649 -
08215 -pf00312 -
08220 -pf00753pf07521 -
08225 -# -
BMC-P
BMC-H
BMC-T
Aldehyde dehydrogenase
PTAC
Signature enzyme
Alcohol dehydrogenase
Regulator

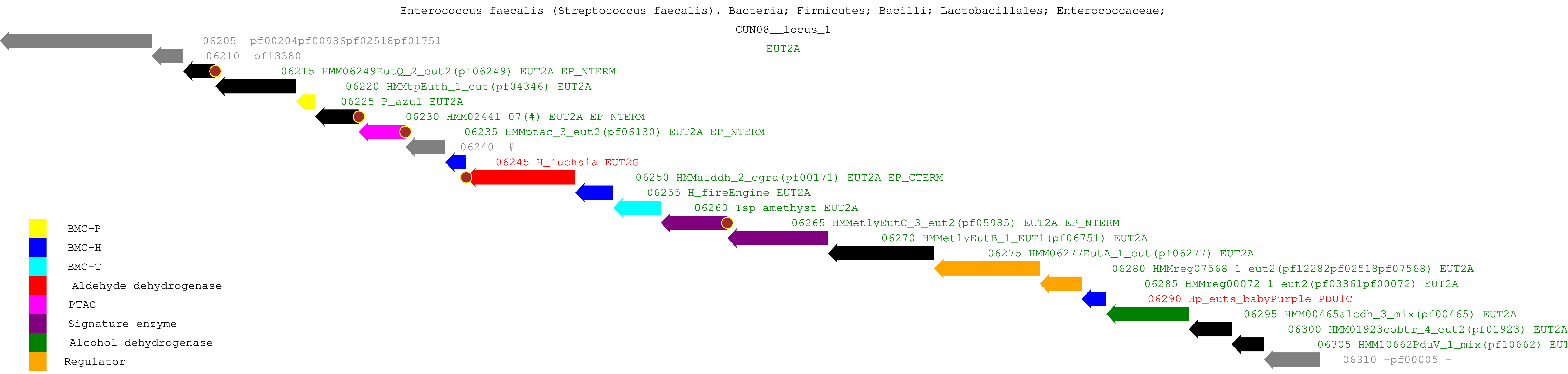

Enterococcus faecalis (Streptococcus faecalis). Bacteria; Firmicutes; Bacilli; Lactobacillales; Enterococcaceae;
CUN08__locus_1
EUT2A
06205 -pf00204pf00986pf02518pf01751 -
06210 -pf13380 -
06215 HMM06249EutQ_2_eut2(pf06249) EUT2A EP_NTERM
06220 HMMtpEuth_1_eut(pf04346) EUT2A
06225 P_azul EUT2A
06230 HMM02441_07(#) EUT2A EP_NTERM
06235 HMMptac_3_eut2(pf06130) EUT2A EP_NTERM
06240 -# -
06245 H_fuchsia EUT2G
06250 HMMaldh_2_egra(pf00171) EUT2A EP_CTERM
06255 H_fireEngine EUT2A
06260 Tsp_amethyst EUT2A
06265 HMMetlyEutC_3_eut2(pf05985) EUT2A EP_NTERM
06270 HMMetlyEutB_1_EUT1(pf06751) EUT2A
06275 HMM06277EutA_1_eut(pf06277) EUT2A
06280 HMMreg07568_1_eut2(pf12282pf02518pf07568) EUT2A
06285 HMMreg00072_1_eut2(pf03861pf00072) EUT2A
06290 Hp_euts_babyPurple PDU1C
06295 HMM00465alcdh_3_mix(pf00465) EUT2A
06300 HMM01923cobtr_4_eut2(pf01923) EUT2A
06305 HMM10662PduV_1_mix(pf10662) EU1
06310 -pf00005 -
BMC-P
BMC-H
BMC-T
Aldehyde dehydrogenase
PTAC
Signature enzyme
Alcohol dehydrogenase
Regulator

Enterococcus mundtii. Bacteria; Firmicutes; Bacilli; Lactobacillales; Enterococcaceae;

CUS89__locus_2

EUT2A

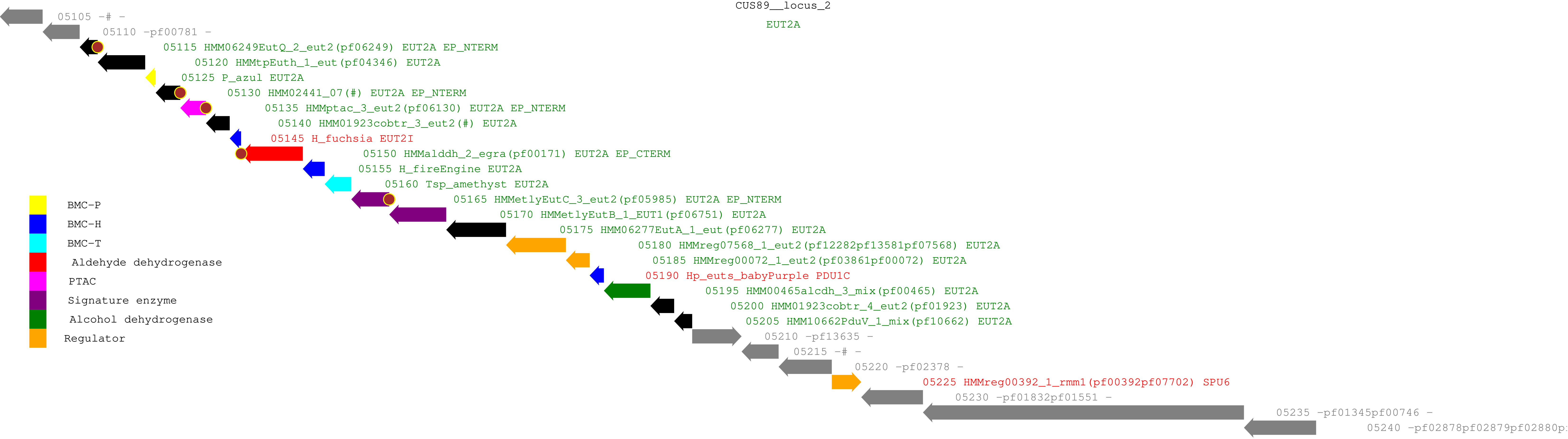

Enterococcus mundtii. Bacteria; Firmicutes; Bacilli; Lactobacillales; Enterococcaceae;

CV769__locus_1

EUT2A

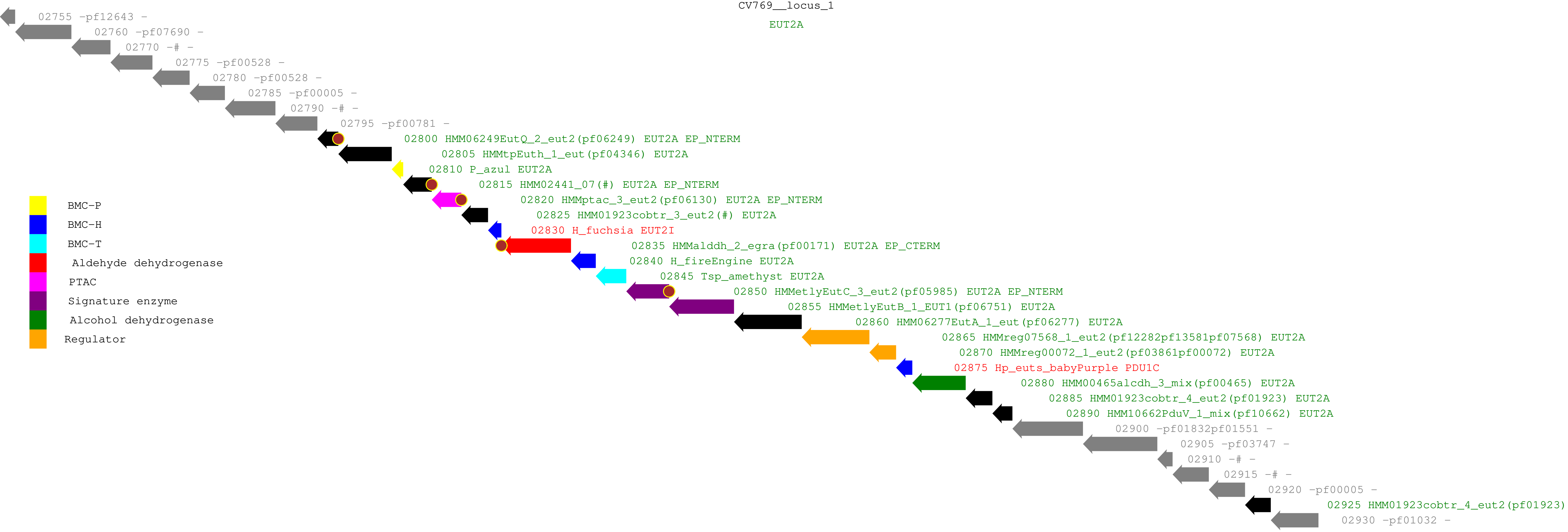

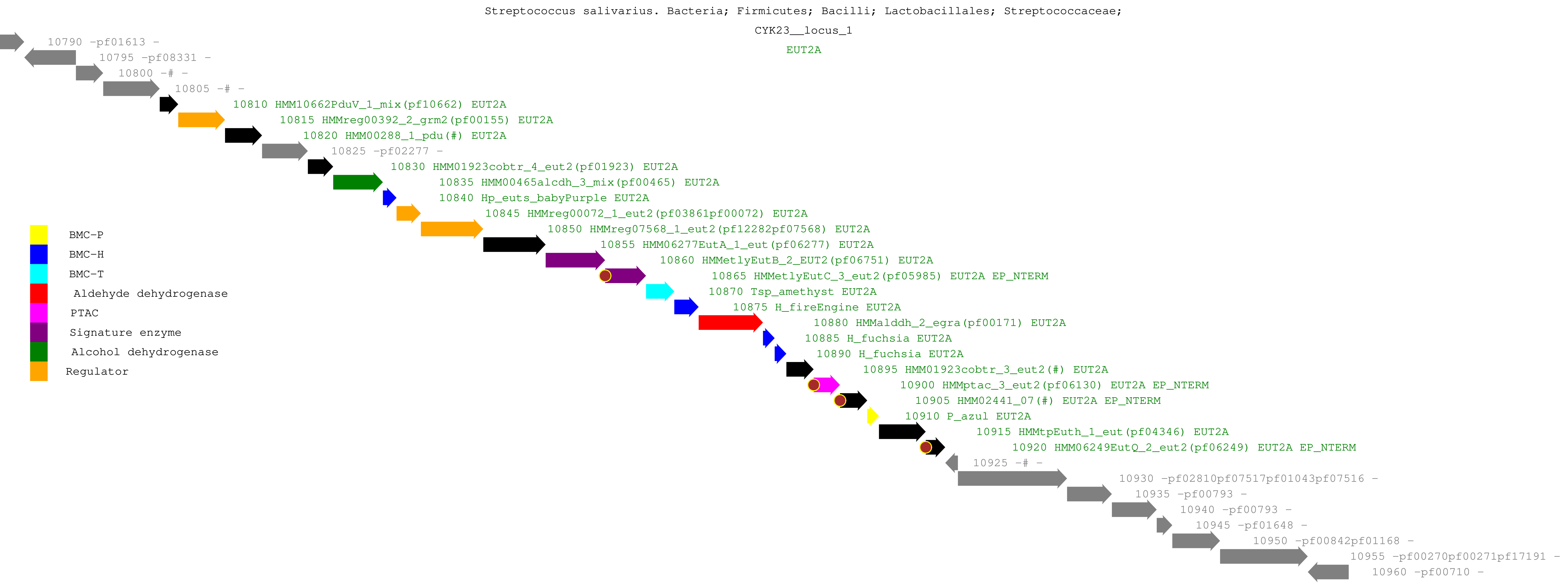
Streptococcus salivarius. Bacteria; Firmicutes; Bacilli; Lactobacillales; Streptococcaceae;
CYK23__locus_1
EUT2A
10790 -pf01613 -
10795 -pf08331 -
10800 -# -
10805 -# -
10810 HMM10662PduV_1_mix(pf10662) EUT2A
10815 HMMreg00392_2_grm2(pf00155) EUT2A
10820 HMM00288_1_pdu(#) EUT2A
10825 -pf02277 -
10830 HMM01923cobtr_4_eut2(pf01923) EUT2A
10835 HMM00465alcdh_3_mix(pf00465) EUT2A
10840 Hp_euts_babyPurple EUT2A
10845 HMMreg00072_1_eut2(pf03861pf00072) EUT2A
10850 HMMreg07568_1_eut2(pf12282pf07568) EUT2A
10855 HMM06277EutA_1_eut(pf06277) EUT2A
10860 HMMetlyEutB_2_EUT2(pf06751) EUT2A
10865 HMMetlyEutC_3_eut2(pf05985) EUT2A EP_NTERM
10870 Tsp_amethyst EUT2A
10875 H_fireEngine EUT2A
10880 HMMalddh_2_egra(pf00171) EUT2A
10885 H_fuchsia EUT2A
10890 H_fuchsia EUT2A
10895 HMM01923cobtr_3_eut2(#) EUT2A
10900 HMMptac_3_eut2(pf06130) EUT2A EP_NTERM
10905 HMM02441_07(#) EUT2A EP_NTERM
10910 P_azul EUT2A
10915 HMMtpEuth_1_eut(pf04346) EUT2A
10920 HMM06249EutQ_2_eut2(pf06249) EUT2A EP_NTERM
10925 -# -
10930 -pf02810pf07517pf01043pf07516 -
10935 -pf00793 -
10940 -pf00793 -
10945 -pf01648 -
10950 -pf00842pf01168 -
10955 -pf00270pf00271pf17191 -
10960 -pf00710 -
BMC-P
BMC-H
BMC-T
Aldehyde dehydrogenase
PTAC
Signature enzyme
Alcohol dehydrogenase
Regulator

Enterococcus faecalis (Streptococcus faecalis). Bacteria; Firmicutes; Bacilli; Lactobacillales; Enterococcaceae;

CYR78__locus_1

EUT2A

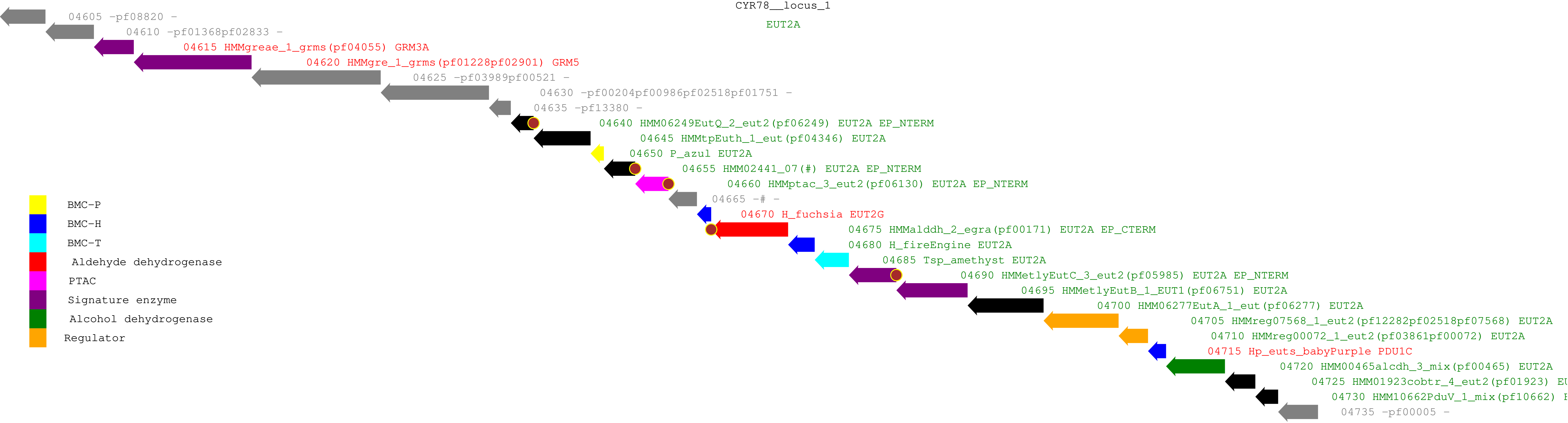

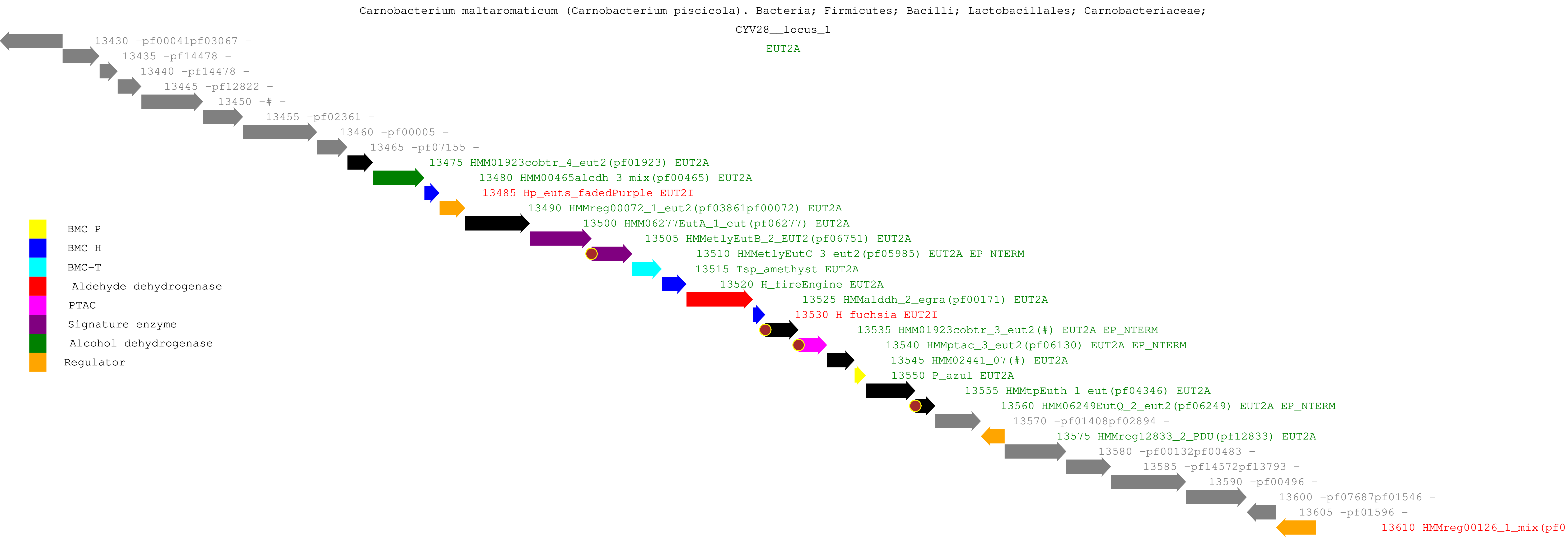
Carnobacterium maltaromaticum (Carnobacterium piscicola). Bacteria; Firmicutes; Bacilli; Lactobacillales; Carnobacteriaceae;
CYV28__locus_1
EUT2A
13430 -pf00041pf03067 -
13435 -pf14478 -
13440 -pf14478 -
13445 -pf12822 -
13450 -# -
13455 -pf02361 -
13460 -pf00005 -
13465 -pf07155 -
13475 HMM01923cobtr_4_eut2(pf01923) EUT2A
13480 HMM00465alcdh_3_mix(pf00465) EUT2A
13485 Hp_euts_fadedPurple EUT2I
13490 HMMreg00072_1_eut2(pf03861pf00072) EUT2A
13500 HMM06277EutA_1_eut(pf06277) EUT2A
13505 HMMetlyEutB_2_EUT2(pf06751) EUT2A
13510 HMMetlyEutC_3_eut2(pf05985) EUT2A EP_NTERM
13515 Tsp_amethyst EUT2A
13520 H_fireEngine EUT2A
13525 HMMaldh_2_egra(pf00171) EUT2A
13530 H_fuchsia EUT2I
13535 HMM01923cobtr_3_eut2(#) EUT2A EP_NTERM
13540 HMMptac_3_eut2(pf06130) EUT2A EP_NTERM
13545 HMM02441_07(#) EUT2A
13550 P_azul EUT2A
13555 HMMtpEuth_1_eut(pf04346) EUT2A
13560 HMM06249EutQ_2_eut2(pf06249) EUT2A EP_NTERM
13570 -pf01408pf02894 -
13575 HMMreg12833_2_PDU(pf12833) EUT2A
13580 -pf00132pf00483 -
13585 -pf14572pf13793 -
13590 -pf00496 -
13600 -pf07687pf01546 -
13605 -pf01596 -
13610 HMMreg00126_1_mix(pf0
BMC-P
BMC-H
BMC-T
Aldehyde dehydrogenase
PTAC
Signature enzyme
Alcohol dehydrogenase
Regulator

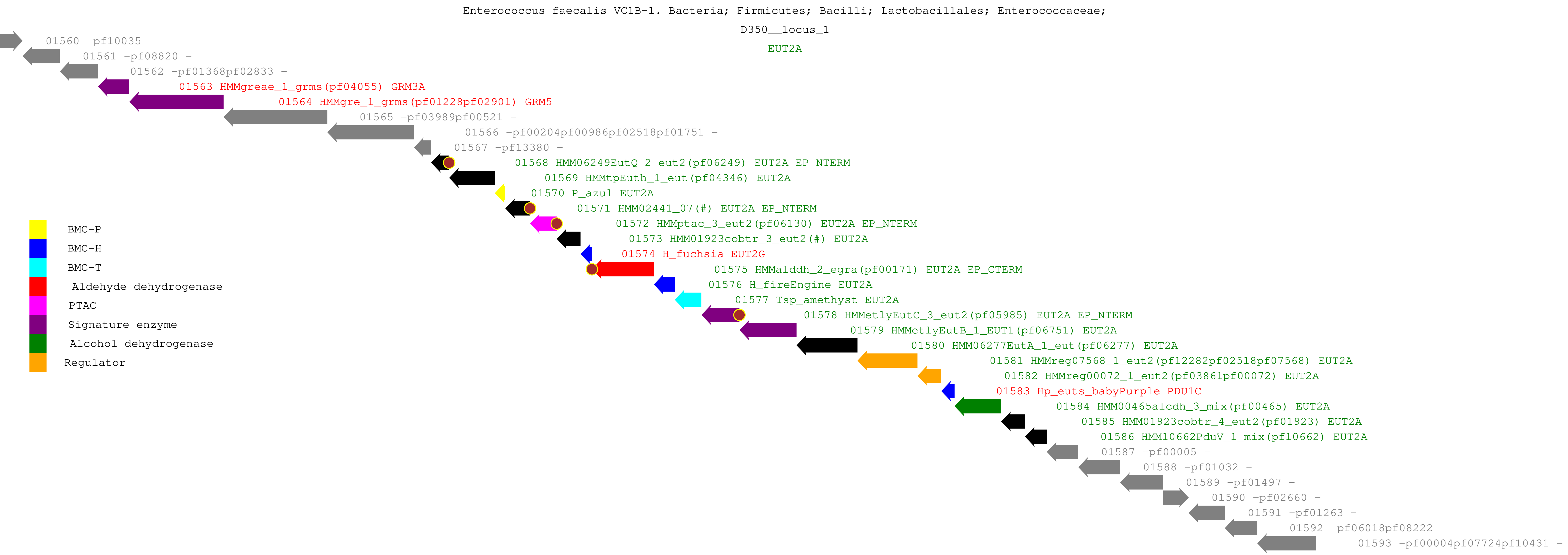
Enterococcus faecalis VC1B-1. Bacteria; Firmicutes; Bacilli; Lactobacillales; Enterococcaceae;
D350__locus_1
EUT2A
01560 -pf10035 -
01561 -pf08820 -
01562 -pf01368pf02833 -
01563 HMMgreae_1_grms(pf04055) GRM3A
01564 HMMgre_1_grms(pf01228pf02901) GRM5
01565 -pf03989pf00521 -
01566 -pf00204pf00986pf02518pf01751 -
01567 -pf13380 -
01568 HMM06249EutQ_2_eut2(pf06249) EUT2A EP_NTERM
01569 HMMtpEuth_1_eut(pf04346) EUT2A
01570 P_azul EUT2A
01571 HMM02441_07(#) EUT2A EP_NTERM
01572 HMMptac_3_eut2(pf06130) EUT2A EP_NTERM
01573 HMM01923cobtr_3_eut2(#) EUT2A
01574 H_fuchsia EUT2G
01575 HMMalddh_2_egra(pf00171) EUT2A EP_CTERM
01576 H_fireEngine EUT2A
01577 Tsp_amethyst EUT2A
01578 HMMetlyEutC_3_eut2(pf05985) EUT2A EP_NTERM
01579 HMMetlyEutB_1_EUT1(pf06751) EUT2A
01580 HMM06277EutA_1_eut(pf06277) EUT2A
01581 HMMreg07568_1_eut2(pf12282pf02518pf07568) EUT2A
01582 HMMreg00072_1_eut2(pf03861pf00072) EUT2A
01583 Hp_euts_babyPurple PDU1C
01584 HMM00465alcdh_3_mix(pf00465) EUT2A
01585 HMM01923cobtr_4_eut2(pf01923) EUT2A
01586 HMM10662PduV_1_mix(pf10662) EUT2A
01587 -pf00005 -
01588 -pf01032 -
01589 -pf01497 -
01590 -pf02660 -
01591 -pf01263 -
01592 -pf06018pf08222 -
01593 -pf00004pf07724pf10431 -
BMC-P
BMC-H
BMC-T
Aldehyde dehydrogenase
PTAC
Signature enzyme
Alcohol dehydrogenase
Regulator

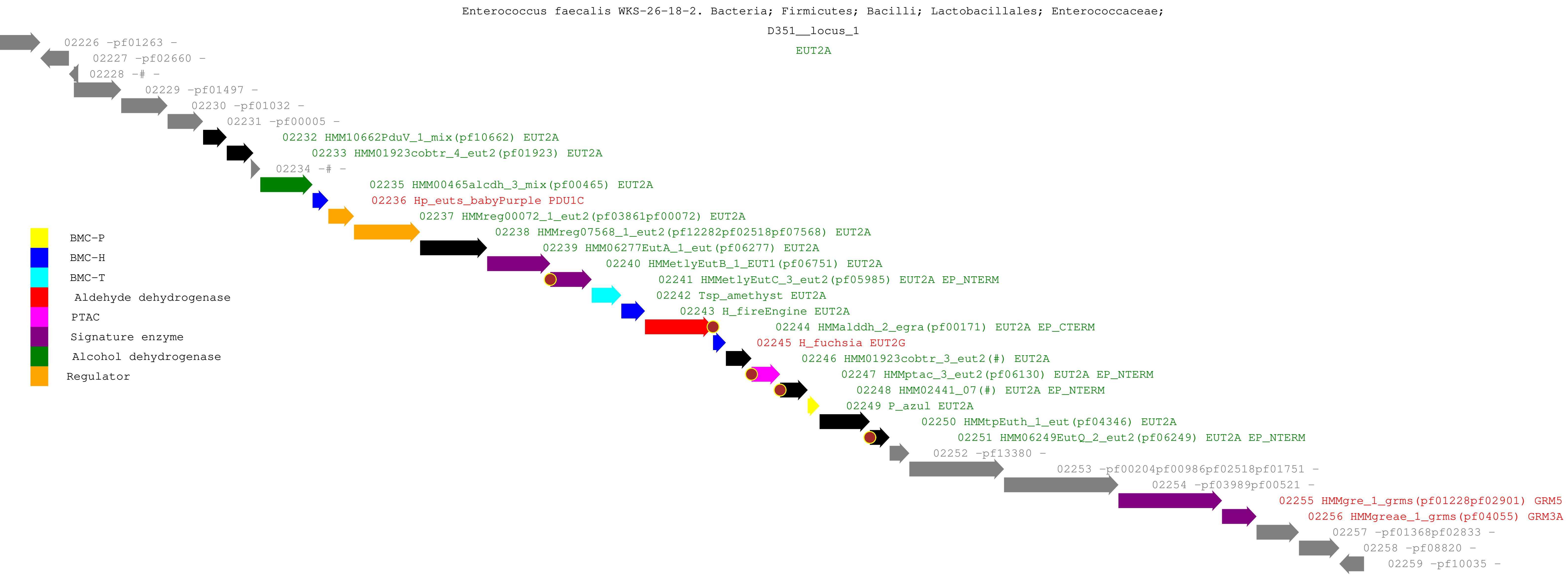

Enterococcus faecalis WKS-26-18-2. Bacteria; Firmicutes; Bacilli; Lactobacillales; Enterococcaceae;
D351__locus_1
EUT2A
02226 -pf01263 -
02227 -pf02660 -
02228 -# -
02229 -pf01497 -
02230 -pf01032 -
02231 -pf00005 -
02232 HMM10662PduV_1_mix(pf10662) EUT2A
02233 HMM01923cobtr_4_eut2(pf01923) EUT2A
02234 -# -
02235 HMM00465alcdh_3_mix(pf00465) EUT2A
02236 Hp_euts_babyPurple PDU1C
02237 HMMreg00072_1_eut2(pf03861pf00072) EUT2A
02238 HMMreg07568_1_eut2(pf12282pf02518pf07568) EUT2A
02239 HMM06277EutA_1_eut(pf06277) EUT2A
02240 HMMetlyEutB_1_EUT1(pf06751) EUT2A
02241 HMMetlyEutC_3_eut2(pf05985) EUT2A EP_NTERM
02242 Tsp_amethyst EUT2A
02243 H_fireEngine EUT2A
02244 HMMalddh_2_egra(pf00171) EUT2A EP_CTERM
02245 H_fuchsia EUT2G
02246 HMM01923cobtr_3_eut2(#) EUT2A
02247 HMMptac_3_eut2(pf06130) EUT2A EP_NTERM
02248 HMM02441_07(#) EUT2A EP_NTERM
02249 P_azul EUT2A
02250 HMMtpEuth_1_eut(pf04346) EUT2A
02251 HMM06249EutQ_2_eut2(pf06249) EUT2A EP_NTERM
02252 -pf13380 -
02253 -pf00204pf00986pf02518pf01751 -
02254 -pf03989pf00521 -
02255 HMMgre_1_grms(pf01228pf02901) GRM5
02256 HMMgreae_1_grms(pf04055) GRM3A
02257 -pf01368pf02833 -
02258 -pf08820 -
02259 -pf10035 -
BMC-P
BMC-H
BMC-T
Aldehyde dehydrogenase
PTAC
Signature enzyme
Alcohol dehydrogenase
Regulator

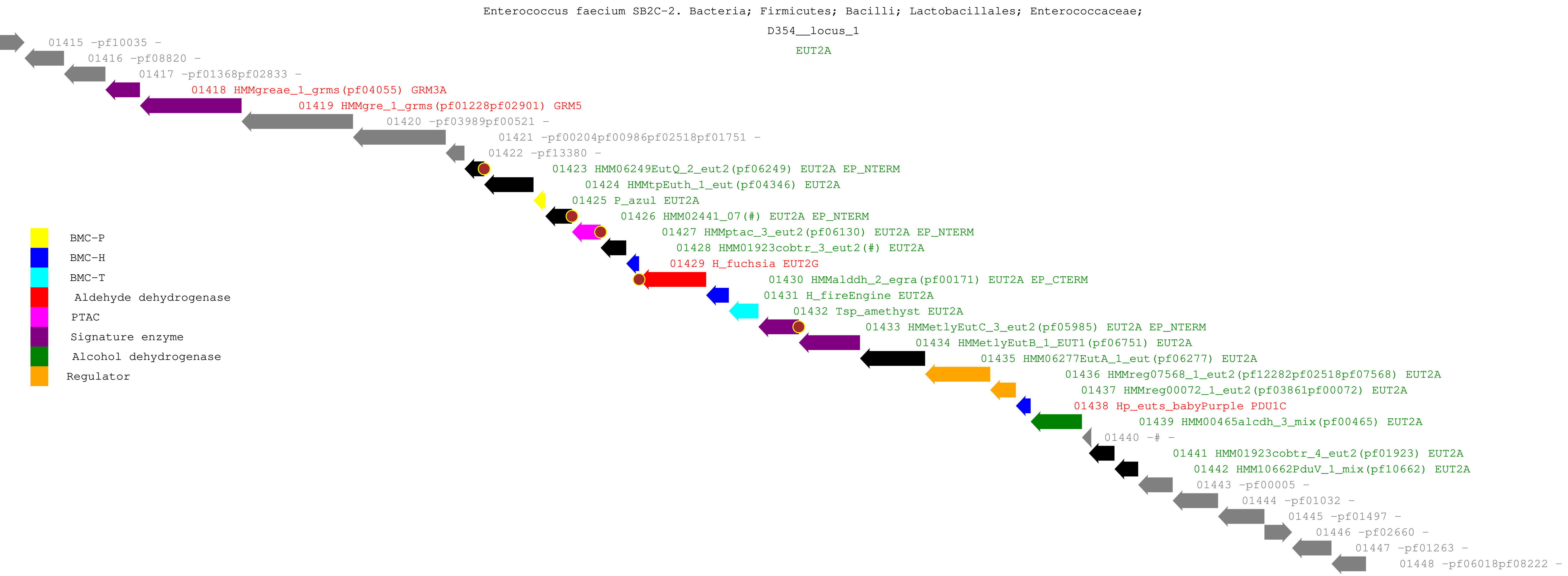
Enterococcus faecium SB2C-2. Bacteria; Firmicutes; Bacilli; Lactobacillales; Enterococcaceae;
D354__locus_1
EUT2A
01415 -pf10035 -
01416 -pf08820 -
01417 -pf01368pf02833 -
01418 HMMgreae_1_grms(pf04055) GRM3A
01419 HMMgre_1_grms(pf01228pf02901) GRM5
01420 -pf03989pf00521 -
01421 -pf00204pf00986pf02518pf01751 -
01422 -pf13380 -
01423 HMM06249EutQ_2_eut2(pf06249) EUT2A EP_NTERM
01424 HMMtpEuth_1_eut(pf04346) EUT2A
01425 P_azul EUT2A
01426 HMM02441_07(#) EUT2A EP_NTERM
01427 HMMptac_3_eut2(pf06130) EUT2A EP_NTERM
01428 HMM01923cobtr_3_eut2(#) EUT2A
01429 H_fuchsia EUT2G
01430 HMMalddh_2_egra(pf00171) EUT2A EP_CTERM
01431 H_fireEngine EUT2A
01432 Tsp_amethyst EUT2A
01433 HMMetlyEutC_3_eut2(pf05985) EUT2A EP_NTERM
01434 HMMetlyEutB_1_EUT1(pf06751) EUT2A
01435 HMM06277EutA_1_eut(pf06277) EUT2A
01436 HMMreg07568_1_eut2(pf12282pf02518pf07568) EUT2A
01437 HMMreg00072_1_eut2(pf03861pf00072) EUT2A
01438 Hp_euts_babyPurple PDU1C
01439 HMM00465alcdh_3_mix(pf00465) EUT2A
01440 -# -
01441 HMM01923cobtr_4_eut2(pf01923) EUT2A
01442 HMM10662PduV_1_mix(pf10662) EUT2A
01443 -pf00005 -
01444 -pf01032 -
01445 -pf01497 -
01446 -pf02660 -
01447 -pf01263 -
01448 -pf06018pf08222 -
BMC-P
BMC-H
BMC-T
Aldehyde dehydrogenase
PTAC
Signature enzyme
Alcohol dehydrogenase
Regulator

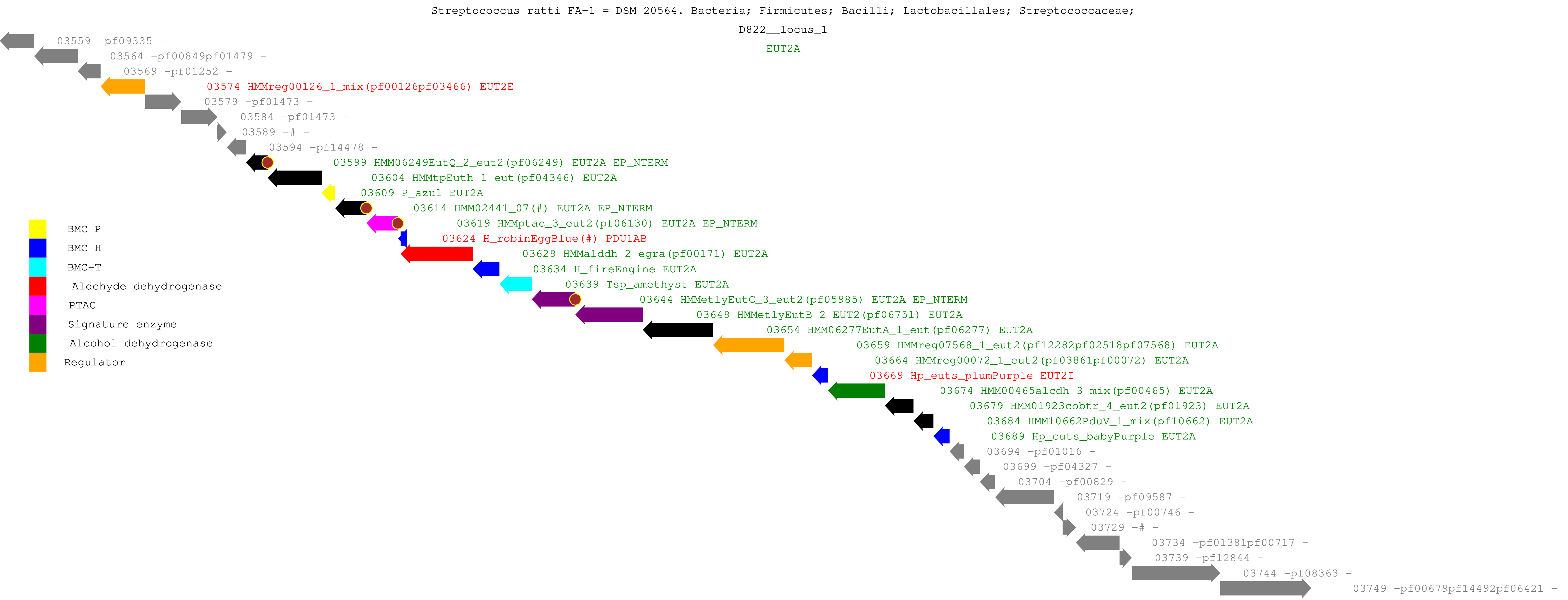

Streptococcus ratti FA-1 = DSM 20564. Bacteria; Firmicutes; Bacilli; Lactobacillales; Streptococcaceae;
D822__locus_1
EUT2A
03559 -pf09335 -
03564 -pf00849pf01479 -
03569 -pf01252 -
03574 HMMreg00126_1_mix(pf00126pf03466) EUT2E
03579 -pf01473 -
03584 -pf01473 -
03589 -# -
03594 -pf14478 -
03599 HMM06249EutQ_2_eut2(pf06249) EUT2A EP_NTERM
03604 HMMtpEuth_1_eut(pf04346) EUT2A
03609 P_azul EUT2A
03614 HMM02441_07(#) EUT2A EP_NTERM
03619 HMMptac_3_eut2(pf06130) EUT2A EP_NTERM
03624 H_robinEggBlue(#) PDU1AB
03629 HMMalddh_2_egra(pf00171) EUT2A
03634 H_fireEngine EUT2A
03639 Tsp_amethyst EUT2A
03644 HMMetlyEutC_3_eut2(pf05985) EUT2A EP_NTERM
03649 HMMetlyEutB_2_EUT2(pf06751) EUT2A
03654 HMM06277EutA_1_eut(pf06277) EUT2A
03659 HMMreg07568_1_eut2(pf12282pf02518pf07568) EUT2A
03664 HMMreg00072_1_eut2(pf03861pf00072) EUT2A
03669 Hp_euts_plumPurple EUT2I
03674 HMM00465alcdh_3_mix(pf00465) EUT2A
03679 HMM01923cobtr_4_eut2(pf01923) EUT2A
03684 HMM10662PduV_1_mix(pf10662) EUT2A
03689 Hp_euts_babyPurple EUT2A
03694 -pf01016 -
03699 -pf04327 -
03704 -pf00829 -
03719 -pf09587 -
03724 -pf00746 -
03729 -# -
03734 -pf01381pf00717 -
03739 -pf12844 -
03744 -pf08363 -
03749 -pf00679pf14492pf06421 -
BMC-P
BMC-H
BMC-T
Aldehyde dehydrogenase
PTAC
Signature enzyme
Alcohol dehydrogenase
Regulator

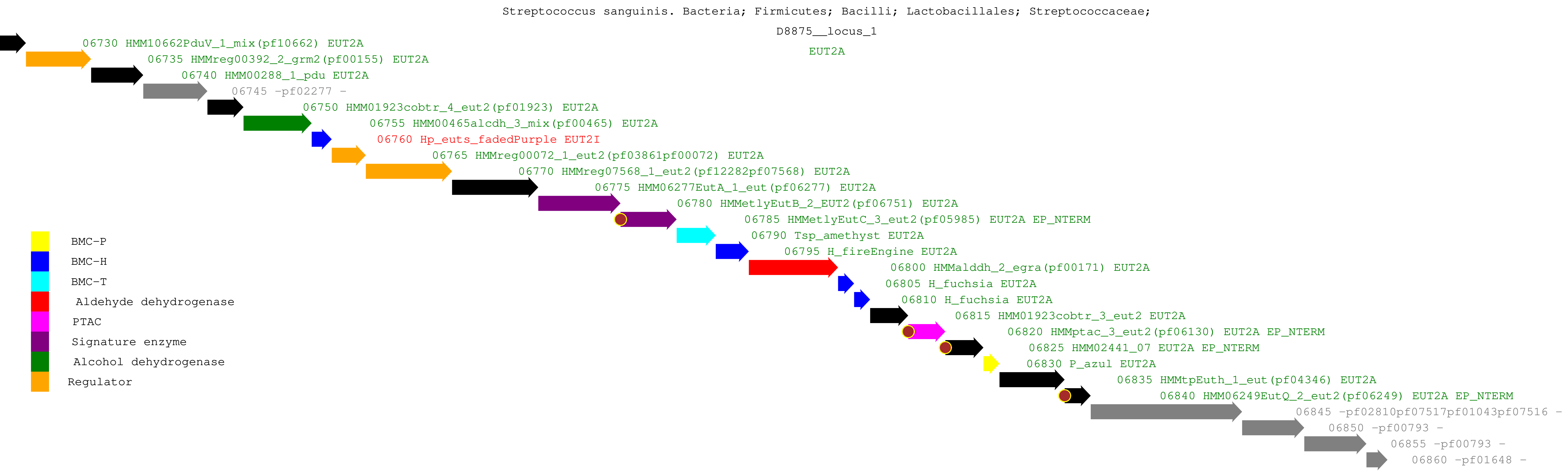
Streptococcus sanguinis. Bacteria; Firmicutes; Bacilli; Lactobacillales; Streptococcaceae;
D8875__locus_1
EUT2A
06730 HMM10662PduV_1_mix(pf10662) EUT2A
06735 HMMreg00392_2_grm2(pf00155) EUT2A
06740 HMM00288_1_pdu EUT2A
06745 -pf02277 -
06750 HMM01923cobtr_4_eut2(pf01923) EUT2A
06755 HMM00465alcdh_3_mix(pf00465) EUT2A
06760 Hp_euts_fadedPurple EUT2I
06765 HMMreg00072_1_eut2(pf03861pf00072) EUT2A
06770 HMMreg07568_1_eut2(pf12282pf07568) EUT2A
06775 HMM06277EutA_1_eut(pf06277) EUT2A
06780 HMMetlyEutB_2_EUT2(pf06751) EUT2A
06785 HMMetlyEutC_3_eut2(pf05985) EUT2A EP_NTERM
06790 Tsp_amethyst EUT2A
06795 H_fireEngine EUT2A
06800 HMMalddh_2_egra(pf00171) EUT2A
06805 H_fuchsia EUT2A
06810 H_fuchsia EUT2A
06815 HMM01923cobtr_3_eut2 EUT2A
06820 HMMptac_3_eut2(pf06130) EUT2A EP_NTERM
06825 HMM02441_07 EUT2A EP_NTERM
06830 P_azul EUT2A
06835 HMMtpEuth_1_eut(pf04346) EUT2A
06840 HMM06249EutQ_2_eut2(pf06249) EUT2A EP_NTERM
06845 -pf02810pf07517pf01043pf07516 -
06850 -pf00793 -
06855 -pf00793 -
06860 -pf01648 -
BMC-P
BMC-H
BMC-T
Aldehyde dehydrogenase
PTAC
Signature enzyme
Alcohol dehydrogenase
Regulator

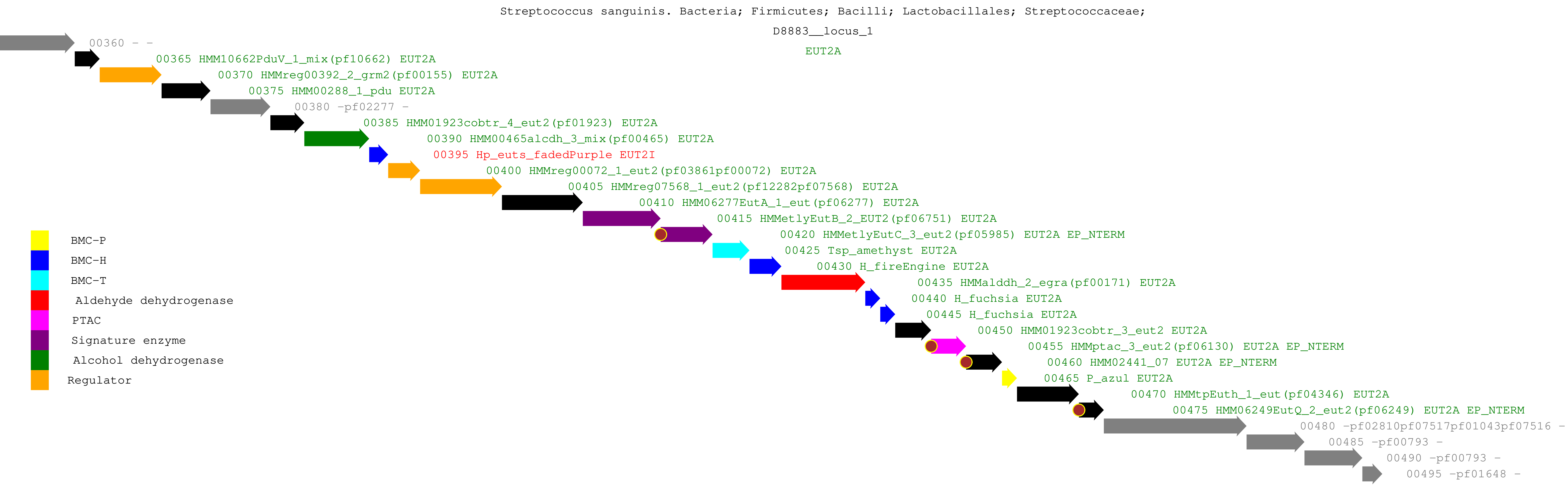

Streptococcus sanguinis. Bacteria; Firmicutes; Bacilli; Lactobacillales; Streptococcaceae;
D8883__locus_1
EUT2A
00360 - -
00365 HMM10662PduV_1_mix(pf10662) EUT2A
00370 HMMreg00392_2_grm2(pf00155) EUT2A
00375 HMM00288_1_pdu EUT2A
00380 -pf02277 -
00385 HMM01923cobtr_4_eut2(pf01923) EUT2A
00390 HMM00465alcdh_3_mix(pf00465) EUT2A
00395 Hp_euts_fadedPurple EUT2I
00400 HMMreg00072_1_eut2(pf03861pf00072) EUT2A
00405 HMMreg07568_1_eut2(pf12282pf07568) EUT2A
00410 HMM06277EutA_1_eut(pf06277) EUT2A
00415 HMMetlyEutB_2_EUT2(pf06751) EUT2A
00420 HMMetlyEutC_3_eut2(pf05985) EUT2A EP_NTERM
00425 Tsp_amethyst EUT2A
00430 H_fireEngine EUT2A
00435 HMMalddh_2_egra(pf00171) EUT2A
00440 H_fuchsia EUT2A
00445 H_fuchsia EUT2A
00450 HMM01923cobtr_3_eut2 EUT2A
00455 HMMptac_3_eut2(pf06130) EUT2A EP_NTERM
00460 HMM02441_07 EUT2A EP_NTERM
00465 P_azul EUT2A
00470 HMMtpEuth_1_eut(pf04346) EUT2A
00475 HMM06249EutQ_2_eut2(pf06249) EUT2A EP_NTERM
00480 -pf02810pf07517pf01043pf07516 -
00485 -pf00793 -
00490 -pf00793 -
00495 -pf01648 -
BMC-P
BMC-H
BMC-T
Aldehyde dehydrogenase
PTAC
Signature enzyme
Alcohol dehydrogenase
Regulator

Streptococcus sp. Bacteria; Firmicutes; Bacilli; Lactobacillales; Streptococcaceae;

D8H99__locus_1_main_sat

EUT2A

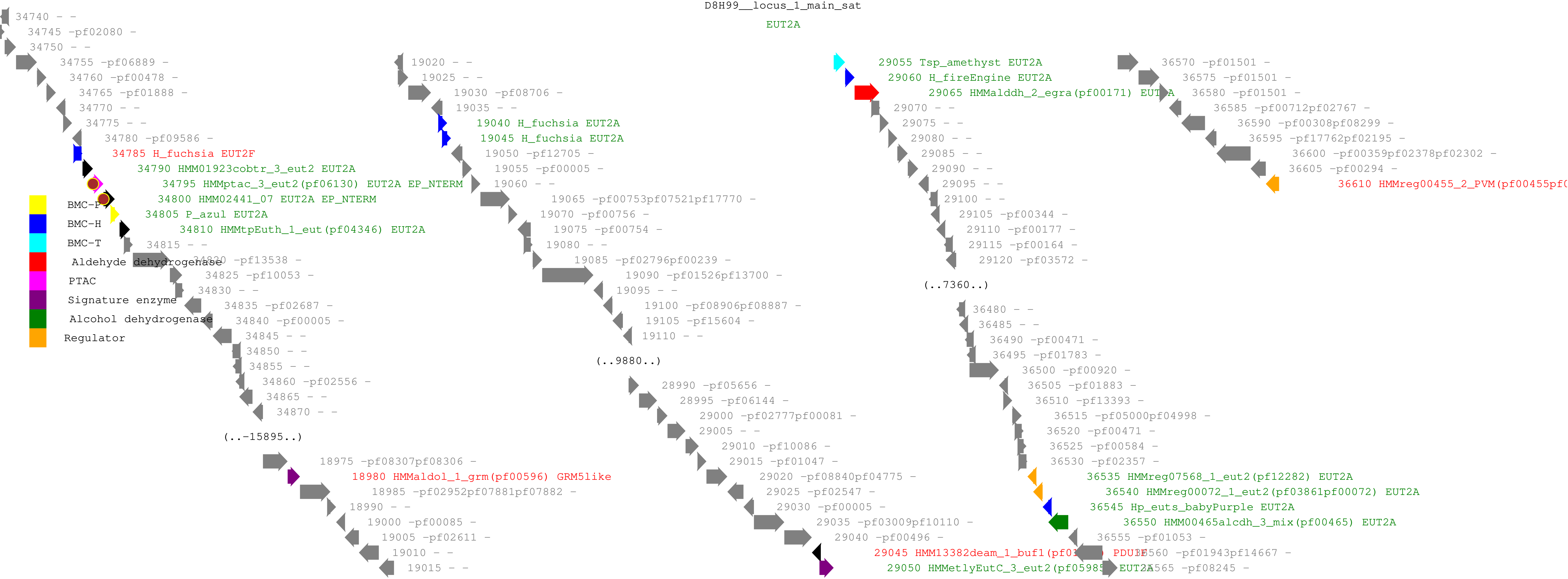

Enterococcus faecalis 02-MB-BW-10. Bacteria; Firmicutes; Bacilli; Lactobacillales; Enterococcaceae;

D927__locus_1

EUT2A

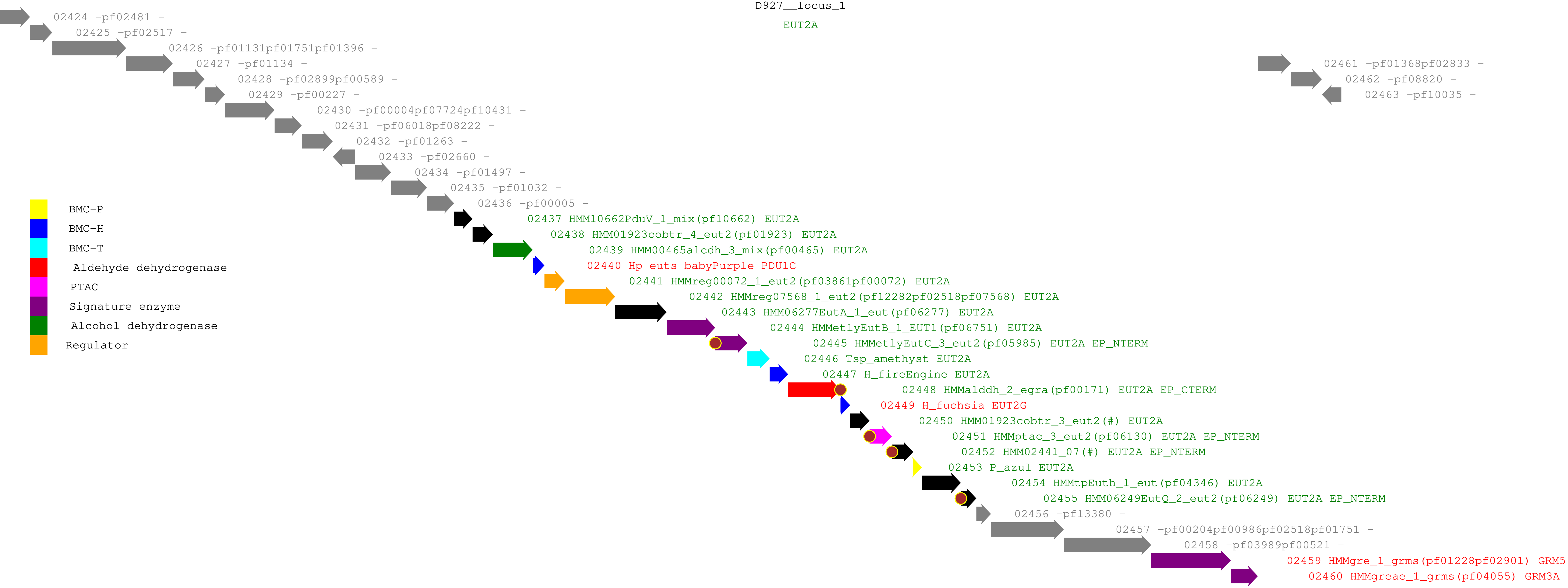

Lactobacillus reuteri. Bacteria; Firmicutes; Bacilli; Lactobacillales; Lactobacillaceae;

DB324__locus_1

EUT2A

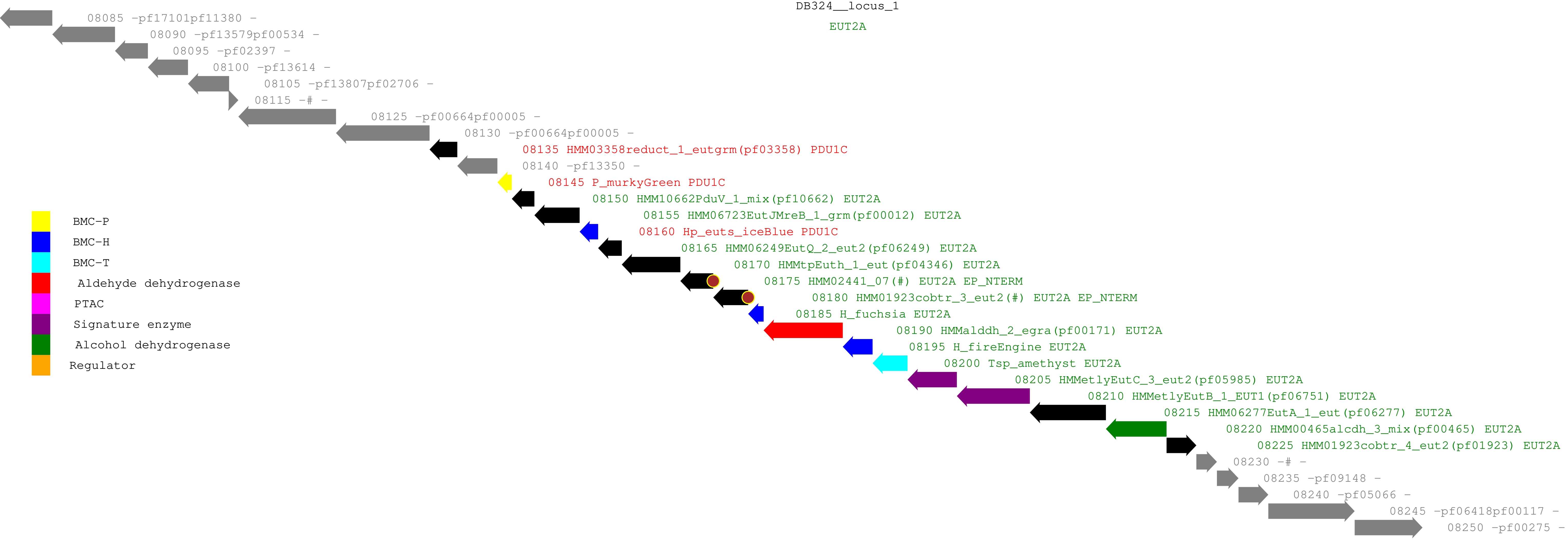

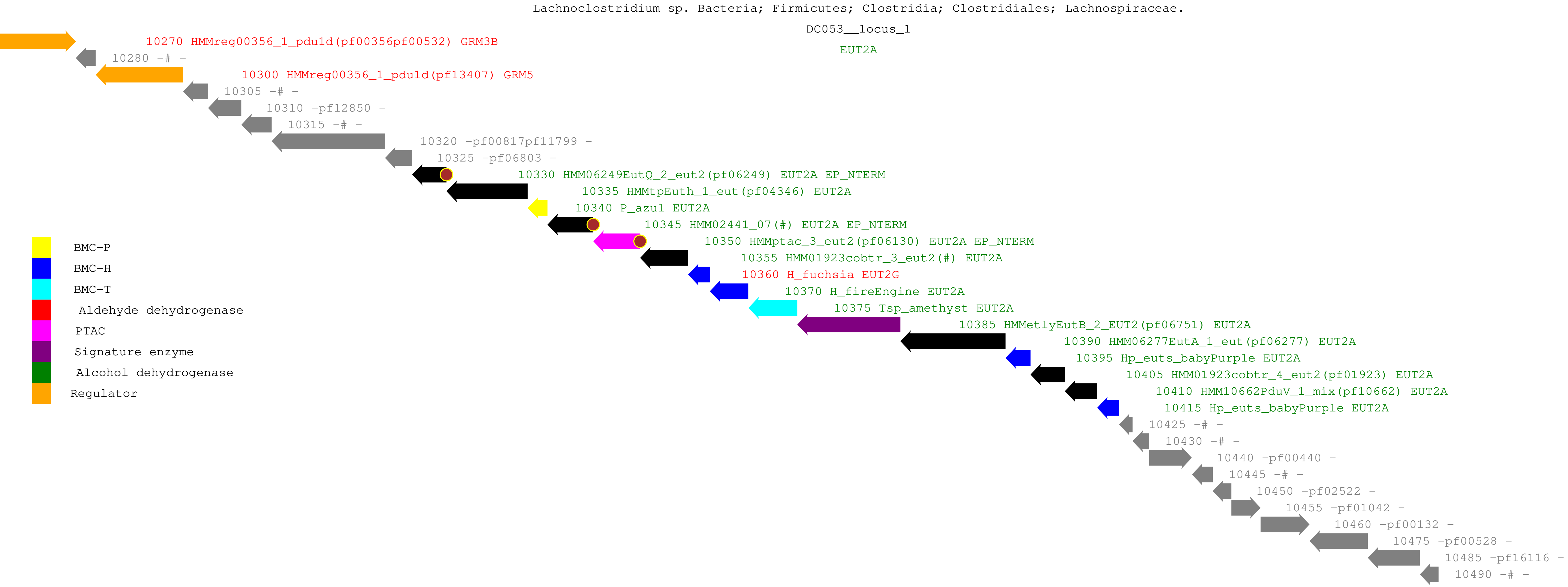
Lachnoclostridium sp. Bacteria; Firmicutes; Clostridia; Clostridiales; Lachnospiraceae.
DC053__locus_1
EUT2A
10270 HMMreg00356_1_pdu1d(pf00356pf00532) GRM3B
10280 -# -
10300 HMMreg00356_1_pdu1d(pf13407) GRM5
10305 -# -
10310 -pf12850 -
10315 -# -
10320 -pf00817pf11799 -
10325 -pf06803 -
10330 HMM06249EutQ_2_eut2(pf06249) EUT2A EP_NTERM
10335 HMMtpEuth_1_eut(pf04346) EUT2A
10340 P_azul EUT2A
10345 HMM02441_07(#) EUT2A EP_NTERM
10350 HMMptac_3_eut2(pf06130) EUT2A EP_NTERM
10355 HMM01923cobtr_3_eut2(#) EUT2A
10360 H_fuchsia EUT2G
10370 H_fireEngine EUT2A
10375 Tsp_amethyst EUT2A
10385 HMMetlyEutB_2_EUT2(pf06751) EUT2A
10390 HMM06277EutA_1_eut(pf06277) EUT2A
10395 Hp_euts_babyPurple EUT2A
10405 HMM01923cobtr_4_eut2(pf01923) EUT2A
10410 HMM10662PduV_1_mix(pf10662) EUT2A
10415 Hp_euts_babyPurple EUT2A
10425 -# -
10430 -# -
10440 -pf00440 -
10445 -# -
10450 -pf02522 -
10455 -pf01042 -
10460 -pf00132 -
10475 -pf00528 -
10485 -pf16116 -
10490 -# -
BMC-P
BMC-H
BMC-T
Aldehyde dehydrogenase
PTAC
Signature enzyme
Alcohol dehydrogenase
Regulator

Clostridiales bacterium. Bacteria; Firmicutes; Clostridia; Clostridiales.

DEF04__locus_1_main_sat

EUT2A

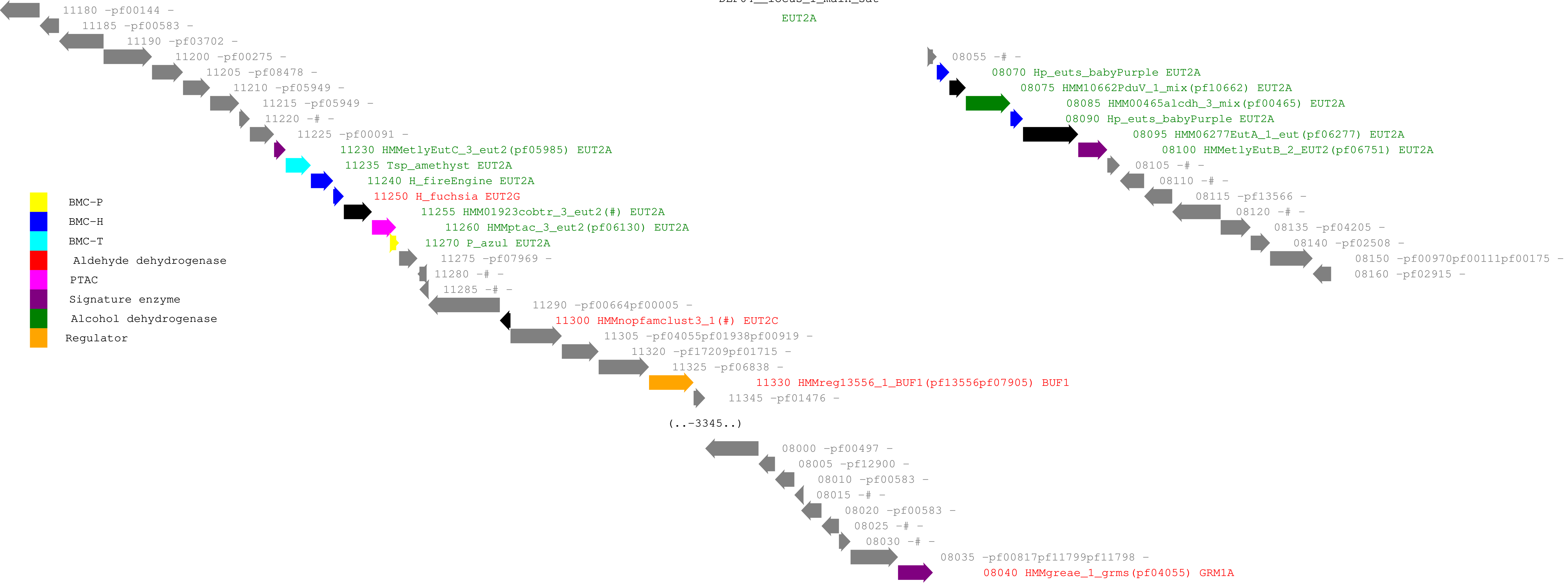

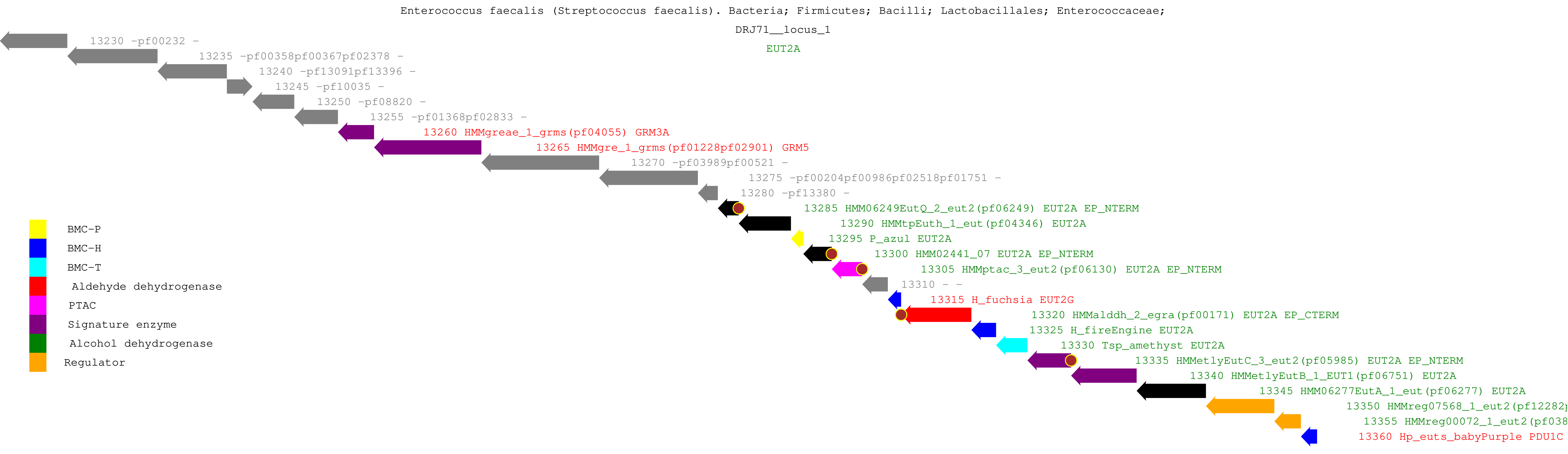

Enterococcus faecalis (Streptococcus faecalis). Bacteria; Firmicutes; Bacilli; Lactobacillales; Enterococcaceae;
DRJ71__locus_1
EUT2A
13230 -pf00232 -
13235 -pf00358pf00367pf02378 -
13240 -pf13091pf13396 -
13245 -pf10035 -
13250 -pf08820 -
13255 -pf01368pf02833 -
13260 HMMgreae_1_grms(pf04055) GRM3A
13265 HMMgre_1_grms(pf01228pf02901) GRM5
13270 -pf03989pf00521 -
13275 -pf00204pf00986pf02518pf01751 -
13280 -pf13380 -
13285 HMM06249EutQ_2_eut2(pf06249) EUT2A EP_NTERM
13290 HMMtpEuth_1_eut(pf04346) EUT2A
13295 P_azul EUT2A
13300 HMM02441_07 EUT2A EP_NTERM
13305 HMMptac_3_eut2(pf06130) EUT2A EP_NTERM
13310 - -
13315 H_fuchsia EUT2G
13320 HMMalddh_2_egra(pf00171) EUT2A EP_CTERM
13325 H_fireEngine EUT2A
13330 Tsp_amethyst EUT2A
13335 HMMetlyEutC_3_eut2(pf05985) EUT2A EP_NTERM
13340 HMMetlyEutB_1_EUT1(pf06751) EUT2A
13345 HMM06277EutA_1_eut(pf06277) EUT2A
13350 HMMreg07568_1_eut2(pf12282p
13355 HMMreg00072_1_eut2(pf038
13360 Hp_euts_babyPurple PDU1C
BMC-P
BMC-H
BMC-T
Aldehyde dehydrogenase
PTAC
Signature enzyme
Alcohol dehydrogenase
Regulator

Lactobacillus sp. Bacteria; Firmicutes; Bacilli; Lactobacillales; Lactobacillaceae;

DUD34__locus_1

EUT2A

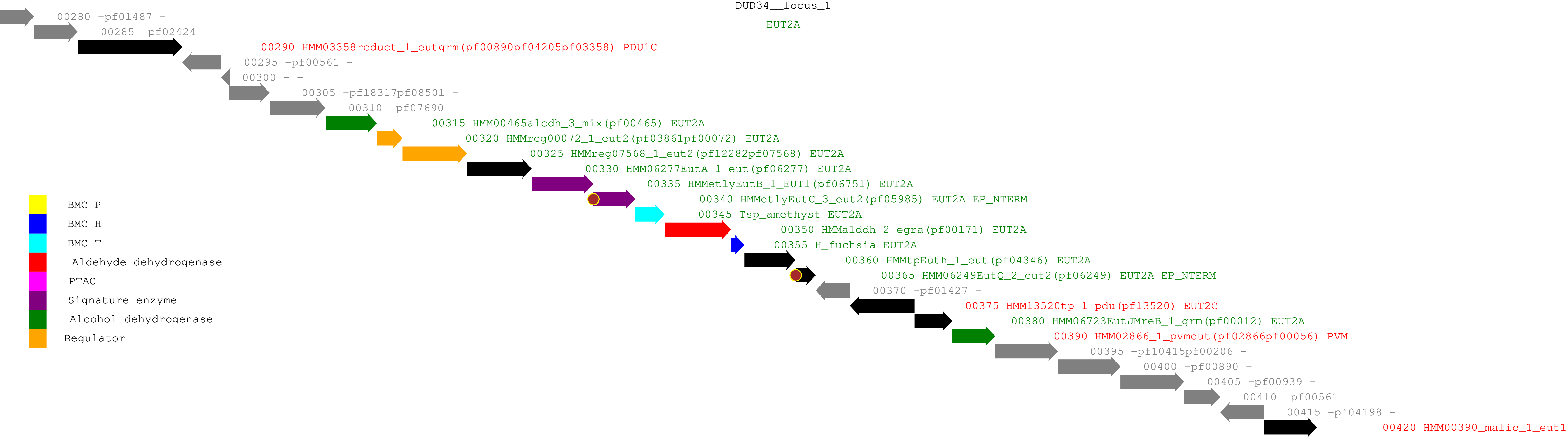

Enterococcus faecalis (Streptococcus faecalis). Bacteria; Firmicutes; Bacilli; Lactobacillales; Enterococcaceae;

DVW78__locus_1

EUT2A

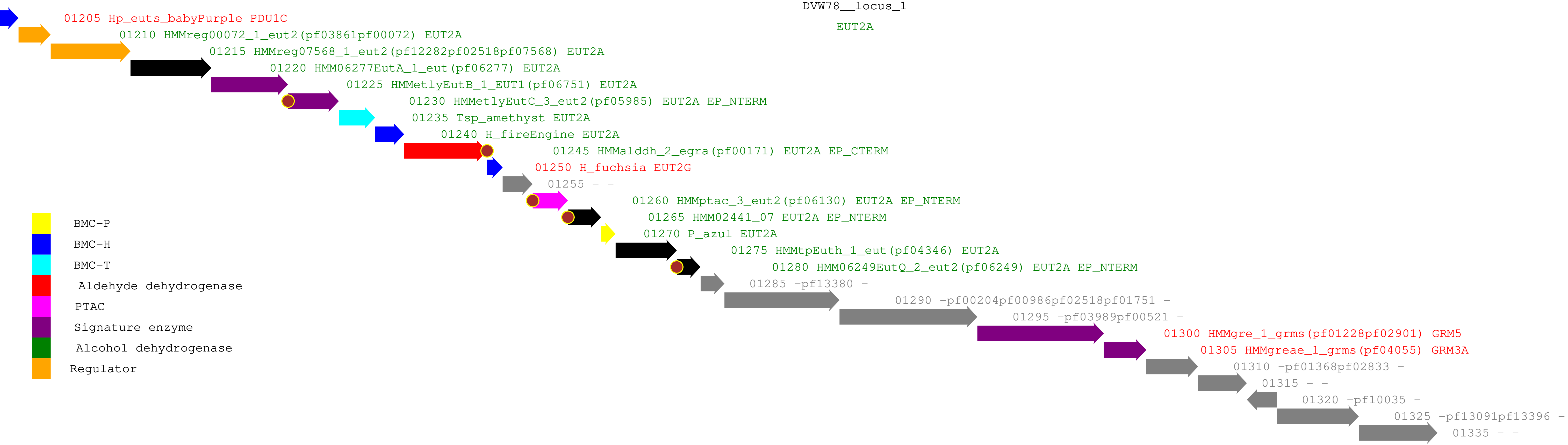

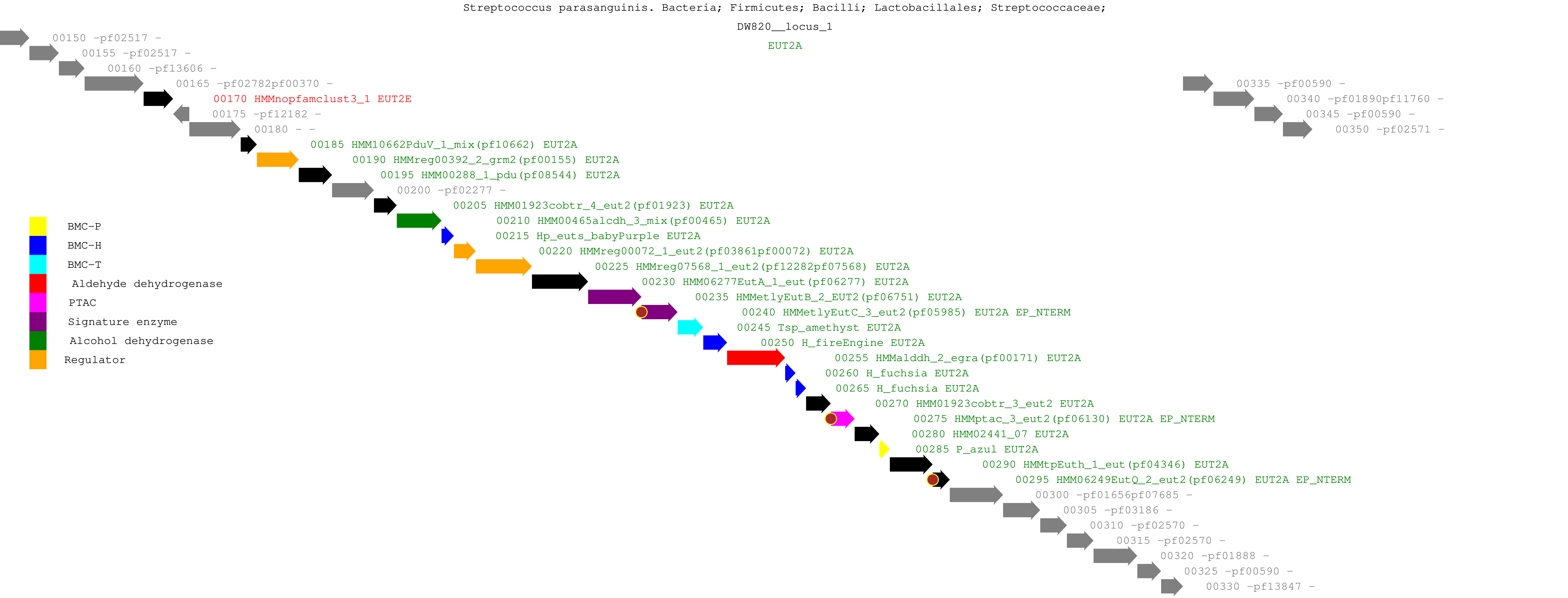

Streptococcus parasanguinis. Bacteria; Firmicutes; Bacilli; Lactobacillales; Streptococcaceae;
DW820__locus_1
EUT2A
00150 -pf02517 -
00155 -pf02517 -
00160 -pf13606 -
00165 -pf02782pf00370 -
00170 HMMnopfamclust3_1 EUT2E
00175 -pf12182 -
00180 - -
00185 HMM10662PduV_1_mix(pf10662) EUT2A
00190 HMMreg00392_2_grm2(pf00155) EUT2A
00195 HMM00288_1_pdu(pf08544) EUT2A
00200 -pf02277 -
00205 HMM01923cobtr_4_eut2(pf01923) EUT2A
00210 HMM00465alcdh_3_mix(pf00465) EUT2A
00215 Hp_euts_babyPurple EUT2A
00220 HMMreg00072_1_eut2(pf03861pf00072) EUT2A
00225 HMMreg07568_1_eut2(pf12282pf07568) EUT2A
00230 HMM06277EutA_1_eut(pf06277) EUT2A
00235 HMMetlyEutB_2_EUT2(pf06751) EUT2A
00240 HMMetlyEutC_3_eut2(pf05985) EUT2A EP_NTERM
00245 Tsp_amethyst EUT2A
00250 H_fireEngine EUT2A
00255 HMMalddh_2_egra(pf00171) EUT2A
00260 H_fuchsia EUT2A
00265 H_fuchsia EUT2A
00270 HMM01923cobtr_3_eut2 EUT2A
00275 HMMptac_3_eut2(pf06130) EUT2A EP_NTERM
00280 HMM02441_07 EUT2A
00285 P_azul EUT2A
00290 HMMtpEuth_1_eut(pf04346) EUT2A
00295 HMM06249EutQ_2_eut2(pf06249) EUT2A EP_NTERM
00300 -pf01656pf07685 -
00305 -pf03186 -
00310 -pf02570 -
00315 -pf02570 -
00320 -pf01888 -
00325 -pf00590 -
00330 -pf13847 -
00335 -pf00590 -
00340 -pf01890pf11760 -
00345 -pf00590 -
00350 -pf02571 -
BMC-P
BMC-H
BMC-T
Aldehyde dehydrogenase
PTAC
Signature enzyme
Alcohol dehydrogenase
Regulator

Listeria monocytogenes serotype 1/2b. Bacteria; Firmicutes; Bacilli; Bacillales; Listeriaceae; Listeria.

E3085__locus_1

EUT2A

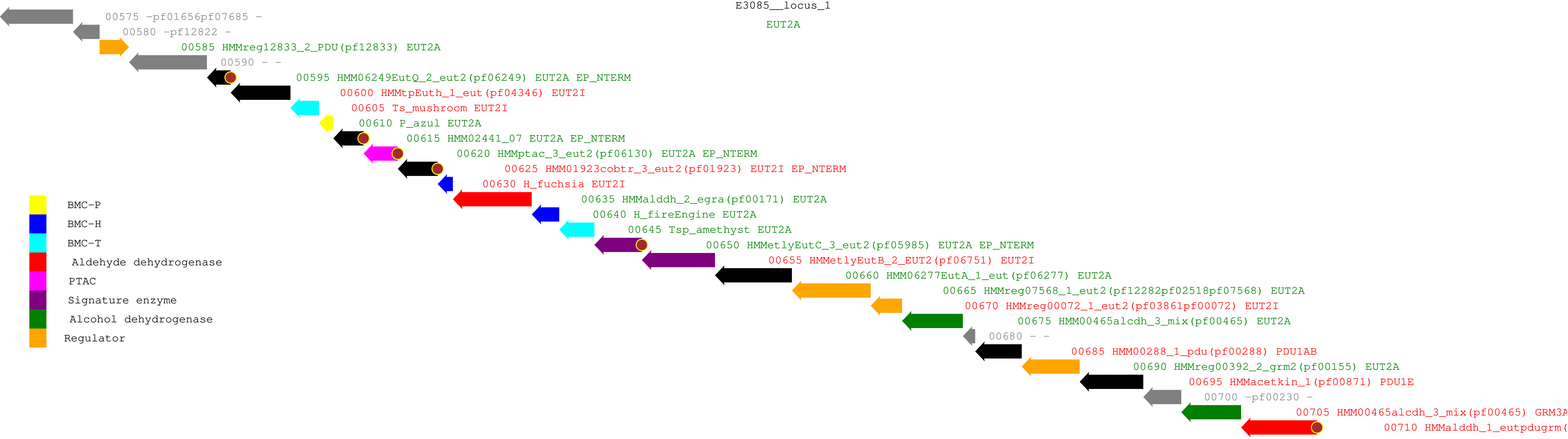

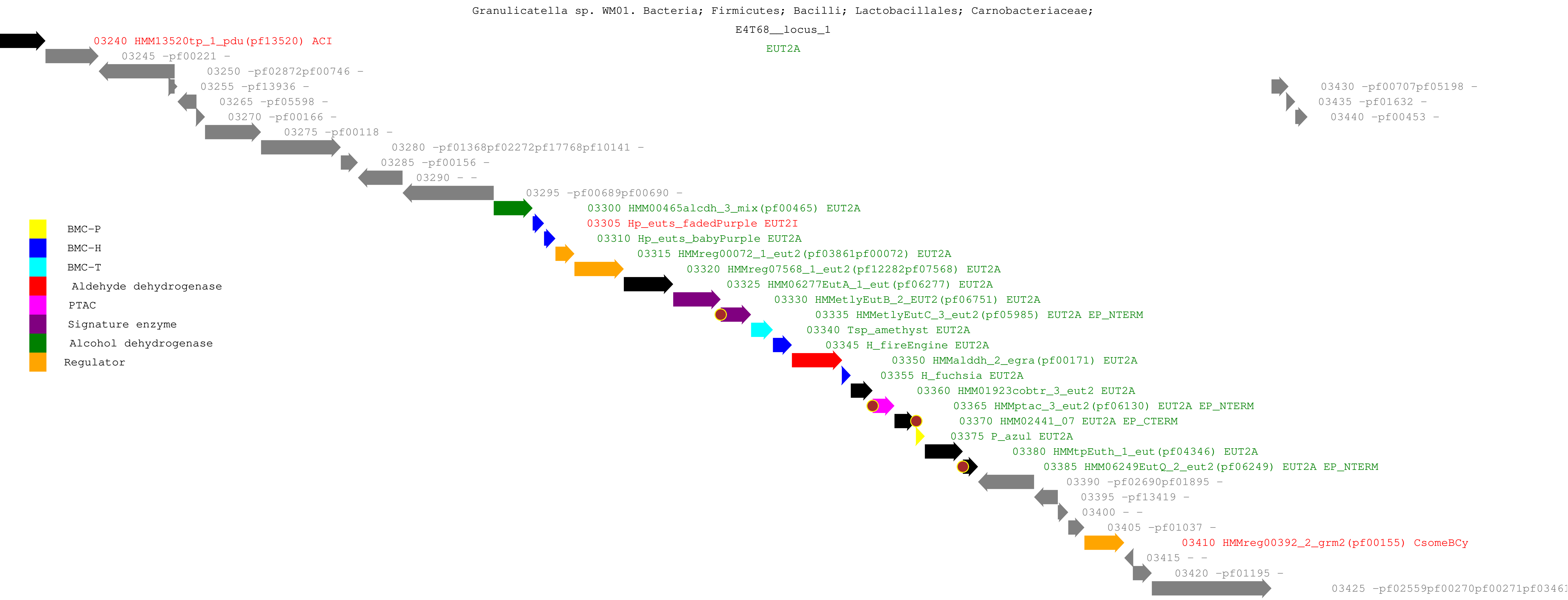
Granulicatella sp. WM01. Bacteria; Firmicutes; Bacilli; Lactobacillales; Carnobacteriaceae;
E4T68__locus_1
EUT2A
03240 HMM13520tp_1_pdu(pf13520) ACI
03245 -pf00221 -
03250 -pf02872pf00746 -
03255 -pf13936 -
03265 -pf05598 -
03270 -pf00166 -
03275 -pf00118 -
03280 -pf01368pf02272pf17768pf10141 -
03285 -pf00156 -
03290 - -
03295 -pf00689pf00690 -
03300 HMM00465alcdh_3_mix(pf00465) EUT2A
03305 Hp_euts_fadedPurple EUT2I
03310 Hp_euts_babyPurple EUT2A
03315 HMMreg00072_1_eut2(pf03861pf00072) EUT2A
03320 HMMreg07568_1_eut2(pf12282pf07568) EUT2A
03325 HMM06277EutA_1_eut(pf06277) EUT2A
03330 HMMetlyEutB_2_EUT2(pf06751) EUT2A
03335 HMMetlyEutC_3_eut2(pf05985) EUT2A EP_NTERM
03340 Tsp_amethyst EUT2A
03345 H_fireEngine EUT2A
03350 HMMalddh_2_egra(pf00171) EUT2A
03355 H_fuchsia EUT2A
03360 HMM01923cobtr_3_eut2 EUT2A
03365 HMMptac_3_eut2(pf06130) EUT2A EP_NTERM
03370 HMM02441_07 EUT2A EP_CTERM
03375 P_azul EUT2A
03380 HMMtpEuth_1_eut(pf04346) EUT2A
03385 HMM06249EutQ_2_eut2(pf06249) EUT2A EP_NTERM
03390 -pf02690pf01895 -
03395 -pf13419 -
03400 - -
03405 -pf01037 -
03410 HMMreg00392_2_grm2(pf00155) CsomeBCy
03415 - -
03420 -pf01195 -
03425 -pf02559pf00270pf00271pf0346
03430 -pf00707pf05198 -
03435 -pf01632 -
03440 -pf00453 -
BMC-P
BMC-H
BMC-T
Aldehyde dehydrogenase
PTAC
Signature enzyme
Alcohol dehydrogenase
Regulator

Streptococcus sp. WM07. Bacteria; Firmicutes; Bacilli; Lactobacillales; Streptococcaceae;

E4T71__locus_1_main_sat

EUT2A

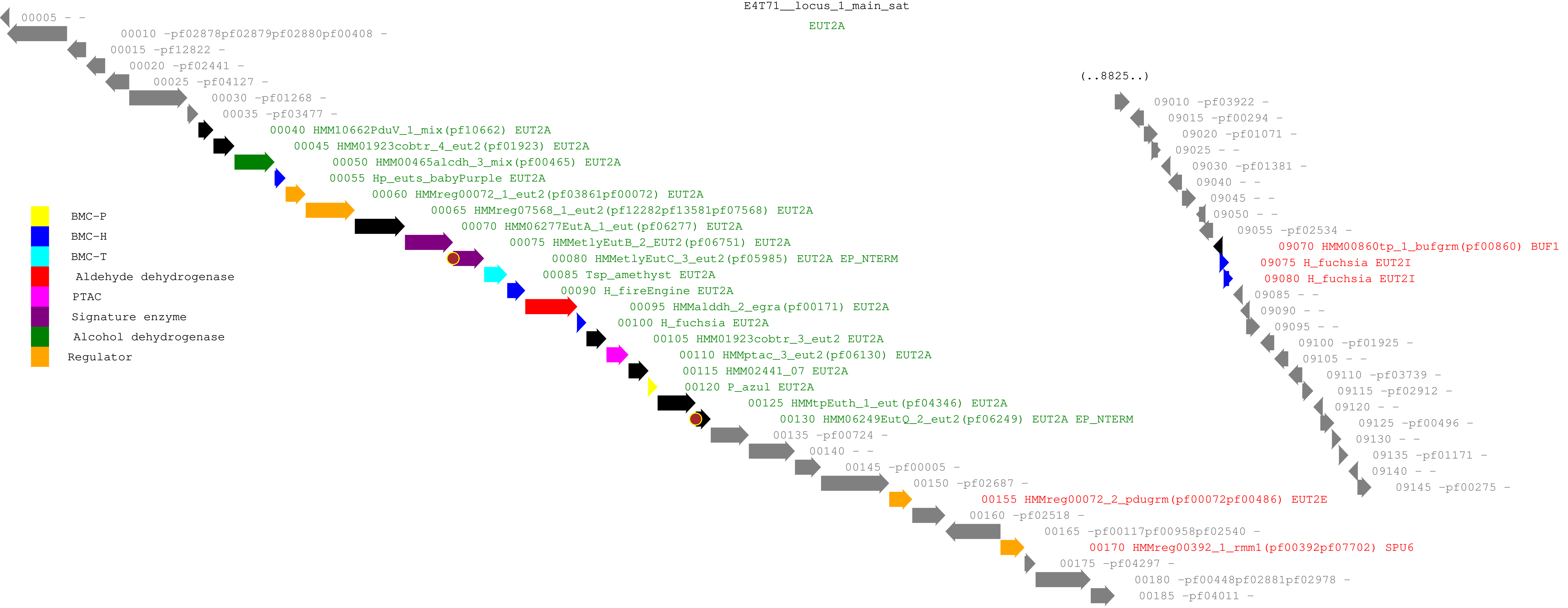

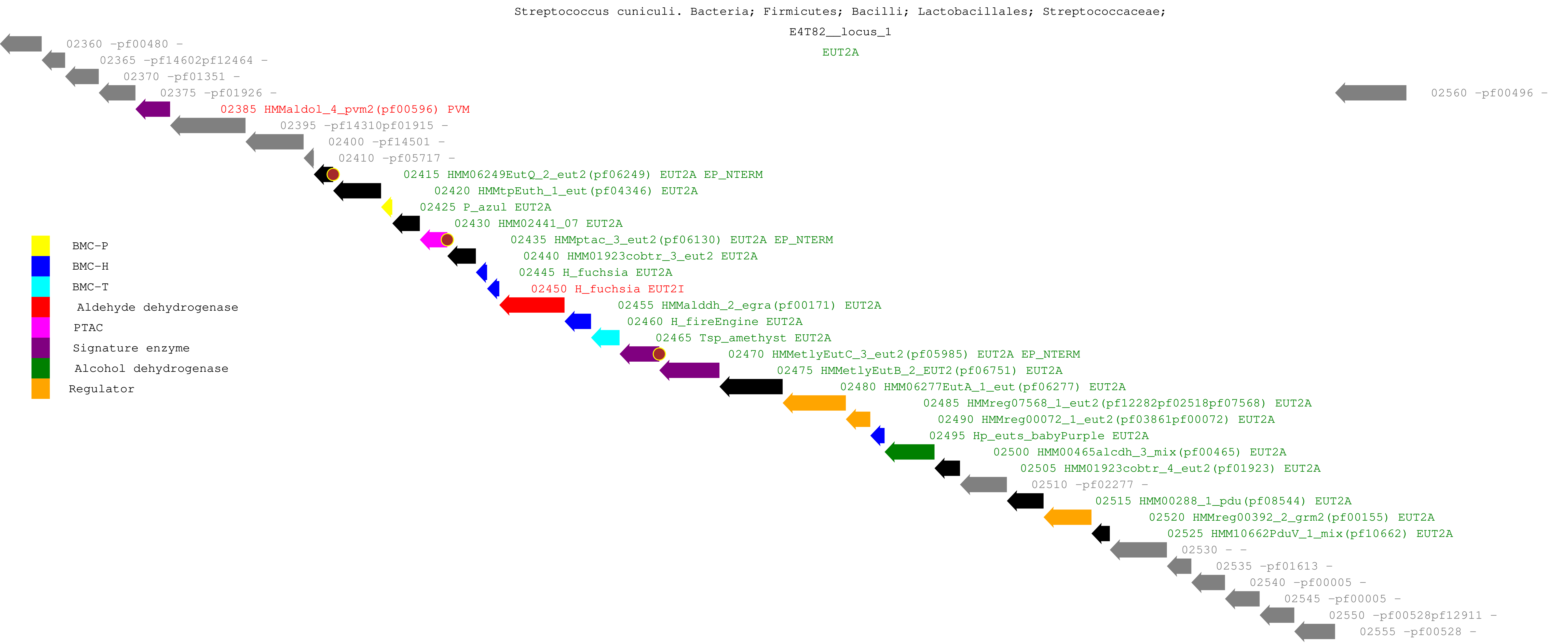
Streptococcus cuniculi. Bacteria; Firmicutes; Bacilli; Lactobacillales; Streptococcaceae;
E4T82__locus_1
EUT2A
02360 -pf00480 -
02365 -pf14602pf12464 -
02370 -pf01351 -
02375 -pf01926 -
02560 -pf00496 -
02385 HMMaldol_4_pvm2(pf00596) PVM
02395 -pf14310pf01915 -
02400 -pf14501 -
02410 -pf05717 -
02415 HMM06249EutQ_2_eut2(pf06249) EUT2A EP_NTERM
02420 HMMtpEuth_1_eut(pf04346) EUT2A
02425 P_azul EUT2A
02430 HMM02441_07 EUT2A
02435 HMMptac_3_eut2(pf06130) EUT2A EP_NTERM
02440 HMM01923cobtr_3_eut2 EUT2A
02445 H_fuchsia EUT2A
02450 H_fuchsia EUT2I
02455 HMMalddh_2_egra(pf00171) EUT2A
02460 H_fireEngine EUT2A
02465 Tsp_amethyst EUT2A
02470 HMMetlyEutC_3_eut2(pf05985) EUT2A EP_NTERM
02475 HMMetlyEutB_2_EUT2(pf06751) EUT2A
02480 HMM06277EutA_1_eut(pf06277) EUT2A
02485 HMMreg07568_1_eut2(pf12282pf02518pf07568) EUT2A
02490 HMMreg00072_1_eut2(pf03861pf00072) EUT2A
02495 Hp_euts_babyPurple EUT2A
02500 HMM00465alcdh_3_mix(pf00465) EUT2A
02505 HMM01923cobtr_4_eut2(pf01923) EUT2A
02510 -pf02277 -
02515 HMM00288_1_pdu(pf08544) EUT2A
02520 HMMreg00392_2_grm2(pf00155) EUT2A
02525 HMM10662PduV_1_mix(pf10662) EUT2A
02530 - -
02535 -pf01613 -
02540 -pf00005 -
02545 -pf00005 -
02550 -pf00528pf12911 -
02555 -pf00528 -
BMC-P
BMC-H
BMC-T
Aldehyde dehydrogenase
PTAC
Signature enzyme
Alcohol dehydrogenase
Regulator

Enterococcus avium (Streptococcus avium). Bacteria; Firmicutes; Bacilli; Lactobacillales; Enterococcaceae;

EASS39__locus_1

EUT2A

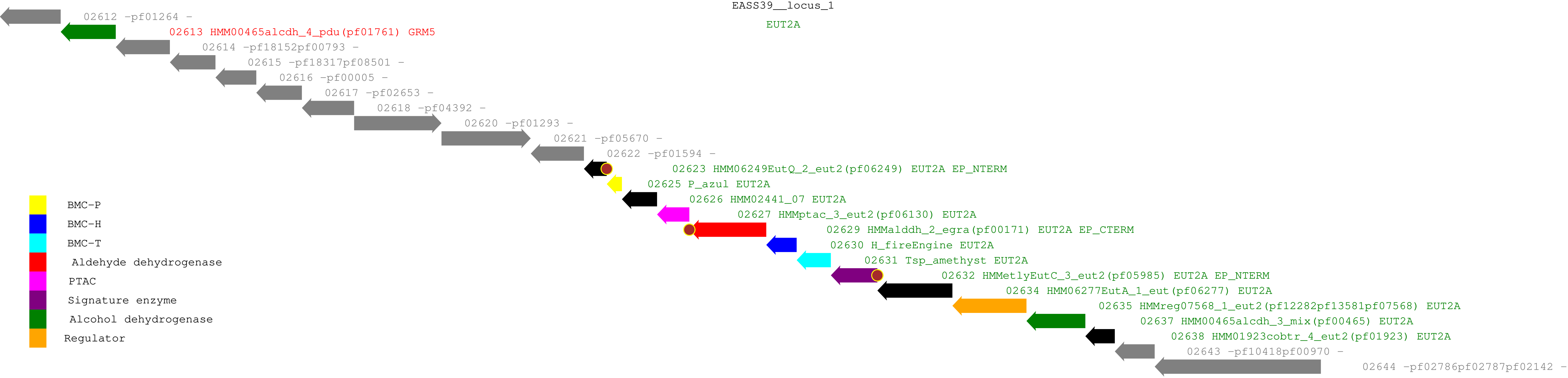

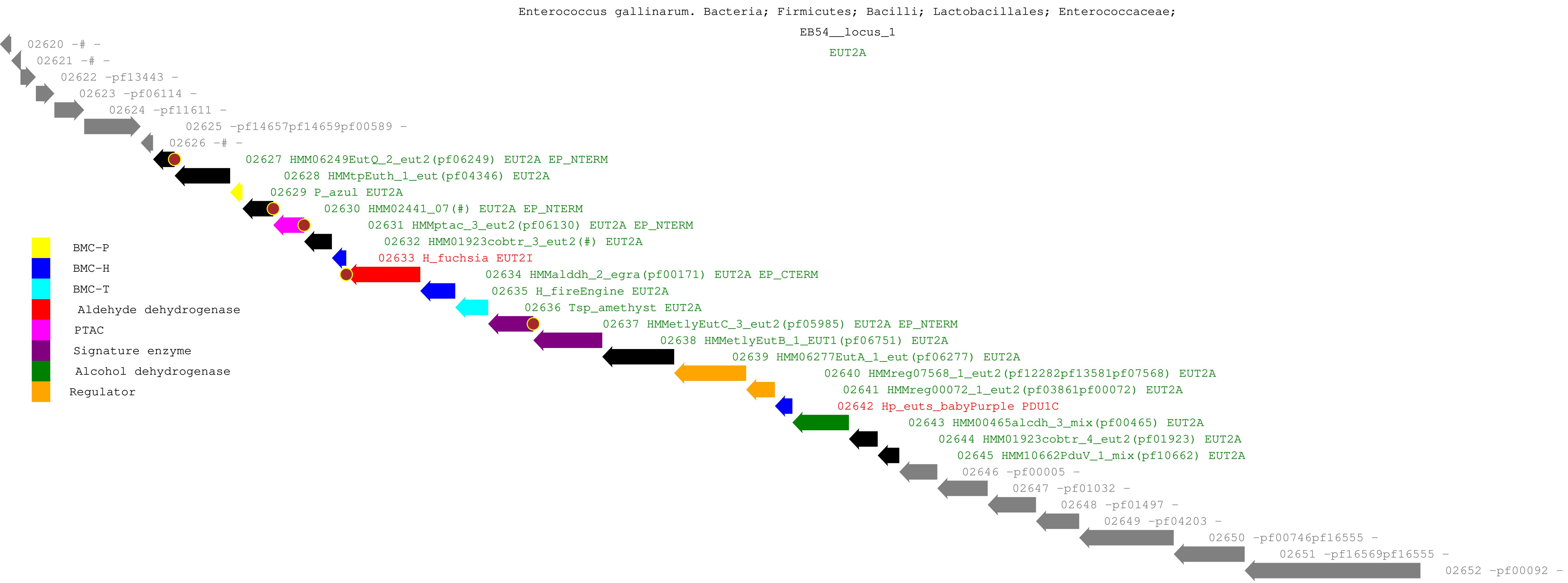
Enterococcus gallinarum. Bacteria; Firmicutes; Bacilli; Lactobacillales; Enterococcaceae;
EB54__locus_1
EUT2A
02620 -# -
02621 -# -
02622 -pf13443 -
02623 -pf06114 -
02624 -pf11611 -
02625 -pf14657pf14659pf00589 -
02626 -# -
02627 HMM06249EutQ_2_eut2(pf06249) EUT2A EP_NTERM
02628 HMMtpEuth_1_eut(pf04346) EUT2A
02629 P_azul EUT2A
02630 HMM02441_07(#) EUT2A EP_NTERM
02631 HMMptac_3_eut2(pf06130) EUT2A EP_NTERM
02632 HMM01923cobtr_3_eut2(#) EUT2A
02633 H_fuchsia EUT2I
02634 HMMalddh_2_egra(pf00171) EUT2A EP_CTERM
02635 H_fireEngine EUT2A
02636 Tsp_amethyst EUT2A
02637 HMMetlyEutC_3_eut2(pf05985) EUT2A EP_NTERM
02638 HMMetlyEutB_1_EUT1(pf06751) EUT2A
02639 HMM06277EutA_1_eut(pf06277) EUT2A
02640 HMMreg07568_1_eut2(pf12282pf13581pf07568) EUT2A
02641 HMMreg00072_1_eut2(pf03861pf00072) EUT2A
02642 Hp_euts_babyPurple PDU1C
02643 HMM00465alcdh_3_mix(pf00465) EUT2A
02644 HMM01923cobtr_4_eut2(pf01923) EUT2A
02645 HMM10662PduV_1_mix(pf10662) EUT2A
02646 -pf00005 -
02647 -pf01032 -
02648 -pf01497 -
02649 -pf04203 -
02650 -pf00746pf16555 -
02651 -pf16569pf16555 -
02652 -pf00092 -
BMC-P
BMC-H
BMC-T
Aldehyde dehydrogenase
PTAC
Signature enzyme
Alcohol dehydrogenase
Regulator

Enterococcus faecalis (Streptococcus faecalis). Bacteria; Firmicutes; Bacilli; Lactobacillales; Enterococcaceae;

EB58__locus_1

EUT2A

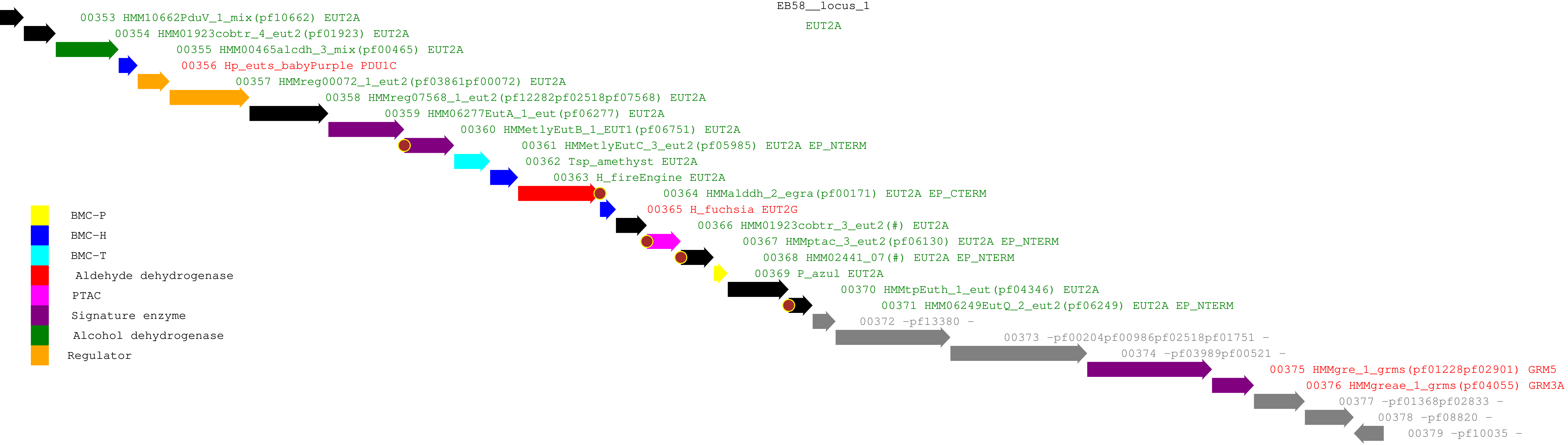

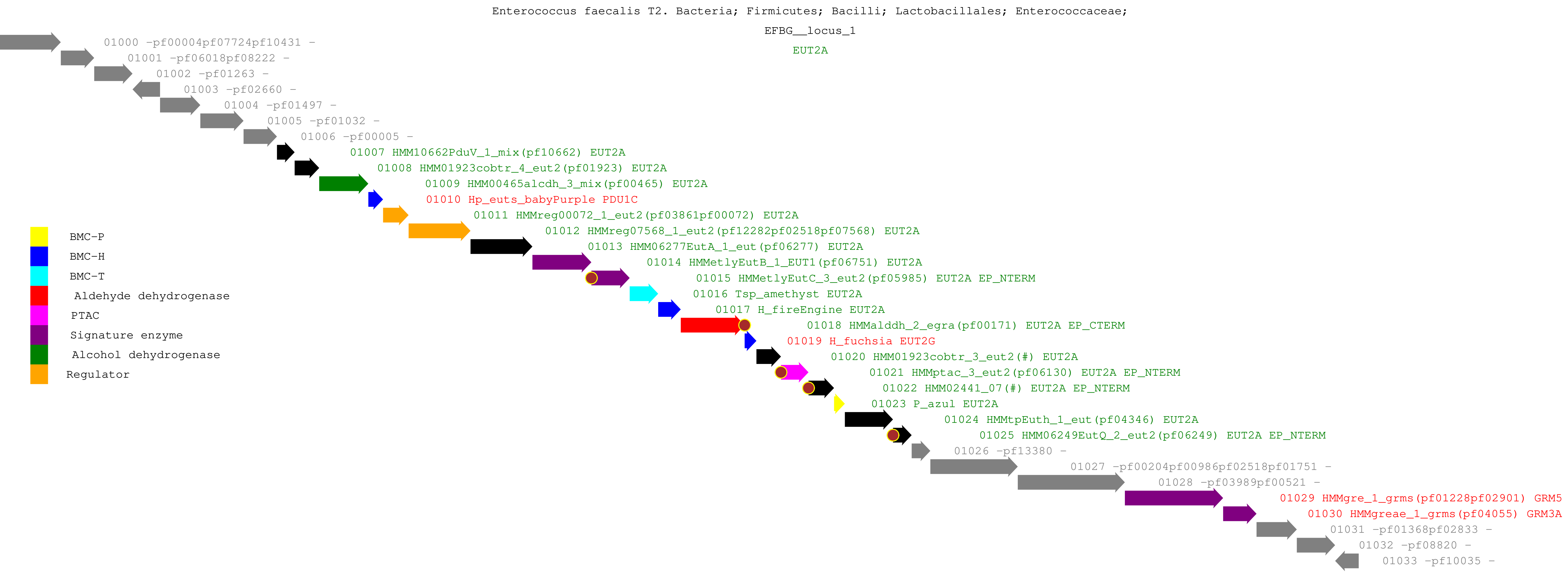
Enterococcus faecalis T2. Bacteria; Firmicutes; Bacilli; Lactobacillales; Enterococcaceae;
EFBG__locus_1
EUT2A
01000 -pf00004pf07724pf10431 -
01001 -pf06018pf08222 -
01002 -pf01263 -
01003 -pf02660 -
01004 -pf01497 -
01005 -pf01032 -
01006 -pf00005 -
01007 HMM10662PduV_1_mix(pf10662) EUT2A
01008 HMM01923cobtr_4_eut2(pf01923) EUT2A
01009 HMM00465alcdh_3_mix(pf00465) EUT2A
01010 Hp_euts_babyPurple PDU1C
01011 HMMreg00072_1_eut2(pf03861pf00072) EUT2A
01012 HMMreg07568_1_eut2(pf12282pf02518pf07568) EUT2A
01013 HMM06277EutA_1_eut(pf06277) EUT2A
01014 HMMetlyEutB_1_EUT1(pf06751) EUT2A
01015 HMMetlyEutC_3_eut2(pf05985) EUT2A EP_NTERM
01016 Tsp_amethyst EUT2A
01017 H_fireEngine EUT2A
01018 HMMalddh_2_egra(pf00171) EUT2A EP_CTERM
01019 H_fuchsia EUT2G
01020 HMM01923cobtr_3_eut2(#) EUT2A
01021 HMMptac_3_eut2(pf06130) EUT2A EP_NTERM
01022 HMM02441_07(#) EUT2A EP_NTERM
01023 P_azul EUT2A
01024 HMMtpEuth_1_eut(pf04346) EUT2A
01025 HMM06249EutQ_2_eut2(pf06249) EUT2A EP_NTERM
01026 -pf13380 -
01027 -pf00204pf00986pf02518pf01751 -
01028 -pf03989pf00521 -
01029 HMMgre_1_grms(pf01228pf02901) GRM5
01030 HMMgreae_1_grms(pf04055) GRM3A
01031 -pf01368pf02833 -
01032 -pf08820 -
01033 -pf10035 -
BMC-P
BMC-H
BMC-T
Aldehyde dehydrogenase
PTAC
Signature enzyme
Alcohol dehydrogenase
Regulator

Enterococcus faecalis Merz96. Bacteria; Firmicutes; Bacilli; Lactobacillales; Enterococcaceae;

EFGG__locus_1

EUT2A

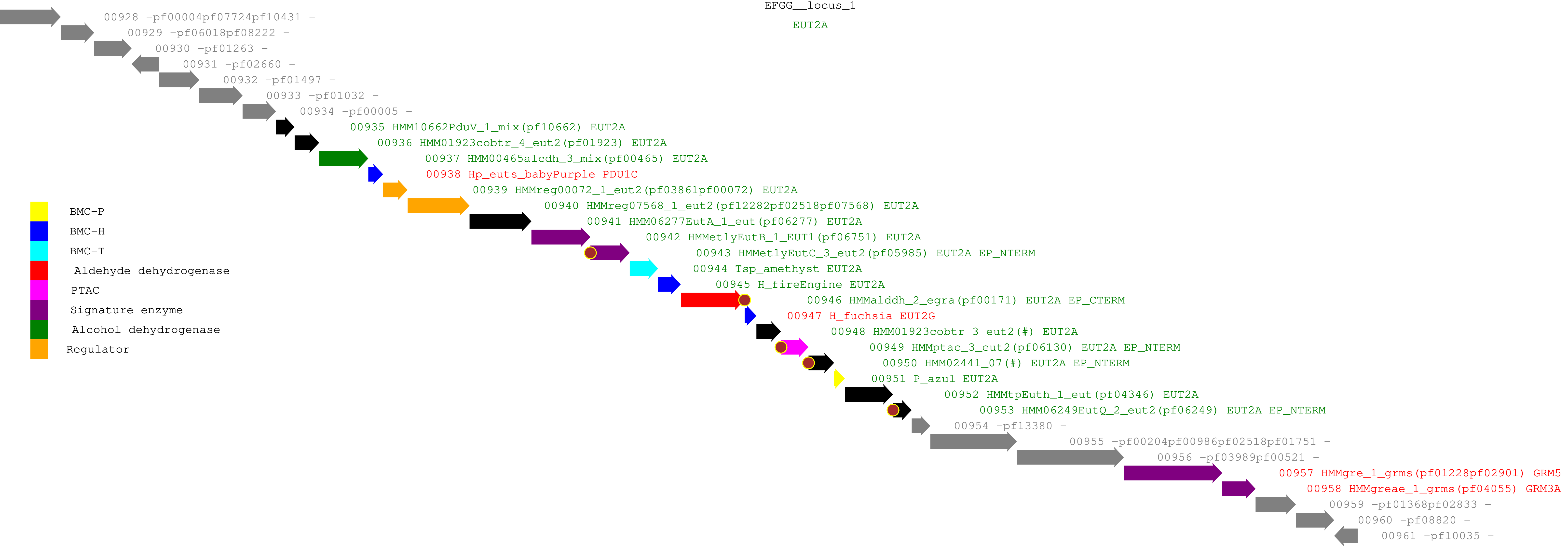

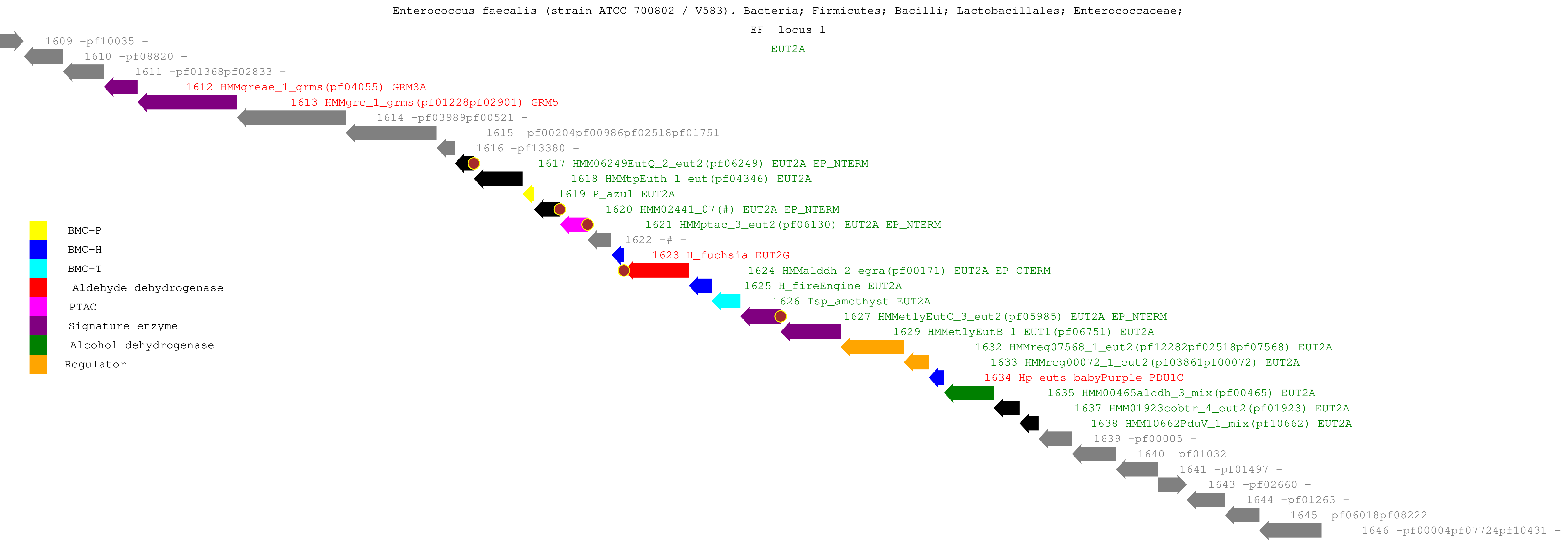
Enterococcus faecalis (strain ATCC 700802 / V583). Bacteria; Firmicutes; Bacilli; Lactobacillales; Enterococcaceae;
EF__locus_1
EUT2A
1609 -pf10035 -
1610 -pf08820 -
1611 -pf01368pf02833 -
1612 HMMgreae_1_grms(pf04055) GRM3A
1613 HMMgre_1_grms(pf01228pf02901) GRM5
1614 -pf03989pf00521 -
1615 -pf00204pf00986pf02518pf01751 -
1616 -pf13380 -
1617 HMM06249EutQ_2_eut2(pf06249) EUT2A EP_NTERM
1618 HMMtpEuth_1_eut(pf04346) EUT2A
1619 P_azul EUT2A
1620 HMM02441_07(#) EUT2A EP_NTERM
1621 HMMptac_3_eut2(pf06130) EUT2A EP_NTERM
1622 -# -
1623 H_fuchsia EUT2G
1624 HMMalddh_2_egra(pf00171) EUT2A EP_CTERM
1625 H_fireEngine EUT2A
1626 Tsp_amethyst EUT2A
1627 HMMetlyEutC_3_eut2(pf05985) EUT2A EP_NTERM
1629 HMMetlyEutB_1_EUT1(pf06751) EUT2A
1632 HMMreg07568_1_eut2(pf12282pf02518pf07568) EUT2A
1633 HMMreg00072_1_eut2(pf03861pf00072) EUT2A
1634 Hp_euts_babyPurple PDU1C
1635 HMM00465alcdh_3_mix(pf00465) EUT2A
1637 HMM01923cobtr_4_eut2(pf01923) EUT2A
1638 HMM10662PduV_1_mix(pf10662) EUT2A
1639 -pf00005 -
1640 -pf01032 -
1641 -pf01497 -
1643 -pf02660 -
1644 -pf01263 -
1645 -pf06018pf08222 -
1646 -pf00004pf07724pf10431 -
BMC-P
BMC-H
BMC-T
Aldehyde dehydrogenase
PTAC
Signature enzyme
Alcohol dehydrogenase
Regulator

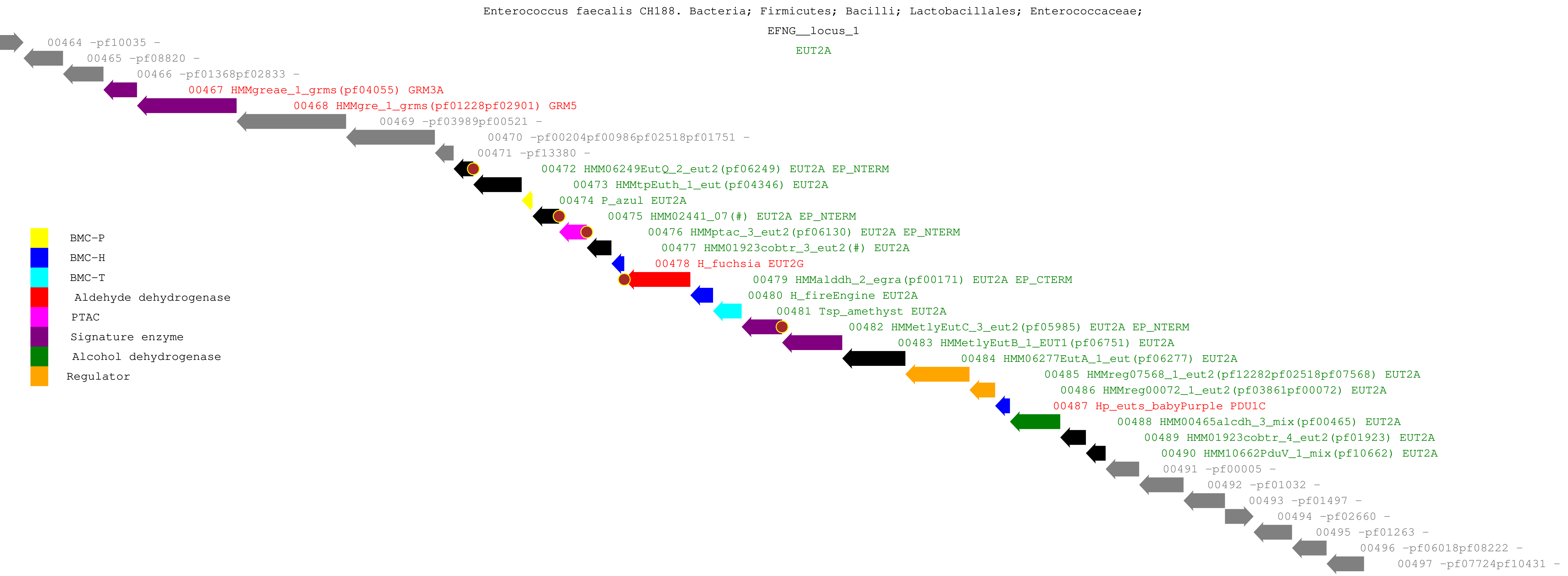

Enterococcus faecalis CH188. Bacteria; Firmicutes; Bacilli; Lactobacillales; Enterococcaceae;
EFNG__locus_1
EUT2A
00464 -pf10035 -
00465 -pf08820 -
00466 -pf01368pf02833 -
00467 HMMgreae_1_grms(pf04055) GRM3A
00468 HMMgre_1_grms(pf01228pf02901) GRM5
00469 -pf03989pf00521 -
00470 -pf00204pf00986pf02518pf01751 -
00471 -pf13380 -
00472 HMM06249EutQ_2_eut2(pf06249) EUT2A EP_NTERM
00473 HMMtpEuth_1_eut(pf04346) EUT2A
00474 P_azul EUT2A
00475 HMM02441_07(#) EUT2A EP_NTERM
00476 HMMptac_3_eut2(pf06130) EUT2A EP_NTERM
00477 HMM01923cobtr_3_eut2(#) EUT2A
00478 H_fuchsia EUT2G
00479 HMMaldh_2_egra(pf00171) EUT2A EP_CTERM
00480 H_fireEngine EUT2A
00481 Tsp_amethyst EUT2A
00482 HMMetlyEutC_3_eut2(pf05985) EUT2A EP_NTERM
00483 HMMetlyEutB_1_EUT1(pf06751) EUT2A
00484 HMM06277EutA_1_eut(pf06277) EUT2A
00485 HMMreg07568_1_eut2(pf12282pf02518pf07568) EUT2A
00486 HMMreg00072_1_eut2(pf03861pf00072) EUT2A
00487 Hp_euts_babyPurple PDU1C
00488 HMM00465alcdh_3_mix(pf00465) EUT2A
00489 HMM01923cobtr_4_eut2(pf01923) EUT2A
00490 HMM10662PduV_1_mix(pf10662) EUT2A
00491 -pf00005 -
00492 -pf01032 -
00493 -pf01497 -
00494 -pf02660 -
00495 -pf01263 -
00496 -pf06018pf08222 -
00497 -pf07724pf10431 -
BMC-P
BMC-H
BMC-T
Aldehyde dehydrogenase
PTAC
Signature enzyme
Alcohol dehydrogenase
Regulator

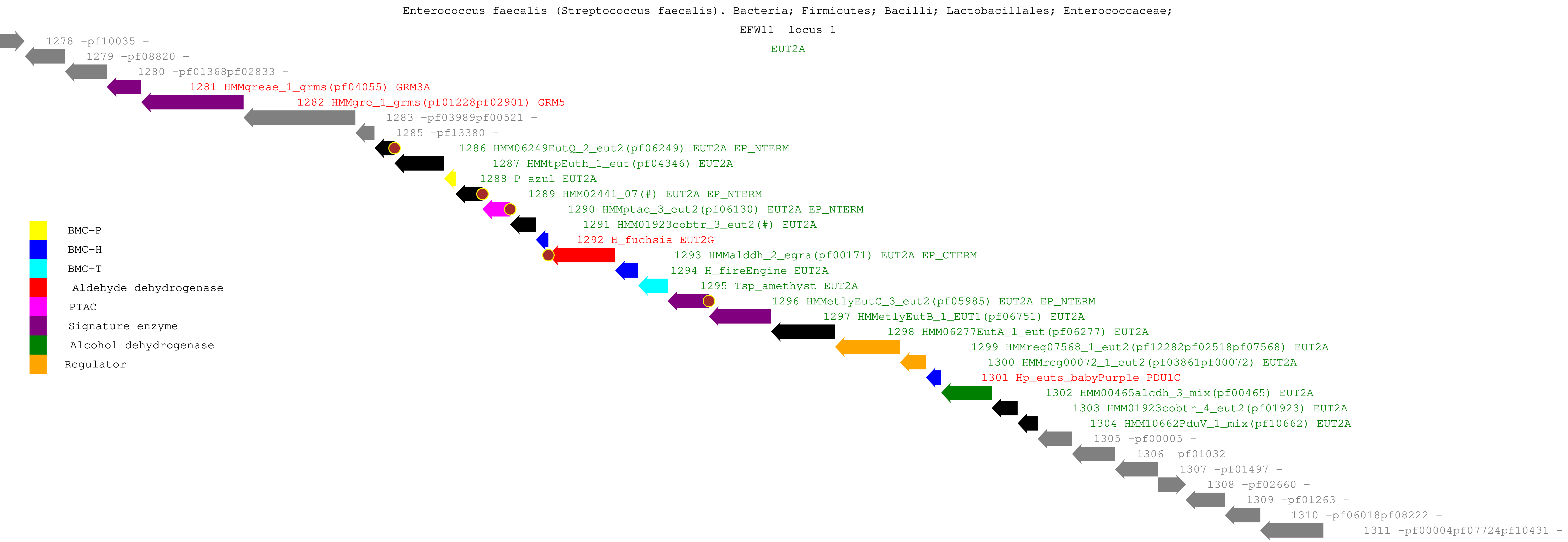
Enterococcus faecalis (Streptococcus faecalis). Bacteria; Firmicutes; Bacilli; Lactobacillales; Enterococcaceae;
EFW11__locus_1
EUT2A
1278 -pf10035 -
1279 -pf08820 -
1280 -pf01368pf02833 -
1281 HMMgreae_1_grms(pf04055) GRM3A
1282 HMMgre_1_grms(pf01228pf02901) GRM5
1283 -pf03989pf00521 -
1285 -pf13380 -
1286 HMM06249EutQ_2_eut2(pf06249) EUT2A EP_NTERM
1287 HMMtpEuth_1_eut(pf04346) EUT2A
1288 P_azul EUT2A
1289 HMM02441_07(#) EUT2A EP_NTERM
1290 HMMptac_3_eut2(pf06130) EUT2A EP_NTERM
1291 HMM01923cobtr_3_eut2(#) EUT2A
1292 H_fuchsia EUT2G
1293 HMMalddh_2_egra(pf00171) EUT2A EP_CTERM
1294 H_fireEngine EUT2A
1295 Tsp_amethyst EUT2A
1296 HMMetlyEutC_3_eut2(pf05985) EUT2A EP_NTERM
1297 HMMetlyEutB_1_EUT1(pf06751) EUT2A
1298 HMM06277EutA_1_eut(pf06277) EUT2A
1299 HMMreg07568_1_eut2(pf12282pf02518pf07568) EUT2A
1300 HMMreg00072_1_eut2(pf03861pf00072) EUT2A
1301 Hp_euts_babyPurple PDU1C
1302 HMM00465alcdh_3_mix(pf00465) EUT2A
1303 HMM01923cobtr_4_eut2(pf01923) EUT2A
1304 HMM10662PduV_1_mix(pf10662) EUT2A
1305 -pf00005 -
1306 -pf01032 -
1307 -pf01497 -
1308 -pf02660 -
1309 -pf01263 -
1310 -pf06018pf08222 -
1311 -pf00004pf07724pf10431 -
BMC-P
BMC-H
BMC-T
Aldehyde dehydrogenase
PTAC
Signature enzyme
Alcohol dehydrogenase
Regulator

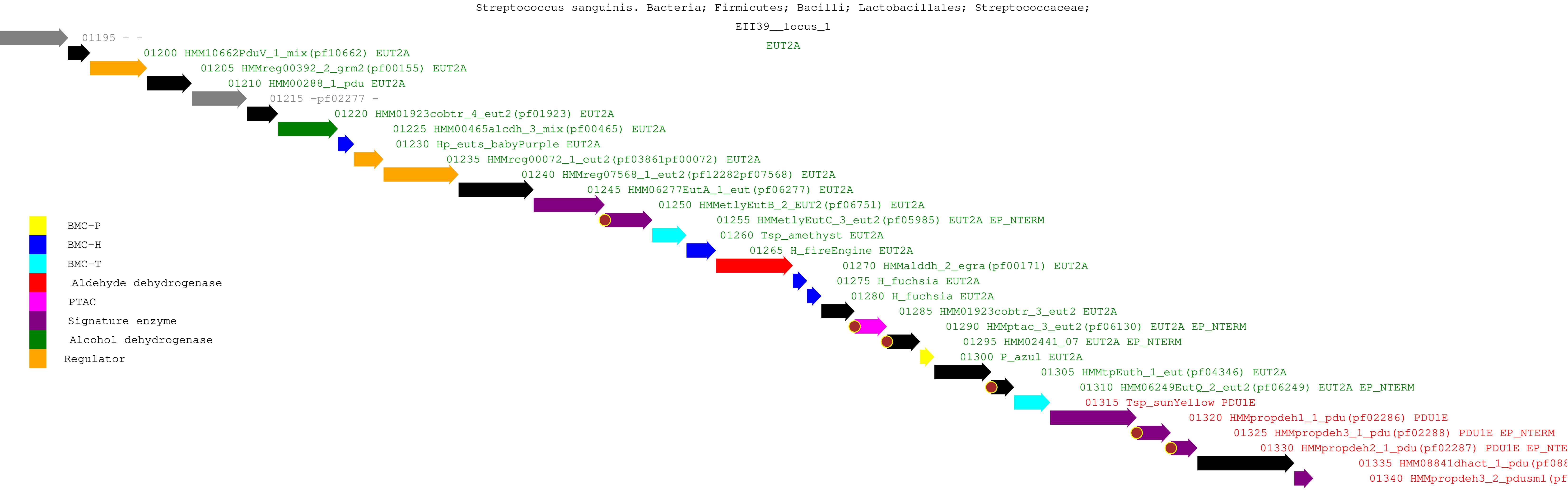

Streptococcus sanguinis. Bacteria; Firmicutes; Bacilli; Lactobacillales; Streptococcaceae;
EII39__locus_1
EUT2A
01195 - -
01200 HMM10662PduV_1_mix(pf10662) EUT2A
01205 HMMreg00392_2_grm2(pf00155) EUT2A
01210 HMM00288_1_pdu EUT2A
01215 -pf02277 -
01220 HMM01923cobtr_4_eut2(pf01923) EUT2A
01225 HMM00465alcdh_3_mix(pf00465) EUT2A
01230 Hp_euts_babyPurple EUT2A
01235 HMMreg00072_1_eut2(pf03861pf00072) EUT2A
01240 HMMreg07568_1_eut2(pf12282pf07568) EUT2A
01245 HMM06277EutA_1_eut(pf06277) EUT2A
01250 HMMetlyEutB_2_EUT2(pf06751) EUT2A
01255 HMMetlyEutC_3_eut2(pf05985) EUT2A EP_NTERM
01260 Tsp_amethyst EUT2A
01265 H_fireEngine EUT2A
01270 HMMalddh_2_egra(pf00171) EUT2A
01275 H_fuchsia EUT2A
01280 H_fuchsia EUT2A
01285 HMM01923cobtr_3_eut2 EUT2A
01290 HMMptac_3_eut2(pf06130) EUT2A EP_NTERM
01295 HMM02441_07 EUT2A EP_NTERM
01300 P_azul EUT2A
01305 HMMtpEuth_1_eut(pf04346) EUT2A
01310 HMM06249EutQ_2_eut2(pf06249) EUT2A EP_NTERM
01315 Tsp_sunYellow PDU1E
01320 HMMpropdeh1_1_pdu(pf02286) PDU1E
01325 HMMpropdeh3_1_pdu(pf02288) PDU1E EP_NTERM
01330 HMMpropdeh2_1_pdu(pf02287) PDU1E EP_NTE
01335 HMM08841dhact_1_pdu(pf088
01340 HMMpropdeh3_2_pdusml(pf
BMC-P
BMC-H
BMC-T
Aldehyde dehydrogenase
PTAC
Signature enzyme
Alcohol dehydrogenase
Regulator

Enterococcus avium (Streptococcus avium). Bacteria; Firmicutes; Bacilli; Lactobacillales; Enterococcaceae;

EK398__locus_1

EUT2A

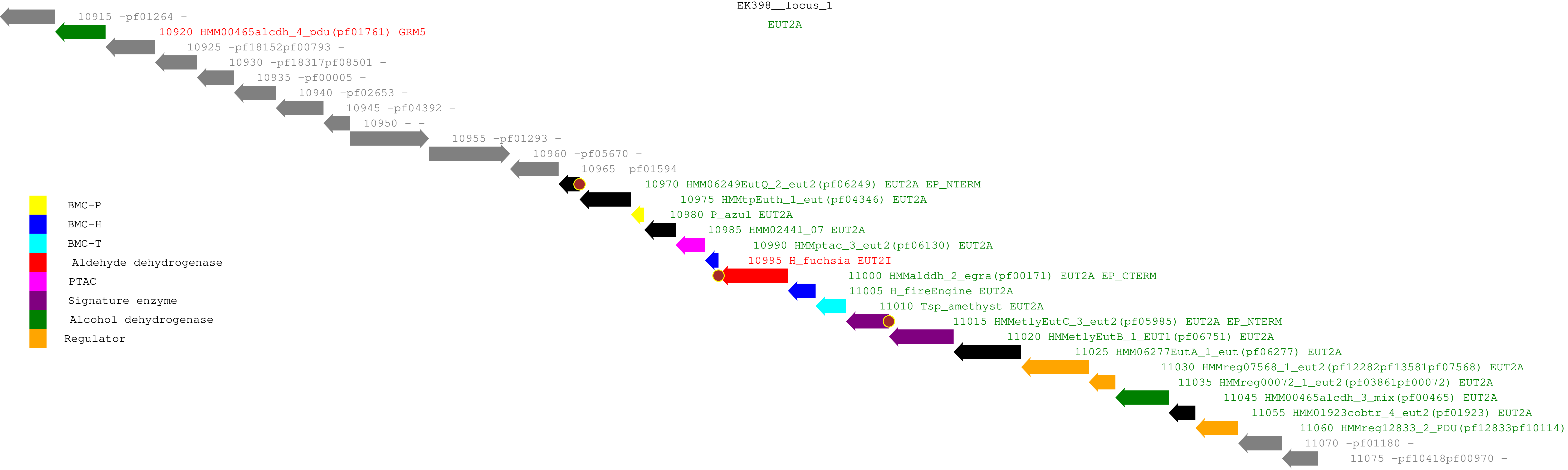

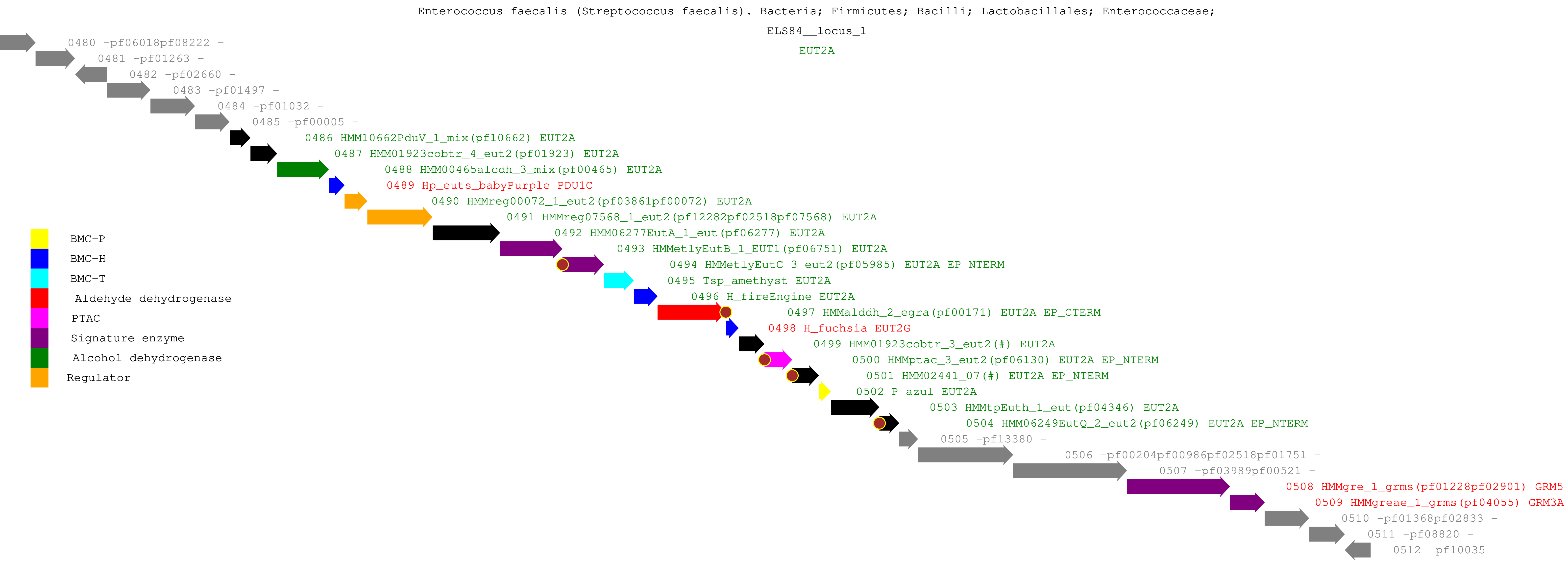
Enterococcus faecalis (Streptococcus faecalis). Bacteria; Firmicutes; Bacilli; Lactobacillales; Enterococcaceae;
ELS84__locus_1
EUT2A
0480 -pf06018pf08222 -
0481 -pf01263 -
0482 -pf02660 -
0483 -pf01497 -
0484 -pf01032 -
0485 -pf00005 -
0486 HMM10662PduV_1_mix(pf10662) EUT2A
0487 HMM01923cobtr_4_eut2(pf01923) EUT2A
0488 HMM00465alcdh_3_mix(pf00465) EUT2A
0489 Hp_euts_babyPurple PDU1C
0490 HMMreg00072_1_eut2(pf03861pf00072) EUT2A
0491 HMMreg07568_1_eut2(pf12282pf02518pf07568) EUT2A
0492 HMM06277EutA_1_eut(pf06277) EUT2A
0493 HMMetlyEutB_1_EUT1(pf06751) EUT2A
0494 HMMetlyEutC_3_eut2(pf05985) EUT2A EP_NTERM
0495 Tsp_amethyst EUT2A
0496 H_fireEngine EUT2A
0497 HMMalddh_2_egra(pf00171) EUT2A EP_CTERM
0498 H_fuchsia EUT2G
0499 HMM01923cobtr_3_eut2(#) EUT2A
0500 HMMptac_3_eut2(pf06130) EUT2A EP_NTERM
0501 HMM02441_07(#) EUT2A EP_NTERM
0502 P_azul EUT2A
0503 HMMtpEuth_1_eut(pf04346) EUT2A
0504 HMM06249EutQ_2_eut2(pf06249) EUT2A EP_NTERM
0505 -pf13380 -
0506 -pf00204pf00986pf02518pf01751 -
0507 -pf03989pf00521 -
0508 HMMgre_1_grms(pf01228pf02901) GRM5
0509 HMMgreae_1_grms(pf04055) GRM3A
0510 -pf01368pf02833 -
0511 -pf08820 -
0512 -pf10035 -
BMC-P
BMC-H
BMC-T
Aldehyde dehydrogenase
PTAC
Signature enzyme
Alcohol dehydrogenase
Regulator

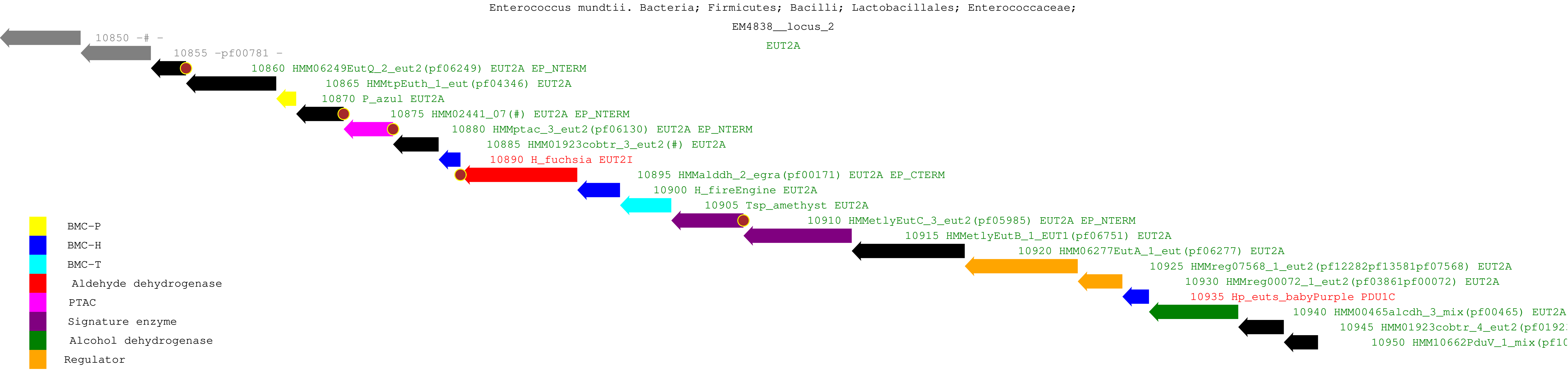
Enterococcus mundtii. Bacteria; Firmicutes; Bacilli; Lactobacillales; Enterococcaceae;
EM4838__locus_2
EUT2A
10850 -# -
10855 -pf00781 -
10860 HMM06249EutQ_2_eut2(pf06249) EUT2A EP_NTERM
10865 HMMtpEuth_1_eut(pf04346) EUT2A
10870 P_azul EUT2A
10875 HMM02441_07(#) EUT2A EP_NTERM
10880 HMMptac_3_eut2(pf06130) EUT2A EP_NTERM
10885 HMM01923cobtr_3_eut2(#) EUT2A
10890 H_fuchsia EUT2I
10895 HMMalddh_2_egra(pf00171) EUT2A EP_CTERM
10900 H_fireEngine EUT2A
10905 Tsp_amethyst EUT2A
10910 HMMetlyEutC_3_eut2(pf05985) EUT2A EP_NTERM
10915 HMMetlyEutB_1_EUT1(pf06751) EUT2A
10920 HMM06277EutA_1_eut(pf06277) EUT2A
10925 HMMreg07568_1_eut2(pf12282pf13581pf07568) EUT2A
10930 HMMreg00072_1_eut2(pf03861pf00072) EUT2A
10935 Hp_euts_babyPurple PDU1C
10940 HMM00465alcdh_3_mix(pf00465) EUT2A
BMC-P
BMC-H
BMC-T
Aldehyde dehydrogenase
PTAC
Signature enzyme
Alcohol dehydrogenase
Regulator

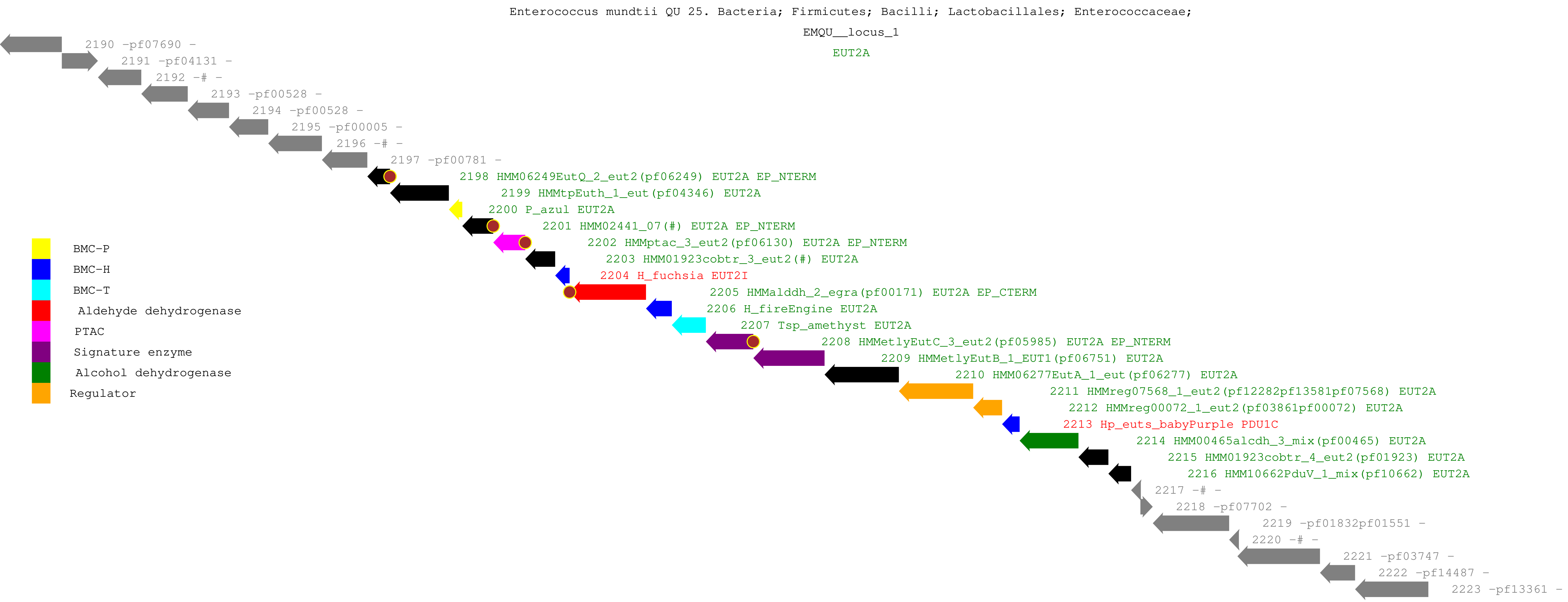

Enterococcus mundtii QU 25. Bacteria; Firmicutes; Bacilli; Lactobacillales; Enterococcaceae;
EMQU__locus_1
EUT2A
2190 -pf07690 -
2191 -pf04131 -
2192 -# -
2193 -pf00528 -
2194 -pf00528 -
2195 -pf00005 -
2196 -# -
2197 -pf00781 -
2198 HMM06249EutQ_2_eut2(pf06249) EUT2A EP_NTERM
2199 HMMtpEuth_1_eut(pf04346) EUT2A
2200 P_azul EUT2A
2201 HMM02441_07(#) EUT2A EP_NTERM
2202 HMMptac_3_eut2(pf06130) EUT2A EP_NTERM
2203 HMM01923cobtr_3_eut2(#) EUT2A
2204 H_fuchsia EUT2I
2205 HMMaldddh_2_egra(pf00171) EUT2A EP_CTERM
2206 H_fireEngine EUT2A
2207 Tsp_amethyst EUT2A
2208 HMMetlyEutC_3_eut2(pf05985) EUT2A EP_NTERM
2209 HMMetlyEutB_1_EUT1(pf06751) EUT2A
2210 HMM06277EutA_1_eut(pf06277) EUT2A
2211 HMMreg07568_1_eut2(pf12282pf13581pf07568) EUT2A
2212 HMMreg00072_1_eut2(pf03861pf00072) EUT2A
2213 Hp_euts_babyPurple PDU1C
2214 HMM00465alcdh_3_mix(pf00465) EUT2A
2215 HMM01923cobtr_4_eut2(pf01923) EUT2A
2216 HMM10662PduV_1_mix(pf10662) EUT2A
2217 -# -
2218 -pf07702 -
2219 -pf01832pf01551 -
2220 -# -
2221 -pf03747 -
2222 -pf14487 -
2223 -pf13361 -
BMC-P
BMC-H
BMC-T
Aldehyde dehydrogenase
PTAC
Signature enzyme
Alcohol dehydrogenase
Regulator

Mycobacterium abscessus. Bacteria; Actinobacteria; Corynebacteriales; Mycobacteriaceae;

ERS075544__locus_1

EUT2A

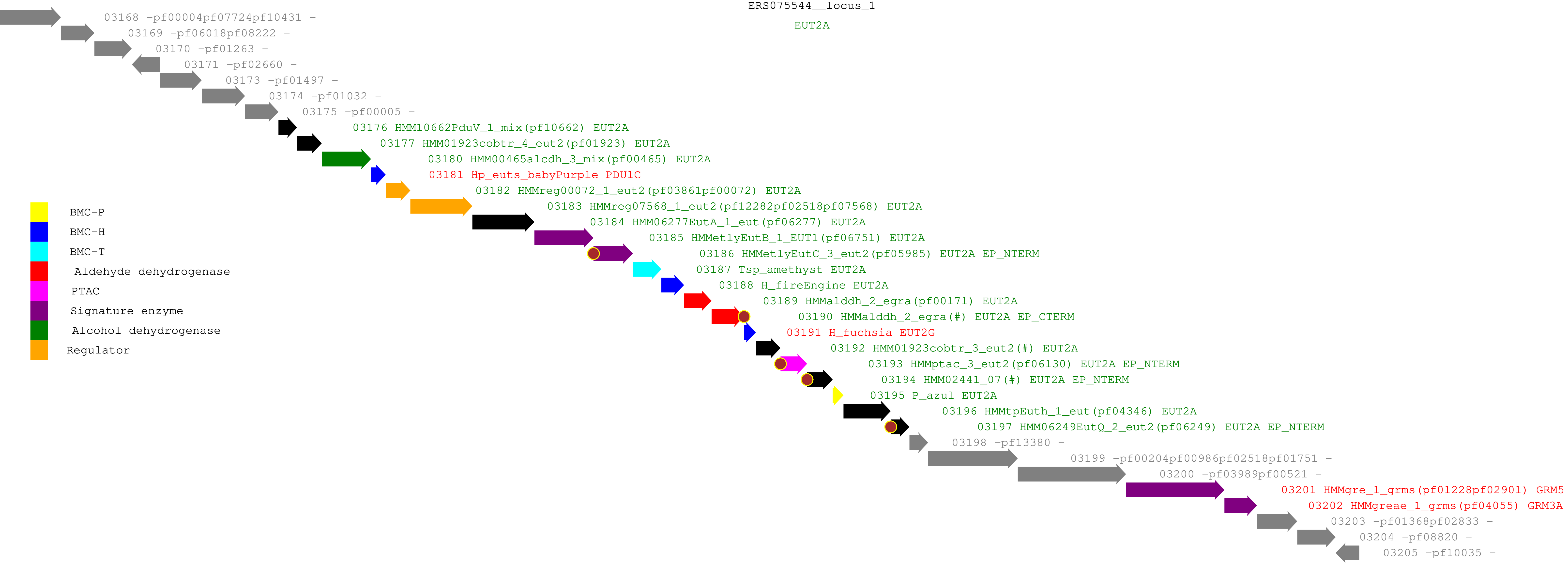

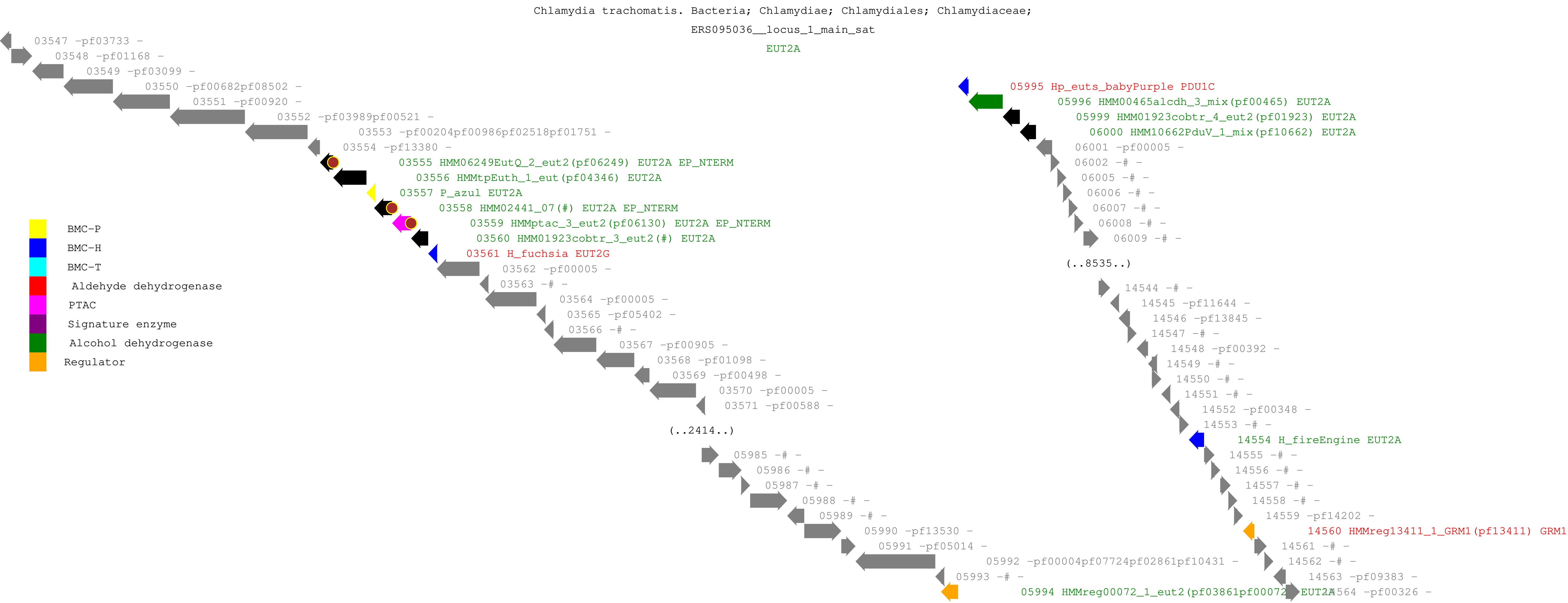
Chlamydia trachomatis. Bacteria; Chlamydiae; Chlamydiales; Chlamydiaceae;
ERS095036__locus_1_main_sat
EUT2A
03547 -pf03733 -
03548 -pf01168 -
03549 -pf03099 -
03550 -pf00682pf08502 -
03551 -pf00920 -
03552 -pf03989pf00521 -
03553 -pf00204pf00986pf02518pf01751 -
03554 -pf13380 -
03555 HMM06249EutQ_2_eut2(pf06249) EUT2A EP_NTERM
03556 HMMtpEuth_1_eut(pf04346) EUT2A
03557 P_azul EUT2A
03558 HMM02441_07(#) EUT2A EP_NTERM
03559 HMMptac_3_eut2(pf06130) EUT2A EP_NTERM
03560 HMM01923cobtr_3_eut2(#) EUT2A
03561 H_fuchsia EUT2G
03562 -pf00005 -
03563 -# -
03564 -pf00005 -
03565 -pf05402 -
03566 -# -
03567 -pf00905 -
03568 -pf01098 -
03569 -pf00498 -
03570 -pf00005 -
03571 -pf00588 -
(..2414..)
05985 -# -
05986 -# -
05987 -# -
05988 -# -
05989 -# -
05990 -pf13530 -
05991 -pf05014 -
05992 -pf00004pf07724pf02861pf10431 -
05993 -# -
05994 HMMreg00072_1_eut2(pf03861pf00072) EUT2A
05995 Hp_euts_babyPurple PDU1C
05996 HMM00465alcdh_3_mix(pf00465) EUT2A
05999 HMM01923cobtr_4_eut2(pf01923) EUT2A
06000 HMM10662PduV_1_mix(pf10662) EUT2A
06001 -pf00005 -
06002 -# -
06005 -# -
06006 -# -
06007 -# -
06008 -# -
06009 -# -
(..8535..)
14544 -# -
14545 -pf11644 -
14546 -pf13845 -
14547 -# -
14548 -pf00392 -
14549 -# -
14550 -# -
14551 -# -
14552 -pf00348 -
14553 -# -
14554 H_fireEngine EUT2A
14555 -# -
14556 -# -
14557 -# -
14558 -# -
14559 -pf14202 -
14560 HMMreg13411_1_GRM1(pf13411) GRM1
14561 -# -
14562 -# -
14563 -pf09383 -
14564 -pf00326 -
BMC-P
BMC-H
BMC-T
Aldehyde dehydrogenase
PTAC
Signature enzyme
Alcohol dehydrogenase
Regulator

Streptococcus pneumoniae. Bacteria; Firmicutes; Bacilli; Lactobacillales; Streptococcaceae;

ERS409405__locus_1

EUT2A

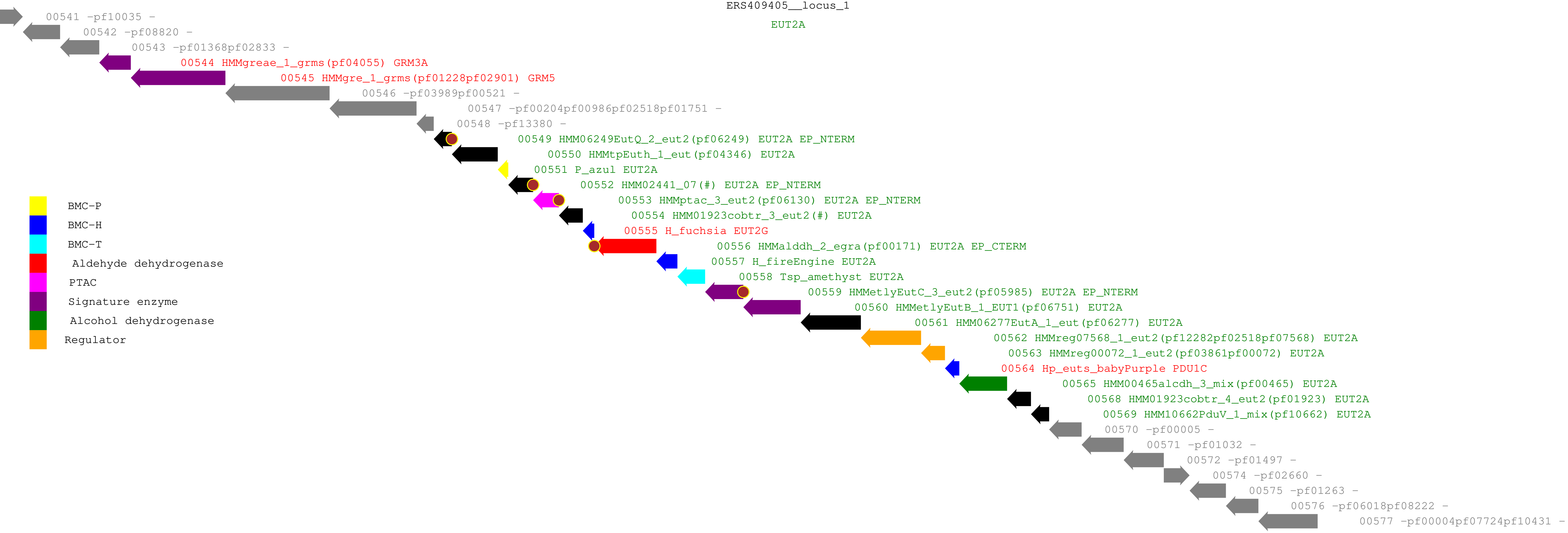

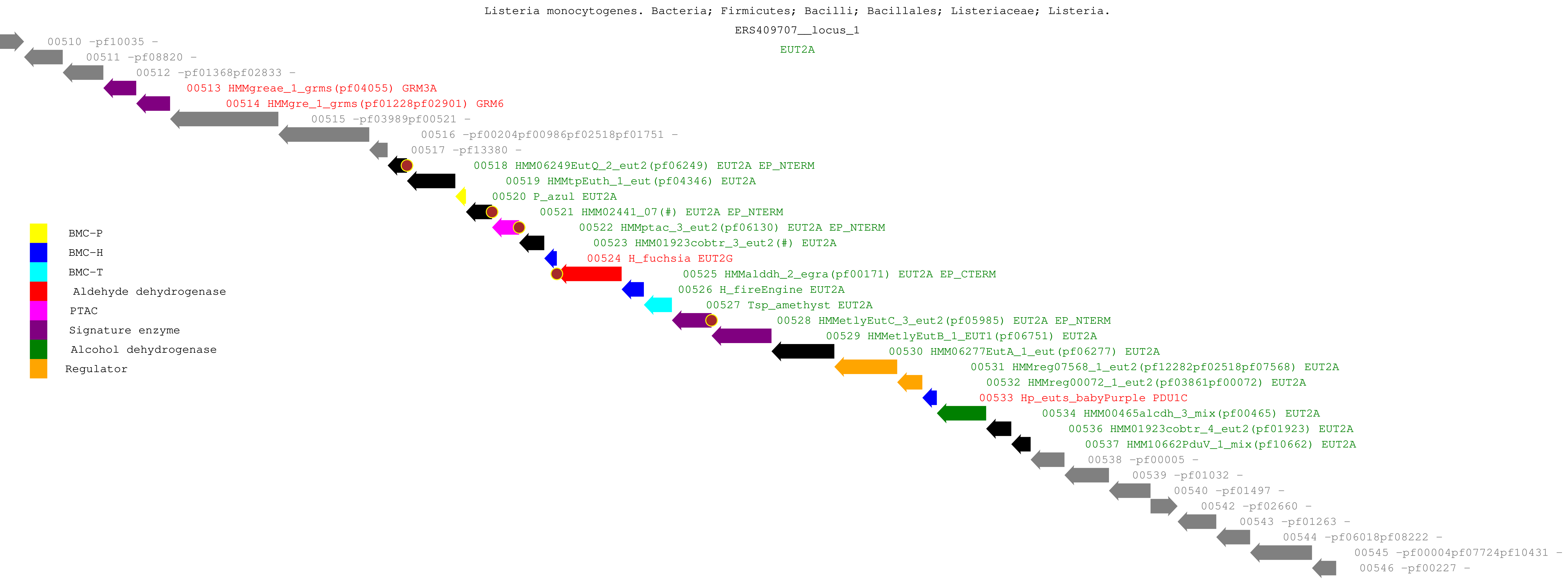
Listeria monocytogenes. Bacteria; Firmicutes; Bacilli; Bacillales; Listeriaceae; Listeria.
ERS409707__locus_1
EUT2A
00510 -pf10035 -
00511 -pf08820 -
00512 -pf01368pf02833 -
00513 HMMgreae_1_grms(pf04055) GRM3A
00514 HMMgre_1_grms(pf01228pf02901) GRM6
00515 -pf03989pf00521 -
00516 -pf00204pf00986pf02518pf01751 -
00517 -pf13380 -
00518 HMM06249EutQ_2_eut2(pf06249) EUT2A EP_NTERM
00519 HMMtpEuth_1_eut(pf04346) EUT2A
00520 P_azul EUT2A
00521 HMM02441_07(#) EUT2A EP_NTERM
00522 HMMptac_3_eut2(pf06130) EUT2A EP_NTERM
00523 HMM01923cobtr_3_eut2(#) EUT2A
00524 H_fuchsia EUT2G
00525 HMMalddh_2_egra(pf00171) EUT2A EP_CTERM
00526 H_fireEngine EUT2A
00527 Tsp_amethyst EUT2A
00528 HMMetlyEutC_3_eut2(pf05985) EUT2A EP_NTERM
00529 HMMetlyEutB_1_EUT1(pf06751) EUT2A
00530 HMM06277EutA_1_eut(pf06277) EUT2A
00531 HMMreg07568_1_eut2(pf12282pf02518pf07568) EUT2A
00532 HMMreg00072_1_eut2(pf03861pf00072) EUT2A
00533 Hp_euts_babyPurple PDU1C
00534 HMM00465alcdh_3_mix(pf00465) EUT2A
00536 HMM01923cobtr_4_eut2(pf01923) EUT2A
00537 HMM10662PduV_1_mix(pf10662) EUT2A
00538 -pf00005 -
00539 -pf01032 -
00540 -pf01497 -
00542 -pf02660 -
00543 -pf01263 -
00544 -pf06018pf08222 -
00545 -pf00004pf07724pf10431 -
00546 -pf00227 -
BMC-P
BMC-H
BMC-T
Aldehyde dehydrogenase
PTAC
Signature enzyme
Alcohol dehydrogenase
Regulator

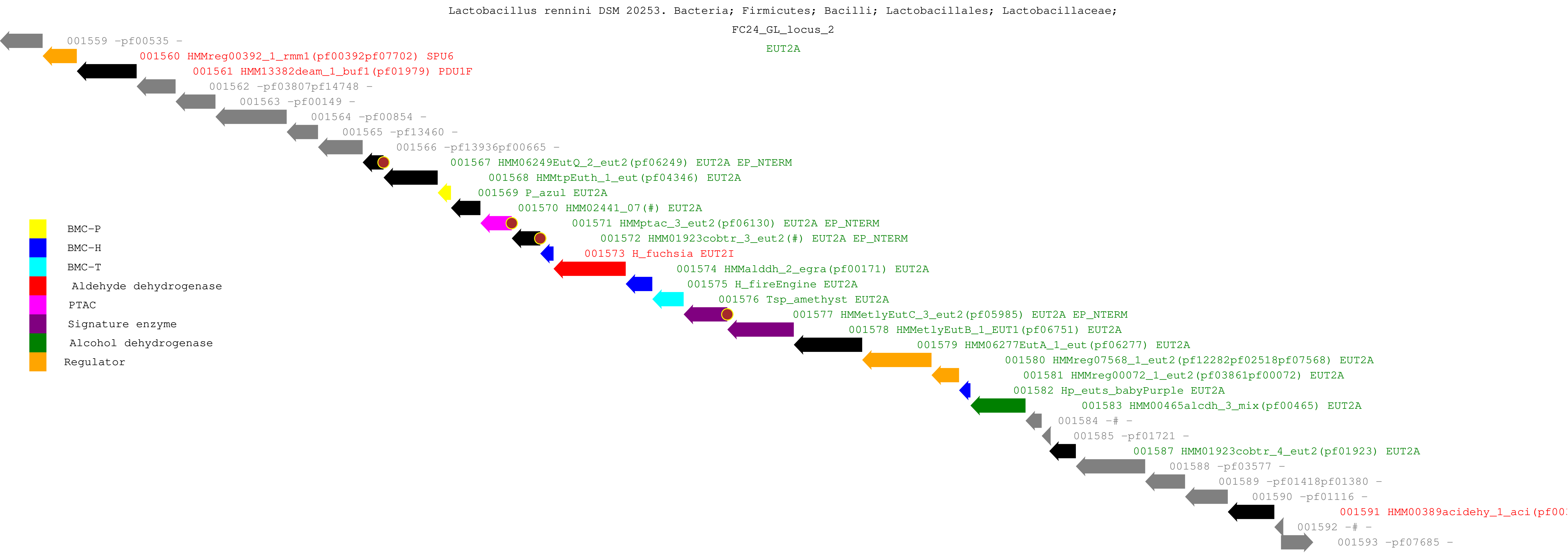

Lactobacillus rennini DSM 20253. Bacteria; Firmicutes; Bacilli; Lactobacillales; Lactobacillaceae;
FC24_GL_locus_2
EUT2A
001559 -pf00535 -
001560 HMMreg00392_1_rmm1(pf00392pf07702) SPU6
001561 HMM13382deam_1_buf1(pf01979) PDU1F
001562 -pf03807pf14748 -
001563 -pf00149 -
001564 -pf00854 -
001565 -pf13460 -
001566 -pf13936pf00665 -
001567 HMM06249EutQ_2_eut2(pf06249) EUT2A EP_NTERM
001568 HMMtpEuth_1_eut(pf04346) EUT2A
001569 P_azul EUT2A
001570 HMM02441_07(#) EUT2A
001571 HMMptac_3_eut2(pf06130) EUT2A EP_NTERM
001572 HMM01923cobtr_3_eut2(#) EUT2A EP_NTERM
001573 H_fuchsia EUT2I
001574 HMMalddh_2_egra(pf00171) EUT2A
001575 H_fireEngine EUT2A
001576 Tsp_amethyst EUT2A
001577 HMMetlyEutC_3_eut2(pf05985) EUT2A EP_NTERM
001578 HMMetlyEutB_1_EUT1(pf06751) EUT2A
001579 HMM06277EutA_1_eut(pf06277) EUT2A
001580 HMMreg07568_1_eut2(pf12282pf02518pf07568) EUT2A
001581 HMMreg00072_1_eut2(pf03861pf00072) EUT2A
001582 Hp_euts_babyPurple EUT2A
001583 HMM00465alcdh_3_mix(pf00465) EUT2A
001584 -# -
001585 -pf01721 -
001587 HMM01923cobtr_4_eut2(pf01923) EUT2A
001588 -pf03577 -
001589 -pf01418pf01380 -
001590 -pf01116 -
001591 HMM00389acidehy_1_aci(pf00
001592 -# -
001593 -pf07685 -
BMC-P
BMC-H
BMC-T
Aldehyde dehydrogenase
PTAC
Signature enzyme
Alcohol dehydrogenase
Regulator

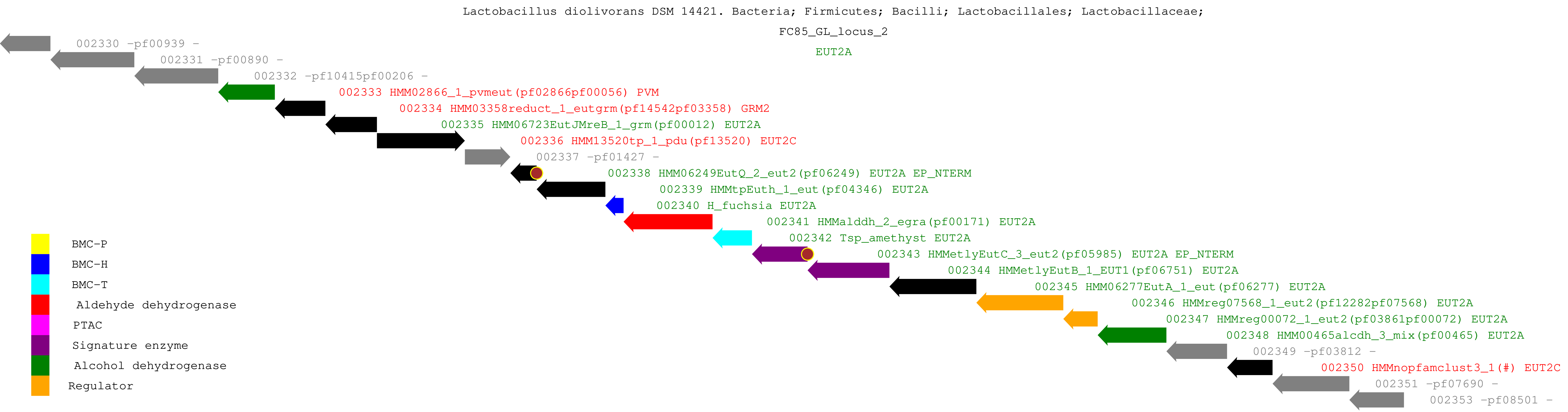

Lactobacillus diolivorans DSM 14421. Bacteria; Firmicutes; Bacilli; Lactobacillales; Lactobacillaceae;
FC85_GL_locus_2
EUT2A
002330 -pf00939 -
002331 -pf00890 -
002332 -pf10415pf00206 -
002333 HMM02866_1_pvmeut(pf02866pf00056) PVM
002334 HMM03358reduct_1_eutgrm(pf14542pf03358) GRM2
002335 HMM06723EutJMreB_1_grm(pf00012) EUT2A
002336 HMM13520tp_1_pdu(pf13520) EUT2C
002337 -pf01427 -
002338 HMM06249EutQ_2_eut2(pf06249) EUT2A EP_NTERM
002339 HMMtpEuth_1_eut(pf04346) EUT2A
002340 H_fuchsia EUT2A
002341 HMMalddh_2_egra(pf00171) EUT2A
002342 Tsp_amethyst EUT2A
002343 HMMetlyEutC_3_eut2(pf05985) EUT2A EP_NTERM
002344 HMMetlyEutB_1_EUT1(pf06751) EUT2A
002345 HMM06277EutA_1_eut(pf06277) EUT2A
002346 HMMreg07568_1_eut2(pf12282pf07568) EUT2A
002347 HMMreg00072_1_eut2(pf03861pf00072) EUT2A
002348 HMM00465alcdh_3_mix(pf00465) EUT2A
002349 -pf03812 -
002350 HMMnopfamclust3_1(#) EUT2C
002351 -pf07690 -
002353 -pf08501 -
BMC-P
BMC-H
BMC-T
Aldehyde dehydrogenase
PTAC
Signature enzyme
Alcohol dehydrogenase
Regulator

Lactobacillus similis DSM 23365 = JCM 2765. Bacteria; Firmicutes; Bacilli; Lactobacillales; Lactobacillaceae;

FD14_GL_locus_1

EUT2A

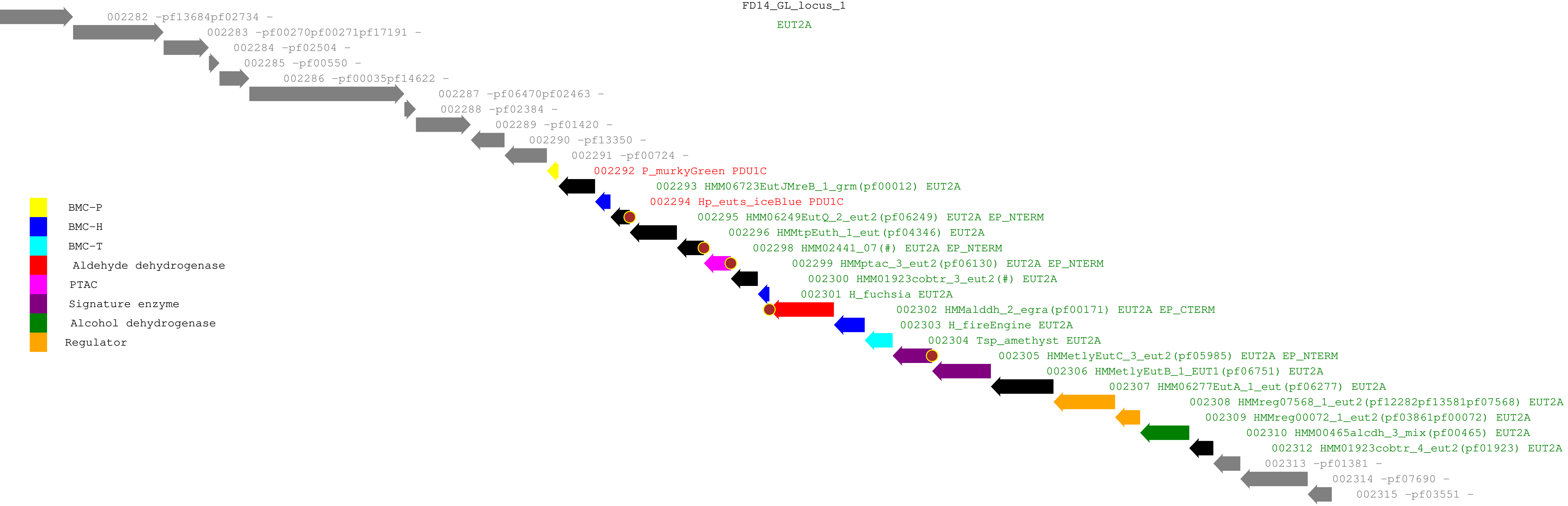

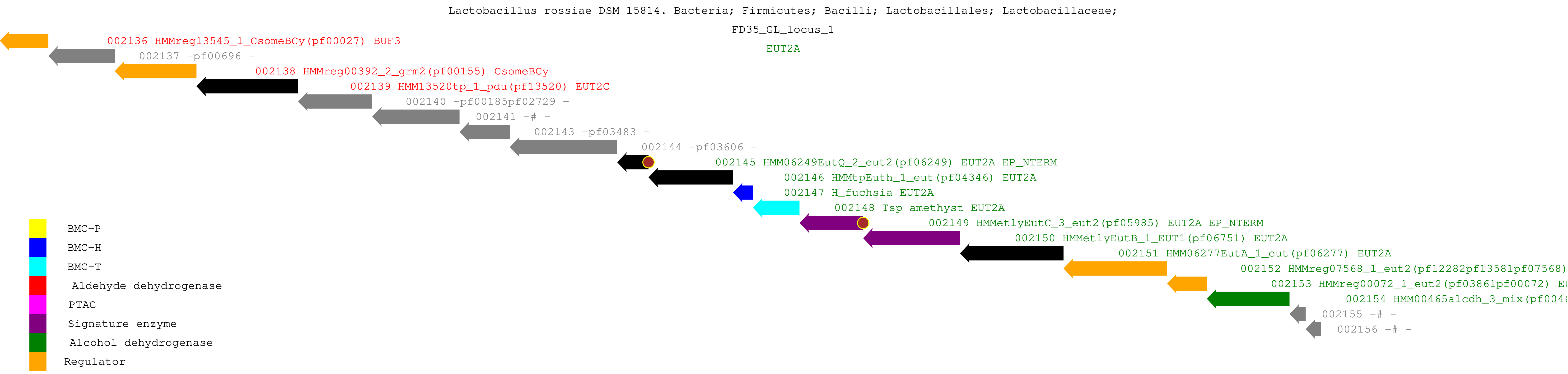

Lactobacillus rossiae DSM 15814. Bacteria; Firmicutes; Bacilli; Lactobacillales; Lactobacillaceae;
FD35_GL_locus_1
EUT2A
002136 HMMreg13545_1_CsomeBCy(pf00027) BUF3
002137 -pf00696 -
002138 HMMreg00392_2_grm2(pf00155) CsomeBCy
002139 HMM13520tp_1_pdu(pf13520) EUT2C
002140 -pf00185pf02729 -
002141 -# -
002143 -pf03483 -
002144 -pf03606 -
002145 HMM06249EutQ_2_eut2(pf06249) EUT2A EP_NTERM
002146 HMMtpEuth_1_eut(pf04346) EUT2A
002147 H_fuchsia EUT2A
002148 Tsp_amethyst EUT2A
002149 HMMetlyEutC_3_eut2(pf05985) EUT2A EP_NTERM
002150 HMMetlyEutB_1_EUT1(pf06751) EUT2A
002151 HMM06277EutA_1_eut(pf06277) EUT2A
002152 HMMreg07568_1_eut2(pf12282pf13581pf07568)
002153 HMMreg00072_1_eut2(pf03861pf00072) E
002154 HMM00465alcdh_3_mix(pf004
002155 -# -
002156 -# -
BMC-P
BMC-H
BMC-T
Aldehyde dehydrogenase
PTAC
Signature enzyme
Alcohol dehydrogenase
Regulator

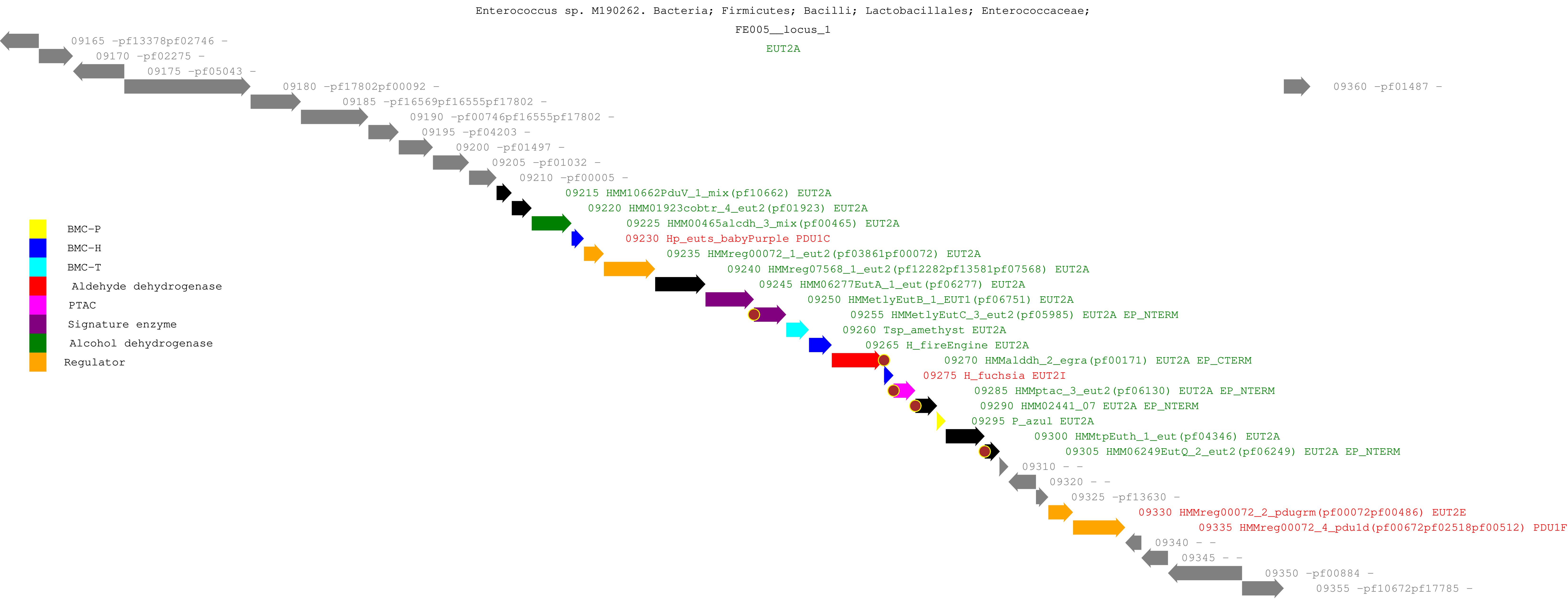

Enterococcus sp. M190262. Bacteria; Firmicutes; Bacilli; Lactobacillales; Enterococcaceae;
FE005__locus_1
EUT2A
09165 -pf13378pf02746 -
09170 -pf02275 -
09175 -pf05043 -
09180 -pf17802pf00092 -
09185 -pf16569pf16555pf17802 -
09190 -pf00746pf16555pf17802 -
09195 -pf04203 -
09200 -pf01497 -
09205 -pf01032 -
09210 -pf00005 -
09215 HMM10662PduV_1_mix(pf10662) EUT2A
09220 HMM01923cobtr_4_eut2(pf01923) EUT2A
09225 HMM00465alcdh_3_mix(pf00465) EUT2A
09230 Hp_euts_babyPurple PDU1C
09235 HMMreg00072_1_eut2(pf03861pf00072) EUT2A
09240 HMMreg07568_1_eut2(pf12282pf13581pf07568) EUT2A
09245 HMM06277EutA_1_eut(pf06277) EUT2A
09250 HMMetlyEutB_1_EUT1(pf06751) EUT2A
09255 HMMetlyEutC_3_eut2(pf05985) EUT2A EP_NTERM
09260 Tsp_amethyst EUT2A
09265 H_fireEngine EUT2A
09270 HMMaldh_2_egra(pf00171) EUT2A EP_CTERM
09275 H_fuchsia EUT2I
09285 HMMptac_3_eut2(pf06130) EUT2A EP_NTERM
09290 HMM02441_07 EUT2A EP_NTERM
09295 P_azul EUT2A
09300 HMMtpEuth_1_eut(pf04346) EUT2A
09305 HMM06249EutQ_2_eut2(pf06249) EUT2A EP_NTERM
09310 - -
09320 - -
09325 -pf13630 -
09330 HMMreg00072_2_pdugrm(pf00072pf00486) EUT2E
09335 HMMreg00072_4_pdu1d(pf00672pf02518pf00512) PDU1F
09340 - -
09345 - -
09350 -pf00884 -
09355 -pf10672pf17785 -
09360 -pf01487 -
BMC-P
BMC-H
BMC-T
Aldehyde dehydrogenase
PTAC
Signature enzyme
Alcohol dehydrogenase
Regulator

Lactobacillus coryniformis. Bacteria; Firmicutes; Bacilli; Lactobacillales; Lactobacillaceae;

FGL77__locus_2

EUT2A

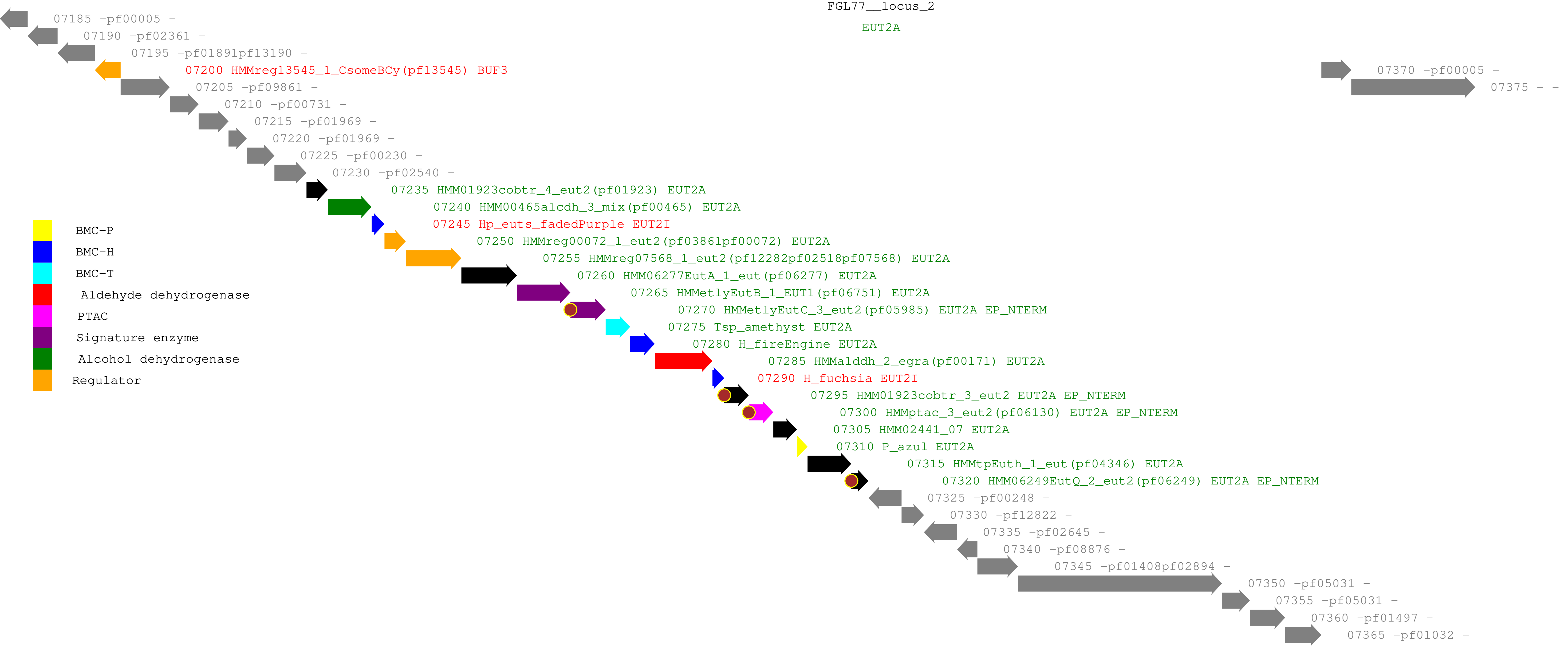

Brachybacterium faecium. Bacteria; Actinobacteria; Micrococcales; Dermabacteraceae;

FM106__locus_1

EUT2A

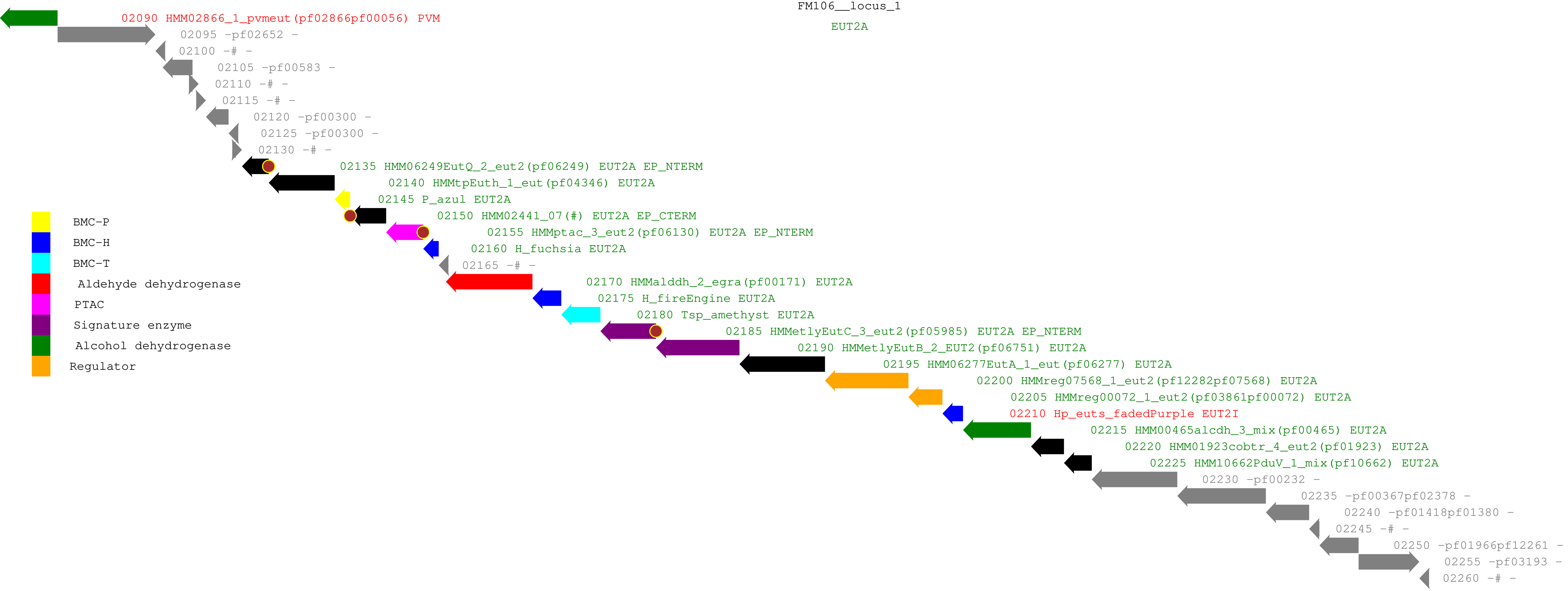

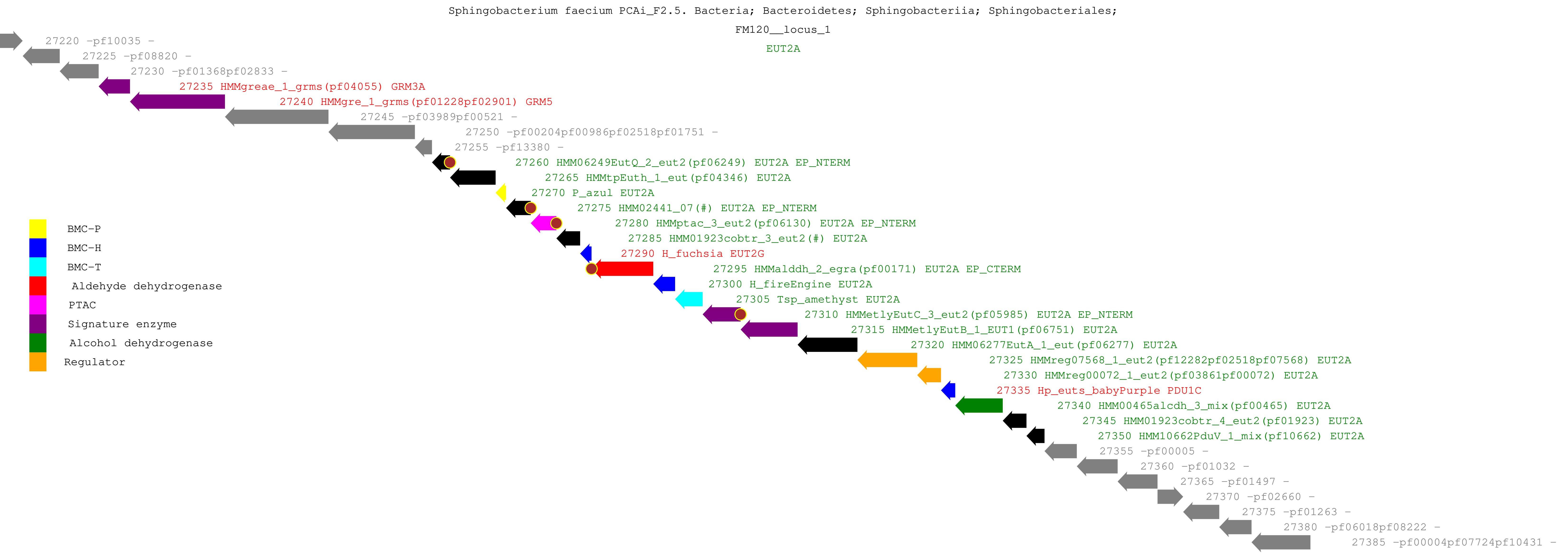
Sphingobacterium faecium PCAi_F2.5. Bacteria; Bacteroidetes; Sphingobacteriia; Sphingobacteriales;
FM120__locus_1
EUT2A
27220 -pf10035 -
27225 -pf08820 -
27230 -pf01368pf02833 -
27235 HMMgreae_1_grms(pf04055) GRM3A
27240 HMMgre_1_grms(pf01228pf02901) GRM5
27245 -pf03989pf00521 -
27250 -pf00204pf00986pf02518pf01751 -
27255 -pf13380 -
27260 HMM06249EutQ_2_eut2(pf06249) EUT2A EP_NTERM
27265 HMMtpEuth_1_eut(pf04346) EUT2A
27270 P_azul EUT2A
27275 HMM02441_07(#) EUT2A EP_NTERM
27280 HMMptac_3_eut2(pf06130) EUT2A EP_NTERM
27285 HMM01923cobtr_3_eut2(#) EUT2A
27290 H_fuchsia EUT2G
27295 HMMalddh_2_egra(pf00171) EUT2A EP_CTERM
27300 H_fireEngine EUT2A
27305 Tsp_amethyst EUT2A
27310 HMMetlyEutC_3_eut2(pf05985) EUT2A EP_NTERM
27315 HMMetlyEutB_1_EUT1(pf06751) EUT2A
27320 HMM06277EutA_1_eut(pf06277) EUT2A
27325 HMMreg07568_1_eut2(pf12282pf02518pf07568) EUT2A
27330 HMMreg00072_1_eut2(pf03861pf00072) EUT2A
27335 Hp_euts_babyPurple PDU1C
27340 HMM00465alcdh_3_mix(pf00465) EUT2A
27345 HMM01923cobtr_4_eut2(pf01923) EUT2A
27350 HMM10662PduV_1_mix(pf10662) EUT2A
27355 -pf00005 -
27360 -pf01032 -
27365 -pf01497 -
27370 -pf02660 -
27375 -pf01263 -
27380 -pf06018pf08222 -
27385 -pf00004pf07724pf10431 -
BMC-P
BMC-H
BMC-T
Aldehyde dehydrogenase
PTAC
Signature enzyme
Alcohol dehydrogenase
Regulator

Vagococcus fluvialis bH819. Bacteria; Firmicutes; Bacilli; Lactobacillales; Enterococcaceae;

FM121__locus_1

EUT2A

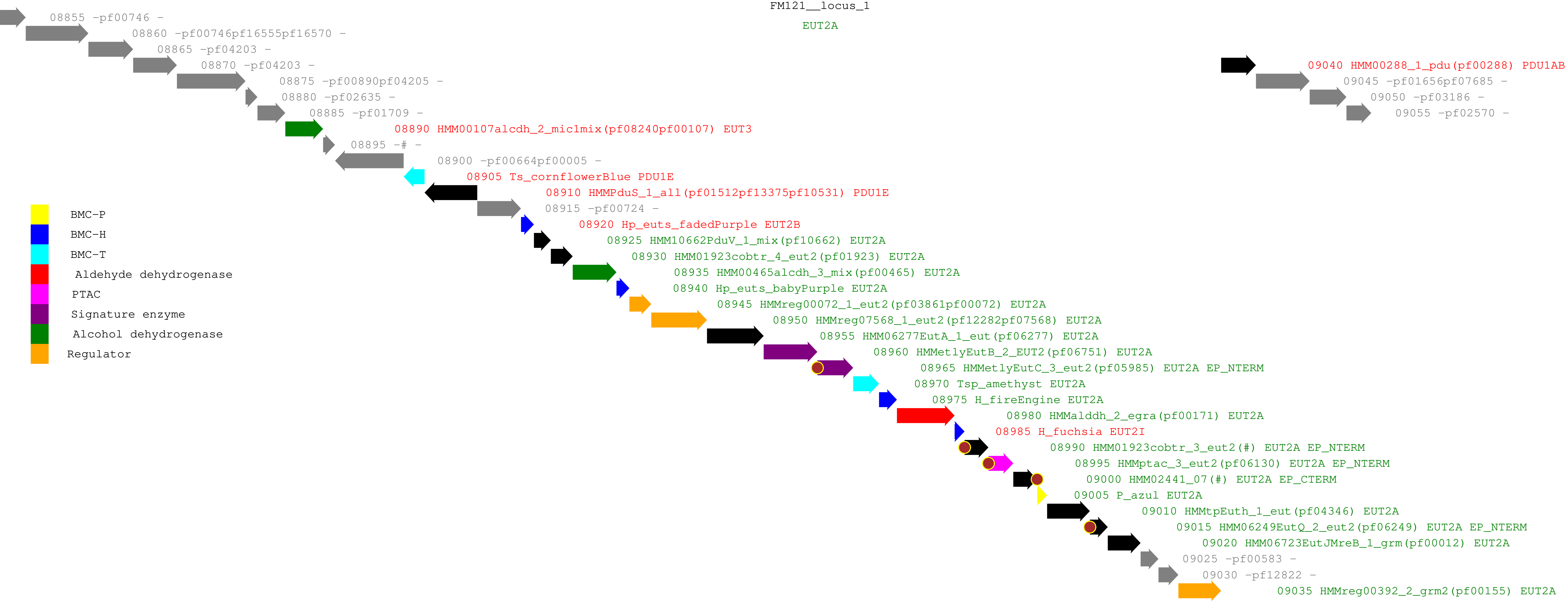

Enterococcus gallinarum. Bacteria; Firmicutes; Bacilli; Lactobacillales; Enterococcaceae;

FSZ06__locus_1_main_sat

EUT2A

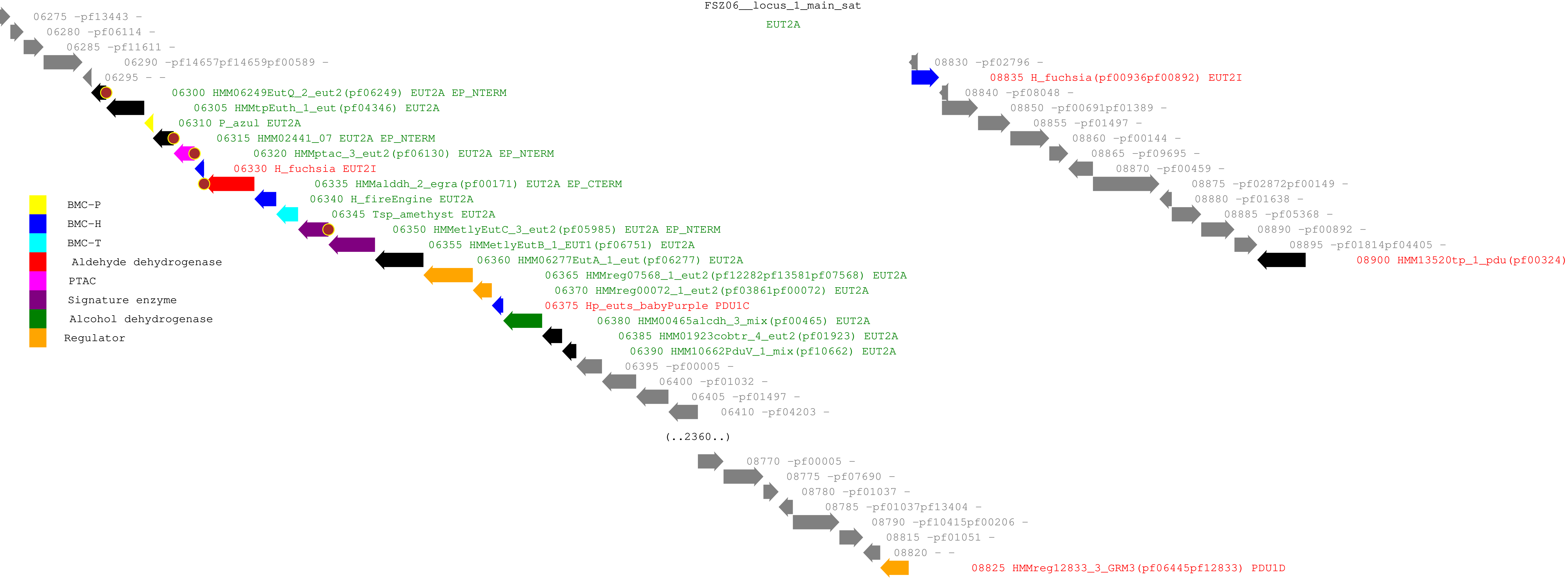

Streptococcus ratti. Bacteria; Firmicutes; Bacilli; Lactobacillales; Streptococcaceae;

FY406__locus_1

EUT2A

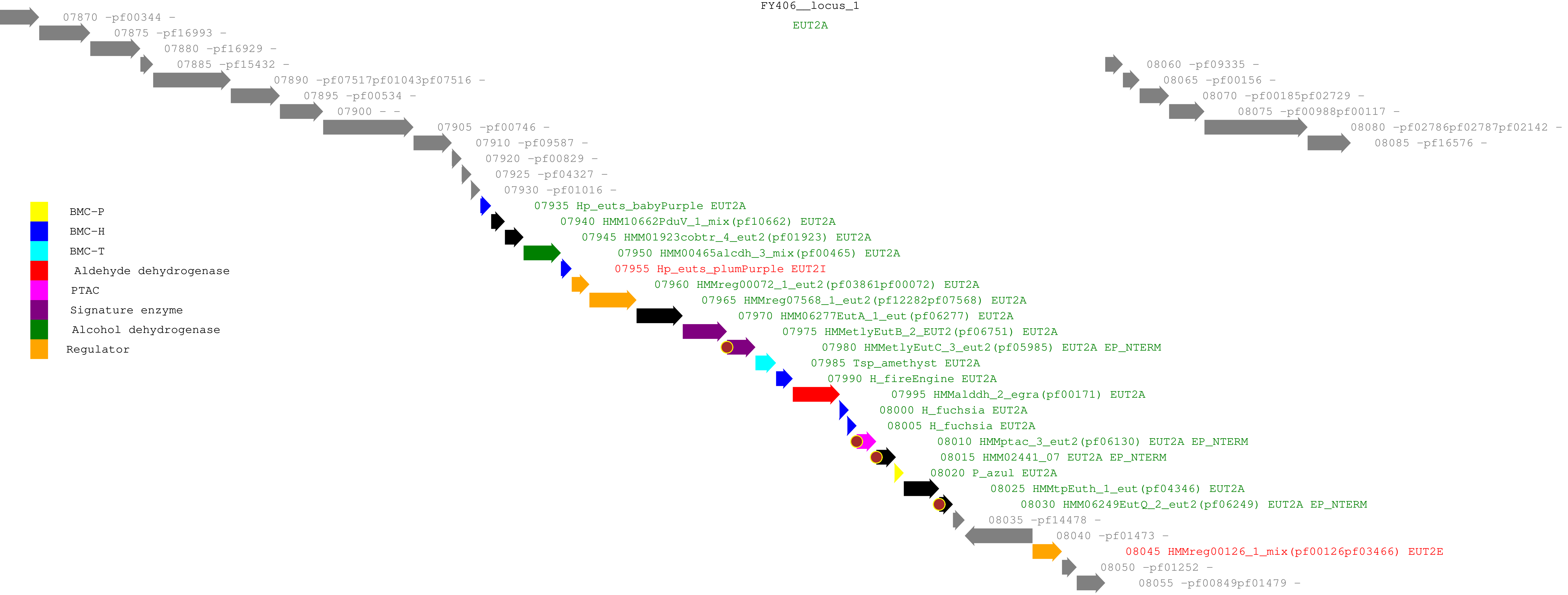

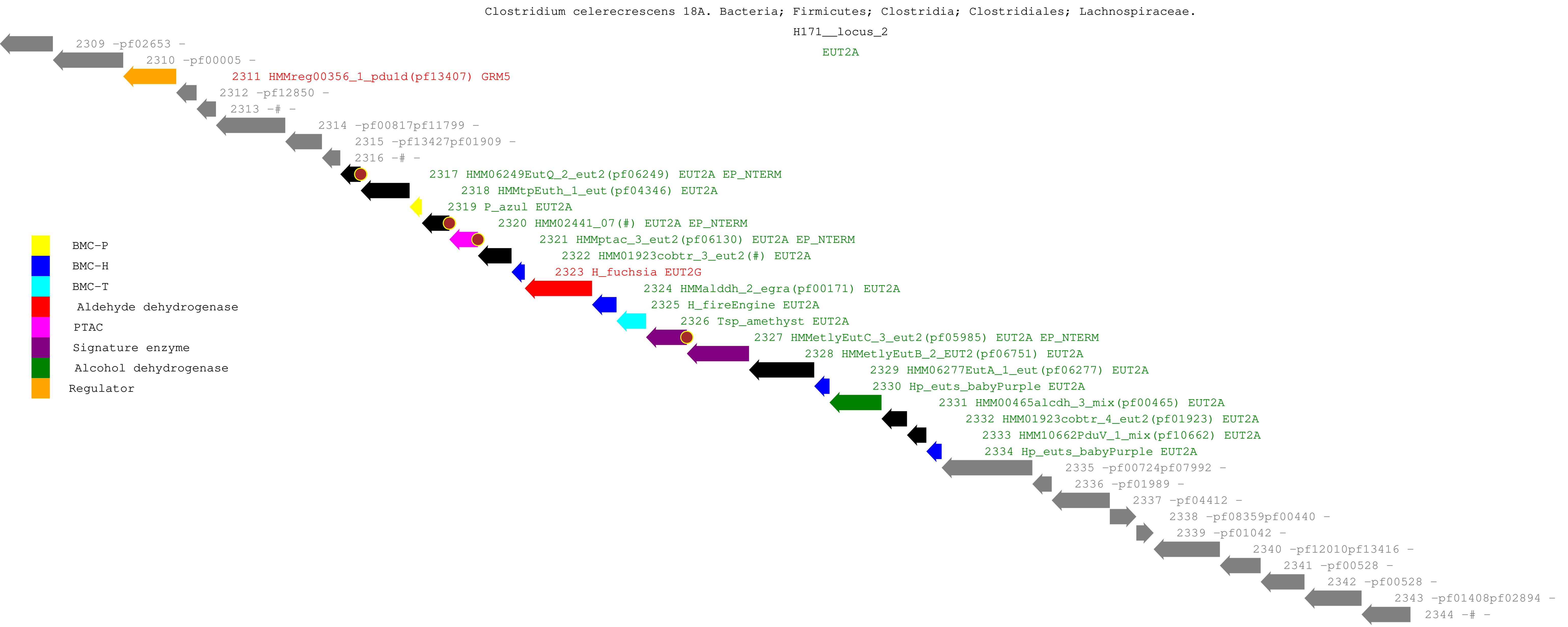
Clostridium celerecrescens 18A. Bacteria; Firmicutes; Clostridia; Clostridiales; Lachnospiraceae.
H171__locus_2
EUT2A
2309 -pf02653 -
2310 -pf00005 -
2311 HMMreg00356_1_pdu1d(pf13407) GRM5
2312 -pf12850 -
2313 -# -
2314 -pf00817pf11799 -
2315 -pf13427pf01909 -
2316 -# -
2317 HMM06249EutQ_2_eut2(pf06249) EUT2A EP_NTERM
2318 HMMtpEuth_1_eut(pf04346) EUT2A
2319 P_azul EUT2A
2320 HMM02441_07(#) EUT2A EP_NTERM
2321 HMMptac_3_eut2(pf06130) EUT2A EP_NTERM
2322 HMM01923cobtr_3_eut2(#) EUT2A
2323 H_fuchsia EUT2G
2324 HMMalddh_2_egra(pf00171) EUT2A
2325 H_fireEngine EUT2A
2326 Tsp_amethyst EUT2A
2327 HMMetlyEutC_3_eut2(pf05985) EUT2A EP_NTERM
2328 HMMetlyEutB_2_EUT2(pf06751) EUT2A
2329 HMM06277EutA_1_eut(pf06277) EUT2A
2330 Hp_euts_babyPurple EUT2A
2331 HMM00465alcdh_3_mix(pf00465) EUT2A
2332 HMM01923cobtr_4_eut2(pf01923) EUT2A
2333 HMM10662PduV_1_mix(pf10662) EUT2A
2334 Hp_euts_babyPurple EUT2A
2335 -pf00724pf07992 -
2336 -pf01989 -
2337 -pf04412 -
2338 -pf08359pf00440 -
2339 -pf01042 -
2340 -pf12010pf13416 -
2341 -pf00528 -
2342 -pf00528 -
2343 -pf01408pf02894 -
2344 -# -
BMC-P
BMC-H
BMC-T
Aldehyde dehydrogenase
PTAC
Signature enzyme
Alcohol dehydrogenase
Regulator

Enterococcus faecalis TX0104. Bacteria; Firmicutes; Bacilli; Lactobacillales; Enterococcaceae;

HMPREF0348__locus_1

EUT2A

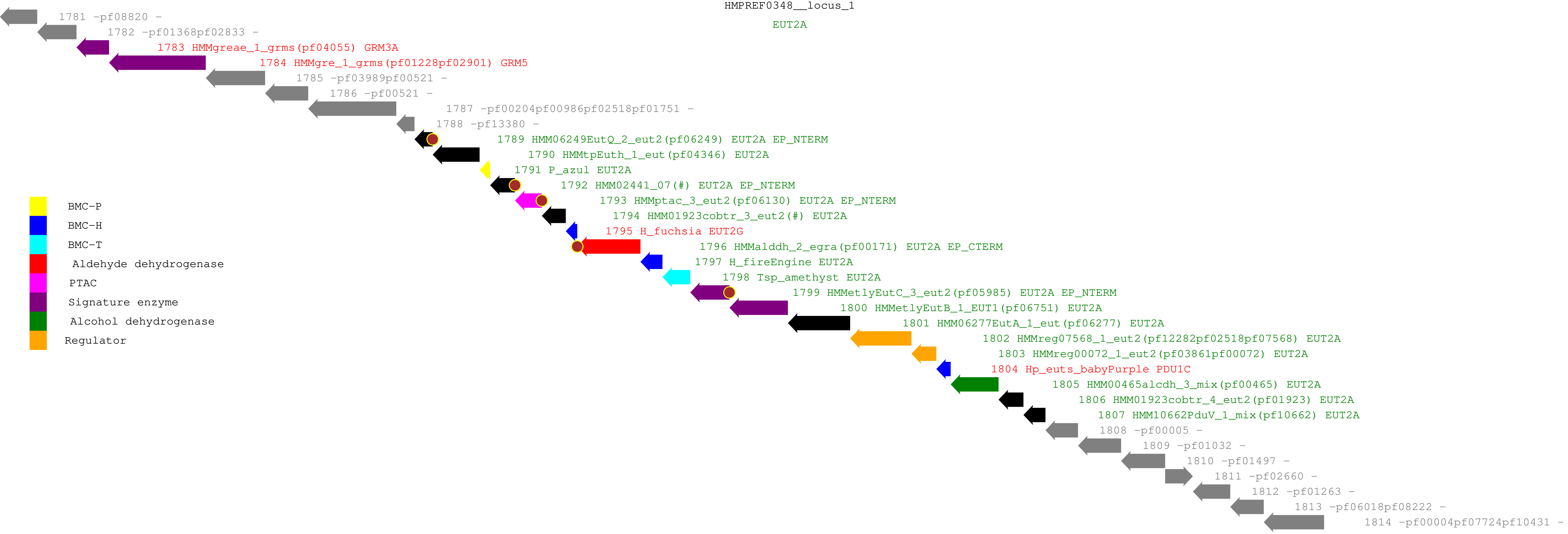

Lactobacillus brevis ATCC 14869 = DSM 20054. Bacteria; Firmicutes; Bacilli; Lactobacillales; Lactobacillaceae;

HMPREF0495__locus_1

EUT2A

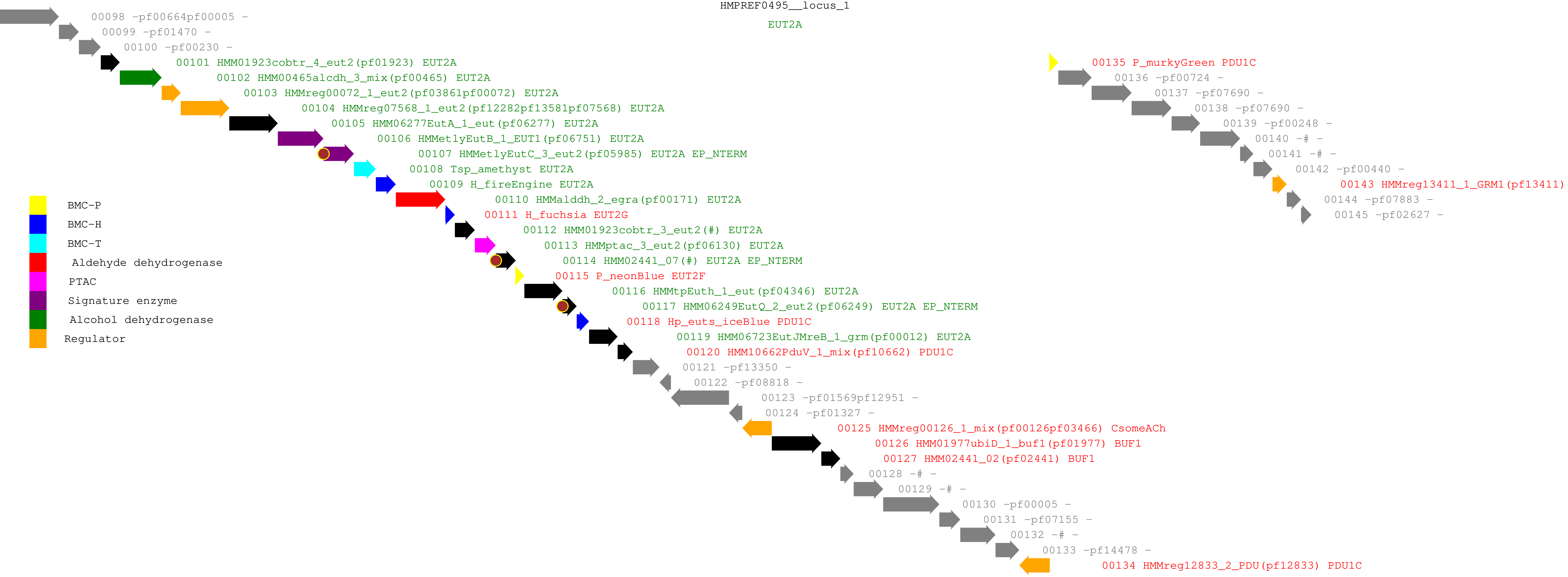

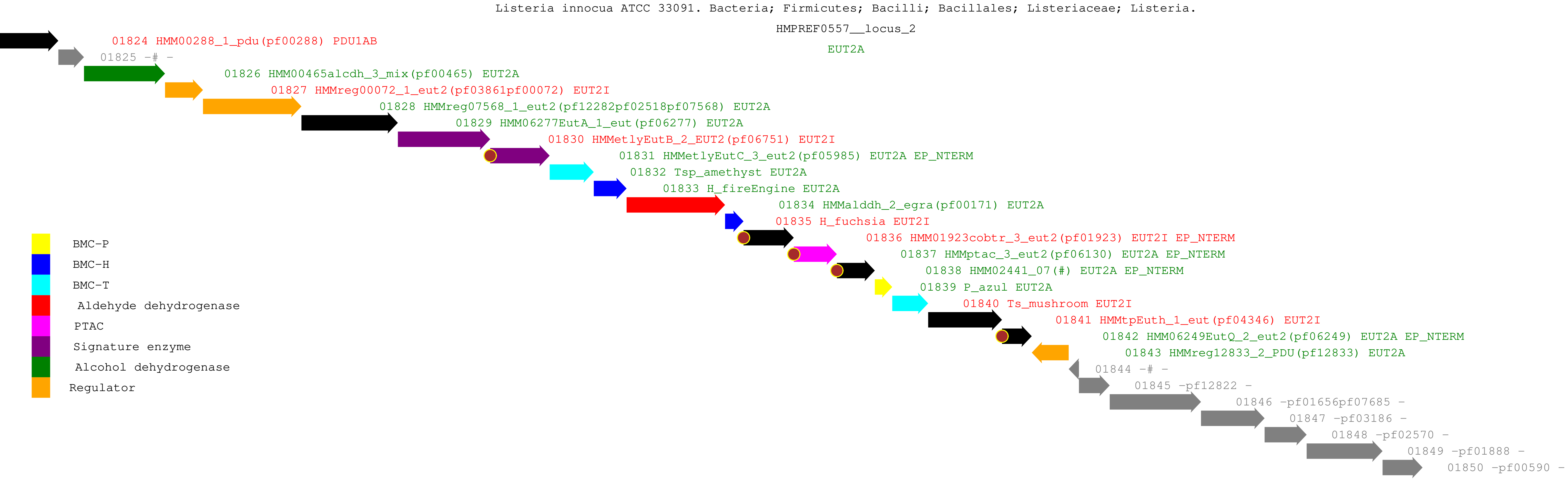

Listeria innocua ATCC 33091. Bacteria; Firmicutes; Bacilli; Bacillales; Listeriaceae; Listeria.
HMPREF0557__locus_2
EUT2A
01824 HMM00288_1_pdu(pf00288) PDU1AB
01825 -# -
01826 HMM00465alcdh_3_mix(pf00465) EUT2A
01827 HMMreg00072_1_eut2(pf03861pf00072) EUT2I
01828 HMMreg07568_1_eut2(pf12282pf02518pf07568) EUT2A
01829 HMM06277EutA_1_eut(pf06277) EUT2A
01830 HMMetlyEutB_2_EUT2(pf06751) EUT2I
01831 HMMetlyEutC_3_eut2(pf05985) EUT2A EP_NTERM
01832 Tsp_amethyst EUT2A
01833 H_fireEngine EUT2A
01834 HMMalddh_2_egra(pf00171) EUT2A
01835 H_fuchsia EUT2I
01836 HMM01923cobtr_3_eut2(pf01923) EUT2I EP_NTERM
01837 HMMptac_3_eut2(pf06130) EUT2A EP_NTERM
01838 HMM02441_07(#) EUT2A EP_NTERM
01839 P_azul EUT2A
01840 Ts_mushroom EUT2I
01841 HMMtpEuth_1_eut(pf04346) EUT2I
01842 HMM06249EutQ_2_eut2(pf06249) EUT2A EP_NTERM
01843 HMMreg12833_2_PDU(pf12833) EUT2A
01844 -# -
01845 -pf12822 -
01846 -pf01656pf07685 -
01847 -pf03186 -
01848 -pf02570 -
01849 -pf01888 -
01850 -pf00590 -
BMC-P
BMC-H
BMC-T
Aldehyde dehydrogenase
PTAC
Signature enzyme
Alcohol dehydrogenase
Regulator

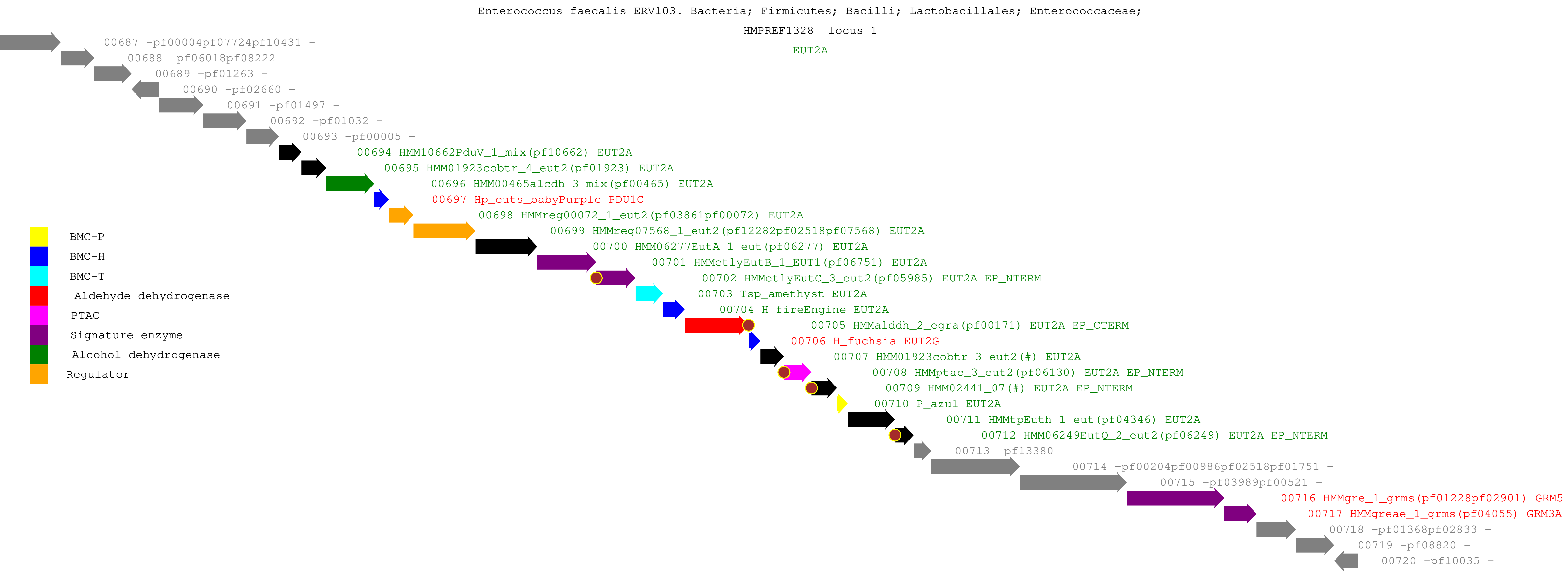
Enterococcus faecalis ERV103. Bacteria; Firmicutes; Bacilli; Lactobacillales; Enterococcaceae;
HMPREF1328__locus_1
EUT2A
00687 -pf00004pf07724pf10431 -
00688 -pf06018pf08222 -
00689 -pf01263 -
00690 -pf02660 -
00691 -pf01497 -
00692 -pf01032 -
00693 -pf00005 -
00694 HMM10662PduV_1_mix(pf10662) EUT2A
00695 HMM01923cobtr_4_eut2(pf01923) EUT2A
00696 HMM00465alcdh_3_mix(pf00465) EUT2A
00697 Hp_euts_babyPurple PDU1C
00698 HMMreg00072_1_eut2(pf03861pf00072) EUT2A
00699 HMMreg07568_1_eut2(pf12282pf02518pf07568) EUT2A
00700 HMM06277EutA_1_eut(pf06277) EUT2A
00701 HMMetlyEutB_1_EUT1(pf06751) EUT2A
00702 HMMetlyEutC_3_eut2(pf05985) EUT2A EP_NTERM
00703 Tsp_amethyst EUT2A
00704 H_fireEngine EUT2A
00705 HMMalddh_2_egra(pf00171) EUT2A EP_CTERM
00706 H_fuchsia EUT2G
00707 HMM01923cobtr_3_eut2(#) EUT2A
00708 HMMptac_3_eut2(pf06130) EUT2A EP_NTERM
00709 HMM02441_07(#) EUT2A EP_NTERM
00710 P_azul EUT2A
00711 HMMtpEuth_1_eut(pf04346) EUT2A
00712 HMM06249EutQ_2_eut2(pf06249) EUT2A EP_NTERM
00713 -pf13380 -
00714 -pf00204pf00986pf02518pf01751 -
00715 -pf03989pf00521 -
00716 HMMgre_1_grms(pf01228pf02901) GRM5
00717 HMMgreae_1_grms(pf04055) GRM3A
00718 -pf01368pf02833 -
00719 -pf08820 -
00720 -pf10035 -
BMC-P
BMC-H
BMC-T
Aldehyde dehydrogenase
PTAC
Signature enzyme
Alcohol dehydrogenase
Regulator

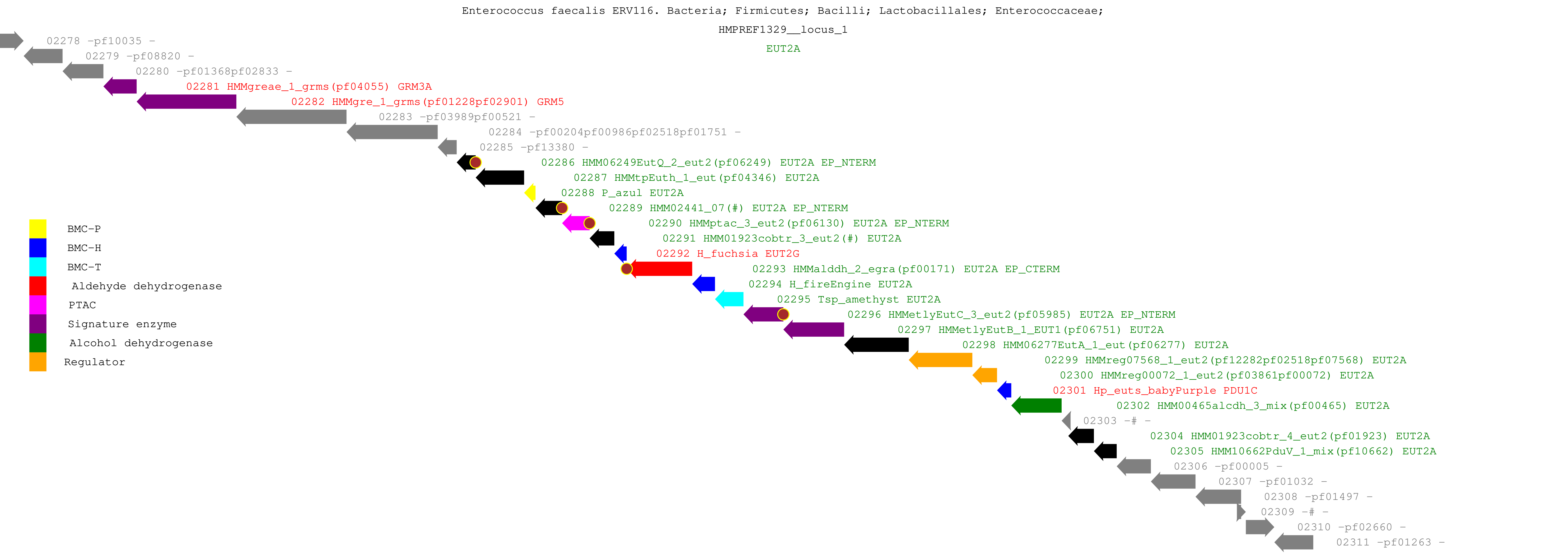

Enterococcus faecalis ERV116. Bacteria; Firmicutes; Bacilli; Lactobacillales; Enterococcaceae;
HMPREF1329__locus_1
EUT2A
02278 -pf10035 -
02279 -pf08820 -
02280 -pf01368pf02833 -
02281 HMMgreae_1_grms(pf04055) GRM3A
02282 HMMgre_1_grms(pf01228pf02901) GRM5
02283 -pf03989pf00521 -
02284 -pf00204pf00986pf02518pf01751 -
02285 -pf13380 -
02286 HMM06249EutQ_2_eut2(pf06249) EUT2A EP_NTERM
02287 HMMtpEuth_1_eut(pf04346) EUT2A
02288 P_azul EUT2A
02289 HMM02441_07(#) EUT2A EP_NTERM
02290 HMMptac_3_eut2(pf06130) EUT2A EP_NTERM
02291 HMM01923cobtr_3_eut2(#) EUT2A
02292 H_fuchsia EUT2G
02293 HMMalddh_2_egra(pf00171) EUT2A EP_CTERM
02294 H_fireEngine EUT2A
02295 Tsp_amethyst EUT2A
02296 HMMetlyEutC_3_eut2(pf05985) EUT2A EP_NTERM
02297 HMMetlyEutB_1_EUT1(pf06751) EUT2A
02298 HMM06277EutA_1_eut(pf06277) EUT2A
02299 HMMreg07568_1_eut2(pf12282pf02518pf07568) EUT2A
02300 HMMreg00072_1_eut2(pf03861pf00072) EUT2A
02301 Hp_euts_babyPurple PDU1C
02302 HMM00465alcdh_3_mix(pf00465) EUT2A
02303 -# -
02304 HMM01923cobtr_4_eut2(pf01923) EUT2A
02305 HMM10662PduV_1_mix(pf10662) EUT2A
02306 -pf00005 -
02307 -pf01032 -
02308 -pf01497 -
02309 -# -
02310 -pf02660 -
02311 -pf01263 -
BMC-P
BMC-H
BMC-T
Aldehyde dehydrogenase
PTAC
Signature enzyme
Alcohol dehydrogenase
Regulator

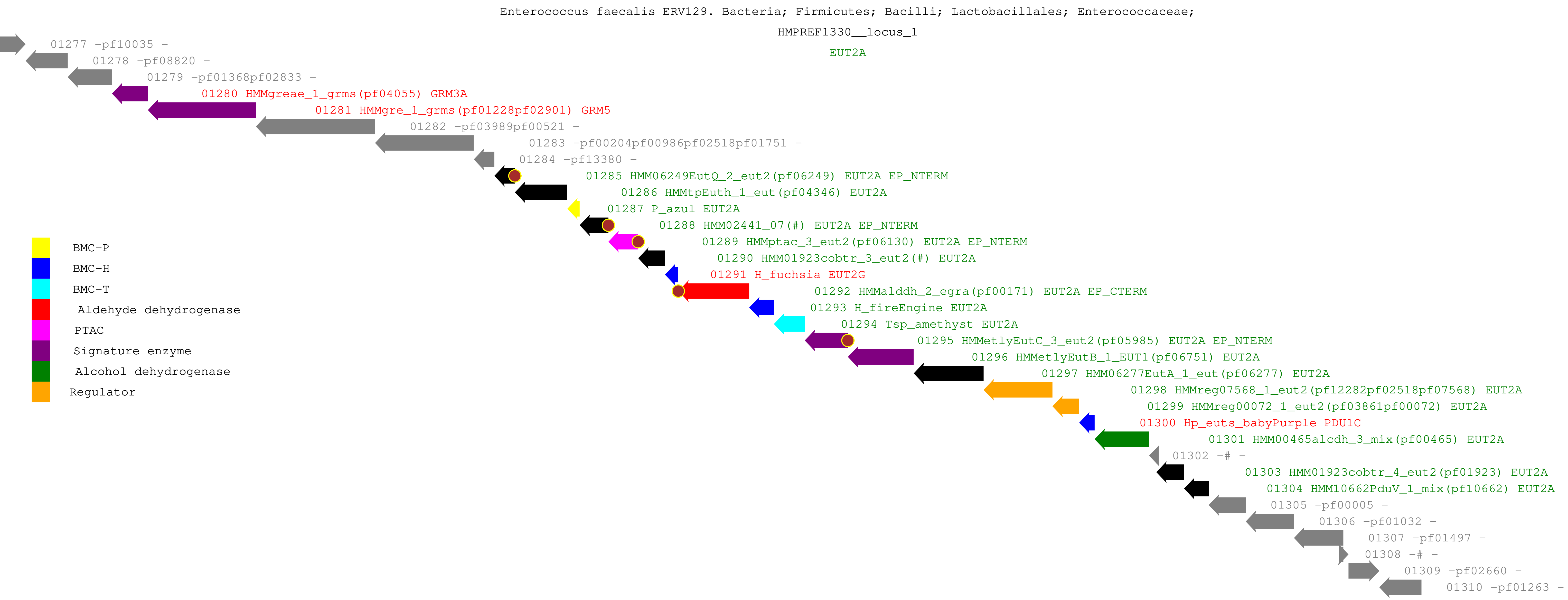
Enterococcus faecalis ERV129. Bacteria; Firmicutes; Bacilli; Lactobacillales; Enterococcaceae;
HMPREF1330__locus_1
EUT2A
01277 -pf10035 -
01278 -pf08820 -
01279 -pf01368pf02833 -
01280 HMMgreae_1_grms(pf04055) GRM3A
01281 HMMgre_1_grms(pf01228pf02901) GRM5
01282 -pf03989pf00521 -
01283 -pf00204pf00986pf02518pf01751 -
01284 -pf13380 -
01285 HMM06249EutQ_2_eut2(pf06249) EUT2A EP_NTERM
01286 HMMtpEuth_1_eut(pf04346) EUT2A
01287 P_azul EUT2A
01288 HMM02441_07(#) EUT2A EP_NTERM
01289 HMMptac_3_eut2(pf06130) EUT2A EP_NTERM
01290 HMM01923cobtr_3_eut2(#) EUT2A
01291 H_fuchsia EUT2G
01292 HMMalddh_2_egra(pf00171) EUT2A EP_CTERM
01293 H_fireEngine EUT2A
01294 Tsp_amethyst EUT2A
01295 HMMetlyEutC_3_eut2(pf05985) EUT2A EP_NTERM
01296 HMMetlyEutB_1_EUT1(pf06751) EUT2A
01297 HMM06277EutA_1_eut(pf06277) EUT2A
01298 HMMreg07568_1_eut2(pf12282pf02518pf07568) EUT2A
01299 HMMreg00072_1_eut2(pf03861pf00072) EUT2A
01300 Hp_euts_babyPurple PDU1C
01301 HMM00465alcdh_3_mix(pf00465) EUT2A
01302 -# -
01303 HMM01923cobtr_4_eut2(pf01923) EUT2A
01304 HMM10662PduV_1_mix(pf10662) EUT2A
01305 -pf00005 -
01306 -pf01032 -
01307 -pf01497 -
01308 -# -
01309 -pf02660 -
01310 -pf01263 -
BMC-P
BMC-H
BMC-T
Aldehyde dehydrogenase
PTAC
Signature enzyme
Alcohol dehydrogenase
Regulator

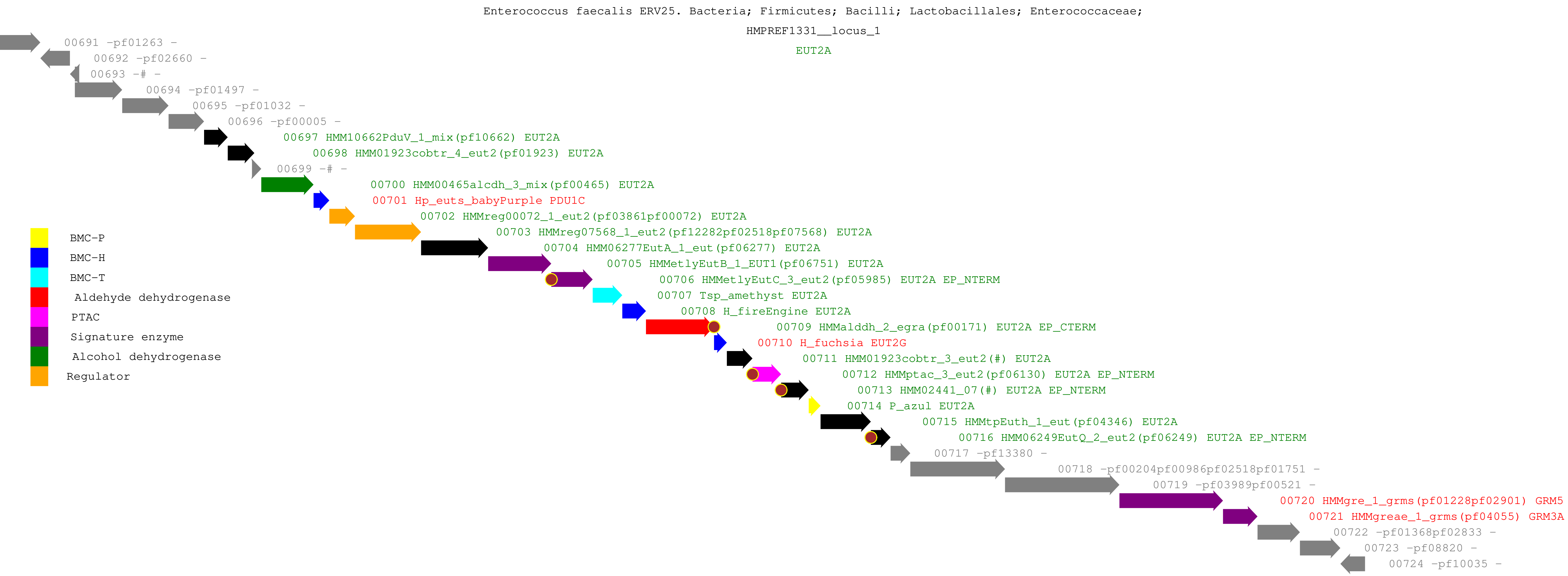
Enterococcus faecalis ERV25. Bacteria; Firmicutes; Bacilli; Lactobacillales; Enterococcaceae;
HMPREF1331__locus_1
EUT2A
00691 -pf01263 -
00692 -pf02660 -
00693 -# -
00694 -pf01497 -
00695 -pf01032 -
00696 -pf00005 -
00697 HMM10662PduV_1_mix(pf10662) EUT2A
00698 HMM01923cobtr_4_eut2(pf01923) EUT2A
00699 -# -
00700 HMM00465alcdh_3_mix(pf00465) EUT2A
00701 Hp_euts_babyPurple PDU1C
00702 HMMreg00072_1_eut2(pf03861pf00072) EUT2A
00703 HMMreg07568_1_eut2(pf12282pf02518pf07568) EUT2A
00704 HMM06277EutA_1_eut(pf06277) EUT2A
00705 HMMetlyEutB_1_EUT1(pf06751) EUT2A
00706 HMMetlyEutC_3_eut2(pf05985) EUT2A EP_NTERM
00707 Tsp_amethyst EUT2A
00708 H_fireEngine EUT2A
00709 HMMalddh_2_egra(pf00171) EUT2A EP_CTERM
00710 H_fuchsia EUT2G
00711 HMM01923cobtr_3_eut2(#) EUT2A
00712 HMMptac_3_eut2(pf06130) EUT2A EP_NTERM
00713 HMM02441_07(#) EUT2A EP_NTERM
00714 P_azul EUT2A
00715 HMMtpEuth_1_eut(pf04346) EUT2A
00716 HMM06249EutQ_2_eut2(pf06249) EUT2A EP_NTERM
00717 -pf13380 -
00718 -pf00204pf00986pf02518pf01751 -
00719 -pf03989pf00521 -
00720 HMMgre_1_grms(pf01228pf02901) GRM5
00721 HMMgreae_1_grms(pf04055) GRM3A
00722 -pf01368pf02833 -
00723 -pf08820 -
00724 -pf10035 -
BMC-P
BMC-H
BMC-T
Aldehyde dehydrogenase
PTAC
Signature enzyme
Alcohol dehydrogenase
Regulator

Enterococcus faecalis ERV31. Bacteria; Firmicutes; Bacilli; Lactobacillales; Enterococcaceae;

HMPREF1332__locus_1

EUT2A

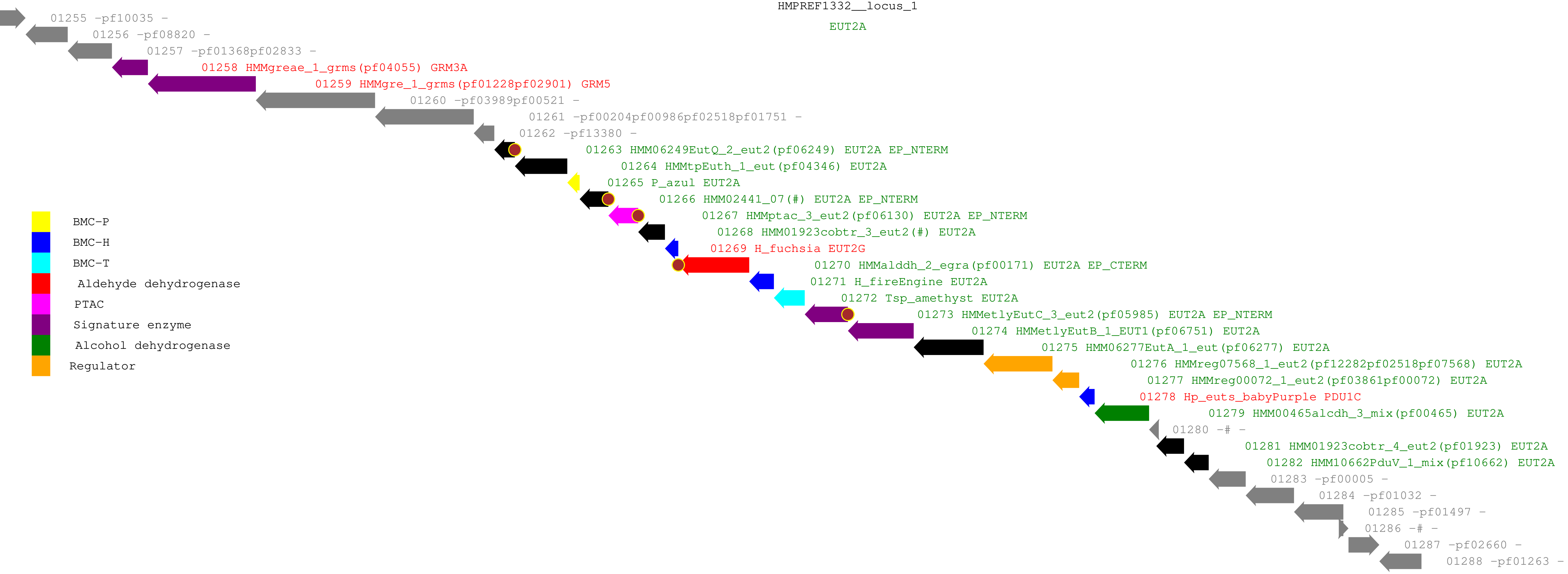

Enterococcus faecalis ERV37. Bacteria; Firmicutes; Bacilli; Lactobacillales; Enterococcaceae;

HMPREF1333__locus_1

EUT2A

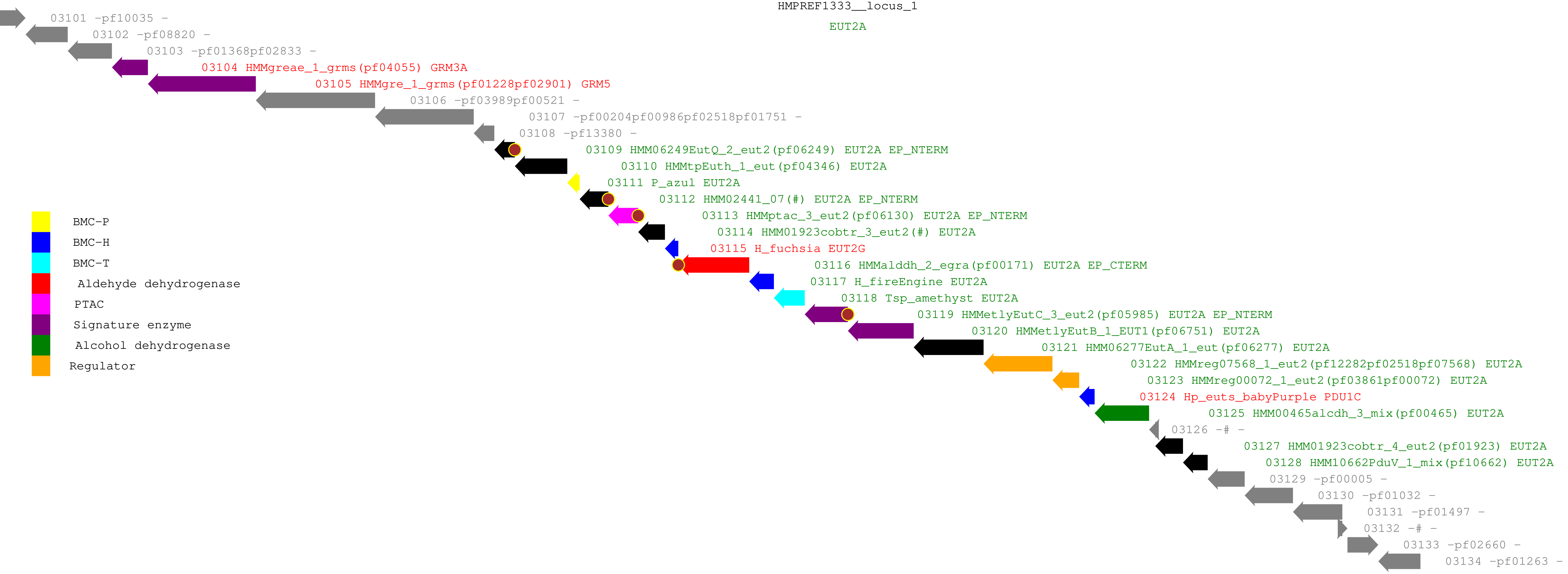

Enterococcus faecalis ERV41. Bacteria; Firmicutes; Bacilli; Lactobacillales; Enterococcaceae;

HMPREF1334__locus_1

EUT2A

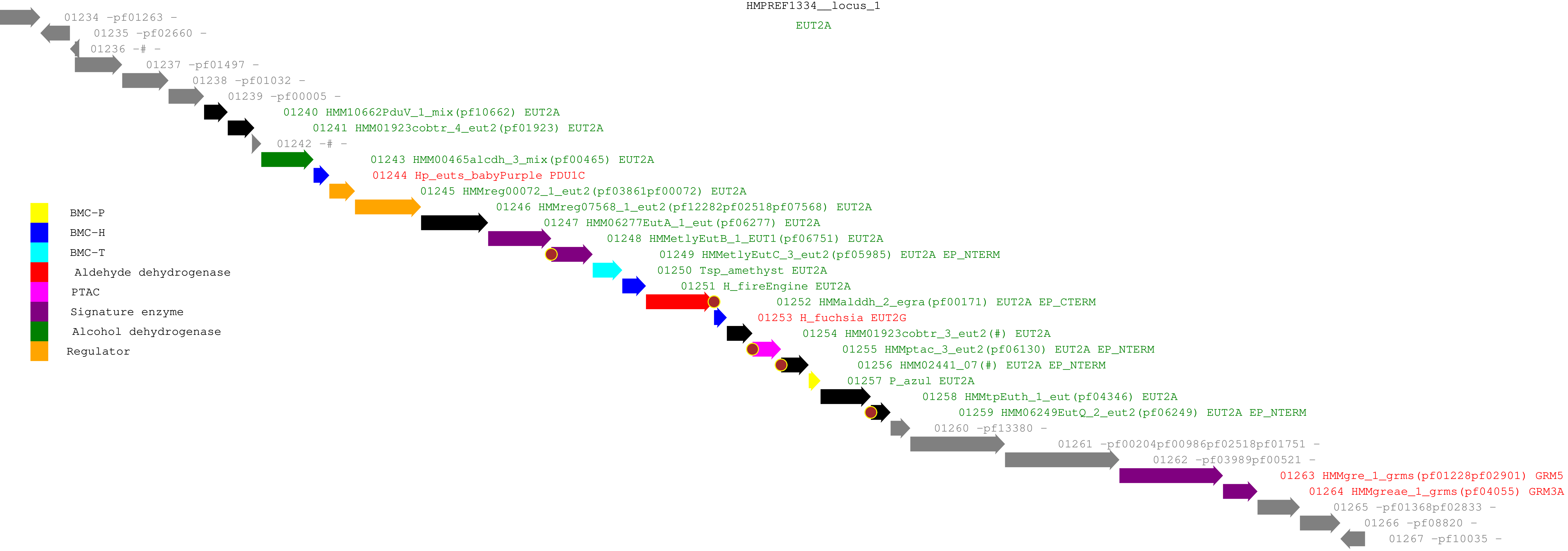

Enterococcus faecalis ERV62. Bacteria; Firmicutes; Bacilli; Lactobacillales; Enterococcaceae;

HMPREF1335__locus_1

EUT2A

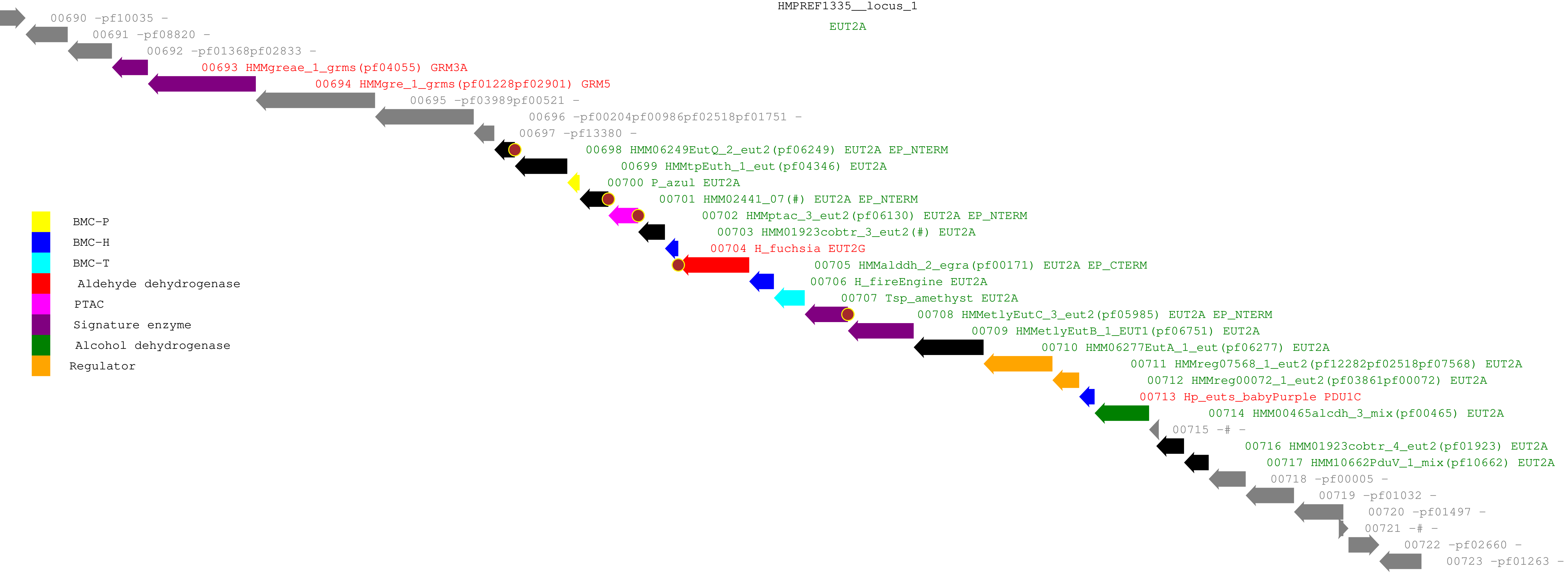

Enterococcus faecalis ERV63. Bacteria; Firmicutes; Bacilli; Lactobacillales; Enterococcaceae;

HMPREF1336__locus_1

EUT2A

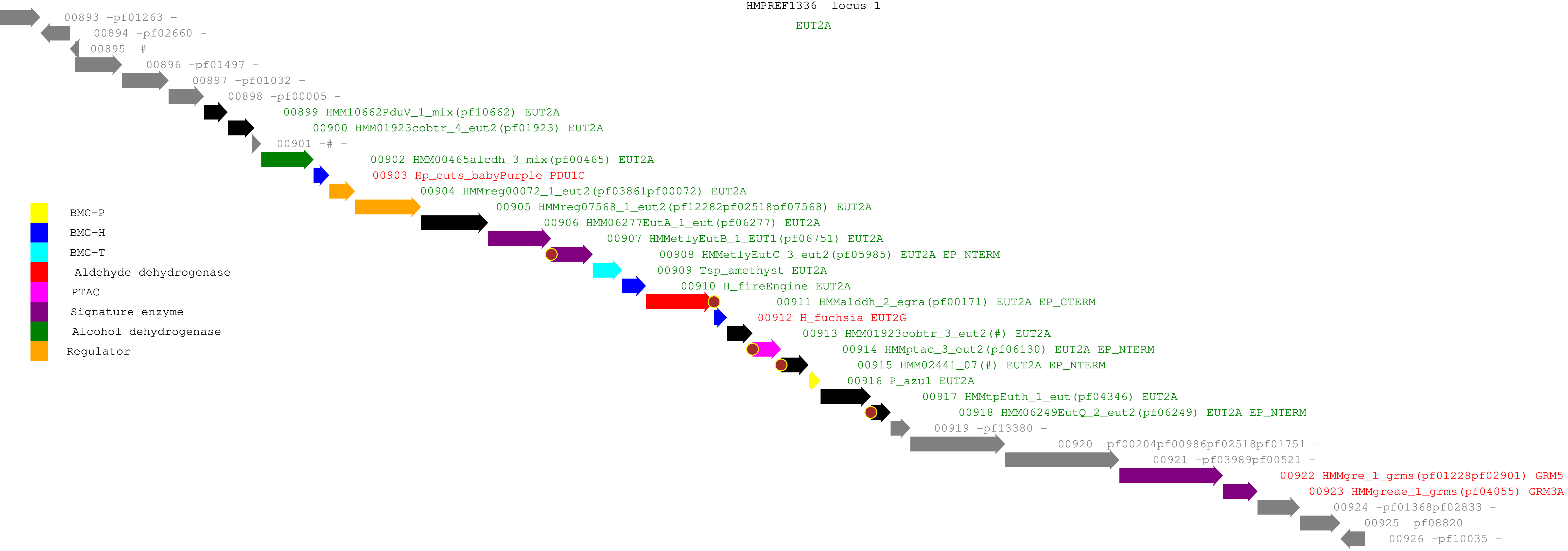

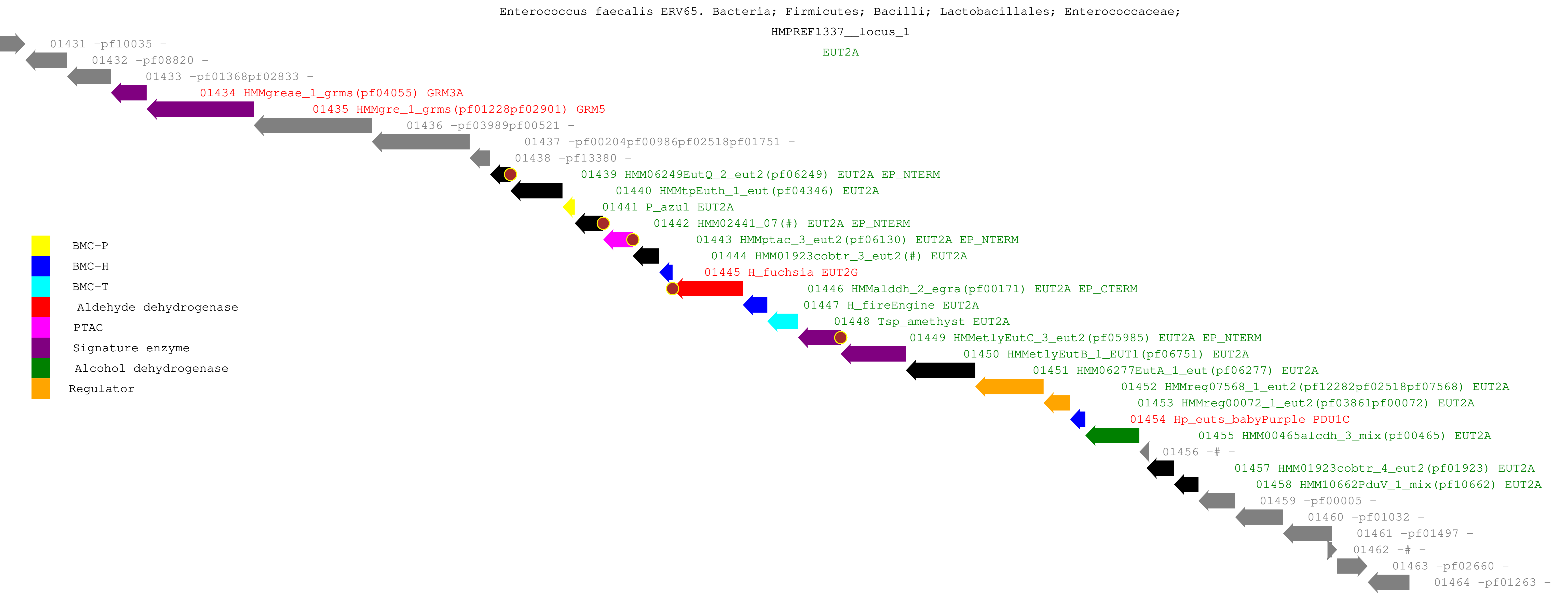
Enterococcus faecalis ERV65. Bacteria; Firmicutes; Bacilli; Lactobacillales; Enterococcaceae;
HMPREF1337__locus_1
EUT2A
01431 -pf10035 -
01432 -pf08820 -
01433 -pf01368pf02833 -
01434 HMMgreae_1_grms(pf04055) GRM3A
01435 HMMgre_1_grms(pf01228pf02901) GRM5
01436 -pf03989pf00521 -
01437 -pf00204pf00986pf02518pf01751 -
01438 -pf13380 -
01439 HMM06249EutQ_2_eut2(pf06249) EUT2A EP_NTERM
01440 HMMtpEuth_1_eut(pf04346) EUT2A
01441 P_azul EUT2A
01442 HMM02441_07(#) EUT2A EP_NTERM
01443 HMMptac_3_eut2(pf06130) EUT2A EP_NTERM
01444 HMM01923cobtr_3_eut2(#) EUT2A
01445 H_fuchsia EUT2G
01446 HMMalddh_2_egra(pf00171) EUT2A EP_CTERM
01447 H_fireEngine EUT2A
01448 Tsp_amethyst EUT2A
01449 HMMetlyEutC_3_eut2(pf05985) EUT2A EP_NTERM
01450 HMMetlyEutB_1_EUT1(pf06751) EUT2A
01451 HMM06277EutA_1_eut(pf06277) EUT2A
01452 HMMreg07568_1_eut2(pf12282pf02518pf07568) EUT2A
01453 HMMreg00072_1_eut2(pf03861pf00072) EUT2A
01454 Hp_euts_babyPurple PDU1C
01455 HMM00465alcdh_3_mix(pf00465) EUT2A
01456 -# -
01457 HMM01923cobtr_4_eut2(pf01923) EUT2A
01458 HMM10662PduV_1_mix(pf10662) EUT2A
01459 -pf00005 -
01460 -pf01032 -
01461 -pf01497 -
01462 -# -
01463 -pf02660 -
01464 -pf01263 -
BMC-P
BMC-H
BMC-T
Aldehyde dehydrogenase
PTAC
Signature enzyme
Alcohol dehydrogenase
Regulator

Enterococcus faecalis ERV68. Bacteria; Firmicutes; Bacilli; Lactobacillales; Enterococcaceae;

HMPREF1338__locus_1

EUT2A

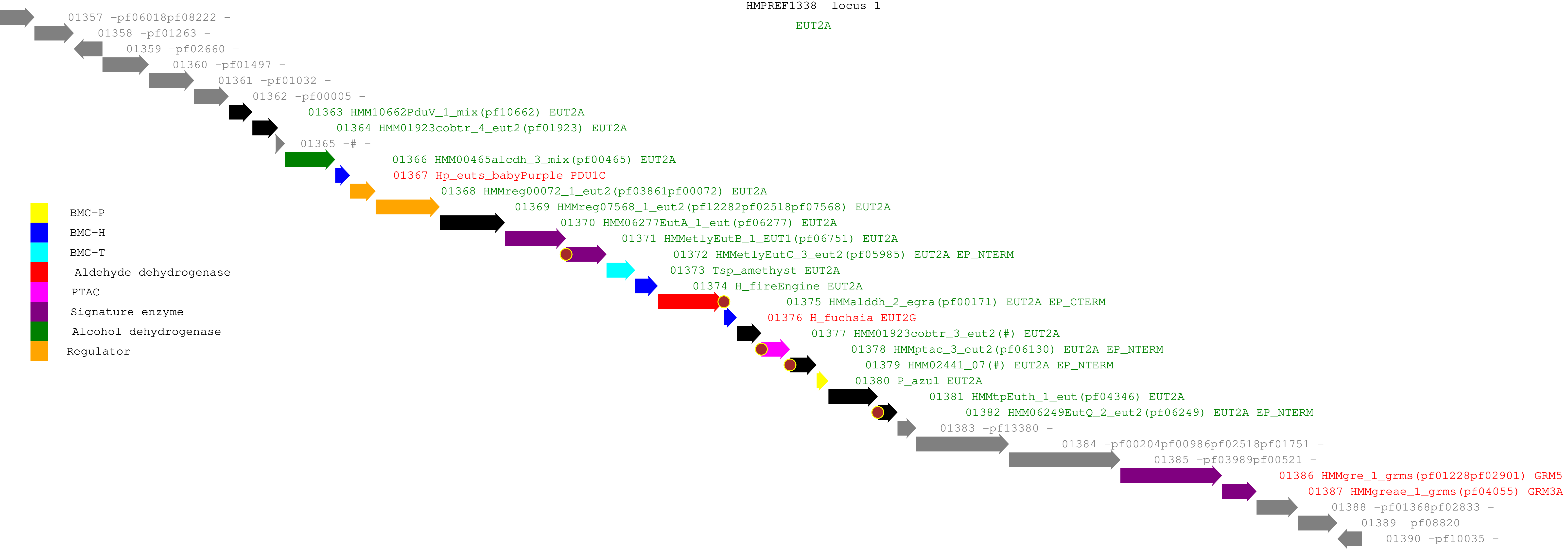

Enterococcus faecalis ERV72. Bacteria; Firmicutes; Bacilli; Lactobacillales; Enterococcaceae;

HMPREF1339__locus_1

EUT2A

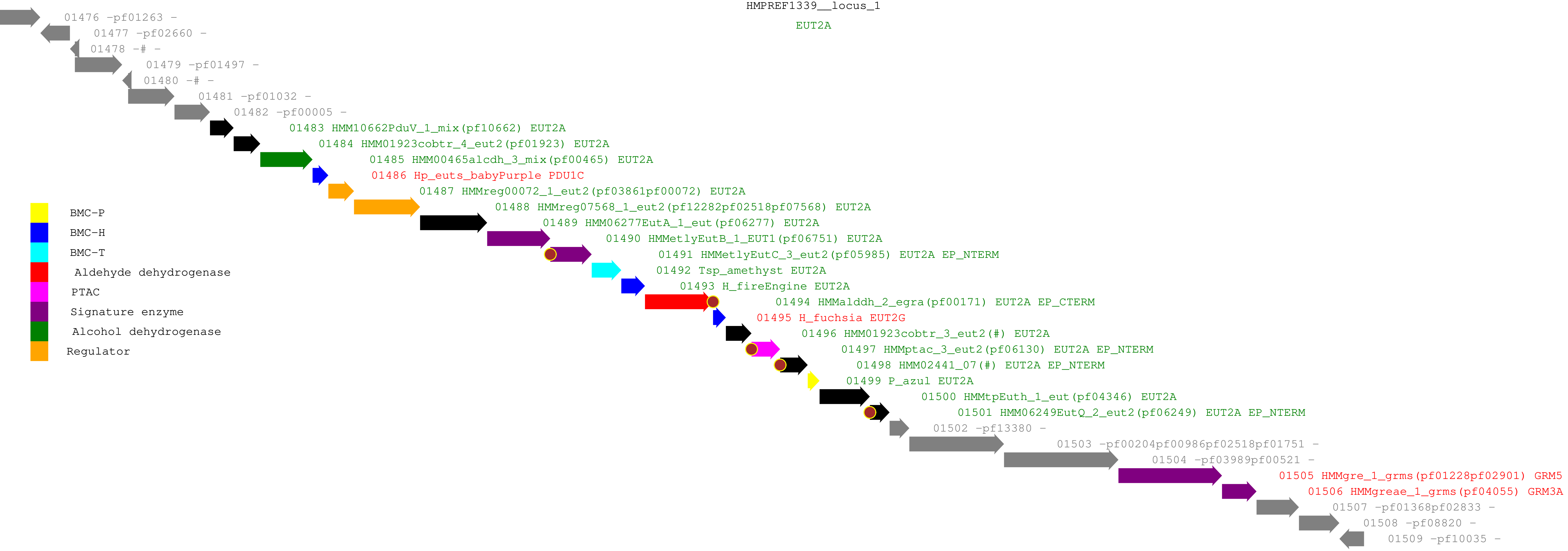

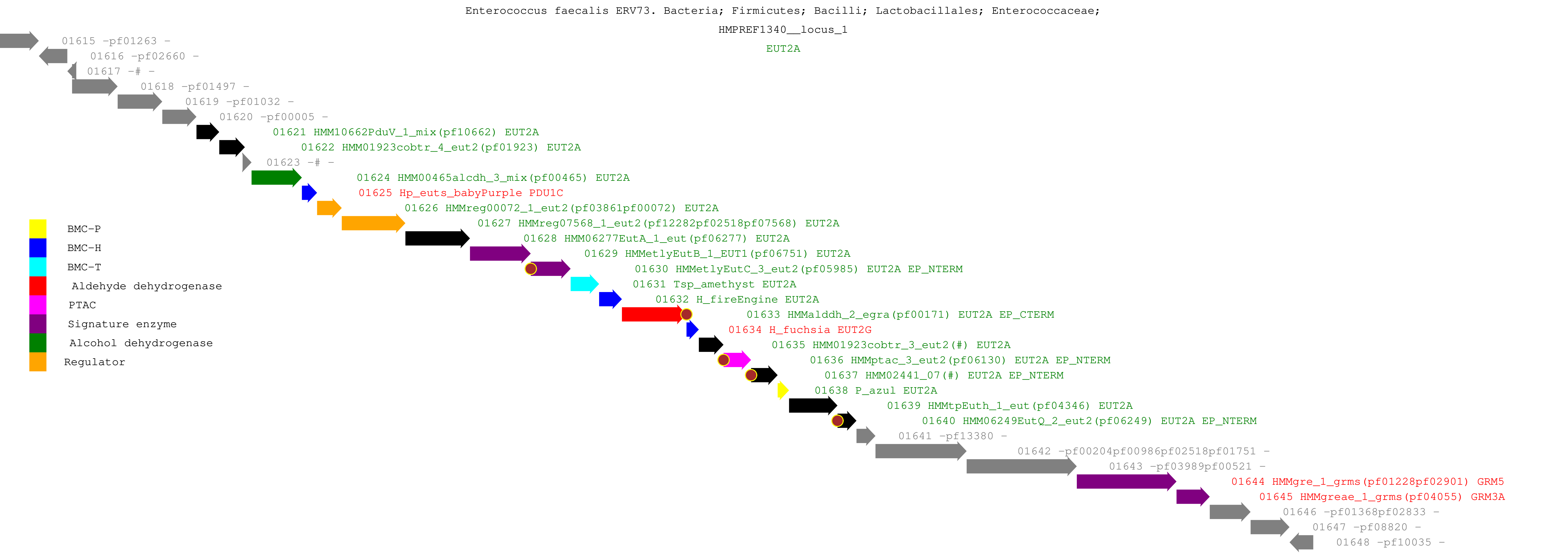

Enterococcus faecalis ERV73. Bacteria; Firmicutes; Bacilli; Lactobacillales; Enterococcaceae;
HMPREF1340__locus_1
EUT2A
01615 -pf01263 -
01616 -pf02660 -
01617 -# -
01618 -pf01497 -
01619 -pf01032 -
01620 -pf00005 -
01621 HMM10662PduV_1_mix(pf10662) EUT2A
01622 HMM01923cobtr_4_eut2(pf01923) EUT2A
01623 -# -
01624 HMM00465alcdh_3_mix(pf00465) EUT2A
01625 Hp_euts_babyPurple PDU1C
01626 HMMreg00072_1_eut2(pf03861pf00072) EUT2A
01627 HMMreg07568_1_eut2(pf12282pf02518pf07568) EUT2A
01628 HMM06277EutA_1_eut(pf06277) EUT2A
01629 HMMetlyEutB_1_EUT1(pf06751) EUT2A
01630 HMMetlyEutC_3_eut2(pf05985) EUT2A EP_NTERM
01631 Tsp_amethyst EUT2A
01632 H_fireEngine EUT2A
01633 HMMalddh_2_egra(pf00171) EUT2A EP_CTERM
01634 H_fuchsia EUT2G
01635 HMM01923cobtr_3_eut2(#) EUT2A
01636 HMMptac_3_eut2(pf06130) EUT2A EP_NTERM
01637 HMM02441_07(#) EUT2A EP_NTERM
01638 P_azul EUT2A
01639 HMMtpEuth_1_eut(pf04346) EUT2A
01640 HMM06249EutQ_2_eut2(pf06249) EUT2A EP_NTERM
01641 -pf13380 -
01642 -pf00204pf00986pf02518pf01751 -
01643 -pf03989pf00521 -
01644 HMMgre_1_grms(pf01228pf02901) GRM5
01645 HMMgreae_1_grms(pf04055) GRM3A
01646 -pf01368pf02833 -
01647 -pf08820 -
01648 -pf10035 -
BMC-P
BMC-H
BMC-T
Aldehyde dehydrogenase
PTAC
Signature enzyme
Alcohol dehydrogenase
Regulator

Enterococcus faecalis ERV81. Bacteria; Firmicutes; Bacilli; Lactobacillales; Enterococcaceae;

HMPREF1341__locus_1

EUT2A

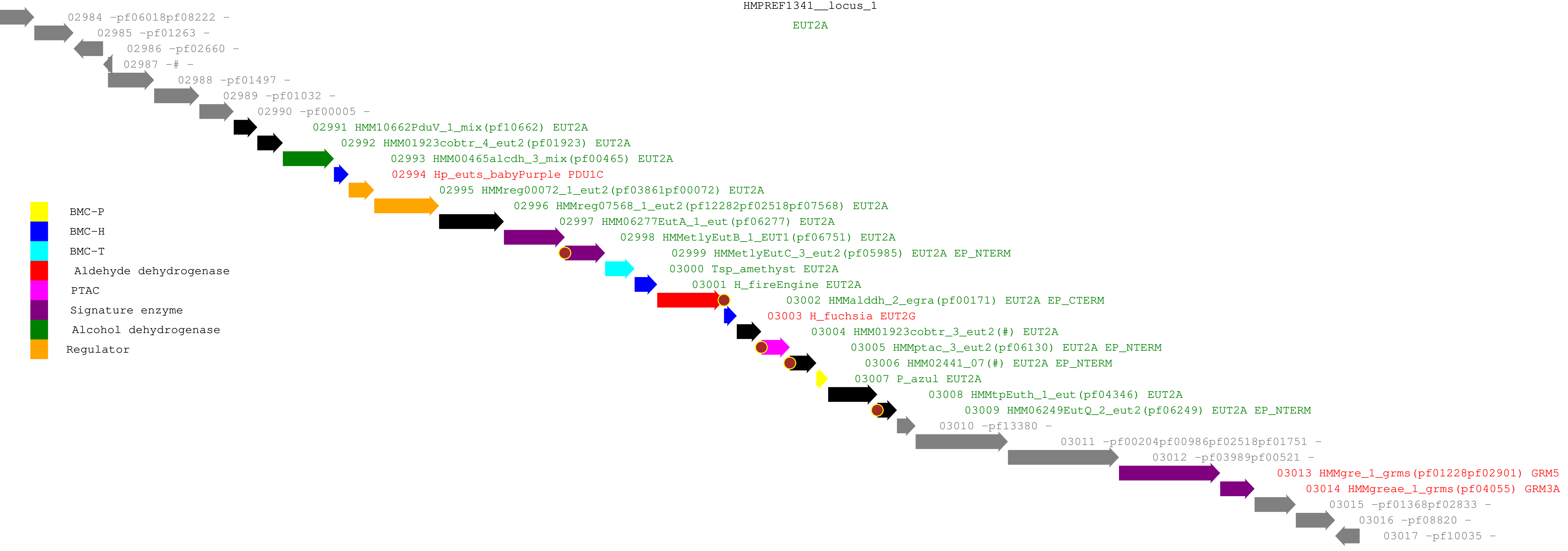

Enterococcus faecalis ERV85. Bacteria; Firmicutes; Bacilli; Lactobacillales; Enterococcaceae;

HMPREF1342__locus_1

EUT2A

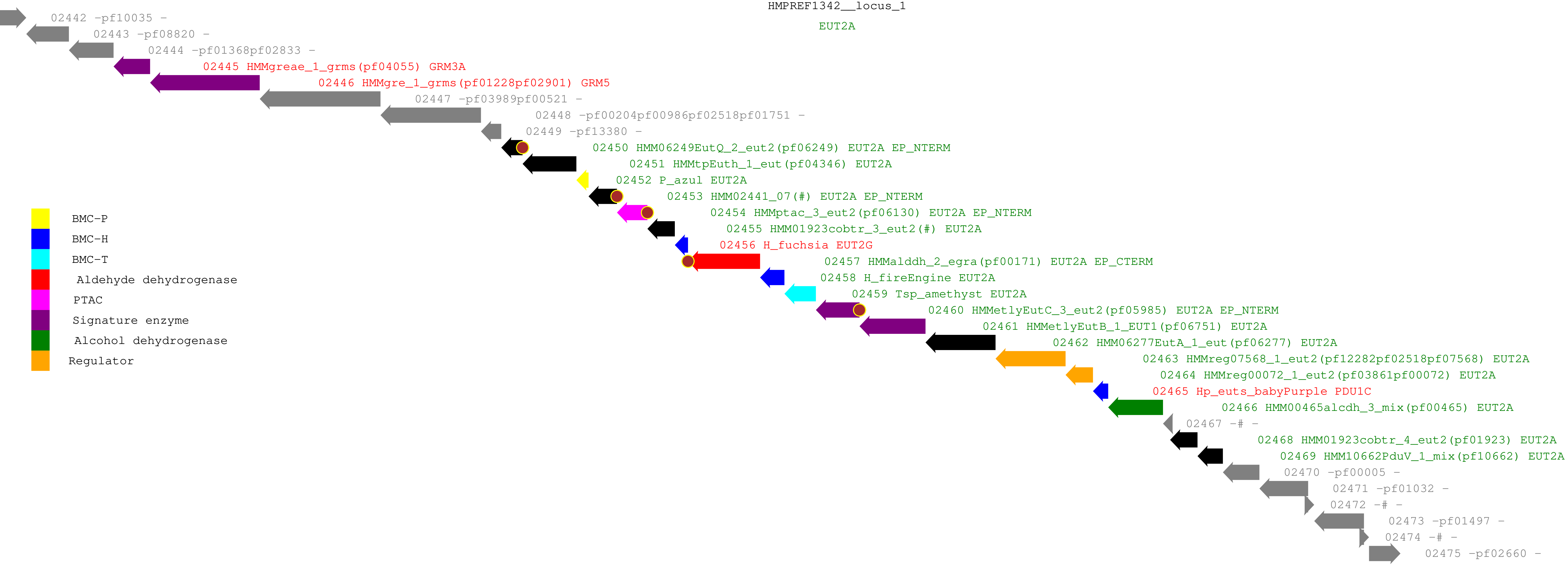

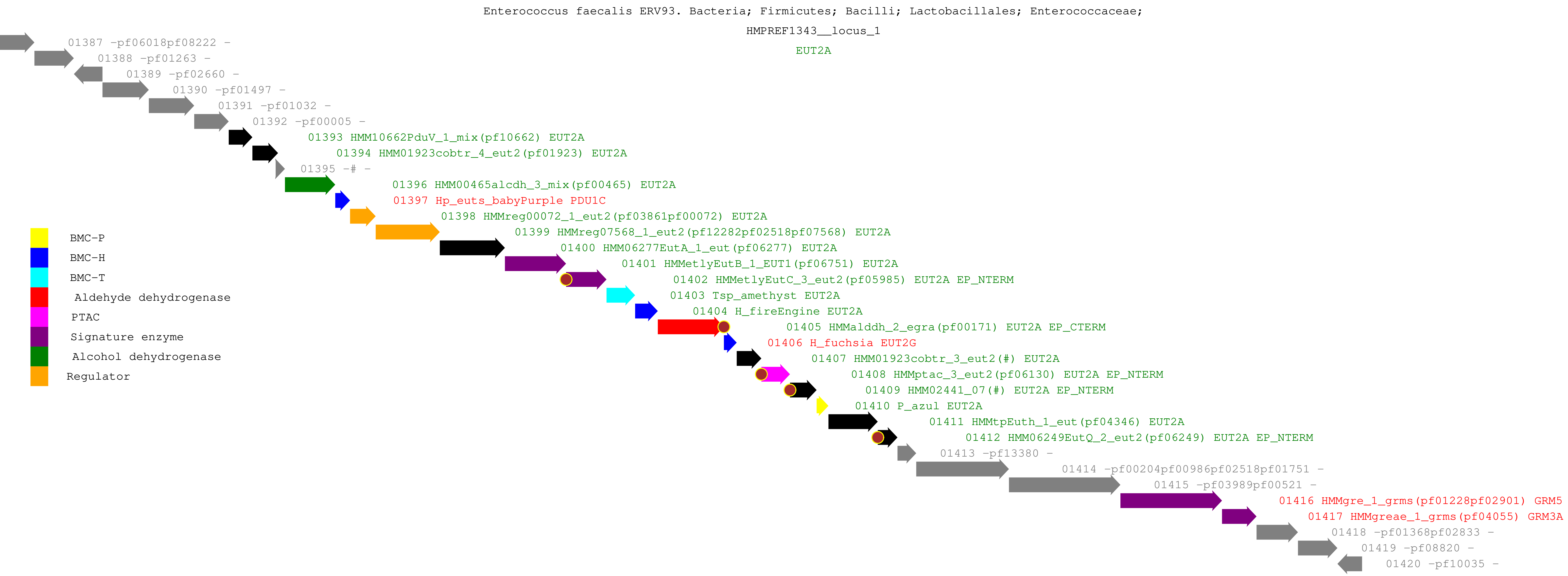

Enterococcus faecalis ERV93. Bacteria; Firmicutes; Bacilli; Lactobacillales; Enterococcaceae;
HMPREF1343__locus_1
EUT2A
01387 -pf06018pf08222 -
01388 -pf01263 -
01389 -pf02660 -
01390 -pf01497 -
01391 -pf01032 -
01392 -pf00005 -
01393 HMM10662PduV_1_mix(pf10662) EUT2A
01394 HMM01923cobtr_4_eut2(pf01923) EUT2A
01395 -# -
01396 HMM00465alcdh_3_mix(pf00465) EUT2A
01397 Hp_euts_babyPurple PDU1C
01398 HMMreg00072_1_eut2(pf03861pf00072) EUT2A
01399 HMMreg07568_1_eut2(pf12282pf02518pf07568) EUT2A
01400 HMM06277EutA_1_eut(pf06277) EUT2A
01401 HMMetlyEutB_1_EUT1(pf06751) EUT2A
01402 HMMetlyEutC_3_eut2(pf05985) EUT2A EP_NTERM
01403 Tsp_amethyst EUT2A
01404 H_fireEngine EUT2A
01405 HMMalddh_2_egra(pf00171) EUT2A EP_CTERM
01406 H_fuchsia EUT2G
01407 HMM01923cobtr_3_eut2(#) EUT2A
01408 HMMptac_3_eut2(pf06130) EUT2A EP_NTERM
01409 HMM02441_07(#) EUT2A EP_NTERM
01410 P_azul EUT2A
01411 HMMtpEuth_1_eut(pf04346) EUT2A
01412 HMM06249EutQ_2_eut2(pf06249) EUT2A EP_NTERM
01413 -pf13380 -
01414 -pf00204pf00986pf02518pf01751 -
01415 -pf03989pf00521 -
01416 HMMgre_1_grms(pf01228pf02901) GRM5
01417 HMMgreae_1_grms(pf04055) GRM3A
01418 -pf01368pf02833 -
01419 -pf08820 -
01420 -pf10035 -
BMC-P
BMC-H
BMC-T
Aldehyde dehydrogenase
PTAC
Signature enzyme
Alcohol dehydrogenase
Regulator

Enterococcus faecalis 918. Bacteria; Firmicutes; Bacilli; Lactobacillales; Enterococcaceae;

HMPREF2097__locus_1

EUT2A

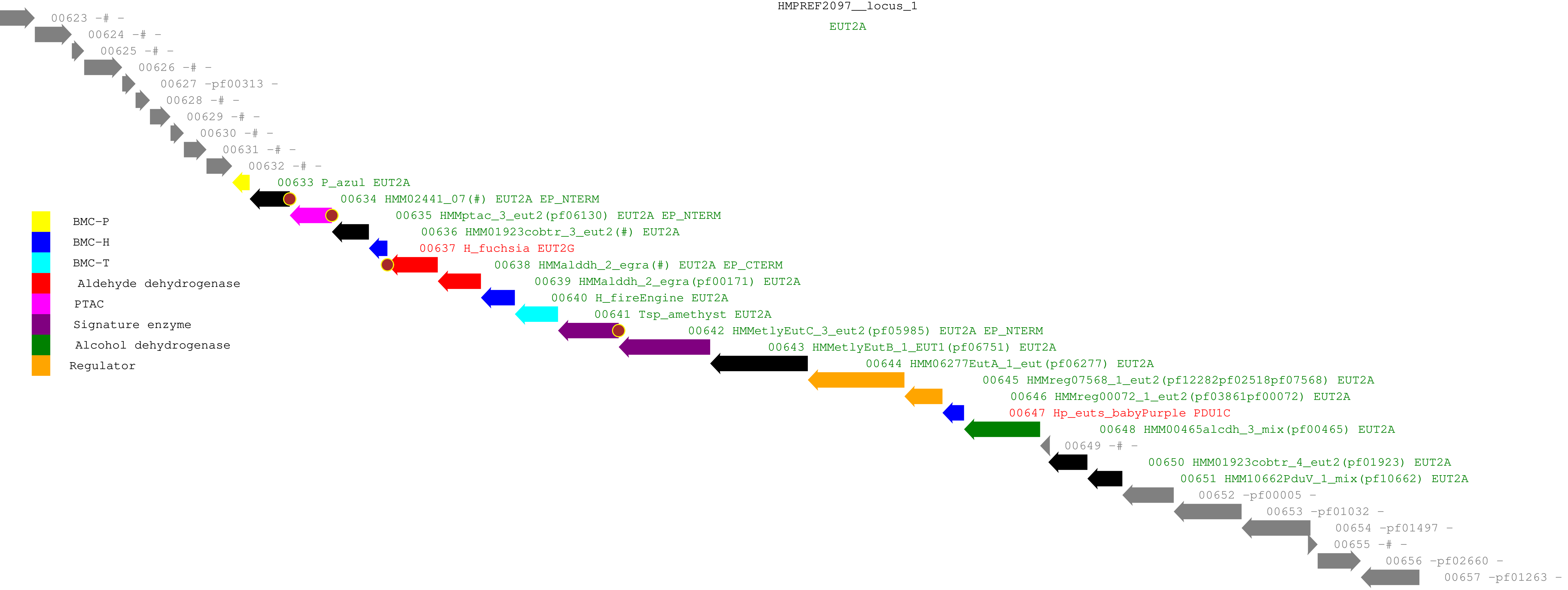

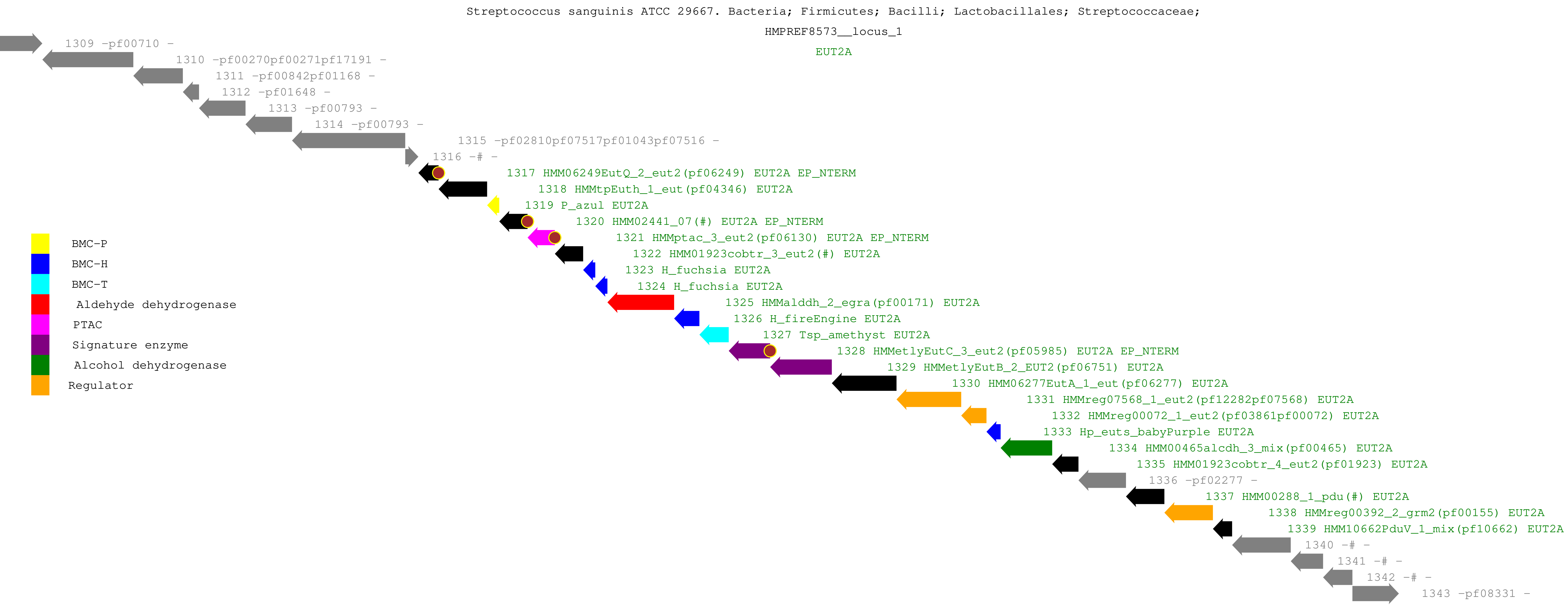

Streptococcus sanguinis ATCC 29667. Bacteria; Firmicutes; Bacilli; Lactobacillales; Streptococcaceae;
HMPREF8573__locus_1
EUT2A
1309 -pf00710 -
1310 -pf00270pf00271pf17191 -
1311 -pf00842pf01168 -
1312 -pf01648 -
1313 -pf00793 -
1314 -pf00793 -
1315 -pf02810pf07517pf01043pf07516 -
1316 -# -
1317 HMM06249EutQ_2_eut2(pf06249) EUT2A EP_NTERM
1318 HMMtpEuth_1_eut(pf04346) EUT2A
1319 P_azul EUT2A
1320 HMM02441_07(#) EUT2A EP_NTERM
1321 HMMptac_3_eut2(pf06130) EUT2A EP_NTERM
1322 HMM01923cobtr_3_eut2(#) EUT2A
1323 H_fuchsia EUT2A
1324 H_fuchsia EUT2A
1325 HMMaldh_2_egra(pf00171) EUT2A
1326 H_fireEngine EUT2A
1327 Tsp_amethyst EUT2A
1328 HMMetlyEutC_3_eut2(pf05985) EUT2A EP_NTERM
1329 HMMetlyEutB_2_EUT2(pf06751) EUT2A
1330 HMM06277EutA_1_eut(pf06277) EUT2A
1331 HMMreg07568_1_eut2(pf12282pf07568) EUT2A
1332 HMMreg00072_1_eut2(pf03861pf00072) EUT2A
1333 Hp_euts_babyPurple EUT2A
1334 HMM00465alcdh_3_mix(pf00465) EUT2A
1335 HMM01923cobtr_4_eut2(pf01923) EUT2A
1336 -pf02277 -
1337 HMM00288_1_pdu(#) EUT2A
1338 HMMreg00392_2_grm2(pf00155) EUT2A
1339 HMM10662PduV_1_mix(pf10662) EUT2A
1340 -# -
1341 -# -
1342 -# -
1343 -pf08331 -
BMC-P
BMC-H
BMC-T
Aldehyde dehydrogenase
PTAC
Signature enzyme
Alcohol dehydrogenase
Regulator

Streptococcus parasanguinis ATCC 903. Bacteria; Firmicutes; Bacilli; Lactobacillales; Streptococcaceae;

HMPREF8577__locus_1

EUT2A

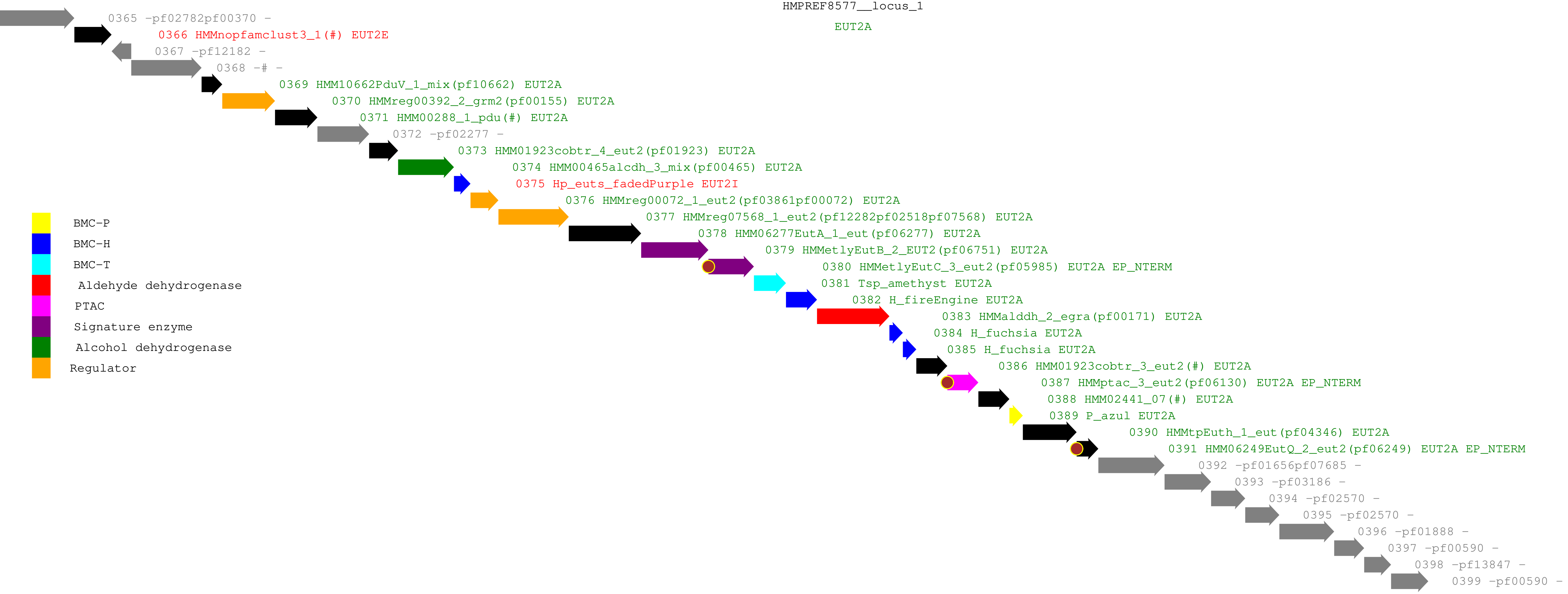

Enterococcus faecalis S613. Bacteria; Firmicutes; Bacilli; Lactobacillales; Enterococcaceae;

HMPREF9376__locus_1

EUT2A

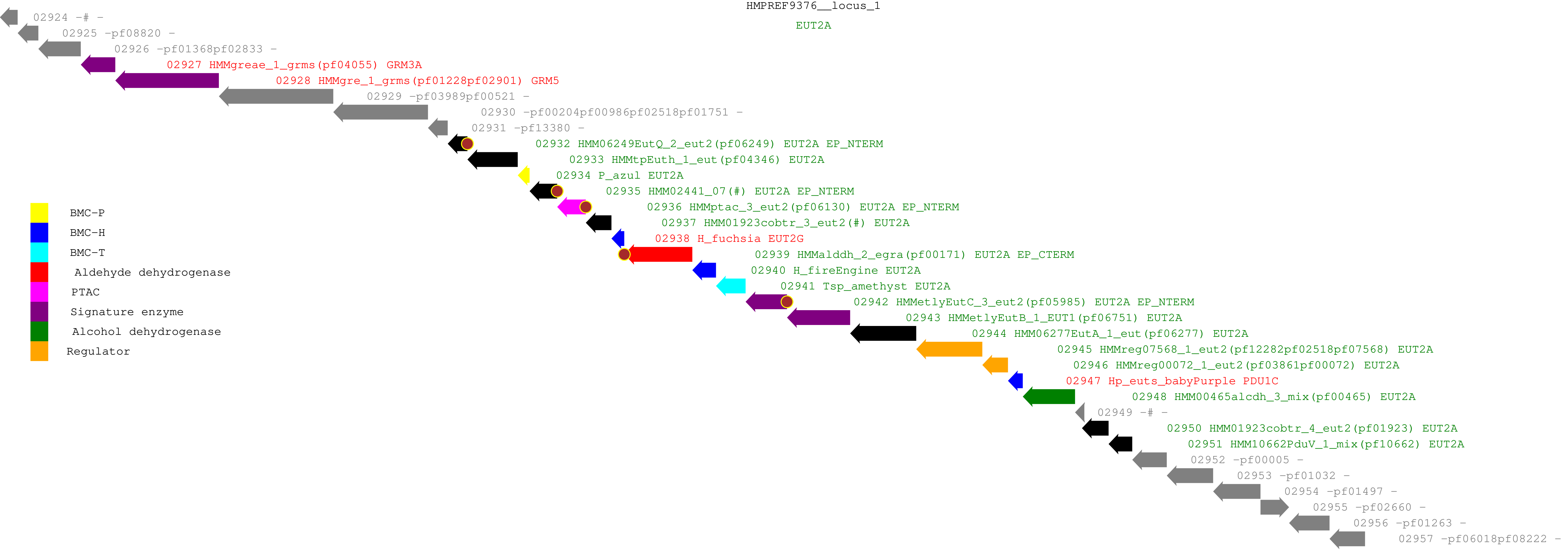

Enterococcus faecalis R712. Bacteria; Firmicutes; Bacilli; Lactobacillales; Enterococcaceae;

HMPREF9377__locus_1

EUT2A

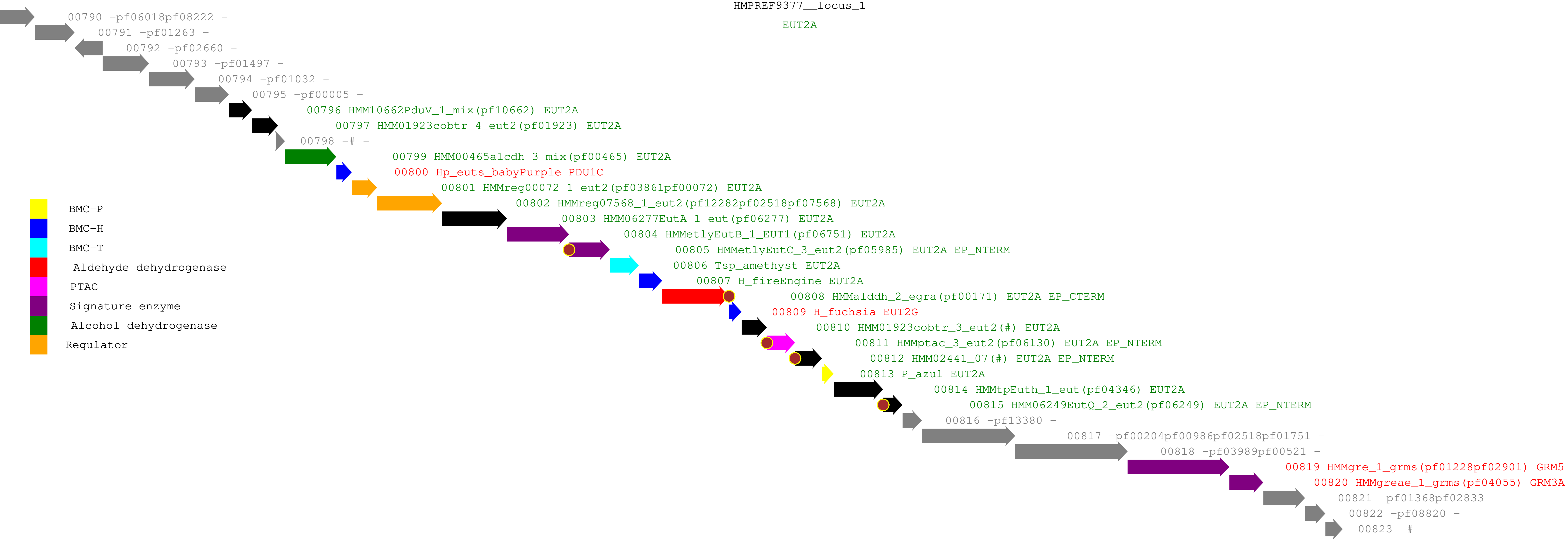

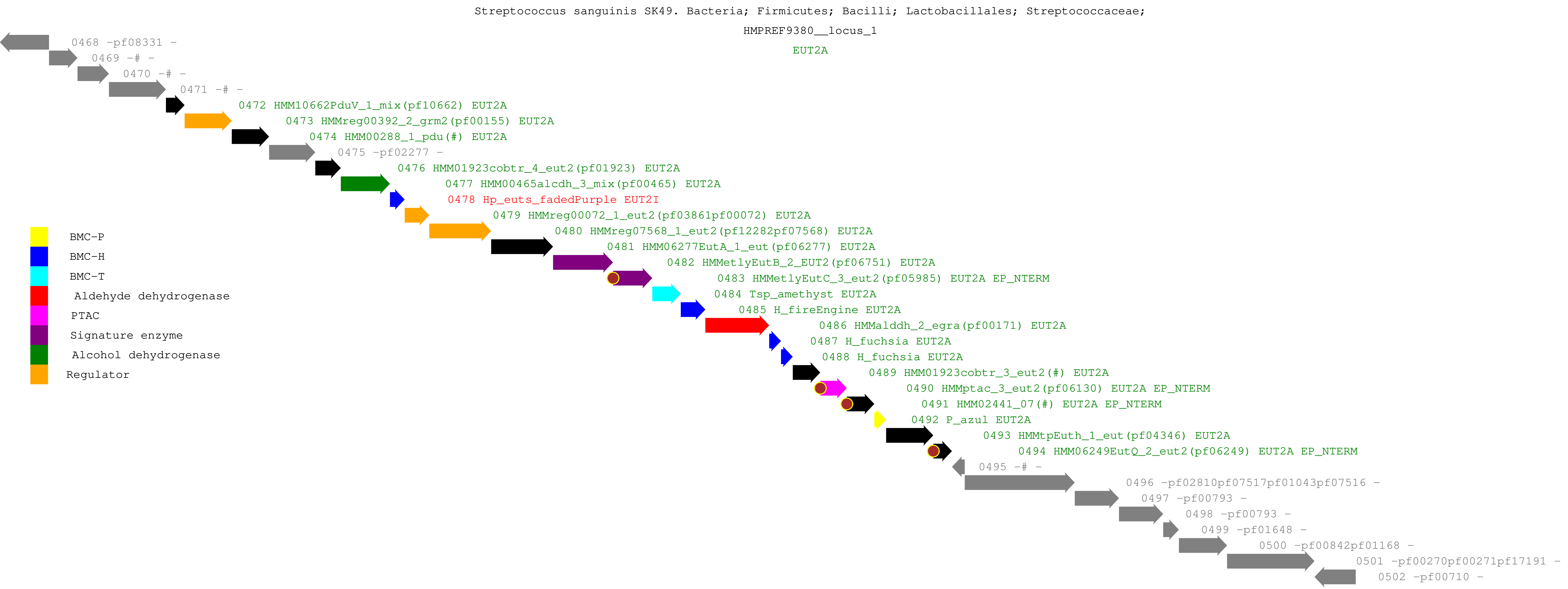
Streptococcus sanguinis SK49. Bacteria; Firmicutes; Bacilli; Lactobacillales; Streptococcaceae;
HMPREF9380__locus_1
EUT2A
0468 -pf08331 -
0469 -# -
0470 -# -
0471 -# -
0472 HMM10662PduV_1_mix(pf10662) EUT2A
0473 HMMreg00392_2_grm2(pf00155) EUT2A
0474 HMM00288_1_pdu(#) EUT2A
0475 -pf02277 -
0476 HMM01923cobtr_4_eut2(pf01923) EUT2A
0477 HMM00465alcdh_3_mix(pf00465) EUT2A
0478 Hp_euts_fadedPurple EUT2I
0479 HMMreg00072_1_eut2(pf03861pf00072) EUT2A
0480 HMMreg07568_1_eut2(pf12282pf07568) EUT2A
0481 HMM06277EutA_1_eut(pf06277) EUT2A
0482 HMMetlyEutB_2_EUT2(pf06751) EUT2A
0483 HMMetlyEutC_3_eut2(pf05985) EUT2A EP_NTERM
0484 Tsp_amethyst EUT2A
0485 H_fireEngine EUT2A
0486 HMMaldh_2_egra(pf00171) EUT2A
0487 H_fuchsia EUT2A
0488 H_fuchsia EUT2A
0489 HMM01923cobtr_3_eut2(#) EUT2A
0490 HMMptac_3_eut2(pf06130) EUT2A EP_NTERM
0491 HMM02441_07(#) EUT2A EP_NTERM
0492 P_azul EUT2A
0493 HMMtpEuth_1_eut(pf04346) EUT2A
0494 HMM06249EutQ_2_eut2(pf06249) EUT2A EP_NTERM
0495 -# -
0496 -pf02810pf07517pf01043pf07516 -
0497 -pf00793 -
0498 -pf00793 -
0499 -pf01648 -
0500 -pf00842pf01168 -
0501 -pf00270pf00271pf17191 -
0502 -pf00710 -
BMC-P
BMC-H
BMC-T
Aldehyde dehydrogenase
PTAC
Signature enzyme
Alcohol dehydrogenase
Regulator

Streptococcus sanguinis SK160. Bacteria; Firmicutes; Bacilli; Lactobacillales; Streptococcaceae;

HMPREF9384__locus_1

EUT2A

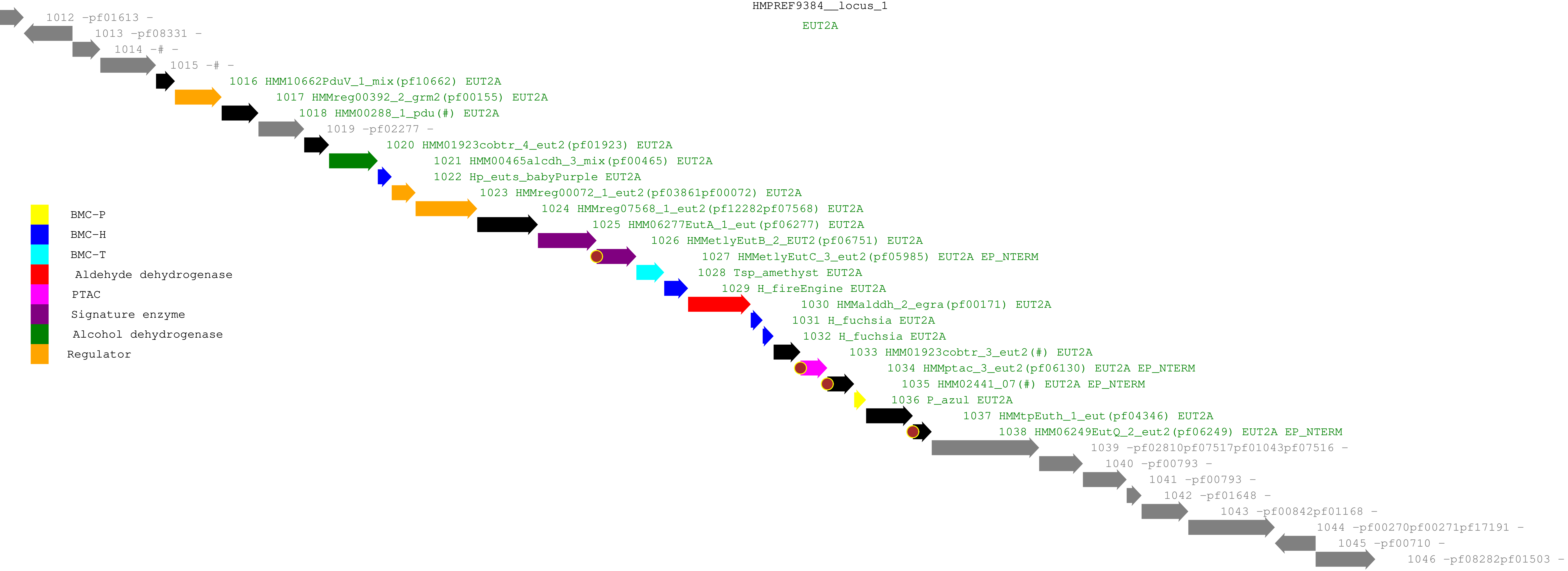

Streptococcus sanguinis SK408. Bacteria; Firmicutes; Bacilli; Lactobacillales; Streptococcaceae;

HMPREF9391__locus_1

EUT2A

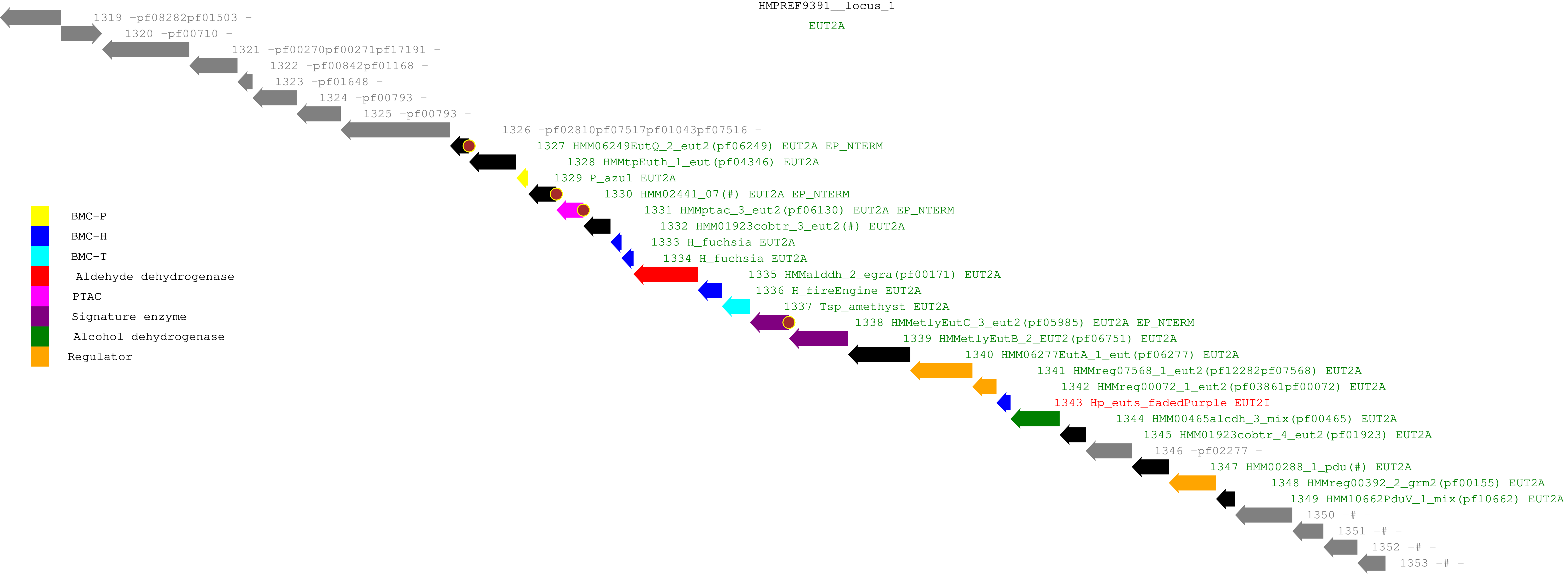

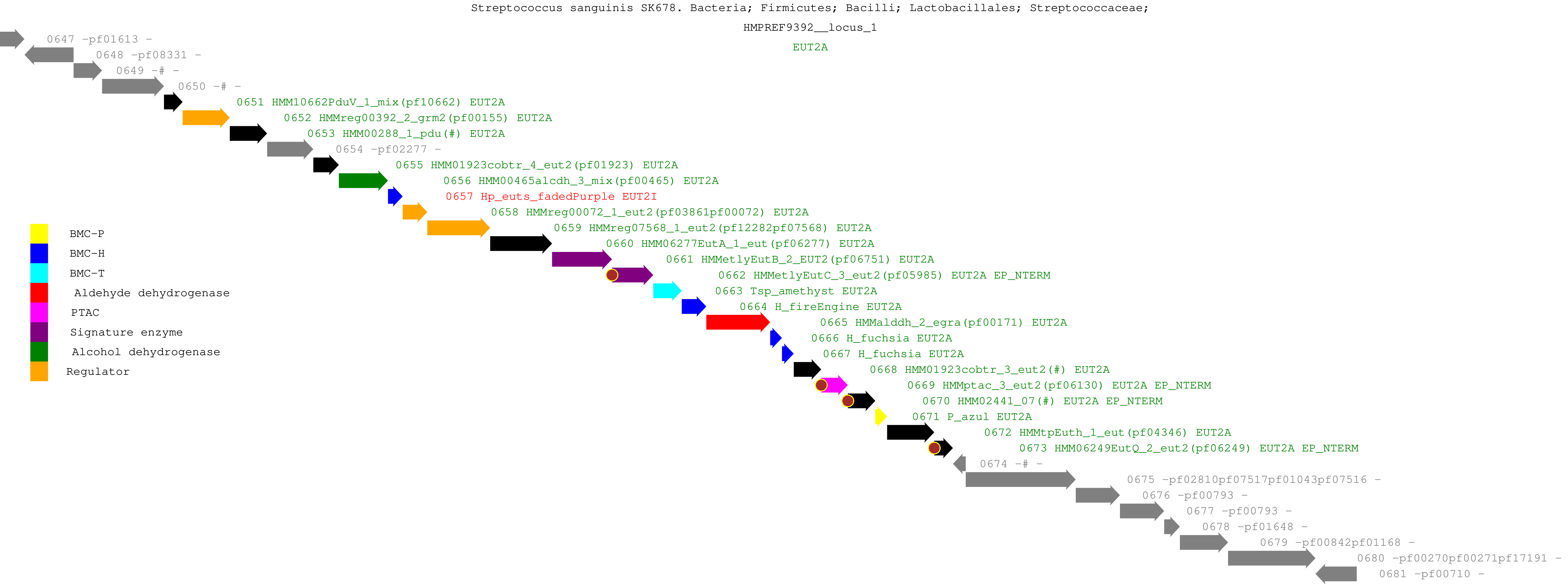
Streptococcus sanguinis SK678. Bacteria; Firmicutes; Bacilli; Lactobacillales; Streptococcaceae;
HMPREF9392__locus_1
EUT2A
0647 -pf01613 -
0648 -pf08331 -
0649 -# -
0650 -# -
0651 HMM10662PduV_1_mix(pf10662) EUT2A
0652 HMMreg00392_2_grm2(pf00155) EUT2A
0653 HMM00288_1_pdu(#) EUT2A
0654 -pf02277 -
0655 HMM01923cobtr_4_eut2(pf01923) EUT2A
0656 HMM00465alcdh_3_mix(pf00465) EUT2A
0657 Hp_euts_fadedPurple EUT2I
0658 HMMreg00072_1_eut2(pf03861pf00072) EUT2A
0659 HMMreg07568_1_eut2(pf12282pf07568) EUT2A
0660 HMM06277EutA_1_eut(pf06277) EUT2A
0661 HMMetlyEutB_2_EUT2(pf06751) EUT2A
0662 HMMetlyEutC_3_eut2(pf05985) EUT2A EP_NTERM
0663 Tsp_amethyst EUT2A
0664 H_fireEngine EUT2A
0665 HMMalddh_2_egra(pf00171) EUT2A
0666 H_fuchsia EUT2A
0667 H_fuchsia EUT2A
0668 HMM01923cobtr_3_eut2(#) EUT2A
0669 HMMptac_3_eut2(pf06130) EUT2A EP_NTERM
0670 HMM02441_07(#) EUT2A EP_NTERM
0671 P_azul EUT2A
0672 HMMtpEuth_1_eut(pf04346) EUT2A
0673 HMM06249EutQ_2_eut2(pf06249) EUT2A EP_NTERM
0674 -# -
0675 -pf02810pf07517pf01043pf07516 -
0676 -pf00793 -
0677 -pf00793 -
0678 -pf01648 -
0679 -pf00842pf01168 -
0680 -pf00270pf00271pf17191 -
0681 -pf00710 -
BMC-P
BMC-H
BMC-T
Aldehyde dehydrogenase
PTAC
Signature enzyme
Alcohol dehydrogenase
Regulator

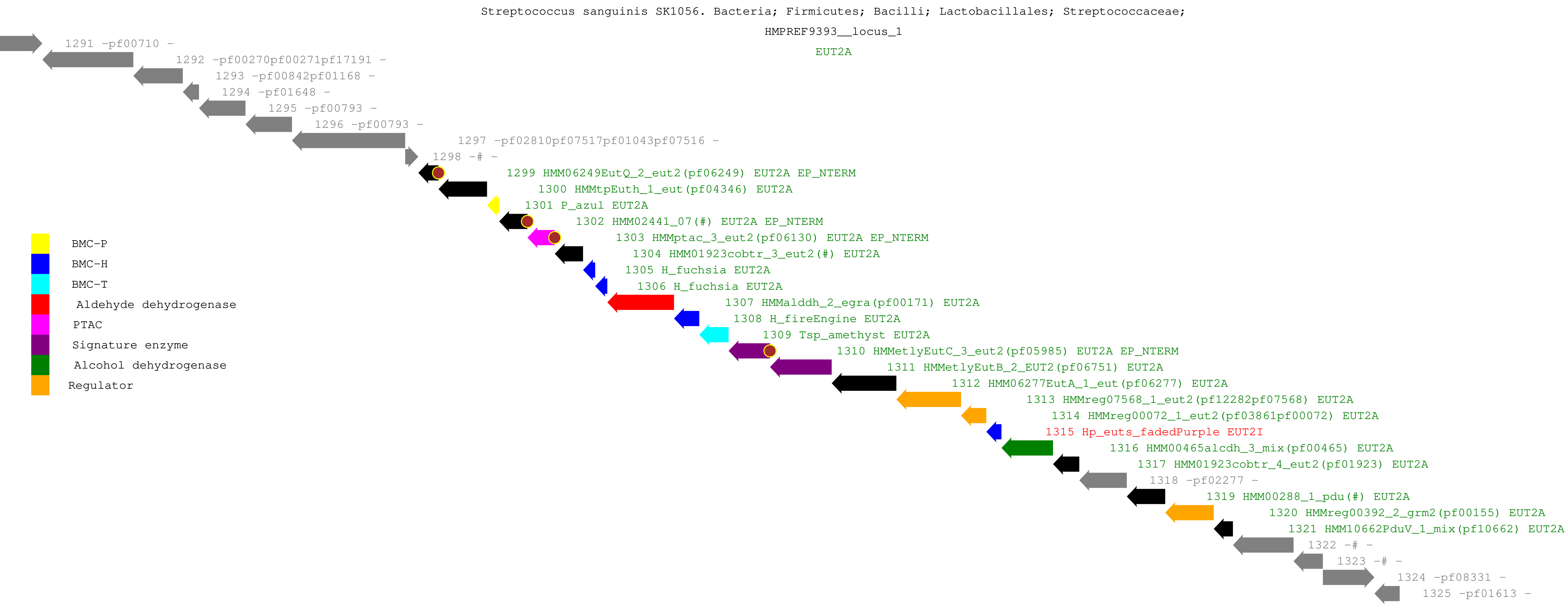

Streptococcus sanguinis SK1056. Bacteria; Firmicutes; Bacilli; Lactobacillales; Streptococcaceae;
HMPREF9393__locus_1
EUT2A
1291 -pf00710 -
1292 -pf00270pf00271pf17191 -
1293 -pf00842pf01168 -
1294 -pf01648 -
1295 -pf00793 -
1296 -pf00793 -
1297 -pf02810pf07517pf01043pf07516 -
1298 -# -
1299 HMM06249EutQ_2_eut2(pf06249) EUT2A EP_NTERM
1300 HMMtpEuth_1_eut(pf04346) EUT2A
1301 P_azul EUT2A
1302 HMM02441_07(#) EUT2A EP_NTERM
1303 HMMptac_3_eut2(pf06130) EUT2A EP_NTERM
1304 HMM01923cobtr_3_eut2(#) EUT2A
1305 H_fuchsia EUT2A
1306 H_fuchsia EUT2A
1307 HMMalddh_2_egra(pf00171) EUT2A
1308 H_fireEngine EUT2A
1309 Tsp_amethyst EUT2A
1310 HMMetlyEutC_3_eut2(pf05985) EUT2A EP_NTERM
1311 HMMetlyEutB_2_EUT2(pf06751) EUT2A
1312 HMM06277EutA_1_eut(pf06277) EUT2A
1313 HMMreg07568_1_eut2(pf12282pf07568) EUT2A
1314 HMMreg00072_1_eut2(pf03861pf00072) EUT2A
1315 Hp_euts_fadedPurple EUT2I
1316 HMM00465alcdh_3_mix(pf00465) EUT2A
1317 HMM01923cobtr_4_eut2(pf01923) EUT2A
1318 -pf02277 -
1319 HMM00288_1_pdu(#) EUT2A
1320 HMMreg00392_2_grm2(pf00155) EUT2A
1321 HMM10662PduV_1_mix(pf10662) EUT2A
1322 -# -
1323 -# -
1324 -pf08331 -
1325 -pf01613 -
BMC-P
BMC-H
BMC-T
Aldehyde dehydrogenase
PTAC
Signature enzyme
Alcohol dehydrogenase
Regulator

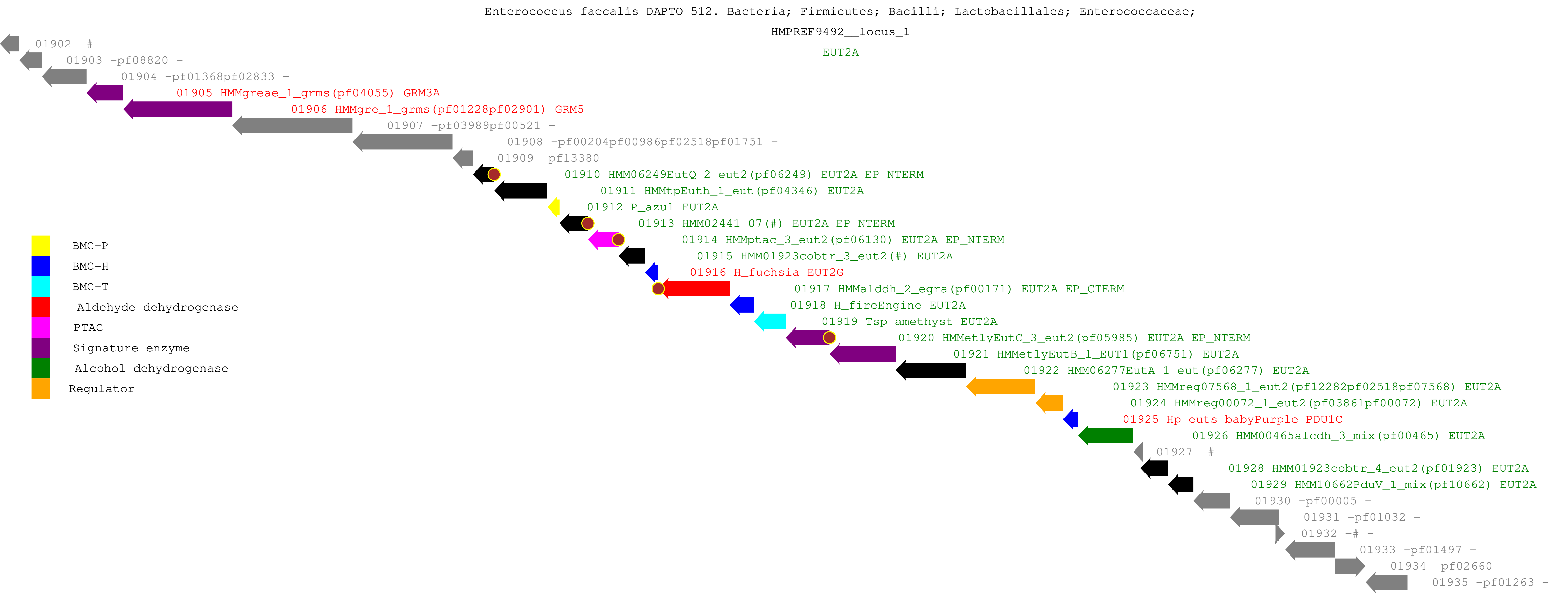
Enterococcus faecalis DAPTO 512. Bacteria; Firmicutes; Bacilli; Lactobacillales; Enterococcaceae;
HMPREF9492__locus_1
EUT2A
01902 -# -
01903 -pf08820 -
01904 -pf01368pf02833 -
01905 HMMgreae_1_grms(pf04055) GRM3A
01906 HMMgre_1_grms(pf01228pf02901) GRM5
01907 -pf03989pf00521 -
01908 -pf00204pf00986pf02518pf01751 -
01909 -pf13380 -
01910 HMM06249EutQ_2_eut2(pf06249) EUT2A EP_NTERM
01911 HMMtpEuth_1_eut(pf04346) EUT2A
01912 P_azul EUT2A
01913 HMM02441_07(#) EUT2A EP_NTERM
01914 HMMptac_3_eut2(pf06130) EUT2A EP_NTERM
01915 HMM01923cobtr_3_eut2(#) EUT2A
01916 H_fuchsia EUT2G
01917 HMMaldh_2_egra(pf00171) EUT2A EP_CTERM
01918 H_fireEngine EUT2A
01919 Tsp_amethyst EUT2A
01920 HMMetlyEutC_3_eut2(pf05985) EUT2A EP_NTERM
01921 HMMetlyEutB_1_EUT1(pf06751) EUT2A
01922 HMM06277EutA_1_eut(pf06277) EUT2A
01923 HMMreg07568_1_eut2(pf12282pf02518pf07568) EUT2A
01924 HMMreg00072_1_eut2(pf03861pf00072) EUT2A
01925 Hp_euts_babyPurple PDU1C
01926 HMM00465alcdh_3_mix(pf00465) EUT2A
01927 -# -
01928 HMM01923cobtr_4_eut2(pf01923) EUT2A
01929 HMM10662PduV_1_mix(pf10662) EUT2A
01930 -pf00005 -
01931 -pf01032 -
01932 -# -
01933 -pf01497 -
01934 -pf02660 -
01935 -pf01263 -
BMC-P
BMC-H
BMC-T
Aldehyde dehydrogenase
PTAC
Signature enzyme
Alcohol dehydrogenase
Regulator

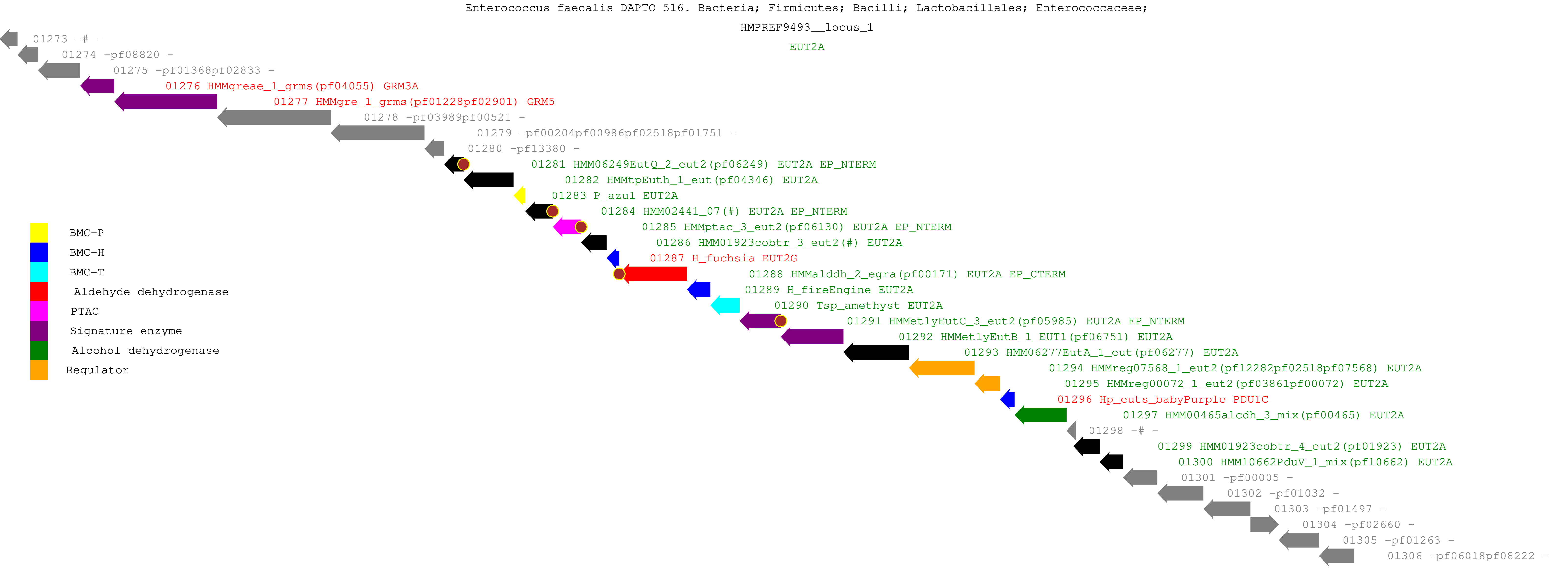
Enterococcus faecalis DAPTO 516. Bacteria; Firmicutes; Bacilli; Lactobacillales; Enterococcaceae;
HMPREF9493__locus_1
EUT2A
01273 -# -
01274 -pf08820 -
01275 -pf01368pf02833 -
01276 HMMgreae_1_grms(pf04055) GRM3A
01277 HMMgre_1_grms(pf01228pf02901) GRM5
01278 -pf03989pf00521 -
01279 -pf00204pf00986pf02518pf01751 -
01280 -pf13380 -
01281 HMM06249EutQ_2_eut2(pf06249) EUT2A EP_NTERM
01282 HMMtpEuth_1_eut(pf04346) EUT2A
01283 P_azul EUT2A
01284 HMM02441_07(#) EUT2A EP_NTERM
01285 HMMptac_3_eut2(pf06130) EUT2A EP_NTERM
01286 HMM01923cobtr_3_eut2(#) EUT2A
01287 H_fuchsia EUT2G
01288 HMMalddh_2_egra(pf00171) EUT2A EP_CTERM
01289 H_fireEngine EUT2A
01290 Tsp_amethyst EUT2A
01291 HMMetlyEutC_3_eut2(pf05985) EUT2A EP_NTERM
01292 HMMetlyEutB_1_EUT1(pf06751) EUT2A
01293 HMM06277EutA_1_eut(pf06277) EUT2A
01294 HMMreg07568_1_eut2(pf12282pf02518pf07568) EUT2A
01295 HMMreg00072_1_eut2(pf03861pf00072) EUT2A
01296 Hp_euts_babyPurple PDU1C
01297 HMM00465alcdh_3_mix(pf00465) EUT2A
01298 -# -
01299 HMM01923cobtr_4_eut2(pf01923) EUT2A
01300 HMM10662PduV_1_mix(pf10662) EUT2A
01301 -pf00005 -
01302 -pf01032 -
01303 -pf01497 -
01304 -pf02660 -
01305 -pf01263 -
01306 -pf06018pf08222 -
BMC-P
BMC-H
BMC-T
Aldehyde dehydrogenase
PTAC
Signature enzyme
Alcohol dehydrogenase
Regulator

Enterococcus faecalis TX4248. Bacteria; Firmicutes; Bacilli; Lactobacillales; Enterococcaceae;

HMPREF9498__locus_1

EUT2A

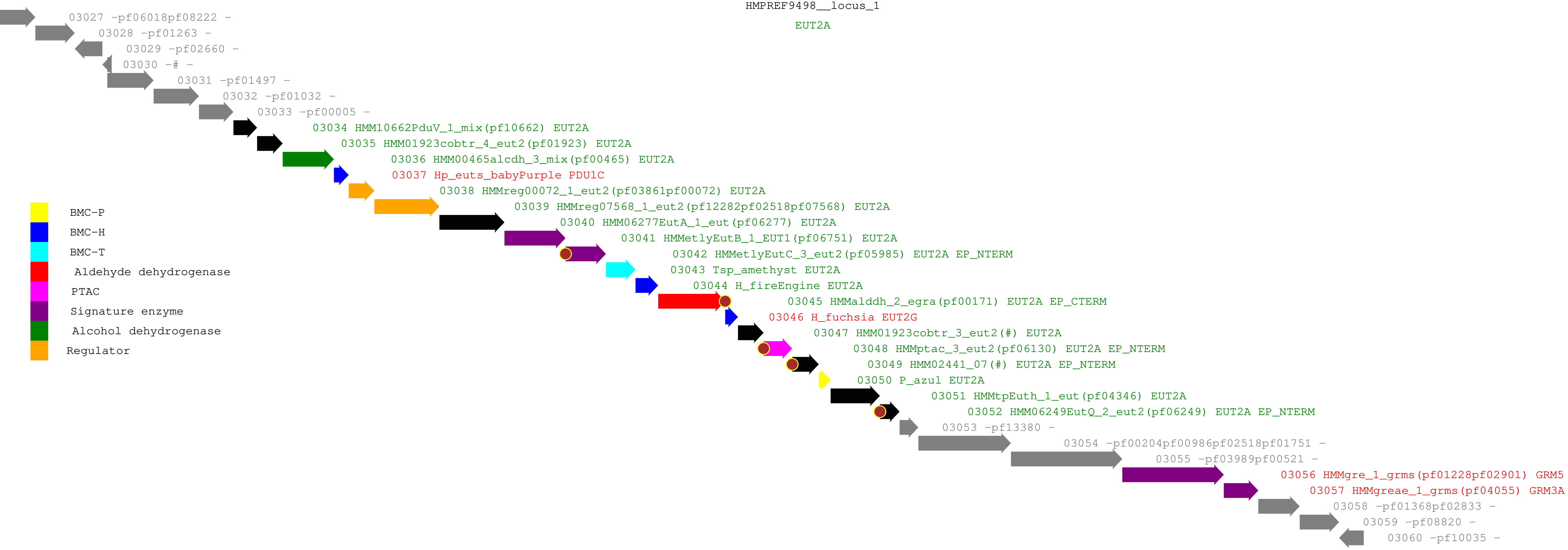

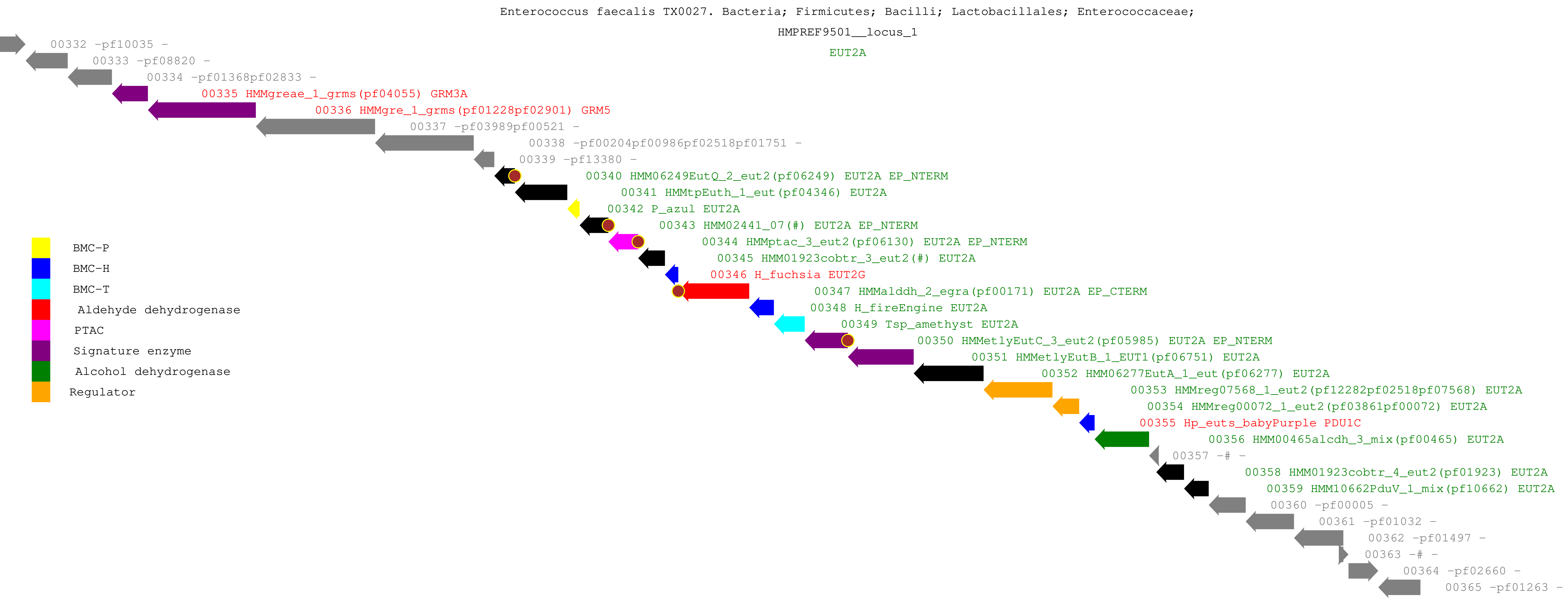
Enterococcus faecalis TX0027. Bacteria; Firmicutes; Bacilli; Lactobacillales; Enterococcaceae;
HMPREF9501__locus_1
EUT2A
00332 -pf10035 -
00333 -pf08820 -
00334 -pf01368pf02833 -
00335 HMMgreae_1_grms(pf04055) GRM3A
00336 HMMgre_1_grms(pf01228pf02901) GRM5
00337 -pf03989pf00521 -
00338 -pf00204pf00986pf02518pf01751 -
00339 -pf13380 -
00340 HMM06249EutQ_2_eut2(pf06249) EUT2A EP_NTERM
00341 HMMtpEuth_1_eut(pf04346) EUT2A
00342 P_azul EUT2A
00343 HMM02441_07(#) EUT2A EP_NTERM
00344 HMMptac_3_eut2(pf06130) EUT2A EP_NTERM
00345 HMM01923cobtr_3_eut2(#) EUT2A
00346 H_fuchsia EUT2G
00347 HMMaldd h_2_egra(pf00171) EUT2A EP_CTERM
00348 H_fireEngine EUT2A
00349 Tsp_amethyst EUT2A
00350 HMMetlyEutC_3_eut2(pf05985) EUT2A EP_NTERM
00351 HMMetlyEutB_1_EUT1(pf06751) EUT2A
00352 HMM06277EutA_1_eut(pf06277) EUT2A
00353 HMMreg07568_1_eut2(pf12282pf02518pf07568) EUT2A
00354 HMMreg00072_1_eut2(pf03861pf00072) EUT2A
00355 Hp_euts_babyPurple PDU1C
00356 HMM00465alcdh_3_mix(pf00465) EUT2A
00357 -# -
00358 HMM01923cobtr_4_eut2(pf01923) EUT2A
00359 HMM10662PduV_1_mix(pf10662) EUT2A
00360 -pf00005 -
00361 -pf01032 -
00362 -pf01497 -
00363 -# -
00364 -pf02660 -
00365 -pf01263 -
BMC-P
BMC-H
BMC-T
Aldehyde dehydrogenase
PTAC
Signature enzyme
Alcohol dehydrogenase
Regulator

Enterococcus faecalis TX0309A. Bacteria; Firmicutes; Bacilli; Lactobacillales; Enterococcaceae;

HMPREF9506__locus_1

EUT2A

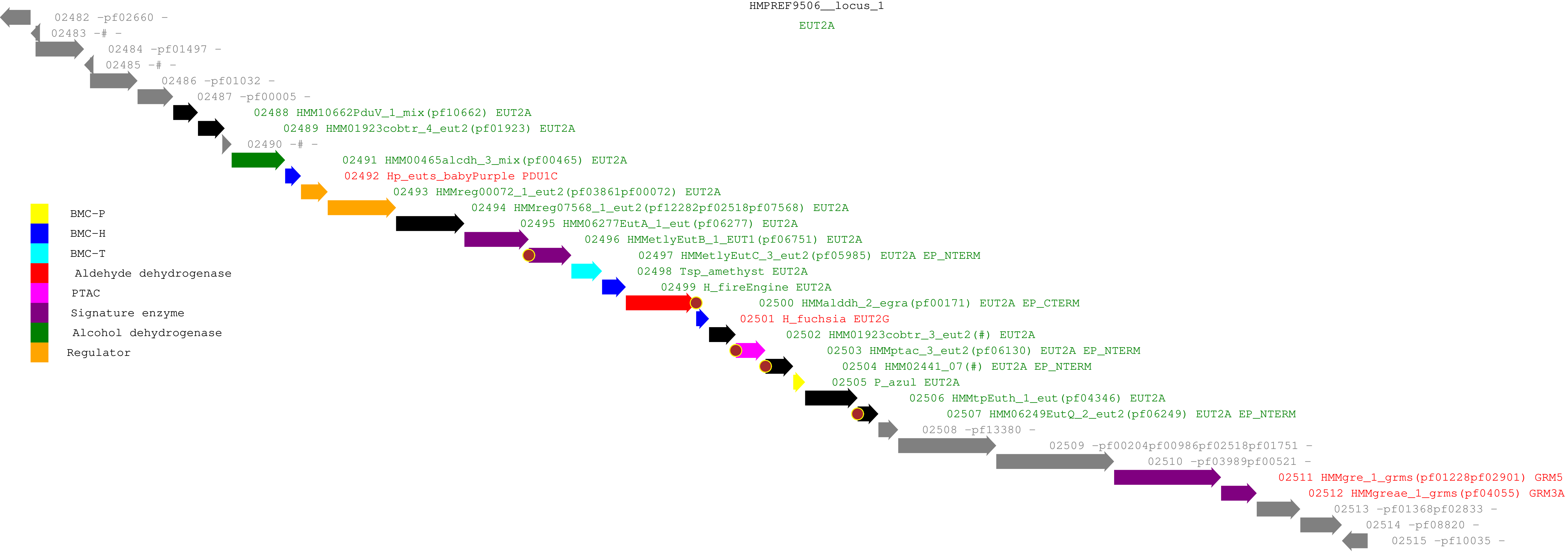

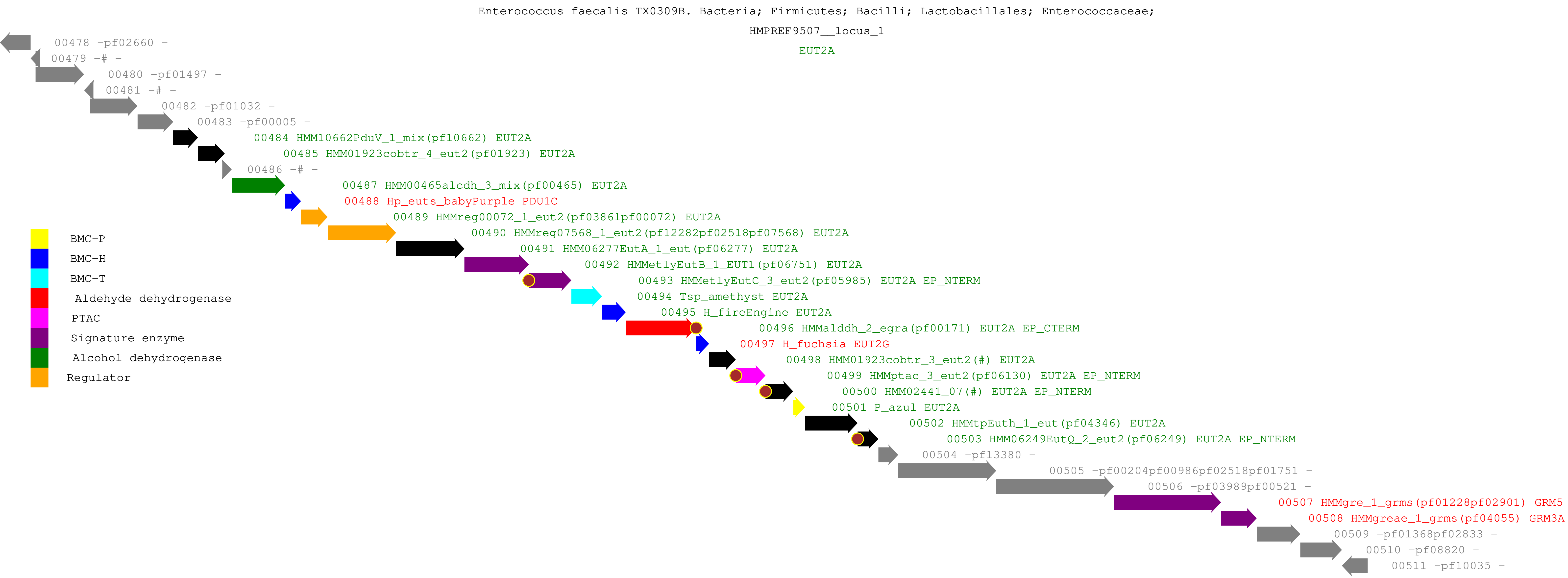
Enterococcus faecalis TX0309B. Bacteria; Firmicutes; Bacilli; Lactobacillales; Enterococcaceae;
HMPREF9507__locus_1
EUT2A
00478 -pf02660 -
00479 -# -
00480 -pf01497 -
00481 -# -
00482 -pf01032 -
00483 -pf00005 -
00484 HMM10662PduV_1_mix(pf10662) EUT2A
00485 HMM01923cobtr_4_eut2(pf01923) EUT2A
00486 -# -
00487 HMM00465alcdh_3_mix(pf00465) EUT2A
00488 Hp_euts_babyPurple PDU1C
00489 HMMreg00072_1_eut2(pf03861pf00072) EUT2A
00490 HMMreg07568_1_eut2(pf12282pf02518pf07568) EUT2A
00491 HMM06277EutA_1_eut(pf06277) EUT2A
00492 HMMetlyEutB_1_EUT1(pf06751) EUT2A
00493 HMMetlyEutC_3_eut2(pf05985) EUT2A EP_NTERM
00494 Tsp_amethyst EUT2A
00495 H_fireEngine EUT2A
00496 HMMaldh_2_egra(pf00171) EUT2A EP_CTERM
00497 H_fuchsia EUT2G
00498 HMM01923cobtr_3_eut2(#) EUT2A
00499 HMMptac_3_eut2(pf06130) EUT2A EP_NTERM
00500 HMM02441_07(#) EUT2A EP_NTERM
00501 P_azul EUT2A
00502 HMMtpEuth_1_eut(pf04346) EUT2A
00503 HMM06249EutQ_2_eut2(pf06249) EUT2A EP_NTERM
00504 -pf13380 -
00505 -pf00204pf00986pf02518pf01751 -
00506 -pf03989pf00521 -
00507 HMMgre_1_grms(pf01228pf02901) GRM5
00508 HMMgreae_1_grms(pf04055) GRM3A
00509 -pf01368pf02833 -
00510 -pf08820 -
00511 -pf10035 -
BMC-P
BMC-H
BMC-T
Aldehyde dehydrogenase
PTAC
Signature enzyme
Alcohol dehydrogenase
Regulator

Enterococcus faecalis TX0411. Bacteria; Firmicutes; Bacilli; Lactobacillales; Enterococcaceae;

HMPREF9509__locus_1

EUT2A

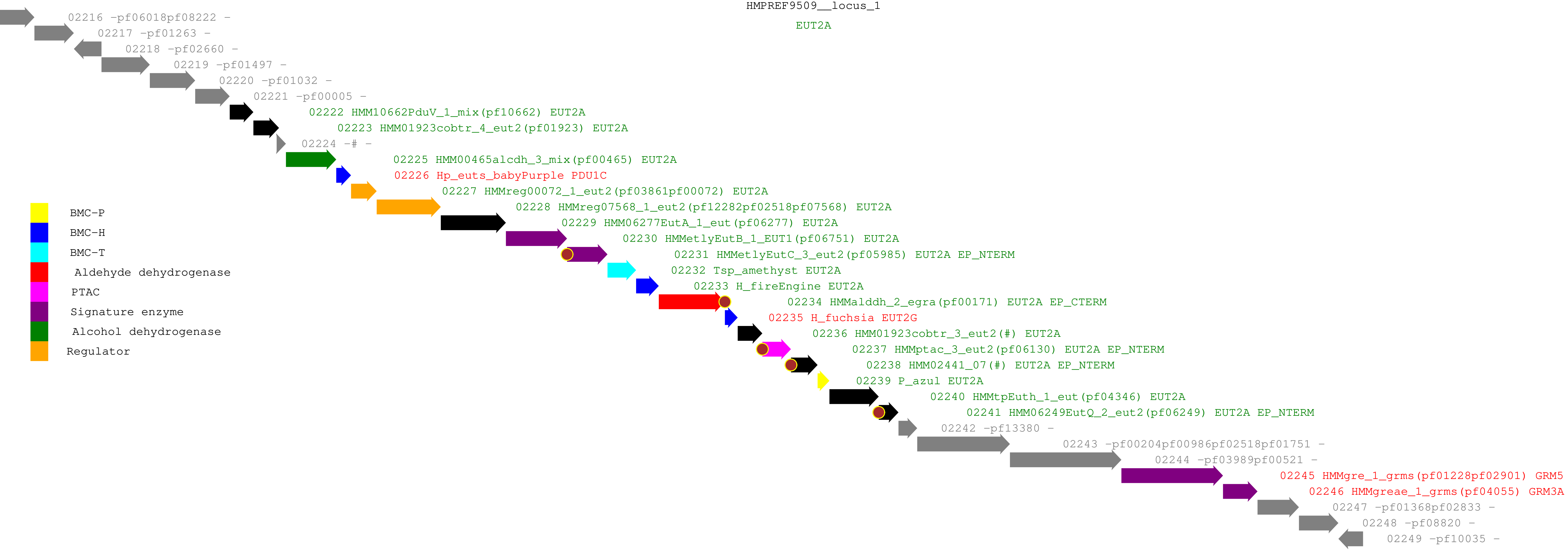

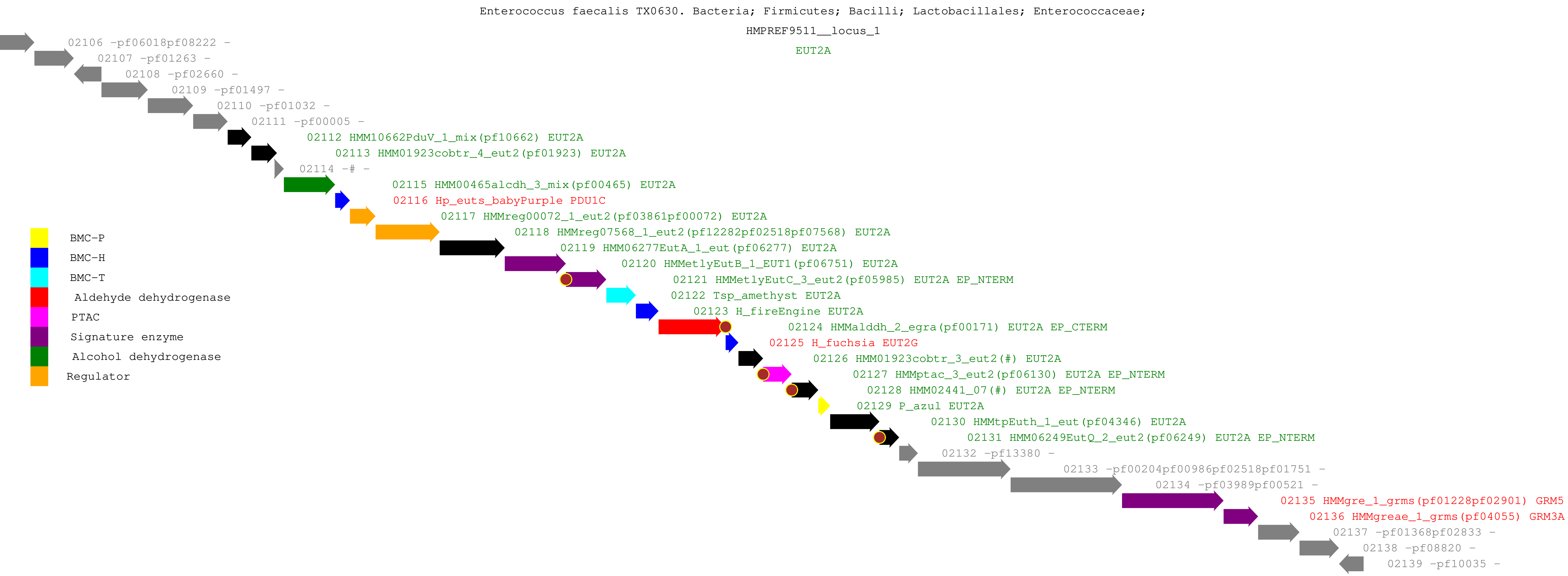
Enterococcus faecalis TX0630. Bacteria; Firmicutes; Bacilli; Lactobacillales; Enterococcaceae;
HMPREF9511__locus_1
EUT2A
02106 -pf06018pf08222 -
02107 -pf01263 -
02108 -pf02660 -
02109 -pf01497 -
02110 -pf01032 -
02111 -pf00005 -
02112 HMM10662PduV_1_mix(pf10662) EUT2A
02113 HMM01923cobtr_4_eut2(pf01923) EUT2A
02114 -# -
02115 HMM00465alcdh_3_mix(pf00465) EUT2A
02116 Hp_euts_babyPurple PDU1C
02117 HMMreg00072_1_eut2(pf03861pf00072) EUT2A
02118 HMMreg07568_1_eut2(pf12282pf02518pf07568) EUT2A
02119 HMM06277EutA_1_eut(pf06277) EUT2A
02120 HMMetlyEutB_1_EUT1(pf06751) EUT2A
02121 HMMetlyEutC_3_eut2(pf05985) EUT2A EP_NTERM
02122 Tsp_amethyst EUT2A
02123 H_fireEngine EUT2A
02124 HMMalddh_2_egra(pf00171) EUT2A EP_CTERM
02125 H_fuchsia EUT2G
02126 HMM01923cobtr_3_eut2(#) EUT2A
02127 HMMptac_3_eut2(pf06130) EUT2A EP_NTERM
02128 HMM02441_07(#) EUT2A EP_NTERM
02129 P_azul EUT2A
02130 HMMtpEuth_1_eut(pf04346) EUT2A
02131 HMM06249EutQ_2_eut2(pf06249) EUT2A EP_NTERM
02132 -pf13380 -
02133 -pf00204pf00986pf02518pf01751 -
02134 -pf03989pf00521 -
02135 HMMgre_1_grms(pf01228pf02901) GRM5
02136 HMMgreae_1_grms(pf04055) GRM3A
02137 -pf01368pf02833 -
02138 -pf08820 -
02139 -pf10035 -
BMC-P
BMC-H
BMC-T
Aldehyde dehydrogenase
PTAC
Signature enzyme
Alcohol dehydrogenase
Regulator

Enterococcus faecalis EnGen0311. Bacteria; Firmicutes; Bacilli; Lactobacillales; Enterococcaceae;

HMPREF9512__locus_1

EUT2A

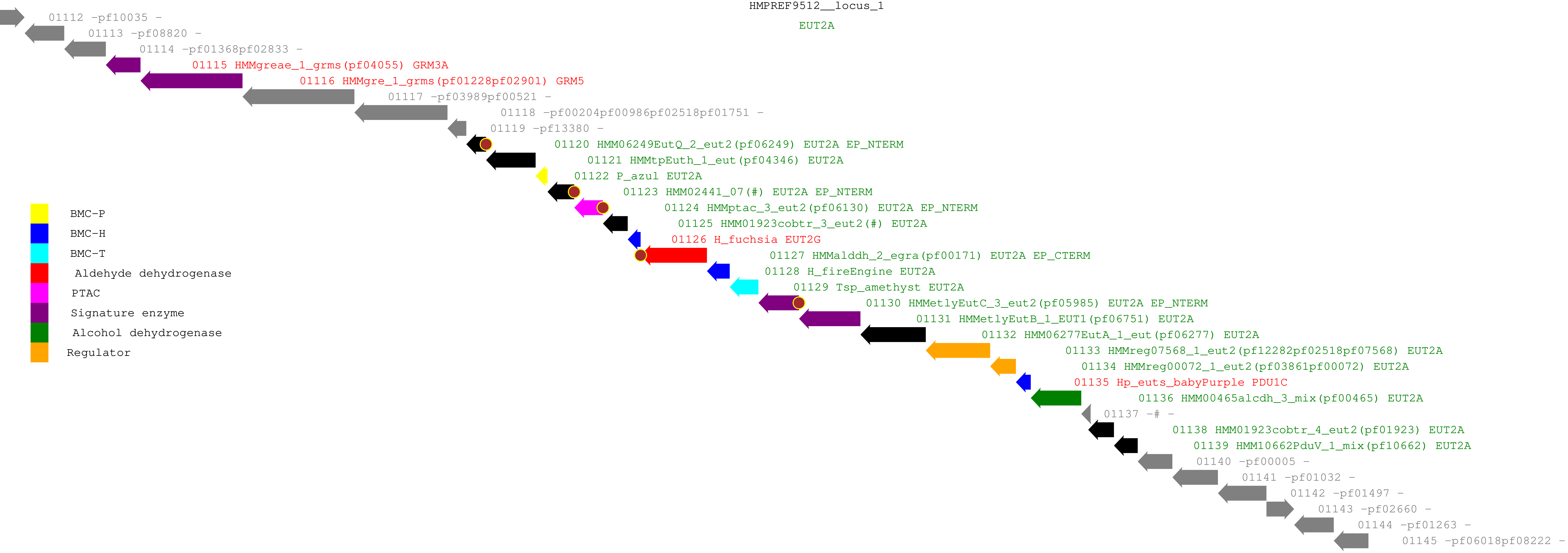

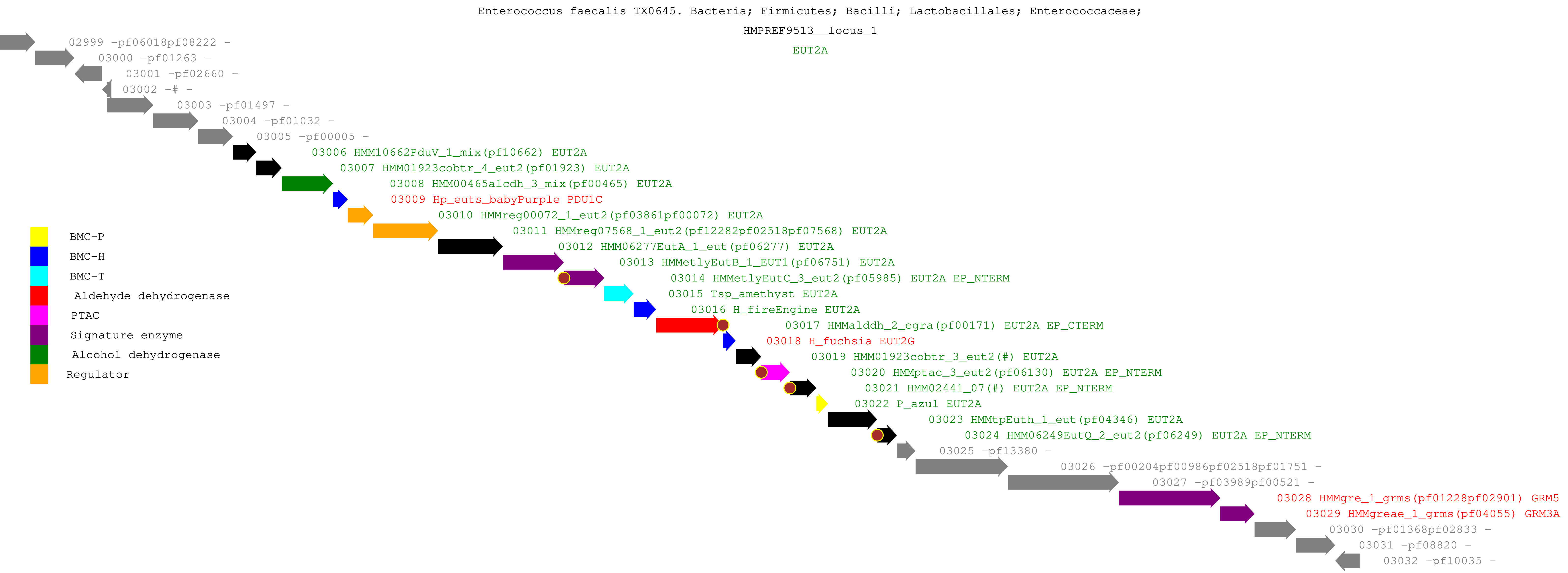
Enterococcus faecalis TX0645. Bacteria; Firmicutes; Bacilli; Lactobacillales; Enterococcaceae;
HMPREF9513__locus_1
EUT2A
02999 -pf06018pf08222 -
03000 -pf01263 -
03001 -pf02660 -
03002 -# -
03003 -pf01497 -
03004 -pf01032 -
03005 -pf00005 -
03006 HMM10662PduV_1_mix(pf10662) EUT2A
03007 HMM01923cobtr_4_eut2(pf01923) EUT2A
03008 HMM00465alcdh_3_mix(pf00465) EUT2A
03009 Hp_euts_babyPurple PDU1C
03010 HMMreg00072_1_eut2(pf03861pf00072) EUT2A
03011 HMMreg07568_1_eut2(pf12282pf02518pf07568) EUT2A
03012 HMM06277EutA_1_eut(pf06277) EUT2A
03013 HMMetlyEutB_1_EUT1(pf06751) EUT2A
03014 HMMetlyEutC_3_eut2(pf05985) EUT2A EP_NTERM
03015 Tsp_amethyst EUT2A
03016 H_fireEngine EUT2A
03017 HMMalddh_2_egra(pf00171) EUT2A EP_CTERM
03018 H_fuchsia EUT2G
03019 HMM01923cobtr_3_eut2(#) EUT2A
03020 HMMptac_3_eut2(pf06130) EUT2A EP_NTERM
03021 HMM02441_07(#) EUT2A EP_NTERM
03022 P_azul EUT2A
03023 HMMtpEuth_1_eut(pf04346) EUT2A
03024 HMM06249EutQ_2_eut2(pf06249) EUT2A EP_NTERM
03025 -pf13380 -
03026 -pf00204pf00986pf02518pf01751 -
03027 -pf03989pf00521 -
03028 HMMgre_1_grms(pf01228pf02901) GRM5
03029 HMMgreae_1_grms(pf04055) GRM3A
03030 -pf01368pf02833 -
03031 -pf08820 -
03032 -pf10035 -
BMC-P
BMC-H
BMC-T
Aldehyde dehydrogenase
PTAC
Signature enzyme
Alcohol dehydrogenase
Regulator

Clostridium celerecrescens. Bacteria; Firmicutes; Clostridia; Clostridiales; Lachnospiraceae.

IO98__locus_2

EUT2A

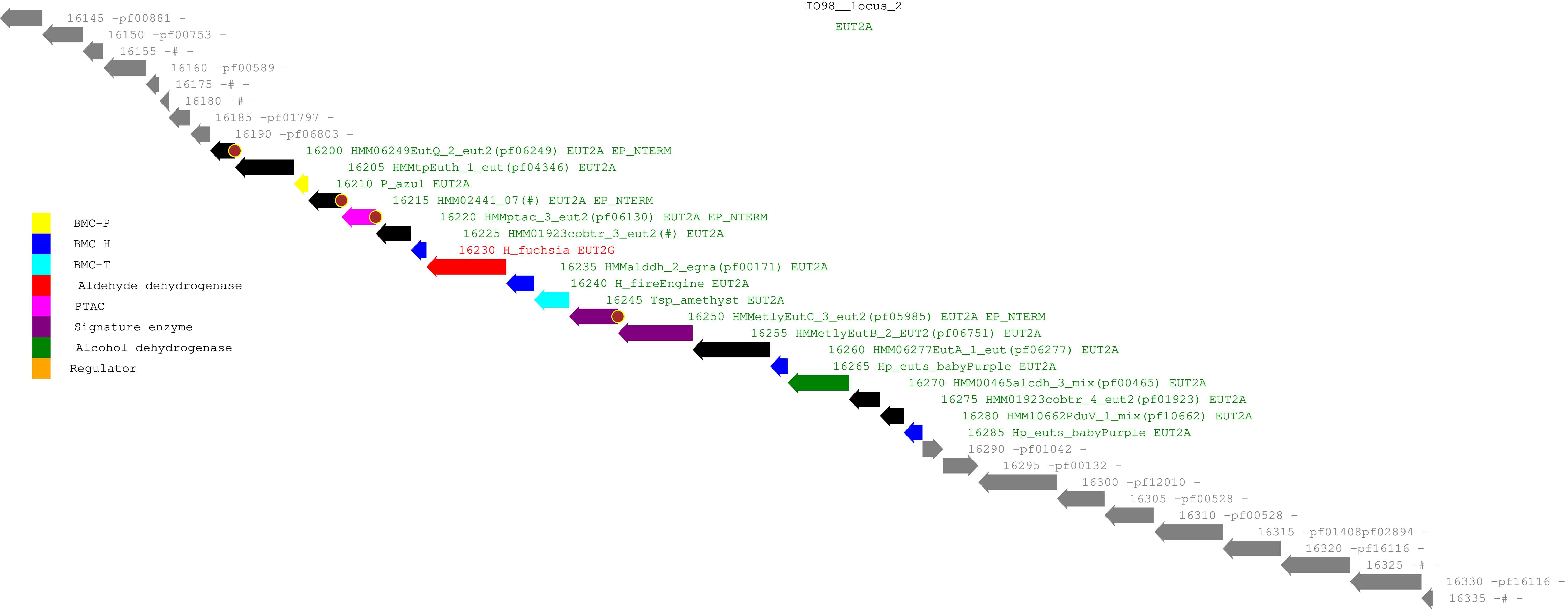

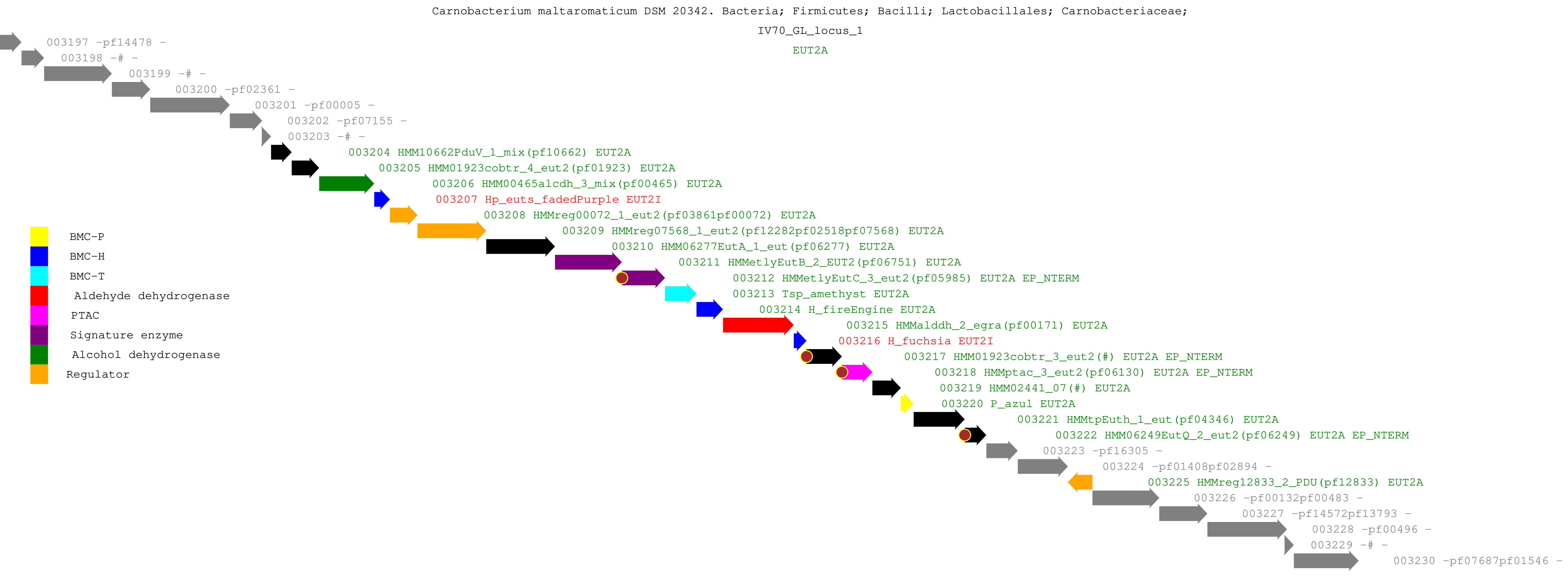

Carnobacterium maltaromaticum DSM 20342. Bacteria; Firmicutes; Bacilli; Lactobacillales; Carnobacteriaceae;
IV70_GL_locus_1
EUT2A
003197 -pf14478 -
003198 -# -
003199 -# -
003200 -pf02361 -
003201 -pf00005 -
003202 -pf07155 -
003203 -# -
003204 HMM10662PduV_1_mix(pf10662) EUT2A
003205 HMM01923cobtr_4_eut2(pf01923) EUT2A
003206 HMM00465alcdh_3_mix(pf00465) EUT2A
003207 Hp_euts_fadedPurple EUT2I
003208 HMMreg00072_1_eut2(pf03861pf00072) EUT2A
003209 HMMreg07568_1_eut2(pf12282pf02518pf07568) EUT2A
003210 HMM06277EutA_1_eut(pf06277) EUT2A
003211 HMMetlyEutB_2_EUT2(pf06751) EUT2A
003212 HMMetlyEutC_3_eut2(pf05985) EUT2A EP_NTERM
003213 Tsp_amethyst EUT2A
003214 H_fireEngine EUT2A
003215 HMMalddh_2_egra(pf00171) EUT2A
003216 H_fuchsia EUT2I
003217 HMM01923cobtr_3_eut2(#) EUT2A EP_NTERM
003218 HMMptac_3_eut2(pf06130) EUT2A EP_NTERM
003219 HMM02441_07(#) EUT2A
003220 P_azul EUT2A
003221 HMMtpEuth_1_eut(pf04346) EUT2A
003222 HMM06249EutQ_2_eut2(pf06249) EUT2A EP_NTERM
003223 -pf16305 -
003224 -pf01408pf02894 -
003225 HMMreg12833_2_PDU(pf12833) EUT2A
003226 -pf00132pf00483 -
003227 -pf14572pf13793 -
003228 -pf00496 -
003229 -# -
003230 -pf07687pf01546 -
BMC-P
BMC-H
BMC-T
Aldehyde dehydrogenase
PTAC
Signature enzyme
Alcohol dehydrogenase
Regulator

Carnobacterium maltaromaticum (Carnobacterium piscicola). Bacteria; Firmicutes; Bacilli; Lactobacillales; Carnobacteriaceae;

IV76_GL_locus_1

EUT2A

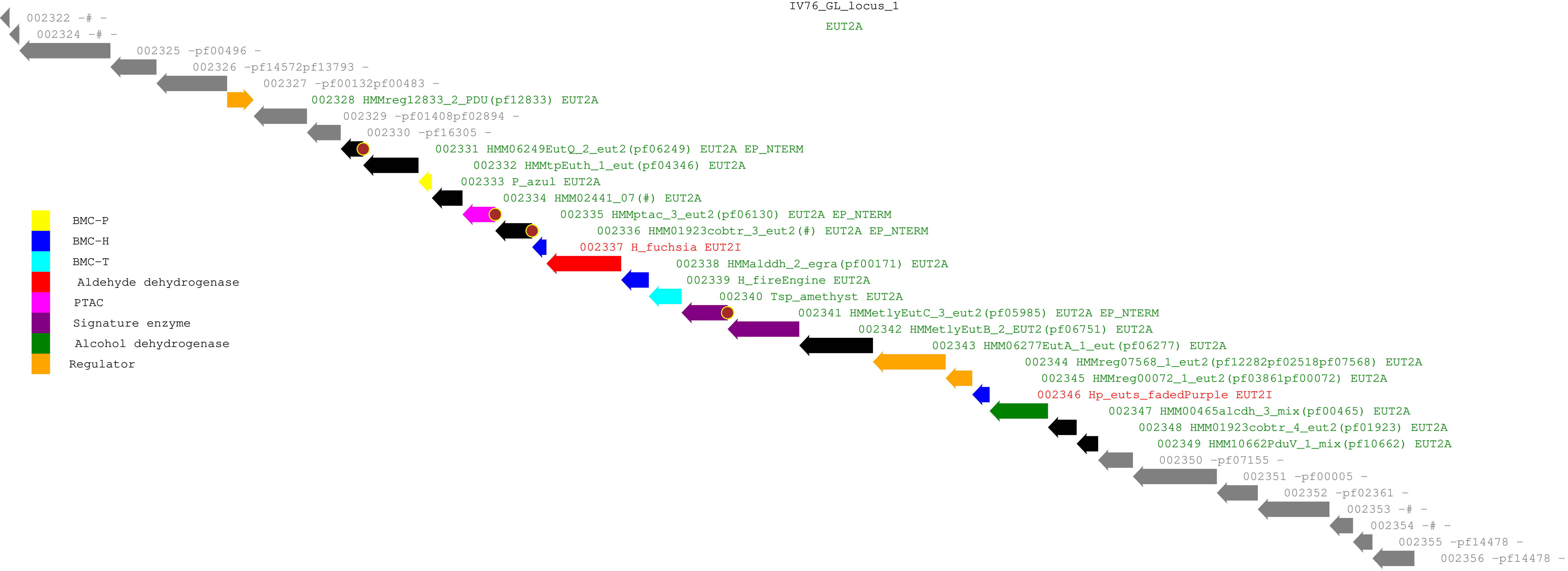

Lactobacillus mellis. Bacteria; Firmicutes; Bacilli; Lactobacillales; Lactobacillaceae;

JG29__locus_1

EUT2A

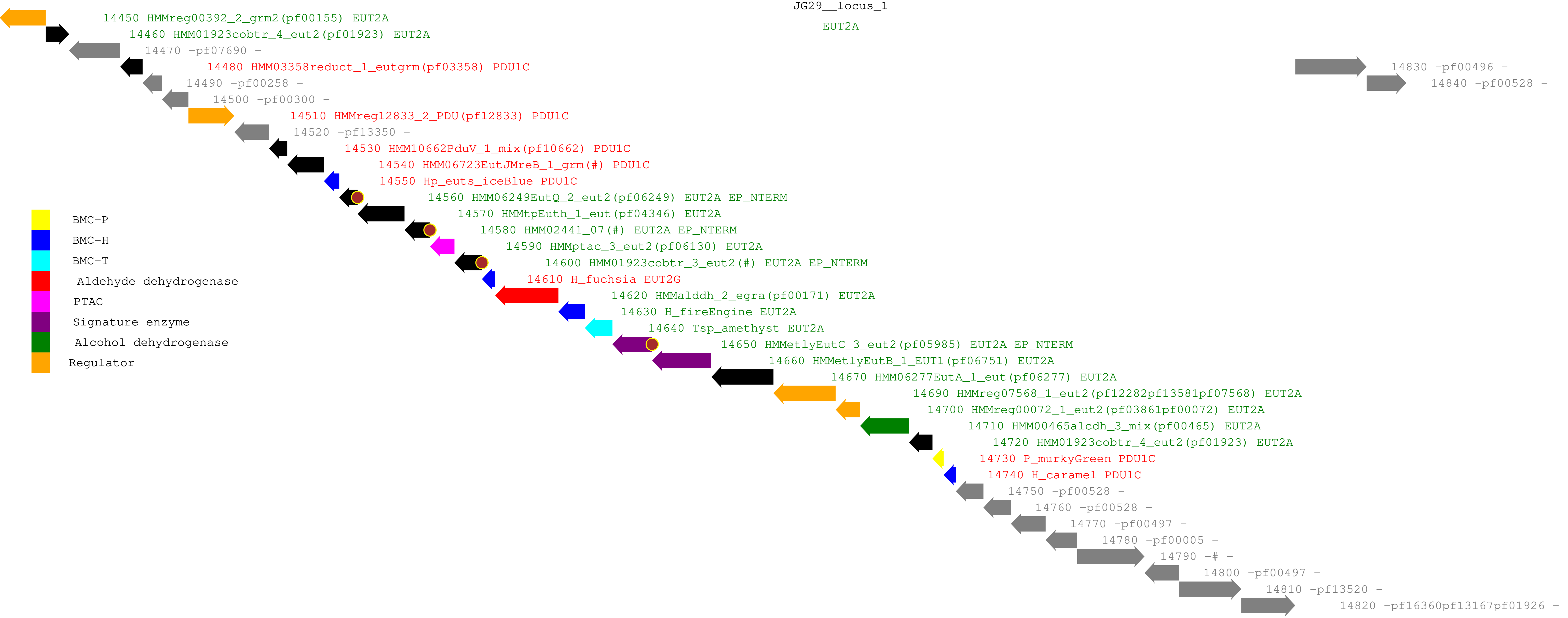

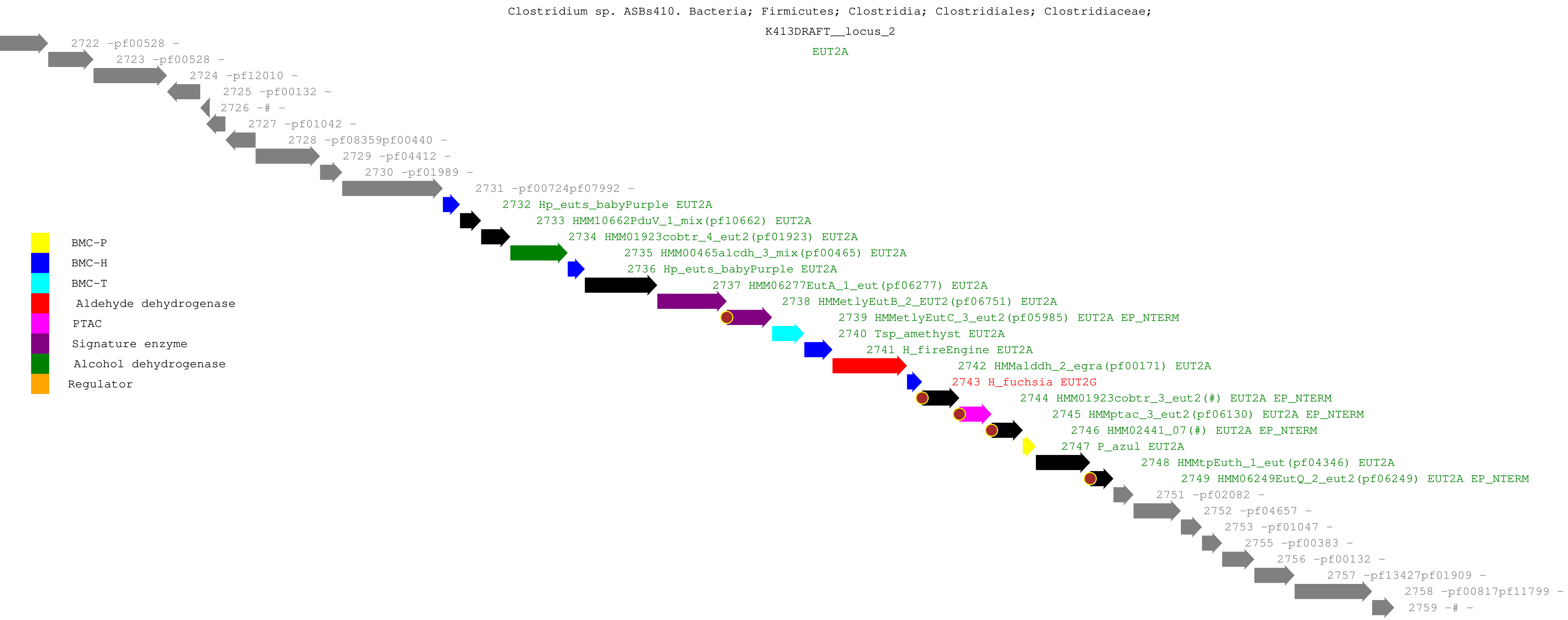
Clostridium sp. ASBs410. Bacteria; Firmicutes; Clostridia; Clostridiales; Clostridiaceae;
K413DRAFT__locus_2
EUT2A
2722 -pf00528 -
2723 -pf00528 -
2724 -pf12010 -
2725 -pf00132 -
2726 -# -
2727 -pf01042 -
2728 -pf08359pf00440 -
2729 -pf04412 -
2730 -pf01989 -
2731 -pf00724pf07992 -
2732 Hp_euts_babyPurple EUT2A
2733 HMM10662PduV_1_mix(pf10662) EUT2A
2734 HMM01923cobtr_4_eut2(pf01923) EUT2A
2735 HMM00465alcdh_3_mix(pf00465) EUT2A
2736 Hp_euts_babyPurple EUT2A
2737 HMM06277EutA_1_eut(pf06277) EUT2A
2738 HMMetlyEutB_2_EUT2(pf06751) EUT2A
2739 HMMetlyEutC_3_eut2(pf05985) EUT2A EP_NTERM
2740 Tsp_amethyst EUT2A
2741 H_fireEngine EUT2A
2742 HMMaldh_2_egra(pf00171) EUT2A
2743 H_fuchsia EUT2G
2744 HMM01923cobtr_3_eut2(#) EUT2A EP_NTERM
2745 HMMptac_3_eut2(pf06130) EUT2A EP_NTERM
2746 HMM02441_07(#) EUT2A EP_NTERM
2747 P_azul EUT2A
2748 HMMtpEuth_1_eut(pf04346) EUT2A
2749 HMM06249EutQ_2_eut2(pf06249) EUT2A EP_NTERM
2751 -pf02082 -
2752 -pf04657 -
2753 -pf01047 -
2755 -pf00383 -
2756 -pf00132 -
2757 -pf13427pf01909 -
2758 -pf00817pf11799 -
2759 -# -
BMC-P
BMC-H
BMC-T
Aldehyde dehydrogenase
PTAC
Signature enzyme
Alcohol dehydrogenase
Regulator

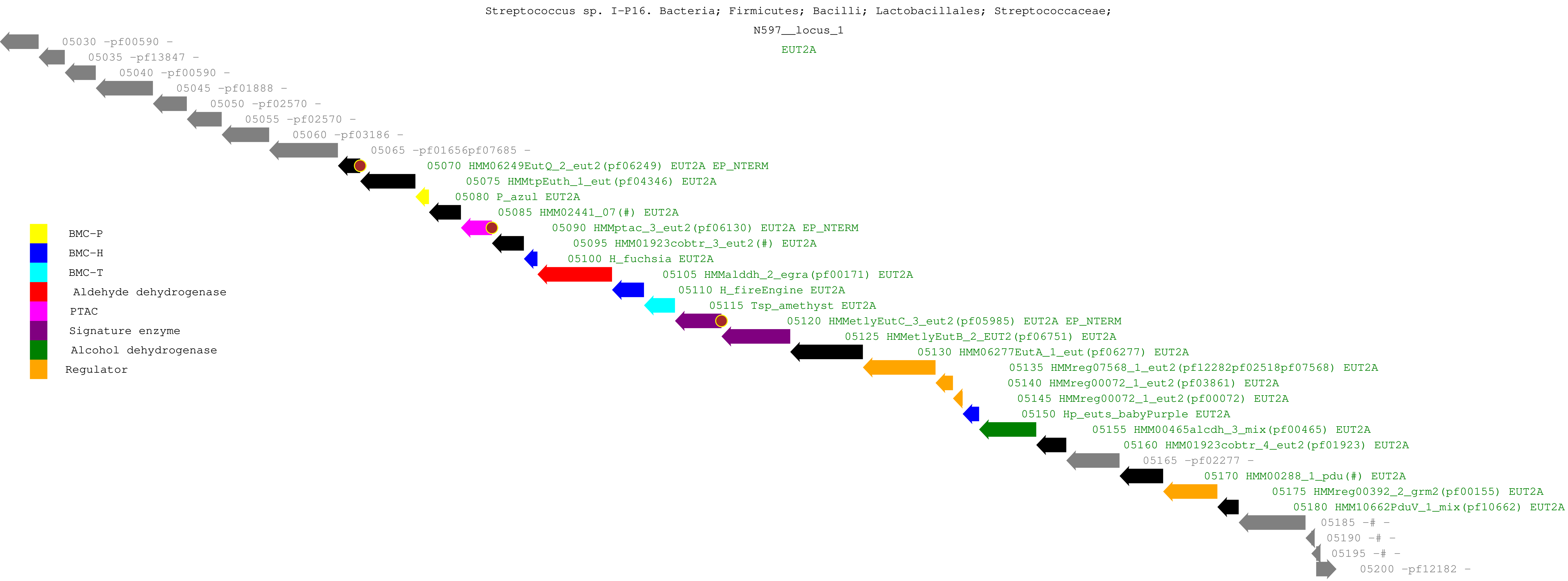

Streptococcus sp. I-P16. Bacteria; Firmicutes; Bacilli; Lactobacillales; Streptococcaceae;
N597__locus_1
EUT2A
05030 -pf00590 -
05035 -pf13847 -
05040 -pf00590 -
05045 -pf01888 -
05050 -pf02570 -
05055 -pf02570 -
05060 -pf03186 -
05065 -pf01656pf07685 -
05070 HMM06249EutQ_2_eut2(pf06249) EUT2A EP_NTERM
05075 HMMtpEuth_1_eut(pf04346) EUT2A
05080 P_azul EUT2A
05085 HMM02441_07(#) EUT2A
05090 HMMptac_3_eut2(pf06130) EUT2A EP_NTERM
05095 HMM01923cobtr_3_eut2(#) EUT2A
05100 H_fuchsia EUT2A
05105 HMMalddh_2_egra(pf00171) EUT2A
05110 H_fireEngine EUT2A
05115 Tsp_amethyst EUT2A
05120 HMMetlyEutC_3_eut2(pf05985) EUT2A EP_NTERM
05125 HMMetlyEutB_2_EUT2(pf06751) EUT2A
05130 HMM06277EutA_1_eut(pf06277) EUT2A
05135 HMMreg07568_1_eut2(pf12282pf02518pf07568) EUT2A
05140 HMMreg00072_1_eut2(pf03861) EUT2A
05145 HMMreg00072_1_eut2(pf00072) EUT2A
05150 Hp_euts_babyPurple EUT2A
05155 HMM00465alcdh_3_mix(pf00465) EUT2A
05160 HMM01923cobtr_4_eut2(pf01923) EUT2A
05165 -pf02277 -
05170 HMM00288_1_pdu(#) EUT2A
05175 HMMreg00392_2_grm2(pf00155) EUT2A
05180 HMM10662PduV_1_mix(pf10662) EUT2A
05185 -# -
05190 -# -
05195 -# -
05200 -pf12182 -
BMC-P
BMC-H
BMC-T
Aldehyde dehydrogenase
PTAC
Signature enzyme
Alcohol dehydrogenase
Regulator

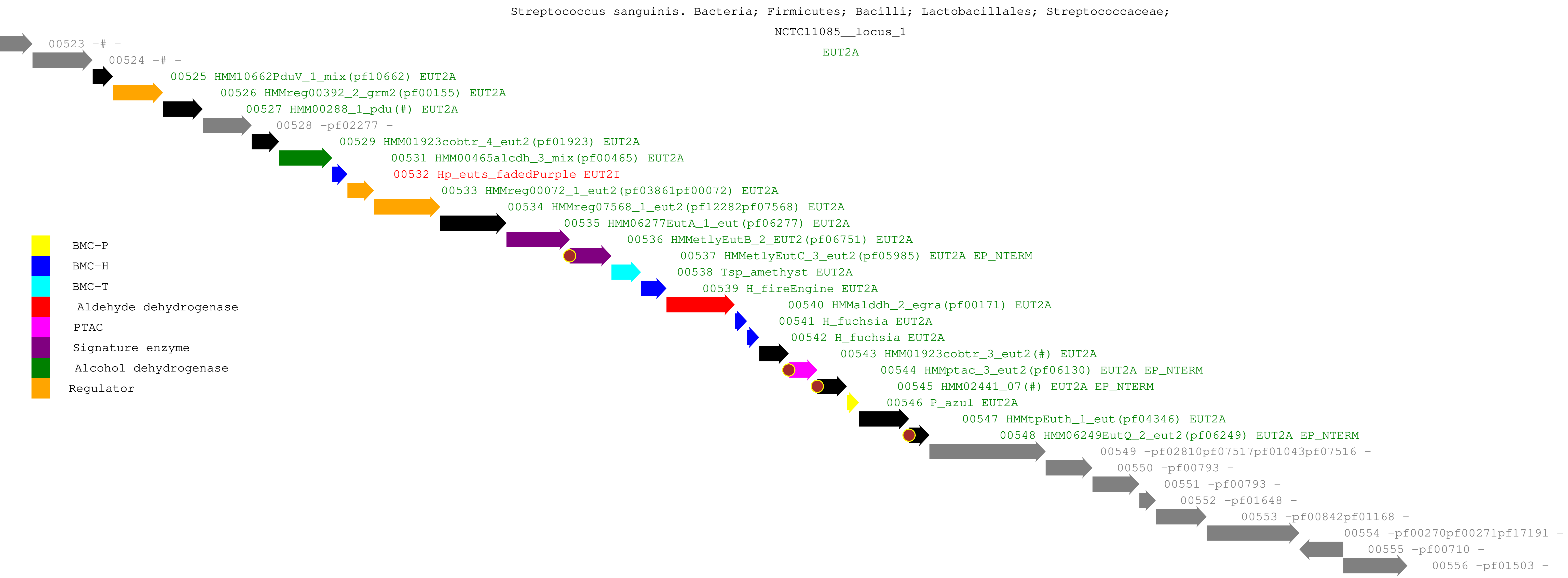
Streptococcus sanguinis. Bacteria; Firmicutes; Bacilli; Lactobacillales; Streptococcaceae;
NCTC11085__locus_1
EUT2A
00523 -# -
00524 -# -
00525 HMM10662PduV_1_mix(pf10662) EUT2A
00526 HMMreg00392_2_grm2(pf00155) EUT2A
00527 HMM00288_1_pdu(#) EUT2A
00528 -pf02277 -
00529 HMM01923cobtr_4_eut2(pf01923) EUT2A
00531 HMM00465alcdh_3_mix(pf00465) EUT2A
00532 Hp_euts_fadedPurple EUT2I
00533 HMMreg00072_1_eut2(pf03861pf00072) EUT2A
00534 HMMreg07568_1_eut2(pf12282pf07568) EUT2A
00535 HMM06277EutA_1_eut(pf06277) EUT2A
00536 HMMetlyEutB_2_EUT2(pf06751) EUT2A
00537 HMMetlyEutC_3_eut2(pf05985) EUT2A EP_NTERM
00538 Tsp_amethyst EUT2A
00539 H_fireEngine EUT2A
00540 HMMaldh_2_egra(pf00171) EUT2A
00541 H_fuchsia EUT2A
00542 H_fuchsia EUT2A
00543 HMM01923cobtr_3_eut2(#) EUT2A
00544 HMMptac_3_eut2(pf06130) EUT2A EP_NTERM
00545 HMM02441_07(#) EUT2A EP_NTERM
00546 P_azul EUT2A
00547 HMMtpEuth_1_eut(pf04346) EUT2A
00548 HMM06249EutQ_2_eut2(pf06249) EUT2A EP_NTERM
00549 -pf02810pf07517pf01043pf07516 -
00550 -pf00793 -
00551 -pf00793 -
00552 -pf01648 -
00553 -pf00842pf01168 -
00554 -pf00270pf00271pf17191 -
00555 -pf00710 -
00556 -pf01503 -
BMC-P
BMC-H
BMC-T
Aldehyde dehydrogenase
PTAC
Signature enzyme
Alcohol dehydrogenase
Regulator

Enterococcus faecalis (Streptococcus faecalis). Bacteria; Firmicutes; Bacilli; Lactobacillales; Enterococcaceae;

NCTC13780__locus_1

EUT2A

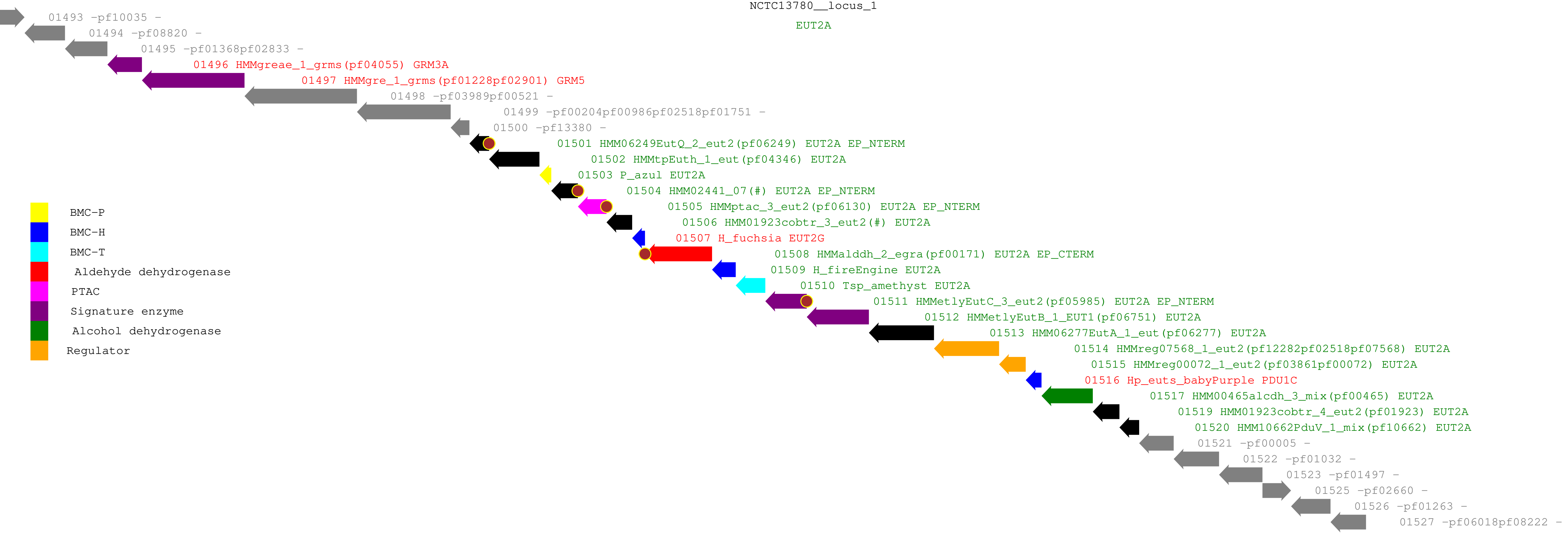

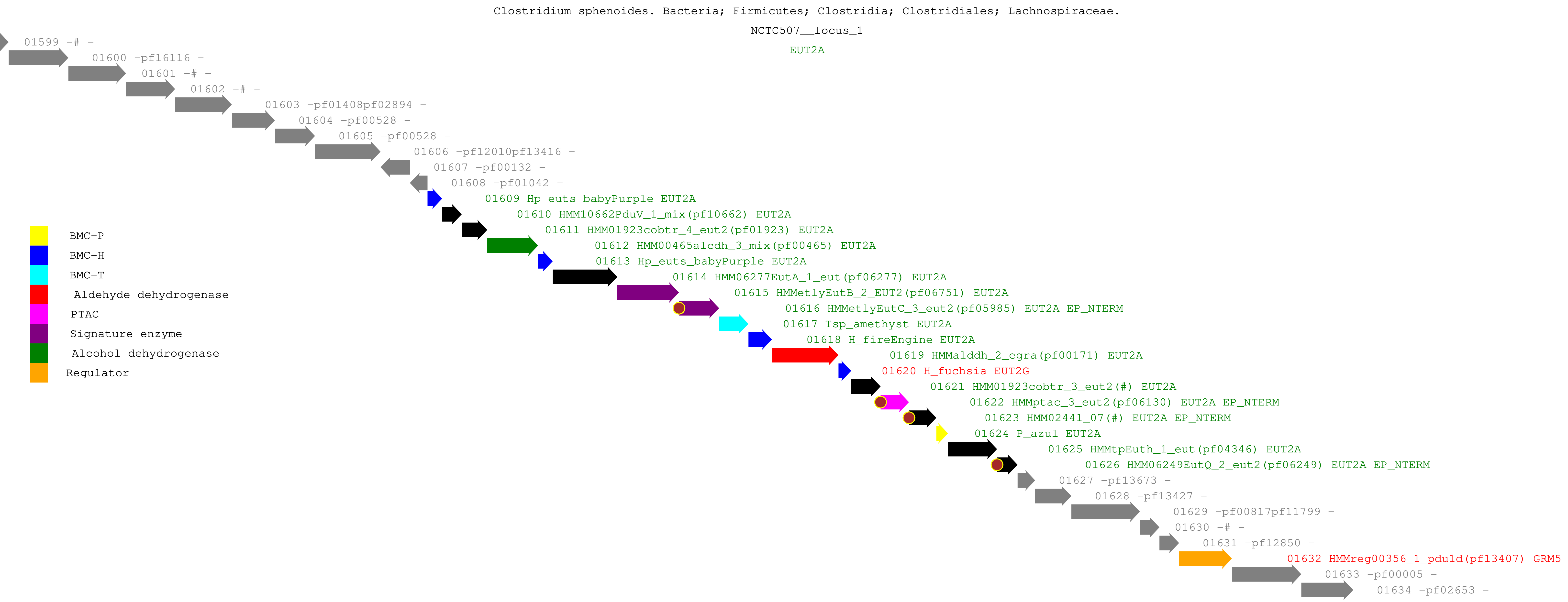

Clostridium sphenoides. Bacteria; Firmicutes; Clostridia; Clostridiales; Lachnospiraceae.
NCTC507__locus_1
EUT2A
01599 -# -
01600 -pf16116 -
01601 -# -
01602 -# -
01603 -pf01408pf02894 -
01604 -pf00528 -
01605 -pf00528 -
01606 -pf12010pf13416 -
01607 -pf00132 -
01608 -pf01042 -
01609 Hp_euts_babyPurple EUT2A
01610 HMM10662PduV_1_mix(pf10662) EUT2A
01611 HMM01923cobtr_4_eut2(pf01923) EUT2A
01612 HMM00465alcdh_3_mix(pf00465) EUT2A
01613 Hp_euts_babyPurple EUT2A
01614 HMM06277EutA_1_eut(pf06277) EUT2A
01615 HMMetlyEutB_2_EUT2(pf06751) EUT2A
01616 HMMetlyEutC_3_eut2(pf05985) EUT2A EP_NTERM
01617 Tsp_amethyst EUT2A
01618 H_fireEngine EUT2A
01619 HMMalddh_2_egra(pf00171) EUT2A
01620 H_fuchsia EUT2G
01621 HMM01923cobtr_3_eut2(#) EUT2A
01622 HMMptac_3_eut2(pf06130) EUT2A EP_NTERM
01623 HMM02441_07(#) EUT2A EP_NTERM
01624 P_azul EUT2A
01625 HMMtpEuth_1_eut(pf04346) EUT2A
01626 HMM06249EutQ_2_eut2(pf06249) EUT2A EP_NTERM
01627 -pf13673 -
01628 -pf13427 -
01629 -pf00817pf11799 -
01630 -# -
01631 -pf12850 -
01632 HMMreg00356_1_pdu1d(pf13407) GRM5
01633 -pf00005 -
01634 -pf02653 -
BMC-P
BMC-H
BMC-T
Aldehyde dehydrogenase
PTAC
Signature enzyme
Alcohol dehydrogenase
Regulator

Enterococcus avium ATCC 14025. Bacteria; Firmicutes; Bacilli; Lactobacillales; Enterococcaceae;

OMU__locus_2

EUT2A

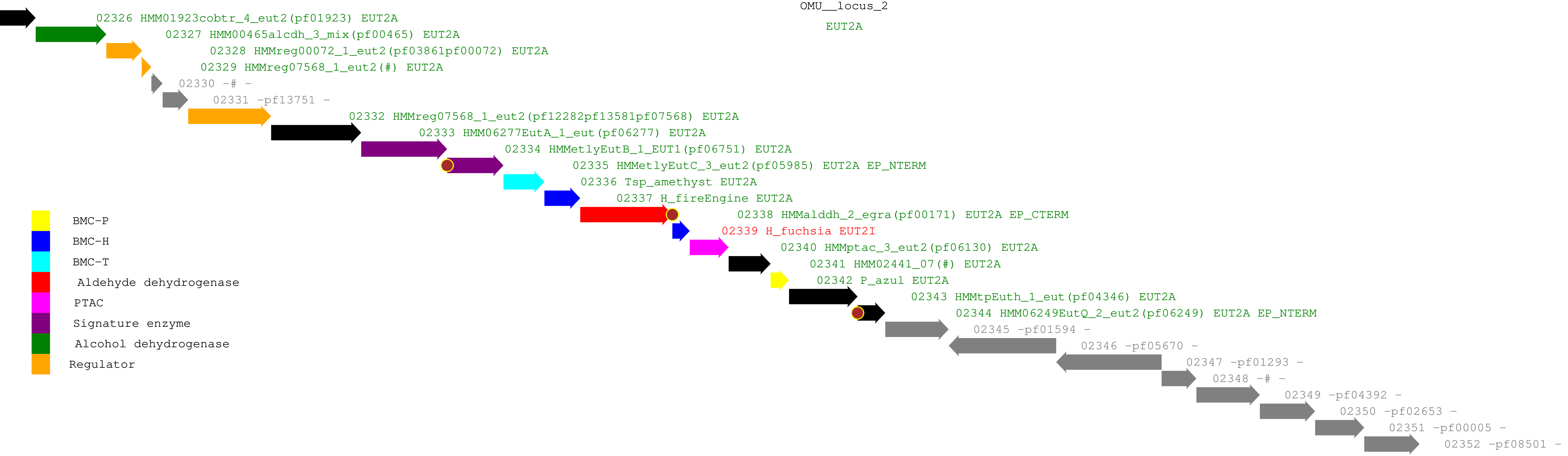

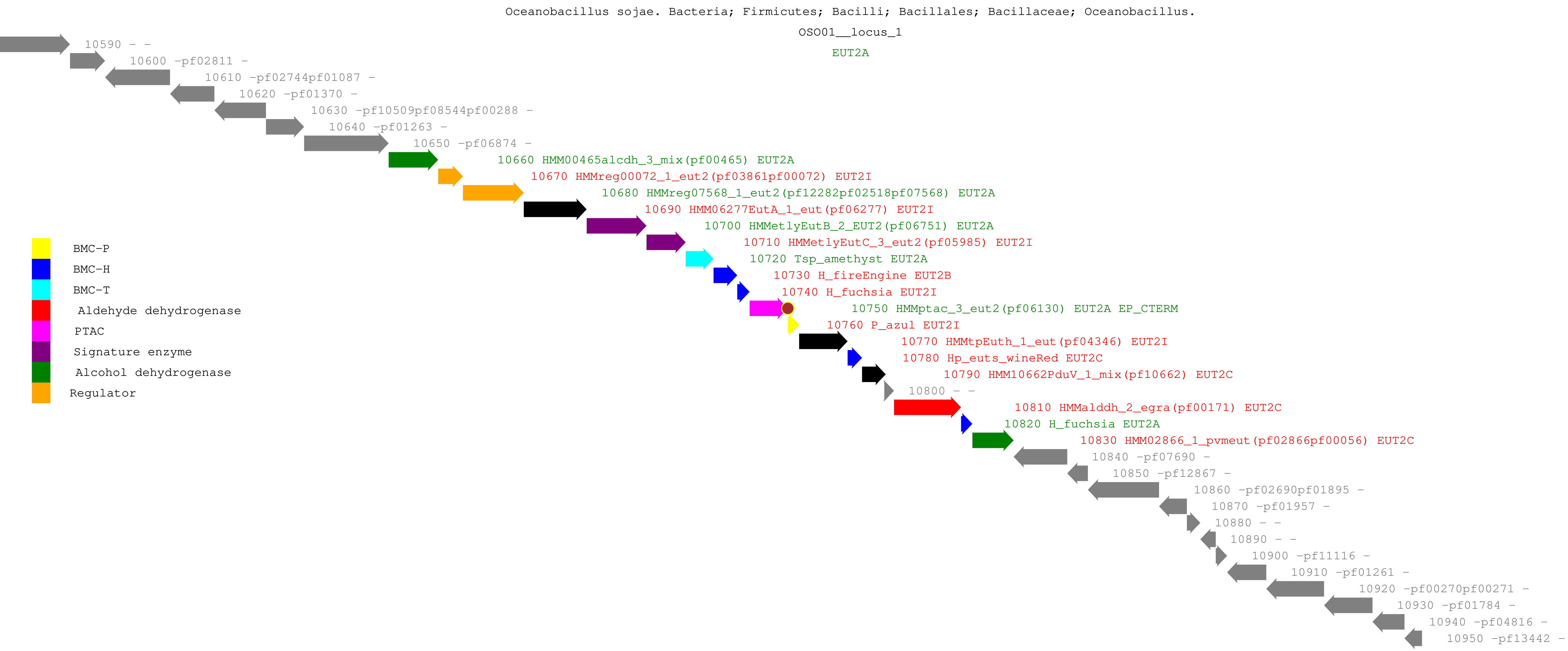
Oceanobacillus sojae. Bacteria; Firmicutes; Bacilli; Bacillales; Bacillaceae; Oceanobacillus.
OSO01__locus_1
EUT2A
10590 - -
10600 -pf02811 -
10610 -pf02744pf01087 -
10620 -pf01370 -
10630 -pf10509pf08544pf00288 -
10640 -pf01263 -
10650 -pf06874 -
10660 HMM00465alcdh_3_mix(pf00465) EUT2A
10670 HMMreg00072_1_eut2(pf03861pf00072) EUT2I
10680 HMMreg07568_1_eut2(pf12282pf02518pf07568) EUT2A
10690 HMM06277EutA_1_eut(pf06277) EUT2I
10700 HMMetlyEutB_2_EUT2(pf06751) EUT2A
10710 HMMetlyEutC_3_eut2(pf05985) EUT2I
10720 Tsp_amethyst EUT2A
10730 H_fireEngine EUT2B
10740 H_fuchsia EUT2I
10750 HMMptac_3_eut2(pf06130) EUT2A EP_CTERM
10760 P_azul EUT2I
10770 HMMtpEuth_1_eut(pf04346) EUT2I
10780 Hp_euts_wineRed EUT2C
10790 HMM10662PduV_1_mix(pf10662) EUT2C
10800 - -
10810 HMMaldh_2_egra(pf00171) EUT2C
10820 H_fuchsia EUT2A
10830 HMM02866_1_pvmeut(pf02866pf00056) EUT2C
10840 -pf07690 -
10850 -pf12867 -
10860 -pf02690pf01895 -
10870 -pf01957 -
10880 - -
10890 - -
10900 -pf11116 -
10910 -pf01261 -
10920 -pf00270pf00271 -
10930 -pf01784 -
10940 -pf04816 -
10950 -pf13442 -
BMC-P
BMC-H
BMC-T
Aldehyde dehydrogenase
PTAC
Signature enzyme
Alcohol dehydrogenase
Regulator

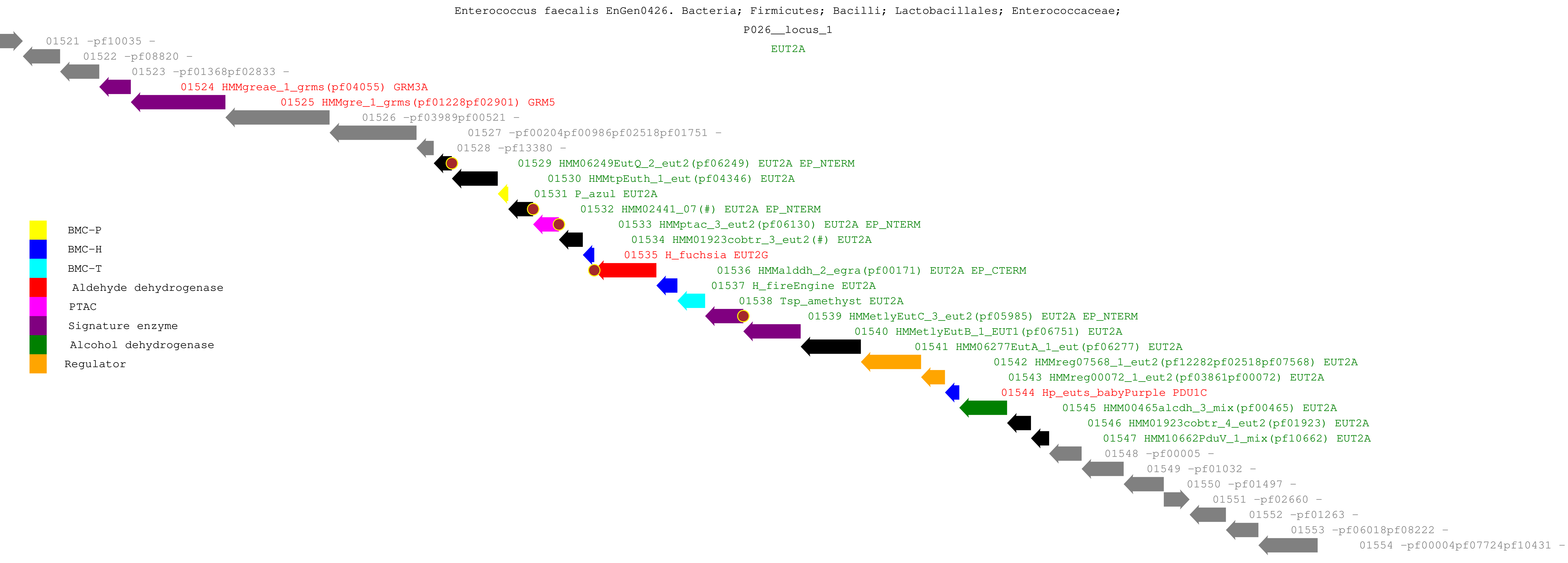
Enterococcus faecalis EnGen0426. Bacteria; Firmicutes; Bacilli; Lactobacillales; Enterococcaceae;
P026__locus_1
EUT2A
01521 -pf10035 -
01522 -pf08820 -
01523 -pf01368pf02833 -
01524 HMMgreae_1_grms(pf04055) GRM3A
01525 HMMgre_1_grms(pf01228pf02901) GRM5
01526 -pf03989pf00521 -
01527 -pf00204pf00986pf02518pf01751 -
01528 -pf13380 -
01529 HMM06249EutQ_2_eut2(pf06249) EUT2A EP_NTERM
01530 HMMtpEuth_1_eut(pf04346) EUT2A
01531 P_azul EUT2A
01532 HMM02441_07(#) EUT2A EP_NTERM
01533 HMMptac_3_eut2(pf06130) EUT2A EP_NTERM
01534 HMM01923cobtr_3_eut2(#) EUT2A
01535 H_fuchsia EUT2G
01536 HMMaldh_2_egra(pf00171) EUT2A EP_CTERM
01537 H_fireEngine EUT2A
01538 Tsp_amethyst EUT2A
01539 HMMetlyEutC_3_eut2(pf05985) EUT2A EP_NTERM
01540 HMMetlyEutB_1_EUT1(pf06751) EUT2A
01541 HMM06277EutA_1_eut(pf06277) EUT2A
01542 HMMreg07568_1_eut2(pf12282pf02518pf07568) EUT2A
01543 HMMreg00072_1_eut2(pf03861pf00072) EUT2A
01544 Hp_euts_babyPurple PDU1C
01545 HMM00465alcdh_3_mix(pf00465) EUT2A
01546 HMM01923cobtr_4_eut2(pf01923) EUT2A
01547 HMM10662PduV_1_mix(pf10662) EUT2A
01548 -pf00005 -
01549 -pf01032 -
01550 -pf01497 -
01551 -pf02660 -
01552 -pf01263 -
01553 -pf06018pf08222 -
01554 -pf00004pf07724pf10431 -
BMC-P
BMC-H
BMC-T
Aldehyde dehydrogenase
PTAC
Signature enzyme
Alcohol dehydrogenase
Regulator

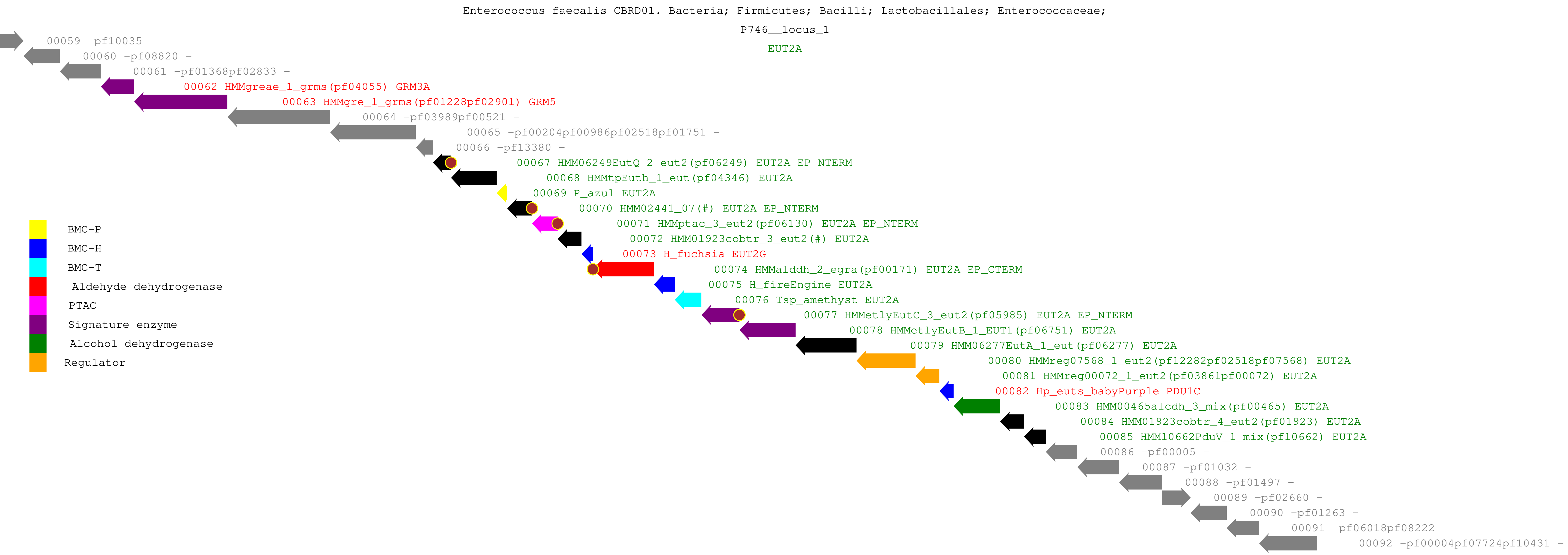
Enterococcus faecalis CBRD01. Bacteria; Firmicutes; Bacilli; Lactobacillales; Enterococcaceae;
P746__locus_1
EUT2A
00059 -pf10035 -
00060 -pf08820 -
00061 -pf01368pf02833 -
00062 HMMgreae_1_grms(pf04055) GRM3A
00063 HMMgre_1_grms(pf01228pf02901) GRM5
00064 -pf03989pf00521 -
00065 -pf00204pf00986pf02518pf01751 -
00066 -pf13380 -
00067 HMM06249EutQ_2_eut2(pf06249) EUT2A EP_NTERM
00068 HMMtpEuth_1_eut(pf04346) EUT2A
00069 P_azul EUT2A
00070 HMM02441_07(#) EUT2A EP_NTERM
00071 HMMptac_3_eut2(pf06130) EUT2A EP_NTERM
00072 HMM01923cobtr_3_eut2(#) EUT2A
00073 H_fuchsia EUT2G
00074 HMMalddh_2_egra(pf00171) EUT2A EP_CTERM
00075 H_fireEngine EUT2A
00076 Tsp_amethyst EUT2A
00077 HMMetlyEutC_3_eut2(pf05985) EUT2A EP_NTERM
00078 HMMetlyEutB_1_EUT1(pf06751) EUT2A
00079 HMM06277EutA_1_eut(pf06277) EUT2A
00080 HMMreg07568_1_eut2(pf12282pf02518pf07568) EUT2A
00081 HMMreg00072_1_eut2(pf03861pf00072) EUT2A
00082 Hp_euts_babyPurple PDU1C
00083 HMM00465alcdh_3_mix(pf00465) EUT2A
00084 HMM01923cobtr_4_eut2(pf01923) EUT2A
00085 HMM10662PduV_1_mix(pf10662) EUT2A
00086 -pf00005 -
00087 -pf01032 -
00088 -pf01497 -
00089 -pf02660 -
00090 -pf01263 -
00091 -pf06018pf08222 -
00092 -pf00004pf07724pf10431 -
BMC-P
BMC-H
BMC-T
Aldehyde dehydrogenase
PTAC
Signature enzyme
Alcohol dehydrogenase
Regulator

Enterococcus canis. Bacteria; Firmicutes; Bacilli; Lactobacillales; Enterococcaceae;

RU97_GL_locus_1

EUT2A

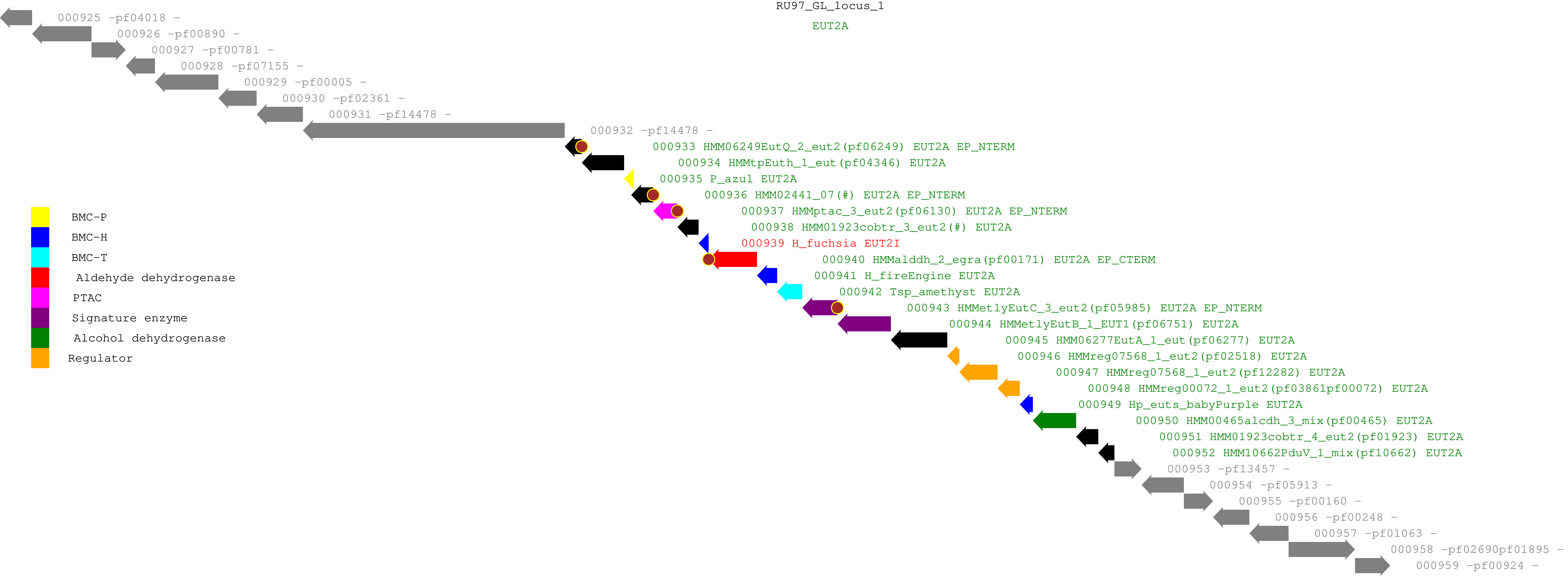

Enterococcus mundtii. Bacteria; Firmicutes; Bacilli; Lactobacillales; Enterococcaceae;

RV08_GL_locus_1

EUT2A

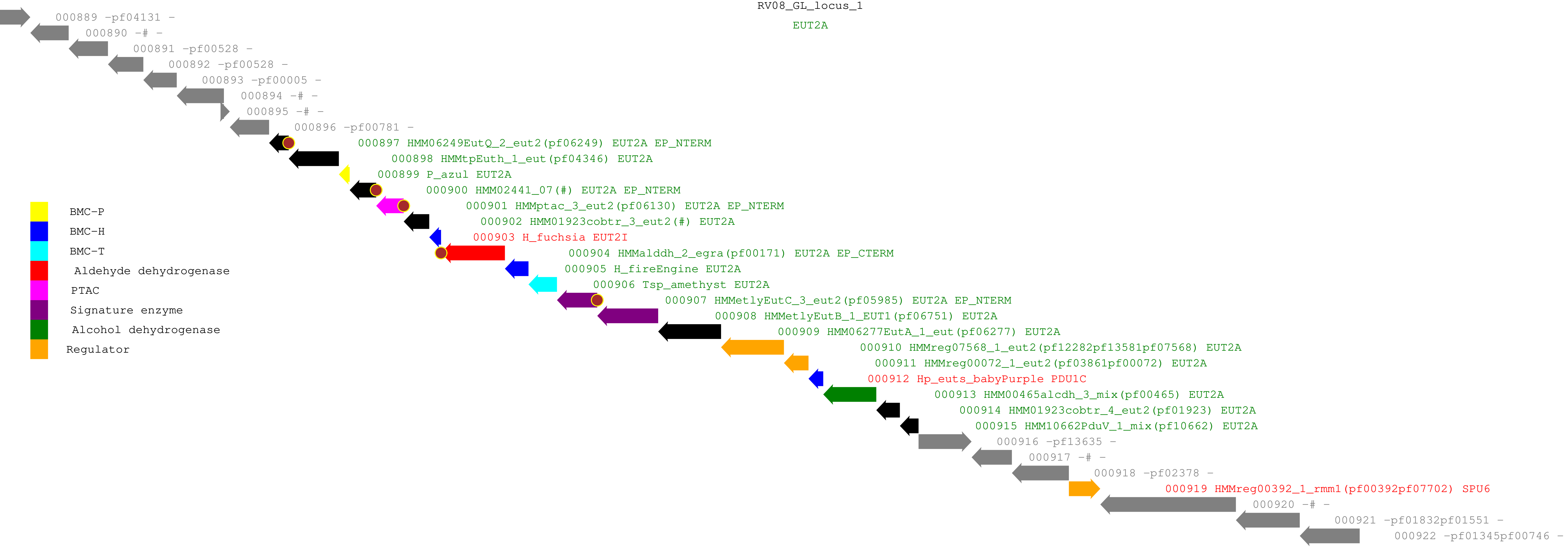

Enterococcus ratti. Bacteria; Firmicutes; Bacilli; Lactobacillales; Enterococcaceae;

RV14_GL_locus_1

EUT2A

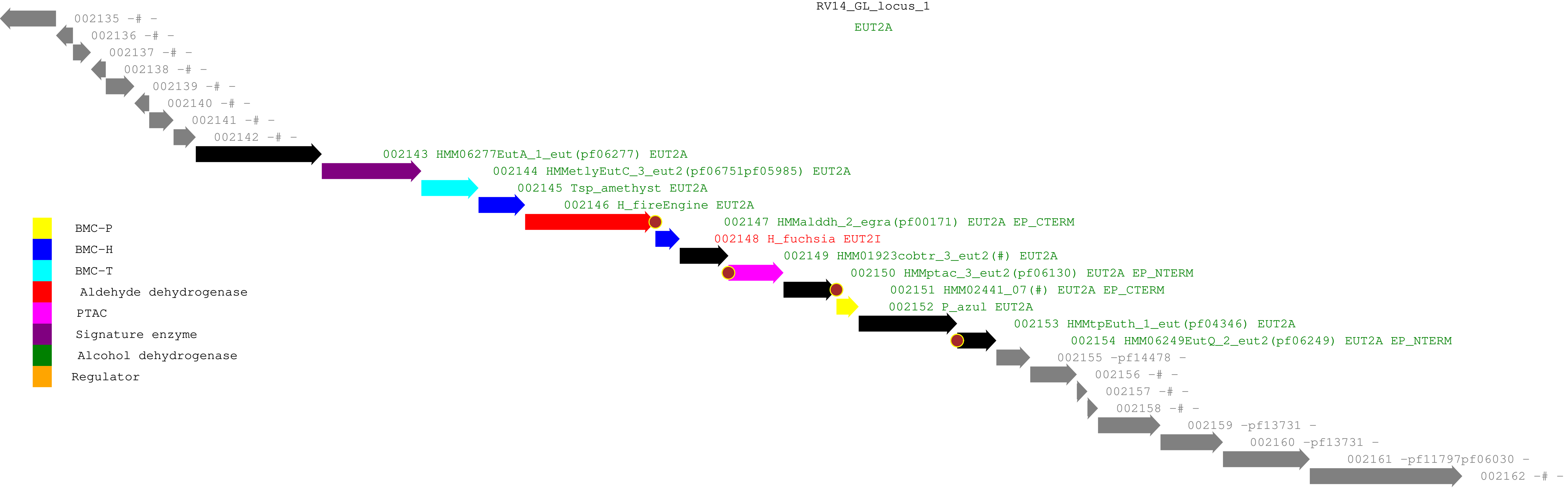

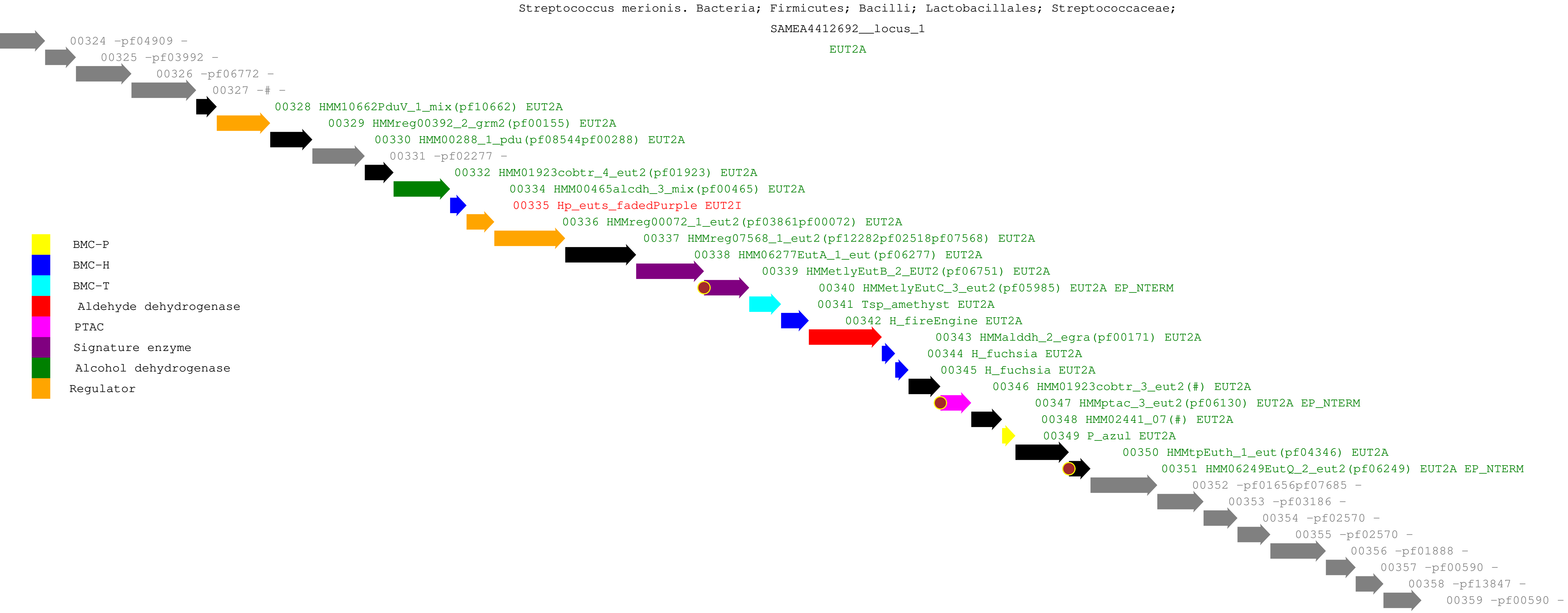

Streptococcus merionis. Bacteria; Firmicutes; Bacilli; Lactobacillales; Streptococcaceae;
SAMEA4412692__locus_1
EUT2A
00324 -pf04909 -
00325 -pf03992 -
00326 -pf06772 -
00327 -# -
00328 HMM10662PduV_1_mix(pf10662) EUT2A
00329 HMMreg00392_2_grm2(pf00155) EUT2A
00330 HMM00288_1_pdu(pf08544pf00288) EUT2A
00331 -pf02277 -
00332 HMM01923cobtr_4_eut2(pf01923) EUT2A
00334 HMM00465alcdh_3_mix(pf00465) EUT2A
00335 Hp_euts_fadedPurple EUT2I
00336 HMMreg00072_1_eut2(pf03861pf00072) EUT2A
00337 HMMreg07568_1_eut2(pf12282pf02518pf07568) EUT2A
00338 HMM06277EutA_1_eut(pf06277) EUT2A
00339 HMMetlyEutB_2_EUT2(pf06751) EUT2A
00340 HMMetlyEutC_3_eut2(pf05985) EUT2A EP_NTERM
00341 Tsp_amethyst EUT2A
00342 H_fireEngine EUT2A
00343 HMMaldh_2_egra(pf00171) EUT2A
00344 H_fuchsia EUT2A
00345 H_fuchsia EUT2A
00346 HMM01923cobtr_3_eut2(#) EUT2A
00347 HMMptac_3_eut2(pf06130) EUT2A EP_NTERM
00348 HMM02441_07(#) EUT2A
00349 P_azul EUT2A
00350 HMMtpEuth_1_eut(pf04346) EUT2A
00351 HMM06249EutQ_2_eut2(pf06249) EUT2A EP_NTERM
00352 -pf01656pf07685 -
00353 -pf03186 -
00354 -pf02570 -
00355 -pf02570 -
00356 -pf01888 -
00357 -pf00590 -
00358 -pf13847 -
00359 -pf00590 -
BMC-P
BMC-H
BMC-T
Aldehyde dehydrogenase
PTAC
Signature enzyme
Alcohol dehydrogenase
Regulator

Pilibacter termitis. Bacteria; Firmicutes; Bacilli; Lactobacillales; Enterococcaceae;

SAMN02745116__locus_1

EUT2A

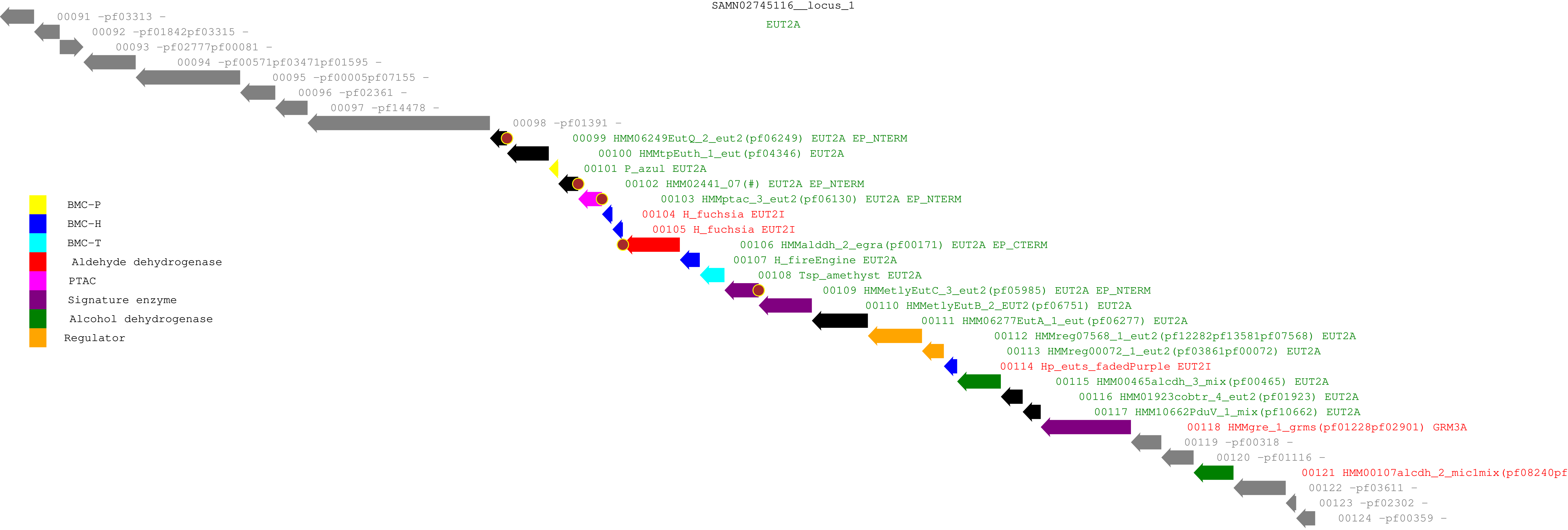

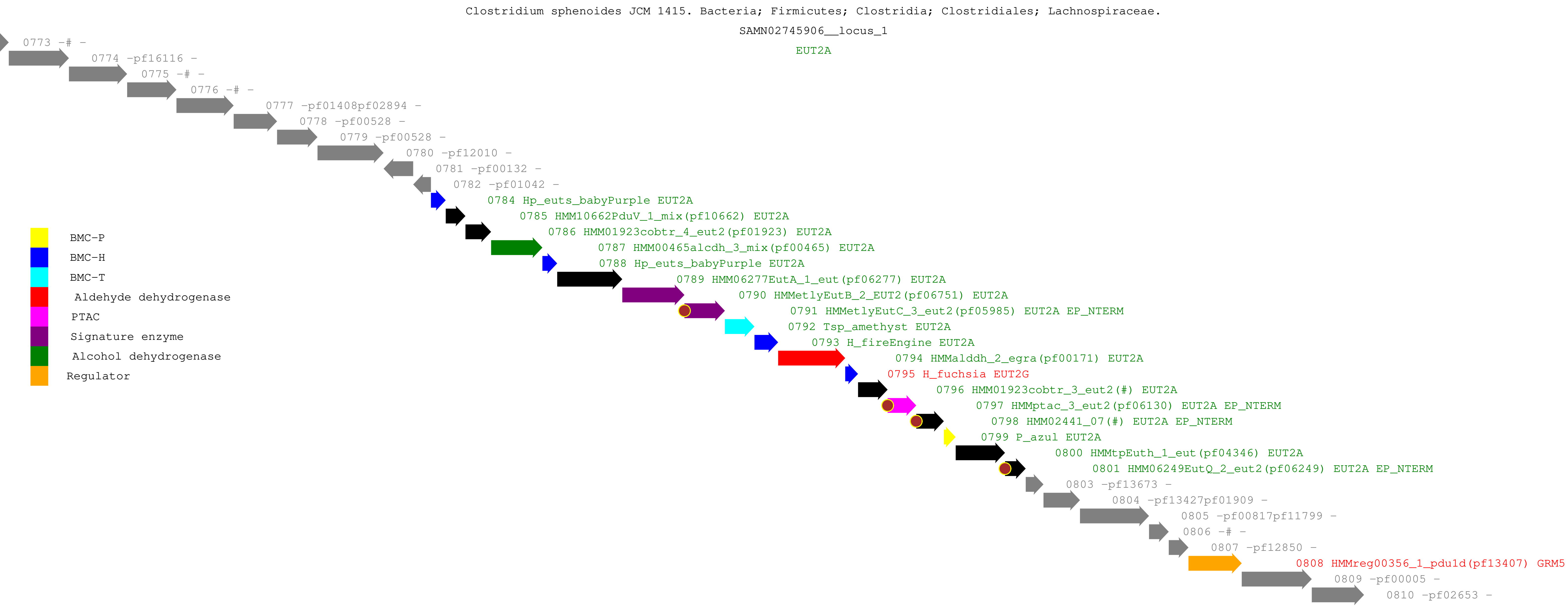

Clostridium sphenoides JCM 1415. Bacteria; Firmicutes; Clostridia; Clostridiales; Lachnospiraceae.
SAMN02745906__locus_1
EUT2A
0773 -# -
0774 -pf16116 -
0775 -# -
0776 -# -
0777 -pf01408pf02894 -
0778 -pf00528 -
0779 -pf00528 -
0780 -pf12010 -
0781 -pf00132 -
0782 -pf01042 -
0784 Hp_euts_babyPurple EUT2A
0785 HMM10662PduV_1_mix(pf10662) EUT2A
0786 HMM01923cobtr_4_eut2(pf01923) EUT2A
0787 HMM00465alcdh_3_mix(pf00465) EUT2A
0788 Hp_euts_babyPurple EUT2A
0789 HMM06277EutA_1_eut(pf06277) EUT2A
0790 HMMetlyEutB_2_EUT2(pf06751) EUT2A
0791 HMMetlyEutC_3_eut2(pf05985) EUT2A EP_NTERM
0792 Tsp_amethyst EUT2A
0793 H_fireEngine EUT2A
0794 HMMalddh_2_egra(pf00171) EUT2A
0795 H_fuchsia EUT2G
0796 HMM01923cobtr_3_eut2(#) EUT2A
0797 HMMptac_3_eut2(pf06130) EUT2A EP_NTERM
0798 HMM02441_07(#) EUT2A EP_NTERM
0799 P_azul EUT2A
0800 HMMtpEuth_1_eut(pf04346) EUT2A
0801 HMM06249EutQ_2_eut2(pf06249) EUT2A EP_NTERM
0803 -pf13673 -
0804 -pf13427pf01909 -
0805 -pf00817pf11799 -
0806 -# -
0807 -pf12850 -
0808 HMMreg00356_1_pdu1d(pf13407) GRM5
0809 -pf00005 -
0810 -pf02653 -
BMC-P
BMC-H
BMC-T
Aldehyde dehydrogenase
PTAC
Signature enzyme
Alcohol dehydrogenase
Regulator

Enterococcus mundtii. Bacteria; Firmicutes; Bacilli; Lactobacillales; Enterococcaceae;

SAMN04487758__locus_1

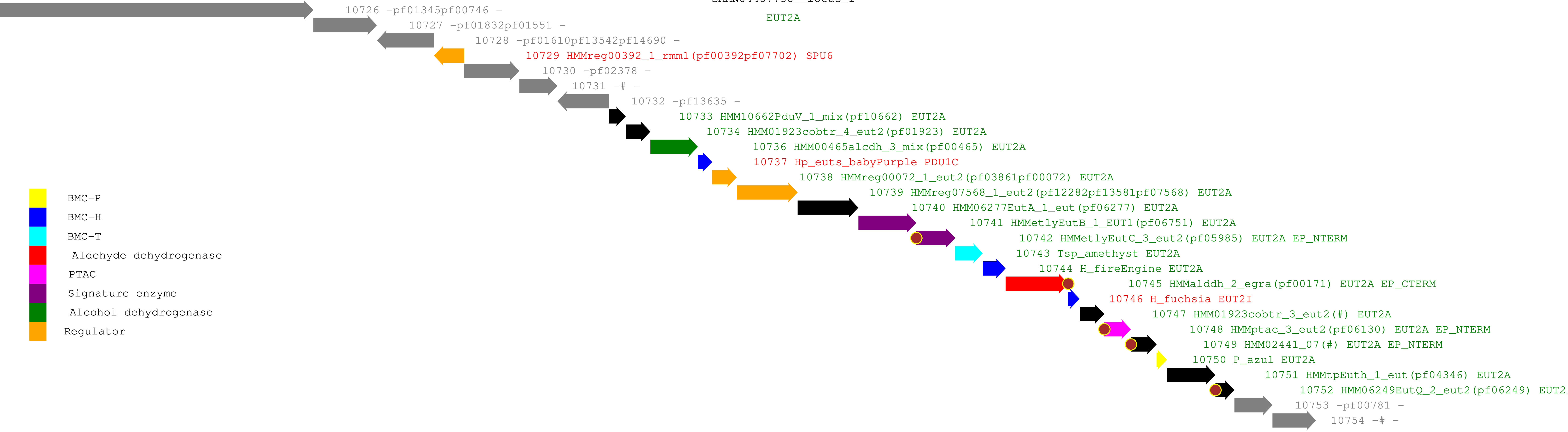

Ignavigranum ruoffiae. Bacteria; Firmicutes; Bacilli; Lactobacillales; Aerococcaceae;

SAMN04488558__locus_1_main_sat

EUT2A

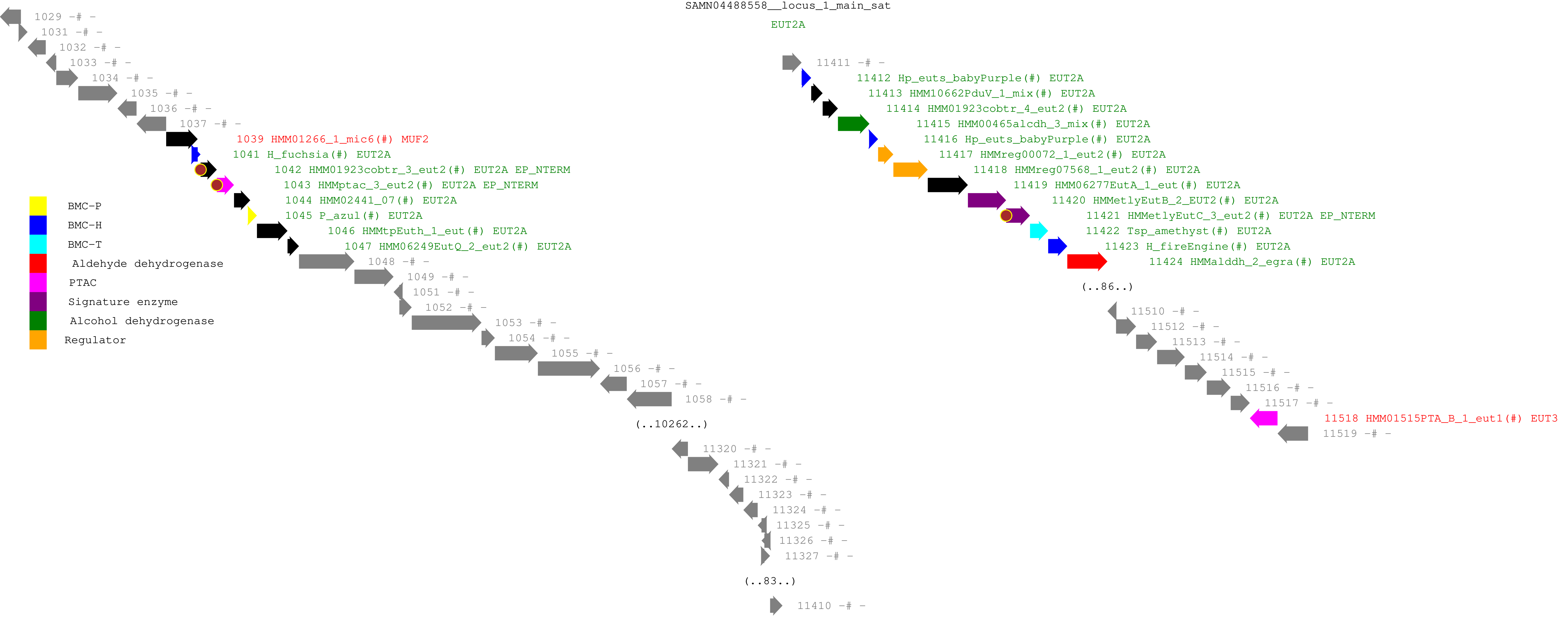

Isobaculum melis. Bacteria; Firmicutes; Bacilli; Lactobacillales; Carnobacteriaceae;

SAMN04488559__locus_1

EUT2A

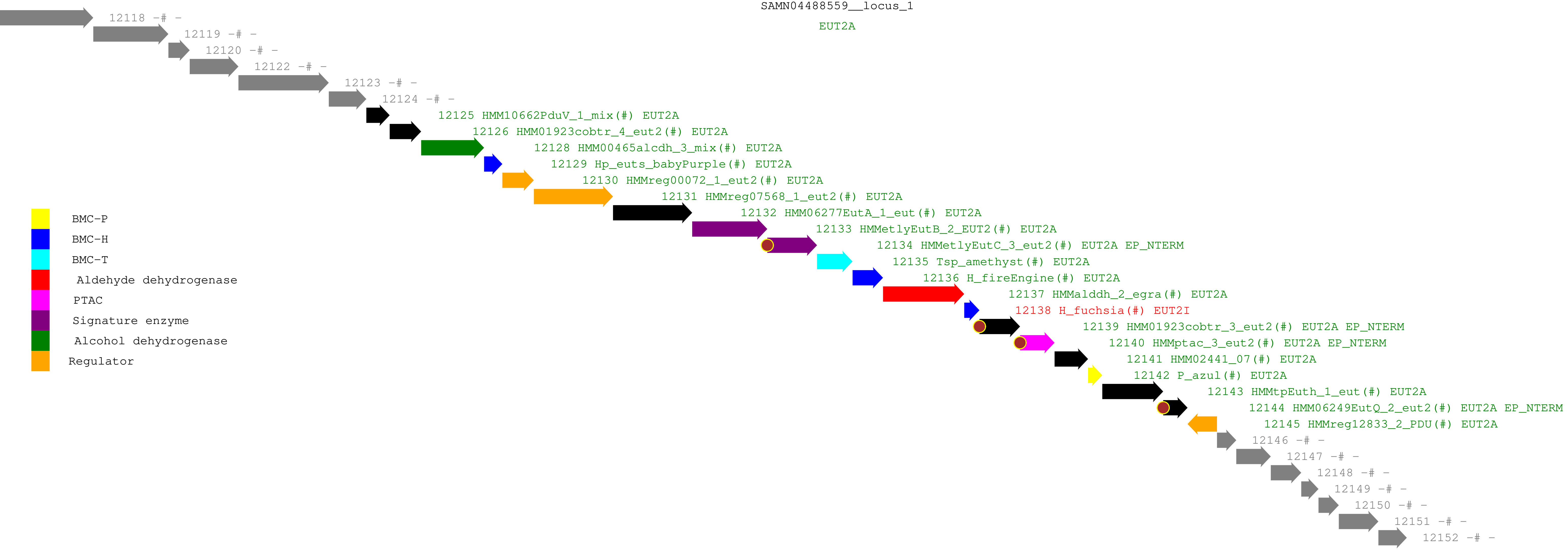

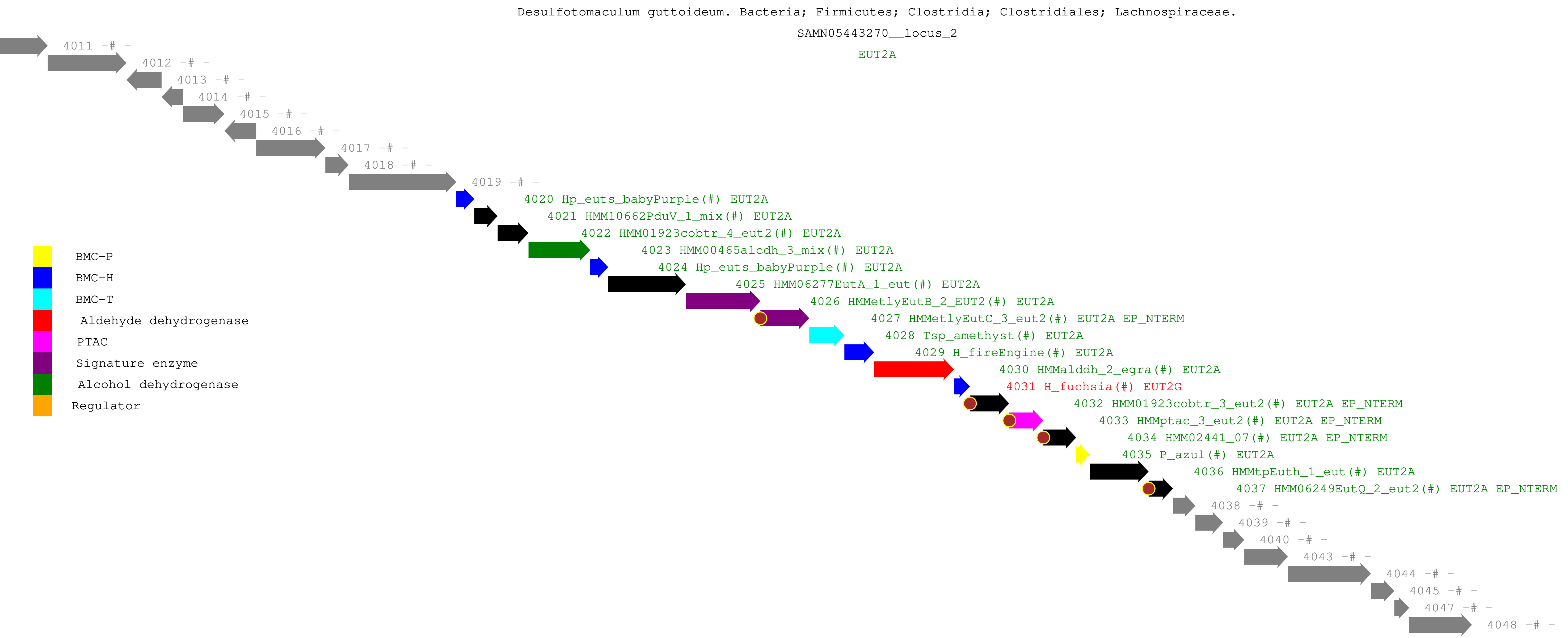

Desulfotomaculum guttoideum. Bacteria; Firmicutes; Clostridia; Clostridiales; Lachnospiraceae.
SAMN05443270__locus_2
EUT2A
4011 -# -
4012 -# -
4013 -# -
4014 -# -
4015 -# -
4016 -# -
4017 -# -
4018 -# -
4019 -# -
4020 Hp_euts_babyPurple(#) EUT2A
4021 HMM10662PduV_1_mix(#) EUT2A
4022 HMM01923cobtr_4_eut2(#) EUT2A
4023 HMM00465alcdh_3_mix(#) EUT2A
4024 Hp_euts_babyPurple(#) EUT2A
4025 HMM06277EutA_1_eut(#) EUT2A
4026 HMMetlyEutB_2_EUT2(#) EUT2A
4027 HMMetlyEutC_3_eut2(#) EUT2A EP_NTERM
4028 Tsp_amethyst(#) EUT2A
4029 H_fireEngine(#) EUT2A
4030 HMMalddh_2_egra(#) EUT2A
4031 H_fuchsia(#) EUT2G
4032 HMM01923cobtr_3_eut2(#) EUT2A EP_NTERM
4033 HMMptac_3_eut2(#) EUT2A EP_NTERM
4034 HMM02441_07(#) EUT2A EP_NTERM
4035 P_azul(#) EUT2A
4036 HMMtpEuth_1_eut(#) EUT2A
4037 HMM06249EutQ_2_eut2(#) EUT2A EP_NTERM
4038 -# -
4039 -# -
4040 -# -
4043 -# -
4044 -# -
4045 -# -
4047 -# -
4048 -# -
BMC-P
BMC-H
BMC-T
Aldehyde dehydrogenase
PTAC
Signature enzyme
Alcohol dehydrogenase
Regulator

Streptococcus pluranimalium. Bacteria; Firmicutes; Bacilli; Lactobacillales; Streptococcaceae;

Sp14A__locus_1

EUT2A

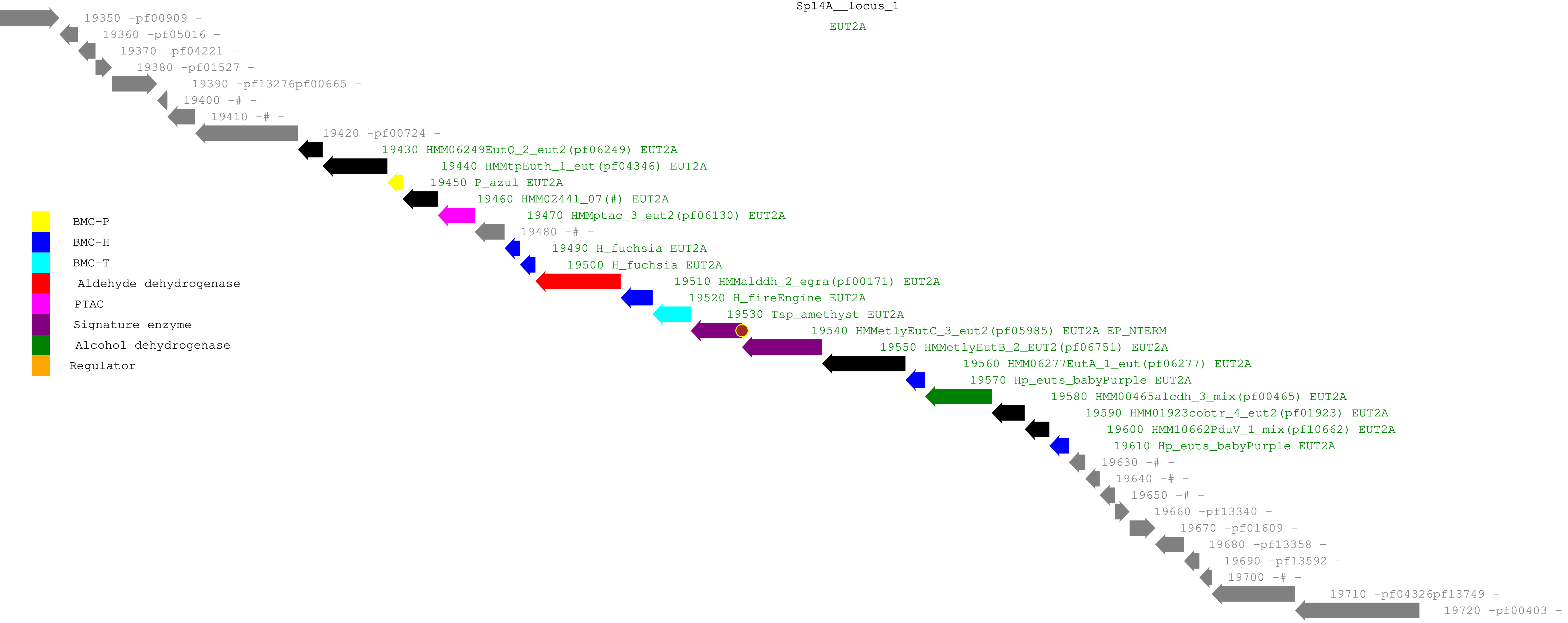

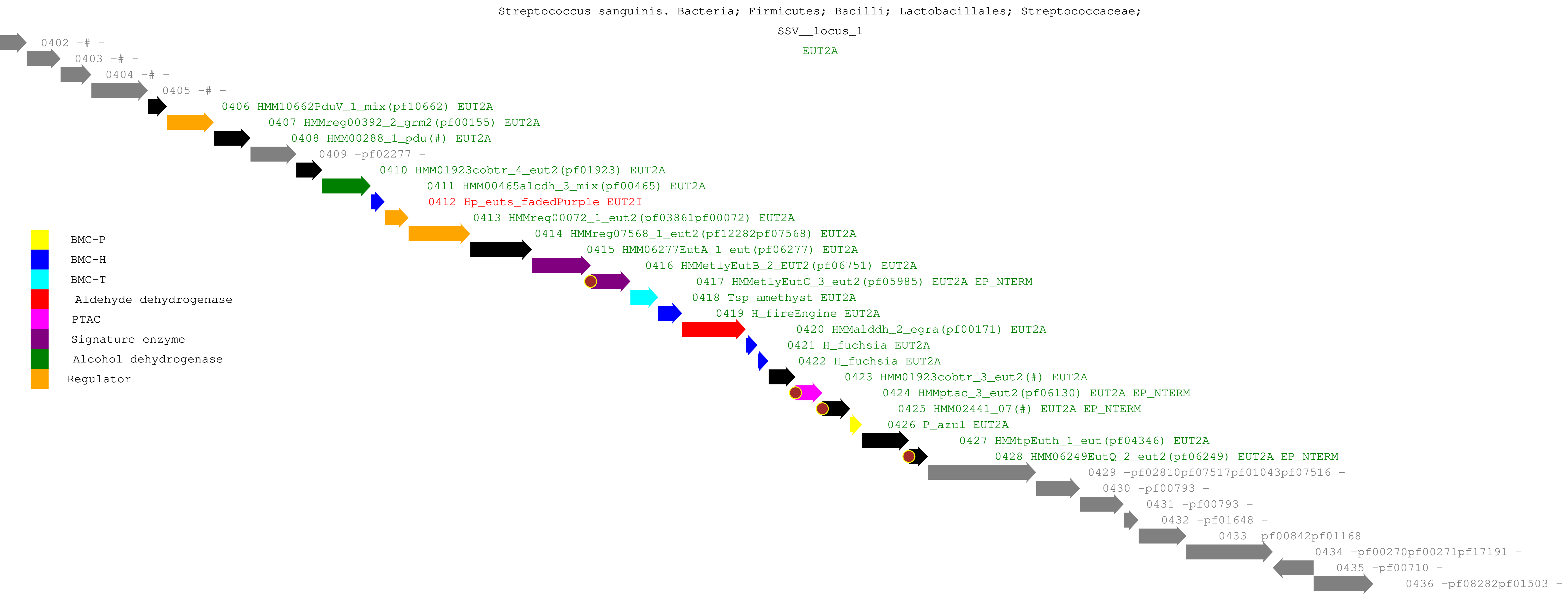
Streptococcus sanguinis. Bacteria; Firmicutes; Bacilli; Lactobacillales; Streptococcaceae;
SSV__locus_1
EUT2A
0402 -# -
0403 -# -
0404 -# -
0405 -# -
0406 HMM10662PduV_1_mix(pf10662) EUT2A
0407 HMMreg00392_2_grm2(pf00155) EUT2A
0408 HMM00288_1_pdu(#) EUT2A
0409 -pf02277 -
0410 HMM01923cobtr_4_eut2(pf01923) EUT2A
0411 HMM00465alcdh_3_mix(pf00465) EUT2A
0412 Hp_euts_fadedPurple EUT2I
0413 HMMreg00072_1_eut2(pf03861pf00072) EUT2A
0414 HMMreg07568_1_eut2(pf12282pf07568) EUT2A
0415 HMM06277EutA_1_eut(pf06277) EUT2A
0416 HMMetlyEutB_2_EUT2(pf06751) EUT2A
0417 HMMetlyEutC_3_eut2(pf05985) EUT2A EP_NTERM
0418 Tsp_amethyst EUT2A
0419 H_fireEngine EUT2A
0420 HMMalddh_2_egra(pf00171) EUT2A
0421 H_fuchsia EUT2A
0422 H_fuchsia EUT2A
0423 HMM01923cobtr_3_eut2(#) EUT2A
0424 HMMptac_3_eut2(pf06130) EUT2A EP_NTERM
0425 HMM02441_07(#) EUT2A EP_NTERM
0426 P_azul EUT2A
0427 HMMtpEuth_1_eut(pf04346) EUT2A
0428 HMM06249EutQ_2_eut2(pf06249) EUT2A EP_NTERM
0429 -pf02810pf07517pf01043pf07516 -
0430 -pf00793 -
0431 -pf00793 -
0432 -pf01648 -
0433 -pf00842pf01168 -
0434 -pf00270pf00271pf17191 -
0435 -pf00710 -
0436 -pf08282pf01503 -
BMC-P
BMC-H
BMC-T
Aldehyde dehydrogenase
PTAC
Signature enzyme
Alcohol dehydrogenase
Regulator

Streptococcus sp. DD11. Bacteria; Firmicutes; Bacilli; Lactobacillales; Streptococcaceae;

STRDD11__locus_1

EUT2A

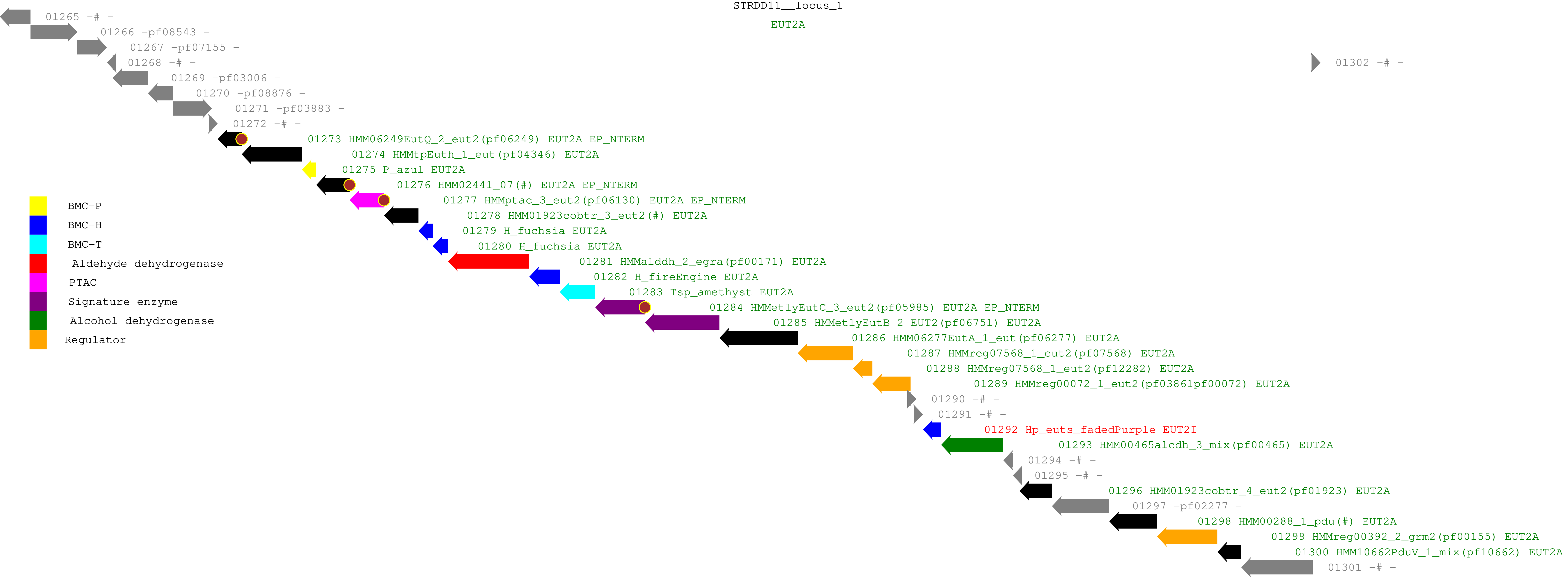

Enterococcus faecalis PF3. Bacteria; Firmicutes; Bacilli; Lactobacillales; Enterococcaceae;

T481__locus_1_main_sat

EUT2A

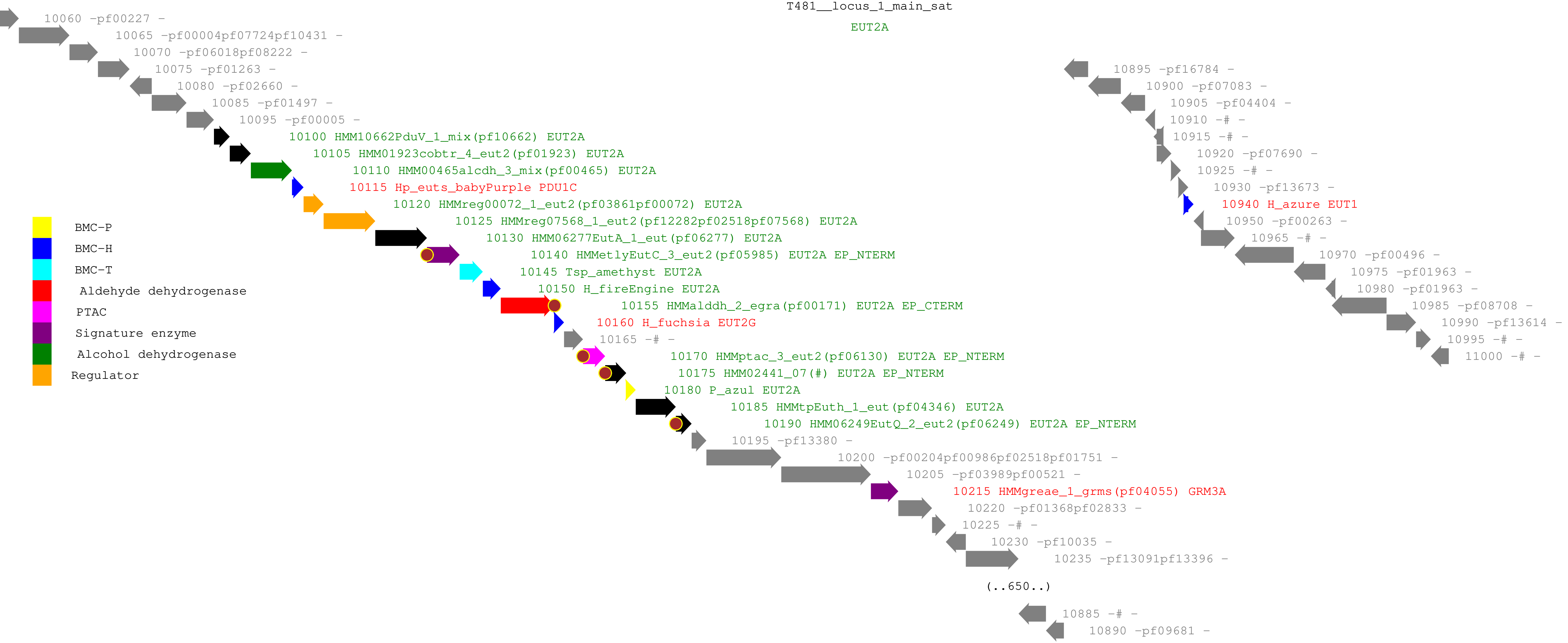

Trichococcus sp. ES5. Bacteria; Firmicutes; Bacilli; Lactobacillales; Carnobacteriaceae;

TES5__locus_1

EUT2A

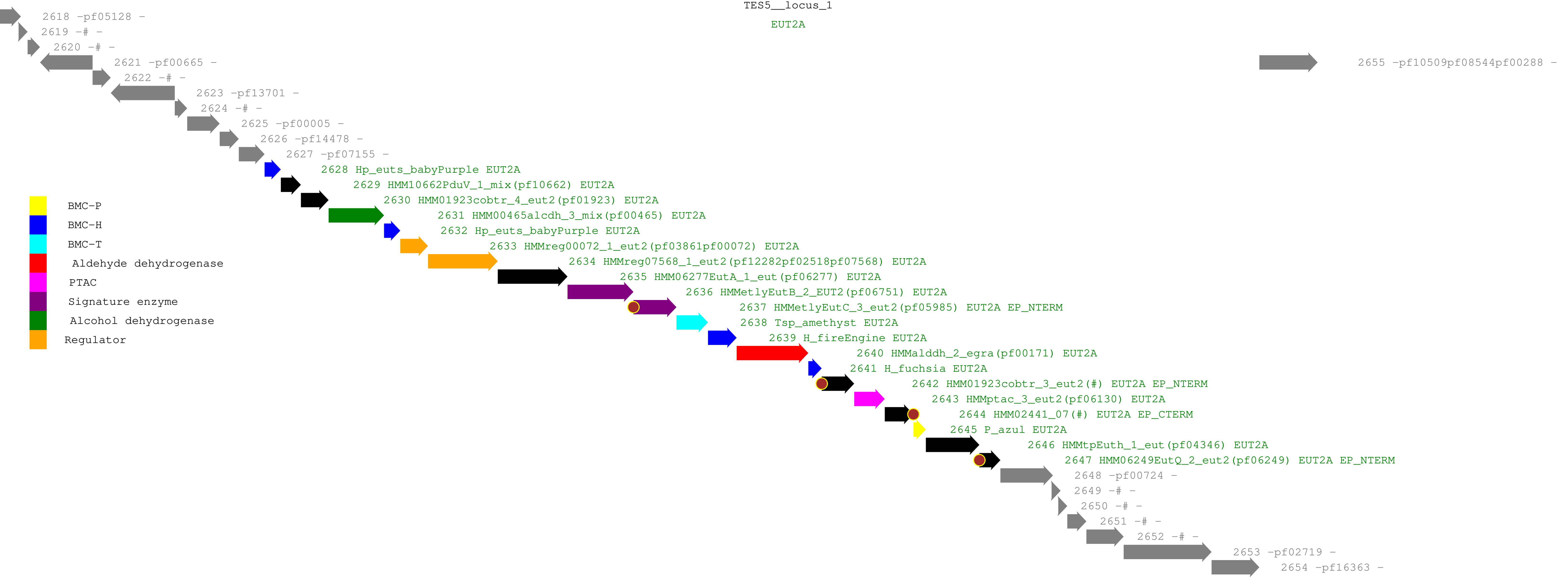

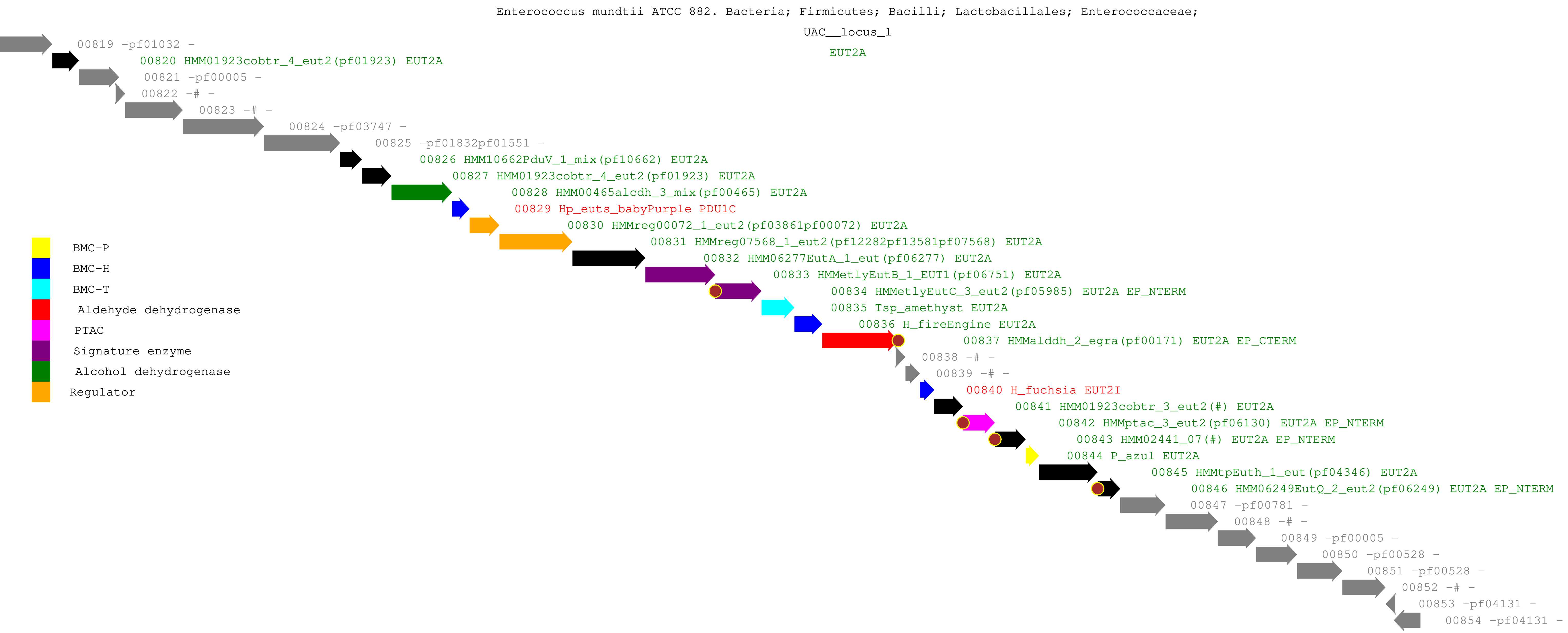

Enterococcus mundtii ATCC 882. Bacteria; Firmicutes; Bacilli; Lactobacillales; Enterococcaceae;
UAC__locus_1
EUT2A
00819 -pf01032 -
00820 HMM01923cobtr_4_eut2(pf01923) EUT2A
00821 -pf00005 -
00822 -# -
00823 -# -
00824 -pf03747 -
00825 -pf01832pf01551 -
00826 HMM10662PduV_1_mix(pf10662) EUT2A
00827 HMM01923cobtr_4_eut2(pf01923) EUT2A
00828 HMM00465alcdh_3_mix(pf00465) EUT2A
00829 Hp_euts_babyPurple PDU1C
00830 HMMreg00072_1_eut2(pf03861pf00072) EUT2A
00831 HMMreg07568_1_eut2(pf12282pf13581pf07568) EUT2A
00832 HMM06277EutA_1_eut(pf06277) EUT2A
00833 HMMetlyEutB_1_EUT1(pf06751) EUT2A
00834 HMMetlyEutC_3_eut2(pf05985) EUT2A EP_NTERM
00835 Tsp_amethyst EUT2A
00836 H_fireEngine EUT2A
00837 HMMalddh_2_egra(pf00171) EUT2A EP_CTERM
00838 -# -
00839 -# -
00840 H_fuchsia EUT2I
00841 HMM01923cobtr_3_eut2(#) EUT2A
00842 HMMptac_3_eut2(pf06130) EUT2A EP_NTERM
00843 HMM02441_07(#) EUT2A EP_NTERM
00844 P_azul EUT2A
00845 HMMtpEuth_1_eut(pf04346) EUT2A
00846 HMM06249EutQ_2_eut2(pf06249) EUT2A EP_NTERM
00847 -pf00781 -
00848 -# -
00849 -pf00005 -
00850 -pf00528 -
00851 -pf00528 -
00852 -# -
00853 -pf04131 -
00854 -pf04131 -
BMC-P
BMC-H
BMC-T
Aldehyde dehydrogenase
PTAC
Signature enzyme
Alcohol dehydrogenase
Regulator

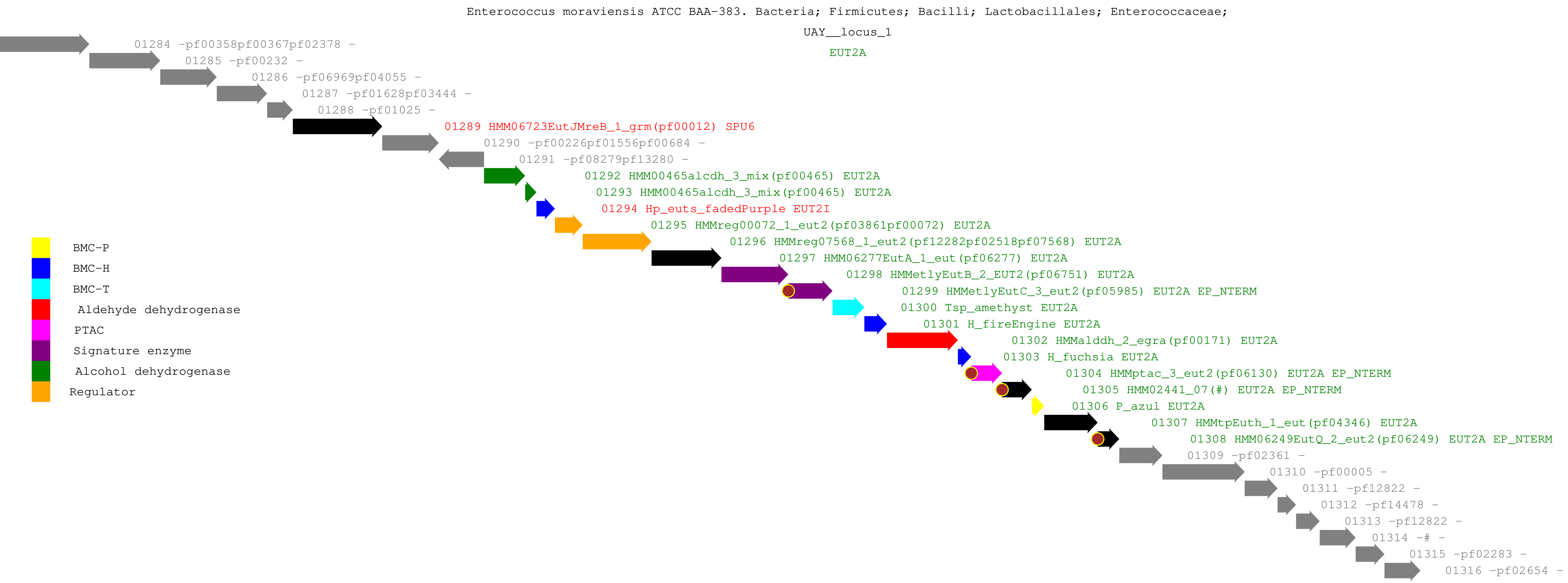

Enterococcus moraviensis ATCC BAA-383. Bacteria; Firmicutes; Bacilli; Lactobacillales; Enterococcaceae;
UAY__locus_1
EUT2A
01284 -pf00358pf00367pf02378 -
01285 -pf00232 -
01286 -pf06969pf04055 -
01287 -pf01628pf03444 -
01288 -pf01025 -
01289 HMM06723EutJMreB_1_grm(pf00012) SPU6
01290 -pf00226pf01556pf00684 -
01291 -pf08279pf13280 -
01292 HMM00465alcdh_3_mix(pf00465) EUT2A
01293 HMM00465alcdh_3_mix(pf00465) EUT2A
01294 Hp_euts_fadedPurple EUT2I
01295 HMMreg00072_1_eut2(pf03861pf00072) EUT2A
01296 HMMreg07568_1_eut2(pf12282pf02518pf07568) EUT2A
01297 HMM06277EutA_1_eut(pf06277) EUT2A
01298 HMMetlyEutB_2_EUT2(pf06751) EUT2A
01299 HMMetlyEutC_3_eut2(pf05985) EUT2A EP_NTERM
01300 Tsp_amethyst EUT2A
01301 H_fireEngine EUT2A
01302 HMMalddh_2_egra(pf00171) EUT2A
01303 H_fuchsia EUT2A
01304 HMMptac_3_eut2(pf06130) EUT2A EP_NTERM
01305 HMM02441_07(#) EUT2A EP_NTERM
01306 P_azul EUT2A
01307 HMMtpEuth_1_eut(pf04346) EUT2A
01308 HMM06249EutQ_2_eut2(pf06249) EUT2A EP_NTERM
01309 -pf02361 -
01310 -pf00005 -
01311 -pf12822 -
01312 -pf14478 -
01313 -pf12822 -
01314 -# -
01315 -pf02283 -
01316 -pf02654 -
BMC-P
BMC-H
BMC-T
Aldehyde dehydrogenase
PTAC
Signature enzyme
Alcohol dehydrogenase
Regulator

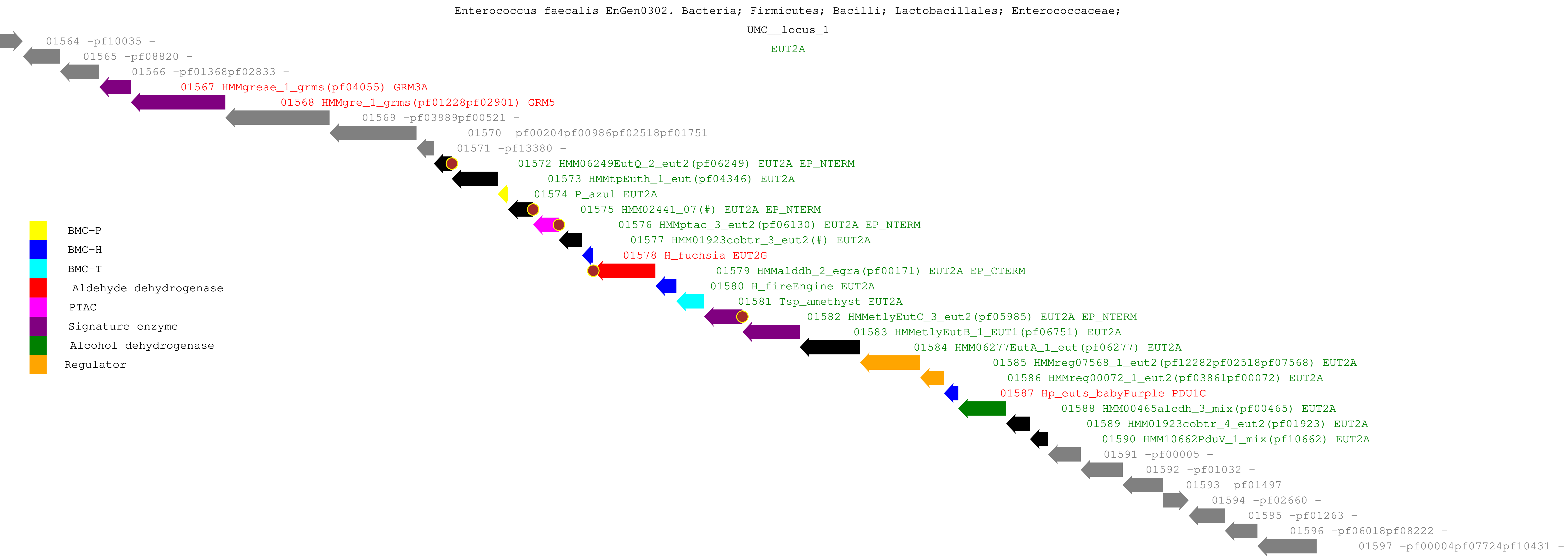
Enterococcus faecalis EnGen0302. Bacteria; Firmicutes; Bacilli; Lactobacillales; Enterococcaceae;
UMC__locus_1
EUT2A
01564 -pf10035 -
01565 -pf08820 -
01566 -pf01368pf02833 -
01567 HMMgreae_1_grms(pf04055) GRM3A
01568 HMMgre_1_grms(pf01228pf02901) GRM5
01569 -pf03989pf00521 -
01570 -pf00204pf00986pf02518pf01751 -
01571 -pf13380 -
01572 HMM06249EutQ_2_eut2(pf06249) EUT2A EP_NTERM
01573 HMMtpEuth_1_eut(pf04346) EUT2A
01574 P_azul EUT2A
01575 HMM02441_07(#) EUT2A EP_NTERM
01576 HMMptac_3_eut2(pf06130) EUT2A EP_NTERM
01577 HMM01923cobtr_3_eut2(#) EUT2A
01578 H_fuchsia EUT2G
01579 HMMalddh_2_egra(pf00171) EUT2A EP_CTERM
01580 H_fireEngine EUT2A
01581 Tsp_amethyst EUT2A
01582 HMMetlyEutC_3_eut2(pf05985) EUT2A EP_NTERM
01583 HMMetlyEutB_1_EUT1(pf06751) EUT2A
01584 HMM06277EutA_1_eut(pf06277) EUT2A
01585 HMMreg07568_1_eut2(pf12282pf02518pf07568) EUT2A
01586 HMMreg00072_1_eut2(pf03861pf00072) EUT2A
01587 Hp_euts_babyPurple PDU1C
01588 HMM00465alcdh_3_mix(pf00465) EUT2A
01589 HMM01923cobtr_4_eut2(pf01923) EUT2A
01590 HMM10662PduV_1_mix(pf10662) EUT2A
01591 -pf00005 -
01592 -pf01032 -
01593 -pf01497 -
01594 -pf02660 -
01595 -pf01263 -
01596 -pf06018pf08222 -
01597 -pf00004pf07724pf10431 -
BMC-P
BMC-H
BMC-T
Aldehyde dehydrogenase
PTAC
Signature enzyme
Alcohol dehydrogenase
Regulator

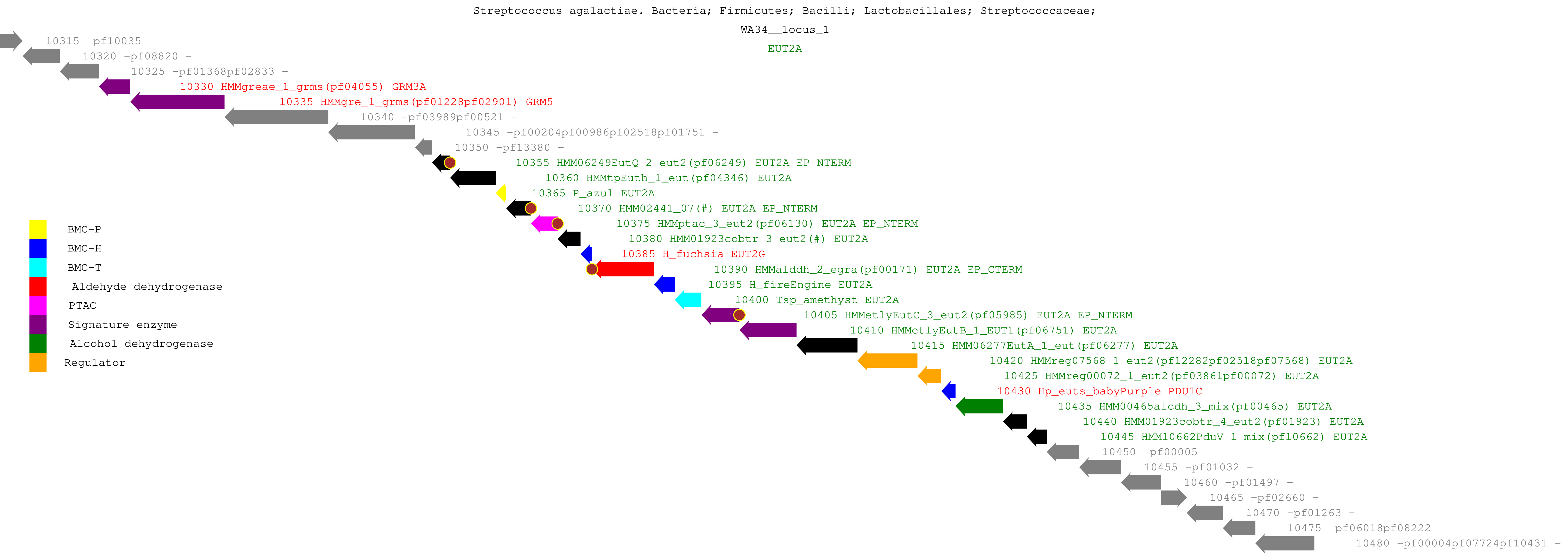

Streptococcus agalactiae. Bacteria; Firmicutes; Bacilli; Lactobacillales; Streptococcaceae;
WA34__locus_1
EUT2A
10315 -pf10035 -
10320 -pf08820 -
10325 -pf01368pf02833 -
10330 HMMgreae_1_grms(pf04055) GRM3A
10335 HMMgre_1_grms(pf01228pf02901) GRM5
10340 -pf03989pf00521 -
10345 -pf00204pf00986pf02518pf01751 -
10350 -pf13380 -
10355 HMM06249EutQ_2_eut2(pf06249) EUT2A EP_NTERM
10360 HMMtpEuth_1_eut(pf04346) EUT2A
10365 P_azul EUT2A
10370 HMM02441_07(#) EUT2A EP_NTERM
10375 HMMptac_3_eut2(pf06130) EUT2A EP_NTERM
10380 HMM01923cobtr_3_eut2(#) EUT2A
10385 H_fuchsia EUT2G
10390 HMMalddh_2_egra(pf00171) EUT2A EP_CTERM
10395 H_fireEngine EUT2A
10400 Tsp_amethyst EUT2A
10405 HMMetlyEutC_3_eut2(pf05985) EUT2A EP_NTERM
10410 HMMetlyEutB_1_EUT1(pf06751) EUT2A
10415 HMM06277EutA_1_eut(pf06277) EUT2A
10420 HMMreg07568_1_eut2(pf12282pf02518pf07568) EUT2A
10425 HMMreg00072_1_eut2(pf03861pf00072) EUT2A
10430 Hp_euts_babyPurple PDU1C
10435 HMM00465alcdh_3_mix(pf00465) EUT2A
10440 HMM01923cobtr_4_eut2(pf01923) EUT2A
10445 HMM10662PduV_1_mix(pf10662) EUT2A
10450 -pf00005 -
10455 -pf01032 -
10460 -pf01497 -
10465 -pf02660 -
10470 -pf01263 -
10475 -pf06018pf08222 -
10480 -pf00004pf07724pf10431 -
BMC-P
BMC-H
BMC-T
Aldehyde dehydrogenase
PTAC
Signature enzyme
Alcohol dehydrogenase
Regulator

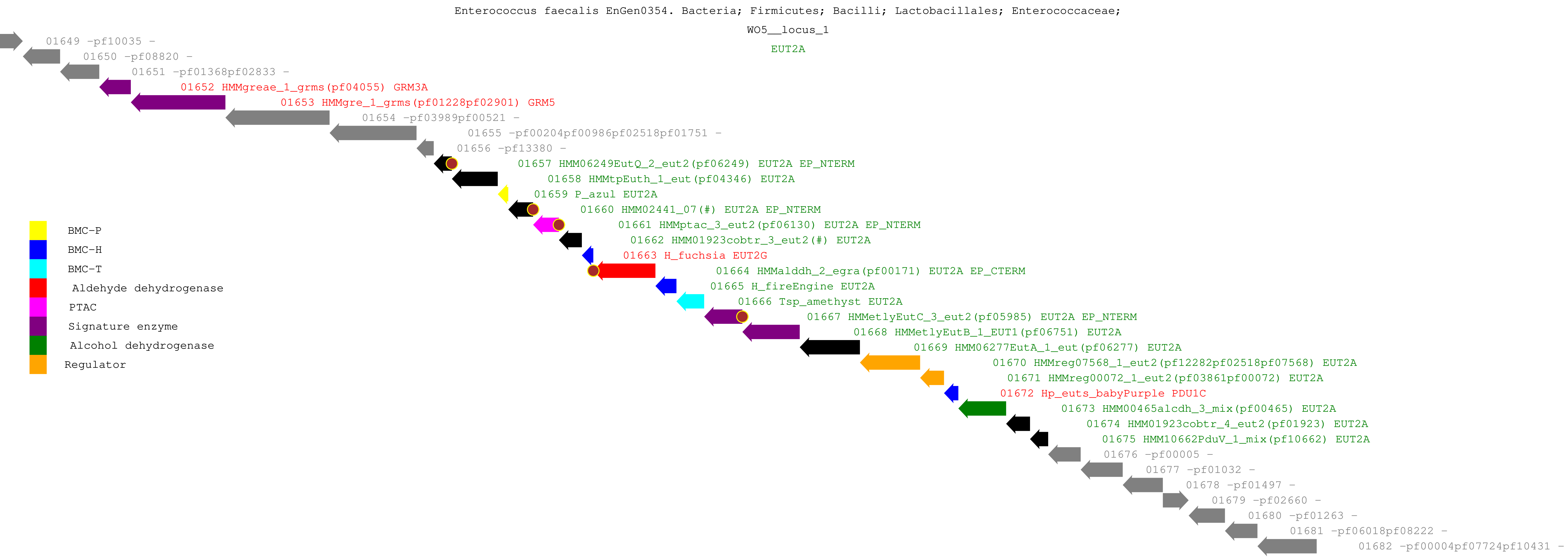
Enterococcus faecalis EnGen0354. Bacteria; Firmicutes; Bacilli; Lactobacillales; Enterococcaceae;
WO5__locus_1
EUT2A
01649 -pf10035 -
01650 -pf08820 -
01651 -pf01368pf02833 -
01652 HMMgreae_1_grms(pf04055) GRM3A
01653 HMMgre_1_grms(pf01228pf02901) GRM5
01654 -pf03989pf00521 -
01655 -pf00204pf00986pf02518pf01751 -
01656 -pf13380 -
01657 HMM06249EutQ_2_eut2(pf06249) EUT2A EP_NTERM
01658 HMMtpEuth_1_eut(pf04346) EUT2A
01659 P_azul EUT2A
01660 HMM02441_07(#) EUT2A EP_NTERM
01661 HMMptac_3_eut2(pf06130) EUT2A EP_NTERM
01662 HMM01923cobtr_3_eut2(#) EUT2A
01663 H_fuchsia EUT2G
01664 HMMalddh_2_egra(pf00171) EUT2A EP_CTERM
01665 H_fireEngine EUT2A
01666 Tsp_amethyst EUT2A
01667 HMMetlyEutC_3_eut2(pf05985) EUT2A EP_NTERM
01668 HMMetlyEutB_1_EUT1(pf06751) EUT2A
01669 HMM06277EutA_1_eut(pf06277) EUT2A
01670 HMMreg07568_1_eut2(pf12282pf02518pf07568) EUT2A
01671 HMMreg00072_1_eut2(pf03861pf00072) EUT2A
01672 Hp_euts_babyPurple PDU1C
01673 HMM00465alcdh_3_mix(pf00465) EUT2A
01674 HMM01923cobtr_4_eut2(pf01923) EUT2A
01675 HMM10662PduV_1_mix(pf10662) EUT2A
01676 -pf00005 -
01677 -pf01032 -
01678 -pf01497 -
01679 -pf02660 -
01680 -pf01263 -
01681 -pf06018pf08222 -
01682 -pf00004pf07724pf10431 -
BMC-P
BMC-H
BMC-T
Aldehyde dehydrogenase
PTAC
Signature enzyme
Alcohol dehydrogenase
Regulator

Enterococcus faecalis EnGen0359. Bacteria; Firmicutes; Bacilli; Lactobacillales; Enterococcaceae;

WOK__locus_1

EUT2A

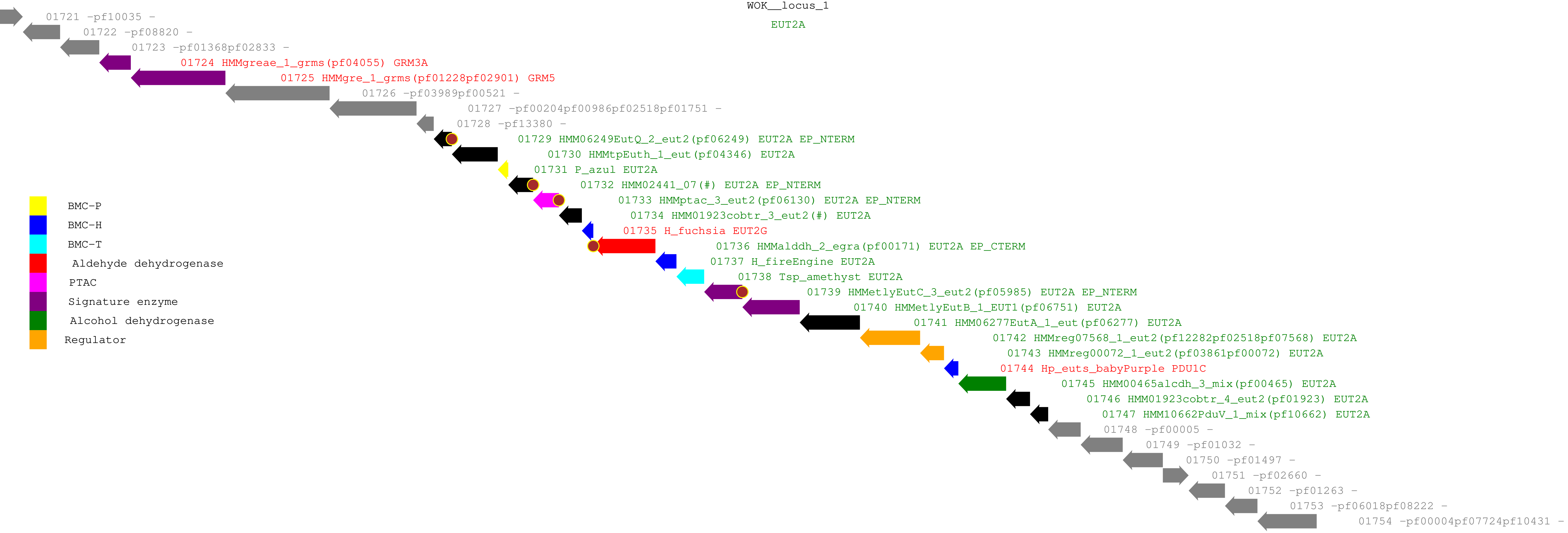

Enterococcus faecalis ATCC 6055. Bacteria; Firmicutes; Bacilli; Lactobacillales; Enterococcaceae;

WOU__locus_1

EUT2A

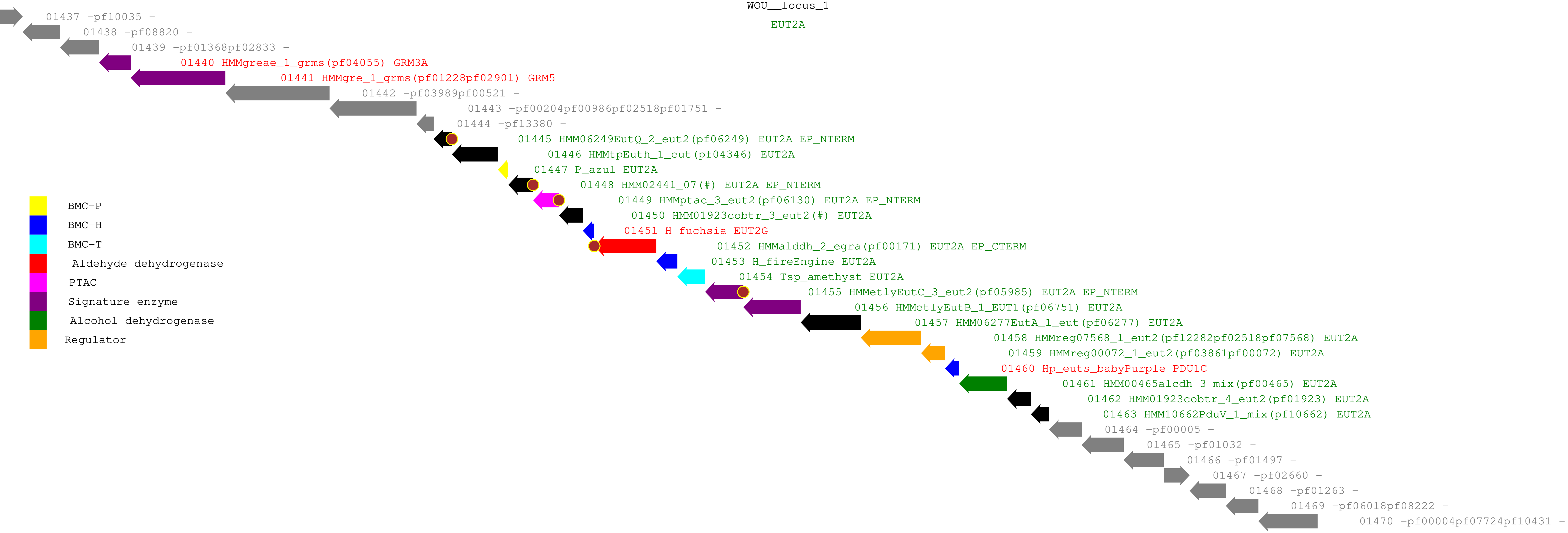

Flavonifractor plautii. Bacteria; Firmicutes; Clostridia; Clostridiales; Flavonifractor.

A4U99__locus_3

EUT2B

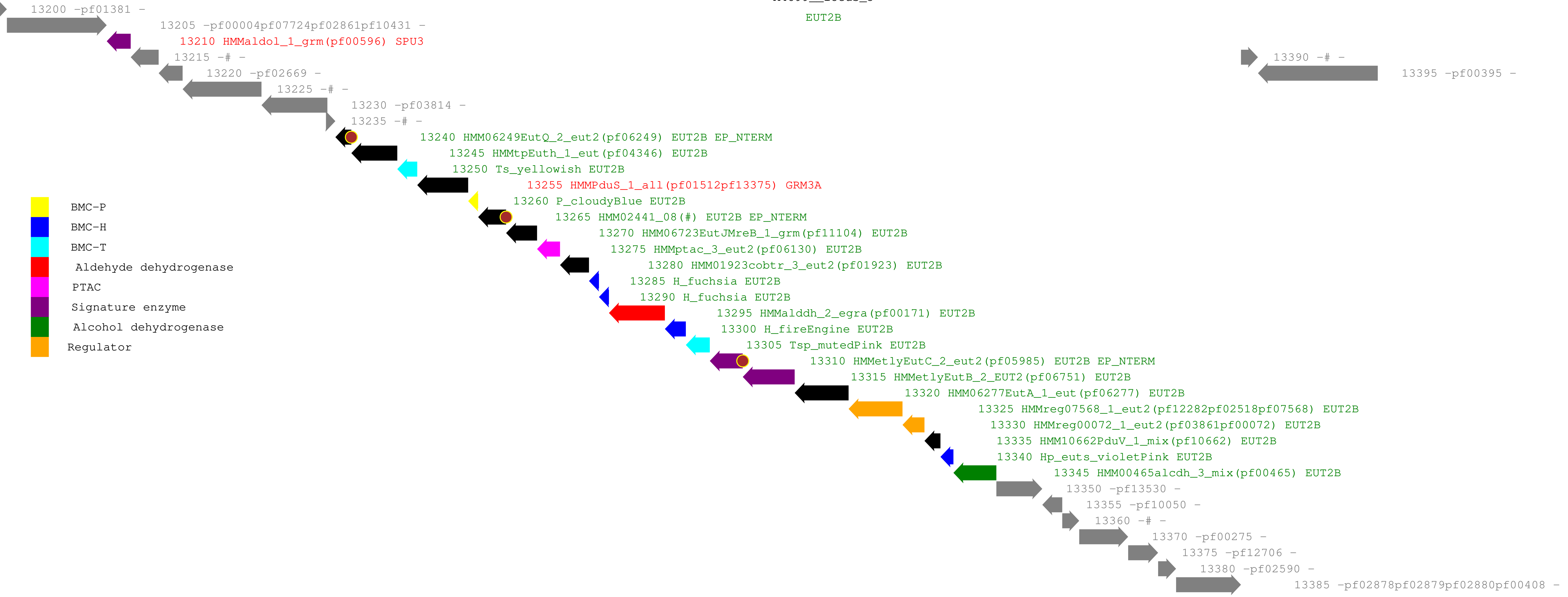

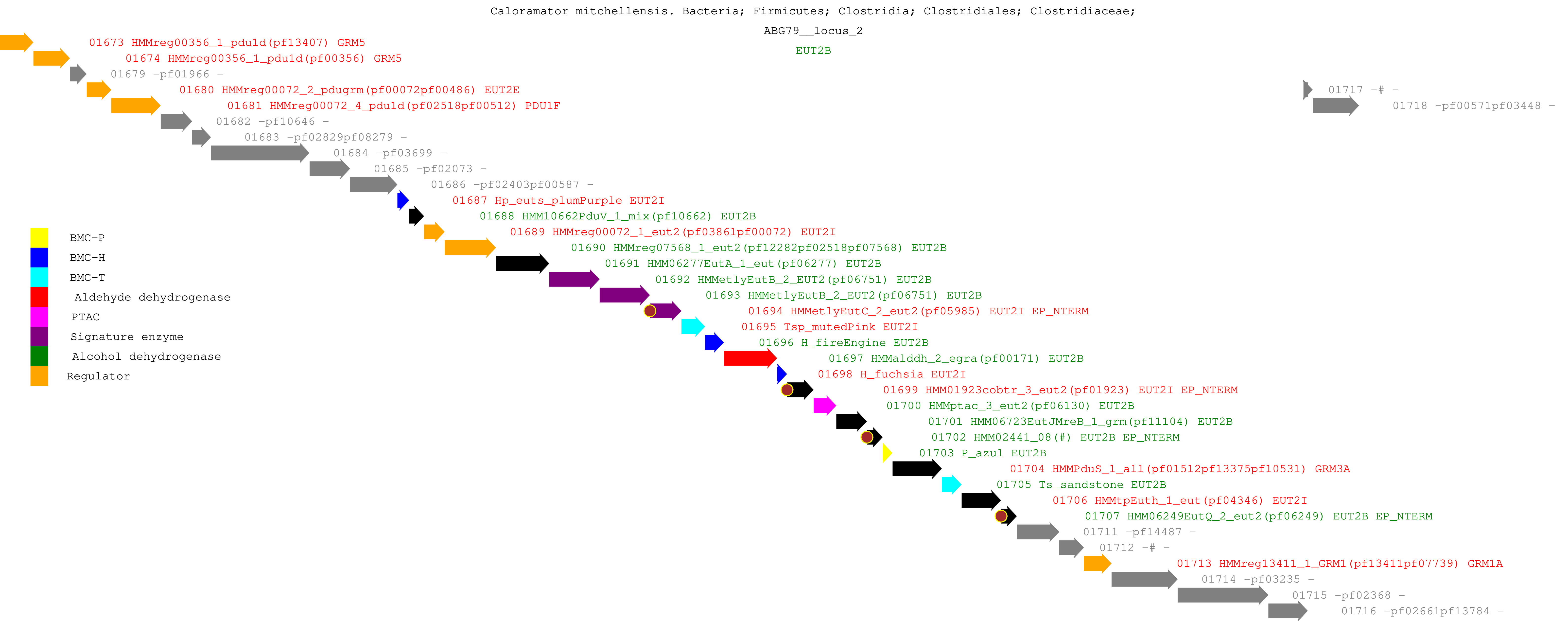
Caloramator mitchellensis. Bacteria; Firmicutes; Clostridia; Clostridiales; Clostridiaceae;
ABG79__locus_2
EUT2B
01673 HMMreg00356_1_pdu1d(pf13407) GRM5
01674 HMMreg00356_1_pdu1d(pf00356) GRM5
01679 -pf01966 -
01680 HMMreg00072_2_pdugrm(pf00072pf00486) EUT2E
01681 HMMreg00072_4_pdu1d(pf02518pf00512) PDU1F
01682 -pf10646 -
01683 -pf02829pf08279 -
01684 -pf03699 -
01685 -pf02073 -
01686 -pf02403pf00587 -
01687 Hp_euts_plumPurple EUT2I
01688 HMM10662PduV_1_mix(pf10662) EUT2B
01689 HMMreg00072_1_eut2(pf03861pf00072) EUT2I
01690 HMMreg07568_1_eut2(pf12282pf02518pf07568) EUT2B
01691 HMM06277EutA_1_eut(pf06277) EUT2B
01692 HMMetlyEutB_2_EUT2(pf06751) EUT2B
01693 HMMetlyEutB_2_EUT2(pf06751) EUT2B
01694 HMMetlyEutC_2_eut2(pf05985) EUT2I EP_NTERM
01695 Tsp_mutedPink EUT2I
01696 H_fireEngine EUT2B
01697 HMMalddh_2_egra(pf00171) EUT2B
01698 H_fuchsia EUT2I
01699 HMM01923cobtr_3_eut2(pf01923) EUT2I EP_NTERM
01700 HMMptac_3_eut2(pf06130) EUT2B
01701 HMM06723EutJMreB_1_grm(pf11104) EUT2B
01702 HMM02441_08(#) EUT2B EP_NTERM
01703 P_azul EUT2B
01704 HMMPduS_1_all(pf01512pf13375pf10531) GRM3A
01705 Ts_sandstone EUT2B
01706 HMMtpEuth_1_eut(pf04346) EUT2I
01707 HMM06249EutQ_2_eut2(pf06249) EUT2B EP_NTERM
01711 -pf14487 -
01712 -# -
01713 HMMreg13411_1_GRM1(pf13411pf07739) GRM1A
01714 -pf03235 -
01715 -pf02368 -
01716 -pf02661pf13784 -
01717 -# -
01718 -pf00571pf03448 -
BMC-P
BMC-H
BMC-T
Aldehyde dehydrogenase
PTAC
Signature enzyme
Alcohol dehydrogenase
Regulator

Acetobacterium bakii. Bacteria; Firmicutes; Clostridia; Clostridiales; Eubacteriaceae;

AKG39__locus_1

EUT2B

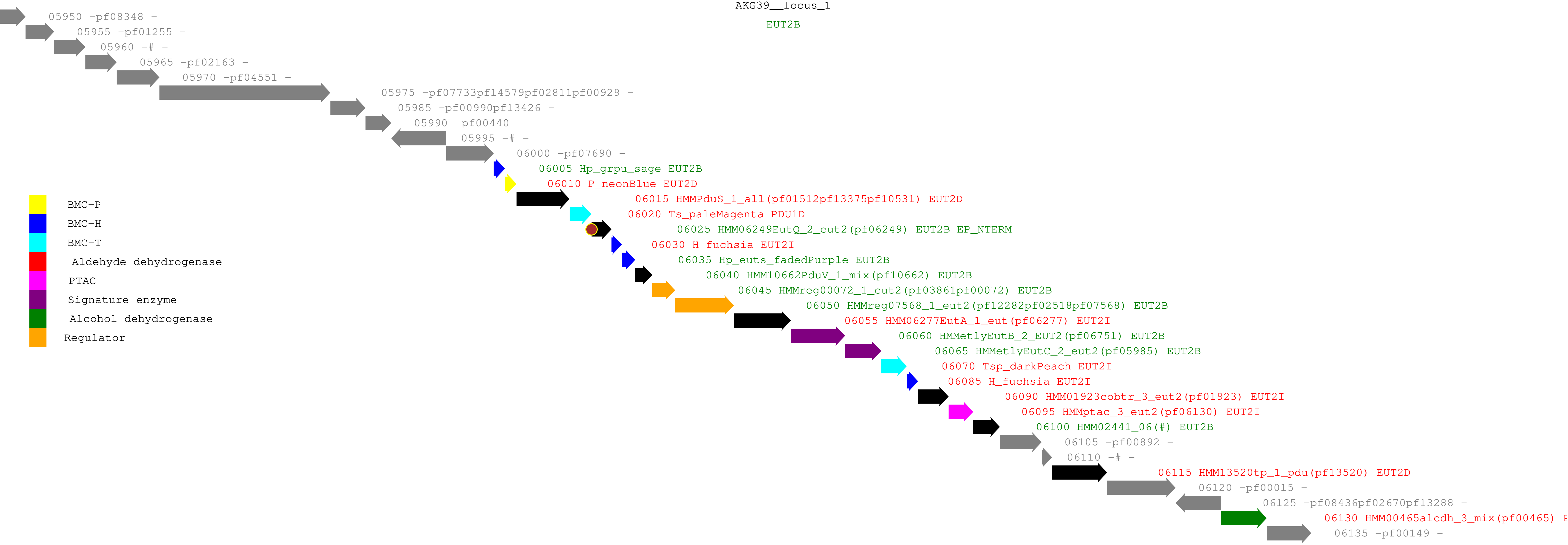

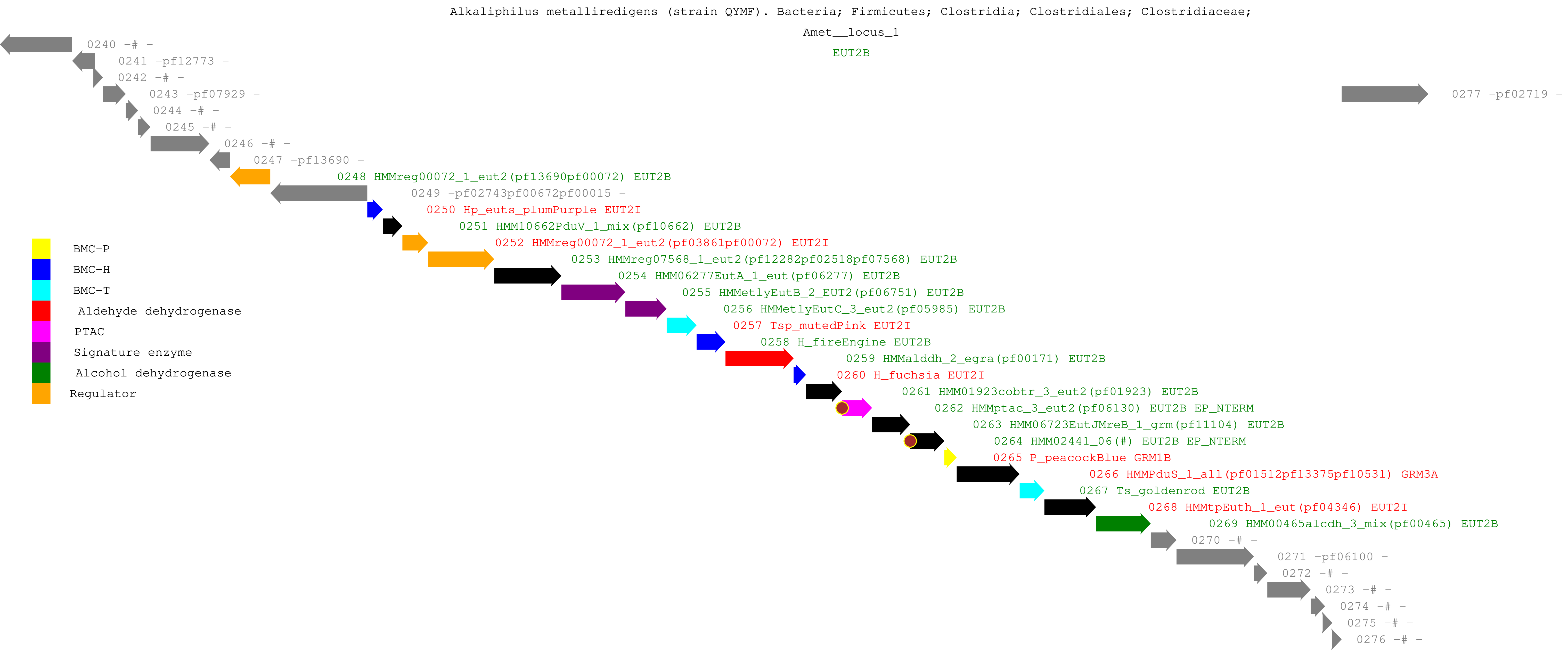

Alkaliphilus metalliredigens (strain QYMF). Bacteria; Firmicutes; Clostridia; Clostridiales; Clostridiaceae;
Amet__locus_1
EUT2B
0240 -# -
0241 -pf12773 -
0242 -# -
0243 -pf07929 -
0244 -# -
0245 -# -
0246 -# -
0247 -pf13690 -
0248 HMMreg00072_1_eut2(pf13690pf00072) EUT2B
0249 -pf02743pf00672pf00015 -
0250 Hp_euts_plumPurple EUT2I
0251 HMM10662PduV_1_mix(pf10662) EUT2B
0252 HMMreg00072_1_eut2(pf03861pf00072) EUT2I
0253 HMMreg07568_1_eut2(pf12282pf02518pf07568) EUT2B
0254 HMM06277EutA_1_eut(pf06277) EUT2B
0255 HMMetlyEutB_2_EUT2(pf06751) EUT2B
0256 HMMetlyEutC_3_eut2(pf05985) EUT2B
0257 Tsp_mutedPink EUT2I
0258 H_fireEngine EUT2B
0259 HMMalddh_2_egra(pf00171) EUT2B
0260 H_fuchsia EUT2I
0261 HMM01923cobtr_3_eut2(pf01923) EUT2B
0262 HMMptac_3_eut2(pf06130) EUT2B EP_NTERM
0263 HMM06723EutJMreB_1_grm(pf11104) EUT2B
0264 HMM02441_06(#) EUT2B EP_NTERM
0265 P_peacockBlue GRM1B
0266 HMMPduS_1_all(pf01512pf13375pf10531) GRM3A
0267 Ts_goldenrod EUT2B
0268 HMMtpEuth_1_eut(pf04346) EUT2I
0269 HMM00465alcdh_3_mix(pf00465) EUT2B
0270 -# -
0271 -pf06100 -
0272 -# -
0273 -# -
0274 -# -
0275 -# -
0276 -# -
0277 -pf02719 -
BMC-P
BMC-H
BMC-T
Aldehyde dehydrogenase
PTAC
Signature enzyme
Alcohol dehydrogenase
Regulator

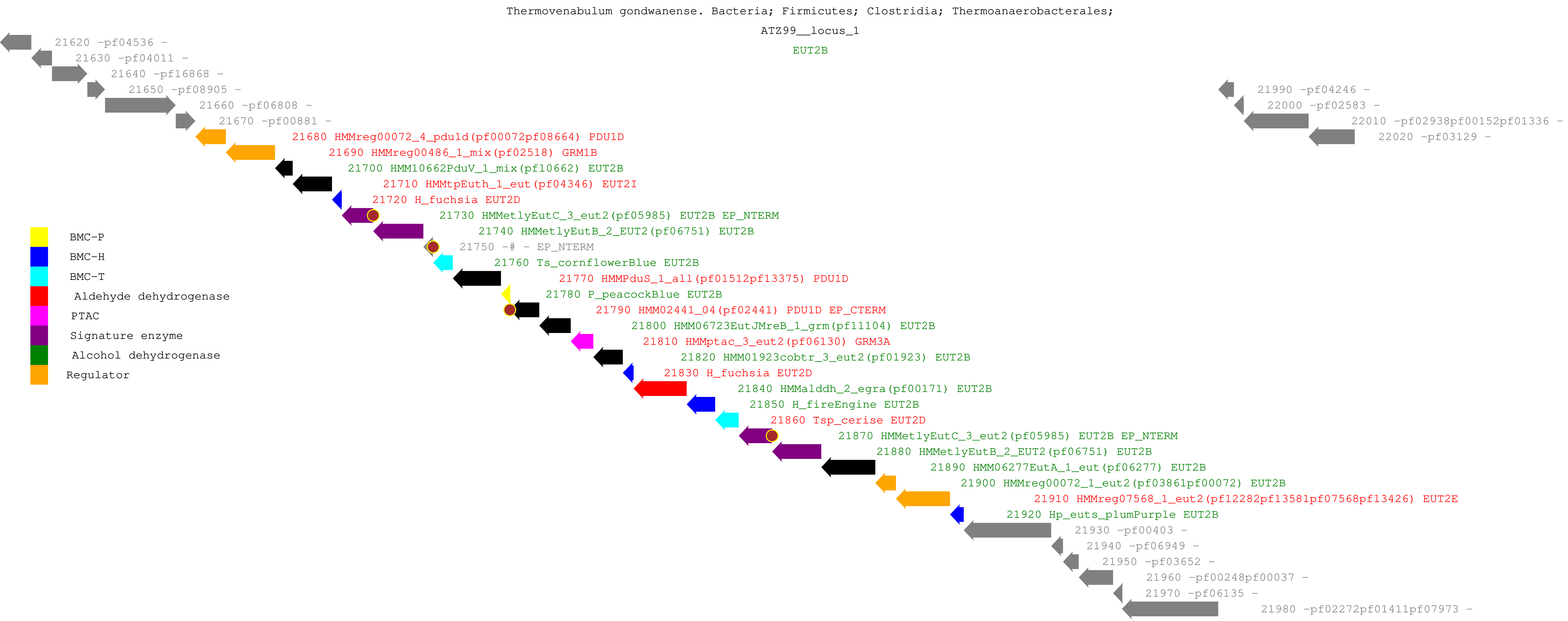

Thermovenabulum gondwanense. Bacteria; Firmicutes; Clostridia; Thermoanaerobacterales;
ATZ99__locus_1
EUT2B
21620 -pf04536 -
21630 -pf04011 -
21640 -pf16868 -
21650 -pf08905 -
21660 -pf06808 -
21670 -pf00881 -
21680 HMMreg00072_4_pdu1d(pf00072pf08664) PDU1D
21690 HMMreg00486_1_mix(pf02518) GRM1B
21700 HMM10662PduV_1_mix(pf10662) EUT2B
21710 HMMtpEuth_1_eut(pf04346) EUT2I
21720 H_fuchsia EUT2D
21730 HMMetlyEutC_3_eut2(pf05985) EUT2B EP_NTERM
21740 HMMetlyEutB_2_EUT2(pf06751) EUT2B
21750 -# - EP_NTERM
21760 Ts_cornflowerBlue EUT2B
21770 HMMPduS_1_all(pf01512pf13375) PDU1D
21780 P_peacockBlue EUT2B
21790 HMM02441_04(pf02441) PDU1D EP_CTERM
21800 HMM06723EutJMreB_1_grm(pf11104) EUT2B
21810 HMMptac_3_eut2(pf06130) GRM3A
21820 HMM01923cobtr_3_eut2(pf01923) EUT2B
21830 H_fuchsia EUT2D
21840 HMMalddh_2_egra(pf00171) EUT2B
21850 H_fireEngine EUT2B
21860 Tsp_cerise EUT2D
21870 HMMetlyEutC_3_eut2(pf05985) EUT2B EP_NTERM
21880 HMMetlyEutB_2_EUT2(pf06751) EUT2B
21890 HMM06277EutA_1_eut(pf06277) EUT2B
21900 HMMreg00072_1_eut2(pf03861pf00072) EUT2B
21910 HMMreg07568_1_eut2(pf12282pf13581pf07568pf13426) EUT2E
21920 Hp_euts_plumPurple EUT2B
21930 -pf00403 -
21940 -pf06949 -
21950 -pf03652 -
21960 -pf00248pf00037 -
21970 -pf06135 -
21980 -pf02272pf01411pf07973 -
21990 -pf04246 -
22000 -pf02583 -
22010 -pf02938pf00152pf01336 -
22020 -pf03129 -
BMC-P
BMC-H
BMC-T
Aldehyde dehydrogenase
PTAC
Signature enzyme
Alcohol dehydrogenase
Regulator

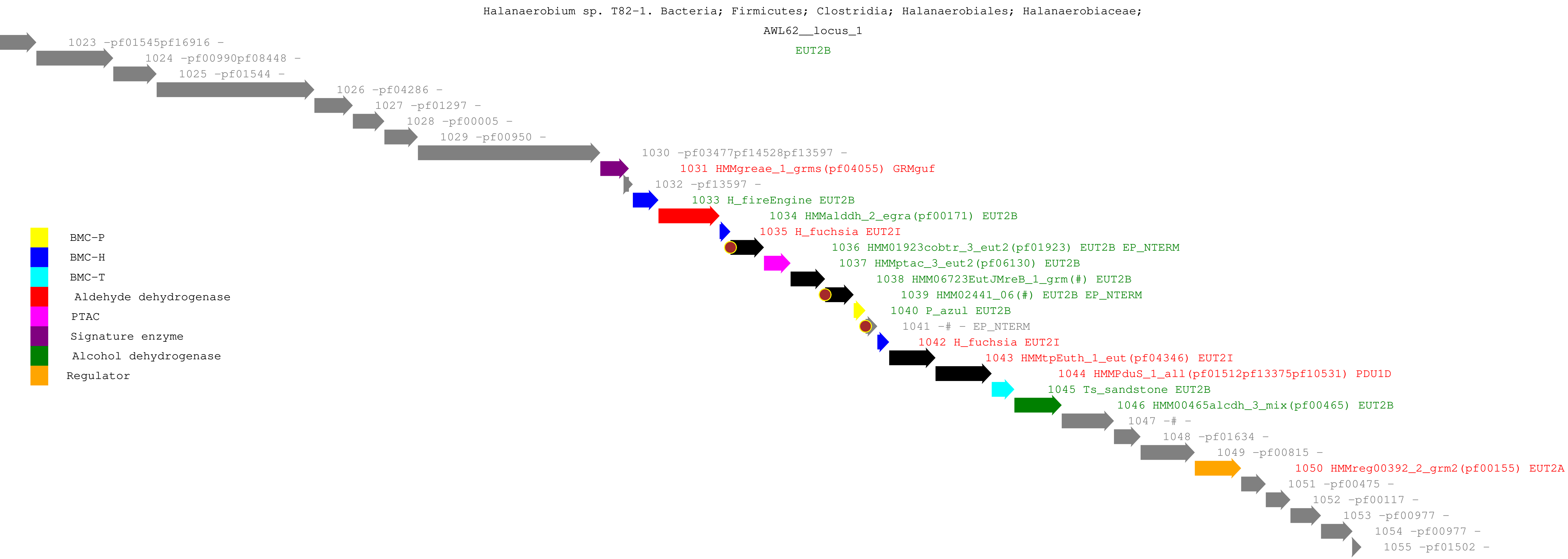
Halanaerobium sp. T82-1. Bacteria; Firmicutes; Clostridia; Halanaerobiales; Halanaerobiaceae;
AWL62__locus_1
EUT2B
1023 -pf01545pf16916 -
1024 -pf00990pf08448 -
1025 -pf01544 -
1026 -pf04286 -
1027 -pf01297 -
1028 -pf00005 -
1029 -pf00950 -
1030 -pf03477pf14528pf13597 -
1031 HMMgreae_1_grms(pf04055) GRMguf
1032 -pf13597 -
1033 H_fireEngine EUT2B
1034 HMMalddh_2_egra(pf00171) EUT2B
1035 H_fuchsia EUT2I
1036 HMM01923cobtr_3_eut2(pf01923) EUT2B EP_NTERM
1037 HMMptac_3_eut2(pf06130) EUT2B
1038 HMM06723EutJMreB_1_grm(#) EUT2B
1039 HMM02441_06(#) EUT2B EP_NTERM
1040 P_azul EUT2B
1041 -# - EP_NTERM
1042 H_fuchsia EUT2I
1043 HMMtpEuth_1_eut(pf04346) EUT2I
1044 HMMPduS_1_all(pf01512pf13375pf10531) PDU1D
1045 Ts_sandstone EUT2B
1046 HMM00465alcdh_3_mix(pf00465) EUT2B
1047 -# -
1048 -pf01634 -
1049 -pf00815 -
1050 HMMreg00392_2_grm2(pf00155) EUT2A
1051 -pf00475 -
1052 -pf00117 -
1053 -pf00977 -
1054 -pf00977 -
1055 -pf01502 -
BMC-P
BMC-H
BMC-T
Aldehyde dehydrogenase
PTAC
Signature enzyme
Alcohol dehydrogenase
Regulator

Eubacterium minutum ATCC 700079. Bacteria; Firmicutes; Clostridia; Clostridiales;

AXF21__locus_1

EUT2B

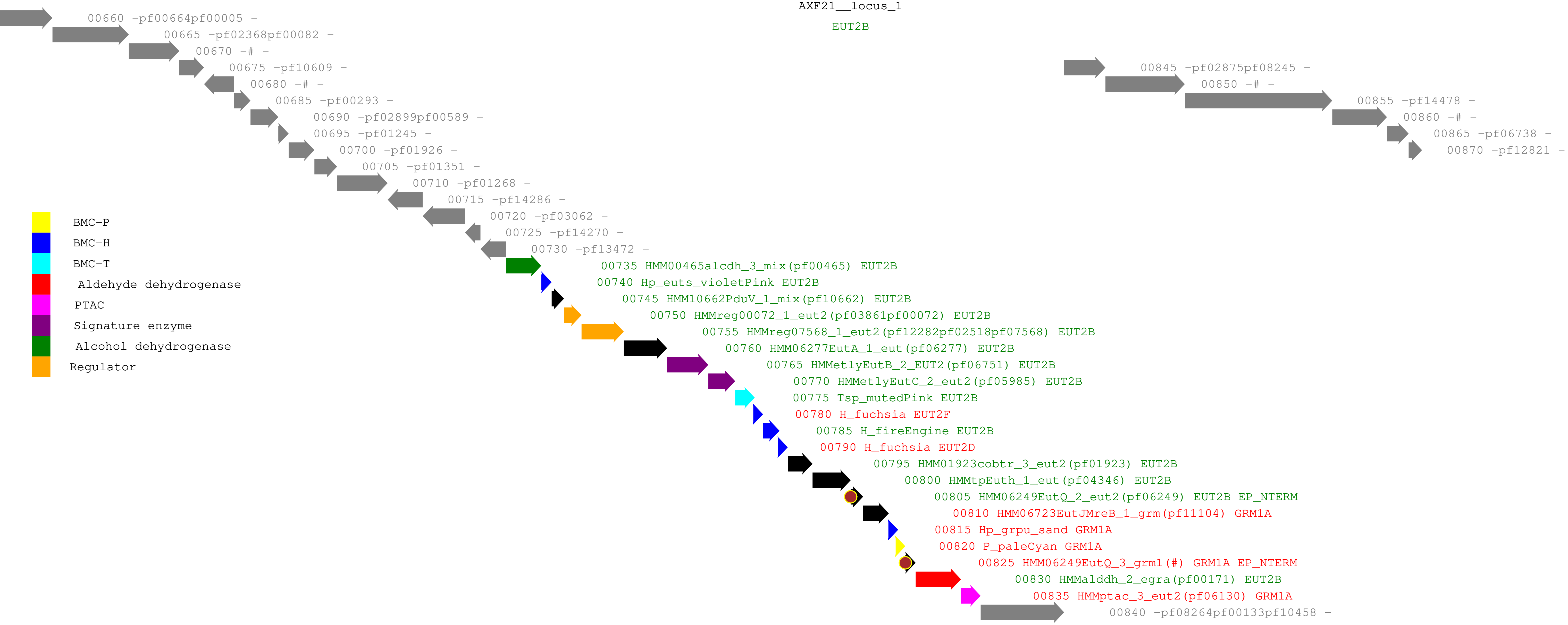

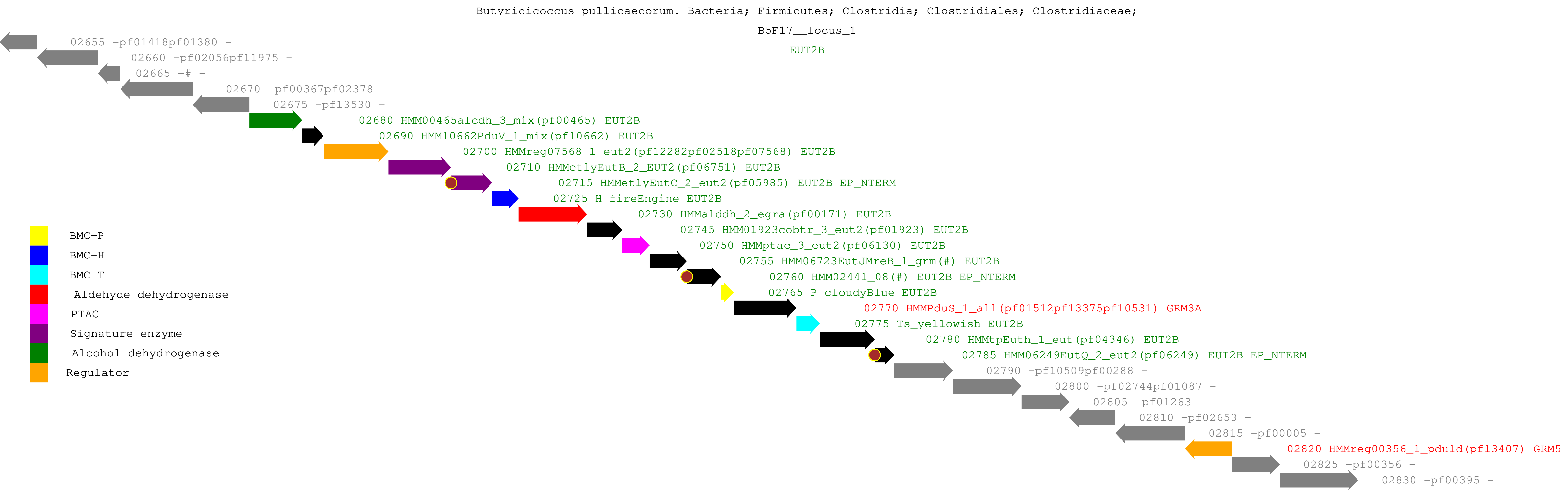

Butyricicoccus pullicaecorum. Bacteria; Firmicutes; Clostridia; Clostridiales; Clostridiaceae;
B5F17__locus_1
EUT2B
02655 -pf01418pf01380 -
02660 -pf02056pf11975 -
02665 -# -
02670 -pf00367pf02378 -
02675 -pf13530 -
02680 HMM00465alcdh_3_mix(pf00465) EUT2B
02690 HMM10662PduV_1_mix(pf10662) EUT2B
02700 HMMreg07568_1_eut2(pf12282pf02518pf07568) EUT2B
02710 HMMetlyEutB_2_EUT2(pf06751) EUT2B
02715 HMMetlyEutC_2_eut2(pf05985) EUT2B EP_NTERM
02725 H_fireEngine EUT2B
02730 HMMaldh_2_egra(pf00171) EUT2B
02745 HMM01923cobtr_3_eut2(pf01923) EUT2B
02750 HMMptac_3_eut2(pf06130) EUT2B
02755 HMM06723EutJMreB_1_grm(#) EUT2B
02760 HMM02441_08(#) EUT2B EP_NTERM
02765 P_cloudyBlue EUT2B
02770 HMMPduS_1_all(pf01512pf13375pf10531) GRM3A
02775 Ts_yellowish EUT2B
02780 HMMtpEuth_1_eut(pf04346) EUT2B
02785 HMM06249EutQ_2_eut2(pf06249) EUT2B EP_NTERM
02790 -pf10509pf00288 -
02800 -pf02744pf01087 -
02805 -pf01263 -
02810 -pf02653 -
02815 -pf00005 -
02820 HMMreg00356_1_pdu1d(pf13407) GRM5
02825 -pf00356 -
02830 -pf00395 -
BMC-P
BMC-H
BMC-T
Aldehyde dehydrogenase
PTAC
Signature enzyme
Alcohol dehydrogenase
Regulator

Lachnoclostridium sp. An196. Bacteria; Firmicutes; Clostridia; Clostridiales; Lachnospiraceae.

B5F29__locus_1_main_sat

EUT2B

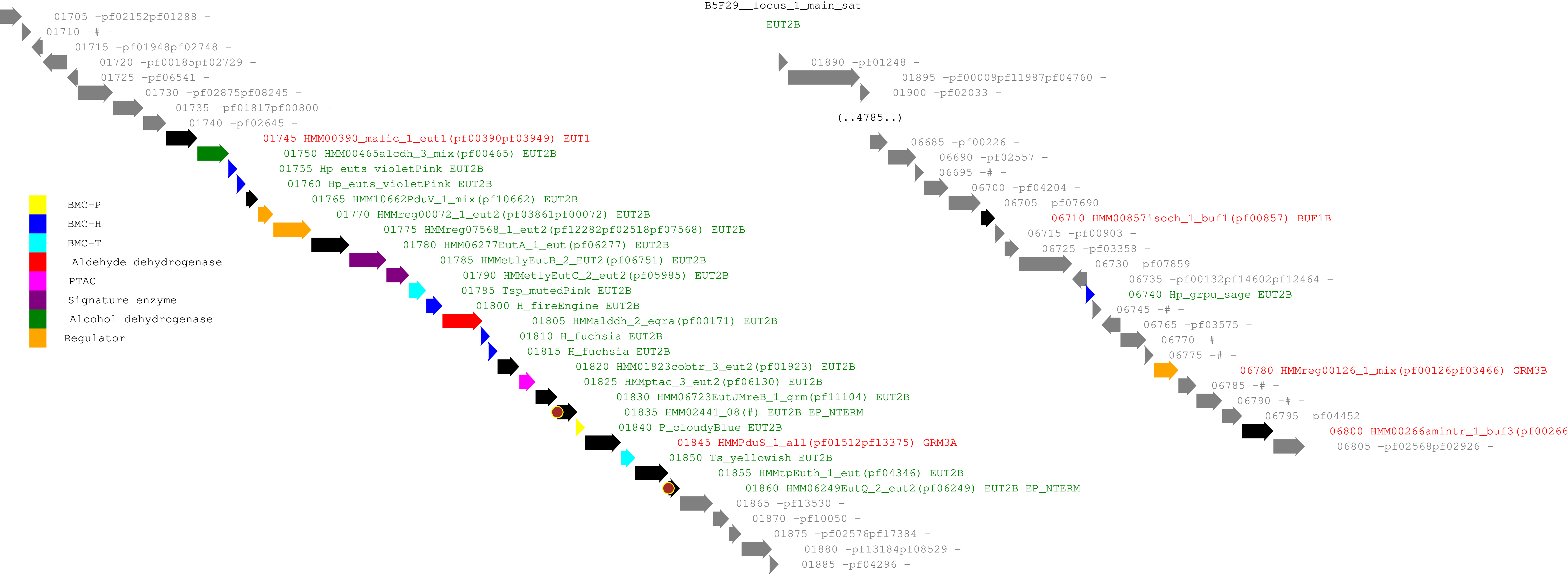

Flavonifractor plautii. Bacteria; Firmicutes; Clostridia; Clostridiales; Flavonifractor.

B5F52__locus_3

EUT2B

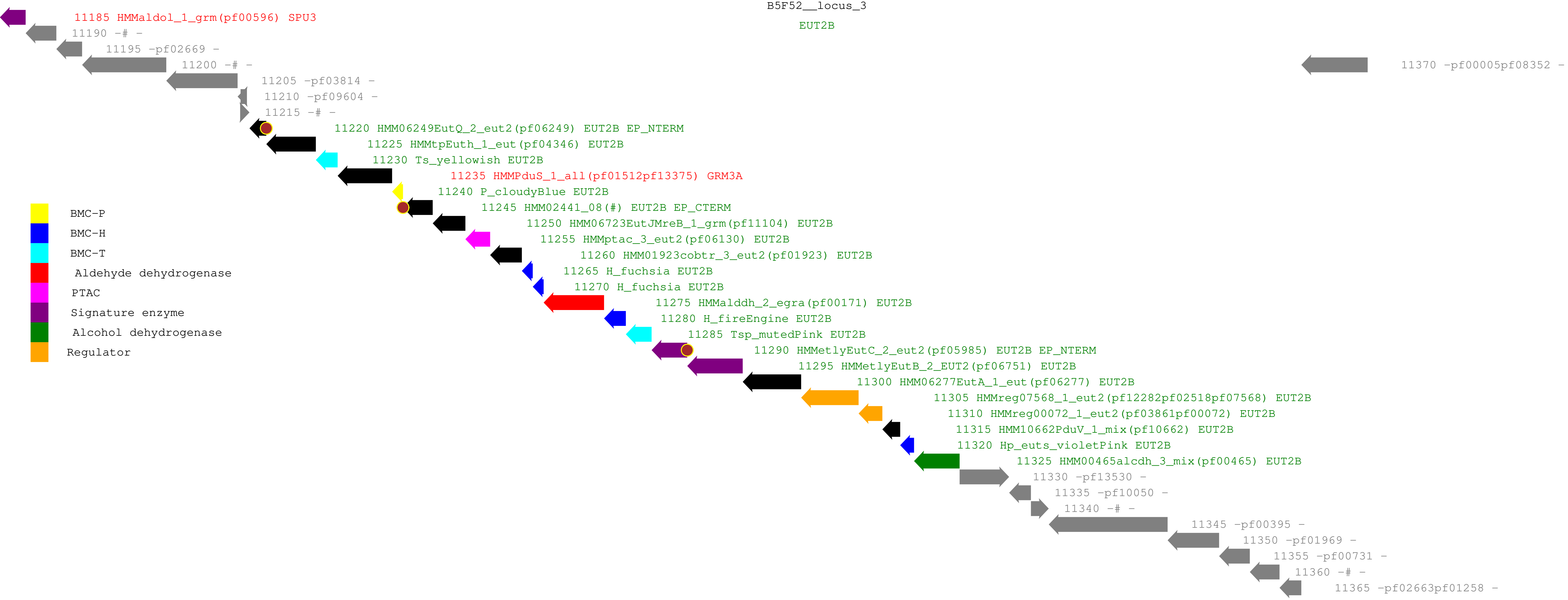

Bacteroidetes bacterium 4572_77. Bacteria; Bacteroidetes.

B7C24__locus_1

EUT2B

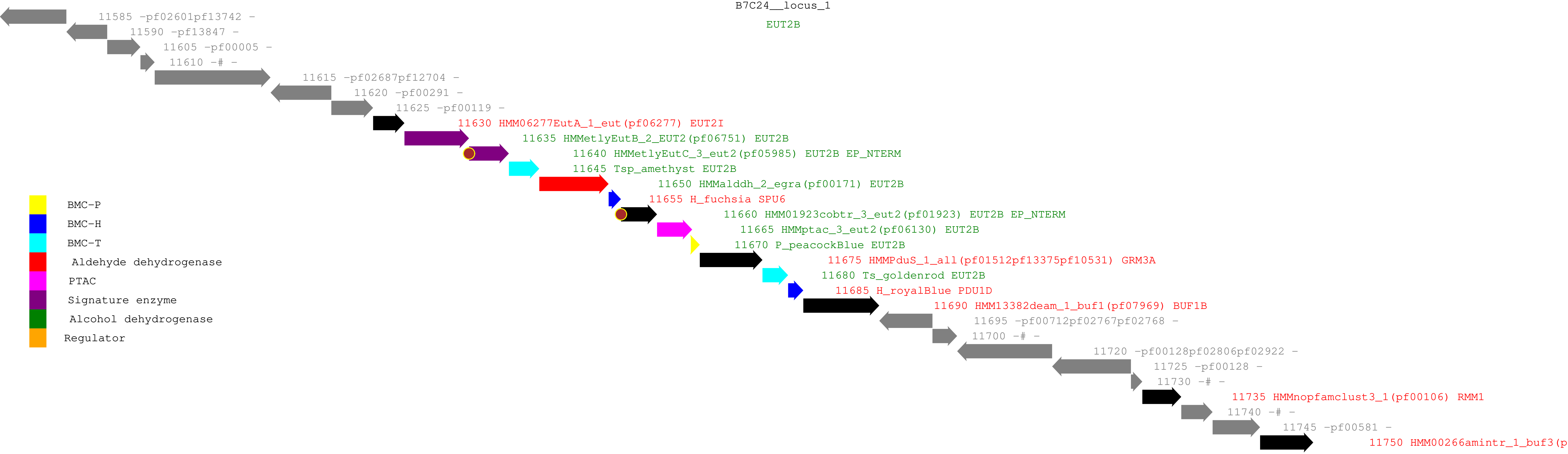

Pseudoflavonifractor capillosus ATCC 29799. Bacteria; Firmicutes; Clostridia; Clostridiales; Pseudoflavonifractor.

BACCAP__locus_2

EUT2B

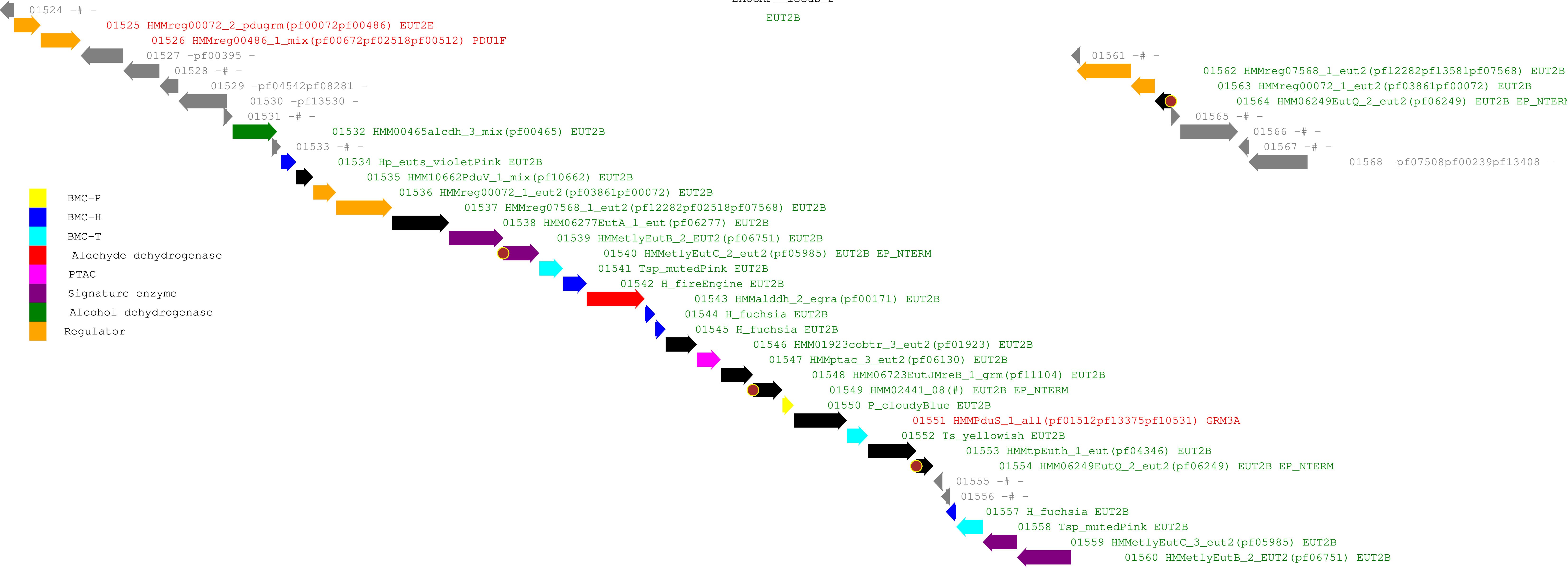

Cloacibacillus porcorum. Bacteria; Synergistetes; Synergistia; Synergistales; Synergistaceae;

BED41__locus_1

EUT2B

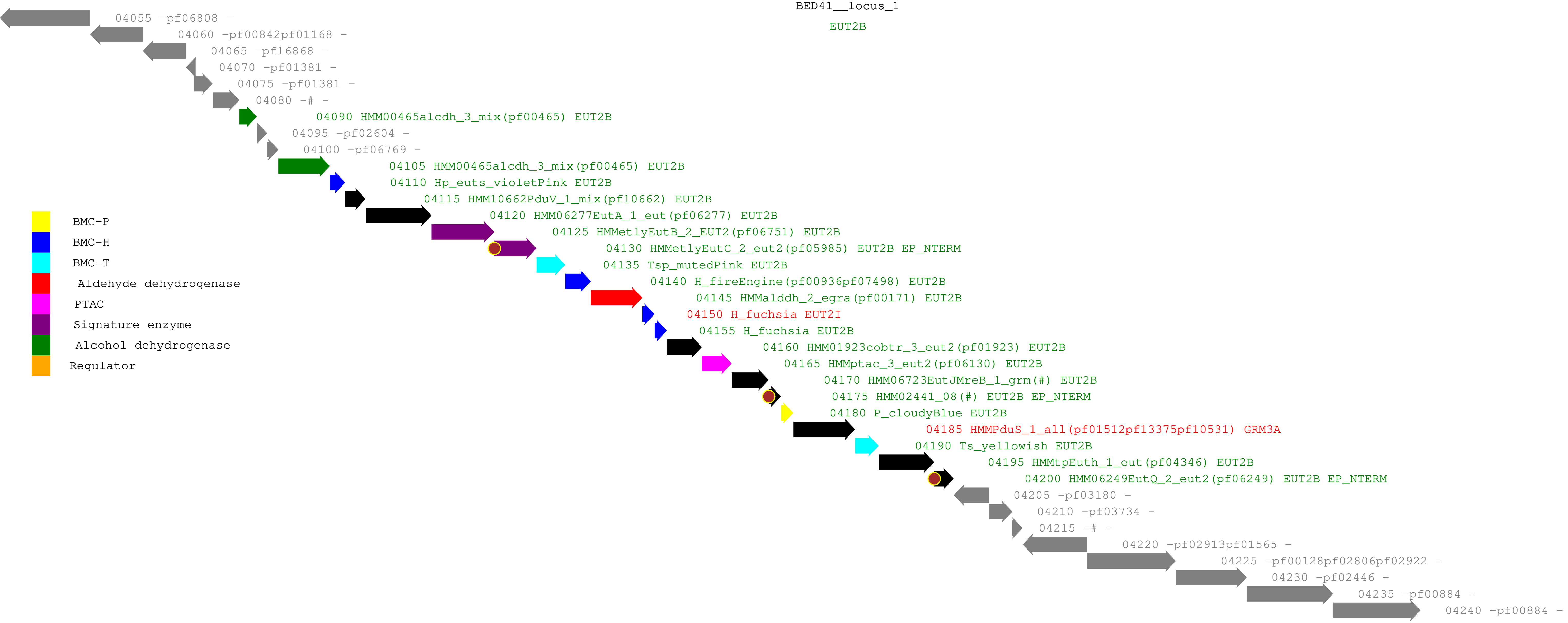

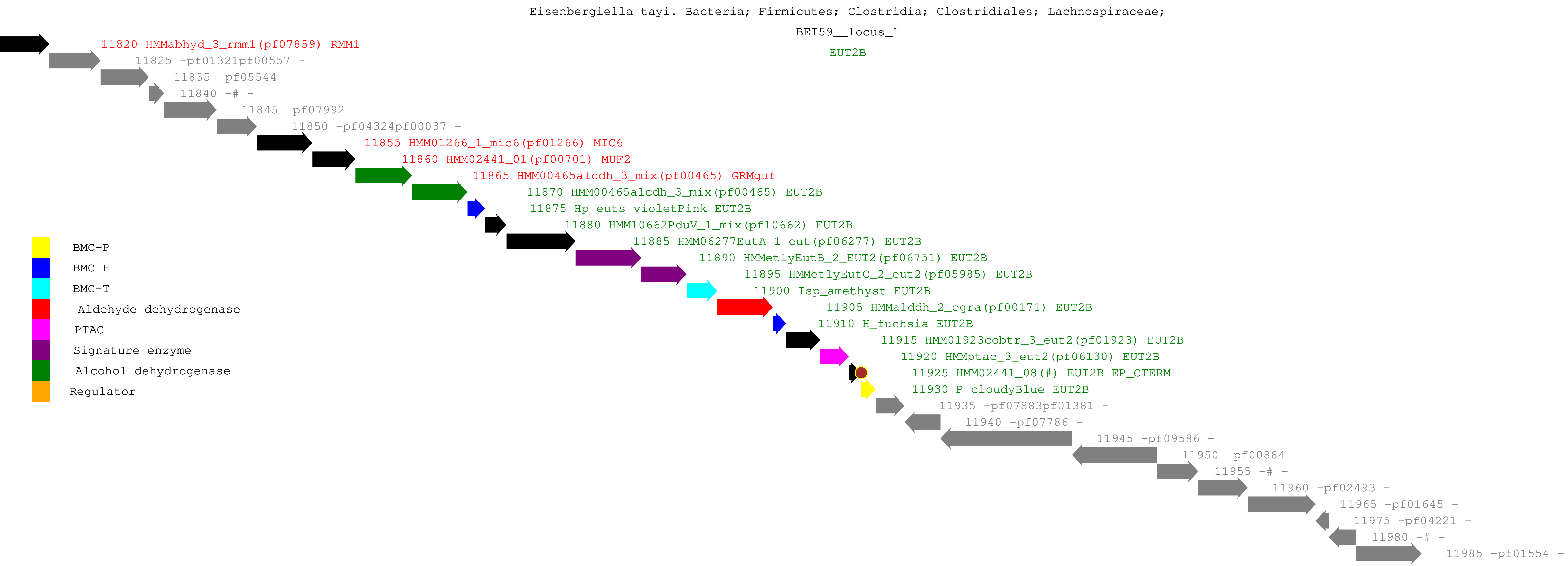
Eisenbergiella tayi. Bacteria; Firmicutes; Clostridia; Clostridiales; Lachnospiraceae;
BEI59__locus_1
EUT2B
11820 HMMabhyd_3_rmm1(pf07859) RMM1
11825 -pf01321pf00557 -
11835 -pf05544 -
11840 -# -
11845 -pf07992 -
11850 -pf04324pf00037 -
11855 HMM01266_1_mic6(pf01266) MIC6
11860 HMM02441_01(pf00701) MUF2
11865 HMM00465alcdh_3_mix(pf00465) GRMguf
11870 HMM00465alcdh_3_mix(pf00465) EUT2B
11875 Hp_euts_violetPink EUT2B
11880 HMM10662PduV_1_mix(pf10662) EUT2B
11885 HMM06277EutA_1_eut(pf06277) EUT2B
11890 HMMetlyEutB_2_EUT2(pf06751) EUT2B
11895 HMMetlyEutC_2_eut2(pf05985) EUT2B
11900 Tsp_amethyst EUT2B
11905 HMMalddh_2_egra(pf00171) EUT2B
11910 H_fuchsia EUT2B
11915 HMM01923cobtr_3_eut2(pf01923) EUT2B
11920 HMMptac_3_eut2(pf06130) EUT2B
11925 HMM02441_08(#) EUT2B EP_CTERM
11930 P_cloudyBlue EUT2B
11935 -pf07883pf01381 -
11940 -pf07786 -
11945 -pf09586 -
11950 -pf00884 -
11955 -# -
11960 -pf02493 -
11965 -pf01645 -
11975 -pf04221 -
11980 -# -
11985 -pf01554 -
BMC-P
BMC-H
BMC-T
Aldehyde dehydrogenase
PTAC
Signature enzyme
Alcohol dehydrogenase
Regulator

Halanaerobium sp. MDAL1. Bacteria; Firmicutes; Clostridia; Halanaerobiales; Halanaerobiaceae;

BHK79__locus_1

EUT2B

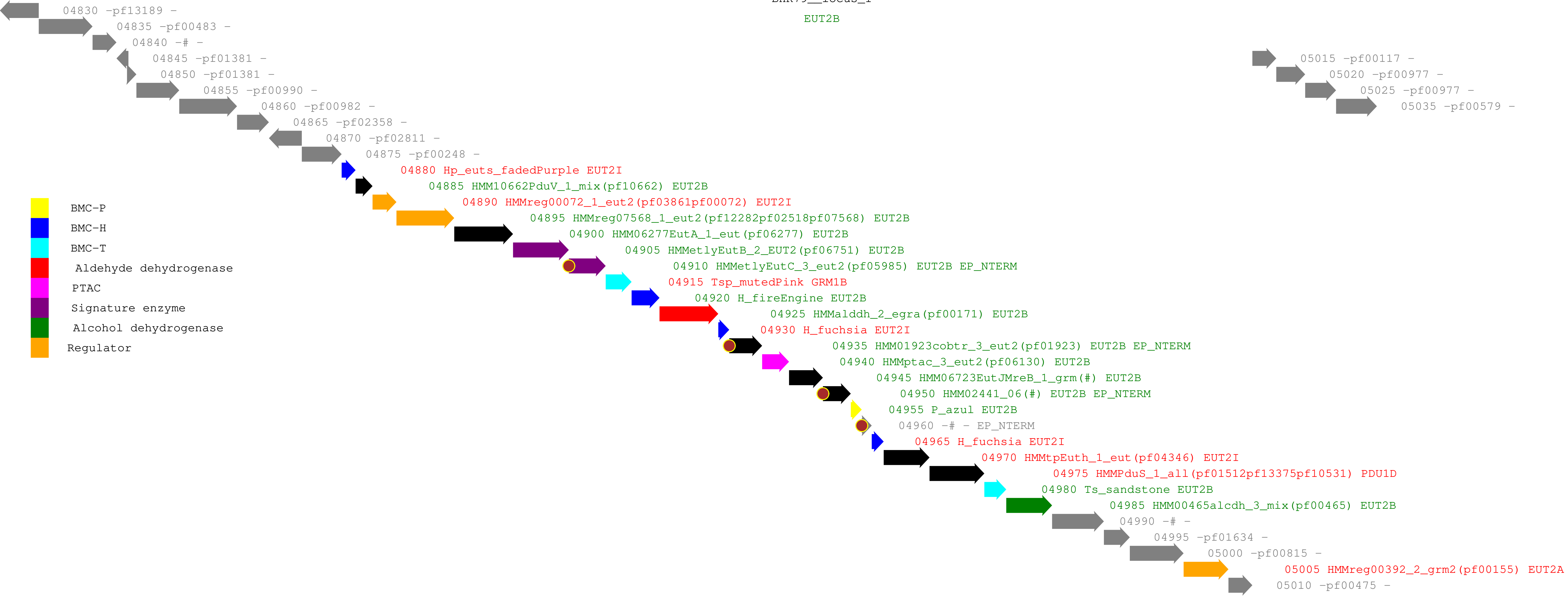

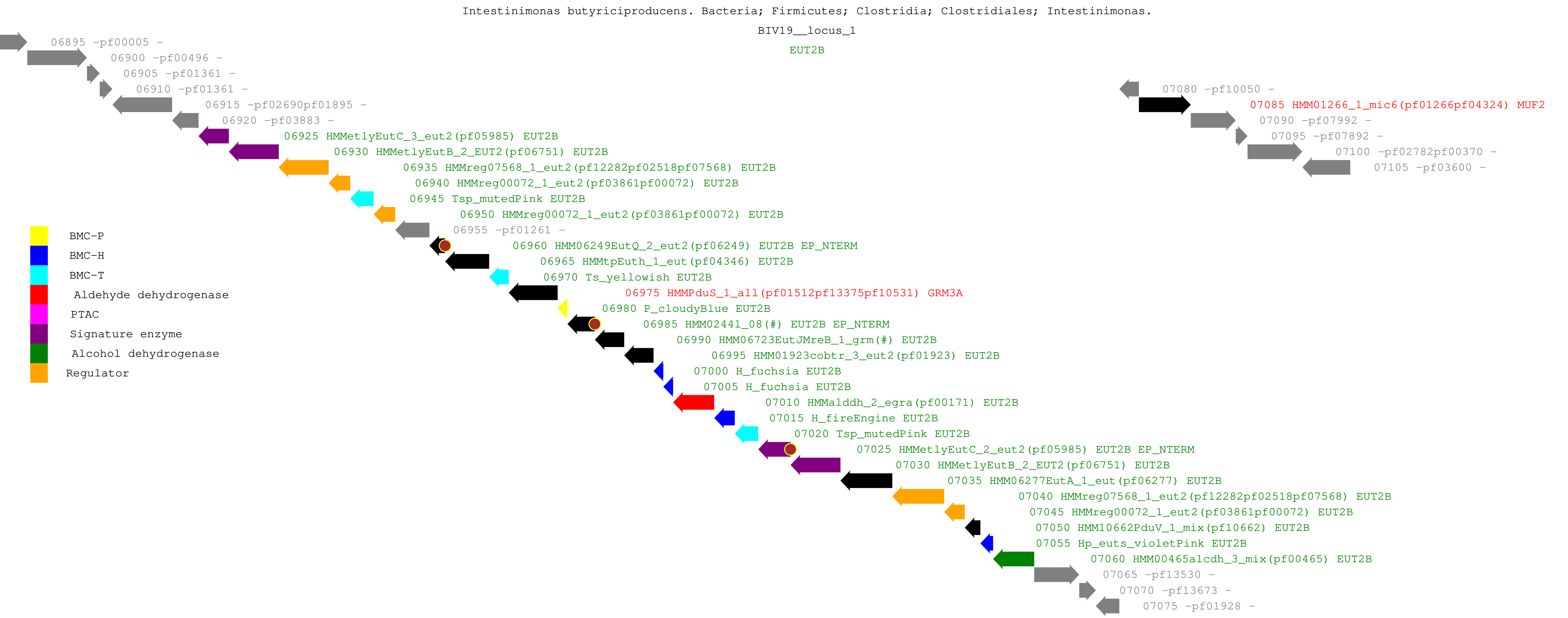
Intestinimonas butyriciproducens. Bacteria; Firmicutes; Clostridia; Clostridiales; Intestinimonas.
BIV19__locus_1
EUT2B
06895 -pf00005 -
06900 -pf00496 -
06905 -pf01361 -
06910 -pf01361 -
06915 -pf02690pf01895 -
06920 -pf03883 -
06925 HMMetlyEutC_3_eut2(pf05985) EUT2B
06930 HMMetlyEutB_2_EUT2(pf06751) EUT2B
06935 HMMreg07568_1_eut2(pf12282pf02518pf07568) EUT2B
06940 HMMreg00072_1_eut2(pf03861pf00072) EUT2B
06945 Tsp_mutedPink EUT2B
06950 HMMreg00072_1_eut2(pf03861pf00072) EUT2B
06955 -pf01261 -
06960 HMM06249EutQ_2_eut2(pf06249) EUT2B EP_NTERM
06965 HMMtpEuth_1_eut(pf04346) EUT2B
06970 Ts_yellowish EUT2B
06975 HMMPduS_1_all(pf01512pf13375pf10531) GRM3A
06980 P_cloudyBlue EUT2B
06985 HMM02441_08(#) EUT2B EP_NTERM
06990 HMM06723EutJMreB_1_grm(#) EUT2B
06995 HMM01923cobtr_3_eut2(pf01923) EUT2B
07000 H_fuchsia EUT2B
07005 H_fuchsia EUT2B
07010 HMMalddh_2_egra(pf00171) EUT2B
07015 H_fireEngine EUT2B
07020 Tsp_mutedPink EUT2B
07025 HMMetlyEutC_2_eut2(pf05985) EUT2B EP_NTERM
07030 HMMetlyEutB_2_EUT2(pf06751) EUT2B
07035 HMM06277EutA_1_eut(pf06277) EUT2B
07040 HMMreg07568_1_eut2(pf12282pf02518pf07568) EUT2B
07045 HMMreg00072_1_eut2(pf03861pf00072) EUT2B
07050 HMM10662PduV_1_mix(pf10662) EUT2B
07055 Hp_euts_violetPink EUT2B
07060 HMM00465alcdh_3_mix(pf00465) EUT2B
07065 -pf13530 -
07070 -pf13673 -
07075 -pf01928 -
07080 -pf10050 -
07085 HMM01266_1_mic6(pf01266pf04324) MUF2
07090 -pf07992 -
07095 -pf07892 -
07100 -pf02782pf00370 -
07105 -pf03600 -
BMC-P
BMC-H
BMC-T
Aldehyde dehydrogenase
PTAC
Signature enzyme
Alcohol dehydrogenase
Regulator

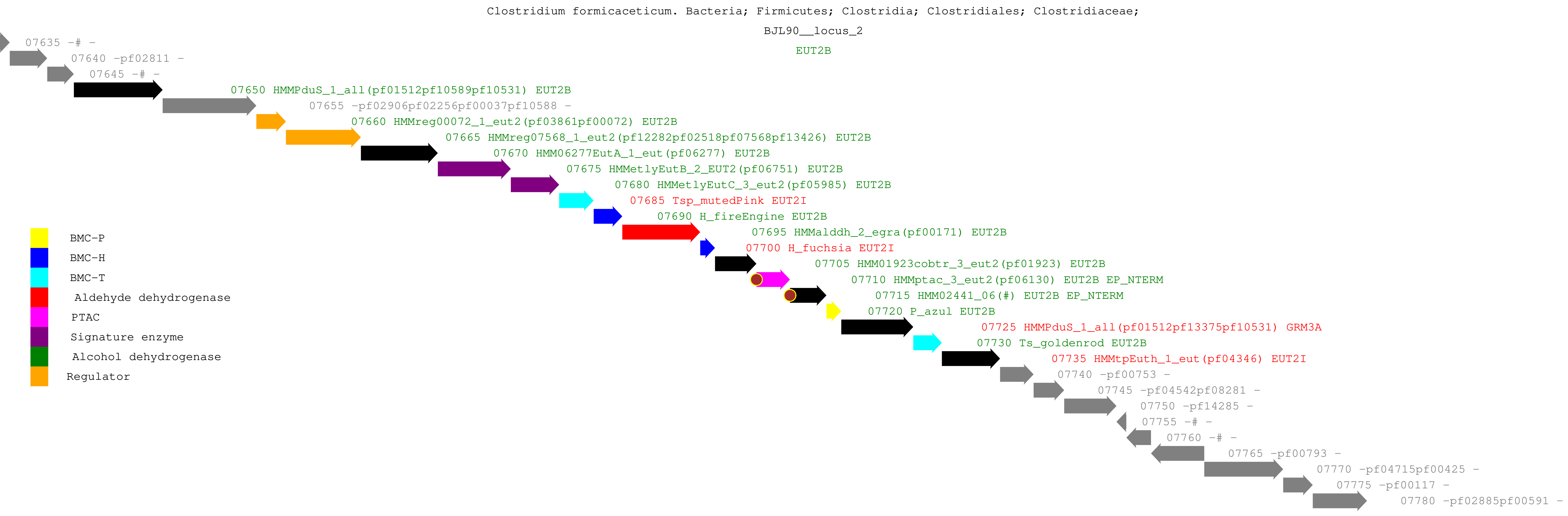

Clostridium formicaceticum. Bacteria; Firmicutes; Clostridia; Clostridiales; Clostridiaceae;
BJL90__locus_2
EUT2B
07635 -# -
07640 -pf02811 -
07645 -# -
07650 HMMPduS_1_all(pf01512pf10589pf10531) EUT2B
07655 -pf02906pf02256pf00037pf10588 -
07660 HMMreg00072_1_eut2(pf03861pf00072) EUT2B
07665 HMMreg07568_1_eut2(pf12282pf02518pf07568pf13426) EUT2B
07670 HMM06277EutA_1_eut(pf06277) EUT2B
07675 HMMetlyEutB_2_EUT2(pf06751) EUT2B
07680 HMMetlyEutC_3_eut2(pf05985) EUT2B
07685 Tsp_mutedPink EUT2I
07690 H_fireEngine EUT2B
07695 HMMalddh_2_egra(pf00171) EUT2B
07700 H_fuchsia EUT2I
07705 HMM01923cobtr_3_eut2(pf01923) EUT2B
07710 HMMptac_3_eut2(pf06130) EUT2B EP_NTERM
07715 HMM02441_06(#) EUT2B EP_NTERM
07720 P_azul EUT2B
07725 HMMPduS_1_all(pf01512pf13375pf10531) GRM3A
07730 Ts_goldenrod EUT2B
07735 HMMtpEuth_1_eut(pf04346) EUT2I
07740 -pf00753 -
07745 -pf04542pf08281 -
07750 -pf14285 -
07755 -# -
07760 -# -
07765 -pf00793 -
07770 -pf04715pf00425 -
07775 -pf00117 -
07780 -pf02885pf00591 -
BMC-P
BMC-H
BMC-T
Aldehyde dehydrogenase
PTAC
Signature enzyme
Alcohol dehydrogenase
Regulator

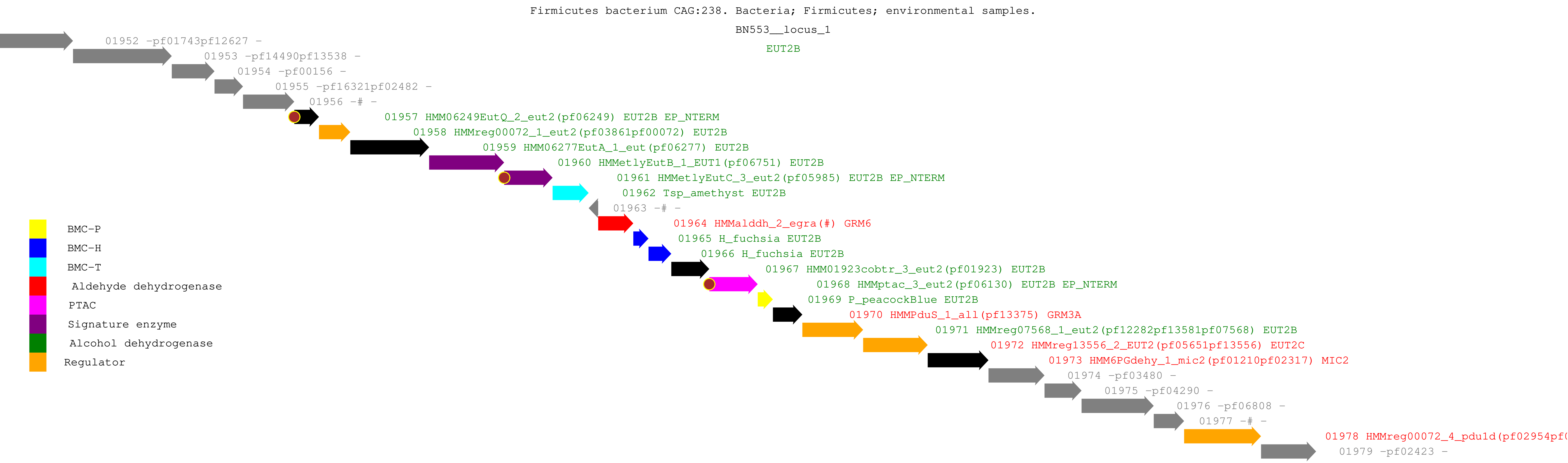

Firmicutes bacterium CAG:238. Bacteria; Firmicutes; environmental samples.
BN553__locus_1
EUT2B
01952 -pf01743pf12627 -
01953 -pf14490pf13538 -
01954 -pf00156 -
01955 -pf16321pf02482 -
01956 -# -
01957 HMM06249EutQ_2_eut2(pf06249) EUT2B EP_NTERM
01958 HMMreg00072_1_eut2(pf03861pf00072) EUT2B
01959 HMM06277EutA_1_eut(pf06277) EUT2B
01960 HMMetlyEutB_1_EUT1(pf06751) EUT2B
01961 HMMetlyEutC_3_eut2(pf05985) EUT2B EP_NTERM
01962 Tsp_amethyst EUT2B
01963 -# -
01964 HMMalddh_2_egra(#) GRM6
01965 H_fuchsia EUT2B
01966 H_fuchsia EUT2B
01967 HMM01923cobtr_3_eut2(pf01923) EUT2B
01968 HMMptac_3_eut2(pf06130) EUT2B EP_NTERM
01969 P_peacockBlue EUT2B
01970 HMMPduS_1_all(pf13375) GRM3A
01971 HMMreg07568_1_eut2(pf12282pf13581pf07568) EUT2B
01972 HMMreg13556_2_EUT2(pf05651pf13556) EUT2C
01973 HMM6PGdehy_1_mic2(pf01210pf02317) MIC2
01974 -pf03480 -
01975 -pf04290 -
01976 -pf06808 -
01977 -# -
01978 HMMreg00072_4_pdu1d(pf02954pf0
01979 -pf02423 -
BMC-P
BMC-H
BMC-T
Aldehyde dehydrogenase
PTAC
Signature enzyme
Alcohol dehydrogenase
Regulator

Marvinbryantia formatexigens DSM 14469. Bacteria; Firmicutes; Clostridia; Clostridiales; Lachnospiraceae;

BRYFOR__locus_1

EUT2B

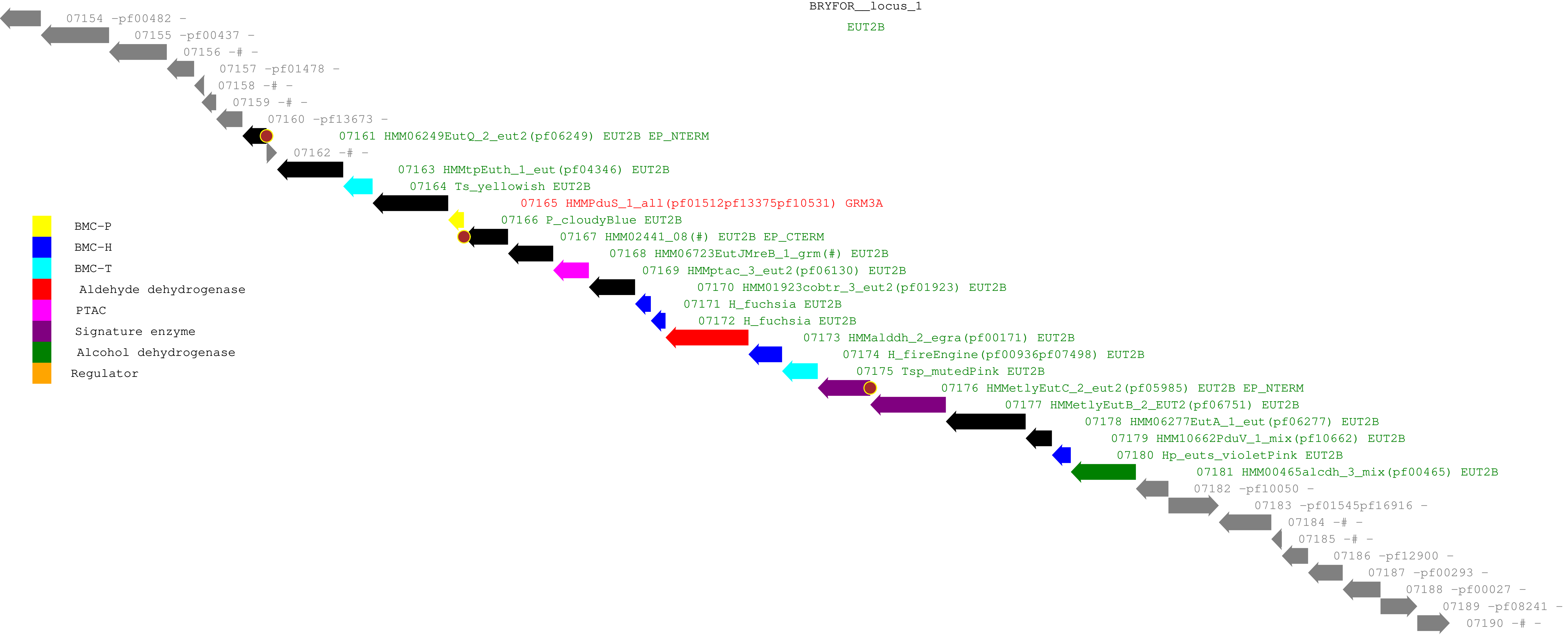

Firmicutes bacterium ADurb.Bin182. Bacteria; Firmicutes.

BWY11__locus_1

EUT2B

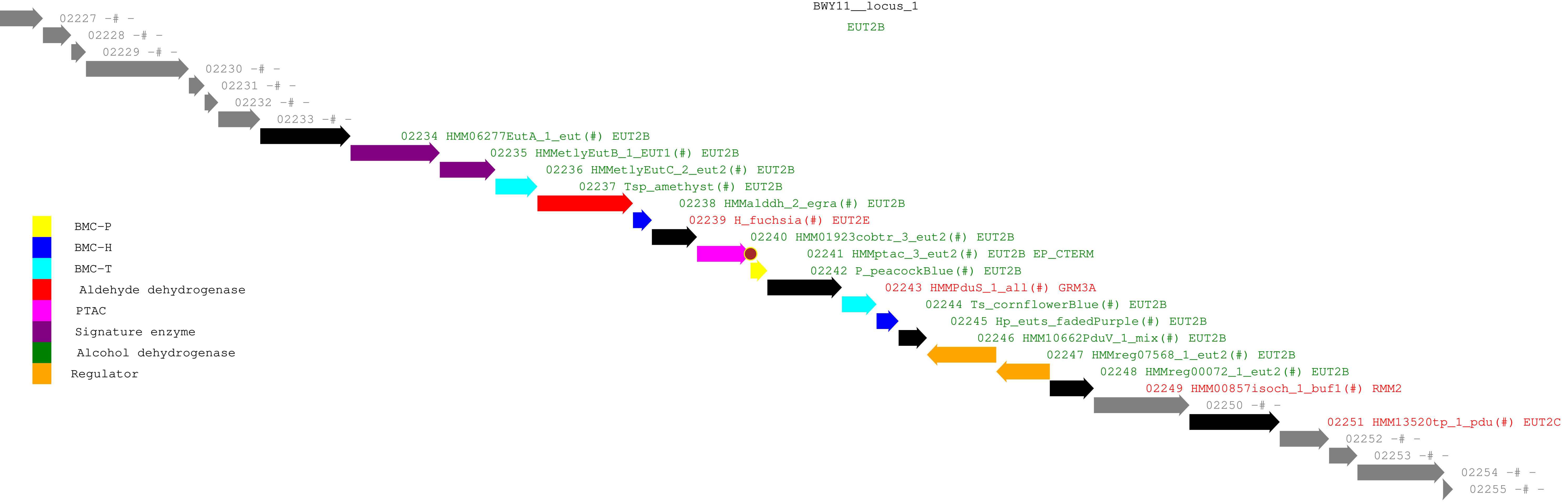

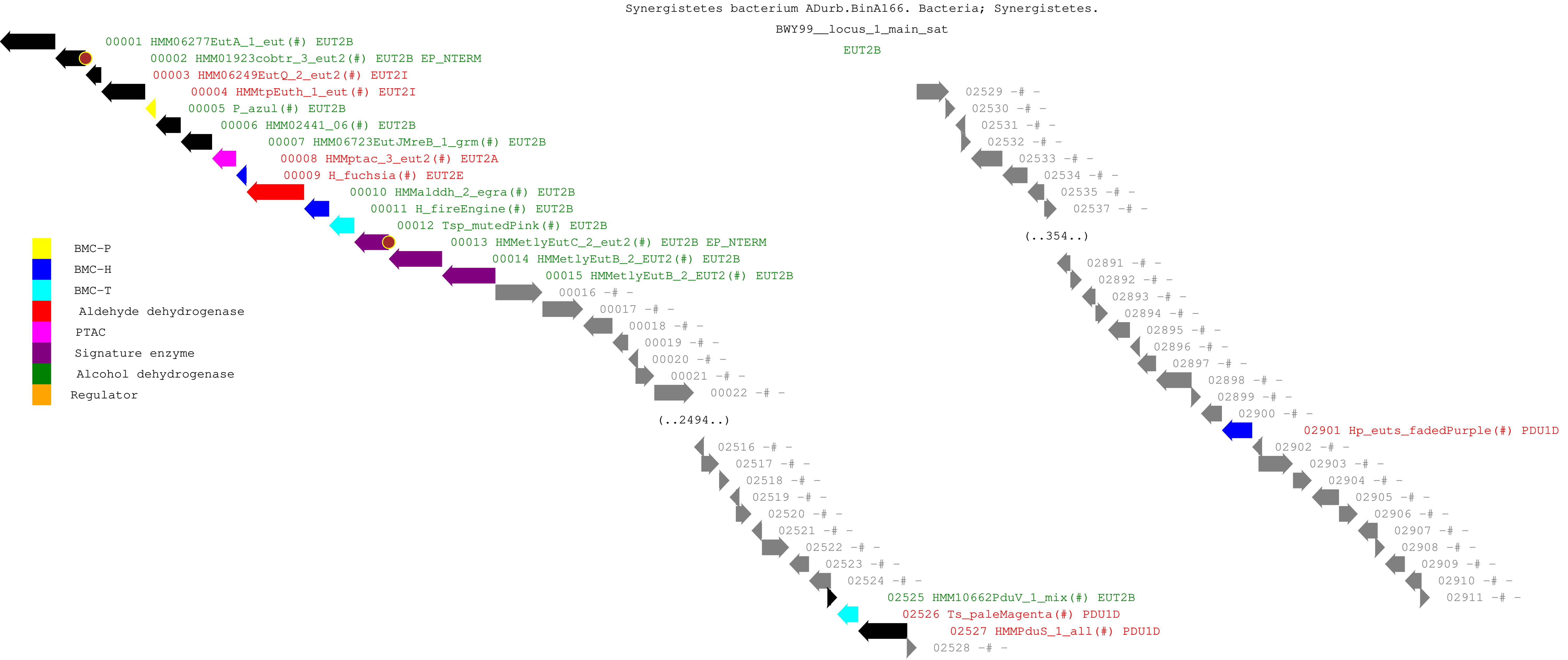

Synergistetes bacterium ADurb.BinA166. Bacteria; Synergistetes.
BWY99__locus_1_main_sat
EUT2B
00001 HMM06277EutA_1_eut(#) EUT2B
00002 HMM01923cobtr_3_eut2(#) EUT2B EP_NTERM
00003 HMM06249EutQ_2_eut2(#) EUT2I
00004 HMMtpEuth_1_eut(#) EUT2I
00005 P_azul(#) EUT2B
00006 HMM02441_06(#) EUT2B
00007 HMM06723EutJMreB_1_grm(#) EUT2B
00008 HMMptac_3_eut2(#) EUT2A
00009 H_fuchsia(#) EUT2E
00010 HMMaldh_2_egra(#) EUT2B
00011 H_fireEngine(#) EUT2B
00012 Tsp_mutedPink(#) EUT2B
00013 HMMetlyEutC_2_eut2(#) EUT2B EP_NTERM
00014 HMMetlyEutB_2_EUT2(#) EUT2B
00015 HMMetlyEutB_2_EUT2(#) EUT2B
00016 -# -
00017 -# -
00018 -# -
00019 -# -
00020 -# -
00021 -# -
00022 -# -
(..2494..)
02516 -# -
02517 -# -
02518 -# -
02519 -# -
02520 -# -
02521 -# -
02522 -# -
02523 -# -
02524 -# -
02525 HMM10662PduV_1_mix(#) EUT2B
02526 Ts_paleMagenta(#) PDU1D
02527 HMMPduS_1_all(#) PDU1D
02528 -# -
02529 -# -
02530 -# -
02531 -# -
02532 -# -
02533 -# -
02534 -# -
02535 -# -
02537 -# -
(..354..)
02891 -# -
02892 -# -
02893 -# -
02894 -# -
02895 -# -
02896 -# -
02897 -# -
02898 -# -
02899 -# -
02900 -# -
02901 Hp_euts_fadedPurple(#) PDU1D
02902 -# -
02903 -# -
02904 -# -
02905 -# -
02906 -# -
02907 -# -
02908 -# -
02909 -# -
02910 -# -
02911 -# -
BMC-P
BMC-H
BMC-T
Aldehyde dehydrogenase
PTAC
Signature enzyme
Alcohol dehydrogenase
Regulator

Clostridium lavalense. Bacteria; Firmicutes; Clostridia; Clostridiales; Lachnospiraceae.

C7256__locus_1

EUT2B

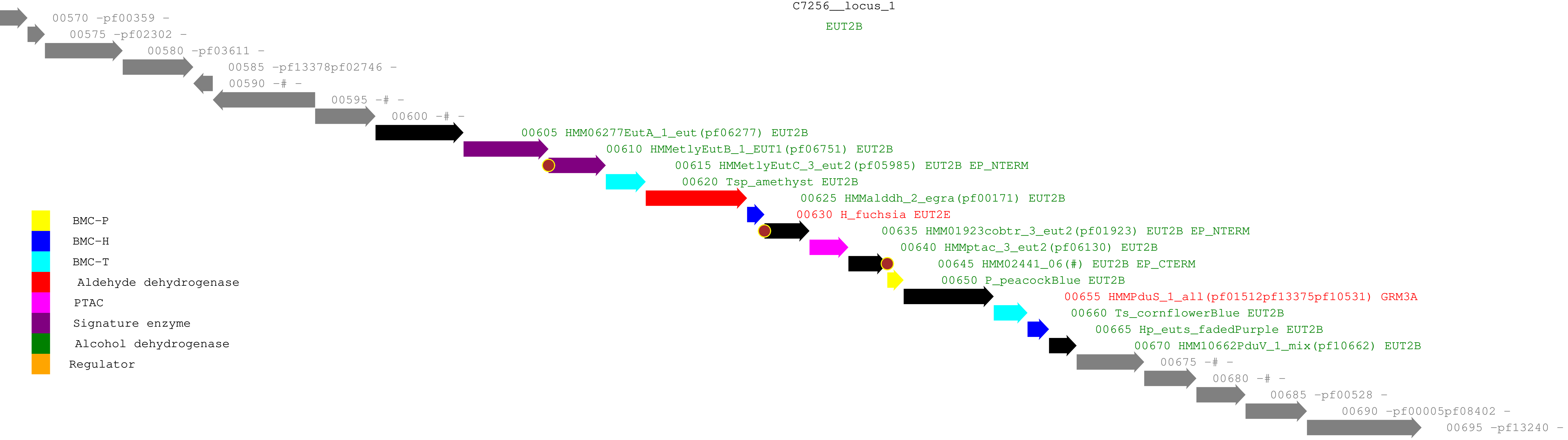

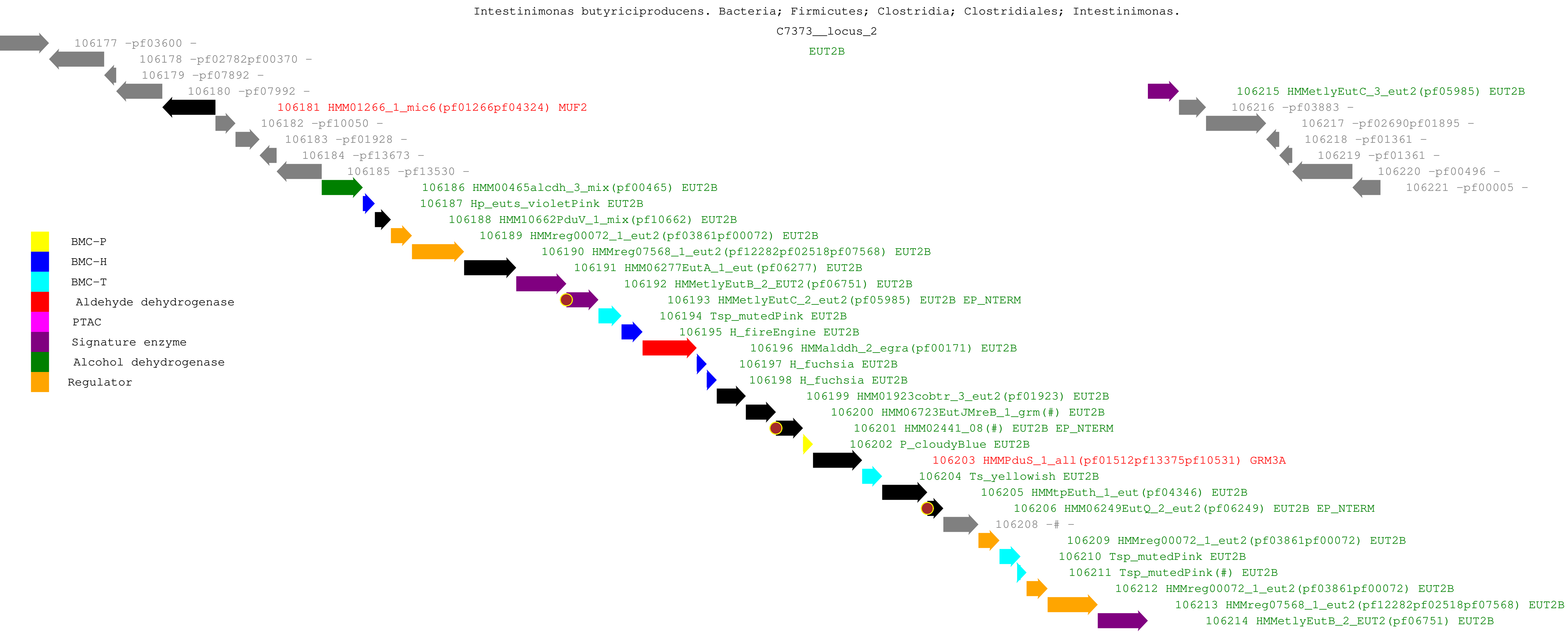

Intestinimonas butyriciproducens. Bacteria; Firmicutes; Clostridia; Clostridiales; Intestinimonas.
C7373__locus_2
EUT2B
106177 -pf03600 -
106178 -pf02782pf00370 -
106179 -pf07892 -
106180 -pf07992 -
106181 HMM01266_1_mic6(pf01266pf04324) MUF2
106182 -pf10050 -
106183 -pf01928 -
106184 -pf13673 -
106185 -pf13530 -
106186 HMM00465alcdh_3_mix(pf00465) EUT2B
106187 Hp_euts_violetPink EUT2B
106188 HMM10662PduV_1_mix(pf10662) EUT2B
106189 HMMreg00072_1_eut2(pf03861pf00072) EUT2B
106190 HMMreg07568_1_eut2(pf12282pf02518pf07568) EUT2B
106191 HMM06277EutA_1_eut(pf06277) EUT2B
106192 HMMetlyEutB_2_EUT2(pf06751) EUT2B
106193 HMMetlyEutC_2_eut2(pf05985) EUT2B EP_NTERM
106194 Tsp_mutedPink EUT2B
106195 H_fireEngine EUT2B
106196 HMMaldh_2_egra(pf00171) EUT2B
106197 H_fuchsia EUT2B
106198 H_fuchsia EUT2B
106199 HMM01923cobtr_3_eut2(pf01923) EUT2B
106200 HMM06723EutJMreB_1_grm(#) EUT2B
106201 HMM02441_08(#) EUT2B EP_NTERM
106202 P_cloudyBlue EUT2B
106203 HMMPduS_1_all(pf01512pf13375pf10531) GRM3A
106204 Ts_yellowish EUT2B
106205 HMMtpEuth_1_eut(pf04346) EUT2B
106206 HMM06249EutQ_2_eut2(pf06249) EUT2B EP_NTERM
106208 -# -
106209 HMMreg00072_1_eut2(pf03861pf00072) EUT2B
106210 Tsp_mutedPink EUT2B
106211 Tsp_mutedPink(#) EUT2B
106212 HMMreg00072_1_eut2(pf03861pf00072) EUT2B
106213 HMMreg07568_1_eut2(pf12282pf02518pf07568) EUT2B
106214 HMMetlyEutB_2_EUT2(pf06751) EUT2B
106215 HMMetlyEutC_3_eut2(pf05985) EUT2B
106216 -pf03883 -
106217 -pf02690pf01895 -
106218 -pf01361 -
106219 -pf01361 -
106220 -pf00496 -
106221 -pf00005 -
BMC-P
BMC-H
BMC-T
Aldehyde dehydrogenase
PTAC
Signature enzyme
Alcohol dehydrogenase
Regulator

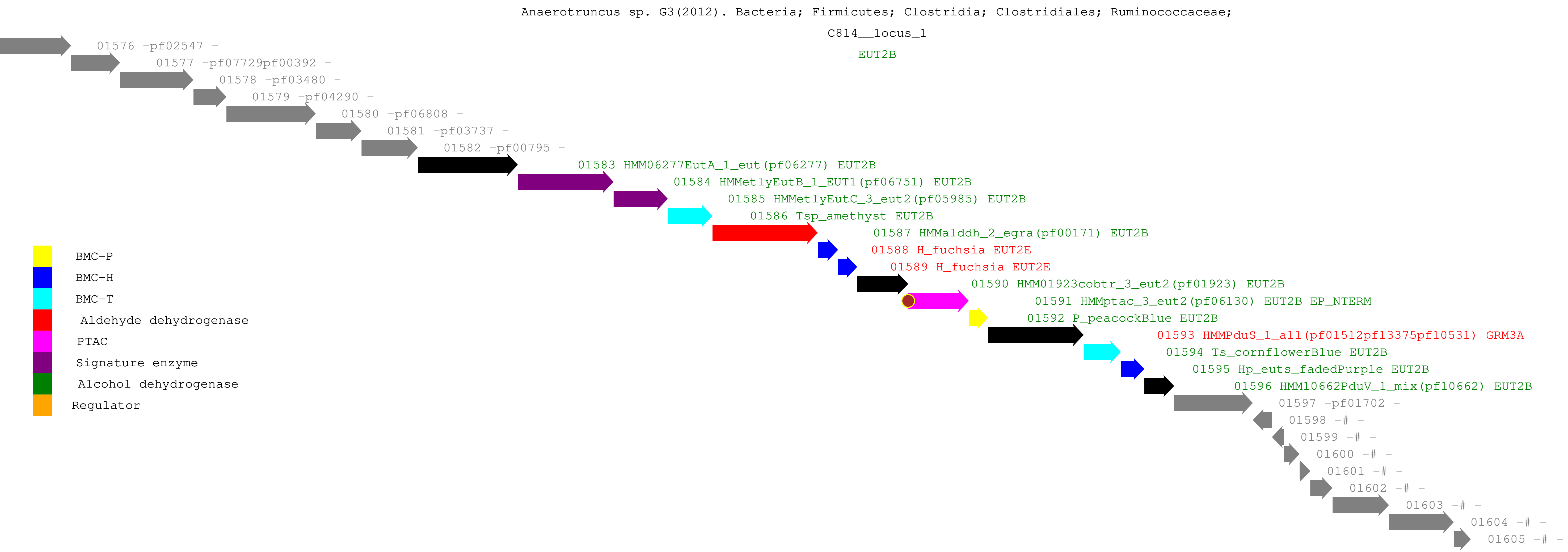
Anaerotruncus sp. G3(2012). Bacteria; Firmicutes; Clostridia; Clostridiales; Ruminococcaceae;
C814__locus_1
EUT2B
01576 -pf02547 -
01577 -pf07729pf00392 -
01578 -pf03480 -
01579 -pf04290 -
01580 -pf06808 -
01581 -pf03737 -
01582 -pf00795 -
01583 HMM06277EutA_1_eut(pf06277) EUT2B
01584 HMMetlyEutB_1_EUT1(pf06751) EUT2B
01585 HMMetlyEutC_3_eut2(pf05985) EUT2B
01586 Tsp_amethyst EUT2B
01587 HMMalddh_2_egra(pf00171) EUT2B
01588 H_fuchsia EUT2E
01589 H_fuchsia EUT2E
01590 HMM01923cobtr_3_eut2(pf01923) EUT2B
01591 HMMptac_3_eut2(pf06130) EUT2B EP_NTERM
01592 P_peacockBlue EUT2B
01593 HMMPduS_1_all(pf01512pf13375pf10531) GRM3A
01594 Ts_cornflowerBlue EUT2B
01595 Hp_euts_fadedPurple EUT2B
01596 HMM10662PduV_1_mix(pf10662) EUT2B
01597 -pf01702 -
01598 -# -
01599 -# -
01600 -# -
01601 -# -
01602 -# -
01603 -# -
01604 -# -
01605 -# -
BMC-P
BMC-H
BMC-T
Aldehyde dehydrogenase
PTAC
Signature enzyme
Alcohol dehydrogenase
Regulator

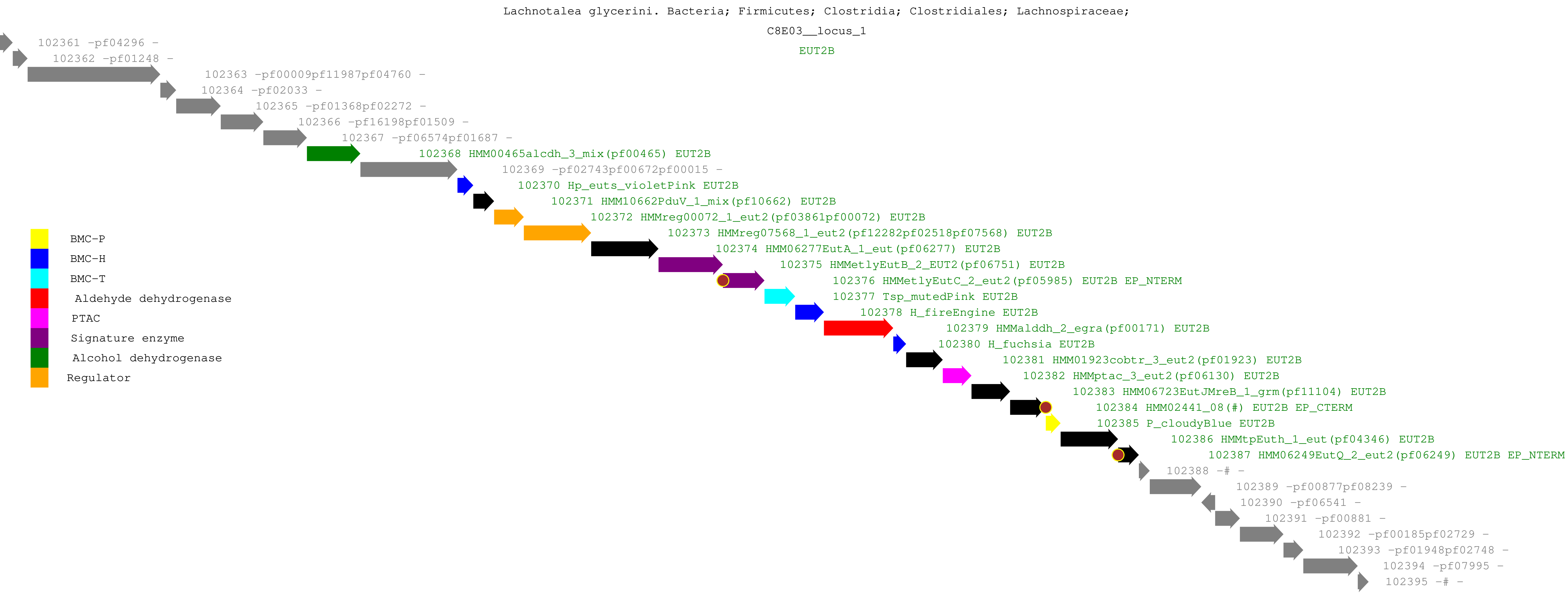
Lachnotalea glycerini. Bacteria; Firmicutes; Clostridia; Clostridiales; Lachnospiraceae;
C8E03__locus_1
EUT2B
102361 -pf04296 -
102362 -pf01248 -
102363 -pf00009pf11987pf04760 -
102364 -pf02033 -
102365 -pf01368pf02272 -
102366 -pf16198pf01509 -
102367 -pf06574pf01687 -
102368 HMM00465alcdh_3_mix(pf00465) EUT2B
102369 -pf02743pf00672pf00015 -
102370 Hp_euts_violetPink EUT2B
102371 HMM10662PduV_1_mix(pf10662) EUT2B
102372 HMMreg00072_1_eut2(pf03861pf00072) EUT2B
102373 HMMreg07568_1_eut2(pf12282pf02518pf07568) EUT2B
102374 HMM06277EutA_1_eut(pf06277) EUT2B
102375 HMMetlyEutB_2_EUT2(pf06751) EUT2B
102376 HMMetlyEutC_2_eut2(pf05985) EUT2B EP_NTERM
102377 Tsp_mutedPink EUT2B
102378 H_fireEngine EUT2B
102379 HMMaldh_2_egra(pf00171) EUT2B
102380 H_fuchsia EUT2B
102381 HMM01923cobtr_3_eut2(pf01923) EUT2B
102382 HMMptac_3_eut2(pf06130) EUT2B
102383 HMM06723EutJMreB_1_grm(pf11104) EUT2B
102384 HMM02441_08(#) EUT2B EP_CTERM
102385 P_cloudyBlue EUT2B
102386 HMMtpEuth_1_eut(pf04346) EUT2B
102387 HMM06249EutQ_2_eut2(pf06249) EUT2B EP_NTERM
102388 -# -
102389 -pf00877pf08239 -
102390 -pf06541 -
102391 -pf00881 -
102392 -pf00185pf02729 -
102393 -pf01948pf02748 -
102394 -pf07995 -
102395 -# -
BMC-P
BMC-H
BMC-T
Aldehyde dehydrogenase
PTAC
Signature enzyme
Alcohol dehydrogenase
Regulator

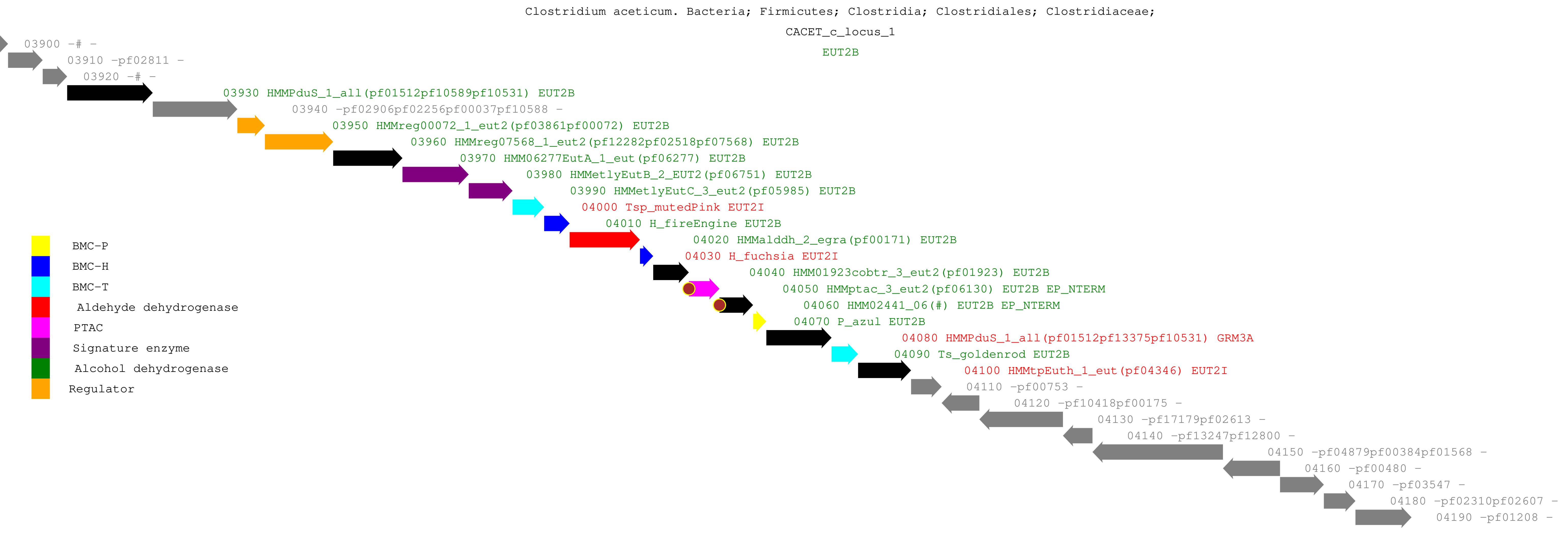

Clostridium aceticum. Bacteria; Firmicutes; Clostridia; Clostridiales; Clostridiaceae;
CACET_c_locus_1
EUT2B
03900 -# -
03910 -pf02811 -
03920 -# -
03930 HMMPduS_1_all(pf01512pf10589pf10531) EUT2B
03940 -pf02906pf02256pf00037pf10588 -
03950 HMMreg00072_1_eut2(pf03861pf00072) EUT2B
03960 HMMreg07568_1_eut2(pf12282pf02518pf07568) EUT2B
03970 HMM06277EutA_1_eut(pf06277) EUT2B
03980 HMMetlyEutB_2_EUT2(pf06751) EUT2B
03990 HMMetlyEutC_3_eut2(pf05985) EUT2B
04000 Tsp_mutedPink EUT2I
04010 H_fireEngine EUT2B
04020 HMMalddh_2_egra(pf00171) EUT2B
04030 H_fuchsia EUT2I
04040 HMM01923cobtr_3_eut2(pf01923) EUT2B
04050 HMMptac_3_eut2(pf06130) EUT2B EP_NTERM
04060 HMM02441_06(#) EUT2B EP_NTERM
04070 P_azul EUT2B
04080 HMMPduS_1_all(pf01512pf13375pf10531) GRM3A
04090 Ts_goldenrod EUT2B
04100 HMMtpEuth_1_eut(pf04346) EUT2I
04110 -pf00753 -
04120 -pf10418pf00175 -
04130 -pf17179pf02613 -
04140 -pf13247pf12800 -
04150 -pf04879pf00384pf01568 -
04160 -pf00480 -
04170 -pf03547 -
04180 -pf02310pf02607 -
04190 -pf01208 -
BMC-P
BMC-H
BMC-T
Aldehyde dehydrogenase
PTAC
Signature enzyme
Alcohol dehydrogenase
Regulator

Coprococcus catus GD/7. Bacteria; Firmicutes; Clostridia; Clostridiales; Lachnospiraceae;

CC1__locus_1

EUT2B

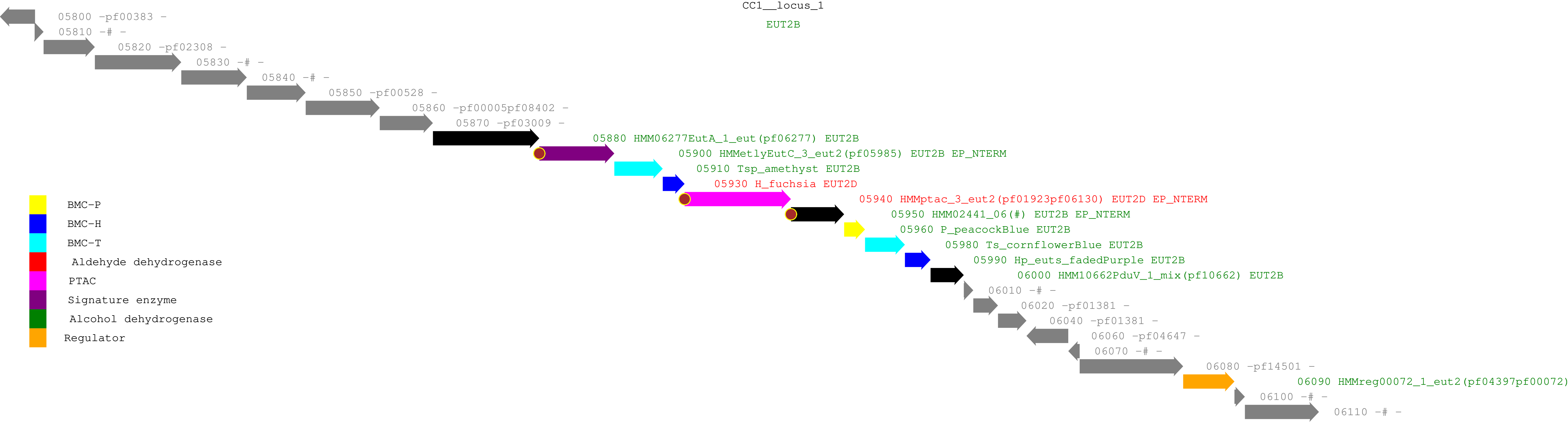

Lachnotalea glycerini. Bacteria; Firmicutes; Clostridia; Clostridiales; Lachnospiraceae;

CG710__locus_1

EUT2B

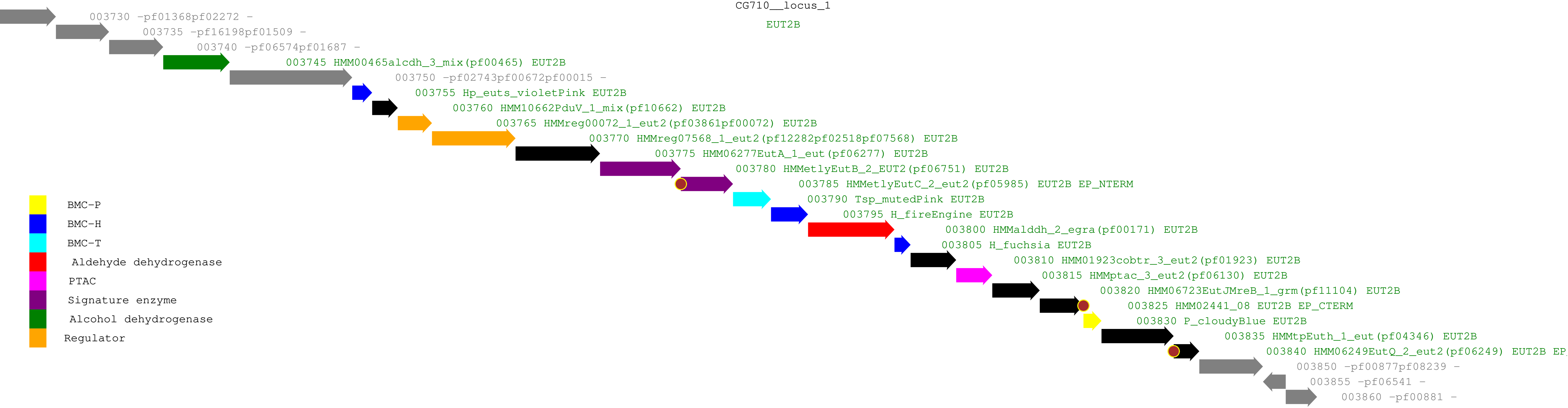

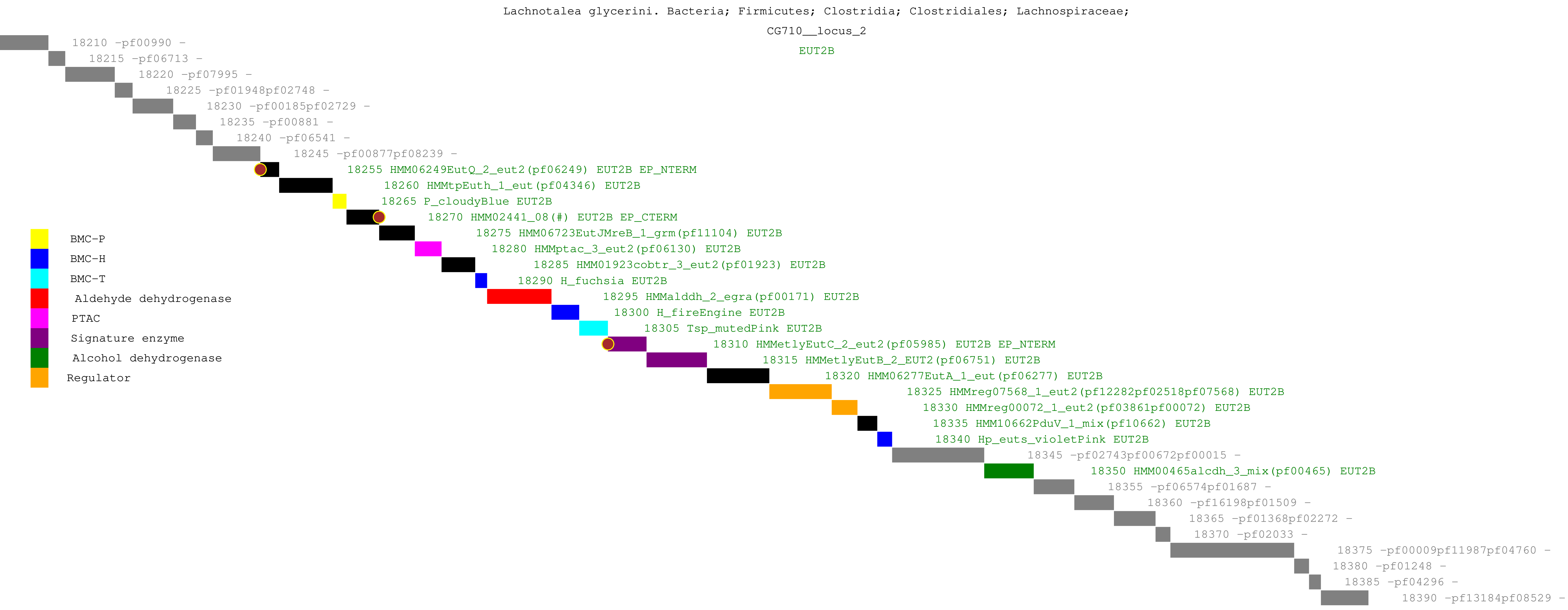

Lachnotalea glycerini. Bacteria; Firmicutes; Clostridia; Clostridiales; Lachnospiraceae;
CG710__locus_2
EUT2B
18210 -pf00990 -
18215 -pf06713 -
18220 -pf07995 -
18225 -pf01948pf02748 -
18230 -pf00185pf02729 -
18235 -pf00881 -
18240 -pf06541 -
18245 -pf00877pf08239 -
18255 HMM06249EutQ_2_eut2(pf06249) EUT2B EP_NTERM
18260 HMMtpEuth_1_eut(pf04346) EUT2B
18265 P_cloudyBlue EUT2B
18270 HMM02441_08(#) EUT2B EP_CTERM
18275 HMM06723EutJMreB_1_grm(pf11104) EUT2B
18280 HMMptac_3_eut2(pf06130) EUT2B
18285 HMM01923cobtr_3_eut2(pf01923) EUT2B
18290 H_fuchsia EUT2B
18295 HMMalddh_2_egra(pf00171) EUT2B
18300 H_fireEngine EUT2B
18305 Tsp_mutedPink EUT2B
18310 HMMetlyEutC_2_eut2(pf05985) EUT2B EP_NTERM
18315 HMMetlyEutB_2_EUT2(pf06751) EUT2B
18320 HMM06277EutA_1_eut(pf06277) EUT2B
18325 HMMreg07568_1_eut2(pf12282pf02518pf07568) EUT2B
18330 HMMreg00072_1_eut2(pf03861pf00072) EUT2B
18335 HMM10662PduV_1_mix(pf10662) EUT2B
18340 Hp_euts_violetPink EUT2B
18345 -pf02743pf00672pf00015 -
18350 HMM00465alcdh_3_mix(pf00465) EUT2B
18355 -pf06574pf01687 -
18360 -pf16198pf01509 -
18365 -pf01368pf02272 -
18370 -pf02033 -
18375 -pf00009pf11987pf04760 -
18380 -pf01248 -
18385 -pf04296 -
18390 -pf13184pf08529 -
BMC-P
BMC-H
BMC-T
Aldehyde dehydrogenase
PTAC
Signature enzyme
Alcohol dehydrogenase
Regulator

Cloacibacillus evryensis DSM 19522. Bacteria; Synergistetes; Synergistia; Synergistales; Synergistaceae;

Cloev__locus_1

EUT2B

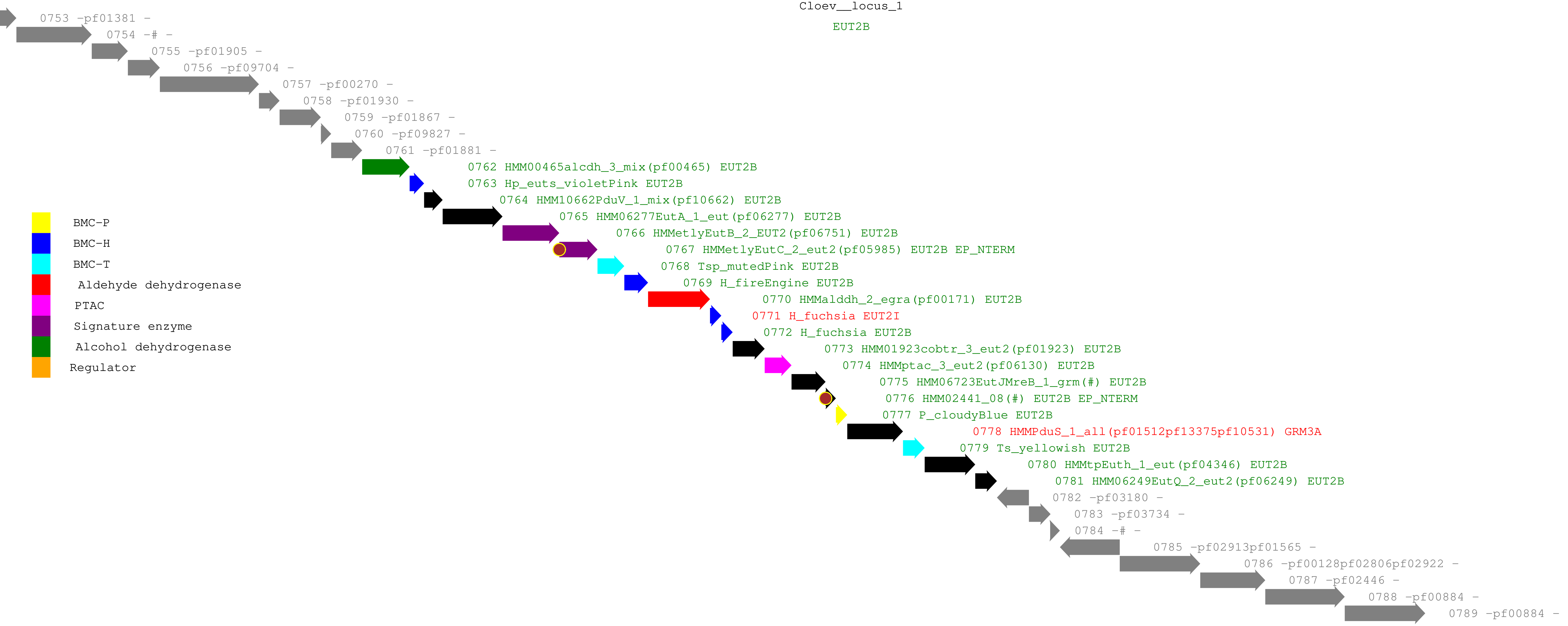

Clostridium asparagiforme DSM 15981. Bacteria; Firmicutes; Clostridia; Clostridiales; Lachnospiraceae.

CLOSTASPAR__locus_1

EUT2B

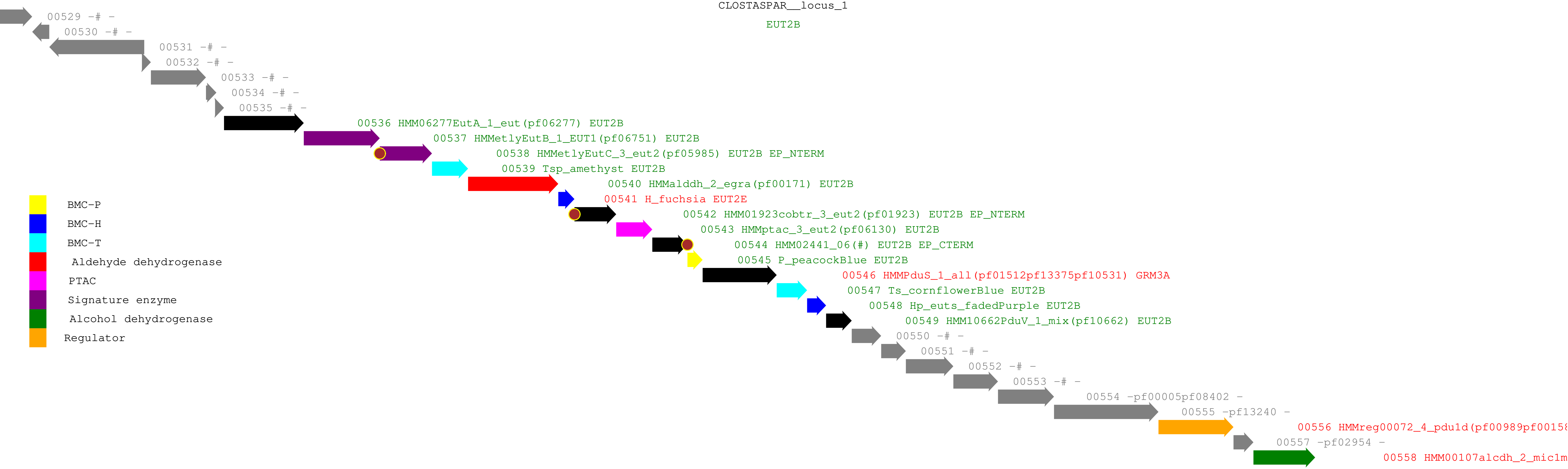

Acetoanaerobium sticklandii (strain ATCC 12662 / DSM 519 / JCM 1433 / Bacteria; Firmicutes; Clostridia; Clostridiales;

CLOST__locus_1

EUT2B

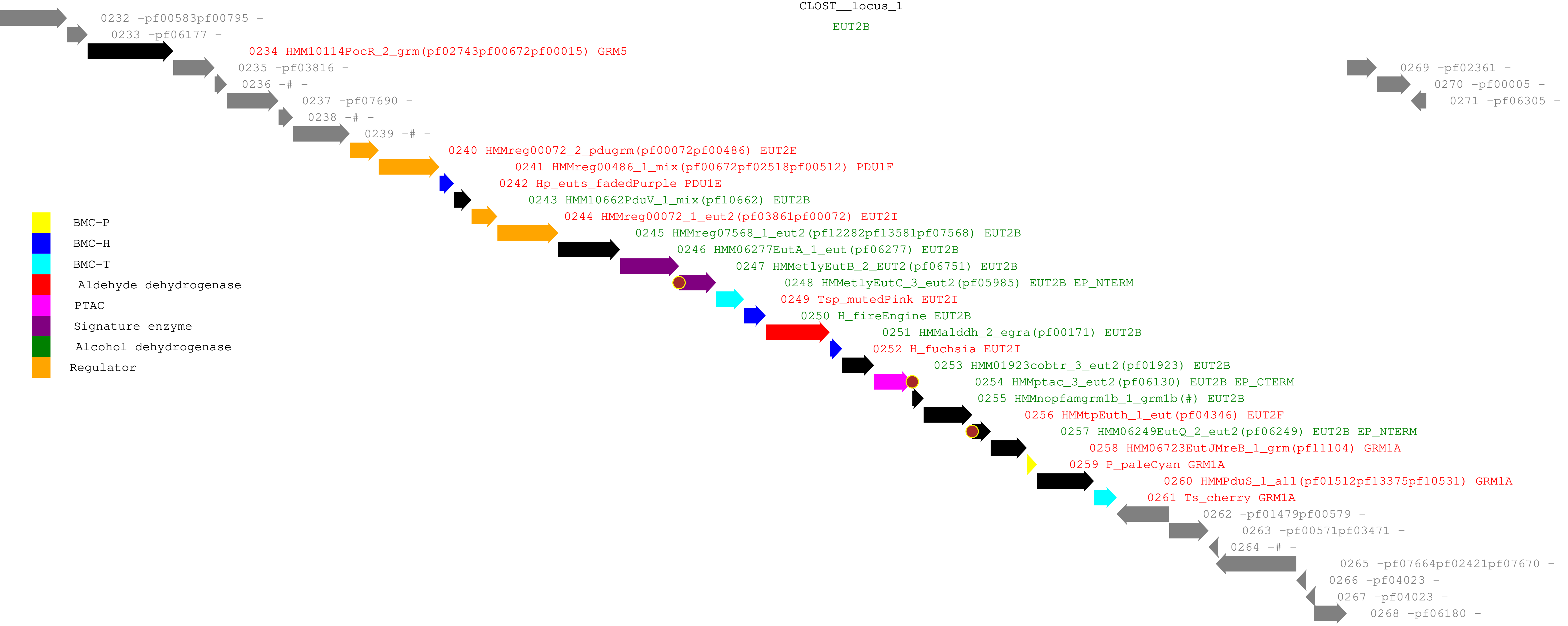

Clostridium symbiosum ATCC 14940. Bacteria; Firmicutes; Clostridia; Clostridiales; Lachnospiraceae.

CLOSYM__locus_1

EUT2B

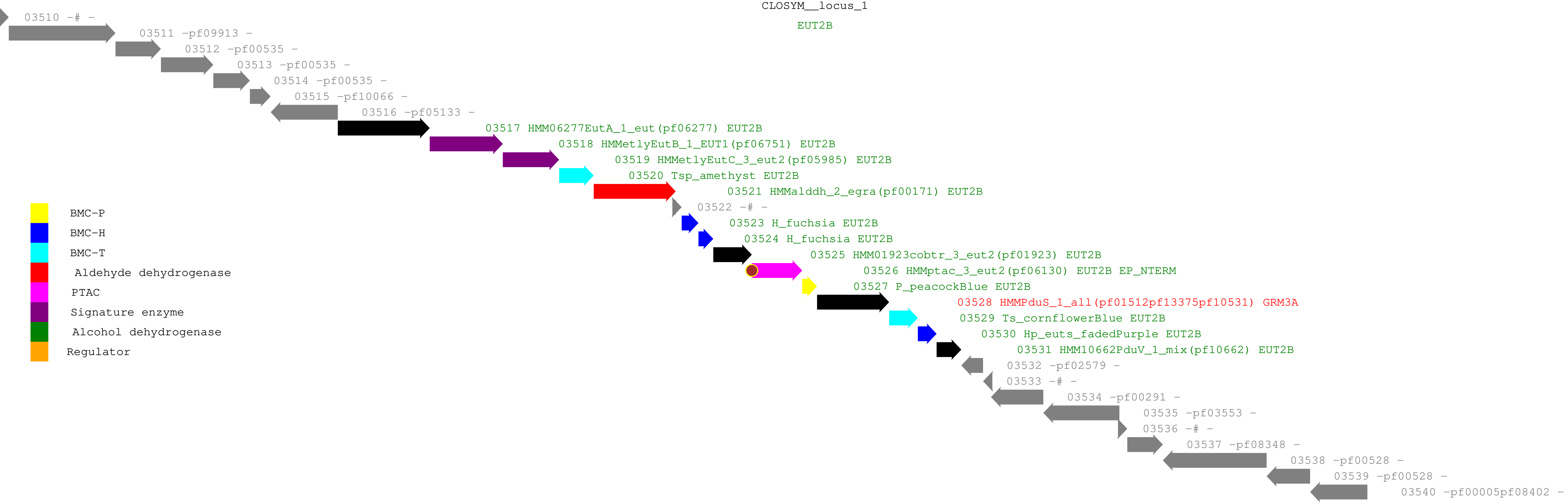

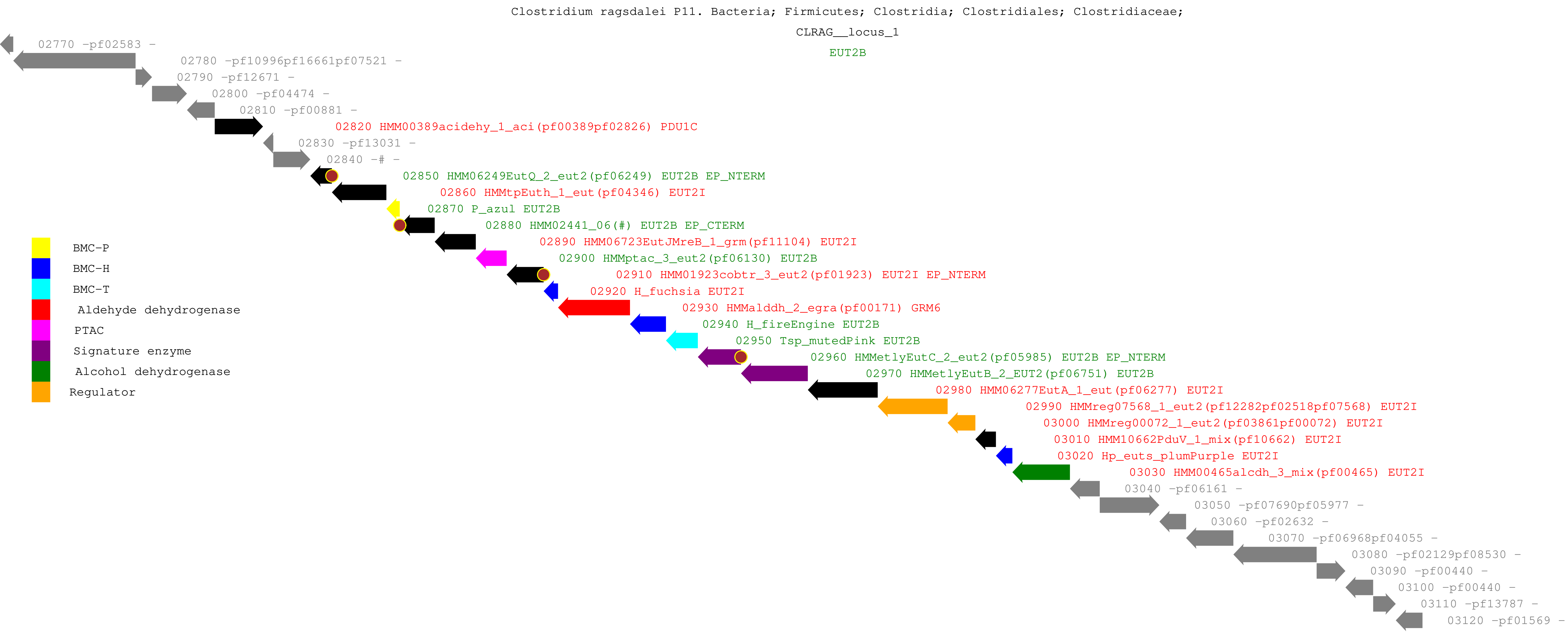
Clostridium ragsdalei P11. Bacteria; Firmicutes; Clostridia; Clostridiales; Clostridiaceae;
CLRAG__locus_1
EUT2B
02770 -pf02583 -
02780 -pf10996pf16661pf07521 -
02790 -pf12671 -
02800 -pf04474 -
02810 -pf00881 -
02820 HMM00389acidehy_1_aci(pf00389pf02826) PDU1C
02830 -pf13031 -
02840 -# -
02850 HMM06249EutQ_2_eut2(pf06249) EUT2B EP_NTERM
02860 HMMtpEuth_1_eut(pf04346) EUT2I
02870 P_azul EUT2B
02880 HMM02441_06(#) EUT2B EP_CTERM
02890 HMM06723EutJMreB_1_grm(pf11104) EUT2I
02900 HMMptac_3_eut2(pf06130) EUT2B
02910 HMM01923cobtr_3_eut2(pf01923) EUT2I EP_NTERM
02920 H_fuchsia EUT2I
02930 HMMalddh_2_egra(pf00171) GRM6
02940 H_fireEngine EUT2B
02950 Tsp_mutedPink EUT2B
02960 HMMetlyEutC_2_eut2(pf05985) EUT2B EP_NTERM
02970 HMMetlyEutB_2_EUT2(pf06751) EUT2B
02980 HMM06277EutA_1_eut(pf06277) EUT2I
02990 HMMreg07568_1_eut2(pf12282pf02518pf07568) EUT2I
03000 HMMreg00072_1_eut2(pf03861pf00072) EUT2I
03010 HMM10662PduV_1_mix(pf10662) EUT2I
03020 Hp_euts_plumPurple EUT2I
03030 HMM00465alcdh_3_mix(pf00465) EUT2I
03040 -pf06161 -
03050 -pf07690pf05977 -
03060 -pf02632 -
03070 -pf06968pf04055 -
03080 -pf02129pf08530 -
03090 -pf00440 -
03100 -pf00440 -
03110 -pf13787 -
03120 -pf01569 -
BMC-P
BMC-H
BMC-T
Aldehyde dehydrogenase
PTAC
Signature enzyme
Alcohol dehydrogenase
Regulator

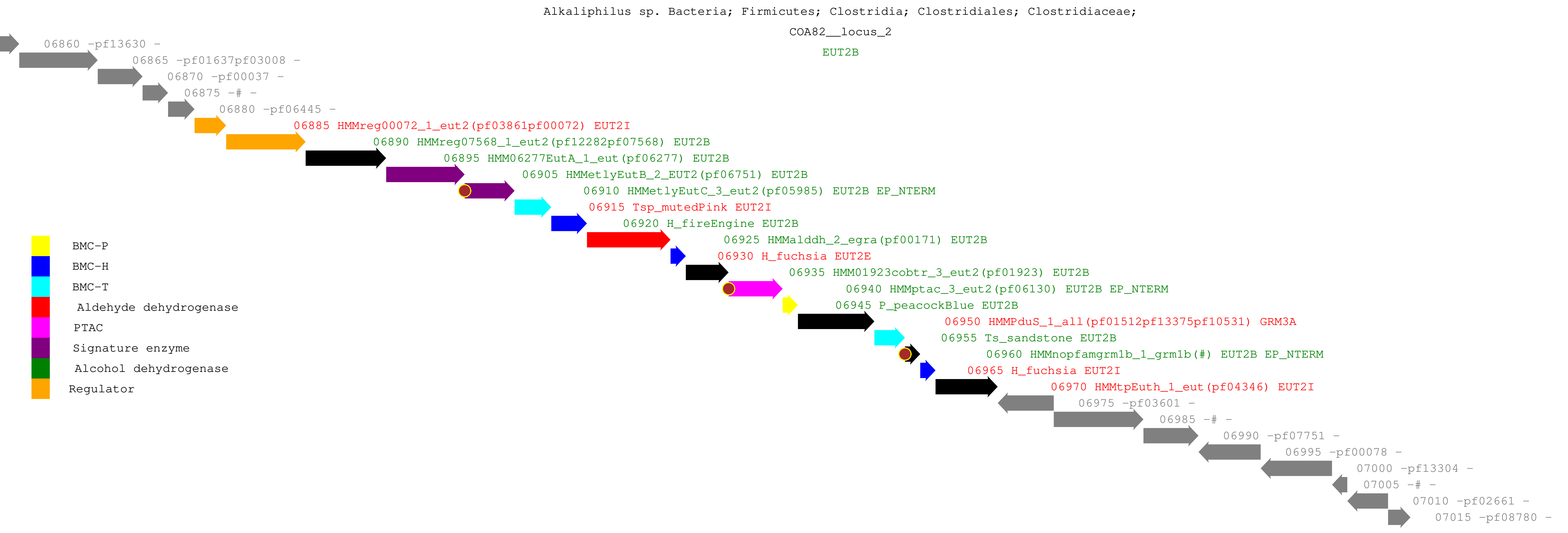

Alkaliphilus sp. Bacteria; Firmicutes; Clostridia; Clostridiales; Clostridiaceae;
COA82__locus_2
EUT2B
06860 -pf13630 -
06865 -pf01637pf03008 -
06870 -pf00037 -
06875 -# -
06880 -pf06445 -
06885 HMMreg00072_1_eut2(pf03861pf00072) EUT2I
06890 HMMreg07568_1_eut2(pf12282pf07568) EUT2B
06895 HMM06277EutA_1_eut(pf06277) EUT2B
06905 HMMetlyEutB_2_EUT2(pf06751) EUT2B
06910 HMMetlyEutC_3_eut2(pf05985) EUT2B EP_NTERM
06915 Tsp_mutedPink EUT2I
06920 H_fireEngine EUT2B
06925 HMMalddh_2_egra(pf00171) EUT2B
06930 H_fuchsia EUT2E
06935 HMM01923cobtr_3_eut2(pf01923) EUT2B
06940 HMMptac_3_eut2(pf06130) EUT2B EP_NTERM
06945 P_peacockBlue EUT2B
06950 HMMPduS_1_all(pf01512pf13375pf10531) GRM3A
06955 Ts_sandstone EUT2B
06960 HMMnopfamgrm1b_1_grm1b(#) EUT2B EP_NTERM
06965 H_fuchsia EUT2I
06970 HMMtpEuth_1_eut(pf04346) EUT2I
06975 -pf03601 -
06985 -# -
06990 -pf07751 -
06995 -pf00078 -
07000 -pf13304 -
07005 -# -
07010 -pf02661 -
07015 -pf08780 -
BMC-P
BMC-H
BMC-T
Aldehyde dehydrogenase
PTAC
Signature enzyme
Alcohol dehydrogenase
Regulator

Clostridium sp. HMb25. Bacteria; Firmicutes; Clostridia; Clostridiales; Clostridiaceae;

CRH03__locus_1

EUT2B

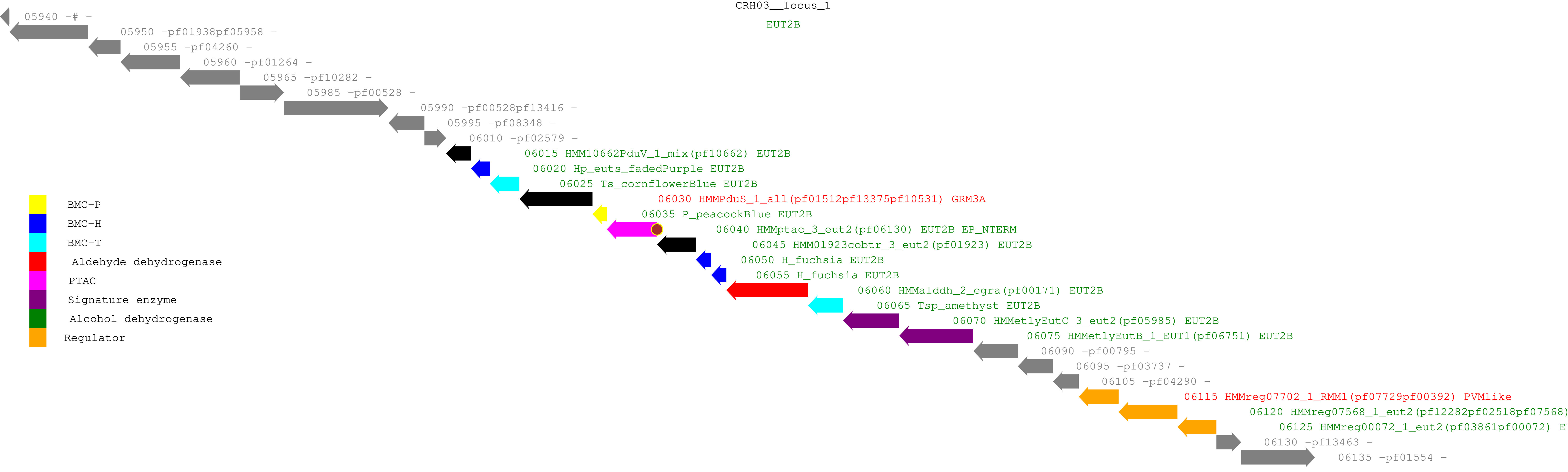

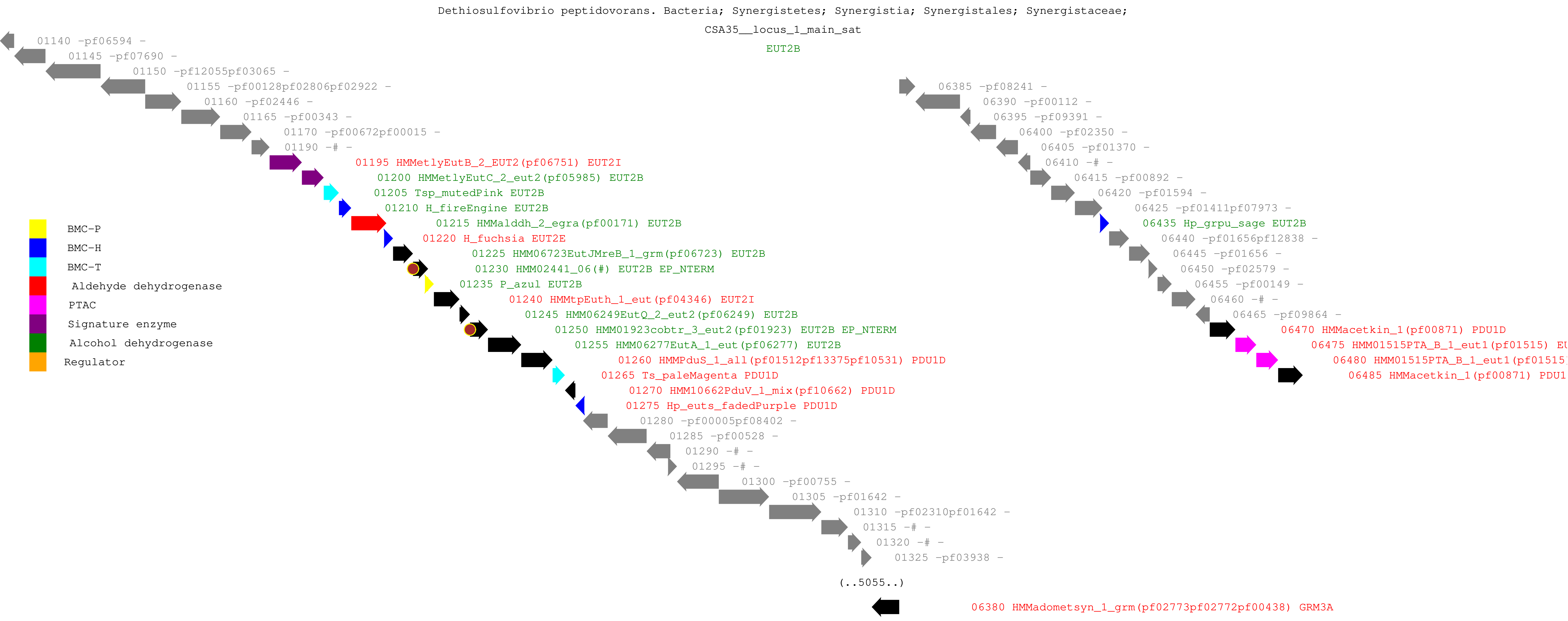
Dethiosulfovibrio peptidovorans. Bacteria; Synergistetes; Synergistia; Synergistales; Synergistaceae;
CSA35__locus_1_main_sat
EUT2B
01140 -pf06594 -
01145 -pf07690 -
01150 -pf12055pf03065 -
01155 -pf00128pf02806pf02922 -
01160 -pf02446 -
01165 -pf00343 -
01170 -pf00672pf00015 -
01190 -# -
01195 HMMetlyEutB_2_EUT2(pf06751) EUT2I
01200 HMMetlyEutC_2_eut2(pf05985) EUT2B
01205 Tsp_mutedPink EUT2B
01210 H_fireEngine EUT2B
01215 HMMaldh_2_egra(pf00171) EUT2B
01220 H_fuchsia EUT2E
01225 HMM06723EutJMreB_1_grm(pf06723) EUT2B
01230 HMM02441_06(#) EUT2B EP_NTERM
01235 P_azul EUT2B
01240 HMMtpEuth_1_eut(pf04346) EUT2I
01245 HMM06249EutQ_2_eut2(pf06249) EUT2B
01250 HMM01923cobtr_3_eut2(pf01923) EUT2B EP_NTERM
01255 HMM06277EutA_1_eut(pf06277) EUT2B
01260 HMMPduS_1_all(pf01512pf13375pf10531) PDU1D
01265 Ts_paleMagenta PDU1D
01270 HMM10662PduV_1_mix(pf10662) PDU1D
01275 Hp_euts_fadedPurple PDU1D
01280 -pf00005pf08402 -
01285 -pf00528 -
01290 -# -
01295 -# -
01300 -pf00755 -
01305 -pf01642 -
01310 -pf02310pf01642 -
01315 -# -
01320 -# -
01325 -pf03938 -
(..5055..)
06380 HMMadometsyn_1_grm(pf02773pf02772pf00438) GRM3A
06385 -pf08241 -
06390 -pf00112 -
06395 -pf09391 -
06400 -pf02350 -
06405 -pf01370 -
06410 -# -
06415 -pf00892 -
06420 -pf01594 -
06425 -pf01411pf07973 -
06435 Hp_grpu_sage EUT2B
06440 -pf01656pf12838 -
06445 -pf01656 -
06450 -pf02579 -
06455 -pf00149 -
06460 -# -
06465 -pf09864 -
06470 HMMacetkin_1(pf00871) PDU1D
06475 HMM01515PTA_B_1_eut1(pf01515) EU
06480 HMM01515PTA_B_1_eut1(pf01515
06485 HMMacetkin_1(pf00871) PDU1
BMC-P
BMC-H
BMC-T
Aldehyde dehydrogenase
PTAC
Signature enzyme
Alcohol dehydrogenase
Regulator

Clostridium ultunense Esp. Bacteria; Firmicutes; Tissierellia.

CUESP1__locus_2

EUT2B

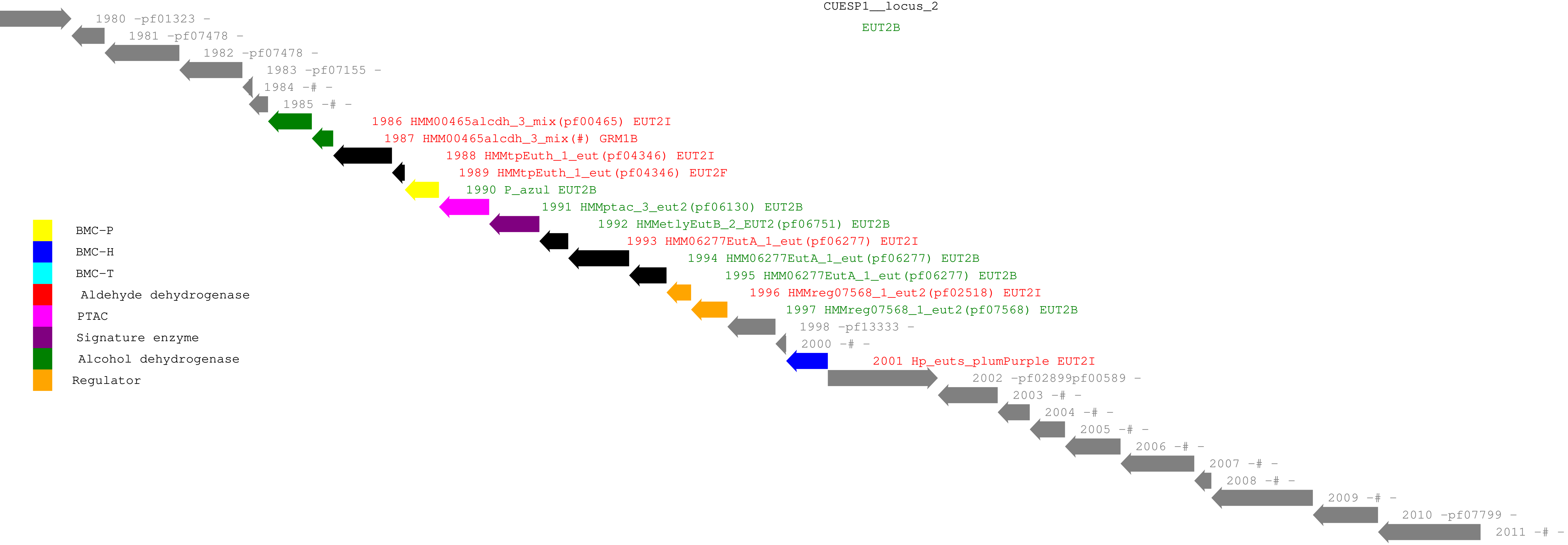

Firmicutes bacterium HGW-Firmicutes-17. Bacteria; Firmicutes.

CVU92__locus_1

EUT2B

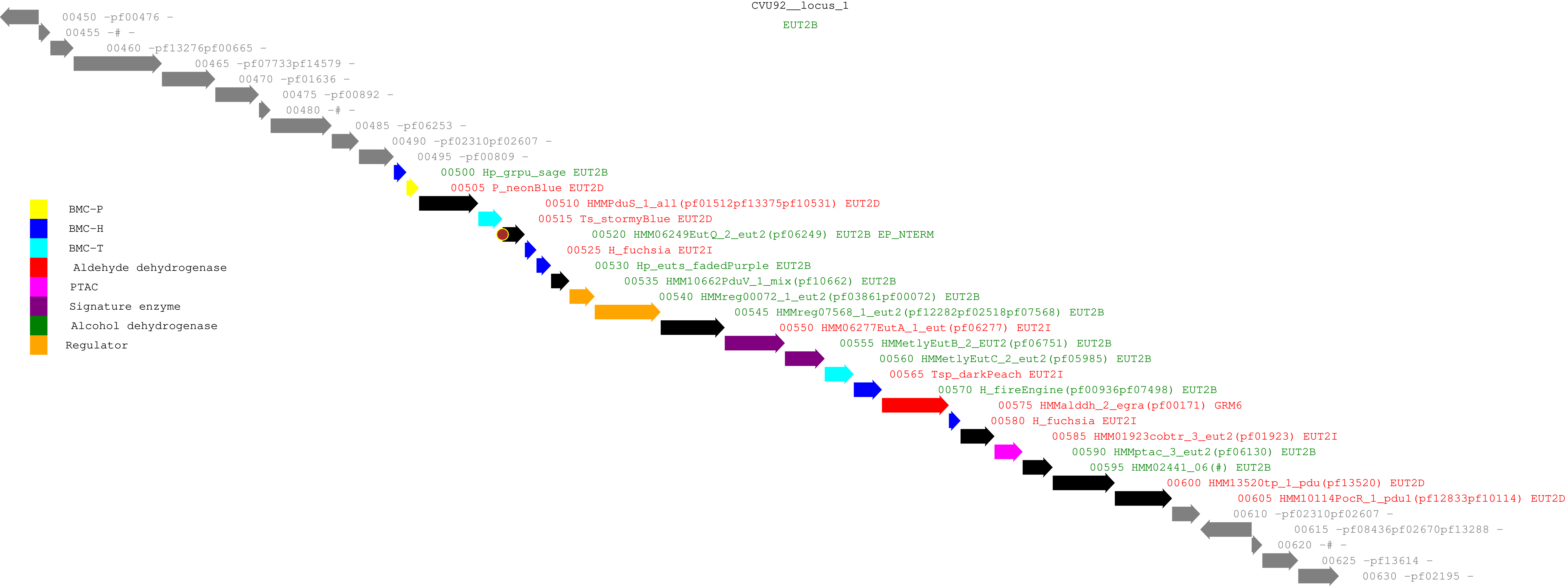

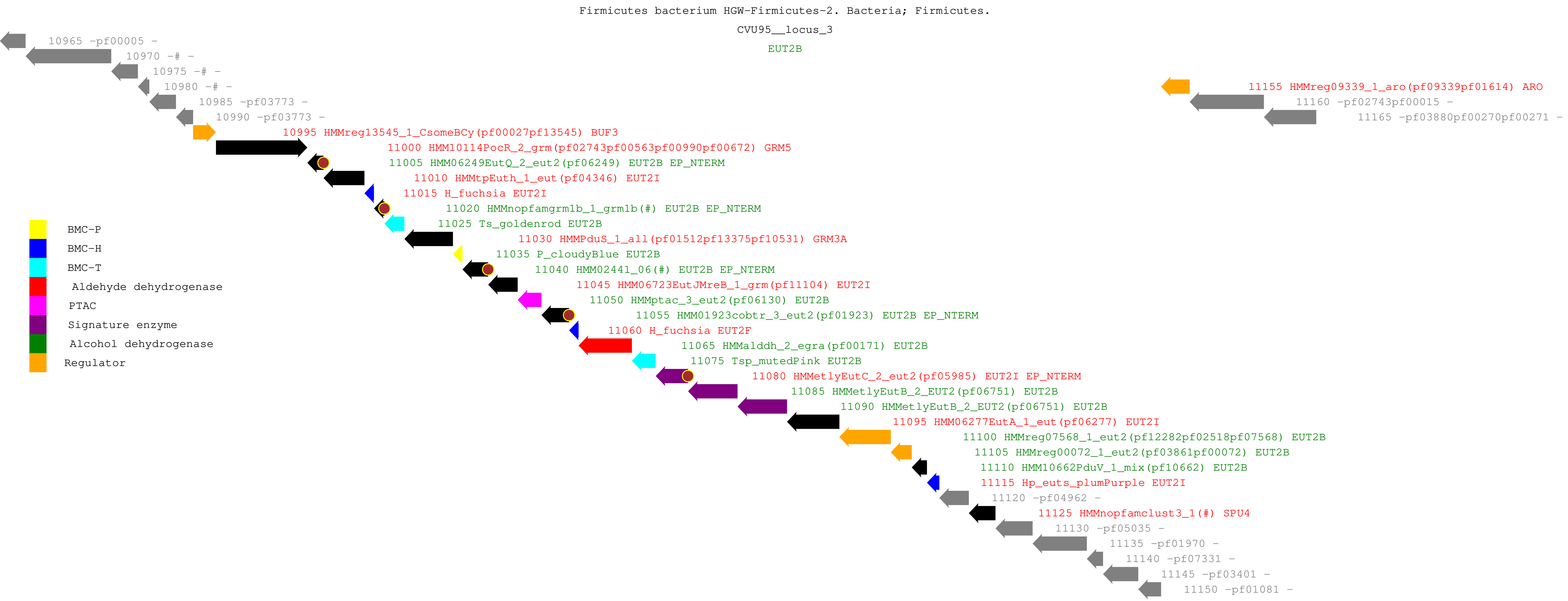

Firmicutes bacterium HGW-Firmicutes-2. Bacteria; Firmicutes.
CVU95__locus_3
EUT2B
10965 -pf00005 -
10970 -# -
10975 -# -
10980 -# -
10985 -pf03773 -
10990 -pf03773 -
10995 HMMreg13545_1_CsomeBCy(pf00027pf13545) BUF3
11000 HMM10114PocR_2_grm(pf02743pf00563pf00990pf00672) GRM5
11005 HMM06249EutQ_2_eut2(pf06249) EUT2B EP_NTERM
11010 HMMtpEuth_1_eut(pf04346) EUT2I
11015 H_fuchsia EUT2I
11020 HMMnopfamgrm1b_1_grm1b(#) EUT2B EP_NTERM
11025 Ts_goldenrod EUT2B
11030 HMMPduS_1_all(pf01512pf13375pf10531) GRM3A
11035 P_cloudyBlue EUT2B
11040 HMM02441_06(#) EUT2B EP_NTERM
11045 HMM06723EutJMreB_1_grm(pf11104) EUT2I
11050 HMMptac_3_eut2(pf06130) EUT2B
11055 HMM01923cobtr_3_eut2(pf01923) EUT2B EP_NTERM
11060 H_fuchsia EUT2F
11065 HMMalddh_2_egra(pf00171) EUT2B
11075 Tsp_mutedPink EUT2B
11080 HMMetlyEutC_2_eut2(pf05985) EUT2I EP_NTERM
11085 HMMetlyEutB_2_EUT2(pf06751) EUT2B
11090 HMMetlyEutB_2_EUT2(pf06751) EUT2B
11095 HMM06277EutA_1_eut(pf06277) EUT2I
11100 HMMreg07568_1_eut2(pf12282pf02518pf07568) EUT2B
11105 HMMreg00072_1_eut2(pf03861pf00072) EUT2B
11110 HMM10662PduV_1_mix(pf10662) EUT2B
11115 Hp_euts_plumPurple EUT2I
11120 -pf04962 -
11125 HMMnopfamclust3_1(#) SPU4
11130 -pf05035 -
11135 -pf01970 -
11140 -pf07331 -
11145 -pf03401 -
11150 -pf01081 -
11155 HMMreg09339_1_aro(pf09339pf01614) ARO
11160 -pf02743pf00015 -
11165 -pf03880pf00270pf00271 -
BMC-P
BMC-H
BMC-T
Aldehyde dehydrogenase
PTAC
Signature enzyme
Alcohol dehydrogenase
Regulator

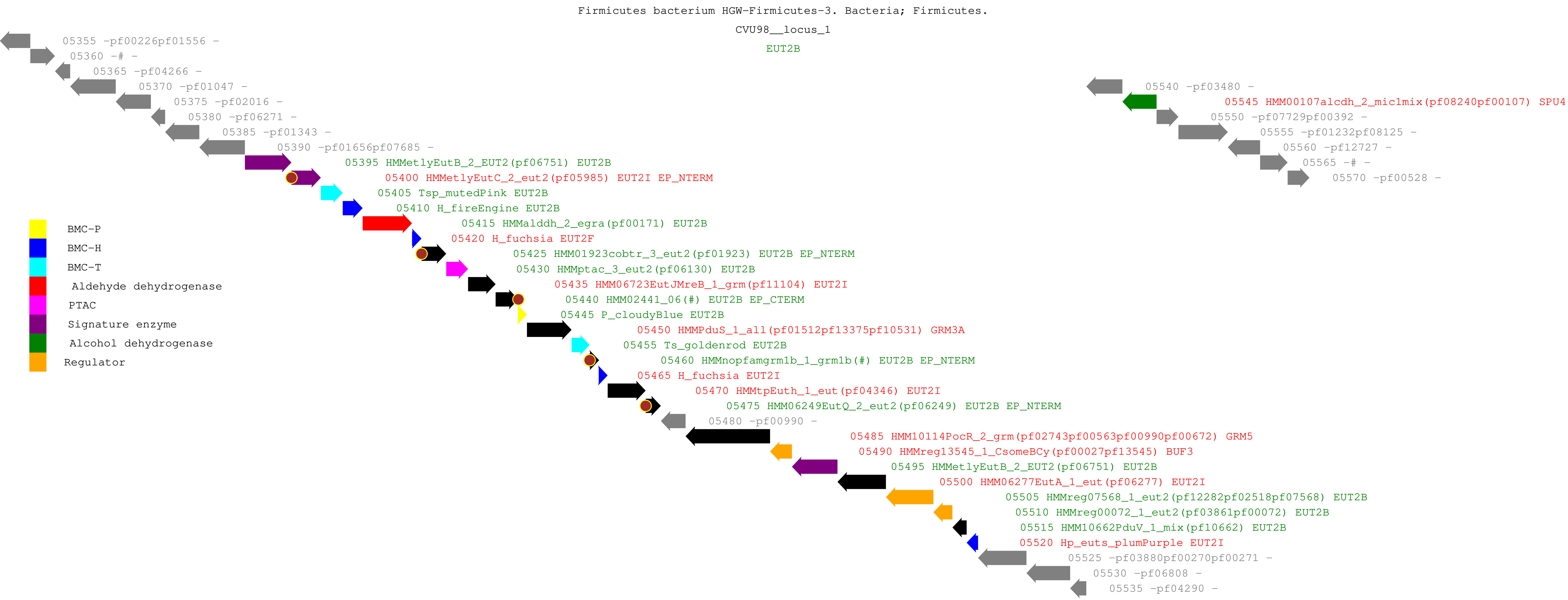

Firmicutes bacterium HGW-Firmicutes-3. Bacteria; Firmicutes.
CVU98__locus_1
EUT2B
05355 -pf00226pf01556 -
05360 -# -
05365 -pf04266 -
05370 -pf01047 -
05375 -pf02016 -
05380 -pf06271 -
05385 -pf01343 -
05390 -pf01656pf07685 -
05395 HMMetlyEutB_2_EUT2(pf06751) EUT2B
05400 HMMetlyEutC_2_eut2(pf05985) EUT2I EP_NTERM
05405 Tsp_mutedPink EUT2B
05410 H_fireEngine EUT2B
05415 HMMalddh_2_egra(pf00171) EUT2B
05420 H_fuchsia EUT2F
05425 HMM01923cobtr_3_eut2(pf01923) EUT2B EP_NTERM
05430 HMMptac_3_eut2(pf06130) EUT2B
05435 HMM06723EutJMreB_1_grm(pf11104) EUT2I
05440 HMM02441_06(#) EUT2B EP_CTERM
05445 P_cloudyBlue EUT2B
05450 HMMPduS_1_all(pf01512pf13375pf10531) GRM3A
05455 Ts_goldenrod EUT2B
05460 HMMnopfamgrm1b_1_grm1b(#) EUT2B EP_NTERM
05465 H_fuchsia EUT2I
05470 HMMtpEuth_1_eut(pf04346) EUT2I
05475 HMM06249EutQ_2_eut2(pf06249) EUT2B EP_NTERM
05480 -pf00990 -
05485 HMM10114PocR_2_grm(pf02743pf00563pf00990pf00672) GRM5
05490 HMMreg13545_1_CsomeBCy(pf00027pf13545) BUF3
05495 HMMetlyEutB_2_EUT2(pf06751) EUT2B
05500 HMM06277EutA_1_eut(pf06277) EUT2I
05505 HMMreg07568_1_eut2(pf12282pf02518pf07568) EUT2B
05510 HMMreg00072_1_eut2(pf03861pf00072) EUT2B
05515 HMM10662PduV_1_mix(pf10662) EUT2B
05520 Hp_euts_plumPurple EUT2I
05525 -pf03880pf00270pf00271 -
05530 -pf06808 -
05535 -pf04290 -
05540 -pf03480 -
05545 HMM00107alcdh_2_mic1mix(pf08240pf00107) SPU4
05550 -pf07729pf00392 -
05555 -pf01232pf08125 -
05560 -pf12727 -
05565 -# -
05570 -pf00528 -
BMC-P
BMC-H
BMC-T
Aldehyde dehydrogenase
PTAC
Signature enzyme
Alcohol dehydrogenase
Regulator

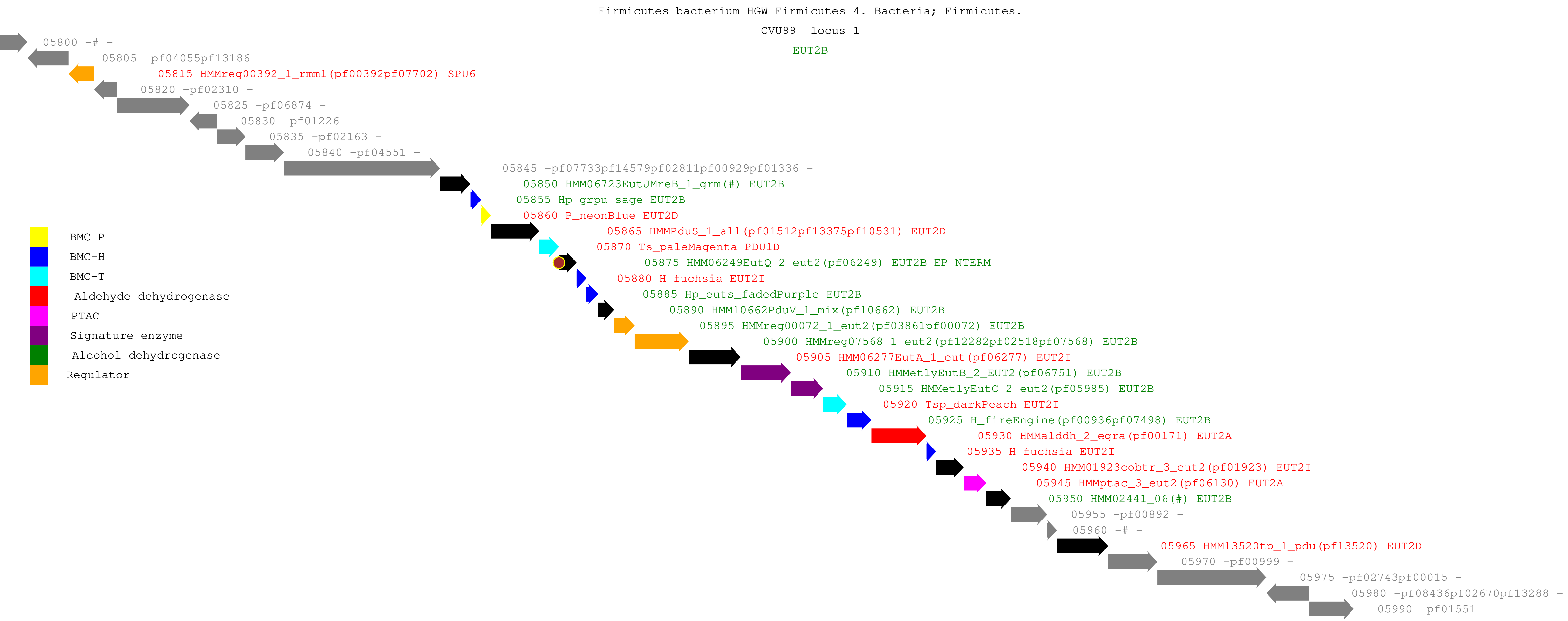
Firmicutes bacterium HGW-Firmicutes-4. Bacteria; Firmicutes.
CVU99__locus_1
EUT2B
05800 -# -
05805 -pf04055pf13186 -
05815 HMMreg00392_1_rmm1(pf00392pf07702) SPU6
05820 -pf02310 -
05825 -pf06874 -
05830 -pf01226 -
05835 -pf02163 -
05840 -pf04551 -
05845 -pf07733pf14579pf02811pf00929pf01336 -
05850 HMM06723EutJMreB_1_grm(#) EUT2B
05855 Hp_grpu_sage EUT2B
05860 P_neonBlue EUT2D
05865 HMMPduS_1_all(pf01512pf13375pf10531) EUT2D
05870 Ts_paleMagenta PDU1D
05875 HMM06249EutQ_2_eut2(pf06249) EUT2B EP_NTERM
05880 H_fuchsia EUT2I
05885 Hp_euts_fadedPurple EUT2B
05890 HMM10662PduV_1_mix(pf10662) EUT2B
05895 HMMreg00072_1_eut2(pf03861pf00072) EUT2B
05900 HMMreg07568_1_eut2(pf12282pf02518pf07568) EUT2B
05905 HMM06277EutA_1_eut(pf06277) EUT2I
05910 HMMetlyEutB_2_EUT2(pf06751) EUT2B
05915 HMMetlyEutC_2_eut2(pf05985) EUT2B
05920 Tsp_darkPeach EUT2I
05925 H_fireEngine(pf00936pf07498) EUT2B
05930 HMMalddh_2_egra(pf00171) EUT2A
05935 H_fuchsia EUT2I
05940 HMM01923cobtr_3_eut2(pf01923) EUT2I
05945 HMMptac_3_eut2(pf06130) EUT2A
05950 HMM02441_06(#) EUT2B
05955 -pf00892 -
05960 -# -
05965 HMM13520tp_1_pdu(pf13520) EUT2D
05970 -pf00999 -
05975 -pf02743pf00015 -
05980 -pf08436pf02670pf13288 -
05990 -pf01551 -
BMC-P
BMC-H
BMC-T
Aldehyde dehydrogenase
PTAC
Signature enzyme
Alcohol dehydrogenase
Regulator

Firmicutes bacterium HGW-Firmicutes-7. Bacteria; Firmicutes.

CVV02__locus_2

EUT2B

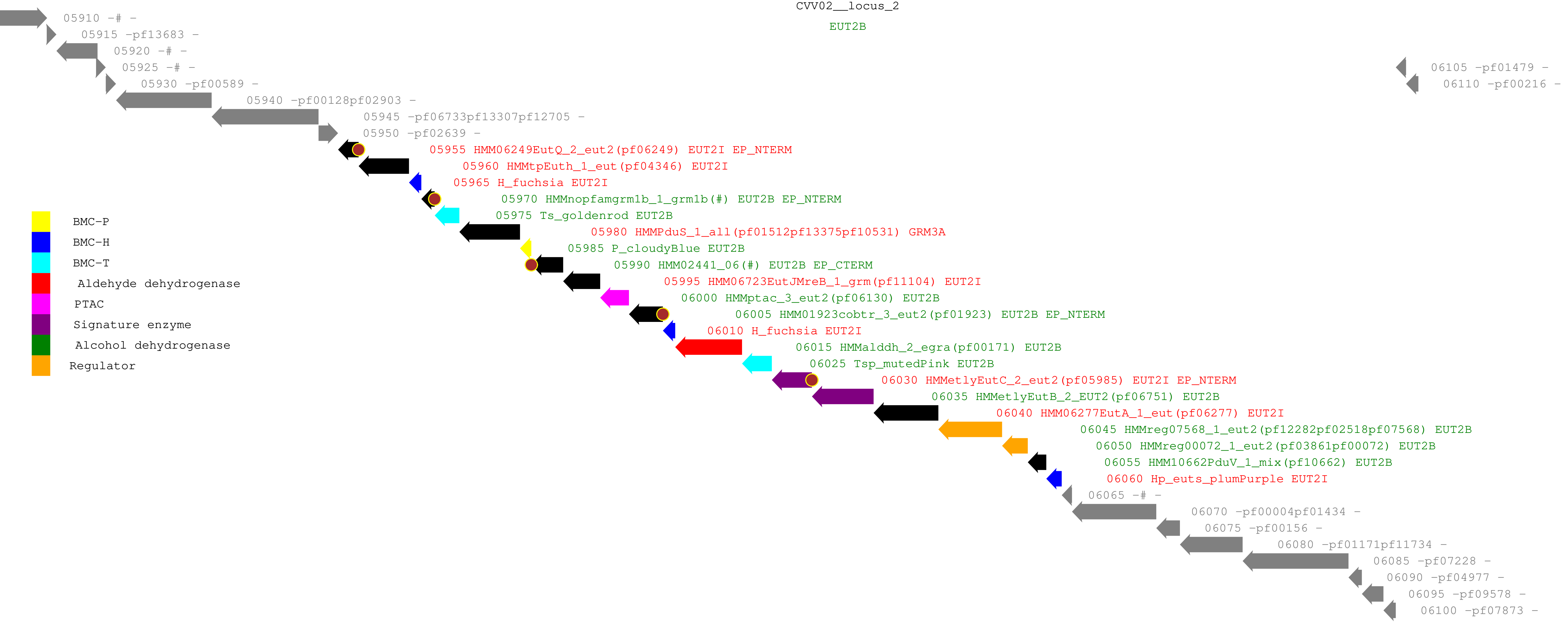

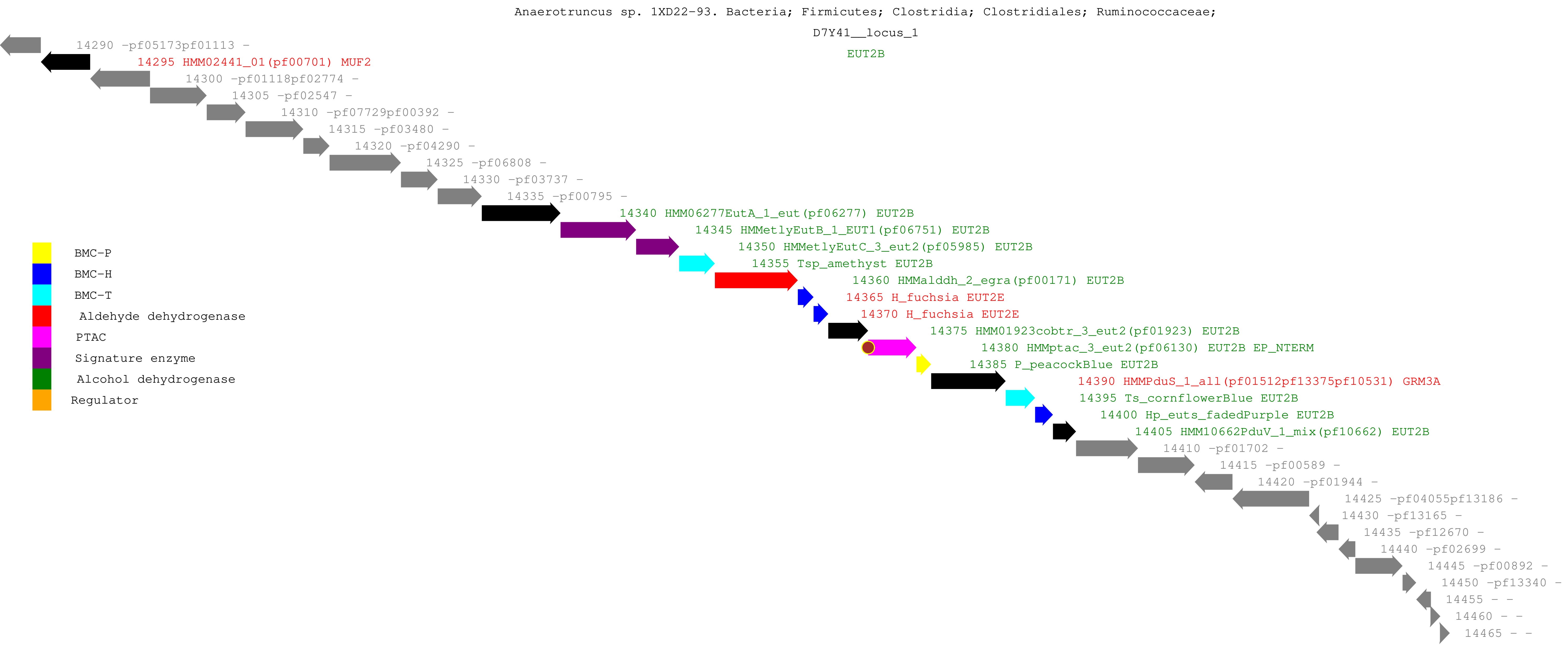

Anaerotruncus sp. 1XD22-93. Bacteria; Firmicutes; Clostridia; Clostridiales; Ruminococcaceae;
D7Y41__locus_1
EUT2B
14290 -pf05173pf01113 -
14295 HMM02441_01(pf00701) MUF2
14300 -pf01118pf02774 -
14305 -pf02547 -
14310 -pf07729pf00392 -
14315 -pf03480 -
14320 -pf04290 -
14325 -pf06808 -
14330 -pf03737 -
14335 -pf00795 -
14340 HMM06277EutA_1_eut(pf06277) EUT2B
14345 HMMetlyEutB_1_EUT1(pf06751) EUT2B
14350 HMMetlyEutC_3_eut2(pf05985) EUT2B
14355 Tsp_amethyst EUT2B
14360 HMMalddh_2_egra(pf00171) EUT2B
14365 H_fuchsia EUT2E
14370 H_fuchsia EUT2E
14375 HMM01923cobtr_3_eut2(pf01923) EUT2B
14380 HMMptac_3_eut2(pf06130) EUT2B EP_NTERM
14385 P_peacockBlue EUT2B
14390 HMMPduS_1_all(pf01512pf13375pf10531) GRM3A
14395 Ts_cornflowerBlue EUT2B
14400 Hp_euts_fadedPurple EUT2B
14405 HMM10662PduV_1_mix(pf10662) EUT2B
14410 -pf01702 -
14415 -pf00589 -
14420 -pf01944 -
14425 -pf04055pf13186 -
14430 -pf13165 -
14435 -pf12670 -
14440 -pf02699 -
14445 -pf00892 -
14450 -pf13340 -
14455 - -
14460 - -
14465 - -
BMC-P
BMC-H
BMC-T
Aldehyde dehydrogenase
PTAC
Signature enzyme
Alcohol dehydrogenase
Regulator

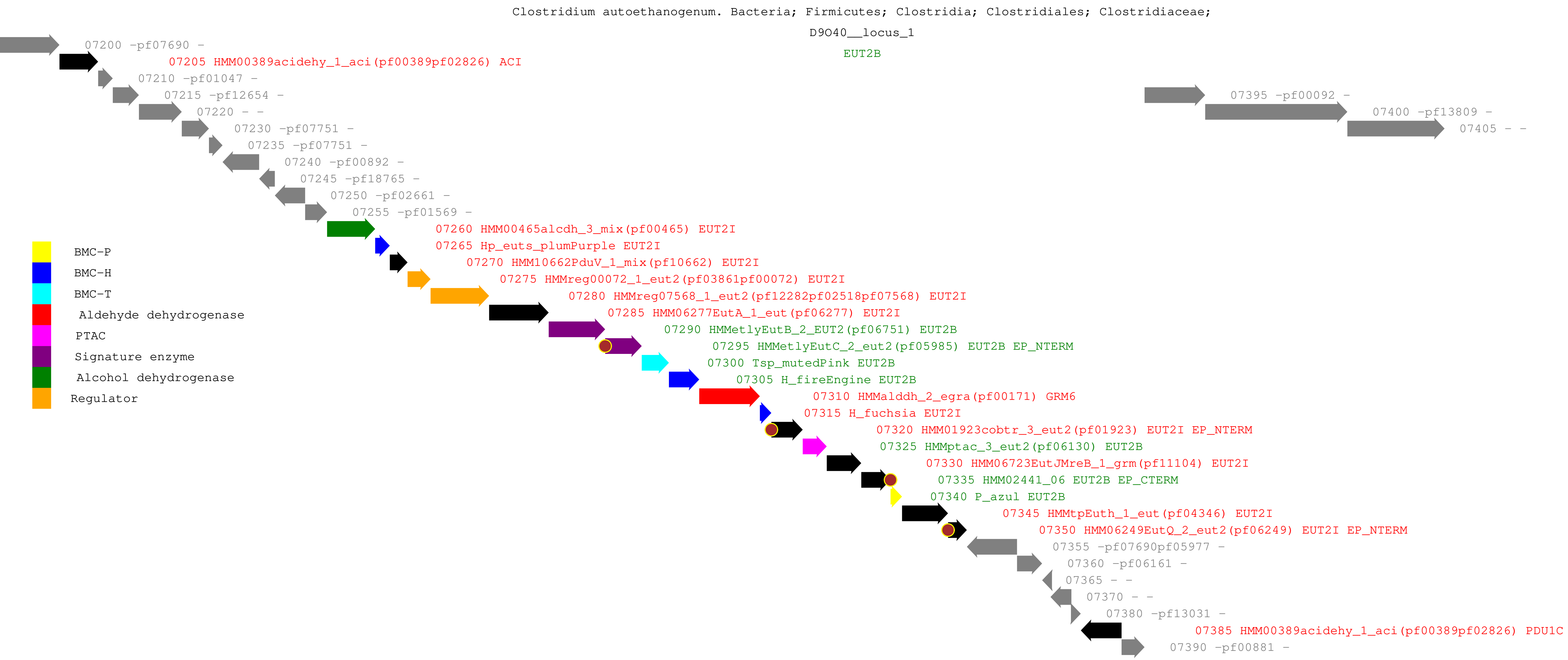

Clostridium autoethanogenum. Bacteria; Firmicutes; Clostridia; Clostridiales; Clostridiaceae;
D9040__locus_1
EUT2B
07200 -pf07690 -
07205 HMM00389acidehy_1_aci(pf00389pf02826) ACI
07210 -pf01047 -
07215 -pf12654 -
07220 - -
07230 -pf07751 -
07235 -pf07751 -
07240 -pf00892 -
07245 -pf18765 -
07250 -pf02661 -
07255 -pf01569 -
07260 HMM00465alcdh_3_mix(pf00465) EUT2I
07265 Hp_euts_plumPurple EUT2I
07270 HMM10662PduV_1_mix(pf10662) EUT2I
07275 HMMreg00072_1_eut2(pf03861pf00072) EUT2I
07280 HMMreg07568_1_eut2(pf12282pf02518pf07568) EUT2I
07285 HMM06277EutA_1_eut(pf06277) EUT2I
07290 HMMetlyEutB_2_EUT2(pf06751) EUT2B
07295 HMMetlyEutC_2_eut2(pf05985) EUT2B EP_NTERM
07300 Tsp_mutedPink EUT2B
07305 H_fireEngine EUT2B
07310 HMMaldh_2_egra(pf00171) GRM6
07315 H_fuchsia EUT2I
07320 HMM01923cobtr_3_eut2(pf01923) EUT2I EP_NTERM
07325 HMMptac_3_eut2(pf06130) EUT2B
07330 HMM06723EutJMreB_1_grm(pf11104) EUT2I
07335 HMM02441_06 EUT2B EP_CTERM
07340 P_azul EUT2B
07345 HMMtpEuth_1_eut(pf04346) EUT2I
07350 HMM06249EutQ_2_eut2(pf06249) EUT2I EP_NTERM
07355 -pf07690pf05977 -
07360 -pf06161 -
07365 - -
07370 - -
07380 -pf13031 -
07385 HMM00389acidehy_1_aci(pf00389pf02826) PDU1C
07390 -pf00881 -
07395 -pf00092 -
07400 -pf13809 -
07405 - -
BMC-P
BMC-H
BMC-T
Aldehyde dehydrogenase
PTAC
Signature enzyme
Alcohol dehydrogenase
Regulator

Clostridiales bacterium. Bacteria; Firmicutes; Clostridia; Clostridiales.

DBX66__locus_1

EUT2B

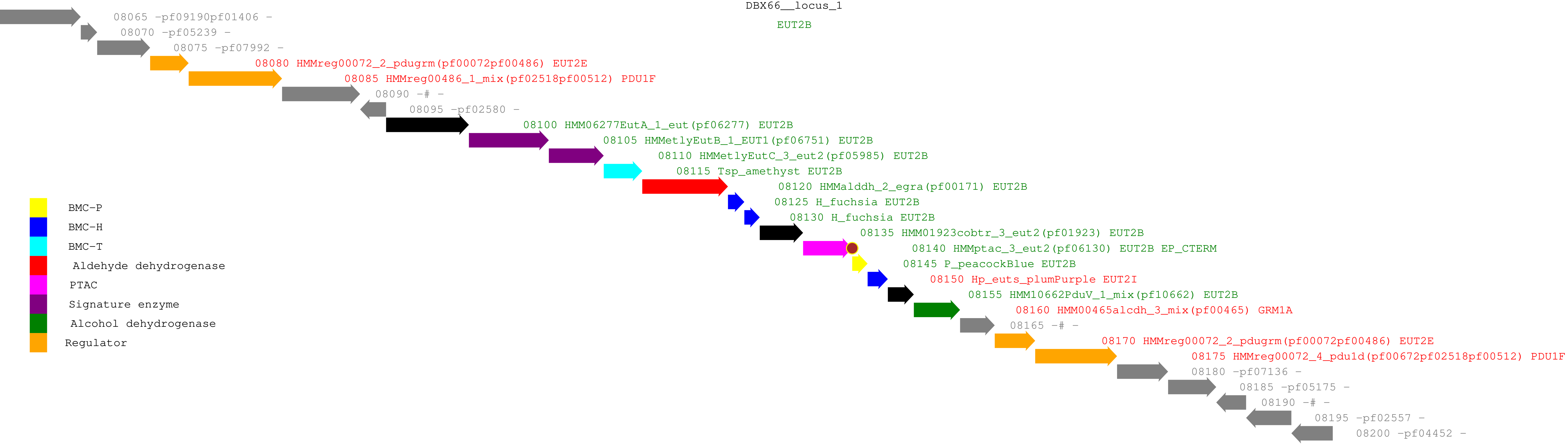

Lachnospiraceae bacterium. Bacteria; Firmicutes; Clostridia; Clostridiales; Lachnospiraceae.

DCR27__locus_1

EUT2B

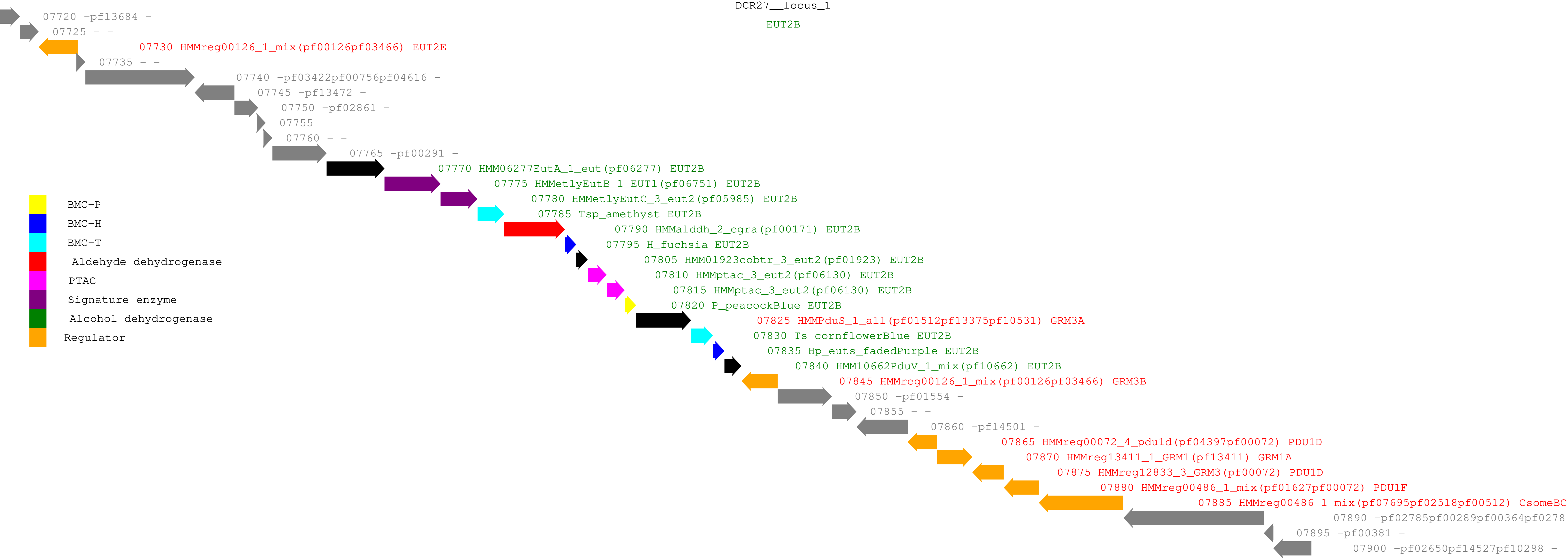

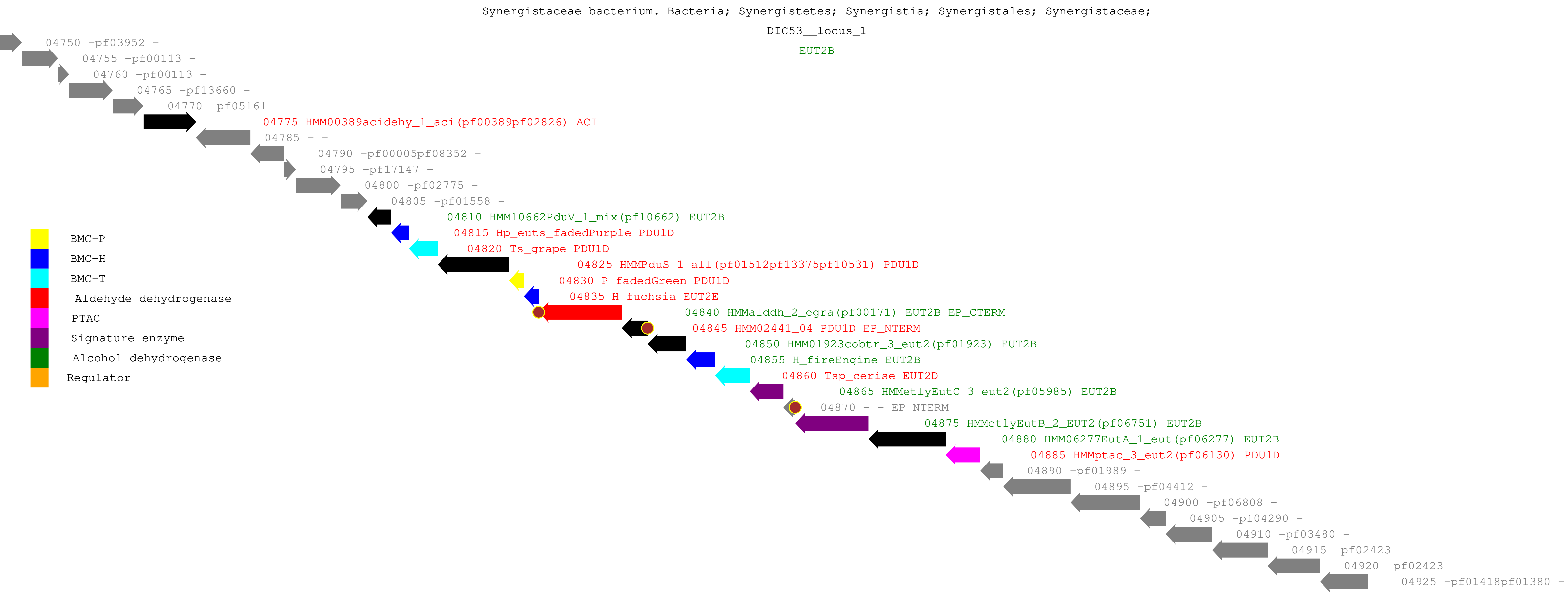
Synergistaceae bacterium. Bacteria; Synergistetes; Synergistia; Synergistales; Synergistaceae;
DIC53__locus_1
EUT2B
04750 -pf03952 -
04755 -pf00113 -
04760 -pf00113 -
04765 -pf13660 -
04770 -pf05161 -
04775 HMM00389acidehy_1_aci(pf00389pf02826) ACI
04785 - -
04790 -pf00005pf08352 -
04795 -pf17147 -
04800 -pf02775 -
04805 -pf01558 -
04810 HMM10662PduV_1_mix(pf10662) EUT2B
04815 Hp_euts_fadedPurple PDU1D
04820 Ts_grape PDU1D
04825 HMMPduS_1_all(pf01512pf13375pf10531) PDU1D
04830 P_fadedGreen PDU1D
04835 H_fuchsia EUT2E
04840 HMMalddh_2_egra(pf00171) EUT2B EP_CTERM
04845 HMM02441_04 PDU1D EP_NTERM
04850 HMM01923cobtr_3_eut2(pf01923) EUT2B
04855 H_fireEngine EUT2B
04860 Tsp_cerise EUT2D
04865 HMMetlyEutC_3_eut2(pf05985) EUT2B
04870 - - EP_NTERM
04875 HMMetlyEutB_2_EUT2(pf06751) EUT2B
04880 HMM06277EutA_1_eut(pf06277) EUT2B
04885 HMMptac_3_eut2(pf06130) PDU1D
04890 -pf01989 -
04895 -pf04412 -
04900 -pf06808 -
04905 -pf04290 -
04910 -pf03480 -
04915 -pf02423 -
04920 -pf02423 -
04925 -pf01418pf01380 -
BMC-P
BMC-H
BMC-T
Aldehyde dehydrogenase
PTAC
Signature enzyme
Alcohol dehydrogenase
Regulator

Clostridium sp. AWRP. Bacteria; Firmicutes; Clostridia; Clostridiales; Clostridiaceae;

DMR38__locus_2

EUT2B

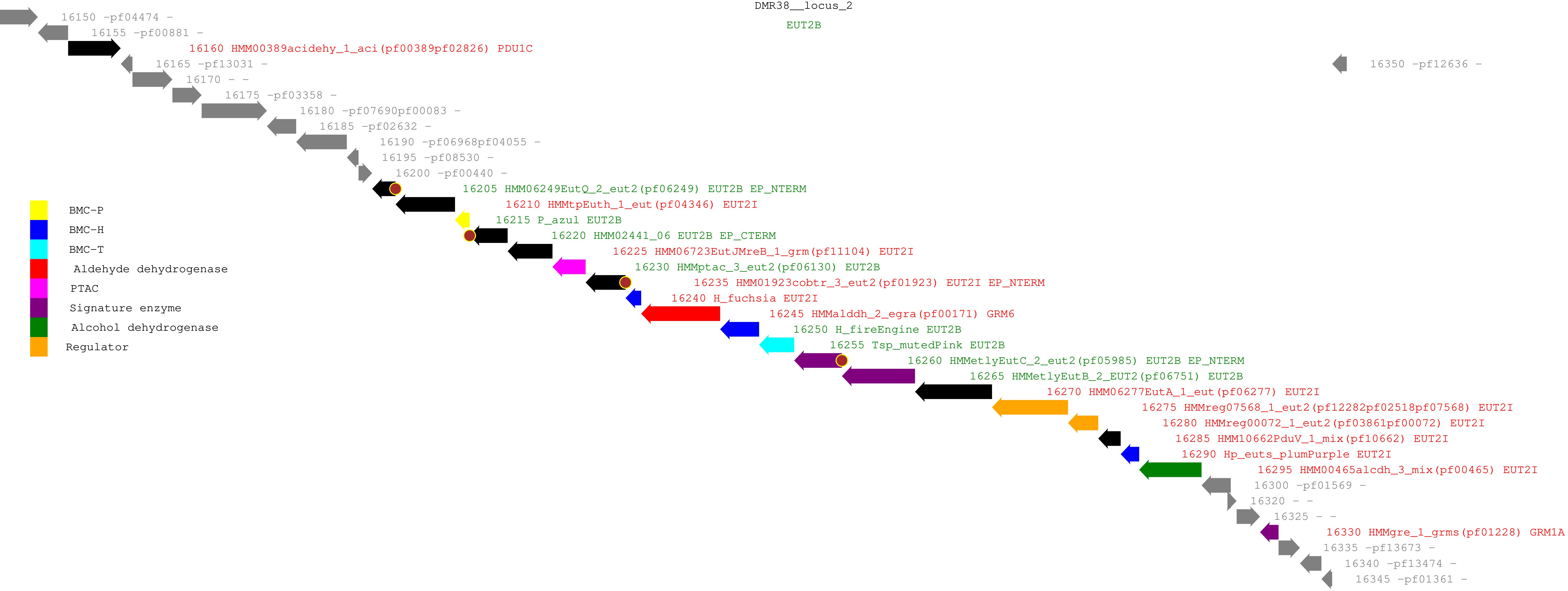

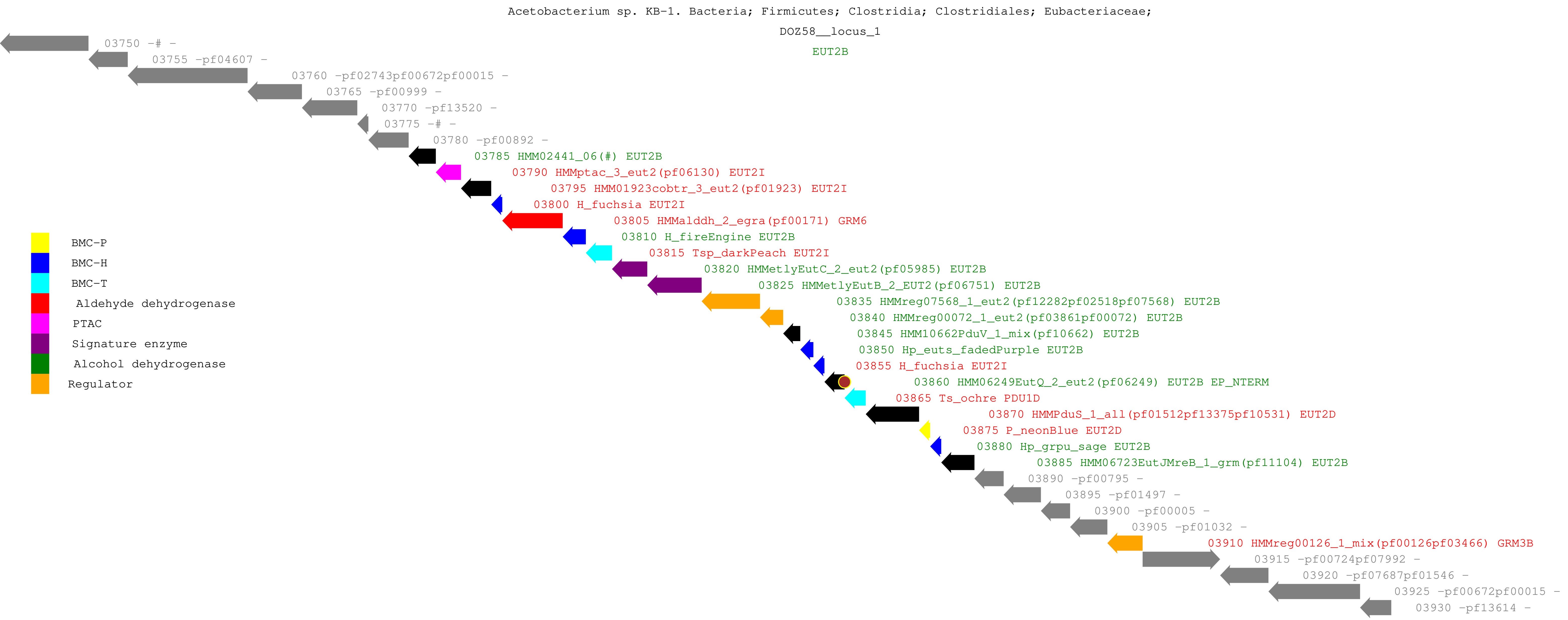
Acetobacterium sp. KB-1. Bacteria; Firmicutes; Clostridia; Clostridiales; Eubacteriaceae;
DOZ58__locus_1
EUT2B
03750 -# -
03755 -pf04607 -
03760 -pf02743pf00672pf00015 -
03765 -pf00999 -
03770 -pf13520 -
03775 -# -
03780 -pf00892 -
03785 HMM02441_06(#) EUT2B
03790 HMMptac_3_eut2(pf06130) EUT2I
03795 HMM01923cobtr_3_eut2(pf01923) EUT2I
03800 H_fuchsia EUT2I
03805 HMMalddh_2_egra(pf00171) GRM6
03810 H_fireEngine EUT2B
03815 Tsp_darkPeach EUT2I
03820 HMMetlyEutC_2_eut2(pf05985) EUT2B
03825 HMMetlyEutB_2_EUT2(pf06751) EUT2B
03835 HMMreg07568_1_eut2(pf12282pf02518pf07568) EUT2B
03840 HMMreg00072_1_eut2(pf03861pf00072) EUT2B
03845 HMM10662PduV_1_mix(pf10662) EUT2B
03850 Hp_euts_fadedPurple EUT2B
03855 H_fuchsia EUT2I
03860 HMM06249EutQ_2_eut2(pf06249) EUT2B EP_NTERM
03865 Ts_ochre PDU1D
03870 HMMPduS_1_all(pf01512pf13375pf10531) EUT2D
03875 P_neonBlue EUT2D
03880 Hp_grpu_sage EUT2B
03885 HMM06723EutJMreB_1_grm(pf11104) EUT2B
03890 -pf00795 -
03895 -pf01497 -
03900 -pf00005 -
03905 -pf01032 -
03910 HMMreg00126_1_mix(pf00126pf03466) GRM3B
03915 -pf00724pf07992 -
03920 -pf07687pf01546 -
03925 -pf00672pf00015 -
03930 -pf13614 -
BMC-P
BMC-H
BMC-T
Aldehyde dehydrogenase
PTAC
Signature enzyme
Alcohol dehydrogenase
Regulator

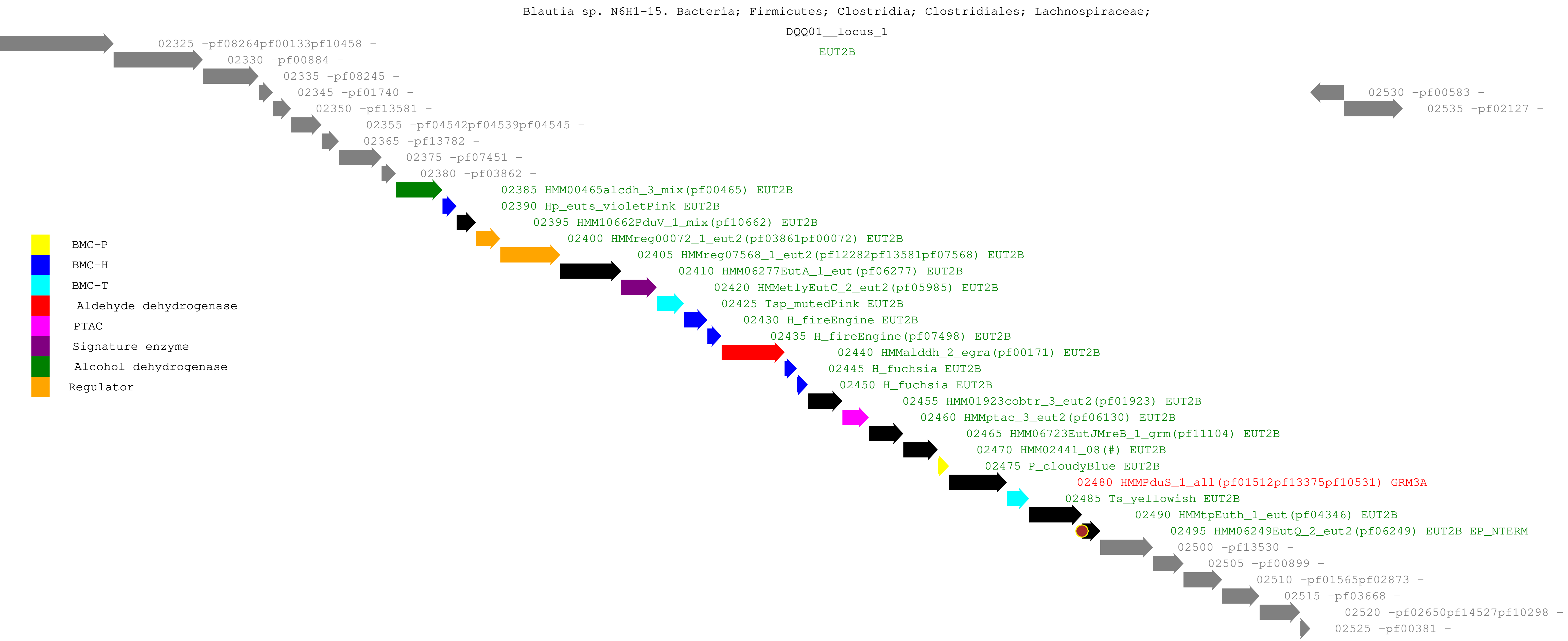

Blautia sp. N6H1-15. Bacteria; Firmicutes; Clostridia; Clostridiales; Lachnospiraceae;
DQQ01__locus_1
EUT2B
02325 -pf08264pf00133pf10458 -
02330 -pf00884 -
02335 -pf08245 -
02345 -pf01740 -
02350 -pf13581 -
02355 -pf04542pf04539pf04545 -
02365 -pf13782 -
02375 -pf07451 -
02380 -pf03862 -
02385 HMM00465alcdh_3_mix(pf00465) EUT2B
02390 Hp_euts_violetPink EUT2B
02395 HMM10662PduV_1_mix(pf10662) EUT2B
02400 HMMreg00072_1_eut2(pf03861pf00072) EUT2B
02405 HMMreg07568_1_eut2(pf12282pf13581pf07568) EUT2B
02410 HMM06277EutA_1_eut(pf06277) EUT2B
02420 HMMetlyEutC_2_eut2(pf05985) EUT2B
02425 Tsp_mutedPink EUT2B
02430 H_fireEngine EUT2B
02435 H_fireEngine(pf07498) EUT2B
02440 HMMalddh_2_egra(pf00171) EUT2B
02445 H_fuchsia EUT2B
02450 H_fuchsia EUT2B
02455 HMM01923cobtr_3_eut2(pf01923) EUT2B
02460 HMMptac_3_eut2(pf06130) EUT2B
02465 HMM06723EutJMreB_1_grm(pf11104) EUT2B
02470 HMM02441_08(#) EUT2B
02475 P_cloudyBlue EUT2B
02480 HMMPduS_1_all(pf01512pf13375pf10531) GRM3A
02485 Ts_yellowish EUT2B
02490 HMMtpEuth_1_eut(pf04346) EUT2B
02495 HMM06249EutQ_2_eut2(pf06249) EUT2B EP_NTERM
02500 -pf13530 -
02505 -pf00899 -
02510 -pf01565pf02873 -
02515 -pf03668 -
02520 -pf02650pf14527pf10298 -
02525 -pf00381 -
02530 -pf00583 -
02535 -pf02127 -
BMC-P
BMC-H
BMC-T
Aldehyde dehydrogenase
PTAC
Signature enzyme
Alcohol dehydrogenase
Regulator

Clostridium sp. AF37-5AT. Bacteria; Firmicutes; Clostridia; Clostridiales; Clostridiaceae;

DW020__locus_1

EUT2B

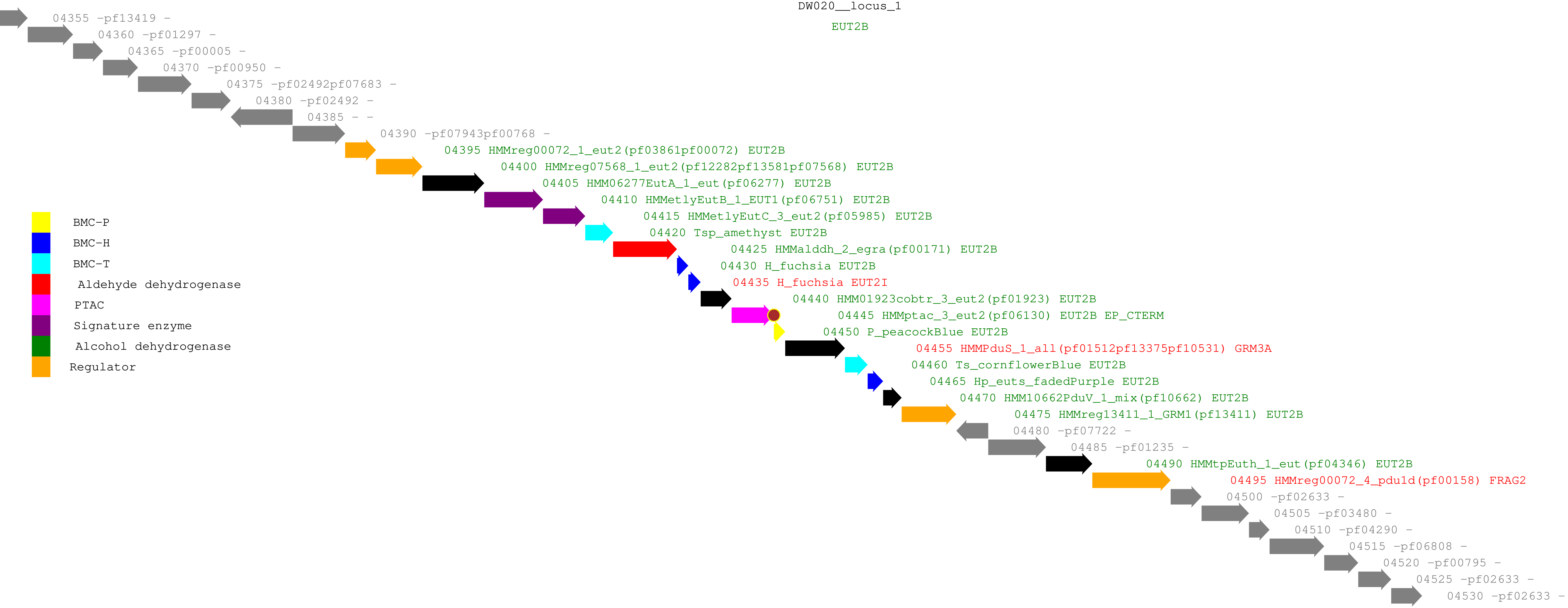

Clostridiaceae bacterium AF42-6. Bacteria; Firmicutes; Clostridia; Clostridiales; Clostridiaceae.

DW058__locus_1

EUT2B

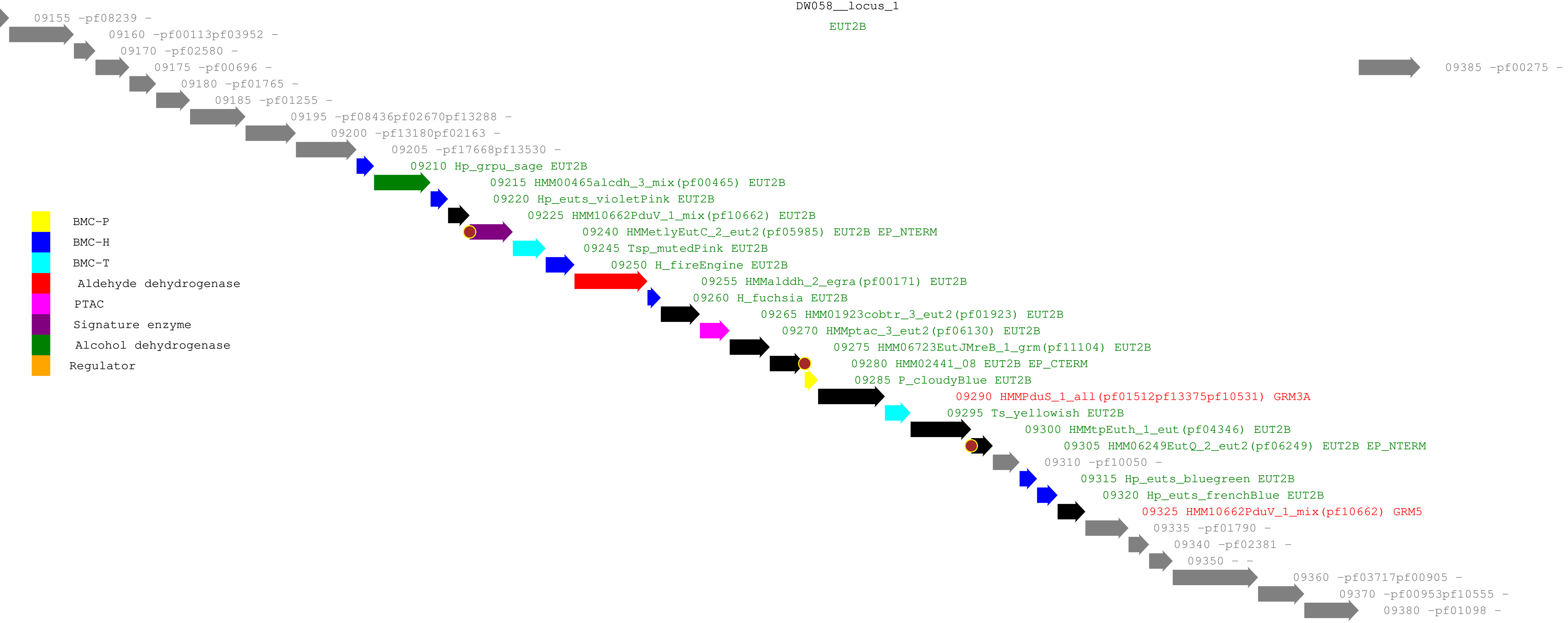

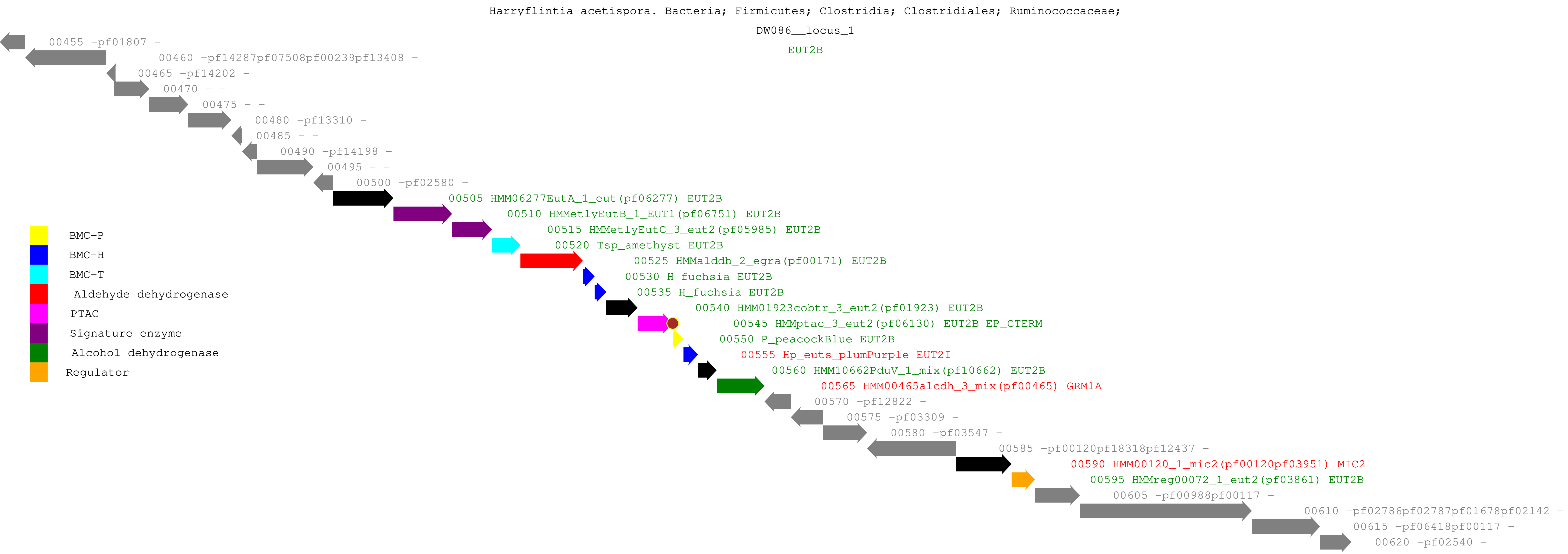
Harryflintia acetispora. Bacteria; Firmicutes; Clostridia; Clostridiales; Ruminococcaceae;
DW086__locus_1
EUT2B
00455 -pf01807 -
00460 -pf14287pf07508pf00239pf13408 -
00465 -pf14202 -
00470 - -
00475 - -
00480 -pf13310 -
00485 - -
00490 -pf14198 -
00495 - -
00500 -pf02580 -
00505 HMM06277EutA_1_eut(pf06277) EUT2B
00510 HMMetlyEutB_1_EUT1(pf06751) EUT2B
00515 HMMetlyEutC_3_eut2(pf05985) EUT2B
00520 Tsp_amethyst EUT2B
00525 HMMalddh_2_egra(pf00171) EUT2B
00530 H_fuchsia EUT2B
00535 H_fuchsia EUT2B
00540 HMM01923cobtr_3_eut2(pf01923) EUT2B
00545 HMMptac_3_eut2(pf06130) EUT2B EP_CTERM
00550 P_peacockBlue EUT2B
00555 Hp_euts_plumPurple EUT2I
00560 HMM10662PduV_1_mix(pf10662) EUT2B
00565 HMM00465alcdh_3_mix(pf00465) GRM1A
00570 -pf12822 -
00575 -pf03309 -
00580 -pf03547 -
00585 -pf00120pf18318pf12437 -
00590 HMM00120_1_mic2(pf00120pf03951) MIC2
00595 HMMreg00072_1_eut2(pf03861) EUT2B
00605 -pf00988pf00117 -
00610 -pf02786pf02787pf01678pf02142 -
00615 -pf06418pf00117 -
00620 -pf02540 -
BMC-P
BMC-H
BMC-T
Aldehyde dehydrogenase
PTAC
Signature enzyme
Alcohol dehydrogenase
Regulator

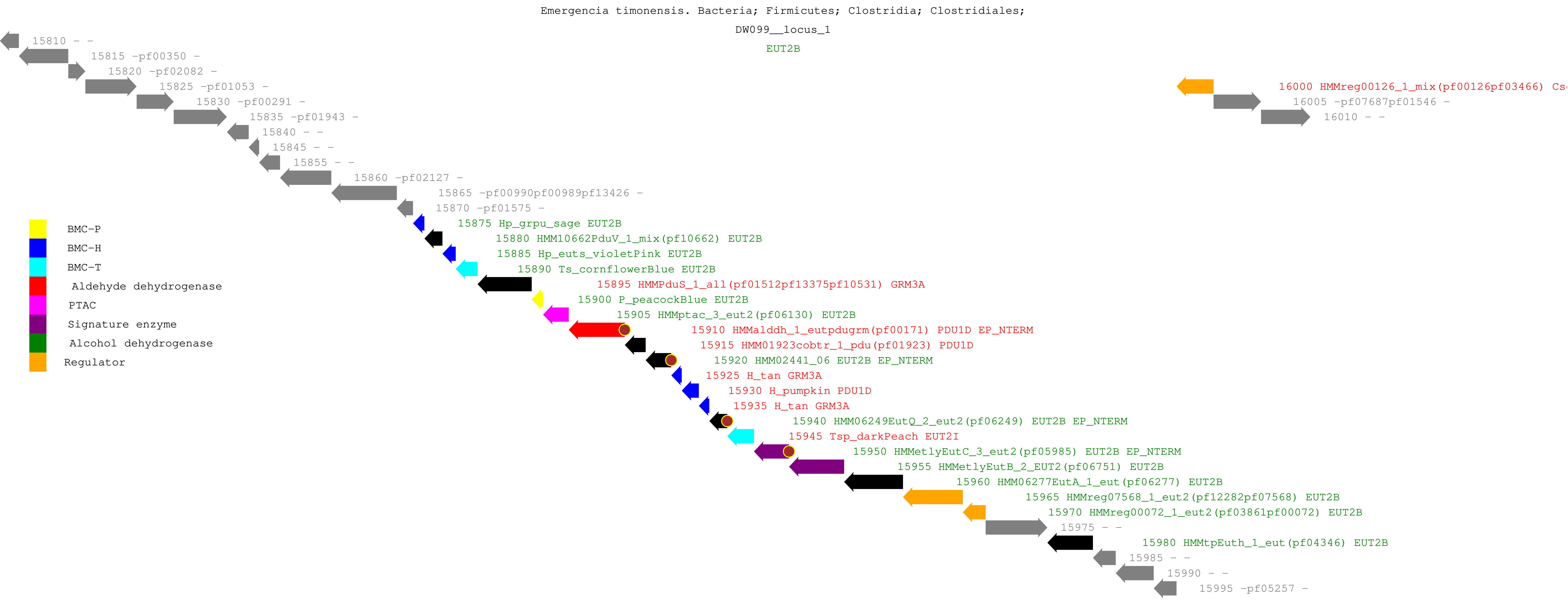
Emergencia timonensis. Bacteria; Firmicutes; Clostridia; Clostridiales;
DW099__locus_1
EUT2B
15810 - -
15815 -pf00350 -
15820 -pf02082 -
15825 -pf01053 -
15830 -pf00291 -
15835 -pf01943 -
15840 - -
15845 - -
15855 - -
15860 -pf02127 -
15865 -pf00990pf00989pf13426 -
15870 -pf01575 -
15875 Hp_grpu_sage EUT2B
15880 HMM10662PduV_1_mix(pf10662) EUT2B
15885 Hp_euts_violetPink EUT2B
15890 Ts_cornflowerBlue EUT2B
15895 HMMPduS_1_all(pf01512pf13375pf10531) GRM3A
15900 P_peacockBlue EUT2B
15905 HMMptac_3_eut2(pf06130) EUT2B
15910 HMMaldh_1_eutpdugrm(pf00171) PDU1D EP_NTERM
15915 HMM01923cobtr_1_pdu(pf01923) PDU1D
15920 HMM02441_06 EUT2B EP_NTERM
15925 H_tan GRM3A
15930 H_pumpkin PDU1D
15935 H_tan GRM3A
15940 HMM06249EutQ_2_eut2(pf06249) EUT2B EP_NTERM
15945 Tsp_darkPeach EUT2I
15950 HMMetlyEutC_3_eut2(pf05985) EUT2B EP_NTERM
15955 HMMetlyEutB_2_EUT2(pf06751) EUT2B
15960 HMM06277EutA_1_eut(pf06277) EUT2B
15965 HMMreg07568_1_eut2(pf12282pf07568) EUT2B
15970 HMMreg00072_1_eut2(pf03861pf00072) EUT2B
15975 - -
15980 HMMtpEuth_1_eut(pf04346) EUT2B
15985 - -
15990 - -
15995 -pf05257 -
16000 HMMreg00126_1_mix(pf00126pf03466) Cs
16005 -pf07687pf01546 -
16010 - -
BMC-P
BMC-H
BMC-T
Aldehyde dehydrogenase
PTAC
Signature enzyme
Alcohol dehydrogenase
Regulator

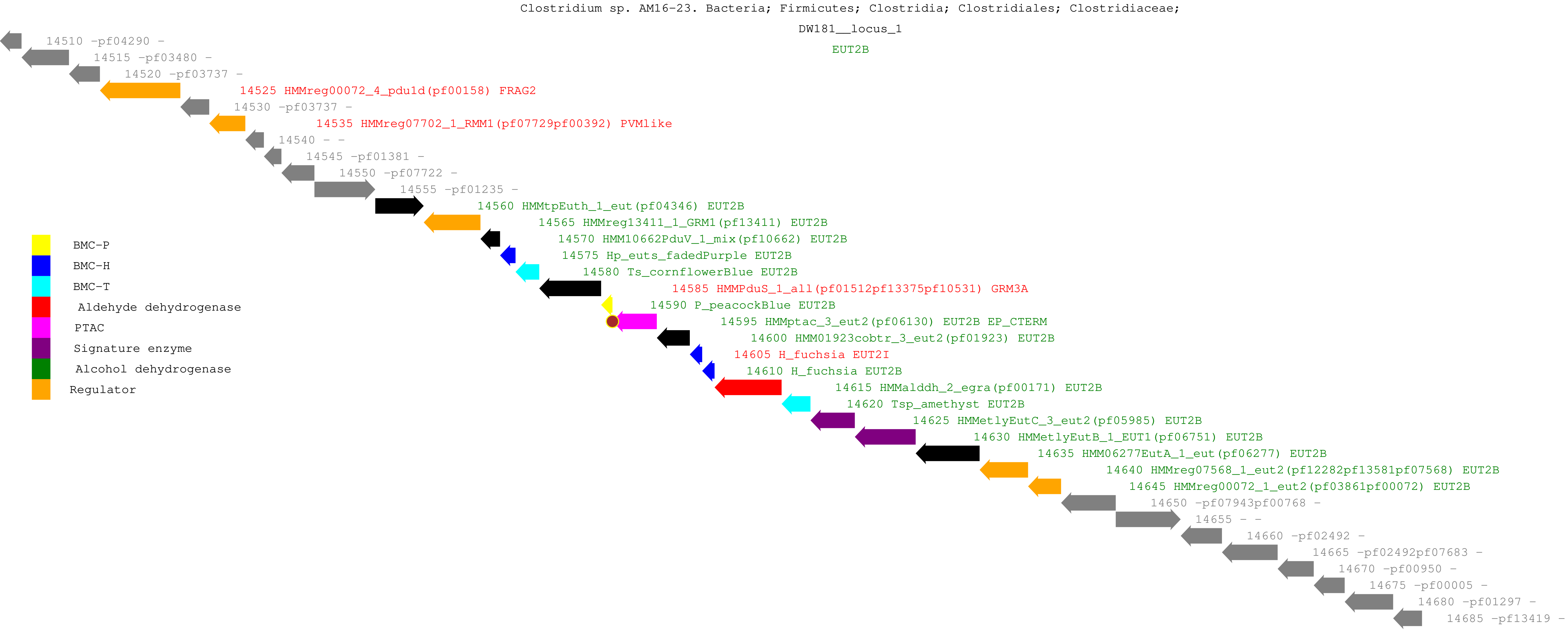
Clostridium sp. AM16-23. Bacteria; Firmicutes; Clostridia; Clostridiales; Clostridiaceae;
DW181__locus_1
EUT2B
14510 -pf04290 -
14515 -pf03480 -
14520 -pf03737 -
14525 HMMreg00072_4_pdu1d(pf00158) FRAG2
14530 -pf03737 -
14535 HMMreg07702_1_RMM1(pf07729pf00392) PVMlike
14540 - -
14545 -pf01381 -
14550 -pf07722 -
14555 -pf01235 -
14560 HMMtpEuth_1_eut(pf04346) EUT2B
14565 HMMreg13411_1_GRM1(pf13411) EUT2B
14570 HMM10662PduV_1_mix(pf10662) EUT2B
14575 Hp_euts_fadedPurple EUT2B
14580 Ts_cornflowerBlue EUT2B
14585 HMMPduS_1_all(pf01512pf13375pf10531) GRM3A
14590 P_peacockBlue EUT2B
14595 HMMptac_3_eut2(pf06130) EUT2B EP_CTERM
14600 HMM01923cobtr_3_eut2(pf01923) EUT2B
14605 H_fuchsia EUT2I
14610 H_fuchsia EUT2B
14615 HMMalddh_2_egra(pf00171) EUT2B
14620 Tsp_amethyst EUT2B
14625 HMMetlyEutC_3_eut2(pf05985) EUT2B
14630 HMMetlyEutB_1_EUT1(pf06751) EUT2B
14635 HMM06277EutA_1_eut(pf06277) EUT2B
14640 HMMreg07568_1_eut2(pf12282pf13581pf07568) EUT2B
14645 HMMreg00072_1_eut2(pf03861pf00072) EUT2B
14650 -pf07943pf00768 -
14655 - -
14660 -pf02492 -
14665 -pf02492pf07683 -
14670 -pf00950 -
14675 -pf00005 -
14680 -pf01297 -
14685 -pf13419 -
BMC-P
BMC-H
BMC-T
Aldehyde dehydrogenase
PTAC
Signature enzyme
Alcohol dehydrogenase
Regulator

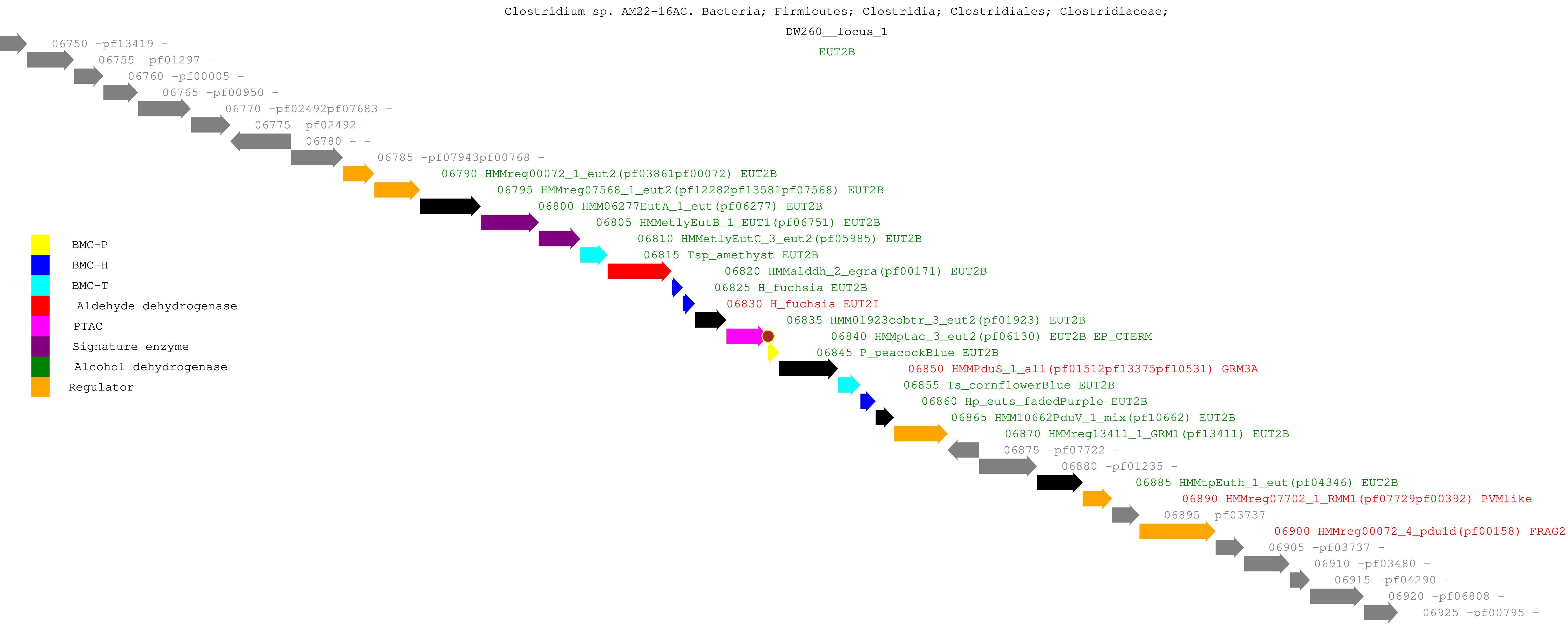
Clostridium sp. AM22-16AC. Bacteria; Firmicutes; Clostridia; Clostridiales; Clostridiaceae;
DW260__locus_1
EUT2B
06750 -pf13419 -
06755 -pf01297 -
06760 -pf00005 -
06765 -pf00950 -
06770 -pf02492pf07683 -
06775 -pf02492 -
06780 - -
06785 -pf07943pf00768 -
06790 HMMreg00072_1_eut2(pf03861pf00072) EUT2B
06795 HMMreg07568_1_eut2(pf12282pf13581pf07568) EUT2B
06800 HMM06277EutA_1_eut(pf06277) EUT2B
06805 HMMetlyEutB_1_EUT1(pf06751) EUT2B
06810 HMMetlyEutC_3_eut2(pf05985) EUT2B
06815 Tsp_amethyst EUT2B
06820 HMMalddh_2_egra(pf00171) EUT2B
06825 H_fuchsia EUT2B
06830 H_fuchsia EUT2I
06835 HMM01923cobtr_3_eut2(pf01923) EUT2B
06840 HMMptac_3_eut2(pf06130) EUT2B EP_CTERM
06845 P_peacockBlue EUT2B
06850 HMMPduS_1_all(pf01512pf13375pf10531) GRM3A
06855 Ts_cornflowerBlue EUT2B
06860 Hp_euts_fadedPurple EUT2B
06865 HMM10662PduV_1_mix(pf10662) EUT2B
06870 HMMreg13411_1_GRM1(pf13411) EUT2B
06875 -pf07722 -
06880 -pf01235 -
06885 HMMtpEuth_1_eut(pf04346) EUT2B
06890 HMMreg07702_1_RMM1(pf07729pf00392) PVMlike
06895 -pf03737 -
06900 HMMreg00072_4_pdu1d(pf00158) FRAG2
06905 -pf03737 -
06910 -pf03480 -
06915 -pf04290 -
06920 -pf06808 -
06925 -pf00795 -
BMC-P
BMC-H
BMC-T
Aldehyde dehydrogenase
PTAC
Signature enzyme
Alcohol dehydrogenase
Regulator

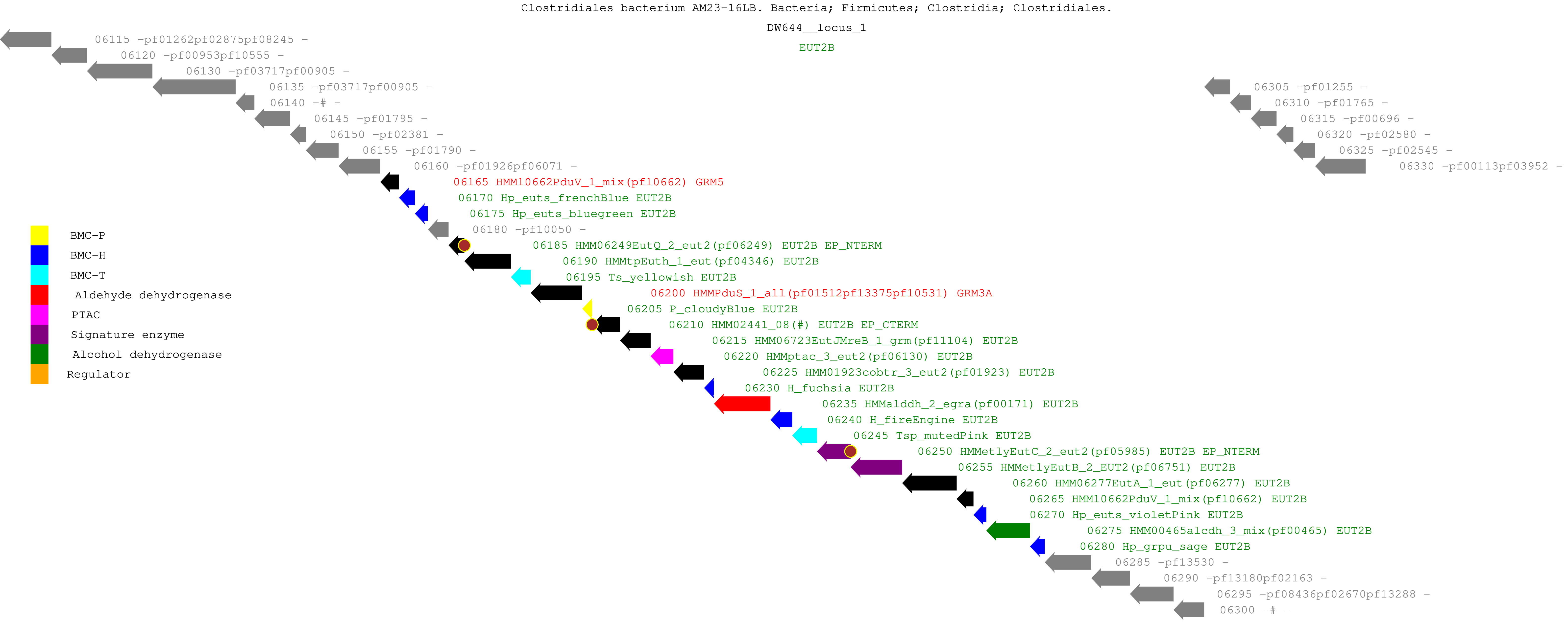

Clostridiales bacterium AM23-16LB. Bacteria; Firmicutes; Clostridia; Clostridiales.
DW644__locus_1
EUT2B
06115 -pf01262pf02875pf08245 -
06120 -pf00953pf10555 -
06130 -pf03717pf00905 -
06135 -pf03717pf00905 -
06140 -# -
06145 -pf01795 -
06150 -pf02381 -
06155 -pf01790 -
06160 -pf01926pf06071 -
06165 HMM10662PduV_1_mix(pf10662) GRM5
06170 Hp_euts_frenchBlue EUT2B
06175 Hp_euts_bluegreen EUT2B
06180 -pf10050 -
06185 HMM06249EutQ_2_eut2(pf06249) EUT2B EP_NTERM
06190 HMMtpEuth_1_eut(pf04346) EUT2B
06195 Ts_yellowish EUT2B
06200 HMMPduS_1_all(pf01512pf13375pf10531) GRM3A
06205 P_cloudyBlue EUT2B
06210 HMM02441_08(#) EUT2B EP_CTERM
06215 HMM06723EutJMreB_1_grm(pf11104) EUT2B
06220 HMMptac_3_eut2(pf06130) EUT2B
06225 HMM01923cobtr_3_eut2(pf01923) EUT2B
06230 H_fuchsia EUT2B
06235 HMMalddh_2_egra(pf00171) EUT2B
06240 H_fireEngine EUT2B
06245 Tsp_mutedPink EUT2B
06250 HMMetlyEutC_2_eut2(pf05985) EUT2B EP_NTERM
06255 HMMetlyEutB_2_EUT2(pf06751) EUT2B
06260 HMM06277EutA_1_eut(pf06277) EUT2B
06265 HMM10662PduV_1_mix(pf10662) EUT2B
06270 Hp_euts_violetPink EUT2B
06275 HMM00465alcdh_3_mix(pf00465) EUT2B
06280 Hp_grpu_sage EUT2B
06285 -pf13530 -
06290 -pf13180pf02163 -
06295 -pf08436pf02670pf13288 -
06300 -# -
06305 -pf01255 -
06310 -pf01765 -
06315 -pf00696 -
06320 -pf02580 -
06325 -pf02545 -
06330 -pf00113pf03952 -
BMC-P
BMC-H
BMC-T
Aldehyde dehydrogenase
PTAC
Signature enzyme
Alcohol dehydrogenase
Regulator

Clostridiaceae bacterium AM27-36LB. Bacteria; Firmicutes; Clostridia; Clostridiales; Clostridiaceae.

DW725__locus_1

EUT2B

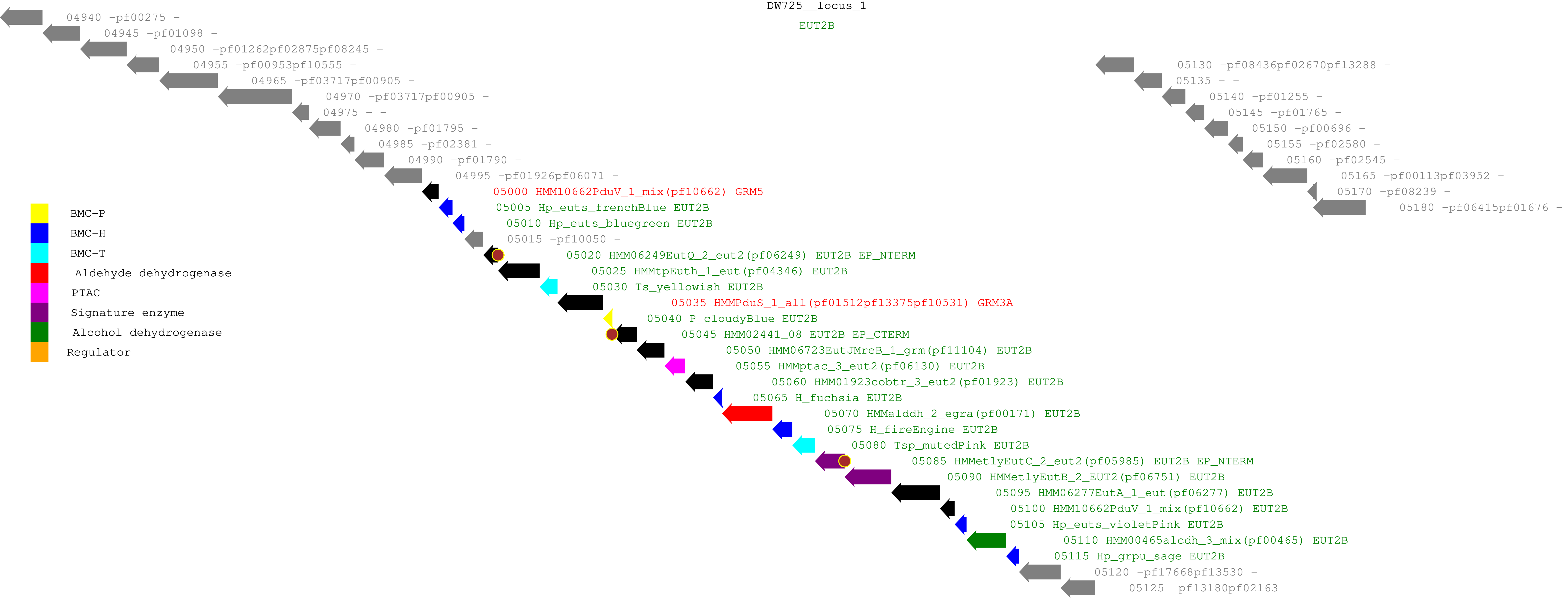

Coprococcus catus. Bacteria; Firmicutes; Clostridia; Clostridiales; Lachnospiraceae;

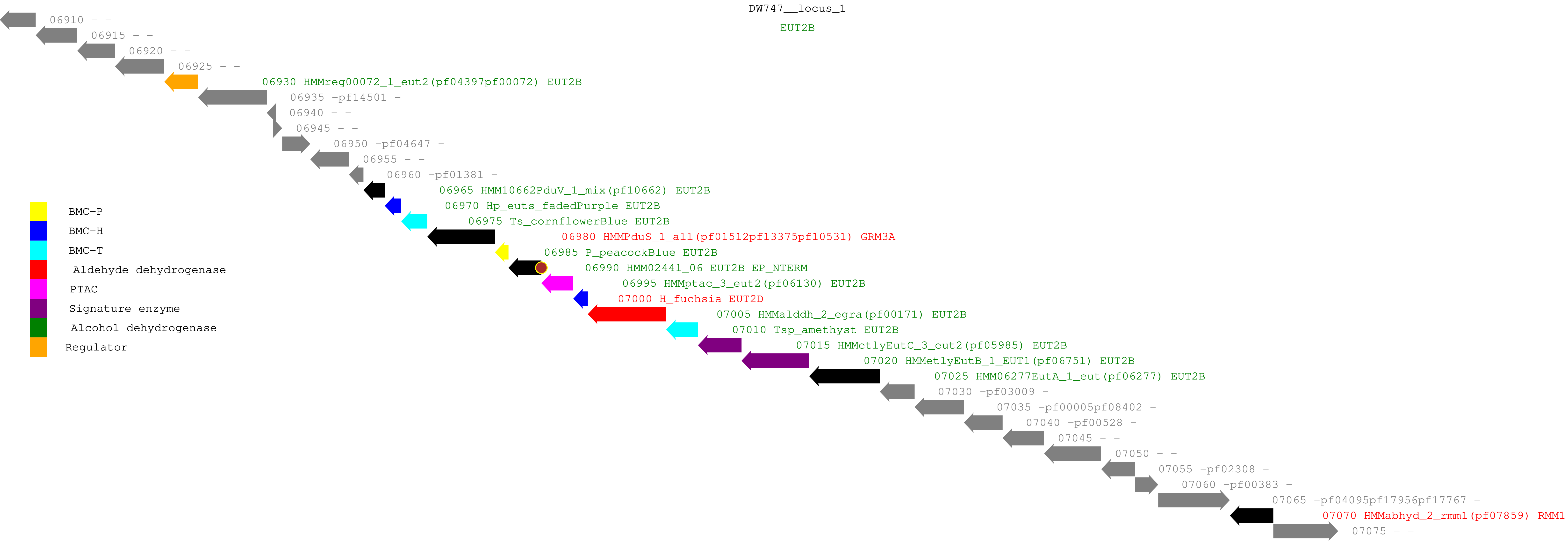

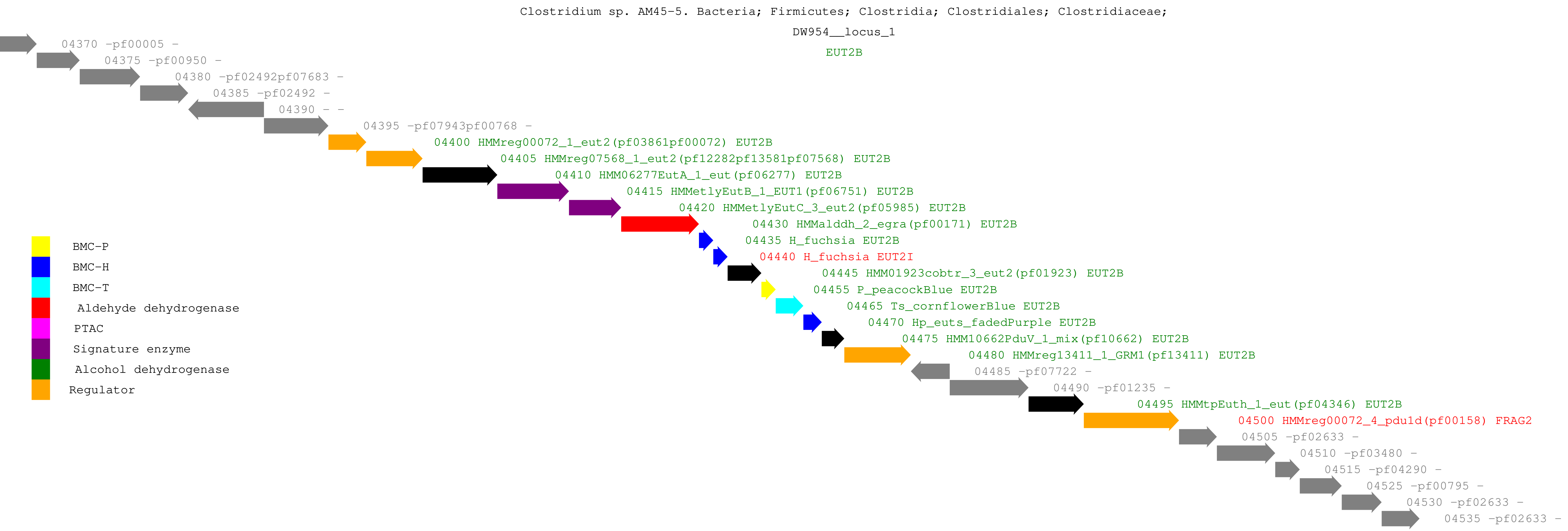
Clostridium sp. AM45-5. Bacteria; Firmicutes; Clostridia; Clostridiales; Clostridiaceae;
DW954__locus_1
EUT2B
04370 -pf00005 -
04375 -pf00950 -
04380 -pf02492pf07683 -
04385 -pf02492 -
04390 - -
04395 -pf07943pf00768 -
04400 HMMreg00072_1_eut2(pf03861pf00072) EUT2B
04405 HMMreg07568_1_eut2(pf12282pf13581pf07568) EUT2B
04410 HMM06277EutA_1_eut(pf06277) EUT2B
04415 HMMetlyEutB_1_EUT1(pf06751) EUT2B
04420 HMMetlyEutC_3_eut2(pf05985) EUT2B
04430 HMMalddh_2_egra(pf00171) EUT2B
04435 H_fuchsia EUT2B
04440 H_fuchsia EUT2I
04445 HMM01923cobtr_3_eut2(pf01923) EUT2B
04455 P_peacockBlue EUT2B
04465 Ts_cornflowerBlue EUT2B
04470 Hp_euts_fadedPurple EUT2B
04475 HMM10662PduV_1_mix(pf10662) EUT2B
04480 HMMreg13411_1_GRM1(pf13411) EUT2B
04485 -pf07722 -
04490 -pf01235 -
04495 HMMtpEuth_1_eut(pf04346) EUT2B
04500 HMMreg00072_4_pdu1d(pf00158) FRAG2
04505 -pf02633 -
04510 -pf03480 -
04515 -pf04290 -
04525 -pf00795 -
04530 -pf02633 -
04535 -pf02633 -
BMC-P
BMC-H
BMC-T
Aldehyde dehydrogenase
PTAC
Signature enzyme
Alcohol dehydrogenase
Regulator

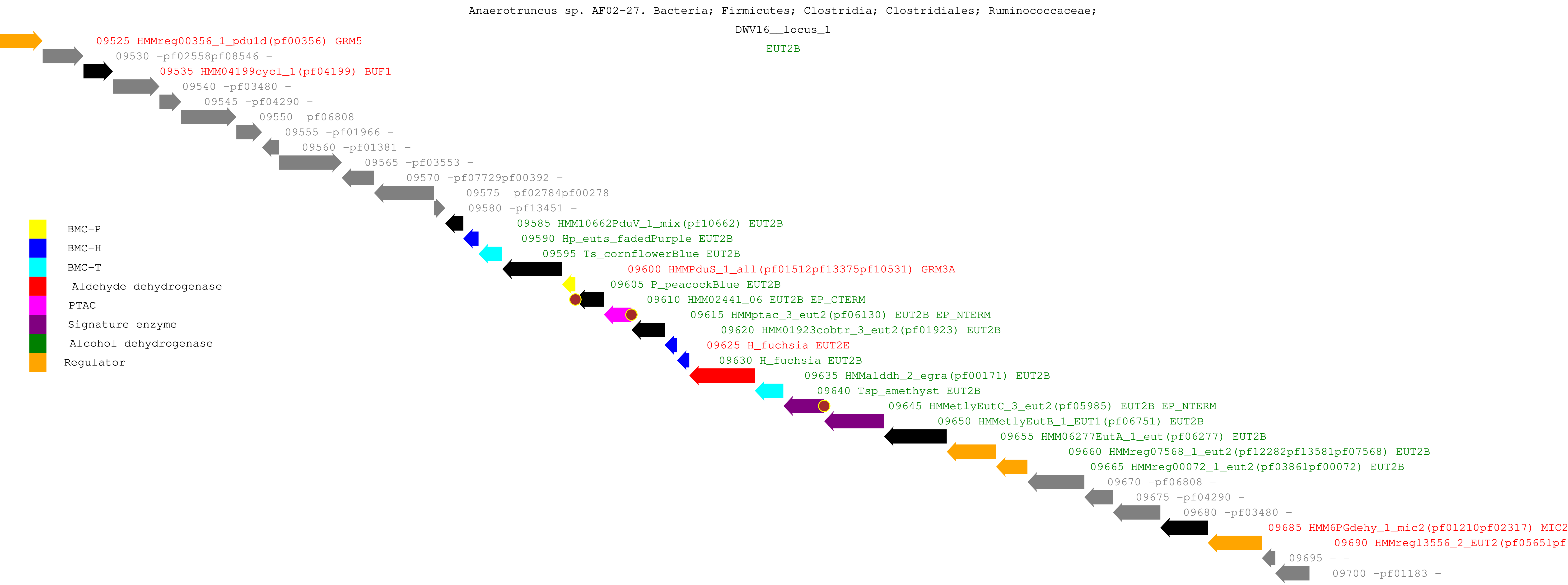
Anaerotruncus sp. AF02-27. Bacteria; Firmicutes; Clostridia; Clostridiales; Ruminococcaceae;
DWV16__locus_1
EUT2B
09525 HMMreg00356_1_pduld(pf00356) GRM5
09530 -pf02558pf08546 -
09535 HMM04199cycl_1(pf04199) BUF1
09540 -pf03480 -
09545 -pf04290 -
09550 -pf06808 -
09555 -pf01966 -
09560 -pf01381 -
09565 -pf03553 -
09570 -pf07729pf00392 -
09575 -pf02784pf00278 -
09580 -pf13451 -
09585 HMM10662PduV_1_mix(pf10662) EUT2B
09590 Hp_euts_fadedPurple EUT2B
09595 Ts_cornflowerBlue EUT2B
09600 HMMPduS_1_all(pf01512pf13375pf10531) GRM3A
09605 P_peacockBlue EUT2B
09610 HMM02441_06 EUT2B EP_CTERM
09615 HMMptac_3_eut2(pf06130) EUT2B EP_NTERM
09620 HMM01923cobtr_3_eut2(pf01923) EUT2B
09625 H_fuchsia EUT2E
09630 H_fuchsia EUT2B
09635 HMMaldh_2_egra(pf00171) EUT2B
09640 Tsp_amethyst EUT2B
09645 HMMetlyEutC_3_eut2(pf05985) EUT2B EP_NTERM
09650 HMMetlyEutB_1_EUT1(pf06751) EUT2B
09655 HMM06277EutA_1_eut(pf06277) EUT2B
09660 HMMreg07568_1_eut2(pf12282pf13581pf07568) EUT2B
09665 HMMreg00072_1_eut2(pf03861pf00072) EUT2B
09670 -pf06808 -
09675 -pf04290 -
09680 -pf03480 -
09685 HMM6PGdehy_1_mic2(pf01210pf02317) MIC2
09690 HMMreg13556_2_EUT2(pf05651pf
09695 - -
09700 -pf01183 -
BMC-P
BMC-H
BMC-T
Aldehyde dehydrogenase
PTAC
Signature enzyme
Alcohol dehydrogenase
Regulator

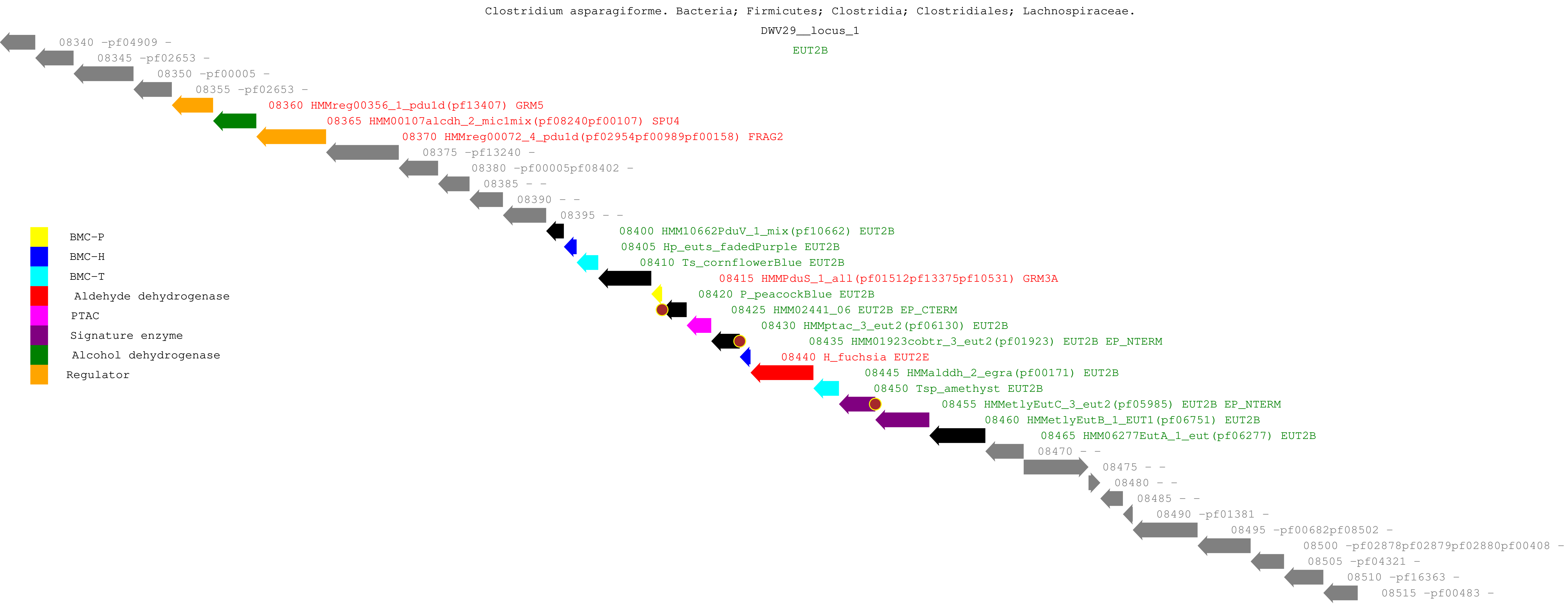
Clostridium asparagiforme. Bacteria; Firmicutes; Clostridia; Clostridiales; Lachnospiraceae.
DWV29__locus_1
EUT2B
08340 -pf04909 -
08345 -pf02653 -
08350 -pf00005 -
08355 -pf02653 -
08360 HMMreg00356_1_pdu1d(pf13407) GRM5
08365 HMM00107alcdh_2_mic1mix(pf08240pf00107) SPU4
08370 HMMreg00072_4_pdu1d(pf02954pf00989pf00158) FRAG2
08375 -pf13240 -
08380 -pf00005pf08402 -
08385 - -
08390 - -
08395 - -
08400 HMM10662PduV_1_mix(pf10662) EUT2B
08405 Hp_euts_fadedPurple EUT2B
08410 Ts_cornflowerBlue EUT2B
08415 HMMPduS_1_all(pf01512pf13375pf10531) GRM3A
08420 P_peacockBlue EUT2B
08425 HMM02441_06 EUT2B EP_CTERM
08430 HMMptac_3_eut2(pf06130) EUT2B
08435 HMM01923cobtr_3_eut2(pf01923) EUT2B EP_NTERM
08440 H_fuchsia EUT2E
08445 HMMalddh_2_egra(pf00171) EUT2B
08450 Tsp_amethyst EUT2B
08455 HMMetlyEutC_3_eut2(pf05985) EUT2B EP_NTERM
08460 HMMetlyEutB_1_EUT1(pf06751) EUT2B
08465 HMM06277EutA_1_eut(pf06277) EUT2B
08470 - -
08475 - -
08480 - -
08485 - -
08490 -pf01381 -
08495 -pf00682pf08502 -
08500 -pf02878pf02879pf02880pf00408 -
08505 -pf04321 -
08510 -pf16363 -
08515 -pf00483 -
BMC-P
BMC-H
BMC-T
Aldehyde dehydrogenase
PTAC
Signature enzyme
Alcohol dehydrogenase
Regulator

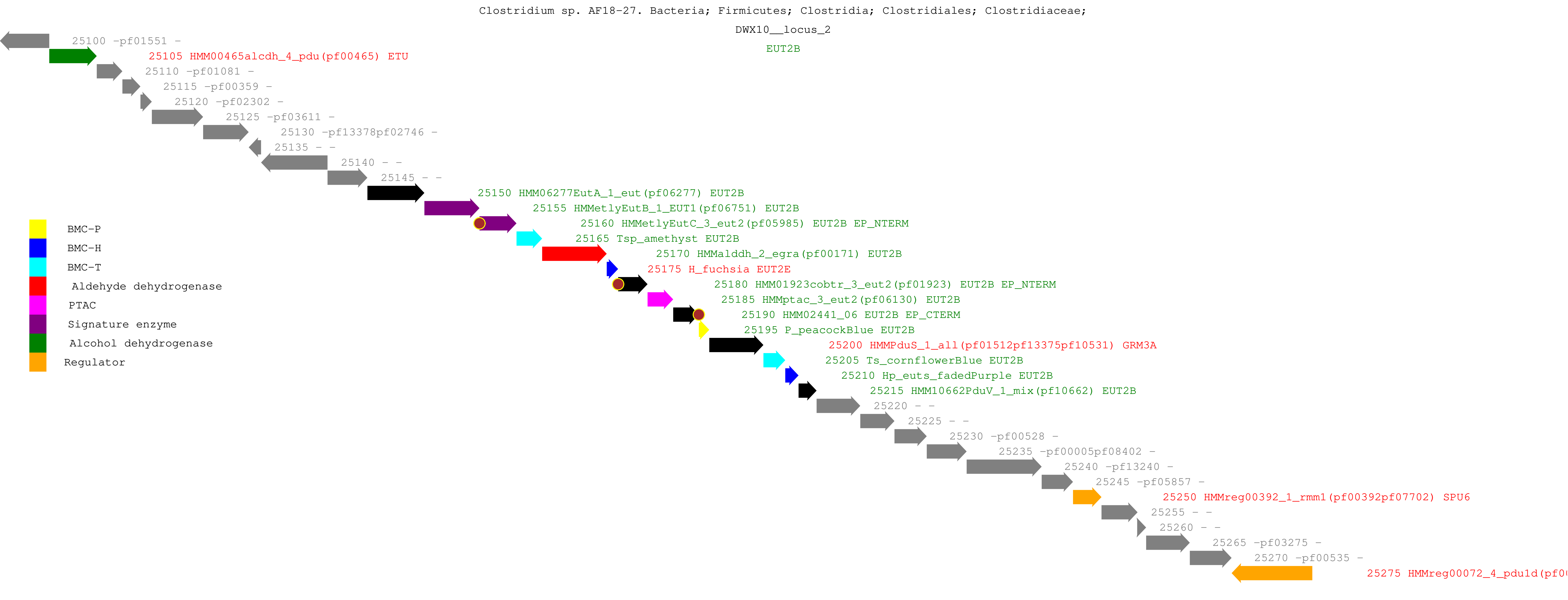

Clostridium sp. AF18-27. Bacteria; Firmicutes; Clostridia; Clostridiales; Clostridiaceae;
DWX10__locus_2
EUT2B
25100 -pf01551 -
25105 HMM00465alcdh_4_pdu(pf00465) ETU
25110 -pf01081 -
25115 -pf00359 -
25120 -pf02302 -
25125 -pf03611 -
25130 -pf13378pf02746 -
25135 - -
25140 - -
25145 - -
25150 HMM06277EutA_1_eut(pf06277) EUT2B
25155 HMMetlyEutB_1_EUT1(pf06751) EUT2B
25160 HMMetlyEutC_3_eut2(pf05985) EUT2B EP_NTERM
25165 Tsp_amethyst EUT2B
25170 HMMalddh_2_egra(pf00171) EUT2B
25175 H_fuchsia EUT2E
25180 HMM01923cobtr_3_eut2(pf01923) EUT2B EP_NTERM
25185 HMMptac_3_eut2(pf06130) EUT2B
25190 HMM02441_06 EUT2B EP_CTERM
25195 P_peacockBlue EUT2B
25200 HMMPduS_1_all(pf01512pf13375pf10531) GRM3A
25205 Ts_cornflowerBlue EUT2B
25210 Hp_euts_fadedPurple EUT2B
25215 HMM10662PduV_1_mix(pf10662) EUT2B
25220 - -
25225 - -
25230 -pf00528 -
25235 -pf00005pf08402 -
25240 -pf13240 -
25245 -pf05857 -
25250 HMMreg00392_1_rmm1(pf00392pf07702) SPU6
25255 - -
25260 - -
25265 -pf03275 -
25270 -pf00535 -
25275 HMMreg00072_4_pdu1d(pf0
BMC-P
BMC-H
BMC-T
Aldehyde dehydrogenase
PTAC
Signature enzyme
Alcohol dehydrogenase
Regulator

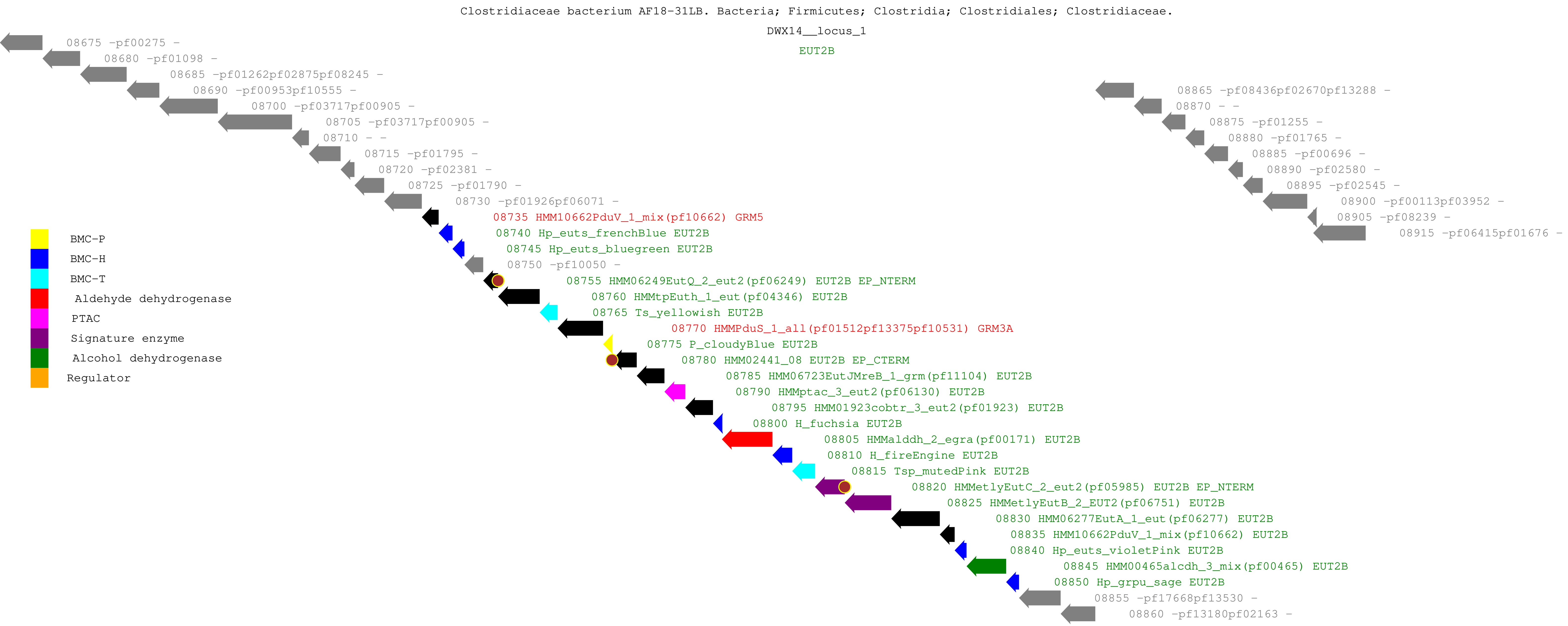
Clostridiaceae bacterium AF18-31LB. Bacteria; Firmicutes; Clostridia; Clostridiales; Clostridiaceae.
DWX14__locus_1
EUT2B
08675 -pf00275 -
08680 -pf01098 -
08685 -pf01262pf02875pf08245 -
08690 -pf00953pf10555 -
08700 -pf03717pf00905 -
08705 -pf03717pf00905 -
08710 - -
08715 -pf01795 -
08720 -pf02381 -
08725 -pf01790 -
08730 -pf01926pf06071 -
08735 HMM10662PduV_1_mix(pf10662) GRM5
08740 Hp_euts_frenchBlue EUT2B
08745 Hp_euts_bluegreen EUT2B
08750 -pf10050 -
08755 HMM06249EutQ_2_eut2(pf06249) EUT2B EP_NTERM
08760 HMMtpEuth_1_eut(pf04346) EUT2B
08765 Ts_yellowish EUT2B
08770 HMMPduS_1_all(pf01512pf13375pf10531) GRM3A
08775 P_cloudyBlue EUT2B
08780 HMM02441_08 EUT2B EP_CTERM
08785 HMM06723EutJMreB_1_grm(pf11104) EUT2B
08790 HMMptac_3_eut2(pf06130) EUT2B
08795 HMM01923cobtr_3_eut2(pf01923) EUT2B
08800 H_fuchsia EUT2B
08805 HMMalddh_2_egra(pf00171) EUT2B
08810 H_fireEngine EUT2B
08815 Tsp_mutedPink EUT2B
08820 HMMetlyEutC_2_eut2(pf05985) EUT2B EP_NTERM
08825 HMMetlyEutB_2_EUT2(pf06751) EUT2B
08830 HMM06277EutA_1_eut(pf06277) EUT2B
08835 HMM10662PduV_1_mix(pf10662) EUT2B
08840 Hp_euts_violetPink EUT2B
08845 HMM00465alcdh_3_mix(pf00465) EUT2B
08850 Hp_grpu_sage EUT2B
08855 -pf17668pf13530 -
08860 -pf13180pf02163 -
08865 -pf08436pf02670pf13288 -
08870 - -
08875 -pf01255 -
08880 -pf01765 -
08885 -pf00696 -
08890 -pf02580 -
08895 -pf02545 -
08900 -pf00113pf03952 -
08905 -pf08239 -
08915 -pf06415pf01676 -
BMC-P
BMC-H
BMC-T
Aldehyde dehydrogenase
PTAC
Signature enzyme
Alcohol dehydrogenase
Regulator

Clostridiaceae bacterium AF29-16BH. Bacteria; Firmicutes; Clostridia; Clostridiales; Clostridiaceae.

DWZ08__locus_1

EUT2B

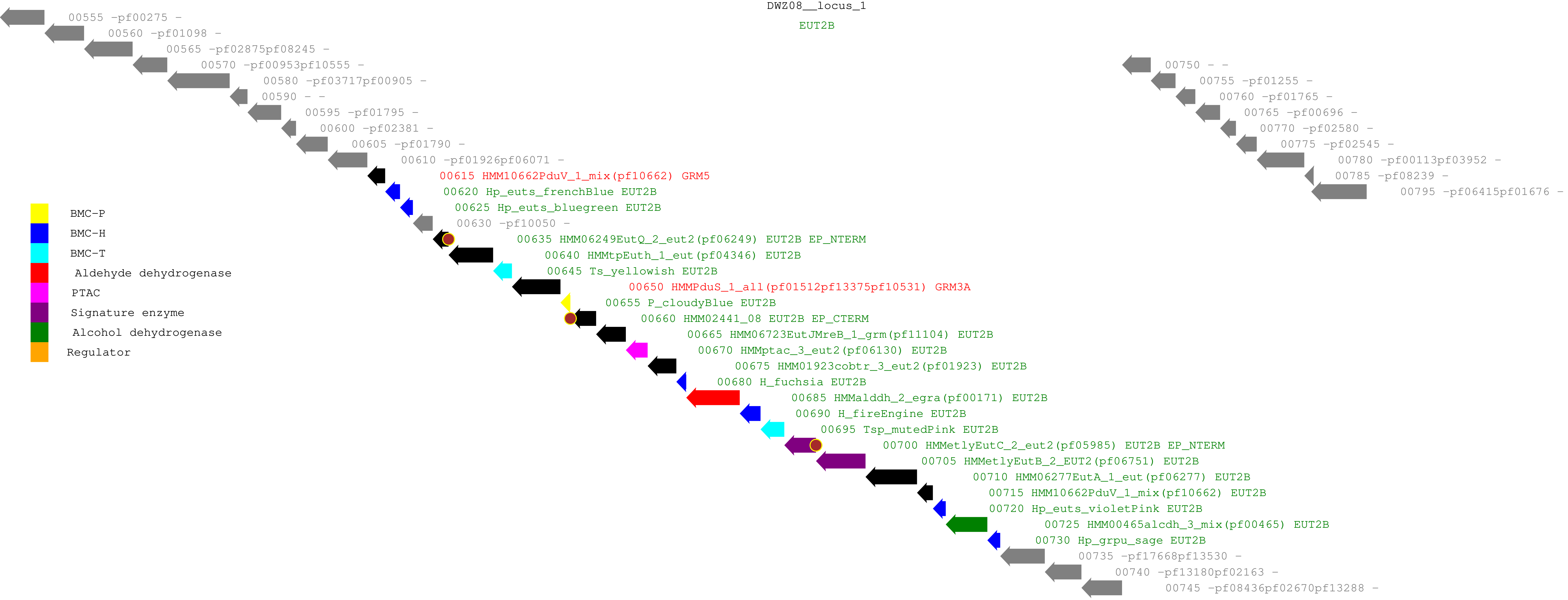

Clostridiaceae bacterium AF31-3BH. Bacteria; Firmicutes; Clostridia; Clostridiales; Clostridiaceae.

DWZ37__locus_1

EUT2B

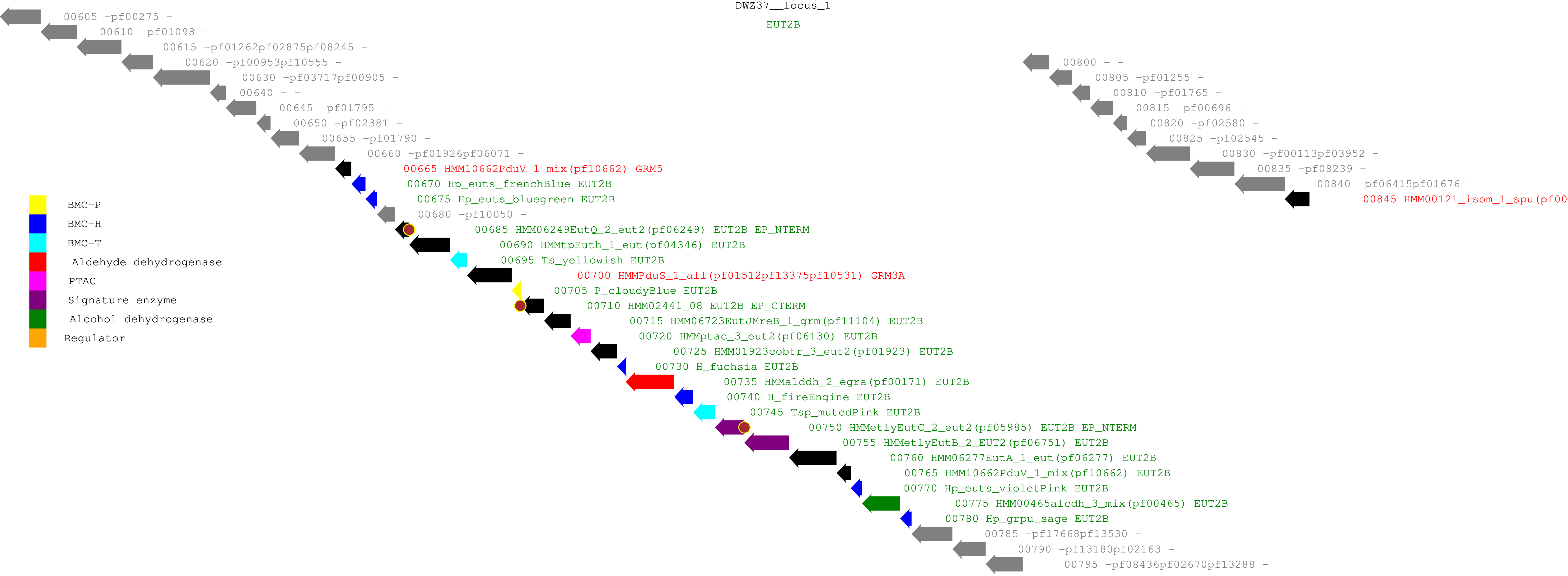

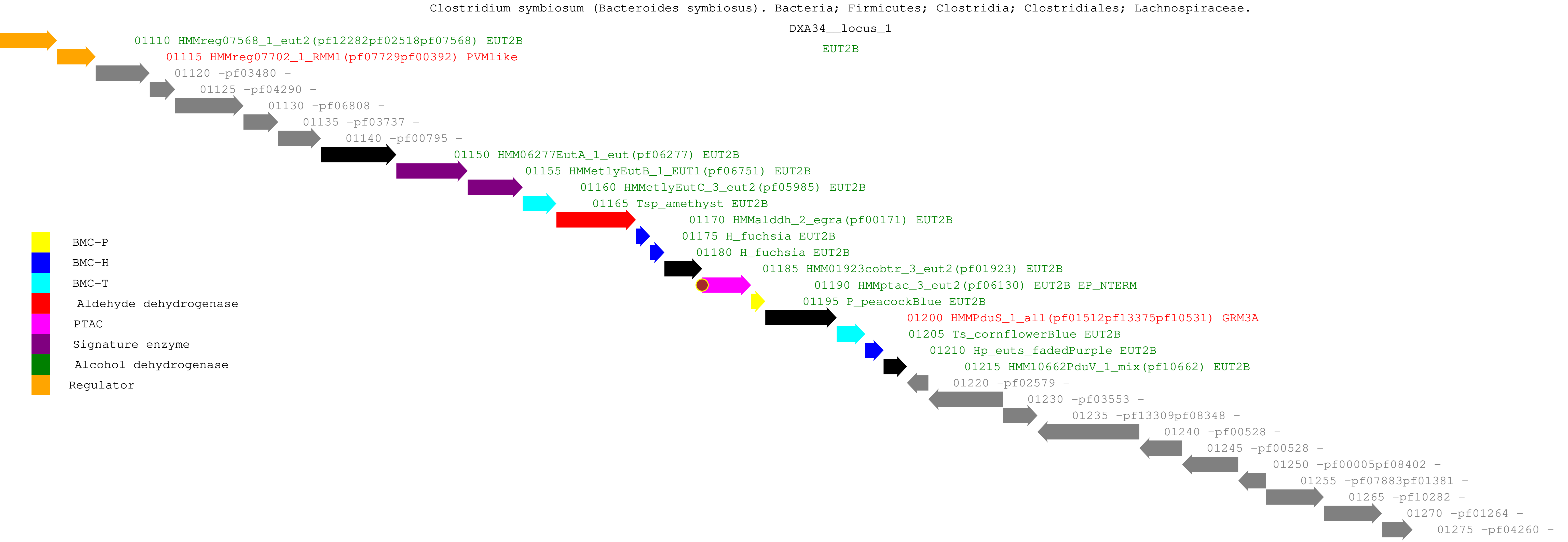
Clostridium symbiosum (Bacteroides symbiosus). Bacteria; Firmicutes; Clostridia; Clostridiales; Lachnospiraceae.
DXA34__locus_1
EUT2B
01110 HMMreg07568_1_eut2(pf12282pf02518pf07568) EUT2B
01115 HMMreg07702_1_RMM1(pf07729pf00392) PVMlike
01120 -pf03480 -
01125 -pf04290 -
01130 -pf06808 -
01135 -pf03737 -
01140 -pf00795 -
01150 HMM06277EutA_1_eut(pf06277) EUT2B
01155 HMMetlyEutB_1_EUT1(pf06751) EUT2B
01160 HMMetlyEutC_3_eut2(pf05985) EUT2B
01165 Tsp_amethyst EUT2B
01170 HMMalddh_2_egra(pf00171) EUT2B
01175 H_fuchsia EUT2B
01180 H_fuchsia EUT2B
01185 HMM01923cobtr_3_eut2(pf01923) EUT2B
01190 HMMptac_3_eut2(pf06130) EUT2B EP_NTERM
01195 P_peacockBlue EUT2B
01200 HMMPduS_1_all(pf01512pf13375pf10531) GRM3A
01205 Ts_cornflowerBlue EUT2B
01210 Hp_euts_fadedPurple EUT2B
01215 HMM10662PduV_1_mix(pf10662) EUT2B
01220 -pf02579 -
01230 -pf03553 -
01235 -pf13309pf08348 -
01240 -pf00528 -
01245 -pf00528 -
01250 -pf00005pf08402 -
01255 -pf07883pf01381 -
01265 -pf10282 -
01270 -pf01264 -
01275 -pf04260 -
BMC-P
BMC-H
BMC-T
Aldehyde dehydrogenase
PTAC
Signature enzyme
Alcohol dehydrogenase
Regulator

Clostridium sp. OF03-18AA. Bacteria; Firmicutes; Clostridia; Clostridiales; Clostridiaceae;

DXA59__locus_1

EUT2B

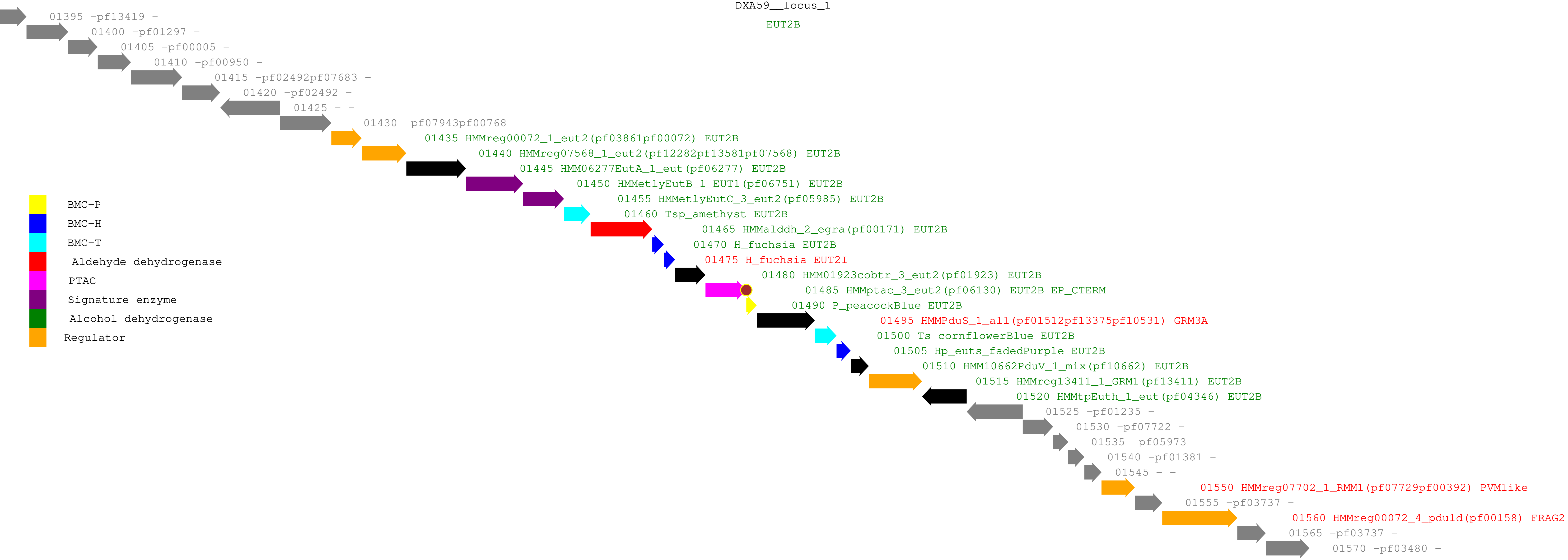

Clostridiaceae bacterium OF09-1. Bacteria; Firmicutes; Clostridia; Clostridiales; Clostridiaceae.

DXA90__locus_1

EUT2B

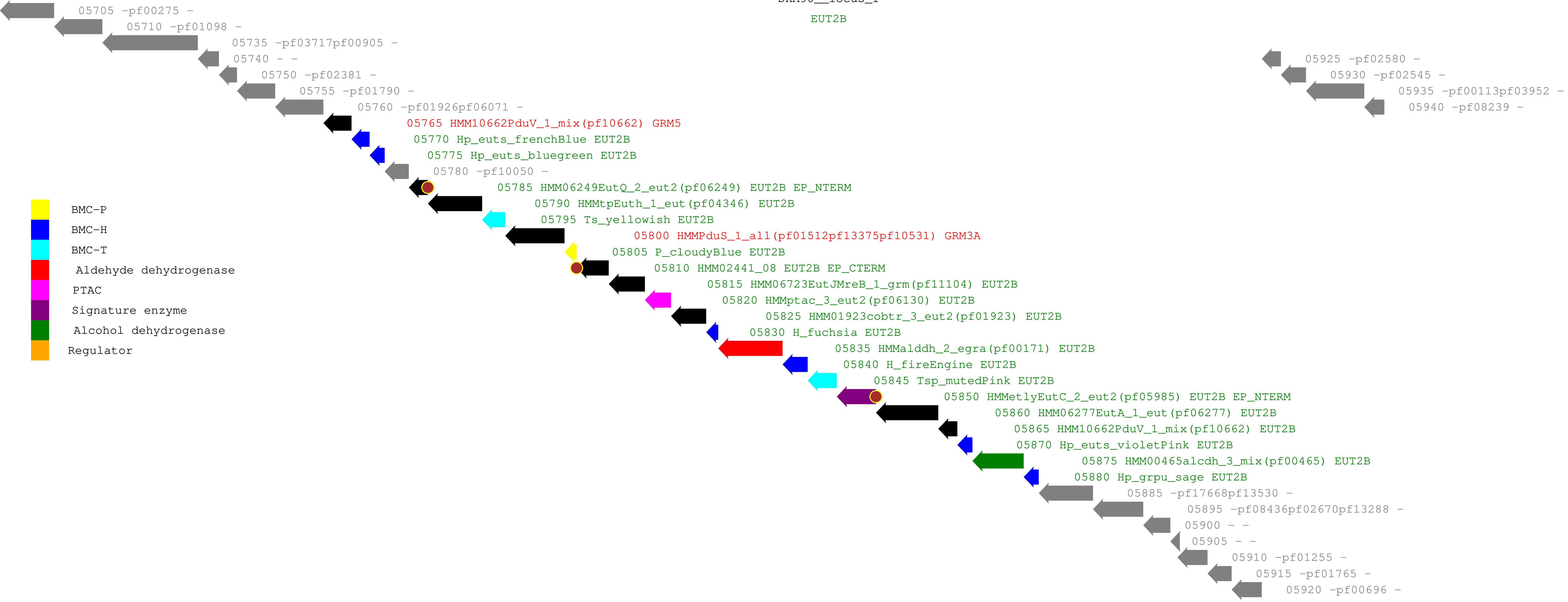

Clostridium sp. OF09-10. Bacteria; Firmicutes; Clostridia; Clostridiales; Clostridiaceae;

DXA91__locus_1

EUT2B

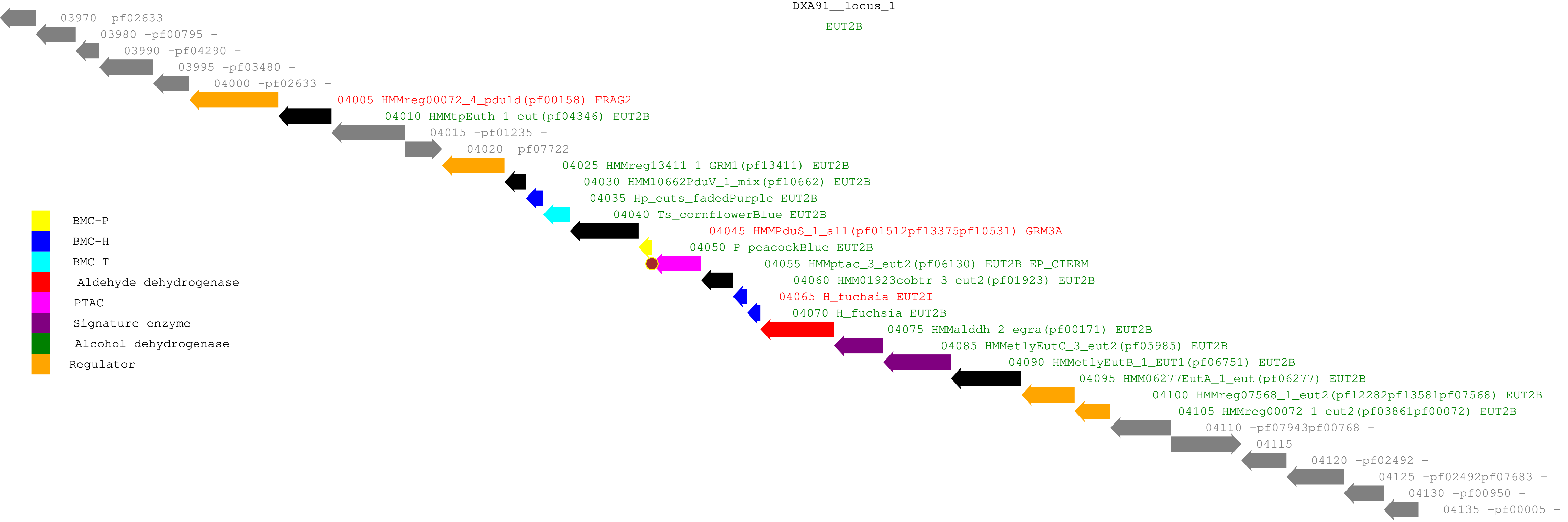

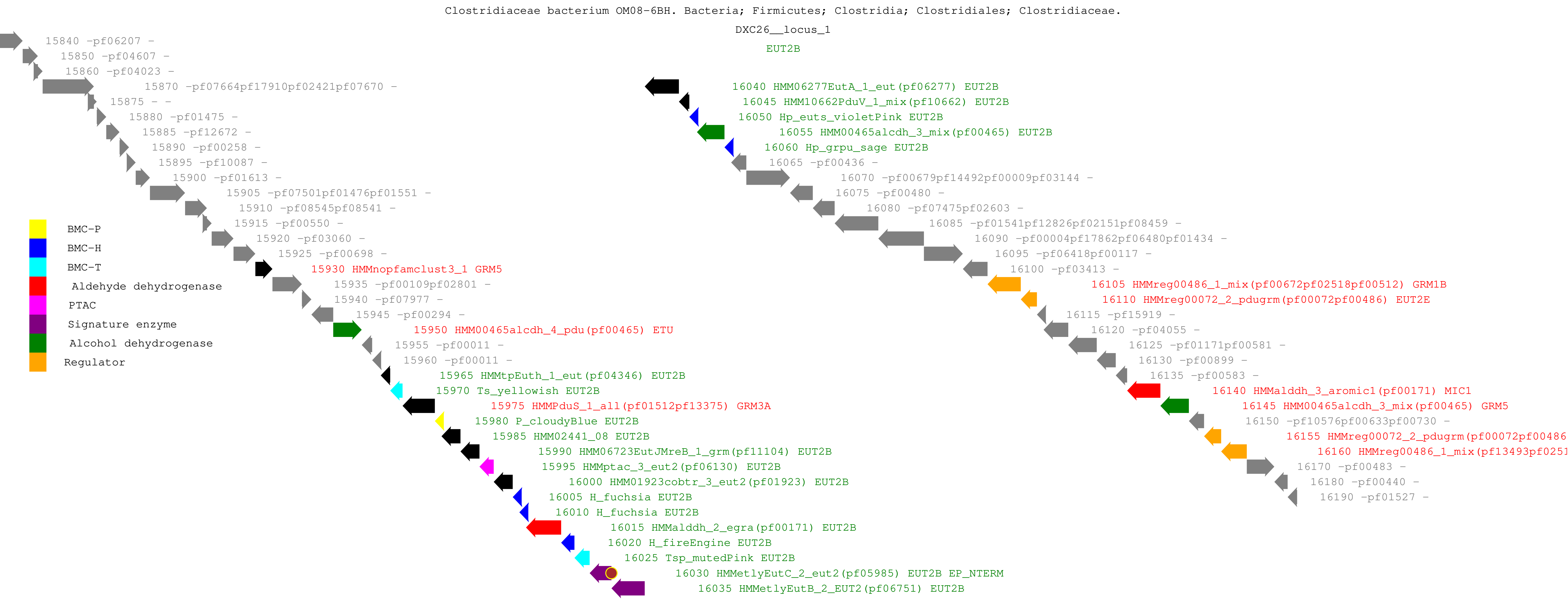

Clostridiaceae bacterium OM08-6BH. Bacteria; Firmicutes; Clostridia; Clostridiales; Clostridiaceae.
DXC26__locus_1
EUT2B
15840 -pf06207 -
15850 -pf04607 -
15860 -pf04023 -
15870 -pf07664pf17910pf02421pf07670 -
15875 - -
15880 -pf01475 -
15885 -pf12672 -
15890 -pf00258 -
15895 -pf10087 -
15900 -pf01613 -
15905 -pf07501pf01476pf01551 -
15910 -pf08545pf08541 -
15915 -pf00550 -
15920 -pf03060 -
15925 -pf00698 -
15930 HMMnopfamclust3_1 GRM5
15935 -pf00109pf02801 -
15940 -pf07977 -
15945 -pf00294 -
15950 HMM00465alcdh_4_pdu(pf00465) ETU
15955 -pf00011 -
15960 -pf00011 -
15965 HMMtpEuth_1_eut(pf04346) EUT2B
15970 Ts_yellowish EUT2B
15975 HMMPduS_1_all(pf01512pf13375) GRM3A
15980 P_cloudyBlue EUT2B
15985 HMM02441_08 EUT2B
15990 HMM06723EutJMreB_1_grm(pf11104) EUT2B
15995 HMMptac_3_eut2(pf06130) EUT2B
16000 HMM01923cobtr_3_eut2(pf01923) EUT2B
16005 H_fuchsia EUT2B
16010 H_fuchsia EUT2B
16015 HMMaldh_2_egra(pf00171) EUT2B
16020 H_fireEngine EUT2B
16025 Tsp_mutedPink EUT2B
16030 HMMetlyEutC_2_eut2(pf05985) EUT2B EP_NTERM
16035 HMMetlyEutB_2_EUT2(pf06751) EUT2B
16040 HMM06277EutA_1_eut(pf06277) EUT2B
16045 HMM10662PduV_1_mix(pf10662) EUT2B
16050 Hp_euts_violetPink EUT2B
16055 HMM00465alcdh_3_mix(pf00465) EUT2B
16060 Hp_grpu_sage EUT2B
16065 -pf00436 -
16070 -pf00679pf14492pf00009pf03144 -
16075 -pf00480 -
16080 -pf07475pf02603 -
16085 -pf01541pf12826pf02151pf08459 -
16090 -pf00004pf17862pf06480pf01434 -
16095 -pf06418pf00117 -
16100 -pf03413 -
16105 HMMreg00486_1_mix(pf00672pf02518pf00512) GRM1B
16110 HMMreg00072_2_pdugrm(pf00072pf00486) EUT2E
16115 -pf15919 -
16120 -pf04055 -
16125 -pf01171pf00581 -
16130 -pf00899 -
16135 -pf00583 -
16140 HMMaldh_3_aromic1(pf00171) MIC1
16145 HMM00465alcdh_3_mix(pf00465) GRM5
16150 -pf10576pf00633pf00730 -
16155 HMMreg00072_2_pdugrm(pf00072pf00486
16160 HMMreg00486_1_mix(pf13493pf0251
16170 -pf00483 -
16180 -pf00440 -
16190 -pf01527 -
BMC-P
BMC-H
BMC-T
Aldehyde dehydrogenase
PTAC
Signature enzyme
Alcohol dehydrogenase
Regulator

Clostridiaceae bacterium OM08-6BH. Bacteria; Firmicutes; Clostridia; Clostridiales; Clostridiaceae.

DXC26__locus_2

EUT2B

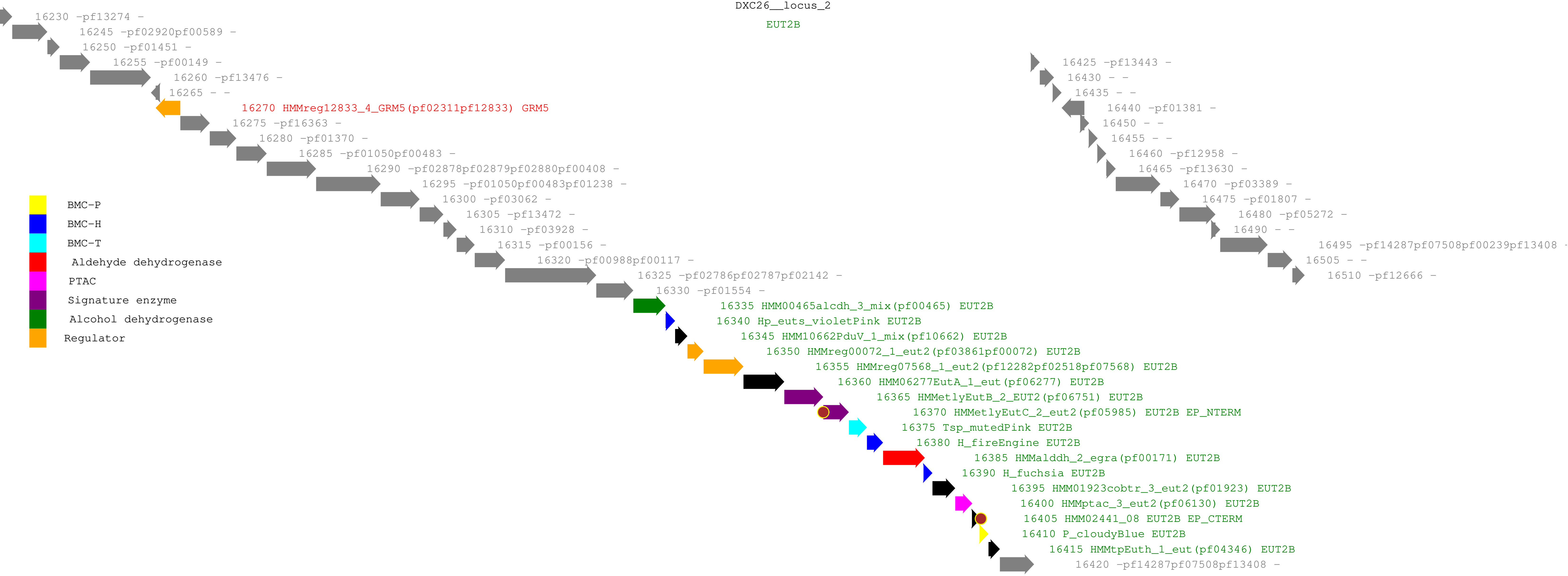

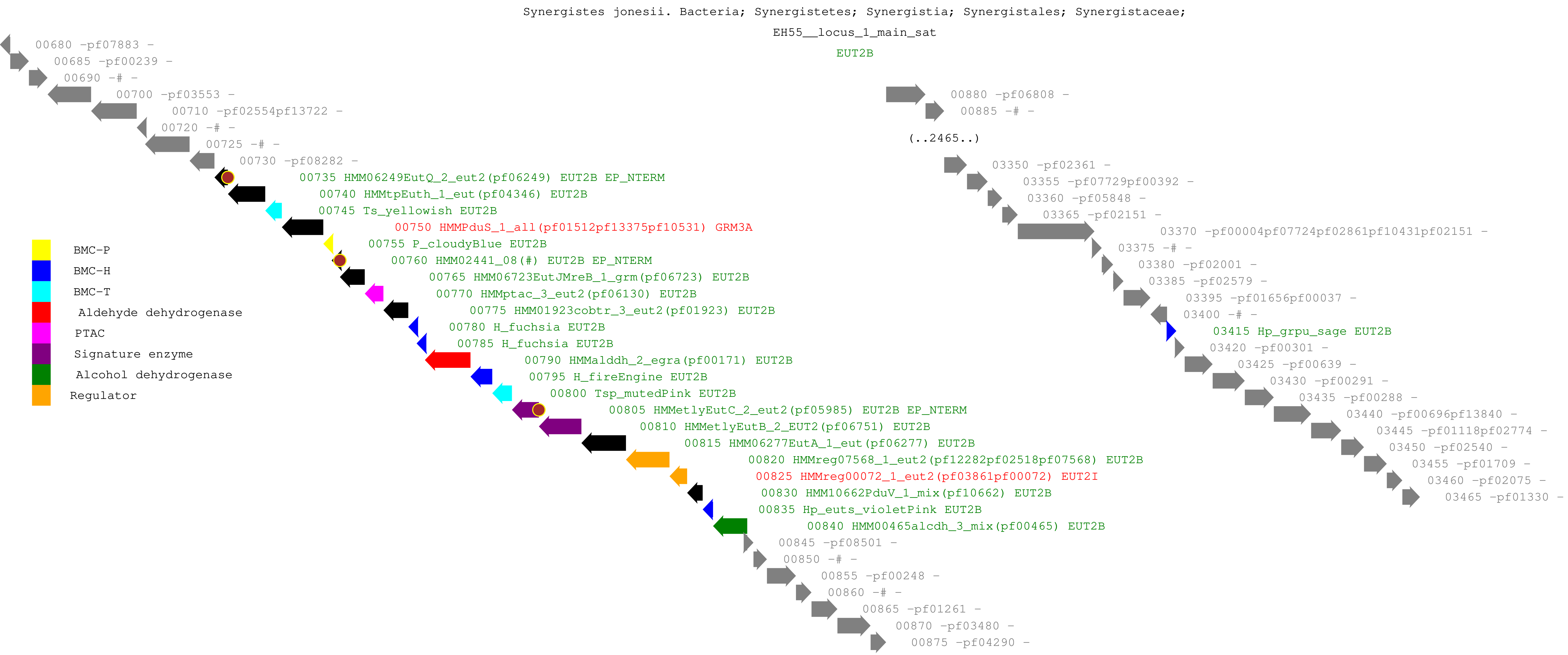
Synergistes jonesii. Bacteria; Synergistetes; Synergistia; Synergistales; Synergistaceae;
EH55__locus_1_main_sat
EUT2B
00680 -pf07883 -
00685 -pf00239 -
00690 -# -
00700 -pf03553 -
00710 -pf02554pf13722 -
00720 -# -
00725 -# -
00730 -pf08282 -
00735 HMM06249EutQ_2_eut2(pf06249) EUT2B EP_NTERM
00740 HMMtpEuth_1_eut(pf04346) EUT2B
00745 Ts_yellowish EUT2B
00750 HMMPduS_1_all(pf01512pf13375pf10531) GRM3A
00755 P_cloudyBlue EUT2B
00760 HMM02441_08(#) EUT2B EP_NTERM
00765 HMM06723EutJMreB_1_grm(pf06723) EUT2B
00770 HMMptac_3_eut2(pf06130) EUT2B
00775 HMM01923cobtr_3_eut2(pf01923) EUT2B
00780 H_fuchsia EUT2B
00785 H_fuchsia EUT2B
00790 HMMaldh_2_egra(pf00171) EUT2B
00795 H_fireEngine EUT2B
00800 Tsp_mutedPink EUT2B
00805 HMMetlyEutC_2_eut2(pf05985) EUT2B EP_NTERM
00810 HMMetlyEutB_2_EUT2(pf06751) EUT2B
00815 HMM06277EutA_1_eut(pf06277) EUT2B
00820 HMMreg07568_1_eut2(pf12282pf02518pf07568) EUT2B
00825 HMMreg00072_1_eut2(pf03861pf00072) EUT2I
00830 HMM10662PduV_1_mix(pf10662) EUT2B
00835 Hp_euts_violetPink EUT2B
00840 HMM00465alcdh_3_mix(pf00465) EUT2B
00845 -pf08501 -
00850 -# -
00855 -pf00248 -
00860 -# -
00865 -pf01261 -
00870 -pf03480 -
00875 -pf04290 -
00880 -pf06808 -
00885 -# -
(..2465..)
03350 -pf02361 -
03355 -pf07729pf00392 -
03360 -pf05848 -
03365 -pf02151 -
03370 -pf00004pf07724pf02861pf10431pf02151 -
03375 -# -
03380 -pf02001 -
03385 -pf02579 -
03395 -pf01656pf00037 -
03400 -# -
03415 Hp_grpu_sage EUT2B
03420 -pf00301 -
03425 -pf00639 -
03430 -pf00291 -
03435 -pf00288 -
03440 -pf00696pf13840 -
03445 -pf01118pf02774 -
03450 -pf02540 -
03455 -pf01709 -
03460 -pf02075 -
03465 -pf01330 -
BMC-P
BMC-H
BMC-T
Aldehyde dehydrogenase
PTAC
Signature enzyme
Alcohol dehydrogenase
Regulator

Fretibacterium sp. OH1220_COT-178. Bacteria; Synergistetes; Synergistia; Synergistales; Synergistaceae;

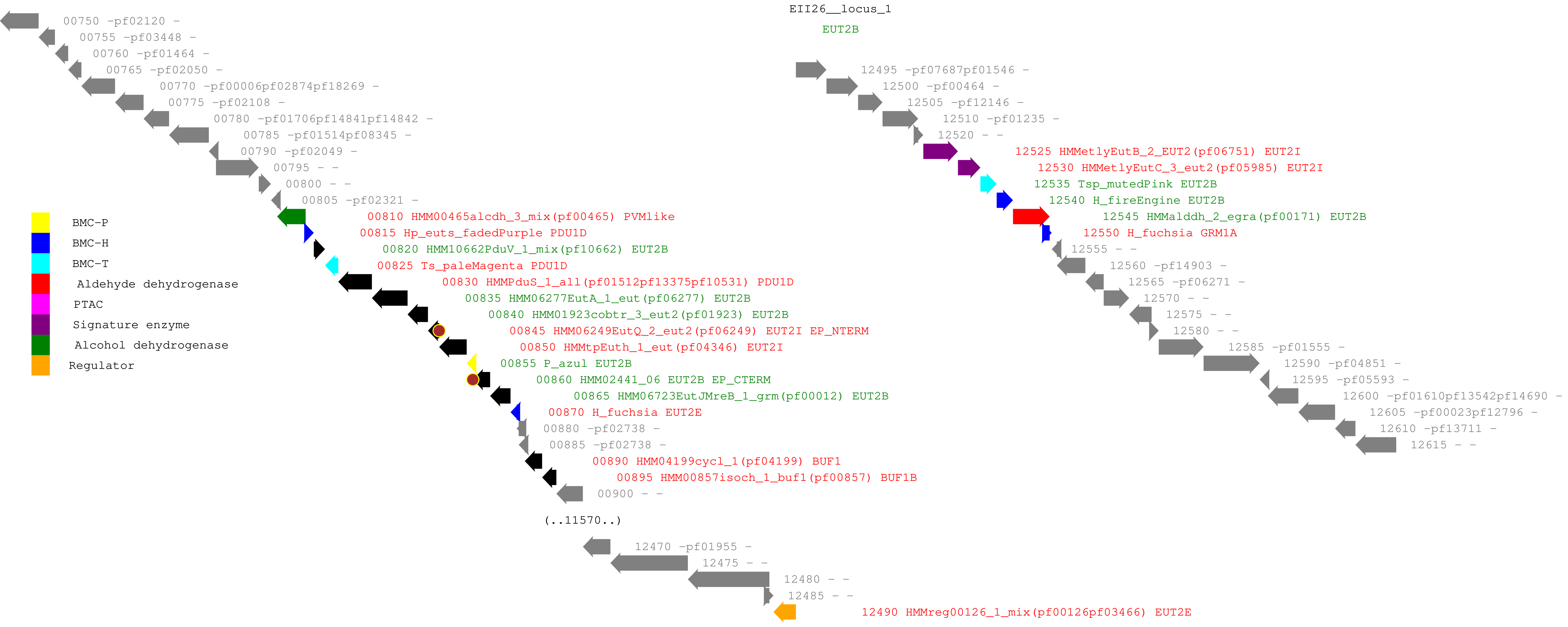

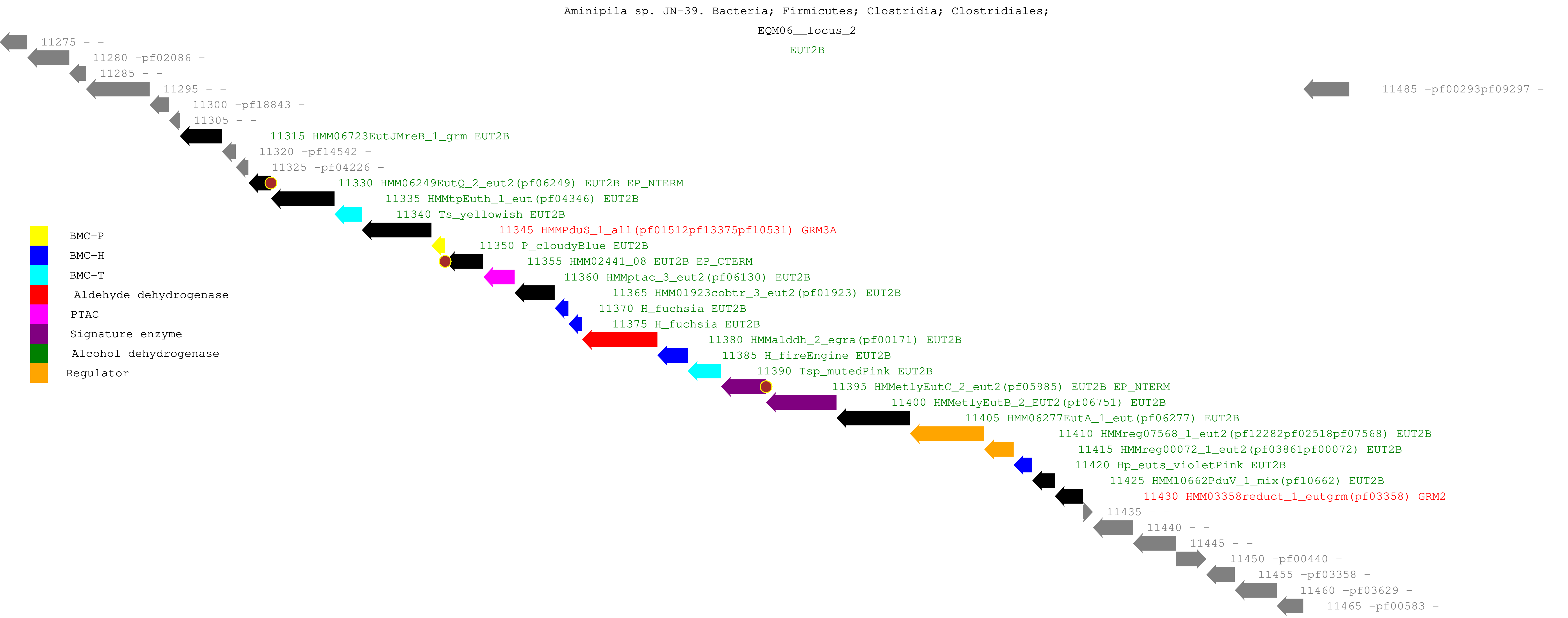

Aminipila sp. JN-39. Bacteria; Firmicutes; Clostridia; Clostridiales;
EQM06__locus_2
EUT2B
11275 - -
11280 -pf02086 -
11285 - -
11295 - -
11300 -pf18843 -
11305 - -
11315 HMM06723EutJMreB_1_grm EUT2B
11320 -pf14542 -
11325 -pf04226 -
11330 HMM06249EutQ_2_eut2(pf06249) EUT2B EP_NTERM
11335 HMMtpEuth_1_eut(pf04346) EUT2B
11340 Ts_yellowish EUT2B
11345 HMMPduS_1_all(pf01512pf13375pf10531) GRM3A
11350 P_cloudyBlue EUT2B
11355 HMM02441_08 EUT2B EP_CTERM
11360 HMMptac_3_eut2(pf06130) EUT2B
11365 HMM01923cobtr_3_eut2(pf01923) EUT2B
11370 H_fuchsia EUT2B
11375 H_fuchsia EUT2B
11380 HMMalddh_2_egra(pf00171) EUT2B
11385 H_fireEngine EUT2B
11390 Tsp_mutedPink EUT2B
11395 HMMetlyEutC_2_eut2(pf05985) EUT2B EP_NTERM
11400 HMMetlyEutB_2_EUT2(pf06751) EUT2B
11405 HMM06277EutA_1_eut(pf06277) EUT2B
11410 HMMreg07568_1_eut2(pf12282pf02518pf07568) EUT2B
11415 HMMreg00072_1_eut2(pf03861pf00072) EUT2B
11420 Hp_euts_violetPink EUT2B
11425 HMM10662PduV_1_mix(pf10662) EUT2B
11430 HMM03358reduct_1_eutgrm(pf03358) GRM2
11435 - -
11440 - -
11445 - -
11450 -pf00440 -
11455 -pf03358 -
11460 -pf03629 -
11465 -pf00583 -
11485 -pf00293pf09297 -
BMC-P
BMC-H
BMC-T
Aldehyde dehydrogenase
PTAC
Signature enzyme
Alcohol dehydrogenase
Regulator

Clostridium symbiosum (Bacteroides symbiosus). Bacteria; Firmicutes; Clostridia; Clostridiales; Lachnospiraceae.

ERS852479__locus_1

EUT2B

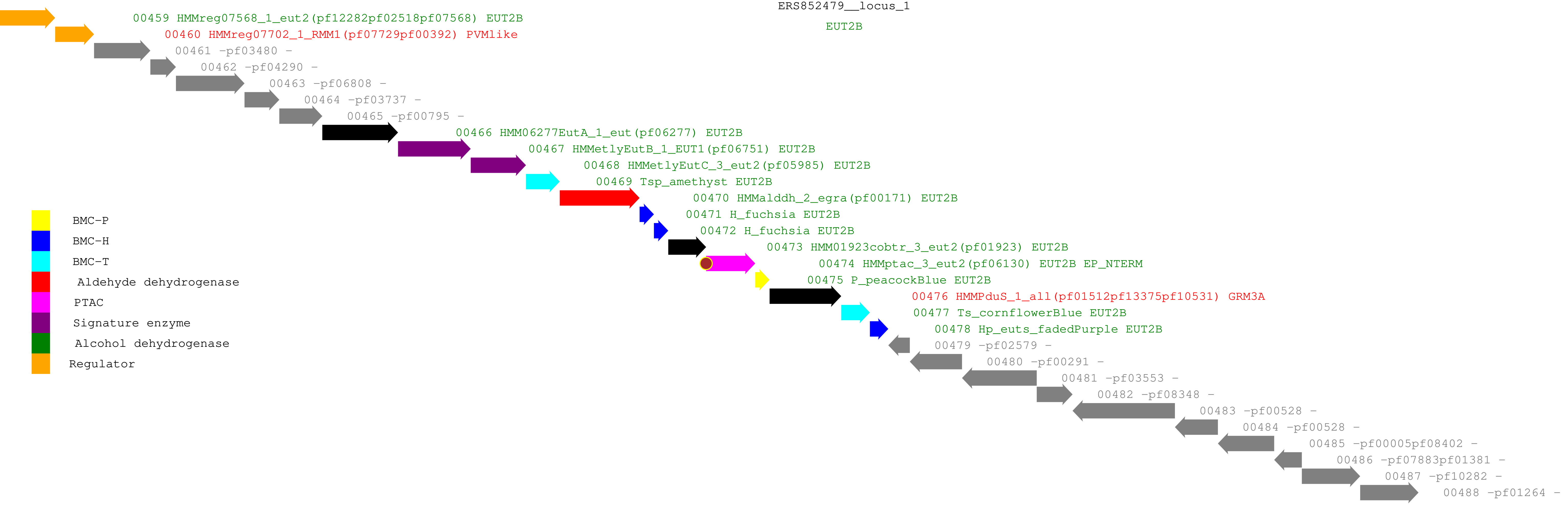

Flavonifractor plautii. Bacteria; Firmicutes; Clostridia; Clostridiales; Flavonifractor.

ERS852504__locus_2

EUT2B

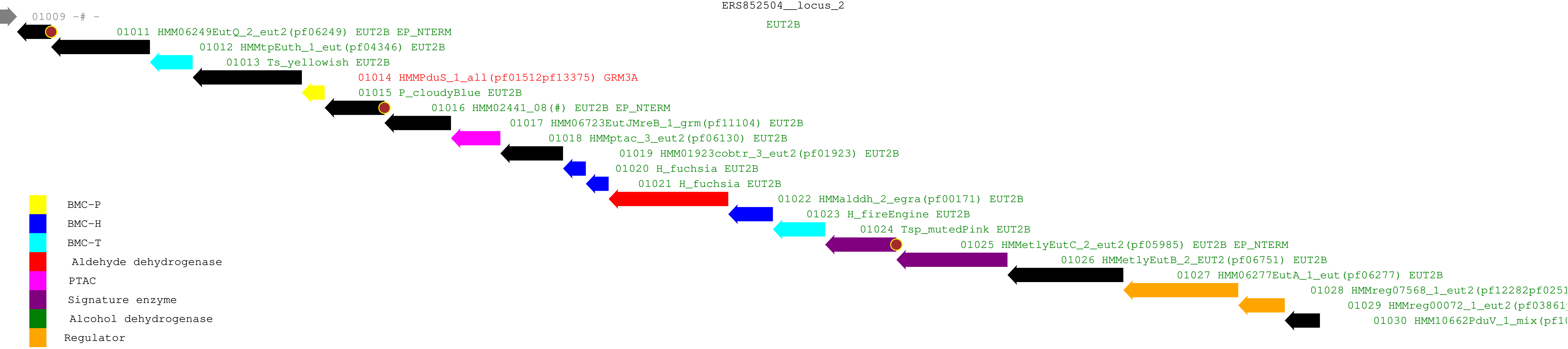

Flavonifractor plautii. Bacteria; Firmicutes; Clostridia; Clostridiales; Flavonifractor.

ERS852518__locus_2

EUT2B

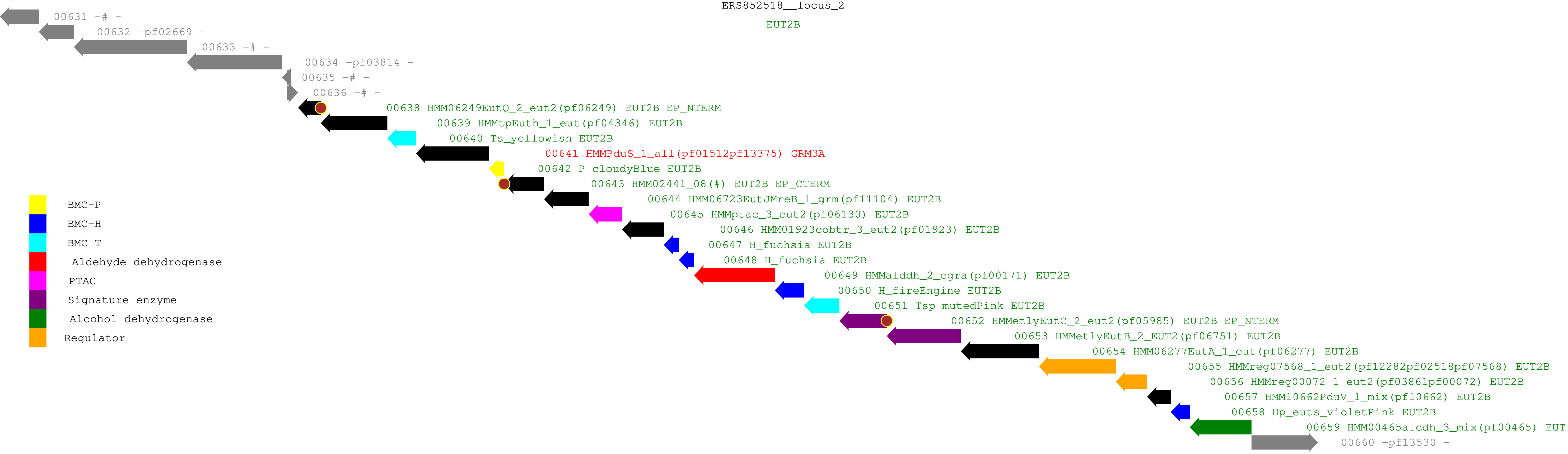

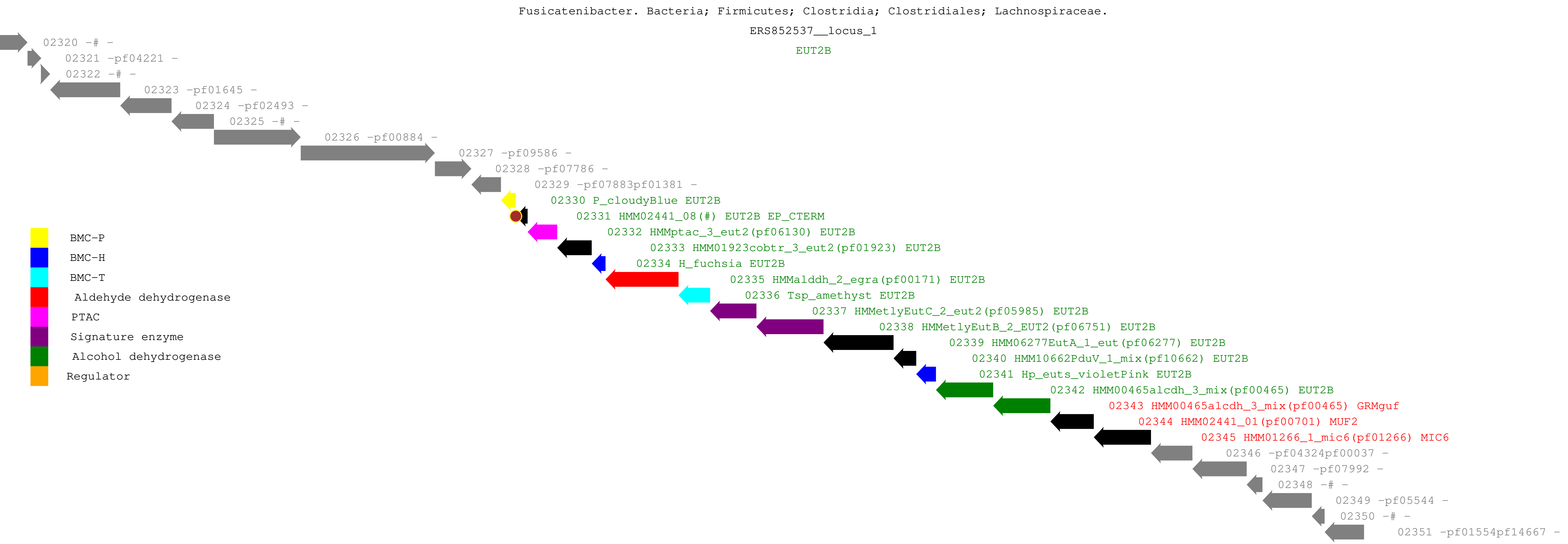
Fusicatenibacter. Bacteria; Firmicutes; Clostridia; Clostridiales; Lachnospiraceae.
ERS852537__locus_1
EUT2B
02320 -# -
02321 -pf04221 -
02322 -# -
02323 -pf01645 -
02324 -pf02493 -
02325 -# -
02326 -pf00884 -
02327 -pf09586 -
02328 -pf07786 -
02329 -pf07883pf01381 -
02330 P_cloudyBlue EUT2B
02331 HMM02441_08(#) EUT2B EP_CTERM
02332 HMMptac_3_eut2(pf06130) EUT2B
02333 HMM01923cobtr_3_eut2(pf01923) EUT2B
02334 H_fuchsia EUT2B
02335 HMMalddh_2_egra(pf00171) EUT2B
02336 Tsp_amethyst EUT2B
02337 HMMetlyEutC_2_eut2(pf05985) EUT2B
02338 HMMetlyEutB_2_EUT2(pf06751) EUT2B
02339 HMM06277EutA_1_eut(pf06277) EUT2B
02340 HMM10662PduV_1_mix(pf10662) EUT2B
02341 Hp_euts_violetPink EUT2B
02342 HMM00465alcdh_3_mix(pf00465) EUT2B
02343 HMM00465alcdh_3_mix(pf00465) GRMguf
02344 HMM02441_01(pf00701) MUF2
02345 HMM01266_1_mic6(pf01266) MIC6
02346 -pf04324pf00037 -
02347 -pf07992 -
02348 -# -
02349 -pf05544 -
02350 -# -
02351 -pf01554pf14667 -
BMC-P
BMC-H
BMC-T
Aldehyde dehydrogenase
PTAC
Signature enzyme
Alcohol dehydrogenase
Regulator

Flavonifractor plautii DSM 6740. Bacteria; Firmicutes; Clostridia; Clostridiales; Ruminococcaceae;

EV206__locus_1

EUT2B

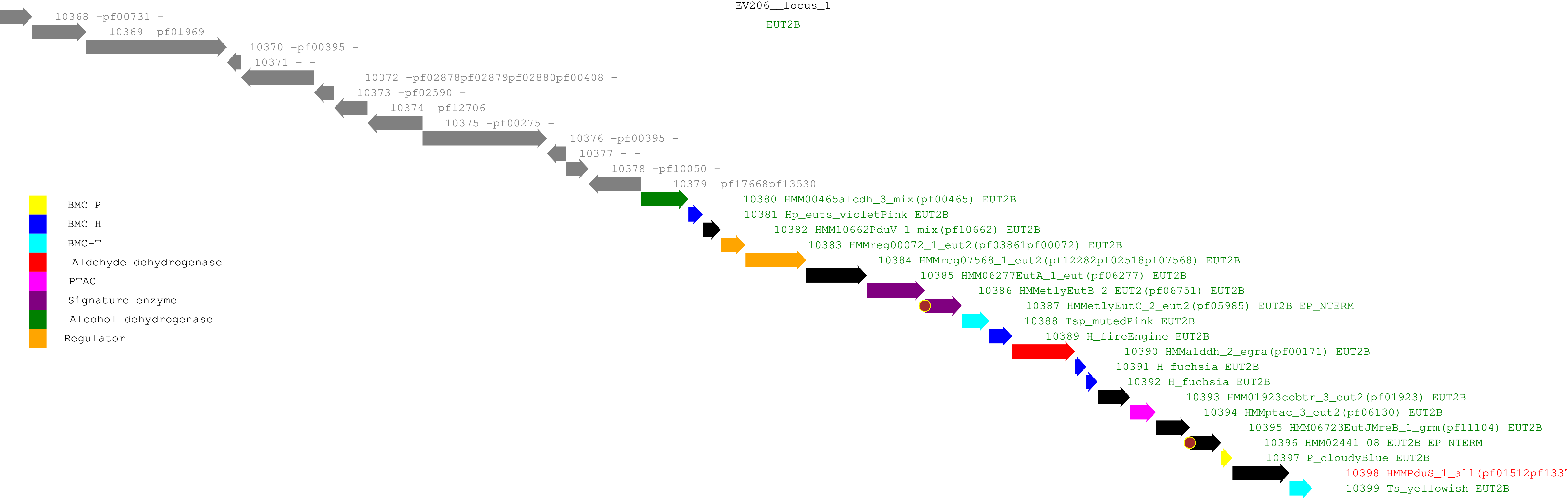

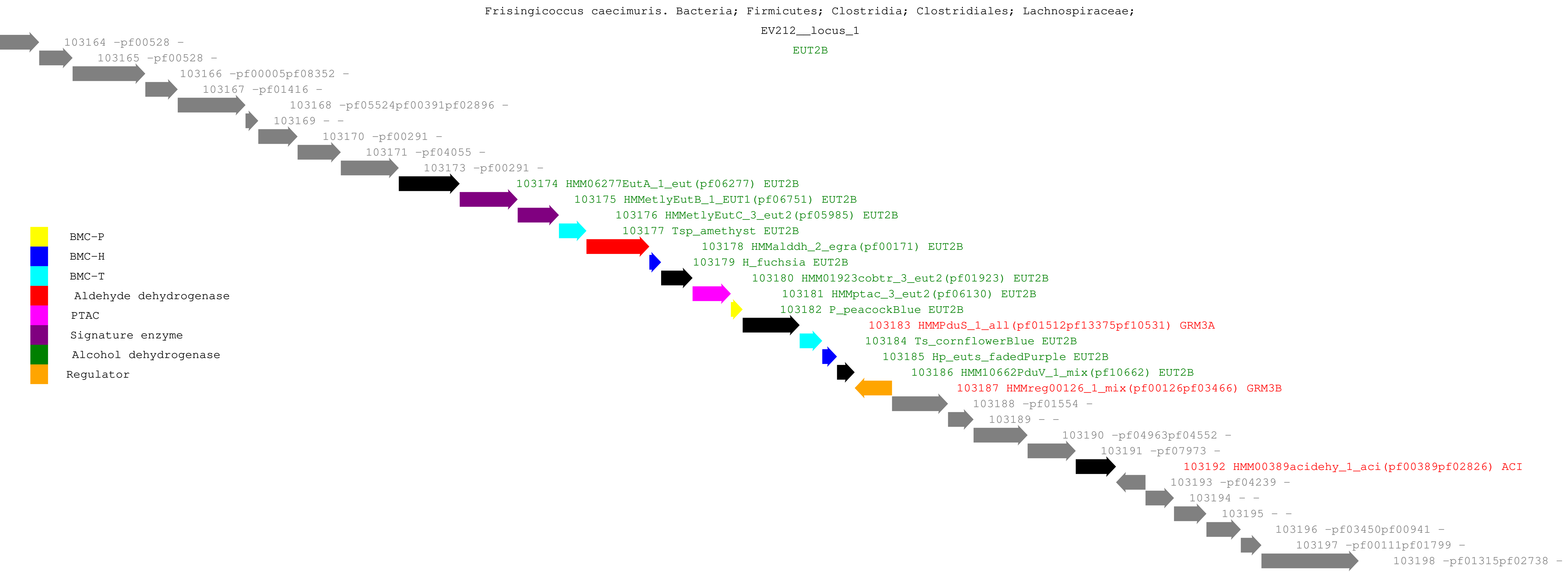
Frisingicoccus caecimuris. Bacteria; Firmicutes; Clostridia; Clostridiales; Lachnospiraceae;
EV212__locus_1
EUT2B
103164 -pf00528 -
103165 -pf00528 -
103166 -pf00005pf08352 -
103167 -pf01416 -
103168 -pf05524pf00391pf02896 -
103169 - -
103170 -pf00291 -
103171 -pf04055 -
103173 -pf00291 -
103174 HMM06277EutA_1_eut(pf06277) EUT2B
103175 HMMetlyEutB_1_EUT1(pf06751) EUT2B
103176 HMMetlyEutC_3_eut2(pf05985) EUT2B
103177 Tsp_amethyst EUT2B
103178 HMMaldd h_2_egra(pf00171) EUT2B
103179 H_fuchsia EUT2B
103180 HMM01923cobtr_3_eut2(pf01923) EUT2B
103181 HMMptac_3_eut2(pf06130) EUT2B
103182 P_peacockBlue EUT2B
103183 HMMPduS_1_all(pf01512pf13375pf10531) GRM3A
103184 Ts_cornflowerBlue EUT2B
103185 Hp_euts_fadedPurple EUT2B
103186 HMM10662PduV_1_mix(pf10662) EUT2B
103187 HMMreg00126_1_mix(pf00126pf03466) GRM3B
103188 -pf01554 -
103189 - -
103190 -pf04963pf04552 -
103191 -pf07973 -
103192 HMM00389acidehy_1_aci(pf00389pf02826) ACI
103193 -pf04239 -
103194 - -
103195 - -
103196 -pf03450pf00941 -
103197 -pf00111pf01799 -
103198 -pf01315pf02738 -
BMC-P
BMC-H
BMC-T
Aldehyde dehydrogenase
PTAC
Signature enzyme
Alcohol dehydrogenase
Regulator

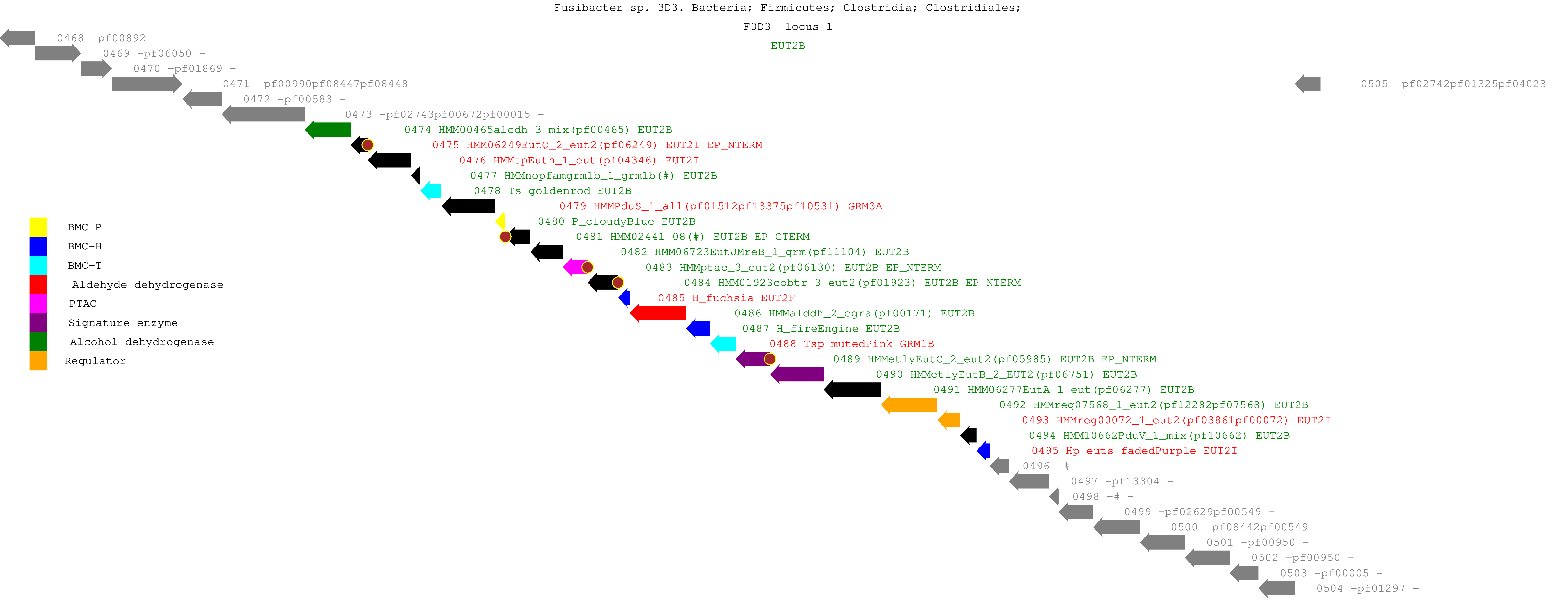

Fusibacter sp. 3D3. Bacteria; Firmicutes; Clostridia; Clostridiales;
F3D3__locus_1
EUT2B
0468 -pf00892 -
0469 -pf06050 -
0470 -pf01869 -
0471 -pf00990pf08447pf08448 -
0472 -pf00583 -
0473 -pf02743pf00672pf00015 -
0474 HMM00465alcdh_3_mix(pf00465) EUT2B
0475 HMM06249EutQ_2_eut2(pf06249) EUT2I EP_NTERM
0476 HMMtpEuth_1_eut(pf04346) EUT2I
0477 HMMnopfamgrm1b_1_grm1b(#) EUT2B
0478 Ts_goldenrod EUT2B
0479 HMMPduS_1_all(pf01512pf13375pf10531) GRM3A
0480 P_cloudyBlue EUT2B
0481 HMM02441_08(#) EUT2B EP_CTERM
0482 HMM06723EutJMreB_1_grm(pf11104) EUT2B
0483 HMMptac_3_eut2(pf06130) EUT2B EP_NTERM
0484 HMM01923cobtr_3_eut2(pf01923) EUT2B EP_NTERM
0485 H_fuchsia EUT2F
0486 HMMalddh_2_egra(pf00171) EUT2B
0487 H_fireEngine EUT2B
0488 Tsp_mutedPink GRM1B
0489 HMMetlyEutC_2_eut2(pf05985) EUT2B EP_NTERM
0490 HMMetlyEutB_2_EUT2(pf06751) EUT2B
0491 HMM06277EutA_1_eut(pf06277) EUT2B
0492 HMMreg07568_1_eut2(pf12282pf07568) EUT2B
0493 HMMreg00072_1_eut2(pf03861pf00072) EUT2I
0494 HMM10662PduV_1_mix(pf10662) EUT2B
0495 Hp_euts_fadedPurple EUT2I
0496 -# -
0497 -pf13304 -
0498 -# -
0499 -pf02629pf00549 -
0500 -pf08442pf00549 -
0501 -pf00950 -
0502 -pf00950 -
0503 -pf00005 -
0504 -pf01297 -
0505 -pf02742pf01325pf04023 -
BMC-P
BMC-H
BMC-T
Aldehyde dehydrogenase
PTAC
Signature enzyme
Alcohol dehydrogenase
Regulator

Crassaminicella sp. SY095. Bacteria; Firmicutes; Clostridia; Clostridiales; Clostridiaceae;

FQB35__locus_2

EUT2B

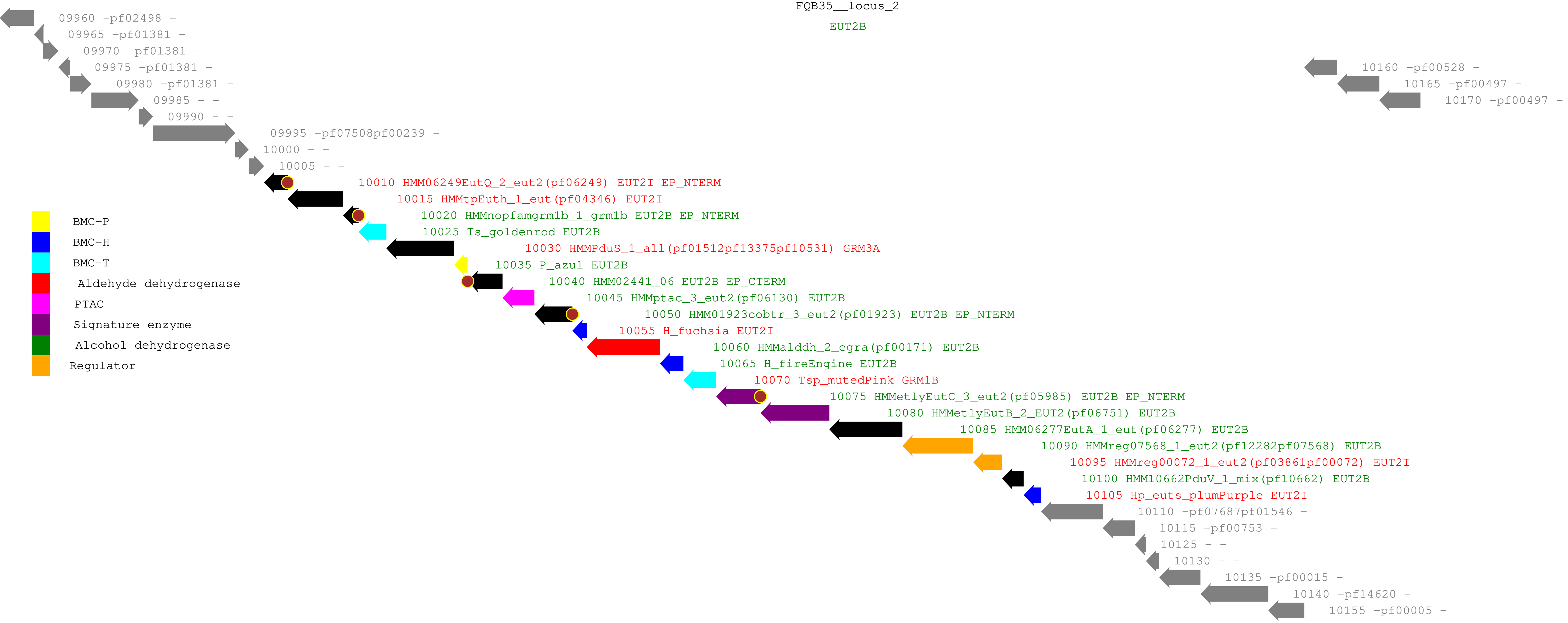

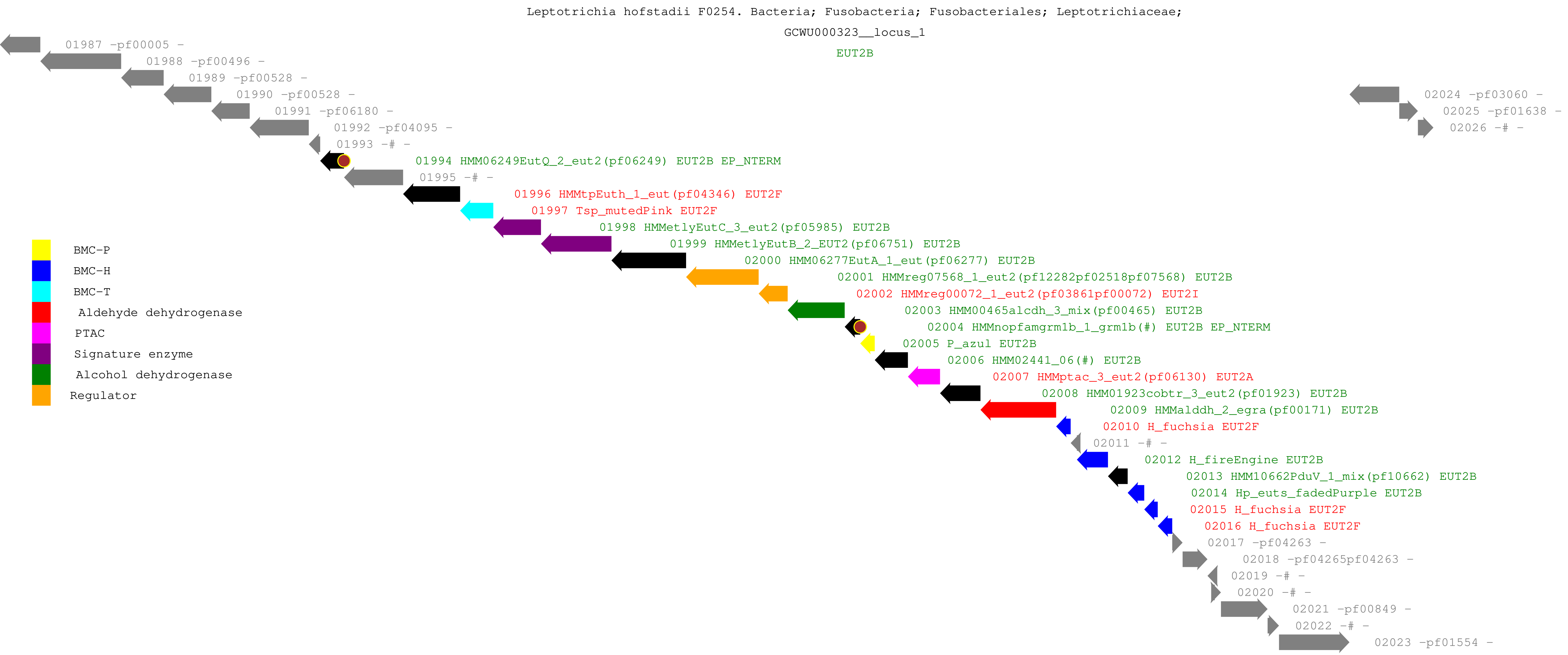
Leptotrichia hofstadii F0254. Bacteria; Fusobacteria; Fusobacteriales; Leptotrichiaceae;
GCWU000323__locus_1
EUT2B
01987 -pf00005 -
01988 -pf00496 -
01989 -pf00528 -
01990 -pf00528 -
01991 -pf06180 -
01992 -pf04095 -
01993 -# -
01994 HMM06249EutQ_2_eut2(pf06249) EUT2B EP_NTERM
01995 -# -
01996 HMMtpEuth_1_eut(pf04346) EUT2F
01997 Tsp_mutedPink EUT2F
01998 HMMetlyEutC_3_eut2(pf05985) EUT2B
01999 HMMetlyEutB_2_EUT2(pf06751) EUT2B
02000 HMM06277EutA_1_eut(pf06277) EUT2B
02001 HMMreg07568_1_eut2(pf12282pf02518pf07568) EUT2B
02002 HMMreg00072_1_eut2(pf03861pf00072) EUT2I
02003 HMM00465alcdh_3_mix(pf00465) EUT2B
02004 HMMnopfamgrm1b_1_grm1b(#) EUT2B EP_NTERM
02005 P_azul EUT2B
02006 HMM02441_06(#) EUT2B
02007 HMMptac_3_eut2(pf06130) EUT2A
02008 HMM01923cobtr_3_eut2(pf01923) EUT2B
02009 HMMaldh_2_egra(pf00171) EUT2B
02010 H_fuchsia EUT2F
02011 -# -
02012 H_fireEngine EUT2B
02013 HMM10662PduV_1_mix(pf10662) EUT2B
02014 Hp_euts_fadedPurple EUT2B
02015 H_fuchsia EUT2F
02016 H_fuchsia EUT2F
02017 -pf04263 -
02018 -pf04265pf04263 -
02019 -# -
02020 -# -
02021 -pf00849 -
02022 -# -
02023 -pf01554 -
02024 -pf03060 -
02025 -pf01638 -
02026 -# -
BMC-P
BMC-H
BMC-T
Aldehyde dehydrogenase
PTAC
Signature enzyme
Alcohol dehydrogenase
Regulator

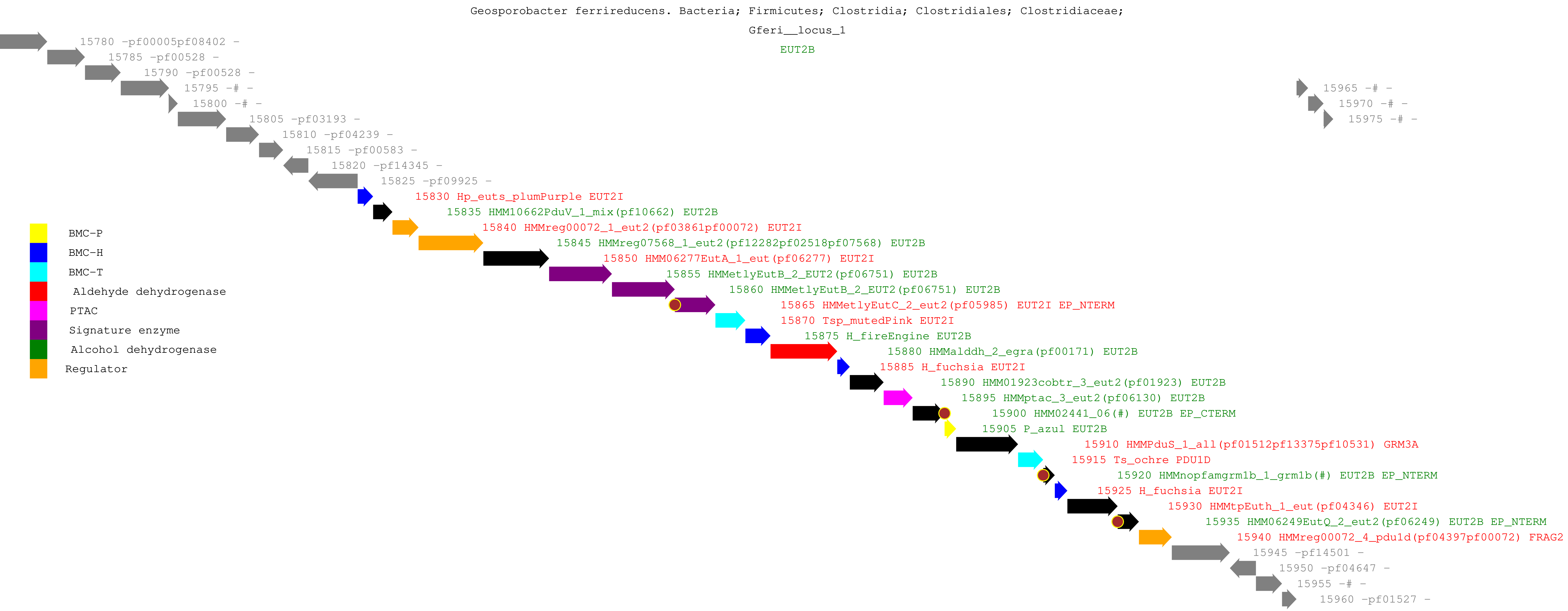

Geosporobacter ferrireducens. Bacteria; Firmicutes; Clostridia; Clostridiales; Clostridiaceae;
Gferi__locus_1
EUT2B
15780 -pf00005pf08402 -
15785 -pf00528 -
15790 -pf00528 -
15795 -# -
15800 -# -
15805 -pf03193 -
15810 -pf04239 -
15815 -pf00583 -
15820 -pf14345 -
15825 -pf09925 -
15830 Hp_euts_plumPurple EUT2I
15835 HMM10662PduV_1_mix(pf10662) EUT2B
15840 HMMreg00072_1_eut2(pf03861pf00072) EUT2I
15845 HMMreg07568_1_eut2(pf12282pf02518pf07568) EUT2B
15850 HMM06277EutA_1_eut(pf06277) EUT2I
15855 HMMetlyEutB_2_EUT2(pf06751) EUT2B
15860 HMMetlyEutB_2_EUT2(pf06751) EUT2B
15865 HMMetlyEutC_2_eut2(pf05985) EUT2I EP_NTERM
15870 Tsp_mutedPink EUT2I
15875 H_fireEngine EUT2B
15880 HMMalddh_2_egra(pf00171) EUT2B
15885 H_fuchsia EUT2I
15890 HMM01923cobtr_3_eut2(pf01923) EUT2B
15895 HMMptac_3_eut2(pf06130) EUT2B
15900 HMM02441_06(#) EUT2B EP_CTERM
15905 P_azul EUT2B
15910 HMMPduS_1_all(pf01512pf13375pf10531) GRM3A
15915 Ts_ochre PDU1D
15920 HMMnopfamgrm1b_1_grm1b(#) EUT2B EP_NTERM
15925 H_fuchsia EUT2I
15930 HMMtpEuth_1_eut(pf04346) EUT2I
15935 HMM06249EutQ_2_eut2(pf06249) EUT2B EP_NTERM
15940 HMMreg00072_4_pdu1d(pf04397pf00072) FRAG2
15945 -pf14501 -
15950 -pf04647 -
15955 -# -
15960 -pf01527 -
15965 -# -
15970 -# -
15975 -# -
BMC-P
BMC-H
BMC-T
Aldehyde dehydrogenase
PTAC
Signature enzyme
Alcohol dehydrogenase
Regulator

Endozoicomonas elysicola. Bacteria; Proteobacteria; Gammaproteobacteria; Oceanospirillales;

GV64__locus_1

EUT2B

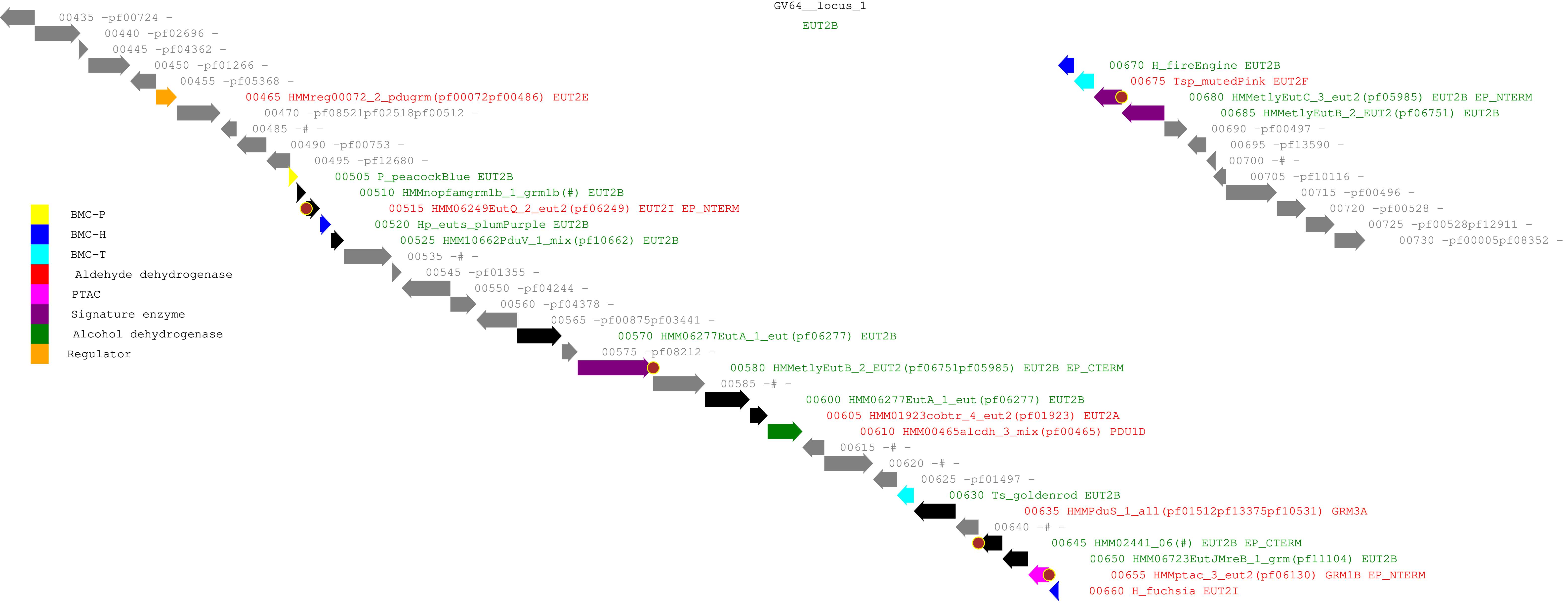

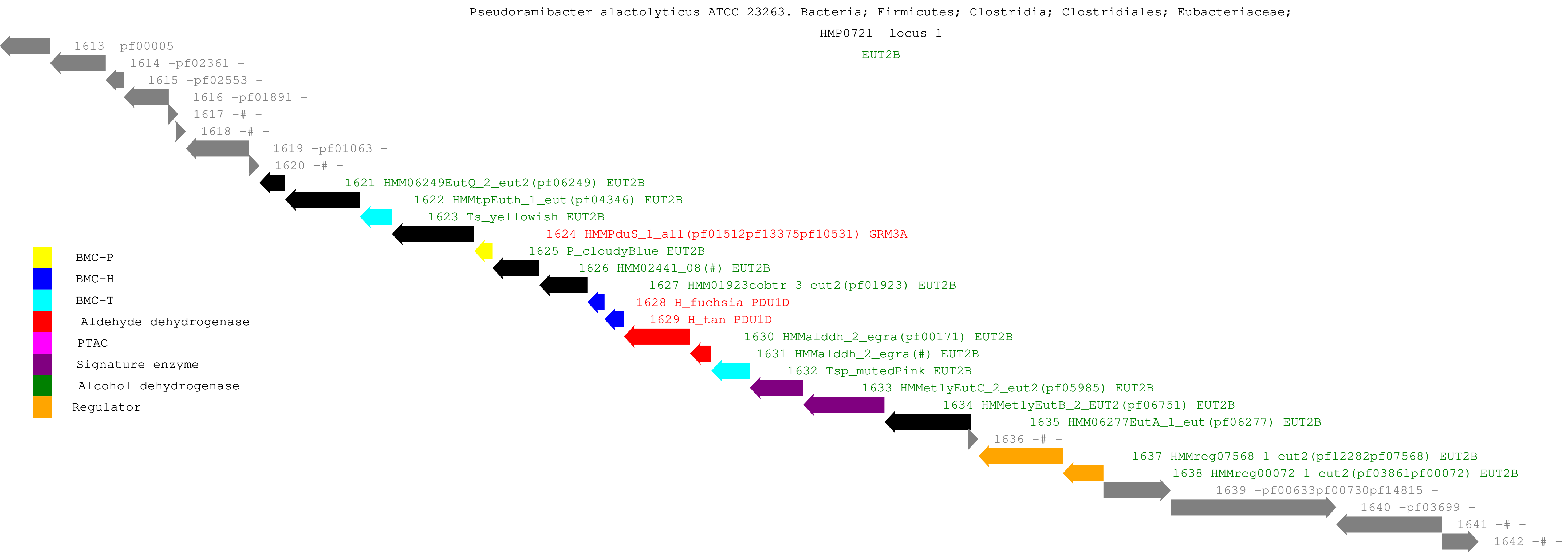

Pseudoramibacter alactolyticus ATCC 23263. Bacteria; Firmicutes; Clostridia; Clostridiales; Eubacteriaceae;
HMP0721__locus_1
EUT2B
1613 -pf00005 -
1614 -pf02361 -
1615 -pf02553 -
1616 -pf01891 -
1617 -# -
1618 -# -
1619 -pf01063 -
1620 -# -
1621 HMM06249EutQ_2_eut2(pf06249) EUT2B
1622 HMMtpEuth_1_eut(pf04346) EUT2B
1623 Ts_yellowish EUT2B
1624 HMMPduS_1_all(pf01512pf13375pf10531) GRM3A
1625 P_cloudyBlue EUT2B
1626 HMM02441_08(#) EUT2B
1627 HMM01923cobtr_3_eut2(pf01923) EUT2B
1628 H_fuchsia PDU1D
1629 H_tan PDU1D
1630 HMMalddh_2_egra(pf00171) EUT2B
1631 HMMalddh_2_egra(#) EUT2B
1632 Tsp_mutedPink EUT2B
1633 HMMetlyEutC_2_eut2(pf05985) EUT2B
1634 HMMetlyEutB_2_EUT2(pf06751) EUT2B
1635 HMM06277EutA_1_eut(pf06277) EUT2B
1636 -# -
1637 HMMreg07568_1_eut2(pf12282pf07568) EUT2B
1638 HMMreg00072_1_eut2(pf03861pf00072) EUT2B
1639 -pf00633pf00730pf14815 -
1640 -pf03699 -
1641 -# -
1642 -# -
BMC-P
BMC-H
BMC-T
Aldehyde dehydrogenase
PTAC
Signature enzyme
Alcohol dehydrogenase
Regulator

Flavonifractor plautii ATCC 29863. Bacteria; Firmicutes; Clostridia; Clostridiales; Flavonifractor.

HMPREF0372__locus_1

EUT2B

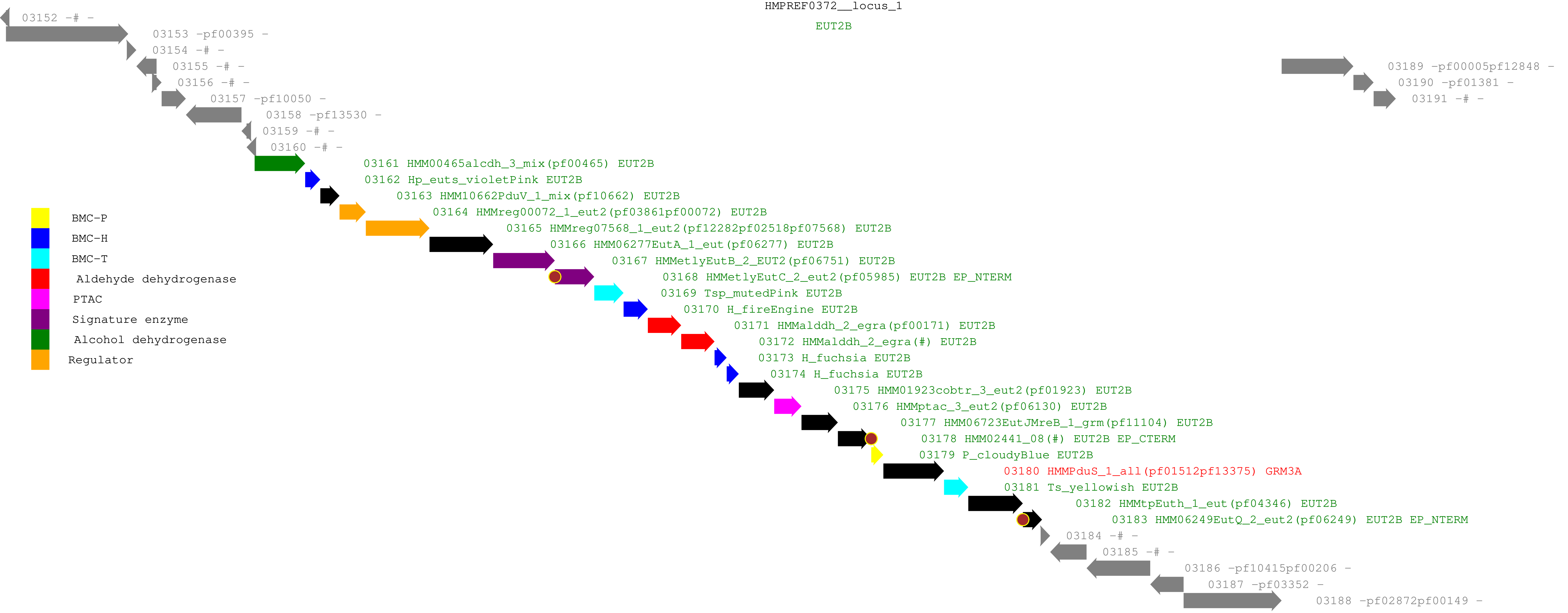

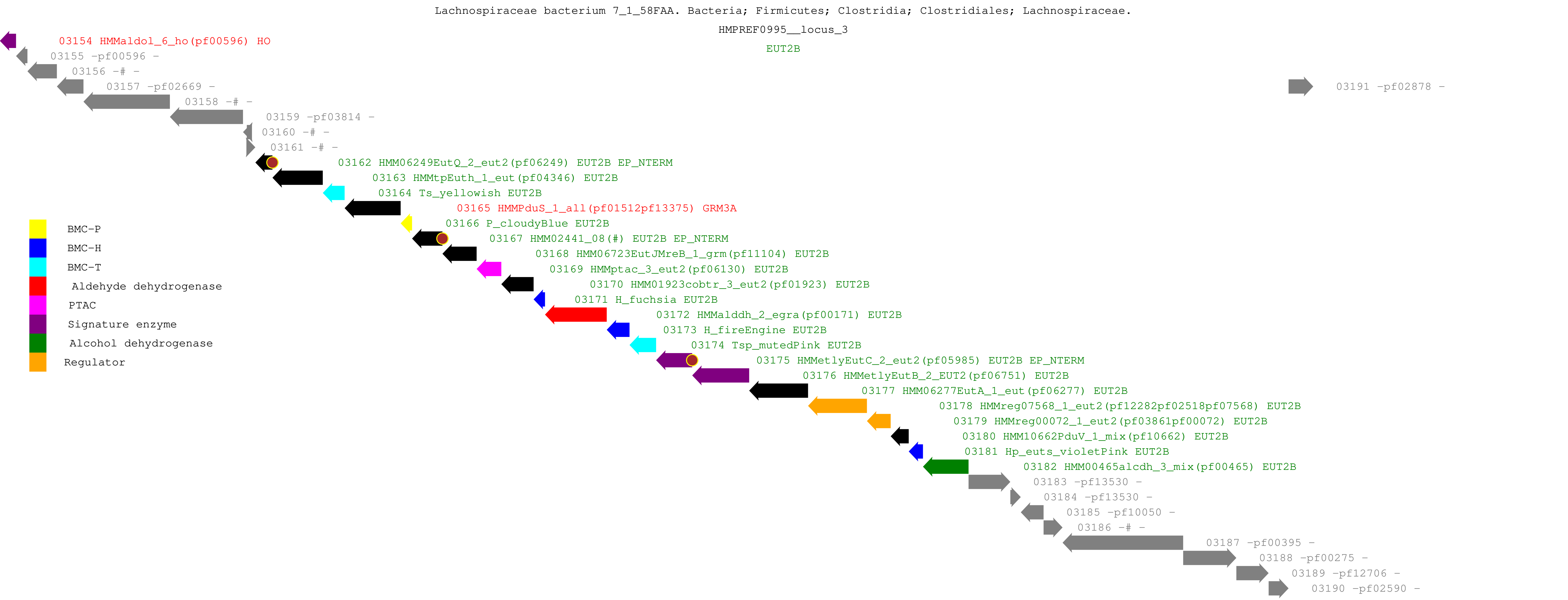
Lachnospiraceae bacterium 7_1_58FAA. Bacteria; Firmicutes; Clostridia; Clostridiales; Lachnospiraceae.
HMPREF0995__locus_3
EUT2B
03154 HMMaldol_6_ho(pf00596) HO
03155 -pf00596 -
03156 -# -
03157 -pf02669 -
03158 -# -
03159 -pf03814 -
03160 -# -
03161 -# -
03162 HMM06249EutQ_2_eut2(pf06249) EUT2B EP_NTERM
03163 HMMtpEuth_1_eut(pf04346) EUT2B
03164 Ts_yellowish EUT2B
03165 HMMPduS_1_all(pf01512pf13375) GRM3A
03166 P_cloudyBlue EUT2B
03167 HMM02441_08(#) EUT2B EP_NTERM
03168 HMM06723EutJMreB_1_grm(pf11104) EUT2B
03169 HMMptac_3_eut2(pf06130) EUT2B
03170 HMM01923cobtr_3_eut2(pf01923) EUT2B
03171 H_fuchsia EUT2B
03172 HMMaldh_2_egra(pf00171) EUT2B
03173 H_fireEngine EUT2B
03174 Tsp_mutedPink EUT2B
03175 HMMetlyEutC_2_eut2(pf05985) EUT2B EP_NTERM
03176 HMMetlyEutB_2_EUT2(pf06751) EUT2B
03177 HMM06277EutA_1_eut(pf06277) EUT2B
03178 HMMreg07568_1_eut2(pf12282pf02518pf07568) EUT2B
03179 HMMreg00072_1_eut2(pf03861pf00072) EUT2B
03180 HMM10662PduV_1_mix(pf10662) EUT2B
03181 Hp_euts_violetPink EUT2B
03182 HMM00465alcdh_3_mix(pf00465) EUT2B
03183 -pf13530 -
03184 -pf13530 -
03185 -pf10050 -
03186 -# -
03187 -pf00395 -
03188 -pf00275 -
03189 -pf12706 -
03190 -pf02590 -
03191 -pf02878 -
BMC-P
BMC-H
BMC-T
Aldehyde dehydrogenase
PTAC
Signature enzyme
Alcohol dehydrogenase
Regulator

Synergistes sp. 3_1_syn1. Bacteria; Synergistetes; Synergistia; Synergistales; Synergistaceae;

HMPREF1006__locus_1

EUT2B

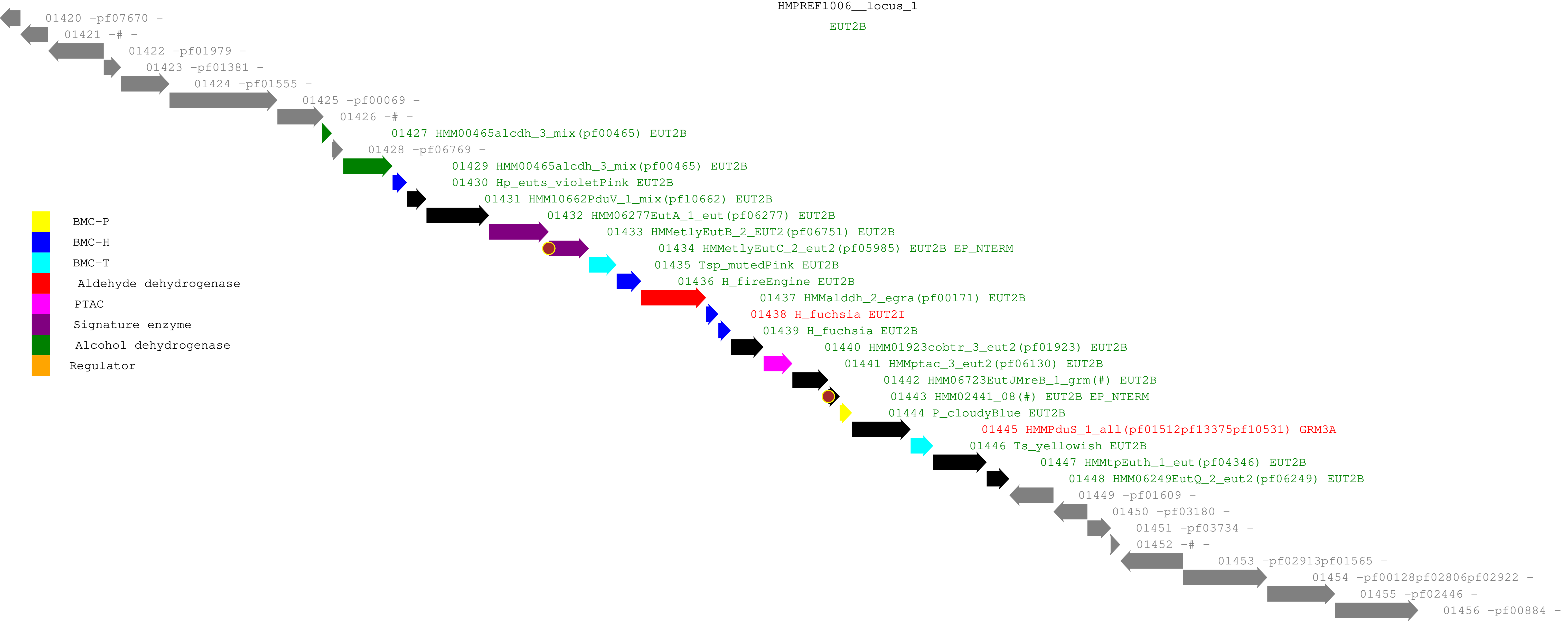

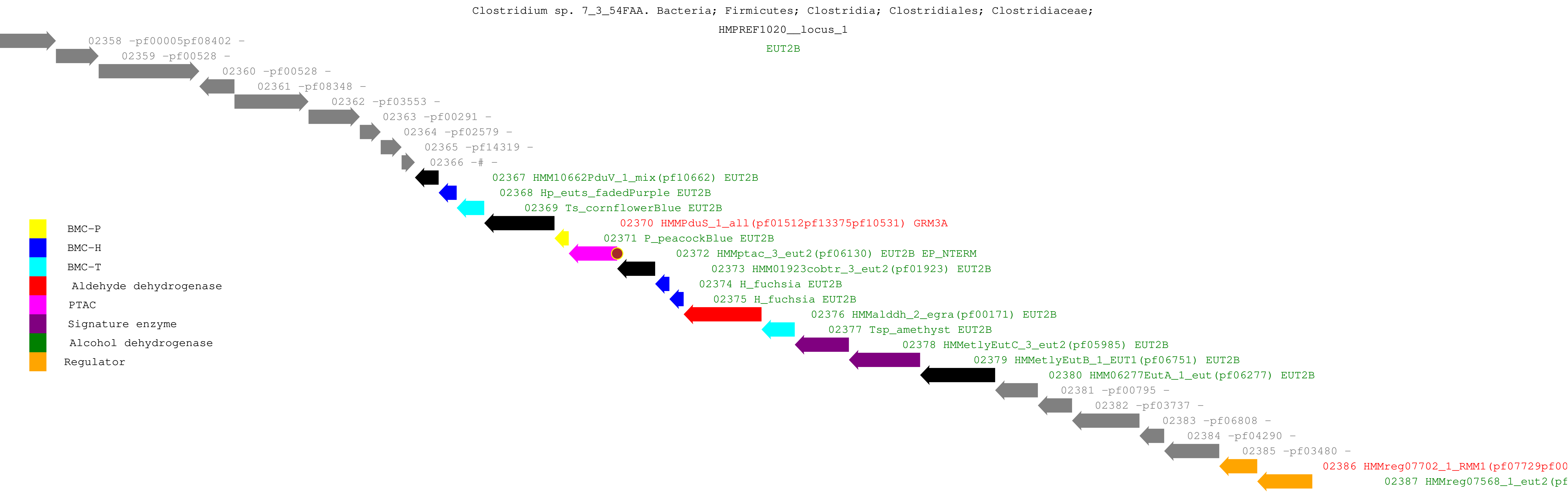
Clostridium sp. 7_3_54FAA. Bacteria; Firmicutes; Clostridia; Clostridiales; Clostridiaceae;
HMPREF1020__locus_1
EUT2B
02358 -pf00005pf08402 -
02359 -pf00528 -
02360 -pf00528 -
02361 -pf08348 -
02362 -pf03553 -
02363 -pf00291 -
02364 -pf02579 -
02365 -pf14319 -
02366 -# -
02367 HMM10662PduV_1_mix(pf10662) EUT2B
02368 Hp_euts_fadedPurple EUT2B
02369 Ts_cornflowerBlue EUT2B
02370 HMMPduS_1_all(pf01512pf13375pf10531) GRM3A
02371 P_peacockBlue EUT2B
02372 HMMptac_3_eut2(pf06130) EUT2B EP_NTERM
02373 HMM01923cobtr_3_eut2(pf01923) EUT2B
02374 H_fuchsia EUT2B
02375 H_fuchsia EUT2B
02376 HMMalddh_2_egra(pf00171) EUT2B
02377 Tsp_amethyst EUT2B
02378 HMMetlyEutC_3_eut2(pf05985) EUT2B
02379 HMMetlyEutB_1_EUT1(pf06751) EUT2B
02380 HMM06277EutA_1_eut(pf06277) EUT2B
02381 -pf00795 -
02382 -pf03737 -
02383 -pf06808 -
02384 -pf04290 -
02385 -pf03480 -
02386 HMMreg07702_1_RMM1(pf07729pf00
02387 HMMreg07568_1_eut2(pf
BMC-P
BMC-H
BMC-T
Aldehyde dehydrogenase
PTAC
Signature enzyme
Alcohol dehydrogenase
Regulator

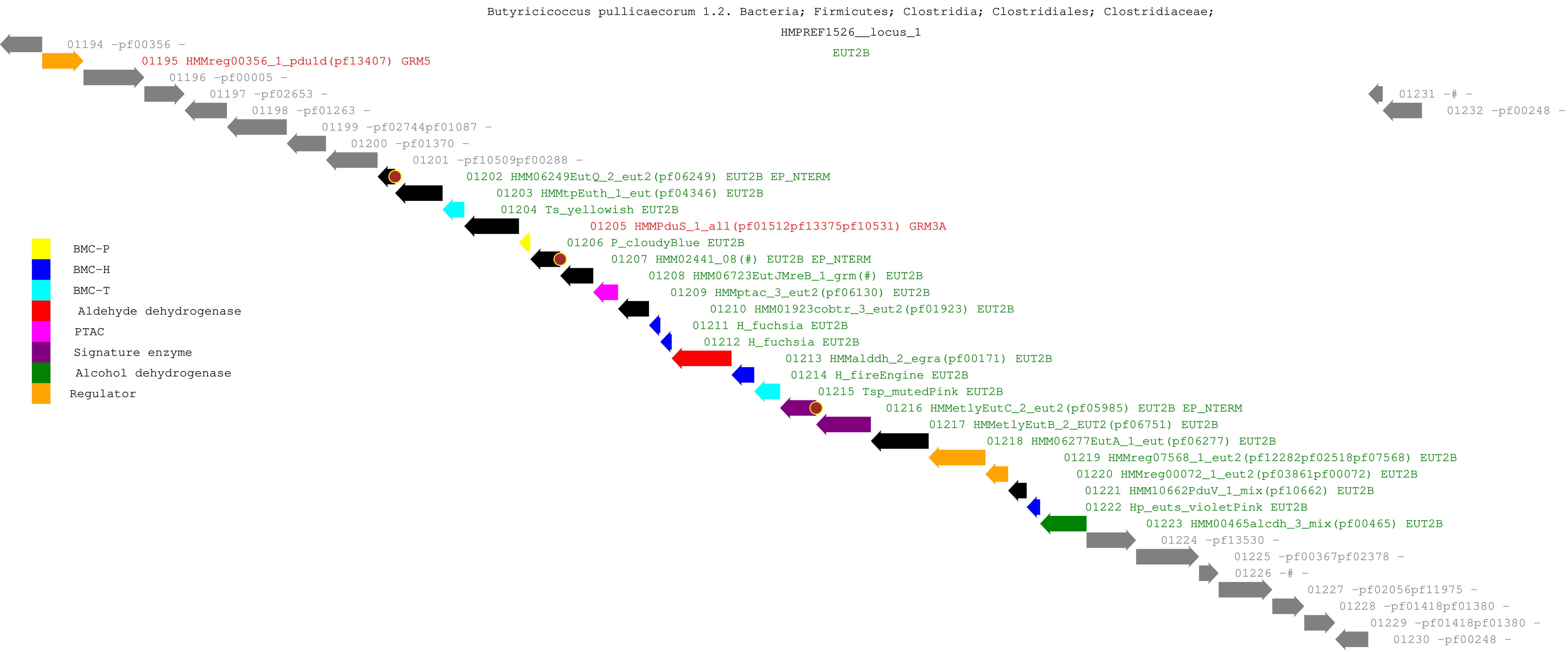
Butyricicoccus pullicaecorum 1.2. Bacteria; Firmicutes; Clostridia; Clostridiales; Clostridiaceae;
HMPREF1526__locus_1
EUT2B
01194 -pf00356 -
01195 HMMreg00356_1_pdu1d(pf13407) GRM5
01196 -pf00005 -
01197 -pf02653 -
01198 -pf01263 -
01199 -pf02744pf01087 -
01200 -pf01370 -
01201 -pf10509pf00288 -
01202 HMM06249EutQ_2_eut2(pf06249) EUT2B EP_NTERM
01203 HMMtpEuth_1_eut(pf04346) EUT2B
01204 Ts_yellowish EUT2B
01205 HMMPduS_1_all(pf01512pf13375pf10531) GRM3A
01206 P_cloudyBlue EUT2B
01207 HMM02441_08(#) EUT2B EP_NTERM
01208 HMM06723EutJMreB_1_grm(#) EUT2B
01209 HMMptac_3_eut2(pf06130) EUT2B
01210 HMM01923cobtr_3_eut2(pf01923) EUT2B
01211 H_fuchsia EUT2B
01212 H_fuchsia EUT2B
01213 HMMalddh_2_egra(pf00171) EUT2B
01214 H_fireEngine EUT2B
01215 Tsp_mutedPink EUT2B
01216 HMMetlyEutC_2_eut2(pf05985) EUT2B EP_NTERM
01217 HMMetlyEutB_2_EUT2(pf06751) EUT2B
01218 HMM06277EutA_1_eut(pf06277) EUT2B
01219 HMMreg07568_1_eut2(pf12282pf02518pf07568) EUT2B
01220 HMMreg00072_1_eut2(pf03861pf00072) EUT2B
01221 HMM10662PduV_1_mix(pf10662) EUT2B
01222 Hp_euts_violetPink EUT2B
01223 HMM00465alcdh_3_mix(pf00465) EUT2B
01224 -pf13530 -
01225 -pf00367pf02378 -
01226 -# -
01227 -pf02056pf11975 -
01228 -pf01418pf01380 -
01229 -pf01418pf01380 -
01230 -pf00248 -
01231 -# -
01232 -pf00248 -
BMC-P
BMC-H
BMC-T
Aldehyde dehydrogenase
PTAC
Signature enzyme
Alcohol dehydrogenase
Regulator

Clostridium sp. KLE 1755. Bacteria; Firmicutes; Clostridia; Clostridiales; Clostridiaceae;

HMPREF1548__locus_2

EUT2B

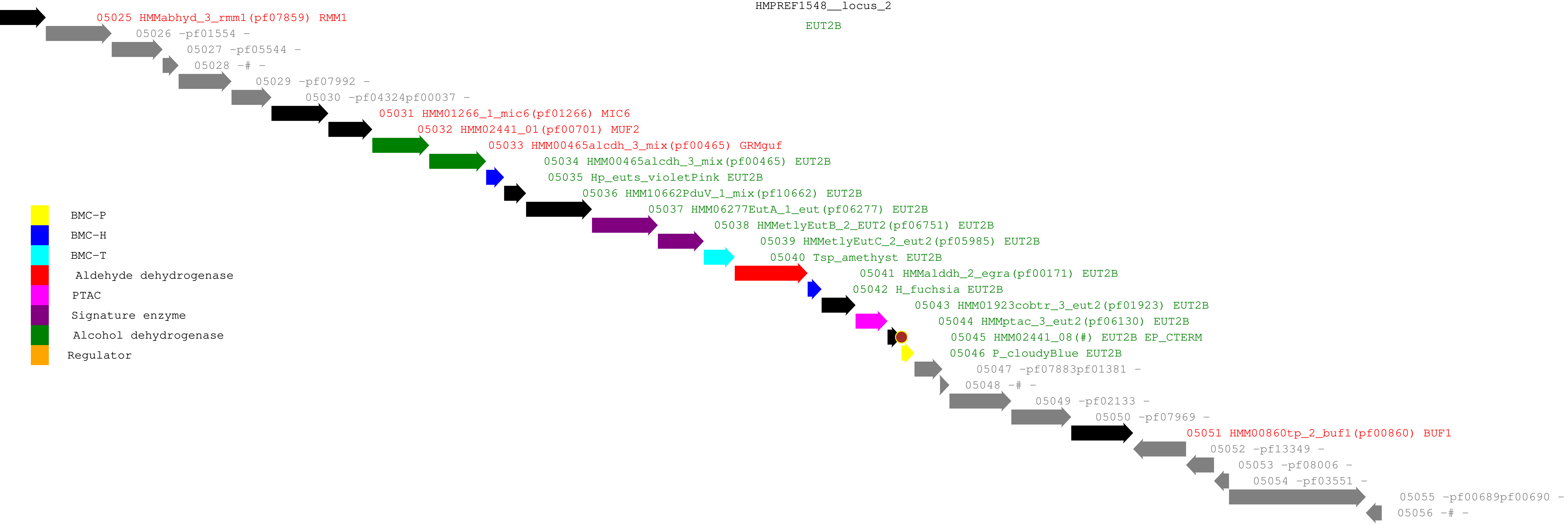

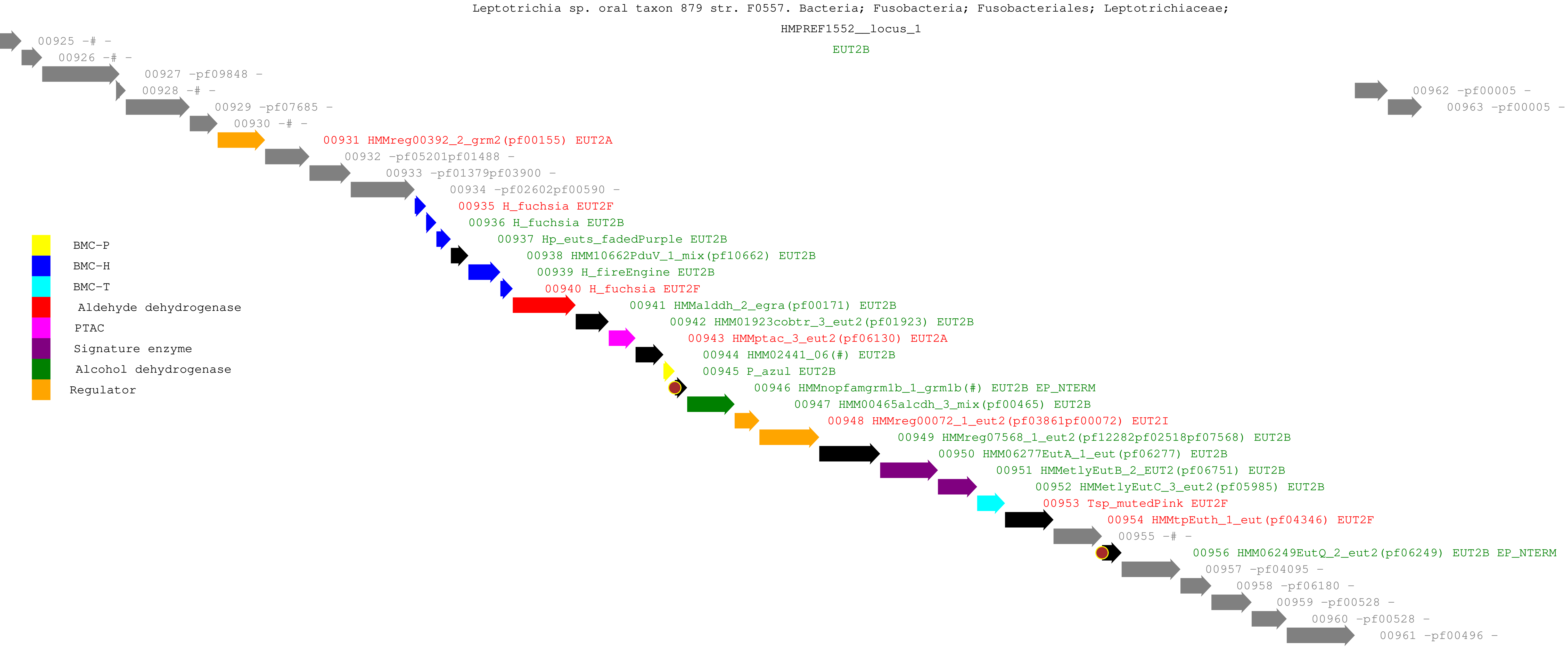
Leptotrichia sp. oral taxon 879 str. F0557. Bacteria; Fusobacteria; Fusobacteriales; Leptotrichiaceae;
HMPREF1552__locus_1
EUT2B
00925 -# -
00926 -# -
00927 -pf09848 -
00928 -# -
00929 -pf07685 -
00930 -# -
00931 HMMreg00392_2_grm2(pf00155) EUT2A
00932 -pf05201pf01488 -
00933 -pf01379pf03900 -
00934 -pf02602pf00590 -
00935 H_fuchsia EUT2F
00936 H_fuchsia EUT2B
00937 Hp_euts_fadedPurple EUT2B
00938 HMM10662PduV_1_mix(pf10662) EUT2B
00939 H_fireEngine EUT2B
00940 H_fuchsia EUT2F
00941 HMMalddh_2_egra(pf00171) EUT2B
00942 HMM01923cobtr_3_eut2(pf01923) EUT2B
00943 HMMptac_3_eut2(pf06130) EUT2A
00944 HMM02441_06(#) EUT2B
00945 P_azul EUT2B
00946 HMMnopfamgrm1b_1_grm1b(#) EUT2B EP_NTERM
00947 HMM00465alcdh_3_mix(pf00465) EUT2B
00948 HMMreg00072_1_eut2(pf03861pf00072) EUT2I
00949 HMMreg07568_1_eut2(pf12282pf02518pf07568) EUT2B
00950 HMM06277EutA_1_eut(pf06277) EUT2B
00951 HMMetlyEutB_2_EUT2(pf06751) EUT2B
00952 HMMetlyEutC_3_eut2(pf05985) EUT2B
00953 Tsp_mutedPink EUT2F
00954 HMMtpEuth_1_eut(pf04346) EUT2F
00955 -# -
00956 HMM06249EutQ_2_eut2(pf06249) EUT2B EP_NTERM
00957 -pf04095 -
00958 -pf06180 -
00959 -pf00528 -
00960 -pf00528 -
00961 -pf00496 -
00962 -pf00005 -
00963 -pf00005 -
BMC-P
BMC-H
BMC-T
Aldehyde dehydrogenase
PTAC
Signature enzyme
Alcohol dehydrogenase
Regulator

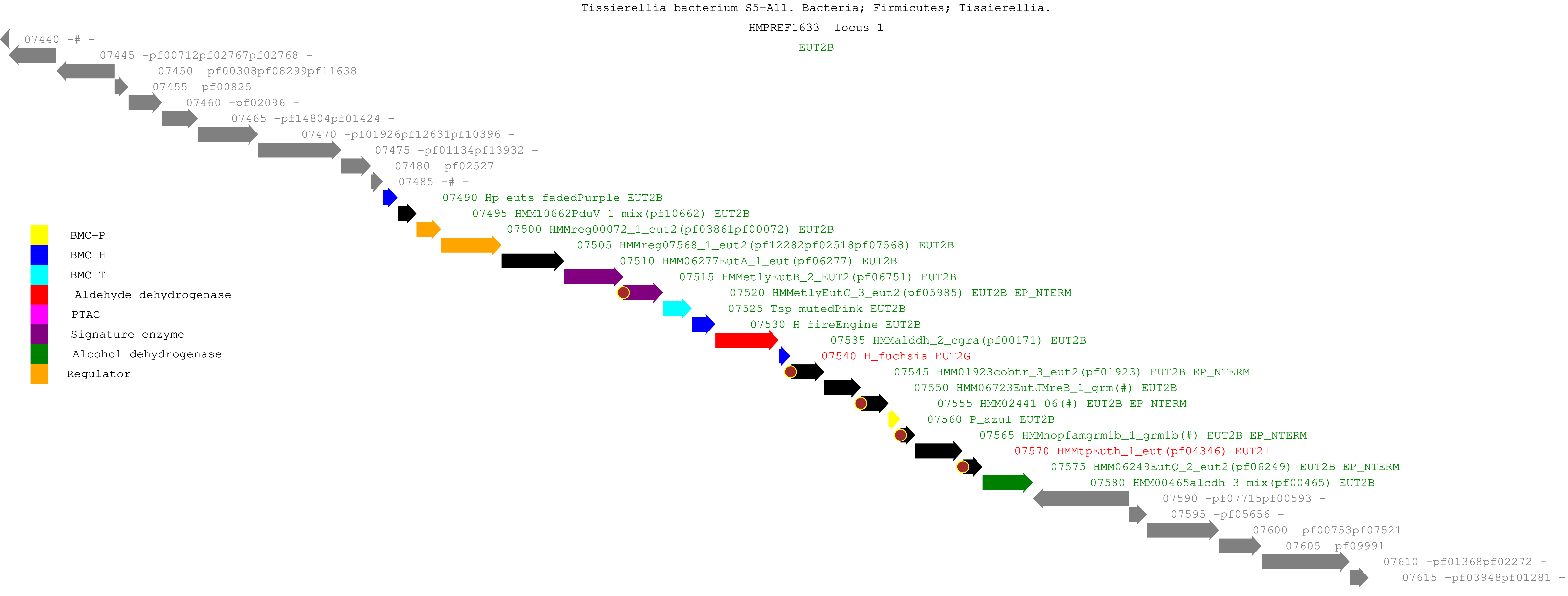

Tissierellia bacterium S5-A11. Bacteria; Firmicutes; Tissierellia.
HMPREF1633__locus_1
EUT2B
07440 -# -
07445 -pf00712pf02767pf02768 -
07450 -pf00308pf08299pf11638 -
07455 -pf00825 -
07460 -pf02096 -
07465 -pf14804pf01424 -
07470 -pf01926pf12631pf10396 -
07475 -pf01134pf13932 -
07480 -pf02527 -
07485 -# -
07490 Hp_euts_fadedPurple EUT2B
07495 HMM10662PduV_1_mix(pf10662) EUT2B
07500 HMMreg00072_1_eut2(pf03861pf00072) EUT2B
07505 HMMreg07568_1_eut2(pf12282pf02518pf07568) EUT2B
07510 HMM06277EutA_1_eut(pf06277) EUT2B
07515 HMMetlyEutB_2_EUT2(pf06751) EUT2B
07520 HMMetlyEutC_3_eut2(pf05985) EUT2B EP_NTERM
07525 Tsp_mutedPink EUT2B
07530 H_fireEngine EUT2B
07535 HMMaldh_2_egra(pf00171) EUT2B
07540 H_fuchsia EUT2G
07545 HMM01923cobtr_3_eut2(pf01923) EUT2B EP_NTERM
07550 HMM06723EutJMreB_1_grm(#) EUT2B
07555 HMM02441_06(#) EUT2B EP_NTERM
07560 P_azul EUT2B
07565 HMMnopfamgrm1b_1_grm1b(#) EUT2B EP_NTERM
07570 HMMtpEuth_1_eut(pf04346) EUT2I
07575 HMM06249EutQ_2_eut2(pf06249) EUT2B EP_NTERM
07580 HMM00465alcdh_3_mix(pf00465) EUT2B
07590 -pf07715pf00593 -
07595 -pf05656 -
07600 -pf00753pf07521 -
07605 -pf09991 -
07610 -pf01368pf02272 -
07615 -pf03948pf01281 -
BMC-P
BMC-H
BMC-T
Aldehyde dehydrogenase
PTAC
Signature enzyme
Alcohol dehydrogenase
Regulator

Leptotrichia sp. oral taxon 225 str. F0581. Bacteria; Fusobacteria; Fusobacteriales; Leptotrichiaceae;

HMPREF9108__locus_1_main_sat

EUT2B

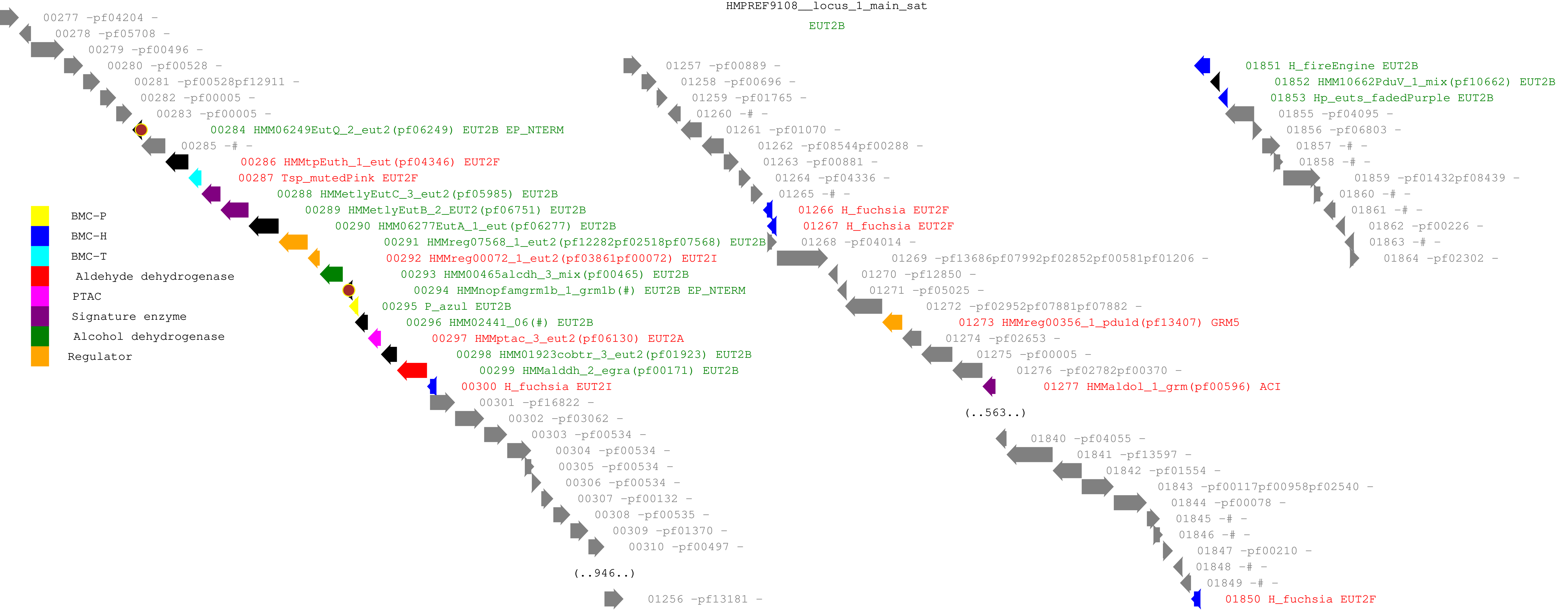

Peptoniphilus sp. oral taxon 375 str. F0436. Bacteria; Firmicutes; Tissierellia; Tissierellales; Peptoniphilaceae;

HMPREF9130__locus_1_main_sat

EUT2B

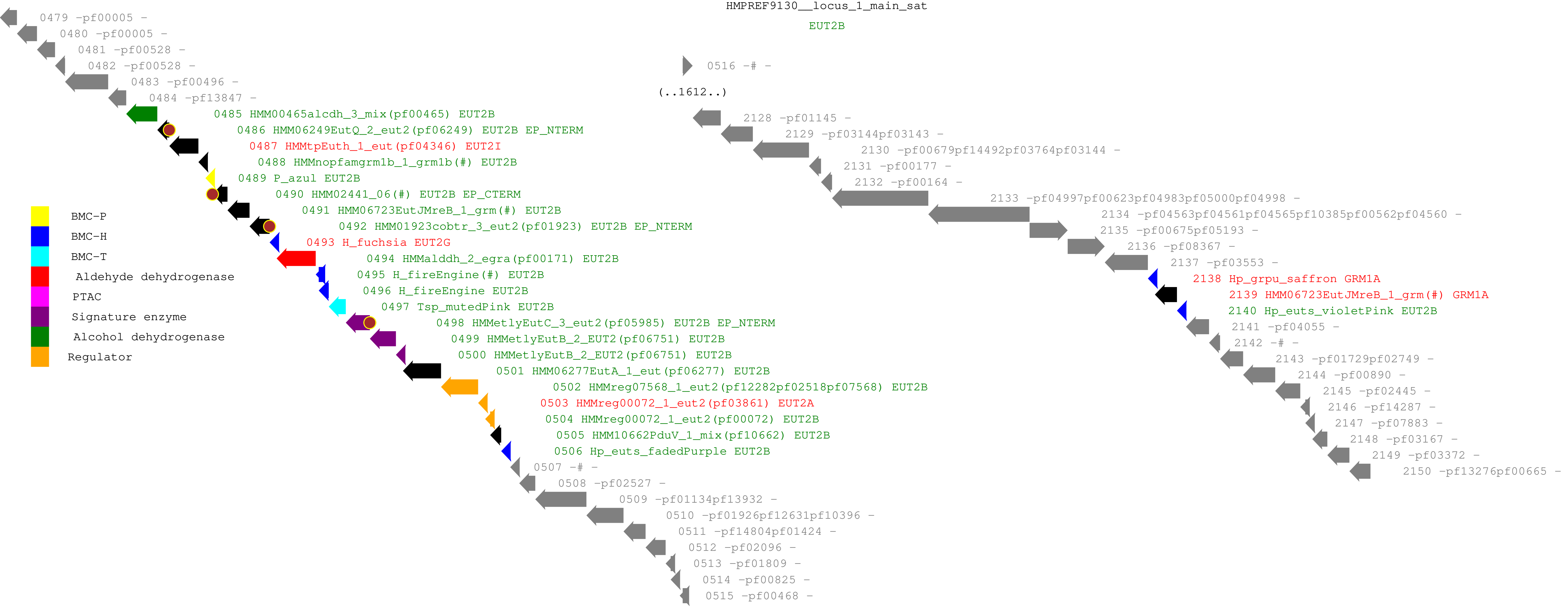

Clostridium orbiscindens 1_3_50AFAA. Bacteria; Firmicutes; Clostridia; Clostridiales; Flavonifractor.

HMPREF9460__locus_3

EUT2B

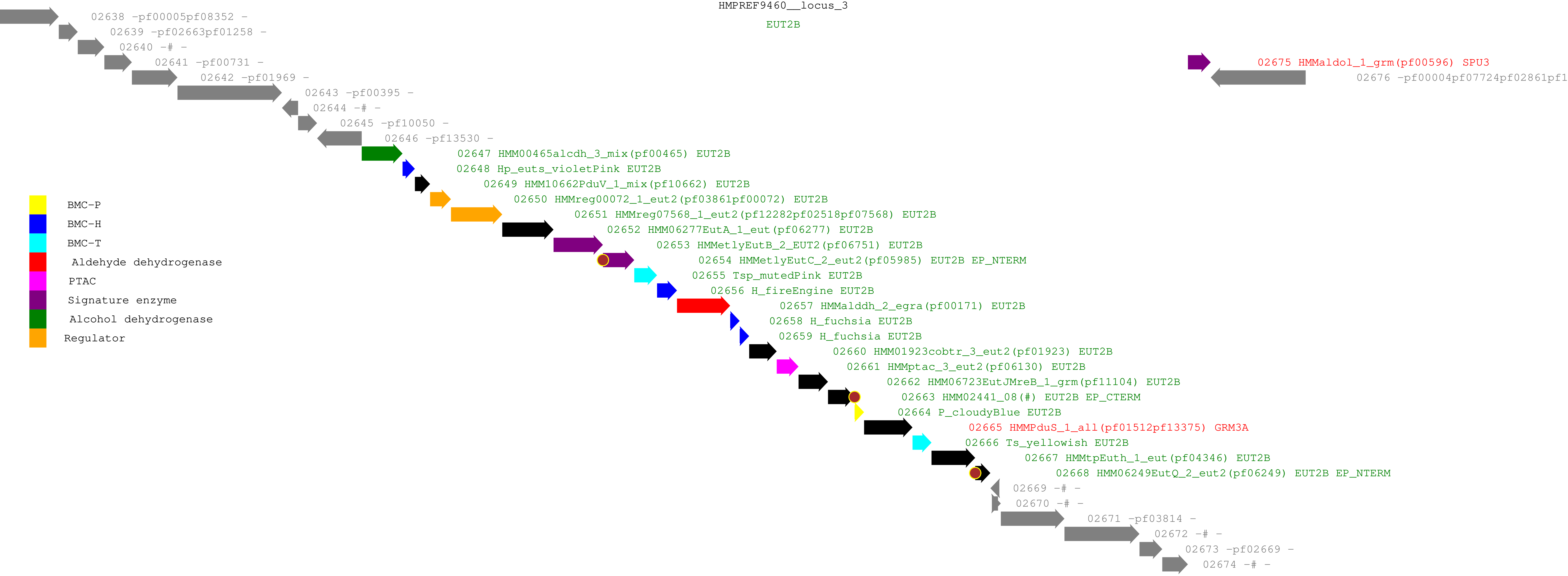

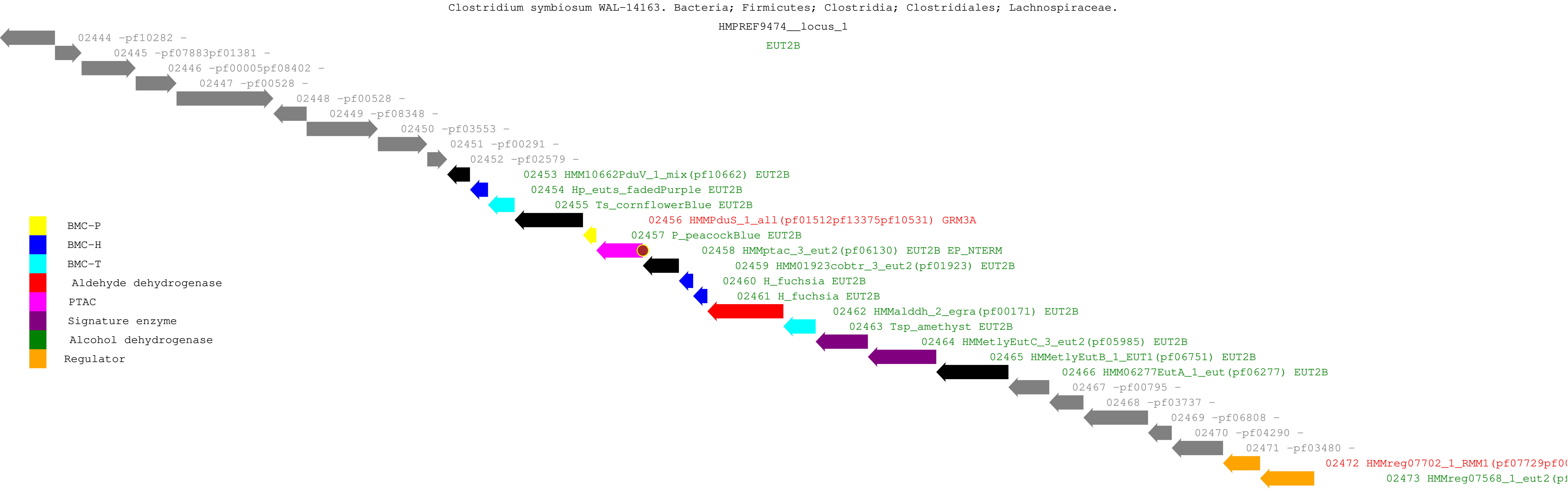
Clostridium symbiosum WAL-14163. Bacteria; Firmicutes; Clostridia; Clostridiales; Lachnospiraceae.
HMPREF9474__locus_1
EUT2B
02444 -pf10282 -
02445 -pf07883pf01381 -
02446 -pf00005pf08402 -
02447 -pf00528 -
02448 -pf00528 -
02449 -pf08348 -
02450 -pf03553 -
02451 -pf00291 -
02452 -pf02579 -
02453 HMM10662PduV_1_mix(pf10662) EUT2B
02454 Hp_euts_fadedPurple EUT2B
02455 Ts_cornflowerBlue EUT2B
02456 HMMPduS_1_all(pf01512pf13375pf10531) GRM3A
02457 P_peacockBlue EUT2B
02458 HMMptac_3_eut2(pf06130) EUT2B EP_NTERM
02459 HMM01923cobtr_3_eut2(pf01923) EUT2B
02460 H_fuchsia EUT2B
02461 H_fuchsia EUT2B
02462 HMMaldh_2_egra(pf00171) EUT2B
02463 Tsp_amethyst EUT2B
02464 HMMetlyEutC_3_eut2(pf05985) EUT2B
02465 HMMetlyEutB_1_EUT1(pf06751) EUT2B
02466 HMM06277EutA_1_eut(pf06277) EUT2B
02467 -pf00795 -
02468 -pf03737 -
02469 -pf06808 -
02470 -pf04290 -
02471 -pf03480 -
BMC-P
BMC-H
BMC-T
Aldehyde dehydrogenase
PTAC
Signature enzyme
Alcohol dehydrogenase
Regulator

Halanaerobium praevalens (strain ATCC 33744 / DSM 2228 / GSL). Bacteria; Firmicutes; Clostridia; Halanaerobiales; Halanaerobiaceae;

Hprae__locus_1

EUT2B

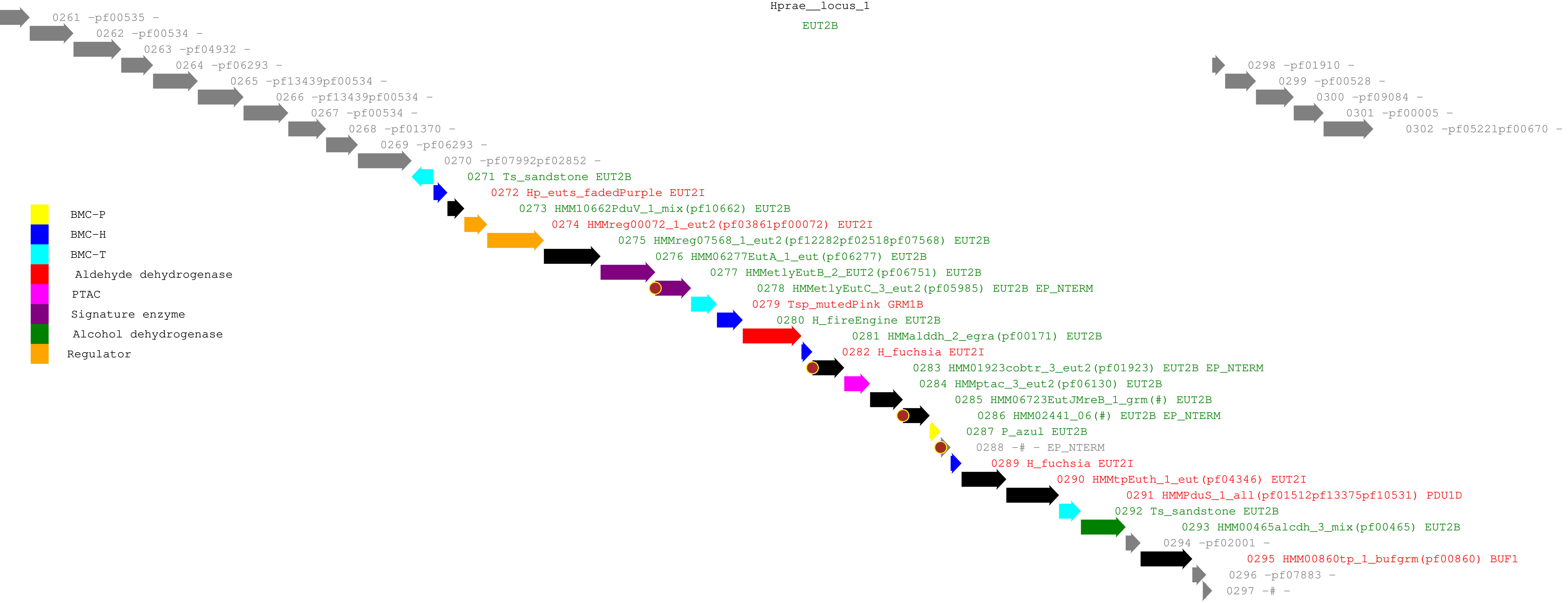

Leptotrichia hofstadii. Bacteria; Fusobacteria; Fusobacteriales; Leptotrichiaceae; Leptotrichia.

JCM16775__locus_1

EUT2B

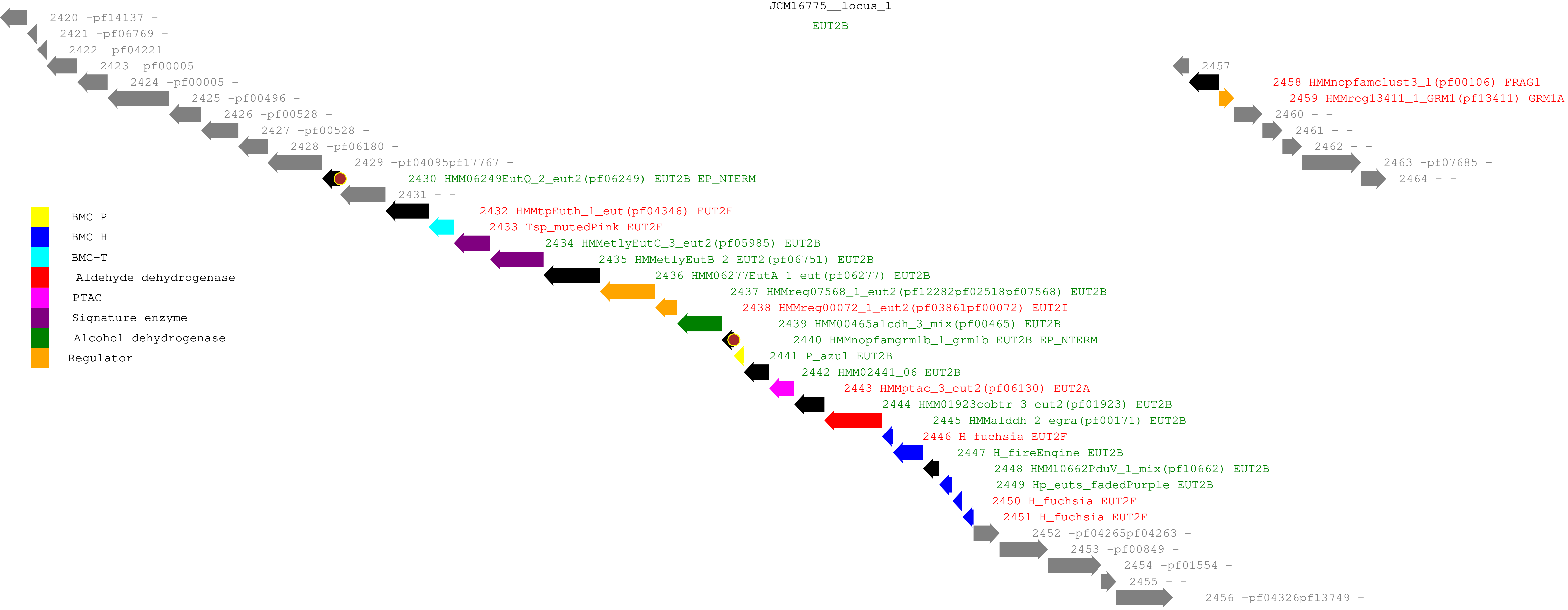

Leptotrichia trevisanii. Bacteria; Fusobacteria; Fusobacteriales; Leptotrichiaceae; Leptotrichia.

JMUB3870__locus_1

EUT2B

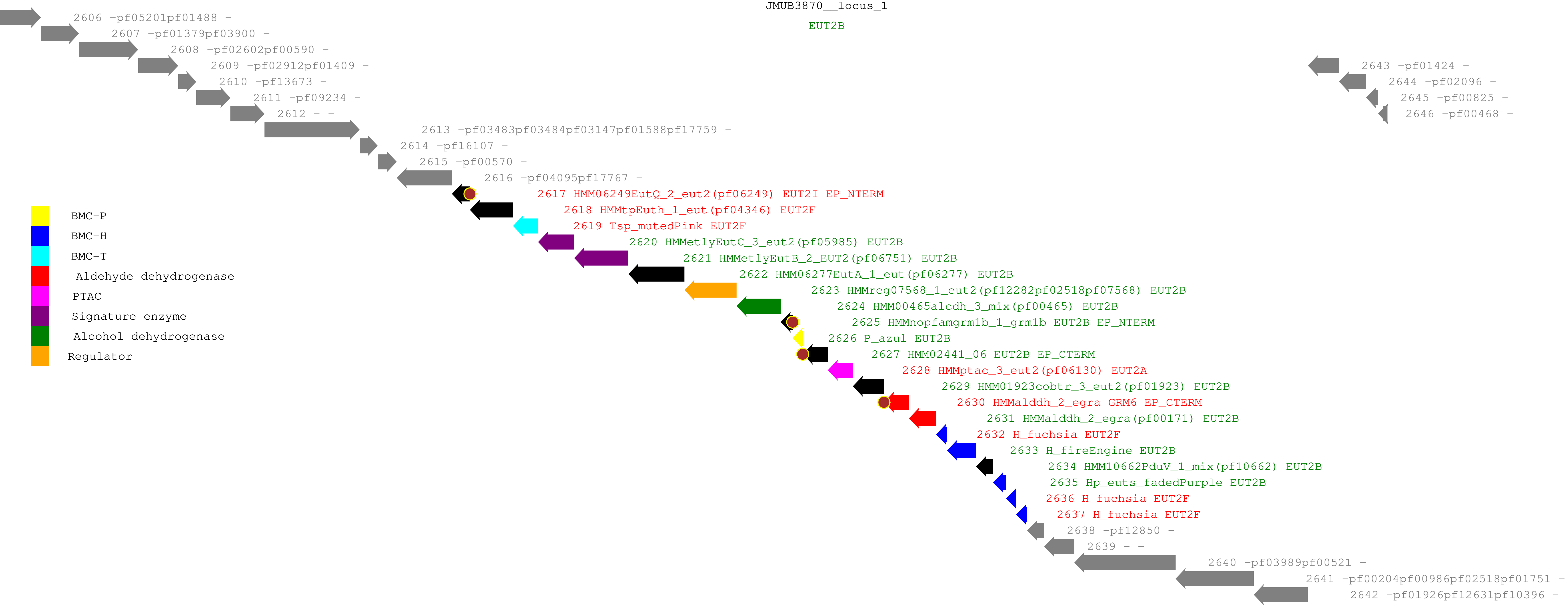

Leptotrichia hongkongensis. Bacteria; Fusobacteria; Fusobacteriales; Leptotrichiaceae; Leptotrichia.

JMUB5056__locus_1

EUT2B

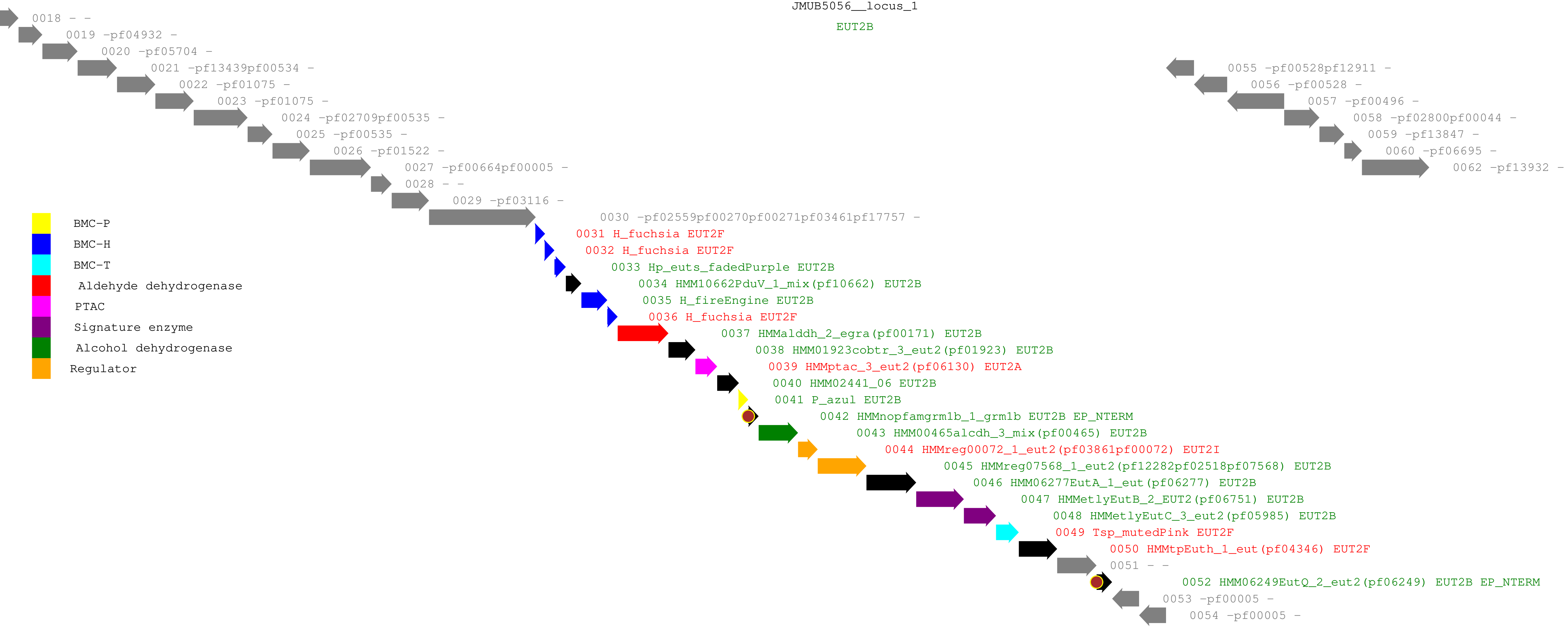

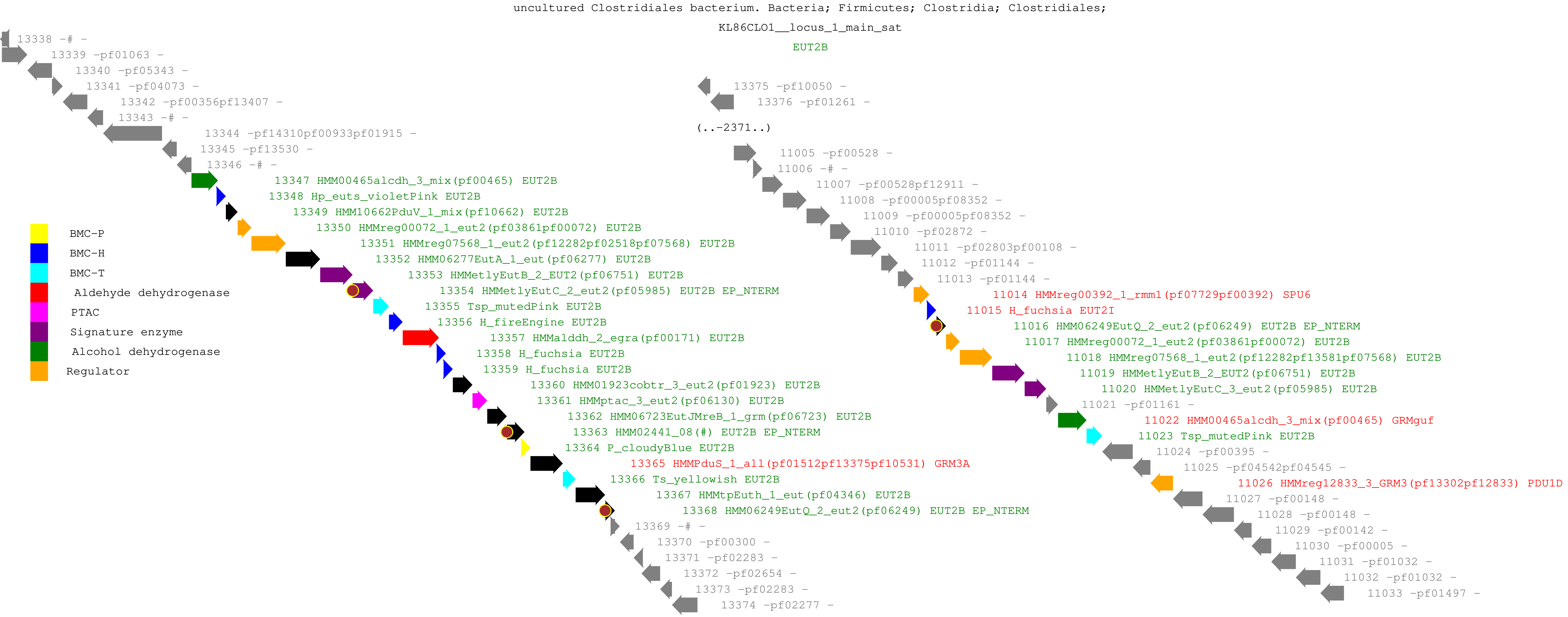

uncultured Clostridiales bacterium. Bacteria; Firmicutes; Clostridia; Clostridiales;
KL86CLO1__locus_1_main_sat
EUT2B
13338 -# -
13339 -pf01063 -
13340 -pf05343 -
13341 -pf04073 -
13342 -pf00356pf13407 -
13343 -# -
13344 -pf14310pf00933pf01915 -
13345 -pf13530 -
13346 -# -
13347 HMM00465alcdh_3_mix(pf00465) EUT2B
13348 Hp_euts_violetPink EUT2B
13349 HMM10662PduV_1_mix(pf10662) EUT2B
13350 HMMreg00072_1_eut2(pf03861pf00072) EUT2B
13351 HMMreg07568_1_eut2(pf12282pf02518pf07568) EUT2B
13352 HMM06277EutA_1_eut(pf06277) EUT2B
13353 HMMetlyEutB_2_EUT2(pf06751) EUT2B
13354 HMMetlyEutC_2_eut2(pf05985) EUT2B EP_NTERM
13355 Tsp_mutedPink EUT2B
13356 H_fireEngine EUT2B
13357 HMMaldh_2_egra(pf00171) EUT2B
13358 H_fuchsia EUT2B
13359 H_fuchsia EUT2B
13360 HMM01923cobtr_3_eut2(pf01923) EUT2B
13361 HMMptac_3_eut2(pf06130) EUT2B
13362 HMM06723EutJMreB_1_grm(pf06723) EUT2B
13363 HMM02441_08(#) EUT2B EP_NTERM
13364 P_cloudyBlue EUT2B
13365 HMMPduS_1_all(pf01512pf13375pf10531) GRM3A
13366 Ts_yellowish EUT2B
13367 HMMtpEuth_1_eut(pf04346) EUT2B
13368 HMM06249EutQ_2_eut2(pf06249) EUT2B EP_NTERM
13369 -# -
13370 -pf00300 -
13371 -pf02283 -
13372 -pf02654 -
13373 -pf02283 -
13374 -pf02277 -
BMC-P
BMC-H
BMC-T
Aldehyde dehydrogenase
PTAC
Signature enzyme
Alcohol dehydrogenase
Regulator
13375 -pf10050 -
13376 -pf01261 -
(..-2371..)
11005 -pf00528 -
11006 -# -
11007 -pf00528pf12911 -
11008 -pf00005pf08352 -
11009 -pf00005pf08352 -
11010 -pf02872 -
11011 -pf02803pf00108 -
11012 -pf01144 -
11013 -pf01144 -
11014 HMMreg00392_1_rmm1(pf07729pf00392) SPU6
11015 H_fuchsia EUT2I
11016 HMM06249EutQ_2_eut2(pf06249) EUT2B EP_NTERM
11017 HMMreg00072_1_eut2(pf03861pf00072) EUT2B
11018 HMMreg07568_1_eut2(pf12282pf13581pf07568) EUT2B
11019 HMMetlyEutB_2_EUT2(pf06751) EUT2B
11020 HMMetlyEutC_3_eut2(pf05985) EUT2B
11021 -pf01161 -
11022 HMM00465alcdh_3_mix(pf00465) GRMguf
11023 Tsp_mutedPink EUT2B
11024 -pf00395 -
11025 -pf04542pf04545 -
11026 HMMreg12833_3_GRM3(pf13302pf12833) PDU1D
11027 -pf00148 -
11028 -pf00148 -
11029 -pf00142 -
11030 -pf00005 -
11031 -pf01032 -
11032 -pf01032 -
11033 -pf01497 -

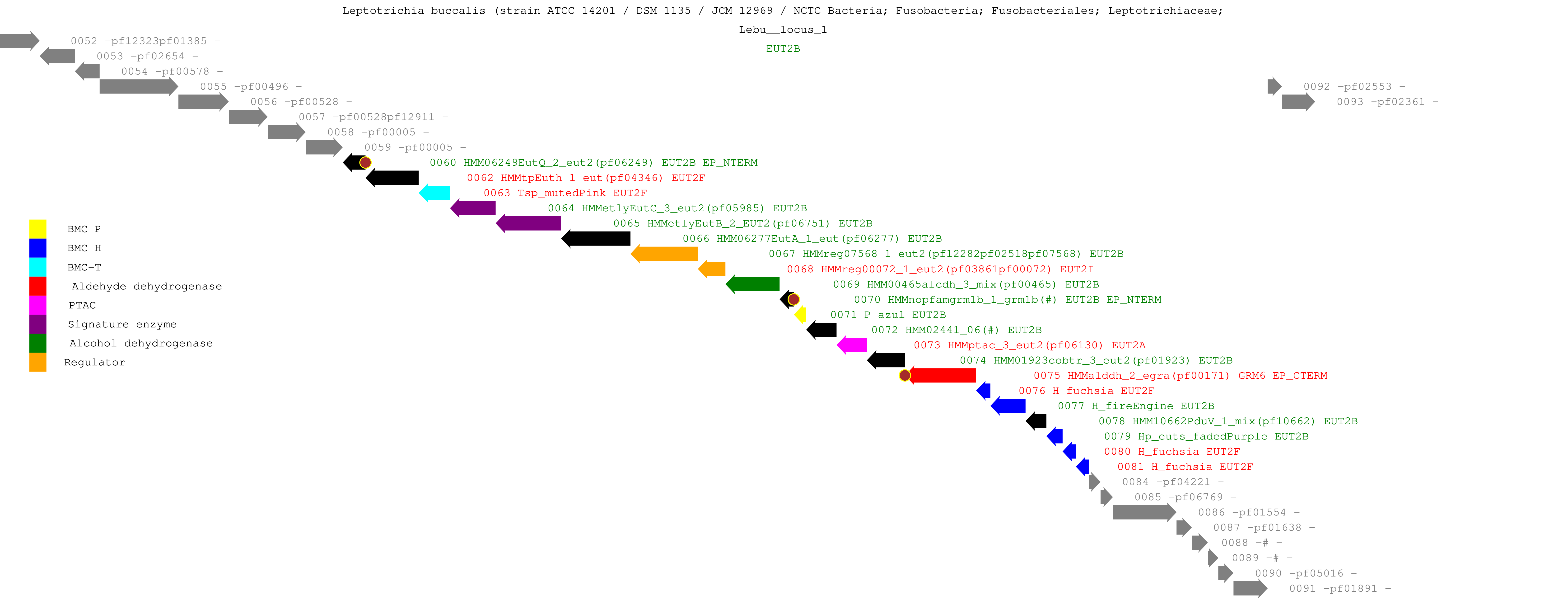

Leptotrichia buccalis (strain ATCC 14201 / DSM 1135 / JCM 12969 / NCTC Bacteria; Fusobacteria; Fusobacteriales; Leptotrichiaceae;
Lebu__locus_1
EUT2B
0052 -pf12323pf01385 -
0053 -pf02654 -
0054 -pf00578 -
0055 -pf00496 -
0056 -pf00528 -
0057 -pf00528pf12911 -
0058 -pf00005 -
0059 -pf00005 -
0060 HMM06249EutQ_2_eut2(pf06249) EUT2B EP_NTERM
0062 HMMtpEuth_1_eut(pf04346) EUT2F
0063 Tsp_mutedPink EUT2F
0064 HMMetlyEutC_3_eut2(pf05985) EUT2B
0065 HMMetlyEutB_2_EUT2(pf06751) EUT2B
0066 HMM06277EutA_1_eut(pf06277) EUT2B
0067 HMMreg07568_1_eut2(pf12282pf02518pf07568) EUT2B
0068 HMMreg00072_1_eut2(pf03861pf00072) EUT2I
0069 HMM00465alcdh_3_mix(pf00465) EUT2B
0070 HMMnopfamgrm1b_1_grm1b(#) EUT2B EP_NTERM
0071 P_azul EUT2B
0072 HMM02441_06(#) EUT2B
0073 HMMptac_3_eut2(pf06130) EUT2A
0074 HMM01923cobtr_3_eut2(pf01923) EUT2B
0075 HMMalddh_2_egra(pf00171) GRM6 EP_CTERM
0076 H_fuchsia EUT2F
0077 H_fireEngine EUT2B
0078 HMM10662PduV_1_mix(pf10662) EUT2B
0079 Hp_euts_fadedPurple EUT2B
0080 H_fuchsia EUT2F
0081 H_fuchsia EUT2F
0084 -pf04221 -
0085 -pf06769 -
0086 -pf01554 -
0087 -pf01638 -
0088 -# -
0089 -# -
0090 -pf05016 -
0091 -pf01891 -
0092 -pf02553 -
0093 -pf02361 -
BMC-P
BMC-H
BMC-T
Aldehyde dehydrogenase
PTAC
Signature enzyme
Alcohol dehydrogenase
Regulator

Anaerococcus prevotii. Bacteria; Firmicutes; Tissierellia; Tissierellales; Peptoniphilaceae;

NCTC13150__locus_2

EUT2B

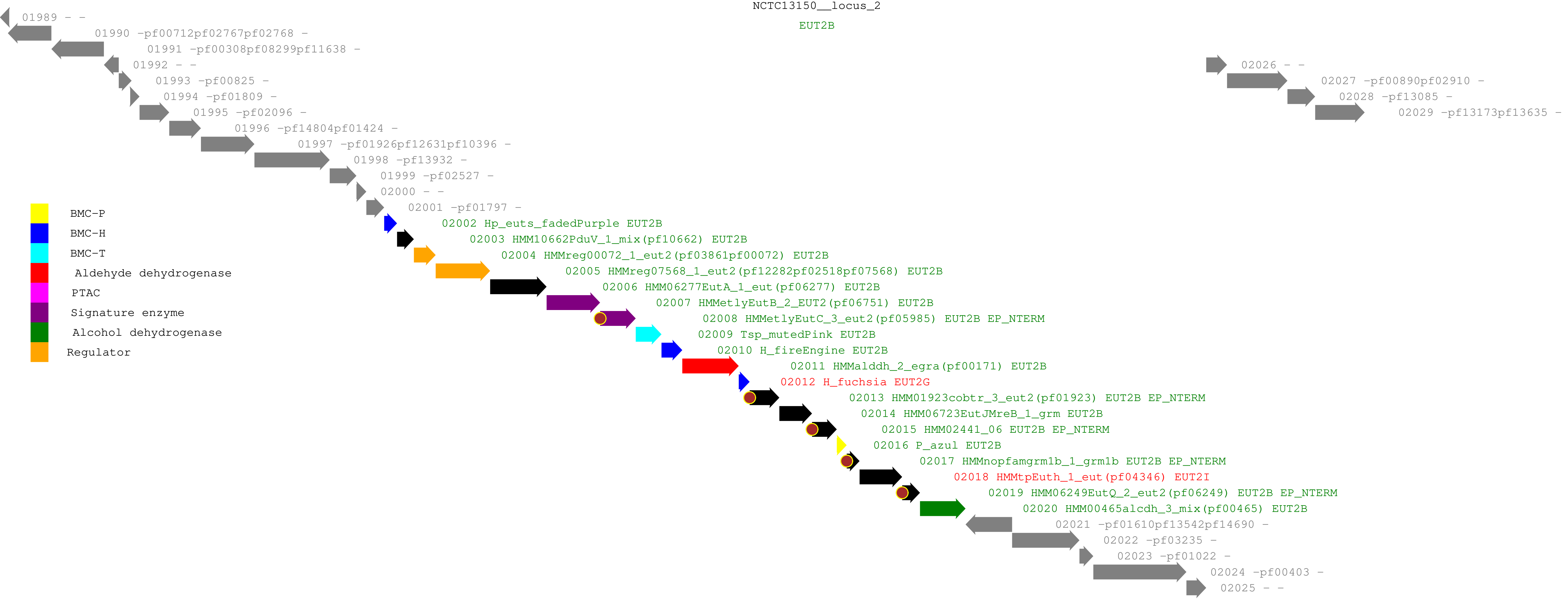

Petrocella atlantisensis. Bacteria; Firmicutes; Clostridia; Clostridiales; Vallitaleaceae;

PATL70BA__locus_1

EUT2B

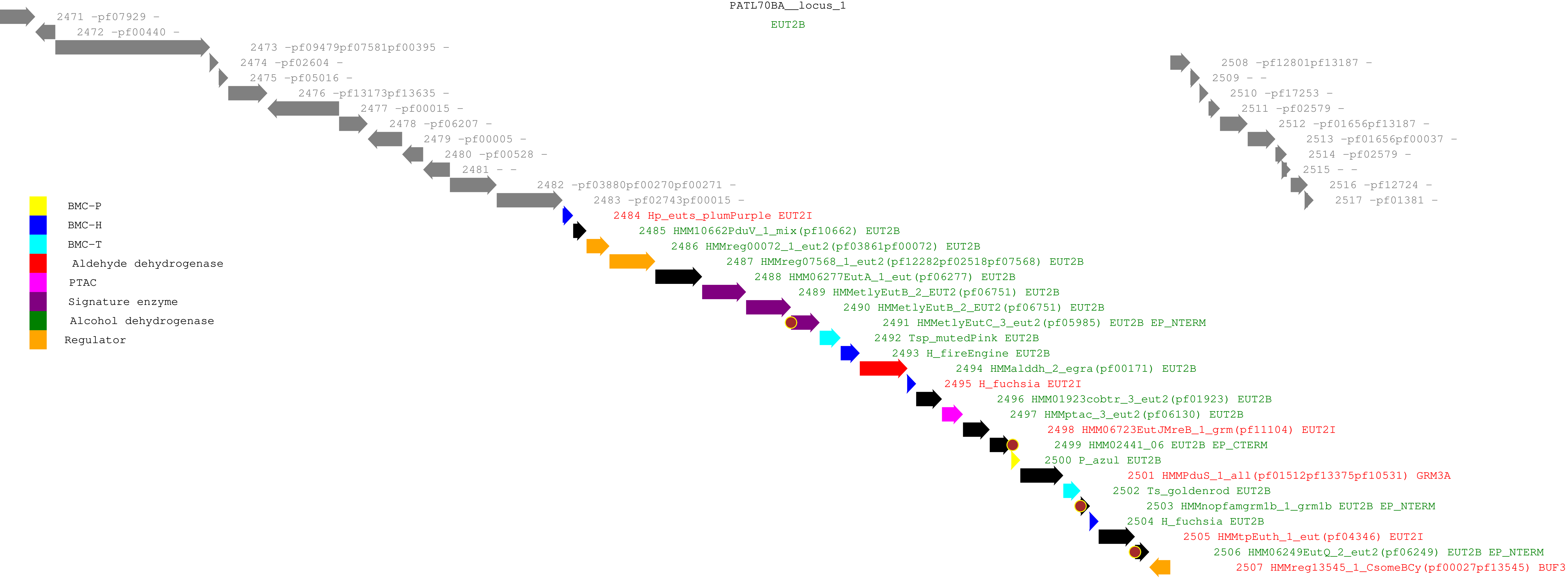

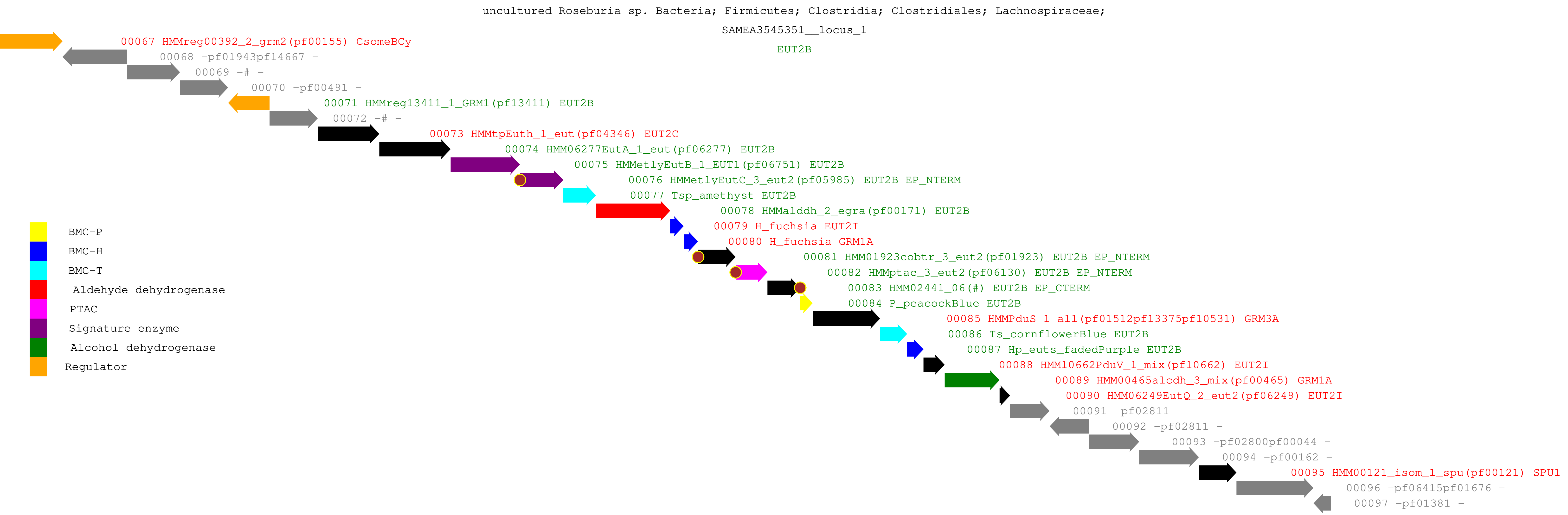

uncultured Roseburia sp. Bacteria; Firmicutes; Clostridia; Clostridiales; Lachnospiraceae;
SAMEA3545351__locus_1
EUT2B
00067 HMMreg00392_2_grm2(pf00155) CsomeBCy
00068 -pf01943pf14667 -
00069 -# -
00070 -pf00491 -
00071 HMMreg13411_1_GRM1(pf13411) EUT2B
00072 -# -
00073 HMMtpEuth_1_eut(pf04346) EUT2C
00074 HMM06277EutA_1_eut(pf06277) EUT2B
00075 HMMetlyEutB_1_EUT1(pf06751) EUT2B
00076 HMMetlyEutC_3_eut2(pf05985) EUT2B EP_NTERM
00077 Tsp_amethyst EUT2B
00078 HMMalddh_2_egra(pf00171) EUT2B
00079 H_fuchsia EUT2I
00080 H_fuchsia GRM1A
00081 HMM01923cobtr_3_eut2(pf01923) EUT2B EP_NTERM
00082 HMMptac_3_eut2(pf06130) EUT2B EP_NTERM
00083 HMM02441_06(#) EUT2B EP_CTERM
00084 P_peacockBlue EUT2B
00085 HMMPduS_1_all(pf01512pf13375pf10531) GRM3A
00086 Ts_cornflowerBlue EUT2B
00087 Hp_euts_fadedPurple EUT2B
00088 HMM10662PduV_1_mix(pf10662) EUT2I
00089 HMM00465alcdh_3_mix(pf00465) GRM1A
00090 HMM06249EutQ_2_eut2(pf06249) EUT2I
00091 -pf02811 -
00092 -pf02811 -
00093 -pf02800pf00044 -
00094 -pf00162 -
00095 HMM00121_isom_1_spu(pf00121) SPU1
00096 -pf06415pf01676 -
00097 -pf01381 -
BMC-P
BMC-H
BMC-T
Aldehyde dehydrogenase
PTAC
Signature enzyme
Alcohol dehydrogenase
Regulator

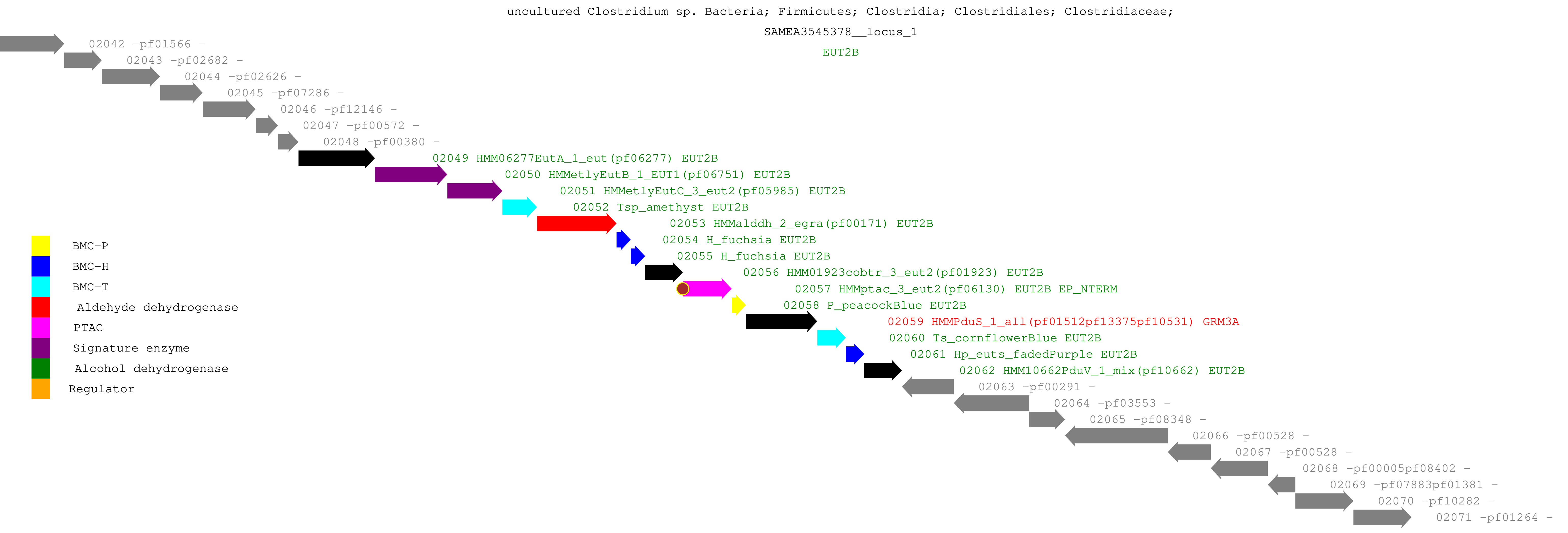
uncultured Clostridium sp. Bacteria; Firmicutes; Clostridia; Clostridiales; Clostridiaceae;
SAMEA3545378__locus_1
EUT2B
02042 -pf01566 -
02043 -pf02682 -
02044 -pf02626 -
02045 -pf07286 -
02046 -pf12146 -
02047 -pf00572 -
02048 -pf00380 -
02049 HMM06277EutA_1_eut(pf06277) EUT2B
02050 HMMetlyEutB_1_EUT1(pf06751) EUT2B
02051 HMMetlyEutC_3_eut2(pf05985) EUT2B
02052 Tsp_amethyst EUT2B
02053 HMMalddh_2_egra(pf00171) EUT2B
02054 H_fuchsia EUT2B
02055 H_fuchsia EUT2B
02056 HMM01923cobtr_3_eut2(pf01923) EUT2B
02057 HMMptac_3_eut2(pf06130) EUT2B EP_NTERM
02058 P_peacockBlue EUT2B
02059 HMMPduS_1_all(pf01512pf13375pf10531) GRM3A
02060 Ts_cornflowerBlue EUT2B
02061 Hp_euts_fadedPurple EUT2B
02062 HMM10662PduV_1_mix(pf10662) EUT2B
02063 -pf00291 -
02064 -pf03553 -
02065 -pf08348 -
02066 -pf00528 -
02067 -pf00528 -
02068 -pf00005pf08402 -
02069 -pf07883pf01381 -
02070 -pf10282 -
02071 -pf01264 -
BMC-P
BMC-H
BMC-T
Aldehyde dehydrogenase
PTAC
Signature enzyme
Alcohol dehydrogenase
Regulator

uncultured Clostridium sp. Bacteria; Firmicutes; Clostridia; Clostridiales; Clostridiaceae;

SAMEA3545387__locus_1

EUT2B

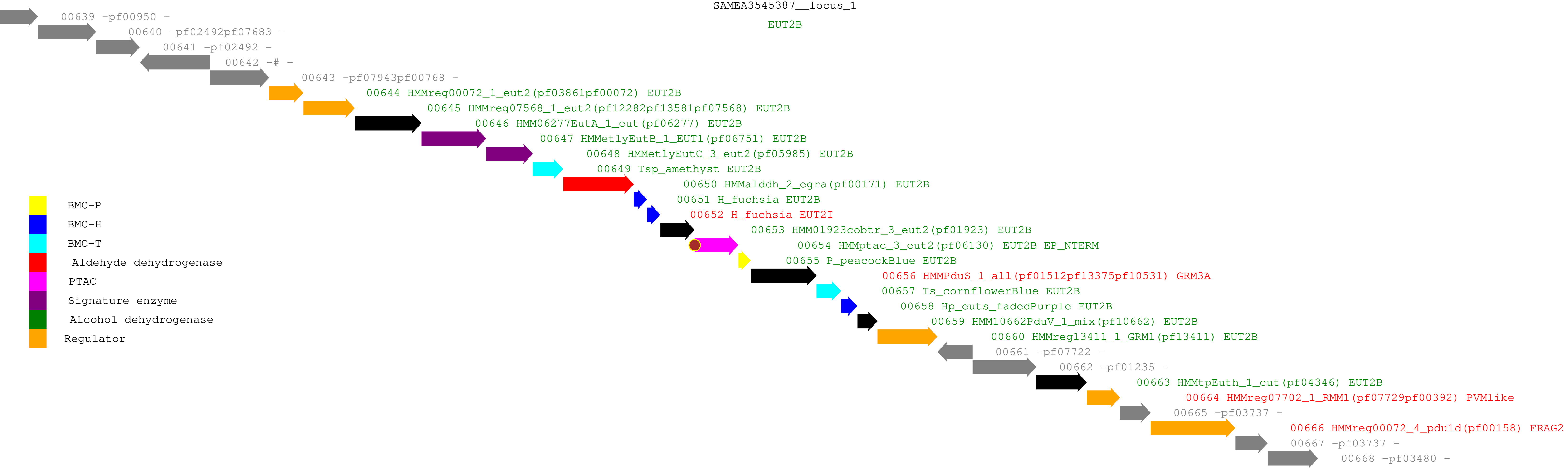

uncultured Butyricicoccus sp. Bacteria; Firmicutes; Clostridia; Clostridiales; Clostridiaceae;

SAMEA3545390__locus_1

EUT2B

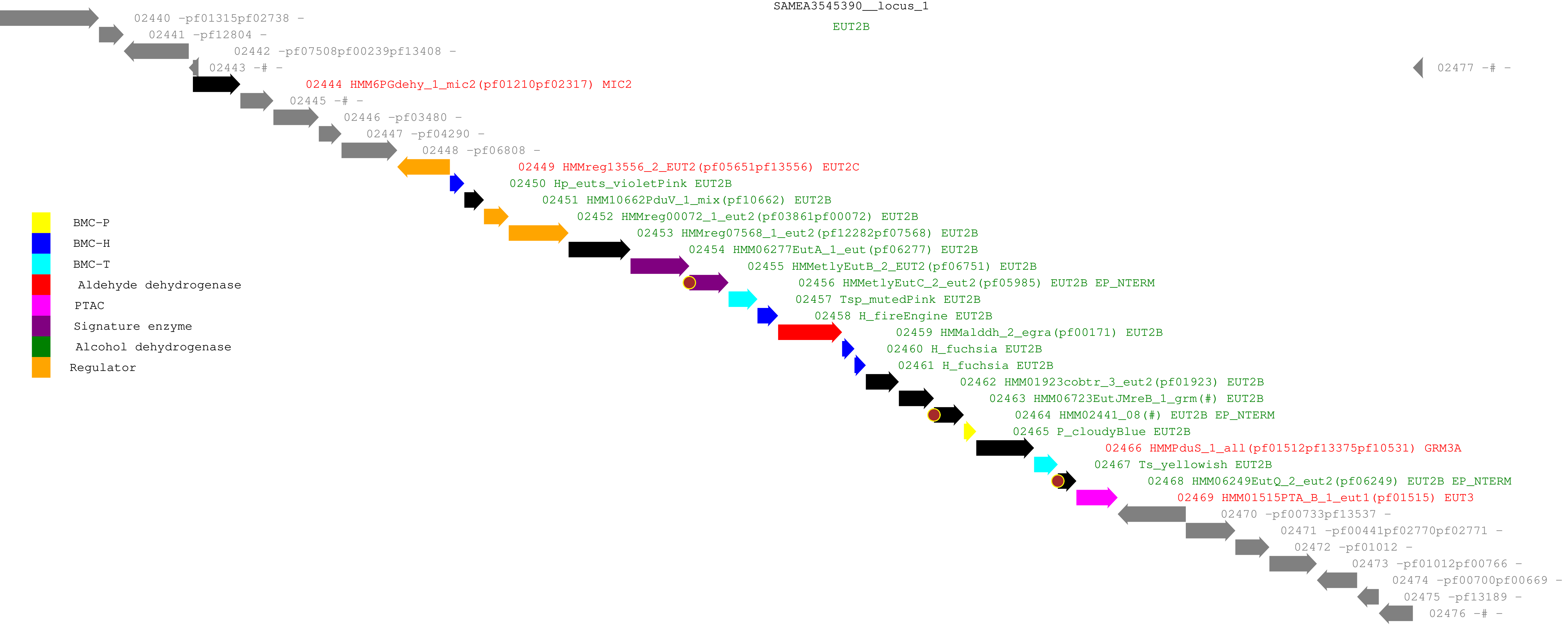

uncultured Flavonifractor sp. Bacteria; Firmicutes; Clostridia; Clostridiales; Flavonifractor;

SAMEA3545396__locus_1

EUT2B

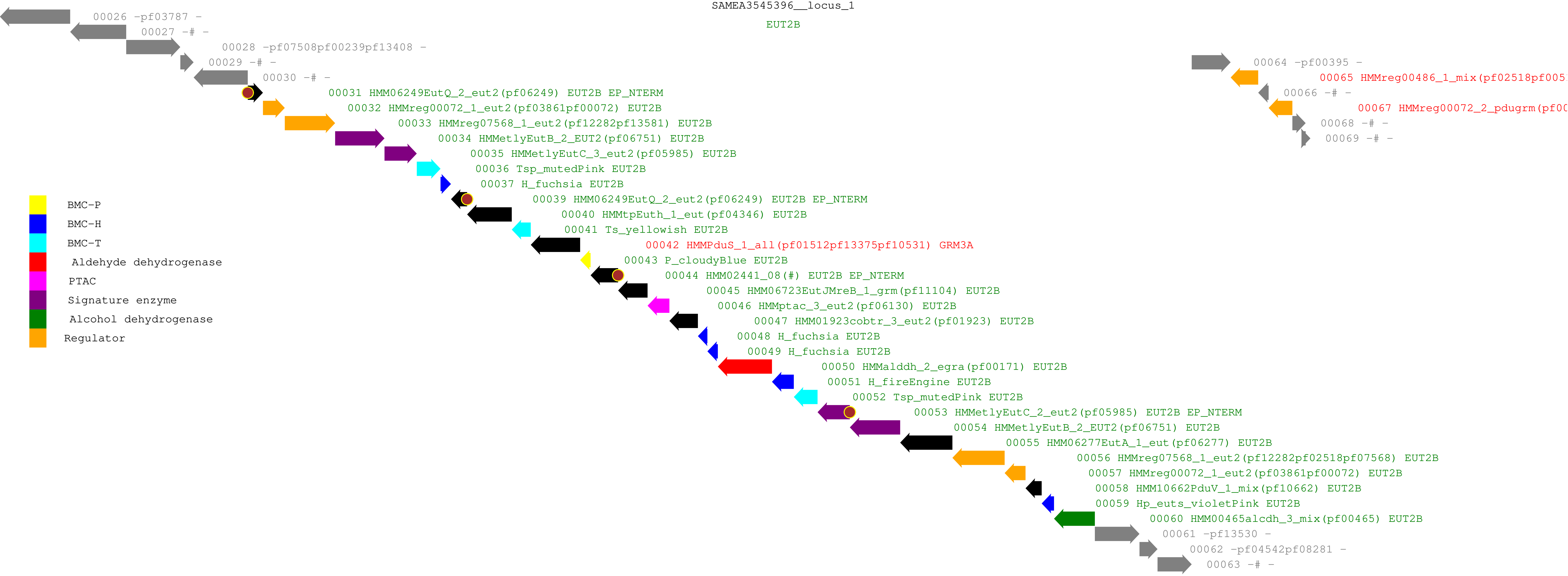

uncultured Clostridium sp. Bacteria; Firmicutes; Clostridia; Clostridiales; Clostridiaceae;

SAMEA3545399__locus_2_main_sat

EUT2B

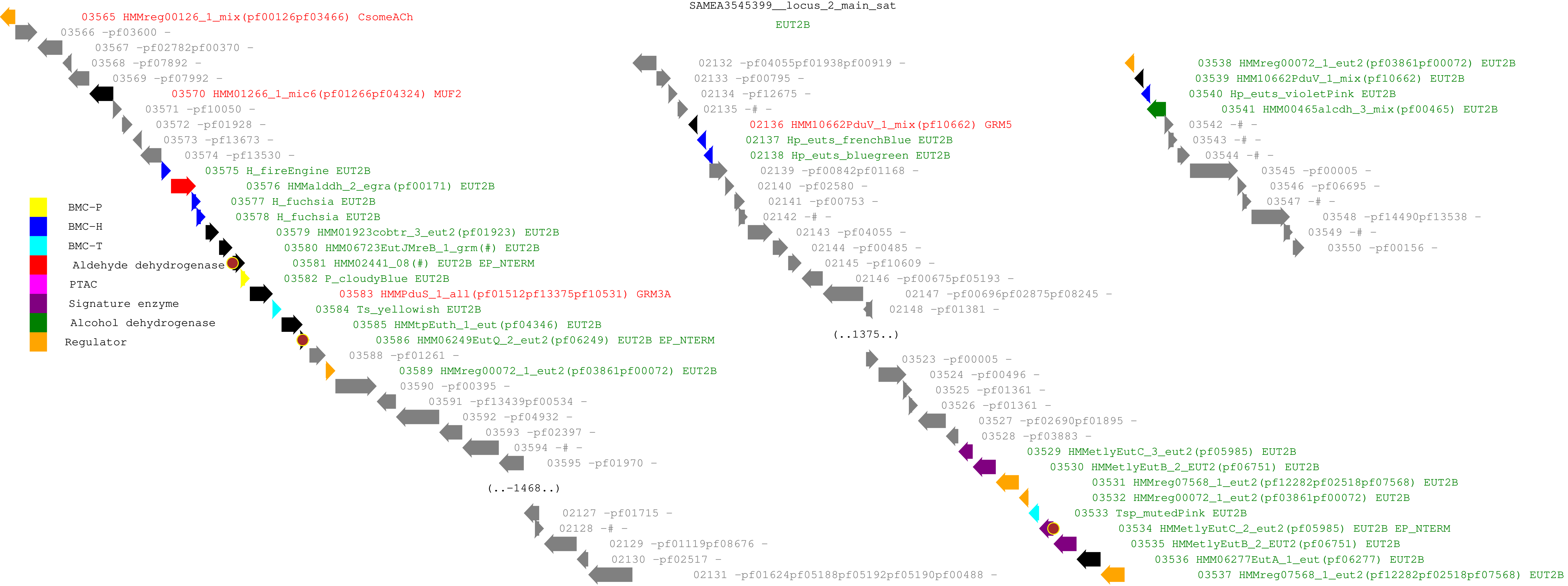

Maledivibacter halophilus. Bacteria; Firmicutes; Clostridia; Clostridiales; Clostridiaceae;

SAMN02194393__locus_2

EUT2B

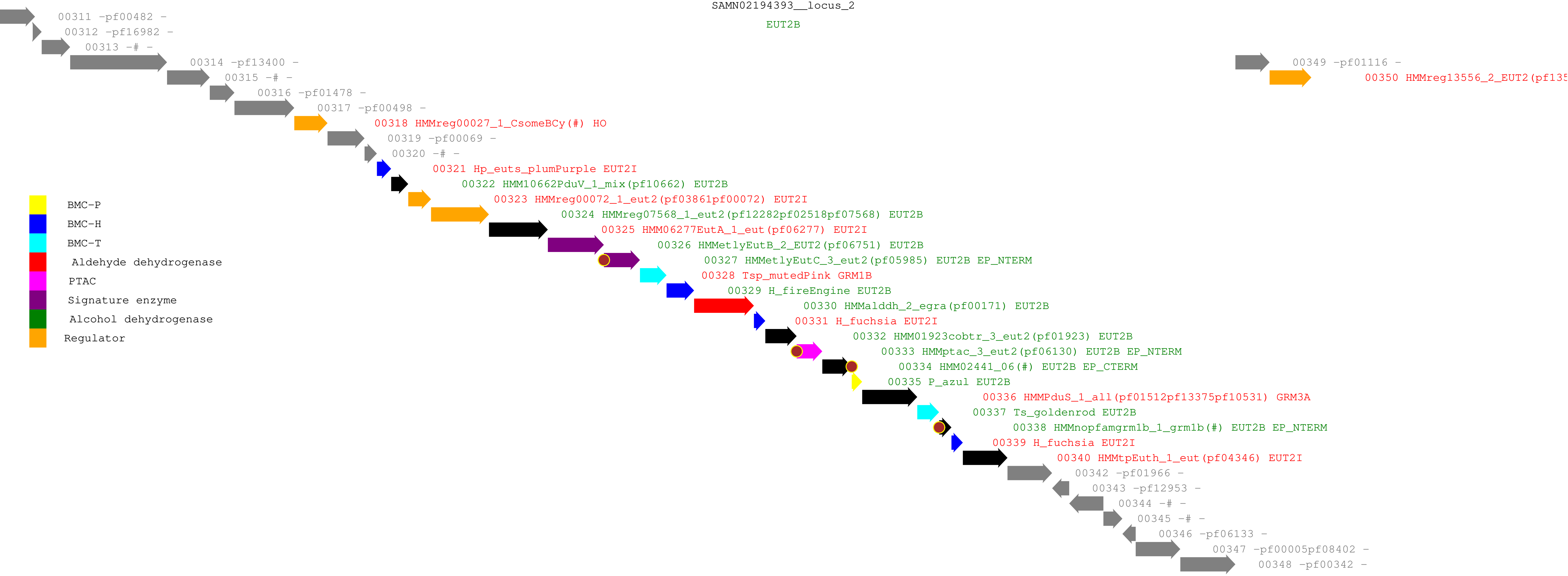

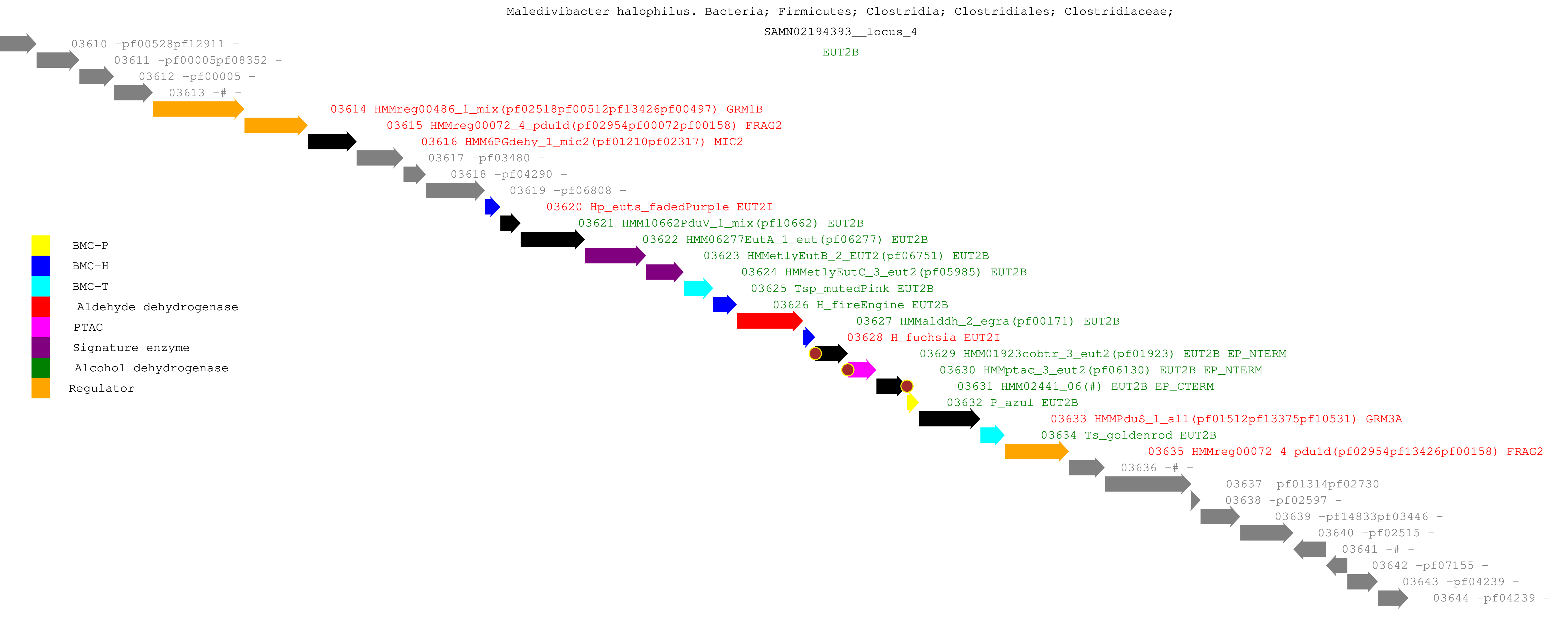
Maledivibacter halophilus. Bacteria; Firmicutes; Clostridia; Clostridiales; Clostridiaceae;
SAMN02194393__locus_4
EUT2B
03610 -pf00528pf12911 -
03611 -pf00005pf08352 -
03612 -pf00005 -
03613 -# -
03614 HMMreg00486_1_mix(pf02518pf00512pf13426pf00497) GRM1B
03615 HMMreg00072_4_pdu1d(pf02954pf00072pf00158) FRAG2
03616 HMM6PGdehy_1_mic2(pf01210pf02317) MIC2
03617 -pf03480 -
03618 -pf04290 -
03619 -pf06808 -
03620 Hp_euts_fadedPurple EUT2I
03621 HMM10662PduV_1_mix(pf10662) EUT2B
03622 HMM06277EutA_1_eut(pf06277) EUT2B
03623 HMMetlyEutB_2_EUT2(pf06751) EUT2B
03624 HMMetlyEutC_3_eut2(pf05985) EUT2B
03625 Tsp_mutedPink EUT2B
03626 H_fireEngine EUT2B
03627 HMMalddh_2_egra(pf00171) EUT2B
03628 H_fuchsia EUT2I
03629 HMM01923cobtr_3_eut2(pf01923) EUT2B EP_NTERM
03630 HMMptac_3_eut2(pf06130) EUT2B EP_NTERM
03631 HMM02441_06(#) EUT2B EP_CTERM
03632 P_azul EUT2B
03633 HMMPduS_1_all(pf01512pf13375pf10531) GRM3A
03634 Ts_goldenrod EUT2B
03635 HMMreg00072_4_pdu1d(pf02954pf13426pf00158) FRAG2
03636 -# -
03637 -pf01314pf02730 -
03638 -pf02597 -
03639 -pf14833pf03446 -
03640 -pf02515 -
03641 -# -
03642 -pf07155 -
03643 -pf04239 -
03644 -pf04239 -
BMC-P
BMC-H
BMC-T
Aldehyde dehydrogenase
PTAC
Signature enzyme
Alcohol dehydrogenase
Regulator

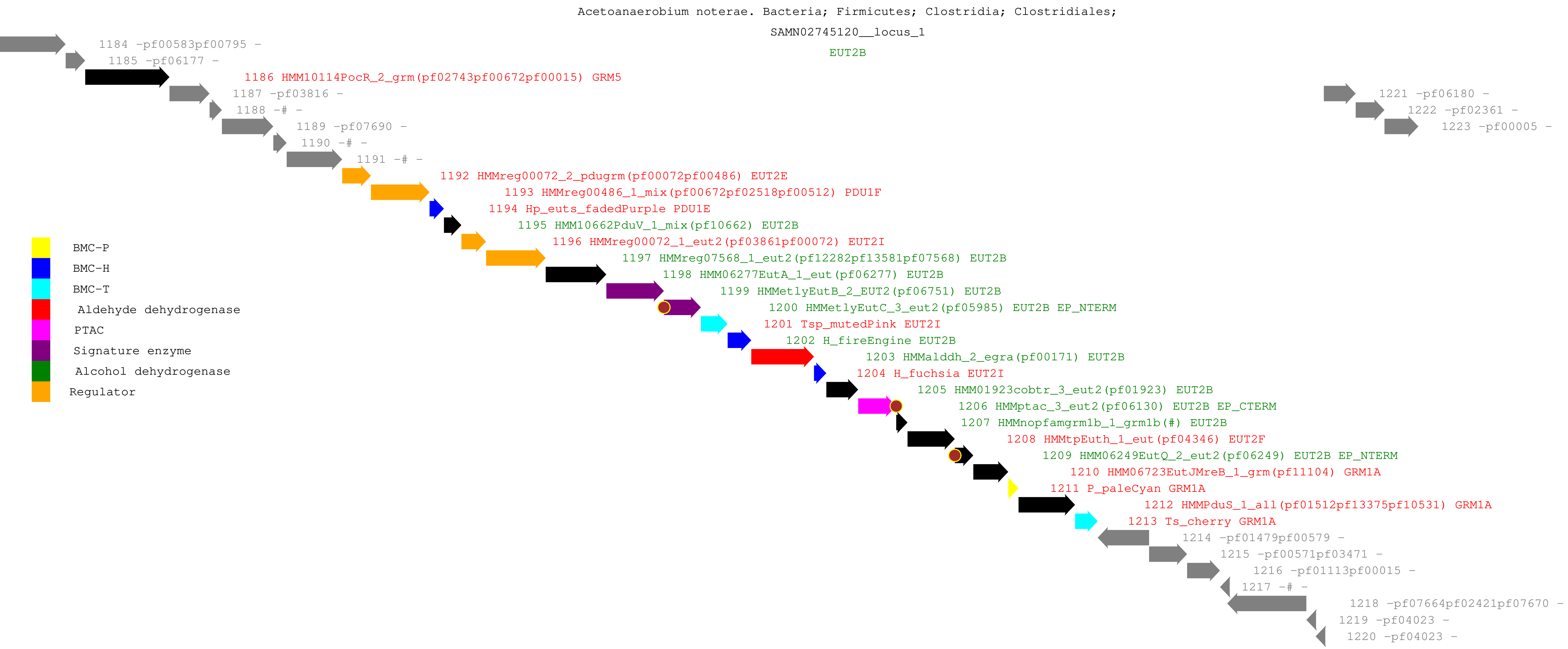

Acetoanaerobium noterae. Bacteria; Firmicutes; Clostridia; Clostridiales;
SAMN02745120__locus_1
EUT2B
1184 -pf00583pf00795 -
1185 -pf06177 -
1186 HMM10114PocR_2_grm(pf02743pf00672pf00015) GRM5
1187 -pf03816 -
1188 -# -
1189 -pf07690 -
1190 -# -
1191 -# -
1192 HMMreg00072_2_pdugrm(pf00072pf00486) EUT2E
1193 HMMreg00486_1_mix(pf00672pf02518pf00512) PDU1F
1194 Hp_euts_fadedPurple PDU1E
1195 HMM10662PduV_1_mix(pf10662) EUT2B
1196 HMMreg00072_1_eut2(pf03861pf00072) EUT2I
1197 HMMreg07568_1_eut2(pf12282pf13581pf07568) EUT2B
1198 HMM06277EutA_1_eut(pf06277) EUT2B
1199 HMMetlyEutB_2_EUT2(pf06751) EUT2B
1200 HMMetlyEutC_3_eut2(pf05985) EUT2B EP_NTERM
1201 Tsp_mutedPink EUT2I
1202 H_fireEngine EUT2B
1203 HMMaldh_2_egra(pf00171) EUT2B
1204 H_fuchsia EUT2I
1205 HMM01923cobtr_3_eut2(pf01923) EUT2B
1206 HMMptac_3_eut2(pf06130) EUT2B EP_CTERM
1207 HMMnopfamgrm1b_1_grm1b(#) EUT2B
1208 HMMtpEuth_1_eut(pf04346) EUT2F
1209 HMM06249EutQ_2_eut2(pf06249) EUT2B EP_NTERM
1210 HMM06723EutJMreB_1_grm(pf11104) GRM1A
1211 P_paleCyan GRM1A
1212 HMMPduS_1_all(pf01512pf13375pf10531) GRM1A
1213 Ts_cherry GRM1A
1214 -pf01479pf00579 -
1215 -pf00571pf03471 -
1216 -pf01113pf00015 -
1217 -# -
1218 -pf07664pf02421pf07670 -
1219 -pf04023 -
1220 -pf04023 -
1221 -pf06180 -
1222 -pf02361 -
1223 -pf00005 -
BMC-P
BMC-H
BMC-T
Aldehyde dehydrogenase
PTAC
Signature enzyme
Alcohol dehydrogenase
Regulator

Lutispora thermophila DSM 19022. Bacteria; Firmicutes; Clostridia; Clostridiales; Clostridiaceae;

SAMN02745176__locus_1

EUT2B

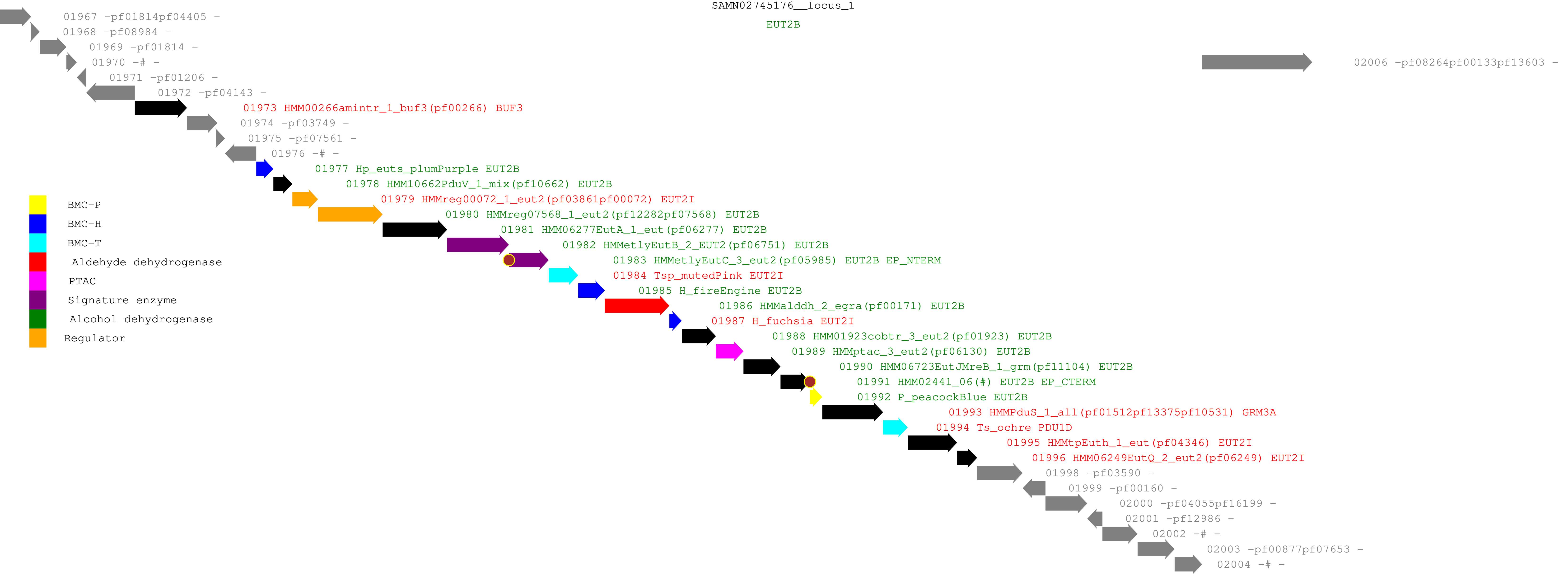

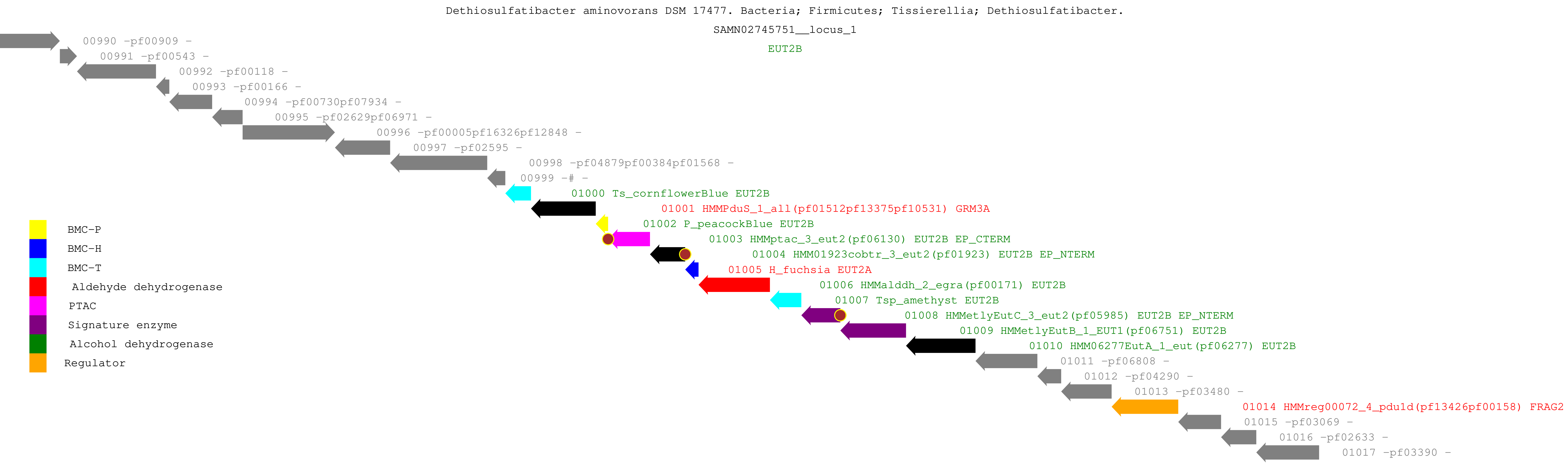

Dethiosulfatibacter aminovorans DSM 17477. Bacteria; Firmicutes; Tissierellia; Dethiosulfatibacter.
SAMN02745751__locus_1
EUT2B
00990 -pf00909 -
00991 -pf00543 -
00992 -pf00118 -
00993 -pf00166 -
00994 -pf00730pf07934 -
00995 -pf02629pf06971 -
00996 -pf00005pf16326pf12848 -
00997 -pf02595 -
00998 -pf04879pf00384pf01568 -
00999 -# -
01000 Ts_cornflowerBlue EUT2B
01001 HMMPduS_1_all(pf01512pf13375pf10531) GRM3A
01002 P_peacockBlue EUT2B
01003 HMMptac_3_eut2(pf06130) EUT2B EP_CTERM
01004 HMM01923cobtr_3_eut2(pf01923) EUT2B EP_NTERM
01005 H_fuchsia EUT2A
01006 HMMalddh_2_egra(pf00171) EUT2B
01007 Tsp_amethyst EUT2B
01008 HMMetlyEutC_3_eut2(pf05985) EUT2B EP_NTERM
01009 HMMetlyEutB_1_EUT1(pf06751) EUT2B
01010 HMM06277EutA_1_eut(pf06277) EUT2B
01011 -pf06808 -
01012 -pf04290 -
01013 -pf03480 -
01014 HMMreg00072_4_pdu1d(pf13426pf00158) FRAG2
01015 -pf03069 -
01016 -pf02633 -
01017 -pf03390 -
BMC-P
BMC-H
BMC-T
Aldehyde dehydrogenase
PTAC
Signature enzyme
Alcohol dehydrogenase
Regulator

Caminicella sporogenes DSM 14501. Bacteria; Firmicutes; Clostridia; Clostridiales; Clostridiaceae;

SAMN02745883__locus_1

EUT2B

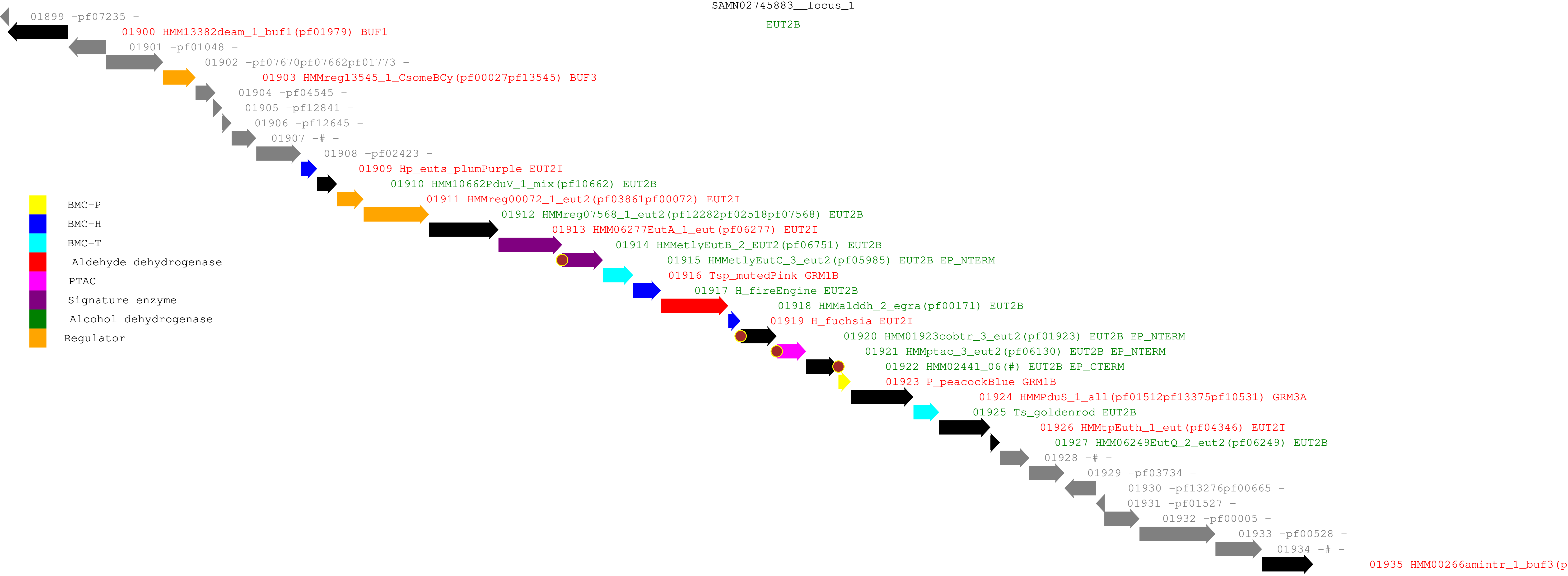

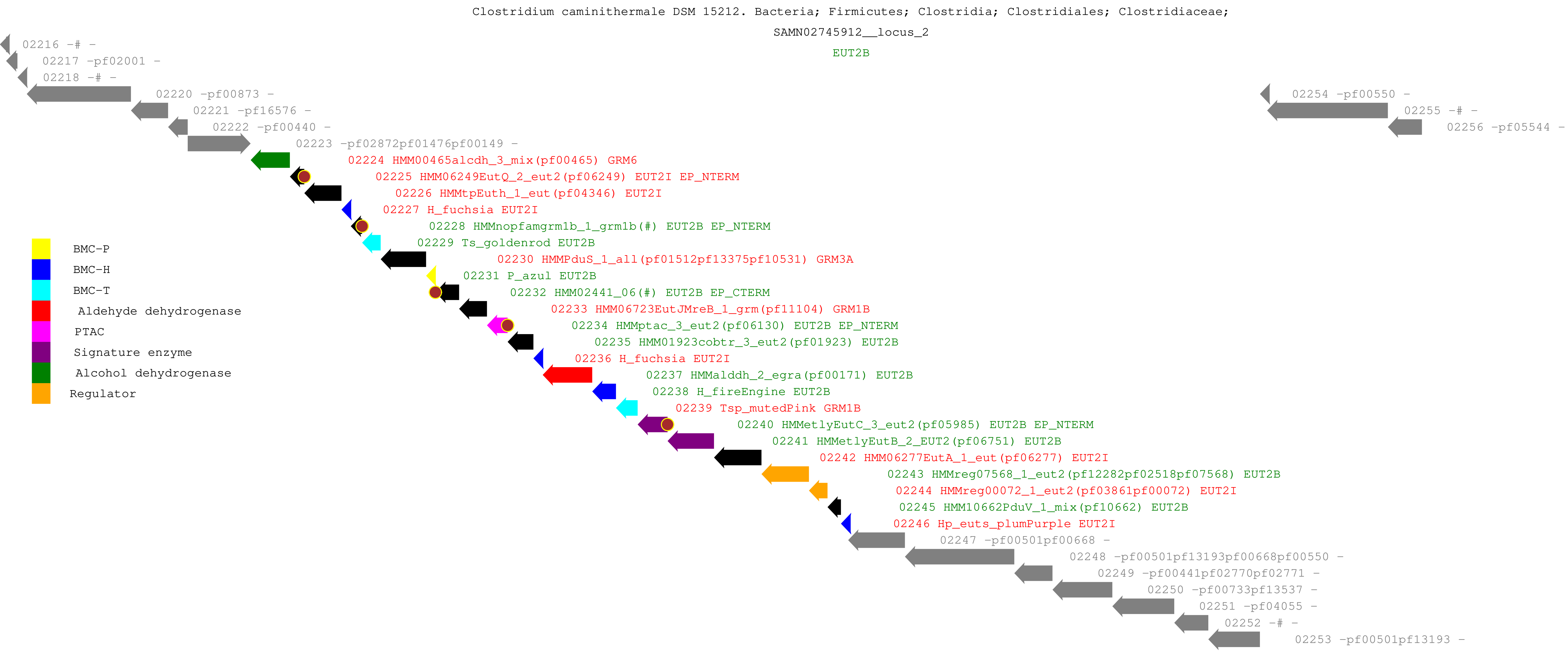

Clostridium caminithermale DSM 15212. Bacteria; Firmicutes; Clostridia; Clostridiales; Clostridiaceae;
SAMN02745912__locus_2
EUT2B
02216 -# -
02217 -pf02001 -
02218 -# -
02220 -pf00873 -
02221 -pf16576 -
02222 -pf00440 -
02223 -pf02872pf01476pf00149 -
02224 HMM00465alcdh_3_mix(pf00465) GRM6
02225 HMM06249EutQ_2_eut2(pf06249) EUT2I EP_NTERM
02226 HMMtpEuth_1_eut(pf04346) EUT2I
02227 H_fuchsia EUT2I
02228 HMMnopfamgrm1b_1_grm1b(#) EUT2B EP_NTERM
02229 Ts_goldenrod EUT2B
02230 HMMPduS_1_all(pf01512pf13375pf10531) GRM3A
02231 P_azul EUT2B
02232 HMM02441_06(#) EUT2B EP_CTERM
02233 HMM06723EutJMreB_1_grm(pf11104) GRM1B
02234 HMMptac_3_eut2(pf06130) EUT2B EP_NTERM
02235 HMM01923cobtr_3_eut2(pf01923) EUT2B
02236 H_fuchsia EUT2I
02237 HMMalddh_2_egra(pf00171) EUT2B
02238 H_fireEngine EUT2B
02239 Tsp_mutedPink GRM1B
02240 HMMetlyEutC_3_eut2(pf05985) EUT2B EP_NTERM
02241 HMMetlyEutB_2_EUT2(pf06751) EUT2B
02242 HMM06277EutA_1_eut(pf06277) EUT2I
02243 HMMreg07568_1_eut2(pf12282pf02518pf07568) EUT2B
02244 HMMreg00072_1_eut2(pf03861pf00072) EUT2I
02245 HMM10662PduV_1_mix(pf10662) EUT2B
02246 Hp_euts_plumPurple EUT2I
02247 -pf00501pf00668 -
02248 -pf00501pf13193pf00668pf00550 -
02249 -pf00441pf02770pf02771 -
02250 -pf00733pf13537 -
02251 -pf04055 -
02252 -# -
02253 -pf00501pf13193 -
02254 -pf00550 -
02255 -# -
02256 -pf05544 -
BMC-P
BMC-H
BMC-T
Aldehyde dehydrogenase
PTAC
Signature enzyme
Alcohol dehydrogenase
Regulator

Geosporobacter subterraneus DSM 17957. Bacteria; Firmicutes; Clostridia; Clostridiales; Clostridiaceae;

SAMN02745975__locus_2

EUT2B

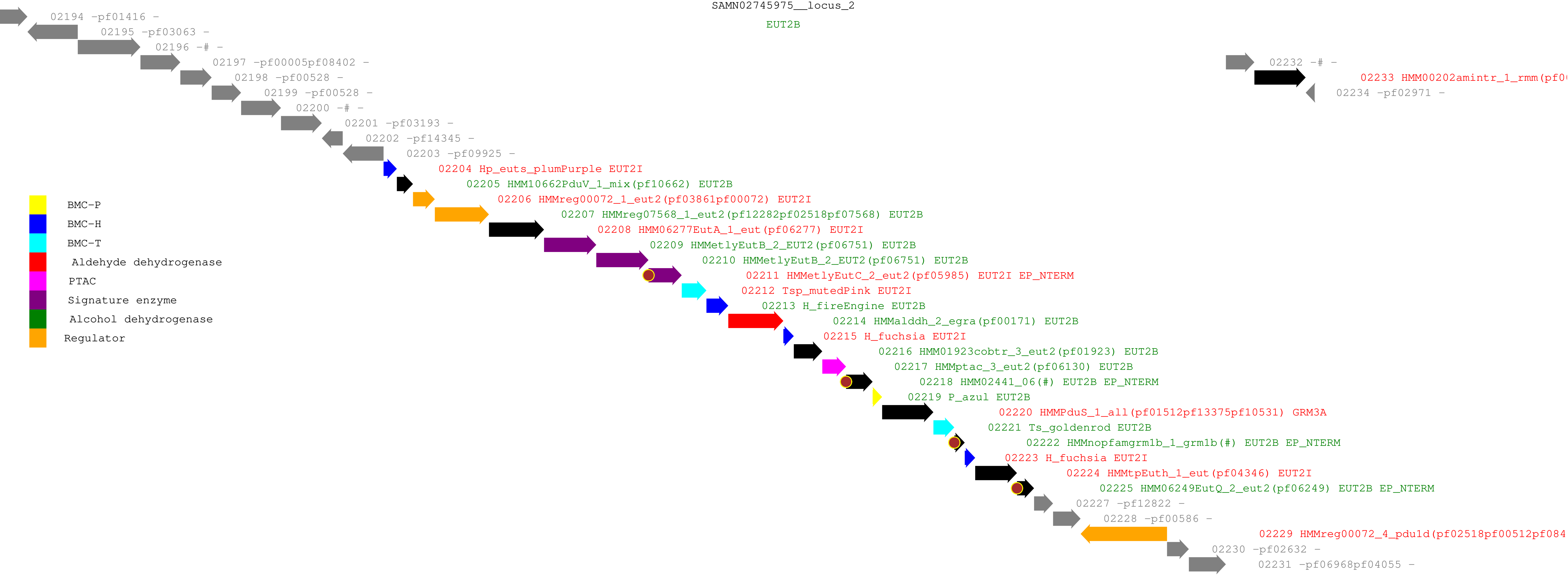

Butyricicoccus pullicaecorum DSM 23266. Bacteria; Firmicutes; Clostridia; Clostridiales; Clostridiaceae;

SAMN02745978__locus_1

EUT2B

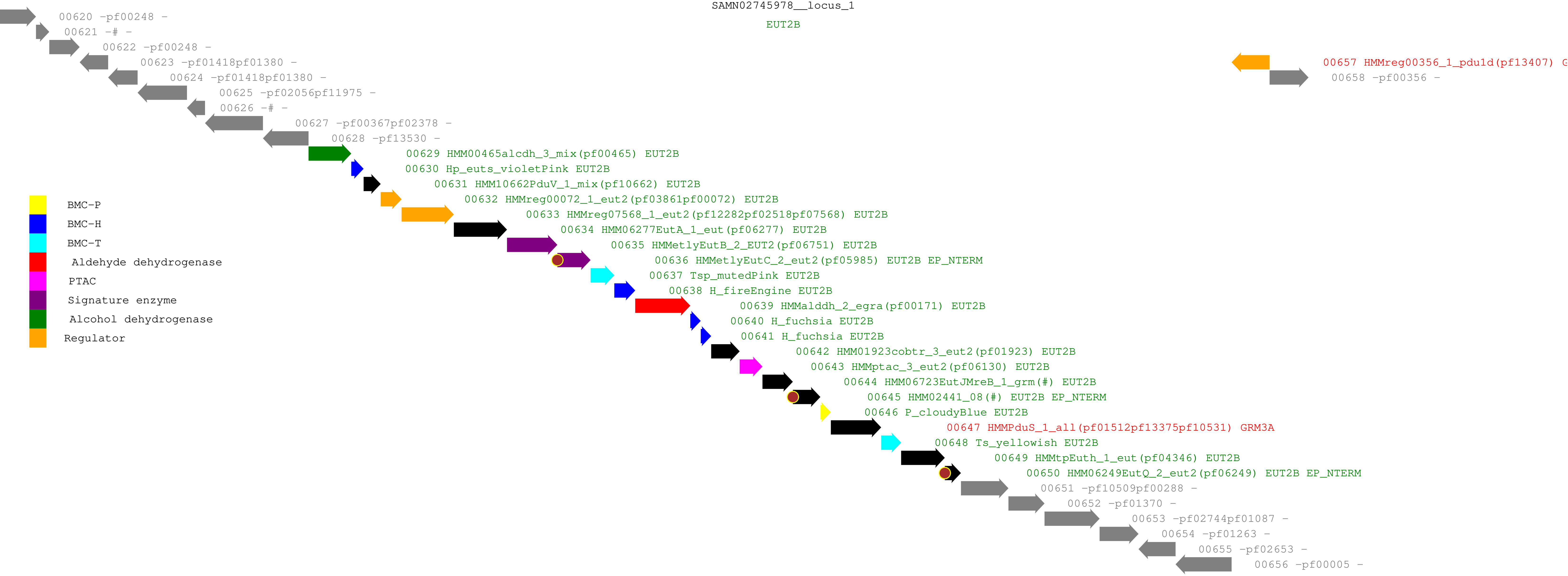

Alkaliphilus peptidifermentans DSM 18978. Bacteria; Firmicutes; Clostridia; Clostridiales; Clostridiaceae;

SAMN03080606__locus_2

EUT2B

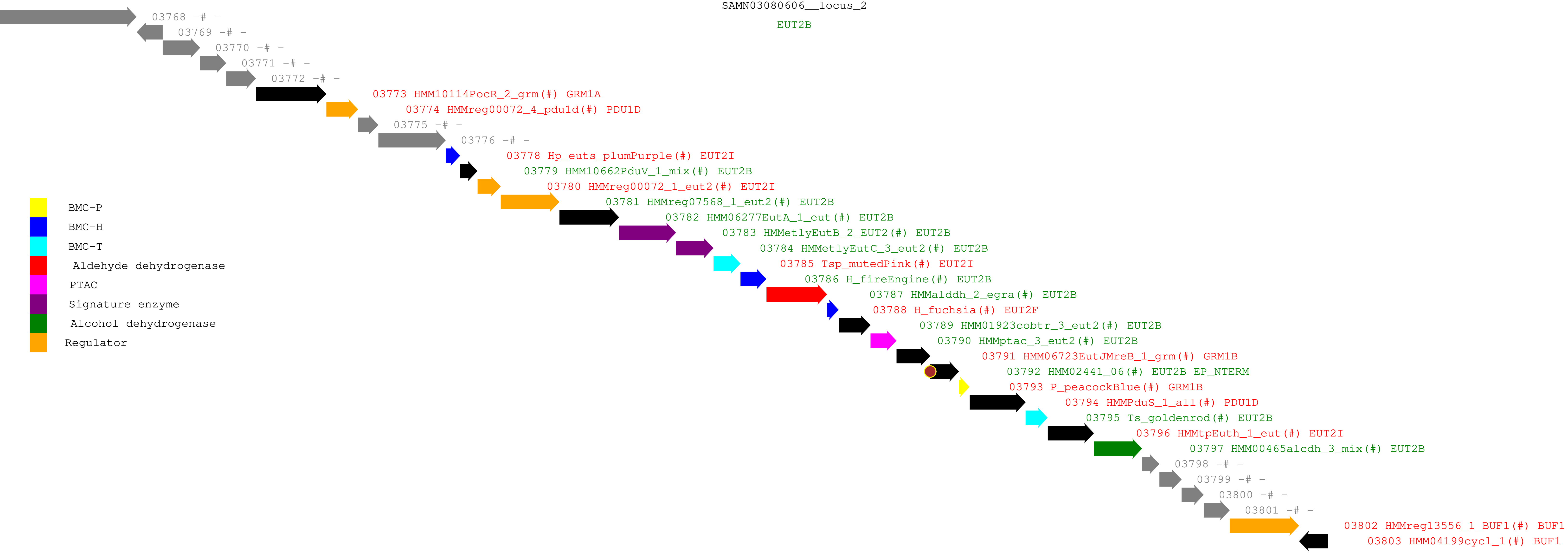

Eubacterium barkeri (Clostridium barkeri). Bacteria; Firmicutes; Clostridia; Clostridiales; Eubacteriaceae;

SAMN04488579__locus_1

EUT2B

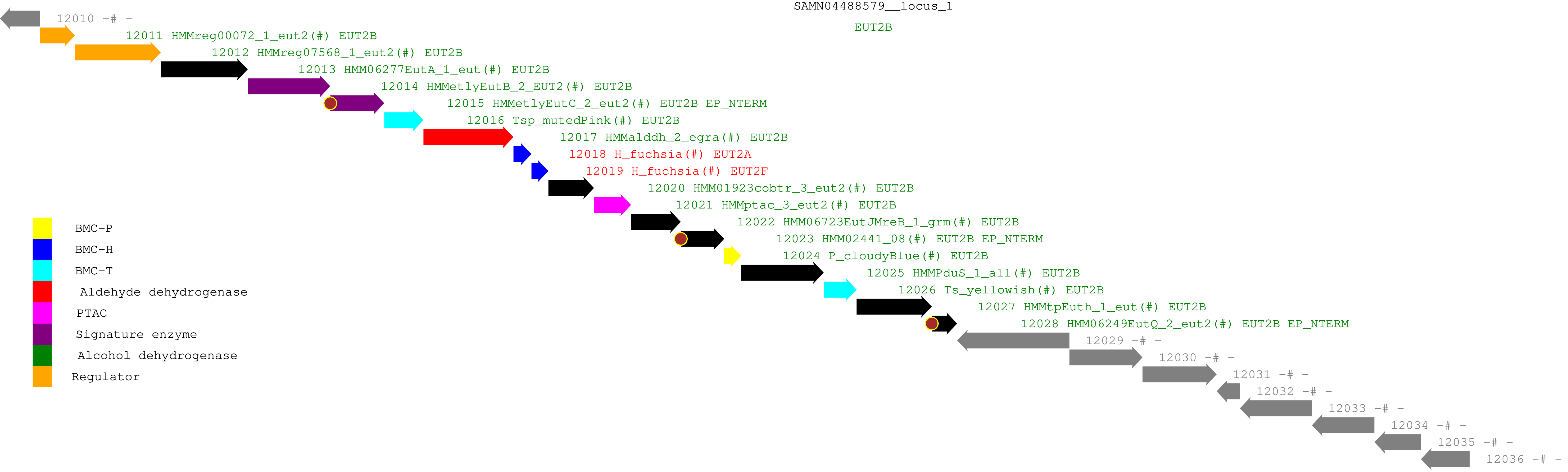

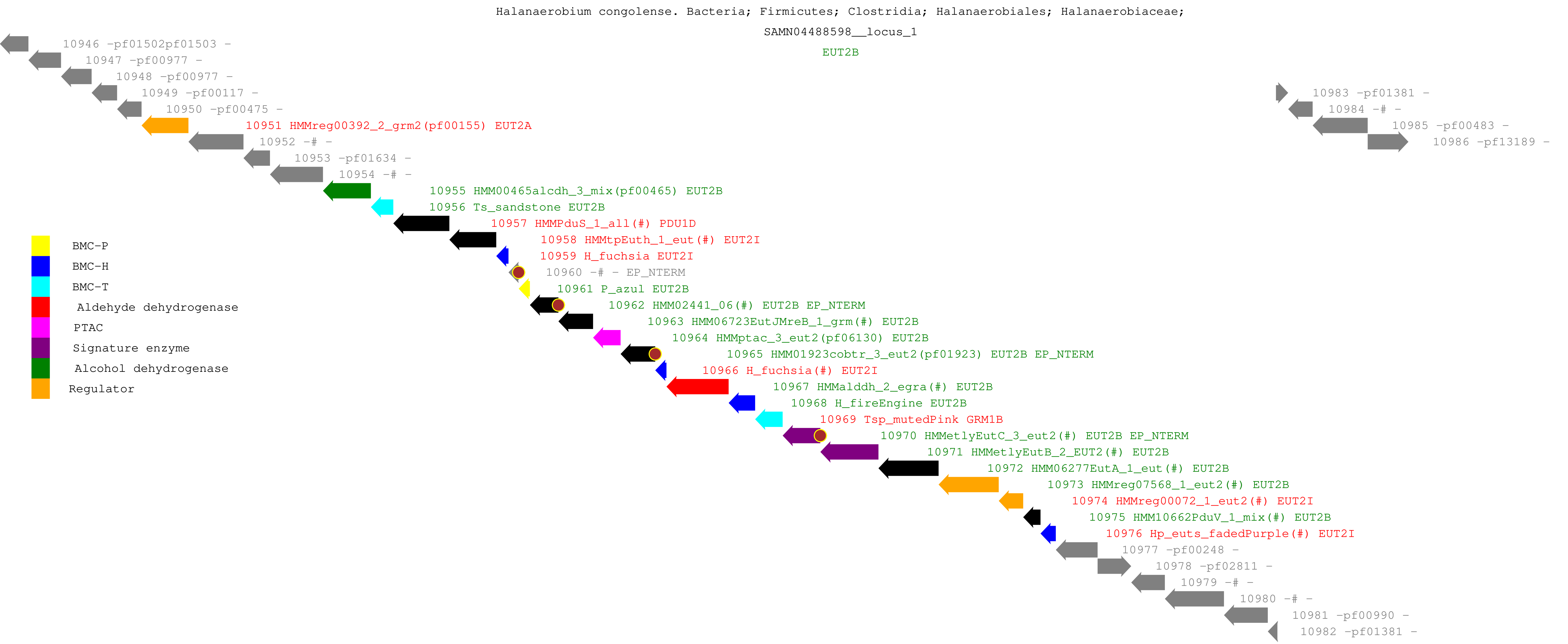
Halanaerobium congolense. Bacteria; Firmicutes; Clostridia; Halanaerobiales; Halanaerobiaceae;
SAMN04488598__locus_1
EUT2B
10946 -pf01502pf01503 -
10947 -pf00977 -
10948 -pf00977 -
10949 -pf00117 -
10950 -pf00475 -
10951 HMMreg00392_2_grm2(pf00155) EUT2A
10952 -# -
10953 -pf01634 -
10954 -# -
10955 HMM00465alcdh_3_mix(pf00465) EUT2B
10956 Ts_sandstone EUT2B
10957 HMMPduS_1_all(#) PDU1D
10958 HMMtpEuth_1_eut(#) EUT2I
10959 H_fuchsia EUT2I
10960 -# - EP_NTERM
10961 P_azul EUT2B
10962 HMM02441_06(#) EUT2B EP_NTERM
10963 HMM06723EutJMreB_1_grm(#) EUT2B
10964 HMMptac_3_eut2(pf06130) EUT2B
10965 HMM01923cobtr_3_eut2(pf01923) EUT2B EP_NTERM
10966 H_fuchsia(#) EUT2I
10967 HMMalddh_2_egra(#) EUT2B
10968 H_fireEngine EUT2B
10969 Tsp_mutedPink GRM1B
10970 HMMetlyEutC_3_eut2(#) EUT2B EP_NTERM
10971 HMMetlyEutB_2_EUT2(#) EUT2B
10972 HMM06277EutA_1_eut(#) EUT2B
10973 HMMreg07568_1_eut2(#) EUT2B
10974 HMMreg00072_1_eut2(#) EUT2I
10975 HMM10662PduV_1_mix(#) EUT2B
10976 Hp_euts_fadedPurple(#) EUT2I
10977 -pf00248 -
10978 -pf02811 -
10979 -# -
10980 -# -
10981 -pf00990 -
10982 -pf01381 -
10983 -pf01381 -
10984 -# -
10985 -pf00483 -
10986 -pf13189 -
BMC-P
BMC-H
BMC-T
Aldehyde dehydrogenase
PTAC
Signature enzyme
Alcohol dehydrogenase
Regulator

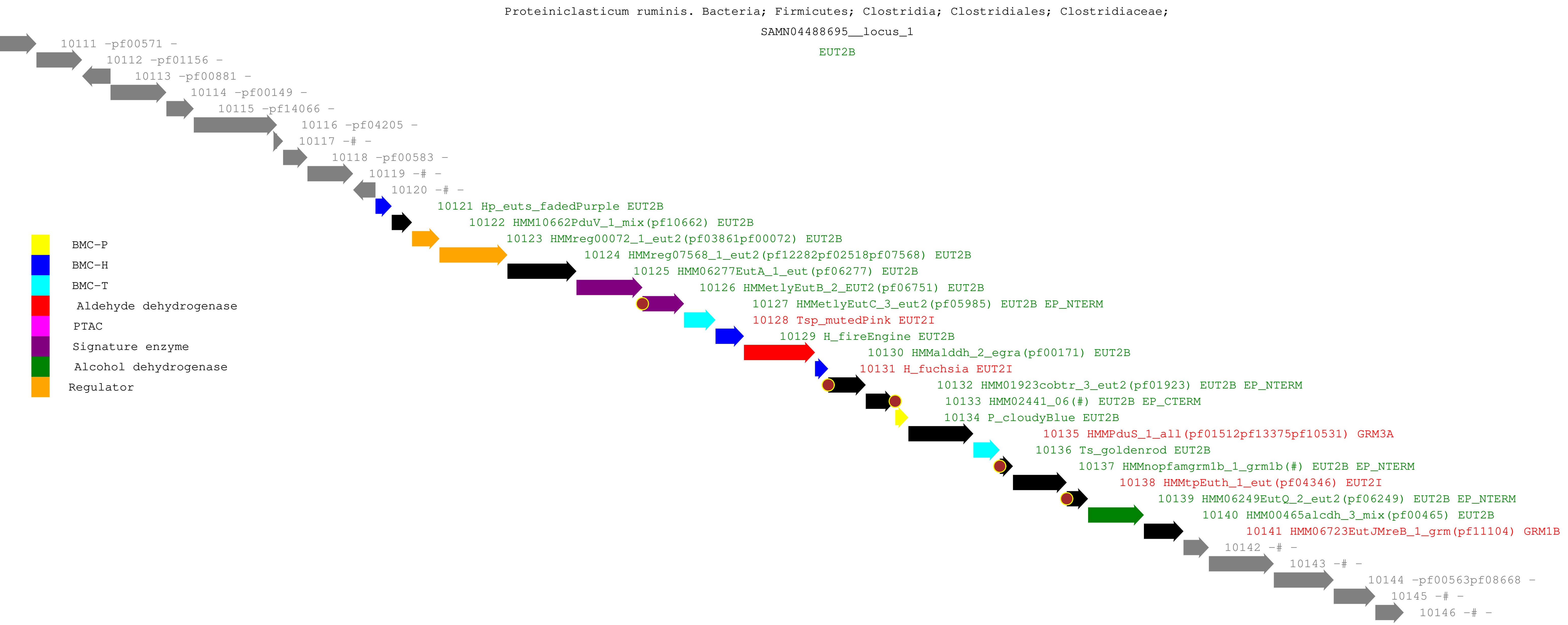
Proteiniclasticum ruminis. Bacteria; Firmicutes; Clostridia; Clostridiales; Clostridiaceae;
SAMN04488695__locus_1
EUT2B
10111 -pf00571 -
10112 -pf01156 -
10113 -pf00881 -
10114 -pf00149 -
10115 -pf14066 -
10116 -pf04205 -
10117 -# -
10118 -pf00583 -
10119 -# -
10120 -# -
10121 Hp_euts_fadedPurple EUT2B
10122 HMM10662PduV_1_mix(pf10662) EUT2B
10123 HMMreg00072_1_eut2(pf03861pf00072) EUT2B
10124 HMMreg07568_1_eut2(pf12282pf02518pf07568) EUT2B
10125 HMM06277EutA_1_eut(pf06277) EUT2B
10126 HMMetlyEutB_2_EUT2(pf06751) EUT2B
10127 HMMetlyEutC_3_eut2(pf05985) EUT2B EP_NTERM
10128 Tsp_mutedPink EUT2I
10129 H_fireEngine EUT2B
10130 HMMalddh_2_egra(pf00171) EUT2B
10131 H_fuchsia EUT2I
10132 HMM01923cobtr_3_eut2(pf01923) EUT2B EP_NTERM
10133 HMM02441_06(#) EUT2B EP_CTERM
10134 P_cloudyBlue EUT2B
10135 HMMPduS_1_all(pf01512pf13375pf10531) GRM3A
10136 Ts_goldenrod EUT2B
10137 HMMnopfamgrm1b_1_grm1b(#) EUT2B EP_NTERM
10138 HMMtpEuth_1_eut(pf04346) EUT2I
10139 HMM06249EutQ_2_eut2(pf06249) EUT2B EP_NTERM
10140 HMM00465alcdh_3_mix(pf00465) EUT2B
10141 HMM06723EutJMreB_1_grm(pf11104) GRM1B
10142 -# -
10143 -# -
10144 -pf00563pf08668 -
10145 -# -
10146 -# -
BMC-P
BMC-H
BMC-T
Aldehyde dehydrogenase
PTAC
Signature enzyme
Alcohol dehydrogenase
Regulator

Halanaerobium congolense. Bacteria; Firmicutes; Clostridia; Halanaerobiales; Halanaerobiaceae;

SAMN04515650__locus_2

EUT2B

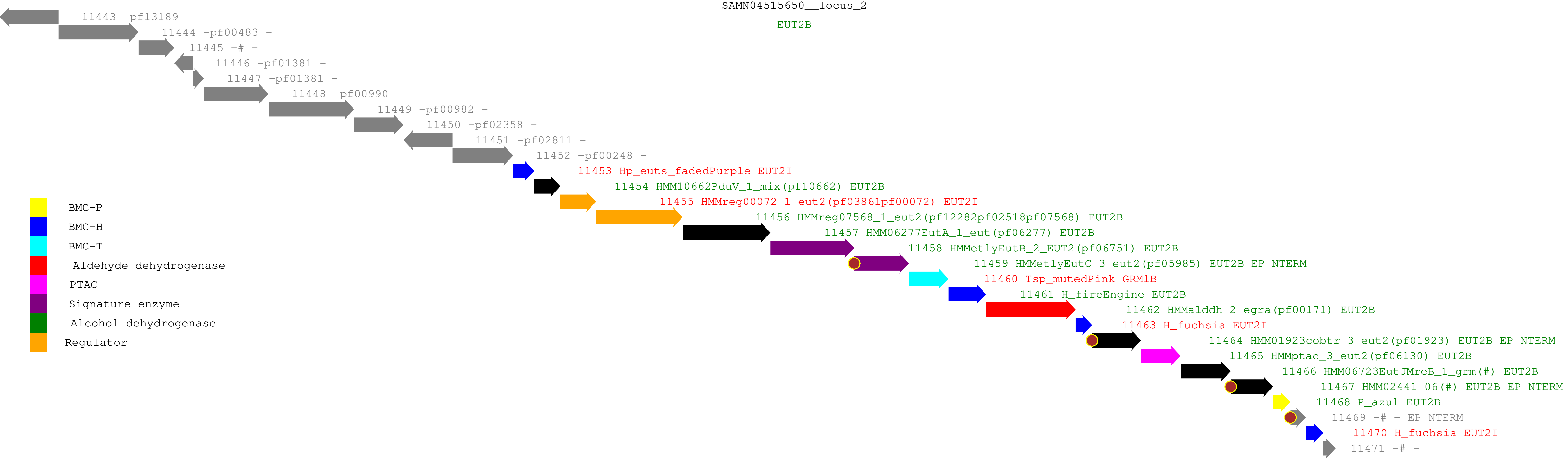

Halanaerobium congolense. Bacteria; Firmicutes; Clostridia; Halanaerobiales; Halanaerobiaceae;

SAMN04515652__locus_1

EUT2B

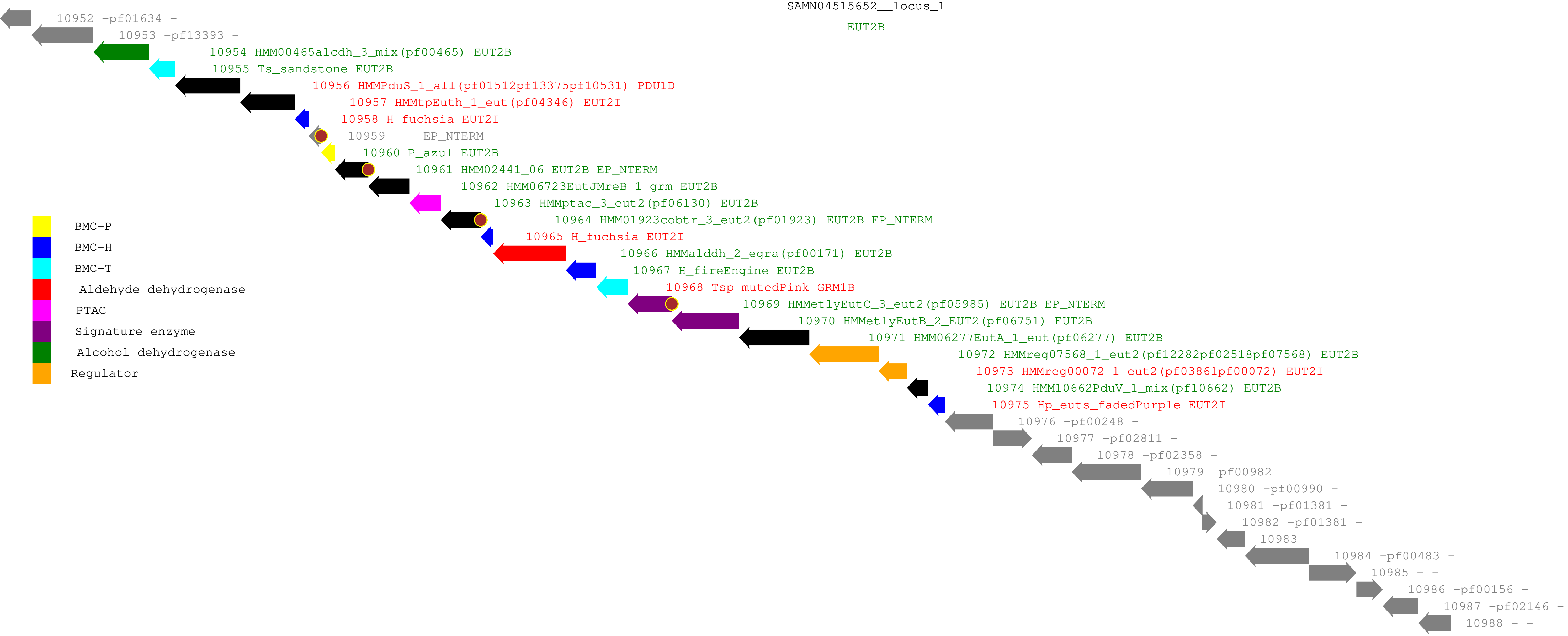

Eubacterium aggregans. Bacteria; Firmicutes; Clostridia; Clostridiales; Eubacteriaceae;

SAMN04515656__locus_2

EUT2B

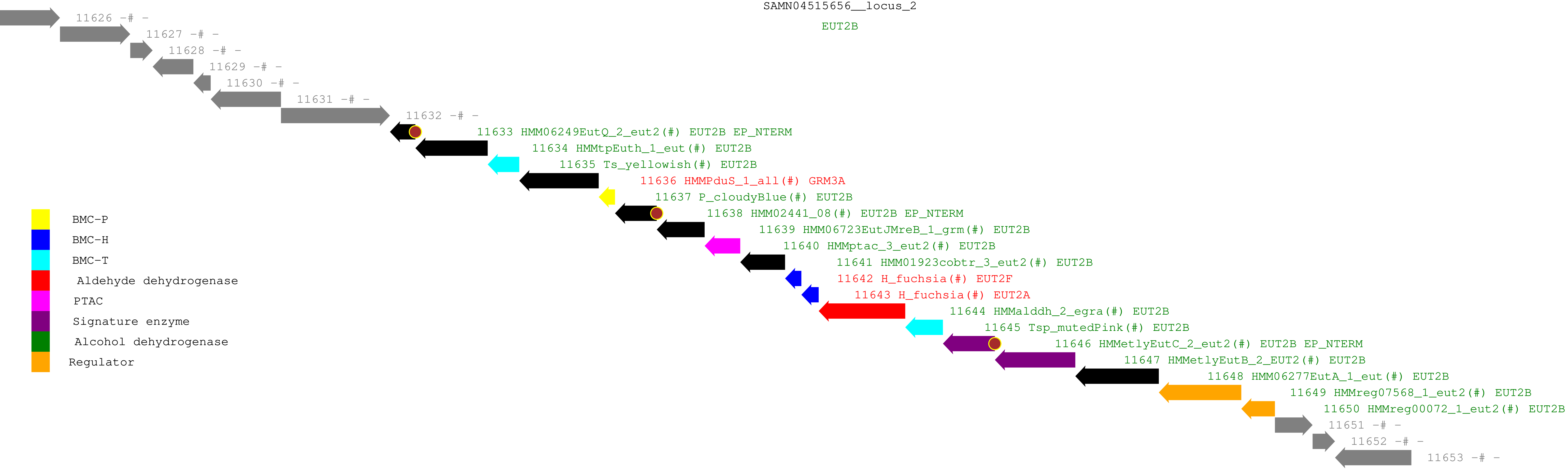

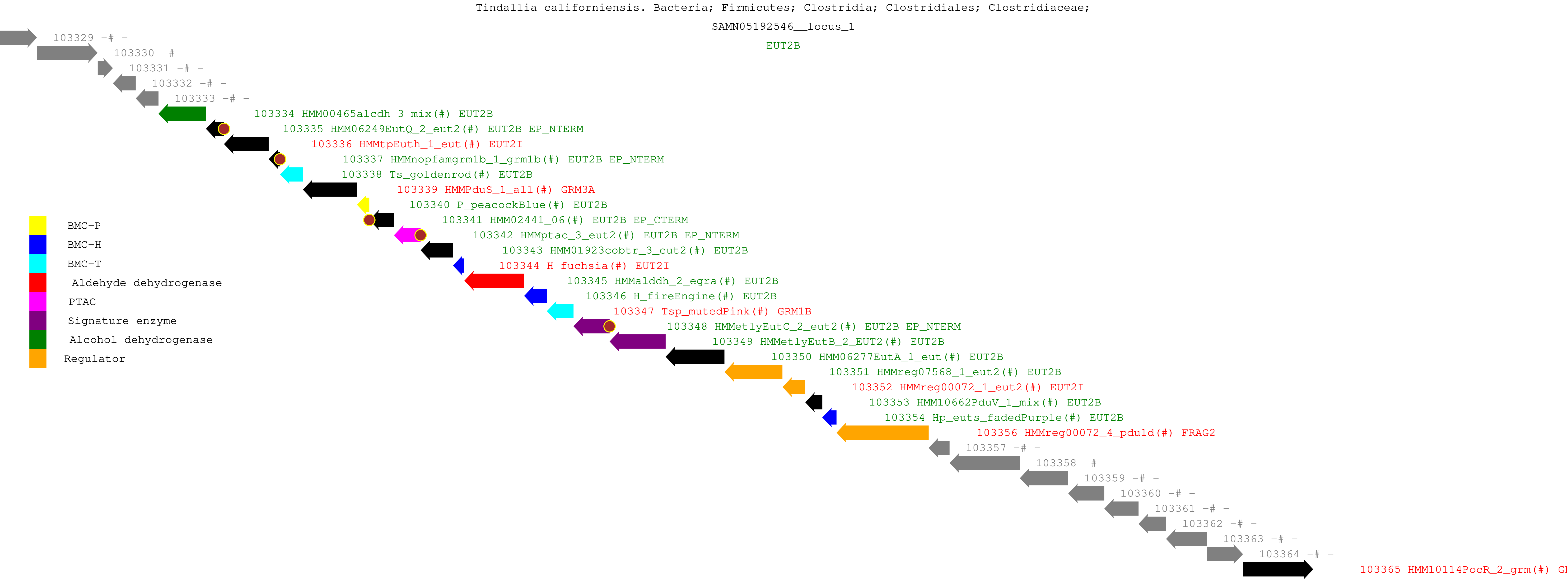

Tindallia californiensis. Bacteria; Firmicutes; Clostridia; Clostridiales; Clostridiaceae;
SAMN05192546__locus_1
EUT2B
103329 -# -
103330 -# -
103331 -# -
103332 -# -
103333 -# -
103334 HMM00465alcdh_3_mix(#) EUT2B
103335 HMM06249EutQ_2_eut2(#) EUT2B EP_NTERM
103336 HMMtpEuth_1_eut(#) EUT2I
103337 HMMnopfamgrm1b_1_grm1b(#) EUT2B EP_NTERM
103338 Ts_goldenrod(#) EUT2B
103339 HMMPduS_1_all(#) GRM3A
103340 P_peacockBlue(#) EUT2B
103341 HMM02441_06(#) EUT2B EP_CTERM
103342 HMMptac_3_eut2(#) EUT2B EP_NTERM
103343 HMM01923cobtr_3_eut2(#) EUT2B
103344 H_fuchsia(#) EUT2I
103345 HMMalddh_2_egra(#) EUT2B
103346 H_fireEngine(#) EUT2B
103347 Tsp_mutedPink(#) GRM1B
103348 HMMetlyEutC_2_eut2(#) EUT2B EP_NTERM
103349 HMMetlyEutB_2_EUT2(#) EUT2B
103350 HMM06277EutA_1_eut(#) EUT2B
103351 HMMreg07568_1_eut2(#) EUT2B
103352 HMMreg00072_1_eut2(#) EUT2I
103353 HMM10662PduV_1_mix(#) EUT2B
103354 Hp_euts_fadedPurple(#) EUT2B
103356 HMMreg00072_4_pdu1d(#) FRAG2
103357 -# -
103358 -# -
103359 -# -
103360 -# -
103361 -# -
103362 -# -
103363 -# -
103364 -# -
103365 HMM10114PocR_2_grm(#) G
BMC-P
BMC-H
BMC-T
Aldehyde dehydrogenase
PTAC
Signature enzyme
Alcohol dehydrogenase
Regulator

Tindallia magadiensis. Bacteria; Firmicutes; Clostridia; Clostridiales; Clostridiaceae;

SAMN05192551__locus_1

EUT2B

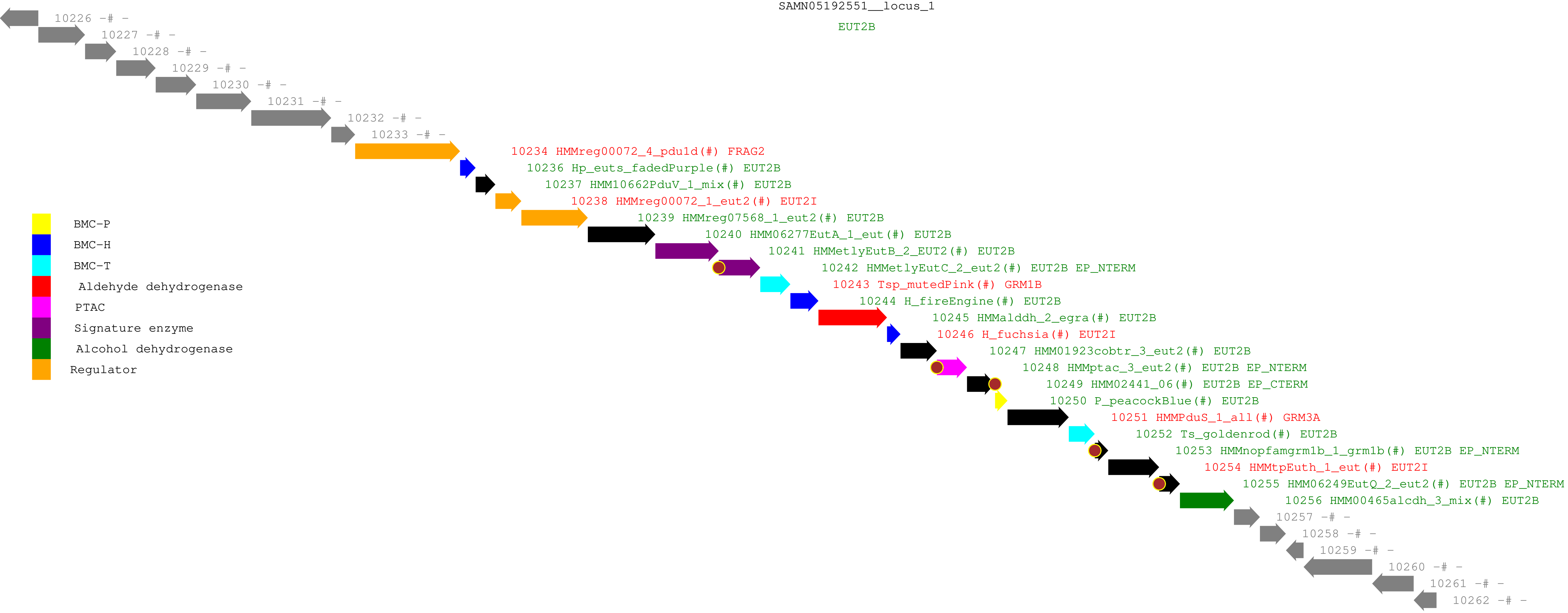

Hydrogenoanaerobacterium saccharovorans. Bacteria; Firmicutes; Clostridia; Clostridiales; Ruminococcaceae;

SAMN05216180__locus_1

EUT2B

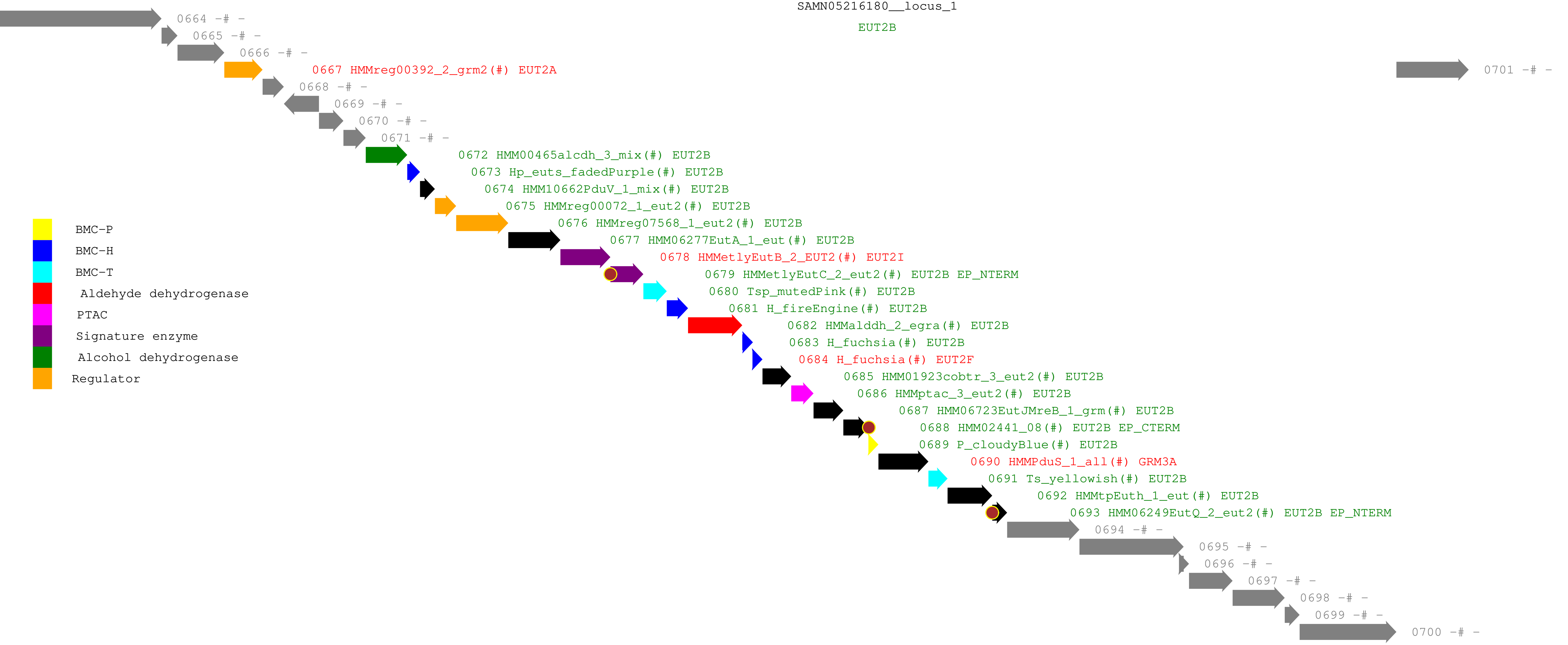

Acetitomaculum ruminis DSM 5522. Bacteria; Firmicutes; Clostridia; Clostridiales; Lachnospiraceae;

SAMN05216249__locus_1

EUT2B

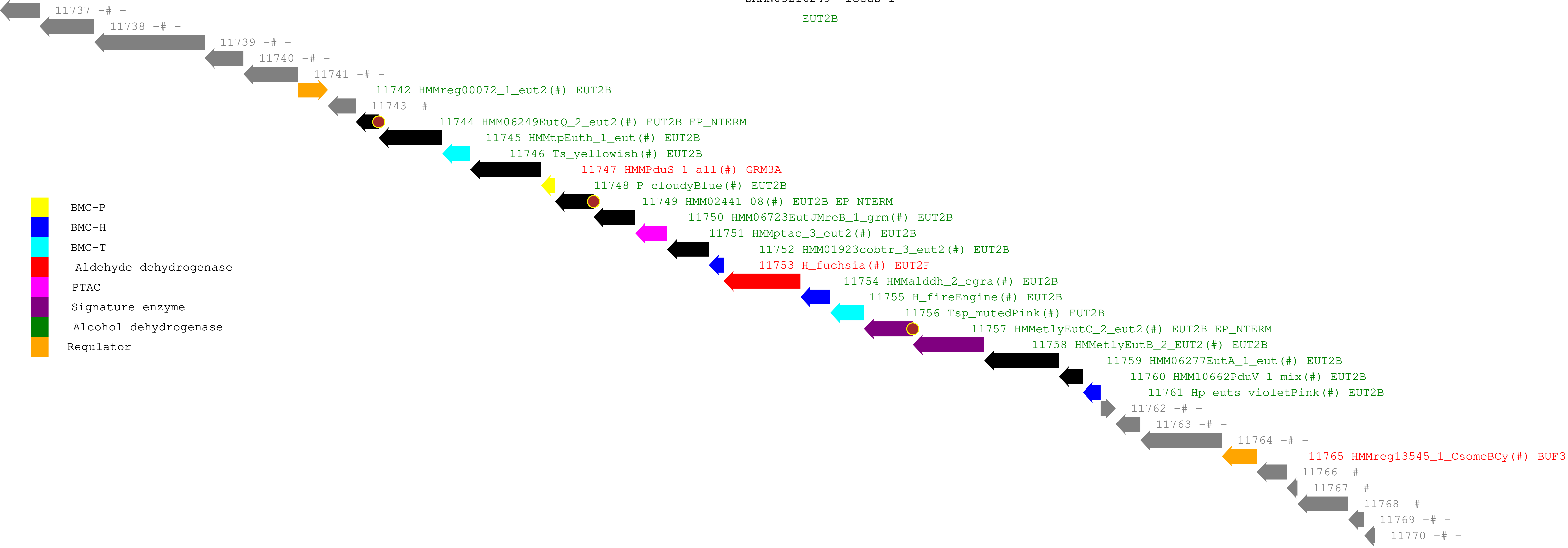

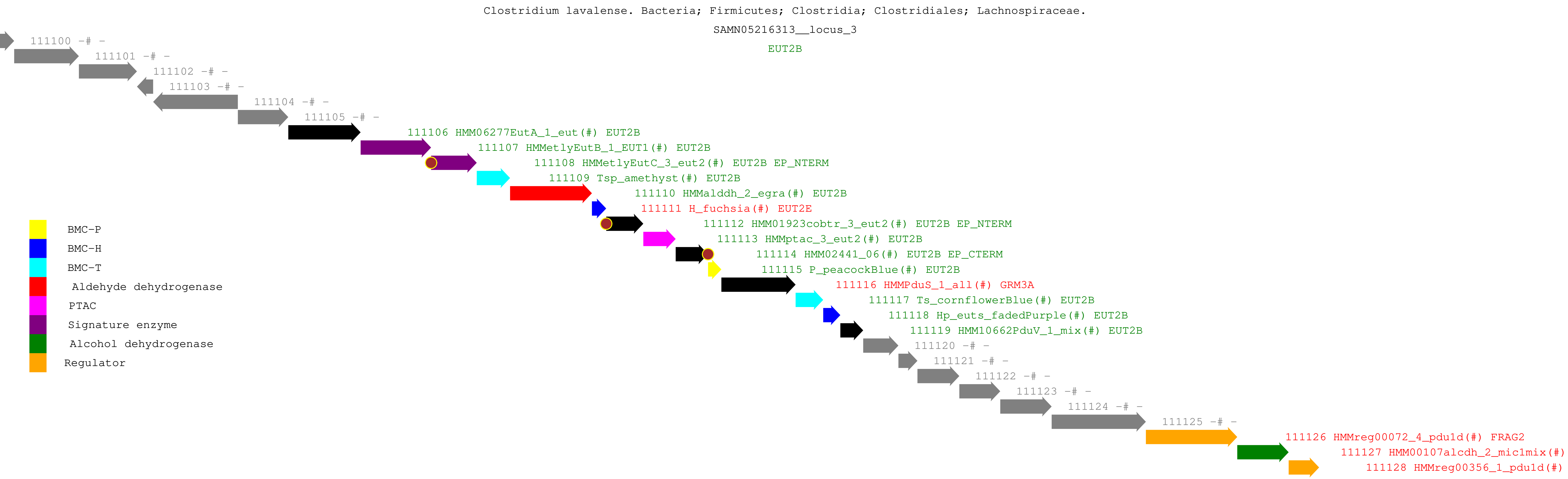

Clostridium lavalense. Bacteria; Firmicutes; Clostridia; Clostridiales; Lachnospiraceae.
SAMN05216313__locus_3
EUT2B
111100 -# -
111101 -# -
111102 -# -
111103 -# -
111104 -# -
111105 -# -
111106 HMM06277EutA_1_eut(#) EUT2B
111107 HMMetlyEutB_1_EUT1(#) EUT2B
111108 HMMetlyEutC_3_eut2(#) EUT2B EP_NTERM
111109 Tsp_amethyst(#) EUT2B
111110 HMMalddh_2_egra(#) EUT2B
111111 H_fuchsia(#) EUT2E
111112 HMM01923cobtr_3_eut2(#) EUT2B EP_NTERM
111113 HMMptac_3_eut2(#) EUT2B
111114 HMM02441_06(#) EUT2B EP_CTERM
111115 P_peacockBlue(#) EUT2B
111116 HMMPduS_1_all(#) GRM3A
111117 Ts_cornflowerBlue(#) EUT2B
111118 Hp_euts_fadedPurple(#) EUT2B
111119 HMM10662PduV_1_mix(#) EUT2B
111120 -# -
111121 -# -
111122 -# -
111123 -# -
111124 -# -
111125 -# -
111126 HMMreg00072_4_pdu1d(#) FRAG2
111127 HMM00107alcdh_2_mic1mix(#)
111128 HMMreg00356_1_pdu1d(#)
BMC-P
BMC-H
BMC-T
Aldehyde dehydrogenase
PTAC
Signature enzyme
Alcohol dehydrogenase
Regulator

Proteiniclasticum ruminis. Bacteria; Firmicutes; Clostridia; Clostridiales; Clostridiaceae;

SAMN05421804__locus_1_main_sat

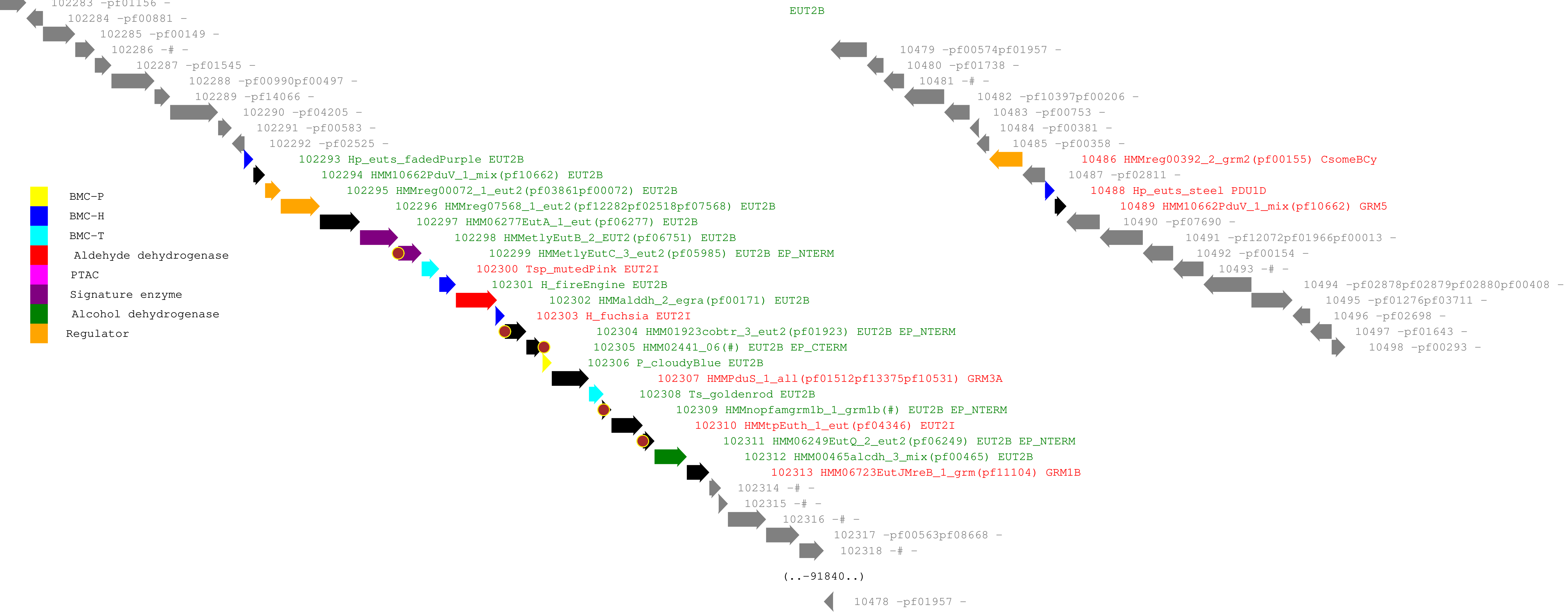

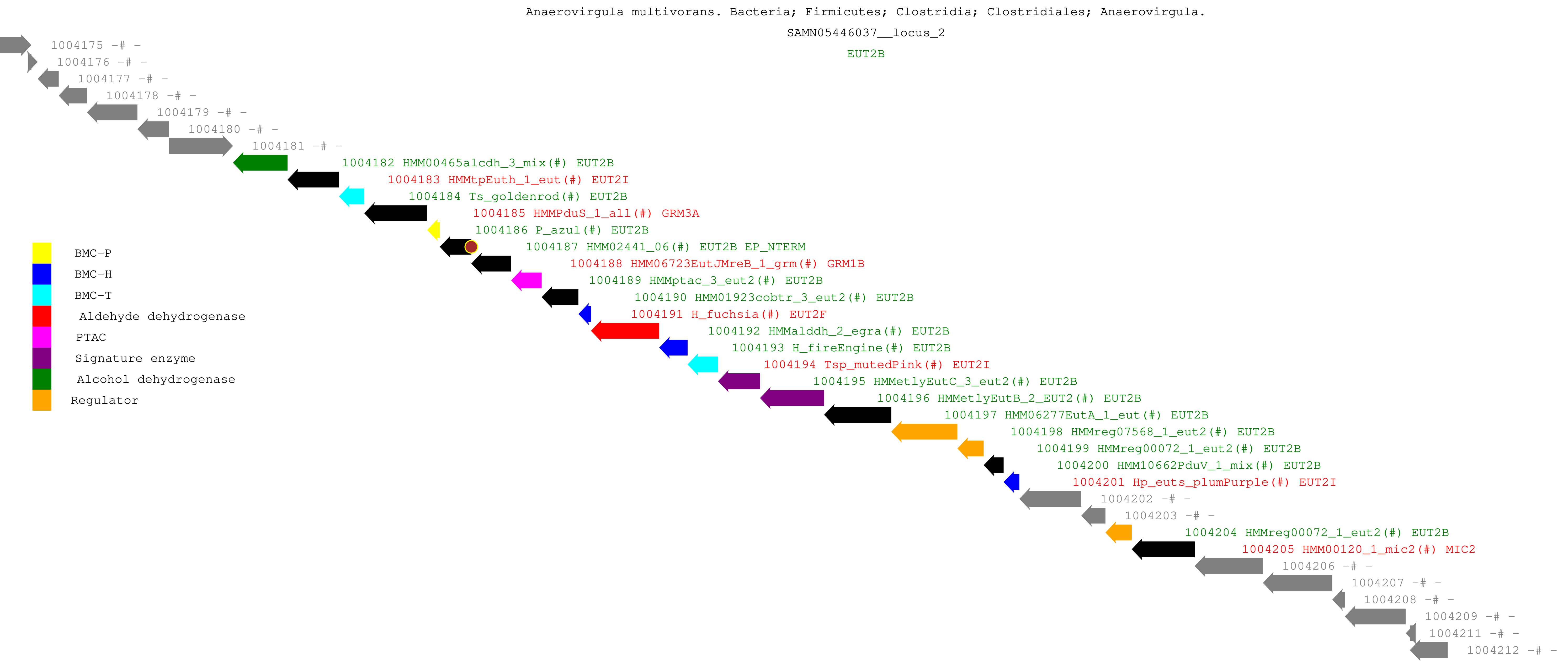
Anaerovirgula multivorans. Bacteria; Firmicutes; Clostridia; Clostridiales; Anaerovirgula.
SAMN05446037__locus_2
EUT2B
1004175 -# -
1004176 -# -
1004177 -# -
1004178 -# -
1004179 -# -
1004180 -# -
1004181 -# -
1004182 HMM00465alcdh_3_mix(#) EUT2B
1004183 HMMtpEuth_1_eut(#) EUT2I
1004184 Ts_goldenrod(#) EUT2B
1004185 HMMPduS_1_all(#) GRM3A
1004186 P_azul(#) EUT2B
1004187 HMM02441_06(#) EUT2B EP_NTERM
1004188 HMM06723EutJMreB_1_grm(#) GRM1B
1004189 HMMptac_3_eut2(#) EUT2B
1004190 HMM01923cobtr_3_eut2(#) EUT2B
1004191 H_fuchsia(#) EUT2F
1004192 HMMalddh_2_egra(#) EUT2B
1004193 H_fireEngine(#) EUT2B
1004194 Tsp_mutedPink(#) EUT2I
1004195 HMMetlyEutC_3_eut2(#) EUT2B
1004196 HMMetlyEutB_2_EUT2(#) EUT2B
1004197 HMM06277EutA_1_eut(#) EUT2B
1004198 HMMreg07568_1_eut2(#) EUT2B
1004199 HMMreg00072_1_eut2(#) EUT2B
1004200 HMM10662PduV_1_mix(#) EUT2B
1004201 Hp_euts_plumPurple(#) EUT2I
1004202 -# -
1004203 -# -
1004204 HMMreg00072_1_eut2(#) EUT2B
1004205 HMM00120_1_mic2(#) MIC2
1004206 -# -
1004207 -# -
1004208 -# -
1004209 -# -
1004211 -# -
1004212 -# -
BMC-P
BMC-H
BMC-T
Aldehyde dehydrogenase
PTAC
Signature enzyme
Alcohol dehydrogenase
Regulator

Natronincola peptidivorans. Bacteria; Firmicutes; Clostridia; Clostridiales; Clostridiaceae;

SAMN05660297__locus_3

EUT2B

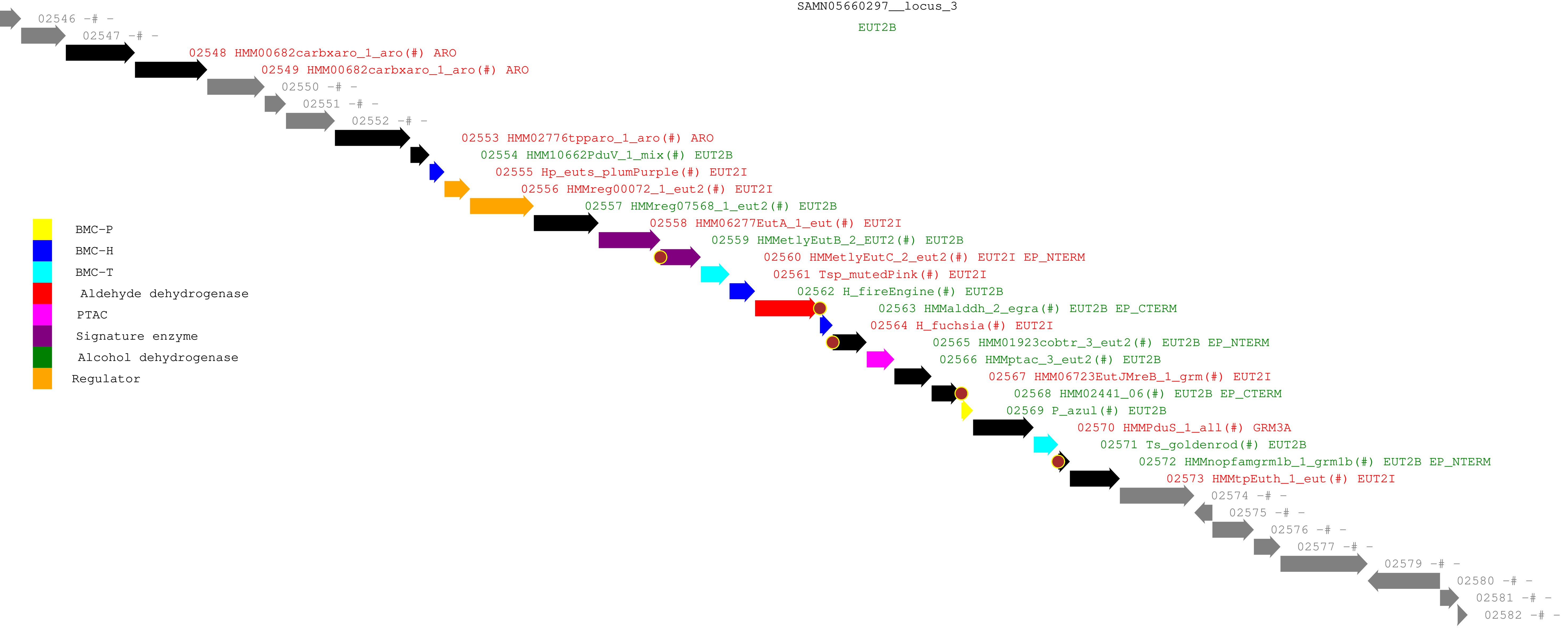

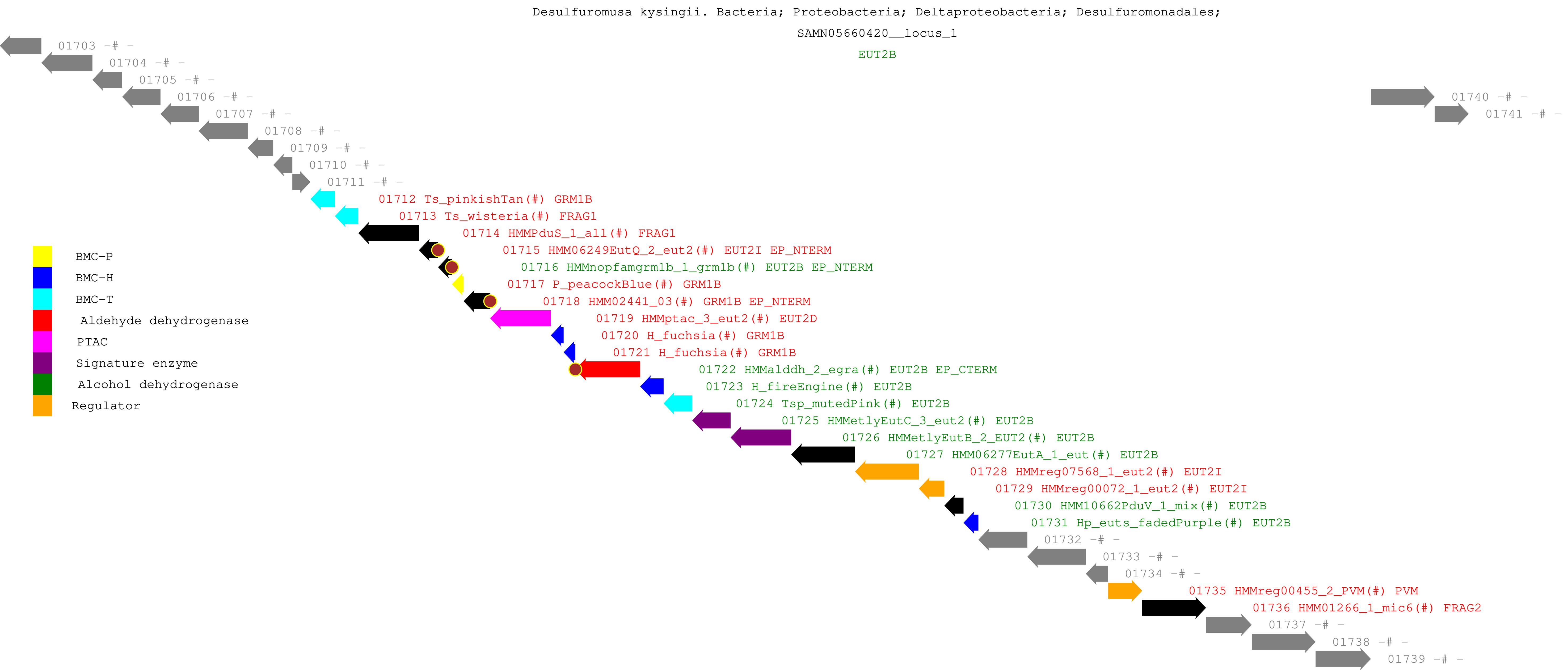
Desulfuromusa kysingii. Bacteria; Proteobacteria; Deltaproteobacteria; Desulfuromonadales;
SAMN05660420__locus_1
EUT2B
01703 -# -
01704 -# -
01705 -# -
01706 -# -
01707 -# -
01708 -# -
01709 -# -
01710 -# -
01711 -# -
01712 Ts_pinkishTan(#) GRM1B
01713 Ts_wisteria(#) FRAG1
01714 HMMPduS_1_all(#) FRAG1
01715 HMM06249EutQ_2_eut2(#) EUT2I EP_NTERM
01716 HMMnopfamgrm1b_1_grm1b(#) EUT2B EP_NTERM
01717 P_peacockBlue(#) GRM1B
01718 HMM02441_03(#) GRM1B EP_NTERM
01719 HMMptac_3_eut2(#) EUT2D
01720 H_fuchsia(#) GRM1B
01721 H_fuchsia(#) GRM1B
01722 HMMalddh_2_egra(#) EUT2B EP_CTERM
01723 H_fireEngine(#) EUT2B
01724 Tsp_mutedPink(#) EUT2B
01725 HMMetlyEutC_3_eut2(#) EUT2B
01726 HMMetlyEutB_2_EUT2(#) EUT2B
01727 HMM06277EutA_1_eut(#) EUT2B
01728 HMMreg07568_1_eut2(#) EUT2I
01729 HMMreg00072_1_eut2(#) EUT2I
01730 HMM10662PduV_1_mix(#) EUT2B
01731 Hp_euts_fadedPurple(#) EUT2B
01732 -# -
01733 -# -
01734 -# -
01735 HMMreg00455_2_PVM(#) PVM
01736 HMM01266_1_mic6(#) FRAG2
01737 -# -
01738 -# -
01739 -# -
01740 -# -
01741 -# -
BMC-P
BMC-H
BMC-T
Aldehyde dehydrogenase
PTAC
Signature enzyme
Alcohol dehydrogenase
Regulator

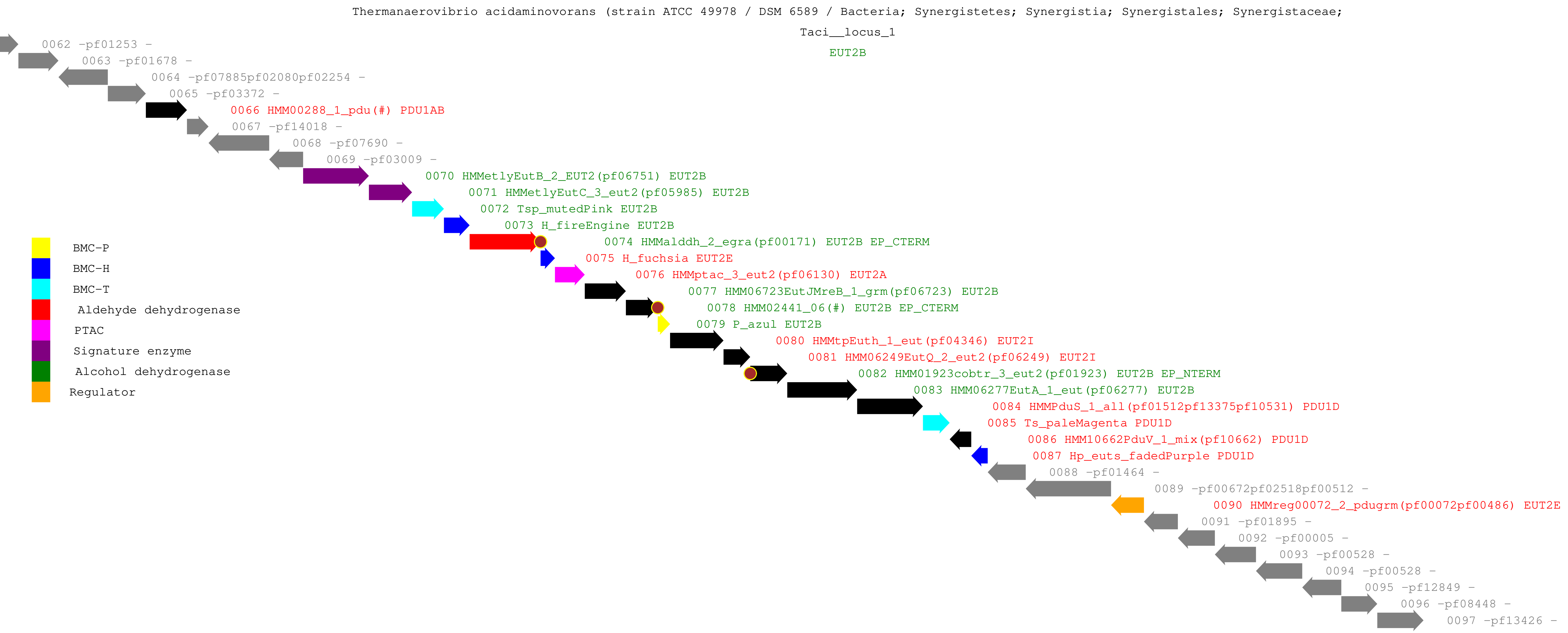
Thermanaerovibrio acidaminovorans (strain ATCC 49978 / DSM 6589 / Bacteria; Synergistetes; Synergistia; Synergistales; Synergistaceae;
Taci__locus_1
EUT2B
0062 -pf01253 -
0063 -pf01678 -
0064 -pf07885pf02080pf02254 -
0065 -pf03372 -
0066 HMM00288_1_pdu(#) PDU1AB
0067 -pf14018 -
0068 -pf07690 -
0069 -pf03009 -
0070 HMMetlyEutB_2_EUT2(pf06751) EUT2B
0071 HMMetlyEutC_3_eut2(pf05985) EUT2B
0072 Tsp_mutedPink EUT2B
0073 H_fireEngine EUT2B
0074 HMMaldh_2_egra(pf00171) EUT2B EP_CTERM
0075 H_fuchsia EUT2E
0076 HMMptac_3_eut2(pf06130) EUT2A
0077 HMM06723EutJMreB_1_grm(pf06723) EUT2B
0078 HMM02441_06(#) EUT2B EP_CTERM
0079 P_azul EUT2B
0080 HMMtpEuth_1_eut(pf04346) EUT2I
0081 HMM06249EutQ_2_eut2(pf06249) EUT2I
0082 HMM01923cobtr_3_eut2(pf01923) EUT2B EP_NTERM
0083 HMM06277EutA_1_eut(pf06277) EUT2B
0084 HMMPduS_1_all(pf01512pf13375pf10531) PDU1D
0085 Ts_paleMagenta PDU1D
0086 HMM10662PduV_1_mix(pf10662) PDU1D
0087 Hp_euts_fadedPurple PDU1D
0088 -pf01464 -
0089 -pf00672pf02518pf00512 -
0090 HMMreg00072_2_pdugrm(pf00072pf00486) EUT2E
0091 -pf01895 -
0092 -pf00005 -
0093 -pf00528 -
0094 -pf00528 -
0095 -pf12849 -
0096 -pf08448 -
0097 -pf13426 -
BMC-P
BMC-H
BMC-T
Aldehyde dehydrogenase
PTAC
Signature enzyme
Alcohol dehydrogenase
Regulator

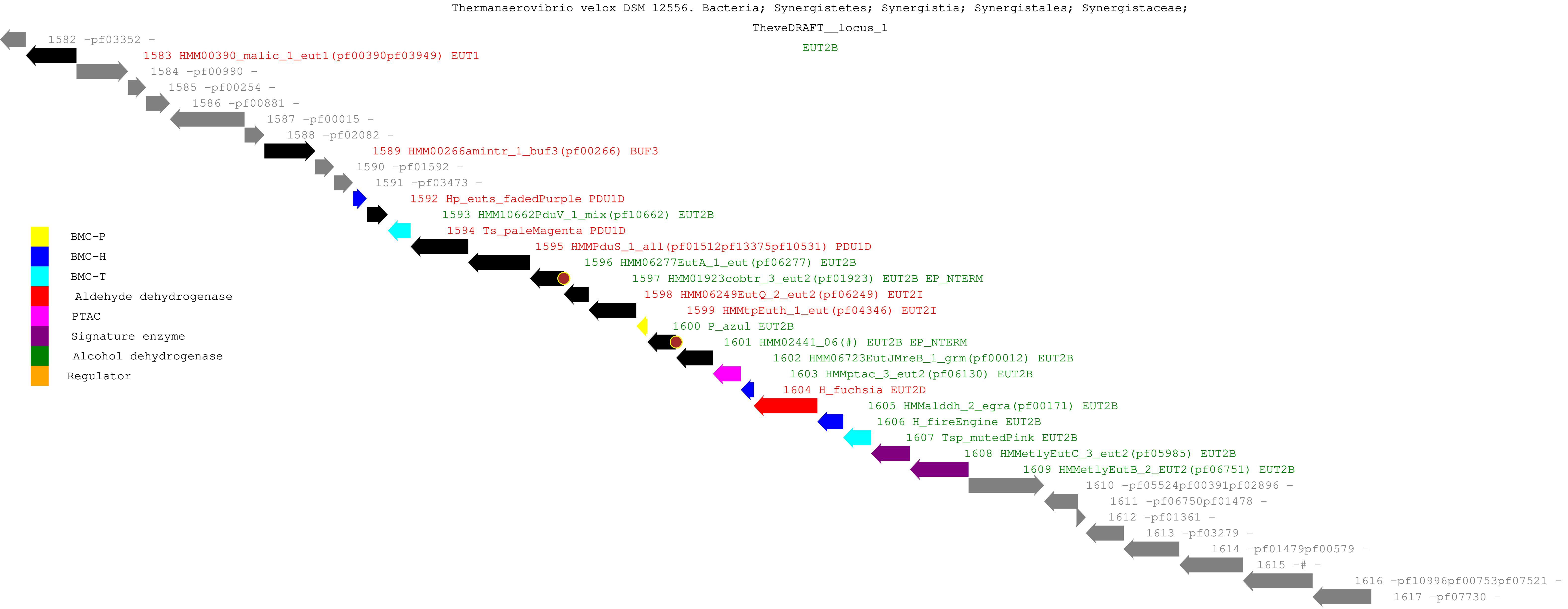
Thermanaerovibrio velox DSM 12556. Bacteria; Synergistetes; Synergistia; Synergistales; Synergistaceae;
TheveDRAFT__locus_1
EUT2B
1582 -pf03352 -
1583 HMM00390_malic_1_eut1(pf00390pf03949) EUT1
1584 -pf00990 -
1585 -pf00254 -
1586 -pf00881 -
1587 -pf00015 -
1588 -pf02082 -
1589 HMM00266amintr_1_buf3(pf00266) BUF3
1590 -pf01592 -
1591 -pf03473 -
1592 Hp_euts_fadedPurple PDU1D
1593 HMM10662PduV_1_mix(pf10662) EUT2B
1594 Ts_paleMagenta PDU1D
1595 HMMPduS_1_all(pf01512pf13375pf10531) PDU1D
1596 HMM06277EutA_1_eut(pf06277) EUT2B
1597 HMM01923cobtr_3_eut2(pf01923) EUT2B EP_NTERM
1598 HMM06249EutQ_2_eut2(pf06249) EUT2I
1599 HMMtpEuth_1_eut(pf04346) EUT2I
1600 P_azul EUT2B
1601 HMM02441_06(#) EUT2B EP_NTERM
1602 HMM06723EutJMreB_1_grm(pf00012) EUT2B
1603 HMMptac_3_eut2(pf06130) EUT2B
1604 H_fuchsia EUT2D
1605 HMMalddh_2_egra(pf00171) EUT2B
1606 H_fireEngine EUT2B
1607 Tsp_mutedPink EUT2B
1608 HMMetlyEutC_3_eut2(pf05985) EUT2B
1609 HMMetlyEutB_2_EUT2(pf06751) EUT2B
1610 -pf05524pf00391pf02896 -
1611 -pf06750pf01478 -
1612 -pf01361 -
1613 -pf03279 -
1614 -pf01479pf00579 -
1615 -# -
1616 -pf10996pf00753pf07521 -
1617 -pf07730 -
BMC-P
BMC-H
BMC-T
Aldehyde dehydrogenase
PTAC
Signature enzyme
Alcohol dehydrogenase
Regulator

Thermacetogenium phaeum (strain ATCC BAA-254 / DSM 12270 / PB). Bacteria; Firmicutes; Clostridia; Thermoanaerobacterales;

Tph_c_locus_1

EUT2B

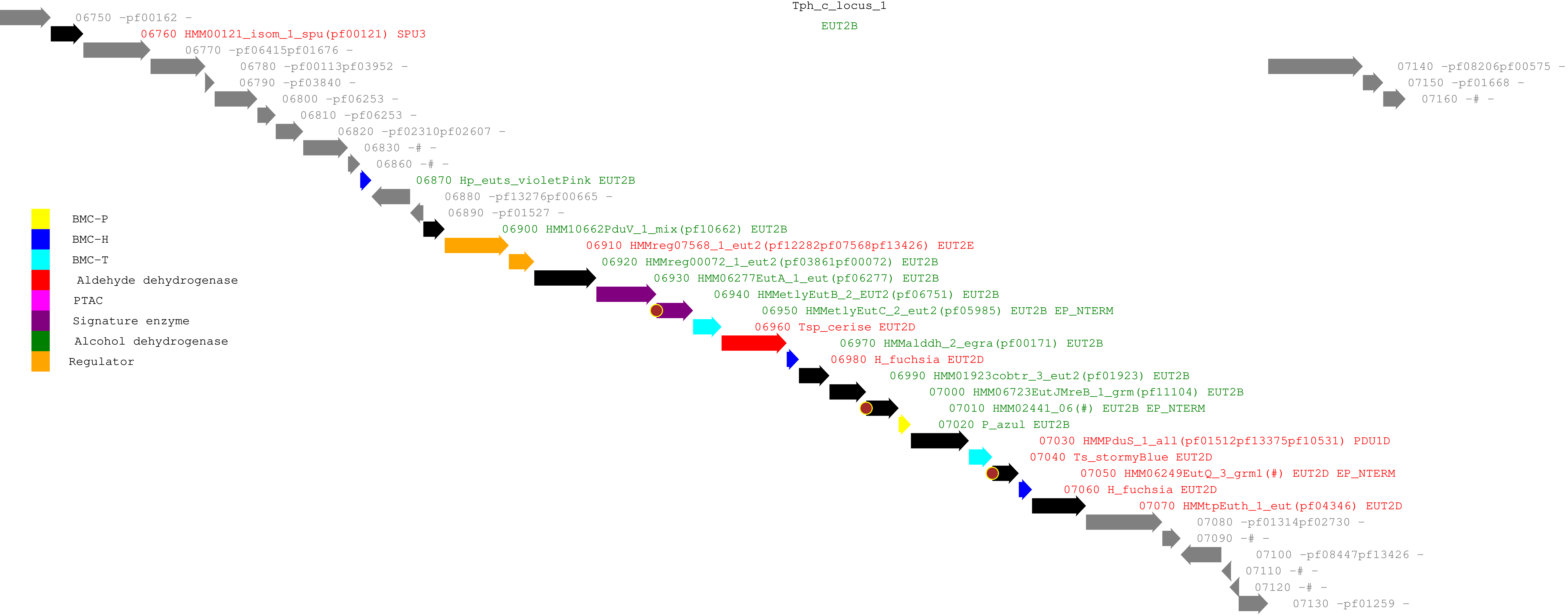

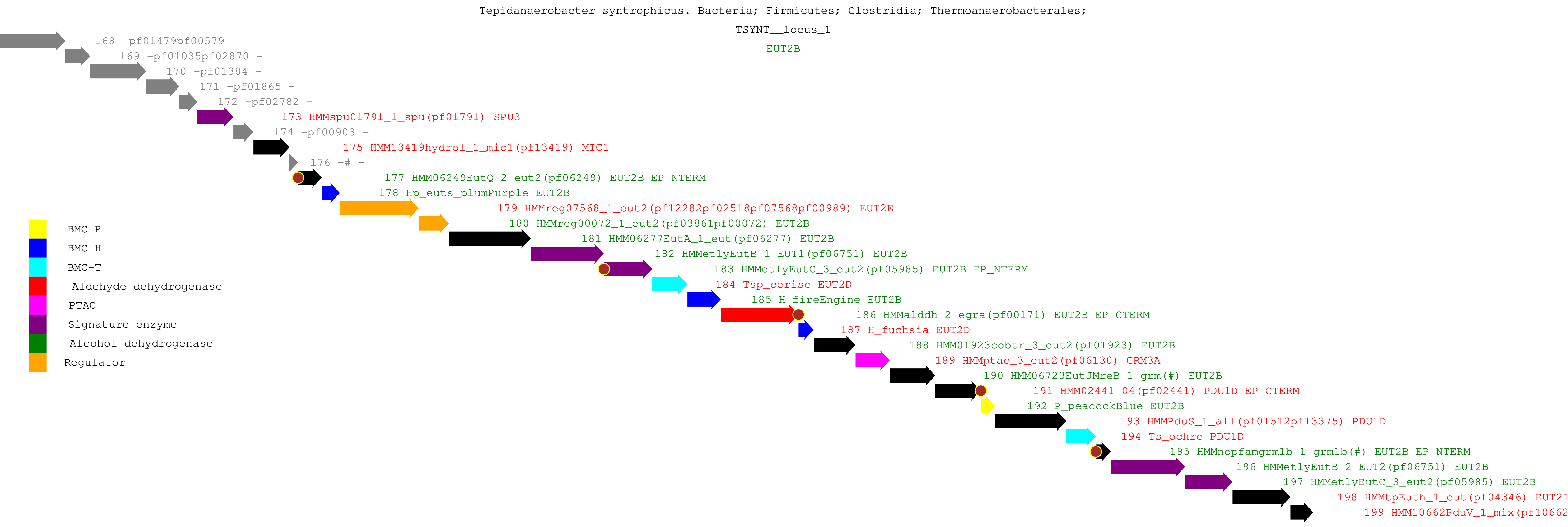
Tepidanaerobacter syntrophicus. Bacteria; Firmicutes; Clostridia; Thermoanaerobacterales;
TSYNT__locus_1
EUT2B
168 -pf01479pf00579 -
169 -pf01035pf02870 -
170 -pf01384 -
171 -pf01865 -
172 -pf02782 -
173 HMMspu01791_1_spu(pf01791) SPU3
174 -pf00903 -
175 HMM13419hydrol_1_mic1(pf13419) MIC1
176 -# -
177 HMM06249EutQ_2_eut2(pf06249) EUT2B EP_NTERM
178 Hp_euts_plumPurple EUT2B
179 HMMreg07568_1_eut2(pf12282pf02518pf07568pf00989) EUT2E
180 HMMreg00072_1_eut2(pf03861pf00072) EUT2B
181 HMM06277EutA_1_eut(pf06277) EUT2B
182 HMMetlyEutB_1_EUT1(pf06751) EUT2B
183 HMMetlyEutC_3_eut2(pf05985) EUT2B EP_NTERM
184 Tsp_cerise EUT2D
185 H_fireEngine EUT2B
186 HMMalddh_2_egra(pf00171) EUT2B EP_CTERM
187 H_fuchsia EUT2D
188 HMM01923cobtr_3_eut2(pf01923) EUT2B
189 HMMptac_3_eut2(pf06130) GRM3A
190 HMM06723EutJMreB_1_grm(#) EUT2B
191 HMM02441_04(pf02441) PDU1D EP_CTERM
192 P_peacockBlue EUT2B
193 HMMPduS_1_all(pf01512pf13375) PDU1D
194 Ts_ochre PDU1D
195 HMMnopfamgrm1b_1_grm1b(#) EUT2B EP_NTERM
196 HMMetlyEutB_2_EUT2(pf06751) EUT2B
197 HMMetlyEutC_3_eut2(pf05985) EUT2B
198 HMMtpEuth_1_eut(pf04346) EUT2I
199 HMM10662PduV_1_mix(pf10662
BMC-P
BMC-H
BMC-T
Aldehyde dehydrogenase
PTAC
Signature enzyme
Alcohol dehydrogenase
Regulator

Clostridium coskatii. Bacteria; Firmicutes; Clostridia; Clostridiales; Clostridiaceae;

WX73__locus_1

EUT2B

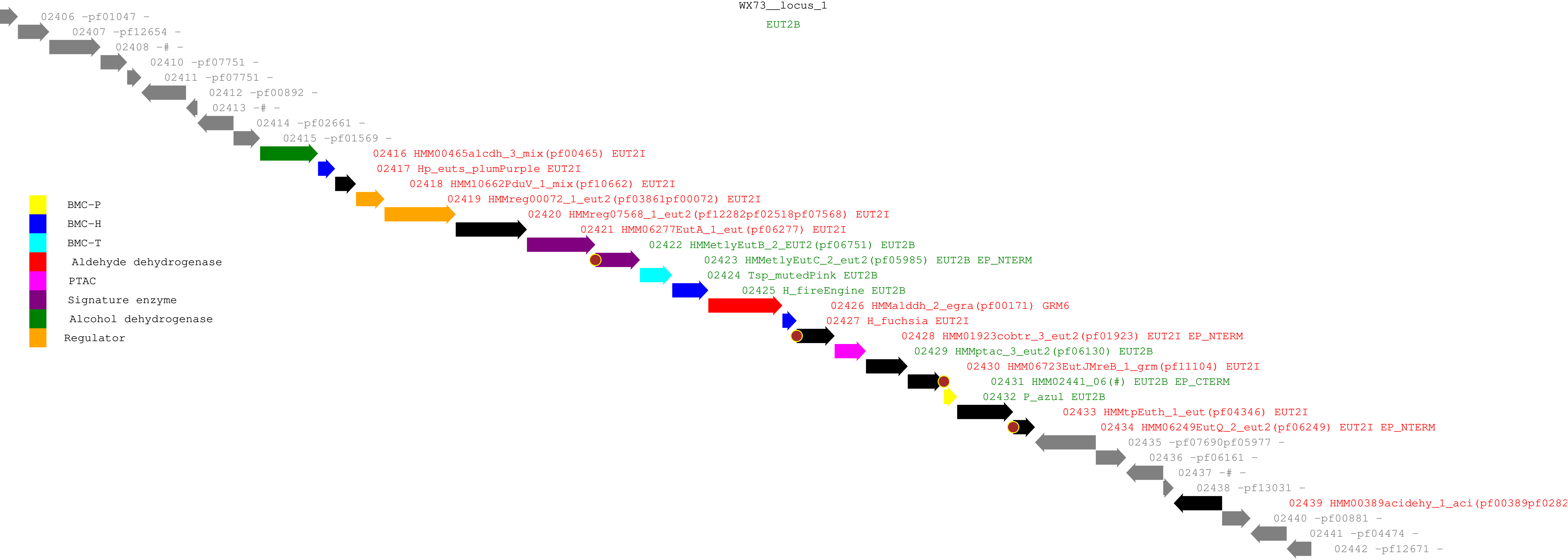

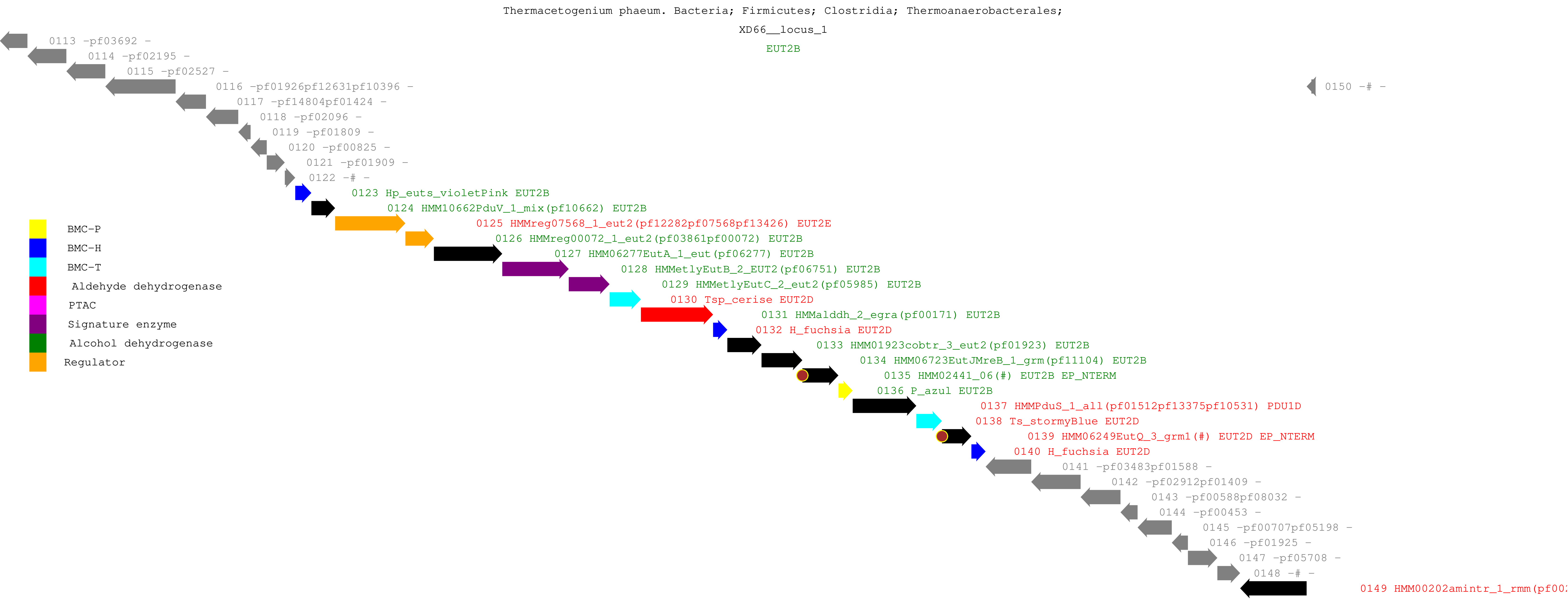
Thermacetogenium phaeum. Bacteria; Firmicutes; Clostridia; Thermoanaerobacterales;
XD66__locus_1
EUT2B
0113 -pf03692 -
0114 -pf02195 -
0115 -pf02527 -
0116 -pf01926pf12631pf10396 -
0117 -pf14804pf01424 -
0118 -pf02096 -
0119 -pf01809 -
0120 -pf00825 -
0121 -pf01909 -
0122 -# -
0123 Hp_euts_violetPink EUT2B
0124 HMM10662PduV_1_mix(pf10662) EUT2B
0125 HMMreg07568_1_eut2(pf12282pf07568pf13426) EUT2E
0126 HMMreg00072_1_eut2(pf03861pf00072) EUT2B
0127 HMM06277EutA_1_eut(pf06277) EUT2B
0128 HMMetlyEutB_2_EUT2(pf06751) EUT2B
0129 HMMetlyEutC_2_eut2(pf05985) EUT2B
0130 Tsp_cerise EUT2D
0131 HMMaldh_2_egra(pf00171) EUT2B
0132 H_fuchsia EUT2D
0133 HMM01923cobtr_3_eut2(pf01923) EUT2B
0134 HMM06723EutJMreB_1_grm(pf11104) EUT2B
0135 HMM02441_06(#) EUT2B EP_NTERM
0136 P_azul EUT2B
0137 HMMPduS_1_all(pf01512pf13375pf10531) PDU1D
0138 Ts_stormyBlue EUT2D
0139 HMM06249EutQ_3_grm1(#) EUT2D EP_NTERM
0140 H_fuchsia EUT2D
0141 -pf03483pf01588 -
0142 -pf02912pf01409 -
0143 -pf00588pf08032 -
0144 -pf00453 -
0145 -pf00707pf05198 -
0146 -pf01925 -
0147 -pf05708 -
0148 -# -
0149 HMM00202amintr_1_rmm(pf00
0150 -# -
BMC-P
BMC-H
BMC-T
Aldehyde dehydrogenase
PTAC
Signature enzyme
Alcohol dehydrogenase
Regulator

Bacillus oceanisediminis 2691. Bacteria; Firmicutes; Bacilli; Bacillales; Bacillaceae; Bacillus.

A361__locus_2

EUT2C

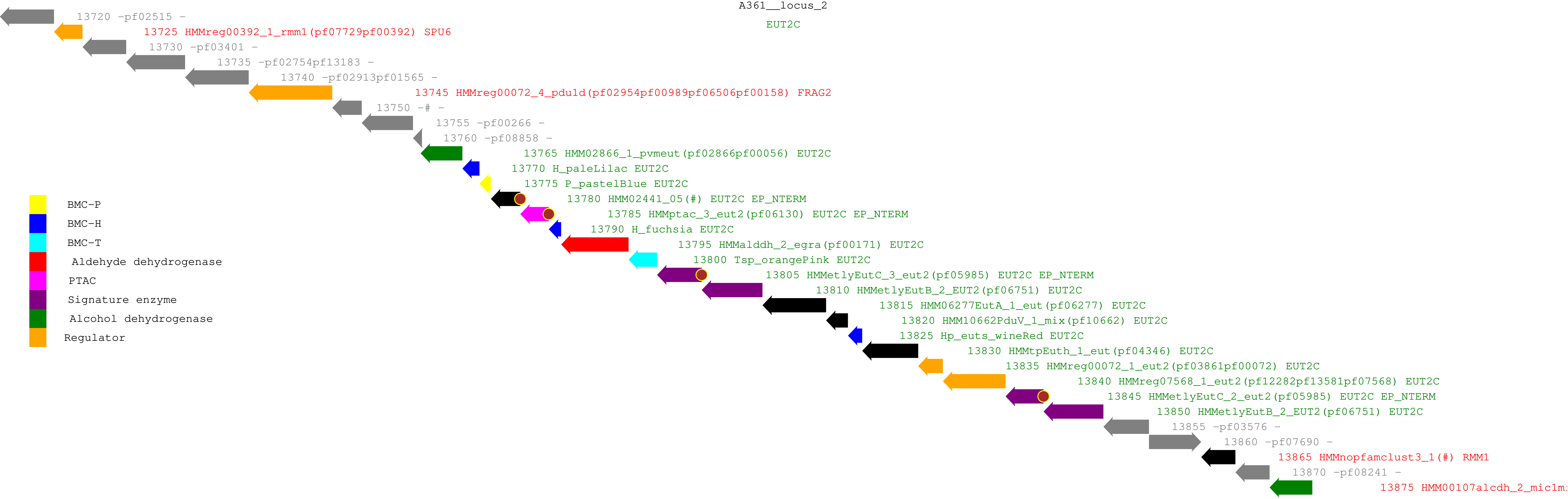

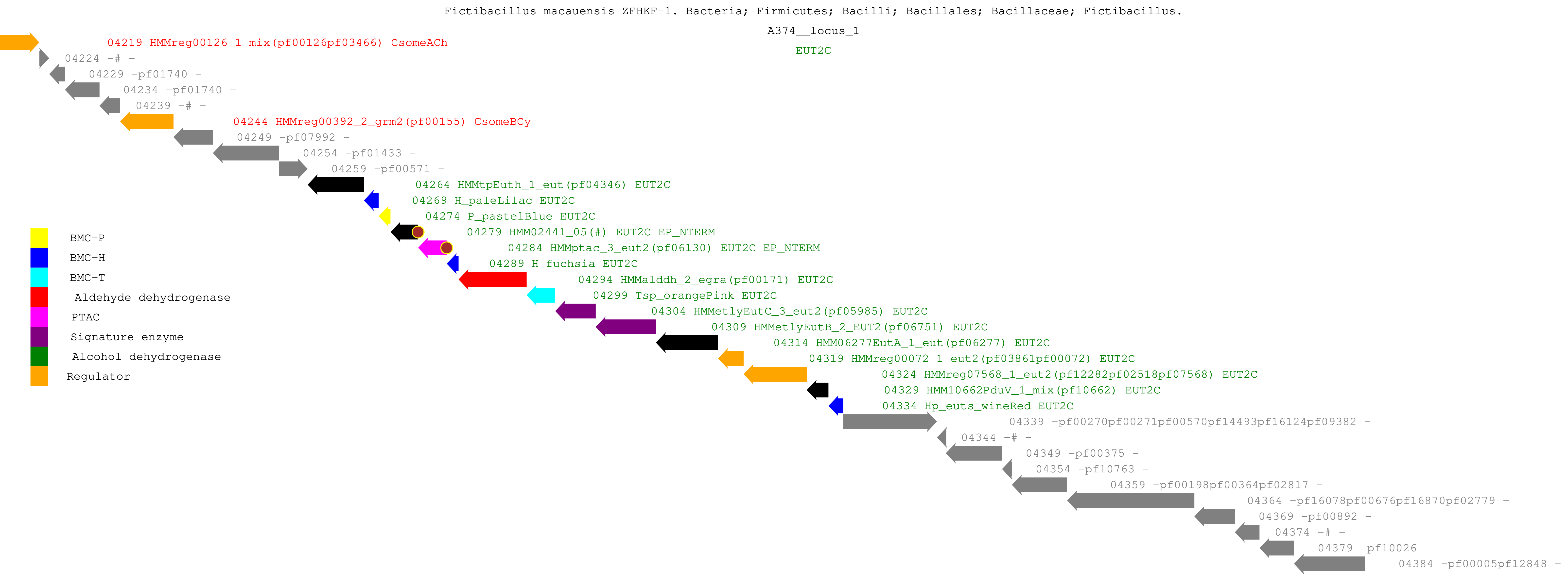

Fictibacillus macauensis ZFHKF-1. Bacteria; Firmicutes; Bacilli; Bacillales; Bacillaceae; Fictibacillus.
A374__locus_1
EUT2C
04219 HMMreg00126_1_mix(pf00126pf03466) CsomeACh
04224 -# -
04229 -pf01740 -
04234 -pf01740 -
04239 -# -
04244 HMMreg00392_2_grm2(pf00155) CsomeBCy
04249 -pf07992 -
04254 -pf01433 -
04259 -pf00571 -
04264 HMMtpEuth_1_eut(pf04346) EUT2C
04269 H_paleLilac EUT2C
04274 P_pastelBlue EUT2C
04279 HMM02441_05(#) EUT2C EP_NTERM
04284 HMMptac_3_eut2(pf06130) EUT2C EP_NTERM
04289 H_fuchsia EUT2C
04294 HMMaldh_2_egra(pf00171) EUT2C
04299 Tsp_orangePink EUT2C
04304 HMMetlyEutC_3_eut2(pf05985) EUT2C
04309 HMMetlyEutB_2_EUT2(pf06751) EUT2C
04314 HMM06277EutA_1_eut(pf06277) EUT2C
04319 HMMreg00072_1_eut2(pf03861pf00072) EUT2C
04324 HMMreg07568_1_eut2(pf12282pf02518pf07568) EUT2C
04329 HMM10662PduV_1_mix(pf10662) EUT2C
04334 Hp_euts_wineRed EUT2C
04339 -pf00270pf00271pf00570pf14493pf16124pf09382 -
04344 -# -
04349 -pf00375 -
04354 -pf10763 -
04359 -pf00198pf00364pf02817 -
04364 -pf16078pf00676pf16870pf02779 -
04369 -pf00892 -
04374 -# -
04379 -pf10026 -
04384 -pf00005pf12848 -
BMC-P
BMC-H
BMC-T
Aldehyde dehydrogenase
PTAC
Signature enzyme
Alcohol dehydrogenase
Regulator

Bacillus badius. Bacteria; Firmicutes; Bacilli; Bacillales; Bacillaceae; Bacillus.

A3781__locus_1

EUT2C

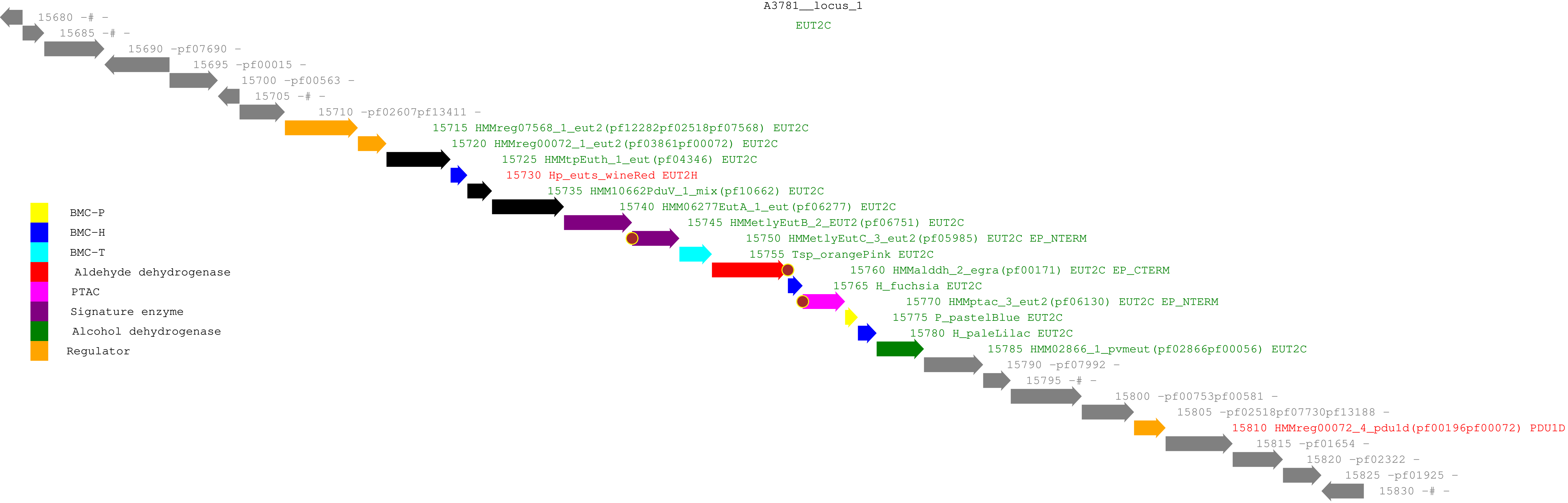

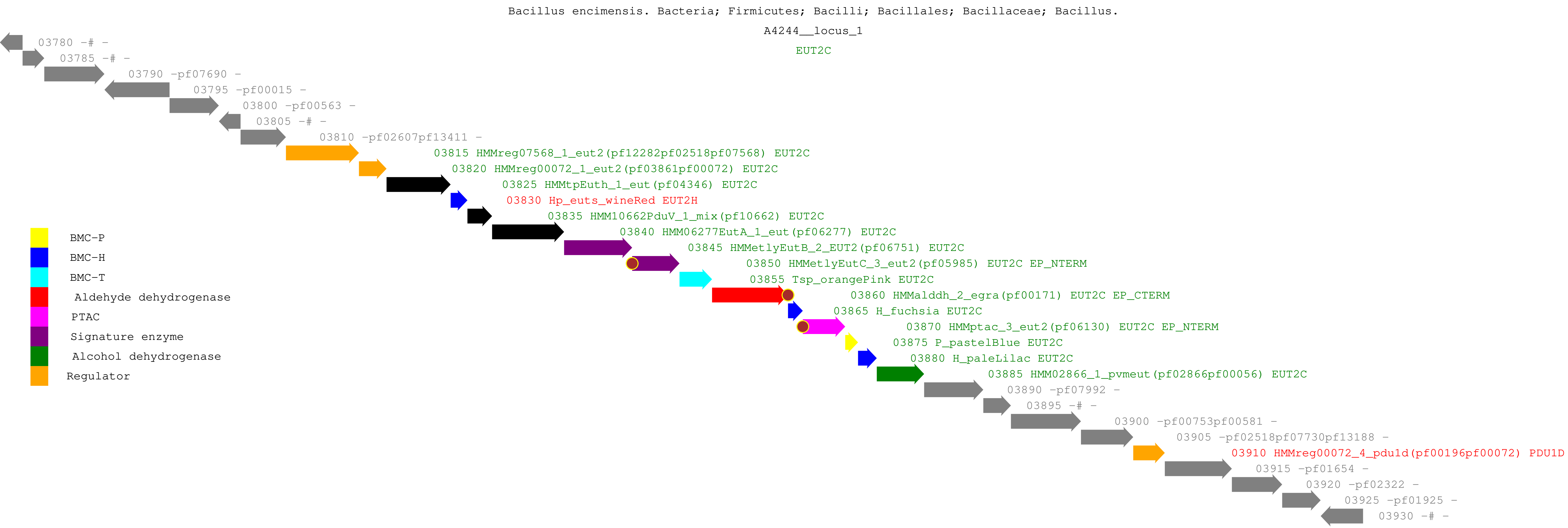

Bacillus encimensis. Bacteria; Firmicutes; Bacilli; Bacillales; Bacillaceae; Bacillus.
A4244__locus_1
EUT2C
03780 -# -
03785 -# -
03790 -pf07690 -
03795 -pf00015 -
03800 -pf00563 -
03805 -# -
03810 -pf02607pf13411 -
03815 HMMreg07568_1_eut2(pf12282pf02518pf07568) EUT2C
03820 HMMreg00072_1_eut2(pf03861pf00072) EUT2C
03825 HMMtpEuth_1_eut(pf04346) EUT2C
03830 Hp_euts_wineRed EUT2H
03835 HMM10662PduV_1_mix(pf10662) EUT2C
03840 HMM06277EutA_1_eut(pf06277) EUT2C
03845 HMMetlyEutB_2_EUT2(pf06751) EUT2C
03850 HMMetlyEutC_3_eut2(pf05985) EUT2C EP_NTERM
03855 Tsp_orangePink EUT2C
03860 HMMaldd h_2_egra(pf00171) EUT2C EP_CTERM
03865 H_fuchsia EUT2C
03870 HMMptac_3_eut2(pf06130) EUT2C EP_NTERM
03875 P_pastelBlue EUT2C
03880 H_paleLilac EUT2C
03885 HMM02866_1_pvmeut(pf02866pf00056) EUT2C
03890 -pf07992 -
03895 -# -
03900 -pf00753pf00581 -
03905 -pf02518pf07730pf13188 -
03910 HMMreg00072_4_pdu1d(pf00196pf00072) PDU1D
03915 -pf01654 -
03920 -pf02322 -
03925 -pf01925 -
03930 -# -
BMC-P
BMC-H
BMC-T
Aldehyde dehydrogenase
PTAC
Signature enzyme
Alcohol dehydrogenase
Regulator

Bacillus sp. FJAT-27215. Bacteria; Firmicutes; Bacilli; Bacillales; Bacillaceae; Bacillus.

A8F95__locus_1

EUT2C

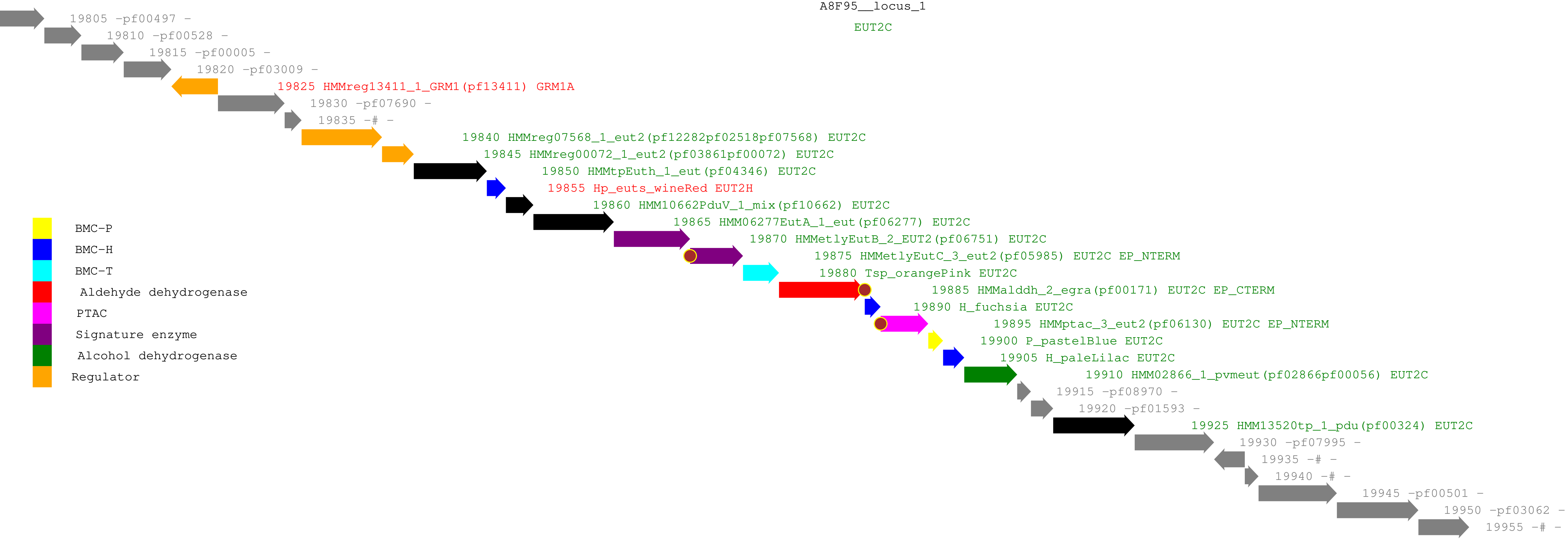

Bacillus sp. FJAT-27231. Bacteria; Firmicutes; Bacilli; Bacillales; Bacillaceae; Bacillus.

AC623__locus_1

EUT2C

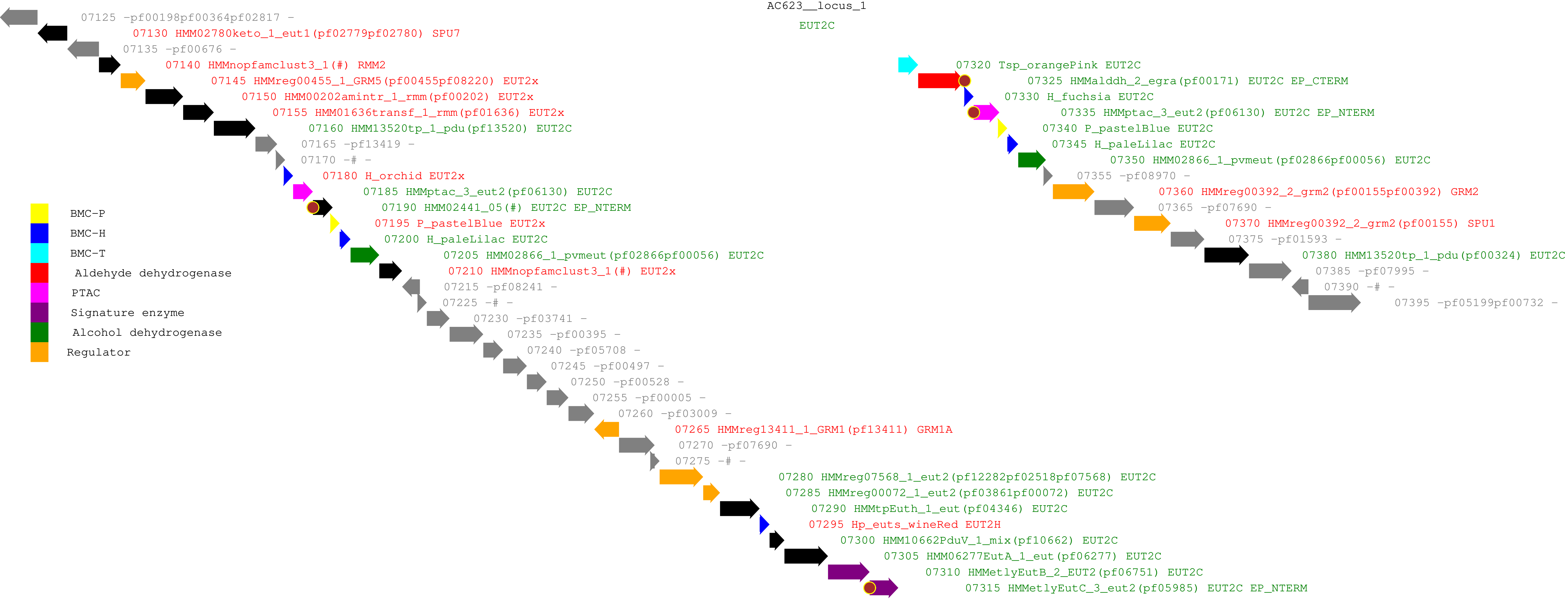

Aneurinibacillus sp. XH2. Bacteria; Firmicutes; Bacilli; Bacillales; Paenibacillaceae;

ACH33__locus_1

EUT2C

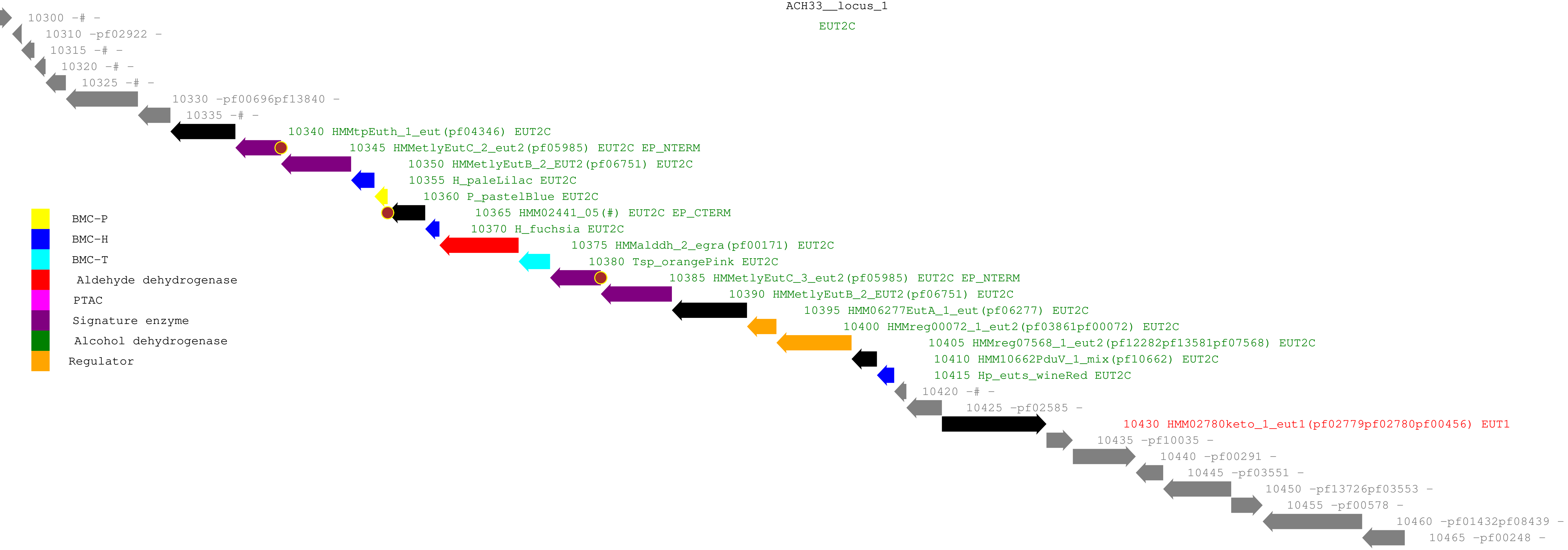

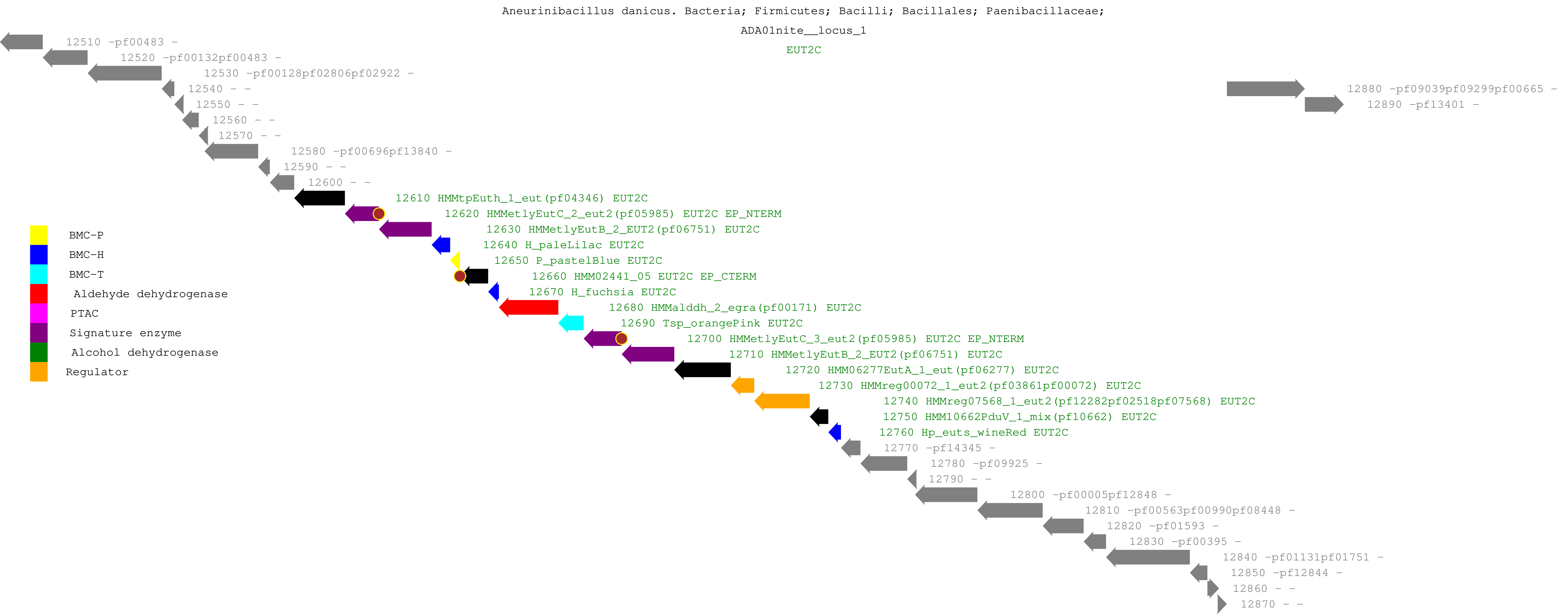
Aneurinibacillus danicus. Bacteria; Firmicutes; Bacilli; Bacillales; Paenibacillaceae;
ADA01nite__locus_1
EUT2C
12510 -pf00483 -
12520 -pf00132pf00483 -
12530 -pf00128pf02806pf02922 -
12540 - -
12550 - -
12560 - -
12570 - -
12580 -pf00696pf13840 -
12590 - -
12600 - -
12610 HMMtpEuth_1_eut(pf04346) EUT2C
12620 HMMetlyEutC_2_eut2(pf05985) EUT2C EP_NTERM
12630 HMMetlyEutB_2_EUT2(pf06751) EUT2C
12640 H_paleLilac EUT2C
12650 P_pastelBlue EUT2C
12660 HMM02441_05 EUT2C EP_CTERM
12670 H_fuchsia EUT2C
12680 HMMalddh_2_egra(pf00171) EUT2C
12690 Tsp_orangePink EUT2C
12700 HMMetlyEutC_3_eut2(pf05985) EUT2C EP_NTERM
12710 HMMetlyEutB_2_EUT2(pf06751) EUT2C
12720 HMM06277EutA_1_eut(pf06277) EUT2C
12730 HMMreg00072_1_eut2(pf03861pf00072) EUT2C
12740 HMMreg07568_1_eut2(pf12282pf02518pf07568) EUT2C
12750 HMM10662PduV_1_mix(pf10662) EUT2C
12760 Hp_euts_wineRed EUT2C
12770 -pf14345 -
12780 -pf09925 -
12790 - -
12800 -pf00005pf12848 -
12810 -pf00563pf00990pf08448 -
12820 -pf01593 -
12830 -pf00395 -
12840 -pf01131pf01751 -
12850 -pf12844 -
12860 - -
12870 - -
12880 -pf09039pf09299pf00665 -
12890 -pf13401 -
BMC-P
BMC-H
BMC-T
Aldehyde dehydrogenase
PTAC
Signature enzyme
Alcohol dehydrogenase
Regulator

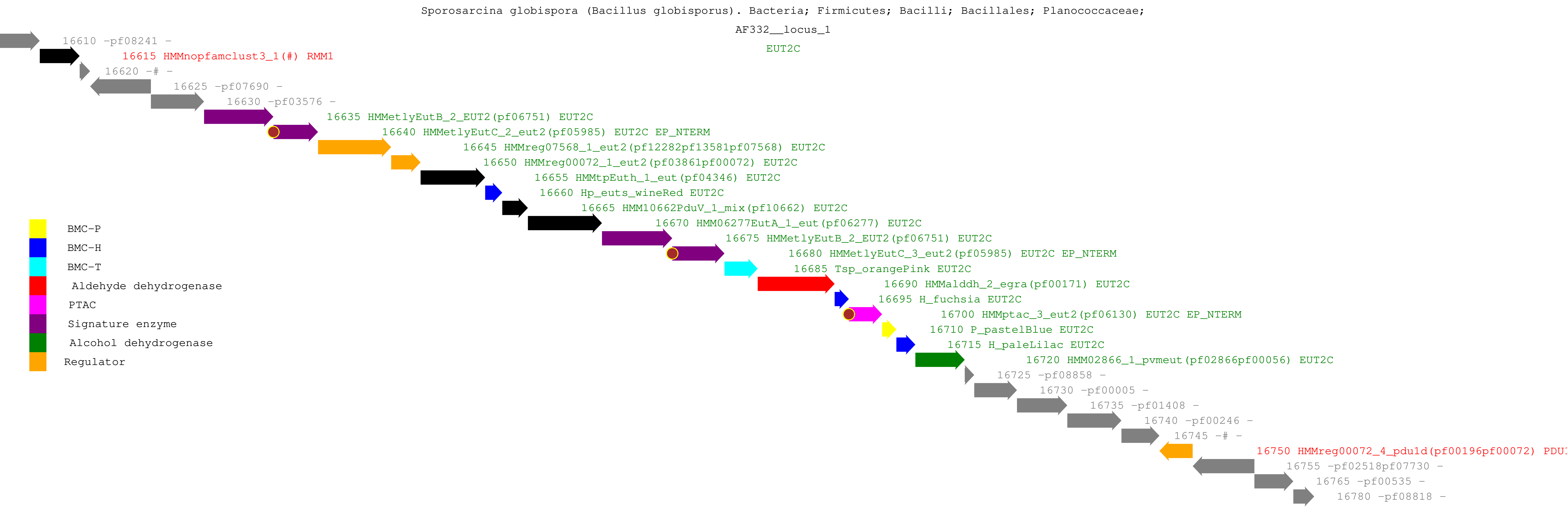
Sporosarcina globispora (Bacillus globisporus). Bacteria; Firmicutes; Bacilli; Bacillales; Planococcaceae;
AF332__locus_1
EUT2C
16610 -pf08241 -
16615 HMMnopfamclust3_1(#) RMM1
16620 -# -
16625 -pf07690 -
16630 -pf03576 -
16635 HMMetlyEutB_2_EUT2(pf06751) EUT2C
16640 HMMetlyEutC_2_eut2(pf05985) EUT2C EP_NTERM
16645 HMMreg07568_1_eut2(pf12282pf13581pf07568) EUT2C
16650 HMMreg00072_1_eut2(pf03861pf00072) EUT2C
16655 HMMtpEuth_1_eut(pf04346) EUT2C
16660 Hp_euts_wineRed EUT2C
16665 HMM10662PduV_1_mix(pf10662) EUT2C
16670 HMM06277EutA_1_eut(pf06277) EUT2C
16675 HMMetlyEutB_2_EUT2(pf06751) EUT2C
16680 HMMetlyEutC_3_eut2(pf05985) EUT2C EP_NTERM
16685 Tsp_orangePink EUT2C
16690 HMMalddh_2_egra(pf00171) EUT2C
16695 H_fuchsia EUT2C
16700 HMMptac_3_eut2(pf06130) EUT2C EP_NTERM
16710 P_pastelBlue EUT2C
16715 H_paleLilac EUT2C
16720 HMM02866_1_pvmeut(pf02866pf00056) EUT2C
16725 -pf08858 -
16730 -pf00005 -
16735 -pf01408 -
16740 -pf00246 -
16745 -# -
16750 HMMreg00072_4_pdu1d(pf00196pf00072) PDU
16755 -pf02518pf07730 -
16765 -pf00535 -
16780 -pf08818 -
BMC-P
BMC-H
BMC-T
Aldehyde dehydrogenase
PTAC
Signature enzyme
Alcohol dehydrogenase
Regulator

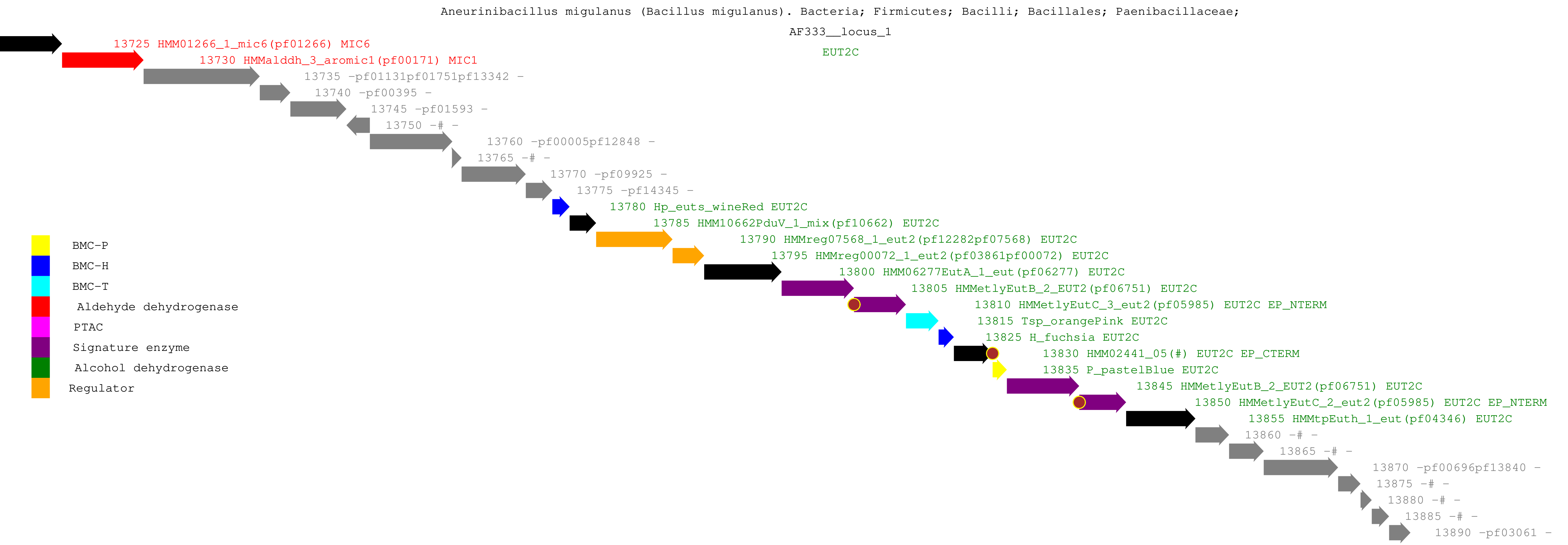
Aneurinibacillus migulanus (Bacillus migulanus). Bacteria; Firmicutes; Bacilli; Bacillales; Paenibacillaceae;
AF333__locus_1
EUT2C
13725 HMM01266_1_mic6(pf01266) MIC6
13730 HMMalddh_3_aromic1(pf00171) MIC1
13735 -pf01131pf01751pf13342 -
13740 -pf00395 -
13745 -pf01593 -
13750 -# -
13760 -pf00005pf12848 -
13765 -# -
13770 -pf09925 -
13775 -pf14345 -
13780 Hp_euts_wineRed EUT2C
13785 HMM10662PduV_1_mix(pf10662) EUT2C
13790 HMMreg07568_1_eut2(pf12282pf07568) EUT2C
13795 HMMreg00072_1_eut2(pf03861pf00072) EUT2C
13800 HMM06277EutA_1_eut(pf06277) EUT2C
13805 HMMetlyEutB_2_EUT2(pf06751) EUT2C
13810 HMMetlyEutC_3_eut2(pf05985) EUT2C EP_NTERM
13815 Tsp_orangePink EUT2C
13825 H_fuchsia EUT2C
13830 HMM02441_05(#) EUT2C EP_CTERM
13835 P_pastelBlue EUT2C
13845 HMMetlyEutB_2_EUT2(pf06751) EUT2C
13850 HMMetlyEutC_2_eut2(pf05985) EUT2C EP_NTERM
13855 HMMtpEuth_1_eut(pf04346) EUT2C
13860 -# -
13865 -# -
13870 -pf00696pf13840 -
13875 -# -
13880 -# -
13885 -# -
13890 -pf03061 -
BMC-P
BMC-H
BMC-T
Aldehyde dehydrogenase
PTAC
Signature enzyme
Alcohol dehydrogenase
Regulator

Bacillus sp. FJAT-21351. Bacteria; Firmicutes; Bacilli; Bacillales; Bacillaceae; Bacillus.

AMS61__locus_1

EUT2C

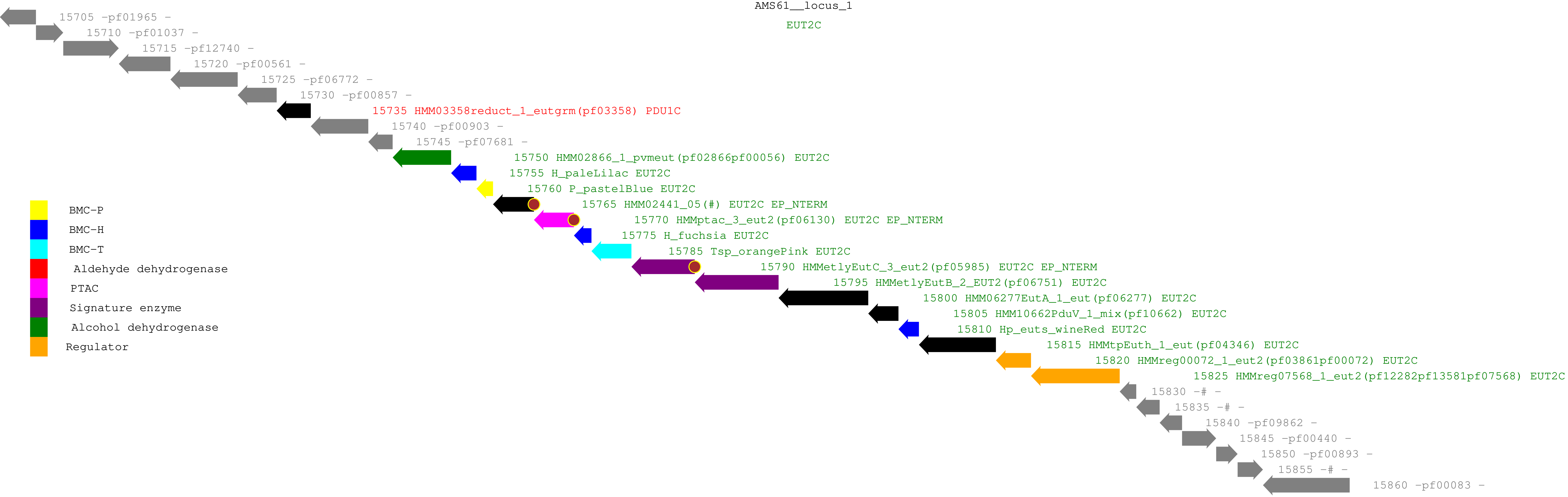

Bacillus sp. Root147. Bacteria; Firmicutes; Bacilli; Bacillales; Bacillaceae; Bacillus.

ASE51__locus_1

EUT2C

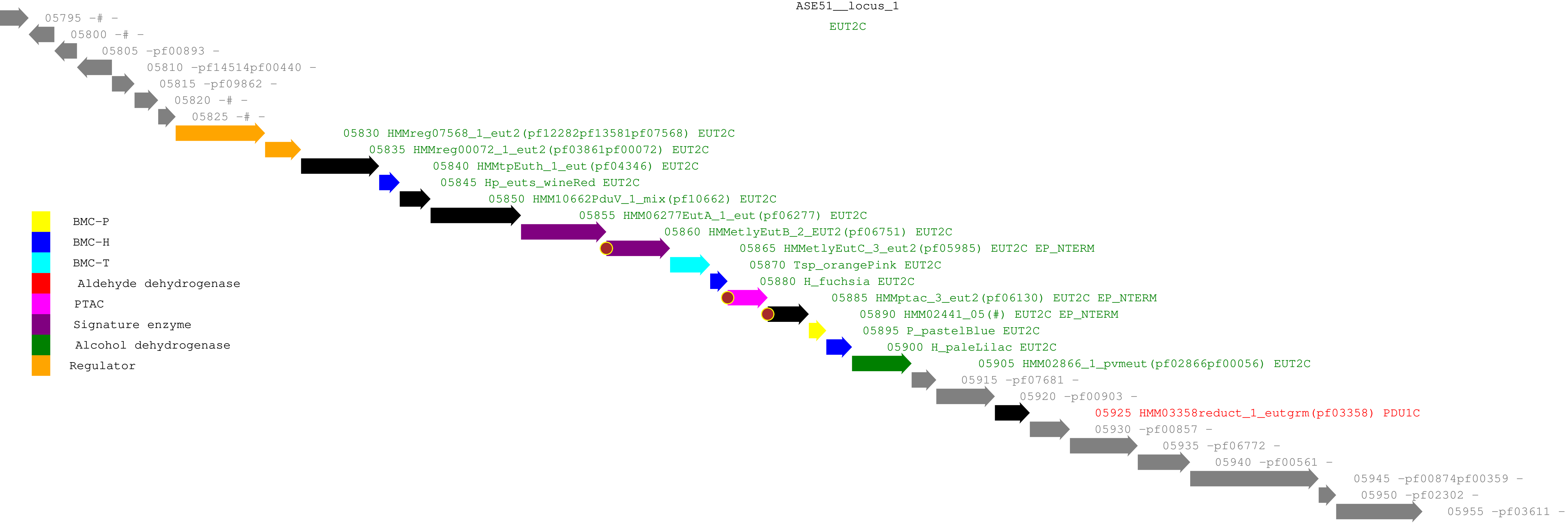

Bacillus sp. Leaf75. Bacteria; Firmicutes; Bacilli; Bacillales; Bacillaceae; Bacillus.

ASG61__locus_1

EUT2C

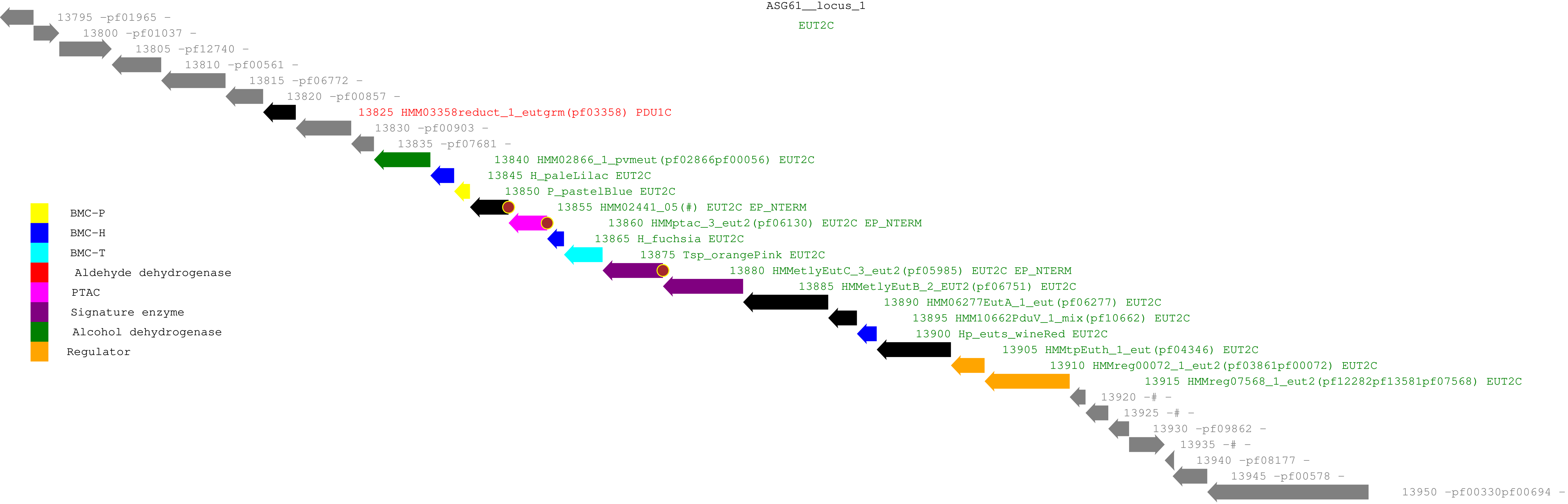

Paenibacillus sp. Soil787. Bacteria; Firmicutes; Bacilli; Bacillales; Paenibacillaceae;

ASG93__locus_1

EUT2C

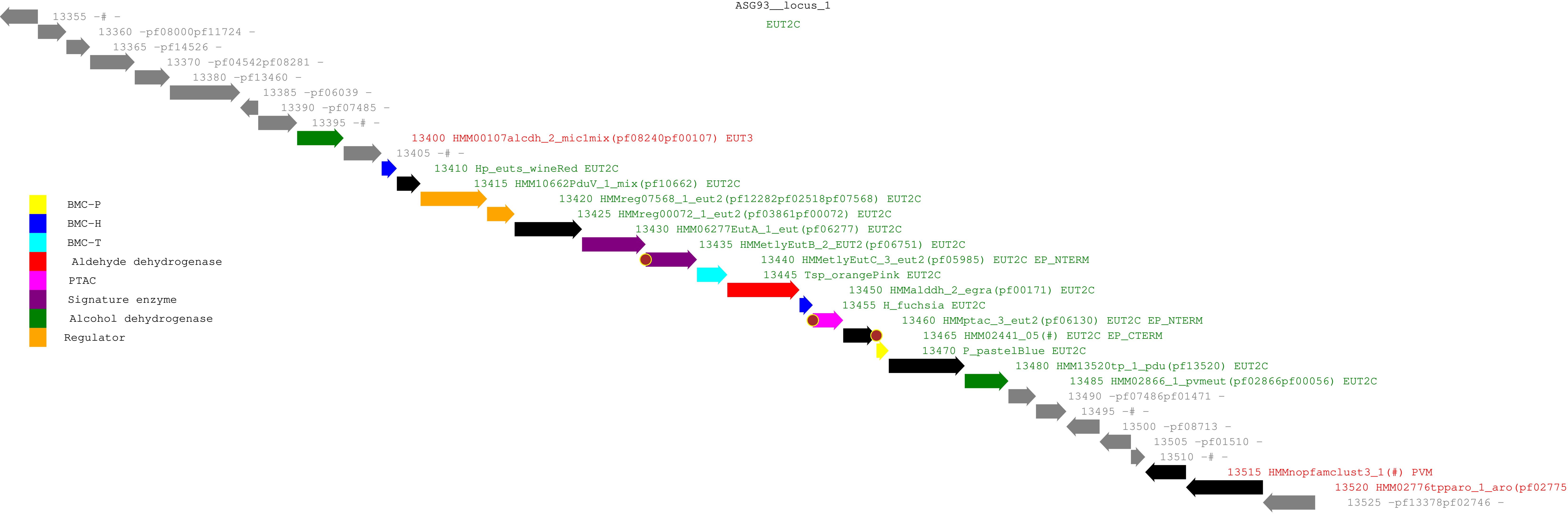

Bacillus sp. Soil531. Bacteria; Firmicutes; Bacilli; Bacillales; Bacillaceae; Bacillus.

ASG98__locus_1

EUT2C

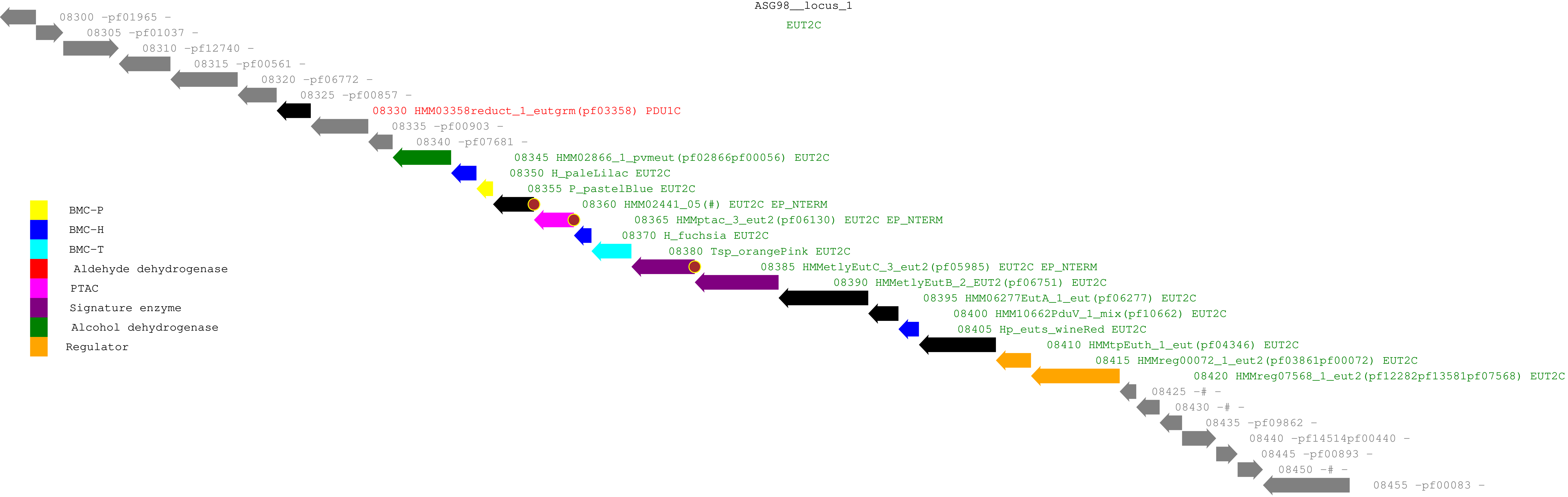

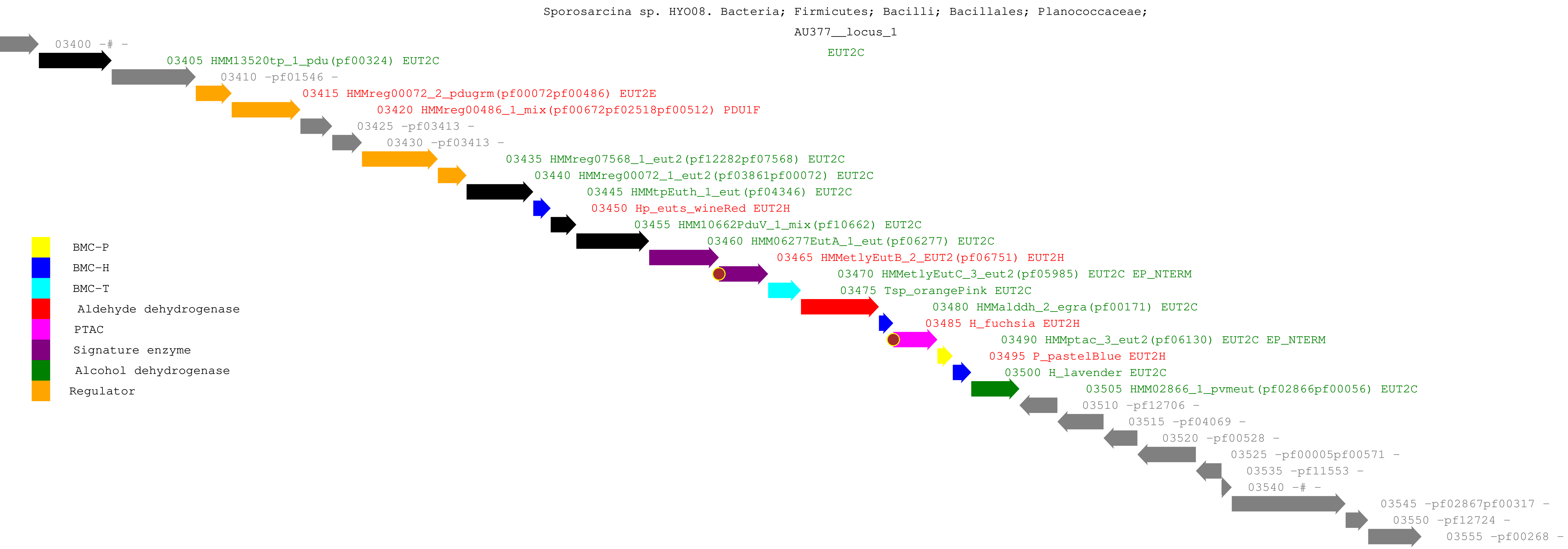

Sporosarcina sp. HYO08. Bacteria; Firmicutes; Bacilli; Bacillales; Planococcaceae;
AU377__locus_1
EUT2C
03400 -# -
03405 HMM13520tp_1_pdu(pf00324) EUT2C
03410 -pf01546 -
03415 HMMreg00072_2_pdugrm(pf00072pf00486) EUT2E
03420 HMMreg00486_1_mix(pf00672pf02518pf00512) PDU1F
03425 -pf03413 -
03430 -pf03413 -
03435 HMMreg07568_1_eut2(pf12282pf07568) EUT2C
03440 HMMreg00072_1_eut2(pf03861pf00072) EUT2C
03445 HMMtpEuth_1_eut(pf04346) EUT2C
03450 Hp_euts_wineRed EUT2H
03455 HMM10662PduV_1_mix(pf10662) EUT2C
03460 HMM06277EutA_1_eut(pf06277) EUT2C
03465 HMMetlyEutB_2_EUT2(pf06751) EUT2H
03470 HMMetlyEutC_3_eut2(pf05985) EUT2C EP_NTERM
03475 Tsp_orangePink EUT2C
03480 HMMalddh_2_egra(pf00171) EUT2C
03485 H_fuchsia EUT2H
03490 HMMptac_3_eut2(pf06130) EUT2C EP_NTERM
03495 P_pastelBlue EUT2H
03500 H_lavender EUT2C
03505 HMM02866_1_pvmeut(pf02866pf00056) EUT2C
03510 -pf12706 -
03515 -pf04069 -
03520 -pf00528 -
03525 -pf00005pf00571 -
03535 -pf11553 -
03540 -# -
03545 -pf02867pf00317 -
03550 -pf12724 -
03555 -pf00268 -
BMC-P
BMC-H
BMC-T
Aldehyde dehydrogenase
PTAC
Signature enzyme
Alcohol dehydrogenase
Regulator

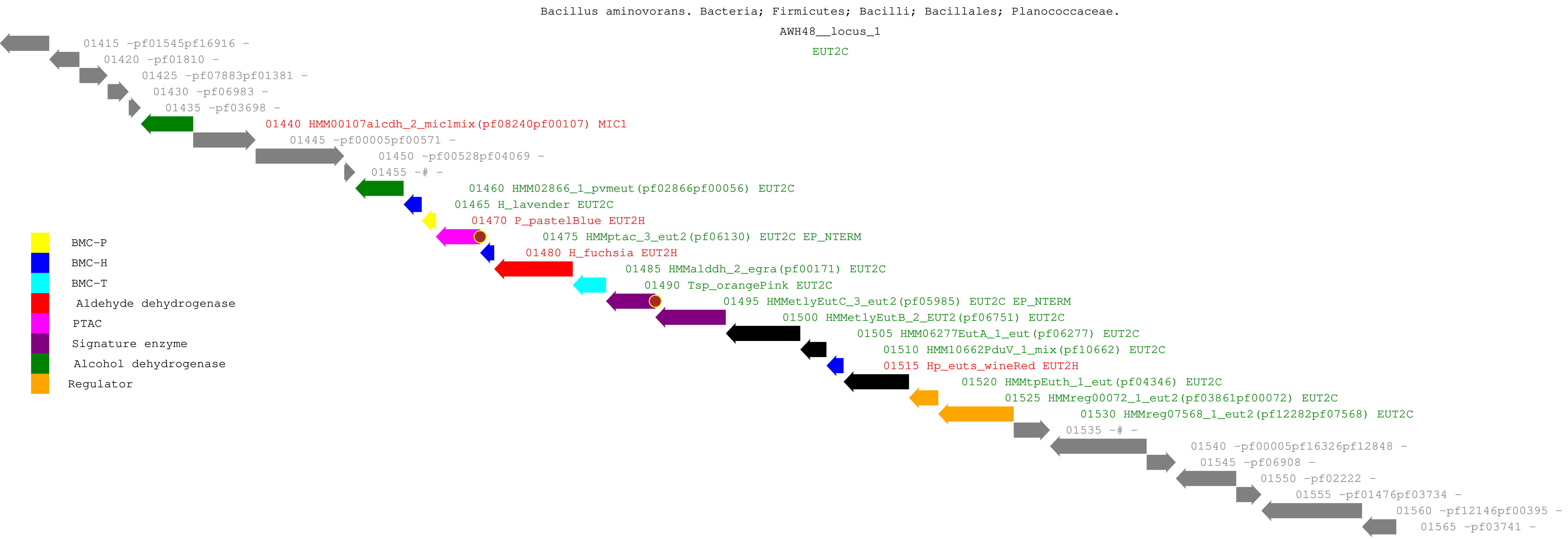
Bacillus aminovorans. Bacteria; Firmicutes; Bacilli; Bacillales; Planococcaceae.
AWH48__locus_1
EUT2C
01415 -pf01545pf16916 -
01420 -pf01810 -
01425 -pf07883pf01381 -
01430 -pf06983 -
01435 -pf03698 -
01440 HMM00107alcdh_2_mic1mix(pf08240pf00107) MIC1
01445 -pf00005pf00571 -
01450 -pf00528pf04069 -
01455 -# -
01460 HMM02866_1_pvmeut(pf02866pf00056) EUT2C
01465 H_lavender EUT2C
01470 P_pastelBlue EUT2H
01475 HMMptac_3_eut2(pf06130) EUT2C EP_NTERM
01480 H_fuchsia EUT2H
01485 HMMalddh_2_egra(pf00171) EUT2C
01490 Tsp_orangePink EUT2C
01495 HMMetlyEutC_3_eut2(pf05985) EUT2C EP_NTERM
01500 HMMetlyEutB_2_EUT2(pf06751) EUT2C
01505 HMM06277EutA_1_eut(pf06277) EUT2C
01510 HMM10662PduV_1_mix(pf10662) EUT2C
01515 Hp_euts_wineRed EUT2H
01520 HMMtpEuth_1_eut(pf04346) EUT2C
01525 HMMreg00072_1_eut2(pf03861pf00072) EUT2C
01530 HMMreg07568_1_eut2(pf12282pf07568) EUT2C
01535 -# -
01540 -pf00005pf16326pf12848 -
01545 -pf06908 -
01550 -pf02222 -
01555 -pf01476pf03734 -
01560 -pf12146pf00395 -
01565 -pf03741 -
BMC-P
BMC-H
BMC-T
Aldehyde dehydrogenase
PTAC
Signature enzyme
Alcohol dehydrogenase
Regulator

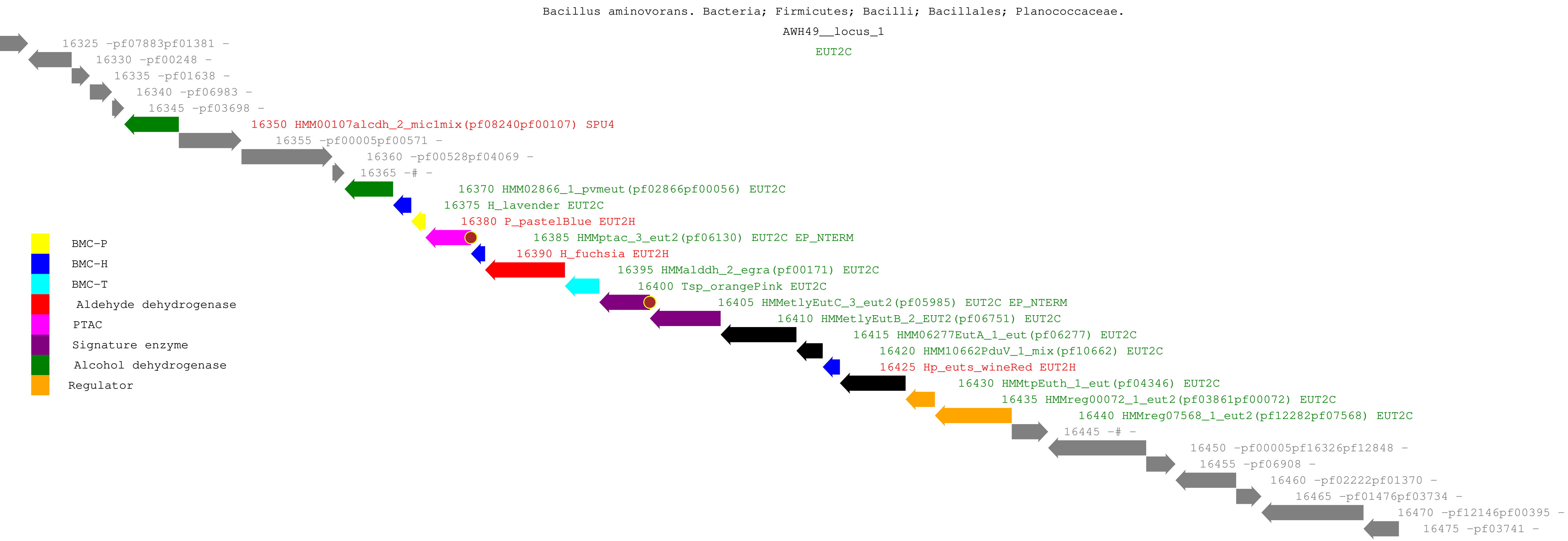

Bacillus aminovorans. Bacteria; Firmicutes; Bacilli; Bacillales; Planococcaceae.
AWH49__locus_1
EUT2C
16325 -pf07883pf01381 -
16330 -pf00248 -
16335 -pf01638 -
16340 -pf06983 -
16345 -pf03698 -
16350 HMM00107alcdh_2_mic1mix(pf08240pf00107) SPU4
16355 -pf00005pf00571 -
16360 -pf00528pf04069 -
16365 -# -
16370 HMM02866_1_pvmeut(pf02866pf00056) EUT2C
16375 H_lavender EUT2C
16380 P_pastelBlue EUT2H
16385 HMMptac_3_eut2(pf06130) EUT2C EP_NTERM
16390 H_fuchsia EUT2H
16395 HMMaldh_2_egra(pf00171) EUT2C
16400 Tsp_orangePink EUT2C
16405 HMMetlyEutC_3_eut2(pf05985) EUT2C EP_NTERM
16410 HMMetlyEutB_2_EUT2(pf06751) EUT2C
16415 HMM06277EutA_1_eut(pf06277) EUT2C
16420 HMM10662PduV_1_mix(pf10662) EUT2C
16425 Hp_euts_wineRed EUT2H
16430 HMMtpEuth_1_eut(pf04346) EUT2C
16435 HMMreg00072_1_eut2(pf03861pf00072) EUT2C
16440 HMMreg07568_1_eut2(pf12282pf07568) EUT2C
16445 -# -
16450 -pf00005pf16326pf12848 -
16455 -pf06908 -
16460 -pf02222pf01370 -
16465 -pf01476pf03734 -
16470 -pf12146pf00395 -
16475 -pf03741 -
BMC-P
BMC-H
BMC-T
Aldehyde dehydrogenase
PTAC
Signature enzyme
Alcohol dehydrogenase
Regulator

Sporosarcina psychrophila (Bacillus psychrophilus). Bacteria; Firmicutes; Bacilli; Bacillales; Planococcaceae;

AZE41__locus_1

EUT2C

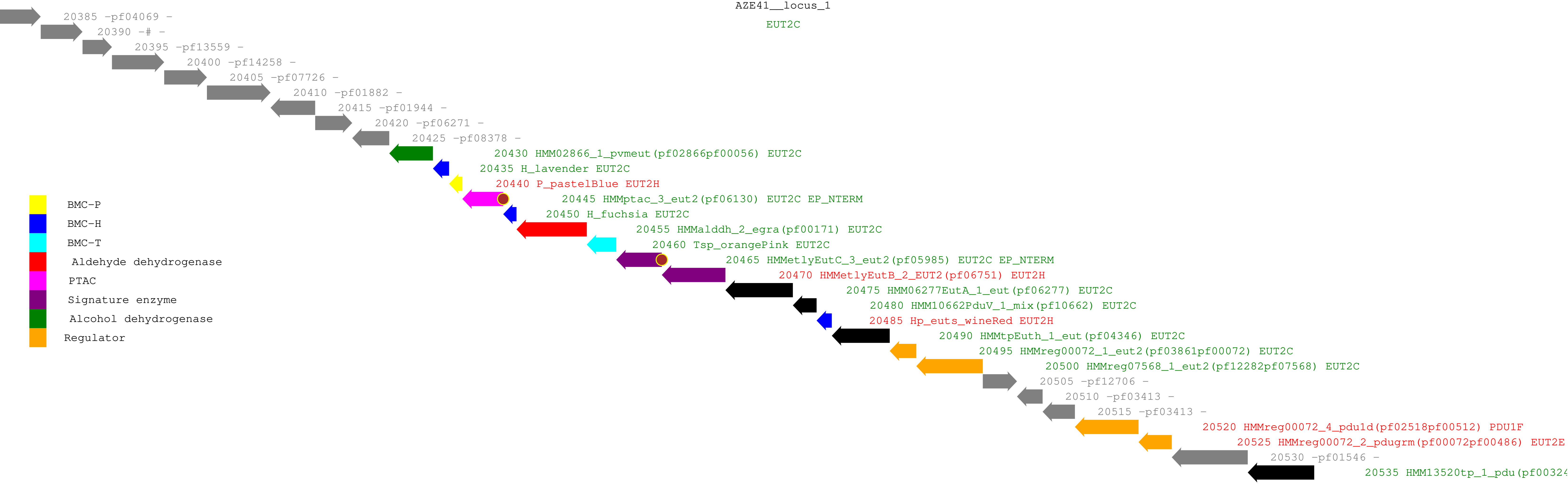

Bacillus asahii. Bacteria; Firmicutes; Bacilli; Bacillales; Bacillaceae; Bacillus.

BAOM__locus_1

EUT2C

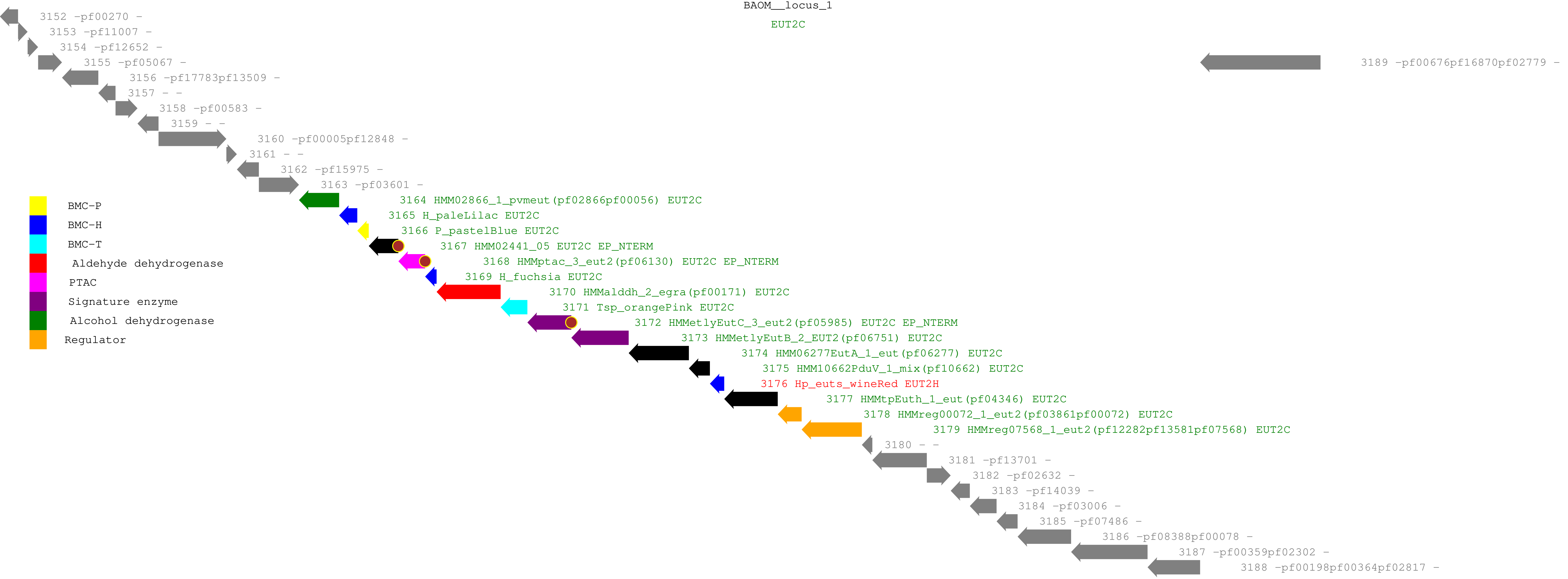

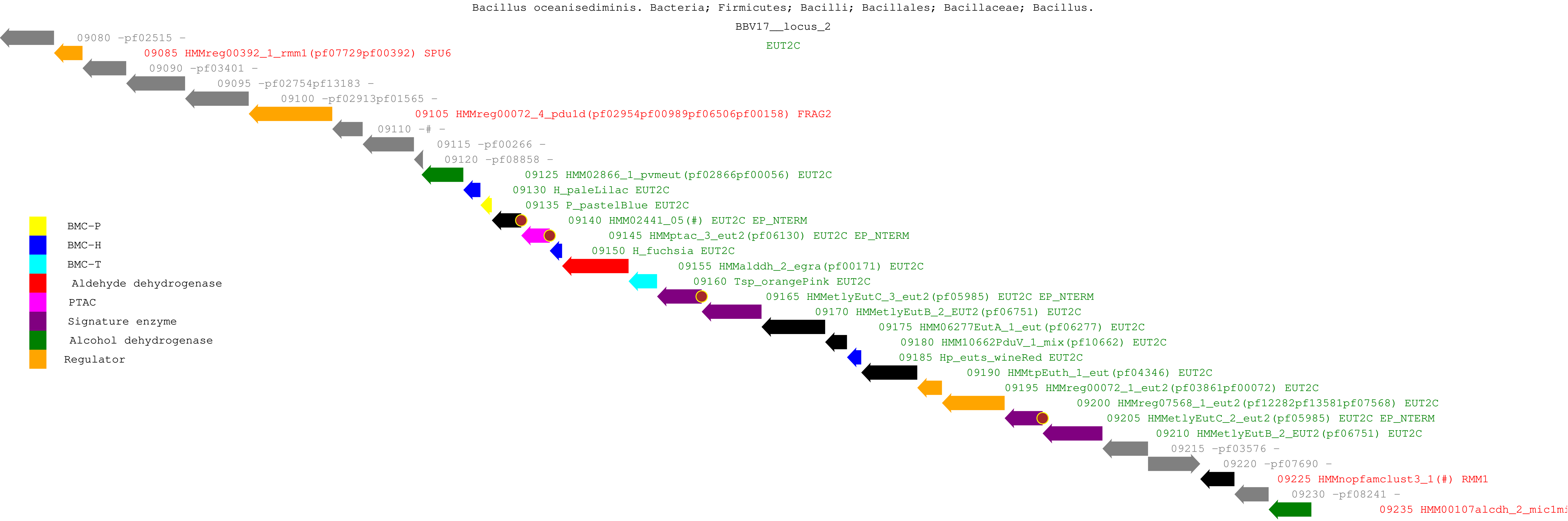

Bacillus oceanisediminis. Bacteria; Firmicutes; Bacilli; Bacillales; Bacillaceae; Bacillus.
BBV17__locus_2
EUT2C
09080 -pf02515 -
09085 HMMreg00392_1_rmm1(pf07729pf00392) SPU6
09090 -pf03401 -
09095 -pf02754pf13183 -
09100 -pf02913pf01565 -
09105 HMMreg00072_4_pdu1d(pf02954pf00989pf06506pf00158) FRAG2
09110 -# -
09115 -pf00266 -
09120 -pf08858 -
09125 HMM02866_1_pvmeut(pf02866pf00056) EUT2C
09130 H_paleLilac EUT2C
09135 P_pastelBlue EUT2C
09140 HMM02441_05(#) EUT2C EP_NTERM
09145 HMMptac_3_eut2(pf06130) EUT2C EP_NTERM
09150 H_fuchsia EUT2C
09155 HMMalddh_2_egra(pf00171) EUT2C
09160 Tsp_orangePink EUT2C
09165 HMMetlyEutC_3_eut2(pf05985) EUT2C EP_NTERM
09170 HMMetlyEutB_2_EUT2(pf06751) EUT2C
09175 HMM06277EutA_1_eut(pf06277) EUT2C
09180 HMM10662PduV_1_mix(pf10662) EUT2C
09185 Hp_euts_wineRed EUT2C
09190 HMMtpEuth_1_eut(pf04346) EUT2C
09195 HMMreg00072_1_eut2(pf03861pf00072) EUT2C
09200 HMMreg07568_1_eut2(pf12282pf13581pf07568) EUT2C
09205 HMMetlyEutC_2_eut2(pf05985) EUT2C EP_NTERM
09210 HMMetlyEutB_2_EUT2(pf06751) EUT2C
09215 -pf03576 -
09220 -pf07690 -
09225 HMMnopfamclust3_1(#) RMM1
09230 -pf08241 -
09235 HMM00107alcdh_2_mic1mi
BMC-P
BMC-H
BMC-T
Aldehyde dehydrogenase
PTAC
Signature enzyme
Alcohol dehydrogenase
Regulator

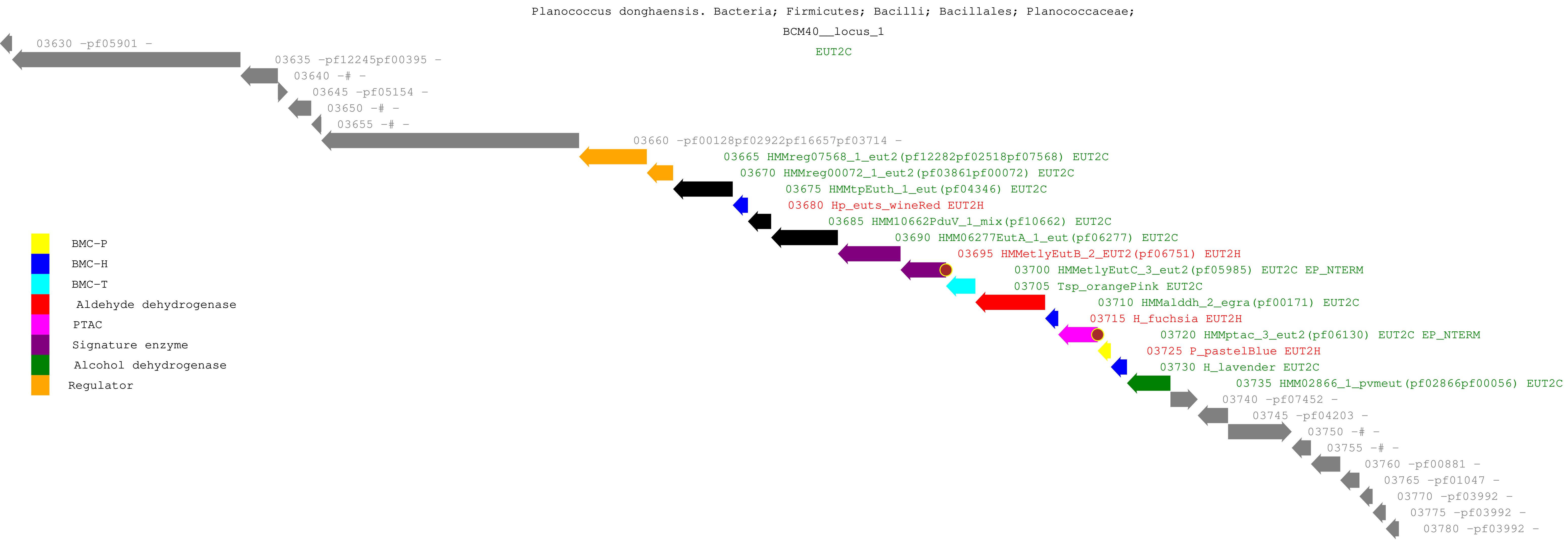

Planococcus donghaensis. Bacteria; Firmicutes; Bacilli; Bacillales; Planococcaceae;
BCM40__locus_1
EUT2C
03630 -pf05901 -
03635 -pf12245pf00395 -
03640 -# -
03645 -pf05154 -
03650 -# -
03655 -# -
03660 -pf00128pf02922pf16657pf03714 -
03665 HMMreg07568_1_eut2(pf12282pf02518pf07568) EUT2C
03670 HMMreg00072_1_eut2(pf03861pf00072) EUT2C
03675 HMMtpEuth_1_eut(pf04346) EUT2C
03680 Hp_euts_wineRed EUT2H
03685 HMM10662PduV_1_mix(pf10662) EUT2C
03690 HMM06277EutA_1_eut(pf06277) EUT2C
03695 HMMetlyEutB_2_EUT2(pf06751) EUT2H
03700 HMMetlyEutC_3_eut2(pf05985) EUT2C EP_NTERM
03705 Tsp_orangePink EUT2C
03710 HMMalddh_2_egra(pf00171) EUT2C
03715 H_fuchsia EUT2H
03720 HMMptac_3_eut2(pf06130) EUT2C EP_NTERM
03725 P_pastelBlue EUT2H
03730 H_lavender EUT2C
03735 HMM02866_1_pvmeut(pf02866pf00056) EUT2C
03740 -pf07452 -
03745 -pf04203 -
03750 -# -
03755 -# -
03760 -pf00881 -
03765 -pf01047 -
03770 -pf03992 -
03775 -pf03992 -
03780 -pf03992 -
BMC-P
BMC-H
BMC-T
Aldehyde dehydrogenase
PTAC
Signature enzyme
Alcohol dehydrogenase
Regulator

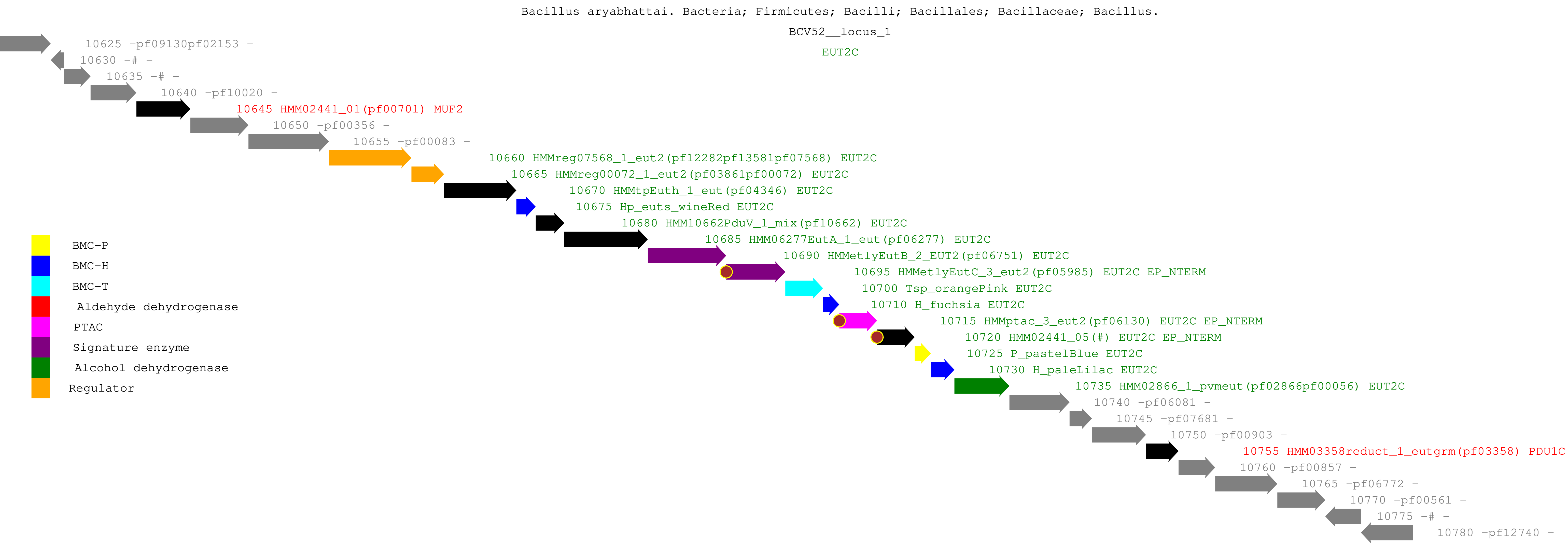

Bacillus aryabhattai. Bacteria; Firmicutes; Bacilli; Bacillales; Bacillaceae; Bacillus.
BCV52__locus_1
EUT2C
10625 -pf09130pf02153 -
10630 -# -
10635 -# -
10640 -pf10020 -
10645 HMM02441_01(pf00701) MUF2
10650 -pf00356 -
10655 -pf00083 -
10660 HMMreg07568_1_eut2(pf12282pf13581pf07568) EUT2C
10665 HMMreg00072_1_eut2(pf03861pf00072) EUT2C
10670 HMMtpEuth_1_eut(pf04346) EUT2C
10675 Hp_euts_wineRed EUT2C
10680 HMM10662PduV_1_mix(pf10662) EUT2C
10685 HMM06277EutA_1_eut(pf06277) EUT2C
10690 HMMetlyEutB_2_EUT2(pf06751) EUT2C
10695 HMMetlyEutC_3_eut2(pf05985) EUT2C EP_NTERM
10700 Tsp_orangePink EUT2C
10710 H_fuchsia EUT2C
10715 HMMptac_3_eut2(pf06130) EUT2C EP_NTERM
10720 HMM02441_05(#) EUT2C EP_NTERM
10725 P_pastelBlue EUT2C
10730 H_paleLilac EUT2C
10735 HMM02866_1_pvmeut(pf02866pf00056) EUT2C
10740 -pf06081 -
10745 -pf07681 -
10750 -pf00903 -
10755 HMM03358reduct_1_eutgrm(pf03358) PDU1C
10760 -pf00857 -
10765 -pf06772 -
10770 -pf00561 -
10775 -# -
10780 -pf12740 -
BMC-P
BMC-H
BMC-T
Aldehyde dehydrogenase
PTAC
Signature enzyme
Alcohol dehydrogenase
Regulator

Bacillus aquimaris. Bacteria; Firmicutes; Bacilli; Bacillales; Bacillaceae; Bacillus.

BHE18__locus_1

EUT2C

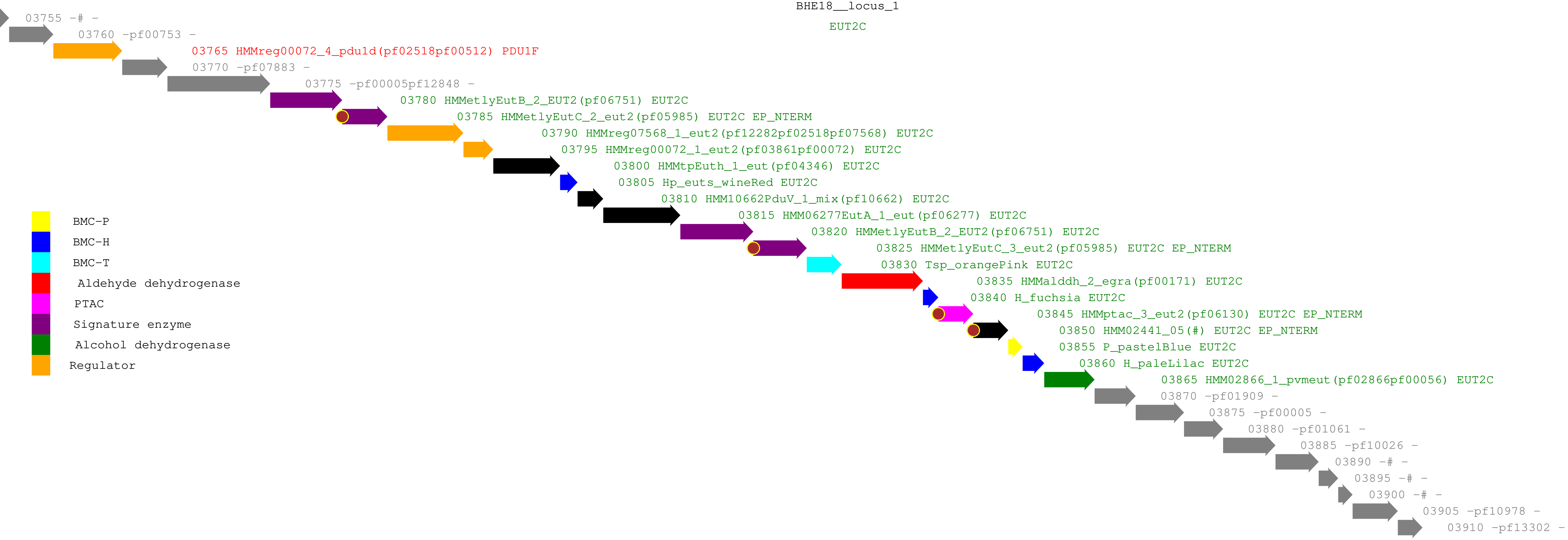

Bacillus sp. MUM 116. Bacteria; Firmicutes; Bacilli; Bacillales; Bacillaceae; Bacillus.

BIV60__locus_3

EUT2C

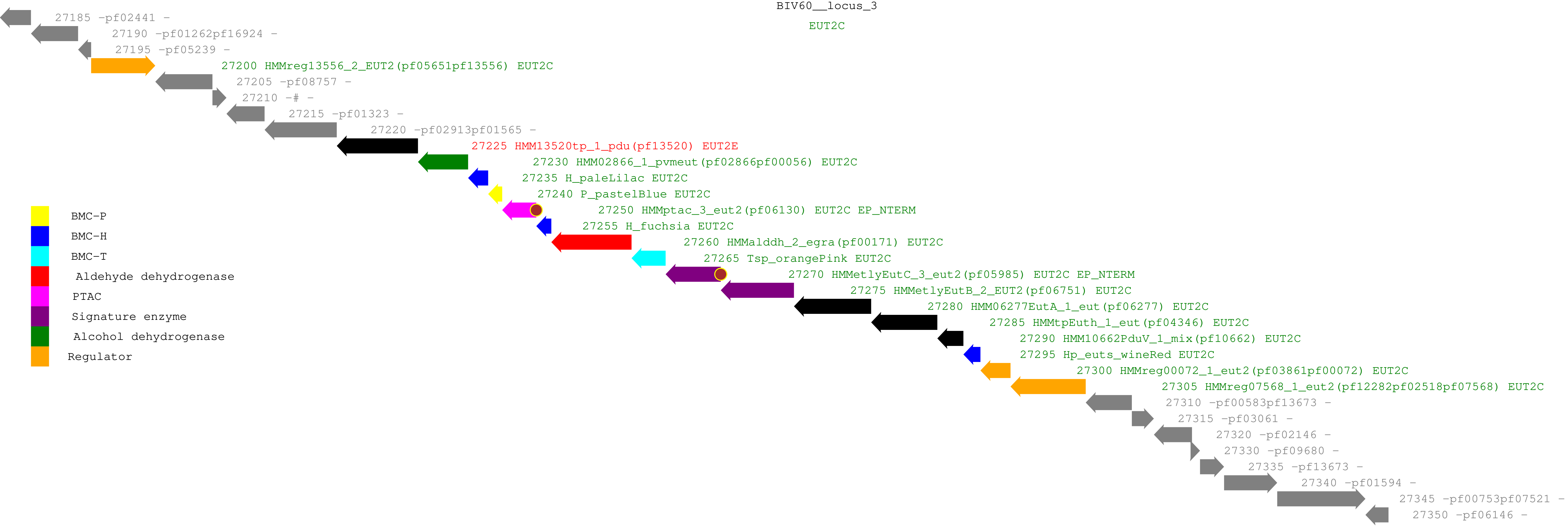

Bacillus megaterium (strain DSM 319). Bacteria; Firmicutes; Bacilli; Bacillales; Bacillaceae; Bacillus.

BMD__locus_1

EUT2C

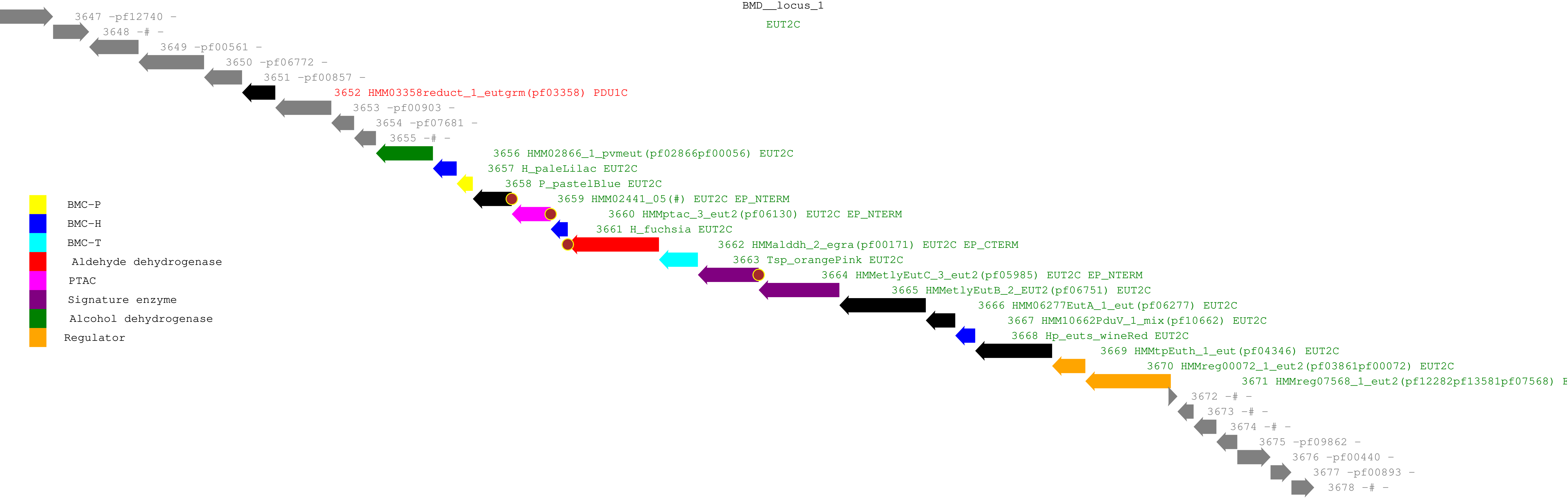

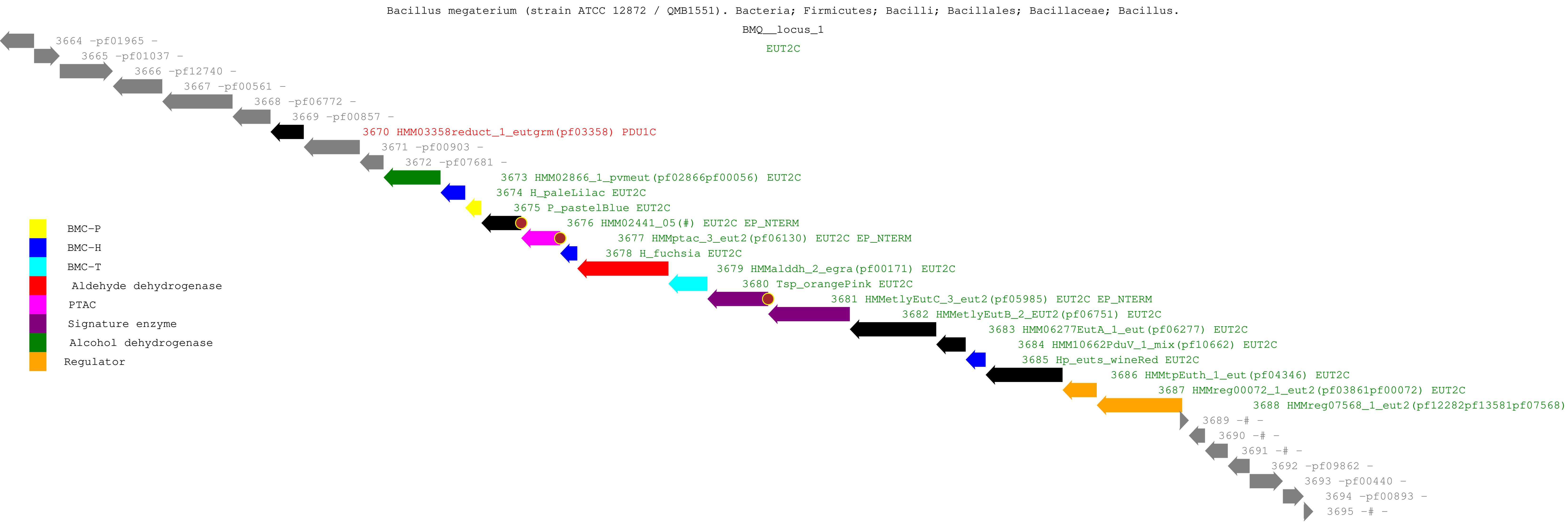
Bacillus megaterium (strain ATCC 12872 / QMB1551). Bacteria; Firmicutes; Bacilli; Bacillales; Bacillaceae; Bacillus.
BMQ__locus_1
EUT2C
3664 -pf01965 -
3665 -pf01037 -
3666 -pf12740 -
3667 -pf00561 -
3668 -pf06772 -
3669 -pf00857 -
3670 HMM03358reduct_1_eutgrm(pf03358) PDU1C
3671 -pf00903 -
3672 -pf07681 -
3673 HMM02866_1_pvmeut(pf02866pf00056) EUT2C
3674 H_paleLilac EUT2C
3675 P_pastelBlue EUT2C
3676 HMM02441_05(#) EUT2C EP_NTERM
3677 HMMptac_3_eut2(pf06130) EUT2C EP_NTERM
3678 H_fuchsia EUT2C
3679 HMMalddh_2_egra(pf00171) EUT2C
3680 Tsp_orangePink EUT2C
3681 HMMetlyEutC_3_eut2(pf05985) EUT2C EP_NTERM
3682 HMMetlyEutB_2_EUT2(pf06751) EUT2C
3683 HMM06277EutA_1_eut(pf06277) EUT2C
3684 HMM10662PduV_1_mix(pf10662) EUT2C
3685 Hp_euts_wineRed EUT2C
3686 HMMtpEuth_1_eut(pf04346) EUT2C
3687 HMMreg00072_1_eut2(pf03861pf00072) EUT2C
3688 HMMreg07568_1_eut2(pf12282pf13581pf07568)
3689 -# -
3690 -# -
3691 -# -
3692 -pf09862 -
3693 -pf00440 -
3694 -pf00893 -
3695 -# -
BMC-P
BMC-H
BMC-T
Aldehyde dehydrogenase
PTAC
Signature enzyme
Alcohol dehydrogenase
Regulator

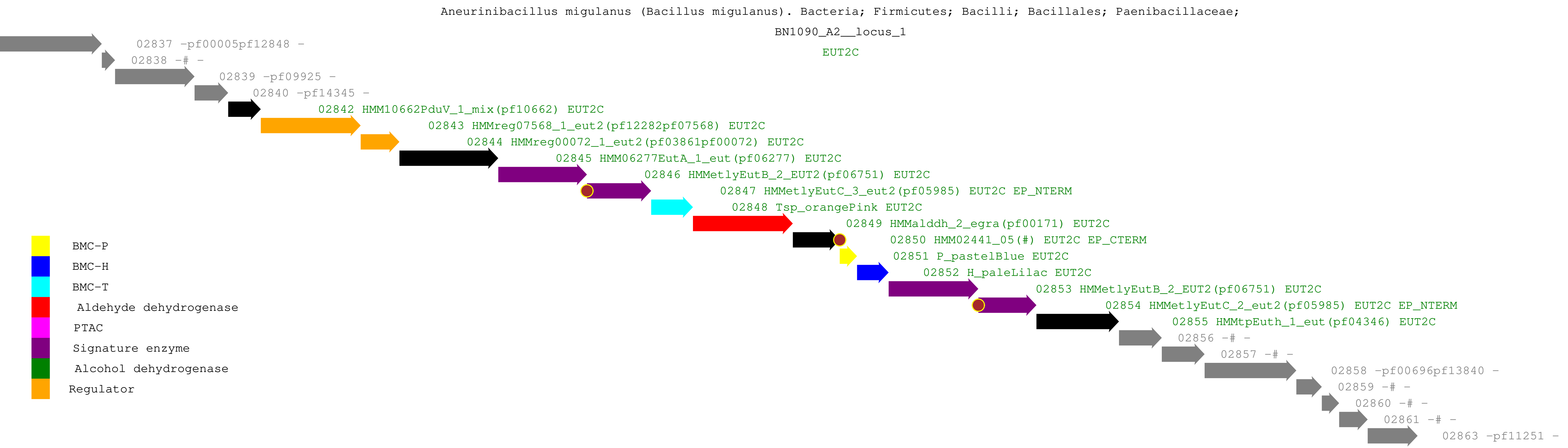
Aneurinibacillus migulanus (Bacillus migulanus). Bacteria; Firmicutes; Bacilli; Bacillales; Paenibacillaceae;
BN1090_A2__locus_1
EUT2C
02837 -pf00005pf12848 -
02838 -# -
02839 -pf09925 -
02840 -pf14345 -
02842 HMM10662PduV_1_mix(pf10662) EUT2C
02843 HMMreg07568_1_eut2(pf12282pf07568) EUT2C
02844 HMMreg00072_1_eut2(pf03861pf00072) EUT2C
02845 HMM06277EutA_1_eut(pf06277) EUT2C
02846 HMMetlyEutB_2_EUT2(pf06751) EUT2C
02847 HMMetlyEutC_3_eut2(pf05985) EUT2C EP_NTERM
02848 Tsp_orangePink EUT2C
02849 HMMalddh_2_egra(pf00171) EUT2C
02850 HMM02441_05(#) EUT2C EP_CTERM
02851 P_pastelBlue EUT2C
02852 H_paleLilac EUT2C
02853 HMMetlyEutB_2_EUT2(pf06751) EUT2C
02854 HMMetlyEutC_2_eut2(pf05985) EUT2C EP_NTERM
02855 HMMtpEuth_1_eut(pf04346) EUT2C
02856 -# -
02857 -# -
02858 -pf00696pf13840 -
02859 -# -
02860 -# -
02861 -# -
02863 -pf11251 -
BMC-P
BMC-H
BMC-T
Aldehyde dehydrogenase
PTAC
Signature enzyme
Alcohol dehydrogenase
Regulator

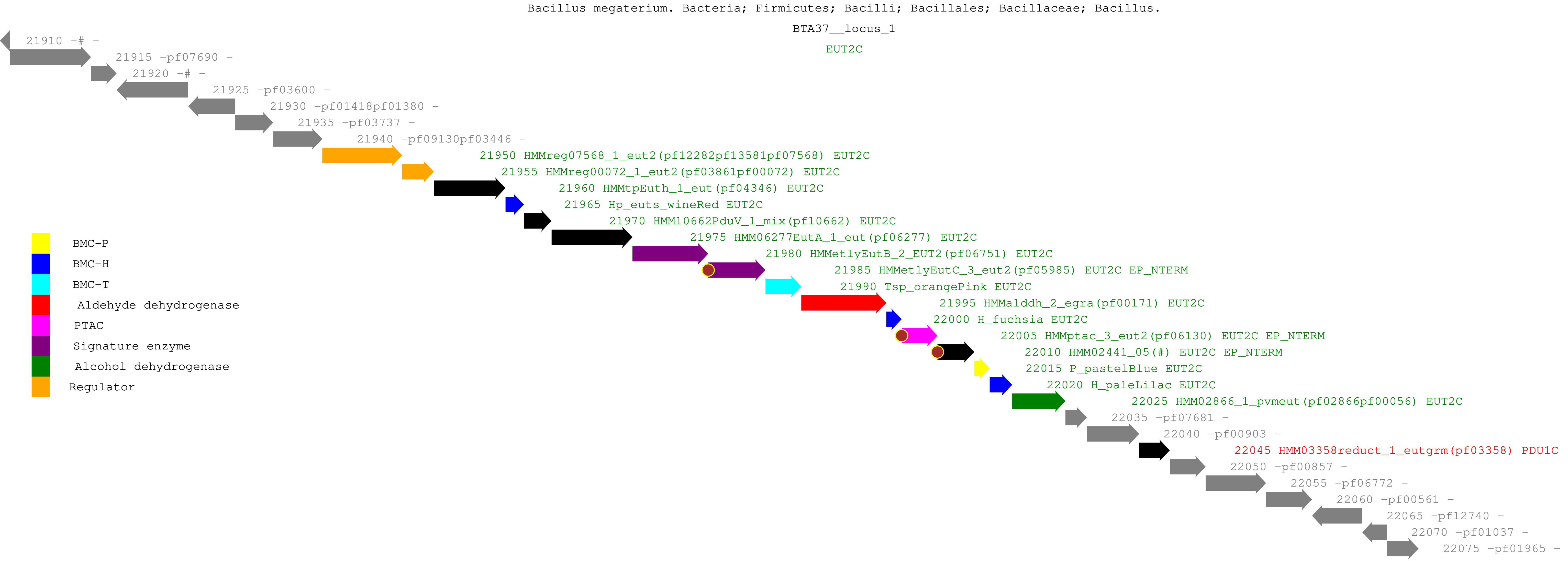

Bacillus megaterium. Bacteria; Firmicutes; Bacilli; Bacillales; Bacillaceae; Bacillus.
BTA37__locus_1
EUT2C
21910 -# -
21915 -pf07690 -
21920 -# -
21925 -pf03600 -
21930 -pf01418pf01380 -
21935 -pf03737 -
21940 -pf09130pf03446 -
21950 HMMreg07568_1_eut2(pf12282pf13581pf07568) EUT2C
21955 HMMreg00072_1_eut2(pf03861pf00072) EUT2C
21960 HMMtpEuth_1_eut(pf04346) EUT2C
21965 Hp_euts_wineRed EUT2C
21970 HMM10662PduV_1_mix(pf10662) EUT2C
21975 HMM06277EutA_1_eut(pf06277) EUT2C
21980 HMMetlyEutB_2_EUT2(pf06751) EUT2C
21985 HMMetlyEutC_3_eut2(pf05985) EUT2C EP_NTERM
21990 Tsp_orangePink EUT2C
21995 HMMaldh_2_egra(pf00171) EUT2C
22000 H_fuchsia EUT2C
22005 HMMptac_3_eut2(pf06130) EUT2C EP_NTERM
22010 HMM02441_05(#) EUT2C EP_NTERM
22015 P_pastelBlue EUT2C
22020 H_paleLilac EUT2C
22025 HMM02866_1_pvmeut(pf02866pf00056) EUT2C
22035 -pf07681 -
22040 -pf00903 -
22045 HMM03358reduct_1_eutgrm(pf03358) PDU1C
22050 -pf00857 -
22055 -pf06772 -
22060 -pf00561 -
22065 -pf12740 -
22070 -pf01037 -
22075 -pf01965 -
BMC-P
BMC-H
BMC-T
Aldehyde dehydrogenase
PTAC
Signature enzyme
Alcohol dehydrogenase
Regulator

Bacillus megaterium. Bacteria; Firmicutes; Bacilli; Bacillales; Bacillaceae; Bacillus.

C2I27__locus_1

EUT2C

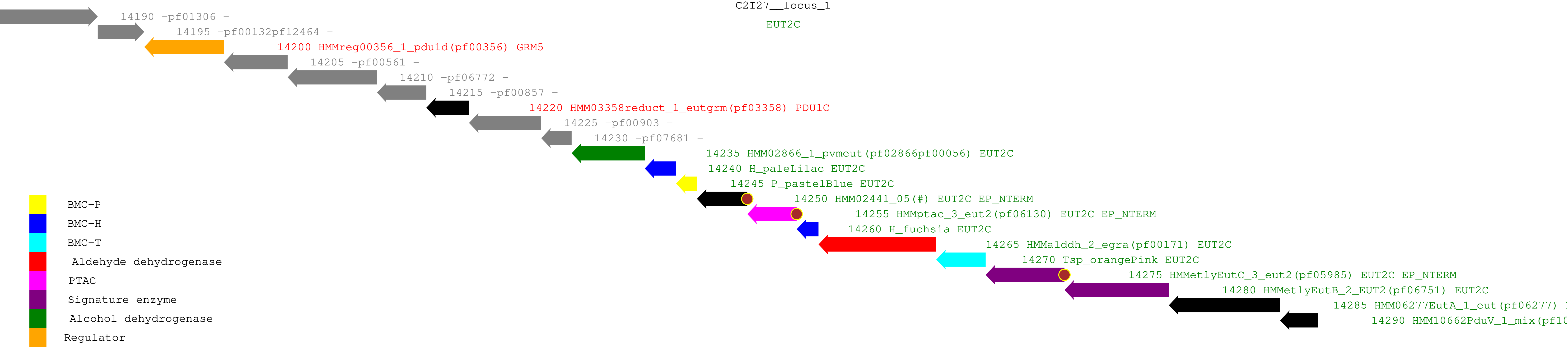

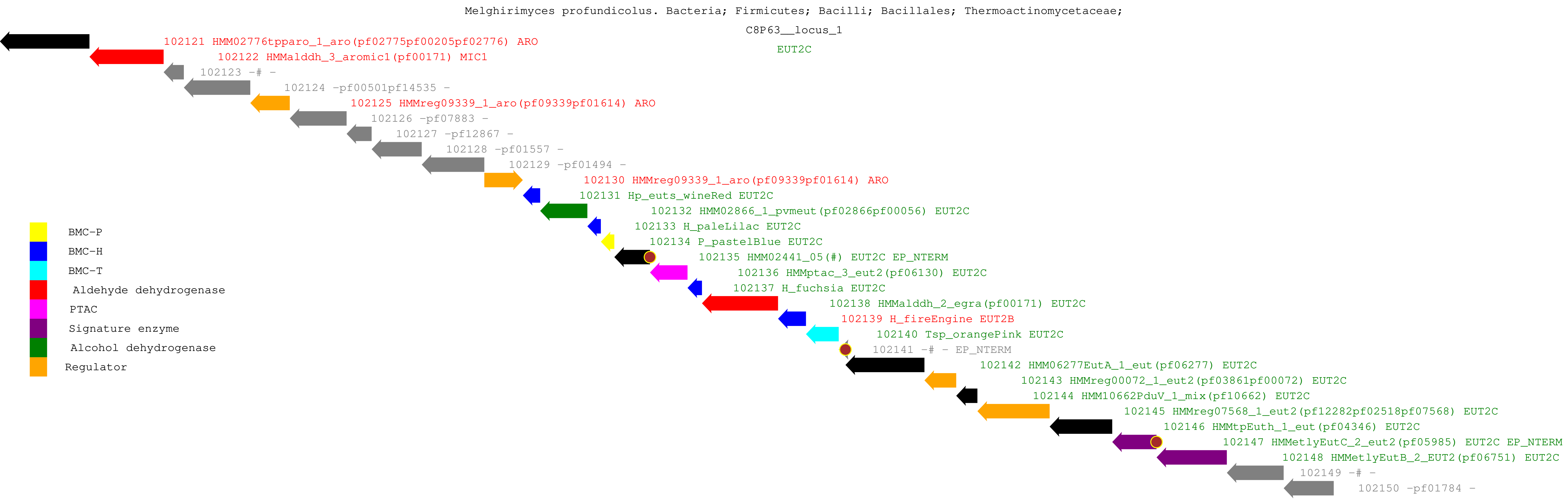

Melghirimyces profundicolus. Bacteria; Firmicutes; Bacilli; Bacillales; Thermoactinomycetaceae;
C8P63__locus_1
EUT2C
102121 HMM02776tpparo_1_aro(pf02775pf00205pf02776) ARO
102122 HMMalddh_3_aromic1(pf00171) MIC1
102123 -# -
102124 -pf00501pf14535 -
102125 HMMreg09339_1_aro(pf09339pf01614) ARO
102126 -pf07883 -
102127 -pf12867 -
102128 -pf01557 -
102129 -pf01494 -
102130 HMMreg09339_1_aro(pf09339pf01614) ARO
102131 Hp_euts_wineRed EUT2C
102132 HMM02866_1_pvmeut(pf02866pf00056) EUT2C
102133 H_paleLilac EUT2C
102134 P_pastelBlue EUT2C
102135 HMM02441_05(#) EUT2C EP_NTERM
102136 HMMptac_3_eut2(pf06130) EUT2C
102137 H_fuchsia EUT2C
102138 HMMalddh_2_egra(pf00171) EUT2C
102139 H_fireEngine EUT2B
102140 Tsp_orangePink EUT2C
102141 -# - EP_NTERM
102142 HMM06277EutA_1_eut(pf06277) EUT2C
102143 HMMreg00072_1_eut2(pf03861pf00072) EUT2C
102144 HMM10662PduV_1_mix(pf10662) EUT2C
102145 HMMreg07568_1_eut2(pf12282pf02518pf07568) EUT2C
102146 HMMtpEuth_1_eut(pf04346) EUT2C
102147 HMMetlyEutC_2_eut2(pf05985) EUT2C EP_NTERM
102148 HMMetlyEutB_2_EUT2(pf06751) EUT2C
102149 -# -
102150 -pf01784 -
BMC-P
BMC-H
BMC-T
Aldehyde dehydrogenase
PTAC
Signature enzyme
Alcohol dehydrogenase
Regulator

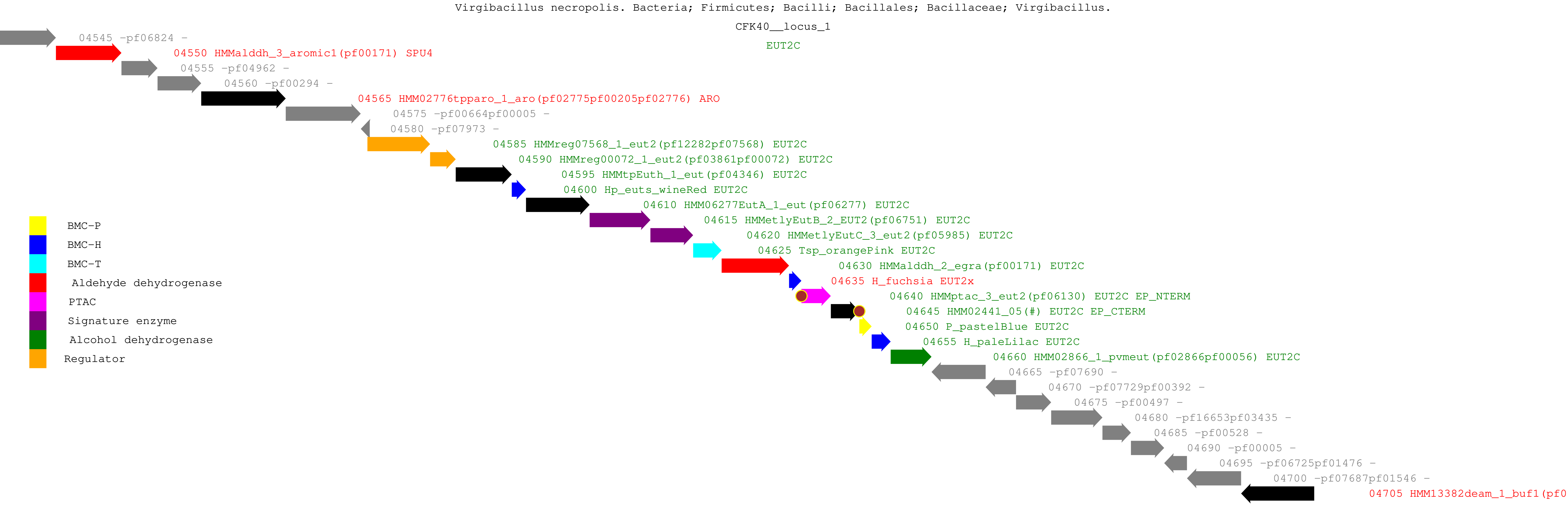

Virgibacillus necropolis. Bacteria; Firmicutes; Bacilli; Bacillales; Bacillaceae; Virgibacillus.
CFK40__locus_1
EUT2C
04545 -pf06824 -
04550 HMMalddh_3_aromic1(pf00171) SPU4
04555 -pf04962 -
04560 -pf00294 -
04565 HMM02776tpparo_1_aro(pf02775pf00205pf02776) ARO
04575 -pf00664pf00005 -
04580 -pf07973 -
04585 HMMreg07568_1_eut2(pf12282pf07568) EUT2C
04590 HMMreg00072_1_eut2(pf03861pf00072) EUT2C
04595 HMMtpEuth_1_eut(pf04346) EUT2C
04600 Hp_euts_wineRed EUT2C
04610 HMM06277EutA_1_eut(pf06277) EUT2C
04615 HMMetlyEutB_2_EUT2(pf06751) EUT2C
04620 HMMetlyEutC_3_eut2(pf05985) EUT2C
04625 Tsp_orangePink EUT2C
04630 HMMalddh_2_egra(pf00171) EUT2C
04635 H_fuchsia EUT2x
04640 HMMptac_3_eut2(pf06130) EUT2C EP_NTERM
04645 HMM02441_05(#) EUT2C EP_CTERM
04650 P_pastelBlue EUT2C
04655 H_paleLilac EUT2C
04660 HMM02866_1_pvmeut(pf02866pf00056) EUT2C
04665 -pf07690 -
04670 -pf07729pf00392 -
04675 -pf00497 -
04680 -pf16653pf03435 -
04685 -pf00528 -
04690 -pf00005 -
04695 -pf06725pf01476 -
04700 -pf07687pf01546 -
04705 HMM13382deam_1_buf1(pf0
BMC-P
BMC-H
BMC-T
Aldehyde dehydrogenase
PTAC
Signature enzyme
Alcohol dehydrogenase
Regulator

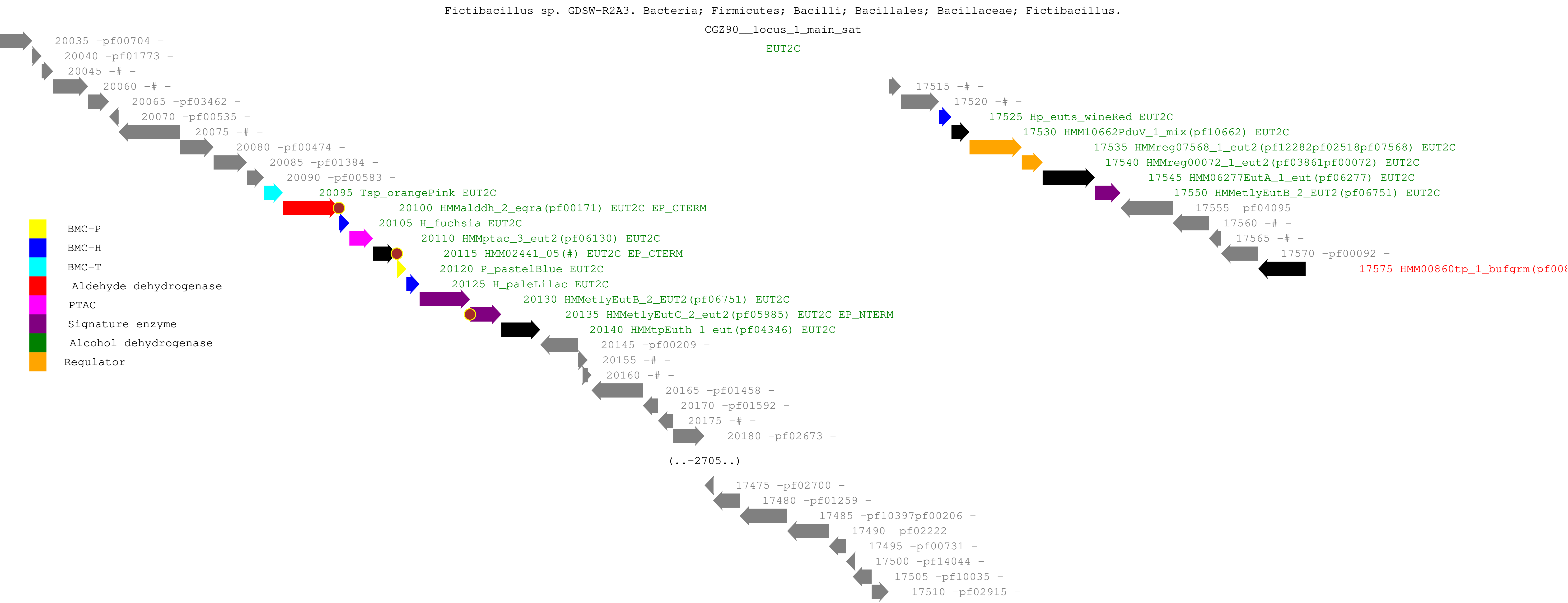

Fictibacillus sp. GDSW-R2A3. Bacteria; Firmicutes; Bacilli; Bacillales; Bacillaceae; Fictibacillus.
CGZ90__locus_1_main_sat
EUT2C
20035 -pf00704 -
20040 -pf01773 -
20045 -# -
20060 -# -
20065 -pf03462 -
20070 -pf00535 -
20075 -# -
20080 -pf00474 -
20085 -pf01384 -
20090 -pf00583 -
20095 Tsp_orangePink EUT2C
20100 HMMalddh_2_egra(pf00171) EUT2C EP_CTERM
20105 H_fuchsia EUT2C
20110 HMMptac_3_eut2(pf06130) EUT2C
20115 HMM02441_05(#) EUT2C EP_CTERM
20120 P_pastelBlue EUT2C
20125 H_paleLilac EUT2C
20130 HMMetlyEutB_2_EUT2(pf06751) EUT2C
20135 HMMetlyEutC_2_eut2(pf05985) EUT2C EP_NTERM
20140 HMMtpEuth_1_eut(pf04346) EUT2C
20145 -pf00209 -
20155 -# -
20160 -# -
20165 -pf01458 -
20170 -pf01592 -
20175 -# -
20180 -pf02673 -
(..-2705..)
17475 -pf02700 -
17480 -pf01259 -
17485 -pf10397pf00206 -
17490 -pf02222 -
17495 -pf00731 -
17500 -pf14044 -
17505 -pf10035 -
17510 -pf02915 -
17515 -# -
17520 -# -
17525 Hp_euts_wineRed EUT2C
17530 HMM10662PduV_1_mix(pf10662) EUT2C
17535 HMMreg07568_1_eut2(pf12282pf02518pf07568) EUT2C
17540 HMMreg00072_1_eut2(pf03861pf00072) EUT2C
17545 HMM06277EutA_1_eut(pf06277) EUT2C
17550 HMMetlyEutB_2_EUT2(pf06751) EUT2C
17555 -pf04095 -
17560 -# -
17565 -# -
17570 -pf00092 -
17575 HMM00860tp_1_bufgrm(pf008
BMC-P
BMC-H
BMC-T
Aldehyde dehydrogenase
PTAC
Signature enzyme
Alcohol dehydrogenase
Regulator

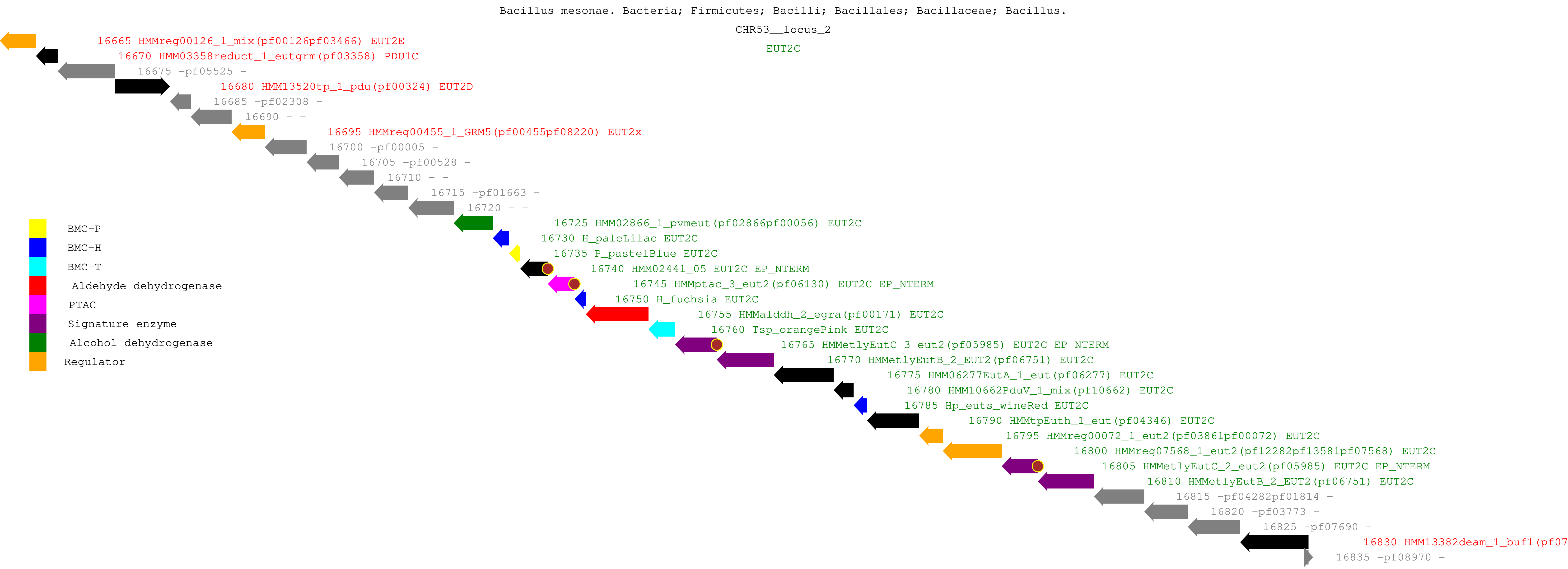
Bacillus mesonae. Bacteria; Firmicutes; Bacilli; Bacillales; Bacillaceae; Bacillus.
CHR53__locus_2
EUT2C
16665 HMMreg00126_1_mix(pf00126pf03466) EUT2E
16670 HMM03358reduct_1_eutgrm(pf03358) PDU1C
16675 -pf05525 -
16680 HMM13520tp_1_pdu(pf00324) EUT2D
16685 -pf02308 -
16690 - -
16695 HMMreg00455_1_GRM5(pf00455pf08220) EUT2x
16700 -pf00005 -
16705 -pf00528 -
16710 - -
16715 -pf01663 -
16720 - -
16725 HMM02866_1_pvmeut(pf02866pf00056) EUT2C
16730 H_paleLilac EUT2C
16735 P_pastelBlue EUT2C
16740 HMM02441_05 EUT2C EP_NTERM
16745 HMMptac_3_eut2(pf06130) EUT2C EP_NTERM
16750 H_fuchsia EUT2C
16755 HMMalddh_2_egra(pf00171) EUT2C
16760 Tsp_orangePink EUT2C
16765 HMMetlyEutC_3_eut2(pf05985) EUT2C EP_NTERM
16770 HMMetlyEutB_2_EUT2(pf06751) EUT2C
16775 HMM06277EutA_1_eut(pf06277) EUT2C
16780 HMM10662PduV_1_mix(pf10662) EUT2C
16785 Hp_euts_wineRed EUT2C
16790 HMMtpEuth_1_eut(pf04346) EUT2C
16795 HMMreg00072_1_eut2(pf03861pf00072) EUT2C
16800 HMMreg07568_1_eut2(pf12282pf13581pf07568) EUT2C
16805 HMMetlyEutC_2_eut2(pf05985) EUT2C EP_NTERM
16810 HMMetlyEutB_2_EUT2(pf06751) EUT2C
16815 -pf04282pf01814 -
16820 -pf03773 -
16825 -pf07690 -
16830 HMM13382deam_1_buf1(pf07
16835 -pf08970 -
BMC-P
BMC-H
BMC-T
Aldehyde dehydrogenase
PTAC
Signature enzyme
Alcohol dehydrogenase
Regulator

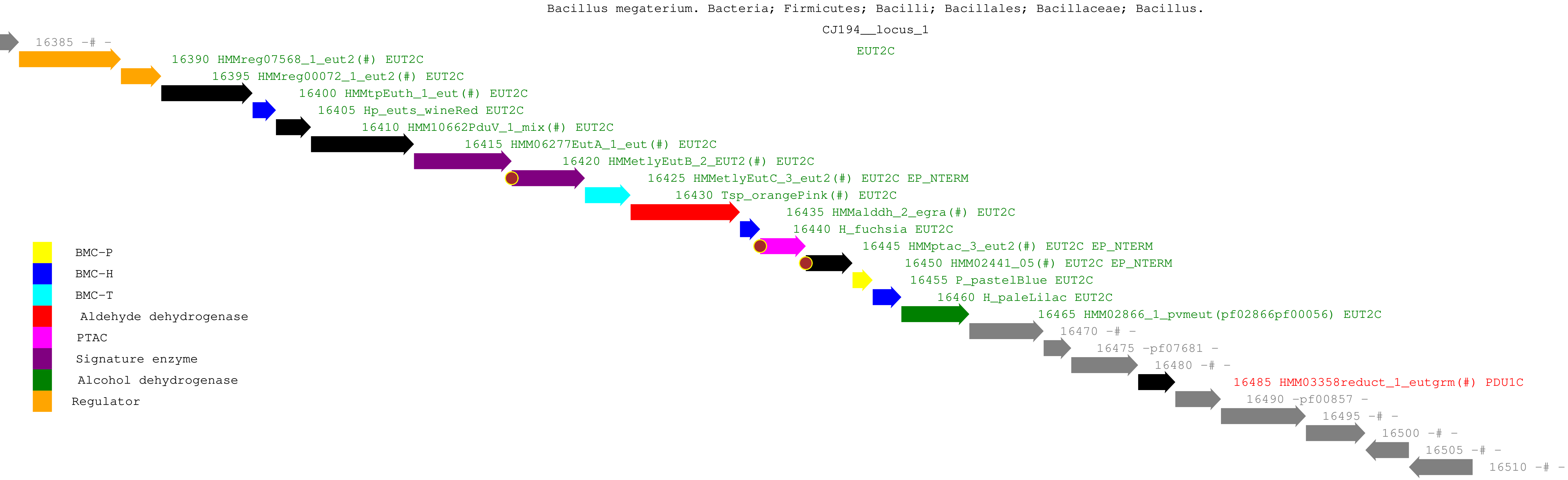
Bacillus megaterium. Bacteria; Firmicutes; Bacilli; Bacillales; Bacillaceae; Bacillus.
CJ194__locus_1
EUT2C
16385 -# -
16390 HMMreg07568_1_eut2(#) EUT2C
16395 HMMreg00072_1_eut2(#) EUT2C
16400 HMMtpEuth_1_eut(#) EUT2C
16405 Hp_euts_wineRed EUT2C
16410 HMM10662PduV_1_mix(#) EUT2C
16415 HMM06277EutA_1_eut(#) EUT2C
16420 HMMetlyEutB_2_EUT2(#) EUT2C
16425 HMMetlyEutC_3_eut2(#) EUT2C EP_NTERM
16430 Tsp_orangePink(#) EUT2C
16435 HMMalddh_2_egra(#) EUT2C
16440 H_fuchsia EUT2C
16445 HMMptac_3_eut2(#) EUT2C EP_NTERM
16450 HMM02441_05(#) EUT2C EP_NTERM
16455 P_pastelBlue EUT2C
16460 H_paleLilac EUT2C
16465 HMM02866_1_pvmeut(pf02866pf00056) EUT2C
16470 -# -
16475 -pf07681 -
16480 -# -
16485 HMM03358reduct_1_eutgrm(#) PDU1C
16490 -pf00857 -
16495 -# -
16500 -# -
16505 -# -
16510 -# -
BMC-P
BMC-H
BMC-T
Aldehyde dehydrogenase
PTAC
Signature enzyme
Alcohol dehydrogenase
Regulator

Bacillus sp. PK3_68. Bacteria; Firmicutes; Bacilli; Bacillales; Bacillaceae; Bacillus.

CJ483__locus_1

EUT2C

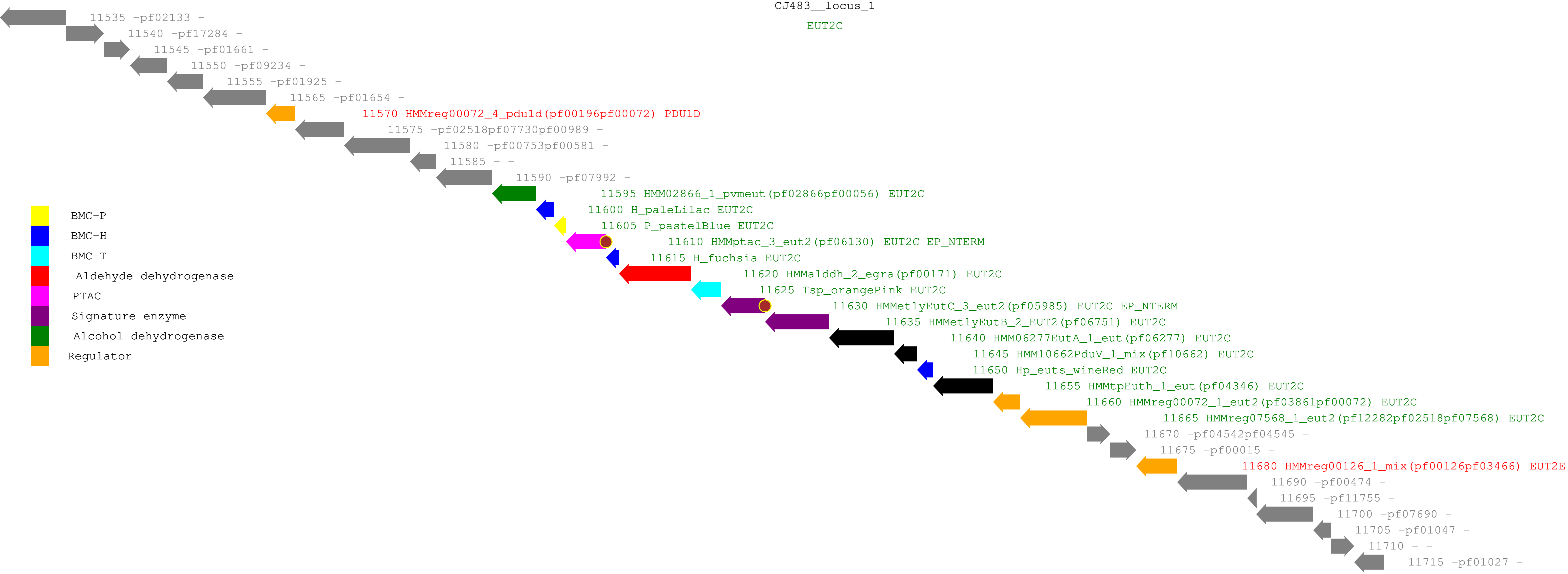

Salimicrobium sp. WN024. Bacteria; Firmicutes; Bacilli; Bacillales; Bacillaceae; Salimicrobium.

CKW00__locus_1

EUT2C

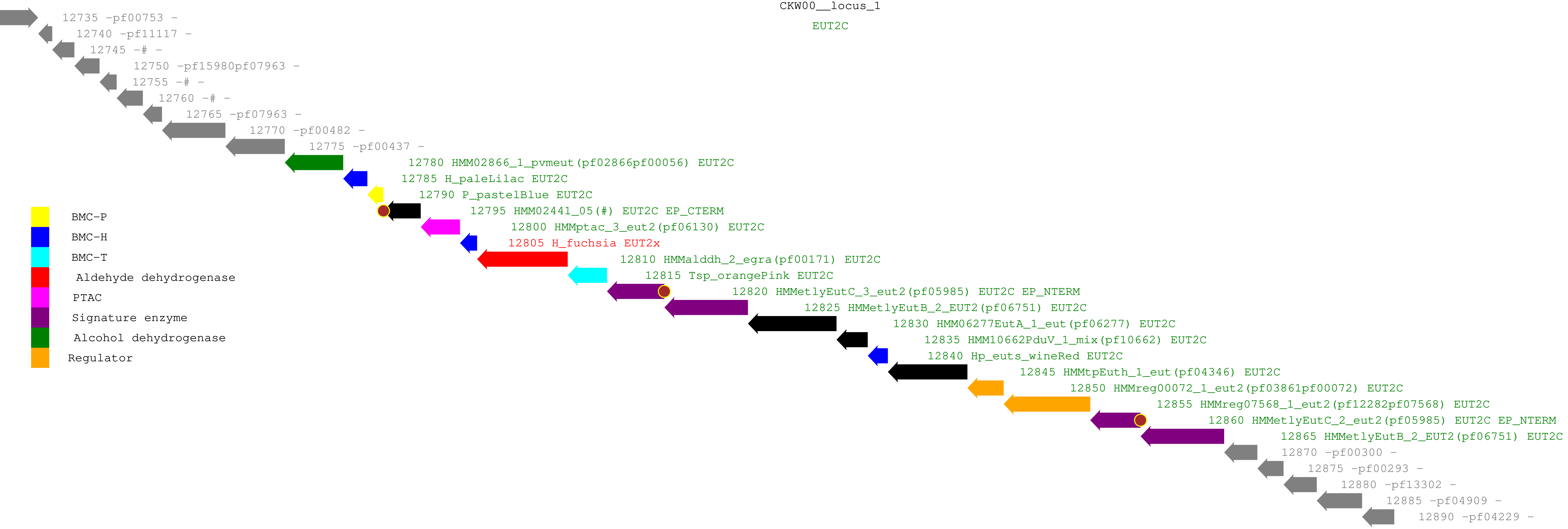

Bacillus megaterium. Bacteria; Firmicutes; Bacilli; Bacillales; Bacillaceae; Bacillus.

COI84__locus_1

EUT2C

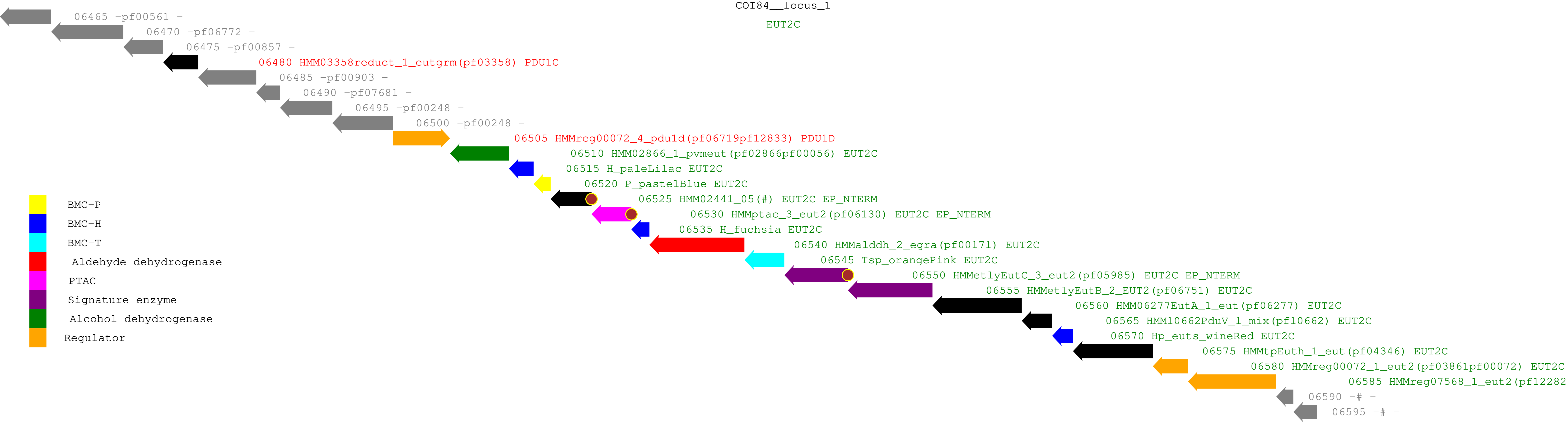

Bacillus megaterium. Bacteria; Firmicutes; Bacilli; Bacillales; Bacillaceae; Bacillus.

COJ21__locus_1

EUT2C

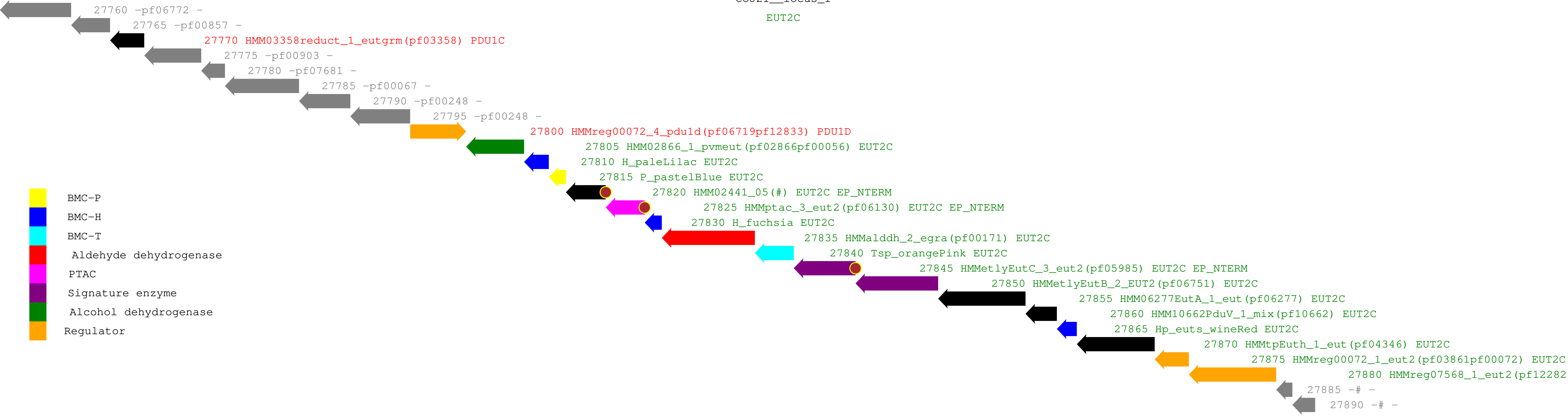

Bacillus megaterium. Bacteria; Firmicutes; Bacilli; Bacillales; Bacillaceae; Bacillus.

COJ23__locus_1

EUT2C

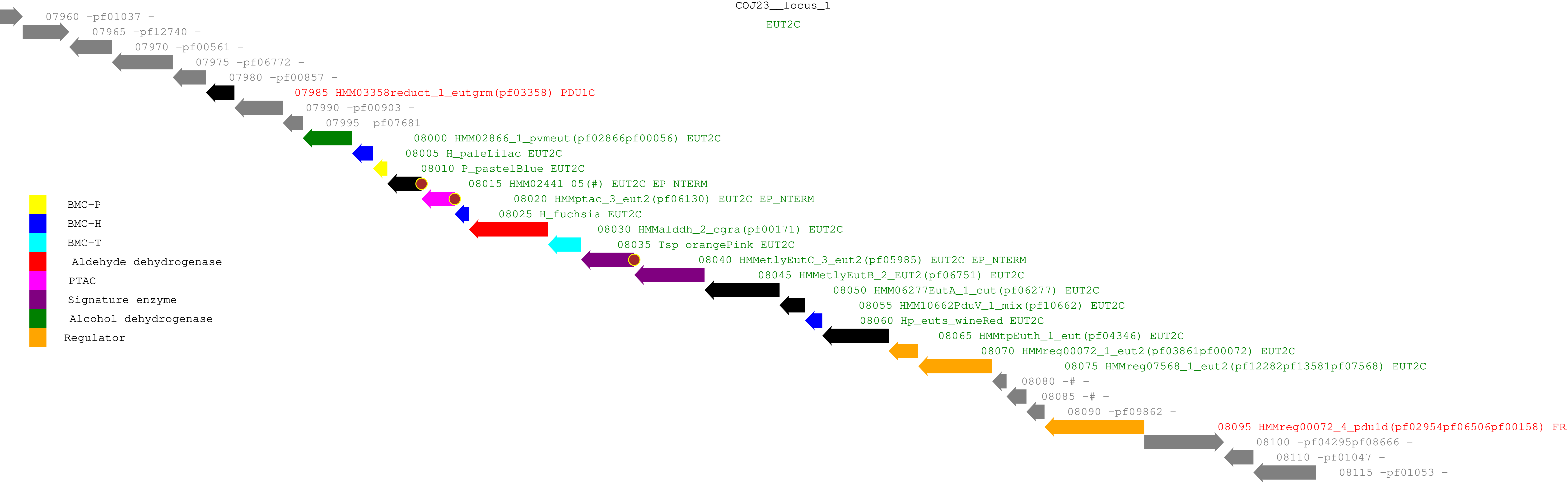

Bacillus megaterium. Bacteria; Firmicutes; Bacilli; Bacillales; Bacillaceae; Bacillus.

COK90__locus_1

EUT2C

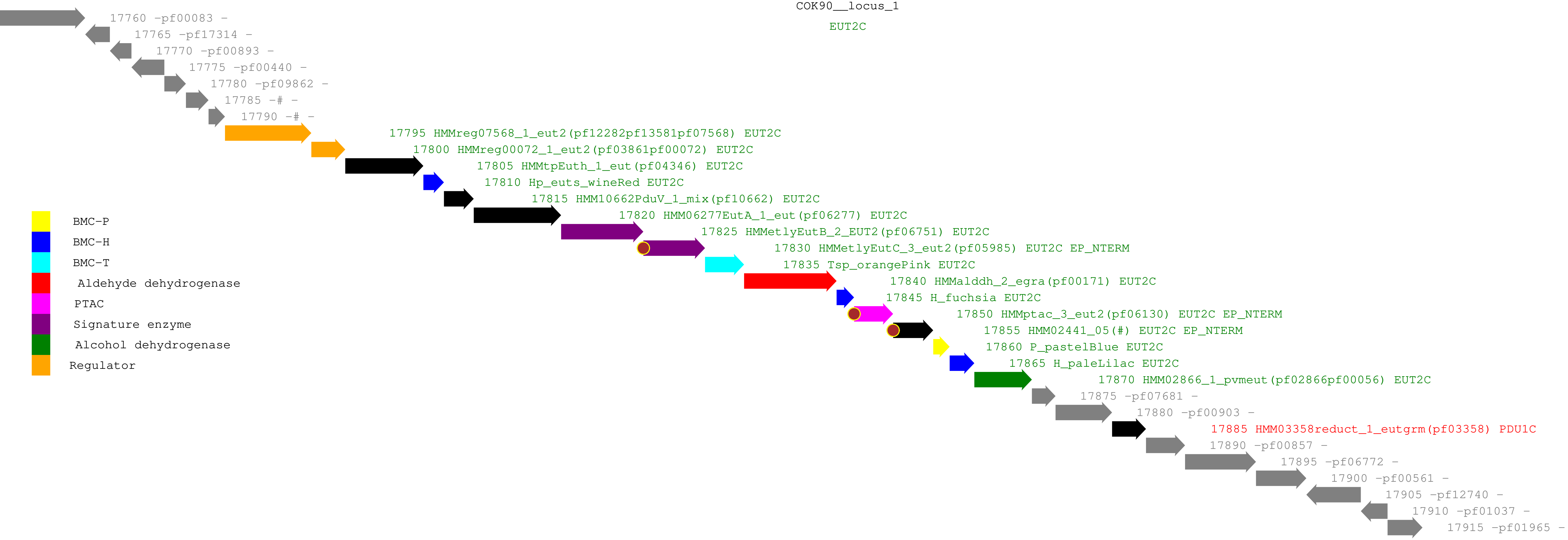

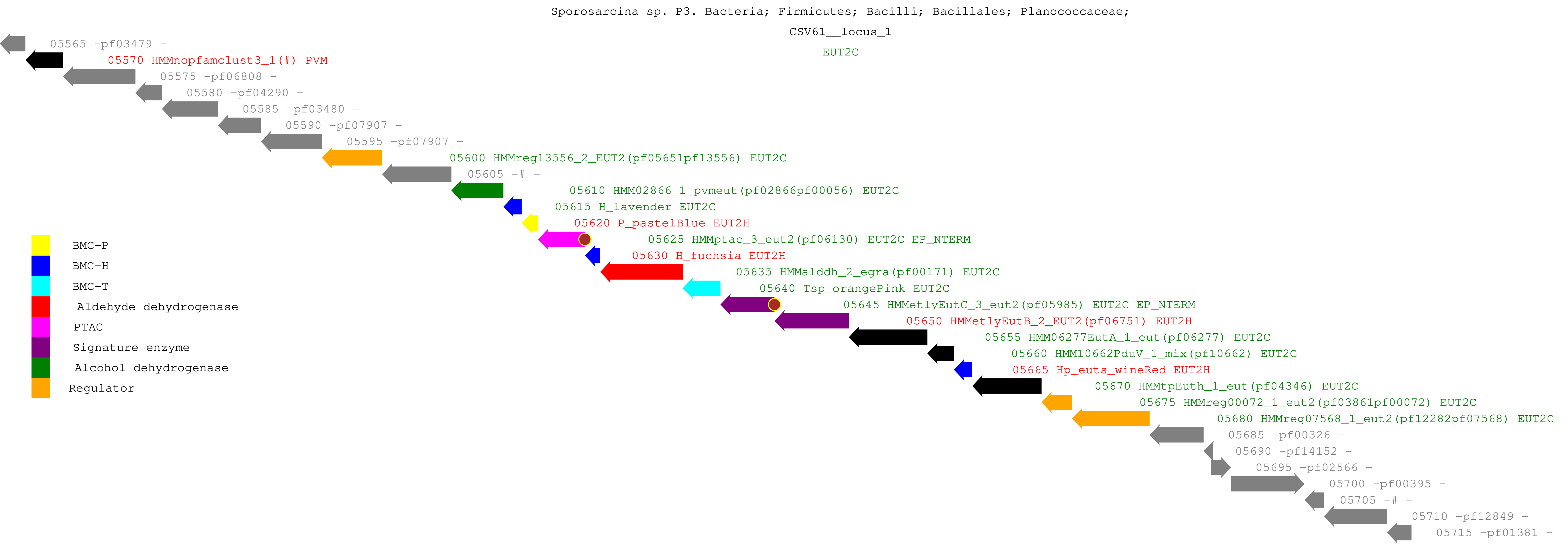
Sporosarcina sp. P3. Bacteria; Firmicutes; Bacilli; Bacillales; Planococcaceae;
CSV61__locus_1
EUT2C
05565 -pf03479 -
05570 HMMnopfamclust3_1(#) PVM
05575 -pf06808 -
05580 -pf04290 -
05585 -pf03480 -
05590 -pf07907 -
05595 -pf07907 -
05600 HMMreg13556_2_EUT2(pf05651pf13556) EUT2C
05605 -# -
05610 HMM02866_1_pvmeut(pf02866pf00056) EUT2C
05615 H_lavender EUT2C
05620 P_pastelBlue EUT2H
05625 HMMptac_3_eut2(pf06130) EUT2C EP_NTERM
05630 H_fuchsia EUT2H
05635 HMMalddh_2_egra(pf00171) EUT2C
05640 Tsp_orangePink EUT2C
05645 HMMetlyEutC_3_eut2(pf05985) EUT2C EP_NTERM
05650 HMMetlyEutB_2_EUT2(pf06751) EUT2H
05655 HMM06277EutA_1_eut(pf06277) EUT2C
05660 HMM10662PduV_1_mix(pf10662) EUT2C
05665 Hp_euts_wineRed EUT2H
05670 HMMtpEuth_1_eut(pf04346) EUT2C
05675 HMMreg00072_1_eut2(pf03861pf00072) EUT2C
05680 HMMreg07568_1_eut2(pf12282pf07568) EUT2C
05685 -pf00326 -
05690 -pf14152 -
05695 -pf02566 -
05700 -pf00395 -
05705 -# -
05710 -pf12849 -
05715 -pf01381 -
BMC-P
BMC-H
BMC-T
Aldehyde dehydrogenase
PTAC
Signature enzyme
Alcohol dehydrogenase
Regulator

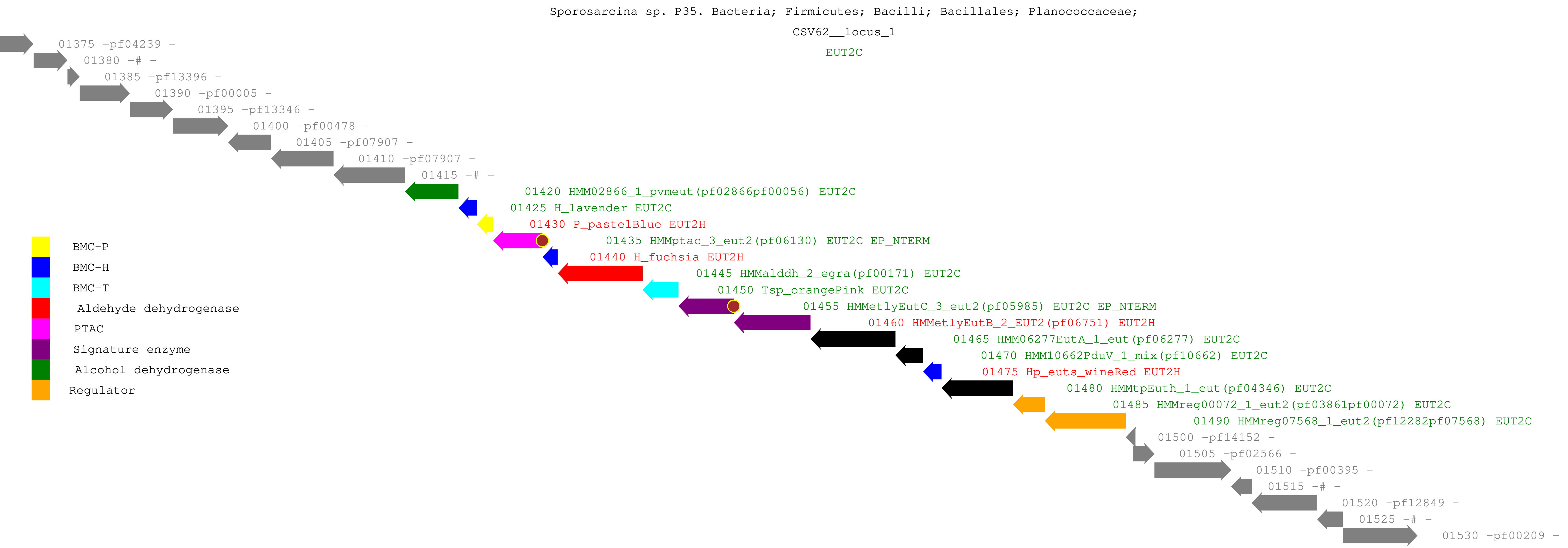

Sporosarcina sp. P35. Bacteria; Firmicutes; Bacilli; Bacillales; Planococcaceae;
CSV62__locus_1
EUT2C
01375 -pf04239 -
01380 -# -
01385 -pf13396 -
01390 -pf00005 -
01395 -pf13346 -
01400 -pf00478 -
01405 -pf07907 -
01410 -pf07907 -
01415 -# -
01420 HMM02866_1_pvmeut(pf02866pf00056) EUT2C
01425 H_lavender EUT2C
01430 P_pastelBlue EUT2H
01435 HMMptac_3_eut2(pf06130) EUT2C EP_NTERM
01440 H_fuchsia EUT2H
01445 HMMaldh_2_egra(pf00171) EUT2C
01450 Tsp_orangePink EUT2C
01455 HMMetlyEutC_3_eut2(pf05985) EUT2C EP_NTERM
01460 HMMetlyEutB_2_EUT2(pf06751) EUT2H
01465 HMM06277EutA_1_eut(pf06277) EUT2C
01470 HMM10662PduV_1_mix(pf10662) EUT2C
01475 Hp_euts_wineRed EUT2H
01480 HMMtpEuth_1_eut(pf04346) EUT2C
01485 HMMreg00072_1_eut2(pf03861pf00072) EUT2C
01490 HMMreg07568_1_eut2(pf12282pf07568) EUT2C
01500 -pf14152 -
01505 -pf02566 -
01510 -pf00395 -
01515 -# -
01520 -pf12849 -
01525 -# -
01530 -pf00209 -
BMC-P
BMC-H
BMC-T
Aldehyde dehydrogenase
PTAC
Signature enzyme
Alcohol dehydrogenase
Regulator

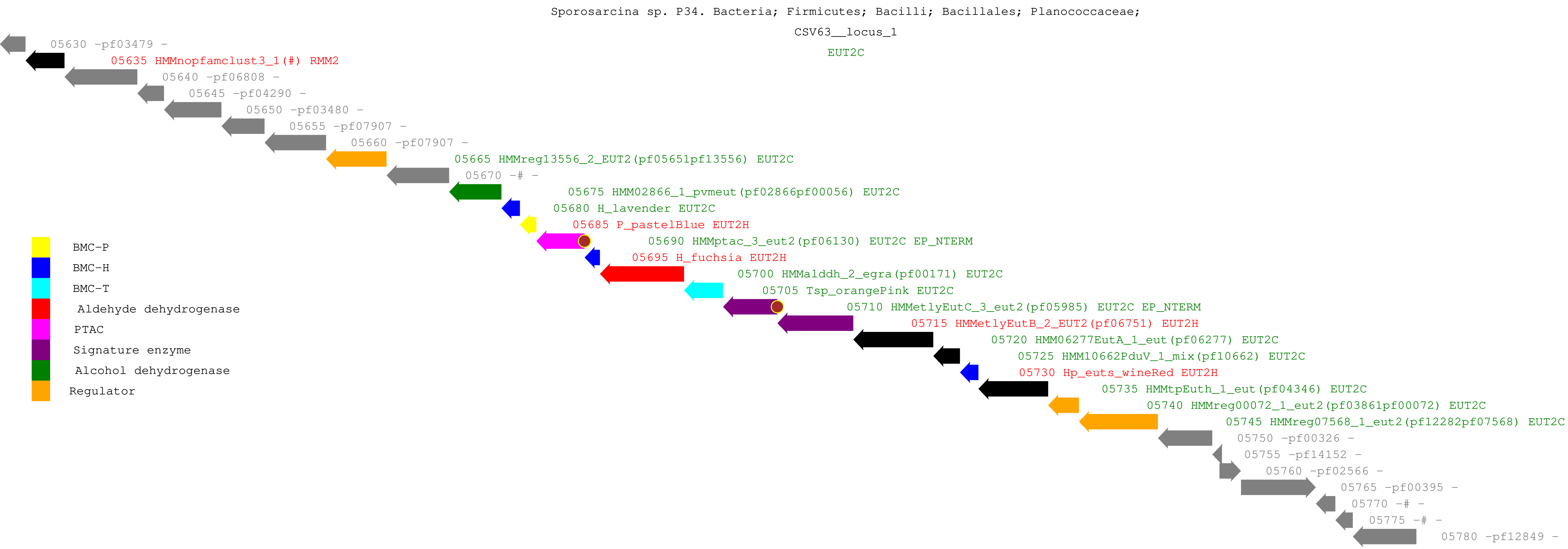

Sporosarcina sp. P34. Bacteria; Firmicutes; Bacilli; Bacillales; Planococcaceae;
CSV63__locus_1
EUT2C
05630 -pf03479 -
05635 HMMnopfamclust3_1(#) RMM2
05640 -pf06808 -
05645 -pf04290 -
05650 -pf03480 -
05655 -pf07907 -
05660 -pf07907 -
05665 HMMreg13556_2_EUT2(pf05651pf13556) EUT2C
05670 -# -
05675 HMM02866_1_pvmeut(pf02866pf00056) EUT2C
05680 H_lavender EUT2C
05685 P_pastelBlue EUT2H
05690 HMMptac_3_eut2(pf06130) EUT2C EP_NTERM
05695 H_fuchsia EUT2H
05700 HMMalddh_2_egra(pf00171) EUT2C
05705 Tsp_orangePink EUT2C
05710 HMMetlyEutC_3_eut2(pf05985) EUT2C EP_NTERM
05715 HMMetlyEutB_2_EUT2(pf06751) EUT2H
05720 HMM06277EutA_1_eut(pf06277) EUT2C
05725 HMM10662PduV_1_mix(pf10662) EUT2C
05730 Hp_euts_wineRed EUT2H
05735 HMMtpEuth_1_eut(pf04346) EUT2C
05740 HMMreg00072_1_eut2(pf03861pf00072) EUT2C
05745 HMMreg07568_1_eut2(pf12282pf07568) EUT2C
05750 -pf00326 -
05755 -pf14152 -
05760 -pf02566 -
05765 -pf00395 -
05770 -# -
05775 -# -
05780 -pf12849 -
BMC-P
BMC-H
BMC-T
Aldehyde dehydrogenase
PTAC
Signature enzyme
Alcohol dehydrogenase
Regulator

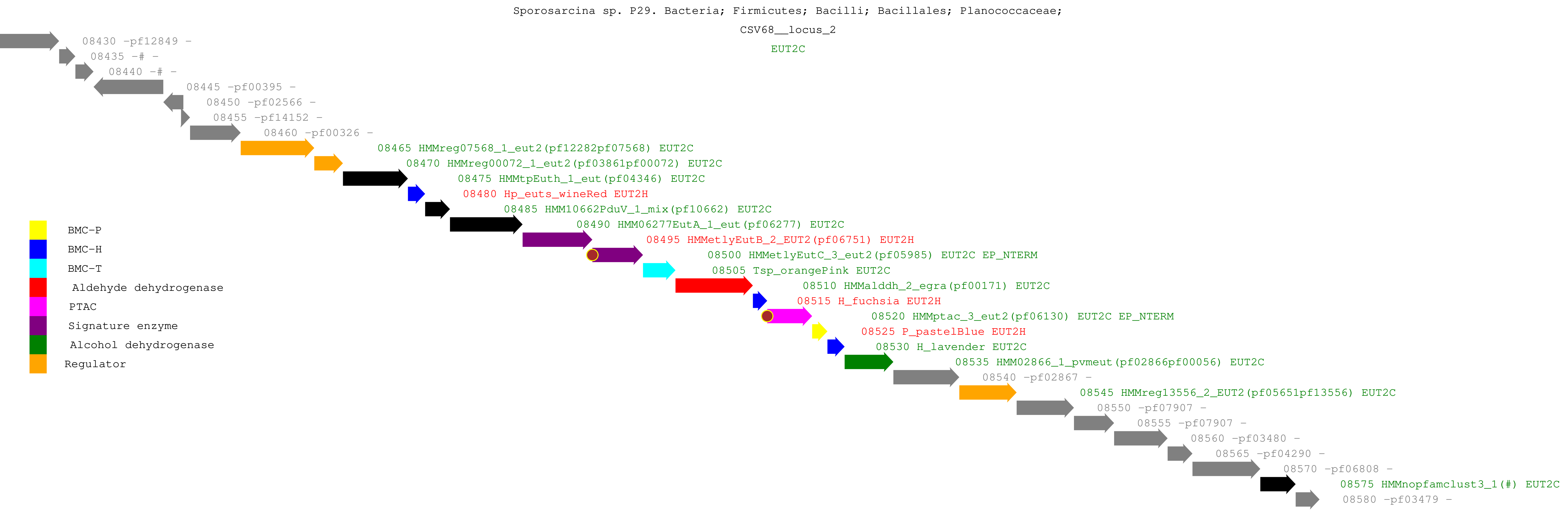

Sporosarcina sp. P29. Bacteria; Firmicutes; Bacilli; Bacillales; Planococcaceae;
CSV68__locus_2
EUT2C
08430 -pf12849 -
08435 -# -
08440 -# -
08445 -pf00395 -
08450 -pf02566 -
08455 -pf14152 -
08460 -pf00326 -
08465 HMMreg07568_1_eut2(pf12282pf07568) EUT2C
08470 HMMreg00072_1_eut2(pf03861pf00072) EUT2C
08475 HMMtpEuth_1_eut(pf04346) EUT2C
08480 Hp_euts_wineRed EUT2H
08485 HMM10662PduV_1_mix(pf10662) EUT2C
08490 HMM06277EutA_1_eut(pf06277) EUT2C
08495 HMMetlyEutB_2_EUT2(pf06751) EUT2H
08500 HMMetlyEutC_3_eut2(pf05985) EUT2C EP_NTERM
08505 Tsp_orangePink EUT2C
08510 HMMaldh_2_egra(pf00171) EUT2C
08515 H_fuchsia EUT2H
08520 HMMptac_3_eut2(pf06130) EUT2C EP_NTERM
08525 P_pastelBlue EUT2H
08530 H_lavender EUT2C
08535 HMM02866_1_pvmeut(pf02866pf00056) EUT2C
08540 -pf02867 -
08545 HMMreg13556_2_EUT2(pf05651pf13556) EUT2C
08550 -pf07907 -
08555 -pf07907 -
08560 -pf03480 -
08565 -pf04290 -
08570 -pf06808 -
08575 HMMnopfamclust3_1(#) EUT2C
08580 -pf03479 -
BMC-P
BMC-H
BMC-T
Aldehyde dehydrogenase
PTAC
Signature enzyme
Alcohol dehydrogenase
Regulator

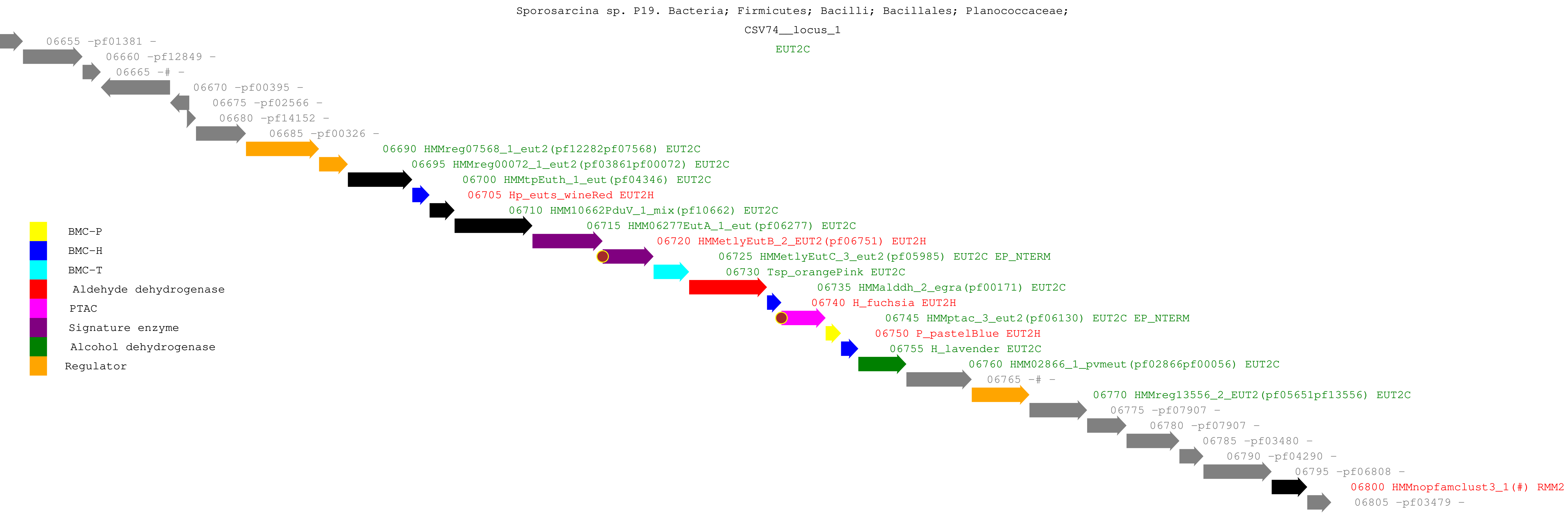

Sporosarcina sp. P19. Bacteria; Firmicutes; Bacilli; Bacillales; Planococcaceae;
CSV74__locus_1
EUT2C
06655 -pf01381 -
06660 -pf12849 -
06665 -# -
06670 -pf00395 -
06675 -pf02566 -
06680 -pf14152 -
06685 -pf00326 -
06690 HMMreg07568_1_eut2(pf12282pf07568) EUT2C
06695 HMMreg00072_1_eut2(pf03861pf00072) EUT2C
06700 HMMtpEuth_1_eut(pf04346) EUT2C
06705 Hp_euts_wineRed EUT2H
06710 HMM10662PduV_1_mix(pf10662) EUT2C
06715 HMM06277EutA_1_eut(pf06277) EUT2C
06720 HMMetlyEutB_2_EUT2(pf06751) EUT2H
06725 HMMetlyEutC_3_eut2(pf05985) EUT2C EP_NTERM
06730 Tsp_orangePink EUT2C
06735 HMMalddh_2_egra(pf00171) EUT2C
06740 H_fuchsia EUT2H
06745 HMMptac_3_eut2(pf06130) EUT2C EP_NTERM
06750 P_pastelBlue EUT2H
06755 H_lavender EUT2C
06760 HMM02866_1_pvmeut(pf02866pf00056) EUT2C
06765 -# -
06770 HMMreg13556_2_EUT2(pf05651pf13556) EUT2C
06775 -pf07907 -
06780 -pf07907 -
06785 -pf03480 -
06790 -pf04290 -
06795 -pf06808 -
06800 HMMnopfamclust3_1(#) RMM2
06805 -pf03479 -
BMC-P
BMC-H
BMC-T
Aldehyde dehydrogenase
PTAC
Signature enzyme
Alcohol dehydrogenase
Regulator

Sporosarcina sp. P17b. Bacteria; Firmicutes; Bacilli; Bacillales; Planococcaceae;

CSV76__locus_1

EUT2C

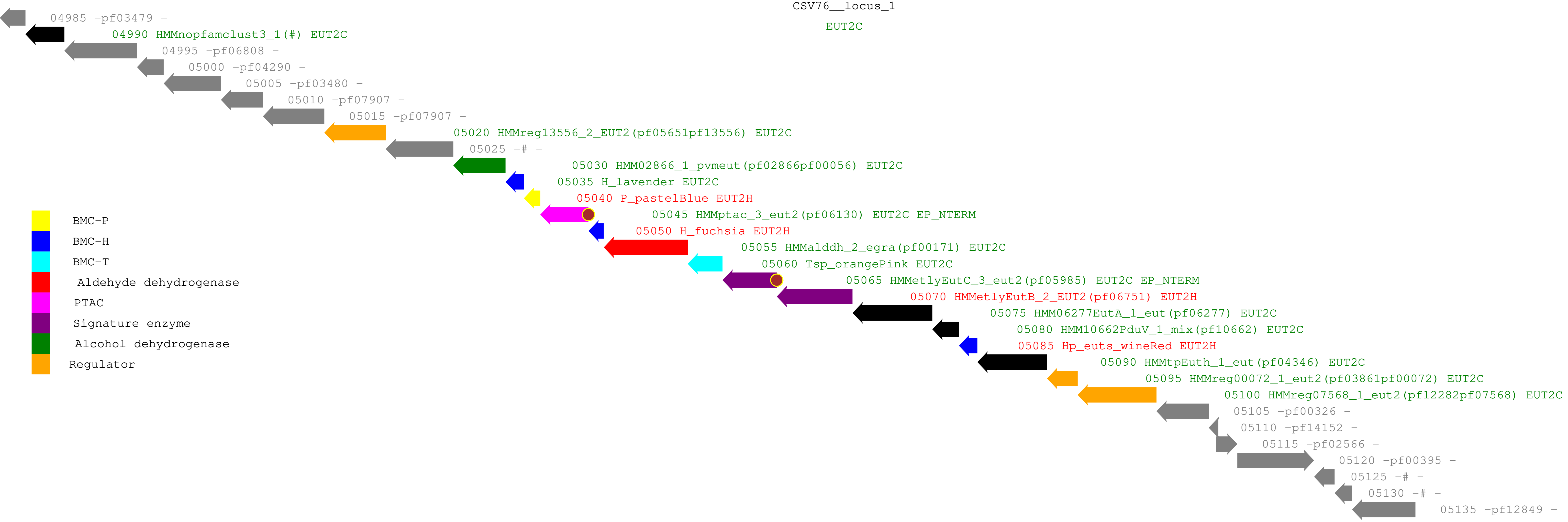

Sporosarcina sp. P16b. Bacteria; Firmicutes; Bacilli; Bacillales; Planococcaceae;

CSV77__locus_2

EUT2C

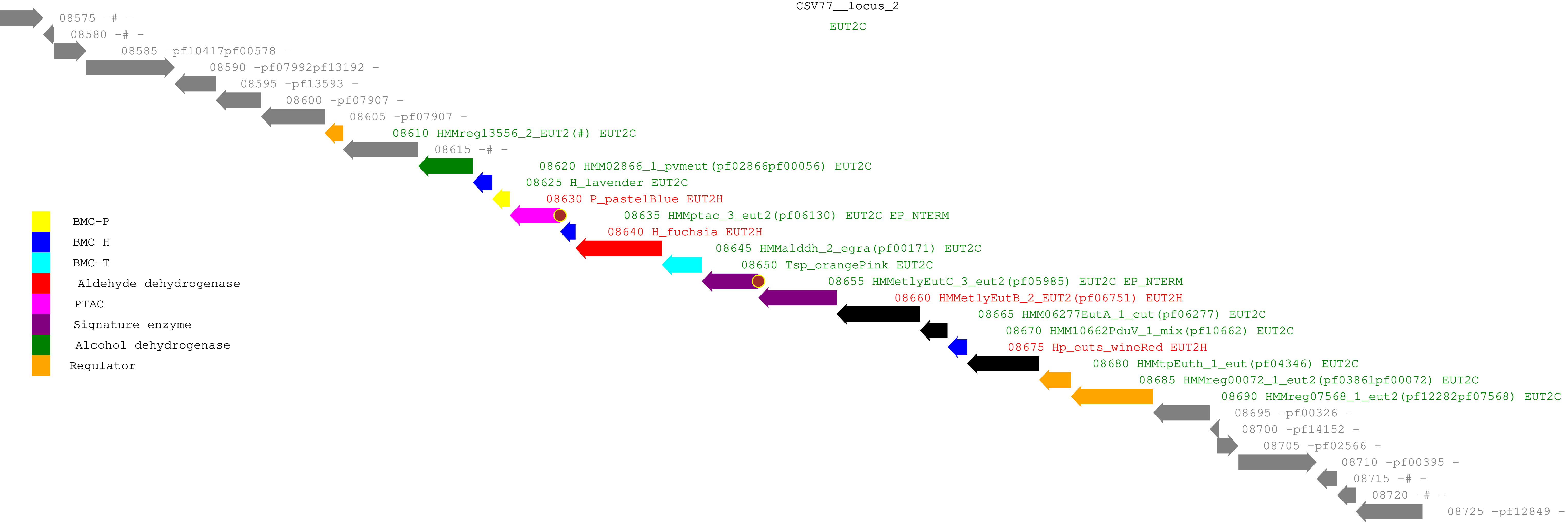

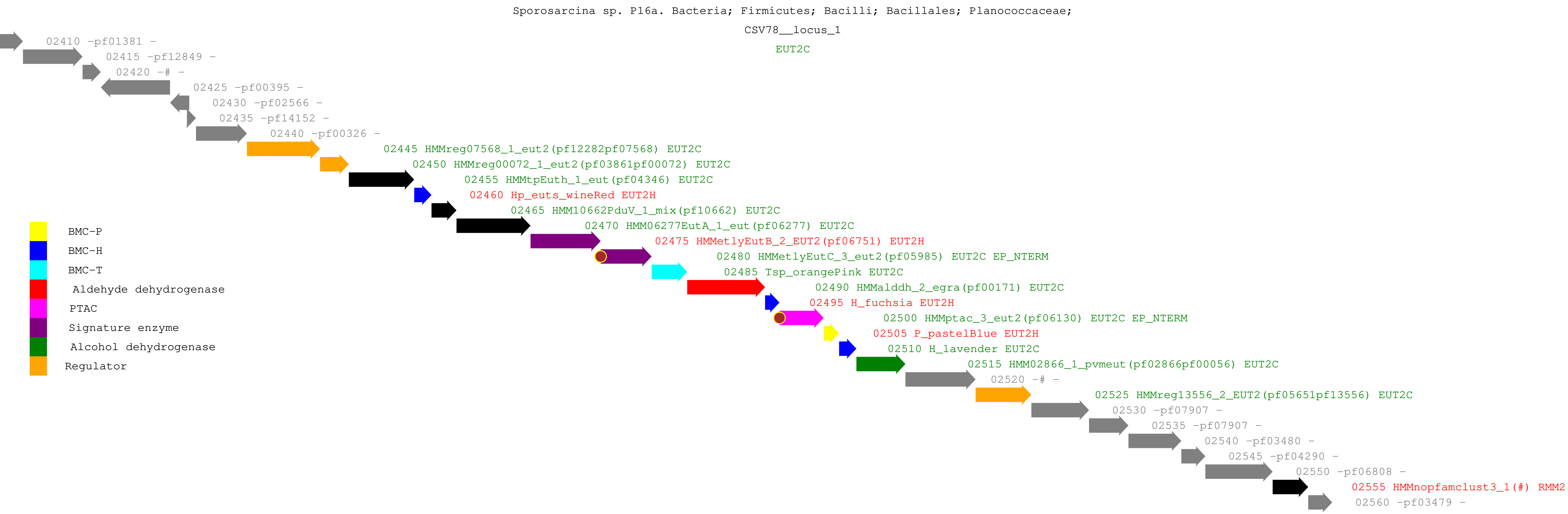
Sporosarcina sp. P16a. Bacteria; Firmicutes; Bacilli; Bacillales; Planococcaceae;
CSV78__locus_1
EUT2C
02410 -pf01381 -
02415 -pf12849 -
02420 -# -
02425 -pf00395 -
02430 -pf02566 -
02435 -pf14152 -
02440 -pf00326 -
02445 HMMreg07568_1_eut2(pf12282pf07568) EUT2C
02450 HMMreg00072_1_eut2(pf03861pf00072) EUT2C
02455 HMMtpEuth_1_eut(pf04346) EUT2C
02460 Hp_euts_wineRed EUT2H
02465 HMM10662PduV_1_mix(pf10662) EUT2C
02470 HMM06277EutA_1_eut(pf06277) EUT2C
02475 HMMetlyEutB_2_EUT2(pf06751) EUT2H
02480 HMMetlyEutC_3_eut2(pf05985) EUT2C EP_NTERM
02485 Tsp_orangePink EUT2C
02490 HMMalddh_2_egra(pf00171) EUT2C
02495 H_fuchsia EUT2H
02500 HMMptac_3_eut2(pf06130) EUT2C EP_NTERM
02505 P_pastelBlue EUT2H
02510 H_lavender EUT2C
02515 HMM02866_1_pvmeut(pf02866pf00056) EUT2C
02520 -# -
02525 HMMreg13556_2_EUT2(pf05651pf13556) EUT2C
02530 -pf07907 -
02535 -pf07907 -
02540 -pf03480 -
02545 -pf04290 -
02550 -pf06808 -
02555 HMMnopfamclust3_1(#) RMM2
02560 -pf03479 -
BMC-P
BMC-H
BMC-T
Aldehyde dehydrogenase
PTAC
Signature enzyme
Alcohol dehydrogenase
Regulator

Sporosarcina sp. P13. Bacteria; Firmicutes; Bacilli; Bacillales; Planococcaceae;

CSV79__locus_1

EUT2C

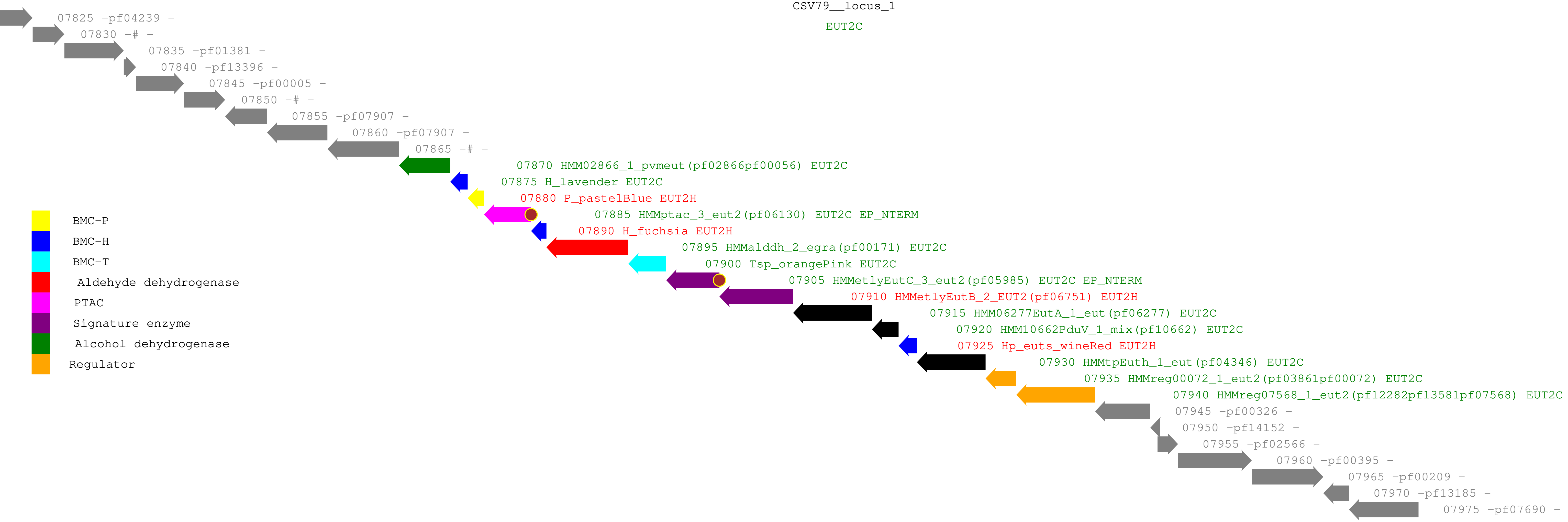

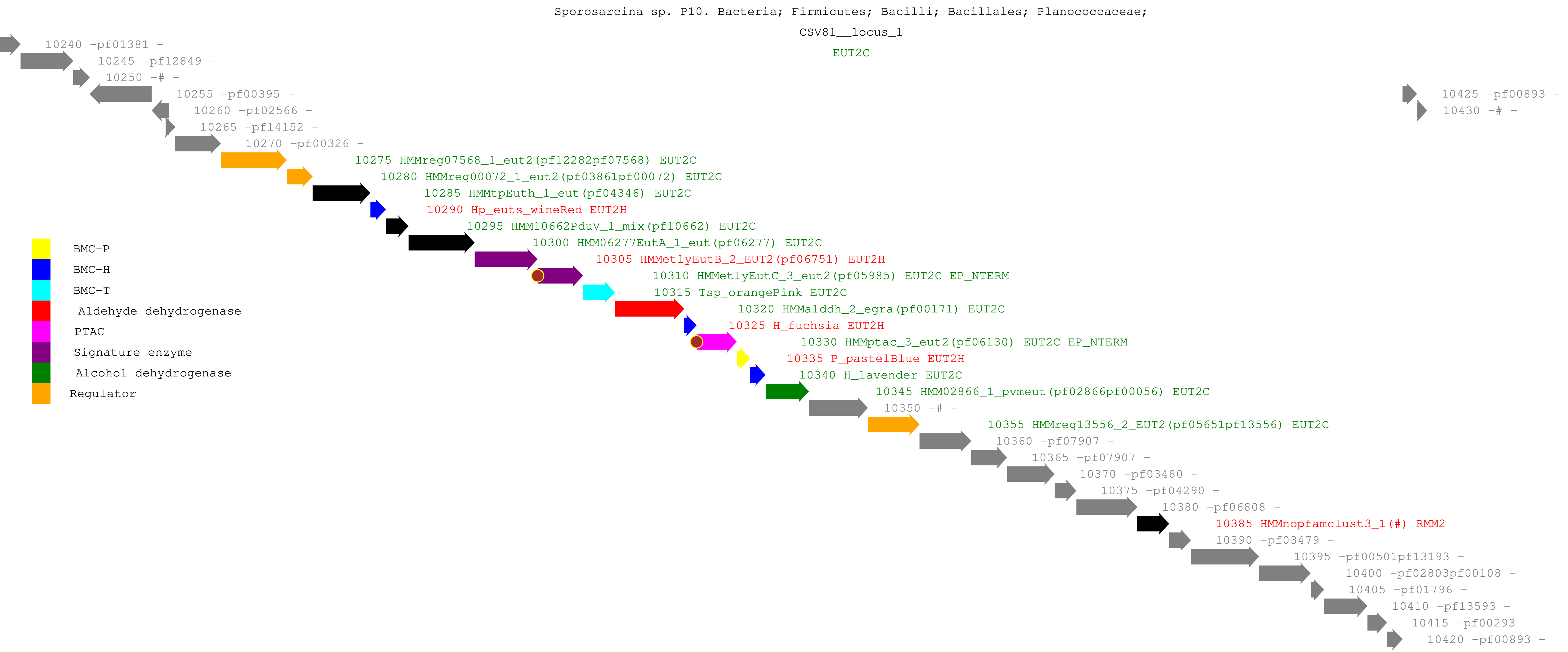

Sporosarcina sp. P10. Bacteria; Firmicutes; Bacilli; Bacillales; Planococcaceae;
CSV81__locus_1
EUT2C
10240 -pf01381 -
10245 -pf12849 -
10250 -# -
10255 -pf00395 -
10260 -pf02566 -
10265 -pf14152 -
10270 -pf00326 -
10275 HMMreg07568_1_eut2(pf12282pf07568) EUT2C
10280 HMMreg00072_1_eut2(pf03861pf00072) EUT2C
10285 HMMtpEuth_1_eut(pf04346) EUT2C
10290 Hp_euts_wineRed EUT2H
10295 HMM10662PduV_1_mix(pf10662) EUT2C
10300 HMM06277EutA_1_eut(pf06277) EUT2C
10305 HMMetlyEutB_2_EUT2(pf06751) EUT2H
10310 HMMetlyEutC_3_eut2(pf05985) EUT2C EP_NTERM
10315 Tsp_orangePink EUT2C
10320 HMMaldh_2_egra(pf00171) EUT2C
10325 H_fuchsia EUT2H
10330 HMMptac_3_eut2(pf06130) EUT2C EP_NTERM
10335 P_pastelBlue EUT2H
10340 H_lavender EUT2C
10345 HMM02866_1_pvmeut(pf02866pf00056) EUT2C
10350 -# -
10355 HMMreg13556_2_EUT2(pf05651pf13556) EUT2C
10360 -pf07907 -
10365 -pf07907 -
10370 -pf03480 -
10375 -pf04290 -
10380 -pf06808 -
10385 HMMnopfamclust3_1(#) RMM2
10390 -pf03479 -
10395 -pf00501pf13193 -
10400 -pf02803pf00108 -
10405 -pf01796 -
10410 -pf13593 -
10415 -pf00293 -
10420 -pf00893 -
10425 -pf00893 -
10430 -# -
BMC-P
BMC-H
BMC-T
Aldehyde dehydrogenase
PTAC
Signature enzyme
Alcohol dehydrogenase
Regulator

Bacillus sp. V5-8f. Bacteria; Firmicutes; Bacilli; Bacillales; Bacillaceae; Bacillus.

CUU64__locus_1

EUT2C

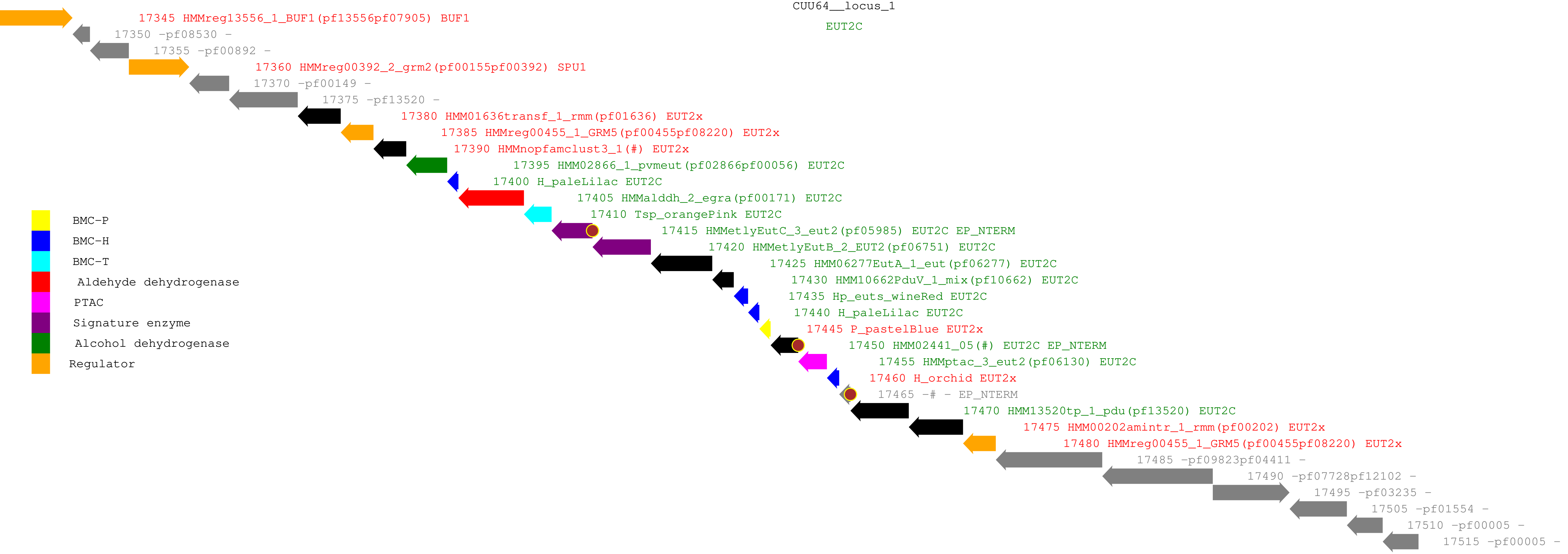

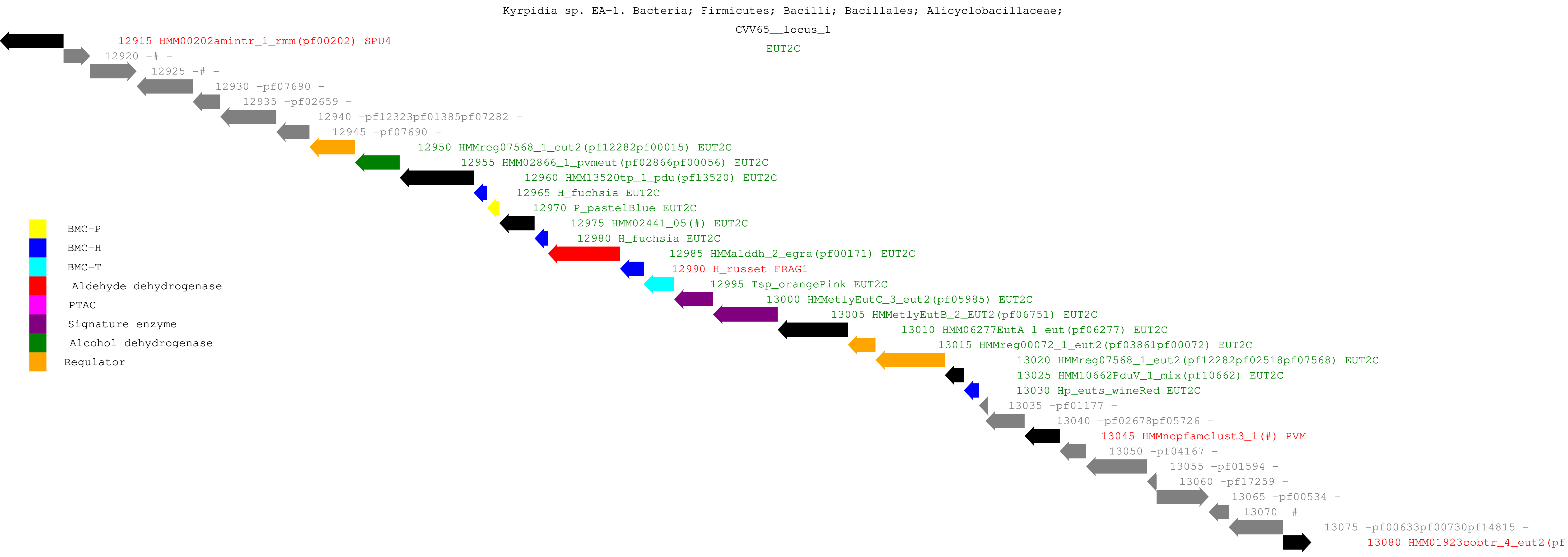

Kyrpidia sp. EA-1. Bacteria; Firmicutes; Bacilli; Bacillales; Alicyclobacillaceae;
CVV65__locus_1
EUT2C
12915 HMM00202amintr_1_rmm(pf00202) SPU4
12920 -# -
12925 -# -
12930 -pf07690 -
12935 -pf02659 -
12940 -pf12323pf01385pf07282 -
12945 -pf07690 -
12950 HMMreg07568_1_eut2(pf12282pf00015) EUT2C
12955 HMM02866_1_pvmeut(pf02866pf00056) EUT2C
12960 HMM13520tp_1_pdu(pf13520) EUT2C
12965 H_fuchsia EUT2C
12970 P_pastelBlue EUT2C
12975 HMM02441_05(#) EUT2C
12980 H_fuchsia EUT2C
12985 HMMalddh_2_egra(pf00171) EUT2C
12990 H_russet FRAG1
12995 Tsp_orangePink EUT2C
13000 HMMetlyEutC_3_eut2(pf05985) EUT2C
13005 HMMetlyEutB_2_EUT2(pf06751) EUT2C
13010 HMM06277EutA_1_eut(pf06277) EUT2C
13015 HMMreg00072_1_eut2(pf03861pf00072) EUT2C
13020 HMMreg07568_1_eut2(pf12282pf02518pf07568) EUT2C
13025 HMM10662PduV_1_mix(pf10662) EUT2C
13030 Hp_euts_wineRed EUT2C
13035 -pf01177 -
13040 -pf02678pf05726 -
13045 HMMnopfamclust3_1(#) PVM
13050 -pf04167 -
13055 -pf01594 -
13060 -pf17259 -
13065 -pf00534 -
13070 -# -
13075 -pf00633pf00730pf14815 -
13080 HMM01923cobtr_4_eut2(pf
BMC-P
BMC-H
BMC-T
Aldehyde dehydrogenase
PTAC
Signature enzyme
Alcohol dehydrogenase
Regulator

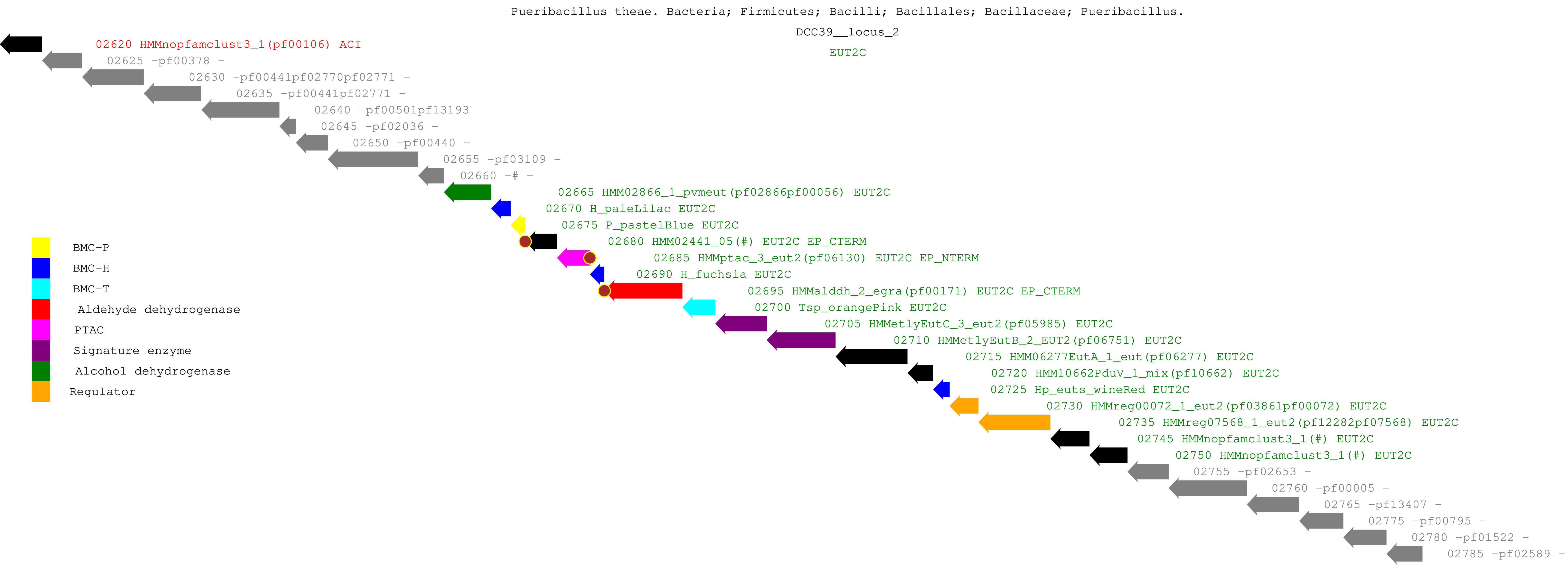
Pueribacillus theae. Bacteria; Firmicutes; Bacilli; Bacillales; Bacillaceae; Pueribacillus.
DCC39__locus_2
EUT2C
02620 HMMnopfamclust3_1(pf00106) ACI
02625 -pf00378 -
02630 -pf00441pf02770pf02771 -
02635 -pf00441pf02771 -
02640 -pf00501pf13193 -
02645 -pf02036 -
02650 -pf00440 -
02655 -pf03109 -
02660 -# -
02665 HMM02866_1_pvmeut(pf02866pf00056) EUT2C
02670 H_paleLilac EUT2C
02675 P_pastelBlue EUT2C
02680 HMM02441_05(#) EUT2C EP_CTERM
02685 HMMptac_3_eut2(pf06130) EUT2C EP_NTERM
02690 H_fuchsia EUT2C
02695 HMMalddh_2_egra(pf00171) EUT2C EP_CTERM
02700 Tsp_orangePink EUT2C
02705 HMMetlyEutC_3_eut2(pf05985) EUT2C
02710 HMMetlyEutB_2_EUT2(pf06751) EUT2C
02715 HMM06277EutA_1_eut(pf06277) EUT2C
02720 HMM10662PduV_1_mix(pf10662) EUT2C
02725 Hp_euts_wineRed EUT2C
02730 HMMreg00072_1_eut2(pf03861pf00072) EUT2C
02735 HMMreg07568_1_eut2(pf12282pf07568) EUT2C
02745 HMMnopfamclust3_1(#) EUT2C
02750 HMMnopfamclust3_1(#) EUT2C
02755 -pf02653 -
02760 -pf00005 -
02765 -pf13407 -
02775 -pf00795 -
02780 -pf01522 -
02785 -pf02589 -
BMC-P
BMC-H
BMC-T
Aldehyde dehydrogenase
PTAC
Signature enzyme
Alcohol dehydrogenase
Regulator

Lysinibacillus sp. 2017. Bacteria; Firmicutes; Bacilli; Bacillales; Bacillaceae;

DCE79__locus_1

EUT2C

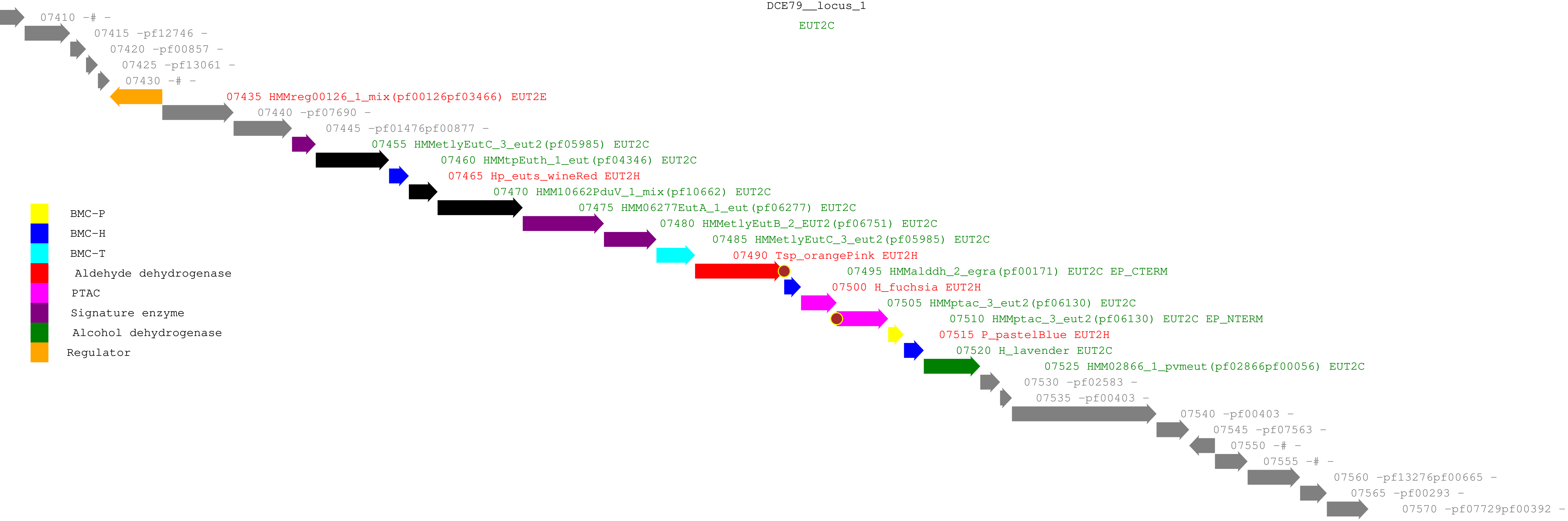

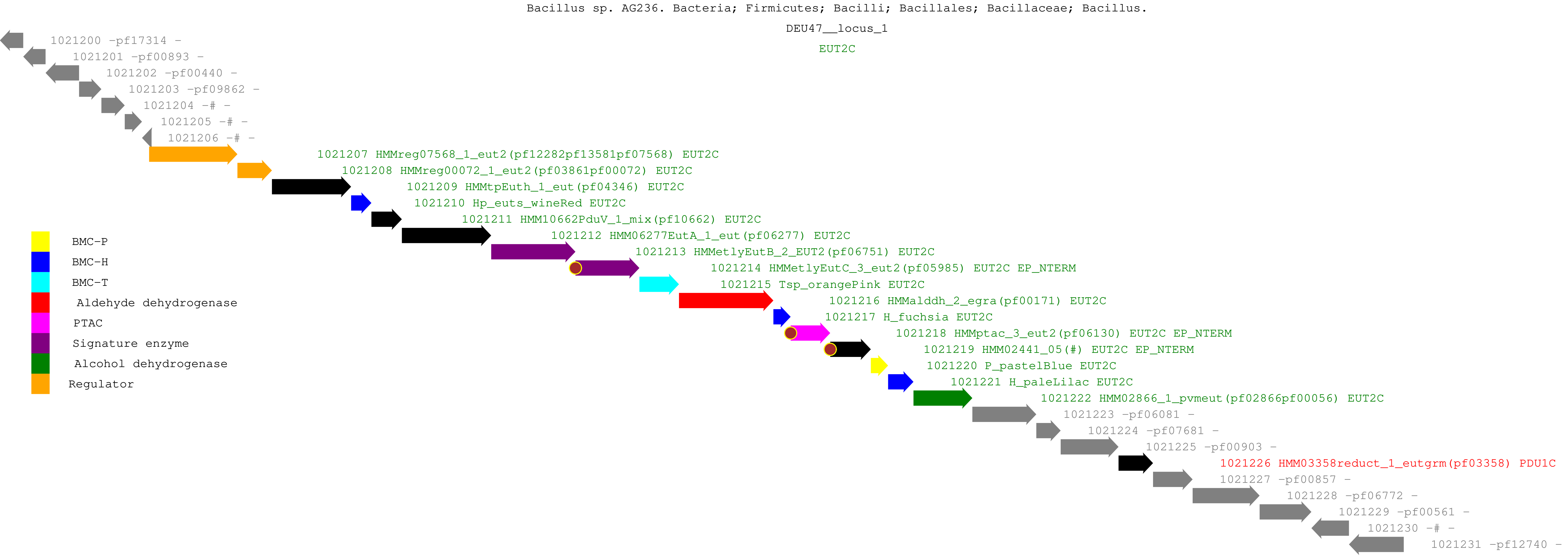
Bacillus sp. AG236. Bacteria; Firmicutes; Bacilli; Bacillales; Bacillaceae; Bacillus.
DEU47__locus_1
EUT2C
1021200 -pf17314 -
1021201 -pf00893 -
1021202 -pf00440 -
1021203 -pf09862 -
1021204 -# -
1021205 -# -
1021206 -# -
1021207 HMMreg07568_1_eut2(pf12282pf13581pf07568) EUT2C
1021208 HMMreg00072_1_eut2(pf03861pf00072) EUT2C
1021209 HMMtpEuth_1_eut(pf04346) EUT2C
1021210 Hp_euts_wineRed EUT2C
1021211 HMM10662PduV_1_mix(pf10662) EUT2C
1021212 HMM06277EutA_1_eut(pf06277) EUT2C
1021213 HMMetlyEutB_2_EUT2(pf06751) EUT2C
1021214 HMMetlyEutC_3_eut2(pf05985) EUT2C EP_NTERM
1021215 Tsp_orangePink EUT2C
1021216 HMMalddh_2_egra(pf00171) EUT2C
1021217 H_fuchsia EUT2C
1021218 HMMptac_3_eut2(pf06130) EUT2C EP_NTERM
1021219 HMM02441_05(#) EUT2C EP_NTERM
1021220 P_pastelBlue EUT2C
1021221 H_paleLilac EUT2C
1021222 HMM02866_1_pvmeut(pf02866pf00056) EUT2C
1021223 -pf06081 -
1021224 -pf07681 -
1021225 -pf00903 -
1021226 HMM03358reduct_1_eutgrm(pf03358) PDU1C
1021227 -pf00857 -
1021228 -pf06772 -
1021229 -pf00561 -
1021230 -# -
1021231 -pf12740 -
BMC-P
BMC-H
BMC-T
Aldehyde dehydrogenase
PTAC
Signature enzyme
Alcohol dehydrogenase
Regulator

Bacillus firmus. Bacteria; Firmicutes; Bacilli; Bacillales; Bacillaceae; Bacillus.

DFO70__locus_2

EUT2C

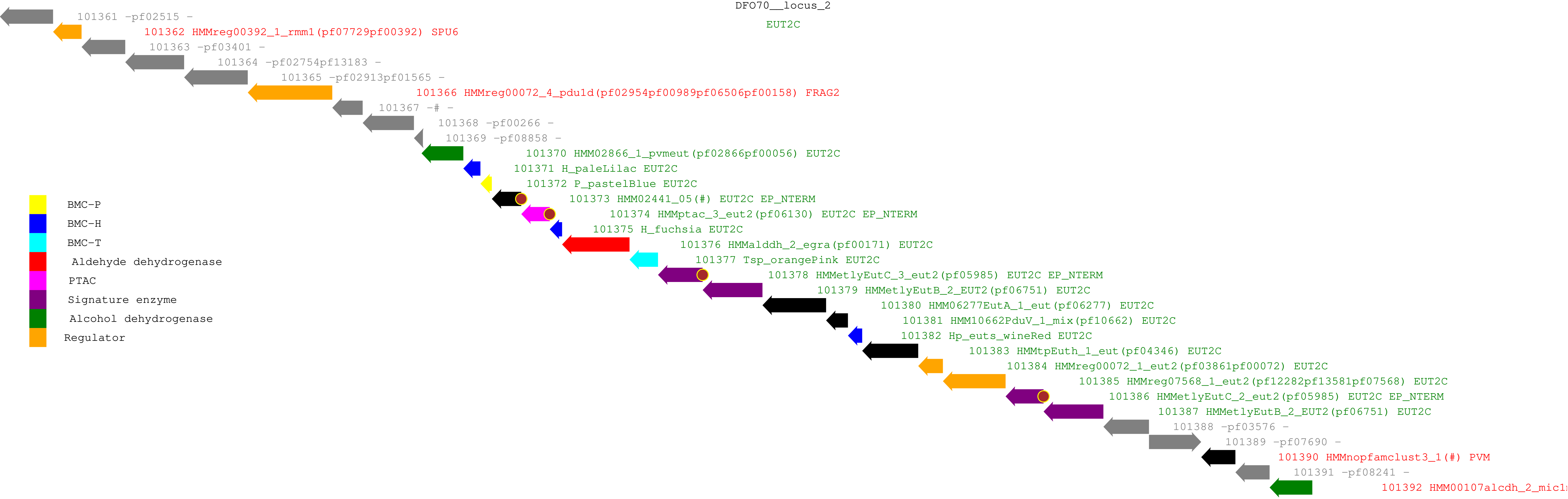

Bacillus oceanisediminis. Bacteria; Firmicutes; Bacilli; Bacillales; Bacillaceae; Bacillus.

DFO72__locus_1

EUT2C

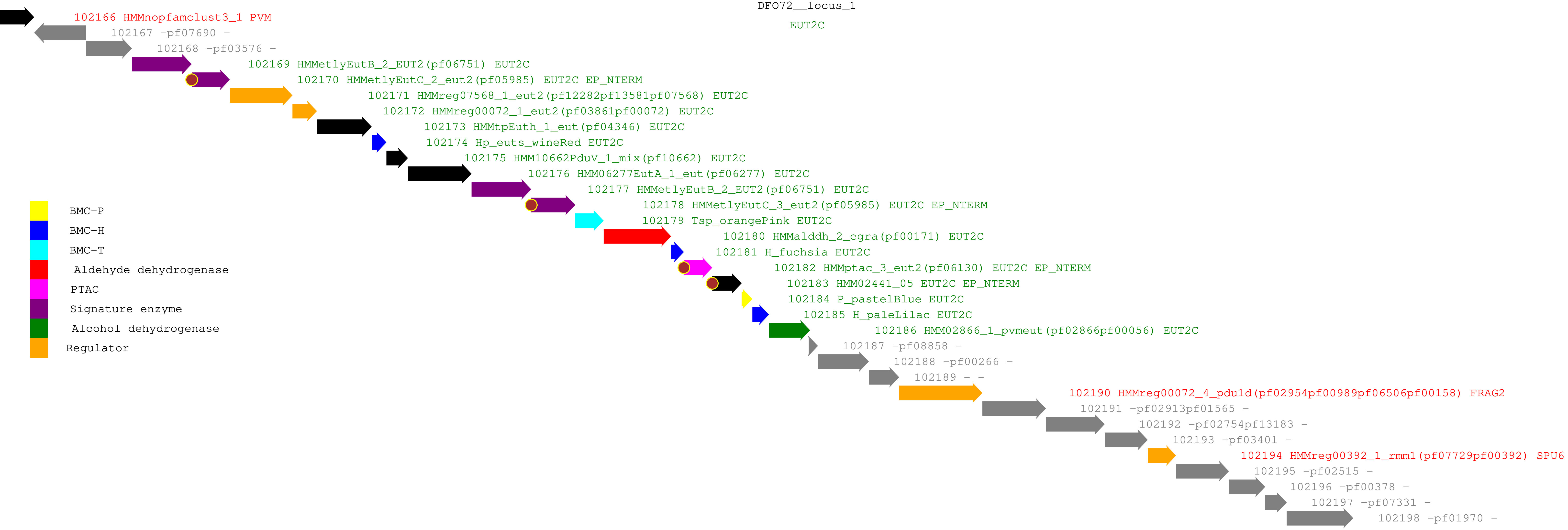

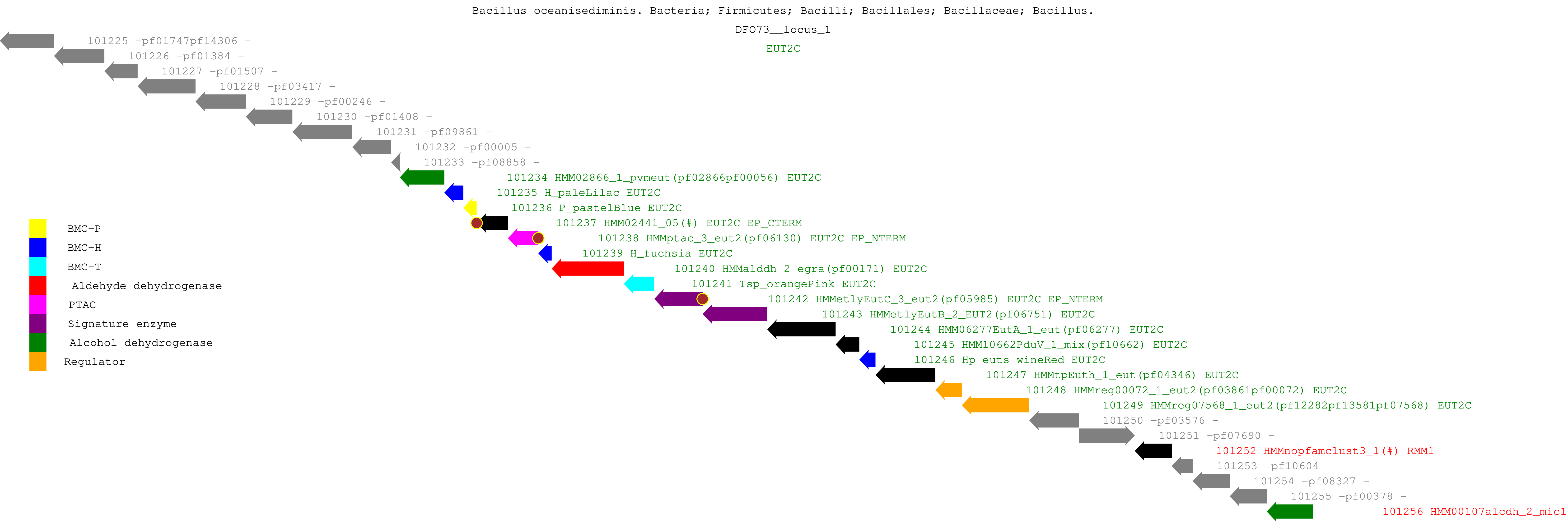

Bacillus oceanisediminis. Bacteria; Firmicutes; Bacilli; Bacillales; Bacillaceae; Bacillus.
DFO73__locus_1
EUT2C
101225 -pf01747pf14306 -
101226 -pf01384 -
101227 -pf01507 -
101228 -pf03417 -
101229 -pf00246 -
101230 -pf01408 -
101231 -pf09861 -
101232 -pf00005 -
101233 -pf08858 -
101234 HMM02866_1_pvmeut(pf02866pf00056) EUT2C
101235 H_paleLilac EUT2C
101236 P_pastelBlue EUT2C
101237 HMM02441_05(#) EUT2C EP_CTERM
101238 HMMptac_3_eut2(pf06130) EUT2C EP_NTERM
101239 H_fuchsia EUT2C
101240 HMMalddh_2_egra(pf00171) EUT2C
101241 Tsp_orangePink EUT2C
101242 HMMetlyEutC_3_eut2(pf05985) EUT2C EP_NTERM
101243 HMMetlyEutB_2_EUT2(pf06751) EUT2C
101244 HMM06277EutA_1_eut(pf06277) EUT2C
101245 HMM10662PduV_1_mix(pf10662) EUT2C
101246 Hp_euts_wineRed EUT2C
101247 HMMtpEuth_1_eut(pf04346) EUT2C
101248 HMMreg00072_1_eut2(pf03861pf00072) EUT2C
101249 HMMreg07568_1_eut2(pf12282pf13581pf07568) EUT2C
101250 -pf03576 -
101251 -pf07690 -
101252 HMMnopfamclust3_1(#) RMM1
101253 -pf10604 -
101254 -pf08327 -
101255 -pf00378 -
101256 HMM00107alcdh_2_mic1
BMC-P
BMC-H
BMC-T
Aldehyde dehydrogenase
PTAC
Signature enzyme
Alcohol dehydrogenase
Regulator

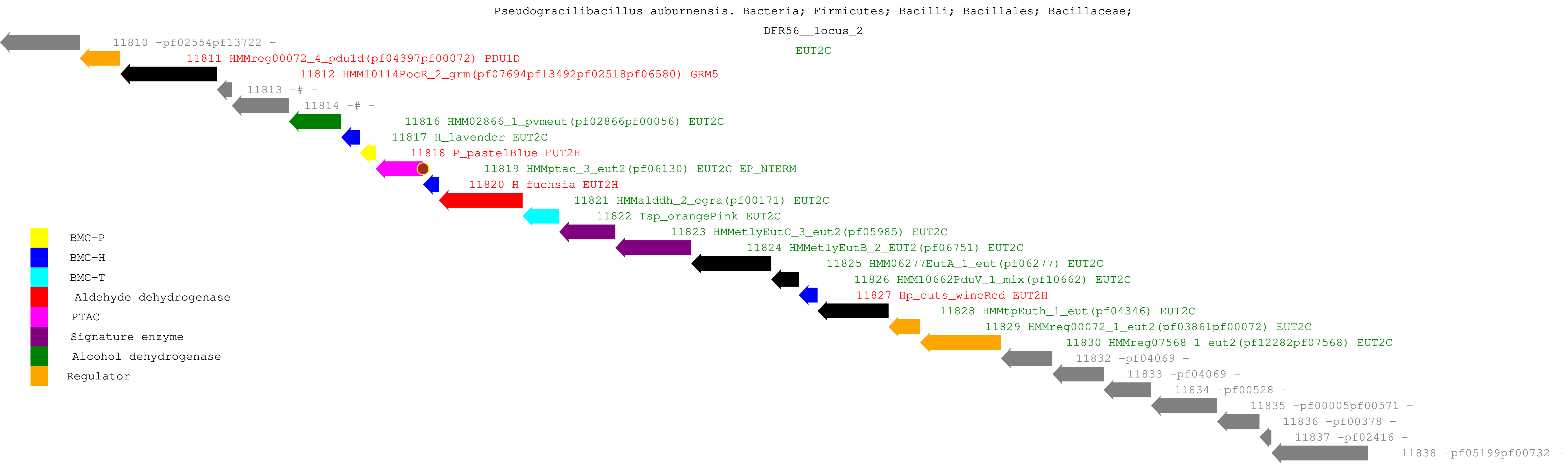

Pseudogracilibacillus auburnensis. Bacteria; Firmicutes; Bacilli; Bacillales; Bacillaceae;
DFR56__locus_2
EUT2C
11810 -pf02554pf13722 -
11811 HMMreg00072_4_pdu1d(pf04397pf00072) PDU1D
11812 HMM10114PocR_2_grm(pf07694pf13492pf02518pf06580) GRM5
11813 -# -
11814 -# -
11816 HMM02866_1_pvmeut(pf02866pf00056) EUT2C
11817 H_lavender EUT2C
11818 P_pastelBlue EUT2H
11819 HMMptac_3_eut2(pf06130) EUT2C EP_NTERM
11820 H_fuchsia EUT2H
11821 HMMalddh_2_egra(pf00171) EUT2C
11822 Tsp_orangePink EUT2C
11823 HMMetlyEutC_3_eut2(pf05985) EUT2C
11824 HMMetlyEutB_2_EUT2(pf06751) EUT2C
11825 HMM06277EutA_1_eut(pf06277) EUT2C
11826 HMM10662PduV_1_mix(pf10662) EUT2C
11827 Hp_euts_wineRed EUT2H
11828 HMMtpEuth_1_eut(pf04346) EUT2C
11829 HMMreg00072_1_eut2(pf03861pf00072) EUT2C
11830 HMMreg07568_1_eut2(pf12282pf07568) EUT2C
11832 -pf04069 -
11833 -pf04069 -
11834 -pf00528 -
11835 -pf00005pf00571 -
11836 -pf00378 -
11837 -pf02416 -
11838 -pf05199pf00732 -
BMC-P
BMC-H
BMC-T
Aldehyde dehydrogenase
PTAC
Signature enzyme
Alcohol dehydrogenase
Regulator

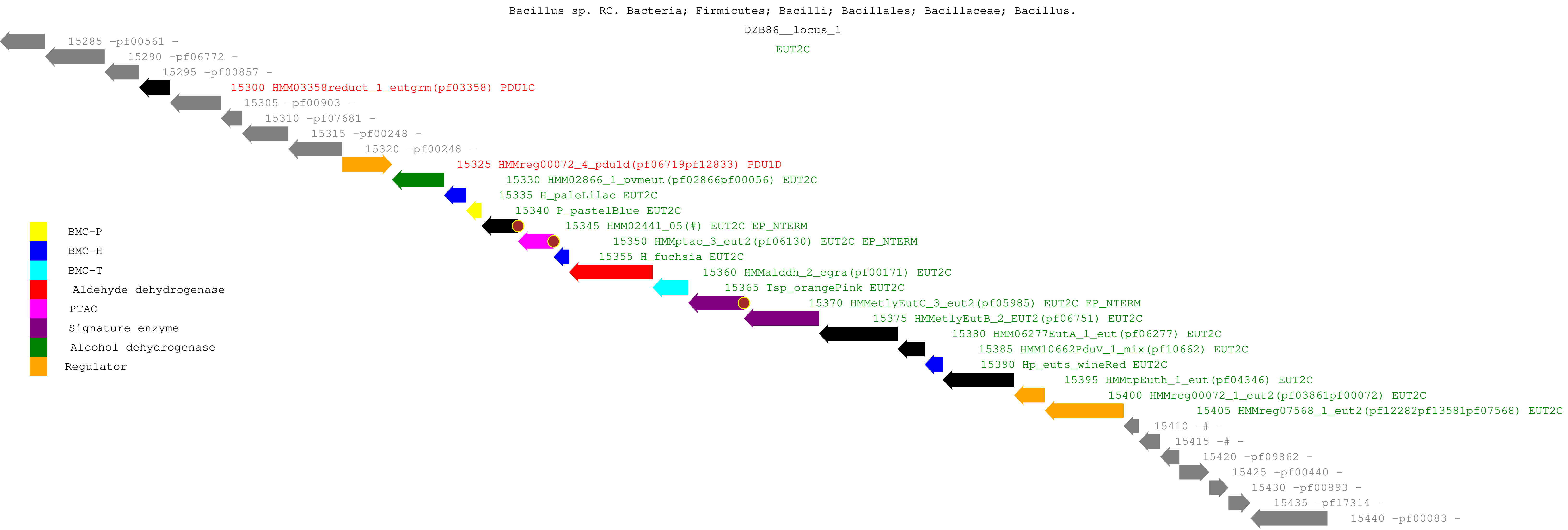

Bacillus sp. RC. Bacteria; Firmicutes; Bacilli; Bacillales; Bacillaceae; Bacillus.
DZB86__locus_1
EUT2C
15285 -pf00561 -
15290 -pf06772 -
15295 -pf00857 -
15300 HMM03358reduct_1_eutgrm(pf03358) PDU1C
15305 -pf00903 -
15310 -pf07681 -
15315 -pf00248 -
15320 -pf00248 -
15325 HMMreg00072_4_pdu1d(pf06719pf12833) PDU1D
15330 HMM02866_1_pvmeut(pf02866pf00056) EUT2C
15335 H_paleLilac EUT2C
15340 P_pastelBlue EUT2C
15345 HMM02441_05(#) EUT2C EP_NTERM
15350 HMMptac_3_eut2(pf06130) EUT2C EP_NTERM
15355 H_fuchsia EUT2C
15360 HMMalddh_2_egra(pf00171) EUT2C
15365 Tsp_orangePink EUT2C
15370 HMMetlyEutC_3_eut2(pf05985) EUT2C EP_NTERM
15375 HMMetlyEutB_2_EUT2(pf06751) EUT2C
15380 HMM06277EutA_1_eut(pf06277) EUT2C
15385 HMM10662PduV_1_mix(pf10662) EUT2C
15390 Hp_euts_wineRed EUT2C
15395 HMMtpEuth_1_eut(pf04346) EUT2C
15400 HMMreg00072_1_eut2(pf03861pf00072) EUT2C
15405 HMMreg07568_1_eut2(pf12282pf13581pf07568) EUT2C
15410 -# -
15415 -# -
15420 -pf09862 -
15425 -pf00440 -
15430 -pf00893 -
15435 -pf17314 -
15440 -pf00083 -
BMC-P
BMC-H
BMC-T
Aldehyde dehydrogenase
PTAC
Signature enzyme
Alcohol dehydrogenase
Regulator

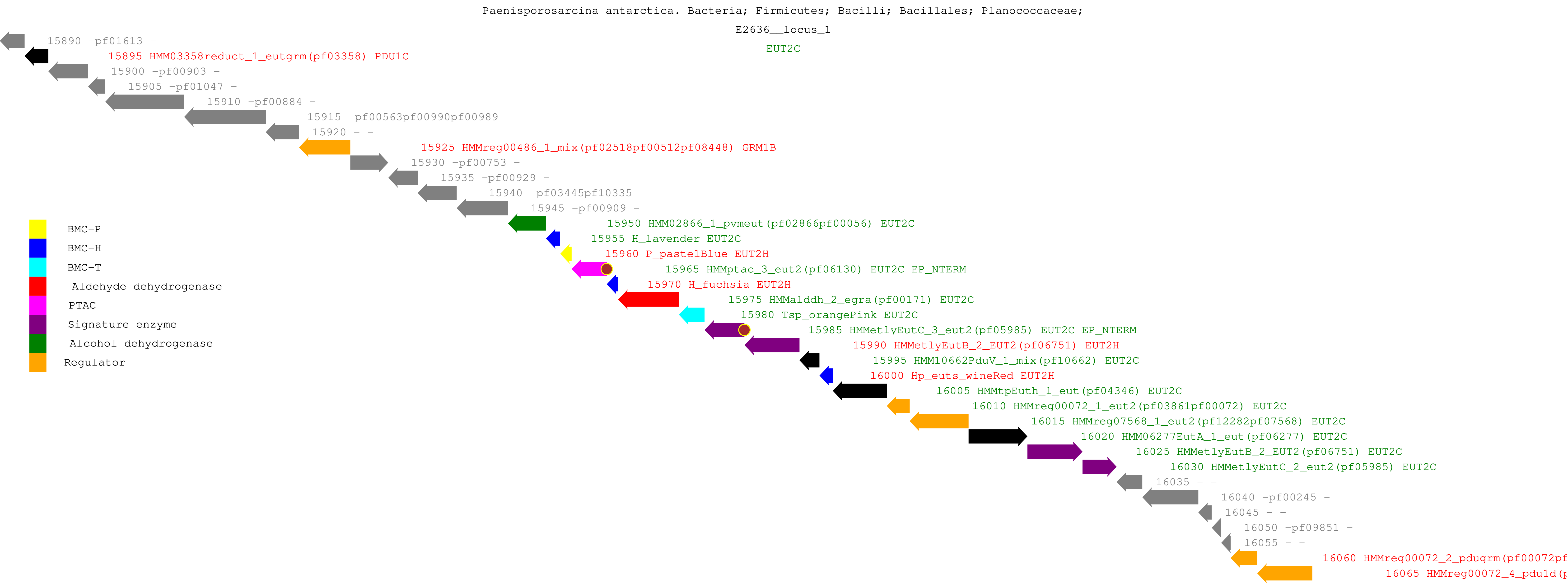

Paenisporosarcina antarctica. Bacteria; Firmicutes; Bacilli; Bacillales; Planococcaceae;
E2636__locus_1
EUT2C
15890 -pf01613 -
15895 HMM03358reduct_1_eutgrm(pf03358) PDU1C
15900 -pf00903 -
15905 -pf01047 -
15910 -pf00884 -
15915 -pf00563pf00990pf00989 -
15920 - -
15925 HMMreg00486_1_mix(pf02518pf00512pf08448) GRM1B
15930 -pf00753 -
15935 -pf00929 -
15940 -pf03445pf10335 -
15945 -pf00909 -
15950 HMM02866_1_pvmeut(pf02866pf00056) EUT2C
15955 H_lavender EUT2C
15960 P_pastelBlue EUT2H
15965 HMMptac_3_eut2(pf06130) EUT2C EP_NTERM
15970 H_fuchsia EUT2H
15975 HMMalddh_2_egra(pf00171) EUT2C
15980 Tsp_orangePink EUT2C
15985 HMMetlyEutC_3_eut2(pf05985) EUT2C EP_NTERM
15990 HMMetlyEutB_2_EUT2(pf06751) EUT2H
15995 HMM10662PduV_1_mix(pf10662) EUT2C
16000 Hp_euts_wineRed EUT2H
16005 HMMtpEuth_1_eut(pf04346) EUT2C
16010 HMMreg00072_1_eut2(pf03861pf00072) EUT2C
16015 HMMreg07568_1_eut2(pf12282pf07568) EUT2C
16020 HMM06277EutA_1_eut(pf06277) EUT2C
16025 HMMetlyEutB_2_EUT2(pf06751) EUT2C
16030 HMMetlyEutC_2_eut2(pf05985) EUT2C
16035 - -
16040 -pf00245 -
16045 - -
16050 -pf09851 -
16055 - -
16060 HMMreg00072_2_pdugrm(pf00072pf
16065 HMMreg00072_4_pdu1d(p
BMC-P
BMC-H
BMC-T
Aldehyde dehydrogenase
PTAC
Signature enzyme
Alcohol dehydrogenase
Regulator

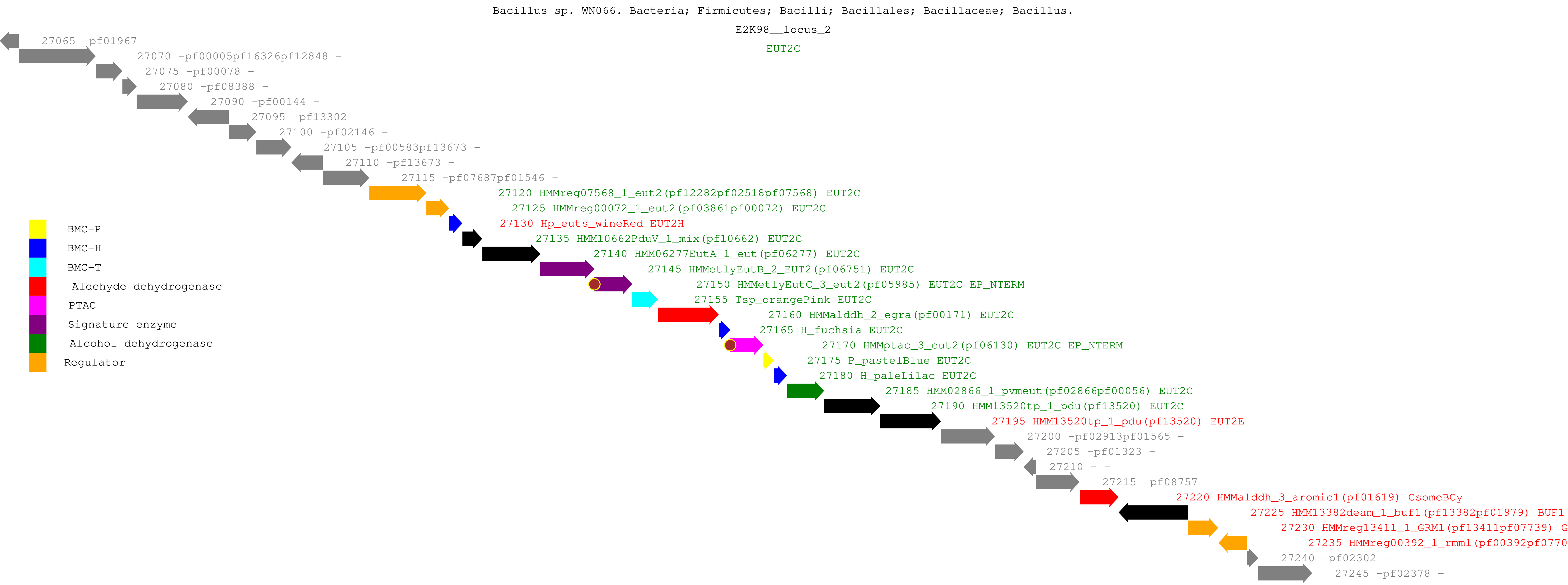

Bacillus sp. WN066. Bacteria; Firmicutes; Bacilli; Bacillales; Bacillaceae; Bacillus.
E2K98__locus_2
EUT2C
27065 -pf01967 -
27070 -pf00005pf16326pf12848 -
27075 -pf00078 -
27080 -pf08388 -
27090 -pf00144 -
27095 -pf13302 -
27100 -pf02146 -
27105 -pf00583pf13673 -
27110 -pf13673 -
27115 -pf07687pf01546 -
27120 HMMreg07568_1_eut2(pf12282pf02518pf07568) EUT2C
27125 HMMreg00072_1_eut2(pf03861pf00072) EUT2C
27130 Hp_euts_wineRed EUT2H
27135 HMM10662PduV_1_mix(pf10662) EUT2C
27140 HMM06277EutA_1_eut(pf06277) EUT2C
27145 HMMetlyEutB_2_EUT2(pf06751) EUT2C
27150 HMMetlyEutC_3_eut2(pf05985) EUT2C EP_NTERM
27155 Tsp_orangePink EUT2C
27160 HMMaldh_2_egra(pf00171) EUT2C
27165 H_fuchsia EUT2C
27170 HMMptac_3_eut2(pf06130) EUT2C EP_NTERM
27175 P_pastelBlue EUT2C
27180 H_paleLilac EUT2C
27185 HMM02866_1_pvmeut(pf02866pf00056) EUT2C
27190 HMM13520tp_1_pdu(pf13520) EUT2C
27195 HMM13520tp_1_pdu(pf13520) EUT2E
27200 -pf02913pf01565 -
27205 -pf01323 -
27210 - -
27215 -pf08757 -
27220 HMMaldh_3_aromic1(pf01619) CsomeBCy
27225 HMM13382deam_1_buf1(pf13382pf01979) BUF1
27230 HMMreg13411_1_GRM1(pf13411pf07739) G
27235 HMMreg00392_1_rmm1(pf00392pf0770
27240 -pf02302 -
27245 -pf02378 -
BMC-P
BMC-H
BMC-T
Aldehyde dehydrogenase
PTAC
Signature enzyme
Alcohol dehydrogenase
Regulator

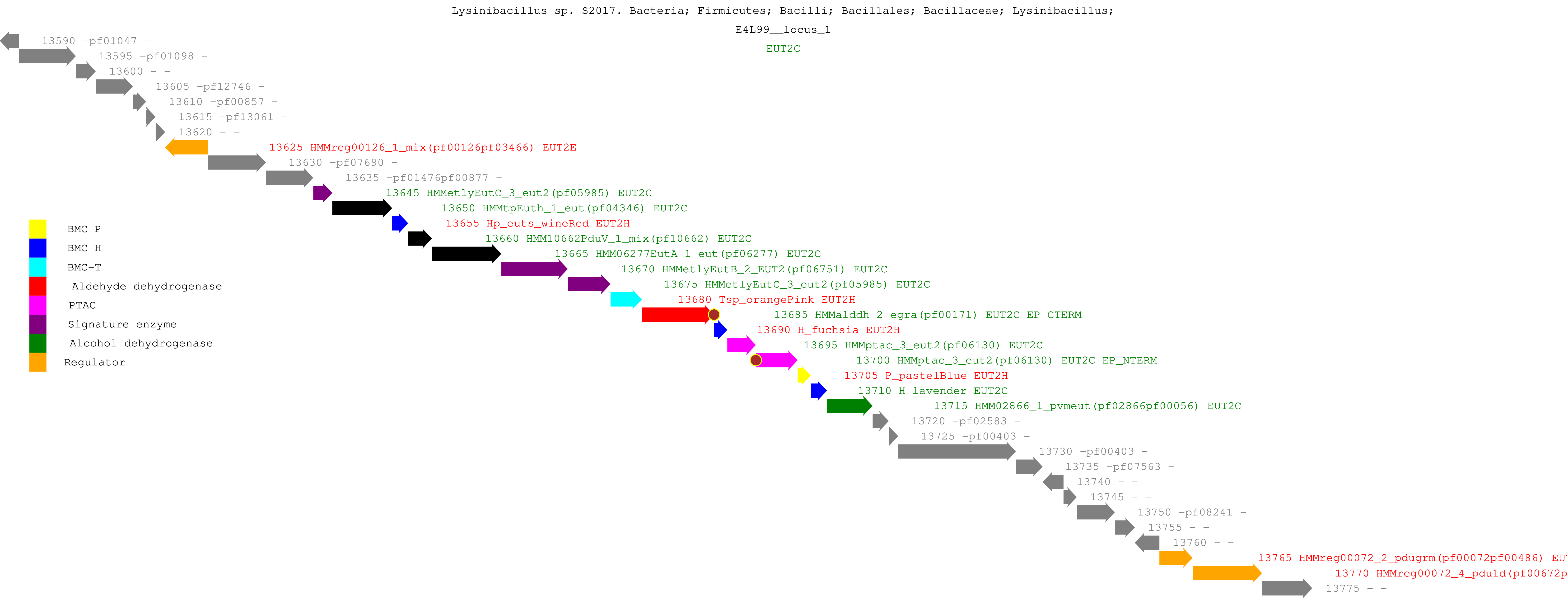

Lysinibacillus sp. S2017. Bacteria; Firmicutes; Bacilli; Bacillales; Bacillaceae; Lysinibacillus;
E4L99__locus_1
EUT2C
13590 -pf01047 -
13595 -pf01098 -
13600 - -
13605 -pf12746 -
13610 -pf00857 -
13615 -pf13061 -
13620 - -
13625 HMMreg00126_1_mix(pf00126pf03466) EUT2E
13630 -pf07690 -
13635 -pf01476pf00877 -
13645 HMMetlyEutC_3_eut2(pf05985) EUT2C
13650 HMMtpEuth_1_eut(pf04346) EUT2C
13655 Hp_euts_wineRed EUT2H
13660 HMM10662PduV_1_mix(pf10662) EUT2C
13665 HMM06277EutA_1_eut(pf06277) EUT2C
13670 HMMetlyEutB_2_EUT2(pf06751) EUT2C
13675 HMMetlyEutC_3_eut2(pf05985) EUT2C
13680 Tsp_orangePink EUT2H
13685 HMMalddh_2_egra(pf00171) EUT2C EP_CTERM
13690 H_fuchsia EUT2H
13695 HMMptac_3_eut2(pf06130) EUT2C
13700 HMMptac_3_eut2(pf06130) EUT2C EP_NTERM
13705 P_pastelBlue EUT2H
13710 H_lavender EUT2C
13715 HMM02866_1_pvmeut(pf02866pf00056) EUT2C
13720 -pf02583 -
13725 -pf00403 -
13730 -pf00403 -
13735 -pf07563 -
13740 - -
13745 - -
13750 -pf08241 -
13755 - -
13760 - -
13765 HMMreg00072_2_pdugrm(pf00072pf00486) EU
13770 HMMreg00072_4_pduld(pf00672p
13775 - -
BMC-P
BMC-H
BMC-T
Aldehyde dehydrogenase
PTAC
Signature enzyme
Alcohol dehydrogenase
Regulator

Paenibacillus sp. N2SHLJ1. Bacteria; Firmicutes; Bacilli; Bacillales; Paenibacillaceae; Paenibacillus.

EYB31__locus_1

EUT2C

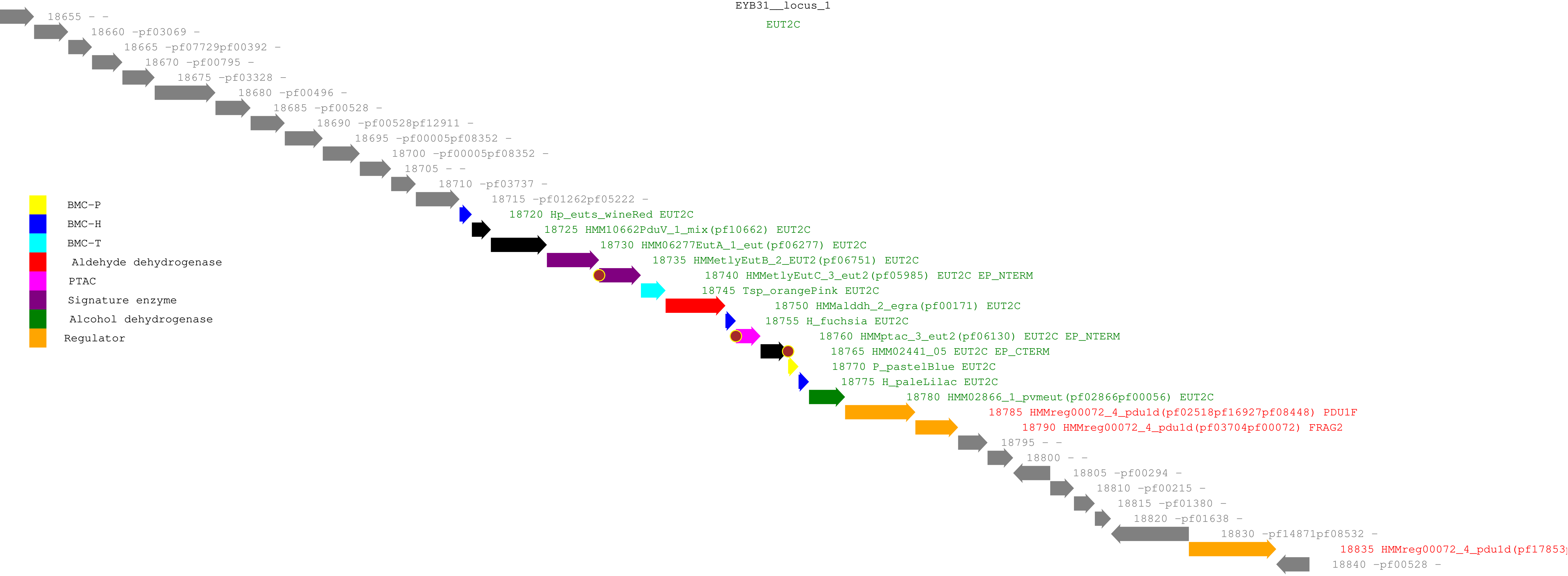

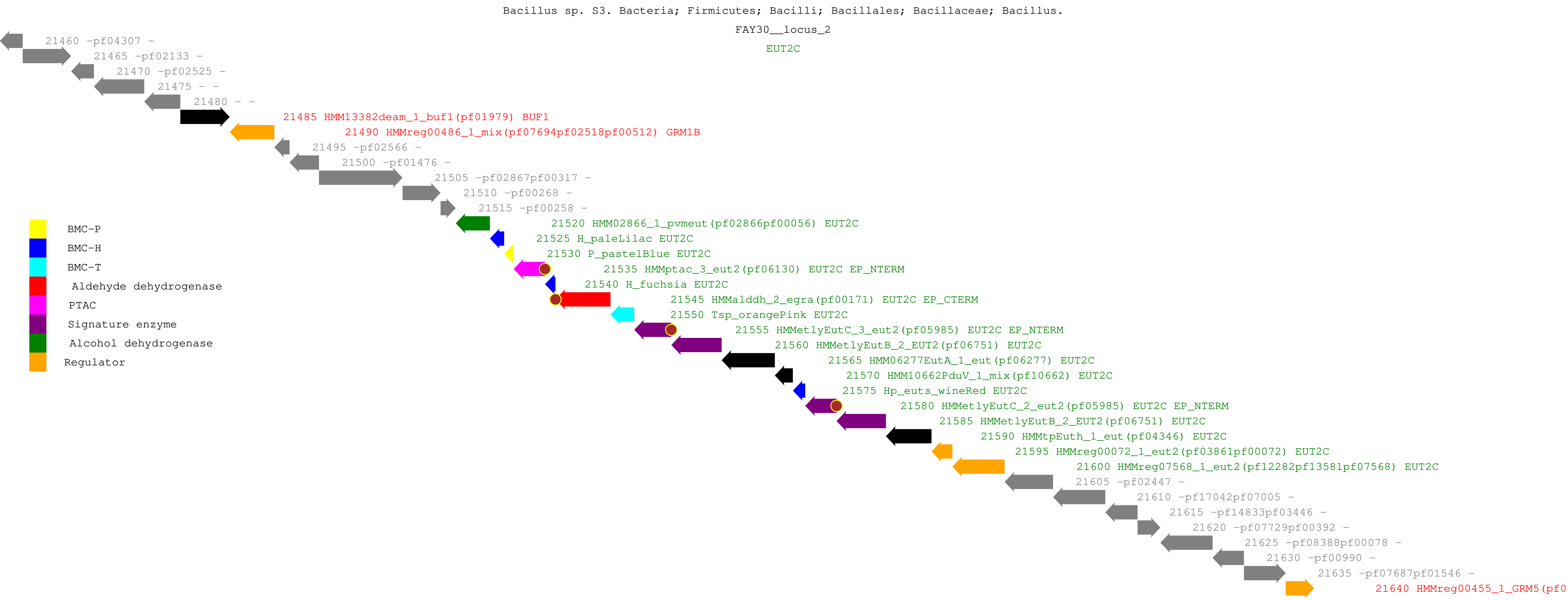

Bacillus sp. S3. Bacteria; Firmicutes; Bacilli; Bacillales; Bacillaceae; Bacillus.
FAY30__locus_2
EUT2C
21460 -pf04307 -
21465 -pf02133 -
21470 -pf02525 -
21475 - -
21480 - -
21485 HMM13382deam_1_buf1(pf01979) BUF1
21490 HMMreg00486_1_mix(pf07694pf02518pf00512) GRM1B
21495 -pf02566 -
21500 -pf01476 -
21505 -pf02867pf00317 -
21510 -pf00268 -
21515 -pf00258 -
21520 HMM02866_1_pvmeut(pf02866pf00056) EUT2C
21525 H_paleLilac EUT2C
21530 P_pastelBlue EUT2C
21535 HMMptac_3_eut2(pf06130) EUT2C EP_NTERM
21540 H_fuchsia EUT2C
21545 HMMaldh_2_egra(pf00171) EUT2C EP_CTERM
21550 Tsp_orangePink EUT2C
21555 HMMetlyEutC_3_eut2(pf05985) EUT2C EP_NTERM
21560 HMMetlyEutB_2_EUT2(pf06751) EUT2C
21565 HMM06277EutA_1_eut(pf06277) EUT2C
21570 HMM10662PduV_1_mix(pf10662) EUT2C
21575 Hp_euts_wineRed EUT2C
21580 HMMetlyEutC_2_eut2(pf05985) EUT2C EP_NTERM
21585 HMMetlyEutB_2_EUT2(pf06751) EUT2C
21590 HMMtpEuth_1_eut(pf04346) EUT2C
21595 HMMreg00072_1_eut2(pf03861pf00072) EUT2C
21600 HMMreg07568_1_eut2(pf12282pf13581pf07568) EUT2C
21605 -pf02447 -
21610 -pf17042pf07005 -
21615 -pf14833pf03446 -
21620 -pf07729pf00392 -
21625 -pf08388pf00078 -
21630 -pf00990 -
21635 -pf07687pf01546 -
21640 HMMreg00455_1_GRM5(pf0
BMC-P
BMC-H
BMC-T
Aldehyde dehydrogenase
PTAC
Signature enzyme
Alcohol dehydrogenase
Regulator

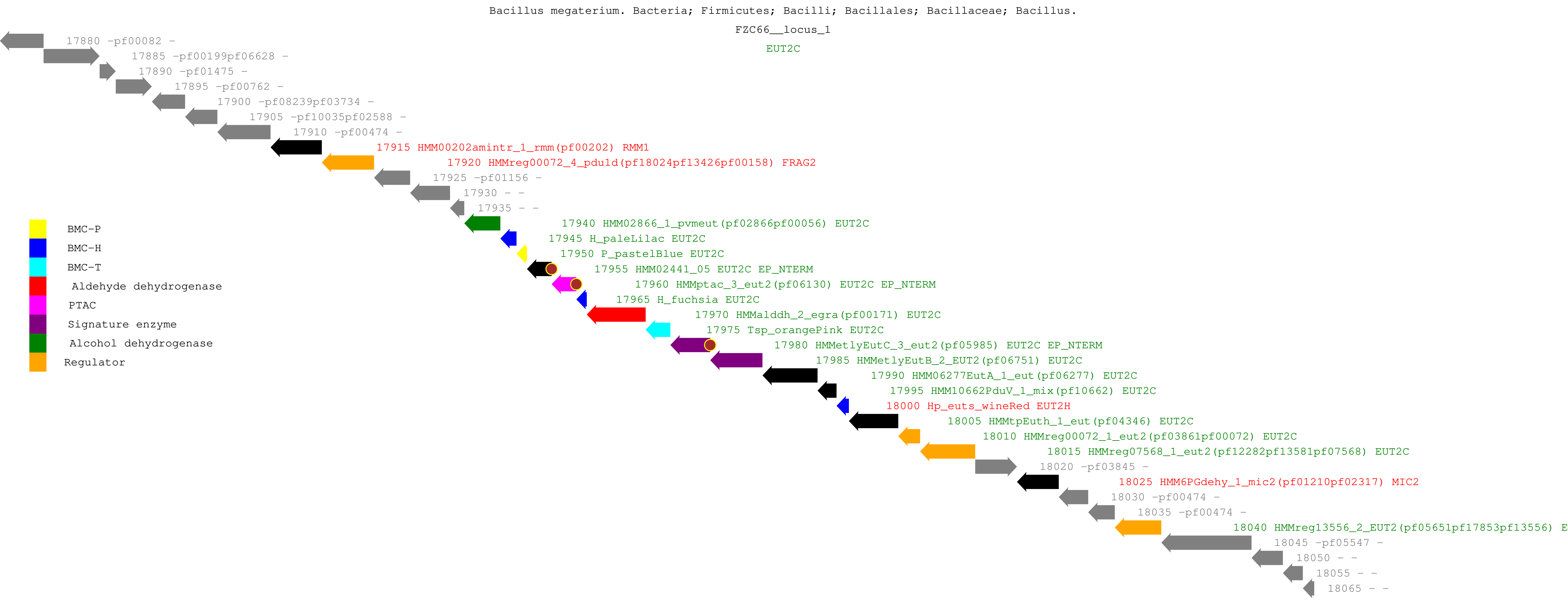

Bacillus megaterium. Bacteria; Firmicutes; Bacilli; Bacillales; Bacillaceae; Bacillus.
FZC66__locus_1
EUT2C
17880 -pf00082 -
17885 -pf00199pf06628 -
17890 -pf01475 -
17895 -pf00762 -
17900 -pf08239pf03734 -
17905 -pf10035pf02588 -
17910 -pf00474 -
17915 HMM00202amintr_1_rmm(pf00202) RMM1
17920 HMMreg00072_4_pdu1d(pf18024pf13426pf00158) FRAG2
17925 -pf01156 -
17930 - -
17935 - -
17940 HMM02866_1_pvmeut(pf02866pf00056) EUT2C
17945 H_paleLilac EUT2C
17950 P_pastelBlue EUT2C
17955 HMM02441_05 EUT2C EP_NTERM
17960 HMMptac_3_eut2(pf06130) EUT2C EP_NTERM
17965 H_fuchsia EUT2C
17970 HMMalddh_2_egra(pf00171) EUT2C
17975 Tsp_orangePink EUT2C
17980 HMMetlyEutC_3_eut2(pf05985) EUT2C EP_NTERM
17985 HMMetlyEutB_2_EUT2(pf06751) EUT2C
17990 HMM06277EutA_1_eut(pf06277) EUT2C
17995 HMM10662PduV_1_mix(pf10662) EUT2C
18000 Hp_euts_wineRed EUT2H
18005 HMMtpEuth_1_eut(pf04346) EUT2C
18010 HMMreg00072_1_eut2(pf03861pf00072) EUT2C
18015 HMMreg07568_1_eut2(pf12282pf13581pf07568) EUT2C
18020 -pf03845 -
18025 HMM6PGdehy_1_mic2(pf01210pf02317) MIC2
18030 -pf00474 -
18035 -pf00474 -
18040 HMMreg13556_2_EUT2(pf05651pf17853pf13556) E
18045 -pf05547 -
18050 - -
18055 - -
18065 - -
BMC-P
BMC-H
BMC-T
Aldehyde dehydrogenase
PTAC
Signature enzyme
Alcohol dehydrogenase
Regulator

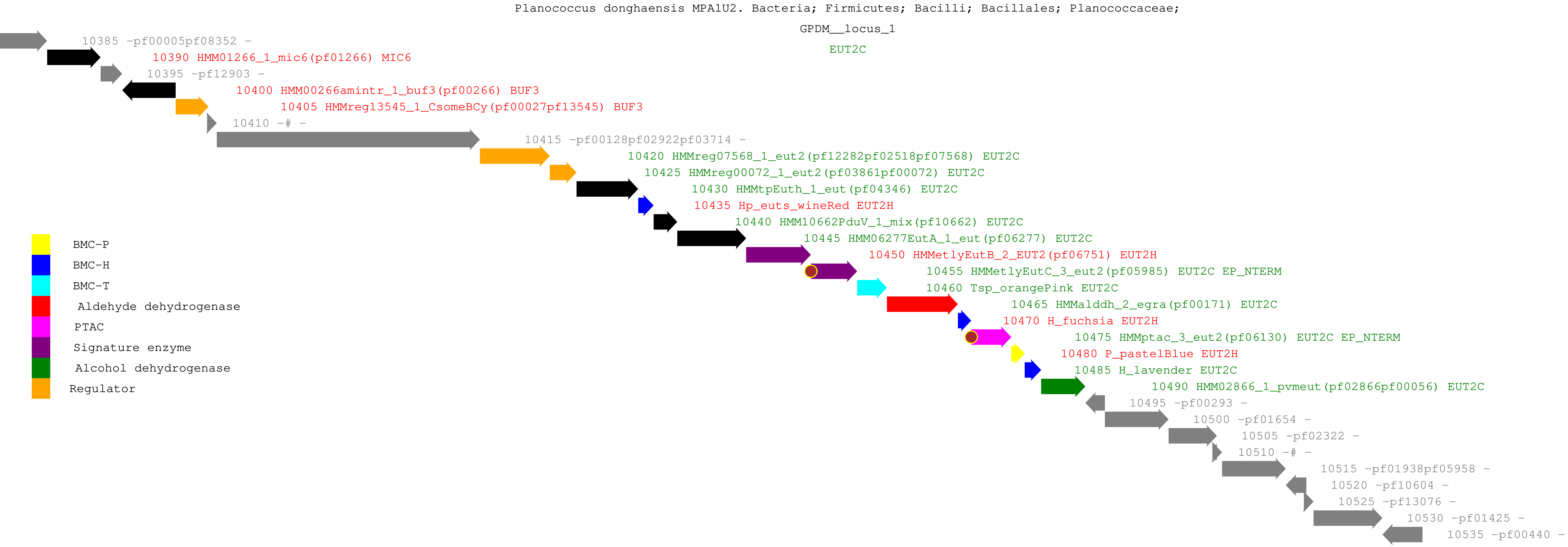

Planococcus donghaensis MPA1U2. Bacteria; Firmicutes; Bacilli; Bacillales; Planococcaceae;
GPDM__locus_1
EUT2C
10385 -pf00005pf08352 -
10390 HMM01266_1_mic6(pf01266) MIC6
10395 -pf12903 -
10400 HMM00266amintr_1_buf3(pf00266) BUF3
10405 HMMreg13545_1_CsomeBCy(pf00027pf13545) BUF3
10410 -# -
10415 -pf00128pf02922pf03714 -
10420 HMMreg07568_1_eut2(pf12282pf02518pf07568) EUT2C
10425 HMMreg00072_1_eut2(pf03861pf00072) EUT2C
10430 HMMtpEuth_1_eut(pf04346) EUT2C
10435 Hp_euts_wineRed EUT2H
10440 HMM10662PduV_1_mix(pf10662) EUT2C
10445 HMM06277EutA_1_eut(pf06277) EUT2C
10450 HMMetlyEutB_2_EUT2(pf06751) EUT2H
10455 HMMetlyEutC_3_eut2(pf05985) EUT2C EP_NTERM
10460 Tsp_orangePink EUT2C
10465 HMMalddh_2_egra(pf00171) EUT2C
10470 H_fuchsia EUT2H
10475 HMMptac_3_eut2(pf06130) EUT2C EP_NTERM
10480 P_pastelBlue EUT2H
10485 H_lavender EUT2C
10490 HMM02866_1_pvmeut(pf02866pf00056) EUT2C
10495 -pf00293 -
10500 -pf01654 -
10505 -pf02322 -
10510 -# -
10515 -pf01938pf05958 -
10520 -pf10604 -
10525 -pf13076 -
10530 -pf01425 -
10535 -pf00440 -
BMC-P
BMC-H
BMC-T
Aldehyde dehydrogenase
PTAC
Signature enzyme
Alcohol dehydrogenase
Regulator

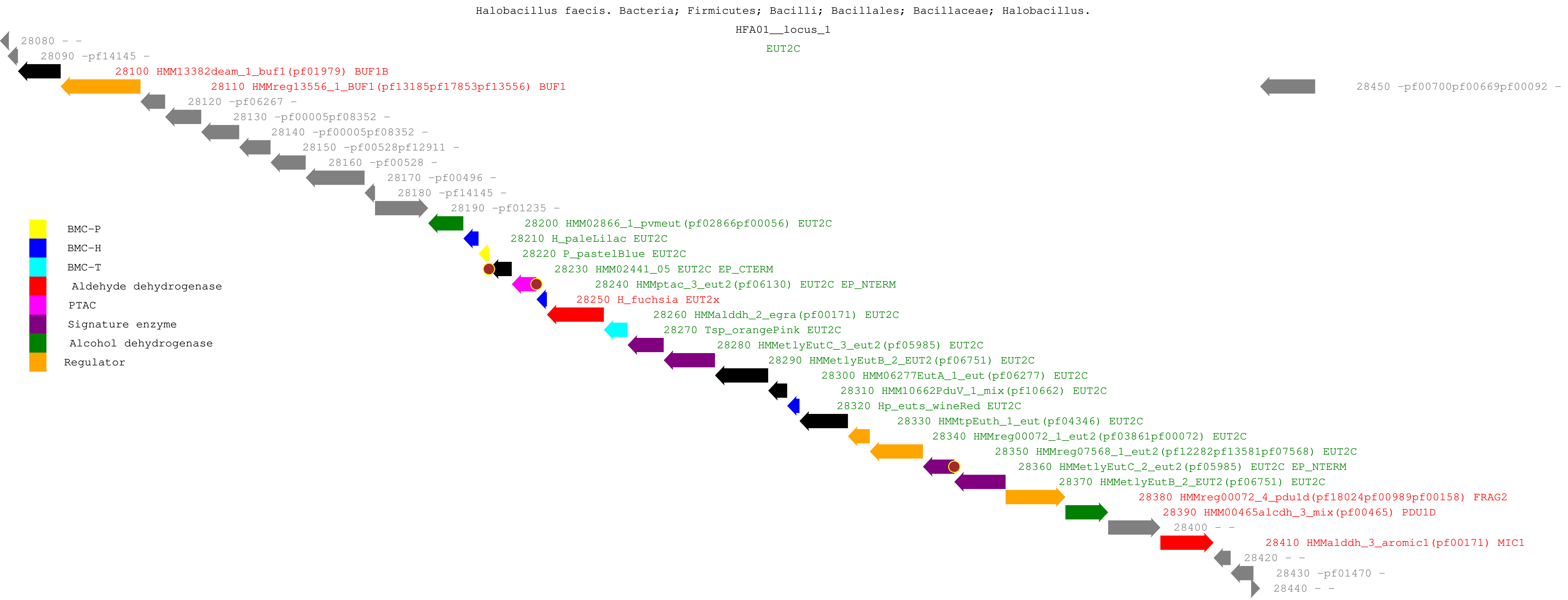
Halobacillus faecis. Bacteria; Firmicutes; Bacilli; Bacillales; Bacillaceae; Halobacillus.
HFA01__locus_1
EUT2C
28080 - -
28090 -pf14145 -
28100 HMM13382deam_1_buf1(pf01979) BUF1B
28110 HMMreg13556_1_BUF1(pf13185pf17853pf13556) BUF1
28450 -pf00700pf00669pf00092 -
28120 -pf06267 -
28130 -pf00005pf08352 -
28140 -pf00005pf08352 -
28150 -pf00528pf12911 -
28160 -pf00528 -
28170 -pf00496 -
28180 -pf14145 -
28190 -pf01235 -
28200 HMM02866_1_pvmeut(pf02866pf00056) EUT2C
28210 H_paleLilac EUT2C
28220 P_pastelBlue EUT2C
28230 HMM02441_05 EUT2C EP_CTERM
28240 HMMptac_3_eut2(pf06130) EUT2C EP_NTERM
28250 H_fuchsia EUT2x
28260 HMMaldh_2_egra(pf00171) EUT2C
28270 Tsp_orangePink EUT2C
28280 HMMetlyEutC_3_eut2(pf05985) EUT2C
28290 HMMetlyEutB_2_EUT2(pf06751) EUT2C
28300 HMM06277EutA_1_eut(pf06277) EUT2C
28310 HMM10662PduV_1_mix(pf10662) EUT2C
28320 Hp_euts_wineRed EUT2C
28330 HMMtpEuth_1_eut(pf04346) EUT2C
28340 HMMreg00072_1_eut2(pf03861pf00072) EUT2C
28350 HMMreg07568_1_eut2(pf12282pf13581pf07568) EUT2C
28360 HMMetlyEutC_2_eut2(pf05985) EUT2C EP_NTERM
28370 HMMetlyEutB_2_EUT2(pf06751) EUT2C
28380 HMMreg00072_4_pdu1d(pf18024pf00989pf00158) FRAG2
28390 HMM00465alcdh_3_mix(pf00465) PDU1D
28400 - -
28410 HMMaldh_3_aromic1(pf00171) MIC1
28420 - -
28430 -pf01470 -
28440 - -
BMC-P
BMC-H
BMC-T
Aldehyde dehydrogenase
PTAC
Signature enzyme
Alcohol dehydrogenase
Regulator

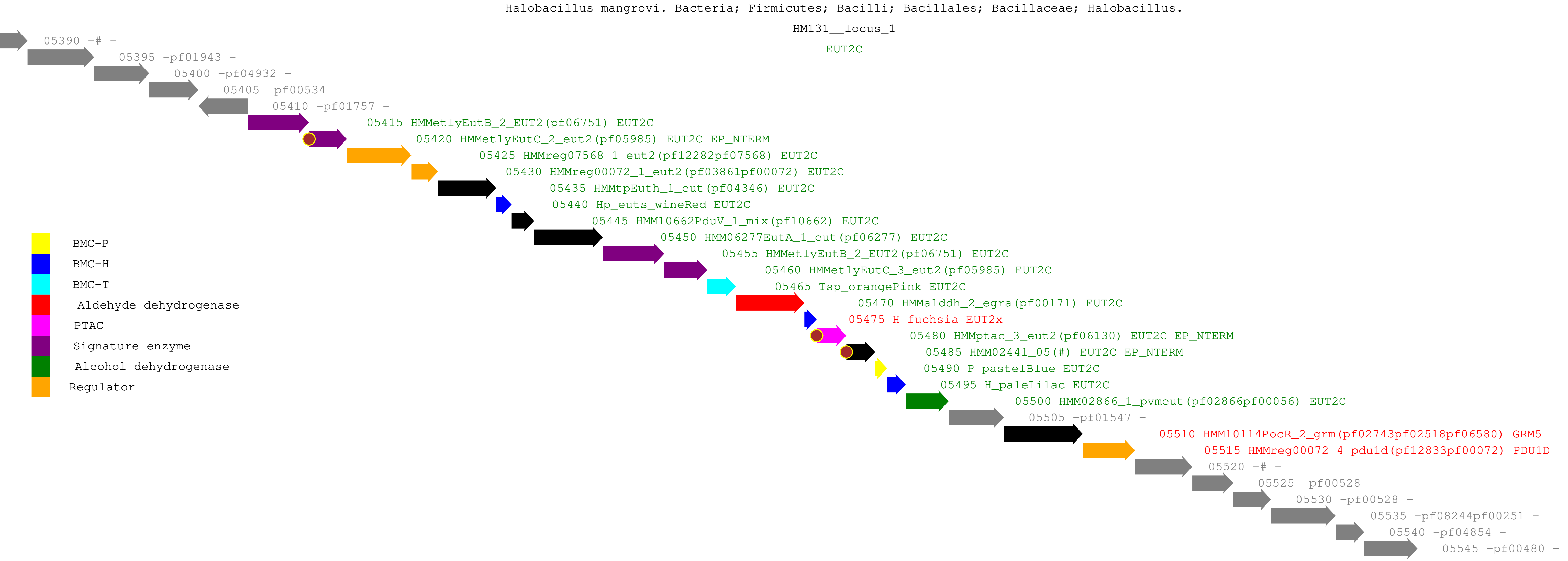
Halobacillus mangrovi. Bacteria; Firmicutes; Bacilli; Bacillales; Bacillaceae; Halobacillus.
HM131__locus_1
EUT2C
05390 -# -
05395 -pf01943 -
05400 -pf04932 -
05405 -pf00534 -
05410 -pf01757 -
05415 HMMetlyEutB_2_EUT2(pf06751) EUT2C
05420 HMMetlyEutC_2_eut2(pf05985) EUT2C EP_NTERM
05425 HMMreg07568_1_eut2(pf12282pf07568) EUT2C
05430 HMMreg00072_1_eut2(pf03861pf00072) EUT2C
05435 HMMtpEuth_1_eut(pf04346) EUT2C
05440 Hp_euts_wineRed EUT2C
05445 HMM10662PduV_1_mix(pf10662) EUT2C
05450 HMM06277EutA_1_eut(pf06277) EUT2C
05455 HMMetlyEutB_2_EUT2(pf06751) EUT2C
05460 HMMetlyEutC_3_eut2(pf05985) EUT2C
05465 Tsp_orangePink EUT2C
05470 HMMalddh_2_egra(pf00171) EUT2C
05475 H_fuchsia EUT2x
05480 HMMptac_3_eut2(pf06130) EUT2C EP_NTERM
05485 HMM02441_05(#) EUT2C EP_NTERM
05490 P_pastelBlue EUT2C
05495 H_paleLilac EUT2C
05500 HMM02866_1_pvmeut(pf02866pf00056) EUT2C
05505 -pf01547 -
05510 HMM10114PocR_2_grm(pf02743pf02518pf06580) GRM5
05515 HMMreg00072_4_pdu1d(pf12833pf00072) PDU1D
05520 -# -
05525 -pf00528 -
05530 -pf00528 -
05535 -pf08244pf00251 -
05540 -pf04854 -
05545 -pf00480 -
BMC-P
BMC-H
BMC-T
Aldehyde dehydrogenase
PTAC
Signature enzyme
Alcohol dehydrogenase
Regulator

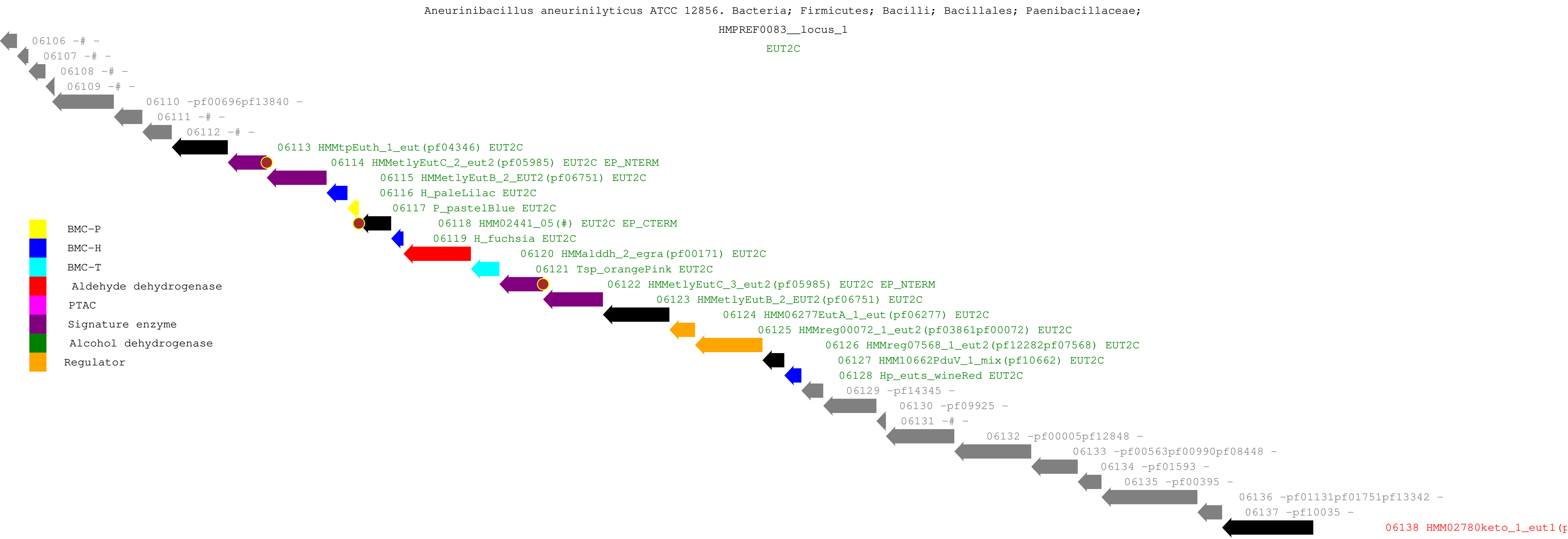

Aneurinibacillus aneurinilyticus ATCC 12856. Bacteria; Firmicutes; Bacilli; Bacillales; Paenibacillaceae;
HMPREF0083__locus_1
EUT2C
06106 -# -
06107 -# -
06108 -# -
06109 -# -
06110 -pf00696pf13840 -
06111 -# -
06112 -# -
06113 HMMtpEuth_1_eut(pf04346) EUT2C
06114 HMMetlyEutC_2_eut2(pf05985) EUT2C EP_NTERM
06115 HMMetlyEutB_2_EUT2(pf06751) EUT2C
06116 H_paleLilac EUT2C
06117 P_pastelBlue EUT2C
06118 HMM02441_05(#) EUT2C EP_CTERM
06119 H_fuchsia EUT2C
06120 HMMalddh_2_egra(pf00171) EUT2C
06121 Tsp_orangePink EUT2C
06122 HMMetlyEutC_3_eut2(pf05985) EUT2C EP_NTERM
06123 HMMetlyEutB_2_EUT2(pf06751) EUT2C
06124 HMM06277EutA_1_eut(pf06277) EUT2C
06125 HMMreg00072_1_eut2(pf03861pf00072) EUT2C
06126 HMMreg07568_1_eut2(pf12282pf07568) EUT2C
06127 HMM10662PduV_1_mix(pf10662) EUT2C
06128 Hp_euts_wineRed EUT2C
06129 -pf14345 -
06130 -pf09925 -
06131 -# -
06132 -pf00005pf12848 -
06133 -pf00563pf00990pf08448 -
06134 -pf01593 -
06135 -pf00395 -
06136 -pf01131pf01751pf13342 -
06137 -pf10035 -
06138 HMM02780keto_1_eut1(p
BMC-P
BMC-H
BMC-T
Aldehyde dehydrogenase
PTAC
Signature enzyme
Alcohol dehydrogenase
Regulator

Bacillus sp. 2_A_57_CT2. Bacteria; Firmicutes; Bacilli; Bacillales; Bacillaceae; Bacillus.

HMPREF1013__locus_1

EUT2C

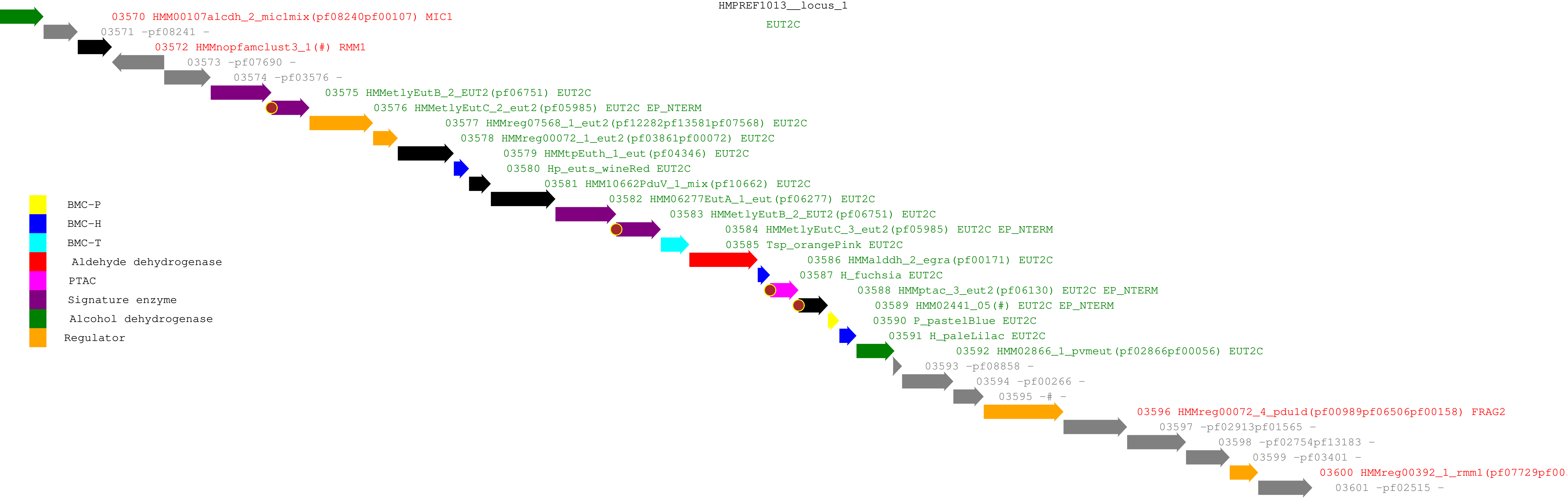

Sporosarcina newyorkensis 2681. Bacteria; Firmicutes; Bacilli; Bacillales; Planococcaceae;

HMPREF9372__locus_1

EUT2C

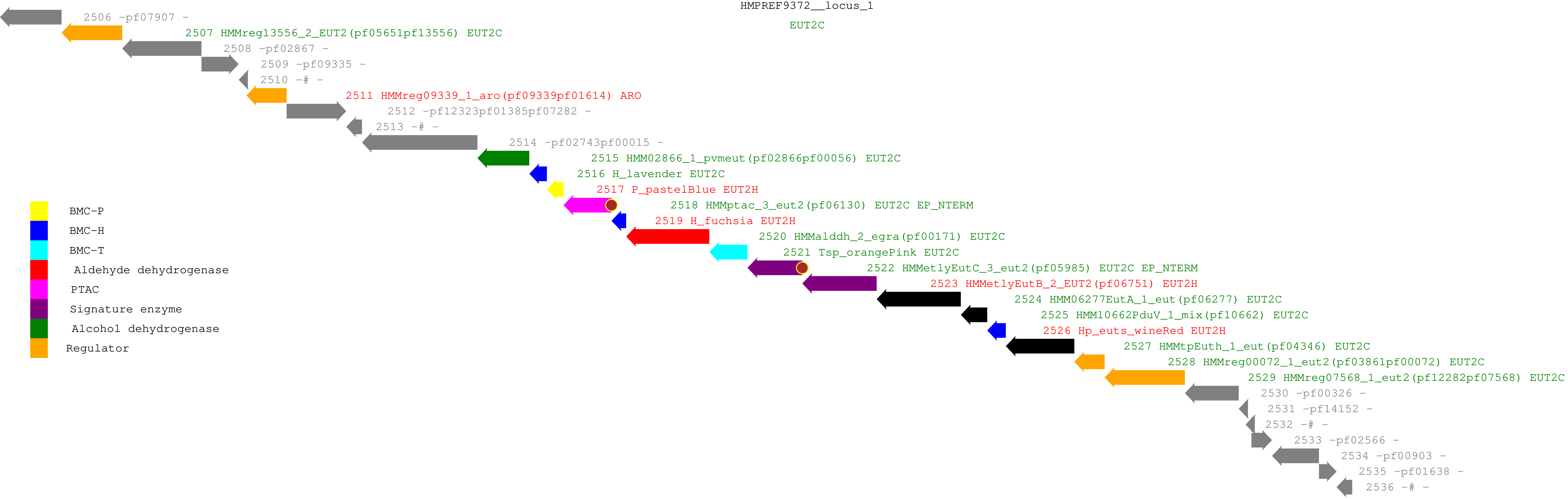

Halobacillus sp. BBL2006. Bacteria; Firmicutes; Bacilli; Bacillales; Bacillaceae; Halobacillus.

LD39__locus_1_main_sat

EUT2C

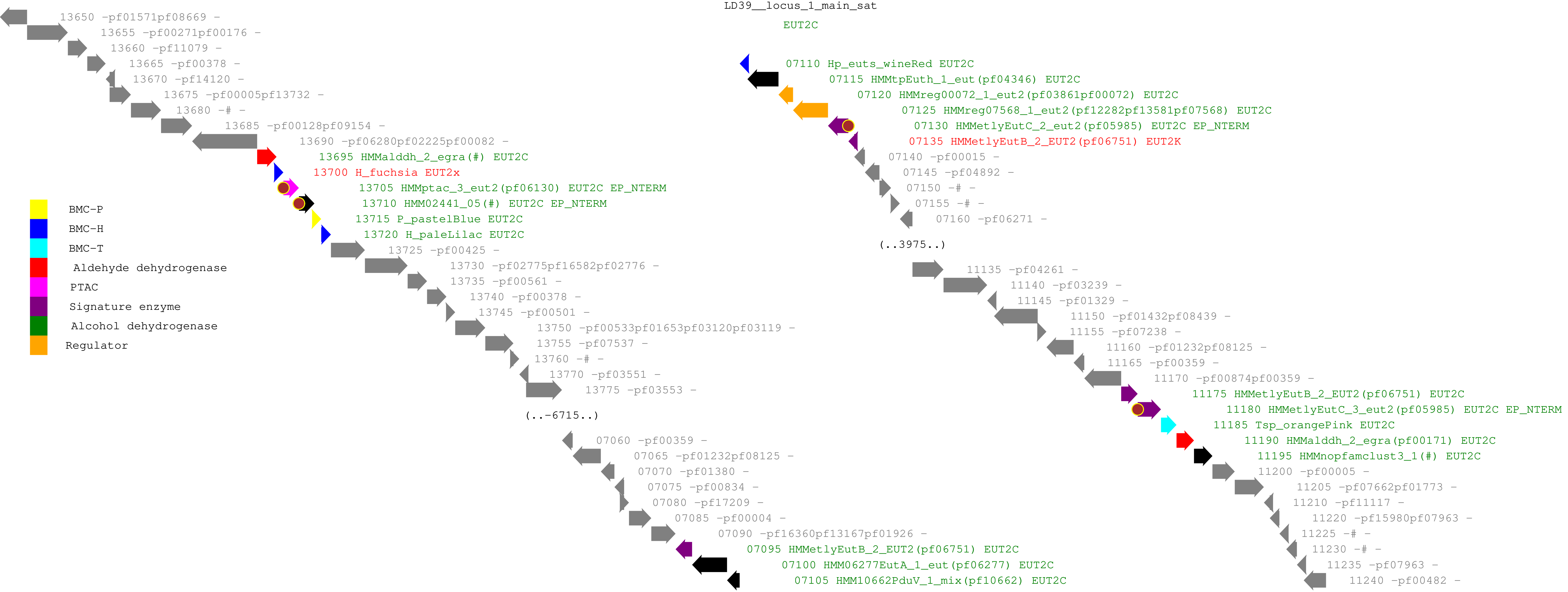

Bacillus sp. (strain OxB-1). Bacteria; Firmicutes; Bacilli; Bacillales; Bacillaceae; Bacillus.

OXB__locus_1

EUT2C

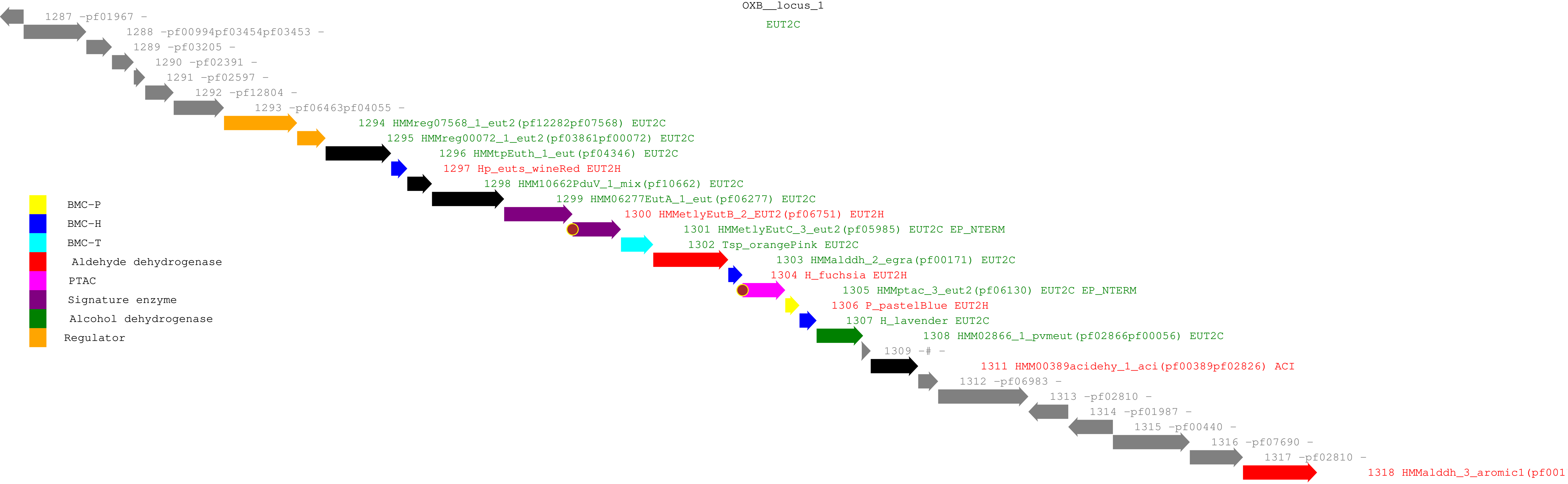

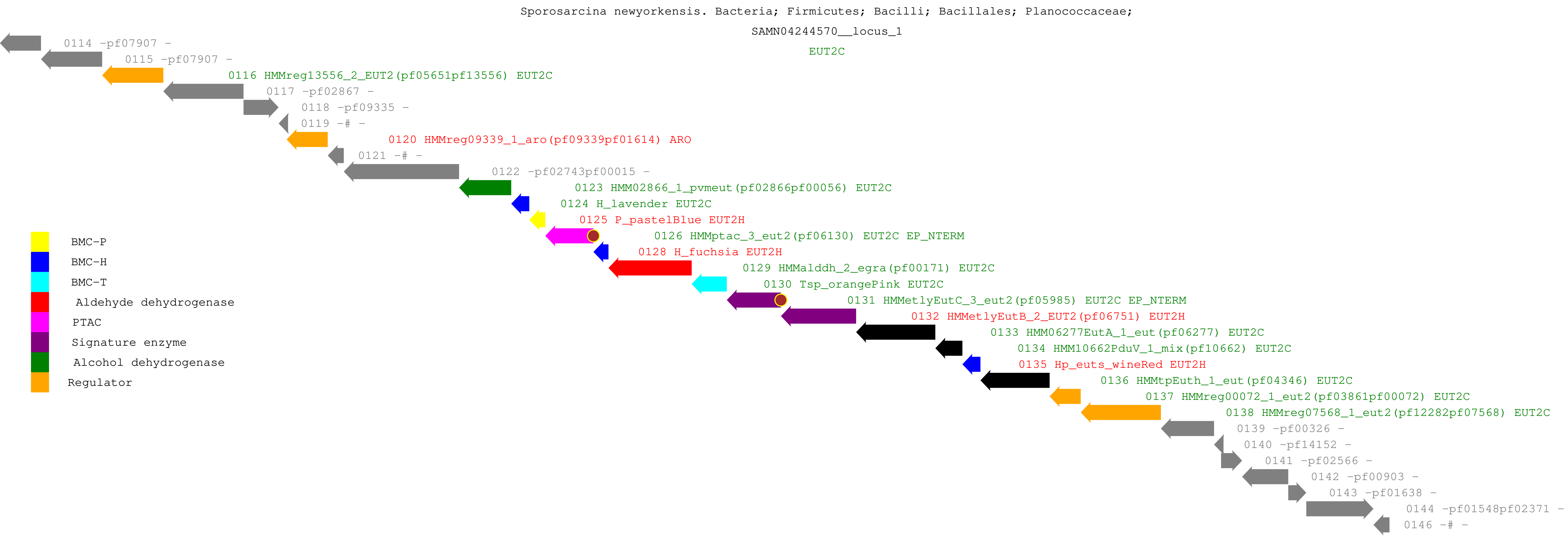
Sporosarcina newyorkensis. Bacteria; Firmicutes; Bacilli; Bacillales; Planococcaceae;
SAMN04244570__locus_1
EUT2C
0114 -pf07907 -
0115 -pf07907 -
0116 HMMreg13556_2_EUT2(pf05651pf13556) EUT2C
0117 -pf02867 -
0118 -pf09335 -
0119 -# -
0120 HMMreg09339_1_aro(pf09339pf01614) ARO
0121 -# -
0122 -pf02743pf00015 -
0123 HMM02866_1_pvmeut(pf02866pf00056) EUT2C
0124 H_lavender EUT2C
0125 P_pastelBlue EUT2H
0126 HMMptac_3_eut2(pf06130) EUT2C EP_NTERM
0128 H_fuchsia EUT2H
0129 HMMalddh_2_egra(pf00171) EUT2C
0130 Tsp_orangePink EUT2C
0131 HMMetlyEutC_3_eut2(pf05985) EUT2C EP_NTERM
0132 HMMetlyEutB_2_EUT2(pf06751) EUT2H
0133 HMM06277EutA_1_eut(pf06277) EUT2C
0134 HMM10662PduV_1_mix(pf10662) EUT2C
0135 Hp_euts_wineRed EUT2H
0136 HMMtpEuth_1_eut(pf04346) EUT2C
0137 HMMreg00072_1_eut2(pf03861pf00072) EUT2C
0138 HMMreg07568_1_eut2(pf12282pf07568) EUT2C
0139 -pf00326 -
0140 -pf14152 -
0141 -pf02566 -
0142 -pf00903 -
0143 -pf01638 -
0144 -pf01548pf02371 -
0146 -# -
BMC-P
BMC-H
BMC-T
Aldehyde dehydrogenase
PTAC
Signature enzyme
Alcohol dehydrogenase
Regulator

Bacillus megaterium. Bacteria; Firmicutes; Bacilli; Bacillales; Bacillaceae; Bacillus.

SAMN04487776__locus_1

EUT2C

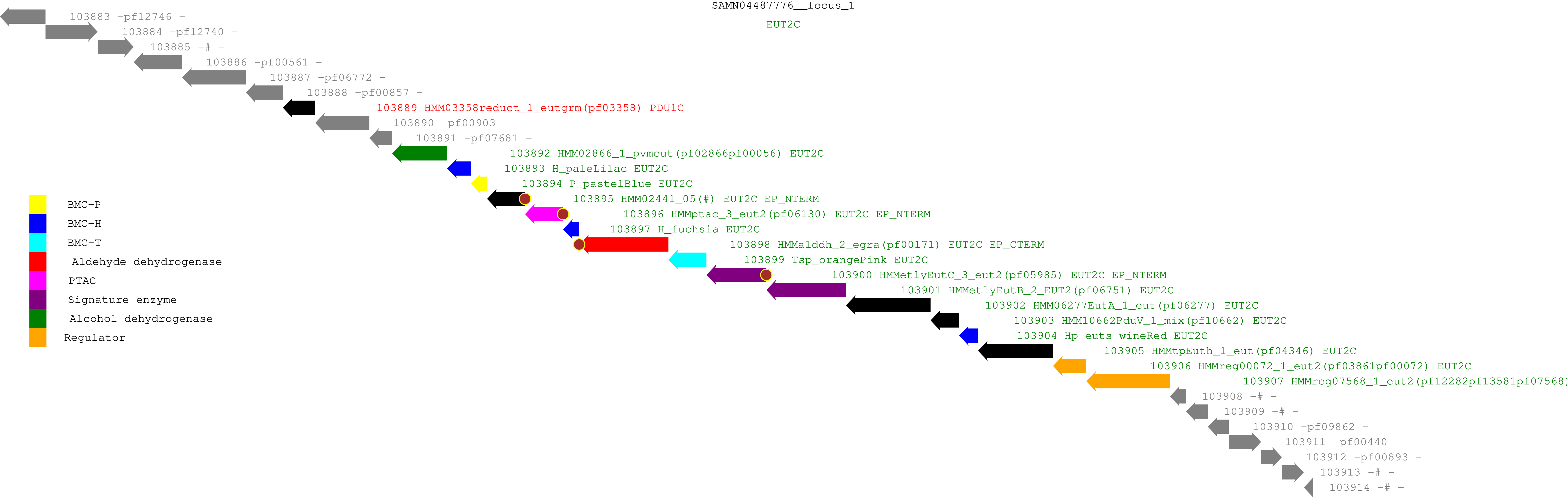

Bacillus aryabhattai B8W22. Bacteria; Firmicutes; Bacilli; Bacillales; Bacillaceae; Bacillus.

SAMN04487777__locus_1

EUT2C

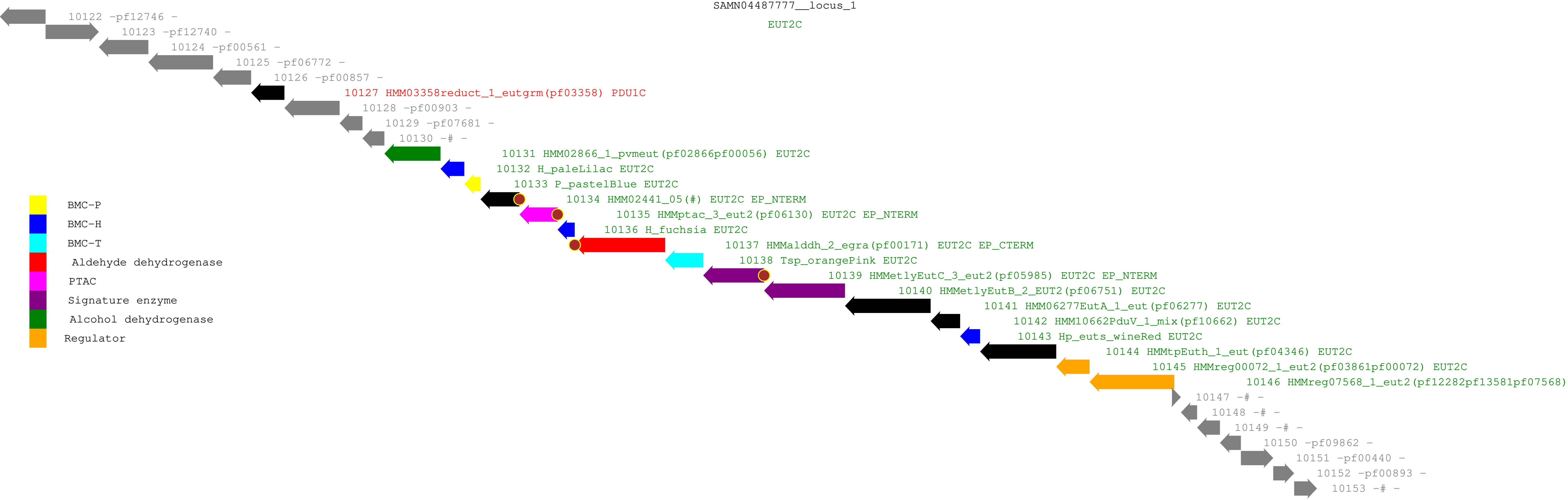

Aneurinibacillus migulanus (Bacillus migulanus). Bacteria; Firmicutes; Bacilli; Bacillales; Paenibacillaceae;

SAMN04487909__locus_1

EUT2C

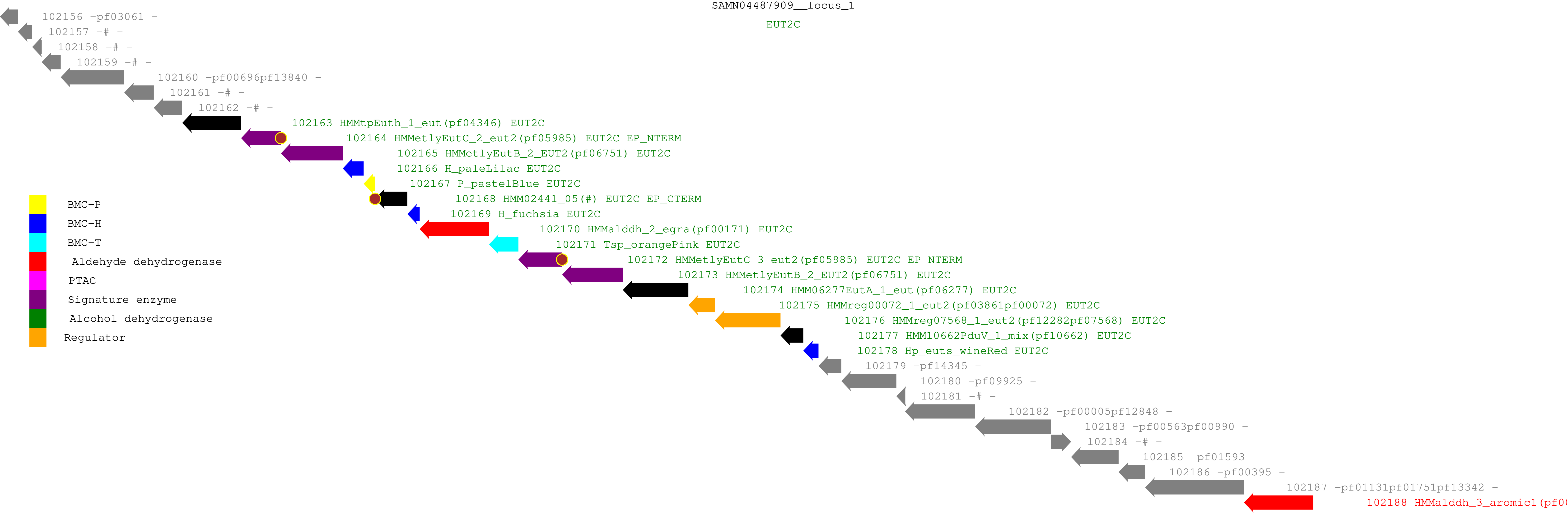

Aneurinibacillus thermoaerophilus. Bacteria; Firmicutes; Bacilli; Bacillales; Paenibacillaceae;

SAMN04489735__locus_1

EUT2C

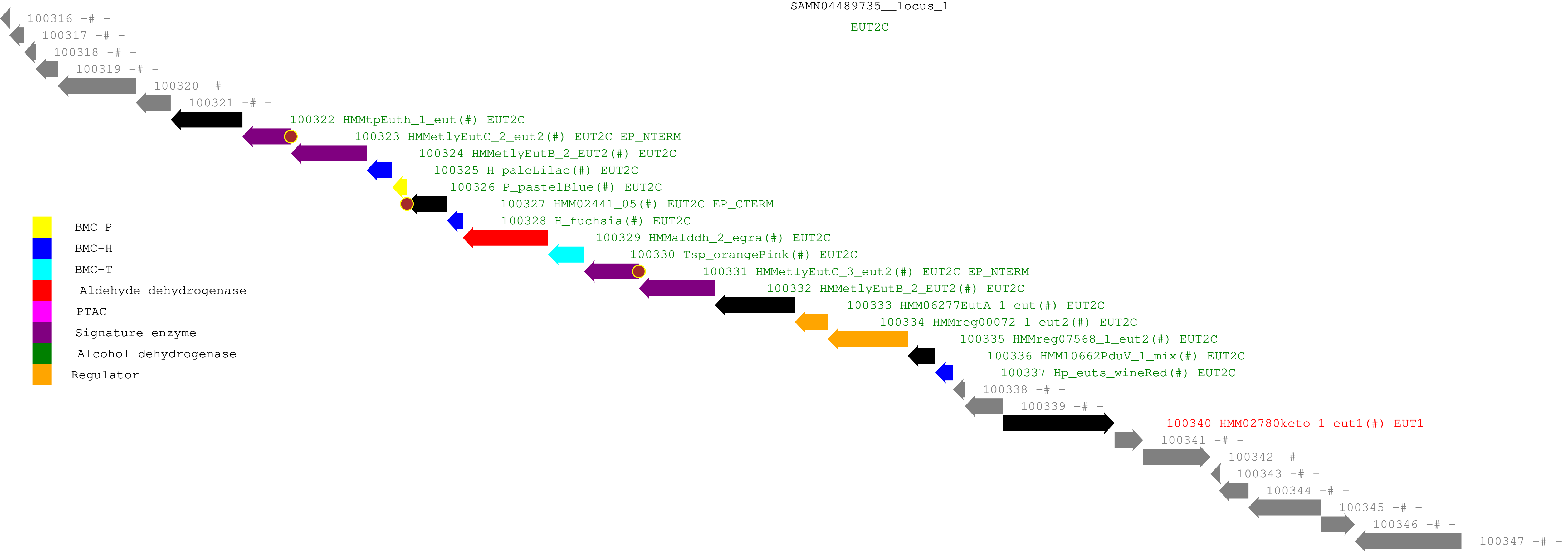

Domibacillus enclensis. Bacteria; Firmicutes; Bacilli; Bacillales; Bacillaceae; Domibacillus.

SAMN05443094__locus_1

EUT2C

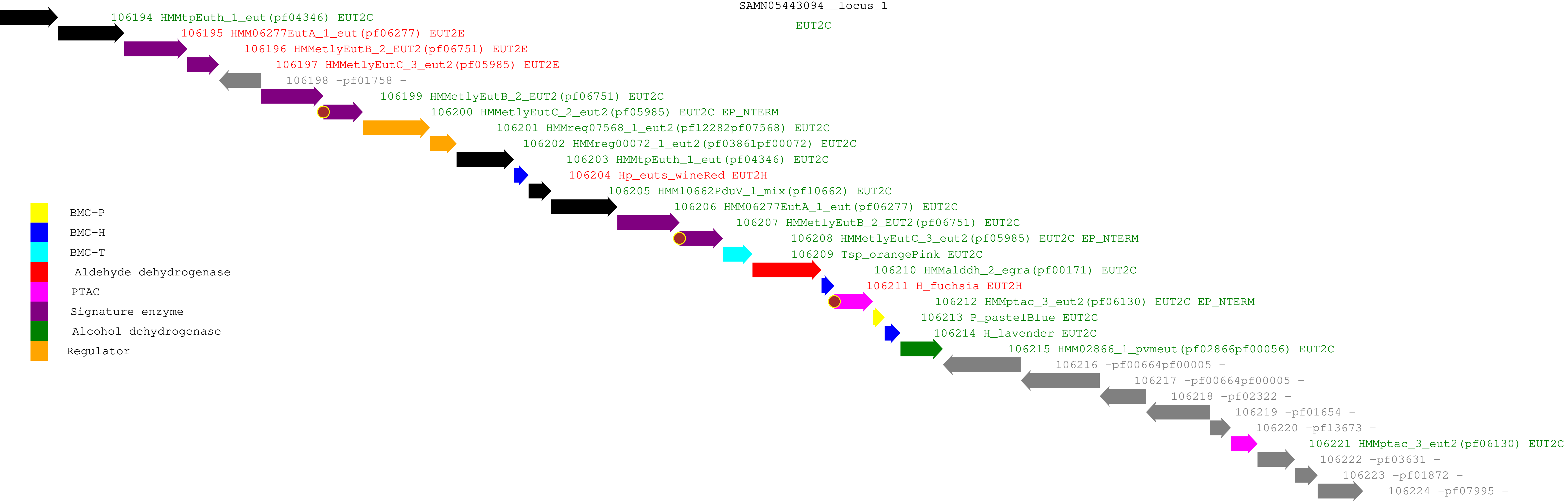

Psychrobacillus sp. OK028. Bacteria; Firmicutes; Bacilli; Bacillales; Bacillaceae;

SAMN05518871__locus_1

EUT2C

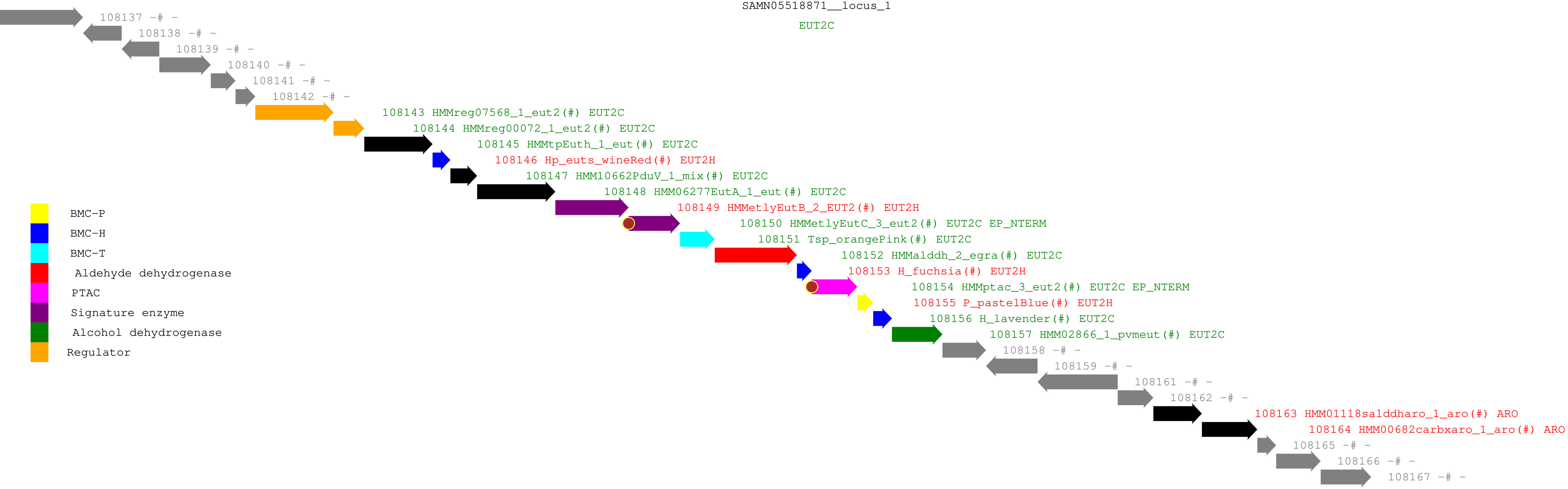

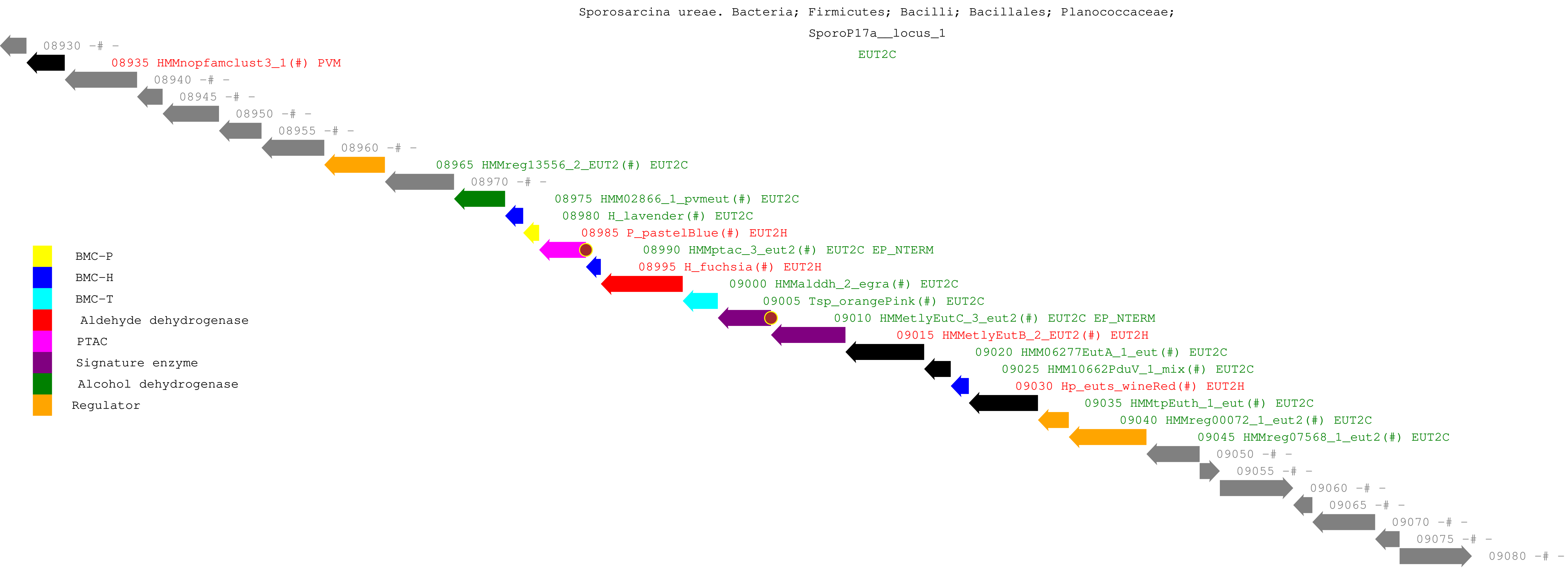
Sporosarcina ureae. Bacteria; Firmicutes; Bacilli; Bacillales; Planococcaceae;
SporoP17a__locus_1
EUT2C
08930 -# -
08935 HMMnopfamclust3_1(#) PVM
08940 -# -
08945 -# -
08950 -# -
08955 -# -
08960 -# -
08965 HMMreg13556_2_EUT2(#) EUT2C
08970 -# -
08975 HMM02866_1_pvmeut(#) EUT2C
08980 H_lavender(#) EUT2C
08985 P_pastelBlue(#) EUT2H
08990 HMMptac_3_eut2(#) EUT2C EP_NTERM
08995 H_fuchsia(#) EUT2H
09000 HMMalddh_2_egra(#) EUT2C
09005 Tsp_orangePink(#) EUT2C
09010 HMMetlyEutC_3_eut2(#) EUT2C EP_NTERM
09015 HMMetlyEutB_2_EUT2(#) EUT2H
09020 HMM06277EutA_1_eut(#) EUT2C
09025 HMM10662PduV_1_mix(#) EUT2C
09030 Hp_euts_wineRed(#) EUT2H
09035 HMMtpEuth_1_eut(#) EUT2C
09040 HMMreg00072_1_eut2(#) EUT2C
09045 HMMreg07568_1_eut2(#) EUT2C
09050 -# -
09055 -# -
09060 -# -
09065 -# -
09070 -# -
09075 -# -
09080 -# -
BMC-P
BMC-H
BMC-T
Aldehyde dehydrogenase
PTAC
Signature enzyme
Alcohol dehydrogenase
Regulator

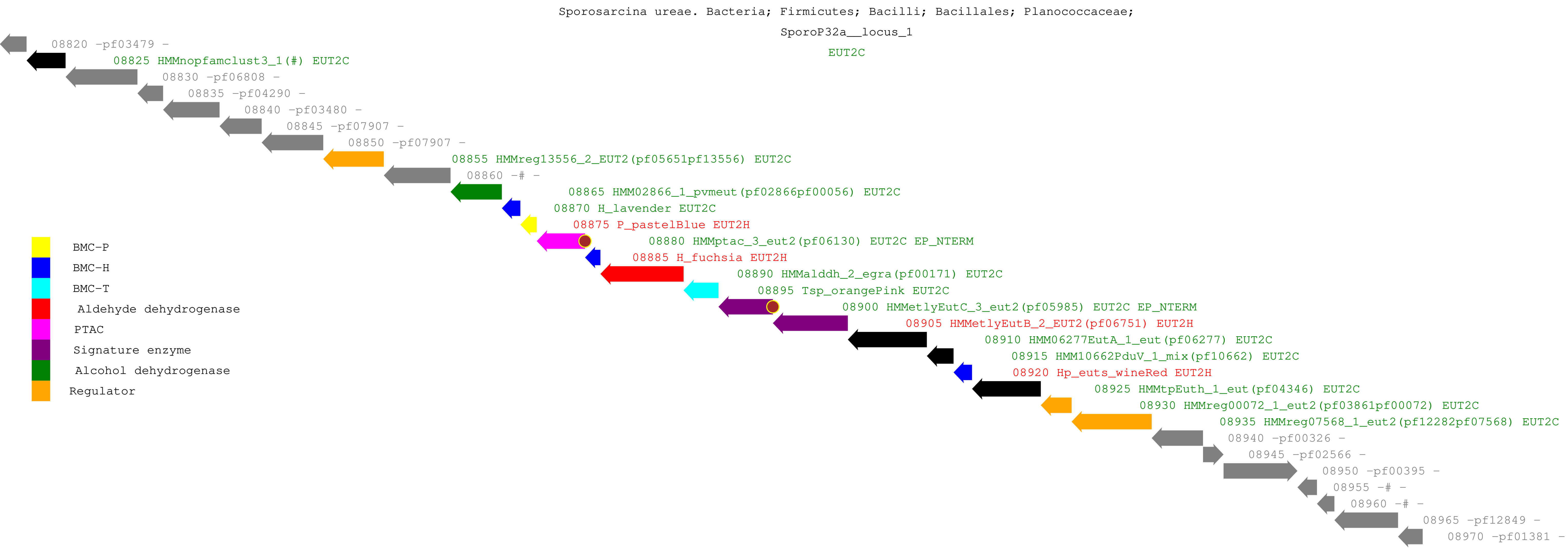

Sporosarcina ureae. Bacteria; Firmicutes; Bacilli; Bacillales; Planococcaceae;
SporoP32a__locus_1
EUT2C
08820 -pf03479 -
08825 HMMnopfamclust3_1(#) EUT2C
08830 -pf06808 -
08835 -pf04290 -
08840 -pf03480 -
08845 -pf07907 -
08850 -pf07907 -
08855 HMMreg13556_2_EUT2(pf05651pf13556) EUT2C
08860 -# -
08865 HMM02866_1_pvmeut(pf02866pf00056) EUT2C
08870 H_lavender EUT2C
08875 P_pastelBlue EUT2H
08880 HMMptac_3_eut2(pf06130) EUT2C EP_NTERM
08885 H_fuchsia EUT2H
08890 HMMalddh_2_egra(pf00171) EUT2C
08895 Tsp_orangePink EUT2C
08900 HMMetlyEutC_3_eut2(pf05985) EUT2C EP_NTERM
08905 HMMetlyEutB_2_EUT2(pf06751) EUT2H
08910 HMM06277EutA_1_eut(pf06277) EUT2C
08915 HMM10662PduV_1_mix(pf10662) EUT2C
08920 Hp_euts_wineRed EUT2H
08925 HMMtpEuth_1_eut(pf04346) EUT2C
08930 HMMreg00072_1_eut2(pf03861pf00072) EUT2C
08935 HMMreg07568_1_eut2(pf12282pf07568) EUT2C
08940 -pf00326 -
08945 -pf02566 -
08950 -pf00395 -
08955 -# -
08960 -# -
08965 -pf12849 -
08970 -pf01381 -
BMC-P
BMC-H
BMC-T
Aldehyde dehydrogenase
PTAC
Signature enzyme
Alcohol dehydrogenase
Regulator

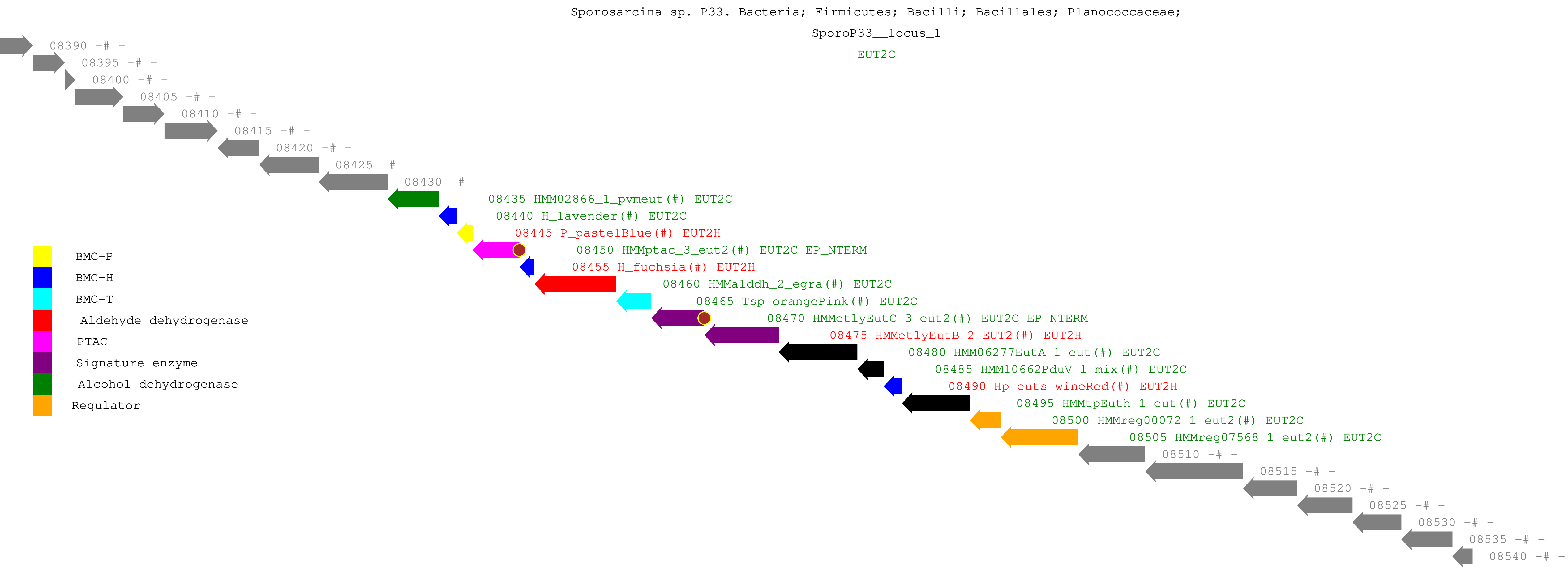
Sporosarcina sp. P33. Bacteria; Firmicutes; Bacilli; Bacillales; Planococcaceae;
SporoP33__locus_1
EUT2C
08390 -# -
08395 -# -
08400 -# -
08405 -# -
08410 -# -
08415 -# -
08420 -# -
08425 -# -
08430 -# -
08435 HMM02866_1_pvmeut(#) EUT2C
08440 H_lavender(#) EUT2C
08445 P_pastelBlue(#) EUT2H
08450 HMMptac_3_eut2(#) EUT2C EP_NTERM
08455 H_fuchsia(#) EUT2H
08460 HMMalddh_2_egra(#) EUT2C
08465 Tsp_orangePink(#) EUT2C
08470 HMMetlyEutC_3_eut2(#) EUT2C EP_NTERM
08475 HMMetlyEutB_2_EUT2(#) EUT2H
08480 HMM06277EutA_1_eut(#) EUT2C
08485 HMM10662PduV_1_mix(#) EUT2C
08490 Hp_euts_wineRed(#) EUT2H
08495 HMMtpEuth_1_eut(#) EUT2C
08500 HMMreg00072_1_eut2(#) EUT2C
08505 HMMreg07568_1_eut2(#) EUT2C
08510 -# -
08515 -# -
08520 -# -
08525 -# -
08530 -# -
08535 -# -
08540 -# -
BMC-P
BMC-H
BMC-T
Aldehyde dehydrogenase
PTAC
Signature enzyme
Alcohol dehydrogenase
Regulator

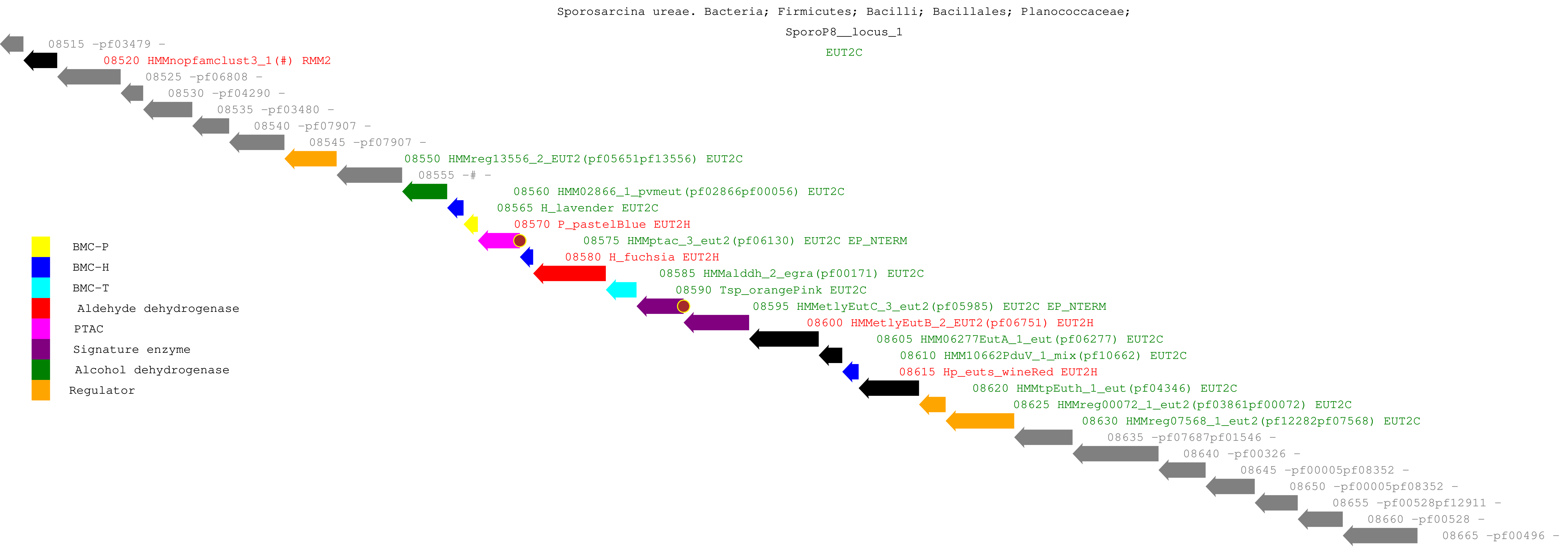

Sporosarcina ureae. Bacteria; Firmicutes; Bacilli; Bacillales; Planococcaceae;
SporoP8__locus_1
EUT2C
08515 -pf03479 -
08520 HMMnopfamclust3_1(#) RMM2
08525 -pf06808 -
08530 -pf04290 -
08535 -pf03480 -
08540 -pf07907 -
08545 -pf07907 -
08550 HMMreg13556_2_EUT2(pf05651pf13556) EUT2C
08555 -# -
08560 HMM02866_1_pvmeut(pf02866pf00056) EUT2C
08565 H_lavender EUT2C
08570 P_pastelBlue EUT2H
08575 HMMptac_3_eut2(pf06130) EUT2C EP_NTERM
08580 H_fuchsia EUT2H
08585 HMMalddh_2_egra(pf00171) EUT2C
08590 Tsp_orangePink EUT2C
08595 HMMetlyEutC_3_eut2(pf05985) EUT2C EP_NTERM
08600 HMMetlyEutB_2_EUT2(pf06751) EUT2H
08605 HMM06277EutA_1_eut(pf06277) EUT2C
08610 HMM10662PduV_1_mix(pf10662) EUT2C
08615 Hp_euts_wineRed EUT2H
08620 HMMtpEuth_1_eut(pf04346) EUT2C
08625 HMMreg00072_1_eut2(pf03861pf00072) EUT2C
08630 HMMreg07568_1_eut2(pf12282pf07568) EUT2C
08635 -pf07687pf01546 -
08640 -pf00326 -
08645 -pf00005pf08352 -
08650 -pf00005pf08352 -
08655 -pf00528pf12911 -
08660 -pf00528 -
08665 -pf00496 -
BMC-P
BMC-H
BMC-T
Aldehyde dehydrogenase
PTAC
Signature enzyme
Alcohol dehydrogenase
Regulator

Sporosarcina ureae. Bacteria; Firmicutes; Bacilli; Bacillales; Planococcaceae;

SporoS204__locus_1

EUT2C

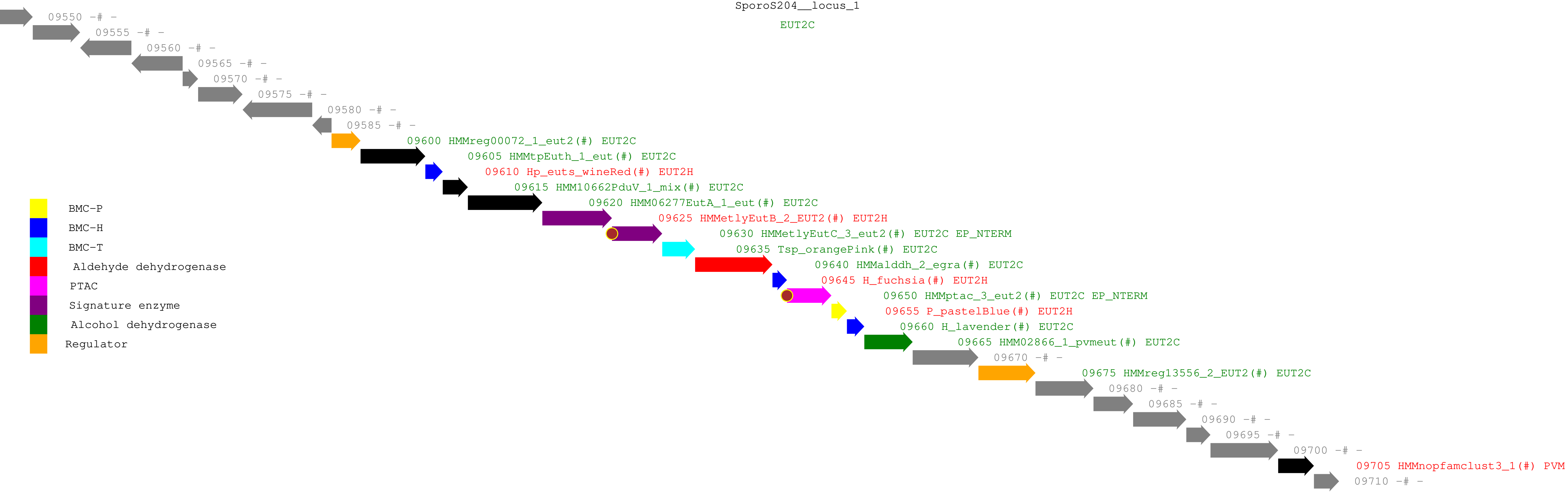

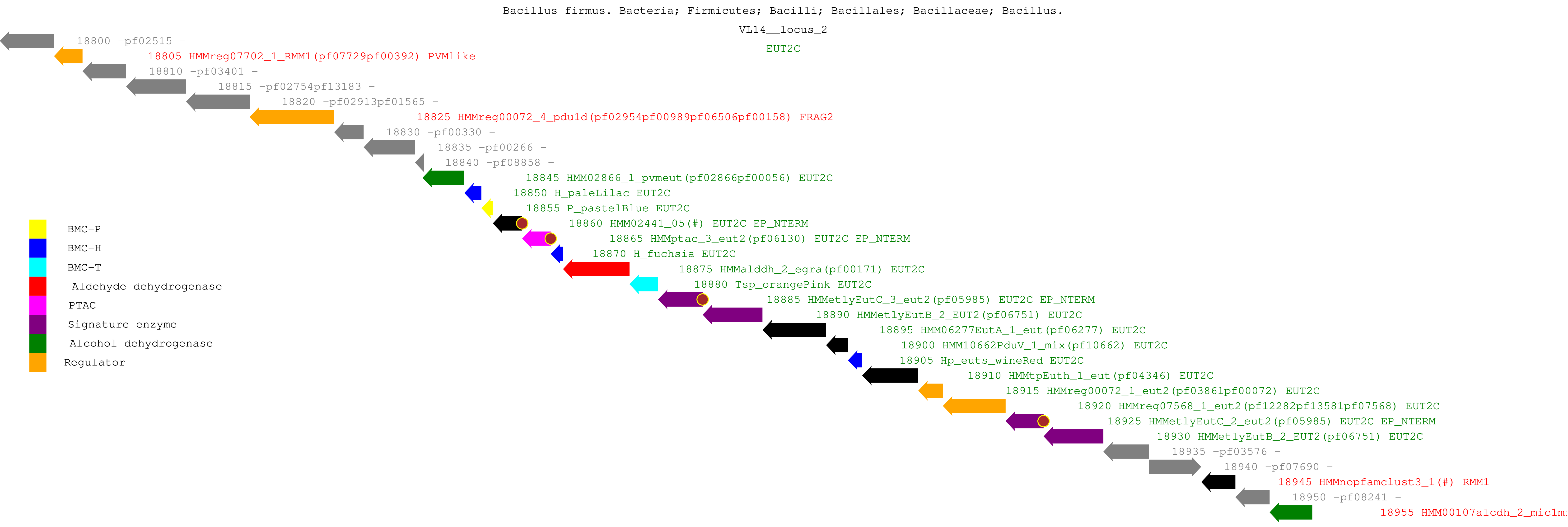
Bacillus firmus. Bacteria; Firmicutes; Bacilli; Bacillales; Bacillaceae; Bacillus.
VL14__locus_2
EUT2C
18800 -pf02515 -
18805 HMMreg07702_1_RMM1(pf07729pf00392) PVMlike
18810 -pf03401 -
18815 -pf02754pf13183 -
18820 -pf02913pf01565 -
18825 HMMreg00072_4_pdu1d(pf02954pf00989pf06506pf00158) FRAG2
18830 -pf00330 -
18835 -pf00266 -
18840 -pf08858 -
18845 HMM02866_1_pvmeut(pf02866pf00056) EUT2C
18850 H_paleLilac EUT2C
18855 P_pastelBlue EUT2C
18860 HMM02441_05(#) EUT2C EP_NTERM
18865 HMMptac_3_eut2(pf06130) EUT2C EP_NTERM
18870 H_fuchsia EUT2C
18875 HMMalddh_2_egra(pf00171) EUT2C
18880 Tsp_orangePink EUT2C
18885 HMMetlyEutC_3_eut2(pf05985) EUT2C EP_NTERM
18890 HMMetlyEutB_2_EUT2(pf06751) EUT2C
18895 HMM06277EutA_1_eut(pf06277) EUT2C
18900 HMM10662PduV_1_mix(pf10662) EUT2C
18905 Hp_euts_wineRed EUT2C
18910 HMMtpEuth_1_eut(pf04346) EUT2C
18915 HMMreg00072_1_eut2(pf03861pf00072) EUT2C
18920 HMMreg07568_1_eut2(pf12282pf13581pf07568) EUT2C
18925 HMMetlyEutC_2_eut2(pf05985) EUT2C EP_NTERM
18930 HMMetlyEutB_2_EUT2(pf06751) EUT2C
18935 -pf03576 -
18940 -pf07690 -
18945 HMMnopfamclust3_1(#) RMM1
18950 -pf08241 -
18955 HMM00107alcdh_2_mic1m
BMC-P
BMC-H
BMC-T
Aldehyde dehydrogenase
PTAC
Signature enzyme
Alcohol dehydrogenase
Regulator

Firmicutes bacterium HGW-Firmicutes-14. Bacteria; Firmicutes.

CVU89__locus_1

EUT2D

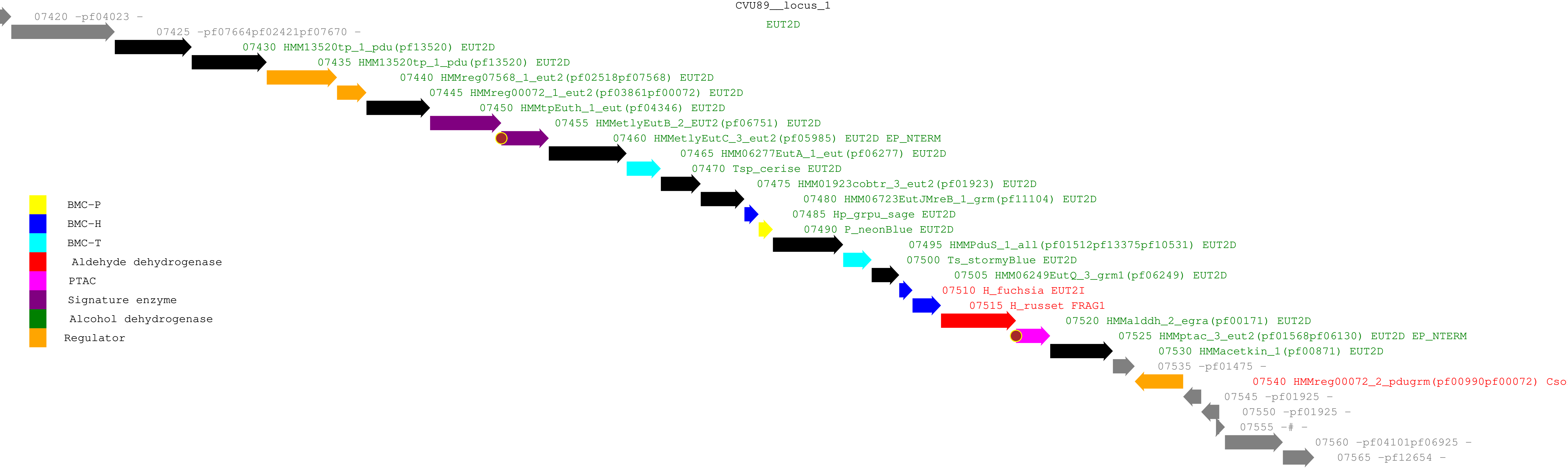

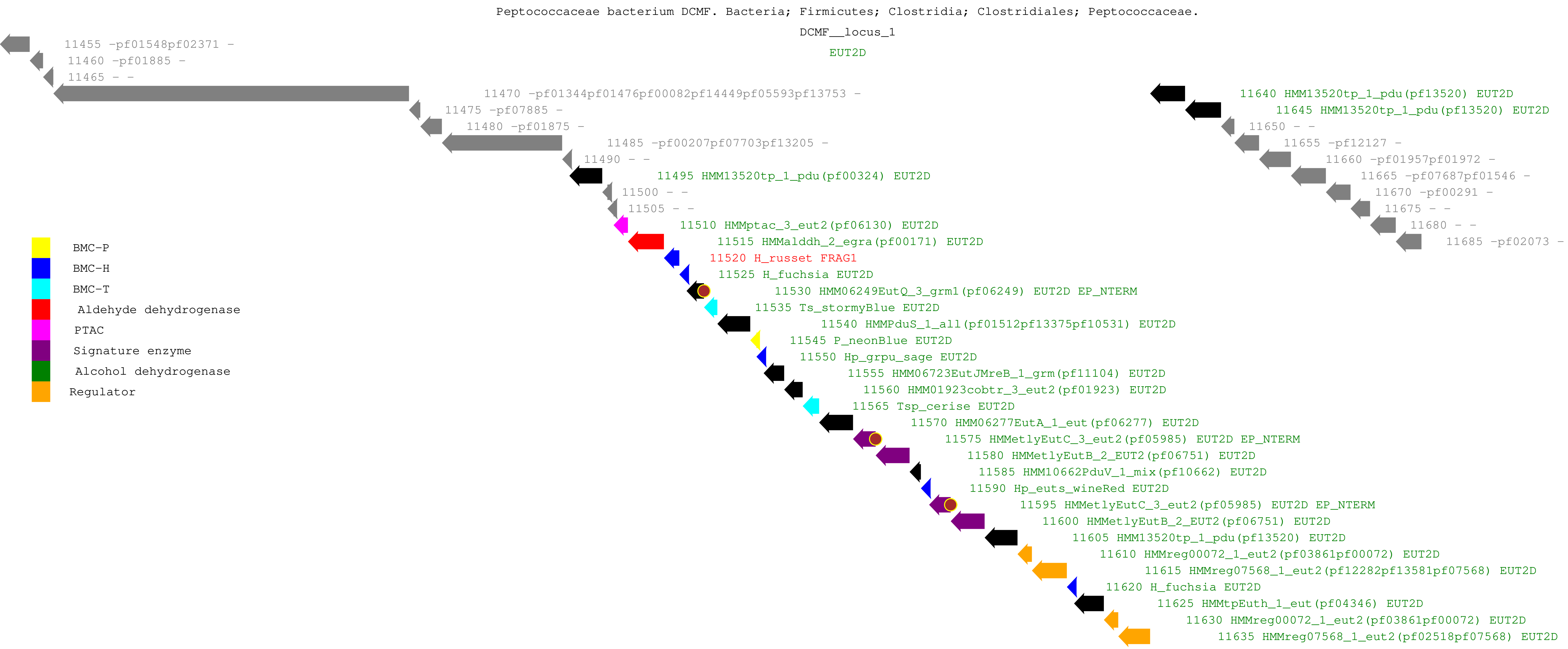
Peptococcaceae bacterium DCMF. Bacteria; Firmicutes; Clostridia; Clostridiales; Peptococcaceae.
DCMF__locus_1
EUT2D
11455 -pf01548pf02371 -
11460 -pf01885 -
11465 - -
11470 -pf01344pf01476pf00082pf14449pf05593pf13753 -
11475 -pf07885 -
11480 -pf01875 -
11485 -pf00207pf07703pf13205 -
11490 - -
11495 HMM13520tp_1_pdu(pf00324) EUT2D
11500 - -
11505 - -
11510 HMMptac_3_eut2(pf06130) EUT2D
11515 HMMaldh_2_egra(pf00171) EUT2D
11520 H_russet FRAG1
11525 H_fuchsia EUT2D
11530 HMM06249EutQ_3_grm1(pf06249) EUT2D EP_NTERM
11535 Ts_stormyBlue EUT2D
11540 HMMPduS_1_all(pf01512pf13375pf10531) EUT2D
11545 P_neonBlue EUT2D
11550 Hp_grpu_sage EUT2D
11555 HMM06723EutJMreB_1_grm(pf11104) EUT2D
11560 HMM01923cobtr_3_eut2(pf01923) EUT2D
11565 Tsp_cerise EUT2D
11570 HMM06277EutA_1_eut(pf06277) EUT2D
11575 HMMetlyEutC_3_eut2(pf05985) EUT2D EP_NTERM
11580 HMMetlyEutB_2_EUT2(pf06751) EUT2D
11585 HMM10662PduV_1_mix(pf10662) EUT2D
11590 Hp_euts_wineRed EUT2D
11595 HMMetlyEutC_3_eut2(pf05985) EUT2D EP_NTERM
11600 HMMetlyEutB_2_EUT2(pf06751) EUT2D
11605 HMM13520tp_1_pdu(pf13520) EUT2D
11610 HMMreg00072_1_eut2(pf03861pf00072) EUT2D
11615 HMMreg07568_1_eut2(pf12282pf13581pf07568) EUT2D
11620 H_fuchsia EUT2D
11625 HMMtpEuth_1_eut(pf04346) EUT2D
11630 HMMreg00072_1_eut2(pf03861pf00072) EUT2D
11635 HMMreg07568_1_eut2(pf02518pf07568) EUT2D
11640 HMM13520tp_1_pdu(pf13520) EUT2D
11645 HMM13520tp_1_pdu(pf13520) EUT2D
11650 - -
11655 -pf12127 -
11660 -pf01957pf01972 -
11665 -pf07687pf01546 -
11670 -pf00291 -
11675 - -
11680 - -
11685 -pf02073 -
BMC-P
BMC-H
BMC-T
Aldehyde dehydrogenase
PTAC
Signature enzyme
Alcohol dehydrogenase
Regulator

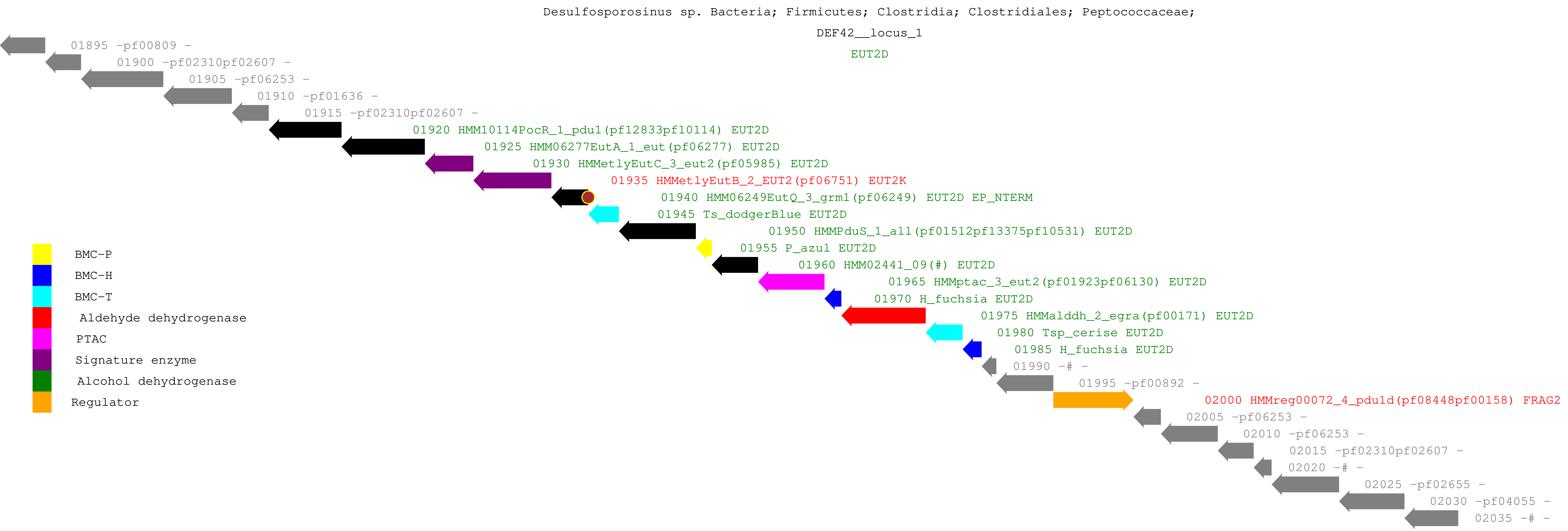
Desulfosporosinus sp. Bacteria; Firmicutes; Clostridia; Clostridiales; Peptococcaceae;
DEF42__locus_1
EUT2D
01895 -pf00809 -
01900 -pf02310pf02607 -
01905 -pf06253 -
01910 -pf01636 -
01915 -pf02310pf02607 -
01920 HMM10114PocR_1_pdu1(pf12833pf10114) EUT2D
01925 HMM06277EutA_1_eut(pf06277) EUT2D
01930 HMMetlyEutC_3_eut2(pf05985) EUT2D
01935 HMMetlyEutB_2_EUT2(pf06751) EUT2K
01940 HMM06249EutQ_3_grm1(pf06249) EUT2D EP_NTERM
01945 Ts_dodgerBlue EUT2D
01950 HMMPduS_1_all(pf01512pf13375pf10531) EUT2D
01955 P_azul EUT2D
01960 HMM02441_09(#) EUT2D
01965 HMMptac_3_eut2(pf01923pf06130) EUT2D
01970 H_fuchsia EUT2D
01975 HMMalddh_2_egra(pf00171) EUT2D
01980 Tsp_cerise EUT2D
01985 H_fuchsia EUT2D
01990 -# -
01995 -pf00892 -
02000 HMMreg00072_4_pdu1d(pf08448pf00158) FRAG2
02005 -pf06253 -
02010 -pf06253 -
02015 -pf02310pf02607 -
02020 -# -
02025 -pf02655 -
02030 -pf04055 -
02035 -# -
BMC-P
BMC-H
BMC-T
Aldehyde dehydrogenase
PTAC
Signature enzyme
Alcohol dehydrogenase
Regulator

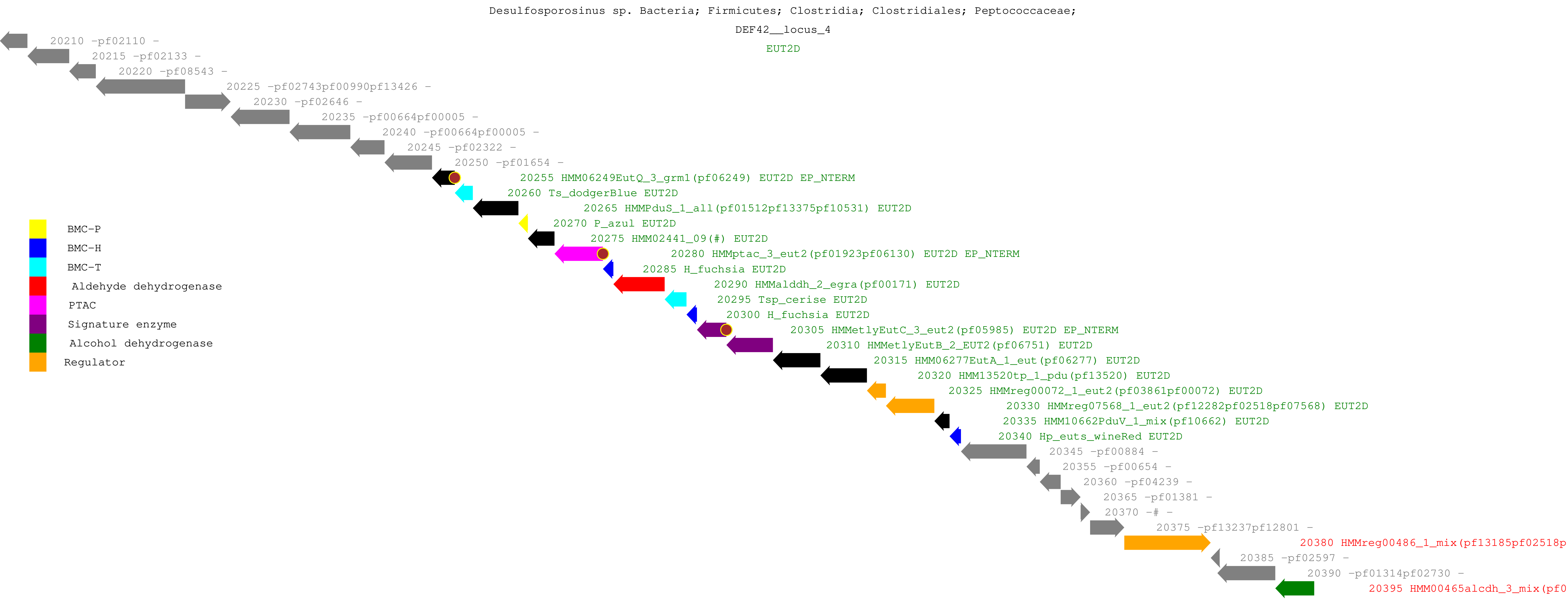
Desulfosporosinus sp. Bacteria; Firmicutes; Clostridia; Clostridiales; Peptococcaceae;
DEF42__locus_4
EUT2D
20210 -pf02110 -
20215 -pf02133 -
20220 -pf08543 -
20225 -pf02743pf00990pf13426 -
20230 -pf02646 -
20235 -pf00664pf00005 -
20240 -pf00664pf00005 -
20245 -pf02322 -
20250 -pf01654 -
20255 HMM06249EutQ_3_grm1(pf06249) EUT2D EP_NTERM
20260 Ts_dodgerBlue EUT2D
20265 HMMPduS_1_all(pf01512pf13375pf10531) EUT2D
20270 P_azul EUT2D
20275 HMM02441_09(#) EUT2D
20280 HMMptac_3_eut2(pf01923pf06130) EUT2D EP_NTERM
20285 H_fuchsia EUT2D
20290 HMMalddh_2_egra(pf00171) EUT2D
20295 Tsp_cerise EUT2D
20300 H_fuchsia EUT2D
20305 HMMetlyEutC_3_eut2(pf05985) EUT2D EP_NTERM
20310 HMMetlyEutB_2_EUT2(pf06751) EUT2D
20315 HMM06277EutA_1_eut(pf06277) EUT2D
20320 HMM13520tp_1_pdu(pf13520) EUT2D
20325 HMMreg00072_1_eut2(pf03861pf00072) EUT2D
20330 HMMreg07568_1_eut2(pf12282pf02518pf07568) EUT2D
20335 HMM10662PduV_1_mix(pf10662) EUT2D
20340 Hp_euts_wineRed EUT2D
20345 -pf00884 -
20355 -pf00654 -
20360 -pf04239 -
20365 -pf01381 -
20370 -# -
20375 -pf13237pf12801 -
20380 HMMreg00486_1_mix(pf13185pf02518p
20385 -pf02597 -
20390 -pf01314pf02730 -
20395 HMM00465alcdh_3_mix(pf0
BMC-P
BMC-H
BMC-T
Aldehyde dehydrogenase
PTAC
Signature enzyme
Alcohol dehydrogenase
Regulator

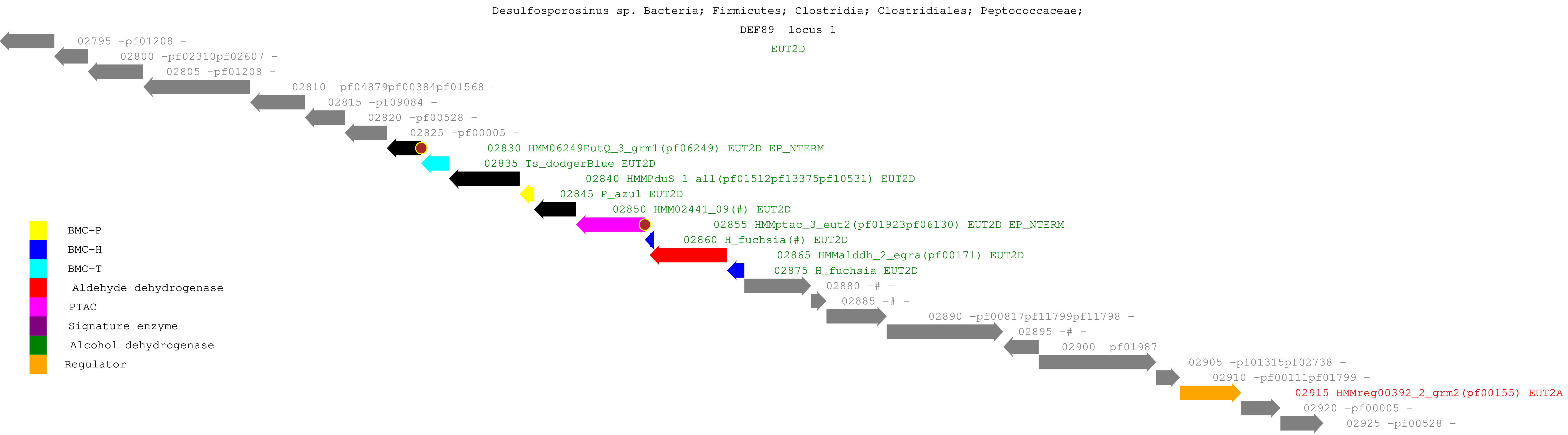
Desulfosporosinus sp. Bacteria; Firmicutes; Clostridia; Clostridiales; Peptococcaceae;
DEF89__locus_1
EUT2D
02795 -pf01208 -
02800 -pf02310pf02607 -
02805 -pf01208 -
02810 -pf04879pf00384pf01568 -
02815 -pf09084 -
02820 -pf00528 -
02825 -pf00005 -
02830 HMM06249EutQ_3_grm1(pf06249) EUT2D EP_NTERM
02835 Ts_dodgerBlue EUT2D
02840 HMMPduS_1_all(pf01512pf13375pf10531) EUT2D
02845 P_azul EUT2D
02850 HMM02441_09(#) EUT2D
02855 HMMptac_3_eut2(pf01923pf06130) EUT2D EP_NTERM
02860 H_fuchsia(#) EUT2D
02865 HMMaldd h_2_egra(pf00171) EUT2D
02875 H_fuchsia EUT2D
02880 -# -
02885 -# -
02890 -pf00817pf11799pf11798 -
02895 -# -
02900 -pf01987 -
02905 -pf01315pf02738 -
02910 -pf00111pf01799 -
02915 HMMreg00392_2_grm2(pf00155) EUT2A
02920 -pf00005 -
02925 -pf00528 -
BMC-P
BMC-H
BMC-T
Aldehyde dehydrogenase
PTAC
Signature enzyme
Alcohol dehydrogenase
Regulator

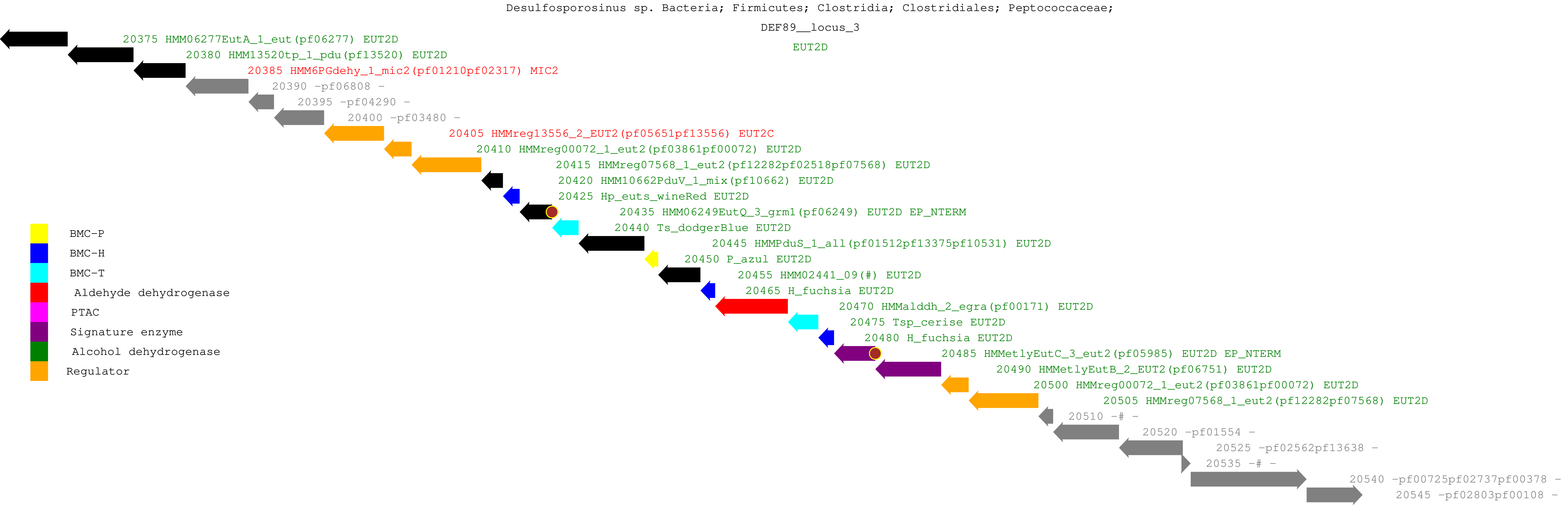
Desulfosporosinus sp. Bacteria; Firmicutes; Clostridia; Clostridiales; Peptococcaceae;
DEF89__locus_3
EUT2D
20375 HMM06277EutA_1_eut(pf06277) EUT2D
20380 HMM13520tp_1_pdu(pf13520) EUT2D
20385 HMM6PGdehy_1_mic2(pf01210pf02317) MIC2
20390 -pf06808 -
20395 -pf04290 -
20400 -pf03480 -
20405 HMMreg13556_2_EUT2(pf05651pf13556) EUT2C
20410 HMMreg00072_1_eut2(pf03861pf00072) EUT2D
20415 HMMreg07568_1_eut2(pf12282pf02518pf07568) EUT2D
20420 HMM10662PduV_1_mix(pf10662) EUT2D
20425 Hp_euts_wineRed EUT2D
20435 HMM06249EutQ_3_grm1(pf06249) EUT2D EP_NTERM
20440 Ts_dodgerBlue EUT2D
20445 HMMPduS_1_all(pf01512pf13375pf10531) EUT2D
20450 P_azul EUT2D
20455 HMM02441_09(#) EUT2D
20465 H_fuchsia EUT2D
20470 HMMalddh_2_egra(pf00171) EUT2D
20475 Tsp_cerise EUT2D
20480 H_fuchsia EUT2D
20485 HMMetlyEutC_3_eut2(pf05985) EUT2D EP_NTERM
20490 HMMetlyEutB_2_EUT2(pf06751) EUT2D
20500 HMMreg00072_1_eut2(pf03861pf00072) EUT2D
20505 HMMreg07568_1_eut2(pf12282pf07568) EUT2D
20510 -# -
20520 -pf01554 -
20525 -pf02562pf13638 -
20535 -# -
20540 -pf00725pf02737pf00378 -
20545 -pf02803pf00108 -
BMC-P
BMC-H
BMC-T
Aldehyde dehydrogenase
PTAC
Signature enzyme
Alcohol dehydrogenase
Regulator

Desulfosporosinus meridiei (strain ATCC BAA-275 / DSM 13257 / NCIMB Bacteria; Firmicutes; Clostridia; Clostridiales; Peptococcaceae;

Desmer__locus_1

EUT2D

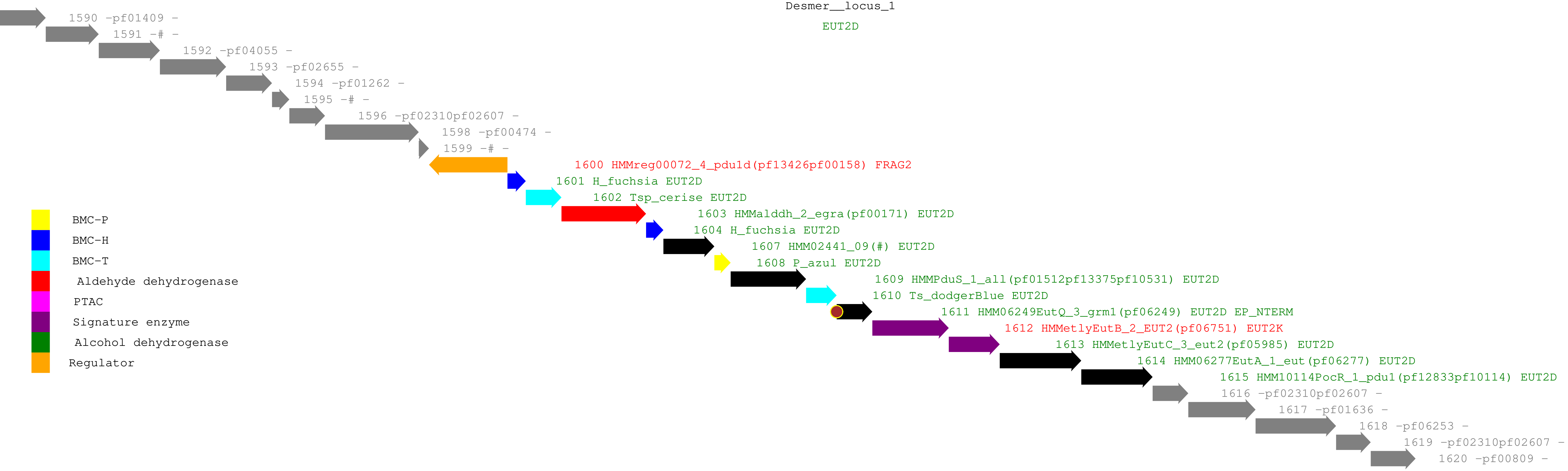

Desulfosporosinus meridiei (strain ATCC BAA-275 / DSM 13257 / NCIMB Bacteria; Firmicutes; Clostridia; Clostridiales; Peptococcaceae;

Desmer__locus_2

EUT2D

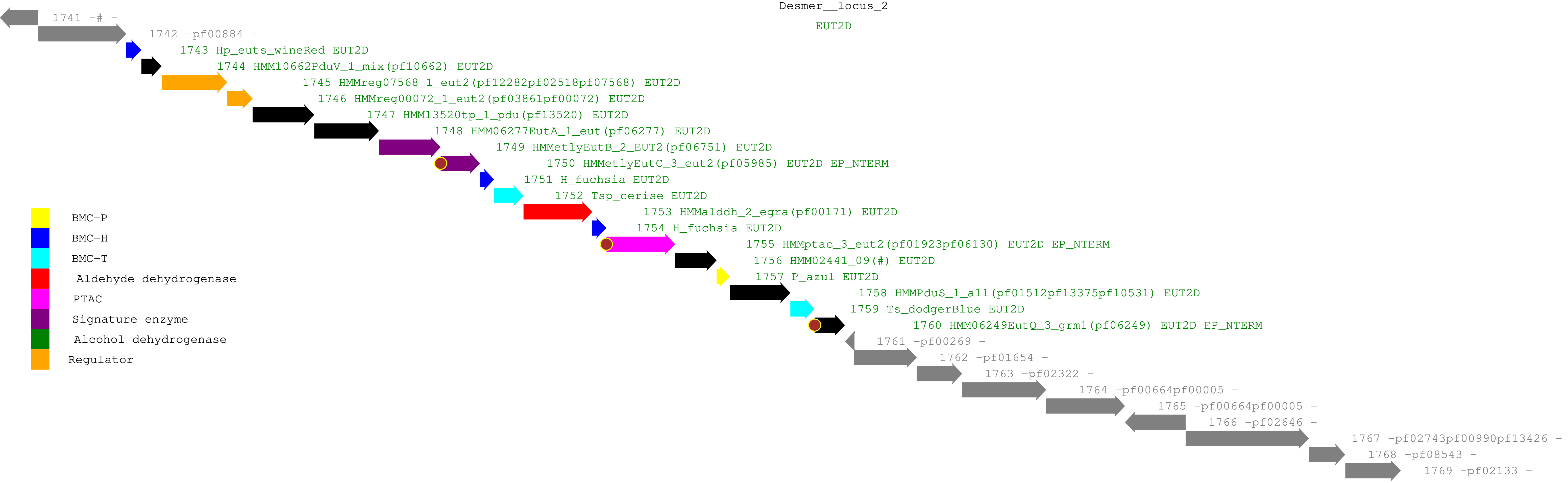

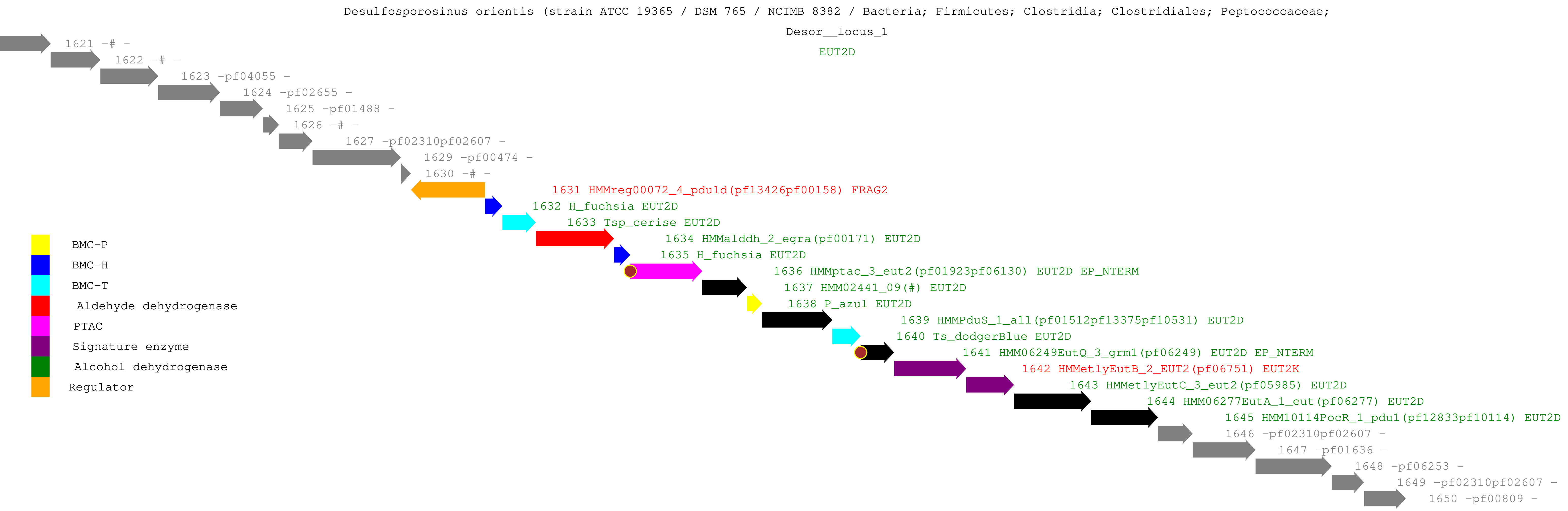
Desulfosporosinus orientis (strain ATCC 19365 / DSM 765 / NCIMB 8382 / Bacteria; Firmicutes; Clostridia; Clostridiales; Peptococcaceae;
Desor__locus_1
EUT2D
1621 -# -
1622 -# -
1623 -pf04055 -
1624 -pf02655 -
1625 -pf01488 -
1626 -# -
1627 -pf02310pf02607 -
1629 -pf00474 -
1630 -# -
1631 HMMreg00072_4_pdu1d(pf13426pf00158) FRAG2
1632 H_fuchsia EUT2D
1633 Tsp_cerise EUT2D
1634 HMMaldh_2_egra(pf00171) EUT2D
1635 H_fuchsia EUT2D
1636 HMMptac_3_eut2(pf01923pf06130) EUT2D EP_NTERM
1637 HMM02441_09(#) EUT2D
1638 P_azul EUT2D
1639 HMMPduS_1_all(pf01512pf13375pf10531) EUT2D
1640 Ts_dodgerBlue EUT2D
1641 HMM06249EutQ_3_grm1(pf06249) EUT2D EP_NTERM
1642 HMMetlyEutB_2_EUT2(pf06751) EUT2K
1643 HMMetlyEutC_3_eut2(pf05985) EUT2D
1644 HMM06277EutA_1_eut(pf06277) EUT2D
1645 HMM10114PocR_1_pdu1(pf12833pf10114) EUT2D
1646 -pf02310pf02607 -
1647 -pf01636 -
1648 -pf06253 -
1649 -pf02310pf02607 -
1650 -pf00809 -
BMC-P
BMC-H
BMC-T
Aldehyde dehydrogenase
PTAC
Signature enzyme
Alcohol dehydrogenase
Regulator

Desulfosporosinus orientis (strain ATCC 19365 / DSM 765 / NCIMB 8382 / Bacteria; Firmicutes; Clostridia; Clostridiales; Peptococcaceae;

Desor__locus_2

EUT2D

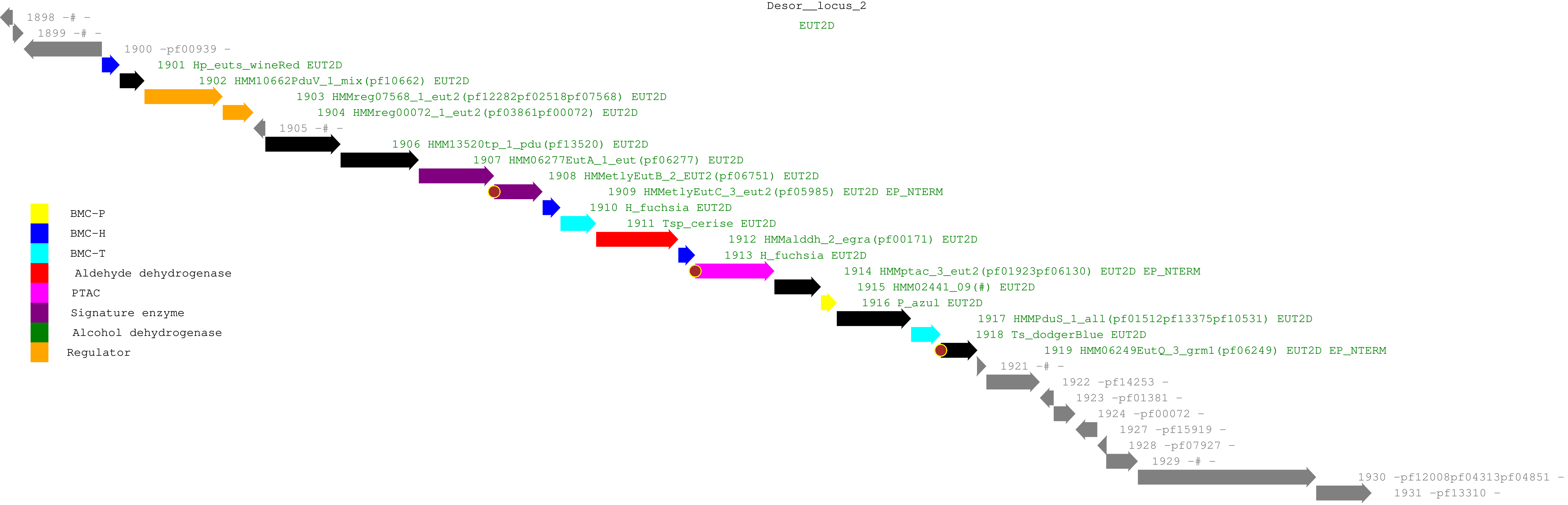

Desulfosporosinus youngiae DSM 17734. Bacteria; Firmicutes; Clostridia; Clostridiales; Peptococcaceae;

DesyoDRAFT__locus_1

EUT2D

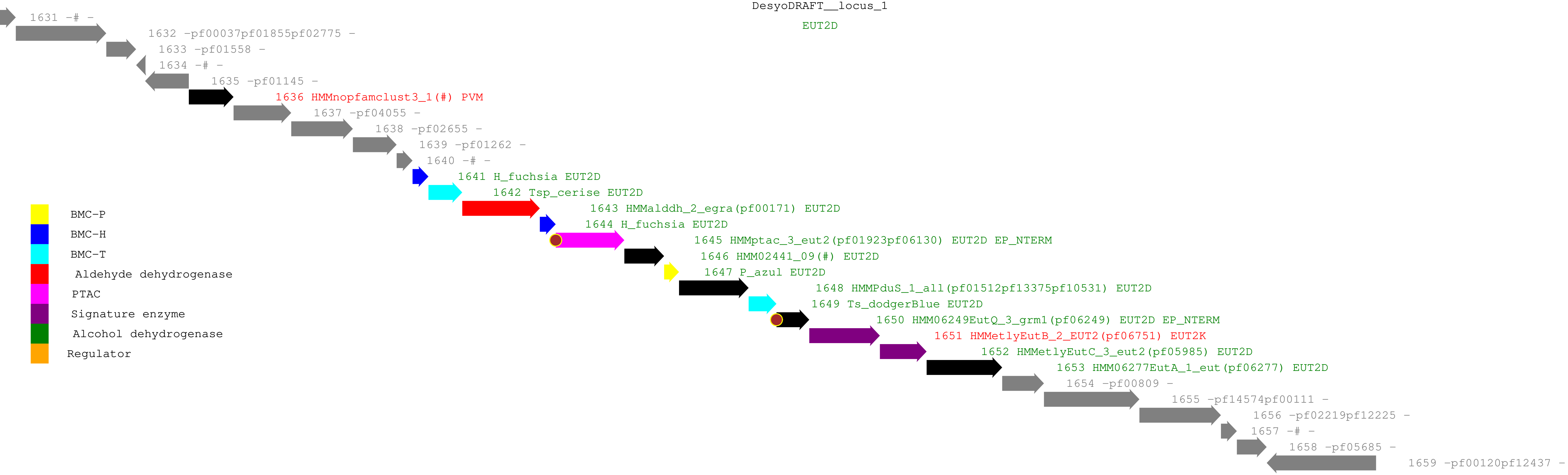

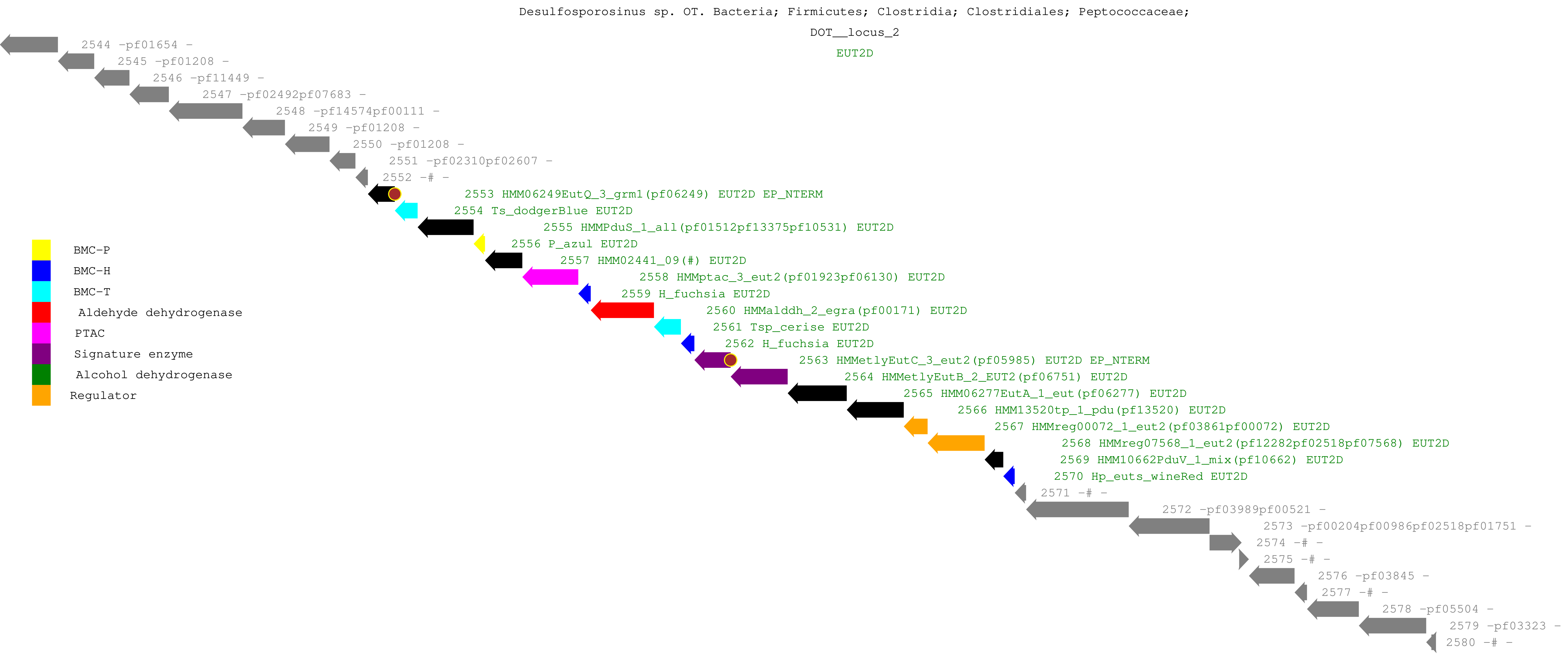
Desulfosporosinus sp. OT. Bacteria; Firmicutes; Clostridia; Clostridiales; Peptococcaceae;
DOT__locus_2
EUT2D
2544 -pf01654 -
2545 -pf01208 -
2546 -pf11449 -
2547 -pf02492pf07683 -
2548 -pf14574pf00111 -
2549 -pf01208 -
2550 -pf01208 -
2551 -pf02310pf02607 -
2552 -# -
2553 HMM06249EutQ_3_grm1(pf06249) EUT2D EP_NTERM
2554 Ts_dodgerBlue EUT2D
2555 HMMPduS_1_all(pf01512pf13375pf10531) EUT2D
2556 P_azul EUT2D
2557 HMM02441_09(#) EUT2D
2558 HMMptac_3_eut2(pf01923pf06130) EUT2D
2559 H_fuchsia EUT2D
2560 HMMalddh_2_egra(pf00171) EUT2D
2561 Tsp_cerise EUT2D
2562 H_fuchsia EUT2D
2563 HMMetlyEutC_3_eut2(pf05985) EUT2D EP_NTERM
2564 HMMetlyEutB_2_EUT2(pf06751) EUT2D
2565 HMM06277EutA_1_eut(pf06277) EUT2D
2566 HMM13520tp_1_pdu(pf13520) EUT2D
2567 HMMreg00072_1_eut2(pf03861pf00072) EUT2D
2568 HMMreg07568_1_eut2(pf12282pf02518pf07568) EUT2D
2569 HMM10662PduV_1_mix(pf10662) EUT2D
2570 Hp_euts_wineRed EUT2D
2571 -# -
2572 -pf03989pf00521 -
2573 -pf00204pf00986pf02518pf01751 -
2574 -# -
2575 -# -
2576 -pf03845 -
2577 -# -
2578 -pf05504 -
2579 -pf03323 -
2580 -# -
BMC-P
BMC-H
BMC-T
Aldehyde dehydrogenase
PTAC
Signature enzyme
Alcohol dehydrogenase
Regulator

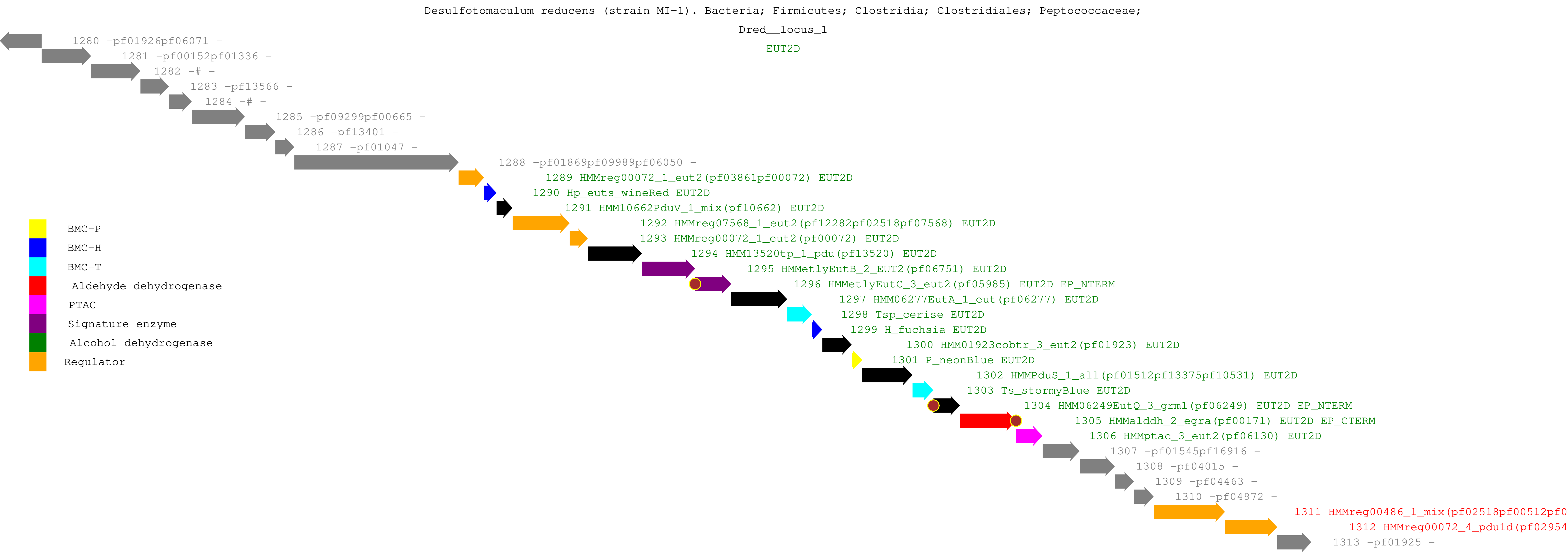
Desulfotomaculum reducens (strain MI-1). Bacteria; Firmicutes; Clostridia; Clostridiales; Peptococcaceae;
Dred__locus_1
EUT2D
1280 -pf01926pf06071 -
1281 -pf00152pf01336 -
1282 -# -
1283 -pf13566 -
1284 -# -
1285 -pf09299pf00665 -
1286 -pf13401 -
1287 -pf01047 -
1288 -pf01869pf09989pf06050 -
1289 HMMreg00072_1_eut2(pf03861pf00072) EUT2D
1290 Hp_euts_wineRed EUT2D
1291 HMM10662PduV_1_mix(pf10662) EUT2D
1292 HMMreg07568_1_eut2(pf12282pf02518pf07568) EUT2D
1293 HMMreg00072_1_eut2(pf00072) EUT2D
1294 HMM13520tp_1_pdu(pf13520) EUT2D
1295 HMMetlyEutB_2_EUT2(pf06751) EUT2D
1296 HMMetlyEutC_3_eut2(pf05985) EUT2D EP_NTERM
1297 HMM06277EutA_1_eut(pf06277) EUT2D
1298 Tsp_cerise EUT2D
1299 H_fuchsia EUT2D
1300 HMM01923cobtr_3_eut2(pf01923) EUT2D
1301 P_neonBlue EUT2D
1302 HMMPduS_1_all(pf01512pf13375pf10531) EUT2D
1303 Ts_stormyBlue EUT2D
1304 HMM06249EutQ_3_grm1(pf06249) EUT2D EP_NTERM
1305 HMMalddh_2_egra(pf00171) EUT2D EP_CTERM
1306 HMMptac_3_eut2(pf06130) EUT2D
1307 -pf01545pf16916 -
1308 -pf04015 -
1309 -pf04463 -
1310 -pf04972 -
1311 HMMreg00486_1_mix(pf02518pf00512pf0
1312 HMMreg00072_4_pdu1d(pf02954
1313 -pf01925 -
BMC-P
BMC-H
BMC-T
Aldehyde dehydrogenase
PTAC
Signature enzyme
Alcohol dehydrogenase
Regulator

Desulfosporosinus sp. OL. Bacteria; Firmicutes; Clostridia; Clostridiales; Peptococcaceae;

DSOL__locus_1_main_sat

EUT2D

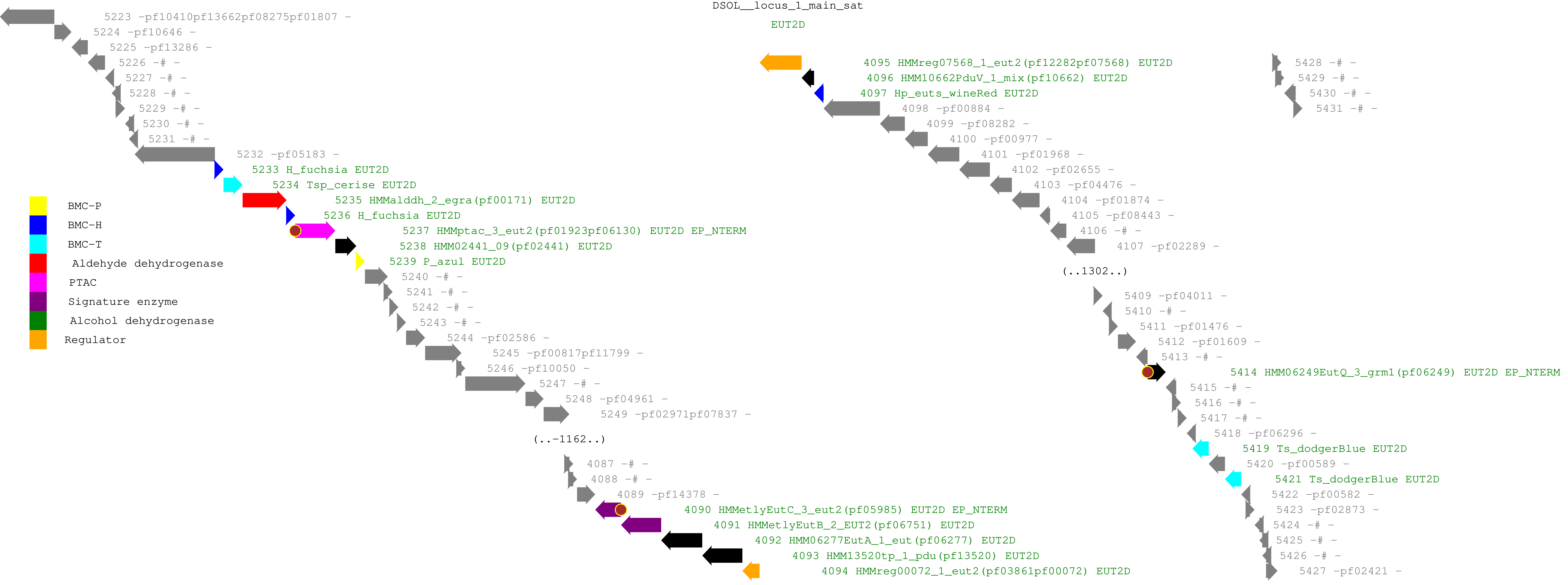

Desulfosporosinus fructosivorans. Bacteria; Firmicutes; Clostridia; Clostridiales; Peptococcaceae;

E4K67__locus_3

EUT2D

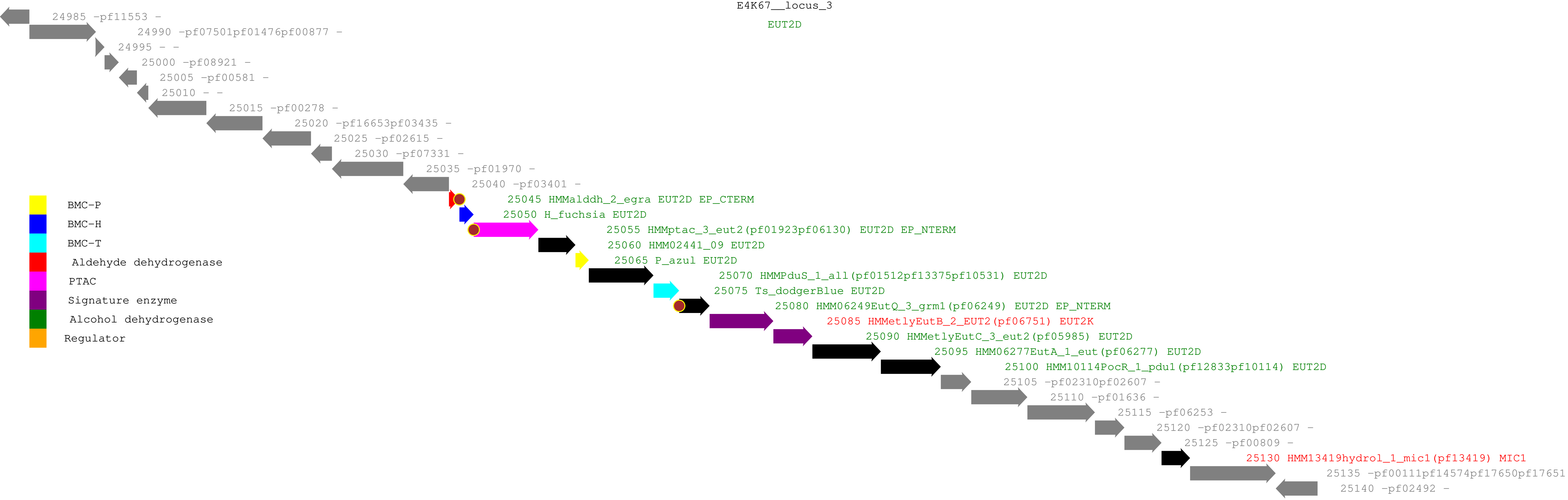

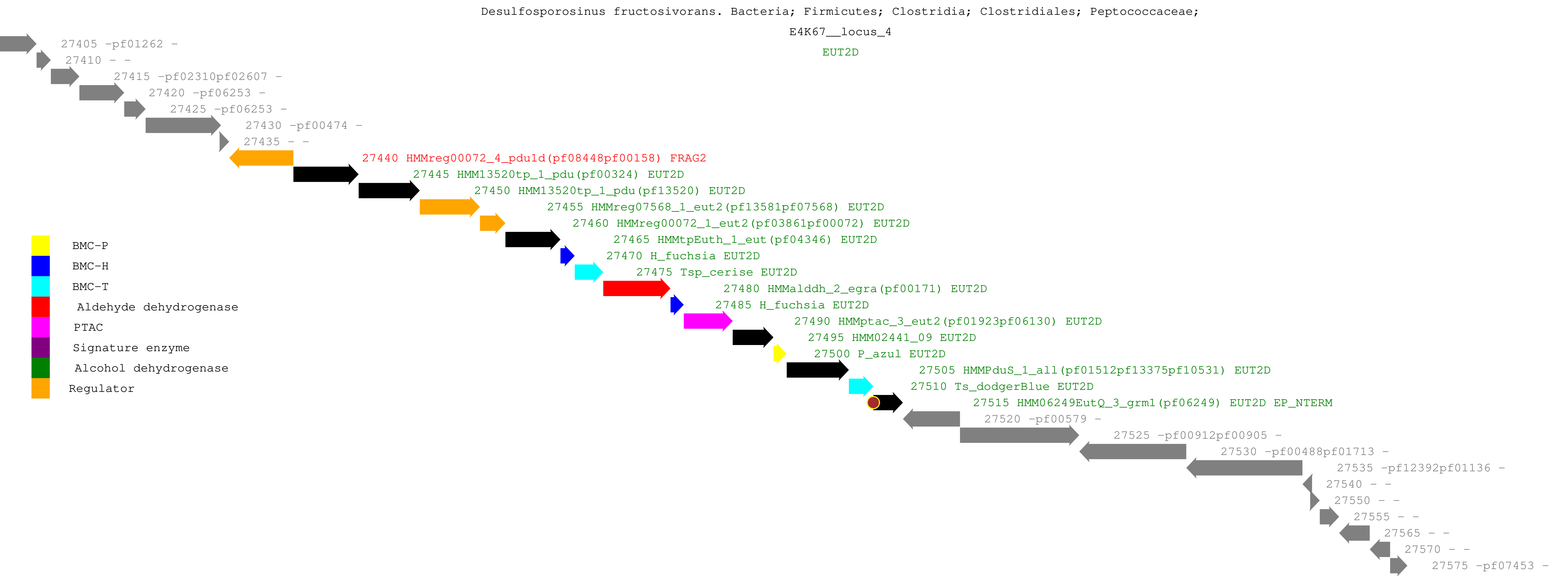
Desulfosporosinus fructosivorans. Bacteria; Firmicutes; Clostridia; Clostridiales; Peptococcaceae;
E4K67__locus_4
EUT2D
27405 -pf01262 -
27410 - -
27415 -pf02310pf02607 -
27420 -pf06253 -
27425 -pf06253 -
27430 -pf00474 -
27435 - -
27440 HMMreg00072_4_pdu1d(pf08448pf00158) FRAG2
27445 HMM13520tp_1_pdu(pf00324) EUT2D
27450 HMM13520tp_1_pdu(pf13520) EUT2D
27455 HMMreg07568_1_eut2(pf13581pf07568) EUT2D
27460 HMMreg00072_1_eut2(pf03861pf00072) EUT2D
27465 HMMtpEuth_1_eut(pf04346) EUT2D
27470 H_fuchsia EUT2D
27475 Tsp_cerise EUT2D
27480 HMMalddh_2_egra(pf00171) EUT2D
27485 H_fuchsia EUT2D
27490 HMMptac_3_eut2(pf01923pf06130) EUT2D
27495 HMM02441_09 EUT2D
27500 P_azul EUT2D
27505 HMMPduS_1_all(pf01512pf13375pf10531) EUT2D
27510 Ts_dodgerBlue EUT2D
27515 HMM06249EutQ_3_grm1(pf06249) EUT2D EP_NTERM
27520 -pf00579 -
27525 -pf00912pf00905 -
27530 -pf00488pf01713 -
27535 -pf12392pf01136 -
27540 - -
27550 - -
27555 - -
27565 - -
27570 - -
27575 -pf07453 -
BMC-P
BMC-H
BMC-T
Aldehyde dehydrogenase
PTAC
Signature enzyme
Alcohol dehydrogenase
Regulator

Clostridia bacterium k32. Bacteria; Firmicutes; Clostridia.

EYS13__locus_1_main_sat

EUT2D

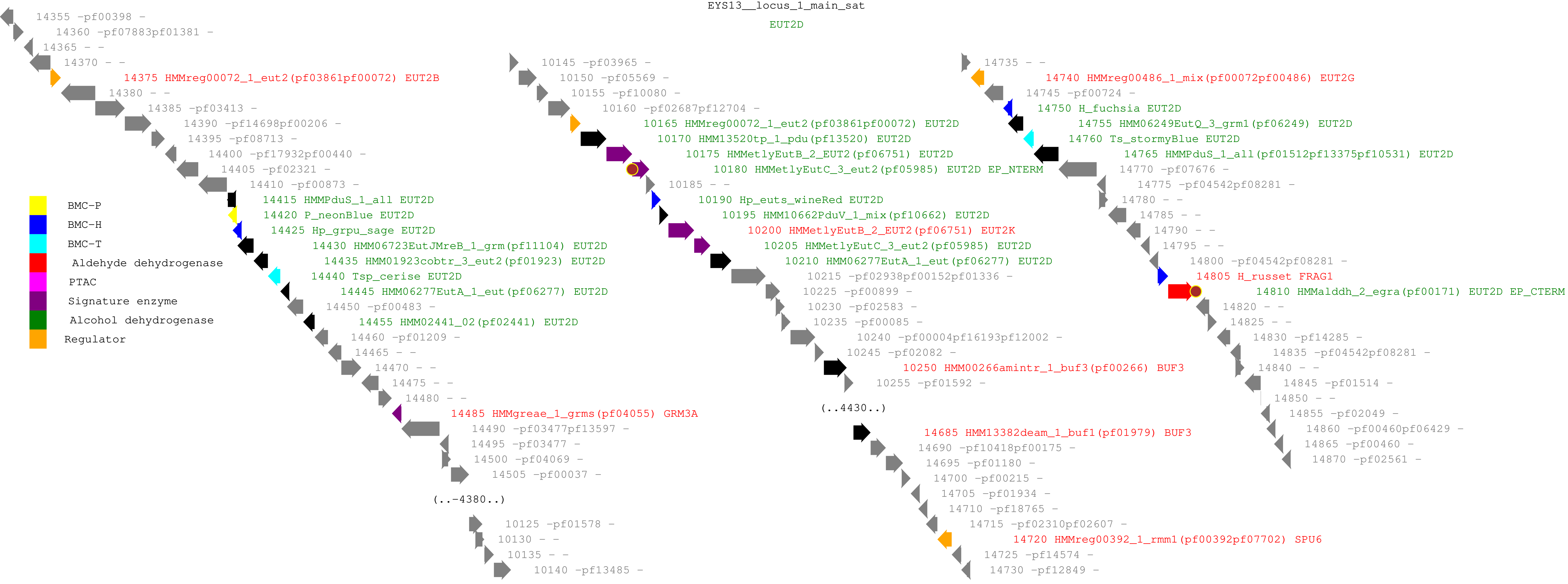

Desulfitobacterium chlororespirans DSM 11544. Bacteria; Firmicutes; Clostridia; Clostridiales; Peptococcaceae;
SAMN02745215__locus_1
EUT2D

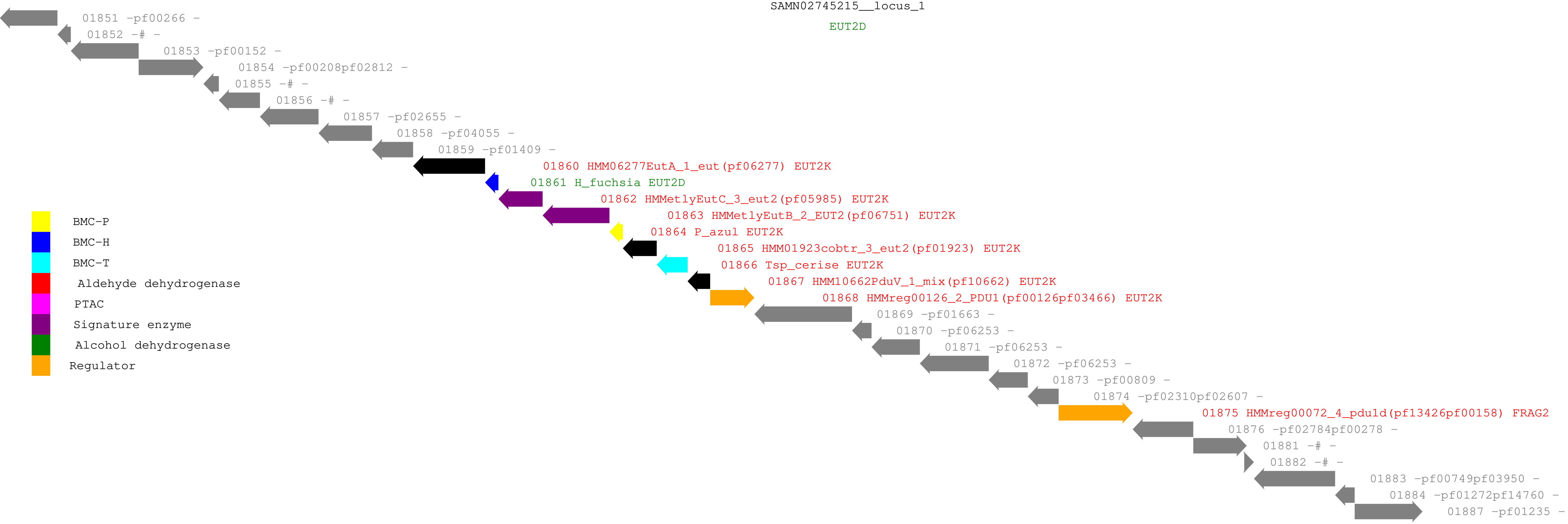

Desulfosporosinus lacus DSM 15449. Bacteria; Firmicutes; Clostridia; Clostridiales; Peptococcaceae;

SAMN02746098__locus_2_main_sat

EUT2D

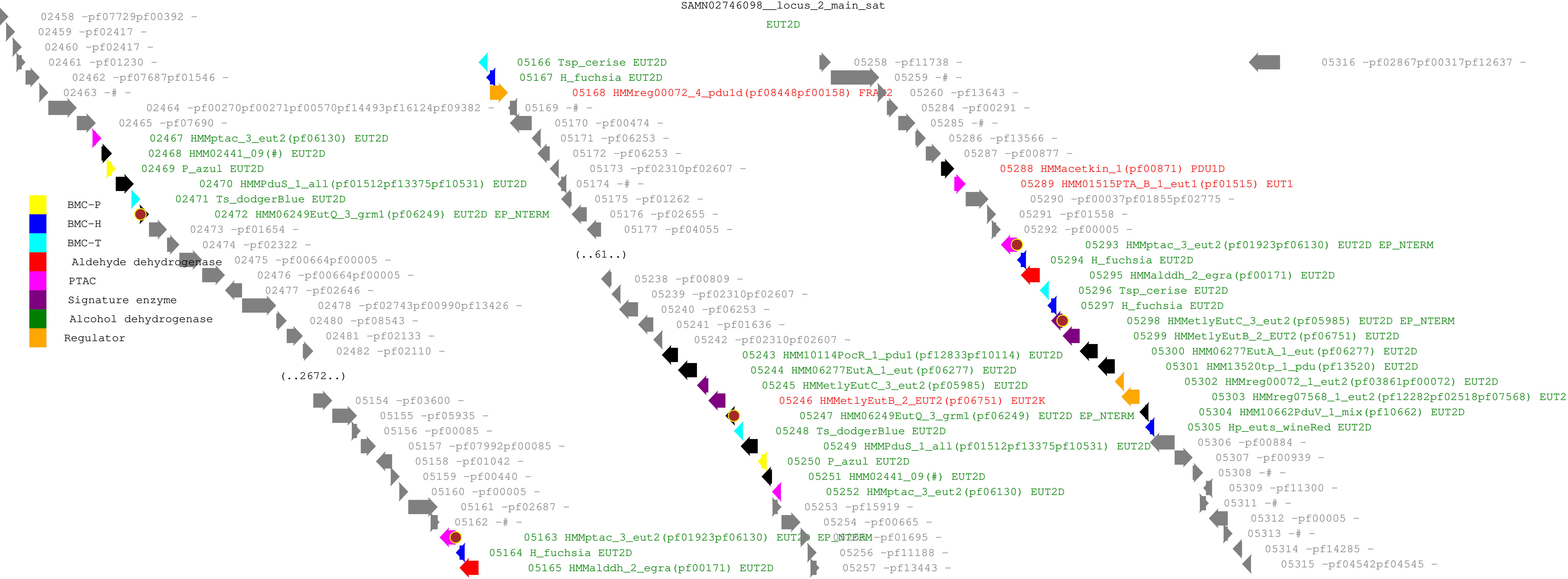

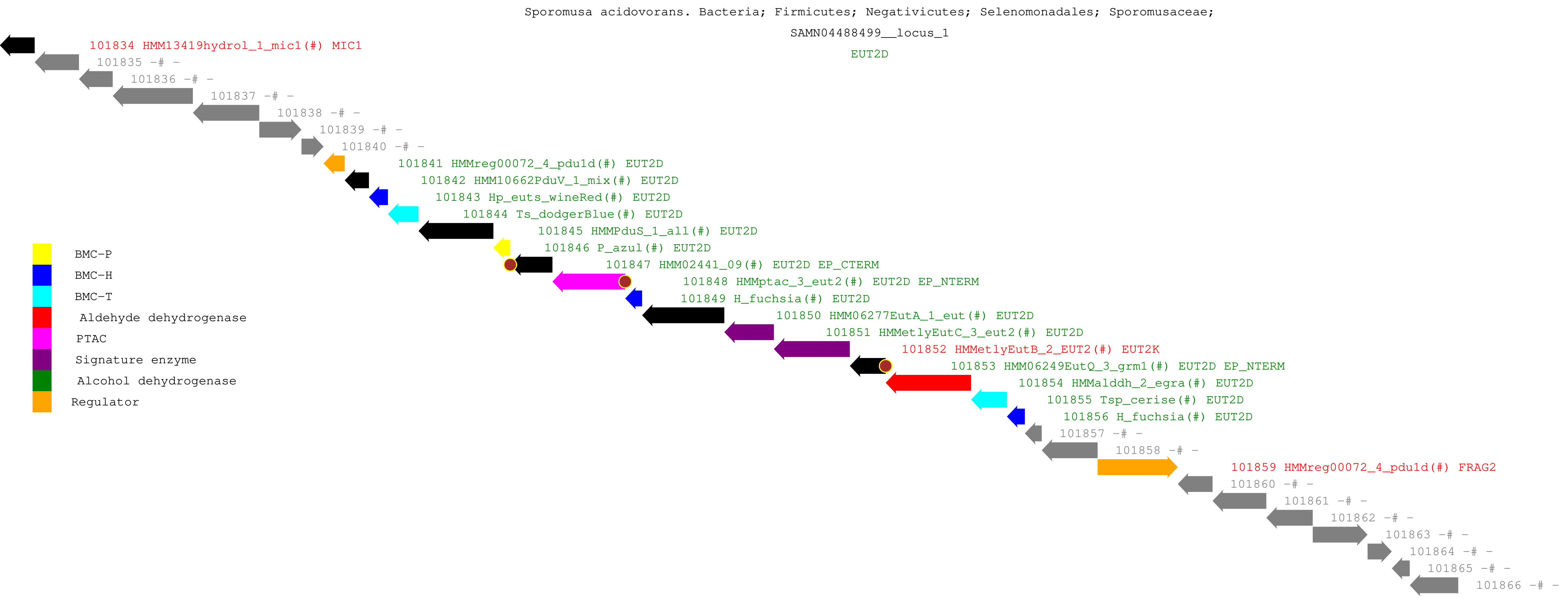

Sporomusa acidovorans. Bacteria; Firmicutes; Negativicutes; Selenomonadales; Sporomusaceae;
SAMN04488499__locus_1
EUT2D
101834 HMM13419hydrol_1_mic1(#) MIC1
101835 -# -
101836 -# -
101837 -# -
101838 -# -
101839 -# -
101840 -# -
101841 HMMreg00072_4_pdu1d(#) EUT2D
101842 HMM10662PduV_1_mix(#) EUT2D
101843 Hp_euts_wineRed(#) EUT2D
101844 Ts_dodgerBlue(#) EUT2D
101845 HMMPduS_1_all(#) EUT2D
101846 P_azul(#) EUT2D
101847 HMM02441_09(#) EUT2D EP_CTERM
101848 HMMptac_3_eut2(#) EUT2D EP_NTERM
101849 H_fuchsia(#) EUT2D
101850 HMM06277EutA_1_eut(#) EUT2D
101851 HMMetlyEutC_3_eut2(#) EUT2D
101852 HMMetlyEutB_2_EUT2(#) EUT2K
101853 HMM06249EutQ_3_grm1(#) EUT2D EP_NTERM
101854 HMMalddh_2_egra(#) EUT2D
101855 Tsp_cerise(#) EUT2D
101856 H_fuchsia(#) EUT2D
101857 -# -
101858 -# -
101859 HMMreg00072_4_pdu1d(#) FRAG2
101860 -# -
101861 -# -
101862 -# -
101863 -# -
101864 -# -
101865 -# -
101866 -# -
BMC-P
BMC-H
BMC-T
Aldehyde dehydrogenase
PTAC
Signature enzyme
Alcohol dehydrogenase
Regulator

Sporomusa acidovorans. Bacteria; Firmicutes; Negativicutes; Selenomonadales; Sporomusaceae;

SAMN04488499__locus_3

EUT2D

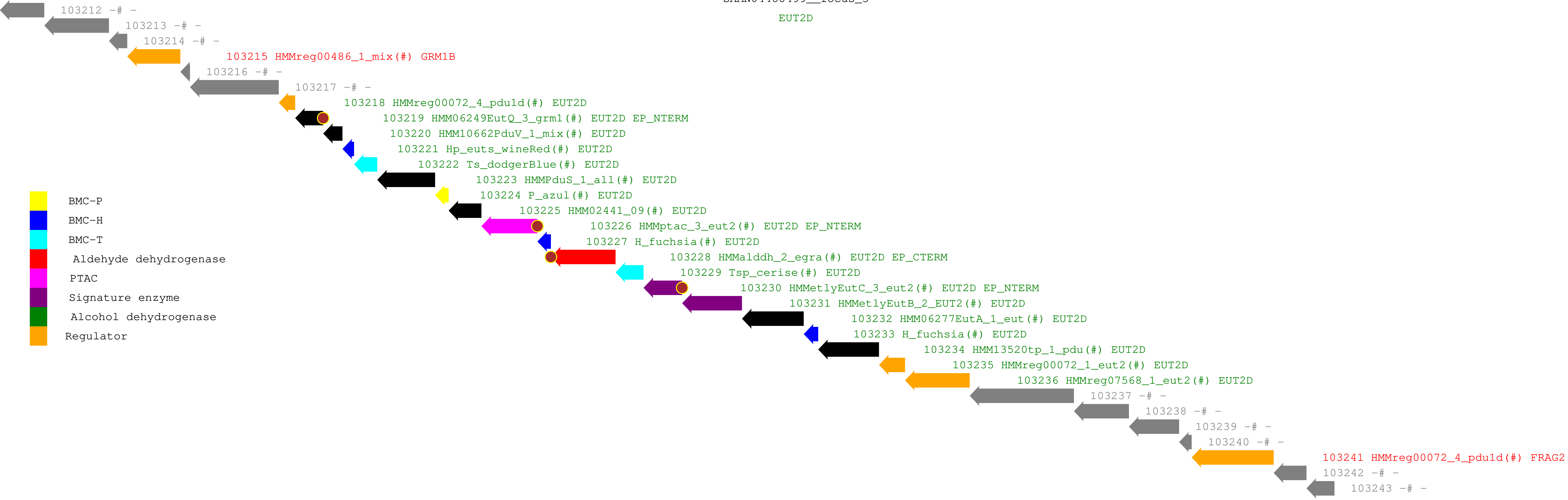

Desulfosporosinus sp. SbF1. Bacteria; Firmicutes; Clostridia; Clostridiales; Peptococcaceae;

SBF1__locus_1

EUT2D

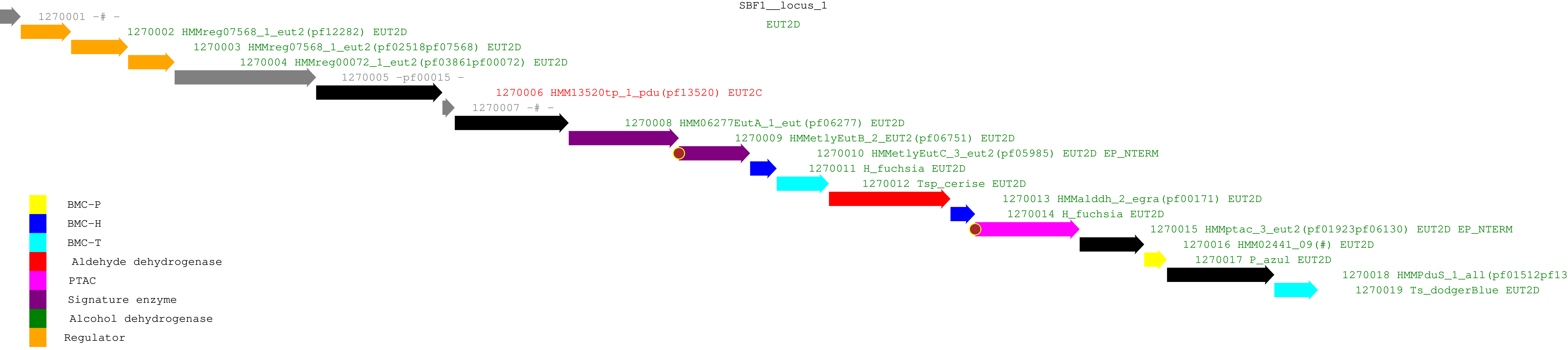

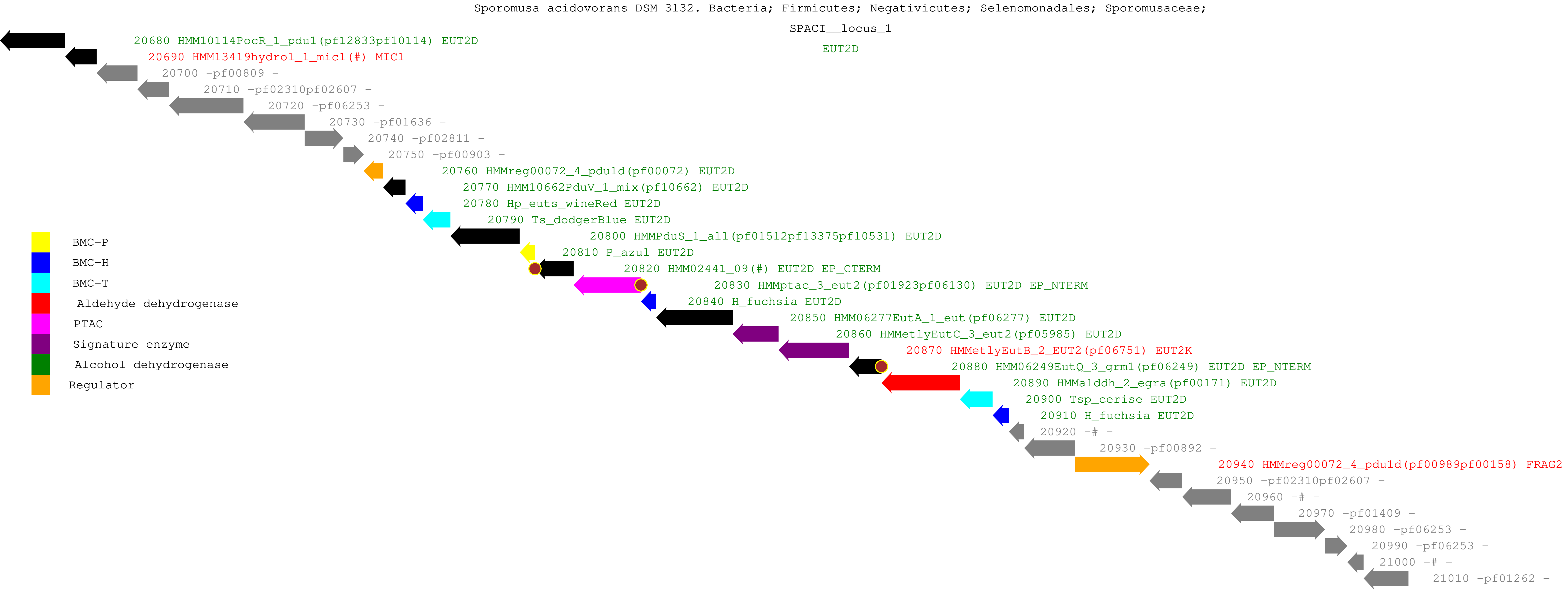
Sporomusa acidovorans DSM 3132. Bacteria; Firmicutes; Negativicutes; Selenomonadales; Sporomusaceae;
SPACI__locus_1
EUT2D
20680 HMM10114PocR_1_pdu1(pf12833pf10114) EUT2D
20690 HMM13419hydrol_1_mic1(#) MIC1
20700 -pf00809 -
20710 -pf02310pf02607 -
20720 -pf06253 -
20730 -pf01636 -
20740 -pf02811 -
20750 -pf00903 -
20760 HMMreg00072_4_pdu1d(pf00072) EUT2D
20770 HMM10662PduV_1_mix(pf10662) EUT2D
20780 Hp_euts_wineRed EUT2D
20790 Ts_dodgerBlue EUT2D
20800 HMMPduS_1_all(pf01512pf13375pf10531) EUT2D
20810 P_azul EUT2D
20820 HMM02441_09(#) EUT2D EP_CTERM
20830 HMMptac_3_eut2(pf01923pf06130) EUT2D EP_NTERM
20840 H_fuchsia EUT2D
20850 HMM06277EutA_1_eut(pf06277) EUT2D
20860 HMMetlyEutC_3_eut2(pf05985) EUT2D
20870 HMMetlyEutB_2_EUT2(pf06751) EUT2K
20880 HMM06249EutQ_3_grm1(pf06249) EUT2D EP_NTERM
20890 HMMaldh_2_egra(pf00171) EUT2D
20900 Tsp_cerise EUT2D
20910 H_fuchsia EUT2D
20920 -# -
20930 -pf00892 -
20940 HMMreg00072_4_pdu1d(pf00989pf00158) FRAG2
20950 -pf02310pf02607 -
20960 -# -
20970 -pf01409 -
20980 -pf06253 -
20990 -pf06253 -
21000 -# -
21010 -pf01262 -
BMC-P
BMC-H
BMC-T
Aldehyde dehydrogenase
PTAC
Signature enzyme
Alcohol dehydrogenase
Regulator

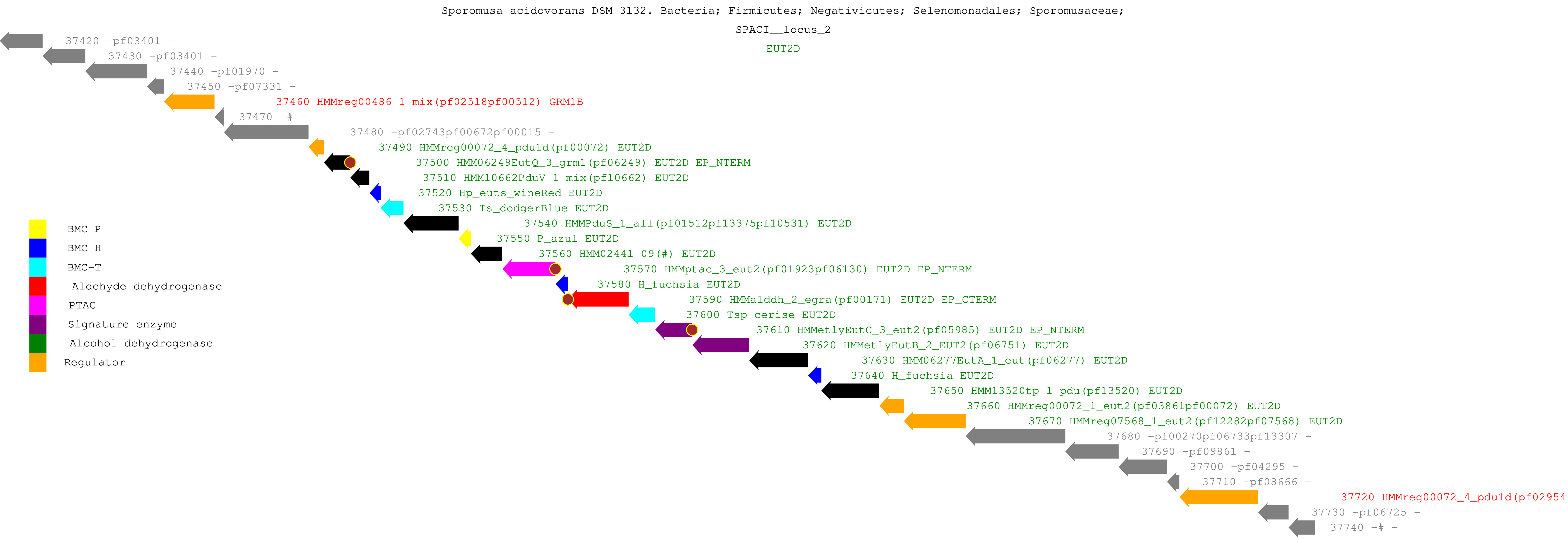
Sporomusa acidovorans DSM 3132. Bacteria; Firmicutes; Negativicutes; Selenomonadales; Sporomusaceae;
SPACI__locus_2
EUT2D
37420 -pf03401 -
37430 -pf03401 -
37440 -pf01970 -
37450 -pf07331 -
37460 HMMreg00486_1_mix(pf02518pf00512) GRM1B
37470 -# -
37480 -pf02743pf00672pf00015 -
37490 HMMreg00072_4_pdu1d(pf00072) EUT2D
37500 HMM06249EutQ_3_grm1(pf06249) EUT2D EP_NTERM
37510 HMM10662PduV_1_mix(pf10662) EUT2D
37520 Hp_euts_wineRed EUT2D
37530 Ts_dodgerBlue EUT2D
37540 HMMPduS_1_all(pf01512pf13375pf10531) EUT2D
37550 P_azul EUT2D
37560 HMM02441_09(#) EUT2D
37570 HMMptac_3_eut2(pf01923pf06130) EUT2D EP_NTERM
37580 H_fuchsia EUT2D
37590 HMMalddh_2_egra(pf00171) EUT2D EP_CTERM
37600 Tsp_cerise EUT2D
37610 HMMetlyEutC_3_eut2(pf05985) EUT2D EP_NTERM
37620 HMMetlyEutB_2_EUT2(pf06751) EUT2D
37630 HMM06277EutA_1_eut(pf06277) EUT2D
37640 H_fuchsia EUT2D
37650 HMM13520tp_1_pdu(pf13520) EUT2D
37660 HMMreg00072_1_eut2(pf03861pf00072) EUT2D
37670 HMMreg07568_1_eut2(pf12282pf07568) EUT2D
37680 -pf00270pf06733pf13307 -
37690 -pf09861 -
37700 -pf04295 -
37710 -pf08666 -
37720 HMMreg00072_4_pdu1d(pf02954
37730 -pf06725 -
37740 -# -
BMC-P
BMC-H
BMC-T
Aldehyde dehydrogenase
PTAC
Signature enzyme
Alcohol dehydrogenase
Regulator

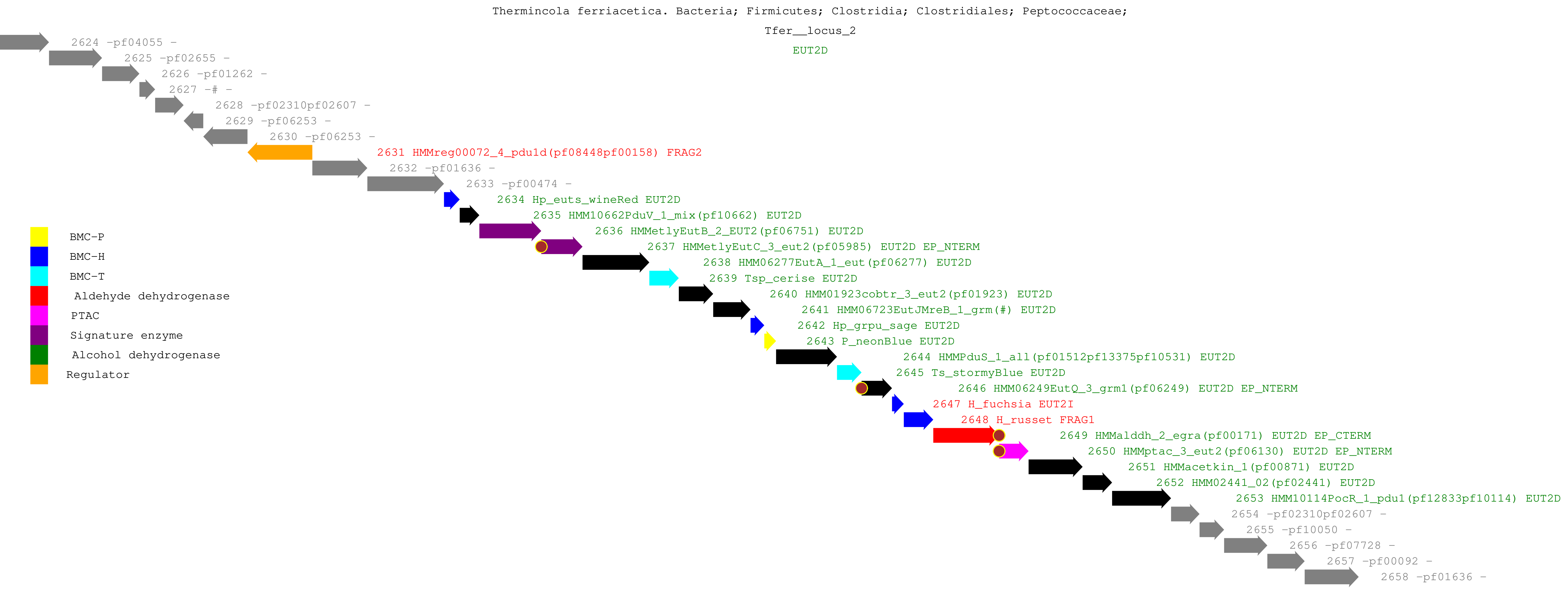
Thermincola ferriacetica. Bacteria; Firmicutes; Clostridia; Clostridiales; Peptococcaceae;
Tfer__locus_2
EUT2D
2624 -pf04055 -
2625 -pf02655 -
2626 -pf01262 -
2627 -# -
2628 -pf02310pf02607 -
2629 -pf06253 -
2630 -pf06253 -
2631 HMMreg00072_4_pdu1d(pf08448pf00158) FRAG2
2632 -pf01636 -
2633 -pf00474 -
2634 Hp_euts_wineRed EUT2D
2635 HMM10662PduV_1_mix(pf10662) EUT2D
2636 HMMetlyEutB_2_EUT2(pf06751) EUT2D
2637 HMMetlyEutC_3_eut2(pf05985) EUT2D EP_NTERM
2638 HMM06277EutA_1_eut(pf06277) EUT2D
2639 Tsp_cerise EUT2D
2640 HMM01923cobtr_3_eut2(pf01923) EUT2D
2641 HMM06723EutJMreB_1_grm(#) EUT2D
2642 Hp_grpu_sage EUT2D
2643 P_neonBlue EUT2D
2644 HMMPduS_1_all(pf01512pf13375pf10531) EUT2D
2645 Ts_stormyBlue EUT2D
2646 HMM06249EutQ_3_grm1(pf06249) EUT2D EP_NTERM
2647 H_fuchsia EUT2I
2648 H_russet FRAG1
2649 HMMalddh_2_egra(pf00171) EUT2D EP_CTERM
2650 HMMptac_3_eut2(pf06130) EUT2D EP_NTERM
2651 HMMacetkin_1(pf00871) EUT2D
2652 HMM02441_02(pf02441) EUT2D
2653 HMM10114PocR_1_pdu1(pf12833pf10114) EUT2D
2654 -pf02310pf02607 -
2655 -pf10050 -
2656 -pf07728 -
2657 -pf00092 -
2658 -pf01636 -
BMC-P
BMC-H
BMC-T
Aldehyde dehydrogenase
PTAC
Signature enzyme
Alcohol dehydrogenase
Regulator

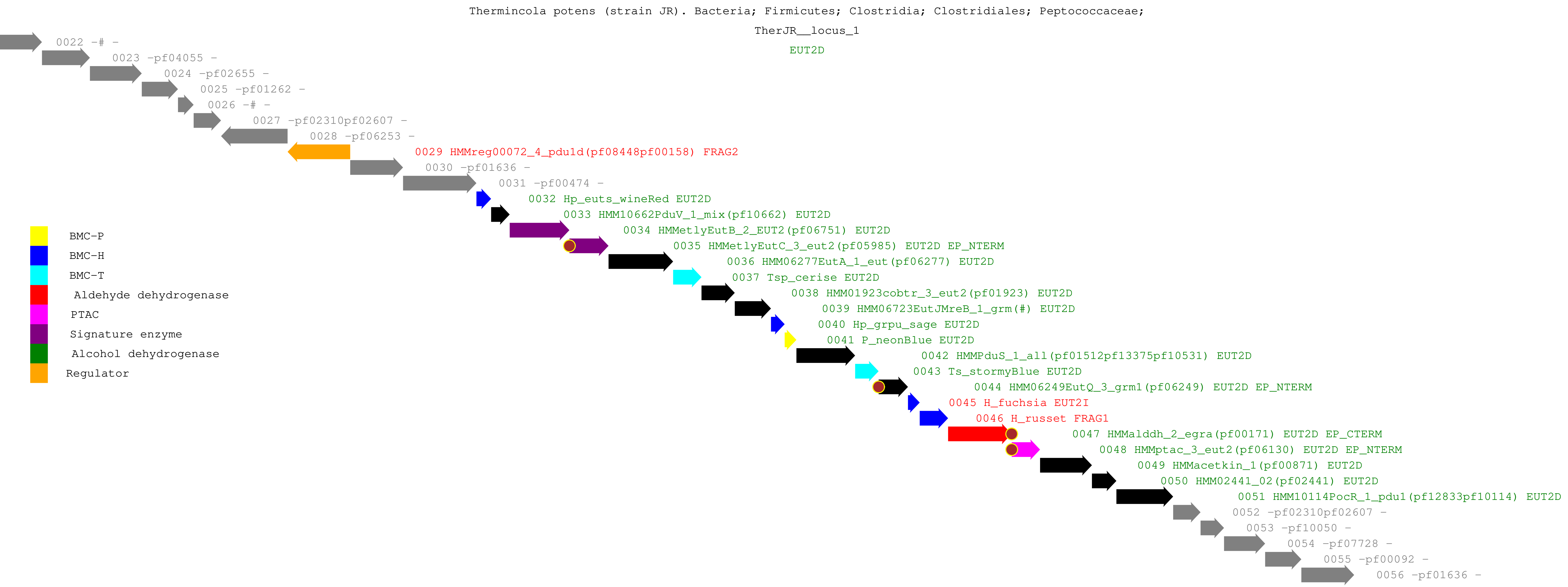

Thermincola potens (strain JR). Bacteria; Firmicutes; Clostridia; Clostridiales; Peptococcaceae;
TherJR__locus_1
EUT2D
0022 -# -
0023 -pf04055 -
0024 -pf02655 -
0025 -pf01262 -
0026 -# -
0027 -pf02310pf02607 -
0028 -pf06253 -
0029 HMMreg00072_4_pdu1d(pf08448pf00158) FRAG2
0030 -pf01636 -
0031 -pf00474 -
0032 Hp_euts_wineRed EUT2D
0033 HMM10662PduV_1_mix(pf10662) EUT2D
0034 HMMetlyEutB_2_EUT2(pf06751) EUT2D
0035 HMMetlyEutC_3_eut2(pf05985) EUT2D EP_NTERM
0036 HMM06277EutA_1_eut(pf06277) EUT2D
0037 Tsp_cerise EUT2D
0038 HMM01923cobtr_3_eut2(pf01923) EUT2D
0039 HMM06723EutJMreB_1_grm(#) EUT2D
0040 Hp_grpu_sage EUT2D
0041 P_neonBlue EUT2D
0042 HMMPduS_1_all(pf01512pf13375pf10531) EUT2D
0043 Ts_stormyBlue EUT2D
0044 HMM06249EutQ_3_grm1(pf06249) EUT2D EP_NTERM
0045 H_fuchsia EUT2I
0046 H_russet FRAG1
0047 HMMalddh_2_egra(pf00171) EUT2D EP_CTERM
0048 HMMptac_3_eut2(pf06130) EUT2D EP_NTERM
0049 HMMacetkin_1(pf00871) EUT2D
0050 HMM02441_02(pf02441) EUT2D
0051 HMM10114PocR_1_pdu1(pf12833pf10114) EUT2D
0052 -pf02310pf02607 -
0053 -pf10050 -
0054 -pf07728 -
0055 -pf00092 -
0056 -pf01636 -
BMC-P
BMC-H
BMC-T
Aldehyde dehydrogenase
PTAC
Signature enzyme
Alcohol dehydrogenase
Regulator

Peptococcaceae bacterium BRH_c23. Bacteria; Firmicutes; Clostridia; Clostridiales; Peptococcaceae.

VR66__locus_1

EUT2D

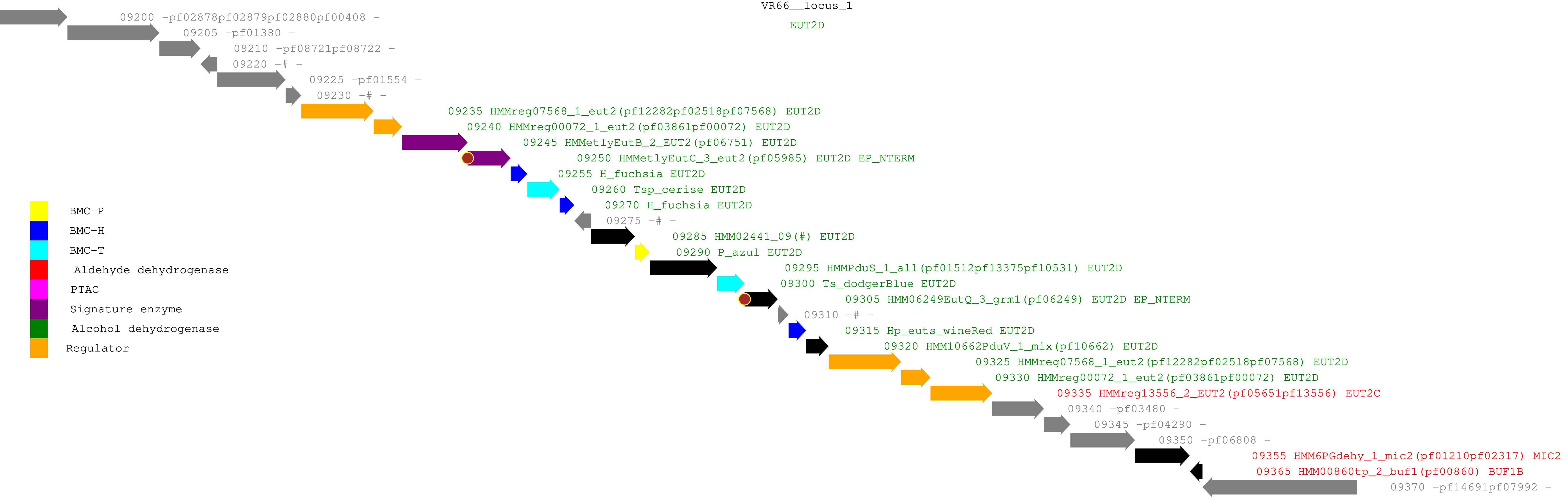

Geobacillus sp. JS12. Bacteria; Firmicutes; Bacilli; Bacillales; Bacillaceae; Geobacillus.

A0V43__locus_1

EUT2E

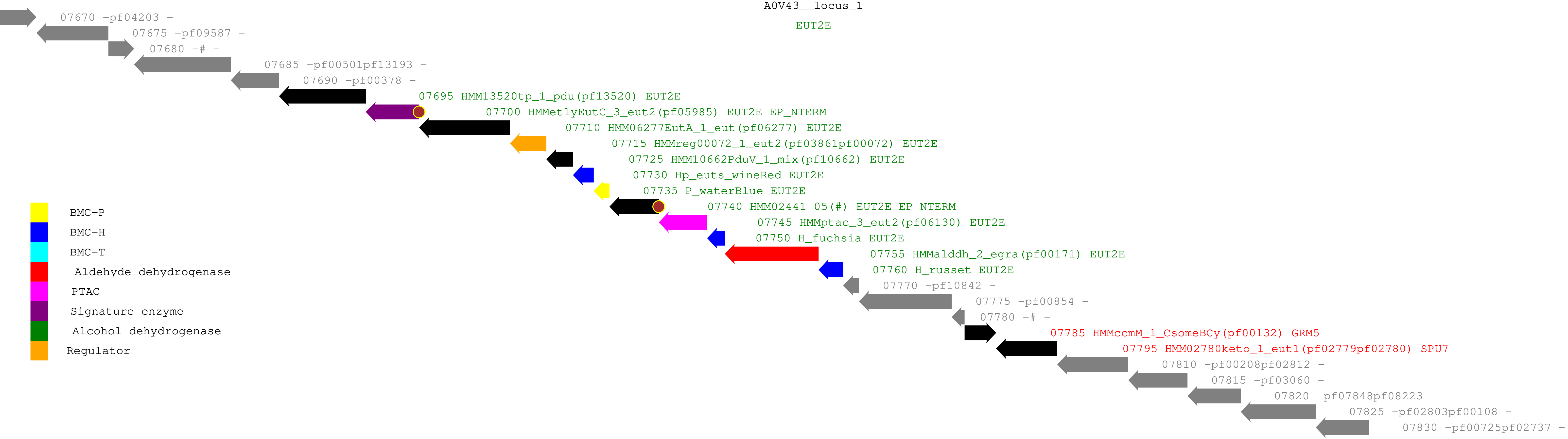

Geobacillus thermoleovorans (Bacillus thermoleovorans). Bacteria; Firmicutes; Bacilli; Bacillales; Bacillaceae; Geobacillus;

A5N86__locus_1_main_sat

EUT2E

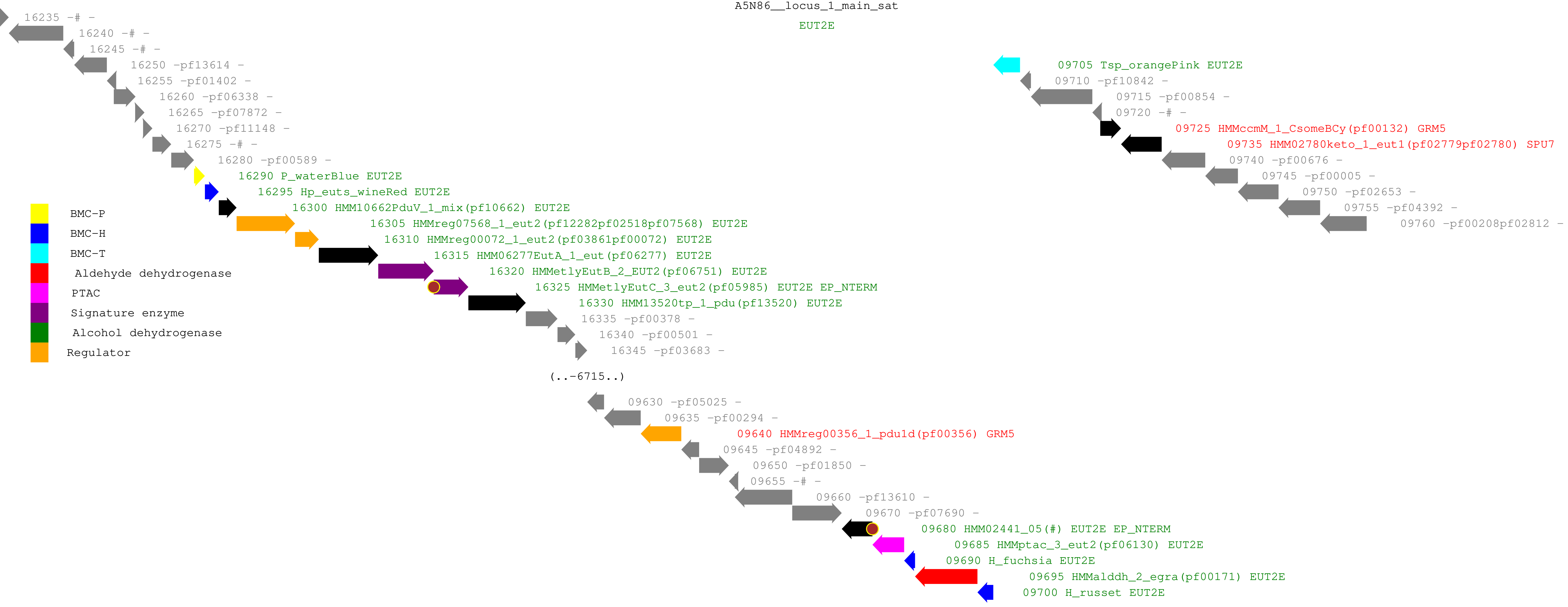

Brevibacillus brevis X23. Bacteria; Firmicutes; Bacilli; Bacillales; Paenibacillaceae;

A616__locus_1

EUT2E

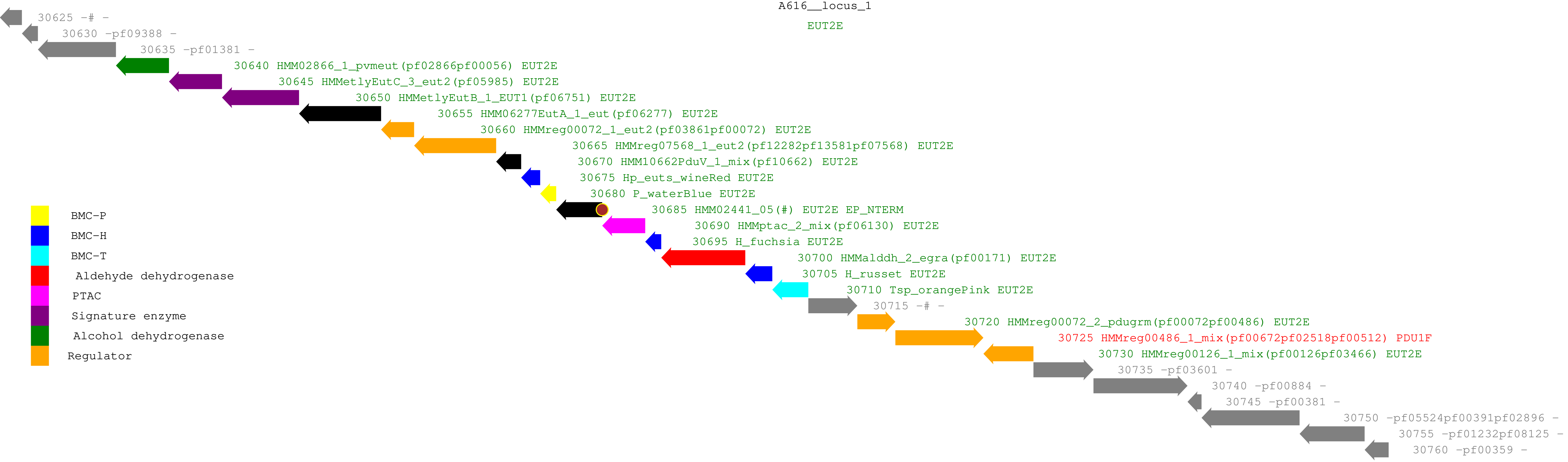

Bacillus sp. FJAT-27215. Bacteria; Firmicutes; Bacilli; Bacillales; Bacillaceae; Bacillus.

A8F95__locus_2

EUT2E

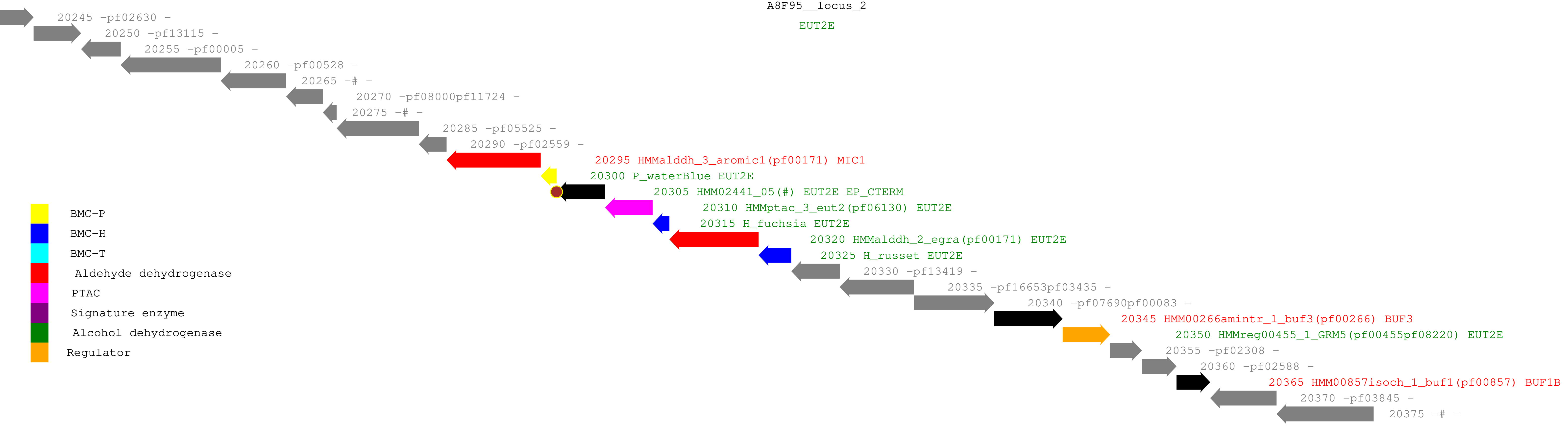

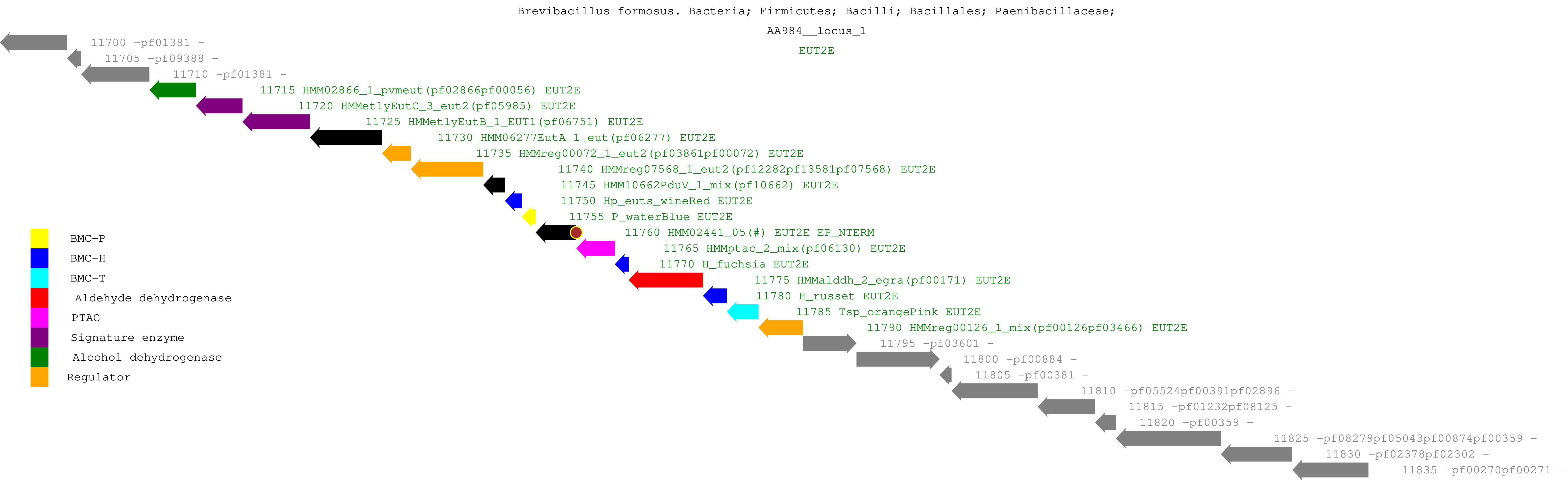
Brevibacillus formosus. Bacteria; Firmicutes; Bacilli; Bacillales; Paenibacillaceae;
AA984__locus_1
EUT2E
11700 -pf01381 -
11705 -pf09388 -
11710 -pf01381 -
11715 HMM02866_1_pvmeut(pf02866pf00056) EUT2E
11720 HMMetlyEutC_3_eut2(pf05985) EUT2E
11725 HMMetlyEutB_1_EUT1(pf06751) EUT2E
11730 HMM06277EutA_1_eut(pf06277) EUT2E
11735 HMMreg00072_1_eut2(pf03861pf00072) EUT2E
11740 HMMreg07568_1_eut2(pf12282pf13581pf07568) EUT2E
11745 HMM10662PduV_1_mix(pf10662) EUT2E
11750 Hp_euts_wineRed EUT2E
11755 P_waterBlue EUT2E
11760 HMM02441_05(#) EUT2E EP_NTERM
11765 HMMptac_2_mix(pf06130) EUT2E
11770 H_fuchsia EUT2E
11775 HMMalddh_2_egra(pf00171) EUT2E
11780 H_russet EUT2E
11785 Tsp_orangePink EUT2E
11790 HMMreg00126_1_mix(pf00126pf03466) EUT2E
11795 -pf03601 -
11800 -pf00884 -
11805 -pf00381 -
11810 -pf05524pf00391pf02896 -
11815 -pf01232pf08125 -
11820 -pf00359 -
11825 -pf08279pf05043pf00874pf00359 -
11830 -pf02378pf02302 -
11835 -pf00270pf00271 -
BMC-P
BMC-H
BMC-T
Aldehyde dehydrogenase
PTAC
Signature enzyme
Alcohol dehydrogenase
Regulator

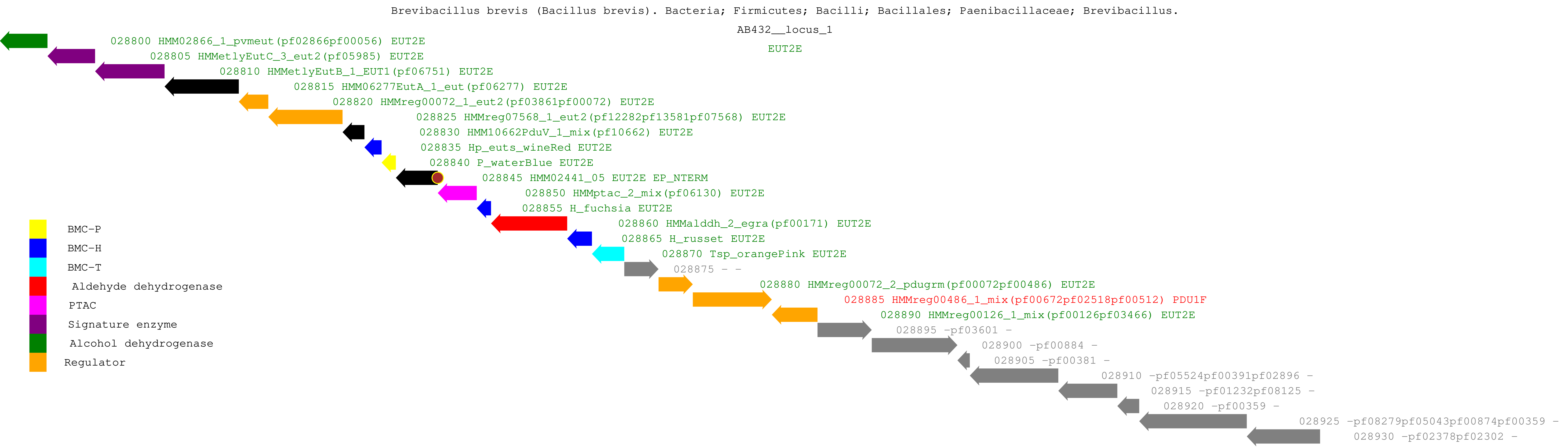
Brevibacillus brevis (Bacillus brevis). Bacteria; Firmicutes; Bacilli; Bacillales; Paenibacillaceae; Brevibacillus.
AB432__locus_1
EUT2E
028800 HMM02866_1_pvmeut(pf02866pf00056) EUT2E
028805 HMMetlyEutC_3_eut2(pf05985) EUT2E
028810 HMMetlyEutB_1_EUT1(pf06751) EUT2E
028815 HMM06277EutA_1_eut(pf06277) EUT2E
028820 HMMreg00072_1_eut2(pf03861pf00072) EUT2E
028825 HMMreg07568_1_eut2(pf12282pf13581pf07568) EUT2E
028830 HMM10662PduV_1_mix(pf10662) EUT2E
028835 Hp_euts_wineRed EUT2E
028840 P_waterBlue EUT2E
028845 HMM02441_05 EUT2E EP_NTERM
028850 HMMptac_2_mix(pf06130) EUT2E
028855 H_fuchsia EUT2E
028860 HMMalddh_2_egra(pf00171) EUT2E
028865 H_russet EUT2E
028870 Tsp_orangePink EUT2E
028875 – –
028880 HMMreg00072_2_pdugrm(pf00072pf00486) EUT2E
028885 HMMreg00486_1_mix(pf00672pf02518pf00512) PDU1F
028890 HMMreg00126_1_mix(pf00126pf03466) EUT2E
028895 –pf03601 –
028900 –pf00884 –
028905 –pf00381 –
028910 –pf05524pf00391pf02896 –
028915 –pf01232pf08125 –
028920 –pf00359 –
028925 –pf08279pf05043pf00874pf00359 –
028930 –pf02378pf02302 –
BMC-P
BMC-H
BMC-T
Aldehyde dehydrogenase
PTAC
Signature enzyme
Alcohol dehydrogenase
Regulator

Geobacillus sp. T6. Bacteria; Firmicutes; Bacilli; Bacillales; Bacillaceae; Geobacillus.

ABH20__locus_1

EUT2E

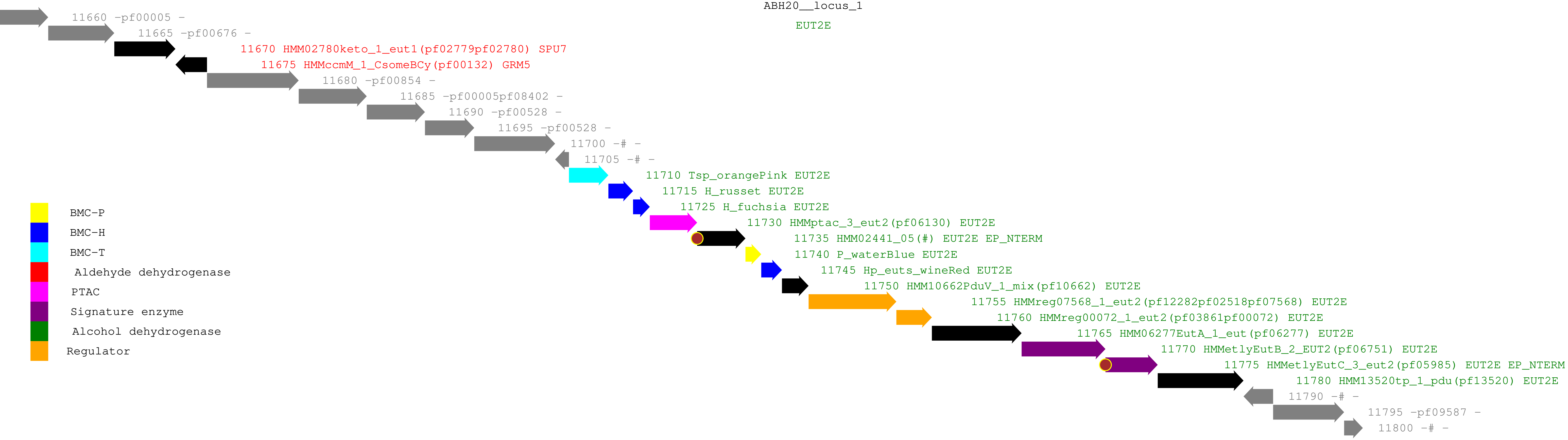

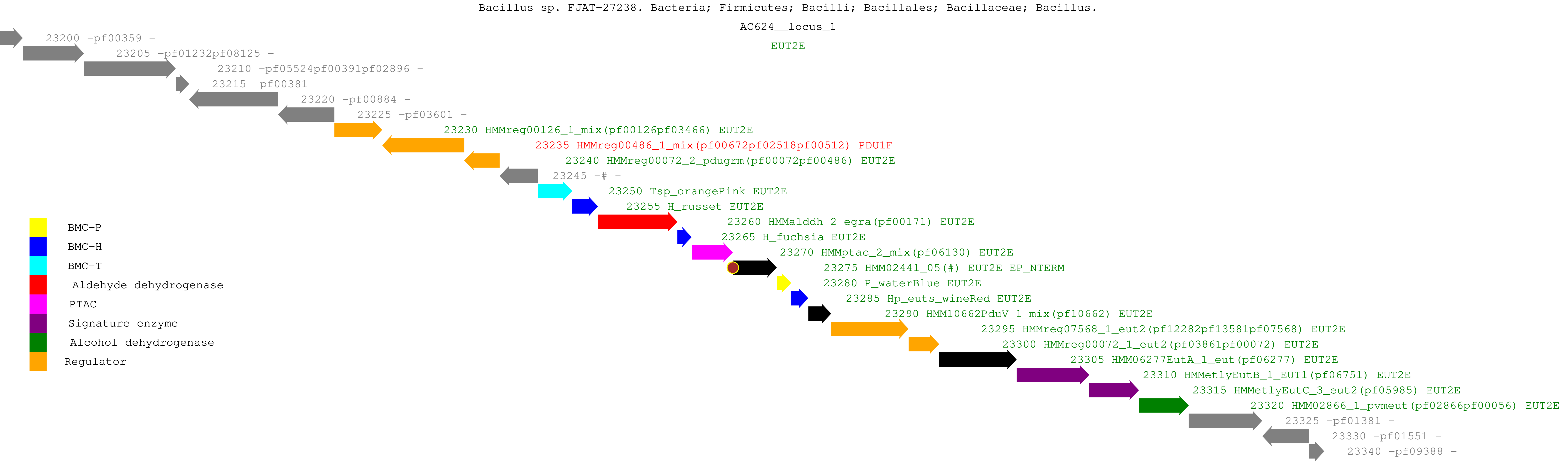
Bacillus sp. FJAT-27238. Bacteria; Firmicutes; Bacilli; Bacillales; Bacillaceae; Bacillus.
AC624__locus_1
EUT2E
23200 -pf00359 -
23205 -pf01232pf08125 -
23210 -pf05524pf00391pf02896 -
23215 -pf00381 -
23220 -pf00884 -
23225 -pf03601 -
23230 HMMreg00126_1_mix(pf00126pf03466) EUT2E
23235 HMMreg00486_1_mix(pf00672pf02518pf00512) PDU1F
23240 HMMreg00072_2_pdugrm(pf00072pf00486) EUT2E
23245 -# -
23250 Tsp_orangePink EUT2E
23255 H_russet EUT2E
23260 HMMalddh_2_egra(pf00171) EUT2E
23265 H_fuchsia EUT2E
23270 HMMptac_2_mix(pf06130) EUT2E
23275 HMM02441_05(#) EUT2E EP_NTERM
23280 P_waterBlue EUT2E
23285 Hp_euts_wineRed EUT2E
23290 HMM10662PduV_1_mix(pf10662) EUT2E
23295 HMMreg07568_1_eut2(pf12282pf13581pf07568) EUT2E
23300 HMMreg00072_1_eut2(pf03861pf00072) EUT2E
23305 HMM06277EutA_1_eut(pf06277) EUT2E
23310 HMMetlyEutB_1_EUT1(pf06751) EUT2E
23315 HMMetlyEutC_3_eut2(pf05985) EUT2E
23320 HMM02866_1_pvmeut(pf02866pf00056) EUT2E
23325 -pf01381 -
23330 -pf01551 -
23340 -pf09388 -
BMC-P
BMC-H
BMC-T
Aldehyde dehydrogenase
PTAC
Signature enzyme
Alcohol dehydrogenase
Regulator

Brevibacillus reuszeri. Bacteria; Firmicutes; Bacilli; Bacillales; Paenibacillaceae;

ADS79__locus_1

EUT2E

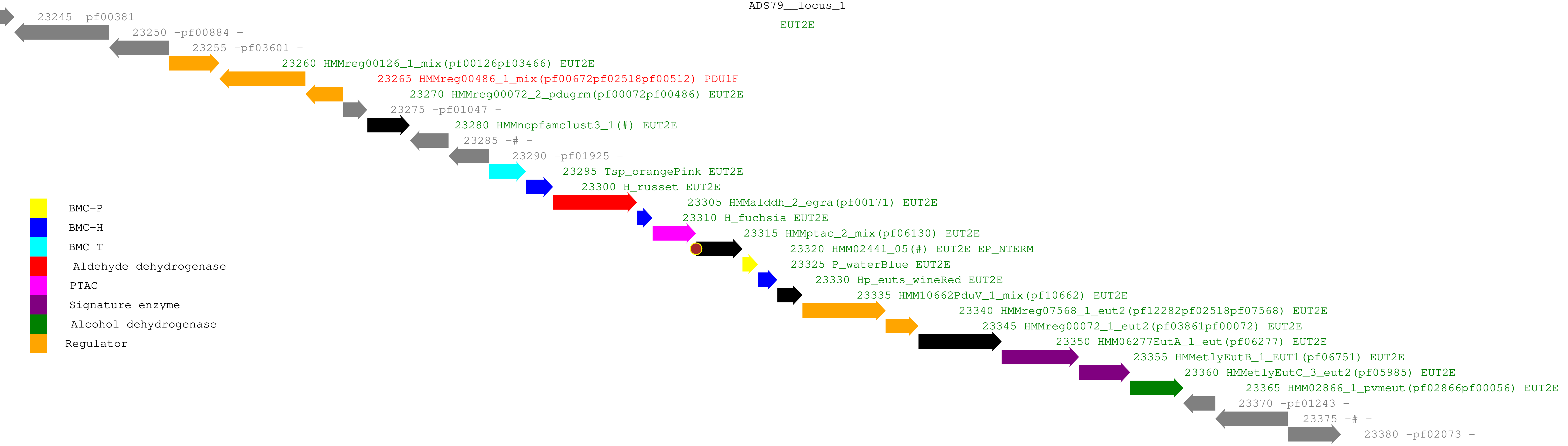

Brevibacillus choshinensis. Bacteria; Firmicutes; Bacilli; Bacillales; Paenibacillaceae;

AN963__locus_2

EUT2E

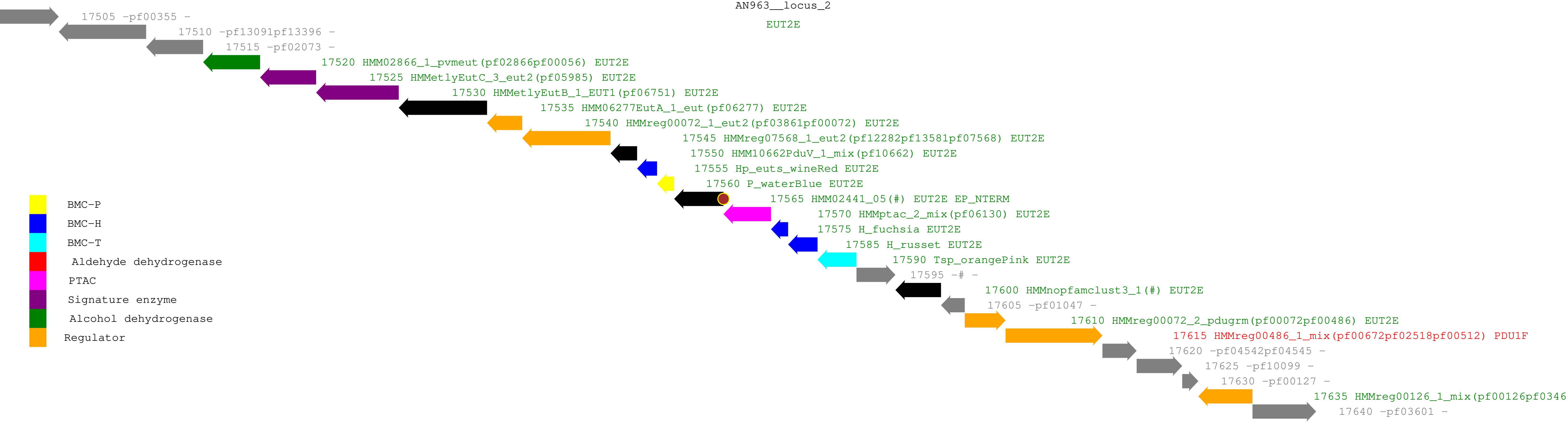

Paenibacillus sp. Root444D2. Bacteria; Firmicutes; Bacilli; Bacillales; Paenibacillaceae;

ASD40__locus_1

EUT2E

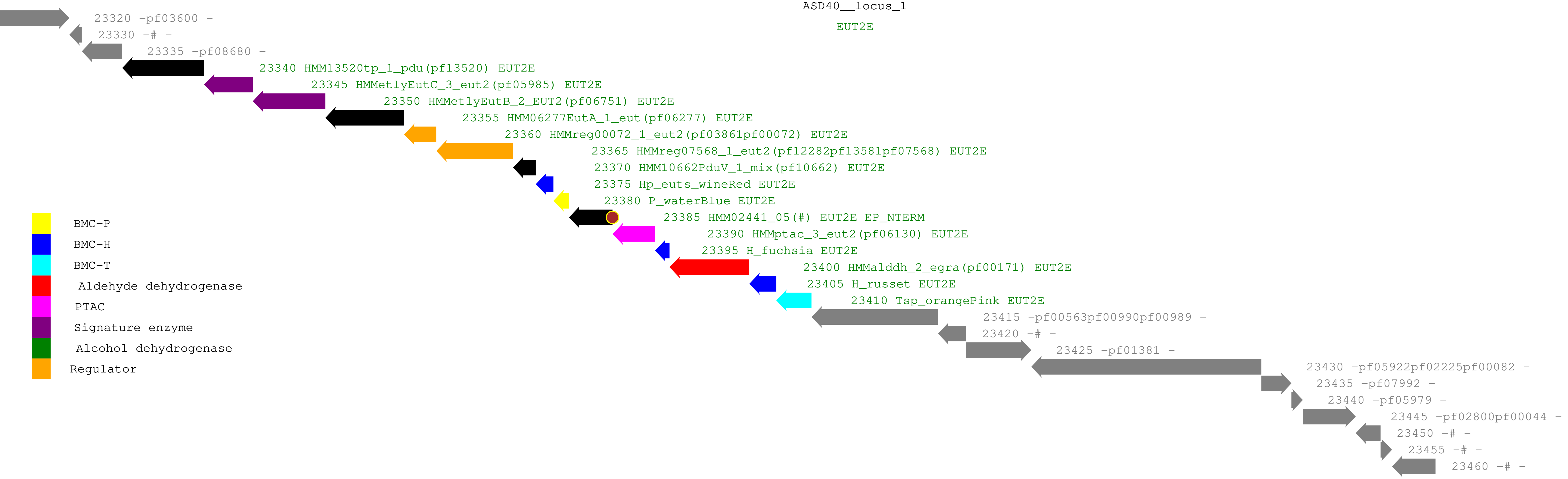

Paenibacillus sp. Soil724D2. Bacteria; Firmicutes; Bacilli; Bacillales; Paenibacillaceae;

ASG85__locus_1

EUT2E

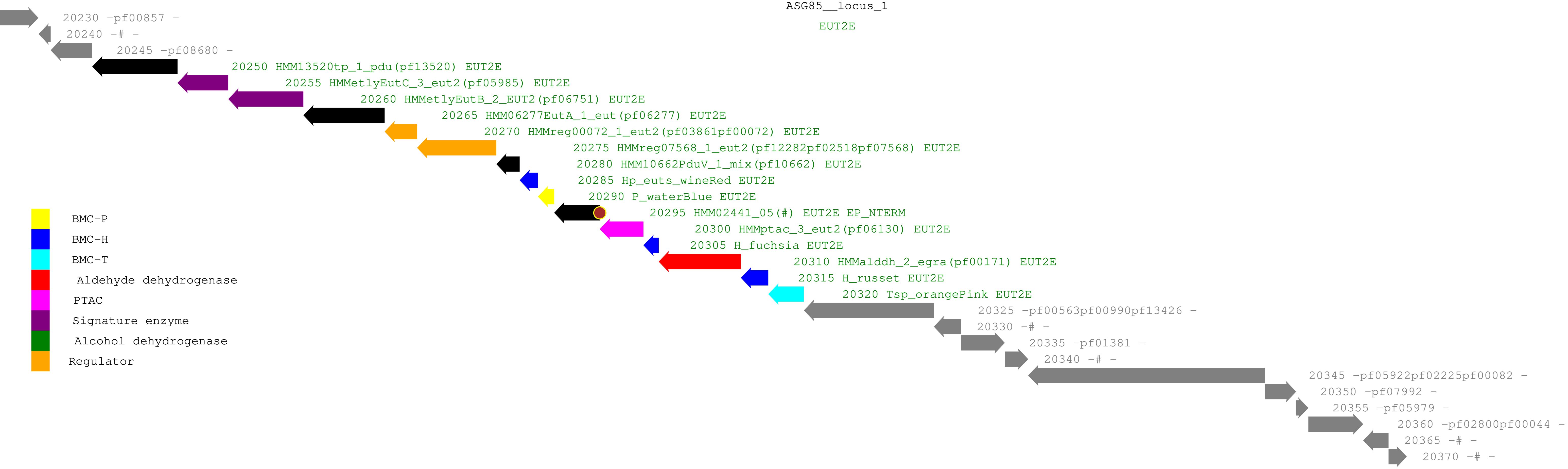

Brevibacillus parabrevis. Bacteria; Firmicutes; Bacilli; Bacillales; Paenibacillaceae;

AV540__locus_1

EUT2E

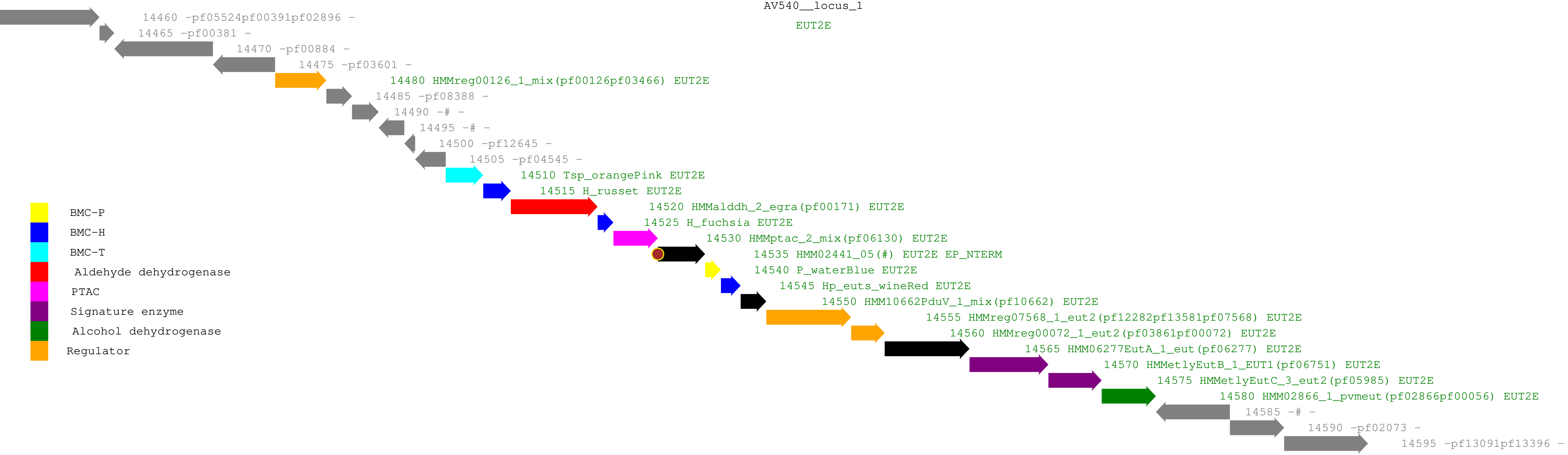

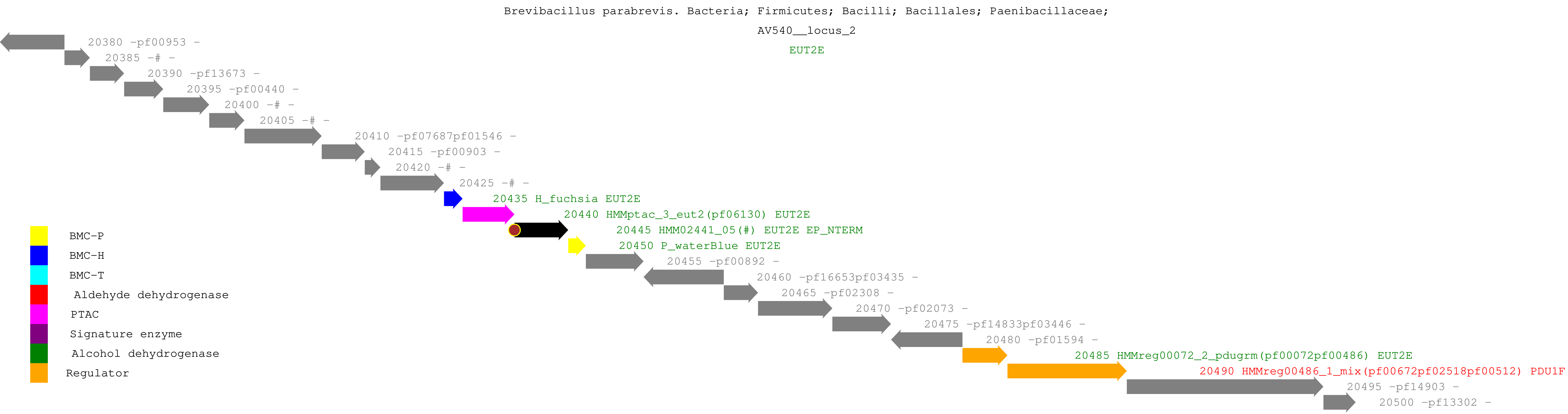

Brevibacillus parabrevis. Bacteria; Firmicutes; Bacilli; Bacillales; Paenibacillaceae;
AV540__locus_2
EUT2E
20380 -pf00953 -
20385 -# -
20390 -pf13673 -
20395 -pf00440 -
20400 -# -
20405 -# -
20410 -pf07687pf01546 -
20415 -pf00903 -
20420 -# -
20425 -# -
20435 H_fuchsia EUT2E
20440 HMMptac_3_eut2(pf06130) EUT2E
20445 HMM02441_05(#) EUT2E EP_NTERM
20450 P_waterBlue EUT2E
20455 -pf00892 -
20460 -pf16653pf03435 -
20465 -pf02308 -
20470 -pf02073 -
20475 -pf14833pf03446 -
20480 -pf01594 -
20485 HMMreg00072_2_pdugrm(pf00072pf00486) EUT2E
20490 HMMreg00486_1_mix(pf00672pf02518pf00512) PDU1F
20495 -pf14903 -
20500 -pf13302 -
BMC-P
BMC-H
BMC-T
Aldehyde dehydrogenase
PTAC
Signature enzyme
Alcohol dehydrogenase
Regulator

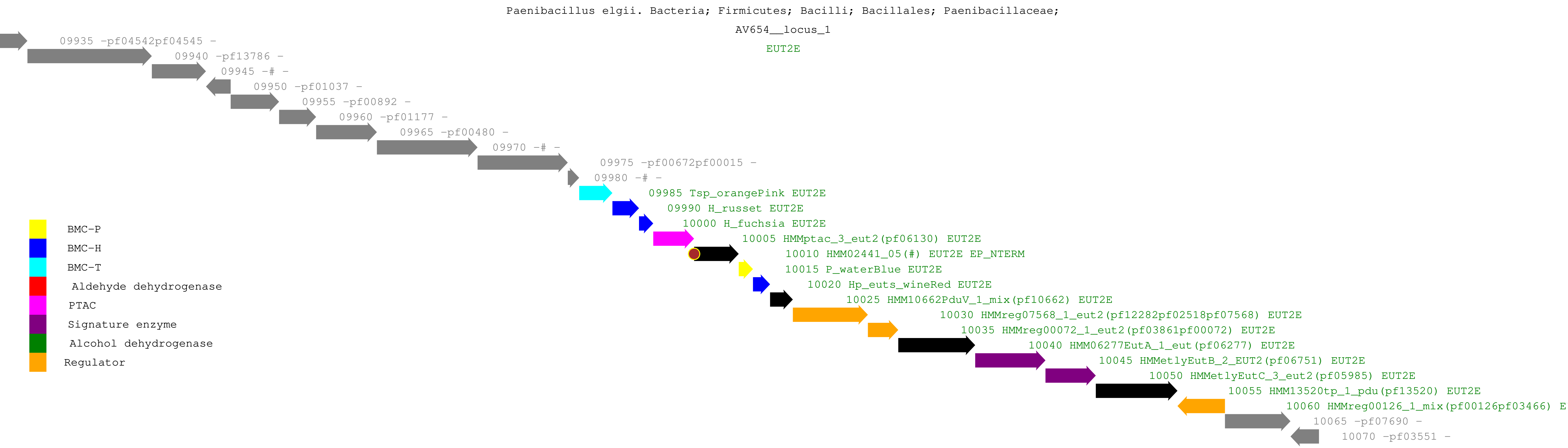
Paenibacillus elgii. Bacteria; Firmicutes; Bacilli; Bacillales; Paenibacillaceae;
AV654__locus_1
EUT2E
09935 -pf04542pf04545 -
09940 -pf13786 -
09945 -# -
09950 -pf01037 -
09955 -pf00892 -
09960 -pf01177 -
09965 -pf00480 -
09970 -# -
09975 -pf00672pf00015 -
09980 -# -
09985 Tsp_orangePink EUT2E
09990 H_russet EUT2E
10000 H_fuchsia EUT2E
10005 HMMptac_3_eut2(pf06130) EUT2E
10010 HMM02441_05(#) EUT2E EP_NTERM
10015 P_waterBlue EUT2E
10020 Hp_euts_wineRed EUT2E
10025 HMM10662PduV_1_mix(pf10662) EUT2E
10030 HMMreg07568_1_eut2(pf12282pf02518pf07568) EUT2E
10035 HMMreg00072_1_eut2(pf03861pf00072) EUT2E
10040 HMM06277EutA_1_eut(pf06277) EUT2E
10045 HMMetlyEutB_2_EUT2(pf06751) EUT2E
10050 HMMetlyEutC_3_eut2(pf05985) EUT2E
10055 HMM13520tp_1_pdu(pf13520) EUT2E
10060 HMMreg00126_1_mix(pf00126pf03466) E
10065 -pf07690 -
10070 -pf03551 -
BMC-P
BMC-H
BMC-T
Aldehyde dehydrogenase
PTAC
Signature enzyme
Alcohol dehydrogenase
Regulator

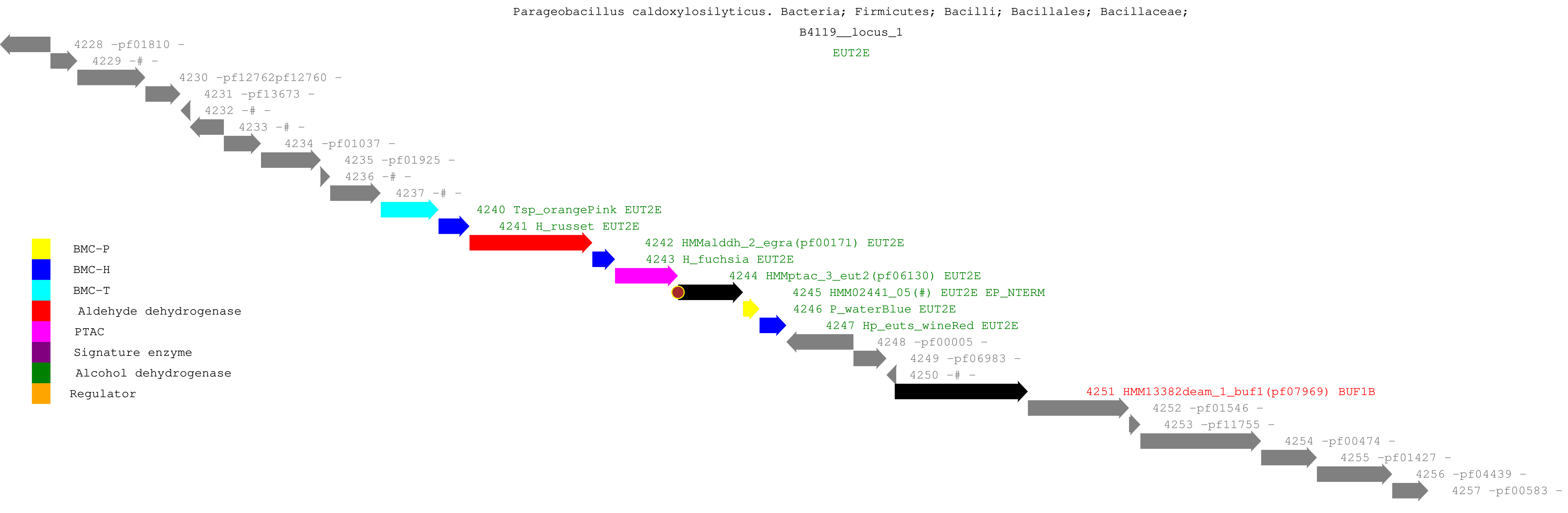

Parageobacillus caldoxylosilyticus. Bacteria; Firmicutes; Bacilli; Bacillales; Bacillaceae;
B4119__locus_1
EUT2E
4228 -pf01810 -
4229 -# -
4230 -pf12762pf12760 -
4231 -pf13673 -
4232 -# -
4233 -# -
4234 -pf01037 -
4235 -pf01925 -
4236 -# -
4237 -# -
4240 Tsp_orangePink EUT2E
4241 H_russet EUT2E
4242 HMMalddh_2_egra(pf00171) EUT2E
4243 H_fuchsia EUT2E
4244 HMMptac_3_eut2(pf06130) EUT2E
4245 HMM02441_05(#) EUT2E EP_NTERM
4246 P_waterBlue EUT2E
4247 Hp_euts_wineRed EUT2E
4248 -pf00005 -
4249 -pf06983 -
4250 -# -
4251 HMM13382deam_1_buf1(pf07969) BUF1B
4252 -pf01546 -
4253 -pf11755 -
4254 -pf00474 -
4255 -pf01427 -
4256 -pf04439 -
4257 -pf00583 -
BMC-P
BMC-H
BMC-T
Aldehyde dehydrogenase
PTAC
Signature enzyme
Alcohol dehydrogenase
Regulator

Brevibacillus brevis (Bacillus brevis). Bacteria; Firmicutes; Bacilli; Bacillales; Paenibacillaceae;

B5G50__locus_1

EUT2E

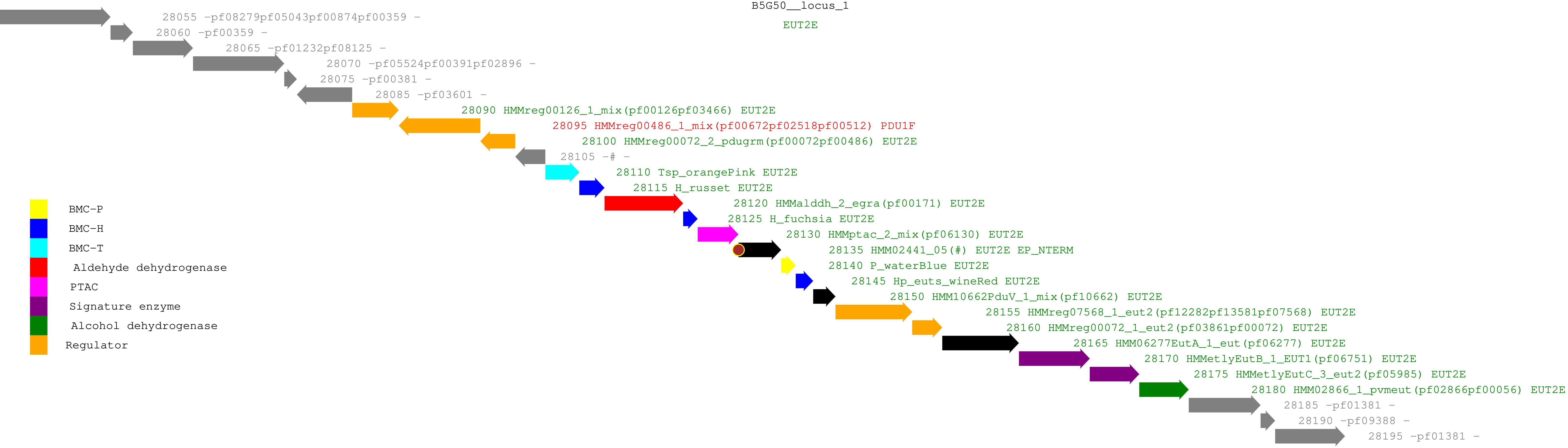

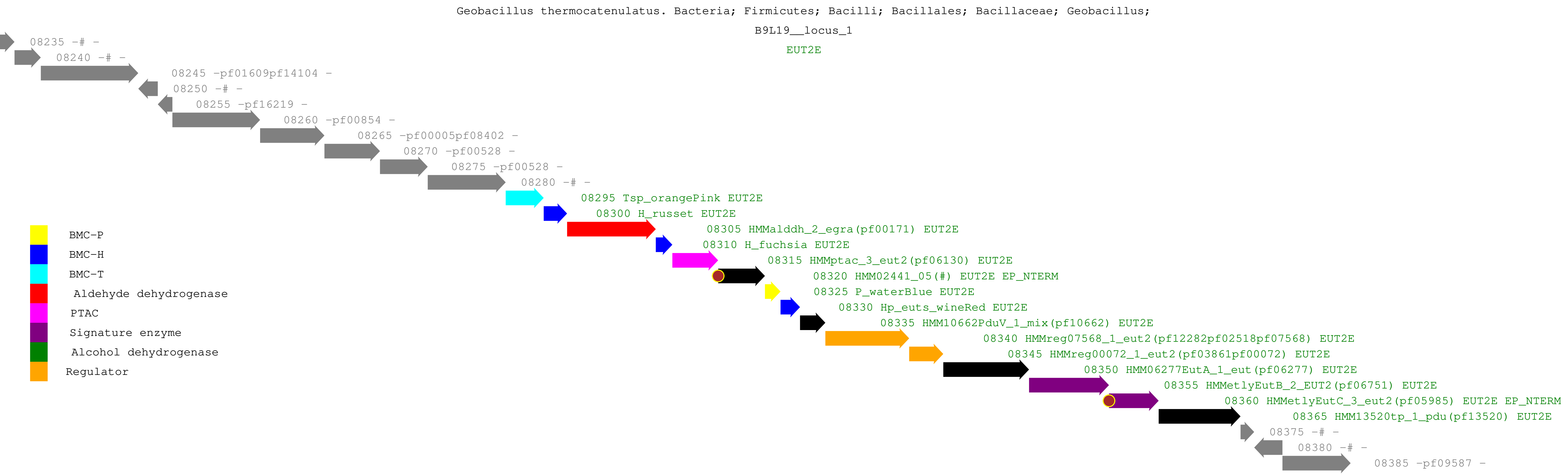
Geobacillus thermocatenulatus. Bacteria; Firmicutes; Bacilli; Bacillales; Bacillaceae; Geobacillus;
B9L19__locus_1
EUT2E
08235 -# -
08240 -# -
08245 -pf01609pf14104 -
08250 -# -
08255 -pf16219 -
08260 -pf00854 -
08265 -pf00005pf08402 -
08270 -pf00528 -
08275 -pf00528 -
08280 -# -
08295 Tsp_orangePink EUT2E
08300 H_russet EUT2E
08305 HMMalddh_2_egra(pf00171) EUT2E
08310 H_fuchsia EUT2E
08315 HMMptac_3_eut2(pf06130) EUT2E
08320 HMM02441_05(#) EUT2E EP_NTERM
08325 P_waterBlue EUT2E
08330 Hp_euts_wineRed EUT2E
08335 HMM10662PduV_1_mix(pf10662) EUT2E
08340 HMMreg07568_1_eut2(pf12282pf02518pf07568) EUT2E
08345 HMMreg00072_1_eut2(pf03861pf00072) EUT2E
08350 HMM06277EutA_1_eut(pf06277) EUT2E
08355 HMMetlyEutB_2_EUT2(pf06751) EUT2E
08360 HMMetlyEutC_3_eut2(pf05985) EUT2E EP_NTERM
08365 HMM13520tp_1_pdu(pf13520) EUT2E
08375 -# -
08380 -# -
08385 -pf09587 -
BMC-P
BMC-H
BMC-T
Aldehyde dehydrogenase
PTAC
Signature enzyme
Alcohol dehydrogenase
Regulator

Paenibacillus baekrokdamisoli. Bacteria; Firmicutes; Bacilli; Bacillales; Paenibacillaceae; Paenibacillus.

Back11__locus_1

EUT2E

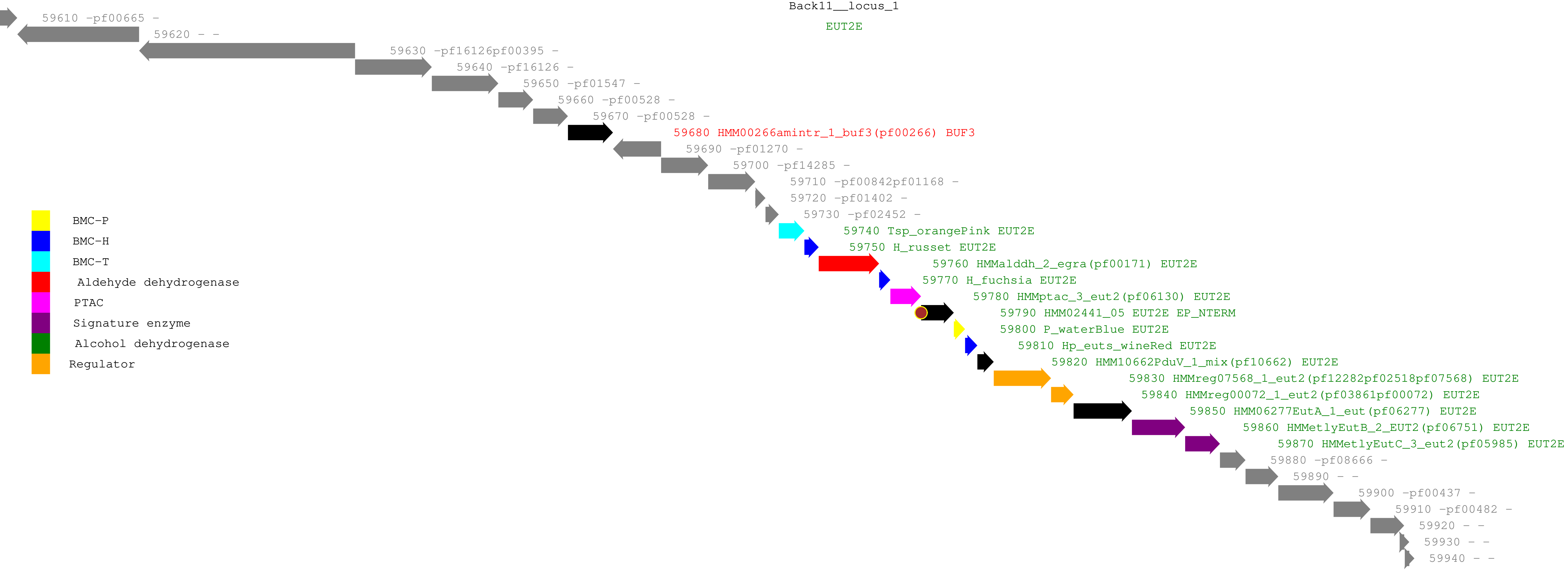

Brevibacillus brevis (strain 47 / JCM 6285 / NBRC 100599). Bacteria; Firmicutes; Bacilli; Bacillales; Paenibacillaceae;

BBR47__locus_1

EUT2E

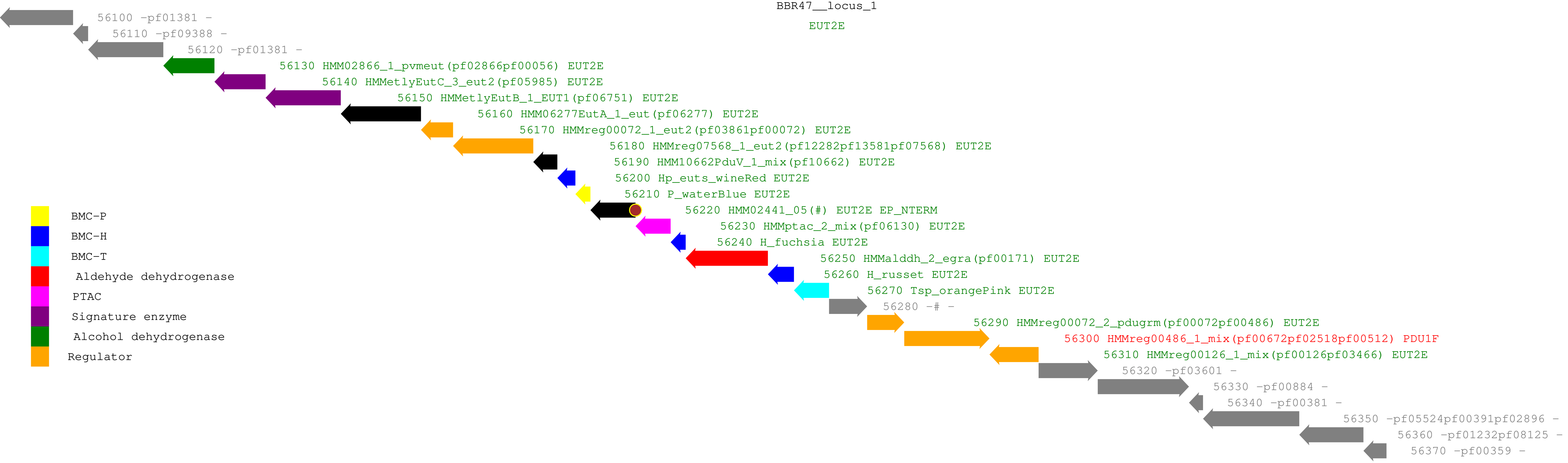

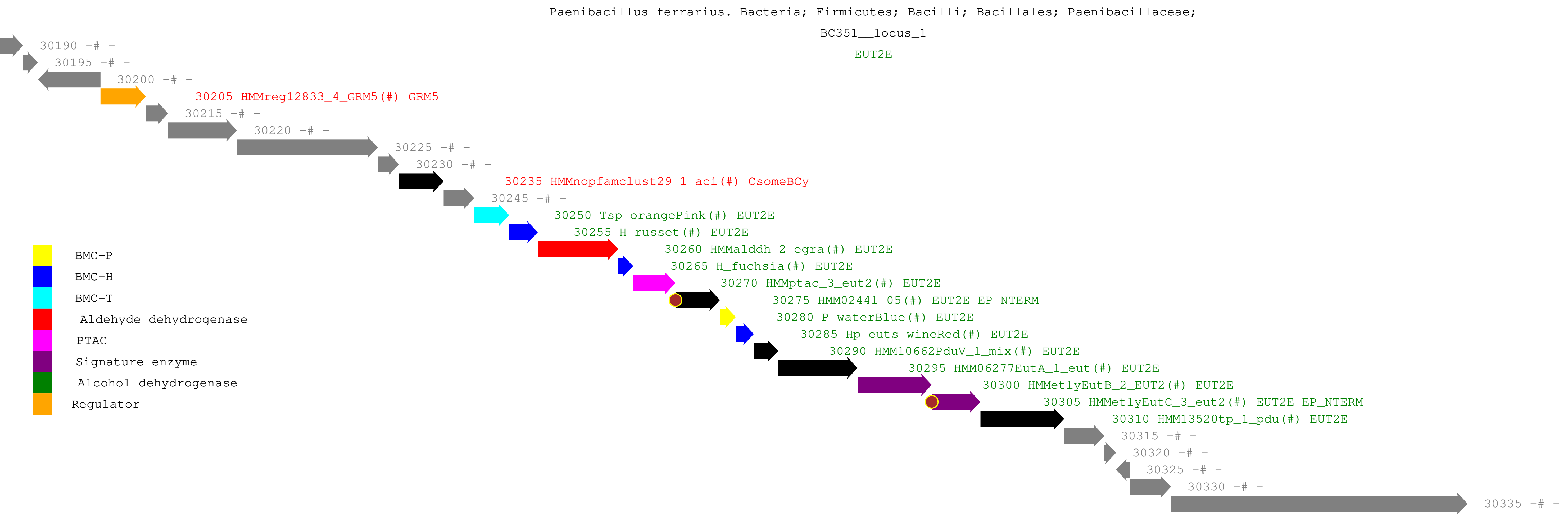

Paenibacillus ferrarius. Bacteria; Firmicutes; Bacilli; Bacillales; Paenibacillaceae;
BC351__locus_1
EUT2E
30190 -# -
30195 -# -
30200 -# -
30205 HMMreg12833_4_GRM5(#) GRM5
30215 -# -
30220 -# -
30225 -# -
30230 -# -
30235 HMMnopfamclust29_1_aci(#) CsomeBCy
30245 -# -
30250 Tsp_orangePink(#) EUT2E
30255 H_russet(#) EUT2E
30260 HMMalddh_2_egra(#) EUT2E
30265 H_fuchsia(#) EUT2E
30270 HMMptac_3_eut2(#) EUT2E
30275 HMM02441_05(#) EUT2E EP_NTERM
30280 P_waterBlue(#) EUT2E
30285 Hp_euts_wineRed(#) EUT2E
30290 HMM10662PduV_1_mix(#) EUT2E
30295 HMM06277EutA_1_eut(#) EUT2E
30300 HMMetlyEutB_2_EUT2(#) EUT2E
30305 HMMetlyEutC_3_eut2(#) EUT2E EP_NTERM
30310 HMM13520tp_1_pdu(#) EUT2E
30315 -# -
30320 -# -
30325 -# -
30330 -# -
30335 -# -
BMC-P
BMC-H
BMC-T
Aldehyde dehydrogenase
PTAC
Signature enzyme
Alcohol dehydrogenase
Regulator

Anoxybacillus amylolyticus. Bacteria; Firmicutes; Bacilli; Bacillales; Bacillaceae; Anoxybacillus.

BDK47__locus_1

EUT2E

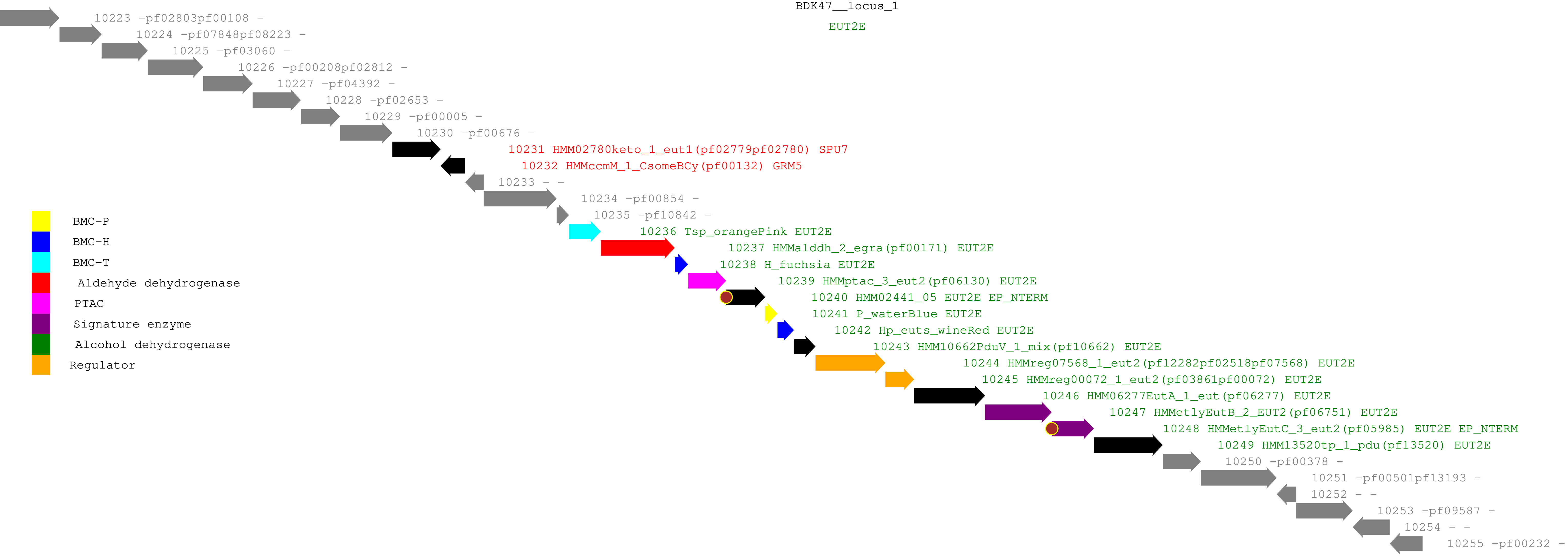

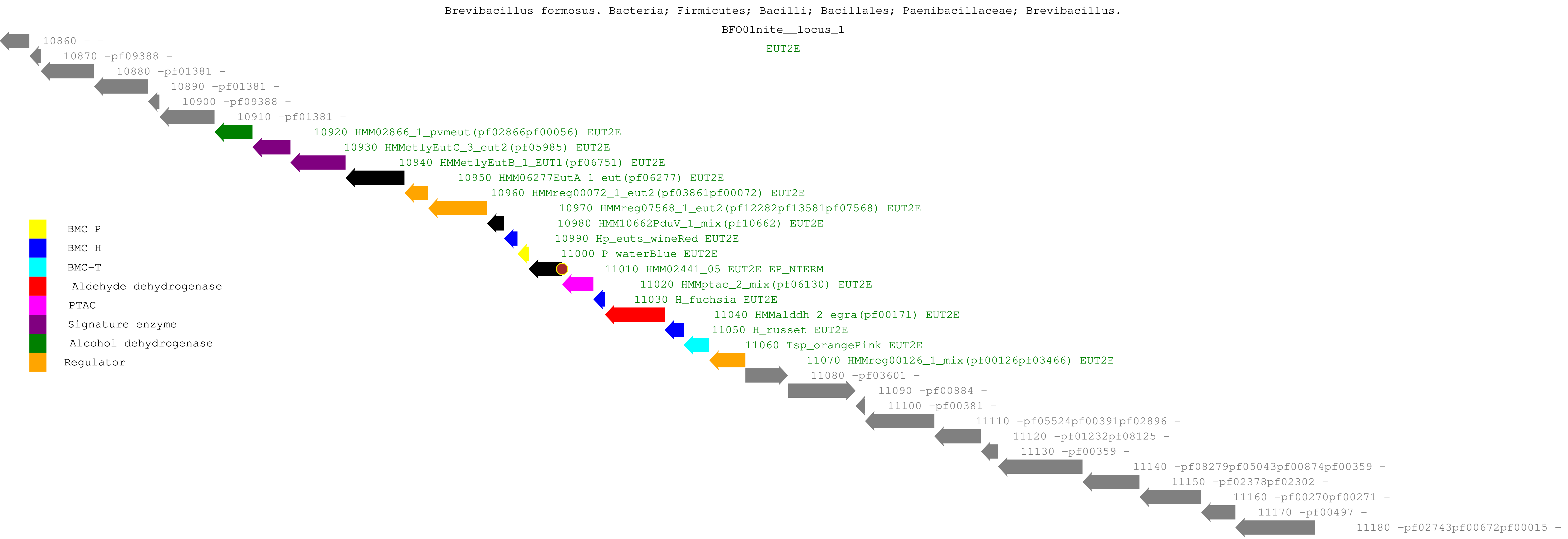

Brevibacillus formosus. Bacteria; Firmicutes; Bacilli; Bacillales; Paenibacillaceae; Brevibacillus.
BFO01nite__locus_1
EUT2E
10860 - -
10870 -pf09388 -
10880 -pf01381 -
10890 -pf01381 -
10900 -pf09388 -
10910 -pf01381 -
10920 HMM02866_1_pvmeut(pf02866pf00056) EUT2E
10930 HMMetlyEutC_3_eut2(pf05985) EUT2E
10940 HMMetlyEutB_1_EUT1(pf06751) EUT2E
10950 HMM06277EutA_1_eut(pf06277) EUT2E
10960 HMMreg00072_1_eut2(pf03861pf00072) EUT2E
10970 HMMreg07568_1_eut2(pf12282pf13581pf07568) EUT2E
10980 HMM10662PduV_1_mix(pf10662) EUT2E
10990 Hp_euts_wineRed EUT2E
11000 P_waterBlue EUT2E
11010 HMM02441_05 EUT2E EP_NTERM
11020 HMMptac_2_mix(pf06130) EUT2E
11030 H_fuchsia EUT2E
11040 HMMalddh_2_egra(pf00171) EUT2E
11050 H_russet EUT2E
11060 Tsp_orangePink EUT2E
11070 HMMreg00126_1_mix(pf00126pf03466) EUT2E
11080 -pf03601 -
11090 -pf00884 -
11100 -pf00381 -
11110 -pf05524pf00391pf02896 -
11120 -pf01232pf08125 -
11130 -pf00359 -
11140 -pf08279pf05043pf00874pf00359 -
11150 -pf02378pf02302 -
11160 -pf00270pf00271 -
11170 -pf00497 -
11180 -pf02743pf00672pf00015 -
BMC-P
BMC-H
BMC-T
Aldehyde dehydrogenase
PTAC
Signature enzyme
Alcohol dehydrogenase
Regulator

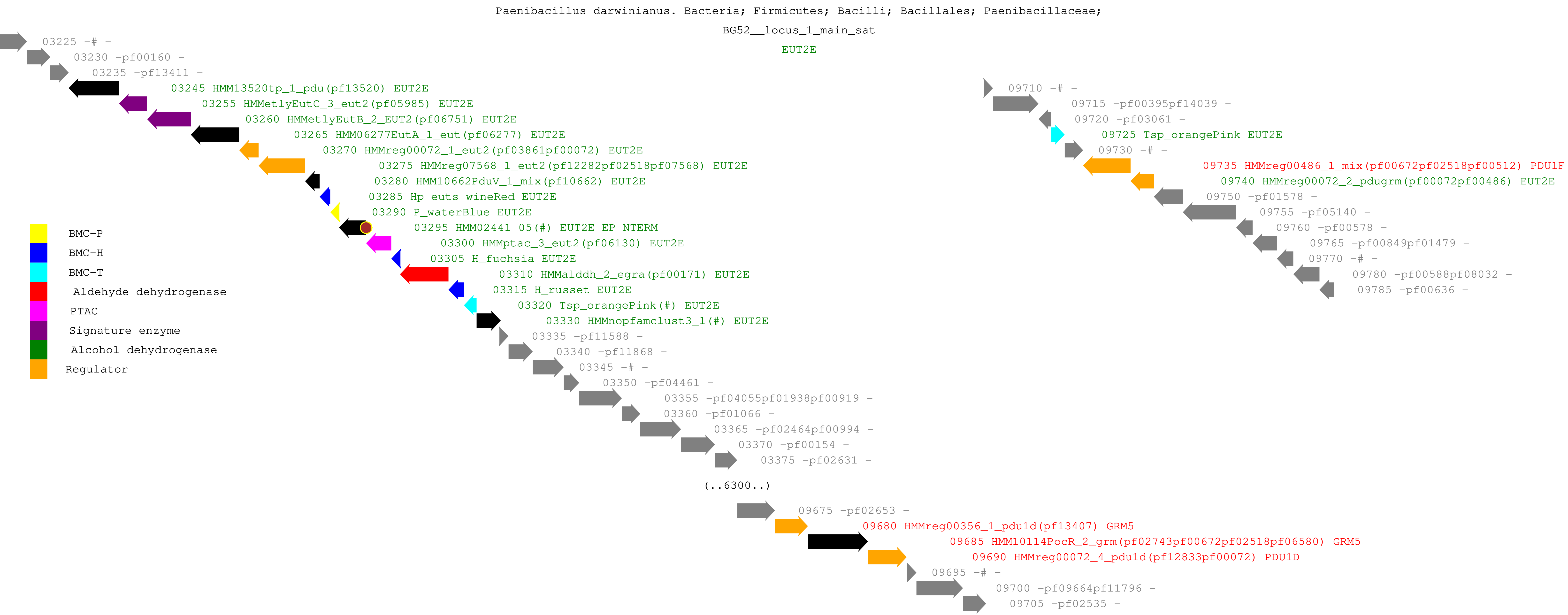
Paenibacillus darwinianus. Bacteria; Firmicutes; Bacilli; Bacillales; Paenibacillaceae;
BG52__locus_1_main_sat
EUT2E
03225 -# -
03230 -pf00160 -
03235 -pf13411 -
03245 HMM13520tp_1_pdu(pf13520) EUT2E
03255 HMMetlyEutC_3_eut2(pf05985) EUT2E
03260 HMMetlyEutB_2_EUT2(pf06751) EUT2E
03265 HMM06277EutA_1_eut(pf06277) EUT2E
03270 HMMreg00072_1_eut2(pf03861pf00072) EUT2E
03275 HMMreg07568_1_eut2(pf12282pf02518pf07568) EUT2E
03280 HMM10662PduV_1_mix(pf10662) EUT2E
03285 Hp_euts_wineRed EUT2E
03290 P_waterBlue EUT2E
03295 HMM02441_05(#) EUT2E EP_NTERM
03300 HMMptac_3_eut2(pf06130) EUT2E
03305 H_fuchsia EUT2E
03310 HMMaldh_2_egra(pf00171) EUT2E
03315 H_russet EUT2E
03320 Tsp_orangePink(#) EUT2E
03330 HMMnopfamclust3_1(#) EUT2E
03335 -pf11588 -
03340 -pf11868 -
03345 -# -
03350 -pf04461 -
03355 -pf04055pf01938pf00919 -
03360 -pf01066 -
03365 -pf02464pf00994 -
03370 -pf00154 -
03375 -pf02631 -
(..6300..)
09675 -pf02653 -
09680 HMMreg00356_1_pdu1d(pf13407) GRM5
09685 HMM10114PocR_2_grm(pf02743pf00672pf02518pf06580) GRM5
09690 HMMreg00072_4_pdu1d(pf12833pf00072) PDU1D
09695 -# -
09700 -pf09664pf11796 -
09705 -pf02535 -
09710 -# -
09715 -pf00395pf14039 -
09720 -pf03061 -
09725 Tsp_orangePink EUT2E
09730 -# -
09735 HMMreg00486_1_mix(pf00672pf02518pf00512) PDU1F
09740 HMMreg00072_2_pdugrm(pf00072pf00486) EUT2E
09750 -pf01578 -
09755 -pf05140 -
09760 -pf00578 -
09765 -pf00849pf01479 -
09770 -# -
09780 -pf00588pf08032 -
09785 -pf00636 -
BMC-P
BMC-H
BMC-T
Aldehyde dehydrogenase
PTAC
Signature enzyme
Alcohol dehydrogenase
Regulator

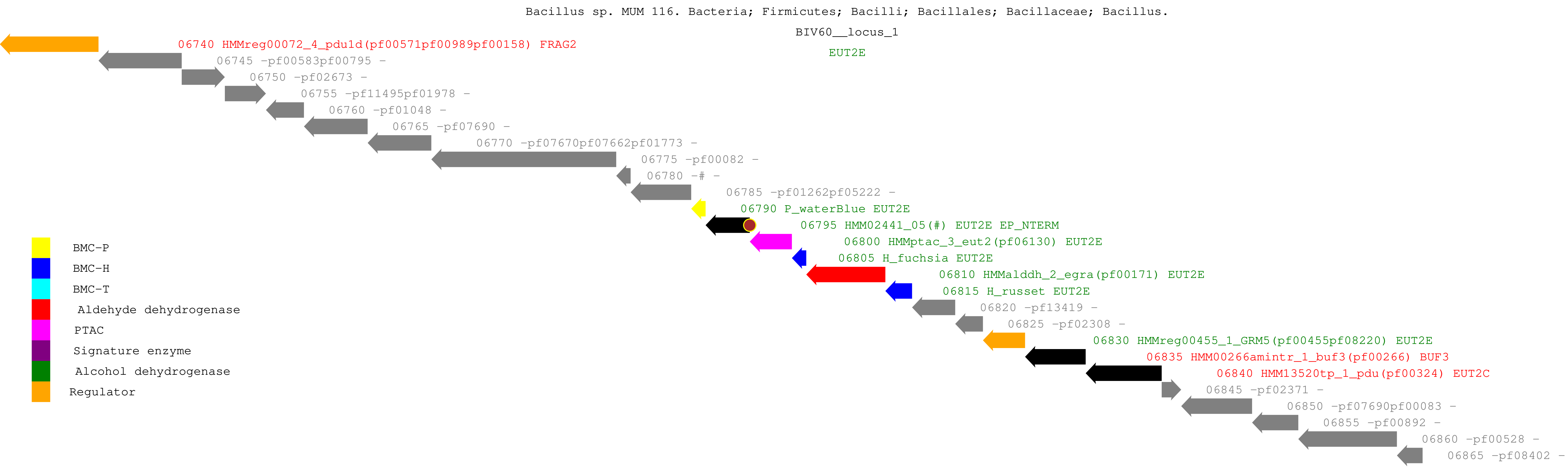
Bacillus sp. MUM 116. Bacteria; Firmicutes; Bacilli; Bacillales; Bacillaceae; Bacillus.
BIV60__locus_1
EUT2E
06740 HMMreg00072_4_pdu1d(pf00571pf00989pf00158) FRAG2
06745 -pf00583pf00795 -
06750 -pf02673 -
06755 -pf11495pf01978 -
06760 -pf01048 -
06765 -pf07690 -
06770 -pf07670pf07662pf01773 -
06775 -pf00082 -
06780 -# -
06785 -pf01262pf05222 -
06790 P_waterBlue EUT2E
06795 HMM02441_05(#) EUT2E EP_NTERM
06800 HMMptac_3_eut2(pf06130) EUT2E
06805 H_fuchsia EUT2E
06810 HMMaldh_2_egra(pf00171) EUT2E
06815 H_russet EUT2E
06820 -pf13419 -
06825 -pf02308 -
06830 HMMreg00455_1_GRM5(pf00455pf08220) EUT2E
06835 HMM00266amintr_1_buf3(pf00266) BUF3
06840 HMM13520tp_1_pdu(pf00324) EUT2C
06845 -pf02371 -
06850 -pf07690pf00083 -
06855 -pf00892 -
06860 -pf00528 -
06865 -pf08402 -
BMC-P
BMC-H
BMC-T
Aldehyde dehydrogenase
PTAC
Signature enzyme
Alcohol dehydrogenase
Regulator

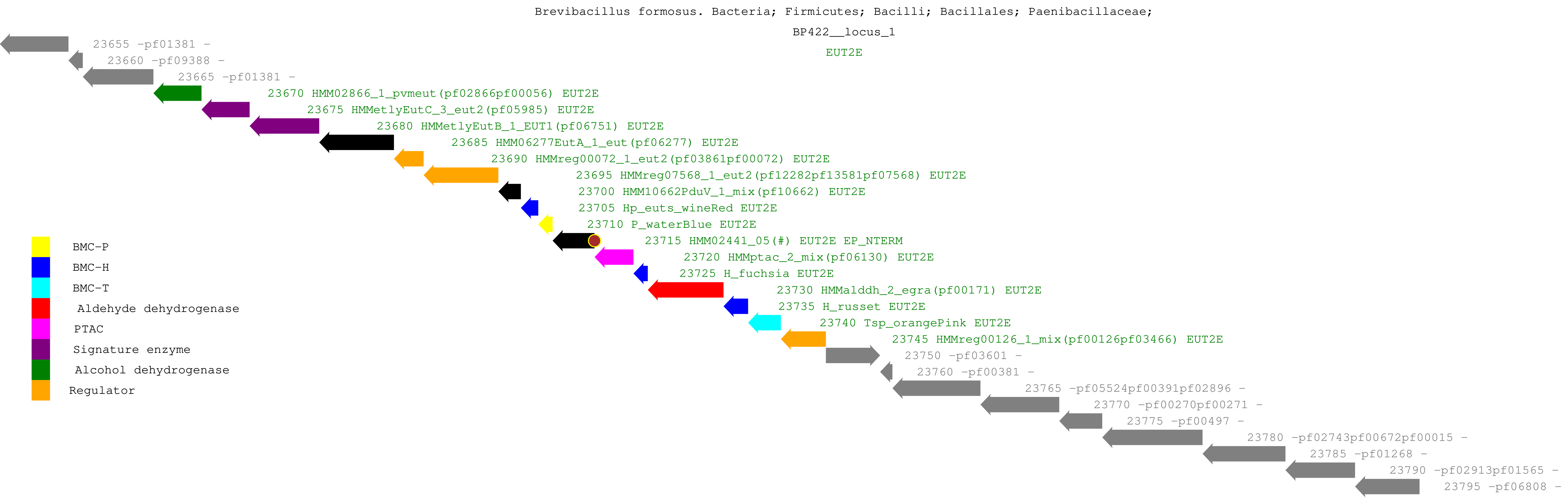
Brevibacillus formosus. Bacteria; Firmicutes; Bacilli; Bacillales; Paenibacillaceae;
BP422__locus_1
EUT2E
23655 -pf01381 -
23660 -pf09388 -
23665 -pf01381 -
23670 HMM02866_1_pvmeut(pf02866pf00056) EUT2E
23675 HMMetlyEutC_3_eut2(pf05985) EUT2E
23680 HMMetlyEutB_1_EUT1(pf06751) EUT2E
23685 HMM06277EutA_1_eut(pf06277) EUT2E
23690 HMMreg00072_1_eut2(pf03861pf00072) EUT2E
23695 HMMreg07568_1_eut2(pf12282pf13581pf07568) EUT2E
23700 HMM10662PduV_1_mix(pf10662) EUT2E
23705 Hp_euts_wineRed EUT2E
23710 P_waterBlue EUT2E
23715 HMM02441_05(#) EUT2E EP_NTERM
23720 HMMptac_2_mix(pf06130) EUT2E
23725 H_fuchsia EUT2E
23730 HMMalddh_2_egra(pf00171) EUT2E
23735 H_russet EUT2E
23740 Tsp_orangePink EUT2E
23745 HMMreg00126_1_mix(pf00126pf03466) EUT2E
23750 -pf03601 -
23760 -pf00381 -
23765 -pf05524pf00391pf02896 -
23770 -pf00270pf00271 -
23775 -pf00497 -
23780 -pf02743pf00672pf00015 -
23785 -pf01268 -
23790 -pf02913pf01565 -
23795 -pf06808 -
BMC-P
BMC-H
BMC-T
Aldehyde dehydrogenase
PTAC
Signature enzyme
Alcohol dehydrogenase
Regulator

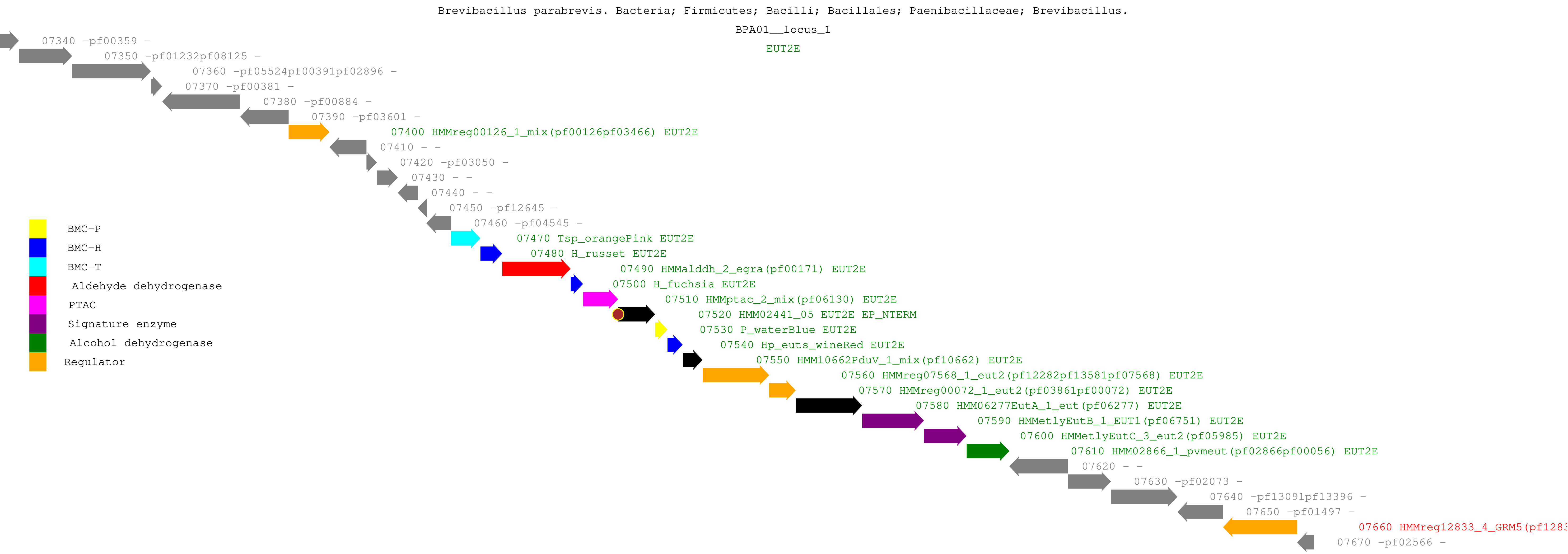

Brevibacillus parabrevis. Bacteria; Firmicutes; Bacilli; Bacillales; Paenibacillaceae; Brevibacillus.
BPA01__locus_1
EUT2E
07340 -pf00359 -
07350 -pf01232pf08125 -
07360 -pf05524pf00391pf02896 -
07370 -pf00381 -
07380 -pf00884 -
07390 -pf03601 -
07400 HMMreg00126_1_mix(pf00126pf03466) EUT2E
07410 - -
07420 -pf03050 -
07430 - -
07440 - -
07450 -pf12645 -
07460 -pf04545 -
07470 Tsp_orangePink EUT2E
07480 H_russet EUT2E
07490 HMMalddh_2_egra(pf00171) EUT2E
07500 H_fuchsia EUT2E
07510 HMMptac_2_mix(pf06130) EUT2E
07520 HMM02441_05 EUT2E EP_NTERM
07530 P_waterBlue EUT2E
07540 Hp_euts_wineRed EUT2E
07550 HMM10662PduV_1_mix(pf10662) EUT2E
07560 HMMreg07568_1_eut2(pf12282pf13581pf07568) EUT2E
07570 HMMreg00072_1_eut2(pf03861pf00072) EUT2E
07580 HMM06277EutA_1_eut(pf06277) EUT2E
07590 HMMetlyEutB_1_EUT1(pf06751) EUT2E
07600 HMMetlyEutC_3_eut2(pf05985) EUT2E
07610 HMM02866_1_pvmeut(pf02866pf00056) EUT2E
07620 - -
07630 -pf02073 -
07640 -pf13091pf13396 -
07650 -pf01497 -
07660 HMMreg12833_4_GRM5(pf1283
07670 -pf02566 -
BMC-P
BMC-H
BMC-T
Aldehyde dehydrogenase
PTAC
Signature enzyme
Alcohol dehydrogenase
Regulator

Brevibacillus parabrevis. Bacteria; Firmicutes; Bacilli; Bacillales; Paenibacillaceae; Brevibacillus.

BPA01__locus_2

EUT2E

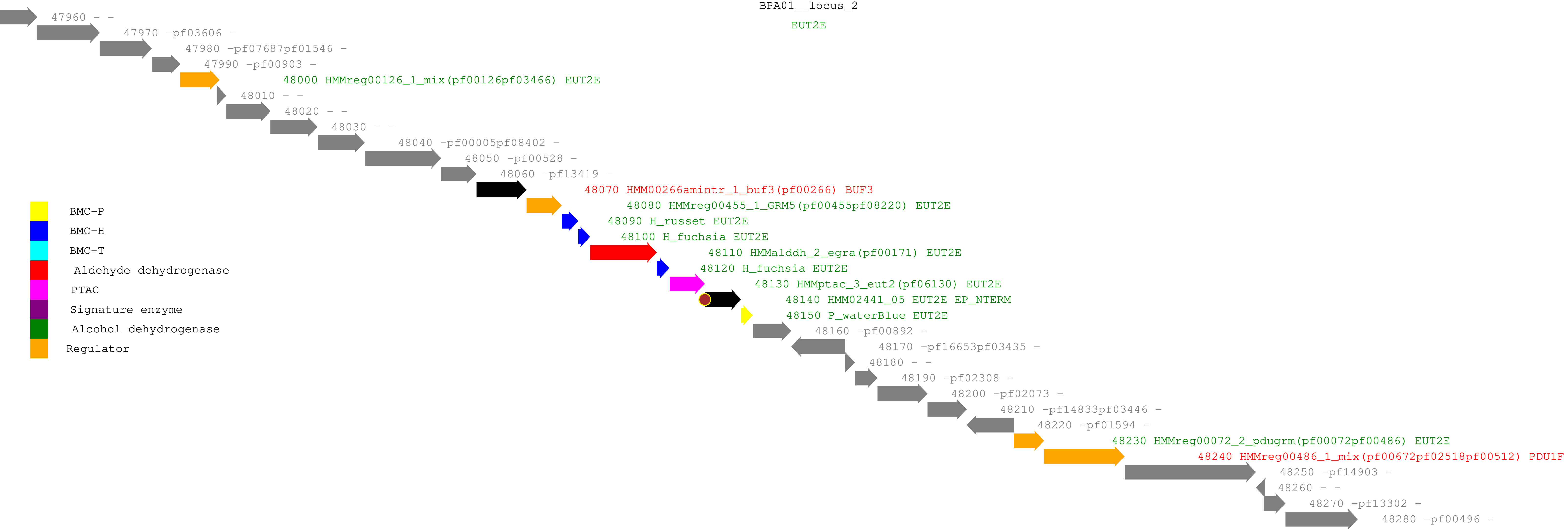

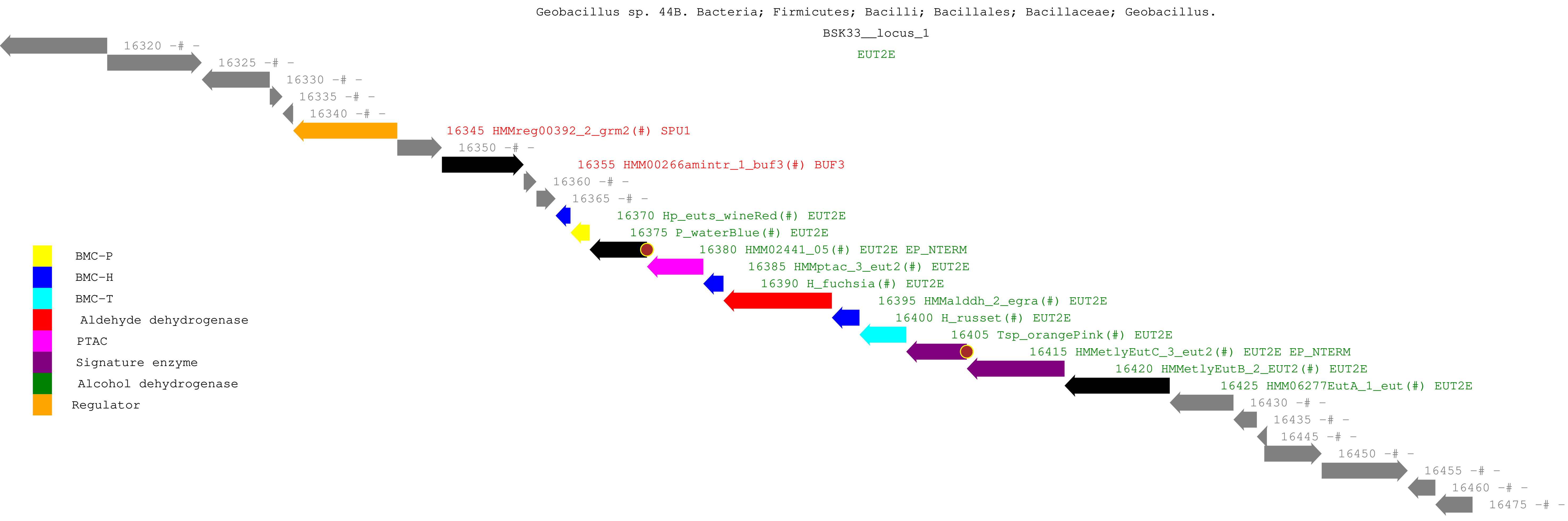
Geobacillus sp. 44B. Bacteria; Firmicutes; Bacilli; Bacillales; Bacillaceae; Geobacillus.
BSK33__locus_1
EUT2E
16320 -# -
16325 -# -
16330 -# -
16335 -# -
16340 -# -
16345 HMMreg00392_2_grm2(#) SPU1
16350 -# -
16355 HMM00266amintr_1_buf3(#) BUF3
16360 -# -
16365 -# -
16370 Hp_euts_wineRed(#) EUT2E
16375 P_waterBlue(#) EUT2E
16380 HMM02441_05(#) EUT2E EP_NTERM
16385 HMMptac_3_eut2(#) EUT2E
16390 H_fuchsia(#) EUT2E
16395 HMMalddh_2_egra(#) EUT2E
16400 H_russet(#) EUT2E
16405 Tsp_orangePink(#) EUT2E
16415 HMMetlyEutC_3_eut2(#) EUT2E EP_NTERM
16420 HMMetlyEutB_2_EUT2(#) EUT2E
16425 HMM06277EutA_1_eut(#) EUT2E
16430 -# -
16435 -# -
16445 -# -
16450 -# -
16455 -# -
16460 -# -
16475 -# -
BMC-P
BMC-H
BMC-T
Aldehyde dehydrogenase
PTAC
Signature enzyme
Alcohol dehydrogenase
Regulator

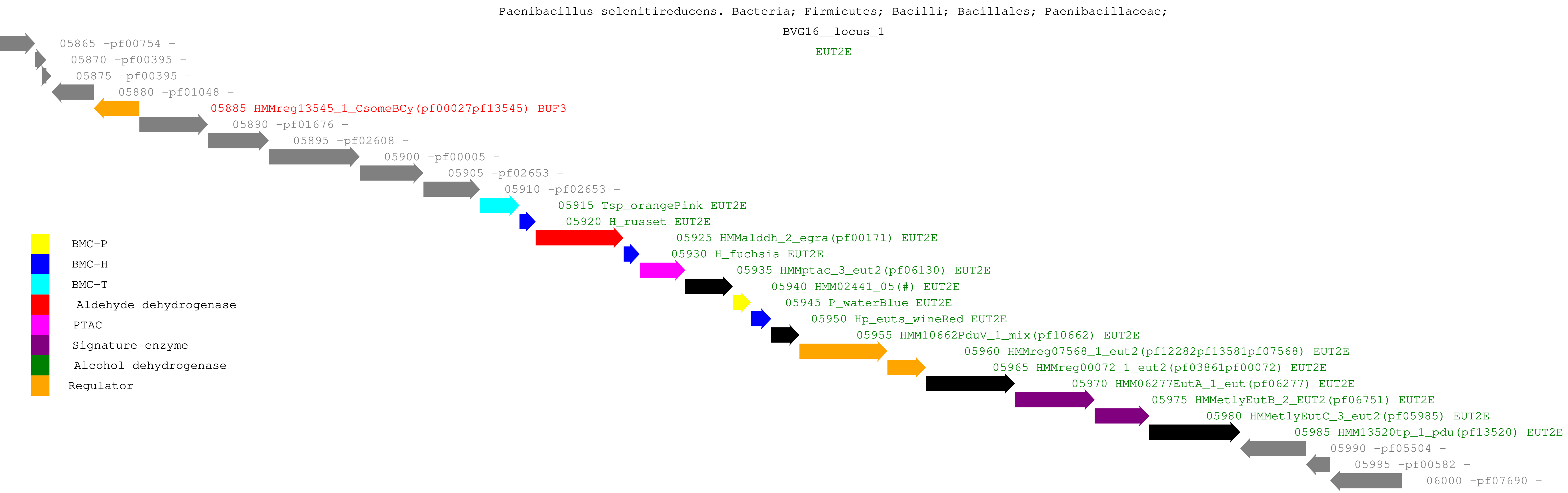

Paenibacillus selenitireducens. Bacteria; Firmicutes; Bacilli; Bacillales; Paenibacillaceae;
BVG16__locus_1
EUT2E
05865 -pf00754 -
05870 -pf00395 -
05875 -pf00395 -
05880 -pf01048 -
05885 HMMreg13545_1_CsomeBCy(pf00027pf13545) BUF3
05890 -pf01676 -
05895 -pf02608 -
05900 -pf00005 -
05905 -pf02653 -
05910 -pf02653 -
05915 Tsp_orangePink EUT2E
05920 H_russet EUT2E
05925 HMMaldh_2_egra(pf00171) EUT2E
05930 H_fuchsia EUT2E
05935 HMMptac_3_eut2(pf06130) EUT2E
05940 HMM02441_05(#) EUT2E
05945 P_waterBlue EUT2E
05950 Hp_euts_wineRed EUT2E
05955 HMM10662PduV_1_mix(pf10662) EUT2E
05960 HMMreg07568_1_eut2(pf12282pf13581pf07568) EUT2E
05965 HMMreg00072_1_eut2(pf03861pf00072) EUT2E
05970 HMM06277EutA_1_eut(pf06277) EUT2E
05975 HMMetlyEutB_2_EUT2(pf06751) EUT2E
05980 HMMetlyEutC_3_eut2(pf05985) EUT2E
05985 HMM13520tp_1_pdu(pf13520) EUT2E
05990 -pf05504 -
05995 -pf00582 -
06000 -pf07690 -
BMC-P
BMC-H
BMC-T
Aldehyde dehydrogenase
PTAC
Signature enzyme
Alcohol dehydrogenase
Regulator

Brevibacillus sp. NRRL B-41110. Bacteria; Firmicutes; Bacilli; Bacillales; Paenibacillaceae;

C7R92__locus_1

EUT2E

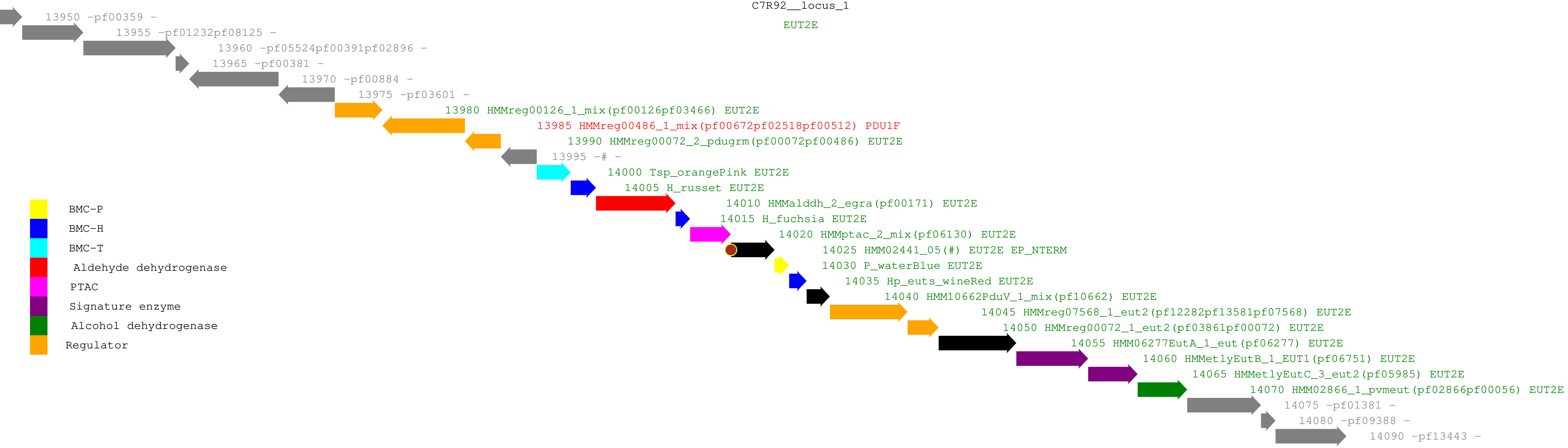

Brevibacillus sp. NRRL NRS-1210. Bacteria; Firmicutes; Bacilli; Bacillales; Paenibacillaceae;

C7R93__locus_1

EUT2E

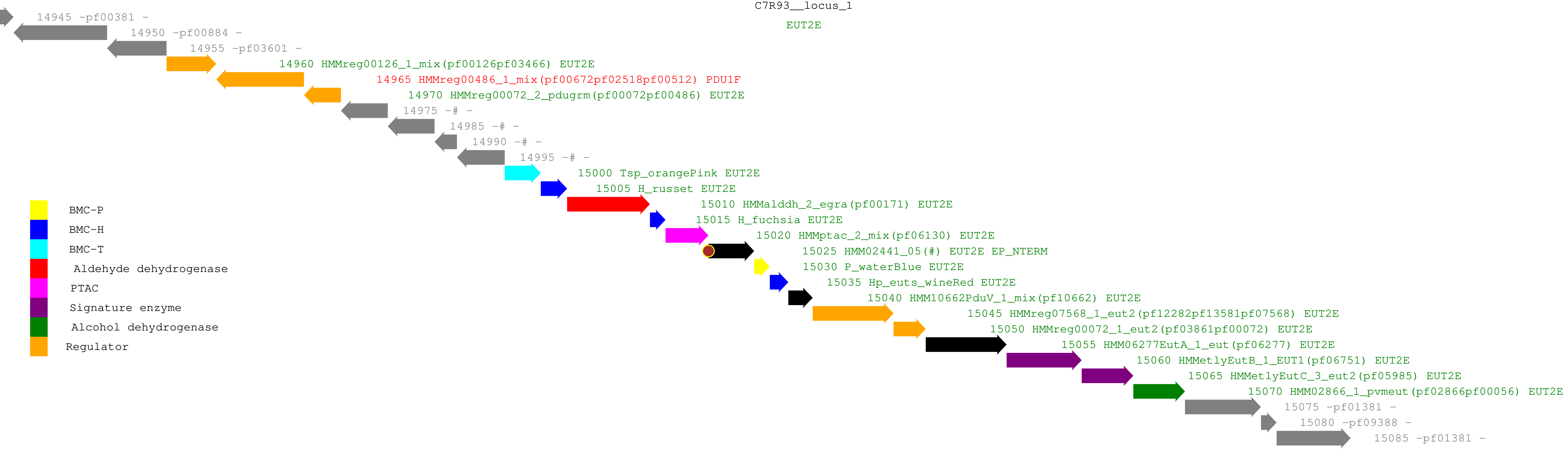

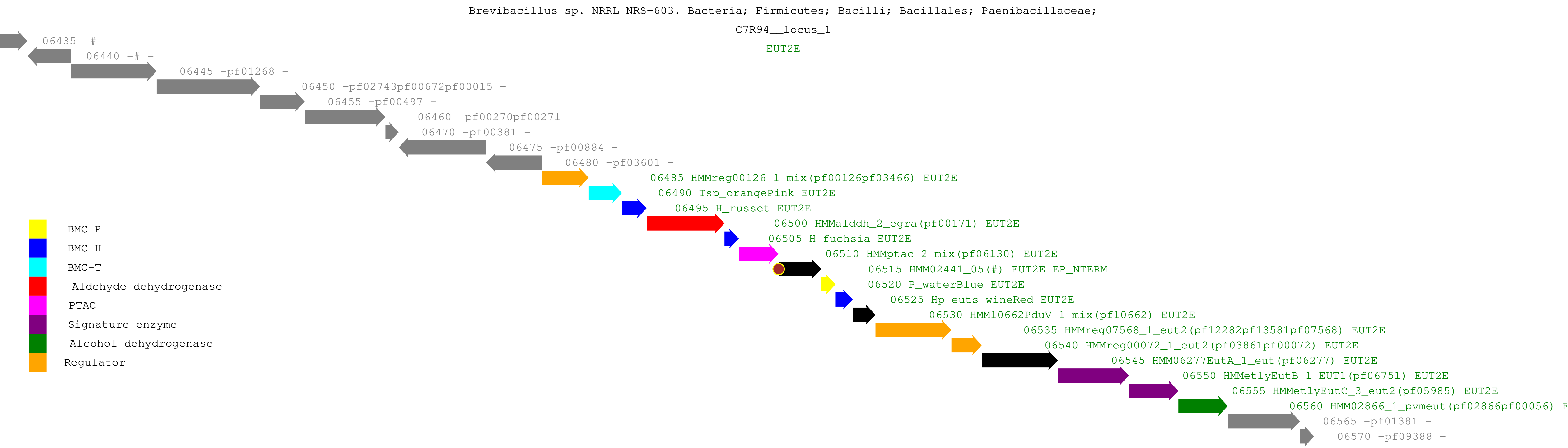

Brevibacillus sp. NRRL NRS-603. Bacteria; Firmicutes; Bacilli; Bacillales; Paenibacillaceae;
C7R94__locus_1
EUT2E
06435 -# -
06440 -# -
06445 -pf01268 -
06450 -pf02743pf00672pf00015 -
06455 -pf00497 -
06460 -pf00270pf00271 -
06470 -pf00381 -
06475 -pf00884 -
06480 -pf03601 -
06485 HMMreg00126_1_mix(pf00126pf03466) EUT2E
06490 Tsp_orangePink EUT2E
06495 H_russet EUT2E
06500 HMMalddh_2_egra(pf00171) EUT2E
06505 H_fuchsia EUT2E
06510 HMMptac_2_mix(pf06130) EUT2E
06515 HMM02441_05(#) EUT2E EP_NTERM
06520 P_waterBlue EUT2E
06525 Hp_euts_wineRed EUT2E
06530 HMM10662PduV_1_mix(pf10662) EUT2E
06535 HMMreg07568_1_eut2(pf12282pf13581pf07568) EUT2E
06540 HMMreg00072_1_eut2(pf03861pf00072) EUT2E
06545 HMM06277EutA_1_eut(pf06277) EUT2E
06550 HMMetlyEutB_1_EUT1(pf06751) EUT2E
06555 HMMetlyEutC_3_eut2(pf05985) EUT2E
06560 HMM02866_1_pvmeut(pf02866pf00056) E
06565 -pf01381 -
06570 -pf09388 -
BMC-P
BMC-H
BMC-T
Aldehyde dehydrogenase
PTAC
Signature enzyme
Alcohol dehydrogenase
Regulator

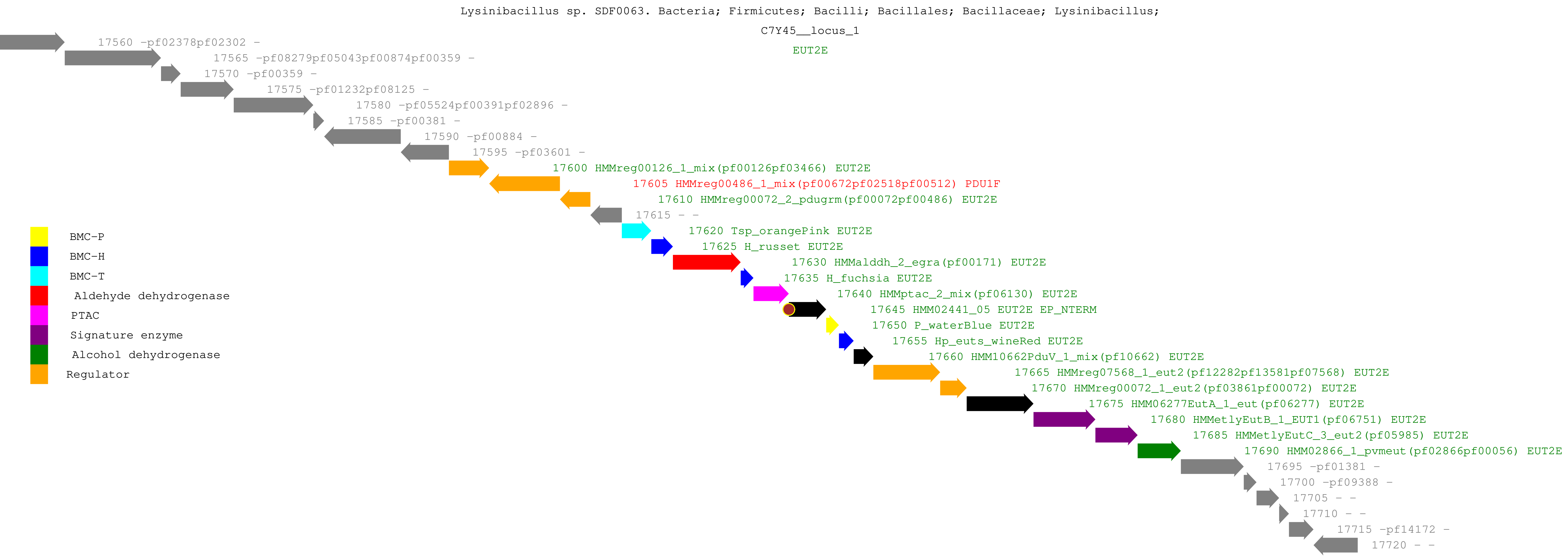
Lysinibacillus sp. SDF0063. Bacteria; Firmicutes; Bacilli; Bacillales; Bacillaceae; Lysinibacillus;
C7Y45__locus_1
EUT2E
17560 -pf02378pf02302 -
17565 -pf08279pf05043pf00874pf00359 -
17570 -pf00359 -
17575 -pf01232pf08125 -
17580 -pf05524pf00391pf02896 -
17585 -pf00381 -
17590 -pf00884 -
17595 -pf03601 -
17600 HMMreg00126_1_mix(pf00126pf03466) EUT2E
17605 HMMreg00486_1_mix(pf00672pf02518pf00512) PDU1F
17610 HMMreg00072_2_pdugrm(pf00072pf00486) EUT2E
17615 - -
17620 Tsp_orangePink EUT2E
17625 H_russet EUT2E
17630 HMMaldh_2_egra(pf00171) EUT2E
17635 H_fuchsia EUT2E
17640 HMMptac_2_mix(pf06130) EUT2E
17645 HMM02441_05 EUT2E EP_NTERM
17650 P_waterBlue EUT2E
17655 Hp_euts_wineRed EUT2E
17660 HMM10662PduV_1_mix(pf10662) EUT2E
17665 HMMreg07568_1_eut2(pf12282pf13581pf07568) EUT2E
17670 HMMreg00072_1_eut2(pf03861pf00072) EUT2E
17675 HMM06277EutA_1_eut(pf06277) EUT2E
17680 HMMetlyEutB_1_EUT1(pf06751) EUT2E
17685 HMMetlyEutC_3_eut2(pf05985) EUT2E
17690 HMM02866_1_pvmeut(pf02866pf00056) EUT2E
17695 -pf01381 -
17700 -pf09388 -
17705 - -
17710 - -
17715 -pf14172 -
17720 - -
BMC-P
BMC-H
BMC-T
Aldehyde dehydrogenase
PTAC
Signature enzyme
Alcohol dehydrogenase
Regulator

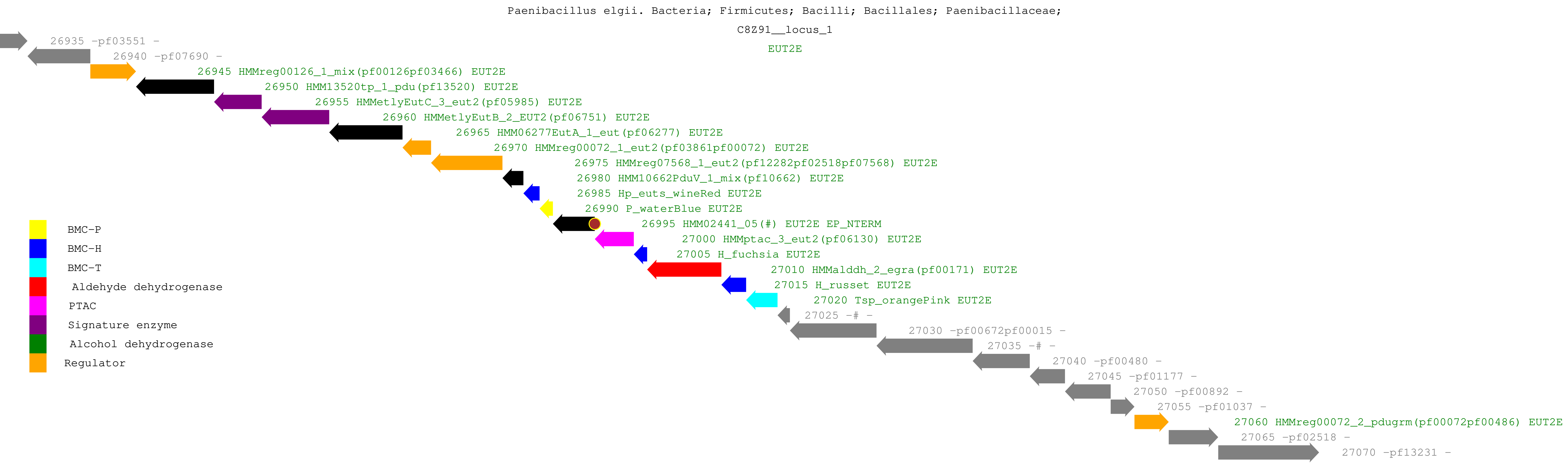
Paenibacillus elgii. Bacteria; Firmicutes; Bacilli; Bacillales; Paenibacillaceae;
C8Z91__locus_1
EUT2E
26935 -pf03551 -
26940 -pf07690 -
26945 HMMreg00126_1_mix(pf00126pf03466) EUT2E
26950 HMM13520tp_1_pdu(pf13520) EUT2E
26955 HMMetlyEutC_3_eut2(pf05985) EUT2E
26960 HMMetlyEutB_2_EUT2(pf06751) EUT2E
26965 HMM06277EutA_1_eut(pf06277) EUT2E
26970 HMMreg00072_1_eut2(pf03861pf00072) EUT2E
26975 HMMreg07568_1_eut2(pf12282pf02518pf07568) EUT2E
26980 HMM10662PduV_1_mix(pf10662) EUT2E
26985 Hp_euts_wineRed EUT2E
26990 P_waterBlue EUT2E
26995 HMM02441_05(#) EUT2E EP_NTERM
27000 HMMptac_3_eut2(pf06130) EUT2E
27005 H_fuchsia EUT2E
27010 HMMalddh_2_egra(pf00171) EUT2E
27015 H_russet EUT2E
27020 Tsp_orangePink EUT2E
27025 -# -
27030 -pf00672pf00015 -
27035 -# -
27040 -pf00480 -
27045 -pf01177 -
27050 -pf00892 -
27055 -pf01037 -
27060 HMMreg00072_2_pdugrm(pf00072pf00486) EUT2E
27065 -pf02518 -
27070 -pf13231 -
BMC-P
BMC-H
BMC-T
Aldehyde dehydrogenase
PTAC
Signature enzyme
Alcohol dehydrogenase
Regulator

Paenibacillus herberti. Bacteria; Firmicutes; Bacilli; Bacillales; Paenibacillaceae;

CGZ75__locus_1

EUT2E

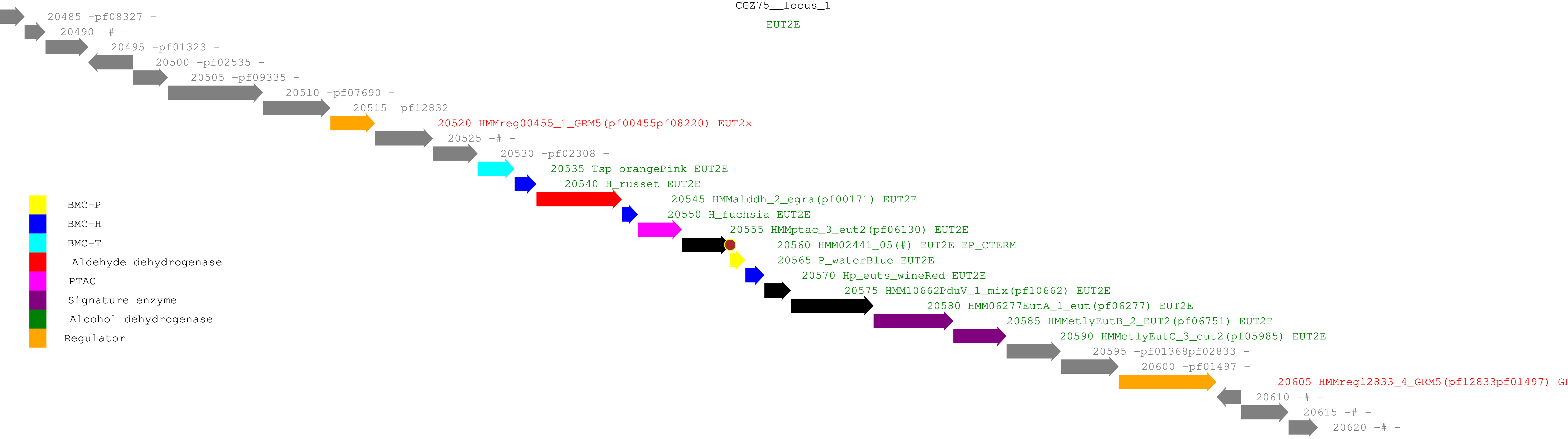

Brevibacillus agri BAB-2500. Bacteria; Firmicutes; Bacilli; Bacillales; Paenibacillaceae;

D478__locus_1

EUT2E

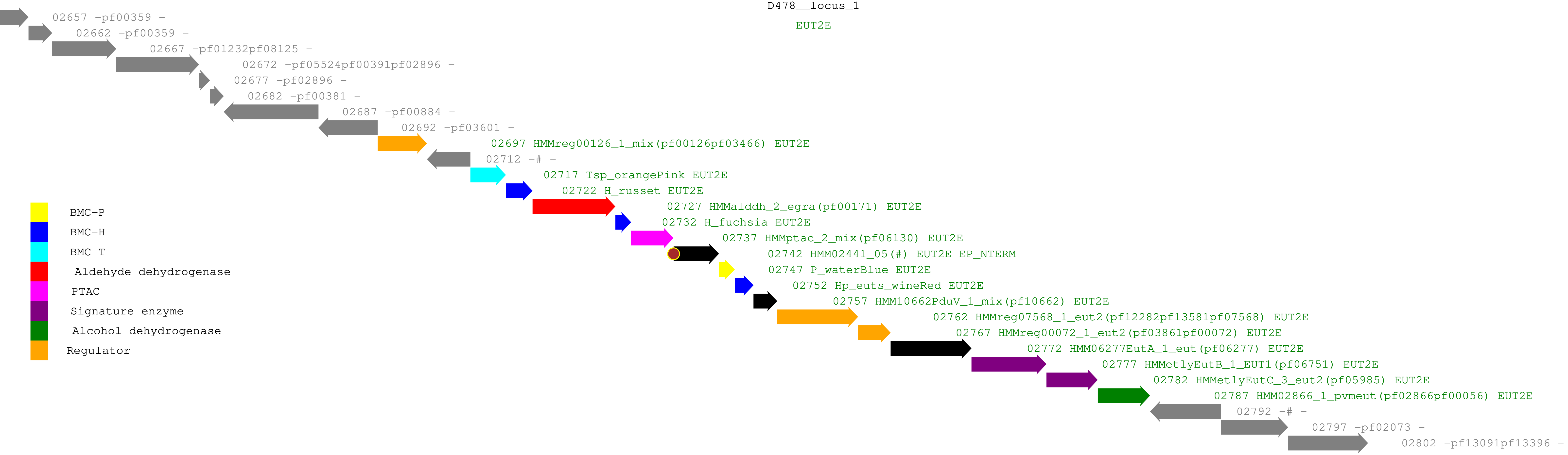

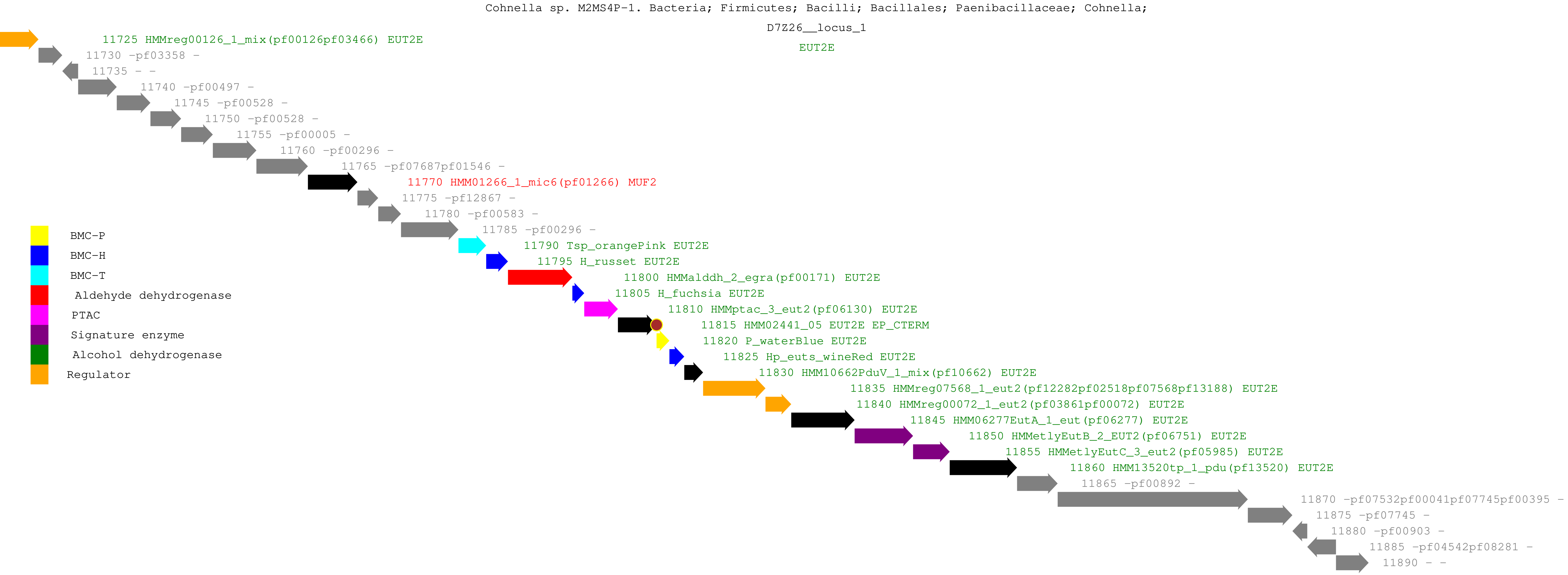

Cohnella sp. M2MS4P-1. Bacteria; Firmicutes; Bacilli; Bacillales; Paenibacillaceae; Cohnella;
D7Z26__locus_1
EUT2E
11725 HMMreg00126_1_mix(pf00126pf03466) EUT2E
11730 -pf03358 -
11735 - -
11740 -pf00497 -
11745 -pf00528 -
11750 -pf00528 -
11755 -pf00005 -
11760 -pf00296 -
11765 -pf07687pf01546 -
11770 HMM01266_1_mic6(pf01266) MUF2
11775 -pf12867 -
11780 -pf00583 -
11785 -pf00296 -
11790 Tsp_orangePink EUT2E
11795 H_russet EUT2E
11800 HMMaldd h_2_egra(pf00171) EUT2E
11805 H_fuchsia EUT2E
11810 HMMptac_3_eut2(pf06130) EUT2E
11815 HMM02441_05 EUT2E EP_CTERM
11820 P_waterBlue EUT2E
11825 Hp_euts_wineRed EUT2E
11830 HMM10662PduV_1_mix(pf10662) EUT2E
11835 HMMreg07568_1_eut2(pf12282pf02518pf07568pf13188) EUT2E
11840 HMMreg00072_1_eut2(pf03861pf00072) EUT2E
11845 HMM06277EutA_1_eut(pf06277) EUT2E
11850 HMMetlyEutB_2_EUT2(pf06751) EUT2E
11855 HMMetlyEutC_3_eut2(pf05985) EUT2E
11860 HMM13520tp_1_pdu(pf13520) EUT2E
11865 -pf00892 -
11870 -pf07532pf00041pf07745pf00395 -
11875 -pf07745 -
11880 -pf00903 -
11885 -pf04542pf08281 -
11890 - -
BMC-P
BMC-H
BMC-T
Aldehyde dehydrogenase
PTAC
Signature enzyme
Alcohol dehydrogenase
Regulator

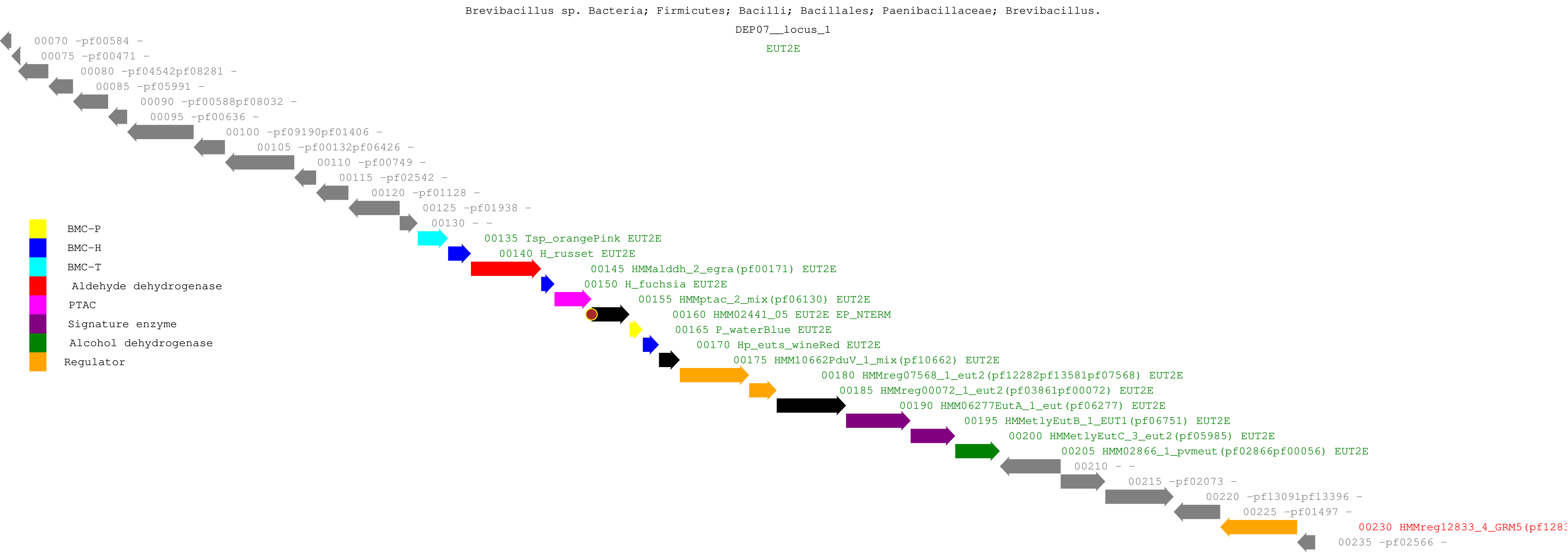
Brevibacillus sp. Bacteria; Firmicutes; Bacilli; Bacillales; Paenibacillaceae; Brevibacillus.
DEP07__locus_1
EUT2E
00070 -pf00584 -
00075 -pf00471 -
00080 -pf04542pf08281 -
00085 -pf05991 -
00090 -pf00588pf08032 -
00095 -pf00636 -
00100 -pf09190pf01406 -
00105 -pf00132pf06426 -
00110 -pf00749 -
00115 -pf02542 -
00120 -pf01128 -
00125 -pf01938 -
00130 - -
00135 Tsp_orangePink EUT2E
00140 H_russet EUT2E
00145 HMMalddh_2_egra(pf00171) EUT2E
00150 H_fuchsia EUT2E
00155 HMMptac_2_mix(pf06130) EUT2E
00160 HMM02441_05 EUT2E EP_NTERM
00165 P_waterBlue EUT2E
00170 Hp_euts_wineRed EUT2E
00175 HMM10662PduV_1_mix(pf10662) EUT2E
00180 HMMreg07568_1_eut2(pf12282pf13581pf07568) EUT2E
00185 HMMreg00072_1_eut2(pf03861pf00072) EUT2E
00190 HMM06277EutA_1_eut(pf06277) EUT2E
00195 HMMetlyEutB_1_EUT1(pf06751) EUT2E
00200 HMMetlyEutC_3_eut2(pf05985) EUT2E
00205 HMM02866_1_pvmeut(pf02866pf00056) EUT2E
00210 - -
00215 -pf02073 -
00220 -pf13091pf13396 -
00225 -pf01497 -
00230 HMMreg12833_4_GRM5(pf1283
00235 -pf02566 -
BMC-P
BMC-H
BMC-T
Aldehyde dehydrogenase
PTAC
Signature enzyme
Alcohol dehydrogenase
Regulator

Brevibacillus sp. Bacteria; Firmicutes; Bacilli; Bacillales; Paenibacillaceae; Brevibacillus.

DEP07__locus_2

EUT2E

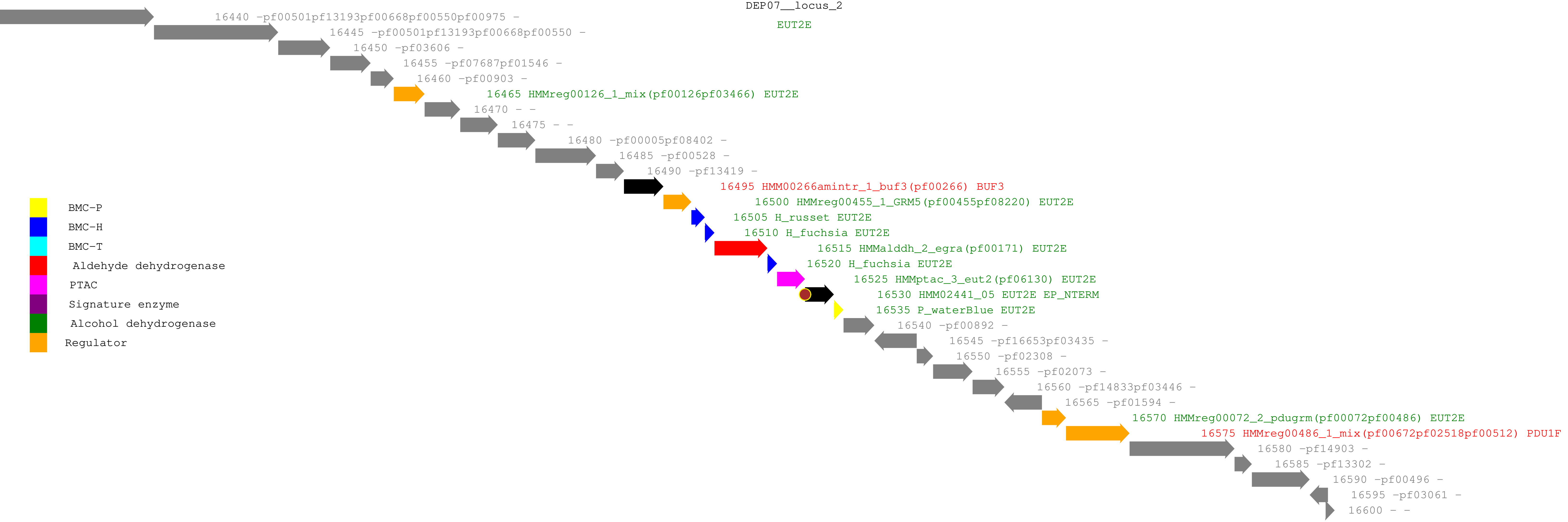

Paenibacillus montanisoli. Bacteria; Firmicutes; Bacilli; Bacillales; Paenibacillaceae;

DL346__locus_1

EUT2E

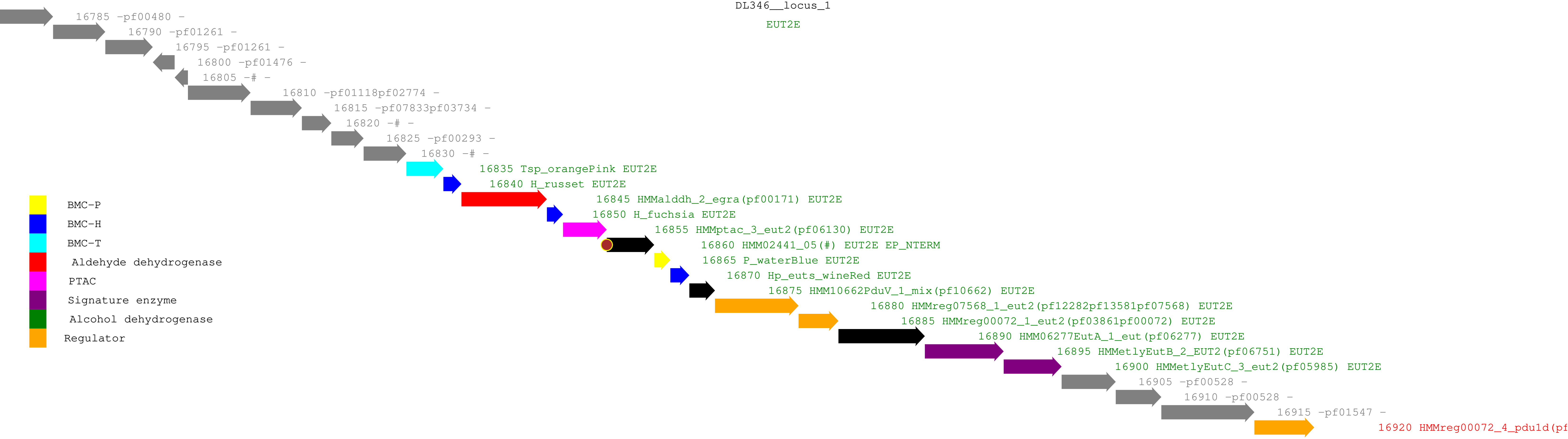

Cohnella luojiensis. Bacteria; Firmicutes; Bacilli; Bacillales; Paenibacillaceae; Cohnella.

E2980__locus_1

EUT2E

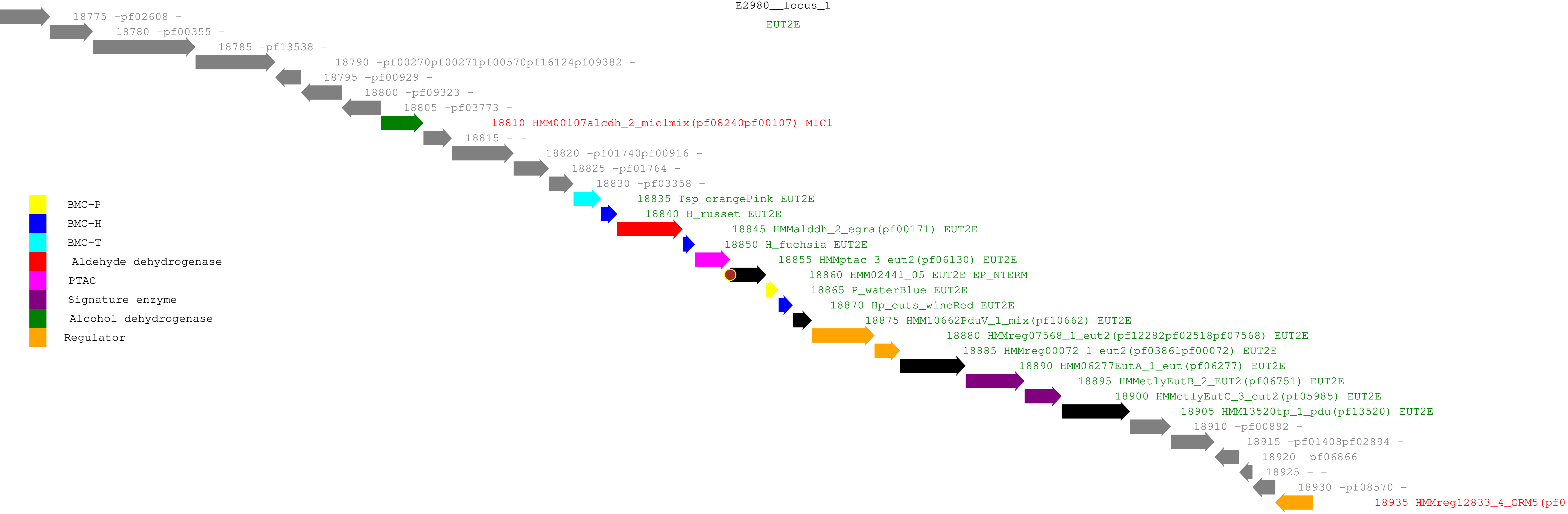

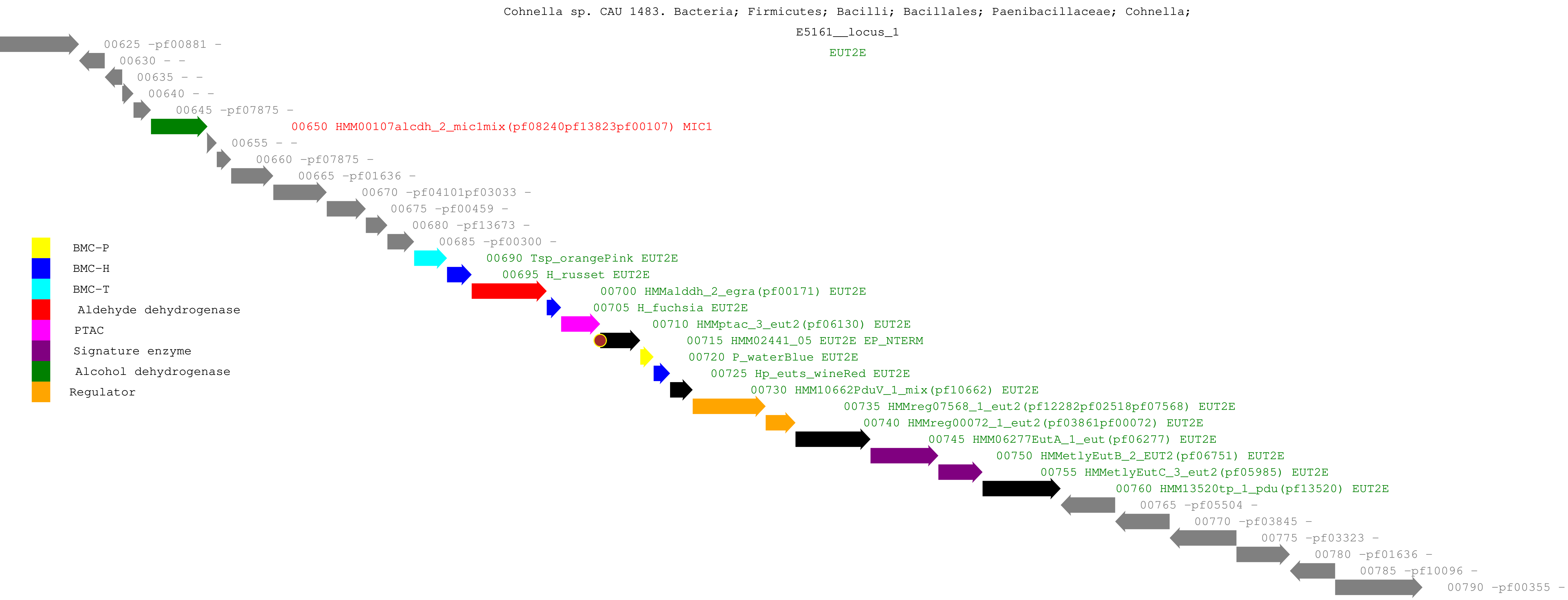
Cohnella sp. CAU 1483. Bacteria; Firmicutes; Bacilli; Bacillales; Paenibacillaceae; Cohnella;
E5161__locus_1
EUT2E
00625 -pf00881 -
00630 - -
00635 - -
00640 - -
00645 -pf07875 -
00650 HMM00107alcdh_2_mic1mix(pf08240pf13823pf00107) MIC1
00655 - -
00660 -pf07875 -
00665 -pf01636 -
00670 -pf04101pf03033 -
00675 -pf00459 -
00680 -pf13673 -
00685 -pf00300 -
00690 Tsp_orangePink EUT2E
00695 H_russet EUT2E
00700 HMMaldh_2_egra(pf00171) EUT2E
00705 H_fuchsia EUT2E
00710 HMMptac_3_eut2(pf06130) EUT2E
00715 HMM02441_05 EUT2E EP_NTERM
00720 P_waterBlue EUT2E
00725 Hp_euts_wineRed EUT2E
00730 HMM10662PduV_1_mix(pf10662) EUT2E
00735 HMMreg07568_1_eut2(pf12282pf02518pf07568) EUT2E
00740 HMMreg00072_1_eut2(pf03861pf00072) EUT2E
00745 HMM06277EutA_1_eut(pf06277) EUT2E
00750 HMMetlyEutB_2_EUT2(pf06751) EUT2E
00755 HMMetlyEutC_3_eut2(pf05985) EUT2E
00760 HMM13520tp_1_pdu(pf13520) EUT2E
00765 -pf05504 -
00770 -pf03845 -
00775 -pf03323 -
00780 -pf01636 -
00785 -pf10096 -
00790 -pf00355 -
BMC-P
BMC-H
BMC-T
Aldehyde dehydrogenase
PTAC
Signature enzyme
Alcohol dehydrogenase
Regulator

Brevibacillus antibioticus. Bacteria; Firmicutes; Bacilli; Bacillales; Paenibacillaceae; Brevibacillus.

E8L90__locus_1

EUT2E

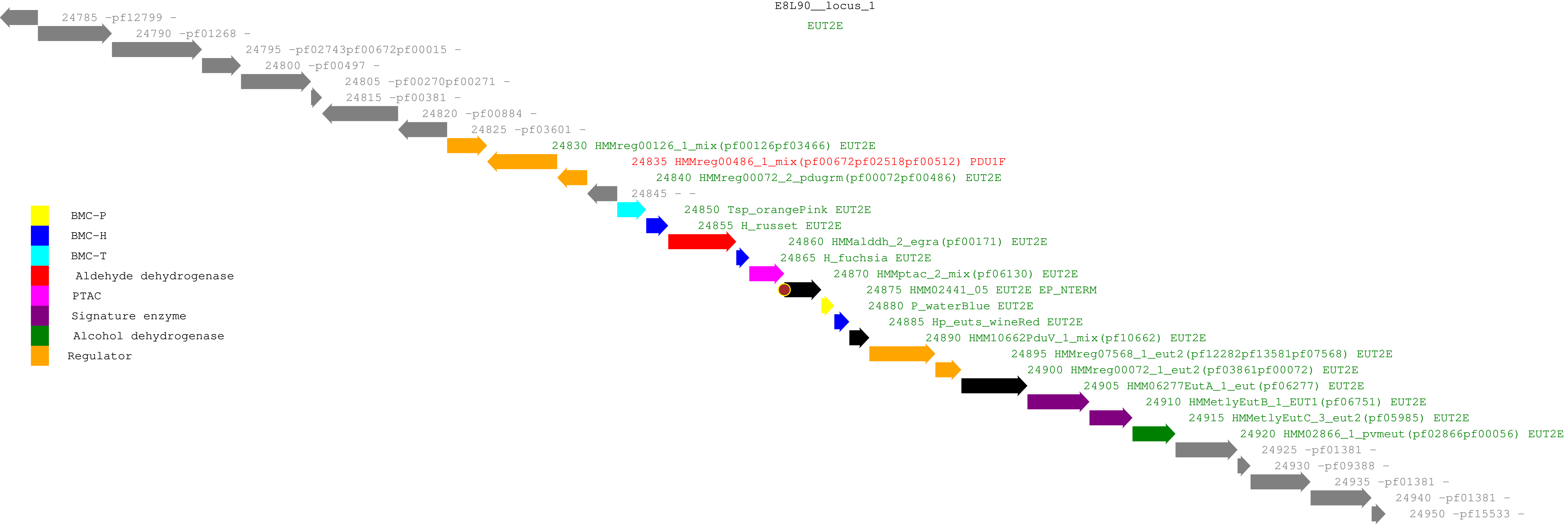

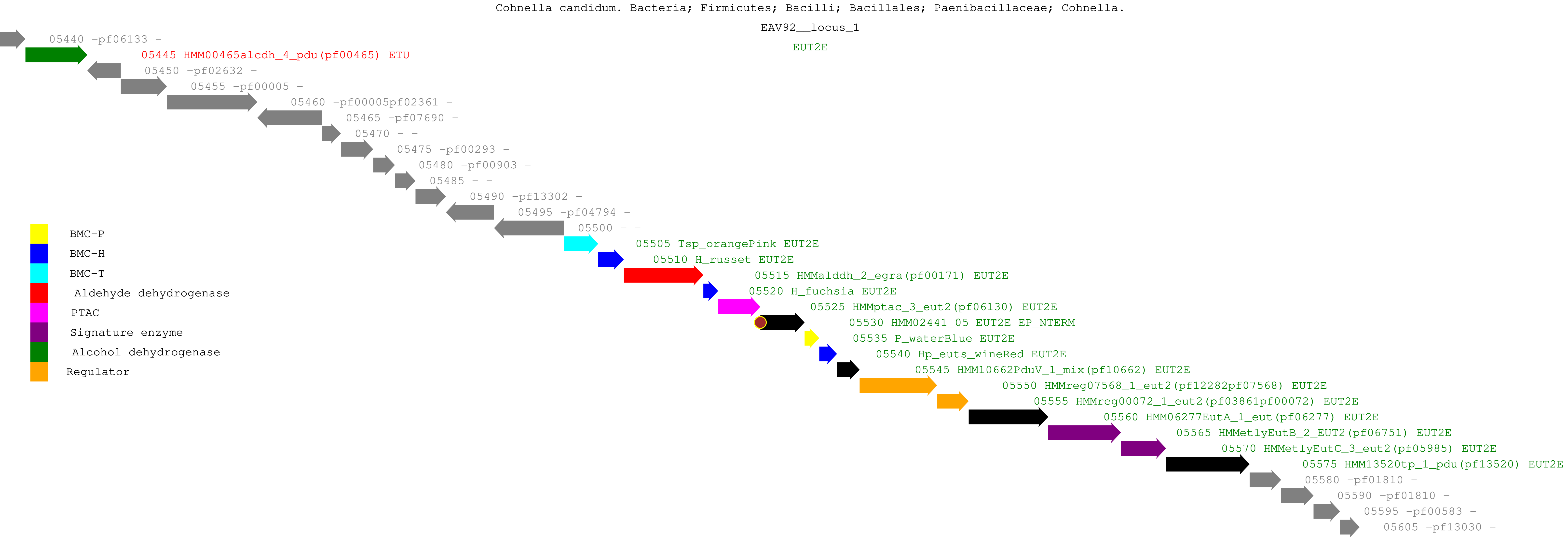

Cohnella candidum. Bacteria; Firmicutes; Bacilli; Bacillales; Paenibacillaceae; Cohnella.
EAV92__locus_1
EUT2E
05440 -pf06133 -
05445 HMM00465alcdh_4_pdu(pf00465) ETU
05450 -pf02632 -
05455 -pf00005 -
05460 -pf00005pf02361 -
05465 -pf07690 -
05470 - -
05475 -pf00293 -
05480 -pf00903 -
05485 - -
05490 -pf13302 -
05495 -pf04794 -
05500 - -
05505 Tsp_orangePink EUT2E
05510 H_russet EUT2E
05515 HMMaldh_2_egra(pf00171) EUT2E
05520 H_fuchsia EUT2E
05525 HMMptac_3_eut2(pf06130) EUT2E
05530 HMM02441_05 EUT2E EP_NTERM
05535 P_waterBlue EUT2E
05540 Hp_euts_wineRed EUT2E
05545 HMM10662PduV_1_mix(pf10662) EUT2E
05550 HMMreg07568_1_eut2(pf12282pf07568) EUT2E
05555 HMMreg00072_1_eut2(pf03861pf00072) EUT2E
05560 HMM06277EutA_1_eut(pf06277) EUT2E
05565 HMMetlyEutB_2_EUT2(pf06751) EUT2E
05570 HMMetlyEutC_3_eut2(pf05985) EUT2E
05575 HMM13520tp_1_pdu(pf13520) EUT2E
05580 -pf01810 -
05590 -pf01810 -
05595 -pf00583 -
05605 -pf13030 -
BMC-P
BMC-H
BMC-T
Aldehyde dehydrogenase
PTAC
Signature enzyme
Alcohol dehydrogenase
Regulator

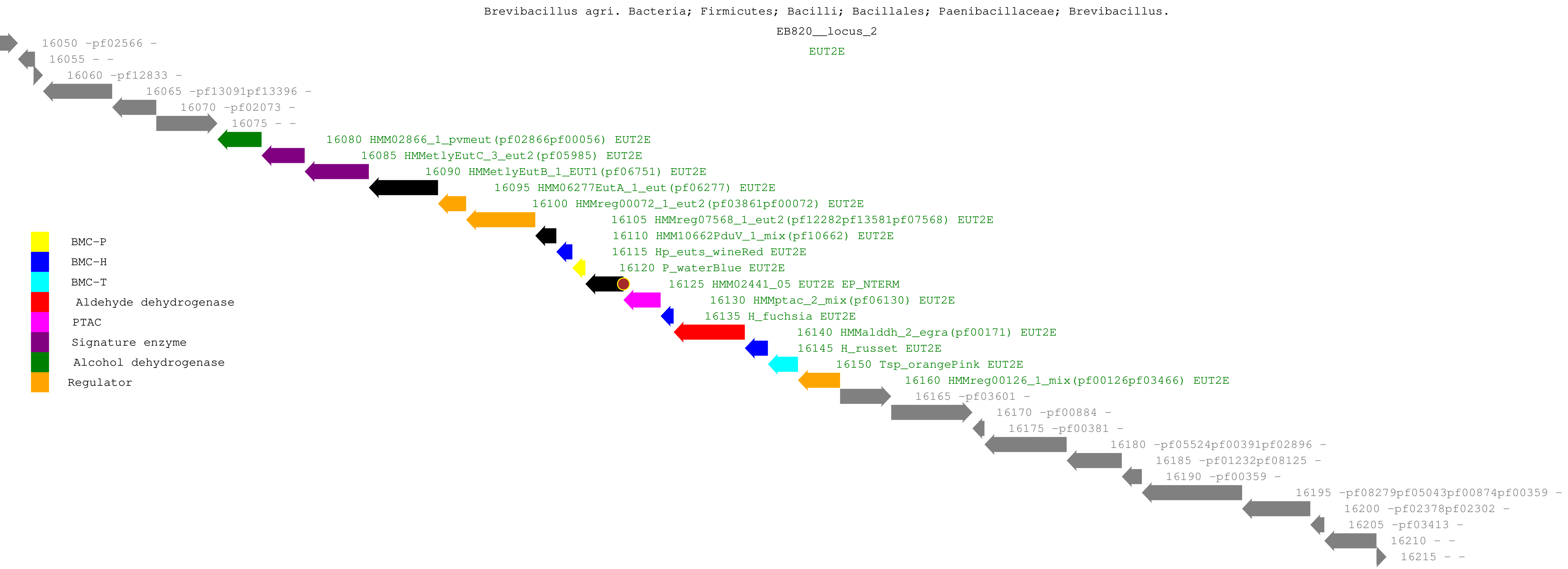

Brevibacillus agri. Bacteria; Firmicutes; Bacilli; Bacillales; Paenibacillaceae; Brevibacillus.
EB820__locus_2
EUT2E
16050 -pf02566 -
16055 - -
16060 -pf12833 -
16065 -pf13091pf13396 -
16070 -pf02073 -
16075 - -
16080 HMM02866_1_pvmeut(pf02866pf00056) EUT2E
16085 HMMetlyEutC_3_eut2(pf05985) EUT2E
16090 HMMetlyEutB_1_EUT1(pf06751) EUT2E
16095 HMM06277EutA_1_eut(pf06277) EUT2E
16100 HMMreg00072_1_eut2(pf03861pf00072) EUT2E
16105 HMMreg07568_1_eut2(pf12282pf13581pf07568) EUT2E
16110 HMM10662PduV_1_mix(pf10662) EUT2E
16115 Hp_euts_wineRed EUT2E
16120 P_waterBlue EUT2E
16125 HMM02441_05 EUT2E EP_NTERM
16130 HMMptac_2_mix(pf06130) EUT2E
16135 H_fuchsia EUT2E
16140 HMMaldh_2_egra(pf00171) EUT2E
16145 H_russet EUT2E
16150 Tsp_orangePink EUT2E
16160 HMMreg00126_1_mix(pf00126pf03466) EUT2E
16165 -pf03601 -
16170 -pf00884 -
16175 -pf00381 -
16180 -pf05524pf00391pf02896 -
16185 -pf01232pf08125 -
16190 -pf00359 -
16195 -pf08279pf05043pf00874pf00359 -
16200 -pf02378pf02302 -
16205 -pf03413 -
16210 - -
16215 - -
BMC-P
BMC-H
BMC-T
Aldehyde dehydrogenase
PTAC
Signature enzyme
Alcohol dehydrogenase
Regulator

Brevibacillus invocatus. Bacteria; Firmicutes; Bacilli; Bacillales; Paenibacillaceae; Brevibacillus.

EDM52__locus_1

EUT2E

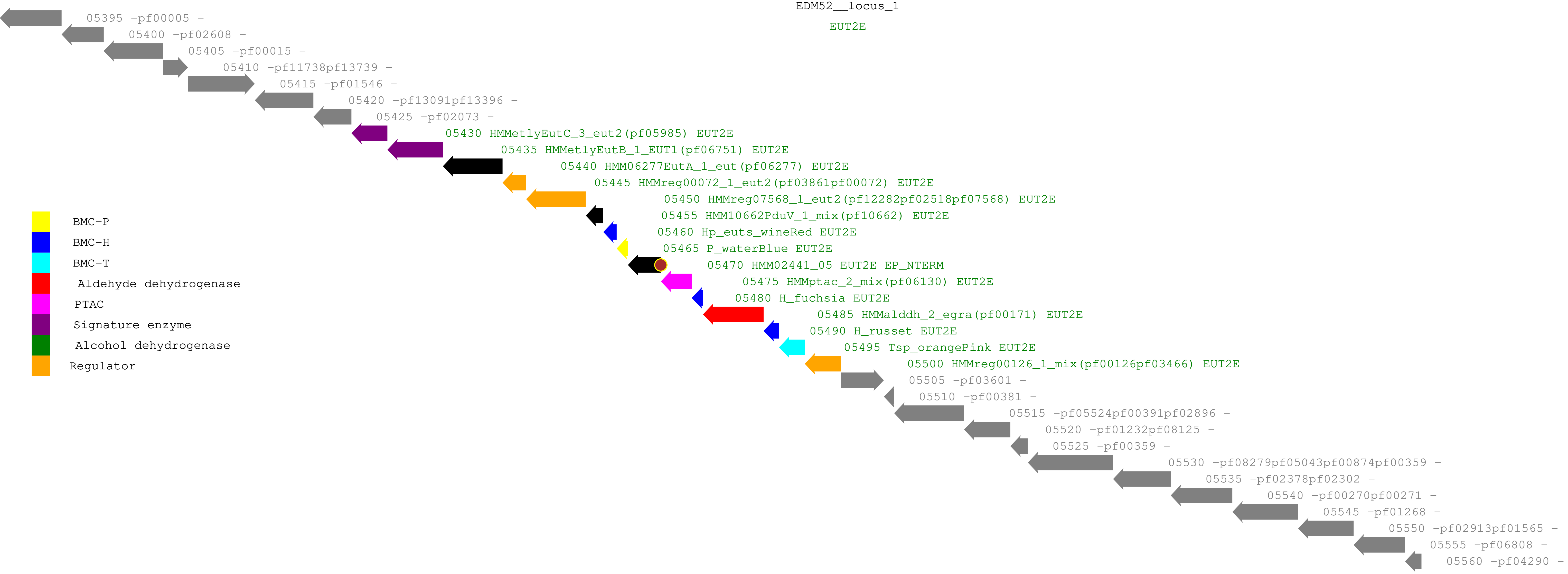

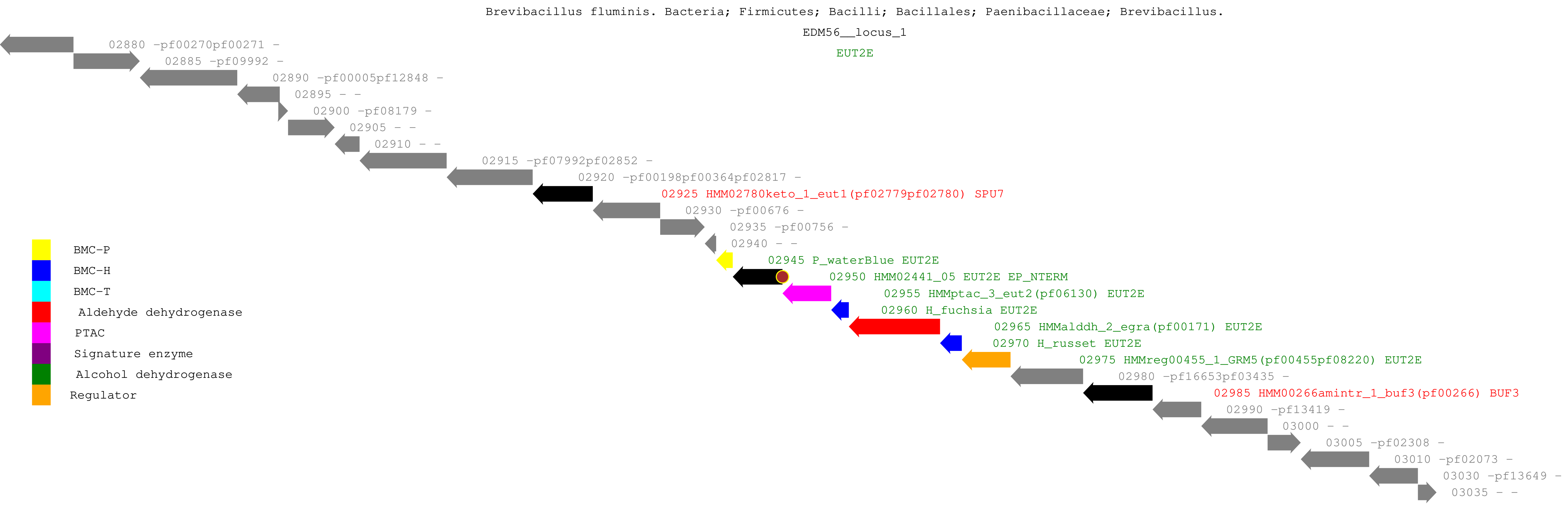

Brevibacillus fluminis. Bacteria; Firmicutes; Bacilli; Bacillales; Paenibacillaceae; Brevibacillus.
EDM56__locus_1
EUT2E
02880 -pf00270pf00271 -
02885 -pf09992 -
02890 -pf00005pf12848 -
02895 - -
02900 -pf08179 -
02905 - -
02910 - -
02915 -pf07992pf02852 -
02920 -pf00198pf00364pf02817 -
02925 HMM02780keto_1_eut1(pf02779pf02780) SPU7
02930 -pf00676 -
02935 -pf00756 -
02940 - -
02945 P_waterBlue EUT2E
02950 HMM02441_05 EUT2E EP_NTERM
02955 HMMptac_3_eut2(pf06130) EUT2E
02960 H_fuchsia EUT2E
02965 HMMalddh_2_egra(pf00171) EUT2E
02970 H_russet EUT2E
02975 HMMreg00455_1_GRM5(pf00455pf08220) EUT2E
02980 -pf16653pf03435 -
02985 HMM00266amintr_1_buf3(pf00266) BUF3
02990 -pf13419 -
03000 - -
03005 -pf02308 -
03010 -pf02073 -
03030 -pf13649 -
03035 - -
BMC-P
BMC-H
BMC-T
Aldehyde dehydrogenase
PTAC
Signature enzyme
Alcohol dehydrogenase
Regulator

Brevibacillus fluminis. Bacteria; Firmicutes; Bacilli; Bacillales; Paenibacillaceae; Brevibacillus.

EDM56__locus_2

EUT2E

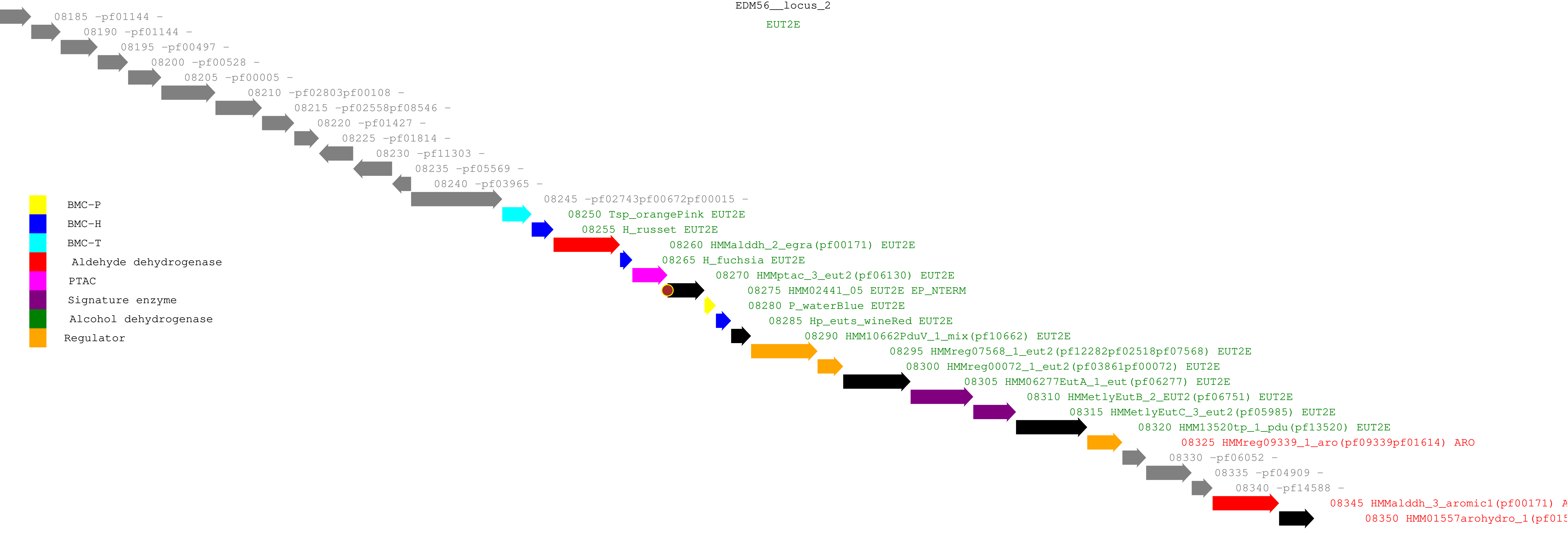

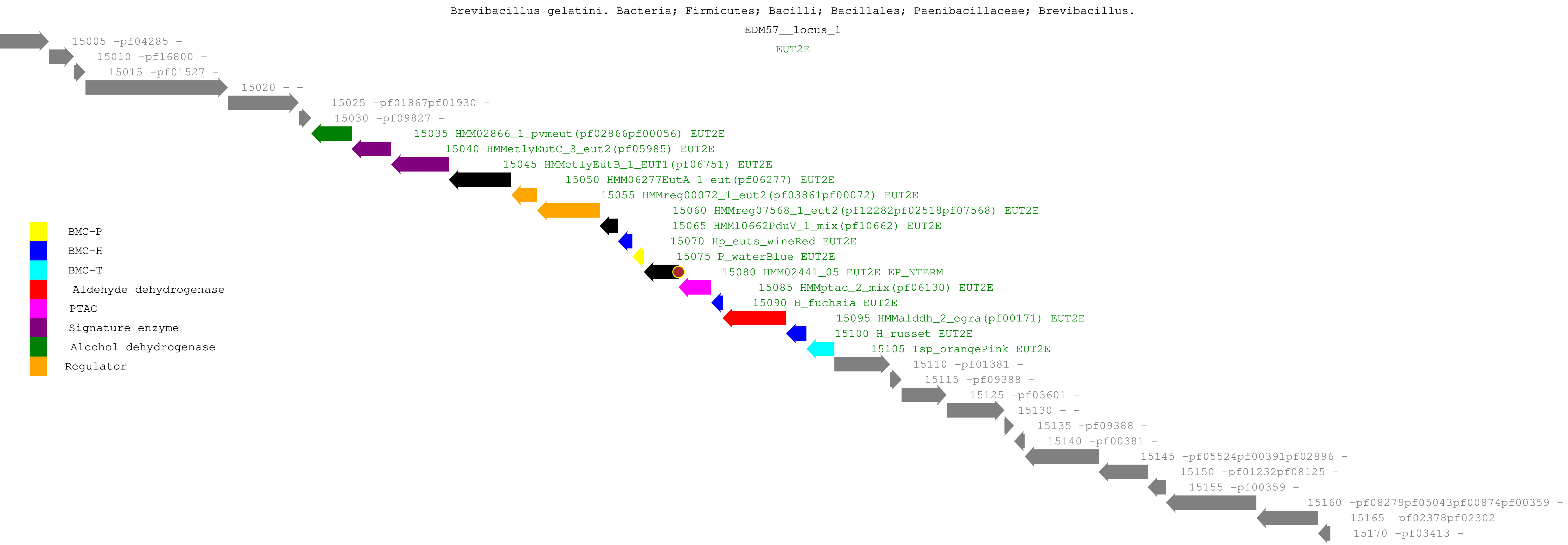

Brevibacillus gelatini. Bacteria; Firmicutes; Bacilli; Bacillales; Paenibacillaceae; Brevibacillus.
EDM57__locus_1
EUT2E
15005 -pf04285 -
15010 -pf16800 -
15015 -pf01527 -
15020 - -
15025 -pf01867pf01930 -
15030 -pf09827 -
15035 HMM02866_1_pvmeut(pf02866pf00056) EUT2E
15040 HMMetlyEutC_3_eut2(pf05985) EUT2E
15045 HMMetlyEutB_1_EUT1(pf06751) EUT2E
15050 HMM06277EutA_1_eut(pf06277) EUT2E
15055 HMMreg00072_1_eut2(pf03861pf00072) EUT2E
15060 HMMreg07568_1_eut2(pf12282pf02518pf07568) EUT2E
15065 HMM10662PduV_1_mix(pf10662) EUT2E
15070 Hp_euts_wineRed EUT2E
15075 P_waterBlue EUT2E
15080 HMM02441_05 EUT2E EP_NTERM
15085 HMMptac_2_mix(pf06130) EUT2E
15090 H_fuchsia EUT2E
15095 HMMalddh_2_egra(pf00171) EUT2E
15100 H_russet EUT2E
15105 Tsp_orangePink EUT2E
15110 -pf01381 -
15115 -pf09388 -
15125 -pf03601 -
15130 - -
15135 -pf09388 -
15140 -pf00381 -
15145 -pf05524pf00391pf02896 -
15150 -pf01232pf08125 -
15155 -pf00359 -
15160 -pf08279pf05043pf00874pf00359 -
15165 -pf02378pf02302 -
15170 -pf03413 -
BMC-P
BMC-H
BMC-T
Aldehyde dehydrogenase
PTAC
Signature enzyme
Alcohol dehydrogenase
Regulator

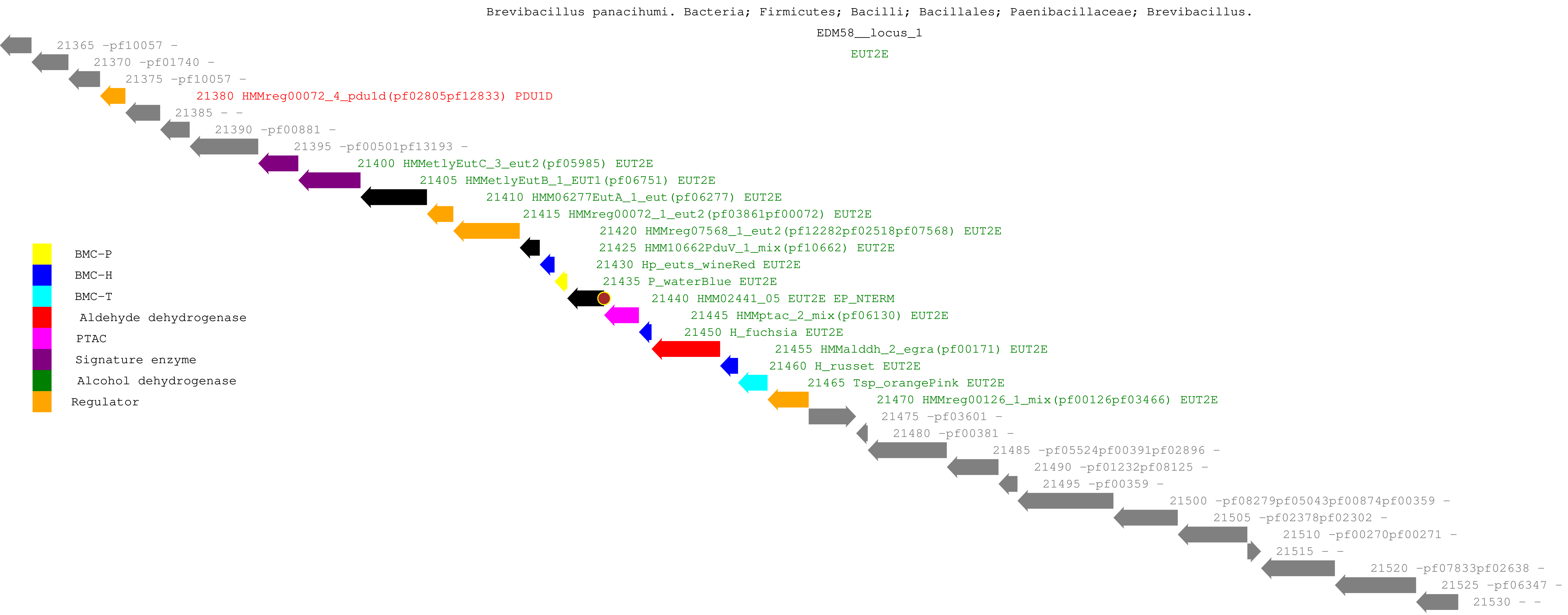

Brevibacillus panacihumi. Bacteria; Firmicutes; Bacilli; Bacillales; Paenibacillaceae; Brevibacillus.
EDM58__locus_1
EUT2E
21365 -pf10057 -
21370 -pf01740 -
21375 -pf10057 -
21380 HMMreg00072_4_pdu1d(pf02805pf12833) PDU1D
21385 - -
21390 -pf00881 -
21395 -pf00501pf13193 -
21400 HMMetlyEutC_3_eut2(pf05985) EUT2E
21405 HMMetlyEutB_1_EUT1(pf06751) EUT2E
21410 HMM06277EutA_1_eut(pf06277) EUT2E
21415 HMMreg00072_1_eut2(pf03861pf00072) EUT2E
21420 HMMreg07568_1_eut2(pf12282pf02518pf07568) EUT2E
21425 HMM10662PduV_1_mix(pf10662) EUT2E
21430 Hp_euts_wineRed EUT2E
21435 P_waterBlue EUT2E
21440 HMM02441_05 EUT2E EP_NTERM
21445 HMMptac_2_mix(pf06130) EUT2E
21450 H_fuchsia EUT2E
21455 HMMalddh_2_egra(pf00171) EUT2E
21460 H_russet EUT2E
21465 Tsp_orangePink EUT2E
21470 HMMreg00126_1_mix(pf00126pf03466) EUT2E
21475 -pf03601 -
21480 -pf00381 -
21485 -pf05524pf00391pf02896 -
21490 -pf01232pf08125 -
21495 -pf00359 -
21500 -pf08279pf05043pf00874pf00359 -
21505 -pf02378pf02302 -
21510 -pf00270pf00271 -
21515 - -
21520 -pf07833pf02638 -
21525 -pf06347 -
21530 - -
BMC-P
BMC-H
BMC-T
Aldehyde dehydrogenase
PTAC
Signature enzyme
Alcohol dehydrogenase
Regulator

Brevibacillus nitrificans. Bacteria; Firmicutes; Bacilli; Bacillales; Paenibacillaceae; Brevibacillus.

EDM59__locus_1

EUT2E

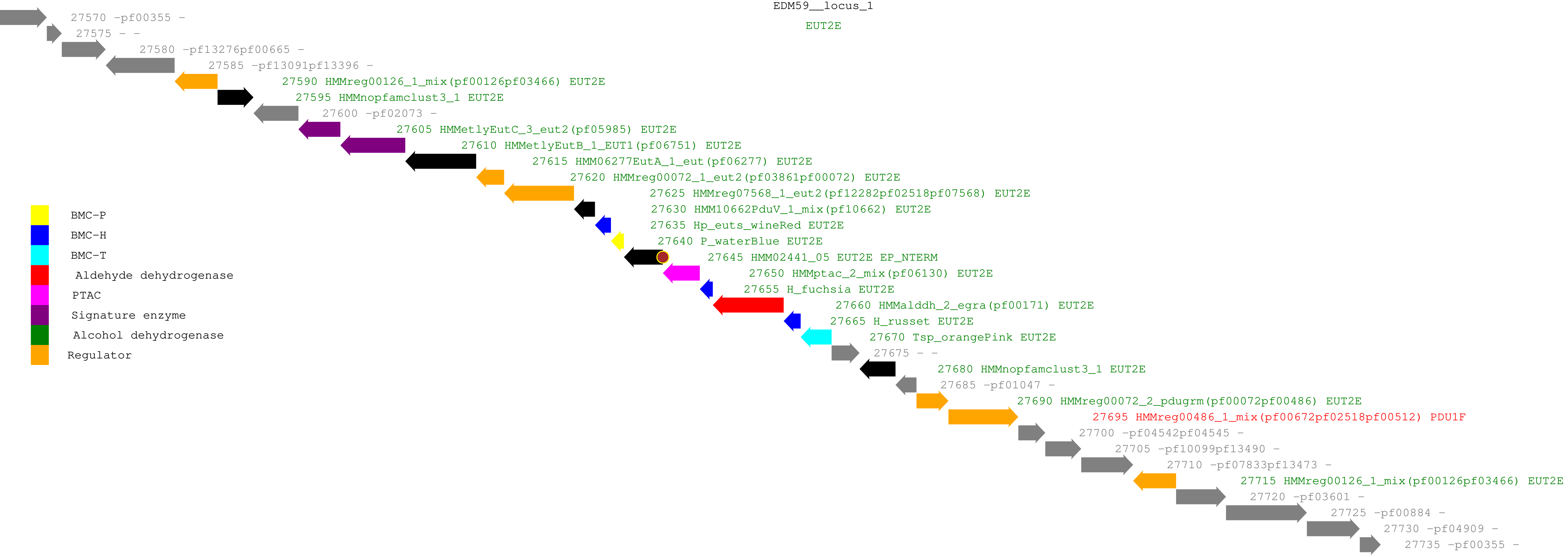

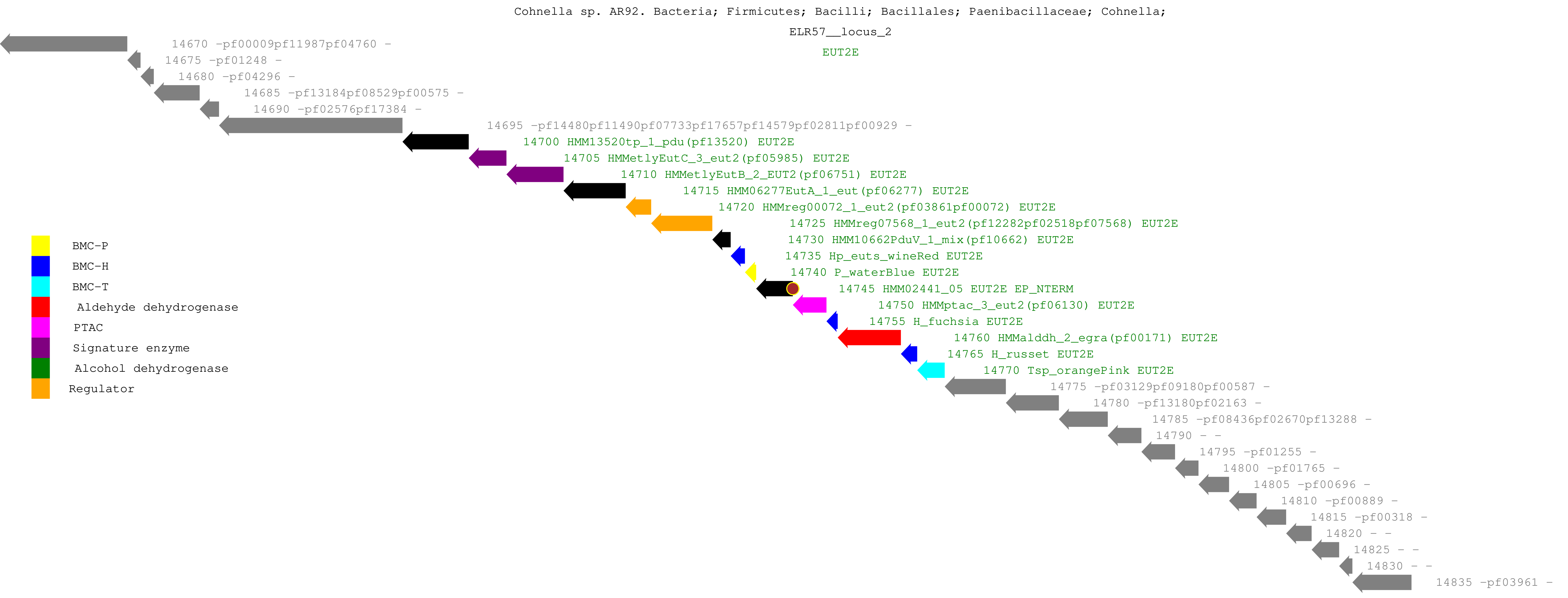
Cohnella sp. AR92. Bacteria; Firmicutes; Bacilli; Bacillales; Paenibacillaceae; Cohnella;
ELR57__locus_2
EUT2E
14670 -pf00009pf11987pf04760 -
14675 -pf01248 -
14680 -pf04296 -
14685 -pf13184pf08529pf00575 -
14690 -pf02576pf17384 -
14695 -pf14480pf11490pf07733pf17657pf14579pf02811pf00929 -
14700 HMM13520tp_1_pdu(pf13520) EUT2E
14705 HMMetlyEutC_3_eut2(pf05985) EUT2E
14710 HMMetlyEutB_2_EUT2(pf06751) EUT2E
14715 HMM06277EutA_1_eut(pf06277) EUT2E
14720 HMMreg00072_1_eut2(pf03861pf00072) EUT2E
14725 HMMreg07568_1_eut2(pf12282pf02518pf07568) EUT2E
14730 HMM10662PduV_1_mix(pf10662) EUT2E
14735 Hp_euts_wineRed EUT2E
14740 P_waterBlue EUT2E
14745 HMM02441_05 EUT2E EP_NTERM
14750 HMMptac_3_eut2(pf06130) EUT2E
14755 H_fuchsia EUT2E
14760 HMMaldh_2_egra(pf00171) EUT2E
14765 H_russet EUT2E
14770 Tsp_orangePink EUT2E
14775 -pf03129pf09180pf00587 -
14780 -pf13180pf02163 -
14785 -pf08436pf02670pf13288 -
14790 - -
14795 -pf01255 -
14800 -pf01765 -
14805 -pf00696 -
14810 -pf00889 -
14815 -pf00318 -
14820 - -
14825 - -
14830 - -
14835 -pf03961 -
BMC-P
BMC-H
BMC-T
Aldehyde dehydrogenase
PTAC
Signature enzyme
Alcohol dehydrogenase
Regulator

Paenibacillus tyrfis. Bacteria; Firmicutes; Bacilli; Bacillales; Paenibacillaceae;

ET33__locus_1

EUT2E

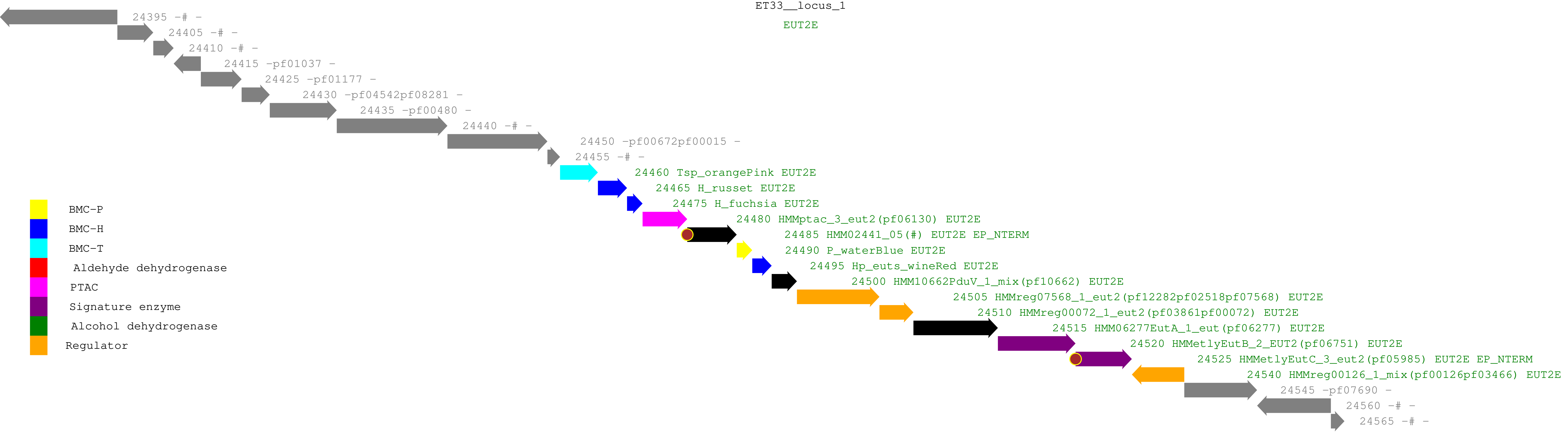

Brevibacillus sp. AG162. Bacteria; Firmicutes; Bacilli; Bacillales; Paenibacillaceae; Brevibacillus.

FB479__locus_1

EUT2E

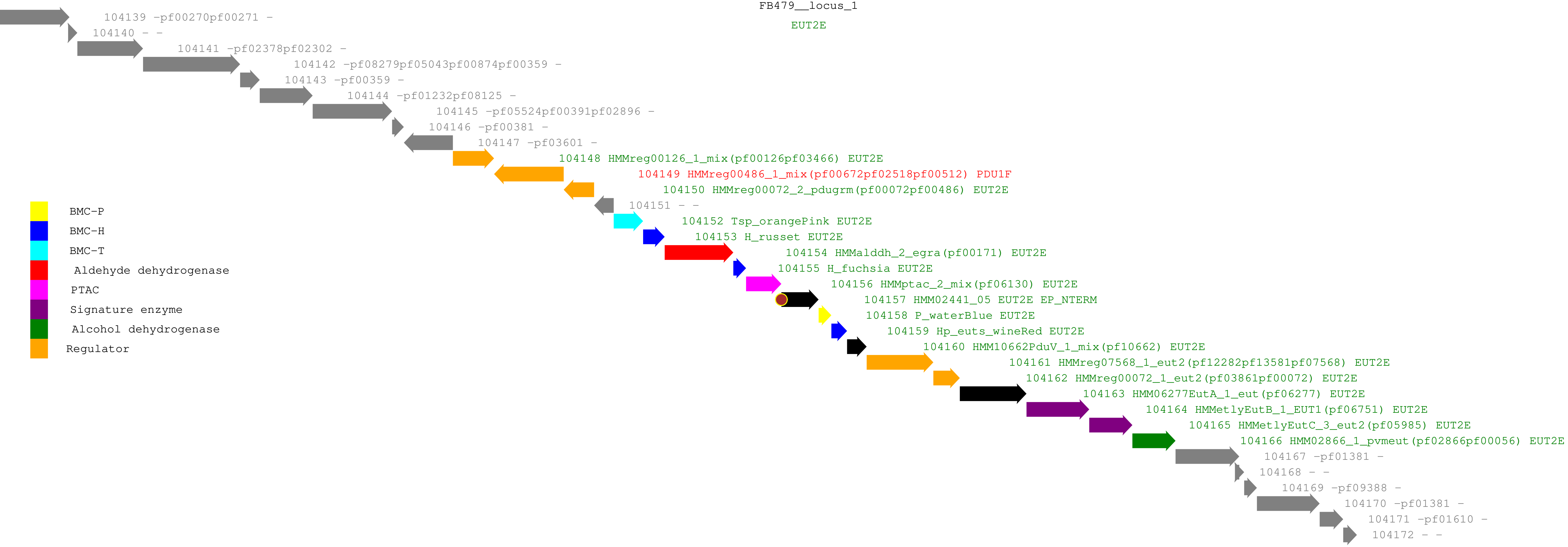

Paenibacillus sp. 597. Bacteria; Firmicutes; Bacilli; Bacillales; Paenibacillaceae; Paenibacillus.

FHW43__locus_1

EUT2E

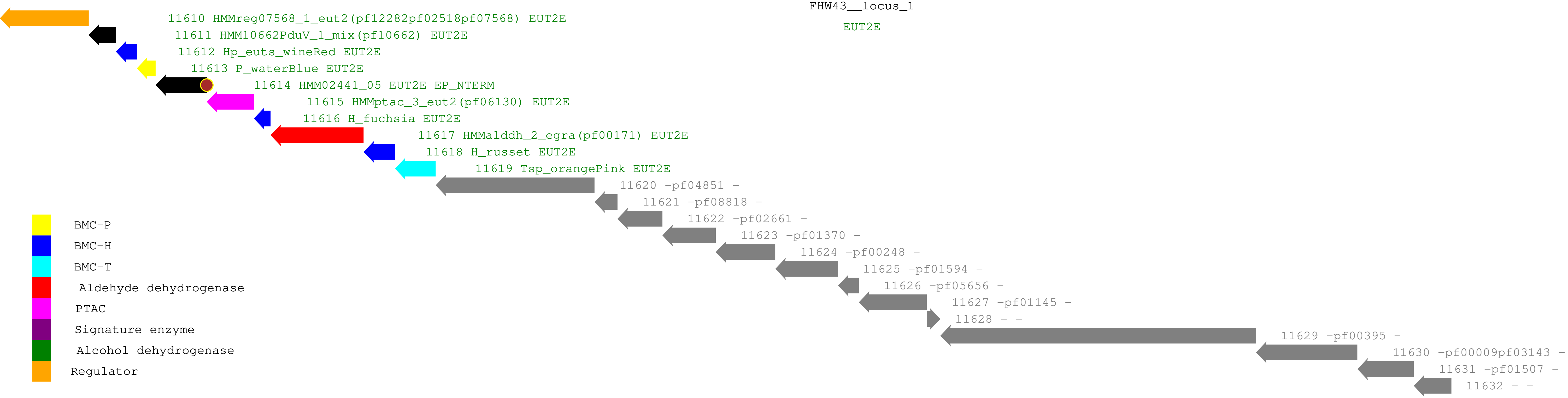

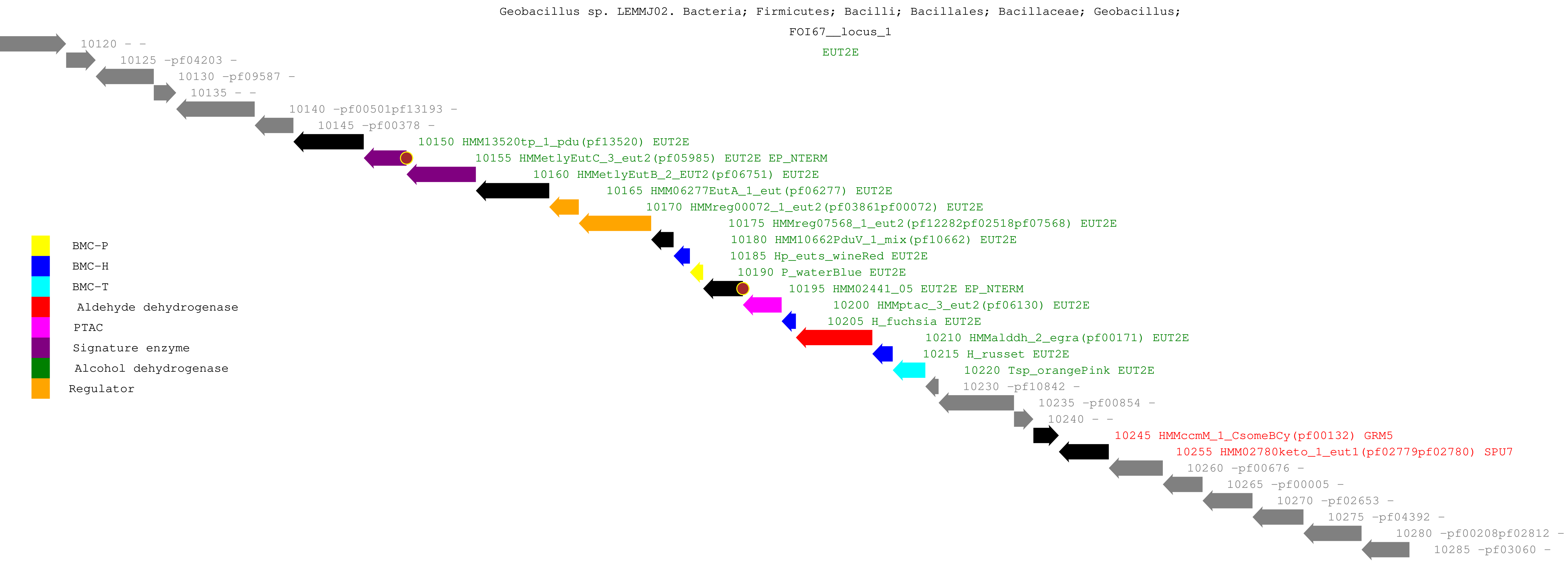

Geobacillus sp. LEMMJ02. Bacteria; Firmicutes; Bacilli; Bacillales; Bacillaceae; Geobacillus;
FOI67__locus_1
EUT2E
10120 - -
10125 -pf04203 -
10130 -pf09587 -
10135 - -
10140 -pf00501pf13193 -
10145 -pf00378 -
10150 HMM13520tp_1_pdu(pf13520) EUT2E
10155 HMMetlyEutC_3_eut2(pf05985) EUT2E EP_NTERM
10160 HMMetlyEutB_2_EUT2(pf06751) EUT2E
10165 HMM06277EutA_1_eut(pf06277) EUT2E
10170 HMMreg00072_1_eut2(pf03861pf00072) EUT2E
10175 HMMreg07568_1_eut2(pf12282pf02518pf07568) EUT2E
10180 HMM10662PduV_1_mix(pf10662) EUT2E
10185 Hp_euts_wineRed EUT2E
10190 P_waterBlue EUT2E
10195 HMM02441_05 EUT2E EP_NTERM
10200 HMMptac_3_eut2(pf06130) EUT2E
10205 H_fuchsia EUT2E
10210 HMMalddh_2_egra(pf00171) EUT2E
10215 H_russet EUT2E
10220 Tsp_orangePink EUT2E
10230 -pf10842 -
10235 -pf00854 -
10240 - -
10245 HMMccmM_1_CsomeBCy(pf00132) GRM5
10255 HMM02780keto_1_eut1(pf02779pf02780) SPU7
10260 -pf00676 -
10265 -pf00005 -
10270 -pf02653 -
10275 -pf04392 -
10280 -pf00208pf02812 -
10285 -pf03060 -
BMC-P
BMC-H
BMC-T
Aldehyde dehydrogenase
PTAC
Signature enzyme
Alcohol dehydrogenase
Regulator

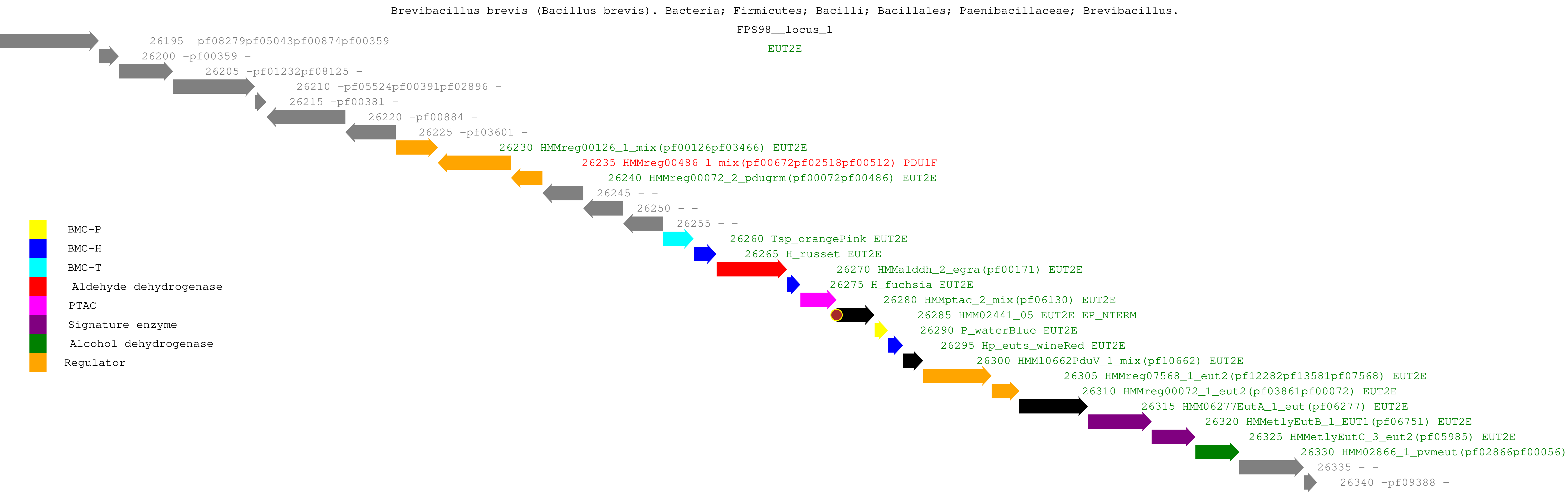
Brevibacillus brevis (Bacillus brevis). Bacteria; Firmicutes; Bacilli; Bacillales; Paenibacillaceae; Brevibacillus.
FPS98__locus_1
EUT2E
26195 -pf08279pf05043pf00874pf00359 -
26200 -pf00359 -
26205 -pf01232pf08125 -
26210 -pf05524pf00391pf02896 -
26215 -pf00381 -
26220 -pf00884 -
26225 -pf03601 -
26230 HMMreg00126_1_mix(pf00126pf03466) EUT2E
26235 HMMreg00486_1_mix(pf00672pf02518pf00512) PDU1F
26240 HMMreg00072_2_pdugrm(pf00072pf00486) EUT2E
26245 - -
26250 - -
26255 - -
26260 Tsp_orangePink EUT2E
26265 H_russet EUT2E
26270 HMMalddh_2_egra(pf00171) EUT2E
26275 H_fuchsia EUT2E
26280 HMMptac_2_mix(pf06130) EUT2E
26285 HMM02441_05 EUT2E EP_NTERM
26290 P_waterBlue EUT2E
26295 Hp_euts_wineRed EUT2E
26300 HMM10662PduV_1_mix(pf10662) EUT2E
26305 HMMreg07568_1_eut2(pf12282pf13581pf07568) EUT2E
26310 HMMreg00072_1_eut2(pf03861pf00072) EUT2E
26315 HMM06277EutA_1_eut(pf06277) EUT2E
26320 HMMetlyEutB_1_EUT1(pf06751) EUT2E
26325 HMMetlyEutC_3_eut2(pf05985) EUT2E
26330 HMM02866_1_pvmeut(pf02866pf00056)
26335 - -
26340 -pf09388 -
BMC-P
BMC-H
BMC-T
Aldehyde dehydrogenase
PTAC
Signature enzyme
Alcohol dehydrogenase
Regulator

Paenibacillus sp. N3.4. Bacteria; Firmicutes; Bacilli; Bacillales; Paenibacillaceae; Paenibacillus.

FU659__locus_1

EUT2E

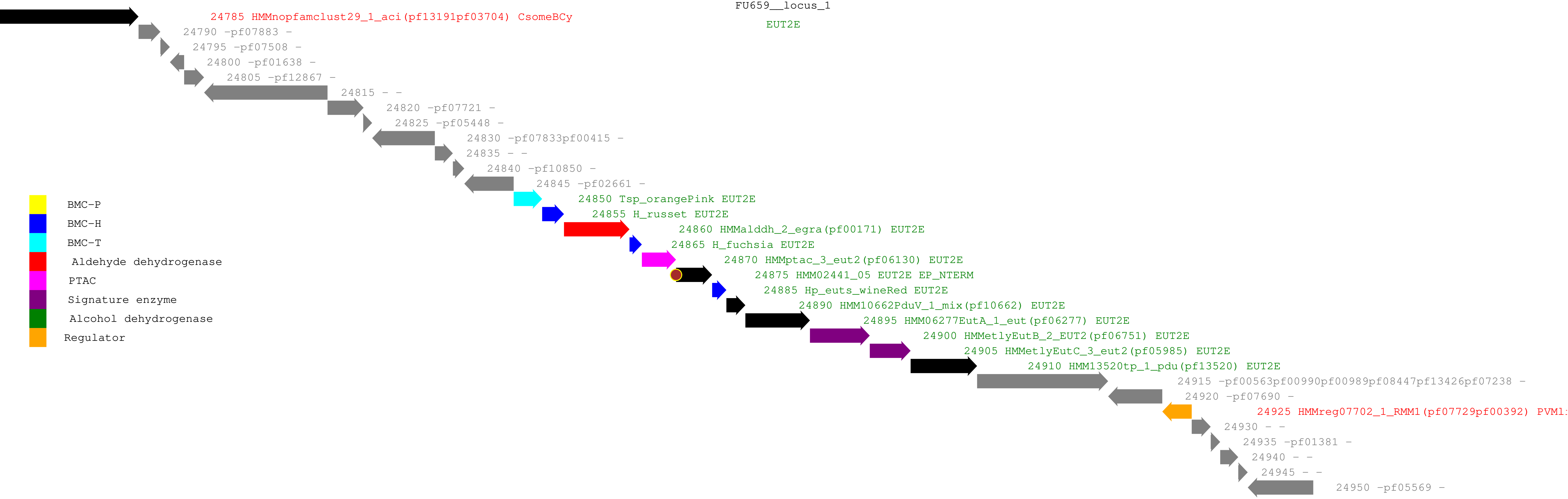

Parageobacillus caldoxylosilyticus NBRC 107762. Bacteria; Firmicutes; Bacilli; Bacillales; Bacillaceae;

GCA01S_059__locus_1

EUT2E

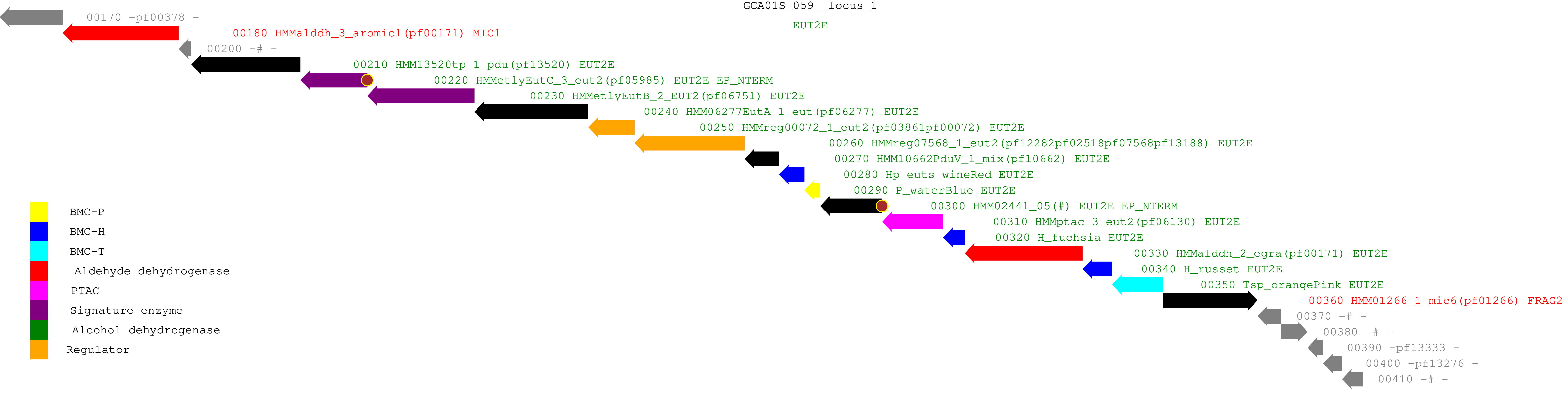

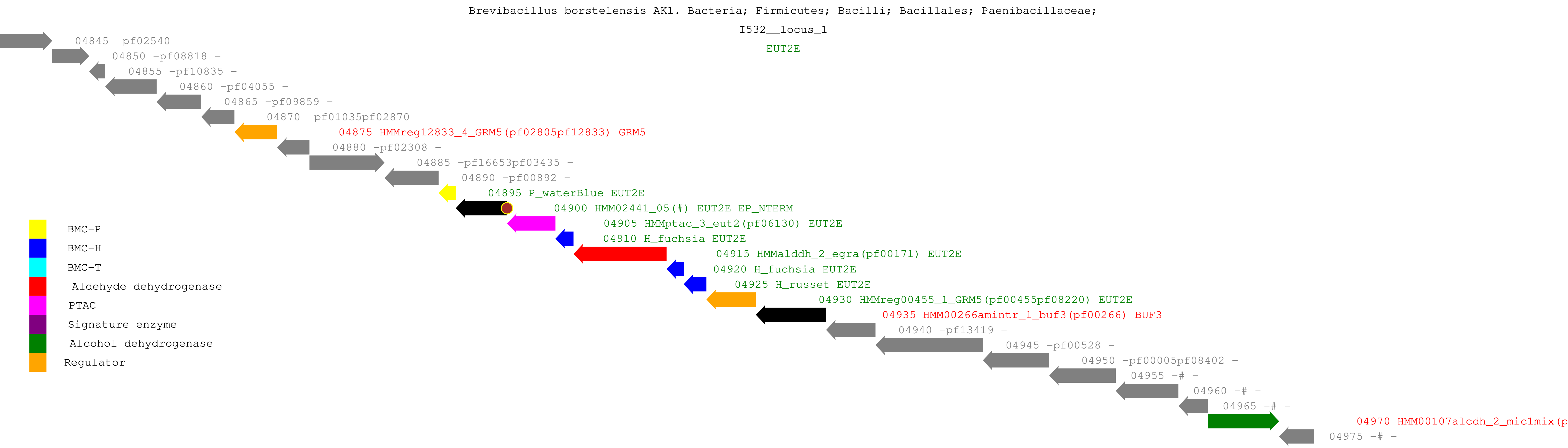

Brevibacillus borstelensis AK1. Bacteria; Firmicutes; Bacilli; Bacillales; Paenibacillaceae;
I532__locus_1
EUT2E
04845 -pf02540 -
04850 -pf08818 -
04855 -pf10835 -
04860 -pf04055 -
04865 -pf09859 -
04870 -pf01035pf02870 -
04875 HMMreg12833_4_GRM5(pf02805pf12833) GRM5
04880 -pf02308 -
04885 -pf16653pf03435 -
04890 -pf00892 -
04895 P_waterBlue EUT2E
04900 HMM02441_05(#) EUT2E EP_NTERM
04905 HMMptac_3_eut2(pf06130) EUT2E
04910 H_fuchsia EUT2E
04915 HMMaldh_2_egra(pf00171) EUT2E
04920 H_fuchsia EUT2E
04925 H_russet EUT2E
04930 HMMreg00455_1_GRM5(pf00455pf08220) EUT2E
04935 HMM00266amintr_1_buf3(pf00266) BUF3
04940 -pf13419 -
04945 -pf00528 -
04950 -pf00005pf08402 -
04955 -# -
04960 -# -
04965 -# -
04970 HMM00107alcdh_2_mic1mix(p
04975 -# -
BMC-P
BMC-H
BMC-T
Aldehyde dehydrogenase
PTAC
Signature enzyme
Alcohol dehydrogenase
Regulator

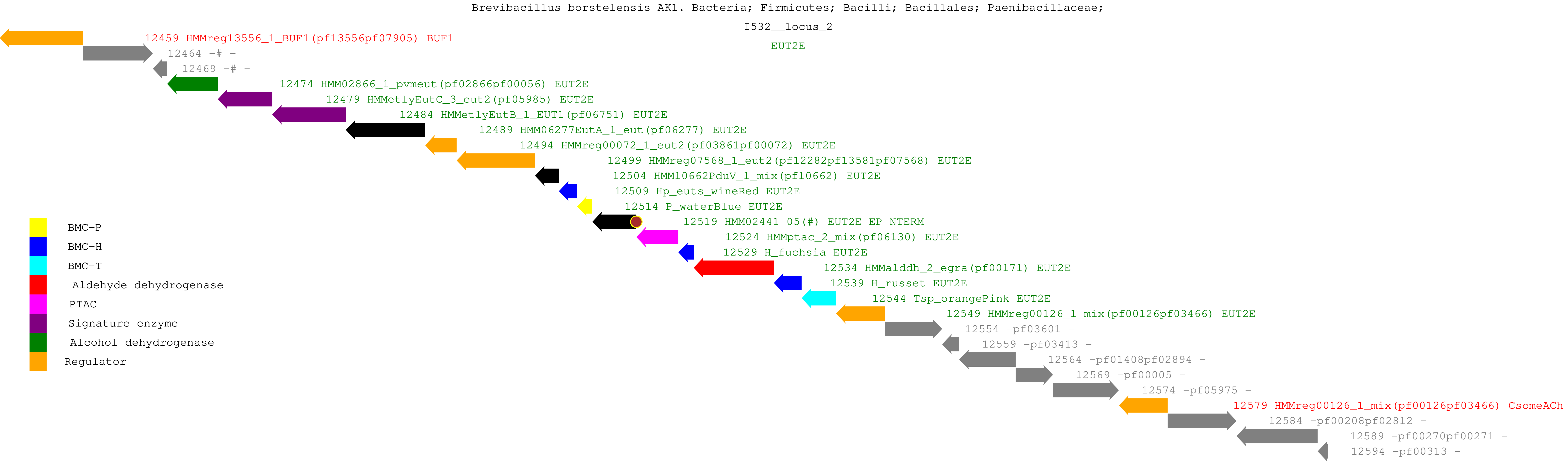
Brevibacillus borstelensis AK1. Bacteria; Firmicutes; Bacilli; Bacillales; Paenibacillaceae;
I532__locus_2
EUT2E
12459 HMMreg13556_1_BUF1(pf13556pf07905) BUF1
12464 -# -
12469 -# -
12474 HMM02866_1_pvmeut(pf02866pf00056) EUT2E
12479 HMMetlyEutC_3_eut2(pf05985) EUT2E
12484 HMMetlyEutB_1_EUT1(pf06751) EUT2E
12489 HMM06277EutA_1_eut(pf06277) EUT2E
12494 HMMreg00072_1_eut2(pf03861pf00072) EUT2E
12499 HMMreg07568_1_eut2(pf12282pf13581pf07568) EUT2E
12504 HMM10662PduV_1_mix(pf10662) EUT2E
12509 Hp_euts_wineRed EUT2E
12514 P_waterBlue EUT2E
12519 HMM02441_05(#) EUT2E EP_NTERM
12524 HMMptac_2_mix(pf06130) EUT2E
12529 H_fuchsia EUT2E
12534 HMMalddh_2_egra(pf00171) EUT2E
12539 H_russet EUT2E
12544 Tsp_orangePink EUT2E
12549 HMMreg00126_1_mix(pf00126pf03466) EUT2E
12554 -pf03601 -
12559 -pf03413 -
12564 -pf01408pf02894 -
12569 -pf00005 -
12574 -pf05975 -
12579 HMMreg00126_1_mix(pf00126pf03466) CsomeACh
12584 -pf00208pf02812 -
12589 -pf00270pf00271 -
12594 -pf00313 -
BMC-P
BMC-H
BMC-T
Aldehyde dehydrogenase
PTAC
Signature enzyme
Alcohol dehydrogenase
Regulator

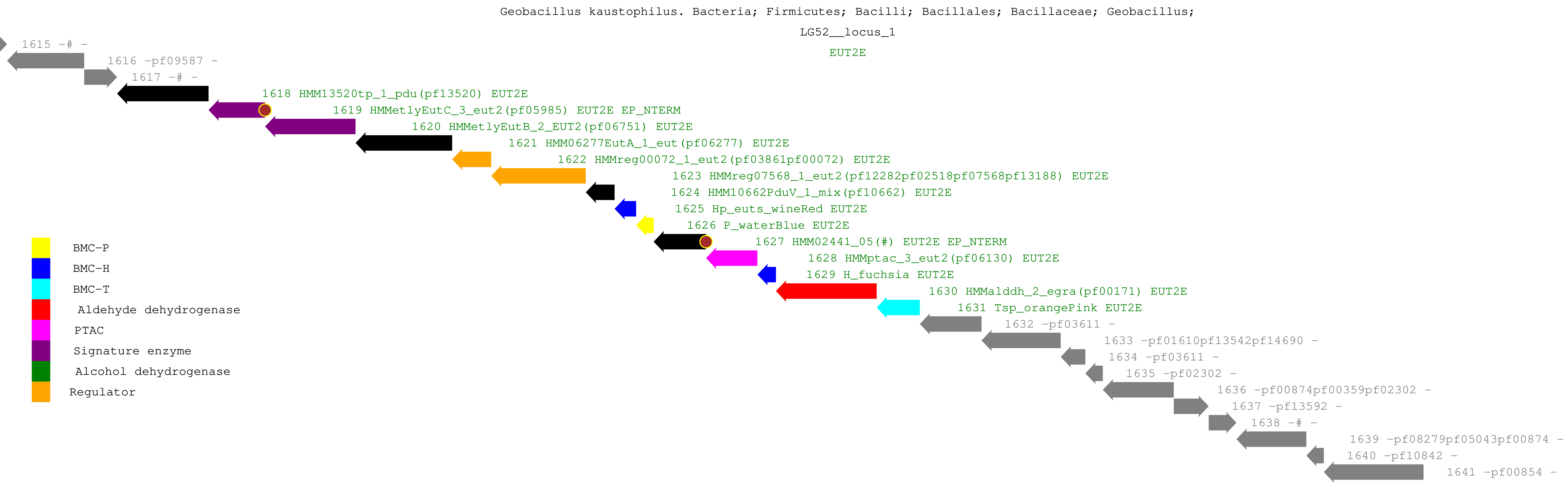

Geobacillus kaustophilus. Bacteria; Firmicutes; Bacilli; Bacillales; Bacillaceae; Geobacillus;
LG52__locus_1
EUT2E
1615 -# -
1616 -pf09587 -
1617 -# -
1618 HMM13520tp_1_pdu(pf13520) EUT2E
1619 HMMetlyEutC_3_eut2(pf05985) EUT2E EP_NTERM
1620 HMMetlyEutB_2_EUT2(pf06751) EUT2E
1621 HMM06277EutA_1_eut(pf06277) EUT2E
1622 HMMreg00072_1_eut2(pf03861pf00072) EUT2E
1623 HMMreg07568_1_eut2(pf12282pf02518pf07568pf13188) EUT2E
1624 HMM10662PduV_1_mix(pf10662) EUT2E
1625 Hp_euts_wineRed EUT2E
1626 P_waterBlue EUT2E
1627 HMM02441_05(#) EUT2E EP_NTERM
1628 HMMptac_3_eut2(pf06130) EUT2E
1629 H_fuchsia EUT2E
1630 HMMalddh_2_egra(pf00171) EUT2E
1631 Tsp_orangePink EUT2E
1632 -pf03611 -
1633 -pf01610pf13542pf14690 -
1634 -pf03611 -
1635 -pf02302 -
1636 -pf00874pf00359pf02302 -
1637 -pf13592 -
1638 -# -
1639 -pf08279pf05043pf00874 -
1640 -pf10842 -
1641 -pf00854 -
BMC-P
BMC-H
BMC-T
Aldehyde dehydrogenase
PTAC
Signature enzyme
Alcohol dehydrogenase
Regulator

Geobacillus sp. BCO2. Bacteria; Firmicutes; Bacilli; Bacillales; Bacillaceae; Geobacillus.

LR69__locus_1

EUT2E

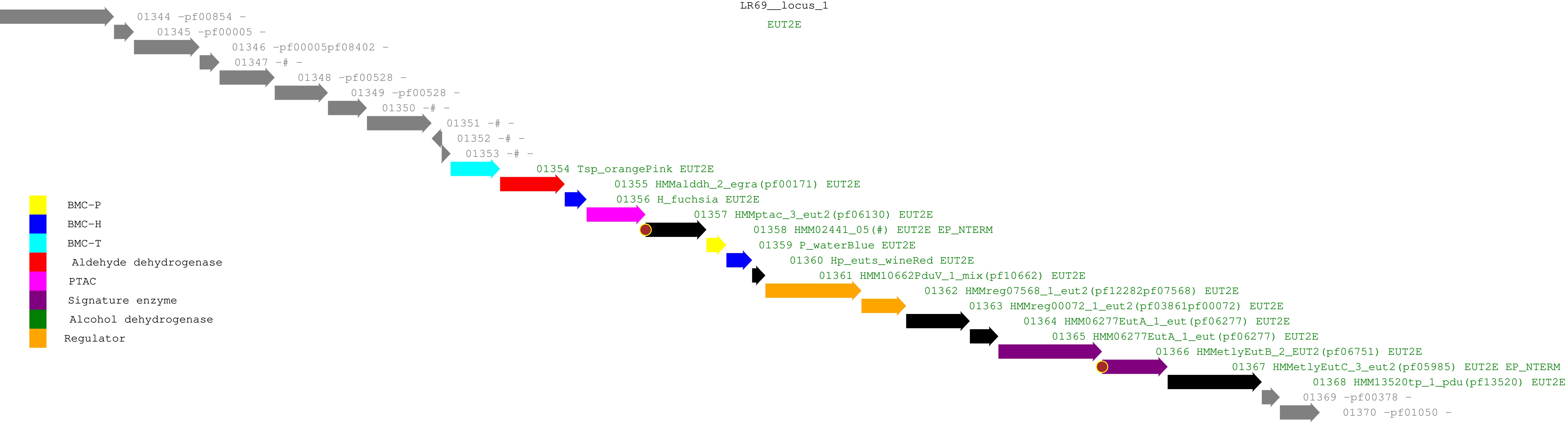

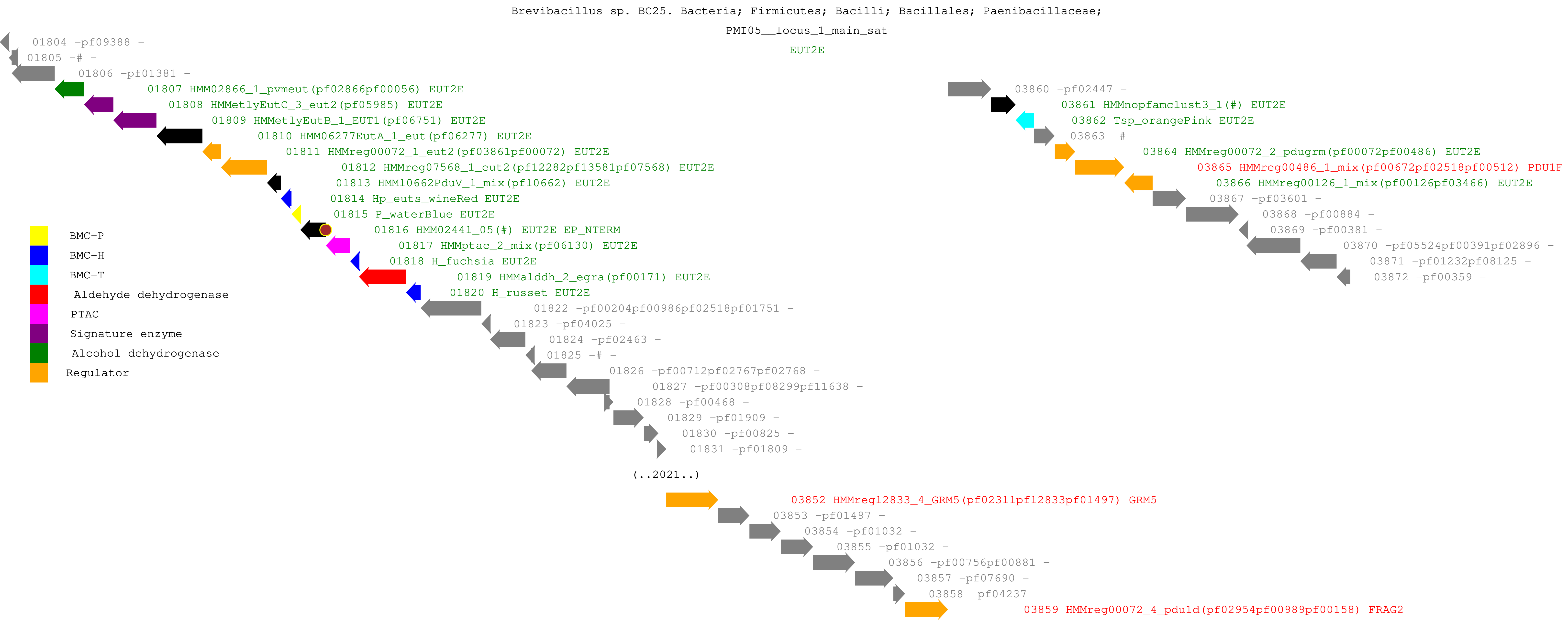
Brevibacillus sp. BC25. Bacteria; Firmicutes; Bacilli; Bacillales; Paenibacillaceae;
PMI05__locus_1_main_sat
EUT2E
01804 -pf09388 -
01805 -# -
01806 -pf01381 -
01807 HMM02866_1_pvmeut(pf02866pf00056) EUT2E
01808 HMMetlyEutC_3_eut2(pf05985) EUT2E
01809 HMMetlyEutB_1_EUT1(pf06751) EUT2E
01810 HMM06277EutA_1_eut(pf06277) EUT2E
01811 HMMreg00072_1_eut2(pf03861pf00072) EUT2E
01812 HMMreg07568_1_eut2(pf12282pf13581pf07568) EUT2E
01813 HMM10662PduV_1_mix(pf10662) EUT2E
01814 Hp_euts_wineRed EUT2E
01815 P_waterBlue EUT2E
01816 HMM02441_05(#) EUT2E EP_NTERM
01817 HMMptac_2_mix(pf06130) EUT2E
01818 H_fuchsia EUT2E
01819 HMMalddh_2_egra(pf00171) EUT2E
01820 H_russet EUT2E
01822 -pf00204pf00986pf02518pf01751 -
01823 -pf04025 -
01824 -pf02463 -
01825 -# -
01826 -pf00712pf02767pf02768 -
01827 -pf00308pf08299pf11638 -
01828 -pf00468 -
01829 -pf01909 -
01830 -pf00825 -
01831 -pf01809 -
(..2021..)
03852 HMMreg12833_4_GRM5(pf02311pf12833pf01497) GRM5
03853 -pf01497 -
03854 -pf01032 -
03855 -pf01032 -
03856 -pf00756pf00881 -
03857 -pf07690 -
03858 -pf04237 -
03859 HMMreg00072_4_pdu1d(pf02954pf00989pf00158) FRAG2
03860 -pf02447 -
03861 HMMnopfamclust3_1(#) EUT2E
03862 Tsp_orangePink EUT2E
03863 -# -
03864 HMMreg00072_2_pdugrm(pf00072pf00486) EUT2E
03865 HMMreg00486_1_mix(pf00672pf02518pf00512) PDU1F
03866 HMMreg00126_1_mix(pf00126pf03466) EUT2E
03867 -pf03601 -
03868 -pf00884 -
03869 -pf00381 -
03870 -pf05524pf00391pf02896 -
03871 -pf01232pf08125 -
03872 -pf00359 -
BMC-P
BMC-H
BMC-T
Aldehyde dehydrogenase
PTAC
Signature enzyme
Alcohol dehydrogenase
Regulator

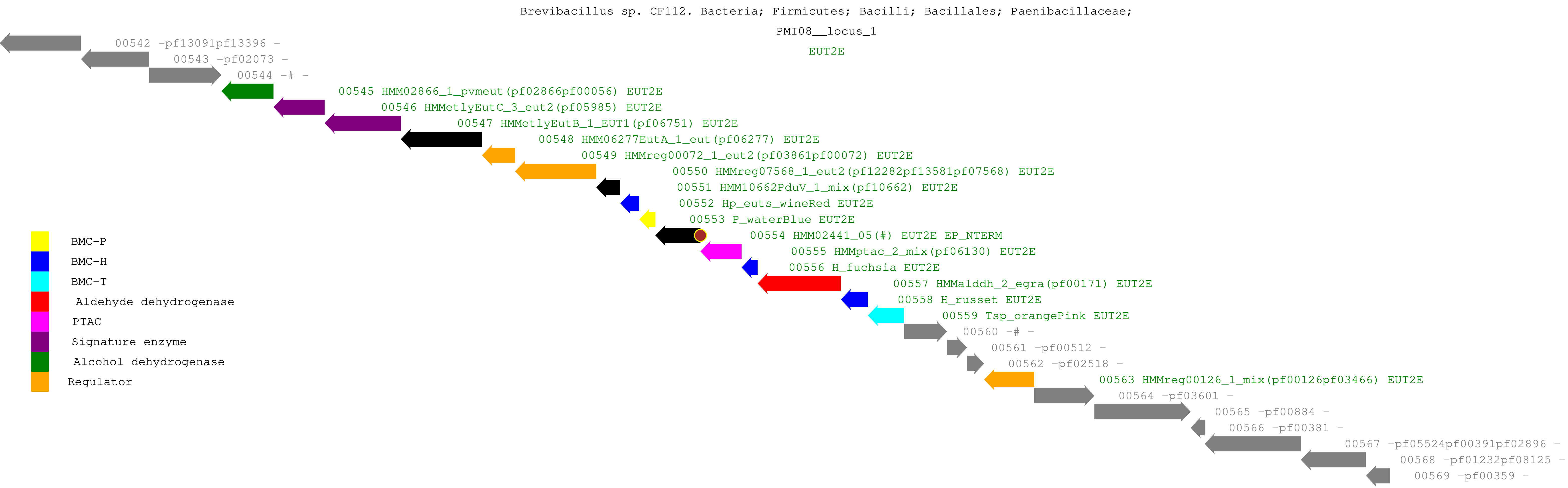

Brevibacillus sp. CF112. Bacteria; Firmicutes; Bacilli; Bacillales; Paenibacillaceae;
PMI08__locus_1
EUT2E
00542 -pf13091pf13396 -
00543 -pf02073 -
00544 -# -
00545 HMM02866_1_pvmeut(pf02866pf00056) EUT2E
00546 HMMetlyEutC_3_eut2(pf05985) EUT2E
00547 HMMetlyEutB_1_EUT1(pf06751) EUT2E
00548 HMM06277EutA_1_eut(pf06277) EUT2E
00549 HMMreg00072_1_eut2(pf03861pf00072) EUT2E
00550 HMMreg07568_1_eut2(pf12282pf13581pf07568) EUT2E
00551 HMM10662PduV_1_mix(pf10662) EUT2E
00552 Hp_euts_wineRed EUT2E
00553 P_waterBlue EUT2E
00554 HMM02441_05(#) EUT2E EP_NTERM
00555 HMMptac_2_mix(pf06130) EUT2E
00556 H_fuchsia EUT2E
00557 HMMalddh_2_egra(pf00171) EUT2E
00558 H_russet EUT2E
00559 Tsp_orangePink EUT2E
00560 -# -
00561 -pf00512 -
00562 -pf02518 -
00563 HMMreg00126_1_mix(pf00126pf03466) EUT2E
00564 -pf03601 -
00565 -pf00884 -
00566 -pf00381 -
00567 -pf05524pf00391pf02896 -
00568 -pf01232pf08125 -
00569 -pf00359 -
BMC-P
BMC-H
BMC-T
Aldehyde dehydrogenase
PTAC
Signature enzyme
Alcohol dehydrogenase
Regulator

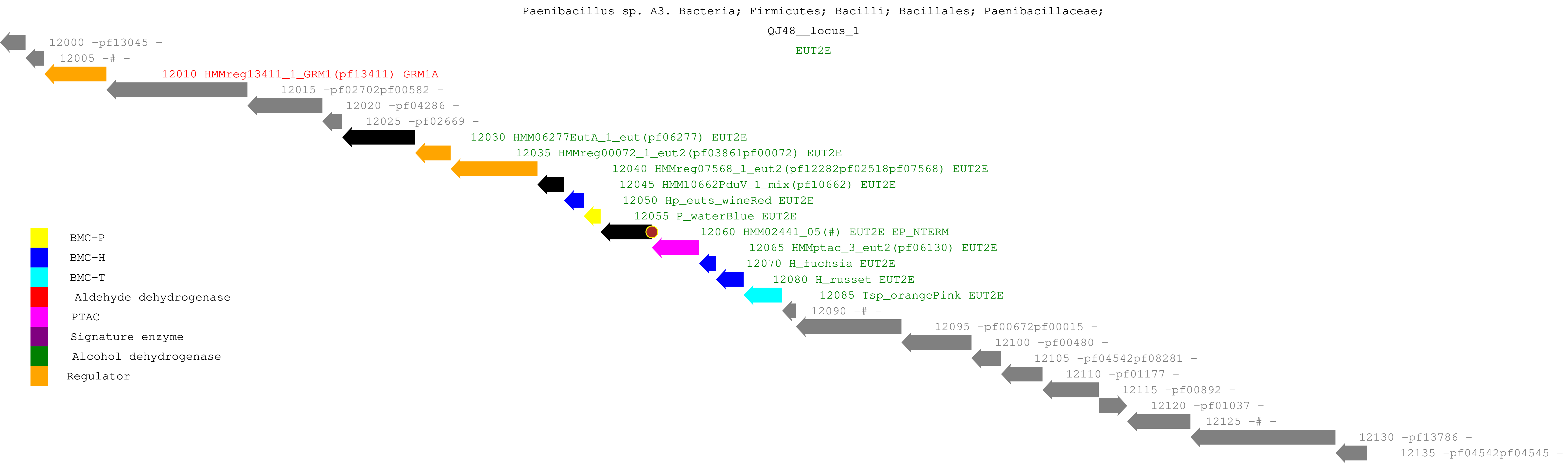

Paenibacillus sp. A3. Bacteria; Firmicutes; Bacilli; Bacillales; Paenibacillaceae;
QJ48__locus_1
EUT2E
12000 -pf13045 -
12005 -# -
12010 HMMreg13411_1_GRM1(pf13411) GRM1A
12015 -pf02702pf00582 -
12020 -pf04286 -
12025 -pf02669 -
12030 HMM06277EutA_1_eut(pf06277) EUT2E
12035 HMMreg00072_1_eut2(pf03861pf00072) EUT2E
12040 HMMreg07568_1_eut2(pf12282pf02518pf07568) EUT2E
12045 HMM10662PduV_1_mix(pf10662) EUT2E
12050 Hp_euts_wineRed EUT2E
12055 P_waterBlue EUT2E
12060 HMM02441_05(#) EUT2E EP_NTERM
12065 HMMptac_3_eut2(pf06130) EUT2E
12070 H_fuchsia EUT2E
12080 H_russet EUT2E
12085 Tsp_orangePink EUT2E
12090 -# -
12095 -pf00672pf00015 -
12100 -pf00480 -
12105 -pf04542pf08281 -
12110 -pf01177 -
12115 -pf00892 -
12120 -pf01037 -
12125 -# -
12130 -pf13786 -
12135 -pf04542pf04545 -
BMC-P
BMC-H
BMC-T
Aldehyde dehydrogenase
PTAC
Signature enzyme
Alcohol dehydrogenase
Regulator

Paenibacillus sp. yr247. Bacteria; Firmicutes; Bacilli; Bacillales; Paenibacillaceae;

SAMN04487897__locus_1

EUT2E

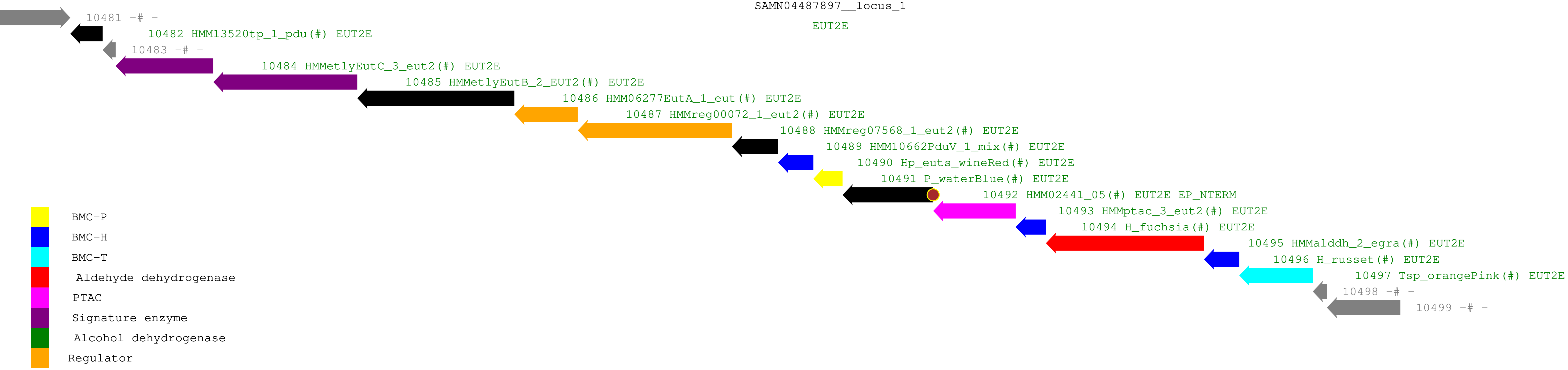

Paenibacillus tianmuensis. Bacteria; Firmicutes; Bacilli; Bacillales; Paenibacillaceae;

SAMN04487970__locus_1

EUT2E

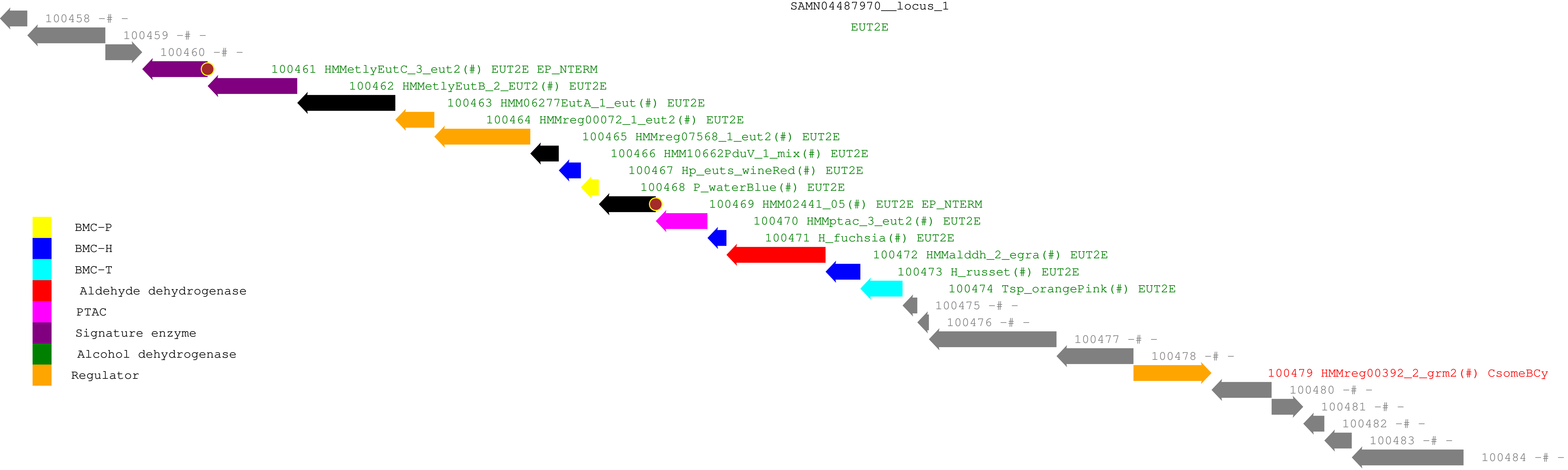

Paenibacillus sp. BC26. Bacteria; Firmicutes; Bacilli; Bacillales; Paenibacillaceae;

SAMN05428962__locus_1

EUT2E

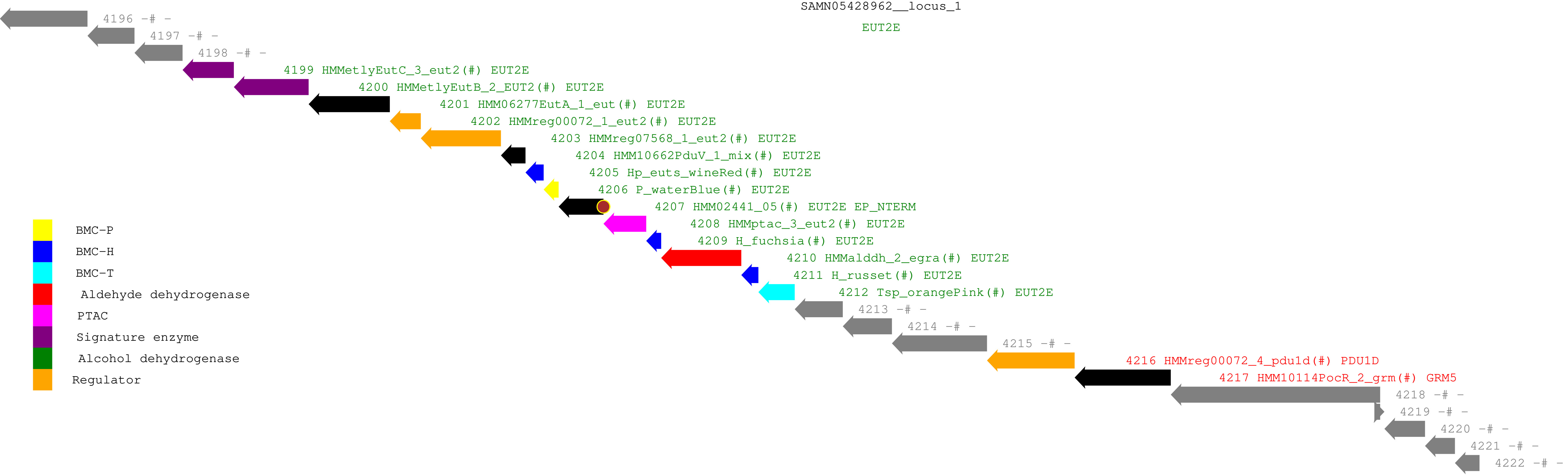

Paenibacillaceae bacterium GAS479. Bacteria; Firmicutes; Bacilli; Bacillales; Paenibacillaceae.

SAMN05444162__locus_1

EUT2E

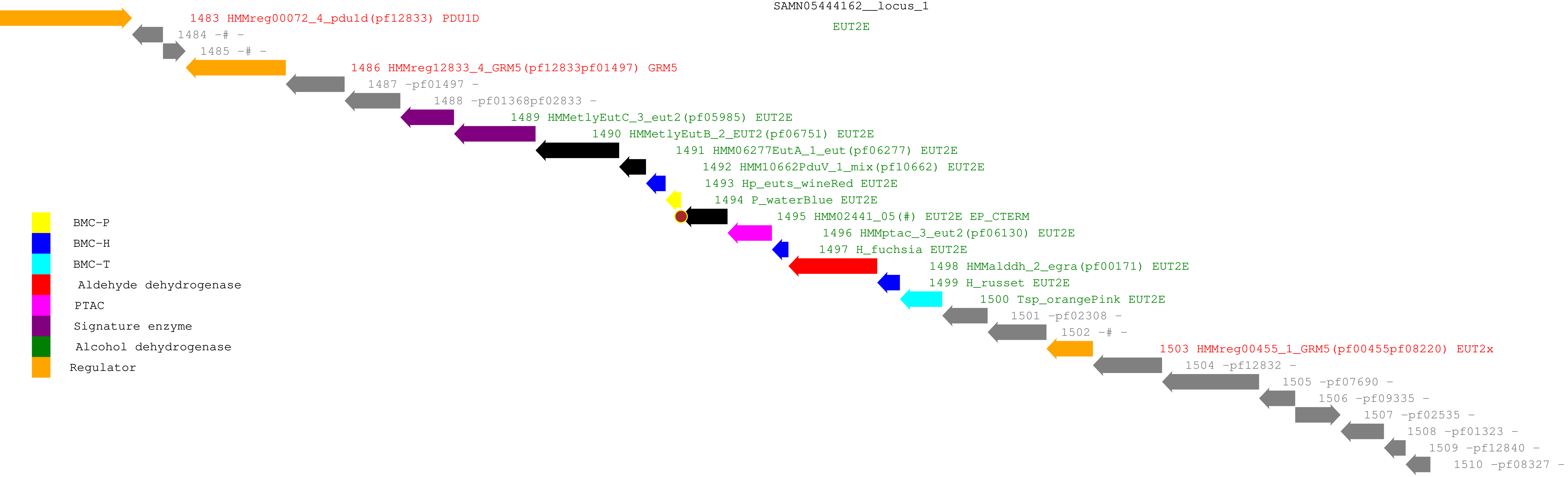

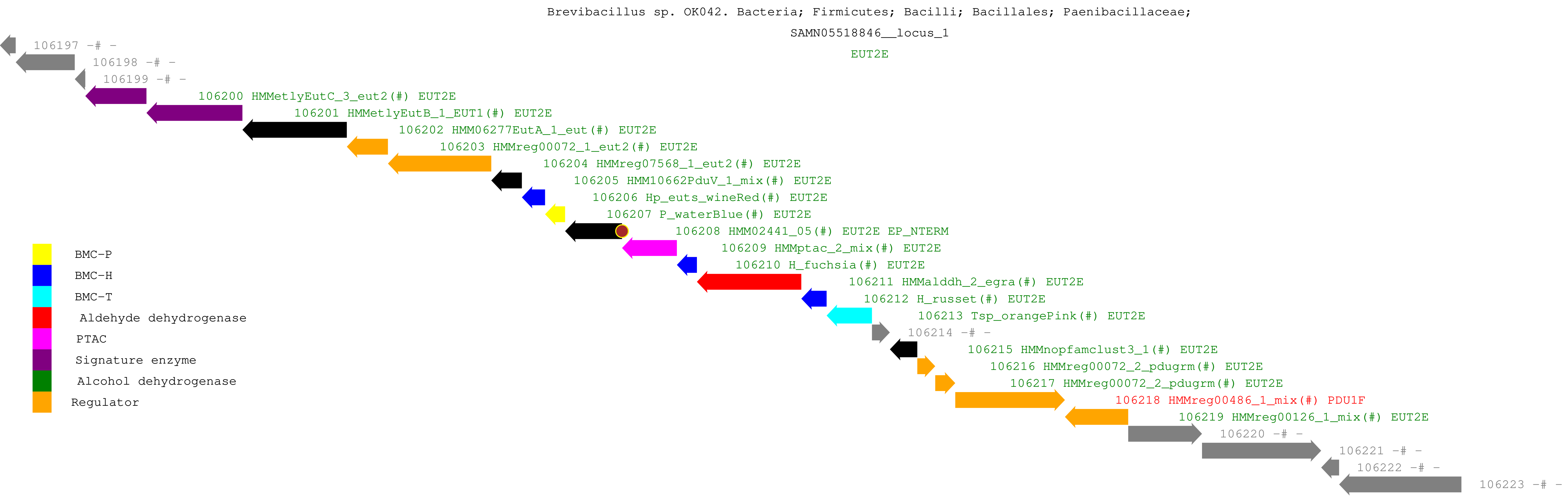
Brevibacillus sp. OK042. Bacteria; Firmicutes; Bacilli; Bacillales; Paenibacillaceae;
SAMN05518846__locus_1
EUT2E
106197 -# -
106198 -# -
106199 -# -
106200 HMMetlyEutC_3_eut2(#) EUT2E
106201 HMMetlyEutB_1_EUT1(#) EUT2E
106202 HMM06277EutA_1_eut(#) EUT2E
106203 HMMreg00072_1_eut2(#) EUT2E
106204 HMMreg07568_1_eut2(#) EUT2E
106205 HMM10662PduV_1_mix(#) EUT2E
106206 Hp_euts_wineRed(#) EUT2E
106207 P_waterBlue(#) EUT2E
106208 HMM02441_05(#) EUT2E EP_NTERM
106209 HMMptac_2_mix(#) EUT2E
106210 H_fuchsia(#) EUT2E
106211 HMMalddh_2_egra(#) EUT2E
106212 H_russet(#) EUT2E
106213 Tsp_orangePink(#) EUT2E
106214 -# -
106215 HMMnopfamclust3_1(#) EUT2E
106216 HMMreg00072_2_pdugrm(#) EUT2E
106217 HMMreg00072_2_pdugrm(#) EUT2E
106218 HMMreg00486_1_mix(#) PDU1F
106219 HMMreg00126_1_mix(#) EUT2E
106220 -# -
106221 -# -
106222 -# -
106223 -# -
BMC-P
BMC-H
BMC-T
Aldehyde dehydrogenase
PTAC
Signature enzyme
Alcohol dehydrogenase
Regulator

Paenibacillus sp. CF384. Bacteria; Firmicutes; Bacilli; Bacillales; Paenibacillaceae;

SAMN05518855__locus_1

EUT2E

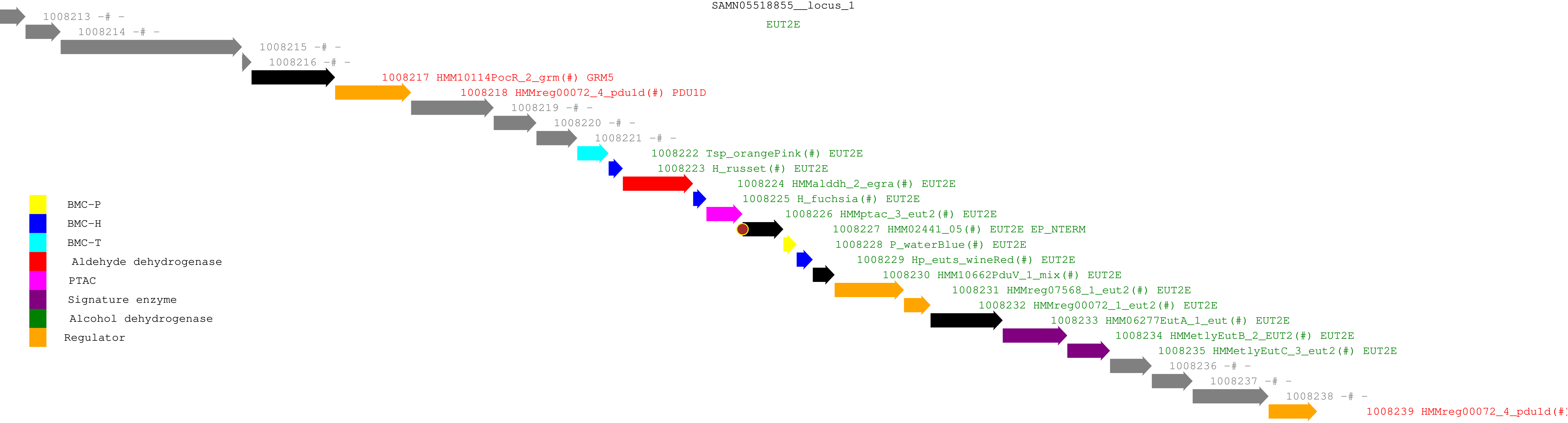

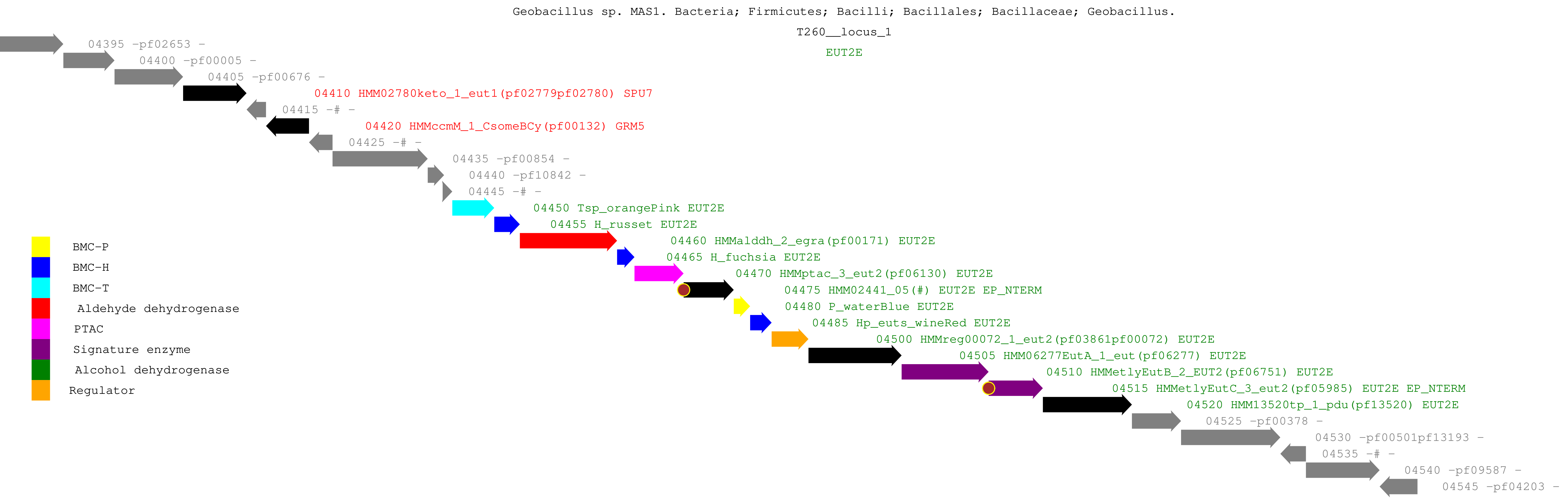

Geobacillus sp. MAS1. Bacteria; Firmicutes; Bacilli; Bacillales; Bacillaceae; Geobacillus.
T260__locus_1
EUT2E
04395 -pf02653 -
04400 -pf00005 -
04405 -pf00676 -
04410 HMM02780keto_1_eut1(pf02779pf02780) SPU7
04415 -# -
04420 HMMccmM_1_CsomeBCy(pf00132) GRM5
04425 -# -
04435 -pf00854 -
04440 -pf10842 -
04445 -# -
04450 Tsp_orangePink EUT2E
04455 H_russet EUT2E
04460 HMMaldh_2_egra(pf00171) EUT2E
04465 H_fuchsia EUT2E
04470 HMMptac_3_eut2(pf06130) EUT2E
04475 HMM02441_05(#) EUT2E EP_NTERM
04480 P_waterBlue EUT2E
04485 Hp_euts_wineRed EUT2E
04500 HMMreg00072_1_eut2(pf03861pf00072) EUT2E
04505 HMM06277EutA_1_eut(pf06277) EUT2E
04510 HMMetlyEutB_2_EUT2(pf06751) EUT2E
04515 HMMetlyEutC_3_eut2(pf05985) EUT2E EP_NTERM
04520 HMM13520tp_1_pdu(pf13520) EUT2E
04525 -pf00378 -
04530 -pf00501pf13193 -
04535 -# -
04540 -pf09587 -
04545 -pf04203 -
BMC-P
BMC-H
BMC-T
Aldehyde dehydrogenase
PTAC
Signature enzyme
Alcohol dehydrogenase
Regulator

Brevibacillus panacihumi W25. Bacteria; Firmicutes; Bacilli; Bacillales; Paenibacillaceae;

T458__locus_1

EUT2E

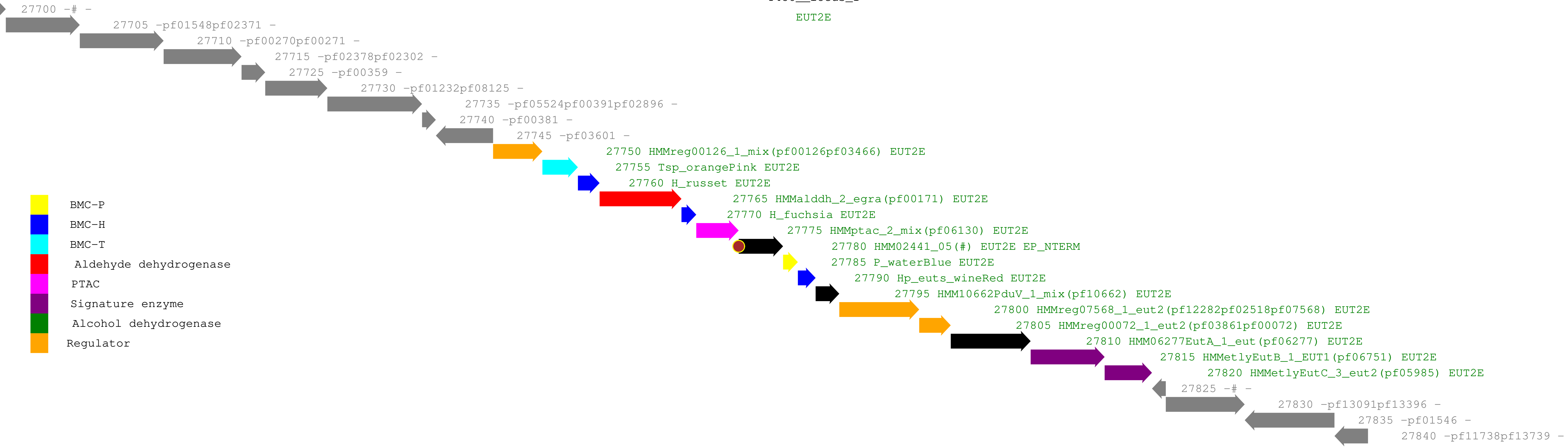

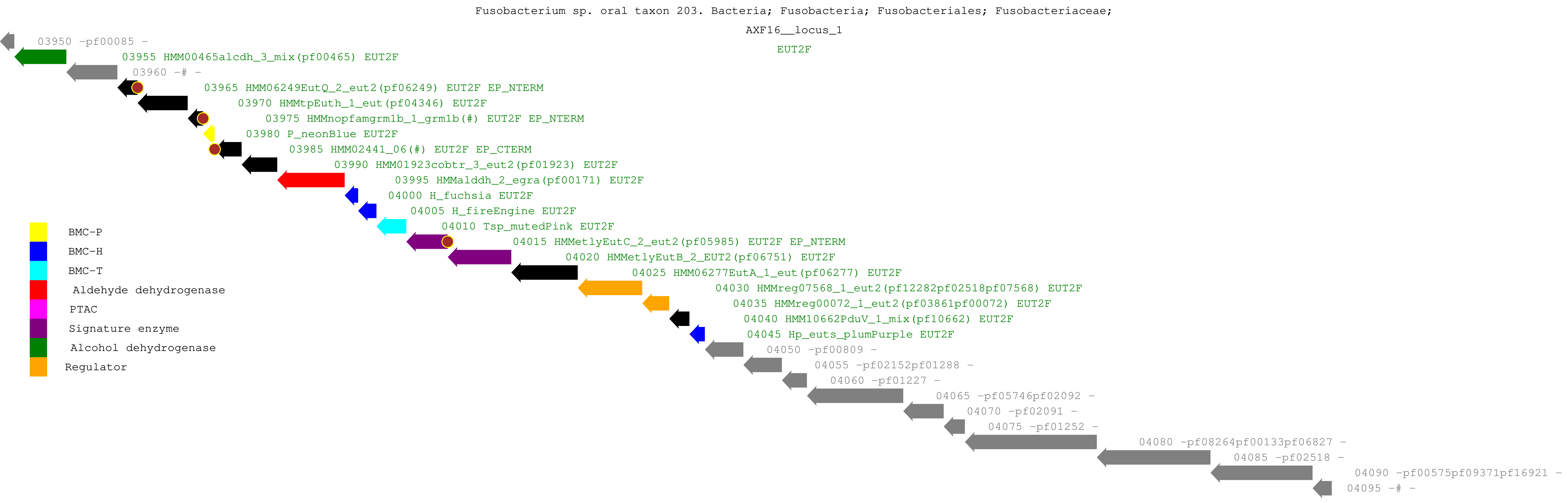
Fusobacterium sp. oral taxon 203. Bacteria; Fusobacteria; Fusobacteriales; Fusobacteriaceae;
AXF16__locus_1
EUT2F
03950 -pf00085 -
03955 HMM00465alcdh_3_mix(pf00465) EUT2F
03960 -# -
03965 HMM06249EutQ_2_eut2(pf06249) EUT2F EP_NTERM
03970 HMMtpEuth_1_eut(pf04346) EUT2F
03975 HMMnopfamgrm1b_1_grm1b(#) EUT2F EP_NTERM
03980 P_neonBlue EUT2F
03985 HMM02441_06(#) EUT2F EP_CTERM
03990 HMM01923cobtr_3_eut2(pf01923) EUT2F
03995 HMMalddh_2_egra(pf00171) EUT2F
04000 H_fuchsia EUT2F
04005 H_fireEngine EUT2F
04010 Tsp_mutedPink EUT2F
04015 HMMetlyEutC_2_eut2(pf05985) EUT2F EP_NTERM
04020 HMMetlyEutB_2_EUT2(pf06751) EUT2F
04025 HMM06277EutA_1_eut(pf06277) EUT2F
04030 HMMreg07568_1_eut2(pf12282pf02518pf07568) EUT2F
04035 HMMreg00072_1_eut2(pf03861pf00072) EUT2F
04040 HMM10662PduV_1_mix(pf10662) EUT2F
04045 Hp_euts_plumPurple EUT2F
04050 -pf00809 -
04055 -pf02152pf01288 -
04060 -pf01227 -
04065 -pf05746pf02092 -
04070 -pf02091 -
04075 -pf01252 -
04080 -pf08264pf00133pf06827 -
04085 -pf02518 -
04090 -pf00575pf09371pf16921 -
04095 -# -
BMC-P
BMC-H
BMC-T
Aldehyde dehydrogenase
PTAC
Signature enzyme
Alcohol dehydrogenase
Regulator

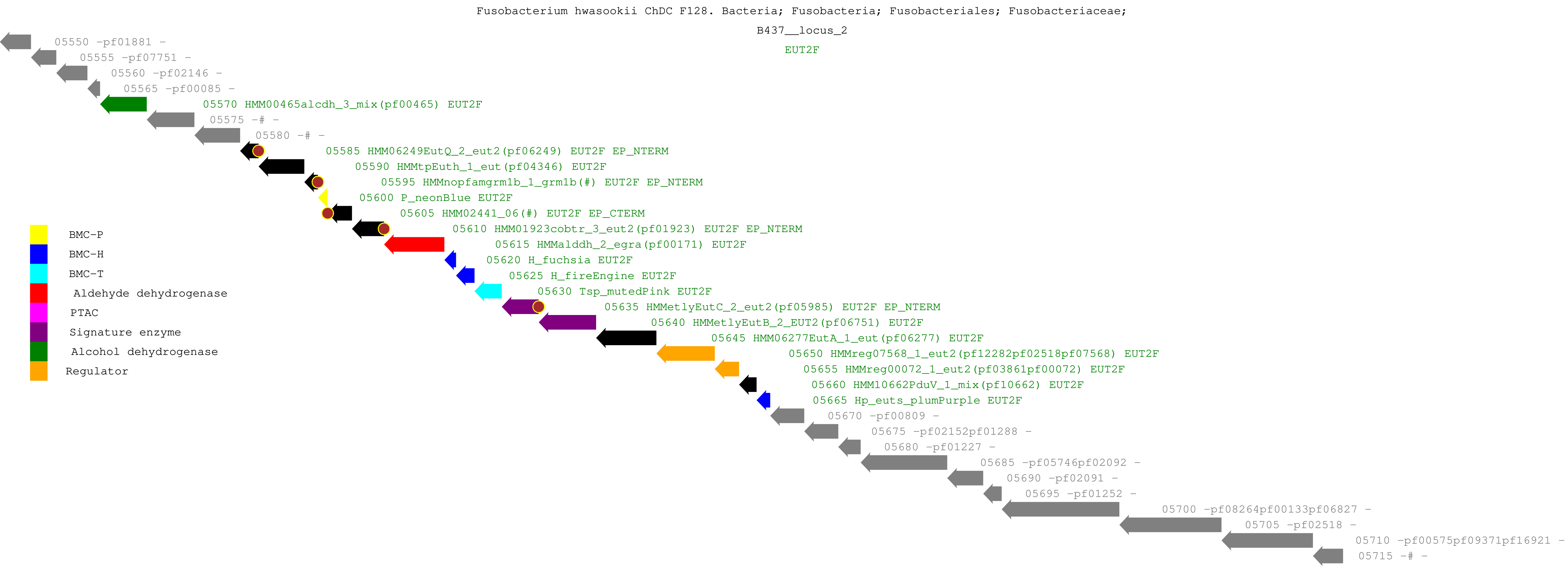

Fusobacterium hwasookii ChDC F128. Bacteria; Fusobacteria; Fusobacteriales; Fusobacteriaceae;
B437__locus_2
EUT2F
05550 -pf01881 -
05555 -pf07751 -
05560 -pf02146 -
05565 -pf00085 -
05570 HMM00465alcdh_3_mix(pf00465) EUT2F
05575 -# -
05580 -# -
05585 HMM06249EutQ_2_eut2(pf06249) EUT2F EP_NTERM
05590 HMMtpEuth_1_eut(pf04346) EUT2F
05595 HMMnopfamgrm1b_1_grm1b(#) EUT2F EP_NTERM
05600 P_neonBlue EUT2F
05605 HMM02441_06(#) EUT2F EP_CTERM
05610 HMM01923cobtr_3_eut2(pf01923) EUT2F EP_NTERM
05615 HMMaldh_2_egra(pf00171) EUT2F
05620 H_fuchsia EUT2F
05625 H_fireEngine EUT2F
05630 Tsp_mutedPink EUT2F
05635 HMMetlyEutC_2_eut2(pf05985) EUT2F EP_NTERM
05640 HMMetlyEutB_2_EUT2(pf06751) EUT2F
05645 HMM06277EutA_1_eut(pf06277) EUT2F
05650 HMMreg07568_1_eut2(pf12282pf02518pf07568) EUT2F
05655 HMMreg00072_1_eut2(pf03861pf00072) EUT2F
05660 HMM10662PduV_1_mix(pf10662) EUT2F
05665 Hp_euts_plumPurple EUT2F
05670 -pf00809 -
05675 -pf02152pf01288 -
05680 -pf01227 -
05685 -pf05746pf02092 -
05690 -pf02091 -
05695 -pf01252 -
05700 -pf08264pf00133pf06827 -
05705 -pf02518 -
05710 -pf00575pf09371pf16921 -
05715 -# -
BMC-P
BMC-H
BMC-T
Aldehyde dehydrogenase
PTAC
Signature enzyme
Alcohol dehydrogenase
Regulator

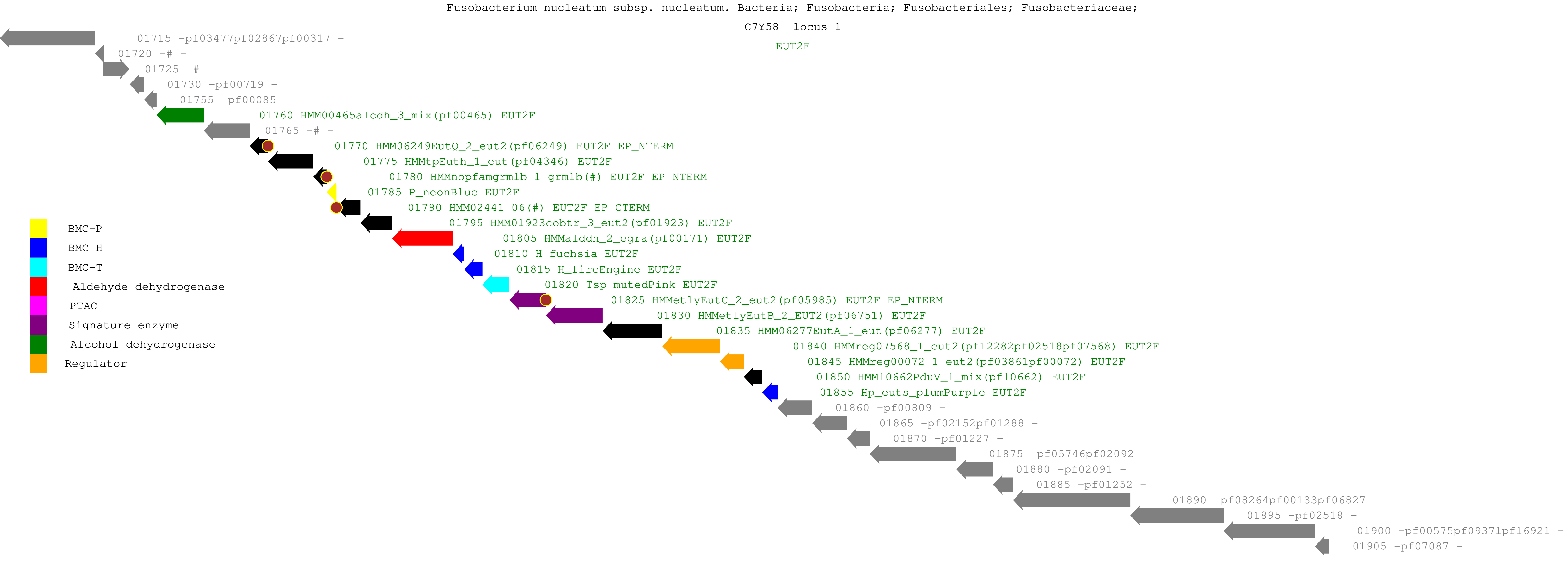
Fusobacterium nucleatum subsp. nucleatum. Bacteria; Fusobacteria; Fusobacteriales; Fusobacteriaceae;
C7Y58__locus_1
EUT2F
01715 -pf03477pf02867pf00317 -
01720 -# -
01725 -# -
01730 -pf00719 -
01755 -pf00085 -
01760 HMM00465alcdh_3_mix(pf00465) EUT2F
01765 -# -
01770 HMM06249EutQ_2_eut2(pf06249) EUT2F EP_NTERM
01775 HMMtpEuth_1_eut(pf04346) EUT2F
01780 HMMnopfamgrm1b_1_grm1b(#) EUT2F EP_NTERM
01785 P_neonBlue EUT2F
01790 HMM02441_06(#) EUT2F EP_CTERM
01795 HMM01923cobtr_3_eut2(pf01923) EUT2F
01805 HMMalddh_2_egra(pf00171) EUT2F
01810 H_fuchsia EUT2F
01815 H_fireEngine EUT2F
01820 Tsp_mutedPink EUT2F
01825 HMMetlyEutC_2_eut2(pf05985) EUT2F EP_NTERM
01830 HMMetlyEutB_2_EUT2(pf06751) EUT2F
01835 HMM06277EutA_1_eut(pf06277) EUT2F
01840 HMMreg07568_1_eut2(pf12282pf02518pf07568) EUT2F
01845 HMMreg00072_1_eut2(pf03861pf00072) EUT2F
01850 HMM10662PduV_1_mix(pf10662) EUT2F
01855 Hp_euts_plumPurple EUT2F
01860 -pf00809 -
01865 -pf02152pf01288 -
01870 -pf01227 -
01875 -pf05746pf02092 -
01880 -pf02091 -
01885 -pf01252 -
01890 -pf08264pf00133pf06827 -
01895 -pf02518 -
01900 -pf00575pf09371pf16921 -
01905 -pf07087 -
BMC-P
BMC-H
BMC-T
Aldehyde dehydrogenase
PTAC
Signature enzyme
Alcohol dehydrogenase
Regulator

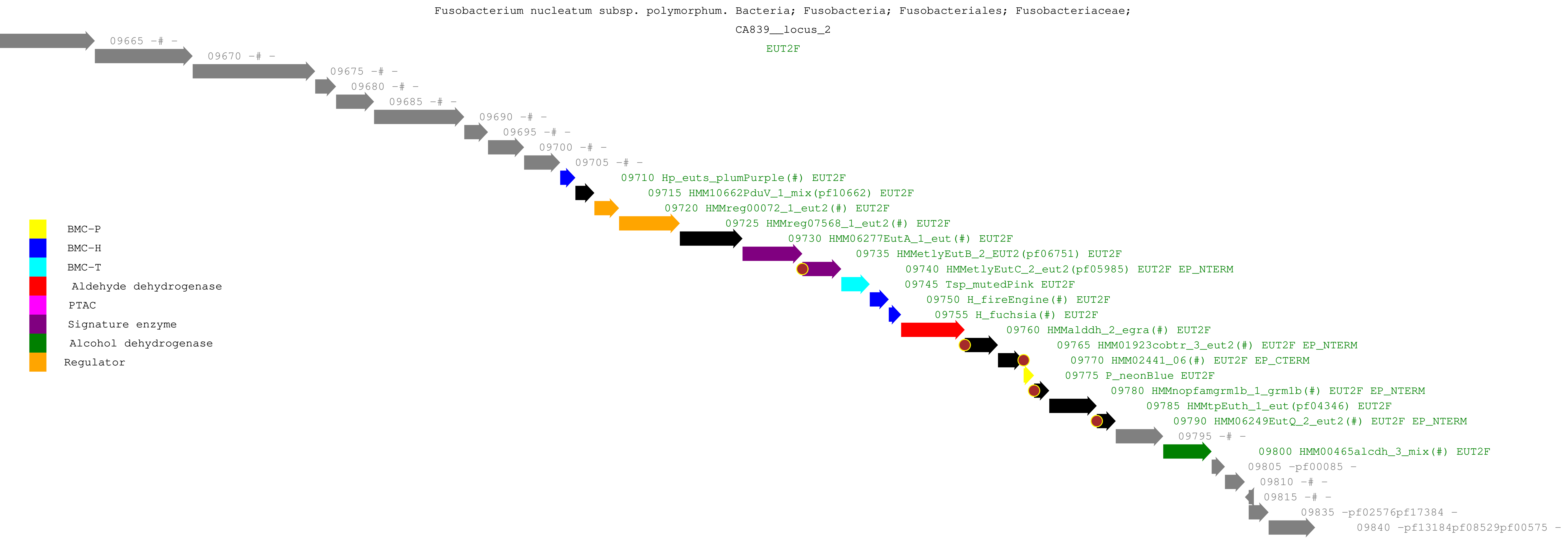
Fusobacterium nucleatum subsp. polymorphum. Bacteria; Fusobacteria; Fusobacteriales; Fusobacteriaceae;
CA839__locus_2
EUT2F
09665 -# -
09670 -# -
09675 -# -
09680 -# -
09685 -# -
09690 -# -
09695 -# -
09700 -# -
09705 -# -
09710 Hp_euts_plumPurple(#) EUT2F
09715 HMM10662PduV_1_mix(pf10662) EUT2F
09720 HMMreg00072_1_eut2(#) EUT2F
09725 HMMreg07568_1_eut2(#) EUT2F
09730 HMM06277EutA_1_eut(#) EUT2F
09735 HMMetlyEutB_2_EUT2(pf06751) EUT2F
09740 HMMetlyEutC_2_eut2(pf05985) EUT2F EP_NTERM
09745 Tsp_mutedPink EUT2F
09750 H_fireEngine(#) EUT2F
09755 H_fuchsia(#) EUT2F
09760 HMMalddh_2_egra(#) EUT2F
09765 HMM01923cobtr_3_eut2(#) EUT2F EP_NTERM
09770 HMM02441_06(#) EUT2F EP_CTERM
09775 P_neonBlue EUT2F
09780 HMMnopfamgrm1b_1_grm1b(#) EUT2F EP_NTERM
09785 HMMtpEuth_1_eut(pf04346) EUT2F
09790 HMM06249EutQ_2_eut2(#) EUT2F EP_NTERM
09795 -# -
09800 HMM00465alcdh_3_mix(#) EUT2F
09805 -pf00085 -
09810 -# -
09815 -# -
09835 -pf02576pf17384 -
09840 -pf13184pf08529pf00575 -
BMC-P
BMC-H
BMC-T
Aldehyde dehydrogenase
PTAC
Signature enzyme
Alcohol dehydrogenase
Regulator

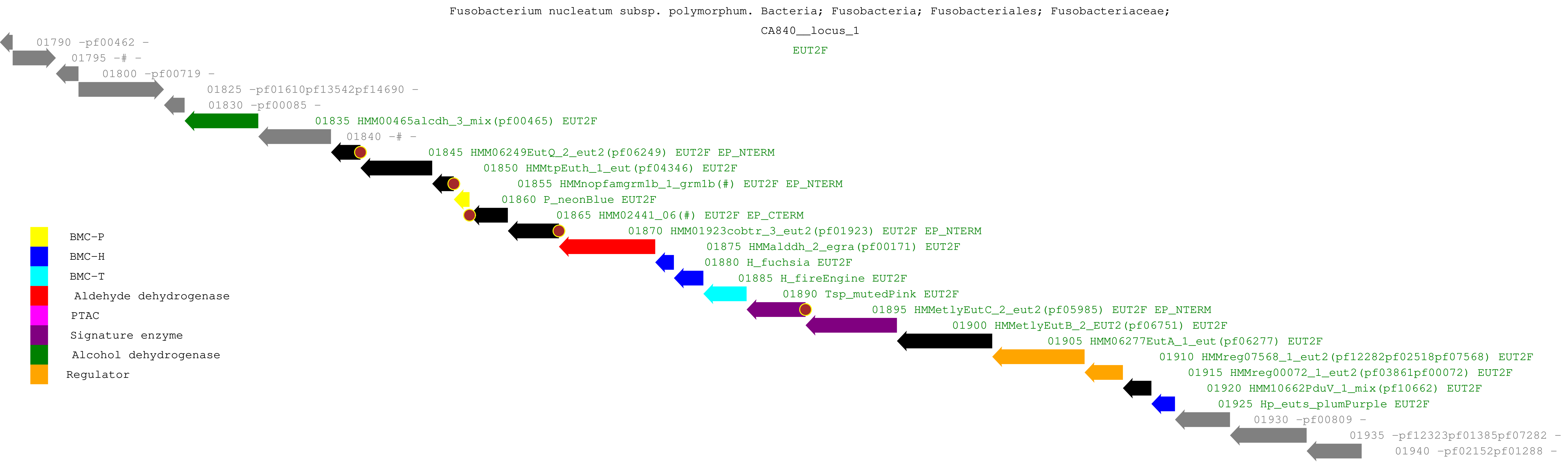

Fusobacterium nucleatum subsp. polymorphum. Bacteria; Fusobacteria; Fusobacteriales; Fusobacteriaceae;
CA840__locus_1
EUT2F
01790 -pf00462 -
01795 -# -
01800 -pf00719 -
01825 -pf01610pf13542pf14690 -
01830 -pf00085 -
01835 HMM00465alcdh_3_mix(pf00465) EUT2F
01840 -# -
01845 HMM06249EutQ_2_eut2(pf06249) EUT2F EP_NTERM
01850 HMMtpEuth_1_eut(pf04346) EUT2F
01855 HMMnopfamgrm1b_1_grm1b(#) EUT2F EP_NTERM
01860 P_neonBlue EUT2F
01865 HMM02441_06(#) EUT2F EP_CTERM
01870 HMM01923cobtr_3_eut2(pf01923) EUT2F EP_NTERM
01875 HMMalddh_2_egra(pf00171) EUT2F
01880 H_fuchsia EUT2F
01885 H_fireEngine EUT2F
01890 Tsp_mutedPink EUT2F
01895 HMMetlyEutC_2_eut2(pf05985) EUT2F EP_NTERM
01900 HMMetlyEutB_2_EUT2(pf06751) EUT2F
01905 HMM06277EutA_1_eut(pf06277) EUT2F
01910 HMMreg07568_1_eut2(pf12282pf02518pf07568) EUT2F
01915 HMMreg00072_1_eut2(pf03861pf00072) EUT2F
01920 HMM10662PduV_1_mix(pf10662) EUT2F
01925 Hp_euts_plumPurple EUT2F
01930 -pf00809 -
01935 -pf12323pf01385pf07282 -
01940 -pf02152pf01288 -
BMC-P
BMC-H
BMC-T
Aldehyde dehydrogenase
PTAC
Signature enzyme
Alcohol dehydrogenase
Regulator

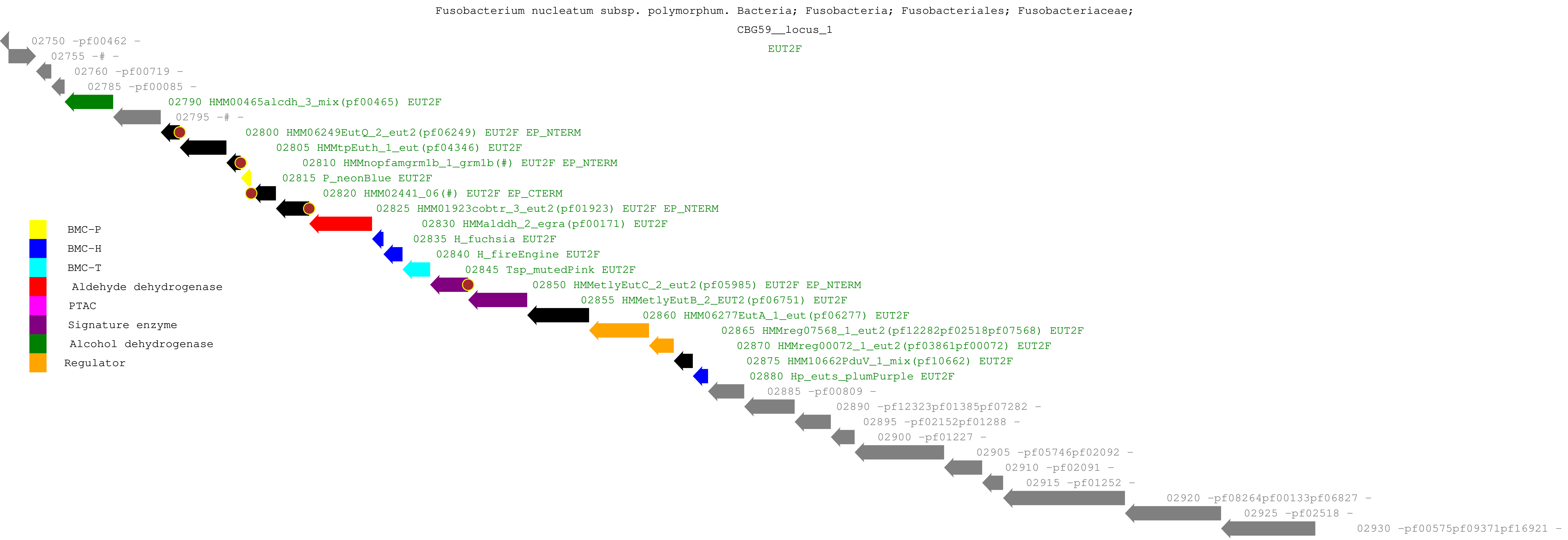

Fusobacterium nucleatum subsp. polymorphum. Bacteria; Fusobacteria; Fusobacteriales; Fusobacteriaceae;
CBG59__locus_1
EUT2F
02750 -pf00462 -
02755 -# -
02760 -pf00719 -
02785 -pf00085 -
02790 HMM00465alcdh_3_mix(pf00465) EUT2F
02795 -# -
02800 HMM06249EutQ_2_eut2(pf06249) EUT2F EP_NTERM
02805 HMMtpEuth_1_eut(pf04346) EUT2F
02810 HMMnopfamgrm1b_1_grm1b(#) EUT2F EP_NTERM
02815 P_neonBlue EUT2F
02820 HMM02441_06(#) EUT2F EP_CTERM
02825 HMM01923cobtr_3_eut2(pf01923) EUT2F EP_NTERM
02830 HMMalddh_2_egra(pf00171) EUT2F
02835 H_fuchsia EUT2F
02840 H_fireEngine EUT2F
02845 Tsp_mutedPink EUT2F
02850 HMMetlyEutC_2_eut2(pf05985) EUT2F EP_NTERM
02855 HMMetlyEutB_2_EUT2(pf06751) EUT2F
02860 HMM06277EutA_1_eut(pf06277) EUT2F
02865 HMMreg07568_1_eut2(pf12282pf02518pf07568) EUT2F
02870 HMMreg00072_1_eut2(pf03861pf00072) EUT2F
02875 HMM10662PduV_1_mix(pf10662) EUT2F
02880 Hp_euts_plumPurple EUT2F
02885 -pf00809 -
02890 -pf12323pf01385pf07282 -
02895 -pf02152pf01288 -
02900 -pf01227 -
02905 -pf05746pf02092 -
02910 -pf02091 -
02915 -pf01252 -
02920 -pf08264pf00133pf06827 -
02925 -pf02518 -
02930 -pf00575pf09371pf16921 -
BMC-P
BMC-H
BMC-T
Aldehyde dehydrogenase
PTAC
Signature enzyme
Alcohol dehydrogenase
Regulator

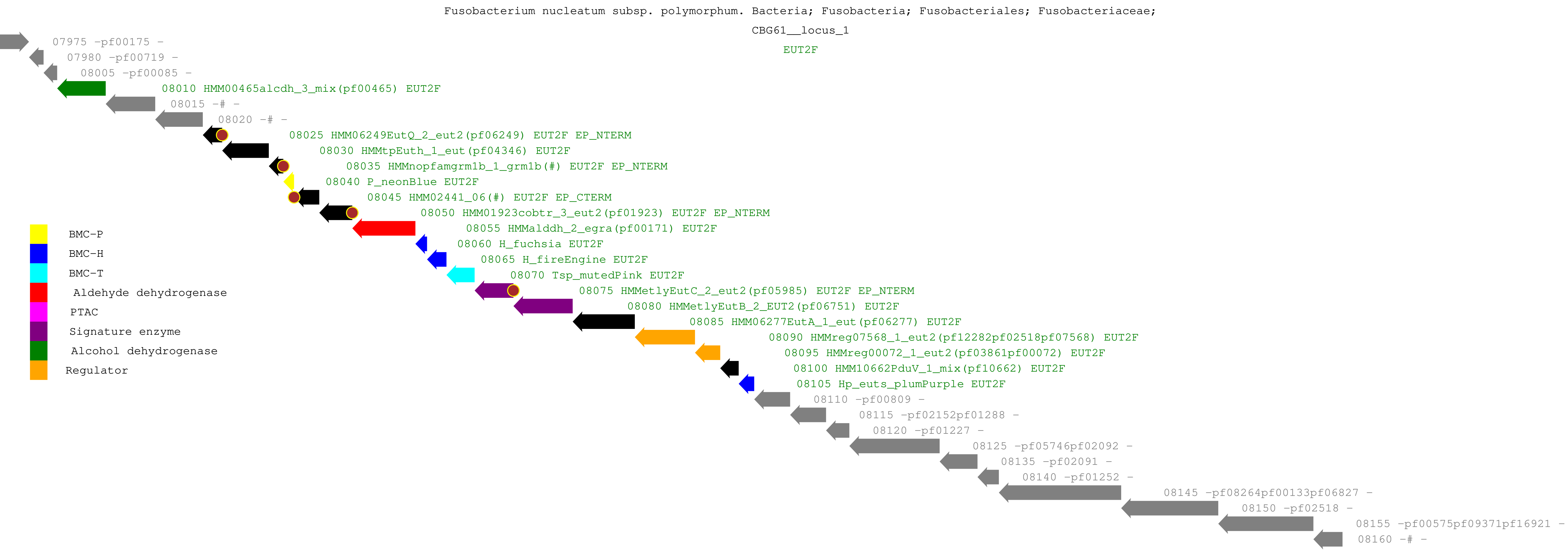
Fusobacterium nucleatum subsp. polymorphum. Bacteria; Fusobacteria; Fusobacteriales; Fusobacteriaceae;
CBG61__locus_1
EUT2F
07975 -pf00175 -
07980 -pf00719 -
08005 -pf00085 -
08010 HMM00465alcdh_3_mix(pf00465) EUT2F
08015 -# -
08020 -# -
08025 HMM06249EutQ_2_eut2(pf06249) EUT2F EP_NTERM
08030 HMMtpEuth_1_eut(pf04346) EUT2F
08035 HMMnopfamgrm1b_1_grm1b(#) EUT2F EP_NTERM
08040 P_neonBlue EUT2F
08045 HMM02441_06(#) EUT2F EP_CTERM
08050 HMM01923cobtr_3_eut2(pf01923) EUT2F EP_NTERM
08055 HMMalddh_2_egra(pf00171) EUT2F
08060 H_fuchsia EUT2F
08065 H_fireEngine EUT2F
08070 Tsp_mutedPink EUT2F
08075 HMMetlyEutC_2_eut2(pf05985) EUT2F EP_NTERM
08080 HMMetlyEutB_2_EUT2(pf06751) EUT2F
08085 HMM06277EutA_1_eut(pf06277) EUT2F
08090 HMMreg07568_1_eut2(pf12282pf02518pf07568) EUT2F
08095 HMMreg00072_1_eut2(pf03861pf00072) EUT2F
08100 HMM10662PduV_1_mix(pf10662) EUT2F
08105 Hp_euts_plumPurple EUT2F
08110 -pf00809 -
08115 -pf02152pf01288 -
08120 -pf01227 -
08125 -pf05746pf02092 -
08135 -pf02091 -
08140 -pf01252 -
08145 -pf08264pf00133pf06827 -
08150 -pf02518 -
08155 -pf00575pf09371pf16921 -
08160 -# -
BMC-P
BMC-H
BMC-T
Aldehyde dehydrogenase
PTAC
Signature enzyme
Alcohol dehydrogenase
Regulator

Fusobacterium nucleatum subsp. animalis. Bacteria; Fusobacteria; Fusobacteriales; Fusobacteriaceae;

CI114__locus_2

EUT2F

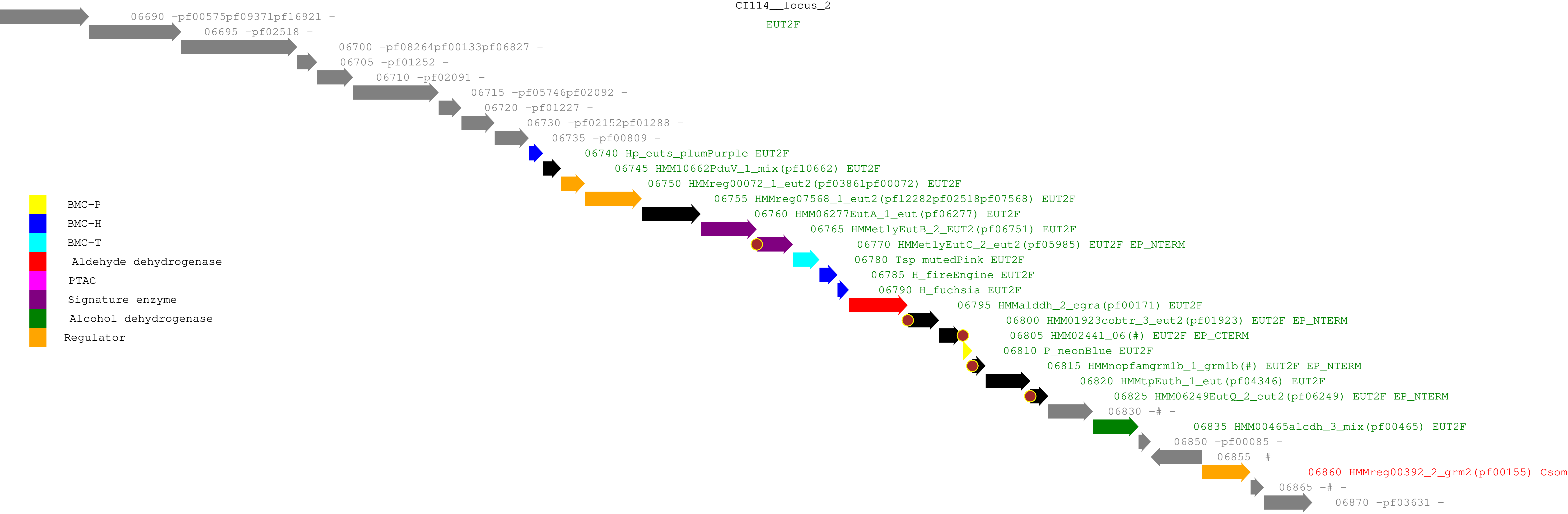

Fusobacterium nucleatum. Bacteria; Fusobacteria; Fusobacteriales; Fusobacteriaceae;

CJ209__locus_2

EUT2F

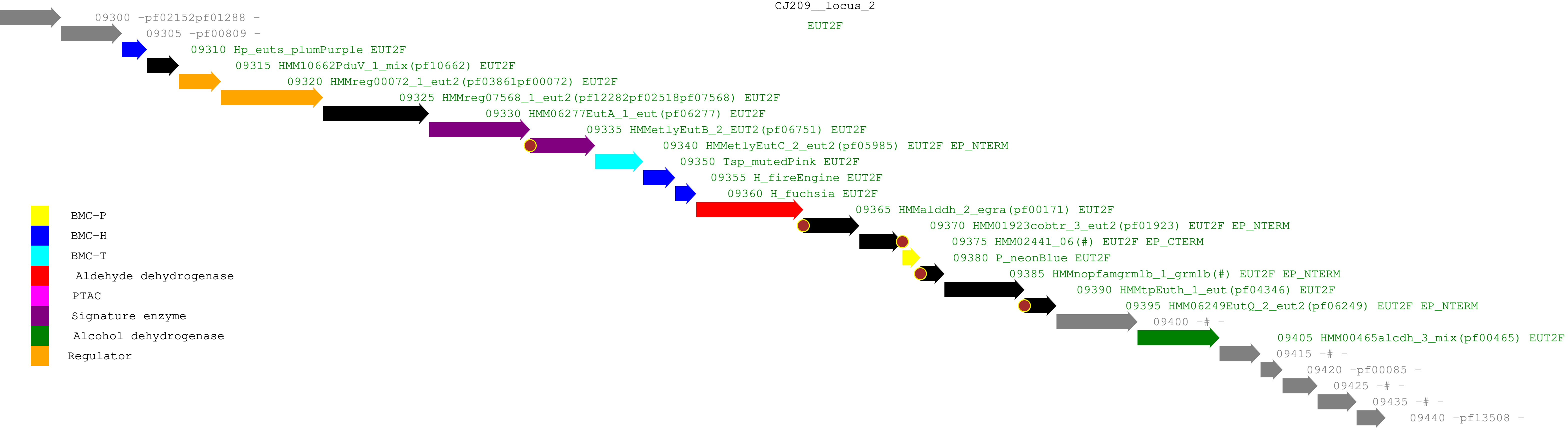

Fusobacterium nucleatum. Bacteria; Fusobacteria; Fusobacteriales; Fusobacteriaceae;

CQA79__locus_2

EUT2F

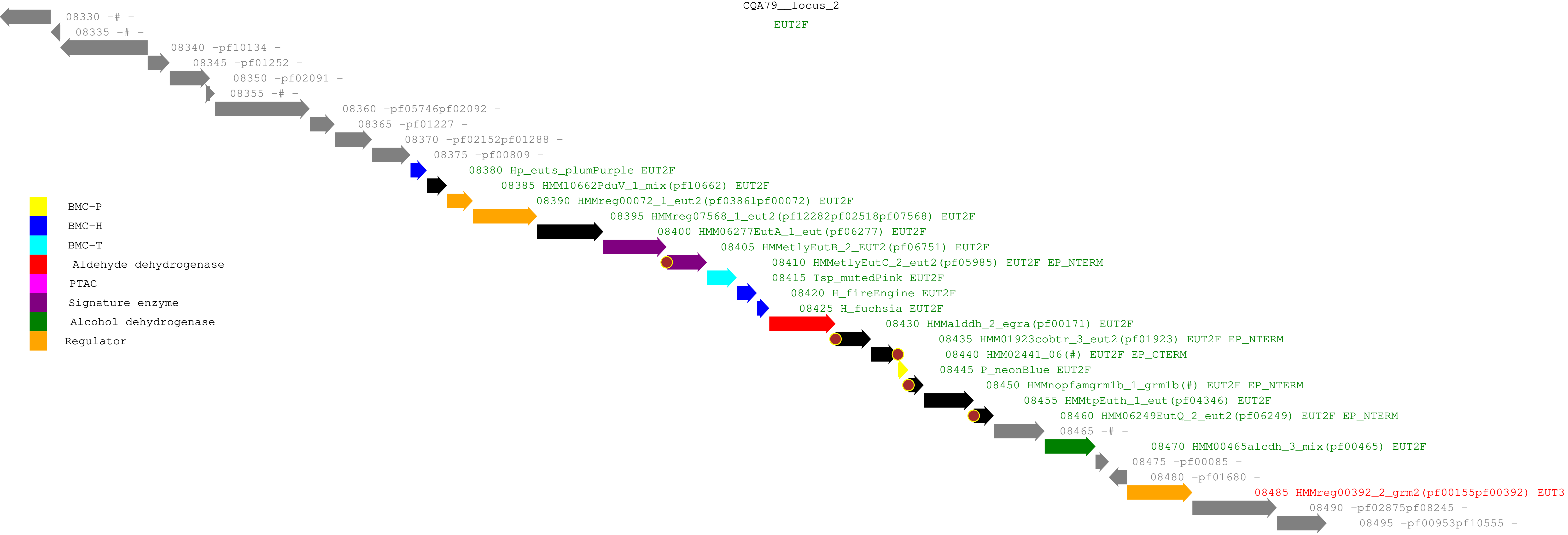

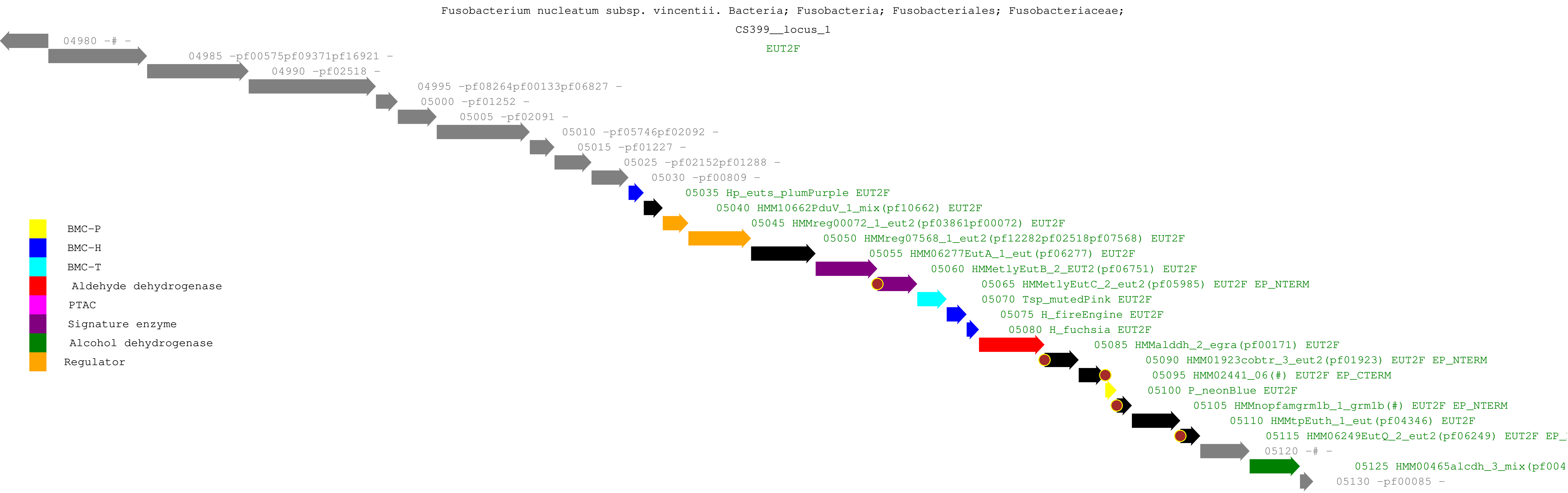

Fusobacterium nucleatum subsp. vincentii. Bacteria; Fusobacteria; Fusobacteriales; Fusobacteriaceae;
CS399__locus_1
EUT2F
04980 -# -
04985 -pf00575pf09371pf16921 -
04990 -pf02518 -
04995 -pf08264pf00133pf06827 -
05000 -pf01252 -
05005 -pf02091 -
05010 -pf05746pf02092 -
05015 -pf01227 -
05025 -pf02152pf01288 -
05030 -pf00809 -
05035 Hp_euts_plumPurple EUT2F
05040 HMM10662PduV_1_mix(pf10662) EUT2F
05045 HMMreg00072_1_eut2(pf03861pf00072) EUT2F
05050 HMMreg07568_1_eut2(pf12282pf02518pf07568) EUT2F
05055 HMM06277EutA_1_eut(pf06277) EUT2F
05060 HMMetlyEutB_2_EUT2(pf06751) EUT2F
05065 HMMetlyEutC_2_eut2(pf05985) EUT2F EP_NTERM
05070 Tsp_mutedPink EUT2F
05075 H_fireEngine EUT2F
05080 H_fuchsia EUT2F
05085 HMMaldh_2_egra(pf00171) EUT2F
05090 HMM01923cobtr_3_eut2(pf01923) EUT2F EP_NTERM
05095 HMM02441_06(#) EUT2F EP_CTERM
05100 P_neonBlue EUT2F
05105 HMMnopfamgrm1b_1_grm1b(#) EUT2F EP_NTERM
05110 HMMtpEuth_1_eut(pf04346) EUT2F
05115 HMM06249EutQ_2_eut2(pf06249) EUT2F EP_
05120 -# -
05125 HMM00465alcdh_3_mix(pf004
05130 -pf00085 -
BMC-P
BMC-H
BMC-T
Aldehyde dehydrogenase
PTAC
Signature enzyme
Alcohol dehydrogenase
Regulator

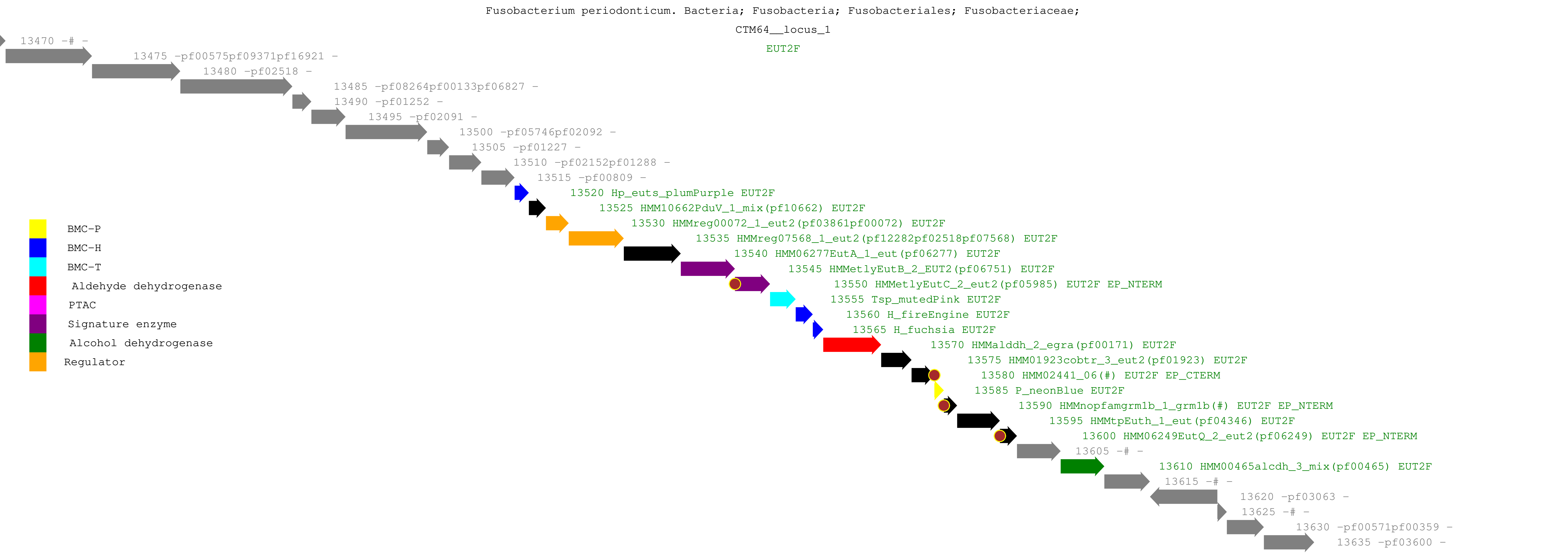

Fusobacterium periodonticum. Bacteria; Fusobacteria; Fusobacteriales; Fusobacteriaceae;
CTM64__locus_1
EUT2F
13470 -# -
13475 -pf00575pf09371pf16921 -
13480 -pf02518 -
13485 -pf08264pf00133pf06827 -
13490 -pf01252 -
13495 -pf02091 -
13500 -pf05746pf02092 -
13505 -pf01227 -
13510 -pf02152pf01288 -
13515 -pf00809 -
13520 Hp_euts_plumPurple EUT2F
13525 HMM10662PduV_1_mix(pf10662) EUT2F
13530 HMMreg00072_1_eut2(pf03861pf00072) EUT2F
13535 HMMreg07568_1_eut2(pf12282pf02518pf07568) EUT2F
13540 HMM06277EutA_1_eut(pf06277) EUT2F
13545 HMMetlyEutB_2_EUT2(pf06751) EUT2F
13550 HMMetlyEutC_2_eut2(pf05985) EUT2F EP_NTERM
13555 Tsp_mutedPink EUT2F
13560 H_fireEngine EUT2F
13565 H_fuchsia EUT2F
13570 HMMalddh_2_egra(pf00171) EUT2F
13575 HMM01923cobtr_3_eut2(pf01923) EUT2F
13580 HMM02441_06(#) EUT2F EP_CTERM
13585 P_neonBlue EUT2F
13590 HMMnopfamgrm1b_1_grm1b(#) EUT2F EP_NTERM
13595 HMMtpEuth_1_eut(pf04346) EUT2F
13600 HMM06249EutQ_2_eut2(pf06249) EUT2F EP_NTERM
13605 -# -
13610 HMM00465alcdh_3_mix(pf00465) EUT2F
13615 -# -
13620 -pf03063 -
13625 -# -
13630 -pf00571pf00359 -
13635 -pf03600 -
BMC-P
BMC-H
BMC-T
Aldehyde dehydrogenase
PTAC
Signature enzyme
Alcohol dehydrogenase
Regulator

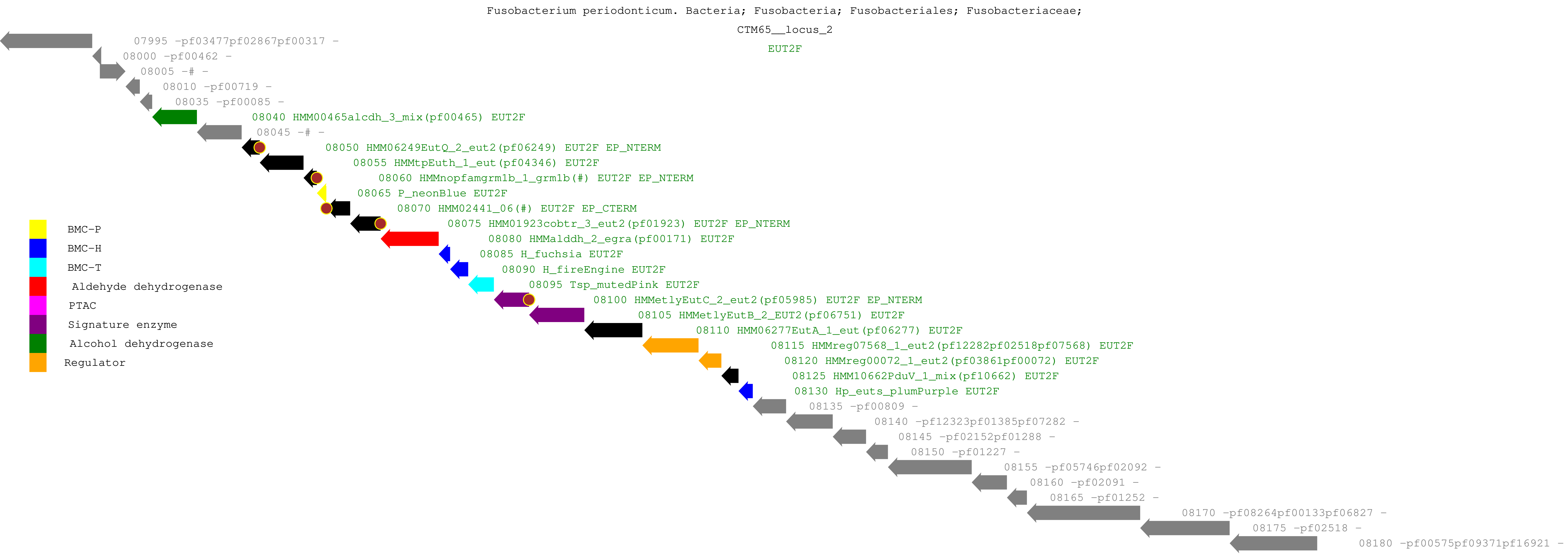
Fusobacterium periodonticum. Bacteria; Fusobacteria; Fusobacteriales; Fusobacteriaceae;
CTM65__locus_2
EUT2F
07995 -pf03477pf02867pf00317 -
08000 -pf00462 -
08005 -# -
08010 -pf00719 -
08035 -pf00085 -
08040 HMM00465alcdh_3_mix(pf00465) EUT2F
08045 -# -
08050 HMM06249EutQ_2_eut2(pf06249) EUT2F EP_NTERM
08055 HMMtpEuth_1_eut(pf04346) EUT2F
08060 HMMnopfamgrm1b_1_grm1b(#) EUT2F EP_NTERM
08065 P_neonBlue EUT2F
08070 HMM02441_06(#) EUT2F EP_CTERM
08075 HMM01923cobtr_3_eut2(pf01923) EUT2F EP_NTERM
08080 HMMalddh_2_egra(pf00171) EUT2F
08085 H_fuchsia EUT2F
08090 H_fireEngine EUT2F
08095 Tsp_mutedPink EUT2F
08100 HMMetlyEutC_2_eut2(pf05985) EUT2F EP_NTERM
08105 HMMetlyEutB_2_EUT2(pf06751) EUT2F
08110 HMM06277EutA_1_eut(pf06277) EUT2F
08115 HMMreg07568_1_eut2(pf12282pf02518pf07568) EUT2F
08120 HMMreg00072_1_eut2(pf03861pf00072) EUT2F
08125 HMM10662PduV_1_mix(pf10662) EUT2F
08130 Hp_euts_plumPurple EUT2F
08135 -pf00809 -
08140 -pf12323pf01385pf07282 -
08145 -pf02152pf01288 -
08150 -pf01227 -
08155 -pf05746pf02092 -
08160 -pf02091 -
08165 -pf01252 -
08170 -pf08264pf00133pf06827 -
08175 -pf02518 -
08180 -pf00575pf09371pf16921 -
BMC-P
BMC-H
BMC-T
Aldehyde dehydrogenase
PTAC
Signature enzyme
Alcohol dehydrogenase
Regulator

Fusobacterium periodonticum. Bacteria; Fusobacteria; Fusobacteriales; Fusobacteriaceae;

CTM69__locus_1

EUT2F

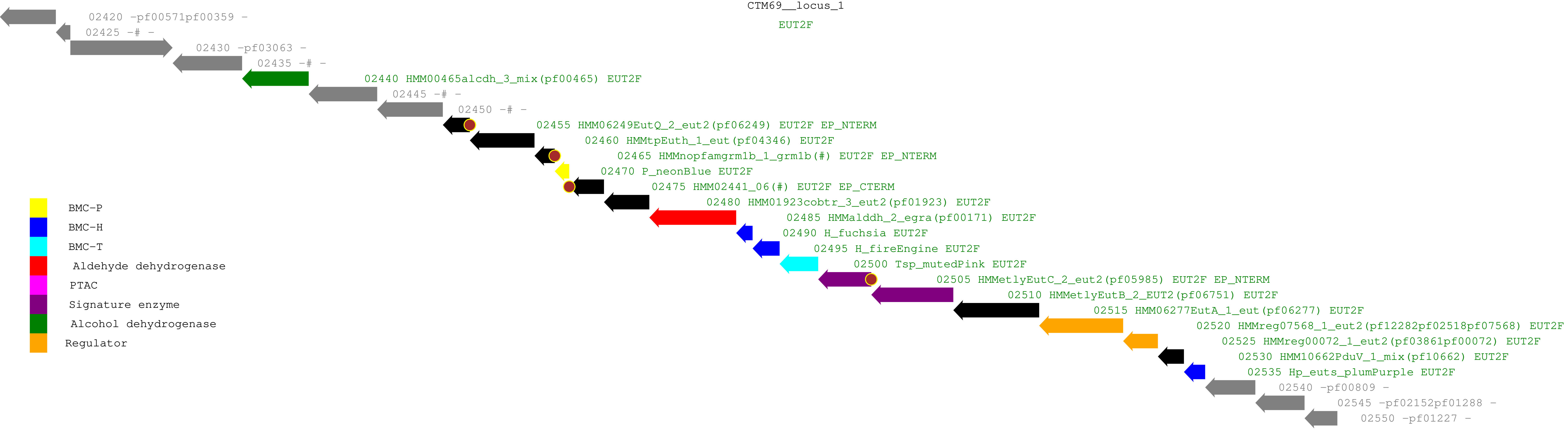

Fusobacterium periodonticum. Bacteria; Fusobacteria; Fusobacteriales; Fusobacteriaceae;

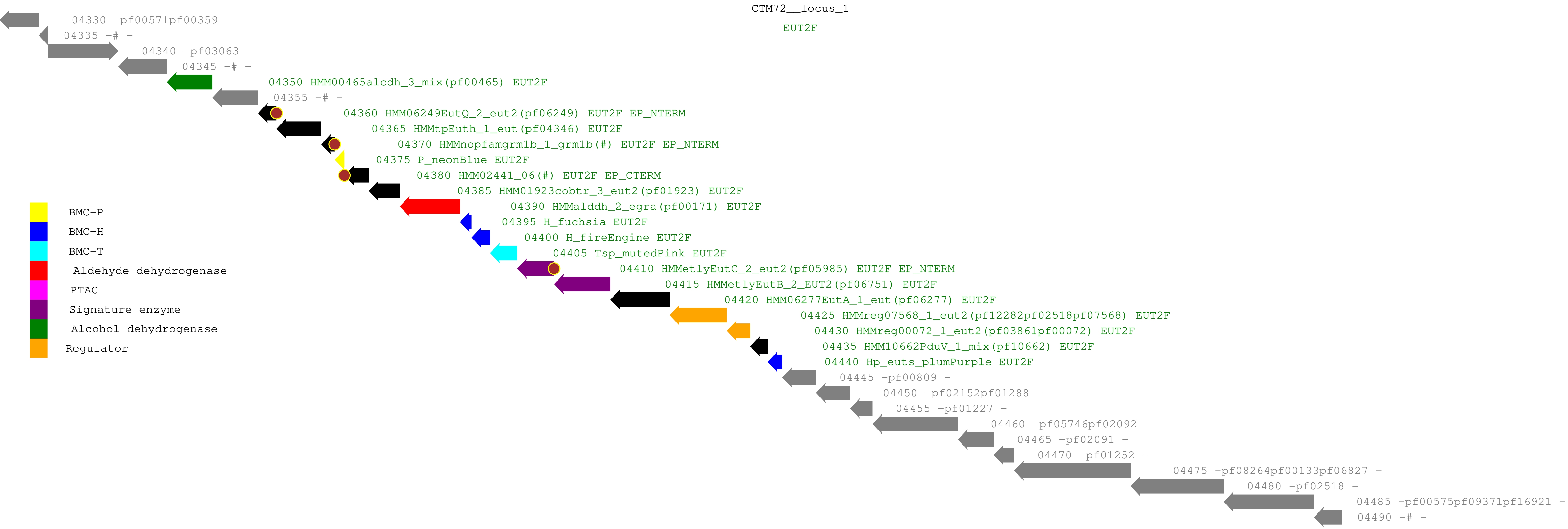

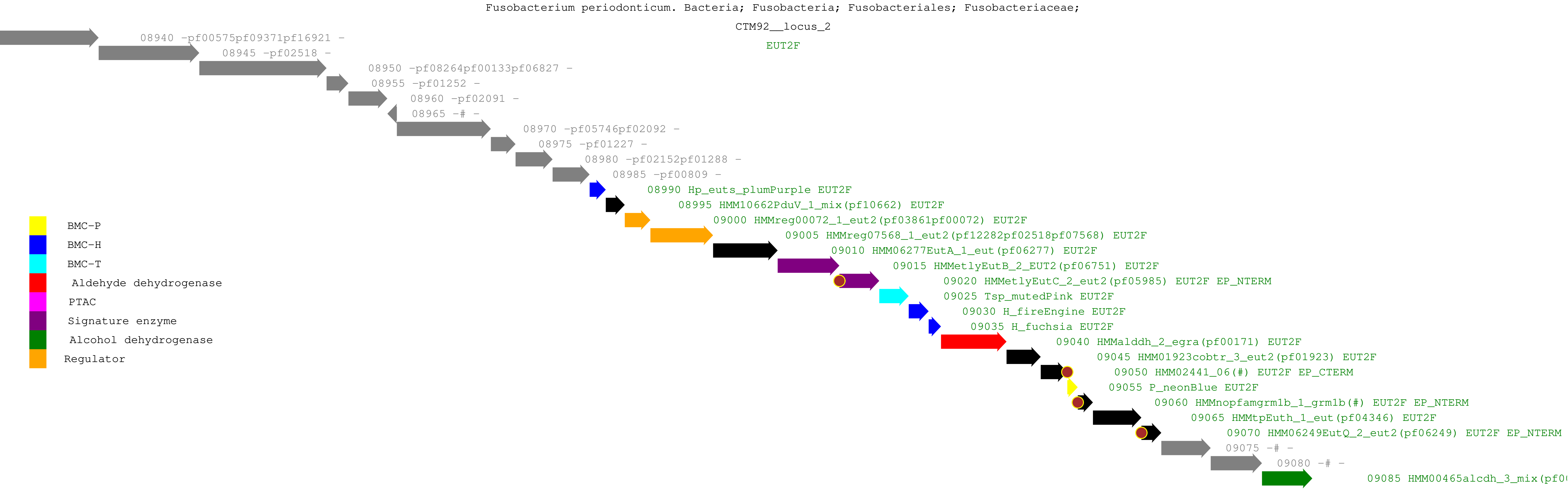

Fusobacterium periodonticum. Bacteria; Fusobacteria; Fusobacteriales; Fusobacteriaceae;
CTM92__locus_2
EUT2F
08940 -pf00575pf09371pf16921 -
08945 -pf02518 -
08950 -pf08264pf00133pf06827 -
08955 -pf01252 -
08960 -pf02091 -
08965 -# -
08970 -pf05746pf02092 -
08975 -pf01227 -
08980 -pf02152pf01288 -
08985 -pf00809 -
08990 Hp_euts_plumPurple EUT2F
08995 HMM10662PduV_1_mix(pf10662) EUT2F
09000 HMMreg00072_1_eut2(pf03861pf00072) EUT2F
09005 HMMreg07568_1_eut2(pf12282pf02518pf07568) EUT2F
09010 HMM06277EutA_1_eut(pf06277) EUT2F
09015 HMMetlyEutB_2_EUT2(pf06751) EUT2F
09020 HMMetlyEutC_2_eut2(pf05985) EUT2F EP_NTERM
09025 Tsp_mutedPink EUT2F
09030 H_fireEngine EUT2F
09035 H_fuchsia EUT2F
09040 HMMalddh_2_egra(pf00171) EUT2F
09045 HMM01923cobtr_3_eut2(pf01923) EUT2F
09050 HMM02441_06(#) EUT2F EP_CTERM
09055 P_neonBlue EUT2F
09060 HMMnopfamgrm1b_1_grm1b(#) EUT2F EP_NTERM
09065 HMMtpEuth_1_eut(pf04346) EUT2F
09070 HMM06249EutQ_2_eut2(pf06249) EUT2F EP_NTERM
09075 -# -
09080 -# -
09085 HMM00465alcdh_3_mix(pf0
BMC-P
BMC-H
BMC-T
Aldehyde dehydrogenase
PTAC
Signature enzyme
Alcohol dehydrogenase
Regulator

Fusobacterium periodonticum. Bacteria; Fusobacteria; Fusobacteriales; Fusobacteriaceae;

CTM98__locus_1

EUT2F

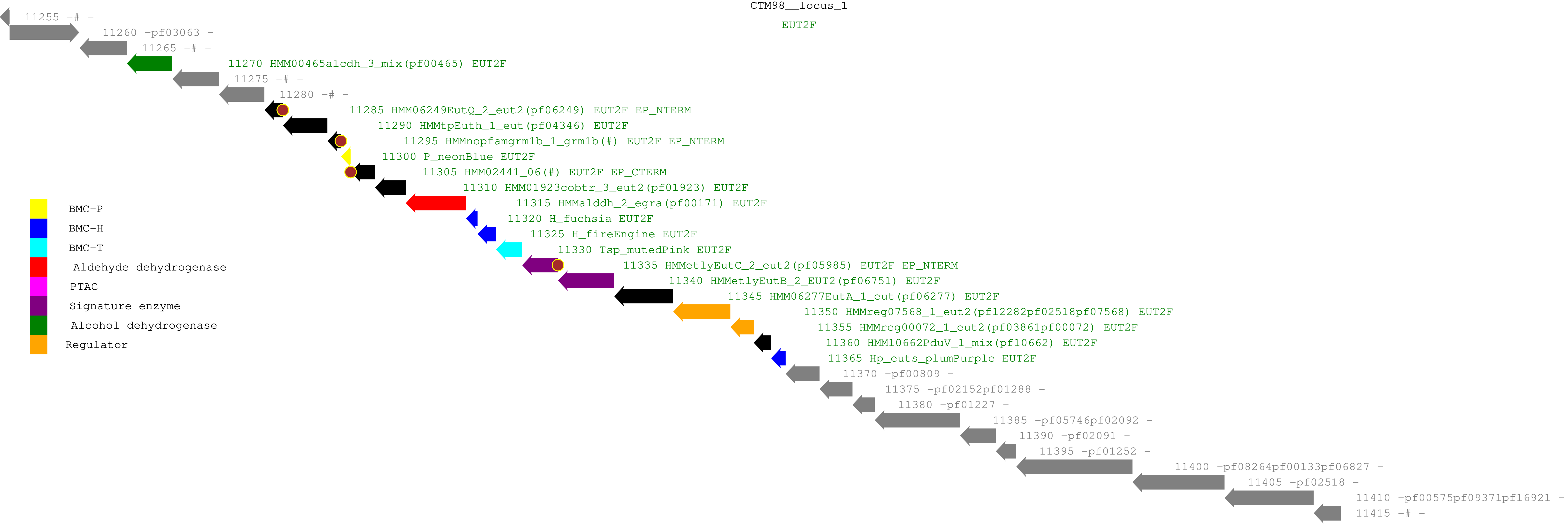

Fusobacterium periodonticum. Bacteria; Fusobacteria; Fusobacteriales; Fusobacteriaceae;

CTN00__locus_1

EUT2F

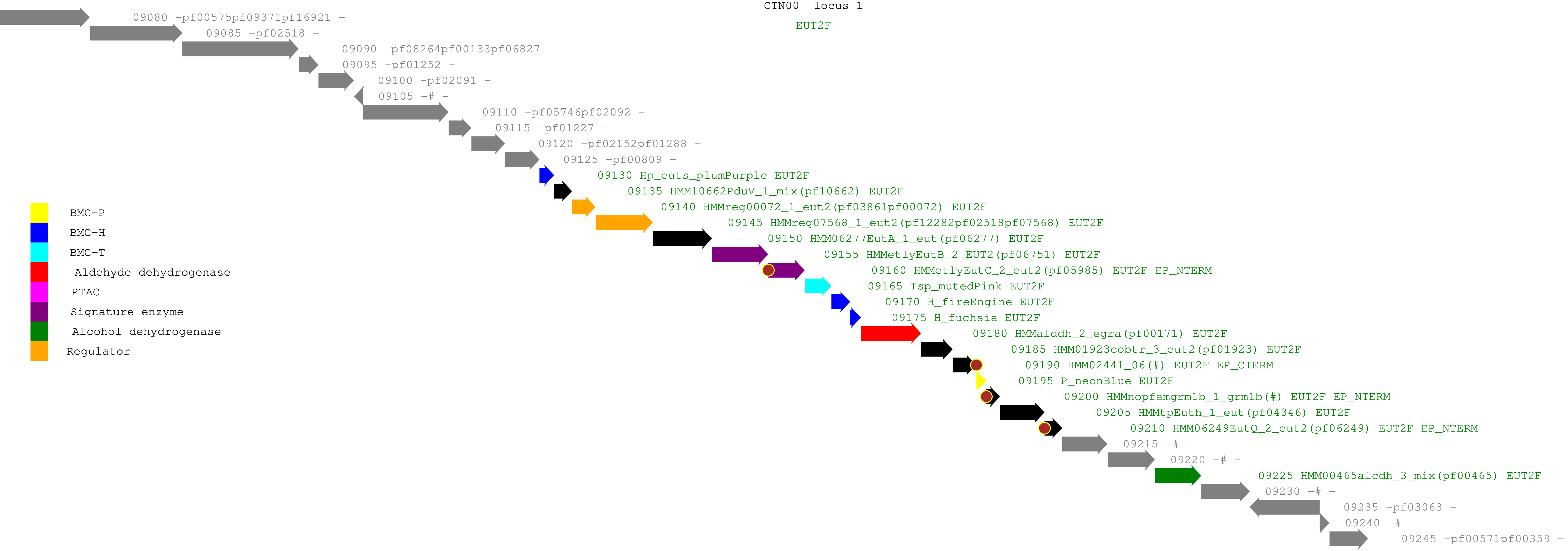

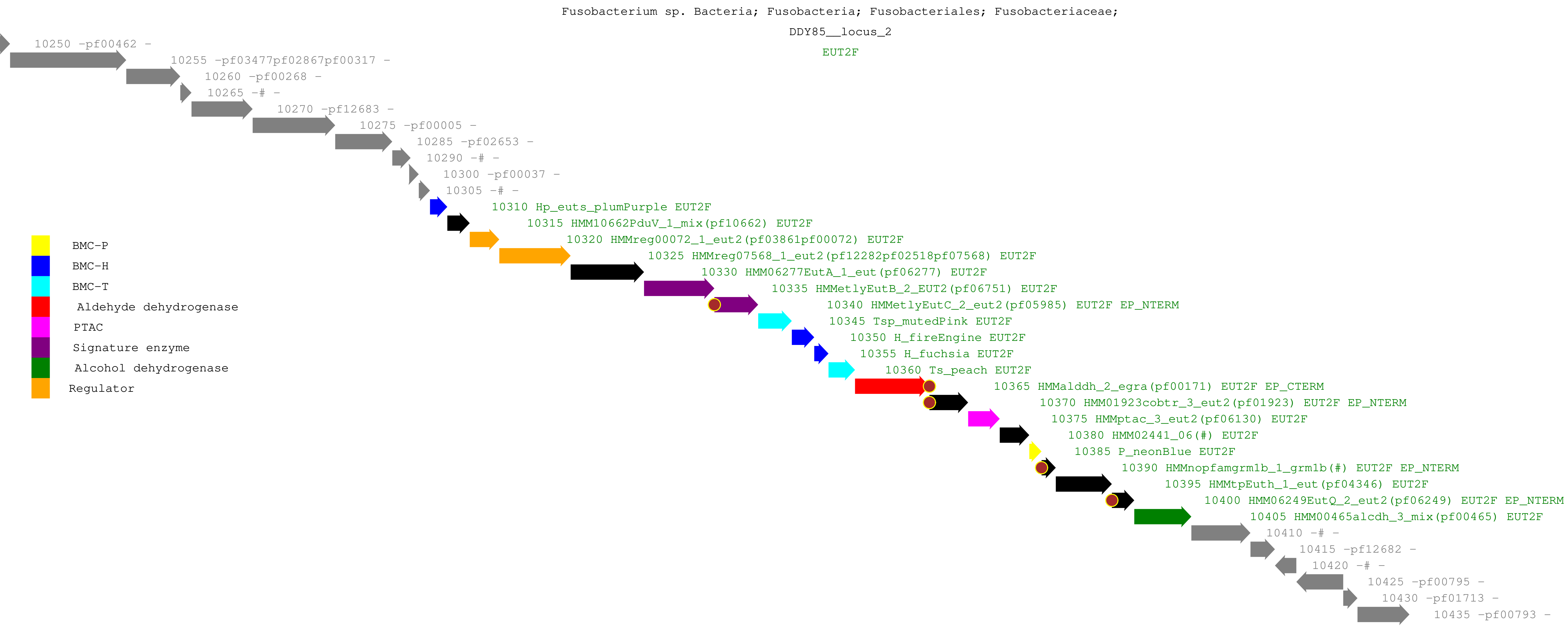
Fusobacterium sp. Bacteria; Fusobacteria; Fusobacteriales; Fusobacteriaceae;
DDY85__locus_2
EUT2F
10250 -pf00462 -
10255 -pf03477pf02867pf00317 -
10260 -pf00268 -
10265 -# -
10270 -pf12683 -
10275 -pf00005 -
10285 -pf02653 -
10290 -# -
10300 -pf00037 -
10305 -# -
10310 Hp_euts_plumPurple EUT2F
10315 HMM10662PduV_1_mix(pf10662) EUT2F
10320 HMMreg00072_1_eut2(pf03861pf00072) EUT2F
10325 HMMreg07568_1_eut2(pf12282pf02518pf07568) EUT2F
10330 HMM06277EutA_1_eut(pf06277) EUT2F
10335 HMMetlyEutB_2_EUT2(pf06751) EUT2F
10340 HMMetlyEutC_2_eut2(pf05985) EUT2F EP_NTERM
10345 Tsp_mutedPink EUT2F
10350 H_fireEngine EUT2F
10355 H_fuchsia EUT2F
10360 Ts_peach EUT2F
10365 HMMaldh_2_egra(pf00171) EUT2F EP_CTERM
10370 HMM01923cobtr_3_eut2(pf01923) EUT2F EP_NTERM
10375 HMMptac_3_eut2(pf06130) EUT2F
10380 HMM02441_06(#) EUT2F
10385 P_neonBlue EUT2F
10390 HMMnopfamgrm1b_1_grm1b(#) EUT2F EP_NTERM
10395 HMMtpEuth_1_eut(pf04346) EUT2F
10400 HMM06249EutQ_2_eut2(pf06249) EUT2F EP_NTERM
10405 HMM00465alcdh_3_mix(pf00465) EUT2F
10410 -# -
10415 -pf12682 -
10420 -# -
10425 -pf00795 -
10430 -pf01713 -
10435 -pf00793 -
BMC-P
BMC-H
BMC-T
Aldehyde dehydrogenase
PTAC
Signature enzyme
Alcohol dehydrogenase
Regulator

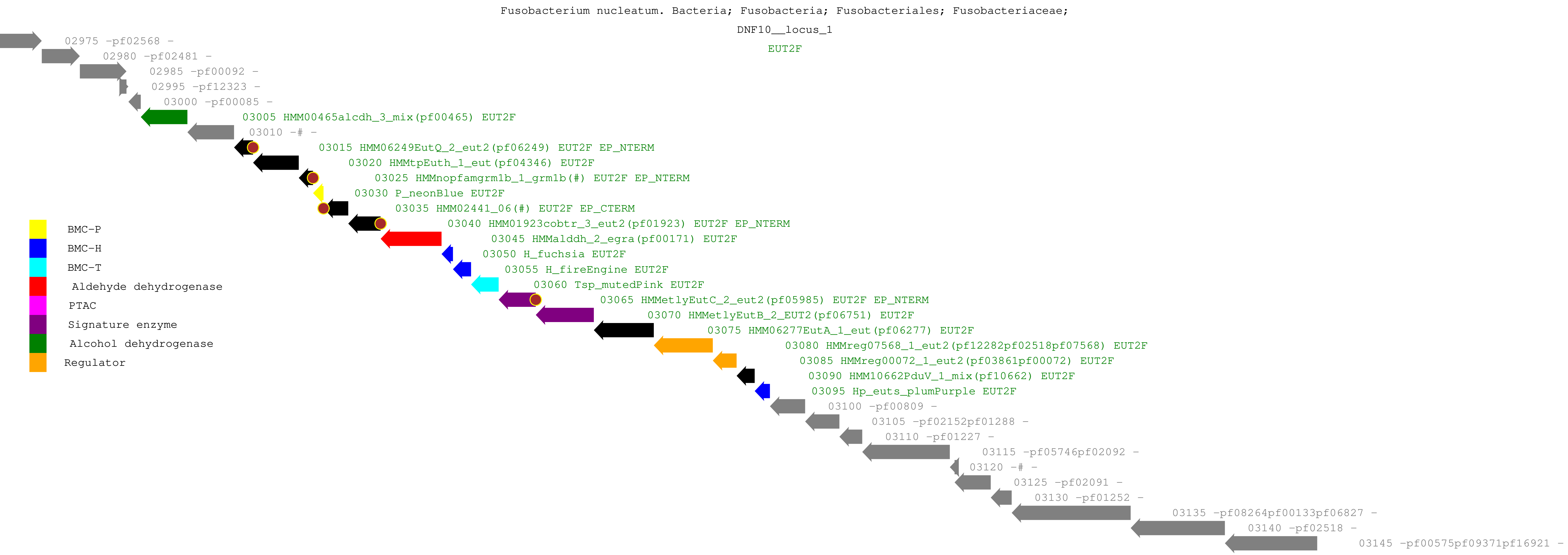

Fusobacterium nucleatum. Bacteria; Fusobacteria; Fusobacteriales; Fusobacteriaceae;
DNF10__locus_1
EUT2F
02975 -pf02568 -
02980 -pf02481 -
02985 -pf00092 -
02995 -pf12323 -
03000 -pf00085 -
03005 HMM00465alcdh_3_mix(pf00465) EUT2F
03010 -# -
03015 HMM06249EutQ_2_eut2(pf06249) EUT2F EP_NTERM
03020 HMMtpEuth_1_eut(pf04346) EUT2F
03025 HMMnopfamgrm1b_1_grm1b(#) EUT2F EP_NTERM
03030 P_neonBlue EUT2F
03035 HMM02441_06(#) EUT2F EP_CTERM
03040 HMM01923cobtr_3_eut2(pf01923) EUT2F EP_NTERM
03045 HMMaldh_2_egra(pf00171) EUT2F
03050 H_fuchsia EUT2F
03055 H_fireEngine EUT2F
03060 Tsp_mutedPink EUT2F
03065 HMMetlyEutC_2_eut2(pf05985) EUT2F EP_NTERM
03070 HMMetlyEutB_2_EUT2(pf06751) EUT2F
03075 HMM06277EutA_1_eut(pf06277) EUT2F
03080 HMMreg07568_1_eut2(pf12282pf02518pf07568) EUT2F
03085 HMMreg00072_1_eut2(pf03861pf00072) EUT2F
03090 HMM10662PduV_1_mix(pf10662) EUT2F
03095 Hp_euts_plumPurple EUT2F
03100 -pf00809 -
03105 -pf02152pf01288 -
03110 -pf01227 -
03115 -pf05746pf02092 -
03120 -# -
03125 -pf02091 -
03130 -pf01252 -
03135 -pf08264pf00133pf06827 -
03140 -pf02518 -
03145 -pf00575pf09371pf16921 -
BMC-P
BMC-H
BMC-T
Aldehyde dehydrogenase
PTAC
Signature enzyme
Alcohol dehydrogenase
Regulator

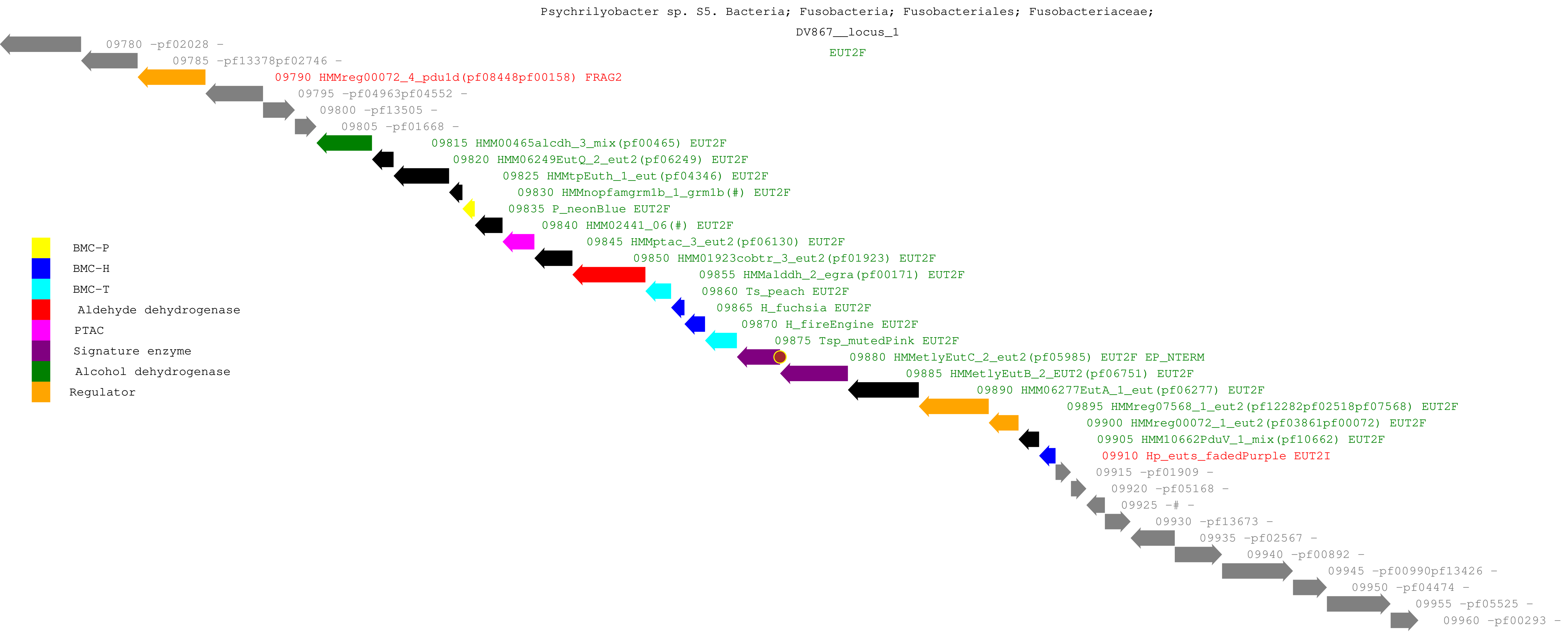
Psychrilyobacter sp. S5. Bacteria; Fusobacteria; Fusobacteriales; Fusobacteriaceae;
DV867__locus_1
EUT2F
09780 -pf02028 -
09785 -pf13378pf02746 -
09790 HMMreg00072_4_pdu1d(pf08448pf00158) FRAG2
09795 -pf04963pf04552 -
09800 -pf13505 -
09805 -pf01668 -
09815 HMM00465alcdh_3_mix(pf00465) EUT2F
09820 HMM06249EutQ_2_eut2(pf06249) EUT2F
09825 HMMtpEuth_1_eut(pf04346) EUT2F
09830 HMMnopfamgrm1b_1_grm1b(#) EUT2F
09835 P_neonBlue EUT2F
09840 HMM02441_06(#) EUT2F
09845 HMMptac_3_eut2(pf06130) EUT2F
09850 HMM01923cobtr_3_eut2(pf01923) EUT2F
09855 HMMalddh_2_egra(pf00171) EUT2F
09860 Ts_peach EUT2F
09865 H_fuchsia EUT2F
09870 H_fireEngine EUT2F
09875 Tsp_mutedPink EUT2F
09880 HMMetlyEutC_2_eut2(pf05985) EUT2F EP_NTERM
09885 HMMetlyEutB_2_EUT2(pf06751) EUT2F
09890 HMM06277EutA_1_eut(pf06277) EUT2F
09895 HMMreg07568_1_eut2(pf12282pf02518pf07568) EUT2F
09900 HMMreg00072_1_eut2(pf03861pf00072) EUT2F
09905 HMM10662PduV_1_mix(pf10662) EUT2F
09910 Hp_euts_fadedPurple EUT2I
09915 -pf01909 -
09920 -pf05168 -
09925 -# -
09930 -pf13673 -
09935 -pf02567 -
09940 -pf00892 -
09945 -pf00990pf13426 -
09950 -pf04474 -
09955 -pf05525 -
09960 -pf00293 -
BMC-P
BMC-H
BMC-T
Aldehyde dehydrogenase
PTAC
Signature enzyme
Alcohol dehydrogenase
Regulator

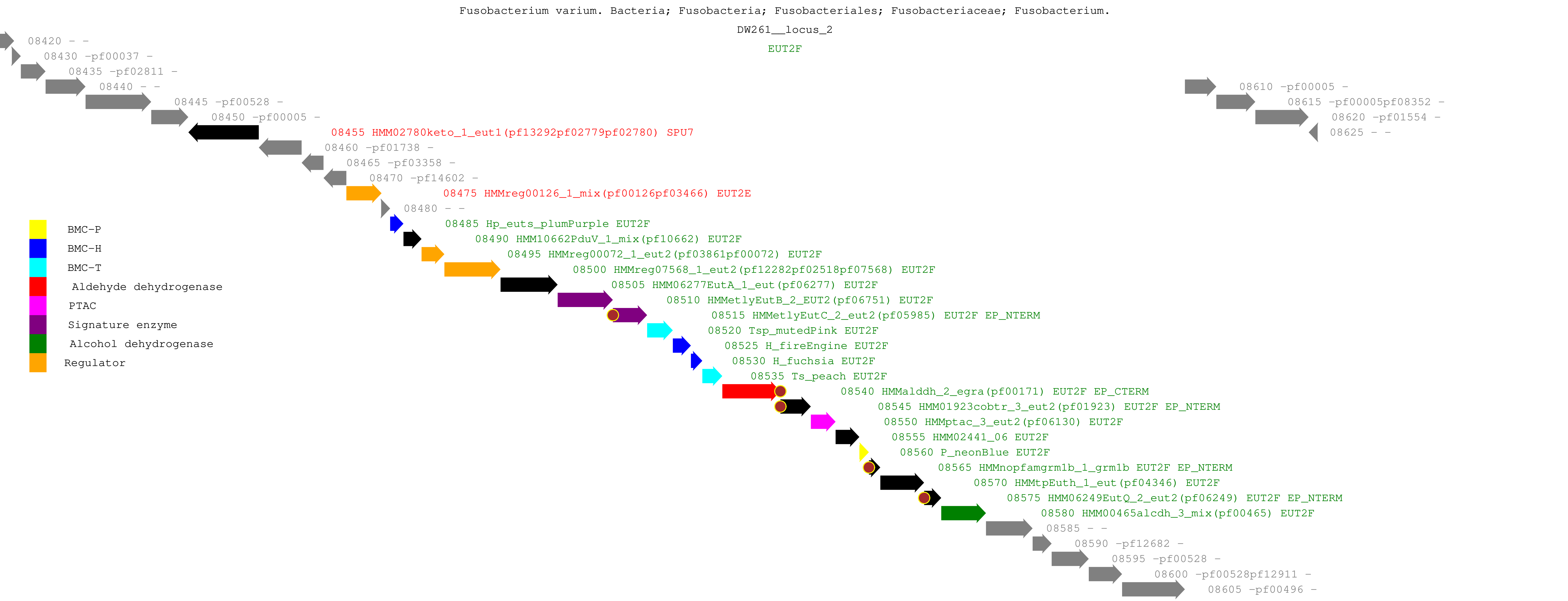
Fusobacterium varium. Bacteria; Fusobacteria; Fusobacteriales; Fusobacteriaceae; Fusobacterium.
DW261__locus_2
EUT2F
08420 - -
08430 -pf00037 -
08435 -pf02811 -
08440 - -
08445 -pf00528 -
08450 -pf00005 -
08455 HMM02780keto_1_eut1(pf13292pf02779pf02780) SPU7
08460 -pf01738 -
08465 -pf03358 -
08470 -pf14602 -
08475 HMMreg00126_1_mix(pf00126pf03466) EUT2E
08480 - -
08485 Hp_euts_plumPurple EUT2F
08490 HMM10662PduV_1_mix(pf10662) EUT2F
08495 HMMreg00072_1_eut2(pf03861pf00072) EUT2F
08500 HMMreg07568_1_eut2(pf12282pf02518pf07568) EUT2F
08505 HMM06277EutA_1_eut(pf06277) EUT2F
08510 HMMetlyEutB_2_EUT2(pf06751) EUT2F
08515 HMMetlyEutC_2_eut2(pf05985) EUT2F EP_NTERM
08520 Tsp_mutedPink EUT2F
08525 H_fireEngine EUT2F
08530 H_fuchsia EUT2F
08535 Ts_peach EUT2F
08540 HMMalddh_2_egra(pf00171) EUT2F EP_CTERM
08545 HMM01923cobtr_3_eut2(pf01923) EUT2F EP_NTERM
08550 HMMptac_3_eut2(pf06130) EUT2F
08555 HMM02441_06 EUT2F
08560 P_neonBlue EUT2F
08565 HMMnopfamgrm1b_1_grm1b EUT2F EP_NTERM
08570 HMMtpEuth_1_eut(pf04346) EUT2F
08575 HMM06249EutQ_2_eut2(pf06249) EUT2F EP_NTERM
08580 HMM00465alcdh_3_mix(pf00465) EUT2F
08585 - -
08590 -pf12682 -
08595 -pf00528 -
08600 -pf00528pf12911 -
08605 -pf00496 -
08610 -pf00005 -
08615 -pf00005pf08352 -
08620 -pf01554 -
08625 - -
BMC-P
BMC-H
BMC-T
Aldehyde dehydrogenase
PTAC
Signature enzyme
Alcohol dehydrogenase
Regulator

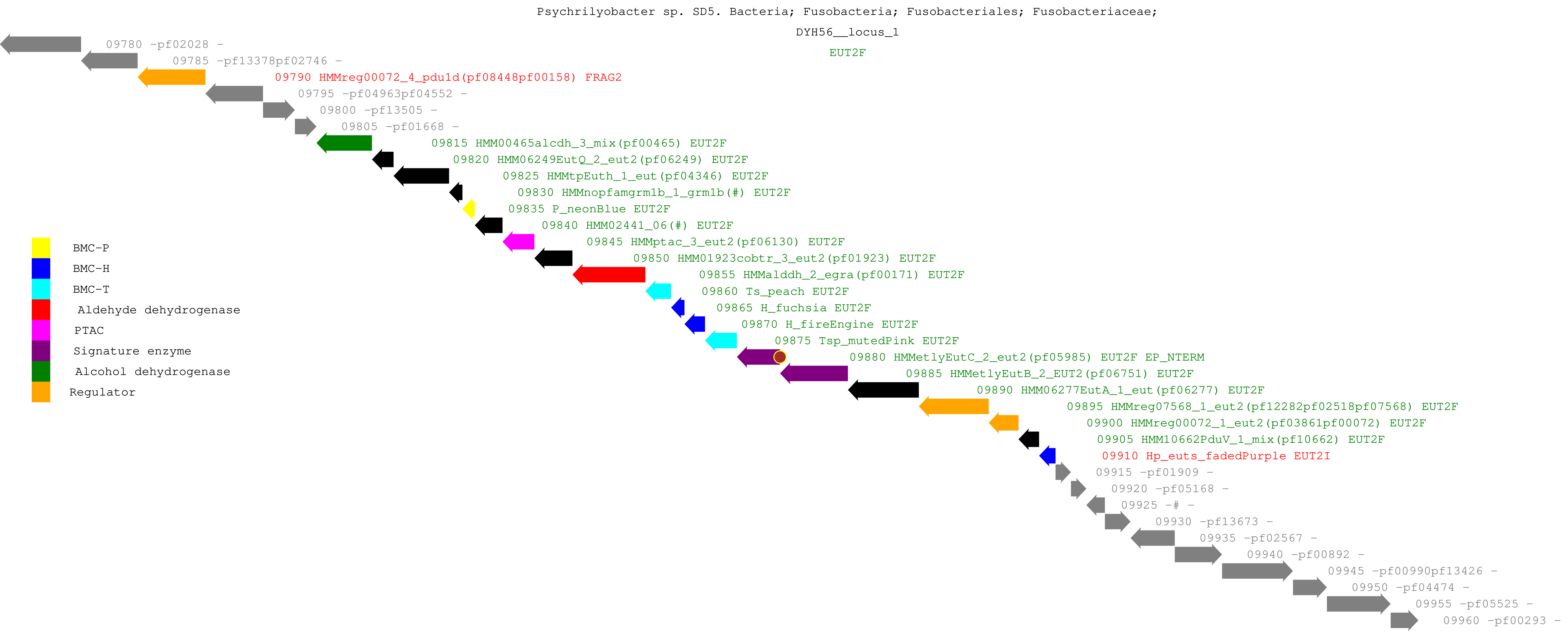
Psychrilyobacter sp. SD5. Bacteria; Fusobacteria; Fusobacteriales; Fusobacteriaceae;
DYH56__locus_1
EUT2F
09780 -pf02028 -
09785 -pf13378pf02746 -
09790 HMMreg00072_4_pdu1d(pf08448pf00158) FRAG2
09795 -pf04963pf04552 -
09800 -pf13505 -
09805 -pf01668 -
09815 HMM00465alcdh_3_mix(pf00465) EUT2F
09820 HMM06249EutQ_2_eut2(pf06249) EUT2F
09825 HMMtpEuth_1_eut(pf04346) EUT2F
09830 HMMnopfamgrm1b_1_grm1b(#) EUT2F
09835 P_neonBlue EUT2F
09840 HMM02441_06(#) EUT2F
09845 HMMptac_3_eut2(pf06130) EUT2F
09850 HMM01923cobtr_3_eut2(pf01923) EUT2F
09855 HMMalddh_2_egra(pf00171) EUT2F
09860 Ts_peach EUT2F
09865 H_fuchsia EUT2F
09870 H_fireEngine EUT2F
09875 Tsp_mutedPink EUT2F
09880 HMMetlyEutC_2_eut2(pf05985) EUT2F EP_NTERM
09885 HMMetlyEutB_2_EUT2(pf06751) EUT2F
09890 HMM06277EutA_1_eut(pf06277) EUT2F
09895 HMMreg07568_1_eut2(pf12282pf02518pf07568) EUT2F
09900 HMMreg00072_1_eut2(pf03861pf00072) EUT2F
09905 HMM10662PduV_1_mix(pf10662) EUT2F
09910 Hp_euts_fadedPurple EUT2I
09915 -pf01909 -
09920 -pf05168 -
09925 -# -
09930 -pf13673 -
09935 -pf02567 -
09940 -pf00892 -
09945 -pf00990pf13426 -
09950 -pf04474 -
09955 -pf05525 -
09960 -pf00293 -
BMC-P
BMC-H
BMC-T
Aldehyde dehydrogenase
PTAC
Signature enzyme
Alcohol dehydrogenase
Regulator

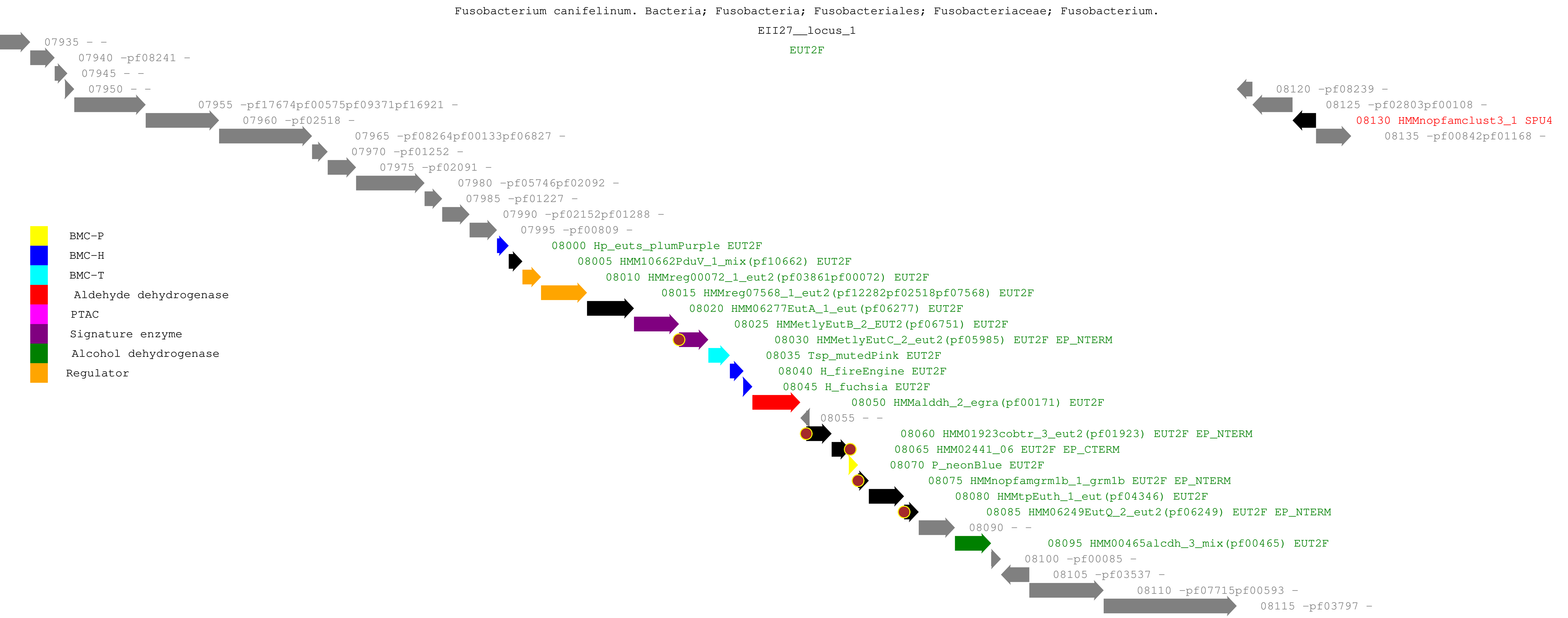
Fusobacterium canifelinum. Bacteria; Fusobacteria; Fusobacteriales; Fusobacteriaceae; Fusobacterium.
EII27__locus_1
EUT2F
07935 - -
07940 -pf08241 -
07945 - -
07950 - -
07955 -pf17674pf00575pf09371pf16921 -
07960 -pf02518 -
07965 -pf08264pf00133pf06827 -
07970 -pf01252 -
07975 -pf02091 -
07980 -pf05746pf02092 -
07985 -pf01227 -
07990 -pf02152pf01288 -
07995 -pf00809 -
08000 Hp_euts_plumPurple EUT2F
08005 HMM10662PduV_1_mix(pf10662) EUT2F
08010 HMMreg00072_1_eut2(pf03861pf00072) EUT2F
08015 HMMreg07568_1_eut2(pf12282pf02518pf07568) EUT2F
08020 HMM06277EutA_1_eut(pf06277) EUT2F
08025 HMMetlyEutB_2_EUT2(pf06751) EUT2F
08030 HMMetlyEutC_2_eut2(pf05985) EUT2F EP_NTERM
08035 Tsp_mutedPink EUT2F
08040 H_fireEngine EUT2F
08045 H_fuchsia EUT2F
08050 HMMalddh_2_egra(pf00171) EUT2F
08055 - -
08060 HMM01923cobtr_3_eut2(pf01923) EUT2F EP_NTERM
08065 HMM02441_06 EUT2F EP_CTERM
08070 P_neonBlue EUT2F
08075 HMMnopfamgrm1b_1_grm1b EUT2F EP_NTERM
08080 HMMtpEuth_1_eut(pf04346) EUT2F
08085 HMM06249EutQ_2_eut2(pf06249) EUT2F EP_NTERM
08090 - -
08095 HMM00465alcdh_3_mix(pf00465) EUT2F
08100 -pf00085 -
08105 -pf03537 -
08110 -pf07715pf00593 -
08115 -pf03797 -
08120 -pf08239 -
08125 -pf02803pf00108 -
08130 HMMnopfamclust3_1 SPU4
08135 -pf00842pf01168 -
BMC-P
BMC-H
BMC-T
Aldehyde dehydrogenase
PTAC
Signature enzyme
Alcohol dehydrogenase
Regulator

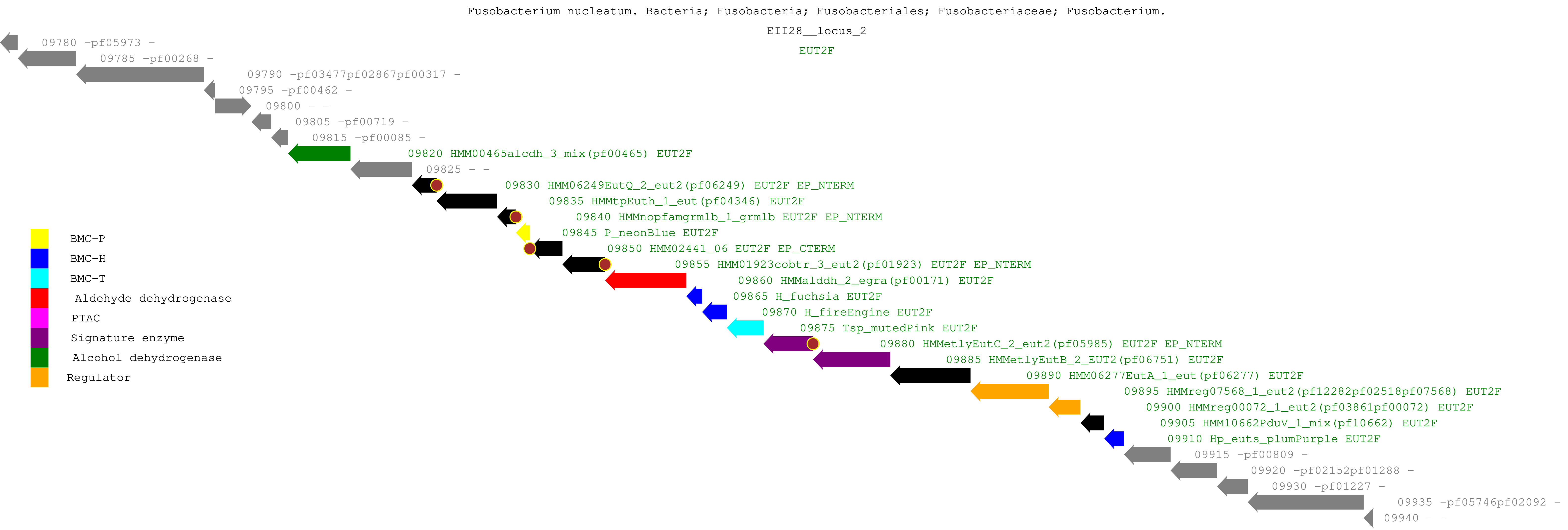
Fusobacterium nucleatum. Bacteria; Fusobacteria; Fusobacteriales; Fusobacteriaceae; Fusobacterium.
EII28__locus_2
EUT2F
09780 -pf05973 -
09785 -pf00268 -
09790 -pf03477pf02867pf00317 -
09795 -pf00462 -
09800 - -
09805 -pf00719 -
09815 -pf00085 -
09820 HMM00465alcdh_3_mix(pf00465) EUT2F
09825 - -
09830 HMM06249EutQ_2_eut2(pf06249) EUT2F EP_NTERM
09835 HMMtpEuth_1_eut(pf04346) EUT2F
09840 HMMnopfamgrm1b_1_grm1b EUT2F EP_NTERM
09845 P_neonBlue EUT2F
09850 HMM02441_06 EUT2F EP_CTERM
09855 HMM01923cobtr_3_eut2(pf01923) EUT2F EP_NTERM
09860 HMMaldh_2_egra(pf00171) EUT2F
09865 H_fuchsia EUT2F
09870 H_fireEngine EUT2F
09875 Tsp_mutedPink EUT2F
09880 HMMetlyEutC_2_eut2(pf05985) EUT2F EP_NTERM
09885 HMMetlyEutB_2_EUT2(pf06751) EUT2F
09890 HMM06277EutA_1_eut(pf06277) EUT2F
09895 HMMreg07568_1_eut2(pf12282pf02518pf07568) EUT2F
09900 HMMreg00072_1_eut2(pf03861pf00072) EUT2F
09905 HMM10662PduV_1_mix(pf10662) EUT2F
09910 Hp_euts_plumPurple EUT2F
09915 -pf00809 -
09920 -pf02152pf01288 -
09930 -pf01227 -
09935 -pf05746pf02092 -
09940 - -
BMC-P
BMC-H
BMC-T
Aldehyde dehydrogenase
PTAC
Signature enzyme
Alcohol dehydrogenase
Regulator

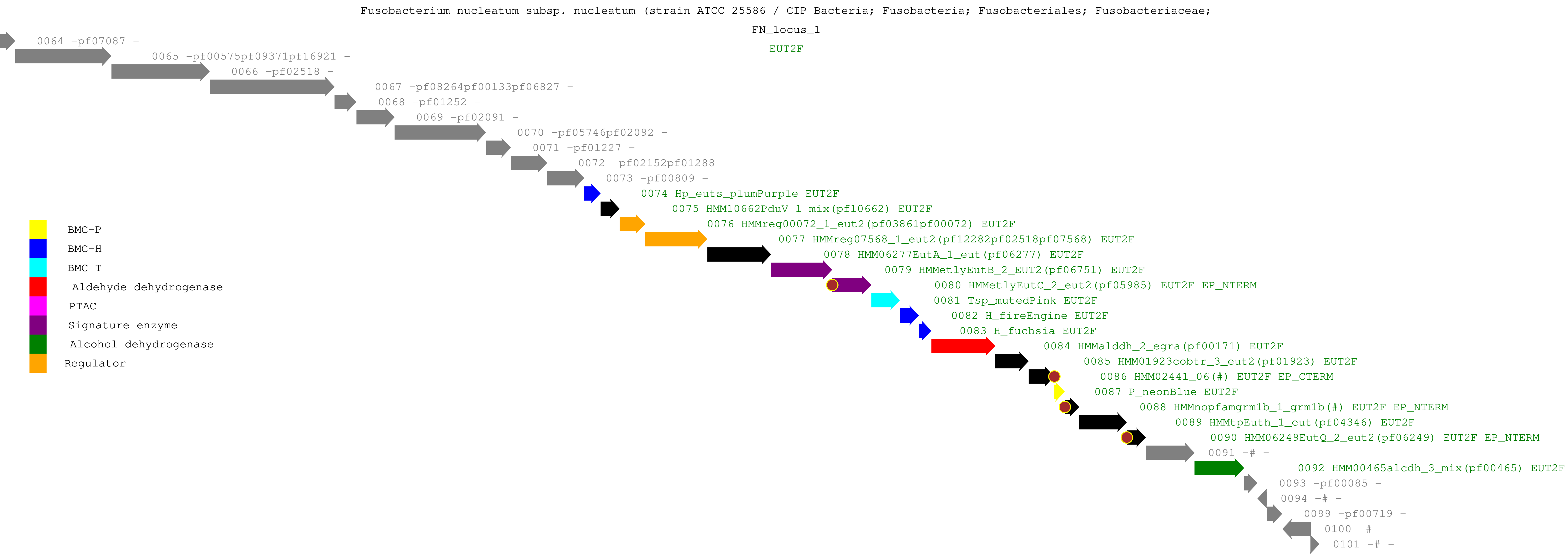
Fusobacterium nucleatum subsp. nucleatum (strain ATCC 25586 / CIP Bacteria; Fusobacteria; Fusobacteriales; Fusobacteriaceae;
FN_locus_1
EUT2F
0064 -pf07087 -
0065 -pf00575pf09371pf16921 -
0066 -pf02518 -
0067 -pf08264pf00133pf06827 -
0068 -pf01252 -
0069 -pf02091 -
0070 -pf05746pf02092 -
0071 -pf01227 -
0072 -pf02152pf01288 -
0073 -pf00809 -
0074 Hp_euts_plumPurple EUT2F
0075 HMM10662PduV_1_mix(pf10662) EUT2F
0076 HMMreg00072_1_eut2(pf03861pf00072) EUT2F
0077 HMMreg07568_1_eut2(pf12282pf02518pf07568) EUT2F
0078 HMM06277EutA_1_eut(pf06277) EUT2F
0079 HMMetlyEutB_2_EUT2(pf06751) EUT2F
0080 HMMetlyEutC_2_eut2(pf05985) EUT2F EP_NTERM
0081 Tsp_mutedPink EUT2F
0082 H_fireEngine EUT2F
0083 H_fuchsia EUT2F
0084 HMMalddh_2_egra(pf00171) EUT2F
0085 HMM01923cobtr_3_eut2(pf01923) EUT2F
0086 HMM02441_06(#) EUT2F EP_CTERM
0087 P_neonBlue EUT2F
0088 HMMnopfamgrm1b_1_grm1b(#) EUT2F EP_NTERM
0089 HMMtpEuth_1_eut(pf04346) EUT2F
0090 HMM06249EutQ_2_eut2(pf06249) EUT2F EP_NTERM
0091 -# -
0092 HMM00465alcdh_3_mix(pf00465) EUT2F
0093 -pf00085 -
0094 -# -
0099 -pf00719 -
0100 -# -
0101 -# -
BMC-P
BMC-H
BMC-T
Aldehyde dehydrogenase
PTAC
Signature enzyme
Alcohol dehydrogenase
Regulator

Fusobacterium nucleatum subsp. polymorphum ATCC 10953. Bacteria; Fusobacteria; Fusobacteriales; Fusobacteriaceae;

FNP__locus_1

EUT2F

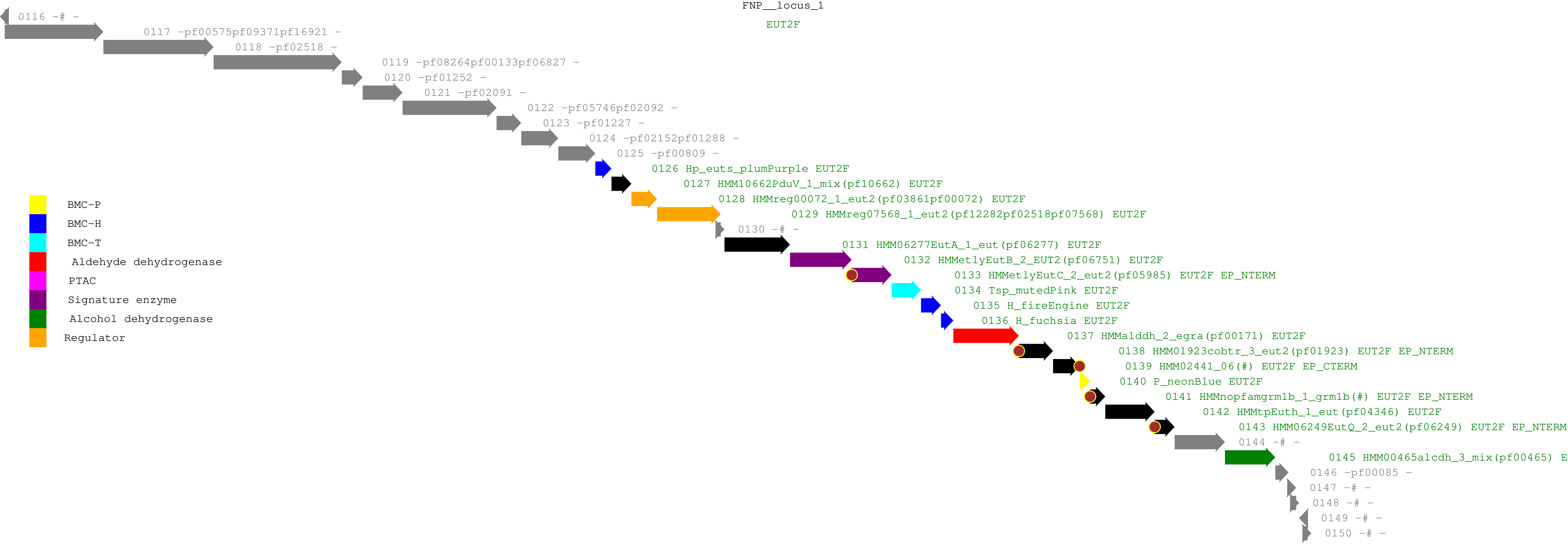

Fusobacterium periodonticum D10. Bacteria; Fusobacteria; Fusobacteriales; Fusobacteriaceae;

FPOG__locus_1

EUT2F

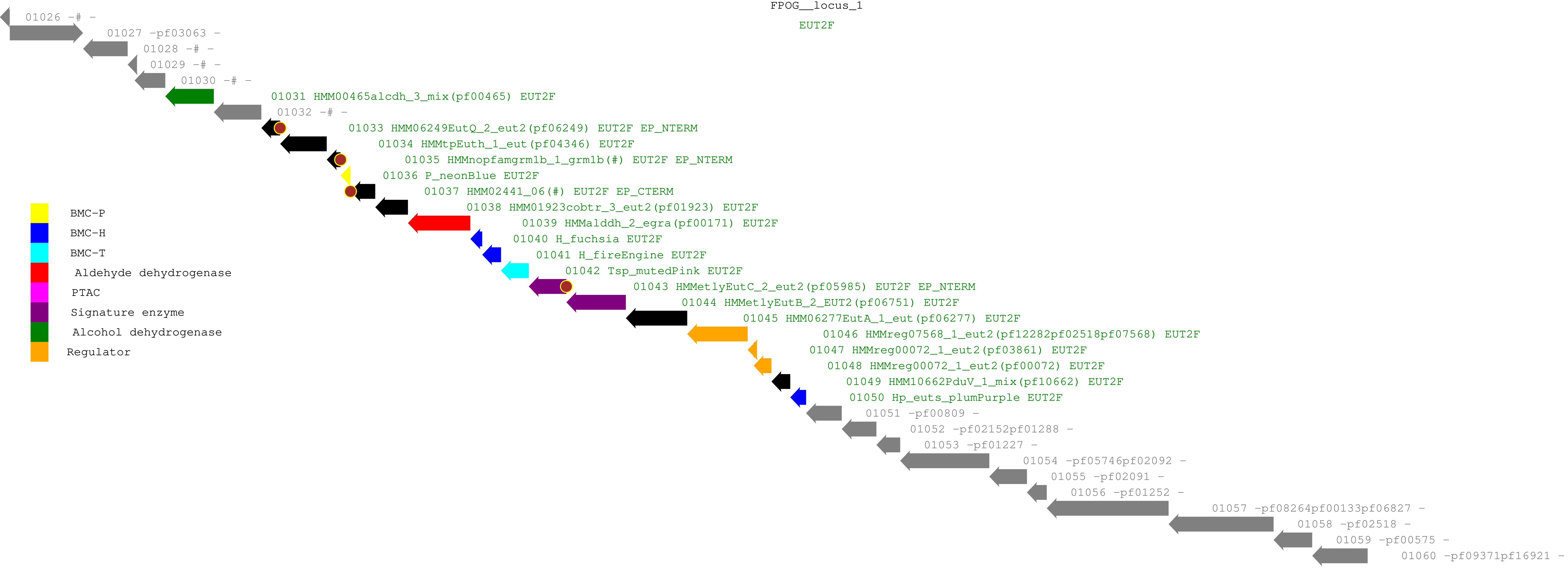

Fusobacterium periodonticum 2_1_31. Bacteria; Fusobacteria; Fusobacteriales; Fusobacteriaceae;

FSAG__locus_1

EUT2F

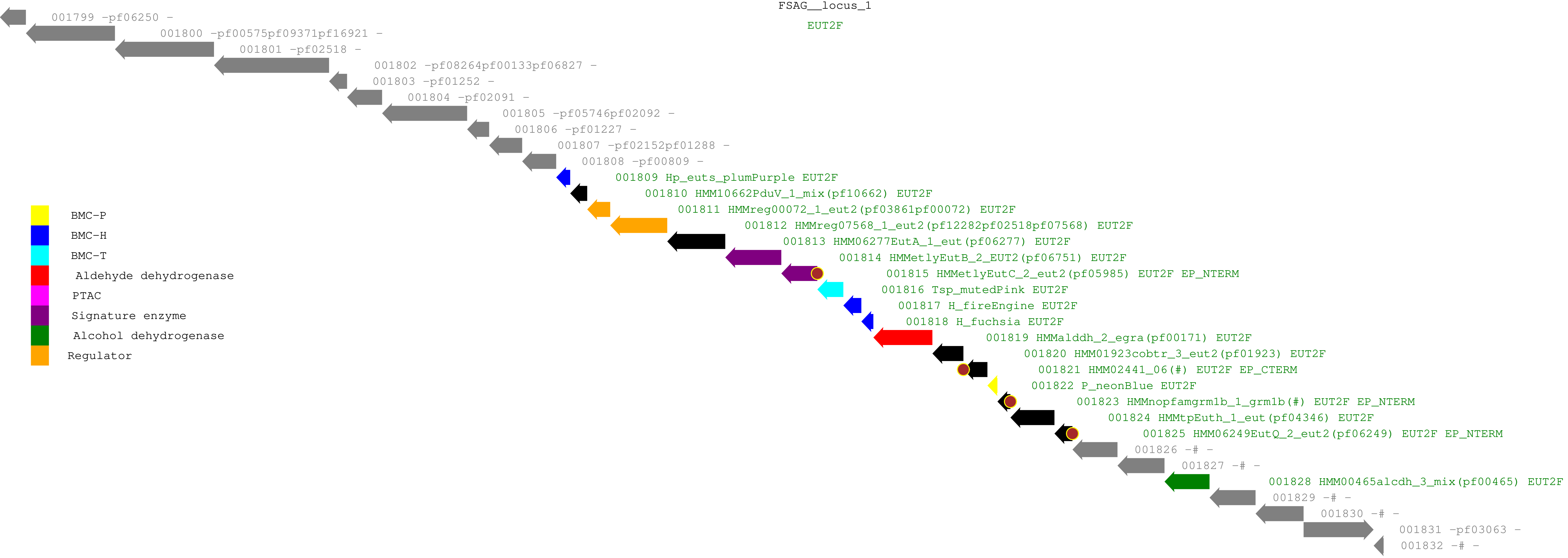

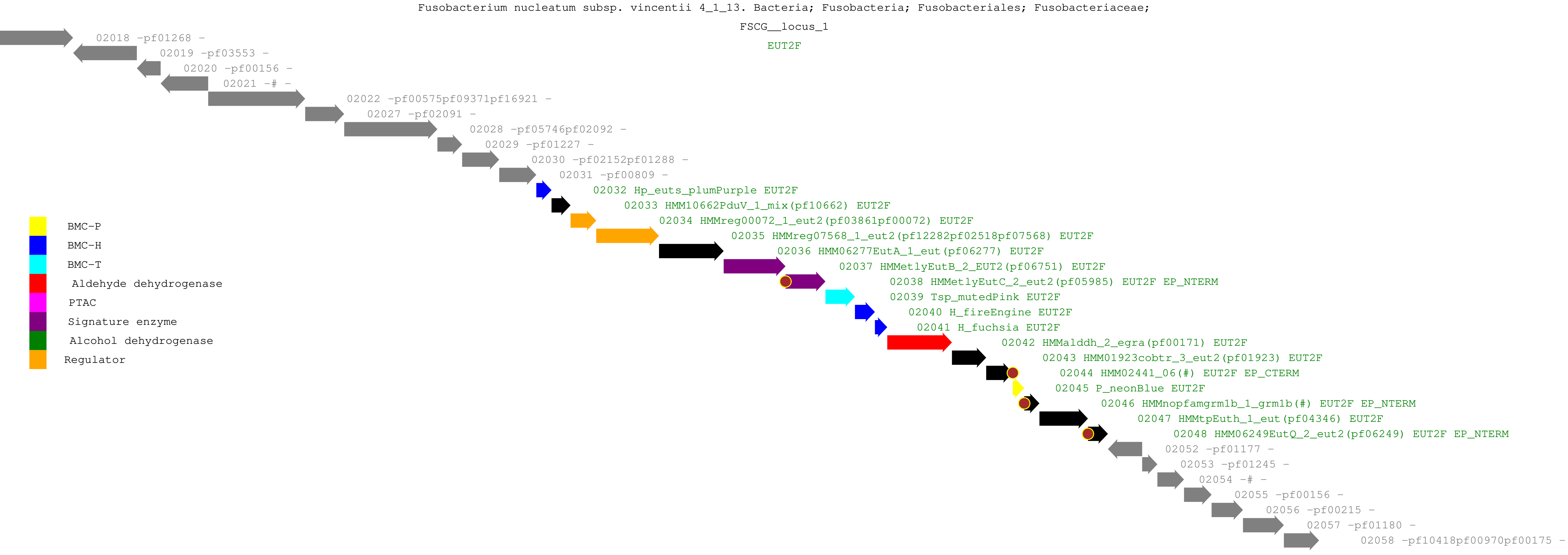

Fusobacterium nucleatum subsp. vincentii 4_1_13. Bacteria; Fusobacteria; Fusobacteriales; Fusobacteriaceae;
FSCG__locus_1
EUT2F
02018 -pf01268 -
02019 -pf03553 -
02020 -pf00156 -
02021 -# -
02022 -pf00575pf09371pf16921 -
02027 -pf02091 -
02028 -pf05746pf02092 -
02029 -pf01227 -
02030 -pf02152pf01288 -
02031 -pf00809 -
02032 Hp_euts_plumPurple EUT2F
02033 HMM10662PduV_1_mix(pf10662) EUT2F
02034 HMMreg00072_1_eut2(pf03861pf00072) EUT2F
02035 HMMreg07568_1_eut2(pf12282pf02518pf07568) EUT2F
02036 HMM06277EutA_1_eut(pf06277) EUT2F
02037 HMMetlyEutB_2_EUT2(pf06751) EUT2F
02038 HMMetlyEutC_2_eut2(pf05985) EUT2F EP_NTERM
02039 Tsp_mutedPink EUT2F
02040 H_fireEngine EUT2F
02041 H_fuchsia EUT2F
02042 HMMalddh_2_egra(pf00171) EUT2F
02043 HMM01923cobtr_3_eut2(pf01923) EUT2F
02044 HMM02441_06(#) EUT2F EP_CTERM
02045 P_neonBlue EUT2F
02046 HMMnopfamgrm1b_1_grm1b(#) EUT2F EP_NTERM
02047 HMMtpEuth_1_eut(pf04346) EUT2F
02048 HMM06249EutQ_2_eut2(pf06249) EUT2F EP_NTERM
02052 -pf01177 -
02053 -pf01245 -
02054 -# -
02055 -pf00156 -
02056 -pf00215 -
02057 -pf01180 -
02058 -pf10418pf00970pf00175 -
BMC-P
BMC-H
BMC-T
Aldehyde dehydrogenase
PTAC
Signature enzyme
Alcohol dehydrogenase
Regulator

Fusobacterium nucleatum subsp. animalis 7_1. Bacteria; Fusobacteria; Fusobacteriales; Fusobacteriaceae;

FSDG__locus_2

EUT2F

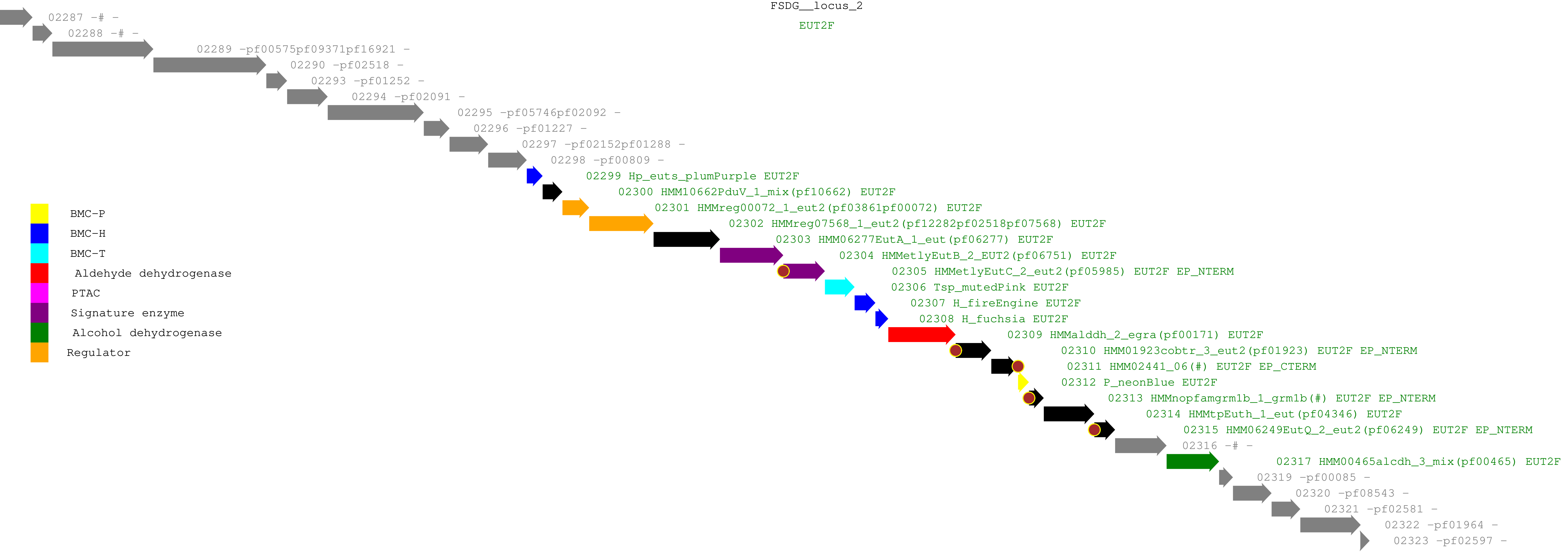

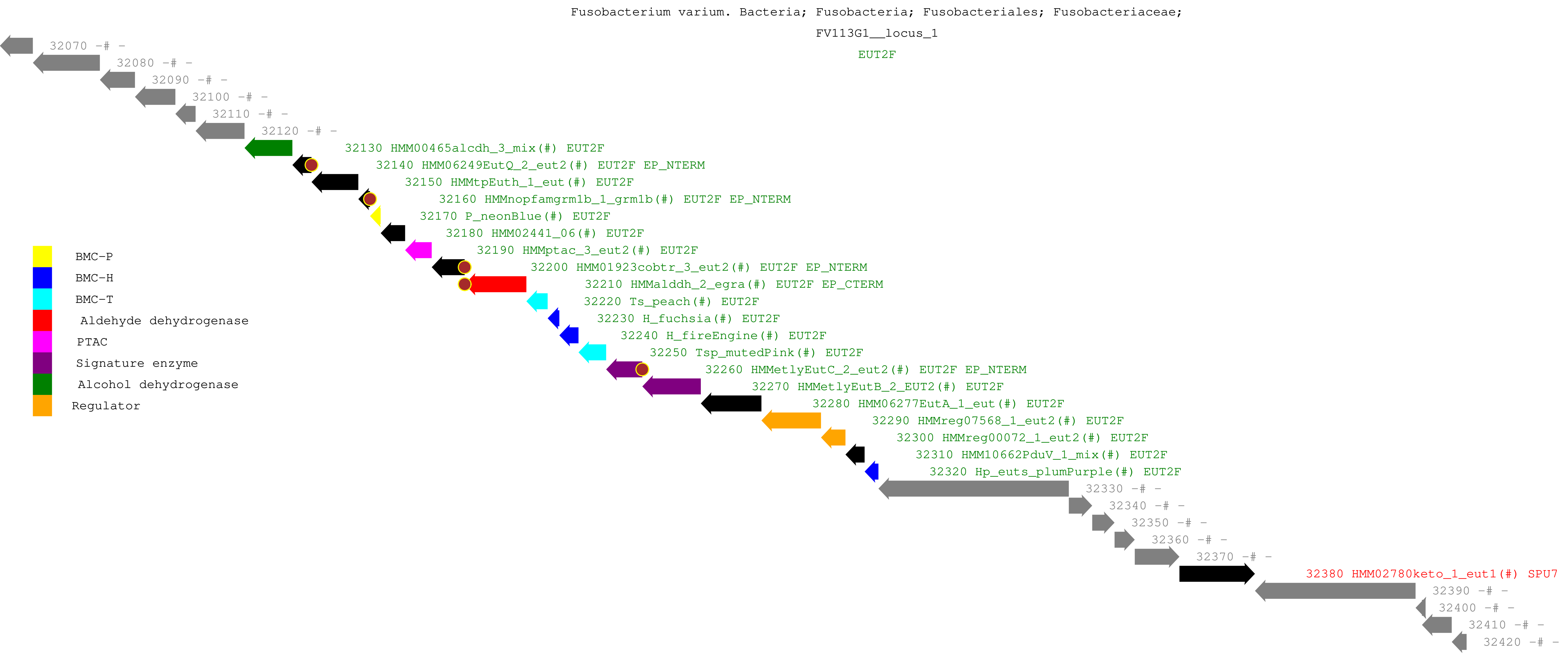
Fusobacterium varium. Bacteria; Fusobacteria; Fusobacteriales; Fusobacteriaceae;
FV113G1__locus_1
EUT2F
32070 -# -
32080 -# -
32090 -# -
32100 -# -
32110 -# -
32120 -# -
32130 HMM00465alcdh_3_mix(#) EUT2F
32140 HMM06249EutQ_2_eut2(#) EUT2F EP_NTERM
32150 HMMtpEuth_1_eut(#) EUT2F
32160 HMMnopfamgrm1b_1_grm1b(#) EUT2F EP_NTERM
32170 P_neonBlue(#) EUT2F
32180 HMM02441_06(#) EUT2F
32190 HMMptac_3_eut2(#) EUT2F
32200 HMM01923cobtr_3_eut2(#) EUT2F EP_NTERM
32210 HMMalddh_2_egra(#) EUT2F EP_CTERM
32220 Ts_peach(#) EUT2F
32230 H_fuchsia(#) EUT2F
32240 H_fireEngine(#) EUT2F
32250 Tsp_mutedPink(#) EUT2F
32260 HMMetlyEutC_2_eut2(#) EUT2F EP_NTERM
32270 HMMetlyEutB_2_EUT2(#) EUT2F
32280 HMM06277EutA_1_eut(#) EUT2F
32290 HMMreg07568_1_eut2(#) EUT2F
32300 HMMreg00072_1_eut2(#) EUT2F
32310 HMM10662PduV_1_mix(#) EUT2F
32320 Hp_euts_plumPurple(#) EUT2F
32330 -# -
32340 -# -
32350 -# -
32360 -# -
32370 -# -
32380 HMM02780keto_1_eut1(#) SPU7
32390 -# -
32400 -# -
32410 -# -
32420 -# -
BMC-P
BMC-H
BMC-T
Aldehyde dehydrogenase
PTAC
Signature enzyme
Alcohol dehydrogenase
Regulator

Fusobacterium varium ATCC 27725. Bacteria; Fusobacteria; Fusobacteriales; Fusobacteriaceae;

FVAG__locus_2

EUT2F

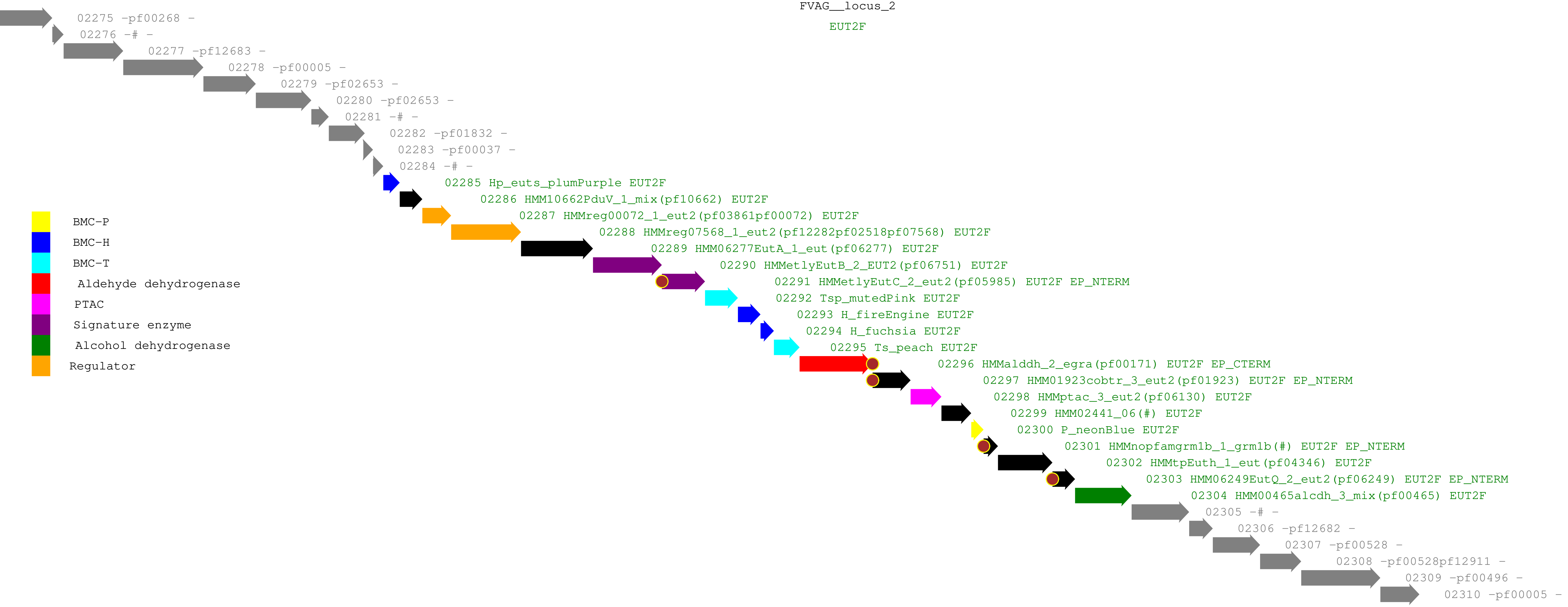

Cetobacterium somerae ATCC BAA-474. Bacteria; Fusobacteria; Fusobacteriales; Fusobacteriaceae;

HMPREF0202__locus_1

EUT2F

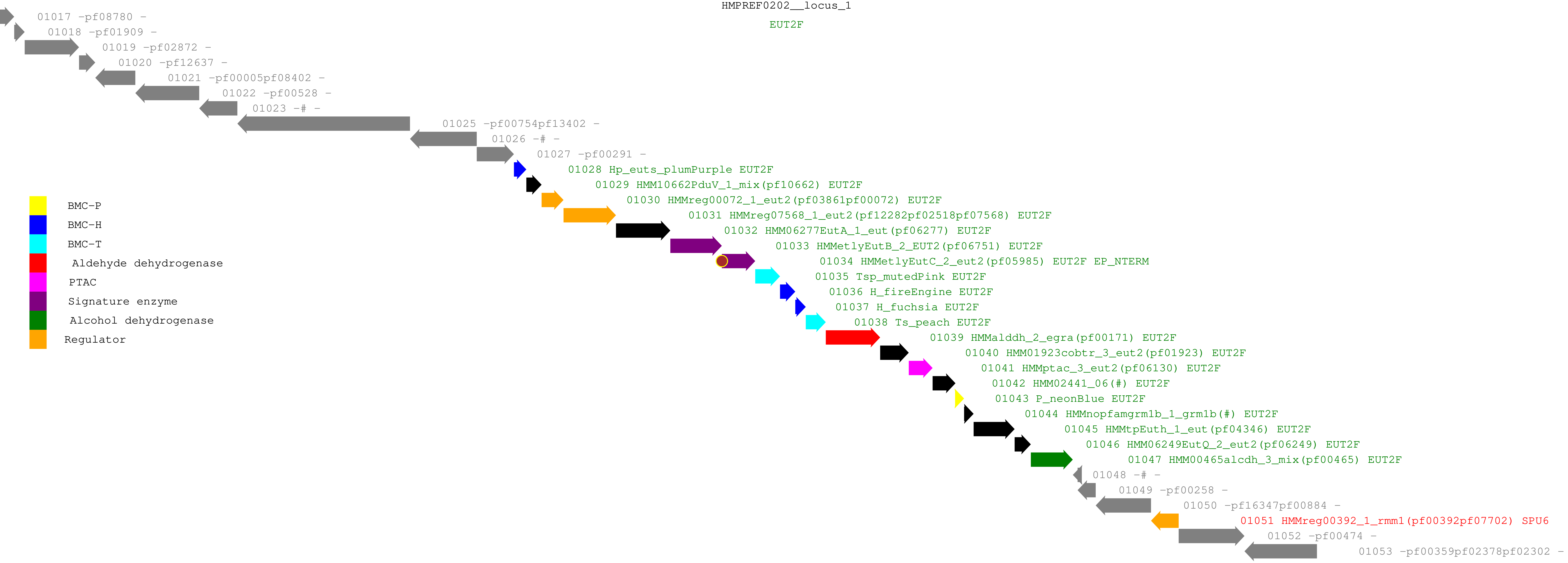

Fusobacterium nucleatum subsp. nucleatum (strain ATCC 23726 / VPI Bacteria; Fusobacteria; Fusobacteriales; Fusobacteriaceae;

HMPREF0397__locus_1

EUT2F

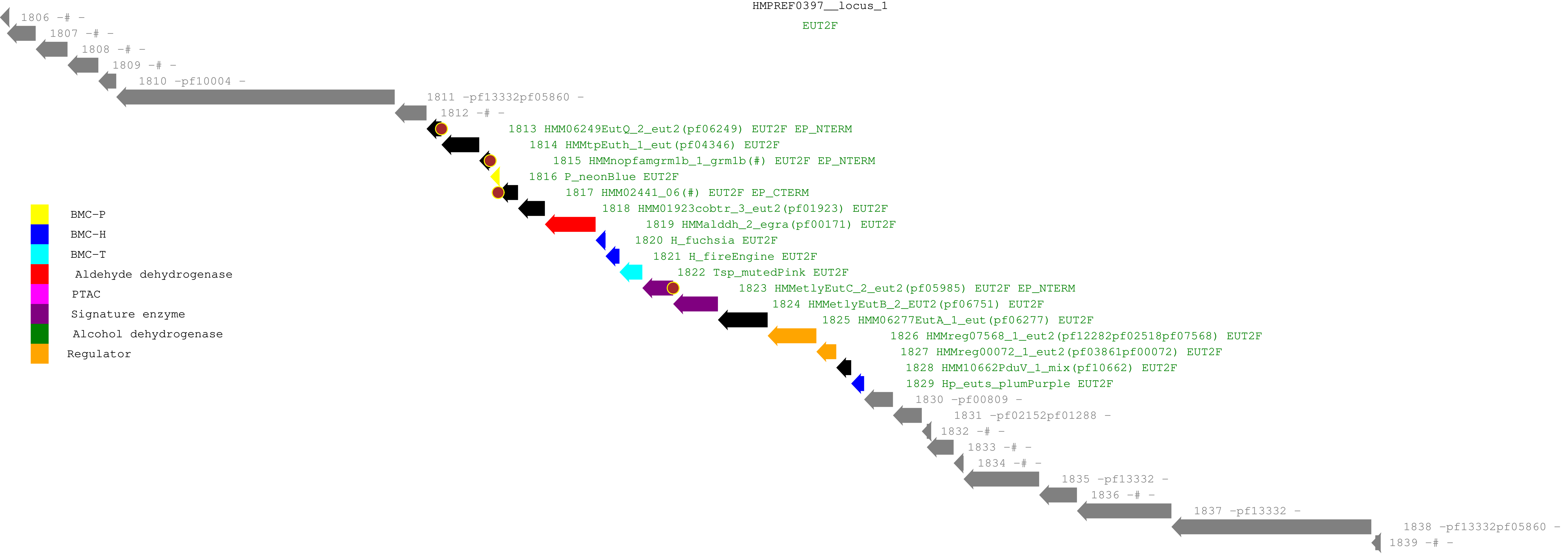

Fusobacterium nucleatum subsp. animalis 11_3_2. Bacteria; Fusobacteria; Fusobacteriales; Fusobacteriaceae;

HMPREF0401__locus_2

EUT2F

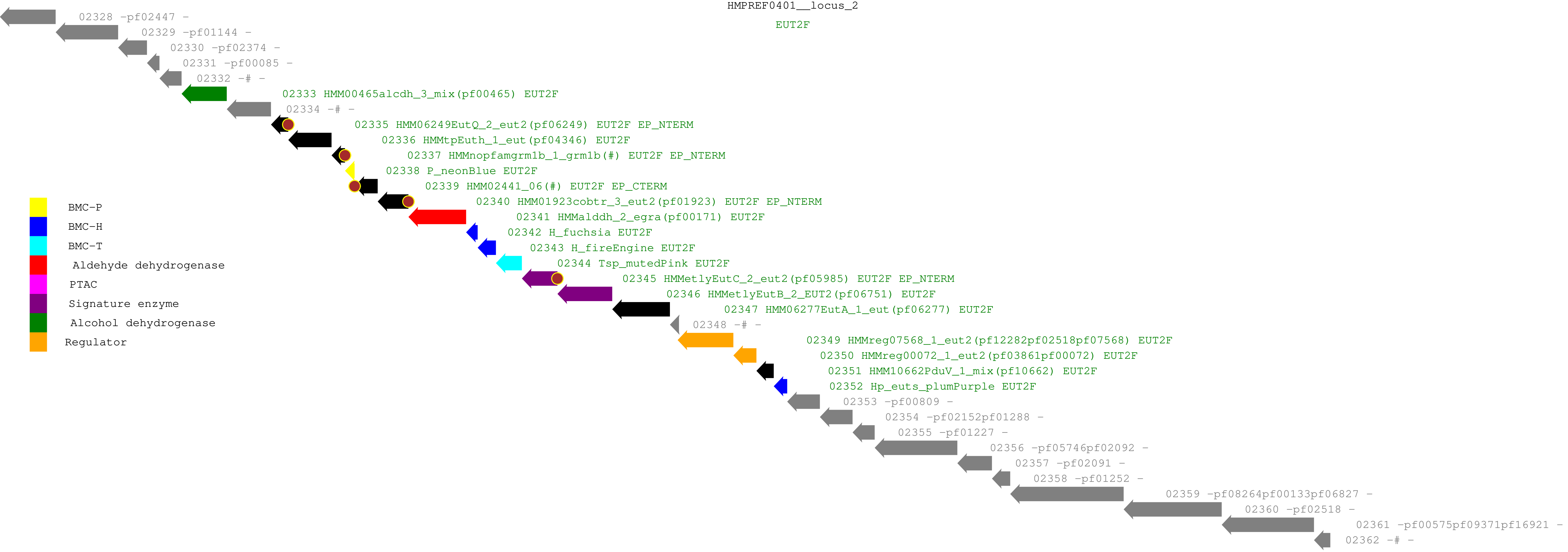

Fusobacterium nucleatum subsp. animalis 4_8. Bacteria; Fusobacteria; Fusobacteriales; Fusobacteriaceae;

HMPREF0409__locus_2

EUT2F

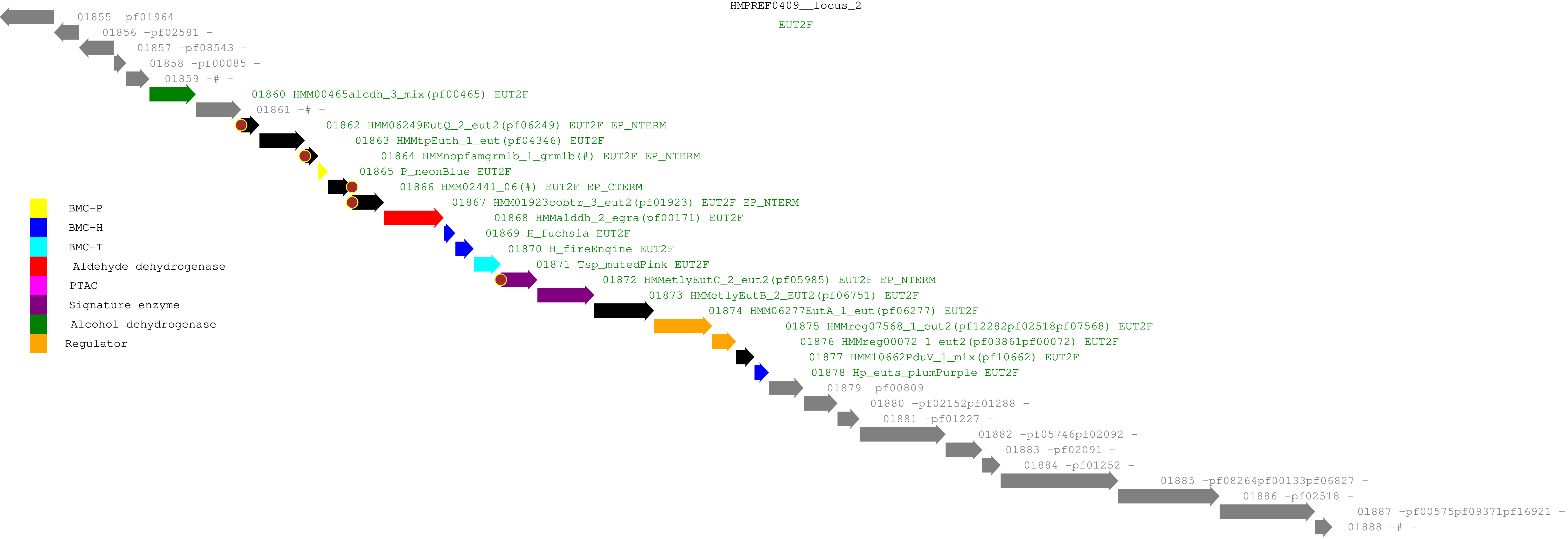

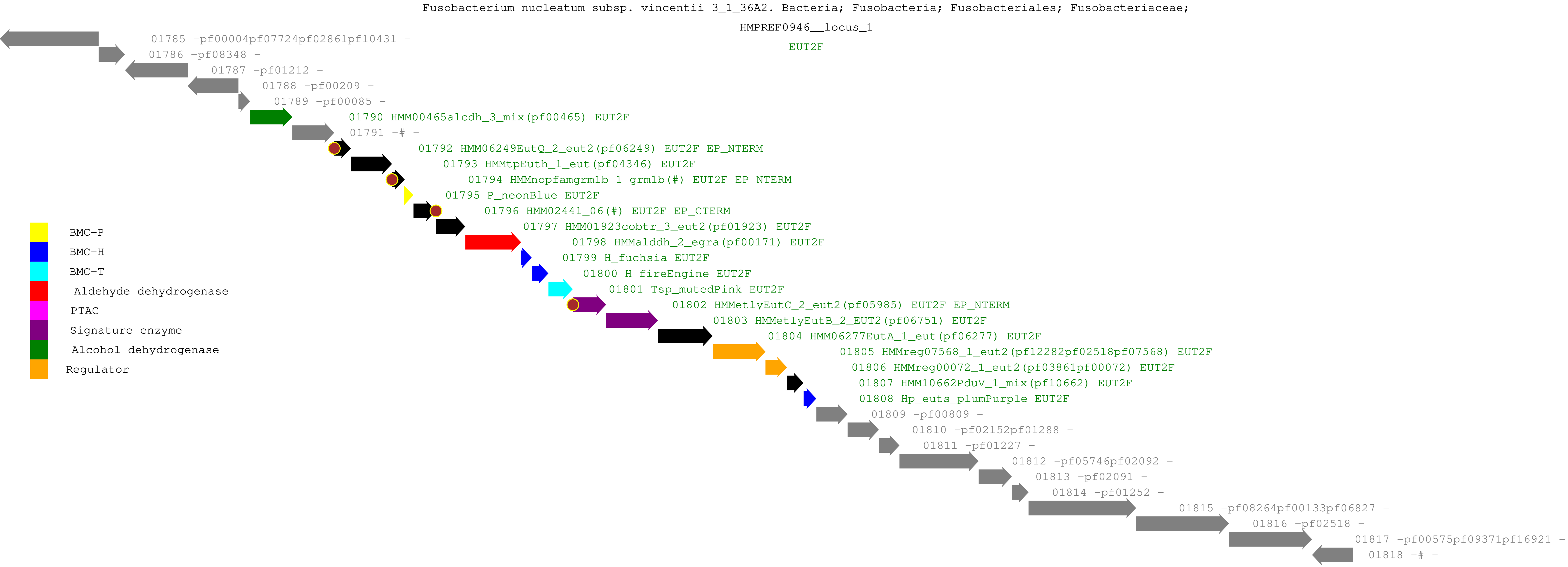

Fusobacterium nucleatum subsp. vincentii 3_1_36A2. Bacteria; Fusobacteria; Fusobacteriales; Fusobacteriaceae;
HMPREF0946__locus_1
EUT2F
01785 -pf00004pf07724pf02861pf10431 -
01786 -pf08348 -
01787 -pf01212 -
01788 -pf00209 -
01789 -pf00085 -
01790 HMM00465alcdh_3_mix(pf00465) EUT2F
01791 -# -
01792 HMM06249EutQ_2_eut2(pf06249) EUT2F EP_NTERM
01793 HMMtpEuth_1_eut(pf04346) EUT2F
01794 HMMnopfamgrm1b_1_grm1b(#) EUT2F EP_NTERM
01795 P_neonBlue EUT2F
01796 HMM02441_06(#) EUT2F EP_CTERM
01797 HMM01923cobtr_3_eut2(pf01923) EUT2F
01798 HMMalddh_2_egra(pf00171) EUT2F
01799 H_fuchsia EUT2F
01800 H_fireEngine EUT2F
01801 Tsp_mutedPink EUT2F
01802 HMMetlyEutC_2_eut2(pf05985) EUT2F EP_NTERM
01803 HMMetlyEutB_2_EUT2(pf06751) EUT2F
01804 HMM06277EutA_1_eut(pf06277) EUT2F
01805 HMMreg07568_1_eut2(pf12282pf02518pf07568) EUT2F
01806 HMMreg00072_1_eut2(pf03861pf00072) EUT2F
01807 HMM10662PduV_1_mix(pf10662) EUT2F
01808 Hp_euts_plumPurple EUT2F
01809 -pf00809 -
01810 -pf02152pf01288 -
01811 -pf01227 -
01812 -pf05746pf02092 -
01813 -pf02091 -
01814 -pf01252 -
01815 -pf08264pf00133pf06827 -
01816 -pf02518 -
01817 -pf00575pf09371pf16921 -
01818 -# -
BMC-P
BMC-H
BMC-T
Aldehyde dehydrogenase
PTAC
Signature enzyme
Alcohol dehydrogenase
Regulator

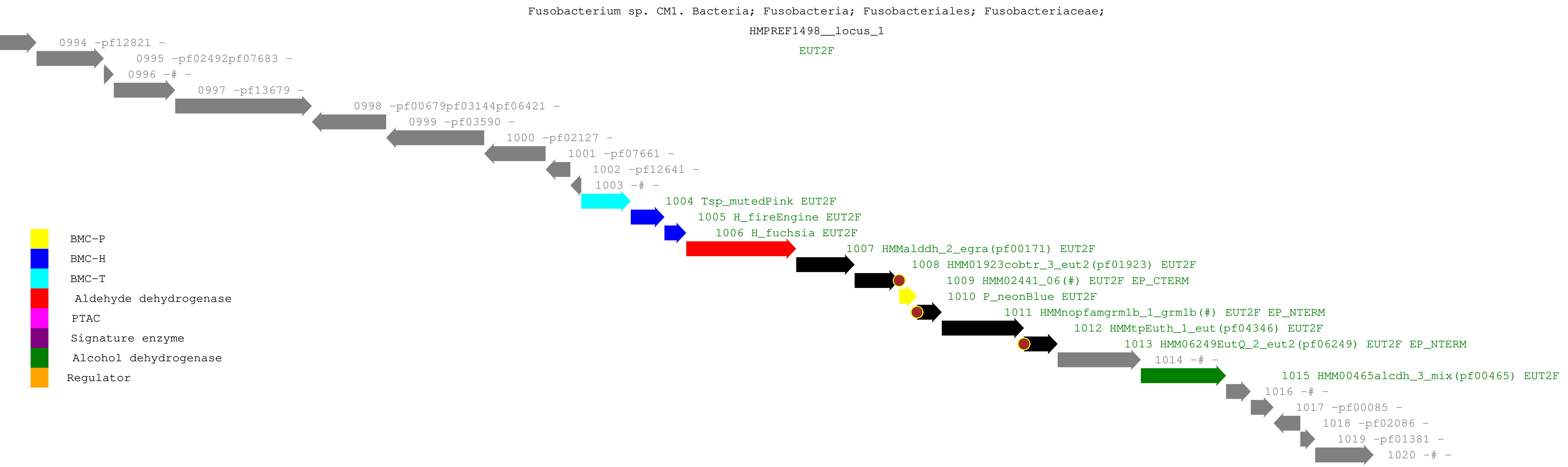
Fusobacterium sp. CM1. Bacteria; Fusobacteria; Fusobacteriales; Fusobacteriaceae;
HMPREF1498__locus_1
EUT2F
0994 -pf12821 -
0995 -pf02492pf07683 -
0996 -# -
0997 -pf13679 -
0998 -pf00679pf03144pf06421 -
0999 -pf03590 -
1000 -pf02127 -
1001 -pf07661 -
1002 -pf12641 -
1003 -# -
1004 Tsp_mutedPink EUT2F
1005 H_fireEngine EUT2F
1006 H_fuchsia EUT2F
1007 HMMalddh_2_egra(pf00171) EUT2F
1008 HMM01923cobtr_3_eut2(pf01923) EUT2F
1009 HMM02441_06(#) EUT2F EP_CTERM
1010 P_neonBlue EUT2F
1011 HMMnopfamgrm1b_1_grm1b(#) EUT2F EP_NTERM
1012 HMMtpEuth_1_eut(pf04346) EUT2F
1013 HMM06249EutQ_2_eut2(pf06249) EUT2F EP_NTERM
1014 -# -
1015 HMM00465alcdh_3_mix(pf00465) EUT2F
1016 -# -
1017 -pf00085 -
1018 -pf02086 -
1019 -pf01381 -
1020 -# -
BMC-P
BMC-H
BMC-T
Aldehyde dehydrogenase
PTAC
Signature enzyme
Alcohol dehydrogenase
Regulator

Fusobacterium sp. CM22. Bacteria; Fusobacteria; Fusobacteriales; Fusobacteriaceae;

HMPREF1500__locus_2

EUT2F

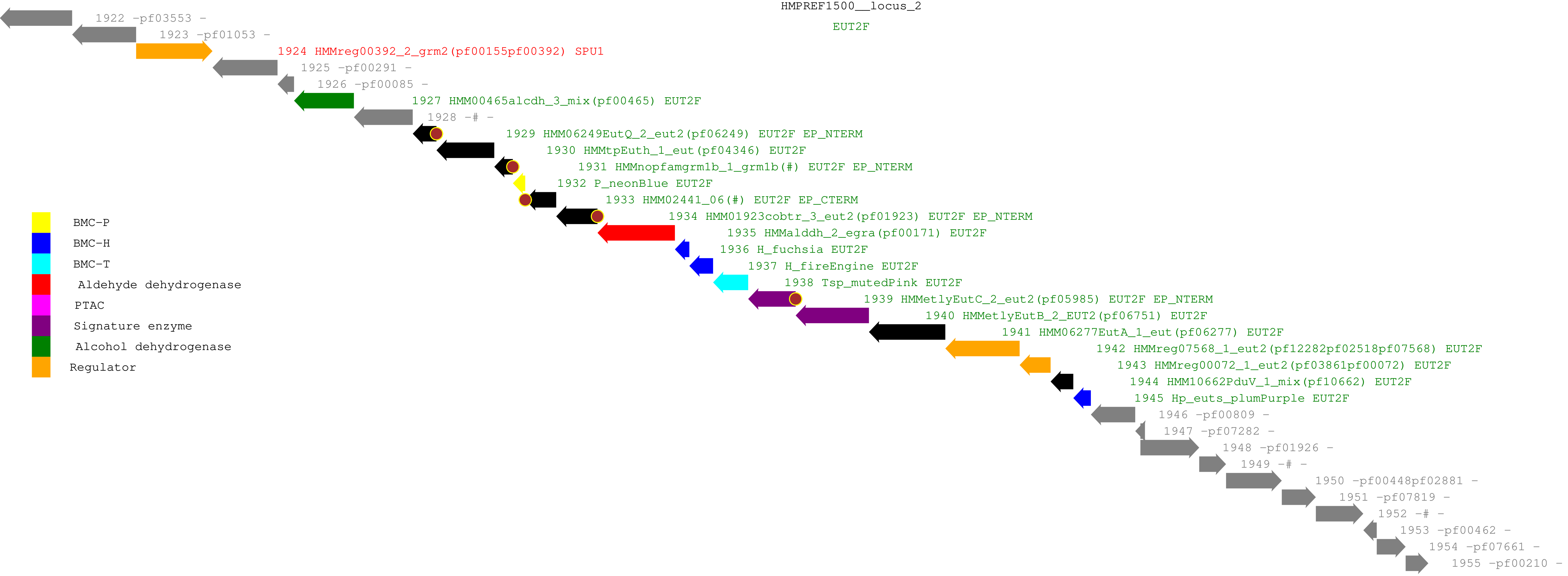

Fusobacterium sp. OBRC1. Bacteria; Fusobacteria; Fusobacteriales; Fusobacteriaceae;

HMPREF1501__locus_1

EUT2F

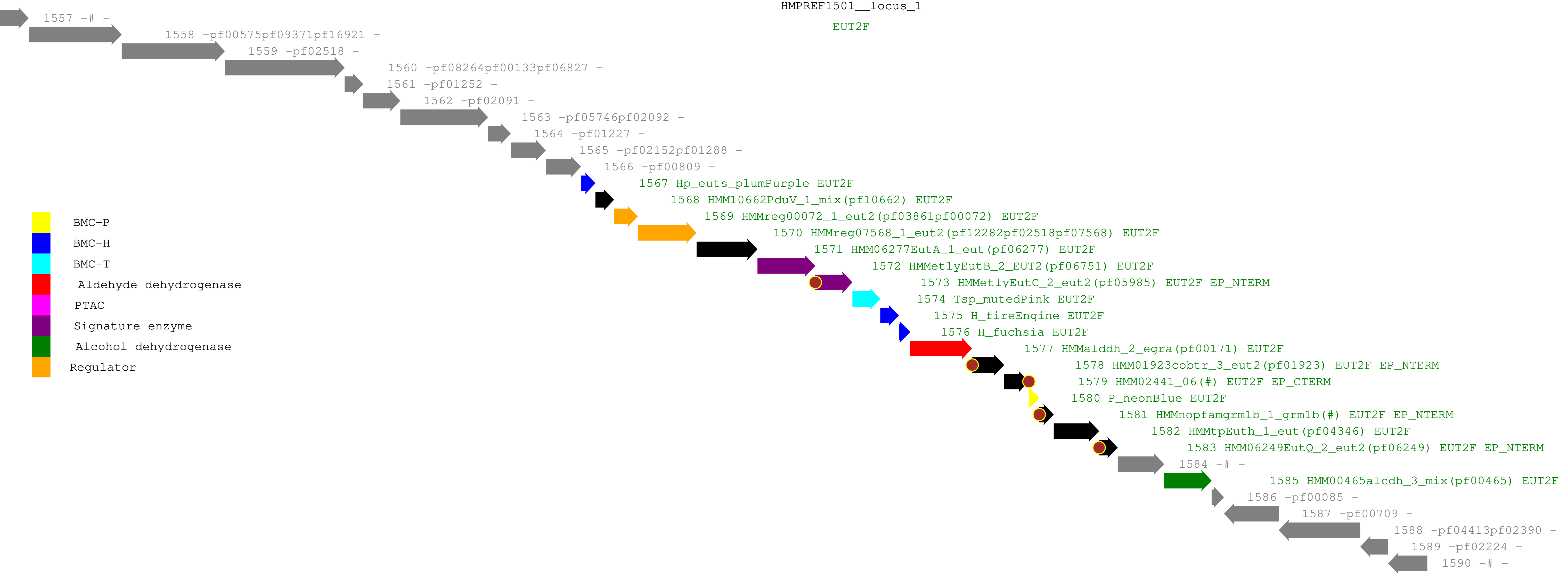

Fusobacterium nucleatum CTI-1. Bacteria; Fusobacteria; Fusobacteriales; Fusobacteriaceae;

HMPREF1538__locus_2

EUT2F

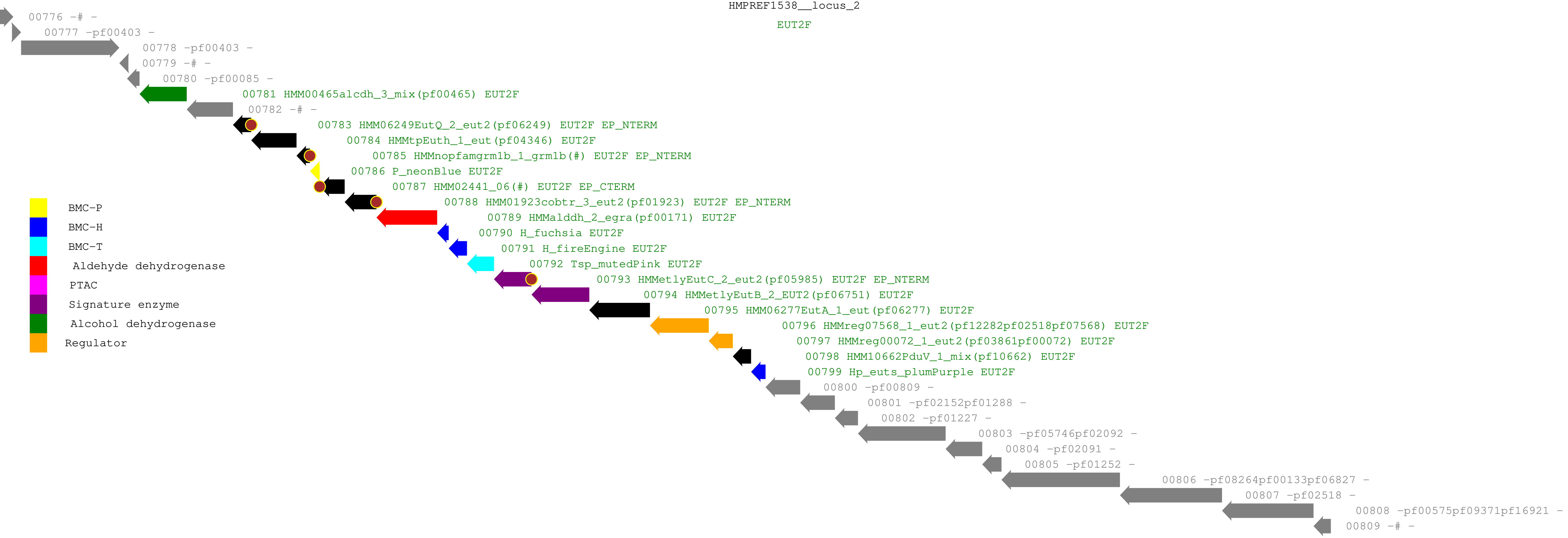

Fusobacterium nucleatum CTI-5. Bacteria; Fusobacteria; Fusobacteriales; Fusobacteriaceae;

HMPREF1766__locus_2

EUT2F

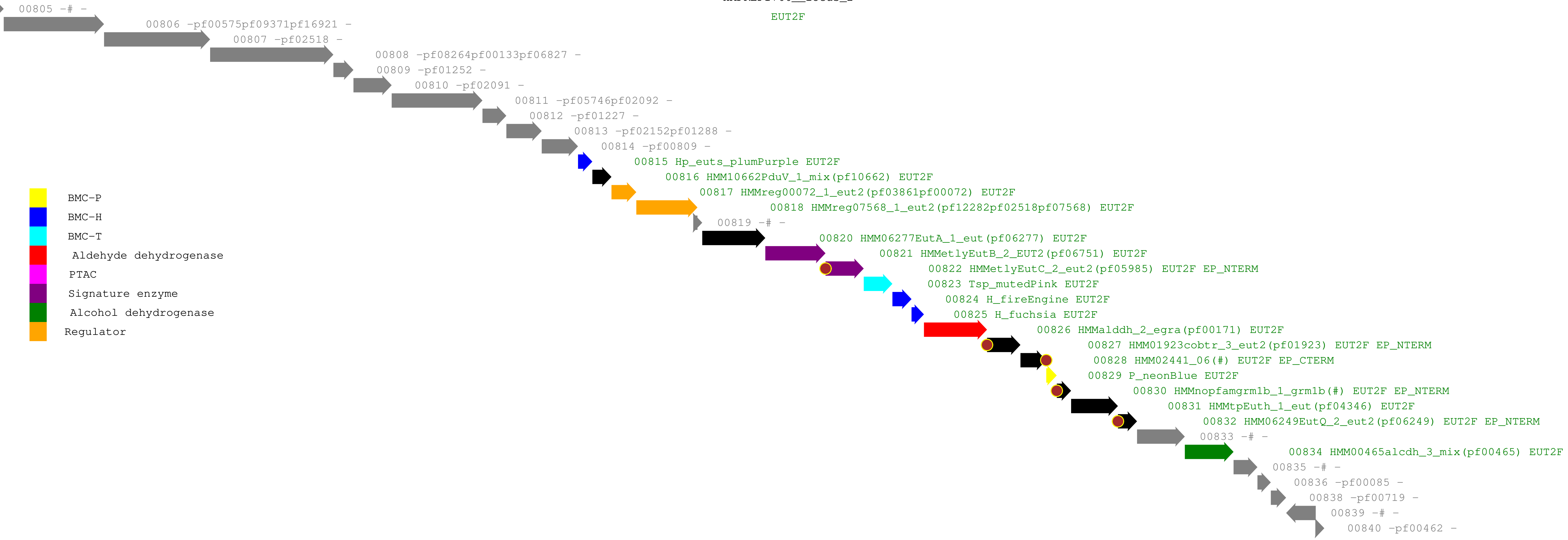

Fusobacterium nucleatum CTI-6. Bacteria; Fusobacteria; Fusobacteriales; Fusobacteriaceae;

HMPREF1767__locus_2

EUT2F

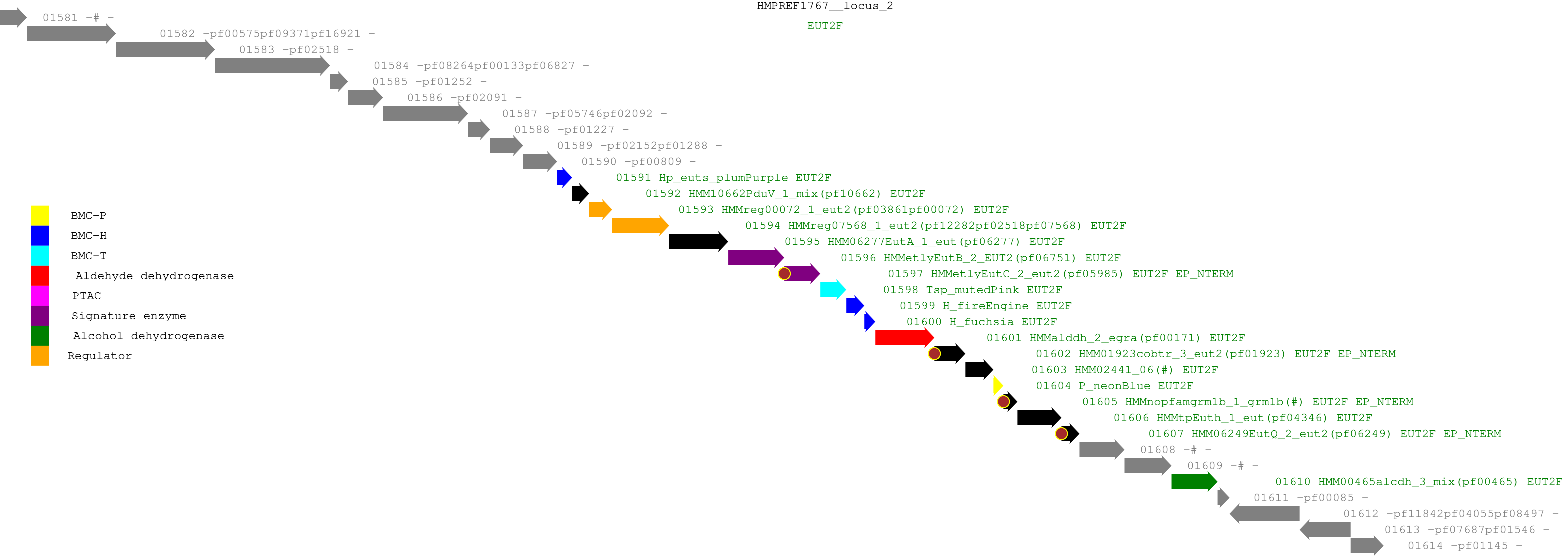

Fusobacterium nucleatum 13_3C. Bacteria; Fusobacteria; Fusobacteriales; Fusobacteriaceae;

HMPREF2085__locus_2

EUT2F

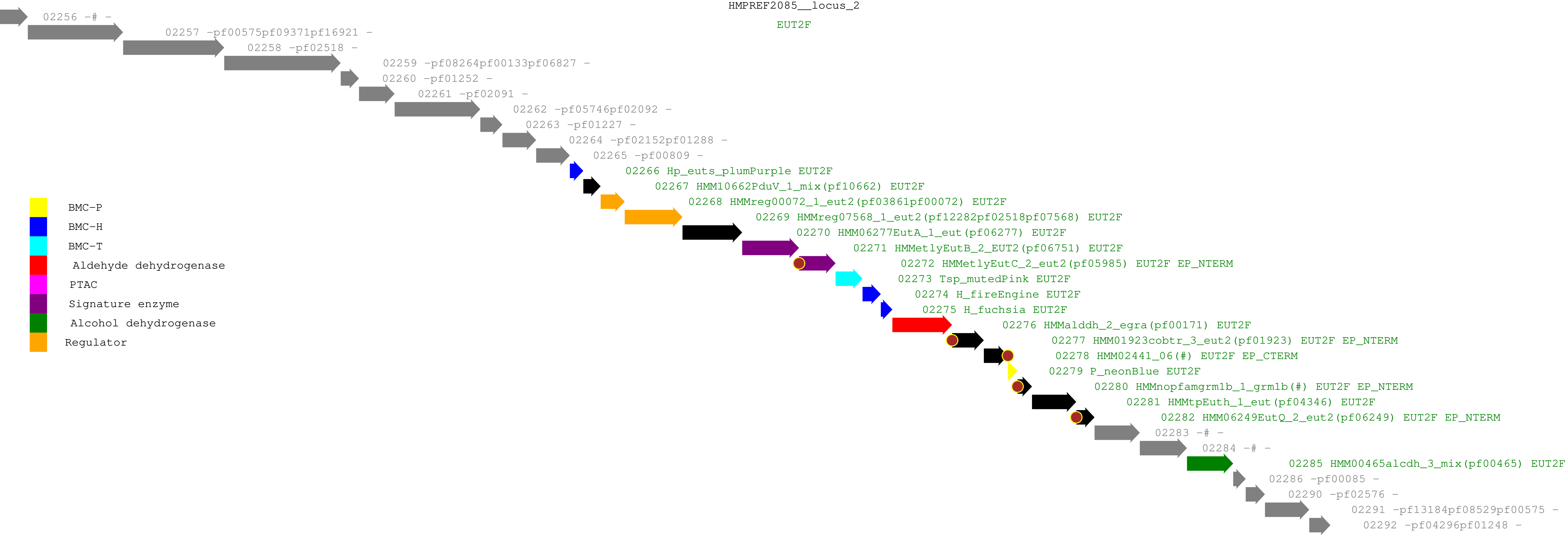

Fusobacterium sp. HMSC073F01. Bacteria; Fusobacteria; Fusobacteriales; Fusobacteriaceae;

HMPREF2747__locus_1

EUT2F

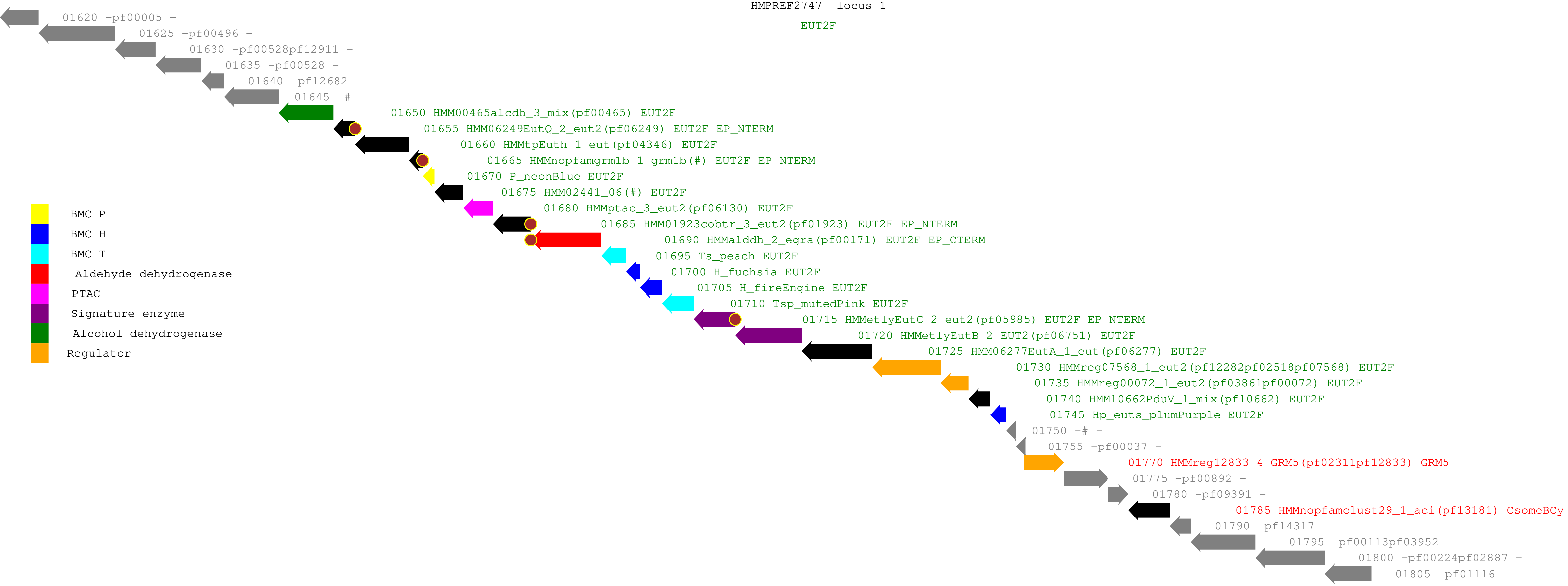

Fusobacterium sp. HMSC064B11. Bacteria; Fusobacteria; Fusobacteriales; Fusobacteriaceae;

HMPREF3051__locus_1

EUT2F

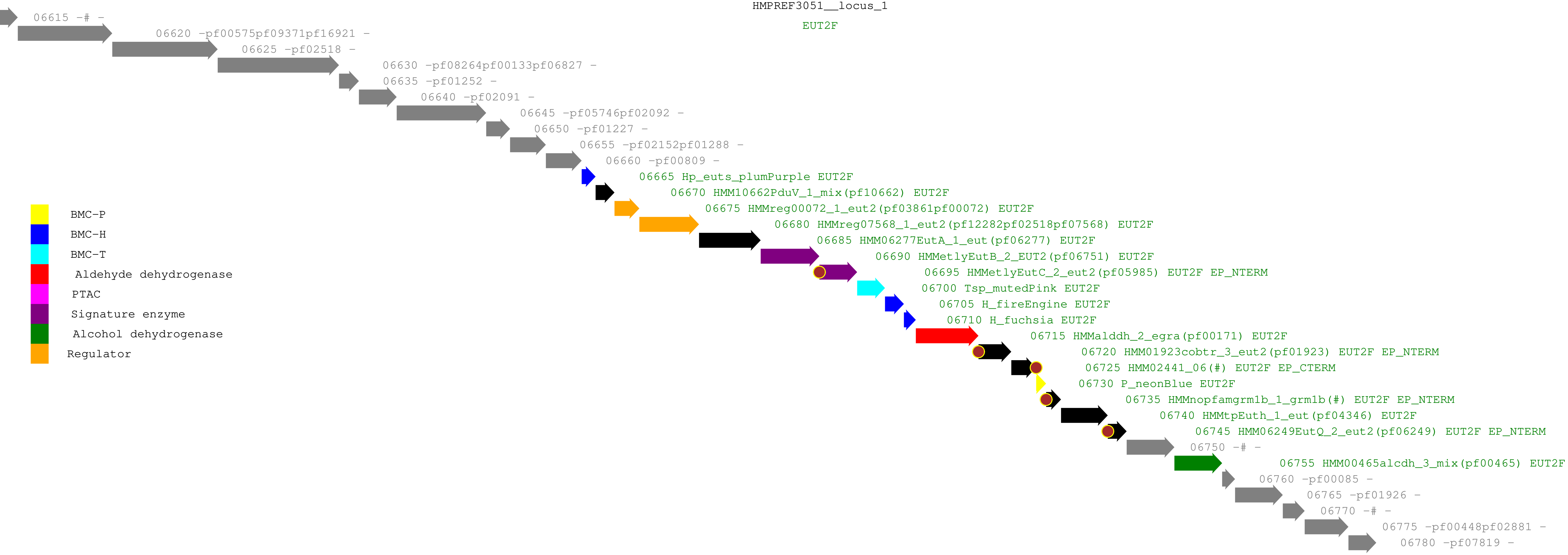

Fusobacterium nucleatum. Bacteria; Fusobacteria; Fusobacteriales; Fusobacteriaceae;

HMPREF3221__locus_1_main_sat

EUT2F

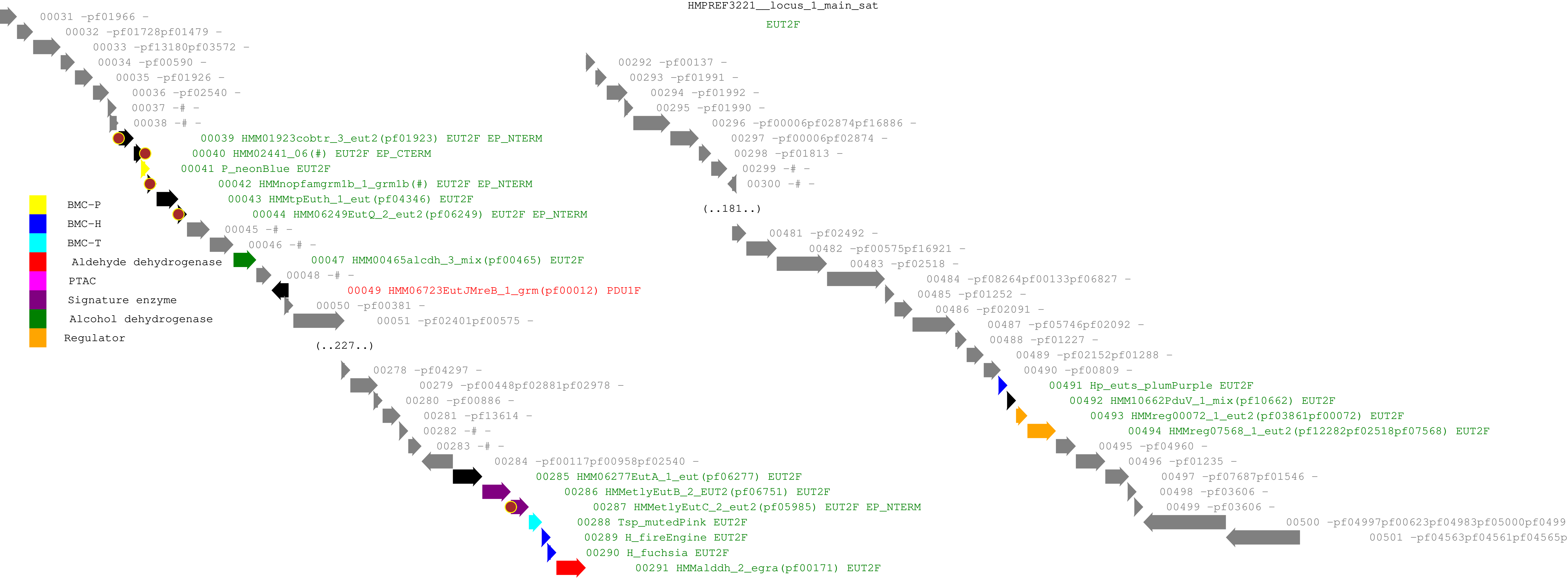

Fusobacterium nucleatum subsp. animalis ATCC 51191. Bacteria; Fusobacteria; Fusobacteriales; Fusobacteriaceae;

HMPREF9094__locus_2

EUT2F

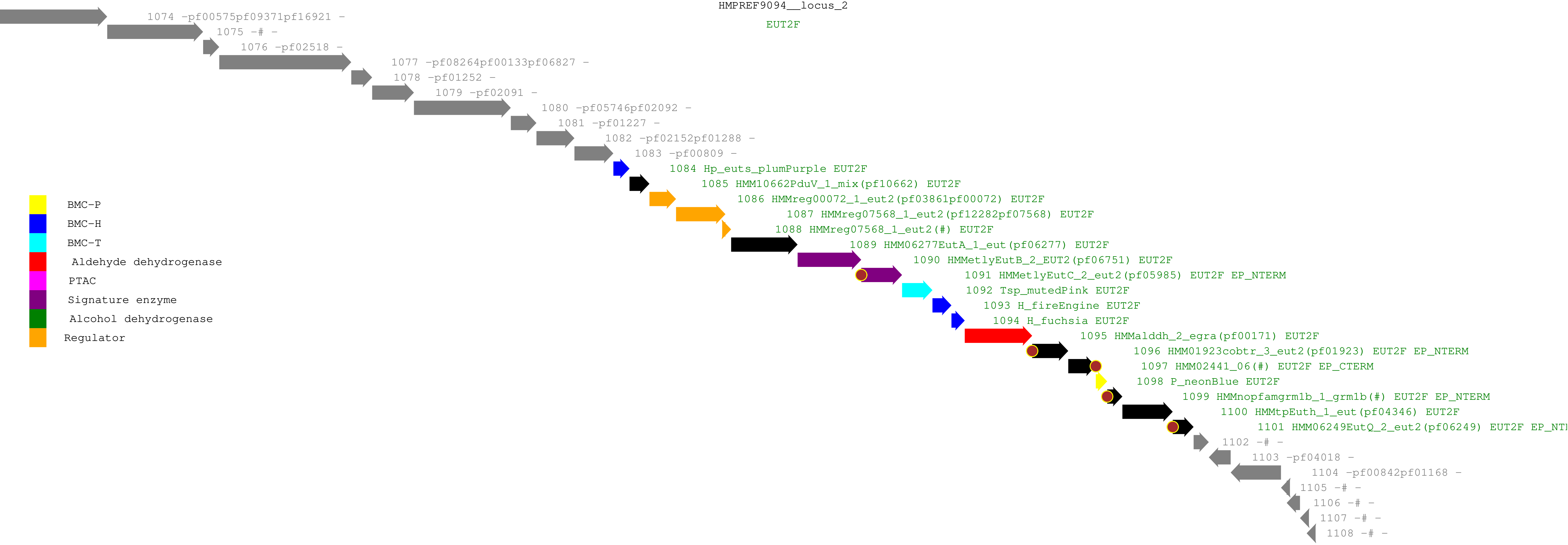

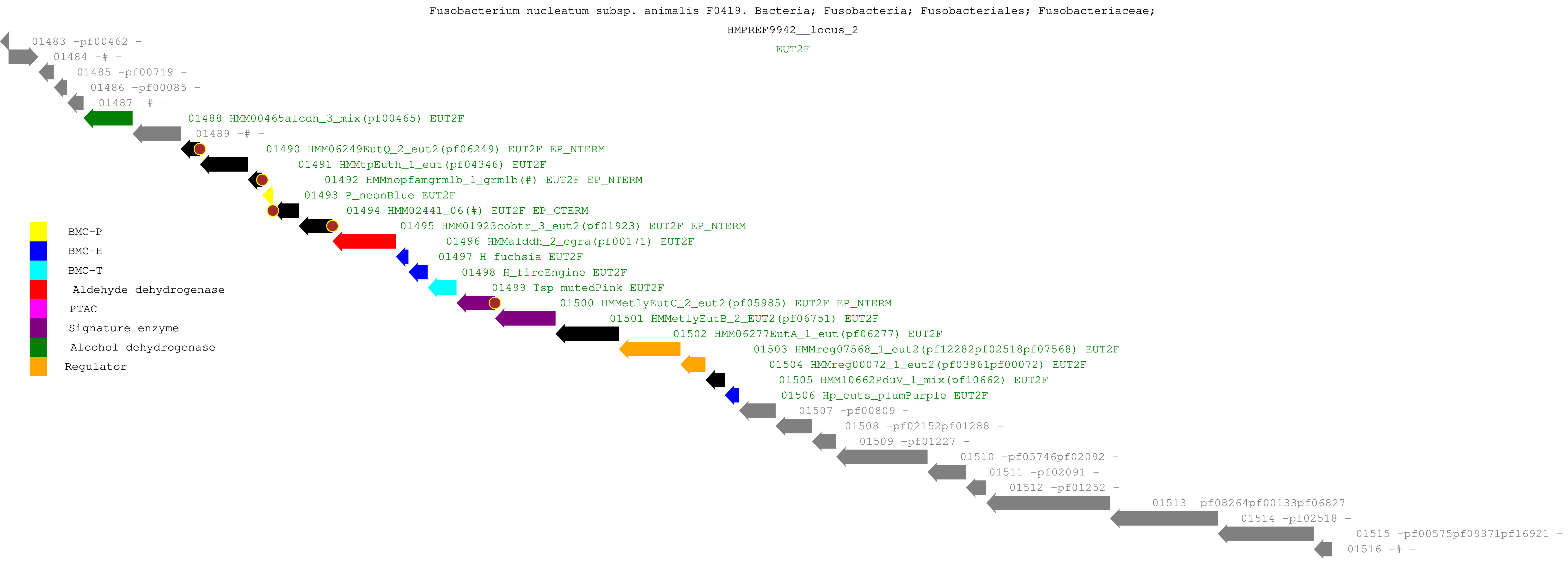
Fusobacterium nucleatum subsp. animalis F0419. Bacteria; Fusobacteria; Fusobacteriales; Fusobacteriaceae;
HMPREF9942__locus_2
EUT2F
01483 -pf00462 -
01484 -# -
01485 -pf00719 -
01486 -pf00085 -
01487 -# -
01488 HMM00465alcdh_3_mix(pf00465) EUT2F
01489 -# -
01490 HMM06249EutQ_2_eut2(pf06249) EUT2F EP_NTERM
01491 HMMtpEuth_1_eut(pf04346) EUT2F
01492 HMMnopfamgrm1b_1_grm1b(#) EUT2F EP_NTERM
01493 P_neonBlue EUT2F
01494 HMM02441_06(#) EUT2F EP_CTERM
01495 HMM01923cobtr_3_eut2(pf01923) EUT2F EP_NTERM
01496 HMMalddh_2_egra(pf00171) EUT2F
01497 H_fuchsia EUT2F
01498 H_fireEngine EUT2F
01499 Tsp_mutedPink EUT2F
01500 HMMetlyEutC_2_eut2(pf05985) EUT2F EP_NTERM
01501 HMMetlyEutB_2_EUT2(pf06751) EUT2F
01502 HMM06277EutA_1_eut(pf06277) EUT2F
01503 HMMreg07568_1_eut2(pf12282pf02518pf07568) EUT2F
01504 HMMreg00072_1_eut2(pf03861pf00072) EUT2F
01505 HMM10662PduV_1_mix(pf10662) EUT2F
01506 Hp_euts_plumPurple EUT2F
01507 -pf00809 -
01508 -pf02152pf01288 -
01509 -pf01227 -
01510 -pf05746pf02092 -
01511 -pf02091 -
01512 -pf01252 -
01513 -pf08264pf00133pf06827 -
01514 -pf02518 -
01515 -pf00575pf09371pf16921 -
01516 -# -
BMC-P
BMC-H
BMC-T
Aldehyde dehydrogenase
PTAC
Signature enzyme
Alcohol dehydrogenase
Regulator

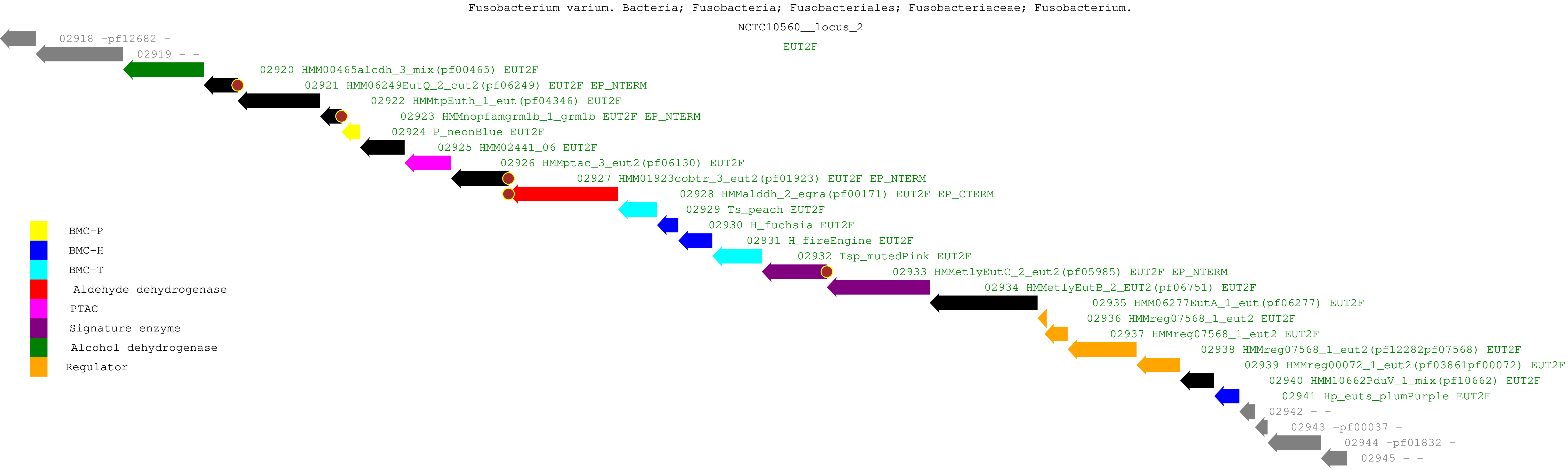

Fusobacterium varium. Bacteria; Fusobacteria; Fusobacteriales; Fusobacteriaceae; Fusobacterium.
NCTC10560__locus_2
EUT2F
02918 -pf12682 -
02919 - -
02920 HMM00465alcdh_3_mix(pf00465) EUT2F
02921 HMM06249EutQ_2_eut2(pf06249) EUT2F EP_NTERM
02922 HMMtpEuth_1_eut(pf04346) EUT2F
02923 HMMnopfamgrm1b_1_grm1b EUT2F EP_NTERM
02924 P_neonBlue EUT2F
02925 HMM02441_06 EUT2F
02926 HMMptac_3_eut2(pf06130) EUT2F
02927 HMM01923cobtr_3_eut2(pf01923) EUT2F EP_NTERM
02928 HMMalddh_2_egra(pf00171) EUT2F EP_CTERM
02929 Ts_peach EUT2F
02930 H_fuchsia EUT2F
02931 H_fireEngine EUT2F
02932 Tsp_mutedPink EUT2F
02933 HMMetlyEutC_2_eut2(pf05985) EUT2F EP_NTERM
02934 HMMetlyEutB_2_EUT2(pf06751) EUT2F
02935 HMM06277EutA_1_eut(pf06277) EUT2F
02936 HMMreg07568_1_eut2 EUT2F
02937 HMMreg07568_1_eut2 EUT2F
02938 HMMreg07568_1_eut2(pf12282pf07568) EUT2F
02939 HMMreg00072_1_eut2(pf03861pf00072) EUT2F
02940 HMM10662PduV_1_mix(pf10662) EUT2F
02941 Hp_euts_plumPurple EUT2F
02942 - -
02943 -pf00037 -
02944 -pf01832 -
02945 - -
BMC-P
BMC-H
BMC-T
Aldehyde dehydrogenase
PTAC
Signature enzyme
Alcohol dehydrogenase
Regulator

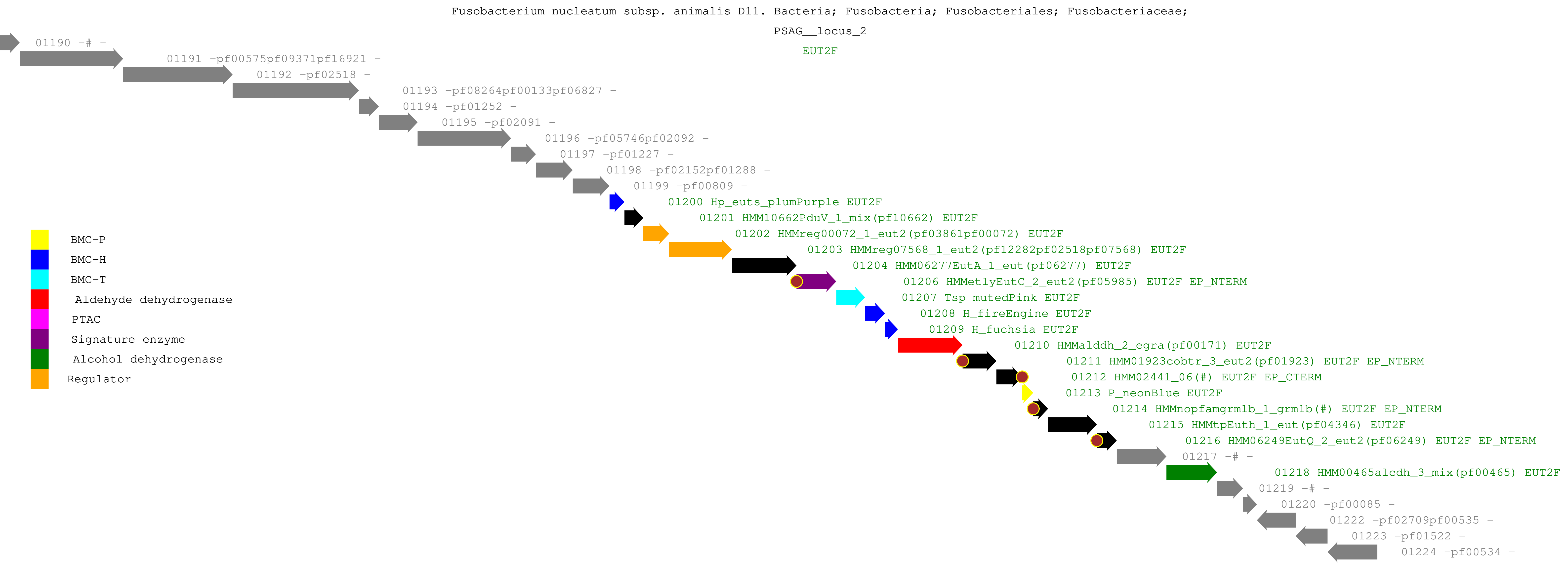

Fusobacterium nucleatum subsp. animalis D11. Bacteria; Fusobacteria; Fusobacteriales; Fusobacteriaceae;
PSAG__locus_2
EUT2F
01190 -# -
01191 -pf00575pf09371pf16921 -
01192 -pf02518 -
01193 -pf08264pf00133pf06827 -
01194 -pf01252 -
01195 -pf02091 -
01196 -pf05746pf02092 -
01197 -pf01227 -
01198 -pf02152pf01288 -
01199 -pf00809 -
01200 Hp_euts_plumPurple EUT2F
01201 HMM10662PduV_1_mix(pf10662) EUT2F
01202 HMMreg00072_1_eut2(pf03861pf00072) EUT2F
01203 HMMreg07568_1_eut2(pf12282pf02518pf07568) EUT2F
01204 HMM06277EutA_1_eut(pf06277) EUT2F
01206 HMMetlyEutC_2_eut2(pf05985) EUT2F EP_NTERM
01207 Tsp_mutedPink EUT2F
01208 H_fireEngine EUT2F
01209 H_fuchsia EUT2F
01210 HMMaldh_2_egra(pf00171) EUT2F
01211 HMM01923cobtr_3_eut2(pf01923) EUT2F EP_NTERM
01212 HMM02441_06(#) EUT2F EP_CTERM
01213 P_neonBlue EUT2F
01214 HMMnopfamgrm1b_1_grm1b(#) EUT2F EP_NTERM
01215 HMMtpEuth_1_eut(pf04346) EUT2F
01216 HMM06249EutQ_2_eut2(pf06249) EUT2F EP_NTERM
01217 -# -
01218 HMM00465alcdh_3_mix(pf00465) EUT2F
01219 -# -
01220 -pf00085 -
01222 -pf02709pf00535 -
01223 -pf01522 -
01224 -pf00534 -
BMC-P
BMC-H
BMC-T
Aldehyde dehydrogenase
PTAC
Signature enzyme
Alcohol dehydrogenase
Regulator

Fusobacterium hwasookii ChDC F174. Bacteria; Fusobacteria; Fusobacteriales; Fusobacteriaceae;

RN87__locus_2

EUT2F

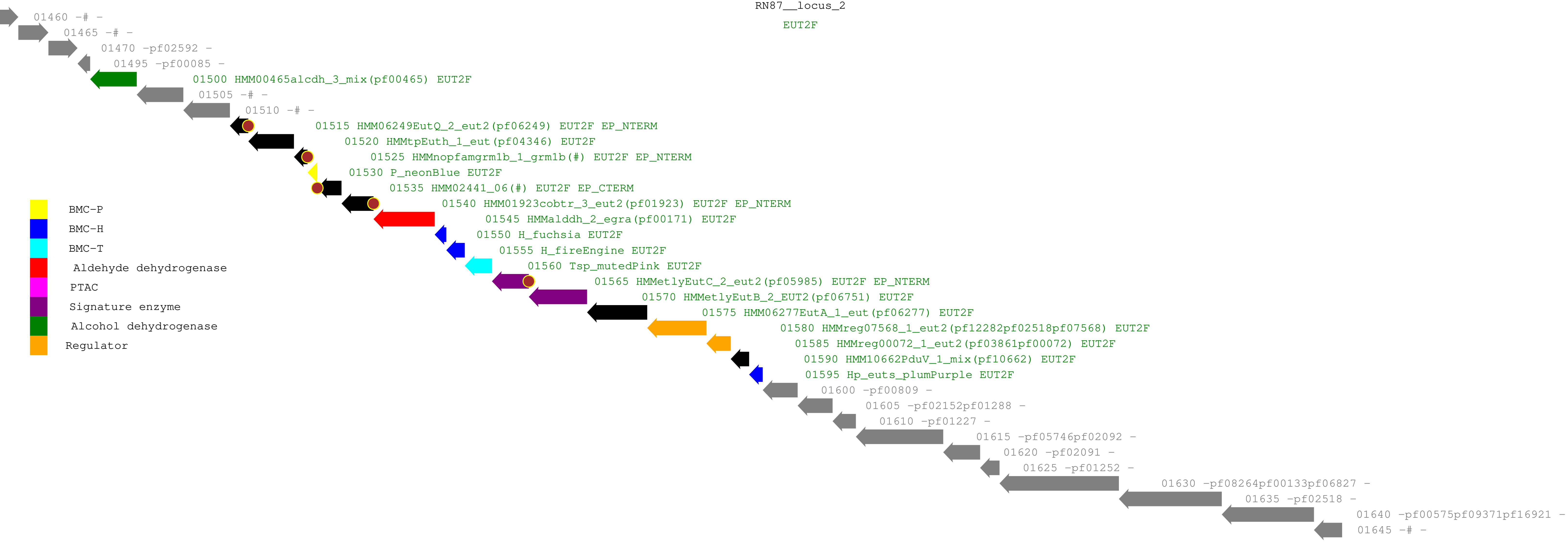

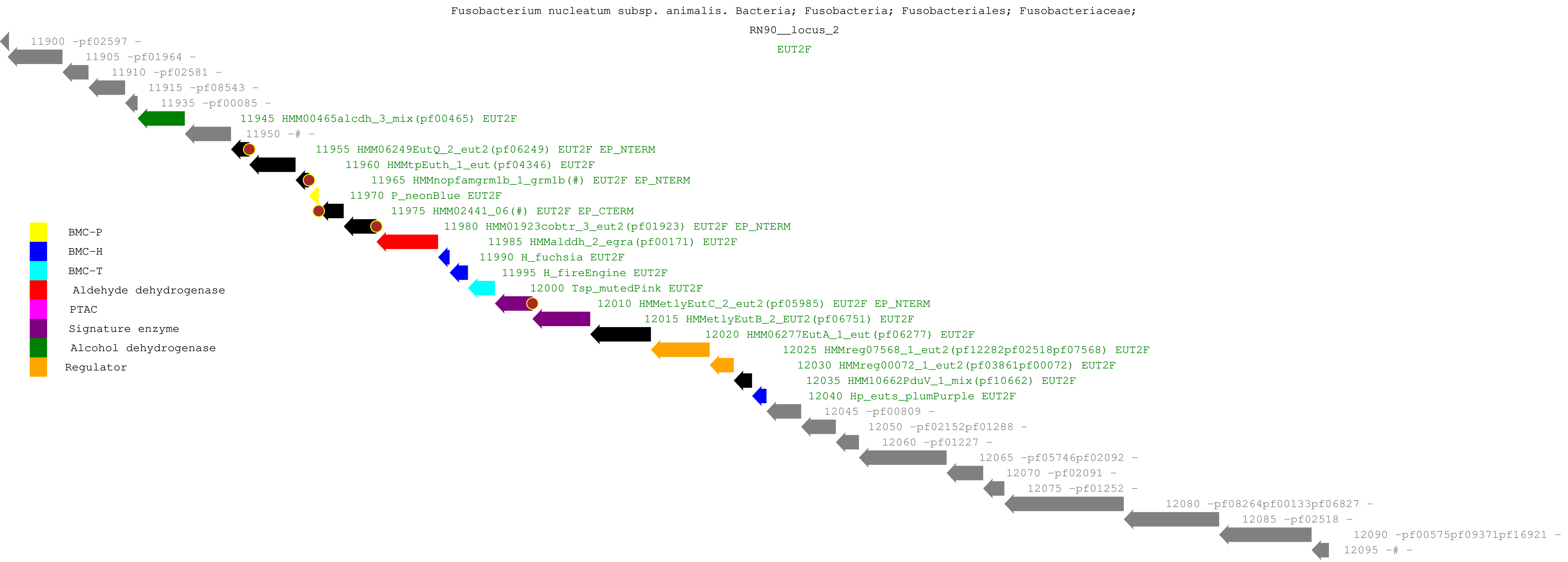
Fusobacterium nucleatum subsp. animalis. Bacteria; Fusobacteria; Fusobacteriales; Fusobacteriaceae;
RN90__locus_2
EUT2F
11900 -pf02597 -
11905 -pf01964 -
11910 -pf02581 -
11915 -pf08543 -
11935 -pf00085 -
11945 HMM00465alcdh_3_mix(pf00465) EUT2F
11950 -# -
11955 HMM06249EutQ_2_eut2(pf06249) EUT2F EP_NTERM
11960 HMMtpEuth_1_eut(pf04346) EUT2F
11965 HMMnopfamgrm1b_1_grm1b(#) EUT2F EP_NTERM
11970 P_neonBlue EUT2F
11975 HMM02441_06(#) EUT2F EP_CTERM
11980 HMM01923cobtr_3_eut2(pf01923) EUT2F EP_NTERM
11985 HMMalddh_2_egra(pf00171) EUT2F
11990 H_fuchsia EUT2F
11995 H_fireEngine EUT2F
12000 Tsp_mutedPink EUT2F
12010 HMMetlyEutC_2_eut2(pf05985) EUT2F EP_NTERM
12015 HMMetlyEutB_2_EUT2(pf06751) EUT2F
12020 HMM06277EutA_1_eut(pf06277) EUT2F
12025 HMMreg07568_1_eut2(pf12282pf02518pf07568) EUT2F
12030 HMMreg00072_1_eut2(pf03861pf00072) EUT2F
12035 HMM10662PduV_1_mix(pf10662) EUT2F
12040 Hp_euts_plumPurple EUT2F
12045 -pf00809 -
12050 -pf02152pf01288 -
12060 -pf01227 -
12065 -pf05746pf02092 -
12070 -pf02091 -
12075 -pf01252 -
12080 -pf08264pf00133pf06827 -
12085 -pf02518 -
12090 -pf00575pf09371pf16921 -
12095 -# -
BMC-P
BMC-H
BMC-T
Aldehyde dehydrogenase
PTAC
Signature enzyme
Alcohol dehydrogenase
Regulator

Fusobacterium nucleatum subsp. polymorphum. Bacteria; Fusobacteria; Fusobacteriales; Fusobacteriaceae;

RN93__locus_1

EUT2F

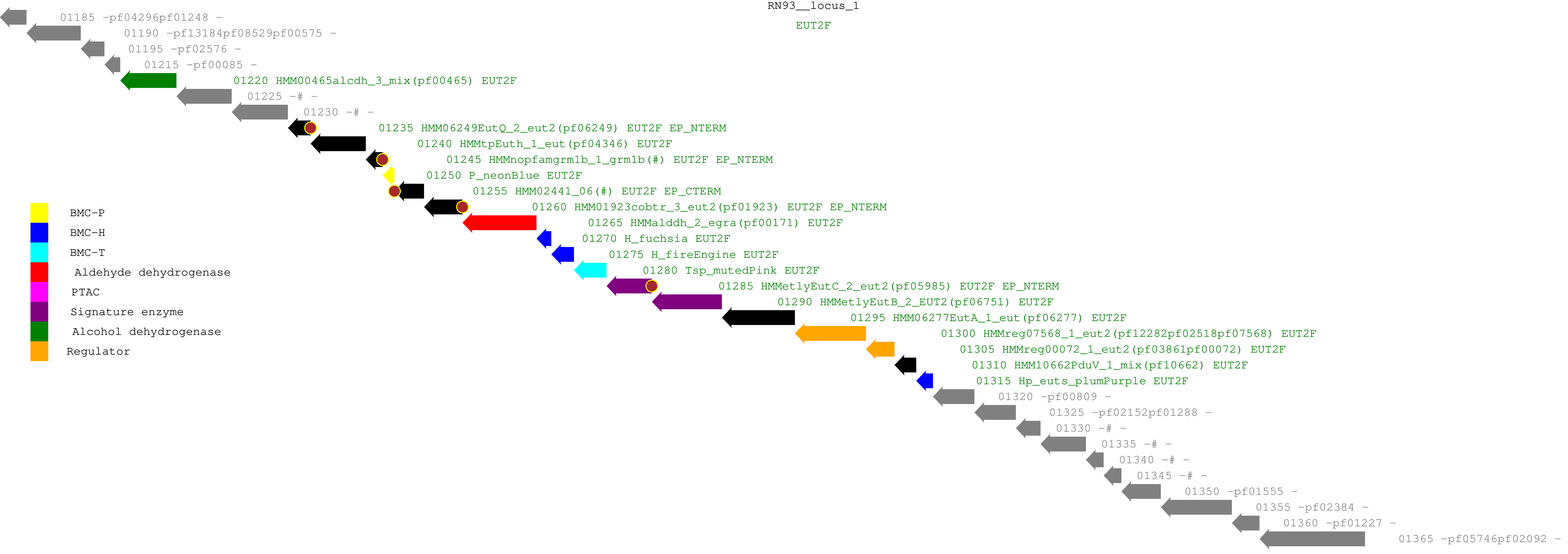

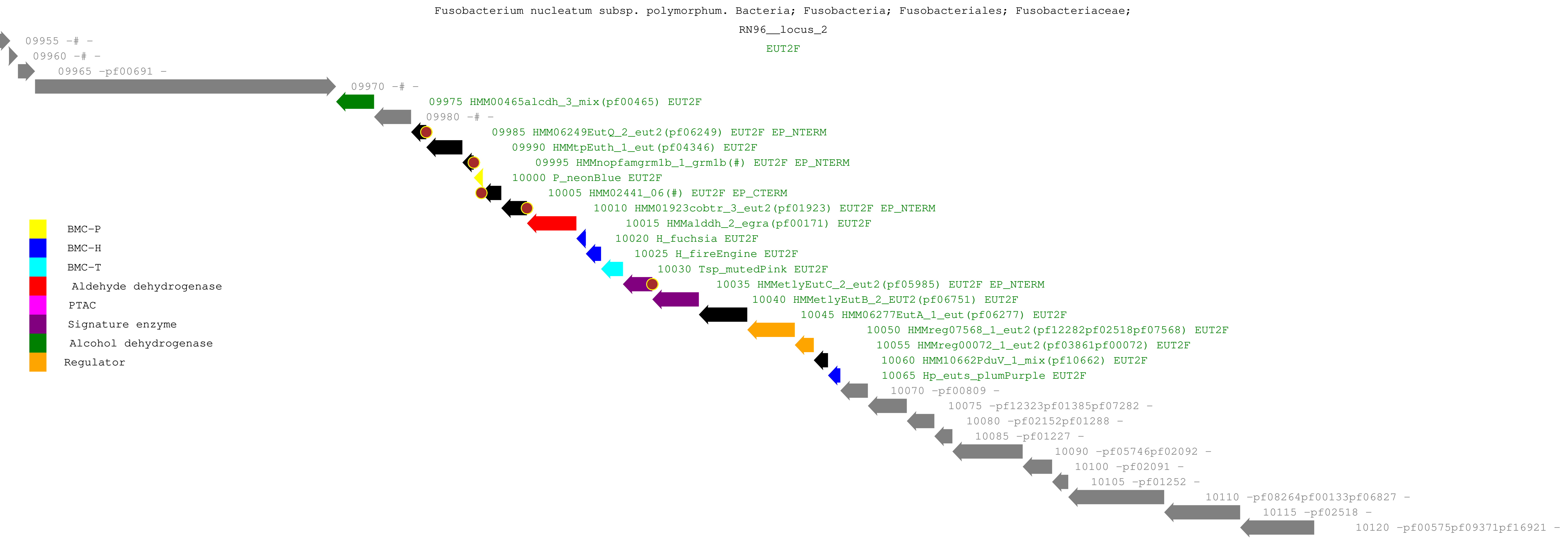
Fusobacterium nucleatum subsp. polymorphum. Bacteria; Fusobacteria; Fusobacteriales; Fusobacteriaceae;
RN96__locus_2
EUT2F
09955 -# -
09960 -# -
09965 -pf00691 -
09970 -# -
09975 HMM00465alcdh_3_mix(pf00465) EUT2F
09980 -# -
09985 HMM06249EutQ_2_eut2(pf06249) EUT2F EP_NTERM
09990 HMMtpEuth_1_eut(pf04346) EUT2F
09995 HMMnopfamgrm1b_1_grm1b(#) EUT2F EP_NTERM
10000 P_neonBlue EUT2F
10005 HMM02441_06(#) EUT2F EP_CTERM
10010 HMM01923cobtr_3_eut2(pf01923) EUT2F EP_NTERM
10015 HMMalddh_2_egra(pf00171) EUT2F
10020 H_fuchsia EUT2F
10025 H_fireEngine EUT2F
10030 Tsp_mutedPink EUT2F
10035 HMMetlyEutC_2_eut2(pf05985) EUT2F EP_NTERM
10040 HMMetlyEutB_2_EUT2(pf06751) EUT2F
10045 HMM06277EutA_1_eut(pf06277) EUT2F
10050 HMMreg07568_1_eut2(pf12282pf02518pf07568) EUT2F
10055 HMMreg00072_1_eut2(pf03861pf00072) EUT2F
10060 HMM10662PduV_1_mix(pf10662) EUT2F
10065 Hp_euts_plumPurple EUT2F
10070 -pf00809 -
10075 -pf12323pf01385pf07282 -
10080 -pf02152pf01288 -
10085 -pf01227 -
10090 -pf05746pf02092 -
10100 -pf02091 -
10105 -pf01252 -
10110 -pf08264pf00133pf06827 -
10115 -pf02518 -
10120 -pf00575pf09371pf16921 -
BMC-P
BMC-H
BMC-T
Aldehyde dehydrogenase
PTAC
Signature enzyme
Alcohol dehydrogenase
Regulator

Fusobacterium hwasookii ChDC F300. Bacteria; Fusobacteria; Fusobacteriales; Fusobacteriaceae;

RN97__locus_1

EUT2F

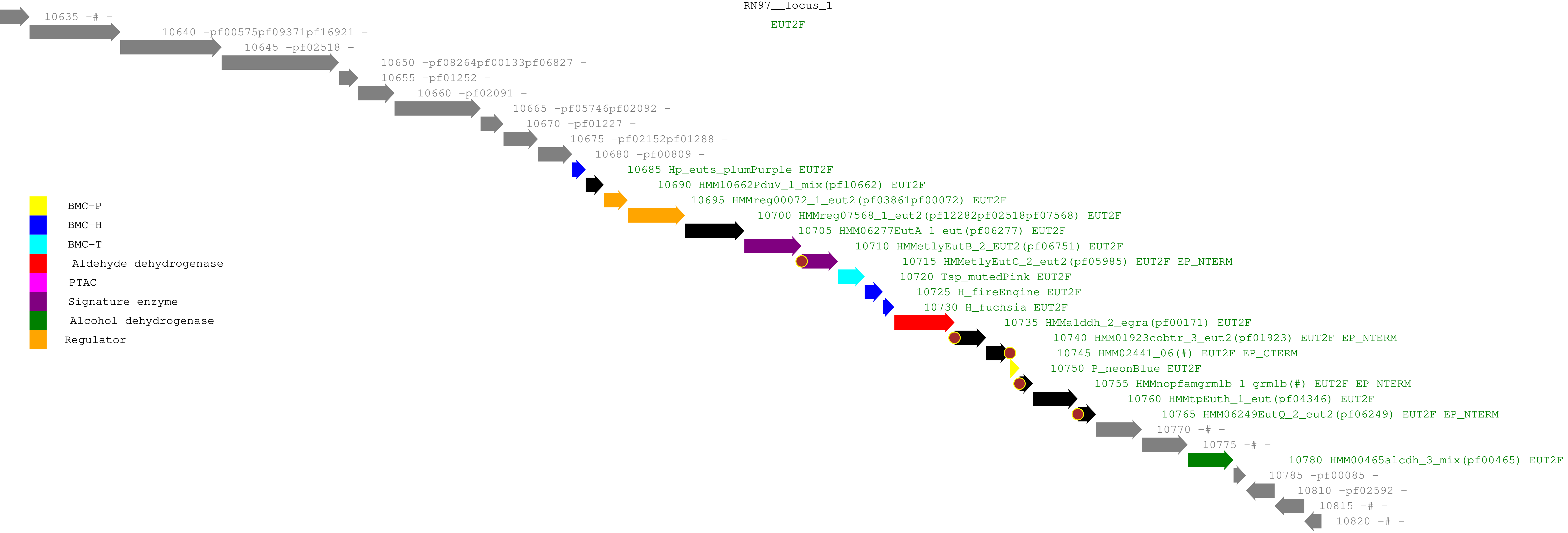

Fusobacterium nucleatum subsp. nucleatum. Bacteria; Fusobacteria; Fusobacteriales; Fusobacteriaceae;

RO03__locus_1

EUT2F

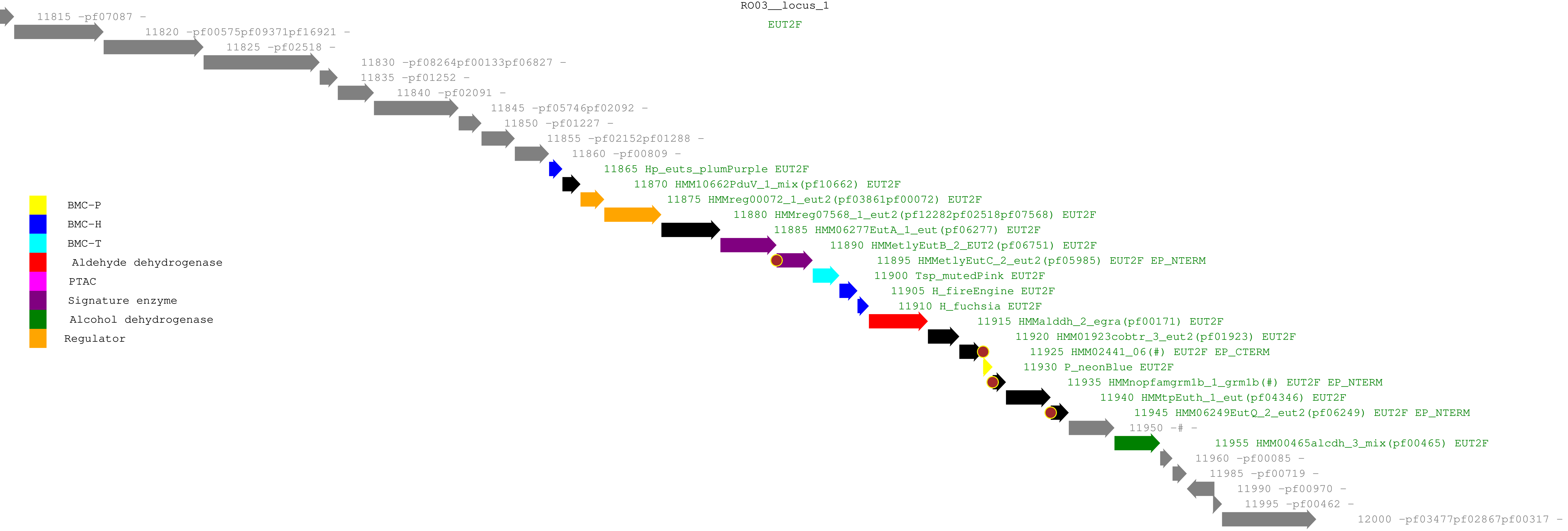

Cetobacterium ceti. Bacteria; Fusobacteria; Fusobacteriales; Fusobacteriaceae;

SAMN02745174__locus_1

EUT2F

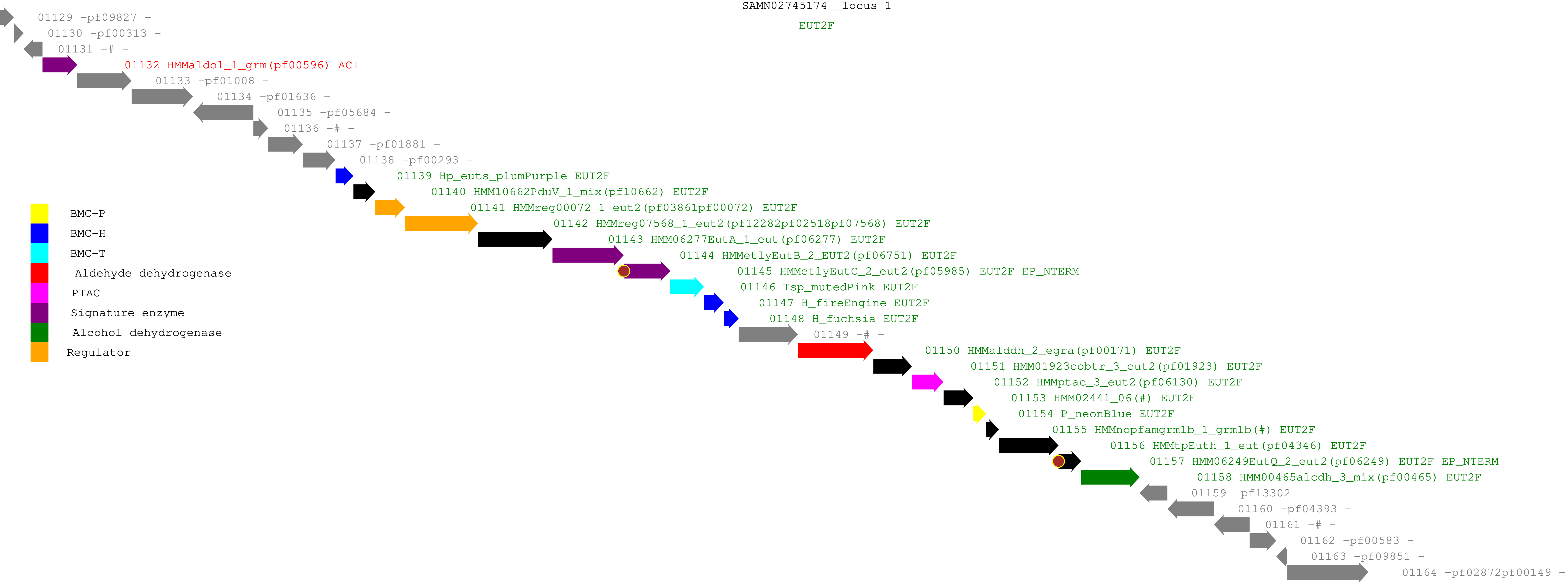

Flavonifractor plautii. Bacteria; Firmicutes; Clostridia; Clostridiales; Flavonifractor.

A4U99__locus_1

EUT2G

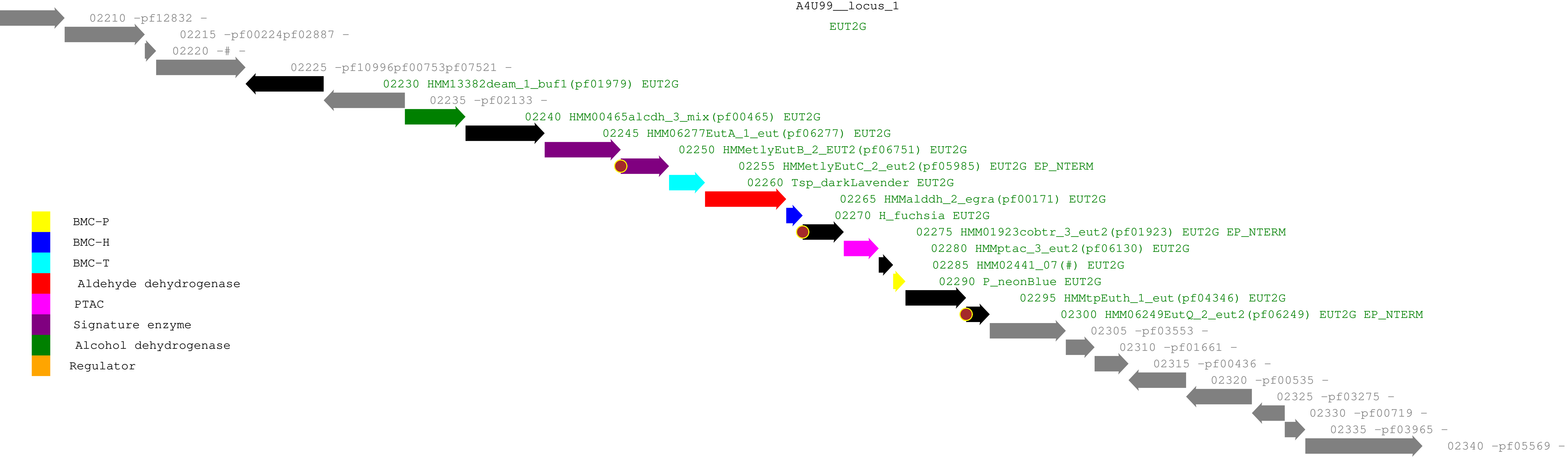

Flavonifractor sp. An10. Bacteria; Firmicutes; Clostridia; Clostridiales; Flavonifractor.

B5E42__locus_1

EUT2G

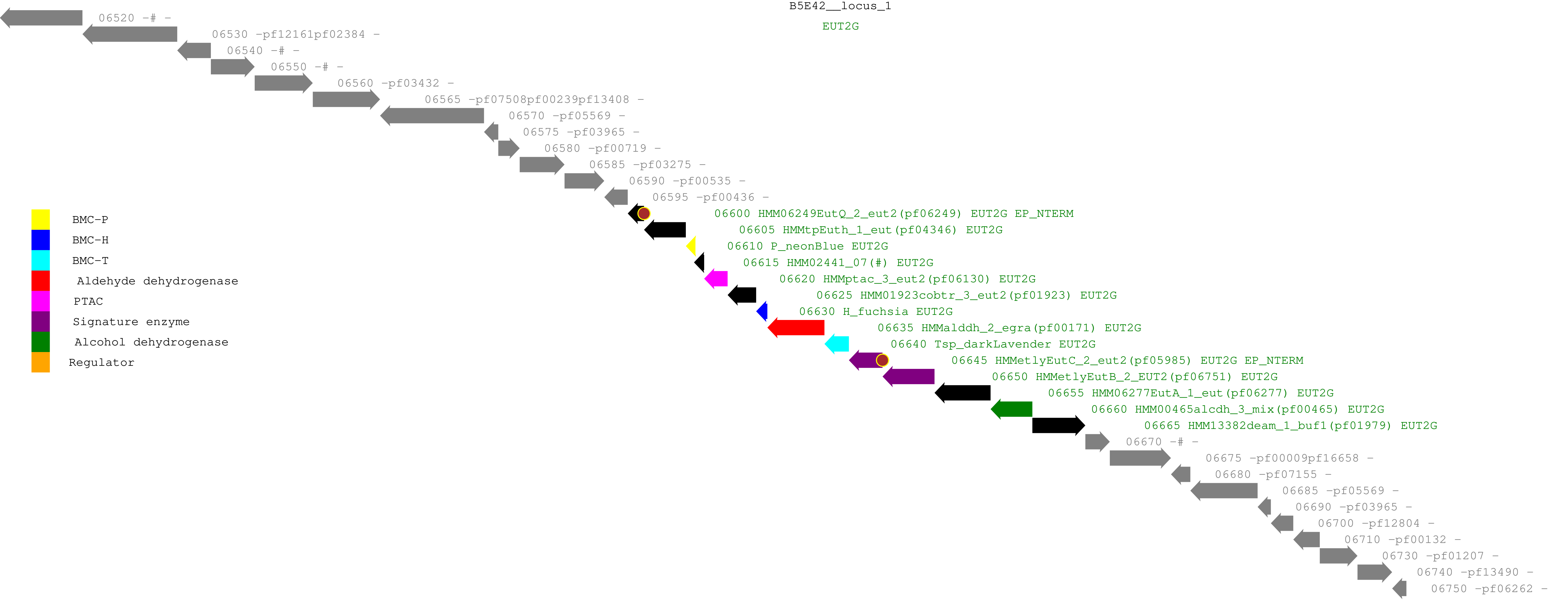

Flavonifractor sp. An112. Bacteria; Firmicutes; Clostridia; Clostridiales; Flavonifractor.

B5E56__locus_1

EUT2G

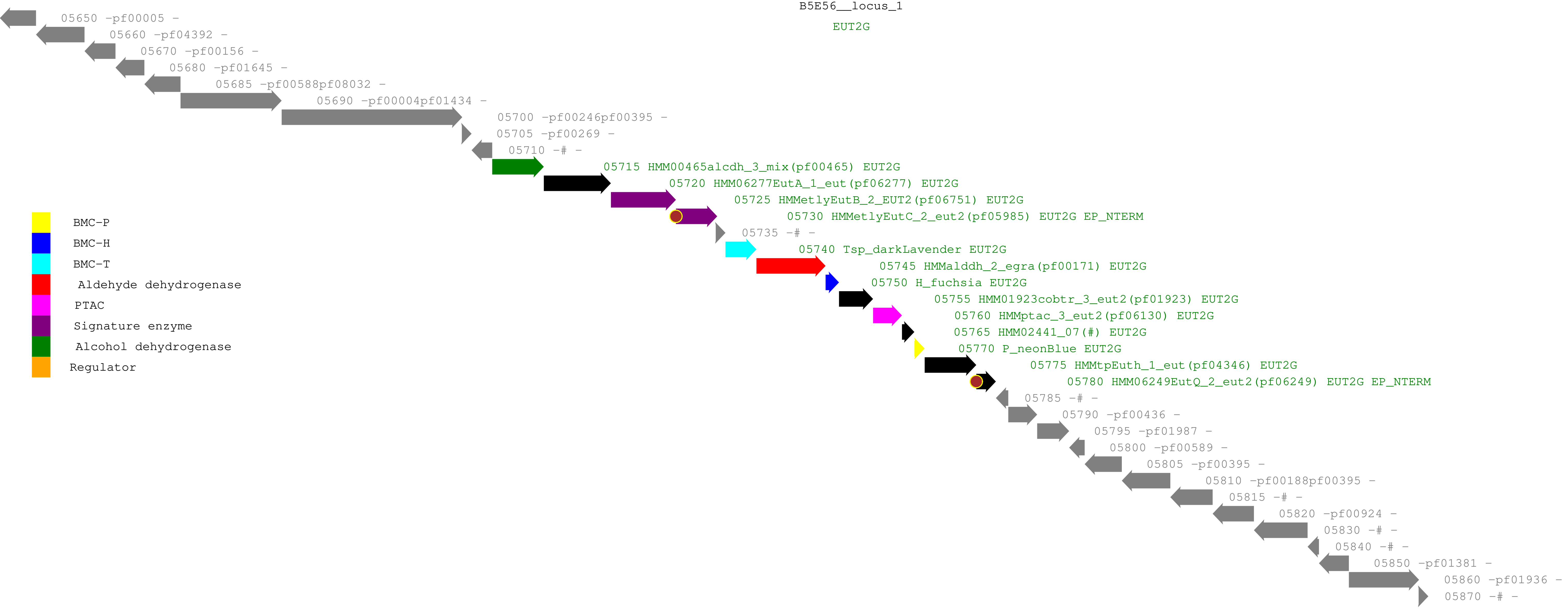

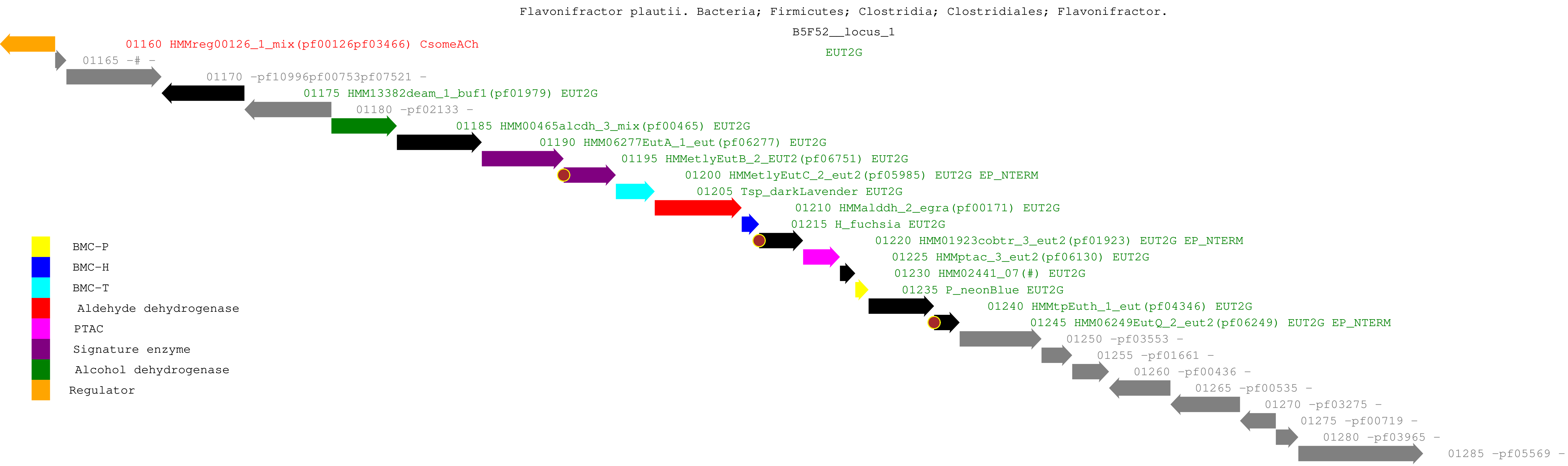
Flavonifractor plautii. Bacteria; Firmicutes; Clostridia; Clostridiales; Flavonifractor.
B5F52__locus_1
EUT2G
01160 HMMreg00126_1_mix(pf00126pf03466) CsomeACh
01165 -# -
01170 -pf10996pf00753pf07521 -
01175 HMM13382deam_1_buf1(pf01979) EUT2G
01180 -pf02133 -
01185 HMM00465alcdh_3_mix(pf00465) EUT2G
01190 HMM06277EutA_1_eut(pf06277) EUT2G
01195 HMMetlyEutB_2_EUT2(pf06751) EUT2G
01200 HMMetlyEutC_2_eut2(pf05985) EUT2G EP_NTERM
01205 Tsp_darkLavender EUT2G
01210 HMMalddh_2_egra(pf00171) EUT2G
01215 H_fuchsia EUT2G
01220 HMM01923cobtr_3_eut2(pf01923) EUT2G EP_NTERM
01225 HMMptac_3_eut2(pf06130) EUT2G
01230 HMM02441_07(#) EUT2G
01235 P_neonBlue EUT2G
01240 HMMtpEuth_1_eut(pf04346) EUT2G
01245 HMM06249EutQ_2_eut2(pf06249) EUT2G EP_NTERM
01250 -pf03553 -
01255 -pf01661 -
01260 -pf00436 -
01265 -pf00535 -
01270 -pf03275 -
01275 -pf00719 -
01280 -pf03965 -
01285 -pf05569 -
BMC-P
BMC-H
BMC-T
Aldehyde dehydrogenase
PTAC
Signature enzyme
Alcohol dehydrogenase
Regulator

Flavonifractor sp. An306. Bacteria; Firmicutes; Clostridia; Clostridiales; Flavonifractor.

B5F88__locus_1

EUT2G

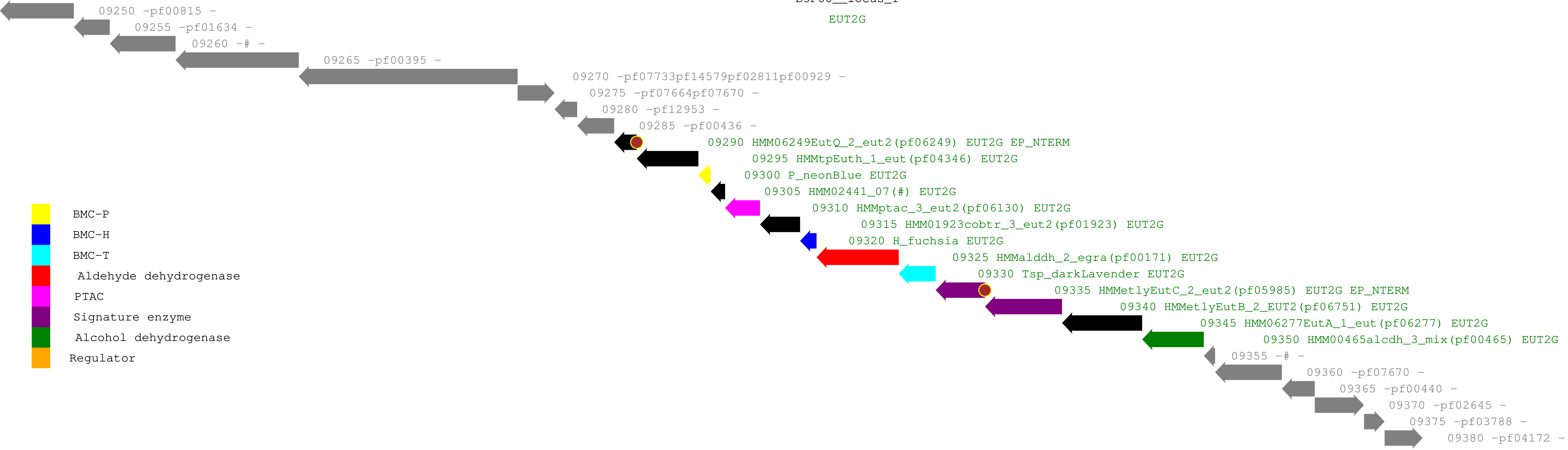

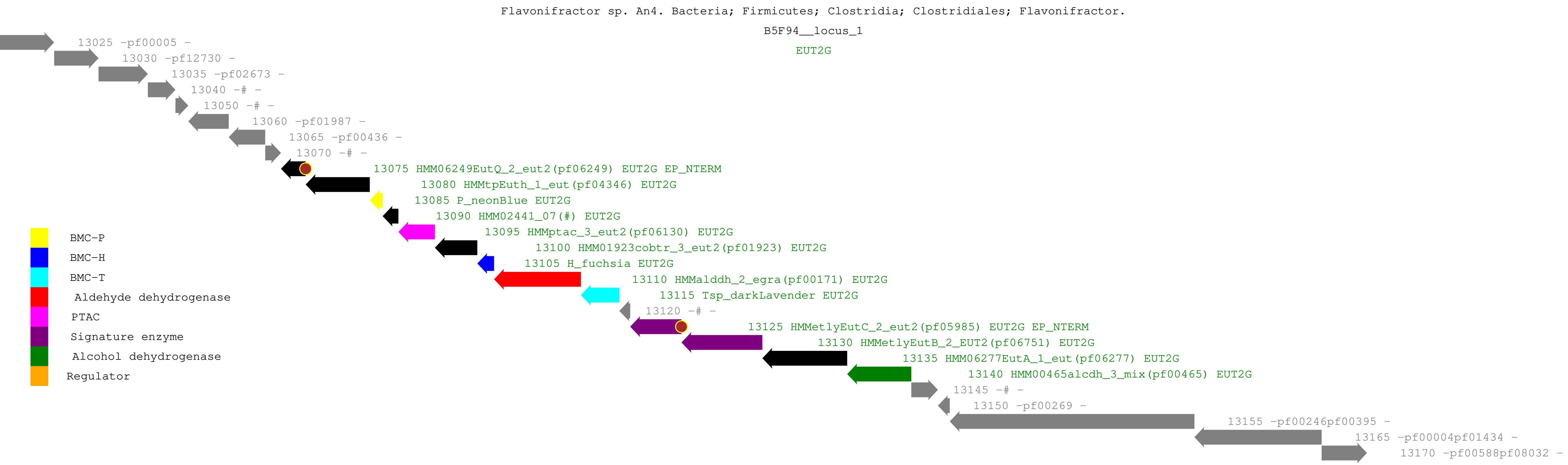
Flavonifractor sp. An4. Bacteria; Firmicutes; Clostridia; Clostridiales; Flavonifractor.
B5F94__locus_1
EUT2G
13025 -pf00005 -
13030 -pf12730 -
13035 -pf02673 -
13040 -# -
13050 -# -
13060 -pf01987 -
13065 -pf00436 -
13070 -# -
13075 HMM06249EutQ_2_eut2(pf06249) EUT2G EP_NTERM
13080 HMMtpEuth_1_eut(pf04346) EUT2G
13085 P_neonBlue EUT2G
13090 HMM02441_07(#) EUT2G
13095 HMMptac_3_eut2(pf06130) EUT2G
13100 HMM01923cobtr_3_eut2(pf01923) EUT2G
13105 H_fuchsia EUT2G
13110 HMMalddh_2_egra(pf00171) EUT2G
13115 Tsp_darkLavender EUT2G
13120 -# -
13125 HMMetlyEutC_2_eut2(pf05985) EUT2G EP_NTERM
13130 HMMetlyEutB_2_EUT2(pf06751) EUT2G
13135 HMM06277EutA_1_eut(pf06277) EUT2G
13140 HMM00465alcdh_3_mix(pf00465) EUT2G
13145 -# -
13150 -pf00269 -
13155 -pf00246pf00395 -
13165 -pf00004pf01434 -
13170 -pf00588pf08032 -
BMC-P
BMC-H
BMC-T
Aldehyde dehydrogenase
PTAC
Signature enzyme
Alcohol dehydrogenase
Regulator

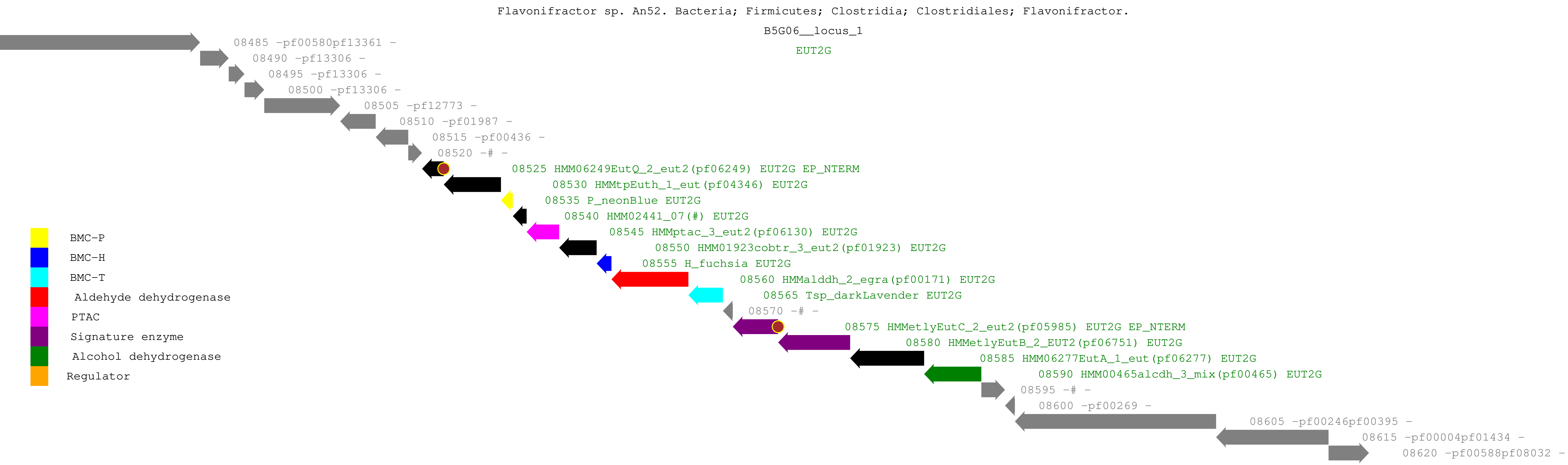
Flavonifractor sp. An52. Bacteria; Firmicutes; Clostridia; Clostridiales; Flavonifractor.
B5G06__locus_1
EUT2G
08485 -pf00580pf13361 -
08490 -pf13306 -
08495 -pf13306 -
08500 -pf13306 -
08505 -pf12773 -
08510 -pf01987 -
08515 -pf00436 -
08520 -# -
08525 HMM06249EutQ_2_eut2(pf06249) EUT2G EP_NTERM
08530 HMMtpEuth_1_eut(pf04346) EUT2G
08535 P_neonBlue EUT2G
08540 HMM02441_07(#) EUT2G
08545 HMMptac_3_eut2(pf06130) EUT2G
08550 HMM01923cobtr_3_eut2(pf01923) EUT2G
08555 H_fuchsia EUT2G
08560 HMMalddh_2_egra(pf00171) EUT2G
08565 Tsp_darkLavender EUT2G
08570 -# -
08575 HMMetlyEutC_2_eut2(pf05985) EUT2G EP_NTERM
08580 HMMetlyEutB_2_EUT2(pf06751) EUT2G
08585 HMM06277EutA_1_eut(pf06277) EUT2G
08590 HMM00465alcdh_3_mix(pf00465) EUT2G
08595 -# -
08600 -pf00269 -
08605 -pf00246pf00395 -
08615 -pf00004pf01434 -
08620 -pf00588pf08032 -
BMC-P
BMC-H
BMC-T
Aldehyde dehydrogenase
PTAC
Signature enzyme
Alcohol dehydrogenase
Regulator

Flavonifractor sp. An82. Bacteria; Firmicutes; Clostridia; Clostridiales; Flavonifractor.

B5G34__locus_1

EUT2G

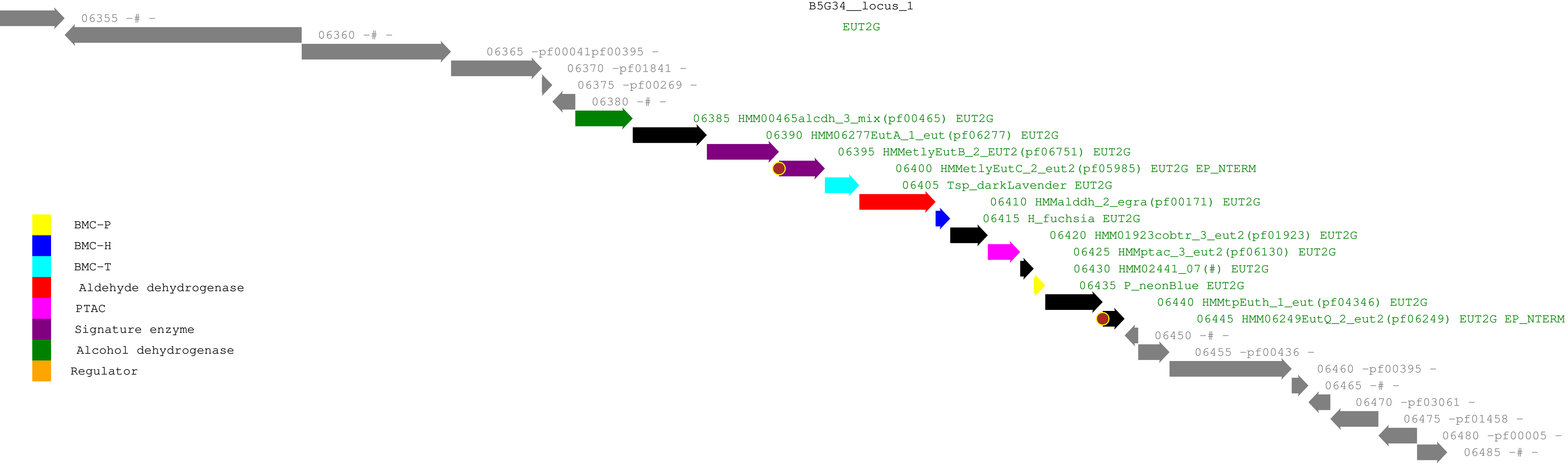

Flavonifractor sp. An9. Bacteria; Firmicutes; Clostridia; Clostridiales; Flavonifractor.

B5G40__locus_1

EUT2G

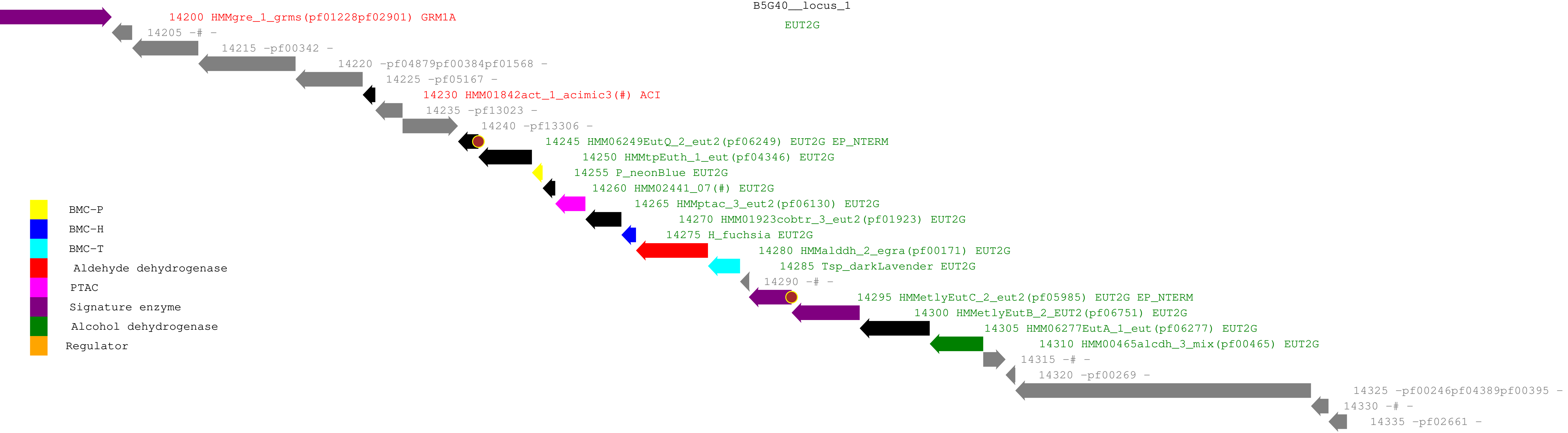

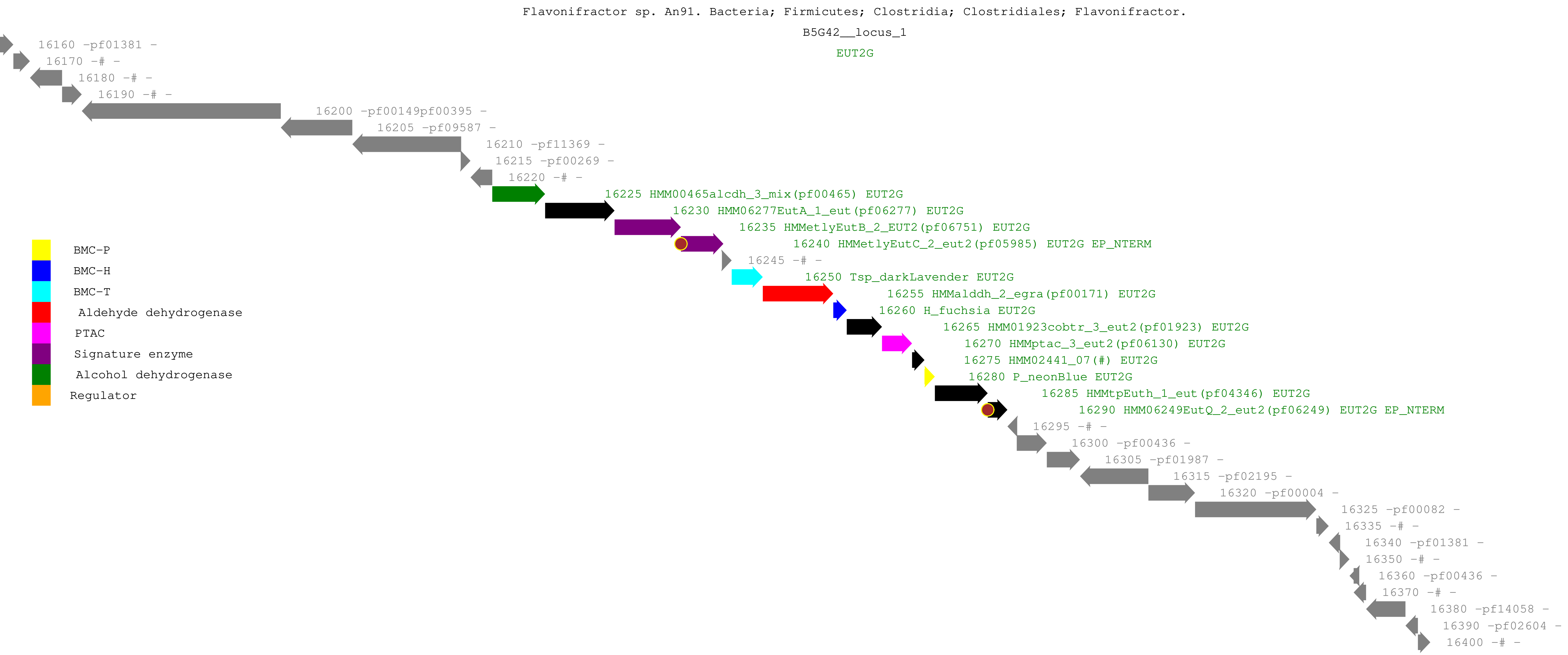
Flavonifractor sp. An91. Bacteria; Firmicutes; Clostridia; Clostridiales; Flavonifractor.
B5G42__locus_1
EUT2G
16160 -pf01381 -
16170 -# -
16180 -# -
16190 -# -
16200 -pf00149pf00395 -
16205 -pf09587 -
16210 -pf11369 -
16215 -pf00269 -
16220 -# -
16225 HMM00465alcdh_3_mix(pf00465) EUT2G
16230 HMM06277EutA_1_eut(pf06277) EUT2G
16235 HMMetlyEutB_2_EUT2(pf06751) EUT2G
16240 HMMetlyEutC_2_eut2(pf05985) EUT2G EP_NTERM
16245 -# -
16250 Tsp_darkLavender EUT2G
16255 HMMalddh_2_egra(pf00171) EUT2G
16260 H_fuchsia EUT2G
16265 HMM01923cobtr_3_eut2(pf01923) EUT2G
16270 HMMptac_3_eut2(pf06130) EUT2G
16275 HMM02441_07(#) EUT2G
16280 P_neonBlue EUT2G
16285 HMMtpEuth_1_eut(pf04346) EUT2G
16290 HMM06249EutQ_2_eut2(pf06249) EUT2G EP_NTERM
16295 -# -
16300 -pf00436 -
16305 -pf01987 -
16315 -pf02195 -
16320 -pf00004 -
16325 -pf00082 -
16335 -# -
16340 -pf01381 -
16350 -# -
16360 -pf00436 -
16370 -# -
16380 -pf14058 -
16390 -pf02604 -
16400 -# -
BMC-P
BMC-H
BMC-T
Aldehyde dehydrogenase
PTAC
Signature enzyme
Alcohol dehydrogenase
Regulator

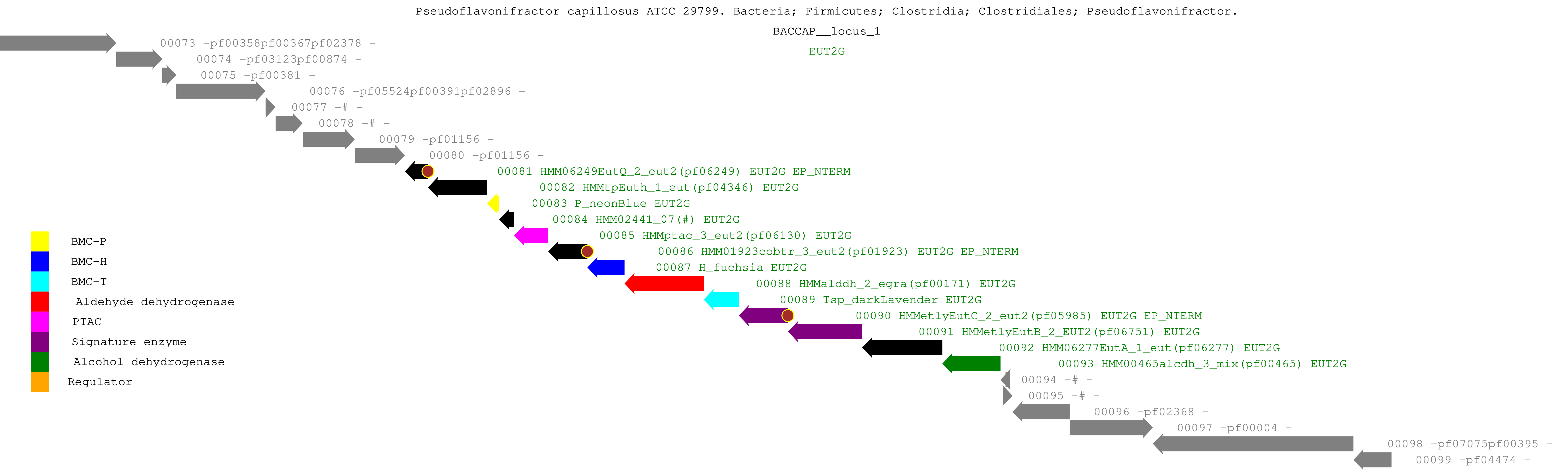
Pseudoflavonifractor capillosus ATCC 29799. Bacteria; Firmicutes; Clostridia; Clostridiales; Pseudoflavonifractor.
BACCAP__locus_1
EUT2G
00073 -pf00358pf00367pf02378 -
00074 -pf03123pf00874 -
00075 -pf00381 -
00076 -pf05524pf00391pf02896 -
00077 -# -
00078 -# -
00079 -pf01156 -
00080 -pf01156 -
00081 HMM06249EutQ_2_eut2(pf06249) EUT2G EP_NTERM
00082 HMMtpEuth_1_eut(pf04346) EUT2G
00083 P_neonBlue EUT2G
00084 HMM02441_07(#) EUT2G
00085 HMMptac_3_eut2(pf06130) EUT2G
00086 HMM01923cobtr_3_eut2(pf01923) EUT2G EP_NTERM
00087 H_fuchsia EUT2G
00088 HMMalddh_2_egra(pf00171) EUT2G
00089 Tsp_darkLavender EUT2G
00090 HMMetlyEutC_2_eut2(pf05985) EUT2G EP_NTERM
00091 HMMetlyEutB_2_EUT2(pf06751) EUT2G
00092 HMM06277EutA_1_eut(pf06277) EUT2G
00093 HMM00465alcdh_3_mix(pf00465) EUT2G
00094 -# -
00095 -# -
00096 -pf02368 -
00097 -pf00004 -
00098 -pf07075pf00395 -
00099 -pf04474 -
BMC-P
BMC-H
BMC-T
Aldehyde dehydrogenase
PTAC
Signature enzyme
Alcohol dehydrogenase
Regulator

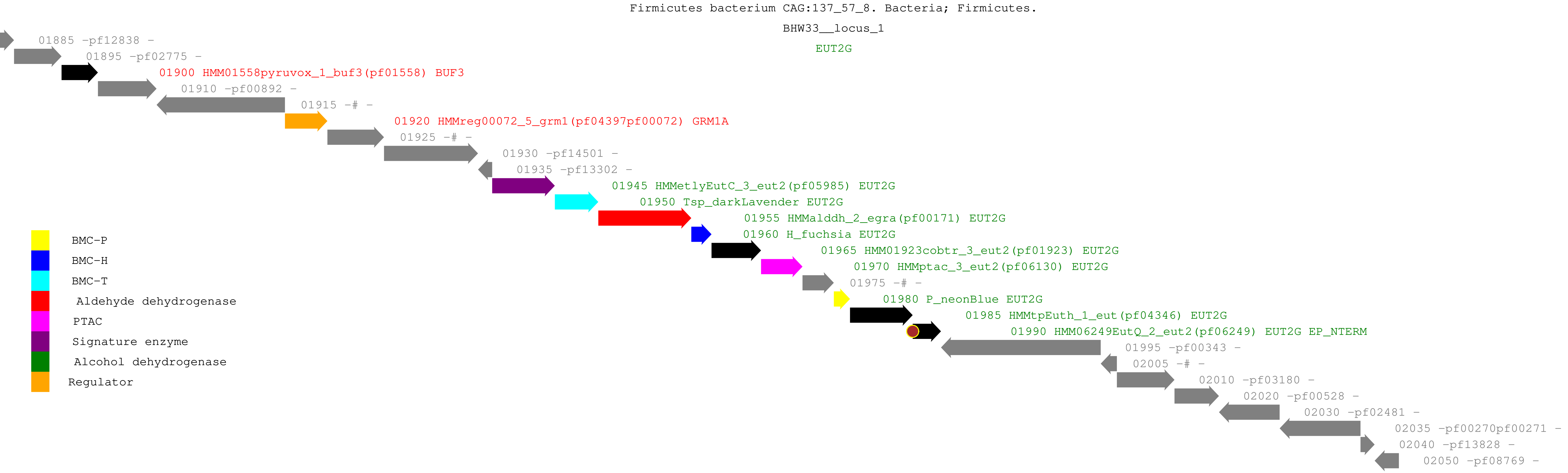
Firmicutes bacterium CAG:137_57_8. Bacteria; Firmicutes.
BHW33__locus_1
EUT2G
01885 -pf12838 -
01895 -pf02775 -
01900 HMM01558pyruvox_1_buf3(pf01558) BUF3
01910 -pf00892 -
01915 -# -
01920 HMMreg00072_5_grm1(pf04397pf00072) GRM1A
01925 -# -
01930 -pf14501 -
01935 -pf13302 -
01945 HMMetlyEutC_3_eut2(pf05985) EUT2G
01950 Tsp_darkLavender EUT2G
01955 HMMaldh_2_egra(pf00171) EUT2G
01960 H_fuchsia EUT2G
01965 HMM01923cobtr_3_eut2(pf01923) EUT2G
01970 HMMptac_3_eut2(pf06130) EUT2G
01975 -# -
01980 P_neonBlue EUT2G
01985 HMMtpEuth_1_eut(pf04346) EUT2G
01990 HMM06249EutQ_2_eut2(pf06249) EUT2G EP_NTERM
01995 -pf00343 -
02005 -# -
02010 -pf03180 -
02020 -pf00528 -
02030 -pf02481 -
02035 -pf00270pf00271 -
02040 -pf13828 -
02050 -pf08769 -
BMC-P
BMC-H
BMC-T
Aldehyde dehydrogenase
PTAC
Signature enzyme
Alcohol dehydrogenase
Regulator

Intestinimonas butyriciproducens. Bacteria; Firmicutes; Clostridia; Clostridiales; Intestinimonas.

BIV19__locus_2

EUT2G

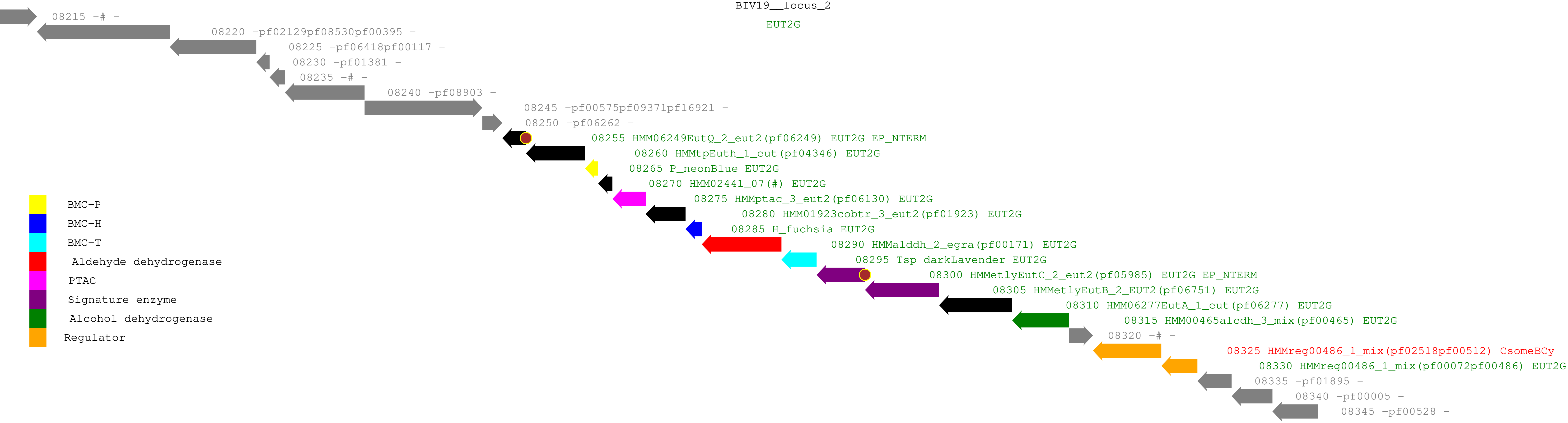

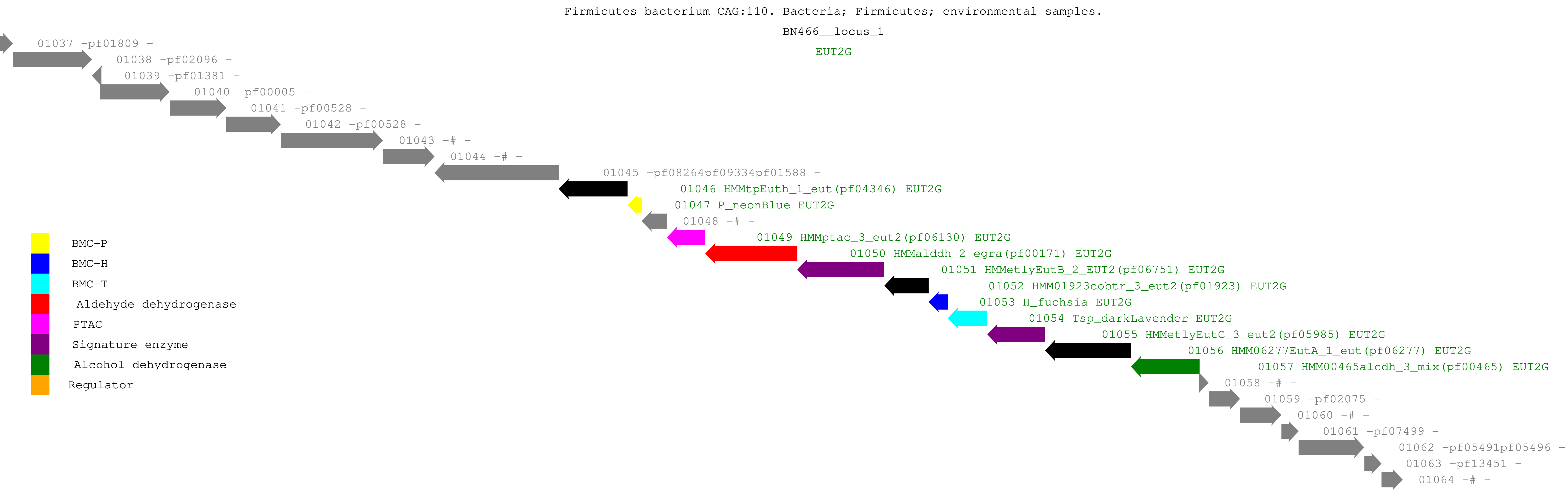
Firmicutes bacterium CAG:110. Bacteria; Firmicutes; environmental samples.
BN466__locus_1
EUT2G
01037 -pf01809 -
01038 -pf02096 -
01039 -pf01381 -
01040 -pf00005 -
01041 -pf00528 -
01042 -pf00528 -
01043 -# -
01044 -# -
01045 -pf08264pf09334pf01588 -
01046 HMMtpEuth_1_eut(pf04346) EUT2G
01047 P_neonBlue EUT2G
01048 -# -
01049 HMMptac_3_eut2(pf06130) EUT2G
01050 HMMalddh_2_egra(pf00171) EUT2G
01051 HMMetlyEutB_2_EUT2(pf06751) EUT2G
01052 HMM01923cobtr_3_eut2(pf01923) EUT2G
01053 H_fuchsia EUT2G
01054 Tsp_darkLavender EUT2G
01055 HMMetlyEutC_3_eut2(pf05985) EUT2G
01056 HMM06277EutA_1_eut(pf06277) EUT2G
01057 HMM00465alcdh_3_mix(pf00465) EUT2G
01058 -# -
01059 -pf02075 -
01060 -# -
01061 -pf07499 -
01062 -pf05491pf05496 -
01063 -pf13451 -
01064 -# -
BMC-P
BMC-H
BMC-T
Aldehyde dehydrogenase
PTAC
Signature enzyme
Alcohol dehydrogenase
Regulator

Firmicutes bacterium CAG:137. Bacteria; Firmicutes; environmental samples.

BN490__locus_1

EUT2G

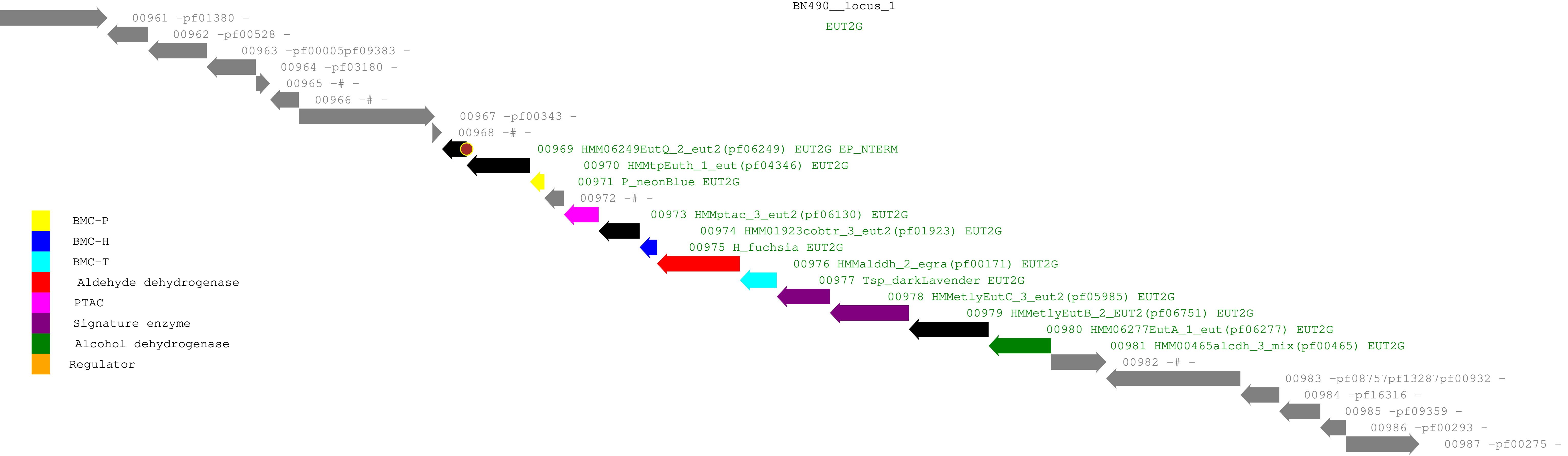

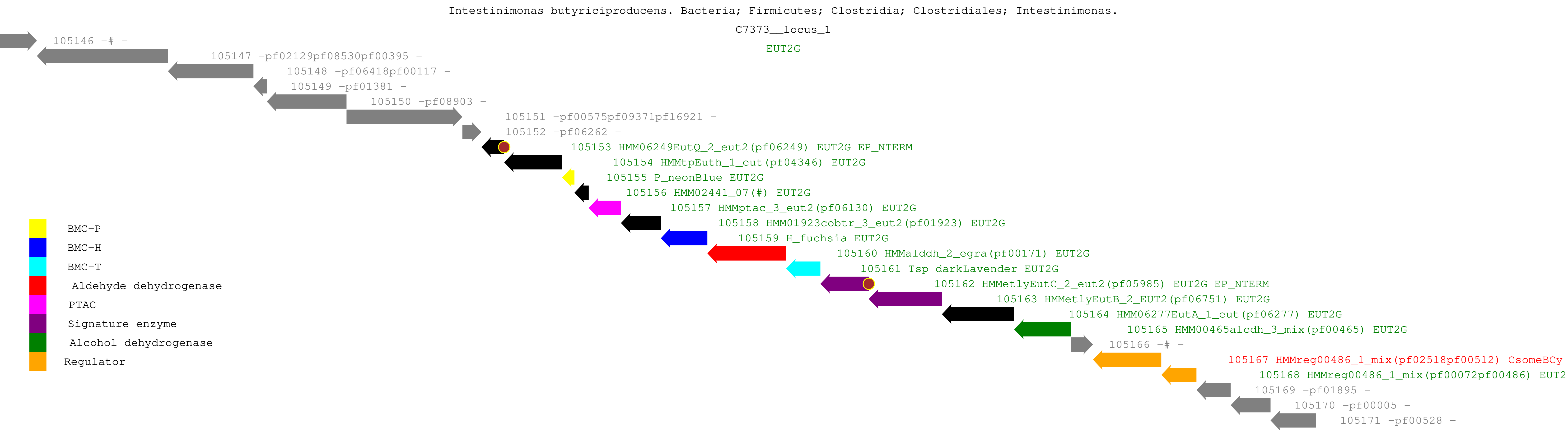

Intestinimonas butyriciproducens. Bacteria; Firmicutes; Clostridia; Clostridiales; Intestinimonas.
C7373__locus_1
EUT2G
105146 -# -
105147 -pf02129pf08530pf00395 -
105148 -pf06418pf00117 -
105149 -pf01381 -
105150 -pf08903 -
105151 -pf00575pf09371pf16921 -
105152 -pf06262 -
105153 HMM06249EutQ_2_eut2(pf06249) EUT2G EP_NTERM
105154 HMMtpEuth_1_eut(pf04346) EUT2G
105155 P_neonBlue EUT2G
105156 HMM02441_07(#) EUT2G
105157 HMMptac_3_eut2(pf06130) EUT2G
105158 HMM01923cobtr_3_eut2(pf01923) EUT2G
105159 H_fuchsia EUT2G
105160 HMMalddh_2_egra(pf00171) EUT2G
105161 Tsp_darkLavender EUT2G
105162 HMMetlyEutC_2_eut2(pf05985) EUT2G EP_NTERM
105163 HMMetlyEutB_2_EUT2(pf06751) EUT2G
105164 HMM06277EutA_1_eut(pf06277) EUT2G
105165 HMM00465alcdh_3_mix(pf00465) EUT2G
105166 -# -
105167 HMMreg00486_1_mix(pf02518pf00512) CsomeBCy
105168 HMMreg00486_1_mix(pf00072pf00486) EUT2
105169 -pf01895 -
105170 -pf00005 -
105171 -pf00528 -
BMC-P
BMC-H
BMC-T
Aldehyde dehydrogenase
PTAC
Signature enzyme
Alcohol dehydrogenase
Regulator

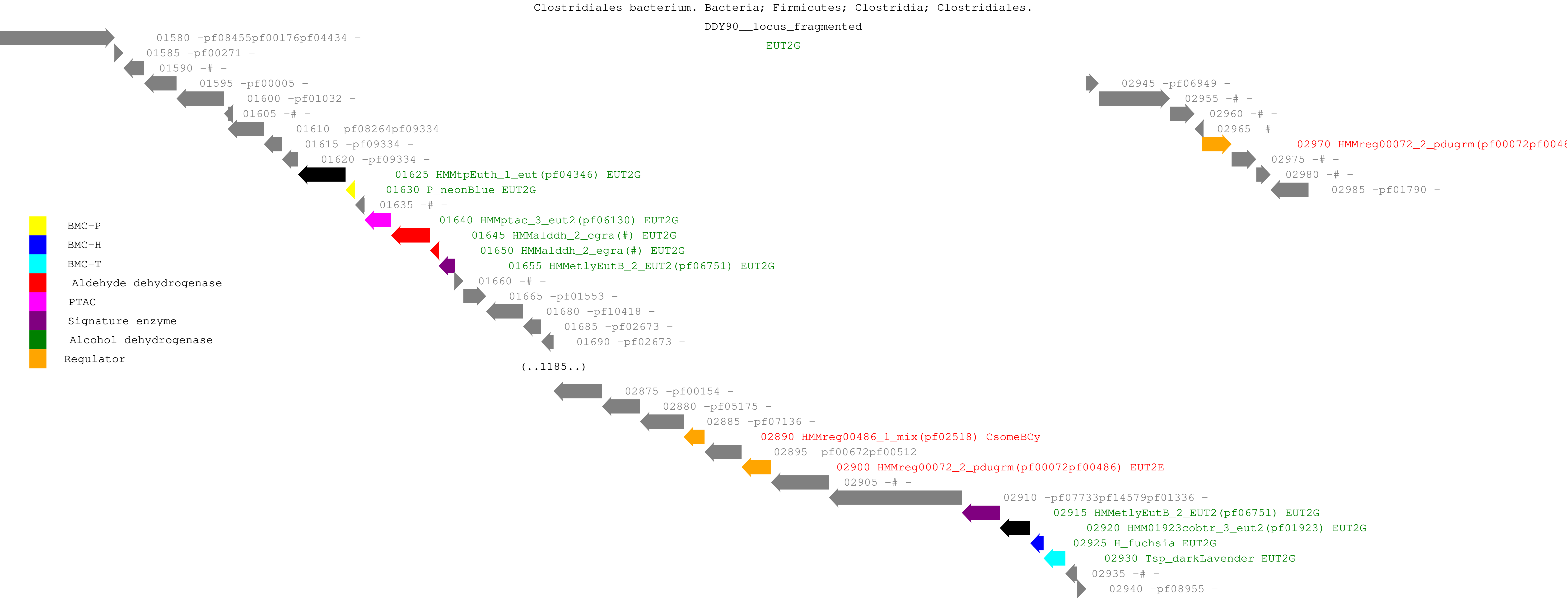
Clostridiales bacterium. Bacteria; Firmicutes; Clostridia; Clostridiales.
DDY90__locus_fragmented
EUT2G
01580 -pf08455pf00176pf04434 -
01585 -pf00271 -
01590 -# -
01595 -pf00005 -
01600 -pf01032 -
01605 -# -
01610 -pf08264pf09334 -
01615 -pf09334 -
01620 -pf09334 -
01625 HMMtpEuth_1_eut(pf04346) EUT2G
01630 P_neonBlue EUT2G
01635 -# -
01640 HMMptac_3_eut2(pf06130) EUT2G
01645 HMMalddh_2_egra(#) EUT2G
01650 HMMalddh_2_egra(#) EUT2G
01655 HMMetlyEutB_2_EUT2(pf06751) EUT2G
01660 -# -
01665 -pf01553 -
01680 -pf10418 -
01685 -pf02673 -
01690 -pf02673 -
(..1185..)
02875 -pf00154 -
02880 -pf05175 -
02885 -pf07136 -
02890 HMMreg00486_1_mix(pf02518) CsomeBCy
02895 -pf00672pf00512 -
02900 HMMreg00072_2_pdugrm(pf00072pf00486) EUT2E
02905 -# -
02910 -pf07733pf14579pf01336 -
02915 HMMetlyEutB_2_EUT2(pf06751) EUT2G
02920 HMM01923cobtr_3_eut2(pf01923) EUT2G
02925 H_fuchsia EUT2G
02930 Tsp_darkLavender EUT2G
02935 -# -
02940 -pf08955 -
02945 -pf06949 -
02955 -# -
02960 -# -
02965 -# -
02970 HMMreg00072_2_pdugrm(pf00072pf004
02975 -# -
02980 -# -
02985 -pf01790 -
BMC-P
BMC-H
BMC-T
Aldehyde dehydrogenase
PTAC
Signature enzyme
Alcohol dehydrogenase
Regulator

Clostridiales bacterium. Bacteria; Firmicutes; Clostridia; Clostridiales.

DFH97__locus_1

EUT2G

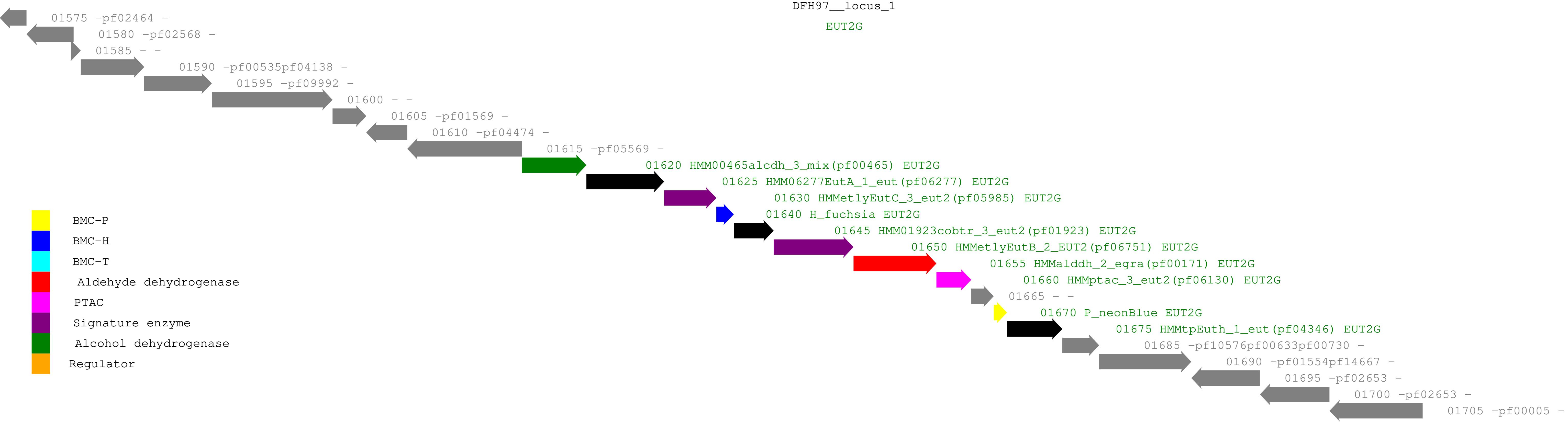

Flavonifractor plautii. Bacteria; Firmicutes; Clostridia; Clostridiales; Flavonifractor.

ERS852504__locus_3

EUT2G

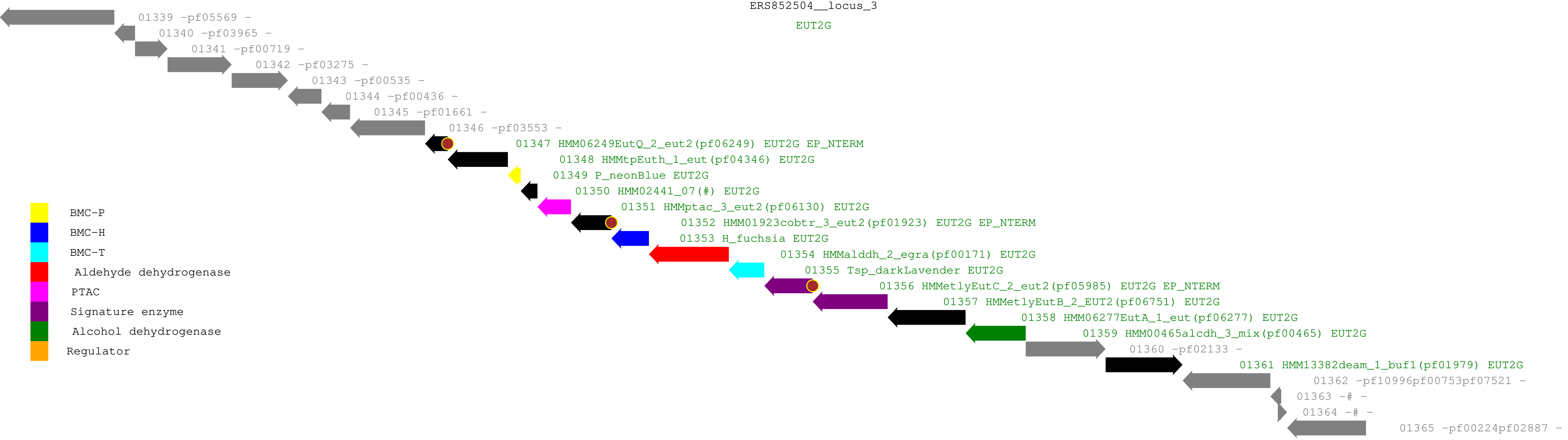

Flavonifractor plautii. Bacteria; Firmicutes; Clostridia; Clostridiales; Flavonifractor.

ERS852518__locus_3

EUT2G

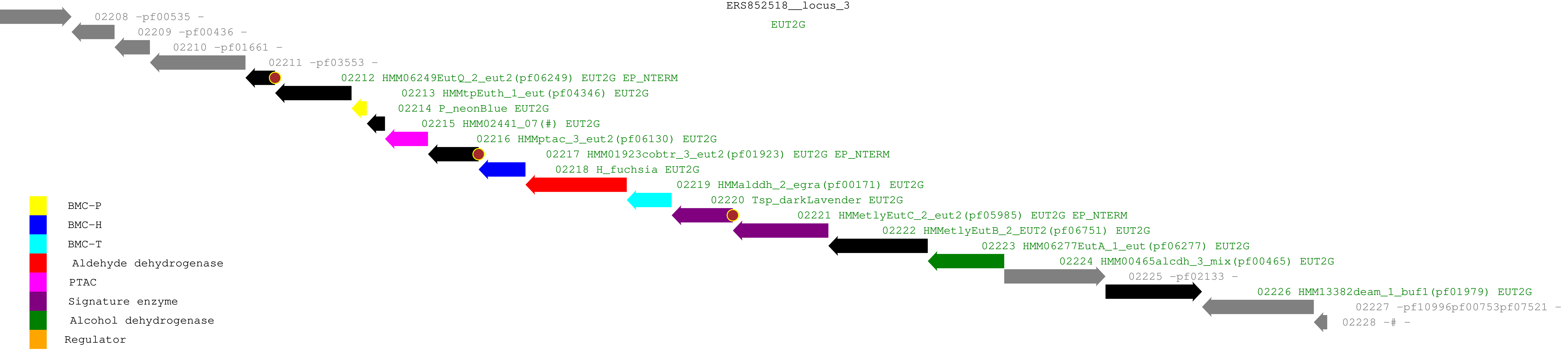

Flavonifractor plautii. Bacteria; Firmicutes; Clostridia; Clostridiales; Flavonifractor.

ERS852518__locus_4

EUT2G

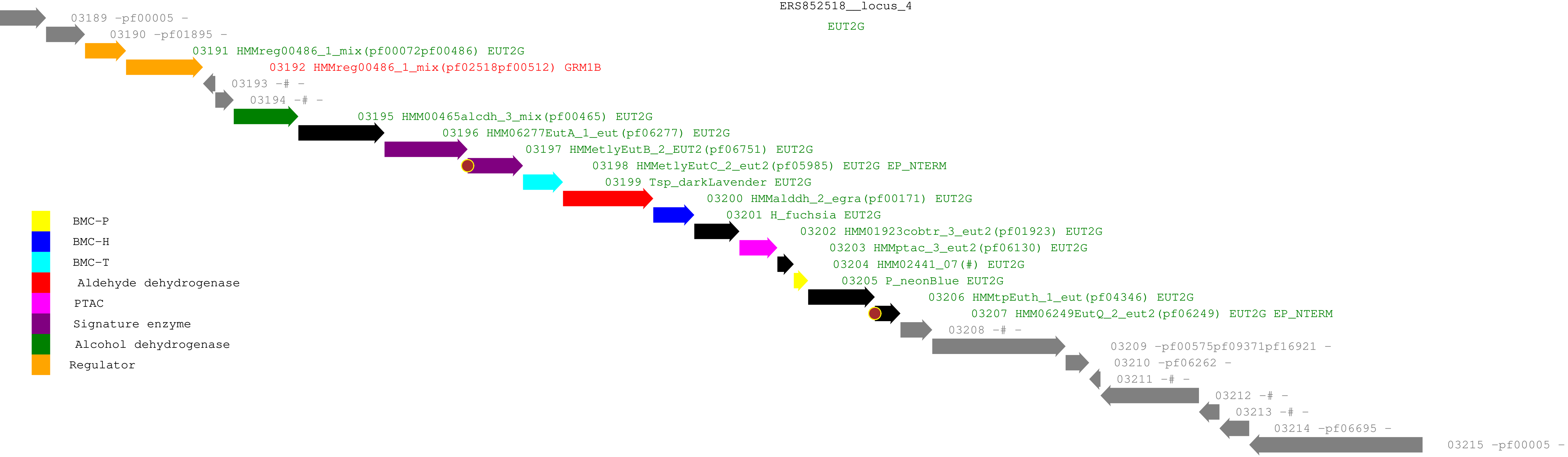

Flavonifractor plautii DSM 6740. Bacteria; Firmicutes; Clostridia; Clostridiales; Ruminococcaceae;

EV206__locus_2

EUT2G

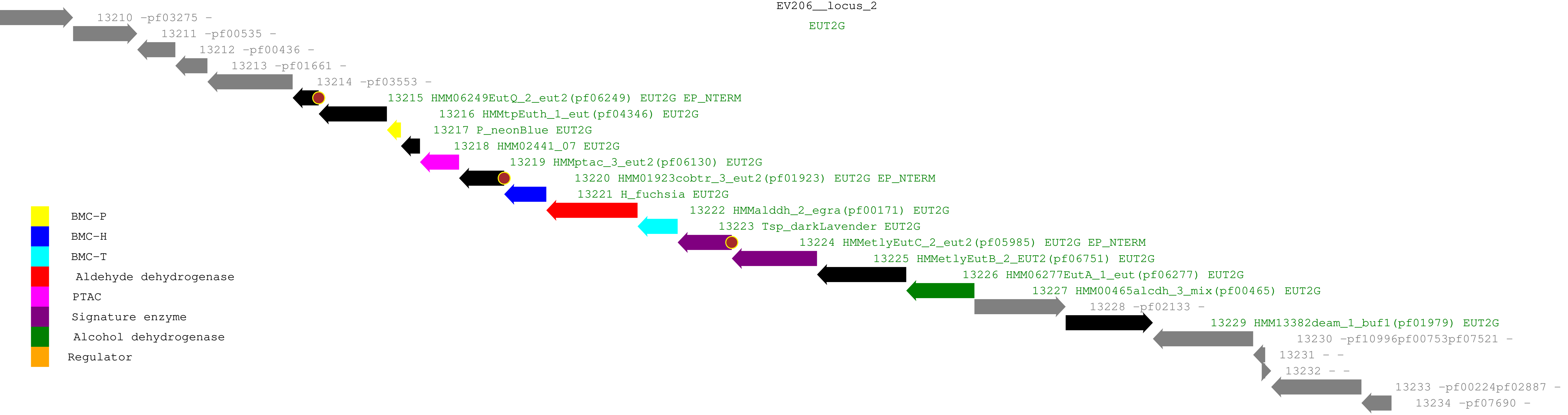

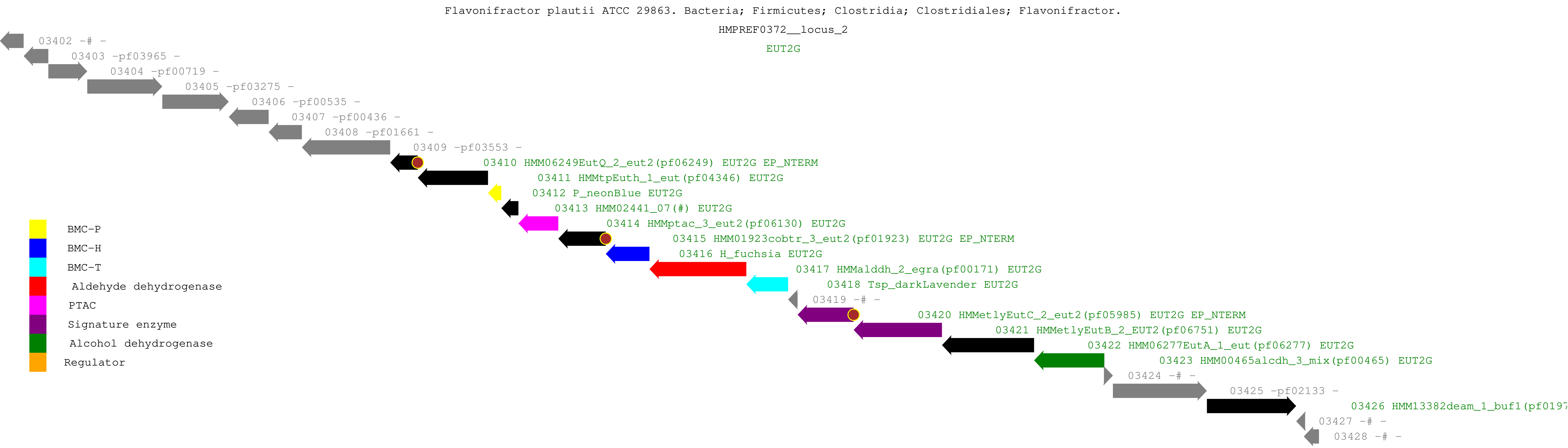
Flavonifractor plautii ATCC 29863. Bacteria; Firmicutes; Clostridia; Clostridiales; Flavonifractor.
HMPREF0372__locus_2
EUT2G
03402 -# -
03403 -pf03965 -
03404 -pf00719 -
03405 -pf03275 -
03406 -pf00535 -
03407 -pf00436 -
03408 -pf01661 -
03409 -pf03553 -
03410 HMM06249EutQ_2_eut2(pf06249) EUT2G EP_NTERM
03411 HMMtpEuth_1_eut(pf04346) EUT2G
03412 P_neonBlue EUT2G
03413 HMM02441_07(#) EUT2G
03414 HMMptac_3_eut2(pf06130) EUT2G
03415 HMM01923cobtr_3_eut2(pf01923) EUT2G EP_NTERM
03416 H_fuchsia EUT2G
03417 HMMalddh_2_egra(pf00171) EUT2G
03418 Tsp_darkLavender EUT2G
03419 -# -
03420 HMMetlyEutC_2_eut2(pf05985) EUT2G EP_NTERM
03421 HMMetlyEutB_2_EUT2(pf06751) EUT2G
03422 HMM06277EutA_1_eut(pf06277) EUT2G
03423 HMM00465alcdh_3_mix(pf00465) EUT2G
03424 -# -
03425 -pf02133 -
03426 HMM13382deam_1_buf1(pf0197
03427 -# -
03428 -# -
BMC-P
BMC-H
BMC-T
Aldehyde dehydrogenase
PTAC
Signature enzyme
Alcohol dehydrogenase
Regulator

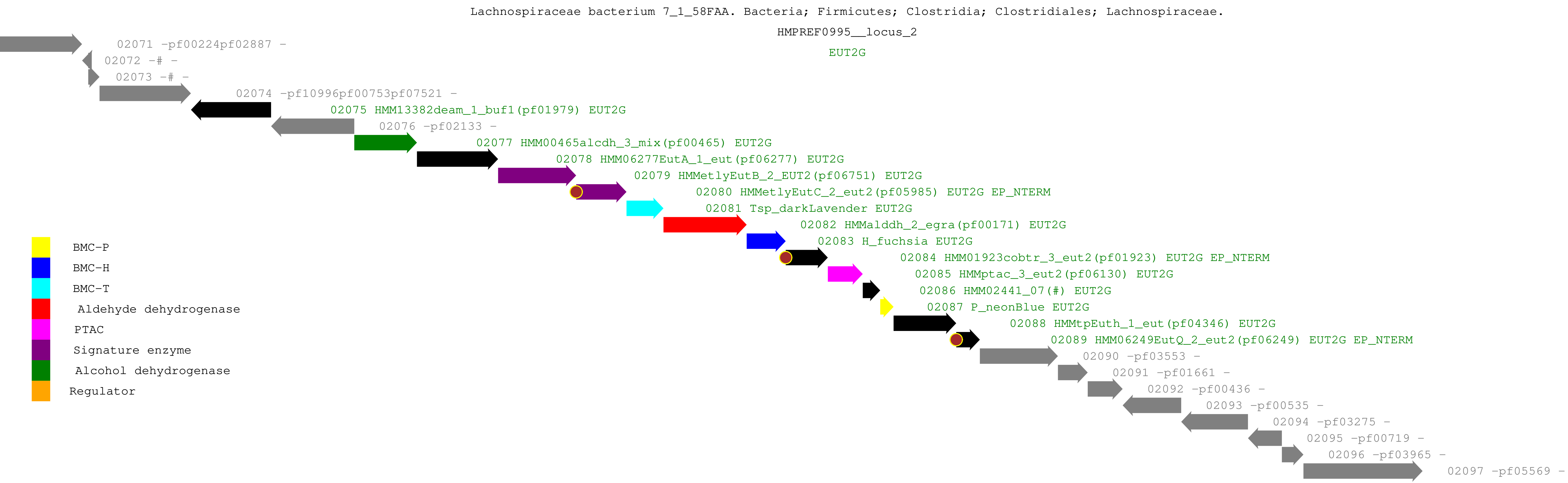

Lachnospiraceae bacterium 7_1_58FAA. Bacteria; Firmicutes; Clostridia; Clostridiales; Lachnospiraceae.
HMPREF0995__locus_2
EUT2G
02071 -pf00224pf02887 -
02072 -# -
02073 -# -
02074 -pf10996pf00753pf07521 -
02075 HMM13382deam_1_buf1(pf01979) EUT2G
02076 -pf02133 -
02077 HMM00465alcdh_3_mix(pf00465) EUT2G
02078 HMM06277EutA_1_eut(pf06277) EUT2G
02079 HMMetlyEutB_2_EUT2(pf06751) EUT2G
02080 HMMetlyEutC_2_eut2(pf05985) EUT2G EP_NTERM
02081 Tsp_darkLavender EUT2G
02082 HMMalddh_2_egra(pf00171) EUT2G
02083 H_fuchsia EUT2G
02084 HMM01923cobtr_3_eut2(pf01923) EUT2G EP_NTERM
02085 HMMptac_3_eut2(pf06130) EUT2G
02086 HMM02441_07(#) EUT2G
02087 P_neonBlue EUT2G
02088 HMMtpEuth_1_eut(pf04346) EUT2G
02089 HMM06249EutQ_2_eut2(pf06249) EUT2G EP_NTERM
02090 -pf03553 -
02091 -pf01661 -
02092 -pf00436 -
02093 -pf00535 -
02094 -pf03275 -
02095 -pf00719 -
02096 -pf03965 -
02097 -pf05569 -
BMC-P
BMC-H
BMC-T
Aldehyde dehydrogenase
PTAC
Signature enzyme
Alcohol dehydrogenase
Regulator

Clostridium orbiscindens 1_3_50AFAA. Bacteria; Firmicutes; Clostridia; Clostridiales; Flavonifractor.

HMPREF9460__locus_2

EUT2G

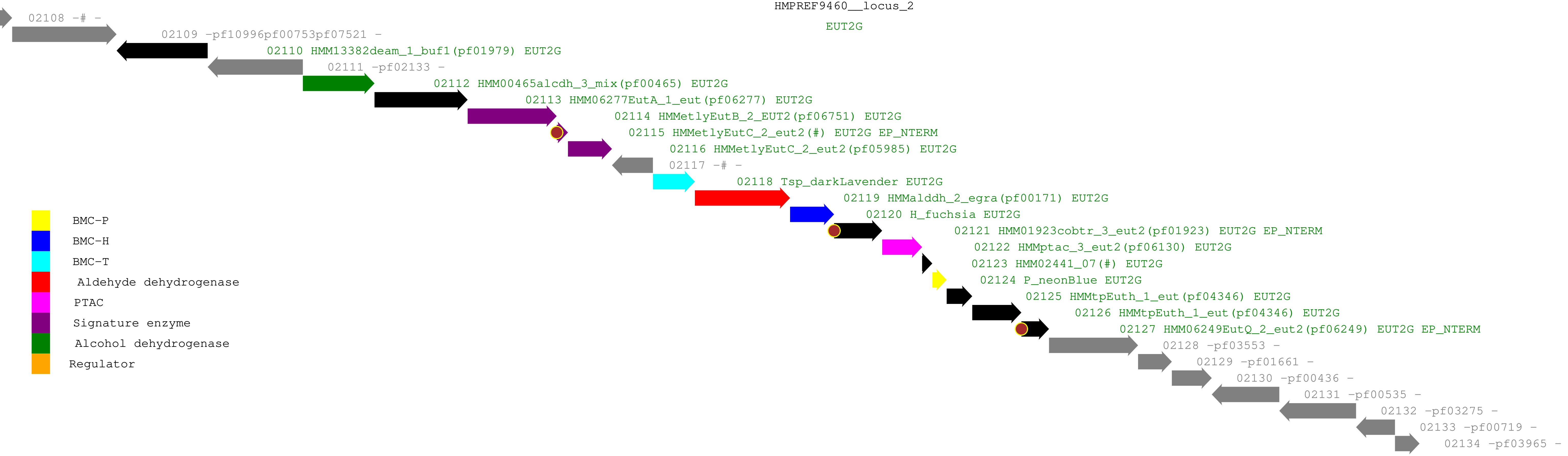

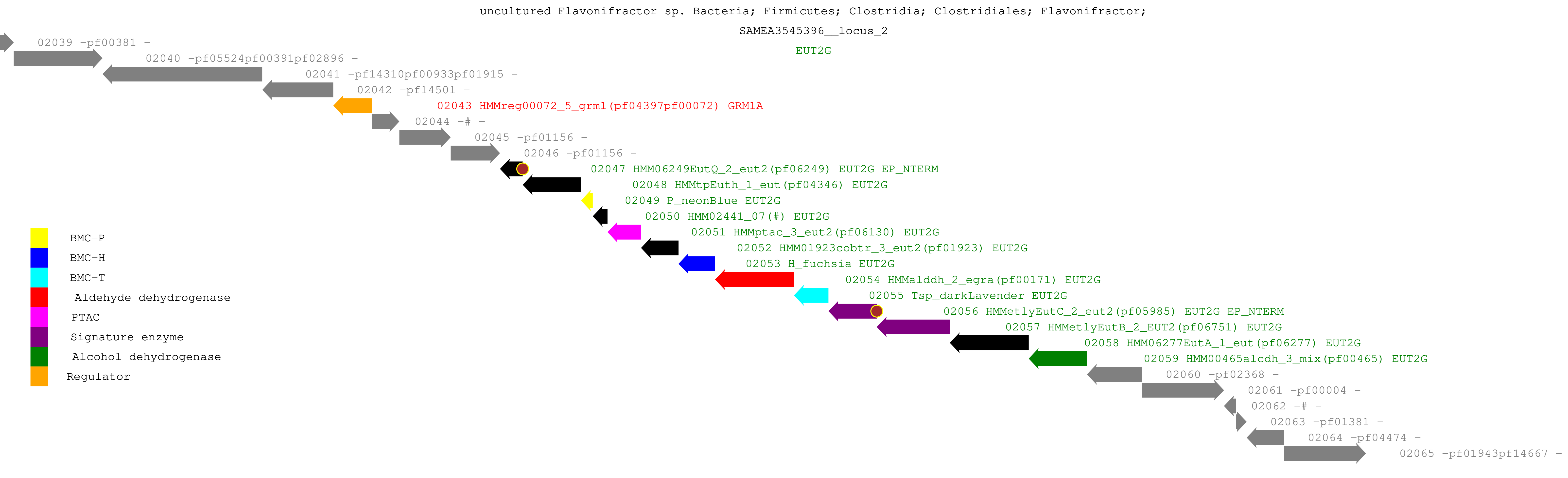

uncultured Flavonifractor sp. Bacteria; Firmicutes; Clostridia; Clostridiales; Flavonifractor;
SAMEA3545396__locus_2
EUT2G
02039 -pf00381 -
02040 -pf05524pf00391pf02896 -
02041 -pf14310pf00933pf01915 -
02042 -pf14501 -
02043 HMMreg00072_5_grm1(pf04397pf00072) GRM1A
02044 -# -
02045 -pf01156 -
02046 -pf01156 -
02047 HMM06249EutQ_2_eut2(pf06249) EUT2G EP_NTERM
02048 HMMtpEuth_1_eut(pf04346) EUT2G
02049 P_neonBlue EUT2G
02050 HMM02441_07(#) EUT2G
02051 HMMptac_3_eut2(pf06130) EUT2G
02052 HMM01923cobtr_3_eut2(pf01923) EUT2G
02053 H_fuchsia EUT2G
02054 HMMalddh_2_egra(pf00171) EUT2G
02055 Tsp_darkLavender EUT2G
02056 HMMetlyEutC_2_eut2(pf05985) EUT2G EP_NTERM
02057 HMMetlyEutB_2_EUT2(pf06751) EUT2G
02058 HMM06277EutA_1_eut(pf06277) EUT2G
02059 HMM00465alcdh_3_mix(pf00465) EUT2G
02060 -pf02368 -
02061 -pf00004 -
02062 -# -
02063 -pf01381 -
02064 -pf04474 -
02065 -pf01943pf14667 -
BMC-P
BMC-H
BMC-T
Aldehyde dehydrogenase
PTAC
Signature enzyme
Alcohol dehydrogenase
Regulator

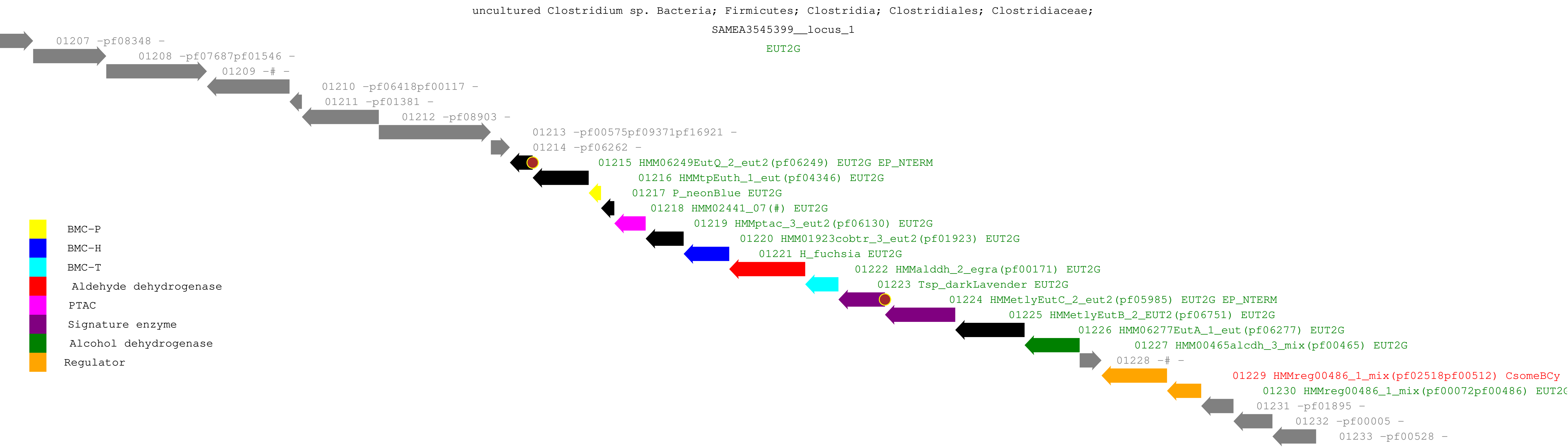

uncultured Clostridium sp. Bacteria; Firmicutes; Clostridia; Clostridiales; Clostridiaceae;
SAMEA3545399__locus_1
EUT2G
01207 -pf08348 -
01208 -pf07687pf01546 -
01209 -# -
01210 -pf06418pf00117 -
01211 -pf01381 -
01212 -pf08903 -
01213 -pf00575pf09371pf16921 -
01214 -pf06262 -
01215 HMM06249EutQ_2_eut2(pf06249) EUT2G EP_NTERM
01216 HMMtpEuth_1_eut(pf04346) EUT2G
01217 P_neonBlue EUT2G
01218 HMM02441_07(#) EUT2G
01219 HMMptac_3_eut2(pf06130) EUT2G
01220 HMM01923cobtr_3_eut2(pf01923) EUT2G
01221 H_fuchsia EUT2G
01222 HMMalddh_2_egra(pf00171) EUT2G
01223 Tsp_darkLavender EUT2G
01224 HMMetlyEutC_2_eut2(pf05985) EUT2G EP_NTERM
01225 HMMetlyEutB_2_EUT2(pf06751) EUT2G
01226 HMM06277EutA_1_eut(pf06277) EUT2G
01227 HMM00465alcdh_3_mix(pf00465) EUT2G
01228 -# -
01229 HMMreg00486_1_mix(pf02518pf00512) CsomeBCy
01230 HMMreg00486_1_mix(pf00072pf00486) EUT2G
01231 -pf01895 -
01232 -pf00005 -
01233 -pf00528 -
BMC-P
BMC-H
BMC-T
Aldehyde dehydrogenase
PTAC
Signature enzyme
Alcohol dehydrogenase
Regulator

uncultured Flavonifractor sp. Bacteria; Firmicutes; Clostridia; Clostridiales; Flavonifractor;

SAMEA3545403__locus_1_main_sat

EUT2G

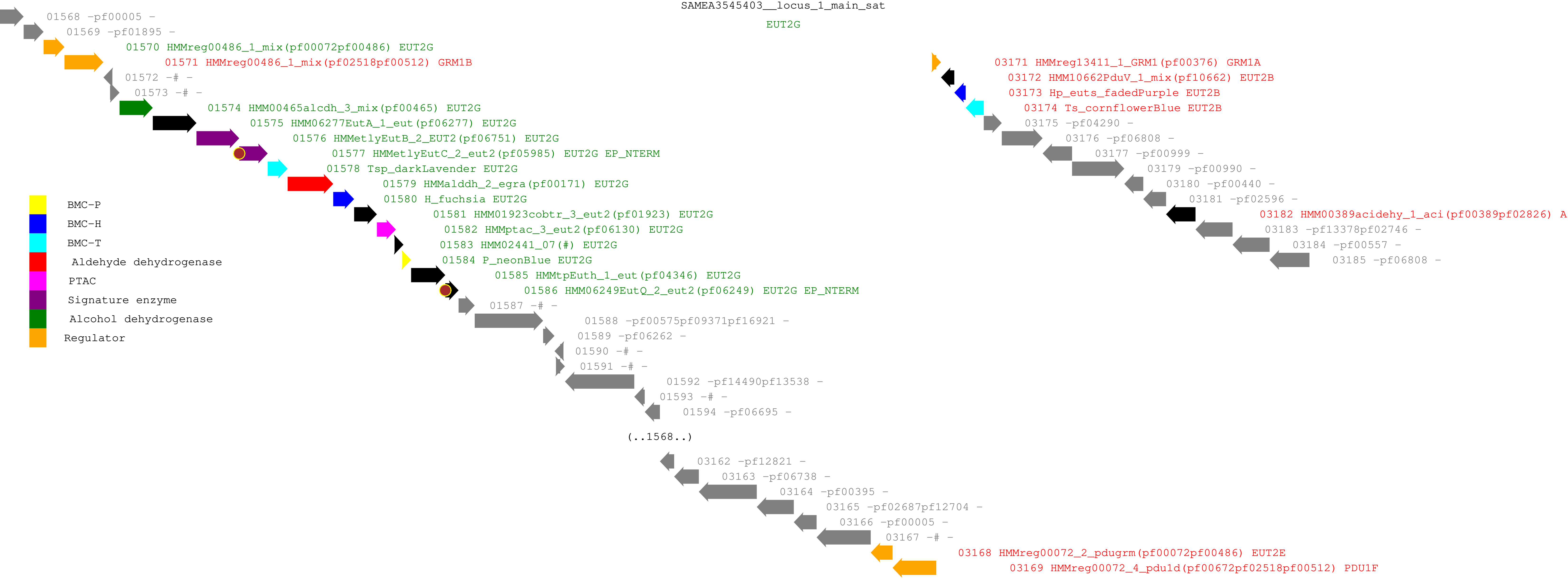

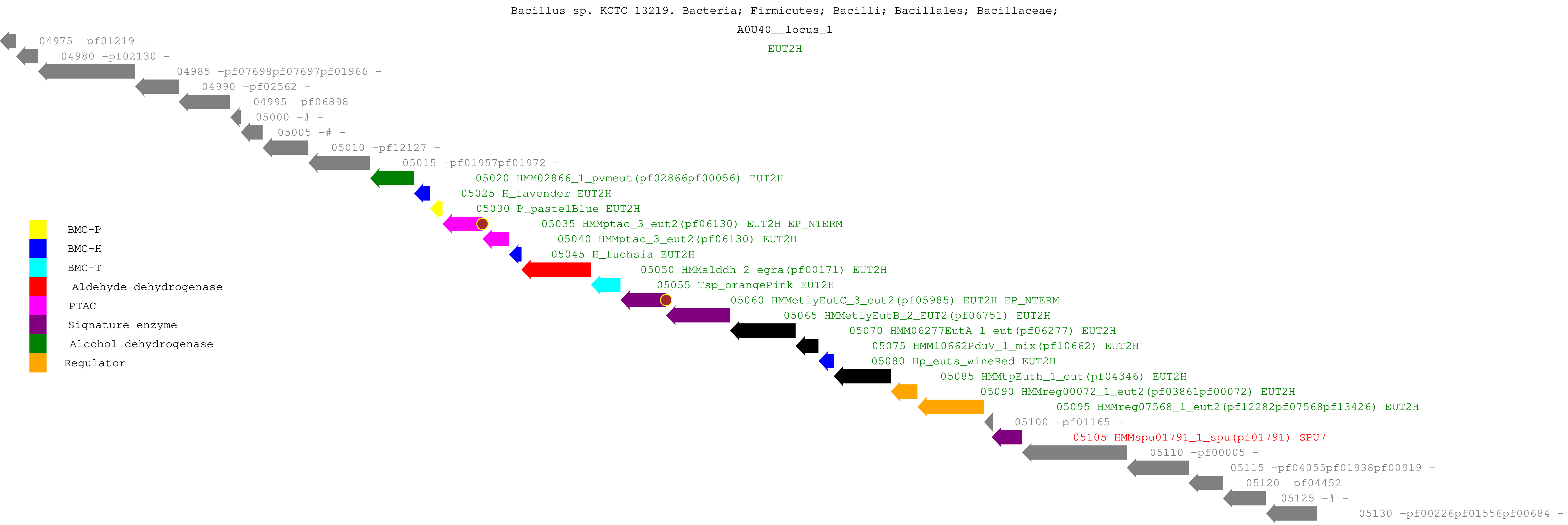

Bacillus sp. KCTC 13219. Bacteria; Firmicutes; Bacilli; Bacillales; Bacillaceae;
A0U40__locus_1
EUT2H
04975 -pf01219 -
04980 -pf02130 -
04985 -pf07698pf07697pf01966 -
04990 -pf02562 -
04995 -pf06898 -
05000 -# -
05005 -# -
05010 -pf12127 -
05015 -pf01957pf01972 -
05020 HMM02866_1_pvmeut(pf02866pf00056) EUT2H
05025 H_lavender EUT2H
05030 P_pastelBlue EUT2H
05035 HMMptac_3_eut2(pf06130) EUT2H EP_NTERM
05040 HMMptac_3_eut2(pf06130) EUT2H
05045 H_fuchsia EUT2H
05050 HMMalddh_2_egra(pf00171) EUT2H
05055 Tsp_orangePink EUT2H
05060 HMMetlyEutC_3_eut2(pf05985) EUT2H EP_NTERM
05065 HMMetlyEutB_2_EUT2(pf06751) EUT2H
05070 HMM06277EutA_1_eut(pf06277) EUT2H
05075 HMM10662PduV_1_mix(pf10662) EUT2H
05080 Hp_euts_wineRed EUT2H
05085 HMMtpEuth_1_eut(pf04346) EUT2H
05090 HMMreg00072_1_eut2(pf03861pf00072) EUT2H
05095 HMMreg07568_1_eut2(pf12282pf07568pf13426) EUT2H
05100 -pf01165 -
05105 HMMspu01791_1_spu(pf01791) SPU7
05110 -pf00005 -
05115 -pf04055pf01938pf00919 -
05120 -pf04452 -
05125 -# -
05130 -pf00226pf01556pf00684 -
BMC-P
BMC-H
BMC-T
Aldehyde dehydrogenase
PTAC
Signature enzyme
Alcohol dehydrogenase
Regulator

Lysinibacillus sp. YR326. Bacteria; Firmicutes; Bacilli; Bacillales; Bacillaceae; Lysinibacillus;

A9A36__locus_1

EUT2H

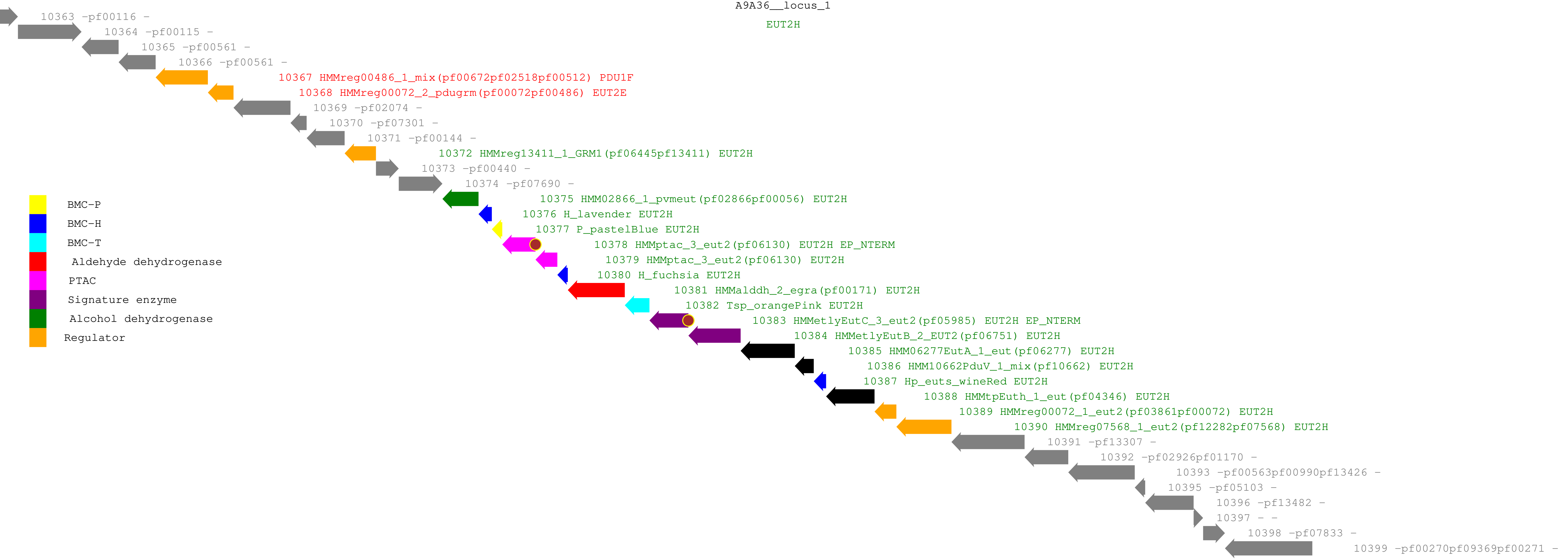

Lysinibacillus xylanilyticus. Bacteria; Firmicutes; Bacilli; Bacillales; Bacillaceae;

ACZ11__locus_1

EUT2H

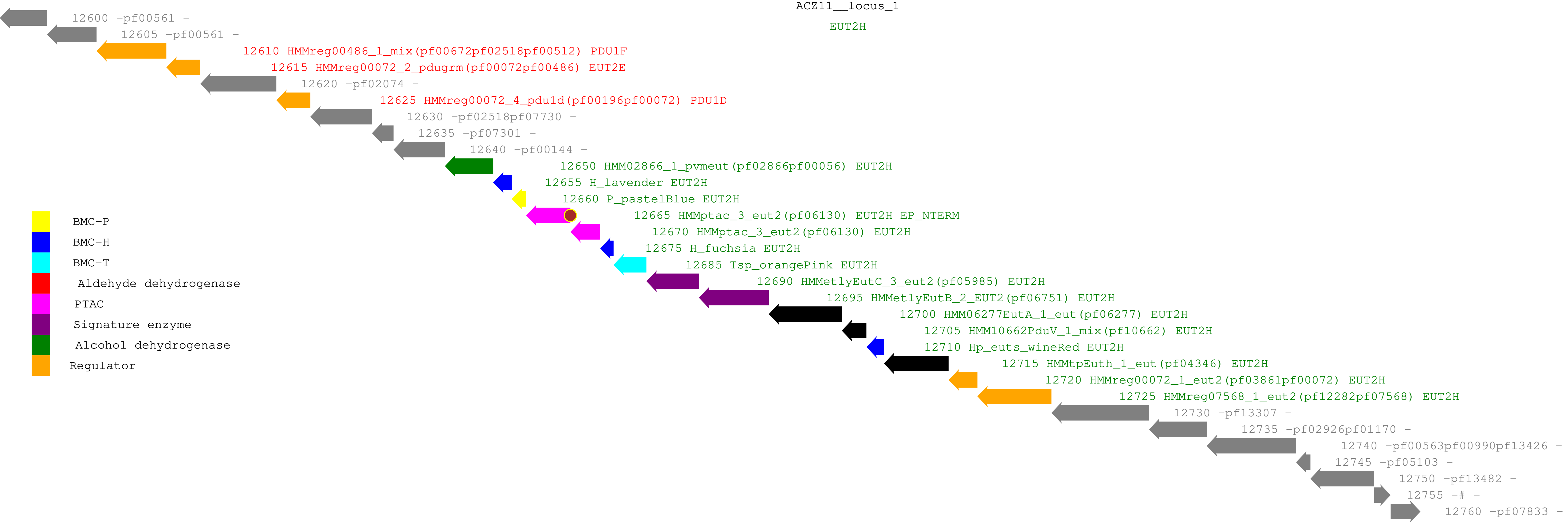

Lysinibacillus macroides. Bacteria; Firmicutes; Bacilli; Bacillales; Bacillaceae;

ADM90__locus_2

EUT2H

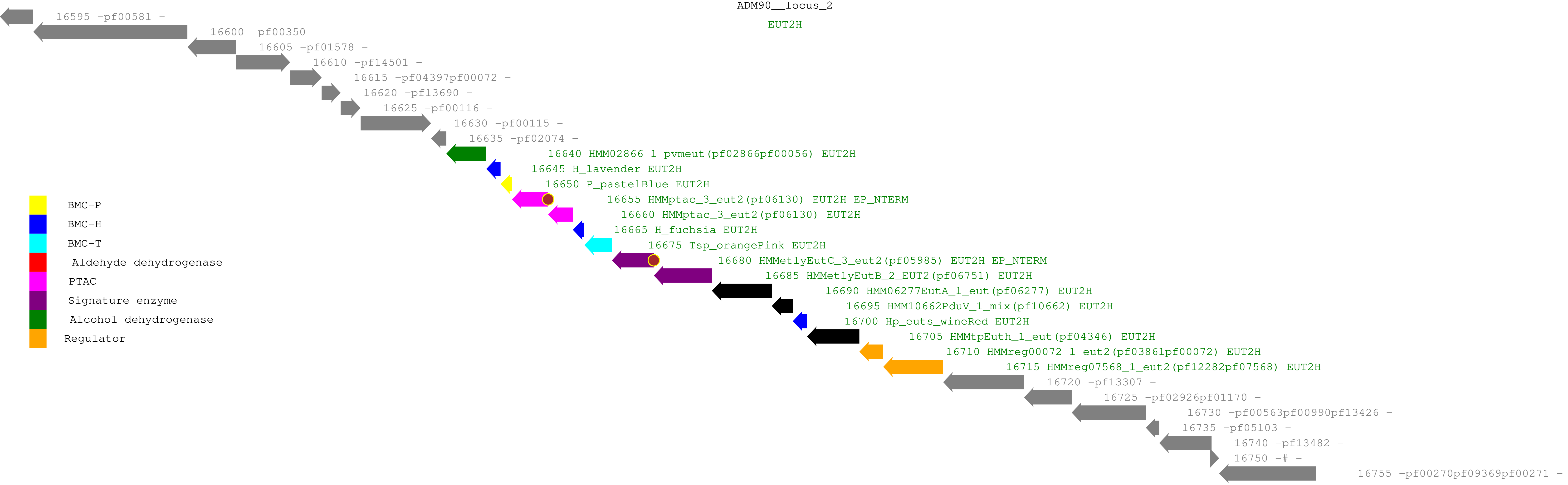

Lysinibacillus sp. F5. Bacteria; Firmicutes; Bacilli; Bacillales; Bacillaceae;

AK833__locus_1

EUT2H

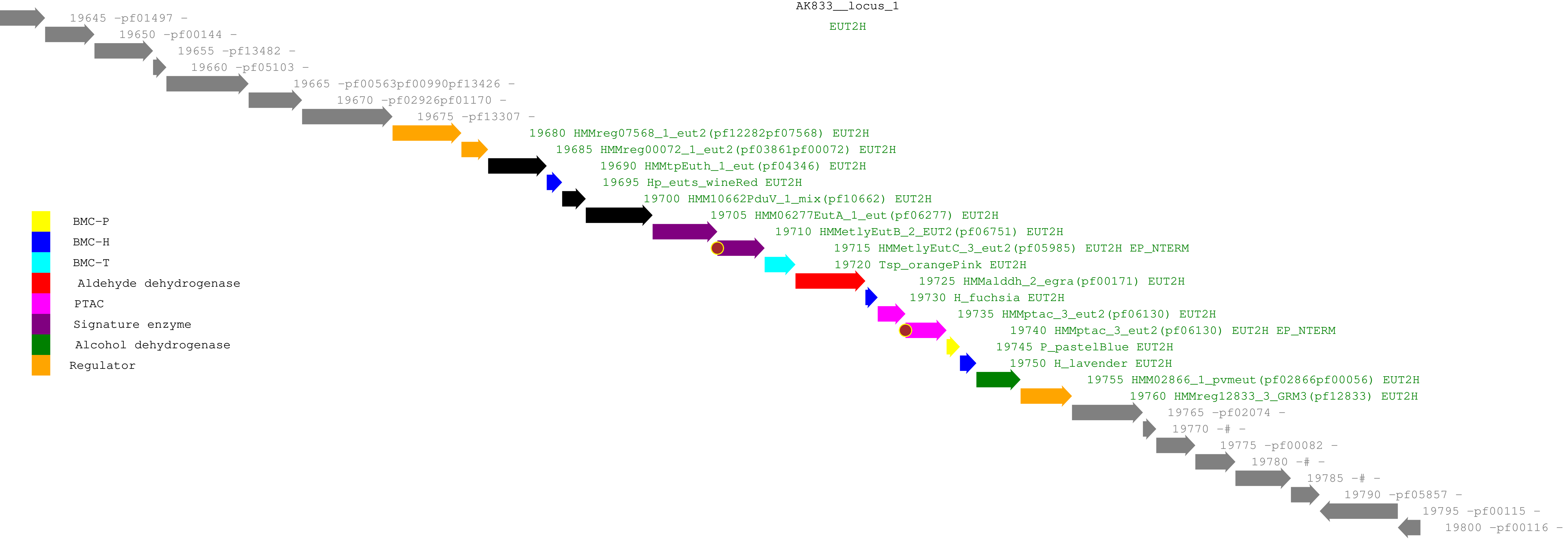

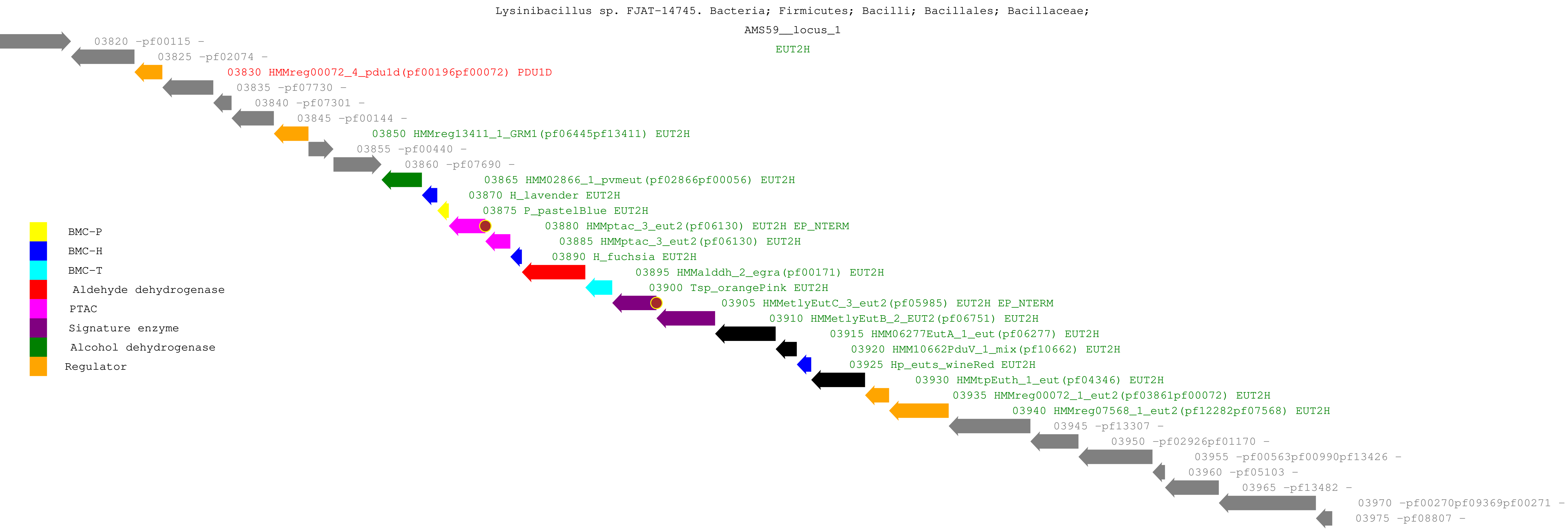
Lysinibacillus sp. FJAT-14745. Bacteria; Firmicutes; Bacilli; Bacillales; Bacillaceae;
AMS59__locus_1
EUT2H
03820 -pf00115 -
03825 -pf02074 -
03830 HMMreg00072_4_pdu1d(pf00196pf00072) PDU1D
03835 -pf07730 -
03840 -pf07301 -
03845 -pf00144 -
03850 HMMreg13411_1_GRM1(pf06445pf13411) EUT2H
03855 -pf00440 -
03860 -pf07690 -
03865 HMM02866_1_pvmeut(pf02866pf00056) EUT2H
03870 H_lavender EUT2H
03875 P_pastelBlue EUT2H
03880 HMMptac_3_eut2(pf06130) EUT2H EP_NTERM
03885 HMMptac_3_eut2(pf06130) EUT2H
03890 H_fuchsia EUT2H
03895 HMMalddh_2_egra(pf00171) EUT2H
03900 Tsp_orangePink EUT2H
03905 HMMetlyEutC_3_eut2(pf05985) EUT2H EP_NTERM
03910 HMMetlyEutB_2_EUT2(pf06751) EUT2H
03915 HMM06277EutA_1_eut(pf06277) EUT2H
03920 HMM10662PduV_1_mix(pf10662) EUT2H
03925 Hp_euts_wineRed EUT2H
03930 HMMtpEuth_1_eut(pf04346) EUT2H
03935 HMMreg00072_1_eut2(pf03861pf00072) EUT2H
03940 HMMreg07568_1_eut2(pf12282pf07568) EUT2H
03945 -pf13307 -
03950 -pf02926pf01170 -
03955 -pf00563pf00990pf13426 -
03960 -pf05103 -
03965 -pf13482 -
03970 -pf00270pf09369pf00271 -
03975 -pf08807 -
BMC-P
BMC-H
BMC-T
Aldehyde dehydrogenase
PTAC
Signature enzyme
Alcohol dehydrogenase
Regulator

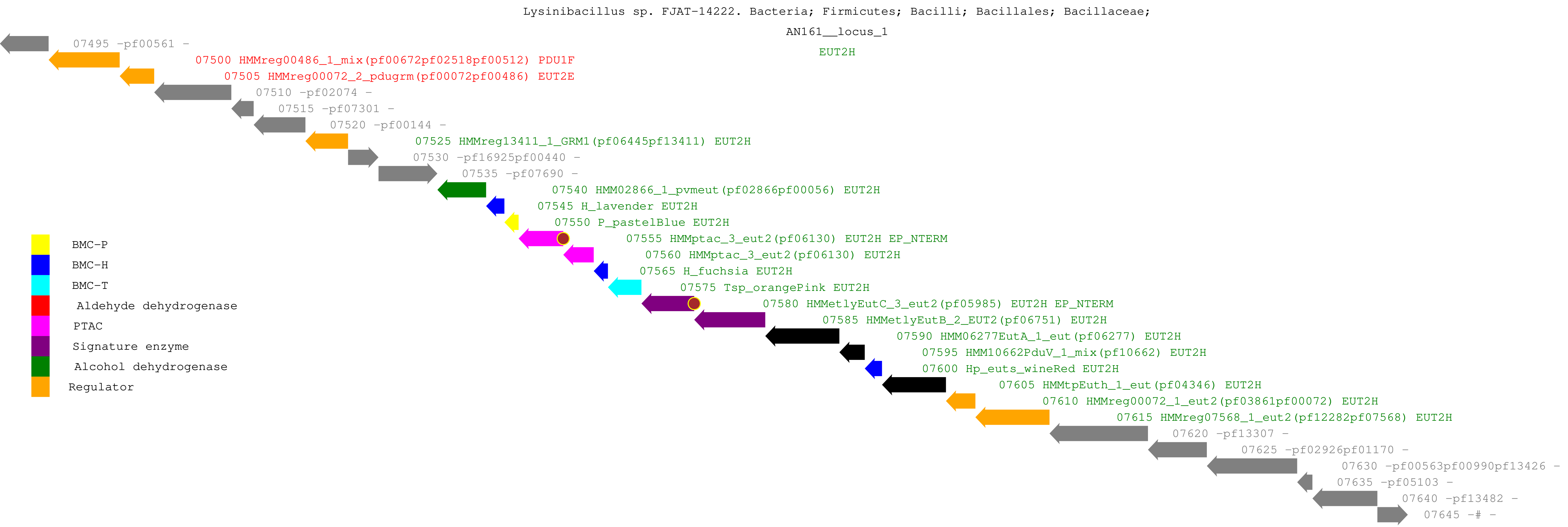
Lysinibacillus sp. FJAT-14222. Bacteria; Firmicutes; Bacilli; Bacillales; Bacillaceae;
AN161__locus_1
EUT2H
07495 -pf00561 -
07500 HMMreg00486_1_mix(pf00672pf02518pf00512) PDU1F
07505 HMMreg00072_2_pdugrm(pf00072pf00486) EUT2E
07510 -pf02074 -
07515 -pf07301 -
07520 -pf00144 -
07525 HMMreg13411_1_GRM1(pf06445pf13411) EUT2H
07530 -pf16925pf00440 -
07535 -pf07690 -
07540 HMM02866_1_pvmeut(pf02866pf00056) EUT2H
07545 H_lavender EUT2H
07550 P_pastelBlue EUT2H
07555 HMMptac_3_eut2(pf06130) EUT2H EP_NTERM
07560 HMMptac_3_eut2(pf06130) EUT2H
07565 H_fuchsia EUT2H
07575 Tsp_orangePink EUT2H
07580 HMMetlyEutC_3_eut2(pf05985) EUT2H EP_NTERM
07585 HMMetlyEutB_2_EUT2(pf06751) EUT2H
07590 HMM06277EutA_1_eut(pf06277) EUT2H
07595 HMM10662PduV_1_mix(pf10662) EUT2H
07600 Hp_euts_wineRed EUT2H
07605 HMMtpEuth_1_eut(pf04346) EUT2H
07610 HMMreg00072_1_eut2(pf03861pf00072) EUT2H
07615 HMMreg07568_1_eut2(pf12282pf07568) EUT2H
07620 -pf13307 -
07625 -pf02926pf01170 -
07630 -pf00563pf00990pf13426 -
07635 -pf05103 -
07640 -pf13482 -
07645 -# -
BMC-P
BMC-H
BMC-T
Aldehyde dehydrogenase
PTAC
Signature enzyme
Alcohol dehydrogenase
Regulator

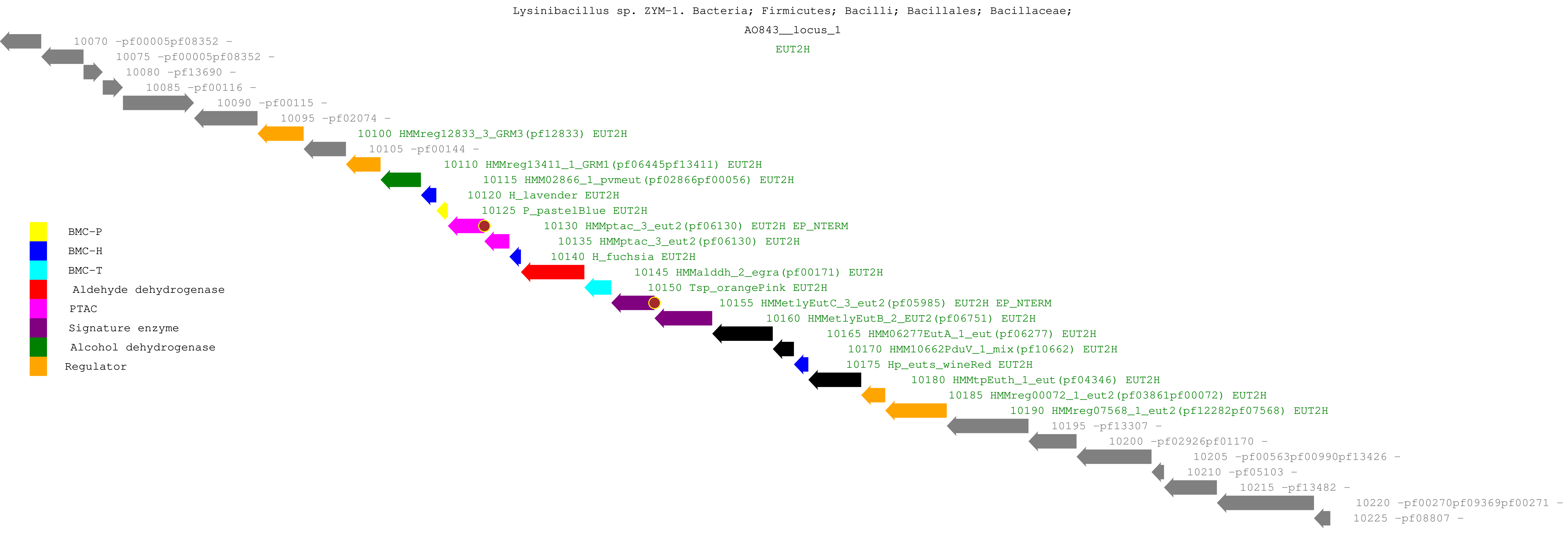
Lysinibacillus sp. ZYM-1. Bacteria; Firmicutes; Bacilli; Bacillales; Bacillaceae;
AO843__locus_1
EUT2H
10070 -pf00005pf08352 -
10075 -pf00005pf08352 -
10080 -pf13690 -
10085 -pf00116 -
10090 -pf00115 -
10095 -pf02074 -
10100 HMMreg12833_3_GRM3(pf12833) EUT2H
10105 -pf00144 -
10110 HMMreg13411_1_GRM1(pf06445pf13411) EUT2H
10115 HMM02866_1_pvmeut(pf02866pf00056) EUT2H
10120 H_lavender EUT2H
10125 P_pastelBlue EUT2H
10130 HMMptac_3_eut2(pf06130) EUT2H EP_NTERM
10135 HMMptac_3_eut2(pf06130) EUT2H
10140 H_fuchsia EUT2H
10145 HMMaldh_2_egra(pf00171) EUT2H
10150 Tsp_orangePink EUT2H
10155 HMMetlyEutC_3_eut2(pf05985) EUT2H EP_NTERM
10160 HMMetlyEutB_2_EUT2(pf06751) EUT2H
10165 HMM06277EutA_1_eut(pf06277) EUT2H
10170 HMM10662PduV_1_mix(pf10662) EUT2H
10175 Hp_euts_wineRed EUT2H
10180 HMMtpEuth_1_eut(pf04346) EUT2H
10185 HMMreg00072_1_eut2(pf03861pf00072) EUT2H
10190 HMMreg07568_1_eut2(pf12282pf07568) EUT2H
10195 -pf13307 -
10200 -pf02926pf01170 -
10205 -pf00563pf00990pf13426 -
10210 -pf05103 -
10215 -pf13482 -
10220 -pf00270pf09369pf00271 -
10225 -pf08807 -
BMC-P
BMC-H
BMC-T
Aldehyde dehydrogenase
PTAC
Signature enzyme
Alcohol dehydrogenase
Regulator

Lysinibacillus sp. KCTC 33748. Bacteria; Firmicutes; Bacilli; Bacillales; Bacillaceae;

B1B04__locus_1

EUT2H

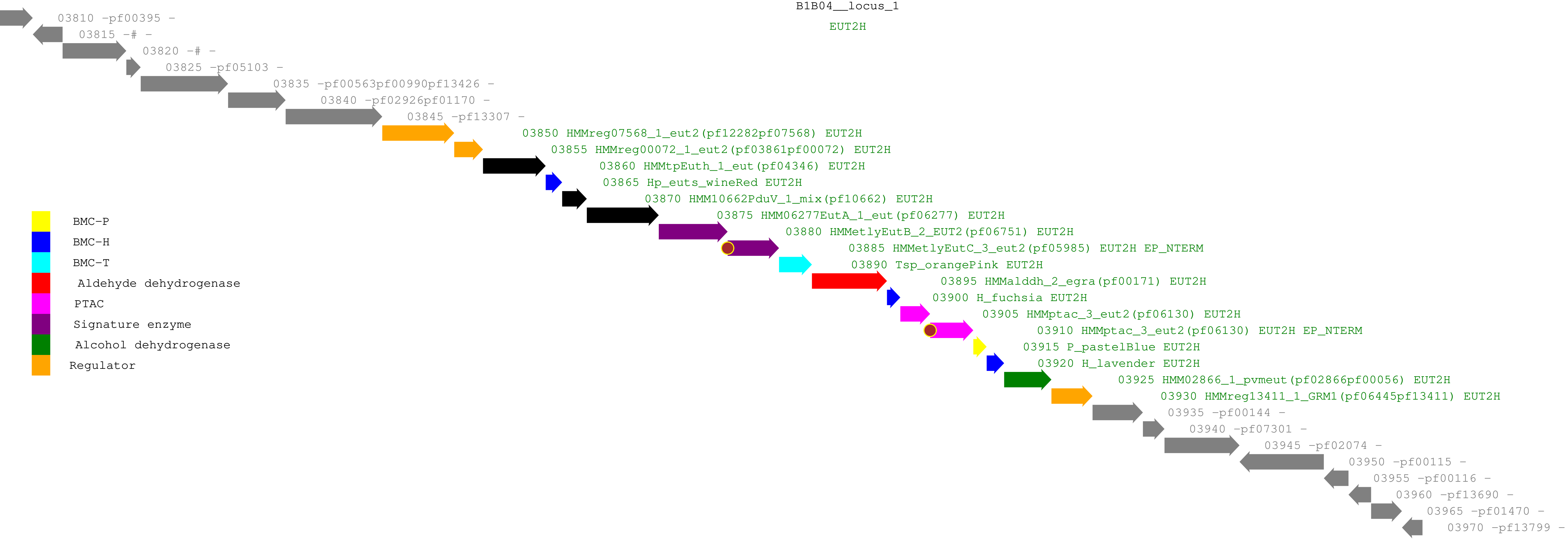

Bacillus sp. B14905. Bacteria; Firmicutes; Bacilli; Bacillales; Bacillaceae; Bacillus.

BB14905__locus_1

EUT2H

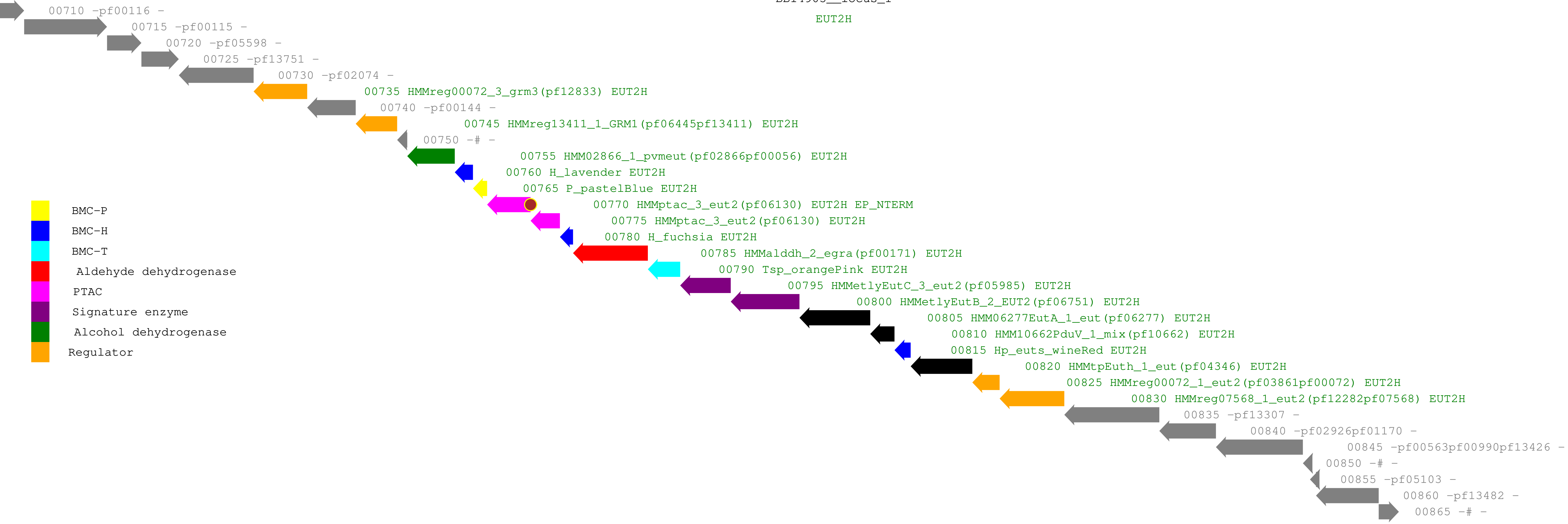

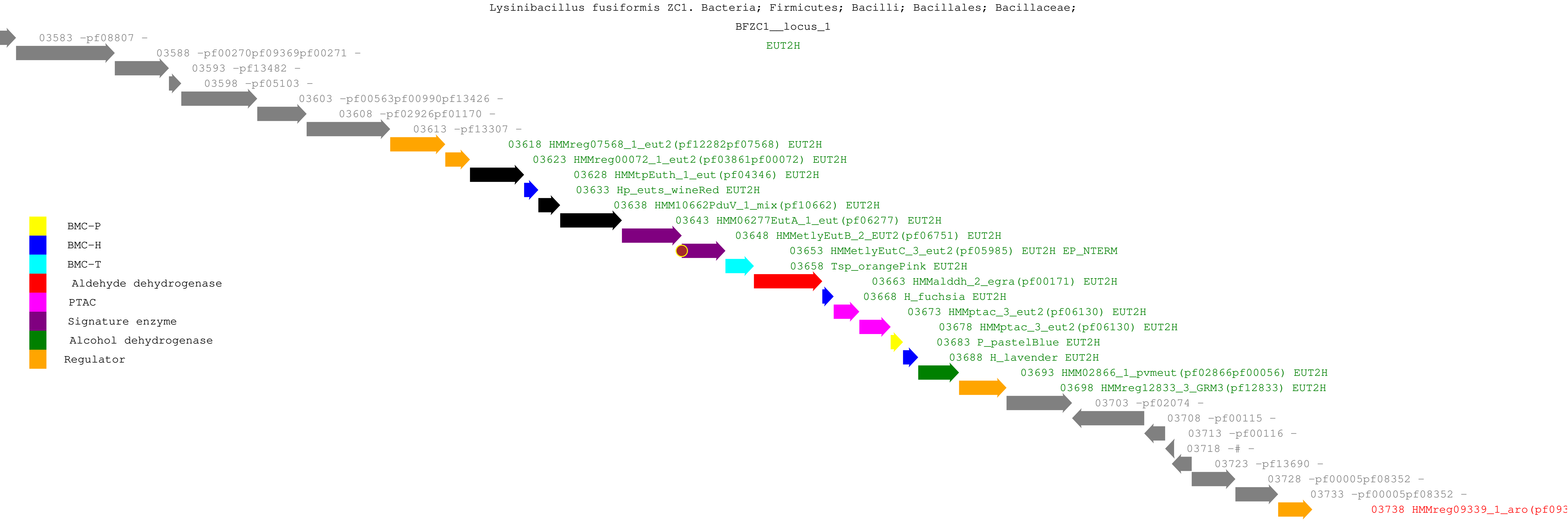

Lysinibacillus fusiformis ZC1. Bacteria; Firmicutes; Bacilli; Bacillales; Bacillaceae;
BFZC1__locus_1
EUT2H
03583 -pf08807 -
03588 -pf00270pf09369pf00271 -
03593 -pf13482 -
03598 -pf05103 -
03603 -pf00563pf00990pf13426 -
03608 -pf02926pf01170 -
03613 -pf13307 -
03618 HMMreg07568_1_eut2(pf12282pf07568) EUT2H
03623 HMMreg00072_1_eut2(pf03861pf00072) EUT2H
03628 HMMtpEuth_1_eut(pf04346) EUT2H
03633 Hp_euts_wineRed EUT2H
03638 HMM10662PduV_1_mix(pf10662) EUT2H
03643 HMM06277EutA_1_eut(pf06277) EUT2H
03648 HMMetlyEutB_2_EUT2(pf06751) EUT2H
03653 HMMetlyEutC_3_eut2(pf05985) EUT2H EP_NTERM
03658 Tsp_orangePink EUT2H
03663 HMMalddh_2_egra(pf00171) EUT2H
03668 H_fuchsia EUT2H
03673 HMMptac_3_eut2(pf06130) EUT2H
03678 HMMptac_3_eut2(pf06130) EUT2H
03683 P_pastelBlue EUT2H
03688 H_lavender EUT2H
03693 HMM02866_1_pvmeut(pf02866pf00056) EUT2H
03698 HMMreg12833_3_GRM3(pf12833) EUT2H
03703 -pf02074 -
03708 -pf00115 -
03713 -pf00116 -
03718 -# -
03723 -pf13690 -
03728 -pf00005pf08352 -
03733 -pf00005pf08352 -
03738 HMMreg09339_1_aro(pf093
BMC-P
BMC-H
BMC-T
Aldehyde dehydrogenase
PTAC
Signature enzyme
Alcohol dehydrogenase
Regulator

Lysinibacillus fusiformis. Bacteria; Firmicutes; Bacilli; Bacillales; Bacillaceae;

BG258__locus_1

EUT2H

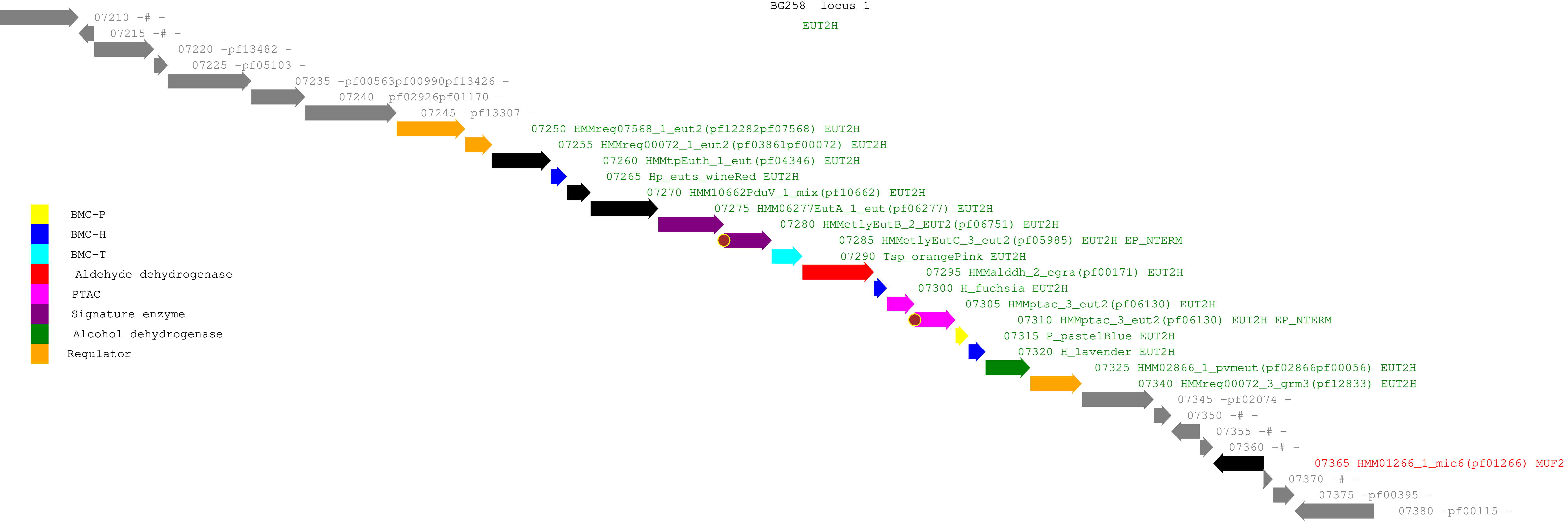

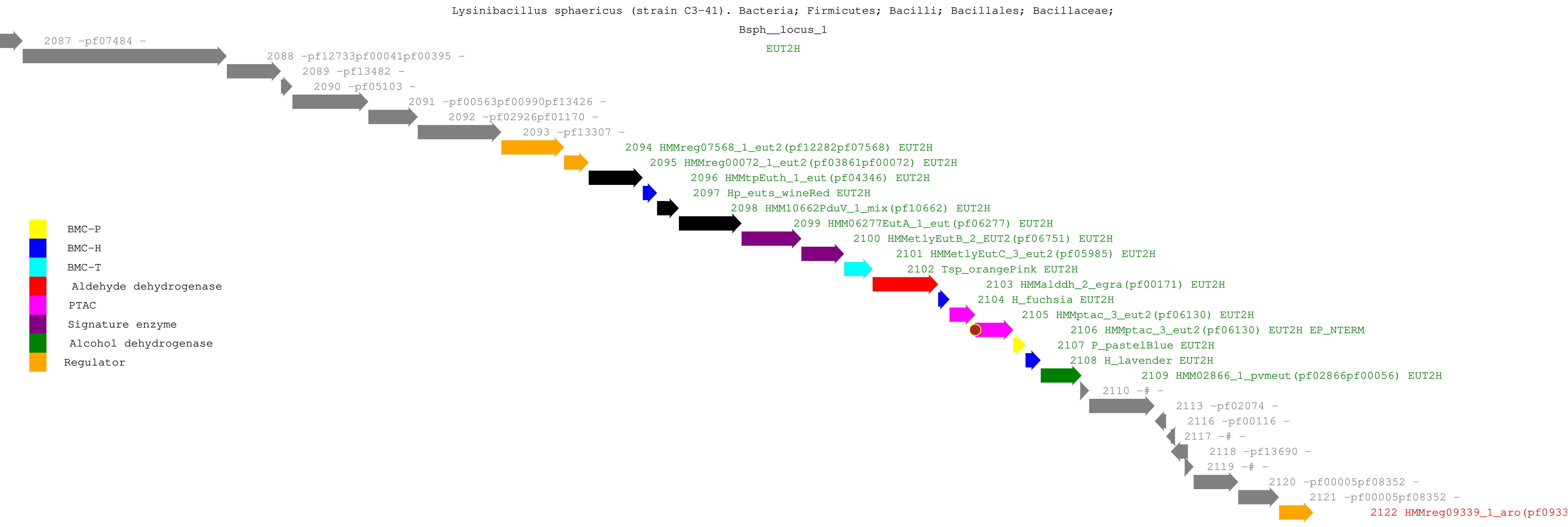

Lysinibacillus sphaericus (strain C3-41). Bacteria; Firmicutes; Bacilli; Bacillales; Bacillaceae;
Bsph__locus_1
EUT2H
2087 -pf07484 -
2088 -pf12733pf00041pf00395 -
2089 -pf13482 -
2090 -pf05103 -
2091 -pf00563pf00990pf13426 -
2092 -pf02926pf01170 -
2093 -pf13307 -
2094 HMMreg07568_1_eut2(pf12282pf07568) EUT2H
2095 HMMreg00072_1_eut2(pf03861pf00072) EUT2H
2096 HMMtpEuth_1_eut(pf04346) EUT2H
2097 Hp_euts_wineRed EUT2H
2098 HMM10662PduV_1_mix(pf10662) EUT2H
2099 HMM06277EutA_1_eut(pf06277) EUT2H
2100 HMMetlyEutB_2_EUT2(pf06751) EUT2H
2101 HMMetlyEutC_3_eut2(pf05985) EUT2H
2102 Tsp_orangePink EUT2H
2103 HMMaldh_2_egra(pf00171) EUT2H
2104 H_fuchsia EUT2H
2105 HMMptac_3_eut2(pf06130) EUT2H
2106 HMMptac_3_eut2(pf06130) EUT2H EP_NTERM
2107 P_pastelBlue EUT2H
2108 H_lavender EUT2H
2109 HMM02866_1_pvmeut(pf02866pf00056) EUT2H
2110 -# -
2113 -pf02074 -
2116 -pf00116 -
2117 -# -
2118 -pf13690 -
2119 -# -
2120 -pf00005pf08352 -
2121 -pf00005pf08352 -
2122 HMMreg09339_1_aro(pf0933
BMC-P
BMC-H
BMC-T
Aldehyde dehydrogenase
PTAC
Signature enzyme
Alcohol dehydrogenase
Regulator

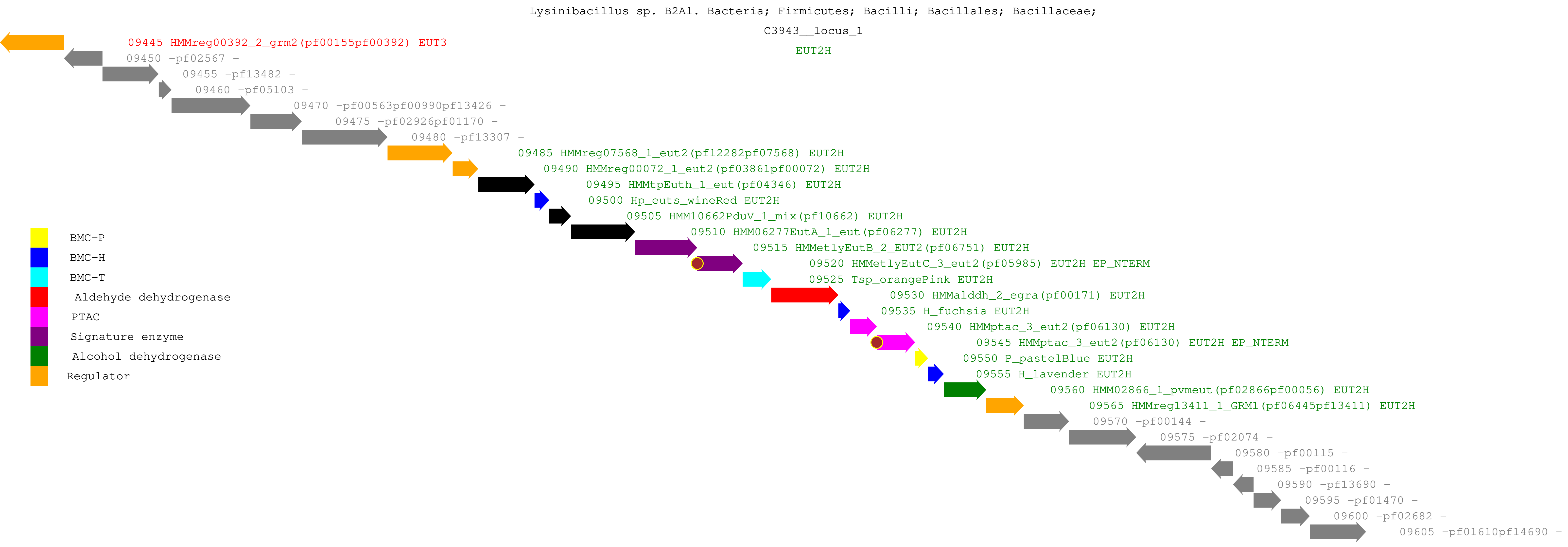
Lysinibacillus sp. B2A1. Bacteria; Firmicutes; Bacilli; Bacillales; Bacillaceae;
C3943__locus_1
EUT2H
09445 HMMreg00392_2_grm2(pf00155pf00392) EUT3
09450 -pf02567 -
09455 -pf13482 -
09460 -pf05103 -
09470 -pf00563pf00990pf13426 -
09475 -pf02926pf01170 -
09480 -pf13307 -
09485 HMMreg07568_1_eut2(pf12282pf07568) EUT2H
09490 HMMreg00072_1_eut2(pf03861pf00072) EUT2H
09495 HMMtpEuth_1_eut(pf04346) EUT2H
09500 Hp_euts_wineRed EUT2H
09505 HMM10662PduV_1_mix(pf10662) EUT2H
09510 HMM06277EutA_1_eut(pf06277) EUT2H
09515 HMMetlyEutB_2_EUT2(pf06751) EUT2H
09520 HMMetlyEutC_3_eut2(pf05985) EUT2H EP_NTERM
09525 Tsp_orangePink EUT2H
09530 HMMalddh_2_egra(pf00171) EUT2H
09535 H_fuchsia EUT2H
09540 HMMptac_3_eut2(pf06130) EUT2H
09545 HMMptac_3_eut2(pf06130) EUT2H EP_NTERM
09550 P_pastelBlue EUT2H
09555 H_lavender EUT2H
09560 HMM02866_1_pvmeut(pf02866pf00056) EUT2H
09565 HMMreg13411_1_GRM1(pf06445pf13411) EUT2H
09570 -pf00144 -
09575 -pf02074 -
09580 -pf00115 -
09585 -pf00116 -
09590 -pf13690 -
09595 -pf01470 -
09600 -pf02682 -
09605 -pf01610pf14690 -
BMC-P
BMC-H
BMC-T
Aldehyde dehydrogenase
PTAC
Signature enzyme
Alcohol dehydrogenase
Regulator

Lysinibacillus sp. SDF0037. Bacteria; Firmicutes; Bacilli; Bacillales; Bacillaceae; Lysinibacillus;

C7Y47__locus_1

EUT2H

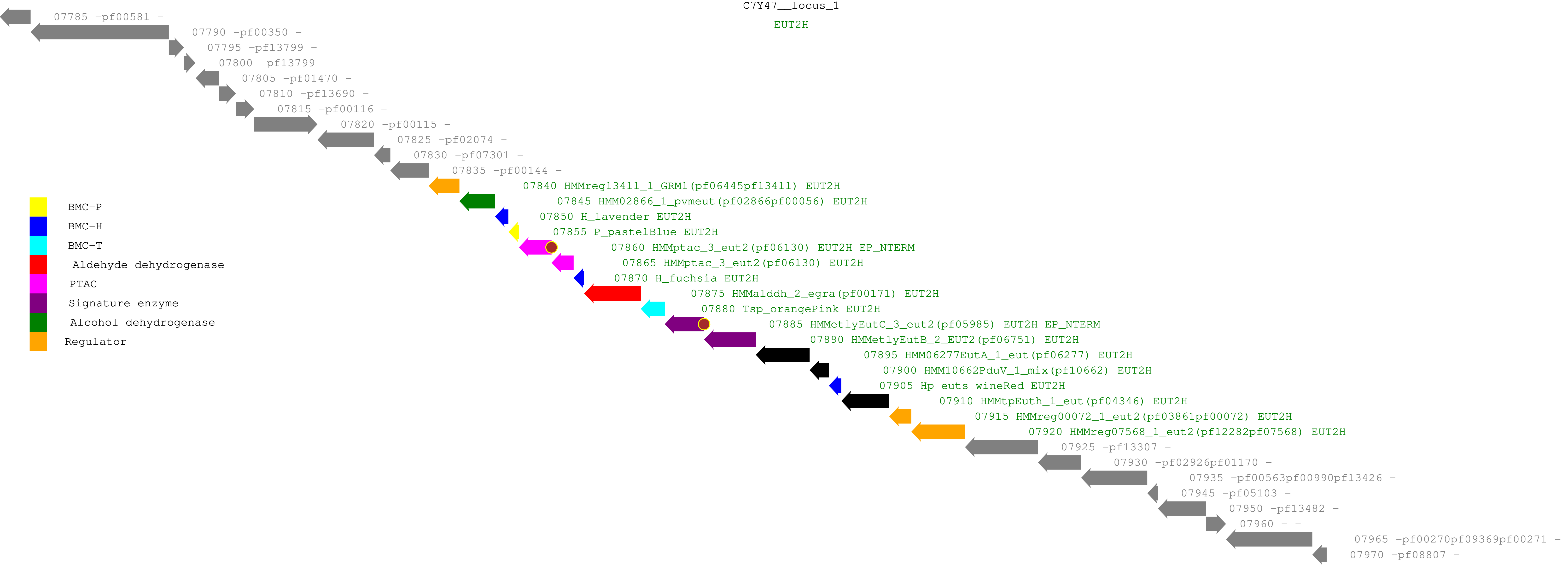

Lysinibacillus boronitolerans JCM 21713 = 10a = NBRC 103108. Bacteria; Firmicutes; Bacilli; Bacillales; Bacillaceae;

CD31__locus_1

EUT2H

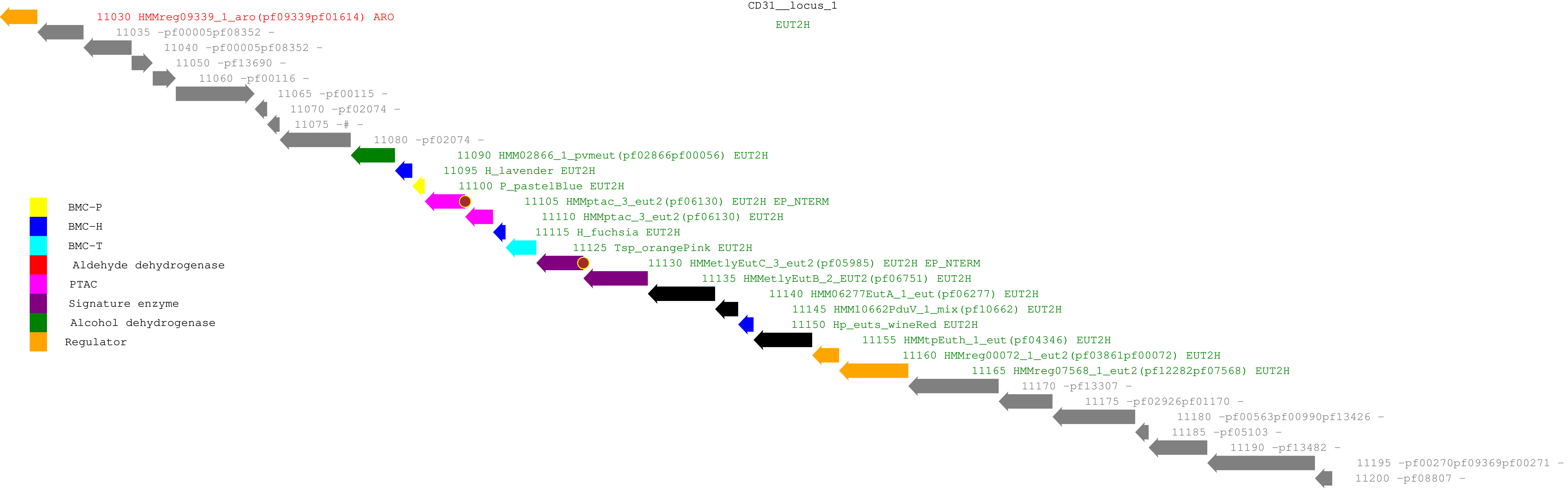

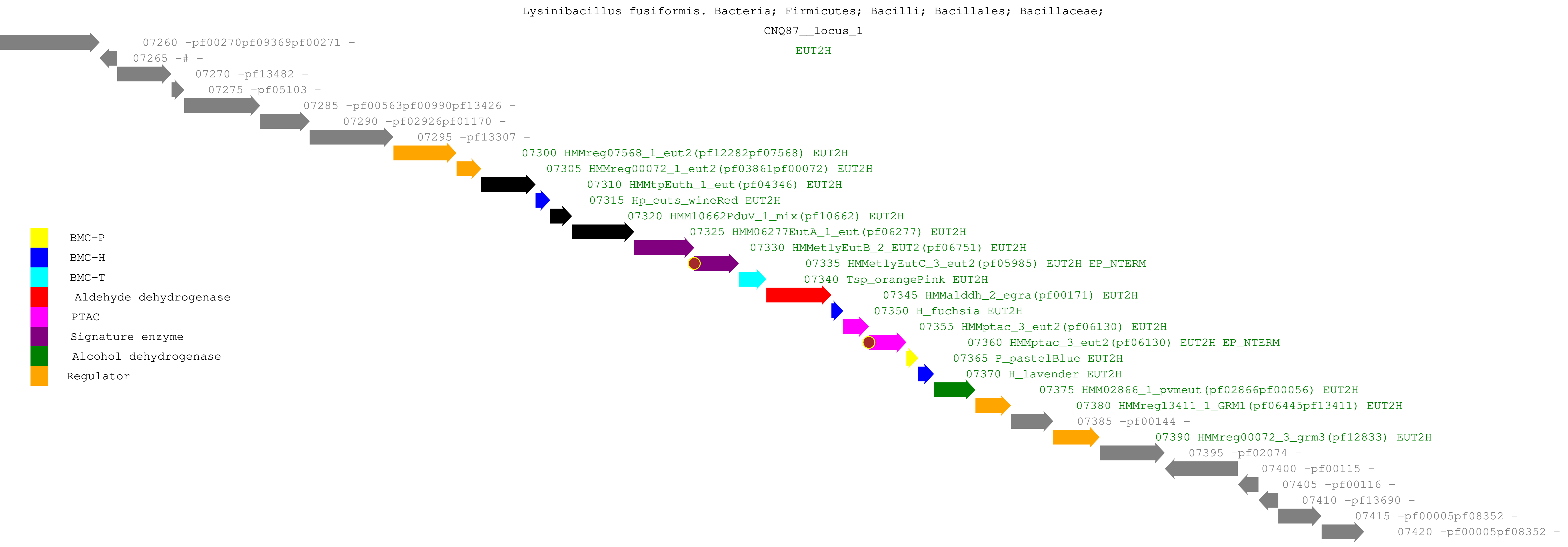
Lysinibacillus fusiformis. Bacteria; Firmicutes; Bacilli; Bacillales; Bacillaceae;
CNQ87__locus_1
EUT2H
07260 -pf00270pf09369pf00271 -
07265 -# -
07270 -pf13482 -
07275 -pf05103 -
07285 -pf00563pf00990pf13426 -
07290 -pf02926pf01170 -
07295 -pf13307 -
07300 HMMreg07568_1_eut2(pf12282pf07568) EUT2H
07305 HMMreg00072_1_eut2(pf03861pf00072) EUT2H
07310 HMMtpEuth_1_eut(pf04346) EUT2H
07315 Hp_euts_wineRed EUT2H
07320 HMM10662PduV_1_mix(pf10662) EUT2H
07325 HMM06277EutA_1_eut(pf06277) EUT2H
07330 HMMetlyEutB_2_EUT2(pf06751) EUT2H
07335 HMMetlyEutC_3_eut2(pf05985) EUT2H EP_NTERM
07340 Tsp_orangePink EUT2H
07345 HMMalddh_2_egra(pf00171) EUT2H
07350 H_fuchsia EUT2H
07355 HMMptac_3_eut2(pf06130) EUT2H
07360 HMMptac_3_eut2(pf06130) EUT2H EP_NTERM
07365 P_pastelBlue EUT2H
07370 H_lavender EUT2H
07375 HMM02866_1_pvmeut(pf02866pf00056) EUT2H
07380 HMMreg13411_1_GRM1(pf06445pf13411) EUT2H
07385 -pf00144 -
07390 HMMreg00072_3_grm3(pf12833) EUT2H
07395 -pf02074 -
07400 -pf00115 -
07405 -pf00116 -
07410 -pf13690 -
07415 -pf00005pf08352 -
07420 -pf00005pf08352 -
BMC-P
BMC-H
BMC-T
Aldehyde dehydrogenase
PTAC
Signature enzyme
Alcohol dehydrogenase
Regulator

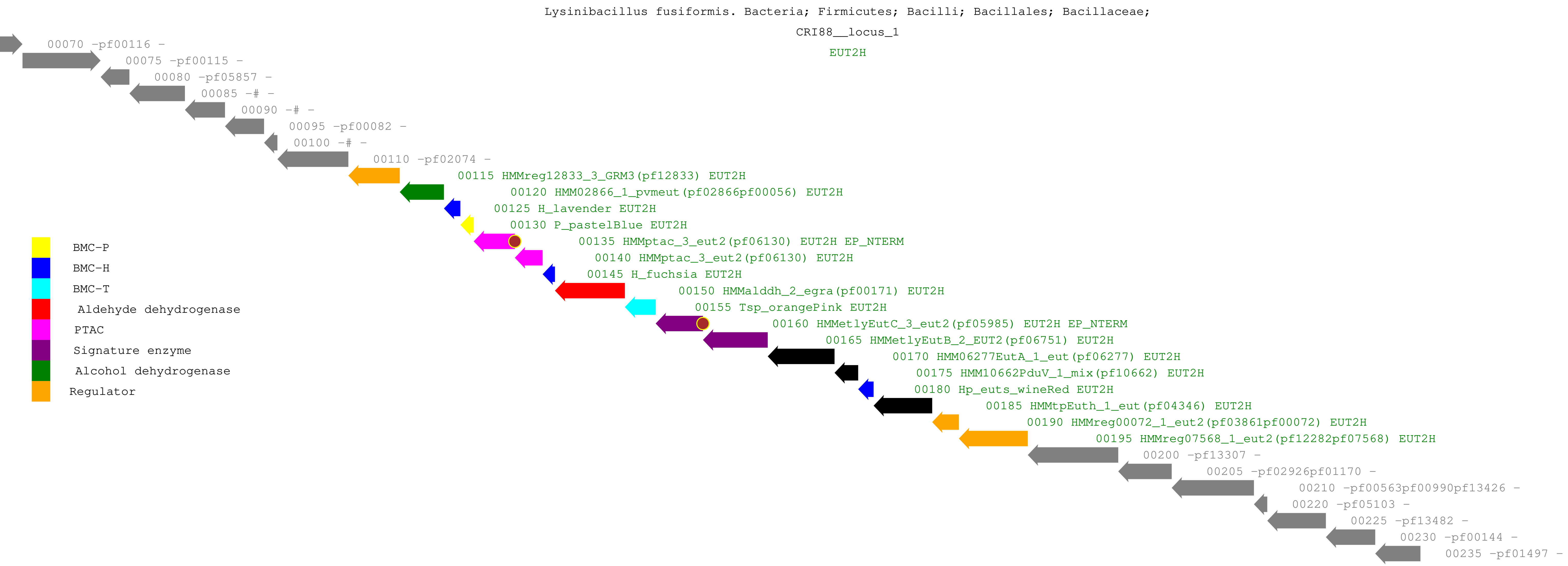
Lysinibacillus fusiformis. Bacteria; Firmicutes; Bacilli; Bacillales; Bacillaceae;
CRI88__locus_1
EUT2H
00070 -pf00116 -
00075 -pf00115 -
00080 -pf05857 -
00085 -# -
00090 -# -
00095 -pf00082 -
00100 -# -
00110 -pf02074 -
00115 HMMreg12833_3_GRM3(pf12833) EUT2H
00120 HMM02866_1_pvmeut(pf02866pf00056) EUT2H
00125 H_lavender EUT2H
00130 P_pastelBlue EUT2H
00135 HMMptac_3_eut2(pf06130) EUT2H EP_NTERM
00140 HMMptac_3_eut2(pf06130) EUT2H
00145 H_fuchsia EUT2H
00150 HMMalddh_2_egra(pf00171) EUT2H
00155 Tsp_orangePink EUT2H
00160 HMMetlyEutC_3_eut2(pf05985) EUT2H EP_NTERM
00165 HMMetlyEutB_2_EUT2(pf06751) EUT2H
00170 HMM06277EutA_1_eut(pf06277) EUT2H
00175 HMM10662PduV_1_mix(pf10662) EUT2H
00180 Hp_euts_wineRed EUT2H
00185 HMMtpEuth_1_eut(pf04346) EUT2H
00190 HMMreg00072_1_eut2(pf03861pf00072) EUT2H
00195 HMMreg07568_1_eut2(pf12282pf07568) EUT2H
00200 -pf13307 -
00205 -pf02926pf01170 -
00210 -pf00563pf00990pf13426 -
00220 -pf05103 -
00225 -pf13482 -
00230 -pf00144 -
00235 -pf01497 -
BMC-P
BMC-H
BMC-T
Aldehyde dehydrogenase
PTAC
Signature enzyme
Alcohol dehydrogenase
Regulator

Lysinibacillus xylanilyticus. Bacteria; Firmicutes; Bacilli; Bacillales; Bacillaceae;

CWD94__locus_1

EUT2H

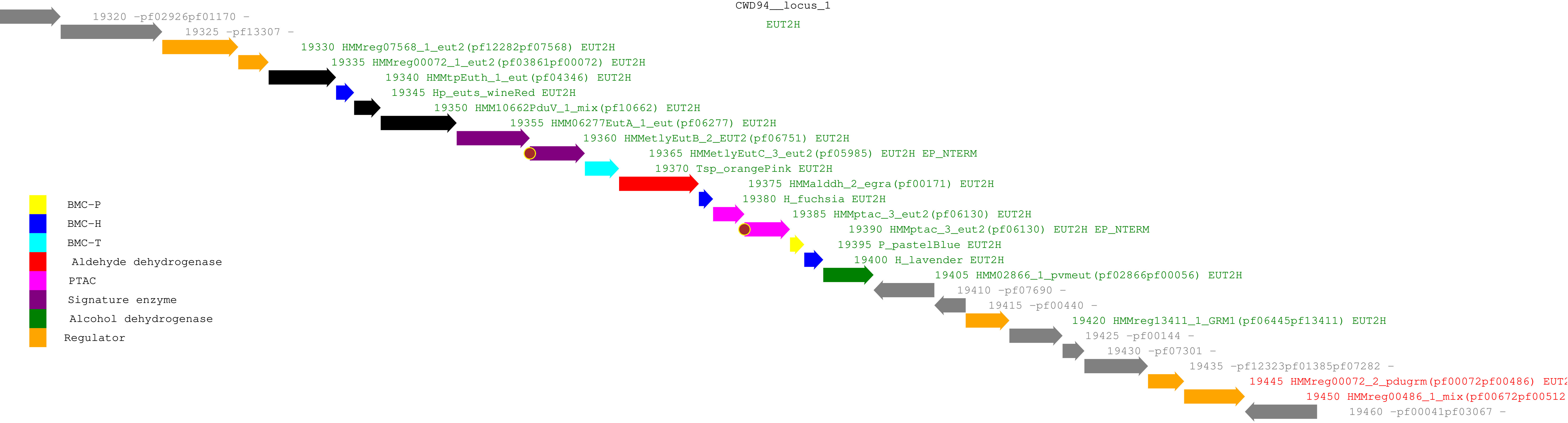

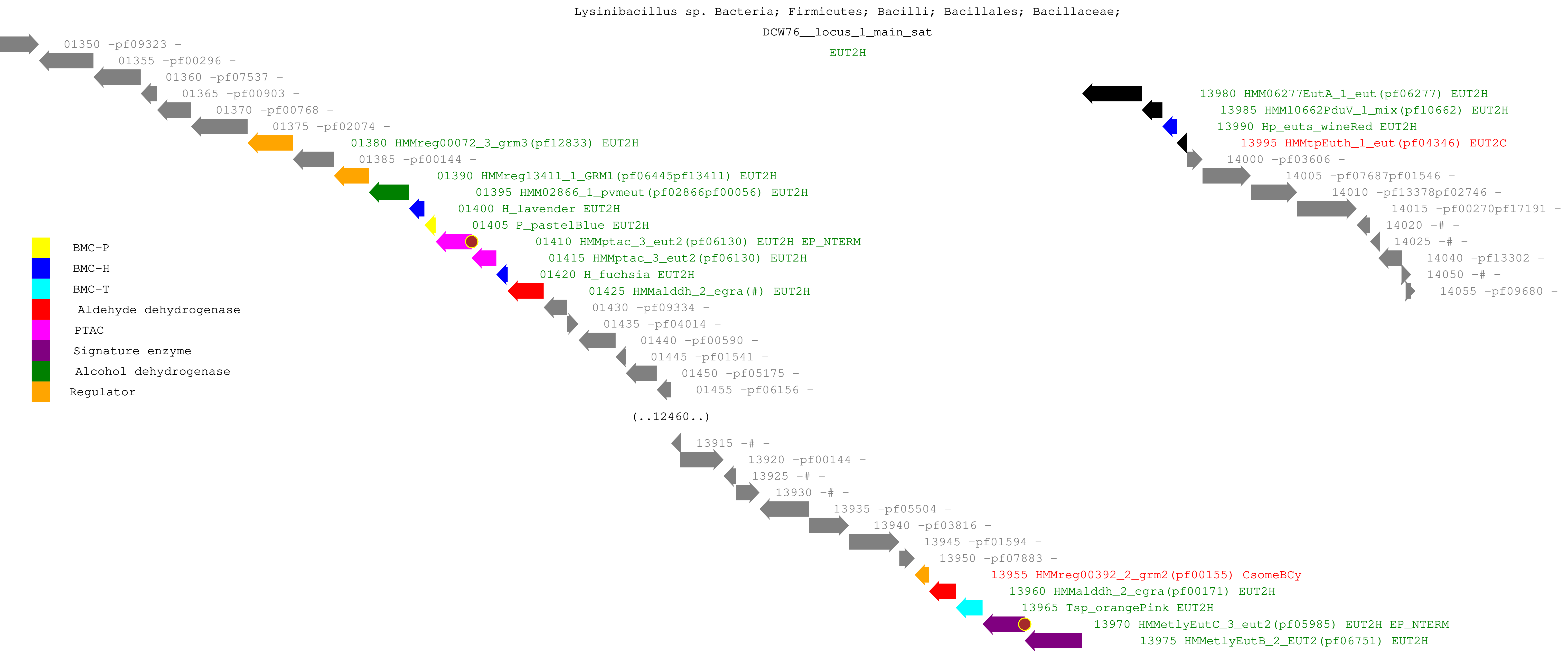
Lysinibacillus sp. Bacteria; Firmicutes; Bacilli; Bacillales; Bacillaceae;
DCW76__locus_1_main_sat
EUT2H
01350 -pf09323 -
01355 -pf00296 -
01360 -pf07537 -
01365 -pf00903 -
01370 -pf00768 -
01375 -pf02074 -
01380 HMMreg00072_3_grm3(pf12833) EUT2H
01385 -pf00144 -
01390 HMMreg13411_1_GRM1(pf06445pf13411) EUT2H
01395 HMM02866_1_pvmeut(pf02866pf00056) EUT2H
01400 H_lavender EUT2H
01405 P_pastelBlue EUT2H
01410 HMMptac_3_eut2(pf06130) EUT2H EP_NTERM
01415 HMMptac_3_eut2(pf06130) EUT2H
01420 H_fuchsia EUT2H
01425 HMMaldh_2_egra(#) EUT2H
01430 -pf09334 -
01435 -pf04014 -
01440 -pf00590 -
01445 -pf01541 -
01450 -pf05175 -
01455 -pf06156 -
(..12460..)
13915 -# -
13920 -pf00144 -
13925 -# -
13930 -# -
13935 -pf05504 -
13940 -pf03816 -
13945 -pf01594 -
13950 -pf07883 -
13955 HMMreg00392_2_grm2(pf00155) CsomeBCy
13960 HMMaldh_2_egra(pf00171) EUT2H
13965 Tsp_orangePink EUT2H
13970 HMMetlyEutC_3_eut2(pf05985) EUT2H EP_NTERM
13975 HMMetlyEutB_2_EUT2(pf06751) EUT2H
13980 HMM06277EutA_1_eut(pf06277) EUT2H
13985 HMM10662PduV_1_mix(pf10662) EUT2H
13990 Hp_euts_wineRed EUT2H
13995 HMMtpEuth_1_eut(pf04346) EUT2C
14000 -pf03606 -
14005 -pf07687pf01546 -
14010 -pf13378pf02746 -
14015 -pf00270pf17191 -
14020 -# -
14025 -# -
14040 -pf13302 -
14050 -# -
14055 -pf09680 -
BMC-P
BMC-H
BMC-T
Aldehyde dehydrogenase
PTAC
Signature enzyme
Alcohol dehydrogenase
Regulator

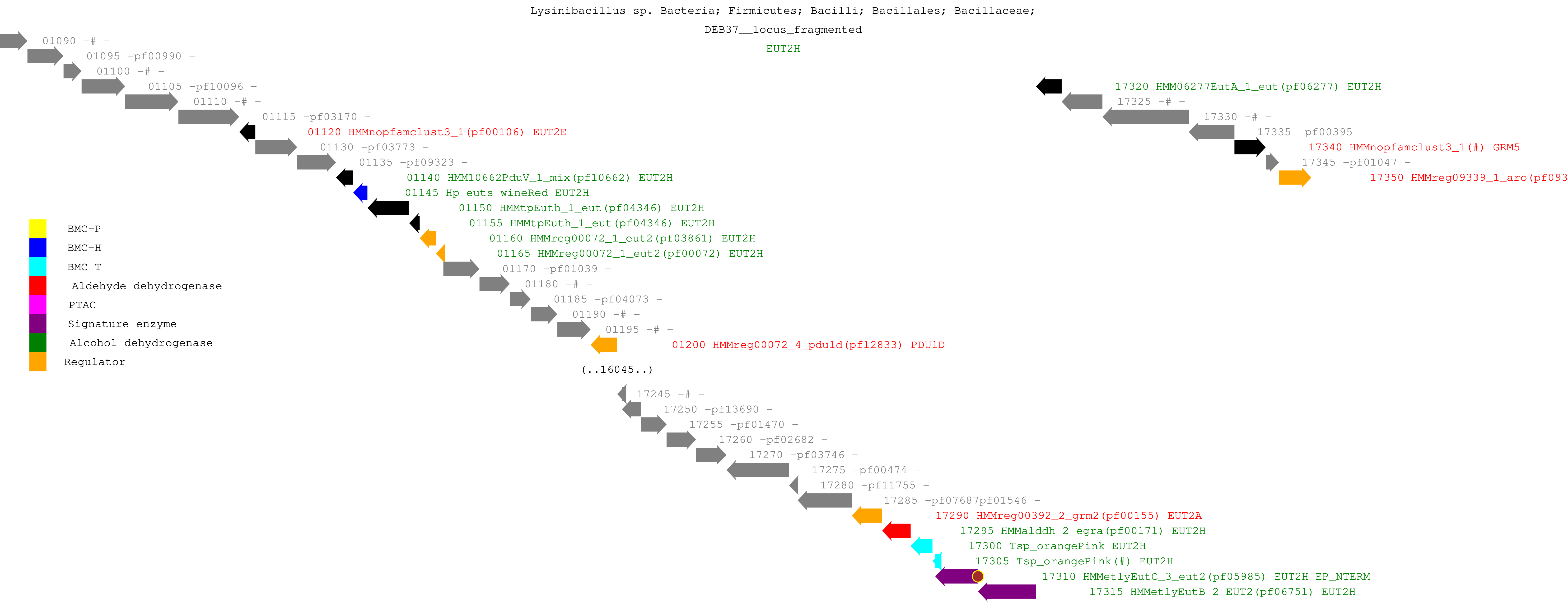
Lysinibacillus sp. Bacteria; Firmicutes; Bacilli; Bacillales; Bacillaceae;
DEB37__locus_fragmented
EUT2H
01090 -# -
01095 -pf00990 -
01100 -# -
01105 -pf10096 -
01110 -# -
01115 -pf03170 -
01120 HMMnopfamclust3_1(pf00106) EUT2E
01130 -pf03773 -
01135 -pf09323 -
01140 HMM10662PduV_1_mix(pf10662) EUT2H
01145 Hp_euts_wineRed EUT2H
01150 HMMtpEuth_1_eut(pf04346) EUT2H
01155 HMMtpEuth_1_eut(pf04346) EUT2H
01160 HMMreg00072_1_eut2(pf03861) EUT2H
01165 HMMreg00072_1_eut2(pf00072) EUT2H
01170 -pf01039 -
01180 -# -
01185 -pf04073 -
01190 -# -
01195 -# -
01200 HMMreg00072_4_pdu1d(pf12833) PDU1D
(..16045..)
17245 -# -
17250 -pf13690 -
17255 -pf01470 -
17260 -pf02682 -
17270 -pf03746 -
17275 -pf00474 -
17280 -pf11755 -
17285 -pf07687pf01546 -
17290 HMMreg00392_2_grm2(pf00155) EUT2A
17295 HMMalddh_2_egra(pf00171) EUT2H
17300 Tsp_orangePink EUT2H
17305 Tsp_orangePink(#) EUT2H
17310 HMMetlyEutC_3_eut2(pf05985) EUT2H EP_NTERM
17315 HMMetlyEutB_2_EUT2(pf06751) EUT2H
17320 HMM06277EutA_1_eut(pf06277) EUT2H
17325 -# -
17330 -# -
17335 -pf00395 -
17340 HMMnopfamclust3_1(#) GRM5
17345 -pf01047 -
17350 HMMreg09339_1_aro(pf093
BMC-P
BMC-H
BMC-T
Aldehyde dehydrogenase
PTAC
Signature enzyme
Alcohol dehydrogenase
Regulator

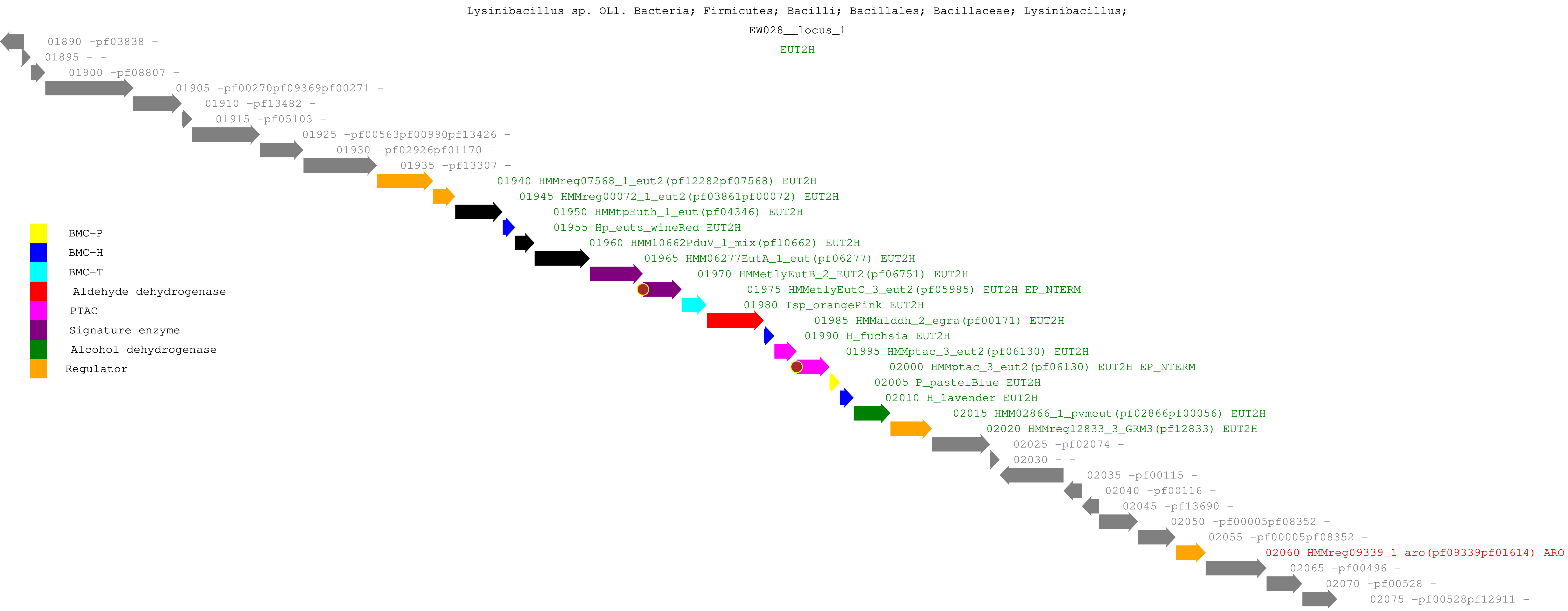
Lysinibacillus sp. OL1. Bacteria; Firmicutes; Bacilli; Bacillales; Bacillaceae; Lysinibacillus;
EW028__locus_1
EUT2H
01890 -pf03838 -
01895 - -
01900 -pf08807 -
01905 -pf00270pf09369pf00271 -
01910 -pf13482 -
01915 -pf05103 -
01925 -pf00563pf00990pf13426 -
01930 -pf02926pf01170 -
01935 -pf13307 -
01940 HMMreg07568_1_eut2(pf12282pf07568) EUT2H
01945 HMMreg00072_1_eut2(pf03861pf00072) EUT2H
01950 HMMtpEuth_1_eut(pf04346) EUT2H
01955 Hp_euts_wineRed EUT2H
01960 HMM10662PduV_1_mix(pf10662) EUT2H
01965 HMM06277EutA_1_eut(pf06277) EUT2H
01970 HMMetlyEutB_2_EUT2(pf06751) EUT2H
01975 HMMetlyEutC_3_eut2(pf05985) EUT2H EP_NTERM
01980 Tsp_orangePink EUT2H
01985 HMMalddh_2_egra(pf00171) EUT2H
01990 H_fuchsia EUT2H
01995 HMMptac_3_eut2(pf06130) EUT2H
02000 HMMptac_3_eut2(pf06130) EUT2H EP_NTERM
02005 P_pastelBlue EUT2H
02010 H_lavender EUT2H
02015 HMM02866_1_pvmeut(pf02866pf00056) EUT2H
02020 HMMreg12833_3_GRM3(pf12833) EUT2H
02025 -pf02074 -
02030 - -
02035 -pf00115 -
02040 -pf00116 -
02045 -pf13690 -
02050 -pf00005pf08352 -
02055 -pf00005pf08352 -
02060 HMMreg09339_1_aro(pf09339pf01614) ARO
02065 -pf00496 -
02070 -pf00528 -
02075 -pf00528pf12911 -
BMC-P
BMC-H
BMC-T
Aldehyde dehydrogenase
PTAC
Signature enzyme
Alcohol dehydrogenase
Regulator

Lysinibacillus varians. Bacteria; Firmicutes; Bacilli; Bacillales; Bacillaceae; Lysinibacillus.

FC752__locus_1

EUT2H

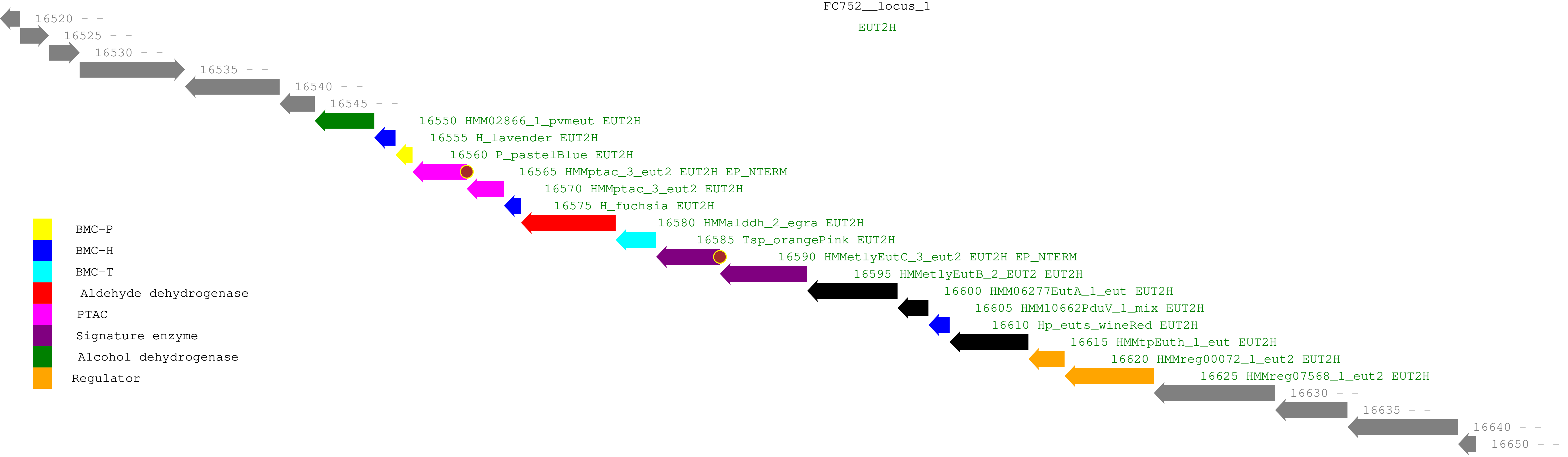

Lysinibacillus mangiferihumi. Bacteria; Firmicutes; Bacilli; Bacillales; Bacillaceae; Lysinibacillus.

FC756__locus_1

EUT2H

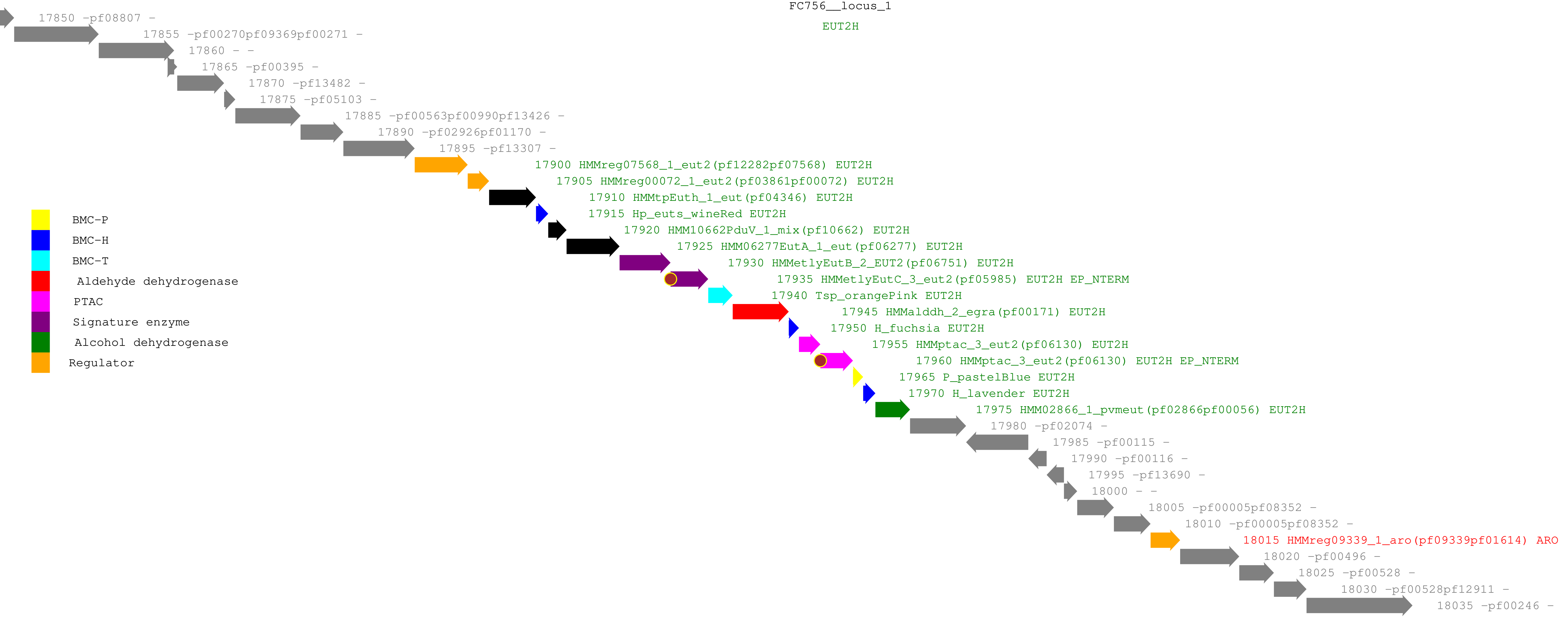

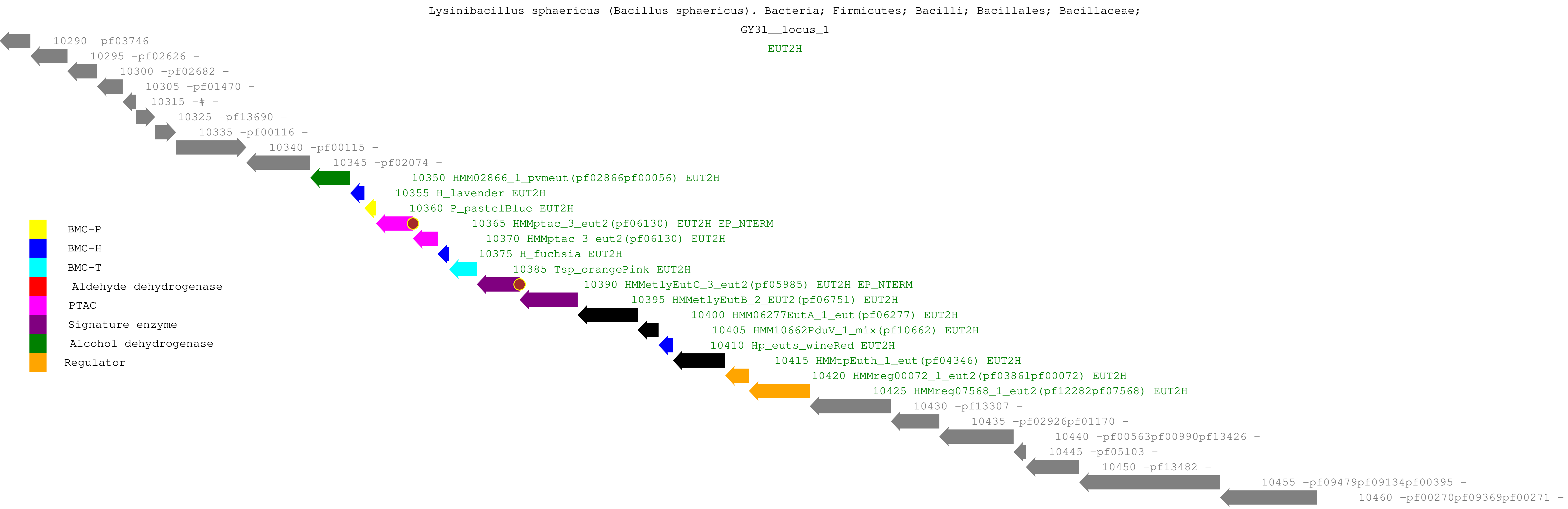
Lysinibacillus sphaericus (Bacillus sphaericus). Bacteria; Firmicutes; Bacilli; Bacillales; Bacillaceae;
GY31__locus_1
EUT2H
10290 -pf03746 -
10295 -pf02626 -
10300 -pf02682 -
10305 -pf01470 -
10315 -# -
10325 -pf13690 -
10335 -pf00116 -
10340 -pf00115 -
10345 -pf02074 -
10350 HMM02866_1_pvmeut(pf02866pf00056) EUT2H
10355 H_lavender EUT2H
10360 P_pastelBlue EUT2H
10365 HMMptac_3_eut2(pf06130) EUT2H EP_NTERM
10370 HMMptac_3_eut2(pf06130) EUT2H
10375 H_fuchsia EUT2H
10385 Tsp_orangePink EUT2H
10390 HMMetlyEutC_3_eut2(pf05985) EUT2H EP_NTERM
10395 HMMetlyEutB_2_EUT2(pf06751) EUT2H
10400 HMM06277EutA_1_eut(pf06277) EUT2H
10405 HMM10662PduV_1_mix(pf10662) EUT2H
10410 Hp_euts_wineRed EUT2H
10415 HMMtpEuth_1_eut(pf04346) EUT2H
10420 HMMreg00072_1_eut2(pf03861pf00072) EUT2H
10425 HMMreg07568_1_eut2(pf12282pf07568) EUT2H
10430 -pf13307 -
10435 -pf02926pf01170 -
10440 -pf00563pf00990pf13426 -
10445 -pf05103 -
10450 -pf13482 -
10455 -pf09479pf09134pf00395 -
10460 -pf00270pf09369pf00271 -
BMC-P
BMC-H
BMC-T
Aldehyde dehydrogenase
PTAC
Signature enzyme
Alcohol dehydrogenase
Regulator

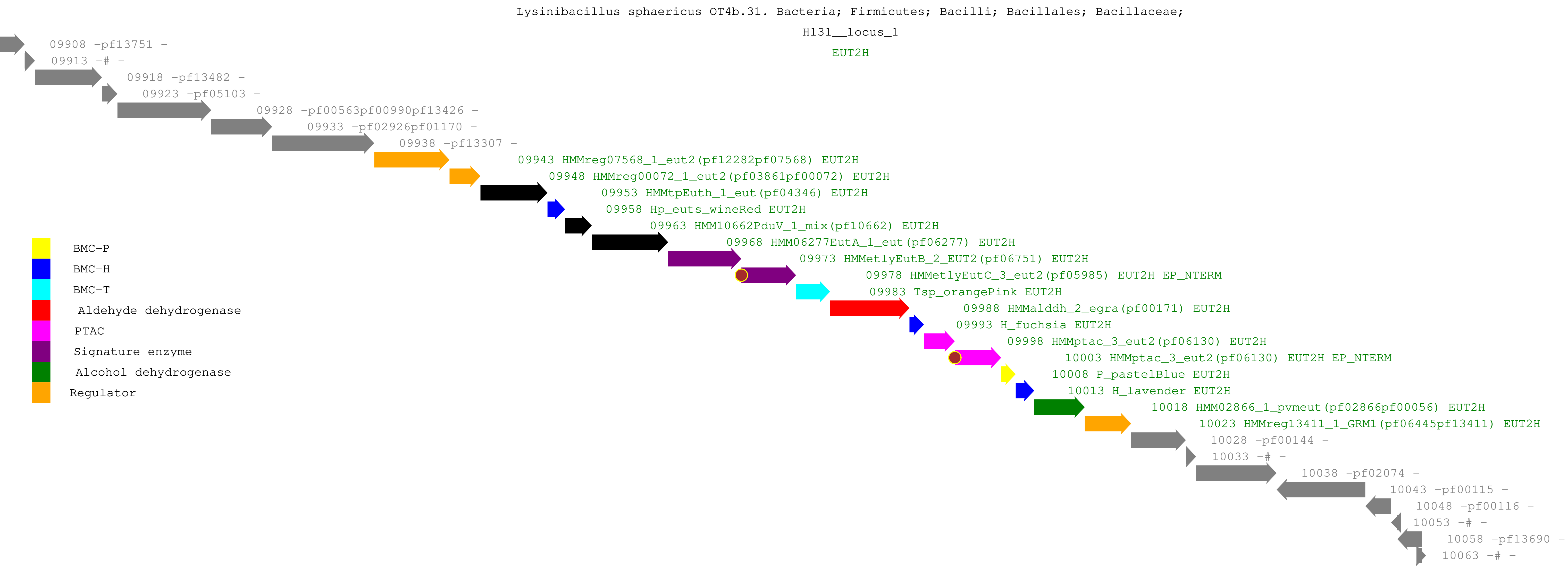
Lysinibacillus sphaericus OT4b.31. Bacteria; Firmicutes; Bacilli; Bacillales; Bacillaceae;
H131__locus_1
EUT2H
09908 -pf13751 -
09913 -# -
09918 -pf13482 -
09923 -pf05103 -
09928 -pf00563pf00990pf13426 -
09933 -pf02926pf01170 -
09938 -pf13307 -
09943 HMMreg07568_1_eut2(pf12282pf07568) EUT2H
09948 HMMreg00072_1_eut2(pf03861pf00072) EUT2H
09953 HMMtpEuth_1_eut(pf04346) EUT2H
09958 Hp_euts_wineRed EUT2H
09963 HMM10662PduV_1_mix(pf10662) EUT2H
09968 HMM06277EutA_1_eut(pf06277) EUT2H
09973 HMMetlyEutB_2_EUT2(pf06751) EUT2H
09978 HMMetlyEutC_3_eut2(pf05985) EUT2H EP_NTERM
09983 Tsp_orangePink EUT2H
09988 HMMalddh_2_egra(pf00171) EUT2H
09993 H_fuchsia EUT2H
09998 HMMptac_3_eut2(pf06130) EUT2H
10003 HMMptac_3_eut2(pf06130) EUT2H EP_NTERM
10008 P_pastelBlue EUT2H
10013 H_lavender EUT2H
10018 HMM02866_1_pvmeut(pf02866pf00056) EUT2H
10023 HMMreg13411_1_GRM1(pf06445pf13411) EUT2H
10028 -pf00144 -
10033 -# -
10038 -pf02074 -
10043 -pf00115 -
10048 -pf00116 -
10053 -# -
10058 -pf13690 -
10063 -# -
BMC-P
BMC-H
BMC-T
Aldehyde dehydrogenase
PTAC
Signature enzyme
Alcohol dehydrogenase
Regulator

Lysinibacillus sp. YS11. Bacteria; Firmicutes; Bacilli; Bacillales; Bacillaceae;

LBYS11__locus_1

EUT2H

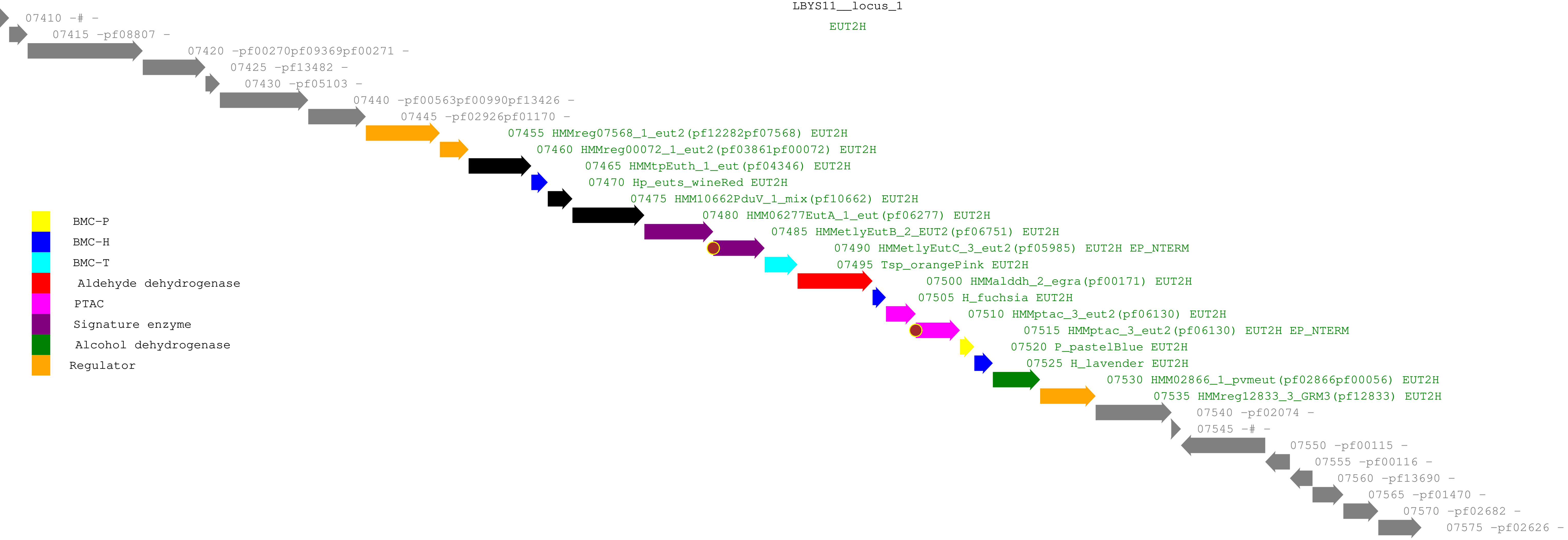

Lysinibacillus fusiformis. Bacteria; Firmicutes; Bacilli; Bacillales; Bacillaceae; Lysinibacillus.

LFU01__locus_1

EUT2H

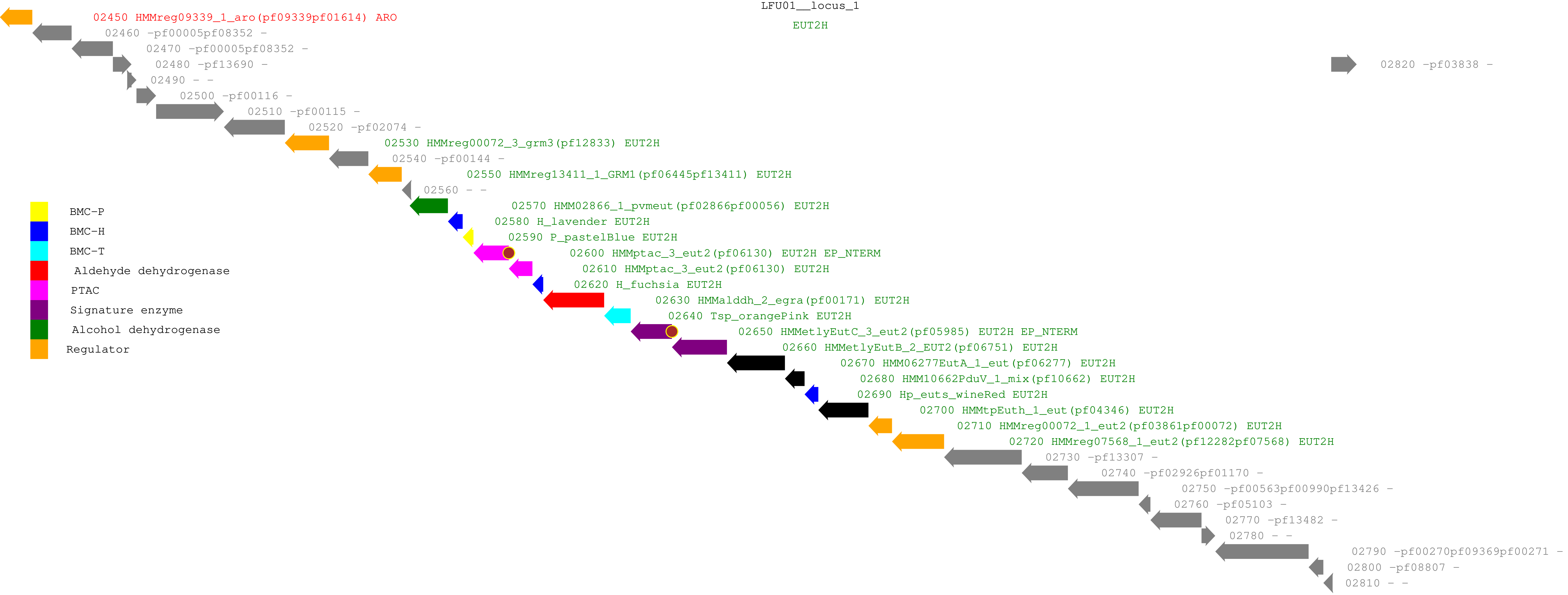

Lysinibacillus sphaericus (Bacillus sphaericus). Bacteria; Firmicutes; Bacilli; Bacillales; Bacillaceae;

LS41612__locus_1

EUT2H

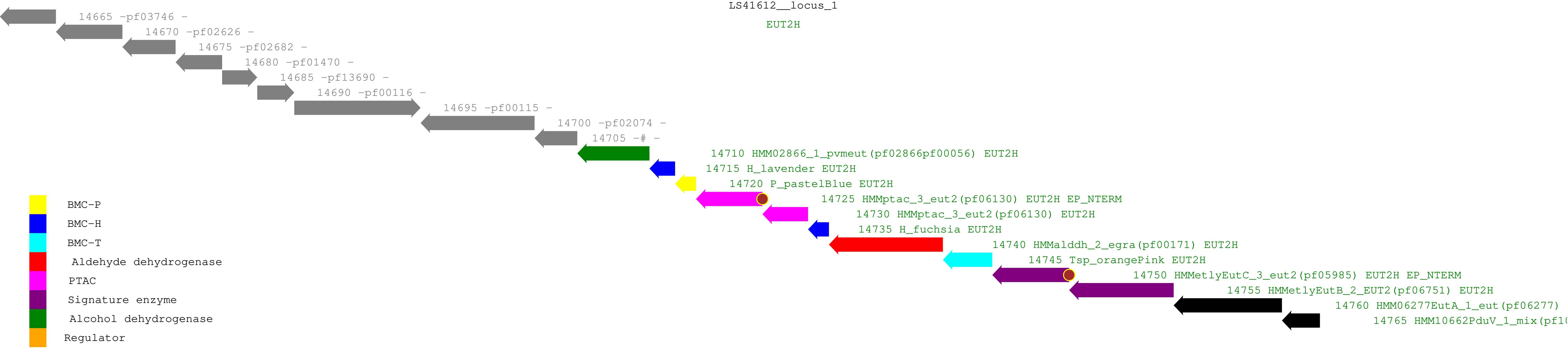

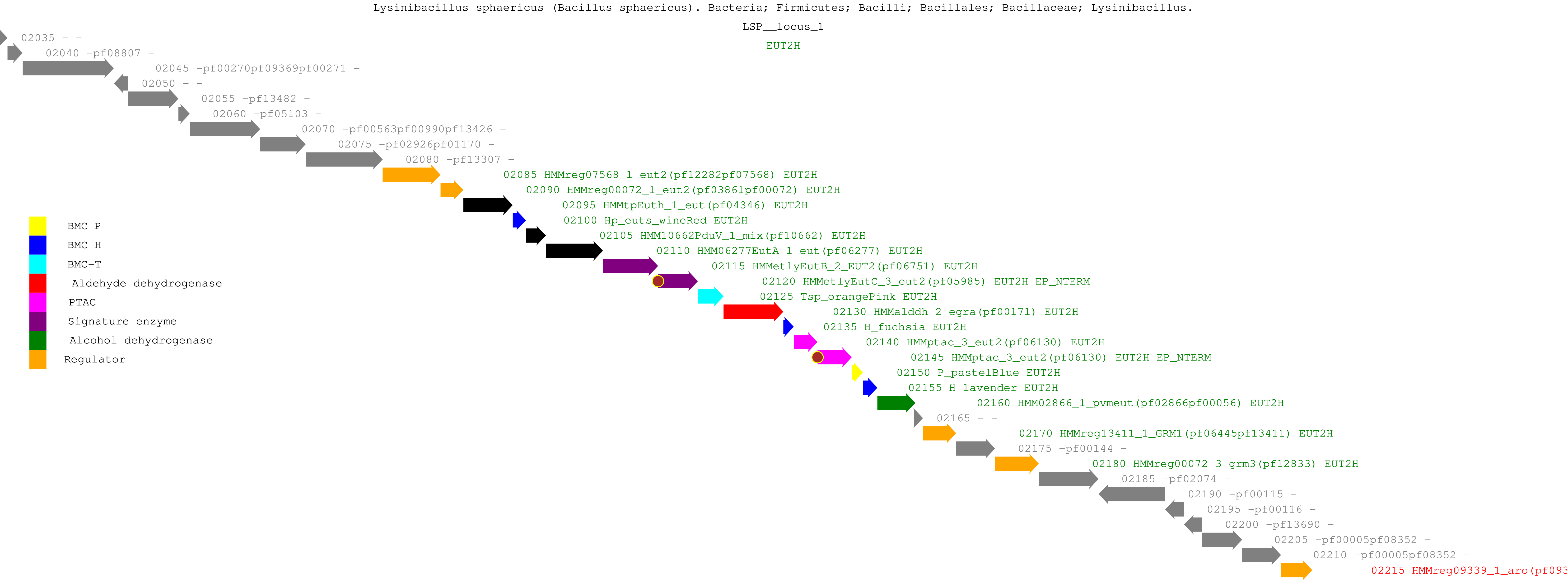
Lysinibacillus sphaericus (Bacillus sphaericus). Bacteria; Firmicutes; Bacilli; Bacillales; Bacillaceae; Lysinibacillus.
LSP__locus_1
EUT2H
02035 - -
02040 -pf08807 -
02045 -pf00270pf09369pf00271 -
02050 - -
02055 -pf13482 -
02060 -pf05103 -
02070 -pf00563pf00990pf13426 -
02075 -pf02926pf01170 -
02080 -pf13307 -
02085 HMMreg07568_1_eut2(pf12282pf07568) EUT2H
02090 HMMreg00072_1_eut2(pf03861pf00072) EUT2H
02095 HMMtpEuth_1_eut(pf04346) EUT2H
02100 Hp_euts_wineRed EUT2H
02105 HMM10662PduV_1_mix(pf10662) EUT2H
02110 HMM06277EutA_1_eut(pf06277) EUT2H
02115 HMMetlyEutB_2_EUT2(pf06751) EUT2H
02120 HMMetlyEutC_3_eut2(pf05985) EUT2H EP_NTERM
02125 Tsp_orangePink EUT2H
02130 HMMalddh_2_egra(pf00171) EUT2H
02135 H_fuchsia EUT2H
02140 HMMptac_3_eut2(pf06130) EUT2H
02145 HMMptac_3_eut2(pf06130) EUT2H EP_NTERM
02150 P_pastelBlue EUT2H
02155 H_lavender EUT2H
02160 HMM02866_1_pvmeut(pf02866pf00056) EUT2H
02165 - -
02170 HMMreg13411_1_GRM1(pf06445pf13411) EUT2H
02175 -pf00144 -
02180 HMMreg00072_3_grm3(pf12833) EUT2H
02185 -pf02074 -
02190 -pf00115 -
02195 -pf00116 -
02200 -pf13690 -
02205 -pf00005pf08352 -
02210 -pf00005pf08352 -
02215 HMMreg09339_1_aro(pf093
BMC-P
BMC-H
BMC-T
Aldehyde dehydrogenase
PTAC
Signature enzyme
Alcohol dehydrogenase
Regulator

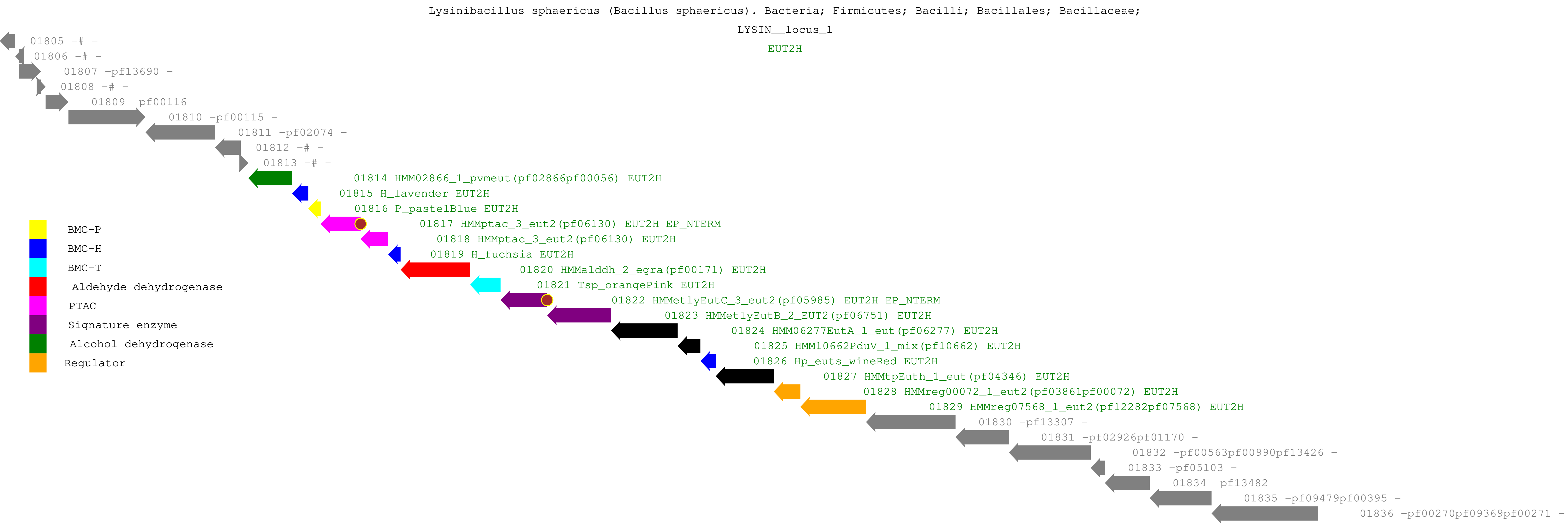

Lysinibacillus sphaericus (Bacillus sphaericus). Bacteria; Firmicutes; Bacilli; Bacillales; Bacillaceae;
LYSIN__locus_1
EUT2H
01805 -# -
01806 -# -
01807 -pf13690 -
01808 -# -
01809 -pf00116 -
01810 -pf00115 -
01811 -pf02074 -
01812 -# -
01813 -# -
01814 HMM02866_1_pvmeut(pf02866pf00056) EUT2H
01815 H_lavender EUT2H
01816 P_pastelBlue EUT2H
01817 HMMptac_3_eut2(pf06130) EUT2H EP_NTERM
01818 HMMptac_3_eut2(pf06130) EUT2H
01819 H_fuchsia EUT2H
01820 HMMalddh_2_egra(pf00171) EUT2H
01821 Tsp_orangePink EUT2H
01822 HMMetlyEutC_3_eut2(pf05985) EUT2H EP_NTERM
01823 HMMetlyEutB_2_EUT2(pf06751) EUT2H
01824 HMM06277EutA_1_eut(pf06277) EUT2H
01825 HMM10662PduV_1_mix(pf10662) EUT2H
01826 Hp_euts_wineRed EUT2H
01827 HMMtpEuth_1_eut(pf04346) EUT2H
01828 HMMreg00072_1_eut2(pf03861pf00072) EUT2H
01829 HMMreg07568_1_eut2(pf12282pf07568) EUT2H
01830 -pf13307 -
01831 -pf02926pf01170 -
01832 -pf00563pf00990pf13426 -
01833 -pf05103 -
01834 -pf13482 -
01835 -pf09479pf00395 -
01836 -pf00270pf09369pf00271 -
BMC-P
BMC-H
BMC-T
Aldehyde dehydrogenase
PTAC
Signature enzyme
Alcohol dehydrogenase
Regulator

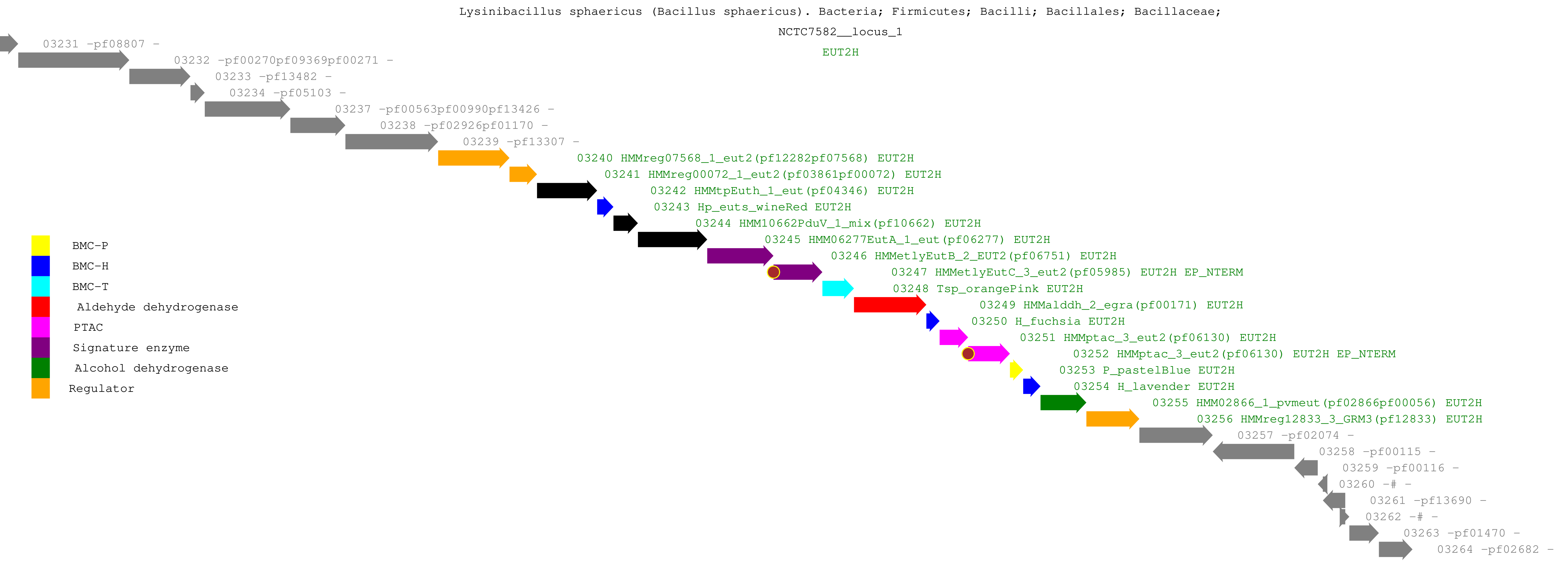
Lysinibacillus sphaericus (Bacillus sphaericus). Bacteria; Firmicutes; Bacilli; Bacillales; Bacillaceae;
NCTC7582__locus_1
EUT2H
03231 -pf08807 -
03232 -pf00270pf09369pf00271 -
03233 -pf13482 -
03234 -pf05103 -
03237 -pf00563pf00990pf13426 -
03238 -pf02926pf01170 -
03239 -pf13307 -
03240 HMMreg07568_1_eut2(pf12282pf07568) EUT2H
03241 HMMreg00072_1_eut2(pf03861pf00072) EUT2H
03242 HMMtpEuth_1_eut(pf04346) EUT2H
03243 Hp_euts_wineRed EUT2H
03244 HMM10662PduV_1_mix(pf10662) EUT2H
03245 HMM06277EutA_1_eut(pf06277) EUT2H
03246 HMMetlyEutB_2_EUT2(pf06751) EUT2H
03247 HMMetlyEutC_3_eut2(pf05985) EUT2H EP_NTERM
03248 Tsp_orangePink EUT2H
03249 HMMalddh_2_egra(pf00171) EUT2H
03250 H_fuchsia EUT2H
03251 HMMptac_3_eut2(pf06130) EUT2H
03252 HMMptac_3_eut2(pf06130) EUT2H EP_NTERM
03253 P_pastelBlue EUT2H
03254 H_lavender EUT2H
03255 HMM02866_1_pvmeut(pf02866pf00056) EUT2H
03256 HMMreg12833_3_GRM3(pf12833) EUT2H
03257 -pf02074 -
03258 -pf00115 -
03259 -pf00116 -
03260 -# -
03261 -pf13690 -
03262 -# -
03263 -pf01470 -
03264 -pf02682 -
BMC-P
BMC-H
BMC-T
Aldehyde dehydrogenase
PTAC
Signature enzyme
Alcohol dehydrogenase
Regulator

Lysinibacillus sphaericus CBAM5. Bacteria; Firmicutes; Bacilli; Bacillales; Bacillaceae;

P799__locus_1

EUT2H

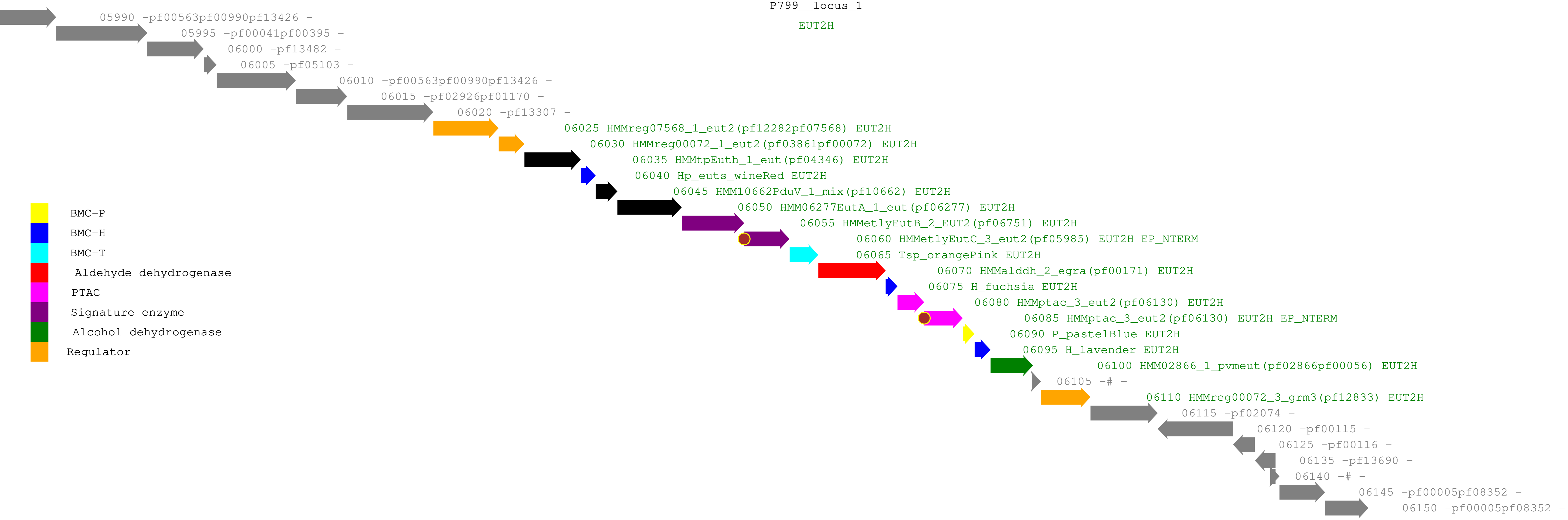

Lysinibacillus sp. A1. Bacteria; Firmicutes; Bacilli; Bacillales; Bacillaceae;

PI85__locus_1

EUT2H

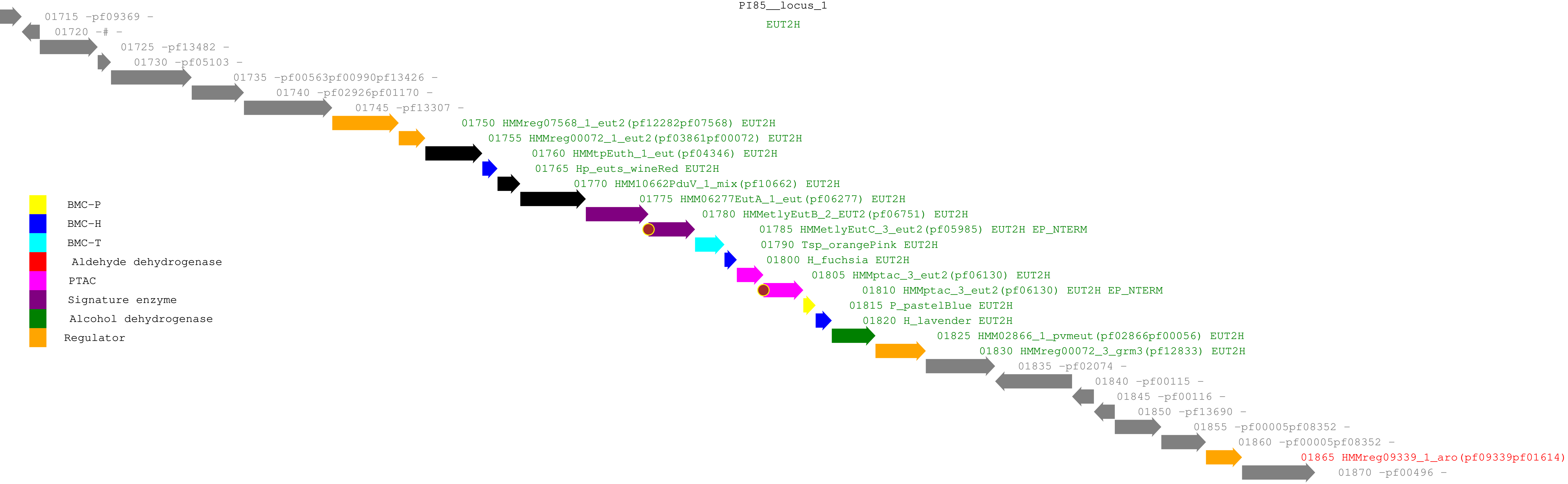

Lysinibacillus sp. SG45. Bacteria; Firmicutes; Bacilli; Bacillales; Bacillaceae;

SAMN02787103__locus_1

EUT2H

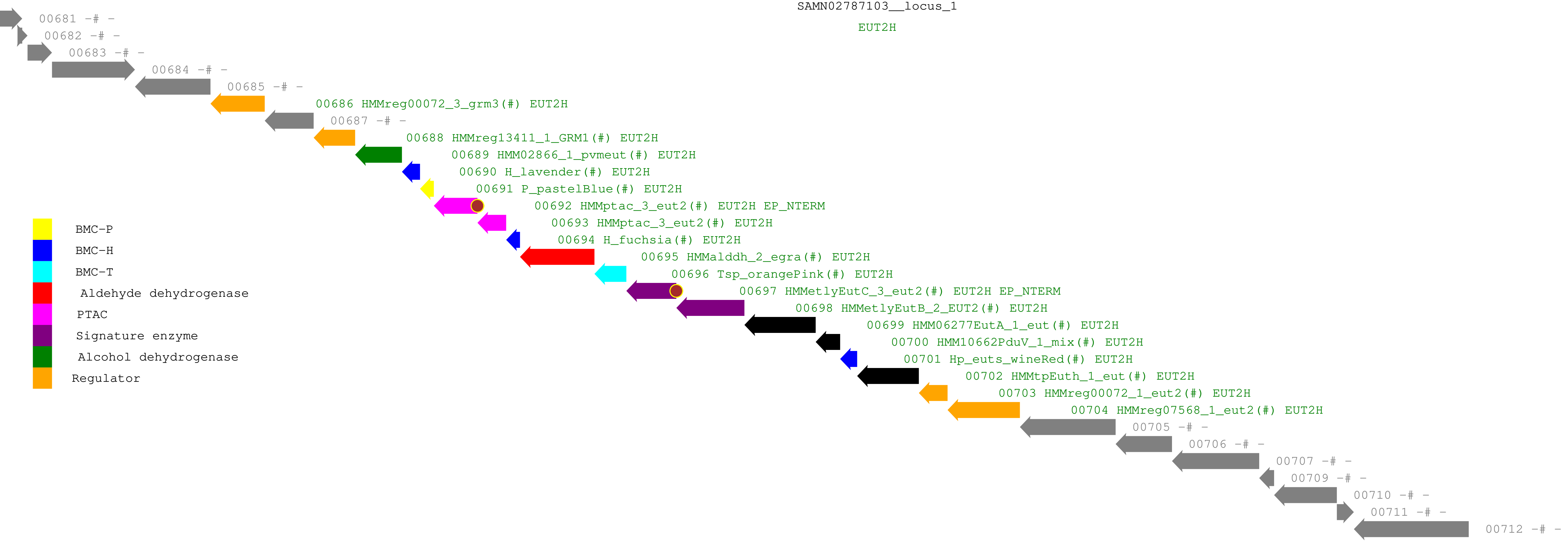

Lysinibacillus sp. AC-3. Bacteria; Firmicutes; Bacilli; Bacillales; Bacillaceae;

SAMN06295926__locus_1

EUT2H

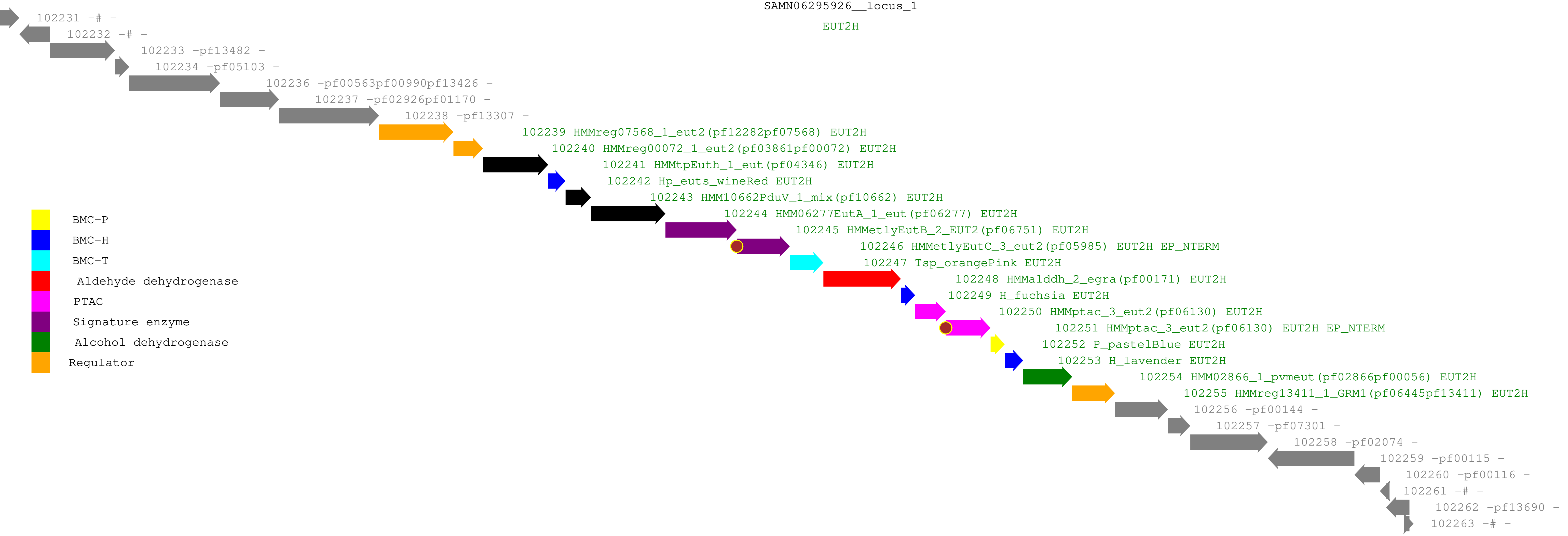

Lysinibacillus varians. Bacteria; Firmicutes; Bacilli; Bacillales; Bacillaceae;

T479__locus_1

EUT2H

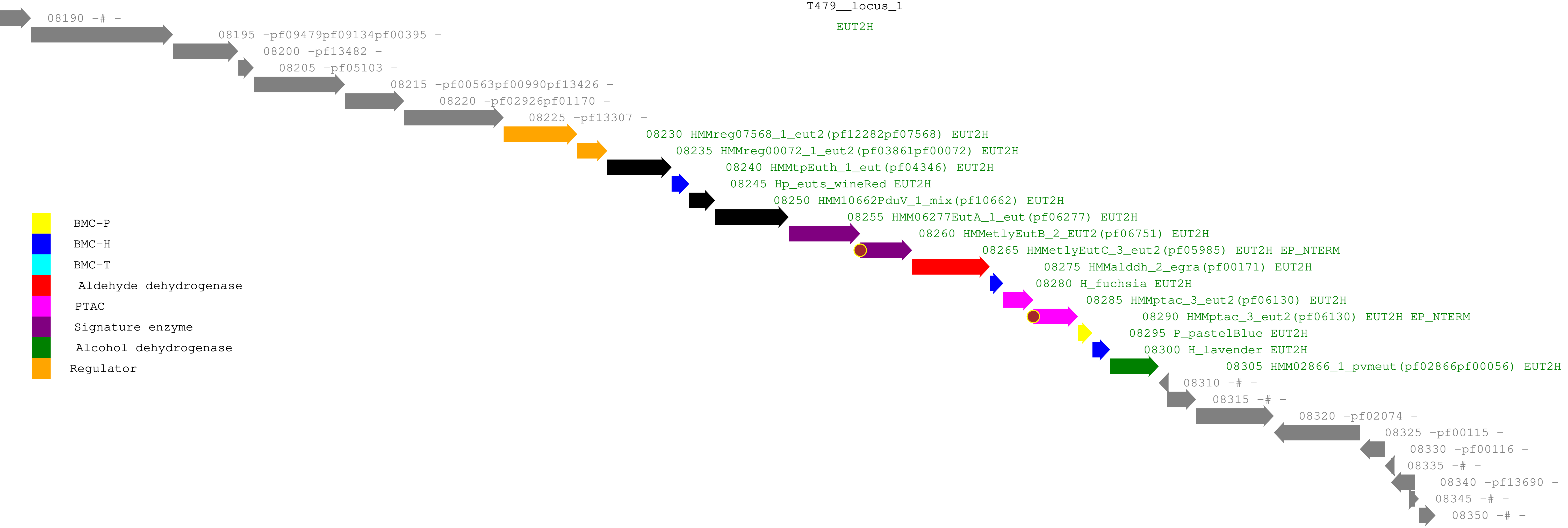

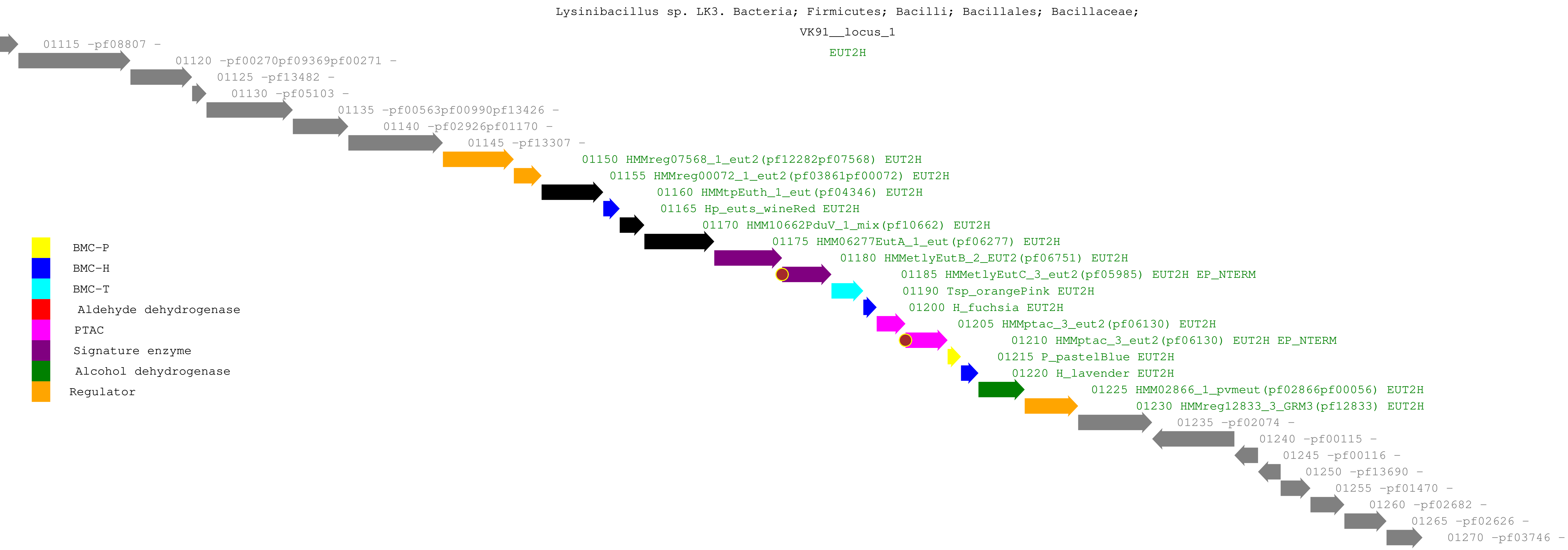
Lysinibacillus sp. LK3. Bacteria; Firmicutes; Bacilli; Bacillales; Bacillaceae;
VK91__locus_1
EUT2H
01115 -pf08807 -
01120 -pf00270pf09369pf00271 -
01125 -pf13482 -
01130 -pf05103 -
01135 -pf00563pf00990pf13426 -
01140 -pf02926pf01170 -
01145 -pf13307 -
01150 HMMreg07568_1_eut2(pf12282pf07568) EUT2H
01155 HMMreg00072_1_eut2(pf03861pf00072) EUT2H
01160 HMMtpEuth_1_eut(pf04346) EUT2H
01165 Hp_euts_wineRed EUT2H
01170 HMM10662PduV_1_mix(pf10662) EUT2H
01175 HMM06277EutA_1_eut(pf06277) EUT2H
01180 HMMetlyEutB_2_EUT2(pf06751) EUT2H
01185 HMMetlyEutC_3_eut2(pf05985) EUT2H EP_NTERM
01190 Tsp_orangePink EUT2H
01200 H_fuchsia EUT2H
01205 HMMptac_3_eut2(pf06130) EUT2H
01210 HMMptac_3_eut2(pf06130) EUT2H EP_NTERM
01215 P_pastelBlue EUT2H
01220 H_lavender EUT2H
01225 HMM02866_1_pvmeut(pf02866pf00056) EUT2H
01230 HMMreg12833_3_GRM3(pf12833) EUT2H
01235 -pf02074 -
01240 -pf00115 -
01245 -pf00116 -
01250 -pf13690 -
01255 -pf01470 -
01260 -pf02682 -
01265 -pf02626 -
01270 -pf03746 -
BMC-P
BMC-H
BMC-T
Aldehyde dehydrogenase
PTAC
Signature enzyme
Alcohol dehydrogenase
Regulator

Blautia sp. YL58. Bacteria; Firmicutes; Clostridia; Clostridiales; Lachnospiraceae;

A4V09__locus_1

EUT2I

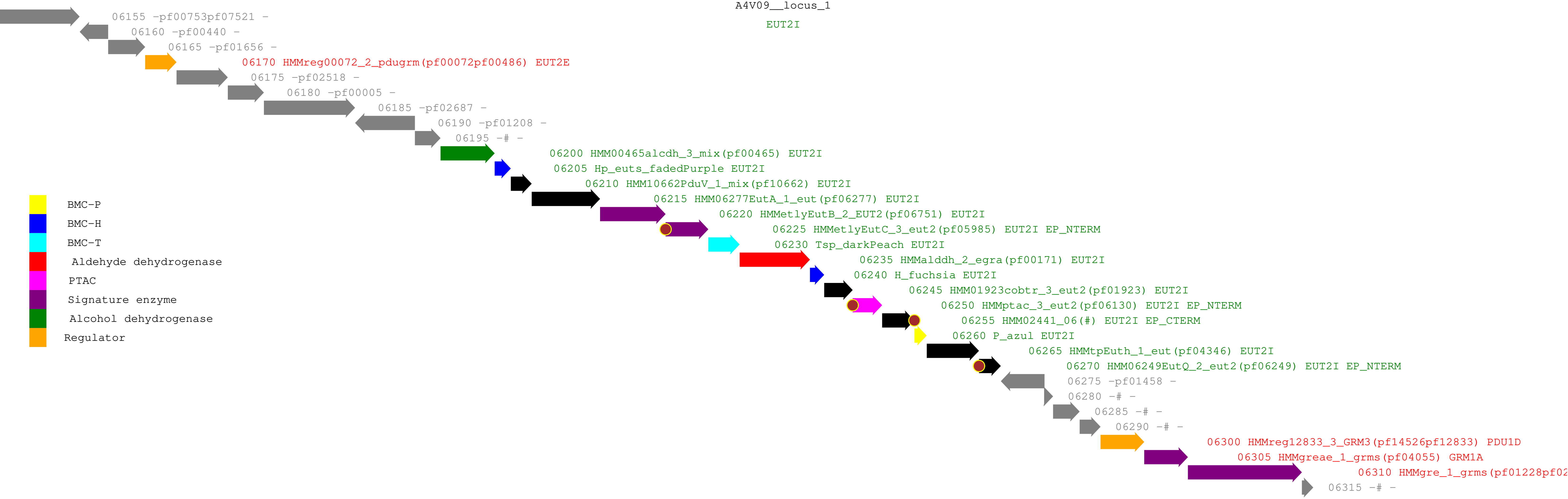

Clostridium perfringens B str. ATCC 3626. Bacteria; Firmicutes; Clostridia; Clostridiales; Clostridiaceae;

AC1__locus_1

EUT2I

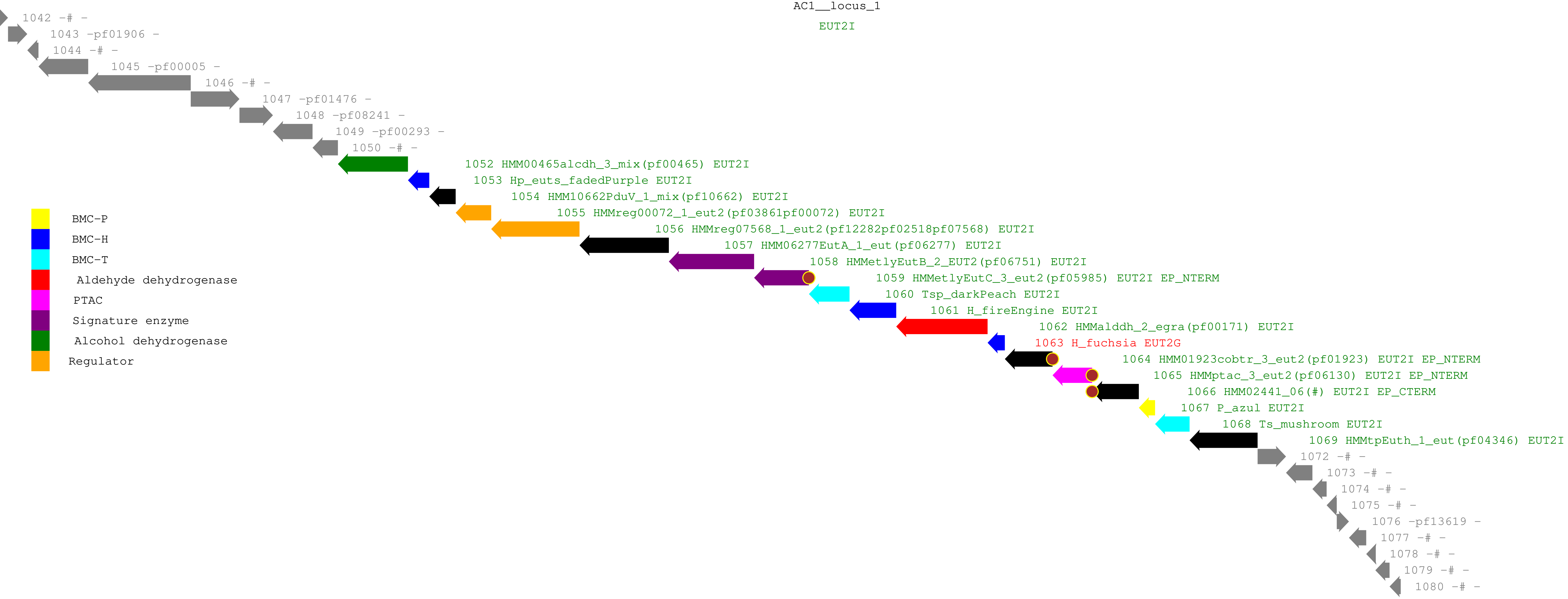

Clostridium perfringens E str. JGS1987. Bacteria; Firmicutes; Clostridia; Clostridiales; Clostridiaceae;

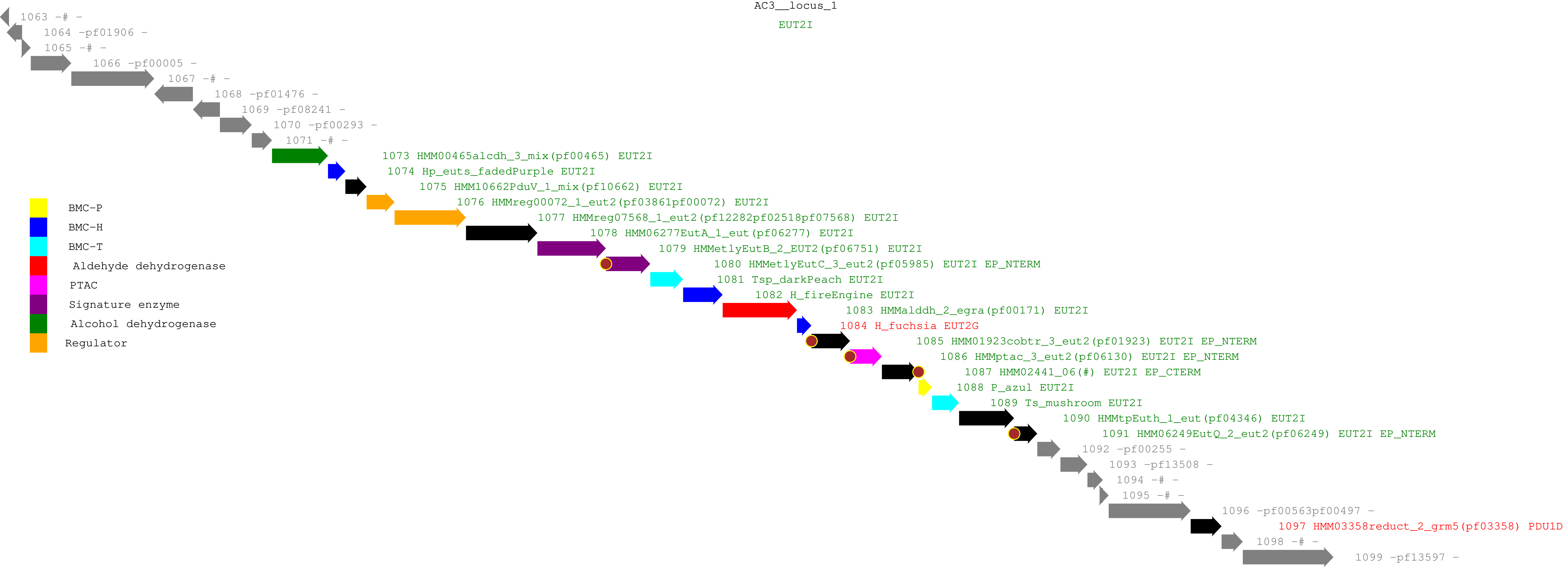

Blautia coccoides. Bacteria; Firmicutes; Clostridia; Clostridiales; Lachnospiraceae;

ADH70__locus_1

EUT2I

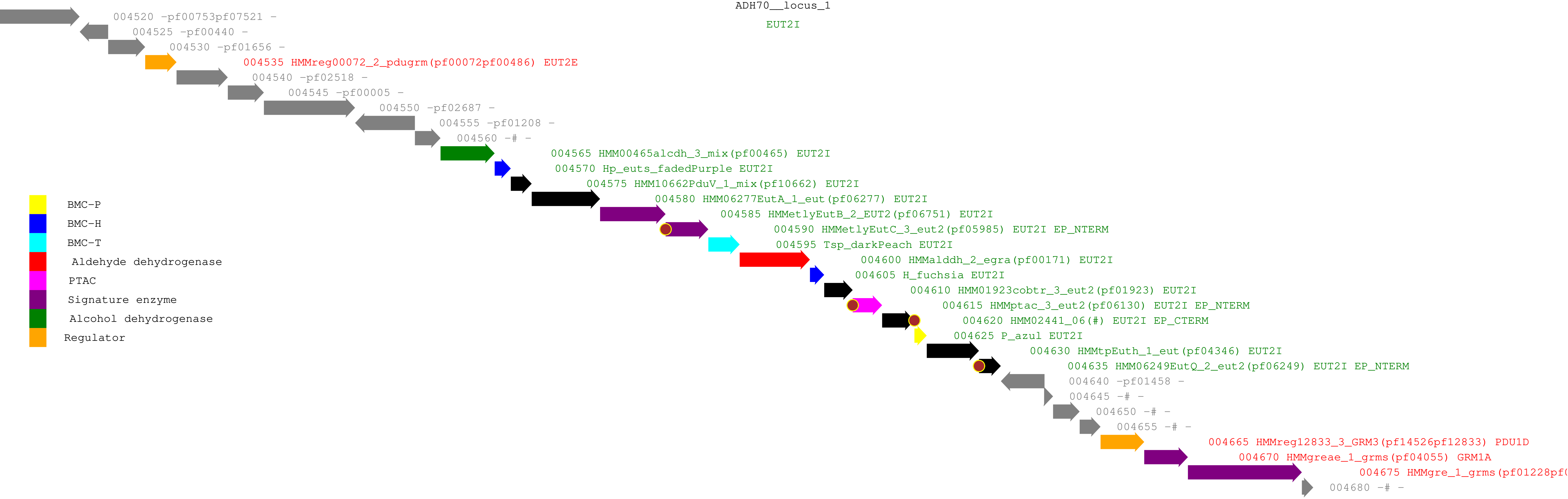

Brevibacillus sp. SKDU10. Bacteria; Firmicutes; Bacilli; Bacillales; Paenibacillaceae;

AYJ08__locus_1_main_sat

EUT2I

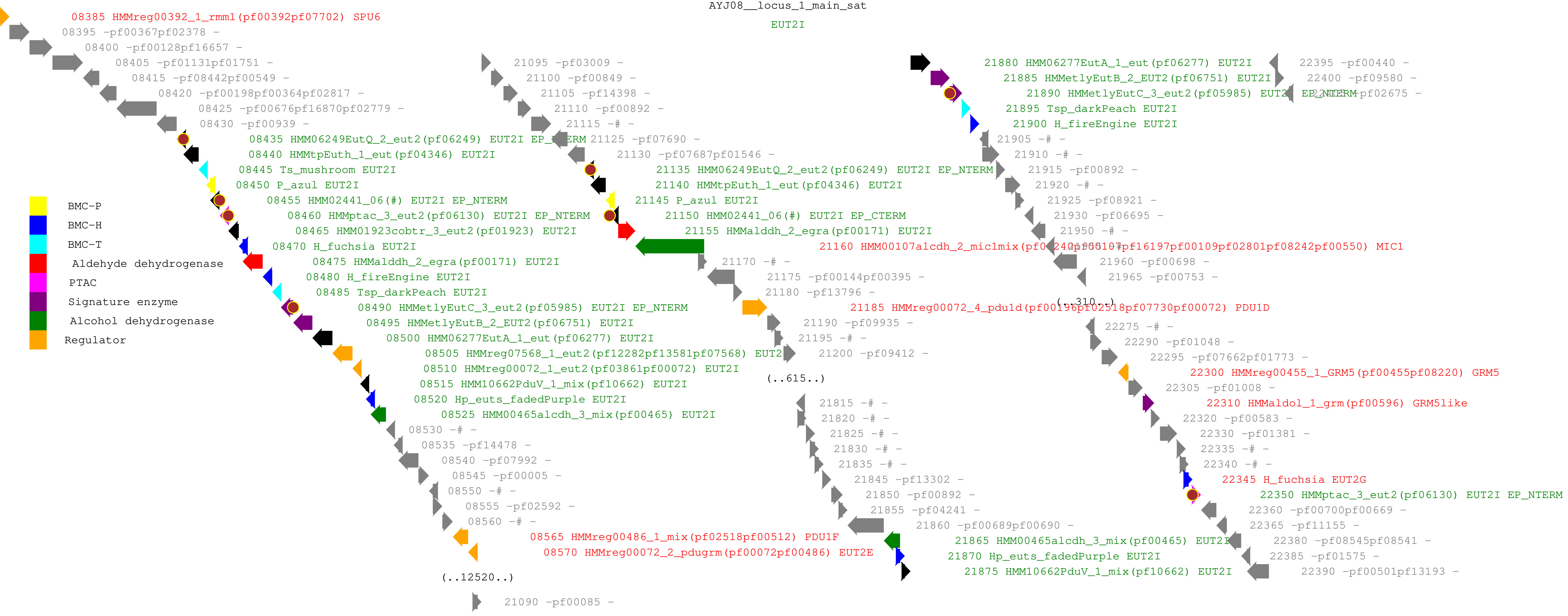

Paraclostridium bifermentans (Clostridium bifermentans). Bacteria; Firmicutes; Clostridia; Clostridiales;

B2H97__locus_1

EUT2I

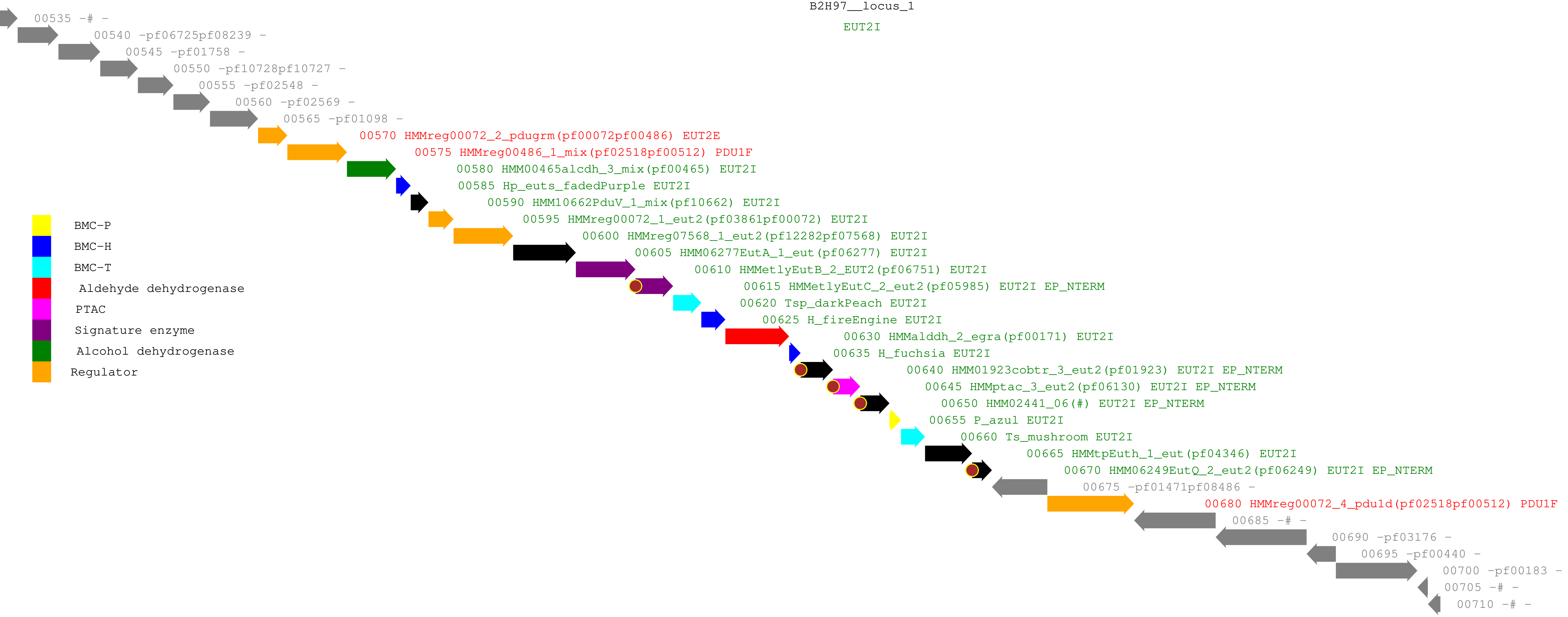

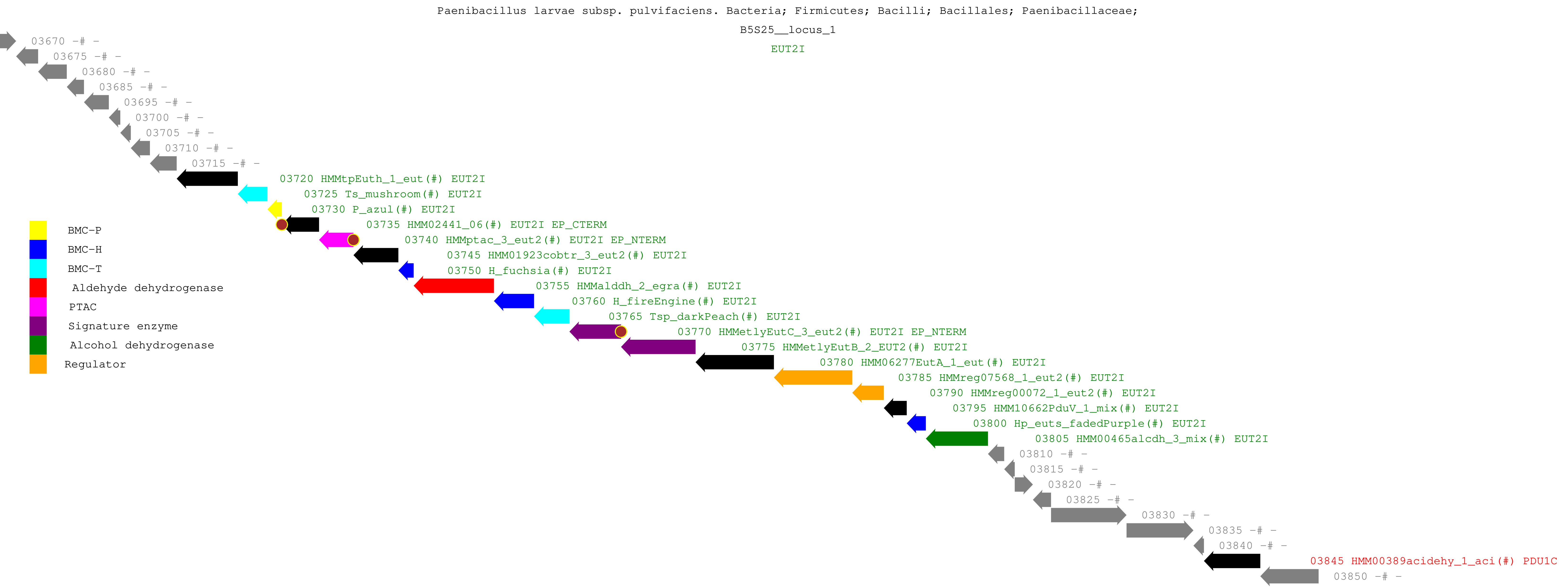

Paenibacillus larvae subsp. pulvifaciens. Bacteria; Firmicutes; Bacilli; Bacillales; Paenibacillaceae;
B5S25__locus_1
EUT2I
03670 -# -
03675 -# -
03680 -# -
03685 -# -
03695 -# -
03700 -# -
03705 -# -
03710 -# -
03715 -# -
03720 HMMtpEuth_1_eut(#) EUT2I
03725 Ts_mushroom(#) EUT2I
03730 P_azul(#) EUT2I
03735 HMM02441_06(#) EUT2I EP_CTERM
03740 HMMptac_3_eut2(#) EUT2I EP_NTERM
03745 HMM01923cobtr_3_eut2(#) EUT2I
03750 H_fuchsia(#) EUT2I
03755 HMMalddh_2_egra(#) EUT2I
03760 H_fireEngine(#) EUT2I
03765 Tsp_darkPeach(#) EUT2I
03770 HMMetlyEutC_3_eut2(#) EUT2I EP_NTERM
03775 HMMetlyEutB_2_EUT2(#) EUT2I
03780 HMM06277EutA_1_eut(#) EUT2I
03785 HMMreg07568_1_eut2(#) EUT2I
03790 HMMreg00072_1_eut2(#) EUT2I
03795 HMM10662PduV_1_mix(#) EUT2I
03800 Hp_euts_fadedPurple(#) EUT2I
03805 HMM00465alcdh_3_mix(#) EUT2I
03810 -# -
03815 -# -
03820 -# -
03825 -# -
03830 -# -
03835 -# -
03840 -# -
03845 HMM00389acidehy_1_aci(#) PDU1C
03850 -# -
BMC-P
BMC-H
BMC-T
Aldehyde dehydrogenase
PTAC
Signature enzyme
Alcohol dehydrogenase
Regulator

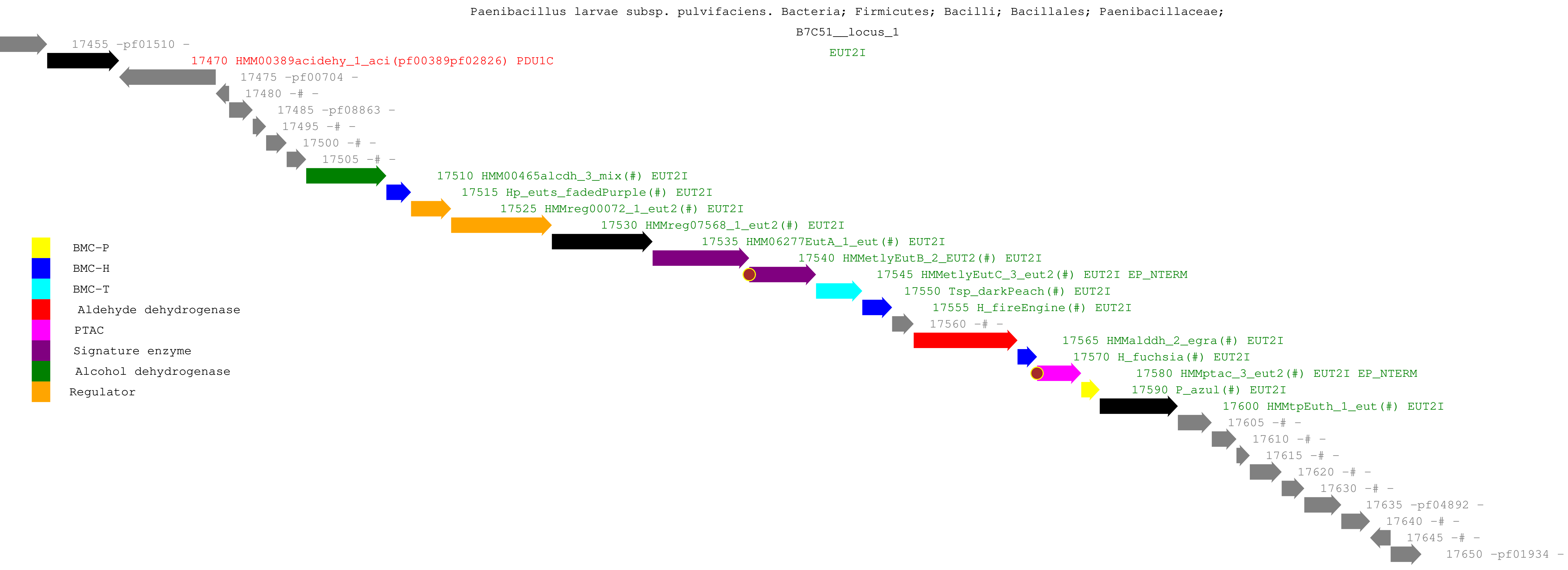
Paenibacillus larvae subsp. pulvifaciens. Bacteria; Firmicutes; Bacilli; Bacillales; Paenibacillaceae;
B7C51__locus_1
EUT2I
17455 -pf01510 -
17470 HMM00389acidehy_1_aci(pf00389pf02826) PDU1C
17475 -pf00704 -
17480 -# -
17485 -pf08863 -
17495 -# -
17500 -# -
17505 -# -
17510 HMM00465alcdh_3_mix(#) EUT2I
17515 Hp_euts_fadedPurple(#) EUT2I
17525 HMMreg00072_1_eut2(#) EUT2I
17530 HMMreg07568_1_eut2(#) EUT2I
[truncated: 348,400 more chars]
